# Investigating the role of gut microbiota in diabetic nephropathy through plasma proteome mediated analysis

**DOI:** 10.1038/s41598-025-90306-7

**Published:** 2025-02-14

**Authors:** Shaojie Fu, Fan Li, Jinyu Yu, Shengjie Ma, Li Zhang, Yanli Cheng

**Affiliations:** 1 Department of Nephrology, The First Hospital of Jilin University, 1 Xinmin Street, Changchun, 130021 Jilin China; 2 Department of Gastroenterology, The First Hospital of Jilin University, Changchun, 130021 Jilin China; 3 Department of Gastric and Colorectal Surgery, General Surgery Center, The First Hospital of Jilin University, Changchun, 130021 Jilin China

**Keywords:** Diabetic nephropathy, Gut microbiota, Plasma proteins, Mendelian randomization analysis, Mediation analysis, Microbiology, Nephrology

## Abstract

Diabetic nephropathy (DN) is the leading cause of end-stage renal disease and poses significant threats to individuals with diabetes. The concept of gut–kidney axis has gained increasing attention in recent years and the in the occurrence and development of DN, alterations in the gut microbiota also plays a crucial and indispensable role. However, the specific causal relationships between various gut microbial communities and DN, as well as the underlying molecular mechanisms, remains unclear. This study utilized data from genome-wide association studies. After screening for qualified instrumental variables, mendelian randomization causal analyses were performed by inverse variance weighting, MR-Egger, weighted median, weighted mode and MR-RAPS methods. Additionally, sensitivity analyses such as heterogeneity, multiplicity, and the direction of the causal effect were carried out to ensure that the results were robust. After identifying significant gut microbiota, protein-proteomics mediation analysis was conducted on potential 3282 plasma proteins to determine those with mediating effects. Finally, Reactome enrichment analysis was performed to ascertain metabolic or signaling pathways with mediating effects. Mendelian randomization analysis indicated associations between 21 gut microbiota and DN. After adjusting significance levels, Catenibacterium and Parasutterella were found to have causal effects on the onset of DN. Subsequently, we identified 22 plasma proteins with mediating effects, along with 27 metabolic or signaling pathways including activated propionic acid metabolism. Increased in the abundance of Catenibacterium and Parasutterella intestinal bacteria are causative factors for DN. More importantly, the underlying mechanism by which the increased abundance of Catenibacterium and Parasutterella intestinal bacteria lead to DN were revealed, providing a blueprint for the involvement of gut–kidney axis in the pathogenesis of DN and paving the way for future studies.

**Supplementary Information:**

The online version contains supplementary material available at 10.1038/s41598-025-90306-7.

## Introduction

The global incidence of diabetes has been steadily increasing, as it is a metabolic disorder characterized by hyperglycemia. As of 2021, the global prevalence of diabetes was 6.1%, with a staggering 537 million affected individuals. It is projected that by 2050, the overall prevalence of diabetes will exceed 10%, with over 13.1 billion people affected, imposing a significant burden worldwide^1–3^. Characterized by the accumulation of extracellular matrix (ECM), mesangial expansion, thickening of the basement membrane, glomerulosclerosis, tubular inflammation, atrophy, and interstitial fibrosis, diabetic nephropathy (DN) stands as the prevailing microvascular complication of diabetes mellitus^4^. DN serves as a major cause of end-stage renal disease (ESRD) globally^5^. Research indicates that ESRD has a profound impact on global health, directly contributing to increased morbidity and mortality rates, as well as serving as a significant risk factor for cardiovascular diseases. Approximately 30–50% of ESRD cases worldwide can be attributed to DN. Although current treatments for DN partially alleviate patients’ conditions, they fail to completely halt the progression of DN to ESRD. The pathogenic mechanisms of DN are still complex and poorly understood, and effective therapeutic approaches remain inadequate. Therefore, there is an urgent need to explore prospective treatment strategies for DN^6^.

In the realm of metabolic diseases, the connection between the gut microbiota and these ailments has emerged as an influential and extensively investigated area of study^7^. Over the past years, a considerable volume of supporting data has underscored a noteworthy link between the gut microbiota and metabolic conditions, encompassing obesity, non-alcoholic fatty liver disease, and diabetes^8–10^. Moreover, recent studies have demonstrated that an imbalance in gut bacteria can impact the progression of chronic kidney disease (CKD) through pathways involving the production of uremic toxins, regulation of inflammation, and modulation of the immune system^11^. In the occurrence and development of DN, alterations in the gut microbiota play a crucial and indispensable role^12^. A profound disparity in the composition of gut microbiota has been unveiled through research, delineating a strong link between gut dysbiosis and the initiation as well as advancement of DN, thereby implying a close correlation between individuals afflicted by DN and those in a state of good health^13^. Additionally, some studies have shown a correlation between specific microbial abundances and clinical indicators of DN, such as urine protein levels and the extent of renal impairment^14^. These findings offer valuable insights that can guide further exploration into the mechanistic roles played by the gut microbiota in the development of DN, warranting deeper investigation. However, a critical question that remains to be answered is how the gut microbiota leads to renal damage in individuals with diabetes. The characteristics of gut microbiota imbalance include disruptions in homeostatic equilibrium and abnormal generation of bacterial metabolites. While certain microbial metabolites (both beneficial and harmful) have been implicated in the pathological progression of DN^15^, the specific causal relationships between various microbial communities and DN are still unclear due to the diverse array of gut microbiota species. The quest to unveil the underlying pathological mechanisms encountered various obstacles, demanding additional research to surmount these current challenges and attain a more holistic comprehension of the intricate correlation between DN and the gut microbiota.

The utilization of genetic variation as instrumental variables characterizes the research approach known as mendelian randomization (MR), facilitating the examination of whether an exposure factor possesses a causative impact on the outcome of interest^16^. Compared to conventional methods, MR could avoid reverse causation bias and attenuates the interference of confounding factors, so it is becoming increasingly popular in epidemiologic studies^17^. In recent years, genome-wide association studies (GWAS) have detected genetic variants associated with plasma proteome levels^18,19^, which provide an opportunity to utilize MR to further reveal the underlying mechanisms behind the causal relationship between exposure and outcome, as many circulating proteins always act as the principal regulators of molecular pathways^20^.

Hence, in this study, we performed a comprehensive two-sample MR analysis to systematically explore the causal relationship between gut microbiota and DN, and further explored the mediating role of plasma proteome between them. Through the elucidation of these mediating effects, we can acquire valuable insights into the mechanistic pathways through which the gut microbiota may exert influence on the risk of DN, thereby opening avenues for potential therapeutic interventions.

## Methods

### Data source

**Fig. 1:**
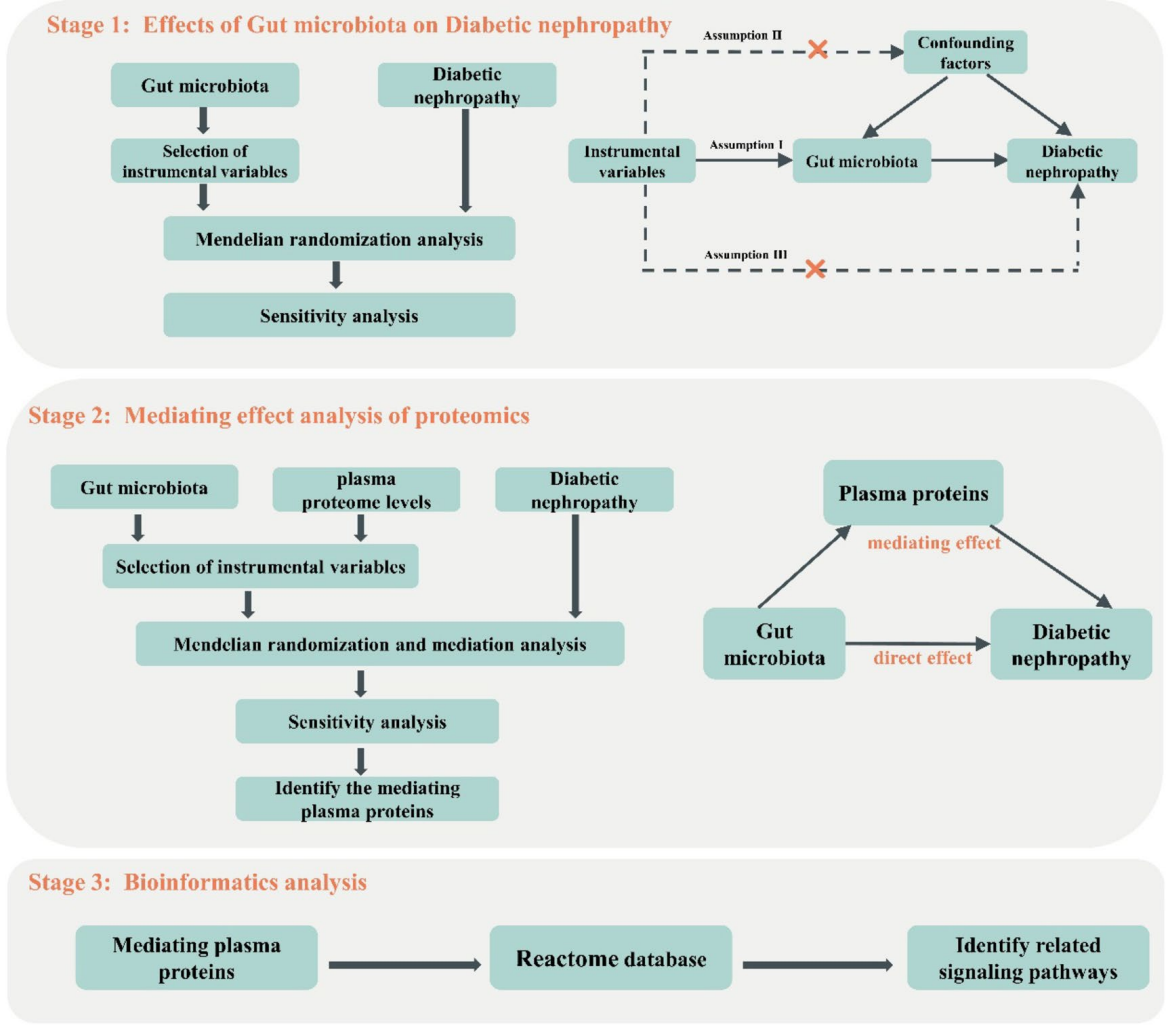
Study design flowchart.

Research design of this study is shown in Fig. 1. The GWAS summary data related to gut microbiome abundance were published by Kurilshikov et al. in 2021. This study conducted a genome-wide association study on 211 types of gut microbiome abundance involving 18,340 participants (14,306 as European population) from 25 cohorts of diverse ethnicities and ages^21^. Genetic variation data related to DN were obtained from FinnGen. The FinnGen study is a large-scale genomics project that analyzed over 500,000 samples from Finnish biobanks, associating genetic variations with health data to understand disease mechanisms and susceptibilities. The project is a collaboration among research organizations, biobanks within Finland, and international industry partners. On May 11, 2023, the database released the R9 version of GWAS data, from which GWAS summary data for 4,111 DN cases and 312,650 controls were obtained based on ICD-10 code “N08.3”^22^. GWAS data based on the plasma proteome were published by Sun et al. in 2018. The study included approximately 50,000 participants in a randomized trial with different blood donation intervals. From mid-2012 to mid-2014, 25 centers of the NHS Blood and Transplant (NHSBT) in England recruited blood donors aged 18 and above, excluding those with a history of major diseases such as myocardial infarction, stroke, cancer, Human immunodefciency virus (HIV), Hepatitis B or C virus, or recent illness or infection. A multiplexed aptamer-based method (SOMAscan assay) was used to measure the relative concentrations of 3,622 plasma proteins or protein complexes, assessed using 4,034 modified aptamers^18^. Prior to analysis, all the aforementioned data underwent a rigorous ethical review and encompassed a diverse range of participants from European populations, encompassing both males and females, thus effectively mitigating the potential for bias resulting from population stratification. The information about the included GWAS datasets is provided in Table 1 and Supplementary Tables 1, and there is no sample overlap in the MR analysis of this study based on the information from the data set sources.

**Table 1:** Details of GWAS datasets included in MR studies.

Trait	Case	Sample size	Year	Author	Sex	Population	NSNP
Abundances of 150 gut microbiota	14,306	14,306	2021	Kurilshikov A	Males and Females	European	5,486,191
Diabetic nephropathy	4111	312,650	2023	FinnGen	Males and Females	European	20,167,370
3282 plasma proteome	3301	3301	2018	Sun BB	Males and Females	European	10,534,735

### Selection of instrumental variables

To perform MR analysis, the instrumental variables must satisfy three assumptions: the relevance assumption, the independence assumption, and the exclusion restriction assumption. Based on these assumptions, we established criteria for selecting instrumental variables. In accordance with the relevance assumption, all eligible single nucleotide polymorphisms (SNPs) must be significantly associated with the exposure. We identified SNPs linked to exposure using a genome-wide significance level as our selection criterion (*P*-value < 5 × 10^−8^). In the mediation MR analysis, due to varying associations between different plasma proteins and genetic variants, extracting too many SNPs can enhance heterogeneity, while too few can result in insufficient explanatory variance, thus a second threshold was set (*P*-value < 1 × 10^−5^). SNPs exhibiting linkage disequilibrium (LD) were excluded based on an r^2^< 0.001 and a window size > 10,000 kb. Data on outcome-related SNPs were extracted based on SNP identifiers within the outcome dataset. During this process, ambiguous SNPs and palindromic SNPs were removed, matching SNPs across both datasets. We calculated the F-statistic for each SNP. The F-statistic, an intermediate measure in variance analysis, gauges the strength of association that instrumental variable SNPs have with exposure^23^. SNPs with an F-statistic less than 10 were considered weak instrumental variables and excluded. To satisfy the independence assumption, i.e., to ensure that SNPs are not associated with any confounders, the MR-PRESSO test, a method used to detect and correct outliers in Mendelian randomization caused by external factors such as pleiotropy of genes, was conducted to identify and exclude potential pleiotropic SNPs. PhenoScanner, an online database for retrieving phenotype information associated with specific genes, was also used to meticulously examine each SNP to assess its potential association with confounders, effectively removing SNPs that demonstrated potential violations of the independence assumption^24^. To fulfill the exclusion restriction assumption, i.e., to ensure that SNPs exert no direct effect on the outcome but only affect it indirectly via the exposure, the MR-Steiger test, a test employed to determine the correct causal direction in Mendelian randomization analyses, was used to verify the causal inference direction of each SNP, excluding those with incorrect directions^25^. Following rigorous screening, the remaining SNPs were deemed valid instrumental variables. In summary, the MR processes for direct and mediated effects were consistent, except for the different extraction thresholds for SNPs.

### Mendelian randomization analysis

Classic MR requires the use of inverse variance weighting (IVW). To evaluate the robustness of the results, we also utilized MR-Egger regression, weighted median (WM), weighted mode approach, and MR robust adjusted profile score (MR-RAPS) to comprehensively assess potential biases. The IVW method employs a form of variance-inverse weighting to combine the causal estimates of the Wald ratio for each SNP^26^. The Wald ratio estimate measures the ratio of the impact of a single SNP on the outcome to its impact on exposures, assuming all associations conform to a logarithmic linear relationship^27^. In the realm of MR analysis, the utilization of MR-Egger regression emerges as an invaluable instrument for establishing a weighted linear regression model that examines the relationship between exposure coefficients and outcomes^28^. Similar to the IVW method, MR-Egger regression allows for the assessment of horizontal pleiotropy through the significance of its intercept. The foundation of the MR-Egger method rests upon the underlying assumption of No Measurement Error (NOME). We also calculated the I^2^statistic to quantify the degree to which MR-Egger violates the NOME assumption. Results should be adjusted when I^2^is less than 90%. When multiple variants are invalid, the results from the above methods may lack robustness; in such cases, the WM method and weighted median approach demonstrate greater robustness. The median weights for individual genetic variants are computed by the WM method, which combines these weights to generate an estimate. Significantly, the WM method can yield dependable estimates of causal effects, provided that at least 50% of the weights utilized in the analysis originate from valid instrumental variables^29^. Even in the presence of some invalid instrumental variables, the WM method can accurately estimate causal relationships and enhance precision. The weighted median method remains robust even with a higher number of invalid instrumental variables. According to the MR analysis guidelines, when pleiotropy is present, MR-Egger regression is employed as the primary analytical method, when heterogeneity is present, IVW random effects model with reference to the weighted median method is employed, and if no heterogeneity is detected, the IVW random effects model is used as the main analysis method. In addition, this study incorporates the recently developed MR-RAPS technique, which employs a random effects distribution to directly model the pleiotropic effects of genetic variants. Compared to traditional Mendelian randomization techniques, this novel strategy offers enhanced robustness. In the analysis of the gut microbiome, to avoid errors due to multiple comparisons (i.e., Type I statistical errors, where the risk of bias increases as the number of comparisons rises, leading to false positives at a *P*-value threshold of 0.05), we applied the false discovery rate (FDR) method to adjust the *P*-values. This approach helps to reduce the false positive rate that may arise from spurious statistical significance when performing numerous statistical tests. Statistical significance was considered when the adjusted *P*-value was < 0.05.

### Sensitivity analysis

Pleiotropy includes horizontal pleiotropy and vertical pleiotropy. Typically, vertical pleiotropy does not affect the reliability of conclusions; however, horizontal pleiotropy should be eliminated. To gauge the degree of horizontal pleiotropy, our primary approach involves the utilization of the MR-Egger method for estimation. If the *P*-value of the MR-Egger intercept is less than 0.05, the instrumental variable is considered to be severely affected by horizontal pleiotropy, rendering the results unreliable. In the context of MR analysis, heterogeneity may still be present among SNPs, even if they are all deemed valid instrumental variables. The presence of substantial heterogeneity can render results unreliable; thus, heterogeneity testing is necessary to enhance the credibility of the outcomes. We employ the IVW method to calculate heterogeneity among SNPs and utilize Cochran’s Q test. If *P*< 0.05, heterogeneity is considered present. Additionally, as per convention, we use the leave-one-out method and have produced a funnel plot. We conducted a comprehensive MR-Steiger test to ensure the correct overall causal direction^30^. Finally, we calculated the statistical power to ascertain the reliability of the negative results. The calculation method has been described in detail in previous literature^31^.

### Proteomics mediation analysis and enrichment analysis

Using GWAS summary data, a two-step mediated MR analysis was conducted to determine whether plasma proteins are intermediary factors in gut microbiota-induced DN, and the process of MR analysis was described as previously. The first step involved a two-sample MR analysis between significant gut microbiota and the plasma proteome, followed by a two-sample MR analysis between the plasma proteome and DN. Proteins significant in all three analyses exhibit partial mediation effects; those significant only in the two-step mediation analysis display complete mediation effects, while proteins not consistently significant across the analyses show no mediation effects. Indirect effects are calculated using the formula β1*β2, while direct effects are determined by subtracting the indirect effects from the total effects. The Reactome database was used to analyze the list of intermediary plasma proteins to understand enriched pathways. The Reactome database serves as a peer-reviewed repository of human biological pathways and reactions. To assess the potential enrichment of specific Reactome pathways within the gene list, over-representation analysis was performed, resulting in the generation of probability scores and significance *P*-values.

### Statistical software and plotting

The conclusions section features various visualizations to depict the findings. For each SNP, scatter plots were generated (Supplementary Fig. 1) to illustrate the associations between exposure factors and outcome effects, accompanied by regression curves to visualize causal estimates. The results of the MR analysis were displayed using a significance heatmap. Funnel plots were employed to assess potential directional effects, pleiotropy, and data distribution (Supplementary Fig. 2). Forest plots were created using the final causal estimates, showcasing the results for each SNP as well as the overall outcomes of the MR analysis (Supplementary Fig. 3). Leave-one-out analysis plots were used to identify whether the SNP was a strong influence utility on the results (Supplementary Fig. 4). The statistical analyses in this study were performed utilizing R (version 4.2.3) and three R packages, namely “Two Sample MR,” “MR-PRESSO,” and “mr.raps.”

## Results

### Datasets information

**Fig. 2:**
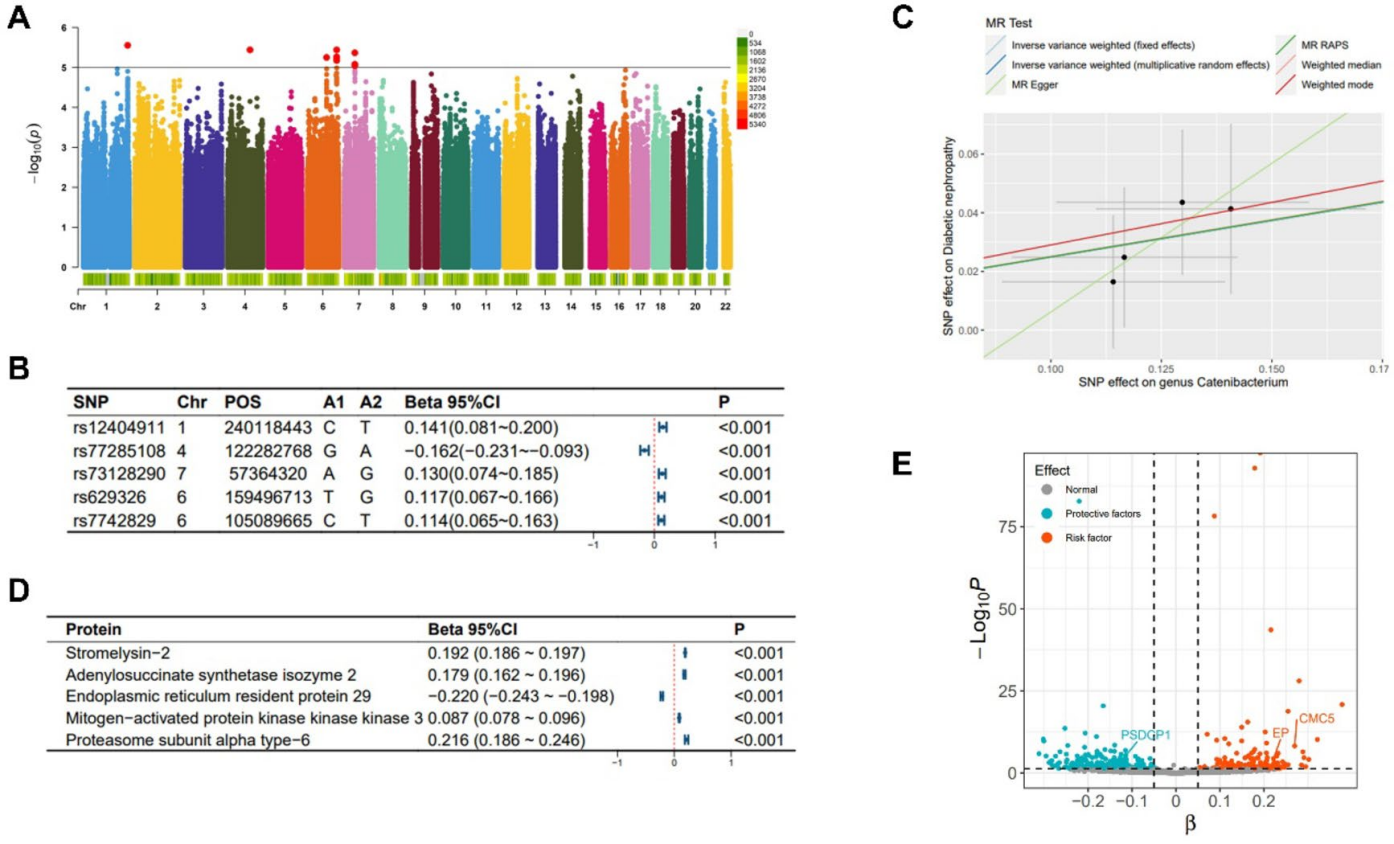
**Analysis related to Catenibacterium.** Note: (**A**) Manhattan plot of the summary data from the genome-wide association study on Catenibacterium; (**B**) The top 5 single nucleotide polymorphisms (SNPs) most significantly associated with Catenibacterium; (**C**) Scatter plot and regression curve of Mendelian randomization exploring the causal association between Catenibacterium and diabetic nephropathy; (**D**) The top 5 plasma proteins most significantly influenced by the abundance of Catenibacterium; (**E**) Scatter plot of the significance level of Catenibacterium’s effects on plasma proteins, with specified proteins indicating mediation effects.

**Fig. 3:**
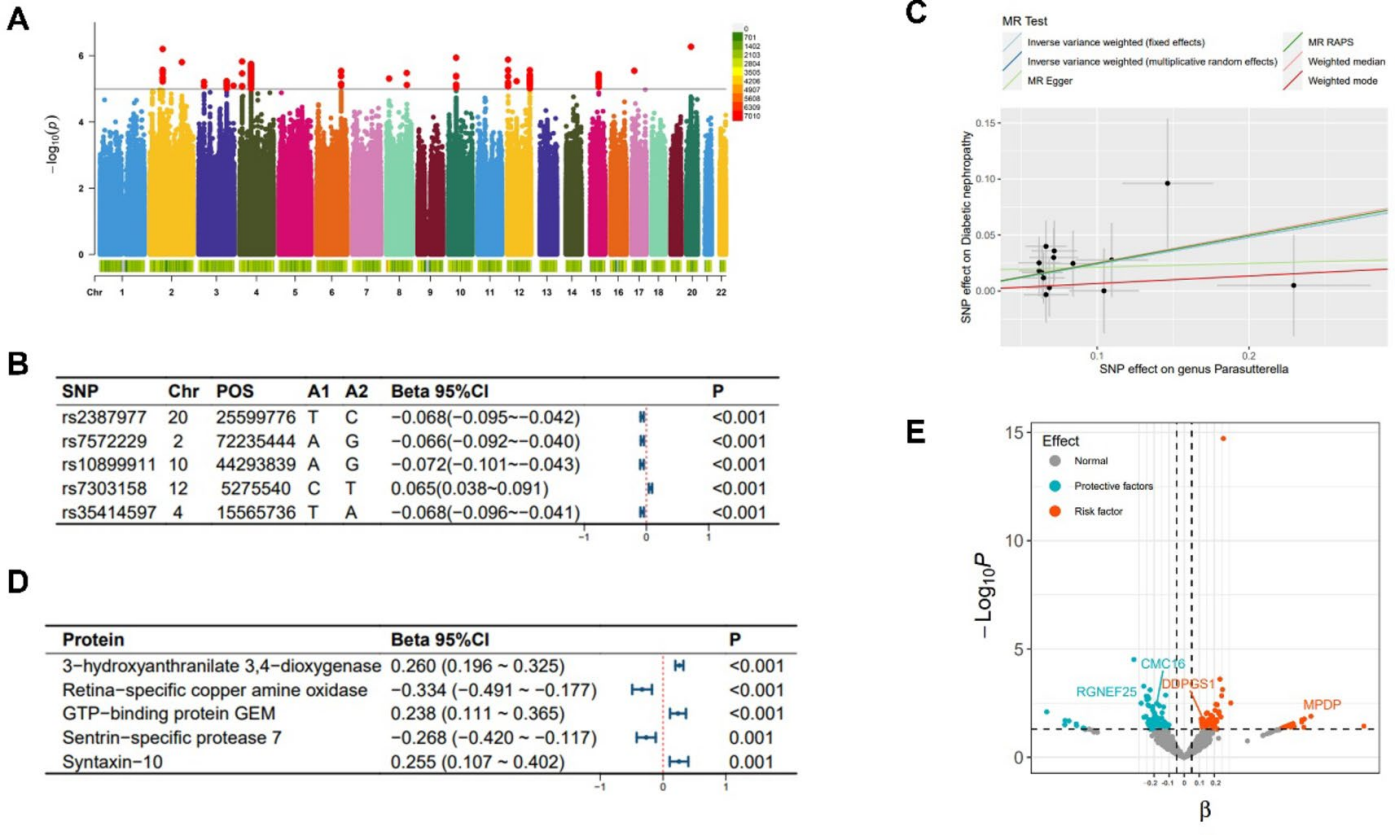
**Analysis related to Parasutterella.** Note: (**A**) Manhattan plot of the summary data from the genome-wide association study on Parasutterella; (**B**) The top 5 single nucleotide polymorphisms (SNPs) most significantly associated with Parasutterella; (**C**) Scatter plot and regression curve of Mendelian randomization exploring the causal association between Parasutterella and diabetic nephropathy; (**D**) The top 5 plasma proteins most significantly influenced by the abundance of Parasutterella; (**E**) Scatter plot of the significance level of Parasutterella’s effects on plasma proteins, with specified proteins indicating mediation effects.

**Fig. 4:**
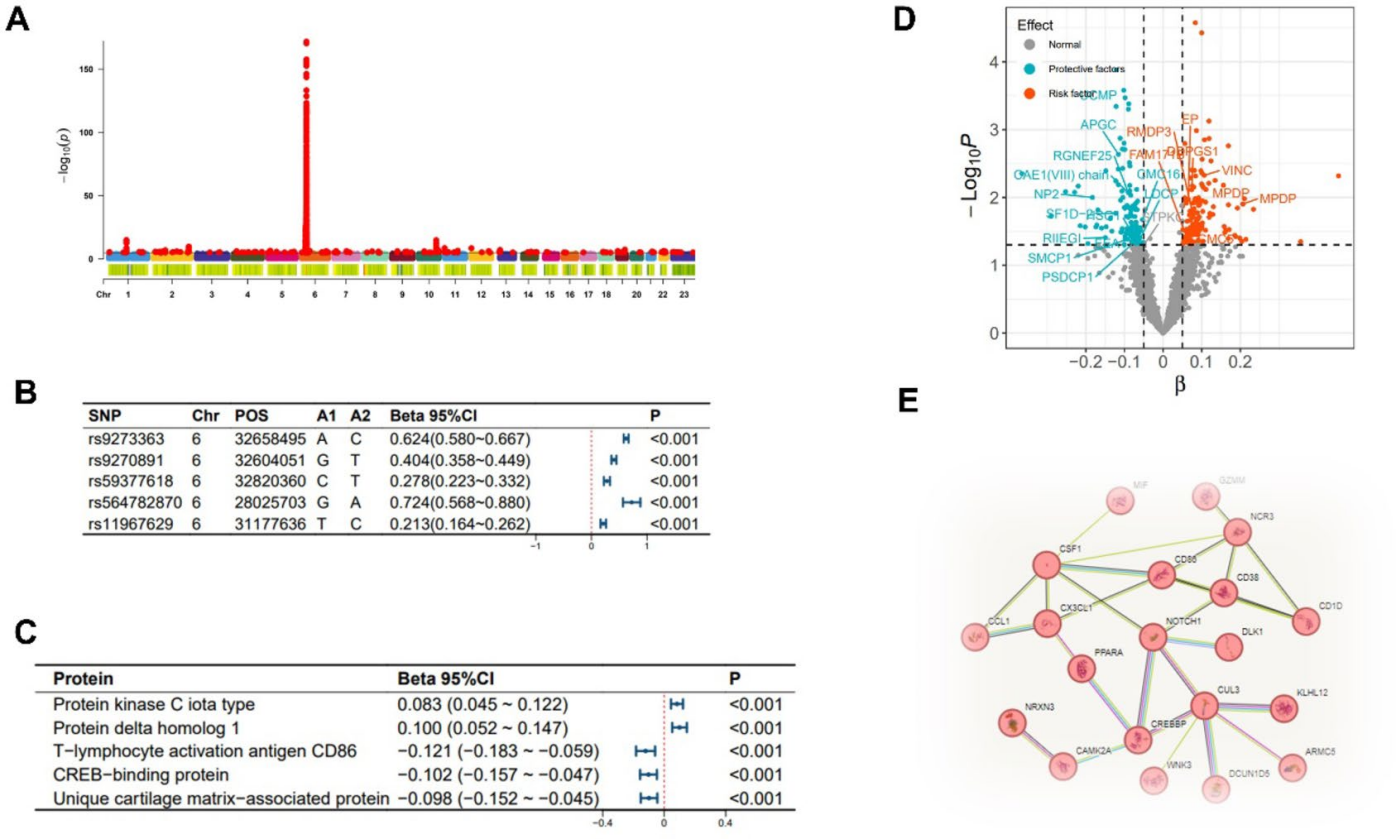
**Analysis related to diabetic nephropathy.** Note: (**A**) Manhattan plot of the summary data from the genome-wide association study on diabetic nephropathy; (**B**) The top 5 single nucleotide polymorphisms (SNPs) most significantly associated with diabetic nephropathy; (**C**) The top 5 plasma proteins most significantly affecting the incidence of diabetic nephropathy; (**D**) Scatter plot of the significance level of the effects of plasma proteins on diabetic nephropathy, with specified proteins indicating mediation effects; (**E**) Schematic diagram of the interaction effects of plasma proteins on the incidence of diabetic nephropathy.

SNPs associated with the abundance of Catenibacterium primarily originate from chromosomes 1/4/6/7 (Fig. 2-A), with the top SNP being rs12404911 (Fig. 2-B). SNPs linked to the abundance of Parasutterella are found across multiple chromosomes (Fig. 3-A). The top SNP is rs2387977 (Fig. 3-B). SNPs associated with DN are predominantly located on chromosome 6 (Fig. 4-A). The top SNP is rs9273363 (Fig. 4-B).

### Screening of instrumental variables

In the MR analysis, initially, a total of 1823 exposure-related SNPs were screened, and no weak instrumental variables were identified; 92 SNPs were excluded from the outcome database due to missing data. After screening with Phenoscanner, a total of 280 SNPs were identified as ambiguous or palindromic SNPs, while 36 SNPs were found to be associated with confounding factors. The MR-PRESSO test revealed no SNPs with horizontal pleiotropy; the MR-Steiger test found no SNPs with incorrect causal direction. After Bonferroni correction, 5 SNPs directly related to the outcome were removed, resulting in 1410 qualified SNPs included in the study. In the mediation MR analysis, initially, 177,296 SNPs associated with exposures were screened, with no weak instrumental variables identified; 15,420 SNPs were removed from the outcome database due to missing data. A total of 20,333 ambiguous or palindromic SNPs were removed during the data merging process (Supplementary Table 2). The MR-PRESSO test revealed no SNPs with horizontal pleiotropy; the MR-Steiger test found no SNPs with incorrect causal direction. After Bonferroni correction, 2,685 SNPs directly related to the outcome were removed, resulting in 138,858 qualified SNPs included in the study. Detailed included SNP data are shown in Supplementary Table 3.

### Mendelian randomization analysis

**Fig. 5:**
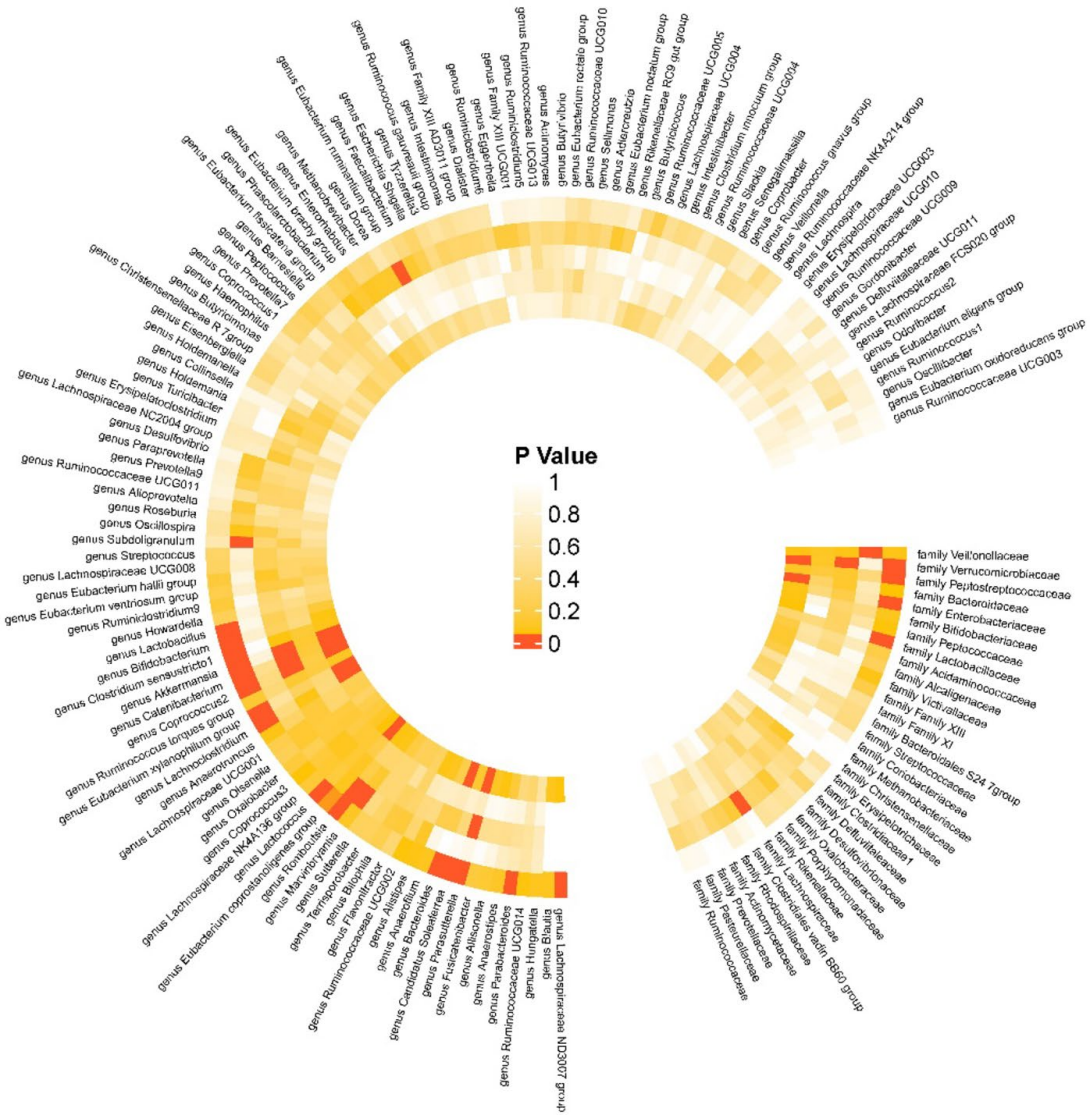
**Heatmap of the significance of the causal relationship between gut microbiota and diabetic nephropathy.** Note: From the outer to the inner layers are the IVW random effects model, MR-Egger model, weighted median model, weighted mode model, and MR-RAPS model.

**Fig. 6:**
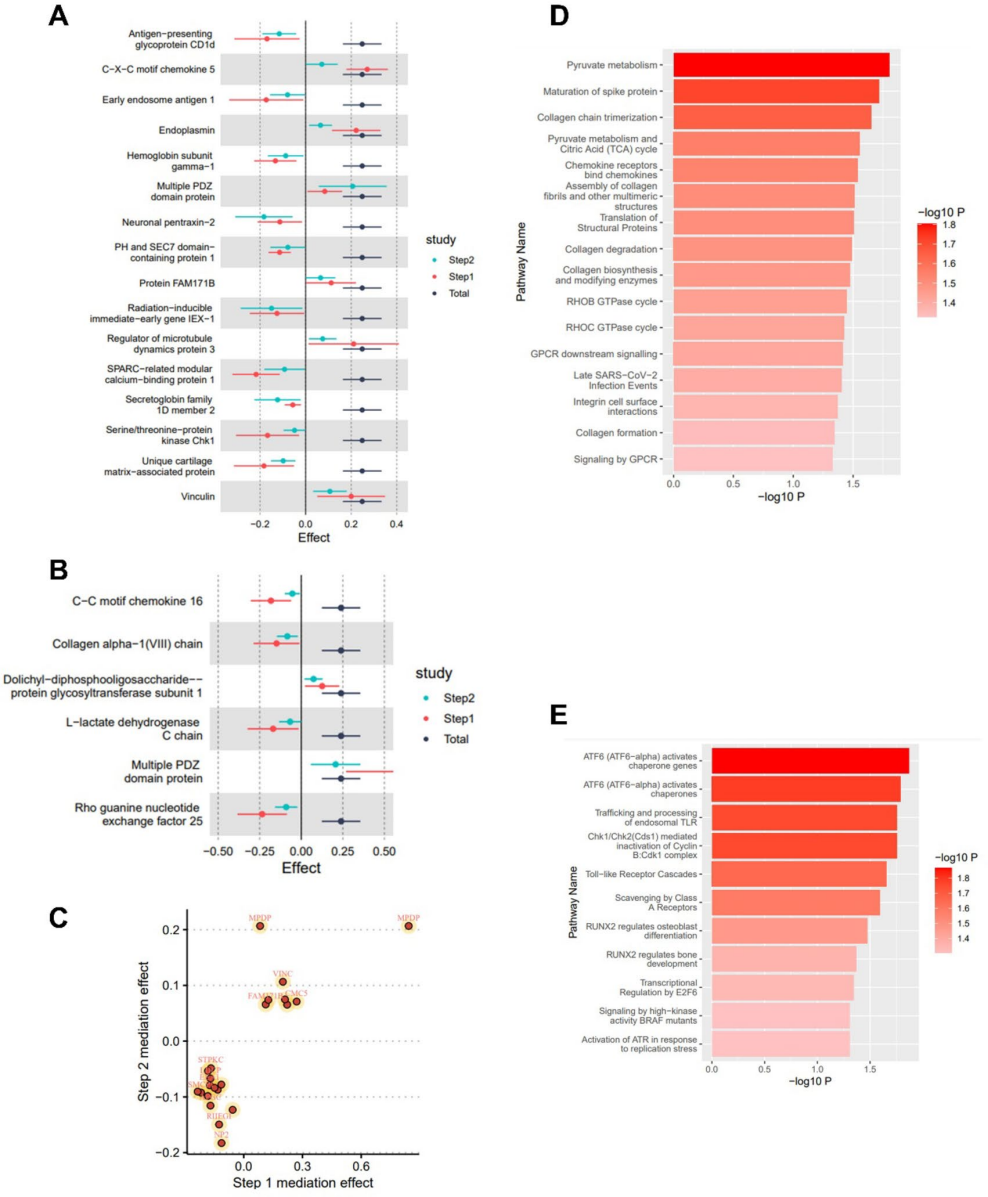
**Results of mediation and enrichment analyses**. Note: (**A**) Forest plot of the total and two-step mediation effects of Catenibacterium; (**B**) Forest plot of the total and two-step mediation effects of Parasutterella; (**C**) Scatter plot of the two-step effects of mediation proteins; (**D**) Pathways of metabolic or signaling transduction mediated by Parasutterella in the incidence of diabetic nephropathy; (**E**) Pathways of metabolic or signaling transduction mediated by Catenibacterium in the incidence of diabetic nephropathy.

As shown in Fig. 5, in the MR analysis, 21 gut microbiotas were found to have a causal relationship with the onset of DN, with 2 remaining significant after adjusting the *P*-values. Specifically, an increased abundance of Catenibacterium [OR (95% CI): 1.282 (1.178 ~ 1.395), *P* = 9.545 × 10^−09^, FDR < 0.001, P_heterogeneity_ = 0.592, P_pleotropy_ = 0.903, P_MR−Steiger_ = 0.733, P_MR−PRESSO_ = 0.910] and Parasutterella [OR (95% CI): 1.270 (1.132 ~ 1.426), *P* = 4.945 × 10^−05^, FDR = 0.004, P_heterogeneity_ = 0.385, P_pleotropy_ = 0.952, P_MR−Steiger_ = 0.501, P_MR−PRESSO_ = 0.945] was both associated with an increased risk of DN, as shown in Figs. 2-C and 3-C respectively. These findings showed no evidence of pleiotropy or heterogeneity. Detailed MR results are presented in Supplementary Table 4. Detailed results of all sensitivity analysis results for this study are presented in Supplementary Tables 5–7. In the mediation MR analysis, causal effects were observed for Catenibacterium on 291 proteins such as Stromelysin-2 after adjusting *P*-values (Fig. 2-D, E), and for Parasutterella on 81 proteins such as 3-hydroxyanthranilate 3,4-dioxygenase (Fig. 3-D, E), and changes in the levels of 253 proteins such as Protein kinase C iota type were causally related to the onset of DN (Fig. 4-C, D). Subsequently, a protein-protein interaction analysis was conducted for plasma proteins inducing DN (Fig. 4-E), and finally, 22 plasma proteins with mediating effects were identified (Table 2; Fig. 6-A, B, C), which mediated the effects of Catenibacterium and Parasutterella on the onset of DN, with mediation effect sizes ranging from 72.9 to 2.8%. Detailed intermediation analysis data are presented in Supplementary Table 8.

**Table 2:** Significant mediating metabolism or signal transduction pathways.

Mediator	Exposure	X-Y			X-M			M-Y			Mediating direction	Mediating effect	Mediating ratio
		OR 95%CI	*P*	Power	OR 95%CI	*P*	Power	OR 95%CI	*P*	Power			
Antigen-presenting glycoprotein CD1d	Catenibacterium	1.282(1.178 ~ 1.395)	< 0.001	100%	0.844(0.732 ~ 0.974)	0.020	100%	0.891(0.827 ~ 0.960)	0.002	88.2%	TRUE	Partial	7.9%
C-X-C motif chemokine 5	Catenibacterium	1.282(1.178 ~ 1.395)	< 0.001	100%	1.310(1.196 ~ 1.435)	< 0.001	100%	1.074(1.001 ~ 1.151)	0.047	50.2%	TRUE	Partial	7.8%
Early endosome antigen 1	Catenibacterium	1.282(1.178 ~ 1.395)	< 0.001	100%	0.841(0.715 ~ 0.990)	0.037	100%	0.924(0.855 ~ 0.998)	0.043	51.5%	TRUE	Partial	5.5%
Endoplasmin	Catenibacterium	1.282(1.178 ~ 1.395)	< 0.001	100%	1.248(1.123 ~ 1.388)	< 0.001	100%	1.068(1.015 ~ 1.123)	0.011	83.9%	TRUE	Partial	5.9%
Hemoglobin subunit gamma-1	Catenibacterium	1.282(1.178 ~ 1.395)	< 0.001	100%	0.876(0.798 ~ 0.961)	0.005	100%	0.916(0.847 ~ 0.991)	0.029	73.8%	TRUE	Partial	4.7%
Multiple PDZ domain protein	Catenibacterium	1.282(1.178 ~ 1.395)	< 0.001	100%	1.087(1.008 ~ 1.173)	0.031	100%	1.230(1.059 ~ 1.427)	0.013	100%	TRUE	Partial	7.0%
Neuronal pentraxin-2	Catenibacterium	1.282(1.178 ~ 1.395)	< 0.001	100%	0.893(0.810 ~ 0.985)	0.023	100%	0.833(0.734 ~ 0.945)	0.010	98.8%	TRUE	Partial	8.3%
PH and Sect. 7 domain-containing protein 1	Catenibacterium	1.282(1.178 ~ 1.395)	< 0.001	100%	0.892(0.850 ~ 0.937)	< 0.001	100%	0.925(0.857 ~ 0.999)	0.047	62.4%	TRUE	Partial	3.6%
Protein FAM171B	Catenibacterium	1.282(1.178 ~ 1.395)	< 0.001	100%	1.119(1.004 ~ 1.247)	0.043	100%	1.068(1.001 ~ 1.140)	0.046	41.3%	TRUE	Partial	3.0%
Radiation-inducible immediate-early gene IEX-1	Catenibacterium	1.282(1.178 ~ 1.395)	< 0.001	100%	0.882(0.783 ~ 0.994)	0.039	100%	0.861(0.752 ~ 0.986)	0.039	98.8%	TRUE	Partial	7.6%
Regulator of microtubule dynamics protein 3	Catenibacterium	1.282(1.178 ~ 1.395)	< 0.001	100%	1.235(1.013 ~ 1.505)	0.037	100%	1.078(1.014 ~ 1.145)	0.015	76.7%	TRUE	Partial	6.4%
Secretoglobin family 1D member 2	Catenibacterium	1.282(1.178 ~ 1.395)	< 0.001	100%	0.945(0.912 ~ 0.980)	0.002	100%	0.884(0.799 ~ 0.978)	0.017	68%	TRUE	Partial	2.8%
Serine/threonine-protein kinase Chk1	Catenibacterium	1.282(1.178 ~ 1.395)	< 0.001	100%	0.846(0.737 ~ 0.972)	0.018	100%	0.953(0.908 ~ 1.000)	0.049	23.9%	TRUE	Partial	3.2%
SPARC-related modular calcium-binding protein 1	Catenibacterium	1.282(1.178 ~ 1.395)	< 0.001	100%	0.805(0.725 ~ 0.893)	< 0.001	100%	0.912(0.834 ~ 0.996)	0.041	74.3%	TRUE	Partial	8.0%
Unique cartilage matrix-associated protein	Catenibacterium	1.282(1.178 ~ 1.395)	< 0.001	100%	0.833(0.730 ~ 0.950)	0.007	100%	0.906(0.859 ~ 0.956)	< 0.001	83.9%	TRUE	Partial	7.3%
Vinculin	Catenibacterium	1.282(1.178 ~ 1.395)	< 0.001	100%	1.221(1.053 ~ 1.417)	0.008	100%	1.112(1.033 ~ 1.198)	0.005	82.2%	TRUE	Partial	8.5%
C-C motif chemokine 16	Parasutterella	1.270(1.132 ~ 1.426)	< 0.001	100%	0.833(0.738 ~ 0.941)	0.003	100%	0.948(0.906 ~ 0.992)	0.021	60.2%	TRUE	Partial	4.1%
Collagen alpha-1(VIII) chain	Parasutterella	1.270(1.132 ~ 1.426)	< 0.001	100%	0.862(0.751 ~ 0.989)	0.035	100%	0.920(0.863 ~ 0.980)	0.009	58.9%	TRUE	Partial	5.2%
Dolichyl-diphosphooligosaccharide–protein glycosyltransferase subunit 1	Parasutterella	1.270(1.132 ~ 1.426)	< 0.001	100%	1.134(1.023 ~ 1.257)	0.017	100%	1.077(1.020 ~ 1.137)	0.008	59.1%	TRUE	Partial	3.9%
L-lactate dehydrogenase C chain	Parasutterella	1.270(1.132 ~ 1.426)	< 0.001	100%	0.844(0.724 ~ 0.985)	0.031	100%	0.935(0.875 ~ 1.000)	0.049	44.9%	TRUE	Partial	4.8%
Multiple PDZ domain protein	Parasutterella	1.270(1.132 ~ 1.426)	< 0.001	100%	2.321(1.309 ~ 4.114)	0.013	100%	1.230(1.059 ~ 1.427)	0.013	100%	TRUE	Partial	72.9%
Rho guanine nucleotide exchange factor 25	Parasutterella	1.270(1.132 ~ 1.426)	< 0.001	100%	0.791(0.681 ~ 0.917)	0.002	100%	0.914(0.854 ~ 0.977)	0.008	72.4%	TRUE	Partial	8.8%

### Enrichment analysis

In the enrichment analysis, 27 significant pathways mediating the effects of Catenibacterium and Parasutterella on the onset of DN were identified. Specifically, 11 significant pathways were associated with the pathogenesis of DN due to Catenibacterium, with the most significant being activation of transcription factor-6 (ATF6-alpha) activates chaperone genes (Reactions 1/5, Entities 1/10, *P*-value 0.014); 16 significant pathways were linked to the pathogenesis due to Parasutterella, with the most significant being Pyruvate metabolism (Reactions 2/15, Entities 1/31, *P*-value 0.016). Table 3; Fig. 6-D, E display the enriched information on mediating pathways, with detailed data available in the Supplementary Table 9.

**Table 3:** Activated mediating metabolism or signal transduction pathways.

Pathway name	Exposure	Reactions found	Reactions ratio	Entities found	Entities ratio	*P*-value	Mapped proteins
ATF6 (ATF6-alpha) activates chaperone genes	Catenibacterium	1/ 5	0.001	1/ 10	0.001	0.014	Endoplasmin
Pyruvate metabolism	Parasutterella	2/ 15	0.001	1/ 31	0.003	0.016	L-lactate dehydrogenase C chain
ATF6 (ATF6-alpha) activates chaperones	Catenibacterium	1/ 10	0.001	1/ 12	0.001	0.016	Endoplasmin
Trafficking and processing of endosomal TLR	Catenibacterium	2/ 7	0.001	1/ 13	0.001	0.018	Endoplasmin
Chk1/Chk2(Cds1) mediated inactivation of Cyclin B: Cdk1 complex	Catenibacterium	1/ 5	0.001	1/ 13	0.001	0.018	Serine/threonine-protein kinase Chk1
Maturation of spike protein	Parasutterella	1/ 12	0.001	1/ 38	0.003	0.019	Dolichyl-diphosphooligosaccharide–protein glycosyltransferase subunit 1
Toll-like Receptor Cascades	Catenibacterium	4/ 198	0.014	2/ 170	0.014	0.022	Endoplasmin; Early endosome antigen 1
Collagen chain trimerization	Parasutterella	1/ 28	0.002	1/ 44	0.004	0.022	Collagen alpha-1(VIII) chain
Scavenging by Class A Receptors	Catenibacterium	2/ 10	0.001	1/ 19	0.002	0.026	Endoplasmin
Pyruvate metabolism and Citric Acid (TCA) cycle	Parasutterella	2/ 36	0.003	1/ 55	0.005	0.028	L-lactate dehydrogenase C chain
Chemokine receptors bind chemokines	Parasutterella	1/ 19	0.001	1/ 57	0.005	0.029	C-C motif chemokine 16
Assembly of collagen fibrils and other multimeric structures	Parasutterella	1/ 26	0.002	1/ 61	0.005	0.031	Collagen alpha-1(VIII) chain
Translation of Structural Proteins	Parasutterella	1/ 46	0.003	1/ 62	0.005	0.031	Dolichyl-diphosphooligosaccharide–protein glycosyltransferase subunit 1
Collagen degradation	Parasutterella	4/ 34	0.002	1/ 64	0.005	0.032	Collagen alpha-1(VIII) chain
RUNX2 regulates osteoblast differentiation	Catenibacterium	1/ 24	0.002	1/ 25	0.002	0.034	Unique cartilage matrix-associated protein
Collagen biosynthesis and modifying enzymes	Parasutterella	19/ 51	0.004	1/ 67	0.006	0.034	Collagen alpha-1(VIII) chain
RHOB GTPase cycle	Parasutterella	1/ 6	0.001	1/ 71	0.006	0.036	Rho guanine nucleotide exchange factor 25
RHOC GTPase cycle	Parasutterella	1/ 6	0.001	1/ 75	0.006	0.038	Rho guanine nucleotide exchange factor 25
GPCR downstream signalling	Parasutterella	4/ 175	0.012	2/ 638	0.054	0.038	Rho guanine nucleotide exchange factor 25;C-C motif chemokine 16
Late SARS-CoV-2 Infection Events	Parasutterella	1/ 69	0.005	1/ 79	0.007	0.040	Dolichyl-diphosphooligosaccharide–protein glycosyltransferase subunit 1
Integrin cell surface interactions	Parasutterella	1/ 55	0.004	1/ 85	0.007	0.043	Collagen alpha-1(VIII) chain
RUNX2 regulates bone development	Catenibacterium	1/ 32	0.002	1/ 32	0.003	0.043	Unique cartilage matrix-associated protein
Collagen formation	Parasutterella	20/ 77	0.005	1/ 90	0.008	0.045	Collagen alpha-1(VIII) chain
Transcriptional Regulation by E2F6	Catenibacterium	3/ 33	0.002	1/ 34	0.003	0.045	Serine/threonine-protein kinase Chk1
Signaling by GPCR	Parasutterella	5/ 392	0.027	2/ 713	0.061	0.047	Rho guanine nucleotide exchange factor 25;C-C motif chemokine 16
Signaling by high-kinase activity BRAF mutants	Catenibacterium	4/ 6	0.001	1/ 37	0.003	0.049	Vinculin
Activation of ATR in response to replication stress	Catenibacterium	3/ 9	0.001	1/ 37	0.003	0.049	Serine/threonine-protein kinase Chk1

## Discussion

The pivotal role of gut microbiota in metabolic disease development cannot be overlooked, as disruptions in gut microbiota are intricately linked to various metabolic disorders such as obesity, non-alcoholic fatty liver disease, and diabetes mellitus^8–10^. Consequently, targeting gut microbiota has emerged as a significant intervention strategy for managing diverse ailments^7^. It has been found that there is a strong association between gut microbiota and DN^12^. The imbalance of gut microbiota, changes in the abundance of specific flora and the role of metabolites of gut microbiota have important effects on the occurrence and development of DN^13–15^. Therefore, a systematic study of the causal relationship between gut microbiota and DN as well as the mechanism of intestinal flora disturbance leading to DN is very important for the prevention and treatment of DN. Several recent studies have only explored the gut microbiota that influence the development of DN, without further investigation of the mechanisms behind it^32,33^. Within this study, a thorough two-sample MR analysis was executed, revealing a positive association between the augmentation of Catenibacterium and Parasutterella intestinal bacteria and the heightened risk of DN. Moreover, subsequent intermediate proteomic analysis was carried out to delve deeper into the mechanisms involved. The potential biological mechanism of Catenibacterium and Parasutterella intestinal bacteria causing DN has been explored.

The Catenibacterium genus belongs to the Erysipelotrichaceae family^30^, Parasutterella is a gram-negative, strictly anaerobic genus of micrococcus from the phylum Proteus. To date, the conventional epidemiological investigation of the correlation between these two genera and DN remains limited. The results of this study elucidate that an increased abundance of both bacterial genera in the gut is a risk factor for DN and is associated with an increased risk of DN. Studies have found that Catenibacterium is related to metabolic diseases and can lead to various metabolic disorders^34^. A meta-analysis study on intestinal bacteria related to obesity found that Catenibacterium is significantly higher in obese people than non-obese people, and intake of lactic acid bacteria can significantly reduce the number of the bacterium. It may be one of the treatments for obesity to regulate intestinal microflora through intake of lactic acid bacteria^34,35^. In a study investigating the relationship between microbiota and metabolic markers in African American men (AAM) with prediabetes and vitamin D deficiency, a high abundance of Catenibacterium was found, and the number of the genus changed with worsening blood sugar^36^. Furthermore, a rise in the abundance of this particular genus was observed in stool samples obtained from individuals with ESRD, thereby indicating a plausible association between the levels of this genus and the progression of kidney disease^37^. Parasutterella plays an important role in the development of type 2 diabetes and obesity. In a transformational human study, Parasutterella abundance was found to be positively correlated with BMI and type 2 diabetes, and positively correlated with dietary carbohydrate intake, but not with reduced microbiome α/β diversity and low inflammation in obesity. The association with L-cysteine may be related to the development of type 2 diabetes. And has been linked to fatty acid biosynthetic pathways that lead to weight gain from carbohydrate-rich diets during the development of obesity^38^. In an animal study, Lactobacillus paracasei NL41 was found to reduce the number of Parasutterella by decreasing serum lipopolysaccharide (LPS), free fatty acid (FFA), tumour necrosis factor-α (TNF-α), interleukin (IL)−6, and IL-8, and by increasing the levels of IL-10 modulates the gut microbiota and improves inflammation in type 2 diabetic rats^39^. During the investigation of renal fibrosis, a positive correlation was discovered between the relative abundance of Parasutterella and the disease’s progression. Additionally, the augmentation of Parasutterella appears to facilitate the onset and advancement of renal fibrosis caused by unilateral ureteral occlusion (UUO)^40^. The increased abundance of Parasutterella in UUO mice is consistent with earlier findings in CKD patients^41^, CKD rats^42^, and is associated with chronic intestinal inflammation^43^. Within a two-sample MR investigation examining the relationship between gut microbiota, CKD, and chronic systemic inflammation, a noteworthy discovery emerged. The heightened abundance of Parasutterella was identified as a risk factor for proteinuria, exhibiting a positive association with elevated levels of the urinary albumin: creatinine ratio (UACR)^44^. This finding aligns with earlier studies wherein disruptions in intestinal flora, exemplified by increased Parasutterella abundance, were positively linked to escalated proteinuria in rat models of CKD^42^. The mechanism may involve a enteric-borne uremic toxin, phenyl-sulfate (PS)^45^, which is also one of the causes of DN albuminuria^46^, which can increase albuminuria by damaging podocytes^47^. The association between phenyl sulfate (PS) levels and the urinary albumin: creatinine ratio (UACR) was notably significant among individuals with microalbuminuria. Furthermore, among patients with microalbuminuria, PS emerged as the sole predictive factor for UACR progression over a two-year period^48^. Therefore, intervention on Catenibacterium and Parasutterella intestinal bacteria has the potential to become a new way to prevent and treat DN.

Many circulating proteins are key regulators of molecular pathways, so exploring their potential mediating effect in the association of gut microbiota and DN could help to understand the underlying mechanisms of intestinal flora disorder on DN. In this study, a total of 16 plasma proteins mediating between Catenibacterium and DN were identified, and the highest mediating effects was vinculin. It has been found that vinculin is an important protein in podocytes that is involved in the attachment of extracellular matrix to intracellular actin fibers^49^. Vinculin is able to recruit other proteins in podocytes, such as α-actinin and Arp2/3, thus participating in cell adhesion and force transmission processes^50^. In podocytes, vinculin plays an important role in maintaining the normal morphology of foot processes^51,52^, and this process may be affected by palladin. Palladin, a ubiquitously expressed actin-related protein, is a key component involved in cytoskeleton construction in cells. Vinculin expression is significantly downregulated when palladin is inhibited, and palladin may affect podocyte adhesion and extracellular signaling by regulating vinculin interactions with these proteins^53,54^. DN is associated with podocyte injury and dysfunction^55^. It has been found that the induction of aptamers and cytoskeletal proteins such as vinculin in podocytes is inhibited under diabetic conditions, the levels of focal adhesion components are decreased in DN, however, phosphorylated vinculin is upregulated under hyperglycemic conditions, and podocyte adhesion to the basement membrane is impaired. DN is also associated with the activation of the Rho-ROCK signaling pathway, which is involved in the regulation of the cytoskeleton, and its inhibition prevents proteinuria and renal failure. Increased GSK3β induction and decreased vinculin induction under diabetic conditions suggest that GSK3β stimulates disordered adhesion and remodeling of the cytoskeleton in podocytes through the Rho A-GSK3β pathway^56^. Vinculin expression is increased in patients with DN who have type 1 diabetes^57^. Consistent with our study, the increased abundance of Catenibacterium increases the plasma protein vinculin, which contributes to the increased risk of DN. In addition, vinculin may be involved in the regulation of renal inflammation through several pathways, including increased vinculin strengthening the connection between renal tubular epithelial cells and interstitial cells, thereby driving renal interstitial inflammation^58^. Cyclosporine A can cause an increase in vinculin expression in the mouse kidney, and this increase is positively correlated with the severity of cyclosporine A-induced kidney injury^59^. By establishing a 3D in vitro tissue model, Cha et al. found that intracellular and extracellular fibronectin can regulate the polarization state of macrophages through vinculin, thereby affecting the inflammatory response^60^. Porst et al. found that cellulose-1 can affect the function of mesangial cells by regulating the attachment, spreading, and proliferation of interstitial cells, and vinculin is involved in this process^61^. Bains et al. quantitatively showed increased expression of cell-junctional proteins such as vinculin in glomerular cells in cases of proteinuria, suggesting their involvement in renal pathology^62^. Oxidative stress can also lead to renal cell injury and apoptosis, and vinculin, a member of the cell-matrix junction component, may also be involved in oxidative stress-induced apoptosis because of its role in maintaining cytoskeletal structure^63^. Studies have shown that the intercellular junction protein vinculin can promote the development of renal fibrosis by regulating the EMT process of renal tubular epithelial cells, and the mechanism involves the activation of p38 signaling pathway induced by acetic acid^58^, the action of angiotensin II (Ang II)^64^, and T cell death-associated genes 51 (TDAG51) on vinculin regulation^65^and other levels. As a pivotal cytoskeletal protein, vinculin plays an indispensable role in maintaining cellular morphological and structural integrity. Dysregulation of vinculin’s biological function may be intricately associated with inflammatory responses, apoptosis, oxidative stress-induced injury, and renal fibrosis in DN. Existing research has demonstrated that vinculin represents a promising therapeutic target for conditions such as age-related heart failure, impaired angiogenesis due to dysfunctional high-density lipoprotein (dHDL), atherosclerosis, and other diseases^66–68^. This insight offers a novel research avenue for elucidating the molecular mechanisms underlying DN and holds potential as a significant therapeutic target for DN.

A total of 6 plasma proteins mediating between Parasutterella and DN were identified, and the highest mediating effects was Multiple PDZ domain protein (MPDZ also named MUPP1). MUPP1 is an osmotic response protein in kidney cells. Under hypertonic conditions induced by sucrose and mannitol, the tight junction protein MUPP1 is significantly up-regulated and plays an important role in the osmotic stress response of renal cells^69^. This up-regulation was found to be necessary for the maintenance of tight epithelial properties in these cells. In line with our study, Parasutterella increased plasma MUPP1 abundance in hyperosmolar hyperglycemia, which contributed to the increased risk of DN. The role of MUPP1 in the kidney may be mediated by its interaction with Cldn4, MUPP1 contributes to the maintenance of a tight epithelium in the medulla of the kidney under hypertonic stress by correctly localizing Cldn4 to the tight junctions^70^. MUPP1 could interact with renal tubule-associated K (+) channel Kir4.2, and the interaction played an important role in the regulation of K (+) transport in renal tubules. The study found that, MUPP1 can bind to Kir4.2 and reduce its cell membrane expression. Thus MUPP1 and Kir4.2 may participate in a protein complex in the nephron that could regulate transport of K (+) as well as other ions^71^. In addition, Hepatocyte growth factor activator inhibitor-1 (HAI-1) is a Kunitz-type serine protease inhibitor. HAI-1 led to apoptosis through a reduction in the levels of MUPP1 in cervical cancer cell lines^72^. MUPP1 plays a crucial role in establishing cell polarity, stabilizing gap junctions, and maintaining its physiological functions in the kidney by regulating the localization and function of various associated proteins. Consequently, therapeutic strategies targeting MUPP1 may hold potential value in treating or delaying the progression of DN through the restoration of intercellular junctions, repair of the glomerular filtration barrier, and anti-inflammatory and anti-fibrotic effects. However, current research on MUPP1 in DN remains in its preliminary stages, with its specific mechanisms of action yet to be fully elucidated. Further in-depth studies are urgently required to validate its clinical application potential. Investigating the mechanisms of MUPP1 in DN will facilitate its clinical translation as a therapeutic target and holds significant research and clinical application value.

In this study, we conducted an enrichment analysis of plasma proteins associated with intestinal microflora in DN patients. Our findings suggest that 11 pathways may mediate the effects of Catenibacterium on DN, including the ATF6 (ATF6-alpha) activated chaperone genes pathway. The ATF6 pathway is a critical component of the endoplasmic reticulum (ER) stress response mechanism. Previous research has established a close association between ER stress and the development of DN^73^. Over-activation of the ATF6 pathway may impair renal cell function and survival through the following mechanisms: (1) Exacerbation of inflammatory responses: Zhu et al. demonstrated that (+)-catechin reduces the activation of ER stress-related NLRP3 inflammasome by downregulating ATF6 and other ER stress-related factors, thereby alleviating DN injury^74^. (2) Induction of apoptosis: Studies have shown that lipotoxicity-induced protein arginine methyltransferase 1 (PRMT1) increases mesangial cell apoptosis and promotes DN progression by activating the PERK and ATF6-mediated ER stress pathway^75^. Additionally, ursodeoxycholic acid (UDCA) and 4-phenylbutyric acid (4-PBA) mitigated renal lesions in diabetic mice by reducing macrophage injury and renal tubular epithelial cell apoptosis, achieved through inhibition of the ATF6 signaling pathway and prevention of ER stress activation^76^. Given its involvement in DN pathogenesis, inhibiting ATF6 activity may help alleviate renal injury^77^. In this study, enrichment analysis revealed association between Catenibacterium and ATF6 pathway activation, suggesting that Catenibacterium may play a crucial role in DN development via ATF6 pathway activation. Therefore, inhibiting Catenibacterium could offer a novel therapeutic strategy for DN.

In the enrichment analysis conducted in this study, we identified that Parasutterella influences DN via 16 pathways, including pyruvate metabolism and the citric acid cycle (TCA cycle). Pyruvate, as an end product of glycolysis, plays a pivotal role in cellular energy metabolism. The onset and progression of DN are closely associated with impaired glucose metabolism. Research has demonstrated that the enzymatic activity of the glycolytic pathway in kidney tissues of DN patients is diminished, leading to reduced pyruvate production. Concurrently, the enzymatic activity of the TCA cycle is also suppressed, hindering the oxidative metabolism of pyruvate. These abnormalities in glucose metabolism result in inadequate energy supply to renal cells, triggering pathological processes such as cell dysfunction, inflammatory responses, and apoptosis, ultimately promoting the development and progression of DN^78^. Pyruvate kinase M2 (PKM2), a key enzyme in pyruvate metabolism, exhibits altered expression and activity in DN, potentially contributing to its pathogenesis through the following mechanisms: (1) Increased glycolytic flux: PKM2 activation can enhance the glycolytic pathway, reducing intracellular accumulation of free glucose and other metabolites, thereby mitigating high glucose toxicity and inhibiting glomerular pathology^79^; (2) Maintenance of mitochondrial function: PKM2 expression and activity can influence mitochondrial morphology and function, increasing ATP production and sustaining energy metabolism and normal kidney cell function^80^; (3) Improvement of glomerular function and structure: PKM2 activation can reduce glomerular hypertrophy, improve glomerular structure, and increase glomerular filtration rate, thus delaying renal function decline^81^. In this study, Parasutterella was identified as being associated with the pyruvate metabolism pathway, indicating its potential role in the pathogenesis of DN through the modulation of pyruvate metabolism. Intervention in this pathway may offer a novel therapeutic strategy for the treatment of DN^82^.

By employing rigorous criteria, our two-sample MR analysis offers compelling insights into the complex interplay between gut microbiota and DN. The findings suggest that an augmentation in the abundance of intestinal bacteria, specifically Catenibacterium and Parasutterella, may heighten the risk of developing DN. Furthermore, our mediation analysis revealed the important role of plasma proteome as mediators, depicting the causal trajectories from the abundance of gut microbiota increases to the progression of DN, which has profound significance in constructing a blueprint from the abundance of gut microbiota in vivo to the pathogenesis of DN, thereby effectively guiding the prevention of DN and delaying its progression.

However, it is crucial to acknowledge certain limitations in our study. First, the genetic analyses in this study were performed on individuals of European ancestry, but the fact that the gut microbiota varies significantly across geographic and ethnographic groups challenges the generalizability of our finding^83^. For example, Asian populations consume more fermented foods such as yoghurt and kimchi, which contributes to an increase in the abundance of lactic acid bacteria (e.g. Lactobacillus and Bifidobacterium) in the gut. Whereas in Europe, although fermented foods are also consumed, they are usually dominated by cheese and beer, which are more likely to affect changes in Lactobacillus casei, and Saccharomyces cerevisiae. This diversity means that the relationship between gut microbiota and DN that we obtained based on European populations may be limited when generalized to other populations, suggesting that future studies should consider diverse sample sources to explore the characteristics of gut microbiota and their impact on DN in different ethnic and geographical contexts. Second, our data were derived from publicly available aggregated statistics and the raw clinical outcome data for each individual were not available, thus preventing further population stratification analyses. Lastly, we did not perform experimental validations for the identified mediating proteins and potential mechanisms by which gut microbiota affects DN.

## Conclusions

Through our extensive MR analysis, a multifaceted association between gut microbiota and DN has been uncovered. Remarkably, we have identified the augmentation of Catenibacterium and Parasutterella intestinal bacteria as causal contributors to the development of DN. More importantly, we reveal the underlying mechanism by which the increased abundance of Catenibacterium and Parasutterella intestinal bacteria lead to DN, providing a blueprint for the involvement of gut–kidney axis in the pathogenesis of DN and paving the way for future studies.

## Electronic supplementary material

Below is the link to the electronic supplementary material.

Supplementary Material 1

Supplementary Material 2

Supplementary Material 3

Supplementary Material 4

Supplementary Material 5

Supplementary Material 6

## Data Availability

GWAS data on gut microbiota can be obtained from the following website: https://mibiogen.gcc.rug.nl/. GWAS data on plasma proteome can be accessed from the following website: https://www.ebi.ac.uk/gwas/downloads/summary-statistics18. GWAS data on diabetic nephropathy can be retrieved from the following website: https://www.finngen.fi/en/access_results.

## References

[1] Collaborators, G. D. Global, regional, and national burden of diabetes from 1990 to 2021, with projections of prevalence to 2050: a systematic analysis for the global burden of Disease Study 2021. *Lancet (London England)*. **402**, 203–234 (2023). 37356446 10.1016/S0140-6736(23)01301-6 PMC10364581

[2] Gregg, E. W. et al. Improving health outcomes of people with diabetes: target setting for the WHO Global Diabetes Compact. *Lancet (London England)*. **401**, 1302–1312 (2023). 36931289 10.1016/S0140-6736(23)00001-6 PMC10420388

[3] Lemp, J. M. et al. Quasi-experimental evaluation of a nationwide diabetes prevention programme. *Nature* **624**, 138–144 (2023). 37968391 10.1038/s41586-023-06756-4 PMC12580629

[4] Petrazzuolo, A. et al. Broadening horizons in mechanisms, management, and treatment of diabetic kidney disease. *Pharmacol. Res.* **190**, 106710 (2023). 36871895 10.1016/j.phrs.2023.106710

[5] Lyssenko, V. & Vaag, A. Genetics of diabetes-associated microvascular complications. *Diabetologia* **66**, 1601–1613 (2023). 37452207 10.1007/s00125-023-05964-x PMC10390394

[6] Collaboration, G. C. K. D. Global, regional, and national burden of chronic kidney disease, 1990–2017: a systematic analysis for the global burden of Disease Study 2017. *Lancet (London England)*. **395**, 709–733 (2020). 32061315 10.1016/S0140-6736(20)30045-3 PMC7049905

[7] Aron-Wisnewsky, J., Warmbrunn, M. V., Nieuwdorp, M. & Clément, K. Metabolism and metabolic disorders and the Microbiome: the intestinal microbiota Associated with obesity, lipid metabolism, and Metabolic Health-Pathophysiology and therapeutic strategies. *Gastroenterology* **160**, 573–599 (2021). 33253685 10.1053/j.gastro.2020.10.057

[8] Van Hul, M. & Cani, P. D. The gut microbiota in obesity and weight management: microbes as friends or foe? *Nat. Rev. Endocrinol.* **19**, 258–271 (2023). 36650295 10.1038/s41574-022-00794-0

[9] Canfora, E. E., Meex, R. C. R., Venema, K. & Blaak, E. E. Gut microbial metabolites in obesity, NAFLD and T2DM. *Nat. Rev. Endocrinol.* **15**, 261–273 (2019). 30670819 10.1038/s41574-019-0156-z

[10] Aron-Wisnewsky, J. et al. Gut microbiota and human NAFLD: disentangling microbial signatures from metabolic disorders. *Nat. Rev. Gastroenterol. Hepatol.* **17**, 279–297 (2020). 32152478 10.1038/s41575-020-0269-9

[11] Ondrussek-Sekac, M., Navas-Carrillo, D. & Orenes-Piñero, E. Intestinal microbiota alterations in chronic kidney disease and the influence of dietary components. *Crit. Rev. Food Sci. Nutr.* **61**, 1490–1502 (2021). 32393049 10.1080/10408398.2020.1761771

[12] Vaziri, N. D., Zhao, Y. Y. & Pahl, M. V. Altered intestinal microbial flora and impaired epithelial barrier structure and function in CKD: the nature, mechanisms, consequences and potential treatment. *Nephrol. Dial Transpl.* **31**, 737–746 (2016). 10.1093/ndt/gfv095 25883197

[13] Fernandes, R., Viana, S. D., Nunes, S. & Reis, F. Diabetic gut microbiota dysbiosis as an inflammaging and immunosenescence condition that fosters progression of retinopathy and nephropathy. *Biochim. Biophys. Acta Mol. Basis Dis.* **1865**, 1876–1897 (2019). 30287404 10.1016/j.bbadis.2018.09.032

[14] Wang, X. et al. Aberrant gut microbiota alters host metabolome and impacts renal failure in humans and rodents. *Gut* **69**, 2131–2142 (2020). 32241904 10.1136/gutjnl-2019-319766 PMC7677483

[15] Fang, Q. et al. Roles of Gut Microbial metabolites in Diabetic kidney disease. *Front. Endocrinol. (Lausanne)*. **12**, 636175 (2021). 34093430 10.3389/fendo.2021.636175 PMC8173181

[16] Emdin, C. A., Khera, A. V. & Kathiresan, S. *Mendelian Randomization Jama* **318**, 1925–1926 (2017). 29164242 10.1001/jama.2017.17219

[17] Yuan, S. et al. Plasma proteins and onset of type 2 diabetes and diabetic complications: Proteome-wide mendelian randomization and colocalization analyses. *Cell. Rep. Med.* **4**, 101174 (2023). 37652020 10.1016/j.xcrm.2023.101174 PMC10518626

[18] Sun, B. B. et al. Genomic atlas of the human plasma proteome. *Nature* **558**, 73–79 (2018). 29875488 10.1038/s41586-018-0175-2 PMC6697541

[19] Ferkingstad, E. et al. Large-scale integration of the plasma proteome with genetics and disease. *Nat. Genet.* **53**, 1712–1721 (2021). 34857953 10.1038/s41588-021-00978-w

[20] Finan, C. et al., *The druggable genome and support for target identification and validation in drug development. Sci. Transl Med.* ***9*** (2017). 10.1126/scitranslmed.aag1166 PMC6321762 28356508

[21] Kurilshikov, A. et al. Large-scale association analyses identify host factors influencing human gut microbiome composition. *Nat. Genet.* **53**, 156–165 (2021). 33462485 10.1038/s41588-020-00763-1 PMC8515199

[22] Kurki, M. I. et al. FinnGen provides genetic insights from a well-phenotyped isolated population. *Nature* **613**, 508–518 (2023). 36653562 10.1038/s41586-022-05473-8 PMC9849126

[23] Burgess, S., Small, D. S. & Thompson, S. G. A review of instrumental variable estimators for mendelian randomization. *Stat. Methods Med. Res.* **26**, 2333–2355 (2017). 26282889 10.1177/0962280215597579 PMC5642006

[24] Li, F. et al. A mendelian randomization study with populations of European ancestry rules out a causal relationship between inflammatory bowel disease and colorectal cancer. *Front. Genet.* **13**, 949325 (2022). 36092900 10.3389/fgene.2022.949325 PMC9449310

[25] Staley, J. R. et al. PhenoScanner: a database of human genotype-phenotype associations. *Bioinformatics* **32**, 3207–3209 (2016). 27318201 10.1093/bioinformatics/btw373 PMC5048068

[26] Burgess, S., Butterworth, A. & Thompson, S. G. Mendelian randomization analysis with multiple genetic variants using summarized data. *Genet. Epidemiol.* **37**, 658–665 (2013). 24114802 10.1002/gepi.21758 PMC4377079

[27] Thomas, D. C. & Conti, D. V. Commentary: the concept of ‘Mendelian randomization’. *Int. J. Epidemiol.* **33**, 21–25 (2004). 15075141 10.1093/ije/dyh048

[28] Burgess, S. & Thompson, S. G. Interpreting findings from mendelian randomization using the MR-Egger method. *Eur. J. Epidemiol.* **32**, 377–389 (2017). 28527048 10.1007/s10654-017-0255-x PMC5506233

[29] Bowden, J., Davey Smith, G., Haycock, P. C. & Burgess, S. Consistent estimation in mendelian randomization with some Invalid instruments using a weighted median estimator. *Genet. Epidemiol.* **40**, 304–314 (2016). 27061298 10.1002/gepi.21965 PMC4849733

[30] Ricci, L. et al. Draft genome sequence of a representative strain of the Catenibacterium genus isolated from human feces. *Microbiol. Resour. Announc*. **12**, e0032923 (2023). 37493508 10.1128/MRA.00329-23 PMC10508147

[31] Burgess, S. Sample size and power calculations in mendelian randomization with a single instrumental variable and a binary outcome. *Int. J. Epidemiol.* **43**, 922–929 (2014). 24608958 10.1093/ije/dyu005 PMC4052137

[32] Jin, Y., Han, C., Yang, D. & Gao, S. Association between gut microbiota and diabetic nephropathy: a mendelian randomization study. *Front. Microbiol.* **15**, 1309871 (2024). 38601939 10.3389/fmicb.2024.1309871 PMC11004376

[33] Yan, S., Wang, H., Feng, B., Ye, L. & Chen, A. Causal relationship between gut microbiota and diabetic nephropathy: a two-sample mendelian randomization study. *Front. Immunol.* **15**, 1332757 (2024). 38533501 10.3389/fimmu.2024.1332757 PMC10964483

[34] Burakova, I. et al. The effect of short-term consumption of lactic acid Bacteria on the gut microbiota in obese people. *Nutrients* **14**, 3384 (2022). 36014890 10.3390/nu14163384 PMC9415828

[35] Pinart, M. et al. Gut Microbiome Composition in obese and non-obese persons: a systematic review and Meta-analysis. *Nutrients* **14**, 12 (2022). 10.3390/nu14010012 PMC8746372 35010887

[36] Ciubotaru, I., Green, S. J., Kukreja, S. & Barengolts, E. Significant differences in fecal microbiota are associated with various stages of glucose tolerance in African American male veterans. *Transl Res.* **166**, 401–411 (2015). 26209747 10.1016/j.trsl.2015.06.015 PMC4916963

[37] Vaziri, N. D. et al. Chronic kidney disease alters intestinal microbial flora. *Kidney Int.* **83**, 308–315 (2013). 22992469 10.1038/ki.2012.345

[38] Henneke, L. et al. A dietary carbohydrate - gut Parasutterella - human fatty acid biosynthesis metabolic axis in obesity and type 2 diabetes. *Gut Microbes*. **14**, 2057778 (2022). 35435797 10.1080/19490976.2022.2057778 PMC9037427

[39] Zeng, Z., Guo, X., Zhang, J., Yuan, Q. & Chen, S. Lactobacillus paracasei modulates the gut microbiota and improves inflammation in type 2 diabetic rats. *Food Funct.* **12**, 6809–6820 (2021). 34113945 10.1039/d1fo00515d

[40] Hu, X. et al. Longitudinal analysis of fecal microbiome and metabolome during renal fibrotic progression in a unilateral ureteral obstruction animal model. *Eur. J. Pharmacol.* **886**, 173555 (2020). 32937112 10.1016/j.ejphar.2020.173555

[41] Li, F., Wang, M., Wang, J., Li, R. & Zhang, Y. Alterations to the gut microbiota and their correlation with inflammatory factors in chronic kidney disease. *Front. Cell. Infect. Microbiol.* **9**, 206 (2019). 31245306 10.3389/fcimb.2019.00206 PMC6581668

[42] Feng, Y. L. et al. Microbiome-Metabolomics reveals gut microbiota associated with glycine-conjugated metabolites and polyamine metabolism in chronic kidney disease. *Cell. Mol. Life Sci.* **76**, 4961–4978 (2019). 31147751 10.1007/s00018-019-03155-9 PMC11105293

[43] Chen, Y. J. et al. Parasutterella, in association with irritable bowel syndrome and intestinal chronic inflammation. *J. Gastroenterol. Hepatol.* **33**, 1844–1852 (2018). 29744928 10.1111/jgh.14281

[44] Ren, F. et al. Genetic evidence supporting the causal role of gut microbiota in chronic kidney disease and chronic systemic inflammation in CKD: a bilateral two-sample mendelian randomization study. *Front. Immunol.* **14**, 1287698 (2023). 38022507 10.3389/fimmu.2023.1287698 PMC10652796

[45] Mishima, E. et al. Evaluation of the impact of gut microbiota on uremic solute accumulation by a CE-TOFMS-based metabolomics approach. *Kidney Int.* **92**, 634–645 (2017). 28396122 10.1016/j.kint.2017.02.011

[46] Kikuchi, K. et al. Gut microbiome-derived phenyl sulfate contributes to albuminuria in diabetic kidney disease. *Nat. Commun.* **10**, 1835 (2019). 31015435 10.1038/s41467-019-09735-4 PMC6478834

[47] Brinkkoetter, P. T., Ising, C. & Benzing, T. The role of the podocyte in albumin filtration. *Nat. Rev. Nephrol.* **9**, 328–336 (2013). 23609563 10.1038/nrneph.2013.78

[48] Hoshino, J. et al. A new pathological scoring system by the Japanese classification to predict renal outcome in diabetic nephropathy. *PLoS One*. **13**, e0190923 (2018). 29408865 10.1371/journal.pone.0190923 PMC5800536

[49] Atherton, P., Stutchbury, B., Jethwa, D. & Ballestrem, C. Mechanosensitive components of integrin adhesions: role of vinculin. *Exp. Cell. Res.* **343**, 21–27 (2016). 26607713 10.1016/j.yexcr.2015.11.017 PMC4856733

[50] Carisey, A. et al. Vinculin regulates the recruitment and release of core focal adhesion proteins in a force-dependent manner. *Curr. Biol.* **23**, 271–281 (2013). 23375895 10.1016/j.cub.2013.01.009 PMC3580286

[51] Miao, J. et al. Newly identified cytoskeletal components are associated with dynamic changes of podocyte foot processes. *Nephrol. Dial Transpl.* **24**, 3297–3305 (2009). 10.1093/ndt/gfp338 19617259

[52] Babayeva, S. et al. Plasma from a case of recurrent idiopathic FSGS perturbs non-muscle myosin IIA (MYH9 protein) in human podocytes. *Pediatr. Nephrol.* **26**, 1071–1081 (2011). 21380797 10.1007/s00467-011-1831-z

[53] Artelt, N. et al. The role of Palladin in Podocytes. *J. Am. Soc. Nephrol.* **29**, 1662–1678 (2018). 29720549 10.1681/ASN.2017091039 PMC6054350

[54] Izard, T. & Brown, D. T. Mechanisms and functions of Vinculin interactions with phospholipids at Cell Adhesion sites. *J. Biol. Chem.* **291**, 2548–2555 (2016). 26728462 10.1074/jbc.R115.686493 PMC4742724

[55] Li, J. J. et al., *Podocyte biology in diabetic nephropathy. Kidney Int Suppl, S36-42* (2007). 10.1038/sj.ki.5002384 17653209

[56] Kim, D. Y. et al. Eucalyptol ameliorates dysfunction of actin cytoskeleton formation and Focal Adhesion Assembly in glucose-loaded Podocytes and Diabetic kidney. *Mol. Nutr. Food Res.* **63**, e1900489 (2019). 31483951 10.1002/mnfr.201900489

[57] Millioni, R. et al. Abnormal cytoskeletal protein expression in cultured skin fibroblasts from type 1 diabetes mellitus patients with nephropathy: a proteomic approach. *Proteomics. Clin. Appl.* **2**, 492–503 (2008). 21136853 10.1002/prca.200780112

[58] Schulz, M. C., Kopf, M. & Gekle, M. Crosstalk with renal proximal tubule cells drives acidosis-induced inflammatory response and dedifferentiation of fibroblasts via p38-singaling. *Cell. Communication Signaling: CCS*. **22**, 148 (2024). 38395872 10.1186/s12964-024-01527-8 PMC10893741

[59] O’Connell, S., Slattery, C., Ryan, M. P. & McMorrow, T. Identification of novel indicators of cyclosporine A nephrotoxicity in a CD-1 mouse model. *Toxicol. Appl. Pharmacol.* **252**, 201–210 (2011). 21354196 10.1016/j.taap.2011.02.015

[60] Cha, B. H. et al., *Integrin-Mediated Interactions Control Macrophage Polarization in 3D Hydrogels. Adv. Healthc. Mater.* ***6*** (2017). 10.1002/adhm.201700289 PMC5677560 28782184

[61] Porst, M. et al. Fibrillin-1 regulates mesangial cell attachment, spreading, migration and proliferation. *Kidney Int.* **69**, 450–456 (2006). 16395273 10.1038/sj.ki.5000030

[62] Bains, R., Furness, P. N. & Critchley, D. R. A quantitative immunofluorescence study of glomerular cell adhesion proteins in proteinuric states. *J. Pathol.* **183**, 272–280 (1997). 9422981 10.1002/(SICI)1096-9896(199711)183:3<272::AID-PATH914>3.0.CO;2-U

[63] Wang, Y. K., Lin, H. H. & Tang, M. J. Collagen gel overlay induces two phases of apoptosis in MDCK cells. *Am. J. Physiol. Cell Physiol.* **280**, C1440–1448 (2001). 11350739 10.1152/ajpcell.2001.280.6.C1440

[64] Costantino, V. V. et al. Losartan through Hsp70 avoids Angiotensin II Induced Mesenchymal epithelial transition in proximal tubule cells from spontaneously hypertensive rats. *Cell. Physiol. Biochem.* **53**, 713–730 (2019). 31599538 10.33594/000000167

[65] Carlisle, R. E. et al. TDAG51 mediates epithelial-to-mesenchymal transition in human proximal tubular epithelium. *Am. J. Physiol. Ren. Physiol.* **303**, F467–481 (2012). 10.1152/ajprenal.00481.2011 22592641

[66] Kaushik, G. et al., *Vinculin network–mediated cytoskeletal remodeling regulates contractile function in the aging heart. Sci. Transl. Med.* ***7*** (2015). 10.1126/scitranslmed.aaa5843 PMC4507505 26084806

[67] Li, H. M. et al., *Angiogenic and Antiangiogenic mechanisms of high density lipoprotein from healthy subjects and coronary artery diseases patients. Redox Biol.* ***36*** (2020). 10.1016/j.redox.2020.101642 PMC7364160 32863238

[68] Shih, Y. T. et al. Vinculin phosphorylation impairs vascular endothelial junctions promoting atherosclerosis. *Eur. Heart J.* **44**, 304–318 (2023). 36380599 10.1093/eurheartj/ehac647 PMC10202442

[69] Lanaspa, M. A. et al. The tight junction protein, MUPP1, is up-regulated by hypertonicity and is important in the osmotic stress response in kidney cells. *Proc. Natl. Acad. Sci. U.S.A.* **104**, 13672–13677 (2007). 17690246 10.1073/pnas.0702752104 PMC1959440

[70] Lanaspa, M. A., Andres-Hernando, A., Rivard, C. J., Dai, Y. & Berl, T. Hypertonic stress increases claudin-4 expression and tight junction integrity in association with MUPP1 in IMCD3 cells. *Proc. Natl. Acad. Sci. U.S.A.* **105**, 15797–15802 (2008). 18840681 10.1073/pnas.0805761105 PMC2572948

[71] Sindic, A. et al. MUPP1 complexes renal K + channels to alter cell surface expression and whole cell currents. *Am. J. Physiol. Renal. Physiol.* **297**, F36–45 (2009). 19420109 10.1152/ajprenal.90559.2008 PMC2711712

[72] Nakamura, K. et al. The role of hepatocyte growth factor activator inhibitor-1 (HAI-1) as a prognostic indicator in cervical cancer. *Int. J. Oncol.* **35**, 239–248 (2009). 19578736

[73] Madhusudhan, T. et al. Defective podocyte insulin signalling through p85-XBP1 promotes ATF6-dependent maladaptive ER-stress response in diabetic nephropathy. *Nat. Commun.* **6**, 6496 (2015). 25754093 10.1038/ncomms7496 PMC4366504

[74] Zhang, X. et al., *(+)-Catechin ameliorates diabetic nephropathy injury by inhibiting endoplasmic reticulum stress-related NLRP3-mediated inflammation. Food Funct.* (2024). 10.1039/d3fo05400d 38687305

[75] Park, M. J., Han, H. J. & Kim, D. I. Lipotoxicity-Induced PRMT1 exacerbates Mesangial Cell apoptosis via endoplasmic reticulum stress. *Int. J. Mol. Sci.* **18**, 1421 (2017). 28671608 10.3390/ijms18071421 PMC5535913

[76] Cao, A. L. et al. Ursodeoxycholic acid and 4-phenylbutyrate prevent endoplasmic reticulum stress-induced podocyte apoptosis in diabetic nephropathy. *Lab. Invest.* **96**, 610–622 (2016). 26999661 10.1038/labinvest.2016.44

[77] Kan, S. et al. Inhibition of HDAC6 with CAY10603 alleviates acute and chronic kidney injury by suppressing the ATF6 branch of UPR. *Arch. Biochem. Biophys.* **756**, 110009 (2024). 38642631 10.1016/j.abb.2024.110009

[78] Lee, J. Exploring renal pyruvate metabolism as a Therapeutic Avenue for Diabetic kidney Injury. *Diabetes Metabolism J.* **48**, 385–386 (2024). 10.4093/dmj.2024.0210 PMC11140400 38802117

[79] Qi, W. et al. Pyruvate kinase M2 activation may protect against the progression of diabetic glomerular pathology and mitochondrial dysfunction. *Nat. Med.* **23**, 753–762 (2017). 28436957 10.1038/nm.4328 PMC5575773

[80] Fu, J. et al., *Regeneration of glomerular metabolism and function by podocyte pyruvate kinase M2 in diabetic nephropathy. JCI Insight* ***7*** (2022). 10.1172/jci.insight.155260 PMC8983139 35133981

[81] Gordin, D. et al. Characterization of Glycolytic Enzymes and pyruvate kinase M2 in type 1 and 2 Diabetic Nephropathy. *Diabetes Care*. **42**, 1263–1273 (2019). 31076418 10.2337/dc18-2585 PMC6609957

[82] Chen, Z. et al. Reduction of anaerobic glycolysis contributes to angiotensin II-induced podocyte injury with foot process effacement. *Kidney Int.* **103**, 735–748 (2023). 36731609 10.1016/j.kint.2023.01.007

[83] Núñez Casal, A. Race and indigeneity in human microbiome science: microbiomisation and the historiality of otherness. *Hist. Philos. Life Sci.* **46**, 17 (2024). 38565750 10.1007/s40656-024-00614-w PMC10987353

