# Supplementary material for: Investigating the role of gut microbiota in diabetic nephropathy through plasma proteome mediated analysis
Source: Sci Rep. 2025 Feb 14;15:5457. doi: 10.1038/s41598-025-90306-7 (PMC11828962; doi:10.1038/s41598-025-90306-7)
Supplement: Supplementary file 6 — Supplementary Material 6 [file 41598_2025_90306_MOESM6_ESM.docx]

**R code for performing sample size and power calculations**

We here provide R code for performing sample size and power calculations. The code requires the proposed values of the causal effect (β_1_, a log odds ratio) and squared correlation (ρ^2^_GX_), and can either provide the sample size (total number of cases and controls) required for a given level of power, or the power for a given sample size.

expit <- function(x) { return(exp(x)/(1+exp(x))) }

rsq = 0.02 # squared correlation

b1 = 0.2 # causal effect (log odds ratio per SD

b1 = log(1.2) # or log of OR per SD)

sig = 0.05 # significance level (alpha)

pow = 0.8 # power level (1-beta)

ratio = 1 # ratio of cases:controls = 1:ratio

cat("Sample size required for ", pow*100, "% power: ",

(qnorm(1-sig/2)+qnorm(pow))^2/b1^2/rsq/(ratio/(1+ratio))/(1/(1+ratio)))

n = 40000 # Sample size

cat("Power of analysis with ", n, "participants: ",

pnorm(sqrt(n*rsq*(ratio/(1+ratio))*(1/(1+ratio)))*b1-qnorm(1-sig/2)))

**Reference**

1. Burgess, S. Sample size and power calculations in Mendelian randomization with a single instrumental variable and a binary outcome. *Int J Epidemiol* **43**, 922-929 (2014).
