# Supplementary material for: Investigating the role of gut microbiota in diabetic nephropathy through plasma proteome mediated analysis
Source: Sci Rep. 2025 Feb 14;15:5457. doi: 10.1038/s41598-025-90306-7 (PMC11828962; doi:10.1038/s41598-025-90306-7)
Supplement: Supplementary file 1 — Supplementary Material 1 [file 41598_2025_90306_MOESM1_ESM.pdf]

Supplementary Figure 1: Scatter plots of all MR analyses in this study

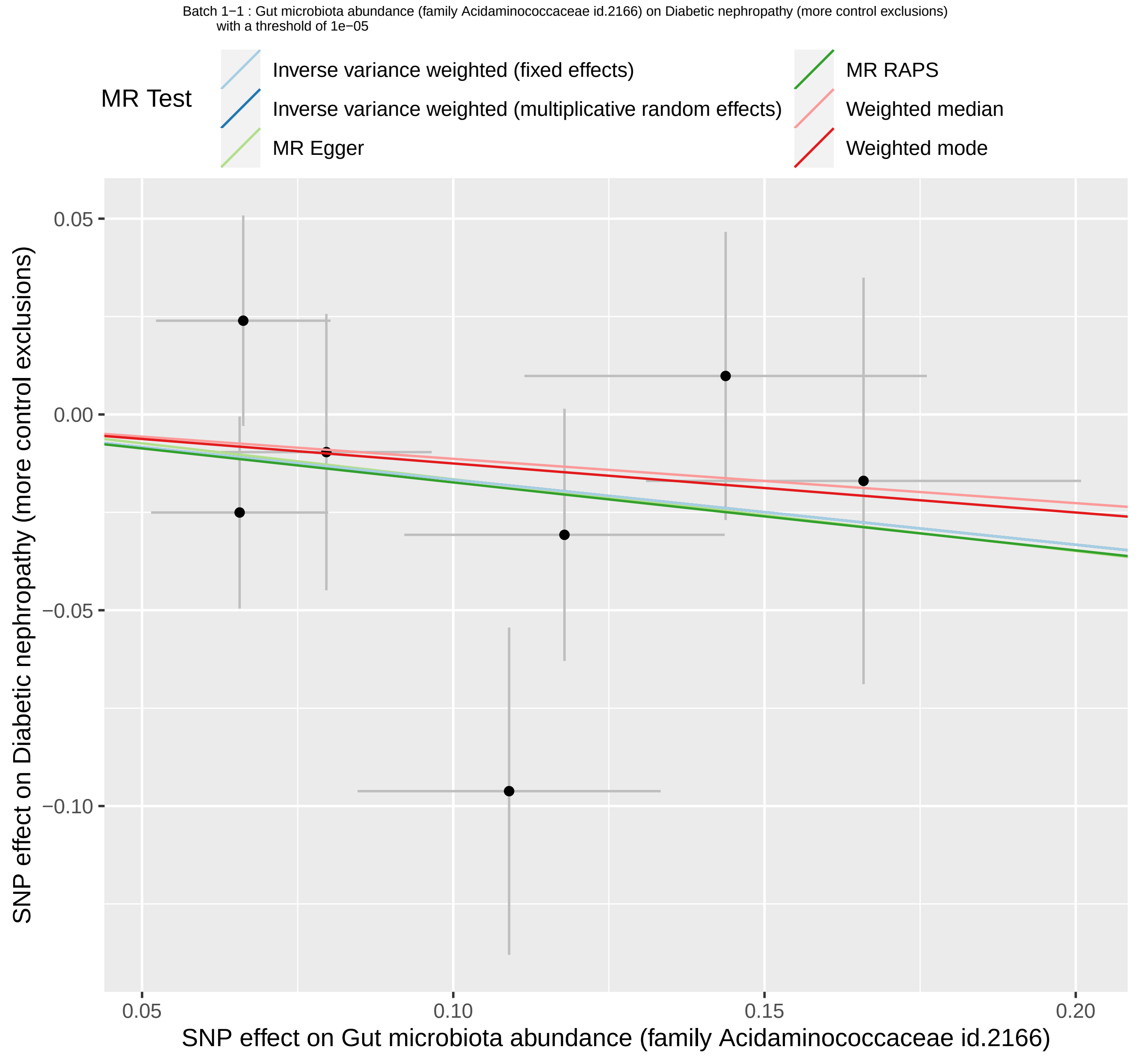

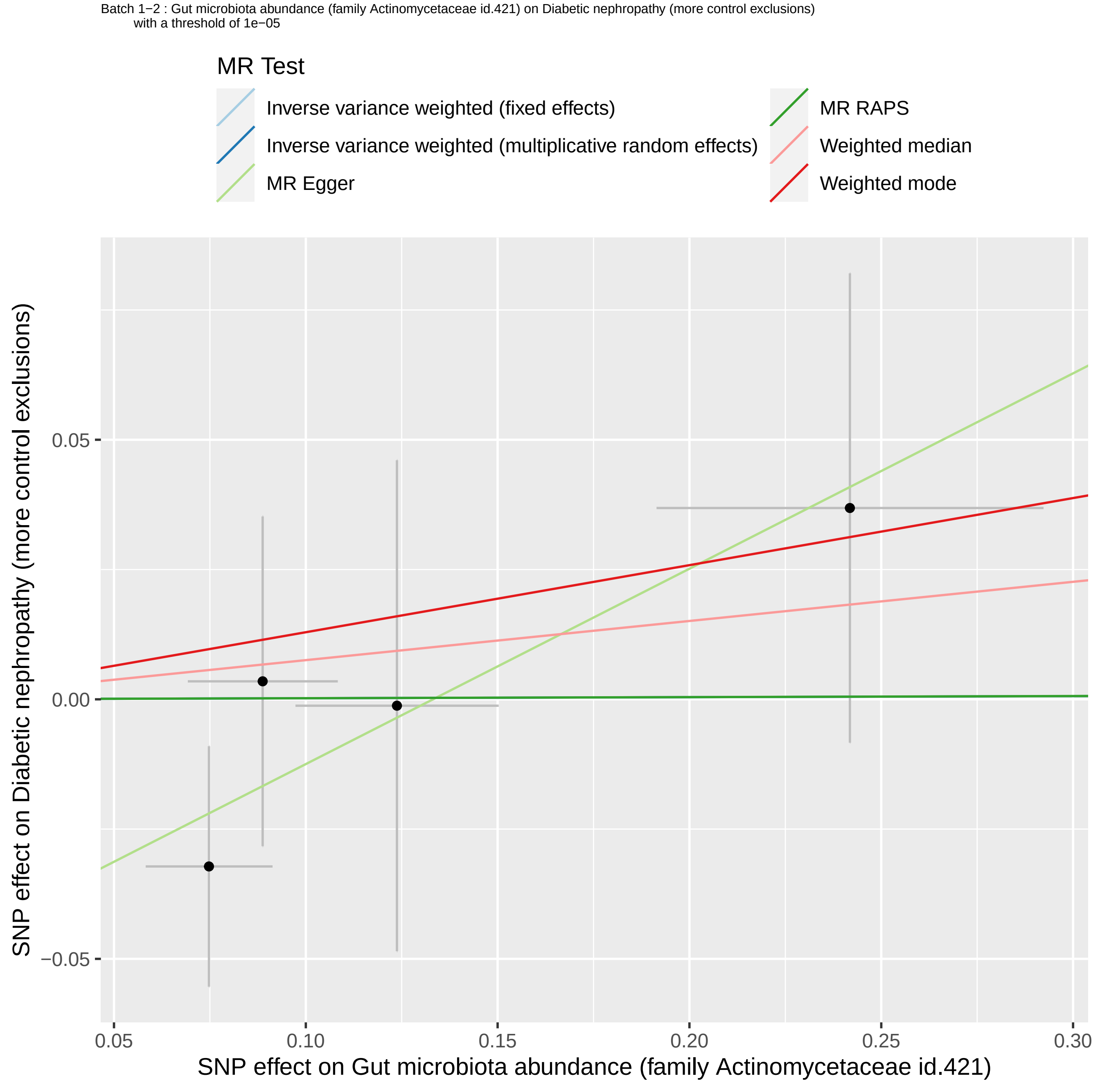
Batch 1−2 : Gut microbiota abundance (family Actinomycetaceae id.421) on Diabetic nephropathy (more control exclusions)
with a threshold of 1e−05
MR Test
Inverse variance weighted (fixed effects)
Inverse variance weighted (multiplicative random effects)
MR Egger
MR RAPS
Weighted median
Weighted mode
0.05
0.00
−0.05
0.05
0.10
0.15
0.20
0.25
0.30
SNP effect on Diabetic nephropathy (more control exclusions)
SNP effect on Gut microbiota abundance (family Actinomycetaceae id.421)

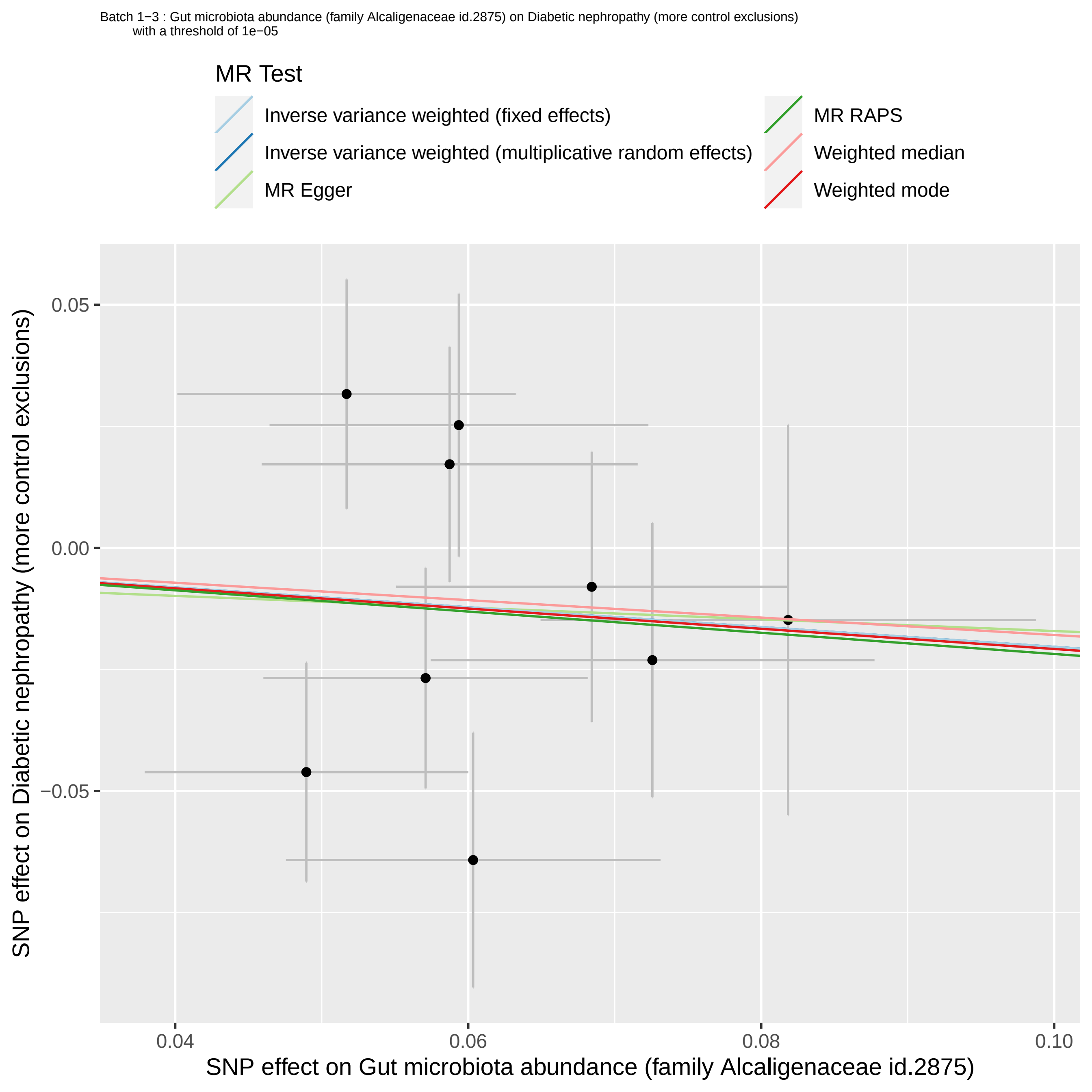
Batch 1−3 : Gut microbiota abundance (family Alcaligenaceae id.2875) on Diabetic nephropathy (more control exclusions)
with a threshold of 1e−05
MR Test
Inverse variance weighted (fixed effects)
Inverse variance weighted (multiplicative random effects)
MR Egger
MR RAPS
Weighted median
Weighted mode
0.05
0.00
−0.05
0.04
0.06
0.08
0.10
SNP effect on Diabetic nephropathy (more control exclusions)
SNP effect on Gut microbiota abundance (family Alcaligenaceae id.2875)

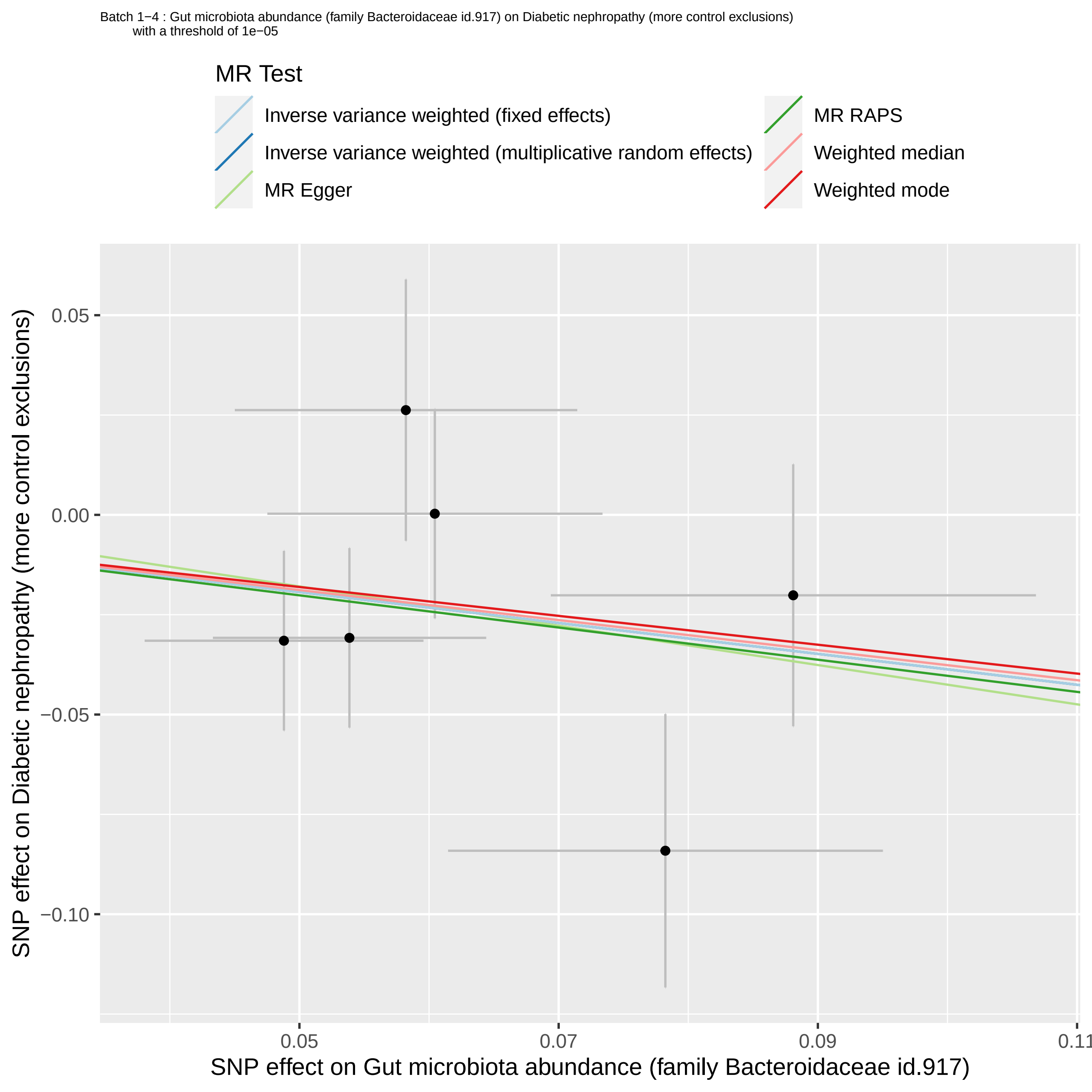
Batch 1−4 : Gut microbiota abundance (family Bacteroidaceae id.917) on Diabetic nephropathy (more control exclusions)
with a threshold of 1e−05
MR Test
Inverse variance weighted (fixed effects)
Inverse variance weighted (multiplicative random effects)
MR Egger
MR RAPS
Weighted median
Weighted mode
0.05
0.00
−0.05
−0.10
0.05
0.07
0.09
0.11
SNP effect on Diabetic nephropathy (more control exclusions)
SNP effect on Gut microbiota abundance (family Bacteroidaceae id.917)

Batch 1−5 : Gut microbiota abundance (family Bacteroidales S24 7group id.11173) on Diabetic nephropathy (more control exclusions) with a threshold of 1e−05

MR Test

- Inverse variance weighted (fixed effects)
- Inverse variance weighted (multiplicative random effects)
- MR Egger
- MR RAPS
- Weighted median
- Weighted mode

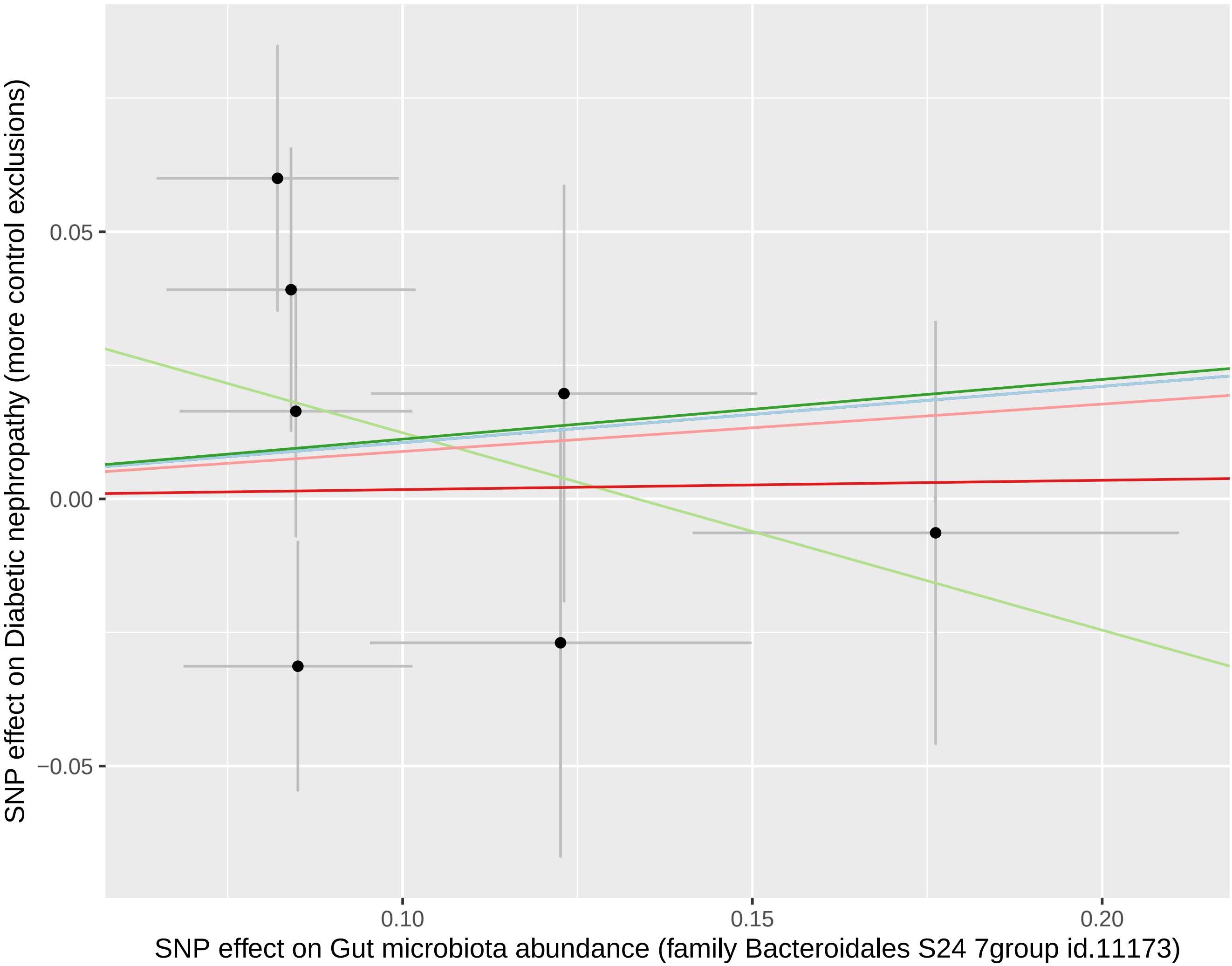

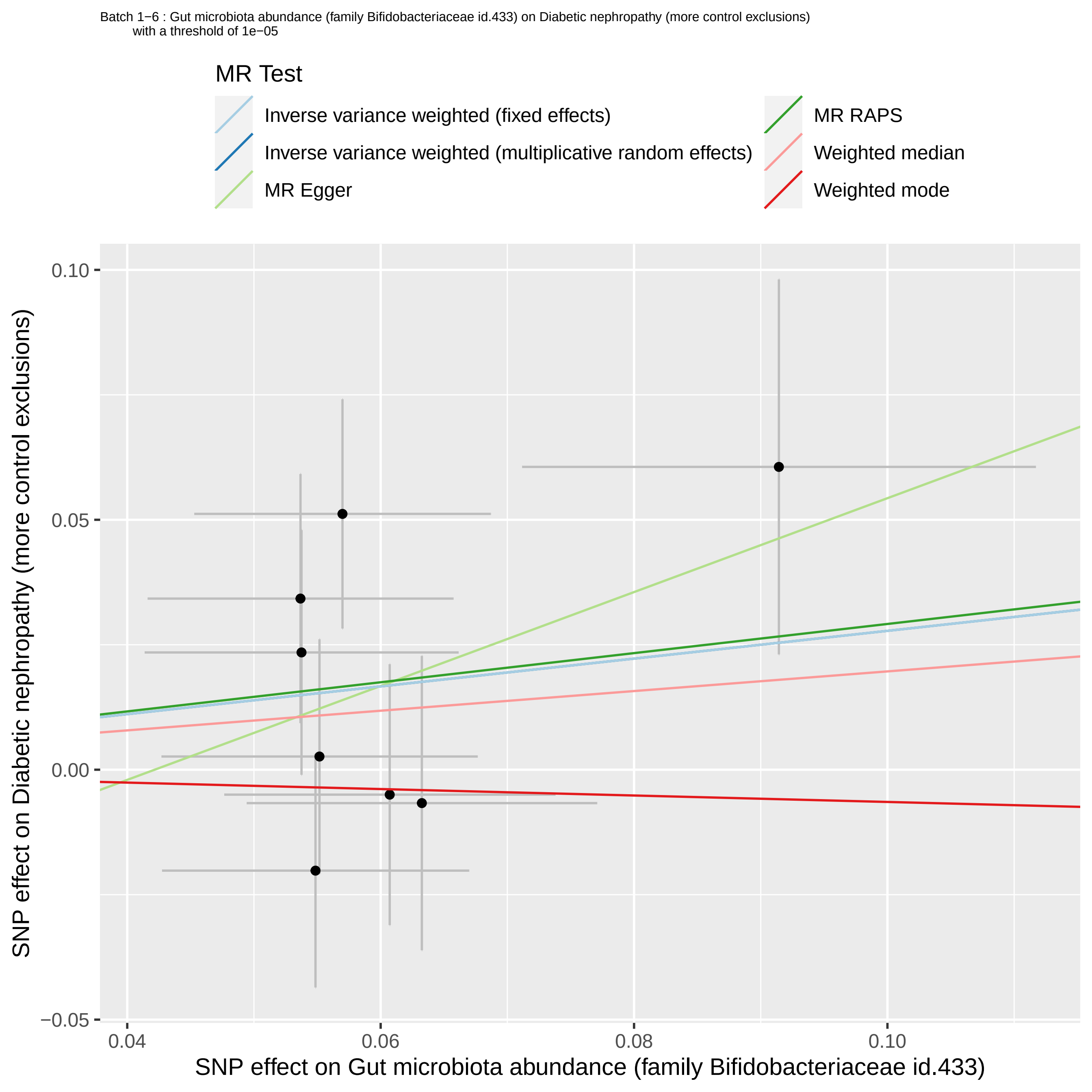
Batch 1−6 : Gut microbiota abundance (family Bifidobacteriaceae id.433) on Diabetic nephropathy (more control exclusions)
with a threshold of 1e−05
MR Test
Inverse variance weighted (fixed effects)
Inverse variance weighted (multiplicative random effects)
MR Egger
MR RAPS
Weighted median
Weighted mode
0.10
0.05
0.00
−0.05
0.04
0.06
0.08
0.10
SNP effect on Diabetic nephropathy (more control exclusions)
SNP effect on Gut microbiota abundance (family Bifidobacteriaceae id.433)

Batch 1−8 : Gut microbiota abundance (family Clostridiaceae1 id.1869) on Diabetic nephropathy (more control exclusions)
with a threshold of 1e−05

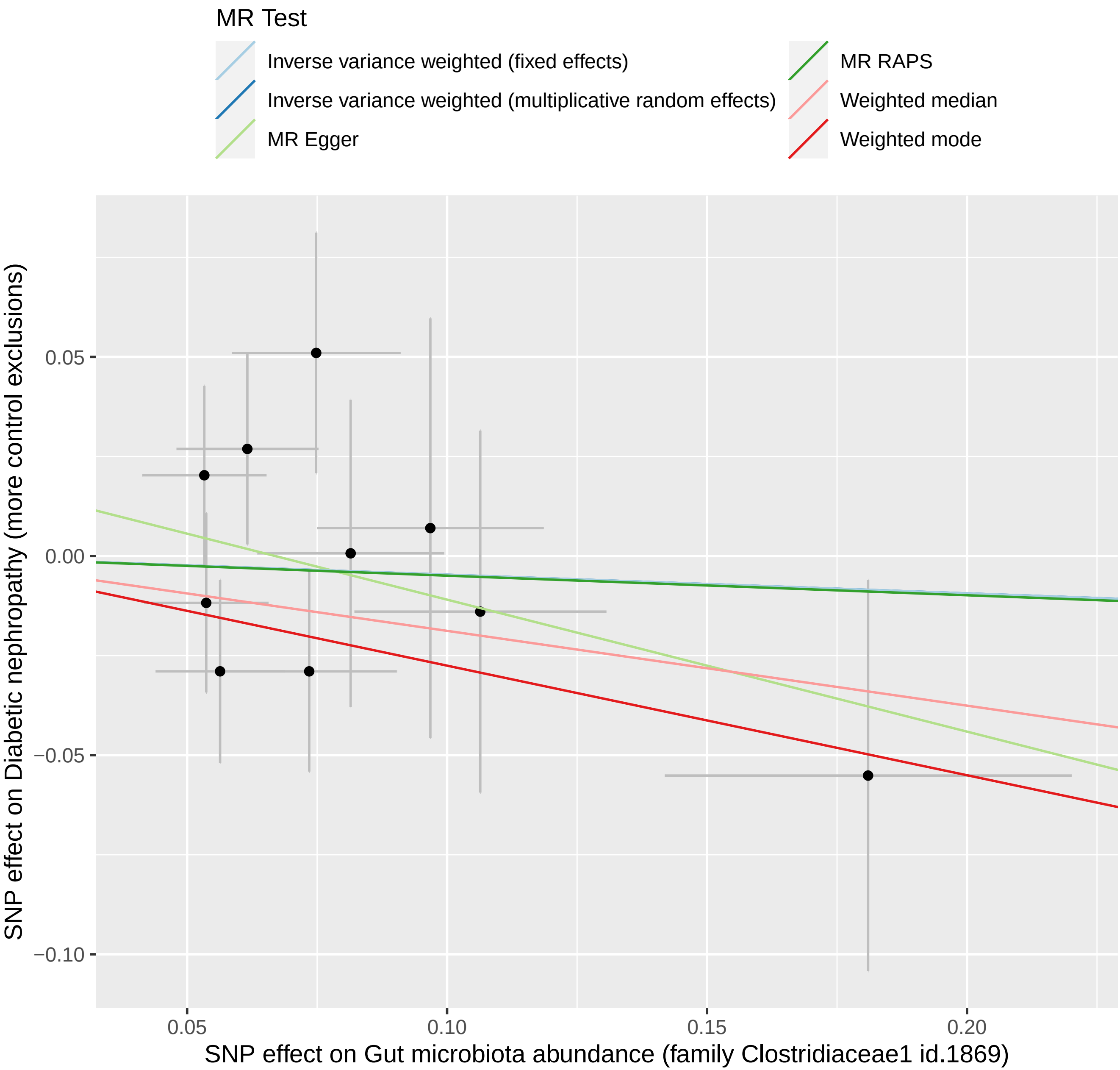

Batch 1−9 : Gut microbiota abundance (family Clostridiales vadin BB60 group id.11286) on Diabetic nephropathy (more control exclusions) with a threshold of 1e−05

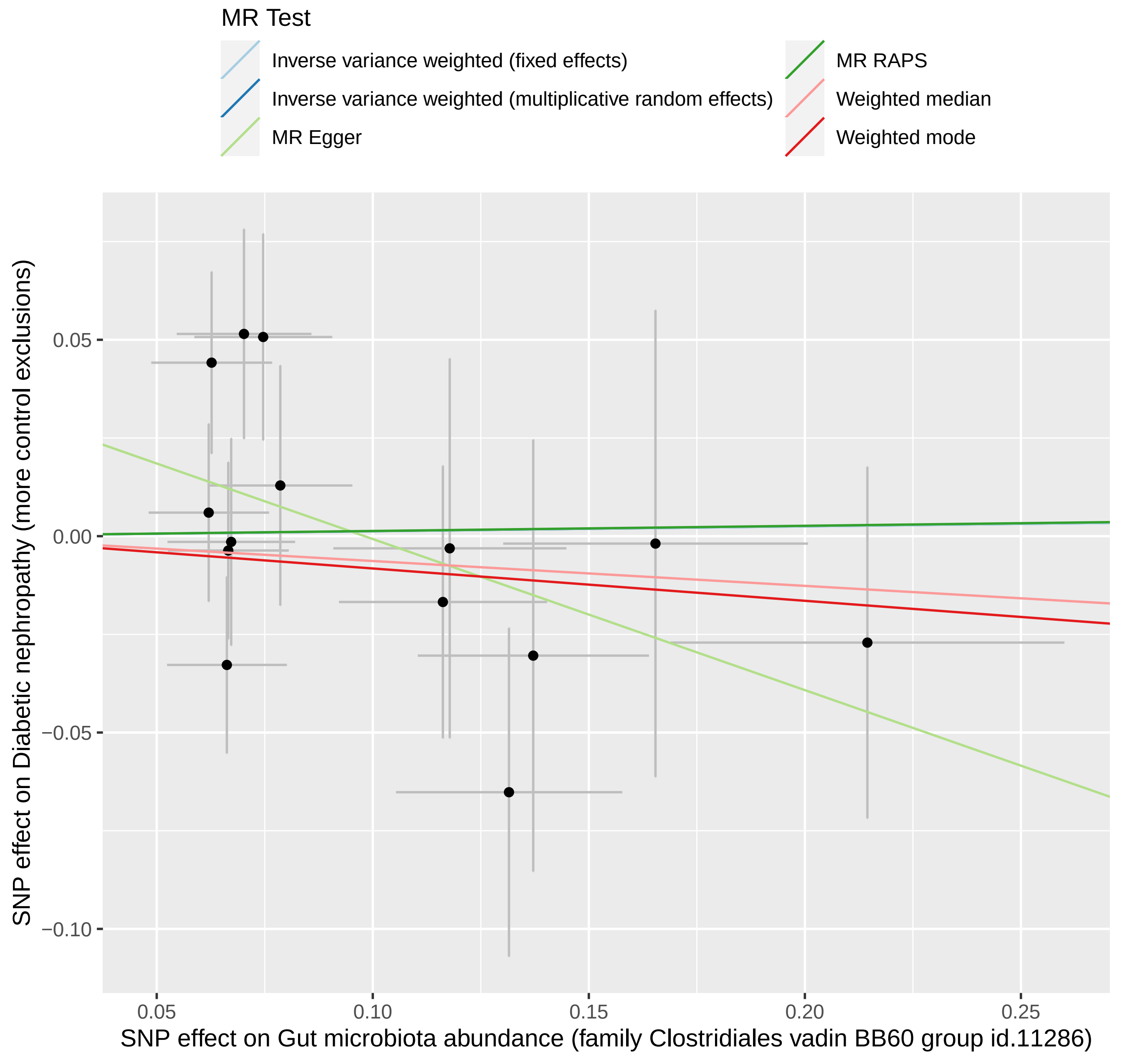

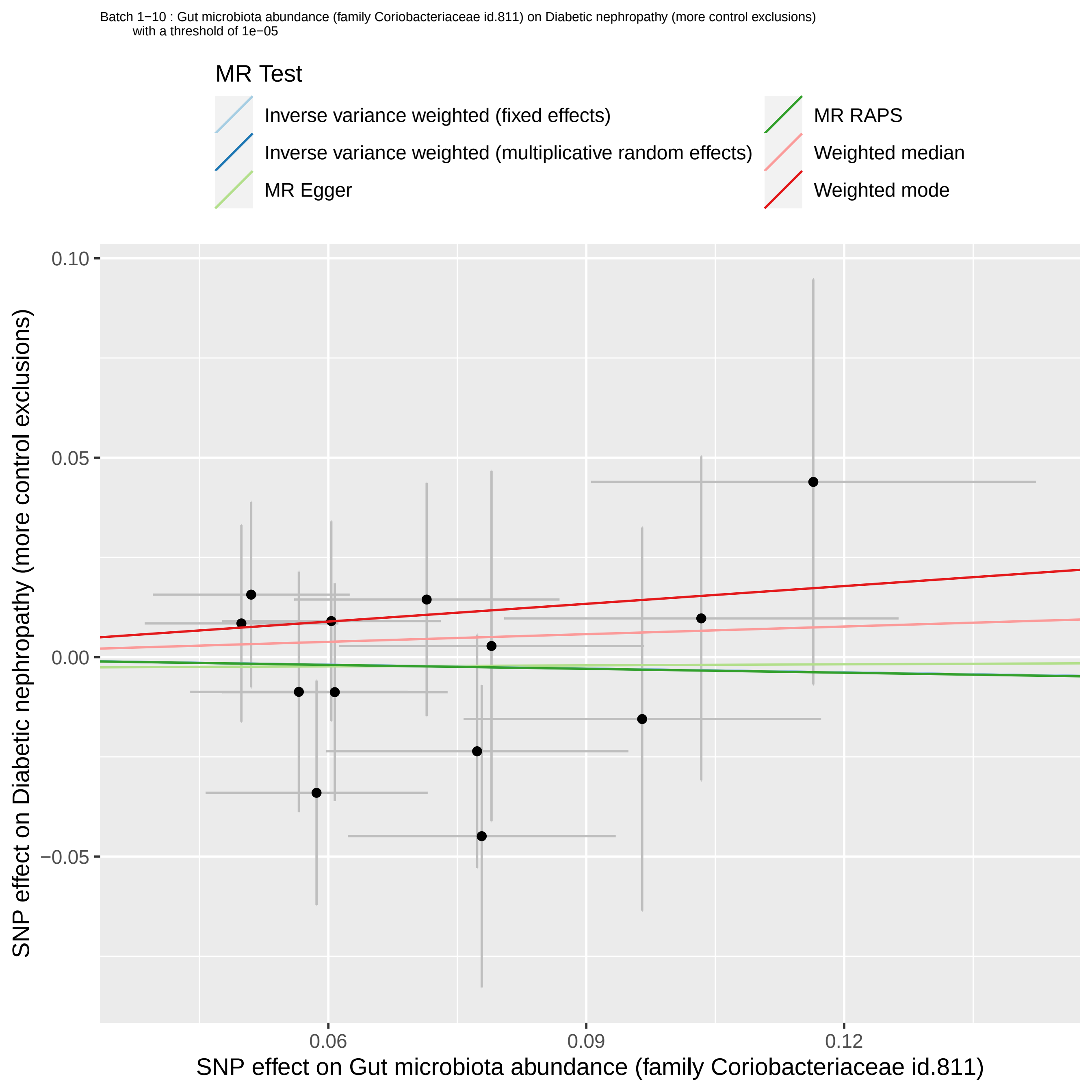
Batch 1−10 : Gut microbiota abundance (family Coriobacteriaceae id.811) on Diabetic nephropathy (more control exclusions)
with a threshold of 1e−05
MR Test
Inverse variance weighted (fixed effects)
Inverse variance weighted (multiplicative random effects)
MR Egger
MR RAPS
Weighted median
Weighted mode
0.10
0.05
0.00
−0.05
0.06
0.09
0.12
SNP effect on Diabetic nephropathy (more control exclusions)
SNP effect on Gut microbiota abundance (family Coriobacteriaceae id.811)

Batch 1−11 : Gut microbiota abundance (family Defluviitaleaceae id.1924) on Diabetic nephropathy (more control exclusions) with a threshold of 1e−05

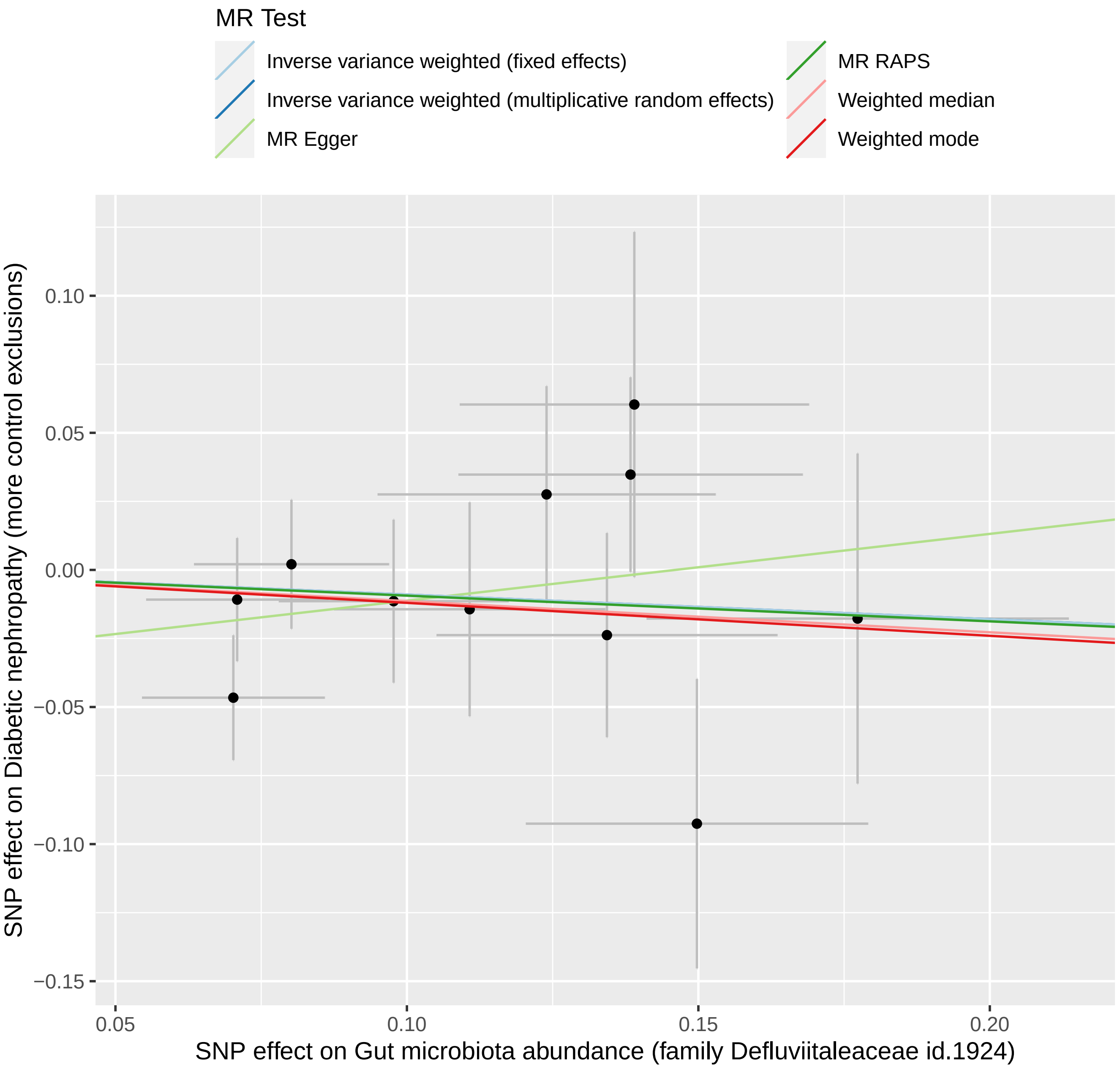

Batch 1−12 : Gut microbiota abundance (family Desulfovibrionaceae id.3169) on Diabetic nephropathy (more control exclusions)
with a threshold of 1e−05

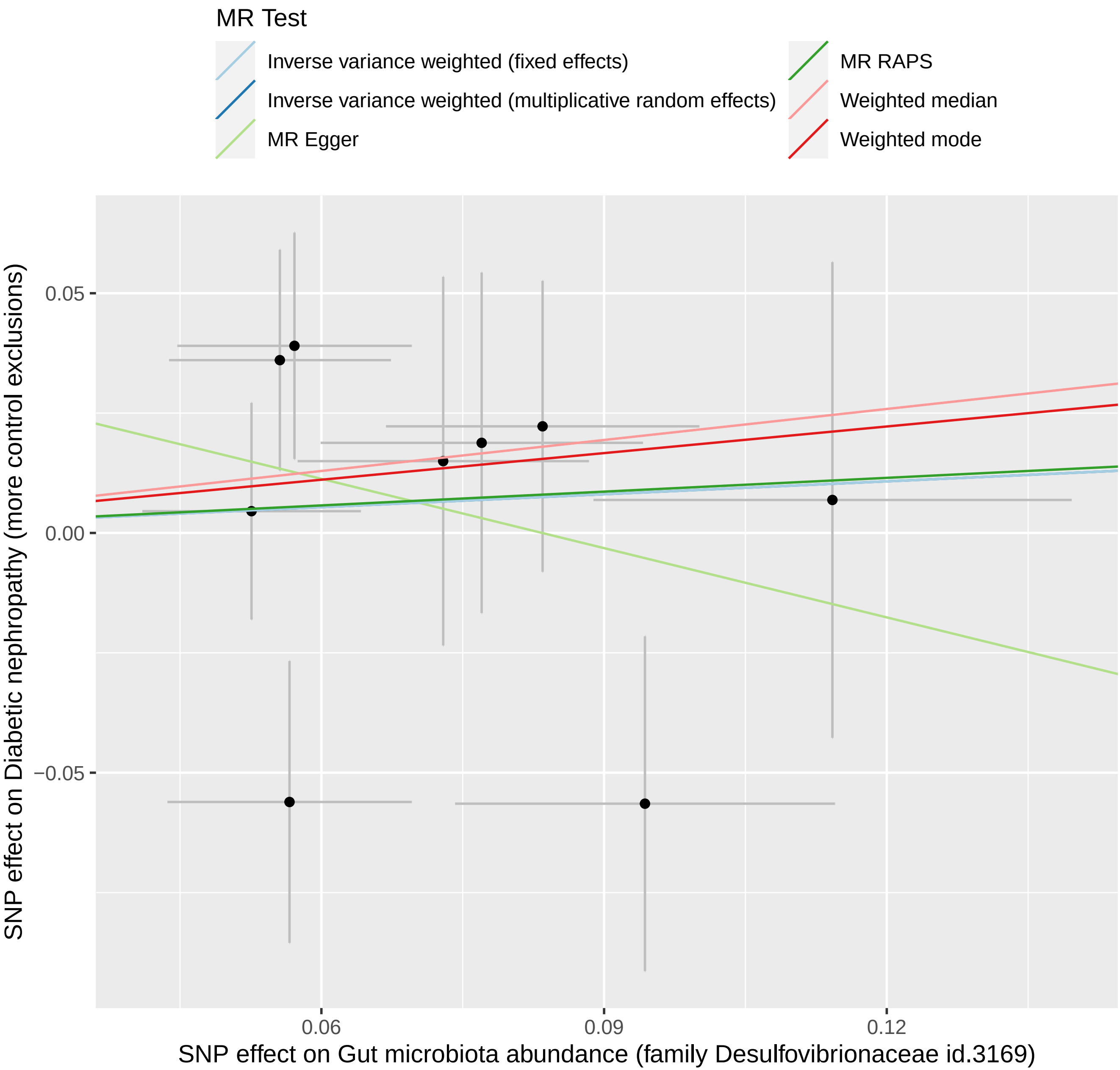

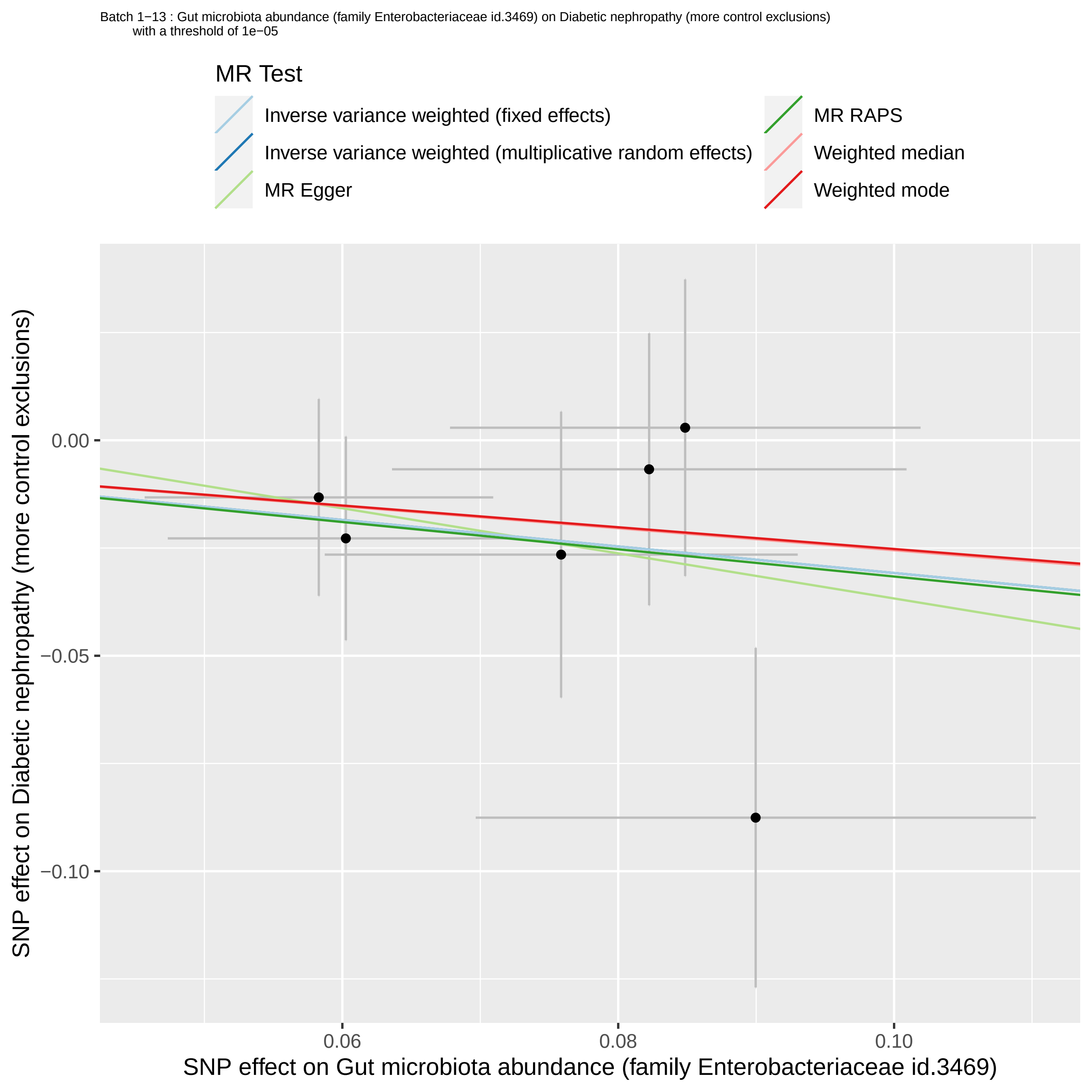
Batch 1−13 : Gut microbiota abundance (family Enterobacteriaceae id.3469) on Diabetic nephropathy (more control exclusions) with a threshold of 1e−05
MR Test
Inverse variance weighted (fixed effects)
Inverse variance weighted (multiplicative random effects)
MR Egger
MR RAPS
Weighted median
Weighted mode
0.00
−0.05
−0.10
0.06
0.08
0.10
SNP effect on Diabetic nephropathy (more control exclusions)
SNP effect on Gut microbiota abundance (family Enterobacteriaceae id.3469)

Batch 1−14 : Gut microbiota abundance (family Erysipelotrichaceae id.2149) on Diabetic nephropathy (more control exclusions) with a threshold of 1e−05

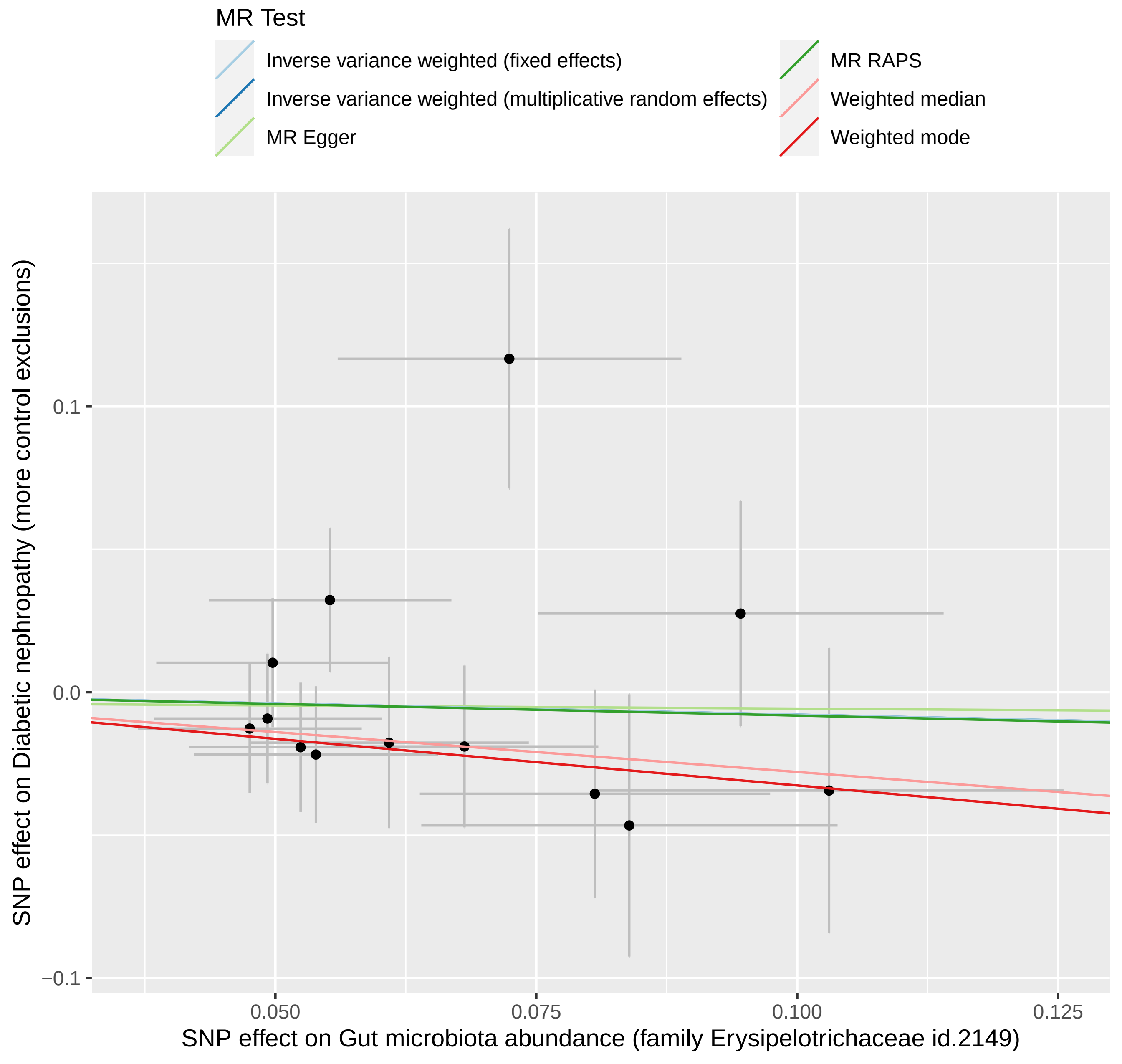

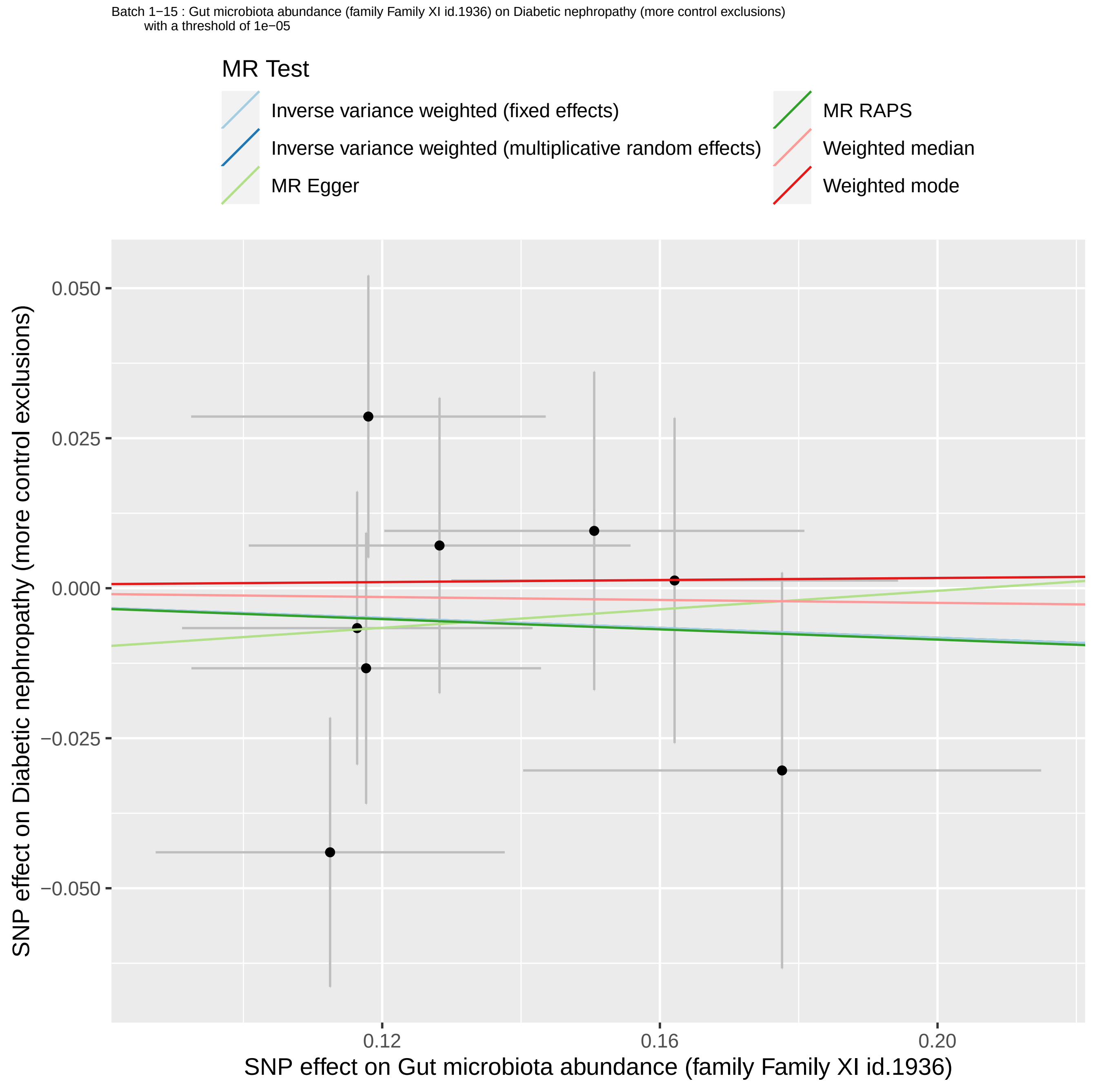
Batch 1−15 : Gut microbiota abundance (family Family XI id.1936) on Diabetic nephropathy (more control exclusions)
with a threshold of 1e−05
MR Test
Inverse variance weighted (fixed effects)
Inverse variance weighted (multiplicative random effects)
MR Egger
MR RAPS
Weighted median
Weighted mode
0.050
0.025
0.000
−0.025
−0.050
0.12
0.16
0.20
SNP effect on Diabetic nephropathy (more control exclusions)
SNP effect on Gut microbiota abundance (family Family XI id.1936)

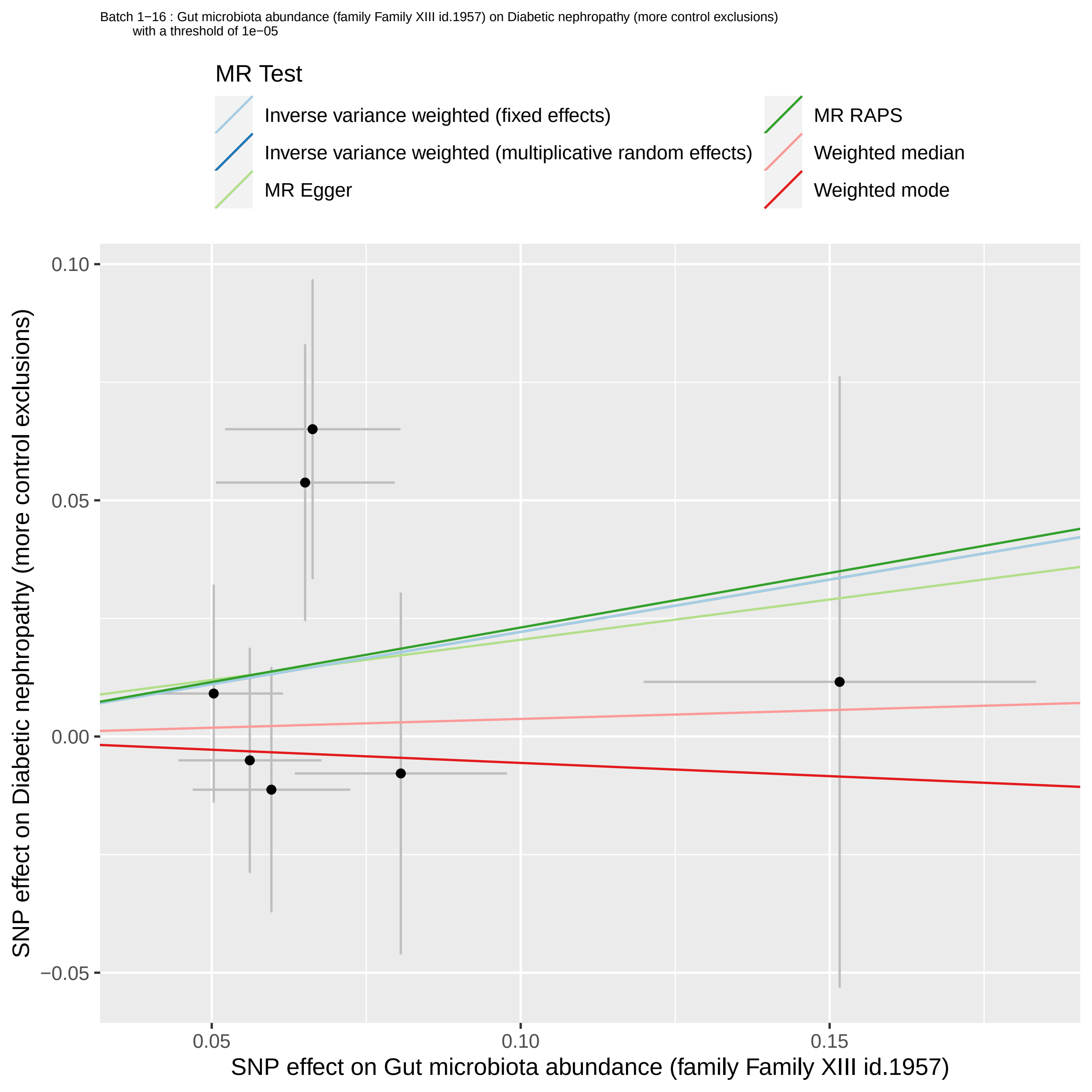

Batch 1−16 : Gut microbiota abundance (family Family XIII id.1957) on Diabetic nephropathy (more control exclusions) with a threshold of 1e−05
MR Test
Inverse variance weighted (fixed effects)
Inverse variance weighted (multiplicative random effects)
MR Egger
MR RAPS
Weighted median
Weighted mode
0.10
0.05
0.00
−0.05
0.05
0.10
0.15
SNP effect on Diabetic nephropathy (more control exclusions)
SNP effect on Gut microbiota abundance (family Family XIII id.1957)

Batch 1−17 : Gut microbiota abundance (family Lachnospiraceae id.1987) on Diabetic nephropathy (more control exclusions)
with a threshold of 1e−05

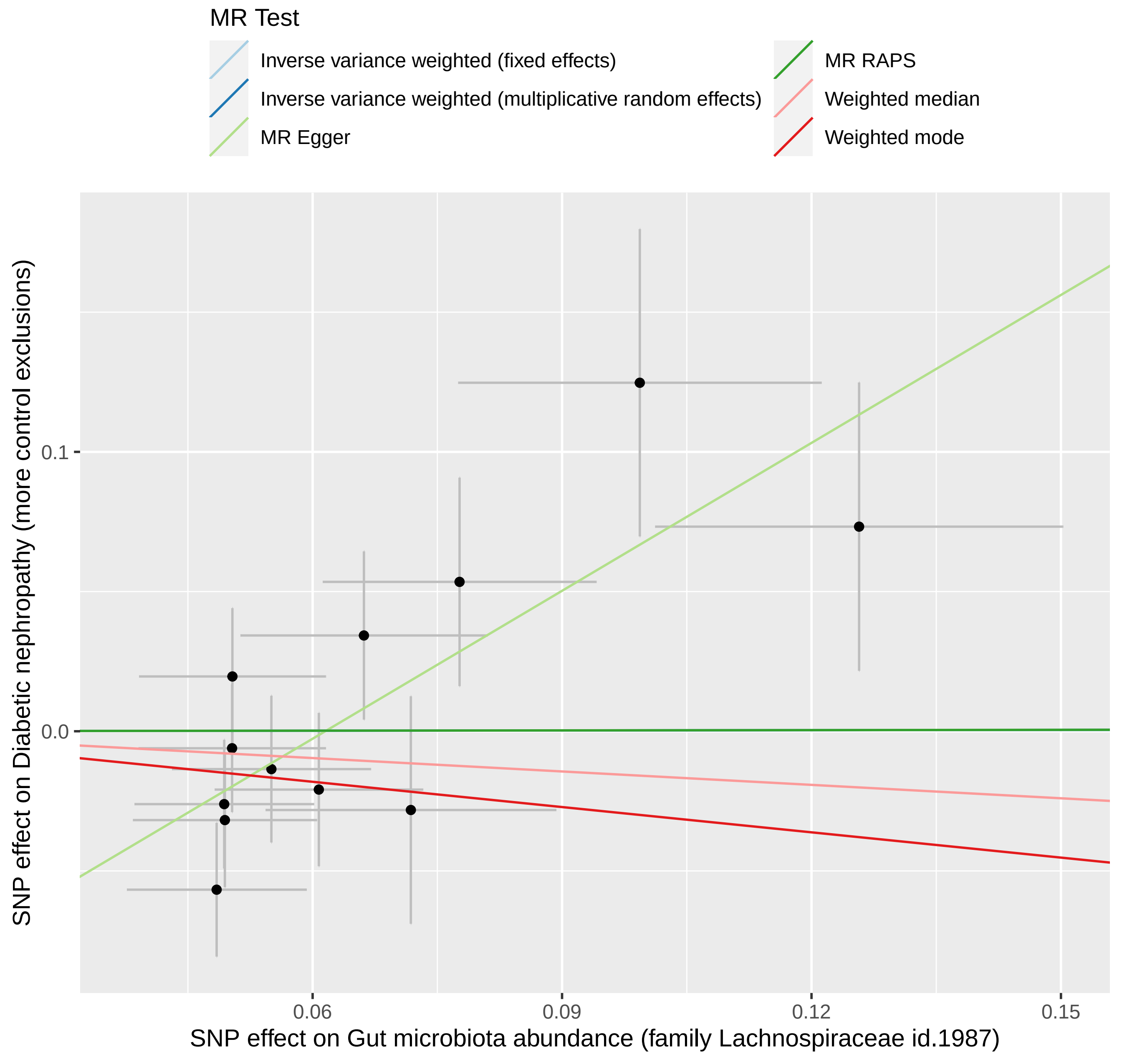

Batch 1−18 : Gut microbiota abundance (family Lactobacillaceae id.1836) on Diabetic nephropathy (more control exclusions) with a threshold of 1e−05

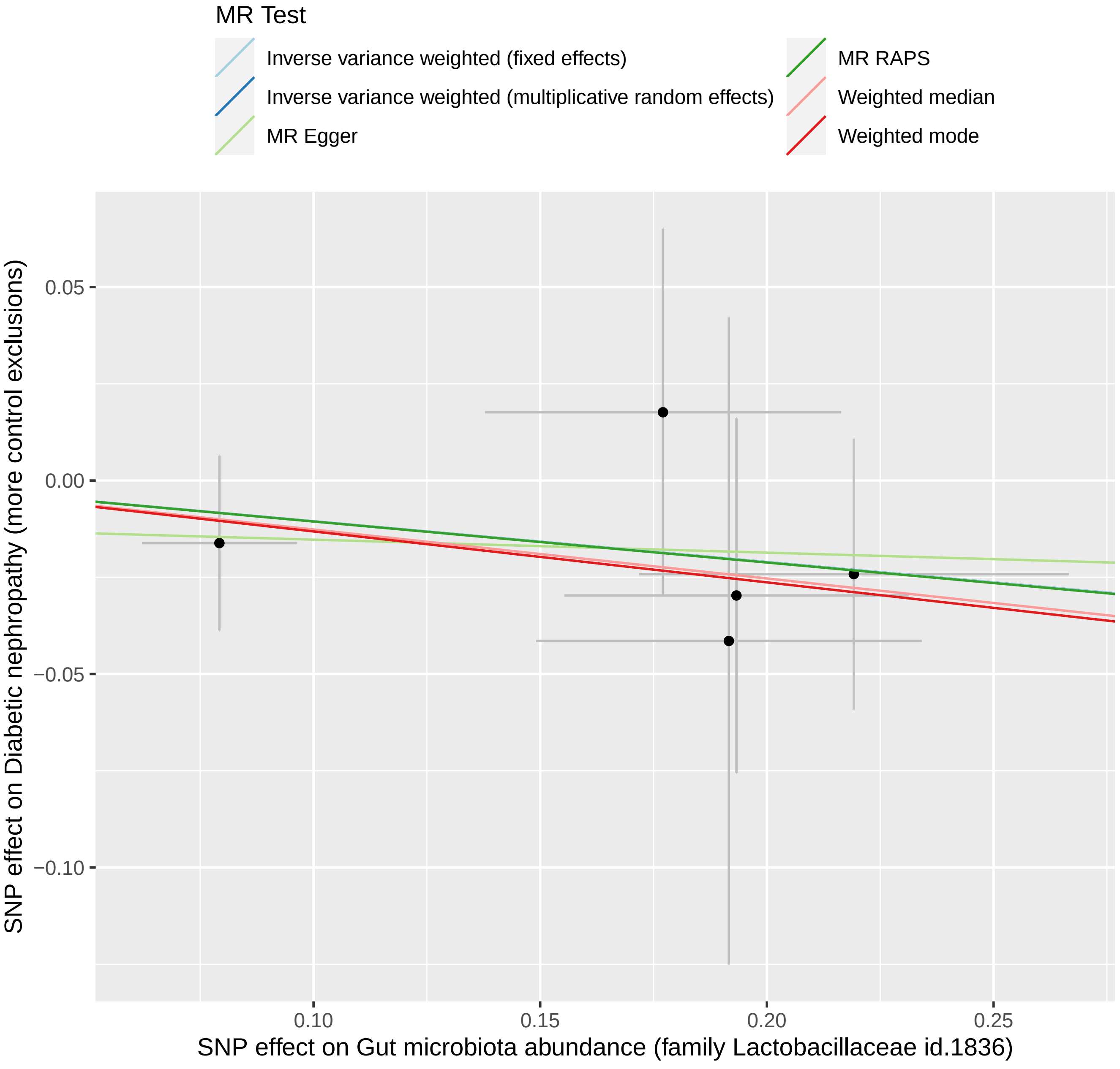

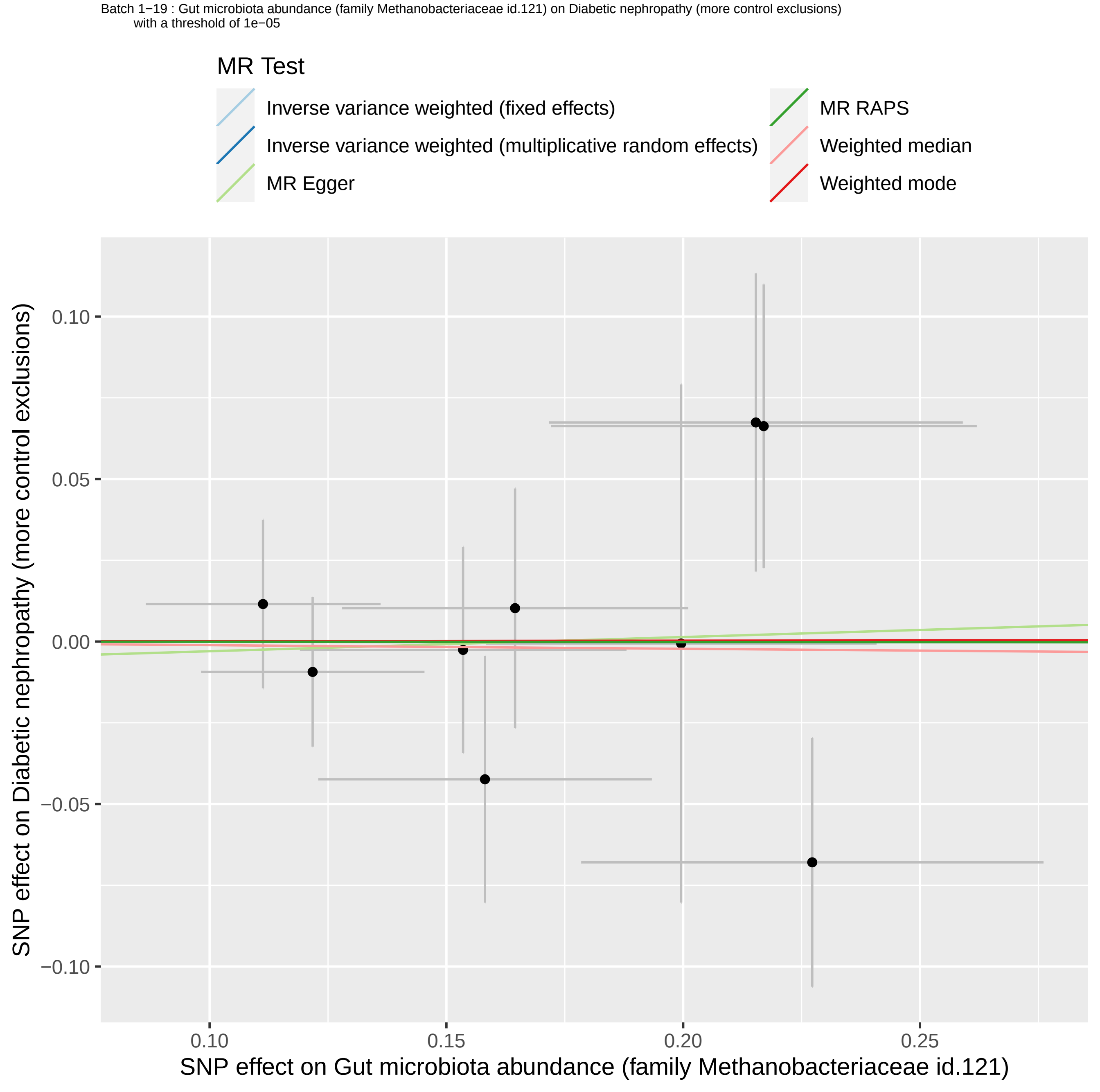

Batch 1−19 : Gut microbiota abundance (family Methanobacteriaceae id.121) on Diabetic nephropathy (more control exclusions) with a threshold of 1e−05
MR Test
Inverse variance weighted (fixed effects)
Inverse variance weighted (multiplicative random effects)
MR Egger
MR RAPS
Weighted median
Weighted mode
0.10
0.05
0.00
−0.05
−0.10
0.10
0.15
0.20
0.25
SNP effect on Diabetic nephropathy (more control exclusions)
SNP effect on Gut microbiota abundance (family Methanobacteriaceae id.121)

Batch 1−20 : Gut microbiota abundance (family Oxalobacteraceae id.2966) on Diabetic nephropathy (more control exclusions)
with a threshold of 1e−05

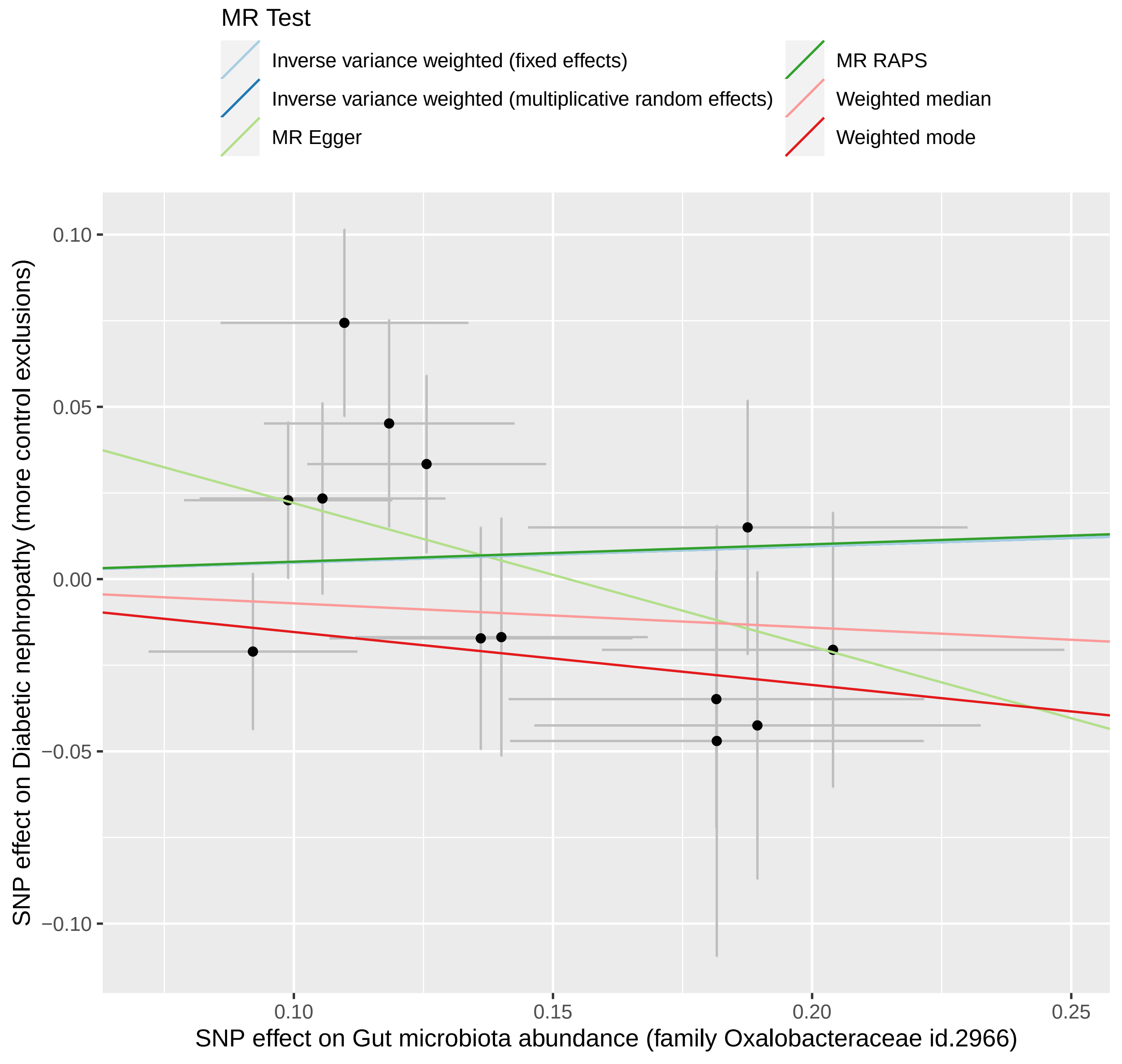

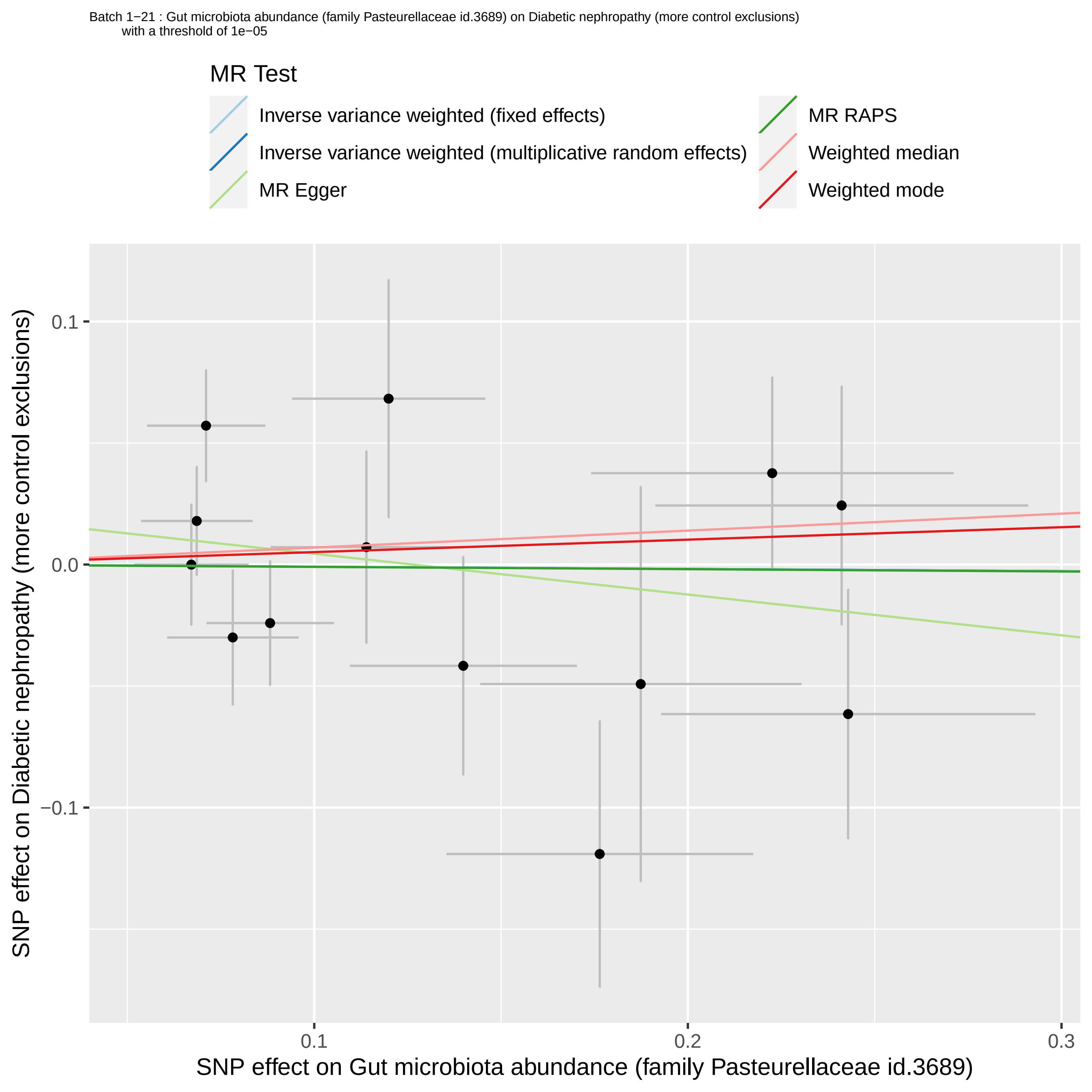
Batch 1−21 : Gut microbiota abundance (family Pasteurellaceae id.3689) on Diabetic nephropathy (more control exclusions)
with a threshold of 1e−05
MR Test
Inverse variance weighted (fixed effects)
Inverse variance weighted (multiplicative random effects)
MR Egger
MR RAPS
Weighted median
Weighted mode
0.1
0.0
−0.1
0.1
0.2
0.3
SNP effect on Diabetic nephropathy (more control exclusions)
SNP effect on Gut microbiota abundance (family Pasteurellaceae id.3689)

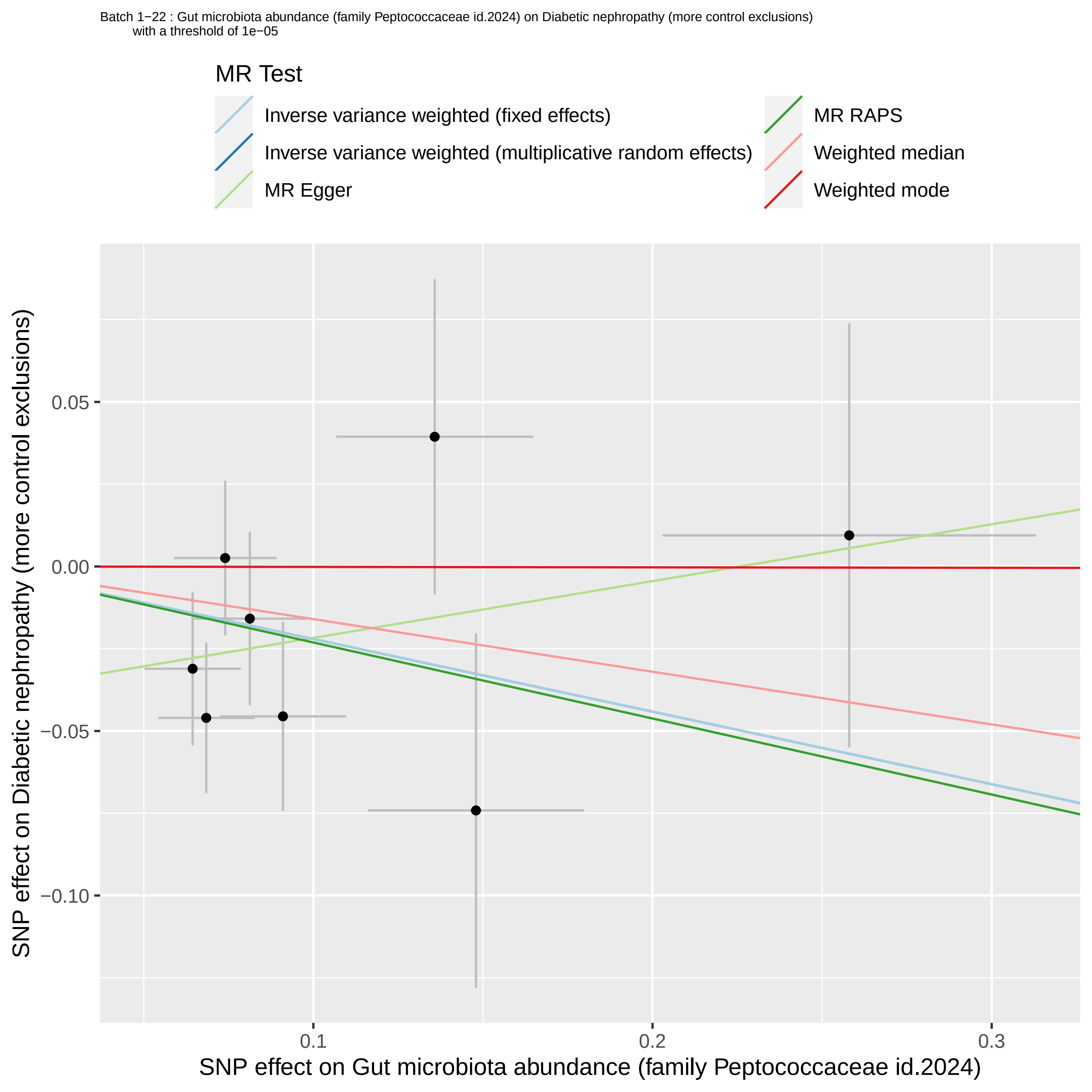
Batch 1−22 : Gut microbiota abundance (family Peptococcaceae id.2024) on Diabetic nephropathy (more control exclusions)
with a threshold of 1e−05
MR Test
Inverse variance weighted (fixed effects)
Inverse variance weighted (multiplicative random effects)
MR Egger
MR RAPS
Weighted median
Weighted mode
0.05
0.00
−0.05
−0.10
0.1
0.2
0.3
SNP effect on Diabetic nephropathy (more control exclusions)
SNP effect on Gut microbiota abundance (family Peptococcaceae id.2024)

Batch 1−23 : Gut microbiota abundance (family Peptostreptococcaceae id.2042) on Diabetic nephropathy (more control exclusions)
with a threshold of 1e−05

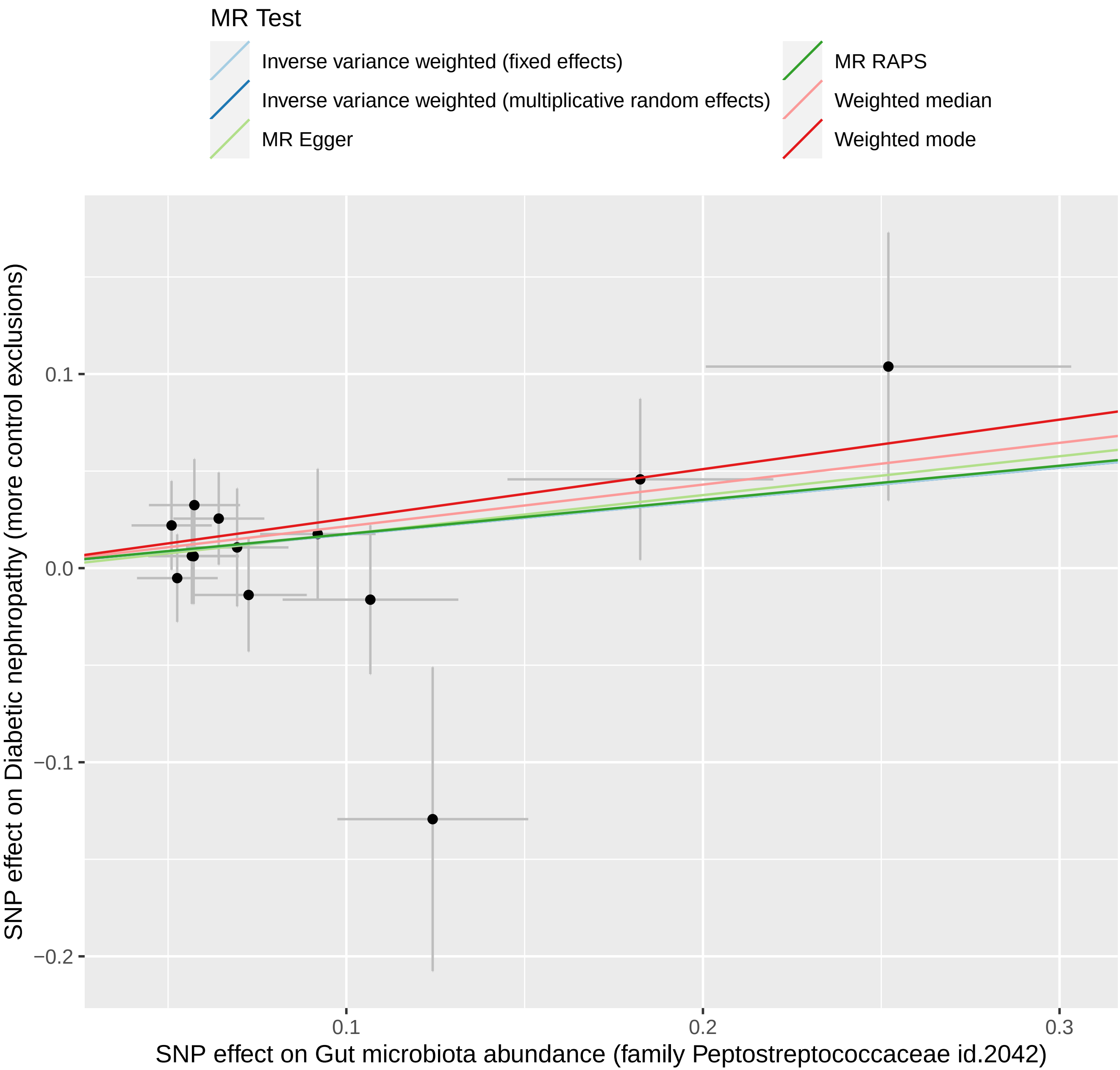

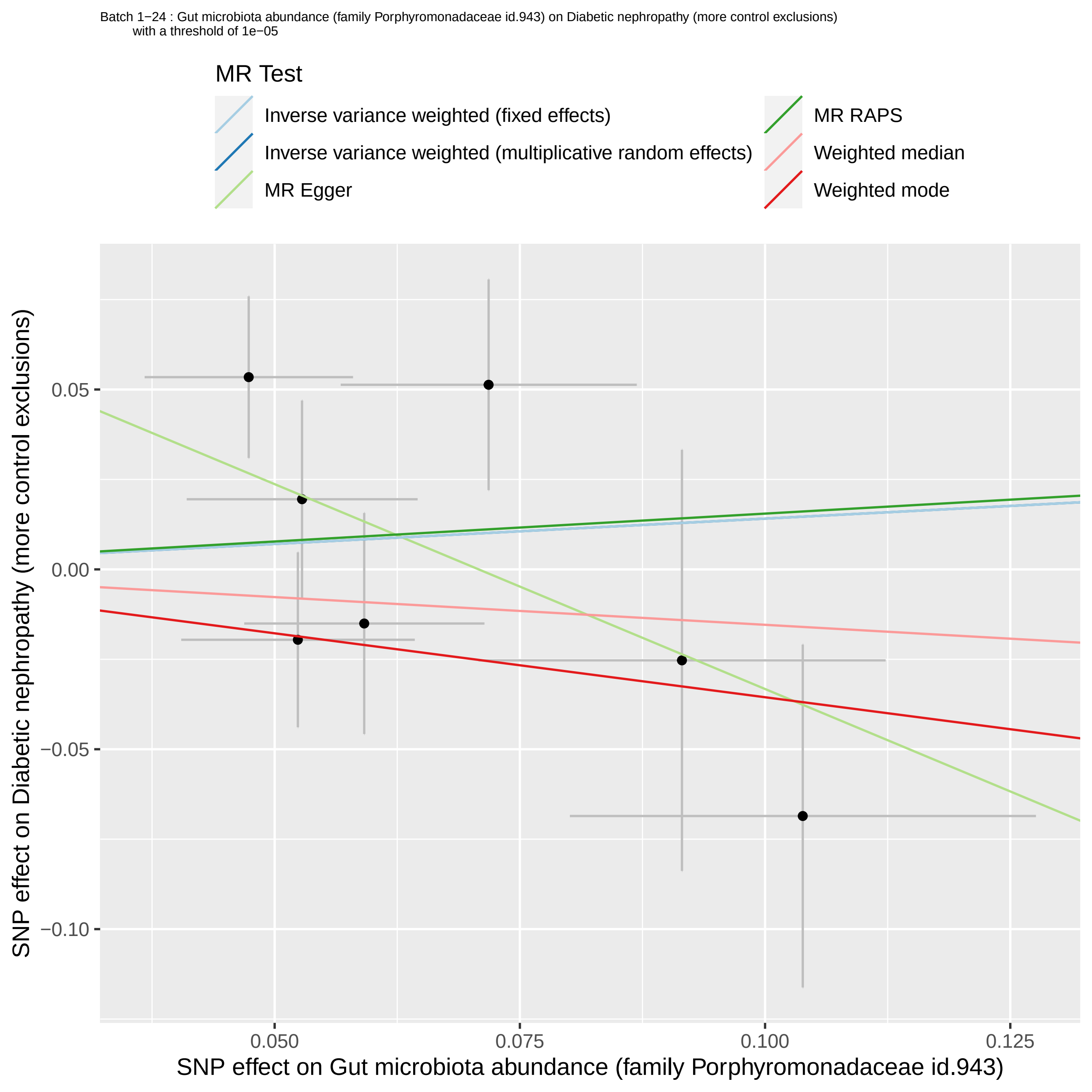
Batch 1−24 : Gut microbiota abundance (family Porphyromonadaceae id.943) on Diabetic nephropathy (more control exclusions)
with a threshold of 1e−05
MR Test
Inverse variance weighted (fixed effects)
Inverse variance weighted (multiplicative random effects)
MR Egger
MR RAPS
Weighted median
Weighted mode
0.05
0.00
−0.05
−0.10
0.050
0.075
0.100
0.125
SNP effect on Gut microbiota abundance (family Porphyromonadaceae id.943)
SNP effect on Diabetic nephropathy (more control exclusions)

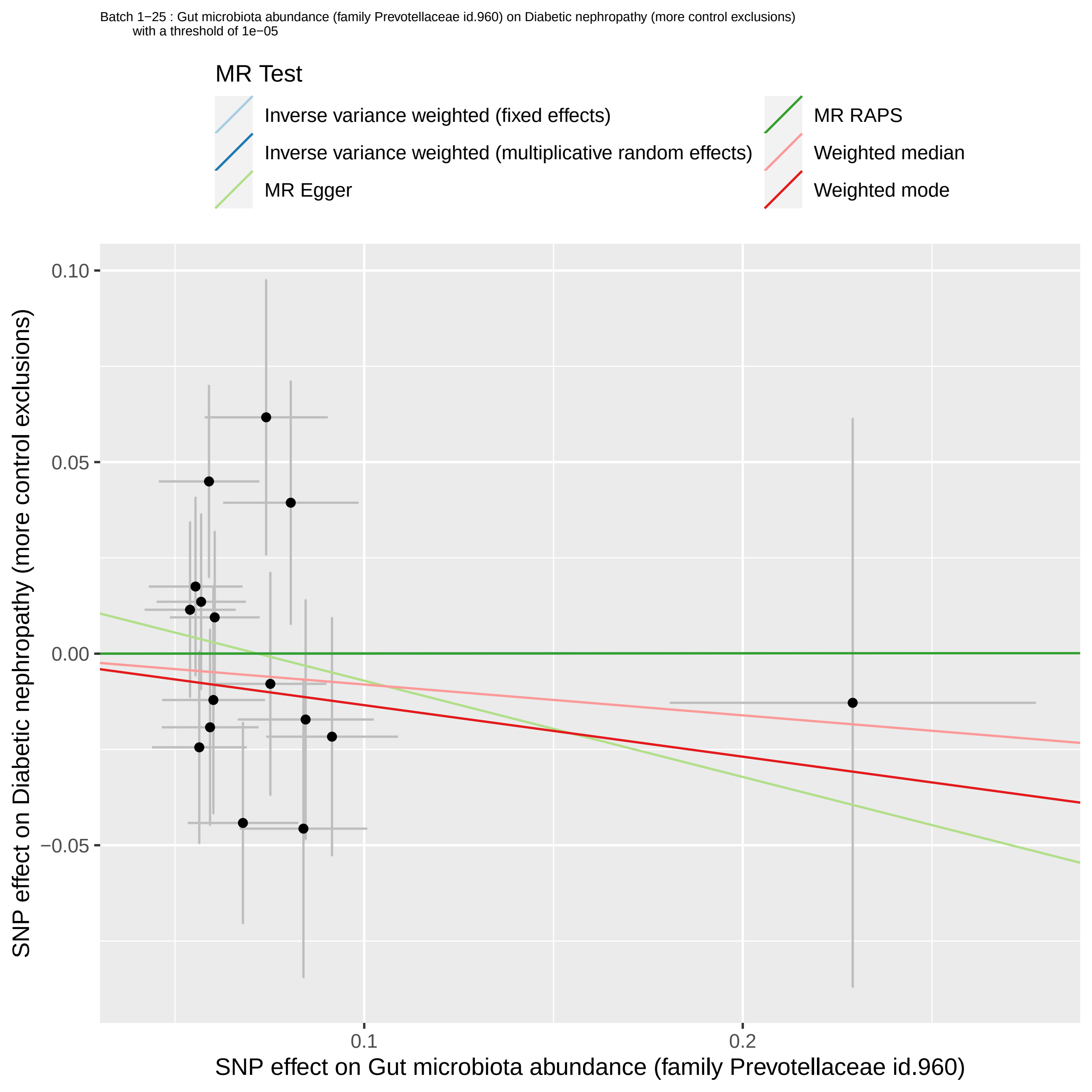
Batch 1−25 : Gut microbiota abundance (family Prevotellaceae id.960) on Diabetic nephropathy (more control exclusions)
with a threshold of 1e−05
MR Test
Inverse variance weighted (fixed effects)
Inverse variance weighted (multiplicative random effects)
MR Egger
MR RAPS
Weighted median
Weighted mode
0.10
0.05
0.00
−0.05
0.1
0.2
SNP effect on Diabetic nephropathy (more control exclusions)
SNP effect on Gut microbiota abundance (family Prevotellaceae id.960)

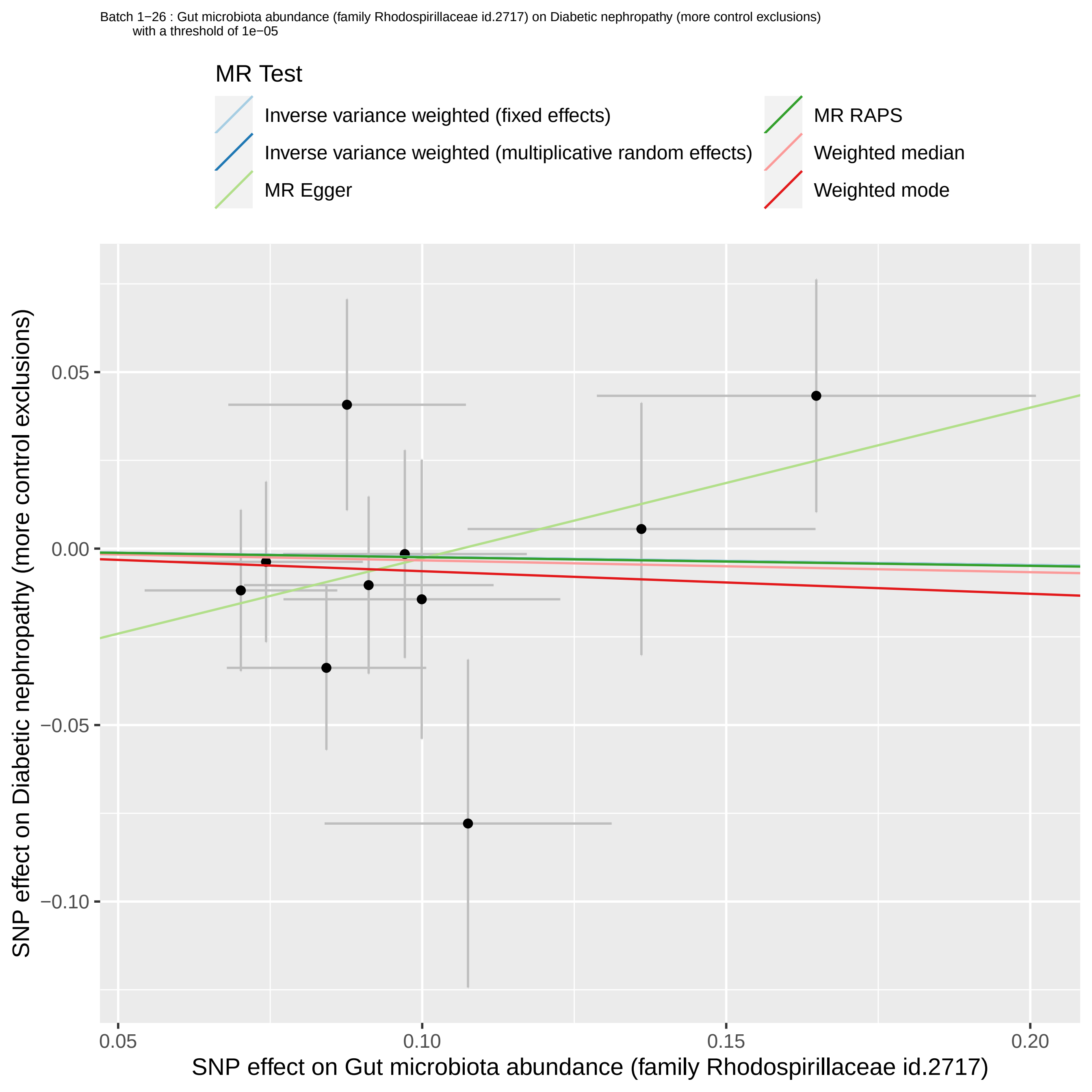

Batch 1−26 : Gut microbiota abundance (family Rhodospirillaceae id.2717) on Diabetic nephropathy (more control exclusions) with a threshold of 1e−05
MR Test
Inverse variance weighted (fixed effects)
Inverse variance weighted (multiplicative random effects)
MR Egger
MR RAPS
Weighted median
Weighted mode
0.05
0.00
−0.05
−0.10
0.05
0.10
0.15
0.20
SNP effect on Diabetic nephropathy (more control exclusions)
SNP effect on Gut microbiota abundance (family Rhodospirillaceae id.2717)

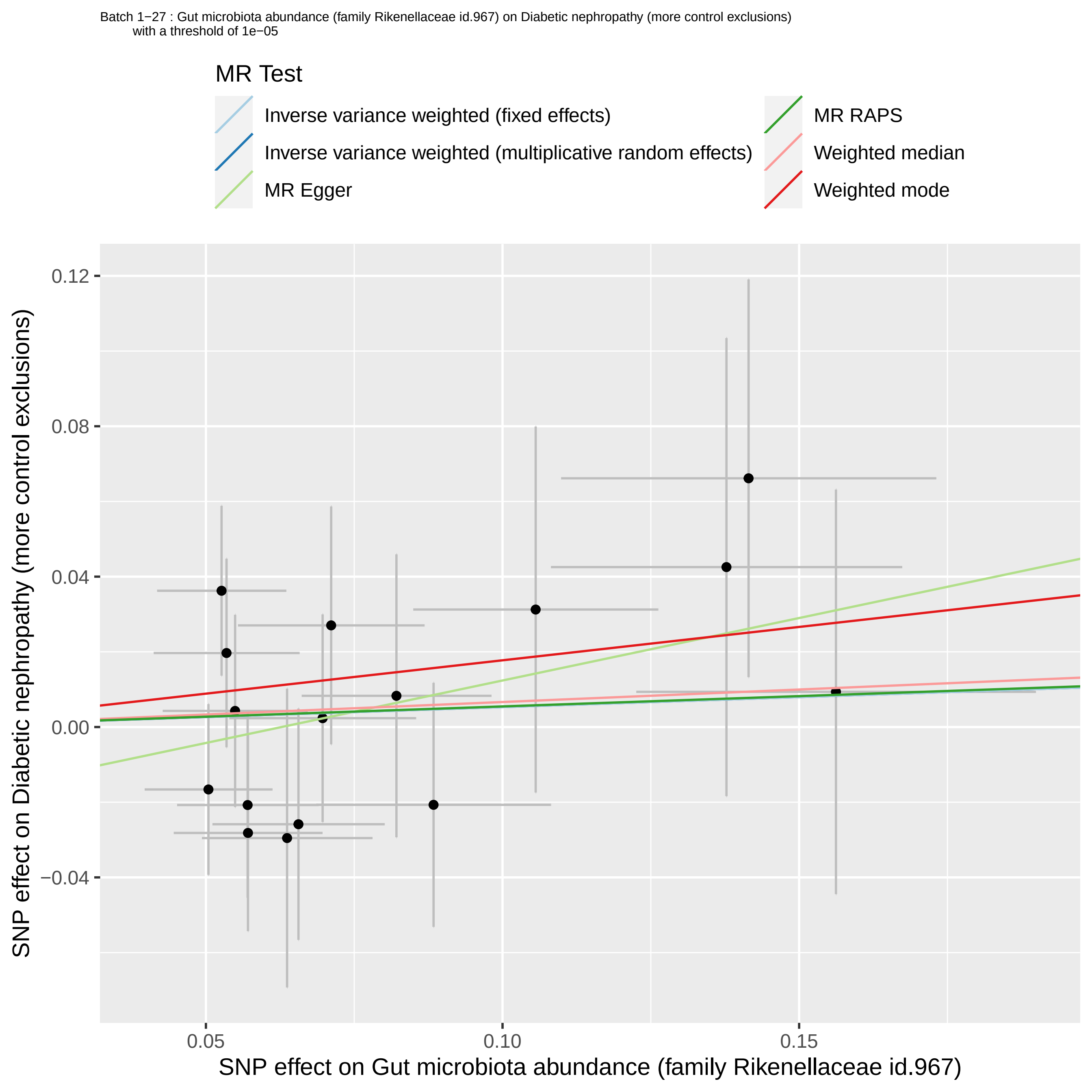
Batch 1−27 : Gut microbiota abundance (family Rikenellaceae id.967) on Diabetic nephropathy (more control exclusions)
with a threshold of 1e−05
MR Test
Inverse variance weighted (fixed effects)
Inverse variance weighted (multiplicative random effects)
MR Egger
MR RAPS
Weighted median
Weighted mode
0.12
0.08
0.04
0.00
−0.04
0.05
0.10
0.15
SNP effect on Diabetic nephropathy (more control exclusions)
SNP effect on Gut microbiota abundance (family Rikenellaceae id.967)

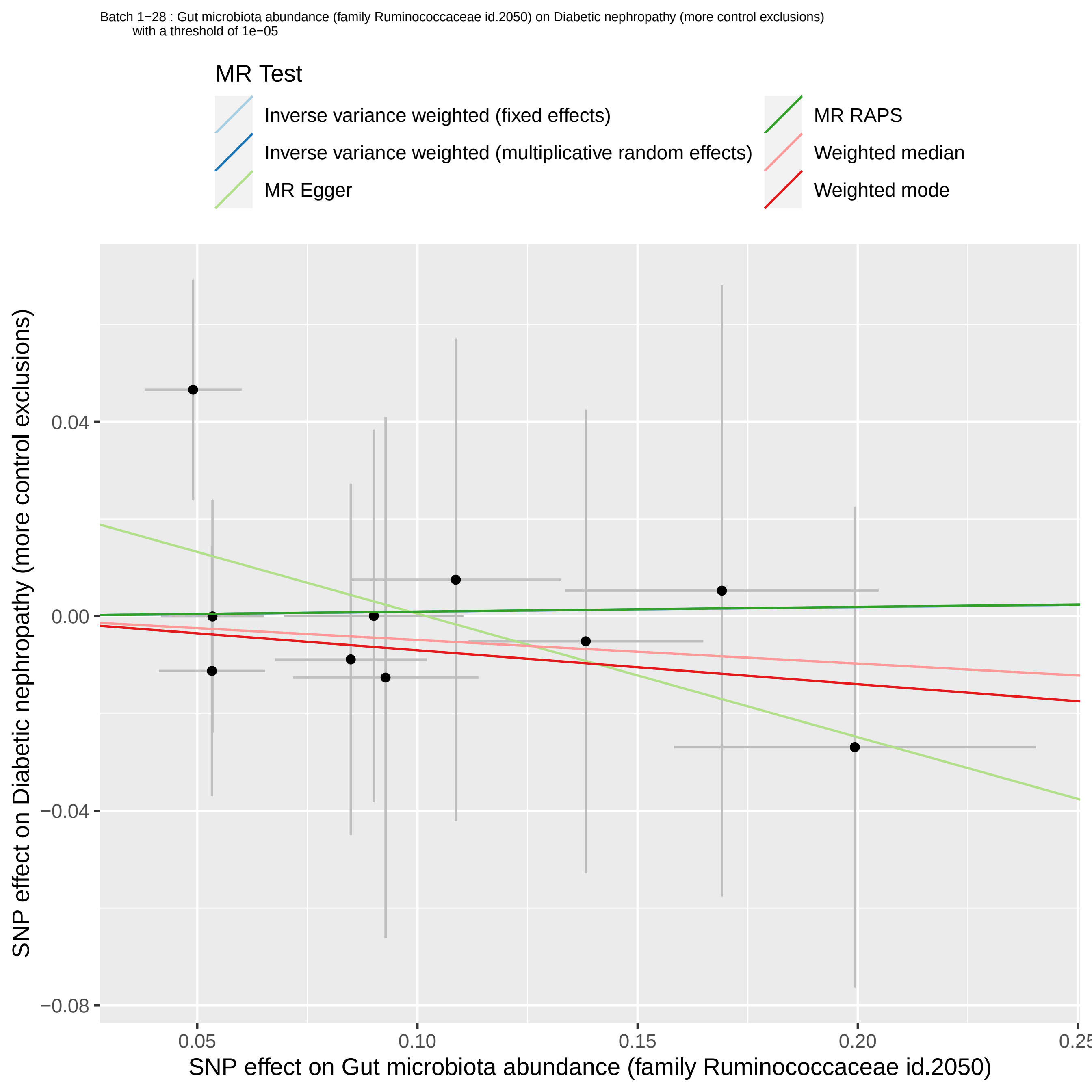
Batch 1−28 : Gut microbiota abundance (family Ruminococcaceae id.2050) on Diabetic nephropathy (more control exclusions)
with a threshold of 1e−05
MR Test
Inverse variance weighted (fixed effects)
Inverse variance weighted (multiplicative random effects)
MR Egger
MR RAPS
Weighted median
Weighted mode
SNP effect on Diabetic nephropathy (more control exclusions)
0.04
0.00
−0.04
−0.08
0.05
0.10
0.15
0.20
0.25
SNP effect on Gut microbiota abundance (family Ruminococcaceae id.2050)

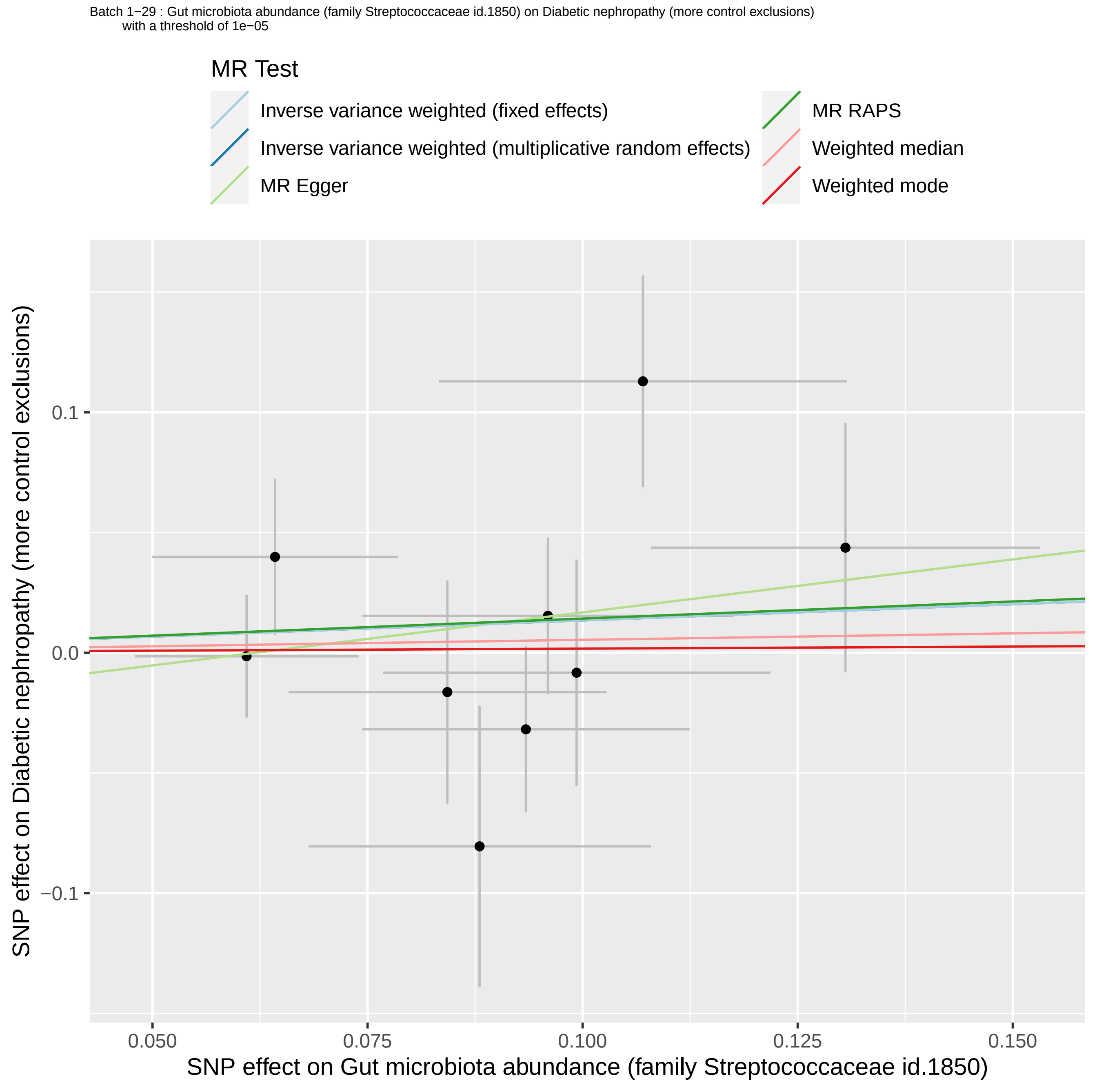

Batch 1−29 : Gut microbiota abundance (family Streptococcaceae id.1850) on Diabetic nephropathy (more control exclusions)
with a threshold of 1e−05
MR Test
Inverse variance weighted (fixed effects)
Inverse variance weighted (multiplicative random effects)
MR Egger
MR RAPS
Weighted median
Weighted mode
0.1
0.0
−0.1
0.050
0.075
0.100
0.125
0.150
SNP effect on Diabetic nephropathy (more control exclusions)
SNP effect on Gut microbiota abundance (family Streptococcaceae id.1850)

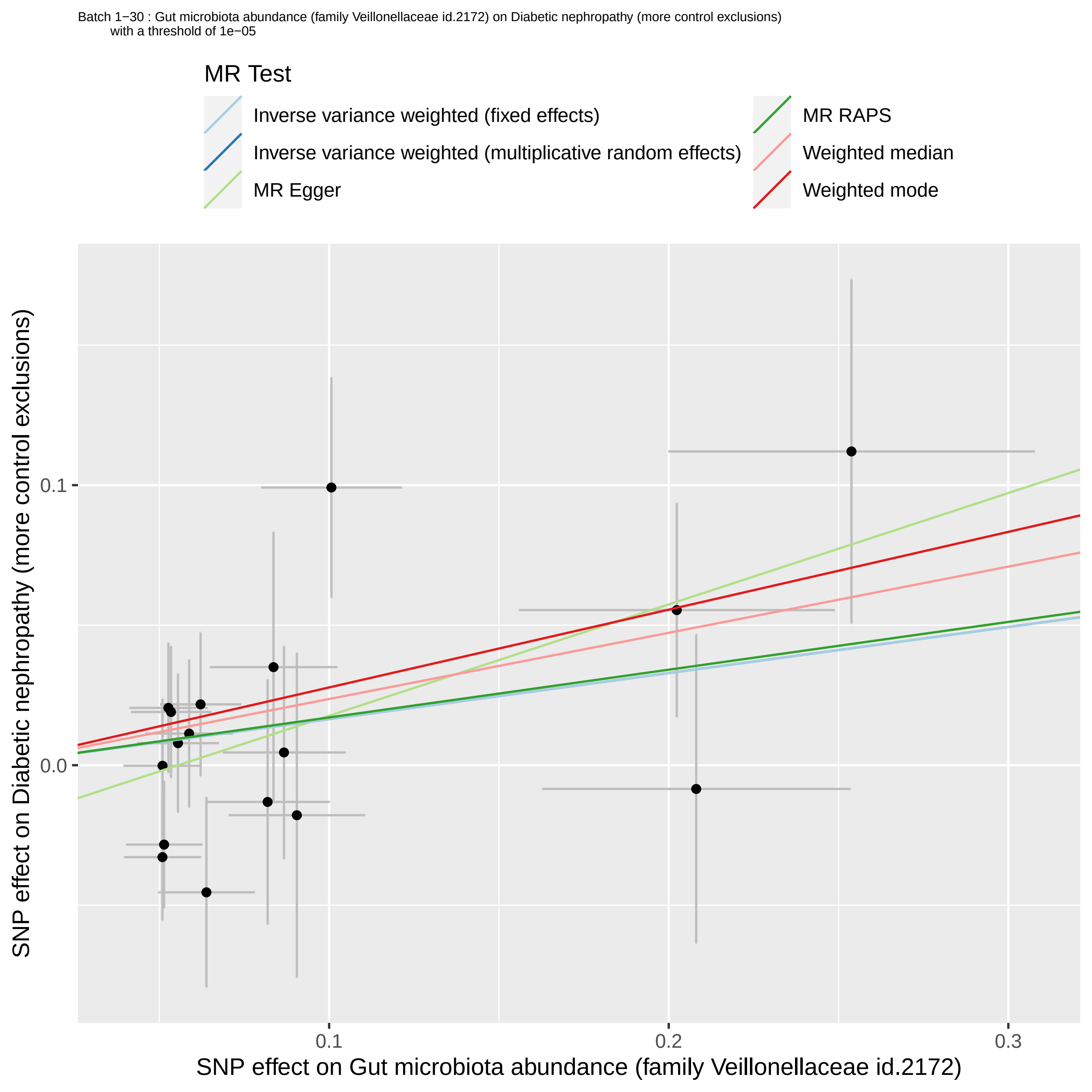

Batch 1−30 : Gut microbiota abundance (family Veillonellaceae id.2172) on Diabetic nephropathy (more control exclusions)
with a threshold of 1e−05
MR Test
Inverse variance weighted (fixed effects)
Inverse variance weighted (multiplicative random effects)
MR Egger
MR RAPS
Weighted median
Weighted mode
SNP effect on Diabetic nephropathy (more control exclusions)
0.1
0.0
0.1
0.2
0.3
SNP effect on Gut microbiota abundance (family Veillonellaceae id.2172)

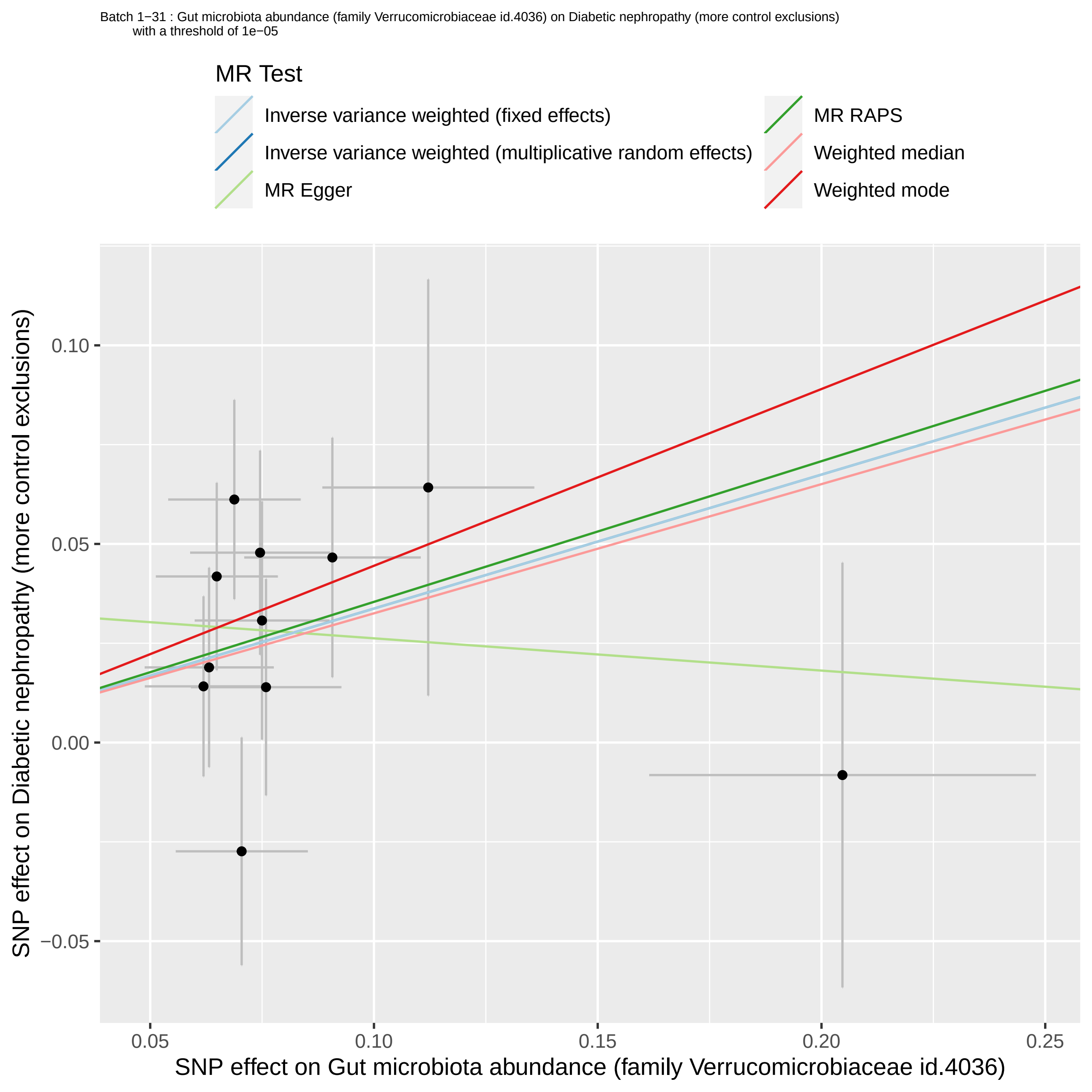
Batch 1−31 : Gut microbiota abundance (family Verrucomicrobiaceae id.4036) on Diabetic nephropathy (more control exclusions) with a threshold of 1e−05
MR Test
Inverse variance weighted (fixed effects)
Inverse variance weighted (multiplicative random effects)
MR Egger
MR RAPS
Weighted median
Weighted mode
SNP effect on Diabetic nephropathy (more control exclusions)
0.10
0.05
0.00
−0.05
0.05
0.10
0.15
0.20
0.25
SNP effect on Gut microbiota abundance (family Verrucomicrobiaceae id.4036)

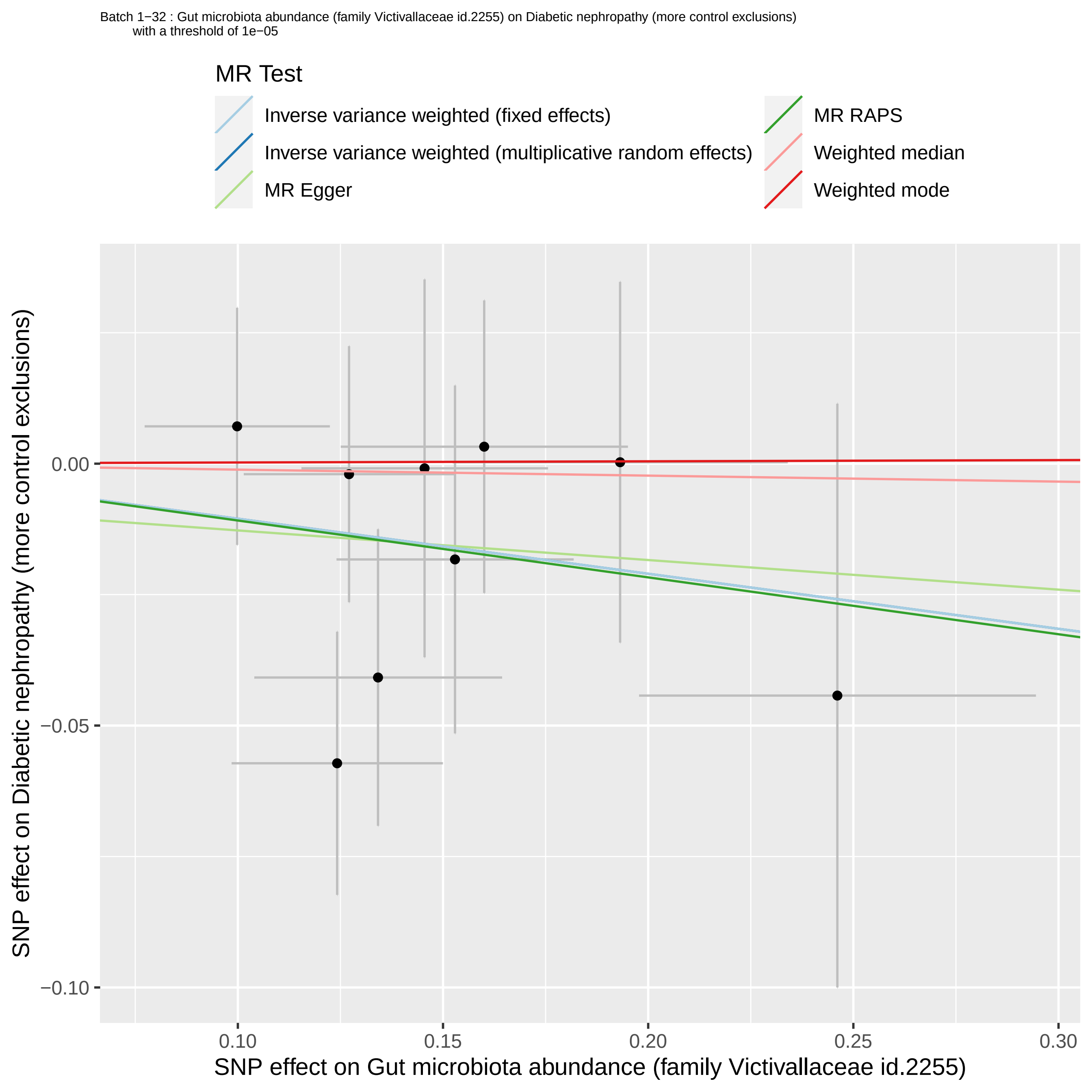

Batch 1−32 : Gut microbiota abundance (family Victivallaceae id.2255) on Diabetic nephropathy (more control exclusions)
with a threshold of 1e−05
MR Test
Inverse variance weighted (fixed effects)
Inverse variance weighted (multiplicative random effects)
MR Egger
MR RAPS
Weighted median
Weighted mode
SNP effect on Diabetic nephropathy (more control exclusions)
0.00
−0.05
−0.10
0.10
0.15
0.20
0.25
0.30
SNP effect on Gut microbiota abundance (family Victivallaceae id.2255)

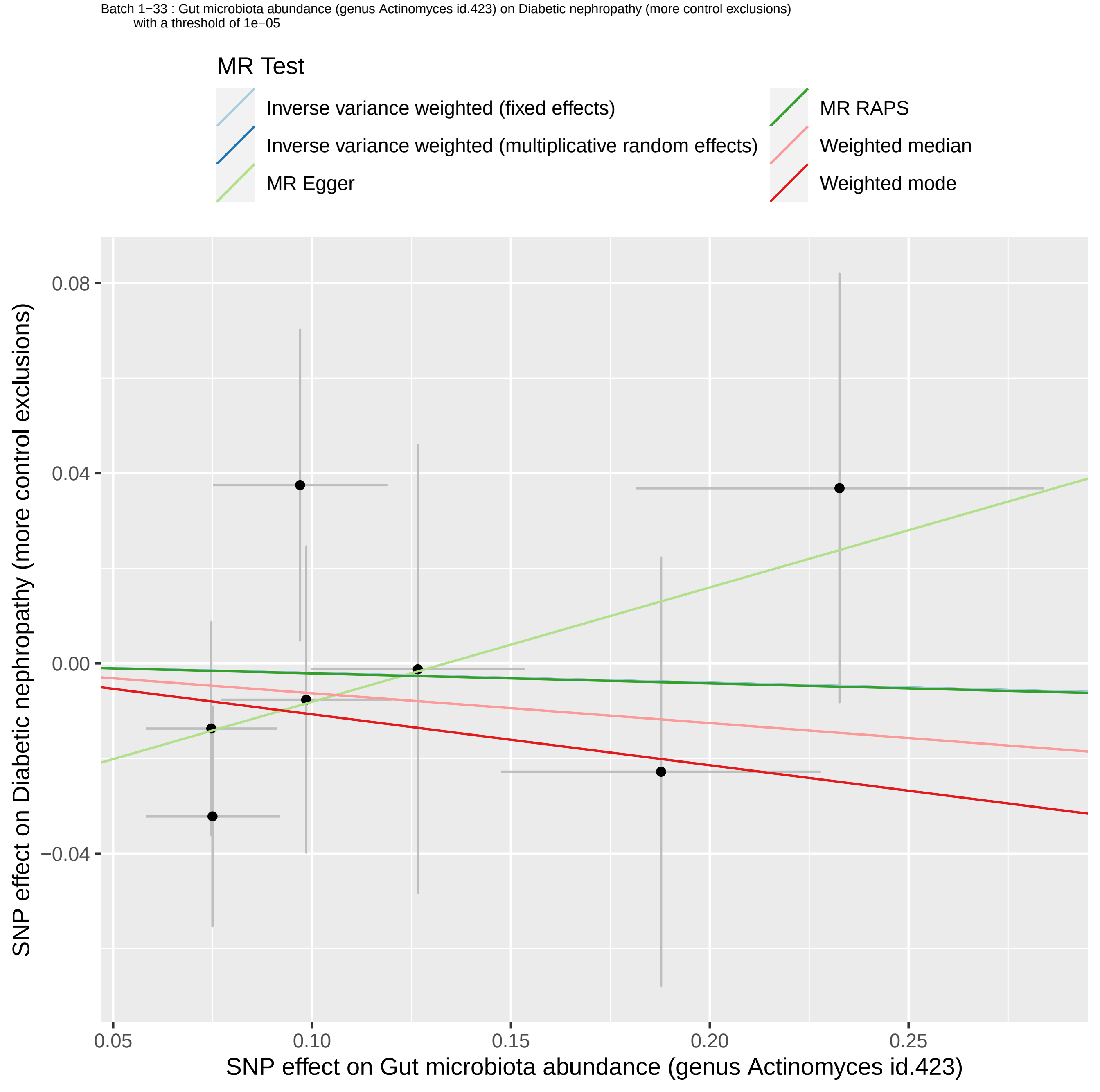

Batch 1−33 : Gut microbiota abundance (genus Actinomyces id.423) on Diabetic nephropathy (more control exclusions)
with a threshold of 1e−05
MR Test
Inverse variance weighted (fixed effects)
Inverse variance weighted (multiplicative random effects)
MR Egger
MR RAPS
Weighted median
Weighted mode
0.08
0.04
0.00
−0.04
0.05
0.10
0.15
0.20
0.25
SNP effect on Diabetic nephropathy (more control exclusions)
SNP effect on Gut microbiota abundance (genus Actinomyces id.423)

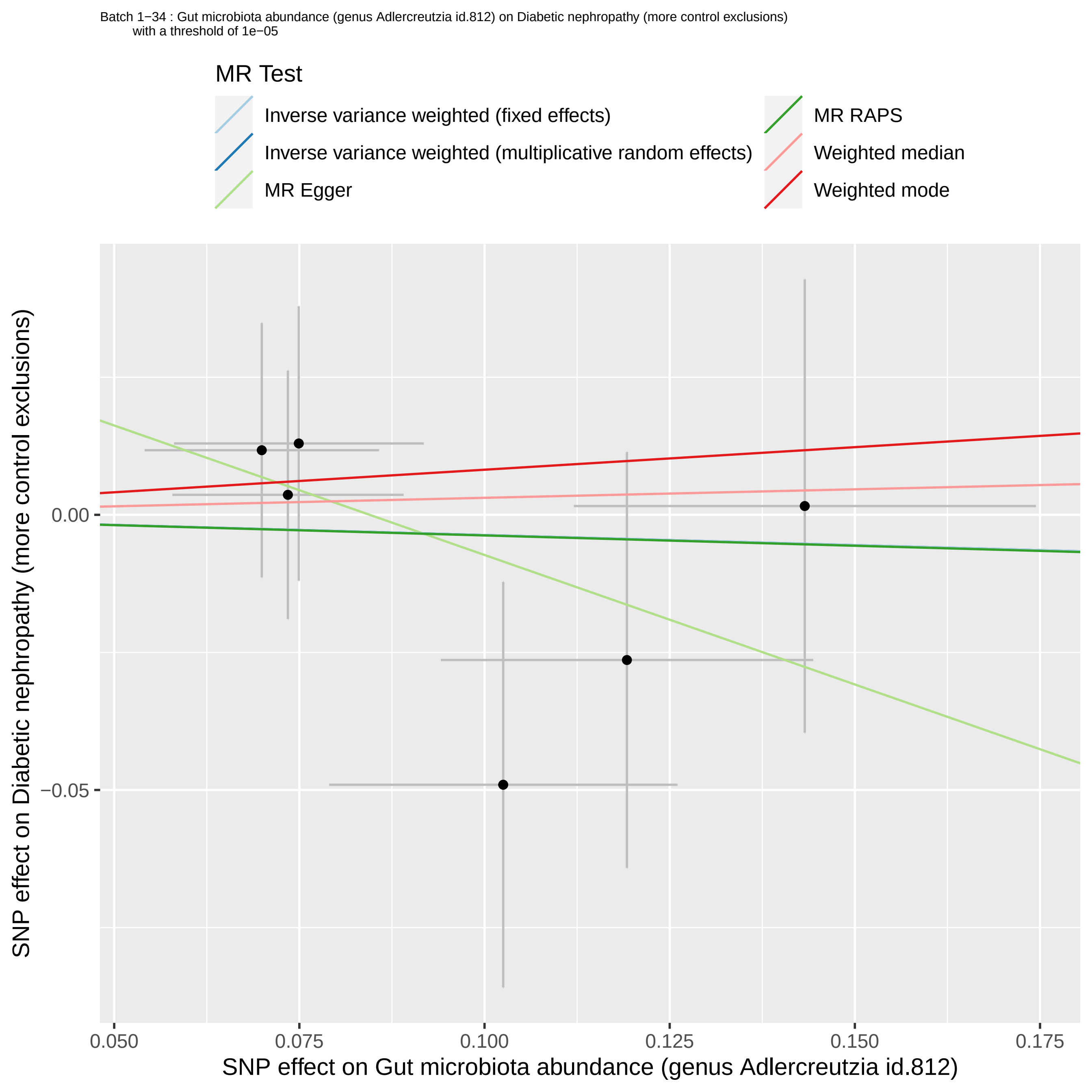

Batch 1−34 : Gut microbiota abundance (genus Adlercreutzia id.812) on Diabetic nephropathy (more control exclusions) with a threshold of 1e−05
MR Test
Inverse variance weighted (fixed effects)
Inverse variance weighted (multiplicative random effects)
MR Egger
MR RAPS
Weighted median
Weighted mode
0.00
−0.05
0.050
0.075
0.100
0.125
0.150
0.175
SNP effect on Gut microbiota abundance (genus Adlercreutzia id.812)
SNP effect on Diabetic nephropathy (more control exclusions)

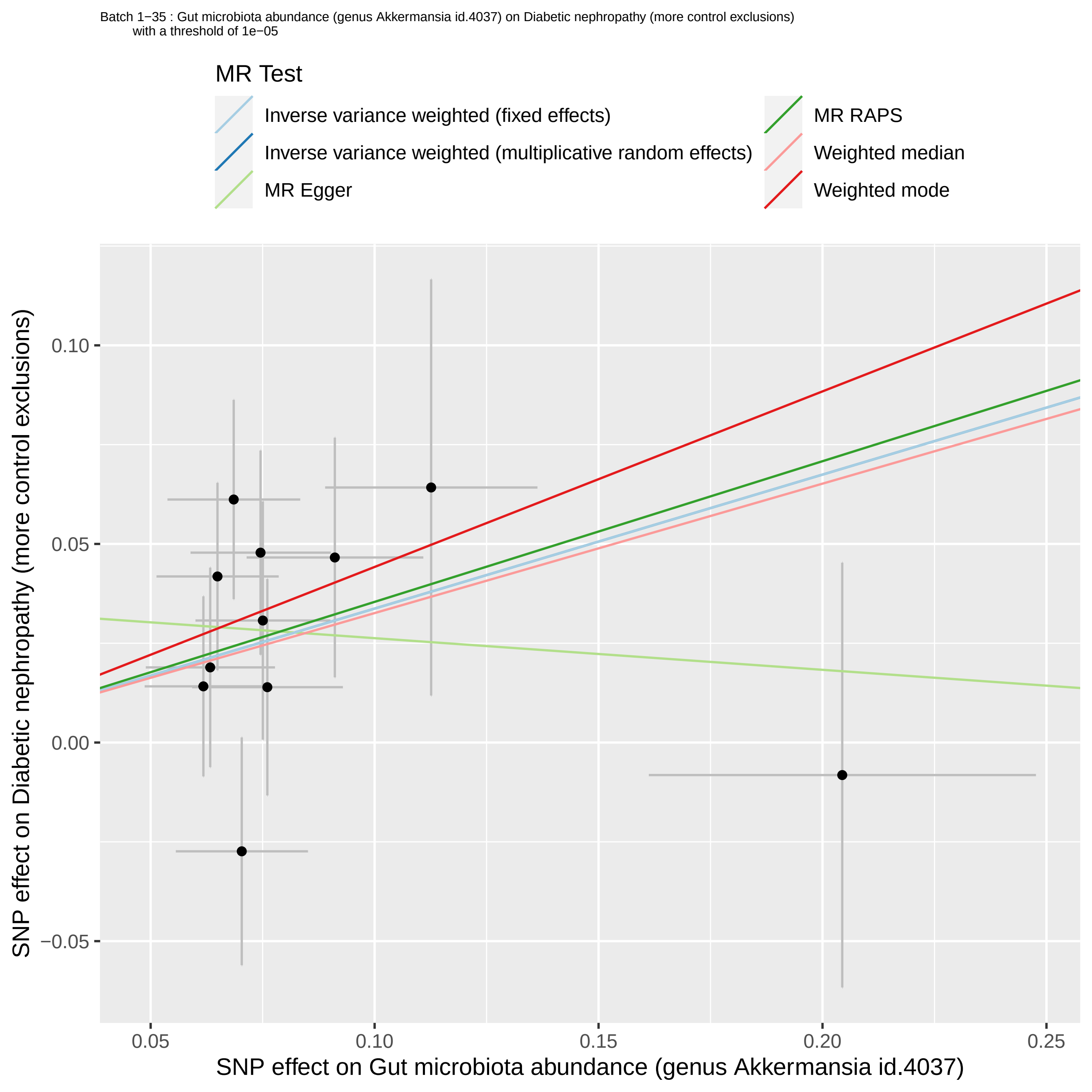

Batch 1−35 : Gut microbiota abundance (genus Akkermansia id.4037) on Diabetic nephropathy (more control exclusions) with a threshold of 1e−05
MR Test
Inverse variance weighted (fixed effects)
Inverse variance weighted (multiplicative random effects)
MR Egger
MR RAPS
Weighted median
Weighted mode
SNP effect on Diabetic nephropathy (more control exclusions)
0.10
0.05
0.00
−0.05
0.05
0.10
0.15
0.20
0.25
SNP effect on Gut microbiota abundance (genus Akkermansia id.4037)

Batch 1−36 : Gut microbiota abundance (genus Alistipes id.968) on Diabetic nephropathy (more control exclusions) with a threshold of 1e−05

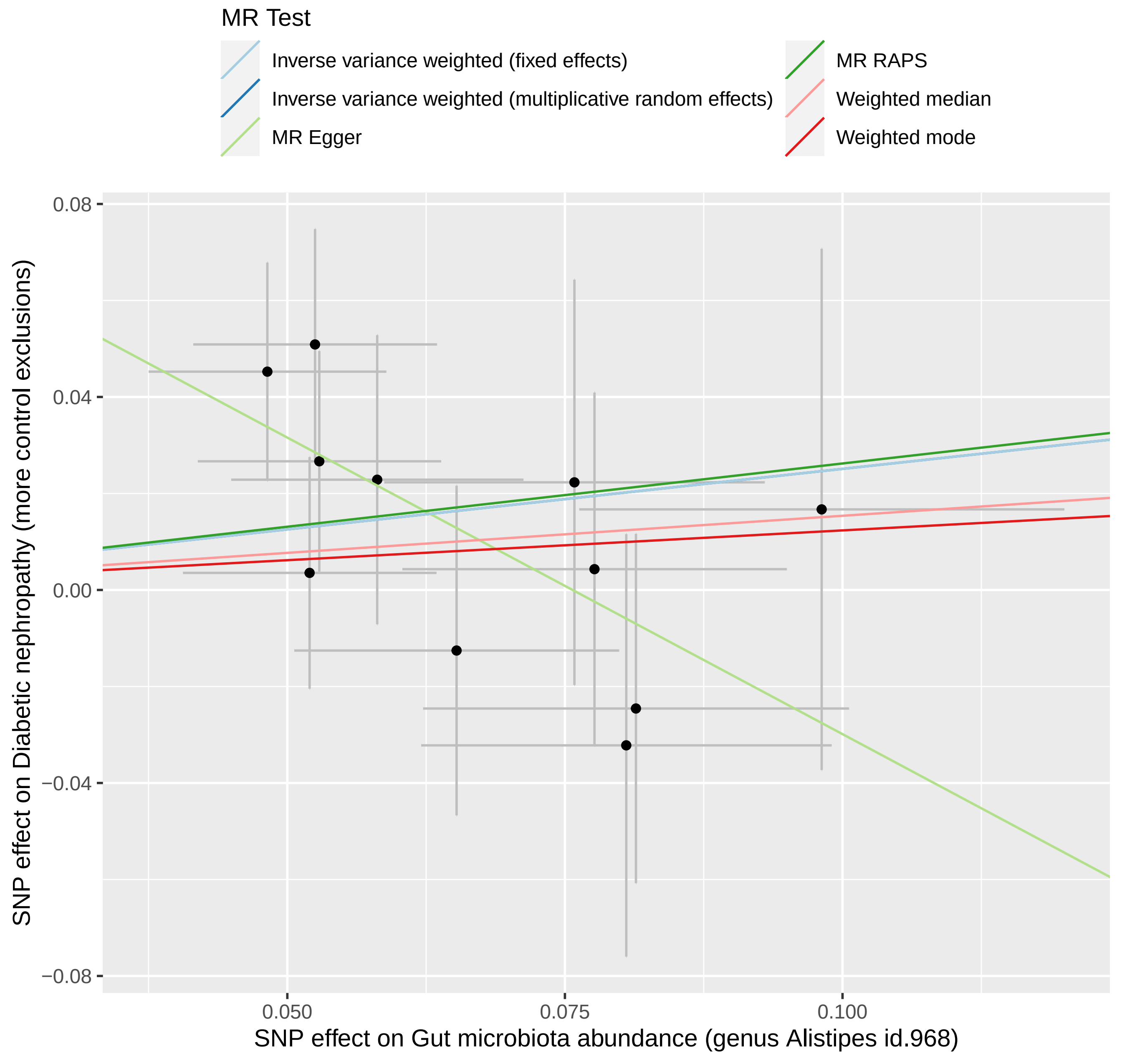

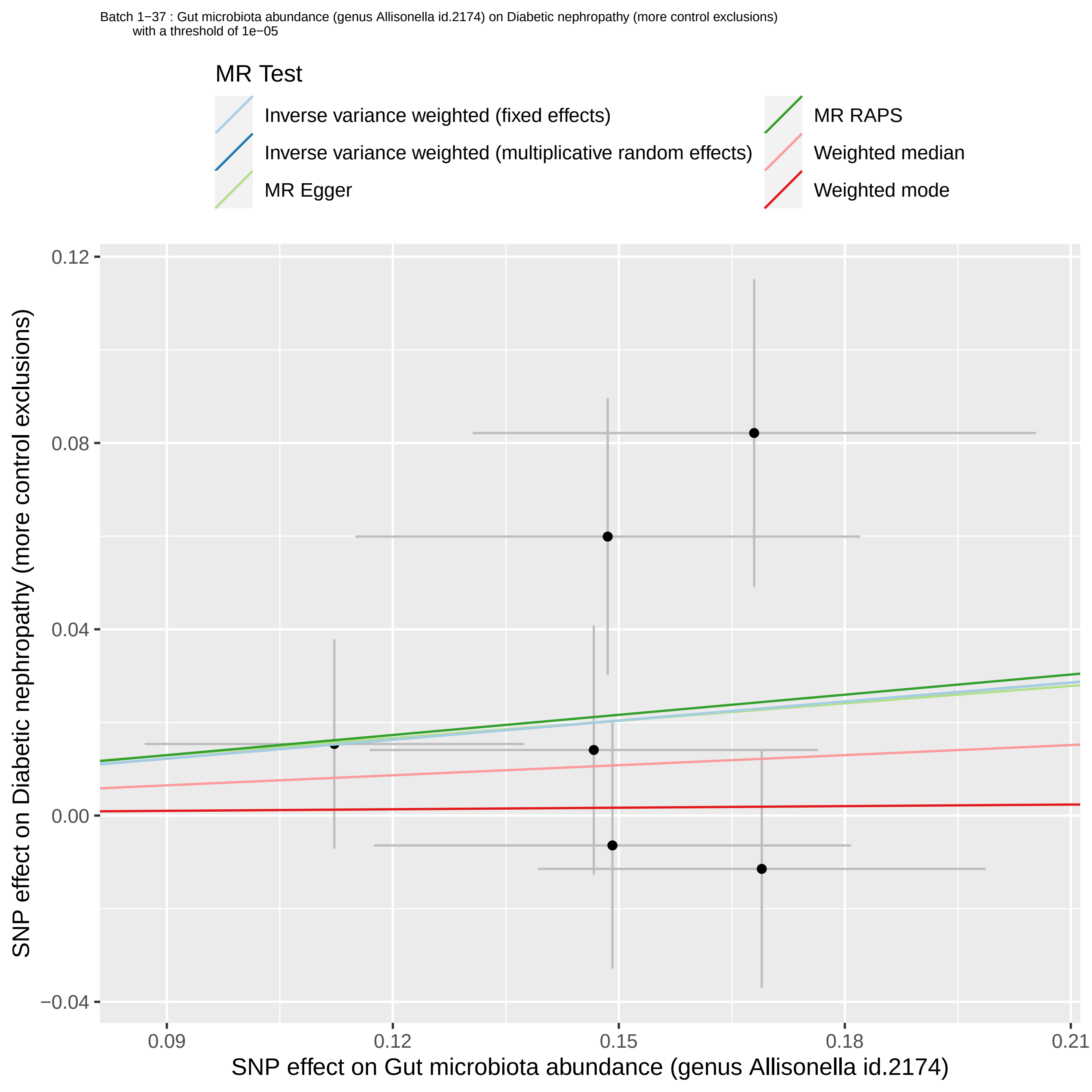

Batch 1−37 : Gut microbiota abundance (genus Allisonella id.2174) on Diabetic nephropathy (more control exclusions)
with a threshold of 1e−05
MR Test
Inverse variance weighted (fixed effects)
Inverse variance weighted (multiplicative random effects)
MR Egger
MR RAPS
Weighted median
Weighted mode
0.12
0.08
0.04
0.00
−0.04
0.09
0.12
0.15
0.18
0.21
SNP effect on Diabetic nephropathy (more control exclusions)
SNP effect on Gut microbiota abundance (genus Allisonella id.2174)

Batch 1−38 : Gut microbiota abundance (genus Alloprevotella id.961) on Diabetic nephropathy (more control exclusions) with a threshold of 1e−05

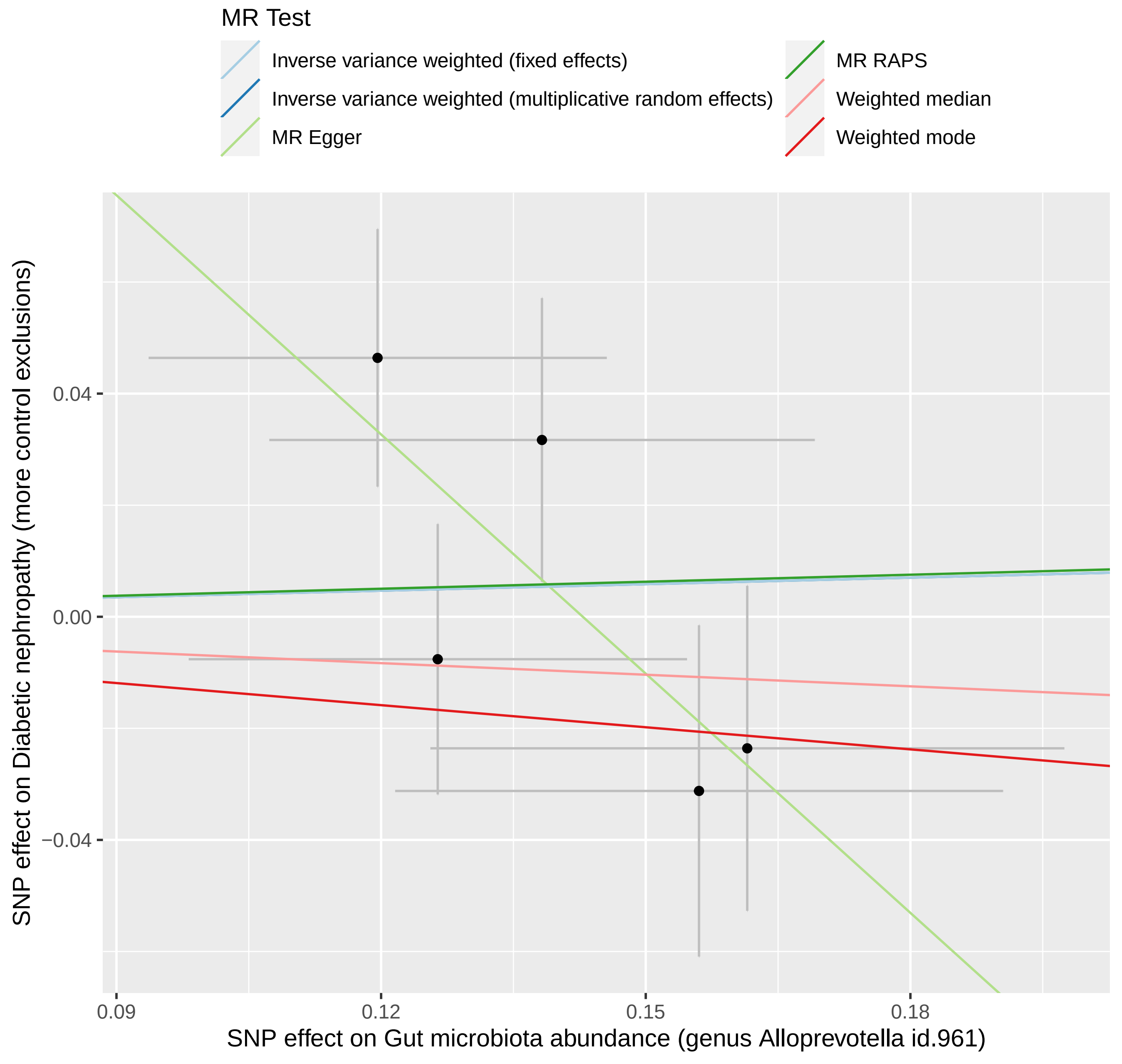

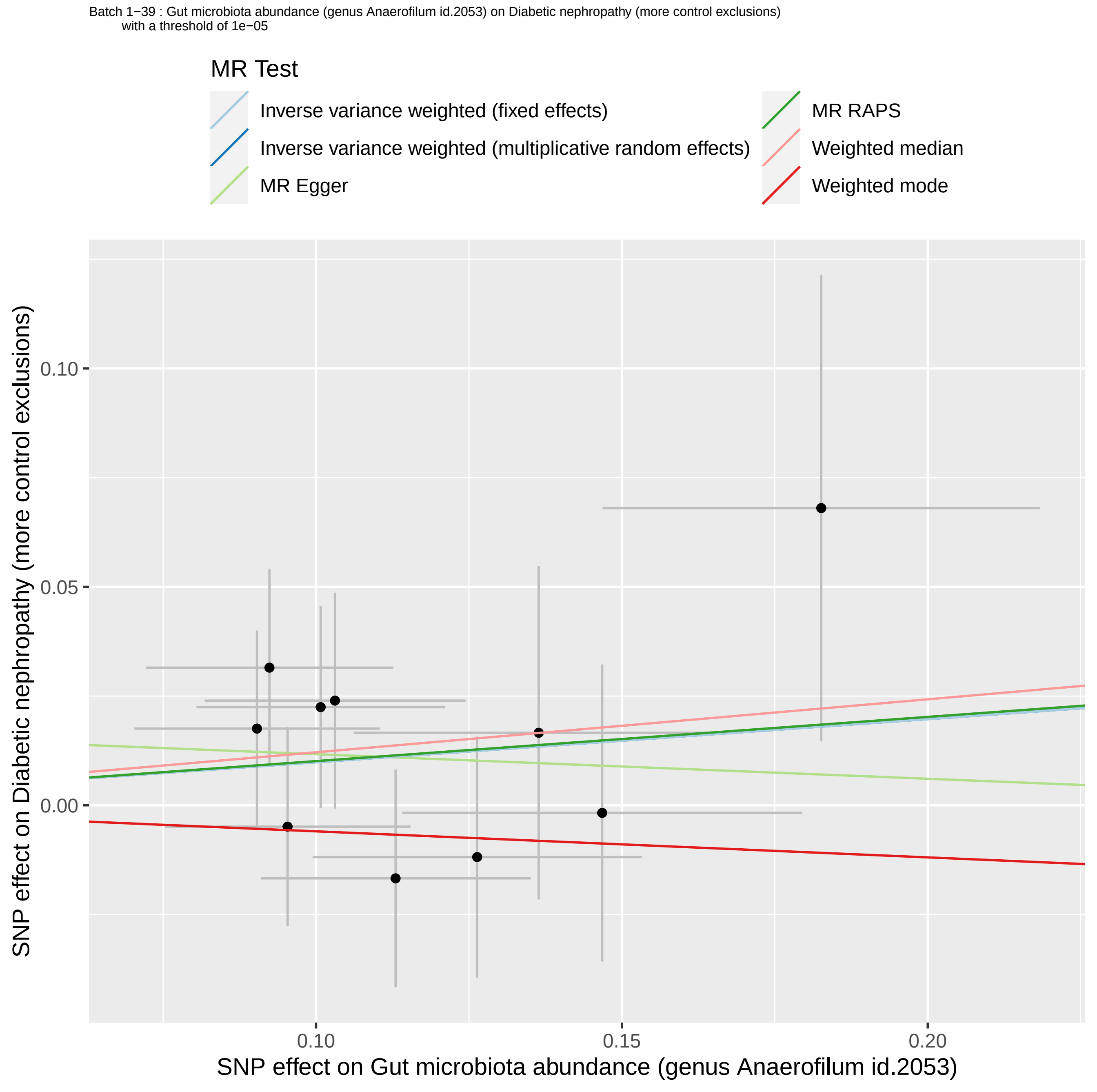

Batch 1−39 : Gut microbiota abundance (genus Anaerofilum id.2053) on Diabetic nephropathy (more control exclusions) with a threshold of 1e−05
MR Test
Inverse variance weighted (fixed effects)
Inverse variance weighted (multiplicative random effects)
MR Egger
MR RAPS
Weighted median
Weighted mode
0.10
0.05
0.00
0.10
0.15
0.20
SNP effect on Diabetic nephropathy (more control exclusions)
SNP effect on Gut microbiota abundance (genus Anaerofilum id.2053)

Batch 1−40 : Gut microbiota abundance (genus Anaerostipes id.1991) on Diabetic nephropathy (more control exclusions) with a threshold of 1e−05

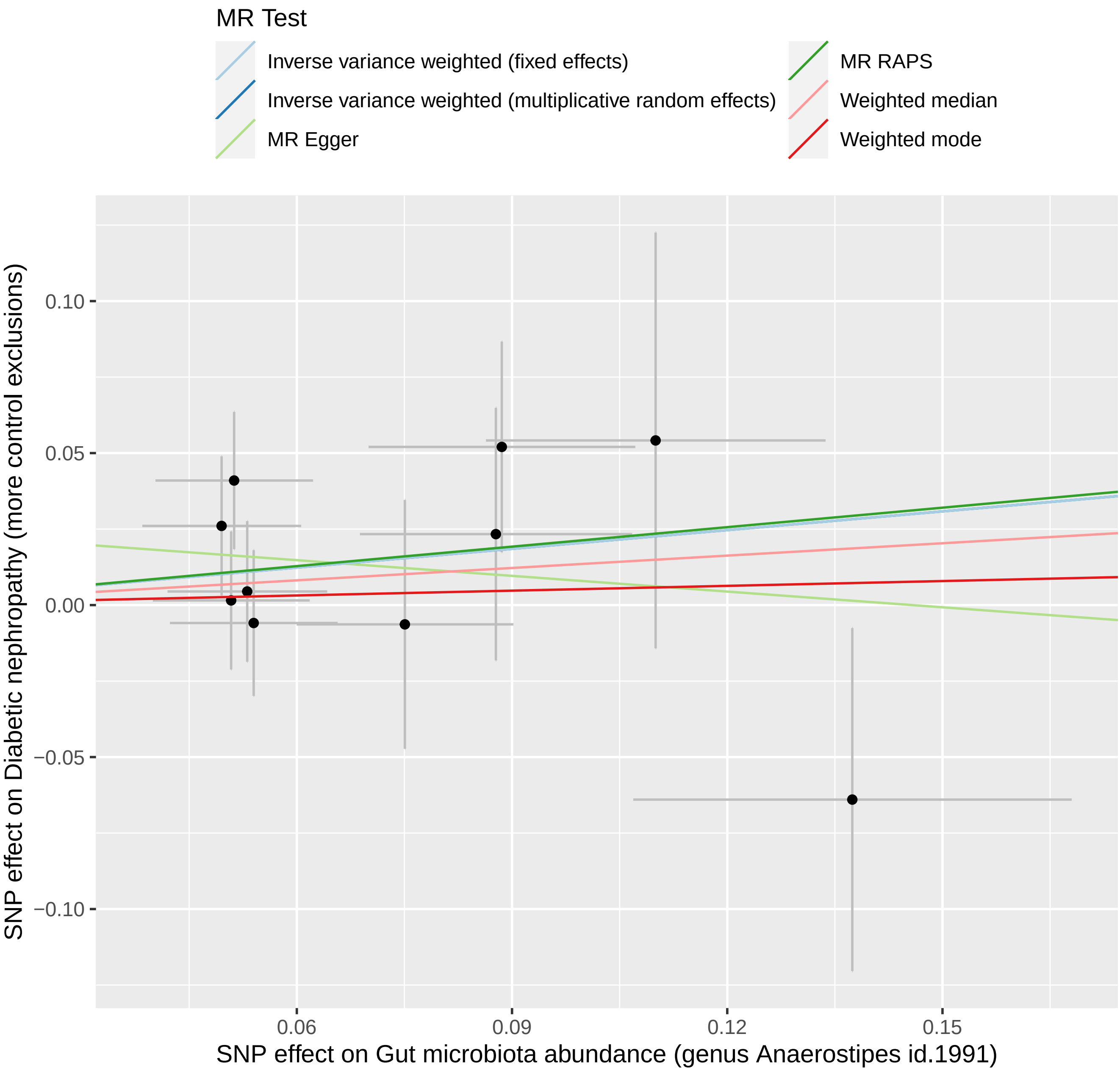

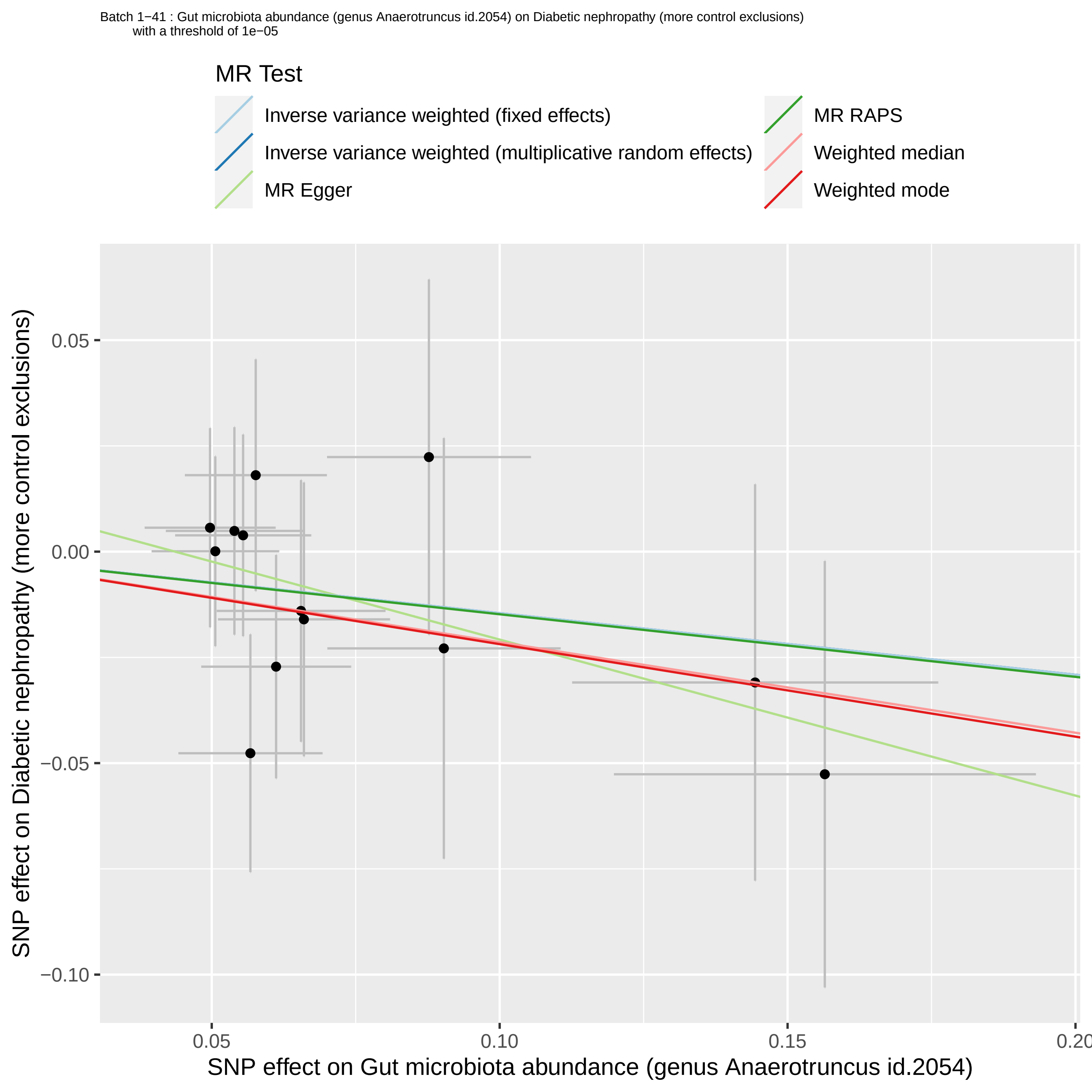

Batch 1−41 : Gut microbiota abundance (genus Anaerotruncus id.2054) on Diabetic nephropathy (more control exclusions)
with a threshold of 1e−05
MR Test
Inverse variance weighted (fixed effects)
Inverse variance weighted (multiplicative random effects)
MR Egger
MR RAPS
Weighted median
Weighted mode
0.05
0.00
−0.05
−0.10
0.05
0.10
0.15
0.20
SNP effect on Diabetic nephropathy (more control exclusions)
SNP effect on Gut microbiota abundance (genus Anaerotruncus id.2054)

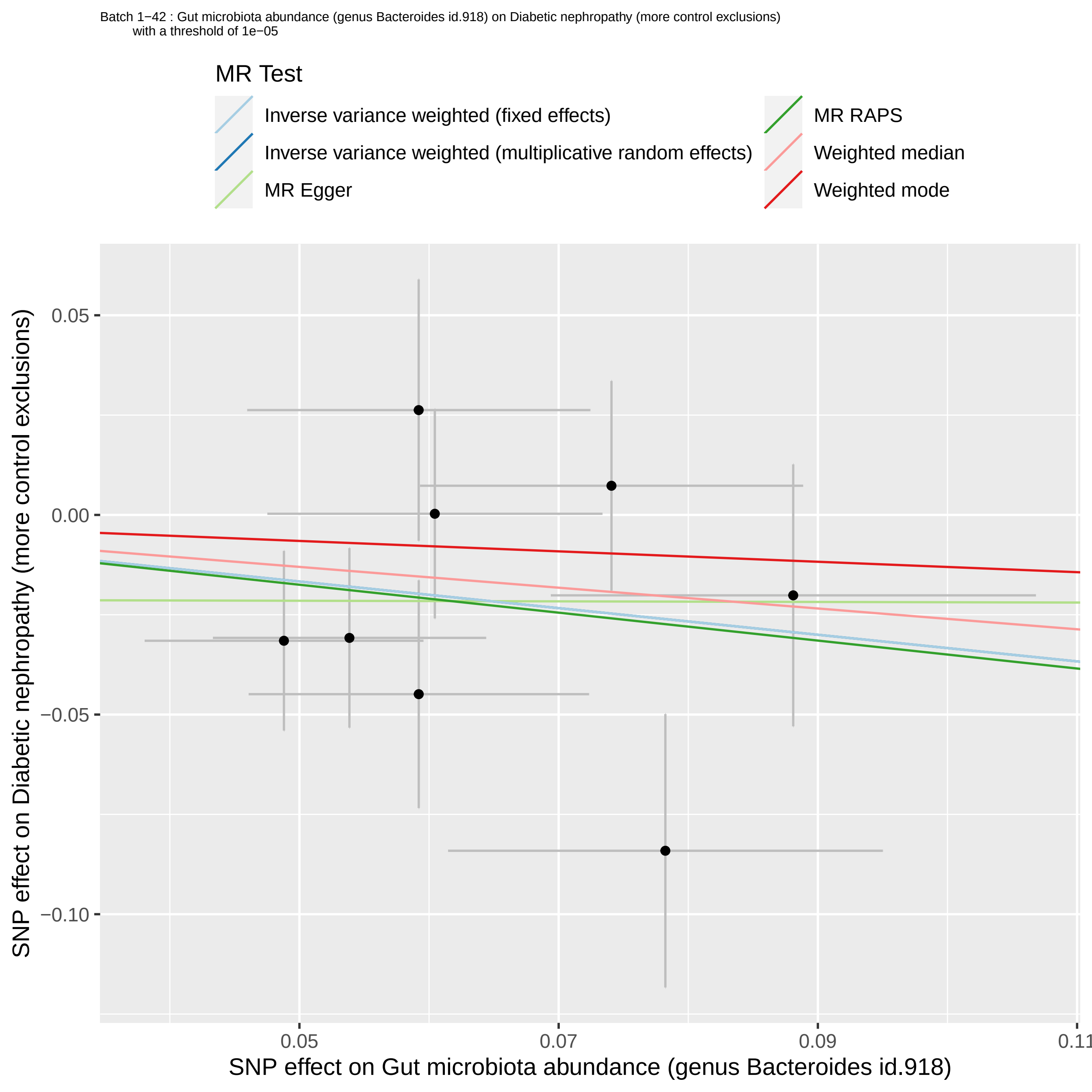
Batch 1−42 : Gut microbiota abundance (genus Bacteroides id.918) on Diabetic nephropathy (more control exclusions)
with a threshold of 1e−05
MR Test
Inverse variance weighted (fixed effects)
Inverse variance weighted (multiplicative random effects)
MR Egger
MR RAPS
Weighted median
Weighted mode
0.05
0.00
−0.05
−0.10
0.05
0.07
0.09
0.11
SNP effect on Diabetic nephropathy (more control exclusions)
SNP effect on Gut microbiota abundance (genus Bacteroides id.918)

Batch 1−43 : Gut microbiota abundance (genus Barnesiella id.944) on Diabetic nephropathy (more control exclusions)
with a threshold of 1e−05

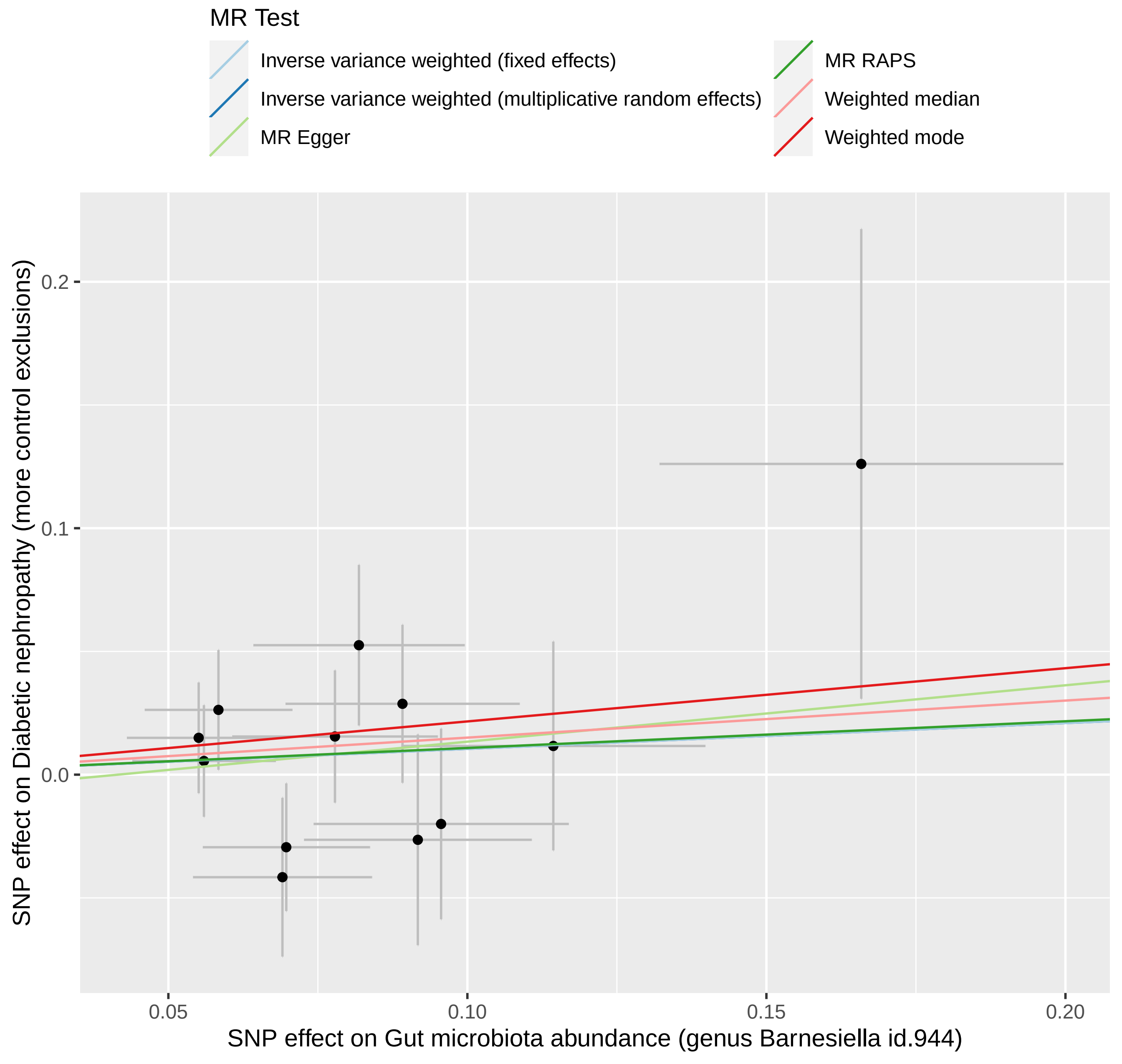

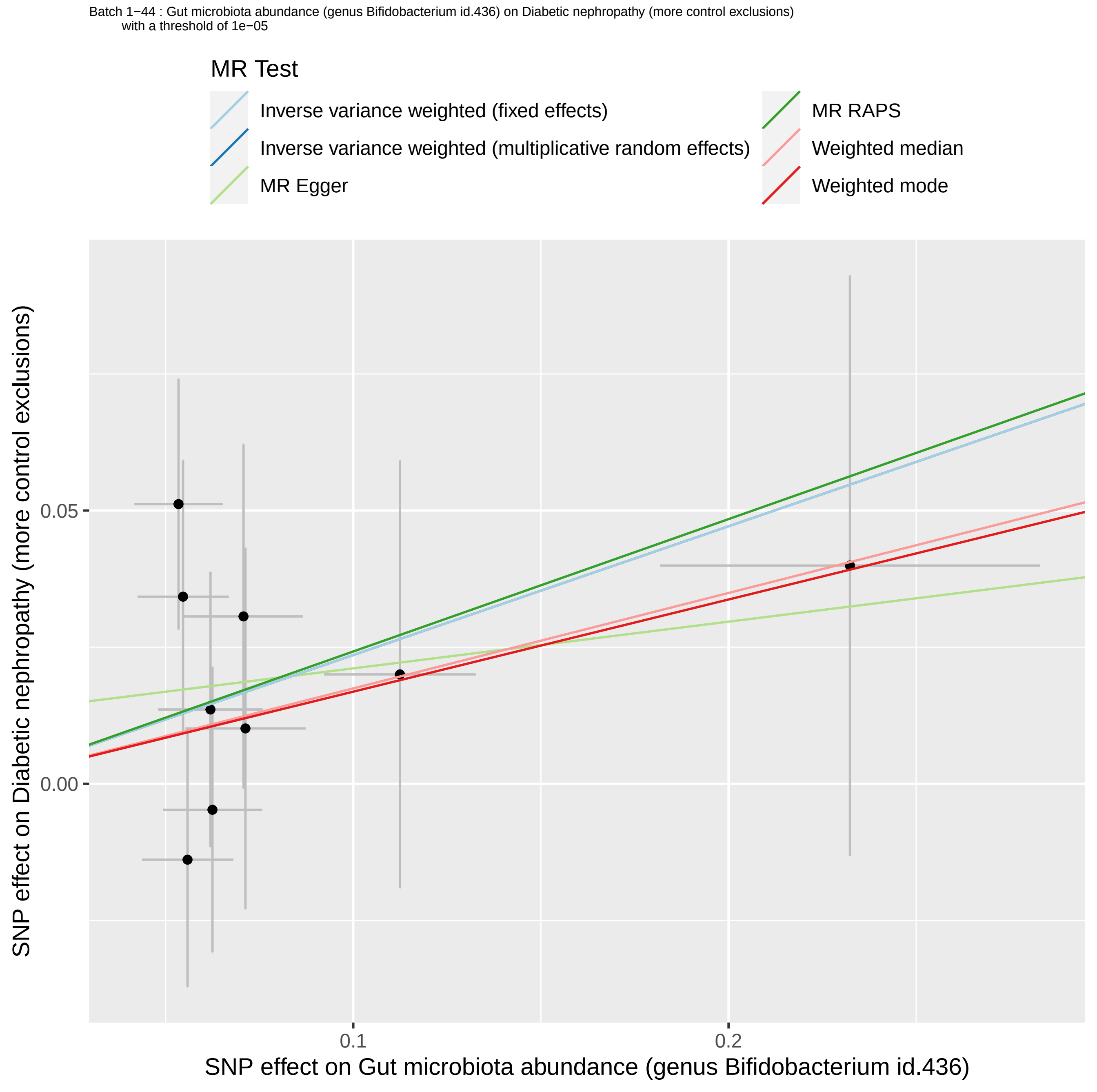
Batch 1−44 : Gut microbiota abundance (genus Bifidobacterium id.436) on Diabetic nephropathy (more control exclusions)
with a threshold of 1e−05
MR Test
Inverse variance weighted (fixed effects)
Inverse variance weighted (multiplicative random effects)
MR Egger
MR RAPS
Weighted median
Weighted mode
SNP effect on Diabetic nephropathy (more control exclusions)
0.05
0.00
0.1
0.2
SNP effect on Gut microbiota abundance (genus Bifidobacterium id.436)

Batch 1−45 : Gut microbiota abundance (genus Bilophila id.3170) on Diabetic nephropathy (more control exclusions)
with a threshold of 1e−05

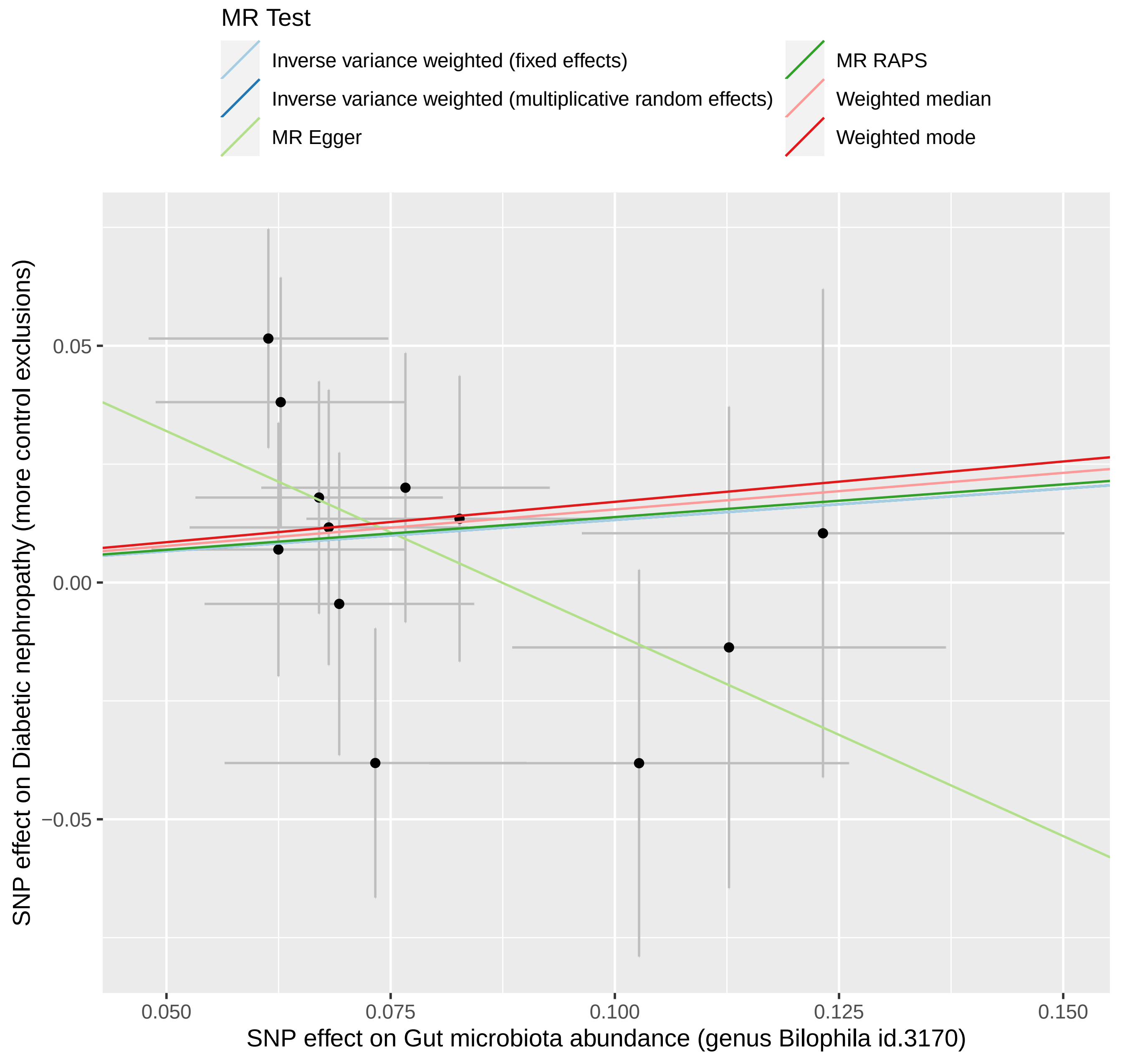

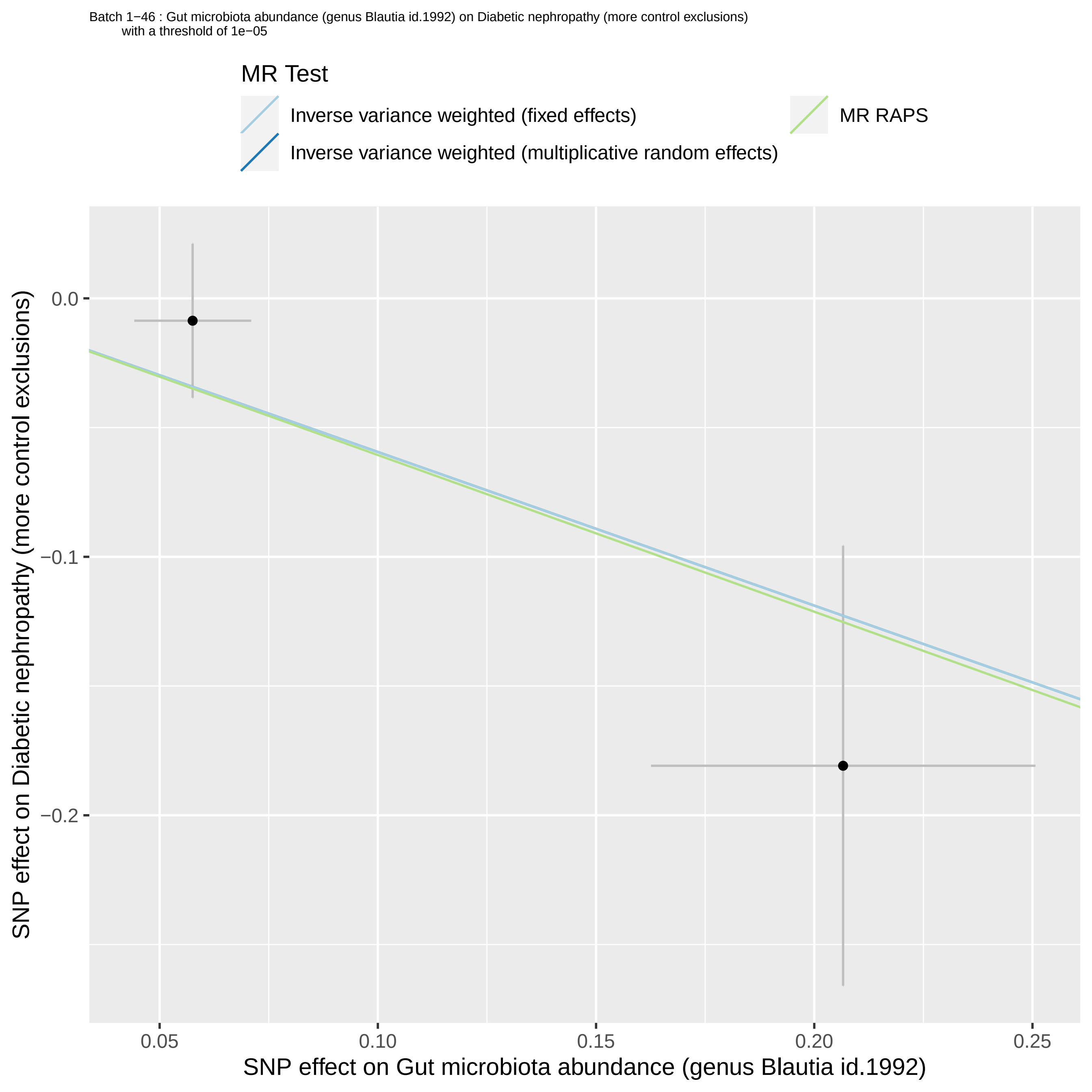

Batch 1−46 : Gut microbiota abundance (genus Blautia id.1992) on Diabetic nephropathy (more control exclusions)
with a threshold of 1e−05
MR Test
Inverse variance weighted (fixed effects)
Inverse variance weighted (multiplicative random effects)
MR RAPS
0.0
−0.1
−0.2
SNP effect on Diabetic nephropathy (more control exclusions)
0.05
0.10
0.15
0.20
0.25
SNP effect on Gut microbiota abundance (genus Blautia id.1992)

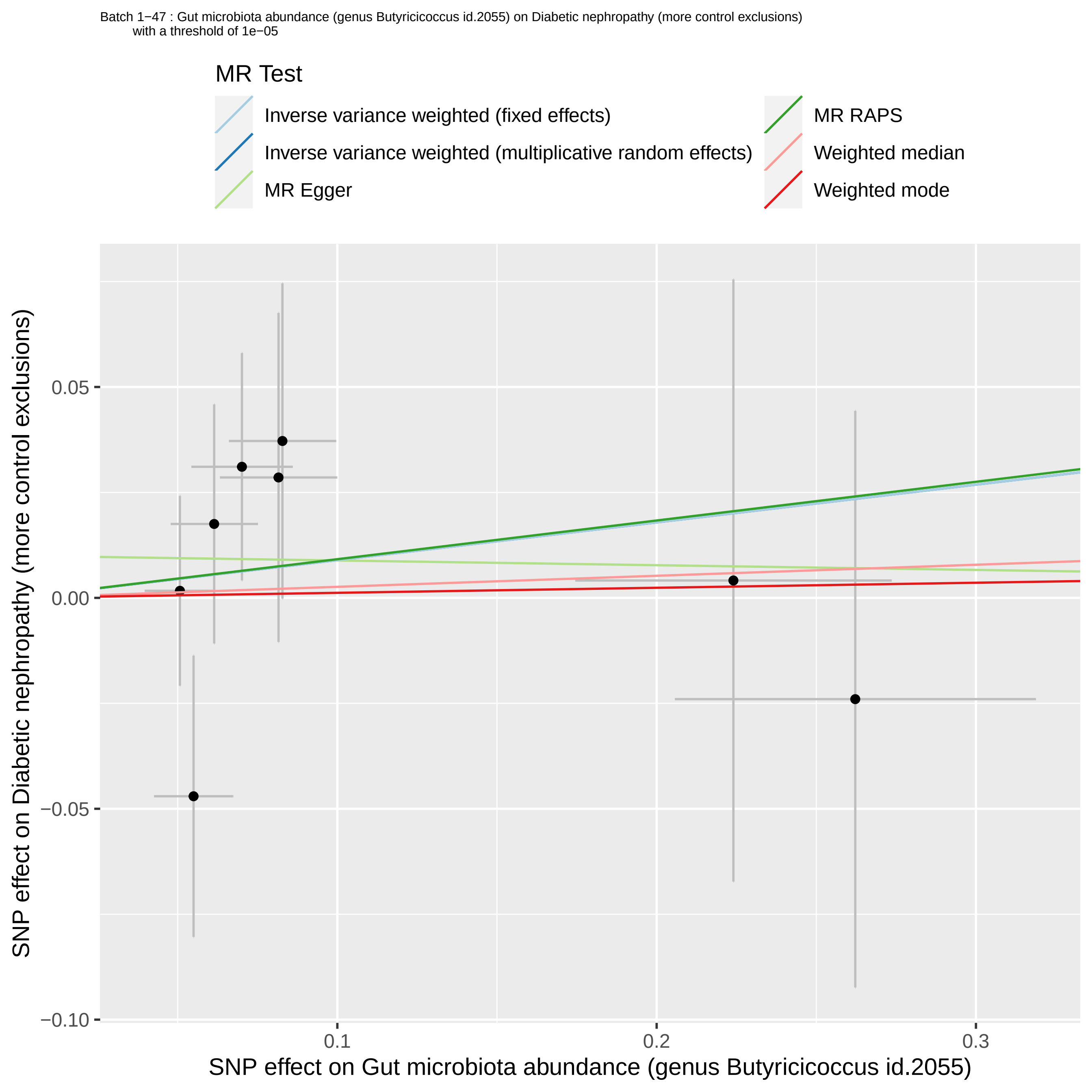
Batch 1−47 : Gut microbiota abundance (genus Butyricicoccus id.2055) on Diabetic nephropathy (more control exclusions) with a threshold of 1e−05
MR Test
Inverse variance weighted (fixed effects)
Inverse variance weighted (multiplicative random effects)
MR Egger
MR RAPS
Weighted median
Weighted mode
0.05
0.00
−0.05
−0.10
0.1
0.2
0.3
SNP effect on Diabetic nephropathy (more control exclusions)
SNP effect on Gut microbiota abundance (genus Butyricicoccus id.2055)

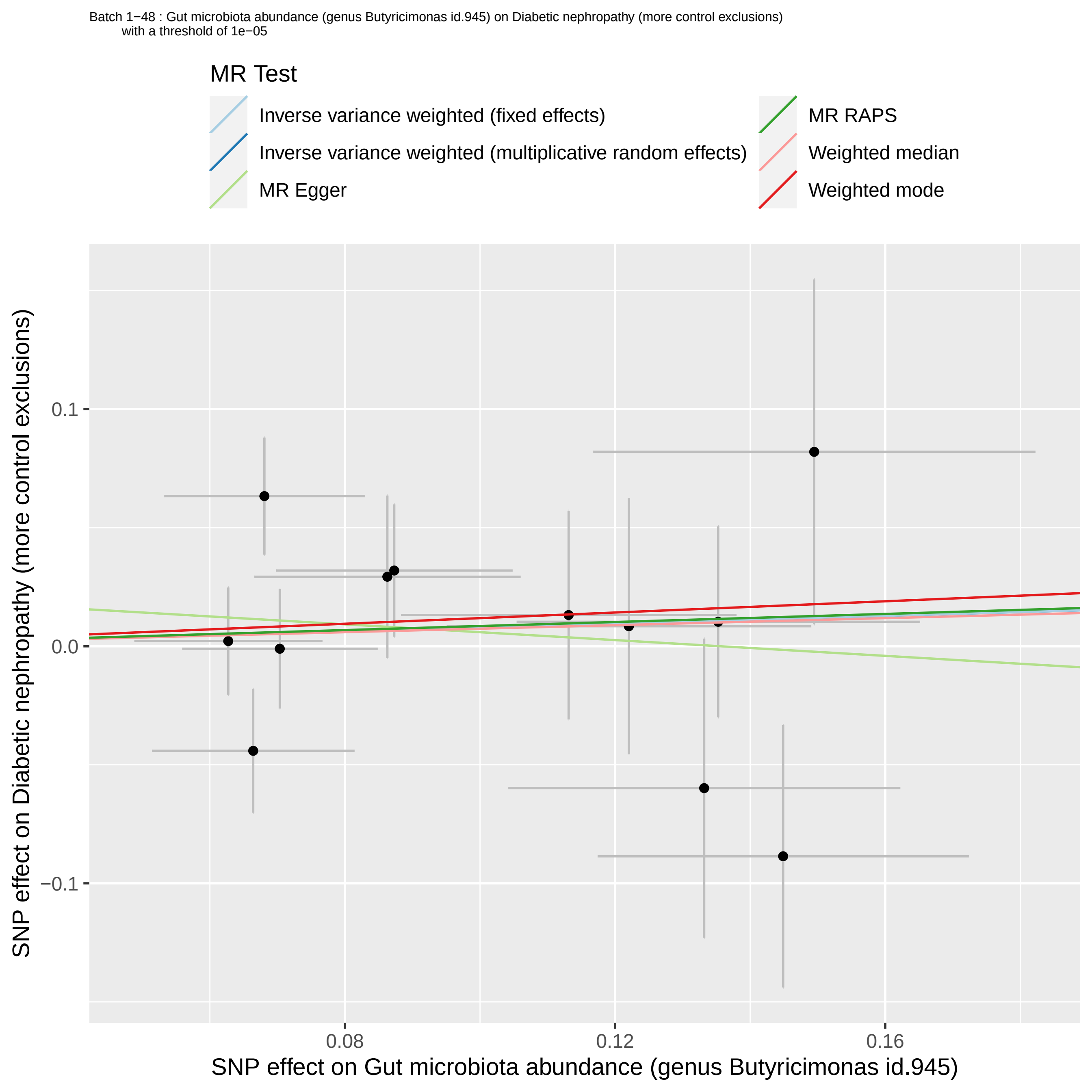
Batch 1−48 : Gut microbiota abundance (genus Butyricimonas id.945) on Diabetic nephropathy (more control exclusions) with a threshold of 1e−05
MR Test
Inverse variance weighted (fixed effects)
Inverse variance weighted (multiplicative random effects)
MR Egger
MR RAPS
Weighted median
Weighted mode
0.1
0.0
−0.1
0.08
0.12
0.16
SNP effect on Diabetic nephropathy (more control exclusions)
SNP effect on Gut microbiota abundance (genus Butyricimonas id.945)

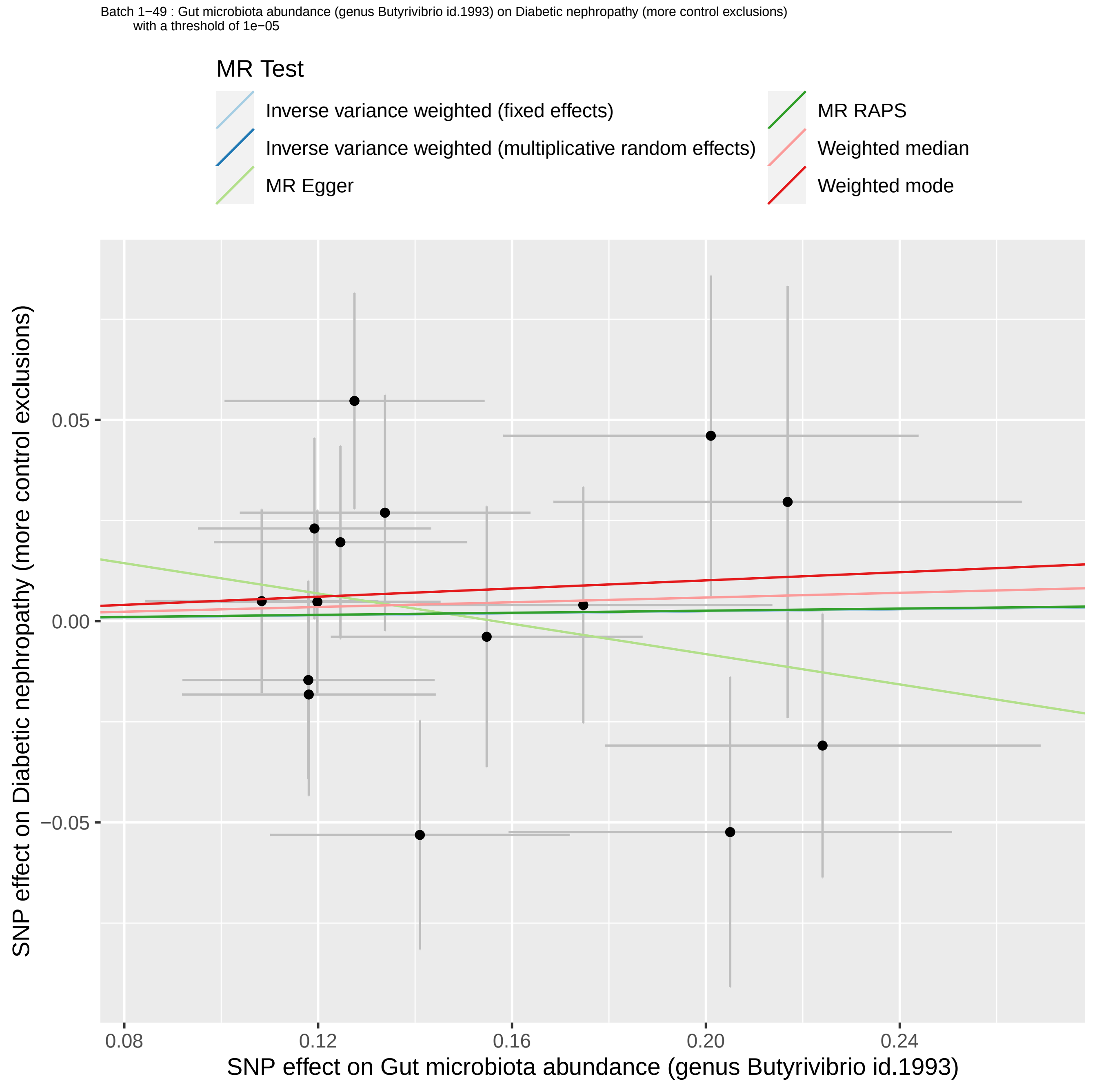

Batch 1−49 : Gut microbiota abundance (genus Butyrivibrio id.1993) on Diabetic nephropathy (more control exclusions)
with a threshold of 1e−05
MR Test
Inverse variance weighted (fixed effects)
Inverse variance weighted (multiplicative random effects)
MR Egger
MR RAPS
Weighted median
Weighted mode
0.05
0.00
−0.05
0.08
0.12
0.16
0.20
0.24
SNP effect on Gut microbiota abundance (genus Butyrivibrio id.1993)
SNP effect on Diabetic nephropathy (more control exclusions)

Batch 1−50 : Gut microbiota abundance (genus Candidatus Soleaferrea id.11350) on Diabetic nephropathy (more control exclusions) with a threshold of 1e−05

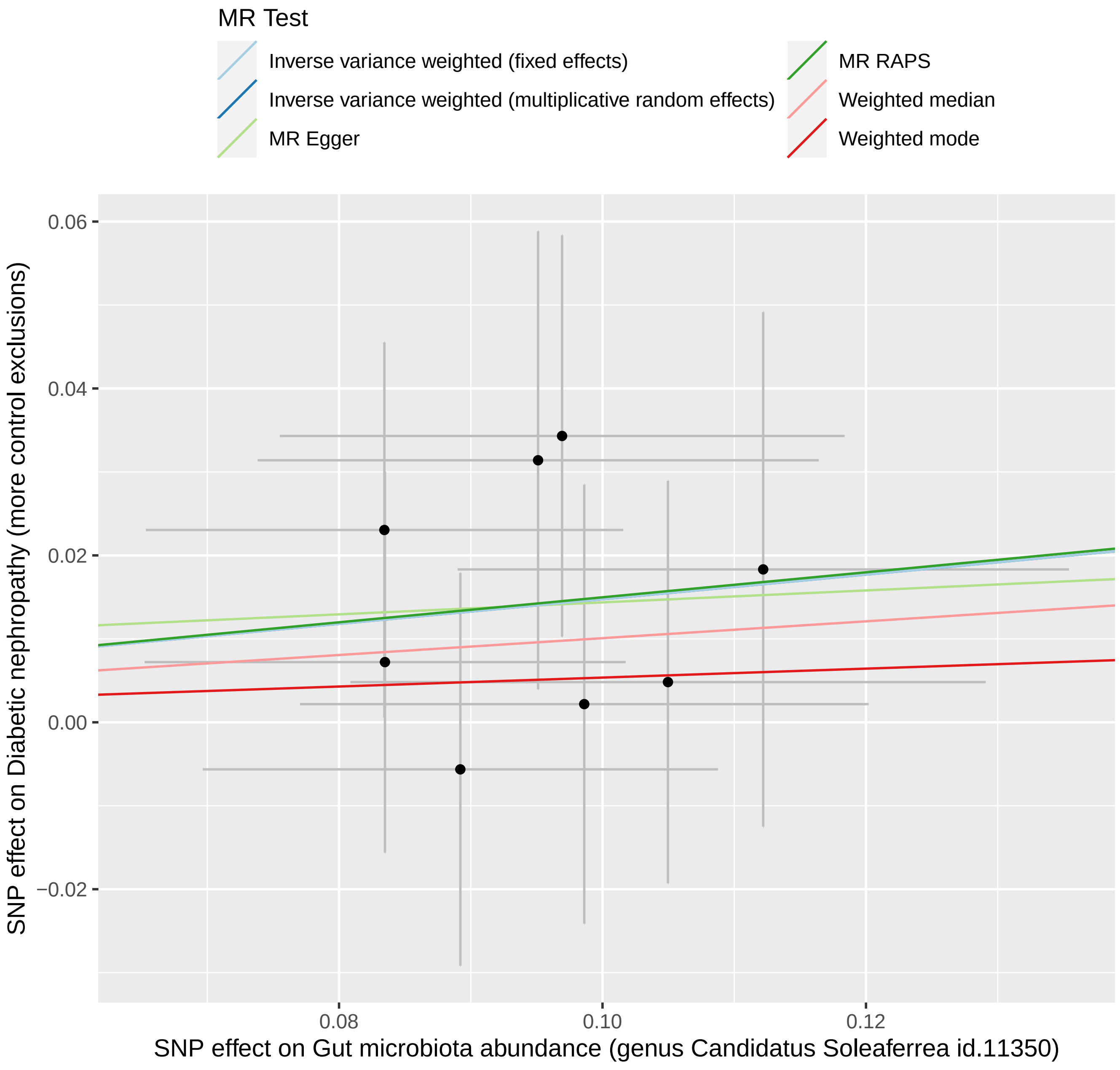

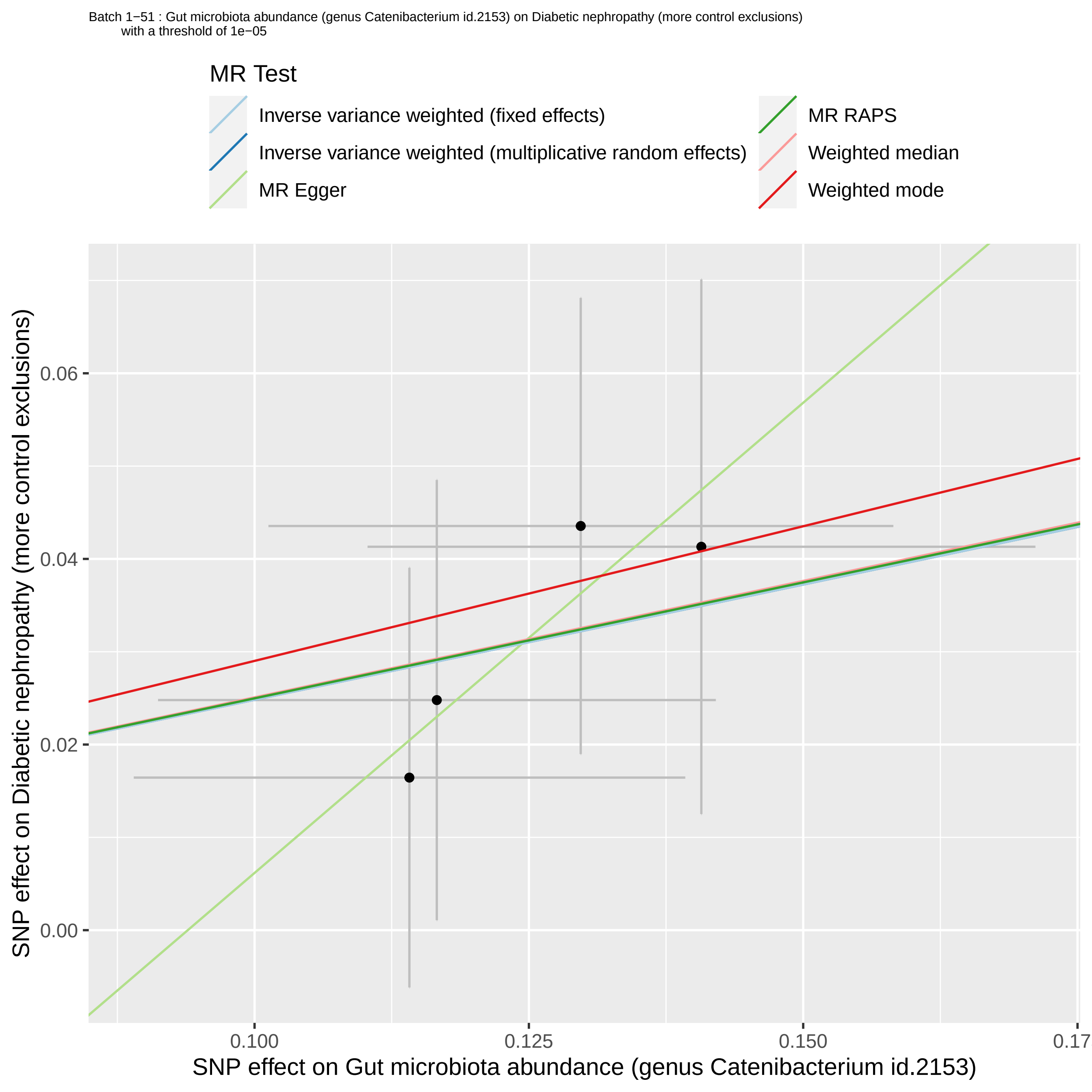

Batch 1−51 : Gut microbiota abundance (genus Catenibacterium id.2153) on Diabetic nephropathy (more control exclusions)
with a threshold of 1e−05
MR Test
Inverse variance weighted (fixed effects)
Inverse variance weighted (multiplicative random effects)
MR Egger
MR RAPS
Weighted median
Weighted mode
SNP effect on Diabetic nephropathy (more control exclusions)
0.06
0.04
0.02
0.00
0.100
0.125
0.150
0.17
SNP effect on Gut microbiota abundance (genus Catenibacterium id.2153)

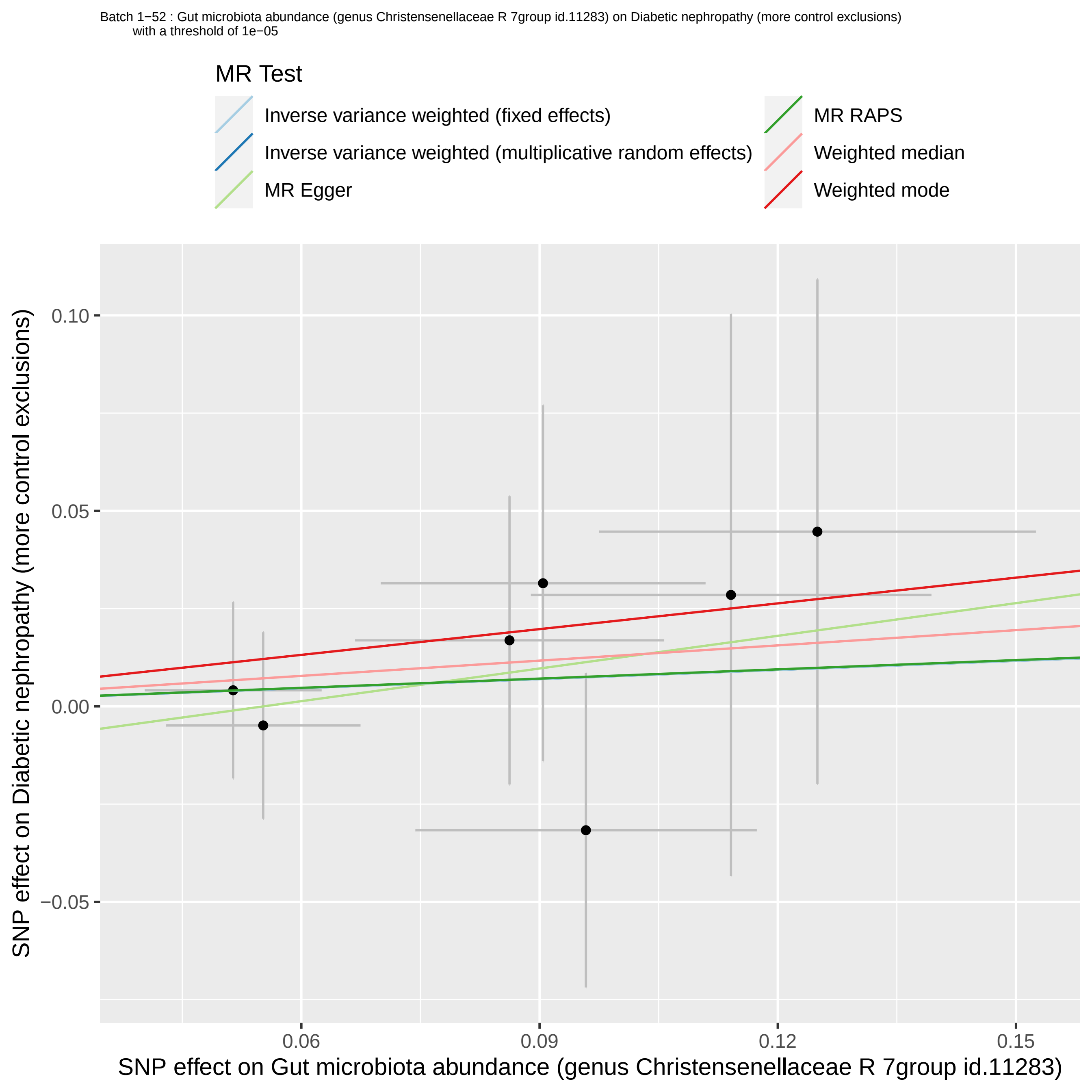

Batch 1−52 : Gut microbiota abundance (genus Christensenellaceae R 7group id.11283) on Diabetic nephropathy (more control exclusions) with a threshold of 1e−05
MR Test
Inverse variance weighted (fixed effects)
Inverse variance weighted (multiplicative random effects)
MR Egger
MR RAPS
Weighted median
Weighted mode
SNP effect on Diabetic nephropathy (more control exclusions)
0.10
0.05
0.00
−0.05
0.06
0.09
0.12
0.15
SNP effect on Gut microbiota abundance (genus Christensenellaceae R 7group id.11283)

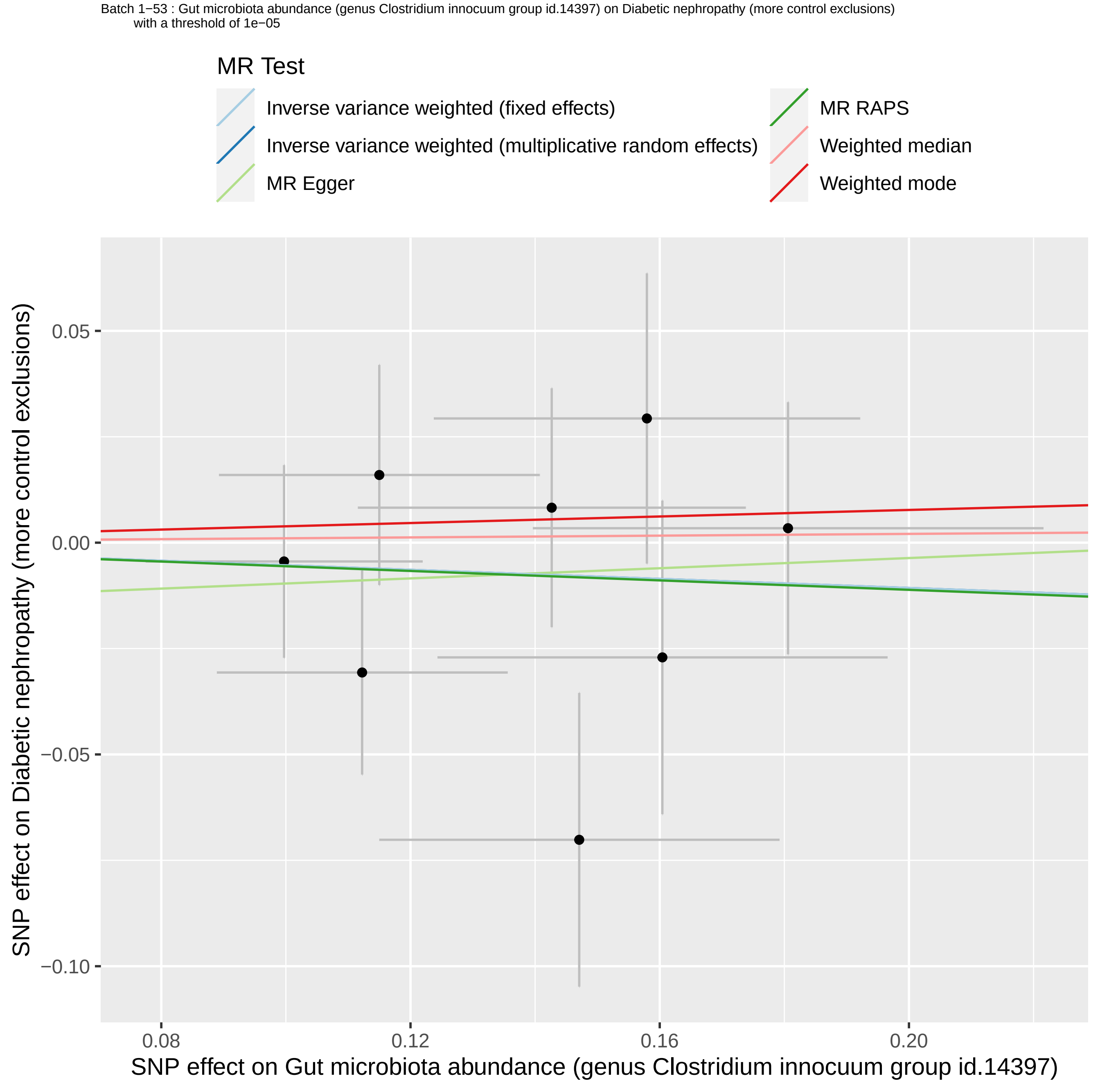
Batch 1−53 : Gut microbiota abundance (genus Clostridium innocuum group id.14397) on Diabetic nephropathy (more control exclusions) with a threshold of 1e−05
MR Test
Inverse variance weighted (fixed effects)
Inverse variance weighted (multiplicative random effects)
MR Egger
MR RAPS
Weighted median
Weighted mode
0.05
0.00
−0.05
−0.10
0.08
0.12
0.16
0.20
SNP effect on Diabetic nephropathy (more control exclusions)
SNP effect on Gut microbiota abundance (genus Clostridium innocuum group id.14397)

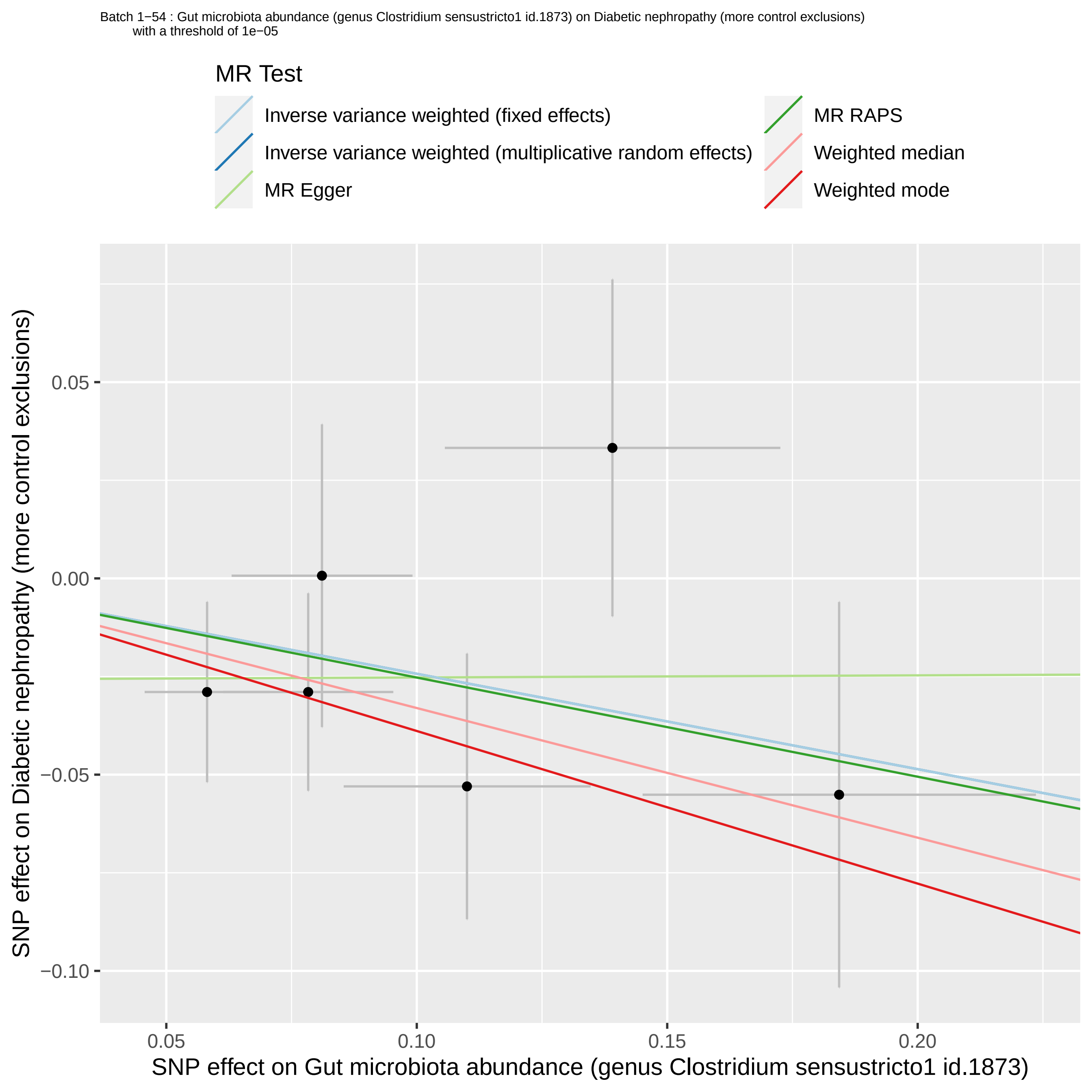

Batch 1−54 : Gut microbiota abundance (genus Clostridium sensustricto1 id.1873) on Diabetic nephropathy (more control exclusions)
with a threshold of 1e−05
MR Test
Inverse variance weighted (fixed effects)
Inverse variance weighted (multiplicative random effects)
MR Egger
MR RAPS
Weighted median
Weighted mode
0.05
0.00
−0.05
−0.10
0.05
0.10
0.15
0.20
SNP effect on Diabetic nephropathy (more control exclusions)
SNP effect on Gut microbiota abundance (genus Clostridium sensustricto1 id.1873)

Batch 1−55 : Gut microbiota abundance (genus Collinsella id.815) on Diabetic nephropathy (more control exclusions) with a threshold of 1e−05

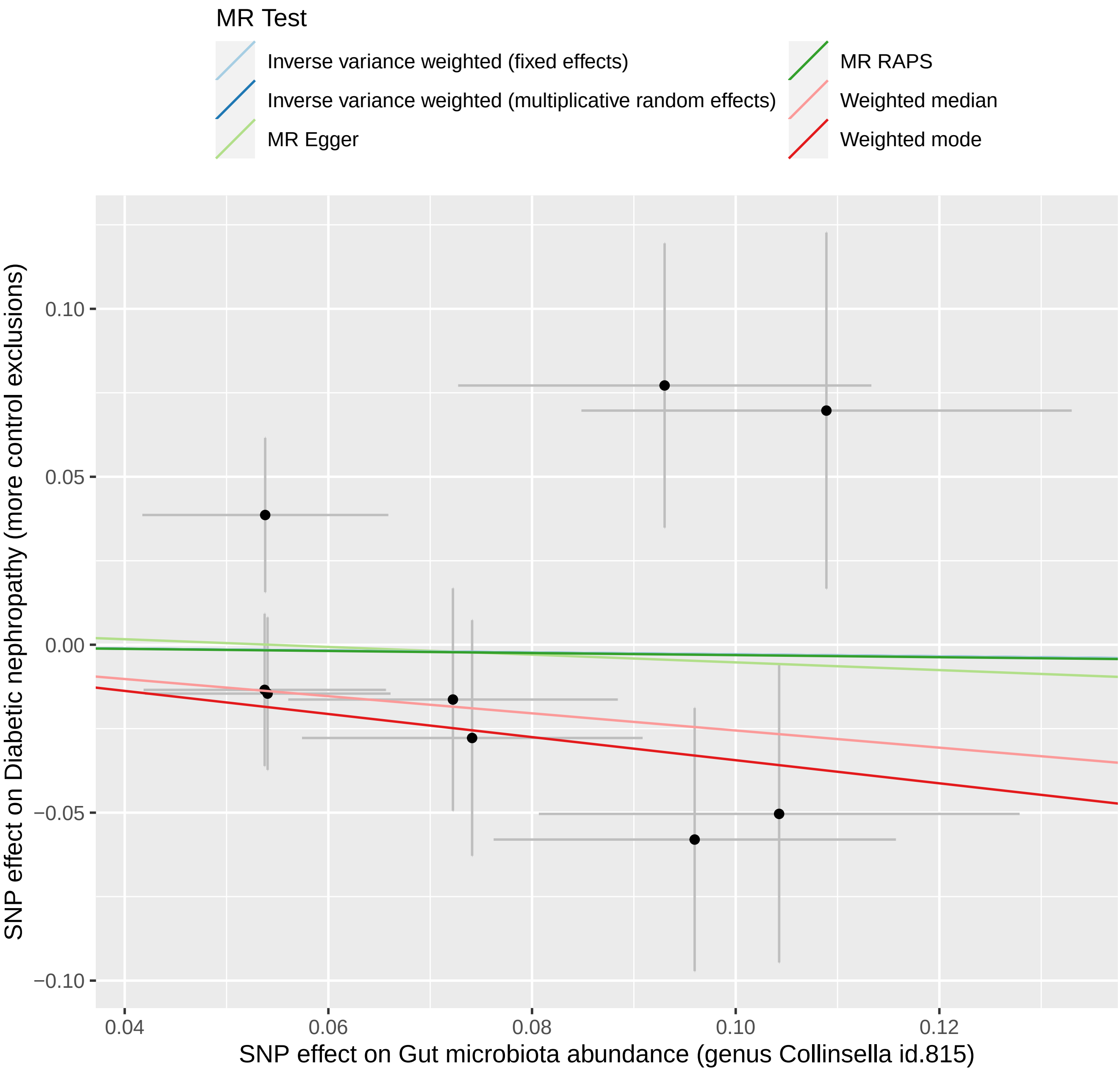

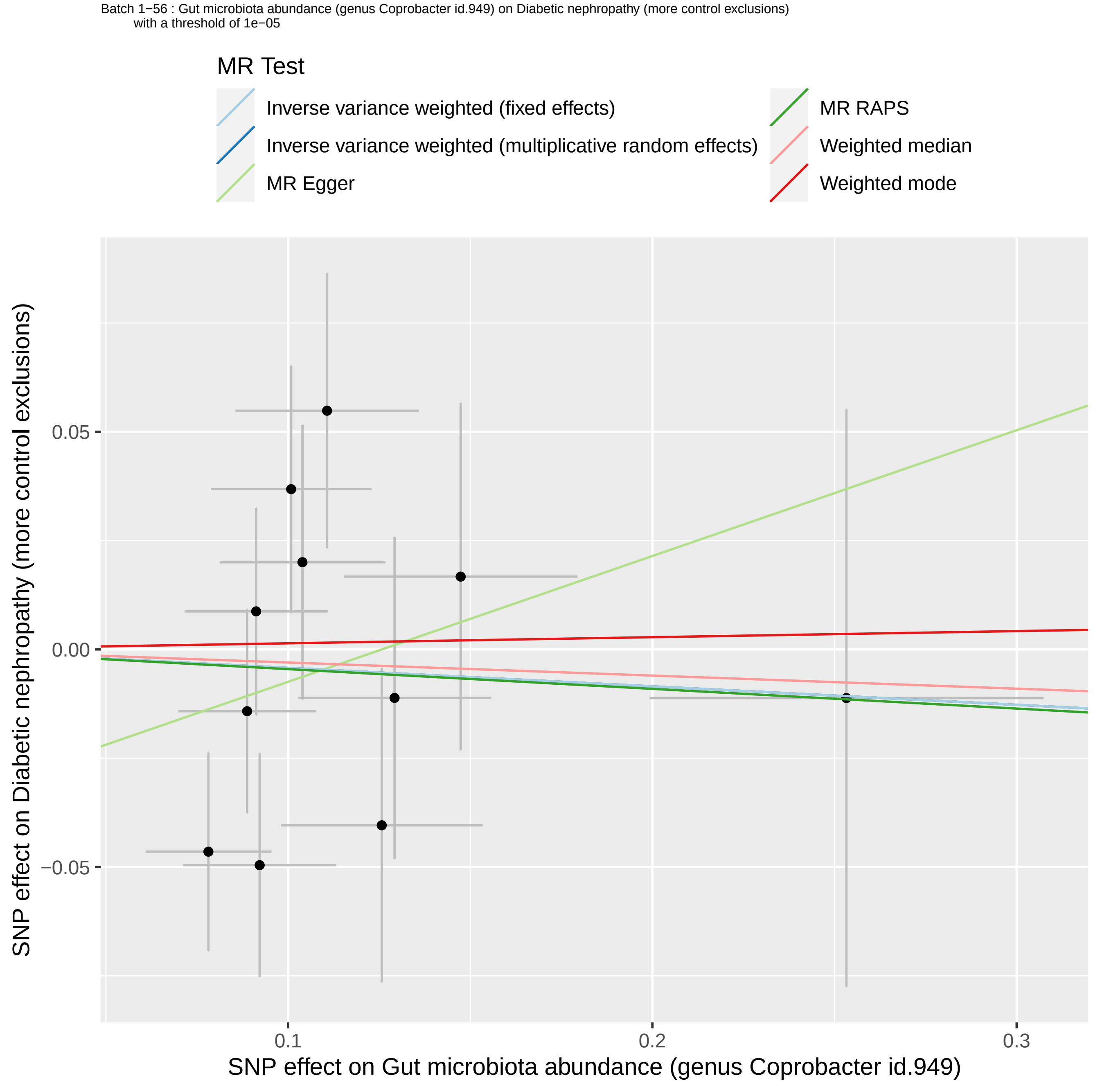

Batch 1−56 : Gut microbiota abundance (genus Coprobacter id.949) on Diabetic nephropathy (more control exclusions)
with a threshold of 1e−05
MR Test
Inverse variance weighted (fixed effects)
Inverse variance weighted (multiplicative random effects)
MR Egger
MR RAPS
Weighted median
Weighted mode
0.05
0.00
−0.05
0.1
0.2
0.3
SNP effect on Diabetic nephropathy (more control exclusions)
SNP effect on Gut microbiota abundance (genus Coprobacter id.949)

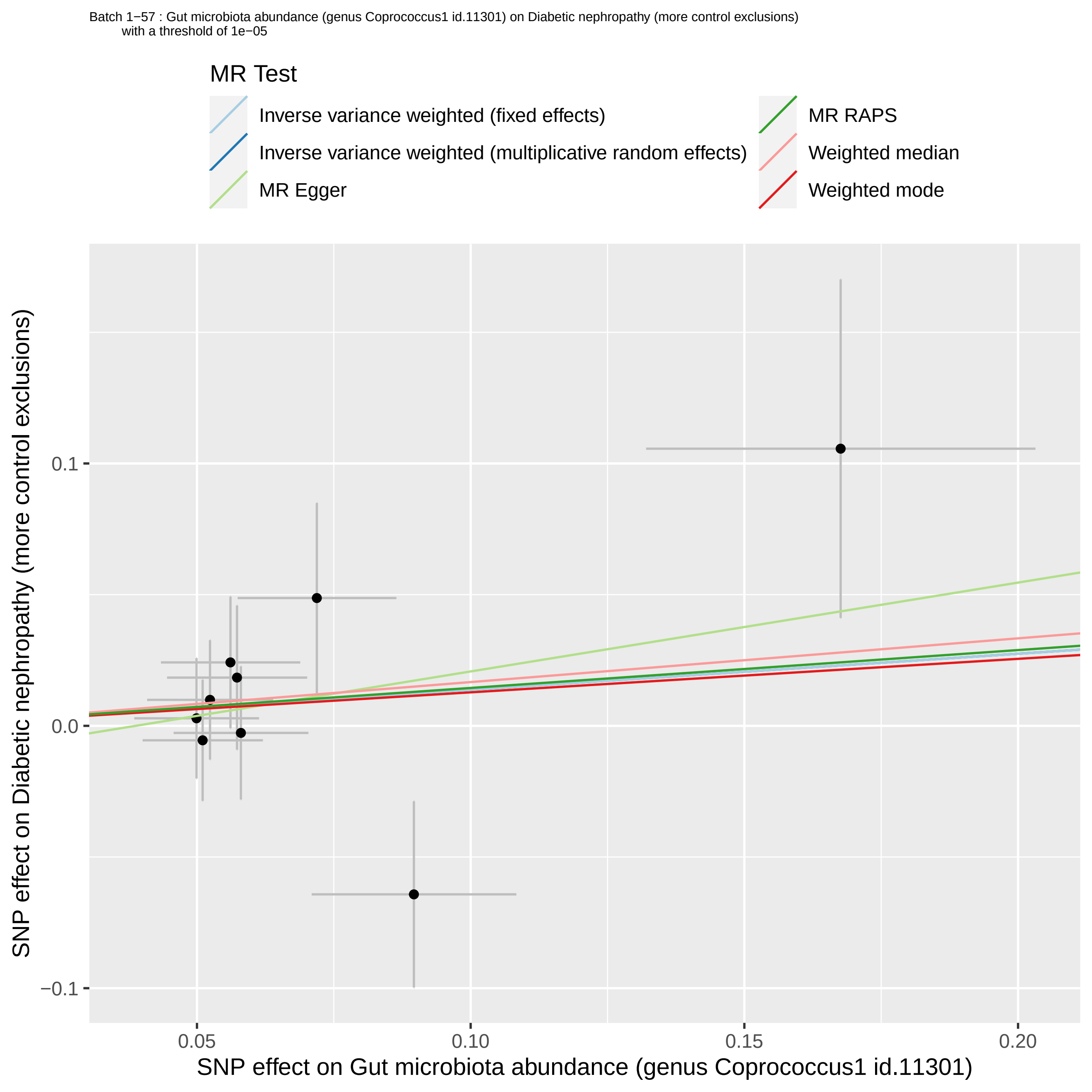
Batch 1−57 : Gut microbiota abundance (genus Coprococcus1 id.11301) on Diabetic nephropathy (more control exclusions)
with a threshold of 1e−05
MR Test
Inverse variance weighted (fixed effects)
Inverse variance weighted (multiplicative random effects)
MR Egger
MR RAPS
Weighted median
Weighted mode
SNP effect on Diabetic nephropathy (more control exclusions)
0.1
0.0
−0.1
0.05
0.10
0.15
0.20
SNP effect on Gut microbiota abundance (genus Coprococcus1 id.11301)

Batch 1−58 : Gut microbiota abundance (genus Coprococcus2 id.11302) on Diabetic nephropathy (more control exclusions)
with a threshold of 1e−05

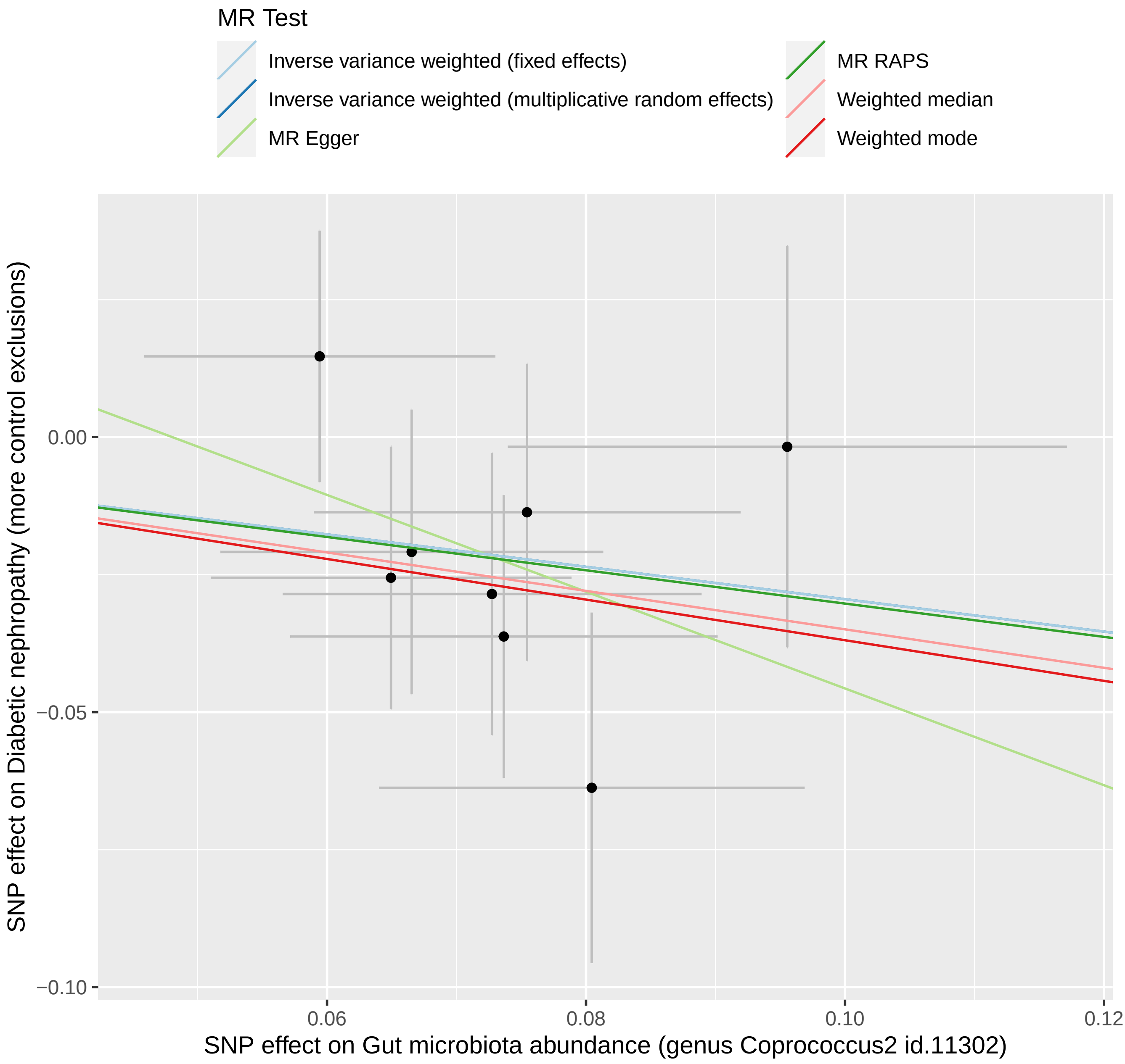

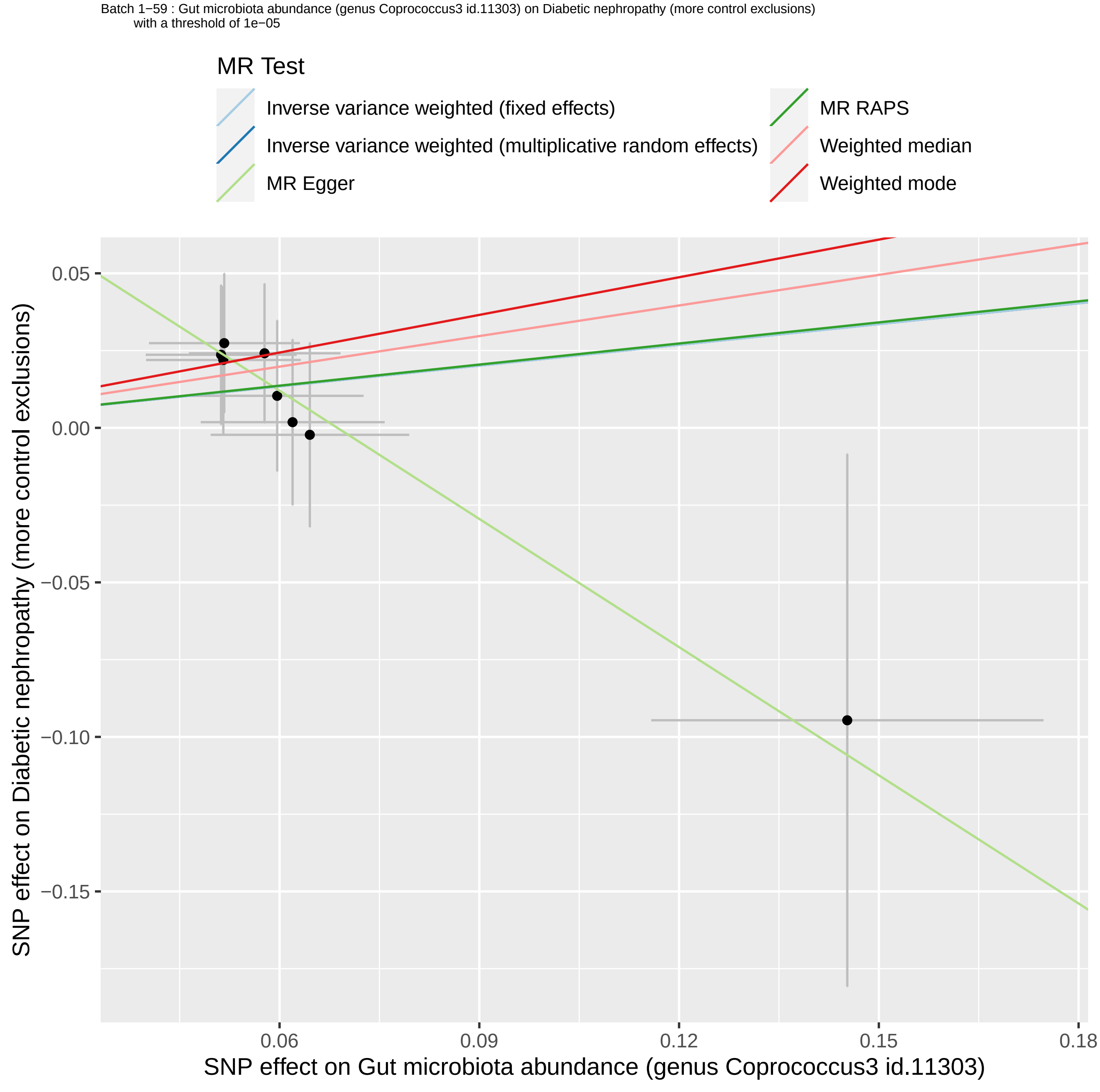

Batch 1−59 : Gut microbiota abundance (genus Coprococcus3 id.11303) on Diabetic nephropathy (more control exclusions)
with a threshold of 1e−05
MR Test
Inverse variance weighted (fixed effects)
Inverse variance weighted (multiplicative random effects)
MR Egger
MR RAPS
Weighted median
Weighted mode
0.05
0.00
−0.05
−0.10
−0.15
0.06
0.09
0.12
0.15
0.18
SNP effect on Diabetic nephropathy (more control exclusions)
SNP effect on Gut microbiota abundance (genus Coprococcus3 id.11303)

Batch 1−60 : Gut microbiota abundance (genus Defluviitaleaceae UCG011 id.11287) on Diabetic nephropathy (more control exclusions) with a threshold of 1e−05

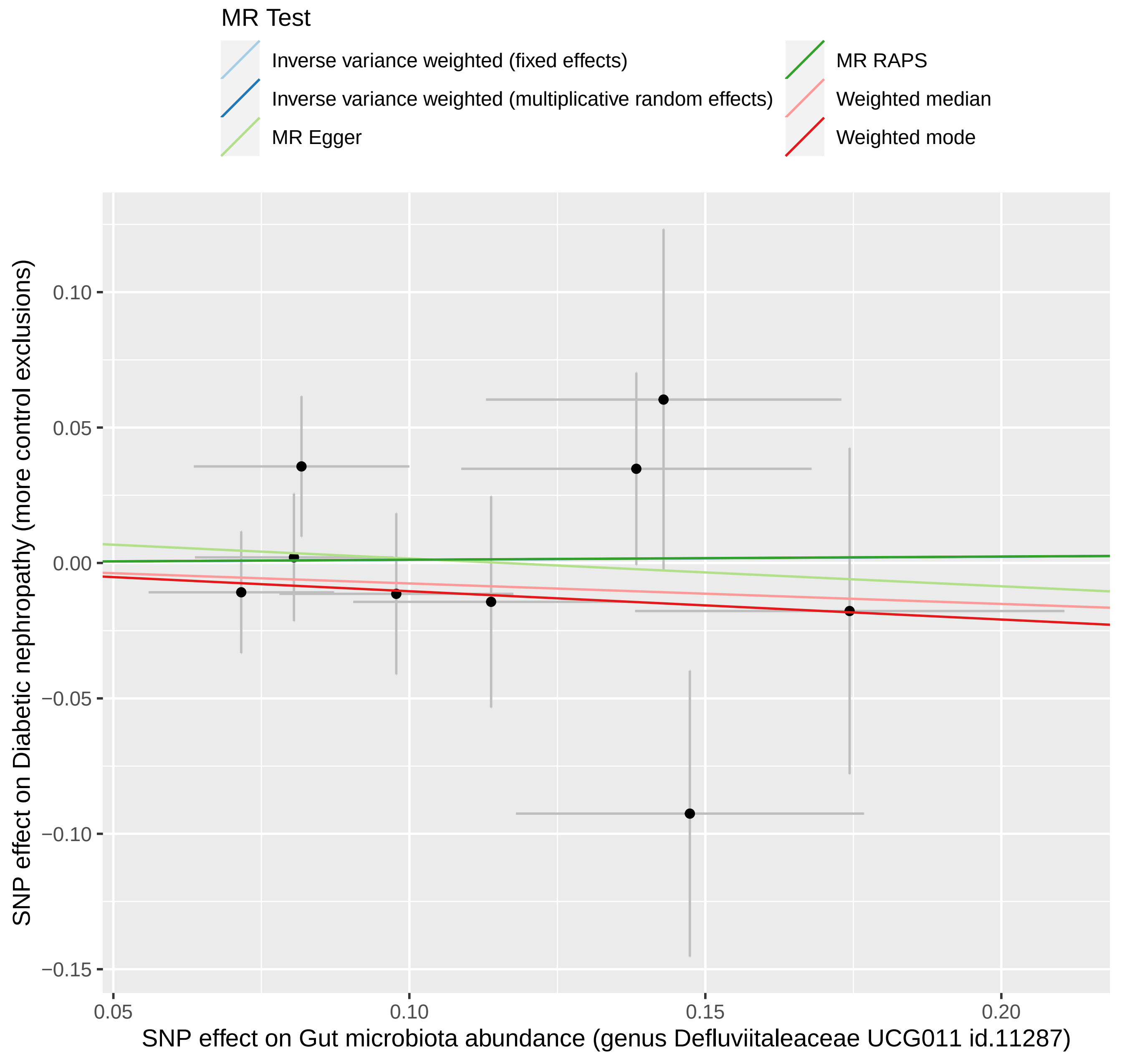

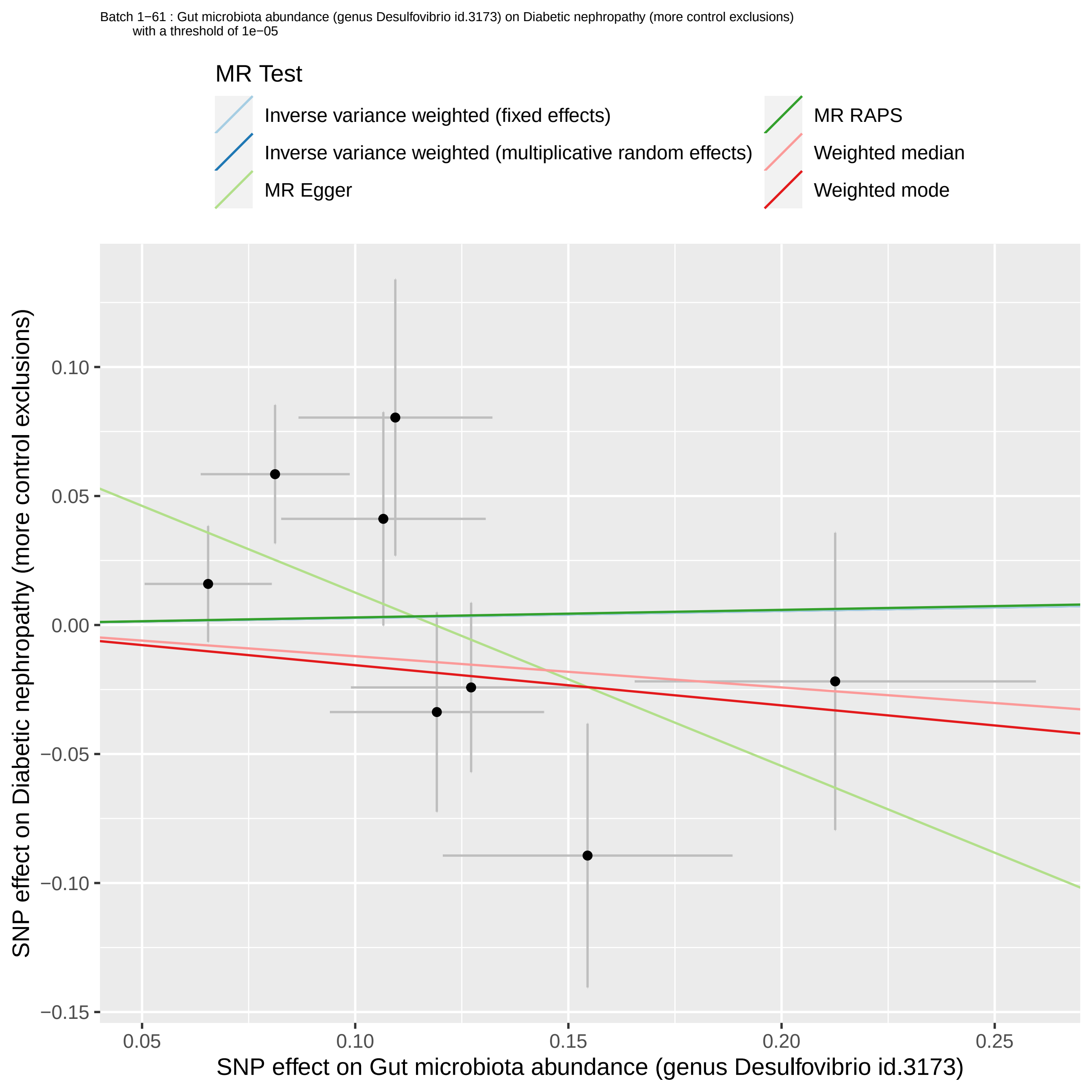
Batch 1−61 : Gut microbiota abundance (genus Desulfovibrio id.3173) on Diabetic nephropathy (more control exclusions) with a threshold of 1e−05
MR Test
Inverse variance weighted (fixed effects)
Inverse variance weighted (multiplicative random effects)
MR Egger
MR RAPS
Weighted median
Weighted mode
0.10
0.05
0.00
−0.05
−0.10
−0.15
0.05
0.10
0.15
0.20
0.25
SNP effect on Diabetic nephropathy (more control exclusions)
SNP effect on Gut microbiota abundance (genus Desulfovibrio id.3173)

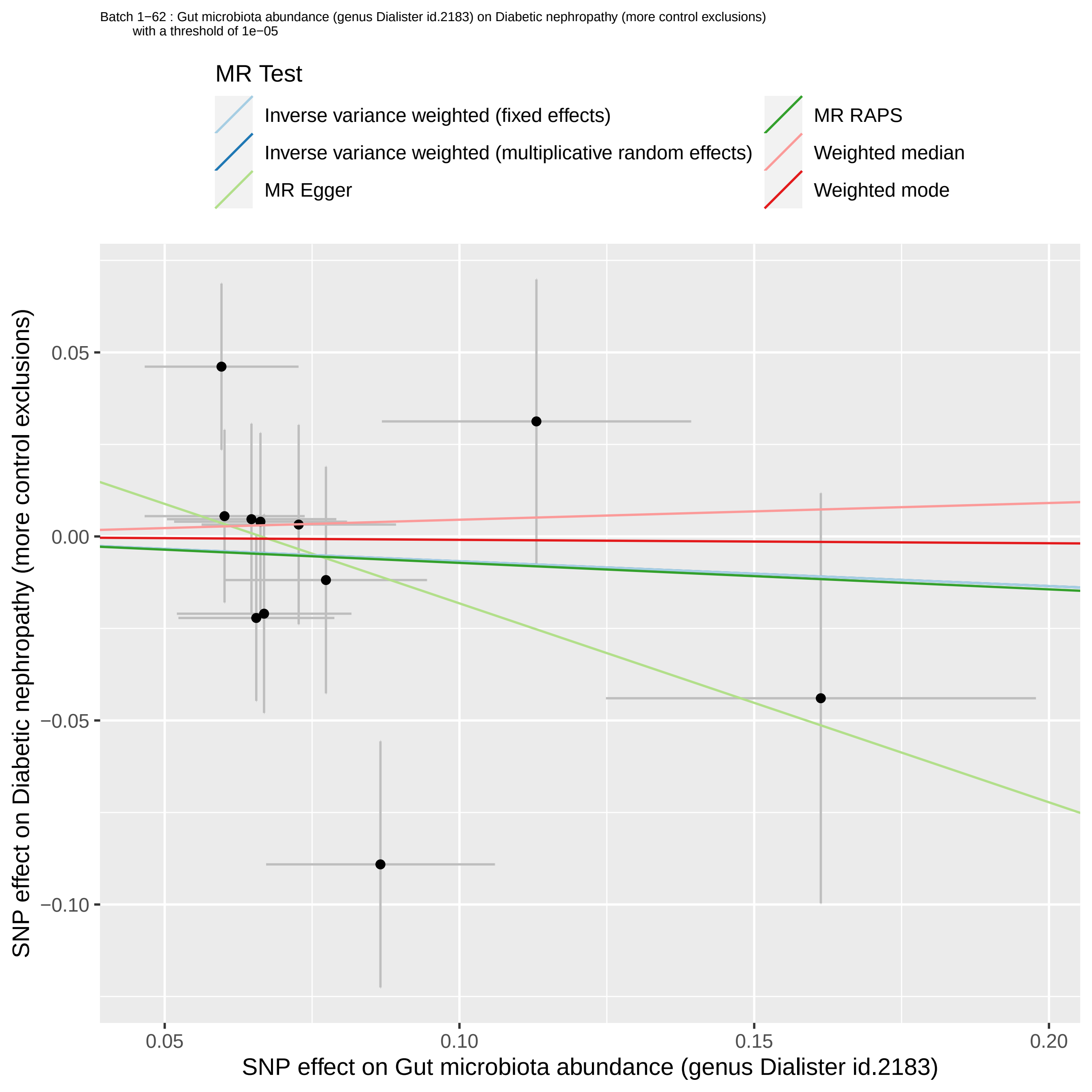
Batch 1−62 : Gut microbiota abundance (genus Dialister id.2183) on Diabetic nephropathy (more control exclusions)
with a threshold of 1e−05
MR Test
Inverse variance weighted (fixed effects)
Inverse variance weighted (multiplicative random effects)
MR Egger
MR RAPS
Weighted median
Weighted mode
SNP effect on Diabetic nephropathy (more control exclusions)
0.05
0.00
−0.05
−0.10
0.05
0.10
0.15
0.20
SNP effect on Gut microbiota abundance (genus Dialister id.2183)

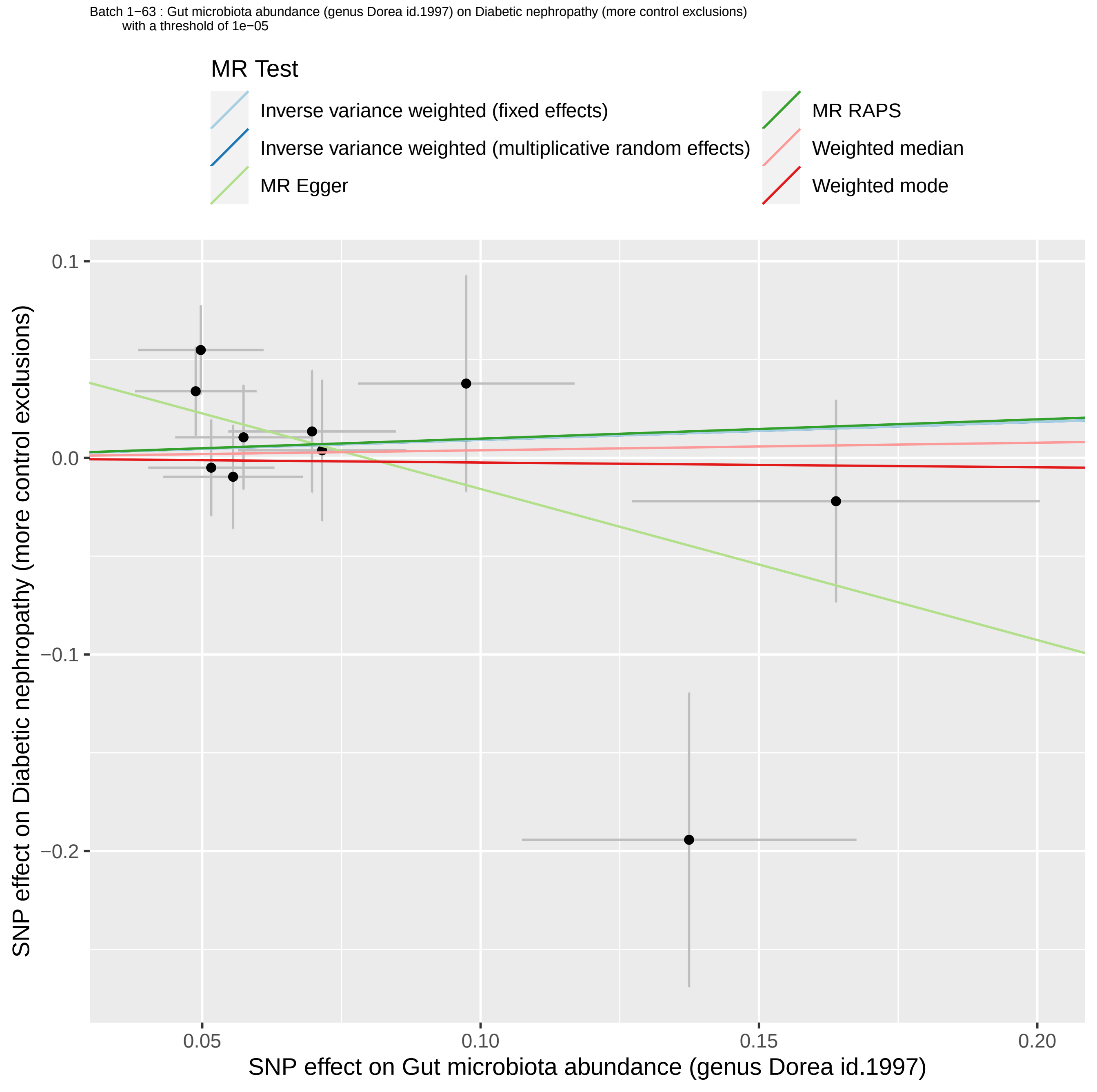
Batch 1−63 : Gut microbiota abundance (genus Dorea id.1997) on Diabetic nephropathy (more control exclusions)
with a threshold of 1e−05
MR Test
Inverse variance weighted (fixed effects)
Inverse variance weighted (multiplicative random effects)
MR Egger
MR RAPS
Weighted median
Weighted mode
0.1
0.0
−0.1
−0.2
0.05
0.10
0.15
0.20
SNP effect on Diabetic nephropathy (more control exclusions)
SNP effect on Gut microbiota abundance (genus Dorea id.1997)

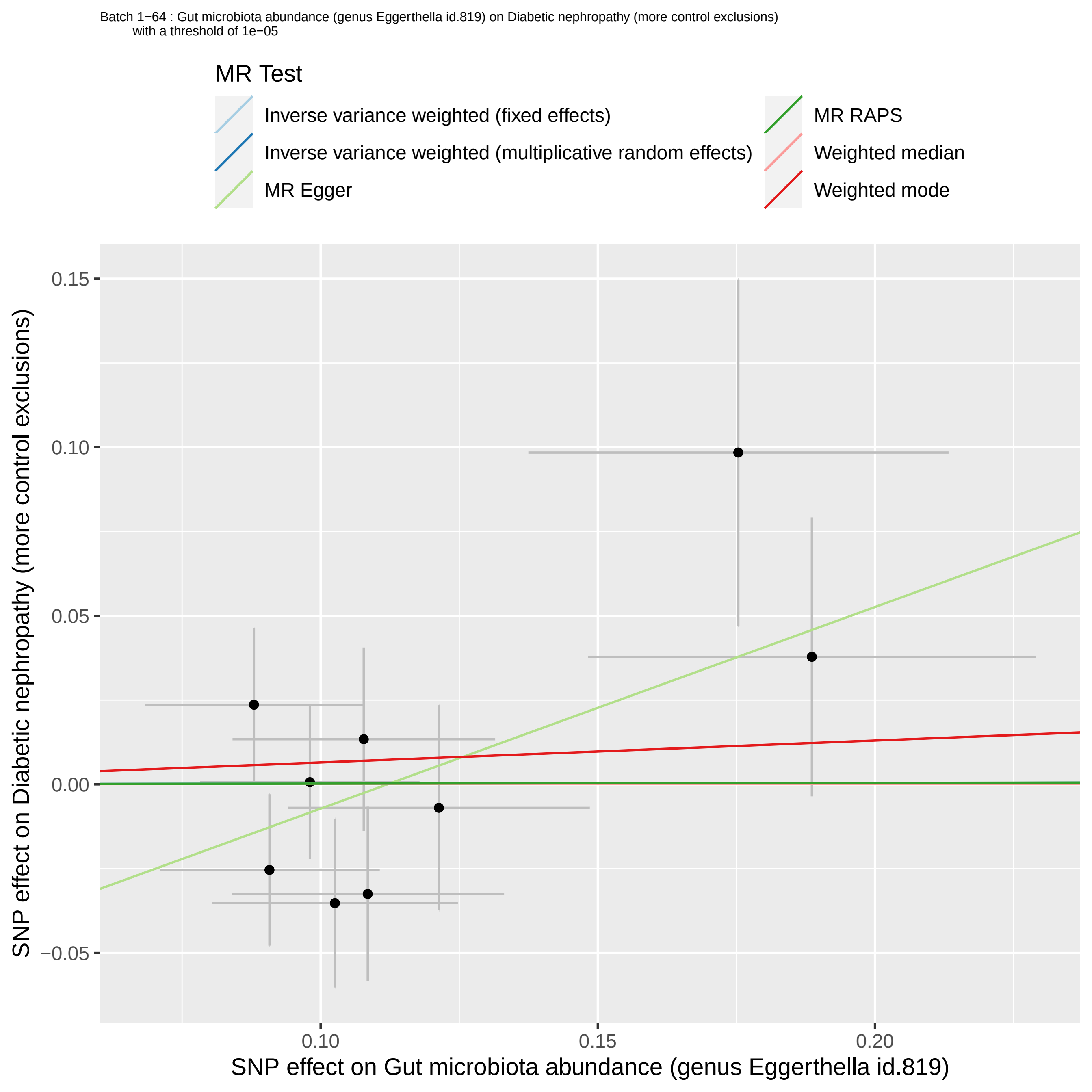

Batch 1−64 : Gut microbiota abundance (genus Eggerthella id.819) on Diabetic nephropathy (more control exclusions)
with a threshold of 1e−05
MR Test
Inverse variance weighted (fixed effects)
Inverse variance weighted (multiplicative random effects)
MR Egger
MR RAPS
Weighted median
Weighted mode
0.15
0.10
0.05
0.00
−0.05
0.10
0.15
0.20
SNP effect on Diabetic nephropathy (more control exclusions)
SNP effect on Gut microbiota abundance (genus Eggerthella id.819)

Batch 1−65 : Gut microbiota abundance (genus Eisenbergiella id.11304) on Diabetic nephropathy (more control exclusions) with a threshold of 1e−05

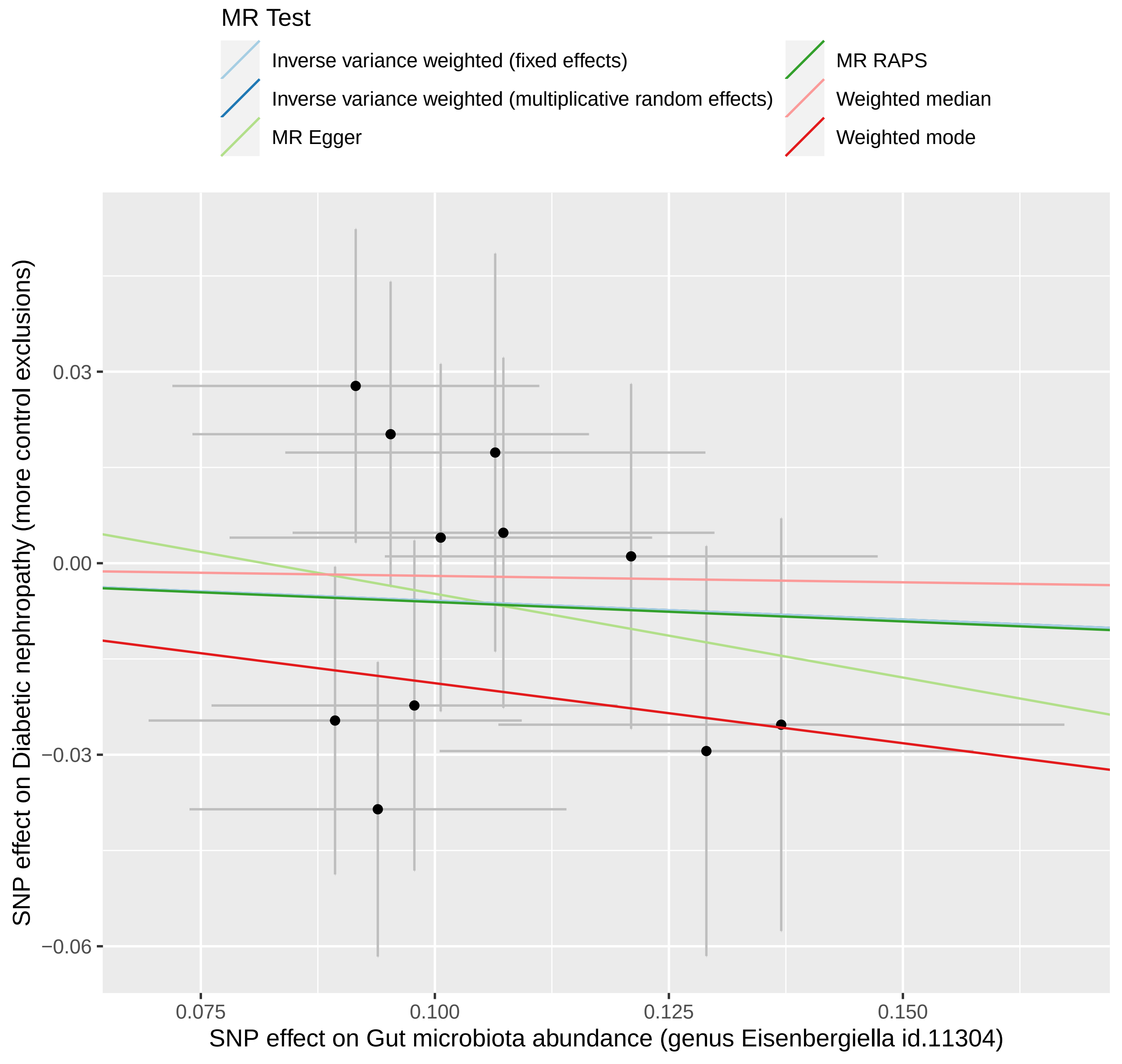

Batch 1−66 : Gut microbiota abundance (genus Enterorhabdus id.820) on Diabetic nephropathy (more control exclusions)
with a threshold of 1e−05

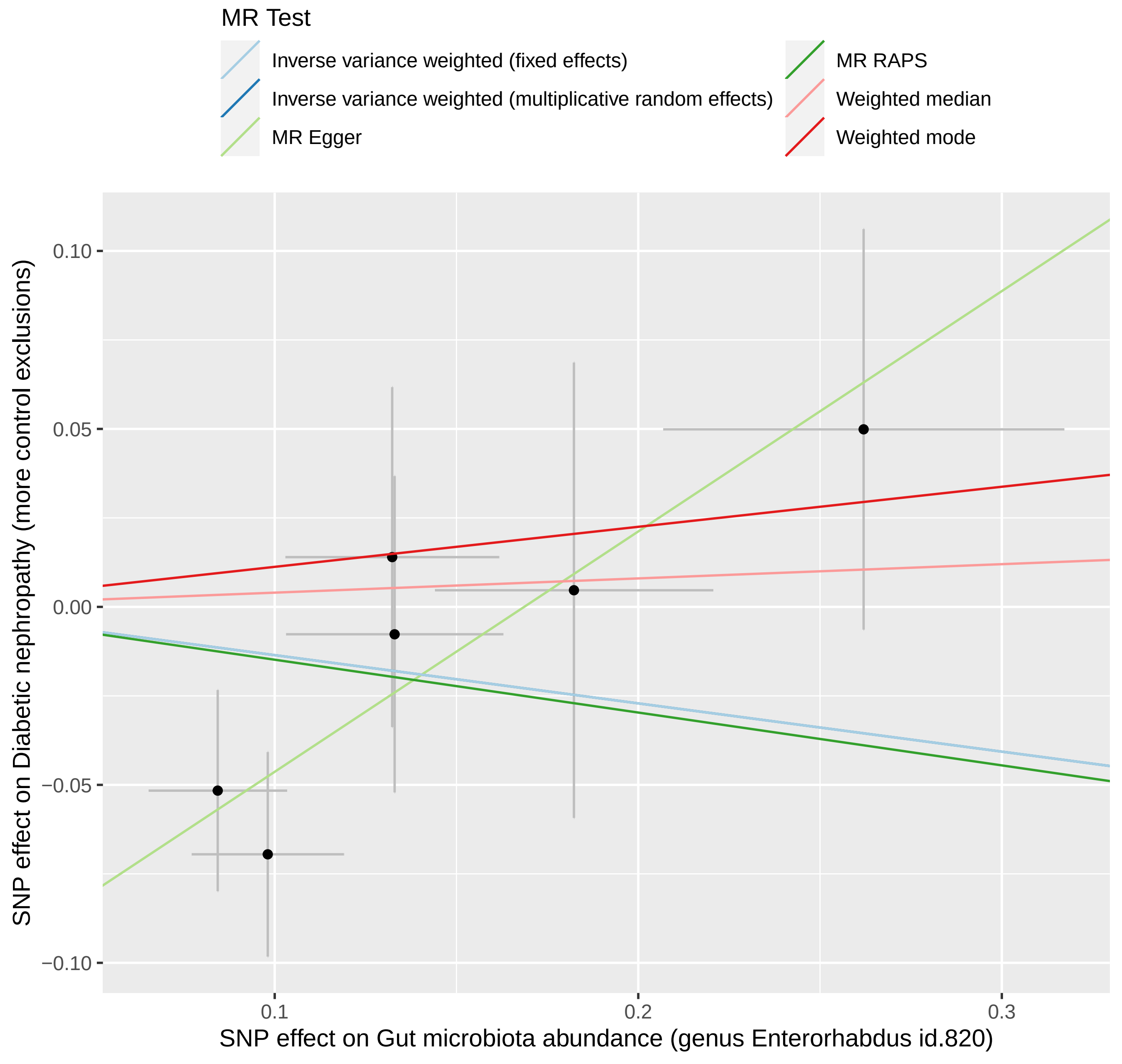

Batch 1−67 : Gut microbiota abundance (genus Erysipelatoclostridium id.11381) on Diabetic nephropathy (more control exclusions) with a threshold of 1e−05

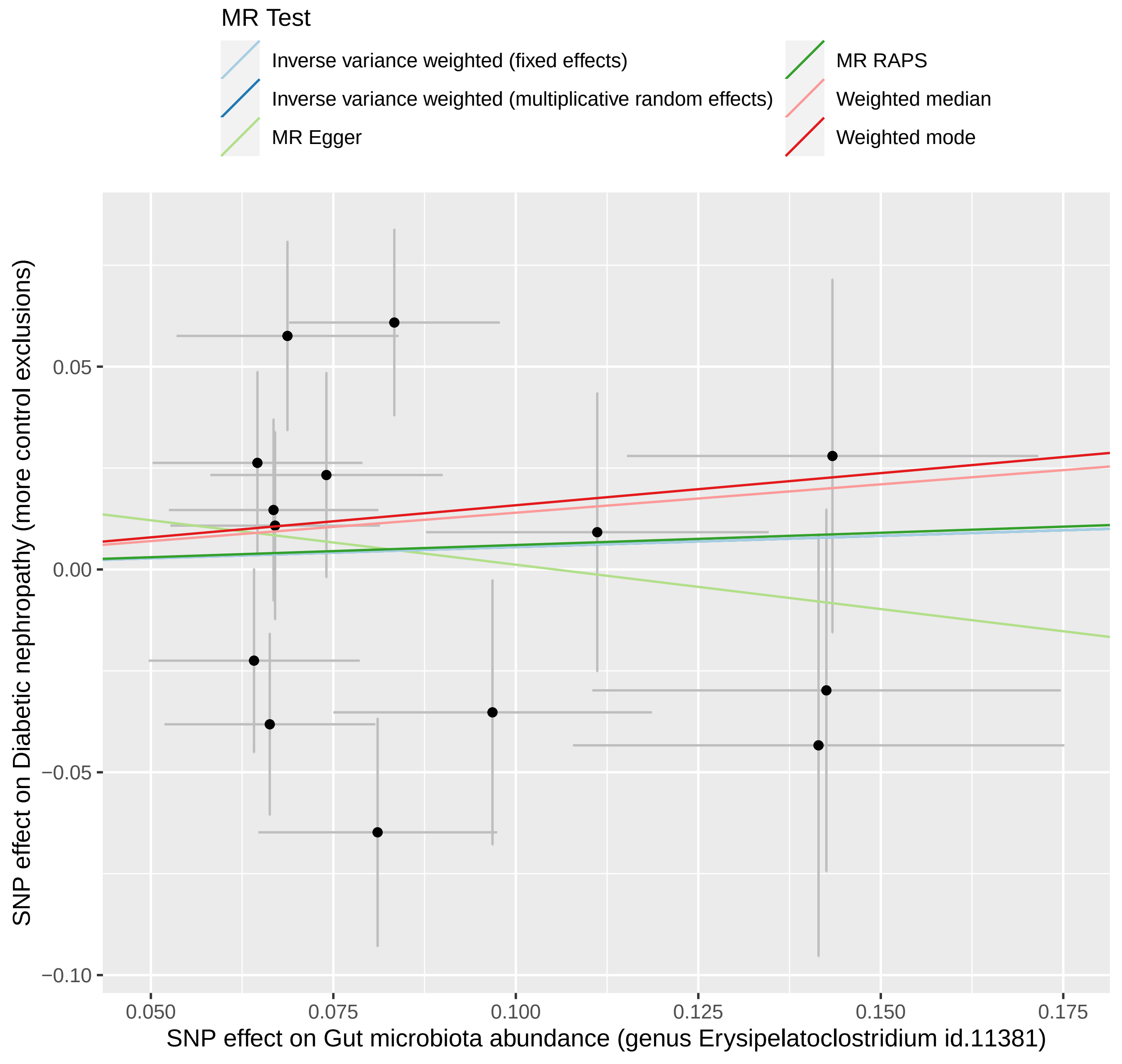

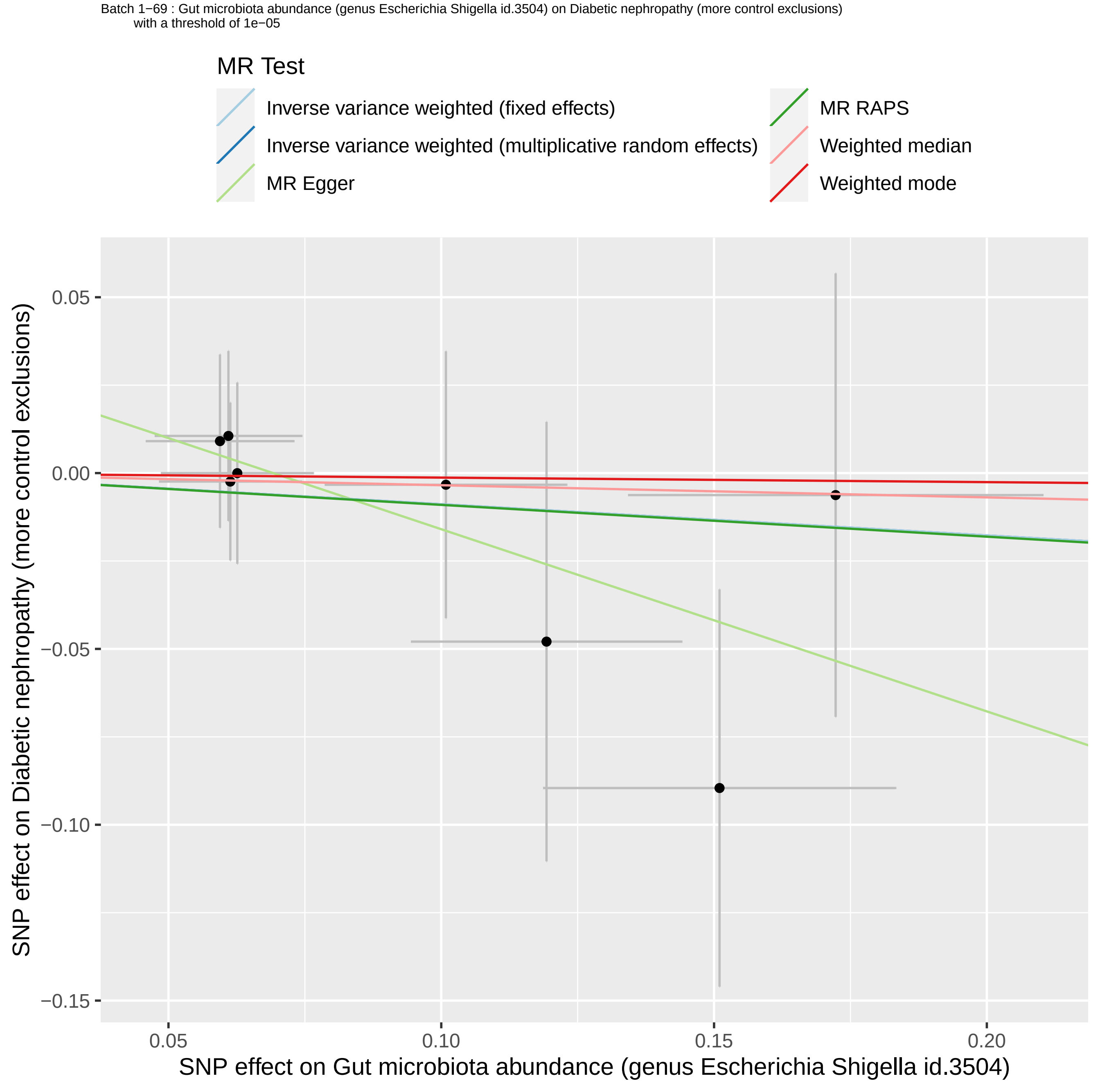
Batch 1−69 : Gut microbiota abundance (genus Escherichia Shigella id.3504) on Diabetic nephropathy (more control exclusions)
with a threshold of 1e−05
MR Test
Inverse variance weighted (fixed effects)
Inverse variance weighted (multiplicative random effects)
MR Egger
MR RAPS
Weighted median
Weighted mode
0.05
0.00
−0.05
−0.10
−0.15
0.05
0.10
0.15
0.20
SNP effect on Diabetic nephropathy (more control exclusions)
SNP effect on Gut microbiota abundance (genus Escherichia Shigella id.3504)

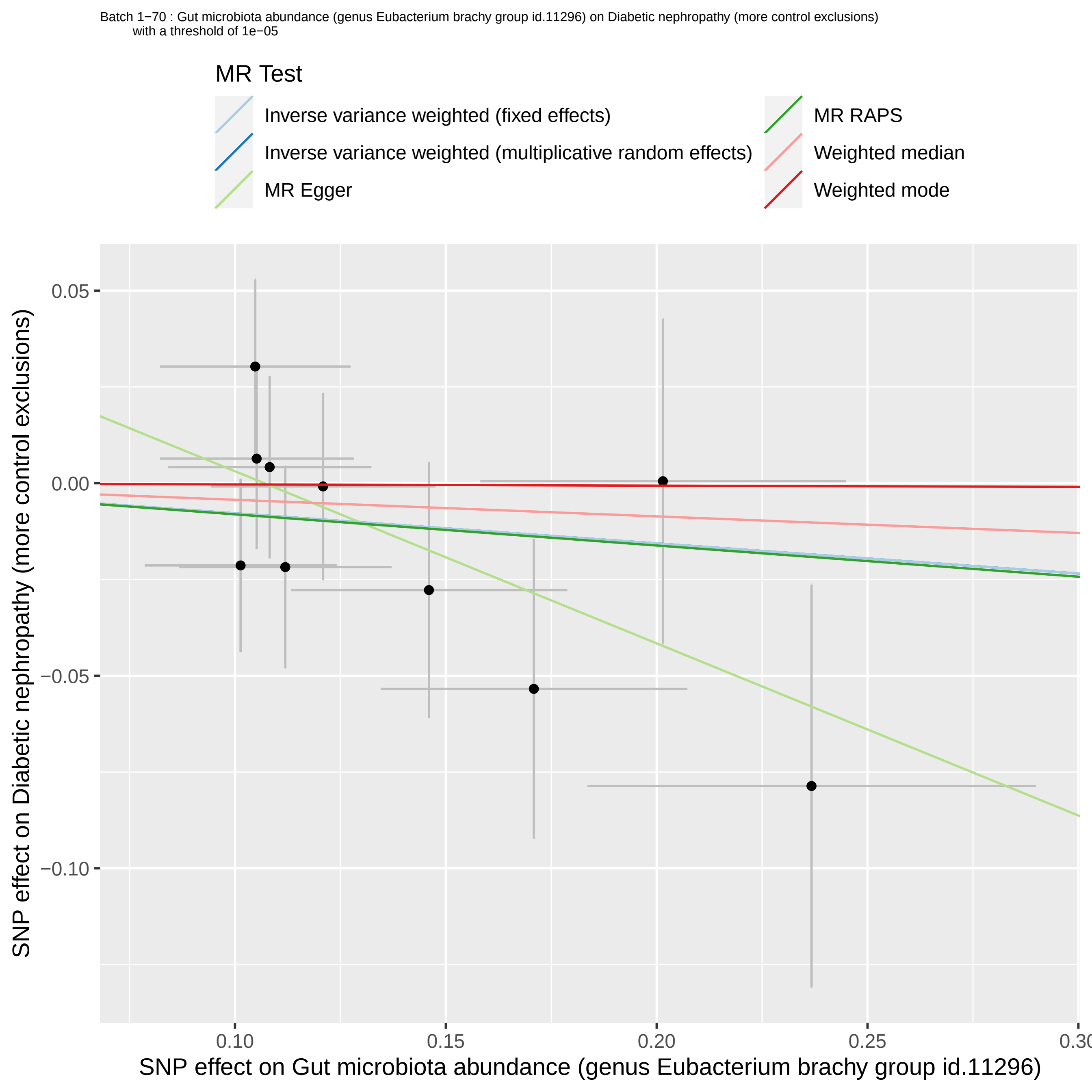

Batch 1−70 : Gut microbiota abundance (genus Eubacterium brachy group id.11296) on Diabetic nephropathy (more control exclusions)
with a threshold of 1e−05
MR Test
Inverse variance weighted (fixed effects)
Inverse variance weighted (multiplicative random effects)
MR Egger
MR RAPS
Weighted median
Weighted mode
0.05
0.00
−0.05
−0.10
0.10
0.15
0.20
0.25
0.30
SNP effect on Diabetic nephropathy (more control exclusions)
SNP effect on Gut microbiota abundance (genus Eubacterium brachy group id.11296)

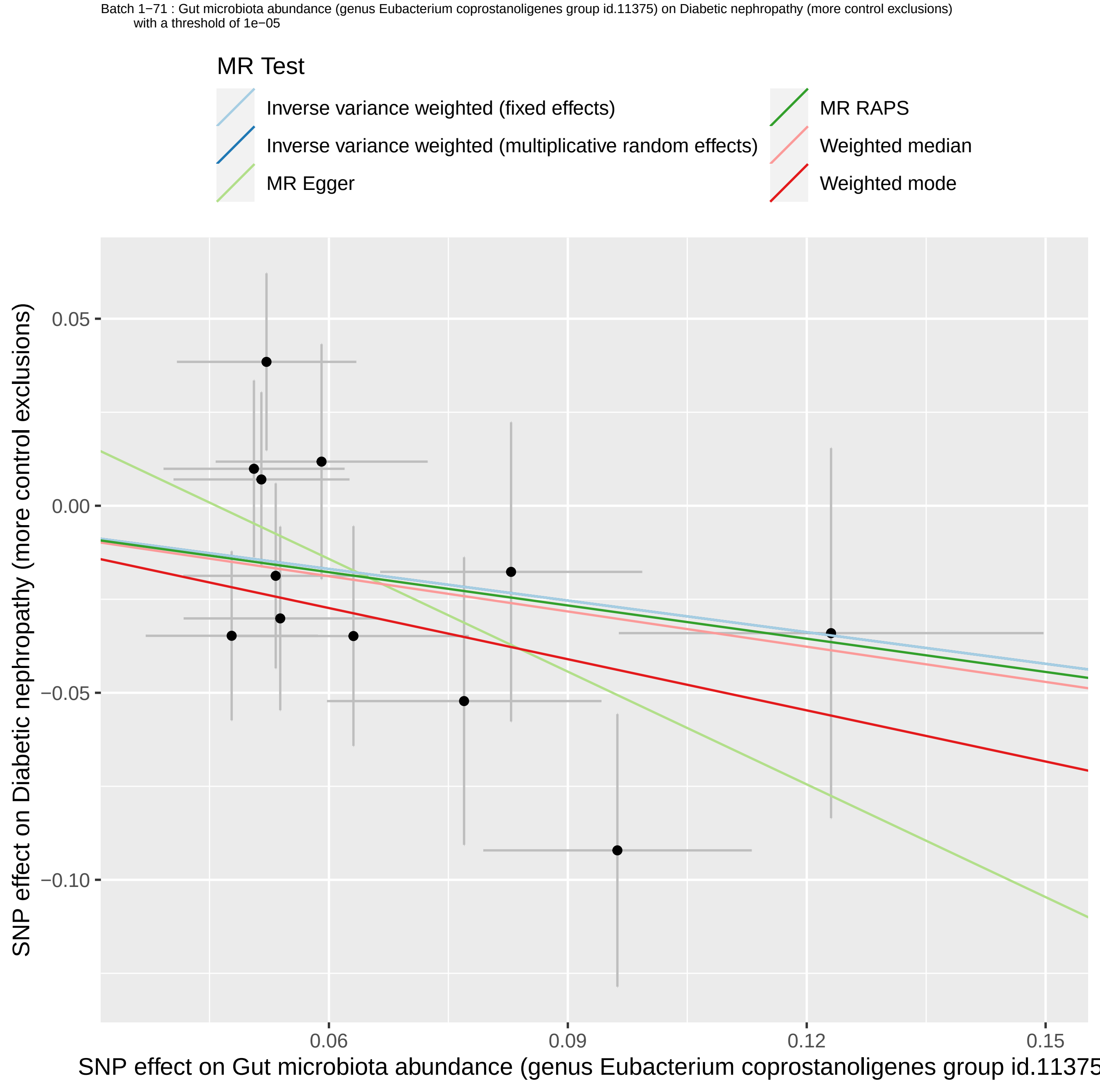

Batch 1−71 : Gut microbiota abundance (genus Eubacterium coprostanoligenes group id.11375) on Diabetic nephropathy (more control exclusions) with a threshold of 1e−05
MR Test
Inverse variance weighted (fixed effects)
Inverse variance weighted (multiplicative random effects)
MR Egger
MR RAPS
Weighted median
Weighted mode
0.05
0.00
−0.05
−0.10
0.06
0.09
0.12
0.15
SNP effect on Diabetic nephropathy (more control exclusions)
SNP effect on Gut microbiota abundance (genus Eubacterium coprostanoligenes group id.11375

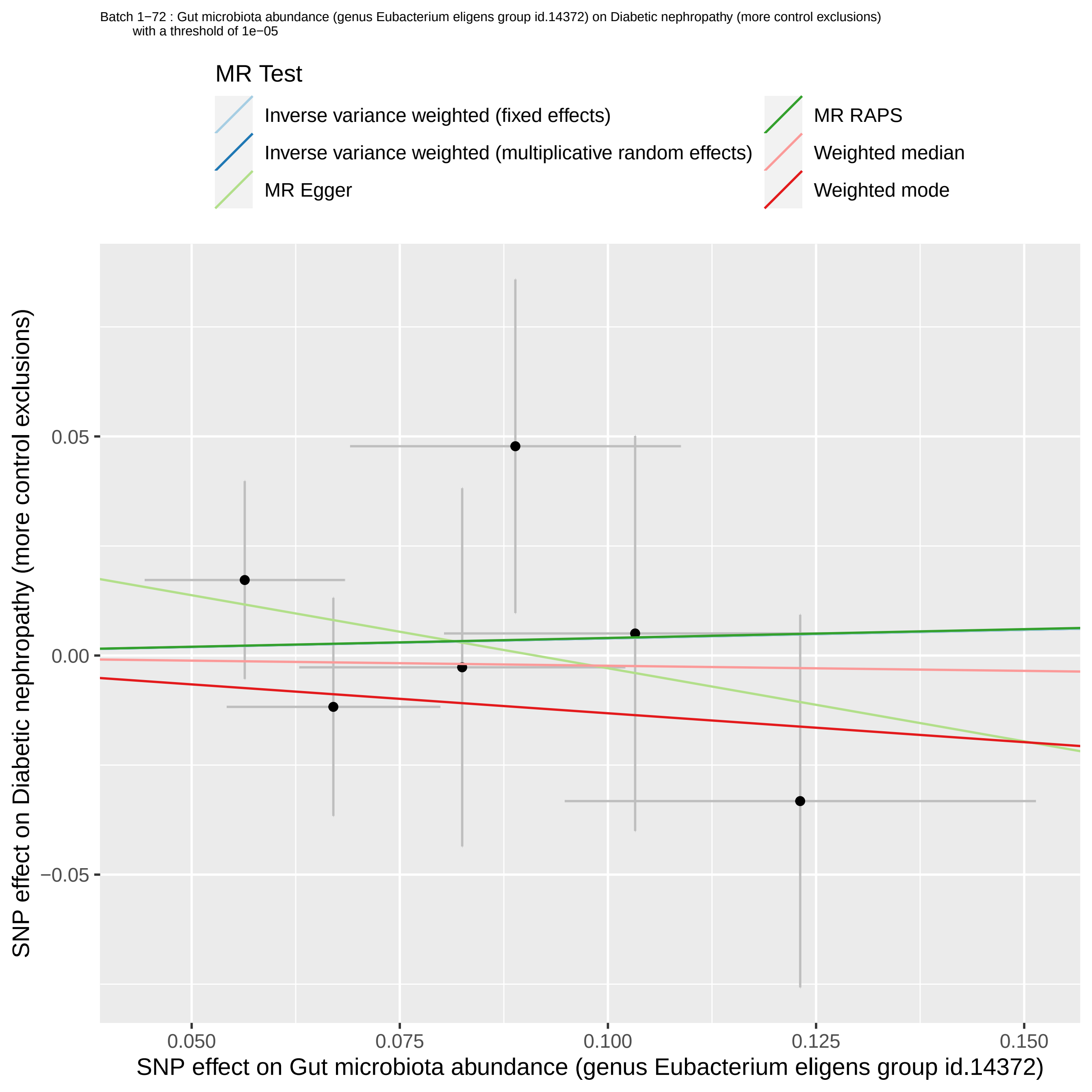

Batch 1−72 : Gut microbiota abundance (genus Eubacterium eligens group id.14372) on Diabetic nephropathy (more control exclusions) with a threshold of 1e−05
MR Test
Inverse variance weighted (fixed effects)
Inverse variance weighted (multiplicative random effects)
MR Egger
MR RAPS
Weighted median
Weighted mode
0.05
0.00
−0.05
0.050
0.075
0.100
0.125
0.150
SNP effect on Diabetic nephropathy (more control exclusions)
SNP effect on Gut microbiota abundance (genus Eubacterium eligens group id.14372)

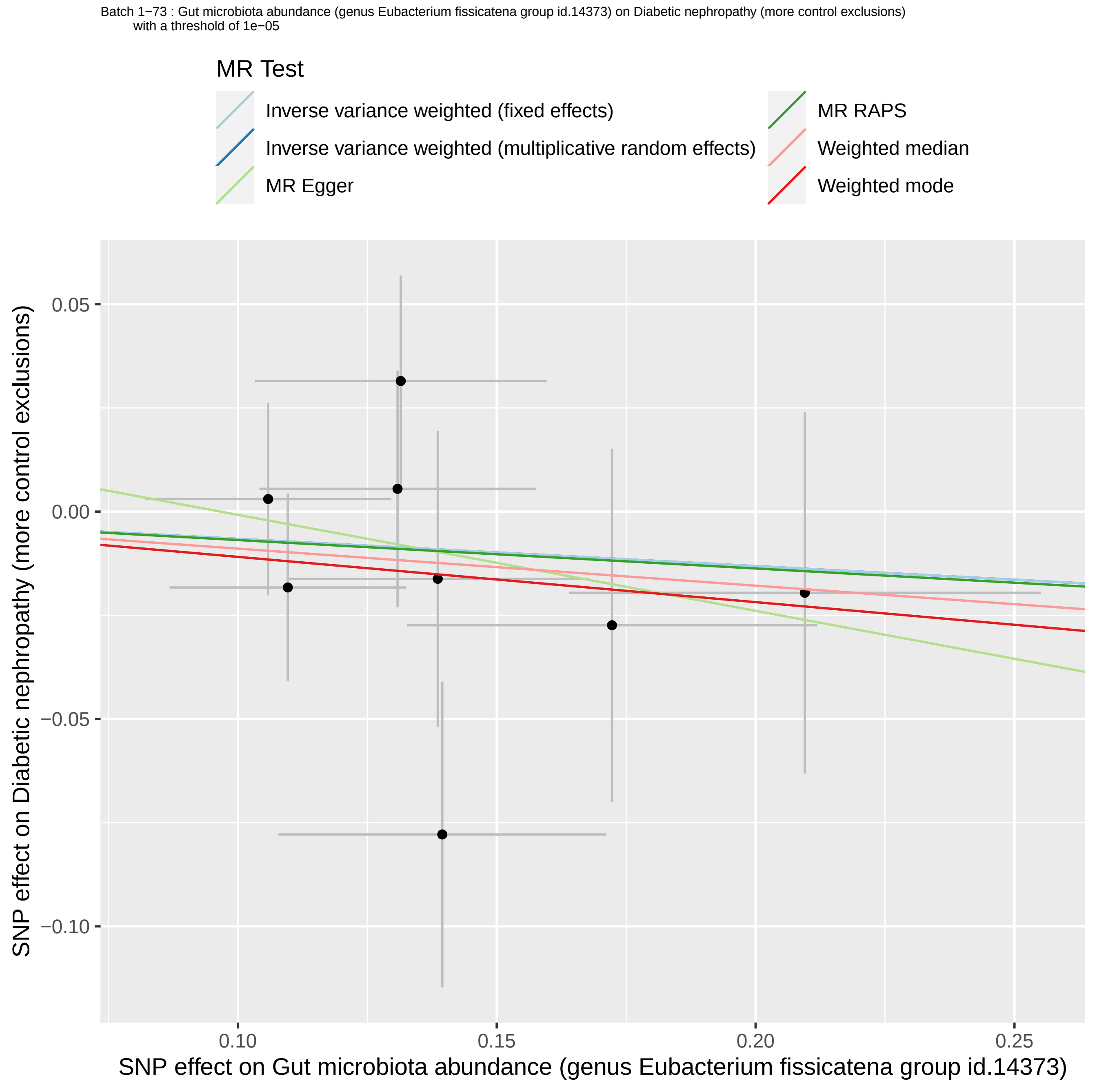
Batch 1−73 : Gut microbiota abundance (genus Eubacterium fissicatena group id.14373) on Diabetic nephropathy (more control exclusions)
with a threshold of 1e−05
MR Test
Inverse variance weighted (fixed effects)
Inverse variance weighted (multiplicative random effects)
MR Egger
MR RAPS
Weighted median
Weighted mode
0.05
0.00
−0.05
−0.10
0.10
0.15
0.20
0.25
SNP effect on Diabetic nephropathy (more control exclusions)
SNP effect on Gut microbiota abundance (genus Eubacterium fissicatena group id.14373)

Batch 1−74 : Gut microbiota abundance (genus Eubacterium hallii group id.11338) on Diabetic nephropathy (more control exclusions) with a threshold of 1e−05

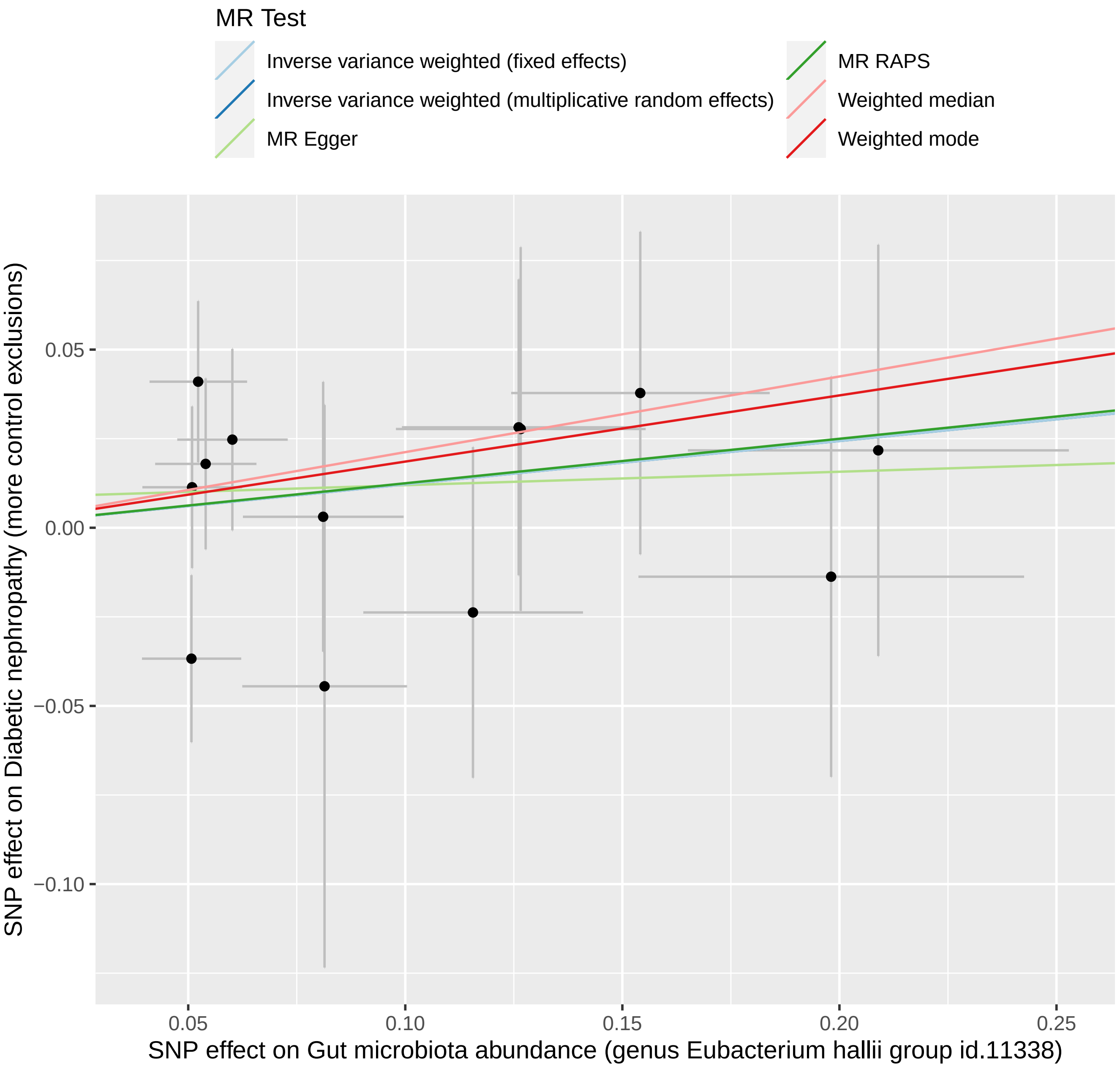

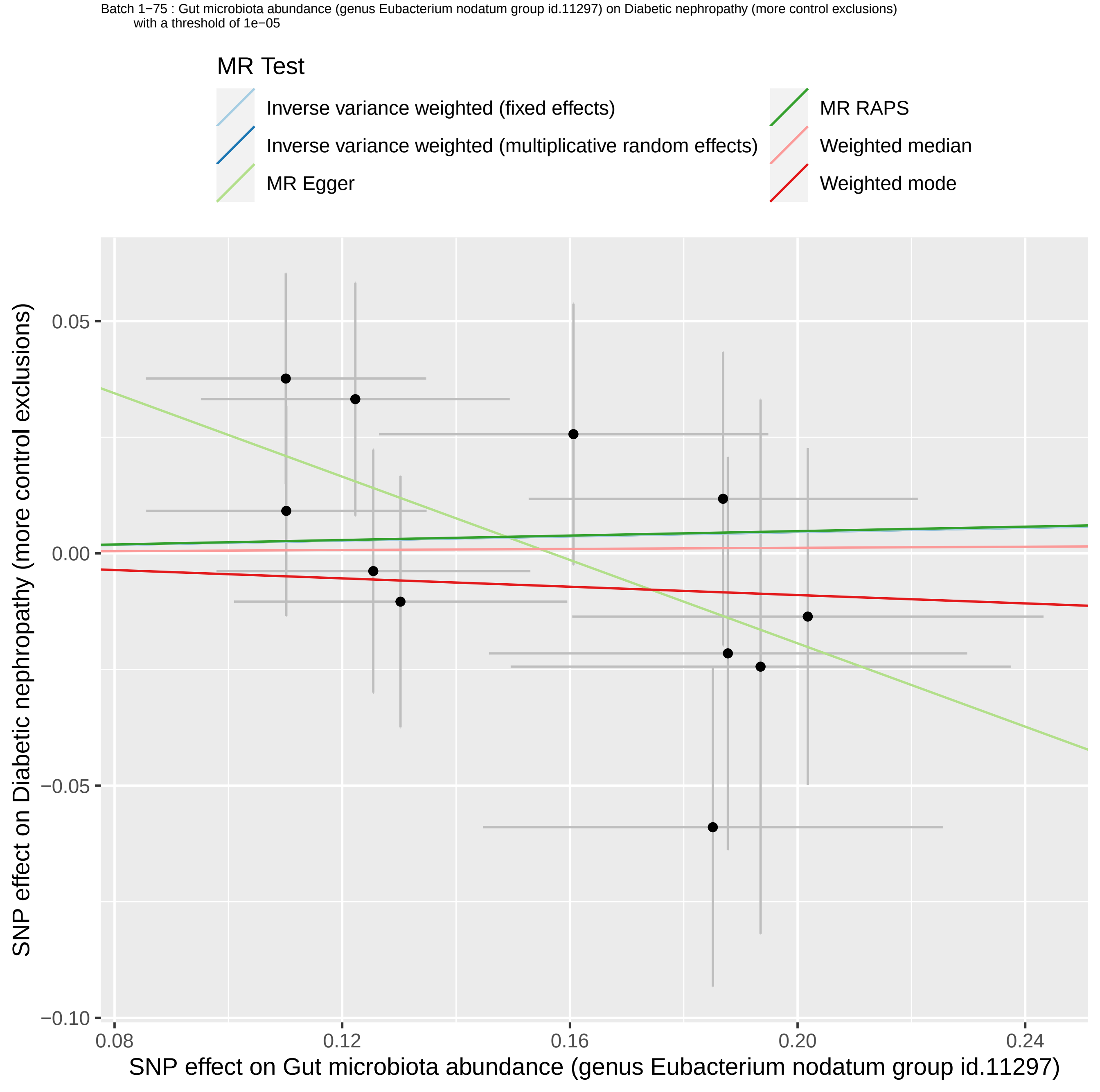

Batch 1−75 : Gut microbiota abundance (genus Eubacterium nodatum group id.11297) on Diabetic nephropathy (more control exclusions) with a threshold of 1e−05
MR Test
Inverse variance weighted (fixed effects)
Inverse variance weighted (multiplicative random effects)
MR Egger
MR RAPS
Weighted median
Weighted mode
0.05
0.00
−0.05
−0.10
0.08
0.12
0.16
0.20
0.24
SNP effect on Diabetic nephropathy (more control exclusions)
SNP effect on Gut microbiota abundance (genus Eubacterium nodatum group id.11297)

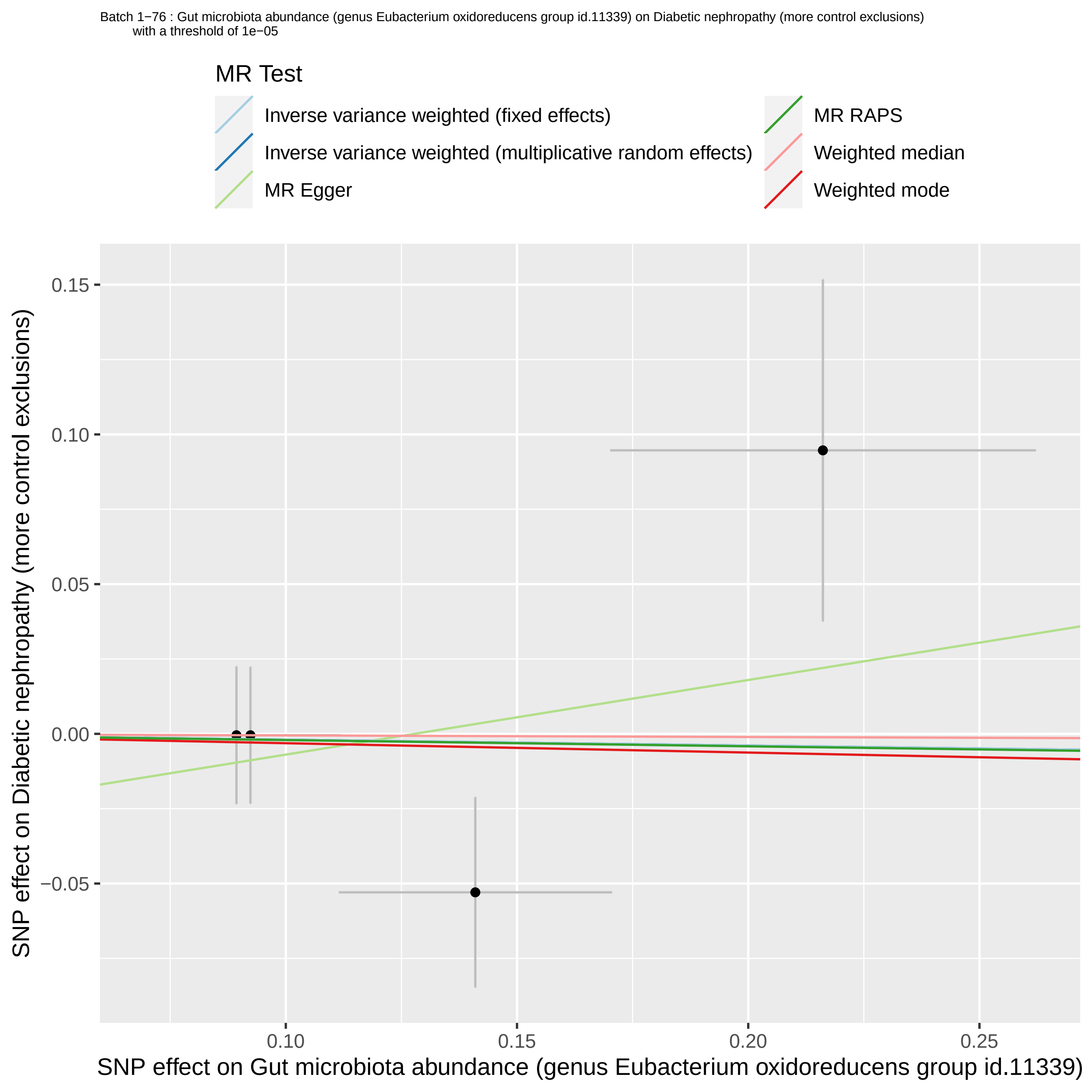

Batch 1−76 : Gut microbiota abundance (genus Eubacterium oxidoreducens group id.11339) on Diabetic nephropathy (more control exclusions)
with a threshold of 1e−05
MR Test
Inverse variance weighted (fixed effects)
Inverse variance weighted (multiplicative random effects)
MR Egger
MR RAPS
Weighted median
Weighted mode
0.15
0.10
0.05
0.00
−0.05
0.10
0.15
0.20
0.25
SNP effect on Diabetic nephropathy (more control exclusions)
SNP effect on Gut microbiota abundance (genus Eubacterium oxidoreducens group id.11339)

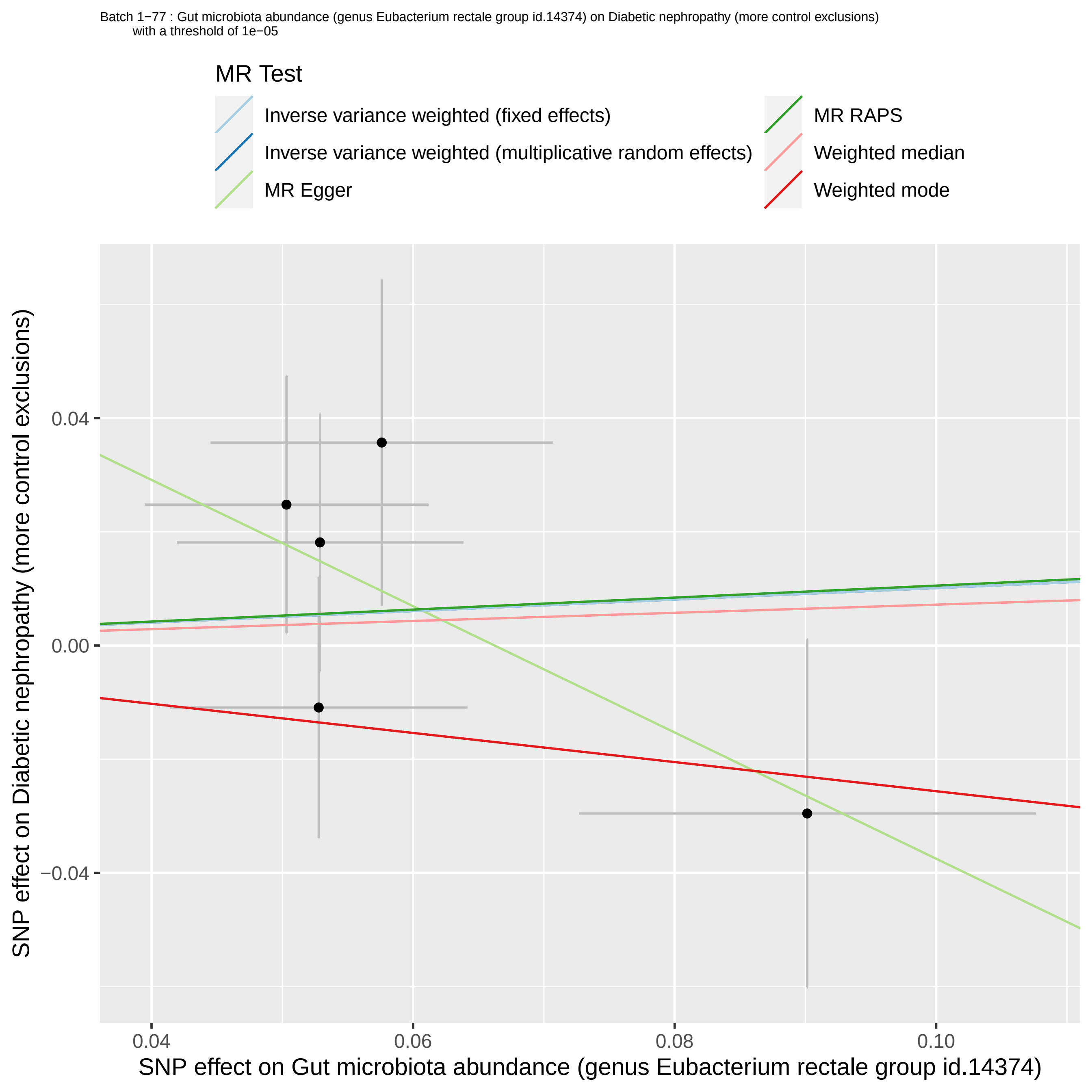

Batch 1−77 : Gut microbiota abundance (genus Eubacterium rectale group id.14374) on Diabetic nephropathy (more control exclusions) with a threshold of 1e−05
MR Test
Inverse variance weighted (fixed effects)
Inverse variance weighted (multiplicative random effects)
MR Egger
MR RAPS
Weighted median
Weighted mode
SNP effect on Diabetic nephropathy (more control exclusions)
0.04
0.00
−0.04
0.04
0.06
0.08
0.10
SNP effect on Gut microbiota abundance (genus Eubacterium rectale group id.14374)

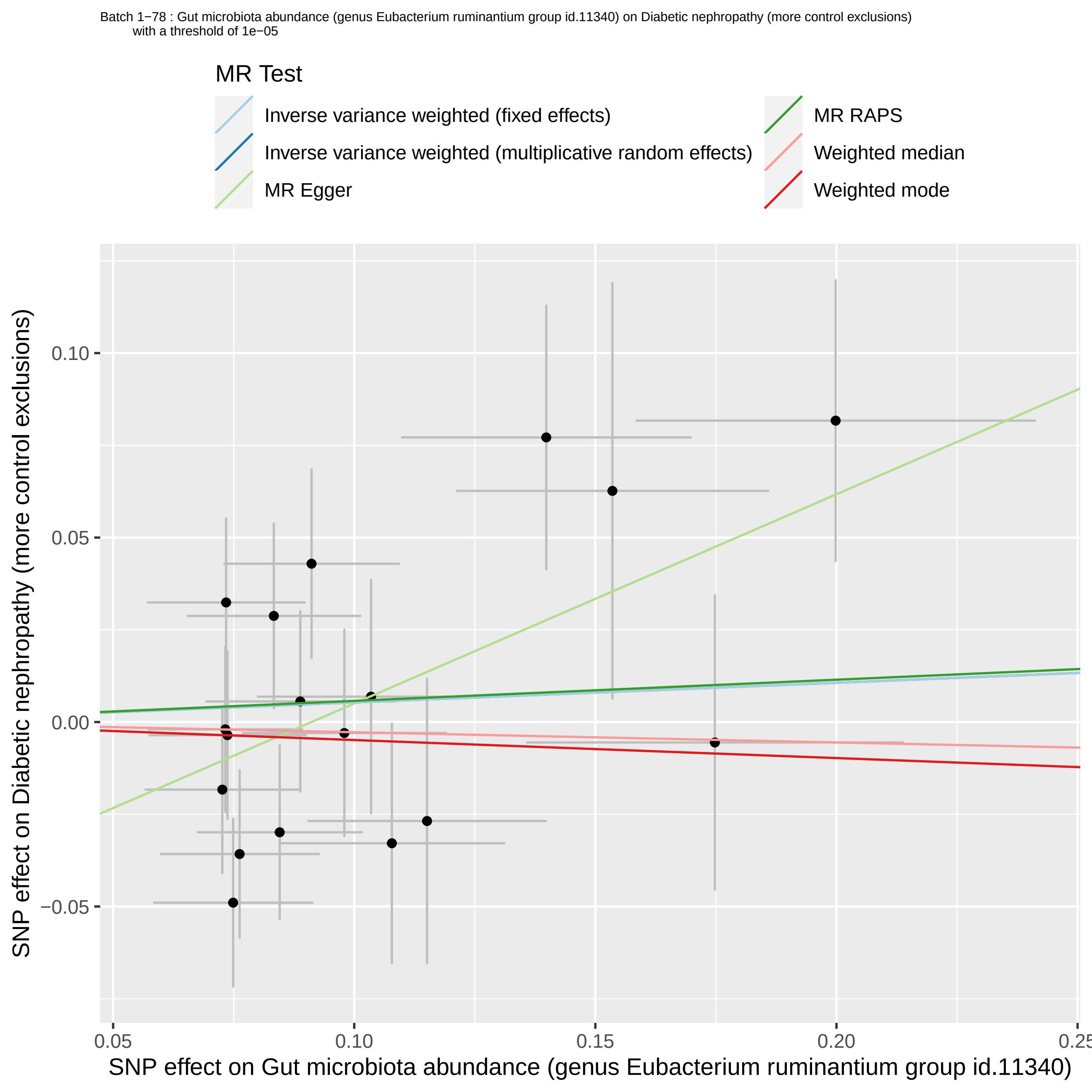

Batch 1−78 : Gut microbiota abundance (genus Eubacterium ruminantium group id.11340) on Diabetic nephropathy (more control exclusions)
with a threshold of 1e−05
MR Test
Inverse variance weighted (fixed effects)
Inverse variance weighted (multiplicative random effects)
MR Egger
MR RAPS
Weighted median
Weighted mode
SNP effect on Diabetic nephropathy (more control exclusions)
0.10
0.05
0.00
−0.05
0.05
0.10
0.15
0.20
0.25
SNP effect on Gut microbiota abundance (genus Eubacterium ruminantium group id.11340)

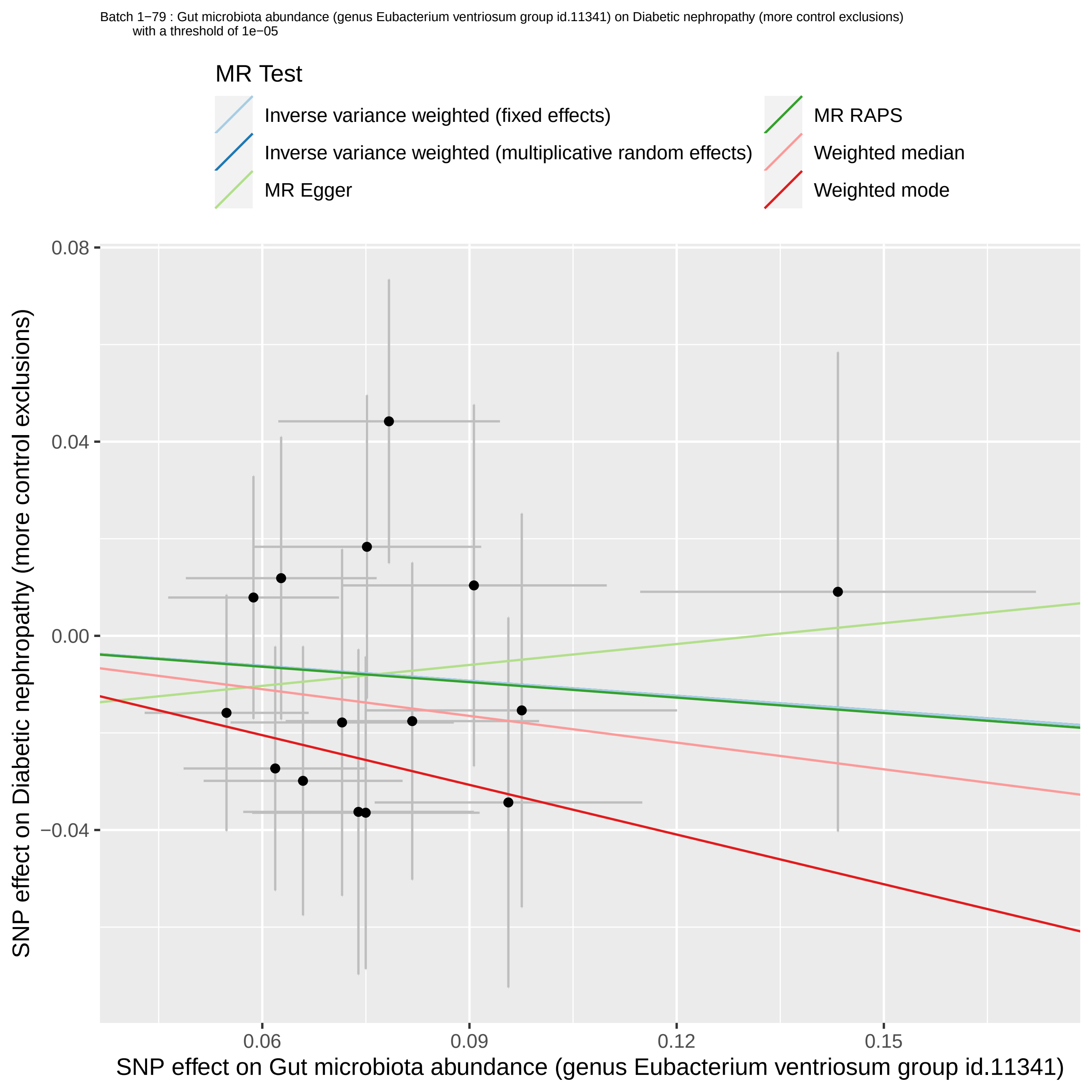

Batch 1−79 : Gut microbiota abundance (genus Eubacterium ventriosum group id.11341) on Diabetic nephropathy (more control exclusions) with a threshold of 1e−05
MR Test
Inverse variance weighted (fixed effects)
Inverse variance weighted (multiplicative random effects)
MR Egger
MR RAPS
Weighted median
Weighted mode
0.08
0.04
0.00
−0.04
0.06
0.09
0.12
0.15
SNP effect on Diabetic nephropathy (more control exclusions)
SNP effect on Gut microbiota abundance (genus Eubacterium ventriosum group id.11341)

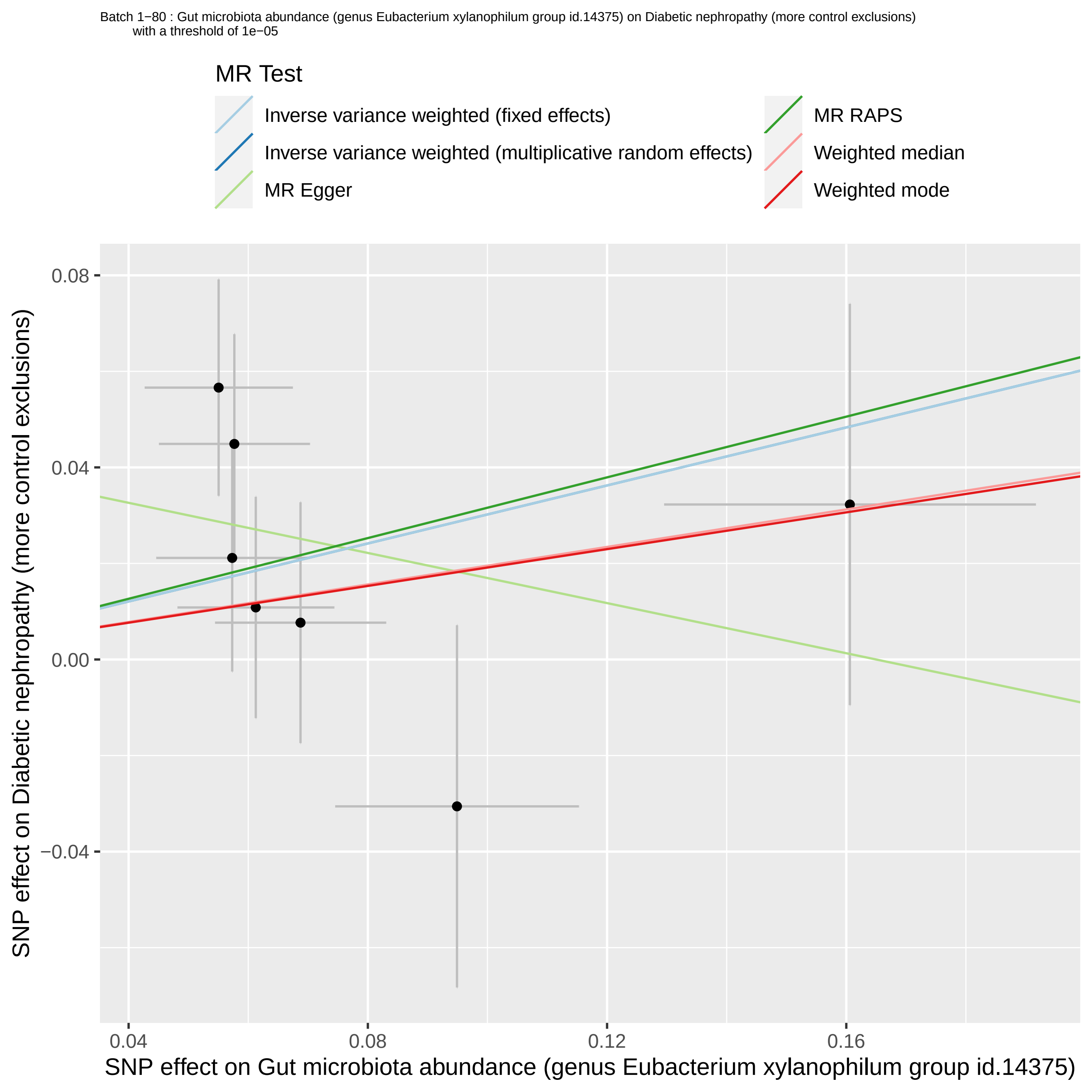
Batch 1−80 : Gut microbiota abundance (genus Eubacterium xylanophilum group id.14375) on Diabetic nephropathy (more control exclusions)
with a threshold of 1e−05
MR Test
Inverse variance weighted (fixed effects)
Inverse variance weighted (multiplicative random effects)
MR Egger
MR RAPS
Weighted median
Weighted mode
0.08
0.04
0.00
−0.04
0.04
0.08
0.12
0.16
SNP effect on Diabetic nephropathy (more control exclusions)
SNP effect on Gut microbiota abundance (genus Eubacterium xylanophilum group id.14375)

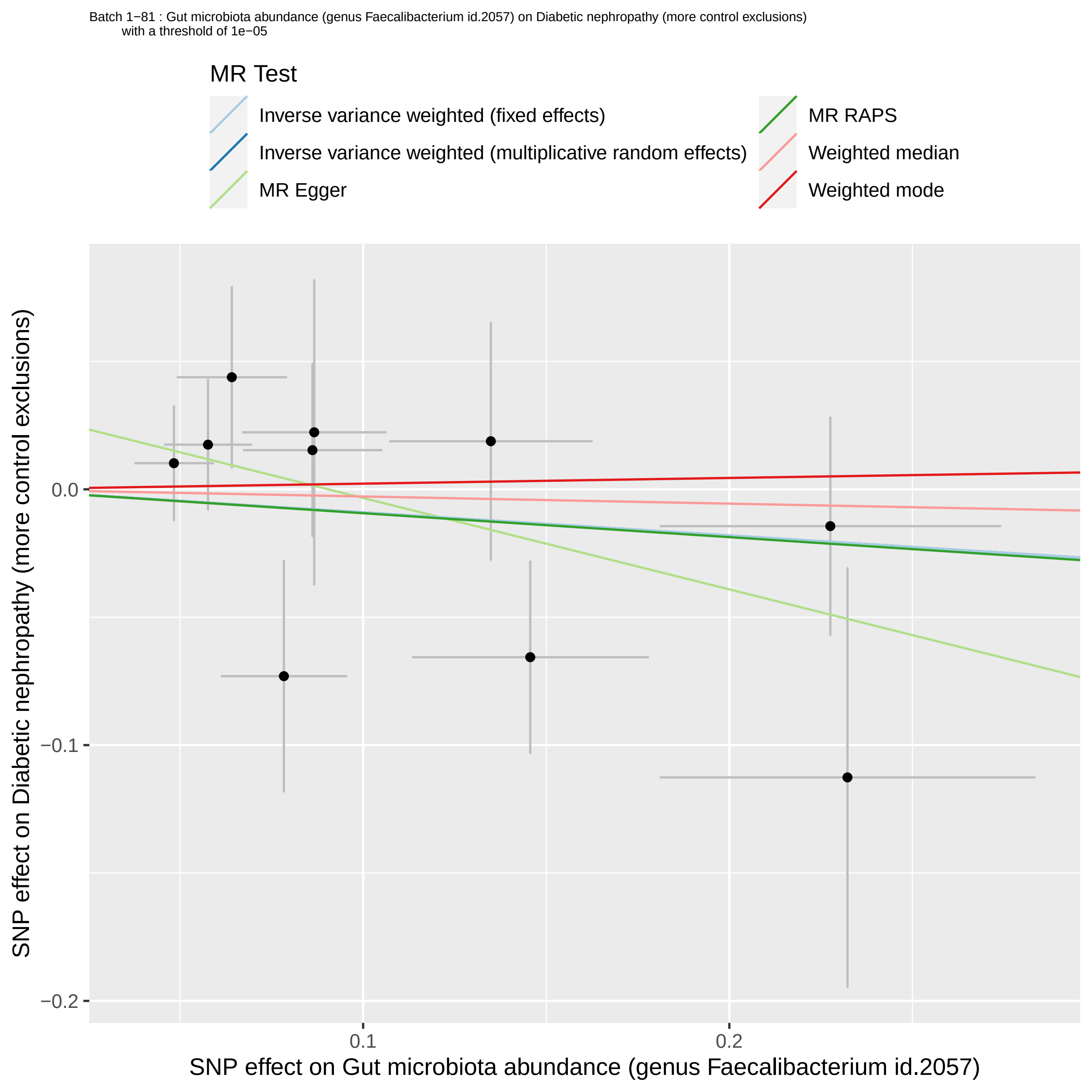

Batch 1−81 : Gut microbiota abundance (genus Faecalibacterium id.2057) on Diabetic nephropathy (more control exclusions)
with a threshold of 1e−05
MR Test
Inverse variance weighted (fixed effects)
Inverse variance weighted (multiplicative random effects)
MR Egger
MR RAPS
Weighted median
Weighted mode
SNP effect on Diabetic nephropathy (more control exclusions)
0.0
−0.1
−0.2
0.1
0.2
SNP effect on Gut microbiota abundance (genus Faecalibacterium id.2057)

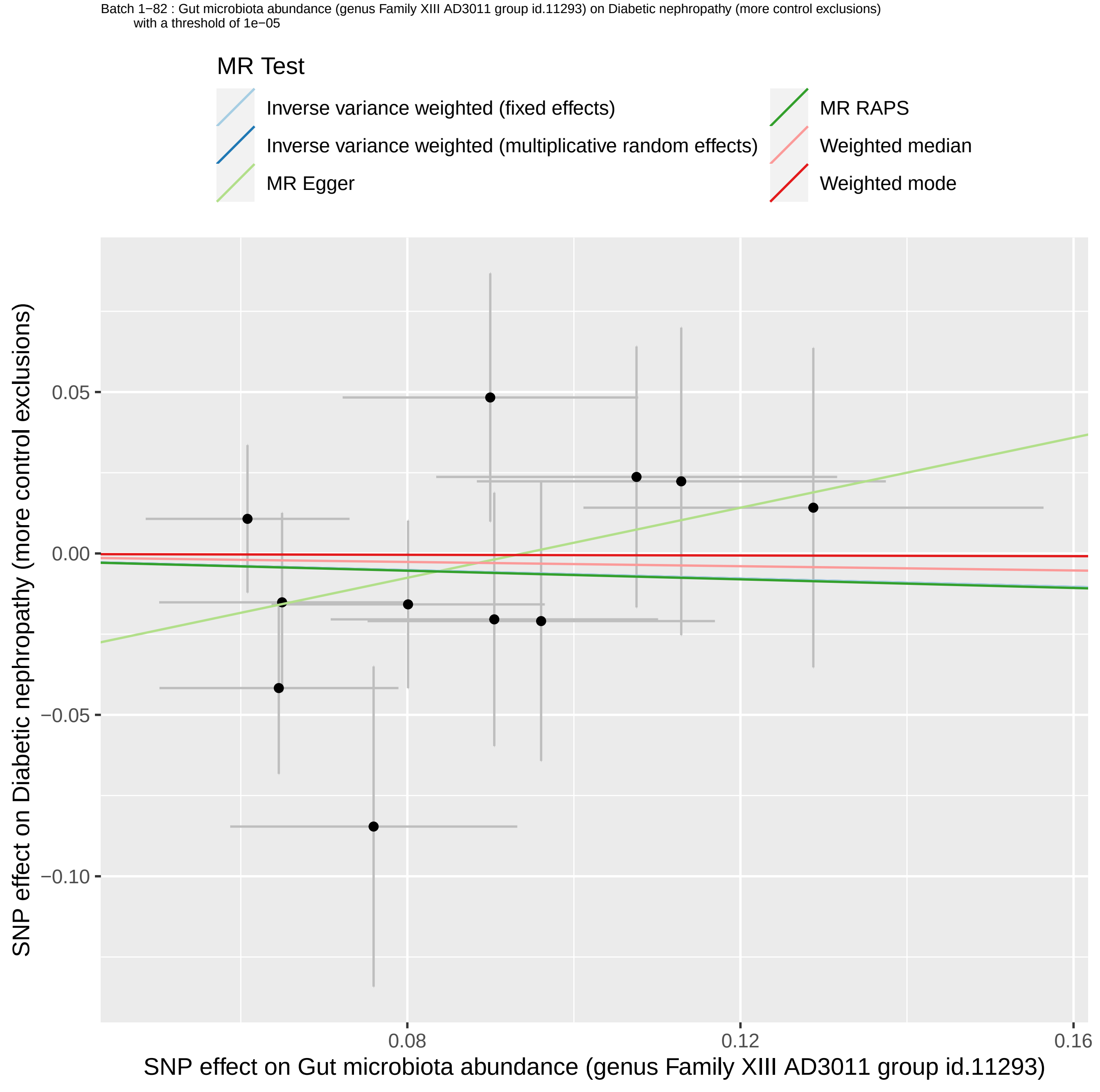
Batch 1−82 : Gut microbiota abundance (genus Family XIII AD3011 group id.11293) on Diabetic nephropathy (more control exclusions) with a threshold of 1e−05
MR Test
Inverse variance weighted (fixed effects)
Inverse variance weighted (multiplicative random effects)
MR Egger
MR RAPS
Weighted median
Weighted mode
0.05
0.00
−0.05
−0.10
0.08
0.12
0.16
SNP effect on Diabetic nephropathy (more control exclusions)
SNP effect on Gut microbiota abundance (genus Family XIII AD3011 group id.11293)

Batch 1−83 : Gut microbiota abundance (genus Family XIII UCG001 id.11294) on Diabetic nephropathy (more control exclusions) with a threshold of 1e−05

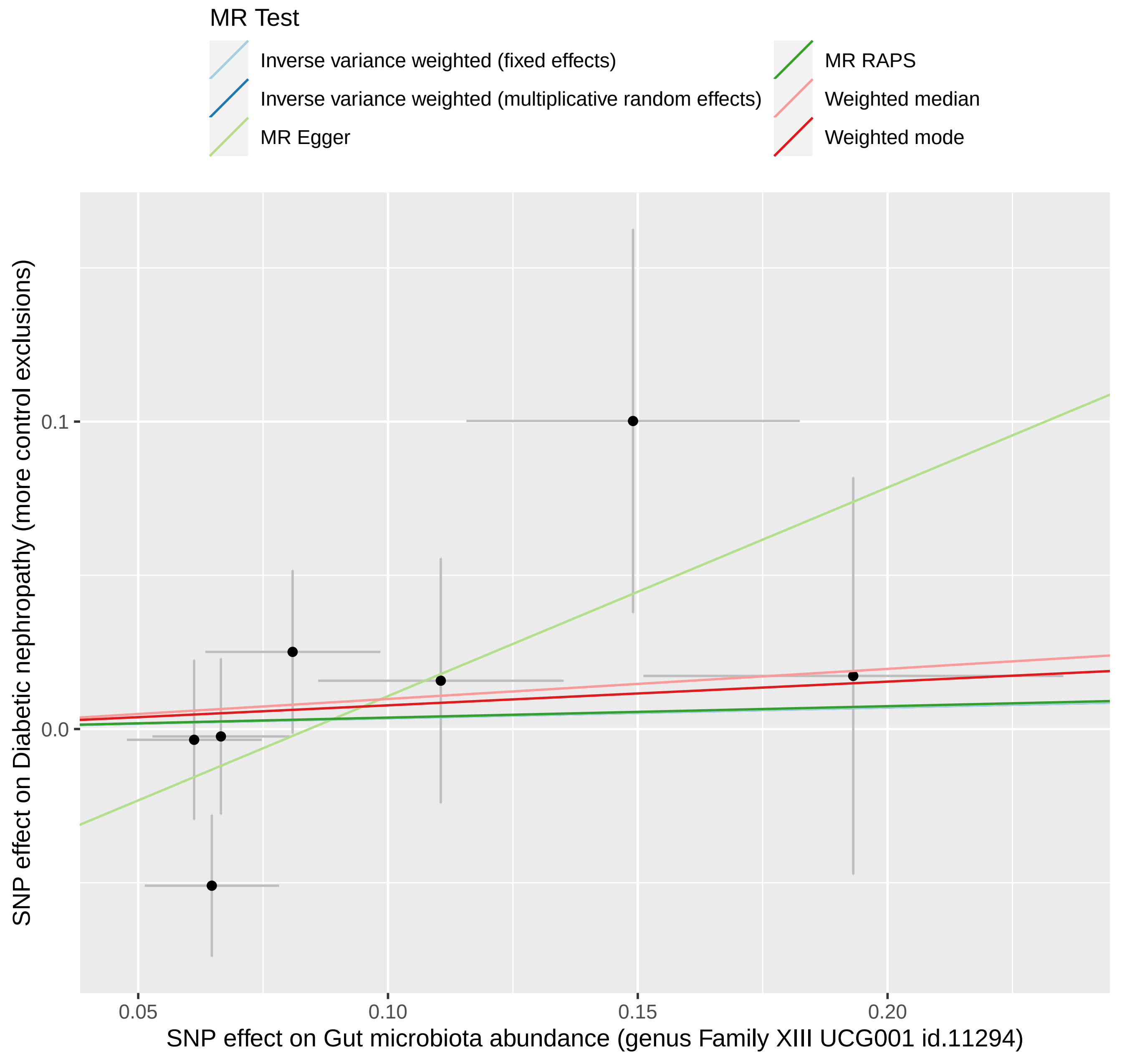

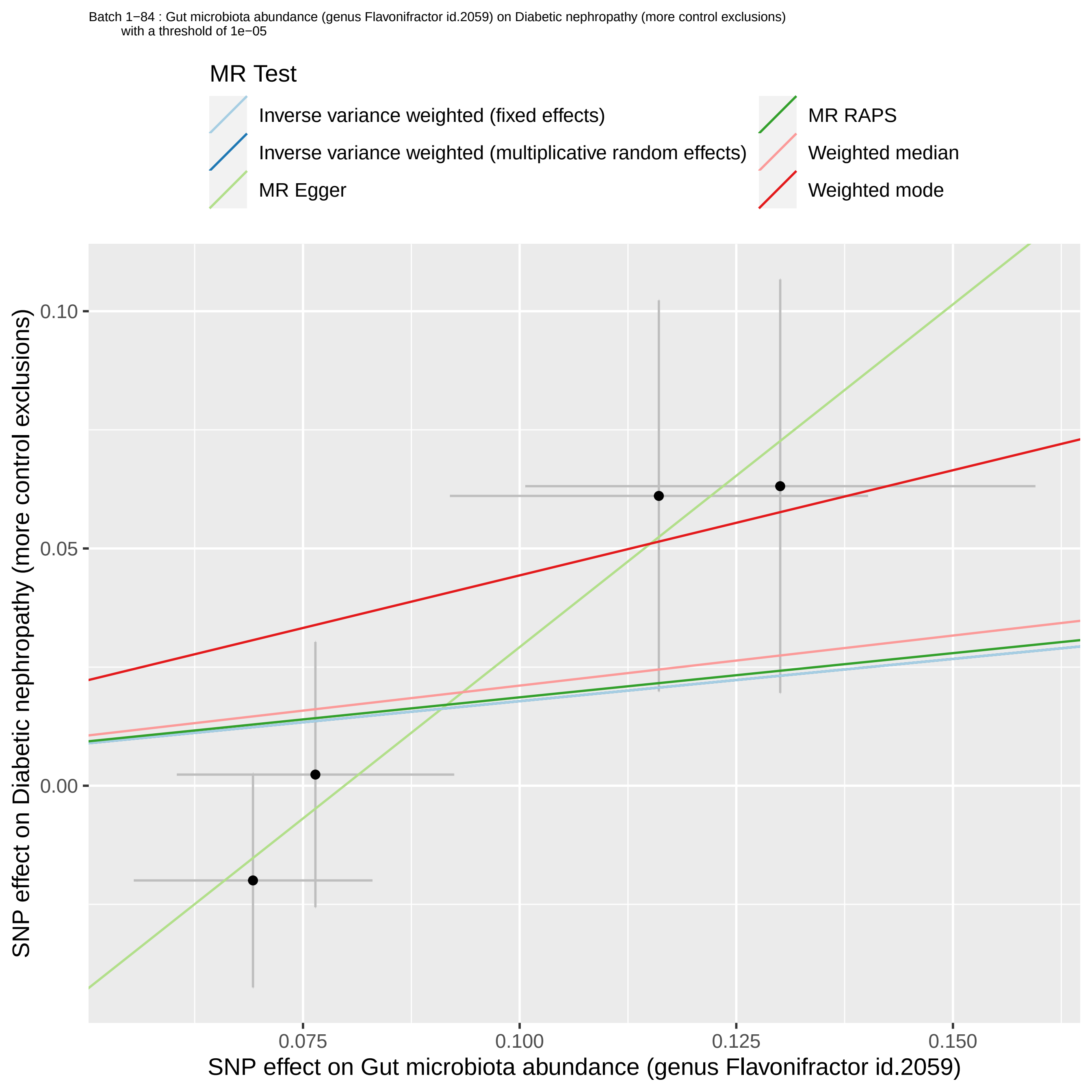
Batch 1−84 : Gut microbiota abundance (genus Flavonifractor id.2059) on Diabetic nephropathy (more control exclusions)
with a threshold of 1e−05
MR Test
Inverse variance weighted (fixed effects)
Inverse variance weighted (multiplicative random effects)
MR Egger
MR RAPS
Weighted median
Weighted mode
0.10
0.05
0.00
0.075
0.100
0.125
0.150
SNP effect on Diabetic nephropathy (more control exclusions)
SNP effect on Gut microbiota abundance (genus Flavonifractor id.2059)

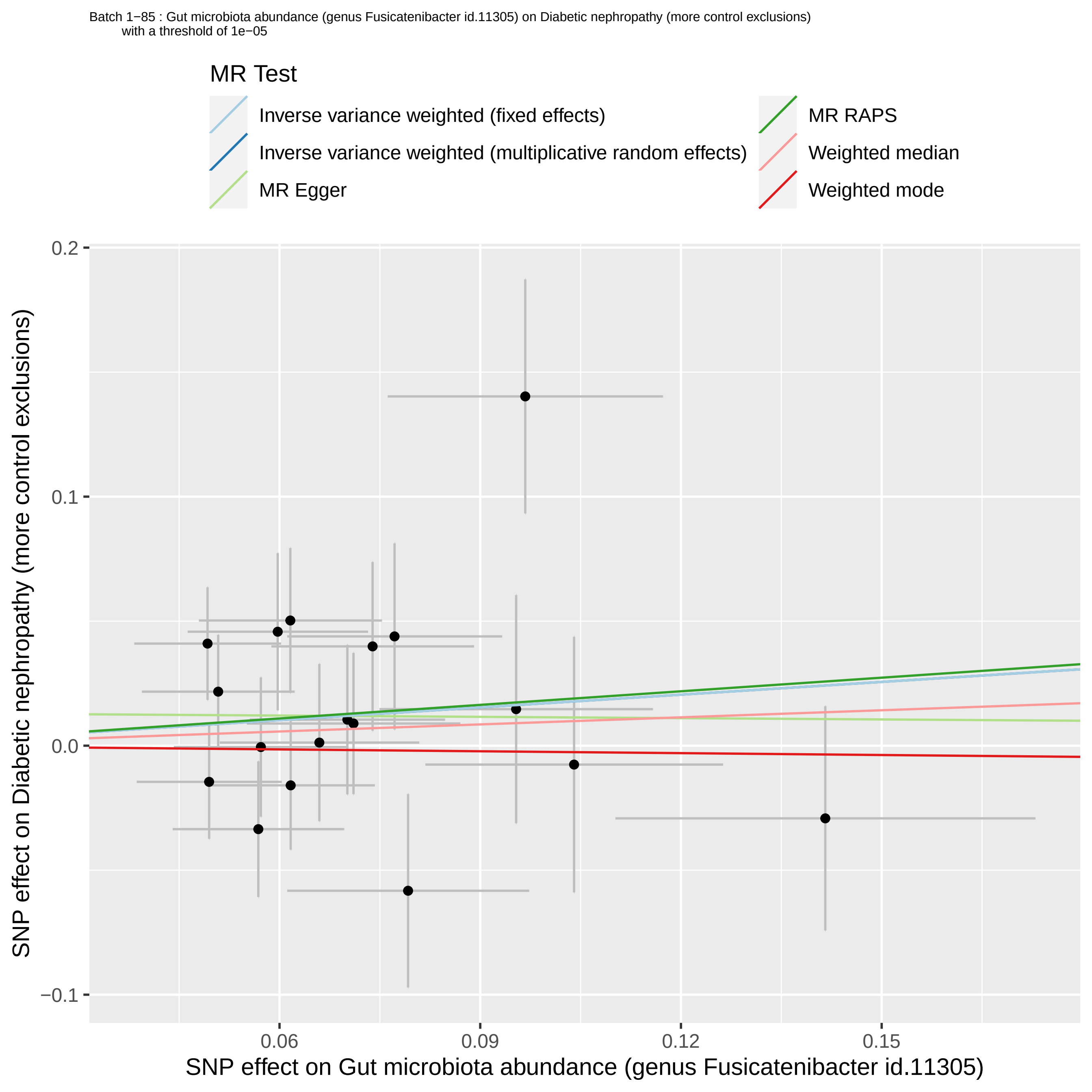

Batch 1−85 : Gut microbiota abundance (genus Fusicatenibacter id.11305) on Diabetic nephropathy (more control exclusions)
with a threshold of 1e−05
MR Test
Inverse variance weighted (fixed effects)
Inverse variance weighted (multiplicative random effects)
MR Egger
MR RAPS
Weighted median
Weighted mode
0.2
0.1
0.0
−0.1
0.06
0.09
0.12
0.15
SNP effect on Diabetic nephropathy (more control exclusions)
SNP effect on Gut microbiota abundance (genus Fusicatenibacter id.11305)

Batch 1−86 : Gut microbiota abundance (genus Gordonibacter id.821) on Diabetic nephropathy (more control exclusions) with a threshold of 1e−05

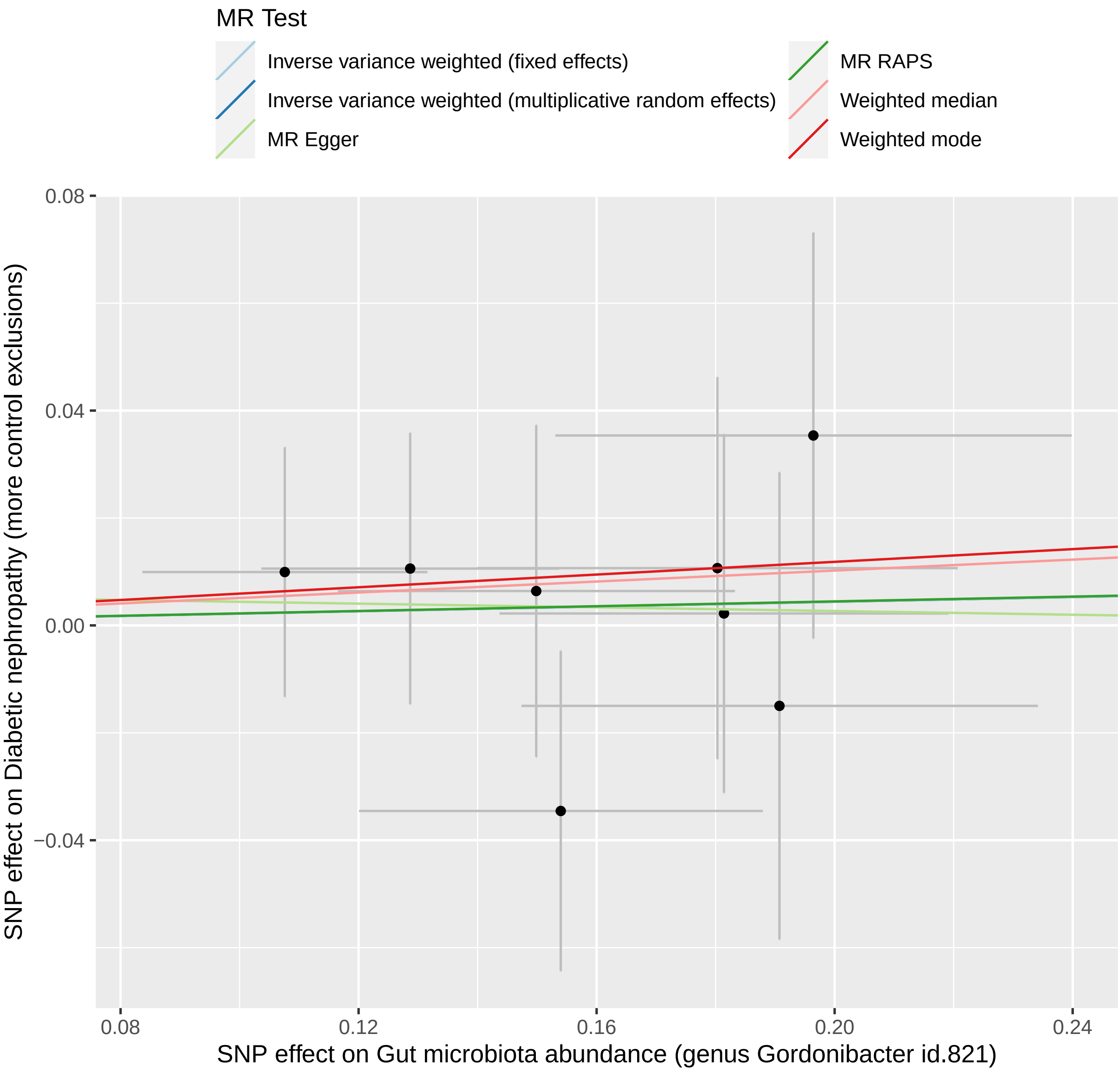

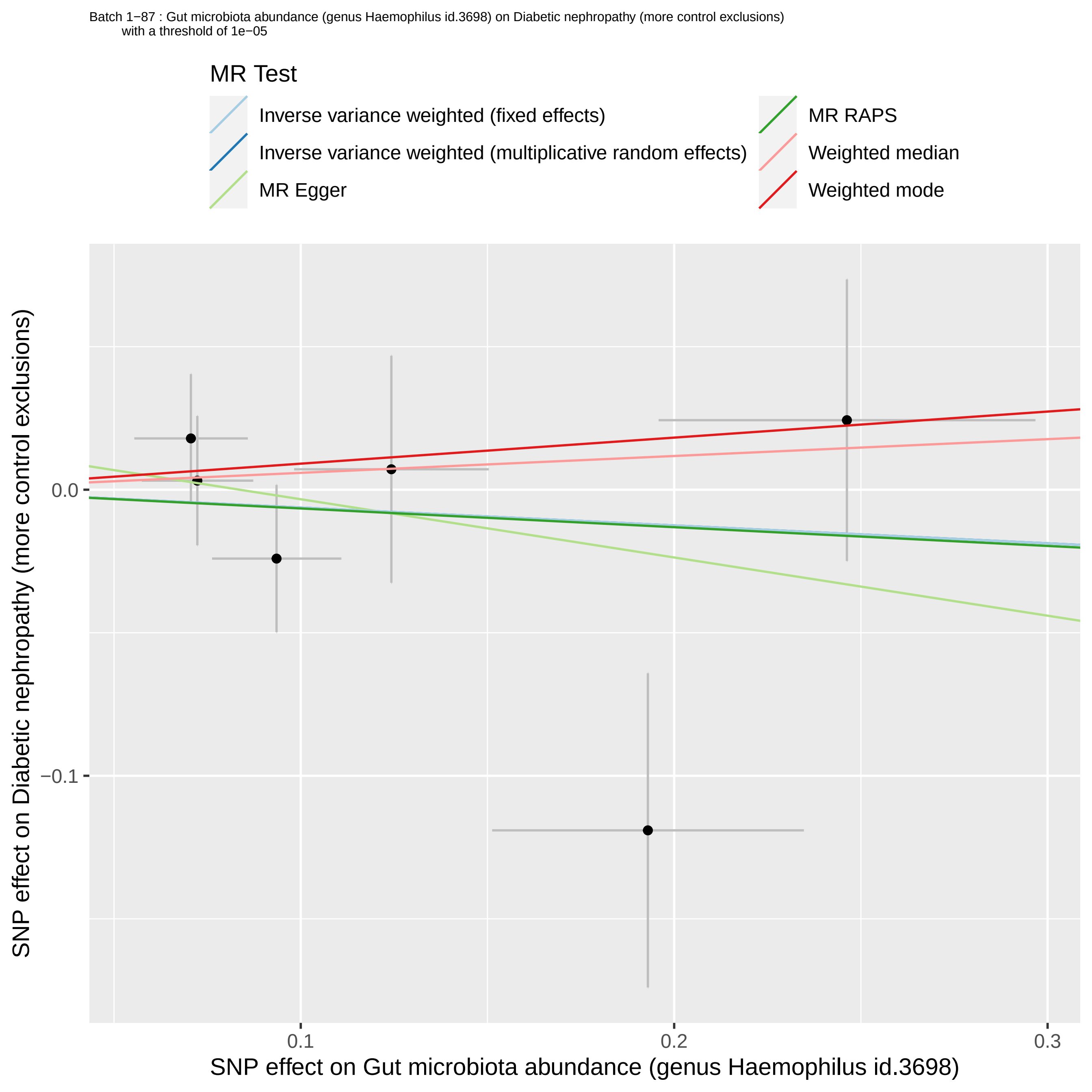
Batch 1−87 : Gut microbiota abundance (genus Haemophilus id.3698) on Diabetic nephropathy (more control exclusions)
with a threshold of 1e−05
MR Test
Inverse variance weighted (fixed effects)
Inverse variance weighted (multiplicative random effects)
MR Egger
MR RAPS
Weighted median
Weighted mode
0.0
−0.1
0.1
0.2
0.3
SNP effect on Diabetic nephropathy (more control exclusions)
SNP effect on Gut microbiota abundance (genus Haemophilus id.3698)

Batch 1−88 : Gut microbiota abundance (genus Holdemanella id.11393) on Diabetic nephropathy (more control exclusions) with a threshold of 1e−05

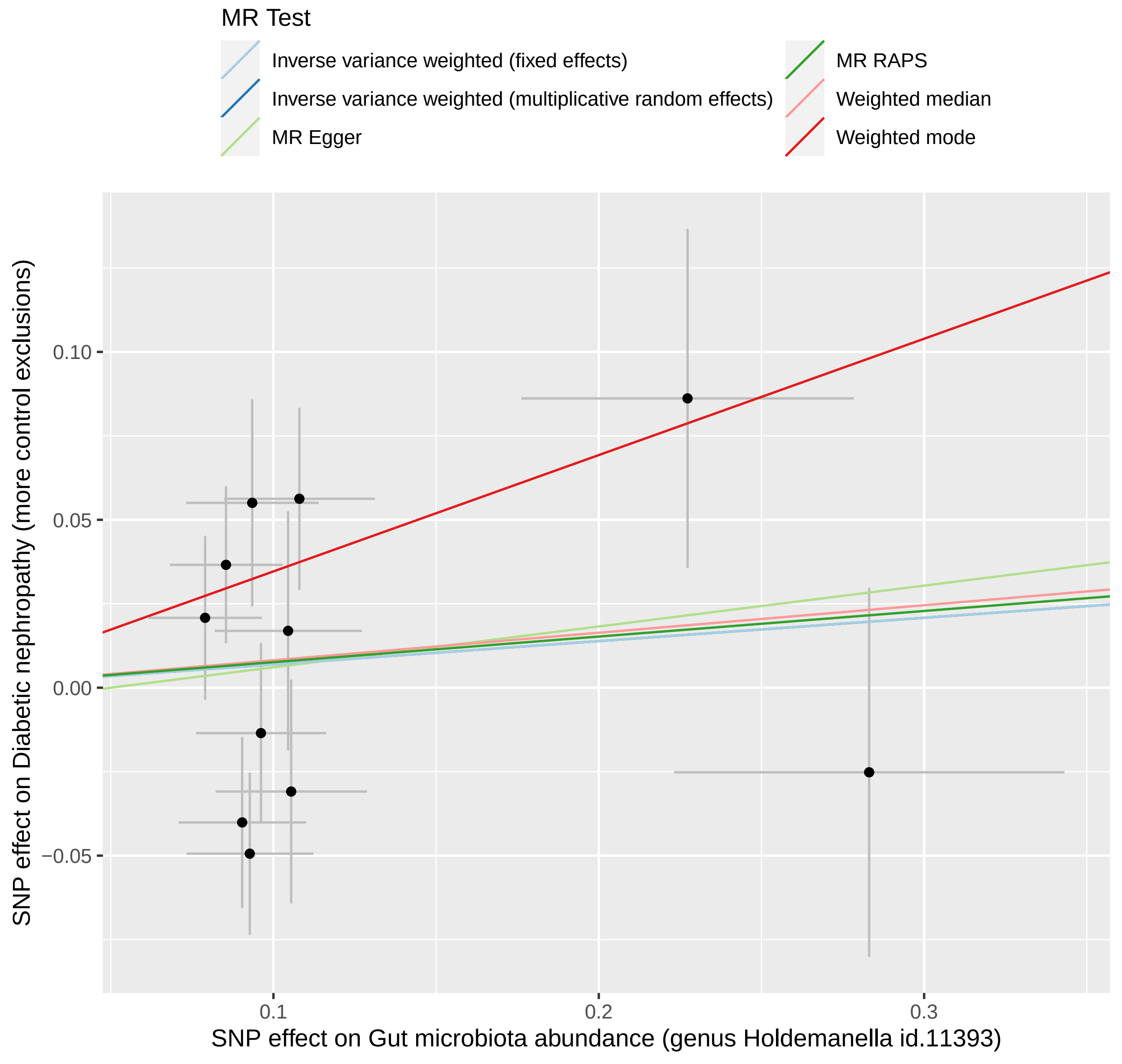

Batch 1−89 : Gut microbiota abundance (genus Holdemania id.2157) on Diabetic nephropathy (more control exclusions)
with a threshold of 1e−05

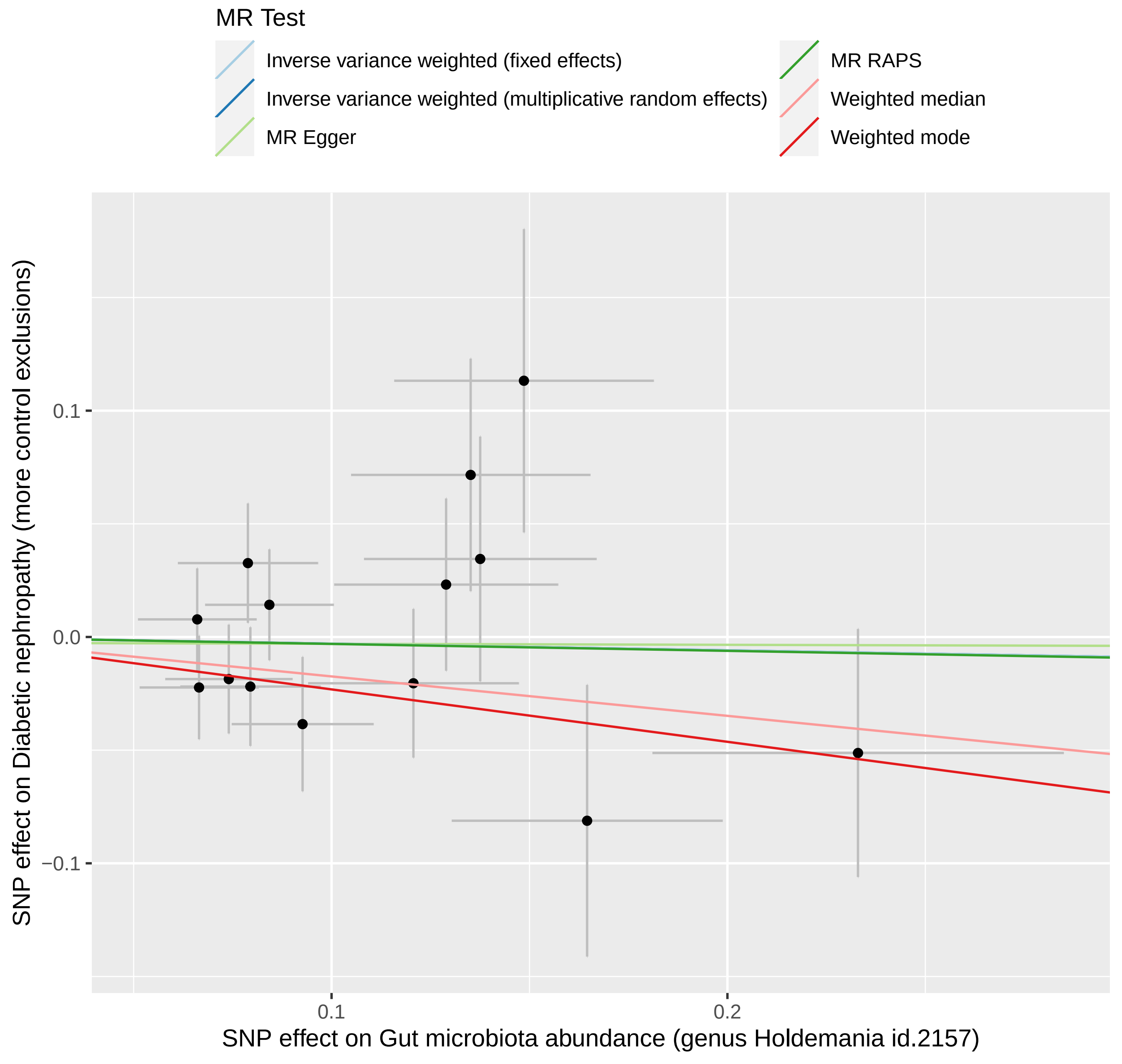

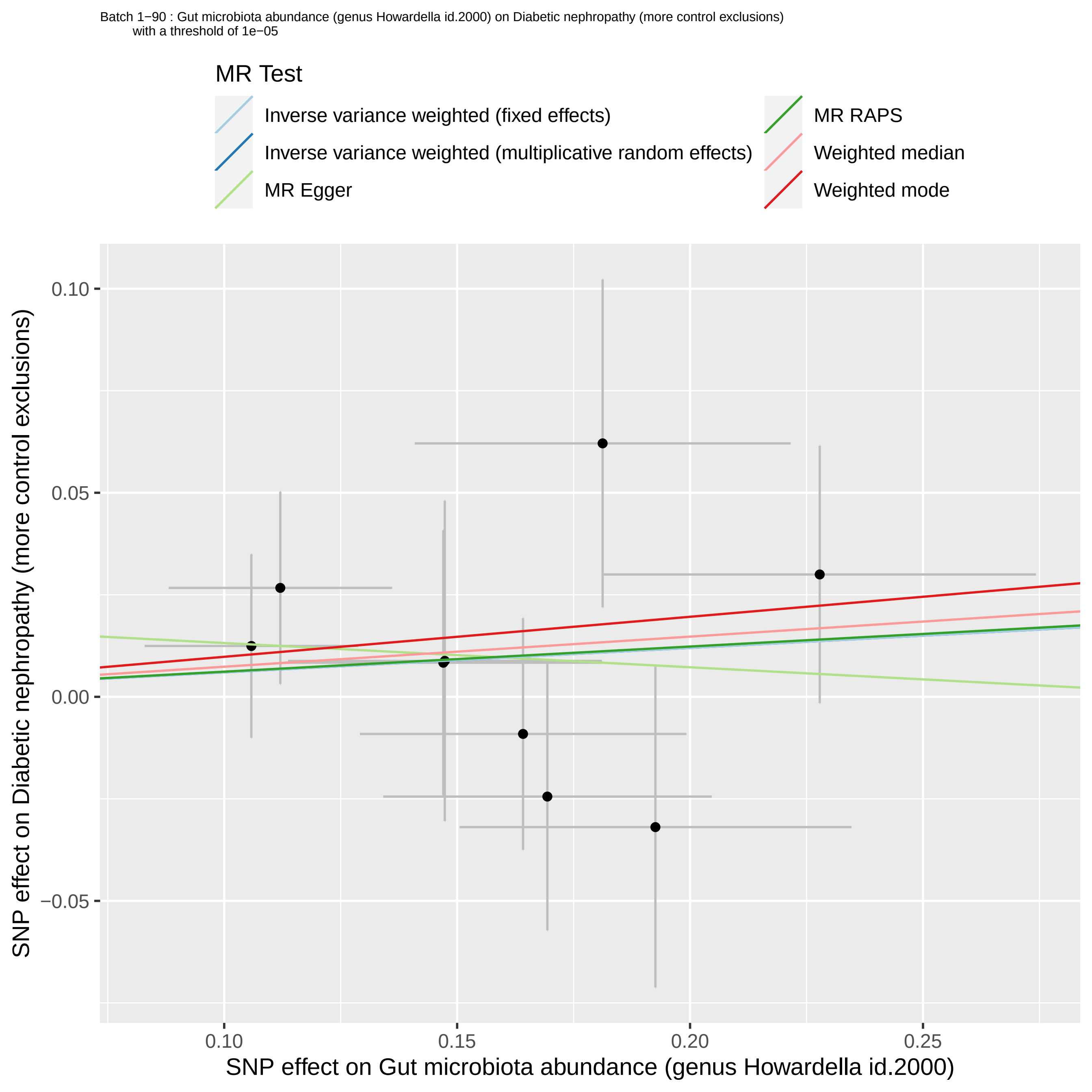

Batch 1−90 : Gut microbiota abundance (genus Howardella id.2000) on Diabetic nephropathy (more control exclusions)
with a threshold of 1e−05
MR Test
Inverse variance weighted (fixed effects)
Inverse variance weighted (multiplicative random effects)
MR Egger
MR RAPS
Weighted median
Weighted mode
0.10
0.05
0.00
−0.05
0.10
0.15
0.20
0.25
SNP effect on Diabetic nephropathy (more control exclusions)
SNP effect on Gut microbiota abundance (genus Howardella id.2000)

Batch 1−91 : Gut microbiota abundance (genus Hungatella id.11306) on Diabetic nephropathy (more control exclusions) with a threshold of 1e−05

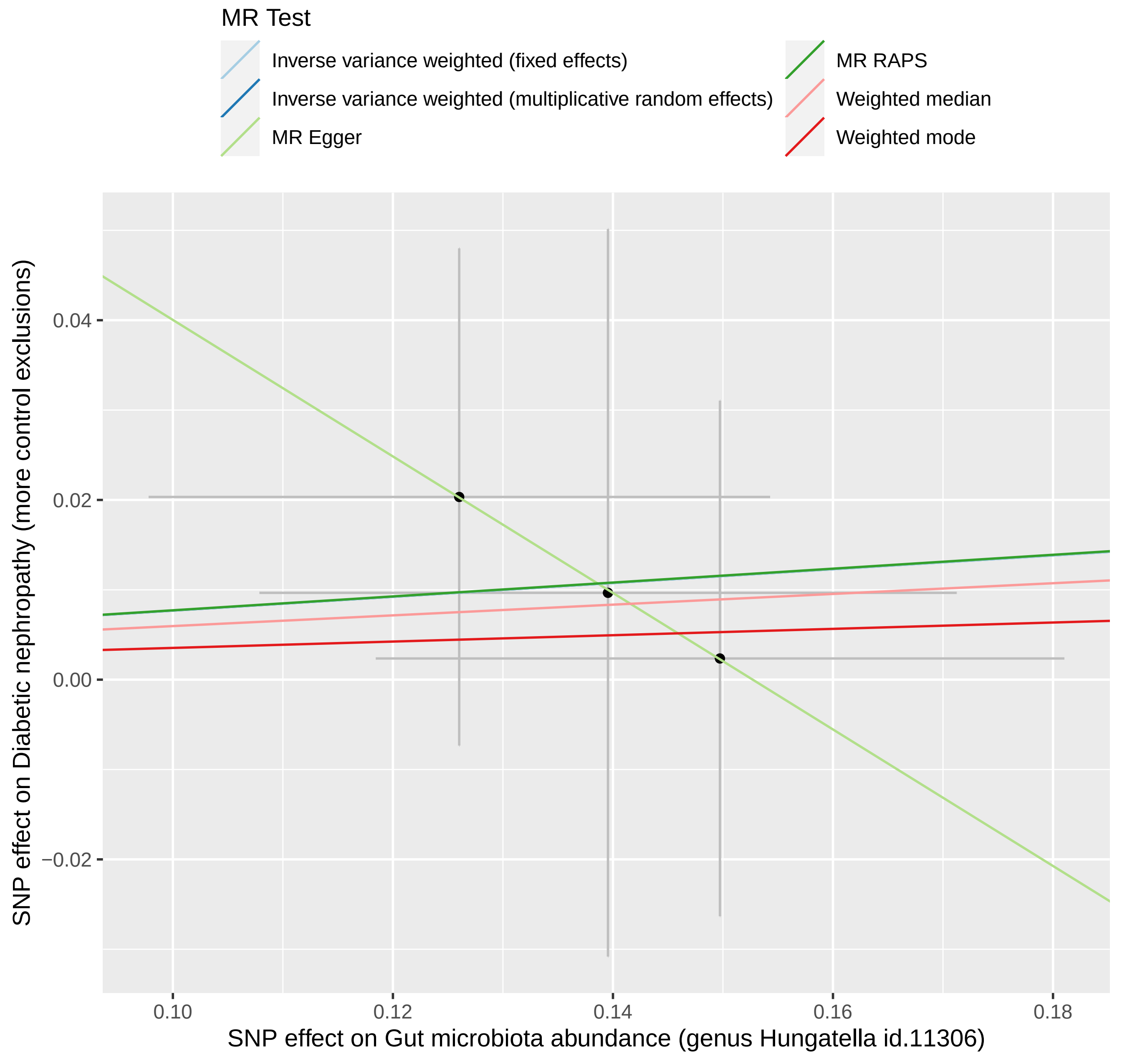

Batch 1−92 : Gut microbiota abundance (genus Intestinibacter id.11345) on Diabetic nephropathy (more control exclusions)
with a threshold of 1e−05

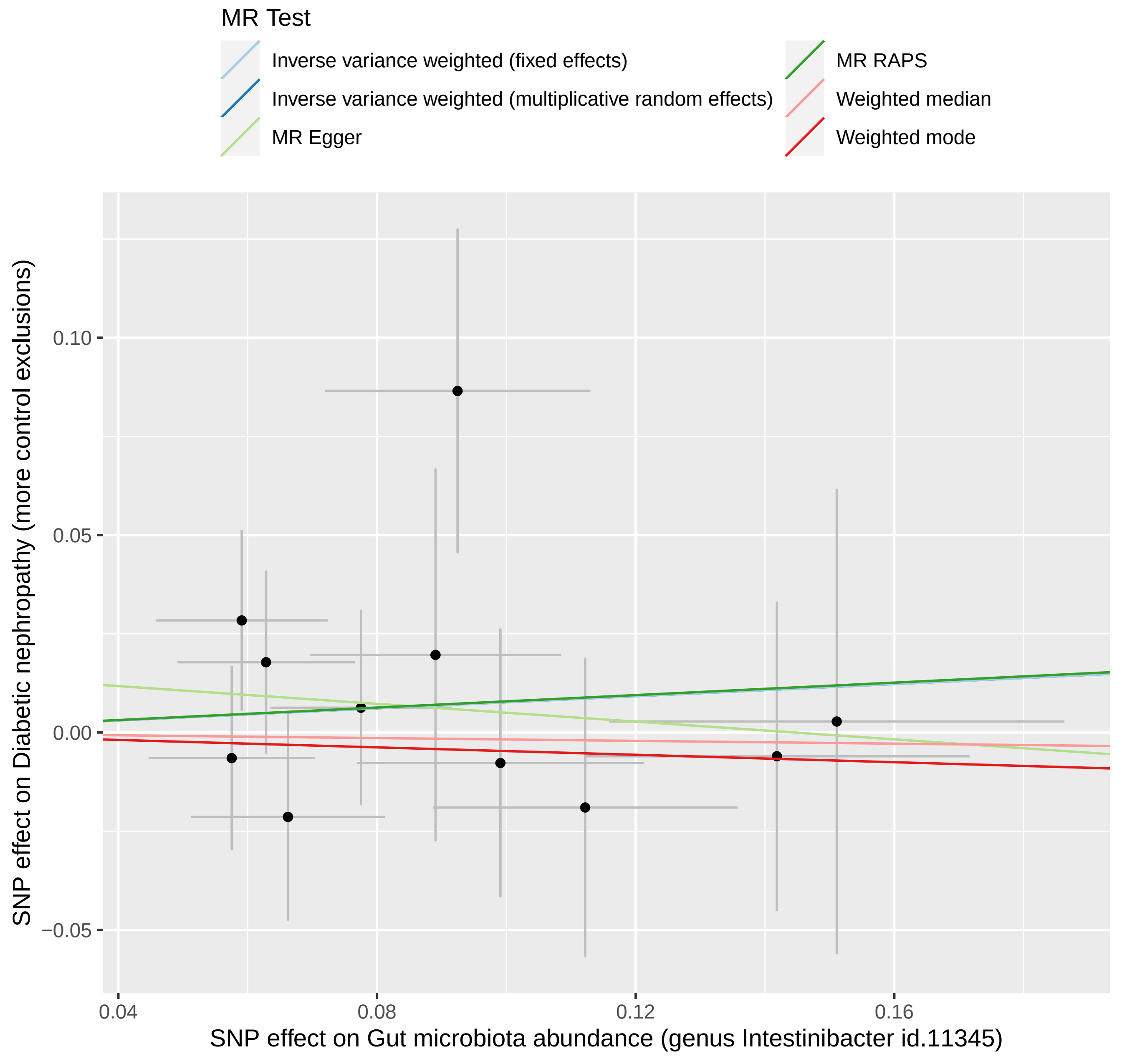

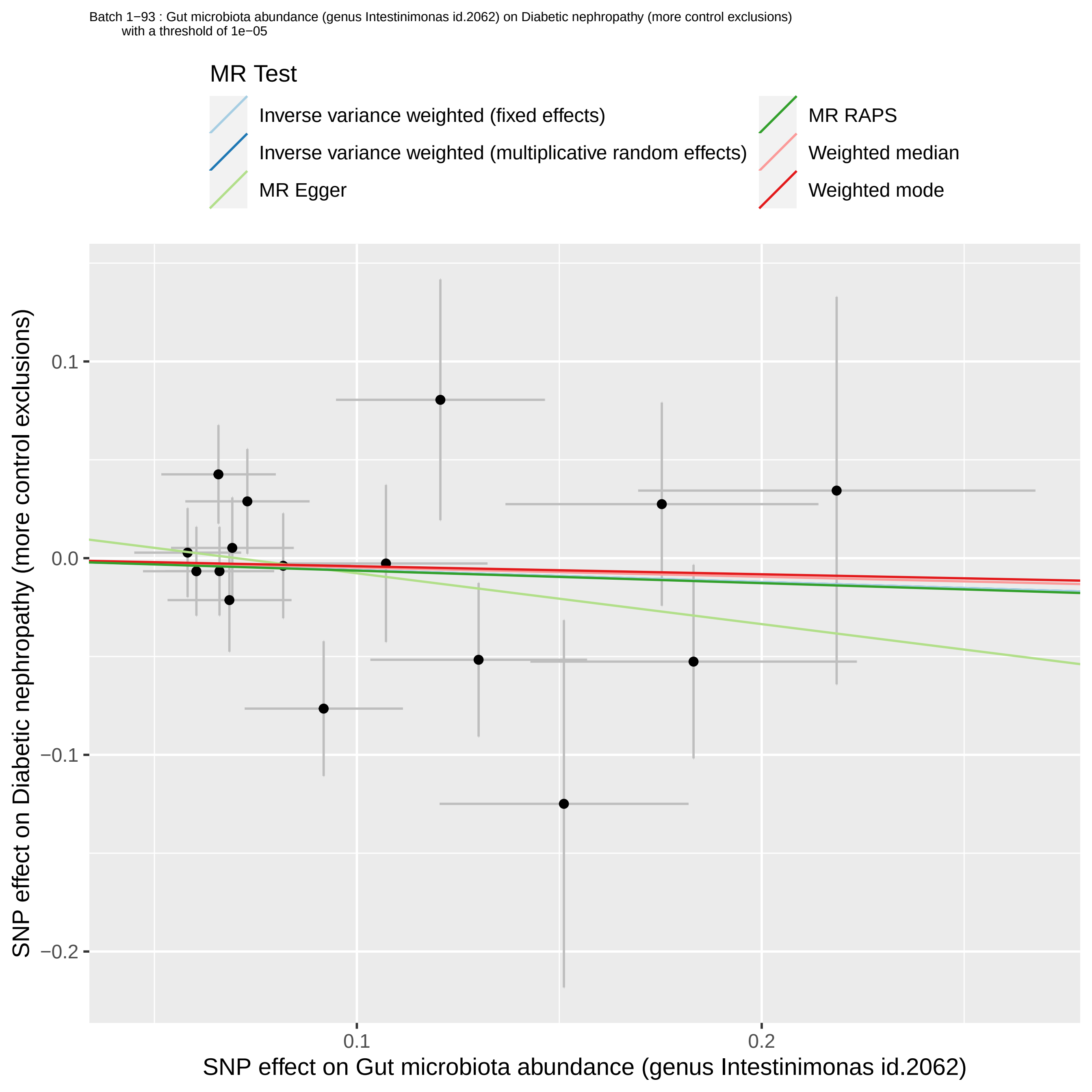

Batch 1−93 : Gut microbiota abundance (genus Intestinimonas id.2062) on Diabetic nephropathy (more control exclusions)
with a threshold of 1e−05
MR Test
Inverse variance weighted (fixed effects)
Inverse variance weighted (multiplicative random effects)
MR Egger
MR RAPS
Weighted median
Weighted mode
0.1
0.0
−0.1
−0.2
SNP effect on Diabetic nephropathy (more control exclusions)
0.1
0.2
SNP effect on Gut microbiota abundance (genus Intestinimonas id.2062)

Batch 1−94 : Gut microbiota abundance (genus Lachnoclostridium id.11308) on Diabetic nephropathy (more control exclusions)
with a threshold of 1e−05

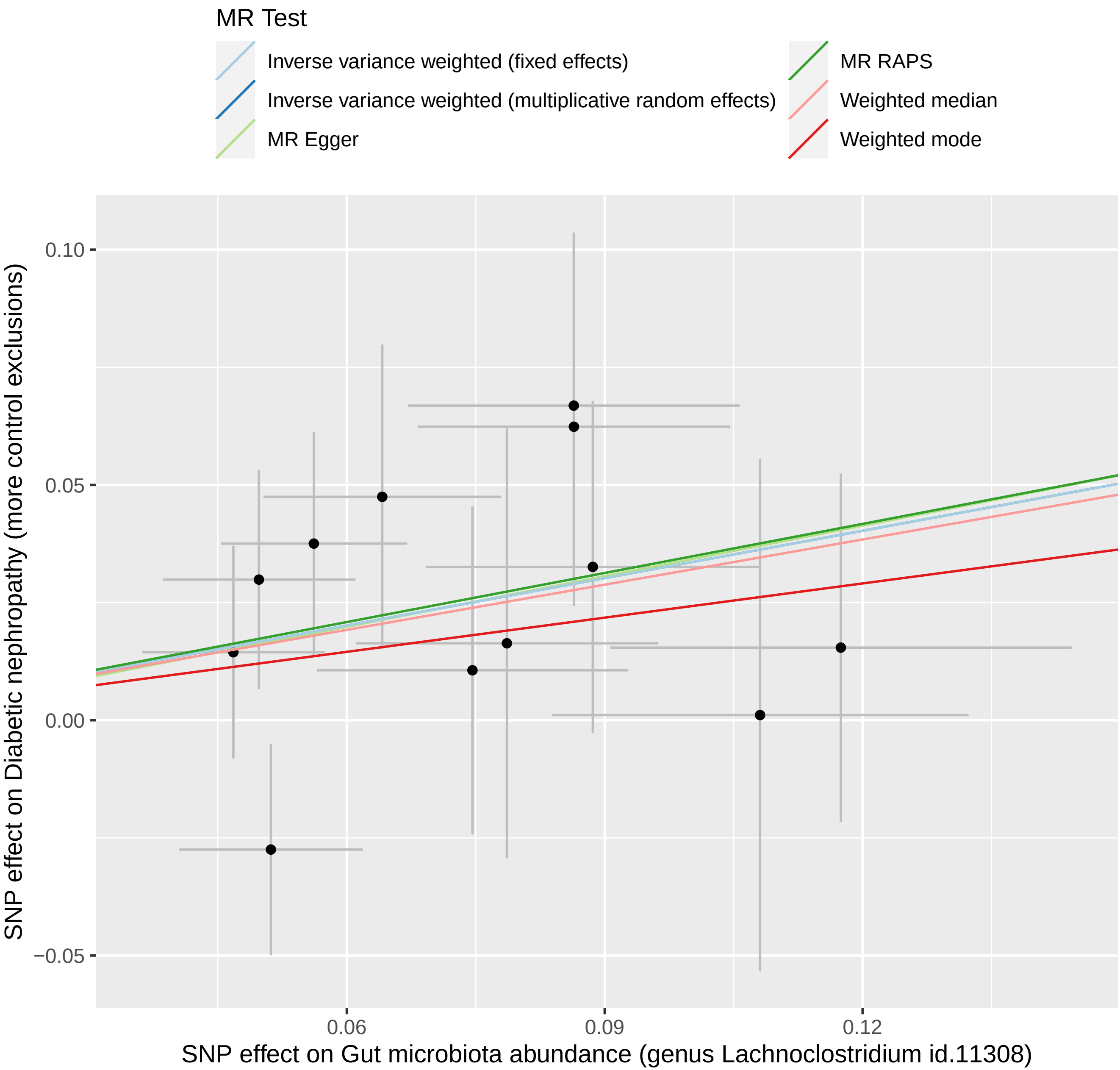

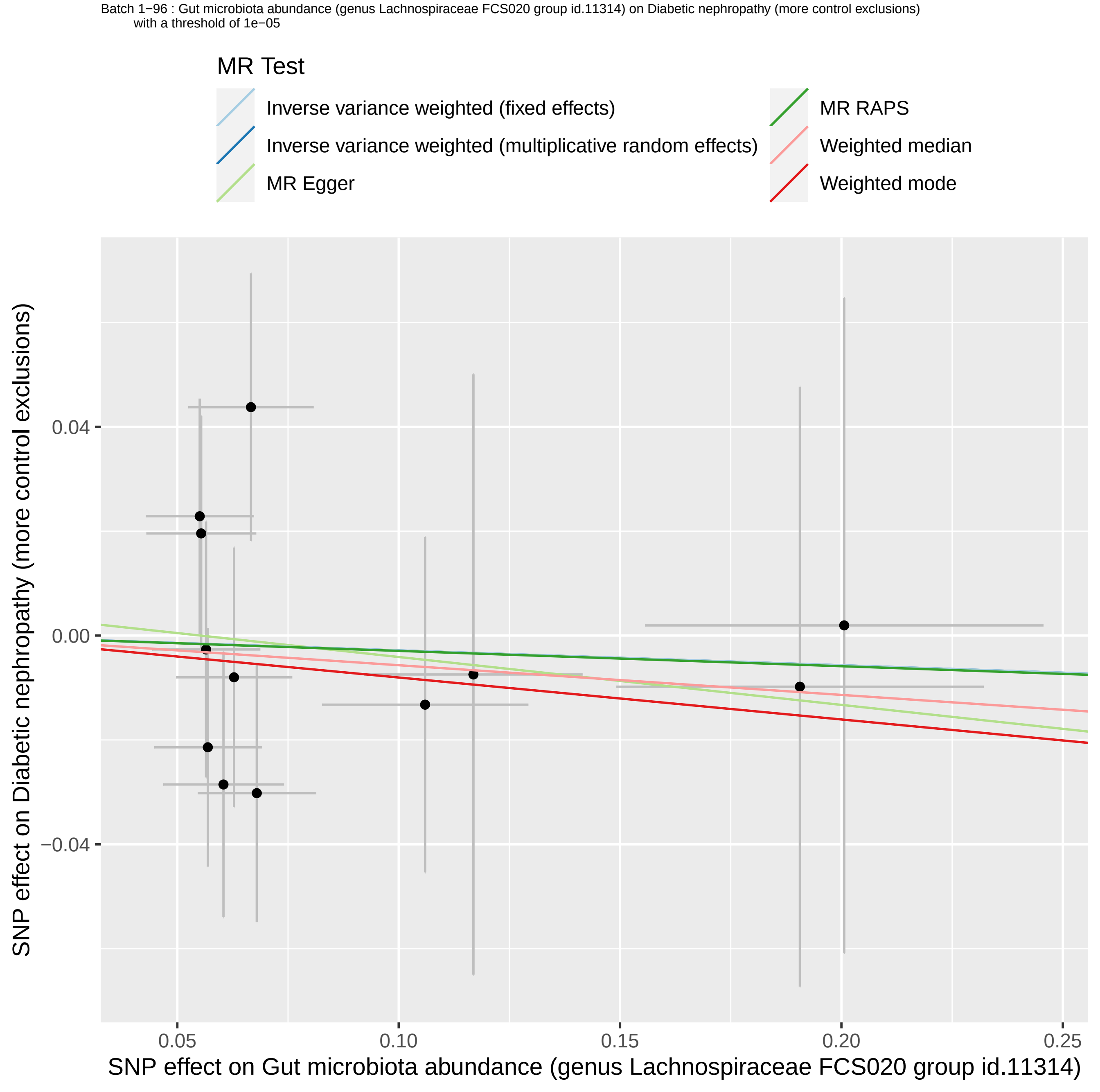
Batch 1−96 : Gut microbiota abundance (genus Lachnospiraceae FCS020 group id.11314) on Diabetic nephropathy (more control exclusions) with a threshold of 1e−05
MR Test
Inverse variance weighted (fixed effects)
Inverse variance weighted (multiplicative random effects)
MR Egger
MR RAPS
Weighted median
Weighted mode
0.04
0.00
−0.04
0.05
0.10
0.15
0.20
0.25
SNP effect on Diabetic nephropathy (more control exclusions)
SNP effect on Gut microbiota abundance (genus Lachnospiraceae FCS020 group id.11314)

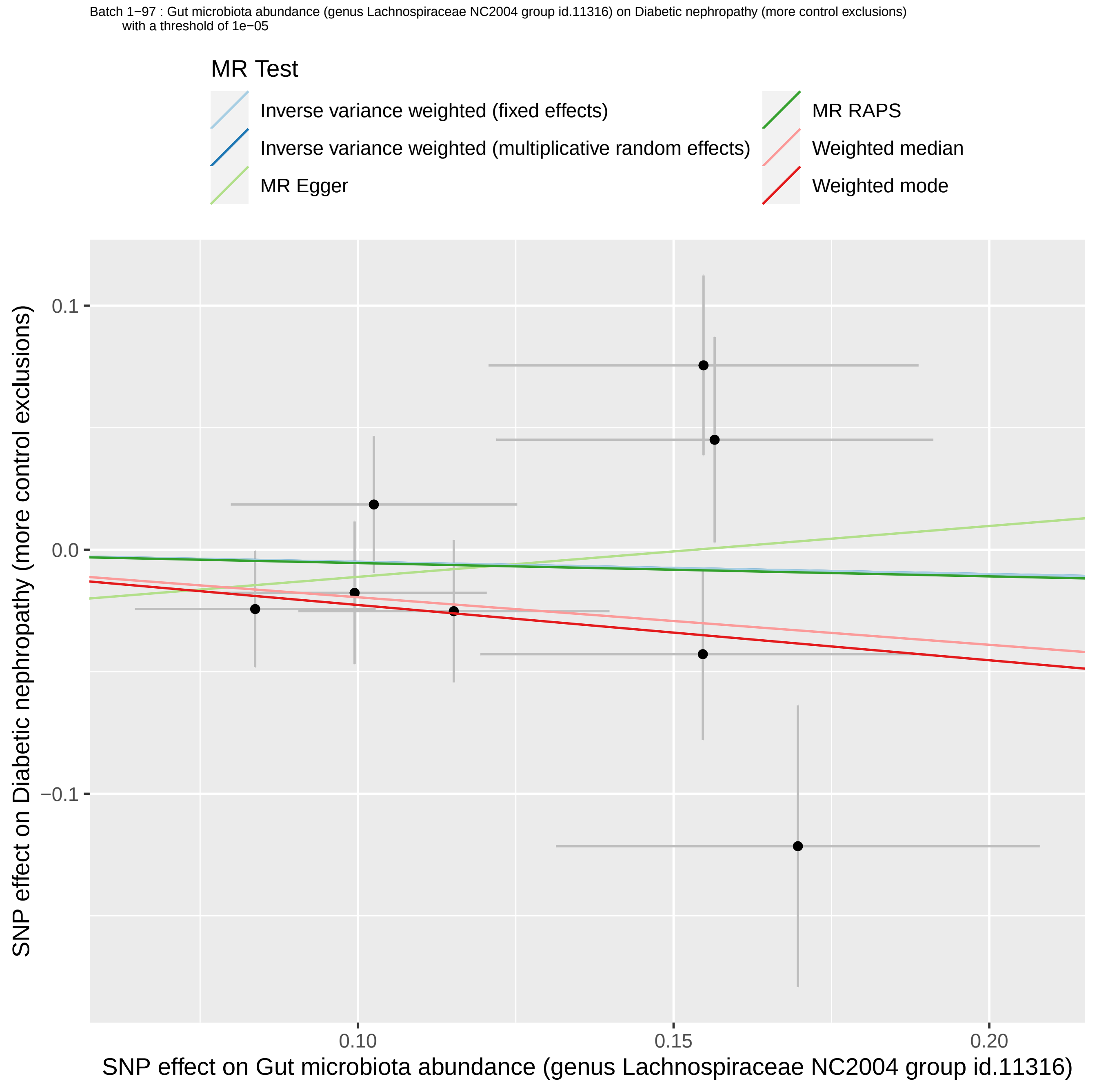

Batch 1−97 : Gut microbiota abundance (genus Lachnospiraceae NC2004 group id.11316) on Diabetic nephropathy (more control exclusions) with a threshold of 1e−05
MR Test
Inverse variance weighted (fixed effects)
Inverse variance weighted (multiplicative random effects)
MR Egger
MR RAPS
Weighted median
Weighted mode
0.1
0.0
−0.1
0.10
0.15
0.20
SNP effect on Diabetic nephropathy (more control exclusions)
SNP effect on Gut microbiota abundance (genus Lachnospiraceae NC2004 group id.11316)

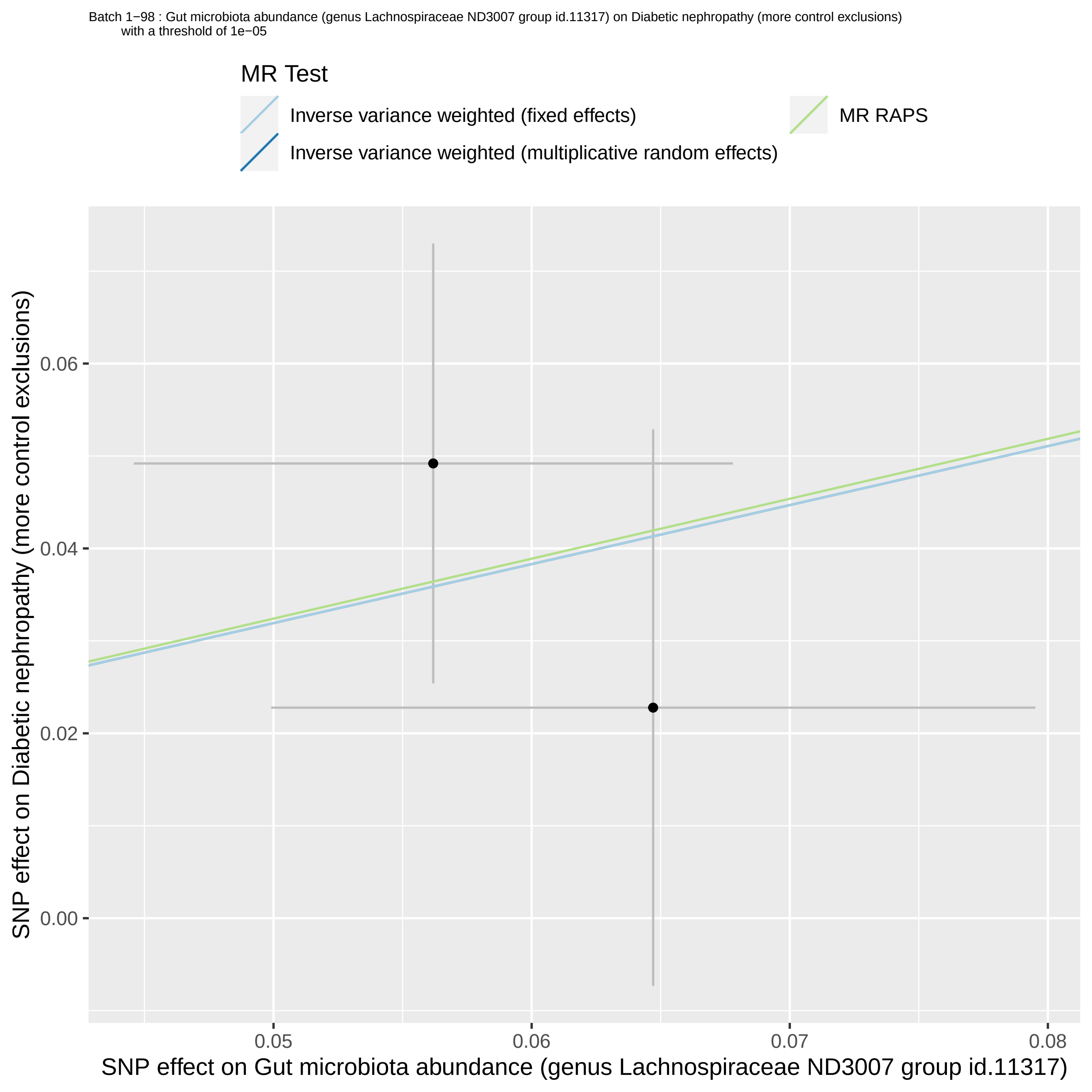
Batch 1−98 : Gut microbiota abundance (genus Lachnospiraceae ND3007 group id.11317) on Diabetic nephropathy (more control exclusions) with a threshold of 1e−05
MR Test
Inverse variance weighted (fixed effects)
Inverse variance weighted (multiplicative random effects)
MR RAPS
0.06
0.04
0.02
0.00
0.05
0.06
0.07
0.08
SNP effect on Diabetic nephropathy (more control exclusions)
SNP effect on Gut microbiota abundance (genus Lachnospiraceae ND3007 group id.11317)

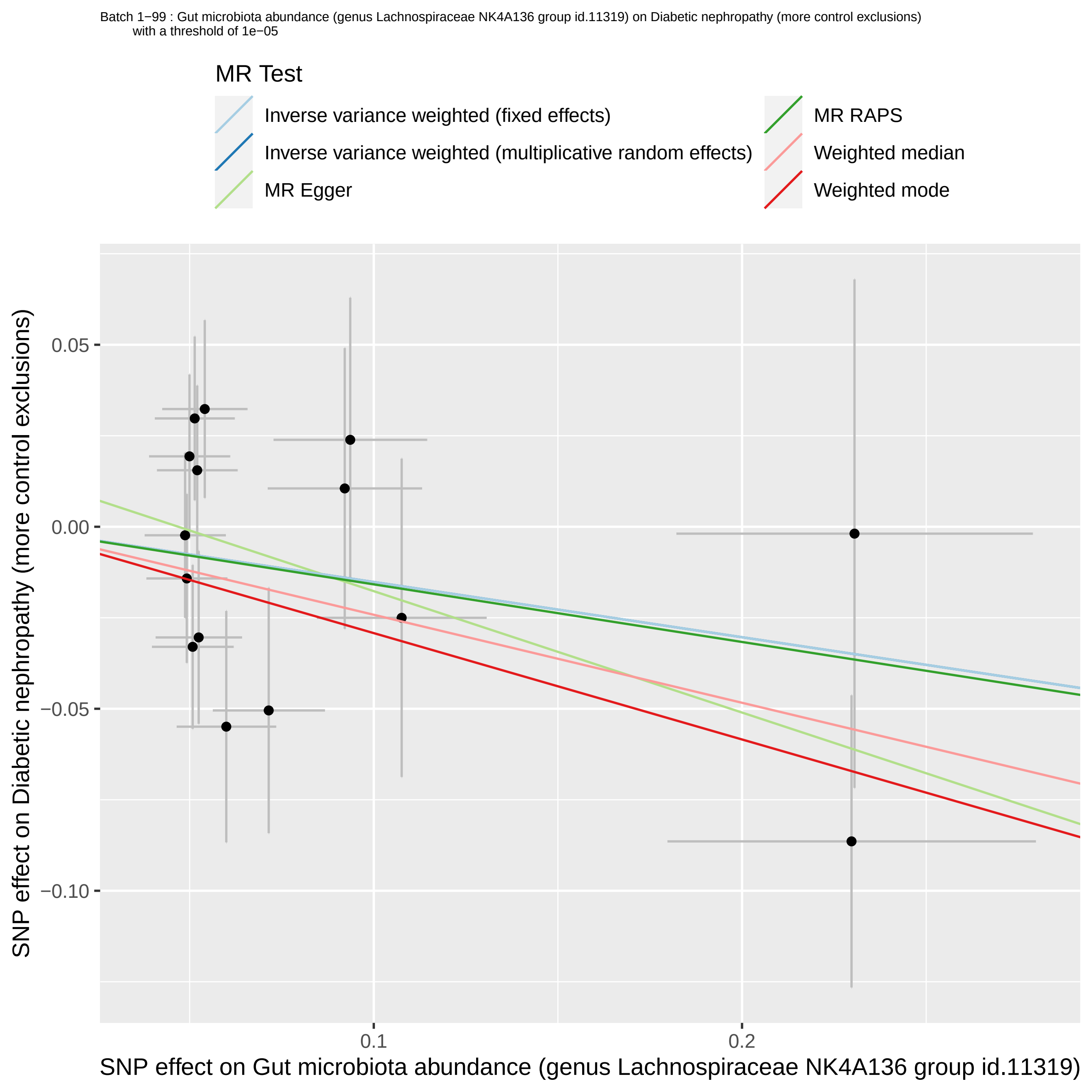

Batch 1−99 : Gut microbiota abundance (genus Lachnospiraceae NK4A136 group id.11319) on Diabetic nephropathy (more control exclusions) with a threshold of 1e−05
MR Test
Inverse variance weighted (fixed effects)
Inverse variance weighted (multiplicative random effects)
MR Egger
MR RAPS
Weighted median
Weighted mode
0.05
0.00
−0.05
−0.10
0.1
0.2
SNP effect on Diabetic nephropathy (more control exclusions)
SNP effect on Gut microbiota abundance (genus Lachnospiraceae NK4A136 group id.11319)

Batch 1−100 : Gut microbiota abundance (genus Lachnospiraceae UCG001 id.11321) on Diabetic nephropathy (more control exclusions)
with a threshold of 1e−05

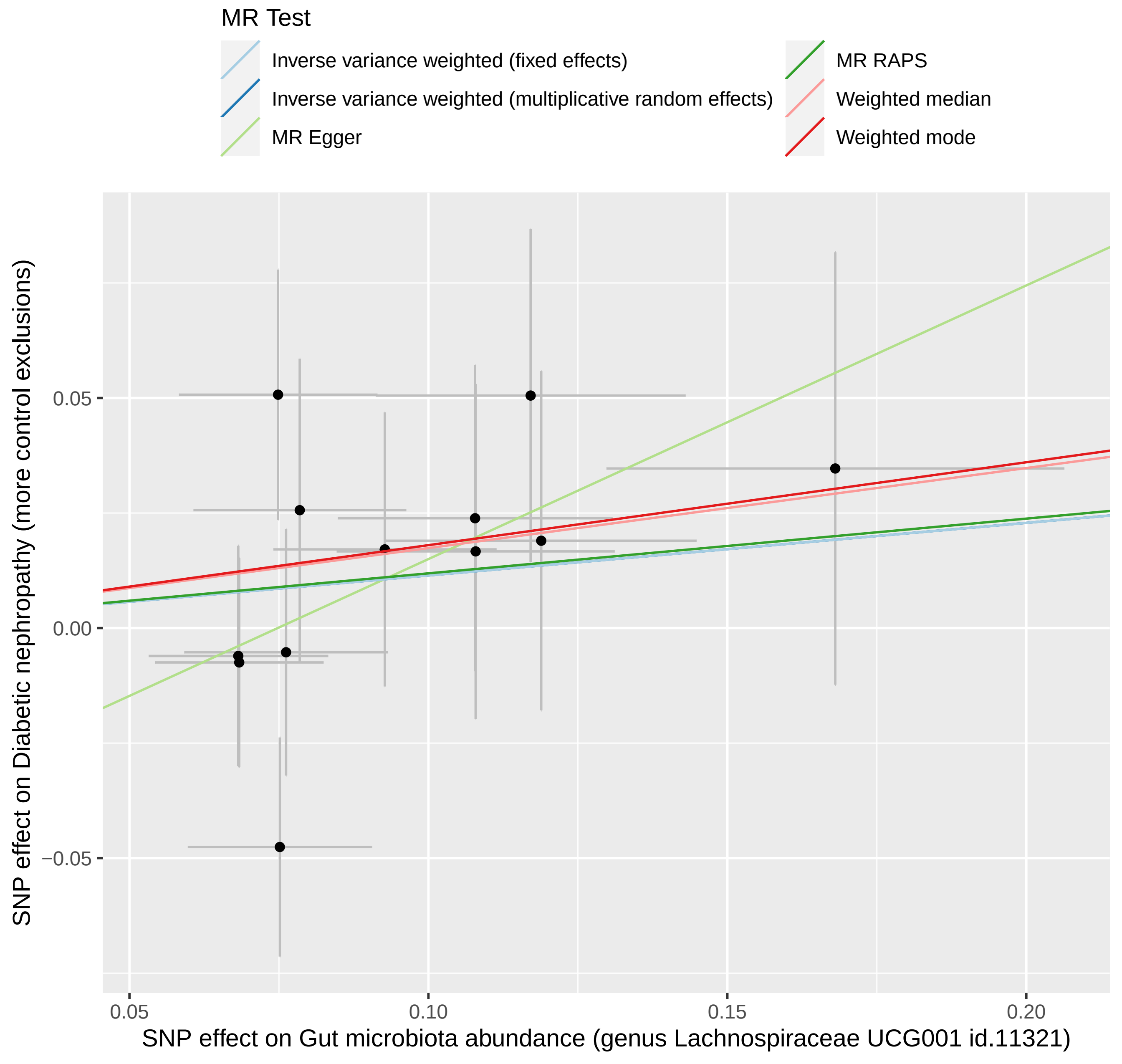

Batch 1−101 : Gut microbiota abundance (genus Lachnospiraceae UCG004 id.11324) on Diabetic nephropathy (more control exclusions) with a threshold of 1e−05

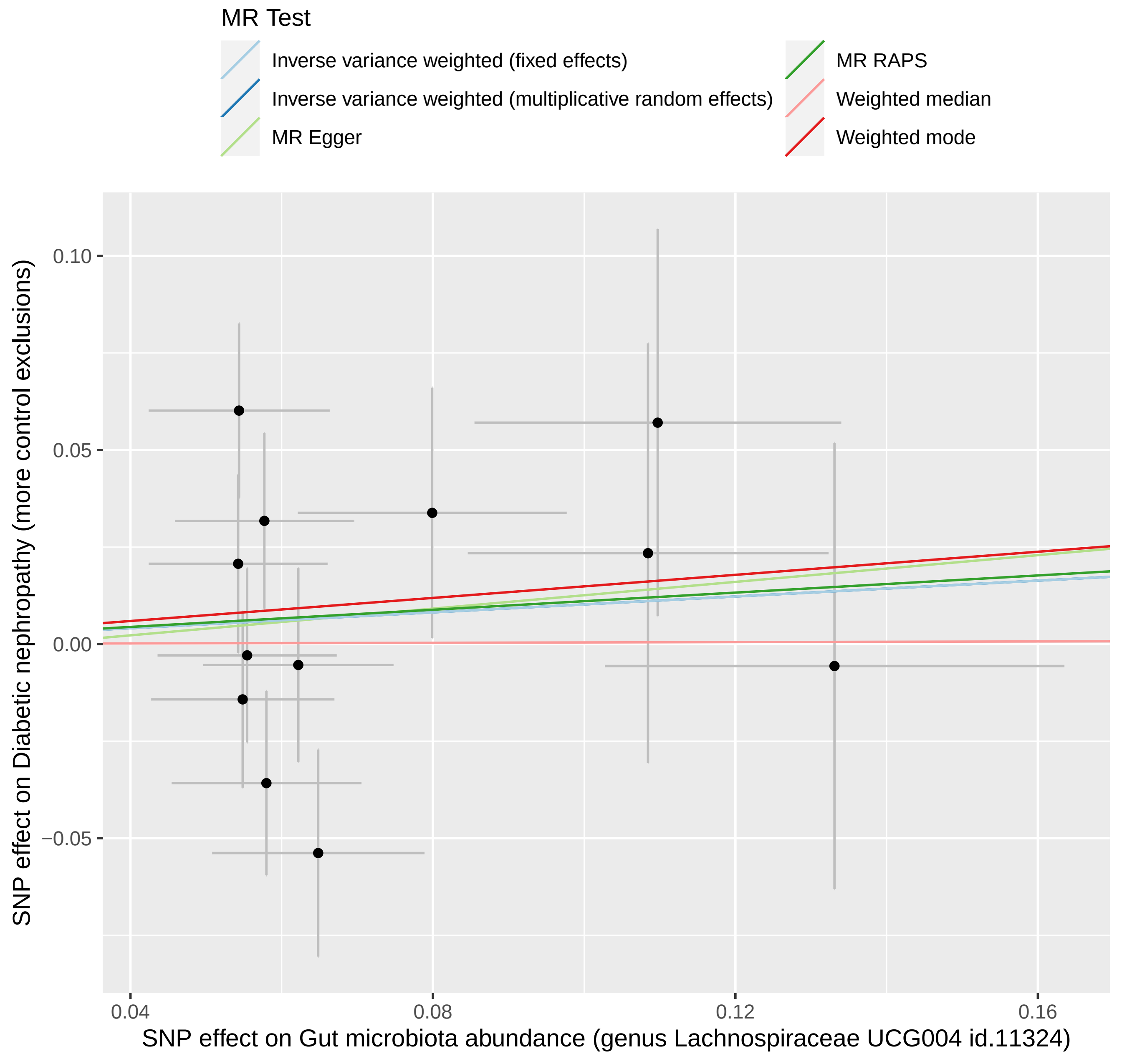

Batch 1−102 : Gut microbiota abundance (genus Lachnospiraceae UCG008 id.11328) on Diabetic nephropathy (more control exclusions) with a threshold of 1e−05

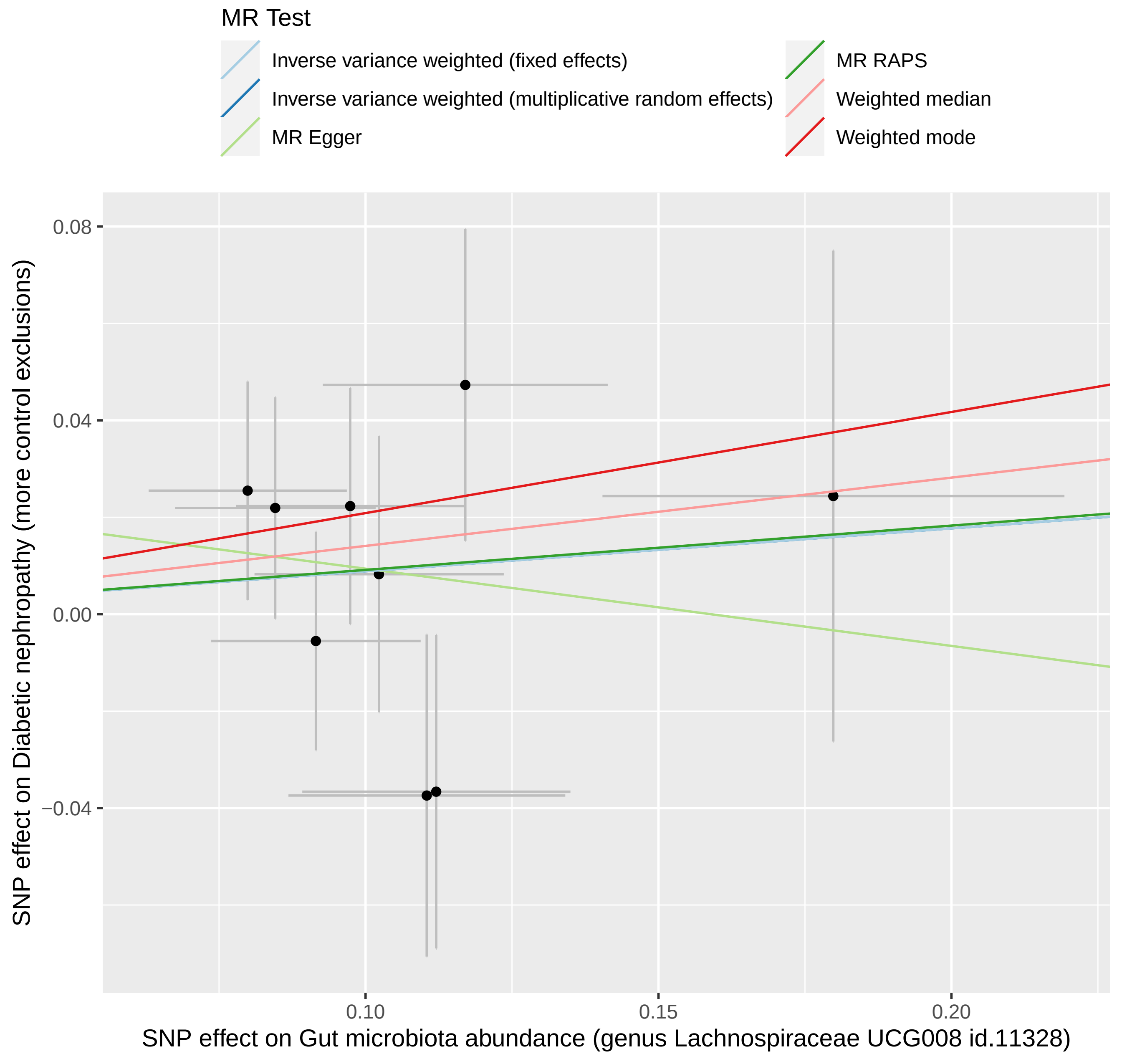

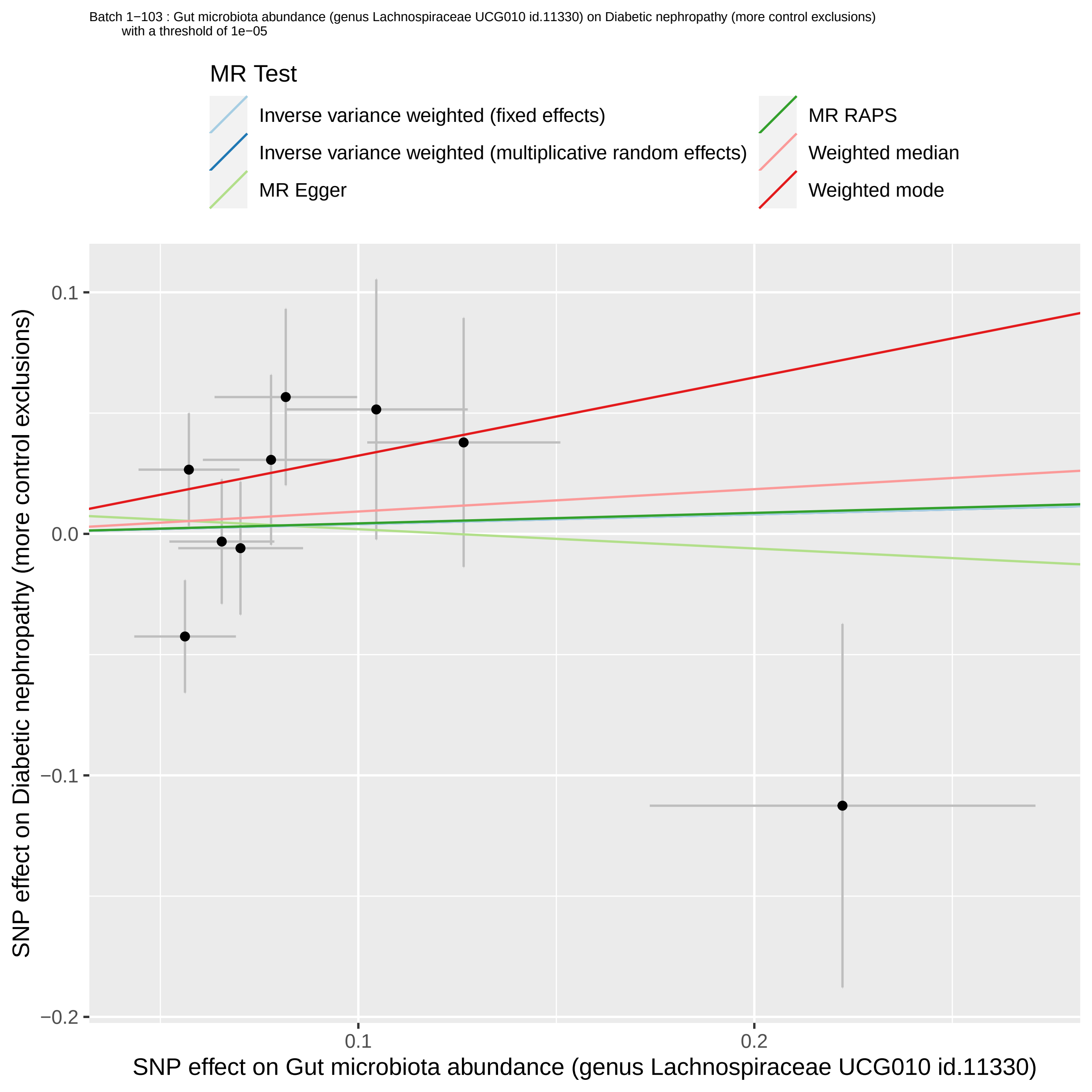
Batch 1−103 : Gut microbiota abundance (genus Lachnospiraceae UCG010 id.11330) on Diabetic nephropathy (more control exclusions)
with a threshold of 1e−05
MR Test
Inverse variance weighted (fixed effects)
Inverse variance weighted (multiplicative random effects)
MR Egger
MR RAPS
Weighted median
Weighted mode
0.1
0.0
−0.1
−0.2
0.1
0.2
SNP effect on Diabetic nephropathy (more control exclusions)
SNP effect on Gut microbiota abundance (genus Lachnospiraceae UCG010 id.11330)

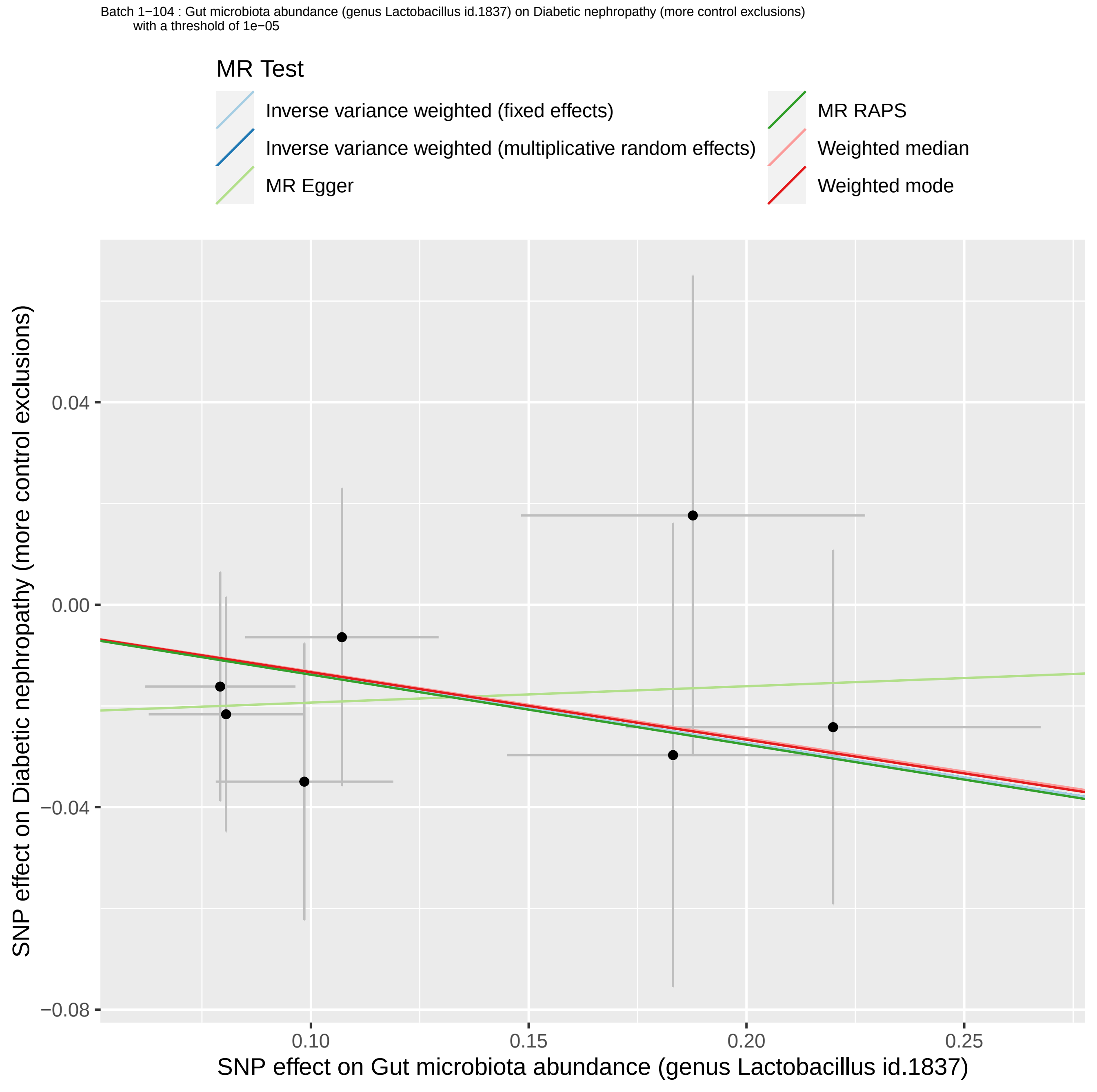

Batch 1−104 : Gut microbiota abundance (genus Lactobacillus id.1837) on Diabetic nephropathy (more control exclusions) with a threshold of 1e−05
MR Test
Inverse variance weighted (fixed effects)
Inverse variance weighted (multiplicative random effects)
MR Egger
MR RAPS
Weighted median
Weighted mode
SNP effect on Diabetic nephropathy (more control exclusions)
0.04
0.00
−0.04
−0.08
0.10
0.15
0.20
0.25
SNP effect on Gut microbiota abundance (genus Lactobacillus id.1837)

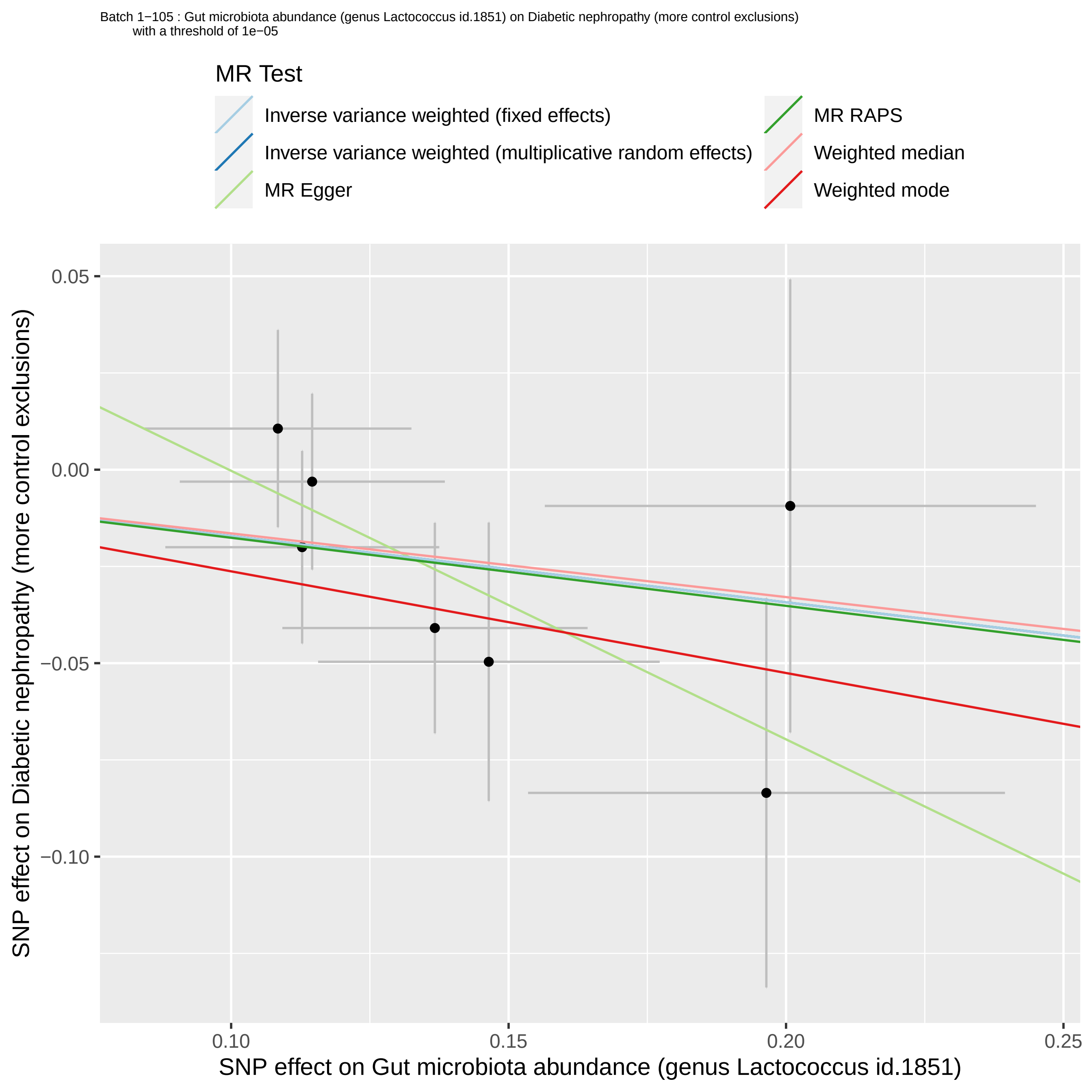
Batch 1−105 : Gut microbiota abundance (genus Lactococcus id.1851) on Diabetic nephropathy (more control exclusions)
with a threshold of 1e−05
MR Test
Inverse variance weighted (fixed effects)
Inverse variance weighted (multiplicative random effects)
MR Egger
MR RAPS
Weighted median
Weighted mode
0.05
0.00
−0.05
−0.10
0.10
0.15
0.20
0.25
SNP effect on Diabetic nephropathy (more control exclusions)
SNP effect on Gut microbiota abundance (genus Lactococcus id.1851)

Batch 1−106 : Gut microbiota abundance (genus Marvinbryantia id.2005) on Diabetic nephropathy (more control exclusions) with a threshold of 1e−05

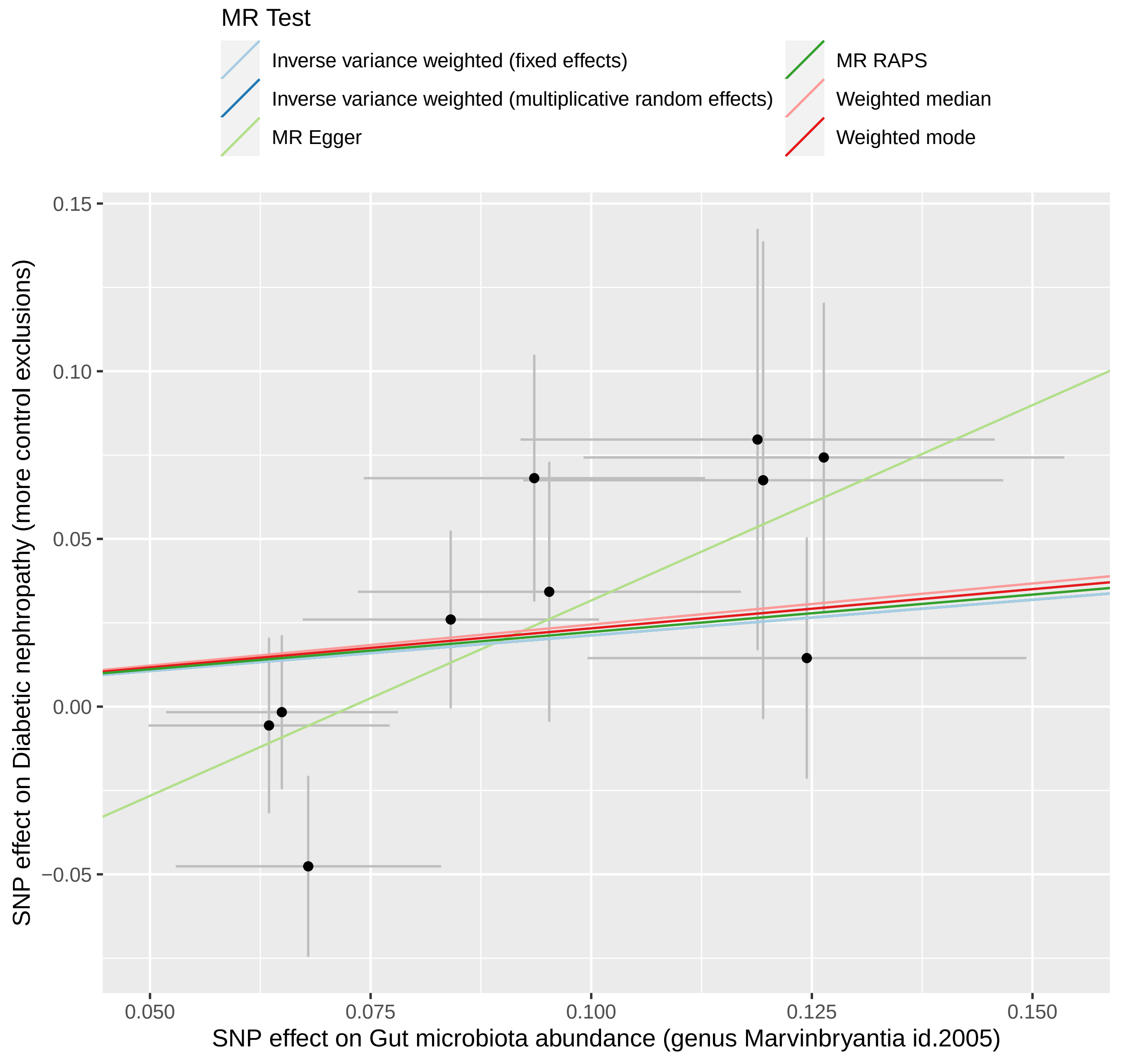

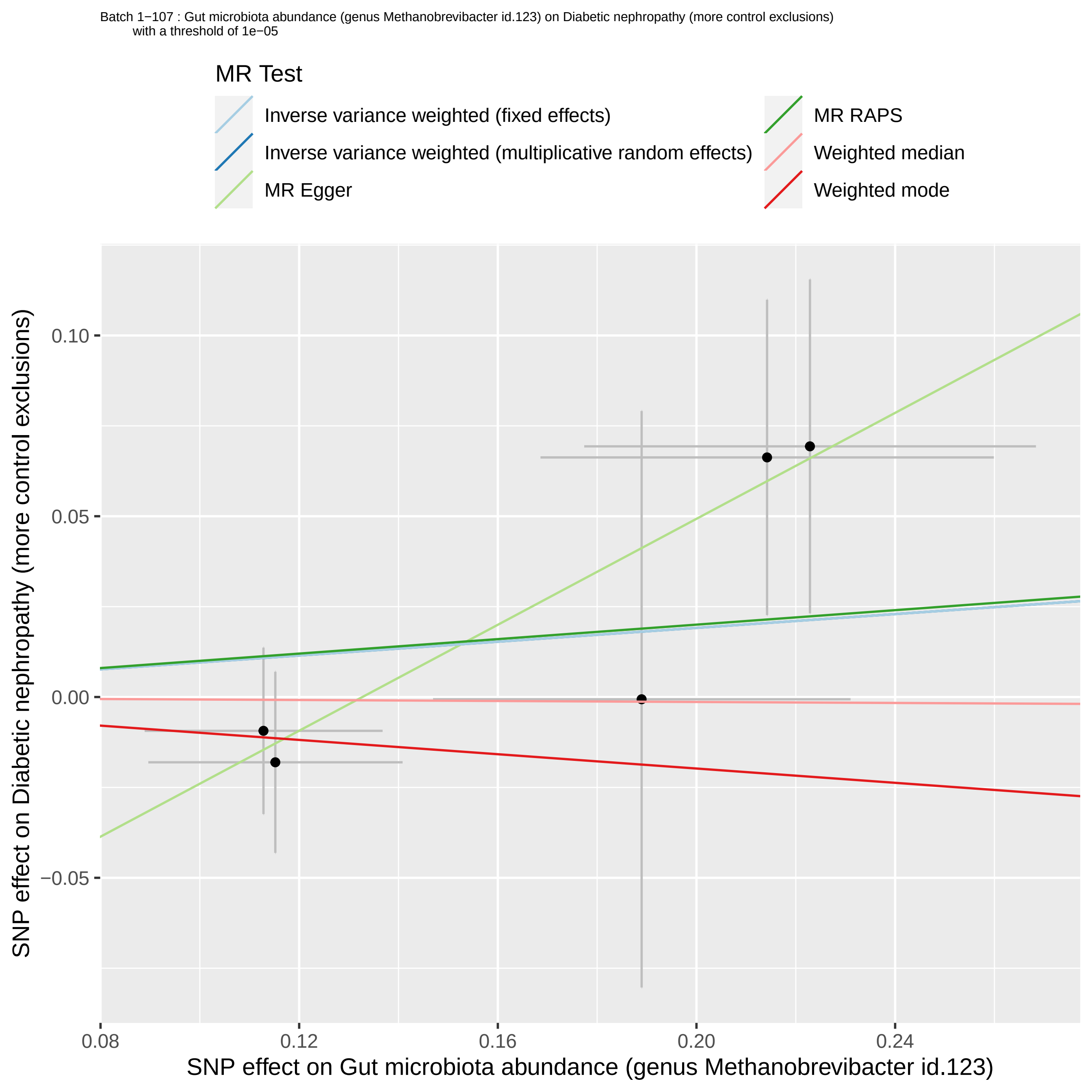

Batch 1−107 : Gut microbiota abundance (genus Methanobrevibacter id.123) on Diabetic nephropathy (more control exclusions) with a threshold of 1e−05
MR Test
Inverse variance weighted (fixed effects)
Inverse variance weighted (multiplicative random effects)
MR Egger
MR RAPS
Weighted median
Weighted mode
0.10
0.05
0.00
−0.05
0.08
0.12
0.16
0.20
0.24
SNP effect on Diabetic nephropathy (more control exclusions)
SNP effect on Gut microbiota abundance (genus Methanobrevibacter id.123)

Batch 1−108 : Gut microbiota abundance (genus Odoribacter id.952) on Diabetic nephropathy (more control exclusions) with a threshold of 1e−05

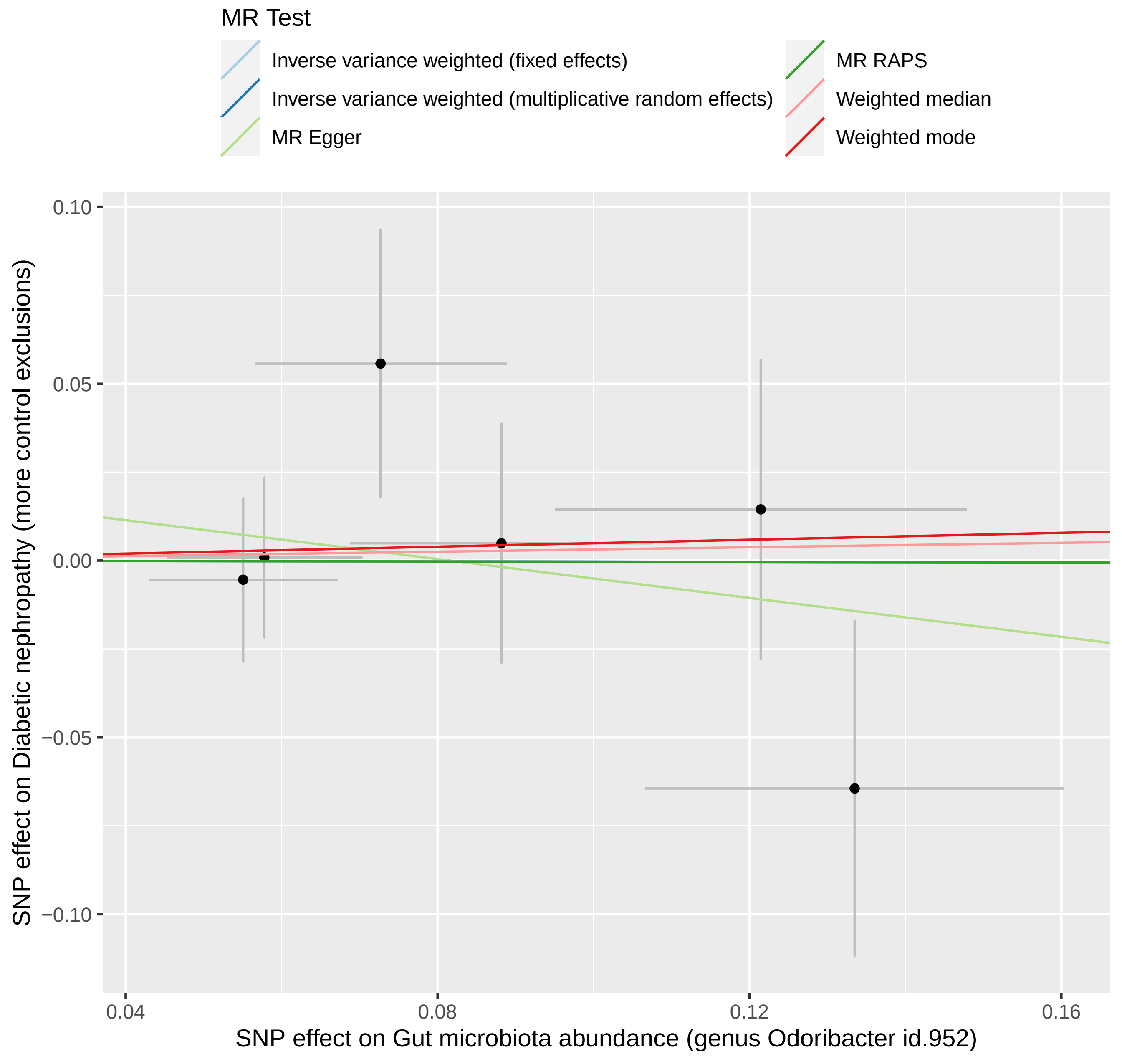

Batch 1−109 : Gut microbiota abundance (genus Olsenella id.822) on Diabetic nephropathy (more control exclusions) with a threshold of 1e−05

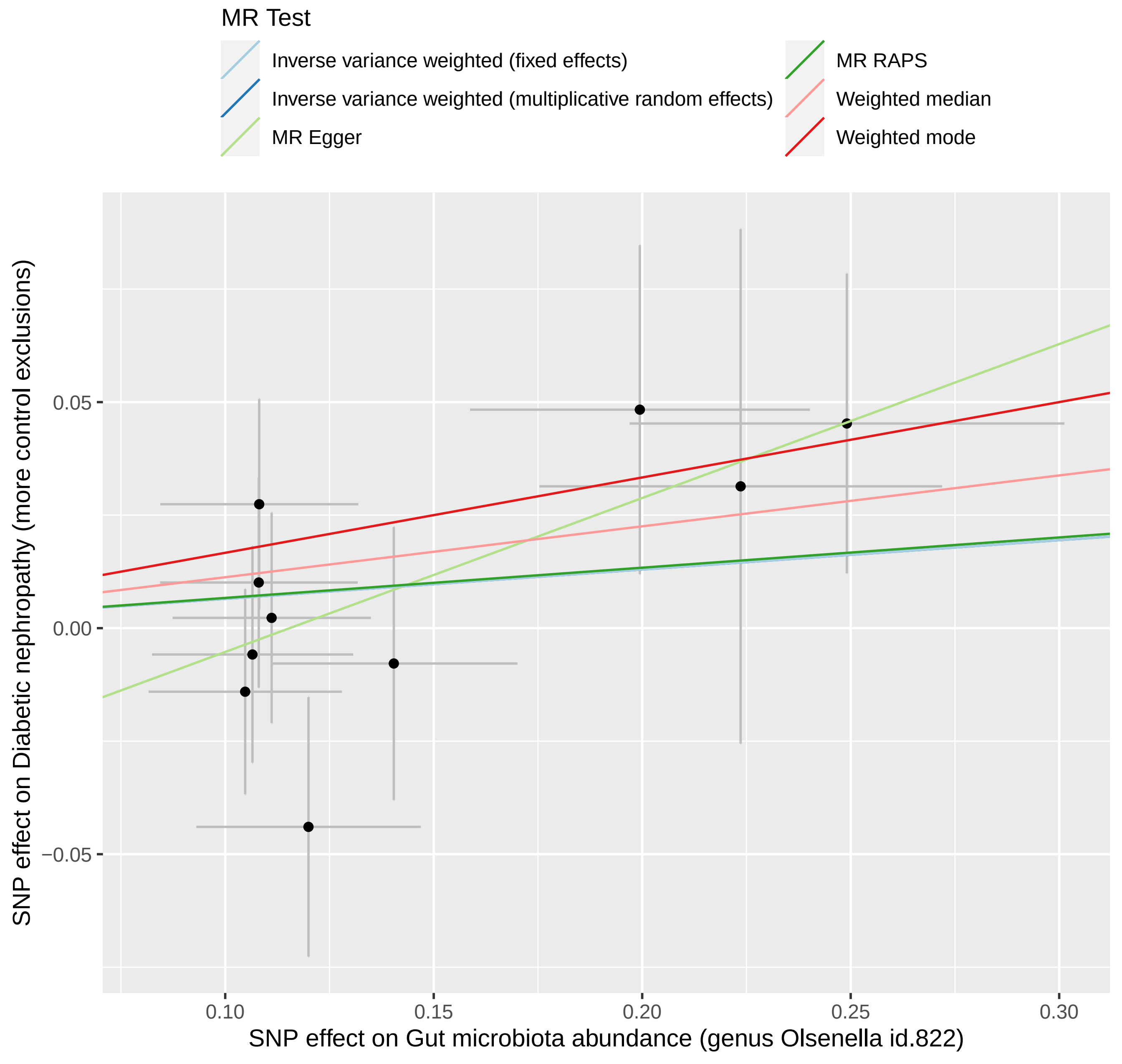

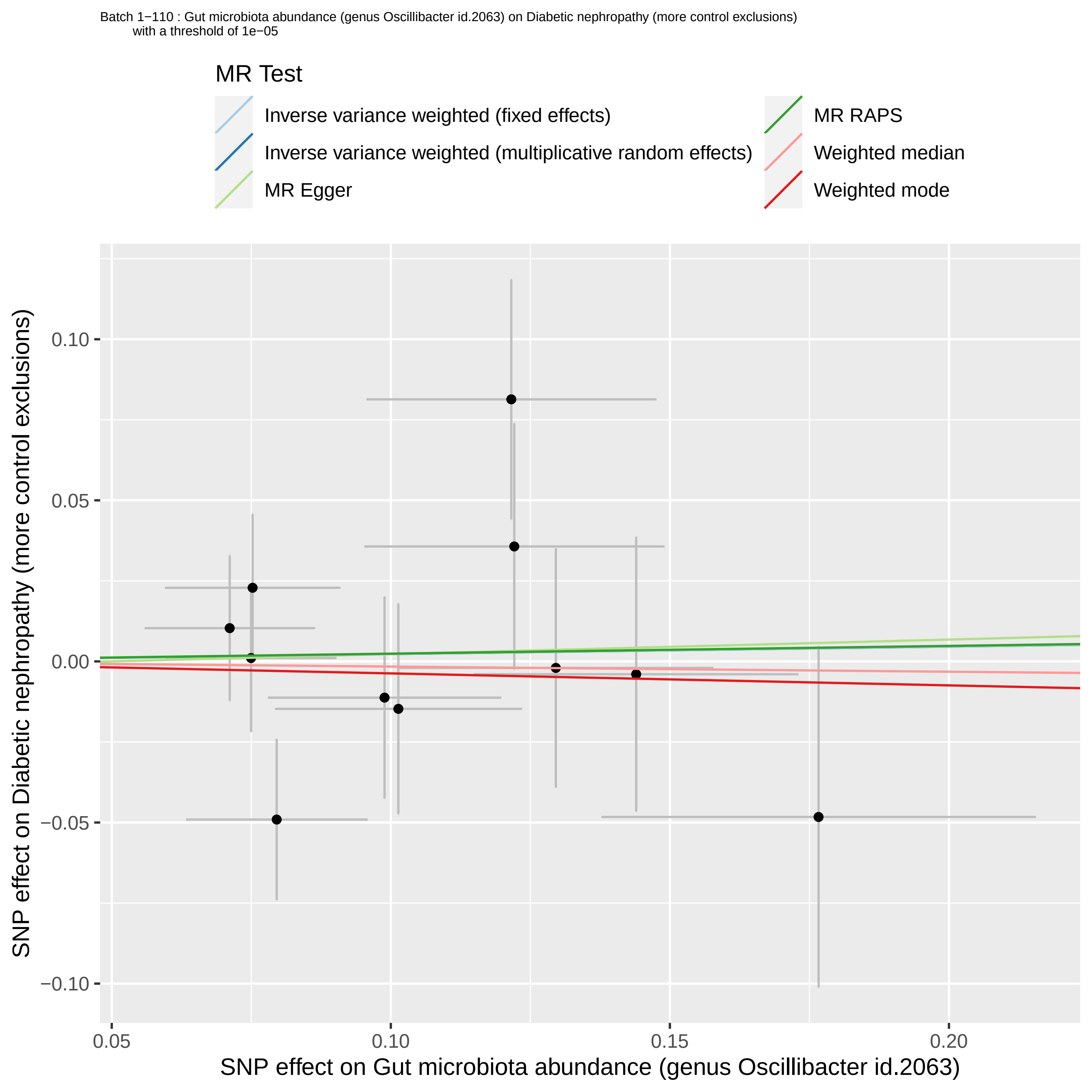
Batch 1−110 : Gut microbiota abundance (genus Oscillibacter id.2063) on Diabetic nephropathy (more control exclusions)
with a threshold of 1e−05
MR Test
Inverse variance weighted (fixed effects)
Inverse variance weighted (multiplicative random effects)
MR Egger
MR RAPS
Weighted median
Weighted mode
0.10
0.05
0.00
−0.05
−0.10
0.05
0.10
0.15
0.20
SNP effect on Diabetic nephropathy (more control exclusions)
SNP effect on Gut microbiota abundance (genus Oscillibacter id.2063)

Batch 1−111 : Gut microbiota abundance (genus Oscillospira id.2064) on Diabetic nephropathy (more control exclusions)
with a threshold of 1e−05

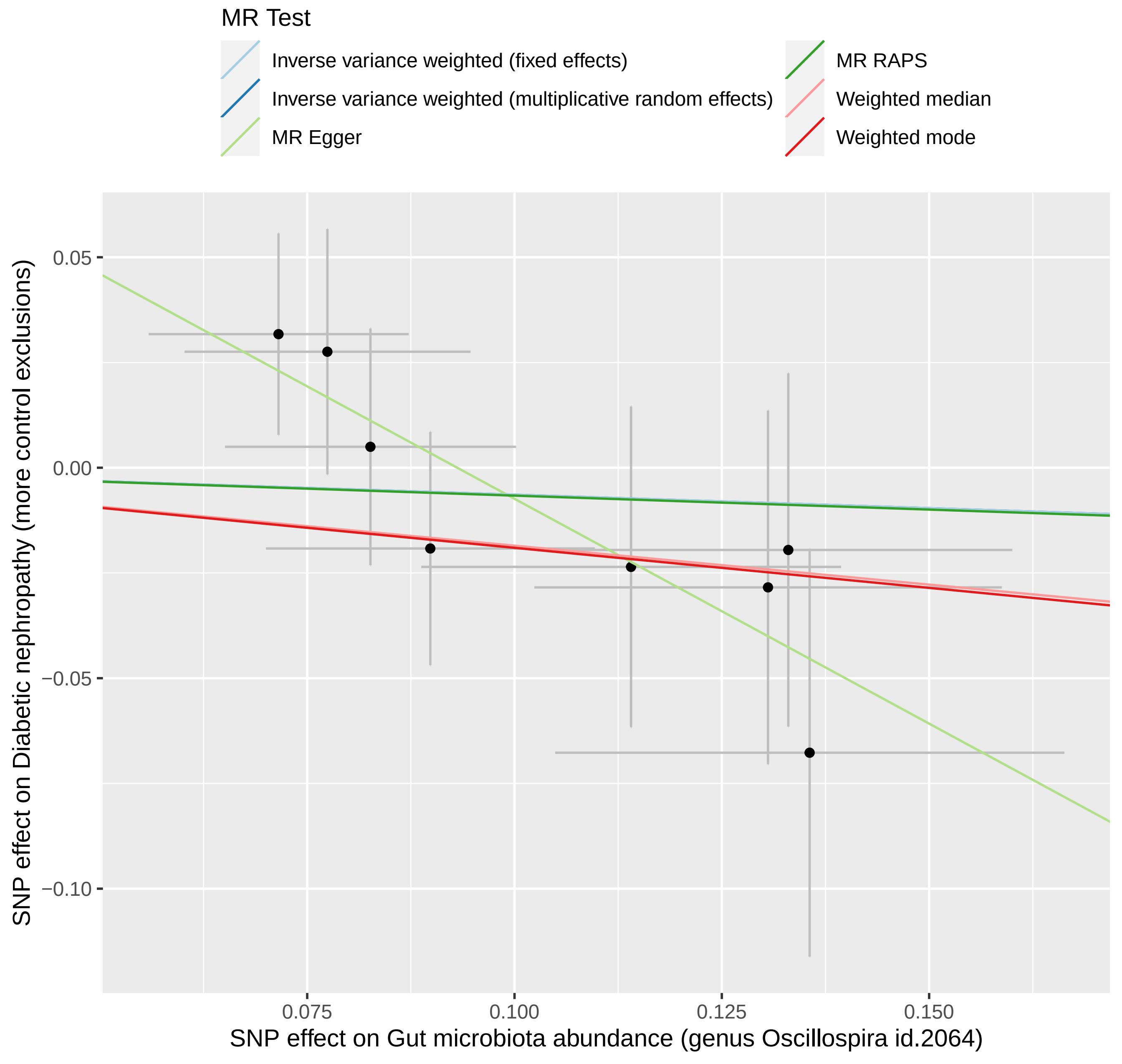

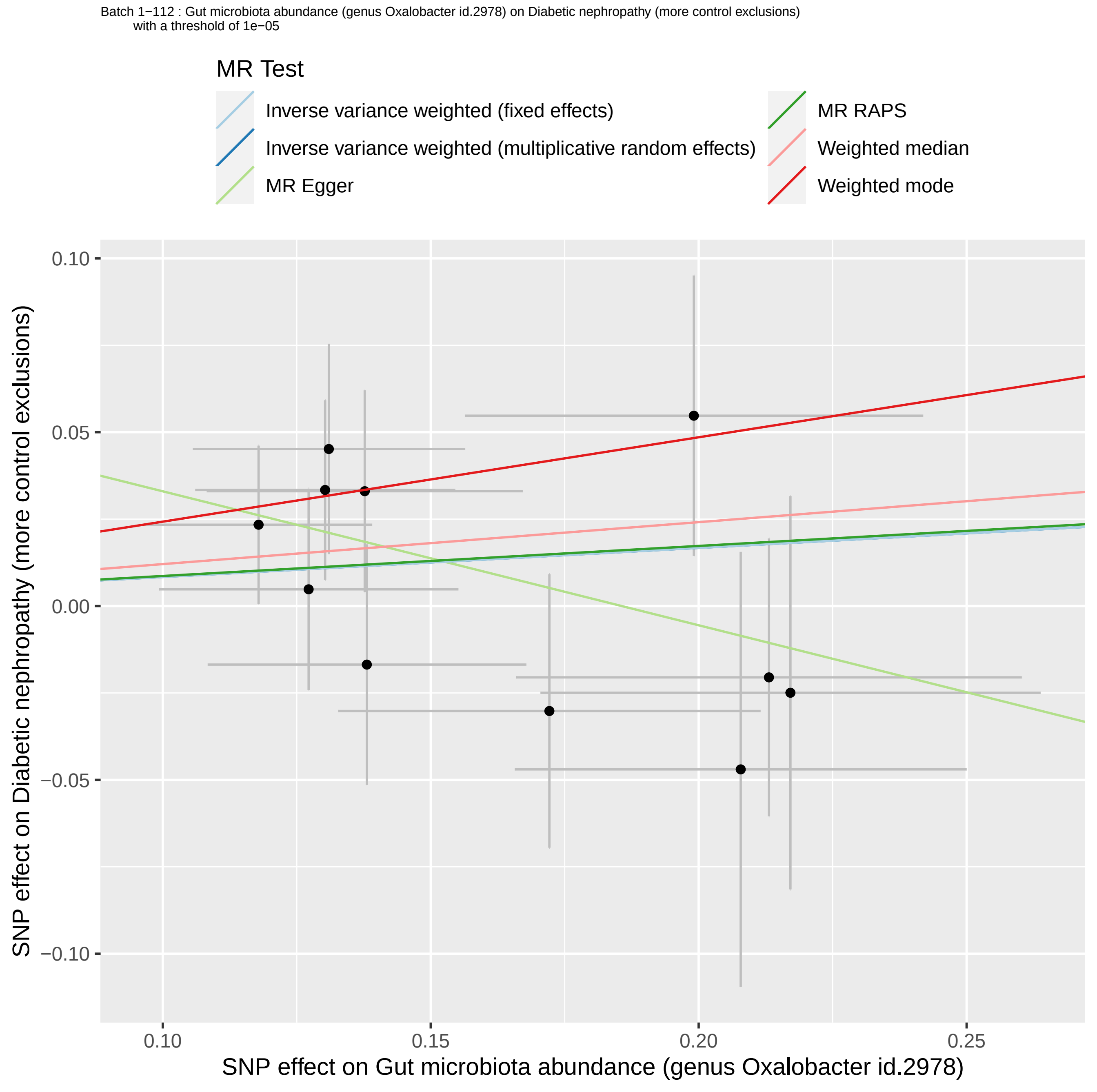
Batch 1−112 : Gut microbiota abundance (genus Oxalobacter id.2978) on Diabetic nephropathy (more control exclusions)
with a threshold of 1e−05
MR Test
Inverse variance weighted (fixed effects)
Inverse variance weighted (multiplicative random effects)
MR Egger
MR RAPS
Weighted median
Weighted mode
0.10
0.05
0.00
−0.05
−0.10
0.10
0.15
0.20
0.25
SNP effect on Diabetic nephropathy (more control exclusions)
SNP effect on Gut microbiota abundance (genus Oxalobacter id.2978)

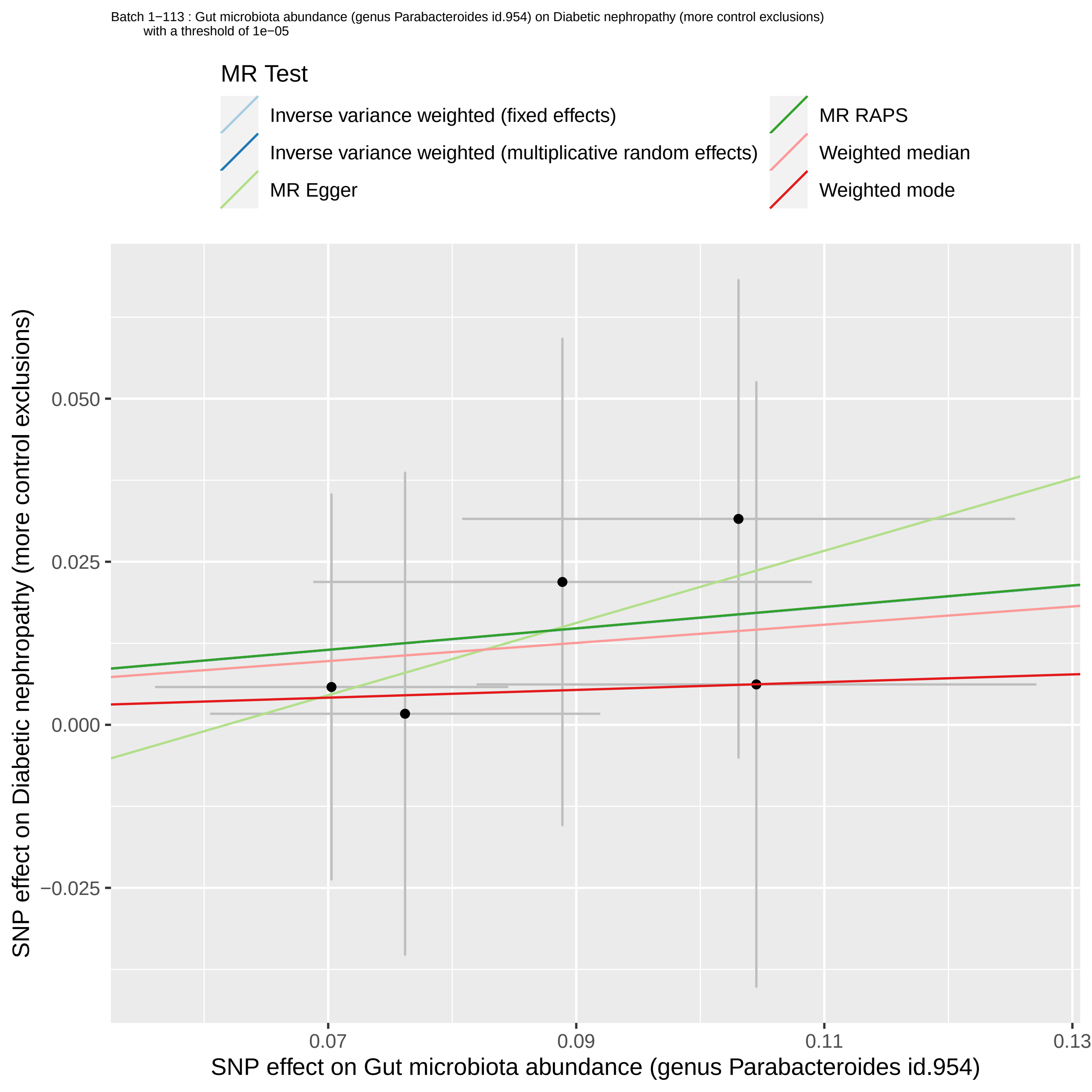

Batch 1−113 : Gut microbiota abundance (genus Parabacteroides id.954) on Diabetic nephropathy (more control exclusions)
with a threshold of 1e−05
MR Test
Inverse variance weighted (fixed effects)
Inverse variance weighted (multiplicative random effects)
MR Egger
MR RAPS
Weighted median
Weighted mode
SNP effect on Diabetic nephropathy (more control exclusions)
0.050
0.025
0.000
−0.025
0.07
0.09
0.11
0.13
SNP effect on Gut microbiota abundance (genus Parabacteroides id.954)

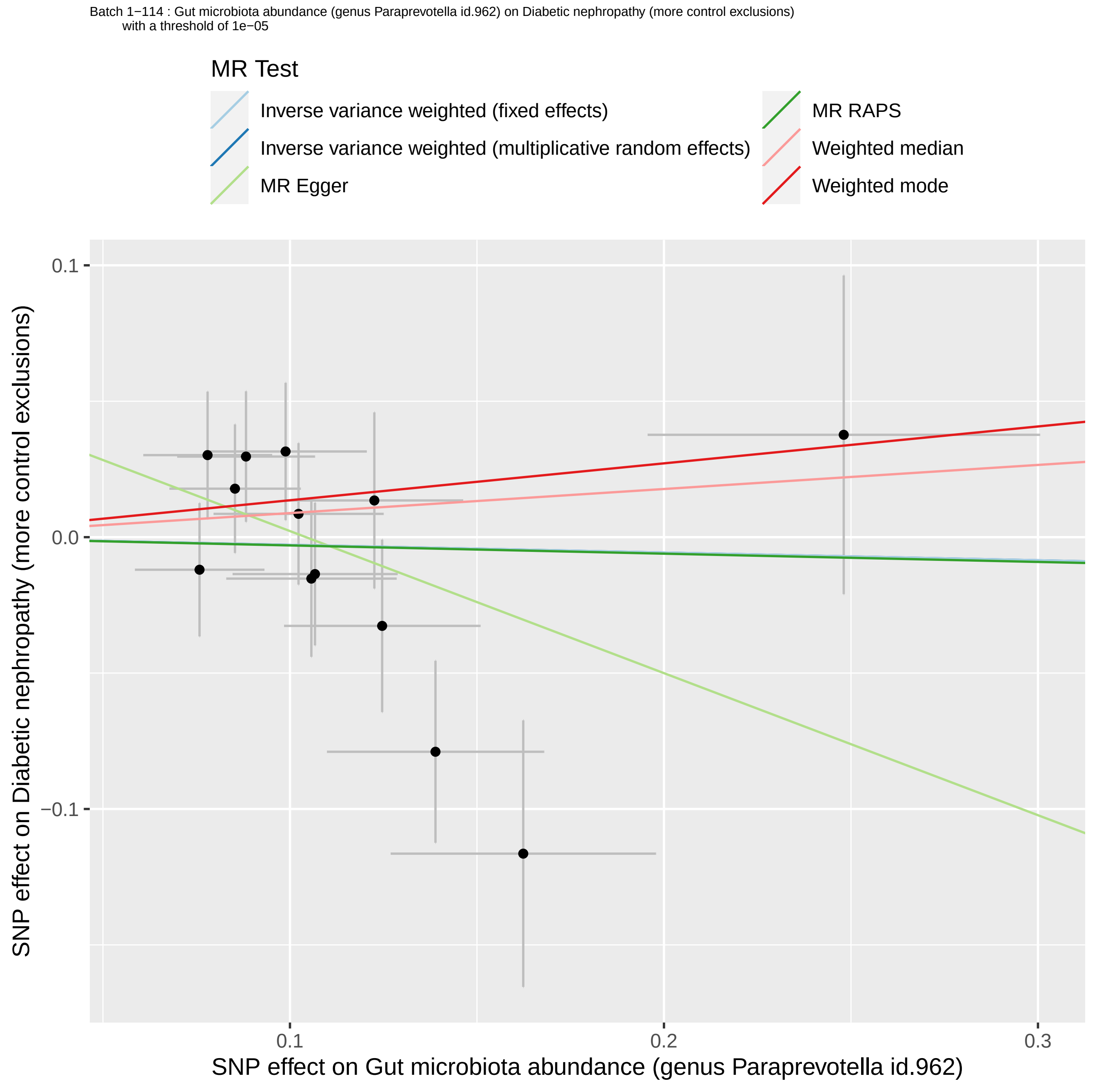
Batch 1−114 : Gut microbiota abundance (genus Paraprevotella id.962) on Diabetic nephropathy (more control exclusions)
with a threshold of 1e−05
MR Test
Inverse variance weighted (fixed effects)
Inverse variance weighted (multiplicative random effects)
MR Egger
MR RAPS
Weighted median
Weighted mode
0.1
0.0
−0.1
0.1
0.2
0.3
SNP effect on Diabetic nephropathy (more control exclusions)
SNP effect on Gut microbiota abundance (genus Paraprevotella id.962)

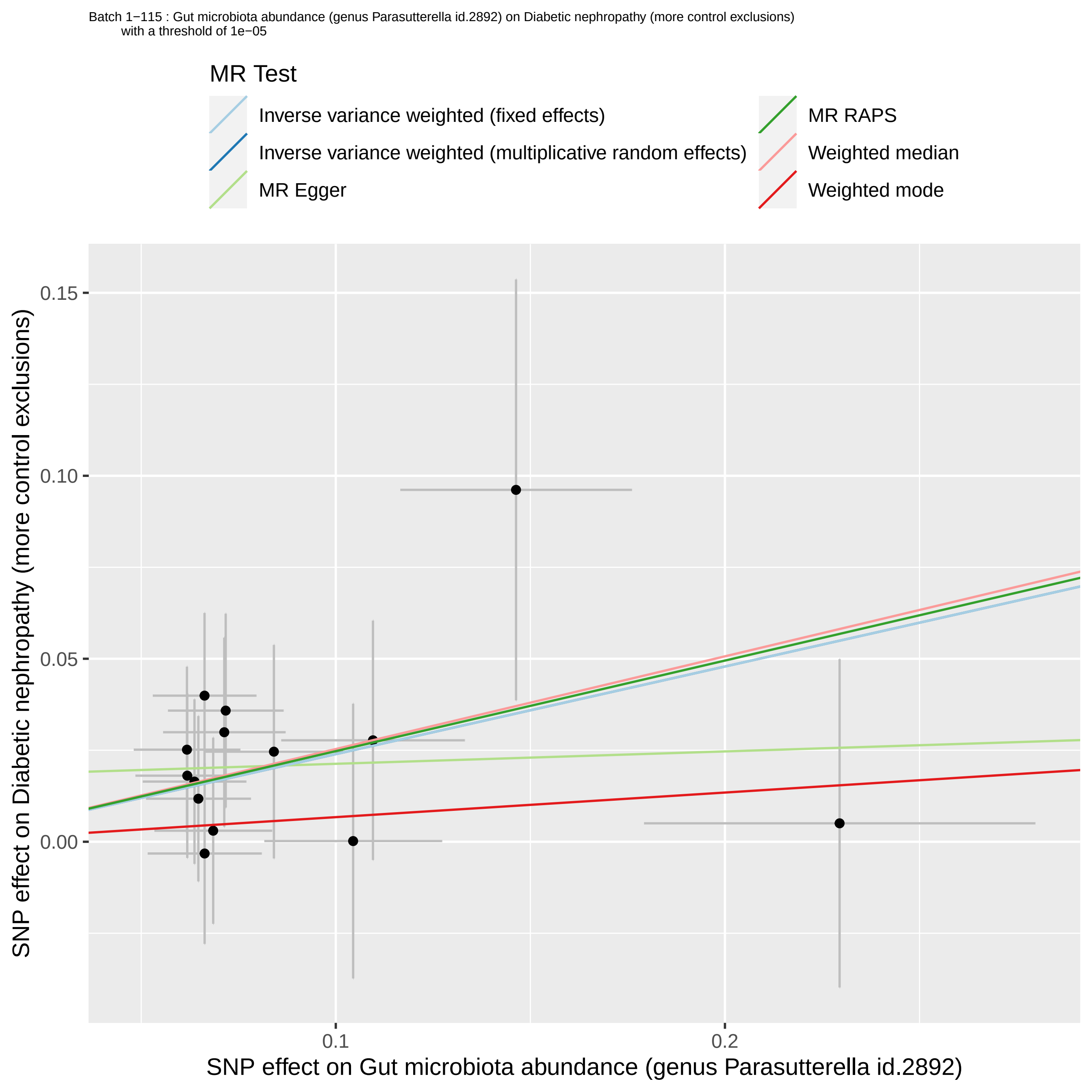
Batch 1−115 : Gut microbiota abundance (genus Parasutterella id.2892) on Diabetic nephropathy (more control exclusions)
with a threshold of 1e−05
MR Test
Inverse variance weighted (fixed effects)
Inverse variance weighted (multiplicative random effects)
MR Egger
MR RAPS
Weighted median
Weighted mode
0.15
0.10
0.05
0.00
0.1
0.2
SNP effect on Diabetic nephropathy (more control exclusions)
SNP effect on Gut microbiota abundance (genus Parasutterella id.2892)

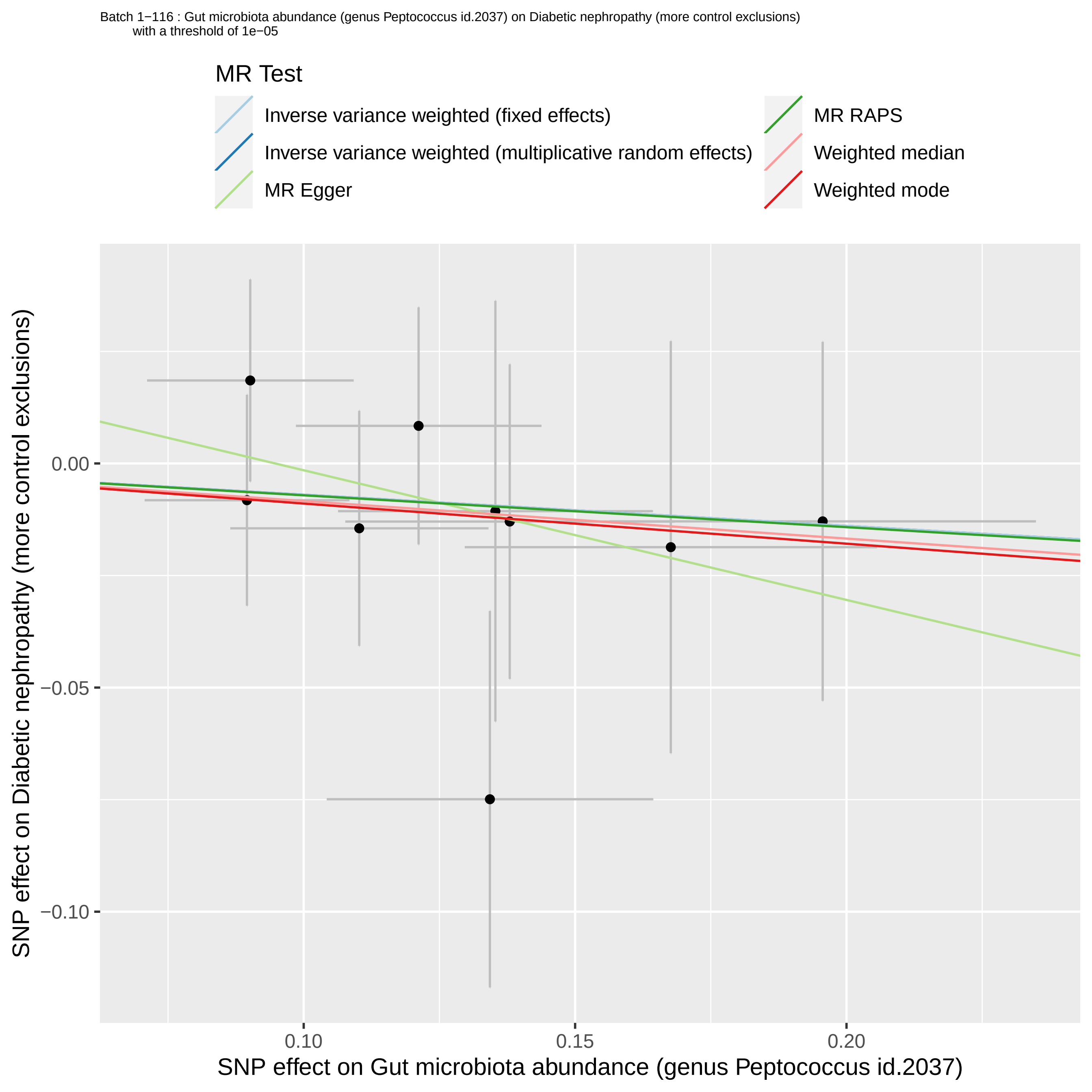

Batch 1−116 : Gut microbiota abundance (genus Peptococcus id.2037) on Diabetic nephropathy (more control exclusions)
with a threshold of 1e−05
MR Test
Inverse variance weighted (fixed effects)
Inverse variance weighted (multiplicative random effects)
MR Egger
MR RAPS
Weighted median
Weighted mode
0.00
−0.05
−0.10
0.10
0.15
0.20
SNP effect on Diabetic nephropathy (more control exclusions)
SNP effect on Gut microbiota abundance (genus Peptococcus id.2037)

Batch 1−117 : Gut microbiota abundance (genus Phascolarctobacterium id.2168) on Diabetic nephropathy (more control exclusions) with a threshold of 1e−05

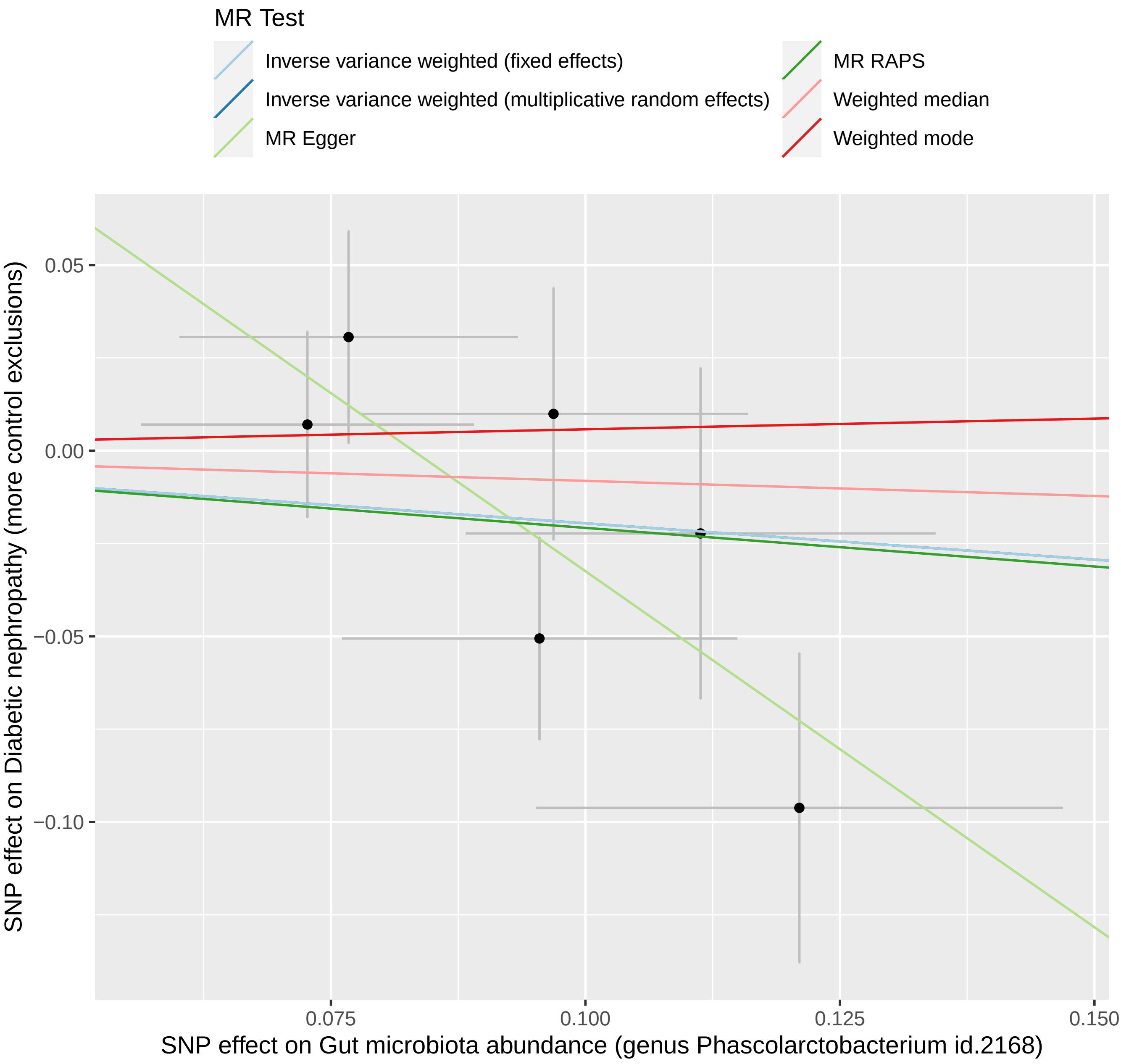

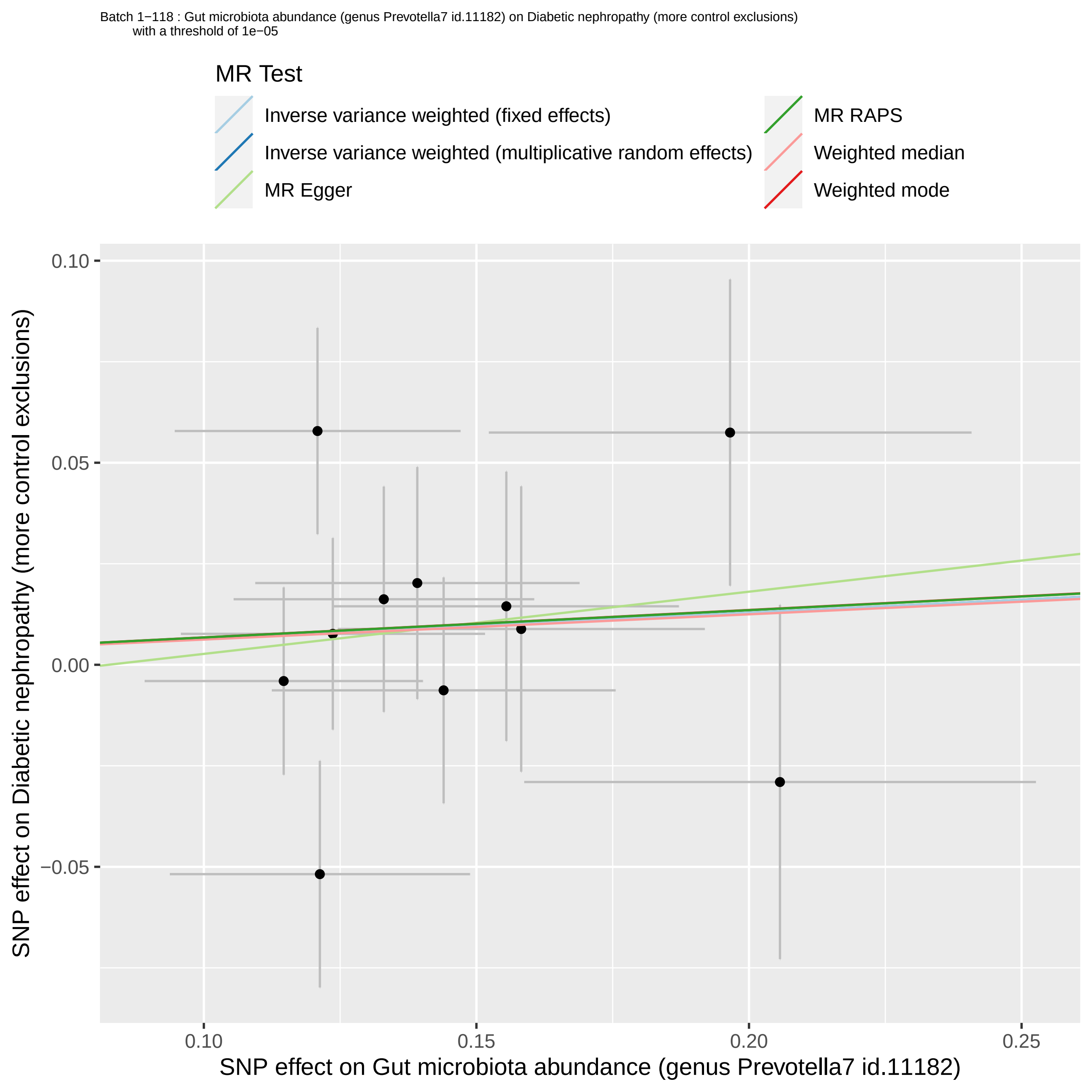
Batch 1−118 : Gut microbiota abundance (genus Prevotella7 id.11182) on Diabetic nephropathy (more control exclusions)
with a threshold of 1e−05
MR Test
Inverse variance weighted (fixed effects)
Inverse variance weighted (multiplicative random effects)
MR Egger
MR RAPS
Weighted median
Weighted mode
0.10
0.05
0.00
−0.05
0.10
0.15
0.20
0.25
SNP effect on Diabetic nephropathy (more control exclusions)
SNP effect on Gut microbiota abundance (genus Prevotella7 id.11182)

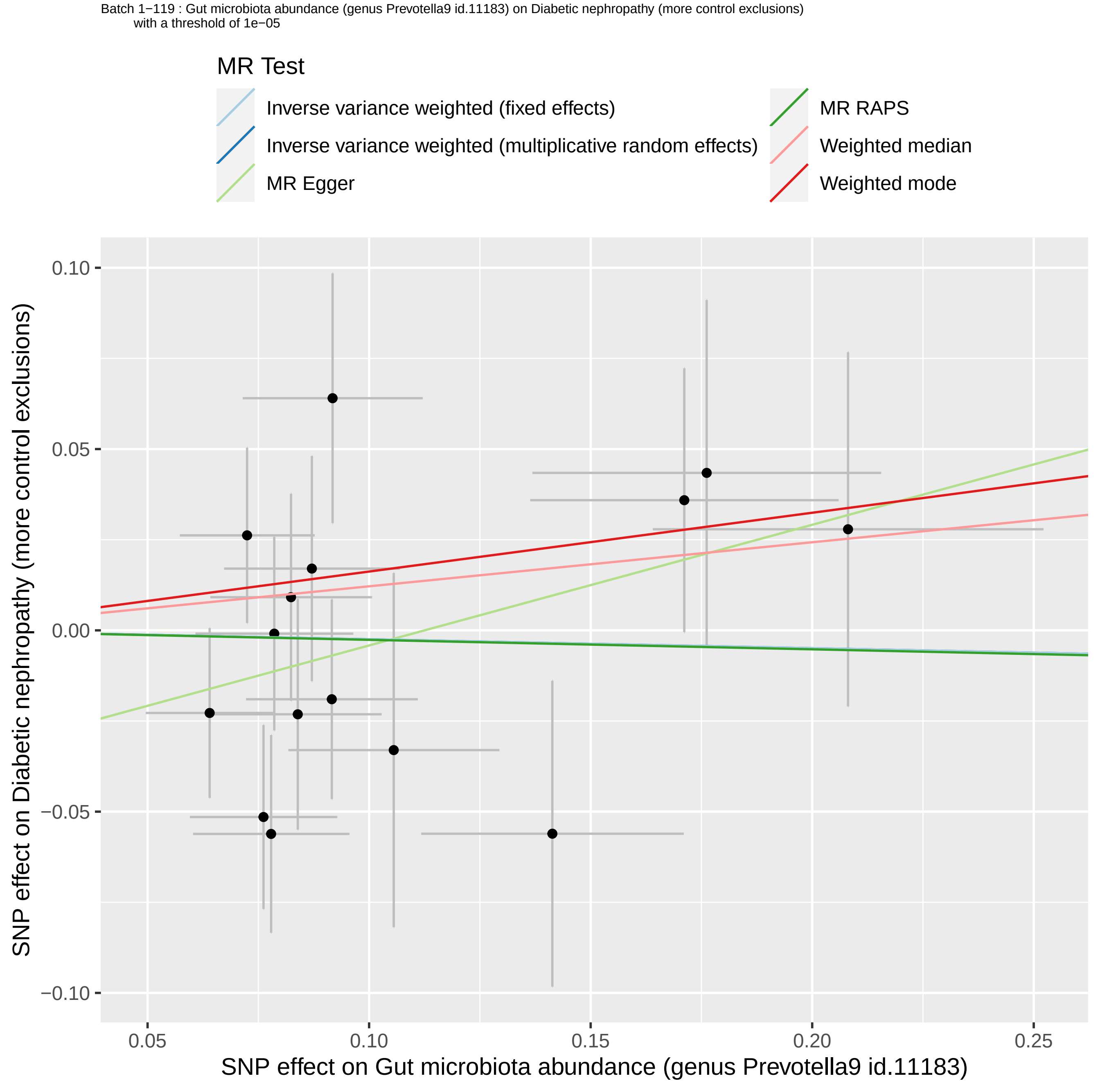
Batch 1−119 : Gut microbiota abundance (genus Prevotella9 id.11183) on Diabetic nephropathy (more control exclusions)
with a threshold of 1e−05
MR Test
Inverse variance weighted (fixed effects)
Inverse variance weighted (multiplicative random effects)
MR Egger
MR RAPS
Weighted median
Weighted mode
0.10
0.05
0.00
−0.05
−0.10
0.05
0.10
0.15
0.20
0.25
SNP effect on Diabetic nephropathy (more control exclusions)
SNP effect on Gut microbiota abundance (genus Prevotella9 id.11183)

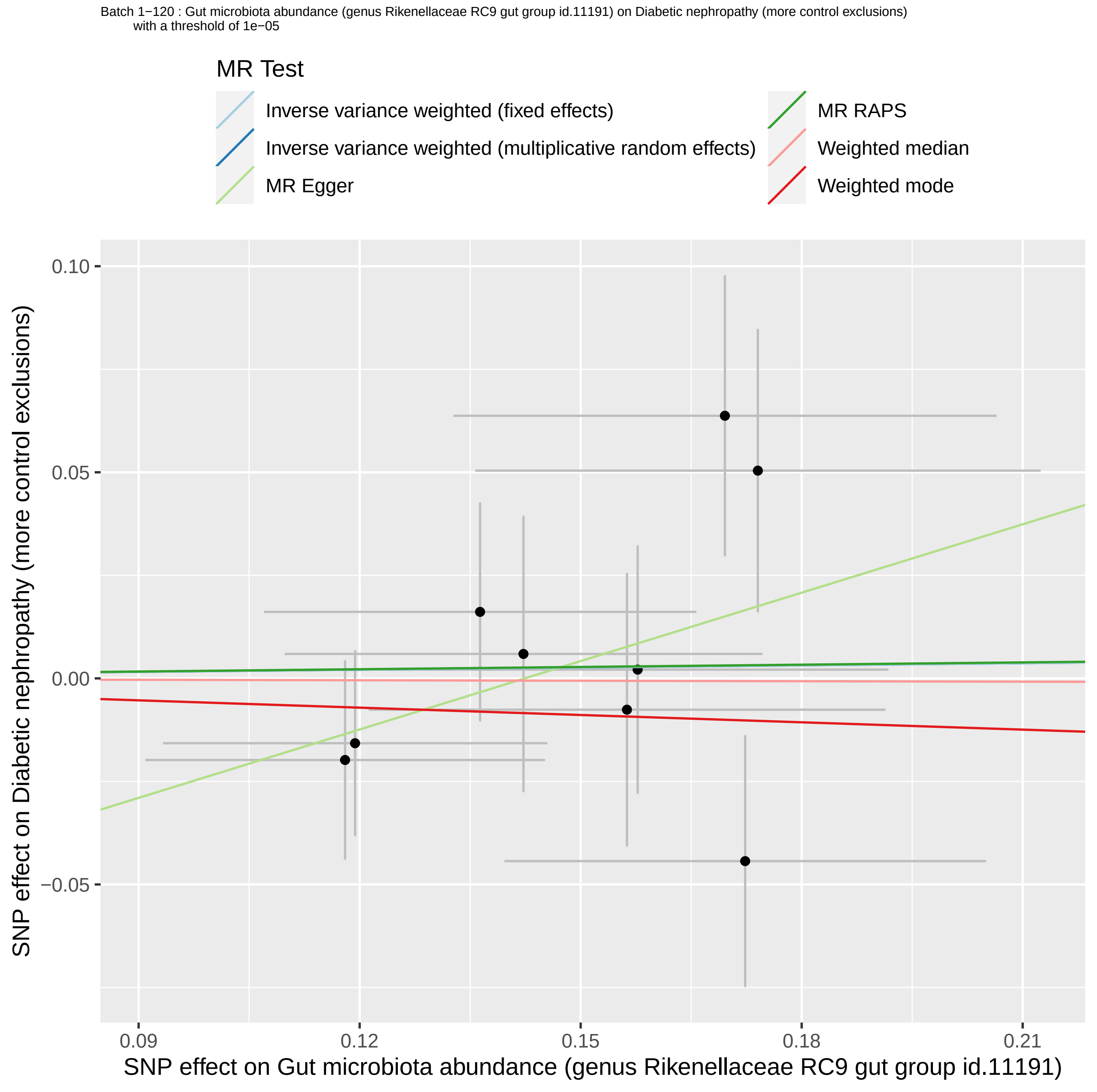

Batch 1−120 : Gut microbiota abundance (genus Rikenellaceae RC9 gut group id.11191) on Diabetic nephropathy (more control exclusions) with a threshold of 1e−05
MR Test
Inverse variance weighted (fixed effects)
Inverse variance weighted (multiplicative random effects)
MR Egger
MR RAPS
Weighted median
Weighted mode
0.10
0.05
0.00
−0.05
0.09
0.12
0.15
0.18
0.21
SNP effect on Diabetic nephropathy (more control exclusions)
SNP effect on Gut microbiota abundance (genus Rikenellaceae RC9 gut group id.11191)

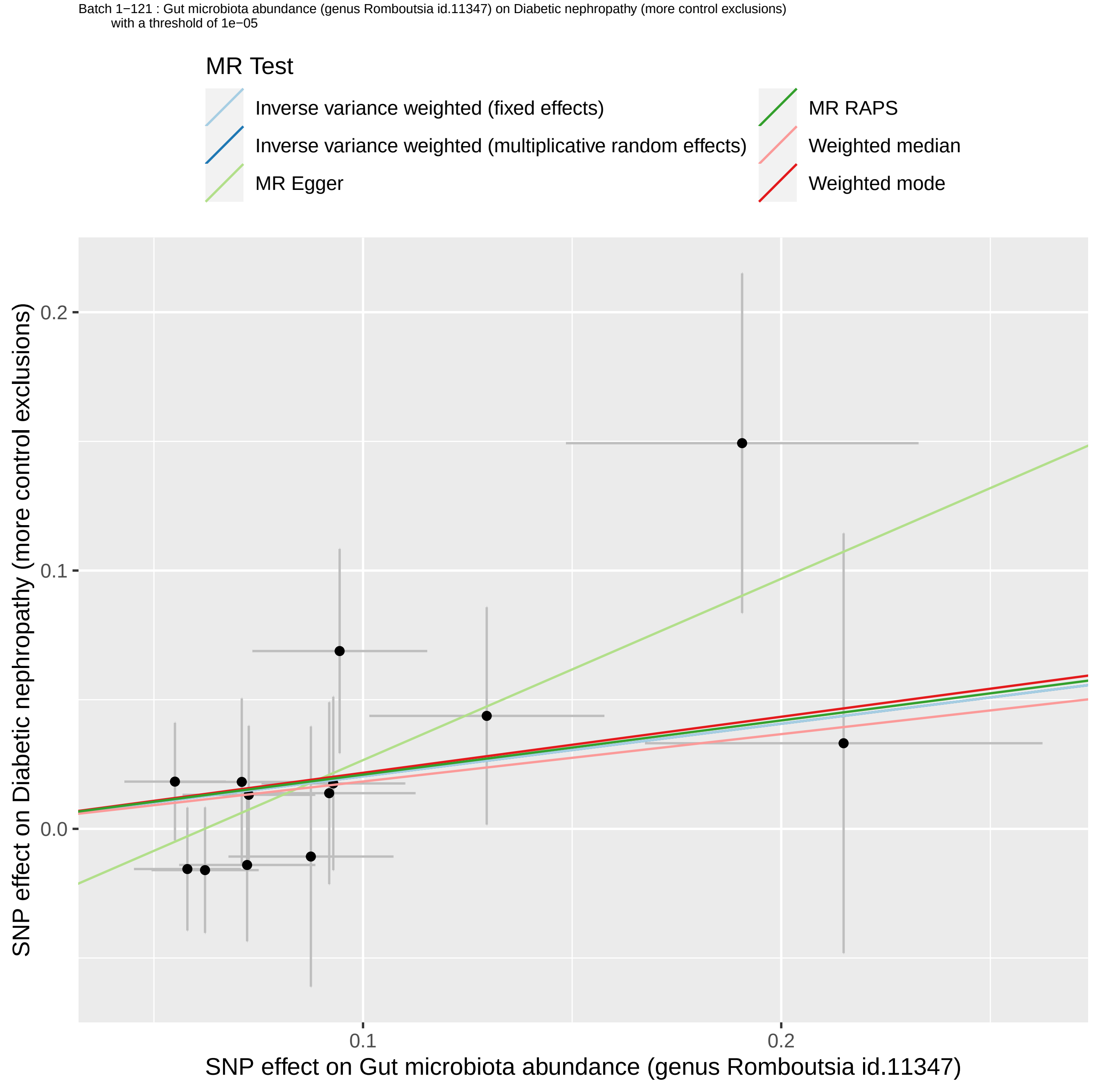

Batch 1−121 : Gut microbiota abundance (genus Romboutsia id.11347) on Diabetic nephropathy (more control exclusions)
with a threshold of 1e−05
MR Test
Inverse variance weighted (fixed effects)
Inverse variance weighted (multiplicative random effects)
MR Egger
MR RAPS
Weighted median
Weighted mode
SNP effect on Diabetic nephropathy (more control exclusions)
0.2
0.1
0.0
0.1
0.2
SNP effect on Gut microbiota abundance (genus Romboutsia id.11347)

Batch 1−122 : Gut microbiota abundance (genus Roseburia id.2012) on Diabetic nephropathy (more control exclusions) with a threshold of 1e−05

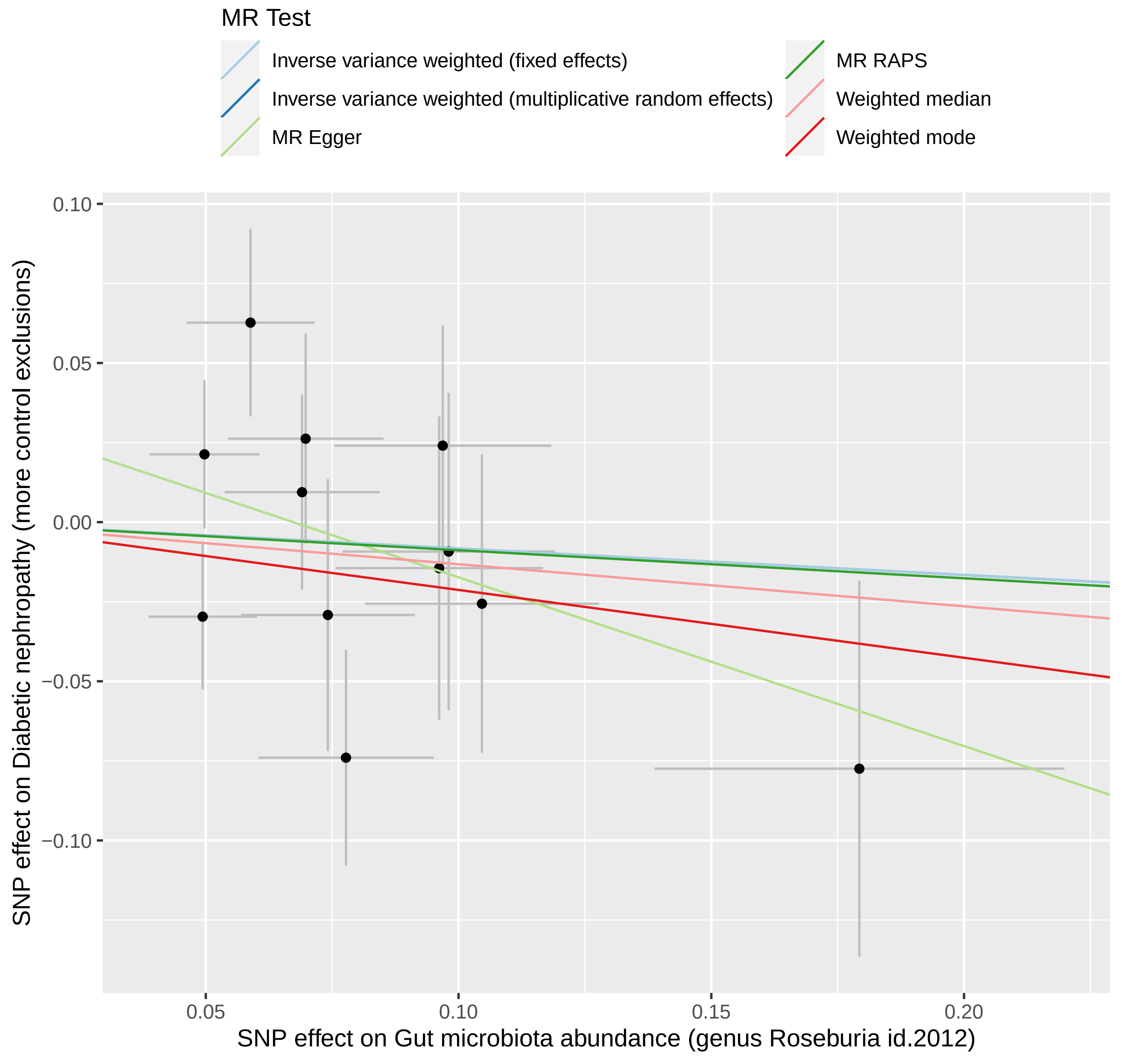

Batch 1−123 : Gut microbiota abundance (genus Ruminiclostridium5 id.11355) on Diabetic nephropathy (more control exclusions) with a threshold of 1e−05

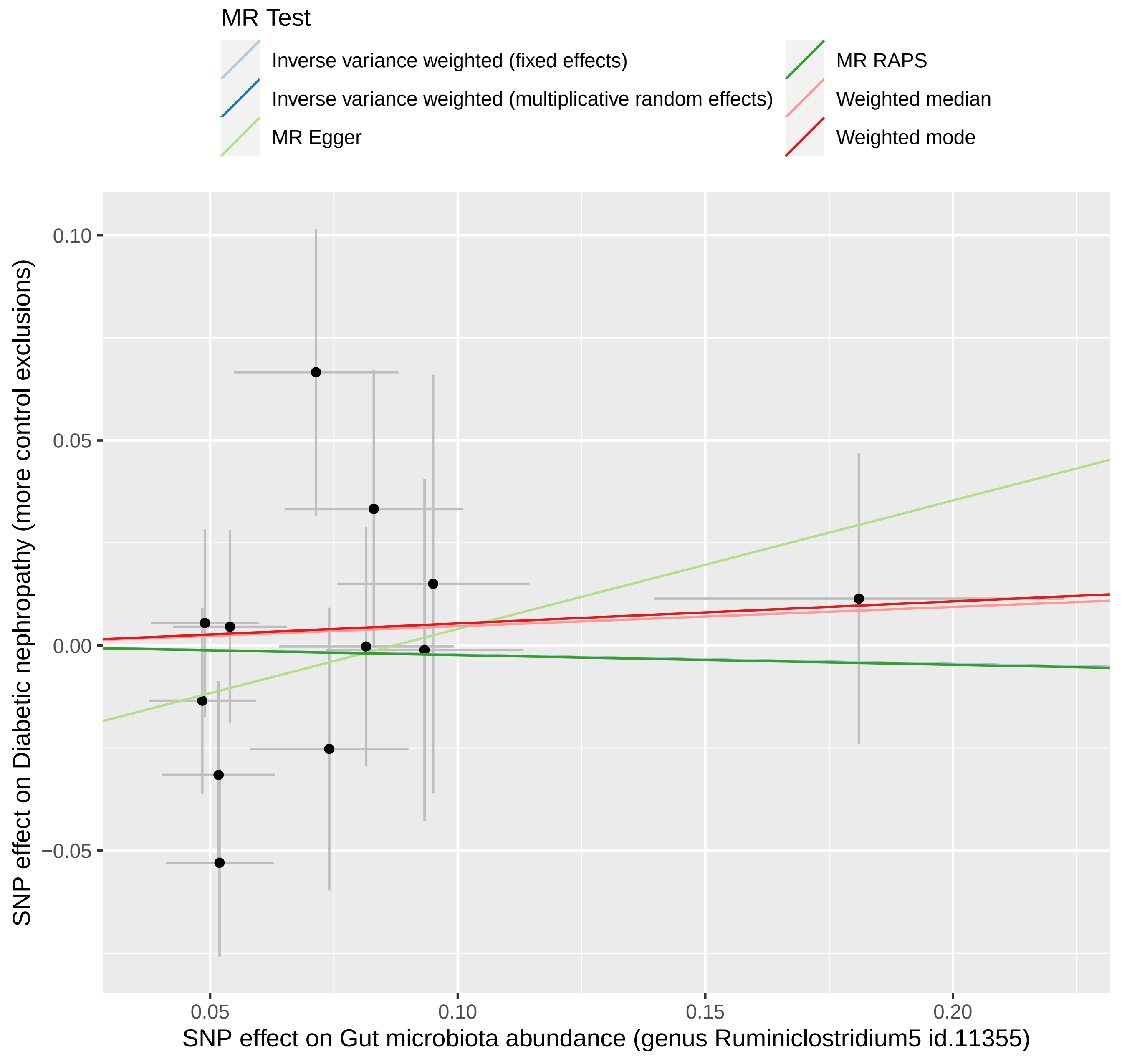

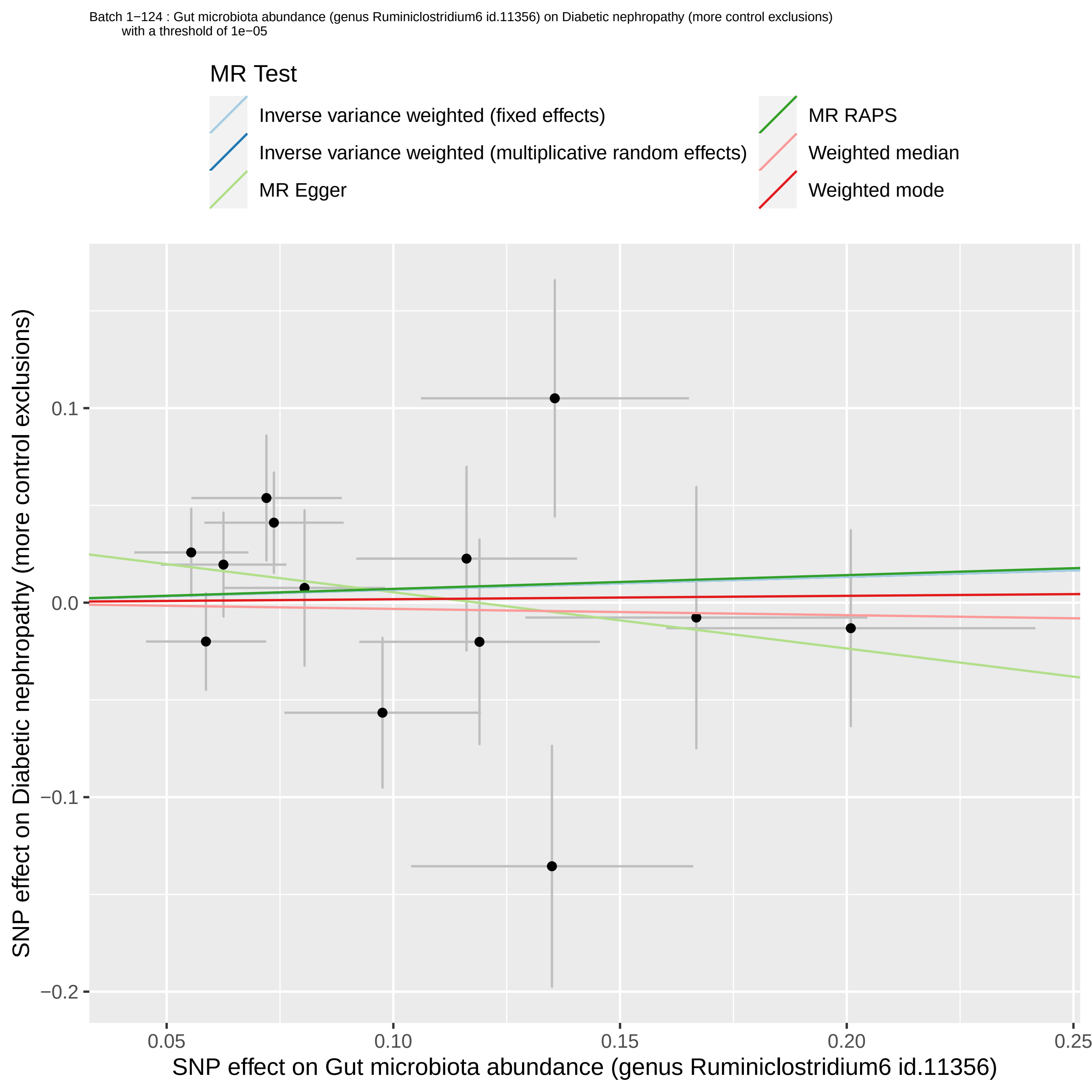

Batch 1−124 : Gut microbiota abundance (genus Ruminiclostridium6 id.11356) on Diabetic nephropathy (more control exclusions) with a threshold of 1e−05
MR Test
Inverse variance weighted (fixed effects)
Inverse variance weighted (multiplicative random effects)
MR Egger
MR RAPS
Weighted median
Weighted mode
SNP effect on Diabetic nephropathy (more control exclusions)
0.1
0.0
−0.1
−0.2
0.05
0.10
0.15
0.20
0.25
SNP effect on Gut microbiota abundance (genus Ruminiclostridium6 id.11356)

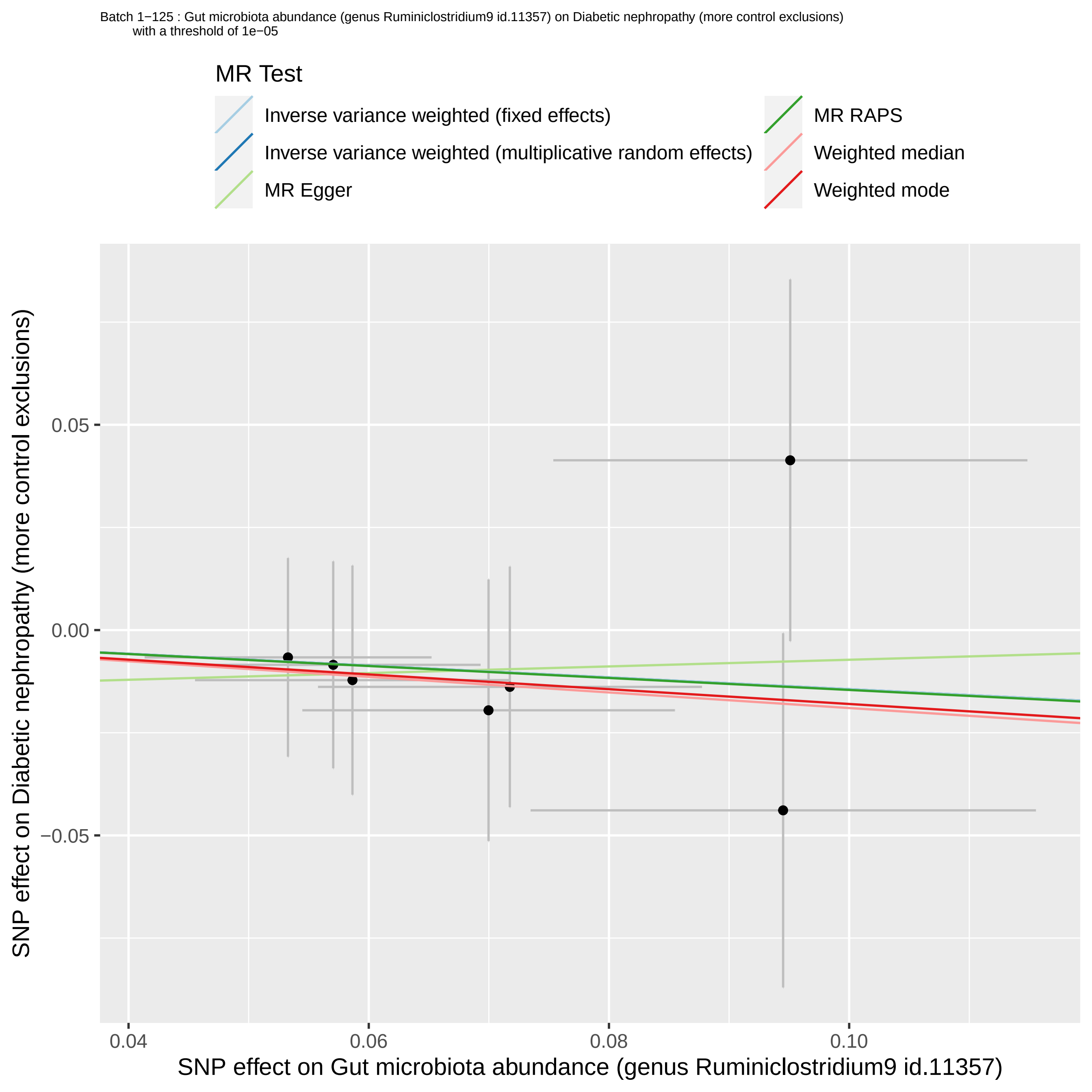
Batch 1−125 : Gut microbiota abundance (genus Ruminiclostridium9 id.11357) on Diabetic nephropathy (more control exclusions) with a threshold of 1e−05
MR Test
Inverse variance weighted (fixed effects)
Inverse variance weighted (multiplicative random effects)
MR Egger
MR RAPS
Weighted median
Weighted mode
0.05
0.00
−0.05
0.04
0.06
0.08
0.10
SNP effect on Diabetic nephropathy (more control exclusions)
SNP effect on Gut microbiota abundance (genus Ruminiclostridium9 id.11357)

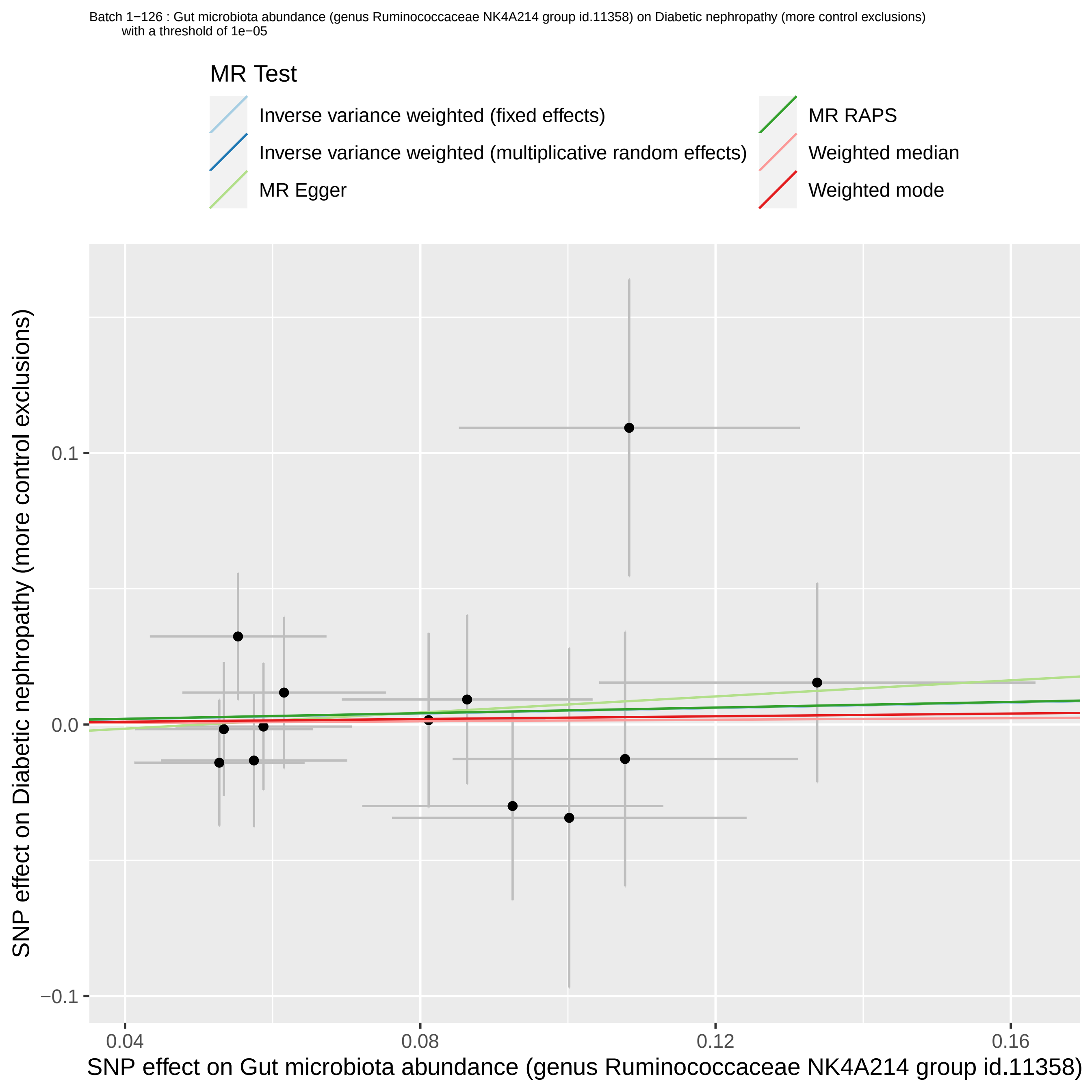
Batch 1−126 : Gut microbiota abundance (genus Ruminococcaceae NK4A214 group id.11358) on Diabetic nephropathy (more control exclusions)
with a threshold of 1e−05
MR Test
Inverse variance weighted (fixed effects)
Inverse variance weighted (multiplicative random effects)
MR Egger
MR RAPS
Weighted median
Weighted mode
0.1
0.0
−0.1
0.04
0.08
0.12
0.16
SNP effect on Diabetic nephropathy (more control exclusions)
SNP effect on Gut microbiota abundance (genus Ruminococcaceae NK4A214 group id.11358)

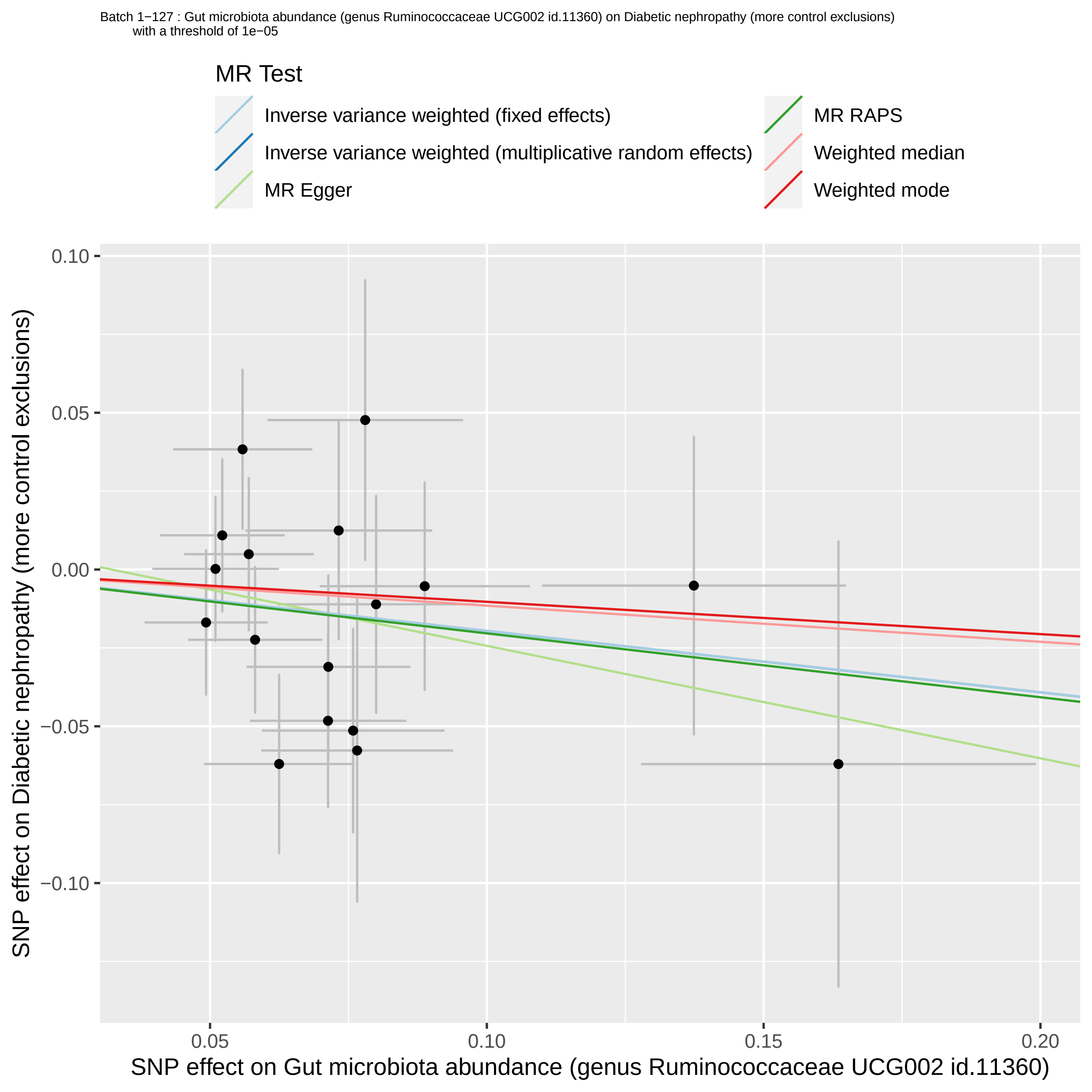

Batch 1−127 : Gut microbiota abundance (genus Ruminococcaceae UCG002 id.11360) on Diabetic nephropathy (more control exclusions) with a threshold of 1e−05
MR Test
Inverse variance weighted (fixed effects)
Inverse variance weighted (multiplicative random effects)
MR Egger
MR RAPS
Weighted median
Weighted mode
0.10
0.05
0.00
−0.05
−0.10
0.05
0.10
0.15
0.20
SNP effect on Diabetic nephropathy (more control exclusions)
SNP effect on Gut microbiota abundance (genus Ruminococcaceae UCG002 id.11360)

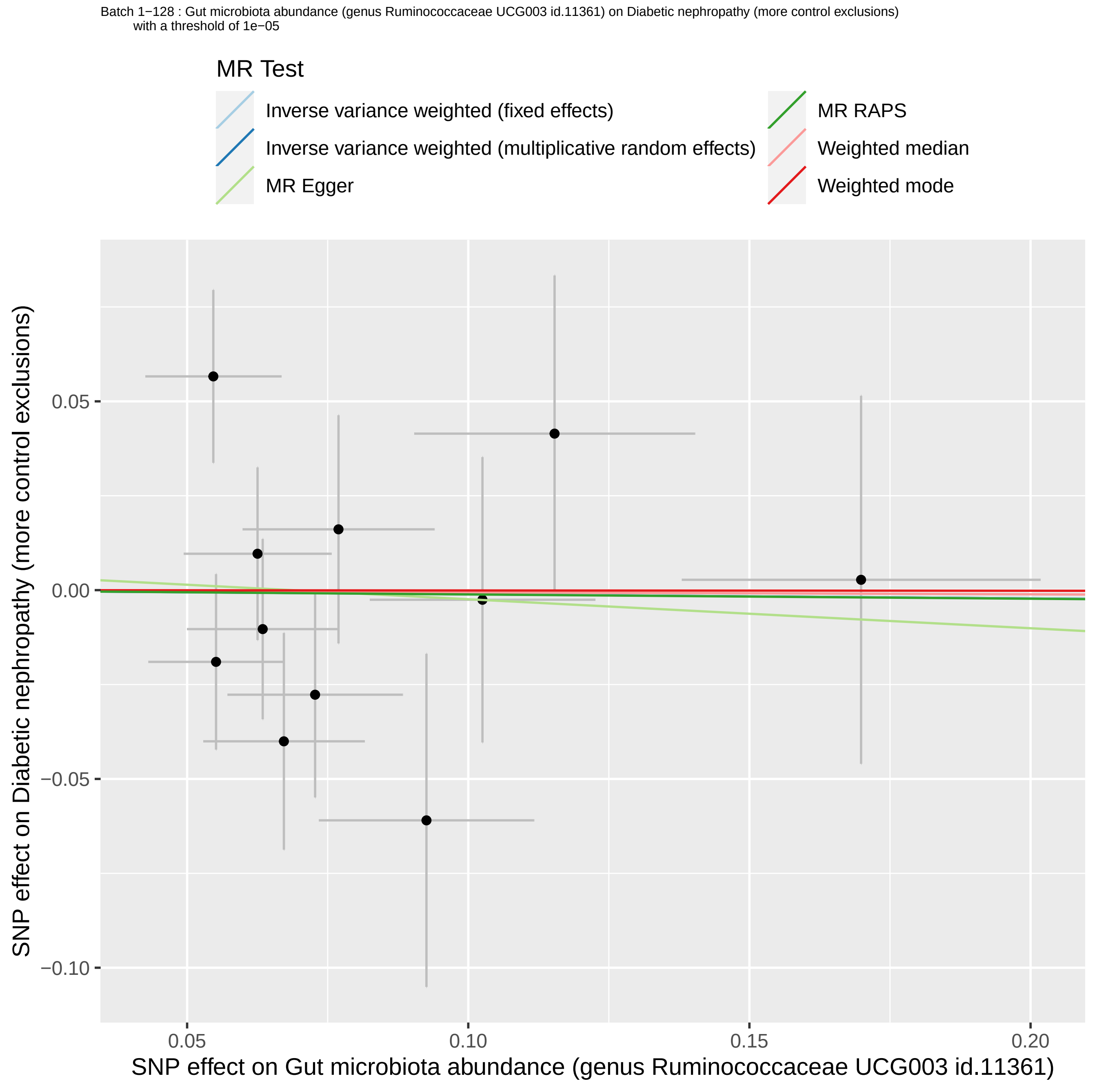
Batch 1−128 : Gut microbiota abundance (genus Ruminococcaceae UCG003 id.11361) on Diabetic nephropathy (more control exclusions)
with a threshold of 1e−05
MR Test
Inverse variance weighted (fixed effects)
Inverse variance weighted (multiplicative random effects)
MR Egger
MR RAPS
Weighted median
Weighted mode
0.05
0.00
−0.05
−0.10
0.05
0.10
0.15
0.20
SNP effect on Diabetic nephropathy (more control exclusions)
SNP effect on Gut microbiota abundance (genus Ruminococcaceae UCG003 id.11361)

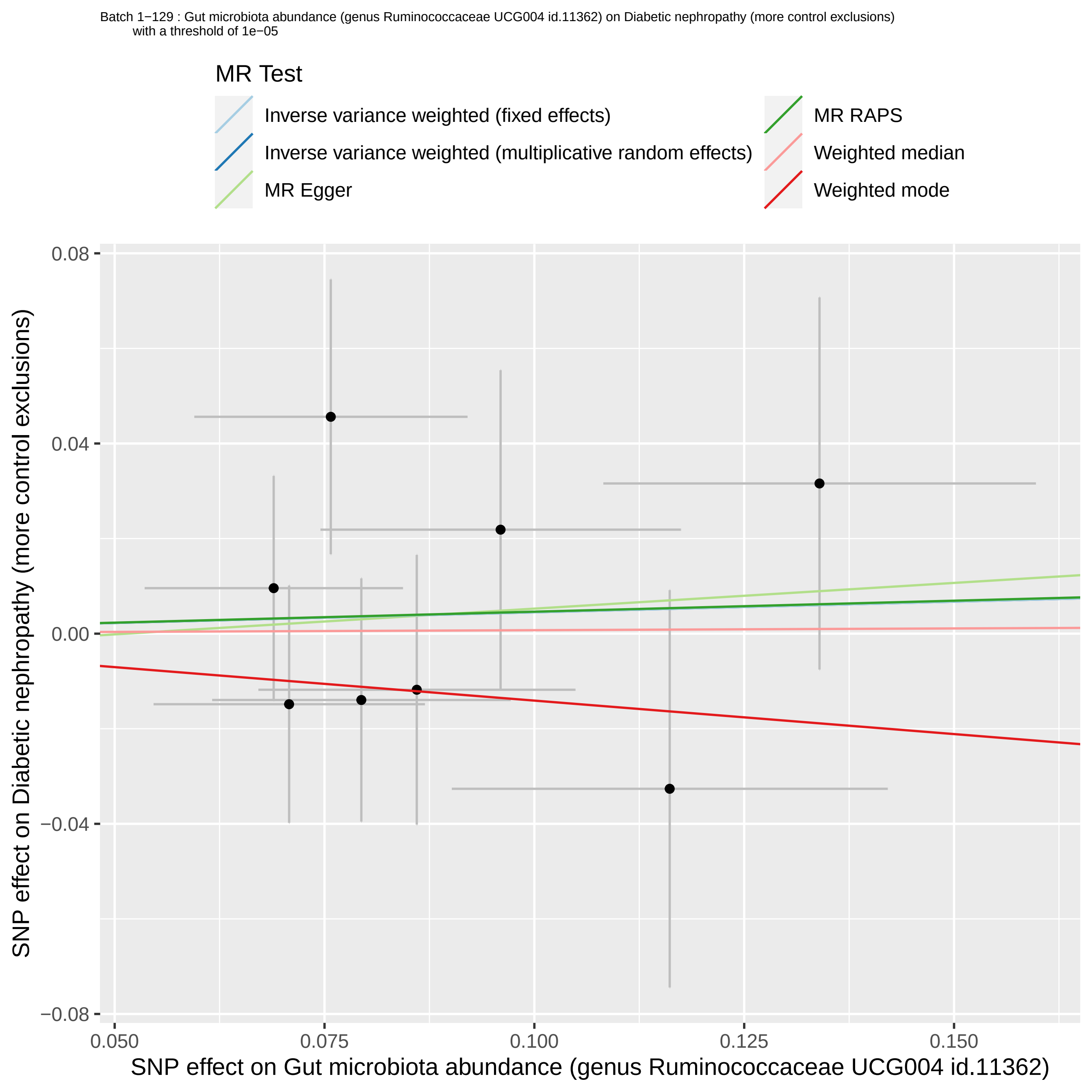

Batch 1−129 : Gut microbiota abundance (genus Ruminococcaceae UCG004 id.11362) on Diabetic nephropathy (more control exclusions) with a threshold of 1e−05
MR Test
Inverse variance weighted (fixed effects)
Inverse variance weighted (multiplicative random effects)
MR Egger
MR RAPS
Weighted median
Weighted mode
0.08
0.04
0.00
−0.04
−0.08
0.050
0.075
0.100
0.125
0.150
SNP effect on Diabetic nephropathy (more control exclusions)
SNP effect on Gut microbiota abundance (genus Ruminococcaceae UCG004 id.11362)

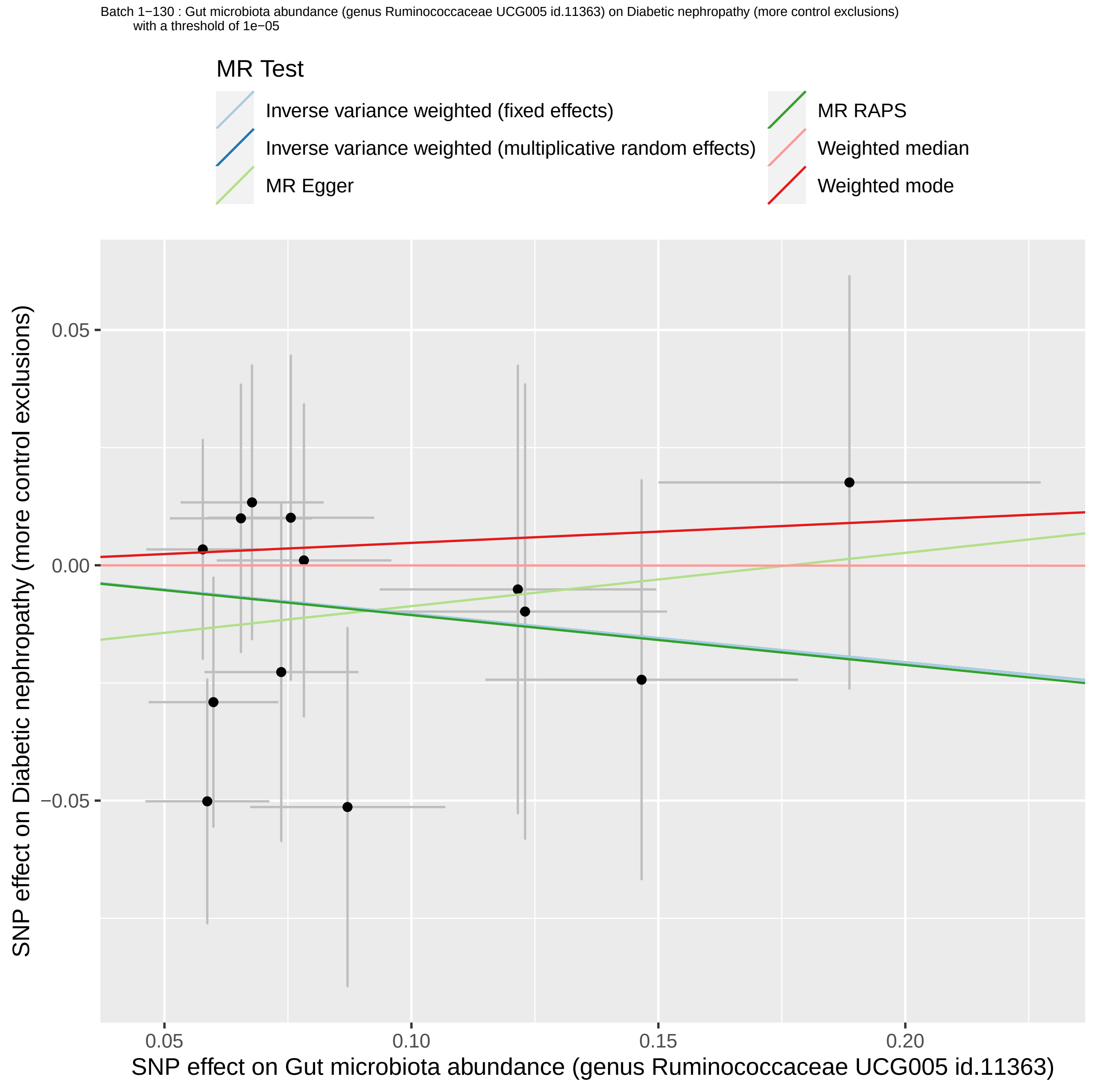
Batch 1−130 : Gut microbiota abundance (genus Ruminococcaceae UCG005 id.11363) on Diabetic nephropathy (more control exclusions)
with a threshold of 1e−05
MR Test
Inverse variance weighted (fixed effects)
Inverse variance weighted (multiplicative random effects)
MR Egger
MR RAPS
Weighted median
Weighted mode
0.05
0.00
−0.05
0.05
0.10
0.15
0.20
SNP effect on Diabetic nephropathy (more control exclusions)
SNP effect on Gut microbiota abundance (genus Ruminococcaceae UCG005 id.11363)

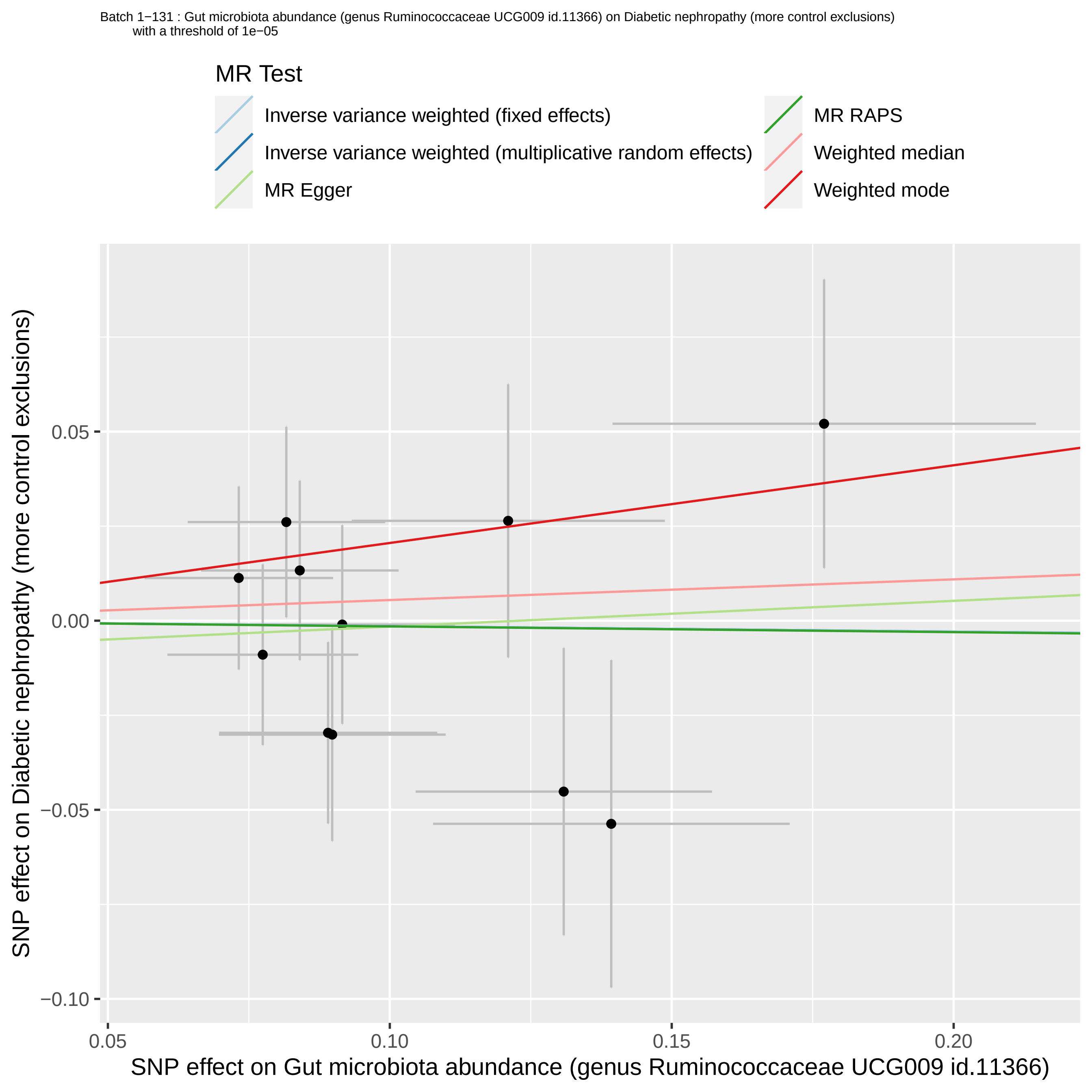
Batch 1−131 : Gut microbiota abundance (genus Ruminococcaceae UCG009 id.11366) on Diabetic nephropathy (more control exclusions)
with a threshold of 1e−05
MR Test
Inverse variance weighted (fixed effects)
Inverse variance weighted (multiplicative random effects)
MR Egger
MR RAPS
Weighted median
Weighted mode
0.05
0.00
−0.05
−0.10
0.05
0.10
0.15
0.20
SNP effect on Diabetic nephropathy (more control exclusions)
SNP effect on Gut microbiota abundance (genus Ruminococcaceae UCG009 id.11366)

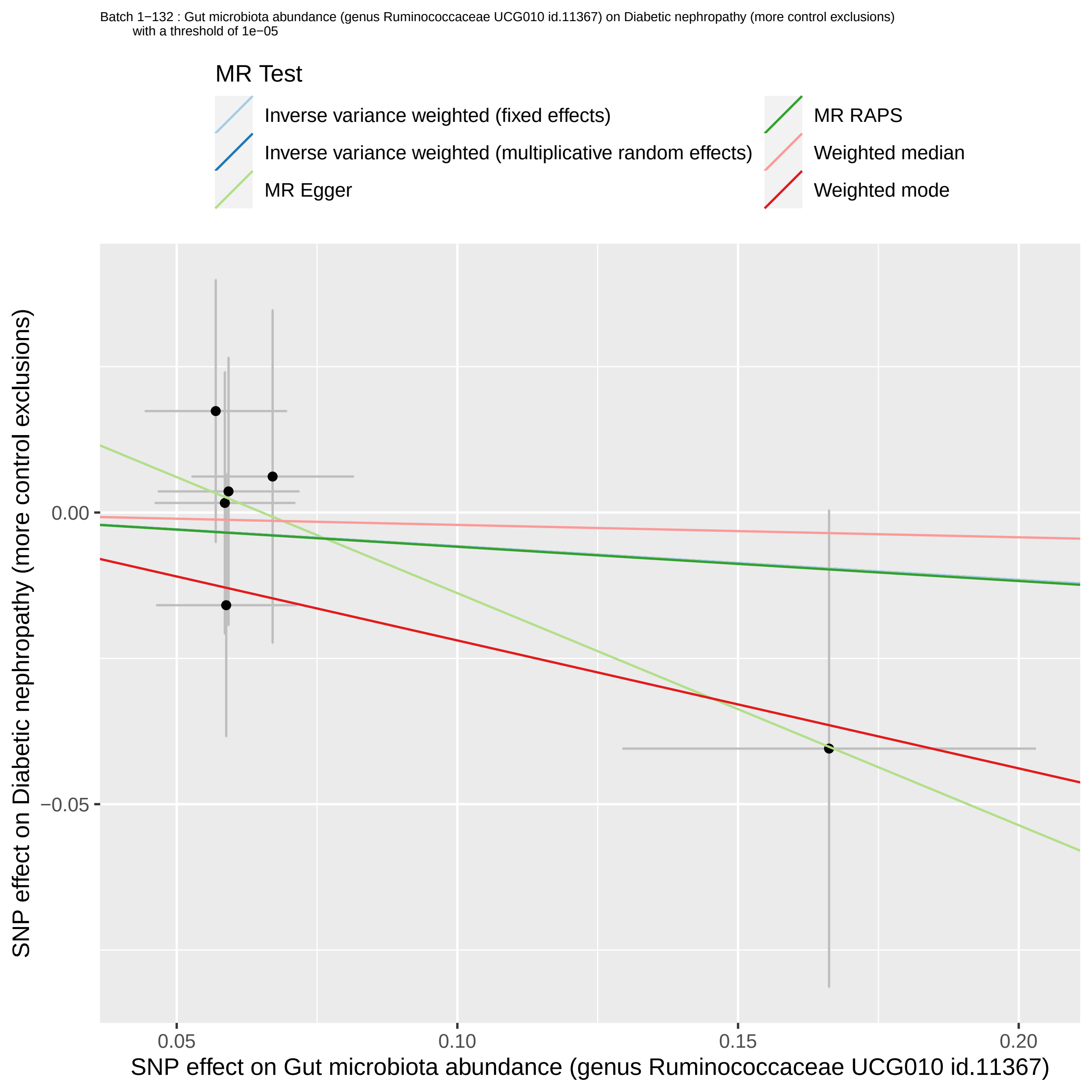
Batch 1−132 : Gut microbiota abundance (genus Ruminococcaceae UCG010 id.11367) on Diabetic nephropathy (more control exclusions)
with a threshold of 1e−05
MR Test
Inverse variance weighted (fixed effects)
Inverse variance weighted (multiplicative random effects)
MR Egger
MR RAPS
Weighted median
Weighted mode
0.00
−0.05
0.05
0.10
0.15
0.20
SNP effect on Diabetic nephropathy (more control exclusions)
SNP effect on Gut microbiota abundance (genus Ruminococcaceae UCG010 id.11367)

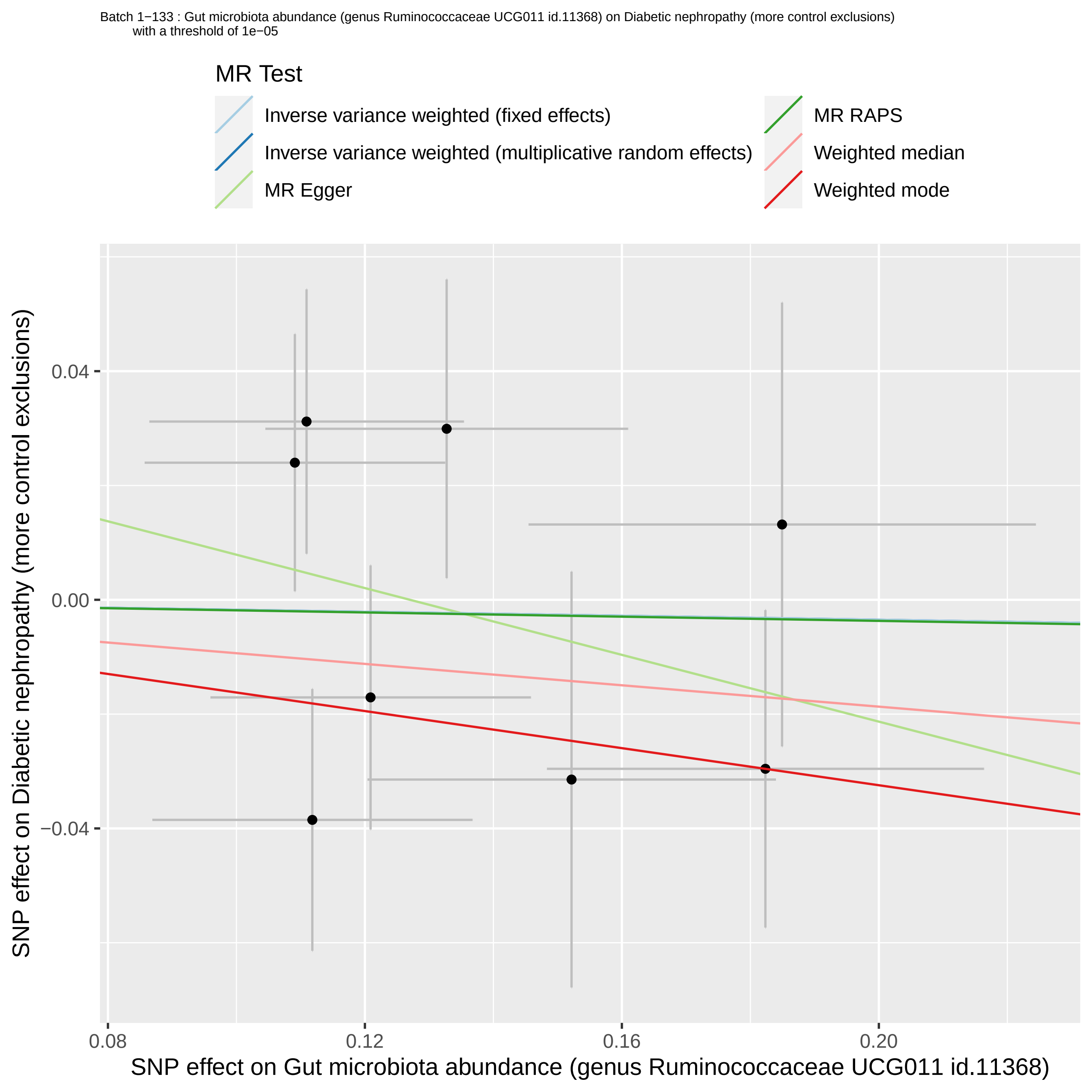

Batch 1−133 : Gut microbiota abundance (genus Ruminococcaceae UCG011 id.11368) on Diabetic nephropathy (more control exclusions)
with a threshold of 1e−05
MR Test
Inverse variance weighted (fixed effects)
Inverse variance weighted (multiplicative random effects)
MR Egger
MR RAPS
Weighted median
Weighted mode
0.04
0.00
−0.04
0.08
0.12
0.16
0.20
SNP effect on Diabetic nephropathy (more control exclusions)
SNP effect on Gut microbiota abundance (genus Ruminococcaceae UCG011 id.11368)

Batch 1−134 : Gut microbiota abundance (genus Ruminococcaceae UCG013 id.11370) on Diabetic nephropathy (more control exclusions) with a threshold of 1e−05

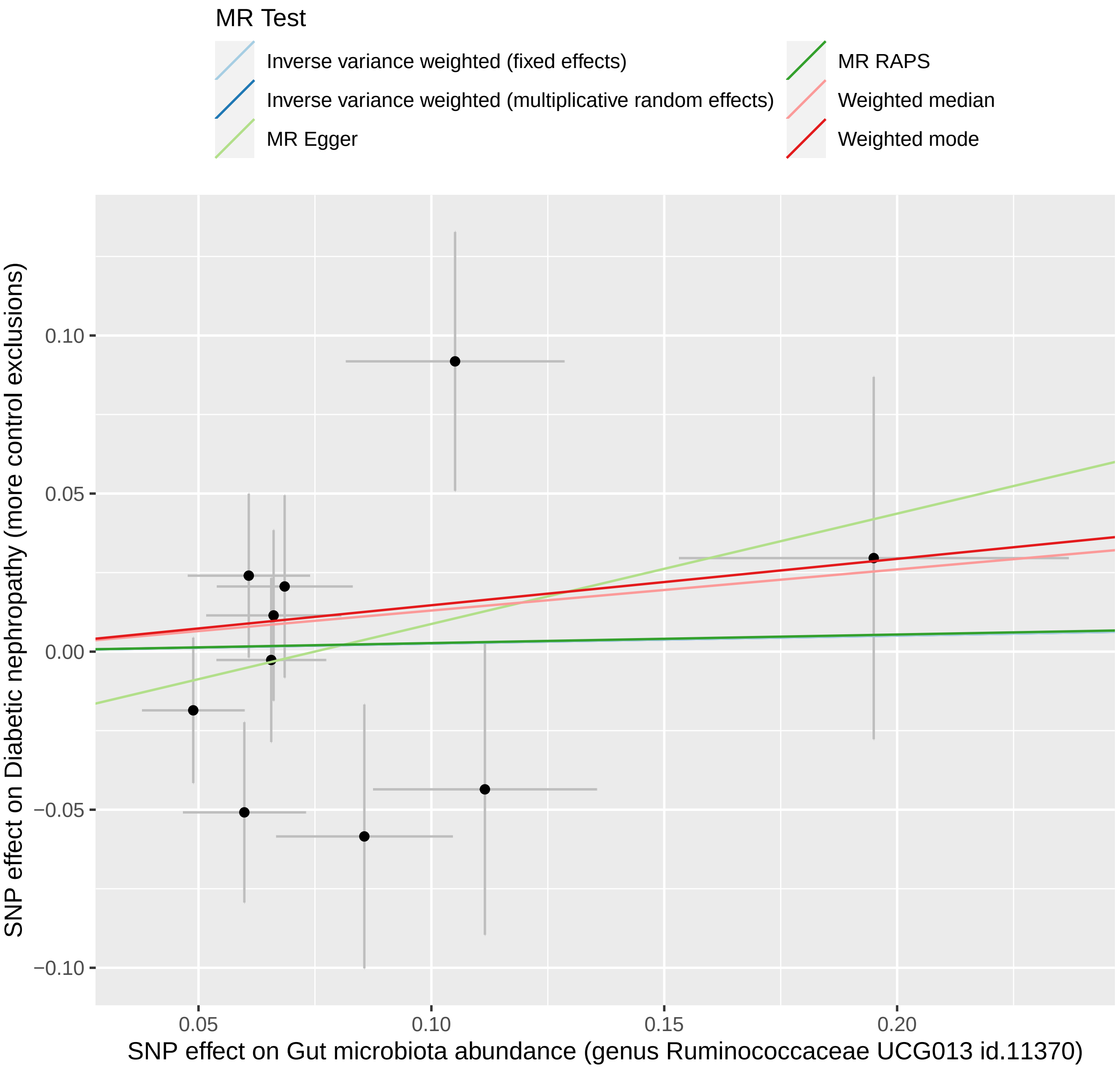

Batch 1−135 : Gut microbiota abundance (genus Ruminococcaceae UCG014 id.11371) on Diabetic nephropathy (more control exclusions) with a threshold of 1e−05

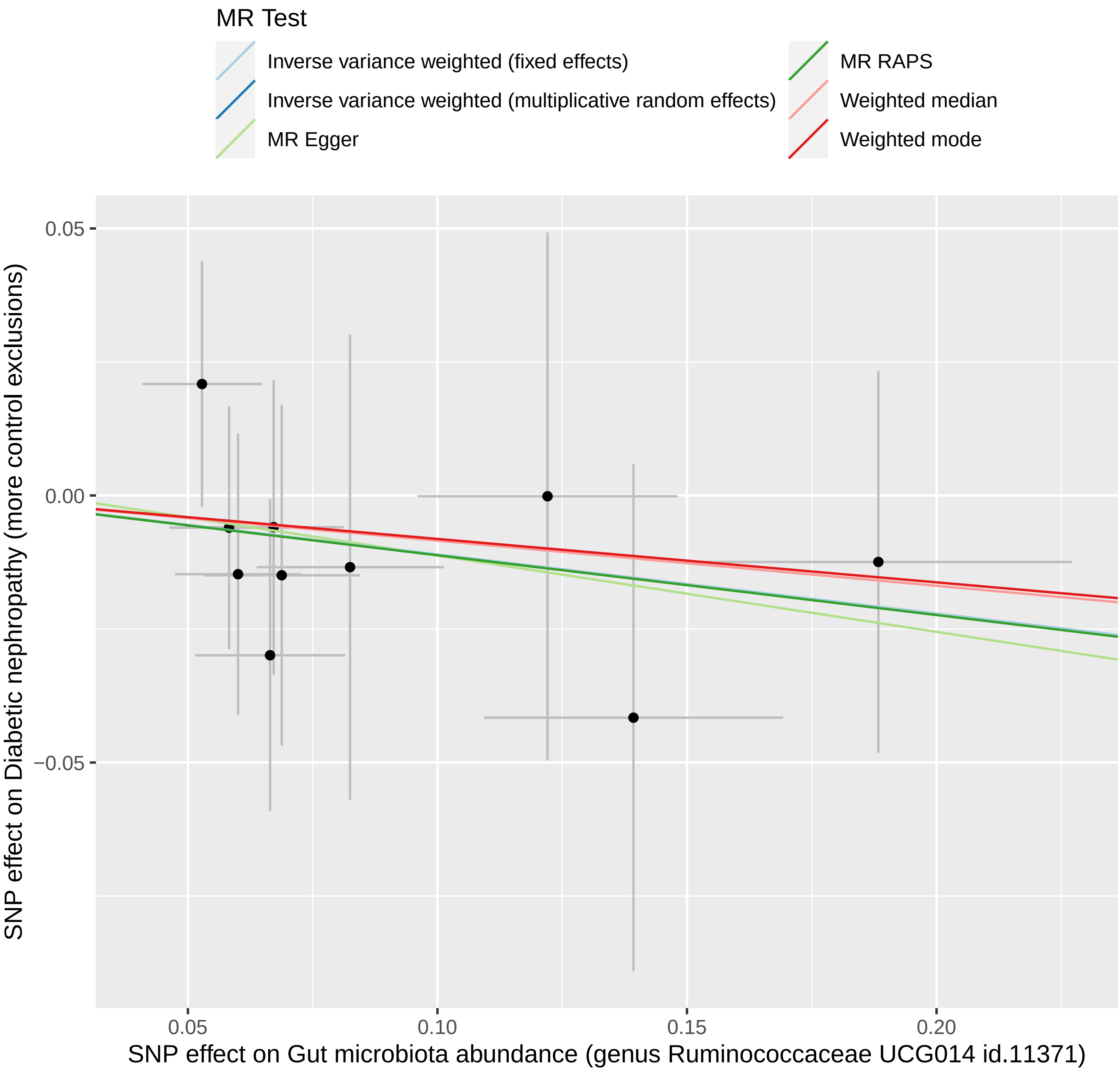

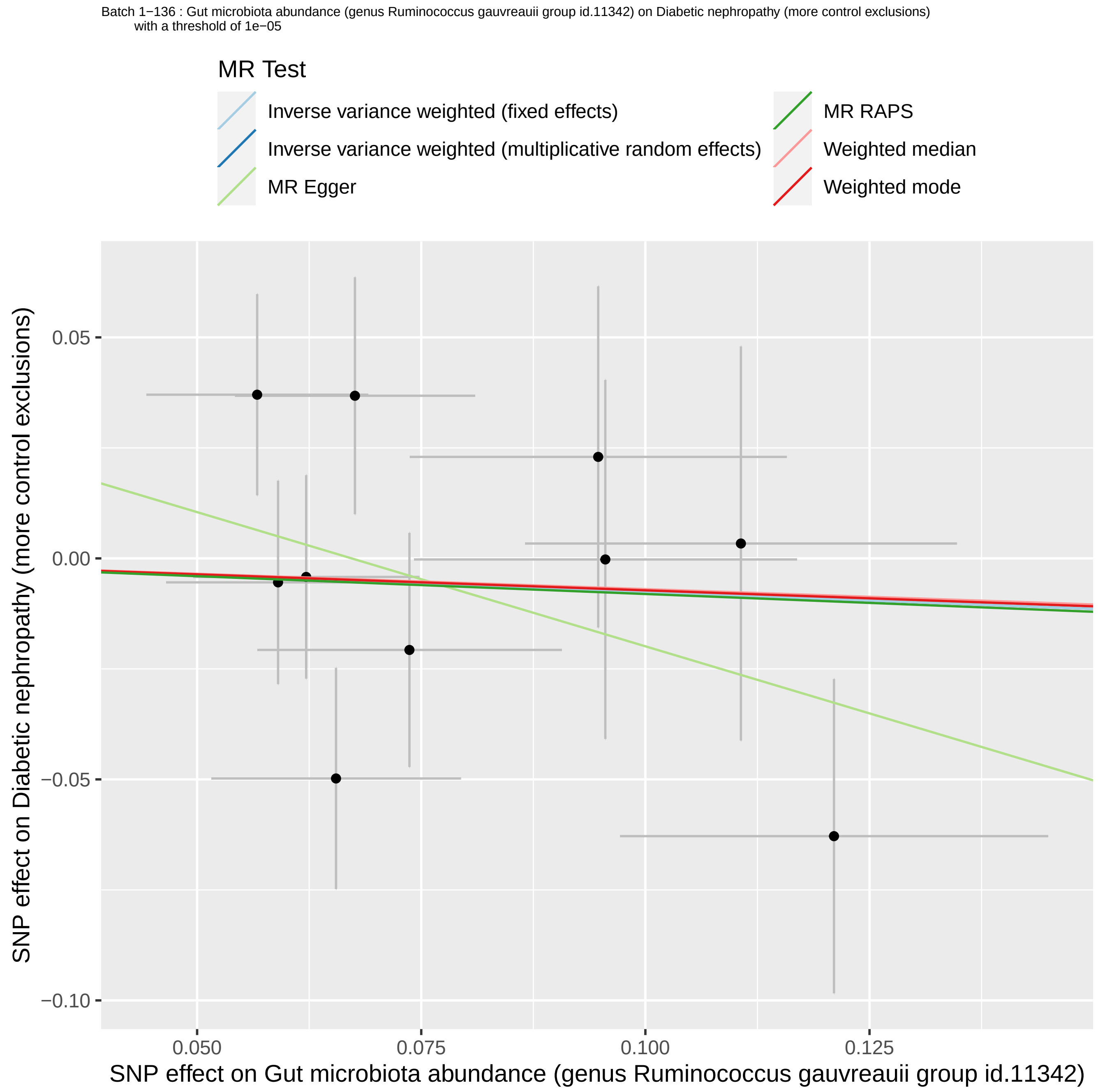
Batch 1−136 : Gut microbiota abundance (genus Ruminococcus gauvreauii group id.11342) on Diabetic nephropathy (more control exclusions) with a threshold of 1e−05
MR Test
Inverse variance weighted (fixed effects)
Inverse variance weighted (multiplicative random effects)
MR Egger
MR RAPS
Weighted median
Weighted mode
0.05
0.00
−0.05
−0.10
0.050
0.075
0.100
0.125
SNP effect on Diabetic nephropathy (more control exclusions)
SNP effect on Gut microbiota abundance (genus Ruminococcus gauvreauii group id.11342)

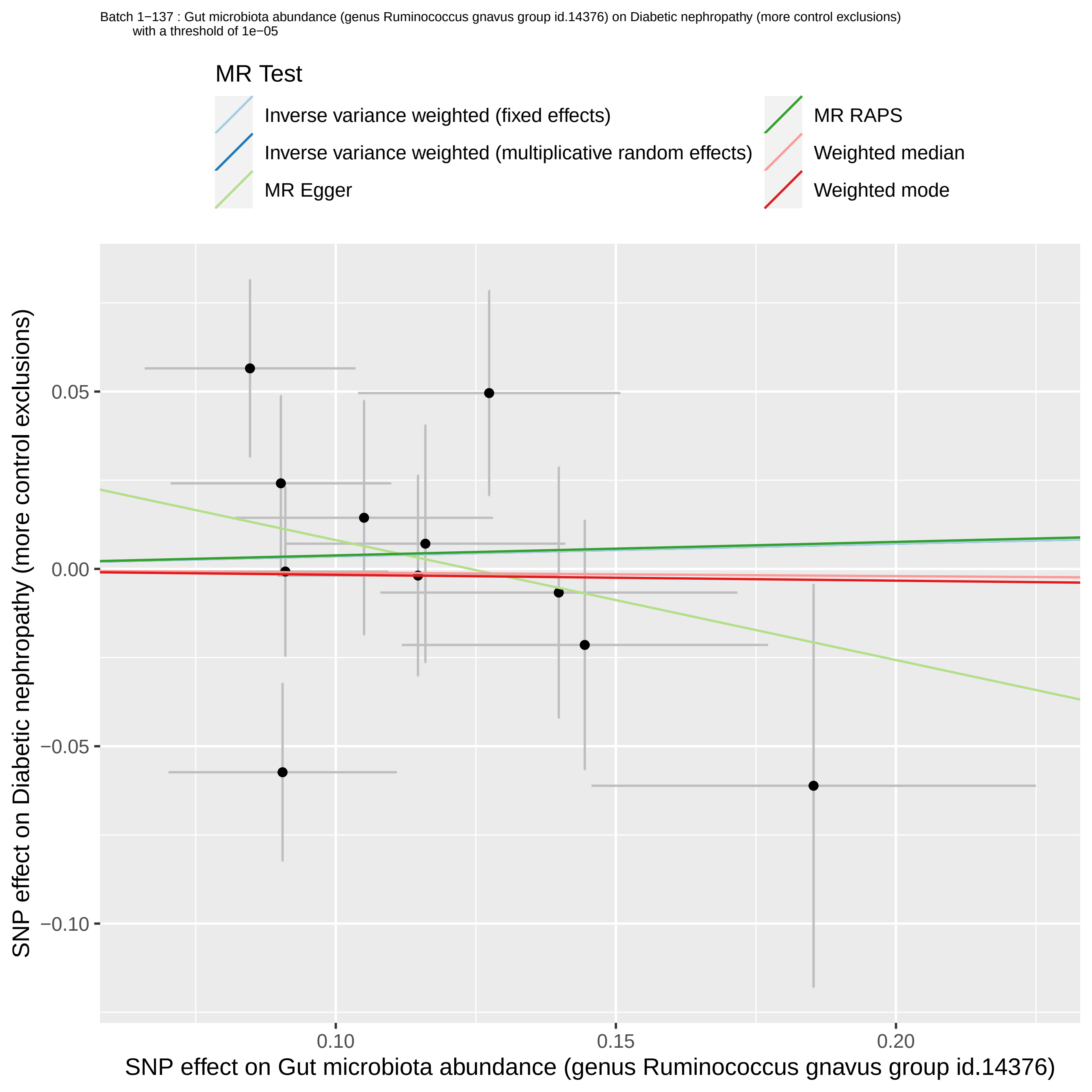
Batch 1−137 : Gut microbiota abundance (genus Ruminococcus gnavus group id.14376) on Diabetic nephropathy (more control exclusions)
with a threshold of 1e−05
MR Test
Inverse variance weighted (fixed effects)
Inverse variance weighted (multiplicative random effects)
MR Egger
MR RAPS
Weighted median
Weighted mode
0.05
0.00
−0.05
−0.10
0.10
0.15
0.20
SNP effect on Diabetic nephropathy (more control exclusions)
SNP effect on Gut microbiota abundance (genus Ruminococcus gnavus group id.14376)

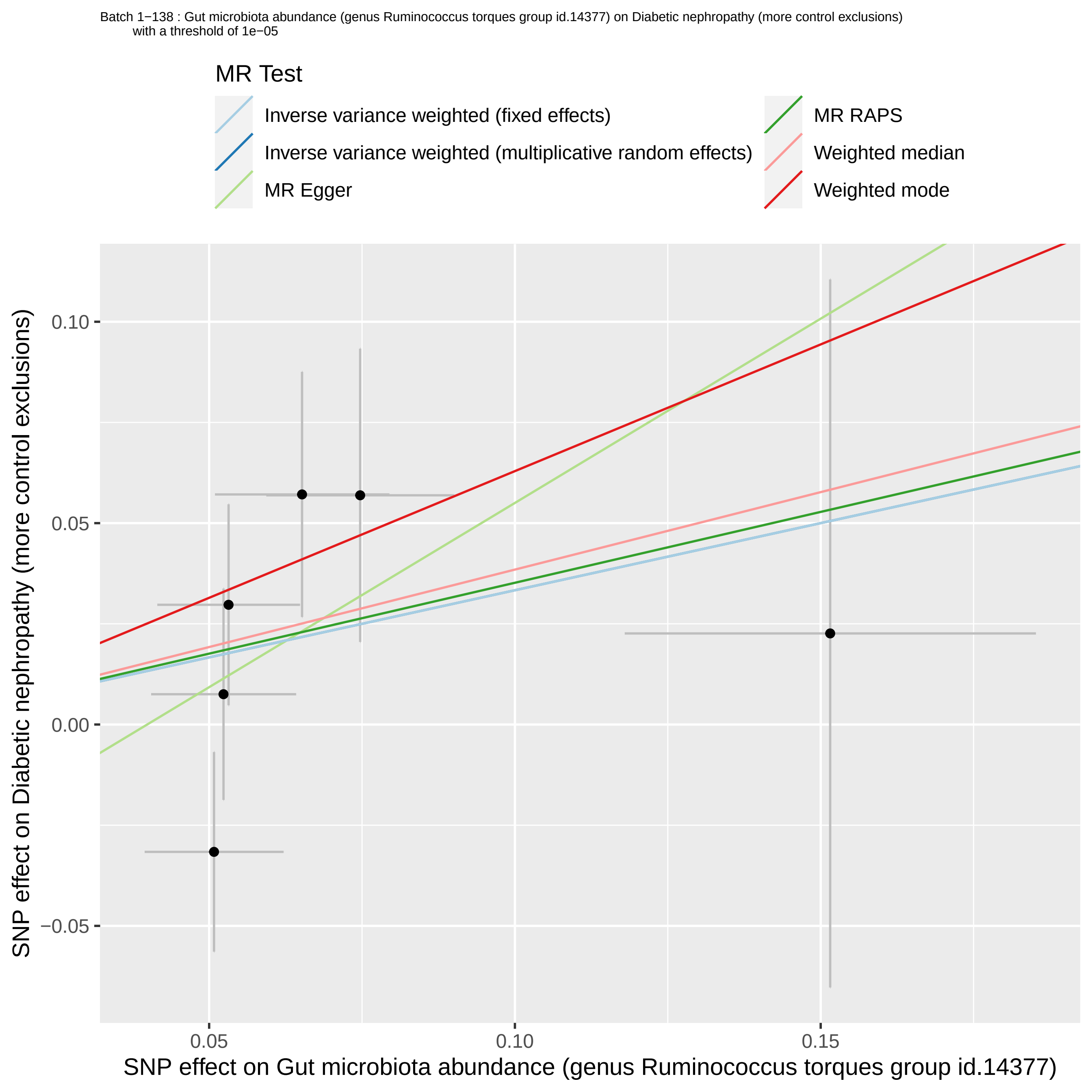

Batch 1−138 : Gut microbiota abundance (genus Ruminococcus torques group id.14377) on Diabetic nephropathy (more control exclusions)
with a threshold of 1e−05
MR Test
Inverse variance weighted (fixed effects)
Inverse variance weighted (multiplicative random effects)
MR Egger
MR RAPS
Weighted median
Weighted mode
0.10
0.05
0.00
−0.05
0.05
0.10
0.15
SNP effect on Diabetic nephropathy (more control exclusions)
SNP effect on Gut microbiota abundance (genus Ruminococcus torques group id.14377)

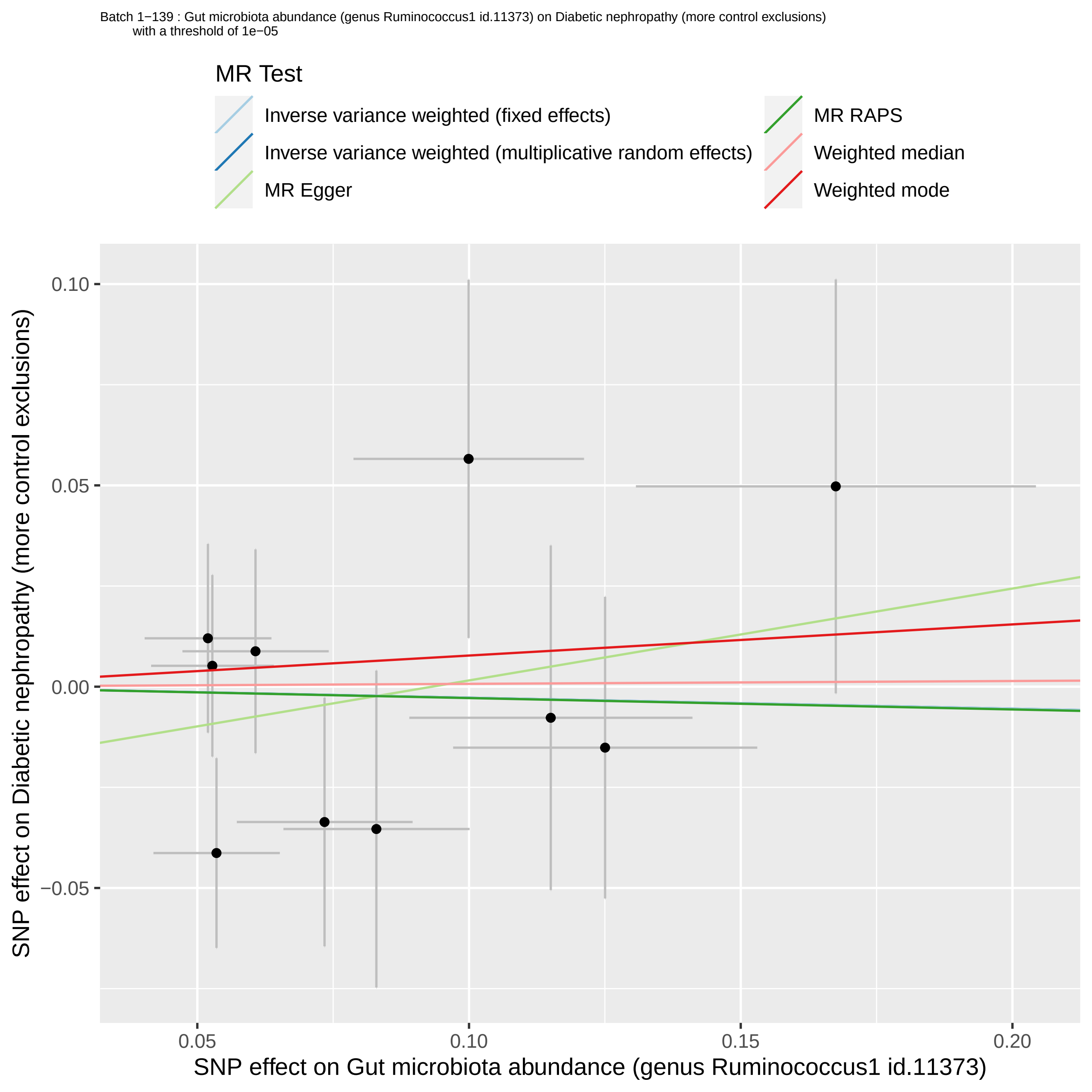

Batch 1−139 : Gut microbiota abundance (genus Ruminococcus1 id.11373) on Diabetic nephropathy (more control exclusions)
with a threshold of 1e−05
MR Test
Inverse variance weighted (fixed effects)
Inverse variance weighted (multiplicative random effects)
MR Egger
MR RAPS
Weighted median
Weighted mode
0.10
0.05
0.00
−0.05
0.05
0.10
0.15
0.20
SNP effect on Diabetic nephropathy (more control exclusions)
SNP effect on Gut microbiota abundance (genus Ruminococcus1 id.11373)

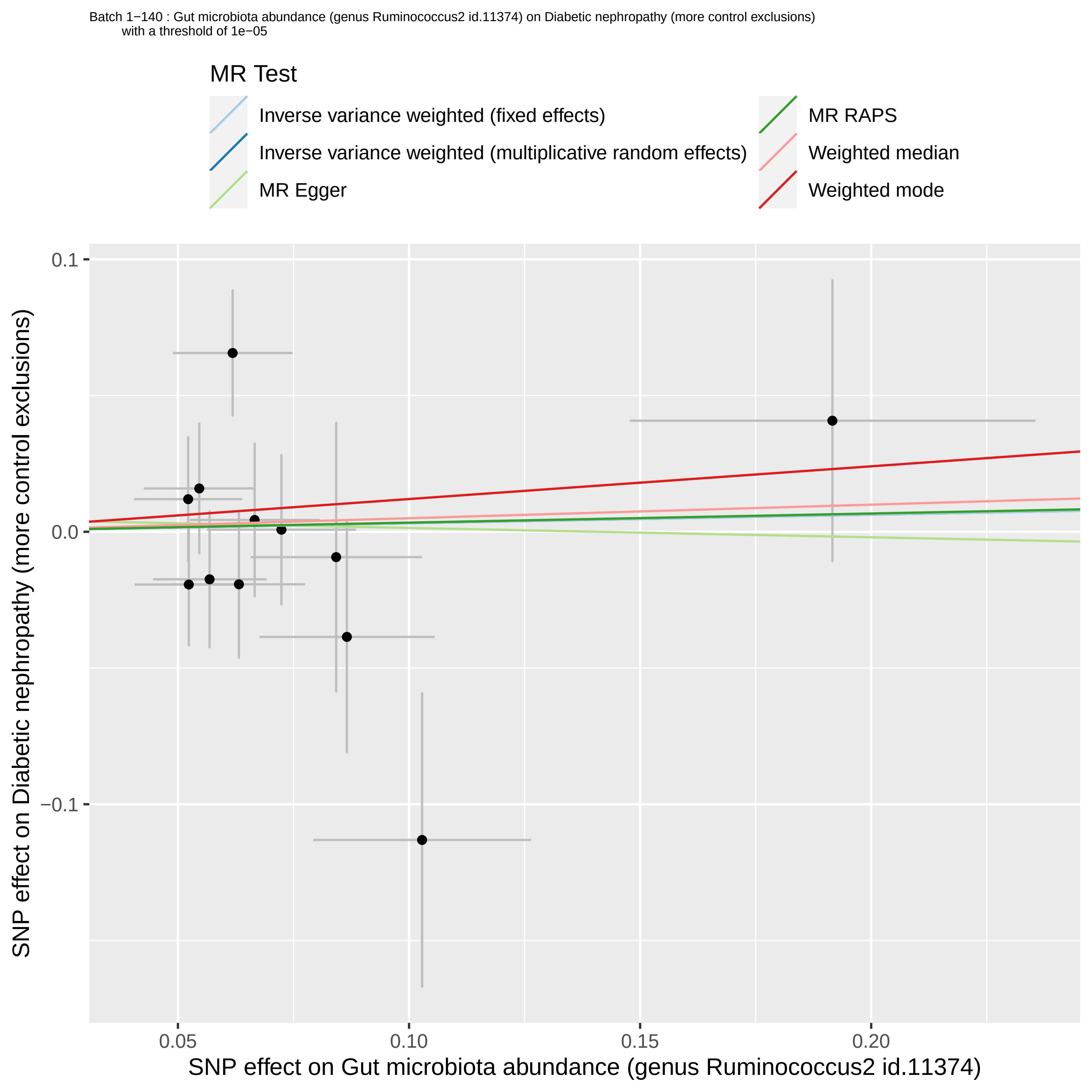
Batch 1−140 : Gut microbiota abundance (genus Ruminococcus2 id.11374) on Diabetic nephropathy (more control exclusions)
with a threshold of 1e−05
MR Test
Inverse variance weighted (fixed effects)
Inverse variance weighted (multiplicative random effects)
MR Egger
MR RAPS
Weighted median
Weighted mode
0.1
0.0
−0.1
0.05
0.10
0.15
0.20
SNP effect on Diabetic nephropathy (more control exclusions)
SNP effect on Gut microbiota abundance (genus Ruminococcus2 id.11374)

Batch 1−141 : Gut microbiota abundance (genus Sellimonas id.14369) on Diabetic nephropathy (more control exclusions) with a threshold of 1e−05

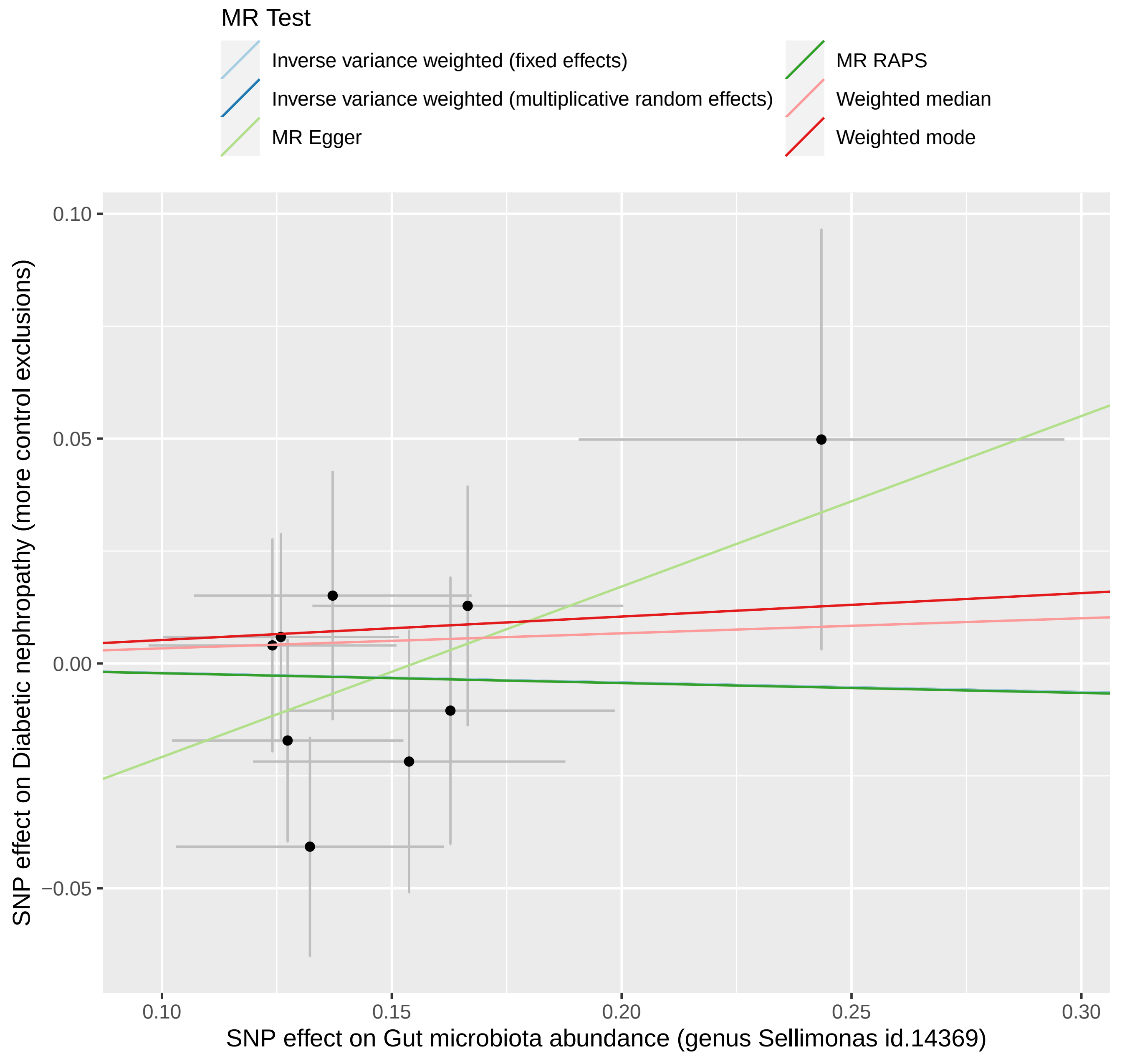

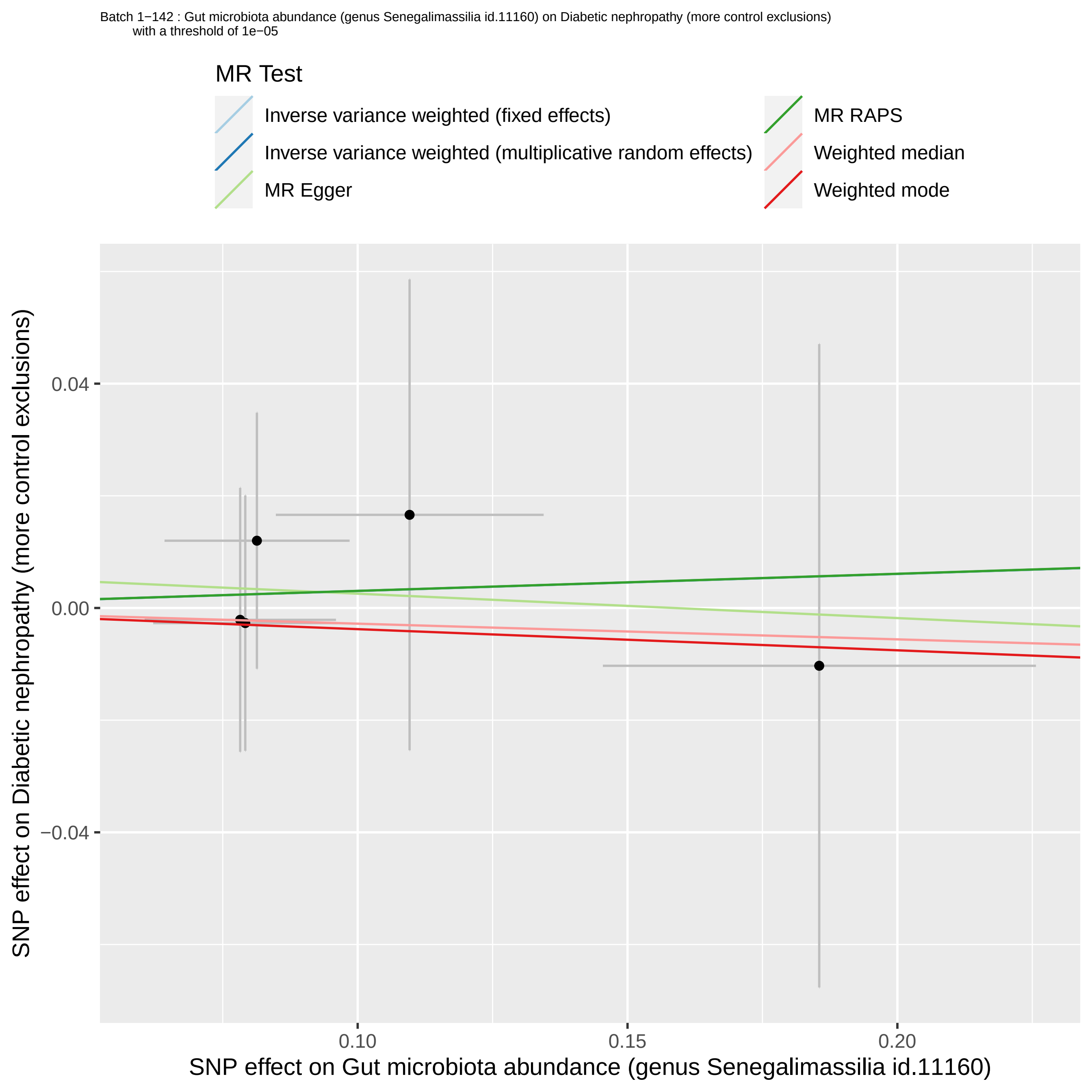

Batch 1−142 : Gut microbiota abundance (genus Senegalimassilia id.11160) on Diabetic nephropathy (more control exclusions)
with a threshold of 1e−05
MR Test
Inverse variance weighted (fixed effects)
Inverse variance weighted (multiplicative random effects)
MR Egger
MR RAPS
Weighted median
Weighted mode
0.04
0.00
−0.04
0.10
0.15
0.20
SNP effect on Diabetic nephropathy (more control exclusions)
SNP effect on Gut microbiota abundance (genus Senegalimassilia id.11160)

Batch 1−143 : Gut microbiota abundance (genus Slackia id.825) on Diabetic nephropathy (more control exclusions) with a threshold of 1e−05

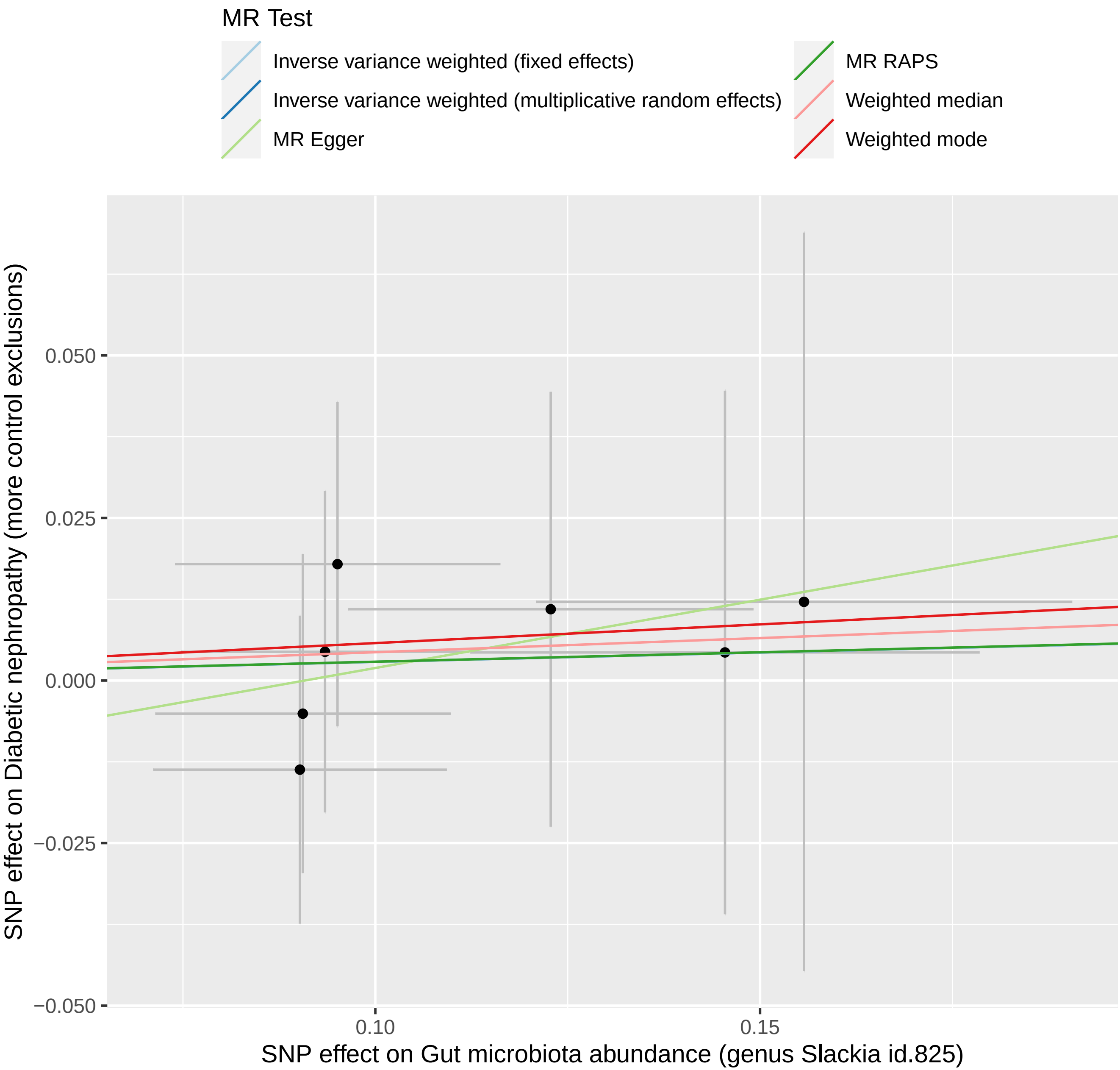

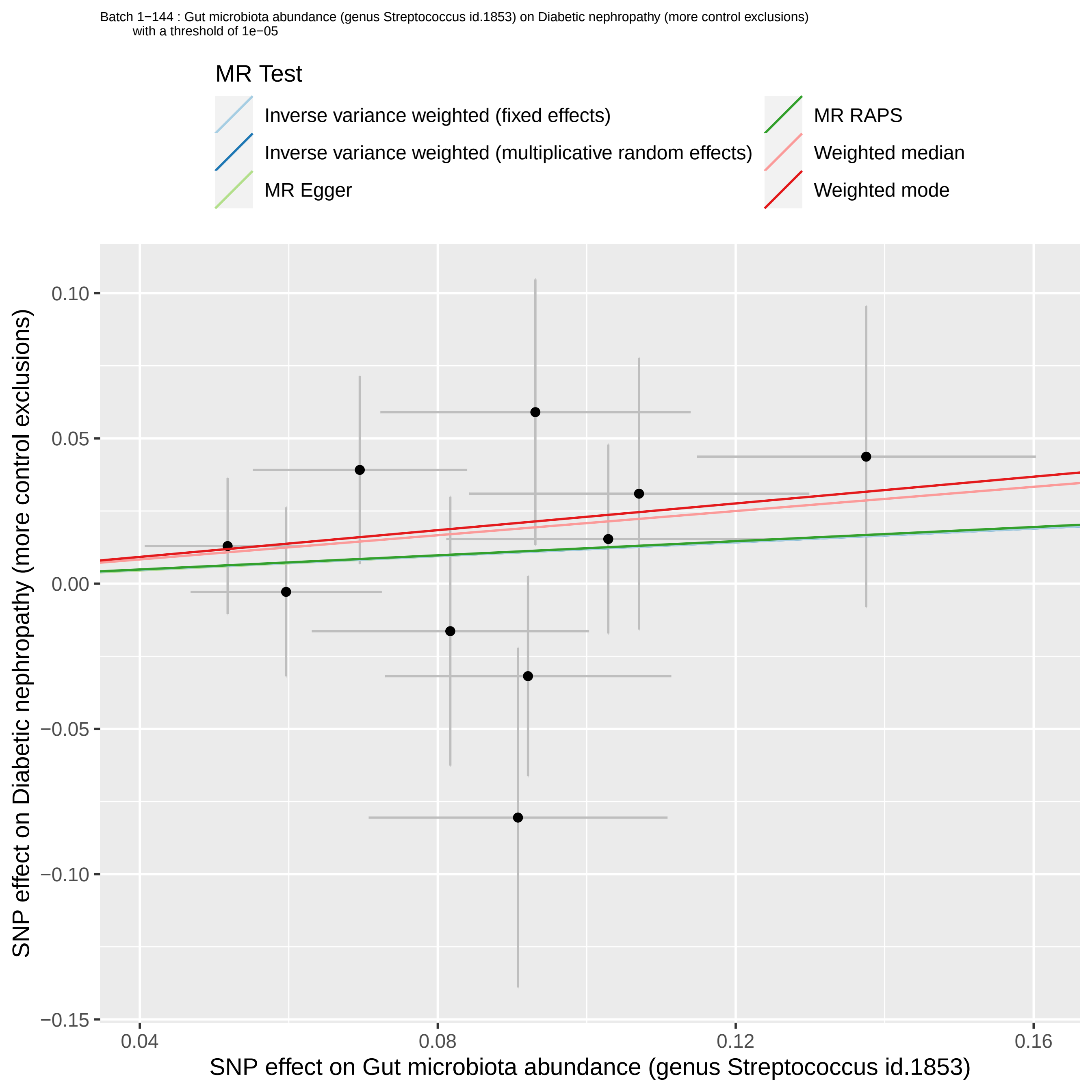

Batch 1−144 : Gut microbiota abundance (genus Streptococcus id.1853) on Diabetic nephropathy (more control exclusions) with a threshold of 1e−05
MR Test
Inverse variance weighted (fixed effects)
Inverse variance weighted (multiplicative random effects)
MR Egger
MR RAPS
Weighted median
Weighted mode
0.10
0.05
0.00
−0.05
−0.10
−0.15
0.04
0.08
0.12
0.16
SNP effect on Diabetic nephropathy (more control exclusions)
SNP effect on Gut microbiota abundance (genus Streptococcus id.1853)

Batch 1−145 : Gut microbiota abundance (genus Subdoligranulum id.2070) on Diabetic nephropathy (more control exclusions) with a threshold of 1e−05

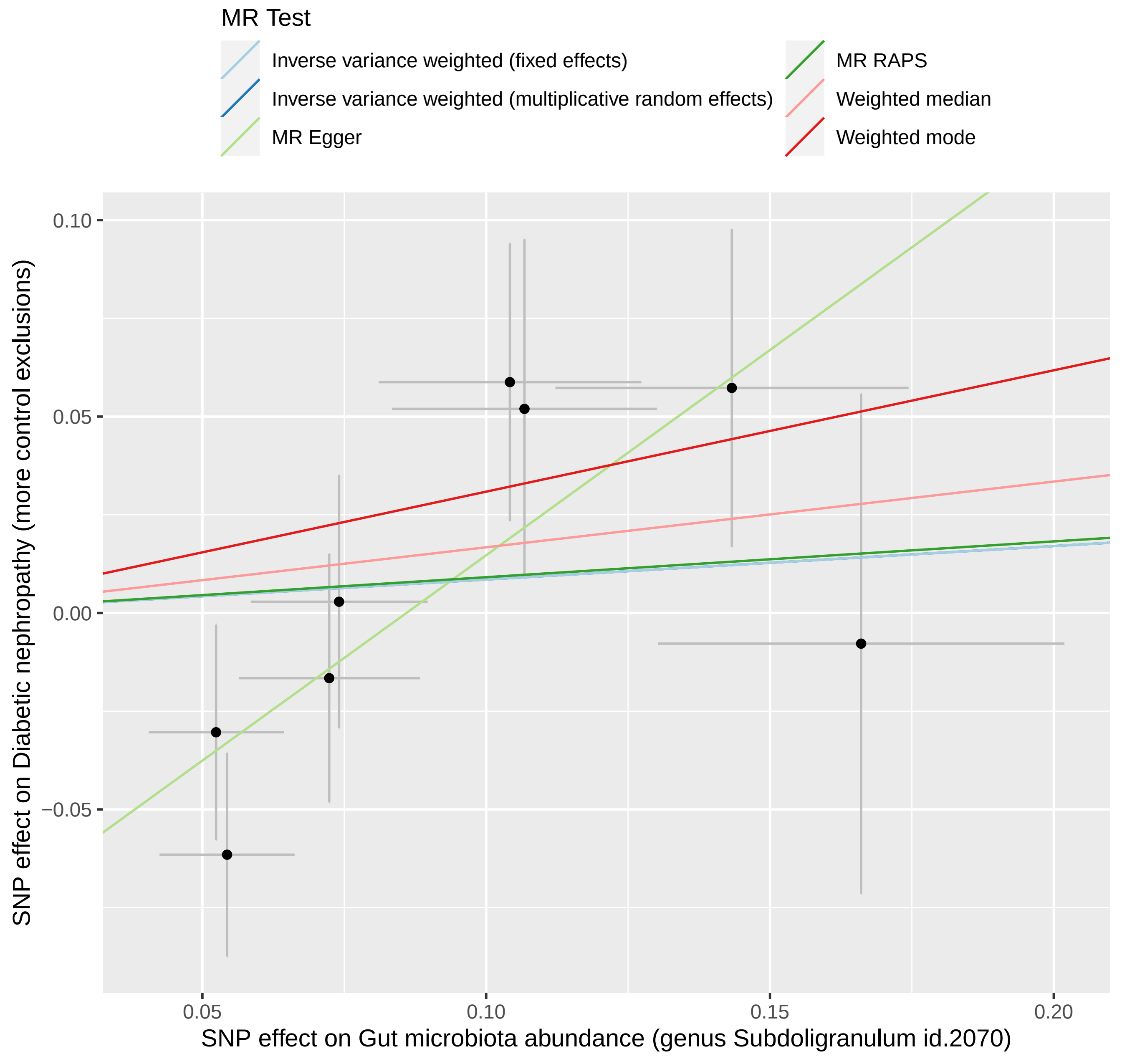

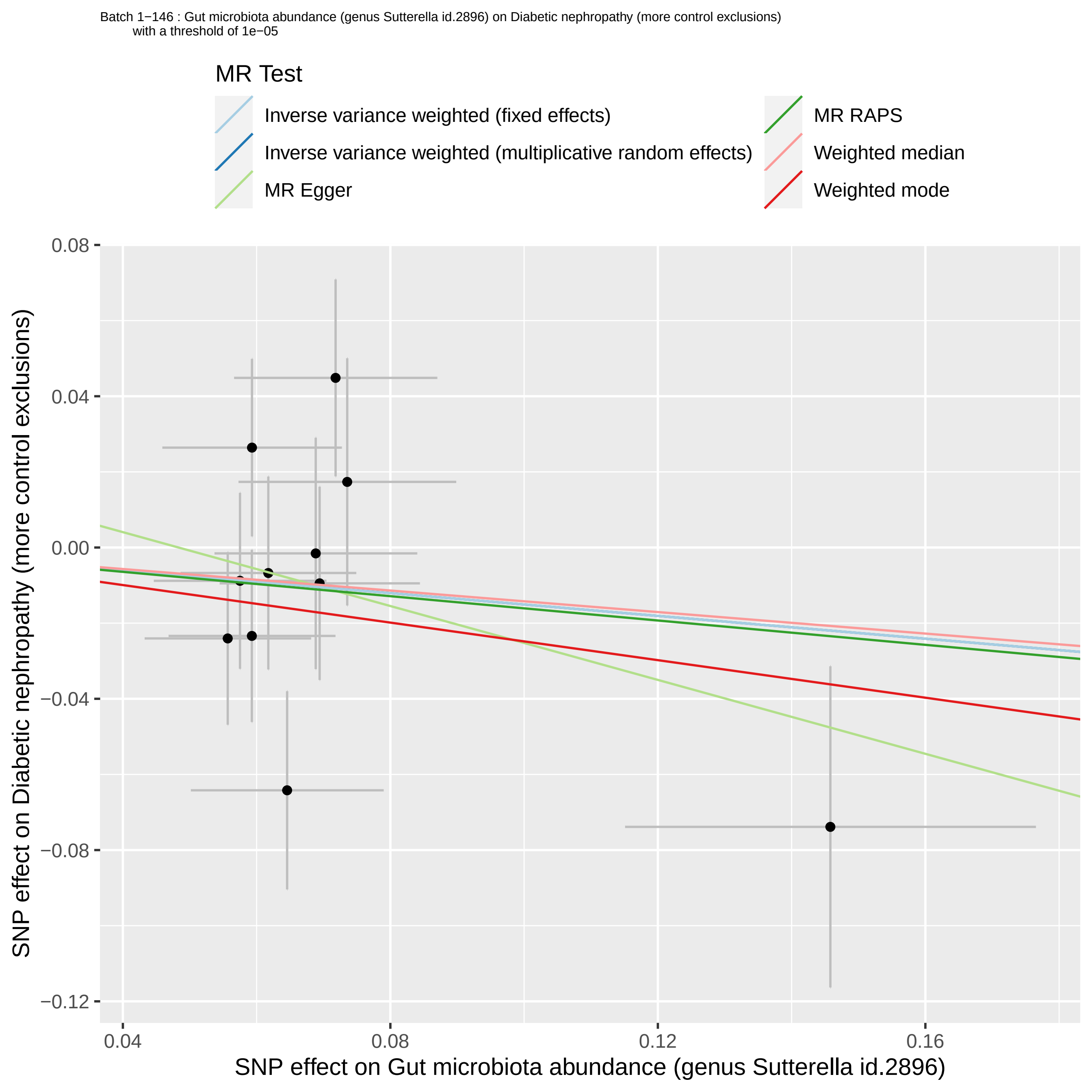
Batch 1−146 : Gut microbiota abundance (genus Sutterella id.2896) on Diabetic nephropathy (more control exclusions) with a threshold of 1e−05
MR Test
Inverse variance weighted (fixed effects)
Inverse variance weighted (multiplicative random effects)
MR Egger
MR RAPS
Weighted median
Weighted mode
0.08
0.04
0.00
−0.04
−0.08
−0.12
0.04
0.08
0.12
0.16
SNP effect on Diabetic nephropathy (more control exclusions)
SNP effect on Gut microbiota abundance (genus Sutterella id.2896)

Batch 1−147 : Gut microbiota abundance (genus Terrisporobacter id.11348) on Diabetic nephropathy (more control exclusions)
with a threshold of 1e−05

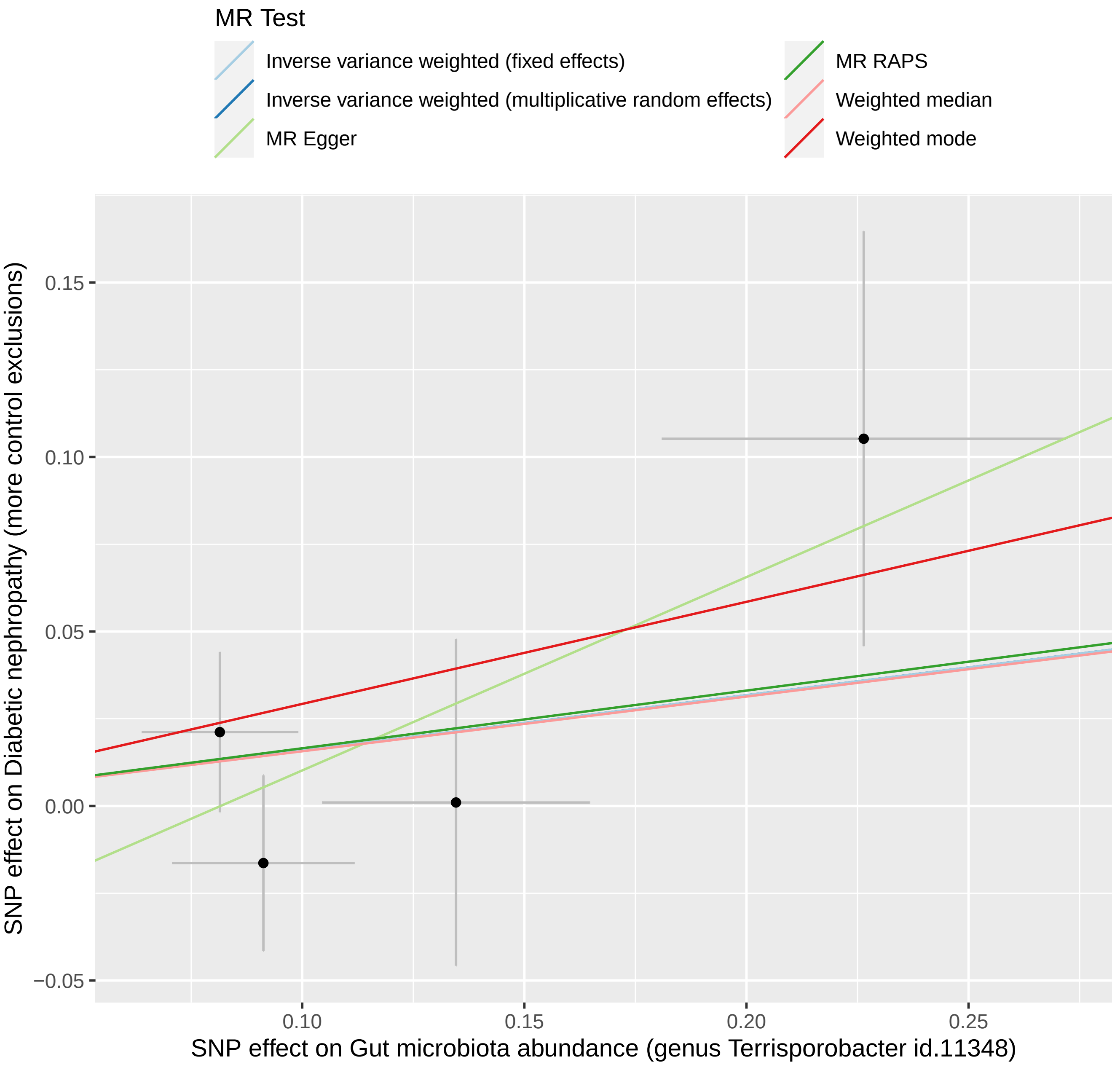

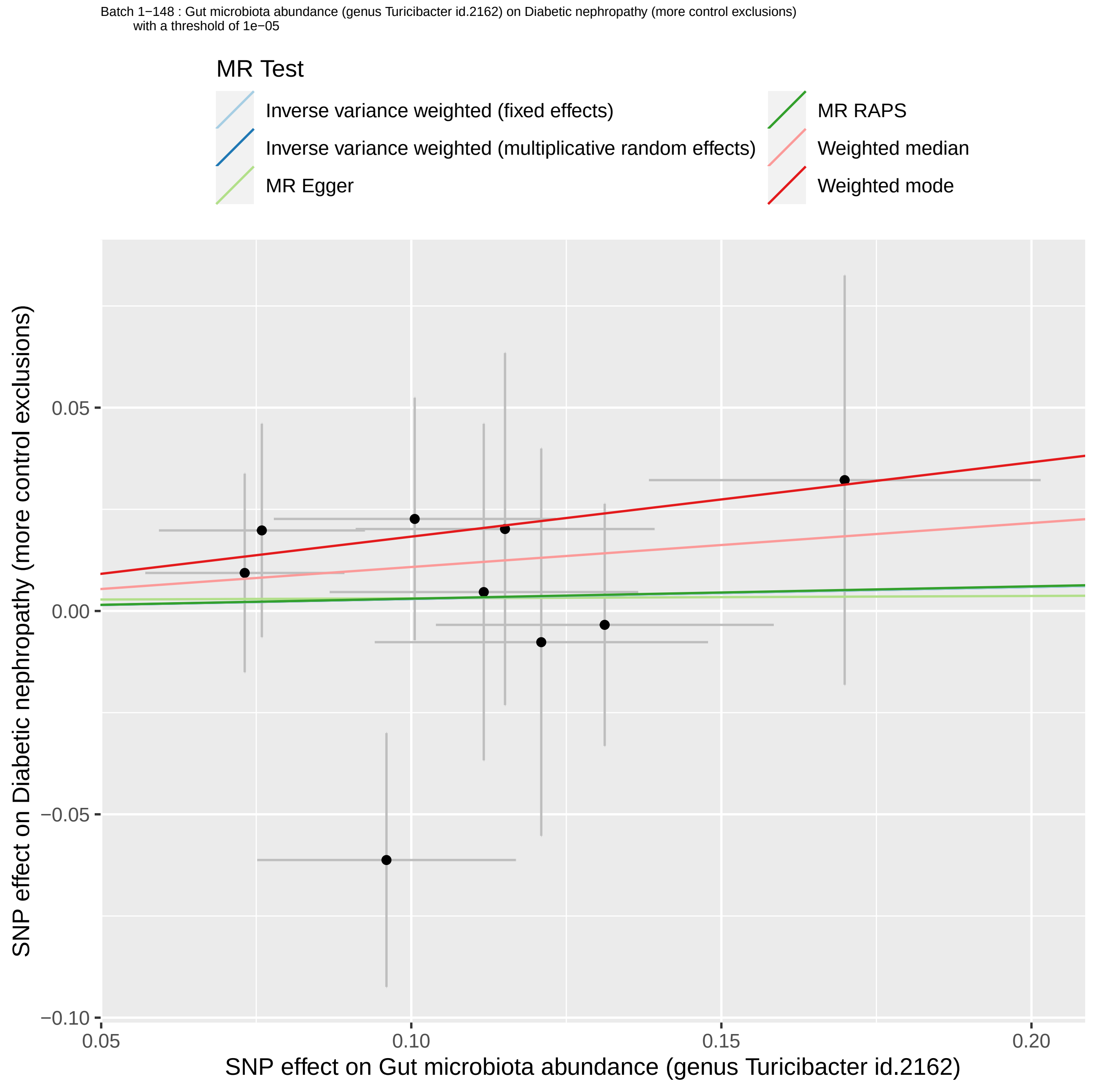

Batch 1−148 : Gut microbiota abundance (genus Turicibacter id.2162) on Diabetic nephropathy (more control exclusions) with a threshold of 1e−05
MR Test
Inverse variance weighted (fixed effects)
Inverse variance weighted (multiplicative random effects)
MR Egger
MR RAPS
Weighted median
Weighted mode
SNP effect on Diabetic nephropathy (more control exclusions)
0.05
0.00
−0.05
−0.10
0.05
0.10
0.15
0.20
SNP effect on Gut microbiota abundance (genus Turicibacter id.2162)

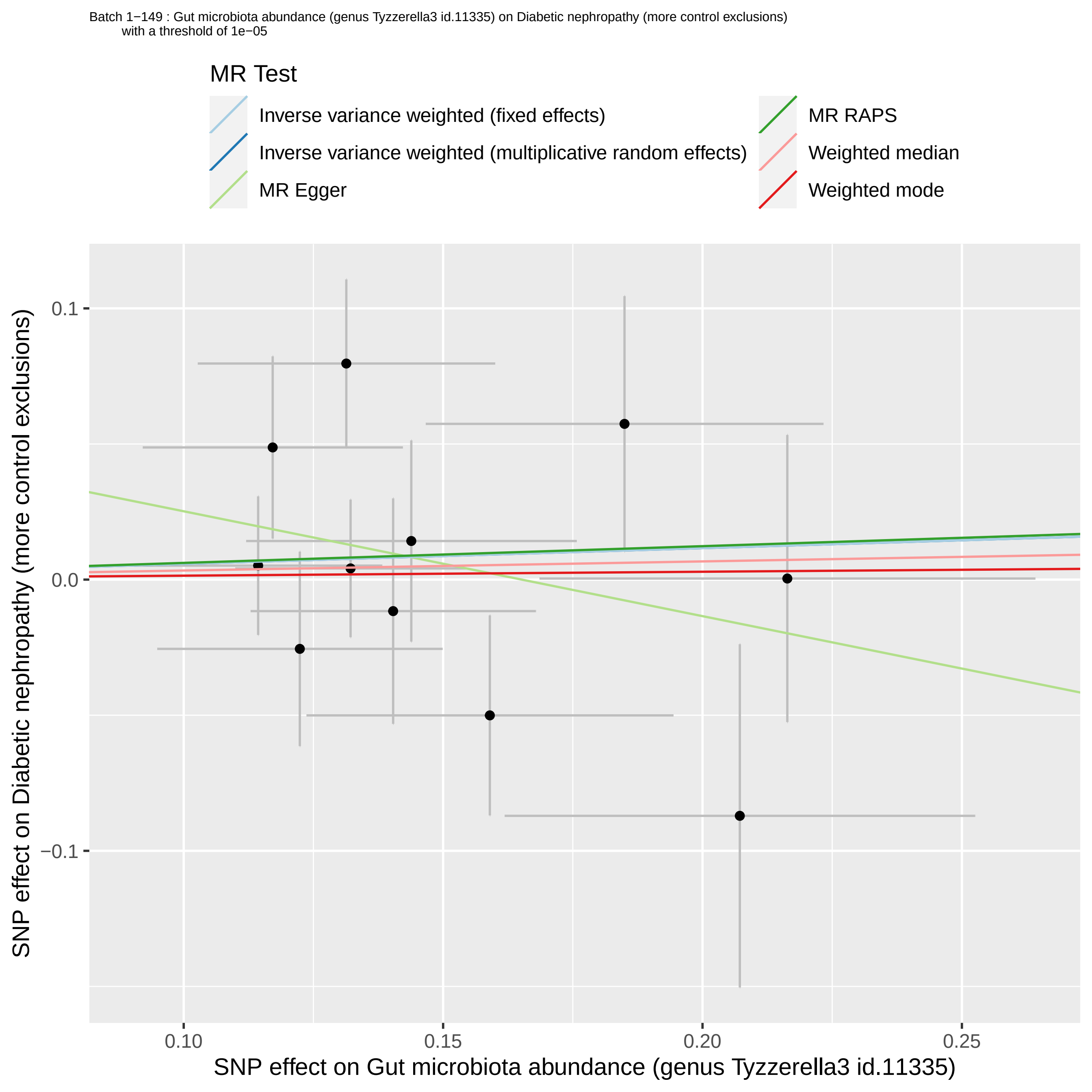

Batch 1−149 : Gut microbiota abundance (genus Tyzzerella3 id.11335) on Diabetic nephropathy (more control exclusions)
with a threshold of 1e−05
MR Test
Inverse variance weighted (fixed effects)
Inverse variance weighted (multiplicative random effects)
MR Egger
MR RAPS
Weighted median
Weighted mode
0.1
0.0
−0.1
0.10
0.15
0.20
0.25
SNP effect on Diabetic nephropathy (more control exclusions)
SNP effect on Gut microbiota abundance (genus Tyzzerella3 id.11335)

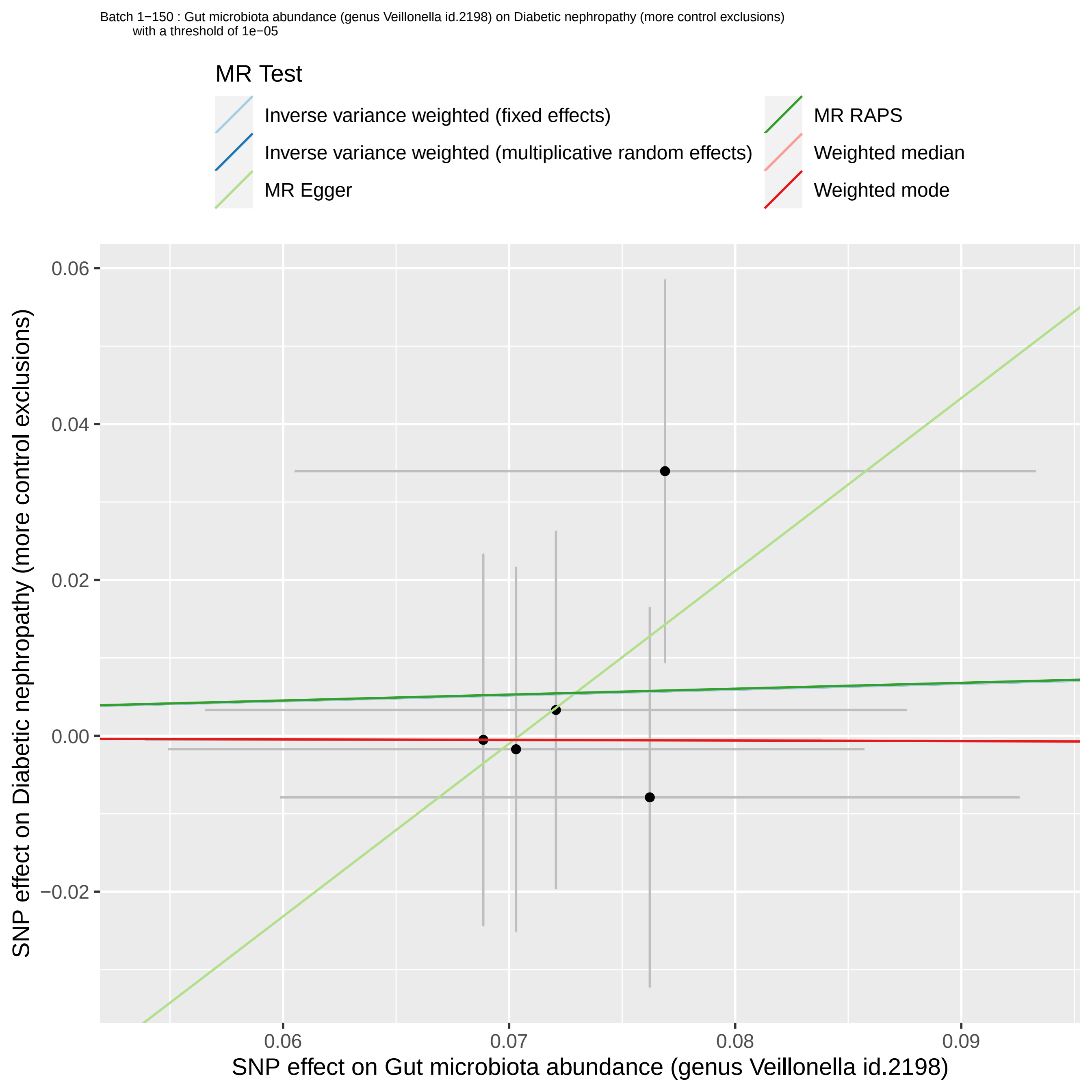

Batch 1−150 : Gut microbiota abundance (genus Veillonella id.2198) on Diabetic nephropathy (more control exclusions)
with a threshold of 1e−05
MR Test
Inverse variance weighted (fixed effects)
Inverse variance weighted (multiplicative random effects)
MR Egger
MR RAPS
Weighted median
Weighted mode
0.06
0.04
0.02
0.00
−0.02
0.06
0.07
0.08
0.09
SNP effect on Diabetic nephropathy (more control exclusions)
SNP effect on Gut microbiota abundance (genus Veillonella id.2198)

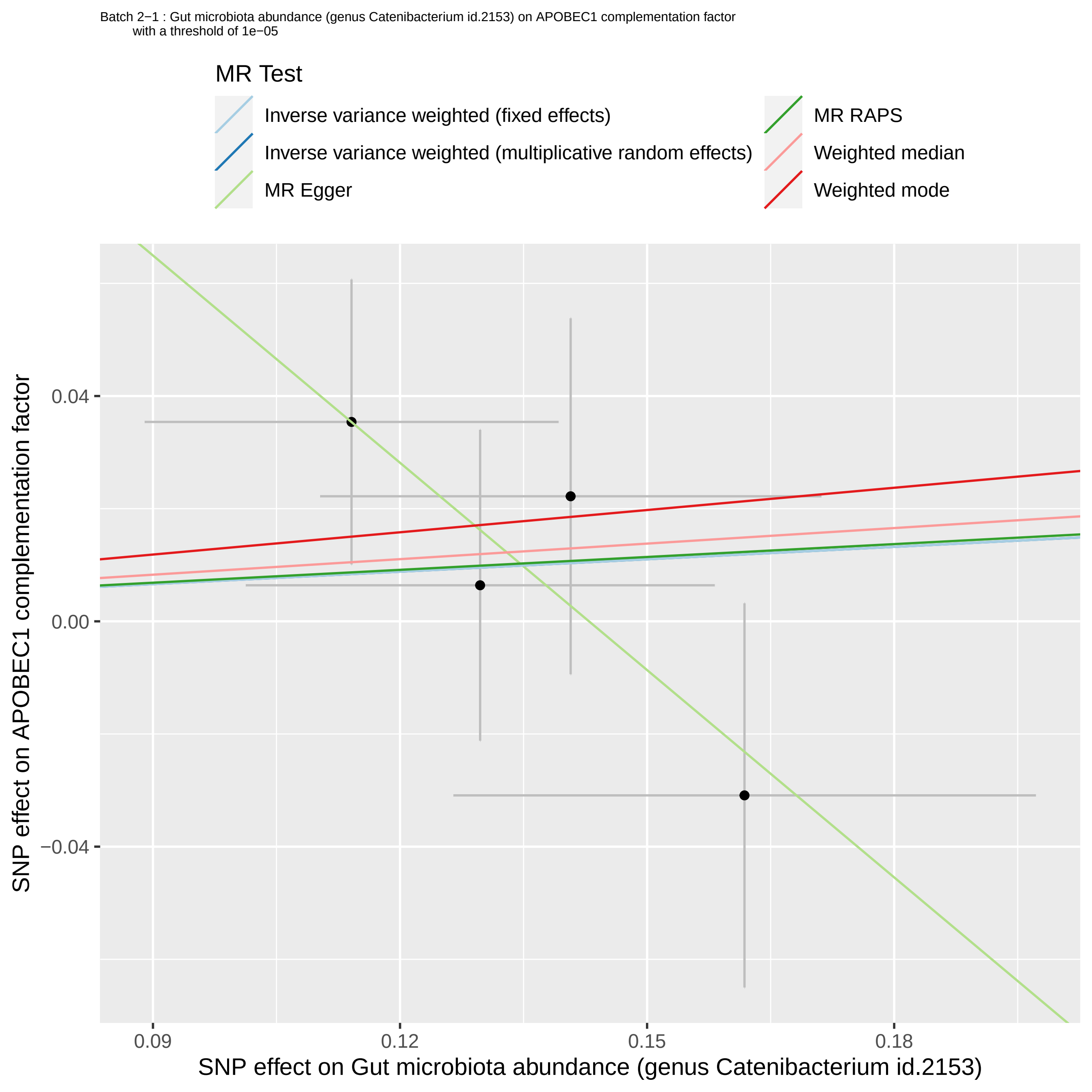

Batch 2−1 : Gut microbiota abundance (genus Catenibacterium id.2153) on APOBEC1 complementation factor
with a threshold of 1e−05
MR Test
Inverse variance weighted (fixed effects)
Inverse variance weighted (multiplicative random effects)
MR Egger
MR RAPS
Weighted median
Weighted mode
0.04
0.00
−0.04
0.09
0.12
0.15
0.18
SNP effect on APOBEC1 complementation factor
SNP effect on Gut microbiota abundance (genus Catenibacterium id.2153)

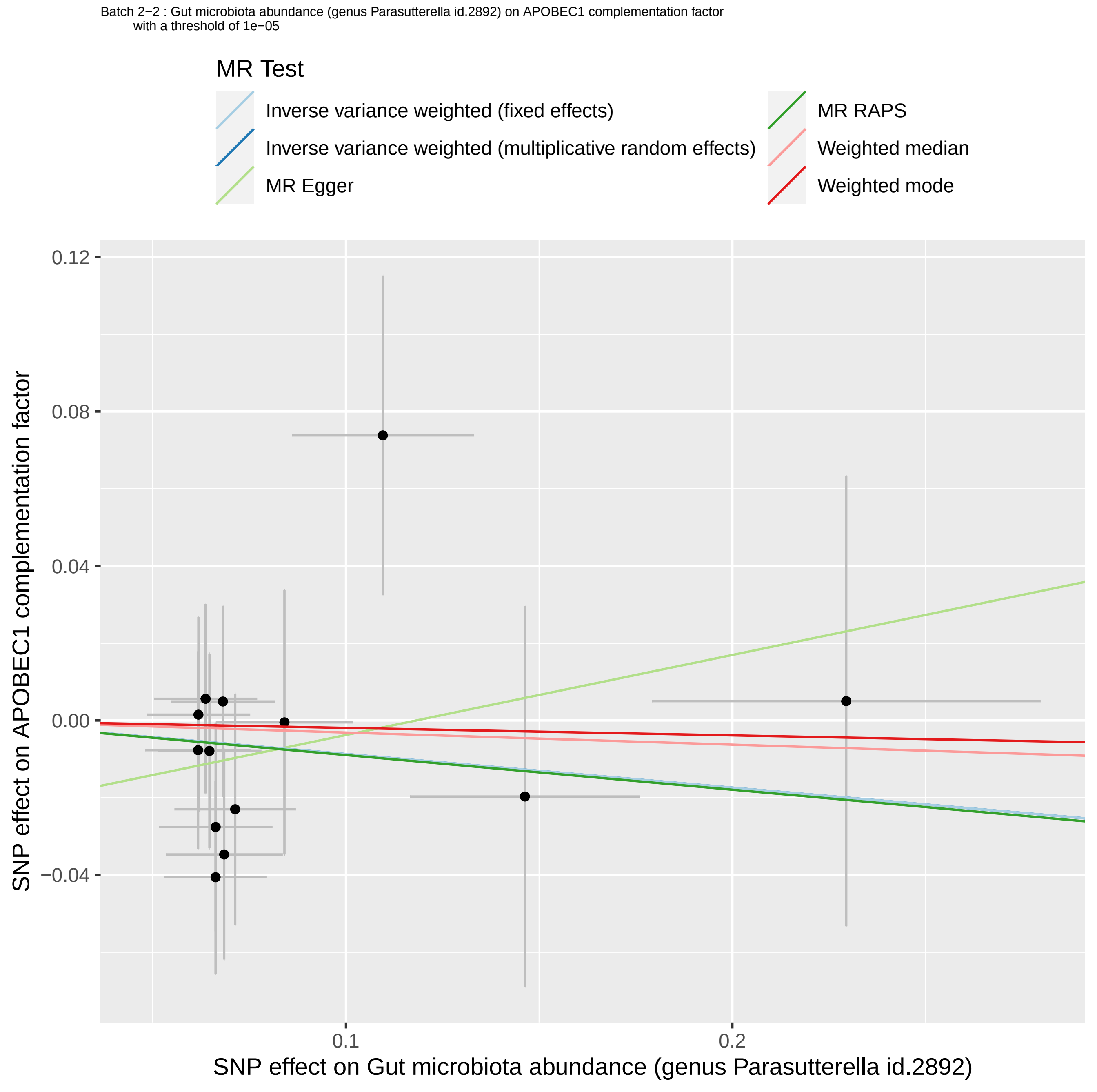
Batch 2−2 : Gut microbiota abundance (genus Parasutterella id.2892) on APOBEC1 complementation factor
with a threshold of 1e−05
MR Test
Inverse variance weighted (fixed effects)
Inverse variance weighted (multiplicative random effects)
MR Egger
MR RAPS
Weighted median
Weighted mode
0.12
0.08
0.04
0.00
−0.04
0.1
0.2
SNP effect on APOBEC1 complementation factor
SNP effect on Gut microbiota abundance (genus Parasutterella id.2892)

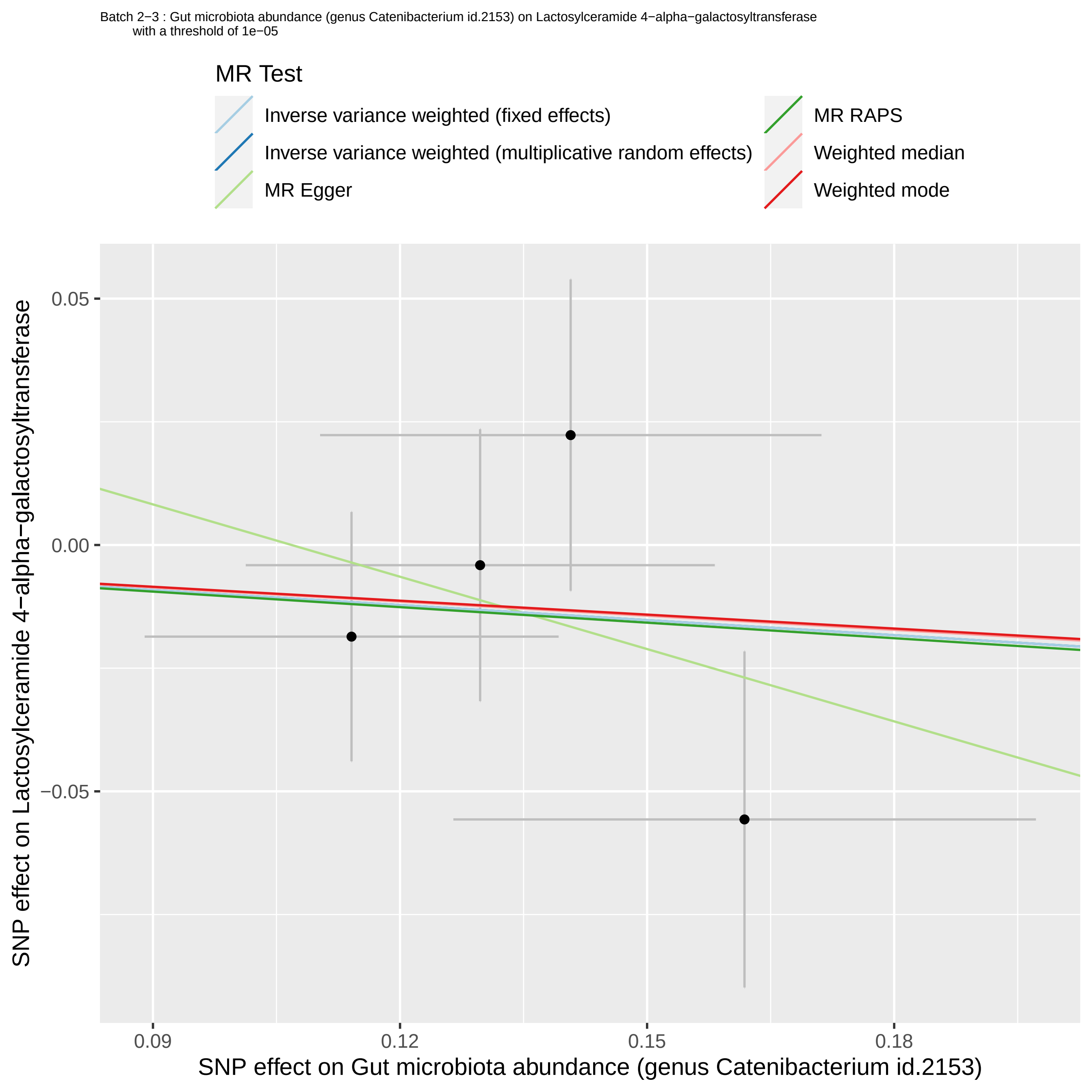

Batch 2−3 : Gut microbiota abundance (genus Catenibacterium id.2153) on Lactosylceramide 4−alpha−galactosyltransferase
with a threshold of 1e−05
MR Test
Inverse variance weighted (fixed effects)
Inverse variance weighted (multiplicative random effects)
MR Egger
MR RAPS
Weighted median
Weighted mode
0.05
0.00
−0.05
SNP effect on Lactosylceramide 4−alpha−galactosyltransferase
0.09
0.12
0.15
0.18
SNP effect on Gut microbiota abundance (genus Catenibacterium id.2153)

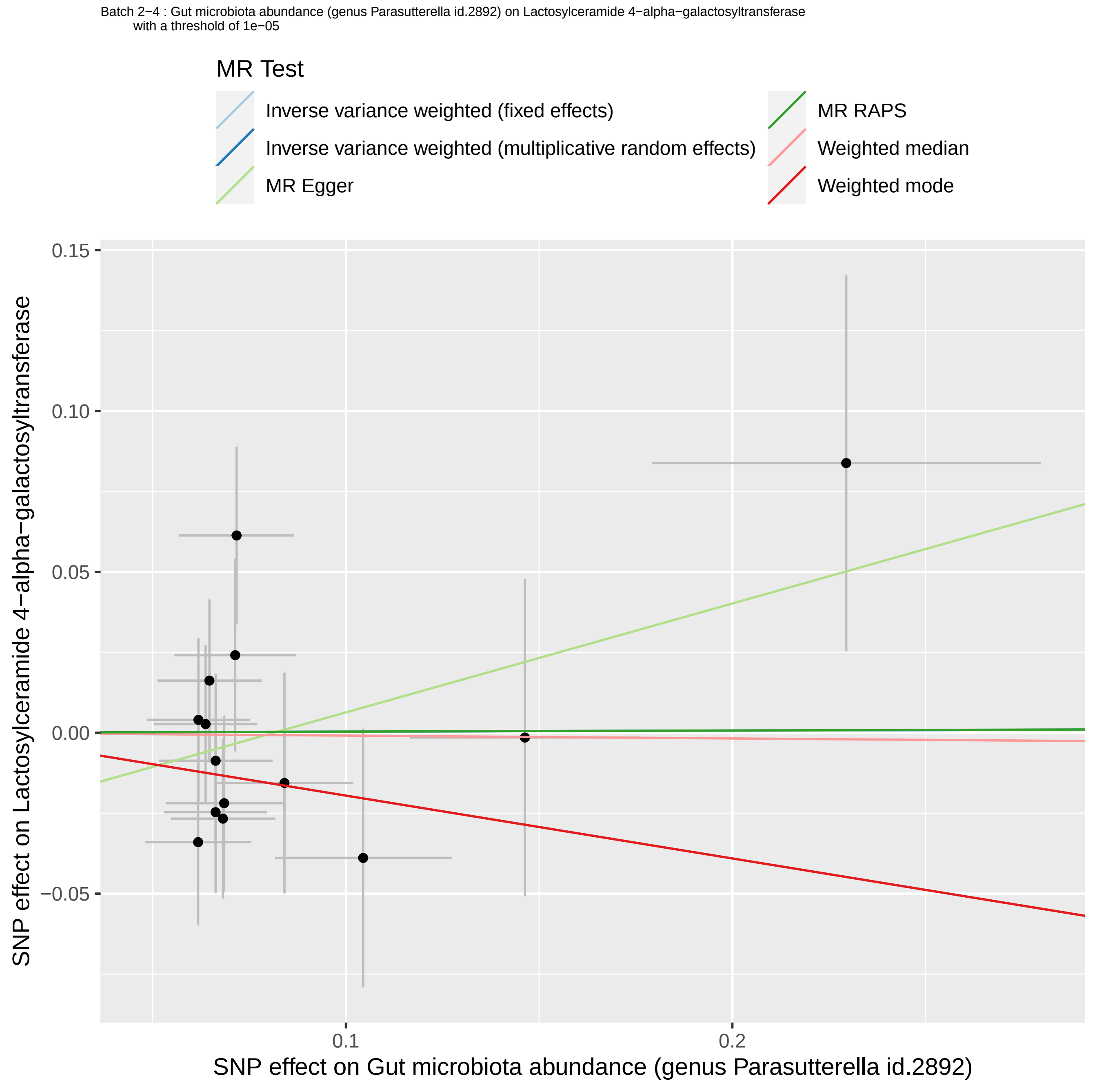

Batch 2−4 : Gut microbiota abundance (genus Parasutterella id.2892) on Lactosylceramide 4−alpha−galactosyltransferase
with a threshold of 1e−05
MR Test
Inverse variance weighted (fixed effects)
Inverse variance weighted (multiplicative random effects)
MR Egger
MR RAPS
Weighted median
Weighted mode
0.15
0.10
0.05
0.00
−0.05
0.1
0.2
SNP effect on Lactosylceramide 4−alpha−galactosyltransferase
SNP effect on Gut microbiota abundance (genus Parasutterella id.2892)

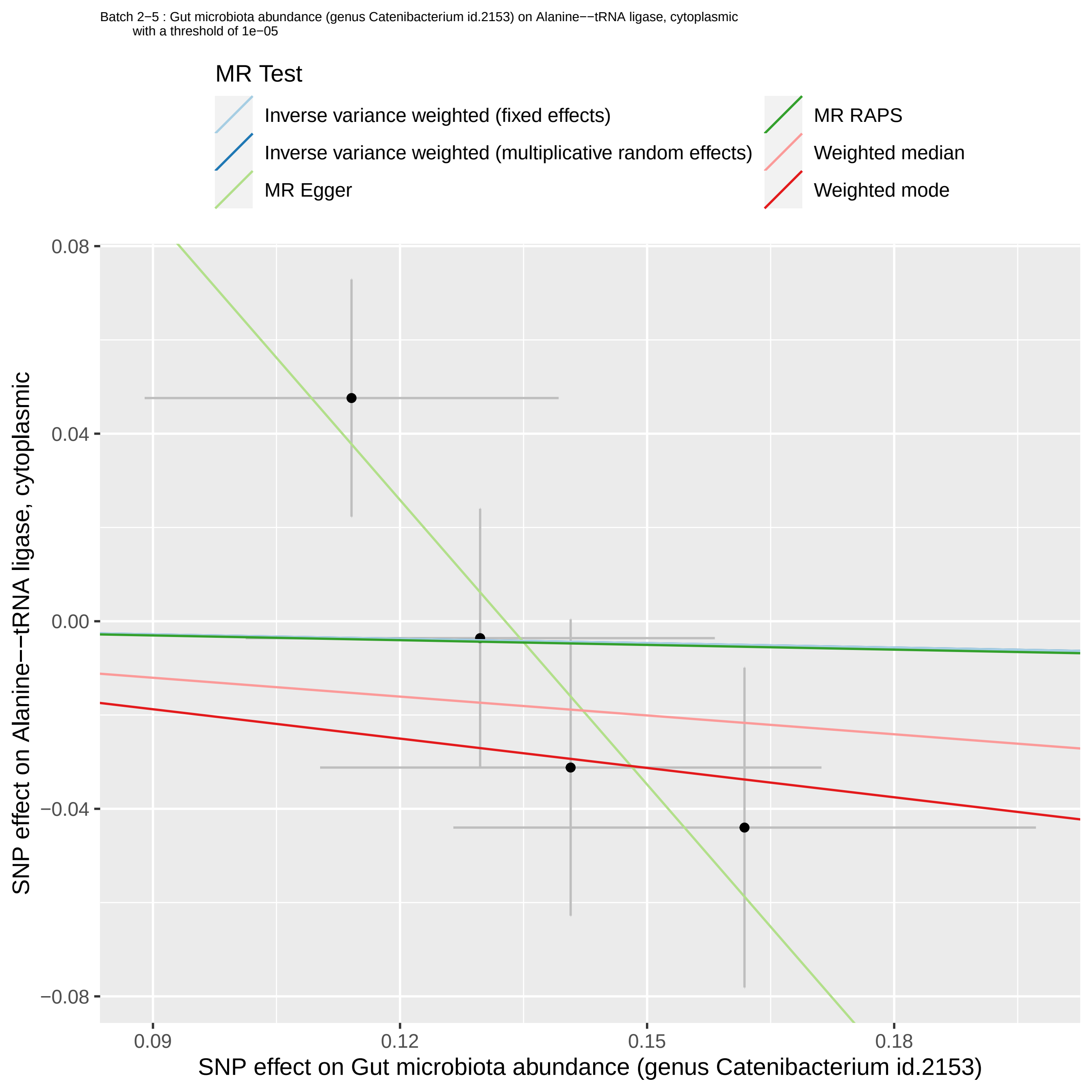

Batch 2−5 : Gut microbiota abundance (genus Catenibacterium id.2153) on Alanine−−tRNA ligase, cytoplasmic
with a threshold of 1e−05
MR Test
Inverse variance weighted (fixed effects)
Inverse variance weighted (multiplicative random effects)
MR Egger
MR RAPS
Weighted median
Weighted mode
0.08
0.04
0.00
−0.04
−0.08
0.09
0.12
0.15
0.18
SNP effect on Alanine−−tRNA ligase, cytoplasmic
SNP effect on Gut microbiota abundance (genus Catenibacterium id.2153)

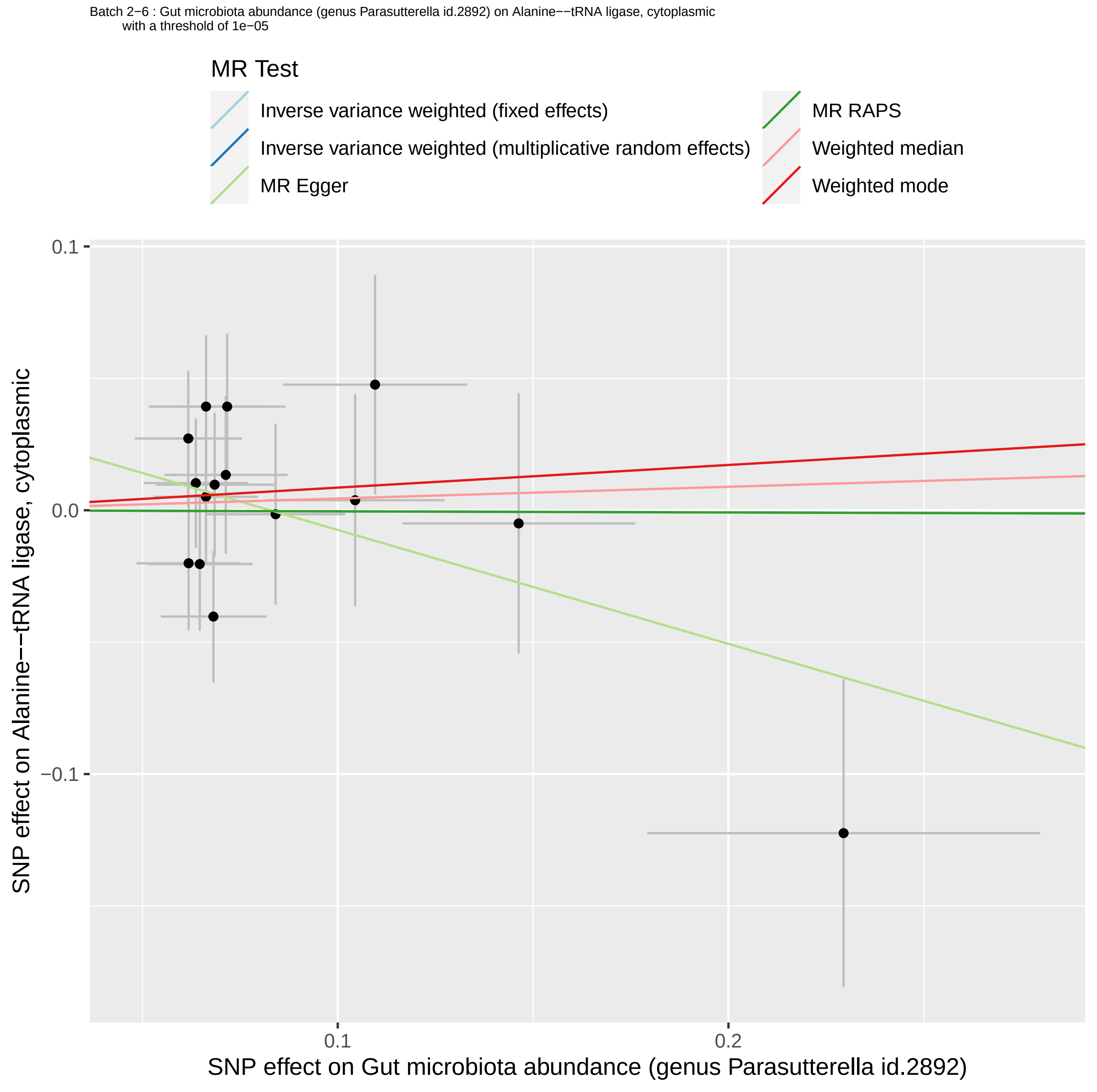
Batch 2−6 : Gut microbiota abundance (genus Parasutterella id.2892) on Alanine−−tRNA ligase, cytoplasmic
with a threshold of 1e−05
MR Test
Inverse variance weighted (fixed effects)
Inverse variance weighted (multiplicative random effects)
MR Egger
MR RAPS
Weighted median
Weighted mode
0.1
0.0
−0.1
0.1
0.2
SNP effect on Alanine−−tRNA ligase, cytoplasmic
SNP effect on Gut microbiota abundance (genus Parasutterella id.2892)

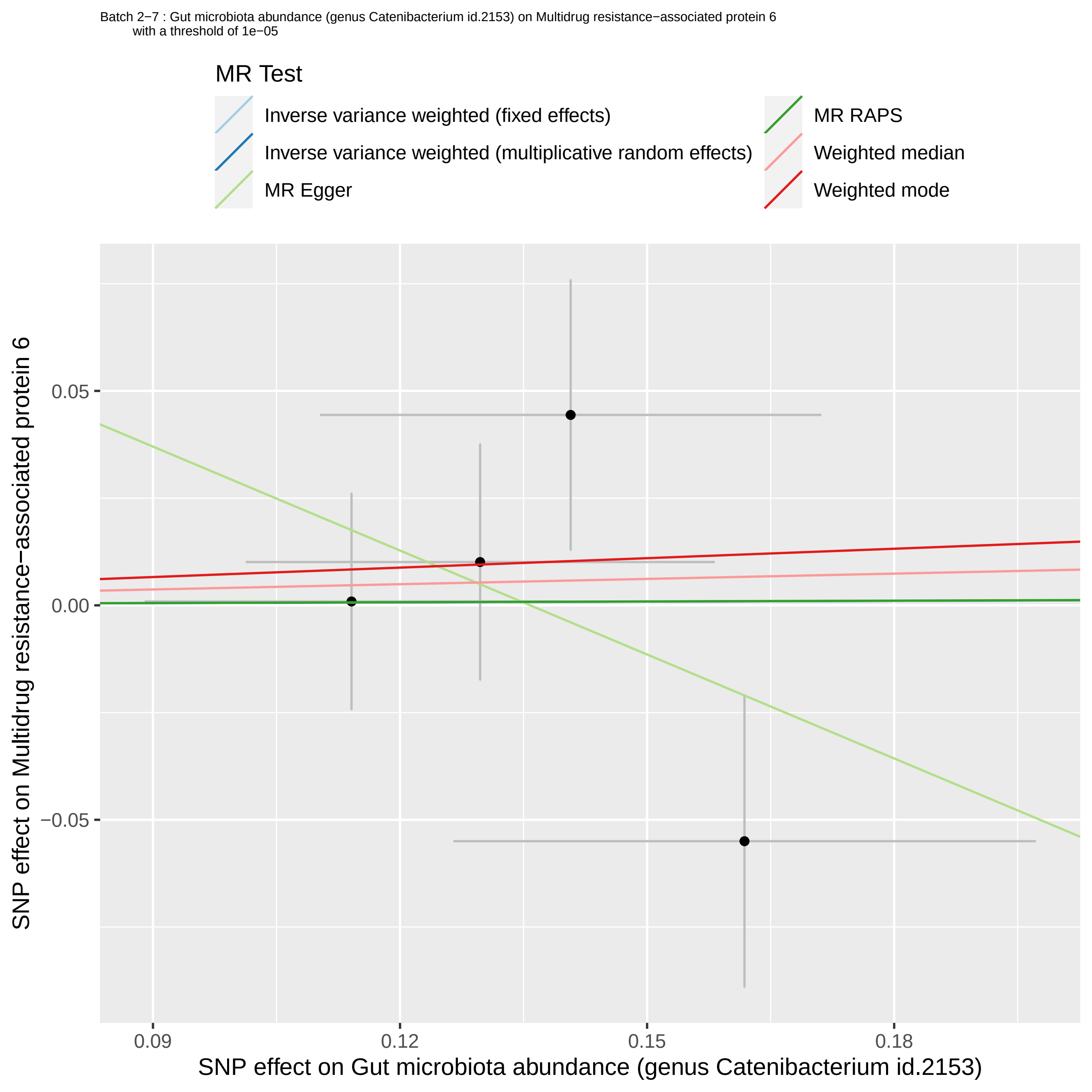

Batch 2−7 : Gut microbiota abundance (genus Catenibacterium id.2153) on Multidrug resistance−associated protein 6
with a threshold of 1e−05
MR Test
Inverse variance weighted (fixed effects)
Inverse variance weighted (multiplicative random effects)
MR Egger
MR RAPS
Weighted median
Weighted mode
0.05
0.00
−0.05
0.09
0.12
0.15
0.18
SNP effect on Multidrug resistance−associated protein 6
SNP effect on Gut microbiota abundance (genus Catenibacterium id.2153)

Batch 2−8 : Gut microbiota abundance (genus Parasutterella id.2892) on Multidrug resistance−associated protein 6
with a threshold of 1e−05

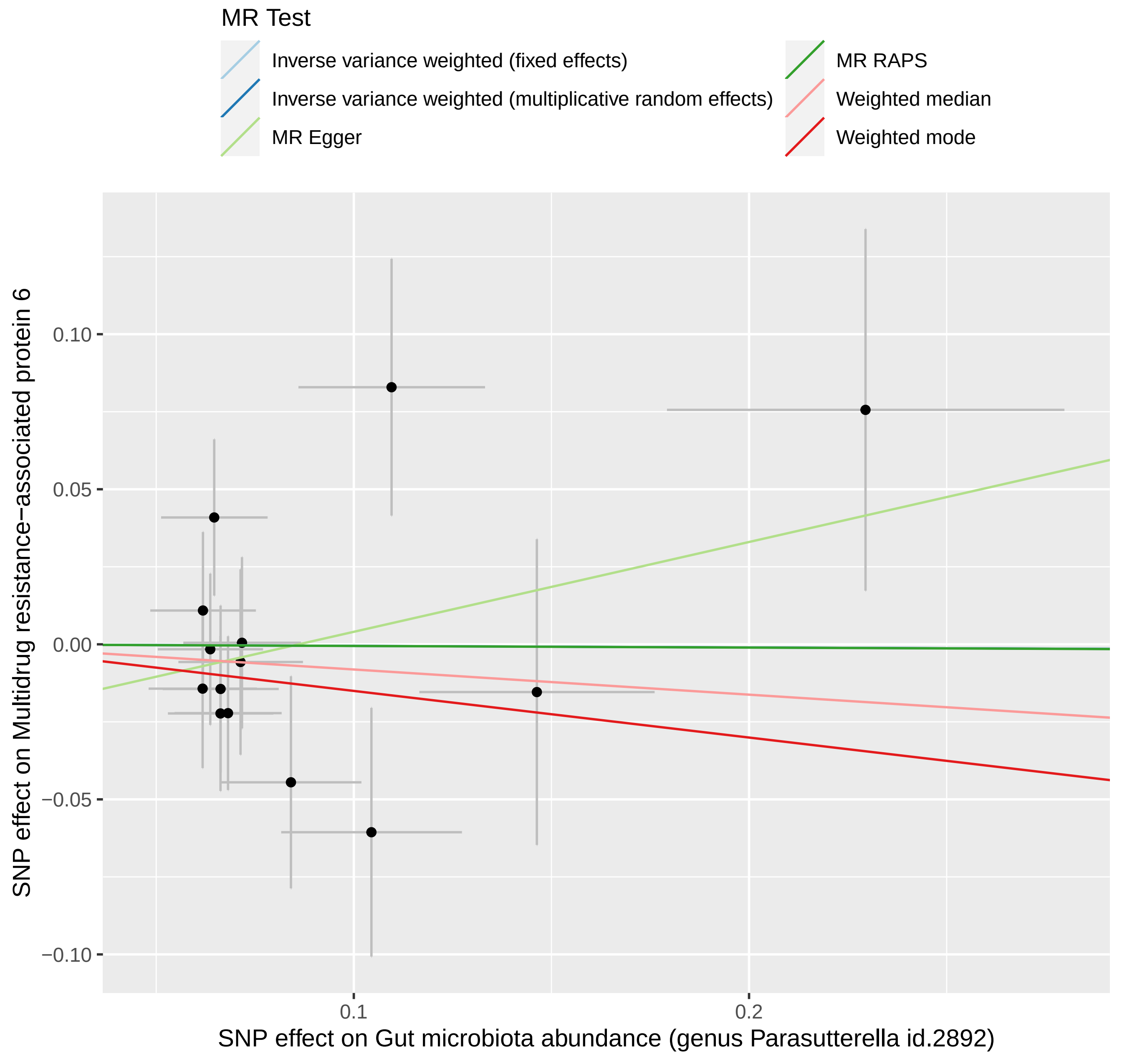

Batch 2−9 : Gut microbiota abundance (genus Catenibacterium id.2153) on Monoacylglycerol lipase ABHD12
with a threshold of 1e−05

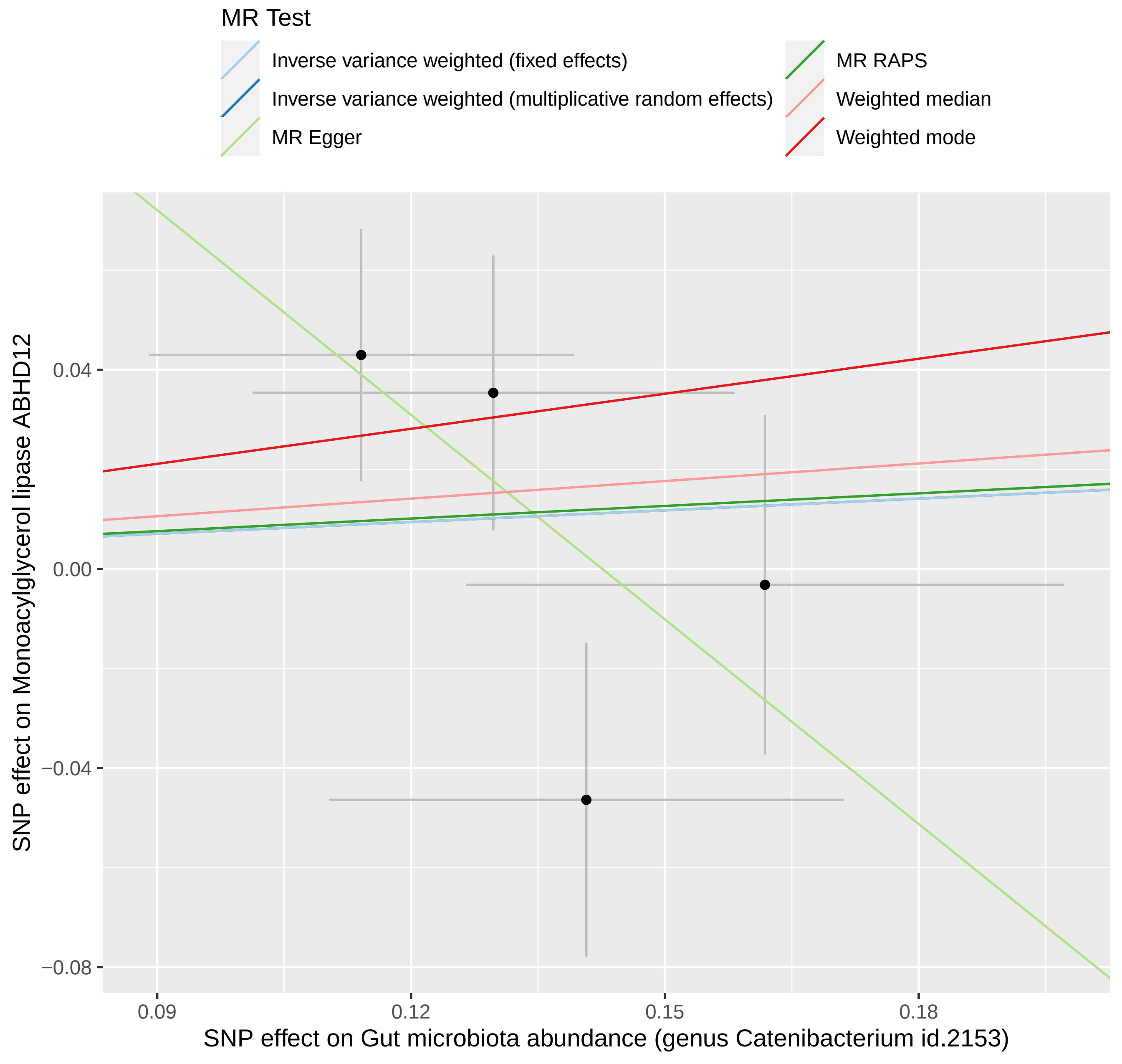

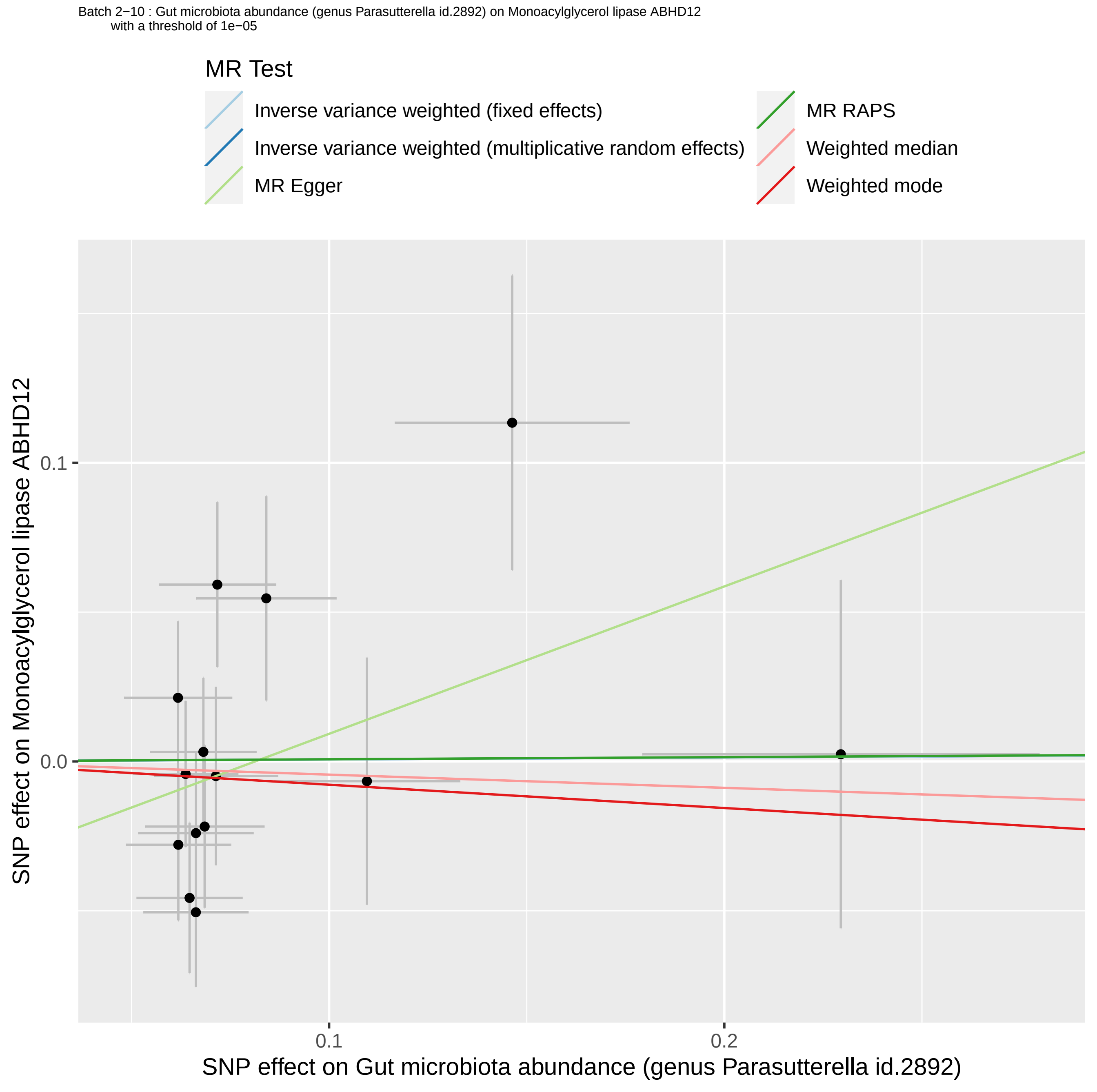

Batch 2−10 : Gut microbiota abundance (genus Parasutterella id.2892) on Monoacylglycerol lipase ABHD12
with a threshold of 1e−05
MR Test
Inverse variance weighted (fixed effects)
Inverse variance weighted (multiplicative random effects)
MR Egger
MR RAPS
Weighted median
Weighted mode
0.1
0.0
0.1
0.2
SNP effect on Monoacylglycerol lipase ABHD12
SNP effect on Gut microbiota abundance (genus Parasutterella id.2892)

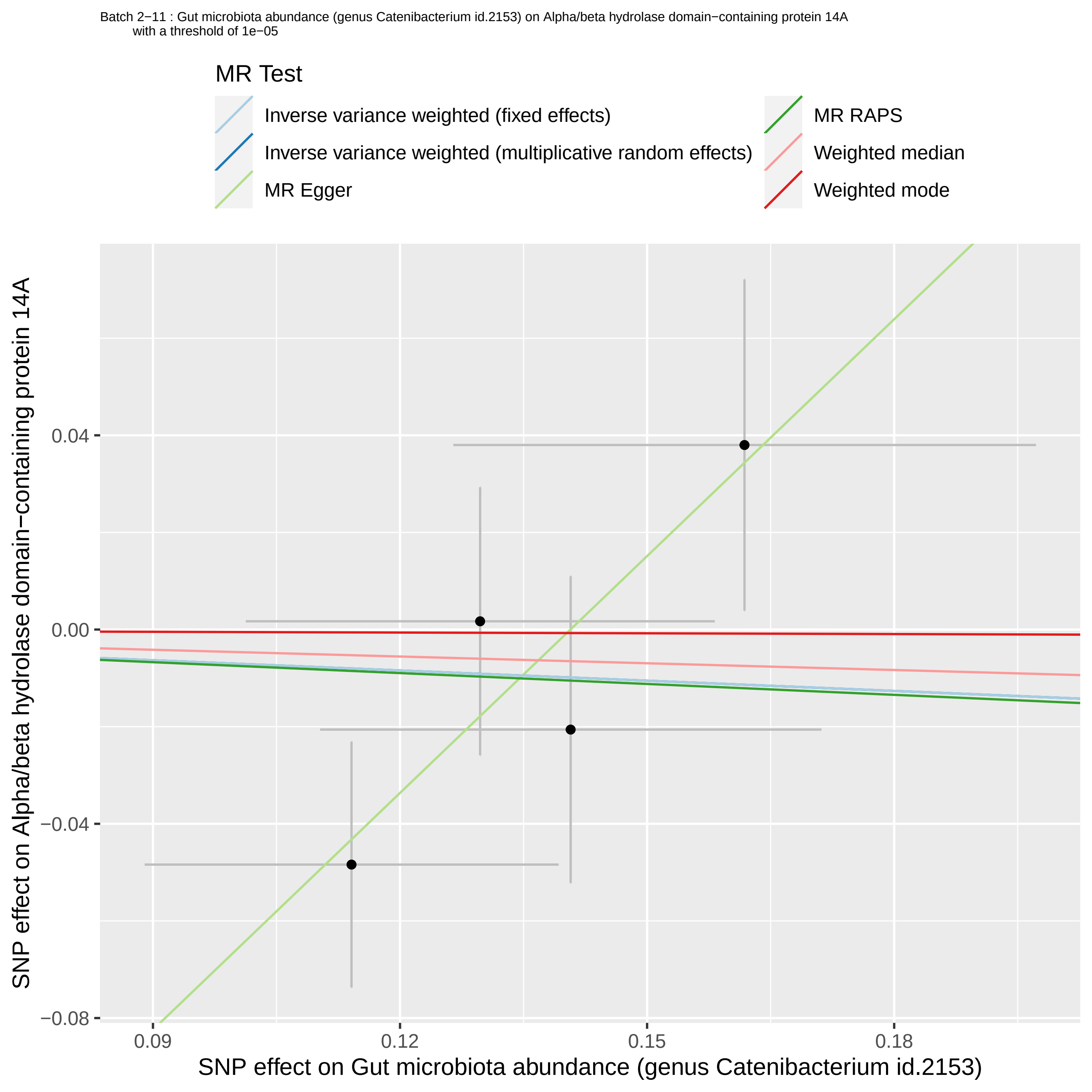

Batch 2−11 : Gut microbiota abundance (genus Catenibacterium id.2153) on Alpha/beta hydrolase domain−containing protein 14A
with a threshold of 1e−05
MR Test
Inverse variance weighted (fixed effects)
Inverse variance weighted (multiplicative random effects)
MR Egger
MR RAPS
Weighted median
Weighted mode
SNP effect on Alpha/beta hydrolase domain−containing protein 14A
0.04
0.00
−0.04
−0.08
0.09
0.12
0.15
0.18
SNP effect on Gut microbiota abundance (genus Catenibacterium id.2153)

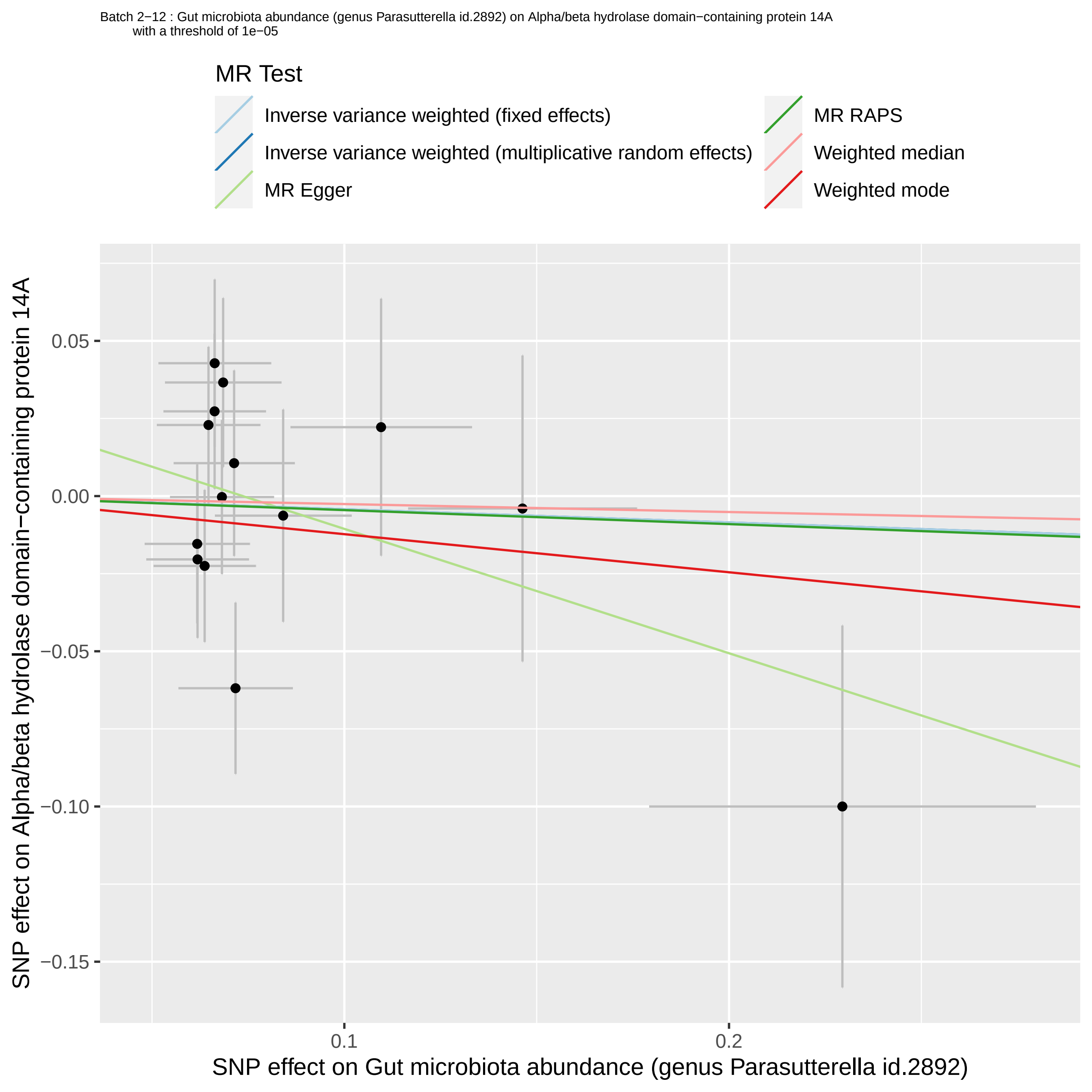

Batch 2−12 : Gut microbiota abundance (genus Parasutterella id.2892) on Alpha/beta hydrolase domain−containing protein 14A
with a threshold of 1e−05
MR Test
Inverse variance weighted (fixed effects)
Inverse variance weighted (multiplicative random effects)
MR Egger
MR RAPS
Weighted median
Weighted mode
0.05
0.00
−0.05
−0.10
−0.15
0.1
0.2
SNP effect on Alpha/beta hydrolase domain−containing protein 14A
SNP effect on Gut microbiota abundance (genus Parasutterella id.2892)

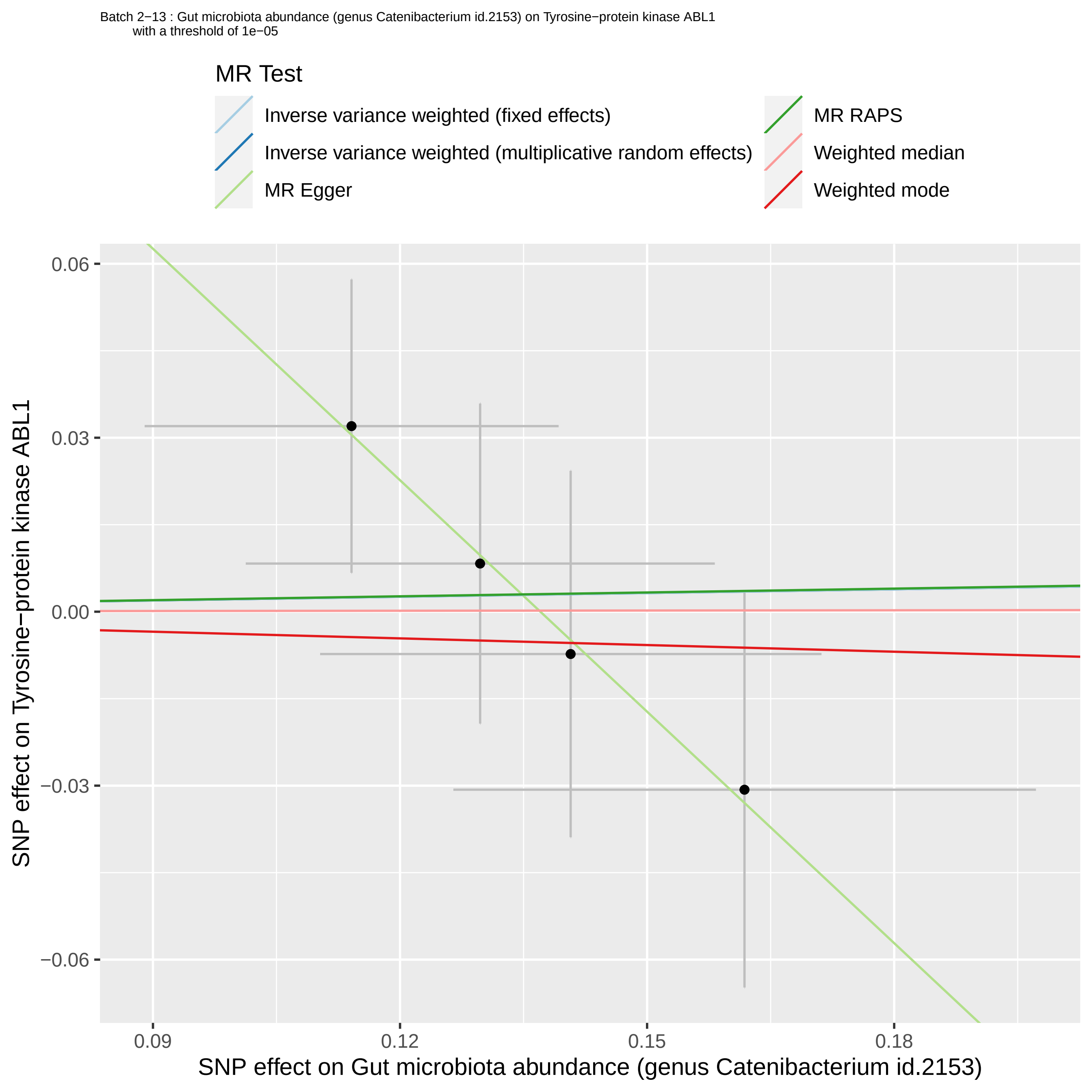

Batch 2−13 : Gut microbiota abundance (genus Catenibacterium id.2153) on Tyrosine−protein kinase ABL1
with a threshold of 1e−05
MR Test
Inverse variance weighted (fixed effects)
Inverse variance weighted (multiplicative random effects)
MR Egger
MR RAPS
Weighted median
Weighted mode
SNP effect on Tyrosine−protein kinase ABL1
0.06
0.03
0.00
−0.03
−0.06
0.09
0.12
0.15
0.18
SNP effect on Gut microbiota abundance (genus Catenibacterium id.2153)

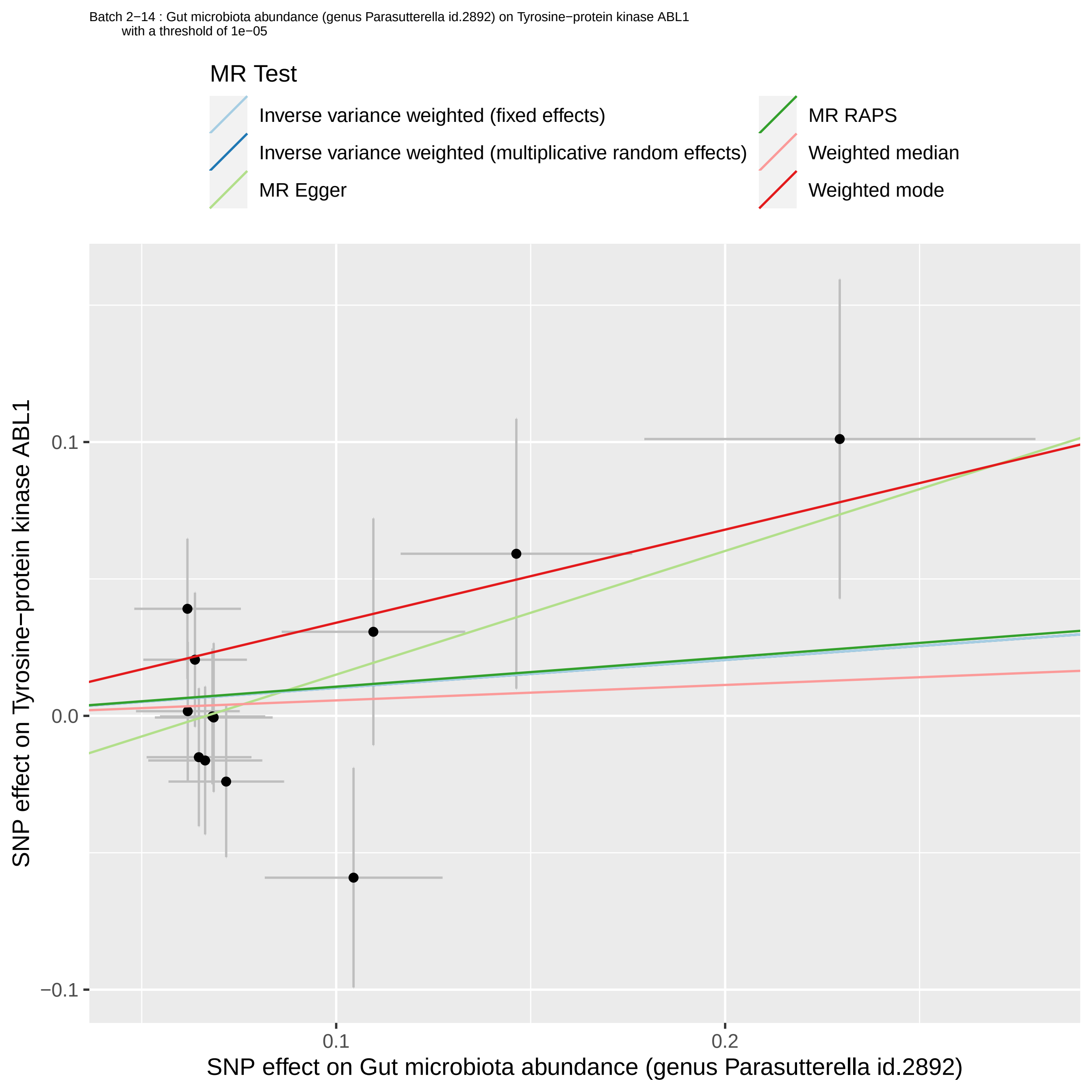

Batch 2−14 : Gut microbiota abundance (genus Parasutterella id.2892) on Tyrosine−protein kinase ABL1
with a threshold of 1e−05
MR Test
Inverse variance weighted (fixed effects)
Inverse variance weighted (multiplicative random effects)
MR Egger
MR RAPS
Weighted median
Weighted mode
0.1
0.0
−0.1
0.1
0.2
SNP effect on Tyrosine−protein kinase ABL1
SNP effect on Gut microbiota abundance (genus Parasutterella id.2892)

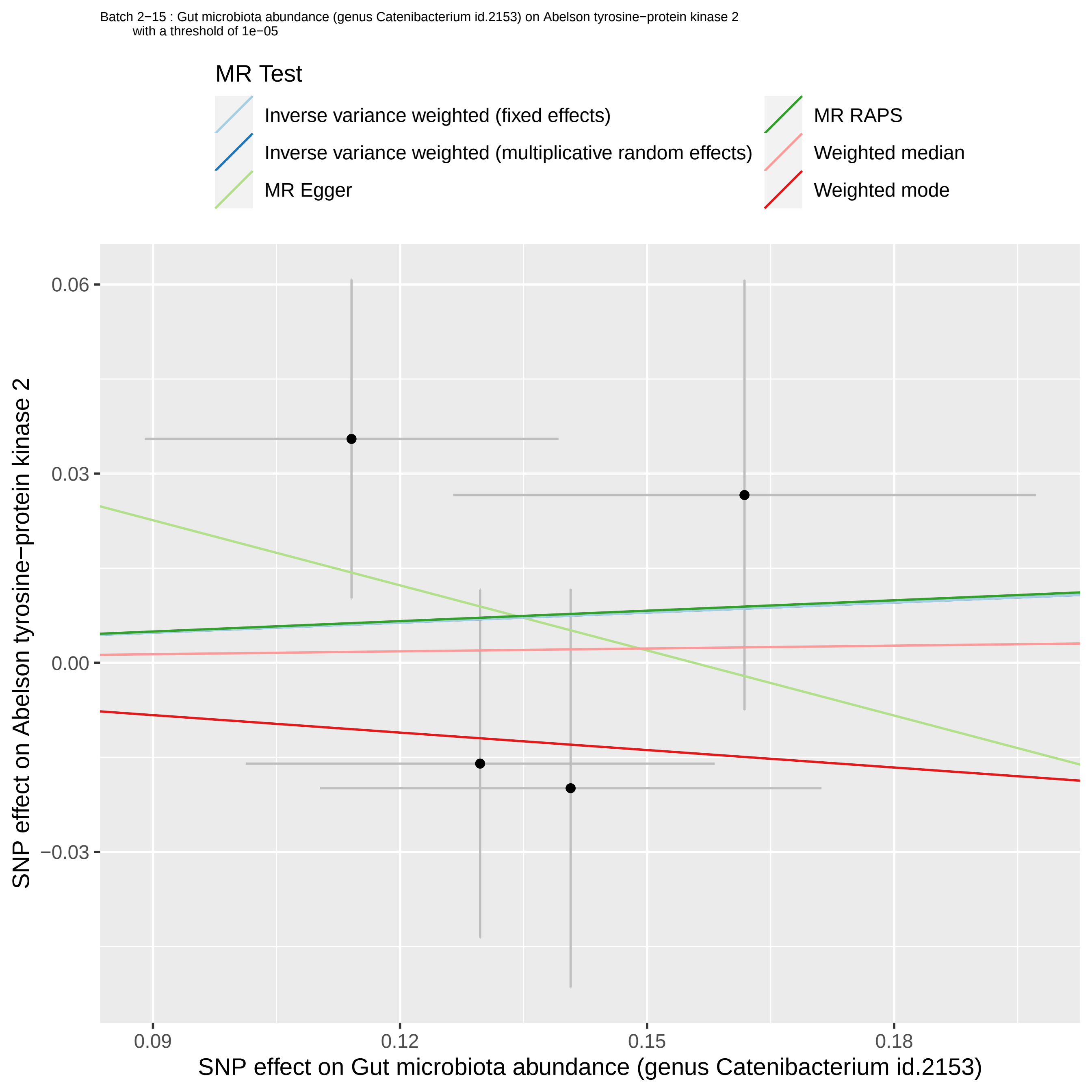

Batch 2−15 : Gut microbiota abundance (genus Catenibacterium id.2153) on Abelson tyrosine−protein kinase 2
with a threshold of 1e−05
MR Test
Inverse variance weighted (fixed effects)
Inverse variance weighted (multiplicative random effects)
MR Egger
MR RAPS
Weighted median
Weighted mode
0.06
0.03
0.00
−0.03
0.09
0.12
0.15
0.18
SNP effect on Abelson tyrosine−protein kinase 2
SNP effect on Gut microbiota abundance (genus Catenibacterium id.2153)

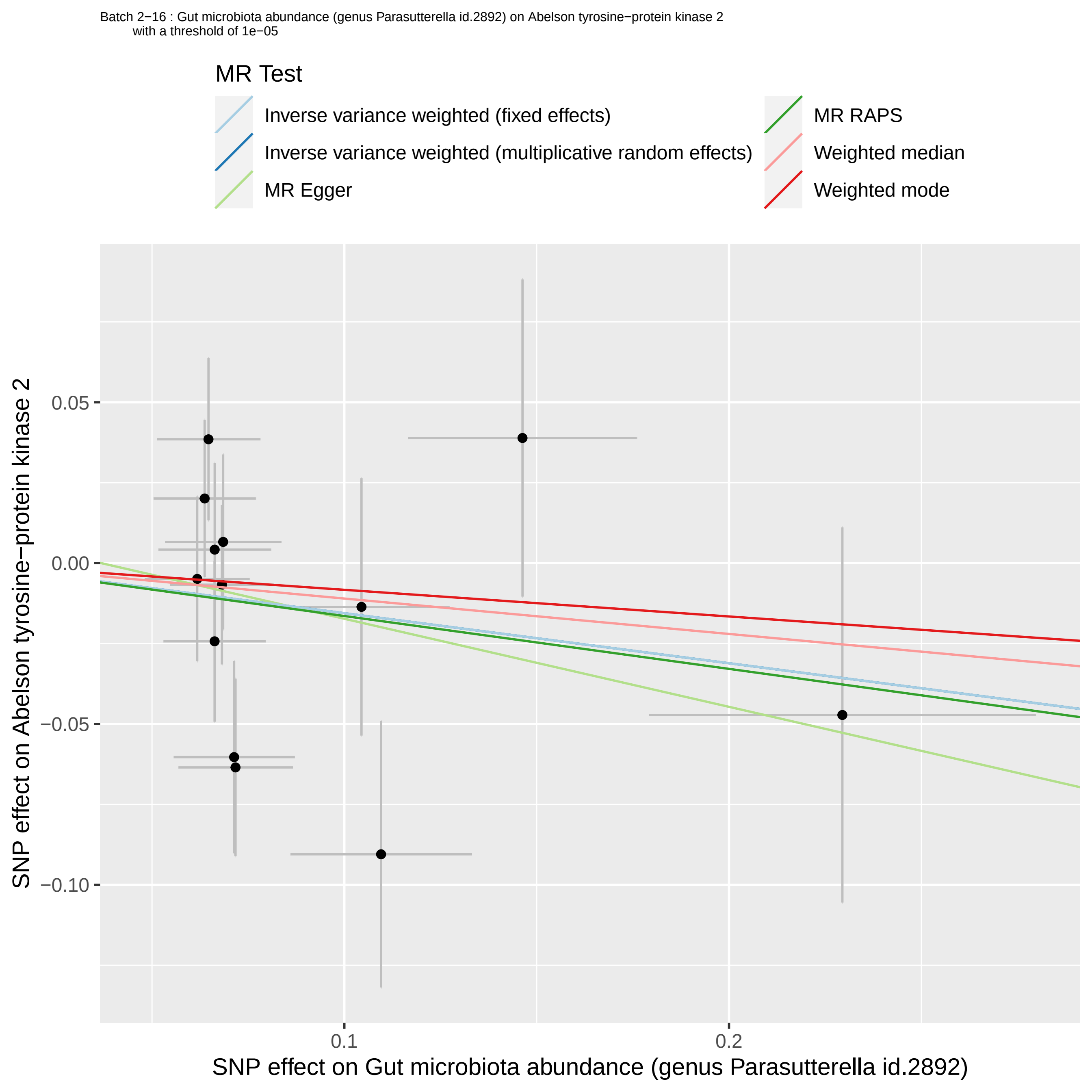

Batch 2−16 : Gut microbiota abundance (genus Parasutterella id.2892) on Abelson tyrosine−protein kinase 2
with a threshold of 1e−05
MR Test
Inverse variance weighted (fixed effects)
Inverse variance weighted (multiplicative random effects)
MR Egger
MR RAPS
Weighted median
Weighted mode
0.05
0.00
−0.05
−0.10
0.1
0.2
SNP effect on Abelson tyrosine−protein kinase 2
SNP effect on Gut microbiota abundance (genus Parasutterella id.2892)

Batch 2−17 : Gut microbiota abundance (genus Catenibacterium id.2153) on Actin−binding LIM protein 3
with a threshold of 1e−05

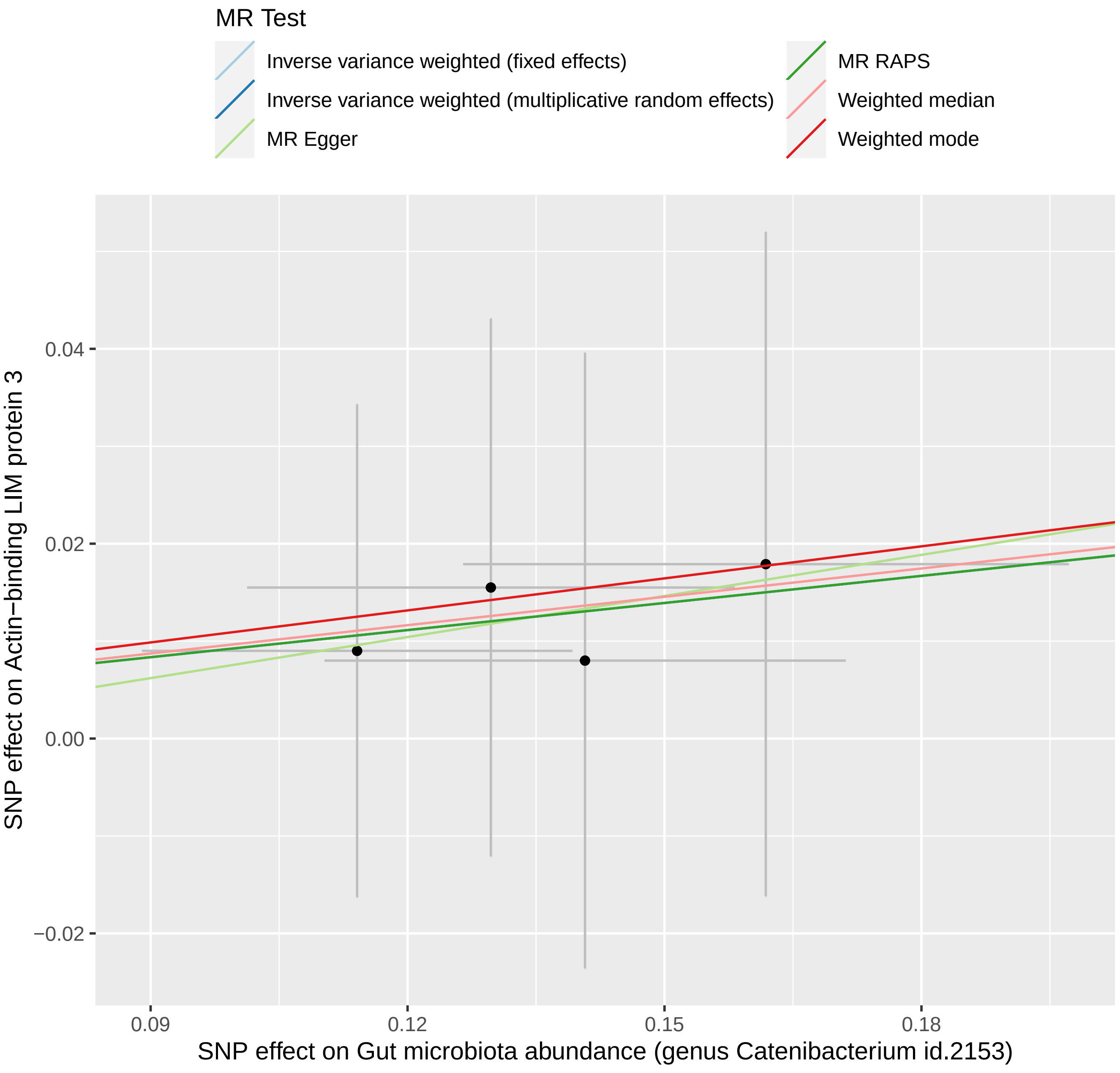

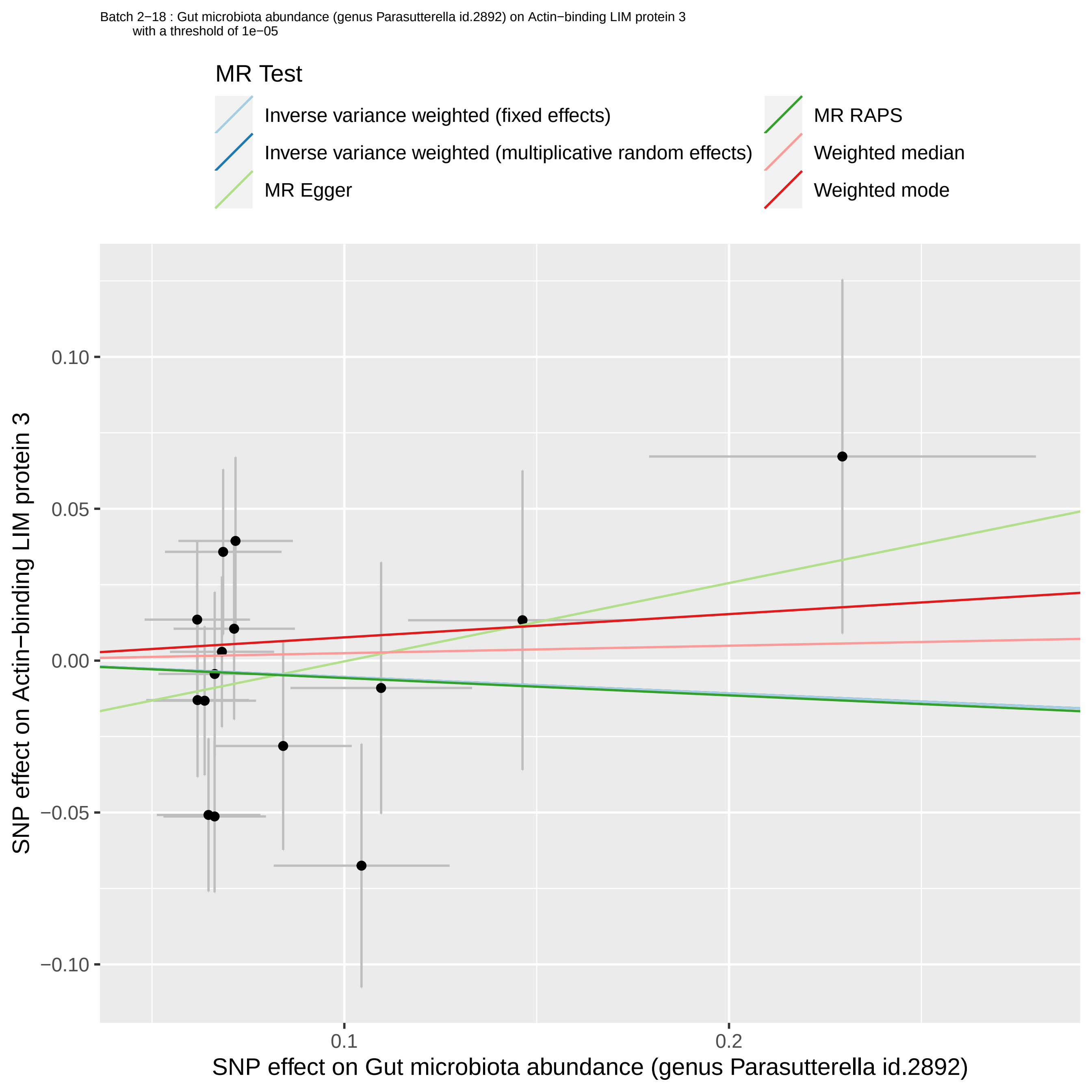

Batch 2−18 : Gut microbiota abundance (genus Parasutterella id.2892) on Actin−binding LIM protein 3
with a threshold of 1e−05
MR Test
Inverse variance weighted (fixed effects)
Inverse variance weighted (multiplicative random effects)
MR Egger
MR RAPS
Weighted median
Weighted mode
0.10
0.05
0.00
−0.05
−0.10
0.1
0.2
SNP effect on Actin−binding LIM protein 3
SNP effect on Gut microbiota abundance (genus Parasutterella id.2892)

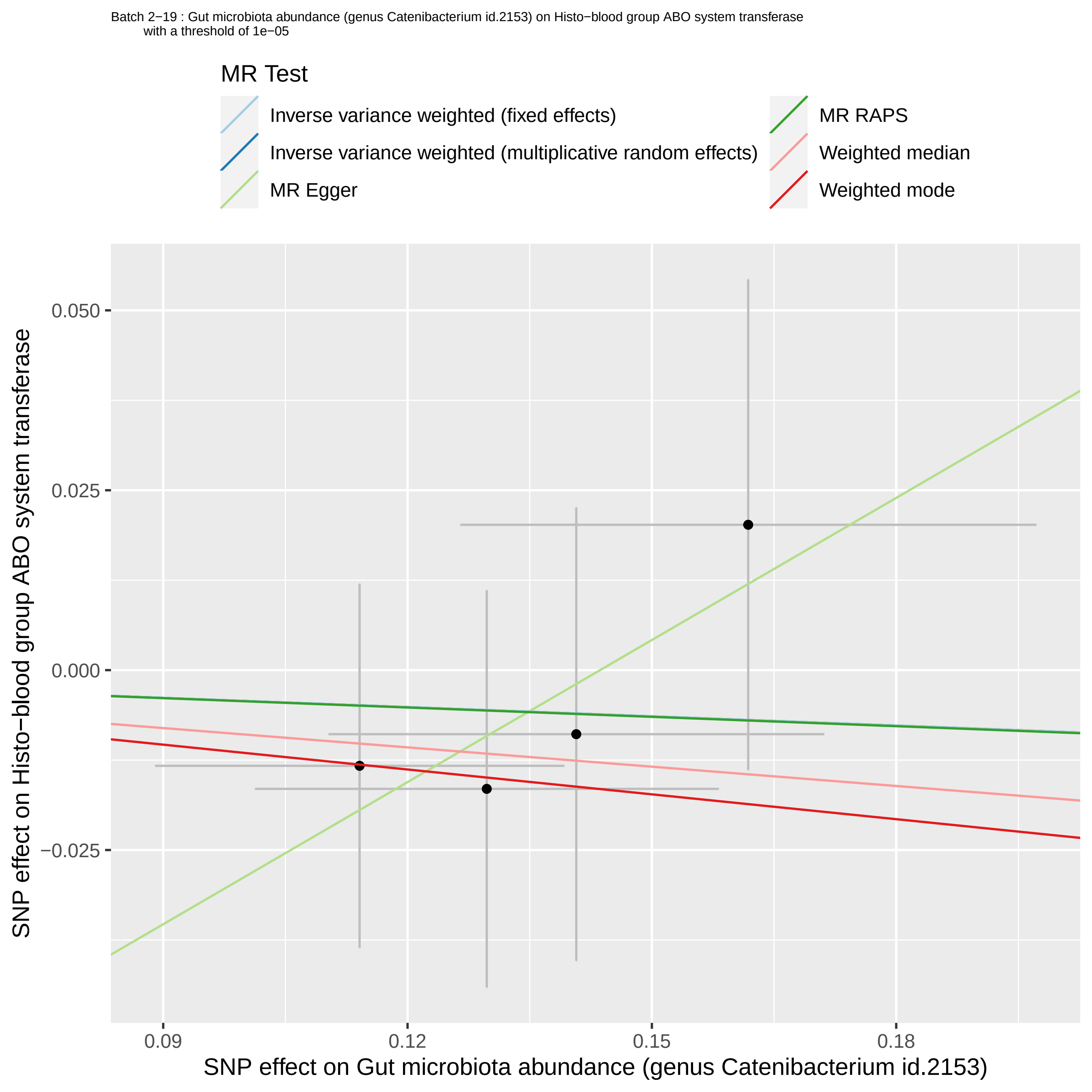

Batch 2−19 : Gut microbiota abundance (genus Catenibacterium id.2153) on Histo−blood group ABO system transferase
with a threshold of 1e−05
MR Test
Inverse variance weighted (fixed effects)
Inverse variance weighted (multiplicative random effects)
MR Egger
MR RAPS
Weighted median
Weighted mode
0.050
0.025
0.000
−0.025
0.09
0.12
0.15
0.18
SNP effect on Histo−blood group ABO system transferase
SNP effect on Gut microbiota abundance (genus Catenibacterium id.2153)

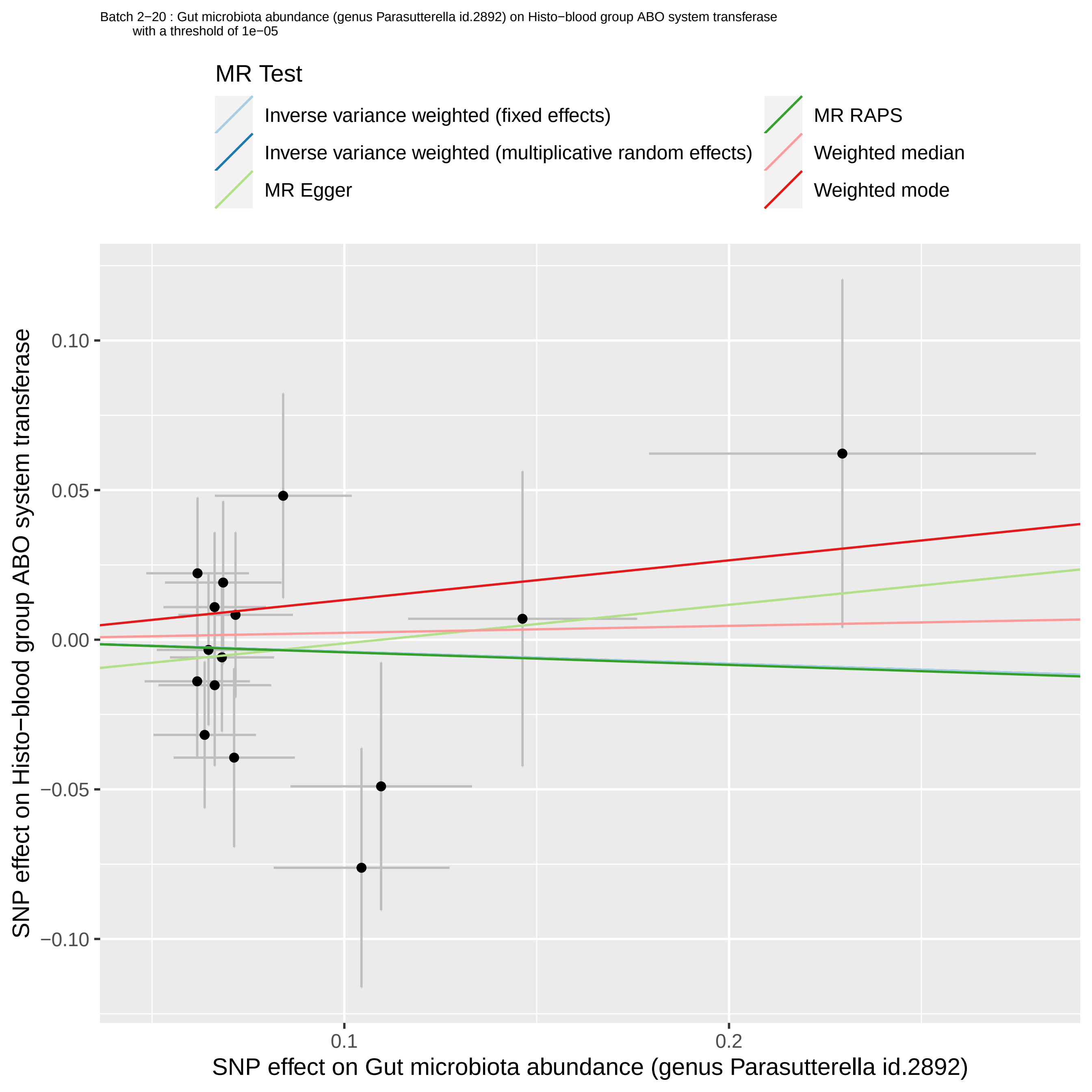
Batch 2−20 : Gut microbiota abundance (genus Parasutterella id.2892) on Histo−blood group ABO system transferase
with a threshold of 1e−05
MR Test
Inverse variance weighted (fixed effects)
Inverse variance weighted (multiplicative random effects)
MR Egger
MR RAPS
Weighted median
Weighted mode
0.10
0.05
0.00
−0.05
−0.10
0.1
0.2
SNP effect on Gut microbiota abundance (genus Parasutterella id.2892)
SNP effect on Histo−blood group ABO system transferase

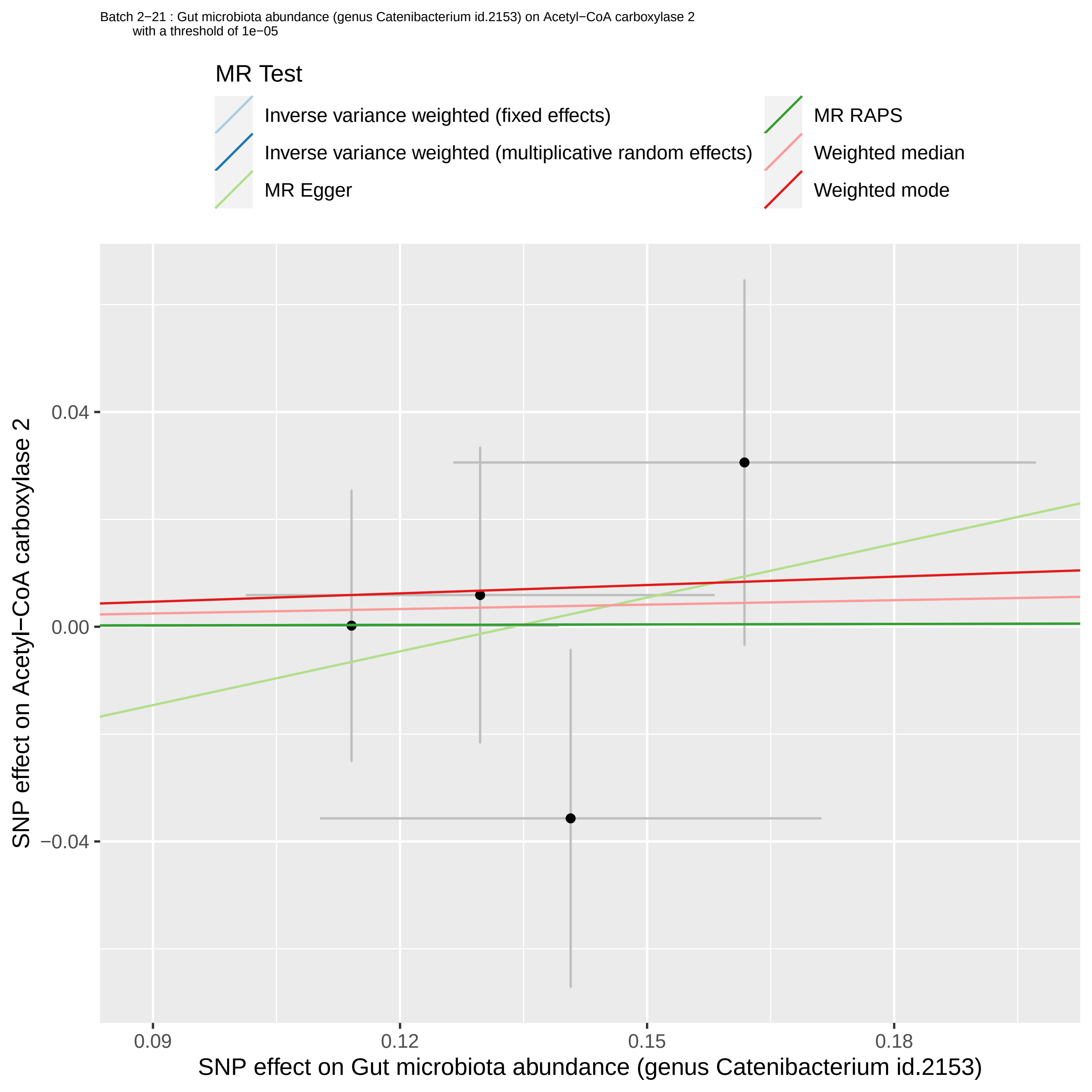
Batch 2−21 : Gut microbiota abundance (genus Catenibacterium id.2153) on Acetyl−CoA carboxylase 2
with a threshold of 1e−05
MR Test
Inverse variance weighted (fixed effects)
Inverse variance weighted (multiplicative random effects)
MR Egger
MR RAPS
Weighted median
Weighted mode
0.04
0.00
−0.04
0.09
0.12
0.15
0.18
SNP effect on Acetyl−CoA carboxylase 2
SNP effect on Gut microbiota abundance (genus Catenibacterium id.2153)

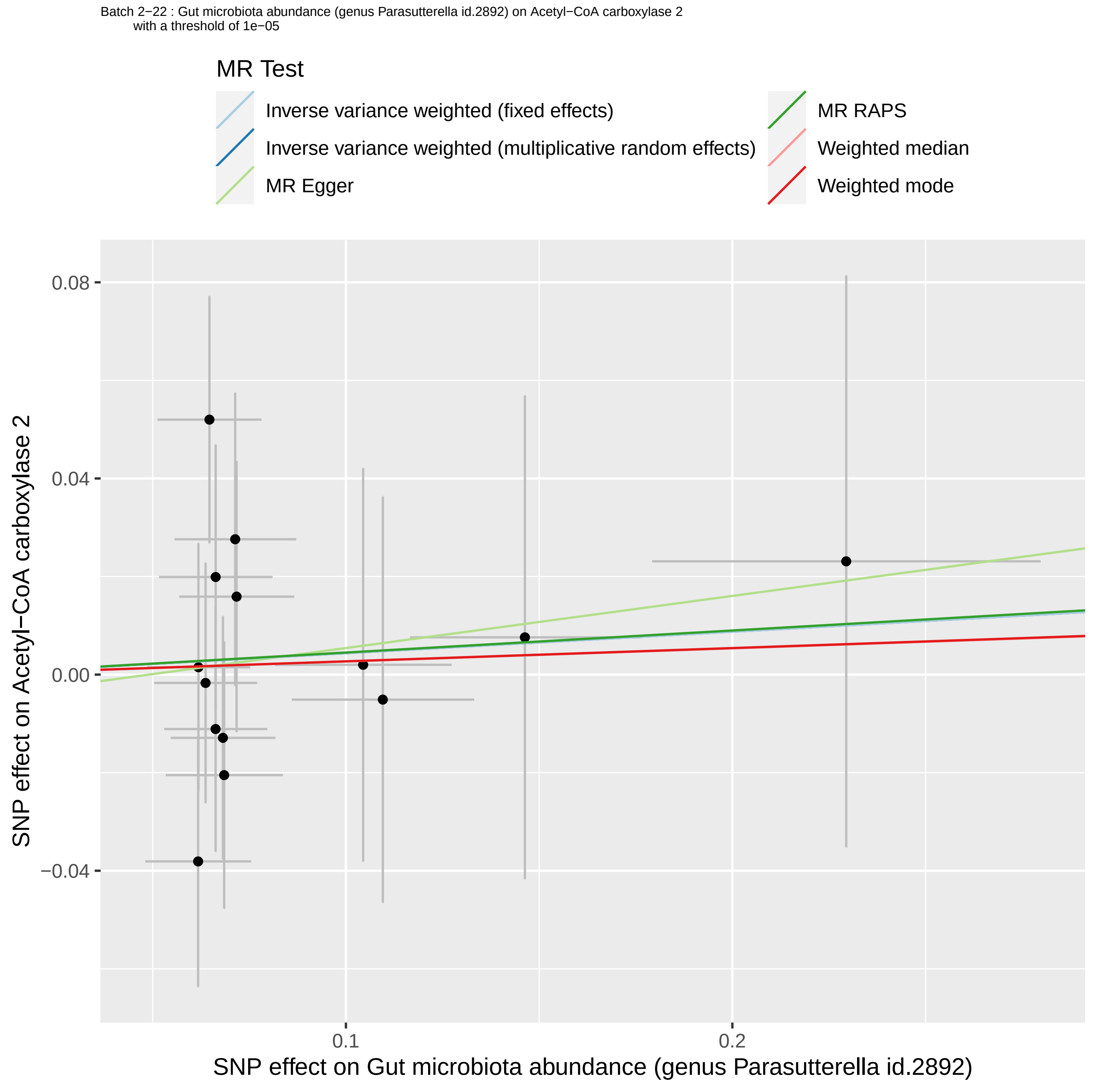

Batch 2−22 : Gut microbiota abundance (genus Parasutterella id.2892) on Acetyl−CoA carboxylase 2
with a threshold of 1e−05
MR Test
Inverse variance weighted (fixed effects)
Inverse variance weighted (multiplicative random effects)
MR Egger
MR RAPS
Weighted median
Weighted mode
0.08
0.04
0.00
−0.04
0.1
0.2
SNP effect on Acetyl−CoA carboxylase 2
SNP effect on Gut microbiota abundance (genus Parasutterella id.2892)

Batch 2−23 : Gut microbiota abundance (genus Catenibacterium id.2153) on Aggrecan core protein
with a threshold of 1e−05

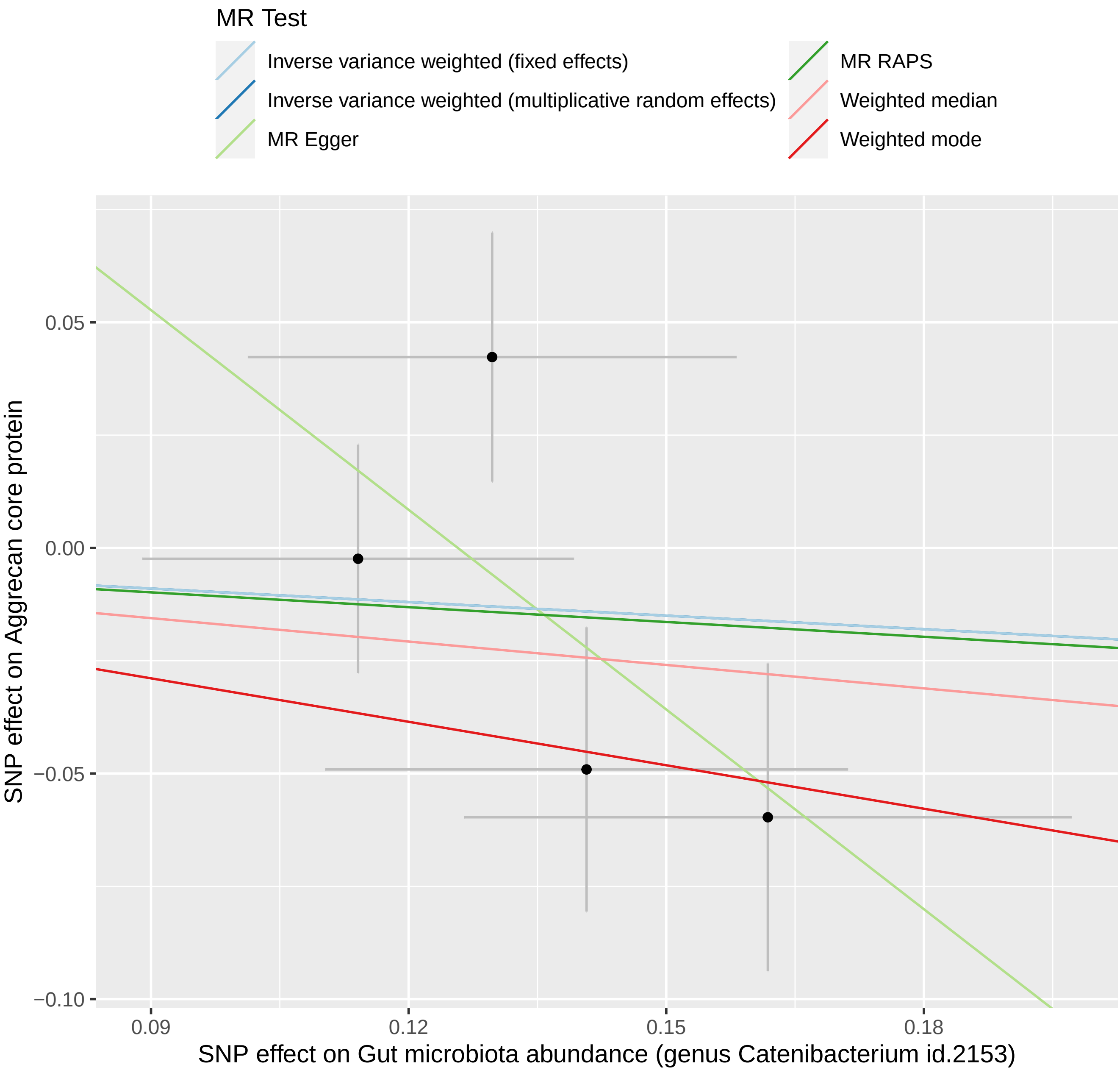

Batch 2−24 : Gut microbiota abundance (genus Parasutterella id.2892) on Aggrecan core protein
with a threshold of 1e−05

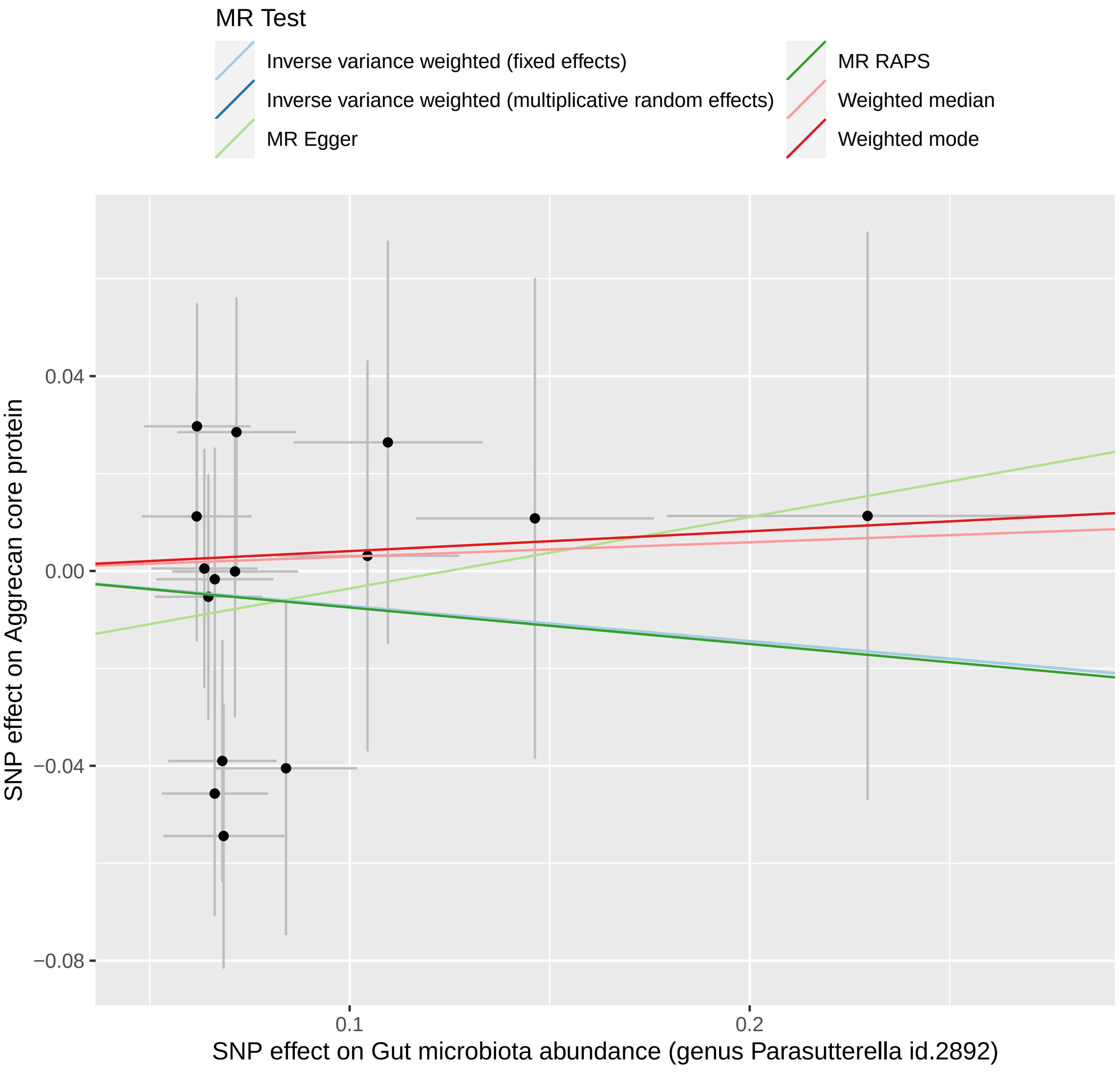

Batch 2−25 : Gut microbiota abundance (genus Catenibacterium id.2153) on Arf−GAP with coiled−coil, ANK repeat and PH domain−containing protein 2 with a threshold of 1e−05

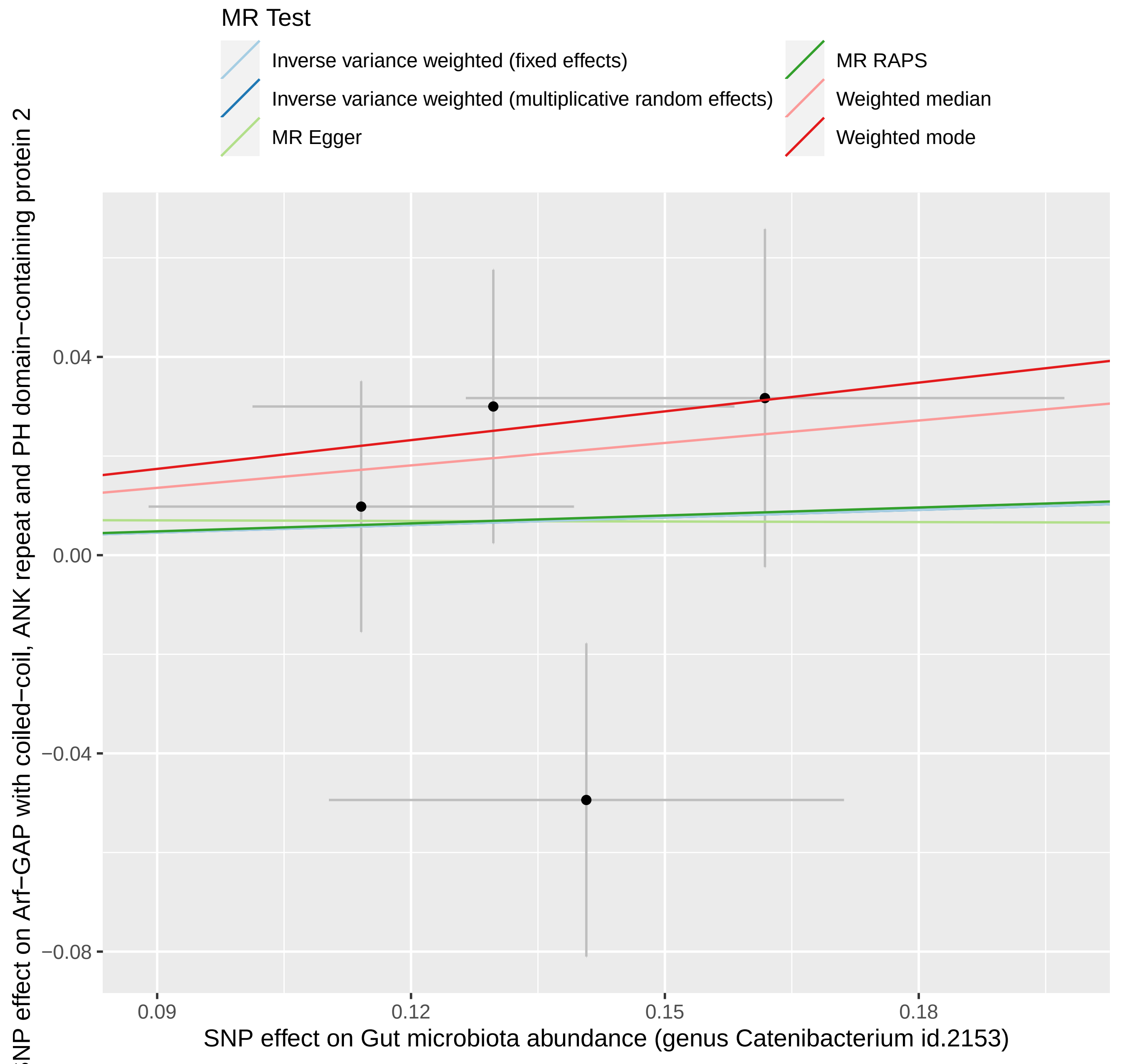

Batch 2−26 : Gut microbiota abundance (genus Parasutterella id.2892) on Arf−GAP with coiled−coil, ANK repeat and PH domain−containing protein 2 with a threshold of 1e−05

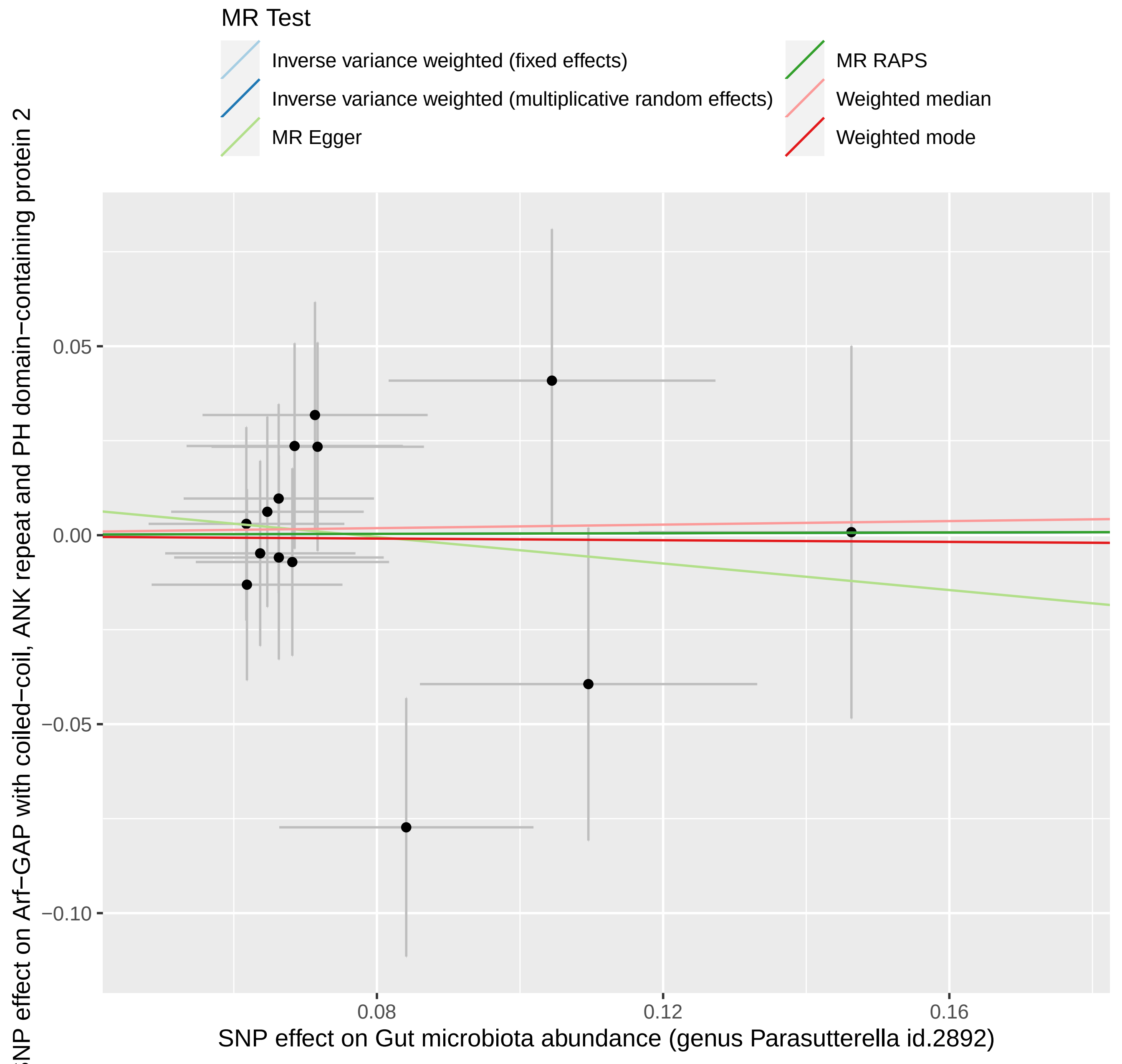

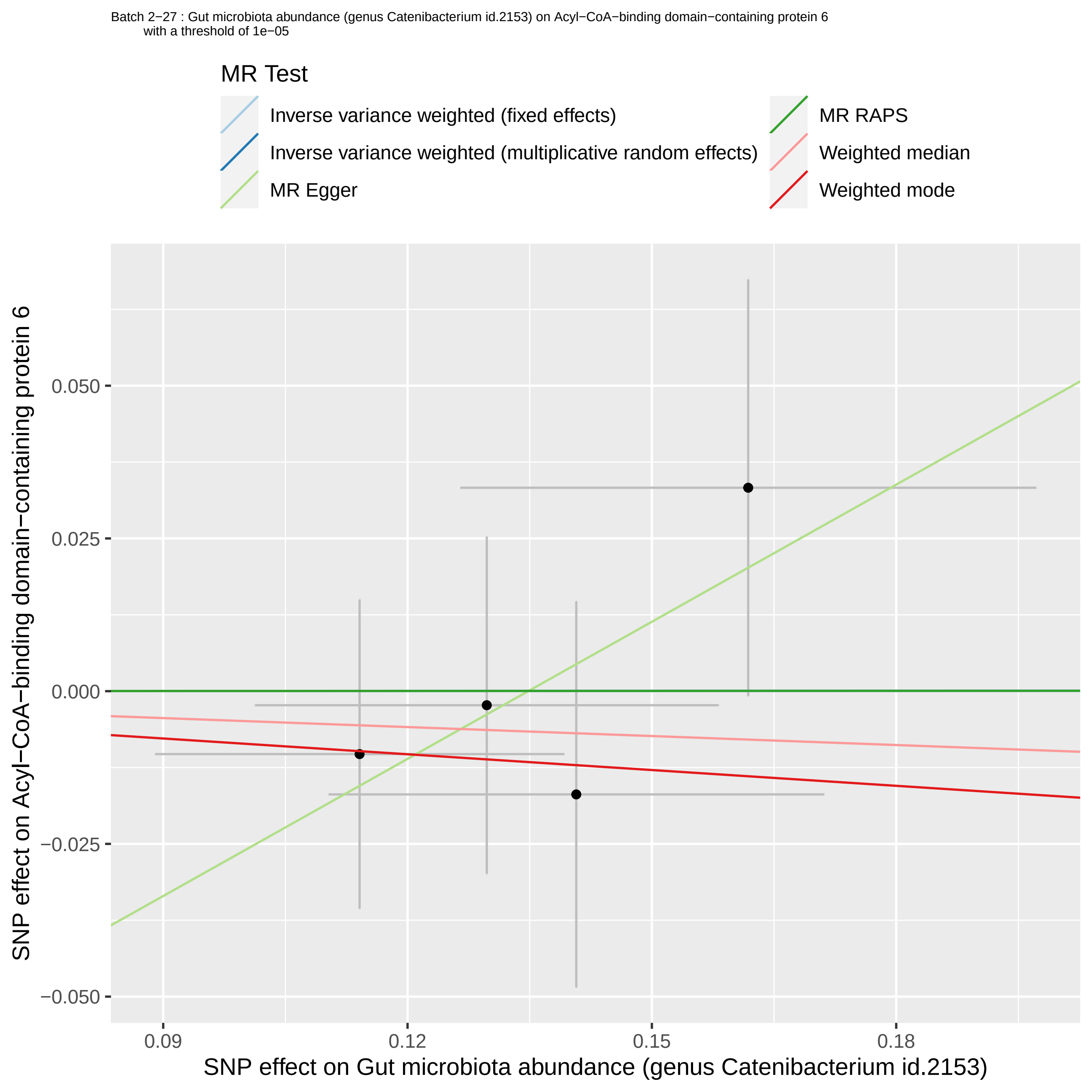

Batch 2−27 : Gut microbiota abundance (genus Catenibacterium id.2153) on Acyl−CoA−binding domain−containing protein 6
with a threshold of 1e−05
MR Test
Inverse variance weighted (fixed effects)
Inverse variance weighted (multiplicative random effects)
MR Egger
MR RAPS
Weighted median
Weighted mode
0.050
0.025
0.000
−0.025
−0.050
0.09
0.12
0.15
0.18
SNP effect on Acyl−CoA−binding domain−containing protein 6
SNP effect on Gut microbiota abundance (genus Catenibacterium id.2153)

Batch 2−28 : Gut microbiota abundance (genus Parasutterella id.2892) on Acyl−CoA−binding domain−containing protein 6
with a threshold of 1e−05

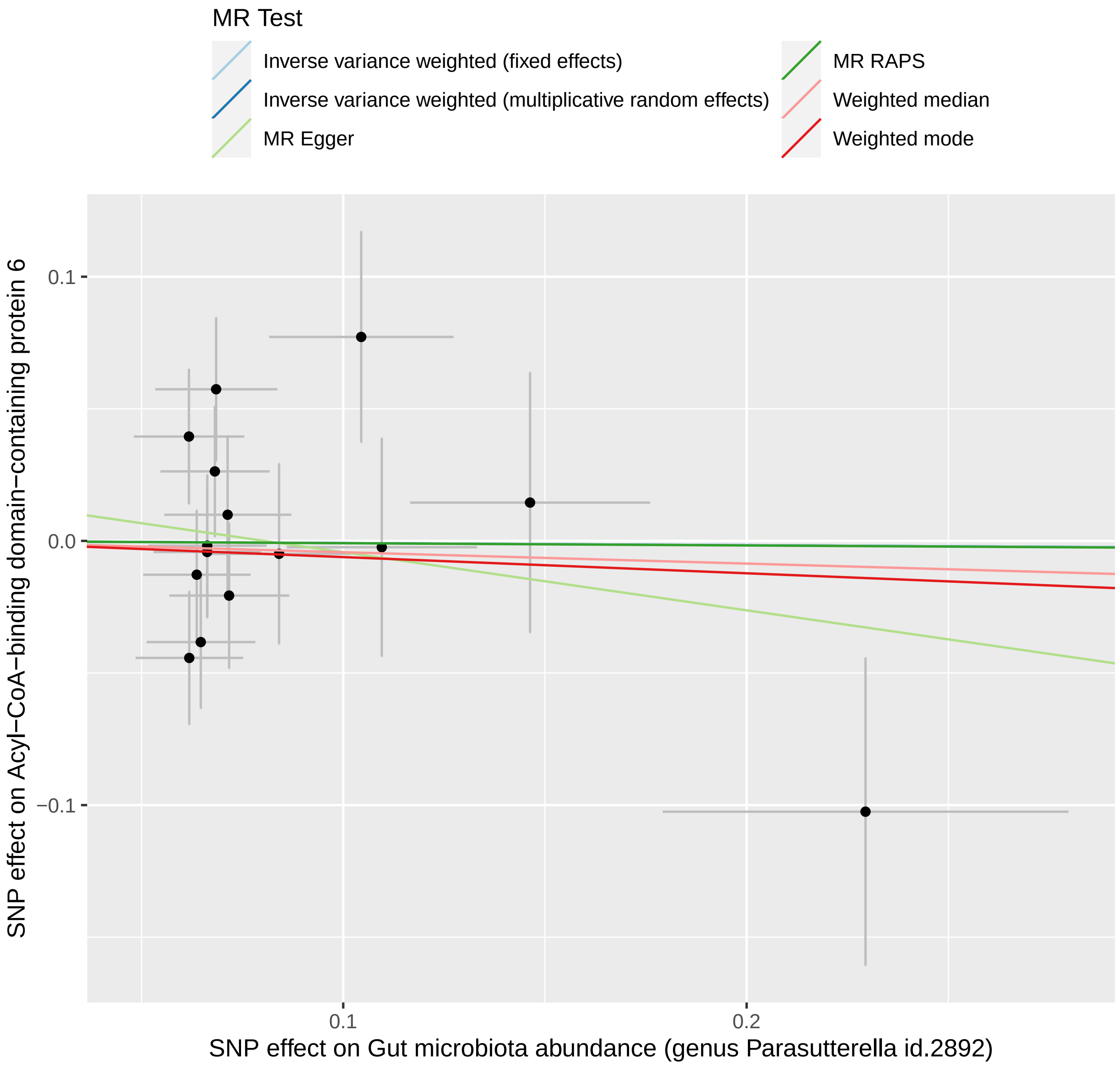

Batch 2−29 : Gut microbiota abundance (genus Catenibacterium id.2153) on Acyl−CoA−binding domain−containing protein 7
with a threshold of 1e−05

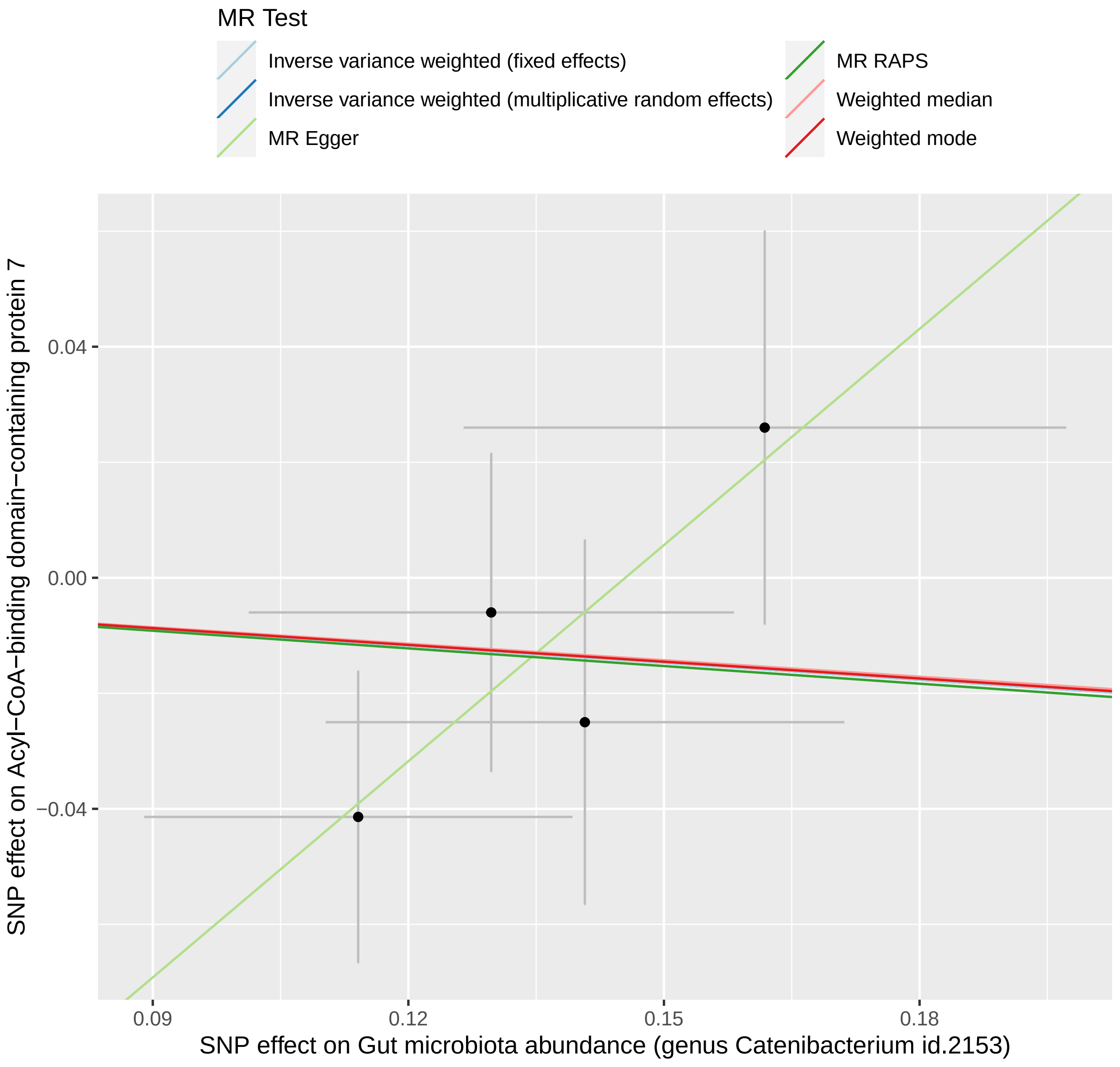

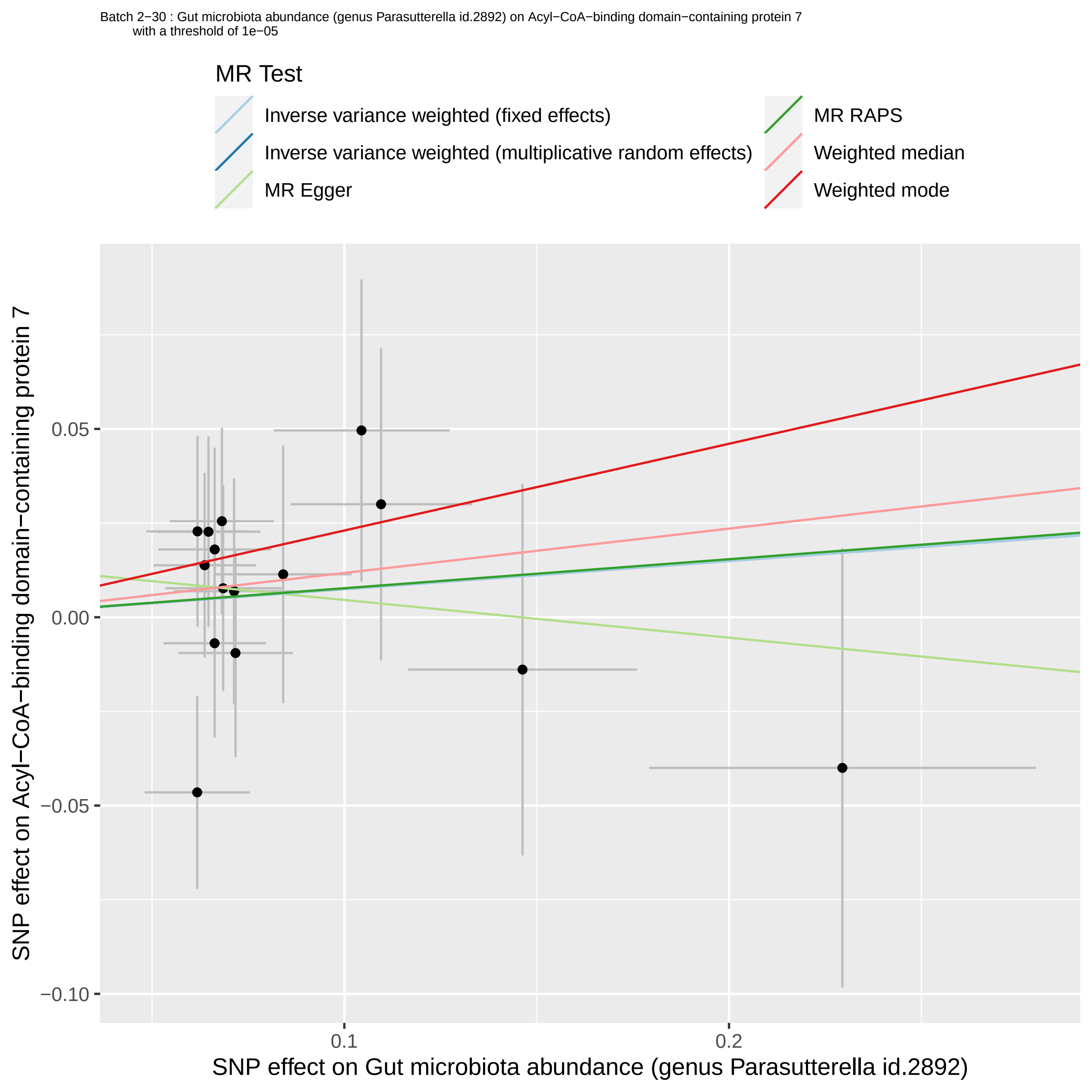
Batch 2−30 : Gut microbiota abundance (genus Parasutterella id.2892) on Acyl−CoA−binding domain−containing protein 7
with a threshold of 1e−05
MR Test
Inverse variance weighted (fixed effects)
Inverse variance weighted (multiplicative random effects)
MR Egger
MR RAPS
Weighted median
Weighted mode
0.05
0.00
−0.05
−0.10
0.1
0.2
SNP effect on Acyl−CoA−binding domain−containing protein 7
SNP effect on Gut microbiota abundance (genus Parasutterella id.2892)

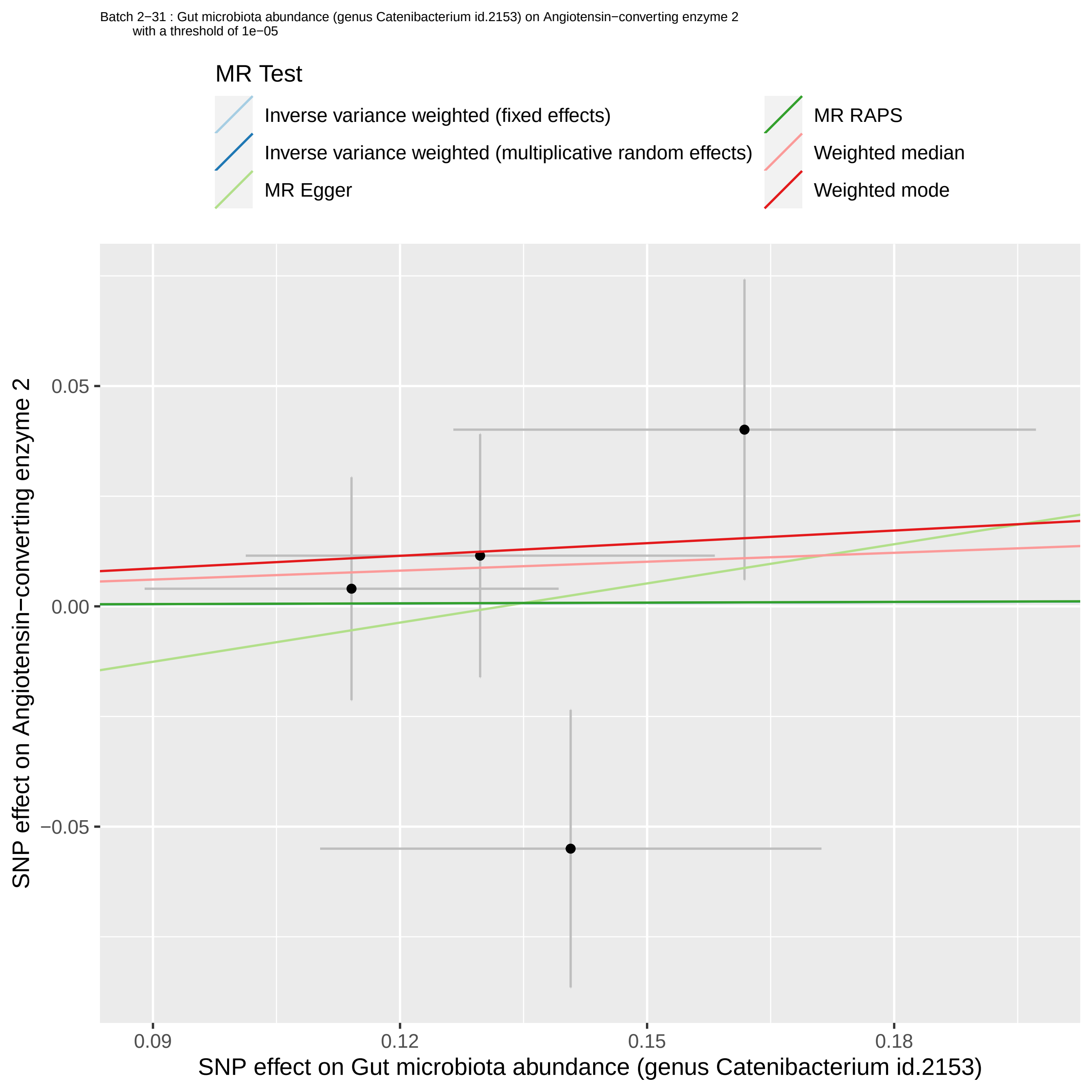

Batch 2−31 : Gut microbiota abundance (genus Catenibacterium id.2153) on Angiotensin−converting enzyme 2
with a threshold of 1e−05
MR Test
Inverse variance weighted (fixed effects)
Inverse variance weighted (multiplicative random effects)
MR Egger
MR RAPS
Weighted median
Weighted mode
0.05
0.00
−0.05
0.09
0.12
0.15
0.18
SNP effect on Angiotensin−converting enzyme 2
SNP effect on Gut microbiota abundance (genus Catenibacterium id.2153)

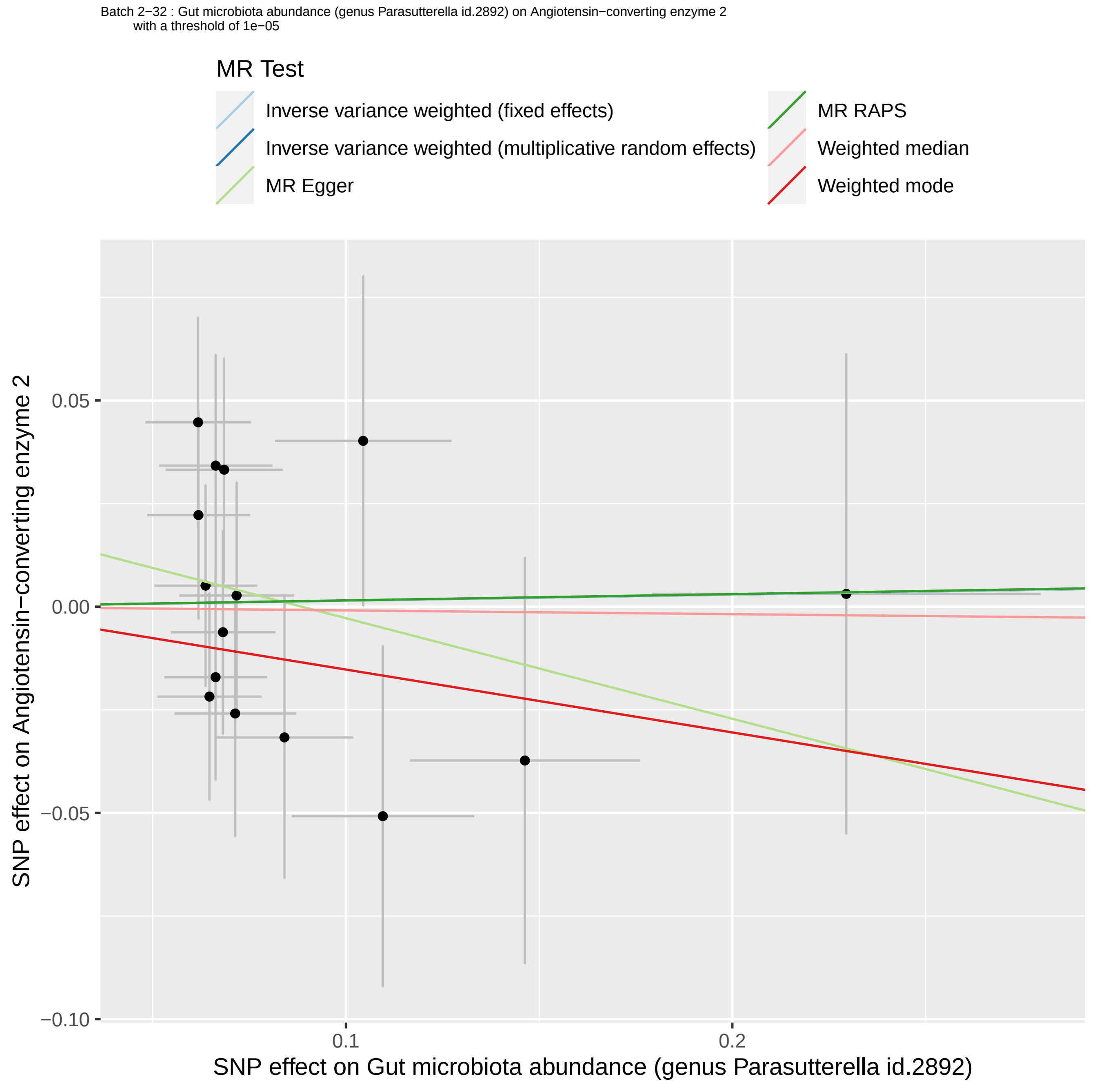

Batch 2−32 : Gut microbiota abundance (genus Parasutterella id.2892) on Angiotensin−converting enzyme 2
with a threshold of 1e−05
MR Test
Inverse variance weighted (fixed effects)
Inverse variance weighted (multiplicative random effects)
MR Egger
MR RAPS
Weighted median
Weighted mode
0.05
0.00
−0.05
−0.10
0.1
0.2
SNP effect on Angiotensin−converting enzyme 2
SNP effect on Gut microbiota abundance (genus Parasutterella id.2892)

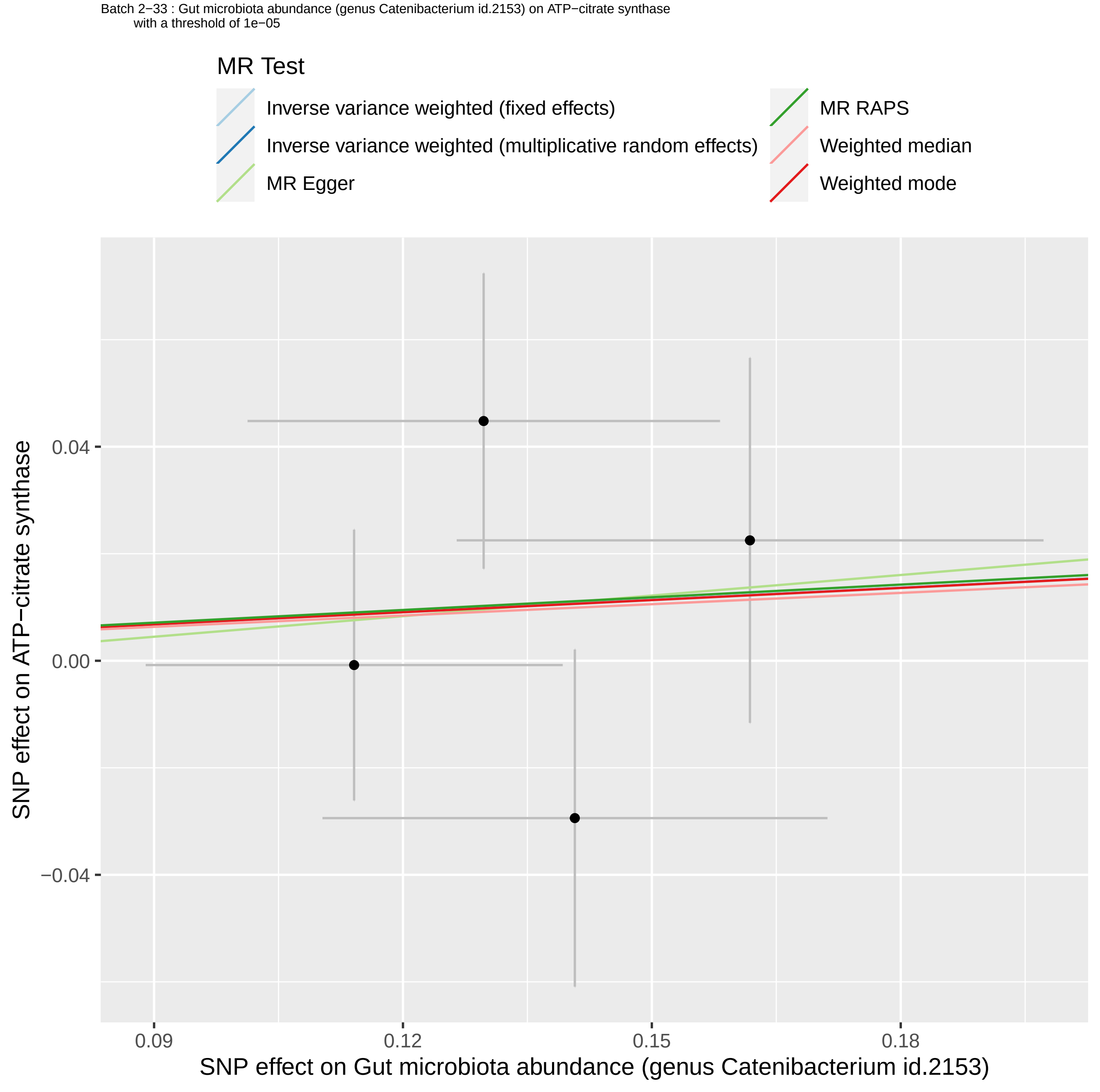

Batch 2−33 : Gut microbiota abundance (genus Catenibacterium id.2153) on ATP−citrate synthase
with a threshold of 1e−05
MR Test
Inverse variance weighted (fixed effects)
Inverse variance weighted (multiplicative random effects)
MR Egger
MR RAPS
Weighted median
Weighted mode
0.04
0.00
−0.04
0.09
0.12
0.15
0.18
SNP effect on ATP−citrate synthase
SNP effect on Gut microbiota abundance (genus Catenibacterium id.2153)

Batch 2−34 : Gut microbiota abundance (genus Parasutterella id.2892) on ATP−citrate synthase
with a threshold of 1e−05

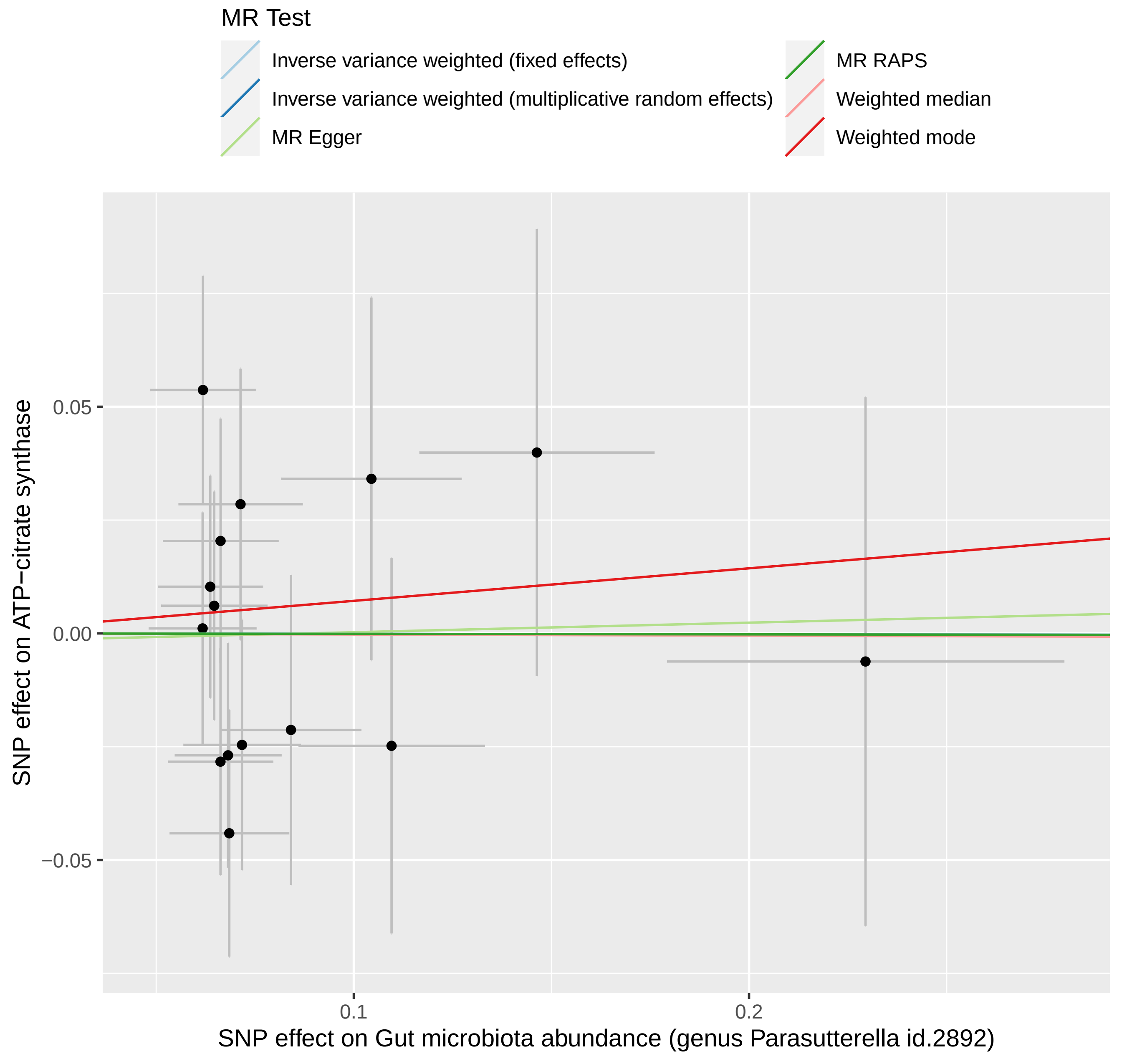

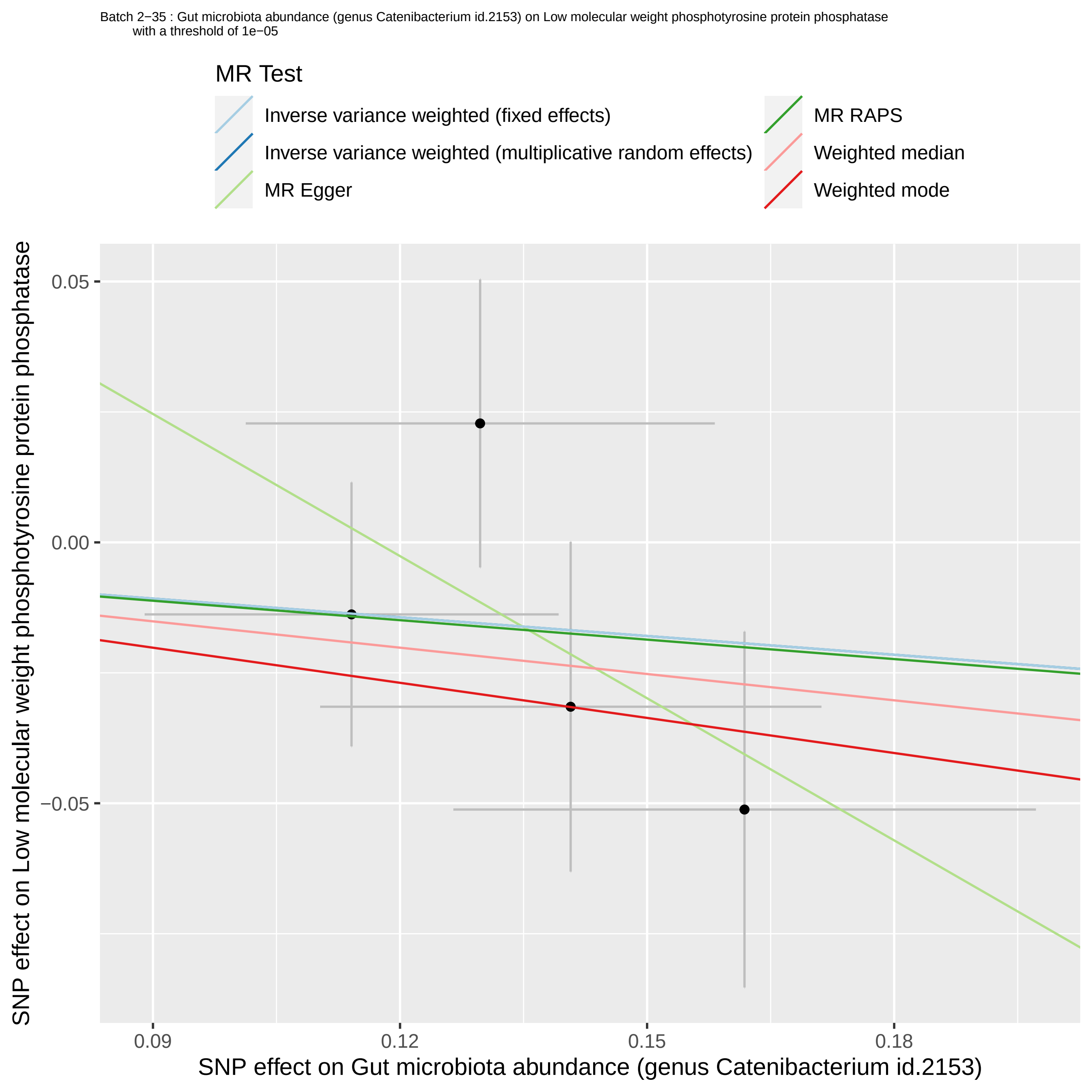

Batch 2−35 : Gut microbiota abundance (genus Catenibacterium id.2153) on Low molecular weight phosphotyrosine protein phosphatase
with a threshold of 1e−05
MR Test
Inverse variance weighted (fixed effects)
Inverse variance weighted (multiplicative random effects)
MR Egger
MR RAPS
Weighted median
Weighted mode
SNP effect on Low molecular weight phosphotyrosine protein phosphatase
0.05
0.00
−0.05
0.09
0.12
0.15
0.18
SNP effect on Gut microbiota abundance (genus Catenibacterium id.2153)

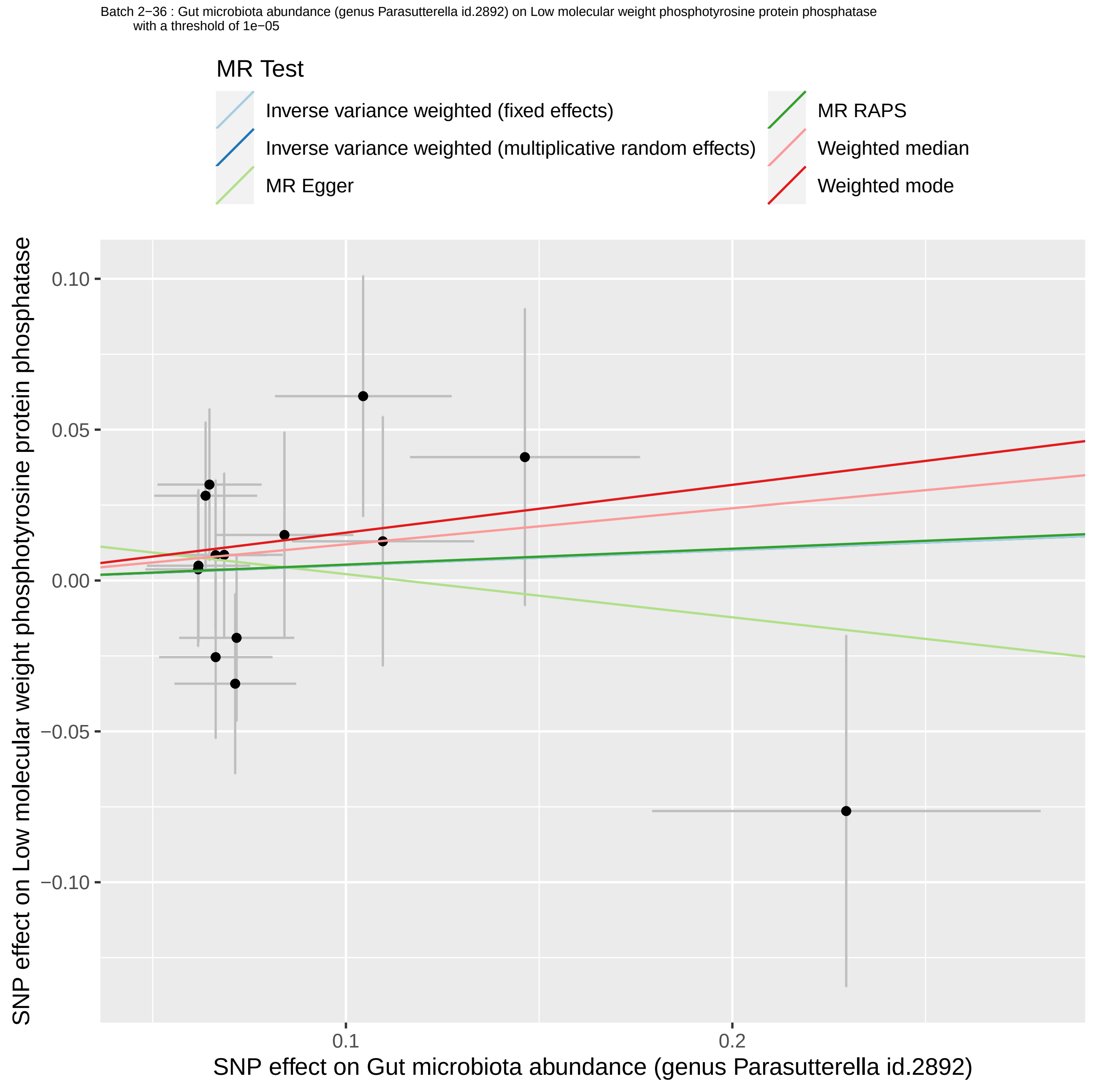
Batch 2−36 : Gut microbiota abundance (genus Parasutterella id.2892) on Low molecular weight phosphotyrosine protein phosphatase
with a threshold of 1e−05
MR Test
Inverse variance weighted (fixed effects)
Inverse variance weighted (multiplicative random effects)
MR Egger
MR RAPS
Weighted median
Weighted mode
SNP effect on Low molecular weight phosphotyrosine protein phosphatase
0.10
0.05
0.00
−0.05
−0.10
0.1
0.2
SNP effect on Gut microbiota abundance (genus Parasutterella id.2892)

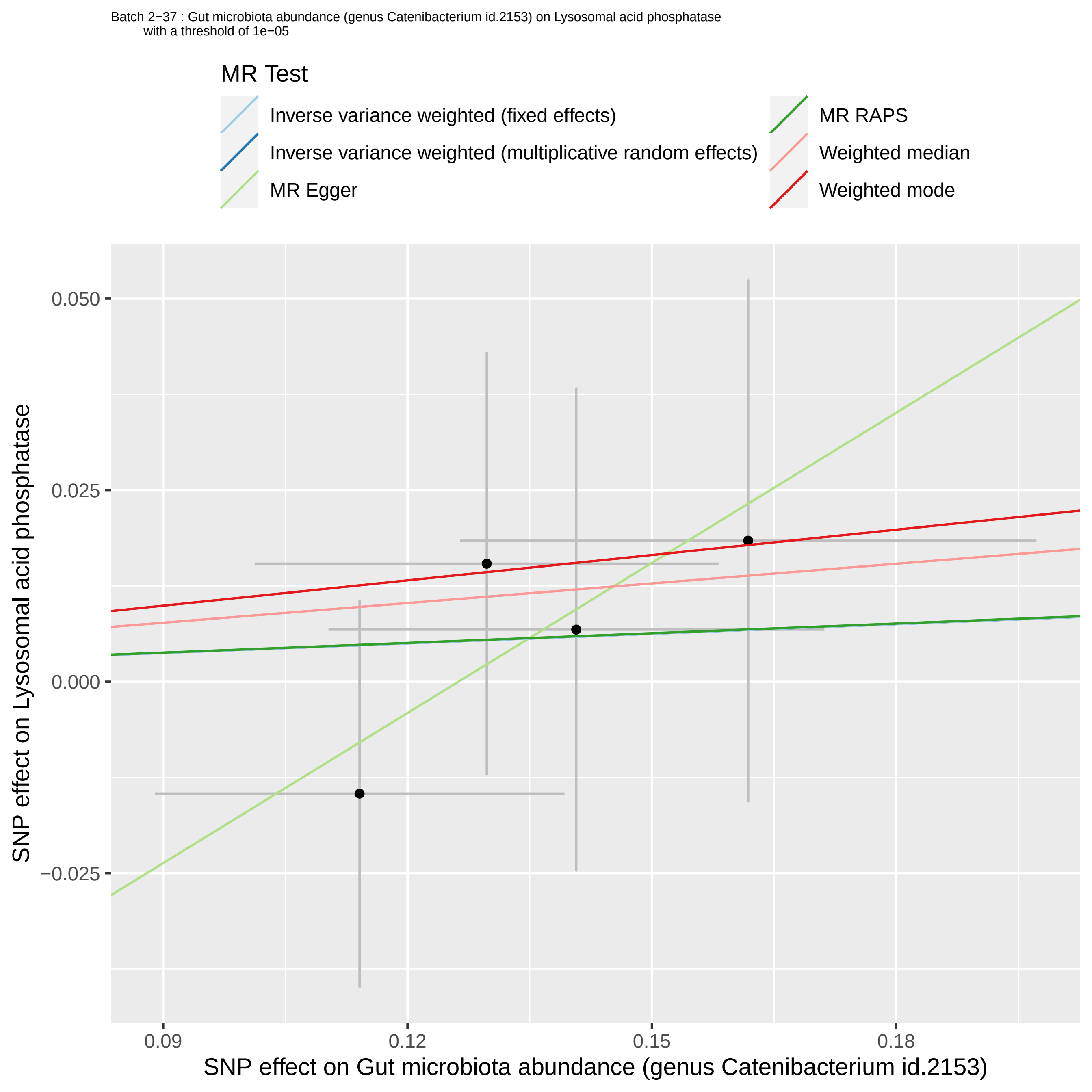

Batch 2−37 : Gut microbiota abundance (genus Catenibacterium id.2153) on Lysosomal acid phosphatase
with a threshold of 1e−05
MR Test
Inverse variance weighted (fixed effects)
Inverse variance weighted (multiplicative random effects)
MR Egger
MR RAPS
Weighted median
Weighted mode
SNP effect on Lysosomal acid phosphatase
0.050
0.025
0.000
−0.025
0.09
0.12
0.15
0.18
SNP effect on Gut microbiota abundance (genus Catenibacterium id.2153)

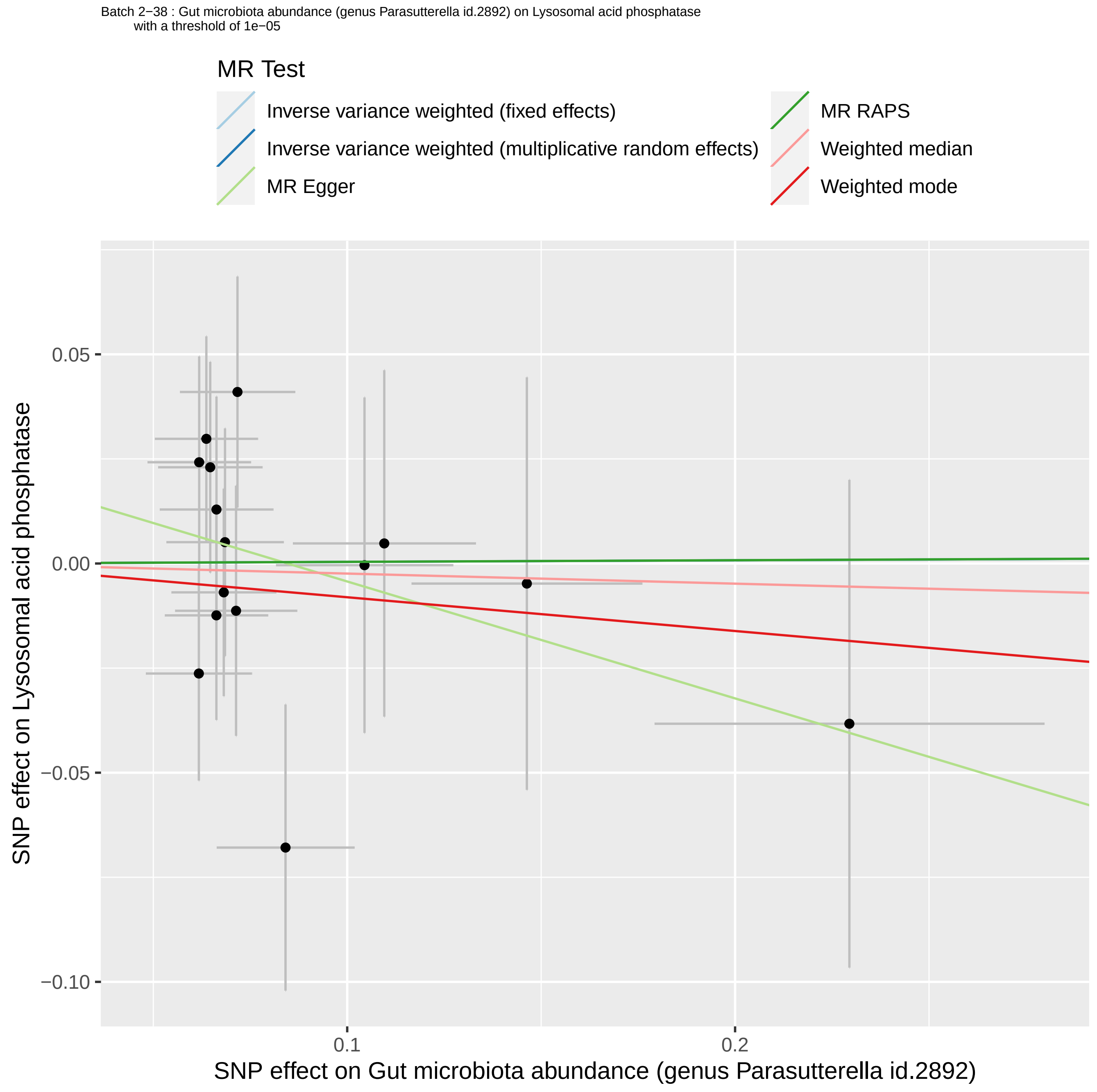
Batch 2−38 : Gut microbiota abundance (genus Parasutterella id.2892) on Lysosomal acid phosphatase
with a threshold of 1e−05
MR Test
Inverse variance weighted (fixed effects)
Inverse variance weighted (multiplicative random effects)
MR Egger
MR RAPS
Weighted median
Weighted mode
0.05
0.00
−0.05
−0.10
0.1
0.2
SNP effect on Lysosomal acid phosphatase
SNP effect on Gut microbiota abundance (genus Parasutterella id.2892)

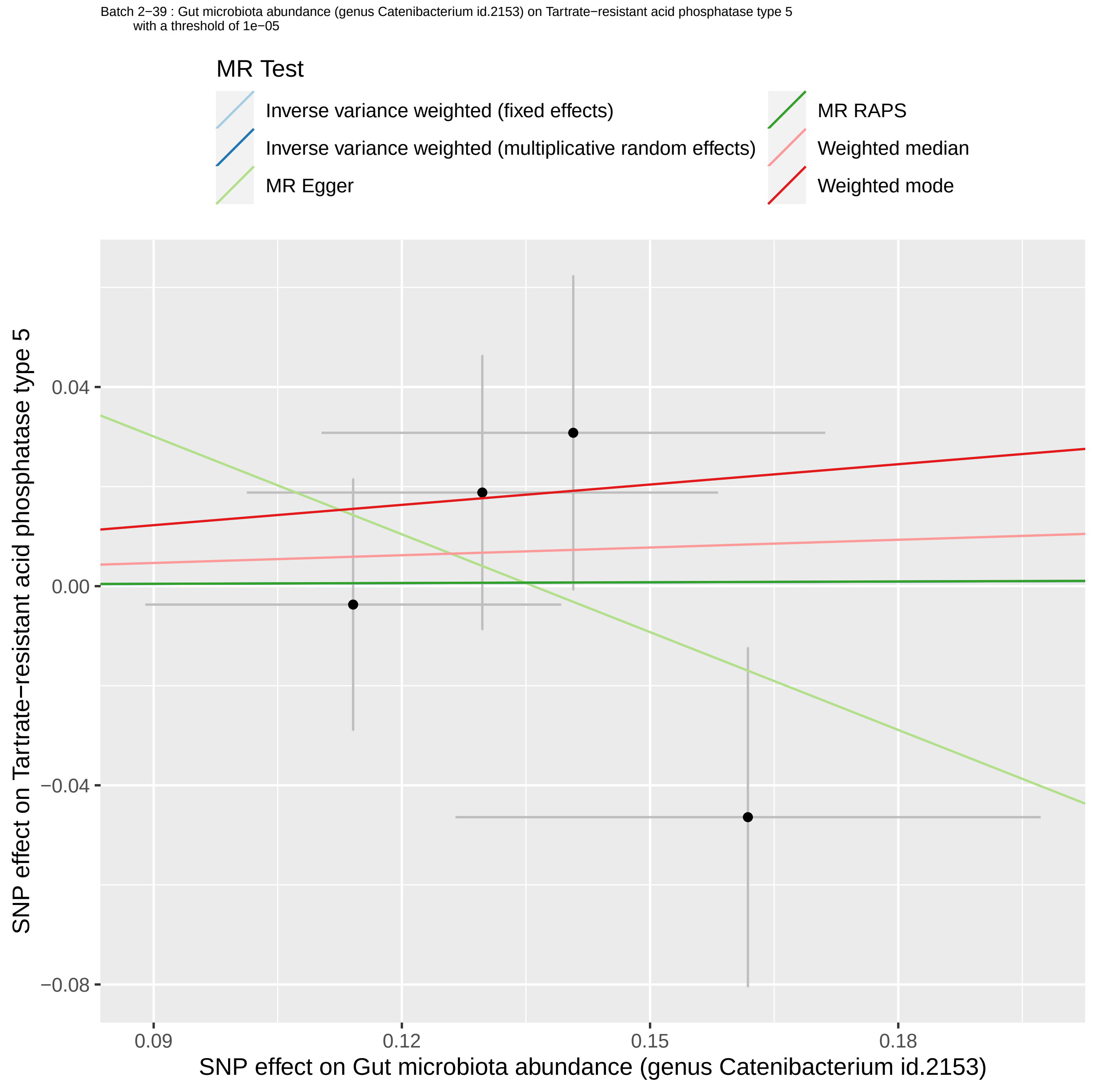
Batch 2−39 : Gut microbiota abundance (genus Catenibacterium id.2153) on Tartrate−resistant acid phosphatase type 5
with a threshold of 1e−05
MR Test
Inverse variance weighted (fixed effects)
Inverse variance weighted (multiplicative random effects)
MR Egger
MR RAPS
Weighted median
Weighted mode
0.04
0.00
−0.04
−0.08
0.09
0.12
0.15
0.18
SNP effect on Tartrate−resistant acid phosphatase type 5
SNP effect on Gut microbiota abundance (genus Catenibacterium id.2153)

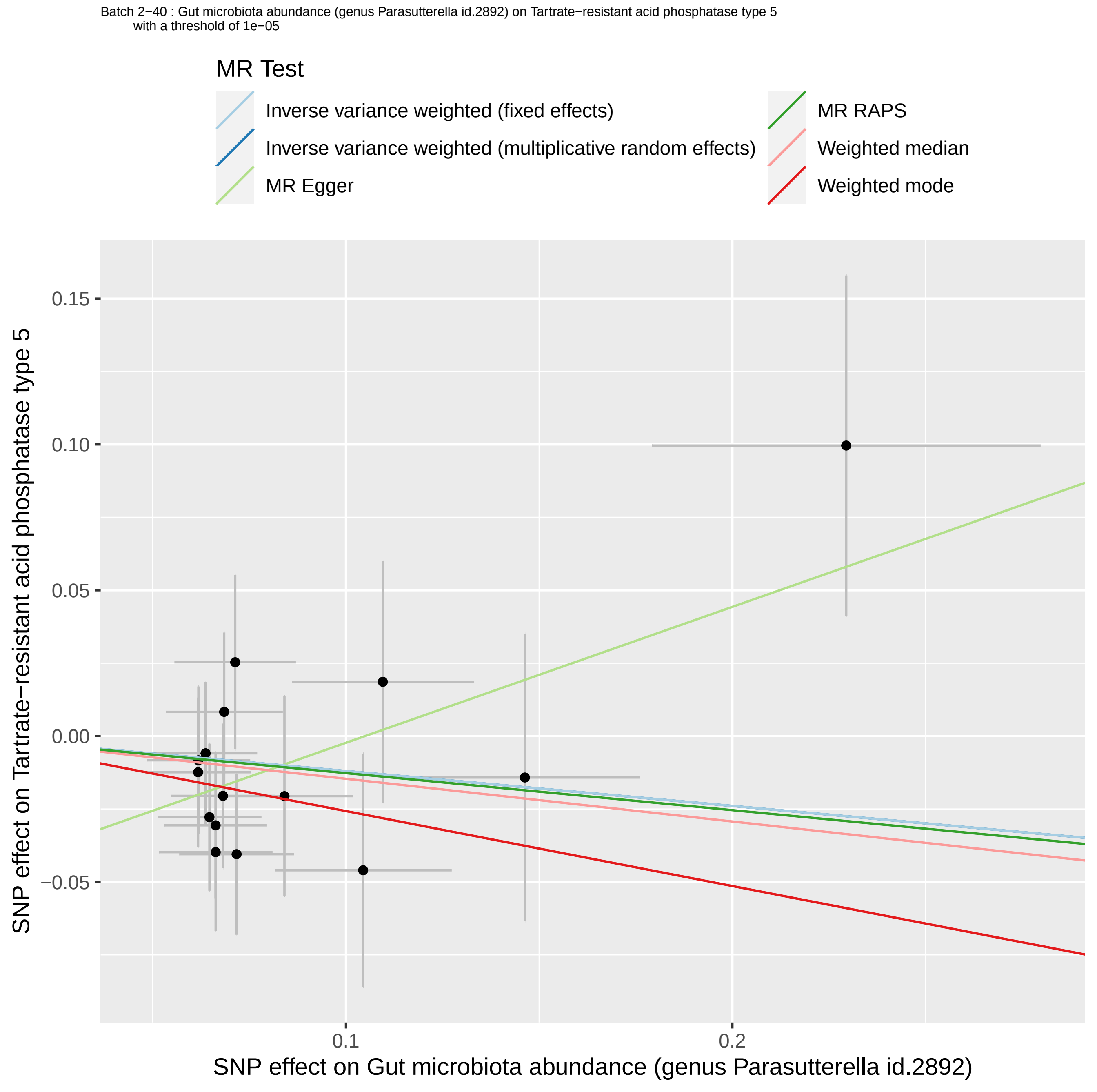

Batch 2−40 : Gut microbiota abundance (genus Parasutterella id.2892) on Tartrate−resistant acid phosphatase type 5
with a threshold of 1e−05
MR Test
Inverse variance weighted (fixed effects)
Inverse variance weighted (multiplicative random effects)
MR Egger
MR RAPS
Weighted median
Weighted mode
0.15
0.10
0.05
0.00
−0.05
0.1
0.2
SNP effect on Tartrate−resistant acid phosphatase type 5
SNP effect on Gut microbiota abundance (genus Parasutterella id.2892)

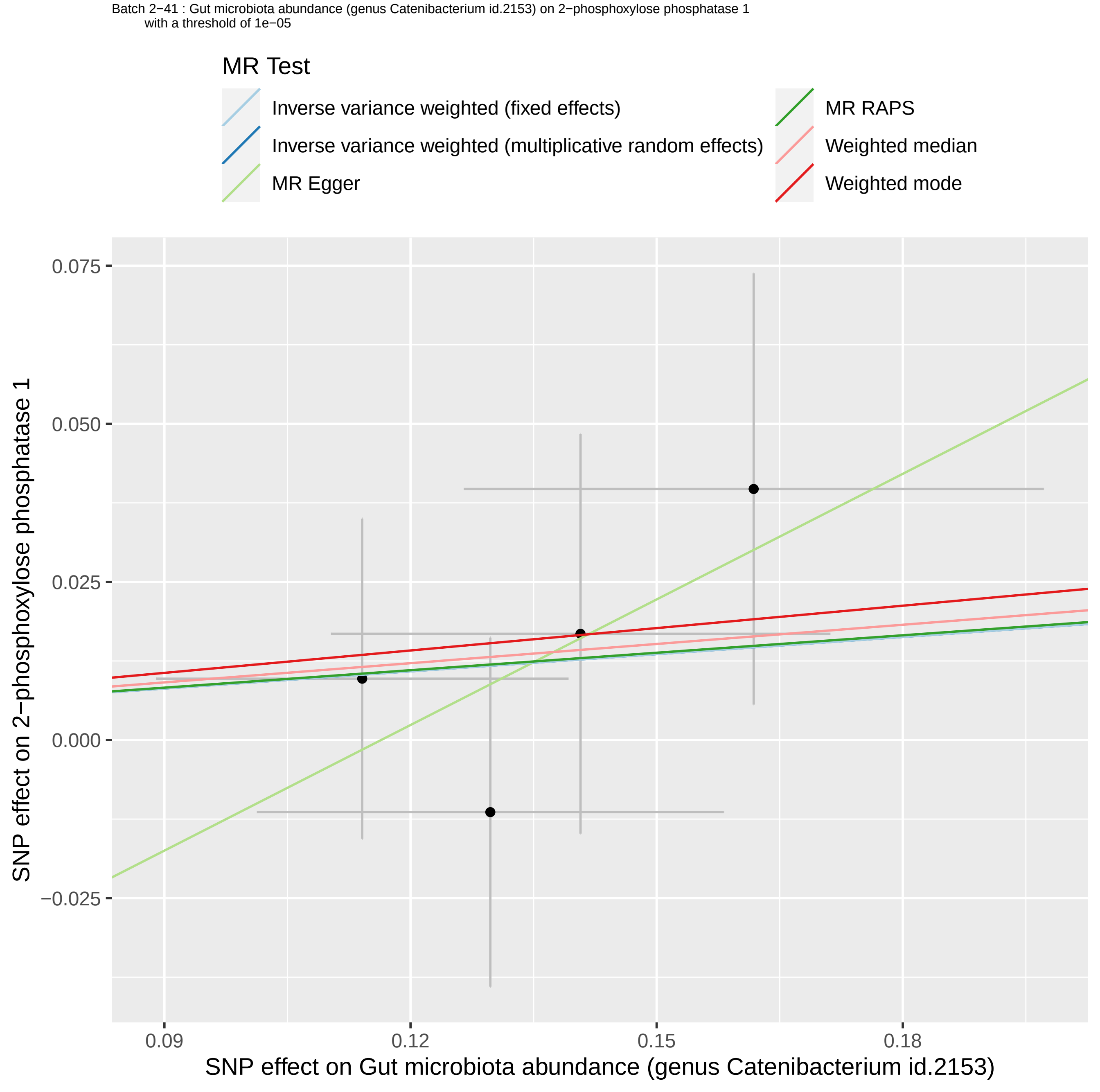

Batch 2−41 : Gut microbiota abundance (genus Catenibacterium id.2153) on 2−phosphoxylose phosphatase 1
with a threshold of 1e−05
MR Test
Inverse variance weighted (fixed effects)
Inverse variance weighted (multiplicative random effects)
MR Egger
MR RAPS
Weighted median
Weighted mode
0.075
0.050
0.025
0.000
−0.025
0.09
0.12
0.15
0.18
SNP effect on 2−phosphoxylose phosphatase 1
SNP effect on Gut microbiota abundance (genus Catenibacterium id.2153)

Batch 2−42 : Gut microbiota abundance (genus Parasutterella id.2892) on 2−phosphoxylose phosphatase 1
with a threshold of 1e−05

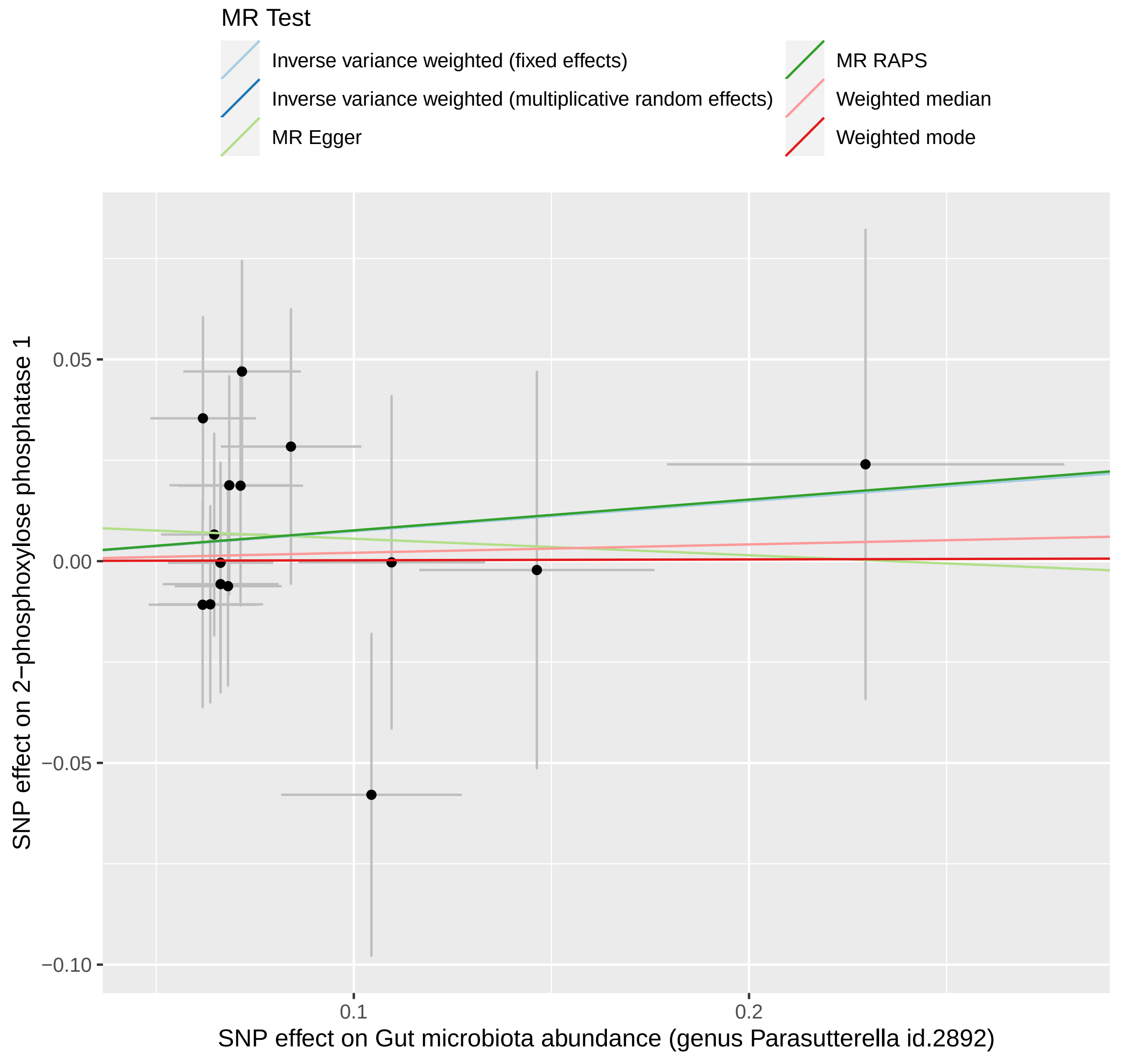

Batch 2−43 : Gut microbiota abundance (genus Catenibacterium id.2153) on Activin receptor type−1B
with a threshold of 1e−05

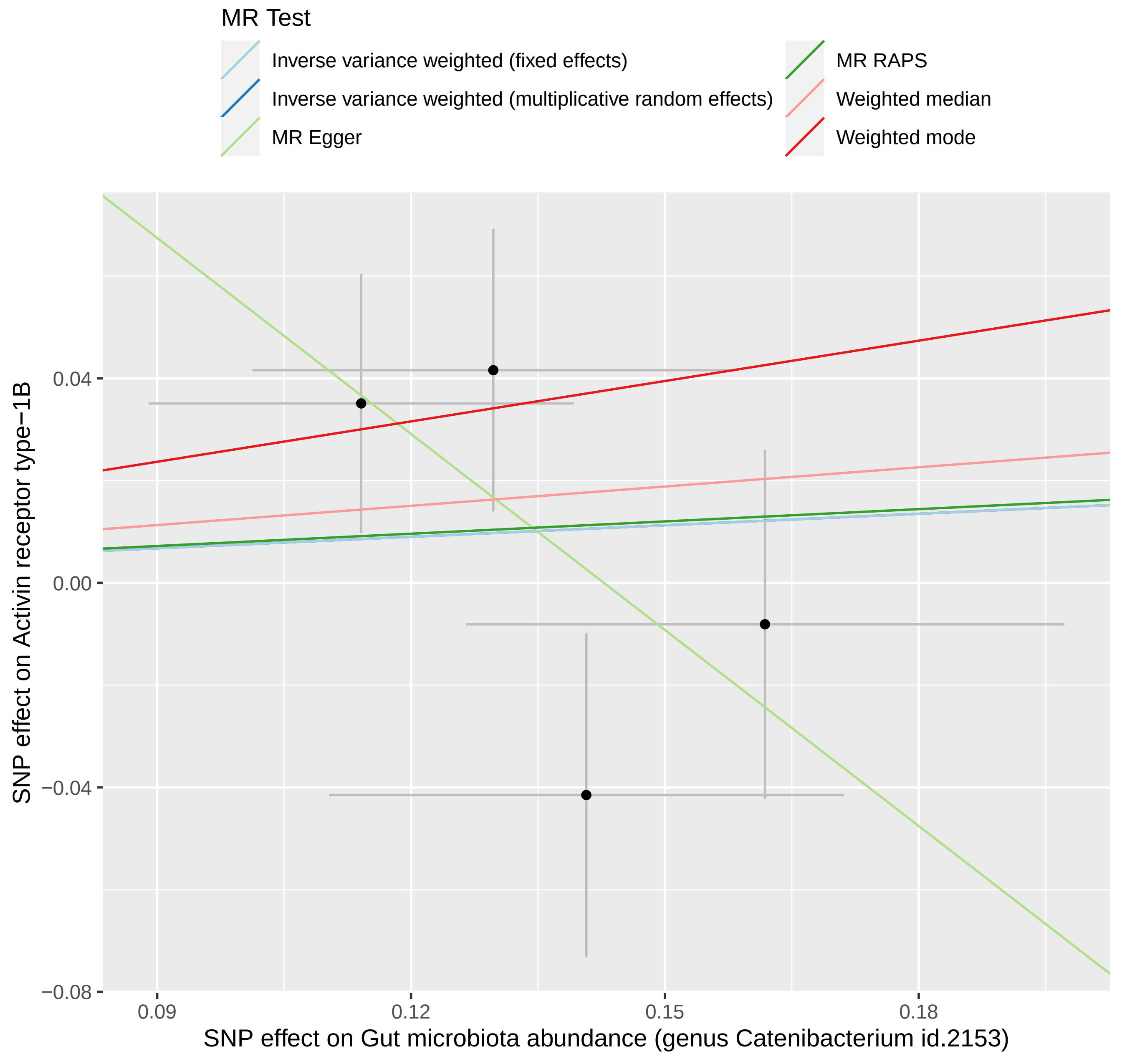

Batch 2−44 : Gut microbiota abundance (genus Parasutterella id.2892) on Activin receptor type−1B
with a threshold of 1e−05

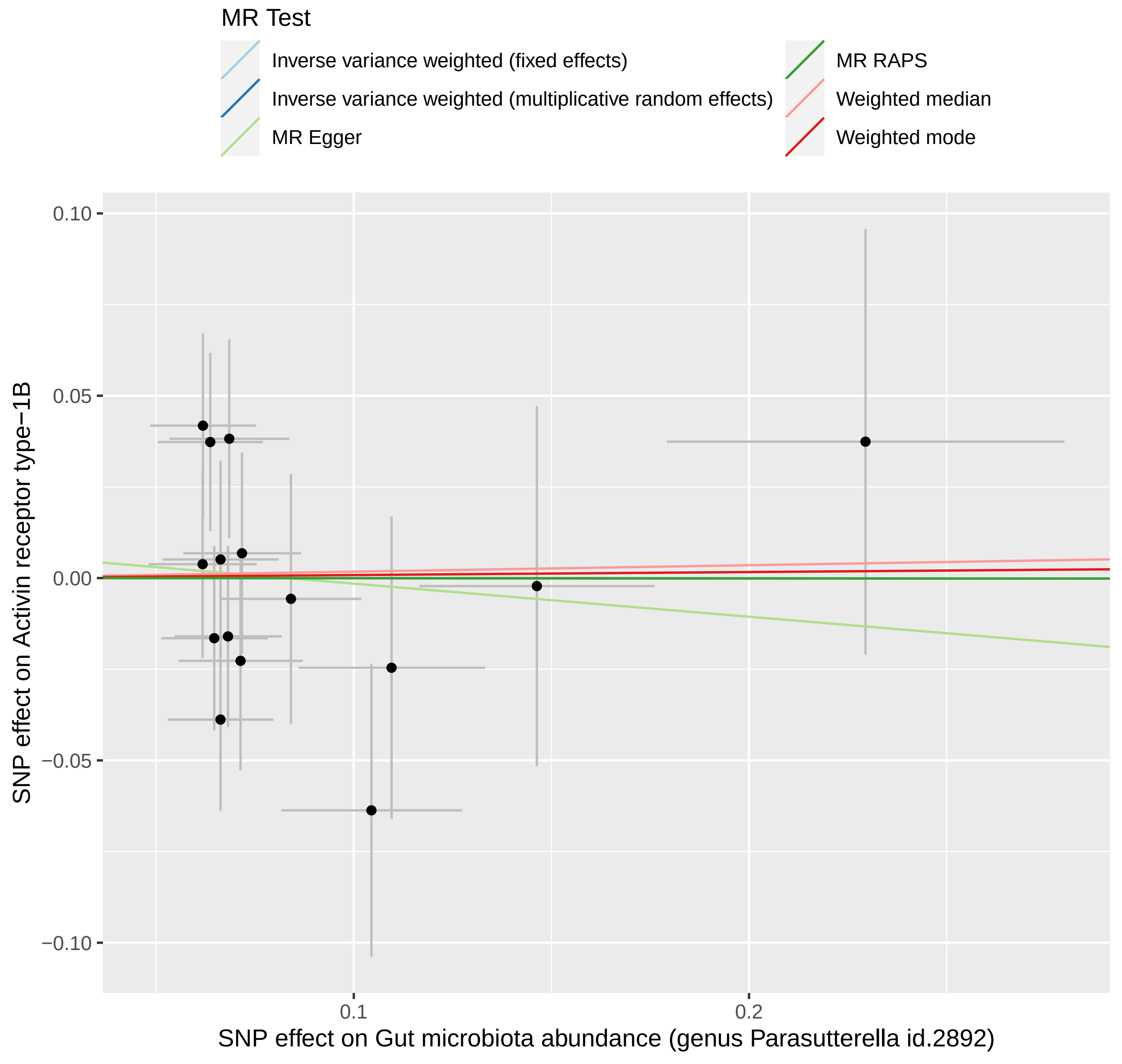

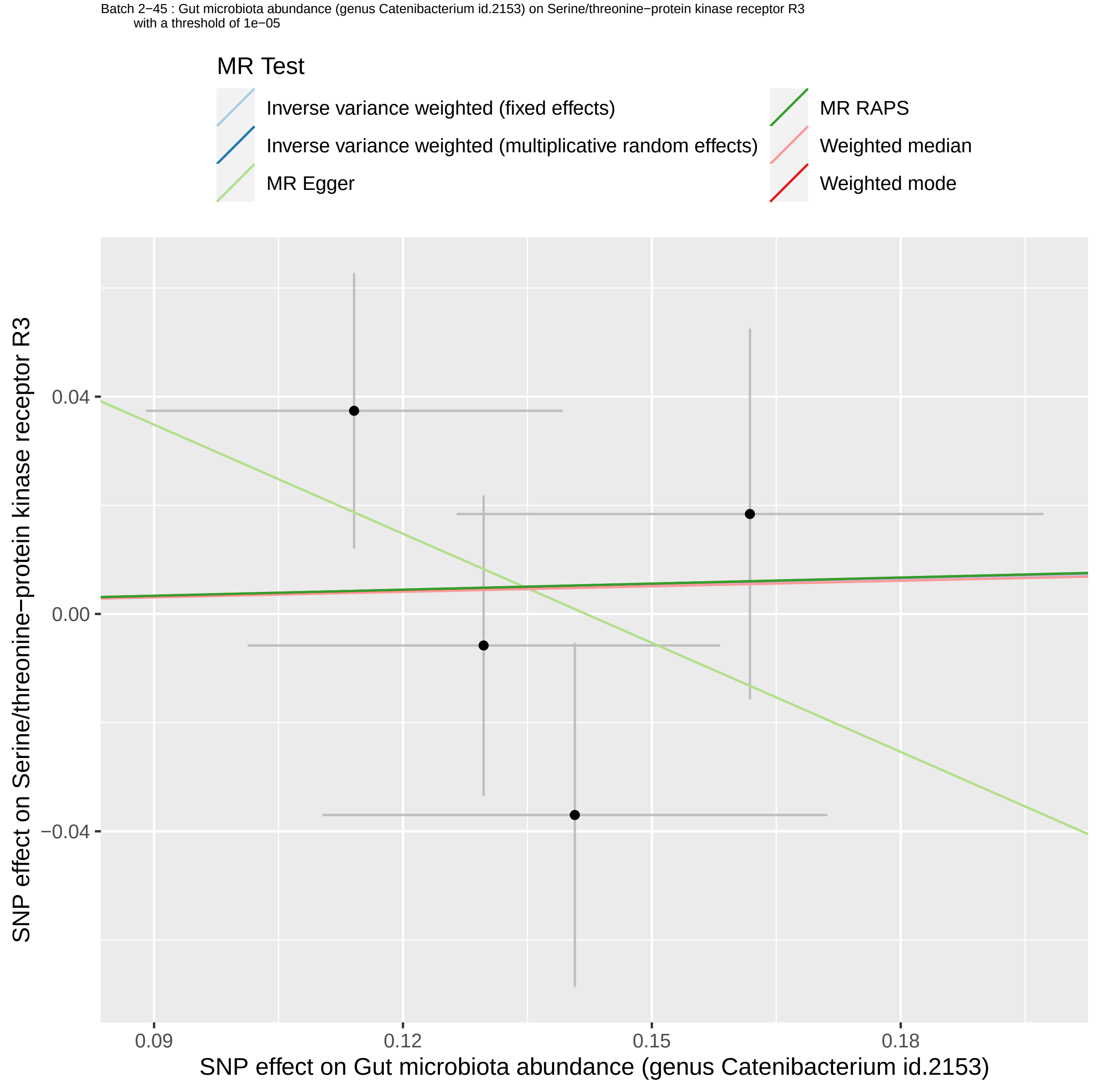
Batch 2−45 : Gut microbiota abundance (genus Catenibacterium id.2153) on Serine/threonine−protein kinase receptor R3
with a threshold of 1e−05
MR Test
Inverse variance weighted (fixed effects)
Inverse variance weighted (multiplicative random effects)
MR Egger
MR RAPS
Weighted median
Weighted mode
0.04
0.00
−0.04
0.09
0.12
0.15
0.18
SNP effect on Serine/threonine−protein kinase receptor R3
SNP effect on Gut microbiota abundance (genus Catenibacterium id.2153)

Batch 2−46 : Gut microbiota abundance (genus Parasutterella id.2892) on Serine/threonine−protein kinase receptor R3
with a threshold of 1e−05

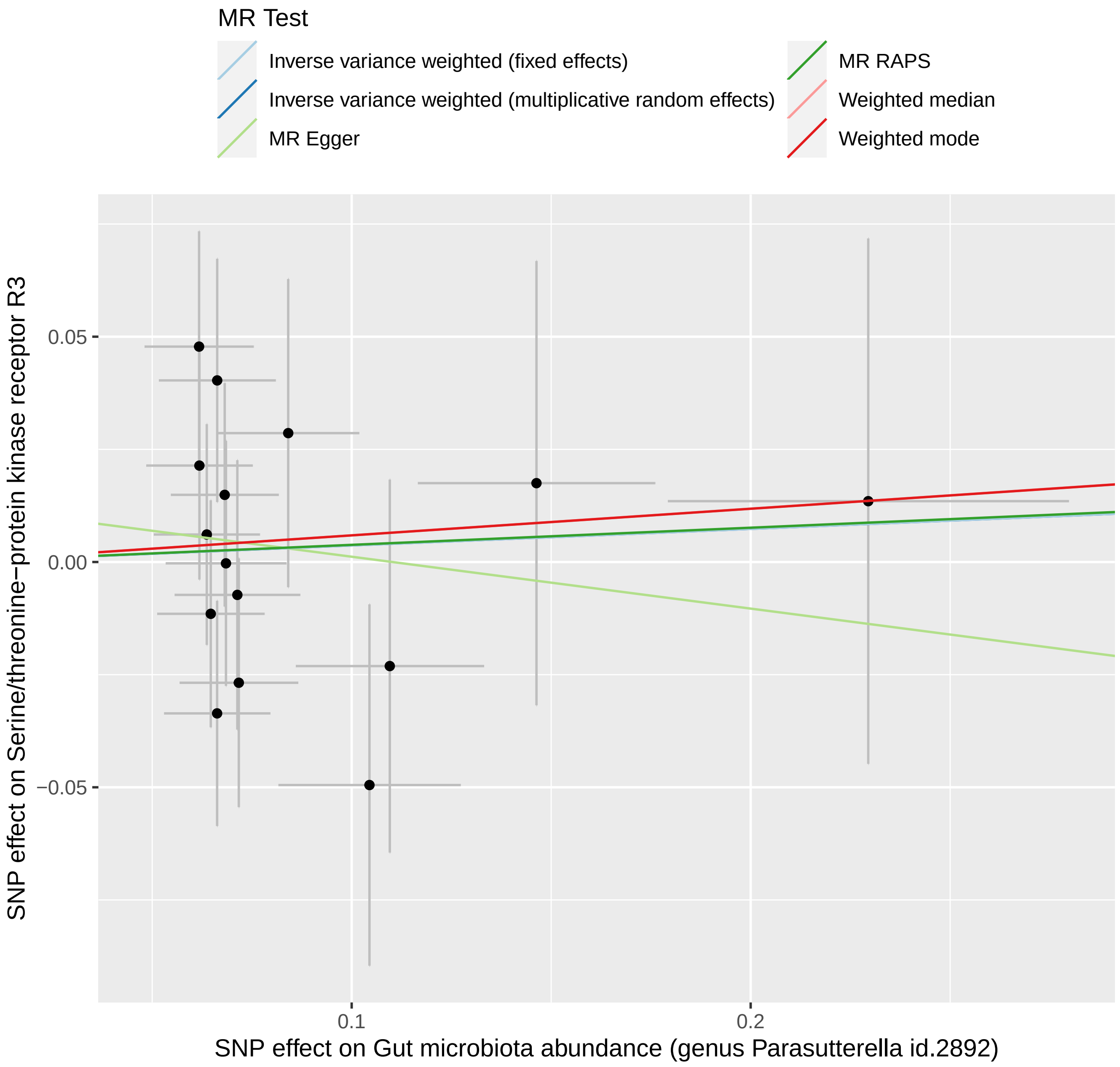

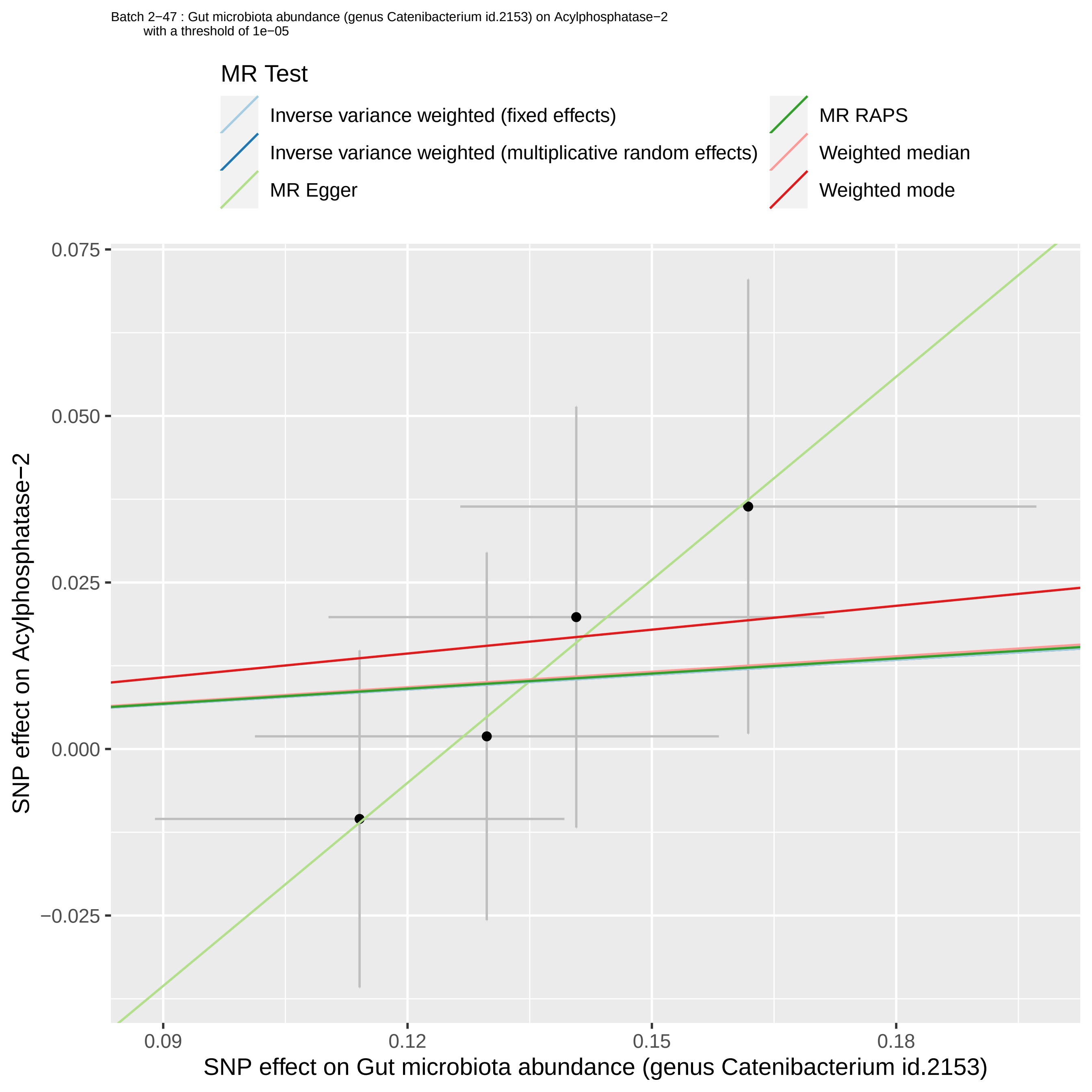

Batch 2−47 : Gut microbiota abundance (genus Catenibacterium id.2153) on Acylphosphatase−2
with a threshold of 1e−05
MR Test
Inverse variance weighted (fixed effects)
Inverse variance weighted (multiplicative random effects)
MR Egger
MR RAPS
Weighted median
Weighted mode
0.075
0.050
0.025
0.000
−0.025
SNP effect on Acylphosphatase−2
0.09
0.12
0.15
0.18
SNP effect on Gut microbiota abundance (genus Catenibacterium id.2153)

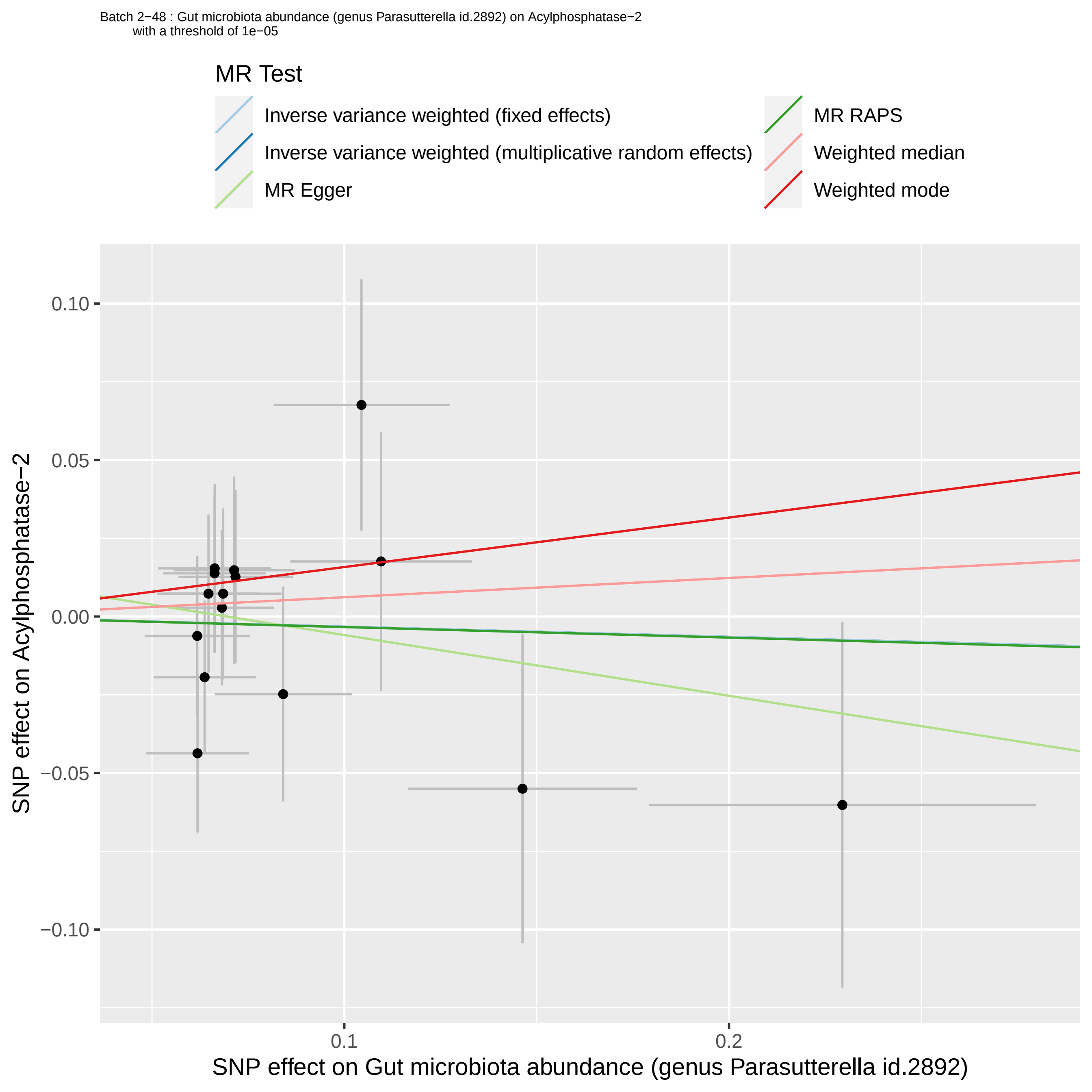
Batch 2−48 : Gut microbiota abundance (genus Parasutterella id.2892) on Acylphosphatase−2
with a threshold of 1e−05
MR Test
Inverse variance weighted (fixed effects)
Inverse variance weighted (multiplicative random effects)
MR Egger
MR RAPS
Weighted median
Weighted mode
0.10
0.05
0.00
−0.05
−0.10
0.1
0.2
SNP effect on Acylphosphatase−2
SNP effect on Gut microbiota abundance (genus Parasutterella id.2892)

Batch 2−49 : Gut microbiota abundance (genus Catenibacterium id.2153) on Disintegrin and metalloproteinase domain−containing protein 11 with a threshold of 1e−05

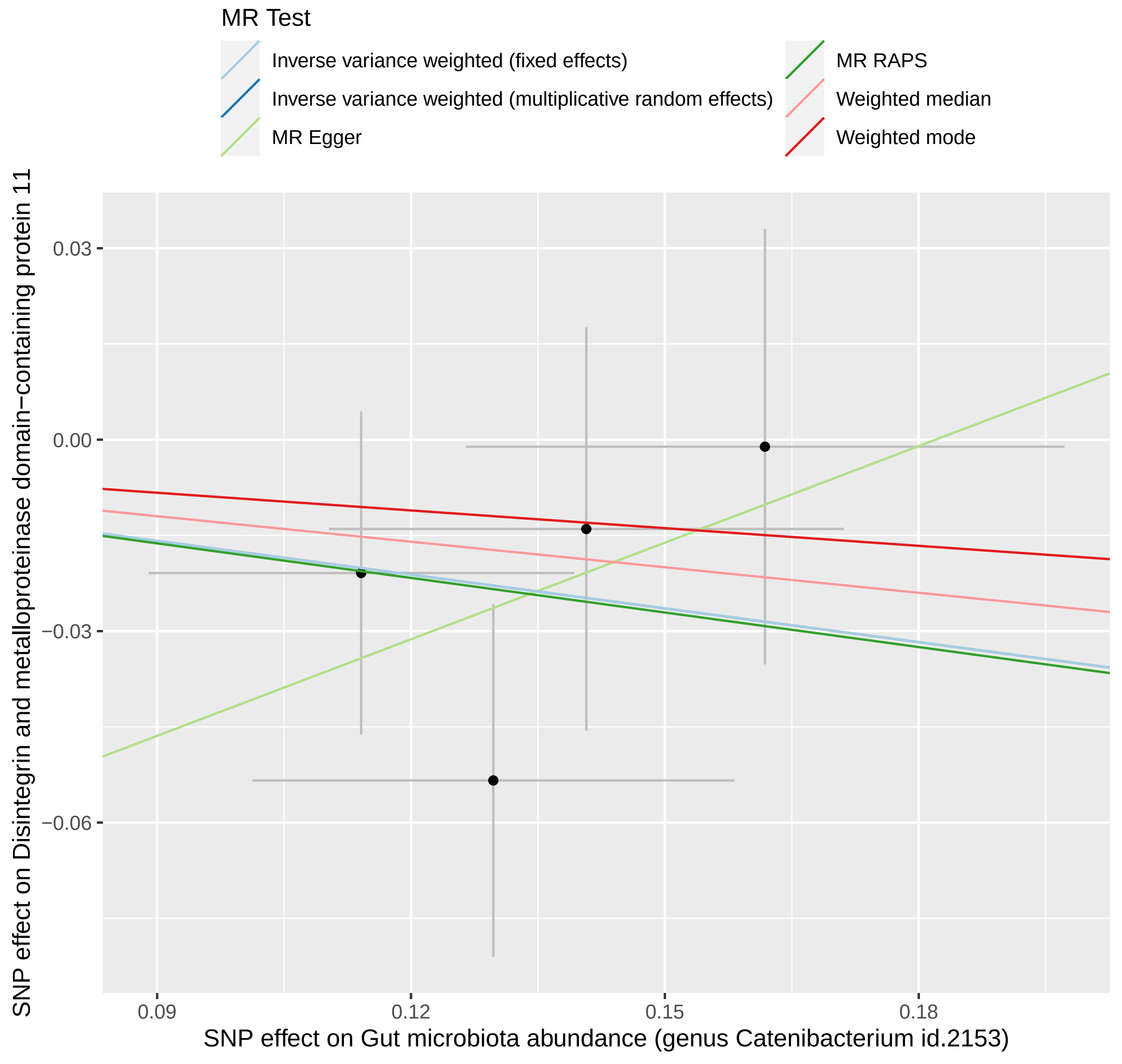

Batch 2−50 : Gut microbiota abundance (genus Parasutterella id.2892) on Disintegrin and metalloproteinase domain−containing protein 11 with a threshold of 1e−05

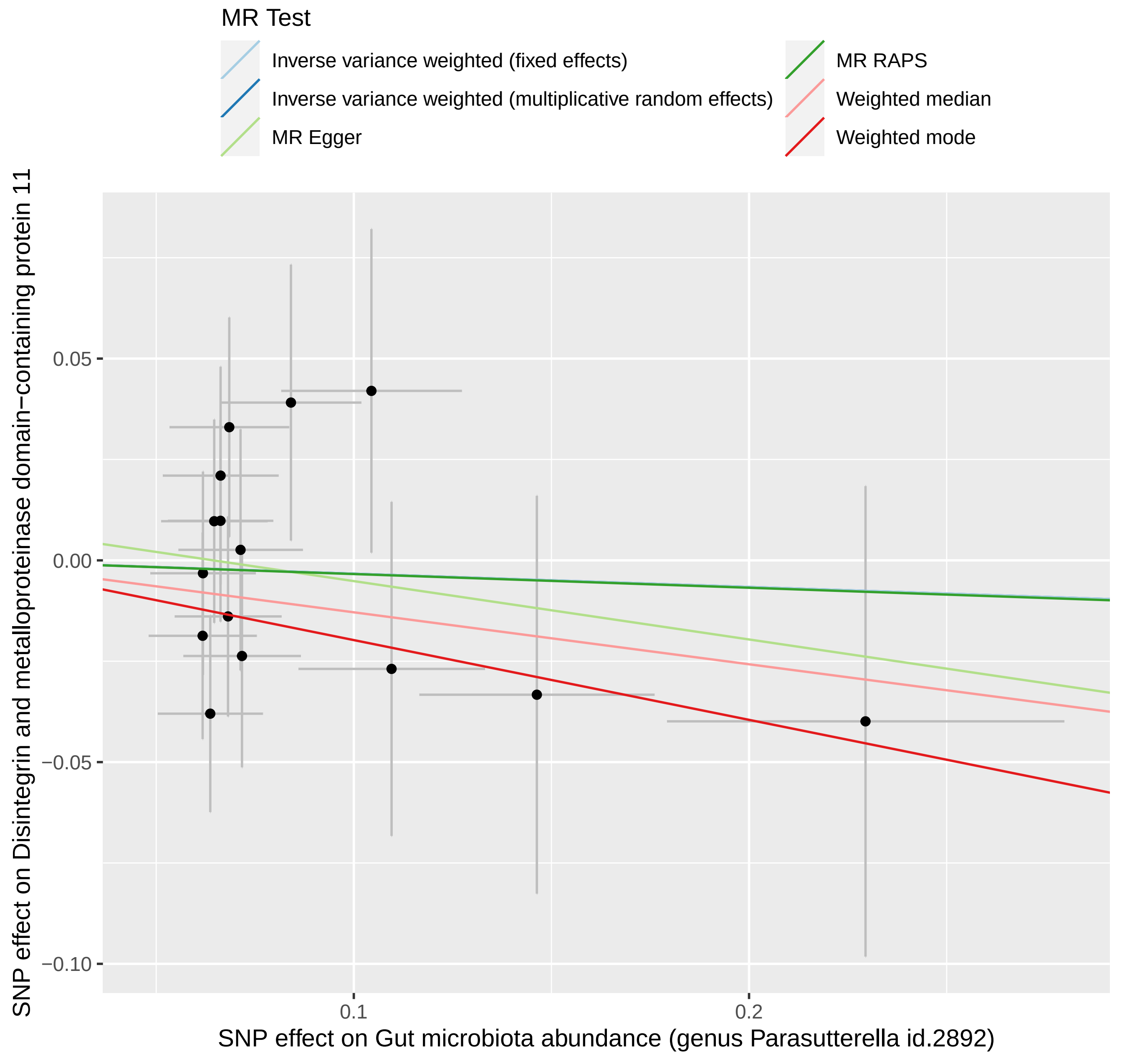

Batch 2−51 : Gut microbiota abundance (genus Catenibacterium id.2153) on Disintegrin and metalloproteinase domain−containing protein 12 with a threshold of 1e−05

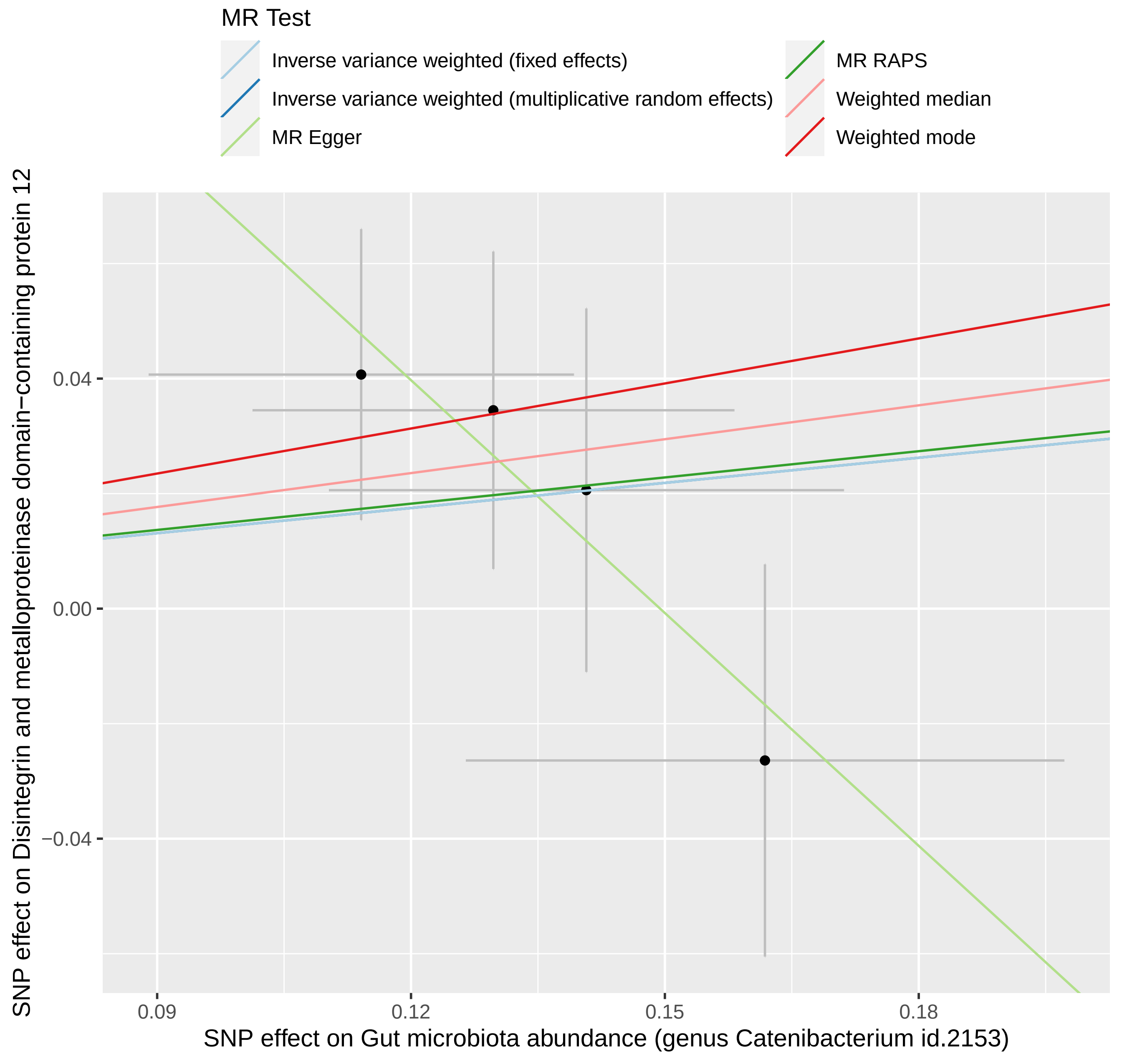

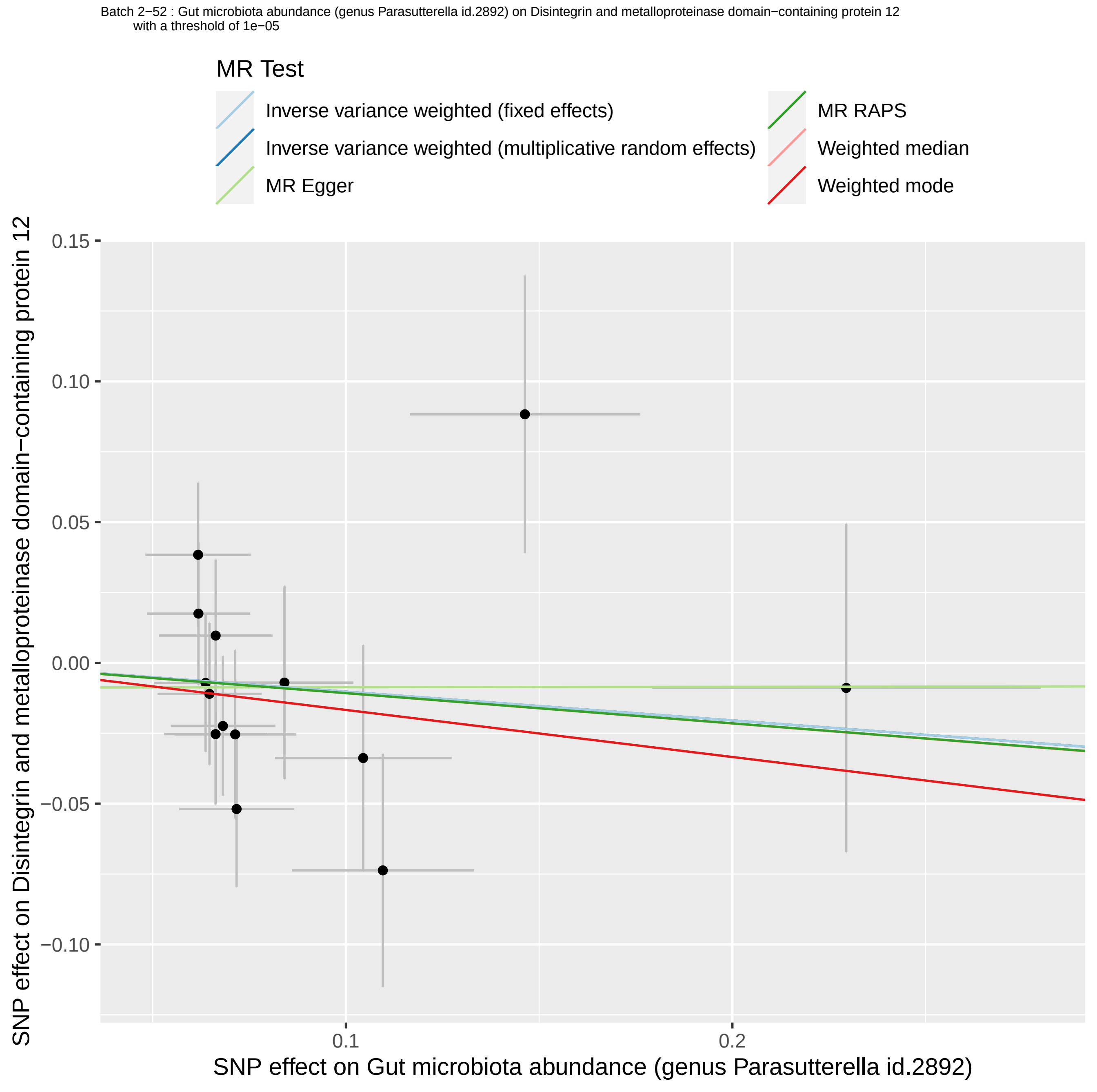

Batch 2−52 : Gut microbiota abundance (genus Parasutterella id.2892) on Disintegrin and metalloproteinase domain−containing protein 12
with a threshold of 1e−05
MR Test
Inverse variance weighted (fixed effects)
Inverse variance weighted (multiplicative random effects)
MR Egger
MR RAPS
Weighted median
Weighted mode
0.15
0.10
0.05
0.00
−0.05
−0.10
0.1
0.2
SNP effect on Disintegrin and metalloproteinase domain−containing protein 12
SNP effect on Gut microbiota abundance (genus Parasutterella id.2892)

Batch 2−53 : Gut microbiota abundance (genus Catenibacterium id.2153) on Disintegrin and metalloproteinase domain−containing protein 19 with a threshold of 1e−05

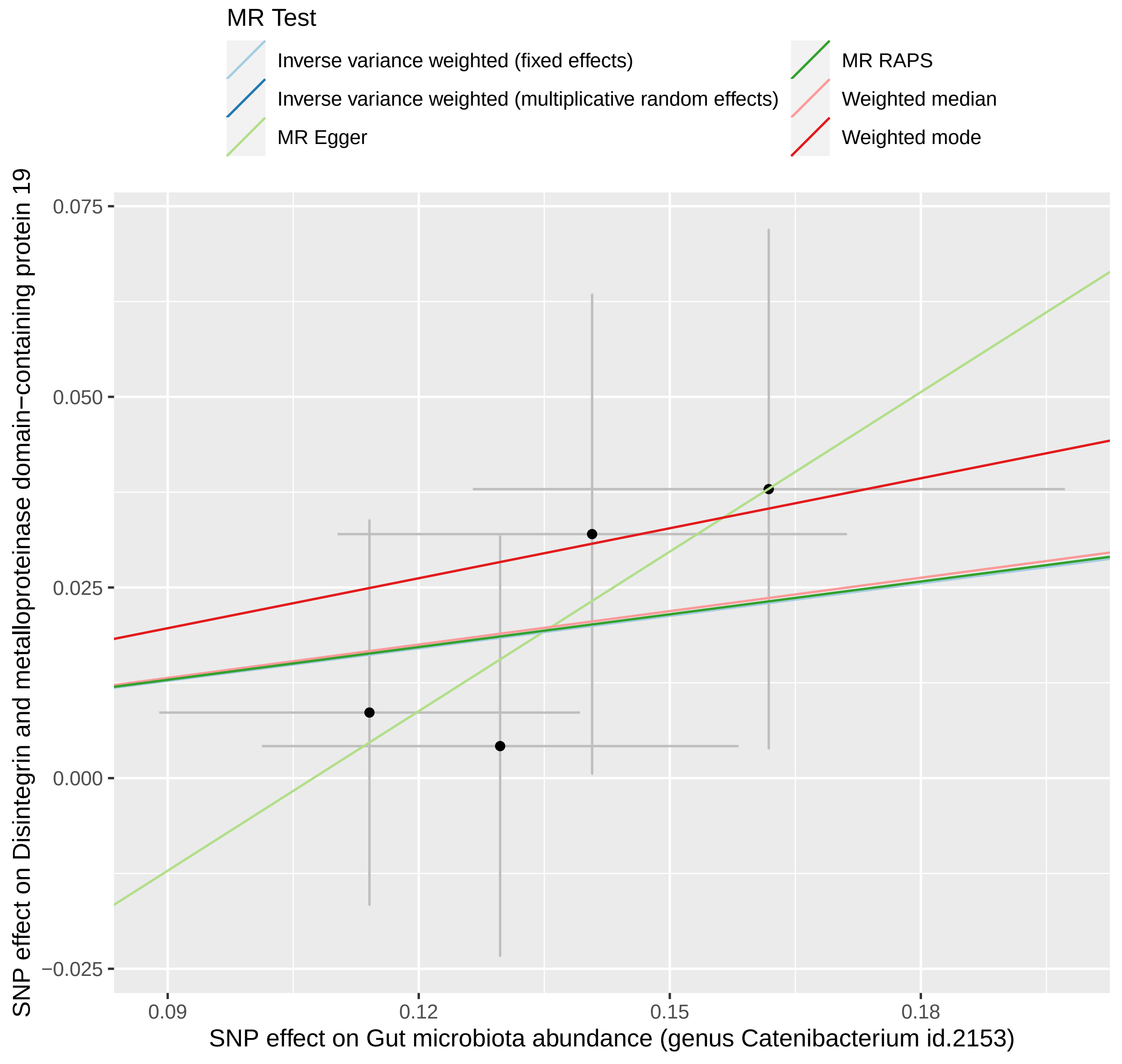

Batch 2−54 : Gut microbiota abundance (genus Parasutterella id.2892) on Disintegrin and metalloproteinase domain−containing protein 19
with a threshold of 1e−05

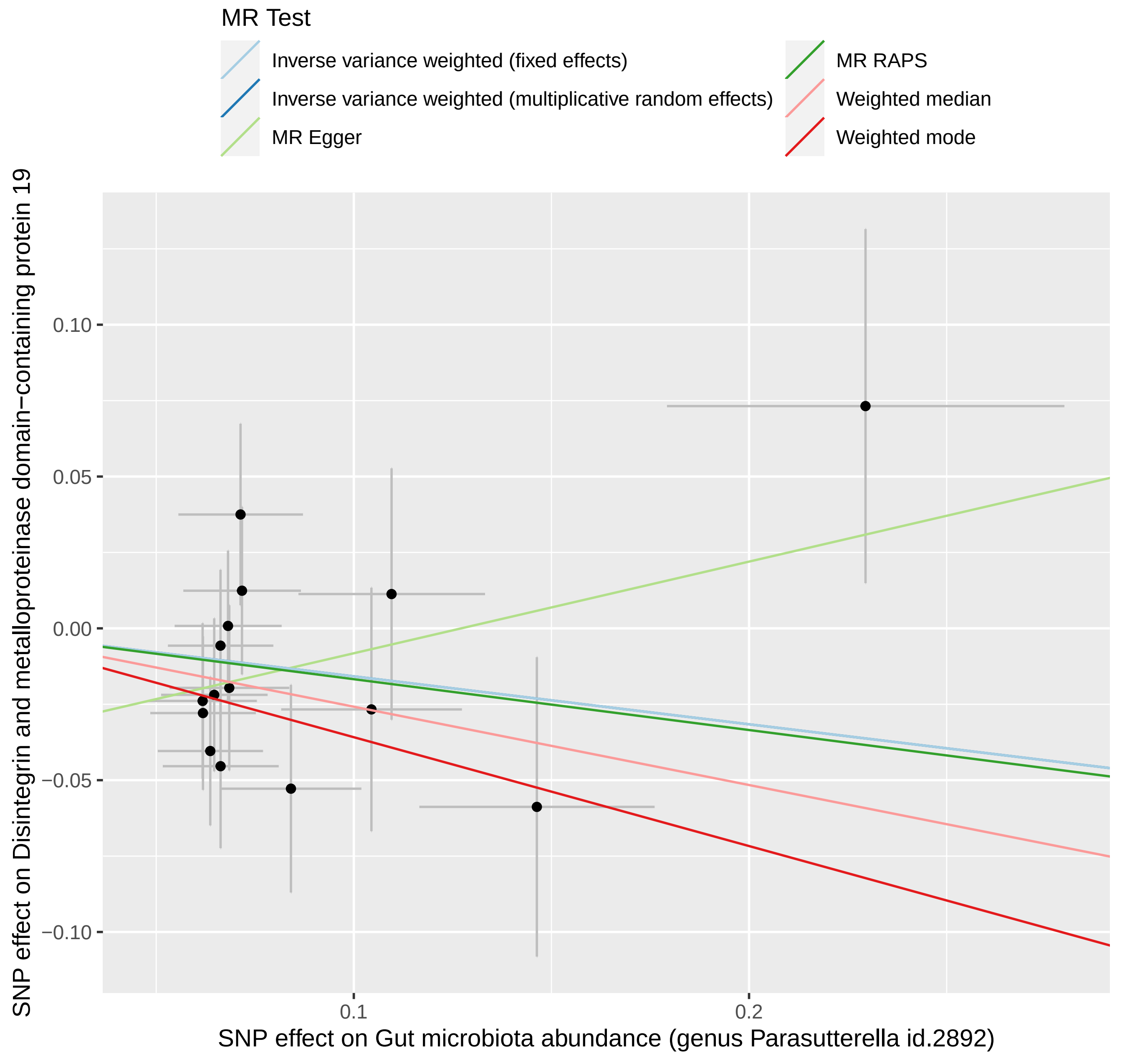

Batch 2−55 : Gut microbiota abundance (genus Catenibacterium id.2153) on Disintegrin and metalloproteinase domain−containing protein 23
with a threshold of 1e−05

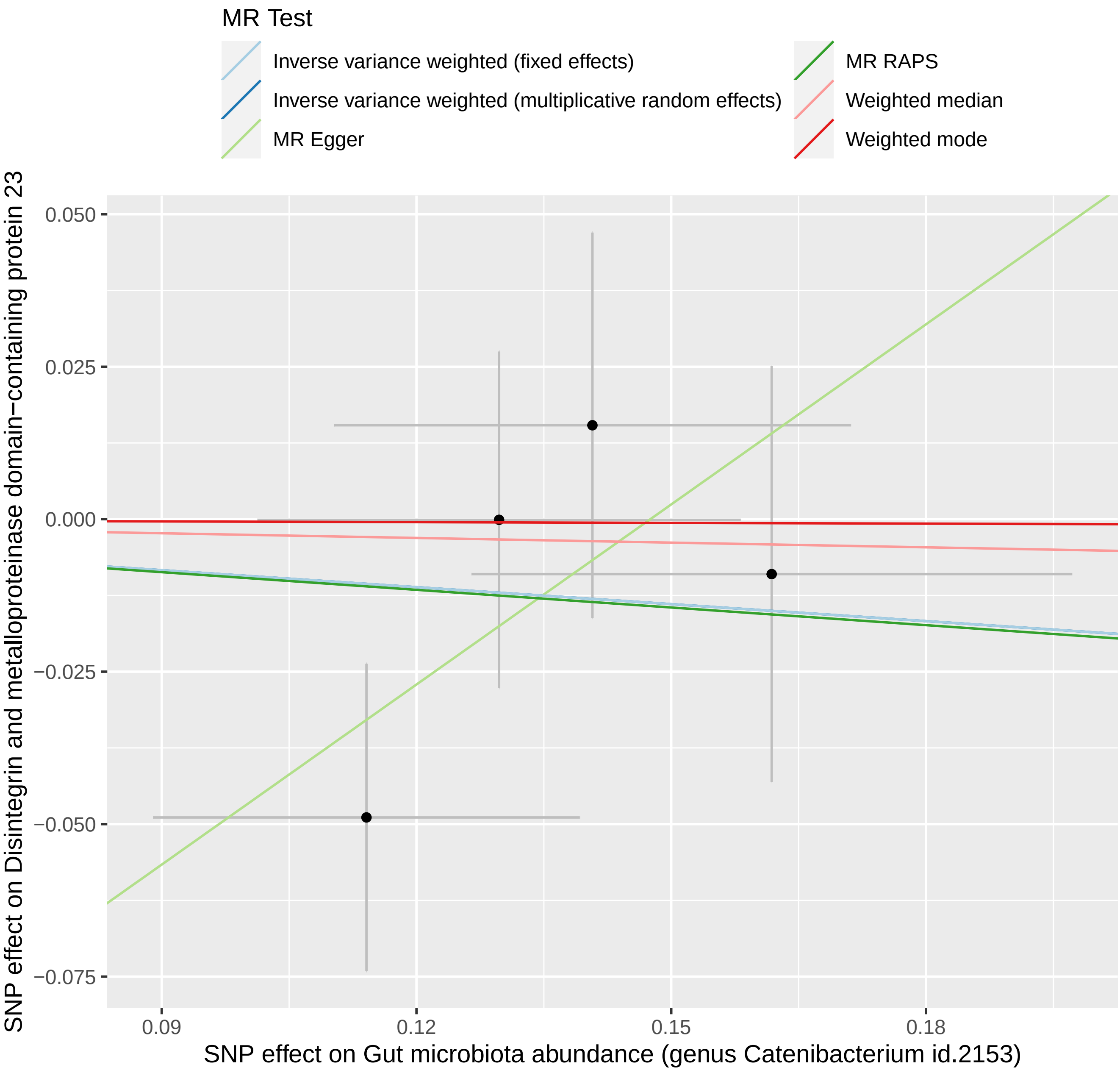

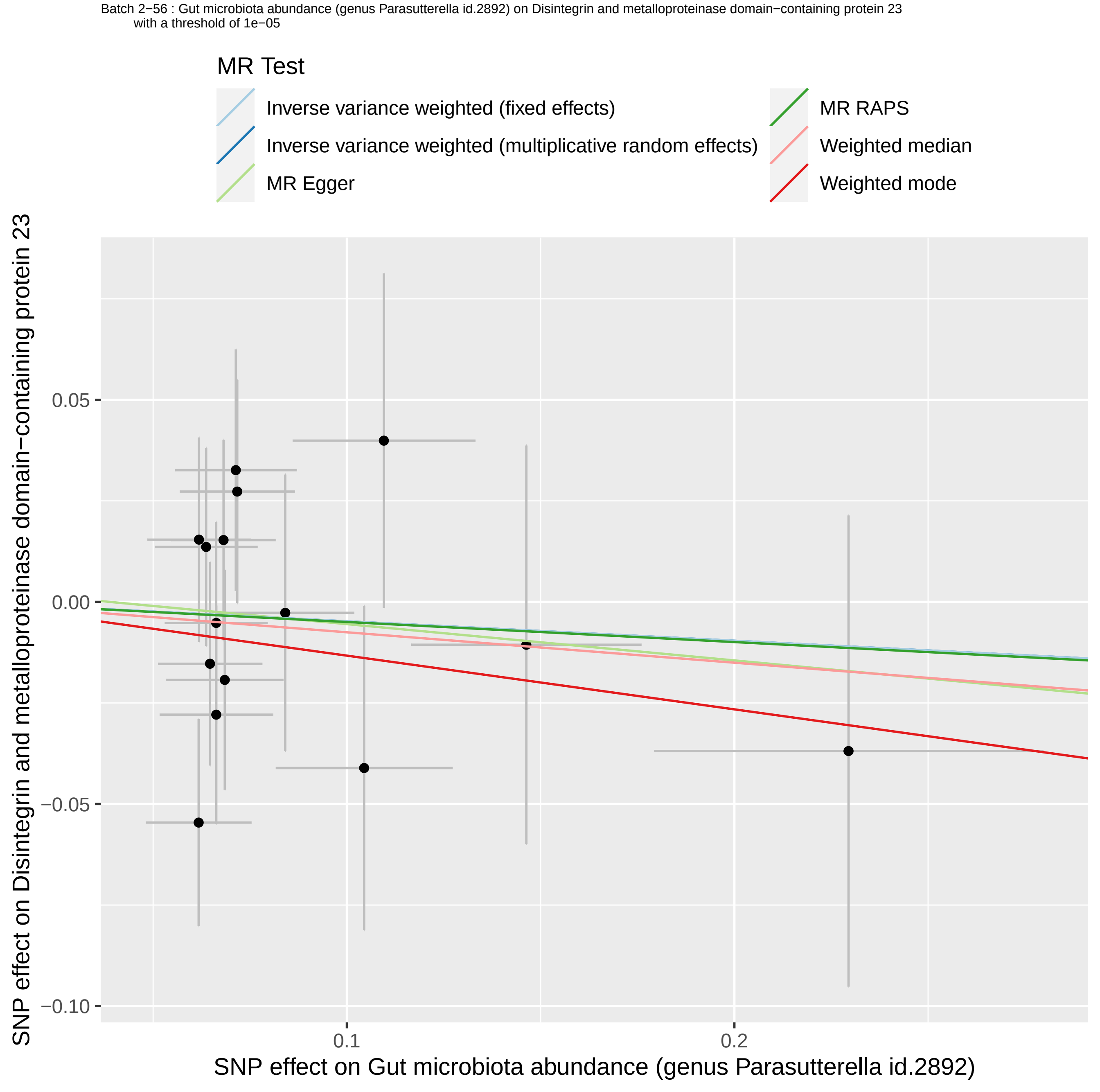

Batch 2−56 : Gut microbiota abundance (genus Parasutterella id.2892) on Disintegrin and metalloproteinase domain−containing protein 23
with a threshold of 1e−05
MR Test
Inverse variance weighted (fixed effects)
Inverse variance weighted (multiplicative random effects)
MR Egger
MR RAPS
Weighted median
Weighted mode
SNP effect on Disintegrin and metalloproteinase domain−containing protein 23
0.05
0.00
−0.05
−0.10
0.1
0.2
SNP effect on Gut microbiota abundance (genus Parasutterella id.2892)

Batch 2−57 : Gut microbiota abundance (genus Catenibacterium id.2153) on Disintegrin and metalloproteinase domain−containing protein 29 with a threshold of 1e−05

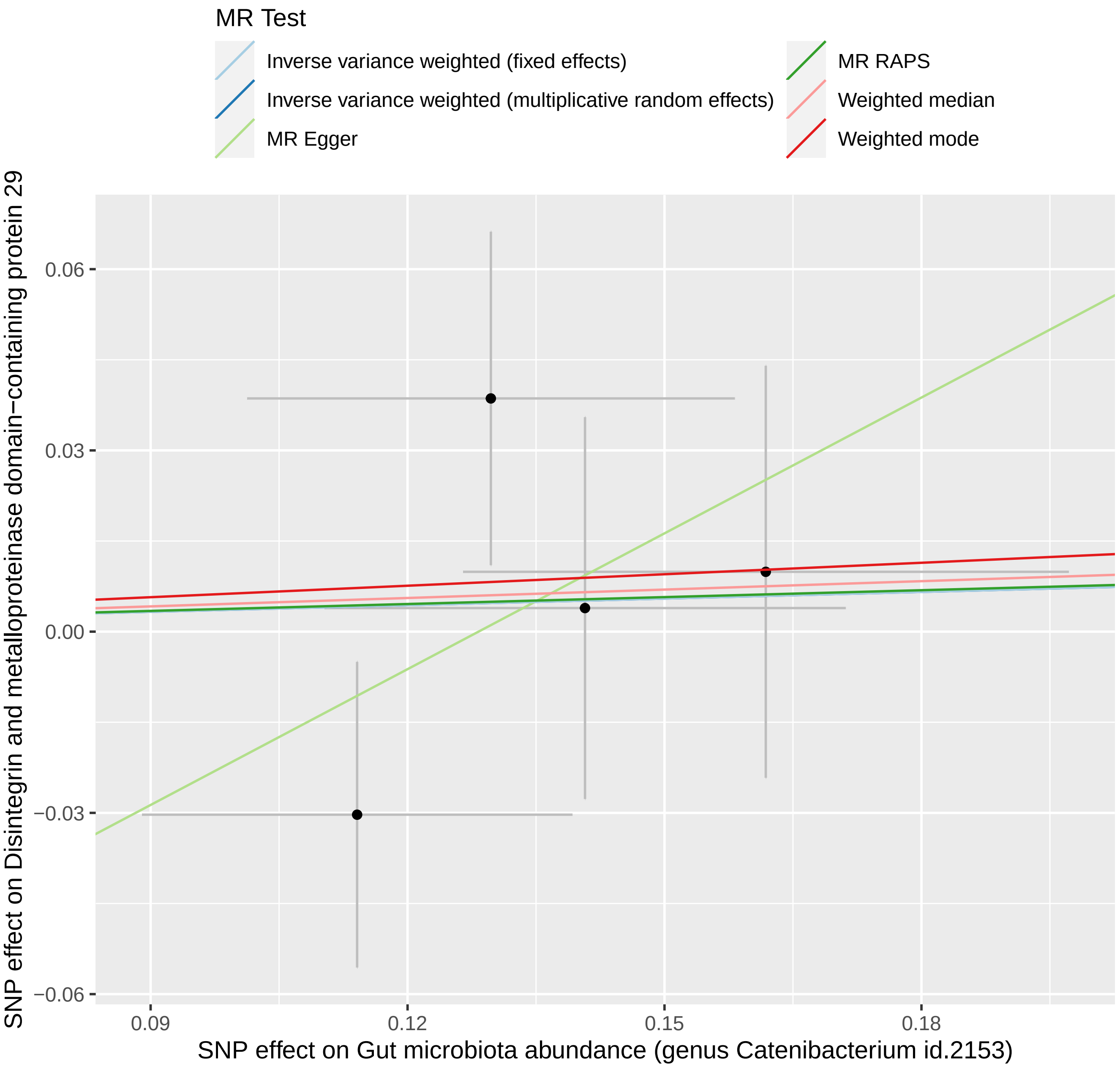

Batch 2−58 : Gut microbiota abundance (genus Parasutterella id.2892) on Disintegrin and metalloproteinase domain−containing protein 29
with a threshold of 1e−05

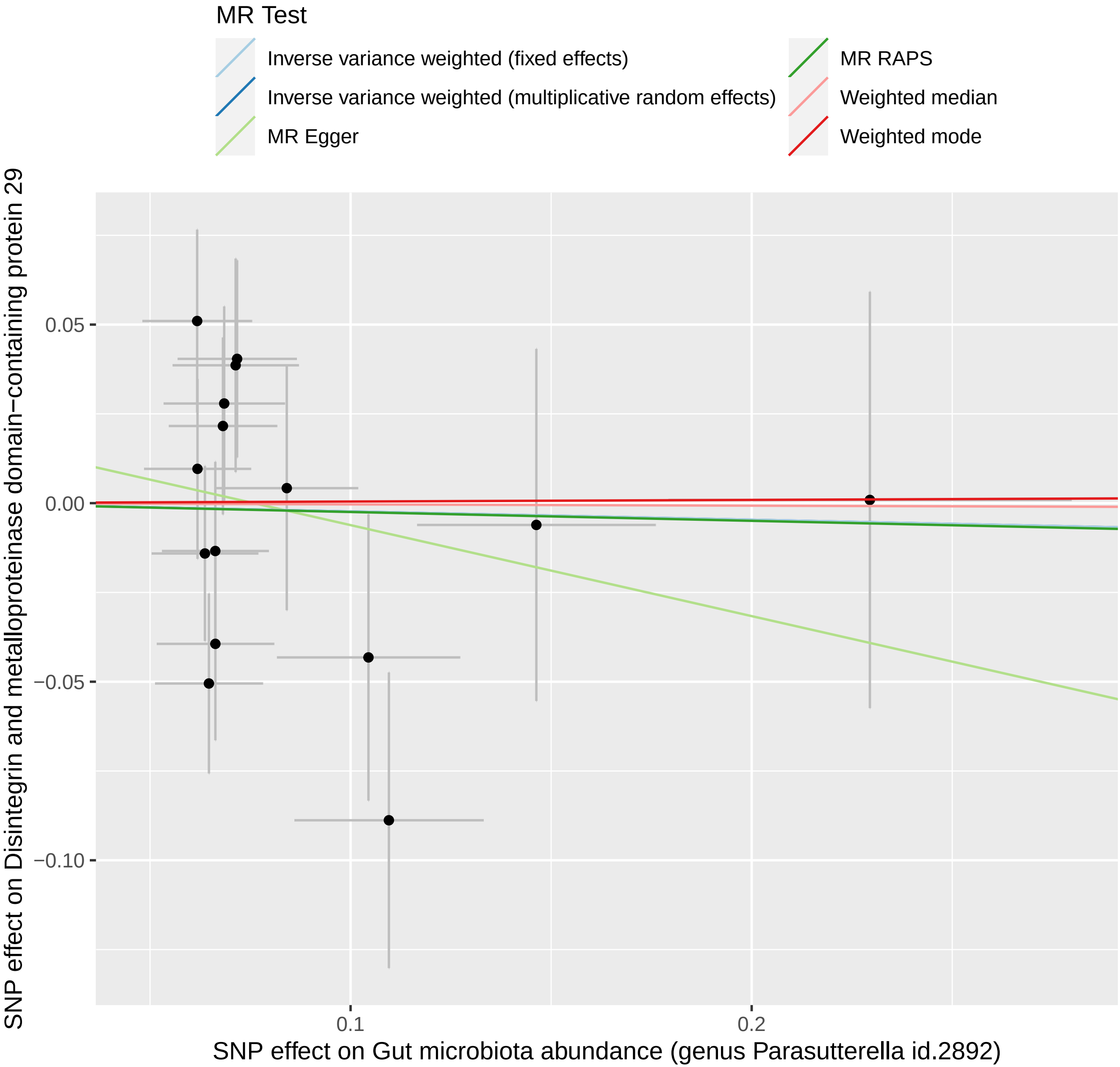

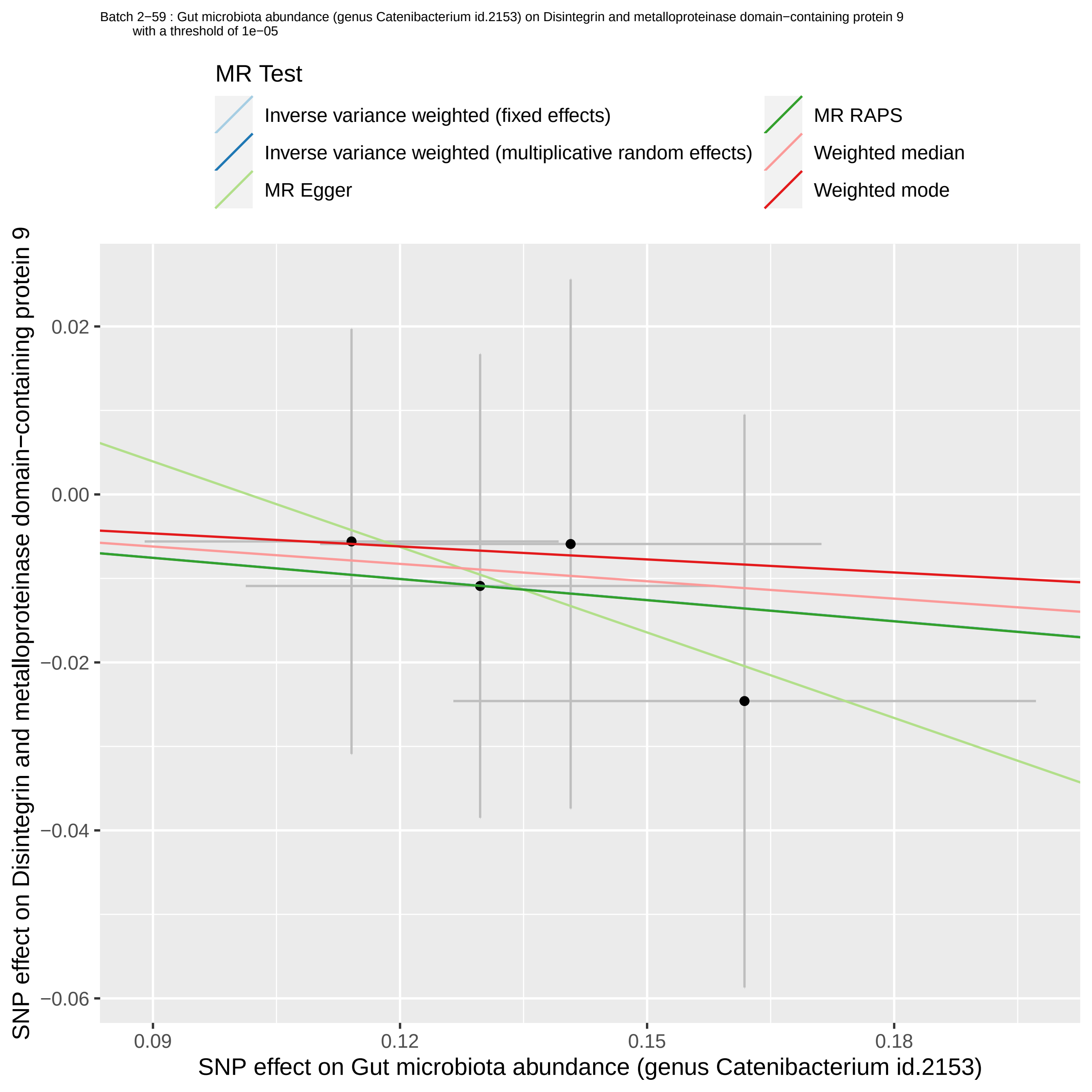

Batch 2−59 : Gut microbiota abundance (genus Catenibacterium id.2153) on Disintegrin and metalloproteinase domain−containing protein 9
with a threshold of 1e−05
MR Test
Inverse variance weighted (fixed effects)
Inverse variance weighted (multiplicative random effects)
MR Egger
MR RAPS
Weighted median
Weighted mode
SNP effect on Disintegrin and metalloproteinase domain−containing protein 9
0.02
0.00
−0.02
−0.04
−0.06
0.09
0.12
0.15
0.18
SNP effect on Gut microbiota abundance (genus Catenibacterium id.2153)

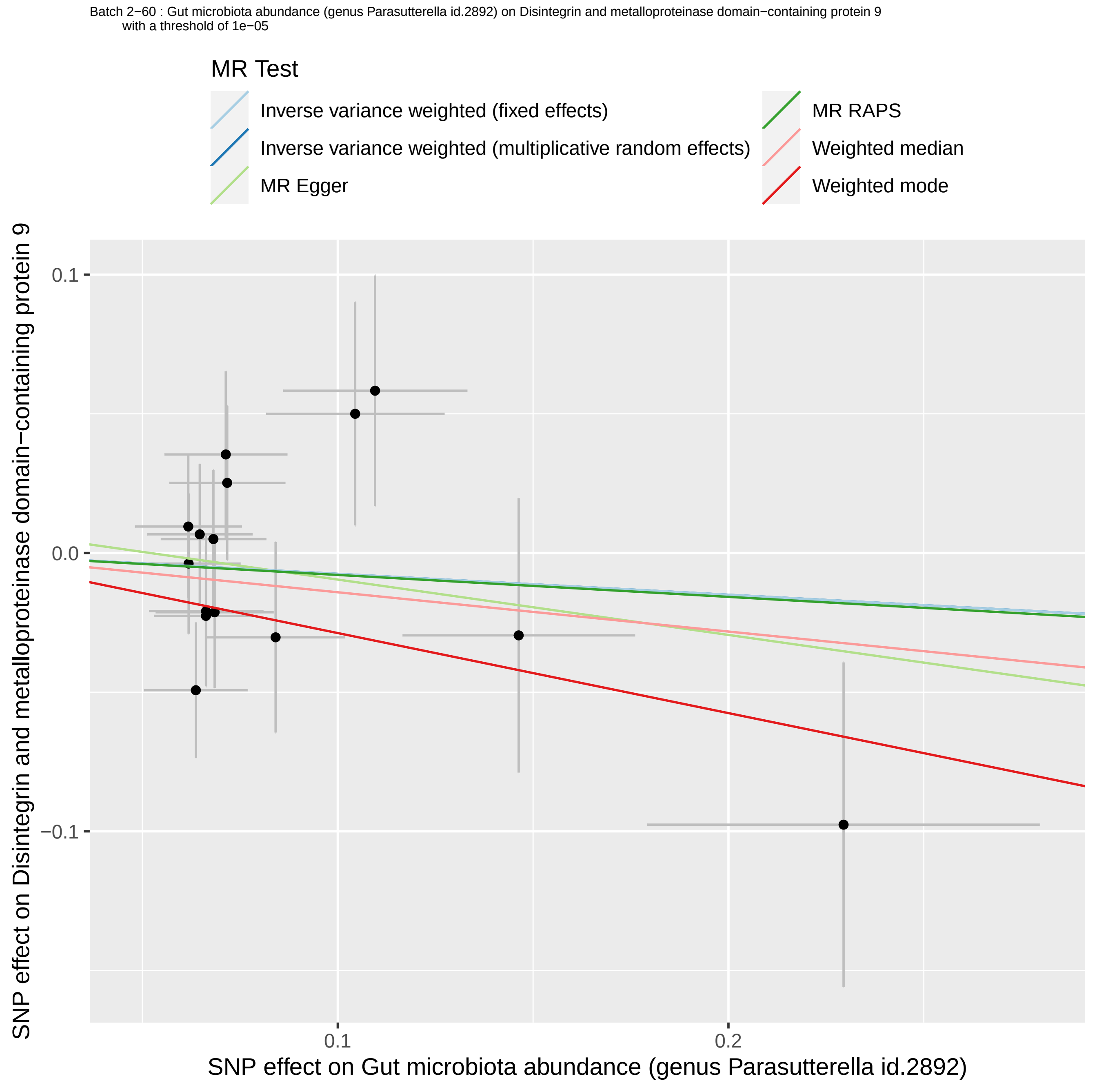

Batch 2−60 : Gut microbiota abundance (genus Parasutterella id.2892) on Disintegrin and metalloproteinase domain−containing protein 9
with a threshold of 1e−05
MR Test
Inverse variance weighted (fixed effects)
Inverse variance weighted (multiplicative random effects)
MR Egger
MR RAPS
Weighted median
Weighted mode
SNP effect on Disintegrin and metalloproteinase domain−containing protein 9
0.1
0.0
−0.1
0.1
0.2
SNP effect on Gut microbiota abundance (genus Parasutterella id.2892)

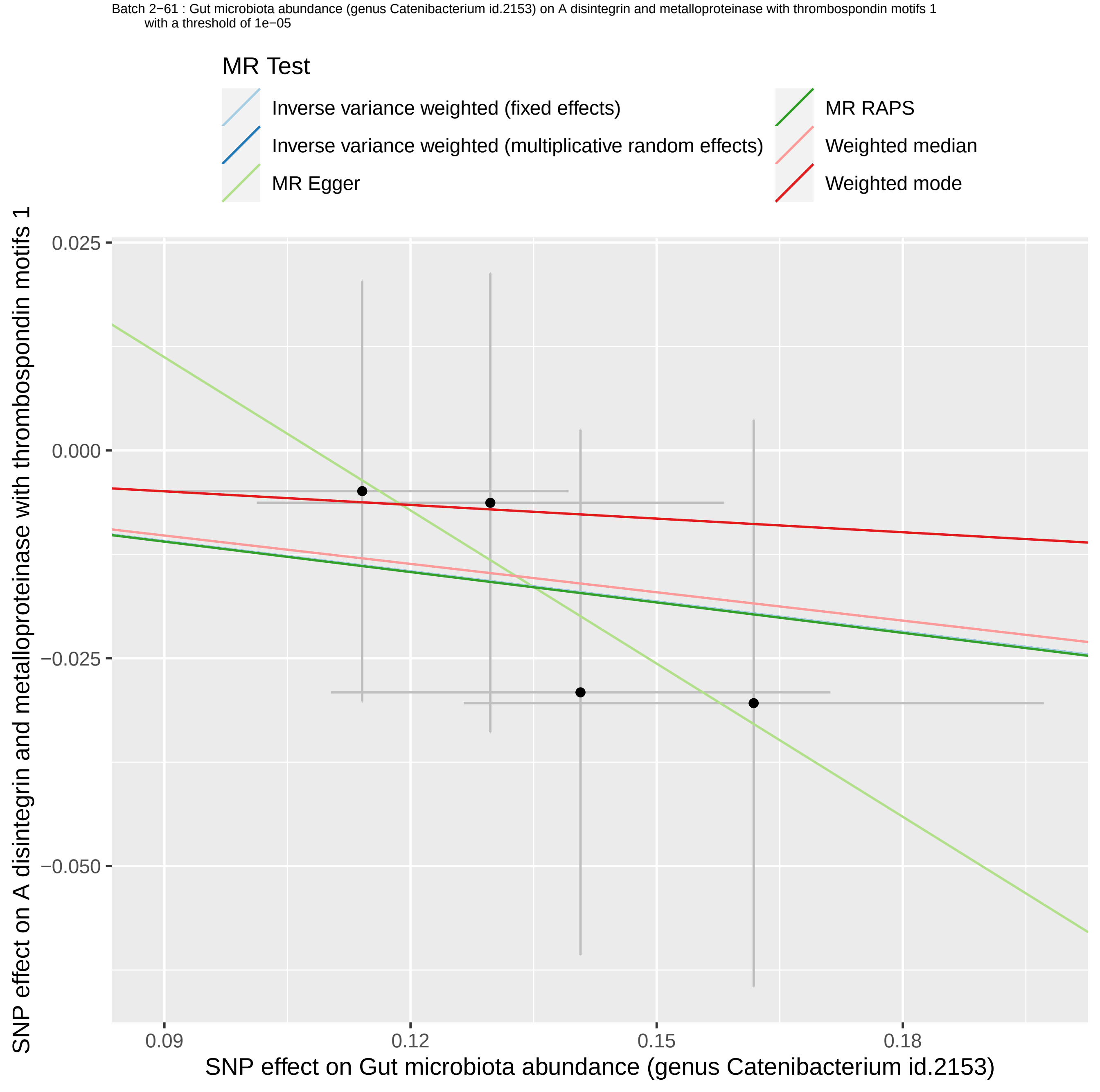

Batch 2−61 : Gut microbiota abundance (genus Catenibacterium id.2153) on A disintegrin and metalloproteinase with thrombospondin motifs 1 with a threshold of 1e−05
MR Test
Inverse variance weighted (fixed effects)
Inverse variance weighted (multiplicative random effects)
MR Egger
MR RAPS
Weighted median
Weighted mode
0.025
0.000
−0.025
−0.050
0.09
0.12
0.15
0.18
SNP effect on A disintegrin and metalloproteinase with thrombospondin motifs 1
SNP effect on Gut microbiota abundance (genus Catenibacterium id.2153)

Batch 2−62 : Gut microbiota abundance (genus Parasutterella id.2892) on A disintegrin and metalloproteinase with thrombospondin motifs 1 with a threshold of 1e−05

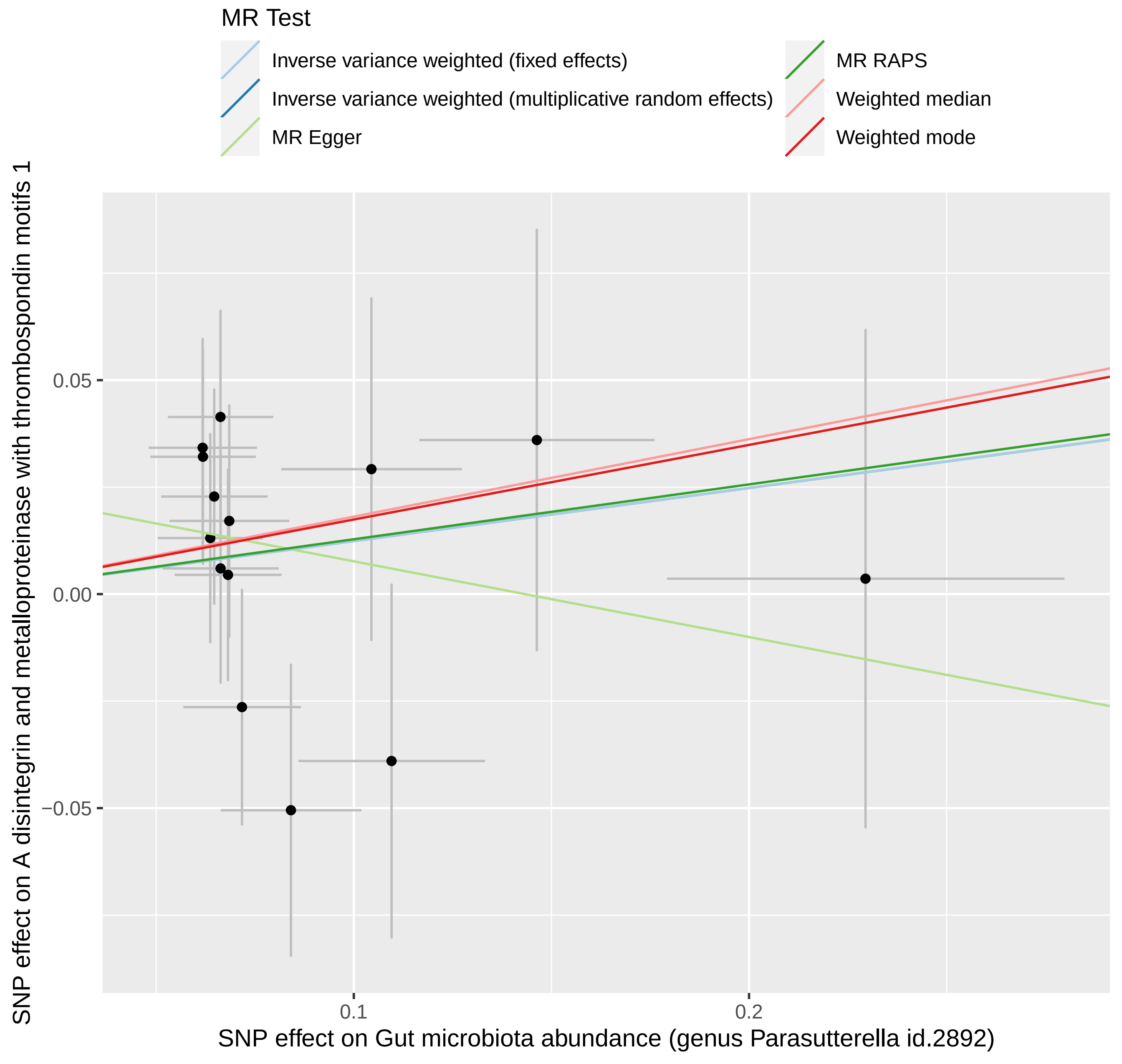

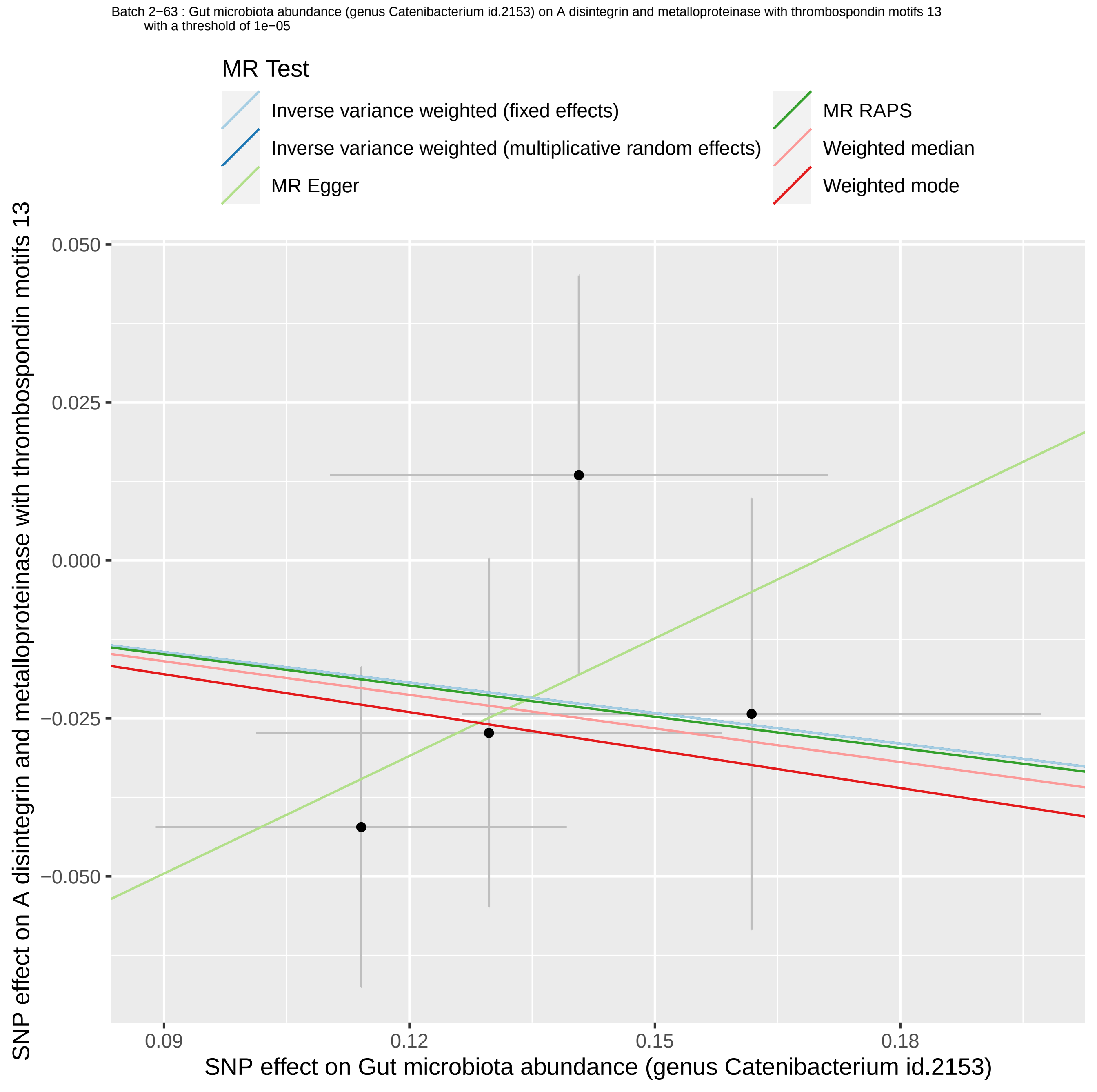

Batch 2−63 : Gut microbiota abundance (genus Catenibacterium id.2153) on A disintegrin and metalloproteinase with thrombospondin motifs 13 with a threshold of 1e−05
MR Test
Inverse variance weighted (fixed effects)
Inverse variance weighted (multiplicative random effects)
MR Egger
MR RAPS
Weighted median
Weighted mode
0.050
0.025
0.000
−0.025
−0.050
0.09
0.12
0.15
0.18
SNP effect on A disintegrin and metalloproteinase with thrombospondin motifs 13
SNP effect on Gut microbiota abundance (genus Catenibacterium id.2153)

Batch 2−64 : Gut microbiota abundance (genus Parasutterella id.2892) on A disintegrin and metalloproteinase with thrombospondin motifs 13 with a threshold of 1e−05

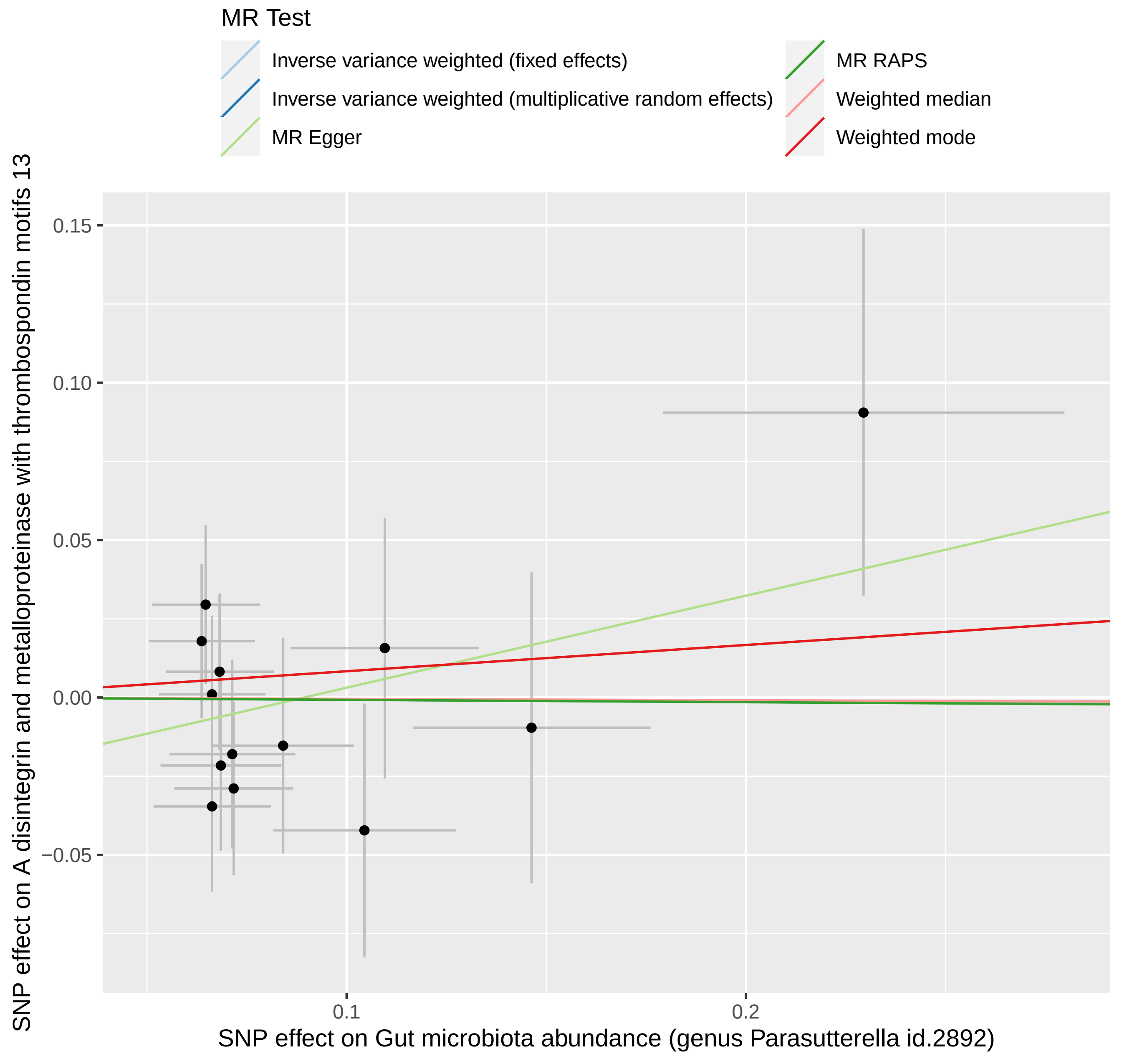

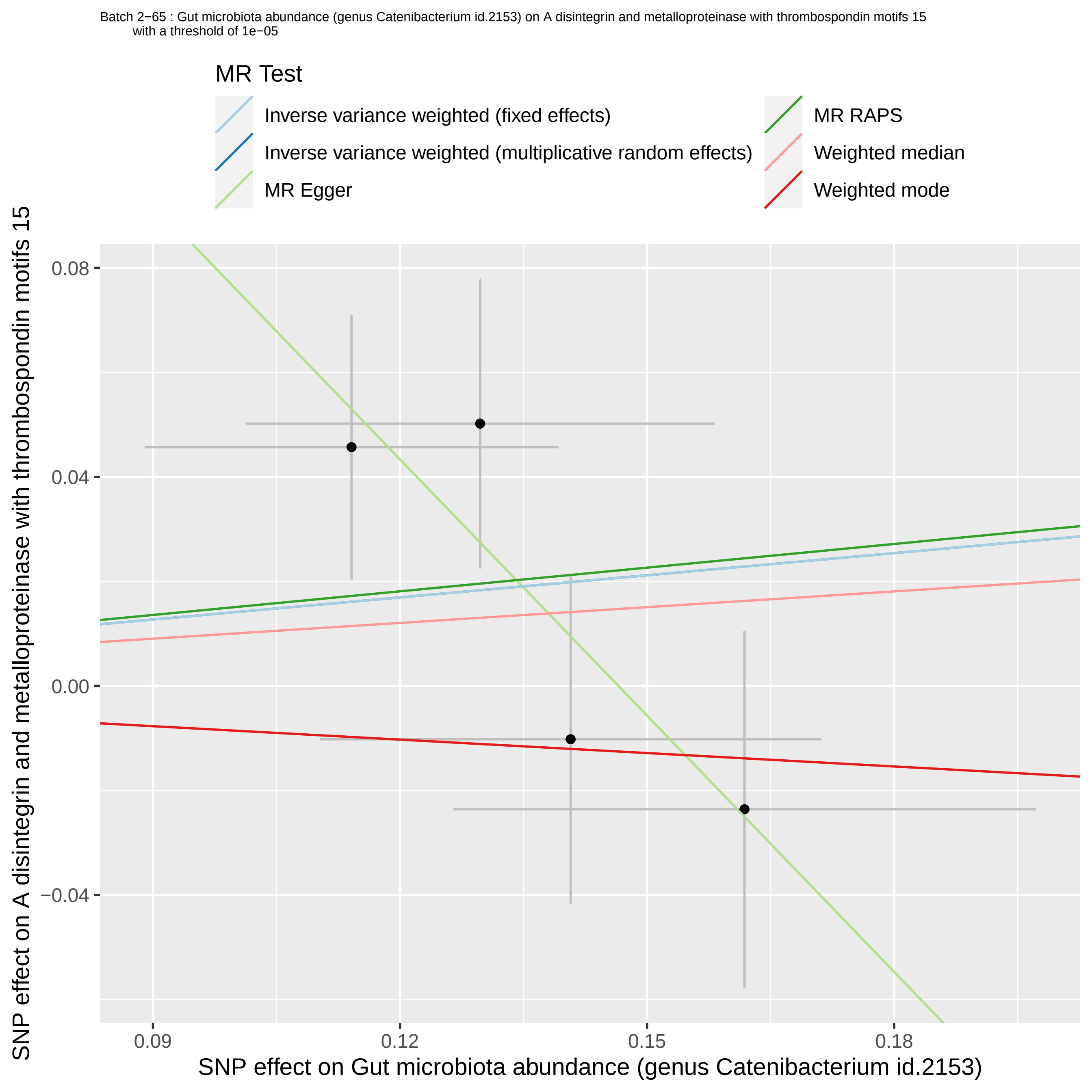

Batch 2−65 : Gut microbiota abundance (genus Catenibacterium id.2153) on A disintegrin and metalloproteinase with thrombospondin motifs 15 with a threshold of 1e−05
MR Test
Inverse variance weighted (fixed effects)
Inverse variance weighted (multiplicative random effects)
MR Egger
MR RAPS
Weighted median
Weighted mode
0.08
0.04
0.00
−0.04
0.09
0.12
0.15
0.18
SNP effect on A disintegrin and metalloproteinase with thrombospondin motifs 15
SNP effect on Gut microbiota abundance (genus Catenibacterium id.2153)

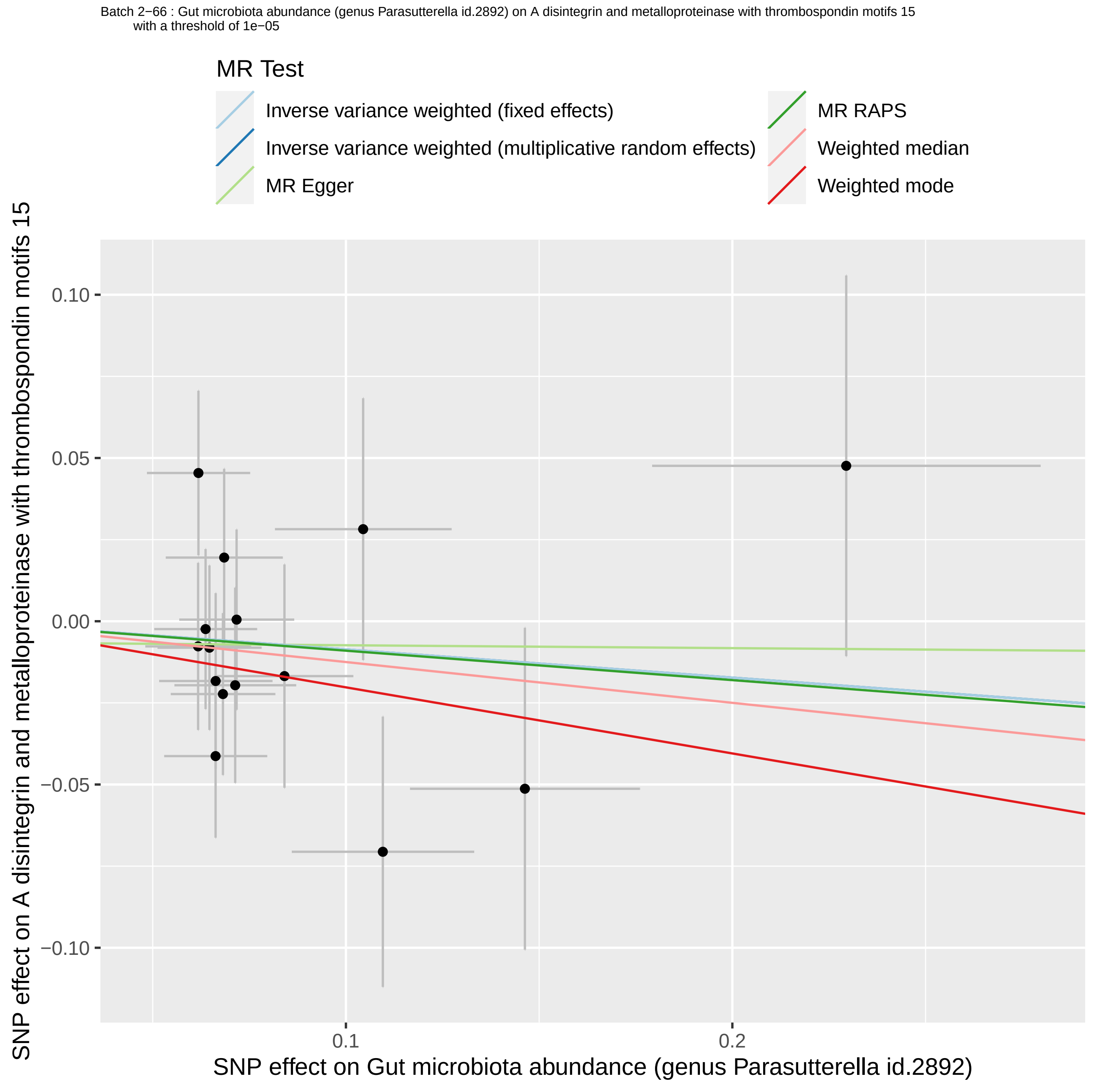

Batch 2−66 : Gut microbiota abundance (genus Parasutterella id.2892) on A disintegrin and metalloproteinase with thrombospondin motifs 15
with a threshold of 1e−05
MR Test
Inverse variance weighted (fixed effects)
Inverse variance weighted (multiplicative random effects)
MR Egger
MR RAPS
Weighted median
Weighted mode
0.10
0.05
0.00
−0.05
−0.10
0.1
0.2
SNP effect on A disintegrin and metalloproteinase with thrombospondin motifs 15
SNP effect on Gut microbiota abundance (genus Parasutterella id.2892)

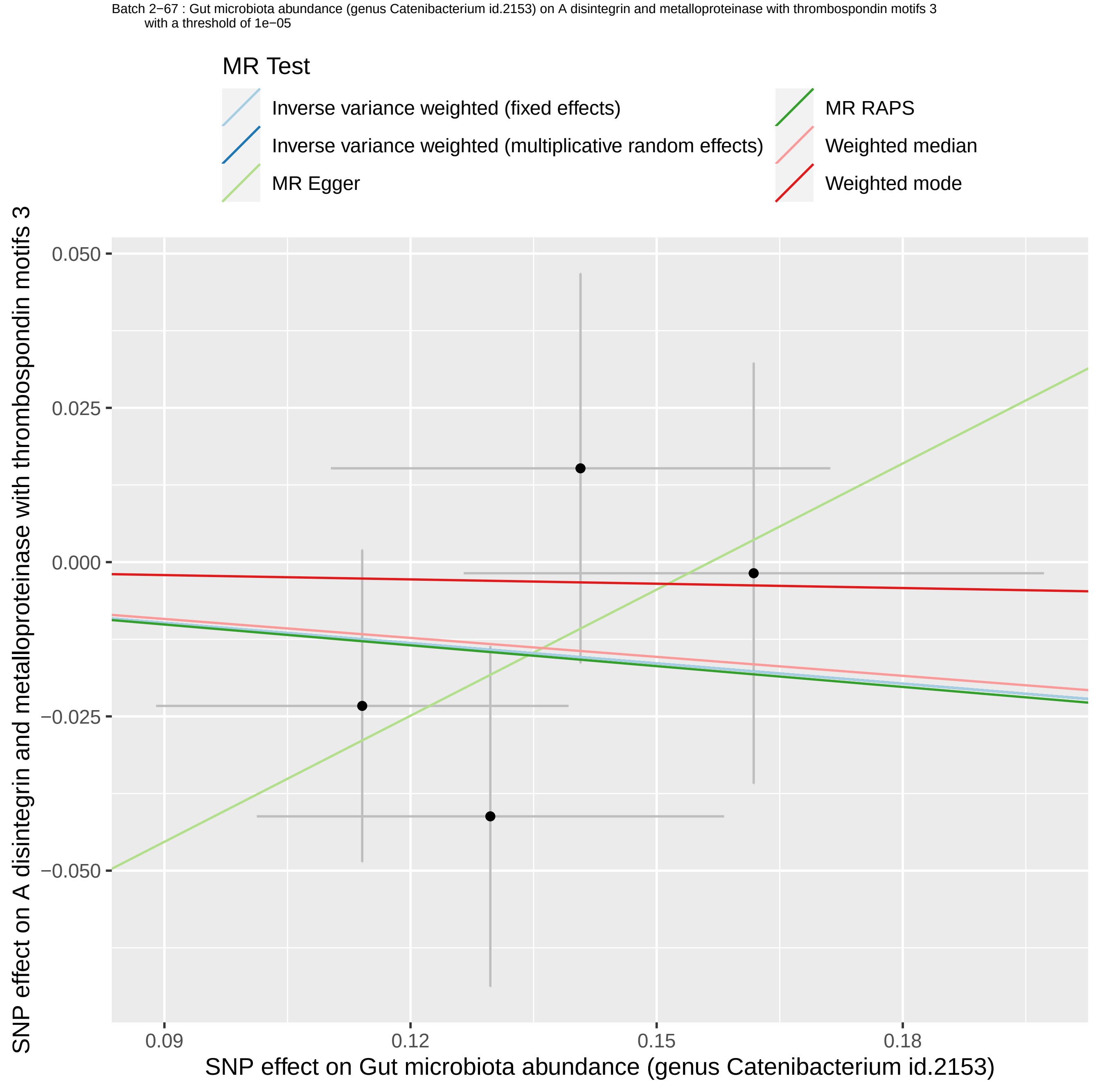
Batch 2−67 : Gut microbiota abundance (genus Catenibacterium id.2153) on A disintegrin and metalloproteinase with thrombospondin motifs 3 with a threshold of 1e−05
MR Test
Inverse variance weighted (fixed effects)
Inverse variance weighted (multiplicative random effects)
MR Egger
MR RAPS
Weighted median
Weighted mode
0.050
0.025
0.000
−0.025
−0.050
0.09
0.12
0.15
0.18
SNP effect on A disintegrin and metalloproteinase with thrombospondin motifs 3
SNP effect on Gut microbiota abundance (genus Catenibacterium id.2153)

Batch 2−68 : Gut microbiota abundance (genus Parasutterella id.2892) on A disintegrin and metalloproteinase with thrombospondin motifs 3 with a threshold of 1e−05

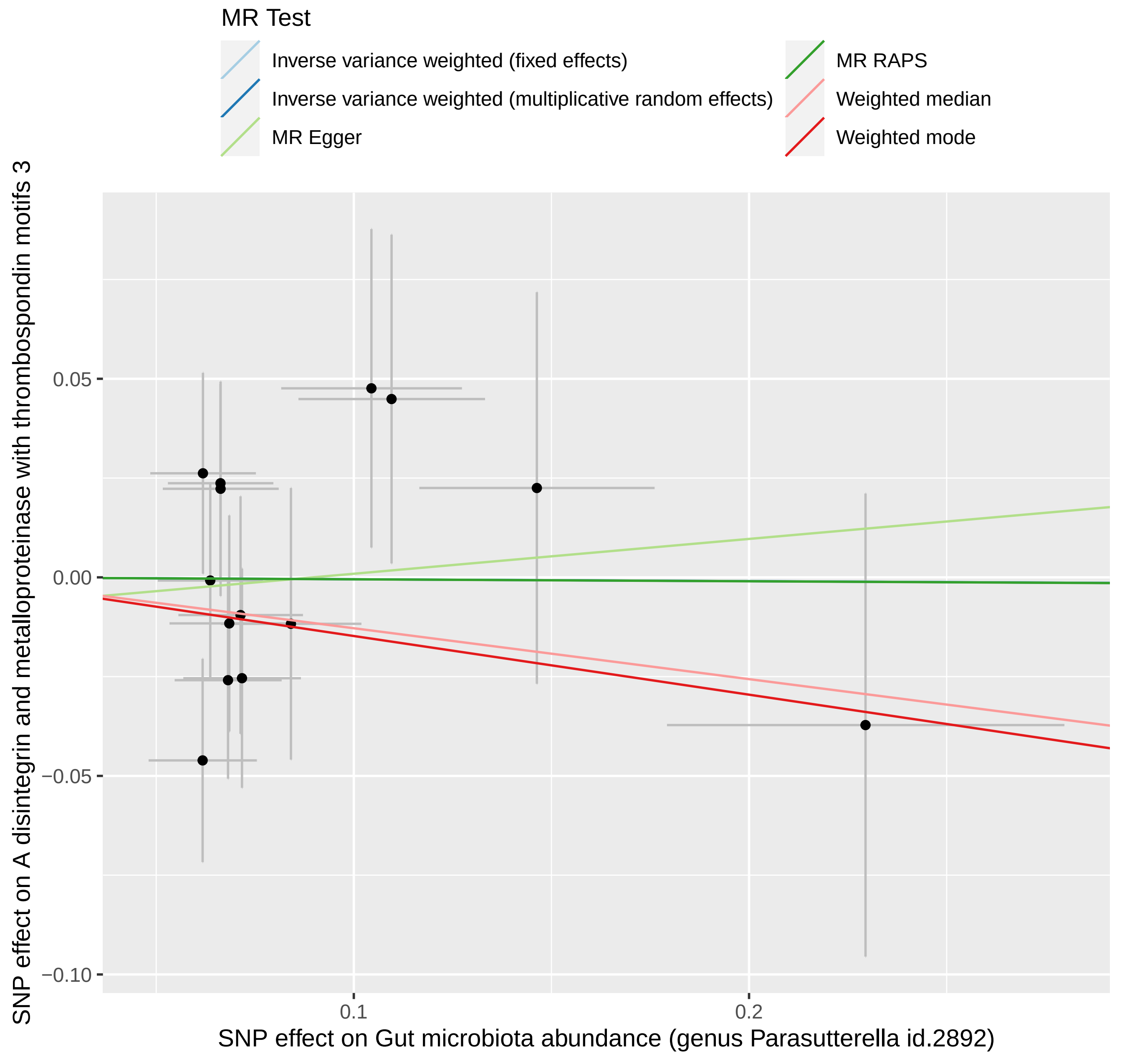

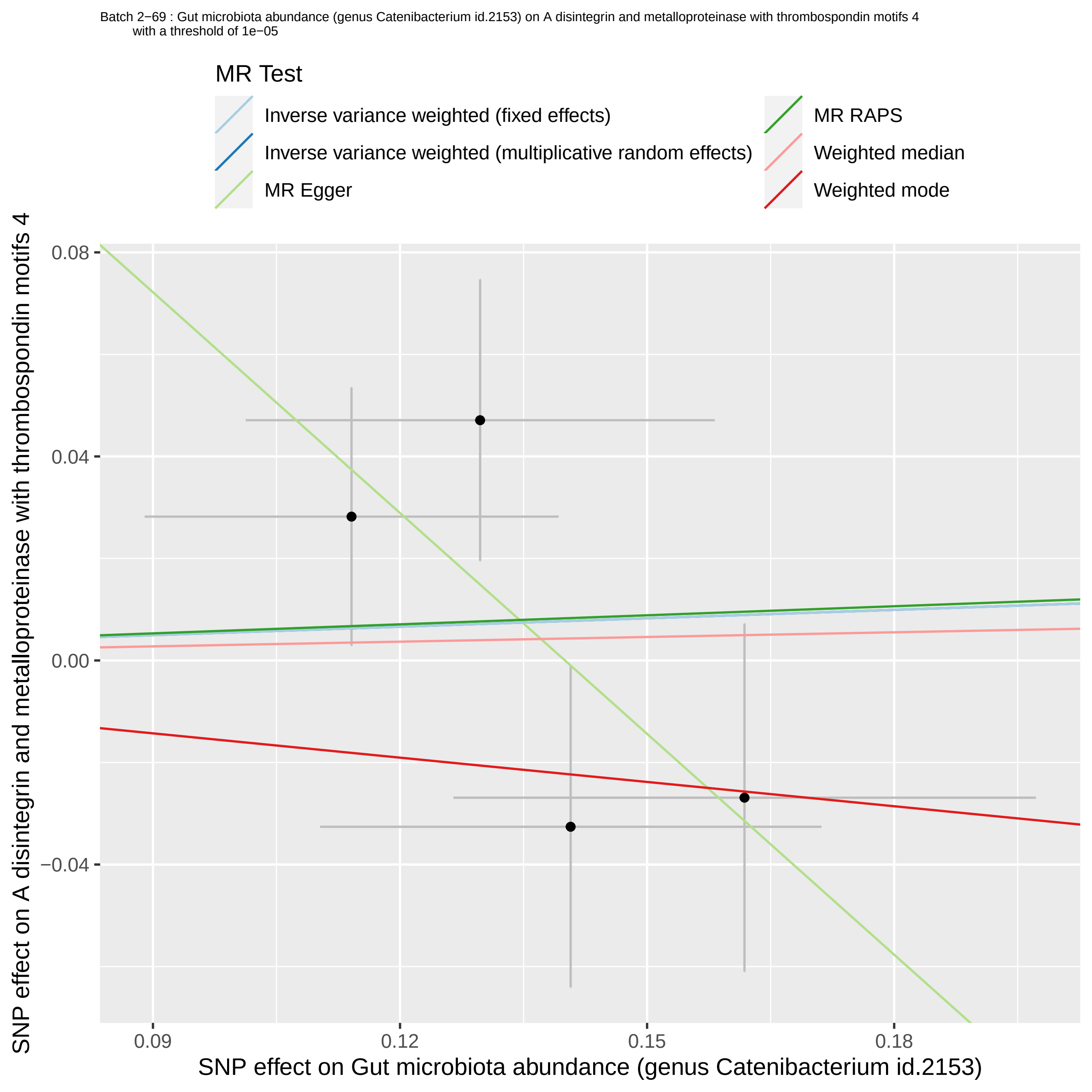

Batch 2−69 : Gut microbiota abundance (genus Catenibacterium id.2153) on A disintegrin and metalloproteinase with thrombospondin motifs 4
with a threshold of 1e−05
MR Test
Inverse variance weighted (fixed effects)
Inverse variance weighted (multiplicative random effects)
MR Egger
MR RAPS
Weighted median
Weighted mode
0.08
0.04
0.00
−0.04
0.09
0.12
0.15
0.18
SNP effect on A disintegrin and metalloproteinase with thrombospondin motifs 4
SNP effect on Gut microbiota abundance (genus Catenibacterium id.2153)

Batch 2−70 : Gut microbiota abundance (genus Parasutterella id.2892) on A disintegrin and metalloproteinase with thrombospondin motifs 4 with a threshold of 1e−05

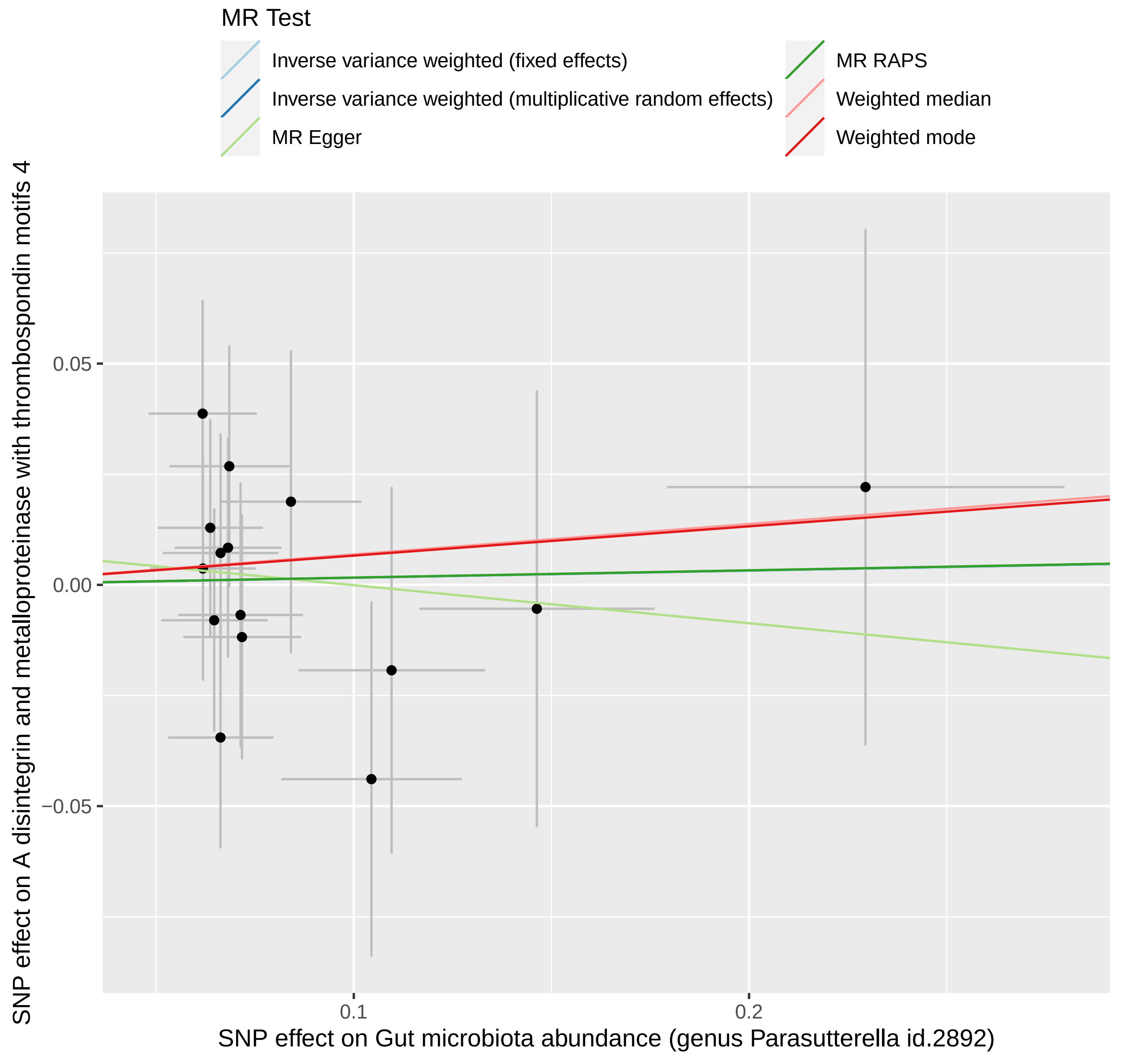

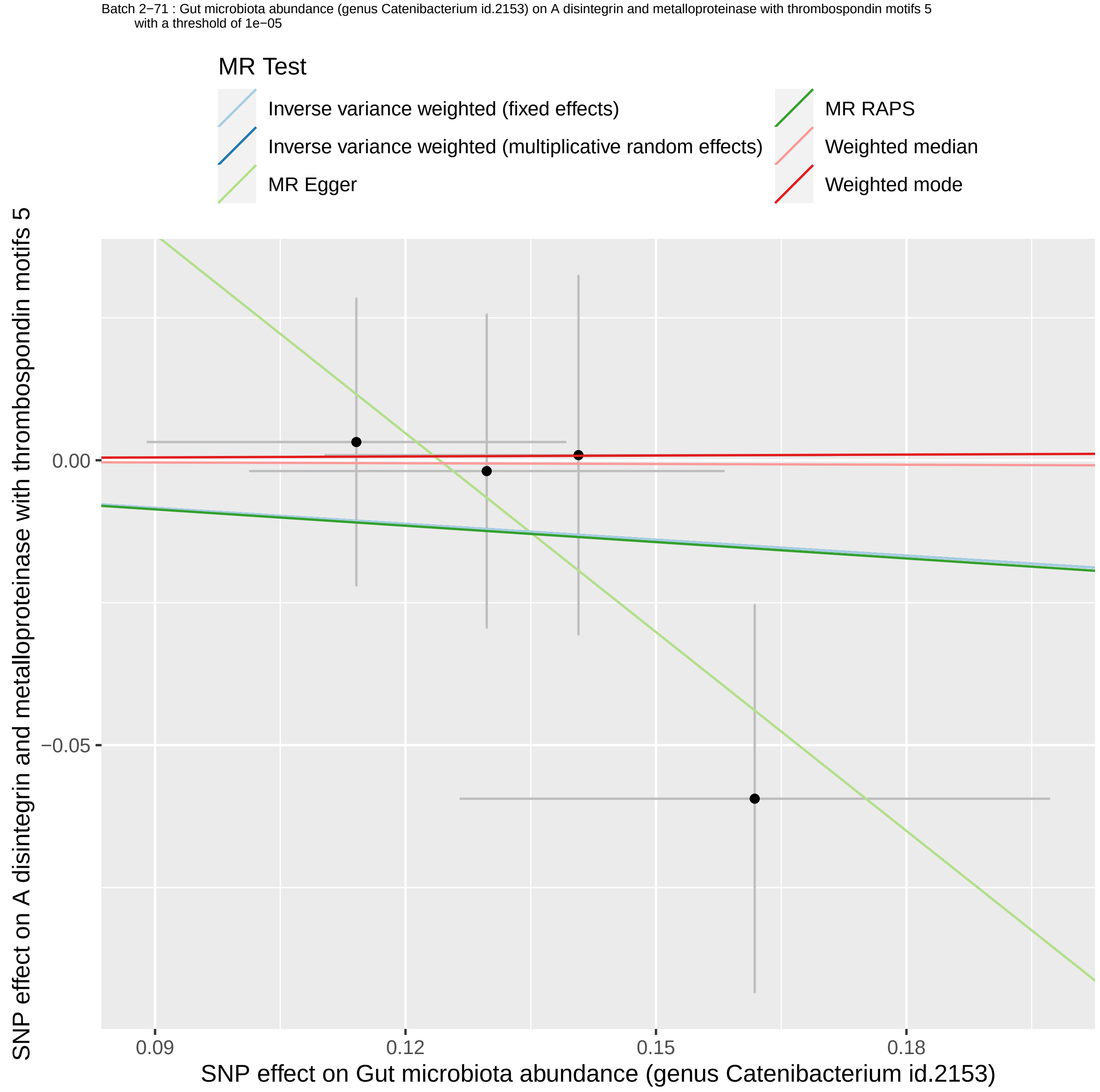

Batch 2−71 : Gut microbiota abundance (genus Catenibacterium id.2153) on A disintegrin and metalloproteinase with thrombospondin motifs 5 with a threshold of 1e−05
MR Test
Inverse variance weighted (fixed effects)
Inverse variance weighted (multiplicative random effects)
MR Egger
MR RAPS
Weighted median
Weighted mode
0.00
−0.05
0.09
0.12
0.15
0.18
SNP effect on A disintegrin and metalloproteinase with thrombospondin motifs 5
SNP effect on Gut microbiota abundance (genus Catenibacterium id.2153)

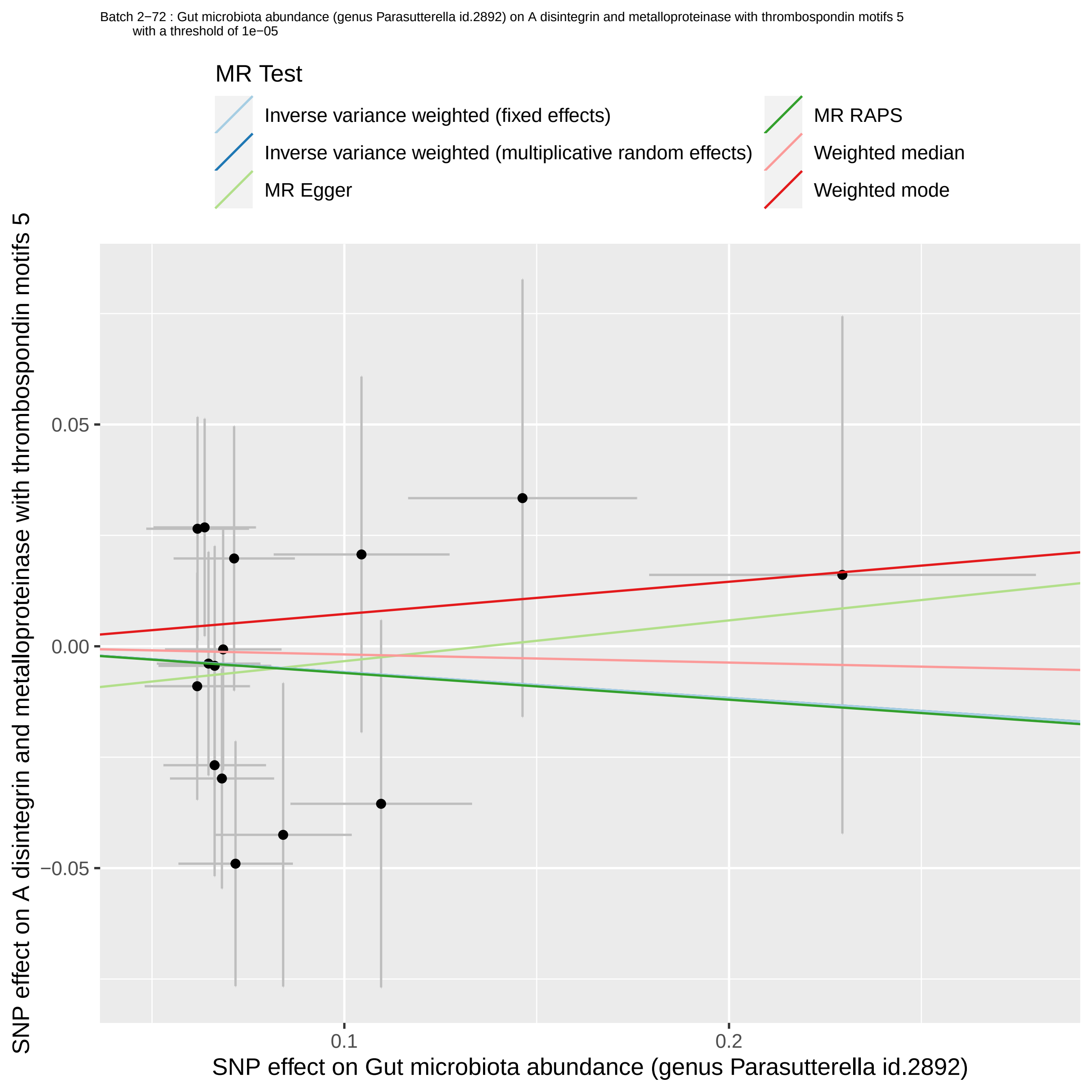

Batch 2−72 : Gut microbiota abundance (genus Parasutterella id.2892) on A disintegrin and metalloproteinase with thrombospondin motifs 5
with a threshold of 1e−05
MR Test
Inverse variance weighted (fixed effects)
Inverse variance weighted (multiplicative random effects)
MR Egger
MR RAPS
Weighted median
Weighted mode
0.05
0.00
−0.05
0.1
0.2
SNP effect on A disintegrin and metalloproteinase with thrombospondin motifs 5
SNP effect on Gut microbiota abundance (genus Parasutterella id.2892)

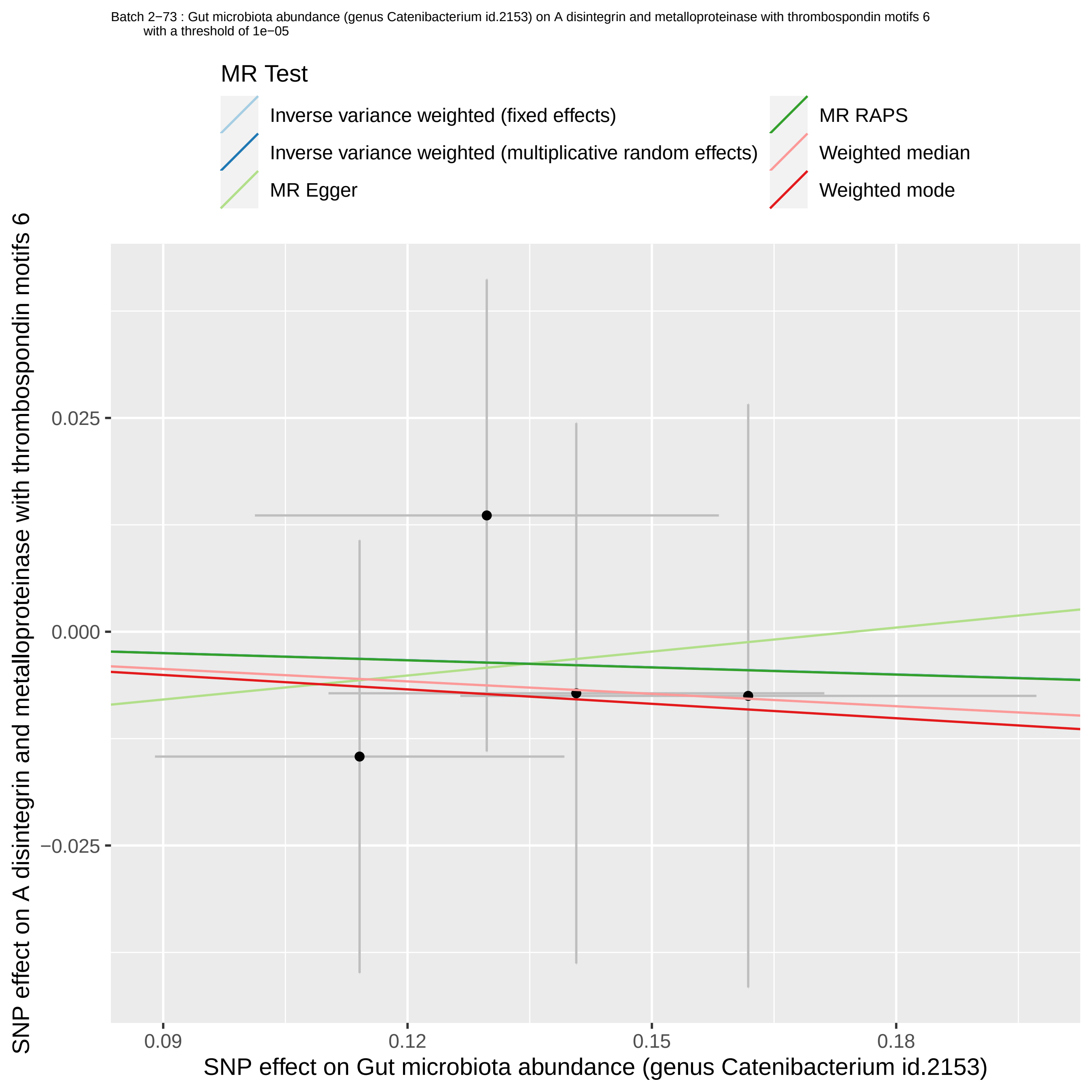
Batch 2−73 : Gut microbiota abundance (genus Catenibacterium id.2153) on A disintegrin and metalloproteinase with thrombospondin motifs 6 with a threshold of 1e−05
MR Test
Inverse variance weighted (fixed effects)
Inverse variance weighted (multiplicative random effects)
MR Egger
MR RAPS
Weighted median
Weighted mode
0.025
0.000
−0.025
0.09
0.12
0.15
0.18
SNP effect on A disintegrin and metalloproteinase with thrombospondin motifs 6
SNP effect on Gut microbiota abundance (genus Catenibacterium id.2153)

Batch 2−74 : Gut microbiota abundance (genus Parasutterella id.2892) on A disintegrin and metalloproteinase with thrombospondin motifs 6 with a threshold of 1e−05

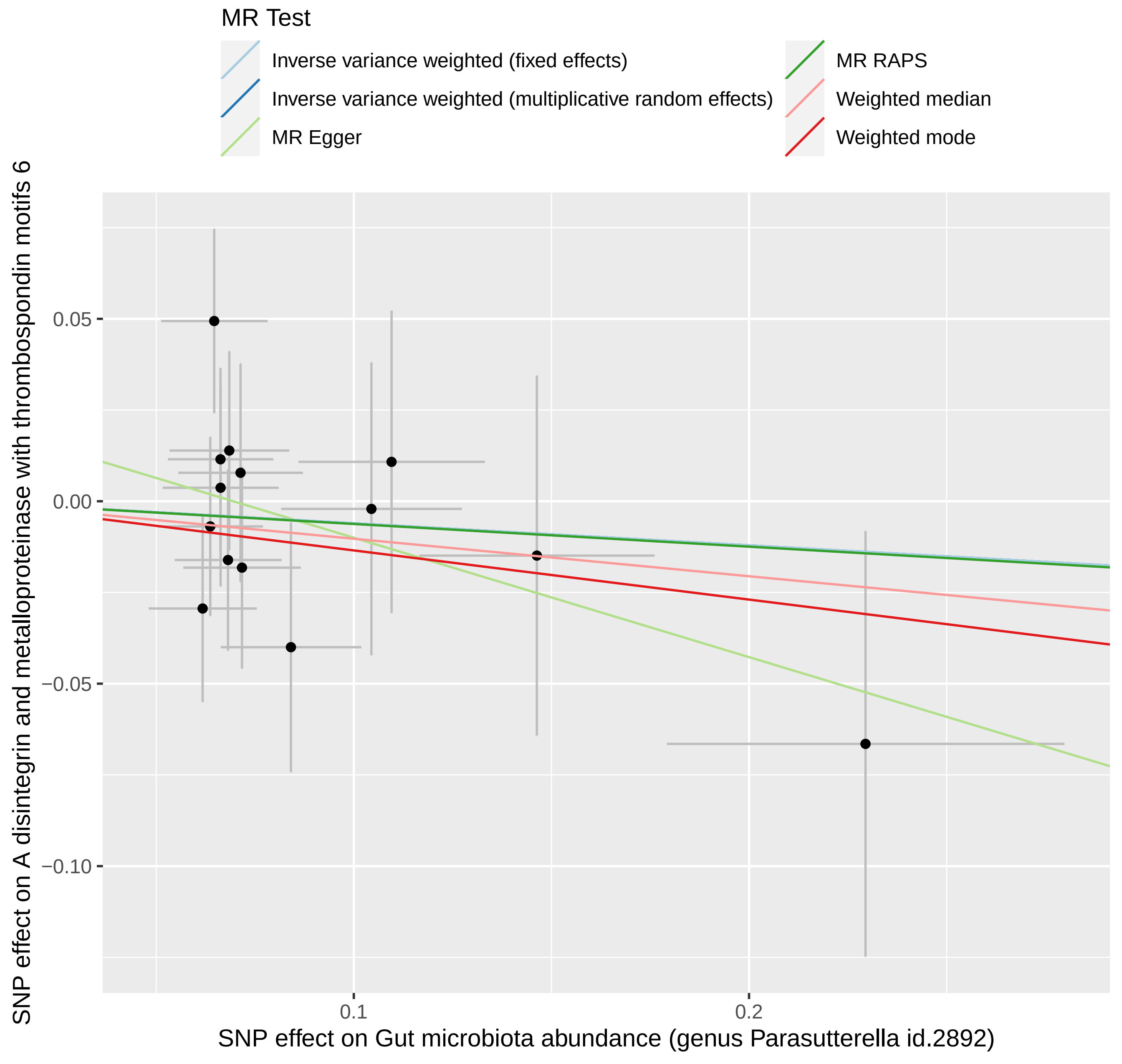

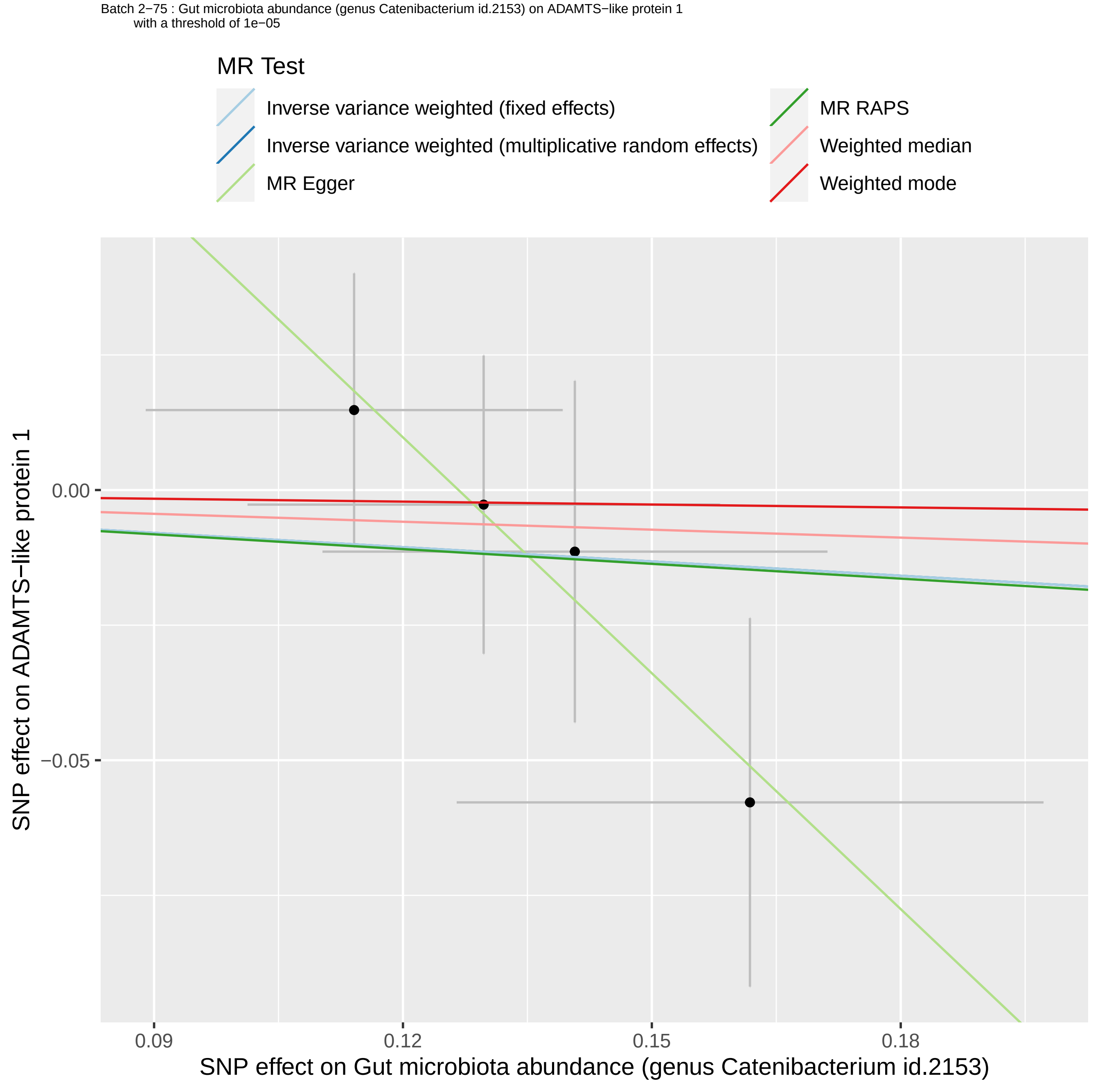

Batch 2−75 : Gut microbiota abundance (genus Catenibacterium id.2153) on ADAMTS−like protein 1
with a threshold of 1e−05
MR Test
Inverse variance weighted (fixed effects)
Inverse variance weighted (multiplicative random effects)
MR Egger
MR RAPS
Weighted median
Weighted mode
SNP effect on ADAMTS−like protein 1
0.00
−0.05
0.09
0.12
0.15
0.18
SNP effect on Gut microbiota abundance (genus Catenibacterium id.2153)

Batch 2−76 : Gut microbiota abundance (genus Parasutterella id.2892) on ADAMTS−like protein 1
with a threshold of 1e−05

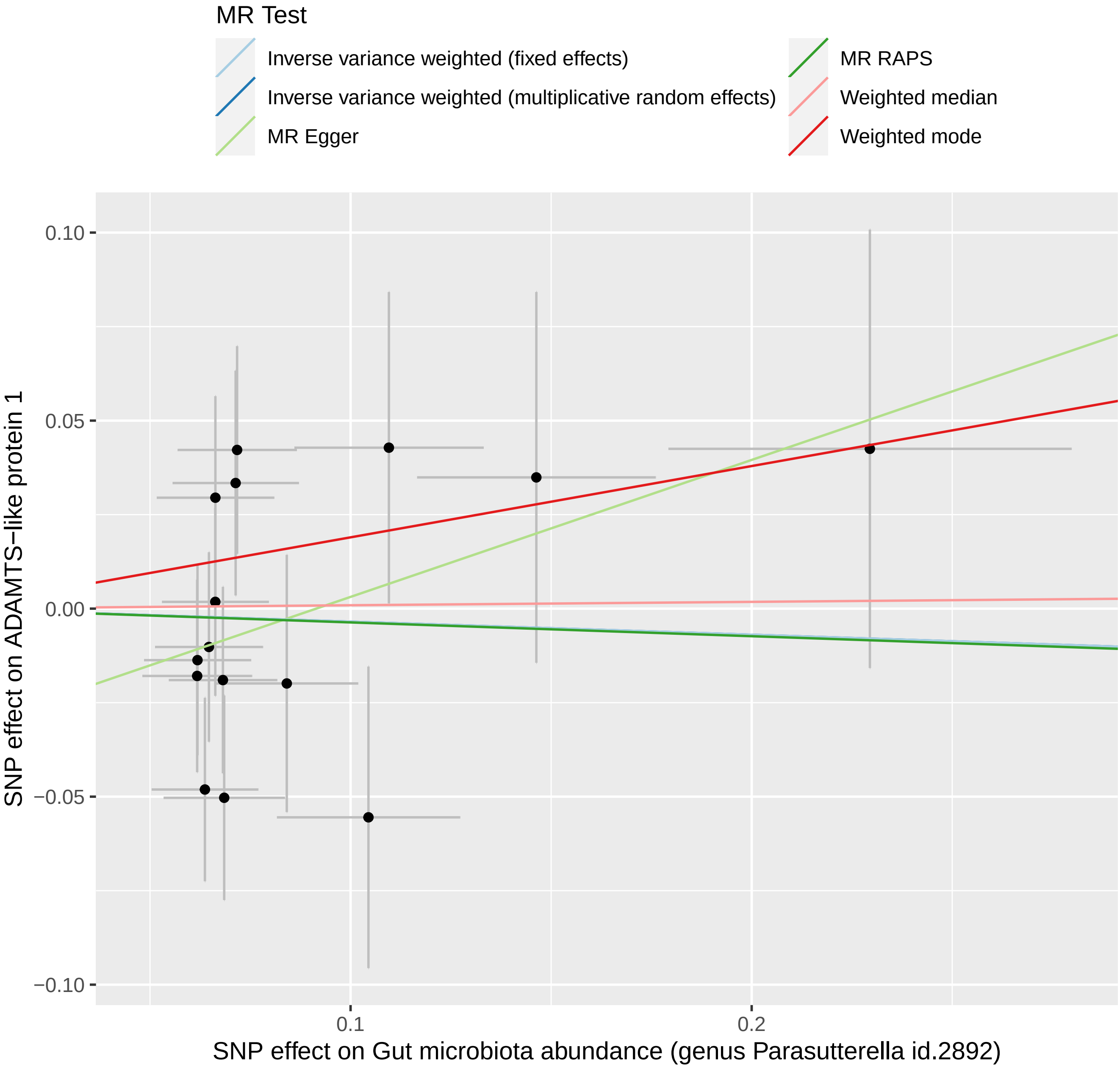

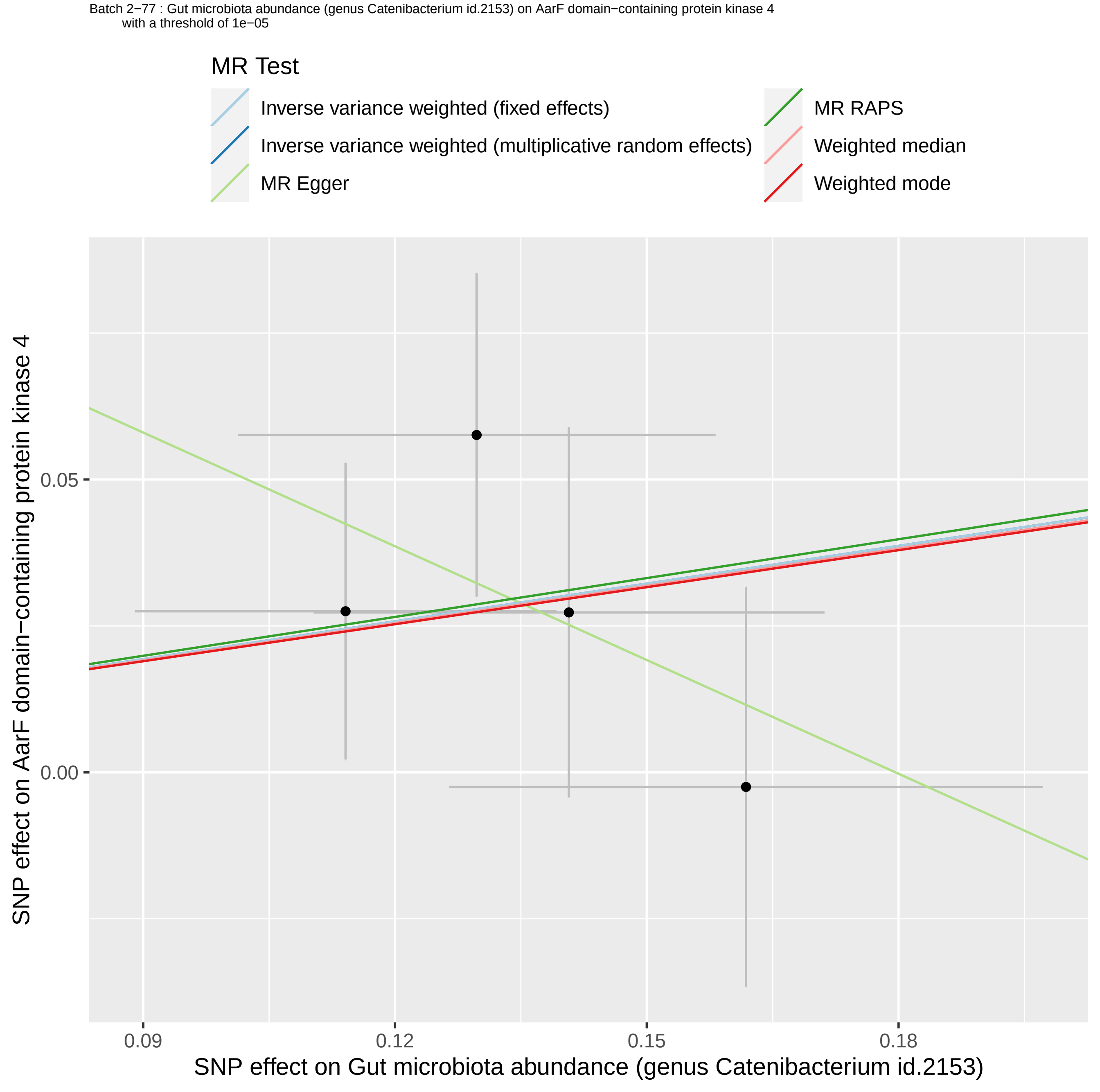

Batch 2−77 : Gut microbiota abundance (genus Catenibacterium id.2153) on AarF domain−containing protein kinase 4
with a threshold of 1e−05
MR Test
Inverse variance weighted (fixed effects)
Inverse variance weighted (multiplicative random effects)
MR Egger
MR RAPS
Weighted median
Weighted mode
SNP effect on AarF domain−containing protein kinase 4
0.05
0.00
0.09
0.12
0.15
0.18
SNP effect on Gut microbiota abundance (genus Catenibacterium id.2153)

Batch 2−78 : Gut microbiota abundance (genus Parasutterella id.2892) on AarF domain−containing protein kinase 4
with a threshold of 1e−05

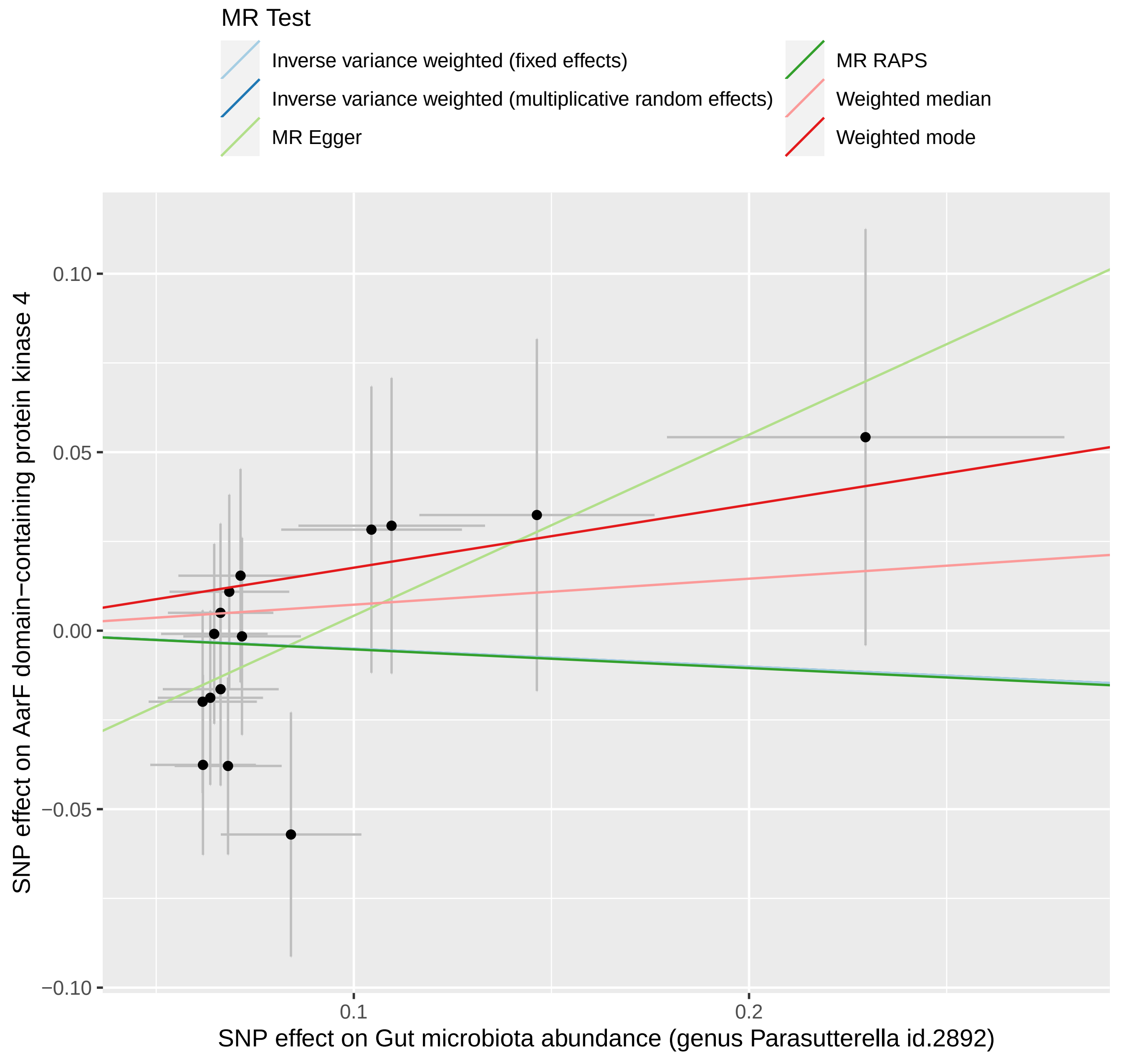

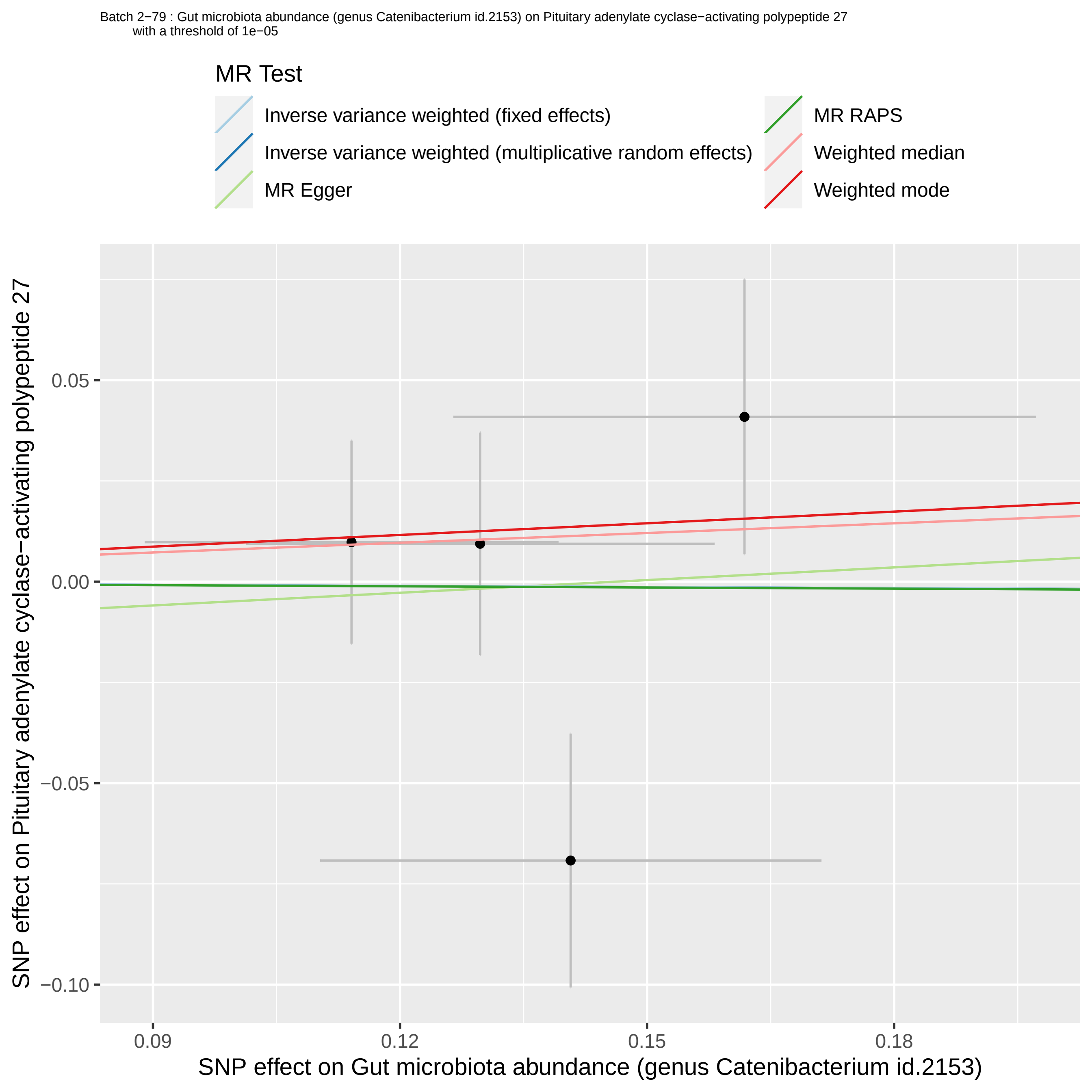

Batch 2−79 : Gut microbiota abundance (genus Catenibacterium id.2153) on Pituitary adenylate cyclase−activating polypeptide 27
with a threshold of 1e−05
MR Test
Inverse variance weighted (fixed effects)
Inverse variance weighted (multiplicative random effects)
MR Egger
MR RAPS
Weighted median
Weighted mode
0.05
0.00
−0.05
−0.10
0.09
0.12
0.15
0.18
SNP effect on Gut microbiota abundance (genus Catenibacterium id.2153)
SNP effect on Pituitary adenylate cyclase−activating polypeptide 27

Batch 2−80 : Gut microbiota abundance (genus Parasutterella id.2892) on Pituitary adenylate cyclase−activating polypeptide 27
with a threshold of 1e−05

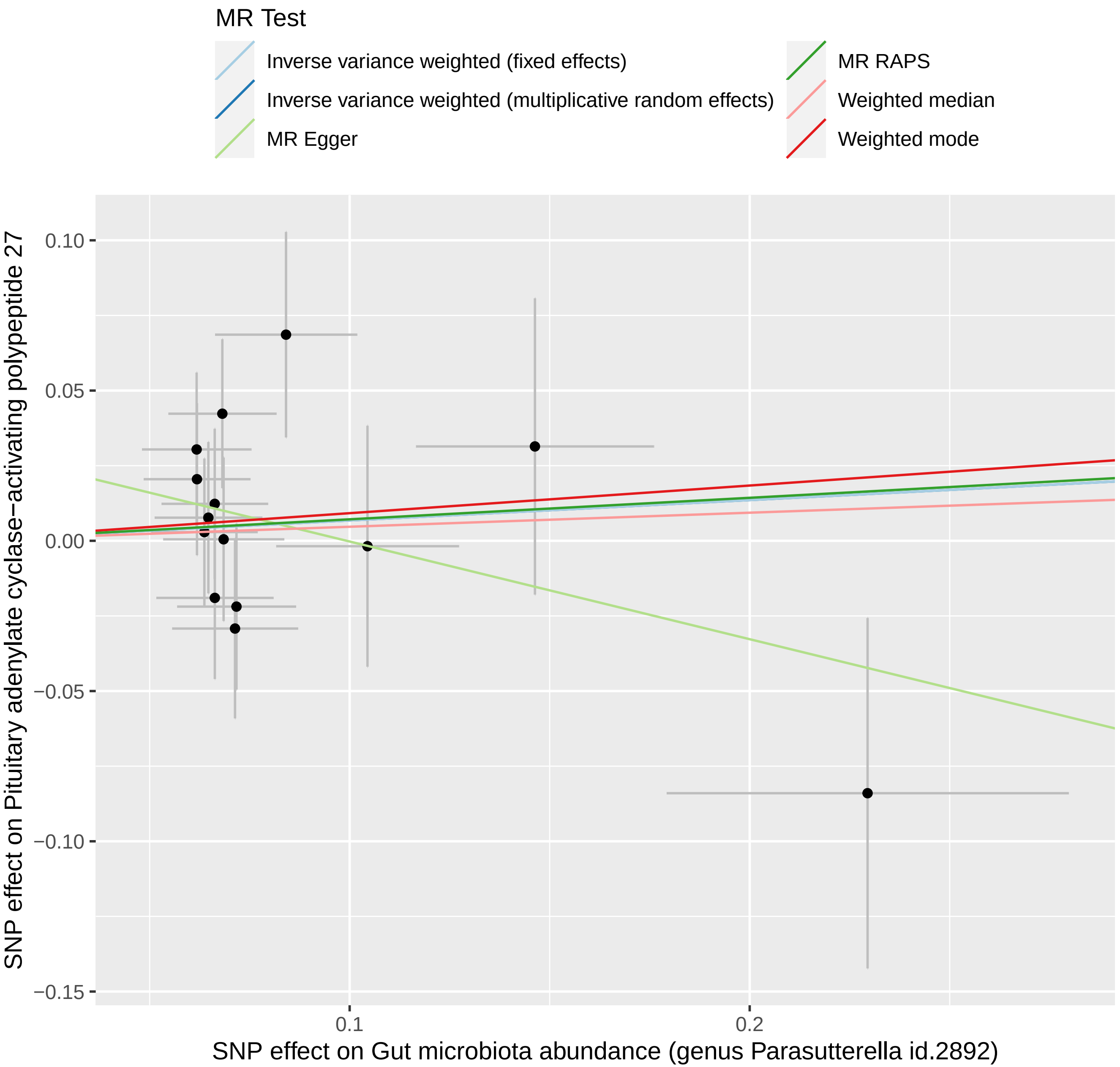

Batch 2−81 : Gut microbiota abundance (genus Catenibacterium id.2153) on Pituitary adenylate cyclase−activating polypeptide 38
with a threshold of 1e−05

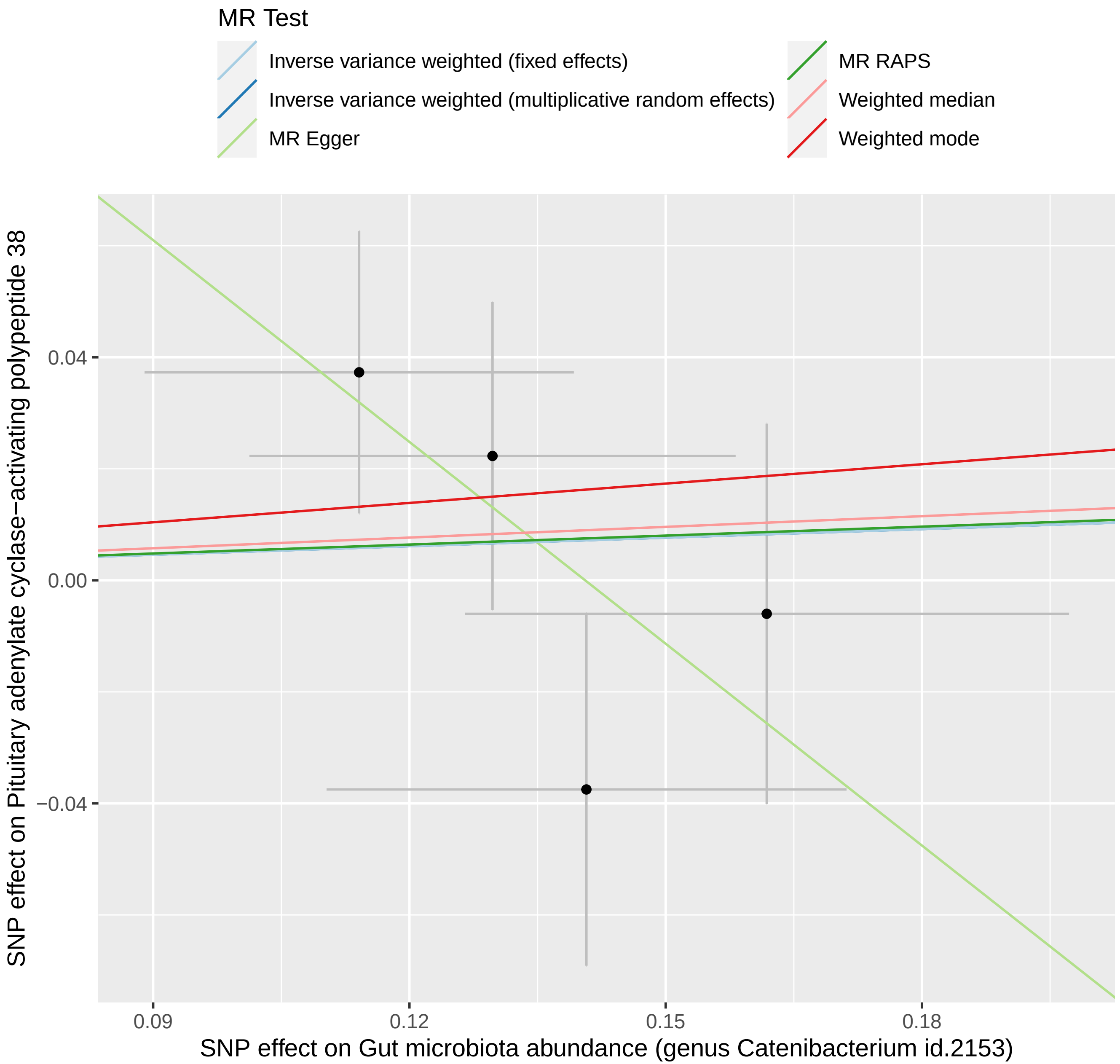

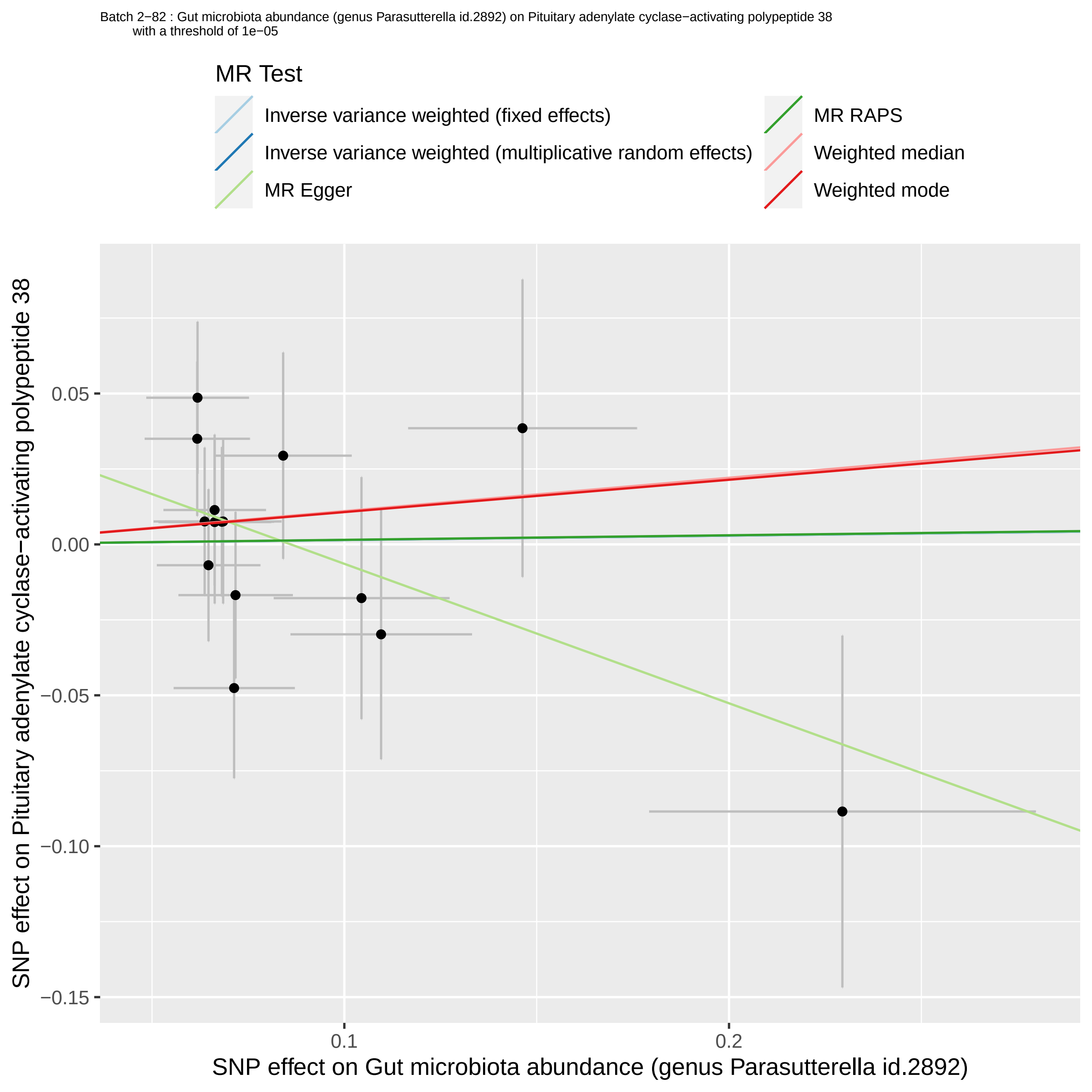

Batch 2−82 : Gut microbiota abundance (genus Parasutterella id.2892) on Pituitary adenylate cyclase−activating polypeptide 38
with a threshold of 1e−05
MR Test
Inverse variance weighted (fixed effects)
Inverse variance weighted (multiplicative random effects)
MR Egger
MR RAPS
Weighted median
Weighted mode
SNP effect on Pituitary adenylate cyclase−activating polypeptide 38
0.05
0.00
−0.05
−0.10
−0.15
0.1
0.2
SNP effect on Gut microbiota abundance (genus Parasutterella id.2892)

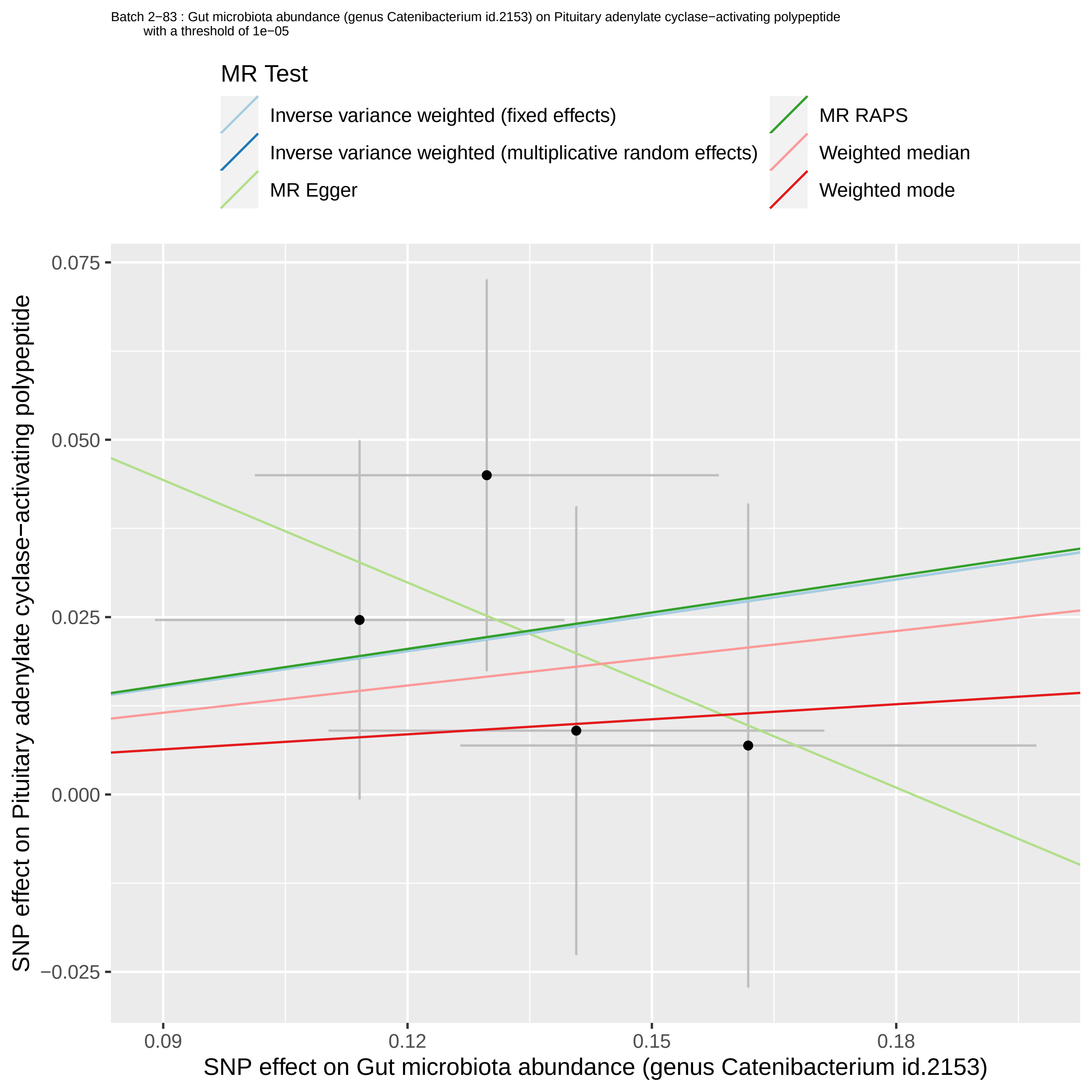
Batch 2−83 : Gut microbiota abundance (genus Catenibacterium id.2153) on Pituitary adenylate cyclase−activating polypeptide
with a threshold of 1e−05
MR Test
Inverse variance weighted (fixed effects)
Inverse variance weighted (multiplicative random effects)
MR Egger
MR RAPS
Weighted median
Weighted mode
0.075
0.050
0.025
0.000
−0.025
0.09
0.12
0.15
0.18
SNP effect on Pituitary adenylate cyclase−activating polypeptide
SNP effect on Gut microbiota abundance (genus Catenibacterium id.2153)

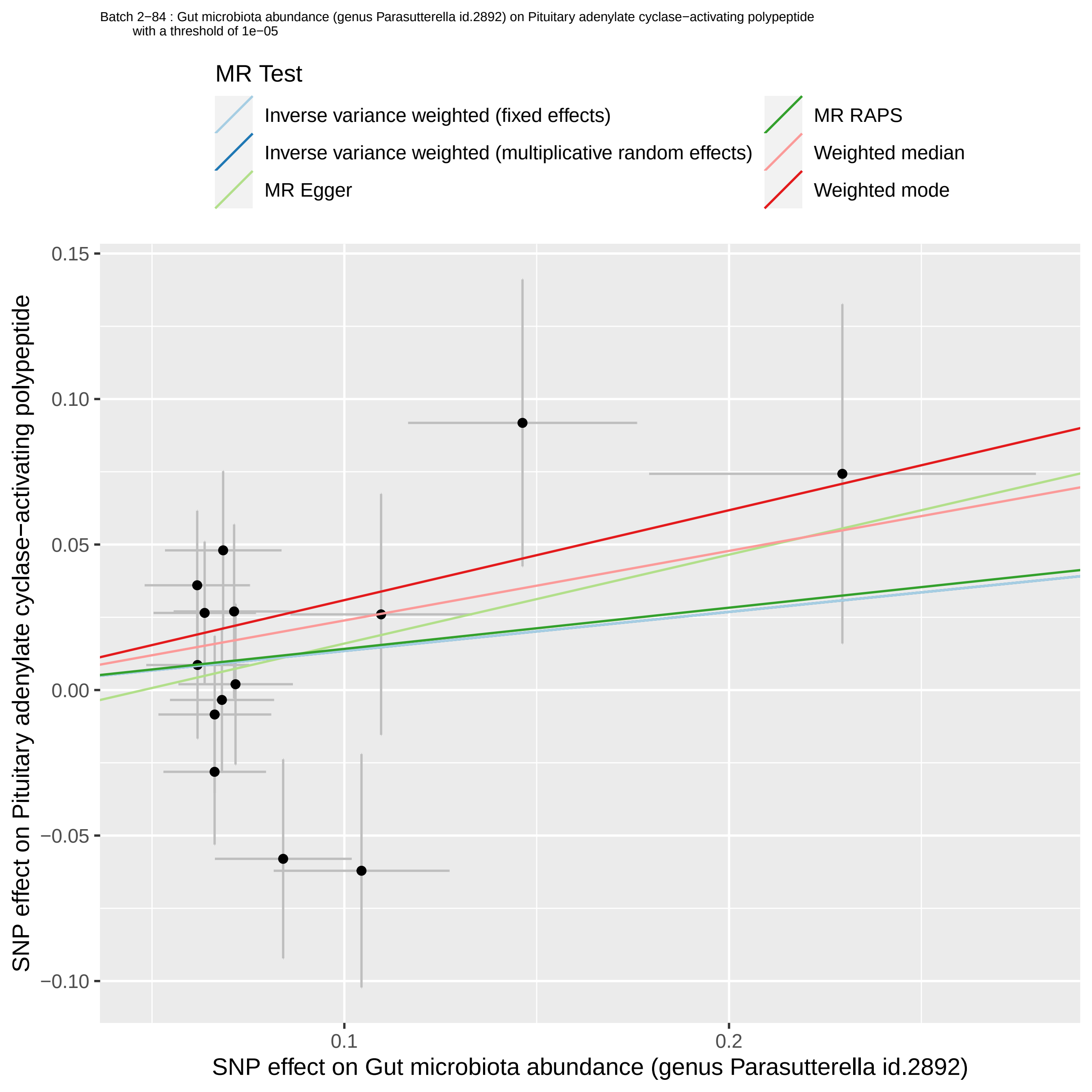

Batch 2−84 : Gut microbiota abundance (genus Parasutterella id.2892) on Pituitary adenylate cyclase−activating polypeptide
with a threshold of 1e−05
MR Test
Inverse variance weighted (fixed effects)
Inverse variance weighted (multiplicative random effects)
MR Egger
MR RAPS
Weighted median
Weighted mode
0.15
0.10
0.05
0.00
−0.05
−0.10
0.1
0.2
SNP effect on Pituitary adenylate cyclase−activating polypeptide
SNP effect on Gut microbiota abundance (genus Parasutterella id.2892)

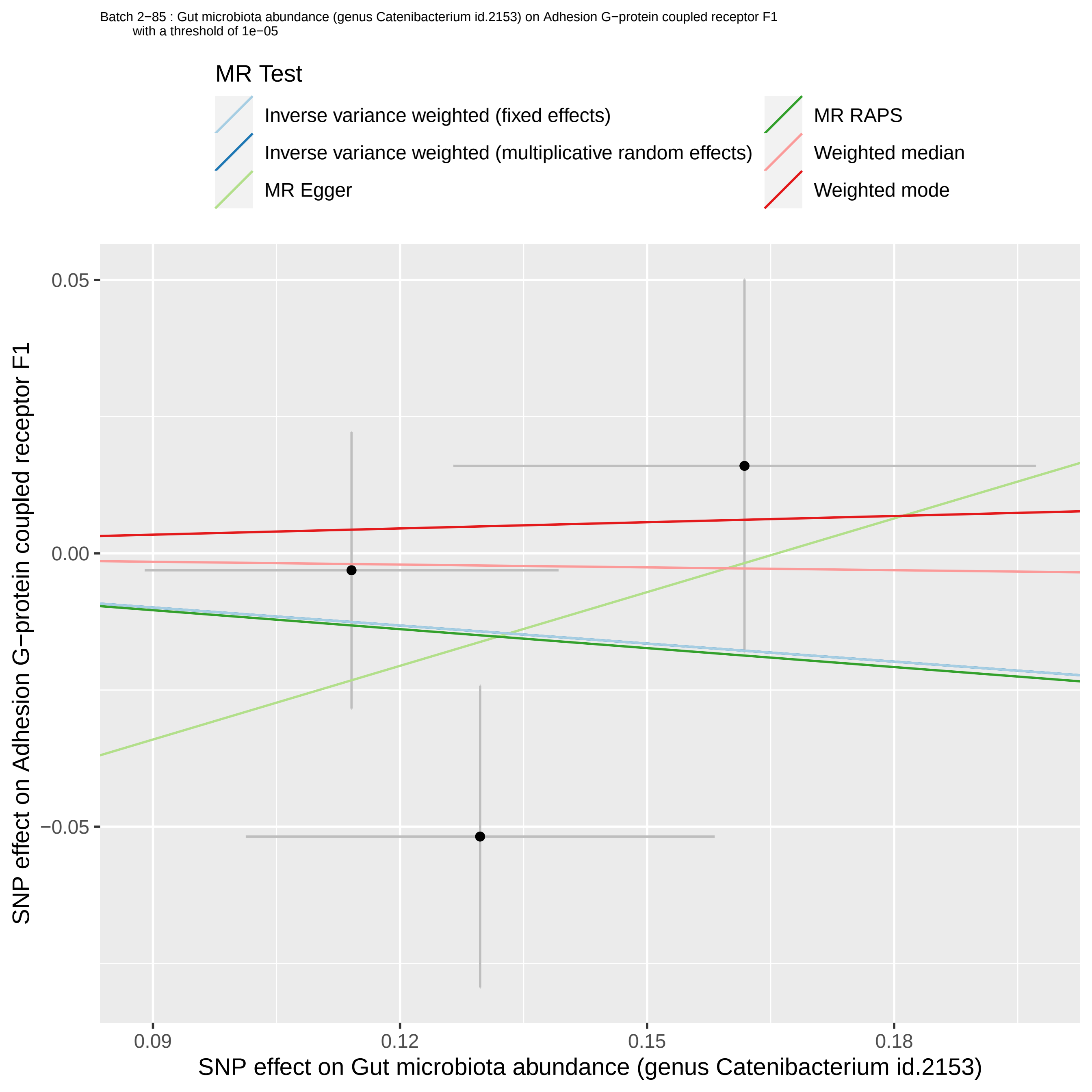

Batch 2−85 : Gut microbiota abundance (genus Catenibacterium id.2153) on Adhesion G−protein coupled receptor F1
with a threshold of 1e−05
MR Test
Inverse variance weighted (fixed effects)
Inverse variance weighted (multiplicative random effects)
MR Egger
MR RAPS
Weighted median
Weighted mode
0.05
0.00
−0.05
0.09
0.12
0.15
0.18
SNP effect on Adhesion G−protein coupled receptor F1
SNP effect on Gut microbiota abundance (genus Catenibacterium id.2153)

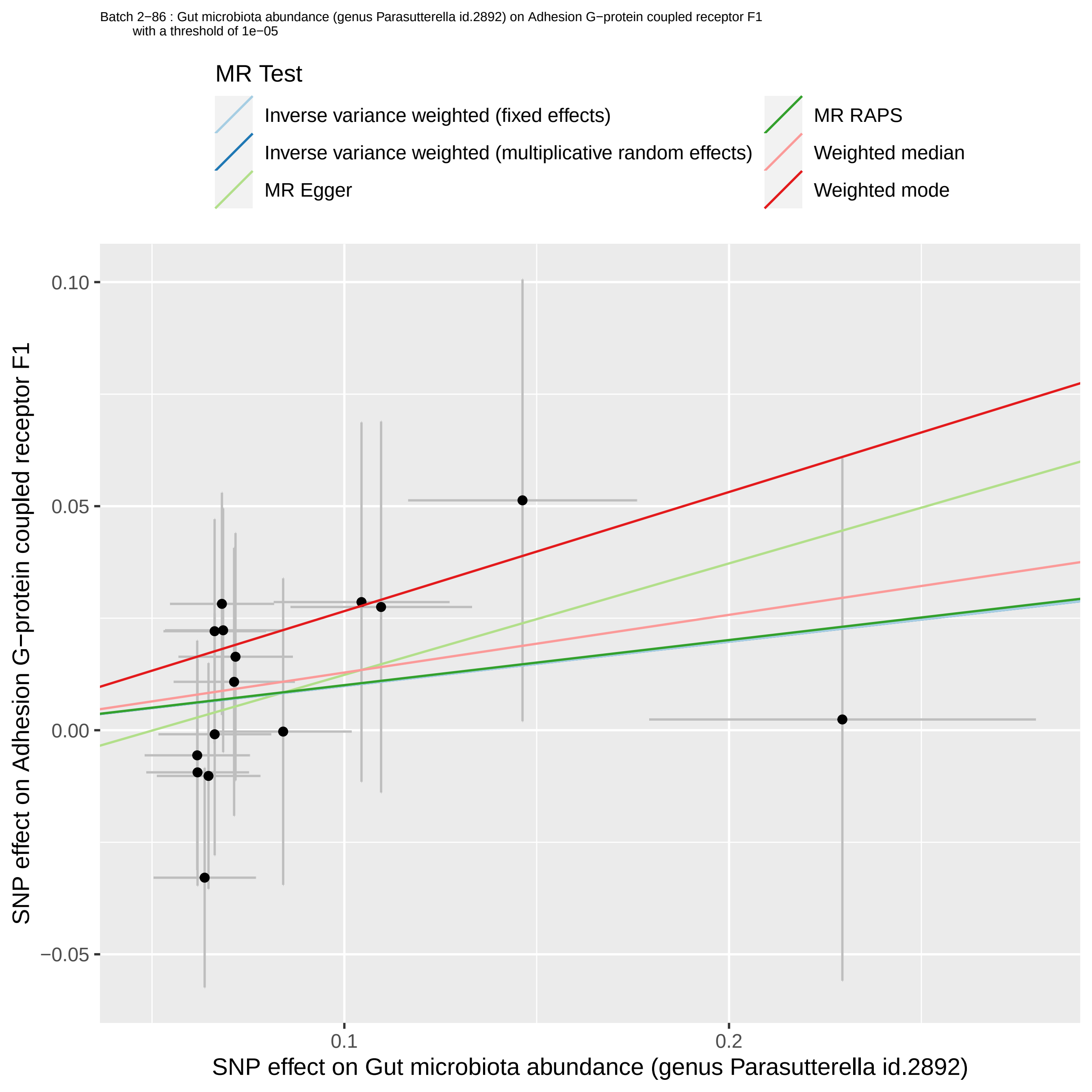
Batch 2−86 : Gut microbiota abundance (genus Parasutterella id.2892) on Adhesion G−protein coupled receptor F1
with a threshold of 1e−05
MR Test
Inverse variance weighted (fixed effects)
Inverse variance weighted (multiplicative random effects)
MR Egger
MR RAPS
Weighted median
Weighted mode
0.10
0.05
0.00
−0.05
0.1
0.2
SNP effect on Adhesion G−protein coupled receptor F1
SNP effect on Gut microbiota abundance (genus Parasutterella id.2892)

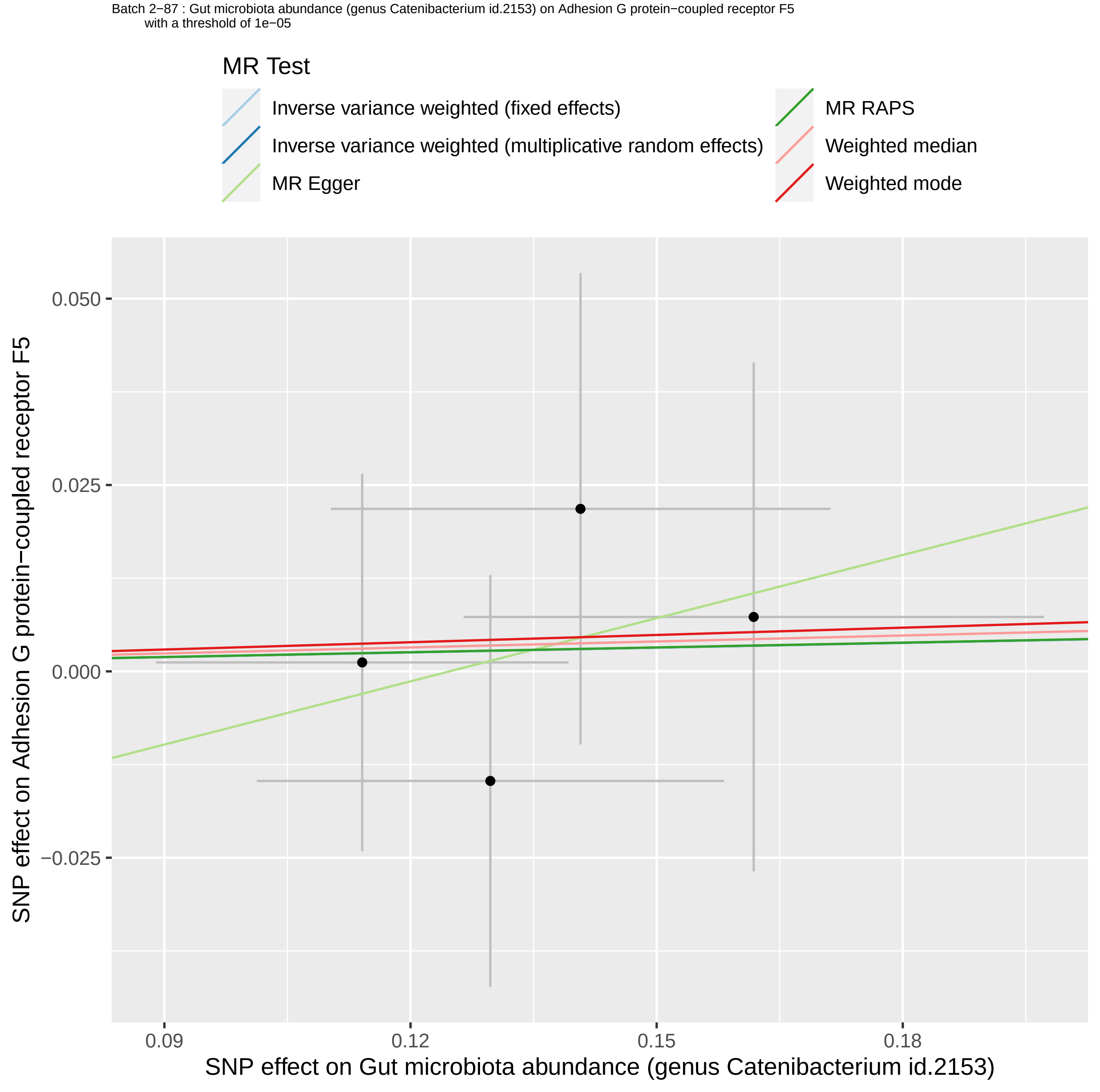
Batch 2−87 : Gut microbiota abundance (genus Catenibacterium id.2153) on Adhesion G protein−coupled receptor F5
with a threshold of 1e−05
MR Test
Inverse variance weighted (fixed effects)
Inverse variance weighted (multiplicative random effects)
MR Egger
MR RAPS
Weighted median
Weighted mode
0.050
0.025
0.000
−0.025
0.09
0.12
0.15
0.18
SNP effect on Adhesion G protein−coupled receptor F5
SNP effect on Gut microbiota abundance (genus Catenibacterium id.2153)

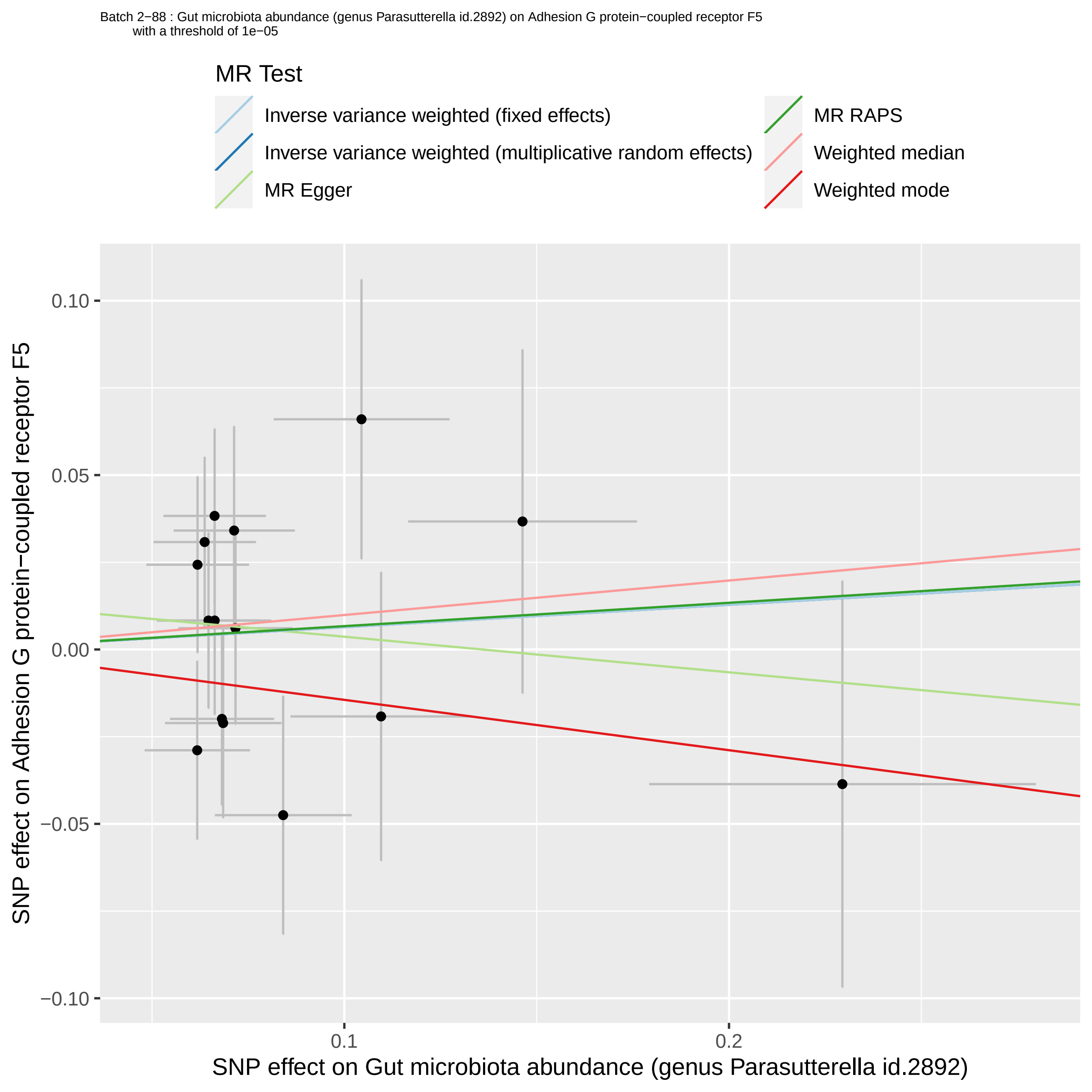
Batch 2−88 : Gut microbiota abundance (genus Parasutterella id.2892) on Adhesion G protein−coupled receptor F5
with a threshold of 1e−05
MR Test
Inverse variance weighted (fixed effects)
Inverse variance weighted (multiplicative random effects)
MR Egger
MR RAPS
Weighted median
Weighted mode
0.10
0.05
0.00
−0.05
−0.10
0.1
0.2
SNP effect on Adhesion G protein−coupled receptor F5
SNP effect on Gut microbiota abundance (genus Parasutterella id.2892)

Batch 2−89 : Gut microbiota abundance (genus Catenibacterium id.2153) on Adhesion G−protein coupled receptor G5
with a threshold of 1e−05

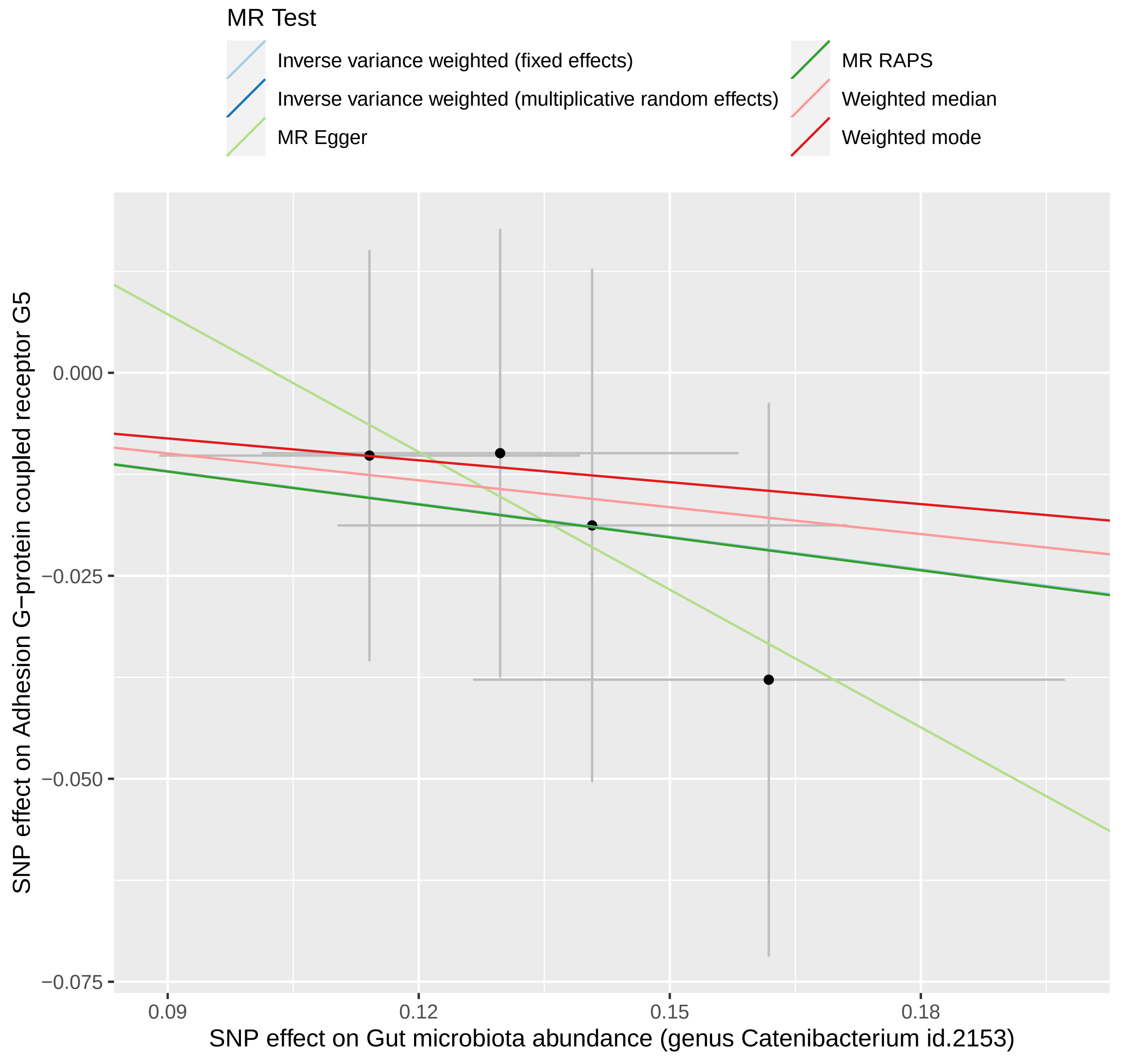

Batch 2−90 : Gut microbiota abundance (genus Parasutterella id.2892) on Adhesion G−protein coupled receptor G5
with a threshold of 1e−05

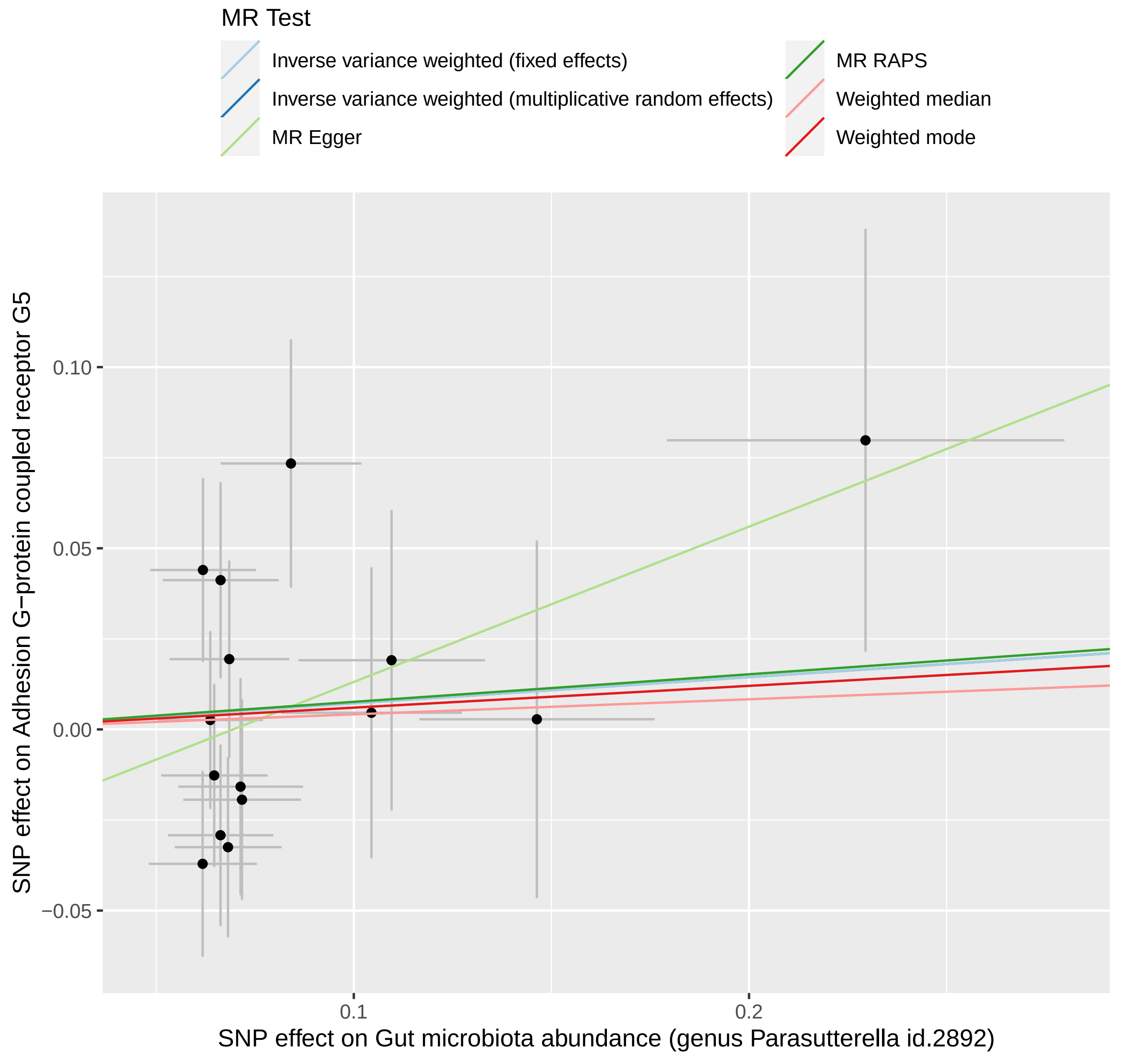

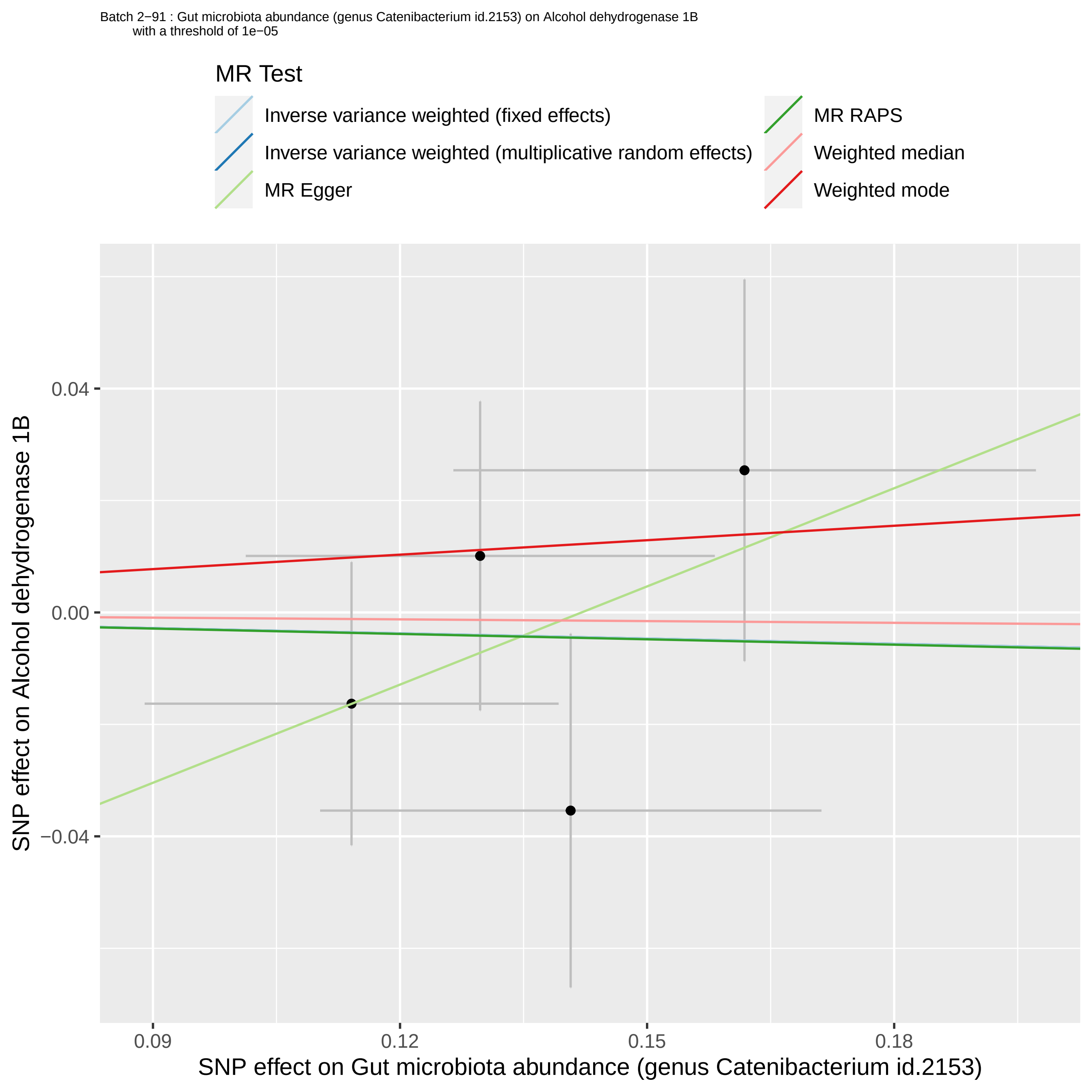
Batch 2−91 : Gut microbiota abundance (genus Catenibacterium id.2153) on Alcohol dehydrogenase 1B
with a threshold of 1e−05
MR Test
Inverse variance weighted (fixed effects)
Inverse variance weighted (multiplicative random effects)
MR Egger
MR RAPS
Weighted median
Weighted mode
0.04
0.00
−0.04
0.09
0.12
0.15
0.18
SNP effect on Alcohol dehydrogenase 1B
SNP effect on Gut microbiota abundance (genus Catenibacterium id.2153)

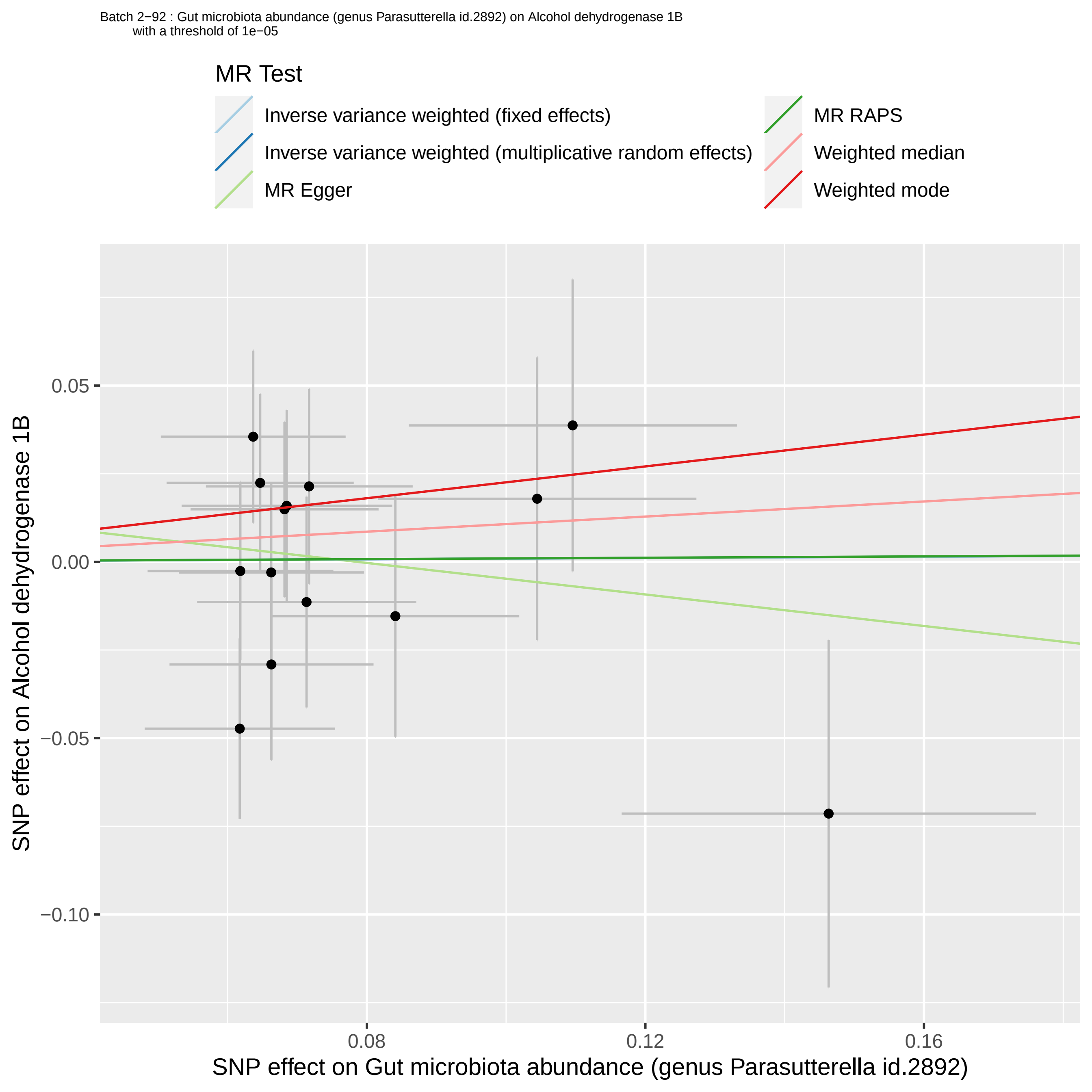

Batch 2−92 : Gut microbiota abundance (genus Parasutterella id.2892) on Alcohol dehydrogenase 1B
with a threshold of 1e−05
MR Test
Inverse variance weighted (fixed effects)
Inverse variance weighted (multiplicative random effects)
MR Egger
MR RAPS
Weighted median
Weighted mode
0.05
0.00
−0.05
−0.10
0.08
0.12
0.16
SNP effect on Alcohol dehydrogenase 1B
SNP effect on Gut microbiota abundance (genus Parasutterella id.2892)

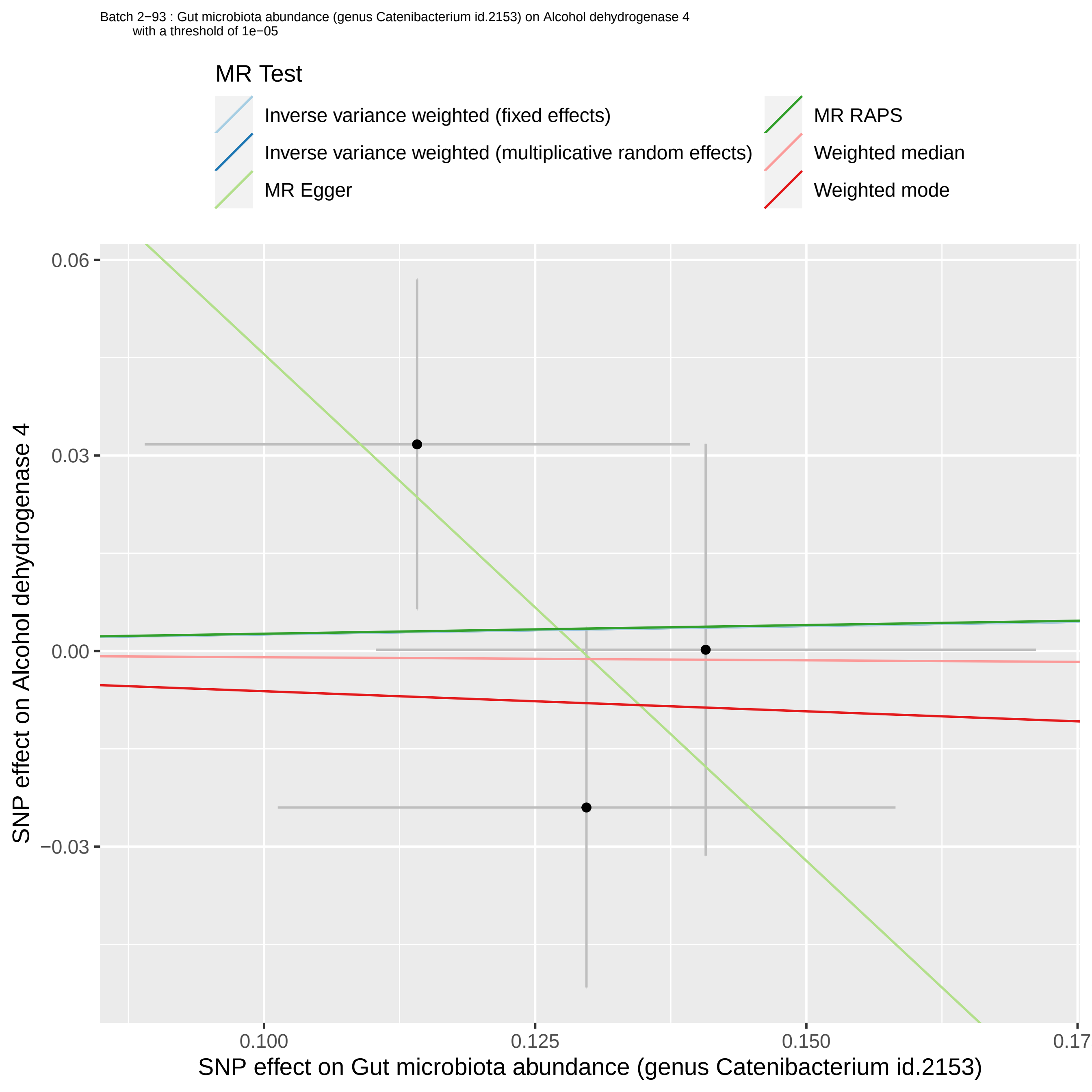
Batch 2−93 : Gut microbiota abundance (genus Catenibacterium id.2153) on Alcohol dehydrogenase 4
with a threshold of 1e−05
MR Test
Inverse variance weighted (fixed effects)
Inverse variance weighted (multiplicative random effects)
MR Egger
MR RAPS
Weighted median
Weighted mode
0.06
0.03
0.00
−0.03
0.100
0.125
0.150
0.17
SNP effect on Alcohol dehydrogenase 4
SNP effect on Gut microbiota abundance (genus Catenibacterium id.2153)

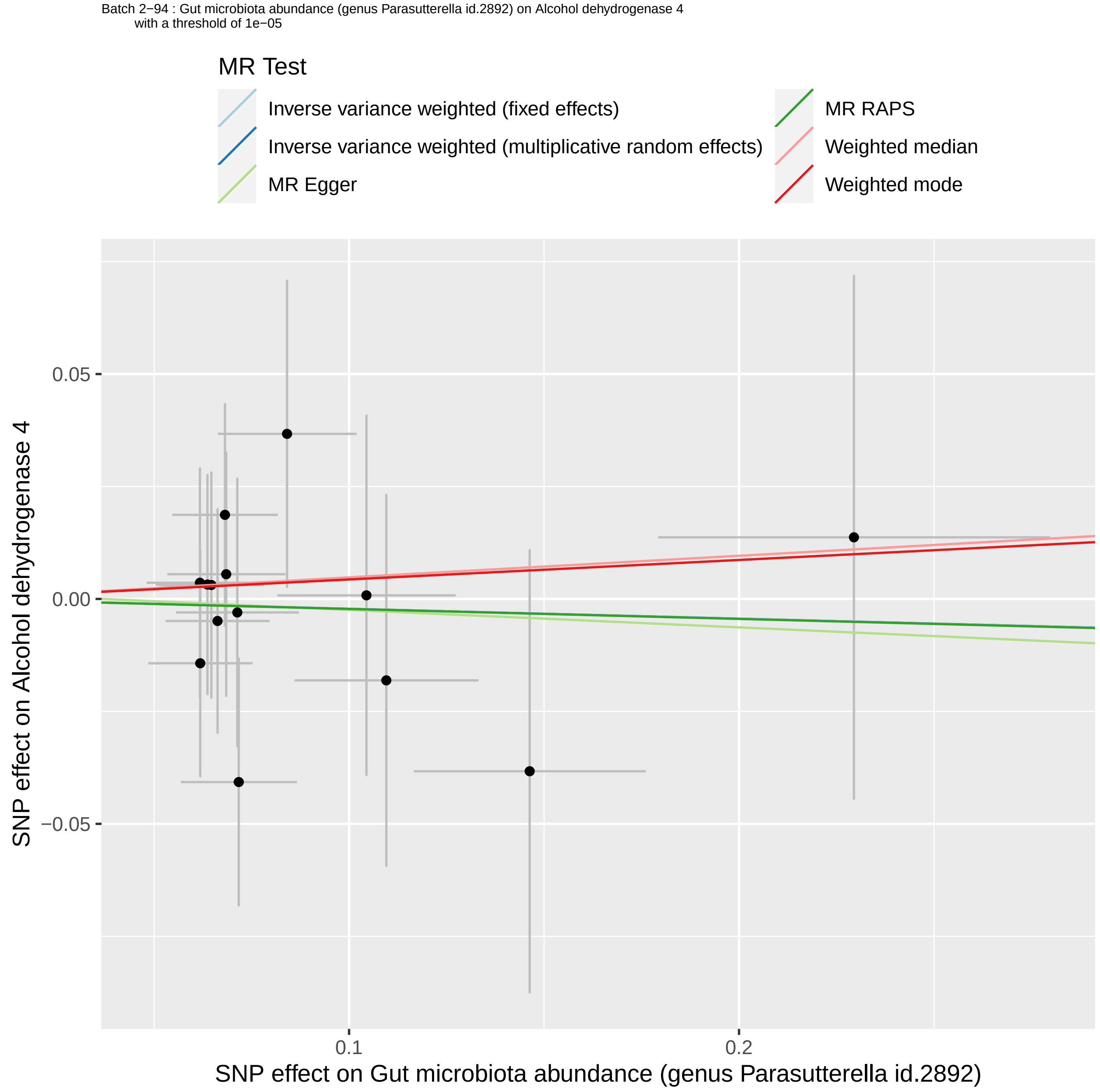
Batch 2−94 : Gut microbiota abundance (genus Parasutterella id.2892) on Alcohol dehydrogenase 4
with a threshold of 1e−05
MR Test
Inverse variance weighted (fixed effects)
Inverse variance weighted (multiplicative random effects)
MR Egger
MR RAPS
Weighted median
Weighted mode
0.05
0.00
−0.05
0.1
0.2
SNP effect on Alcohol dehydrogenase 4
SNP effect on Gut microbiota abundance (genus Parasutterella id.2892)

Batch 2−95 : Gut microbiota abundance (genus Catenibacterium id.2153) on Adrenomedullin
with a threshold of 1e−05

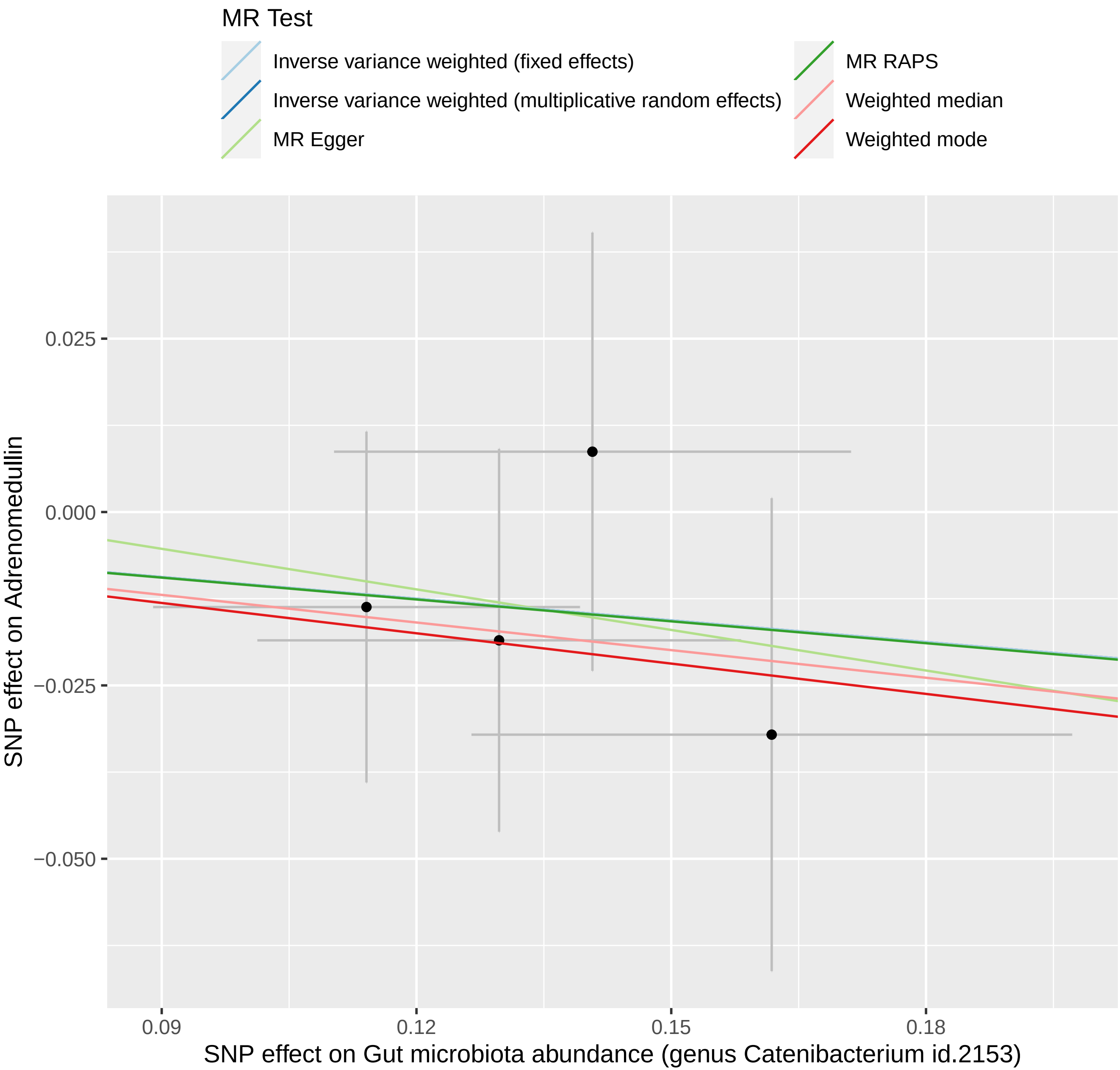

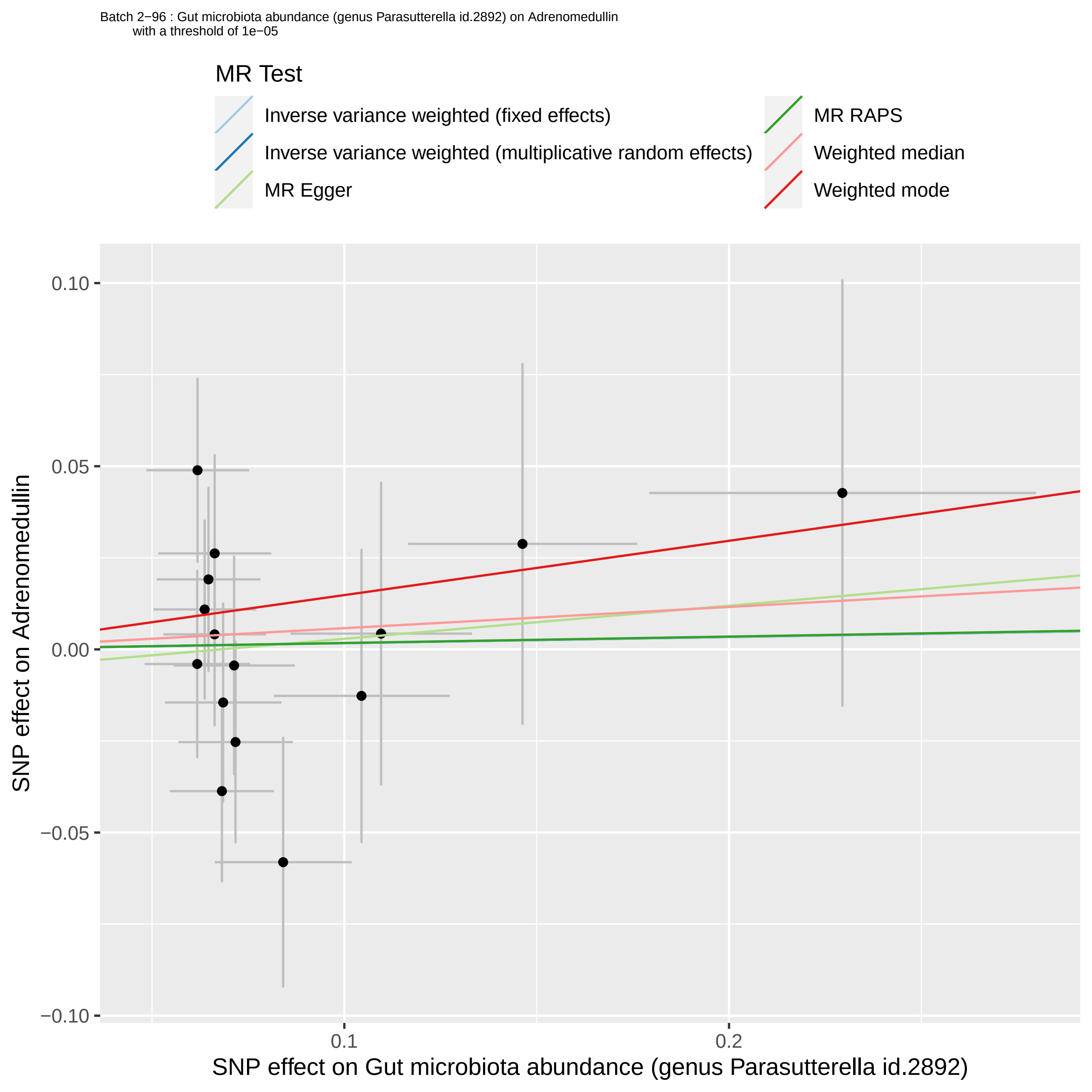

Batch 2−96 : Gut microbiota abundance (genus Parasutterella id.2892) on Adrenomedullin
with a threshold of 1e−05
MR Test
Inverse variance weighted (fixed effects)
Inverse variance weighted (multiplicative random effects)
MR Egger
MR RAPS
Weighted median
Weighted mode
0.10
0.05
0.00
−0.05
−0.10
0.1
0.2
SNP effect on Adrenomedullin
SNP effect on Gut microbiota abundance (genus Parasutterella id.2892)

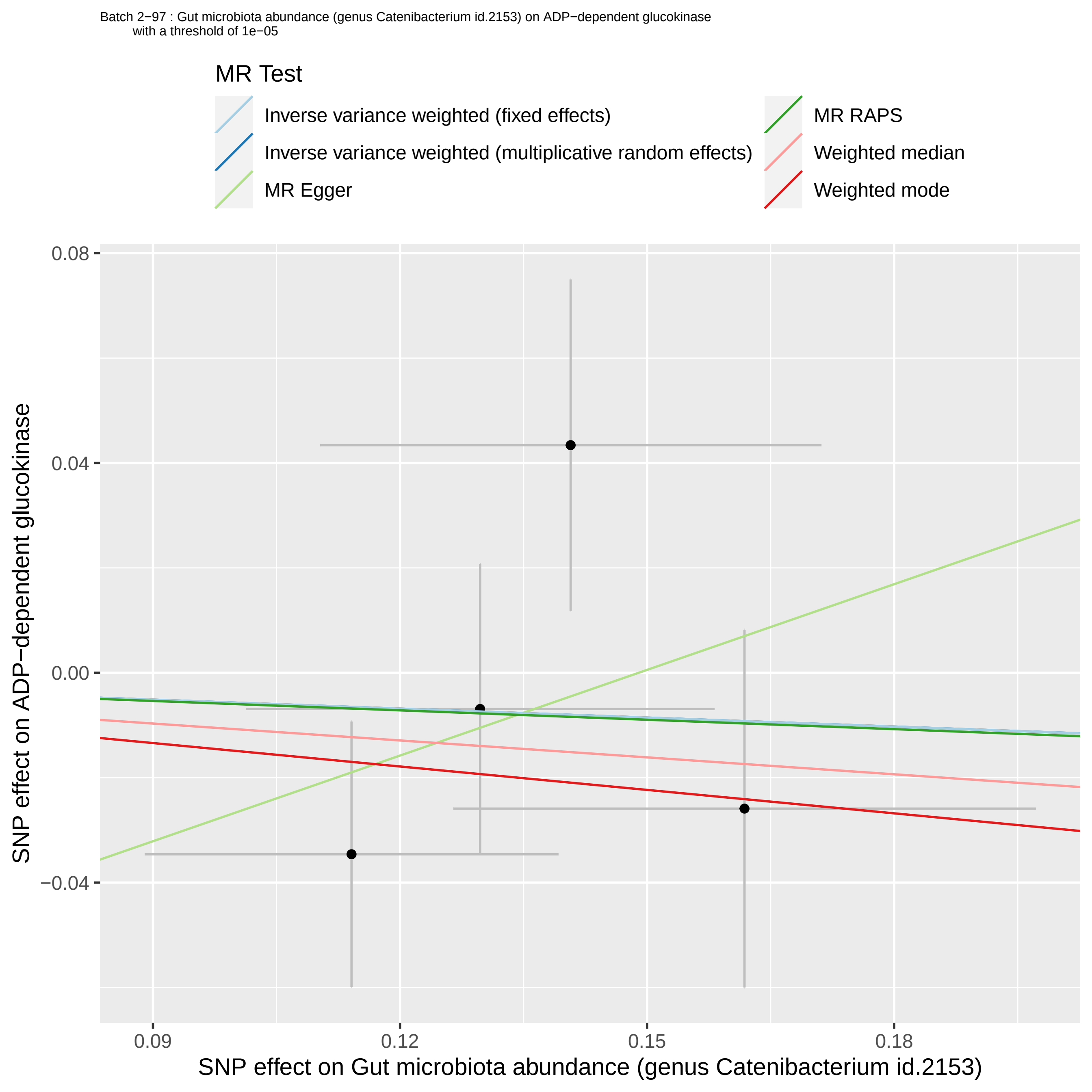

Batch 2−97 : Gut microbiota abundance (genus Catenibacterium id.2153) on ADP−dependent glucokinase
with a threshold of 1e−05
MR Test
Inverse variance weighted (fixed effects)
Inverse variance weighted (multiplicative random effects)
MR Egger
MR RAPS
Weighted median
Weighted mode
0.08
0.04
0.00
−0.04
0.09
0.12
0.15
0.18
SNP effect on ADP−dependent glucokinase
SNP effect on Gut microbiota abundance (genus Catenibacterium id.2153)

Batch 2−98 : Gut microbiota abundance (genus Parasutterella id.2892) on ADP−dependent glucokinase
with a threshold of 1e−05

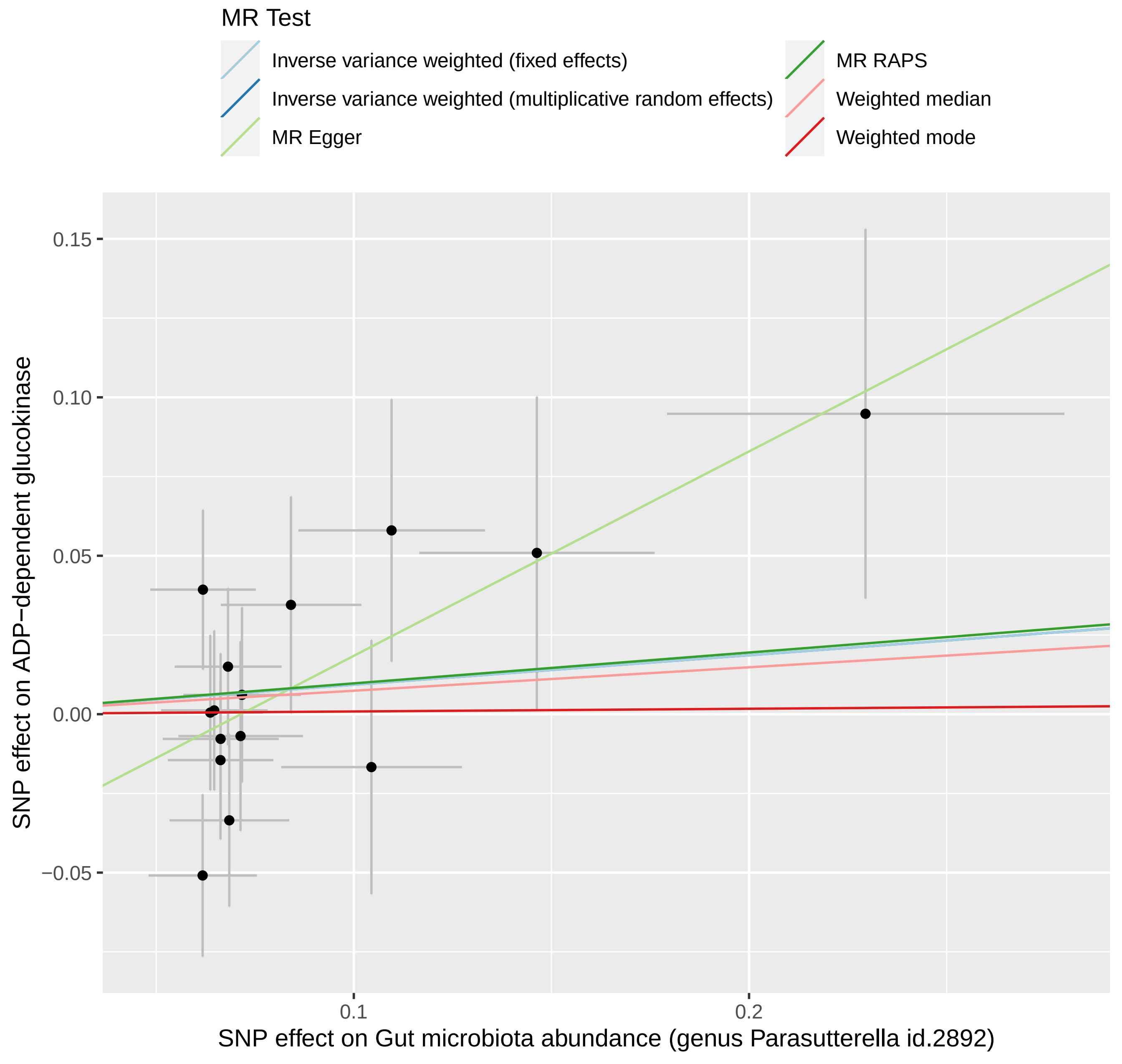

Batch 2−99 : Gut microbiota abundance (genus Catenibacterium id.2153) on beta−adrenergic receptor kinase 1
with a threshold of 1e−05

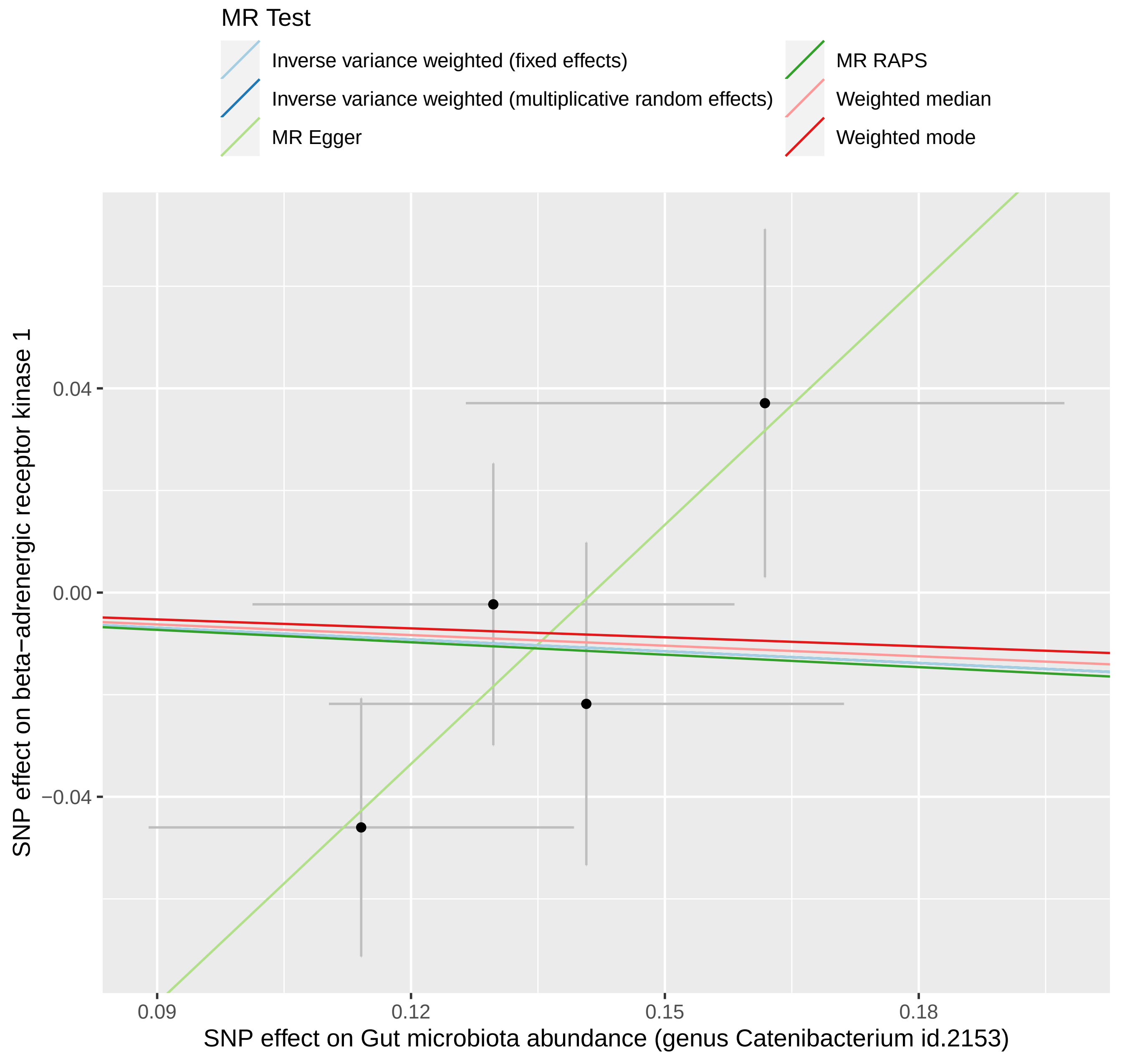

Batch 2−100 : Gut microbiota abundance (genus Parasutterella id.2892) on beta−adrenergic receptor kinase 1
with a threshold of 1e−05

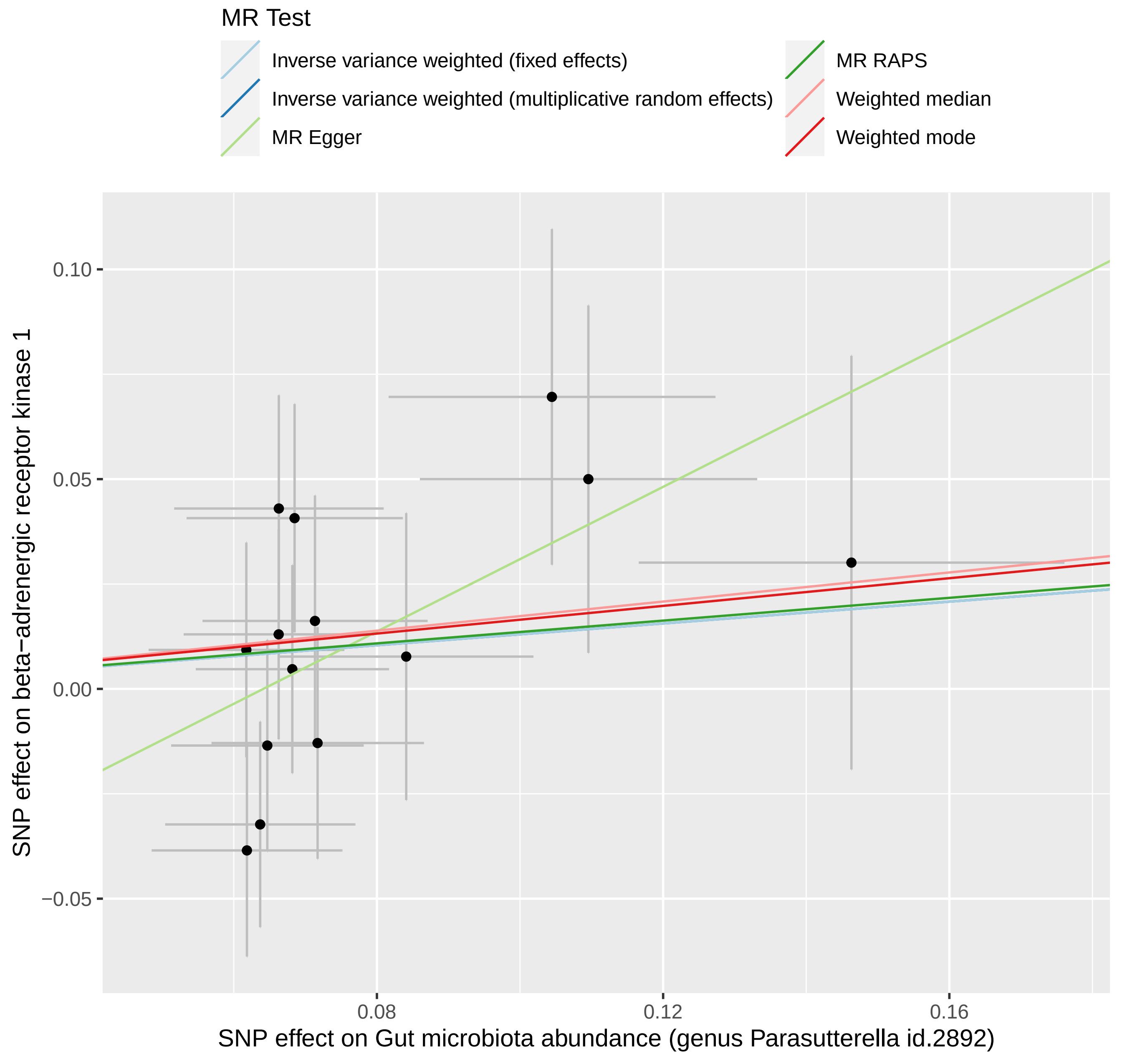

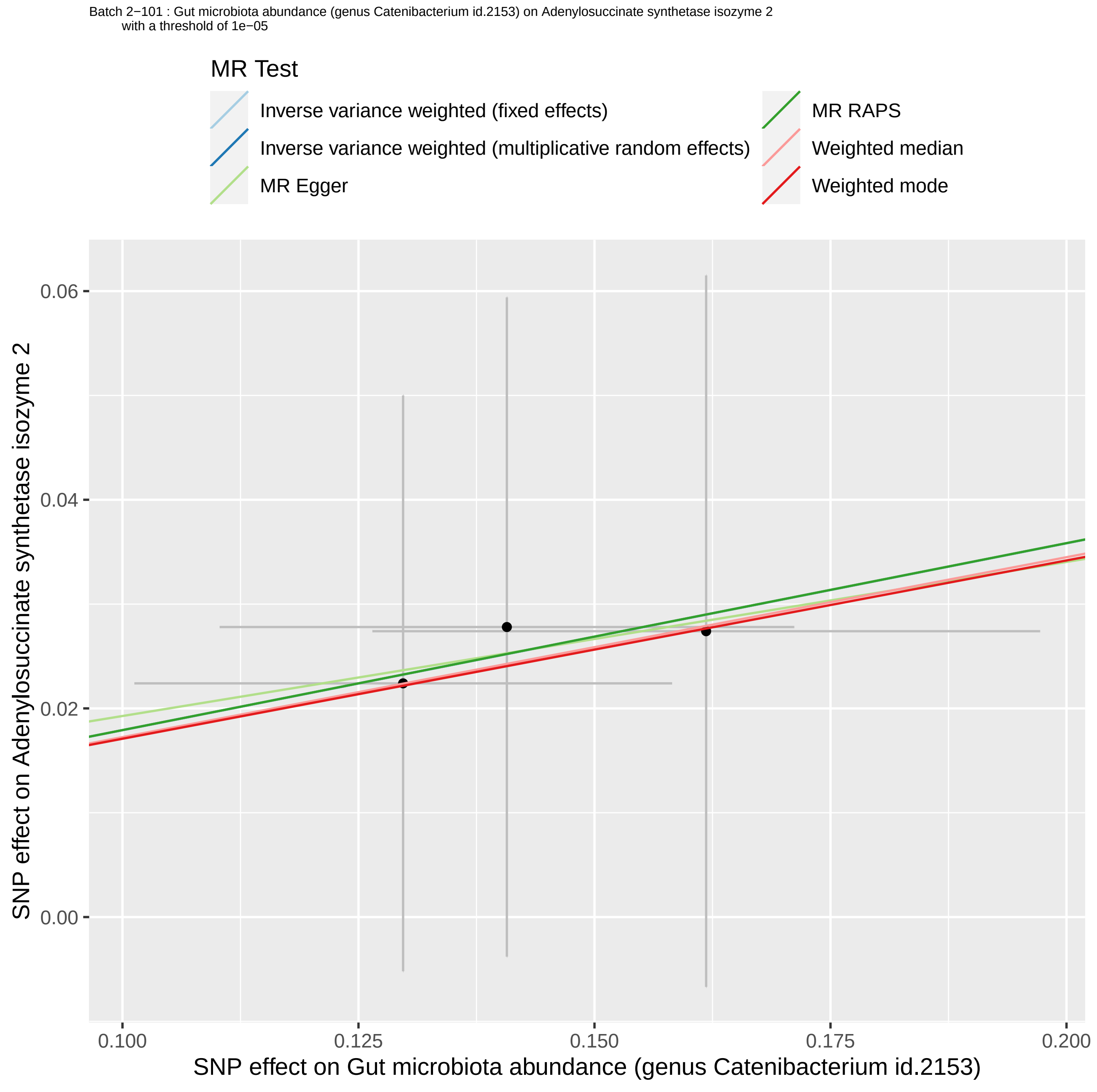
Batch 2−101 : Gut microbiota abundance (genus Catenibacterium id.2153) on Adenylosuccinate synthetase isozyme 2
with a threshold of 1e−05
MR Test
Inverse variance weighted (fixed effects)
Inverse variance weighted (multiplicative random effects)
MR Egger
MR RAPS
Weighted median
Weighted mode
SNP effect on Adenylosuccinate synthetase isozyme 2
0.06
0.04
0.02
0.00
0.100
0.125
0.150
0.175
0.200
SNP effect on Gut microbiota abundance (genus Catenibacterium id.2153)

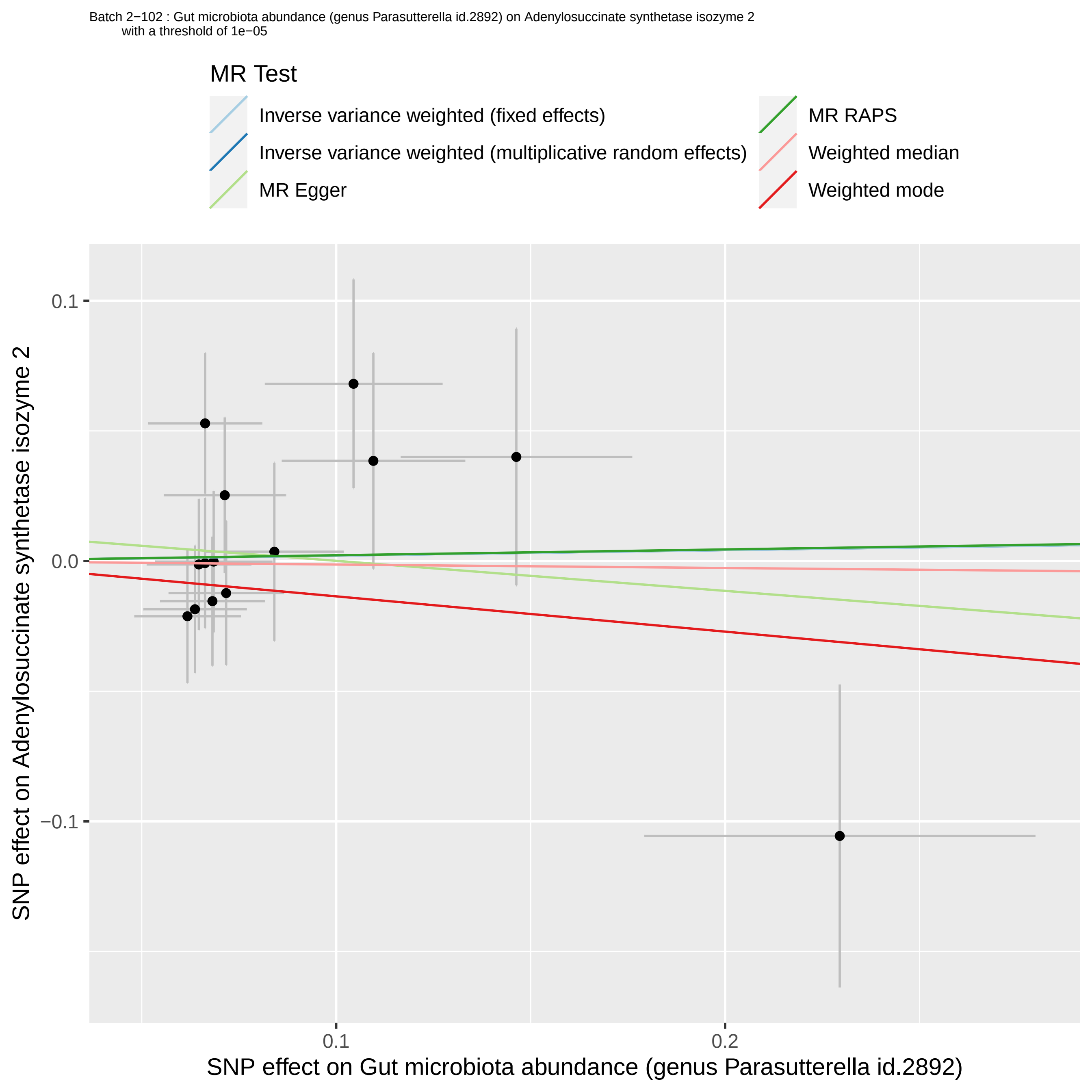
Batch 2−102 : Gut microbiota abundance (genus Parasutterella id.2892) on Adenylosuccinate synthetase isozyme 2
with a threshold of 1e−05
MR Test
Inverse variance weighted (fixed effects)
Inverse variance weighted (multiplicative random effects)
MR Egger
MR RAPS
Weighted median
Weighted mode
0.1
0.0
−0.1
0.1
0.2
SNP effect on Adenylosuccinate synthetase isozyme 2
SNP effect on Gut microbiota abundance (genus Parasutterella id.2892)

Batch 2−103 : Gut microbiota abundance (genus Catenibacterium id.2153) on Adenylosuccinate synthetase isozyme 1
with a threshold of 1e−05

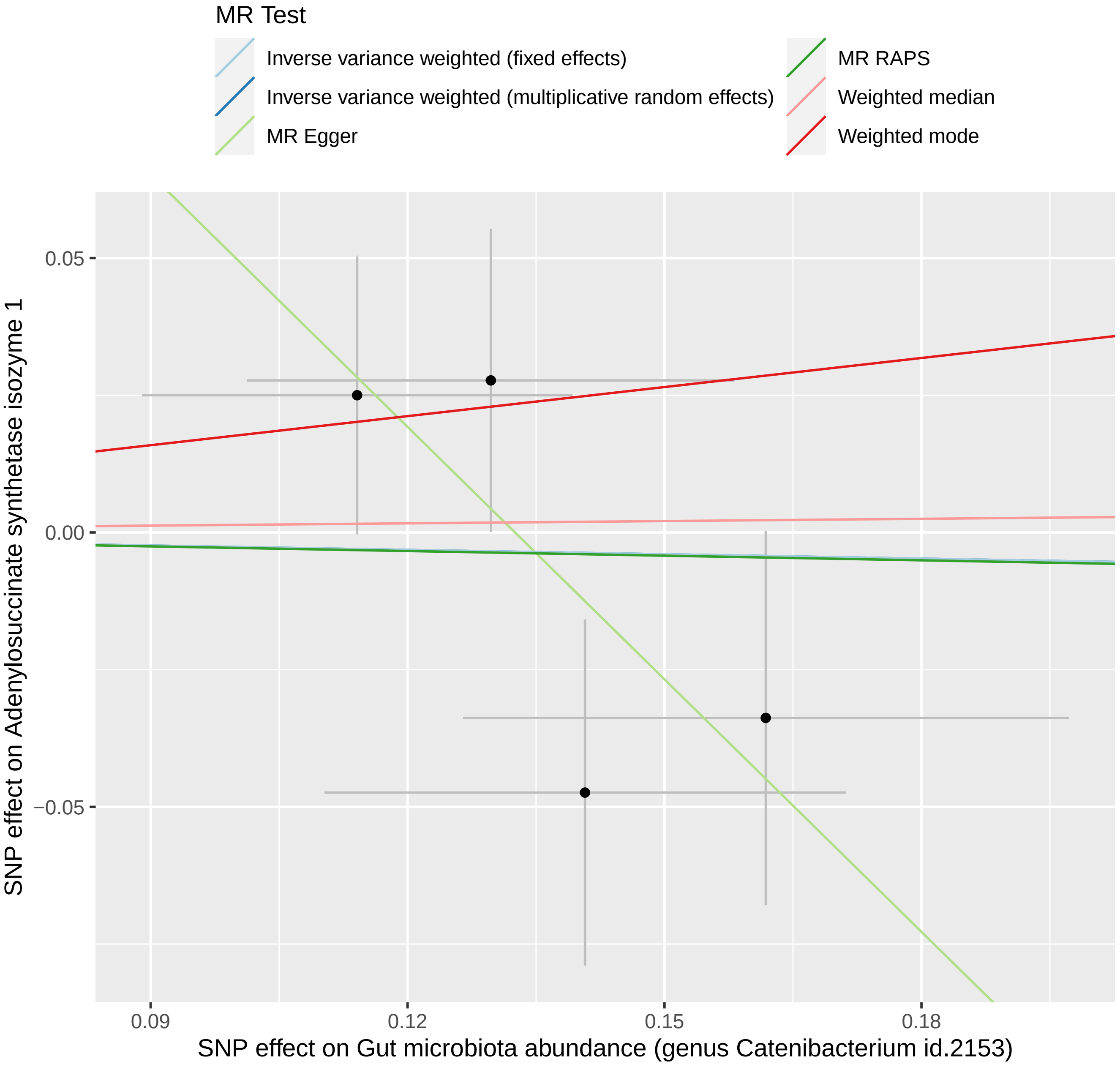

Batch 2−104 : Gut microbiota abundance (genus Parasutterella id.2892) on Adenylosuccinate synthetase isozyme 1
with a threshold of 1e−05

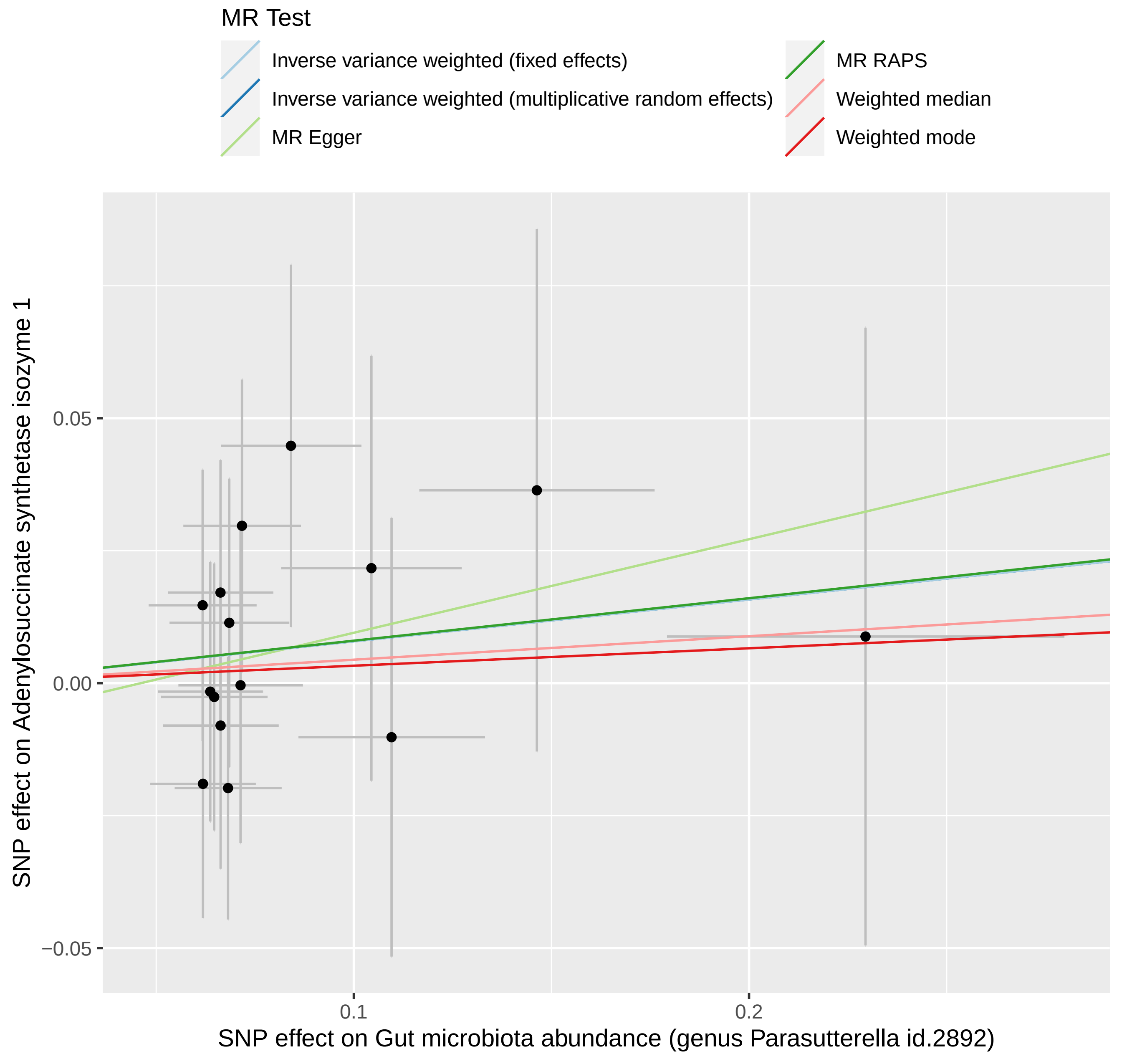

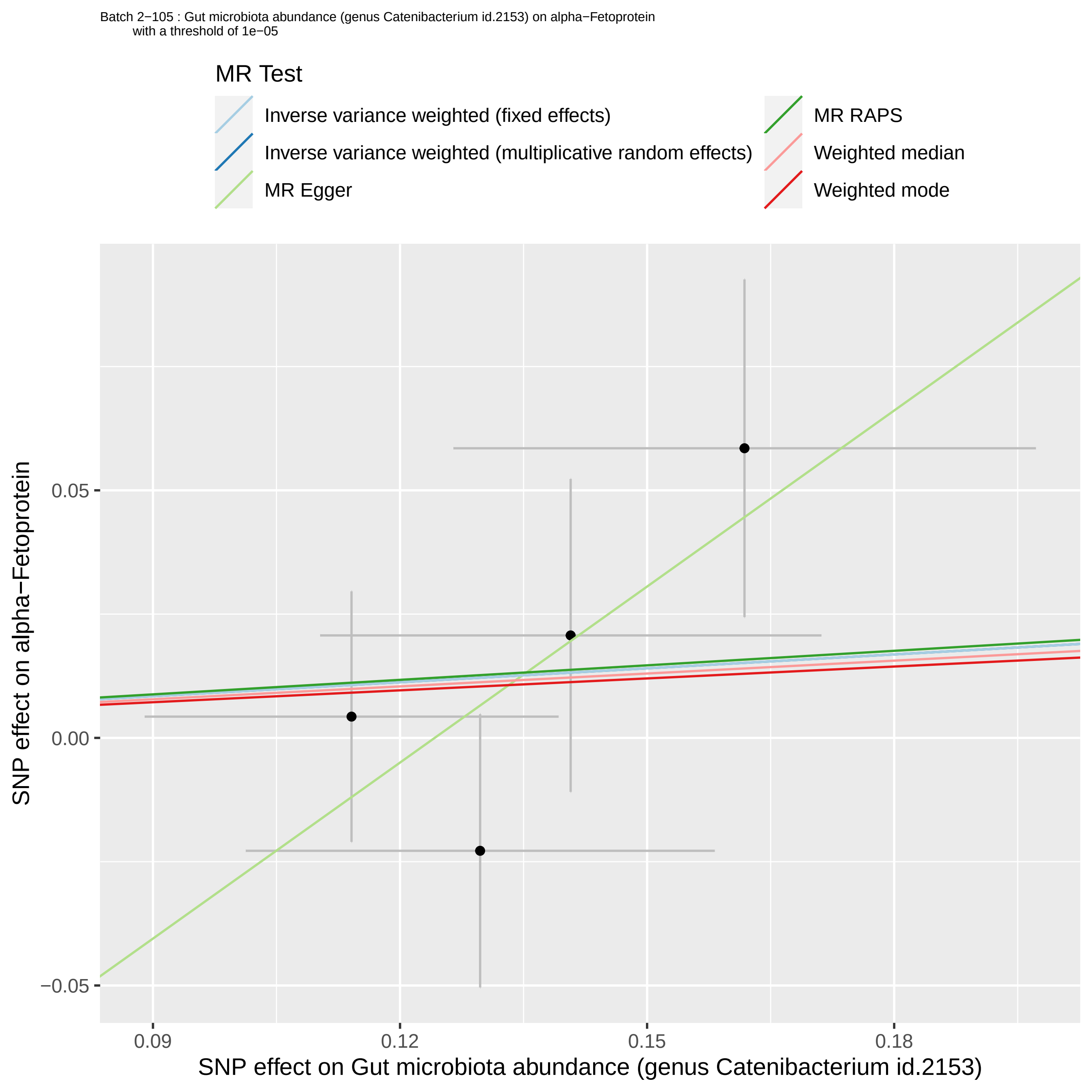
Batch 2−105 : Gut microbiota abundance (genus Catenibacterium id.2153) on alpha−Fetoprotein
with a threshold of 1e−05
MR Test
Inverse variance weighted (fixed effects)
Inverse variance weighted (multiplicative random effects)
MR Egger
MR RAPS
Weighted median
Weighted mode
0.05
0.00
−0.05
0.09
0.12
0.15
0.18
SNP effect on alpha−Fetoprotein
SNP effect on Gut microbiota abundance (genus Catenibacterium id.2153)

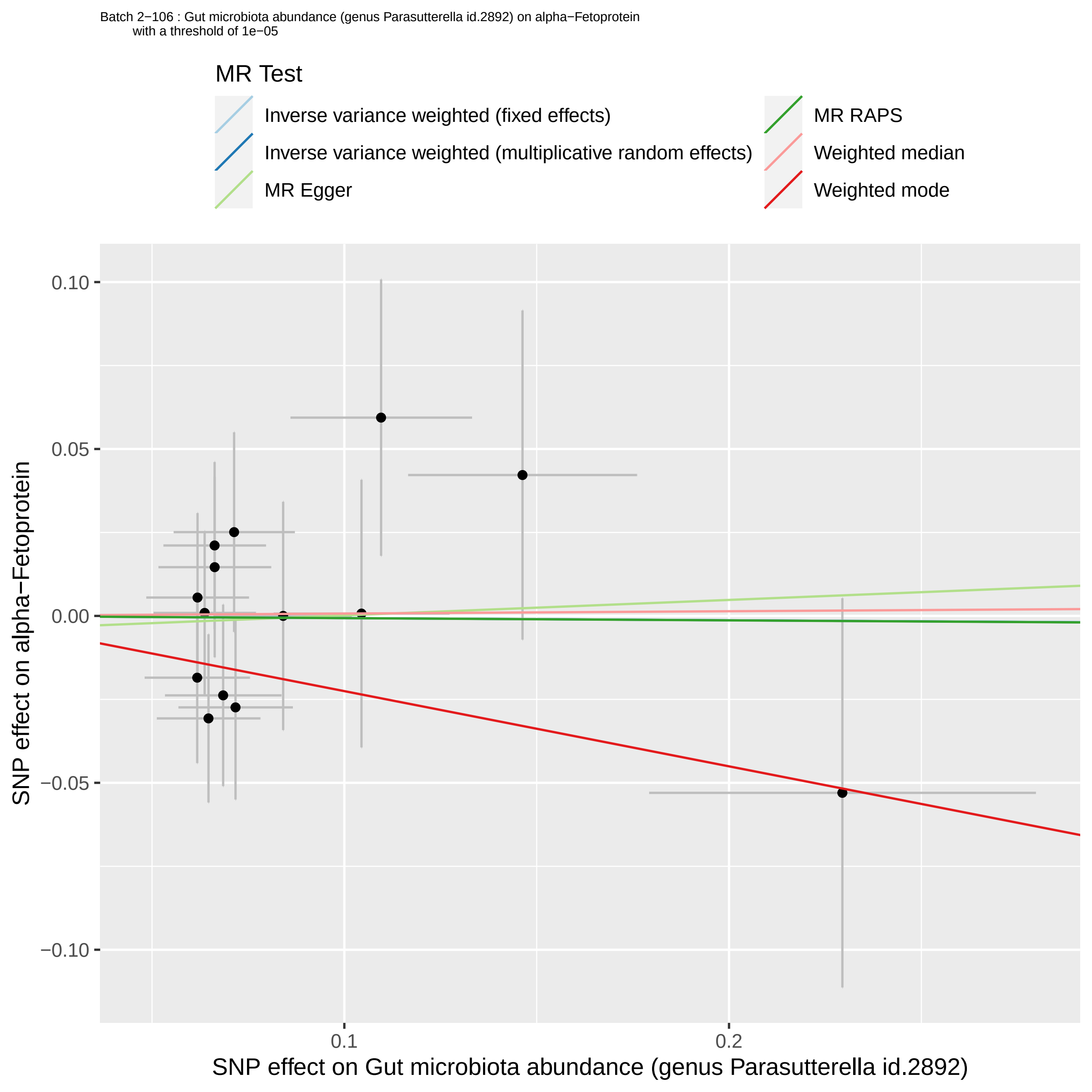

Batch 2−106 : Gut microbiota abundance (genus Parasutterella id.2892) on alpha−Fetoprotein
with a threshold of 1e−05
MR Test
Inverse variance weighted (fixed effects)
Inverse variance weighted (multiplicative random effects)
MR Egger
MR RAPS
Weighted median
Weighted mode
SNP effect on alpha−Fetoprotein
0.10
0.05
0.00
−0.05
−0.10
0.1
0.2
SNP effect on Gut microbiota abundance (genus Parasutterella id.2892)

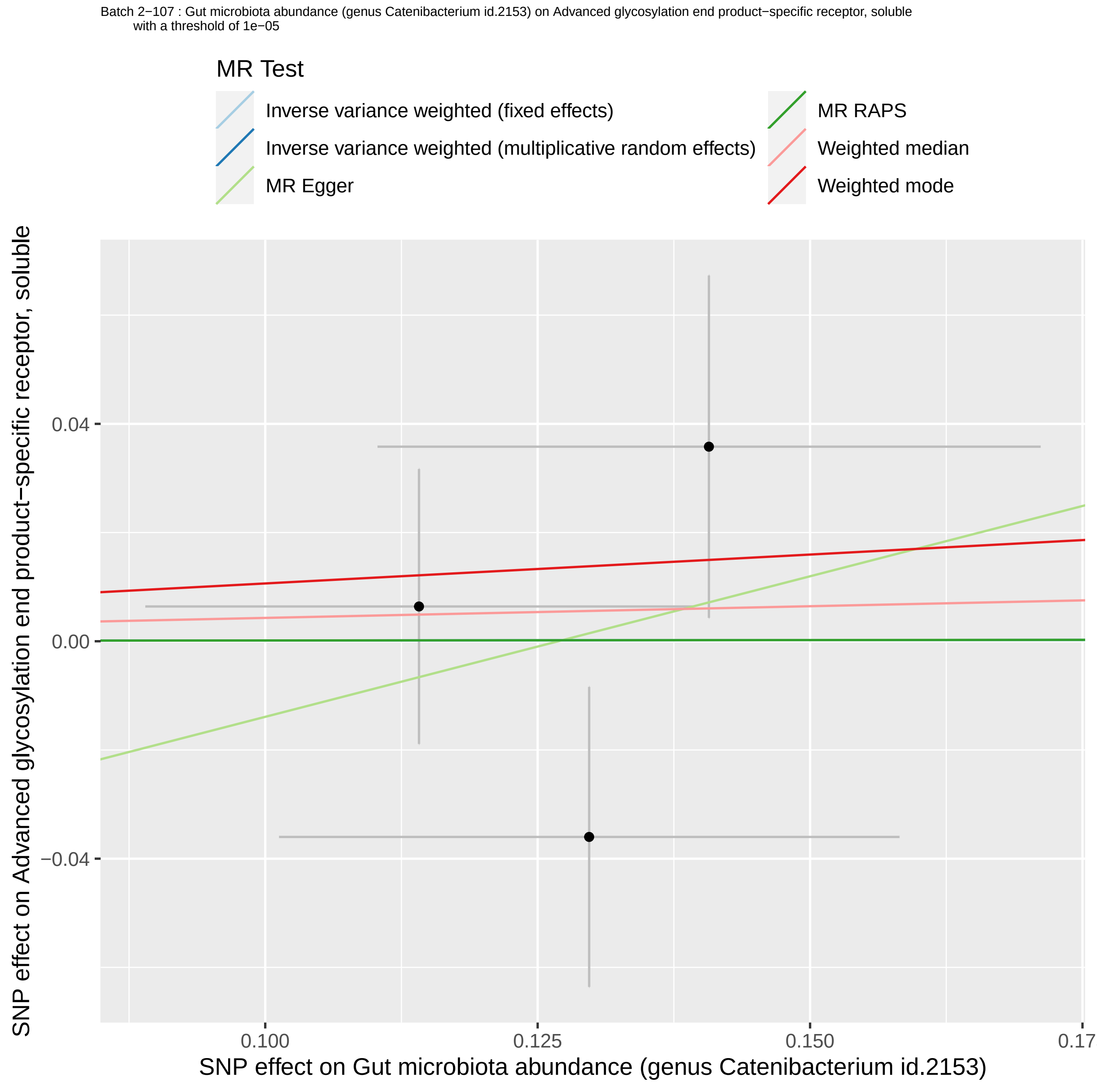
Batch 2−107 : Gut microbiota abundance (genus Catenibacterium id.2153) on Advanced glycosylation end product−specific receptor, soluble
with a threshold of 1e−05
MR Test
Inverse variance weighted (fixed effects)
Inverse variance weighted (multiplicative random effects)
MR Egger
MR RAPS
Weighted median
Weighted mode
SNP effect on Advanced glycosylation end product−specific receptor, soluble
0.04
0.00
−0.04
0.100
0.125
0.150
0.17
SNP effect on Gut microbiota abundance (genus Catenibacterium id.2153)

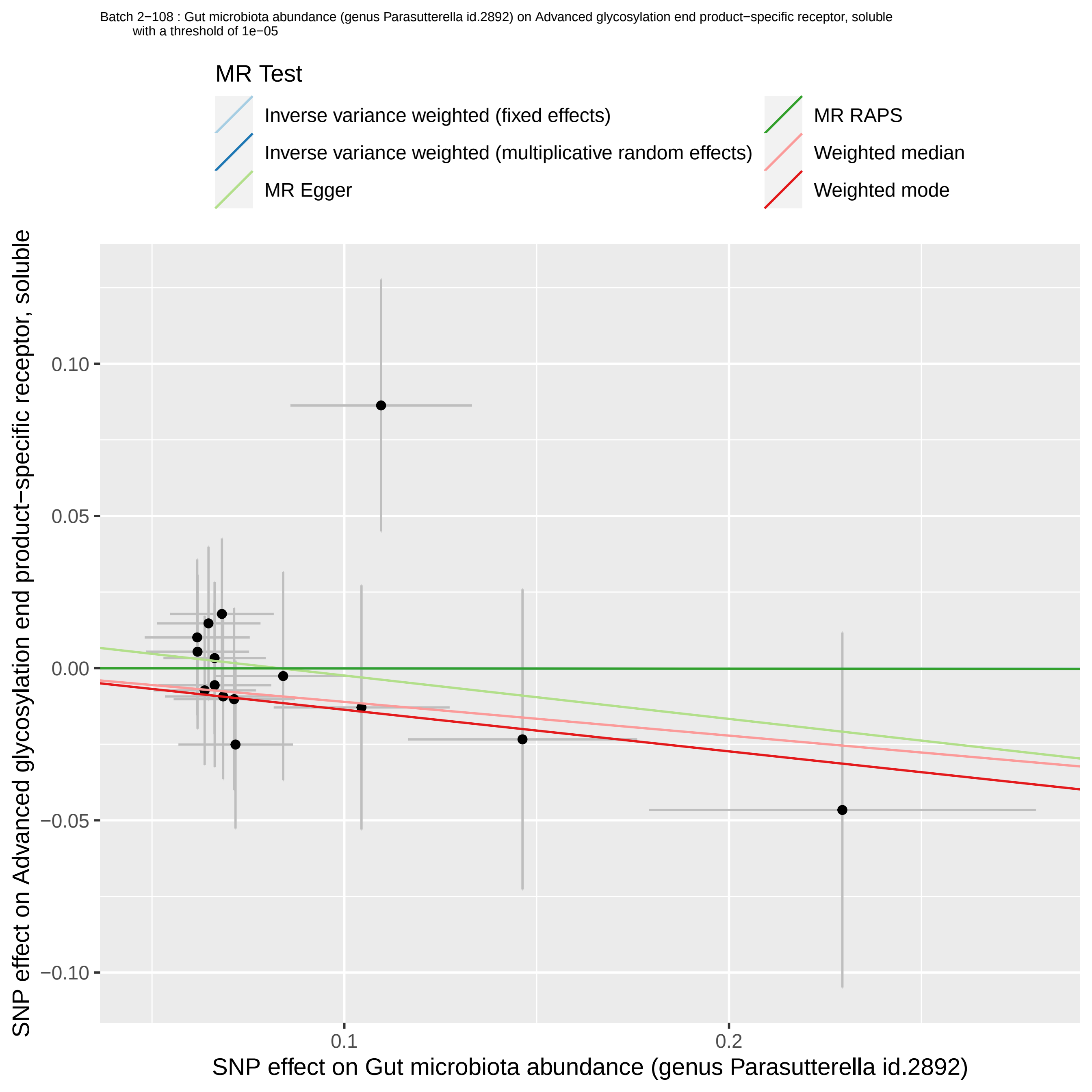

Batch 2−108 : Gut microbiota abundance (genus Parasutterella id.2892) on Advanced glycosylation end product−specific receptor, soluble
with a threshold of 1e−05
MR Test
Inverse variance weighted (fixed effects)
Inverse variance weighted (multiplicative random effects)
MR Egger
MR RAPS
Weighted median
Weighted mode
0.10
0.05
0.00
−0.05
−0.10
0.1
0.2
SNP effect on Advanced glycosylation end product−specific receptor, soluble
SNP effect on Gut microbiota abundance (genus Parasutterella id.2892)

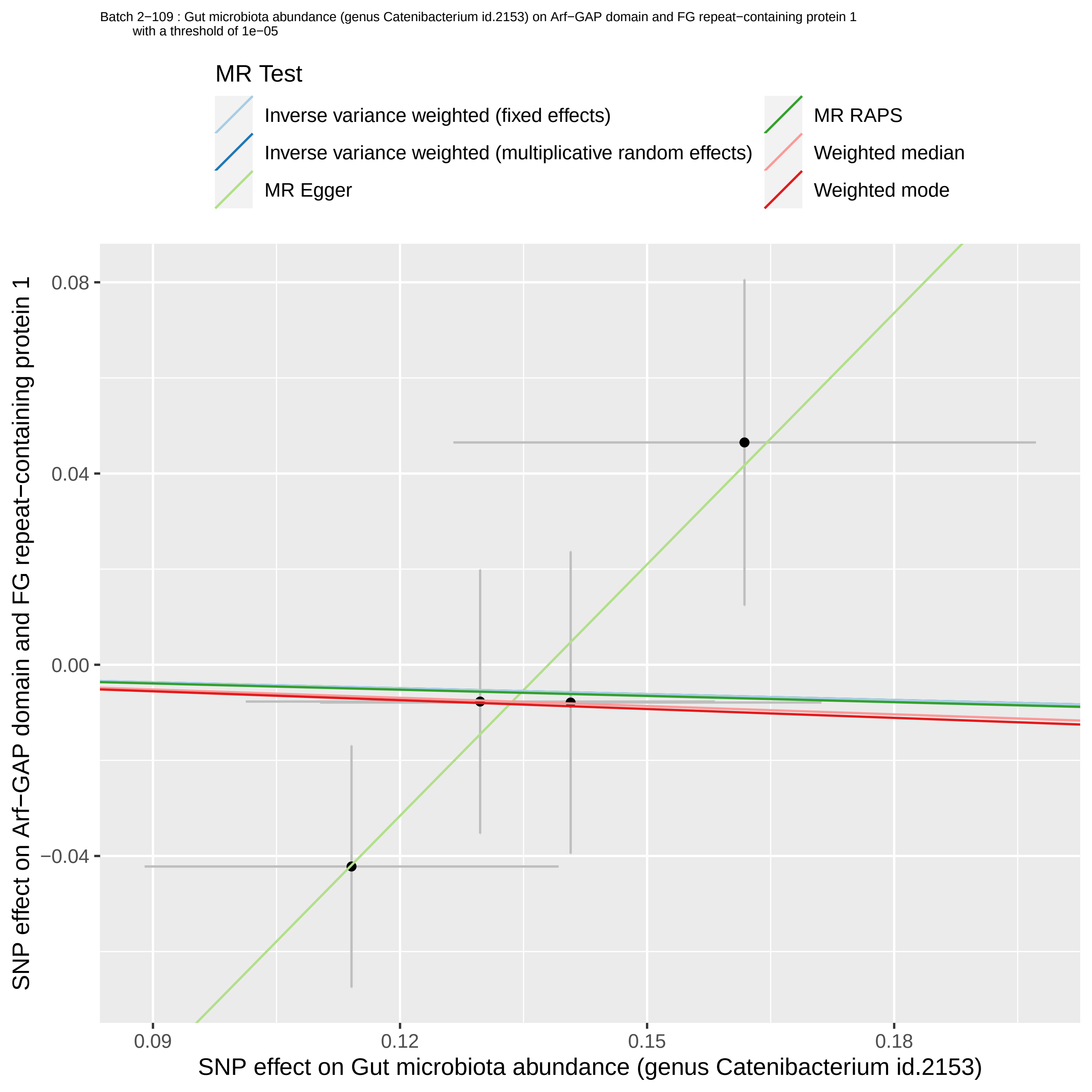
Batch 2−109 : Gut microbiota abundance (genus Catenibacterium id.2153) on Arf−GAP domain and FG repeat−containing protein 1
with a threshold of 1e−05
MR Test
Inverse variance weighted (fixed effects)
Inverse variance weighted (multiplicative random effects)
MR Egger
MR RAPS
Weighted median
Weighted mode
0.08
0.04
0.00
−0.04
0.09
0.12
0.15
0.18
SNP effect on Arf−GAP domain and FG repeat−containing protein 1
SNP effect on Gut microbiota abundance (genus Catenibacterium id.2153)

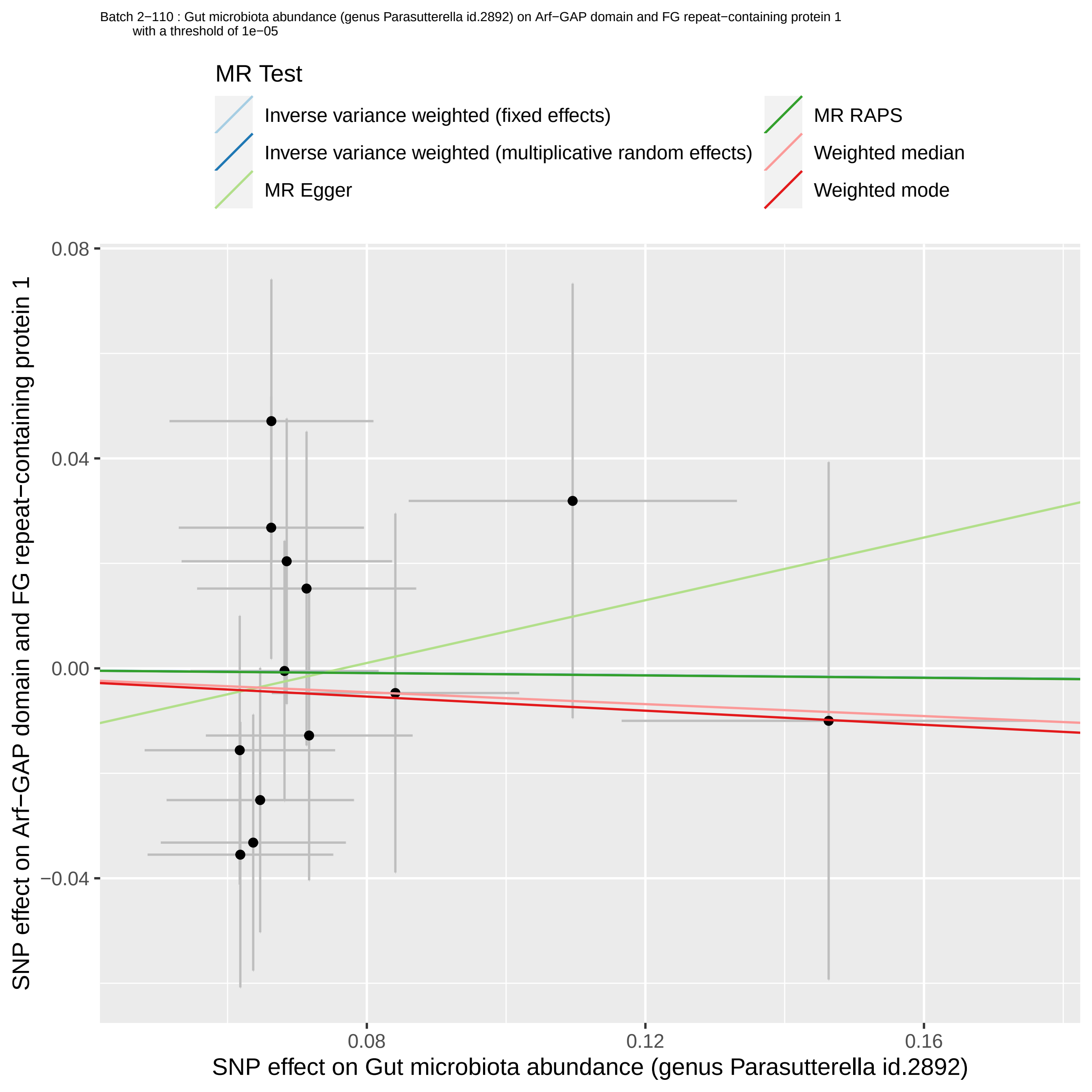
Batch 2−110 : Gut microbiota abundance (genus Parasutterella id.2892) on Arf−GAP domain and FG repeat−containing protein 1
with a threshold of 1e−05
MR Test
Inverse variance weighted (fixed effects)
Inverse variance weighted (multiplicative random effects)
MR Egger
MR RAPS
Weighted median
Weighted mode
SNP effect on Arf−GAP domain and FG repeat−containing protein 1
0.08
0.04
0.00
−0.04
0.08
0.12
0.16
SNP effect on Gut microbiota abundance (genus Parasutterella id.2892)

Batch 2−111 : Gut microbiota abundance (genus Catenibacterium id.2153) on Angiogenic factor with G patch and FHA domains 1
with a threshold of 1e−05

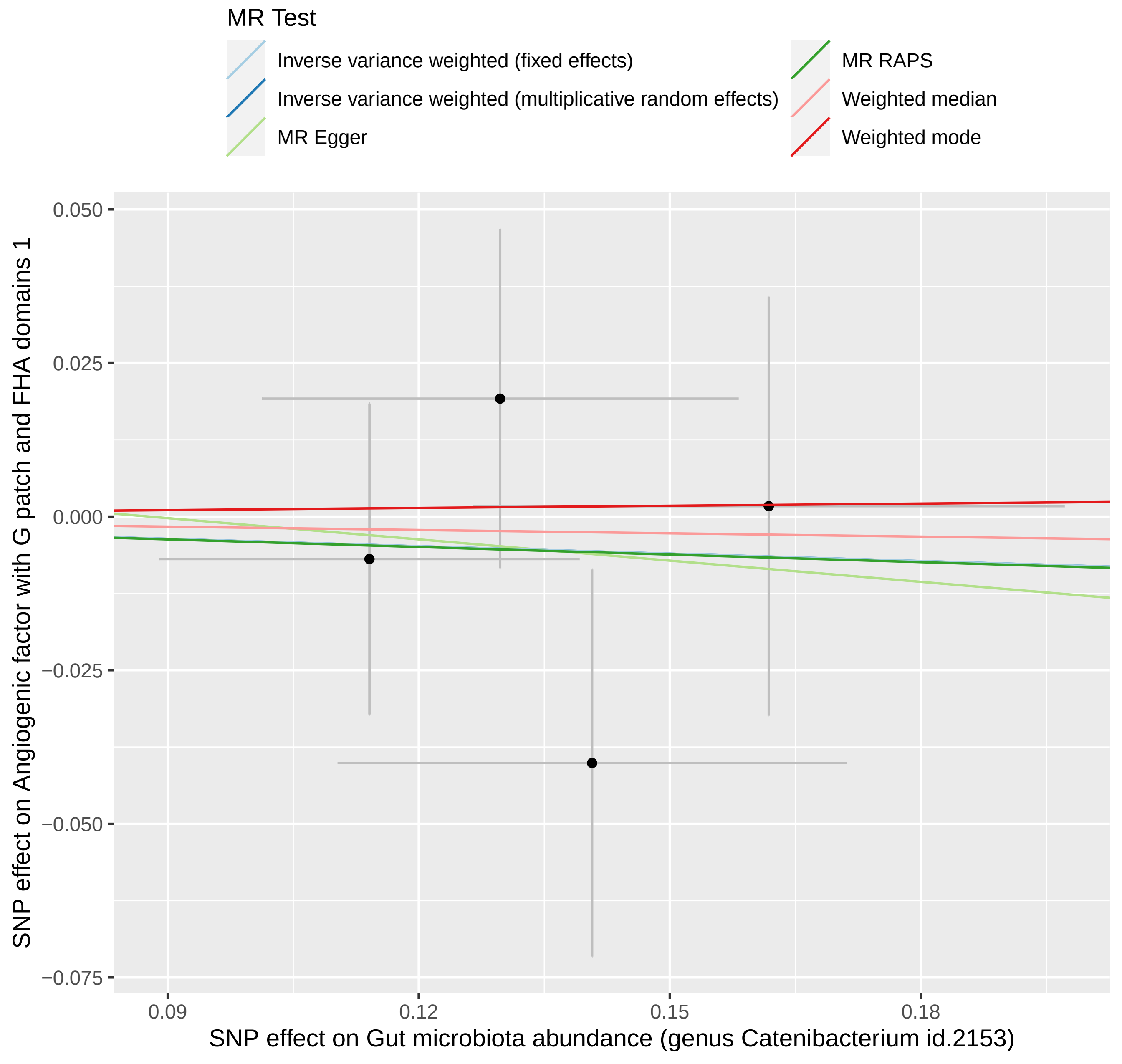

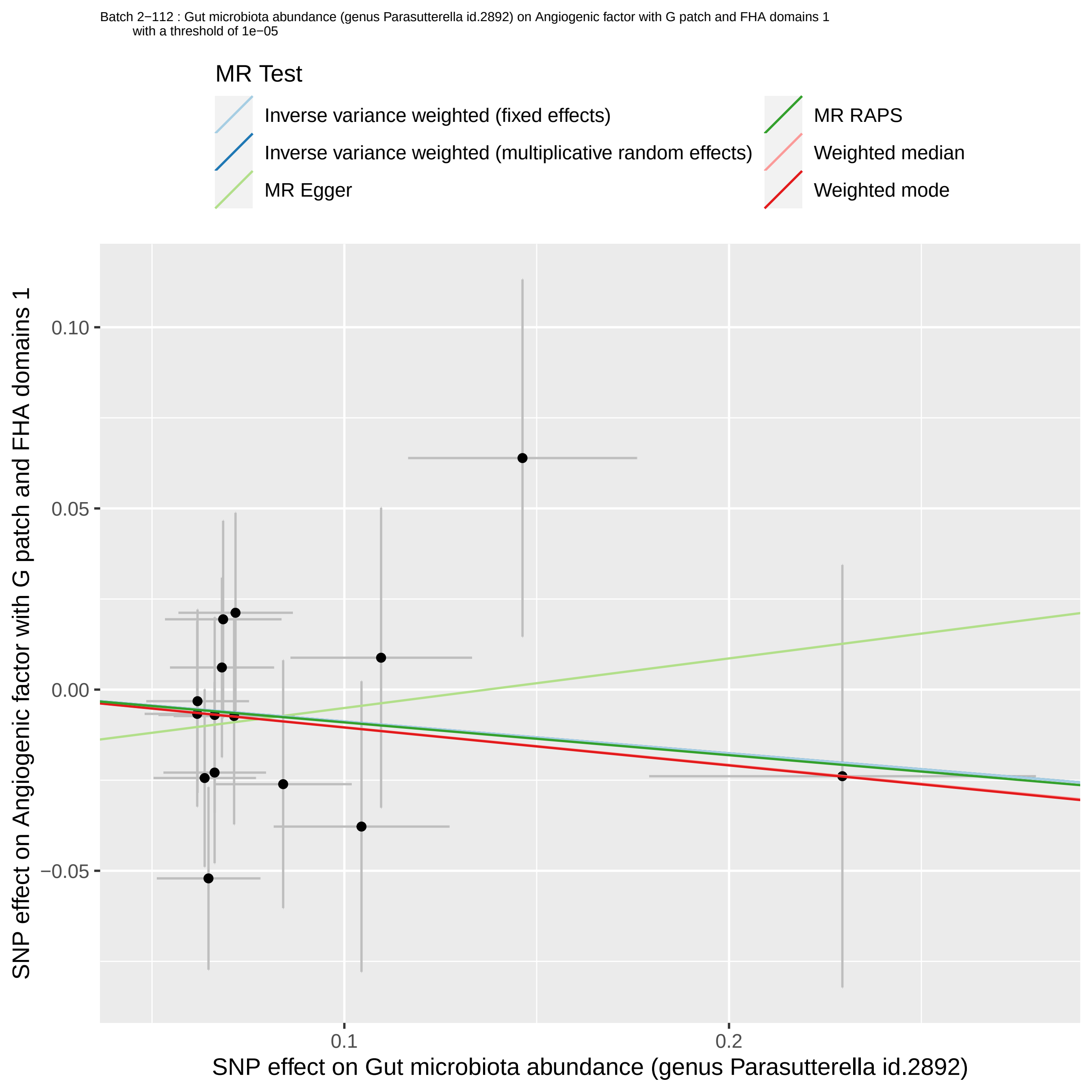

Batch 2−112 : Gut microbiota abundance (genus Parasutterella id.2892) on Angiogenic factor with G patch and FHA domains 1
with a threshold of 1e−05
MR Test
Inverse variance weighted (fixed effects)
Inverse variance weighted (multiplicative random effects)
MR Egger
MR RAPS
Weighted median
Weighted mode
0.10
0.05
0.00
−0.05
0.1
0.2
SNP effect on Angiogenic factor with G patch and FHA domains 1
SNP effect on Gut microbiota abundance (genus Parasutterella id.2892)

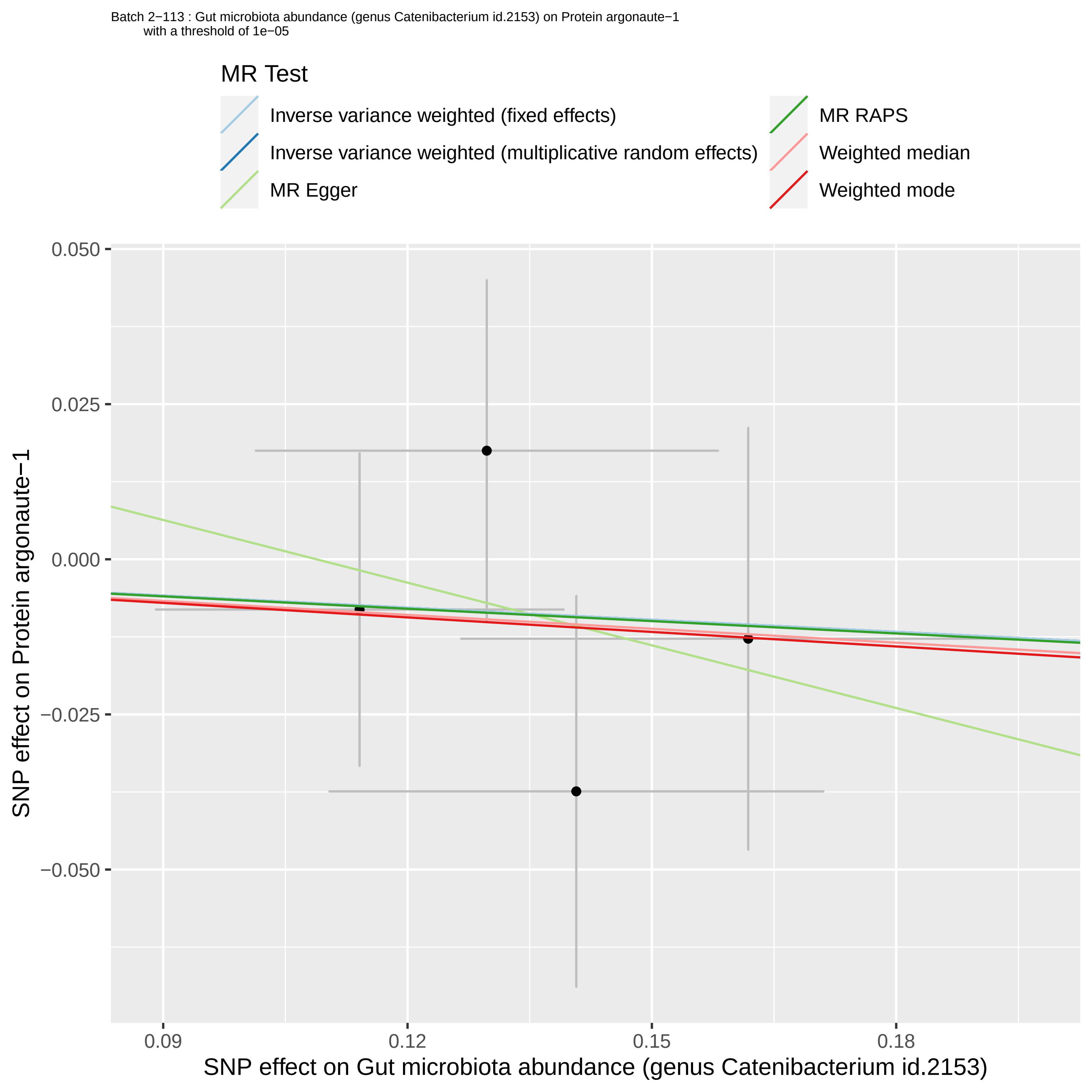

Batch 2−113 : Gut microbiota abundance (genus Catenibacterium id.2153) on Protein argonaute−1
with a threshold of 1e−05
MR Test
Inverse variance weighted (fixed effects)
Inverse variance weighted (multiplicative random effects)
MR Egger
MR RAPS
Weighted median
Weighted mode
0.050
0.025
0.000
−0.025
−0.050
0.09
0.12
0.15
0.18
SNP effect on Protein argonaute−1
SNP effect on Gut microbiota abundance (genus Catenibacterium id.2153)

Batch 2−114 : Gut microbiota abundance (genus Parasutterella id.2892) on Protein argonaute−1
with a threshold of 1e−05

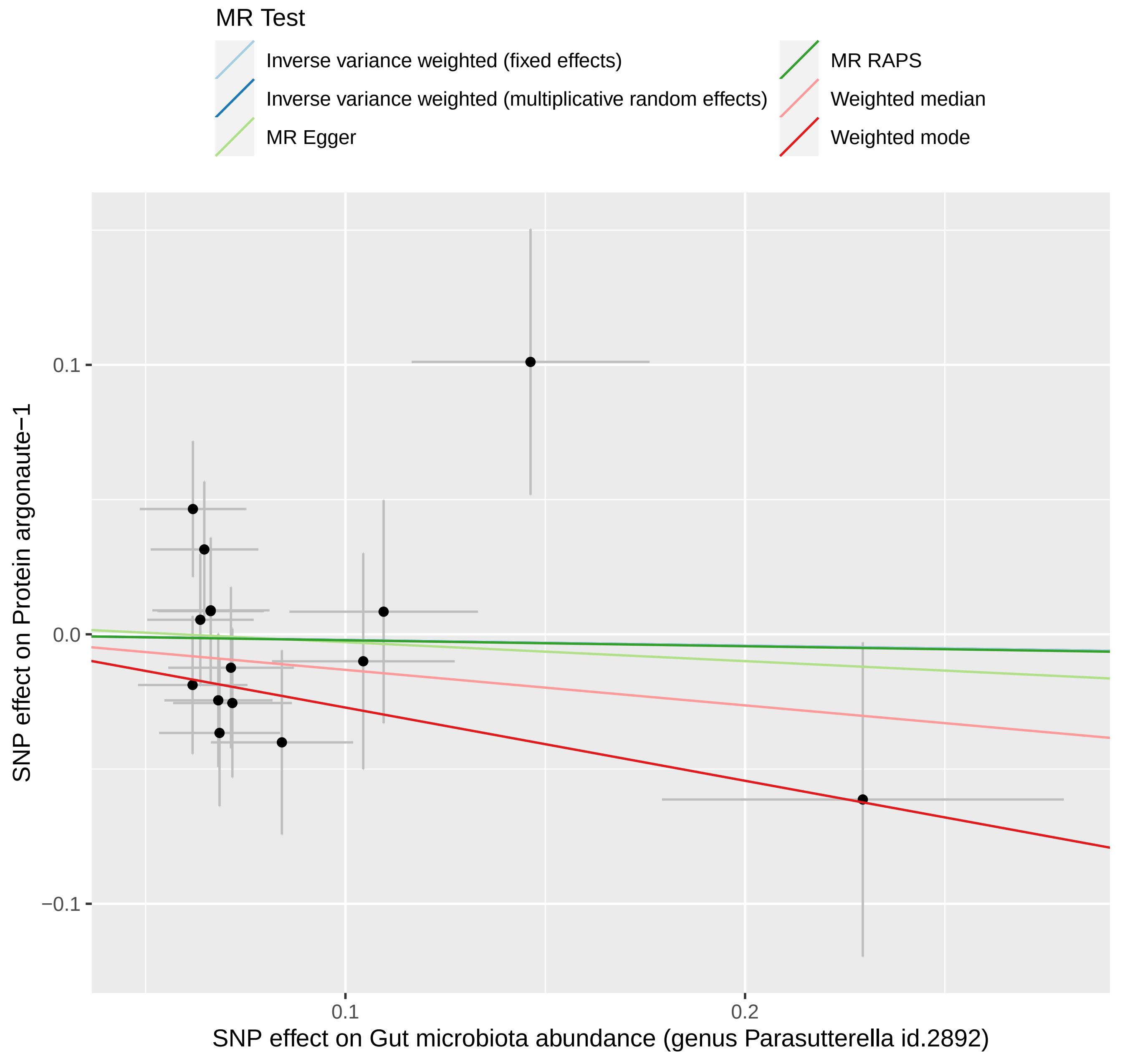

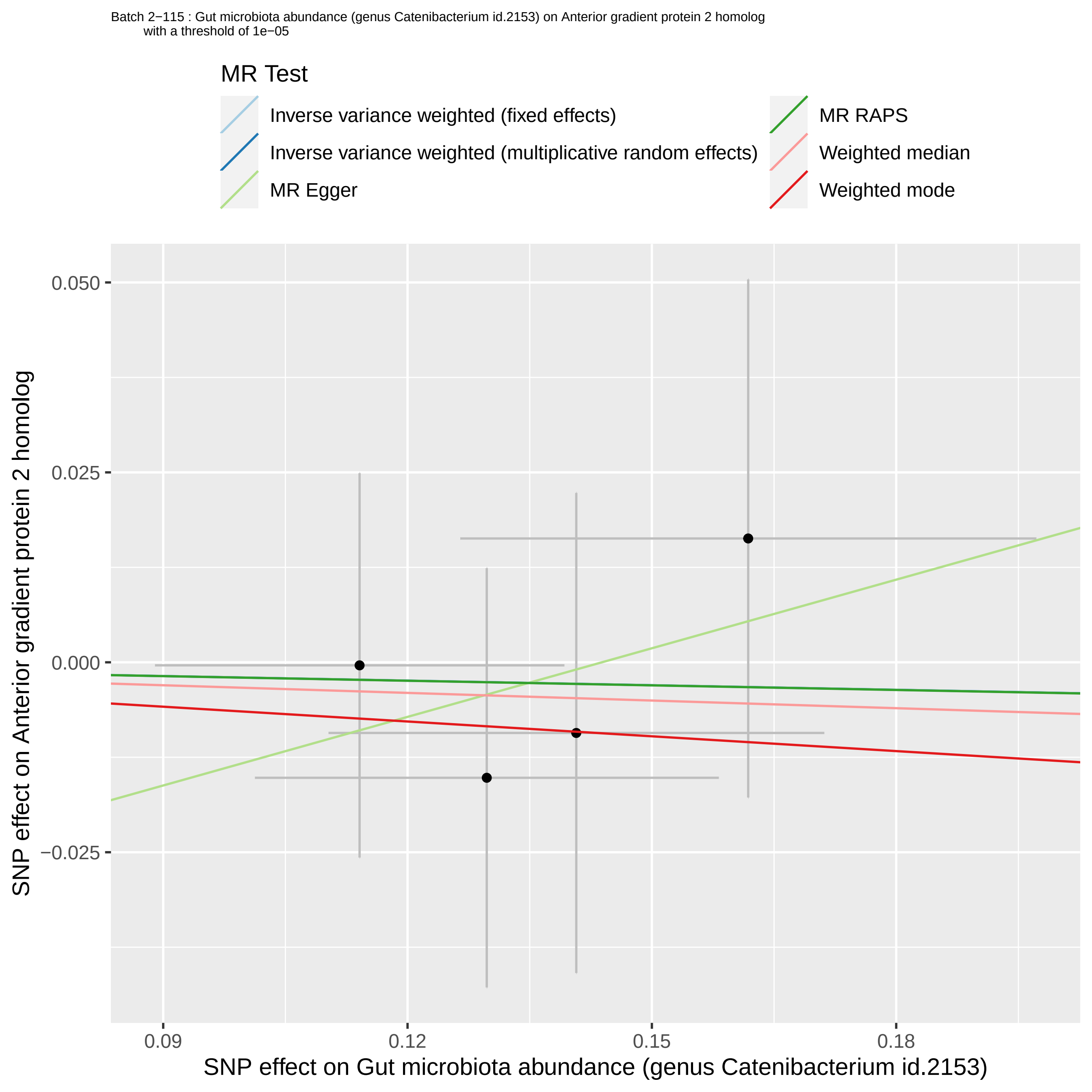
Batch 2−115 : Gut microbiota abundance (genus Catenibacterium id.2153) on Anterior gradient protein 2 homolog
with a threshold of 1e−05
MR Test
Inverse variance weighted (fixed effects)
Inverse variance weighted (multiplicative random effects)
MR Egger
MR RAPS
Weighted median
Weighted mode
0.050
0.025
0.000
−0.025
0.09
0.12
0.15
0.18
SNP effect on Anterior gradient protein 2 homolog
SNP effect on Gut microbiota abundance (genus Catenibacterium id.2153)

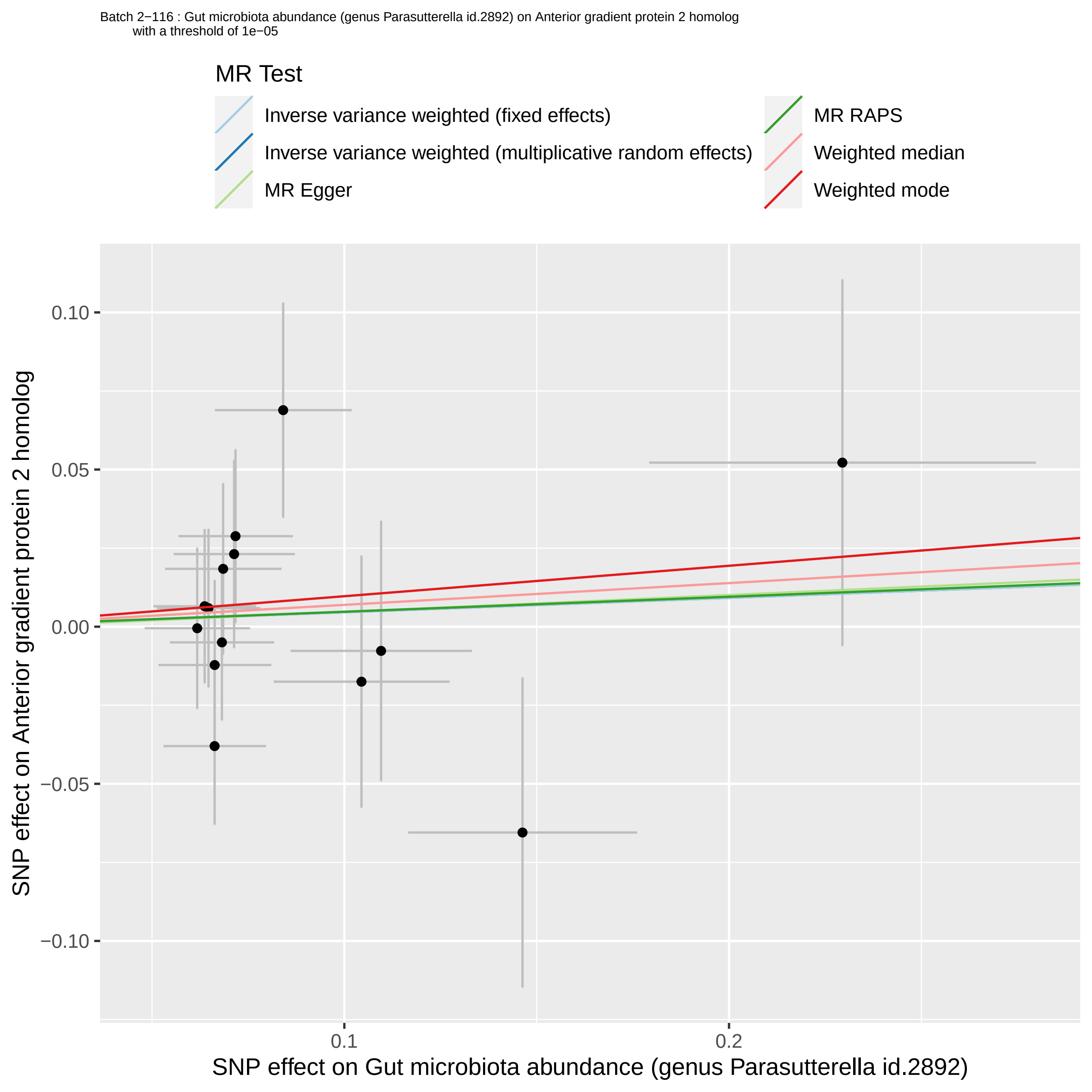

Batch 2−116 : Gut microbiota abundance (genus Parasutterella id.2892) on Anterior gradient protein 2 homolog
with a threshold of 1e−05
MR Test
Inverse variance weighted (fixed effects)
Inverse variance weighted (multiplicative random effects)
MR Egger
MR RAPS
Weighted median
Weighted mode
0.10
0.05
0.00
−0.05
−0.10
0.1
0.2
SNP effect on Anterior gradient protein 2 homolog
SNP effect on Gut microbiota abundance (genus Parasutterella id.2892)

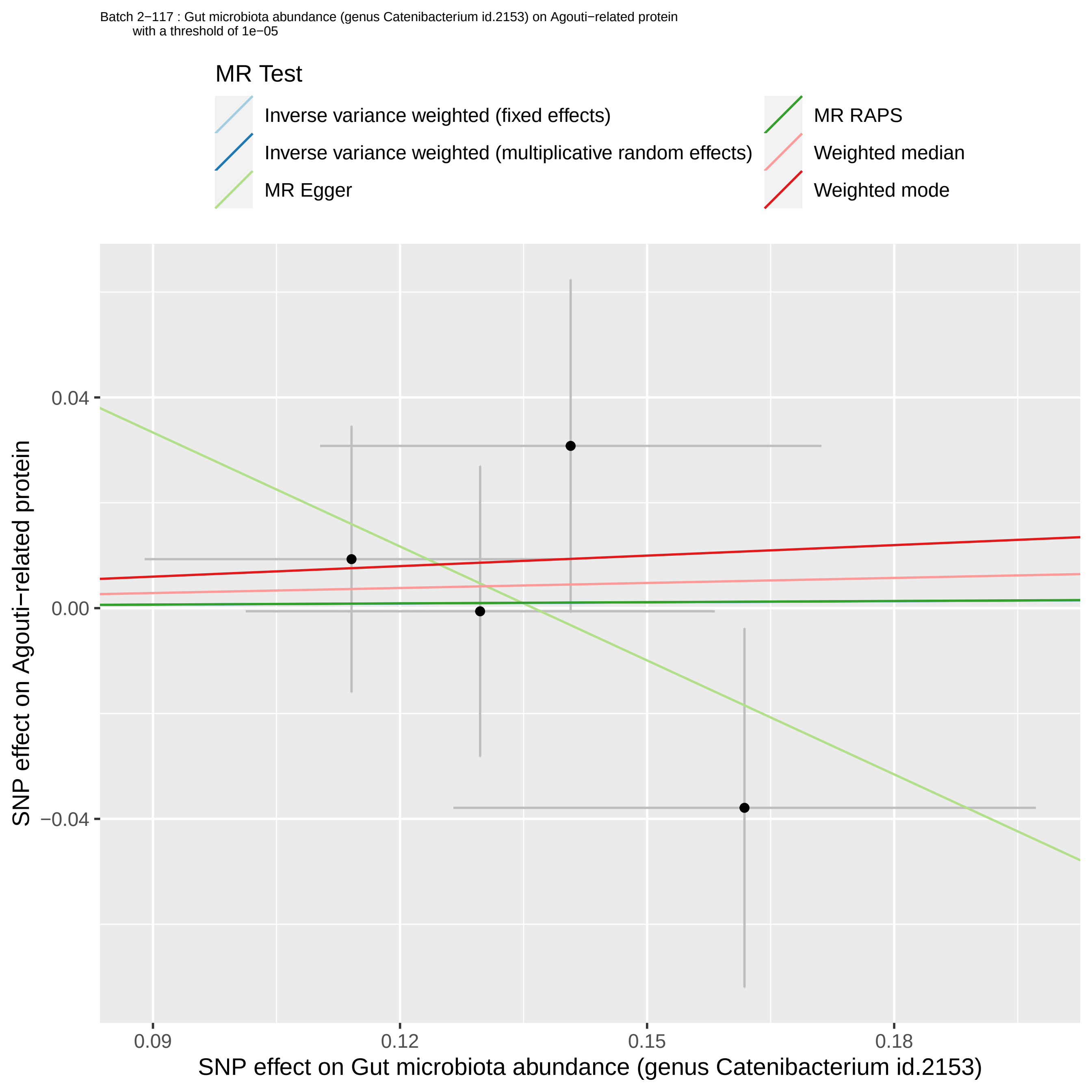
Batch 2−117 : Gut microbiota abundance (genus Catenibacterium id.2153) on Agouti−related protein
with a threshold of 1e−05
MR Test
Inverse variance weighted (fixed effects)
Inverse variance weighted (multiplicative random effects)
MR Egger
MR RAPS
Weighted median
Weighted mode
0.04
0.00
−0.04
0.09
0.12
0.15
0.18
SNP effect on Agouti−related protein
SNP effect on Gut microbiota abundance (genus Catenibacterium id.2153)

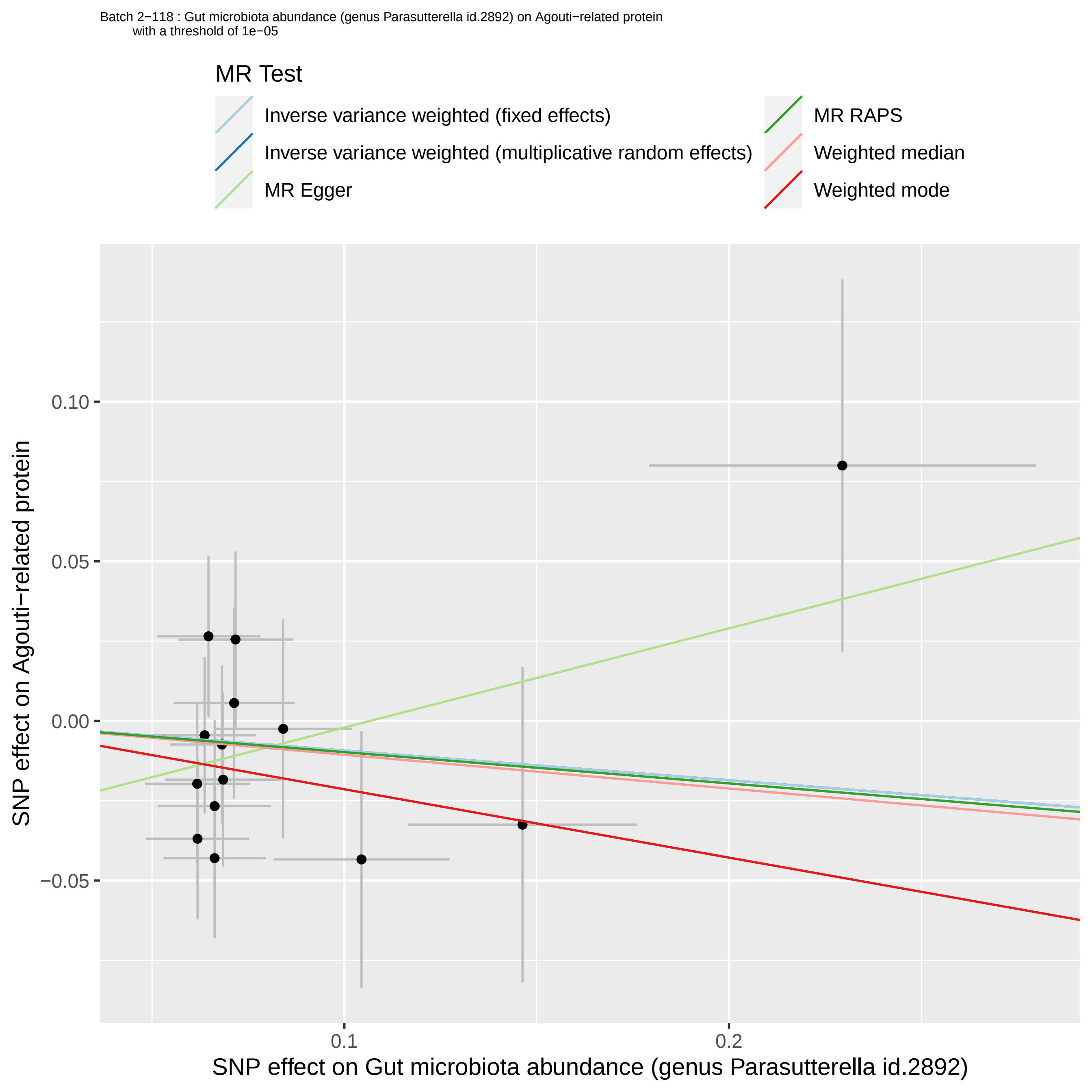
Batch 2−118 : Gut microbiota abundance (genus Parasutterella id.2892) on Agouti−related protein
with a threshold of 1e−05
MR Test
Inverse variance weighted (fixed effects)
Inverse variance weighted (multiplicative random effects)
MR Egger
MR RAPS
Weighted median
Weighted mode
0.10
0.05
0.00
−0.05
0.1
0.2
SNP effect on Agouti−related protein
SNP effect on Gut microbiota abundance (genus Parasutterella id.2892)

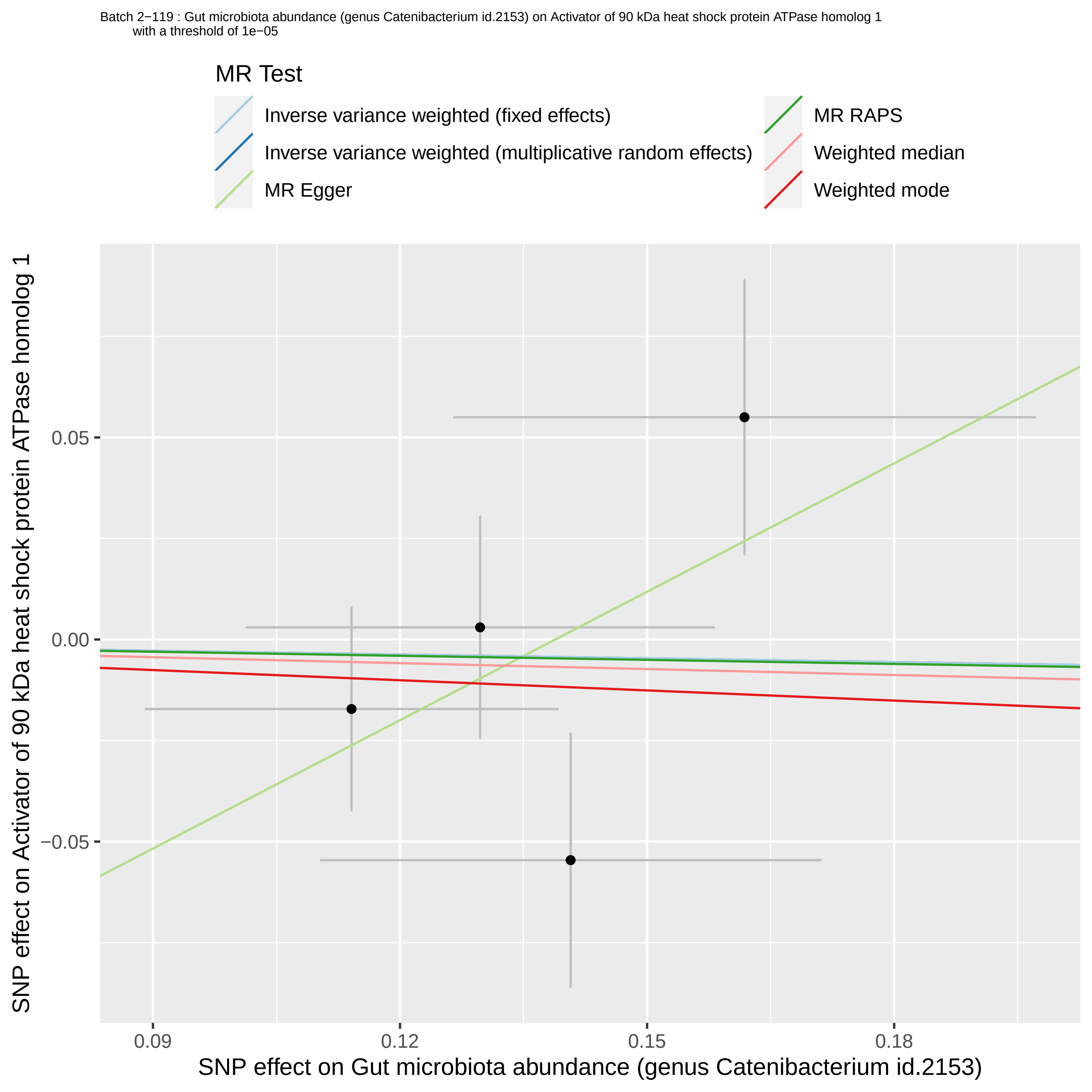
Batch 2−119 : Gut microbiota abundance (genus Catenibacterium id.2153) on Activator of 90 kDa heat shock protein ATPase homolog 1
with a threshold of 1e−05
MR Test
Inverse variance weighted (fixed effects)
Inverse variance weighted (multiplicative random effects)
MR Egger
MR RAPS
Weighted median
Weighted mode
SNP effect on Activator of 90 kDa heat shock protein ATPase homolog 1
0.05
0.00
−0.05
0.09
0.12
0.15
0.18
SNP effect on Gut microbiota abundance (genus Catenibacterium id.2153)

Batch 2−120 : Gut microbiota abundance (genus Parasutterella id.2892) on Activator of 90 kDa heat shock protein ATPase homolog 1 with a threshold of 1e−05

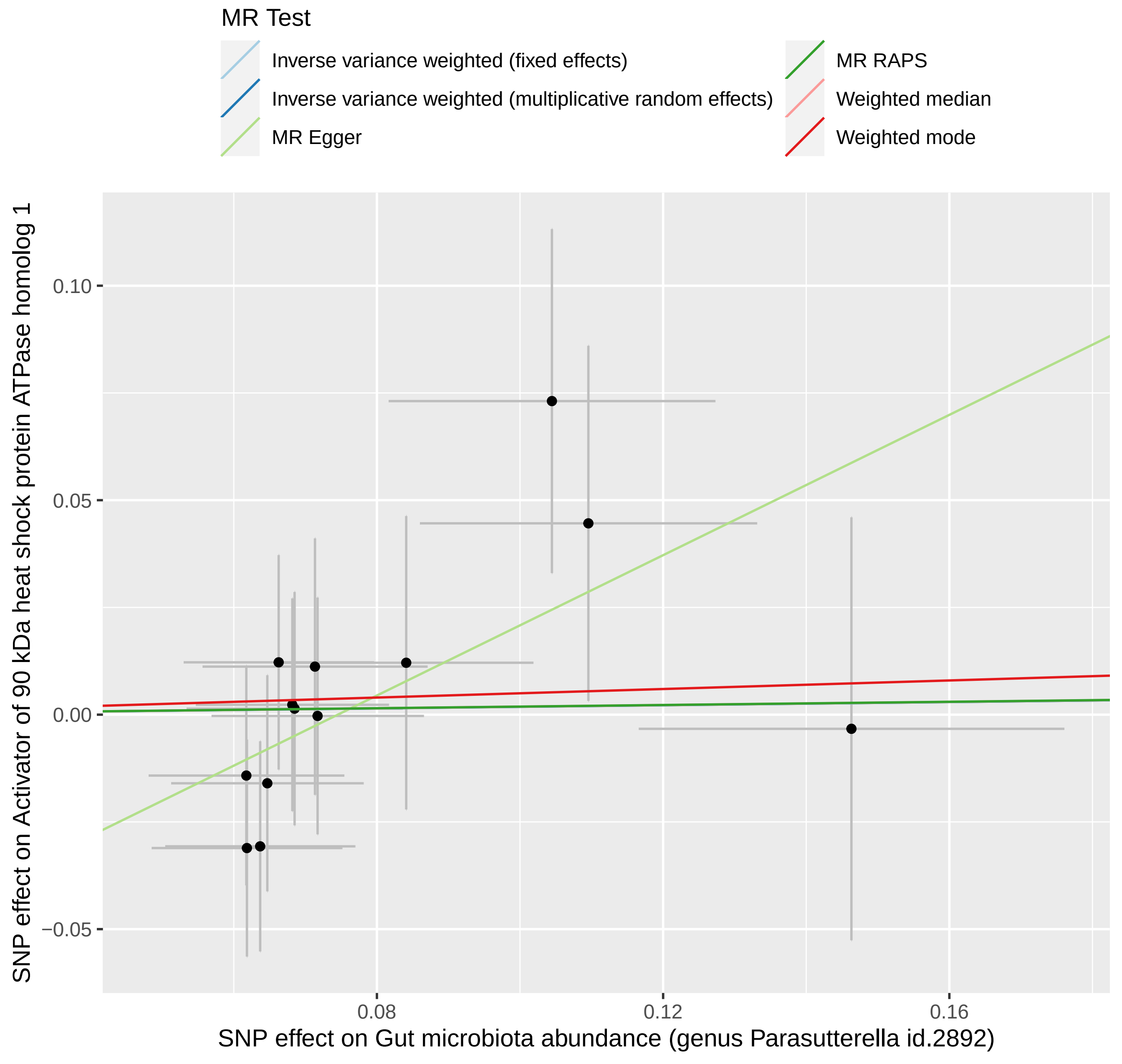

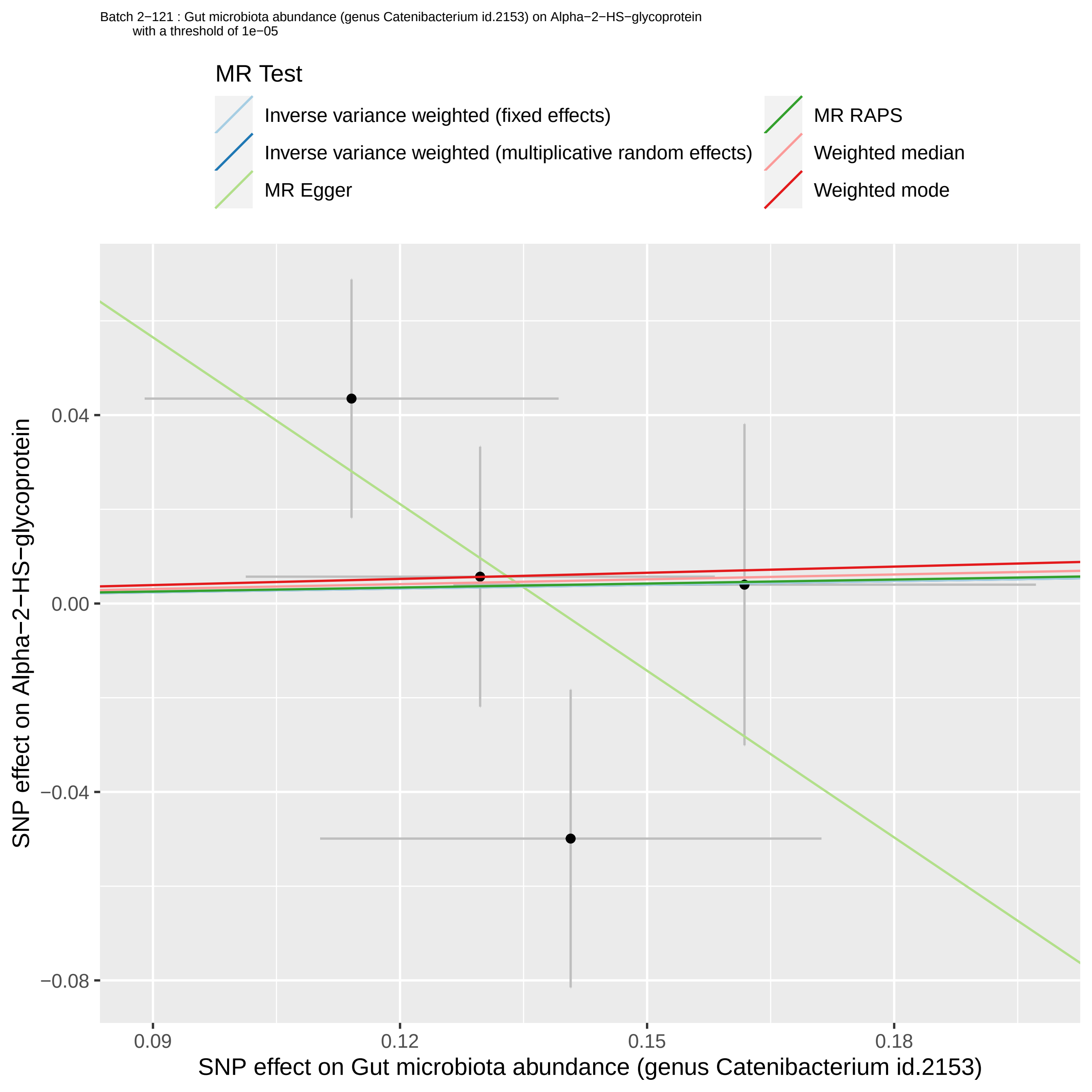
Batch 2−121 : Gut microbiota abundance (genus Catenibacterium id.2153) on Alpha−2−HS−glycoprotein
with a threshold of 1e−05
MR Test
Inverse variance weighted (fixed effects)
Inverse variance weighted (multiplicative random effects)
MR Egger
MR RAPS
Weighted median
Weighted mode
0.04
0.00
−0.04
−0.08
0.09
0.12
0.15
0.18
SNP effect on Alpha−2−HS−glycoprotein
SNP effect on Gut microbiota abundance (genus Catenibacterium id.2153)

Batch 2−122 : Gut microbiota abundance (genus Parasutterella id.2892) on Alpha−2−HS−glycoprotein
with a threshold of 1e−05

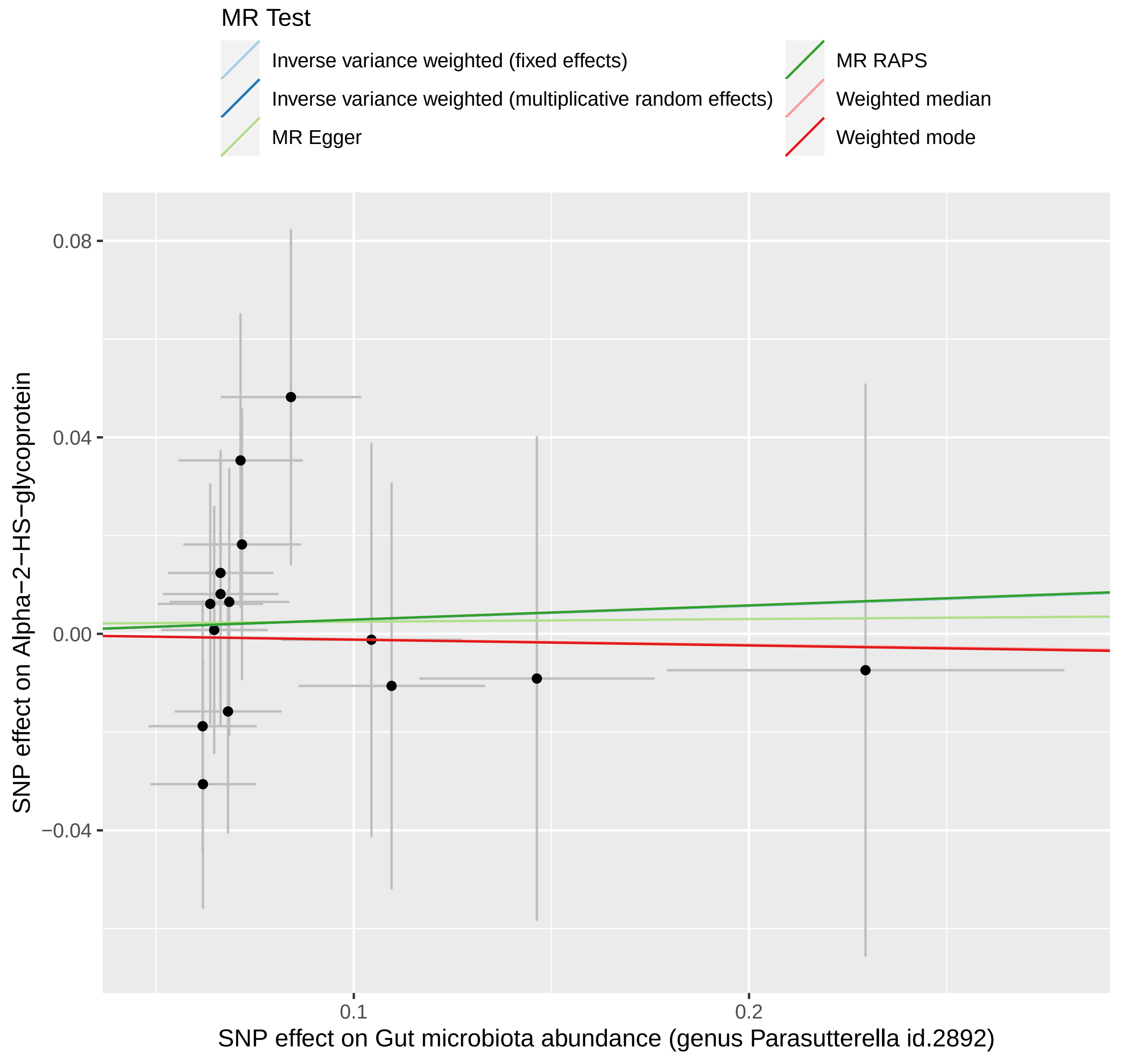

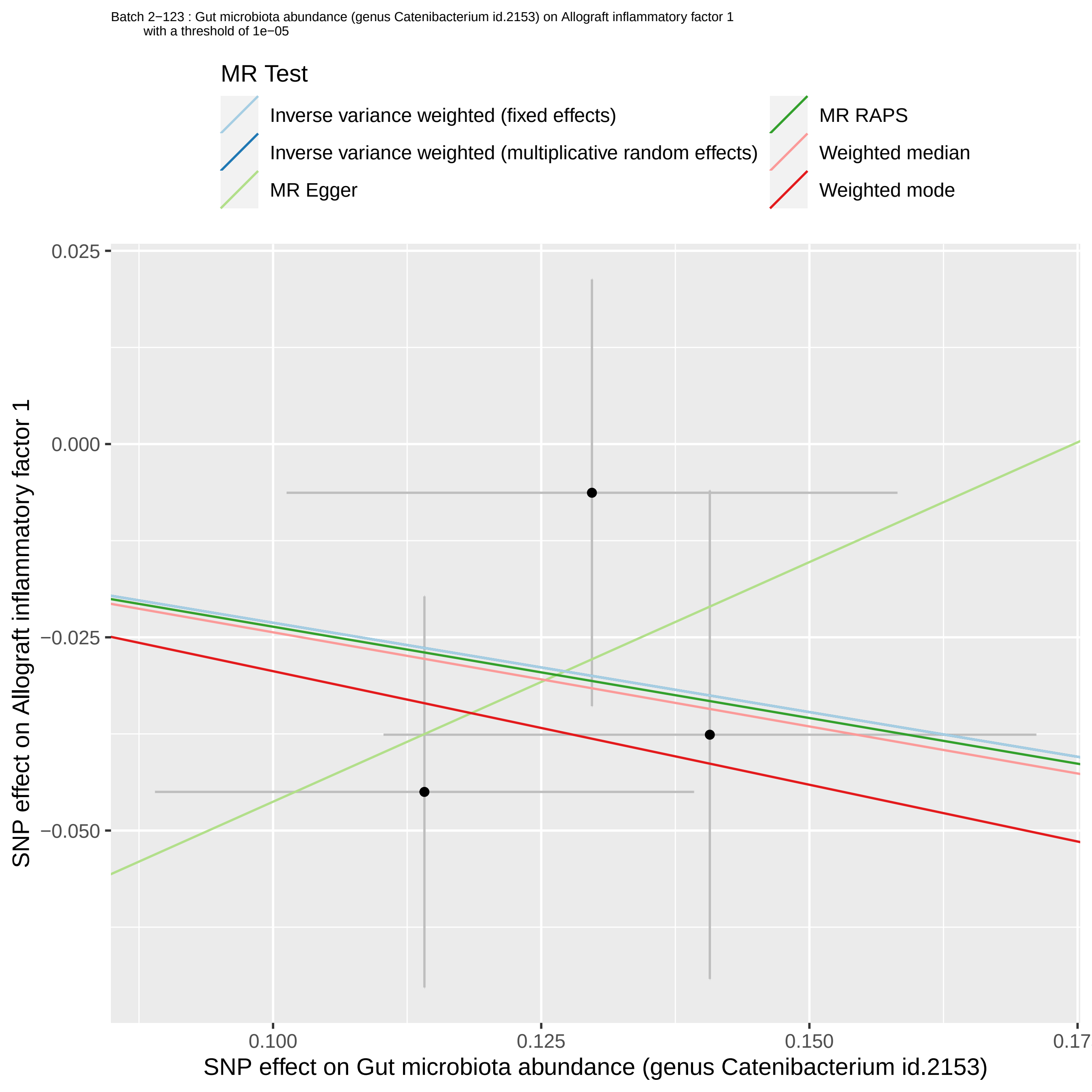

Batch 2−123 : Gut microbiota abundance (genus Catenibacterium id.2153) on Allograft inflammatory factor 1
with a threshold of 1e−05
MR Test
Inverse variance weighted (fixed effects)
Inverse variance weighted (multiplicative random effects)
MR Egger
MR RAPS
Weighted median
Weighted mode
0.025
0.000
−0.025
−0.050
0.100
0.125
0.150
0.17
SNP effect on Allograft inflammatory factor 1
SNP effect on Gut microbiota abundance (genus Catenibacterium id.2153)

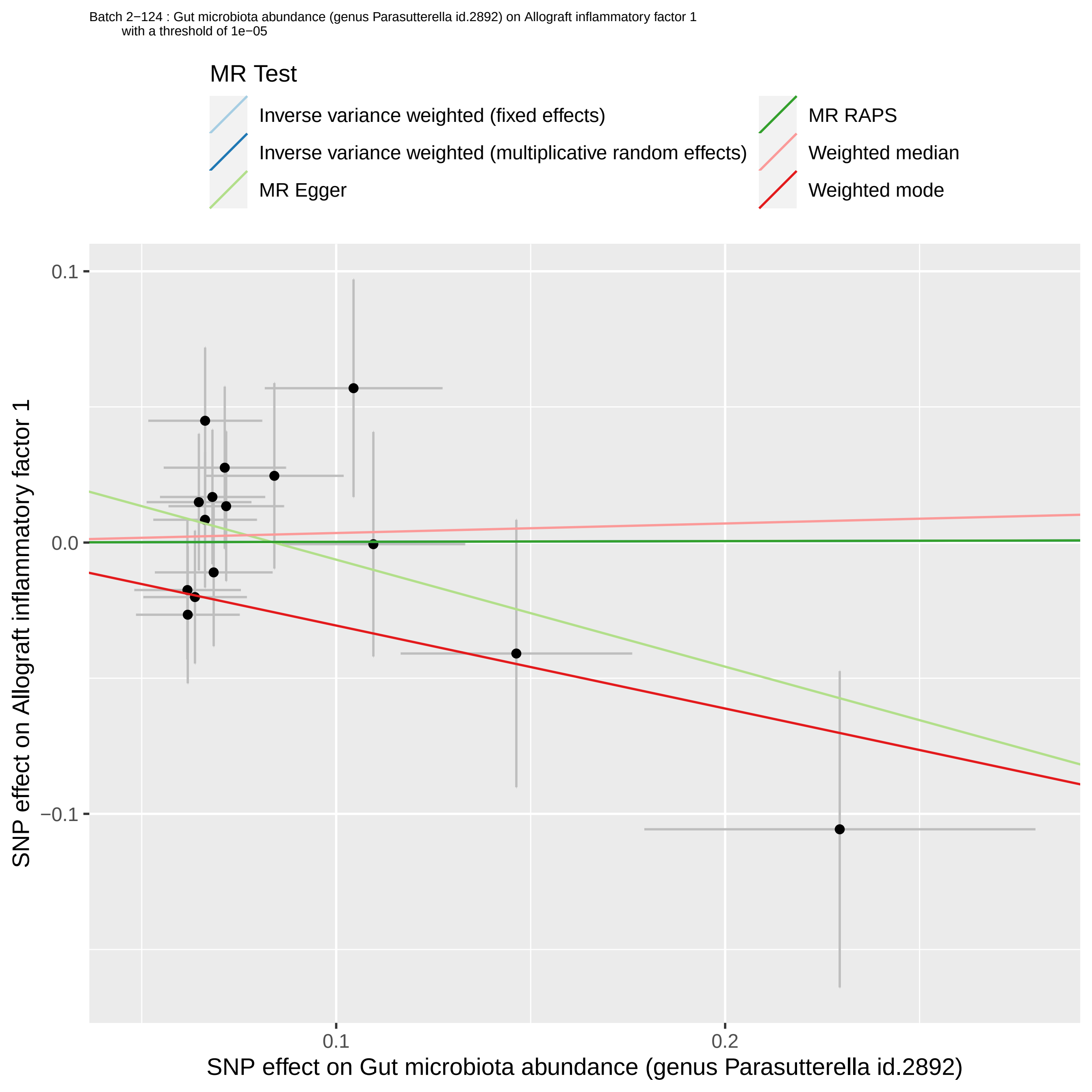

Batch 2−124 : Gut microbiota abundance (genus Parasutterella id.2892) on Allograft inflammatory factor 1
with a threshold of 1e−05
MR Test
Inverse variance weighted (fixed effects)
Inverse variance weighted (multiplicative random effects)
MR Egger
MR RAPS
Weighted median
Weighted mode
0.1
0.0
−0.1
0.1
0.2
SNP effect on Allograft inflammatory factor 1
SNP effect on Gut microbiota abundance (genus Parasutterella id.2892)

Batch 2−125 : Gut microbiota abundance (genus Catenibacterium id.2153) on Apoptosis−inducing factor 1, mitochondrial
with a threshold of 1e−05

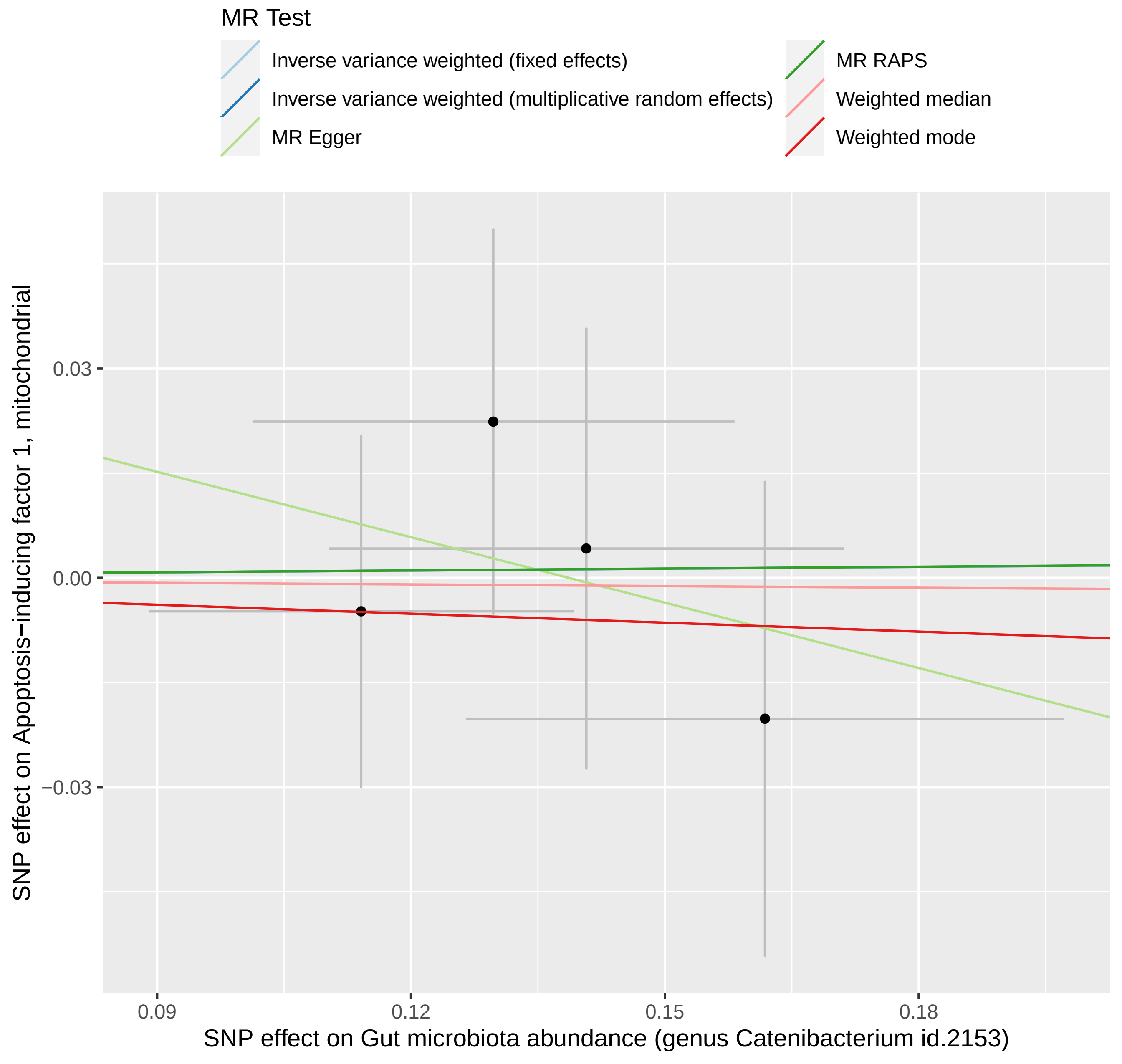

Batch 2−126 : Gut microbiota abundance (genus Parasutterella id.2892) on Apoptosis−inducing factor 1, mitochondrial
with a threshold of 1e−05

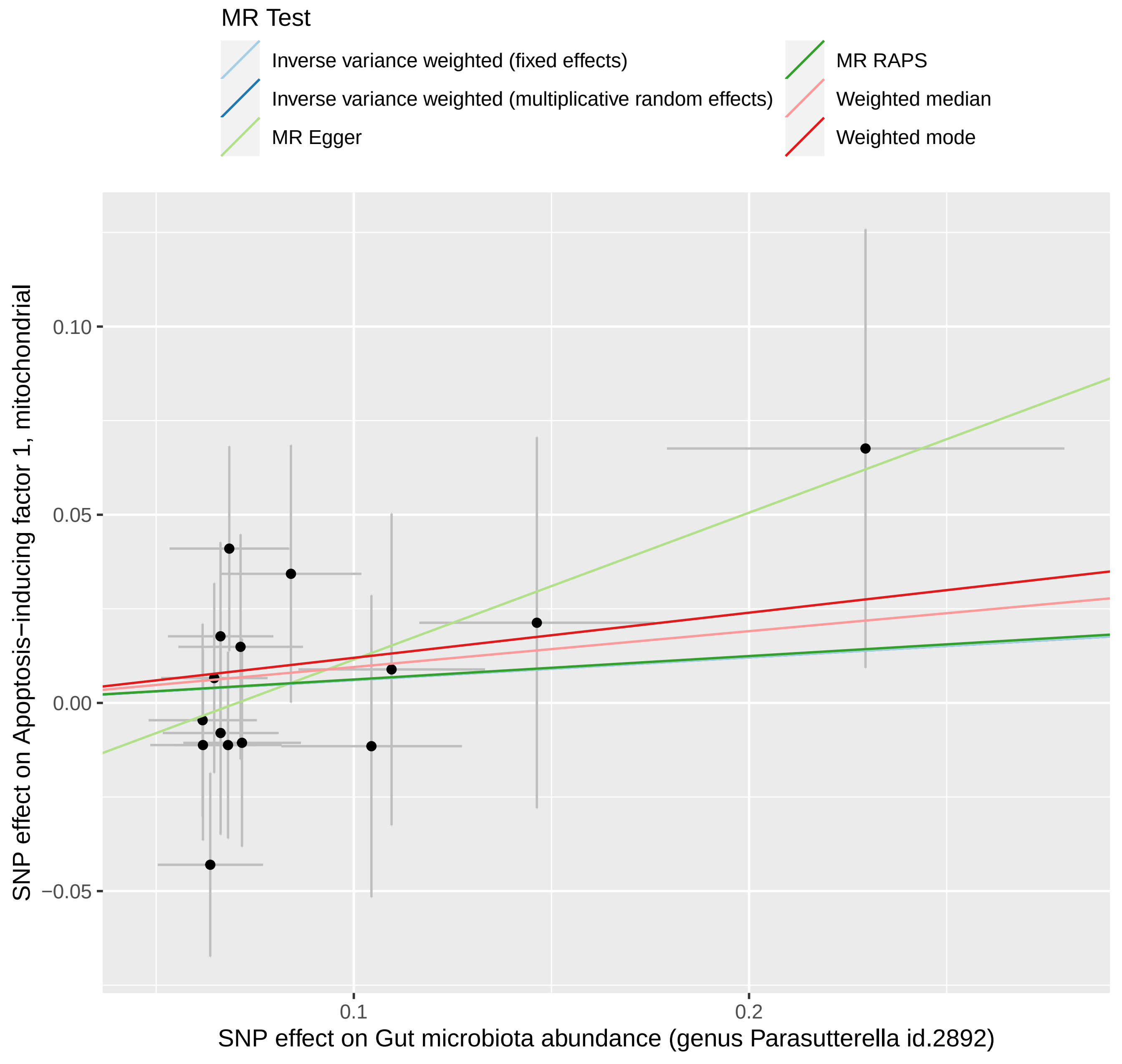

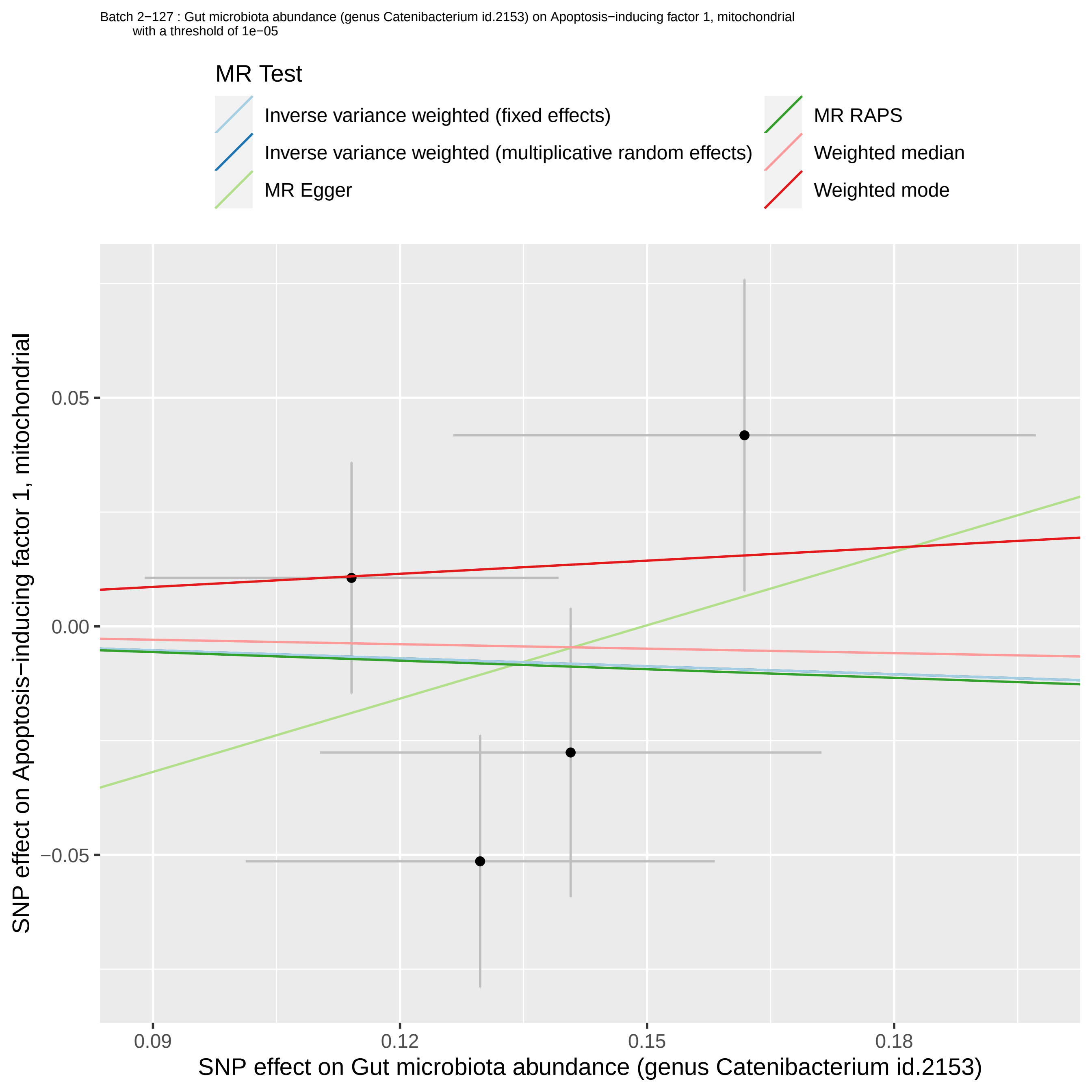
Batch 2−127 : Gut microbiota abundance (genus Catenibacterium id.2153) on Apoptosis−inducing factor 1, mitochondrial
with a threshold of 1e−05
MR Test
Inverse variance weighted (fixed effects)
Inverse variance weighted (multiplicative random effects)
MR Egger
MR RAPS
Weighted median
Weighted mode
0.05
0.00
−0.05
0.09
0.12
0.15
0.18
SNP effect on Apoptosis−inducing factor 1, mitochondrial
SNP effect on Gut microbiota abundance (genus Catenibacterium id.2153)

Batch 2−128 : Gut microbiota abundance (genus Parasutterella id.2892) on Apoptosis−inducing factor 1, mitochondrial
with a threshold of 1e−05

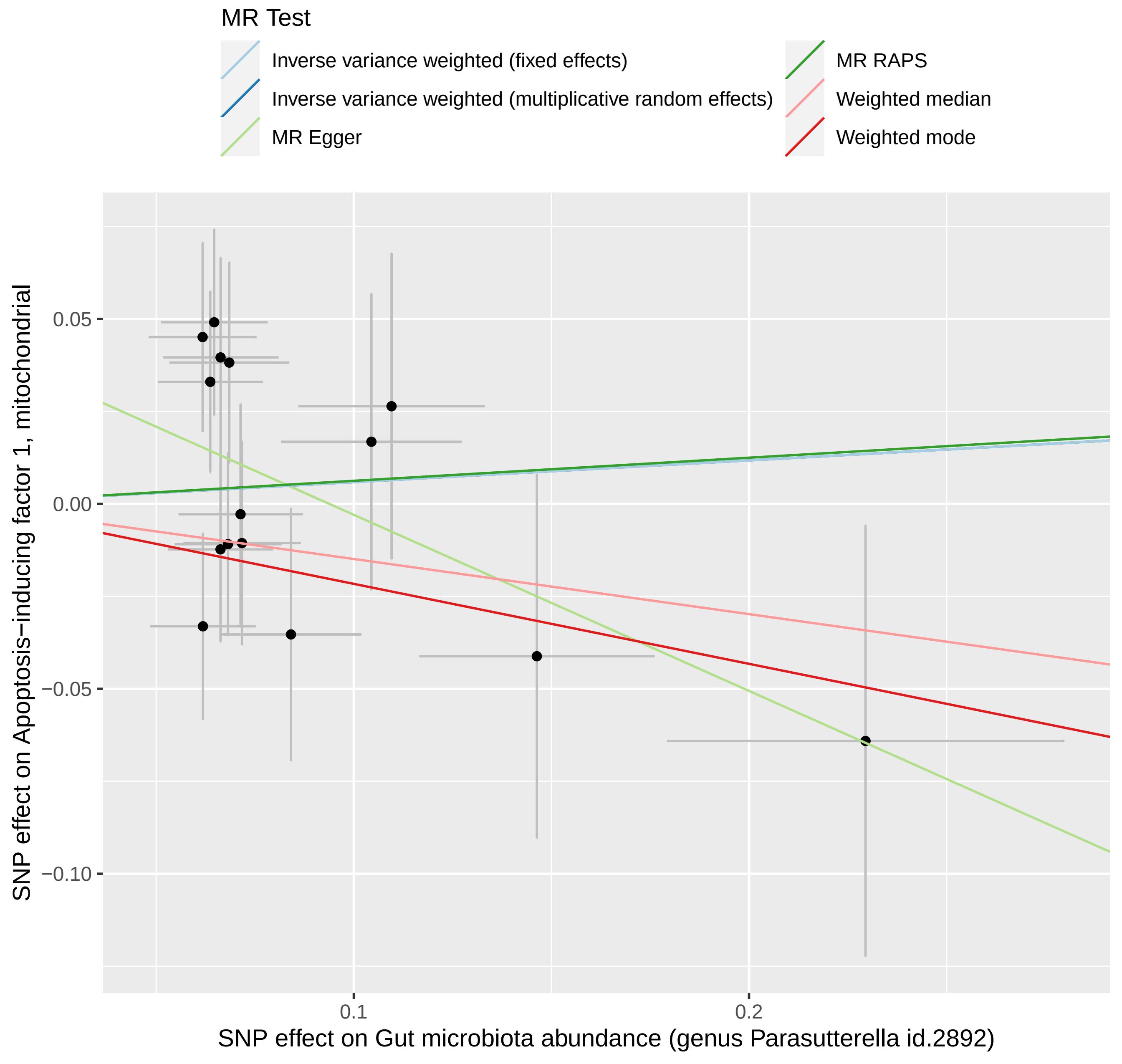

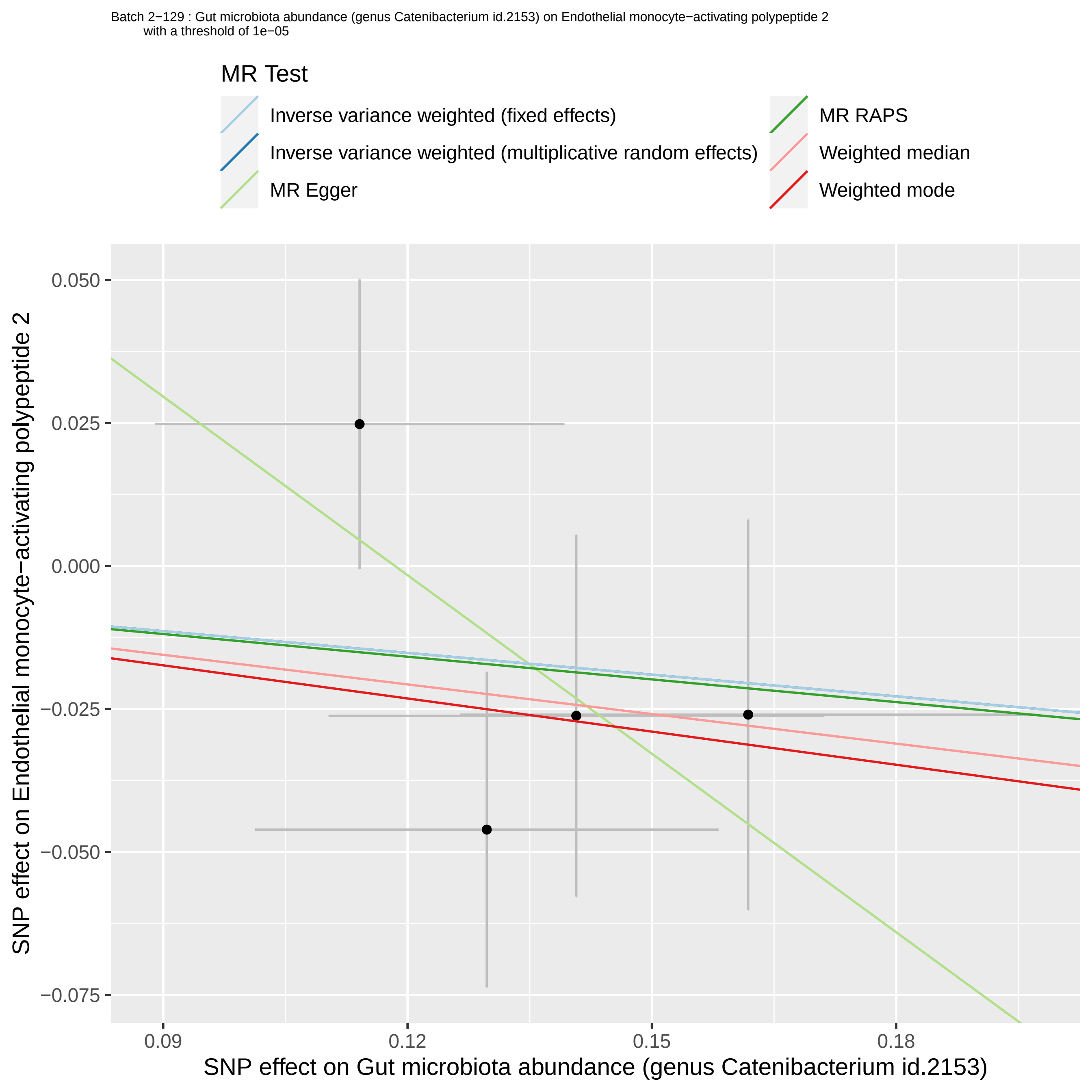
Batch 2−129 : Gut microbiota abundance (genus Catenibacterium id.2153) on Endothelial monocyte−activating polypeptide 2
with a threshold of 1e−05
MR Test
Inverse variance weighted (fixed effects)
Inverse variance weighted (multiplicative random effects)
MR Egger
MR RAPS
Weighted median
Weighted mode
SNP effect on Endothelial monocyte−activating polypeptide 2
0.050
0.025
0.000
−0.025
−0.050
−0.075
0.09
0.12
0.15
0.18
SNP effect on Gut microbiota abundance (genus Catenibacterium id.2153)

Batch 2−130 : Gut microbiota abundance (genus Parasutterella id.2892) on Endothelial monocyte−activating polypeptide 2
with a threshold of 1e−05

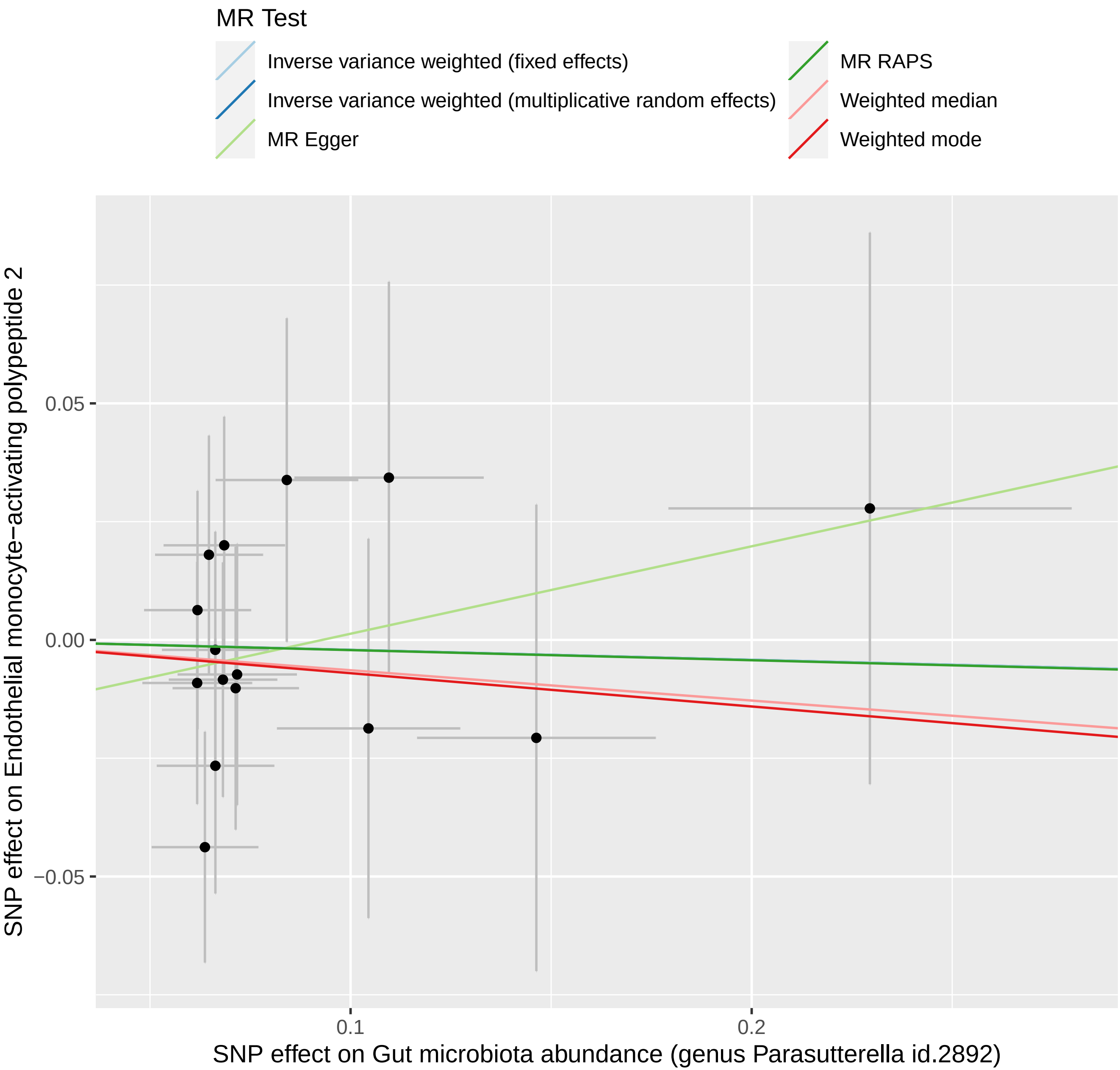

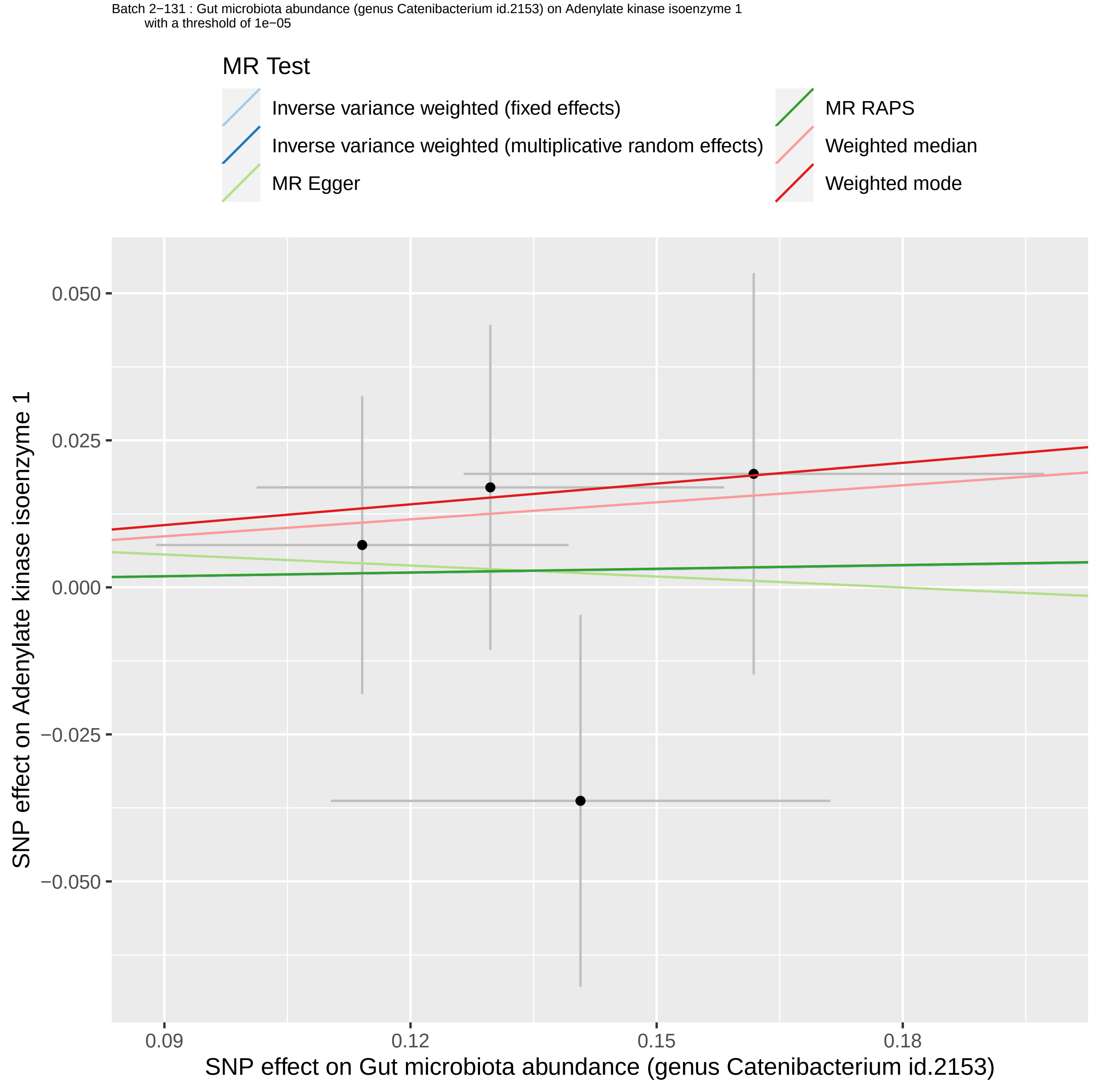

Batch 2−131 : Gut microbiota abundance (genus Catenibacterium id.2153) on Adenylate kinase isoenzyme 1
with a threshold of 1e−05
MR Test
Inverse variance weighted (fixed effects)
Inverse variance weighted (multiplicative random effects)
MR Egger
MR RAPS
Weighted median
Weighted mode
0.050
0.025
0.000
−0.025
−0.050
0.09
0.12
0.15
0.18
SNP effect on Adenylate kinase isoenzyme 1
SNP effect on Gut microbiota abundance (genus Catenibacterium id.2153)

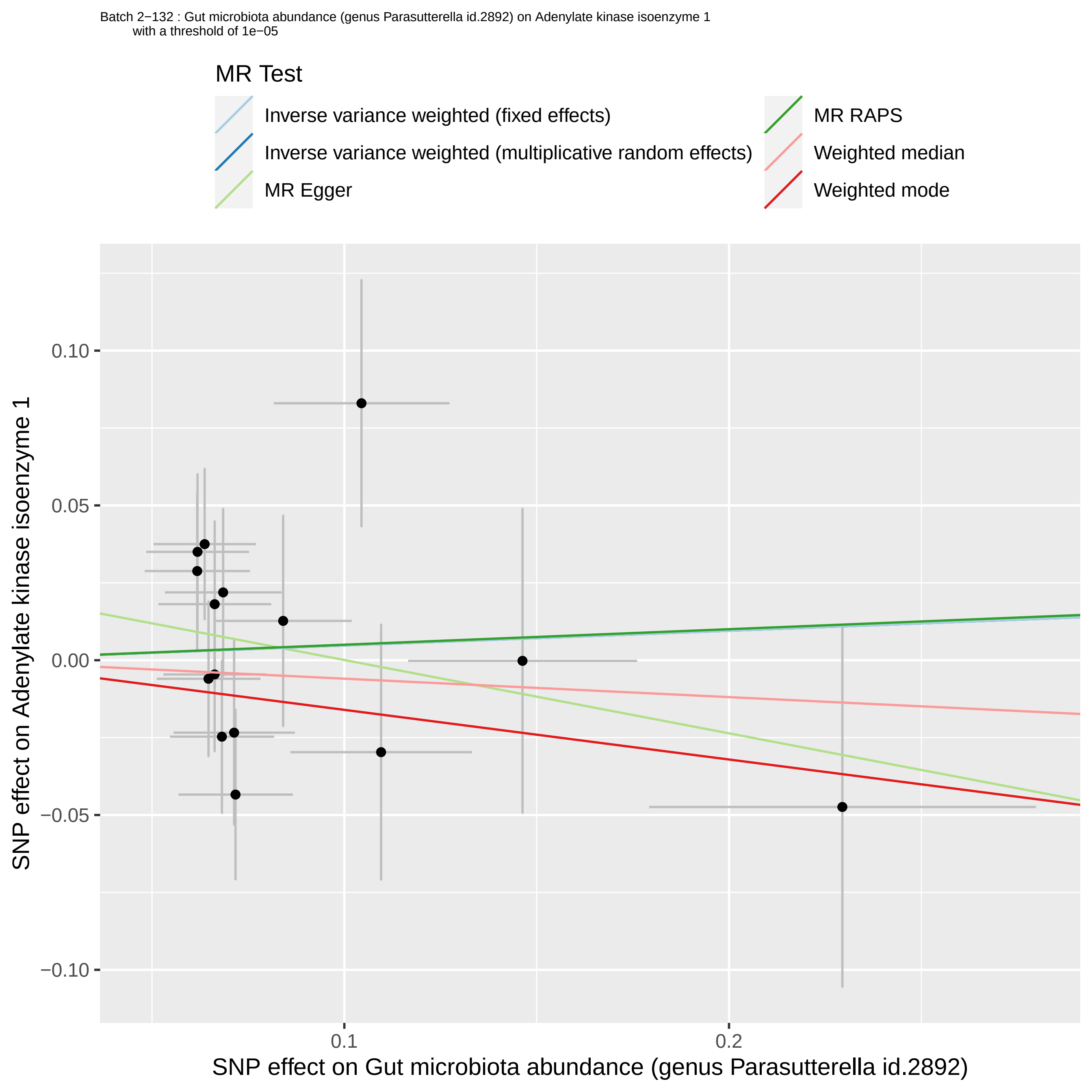

Batch 2−132 : Gut microbiota abundance (genus Parasutterella id.2892) on Adenylate kinase isoenzyme 1
with a threshold of 1e−05
MR Test
Inverse variance weighted (fixed effects)
Inverse variance weighted (multiplicative random effects)
MR Egger
MR RAPS
Weighted median
Weighted mode
0.10
0.05
0.00
−0.05
−0.10
0.1
0.2
SNP effect on Adenylate kinase isoenzyme 1
SNP effect on Gut microbiota abundance (genus Parasutterella id.2892)

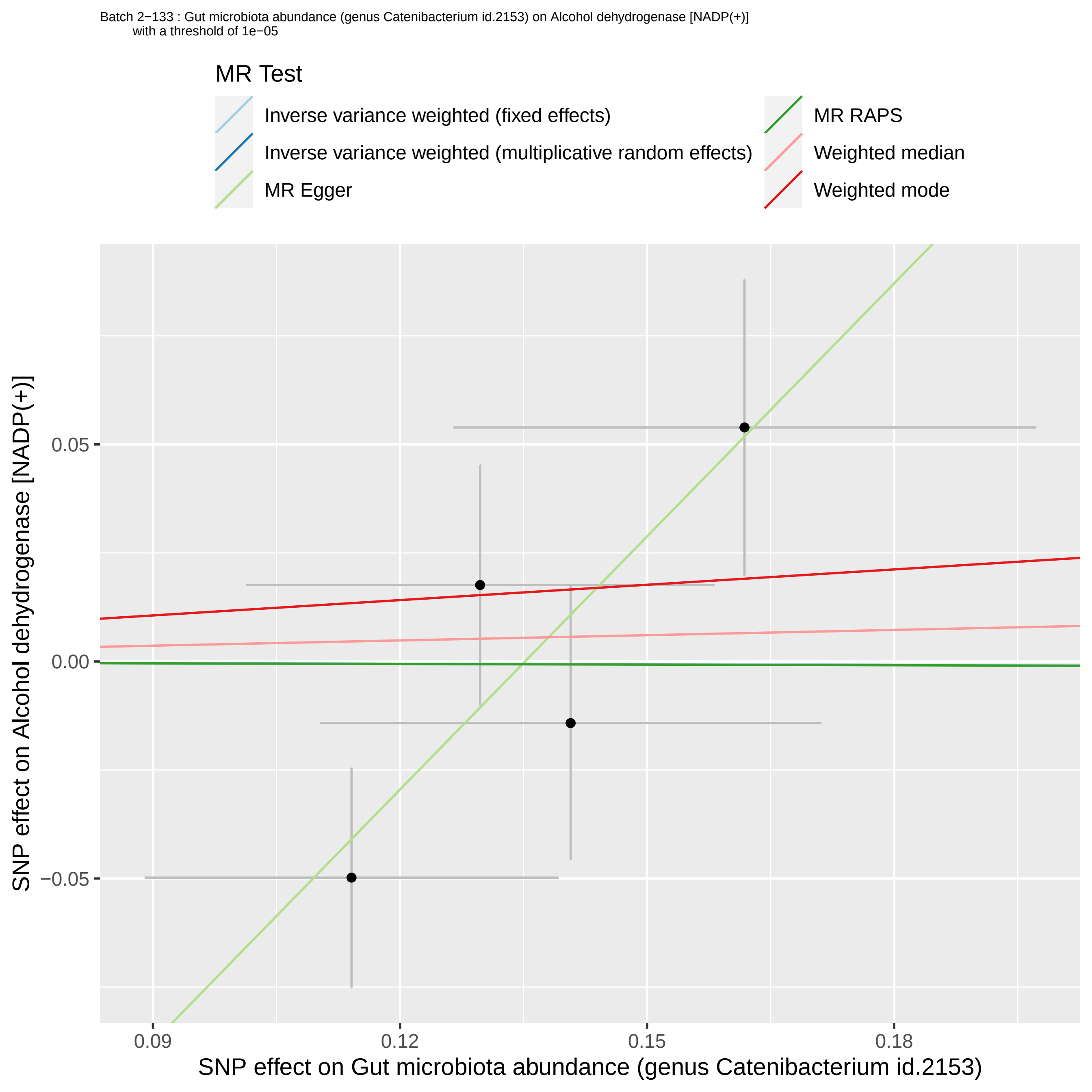
Batch 2−133 : Gut microbiota abundance (genus Catenibacterium id.2153) on Alcohol dehydrogenase [NADP(+)]
with a threshold of 1e−05
MR Test
Inverse variance weighted (fixed effects)
Inverse variance weighted (multiplicative random effects)
MR Egger
MR RAPS
Weighted median
Weighted mode
0.05
0.00
−0.05
0.09
0.12
0.15
0.18
SNP effect on Alcohol dehydrogenase [NADP(+)]
SNP effect on Gut microbiota abundance (genus Catenibacterium id.2153)

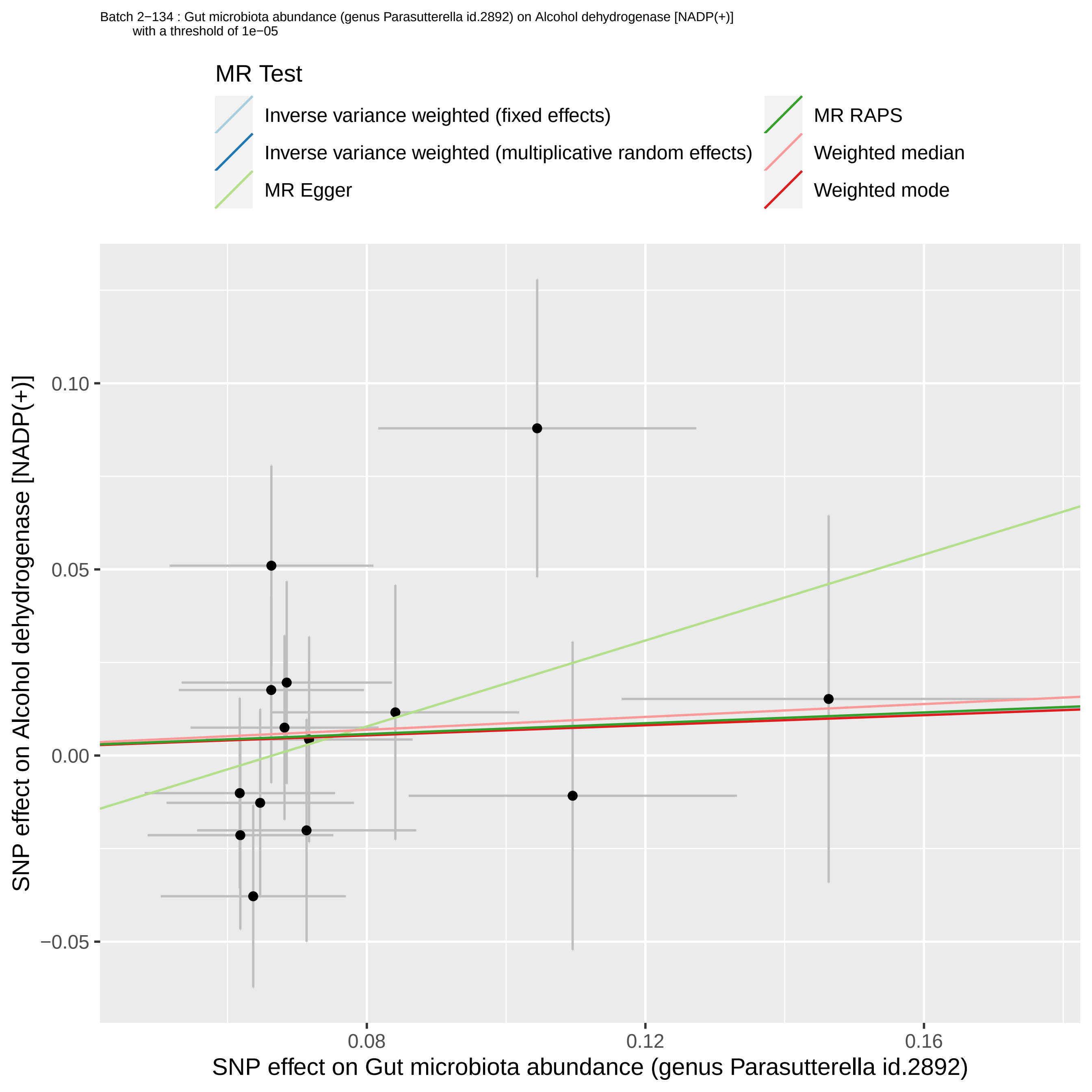
Batch 2−134 : Gut microbiota abundance (genus Parasutterella id.2892) on Alcohol dehydrogenase [NADP(+)]
with a threshold of 1e−05
MR Test
Inverse variance weighted (fixed effects)
Inverse variance weighted (multiplicative random effects)
MR Egger
MR RAPS
Weighted median
Weighted mode
0.10
0.05
0.00
−0.05
0.08
0.12
0.16
SNP effect on Alcohol dehydrogenase [NADP(+)]
SNP effect on Gut microbiota abundance (genus Parasutterella id.2892)

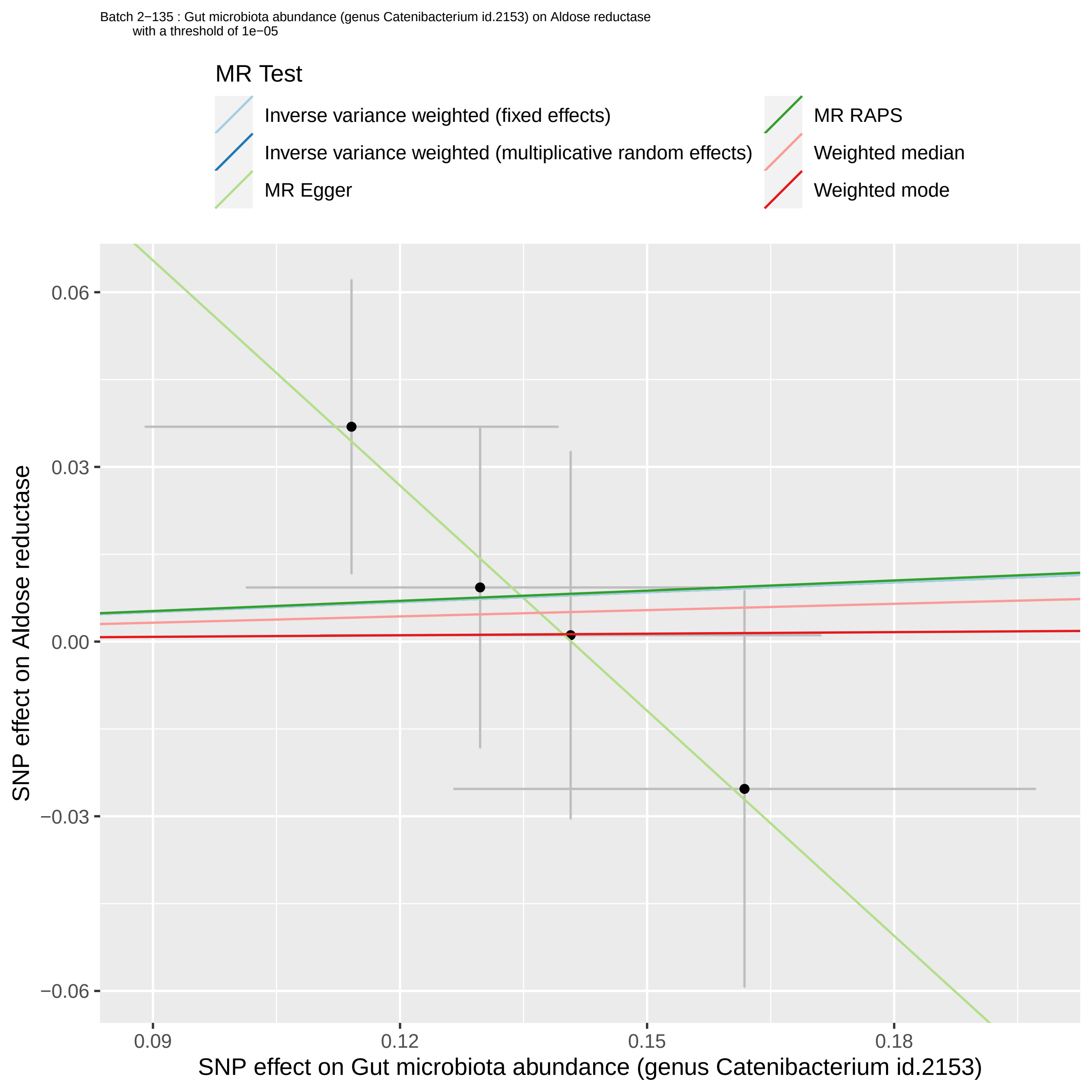
Batch 2−135 : Gut microbiota abundance (genus Catenibacterium id.2153) on Aldose reductase
with a threshold of 1e−05
MR Test
Inverse variance weighted (fixed effects)
Inverse variance weighted (multiplicative random effects)
MR Egger
MR RAPS
Weighted median
Weighted mode
0.06
0.03
0.00
−0.03
−0.06
0.09
0.12
0.15
0.18
SNP effect on Aldose reductase
SNP effect on Gut microbiota abundance (genus Catenibacterium id.2153)

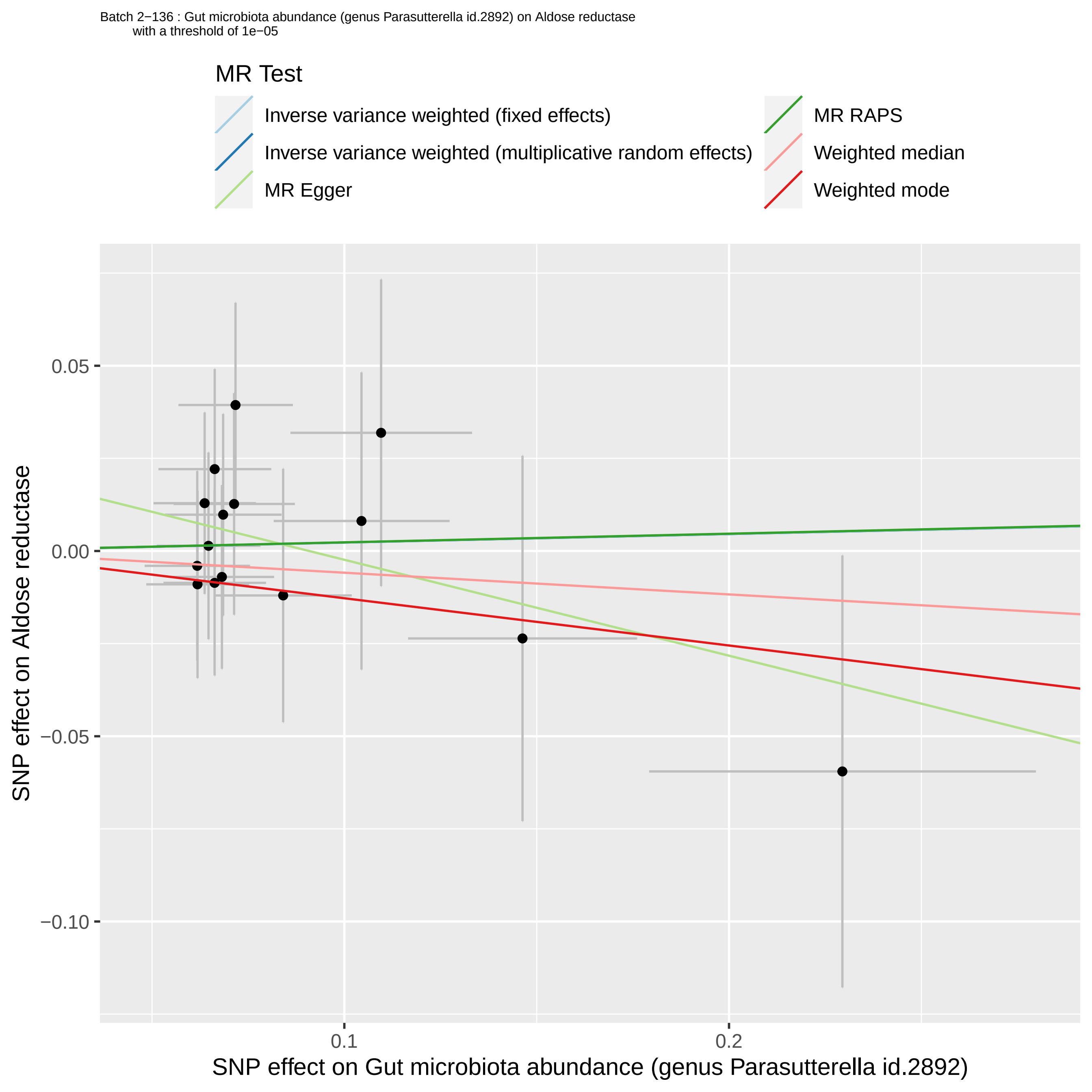

Batch 2−136 : Gut microbiota abundance (genus Parasutterella id.2892) on Aldose reductase
with a threshold of 1e−05
MR Test
Inverse variance weighted (fixed effects)
Inverse variance weighted (multiplicative random effects)
MR Egger
MR RAPS
Weighted median
Weighted mode
0.05
0.00
−0.05
−0.10
0.1
0.2
SNP effect on Aldose reductase
SNP effect on Gut microbiota abundance (genus Parasutterella id.2892)

Batch 2−137 : Gut microbiota abundance (genus Catenibacterium id.2153) on Aldo−keto reductase family 1 member C1
with a threshold of 1e−05

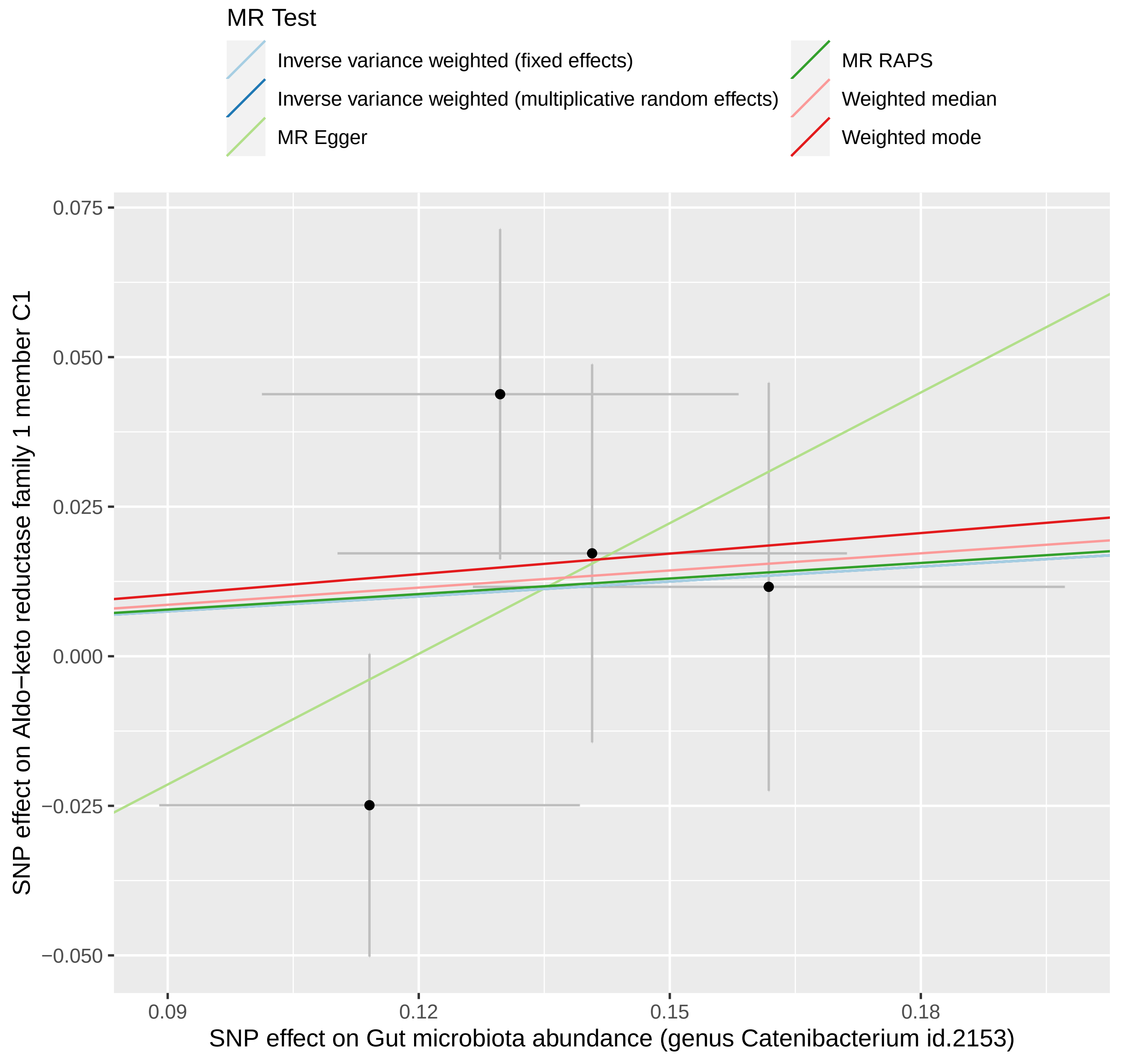

Batch 2−138 : Gut microbiota abundance (genus Parasutterella id.2892) on Aldo−keto reductase family 1 member C1 with a threshold of 1e−05

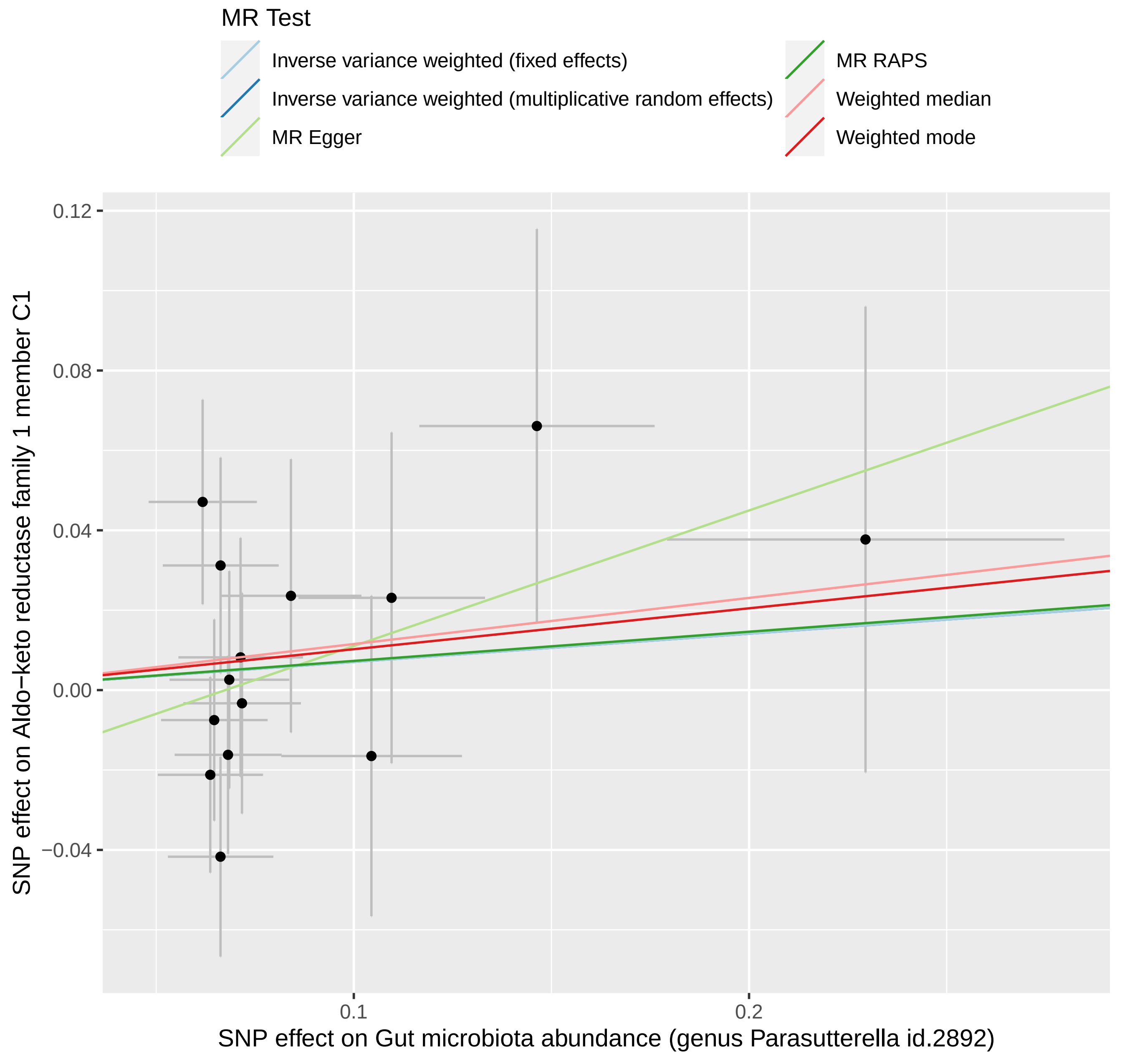

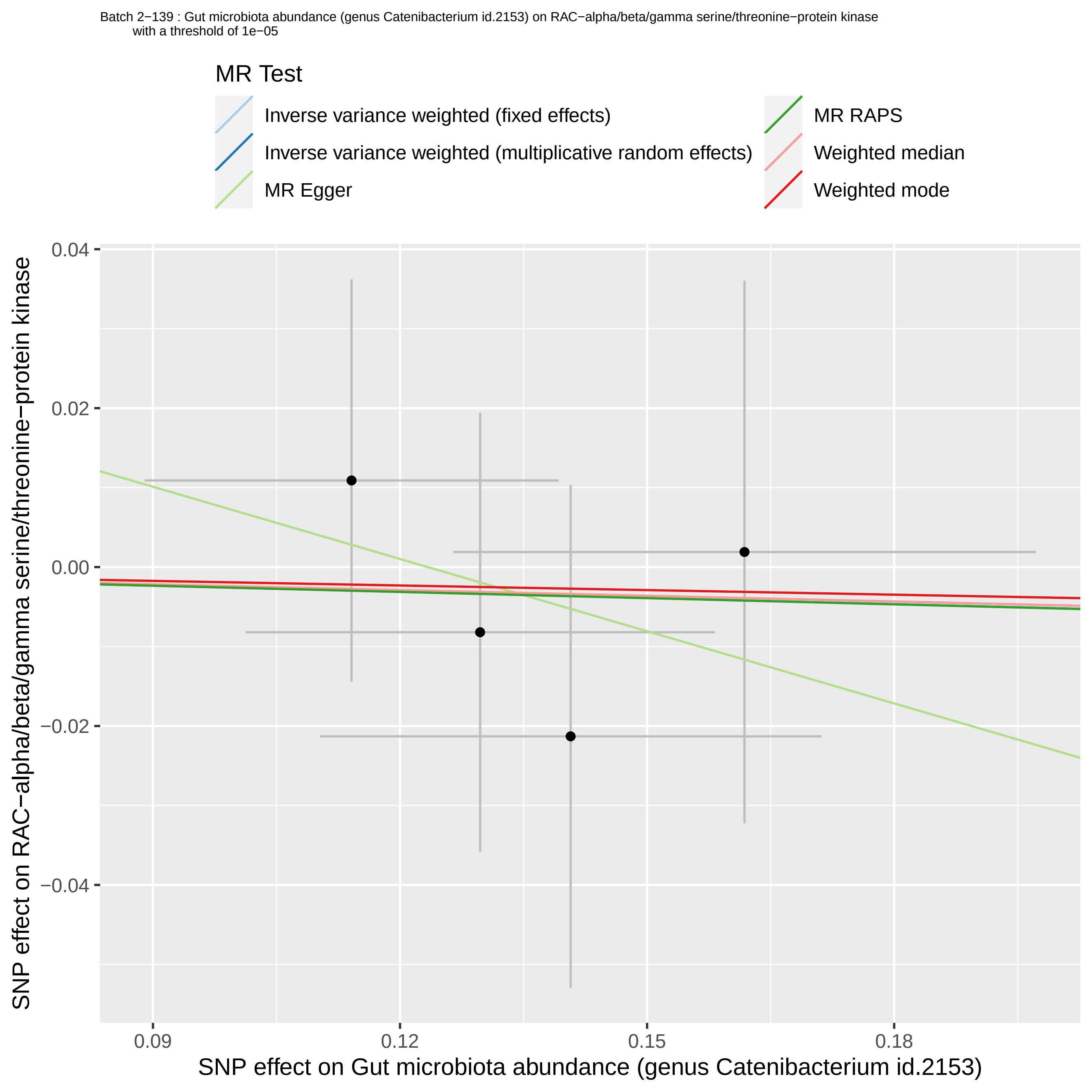
Batch 2−139 : Gut microbiota abundance (genus Catenibacterium id.2153) on RAC−alpha/beta/gamma serine/threonine−protein kinase
with a threshold of 1e−05
MR Test
Inverse variance weighted (fixed effects)
Inverse variance weighted (multiplicative random effects)
MR Egger
MR RAPS
Weighted median
Weighted mode
0.04
0.02
0.00
−0.02
−0.04
0.09
0.12
0.15
0.18
SNP effect on RAC−alpha/beta/gamma serine/threonine−protein kinase
SNP effect on Gut microbiota abundance (genus Catenibacterium id.2153)

Batch 2−140 : Gut microbiota abundance (genus Parasutterella id.2892) on RAC−alpha/beta/gamma serine/threonine−protein kinase
with a threshold of 1e−05

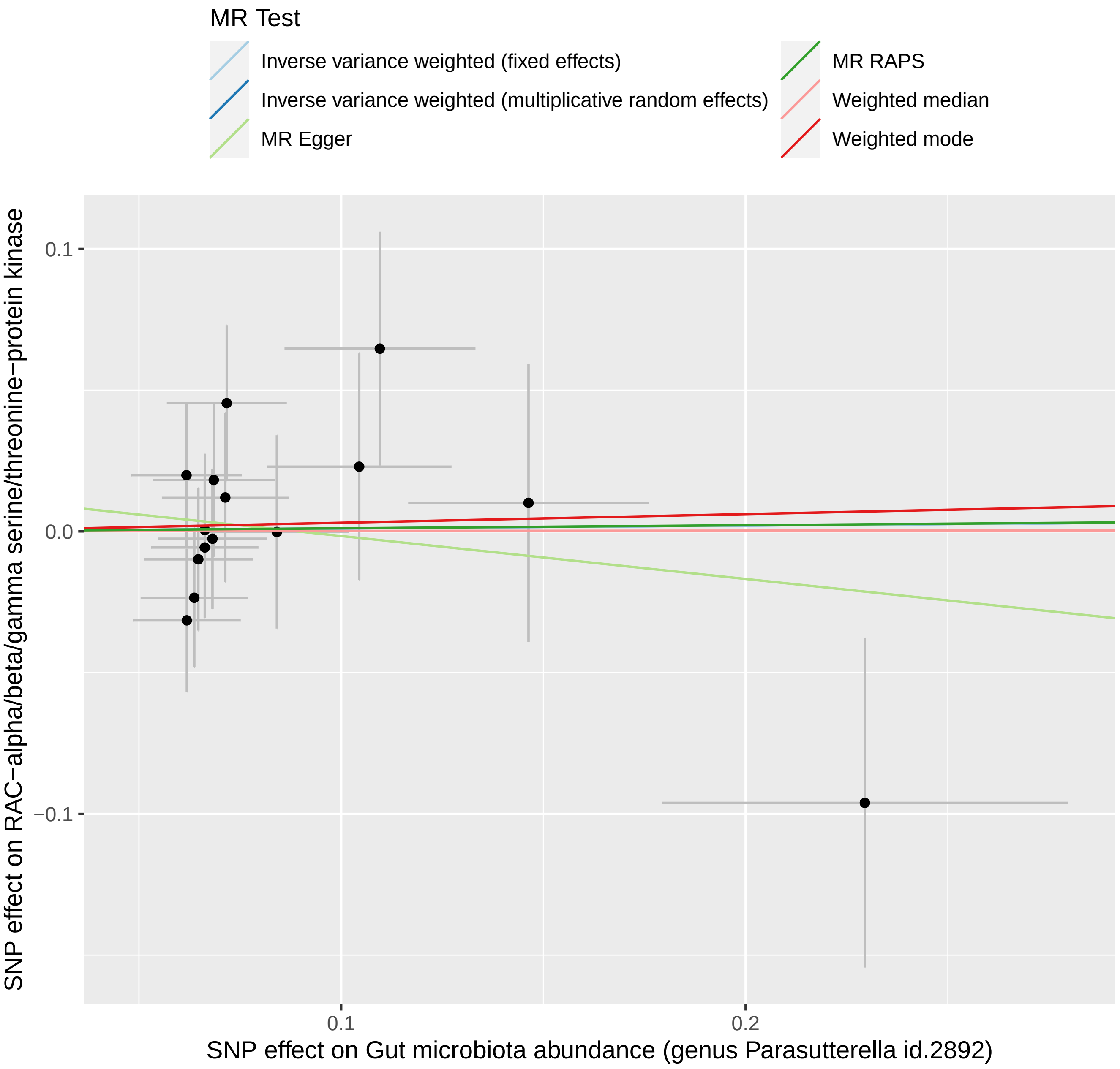

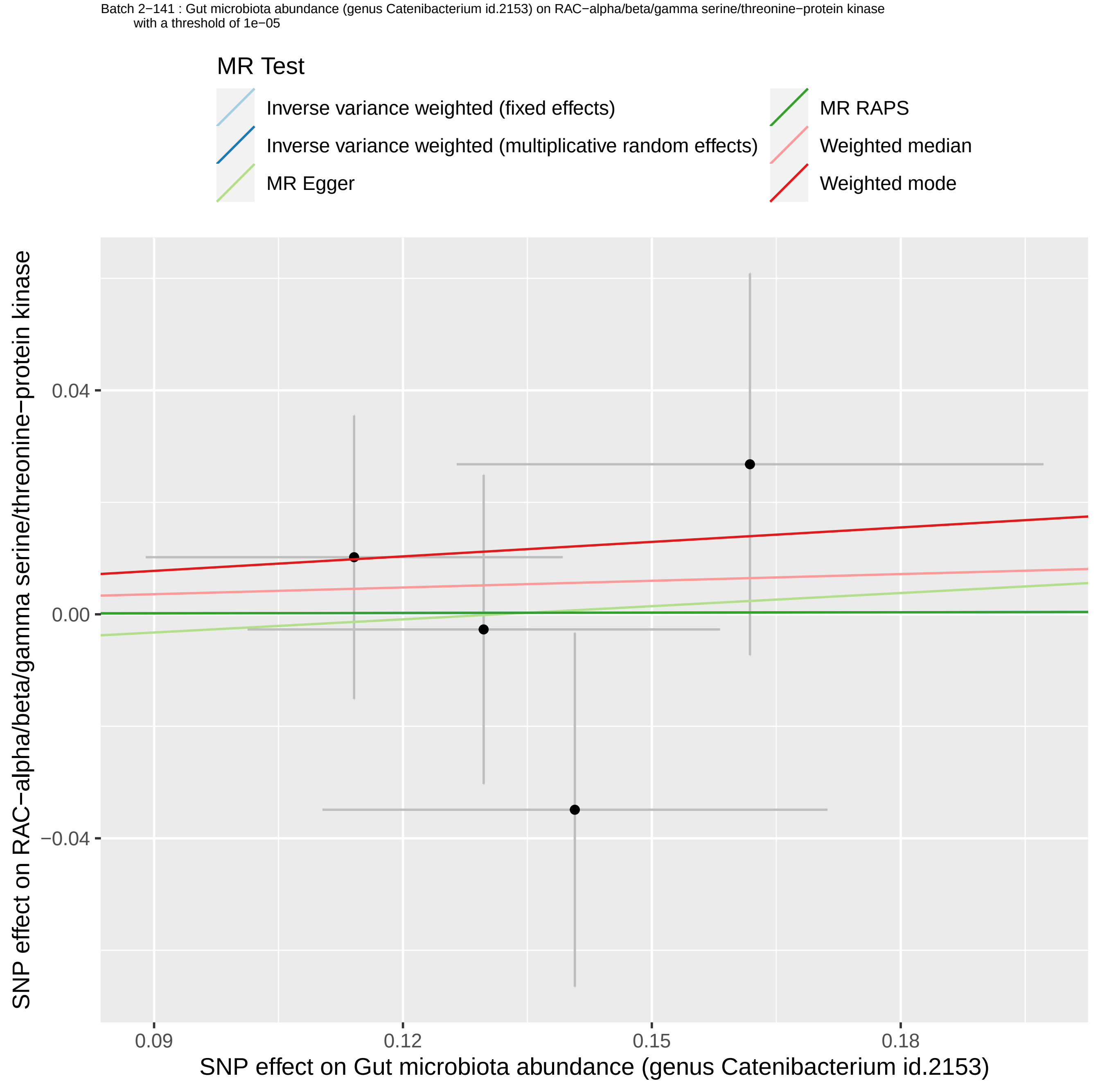

Batch 2−141 : Gut microbiota abundance (genus Catenibacterium id.2153) on RAC−alpha/beta/gamma serine/threonine−protein kinase
with a threshold of 1e−05
MR Test
Inverse variance weighted (fixed effects)
Inverse variance weighted (multiplicative random effects)
MR Egger
MR RAPS
Weighted median
Weighted mode
0.04
0.00
−0.04
0.09
0.12
0.15
0.18
SNP effect on RAC−alpha/beta/gamma serine/threonine−protein kinase
SNP effect on Gut microbiota abundance (genus Catenibacterium id.2153)

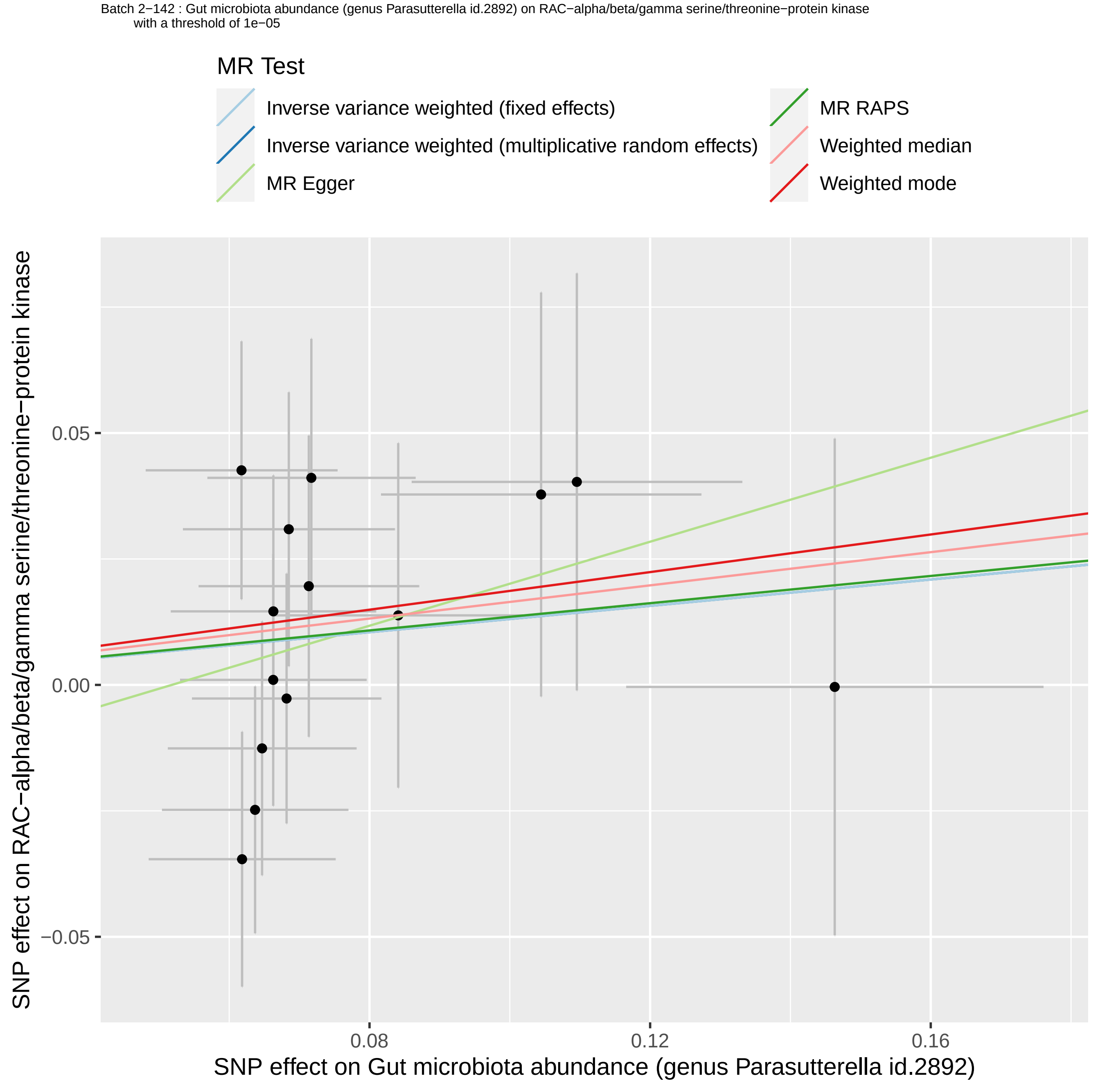

Batch 2−142 : Gut microbiota abundance (genus Parasutterella id.2892) on RAC−alpha/beta/gamma serine/threonine−protein kinase
with a threshold of 1e−05
MR Test
Inverse variance weighted (fixed effects)
Inverse variance weighted (multiplicative random effects)
MR Egger
MR RAPS
Weighted median
Weighted mode
0.05
0.00
−0.05
0.08
0.12
0.16
SNP effect on RAC−alpha/beta/gamma serine/threonine−protein kinase
SNP effect on Gut microbiota abundance (genus Parasutterella id.2892)

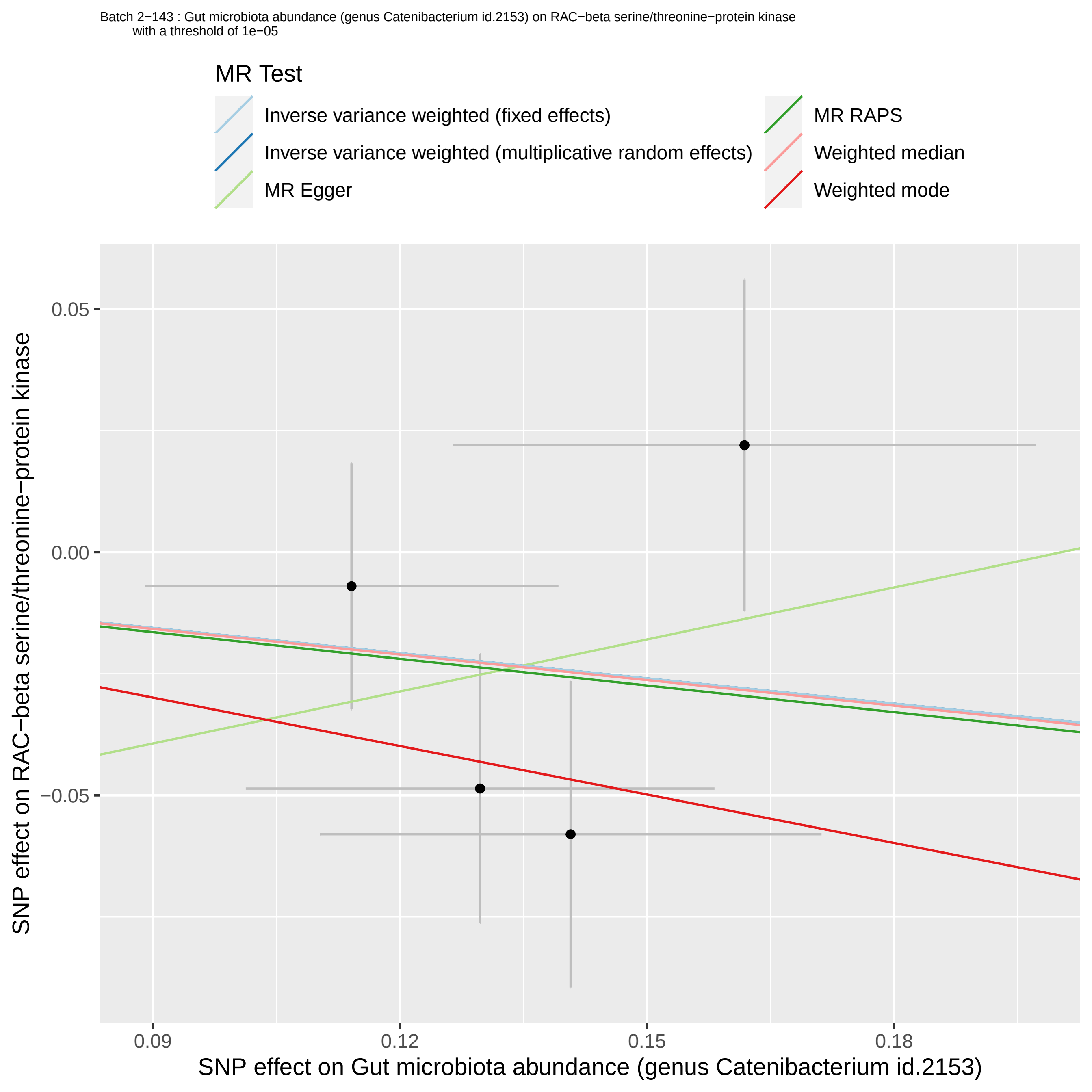
Batch 2−143 : Gut microbiota abundance (genus Catenibacterium id.2153) on RAC−beta serine/threonine−protein kinase
with a threshold of 1e−05
MR Test
Inverse variance weighted (fixed effects)
Inverse variance weighted (multiplicative random effects)
MR Egger
MR RAPS
Weighted median
Weighted mode
0.05
0.00
−0.05
0.09
0.12
0.15
0.18
SNP effect on RAC−beta serine/threonine−protein kinase
SNP effect on Gut microbiota abundance (genus Catenibacterium id.2153)

Batch 2−144 : Gut microbiota abundance (genus Parasutterella id.2892) on RAC−beta serine/threonine−protein kinase
with a threshold of 1e−05

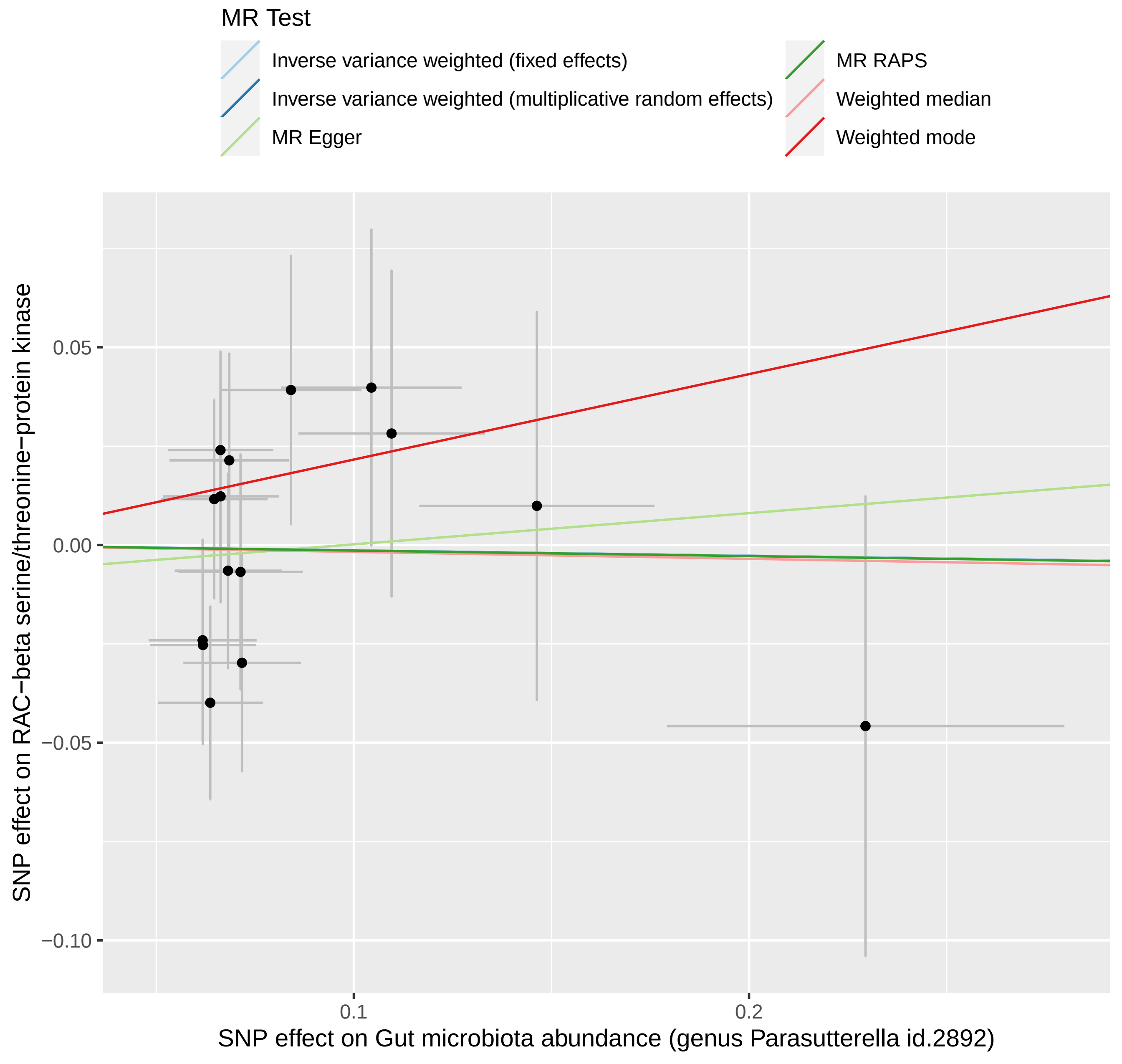

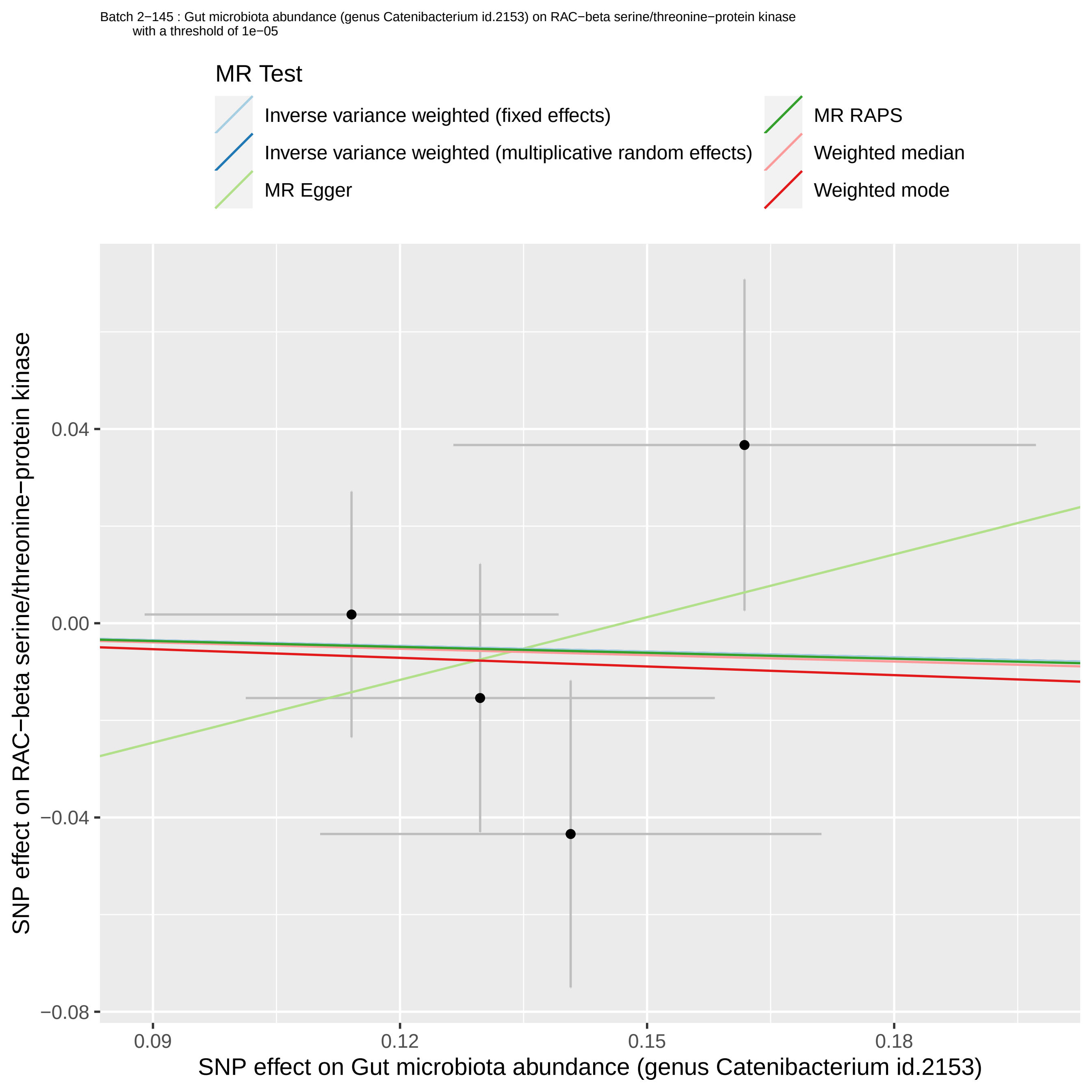

Batch 2−145 : Gut microbiota abundance (genus Catenibacterium id.2153) on RAC−beta serine/threonine−protein kinase
with a threshold of 1e−05
MR Test
Inverse variance weighted (fixed effects)
Inverse variance weighted (multiplicative random effects)
MR Egger
MR RAPS
Weighted median
Weighted mode
SNP effect on RAC−beta serine/threonine−protein kinase
0.04
0.00
−0.04
−0.08
0.09
0.12
0.15
0.18
SNP effect on Gut microbiota abundance (genus Catenibacterium id.2153)

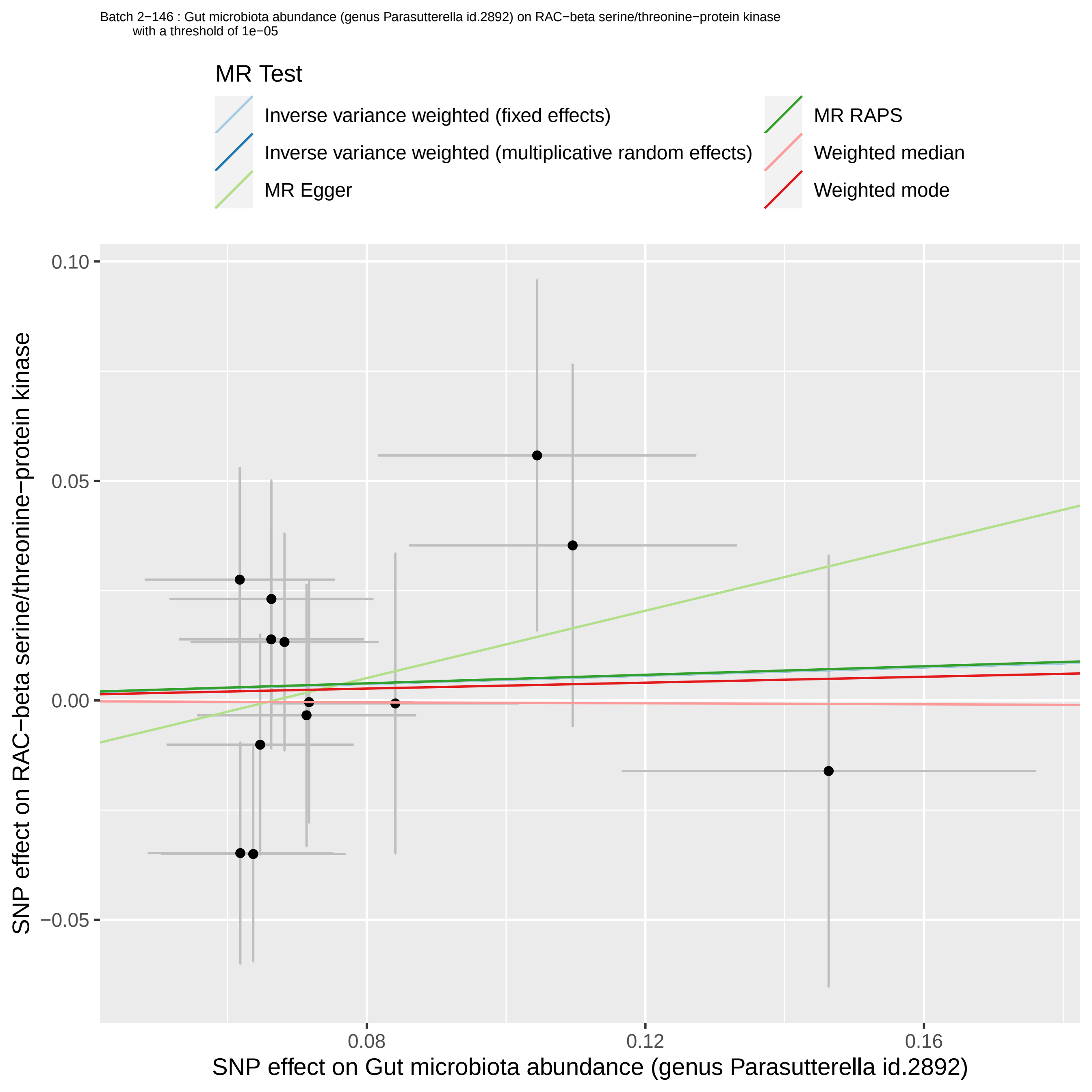

Batch 2−146 : Gut microbiota abundance (genus Parasutterella id.2892) on RAC−beta serine/threonine−protein kinase
with a threshold of 1e−05
MR Test
Inverse variance weighted (fixed effects)
Inverse variance weighted (multiplicative random effects)
MR Egger
MR RAPS
Weighted median
Weighted mode
0.10
0.05
0.00
−0.05
0.08
0.12
0.16
SNP effect on RAC−beta serine/threonine−protein kinase
SNP effect on Gut microbiota abundance (genus Parasutterella id.2892)

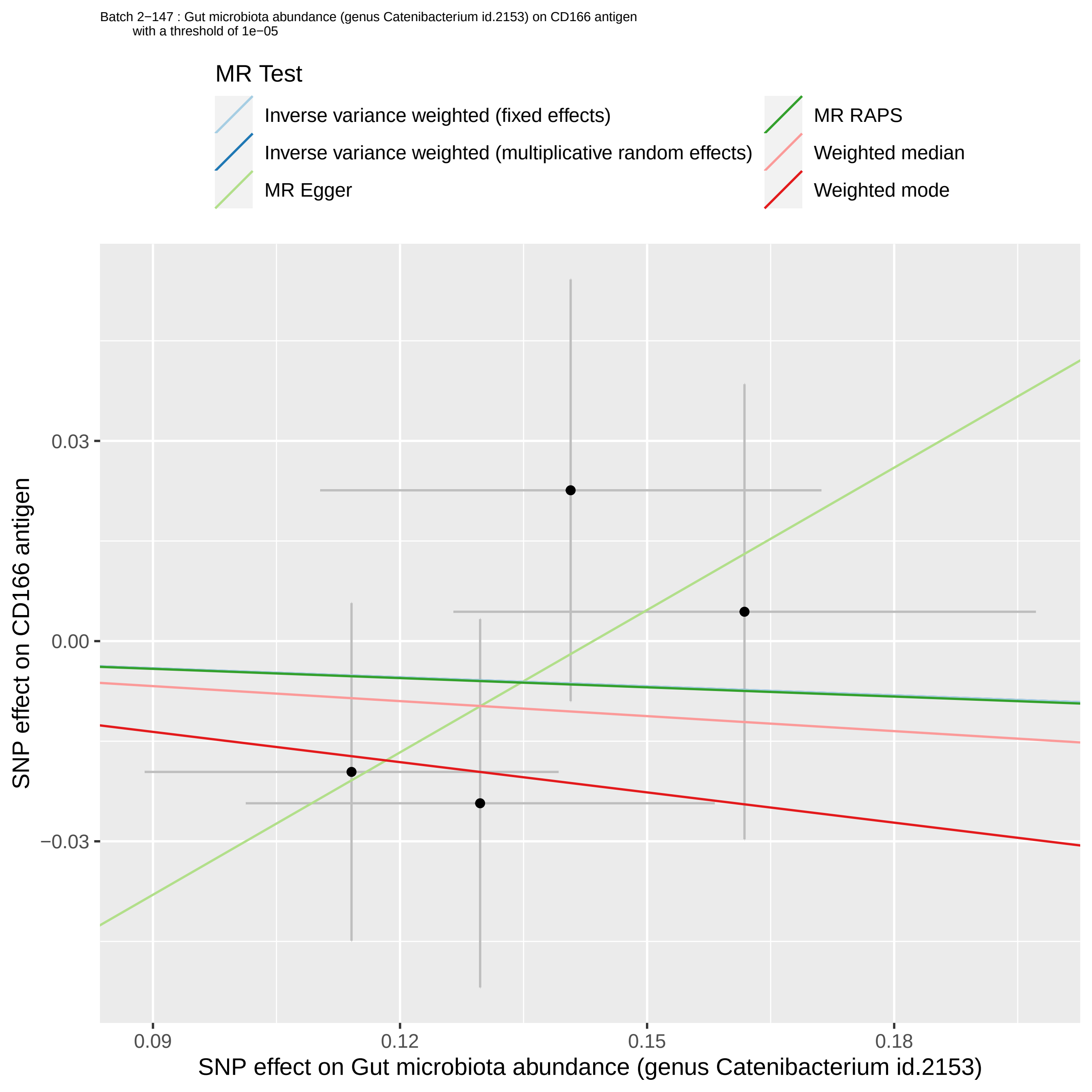

Batch 2−147 : Gut microbiota abundance (genus Catenibacterium id.2153) on CD166 antigen
with a threshold of 1e−05
MR Test
Inverse variance weighted (fixed effects)
Inverse variance weighted (multiplicative random effects)
MR Egger
MR RAPS
Weighted median
Weighted mode
0.03
0.00
−0.03
0.09
0.12
0.15
0.18
SNP effect on CD166 antigen
SNP effect on Gut microbiota abundance (genus Catenibacterium id.2153)

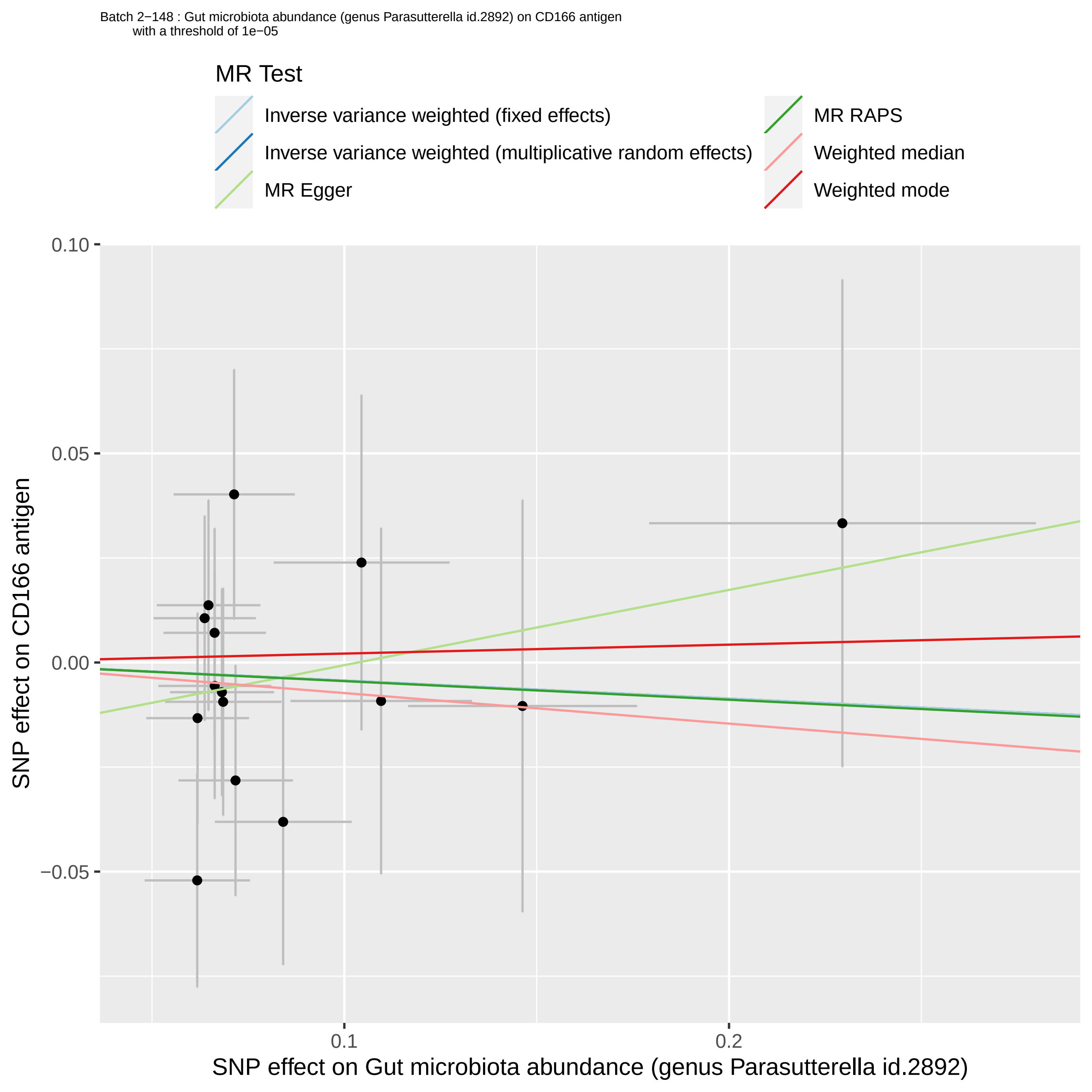
Batch 2−148 : Gut microbiota abundance (genus Parasutterella id.2892) on CD166 antigen
with a threshold of 1e−05
MR Test
Inverse variance weighted (fixed effects)
Inverse variance weighted (multiplicative random effects)
MR Egger
MR RAPS
Weighted median
Weighted mode
0.10
0.05
0.00
−0.05
0.1
0.2
SNP effect on CD166 antigen
SNP effect on Gut microbiota abundance (genus Parasutterella id.2892)

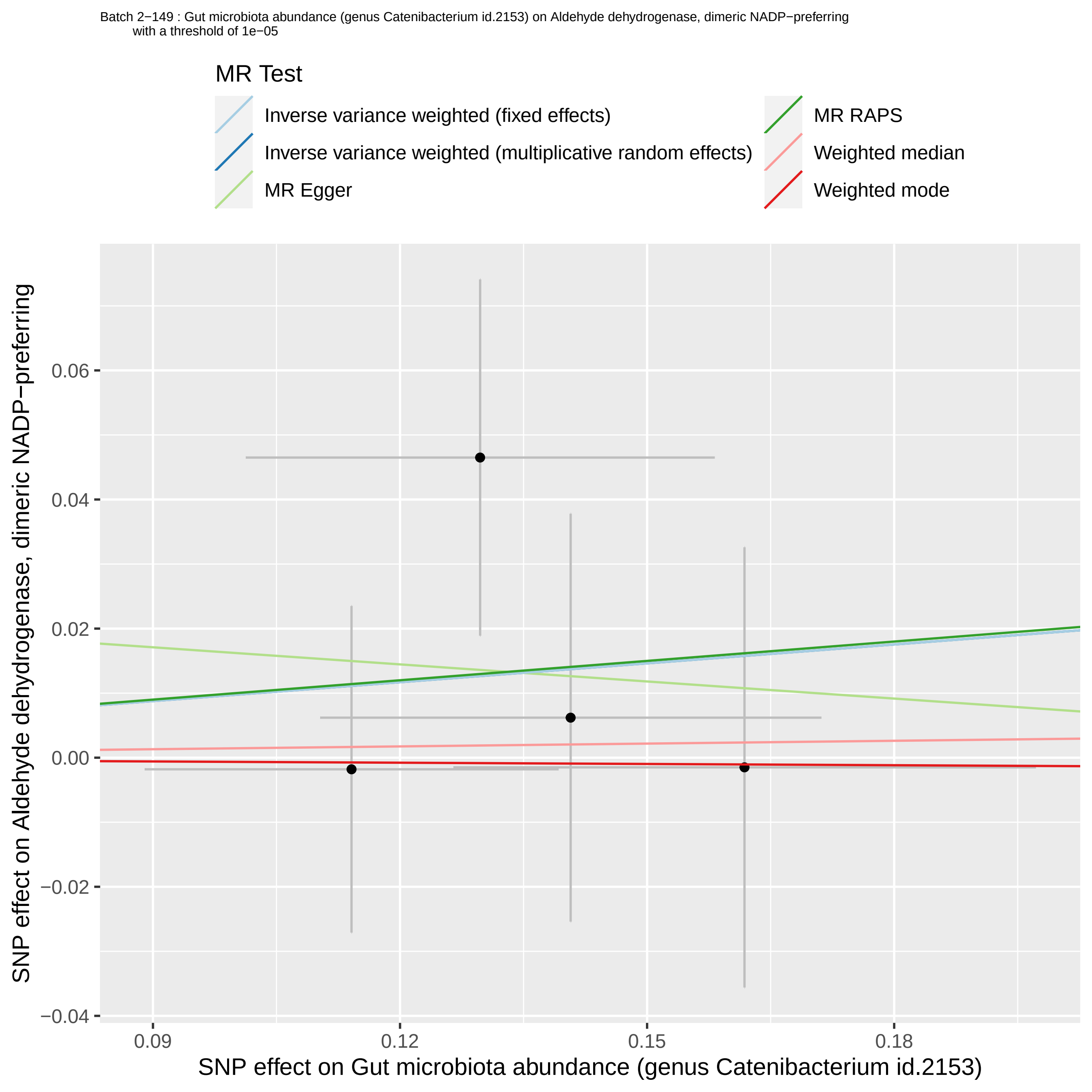

Batch 2−149 : Gut microbiota abundance (genus Catenibacterium id.2153) on Aldehyde dehydrogenase, dimeric NADP−preferring
with a threshold of 1e−05
MR Test
Inverse variance weighted (fixed effects)
Inverse variance weighted (multiplicative random effects)
MR Egger
MR RAPS
Weighted median
Weighted mode
SNP effect on Aldehyde dehydrogenase, dimeric NADP−preferring
0.06
0.04
0.02
0.00
−0.02
−0.04
0.09
0.12
0.15
0.18
SNP effect on Gut microbiota abundance (genus Catenibacterium id.2153)

Batch 2−150 : Gut microbiota abundance (genus Parasutterella id.2892) on Aldehyde dehydrogenase, dimeric NADP−preferring
with a threshold of 1e−05

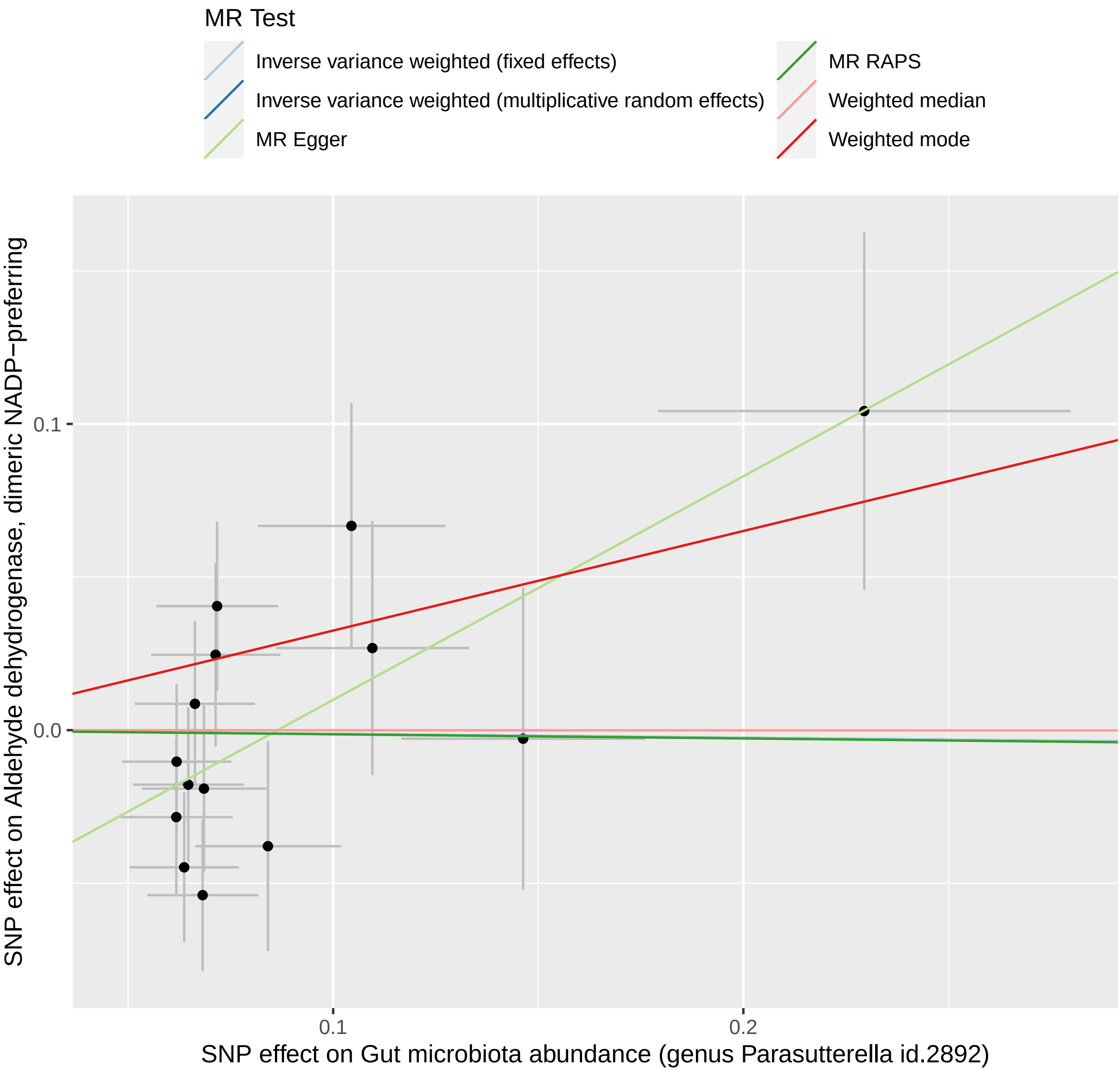

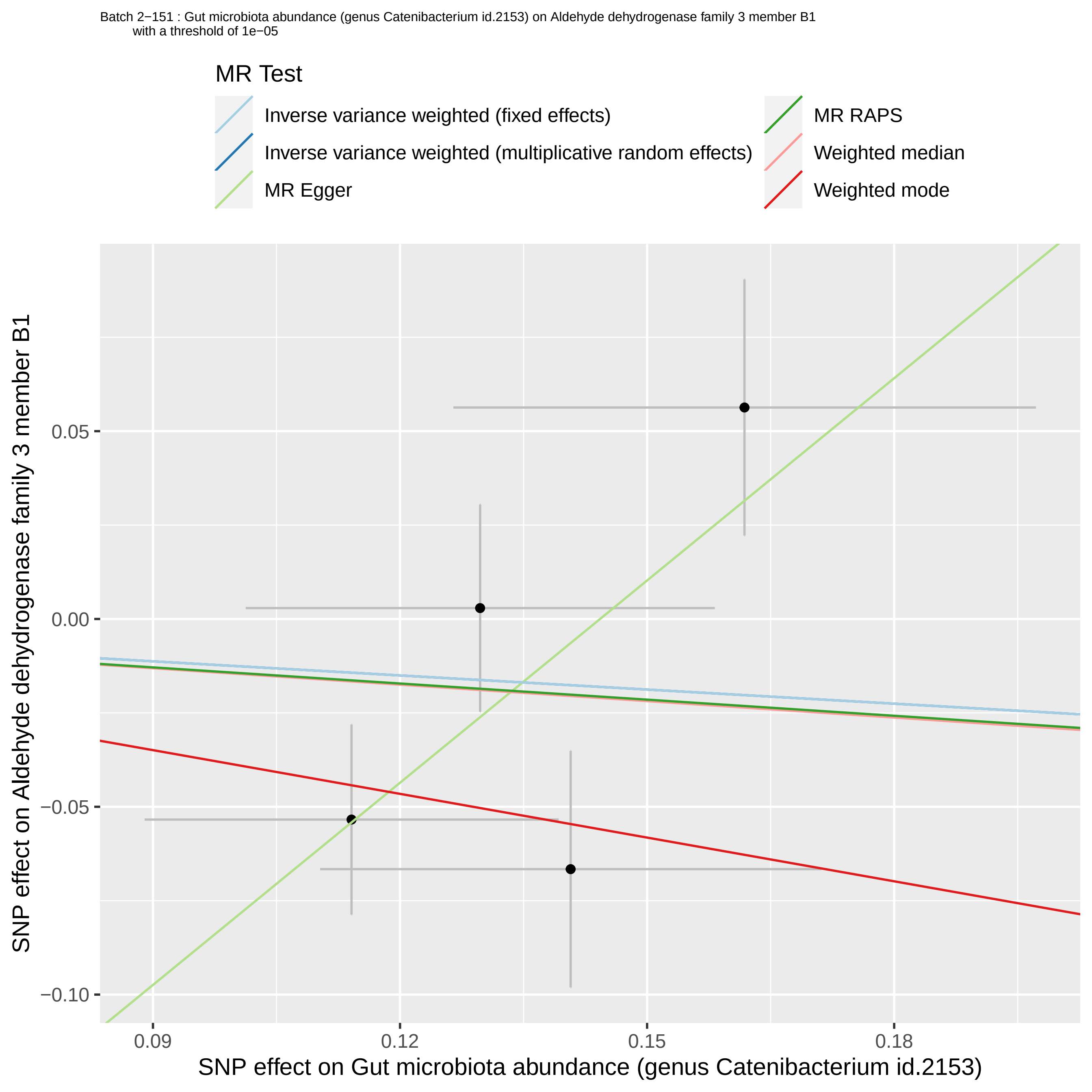

Batch 2−151 : Gut microbiota abundance (genus Catenibacterium id.2153) on Aldehyde dehydrogenase family 3 member B1
with a threshold of 1e−05
MR Test
Inverse variance weighted (fixed effects)
Inverse variance weighted (multiplicative random effects)
MR Egger
MR RAPS
Weighted median
Weighted mode
0.05
0.00
−0.05
−0.10
0.09
0.12
0.15
0.18
SNP effect on Aldehyde dehydrogenase family 3 member B1
SNP effect on Gut microbiota abundance (genus Catenibacterium id.2153)

Batch 2−152 : Gut microbiota abundance (genus Parasutterella id.2892) on Aldehyde dehydrogenase family 3 member B1
with a threshold of 1e−05

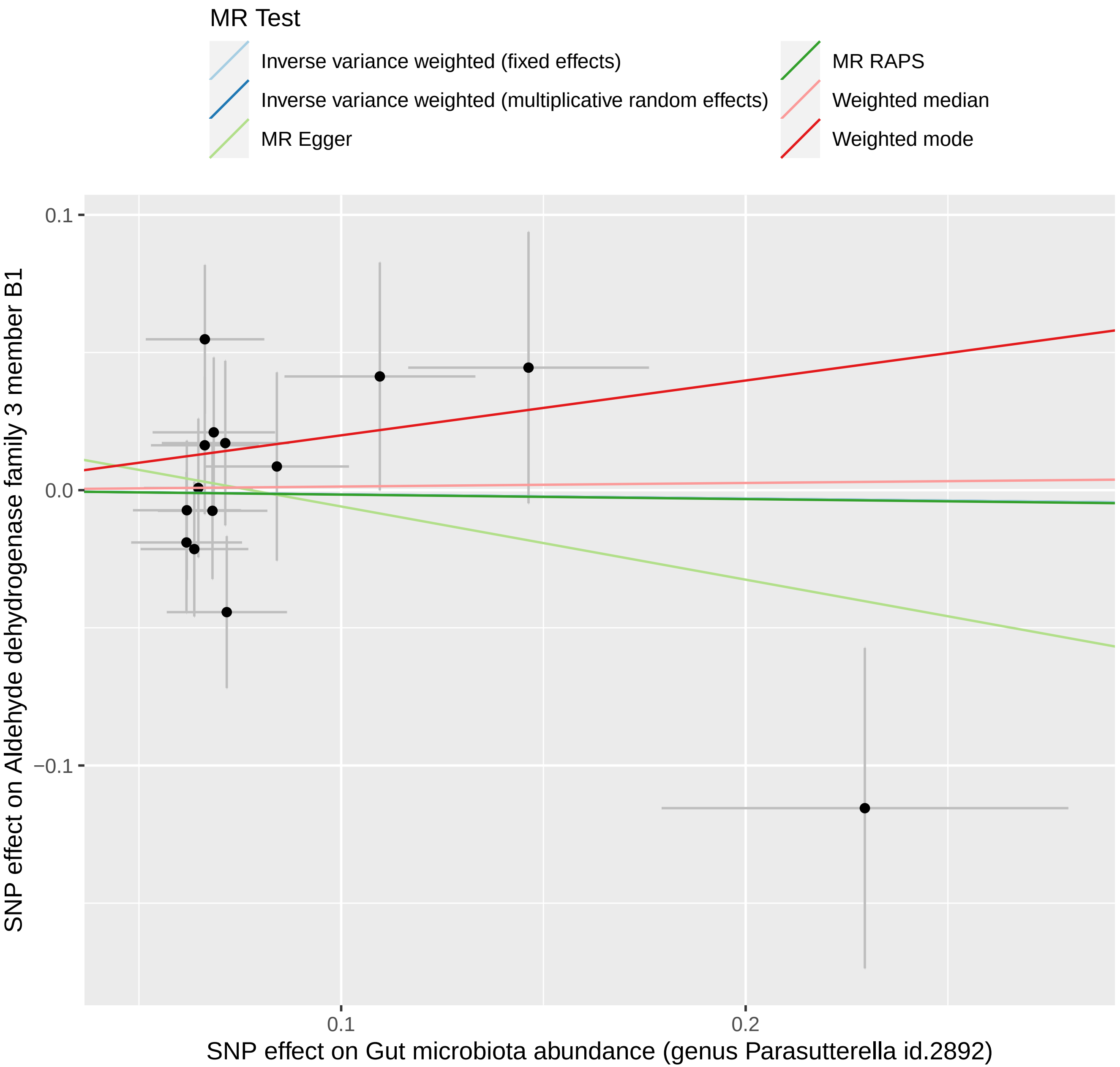

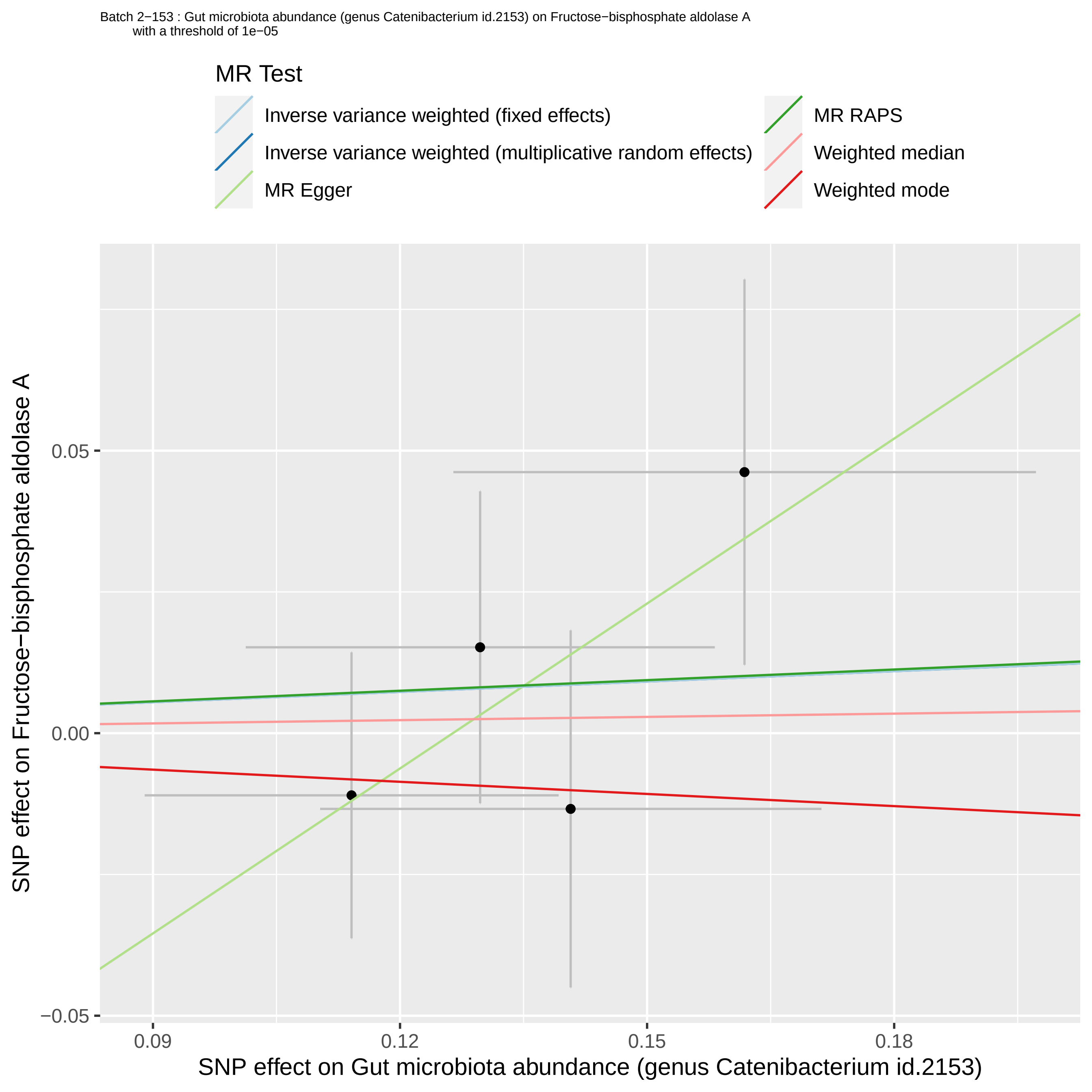

Batch 2−153 : Gut microbiota abundance (genus Catenibacterium id.2153) on Fructose−bisphosphate aldolase A
with a threshold of 1e−05
MR Test
Inverse variance weighted (fixed effects)
Inverse variance weighted (multiplicative random effects)
MR Egger
MR RAPS
Weighted median
Weighted mode
0.05
0.00
−0.05
0.09
0.12
0.15
0.18
SNP effect on Fructose−bisphosphate aldolase A
SNP effect on Gut microbiota abundance (genus Catenibacterium id.2153)

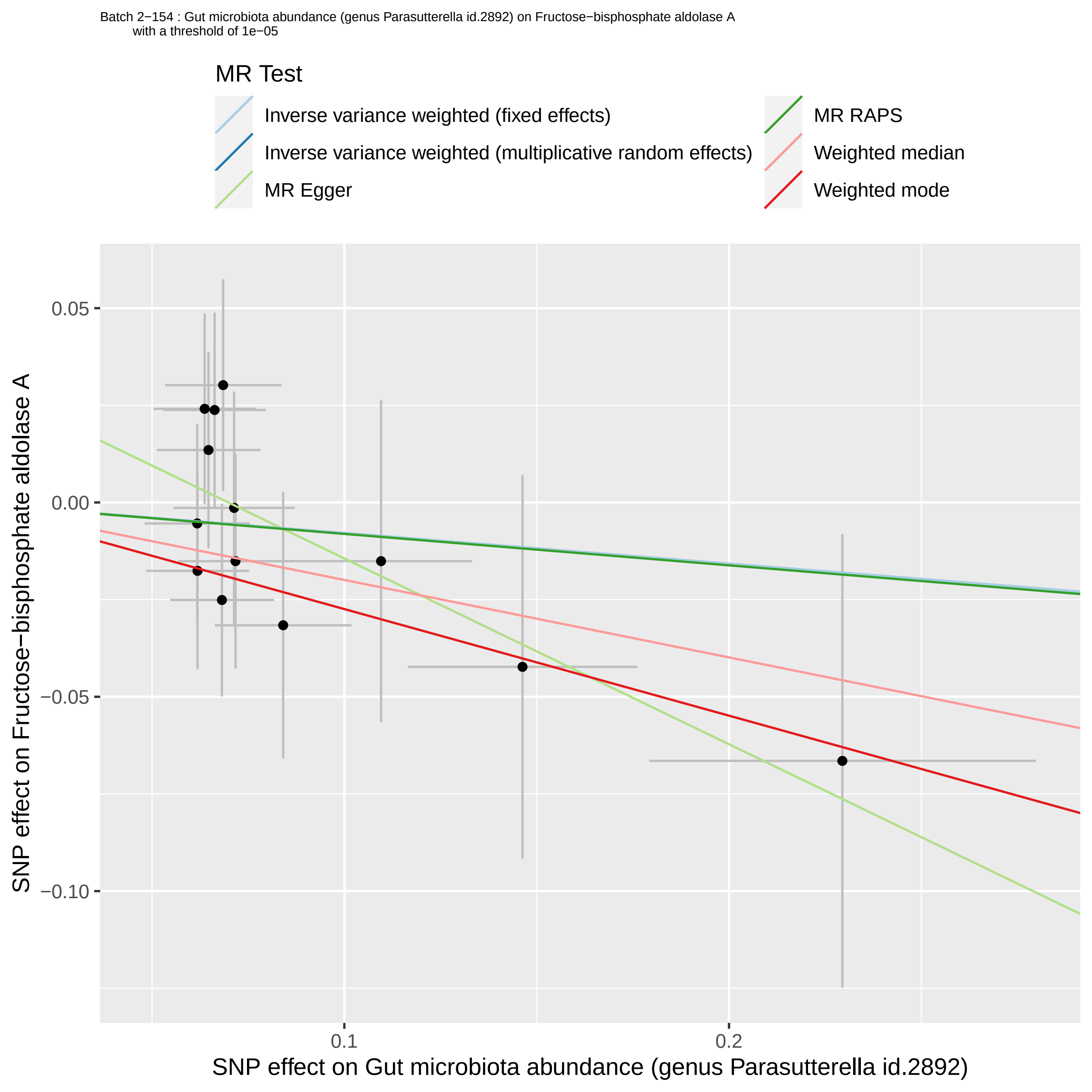

Batch 2−154 : Gut microbiota abundance (genus Parasutterella id.2892) on Fructose−bisphosphate aldolase A
with a threshold of 1e−05
MR Test
Inverse variance weighted (fixed effects)
Inverse variance weighted (multiplicative random effects)
MR Egger
MR RAPS
Weighted median
Weighted mode
0.05
0.00
−0.05
−0.10
0.1
0.2
SNP effect on Fructose−bisphosphate aldolase A
SNP effect on Gut microbiota abundance (genus Parasutterella id.2892)

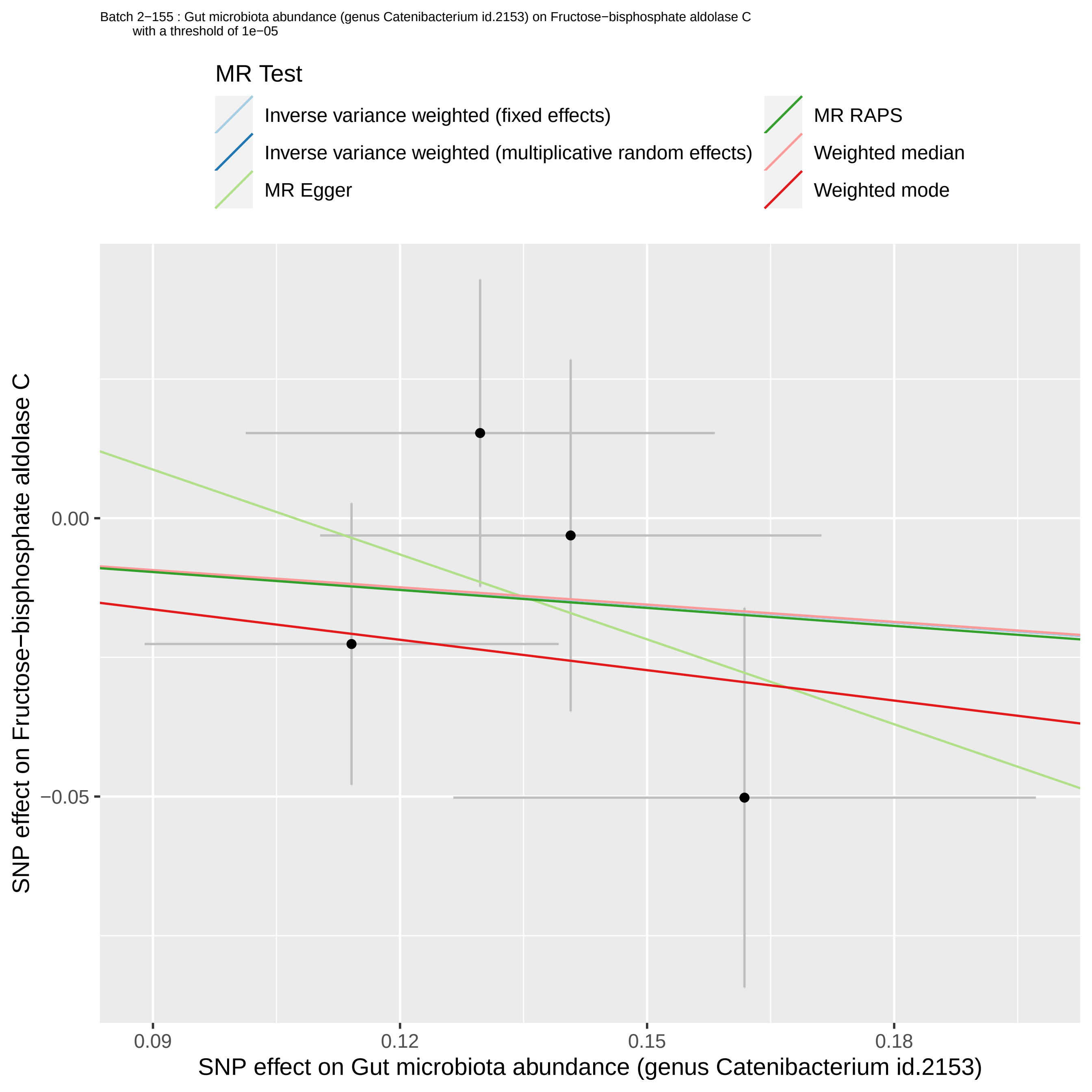
Batch 2−155 : Gut microbiota abundance (genus Catenibacterium id.2153) on Fructose−bisphosphate aldolase C
with a threshold of 1e−05
MR Test
Inverse variance weighted (fixed effects)
Inverse variance weighted (multiplicative random effects)
MR Egger
MR RAPS
Weighted median
Weighted mode
0.00
−0.05
0.09
0.12
0.15
0.18
SNP effect on Fructose−bisphosphate aldolase C
SNP effect on Gut microbiota abundance (genus Catenibacterium id.2153)

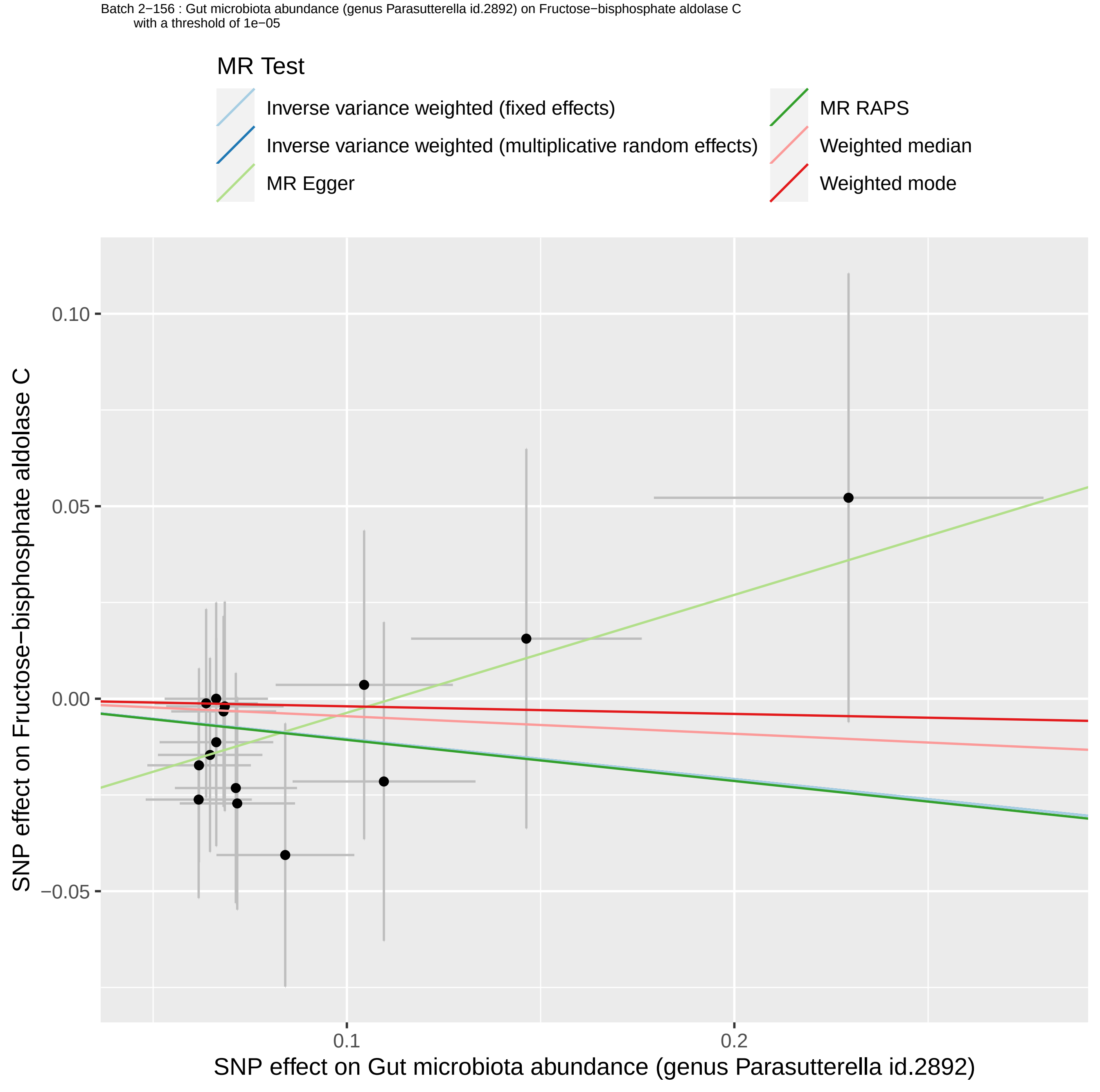
Batch 2−156 : Gut microbiota abundance (genus Parasutterella id.2892) on Fructose−bisphosphate aldolase C
with a threshold of 1e−05
MR Test
Inverse variance weighted (fixed effects)
Inverse variance weighted (multiplicative random effects)
MR Egger
MR RAPS
Weighted median
Weighted mode
0.10
0.05
0.00
−0.05
0.1
0.2
SNP effect on Fructose−bisphosphate aldolase C
SNP effect on Gut microbiota abundance (genus Parasutterella id.2892)

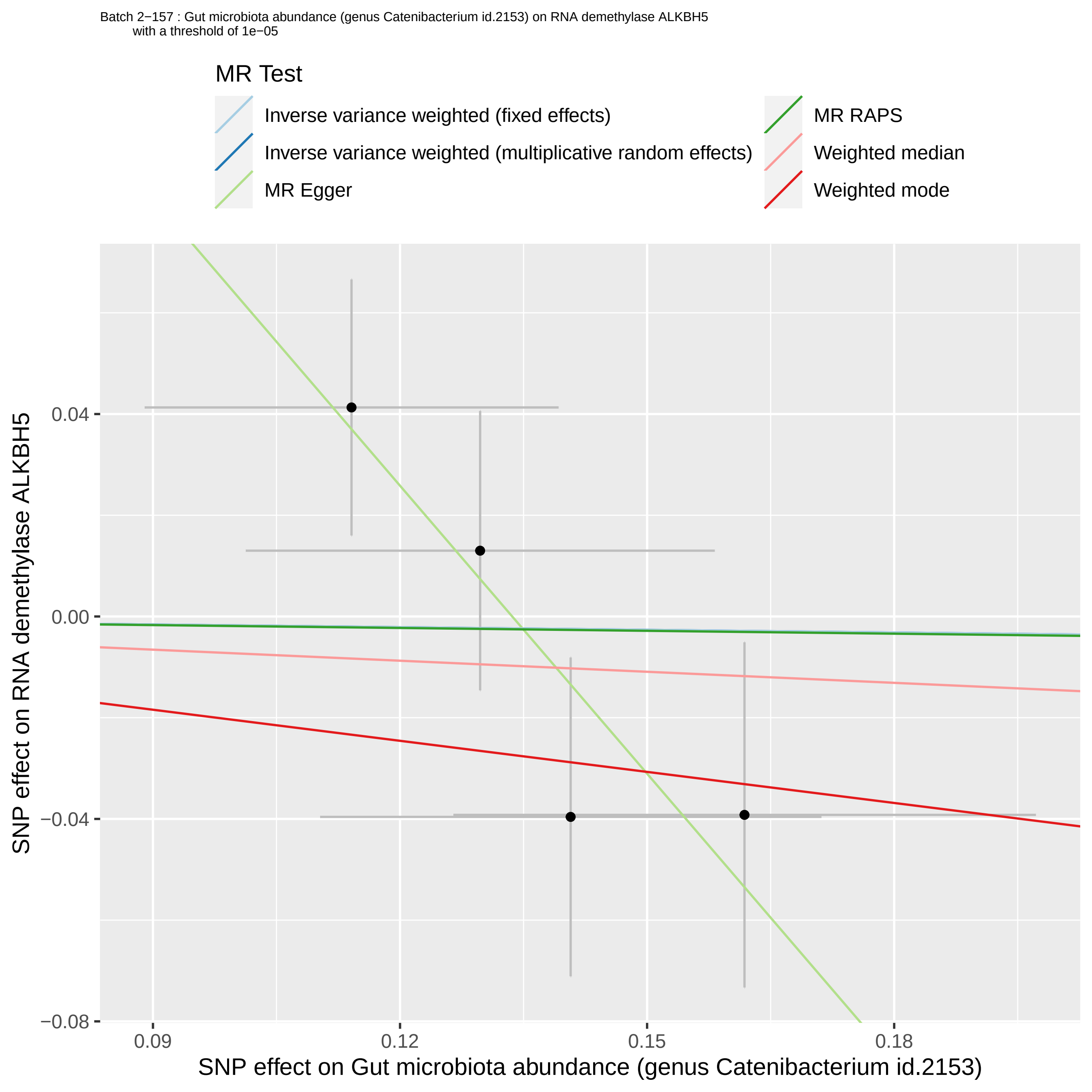

Batch 2−157 : Gut microbiota abundance (genus Catenibacterium id.2153) on RNA demethylase ALKBH5
with a threshold of 1e−05
MR Test
Inverse variance weighted (fixed effects)
Inverse variance weighted (multiplicative random effects)
MR Egger
MR RAPS
Weighted median
Weighted mode
SNP effect on RNA demethylase ALKBH5
0.04
0.00
−0.04
−0.08
0.09
0.12
0.15
0.18
SNP effect on Gut microbiota abundance (genus Catenibacterium id.2153)

Batch 2−158 : Gut microbiota abundance (genus Parasutterella id.2892) on RNA demethylase ALKBH5
with a threshold of 1e−05

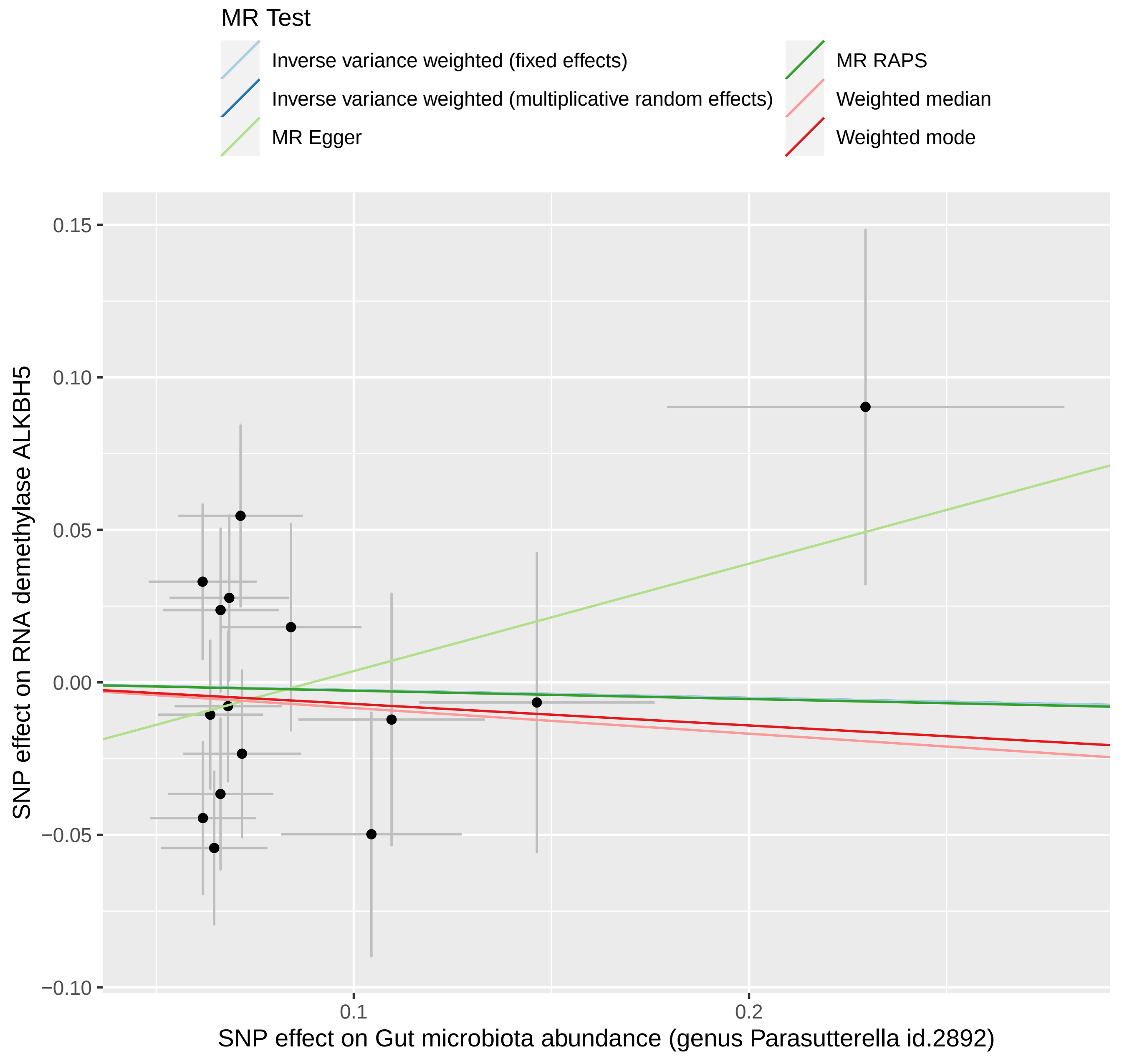

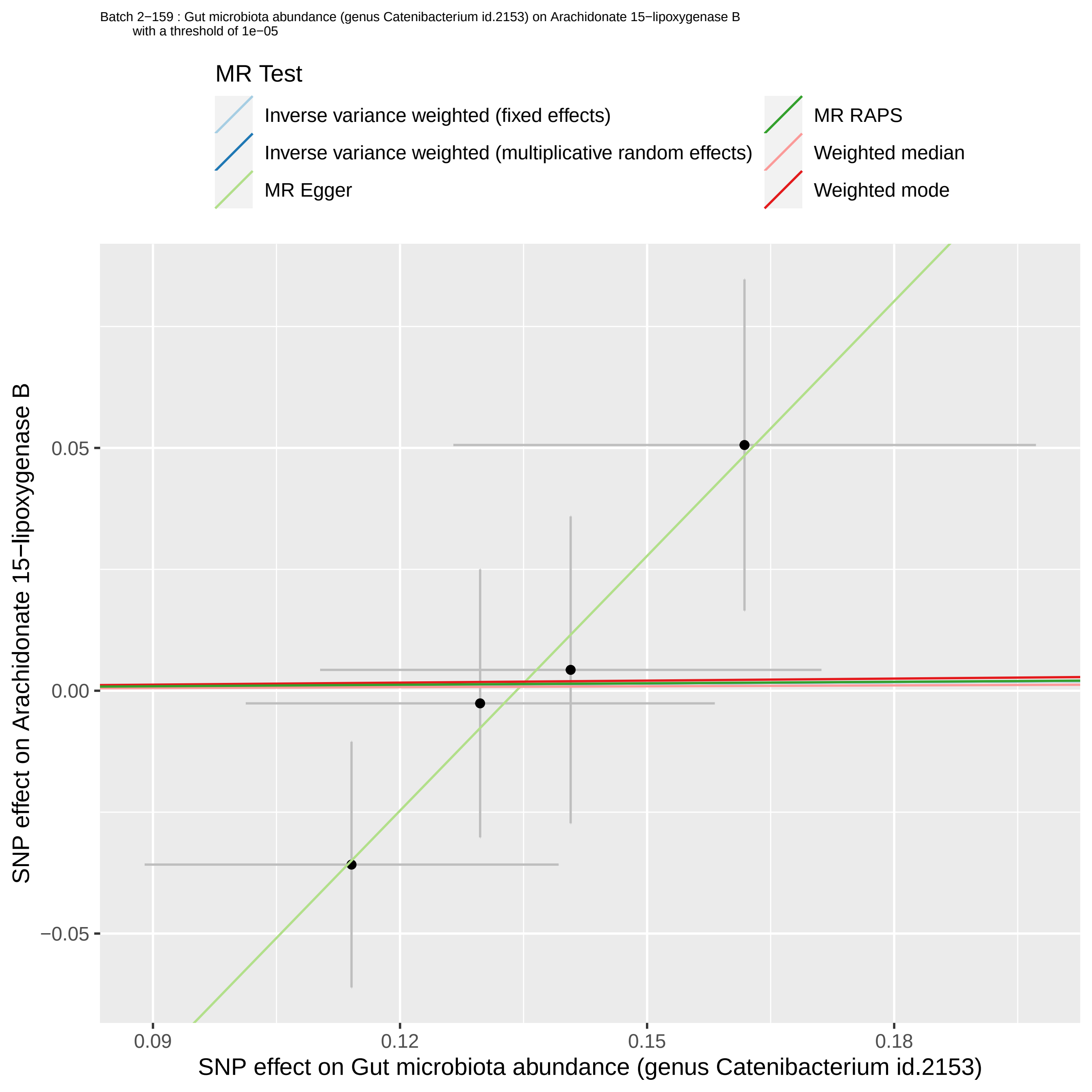
Batch 2−159 : Gut microbiota abundance (genus Catenibacterium id.2153) on Arachidonate 15−lipoxygenase B
with a threshold of 1e−05
MR Test
Inverse variance weighted (fixed effects)
Inverse variance weighted (multiplicative random effects)
MR Egger
MR RAPS
Weighted median
Weighted mode
0.05
0.00
−0.05
0.09
0.12
0.15
0.18
SNP effect on Arachidonate 15−lipoxygenase B
SNP effect on Gut microbiota abundance (genus Catenibacterium id.2153)

Batch 2−160 : Gut microbiota abundance (genus Parasutterella id.2892) on Arachidonate 15−lipoxygenase B
with a threshold of 1e−05

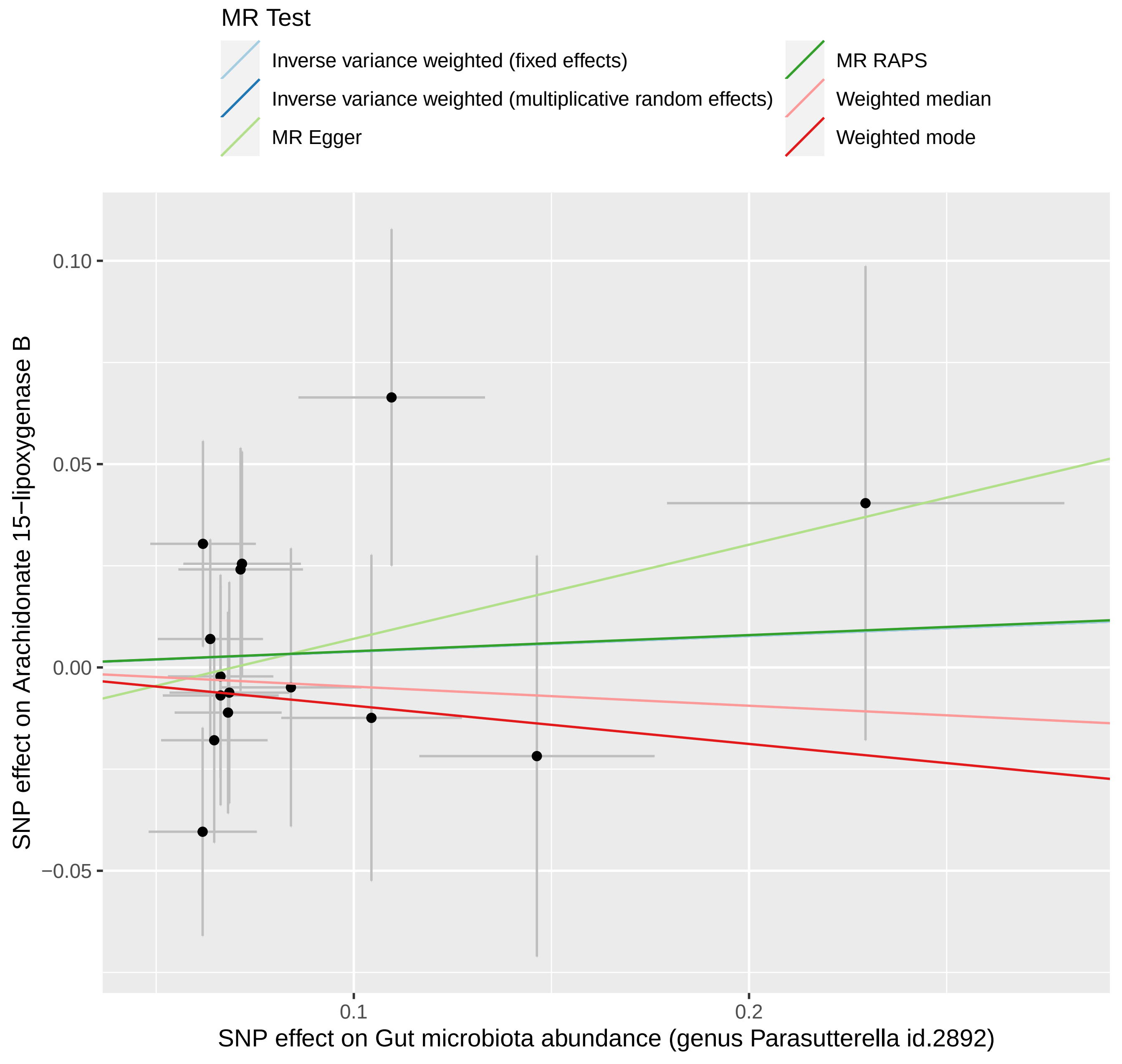

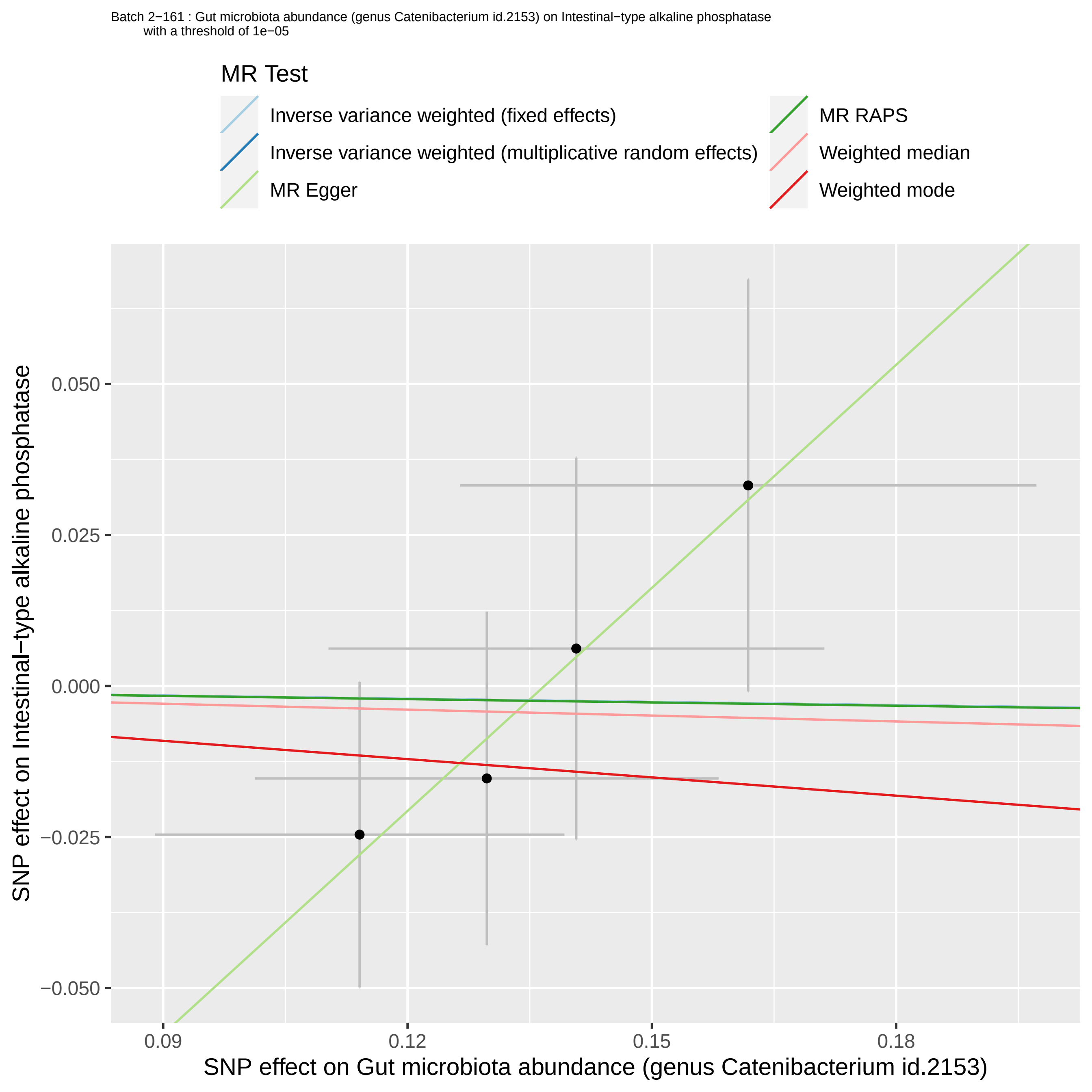
Batch 2−161 : Gut microbiota abundance (genus Catenibacterium id.2153) on Intestinal−type alkaline phosphatase
with a threshold of 1e−05
MR Test
Inverse variance weighted (fixed effects)
Inverse variance weighted (multiplicative random effects)
MR Egger
MR RAPS
Weighted median
Weighted mode
SNP effect on Intestinal−type alkaline phosphatase
0.050
0.025
0.000
−0.025
−0.050
0.09
0.12
0.15
0.18
SNP effect on Gut microbiota abundance (genus Catenibacterium id.2153)

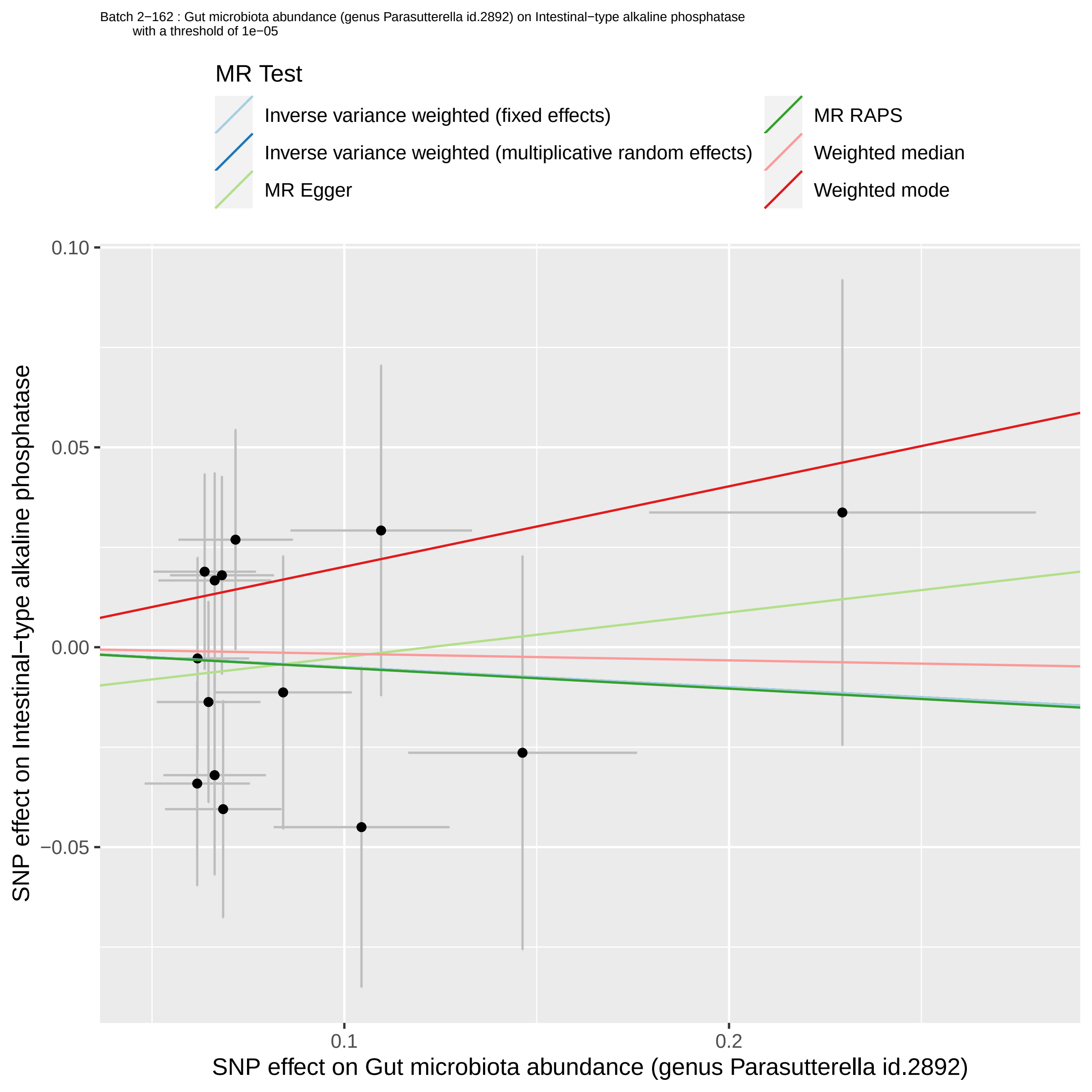

Batch 2−162 : Gut microbiota abundance (genus Parasutterella id.2892) on Intestinal−type alkaline phosphatase
with a threshold of 1e−05
MR Test
Inverse variance weighted (fixed effects)
Inverse variance weighted (multiplicative random effects)
MR Egger
MR RAPS
Weighted median
Weighted mode
0.10
0.05
0.00
−0.05
0.1
0.2
SNP effect on Intestinal−type alkaline phosphatase
SNP effect on Gut microbiota abundance (genus Parasutterella id.2892)

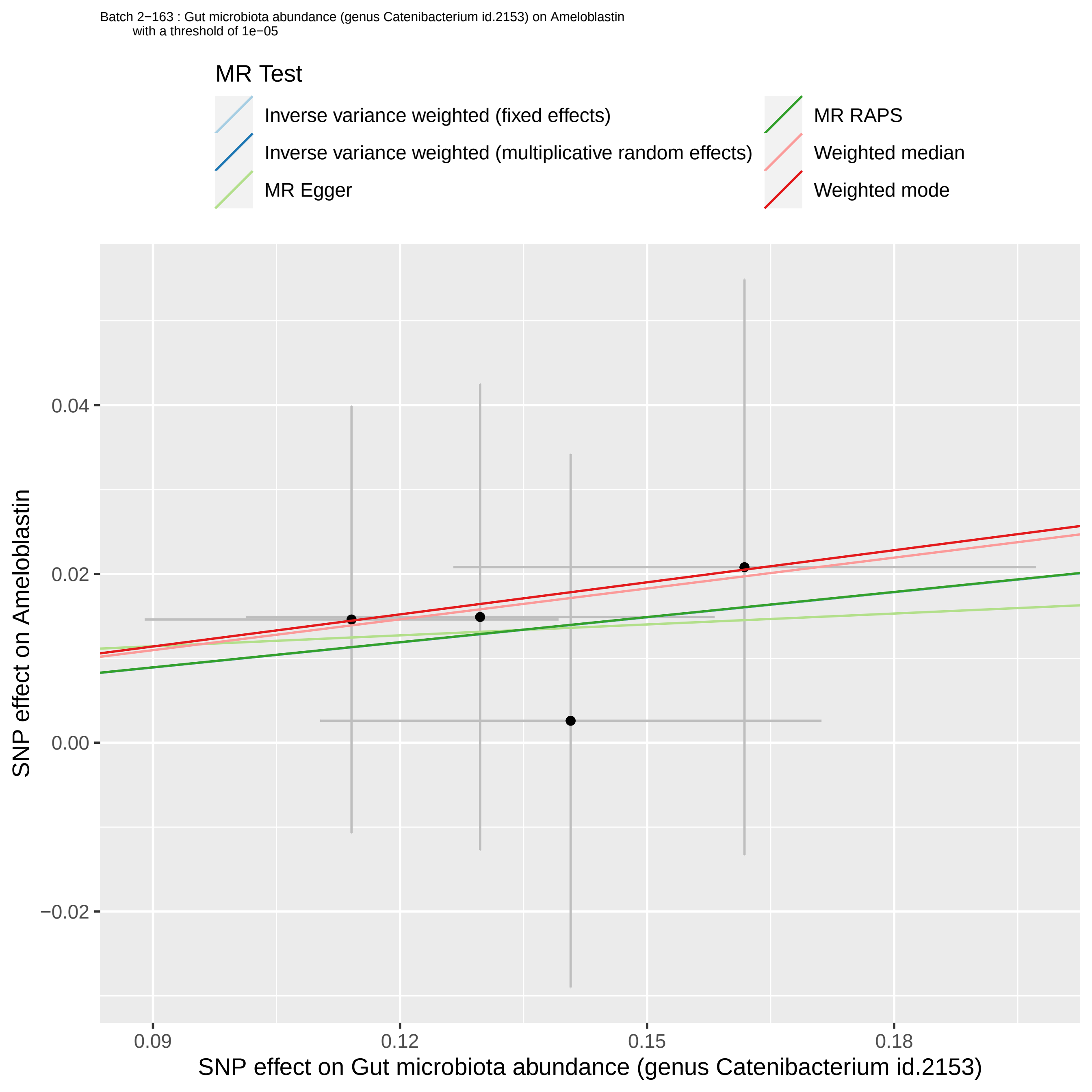

Batch 2−163 : Gut microbiota abundance (genus Catenibacterium id.2153) on Ameloblastin
with a threshold of 1e−05
MR Test
Inverse variance weighted (fixed effects)
Inverse variance weighted (multiplicative random effects)
MR Egger
MR RAPS
Weighted median
Weighted mode
0.04
0.02
0.00
−0.02
0.09
0.12
0.15
0.18
SNP effect on Ameloblastin
SNP effect on Gut microbiota abundance (genus Catenibacterium id.2153)

Batch 2−164 : Gut microbiota abundance (genus Parasutterella id.2892) on Ameloblastin
with a threshold of 1e−05

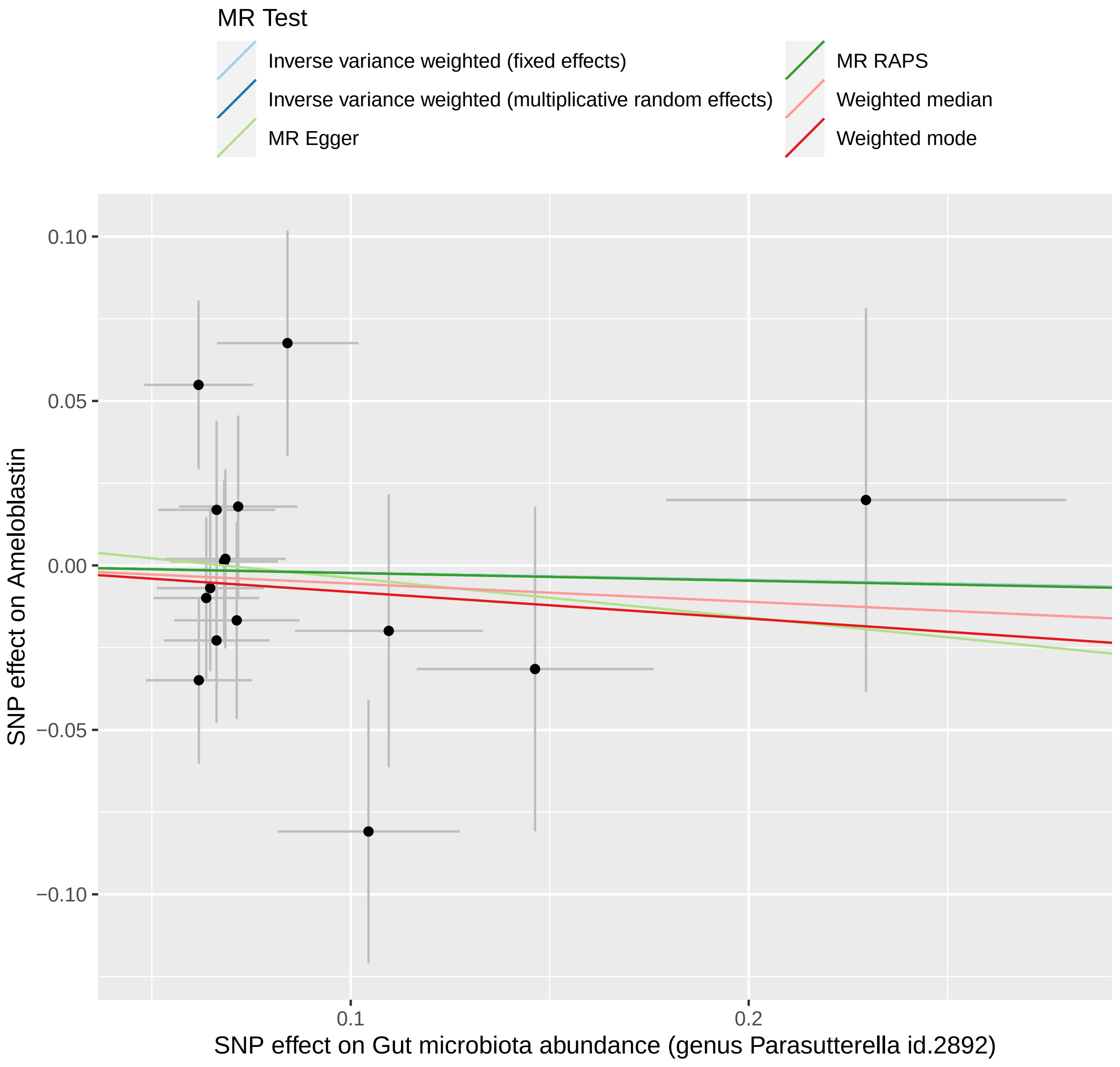

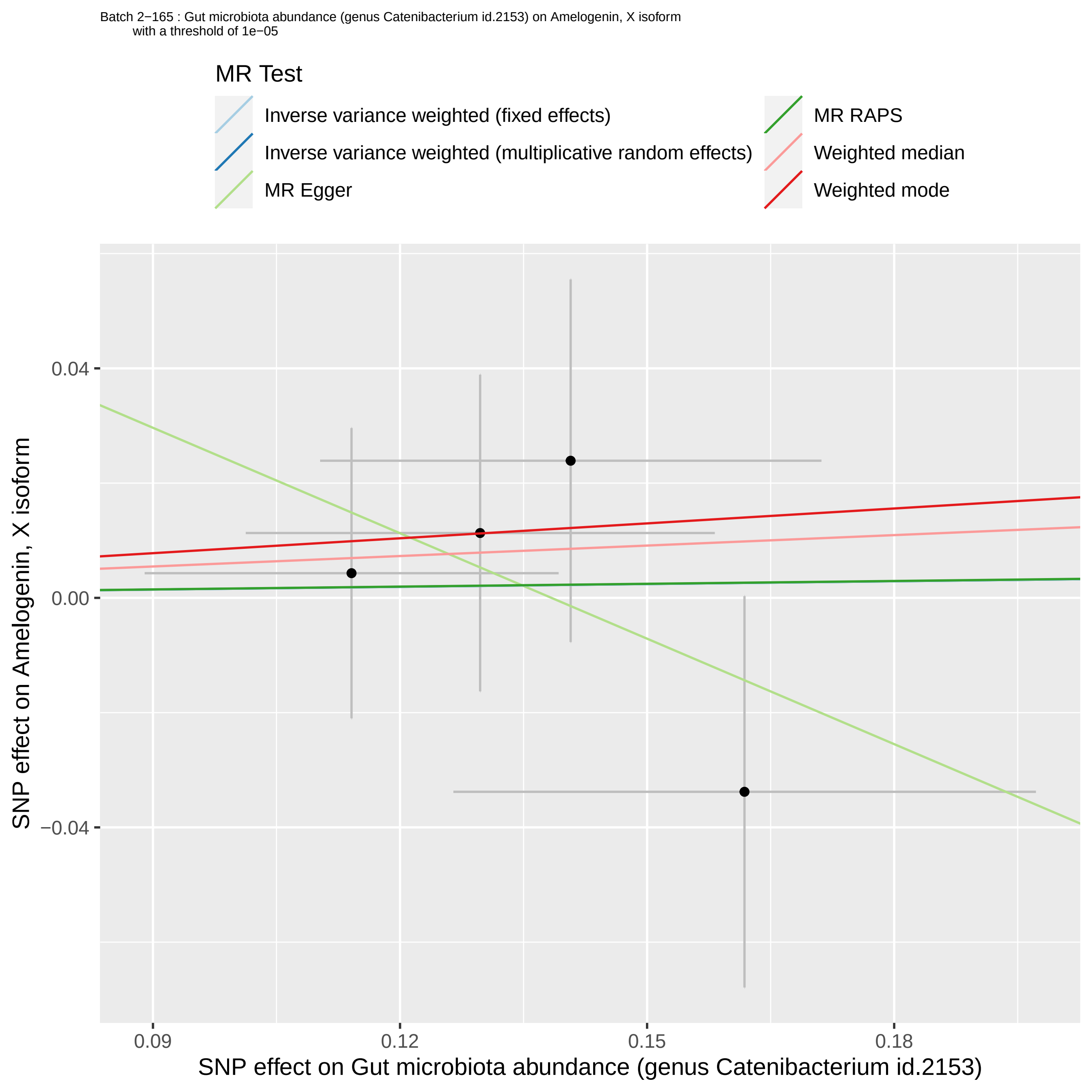

Batch 2−165 : Gut microbiota abundance (genus Catenibacterium id.2153) on Amelogenin, X isoform
with a threshold of 1e−05
MR Test
Inverse variance weighted (fixed effects)
Inverse variance weighted (multiplicative random effects)
MR Egger
MR RAPS
Weighted median
Weighted mode
SNP effect on Amelogenin, X isoform
0.04
0.00
−0.04
0.09
0.12
0.15
0.18
SNP effect on Gut microbiota abundance (genus Catenibacterium id.2153)

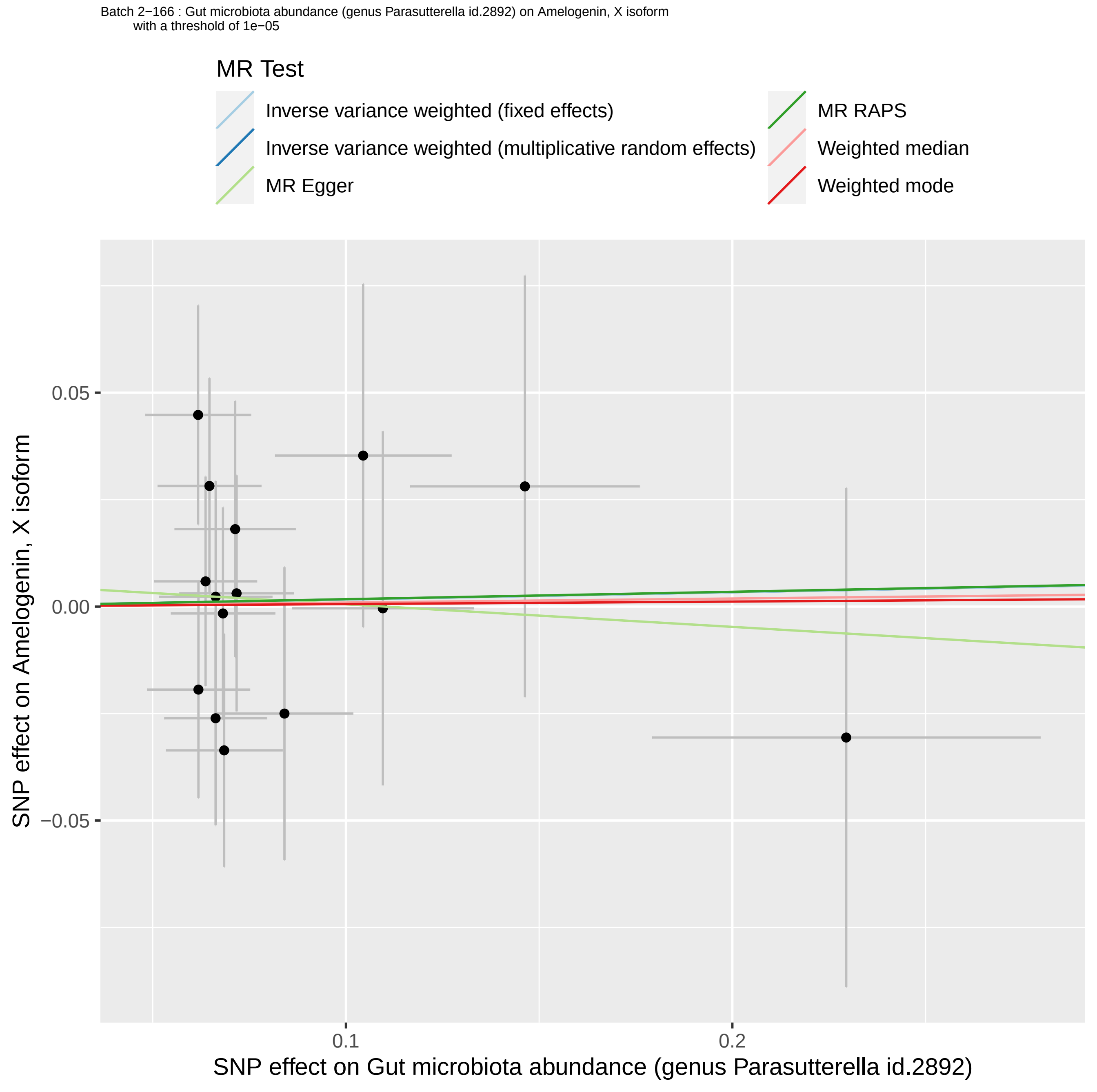
Batch 2−166 : Gut microbiota abundance (genus Parasutterella id.2892) on Amelogenin, X isoform
with a threshold of 1e−05
MR Test
Inverse variance weighted (fixed effects)
Inverse variance weighted (multiplicative random effects)
MR Egger
MR RAPS
Weighted median
Weighted mode
SNP effect on Amelogenin, X isoform
0.05
0.00
−0.05
0.1
0.2
SNP effect on Gut microbiota abundance (genus Parasutterella id.2892)

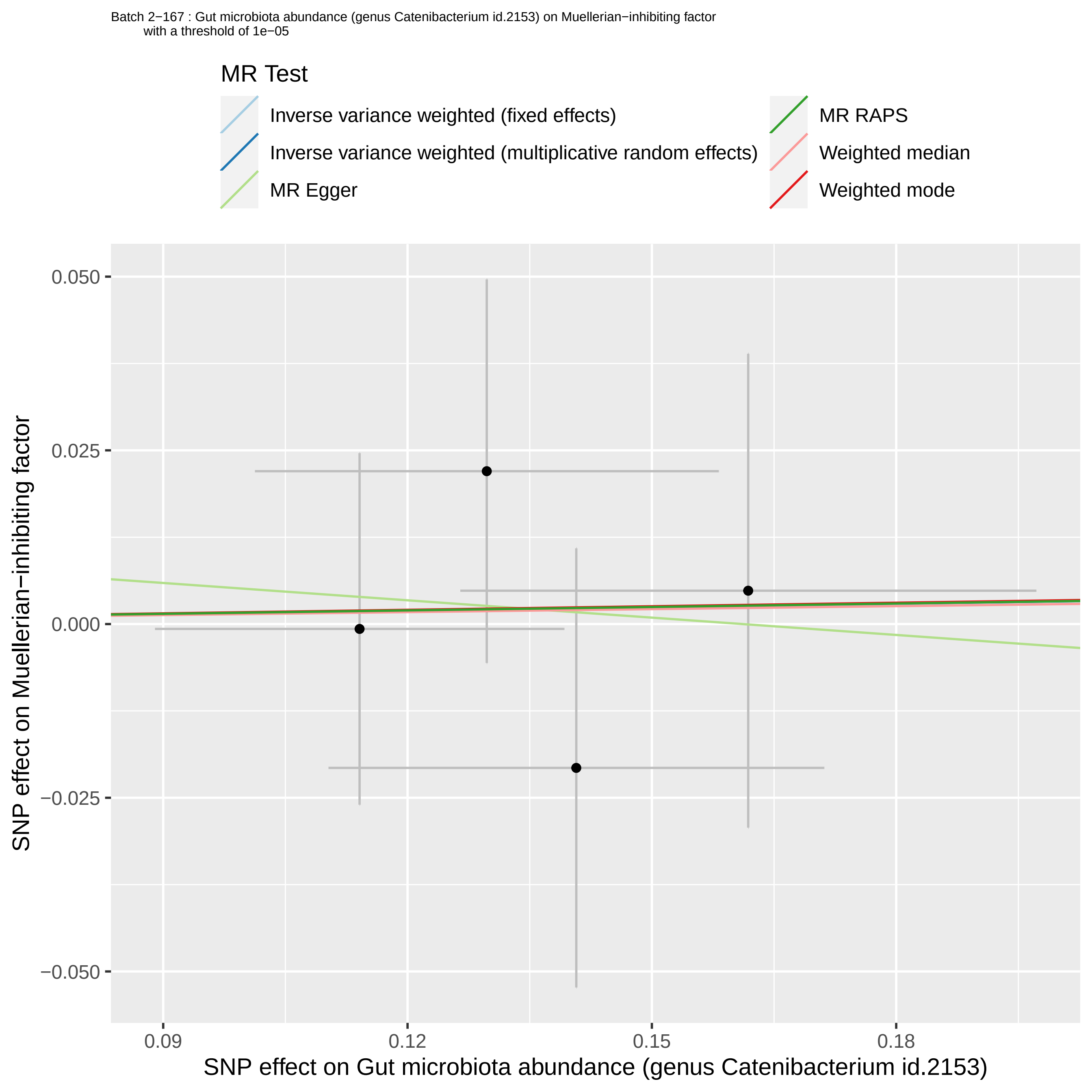
Batch 2−167 : Gut microbiota abundance (genus Catenibacterium id.2153) on Muellerian−inhibiting factor
with a threshold of 1e−05
MR Test
Inverse variance weighted (fixed effects)
Inverse variance weighted (multiplicative random effects)
MR Egger
MR RAPS
Weighted median
Weighted mode
0.050
0.025
0.000
−0.025
−0.050
0.09
0.12
0.15
0.18
SNP effect on Muellerian−inhibiting factor
SNP effect on Gut microbiota abundance (genus Catenibacterium id.2153)

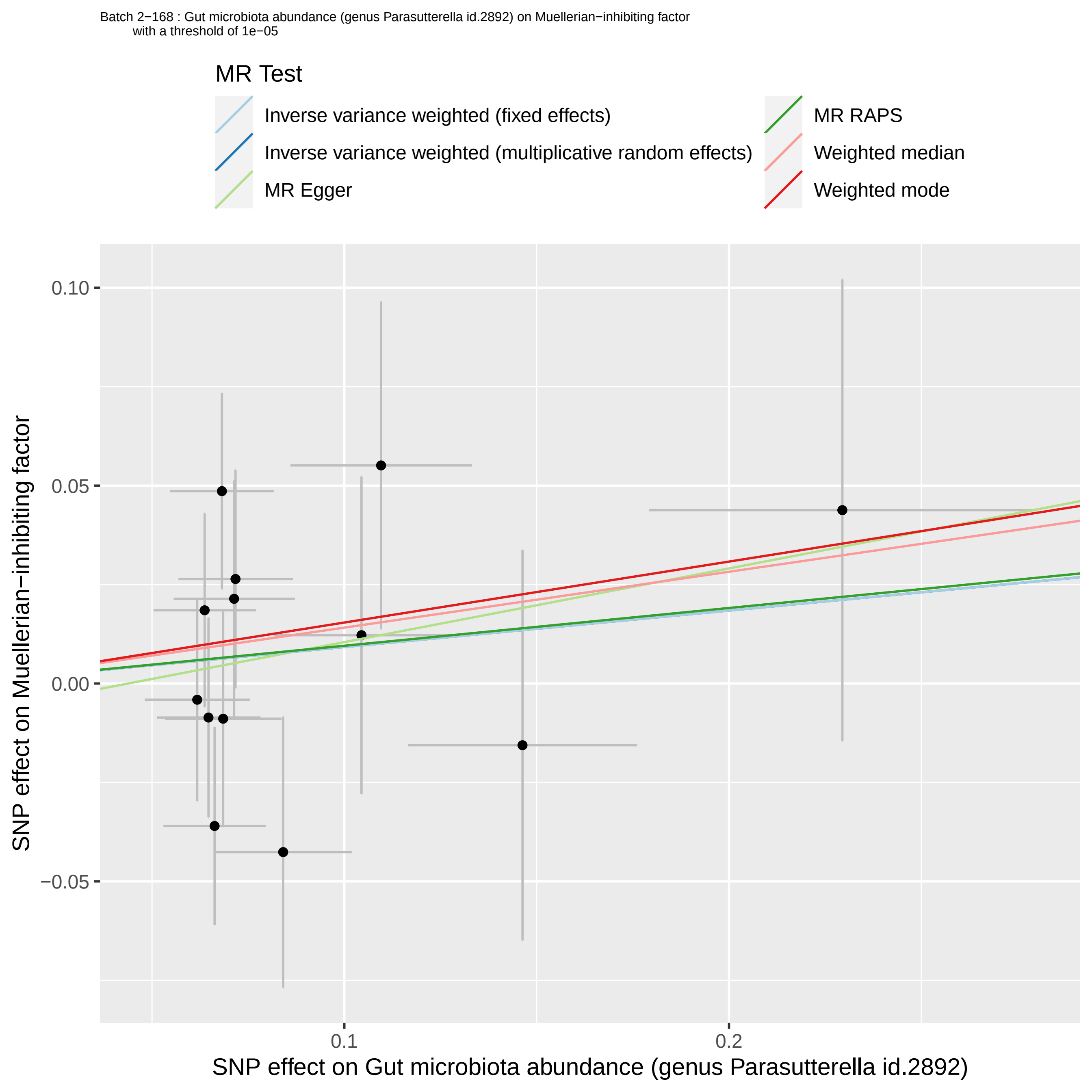

Batch 2−168 : Gut microbiota abundance (genus Parasutterella id.2892) on Muellerian−inhibiting factor
with a threshold of 1e−05
MR Test
Inverse variance weighted (fixed effects)
Inverse variance weighted (multiplicative random effects)
MR Egger
MR RAPS
Weighted median
Weighted mode
0.10
0.05
0.00
−0.05
0.1
0.2
SNP effect on Muellerian−inhibiting factor
SNP effect on Gut microbiota abundance (genus Parasutterella id.2892)

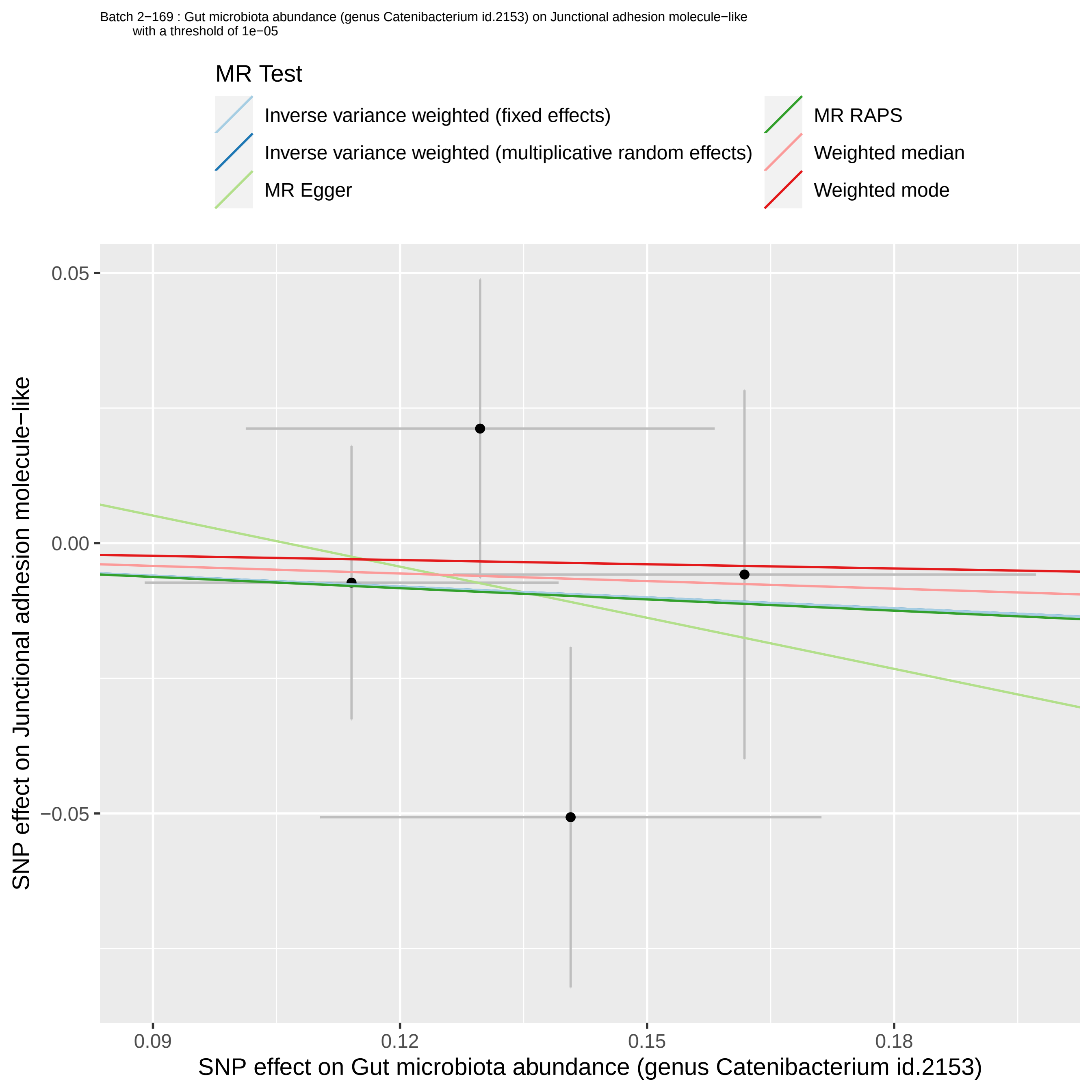
Batch 2−169 : Gut microbiota abundance (genus Catenibacterium id.2153) on Junctional adhesion molecule−like
with a threshold of 1e−05
MR Test
Inverse variance weighted (fixed effects)
Inverse variance weighted (multiplicative random effects)
MR Egger
MR RAPS
Weighted median
Weighted mode
0.05
0.00
−0.05
0.09
0.12
0.15
0.18
SNP effect on Junctional adhesion molecule−like
SNP effect on Gut microbiota abundance (genus Catenibacterium id.2153)

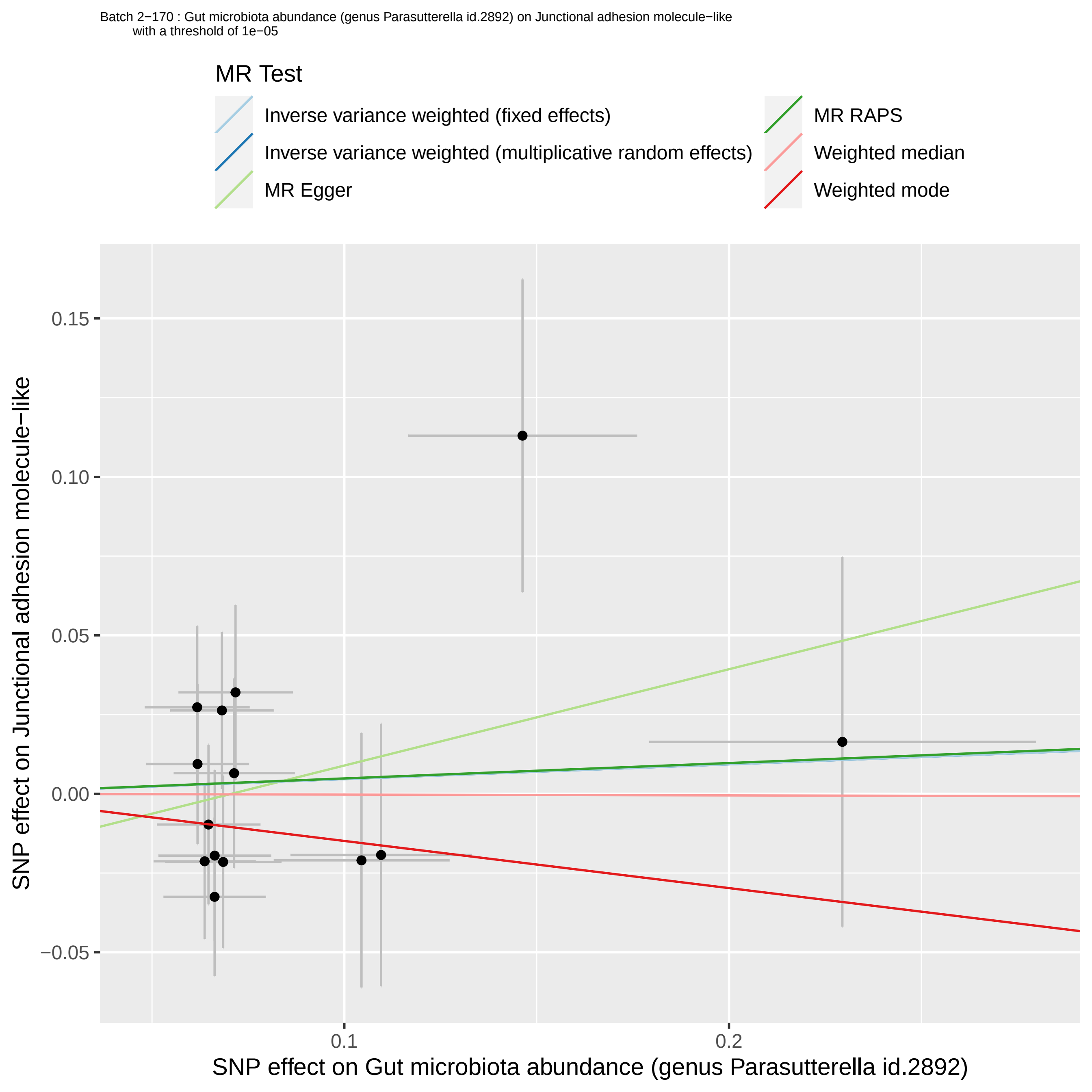
Batch 2−170 : Gut microbiota abundance (genus Parasutterella id.2892) on Junctional adhesion molecule−like
with a threshold of 1e−05
MR Test
Inverse variance weighted (fixed effects)
Inverse variance weighted (multiplicative random effects)
MR Egger
MR RAPS
Weighted median
Weighted mode
0.15
0.10
0.05
0.00
−0.05
0.1
0.2
SNP effect on Junctional adhesion molecule−like
SNP effect on Gut microbiota abundance (genus Parasutterella id.2892)

Batch 2−171 : Gut microbiota abundance (genus Catenibacterium id.2153) on Junctional adhesion molecule−like
with a threshold of 1e−05

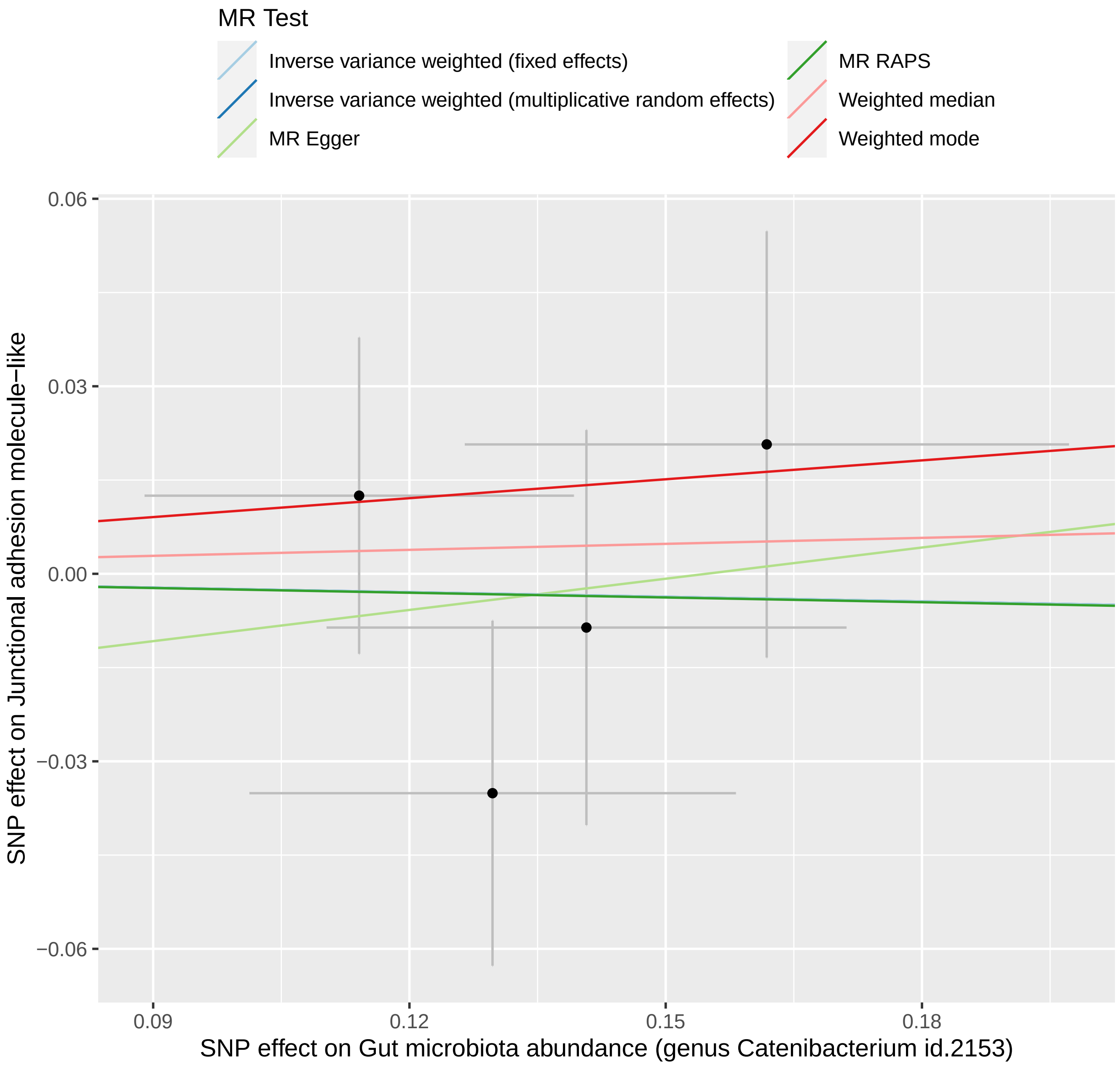

Batch 2−172 : Gut microbiota abundance (genus Parasutterella id.2892) on Junctional adhesion molecule−like
with a threshold of 1e−05

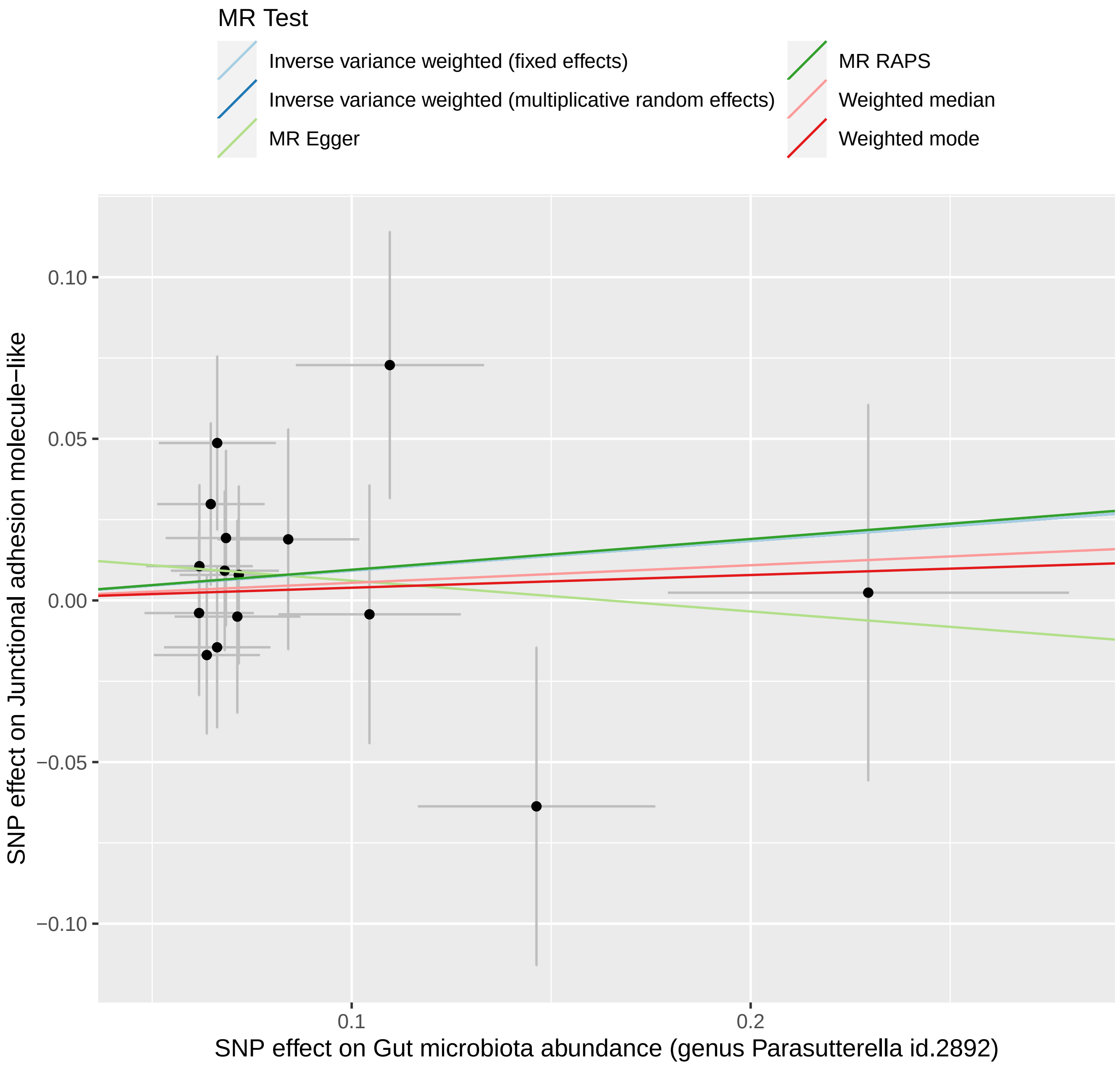

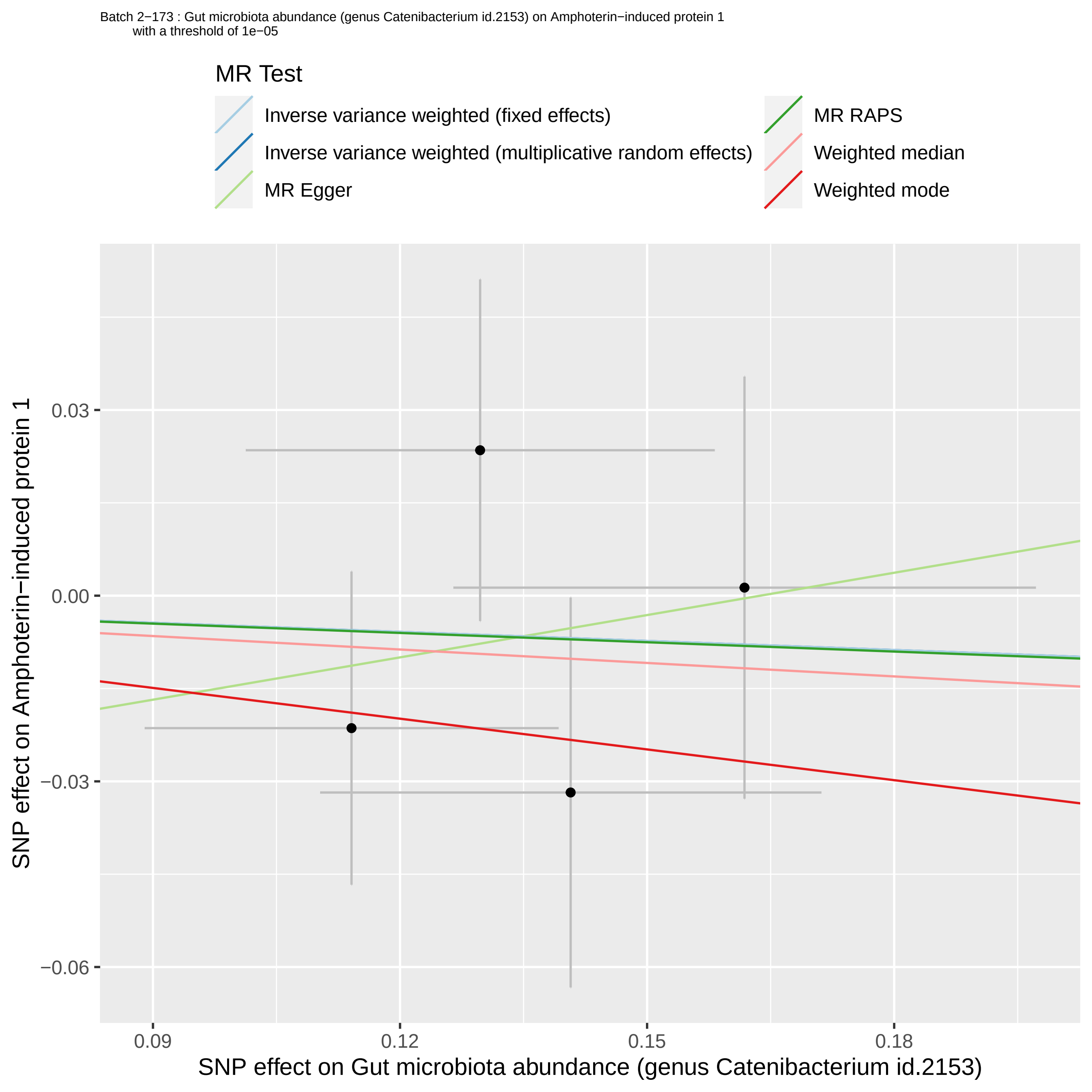
Batch 2−173 : Gut microbiota abundance (genus Catenibacterium id.2153) on Amphoterin−induced protein 1
with a threshold of 1e−05
MR Test
Inverse variance weighted (fixed effects)
Inverse variance weighted (multiplicative random effects)
MR Egger
MR RAPS
Weighted median
Weighted mode
0.03
0.00
−0.03
−0.06
0.09
0.12
0.15
0.18
SNP effect on Amphoterin−induced protein 1
SNP effect on Gut microbiota abundance (genus Catenibacterium id.2153)

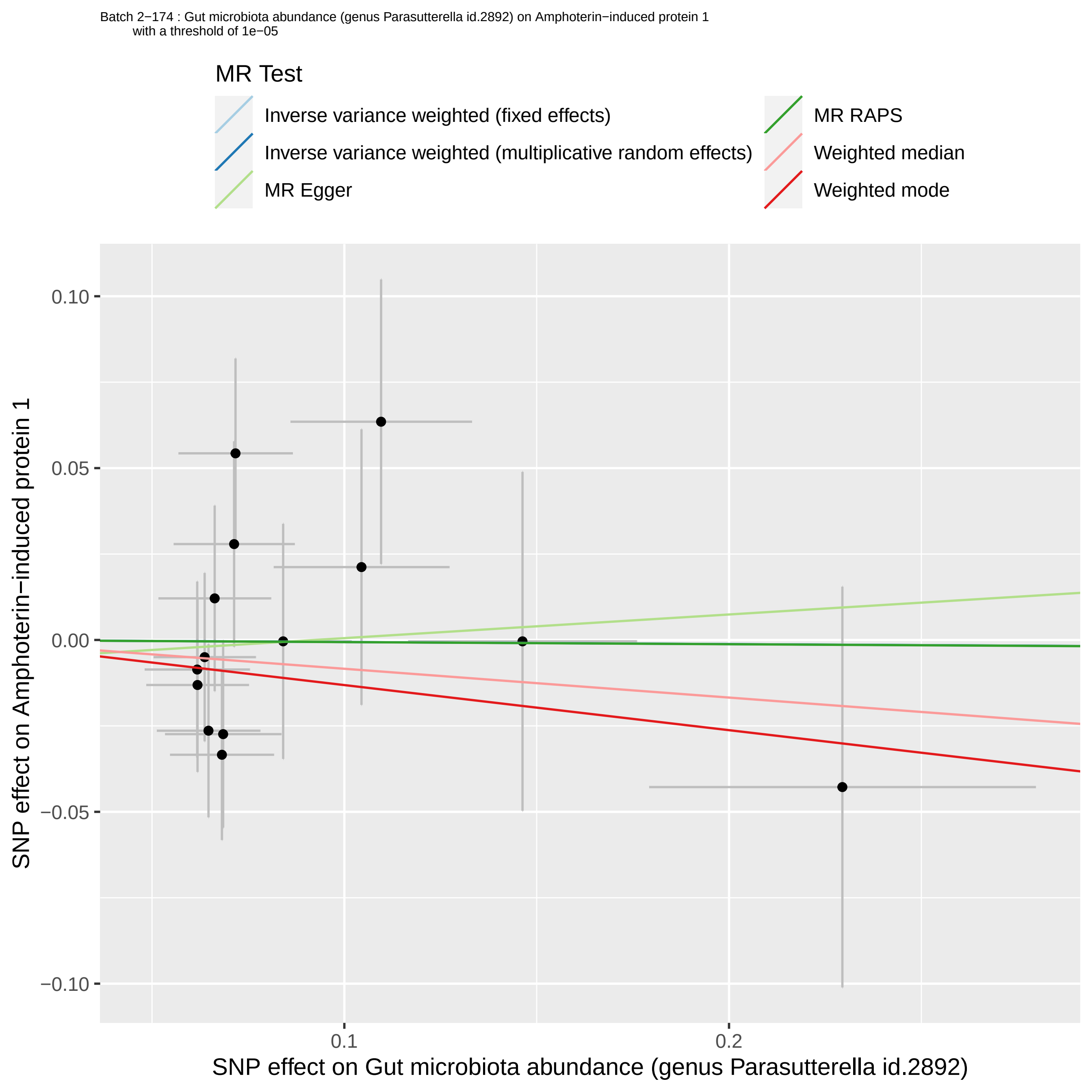
Batch 2−174 : Gut microbiota abundance (genus Parasutterella id.2892) on Amphoterin−induced protein 1
with a threshold of 1e−05
MR Test
Inverse variance weighted (fixed effects)
Inverse variance weighted (multiplicative random effects)
MR Egger
MR RAPS
Weighted median
Weighted mode
SNP effect on Amphoterin−induced protein 1
0.10
0.05
0.00
−0.05
−0.10
0.1
0.2
SNP effect on Gut microbiota abundance (genus Parasutterella id.2892)

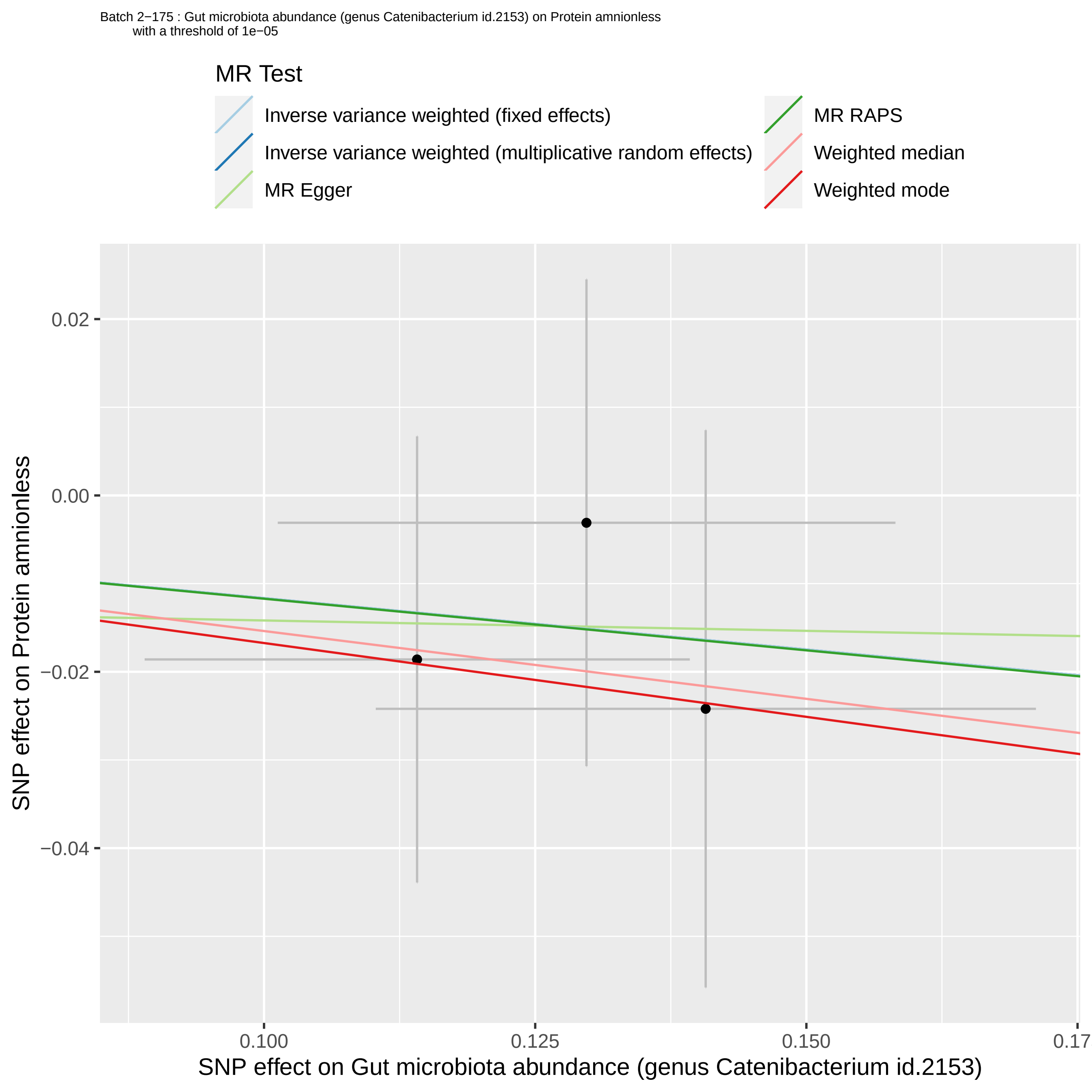
Batch 2−175 : Gut microbiota abundance (genus Catenibacterium id.2153) on Protein amnionless
with a threshold of 1e−05
MR Test
Inverse variance weighted (fixed effects)
Inverse variance weighted (multiplicative random effects)
MR Egger
MR RAPS
Weighted median
Weighted mode
0.02
0.00
−0.02
−0.04
SNP effect on Protein amnionless
0.100
0.125
0.150
0.17
SNP effect on Gut microbiota abundance (genus Catenibacterium id.2153)

Batch 2−176 : Gut microbiota abundance (genus Parasutterella id.2892) on Protein amnionless
with a threshold of 1e−05

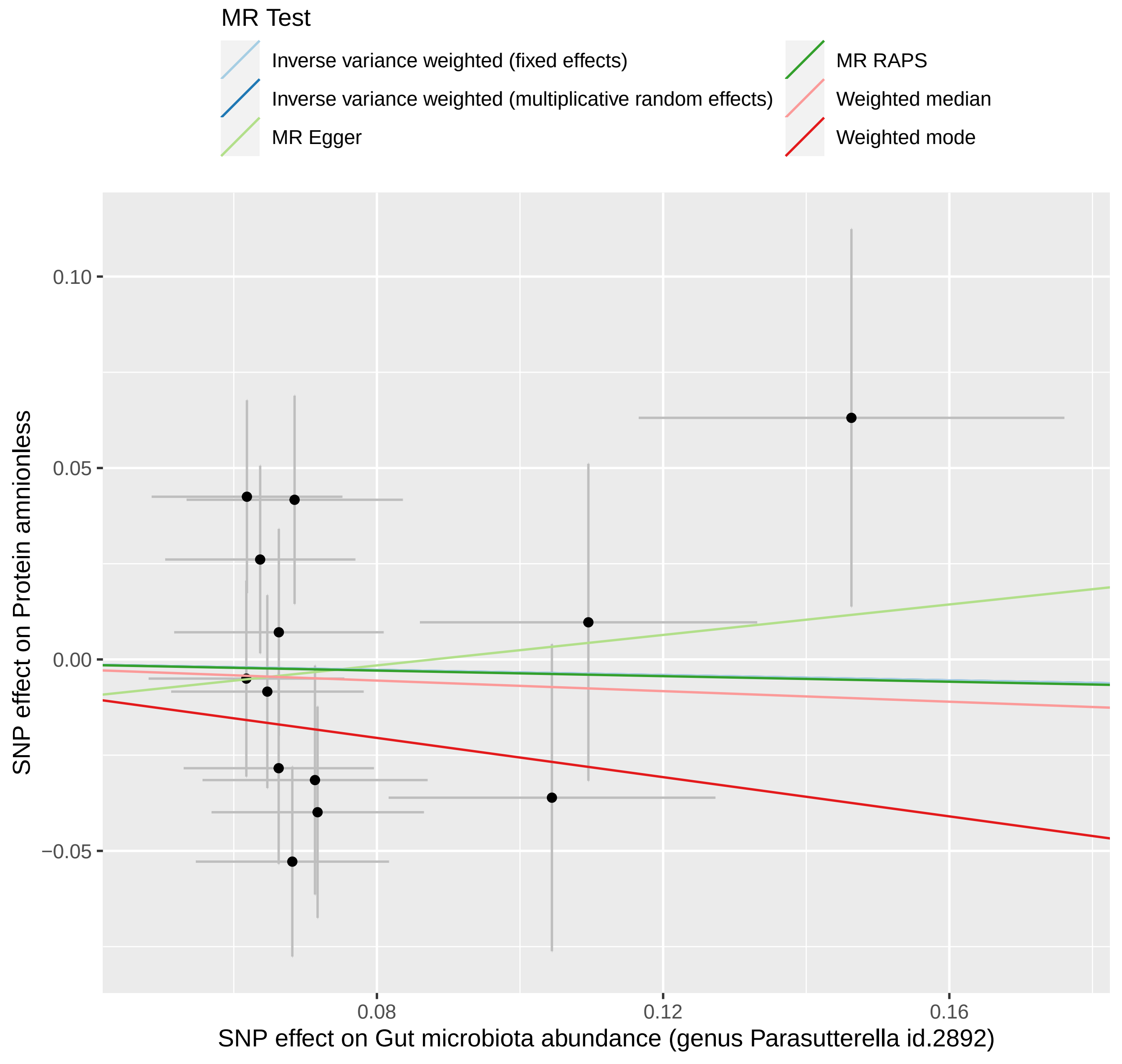

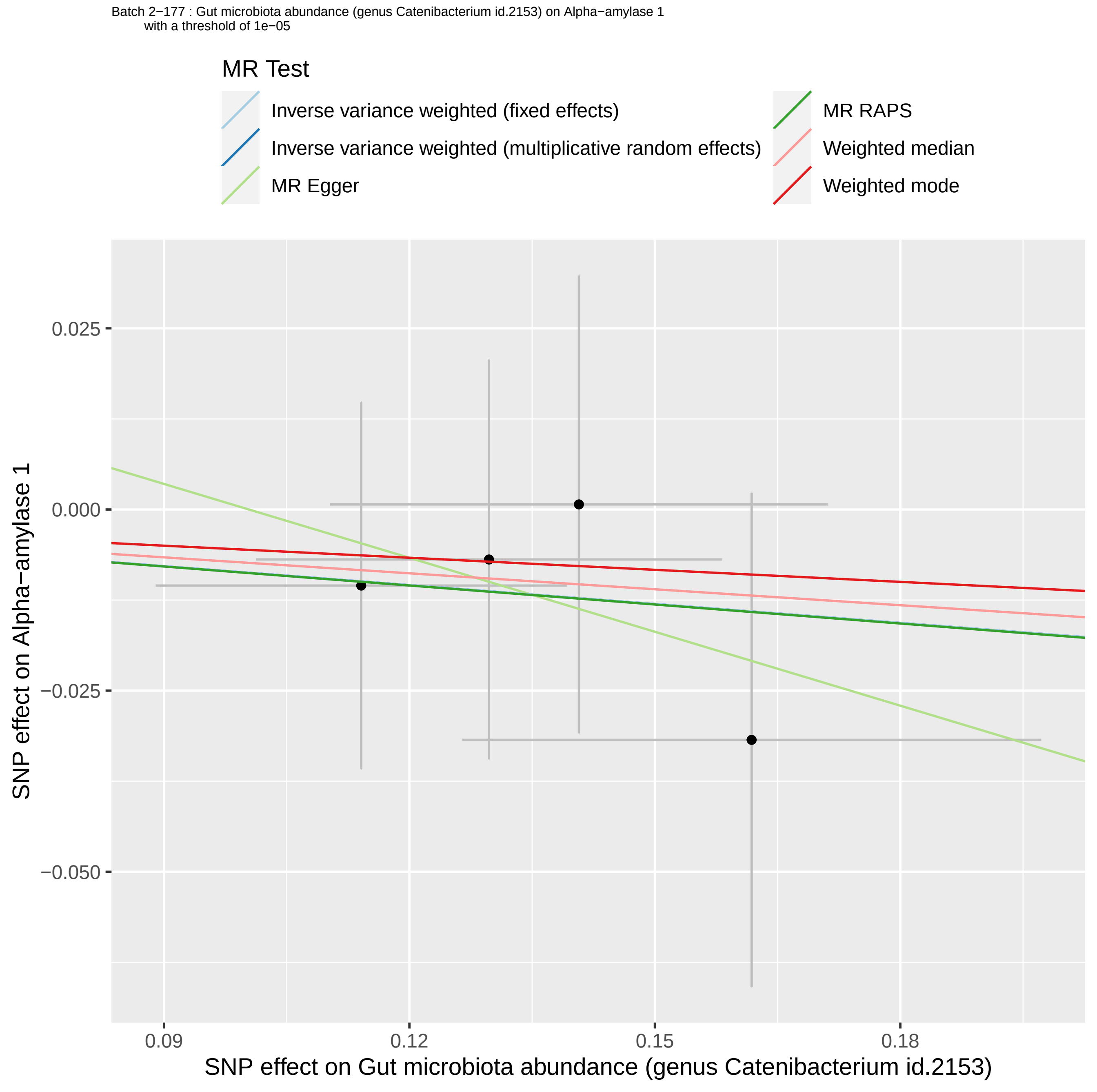
Batch 2−177 : Gut microbiota abundance (genus Catenibacterium id.2153) on Alpha−amylase 1
with a threshold of 1e−05
MR Test
Inverse variance weighted (fixed effects)
Inverse variance weighted (multiplicative random effects)
MR Egger
MR RAPS
Weighted median
Weighted mode
0.025
0.000
−0.025
−0.050
0.09
0.12
0.15
0.18
SNP effect on Alpha−amylase 1
SNP effect on Gut microbiota abundance (genus Catenibacterium id.2153)

Batch 2−178 : Gut microbiota abundance (genus Parasutterella id.2892) on Alpha−amylase 1
with a threshold of 1e−05

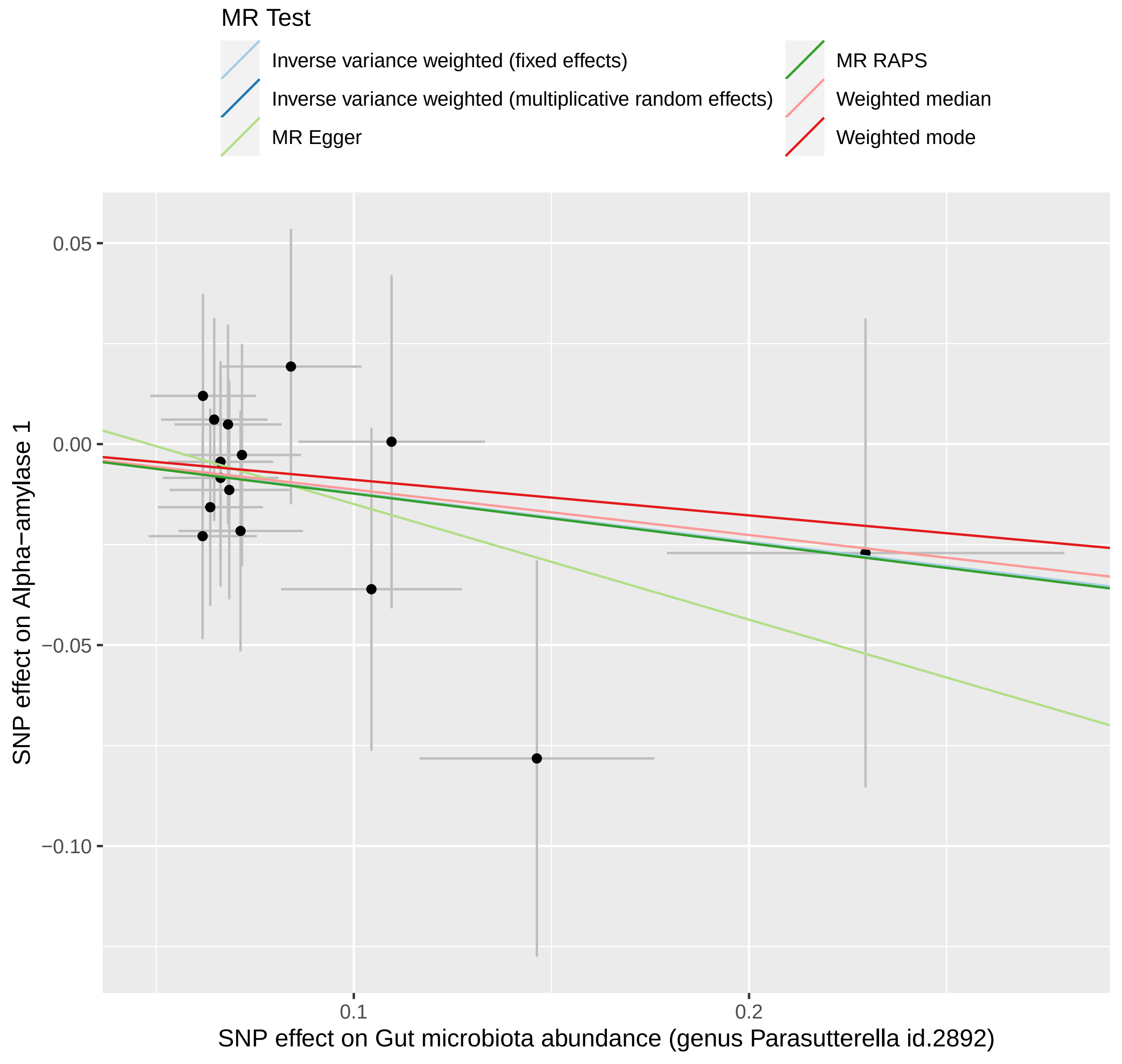

Batch 2−179 : Gut microbiota abundance (genus Catenibacterium id.2153) on Anaphase−promoting complex subunit 10
with a threshold of 1e−05

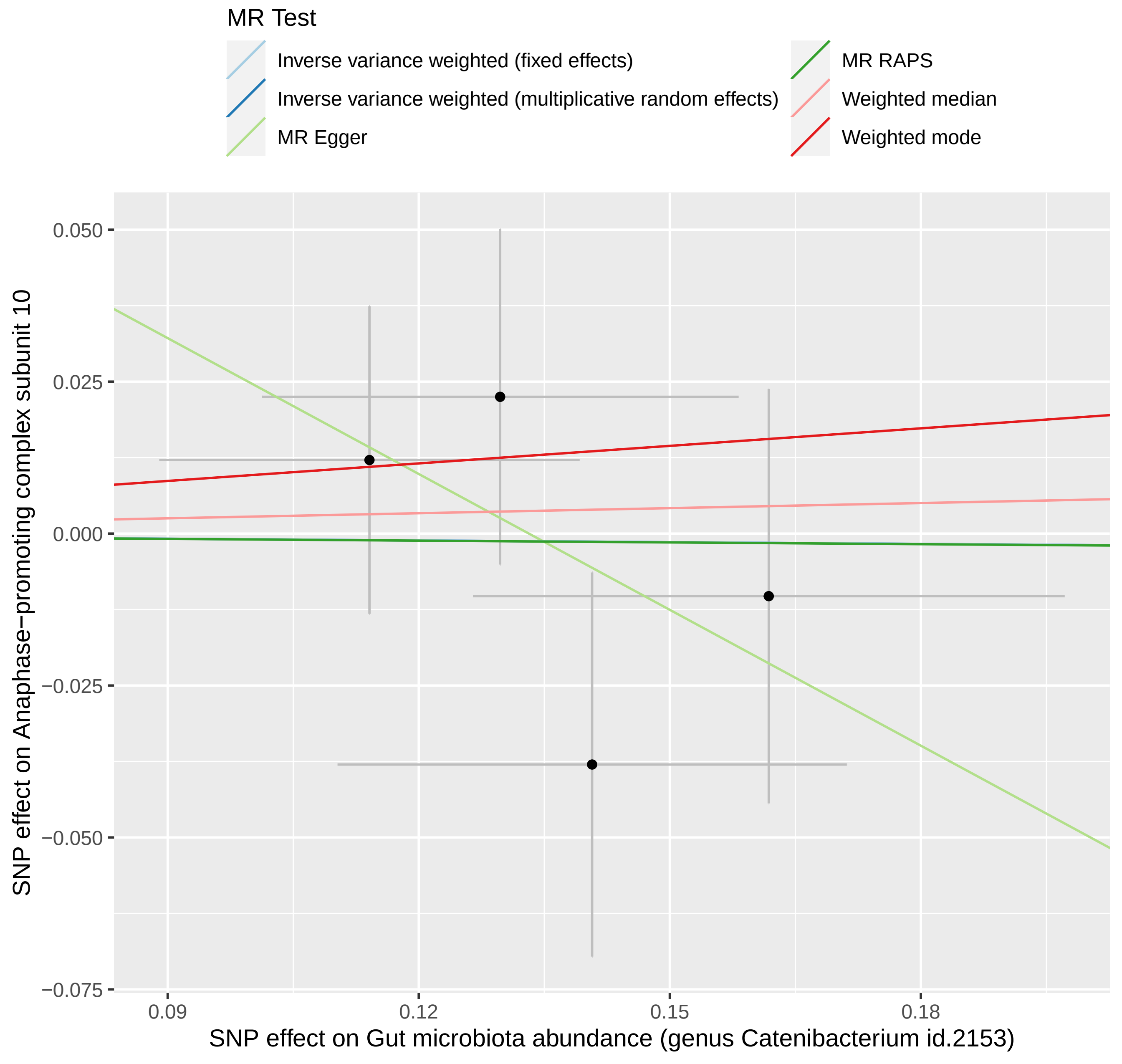

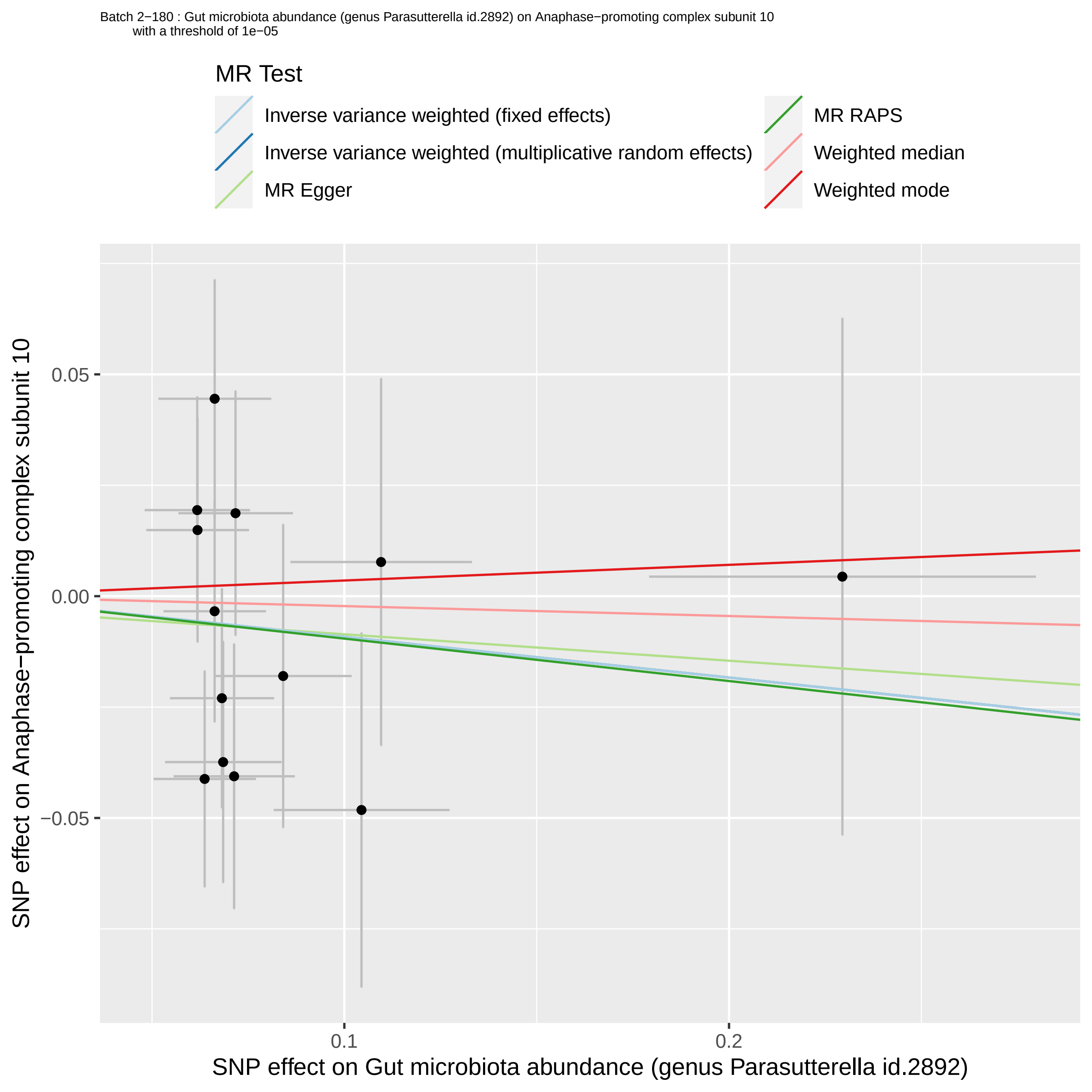
Batch 2−180 : Gut microbiota abundance (genus Parasutterella id.2892) on Anaphase−promoting complex subunit 10
with a threshold of 1e−05
MR Test
Inverse variance weighted (fixed effects)
Inverse variance weighted (multiplicative random effects)
MR Egger
MR RAPS
Weighted median
Weighted mode
0.05
0.00
−0.05
0.1
0.2
SNP effect on Anaphase−promoting complex subunit 10
SNP effect on Gut microbiota abundance (genus Parasutterella id.2892)

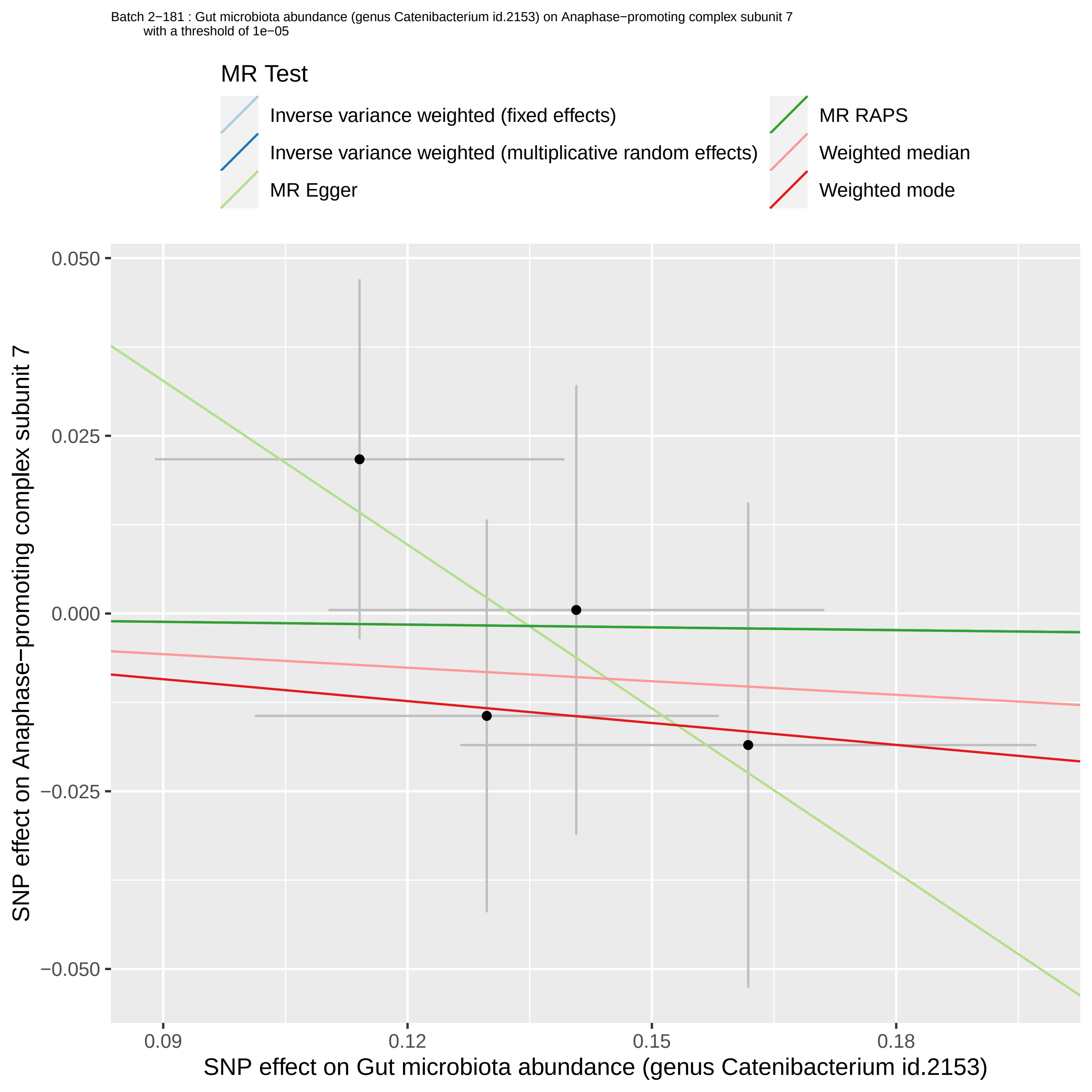

Batch 2−181 : Gut microbiota abundance (genus Catenibacterium id.2153) on Anaphase−promoting complex subunit 7
with a threshold of 1e−05
MR Test
Inverse variance weighted (fixed effects)
Inverse variance weighted (multiplicative random effects)
MR Egger
MR RAPS
Weighted median
Weighted mode
0.050
0.025
0.000
−0.025
−0.050
0.09
0.12
0.15
0.18
SNP effect on Anaphase−promoting complex subunit 7
SNP effect on Gut microbiota abundance (genus Catenibacterium id.2153)

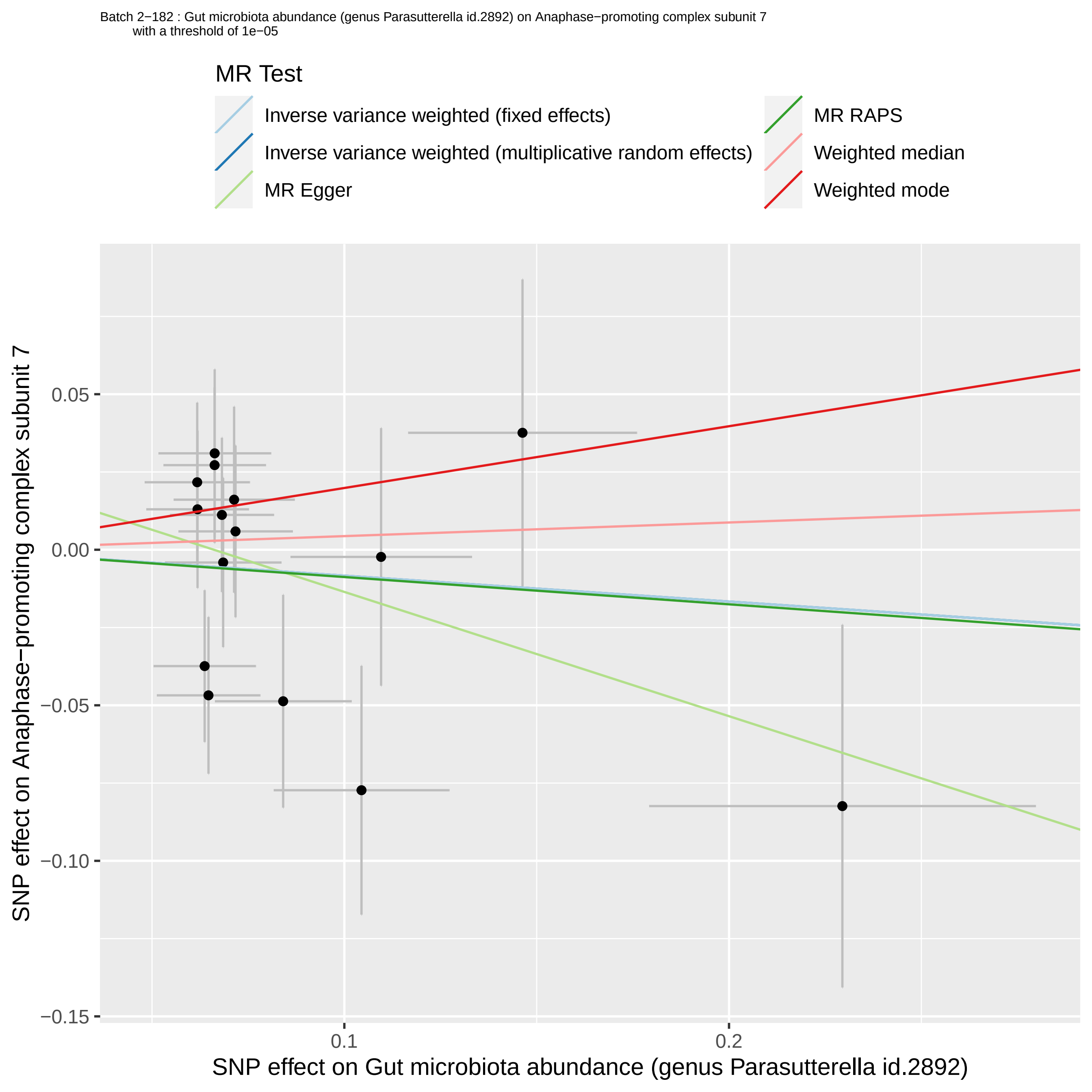

Batch 2−182 : Gut microbiota abundance (genus Parasutterella id.2892) on Anaphase−promoting complex subunit 7
with a threshold of 1e−05
MR Test
Inverse variance weighted (fixed effects)
Inverse variance weighted (multiplicative random effects)
MR Egger
MR RAPS
Weighted median
Weighted mode
SNP effect on Anaphase−promoting complex subunit 7
0.05
0.00
−0.05
−0.10
−0.15
0.1
0.2
SNP effect on Gut microbiota abundance (genus Parasutterella id.2892)

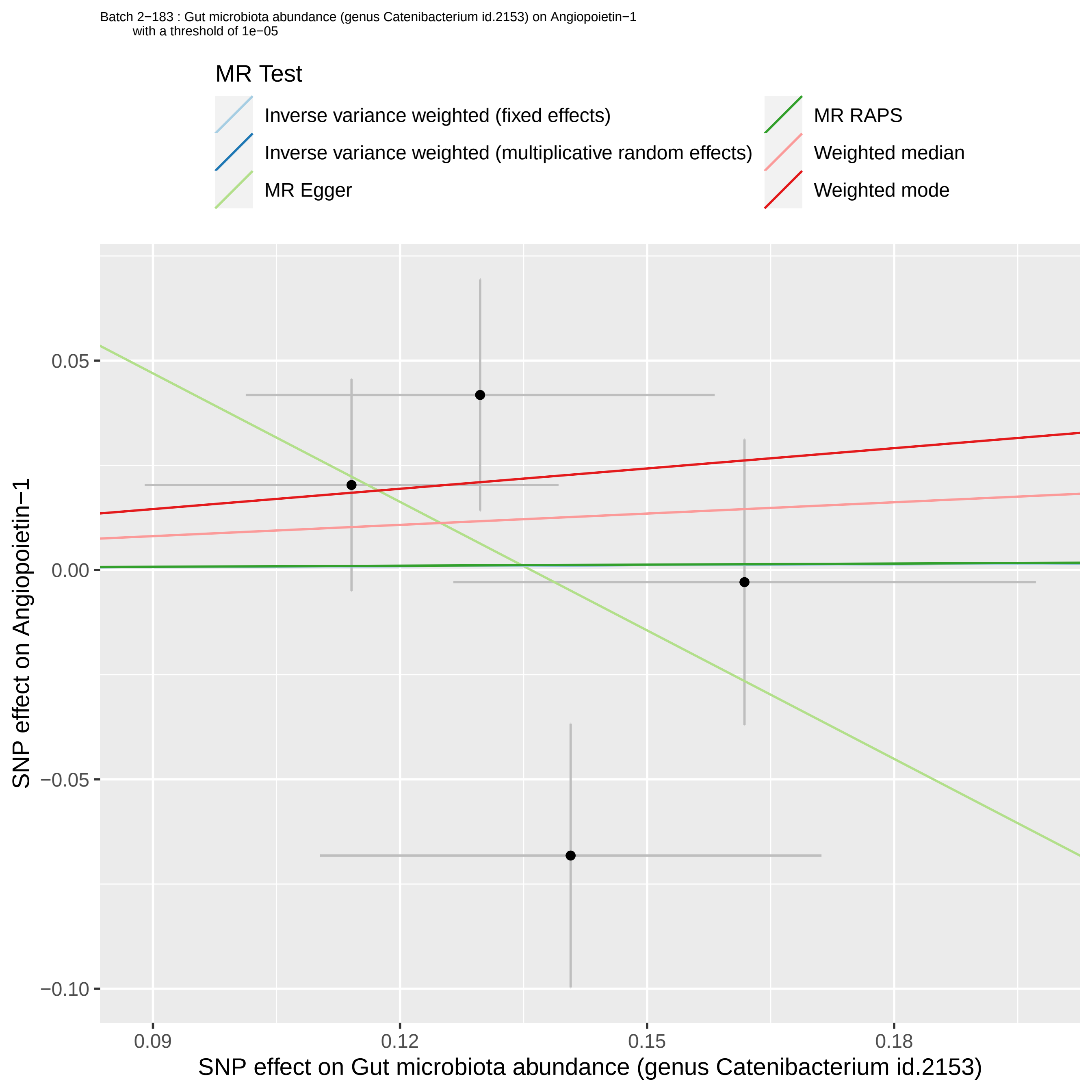

Batch 2−183 : Gut microbiota abundance (genus Catenibacterium id.2153) on Angiopoietin−1
with a threshold of 1e−05
MR Test
Inverse variance weighted (fixed effects)
Inverse variance weighted (multiplicative random effects)
MR Egger
MR RAPS
Weighted median
Weighted mode
0.05
0.00
−0.05
−0.10
0.09
0.12
0.15
0.18
SNP effect on Angiopoietin−1
SNP effect on Gut microbiota abundance (genus Catenibacterium id.2153)

Batch 2−184 : Gut microbiota abundance (genus Parasutterella id.2892) on Angiopoietin−1
with a threshold of 1e−05

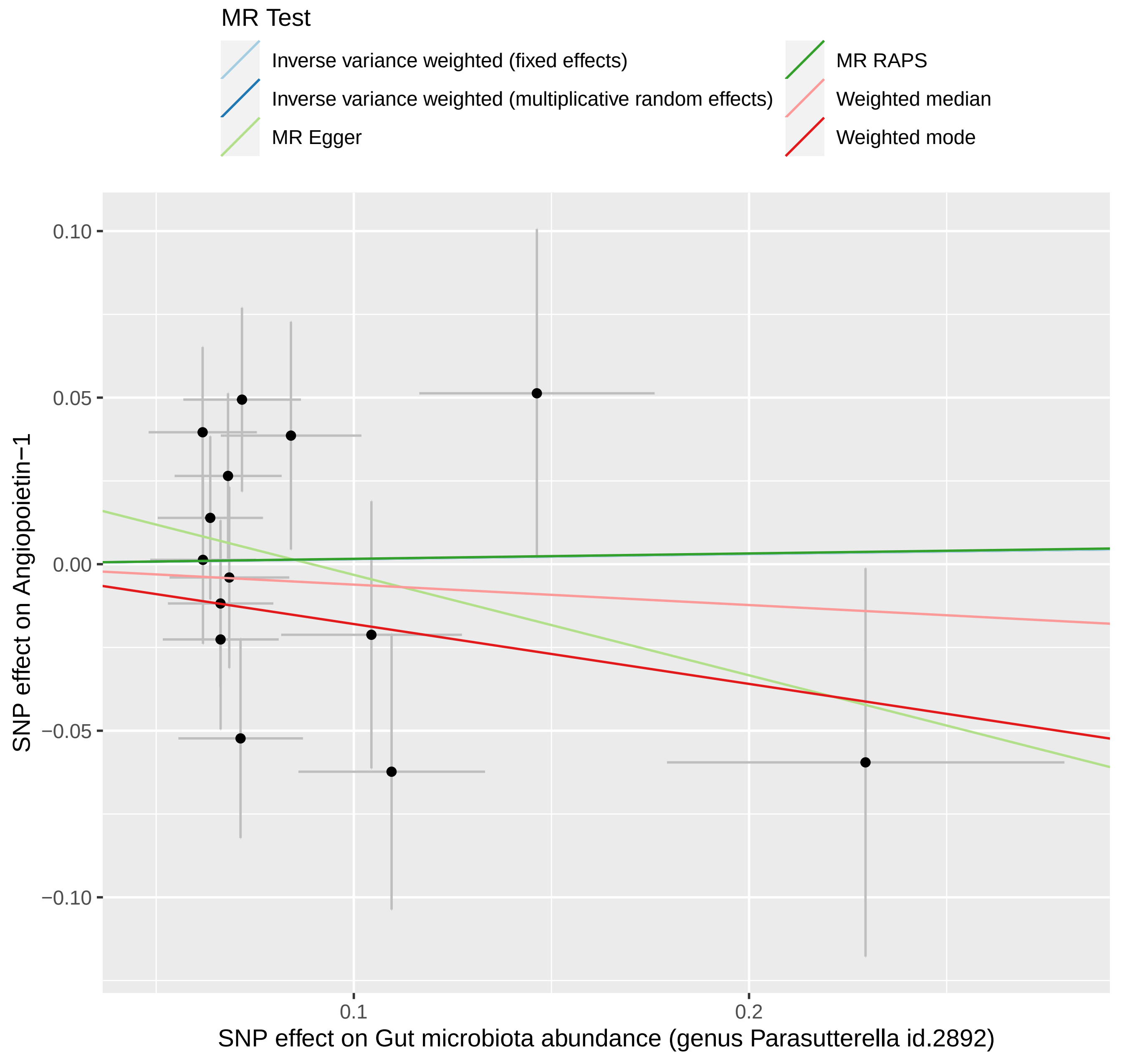

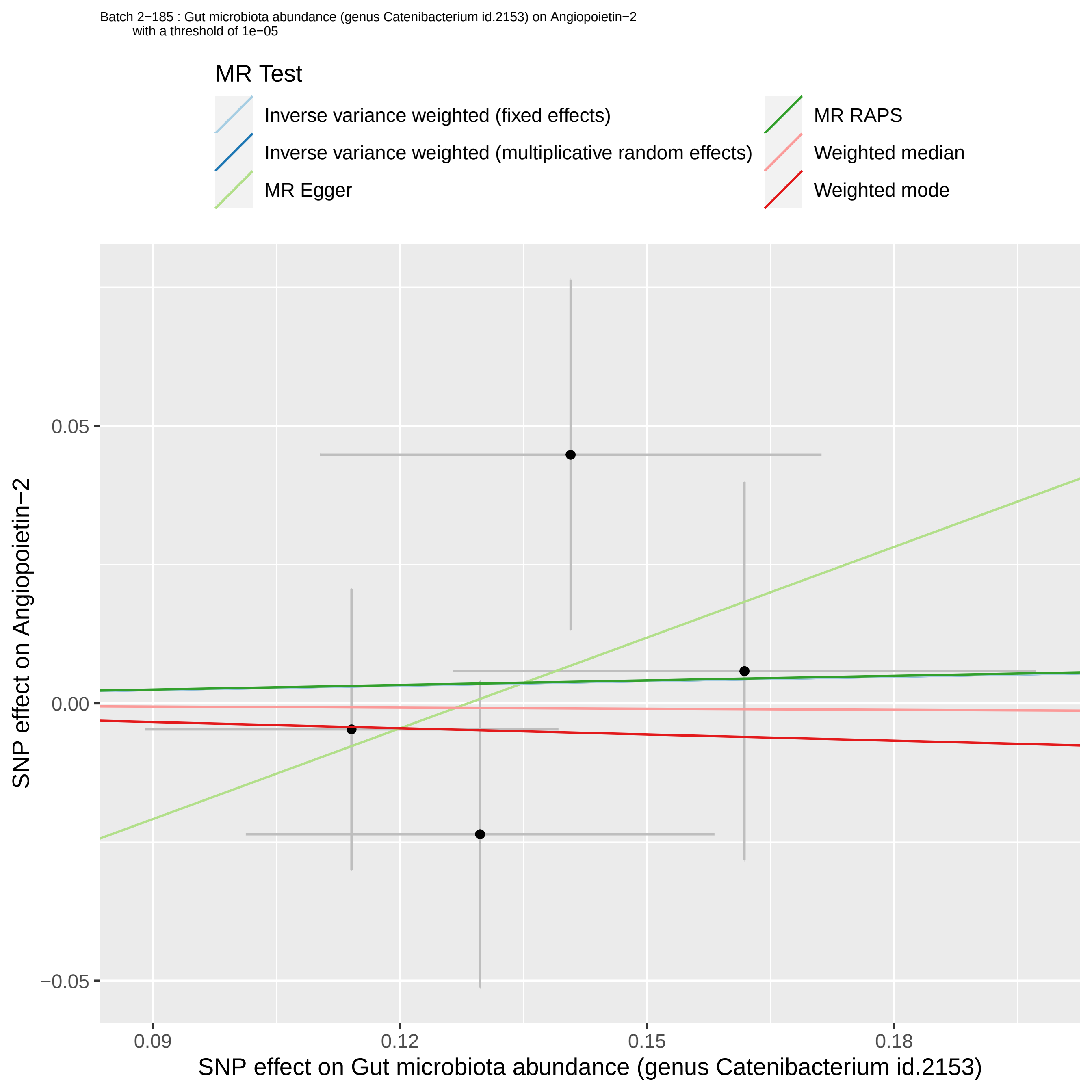
Batch 2−185 : Gut microbiota abundance (genus Catenibacterium id.2153) on Angiopoietin−2
with a threshold of 1e−05
MR Test
Inverse variance weighted (fixed effects)
Inverse variance weighted (multiplicative random effects)
MR Egger
MR RAPS
Weighted median
Weighted mode
0.05
0.00
−0.05
0.09
0.12
0.15
0.18
SNP effect on Angiopoietin−2
SNP effect on Gut microbiota abundance (genus Catenibacterium id.2153)

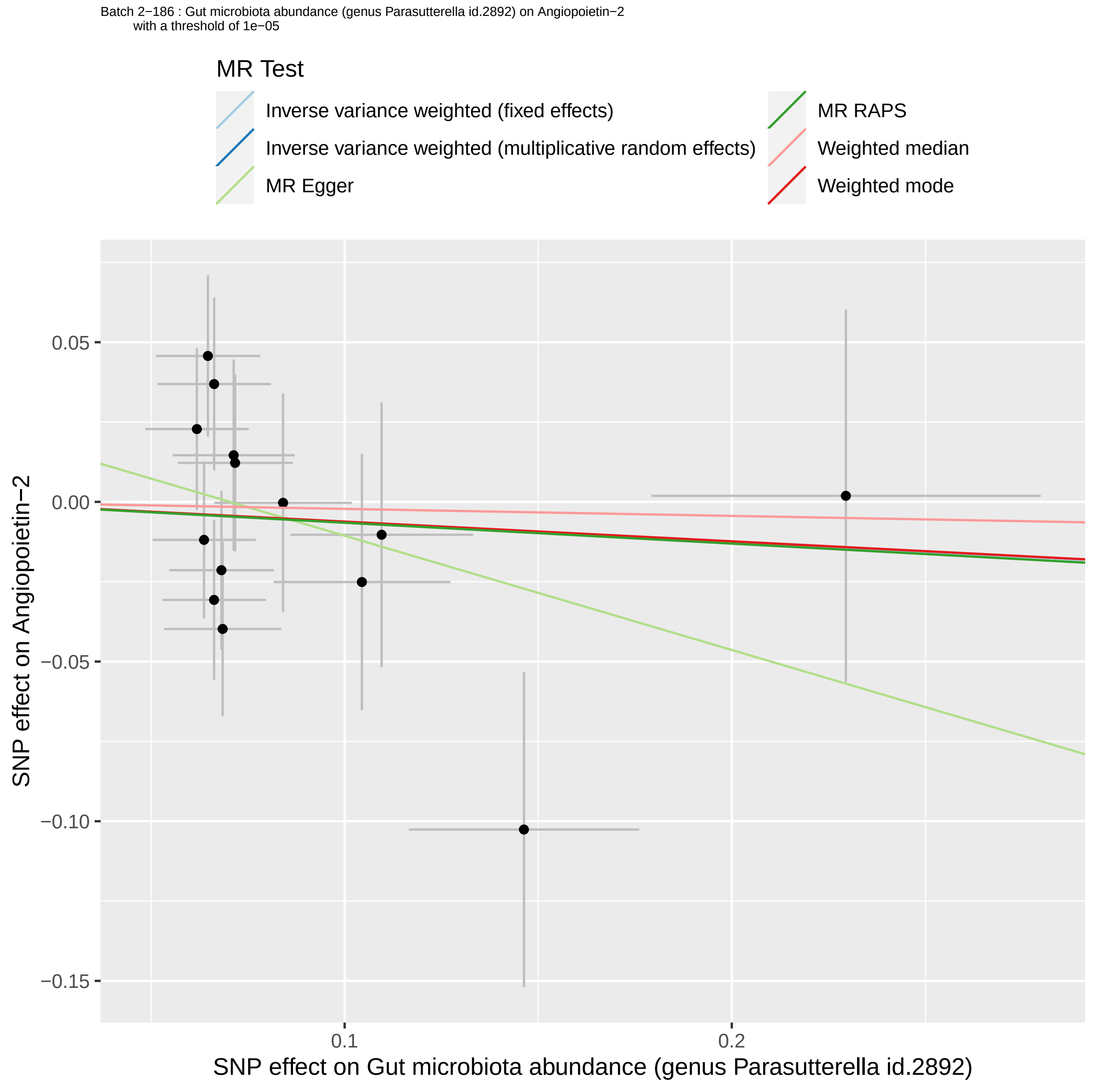

Batch 2−186 : Gut microbiota abundance (genus Parasutterella id.2892) on Angiopoietin−2
with a threshold of 1e−05
MR Test
Inverse variance weighted (fixed effects)
Inverse variance weighted (multiplicative random effects)
MR Egger
MR RAPS
Weighted median
Weighted mode
0.05
0.00
−0.05
−0.10
−0.15
0.1
0.2
SNP effect on Angiopoietin−2
SNP effect on Gut microbiota abundance (genus Parasutterella id.2892)

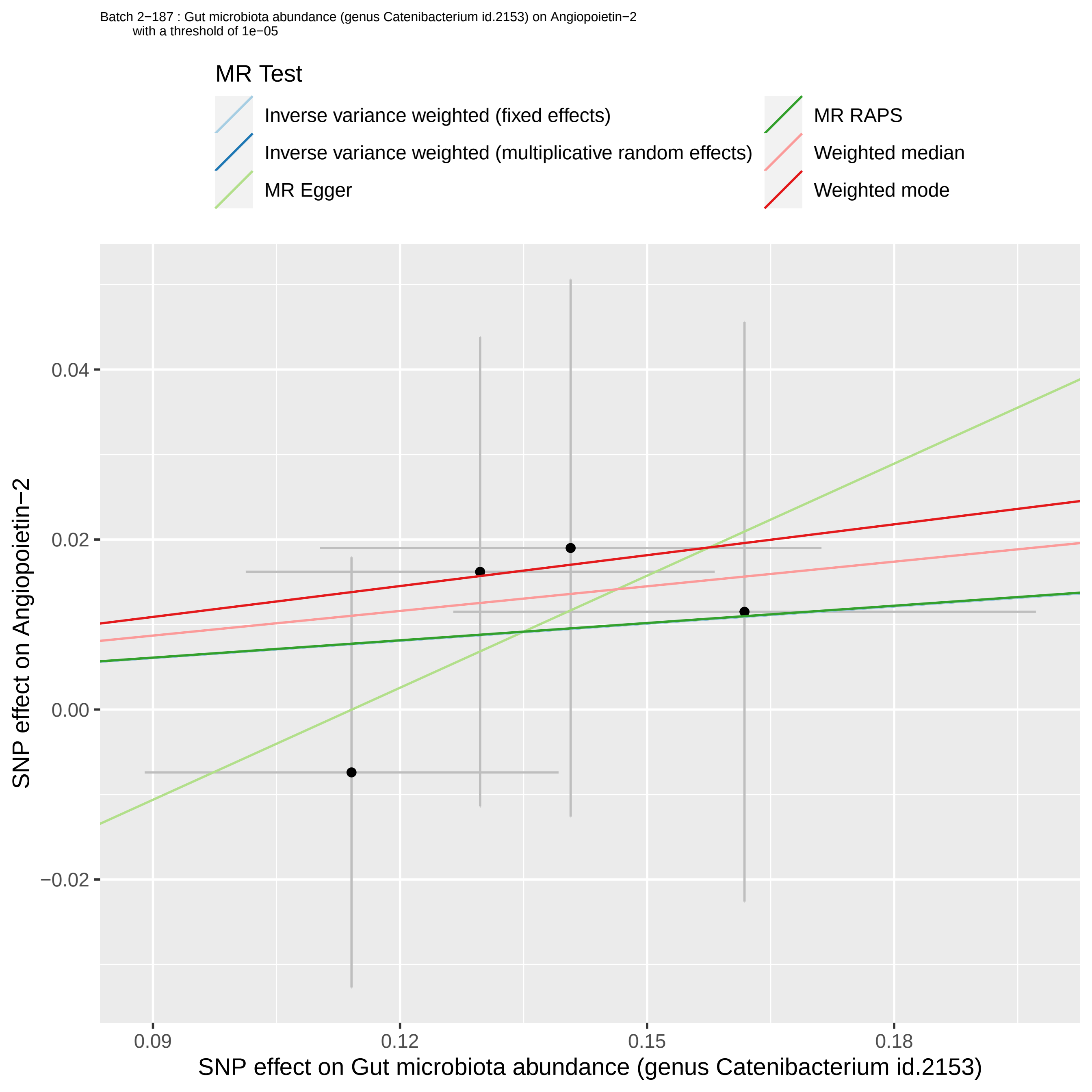

Batch 2−187 : Gut microbiota abundance (genus Catenibacterium id.2153) on Angiopoietin−2
with a threshold of 1e−05
MR Test
Inverse variance weighted (fixed effects)
Inverse variance weighted (multiplicative random effects)
MR Egger
MR RAPS
Weighted median
Weighted mode
0.04
0.02
0.00
−0.02
0.09
0.12
0.15
0.18
SNP effect on Angiopoietin−2
SNP effect on Gut microbiota abundance (genus Catenibacterium id.2153)

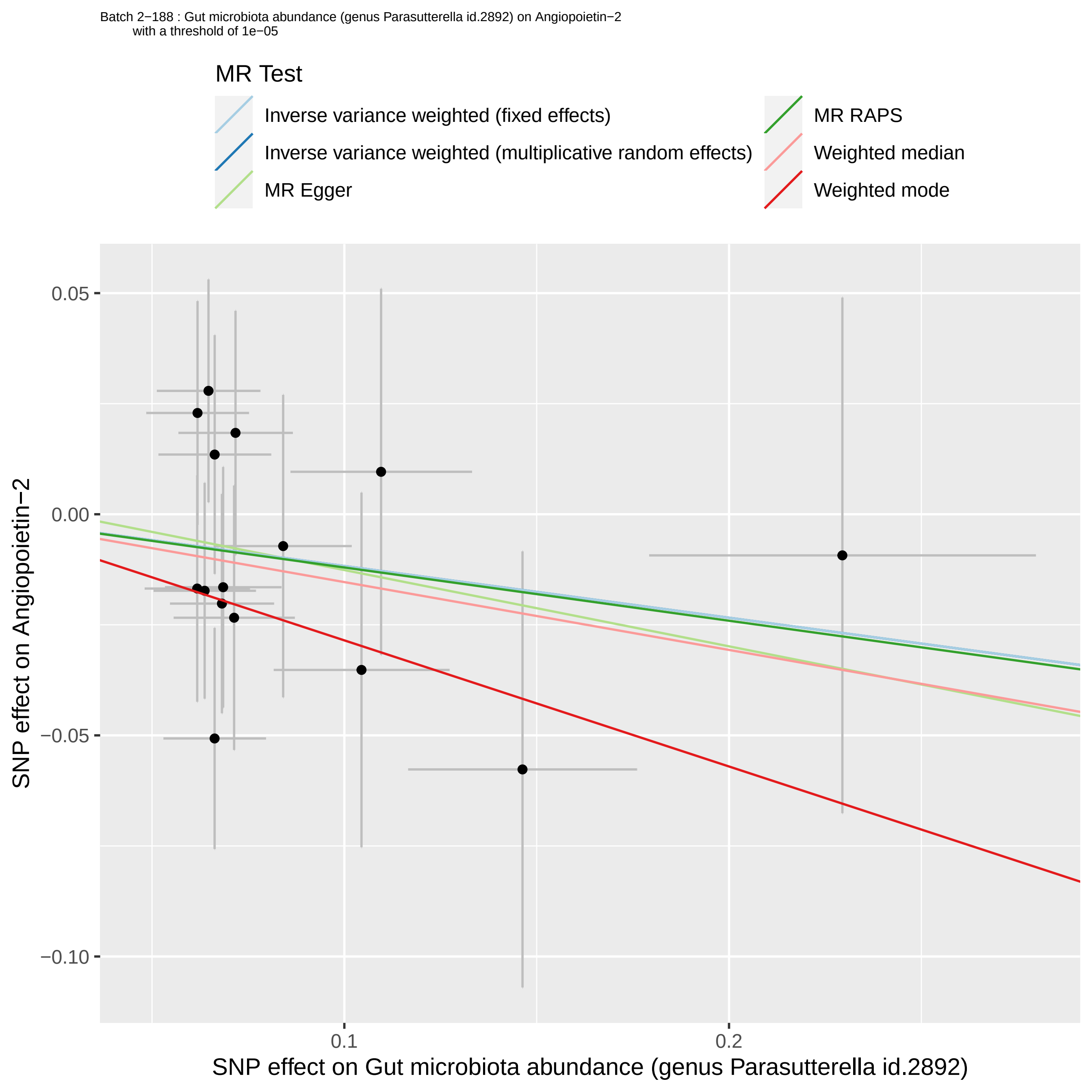

Batch 2−188 : Gut microbiota abundance (genus Parasutterella id.2892) on Angiopoietin−2
with a threshold of 1e−05
MR Test
Inverse variance weighted (fixed effects)
Inverse variance weighted (multiplicative random effects)
MR Egger
MR RAPS
Weighted median
Weighted mode
0.05
0.00
−0.05
−0.10
0.1
0.2
SNP effect on Angiopoietin−2
SNP effect on Gut microbiota abundance (genus Parasutterella id.2892)

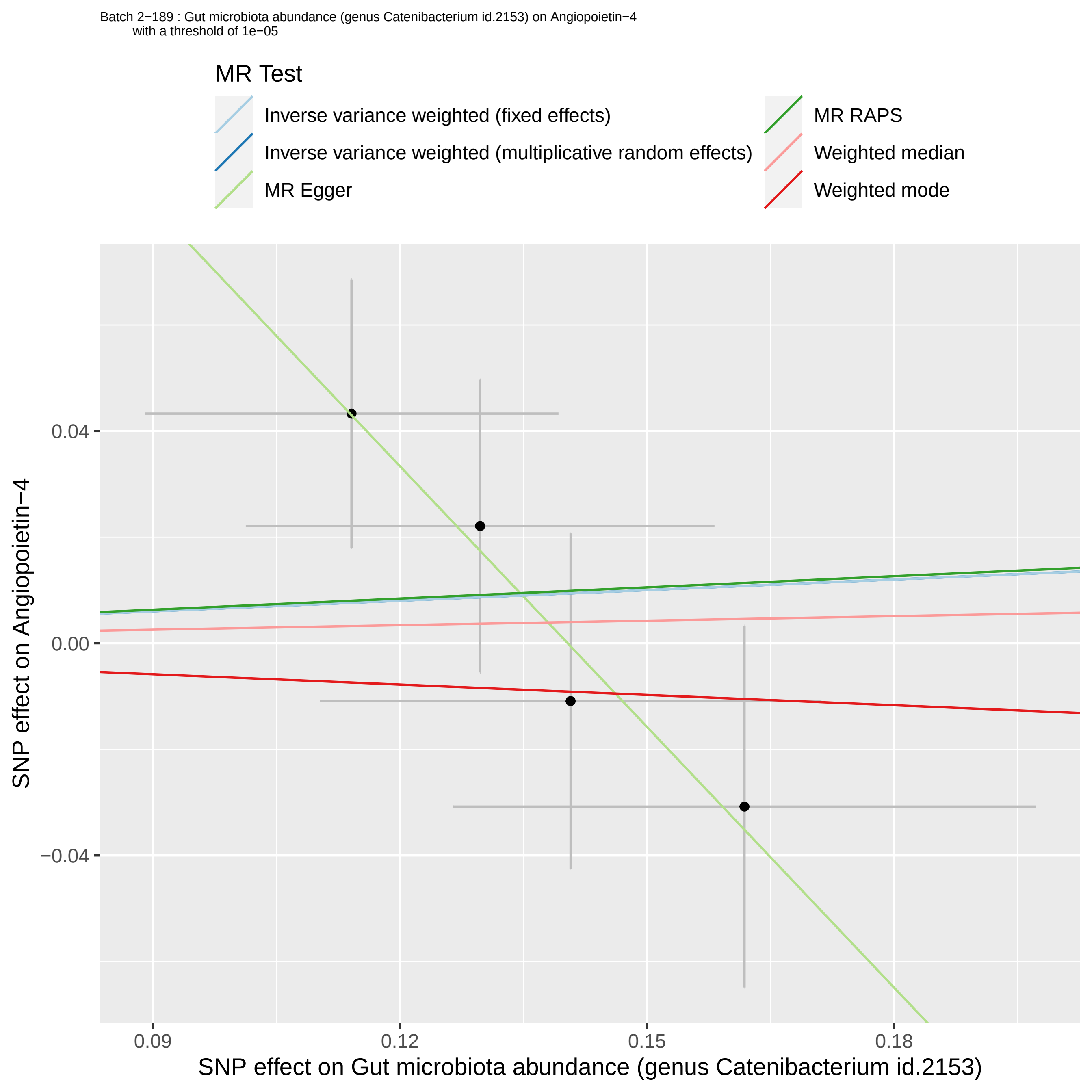
Batch 2−189 : Gut microbiota abundance (genus Catenibacterium id.2153) on Angiopoietin−4
with a threshold of 1e−05
MR Test
Inverse variance weighted (fixed effects)
Inverse variance weighted (multiplicative random effects)
MR Egger
MR RAPS
Weighted median
Weighted mode
SNP effect on Angiopoietin−4
0.04
0.00
−0.04
0.09
0.12
0.15
0.18
SNP effect on Gut microbiota abundance (genus Catenibacterium id.2153)

Batch 2−190 : Gut microbiota abundance (genus Parasutterella id.2892) on Angiopoietin−4
with a threshold of 1e−05

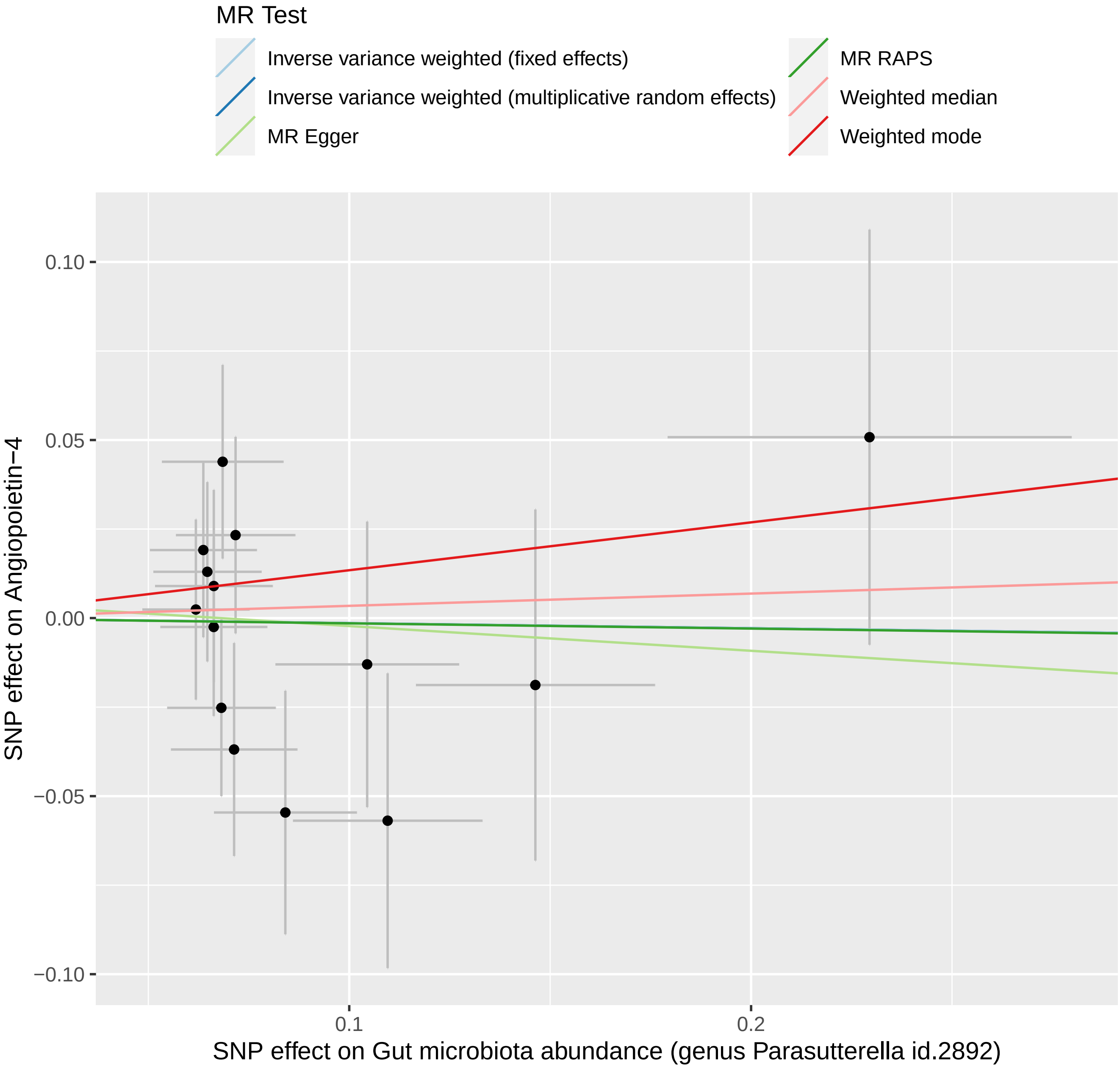

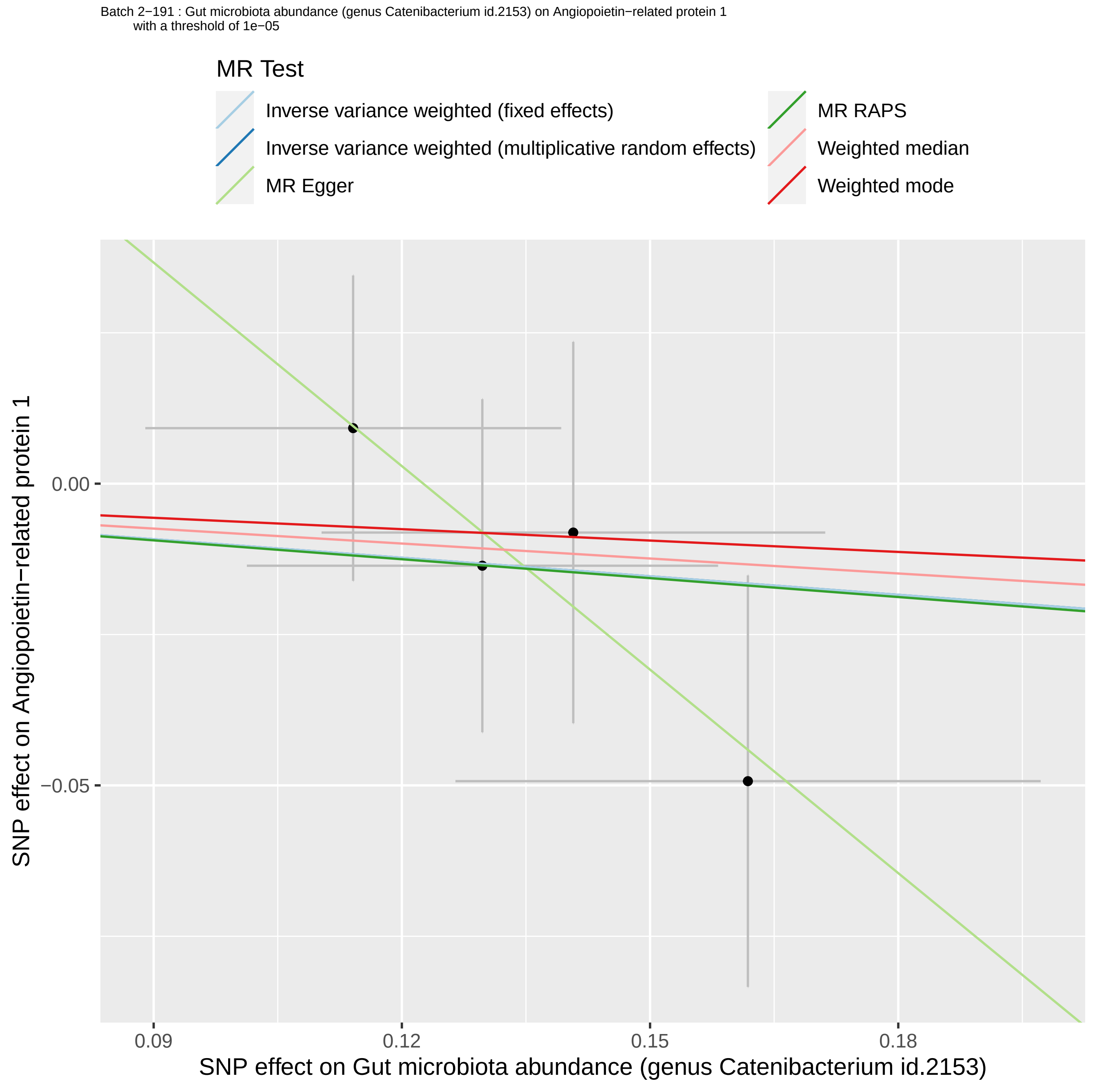
Batch 2−191 : Gut microbiota abundance (genus Catenibacterium id.2153) on Angiopoietin−related protein 1
with a threshold of 1e−05
MR Test
Inverse variance weighted (fixed effects)
Inverse variance weighted (multiplicative random effects)
MR Egger
MR RAPS
Weighted median
Weighted mode
0.00
−0.05
0.09
0.12
0.15
0.18
SNP effect on Angiopoietin−related protein 1
SNP effect on Gut microbiota abundance (genus Catenibacterium id.2153)

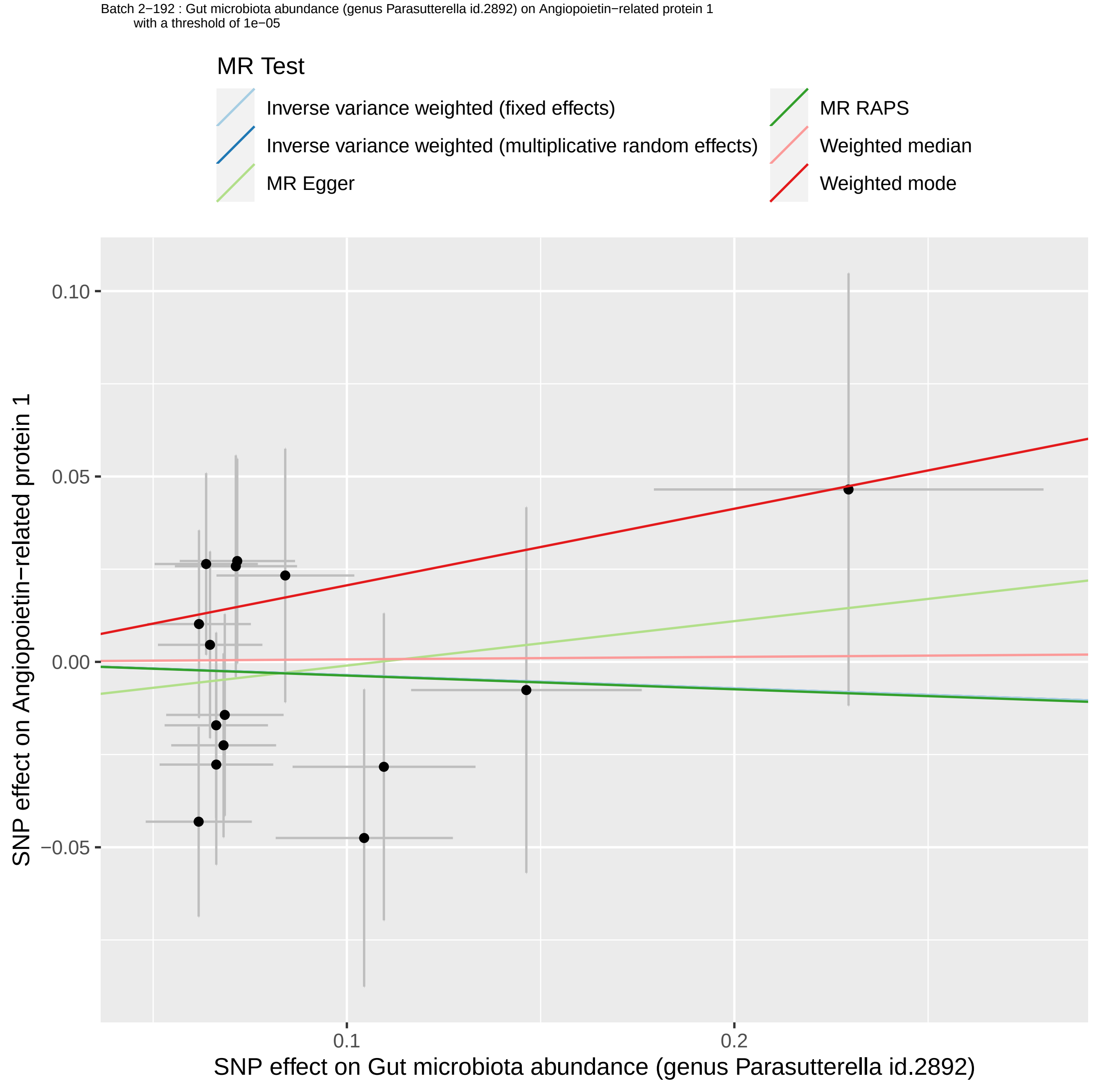
Batch 2−192 : Gut microbiota abundance (genus Parasutterella id.2892) on Angiopoietin−related protein 1
with a threshold of 1e−05
MR Test
Inverse variance weighted (fixed effects)
Inverse variance weighted (multiplicative random effects)
MR Egger
MR RAPS
Weighted median
Weighted mode
0.10
0.05
0.00
−0.05
0.1
0.2
SNP effect on Angiopoietin−related protein 1
SNP effect on Gut microbiota abundance (genus Parasutterella id.2892)

Batch 2−193 : Gut microbiota abundance (genus Catenibacterium id.2153) on Angiopoietin−related protein 1
with a threshold of 1e−05

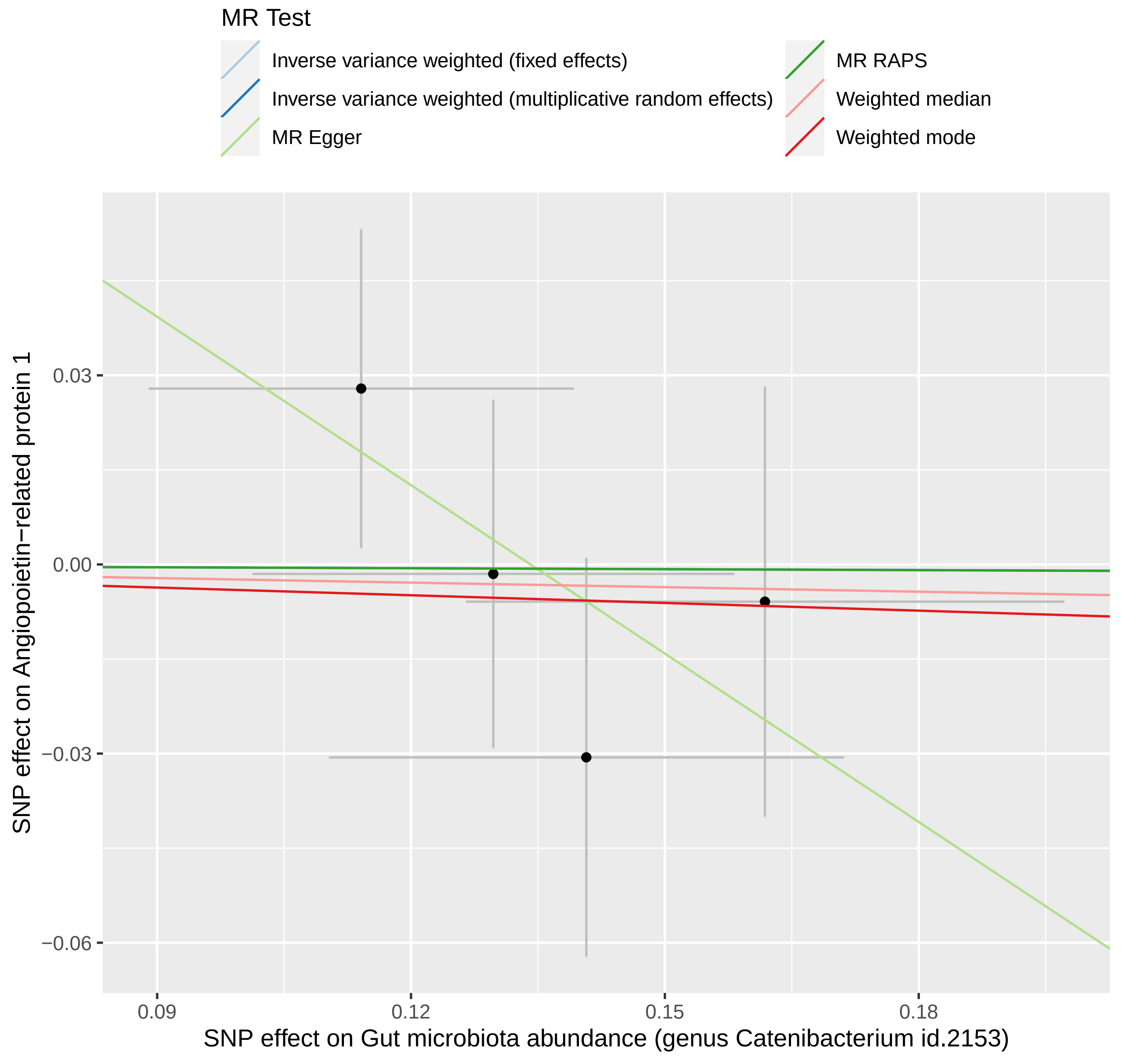

Batch 2−194 : Gut microbiota abundance (genus Parasutterella id.2892) on Angiopoietin−related protein 1
with a threshold of 1e−05

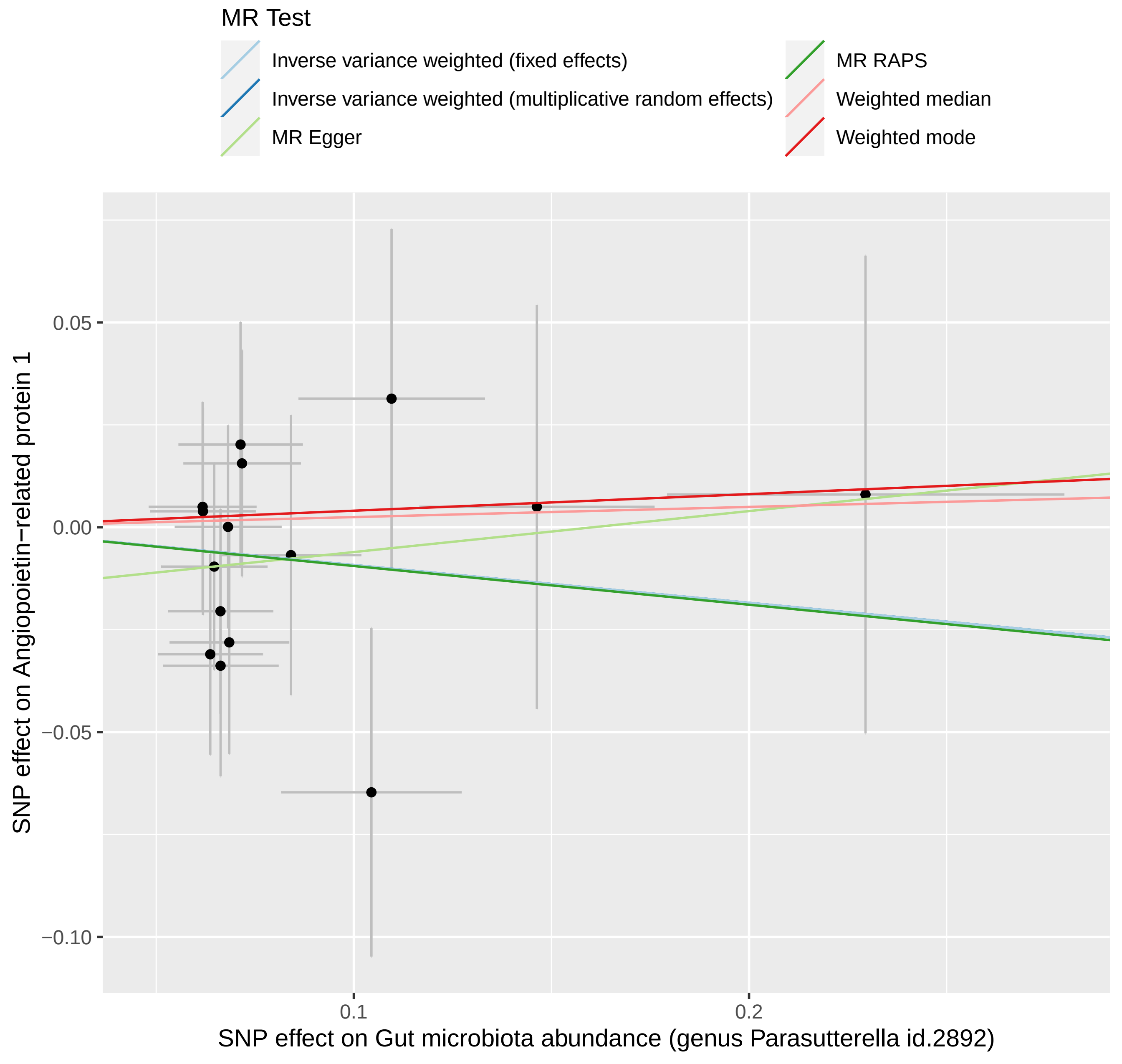

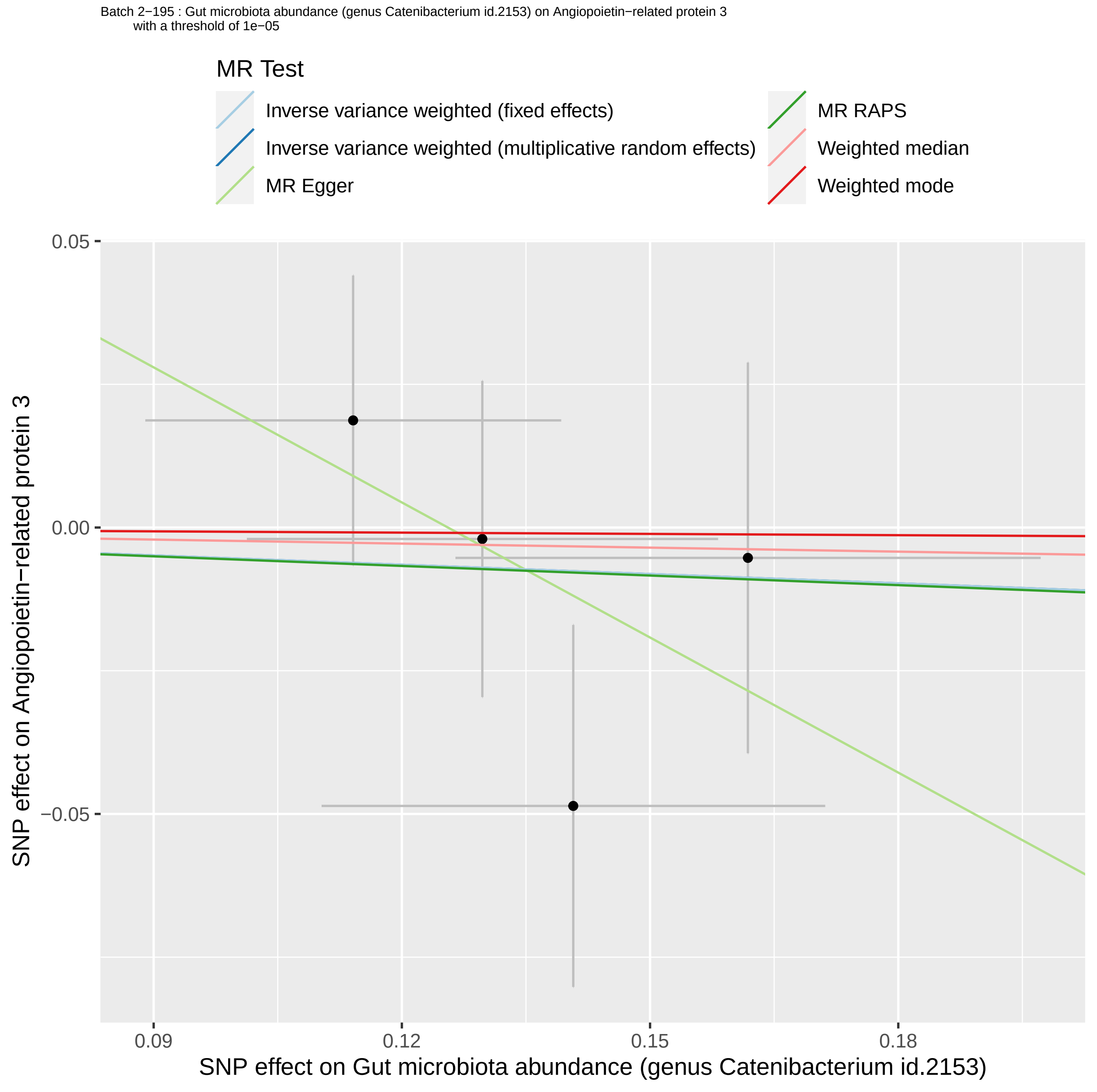
Batch 2−195 : Gut microbiota abundance (genus Catenibacterium id.2153) on Angiopoietin−related protein 3
with a threshold of 1e−05
MR Test
Inverse variance weighted (fixed effects)
Inverse variance weighted (multiplicative random effects)
MR Egger
MR RAPS
Weighted median
Weighted mode
0.05
0.00
−0.05
0.09
0.12
0.15
0.18
SNP effect on Angiopoietin−related protein 3
SNP effect on Gut microbiota abundance (genus Catenibacterium id.2153)

Batch 2−196 : Gut microbiota abundance (genus Parasutterella id.2892) on Angiopoietin−related protein 3
with a threshold of 1e−05

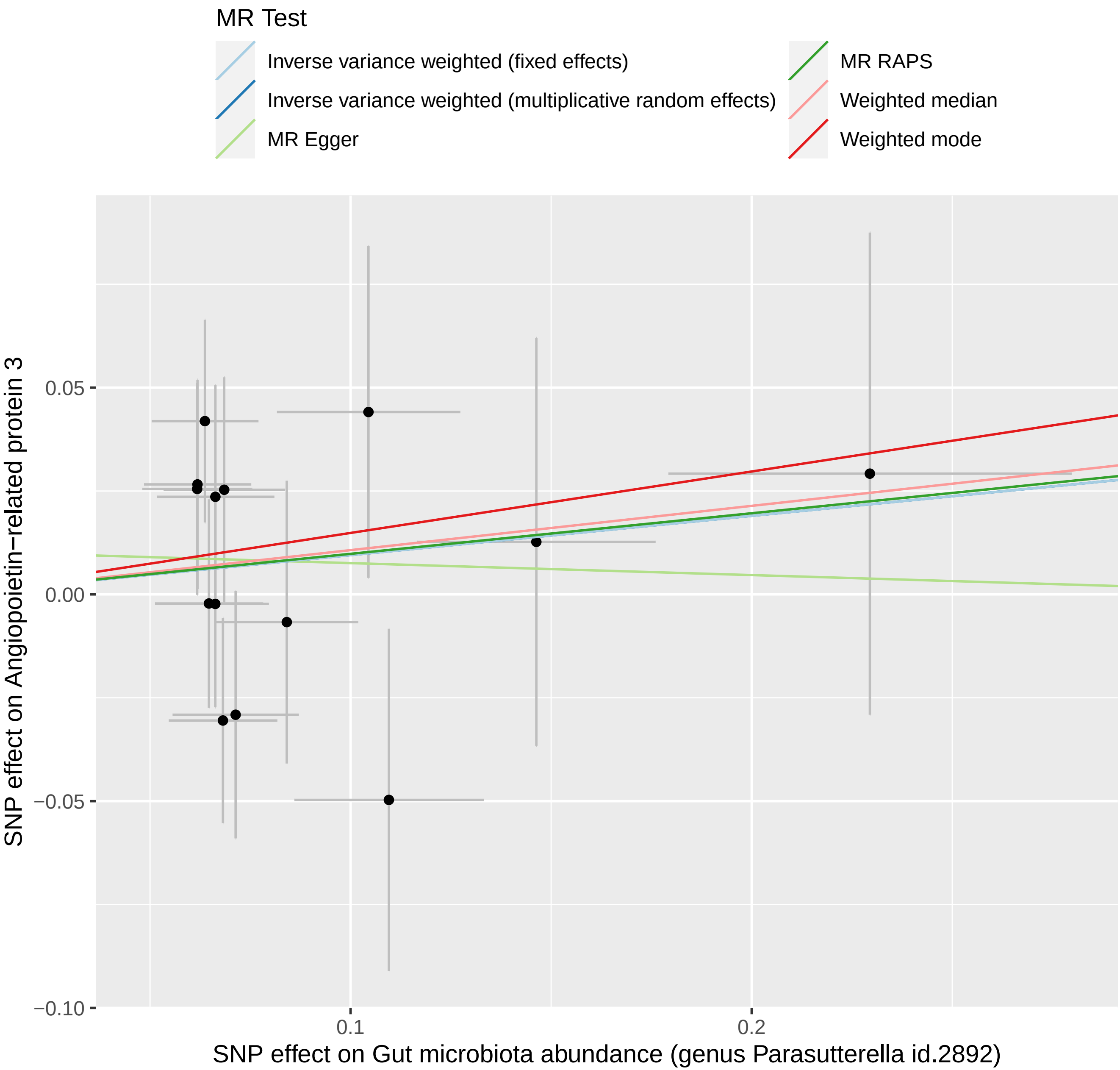

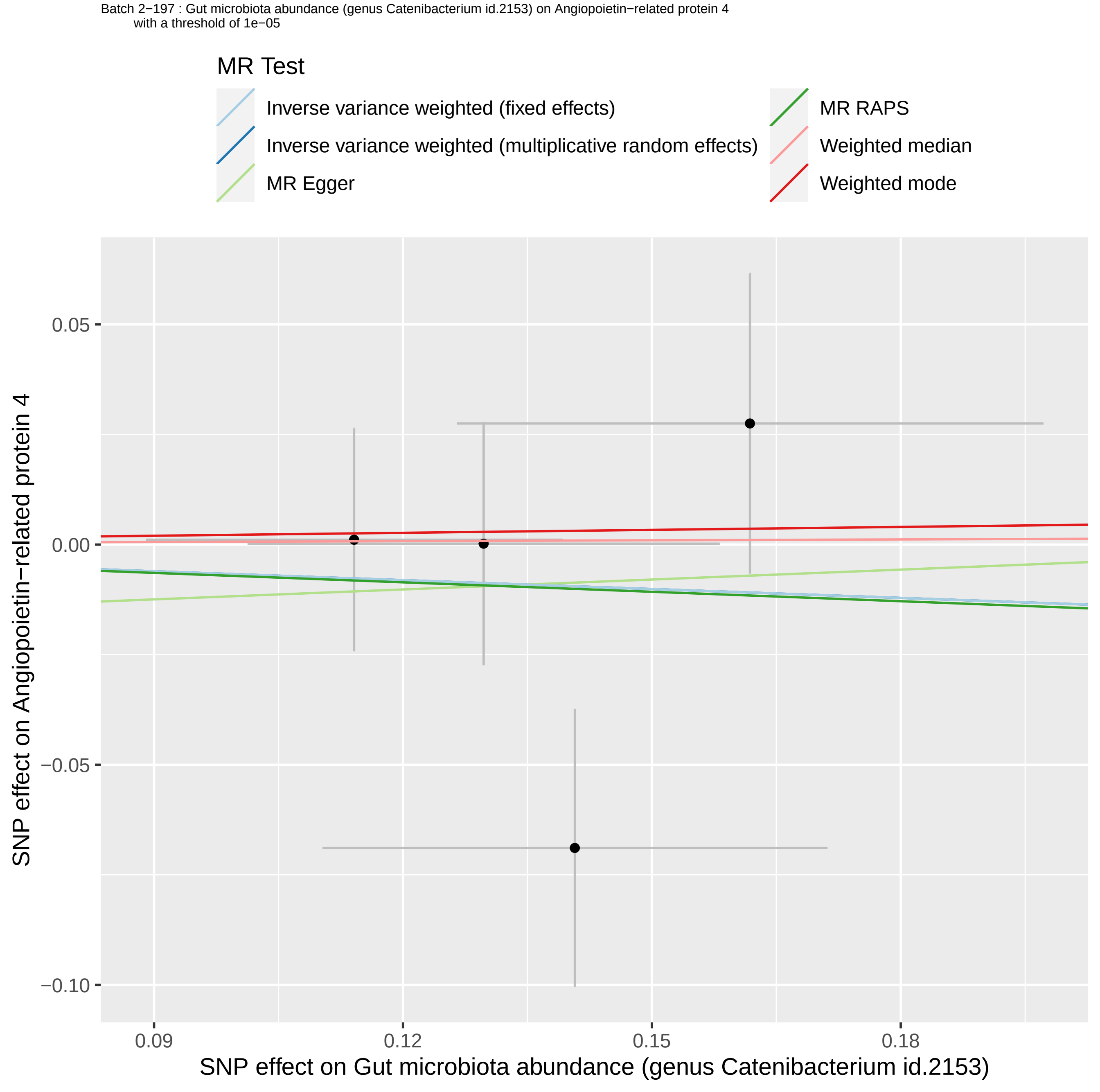

Batch 2−197 : Gut microbiota abundance (genus Catenibacterium id.2153) on Angiopoietin−related protein 4
with a threshold of 1e−05
MR Test
Inverse variance weighted (fixed effects)
Inverse variance weighted (multiplicative random effects)
MR Egger
MR RAPS
Weighted median
Weighted mode
0.05
0.00
−0.05
−0.10
0.09
0.12
0.15
0.18
SNP effect on Angiopoietin−related protein 4
SNP effect on Gut microbiota abundance (genus Catenibacterium id.2153)

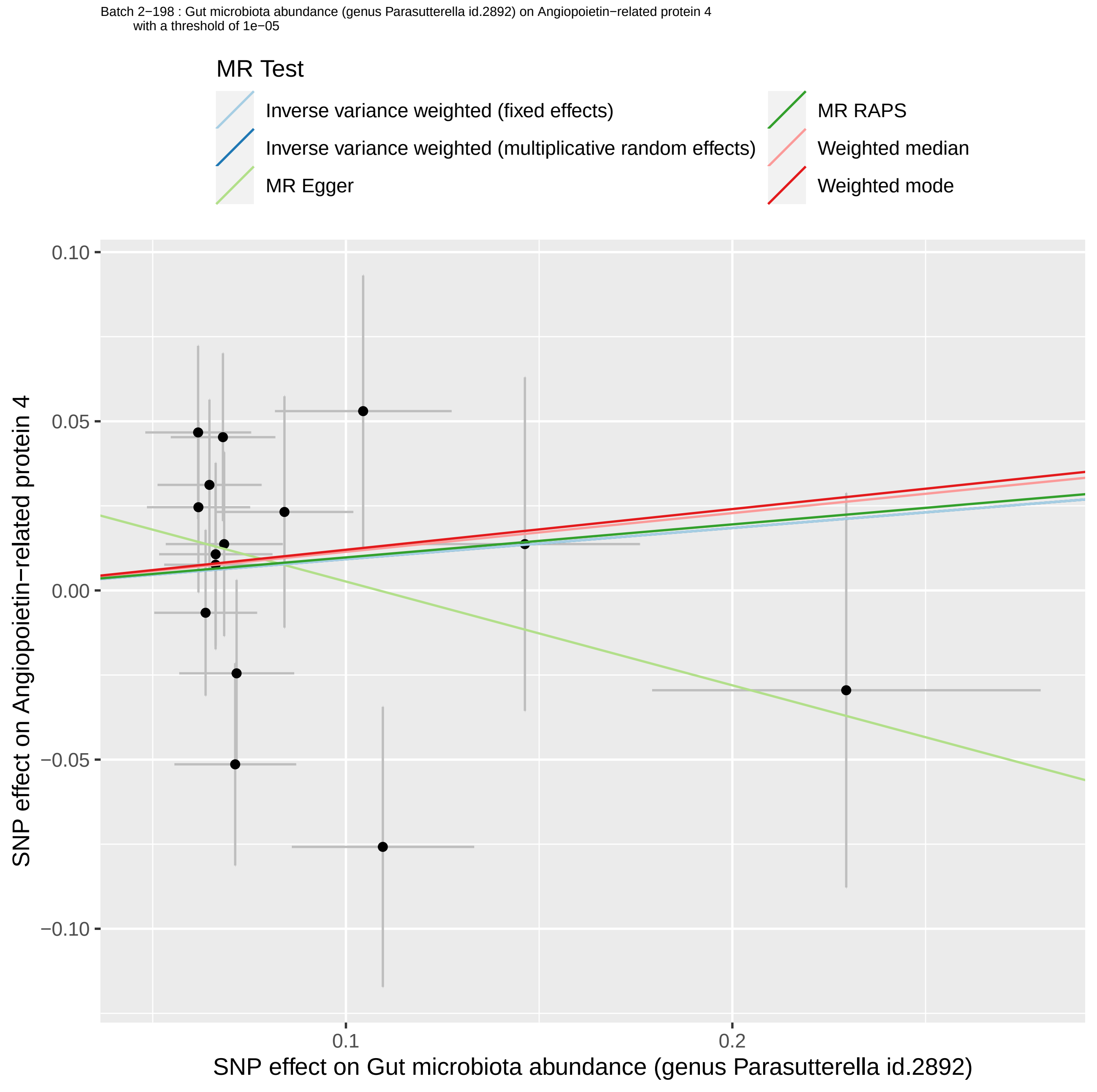

Batch 2−198 : Gut microbiota abundance (genus Parasutterella id.2892) on Angiopoietin−related protein 4
with a threshold of 1e−05
MR Test
Inverse variance weighted (fixed effects)
Inverse variance weighted (multiplicative random effects)
MR Egger
MR RAPS
Weighted median
Weighted mode
0.10
0.05
0.00
−0.05
−0.10
0.1
0.2
SNP effect on Angiopoietin−related protein 4
SNP effect on Gut microbiota abundance (genus Parasutterella id.2892)

Batch 2−199 : Gut microbiota abundance (genus Catenibacterium id.2153) on Angiopoietin−related protein 7
with a threshold of 1e−05

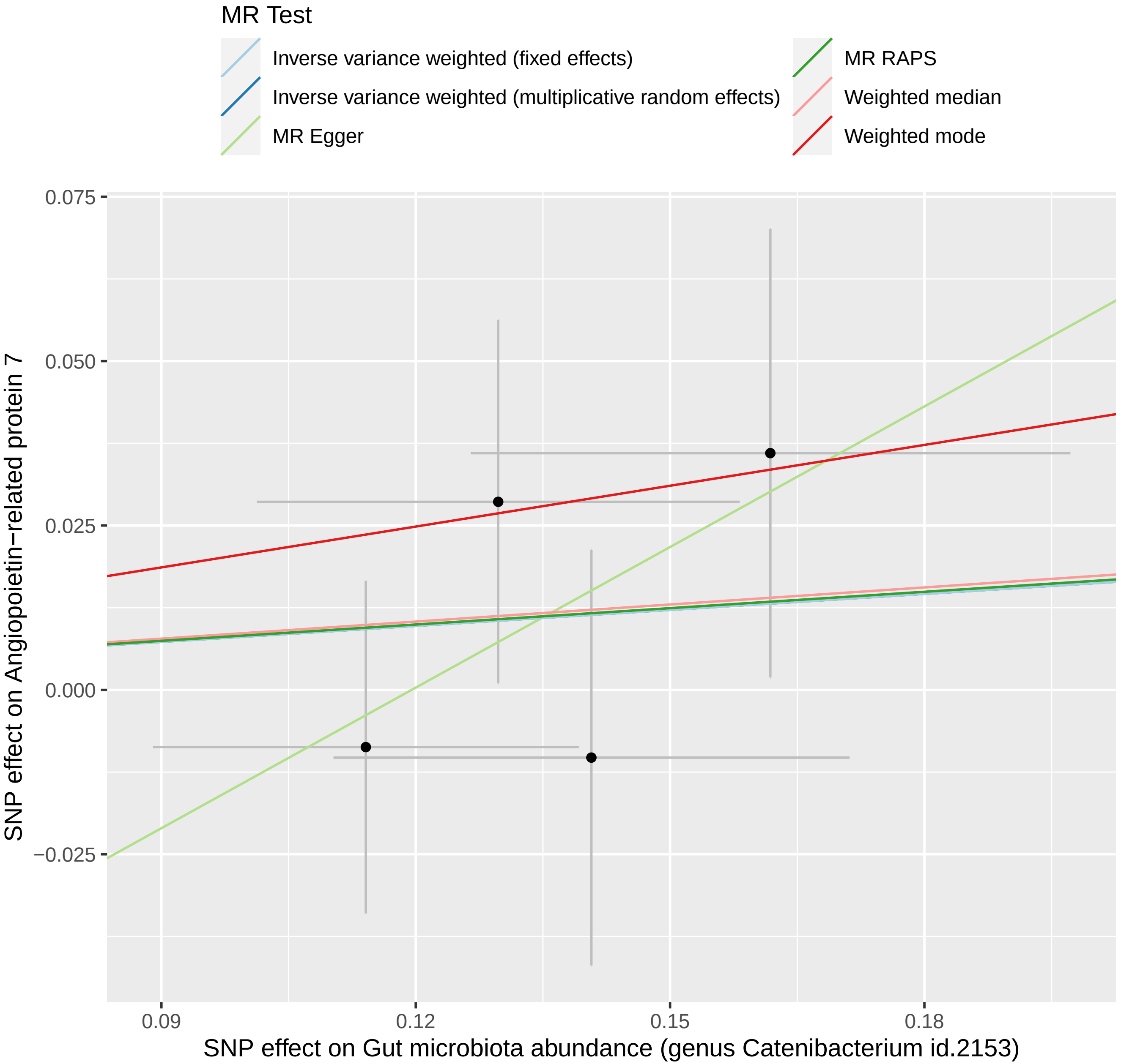

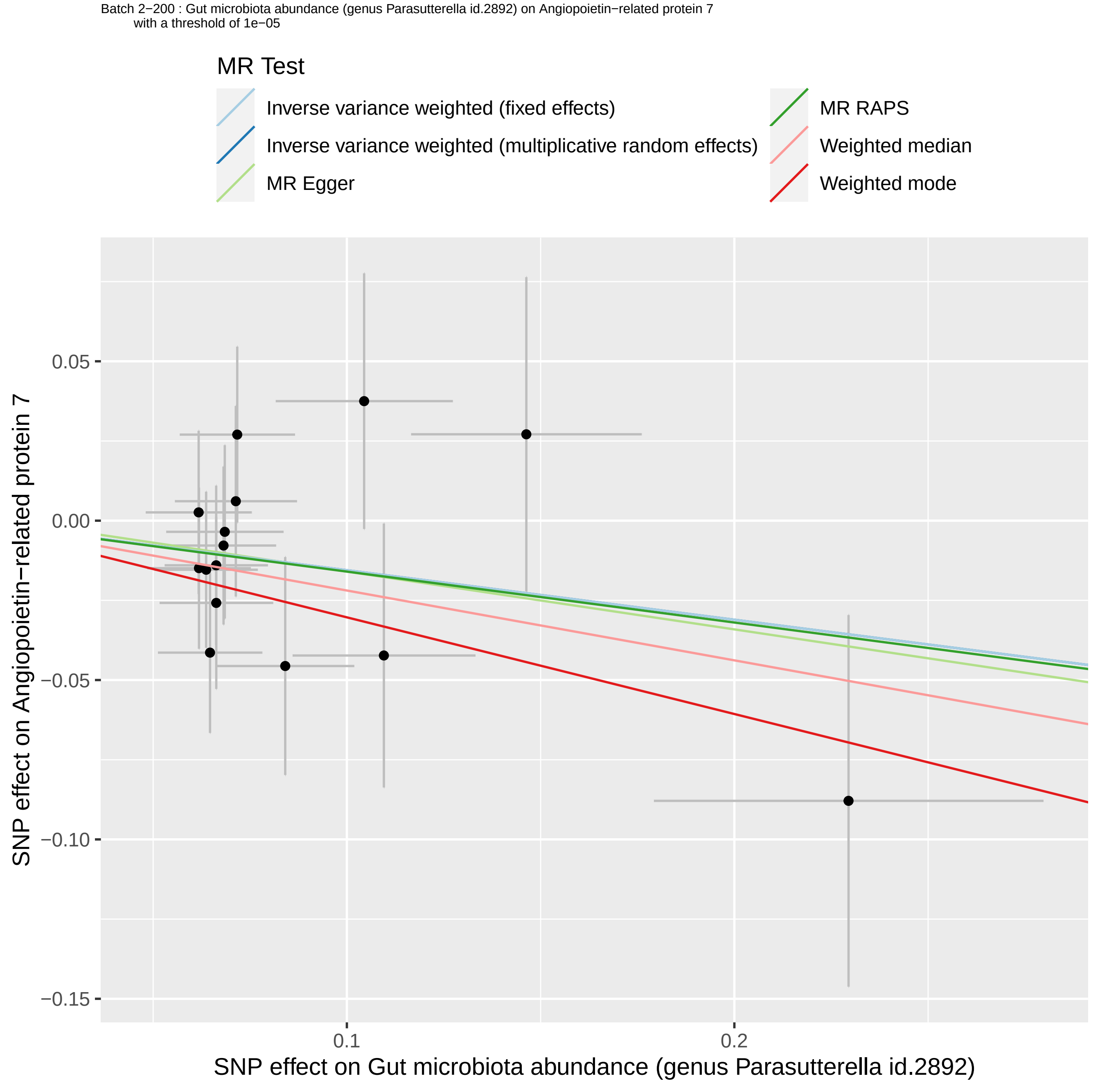
Batch 2−200 : Gut microbiota abundance (genus Parasutterella id.2892) on Angiopoietin−related protein 7
with a threshold of 1e−05
MR Test
Inverse variance weighted (fixed effects)
Inverse variance weighted (multiplicative random effects)
MR Egger
MR RAPS
Weighted median
Weighted mode
0.05
0.00
−0.05
−0.10
−0.15
0.1
0.2
SNP effect on Angiopoietin−related protein 7
SNP effect on Gut microbiota abundance (genus Parasutterella id.2892)

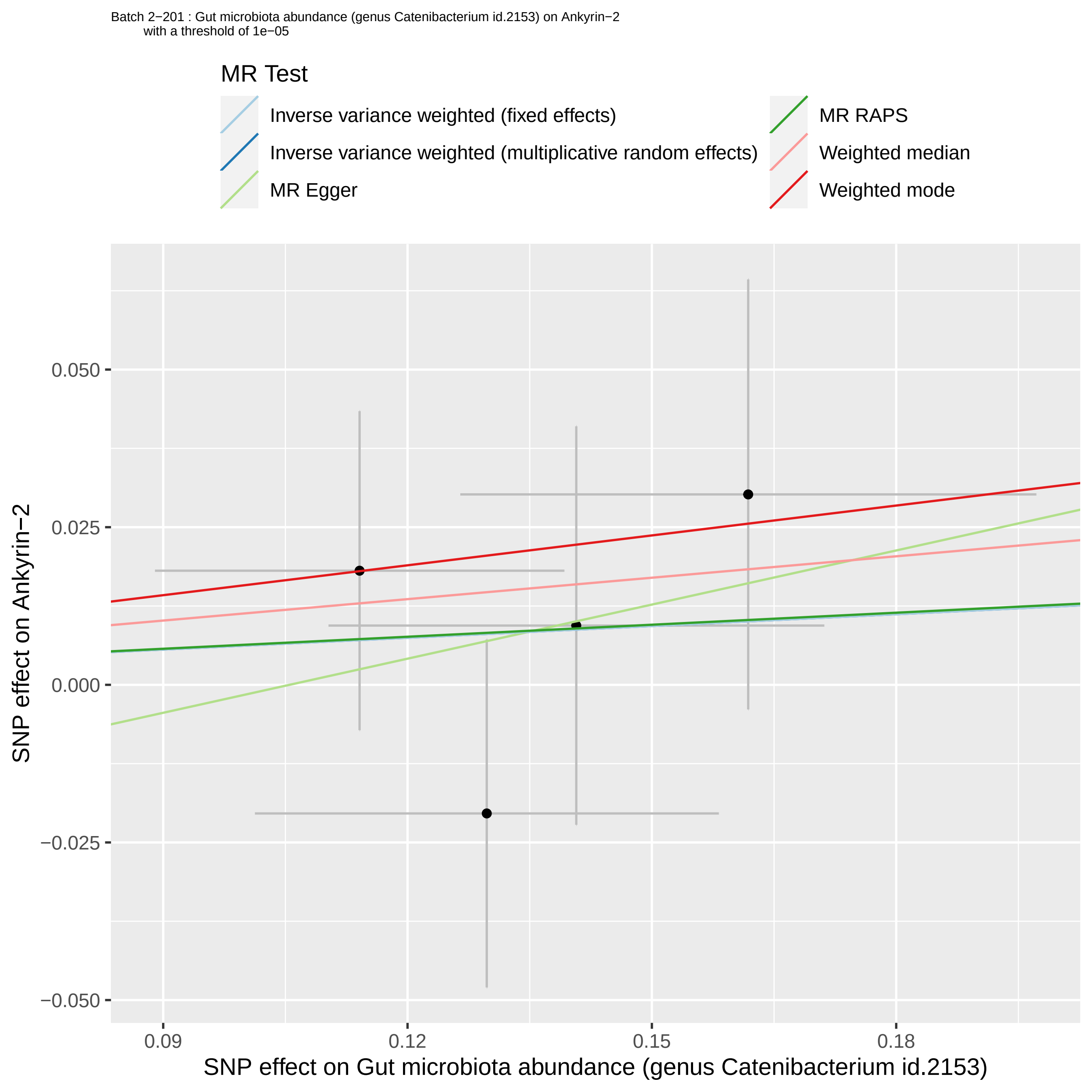

Batch 2−201 : Gut microbiota abundance (genus Catenibacterium id.2153) on Ankyrin−2
with a threshold of 1e−05
MR Test
Inverse variance weighted (fixed effects)
Inverse variance weighted (multiplicative random effects)
MR Egger
MR RAPS
Weighted median
Weighted mode
0.050
0.025
0.000
−0.025
−0.050
SNP effect on Ankyrin−2
0.09
0.12
0.15
0.18
SNP effect on Gut microbiota abundance (genus Catenibacterium id.2153)

Batch 2−202 : Gut microbiota abundance (genus Parasutterella id.2892) on Ankyrin−2
with a threshold of 1e−05

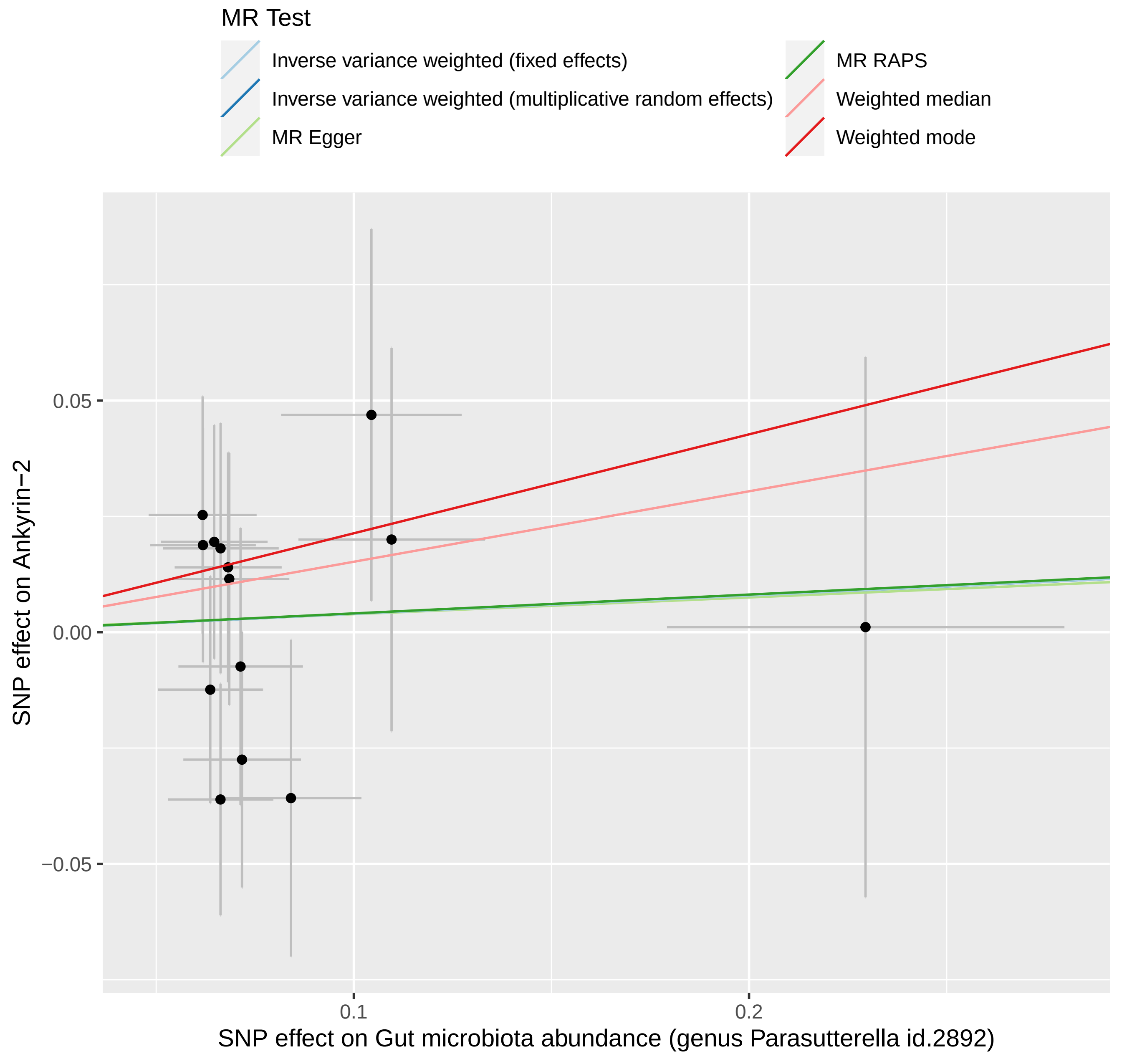

Batch 2−203 : Gut microbiota abundance (genus Catenibacterium id.2153) on Ankyrin repeat domain−containing protein 27 with a threshold of 1e−05

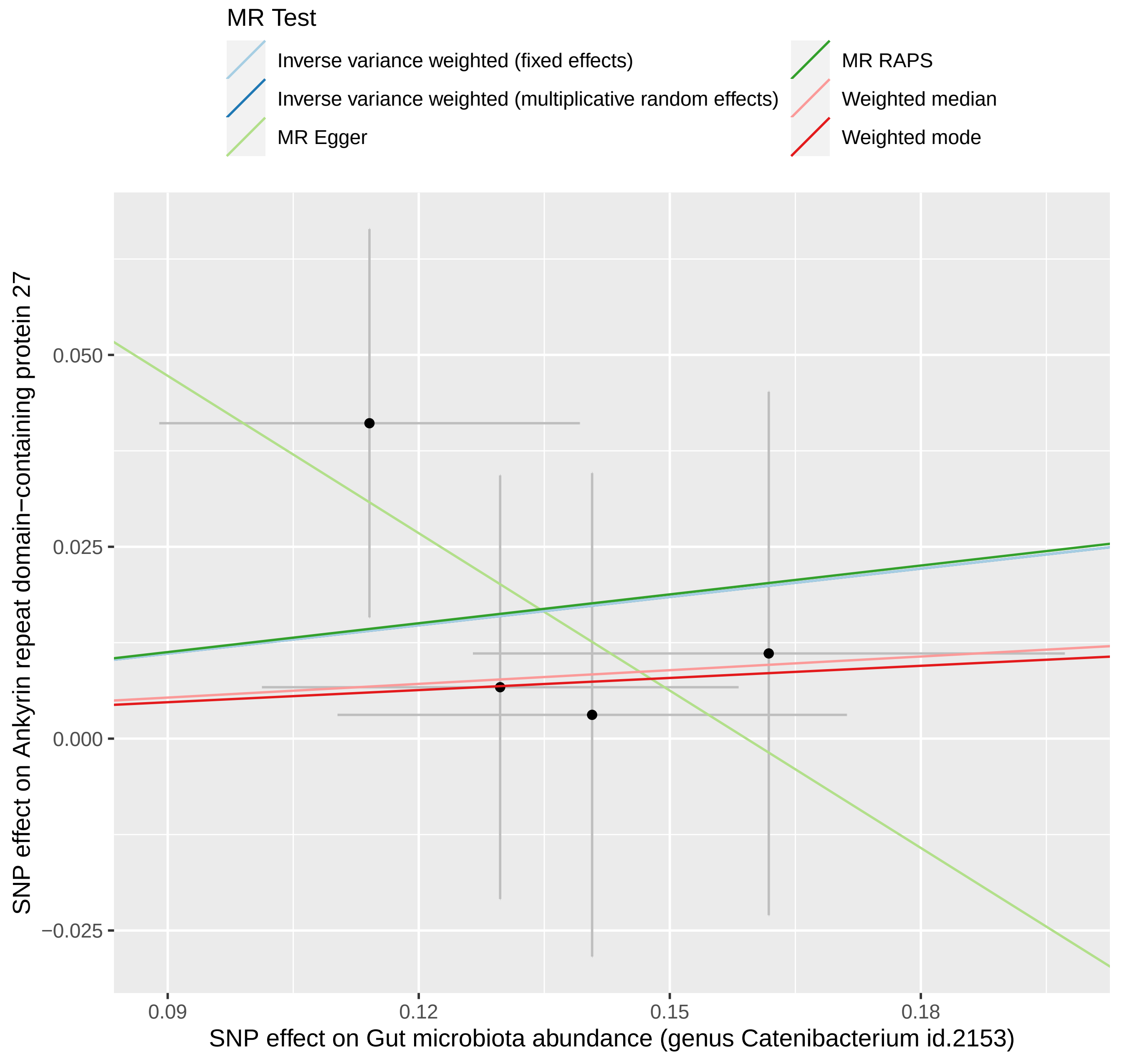

Batch 2−204 : Gut microbiota abundance (genus Parasutterella id.2892) on Ankyrin repeat domain−containing protein 27
with a threshold of 1e−05

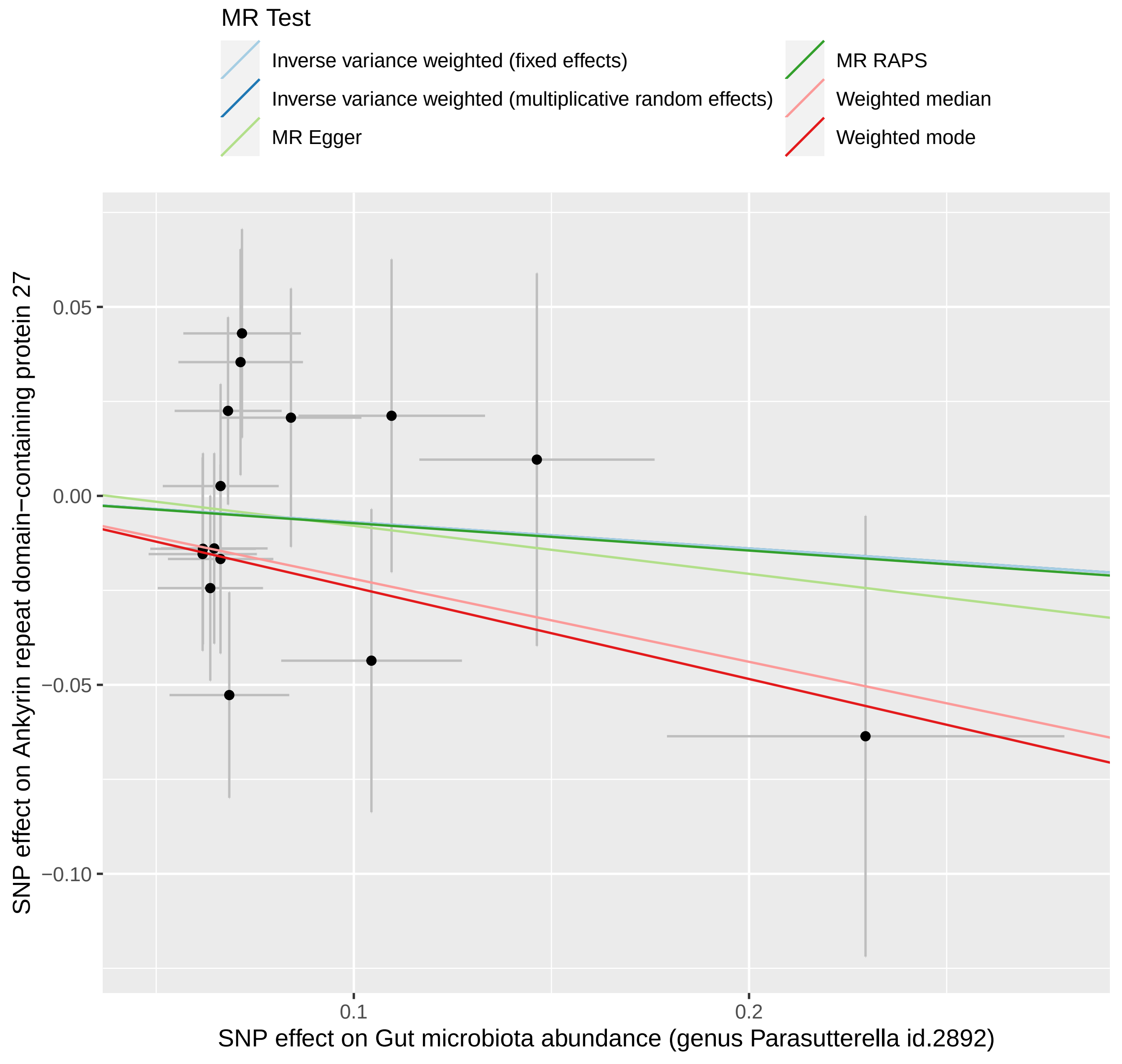

Batch 2−205 : Gut microbiota abundance (genus Catenibacterium id.2153) on Ankyrin repeat domain−containing protein 46
with a threshold of 1e−05

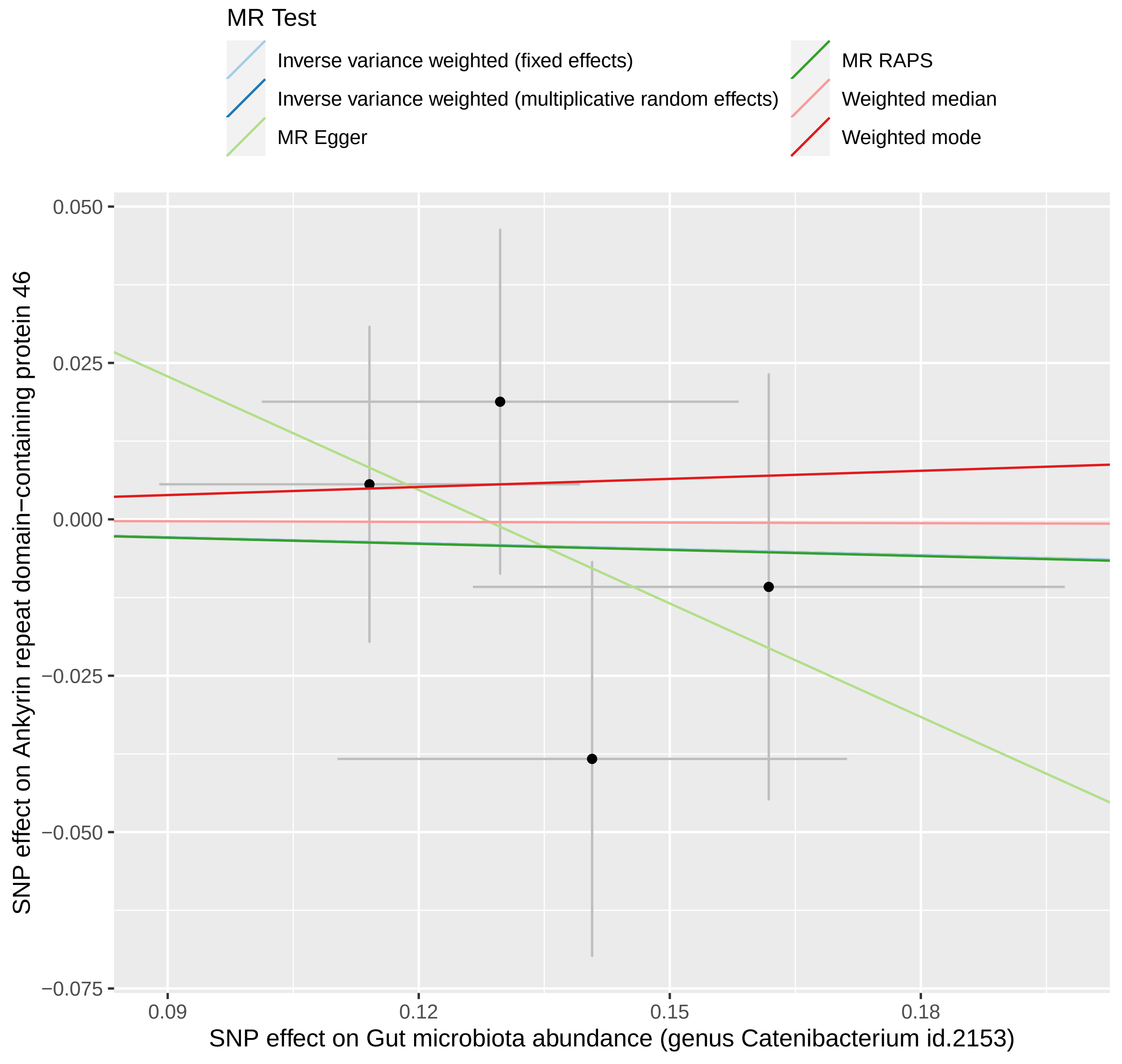

Batch 2−206 : Gut microbiota abundance (genus Parasutterella id.2892) on Ankyrin repeat domain−containing protein 46
with a threshold of 1e−05

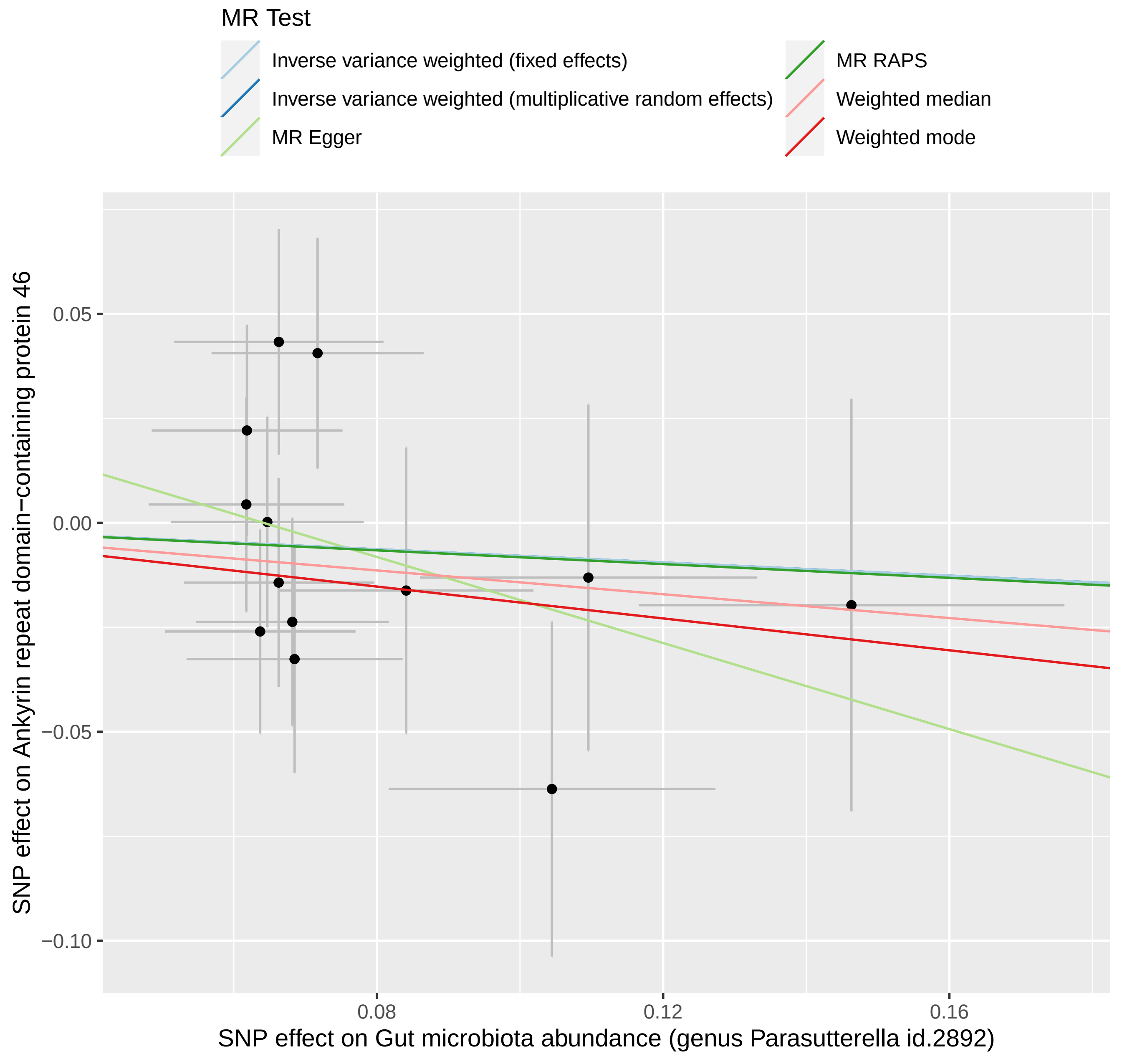

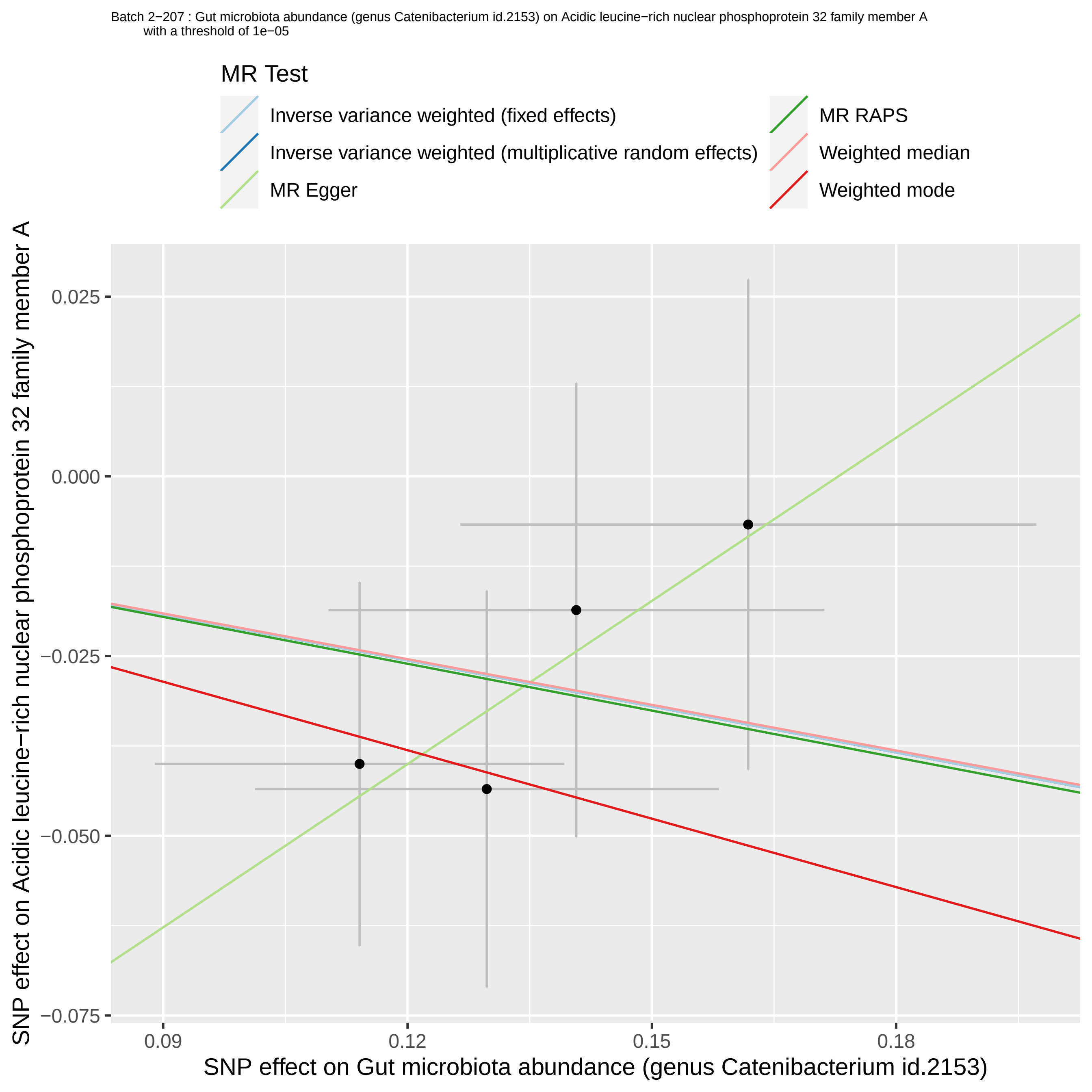
Batch 2−207 : Gut microbiota abundance (genus Catenibacterium id.2153) on Acidic leucine−rich nuclear phosphoprotein 32 family member A
with a threshold of 1e−05
MR Test
Inverse variance weighted (fixed effects)
Inverse variance weighted (multiplicative random effects)
MR Egger
MR RAPS
Weighted median
Weighted mode
0.025
0.000
−0.025
−0.050
−0.075
0.09
0.12
0.15
0.18
SNP effect on Acidic leucine−rich nuclear phosphoprotein 32 family member A
SNP effect on Gut microbiota abundance (genus Catenibacterium id.2153)

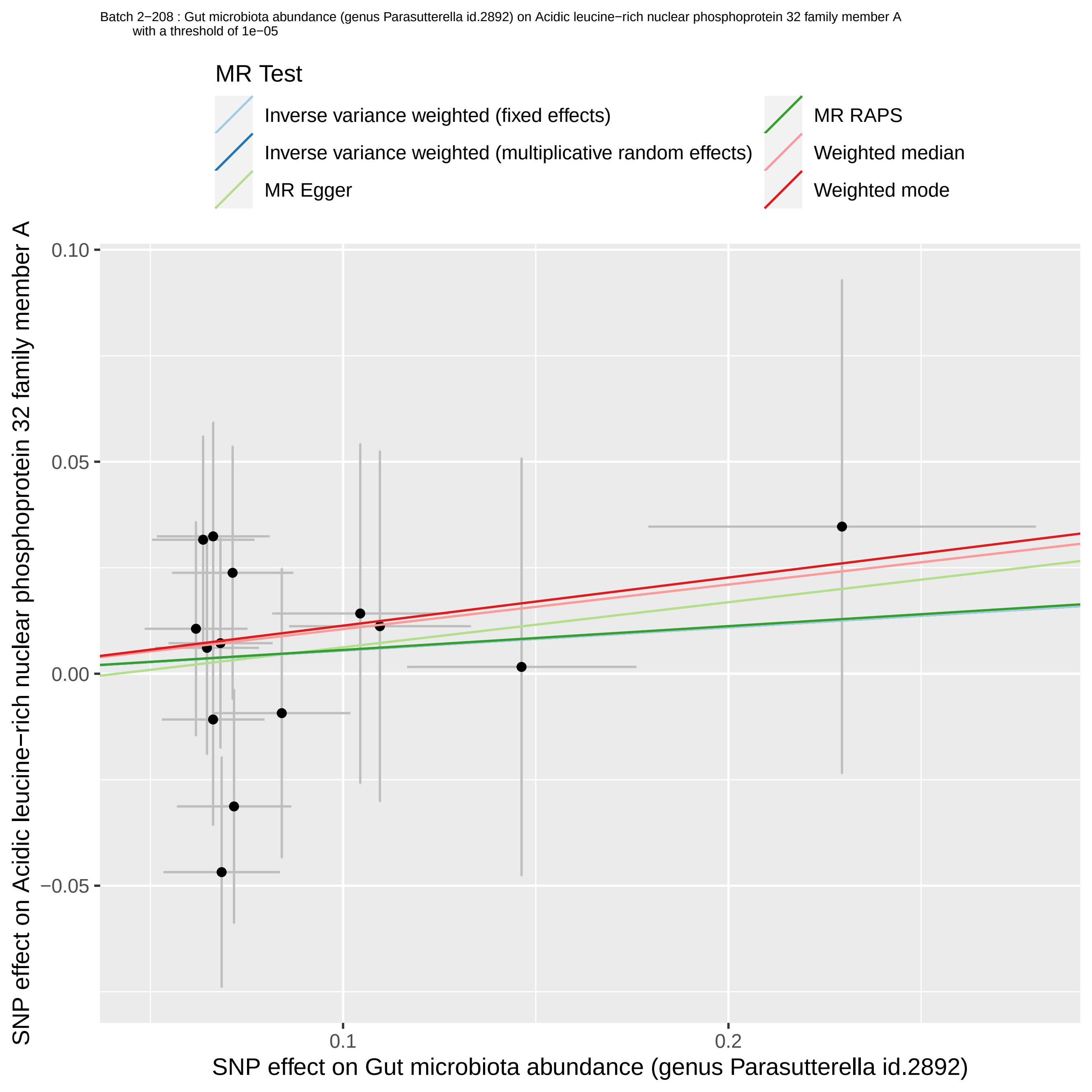
Batch 2−208 : Gut microbiota abundance (genus Parasutterella id.2892) on Acidic leucine−rich nuclear phosphoprotein 32 family member A
with a threshold of 1e−05
MR Test
Inverse variance weighted (fixed effects)
Inverse variance weighted (multiplicative random effects)
MR Egger
MR RAPS
Weighted median
Weighted mode
0.10
0.05
0.00
−0.05
0.1
0.2
SNP effect on Acidic leucine−rich nuclear phosphoprotein 32 family member A
SNP effect on Gut microbiota abundance (genus Parasutterella id.2892)

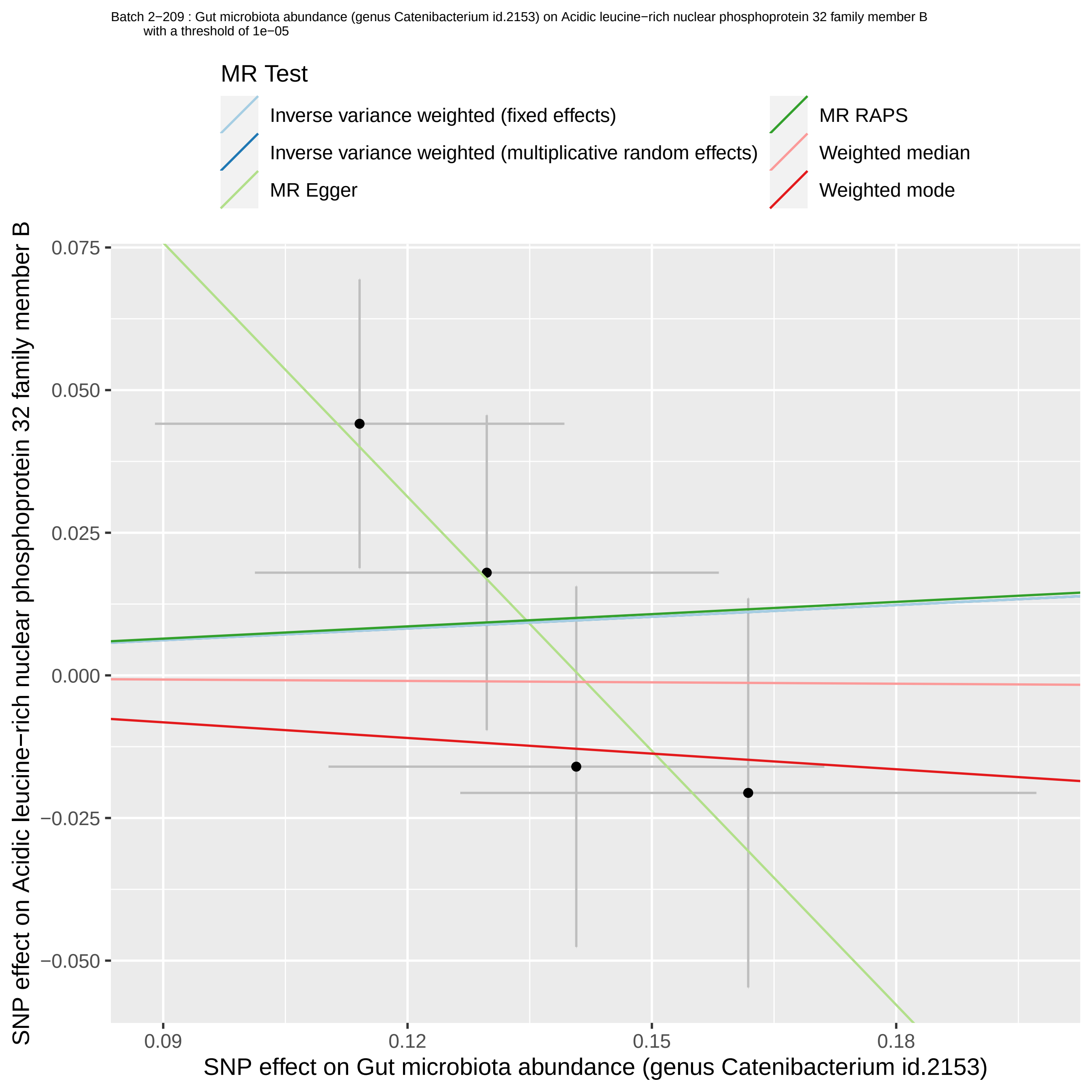
Batch 2−209 : Gut microbiota abundance (genus Catenibacterium id.2153) on Acidic leucine−rich nuclear phosphoprotein 32 family member B
with a threshold of 1e−05
MR Test
Inverse variance weighted (fixed effects)
Inverse variance weighted (multiplicative random effects)
MR Egger
MR RAPS
Weighted median
Weighted mode
SNP effect on Acidic leucine−rich nuclear phosphoprotein 32 family member B
0.075
0.050
0.025
0.000
−0.025
−0.050
0.09
0.12
0.15
0.18
SNP effect on Gut microbiota abundance (genus Catenibacterium id.2153)

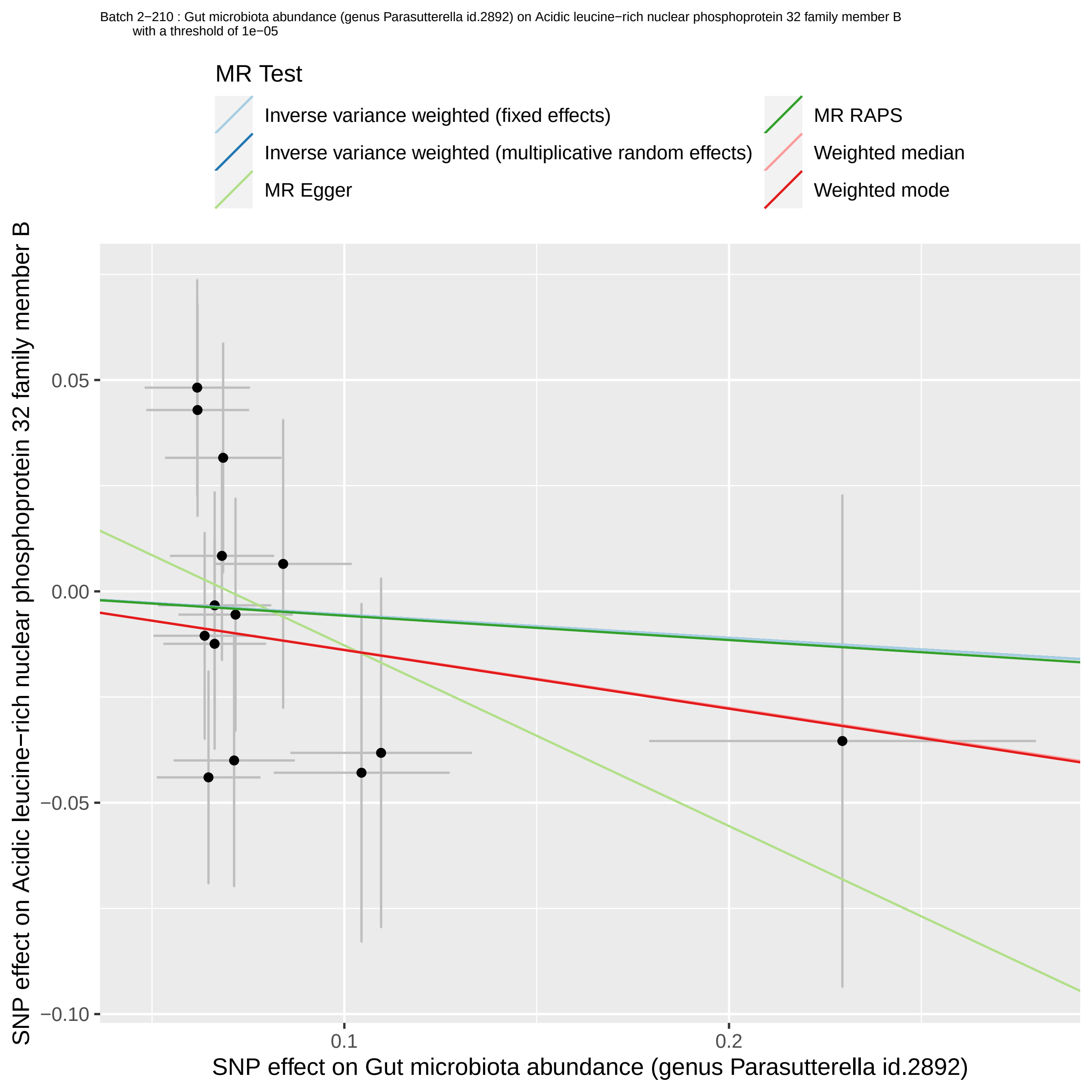
Batch 2−210 : Gut microbiota abundance (genus Parasutterella id.2892) on Acidic leucine−rich nuclear phosphoprotein 32 family member B
with a threshold of 1e−05
MR Test
Inverse variance weighted (fixed effects)
Inverse variance weighted (multiplicative random effects)
MR Egger
MR RAPS
Weighted median
Weighted mode
SNP effect on Acidic leucine−rich nuclear phosphoprotein 32 family member B
0.05
0.00
−0.05
−0.10
0.1
0.2
SNP effect on Gut microbiota abundance (genus Parasutterella id.2892)

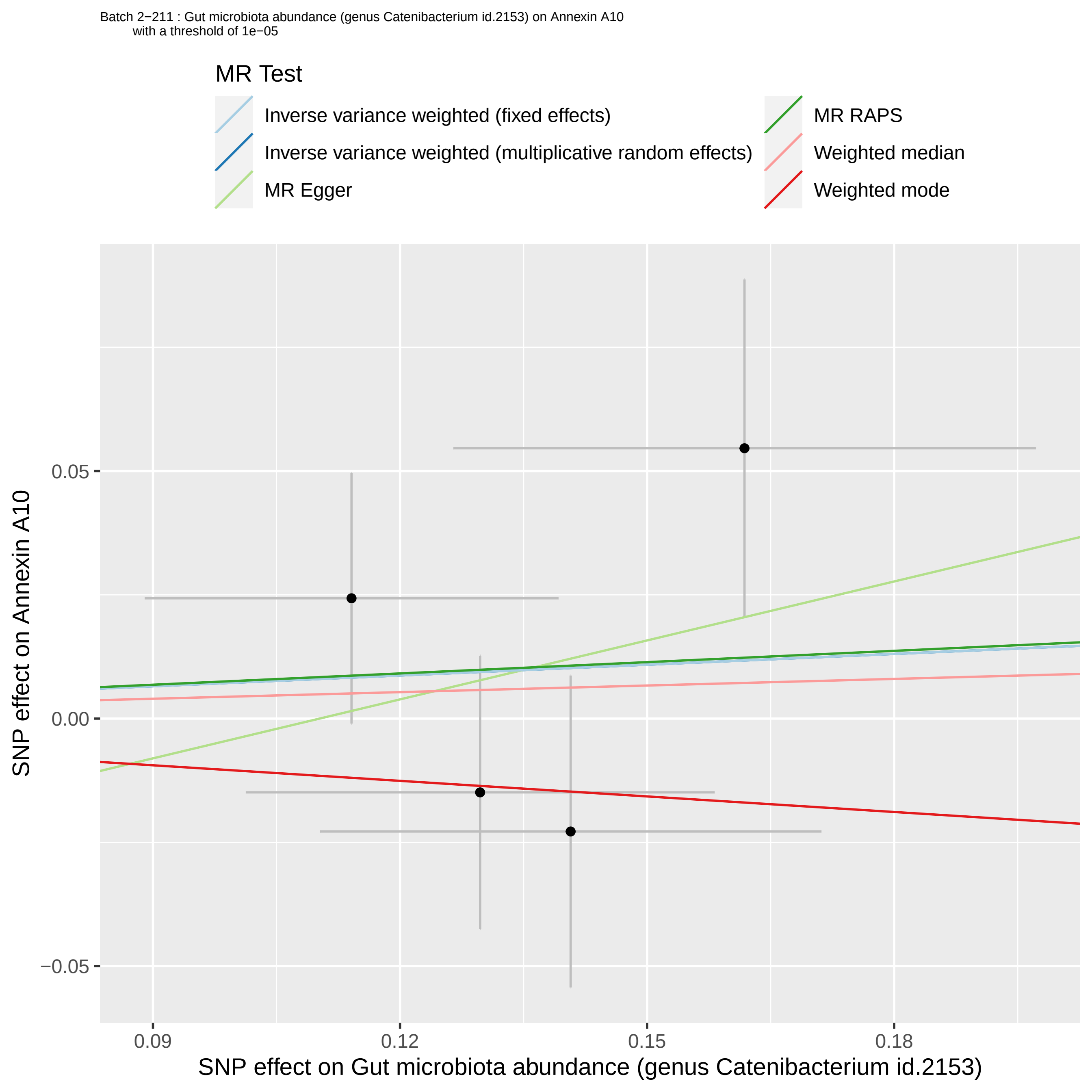

Batch 2−211 : Gut microbiota abundance (genus Catenibacterium id.2153) on Annexin A10
with a threshold of 1e−05
MR Test
Inverse variance weighted (fixed effects)
Inverse variance weighted (multiplicative random effects)
MR Egger
MR RAPS
Weighted median
Weighted mode
0.05
0.00
−0.05
SNP effect on Annexin A10
0.09
0.12
0.15
0.18
SNP effect on Gut microbiota abundance (genus Catenibacterium id.2153)

Batch 2−212 : Gut microbiota abundance (genus Parasutterella id.2892) on Annexin A10
with a threshold of 1e−05

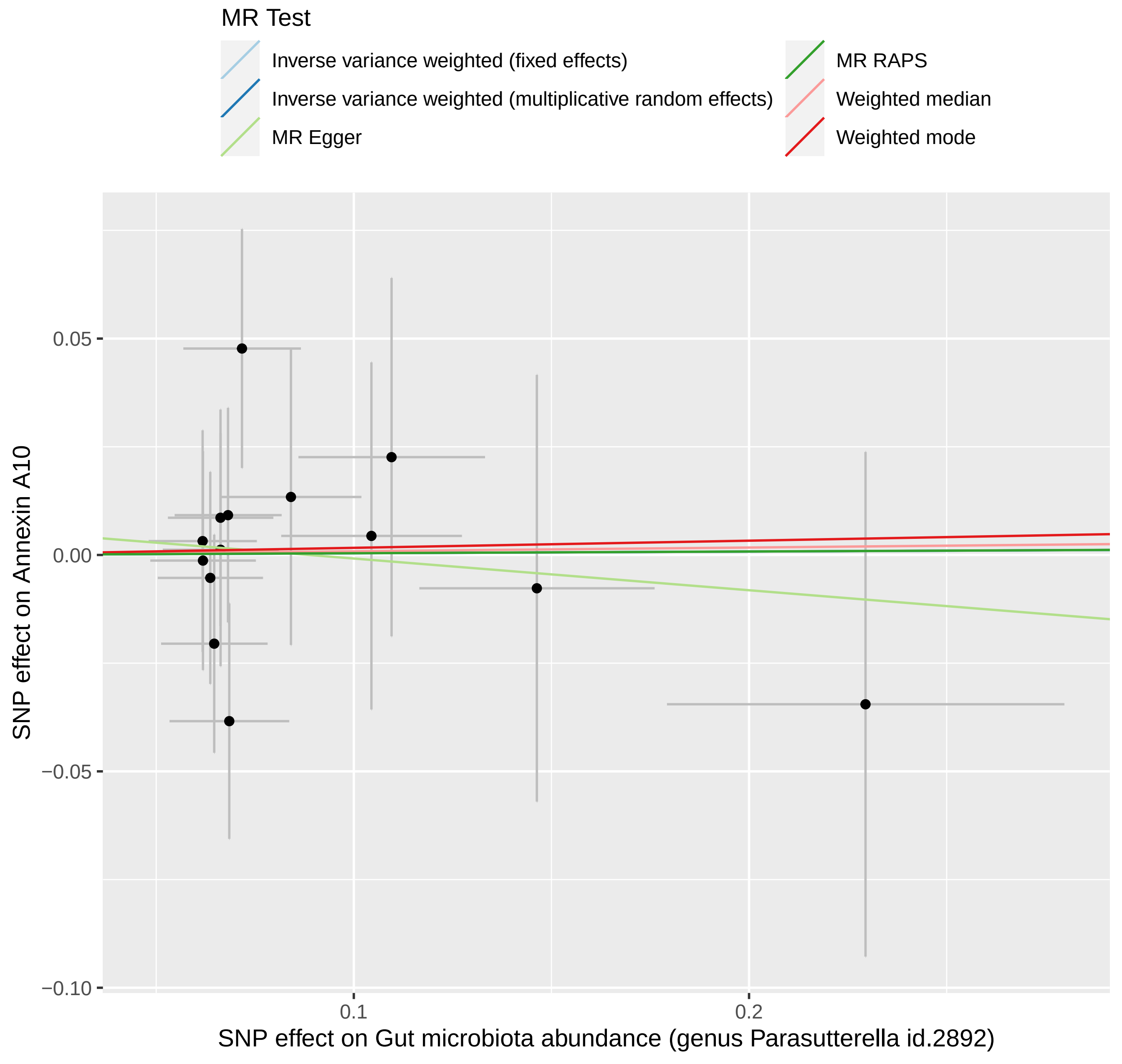

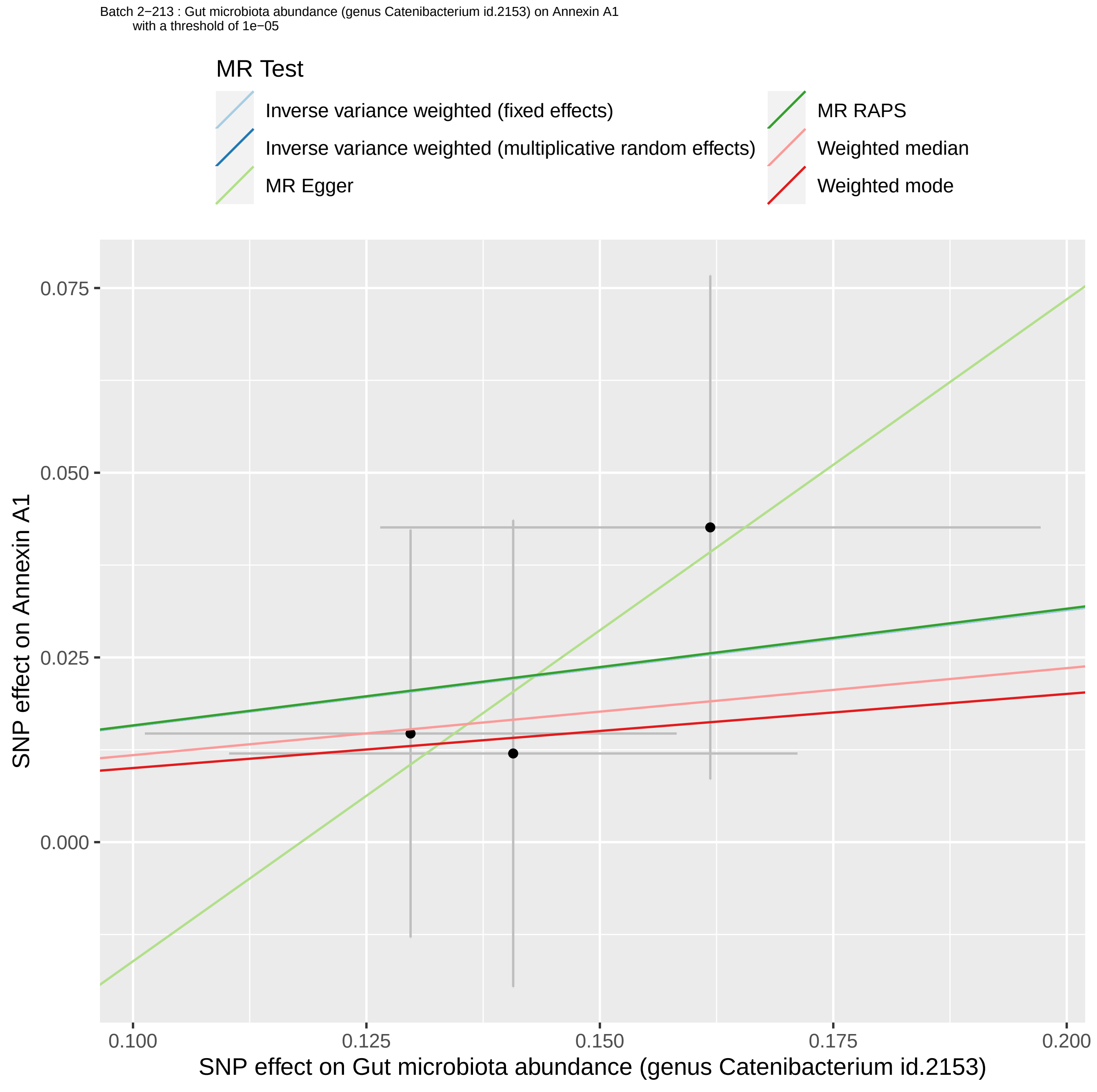

Batch 2−213 : Gut microbiota abundance (genus Catenibacterium id.2153) on Annexin A1
with a threshold of 1e−05
MR Test
Inverse variance weighted (fixed effects)
Inverse variance weighted (multiplicative random effects)
MR Egger
MR RAPS
Weighted median
Weighted mode
0.075
0.050
0.025
0.000
SNP effect on Annexin A1
0.100
0.125
0.150
0.175
0.200
SNP effect on Gut microbiota abundance (genus Catenibacterium id.2153)

Batch 2−214 : Gut microbiota abundance (genus Parasutterella id.2892) on Annexin A1
with a threshold of 1e−05

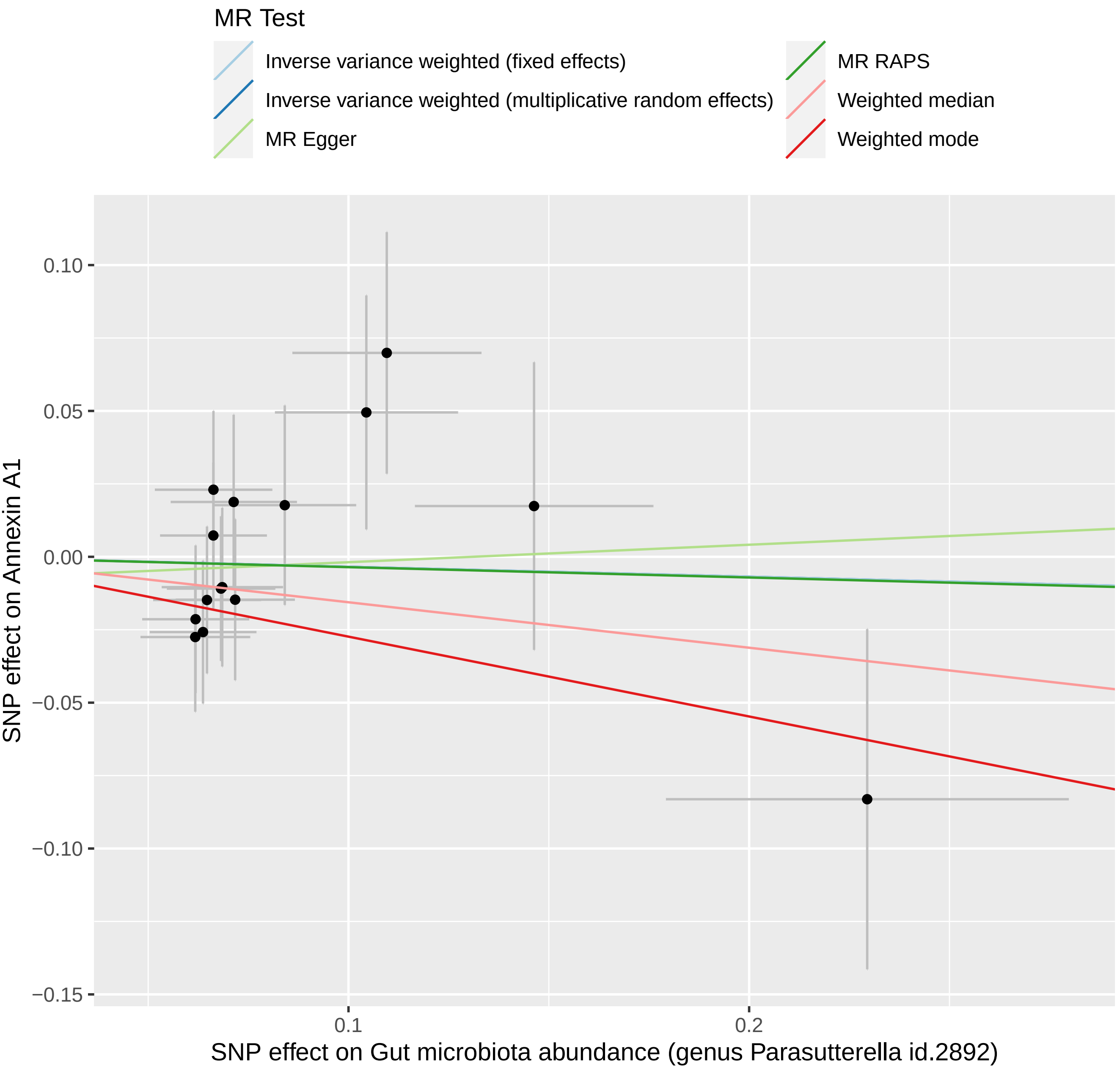

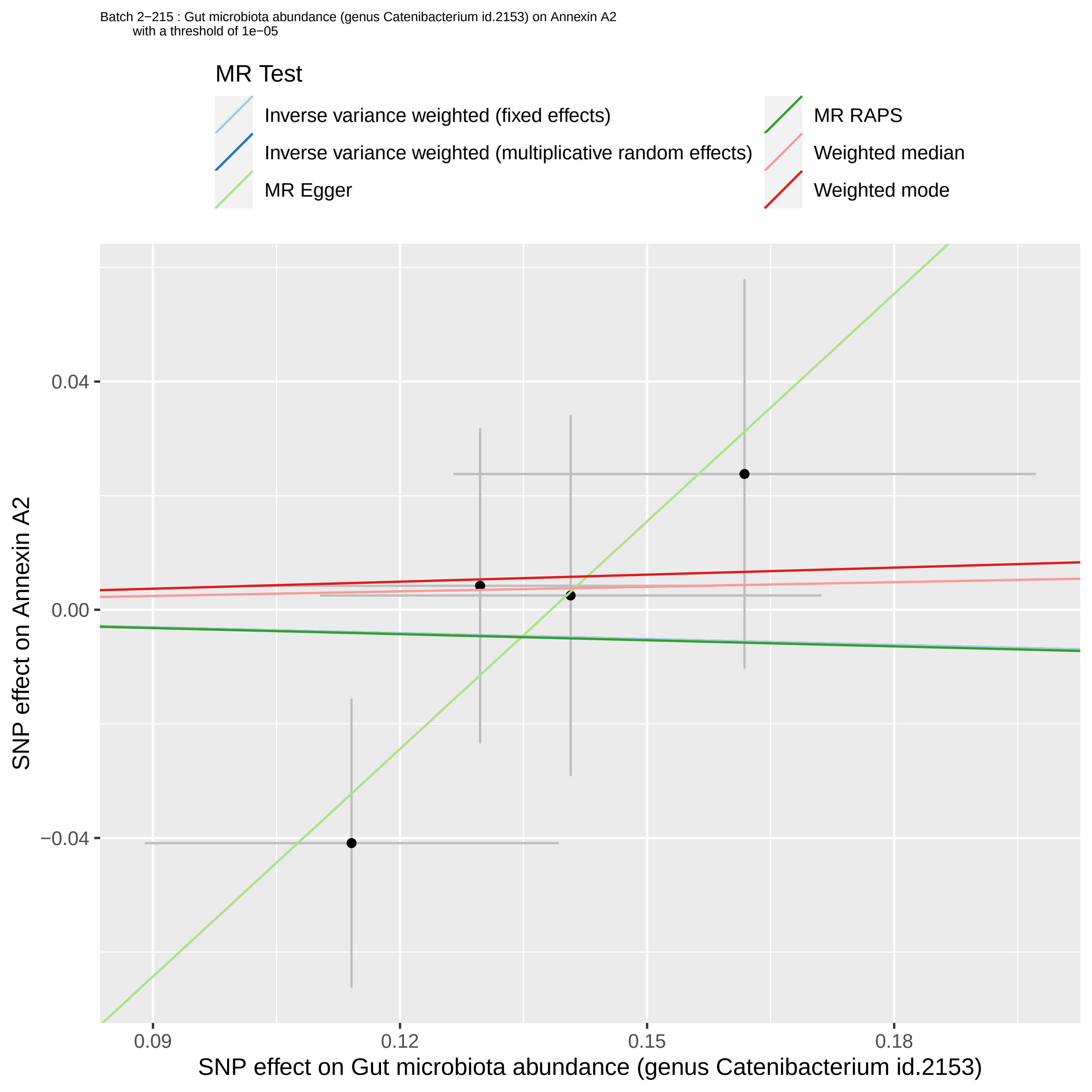
Batch 2−215 : Gut microbiota abundance (genus Catenibacterium id.2153) on Annexin A2
with a threshold of 1e−05
MR Test
Inverse variance weighted (fixed effects)
Inverse variance weighted (multiplicative random effects)
MR Egger
MR RAPS
Weighted median
Weighted mode
0.04
0.00
−0.04
0.09
0.12
0.15
0.18
SNP effect on Annexin A2
SNP effect on Gut microbiota abundance (genus Catenibacterium id.2153)

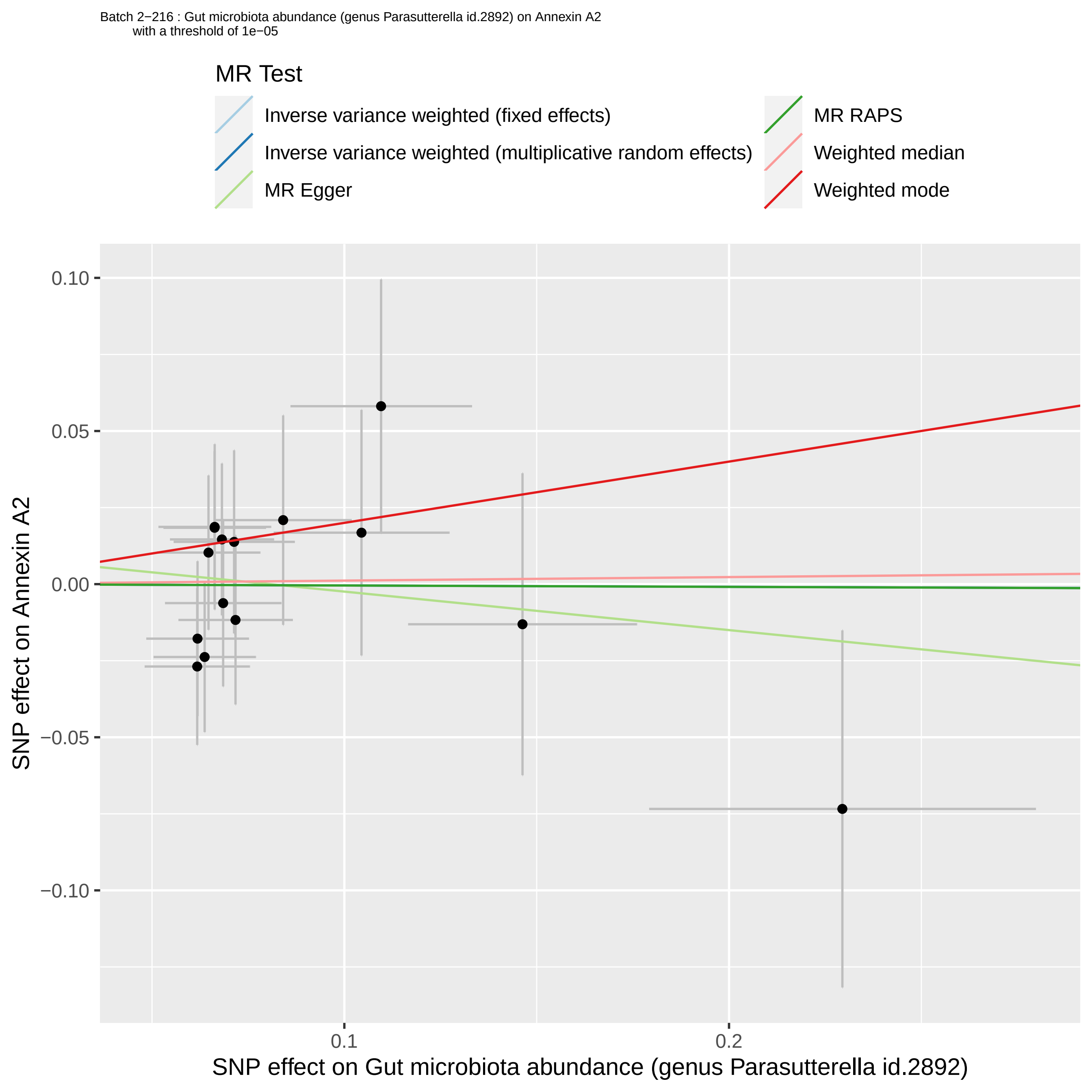
Batch 2−216 : Gut microbiota abundance (genus Parasutterella id.2892) on Annexin A2
with a threshold of 1e−05
MR Test
Inverse variance weighted (fixed effects)
Inverse variance weighted (multiplicative random effects)
MR Egger
MR RAPS
Weighted median
Weighted mode
0.10
0.05
0.00
−0.05
−0.10
0.1
0.2
SNP effect on Annexin A2
SNP effect on Gut microbiota abundance (genus Parasutterella id.2892)

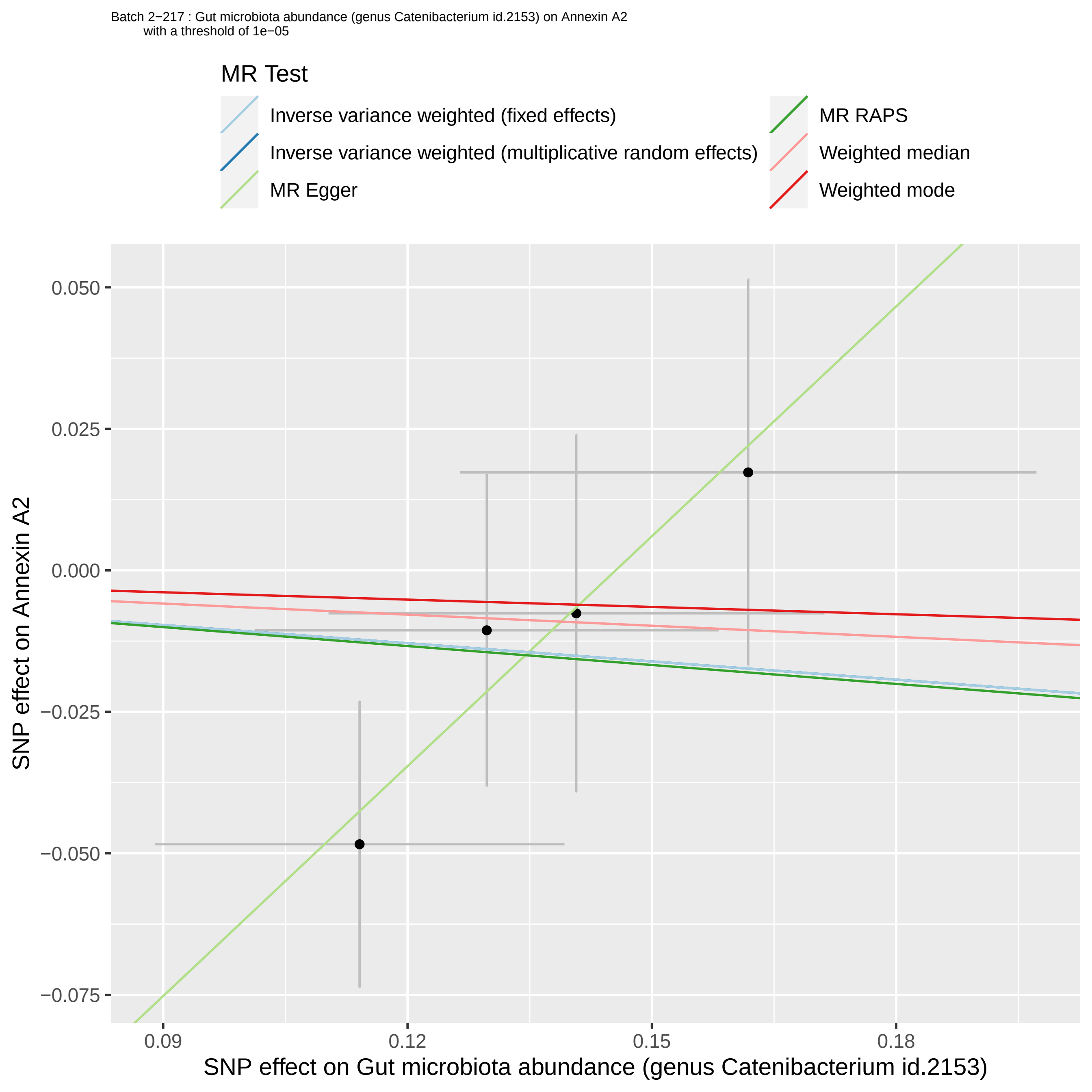

Batch 2−217 : Gut microbiota abundance (genus Catenibacterium id.2153) on Annexin A2
with a threshold of 1e−05
MR Test
Inverse variance weighted (fixed effects)
Inverse variance weighted (multiplicative random effects)
MR Egger
MR RAPS
Weighted median
Weighted mode
0.050
0.025
0.000
−0.025
−0.050
−0.075
0.09
0.12
0.15
0.18
SNP effect on Annexin A2
SNP effect on Gut microbiota abundance (genus Catenibacterium id.2153)

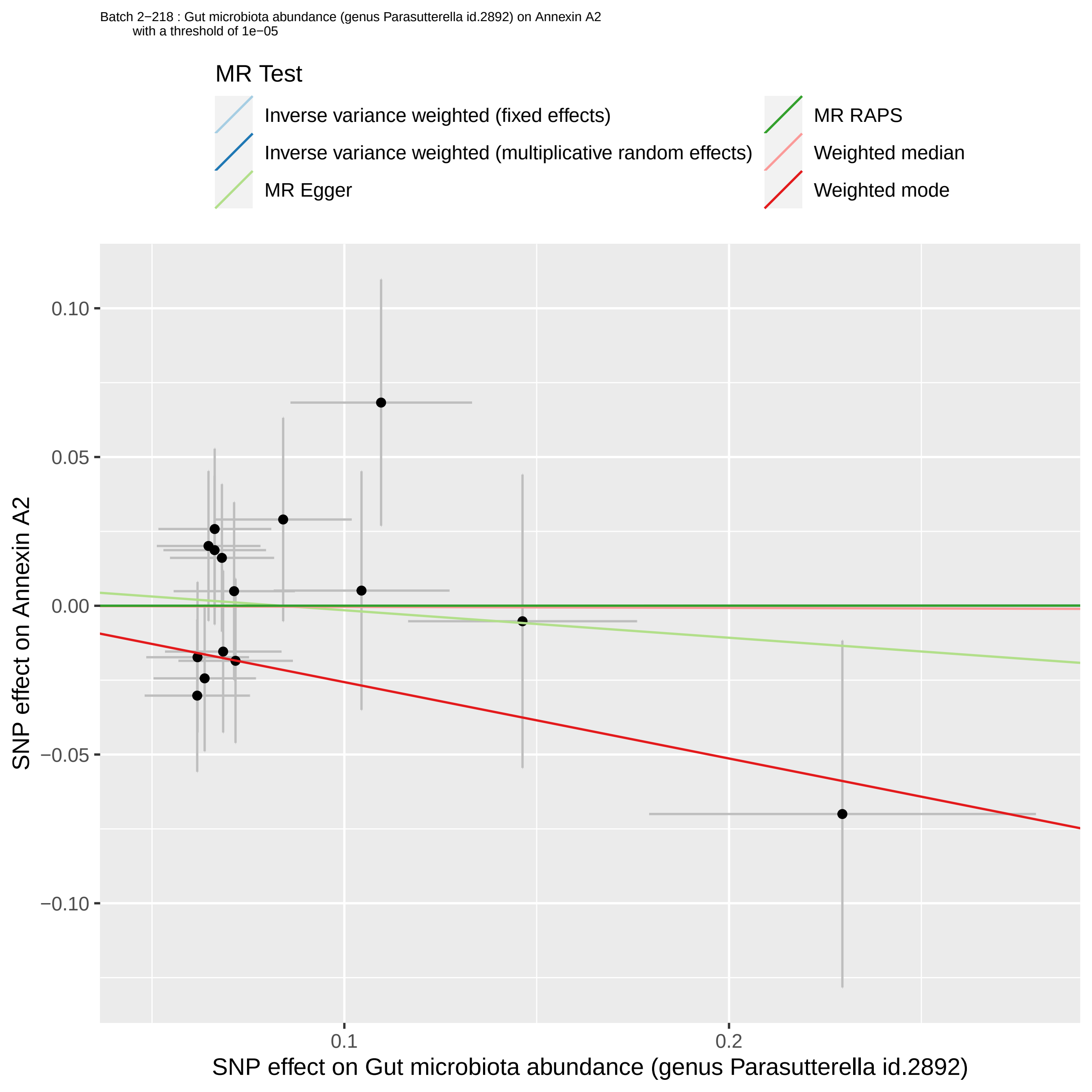
Batch 2−218 : Gut microbiota abundance (genus Parasutterella id.2892) on Annexin A2
with a threshold of 1e−05
MR Test
Inverse variance weighted (fixed effects)
Inverse variance weighted (multiplicative random effects)
MR Egger
MR RAPS
Weighted median
Weighted mode
0.10
0.05
0.00
−0.05
−0.10
0.1
0.2
SNP effect on Annexin A2
SNP effect on Gut microbiota abundance (genus Parasutterella id.2892)

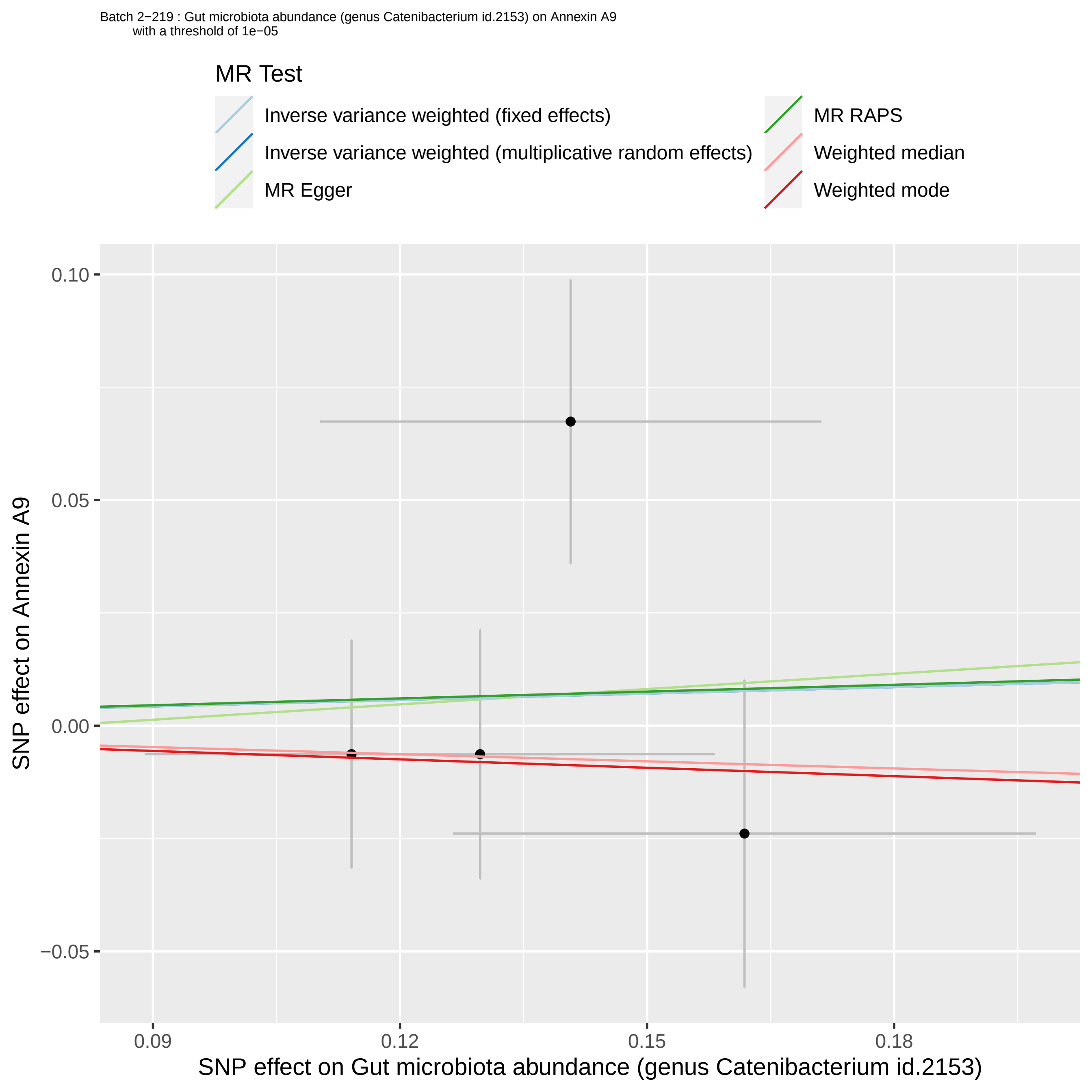

Batch 2−219 : Gut microbiota abundance (genus Catenibacterium id.2153) on Annexin A9
with a threshold of 1e−05
MR Test
Inverse variance weighted (fixed effects)
Inverse variance weighted (multiplicative random effects)
MR Egger
MR RAPS
Weighted median
Weighted mode
0.10
0.05
0.00
−0.05
0.09
0.12
0.15
0.18
SNP effect on Annexin A9
SNP effect on Gut microbiota abundance (genus Catenibacterium id.2153)

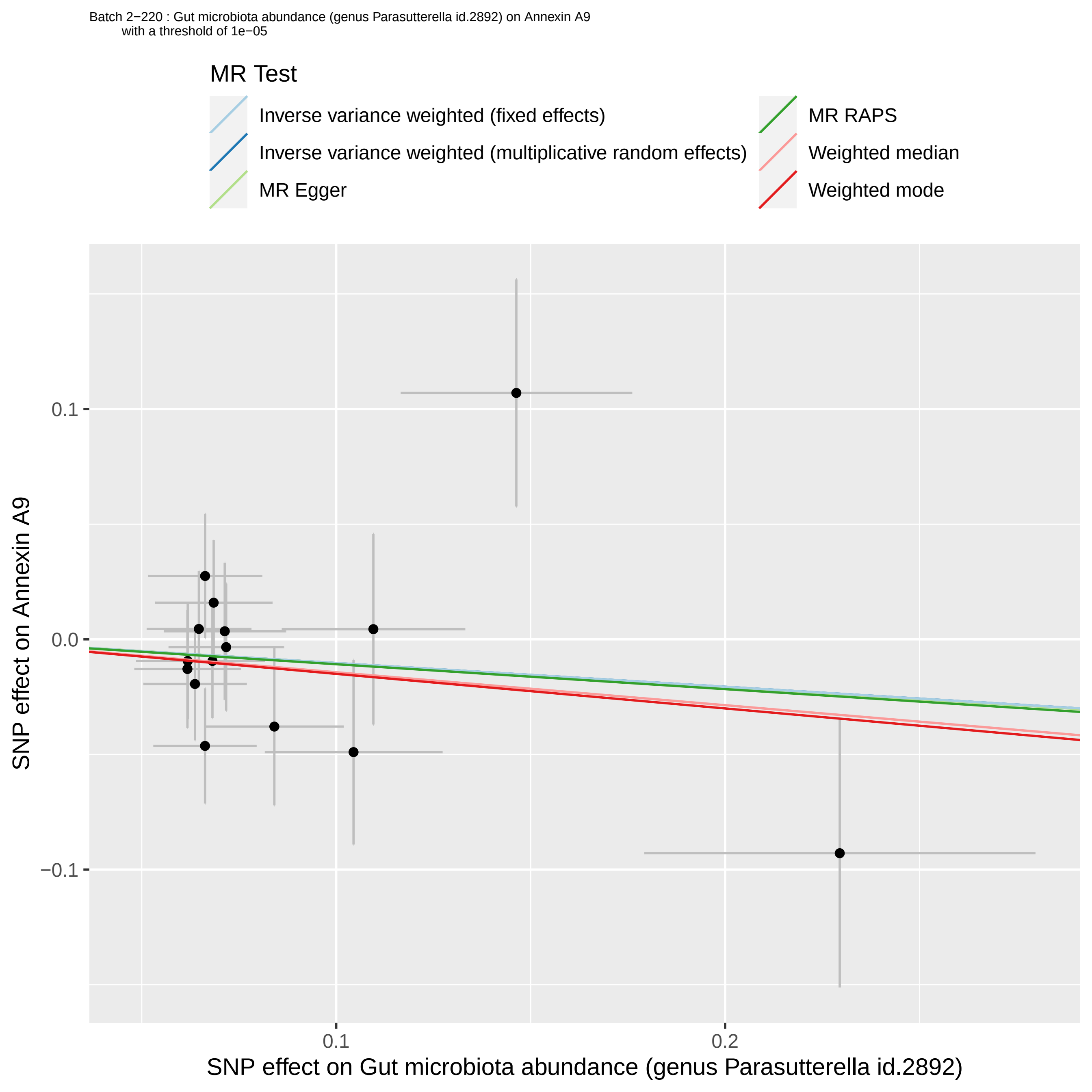

Batch 2−220 : Gut microbiota abundance (genus Parasutterella id.2892) on Annexin A9
with a threshold of 1e−05
MR Test
Inverse variance weighted (fixed effects)
Inverse variance weighted (multiplicative random effects)
MR Egger
MR RAPS
Weighted median
Weighted mode
0.1
0.0
−0.1
0.1
0.2
SNP effect on Annexin A9
SNP effect on Gut microbiota abundance (genus Parasutterella id.2892)

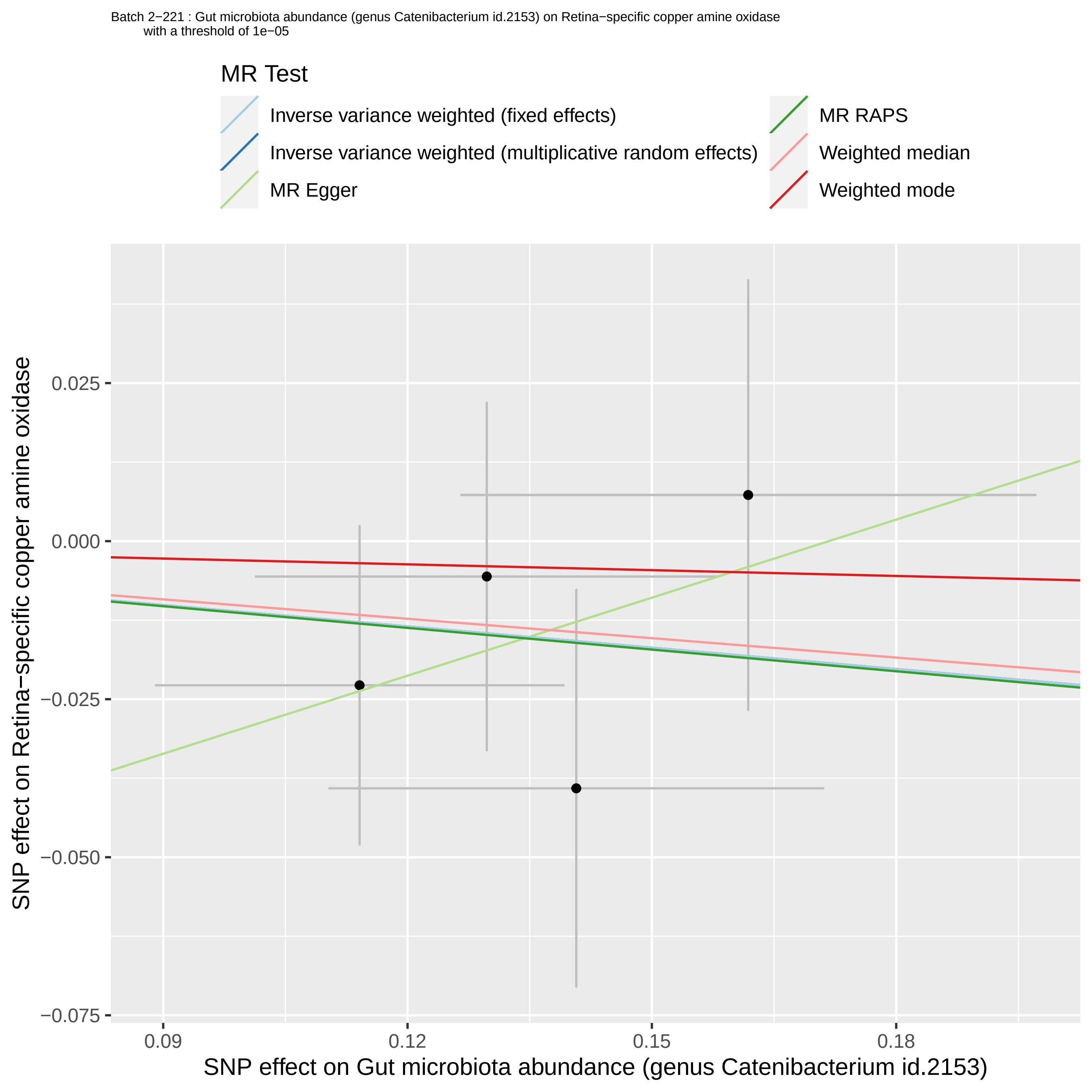
Batch 2−221 : Gut microbiota abundance (genus Catenibacterium id.2153) on Retina−specific copper amine oxidase
with a threshold of 1e−05
MR Test
Inverse variance weighted (fixed effects)
Inverse variance weighted (multiplicative random effects)
MR Egger
MR RAPS
Weighted median
Weighted mode
0.025
0.000
−0.025
−0.050
−0.075
0.09
0.12
0.15
0.18
SNP effect on Retina−specific copper amine oxidase
SNP effect on Gut microbiota abundance (genus Catenibacterium id.2153)

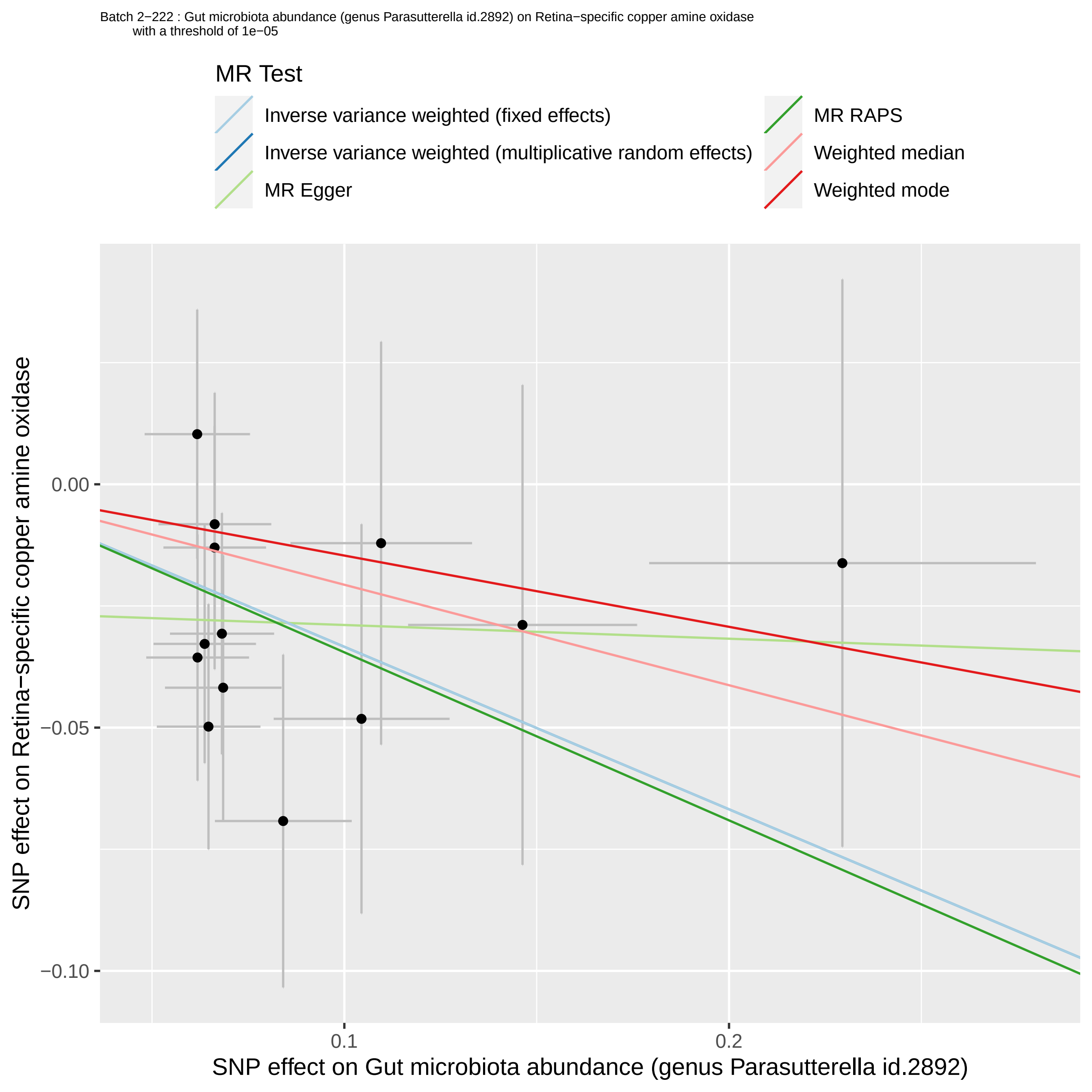
Batch 2−222 : Gut microbiota abundance (genus Parasutterella id.2892) on Retina−specific copper amine oxidase
with a threshold of 1e−05
MR Test
Inverse variance weighted (fixed effects)
Inverse variance weighted (multiplicative random effects)
MR Egger
MR RAPS
Weighted median
Weighted mode
0.00
−0.05
−0.10
0.1
0.2
SNP effect on Retina−specific copper amine oxidase
SNP effect on Gut microbiota abundance (genus Parasutterella id.2892)

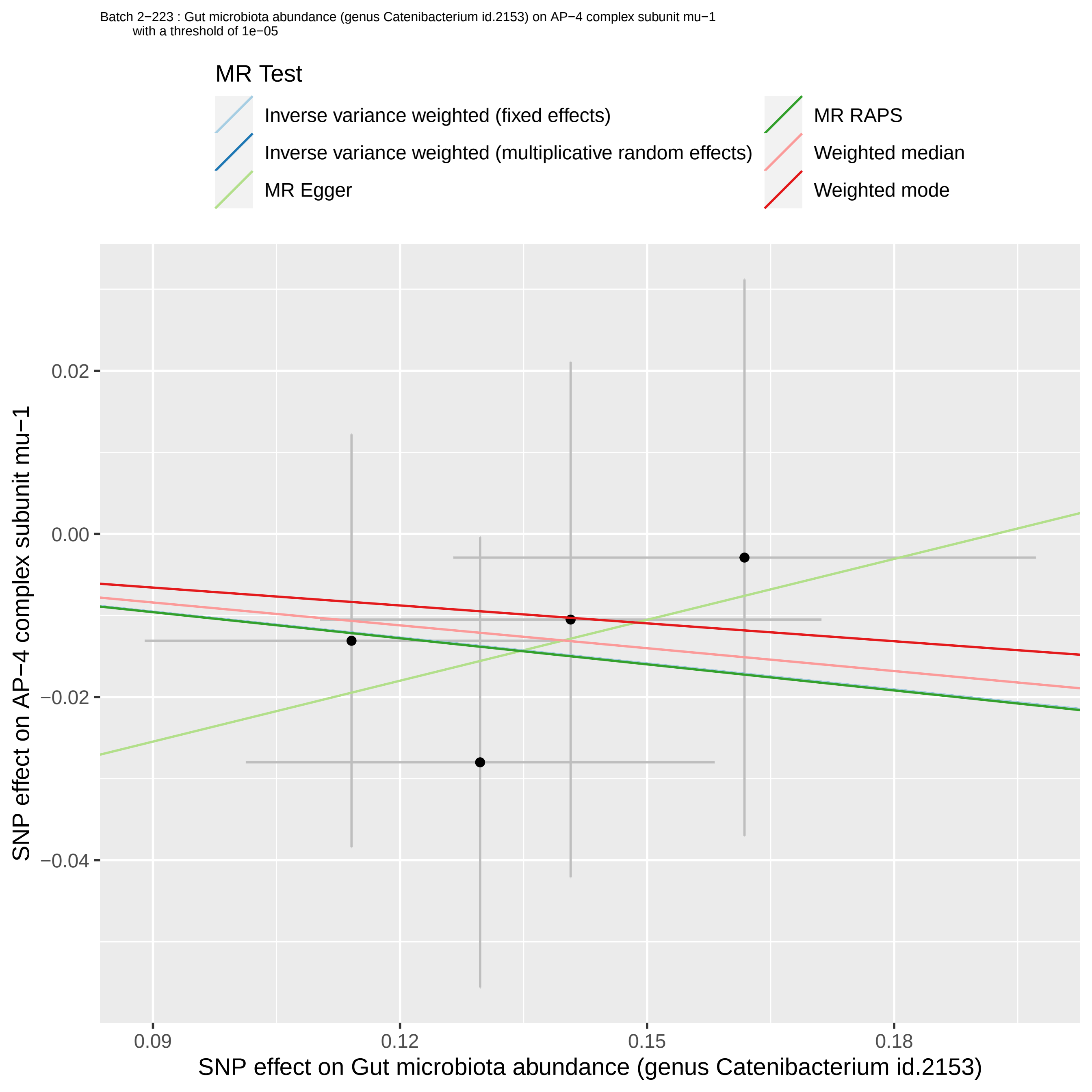
Batch 2−223 : Gut microbiota abundance (genus Catenibacterium id.2153) on AP−4 complex subunit mu−1
with a threshold of 1e−05
MR Test
Inverse variance weighted (fixed effects)
Inverse variance weighted (multiplicative random effects)
MR Egger
MR RAPS
Weighted median
Weighted mode
0.02
0.00
−0.02
−0.04
0.09
0.12
0.15
0.18
SNP effect on AP−4 complex subunit mu−1
SNP effect on Gut microbiota abundance (genus Catenibacterium id.2153)

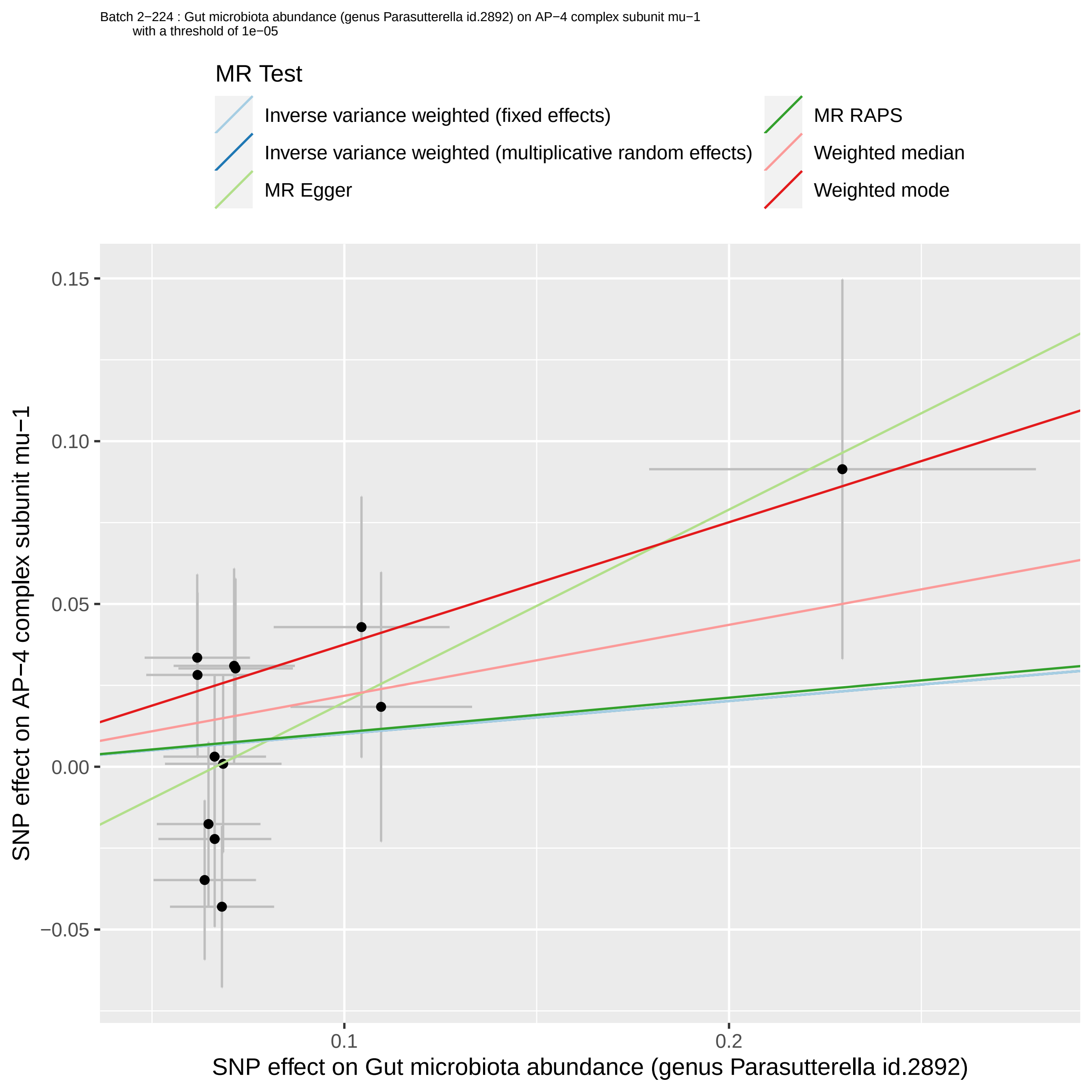
Batch 2−224 : Gut microbiota abundance (genus Parasutterella id.2892) on AP−4 complex subunit mu−1
with a threshold of 1e−05
MR Test
Inverse variance weighted (fixed effects)
Inverse variance weighted (multiplicative random effects)
MR Egger
MR RAPS
Weighted median
Weighted mode
0.15
0.10
0.05
0.00
−0.05
0.1
0.2
SNP effect on AP−4 complex subunit mu−1
SNP effect on Gut microbiota abundance (genus Parasutterella id.2892)

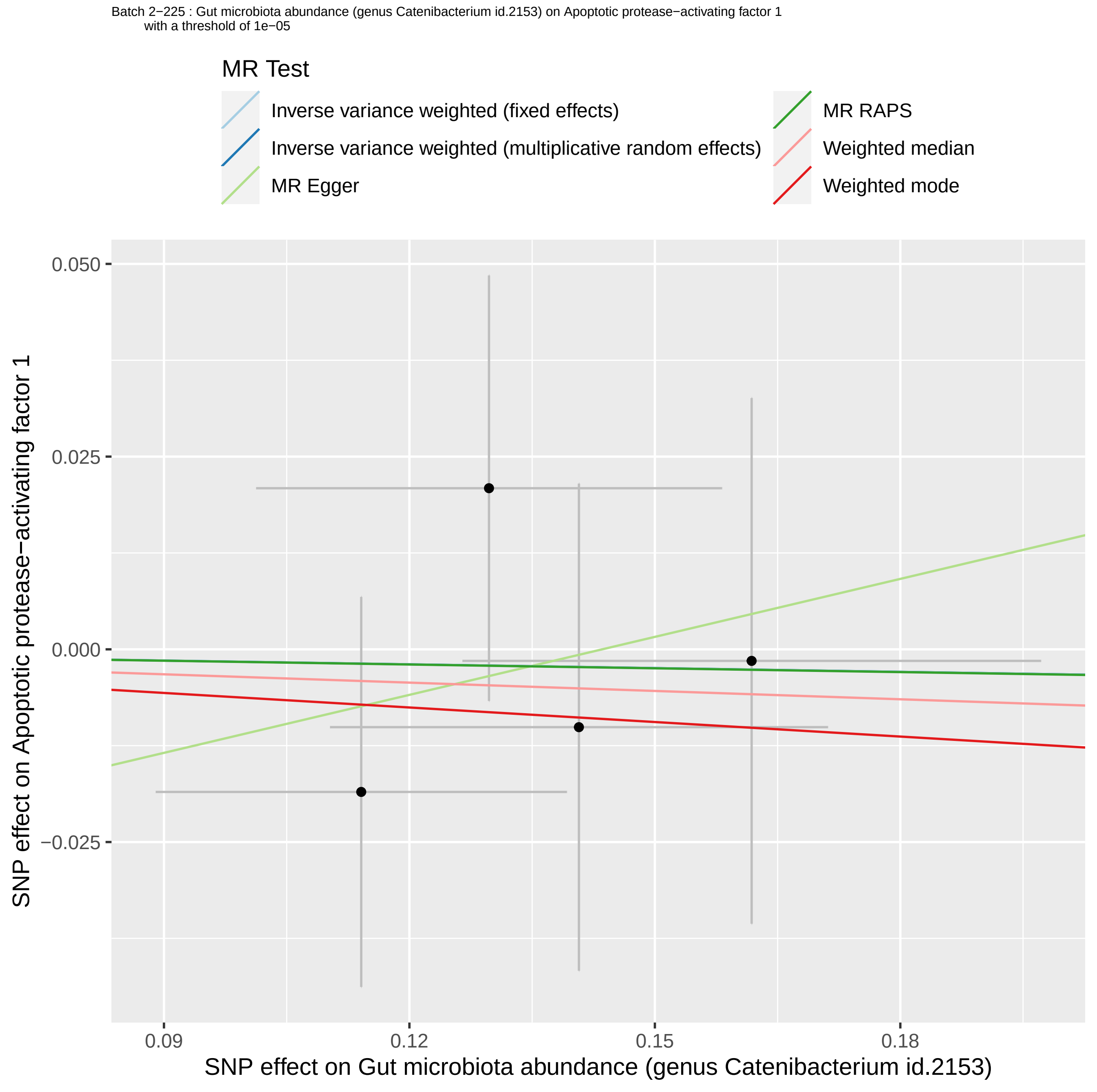

Batch 2−225 : Gut microbiota abundance (genus Catenibacterium id.2153) on Apoptotic protease−activating factor 1
with a threshold of 1e−05
MR Test
Inverse variance weighted (fixed effects)
Inverse variance weighted (multiplicative random effects)
MR Egger
MR RAPS
Weighted median
Weighted mode
0.050
0.025
0.000
−0.025
0.09
0.12
0.15
0.18
SNP effect on Apoptotic protease−activating factor 1
SNP effect on Gut microbiota abundance (genus Catenibacterium id.2153)

Batch 2−226 : Gut microbiota abundance (genus Parasutterella id.2892) on Apoptotic protease−activating factor 1
with a threshold of 1e−05

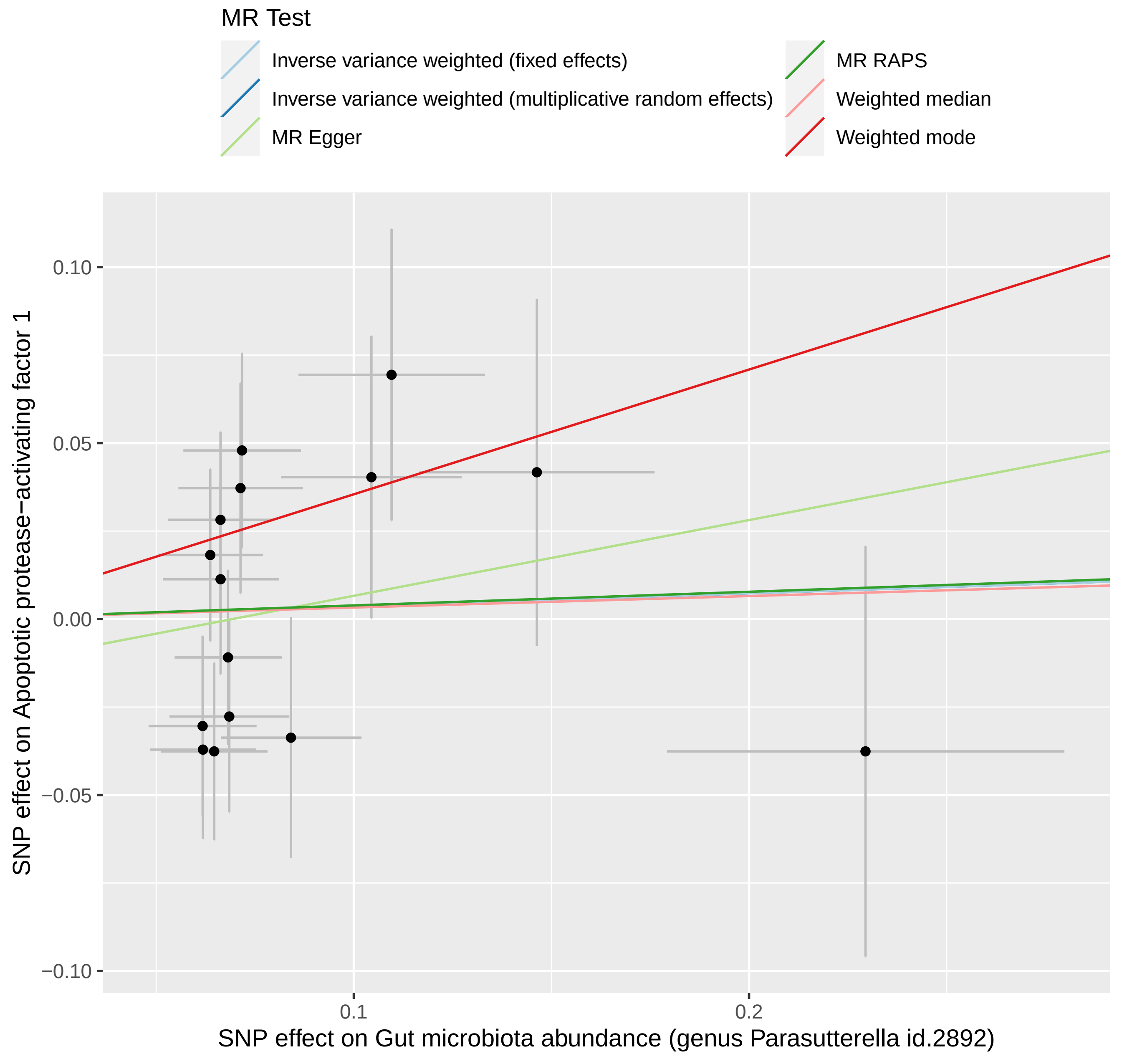

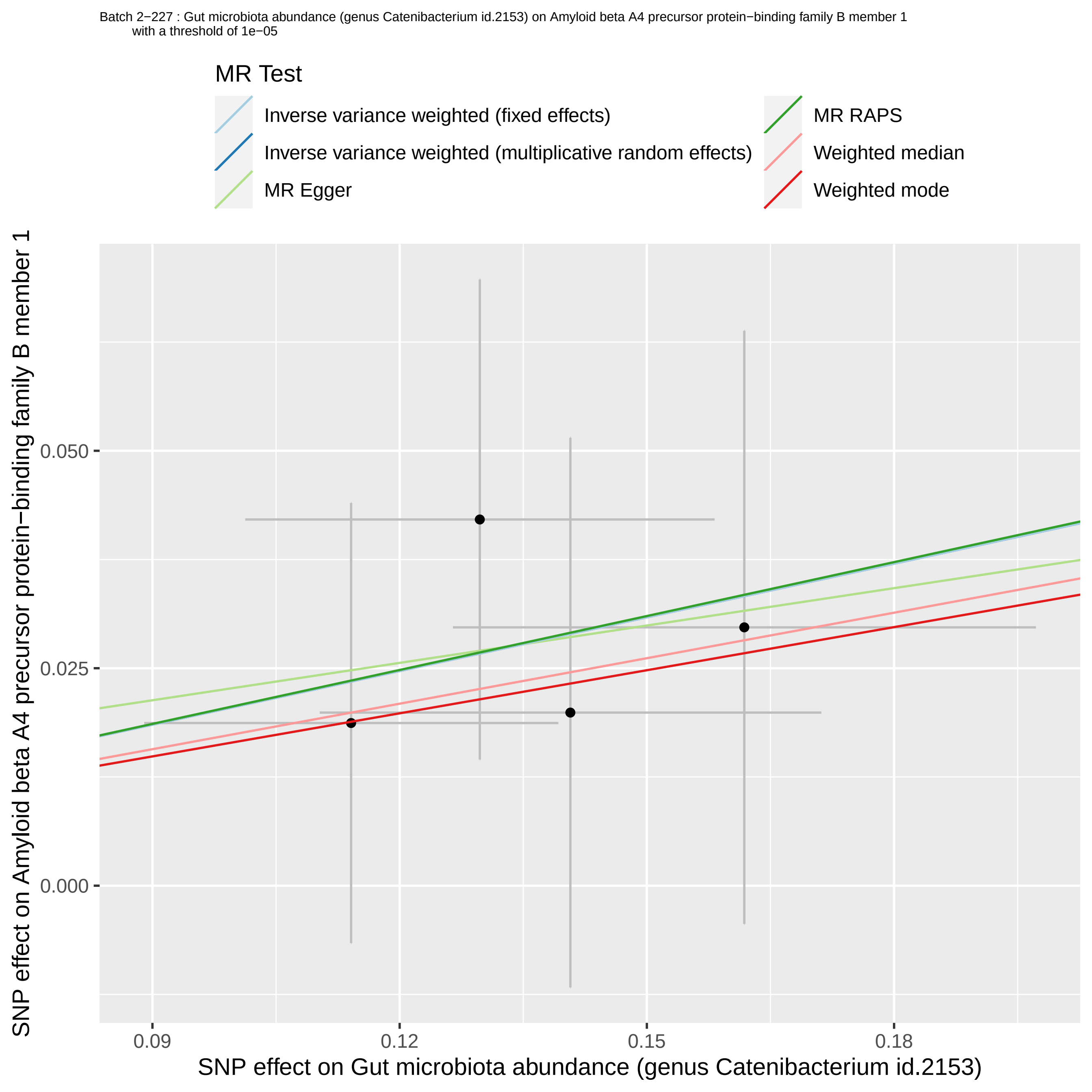

Batch 2−227 : Gut microbiota abundance (genus Catenibacterium id.2153) on Amyloid beta A4 precursor protein−binding family B member 1
with a threshold of 1e−05
MR Test
Inverse variance weighted (fixed effects)
Inverse variance weighted (multiplicative random effects)
MR Egger
MR RAPS
Weighted median
Weighted mode
SNP effect on Amyloid beta A4 precursor protein−binding family B member 1
0.050
0.025
0.000
0.09
0.12
0.15
0.18
SNP effect on Gut microbiota abundance (genus Catenibacterium id.2153)

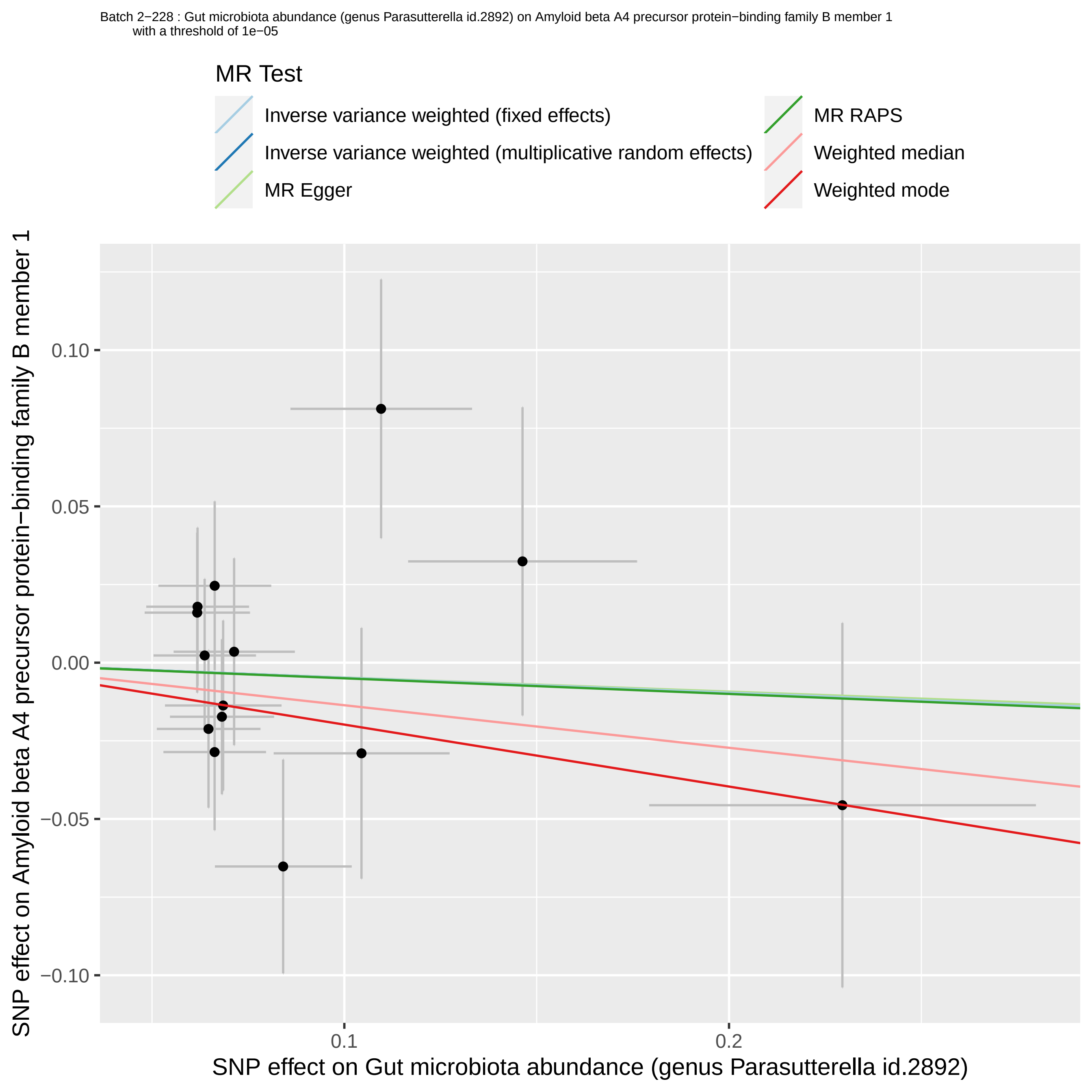
Batch 2−228 : Gut microbiota abundance (genus Parasutterella id.2892) on Amyloid beta A4 precursor protein−binding family B member 1
with a threshold of 1e−05
MR Test
Inverse variance weighted (fixed effects)
Inverse variance weighted (multiplicative random effects)
MR Egger
MR RAPS
Weighted median
Weighted mode
SNP effect on Amyloid beta A4 precursor protein−binding family B member 1
0.10
0.05
0.00
−0.05
−0.10
0.1
0.2
SNP effect on Gut microbiota abundance (genus Parasutterella id.2892)

Batch 2−229 : Gut microbiota abundance (genus Catenibacterium id.2153) on Amyloid beta A4 precursor protein−binding family B member 1
with a threshold of 1e−05

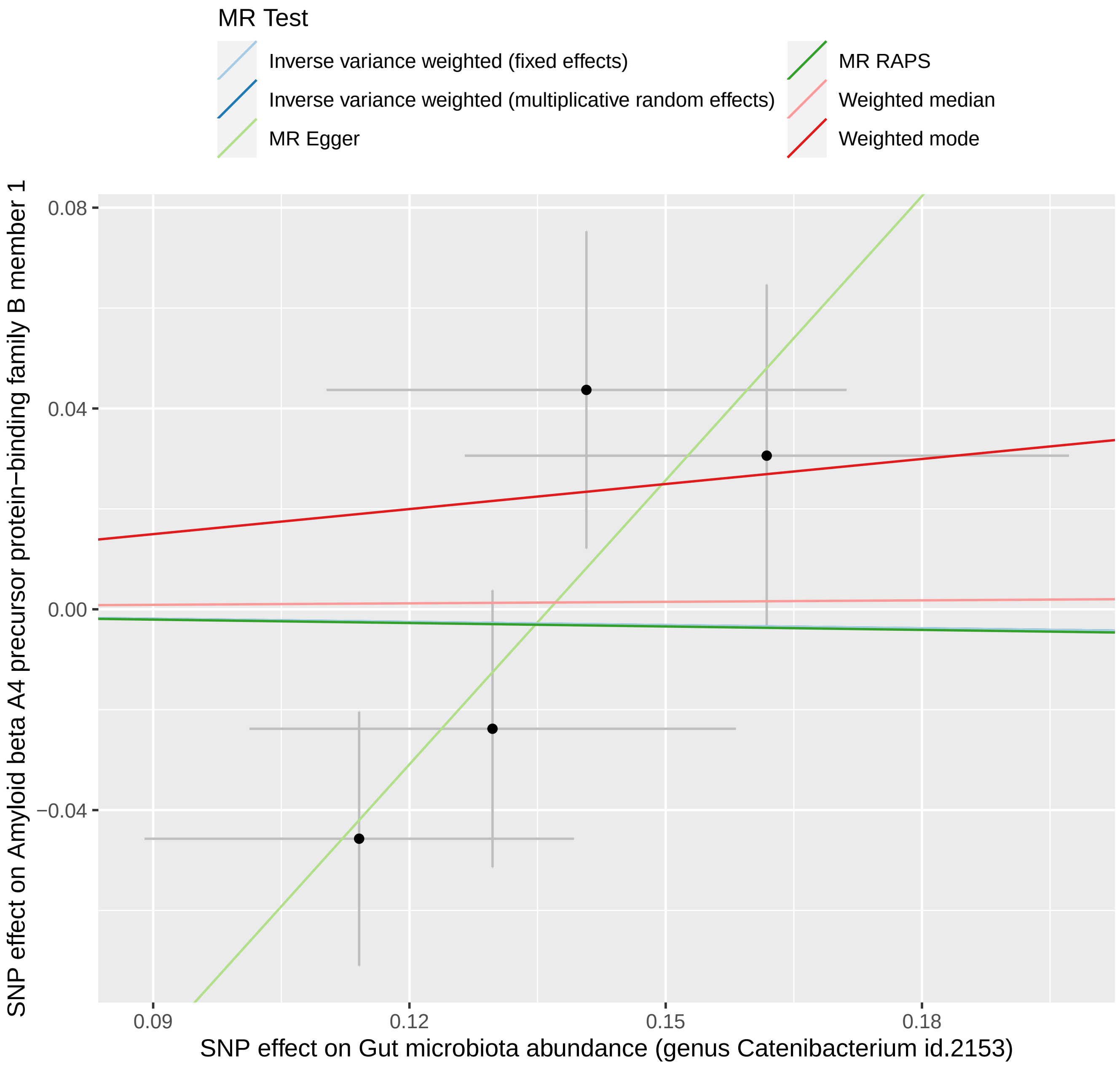

Batch 2−230 : Gut microbiota abundance (genus Parasutterella id.2892) on Amyloid beta A4 precursor protein−binding family B member 1 with a threshold of 1e−05

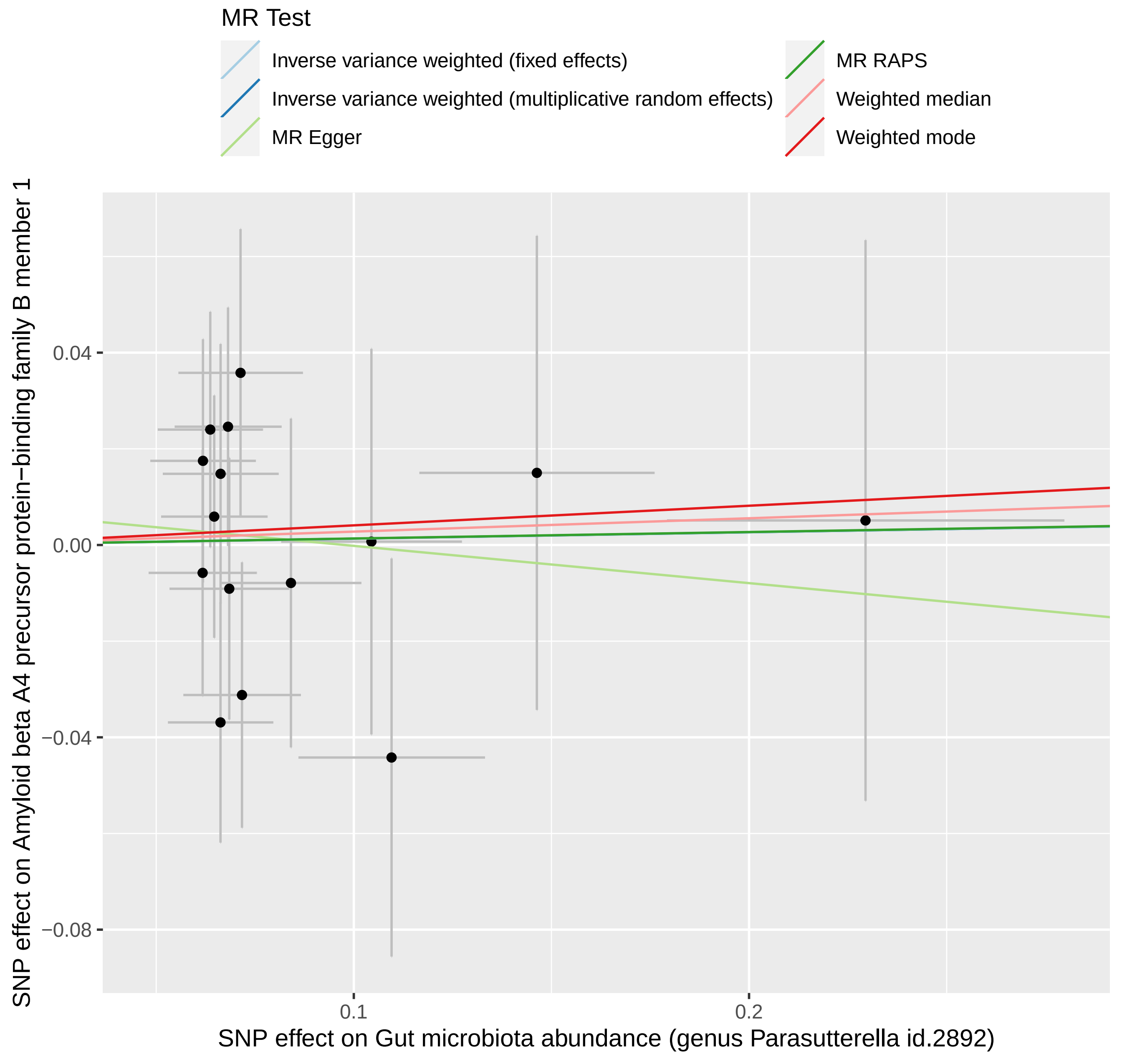

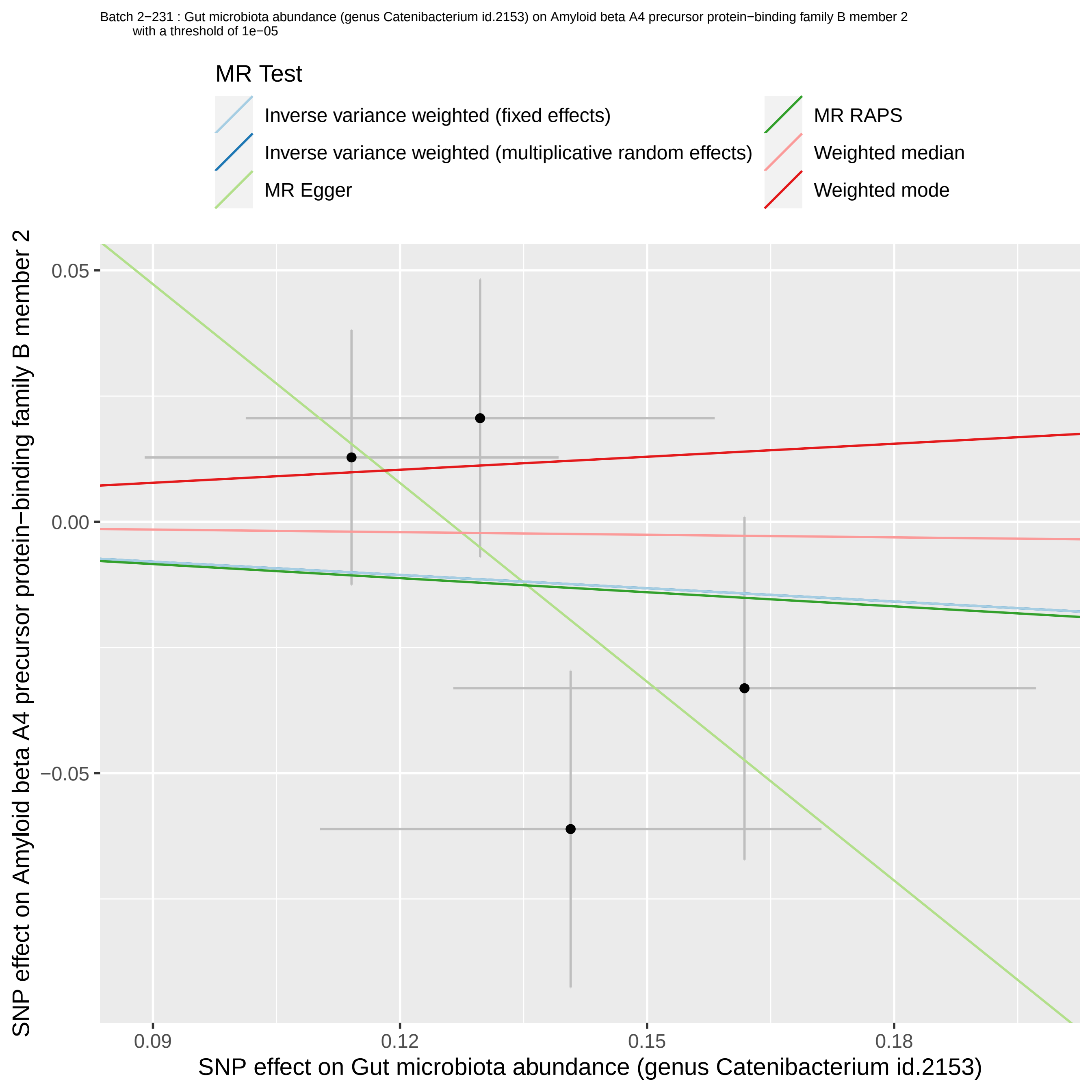
Batch 2−231 : Gut microbiota abundance (genus Catenibacterium id.2153) on Amyloid beta A4 precursor protein−binding family B member 2
with a threshold of 1e−05
MR Test
Inverse variance weighted (fixed effects)
Inverse variance weighted (multiplicative random effects)
MR Egger
MR RAPS
Weighted median
Weighted mode
SNP effect on Amyloid beta A4 precursor protein−binding family B member 2
0.05
0.00
−0.05
0.09
0.12
0.15
0.18
SNP effect on Gut microbiota abundance (genus Catenibacterium id.2153)

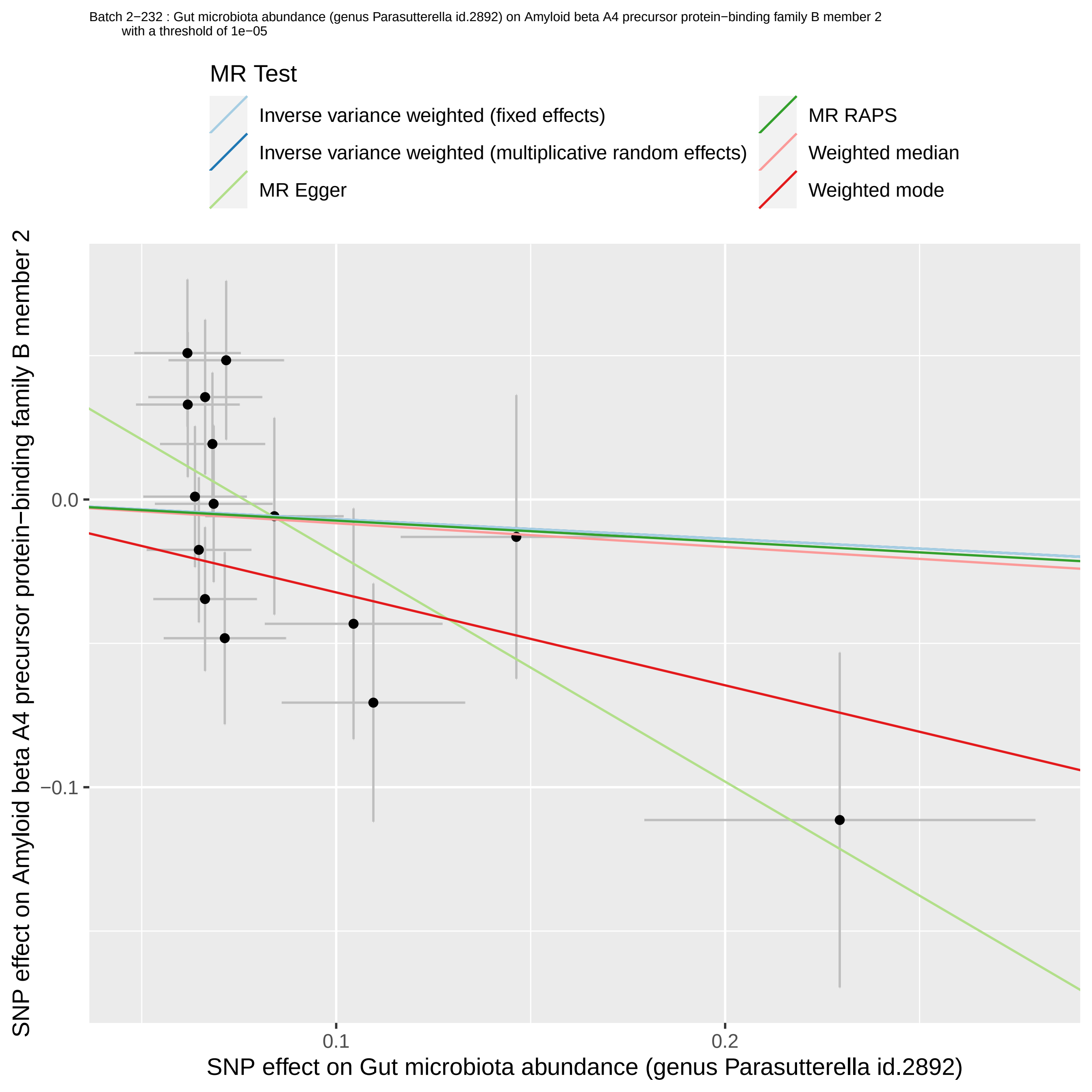
Batch 2−232 : Gut microbiota abundance (genus Parasutterella id.2892) on Amyloid beta A4 precursor protein−binding family B member 2
with a threshold of 1e−05
MR Test
Inverse variance weighted (fixed effects)
Inverse variance weighted (multiplicative random effects)
MR Egger
MR RAPS
Weighted median
Weighted mode
SNP effect on Amyloid beta A4 precursor protein−binding family B member 2
0.0
−0.1
0.1
0.2
SNP effect on Gut microbiota abundance (genus Parasutterella id.2892)

Batch 2−233 : Gut microbiota abundance (genus Catenibacterium id.2153) on Amyloid beta A4 precursor protein−binding family B member 2 with a threshold of 1e−05

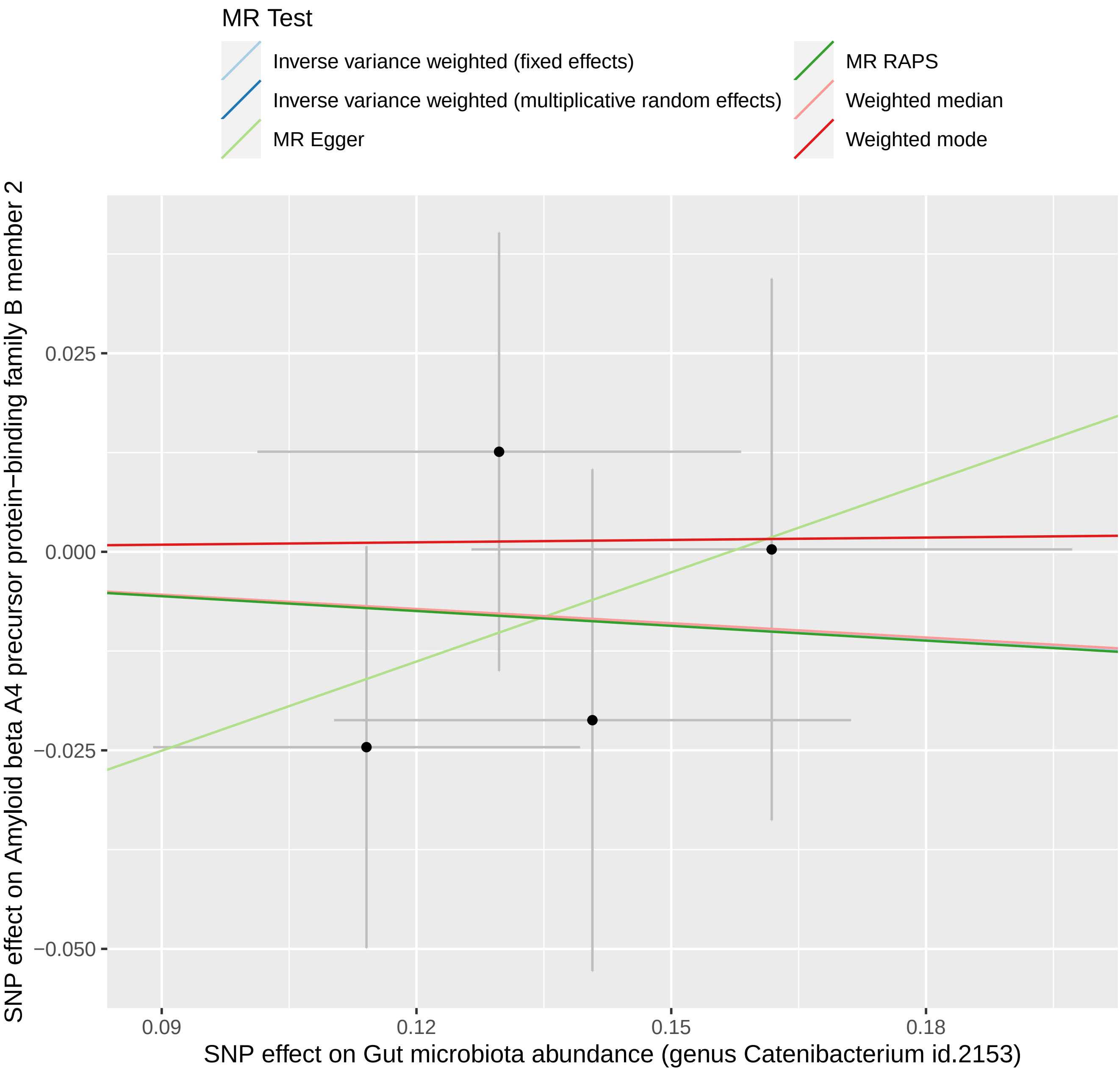

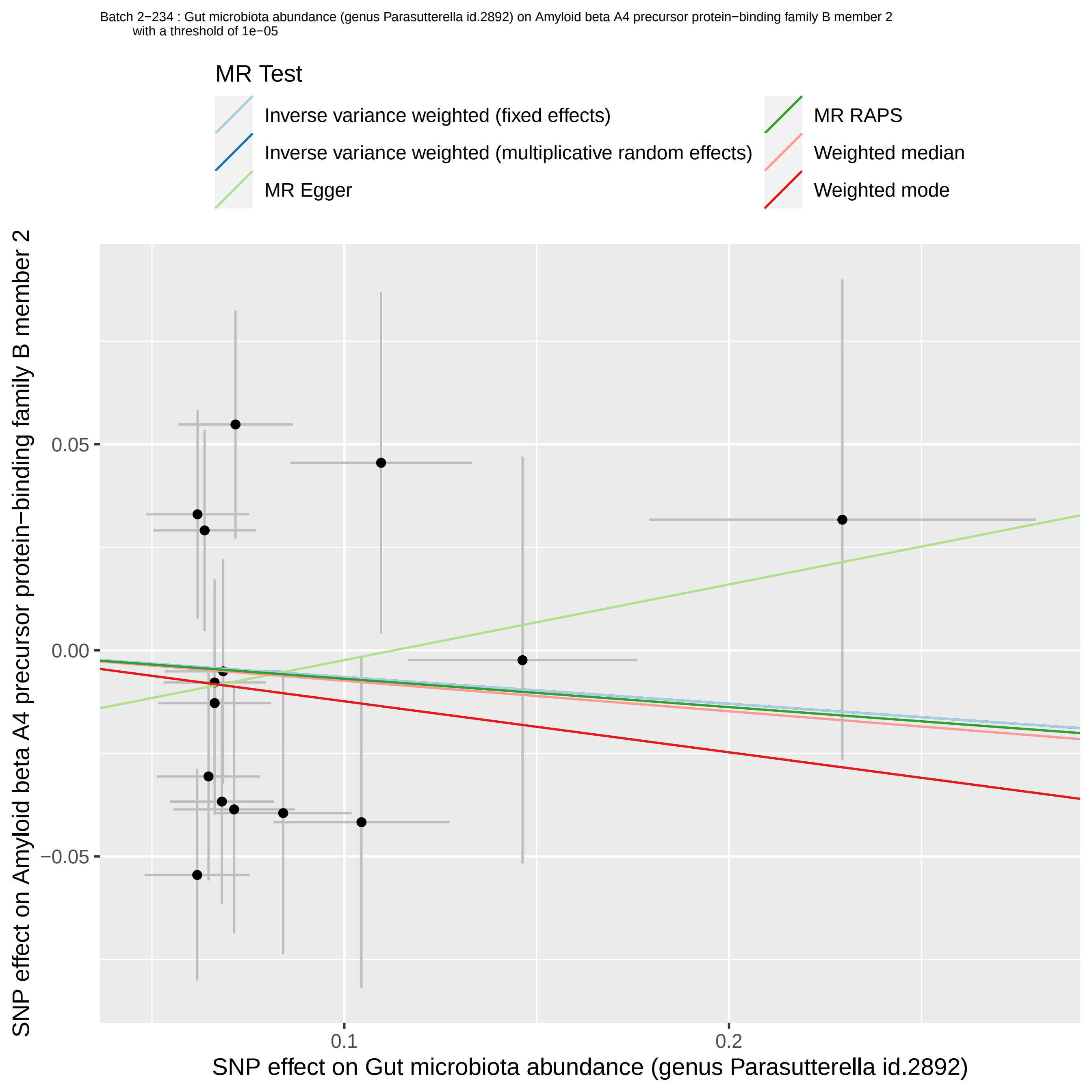

Batch 2−234 : Gut microbiota abundance (genus Parasutterella id.2892) on Amyloid beta A4 precursor protein−binding family B member 2
with a threshold of 1e−05
MR Test
Inverse variance weighted (fixed effects)
Inverse variance weighted (multiplicative random effects)
MR Egger
MR RAPS
Weighted median
Weighted mode
0.05
0.00
−0.05
0.1
0.2
SNP effect on Amyloid beta A4 precursor protein−binding family B member 2
SNP effect on Gut microbiota abundance (genus Parasutterella id.2892)

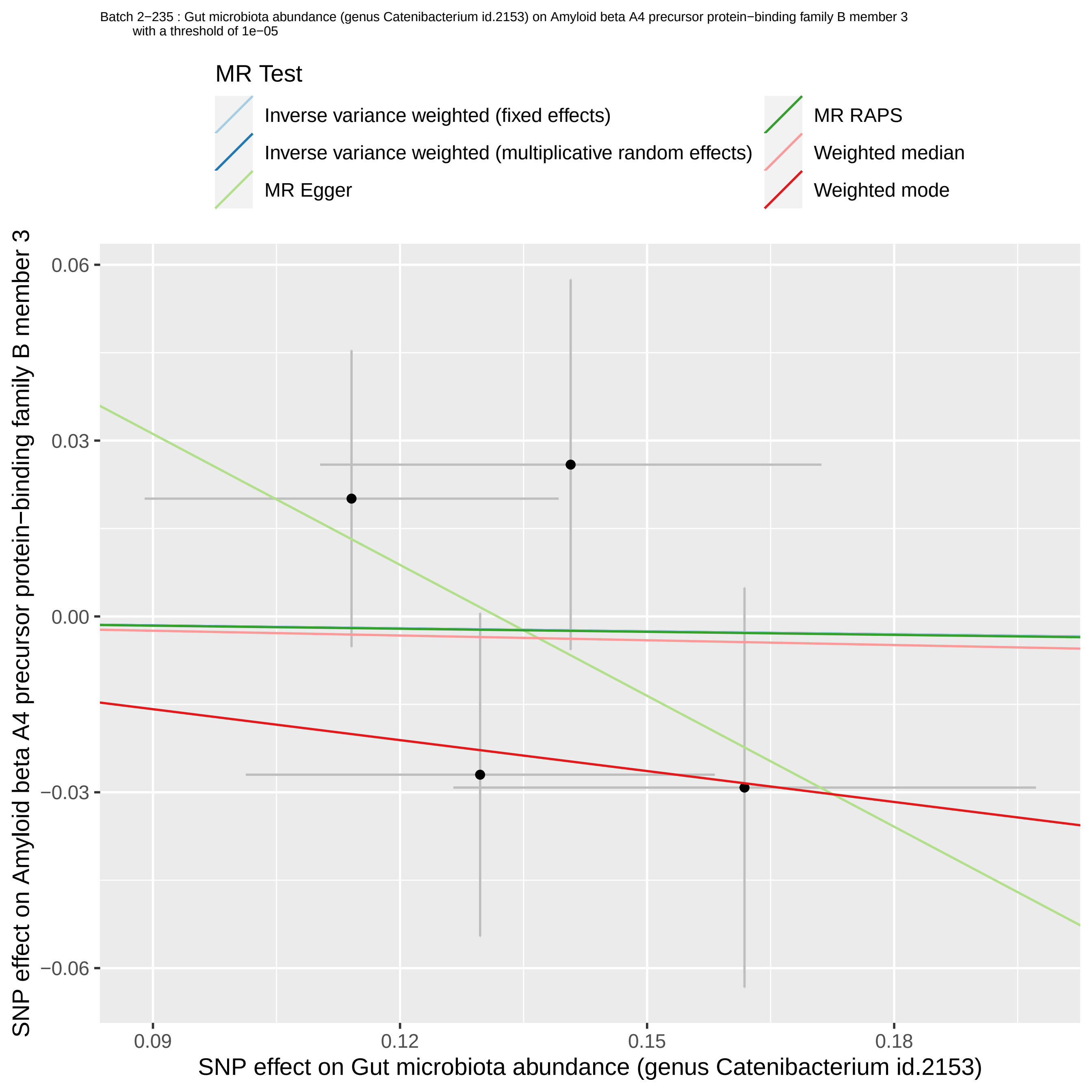

Batch 2−235 : Gut microbiota abundance (genus Catenibacterium id.2153) on Amyloid beta A4 precursor protein−binding family B member 3
with a threshold of 1e−05
MR Test
Inverse variance weighted (fixed effects)
Inverse variance weighted (multiplicative random effects)
MR Egger
MR RAPS
Weighted median
Weighted mode
0.06
0.03
0.00
−0.03
−0.06
0.09
0.12
0.15
0.18
SNP effect on Amyloid beta A4 precursor protein−binding family B member 3
SNP effect on Gut microbiota abundance (genus Catenibacterium id.2153)

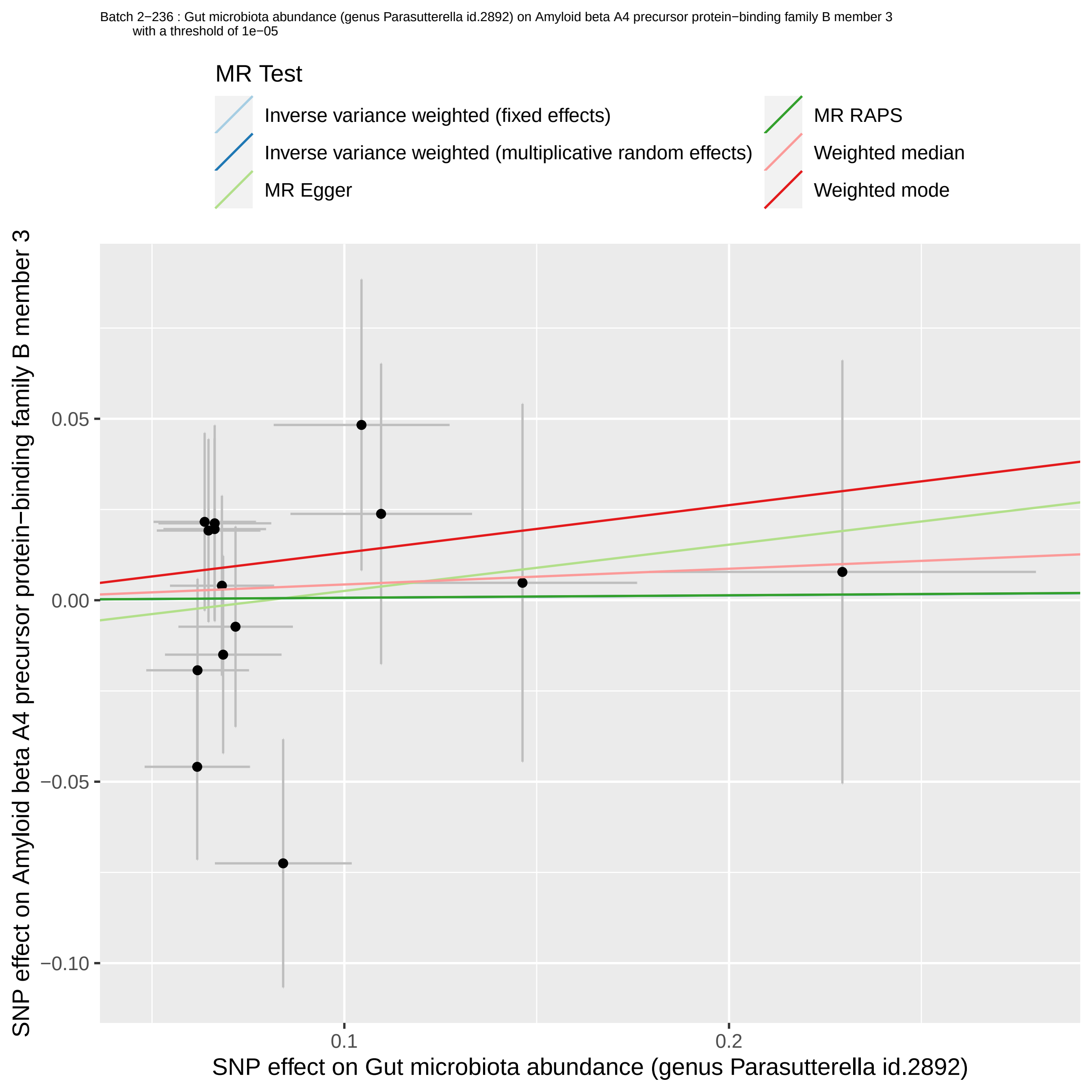

Batch 2−236 : Gut microbiota abundance (genus Parasutterella id.2892) on Amyloid beta A4 precursor protein−binding family B member 3
with a threshold of 1e−05
MR Test
Inverse variance weighted (fixed effects)
Inverse variance weighted (multiplicative random effects)
MR Egger
MR RAPS
Weighted median
Weighted mode
SNP effect on Amyloid beta A4 precursor protein−binding family B member 3
0.05
0.00
−0.05
−0.10
0.1
0.2
SNP effect on Gut microbiota abundance (genus Parasutterella id.2892)

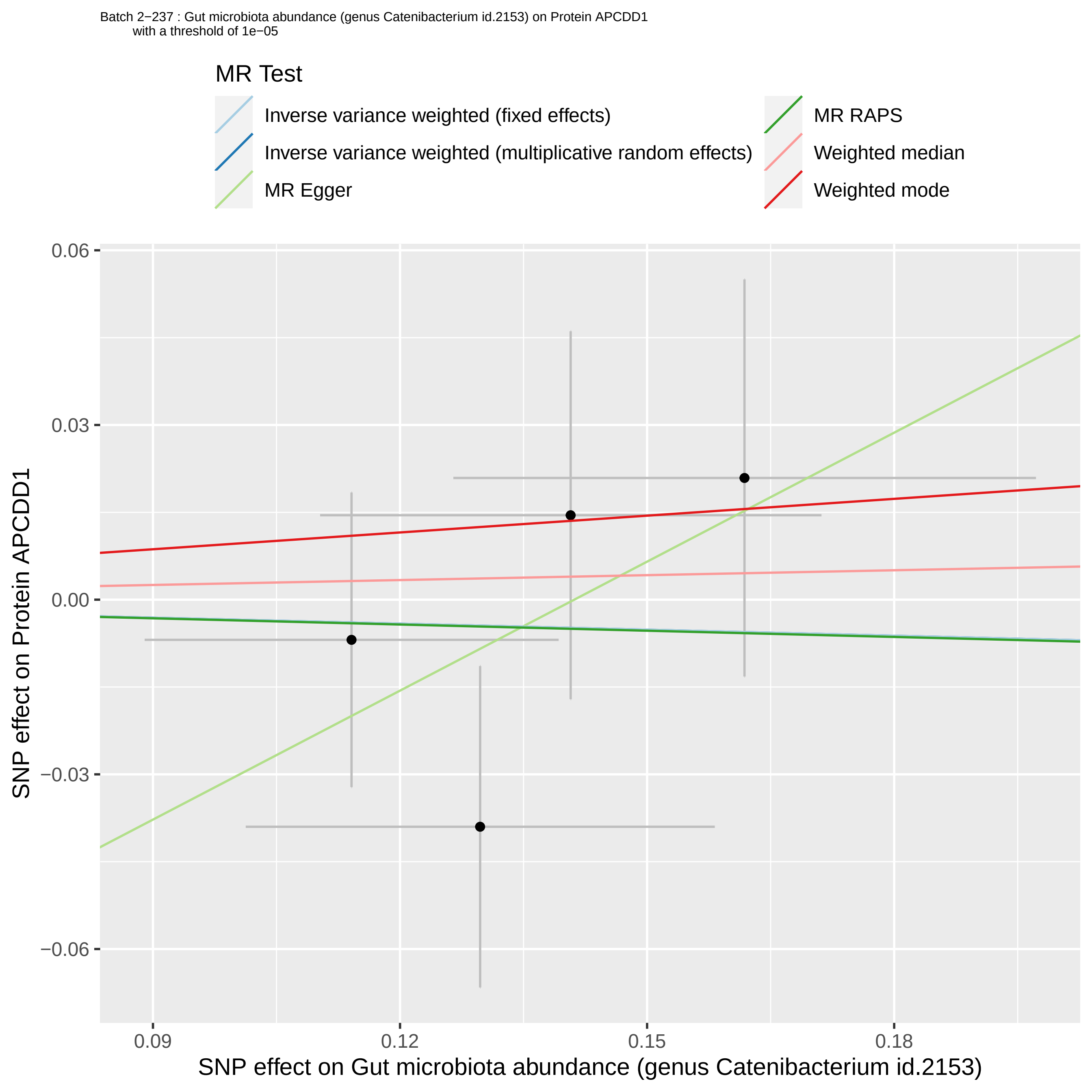

Batch 2−237 : Gut microbiota abundance (genus Catenibacterium id.2153) on Protein APCDD1
with a threshold of 1e−05
MR Test
Inverse variance weighted (fixed effects)
Inverse variance weighted (multiplicative random effects)
MR Egger
MR RAPS
Weighted median
Weighted mode
0.06
0.03
0.00
−0.03
−0.06
0.09
0.12
0.15
0.18
SNP effect on Protein APCDD1
SNP effect on Gut microbiota abundance (genus Catenibacterium id.2153)

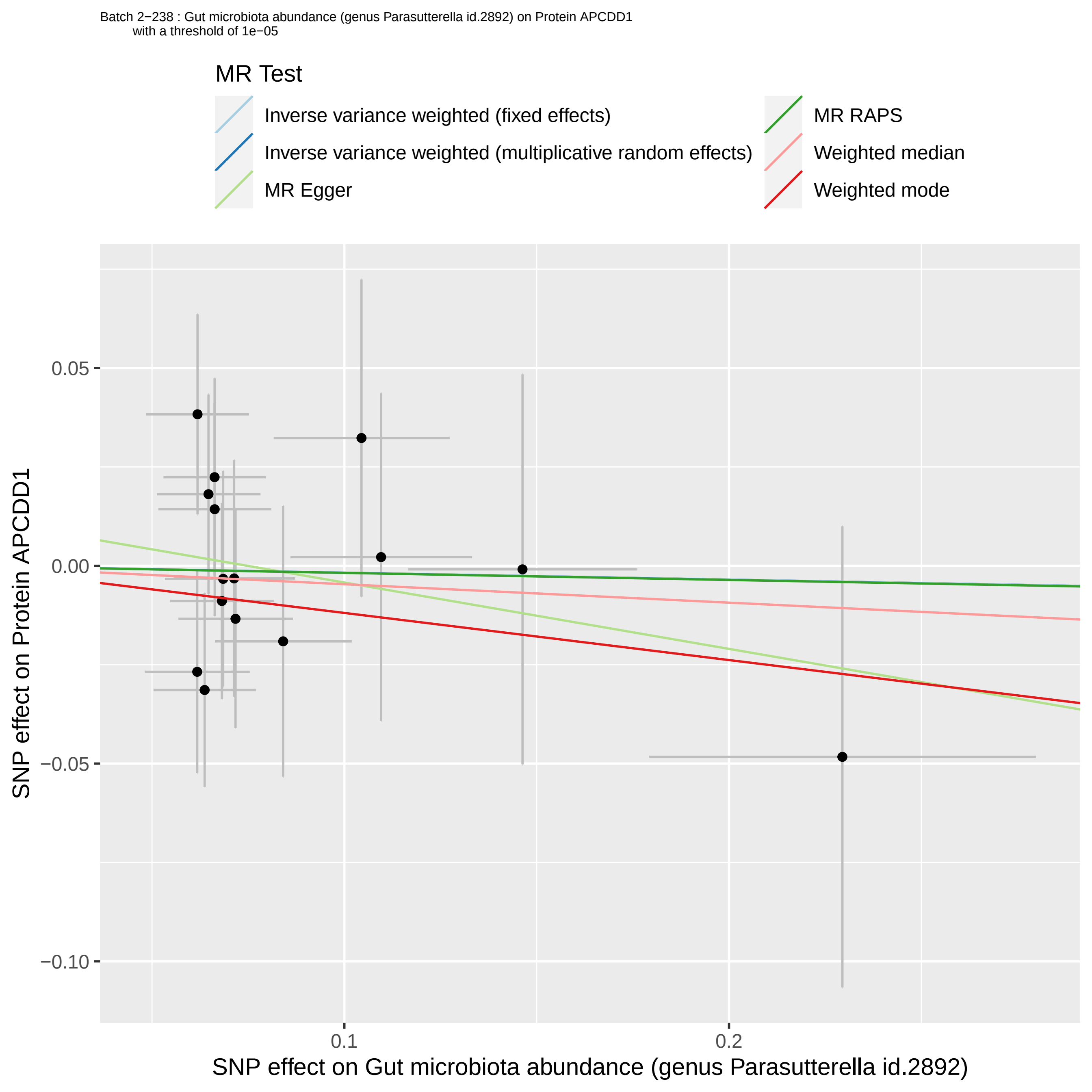

Batch 2−238 : Gut microbiota abundance (genus Parasutterella id.2892) on Protein APCDD1
with a threshold of 1e−05
MR Test
Inverse variance weighted (fixed effects)
Inverse variance weighted (multiplicative random effects)
MR Egger
MR RAPS
Weighted median
Weighted mode
0.05
0.00
−0.05
−0.10
0.1
0.2
SNP effect on Protein APCDD1
SNP effect on Gut microbiota abundance (genus Parasutterella id.2892)

Batch 2−239 : Gut microbiota abundance (genus Catenibacterium id.2153) on Serum amyloid P−component
with a threshold of 1e−05

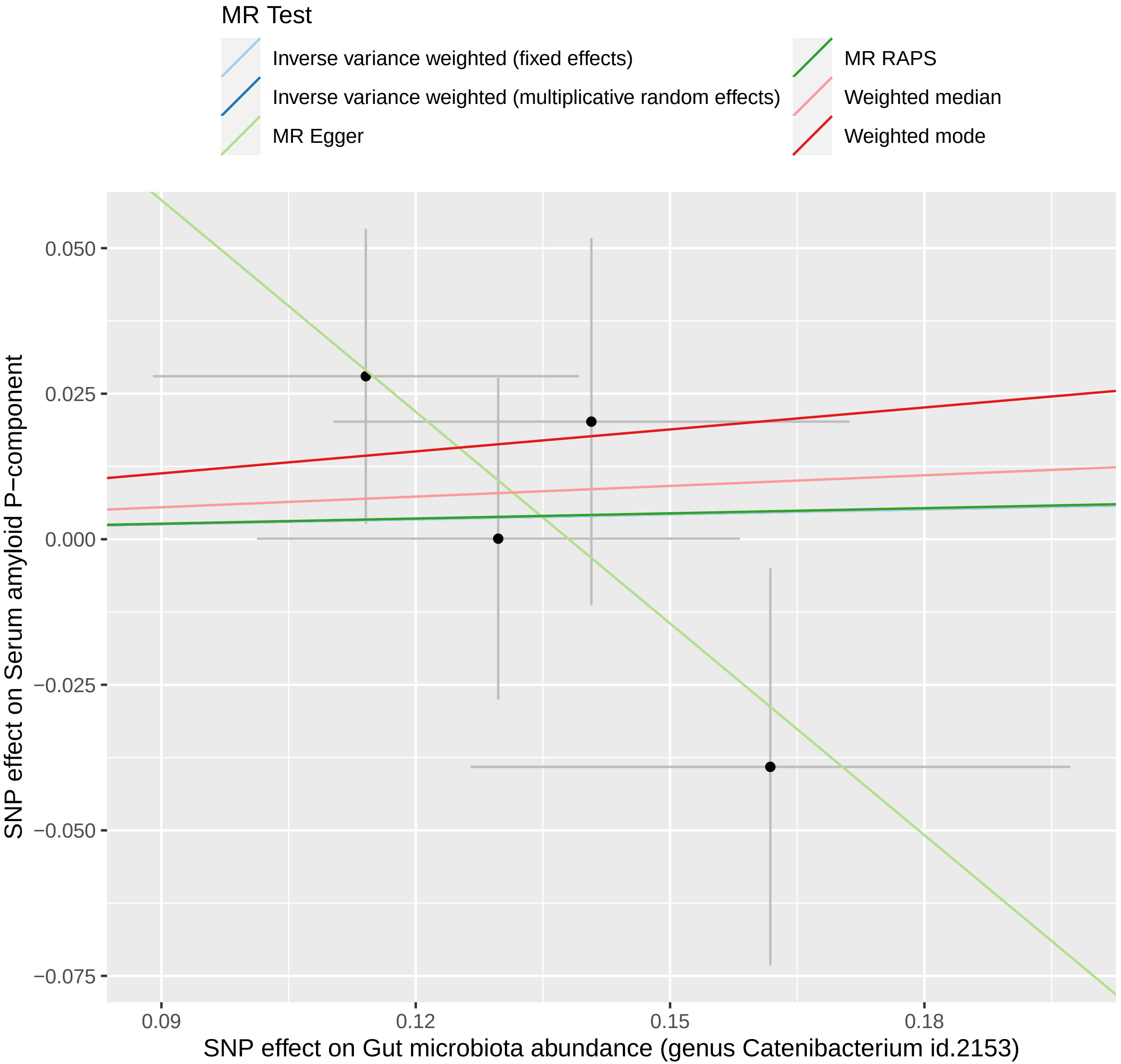

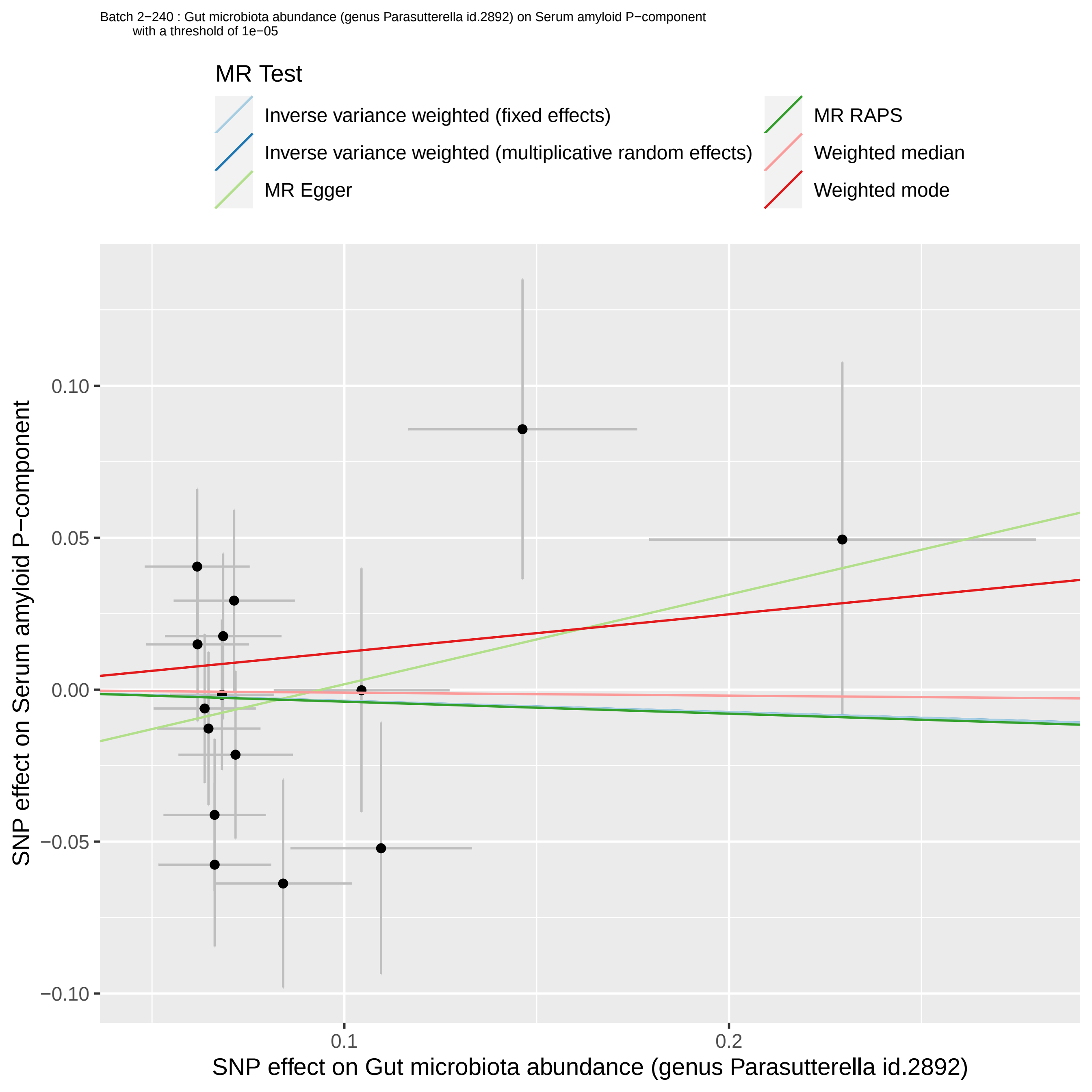

Batch 2−240 : Gut microbiota abundance (genus Parasutterella id.2892) on Serum amyloid P−component
with a threshold of 1e−05
MR Test
Inverse variance weighted (fixed effects)
Inverse variance weighted (multiplicative random effects)
MR Egger
MR RAPS
Weighted median
Weighted mode
0.10
0.05
0.00
−0.05
−0.10
0.1
0.2
SNP effect on Serum amyloid P−component
SNP effect on Gut microbiota abundance (genus Parasutterella id.2892)

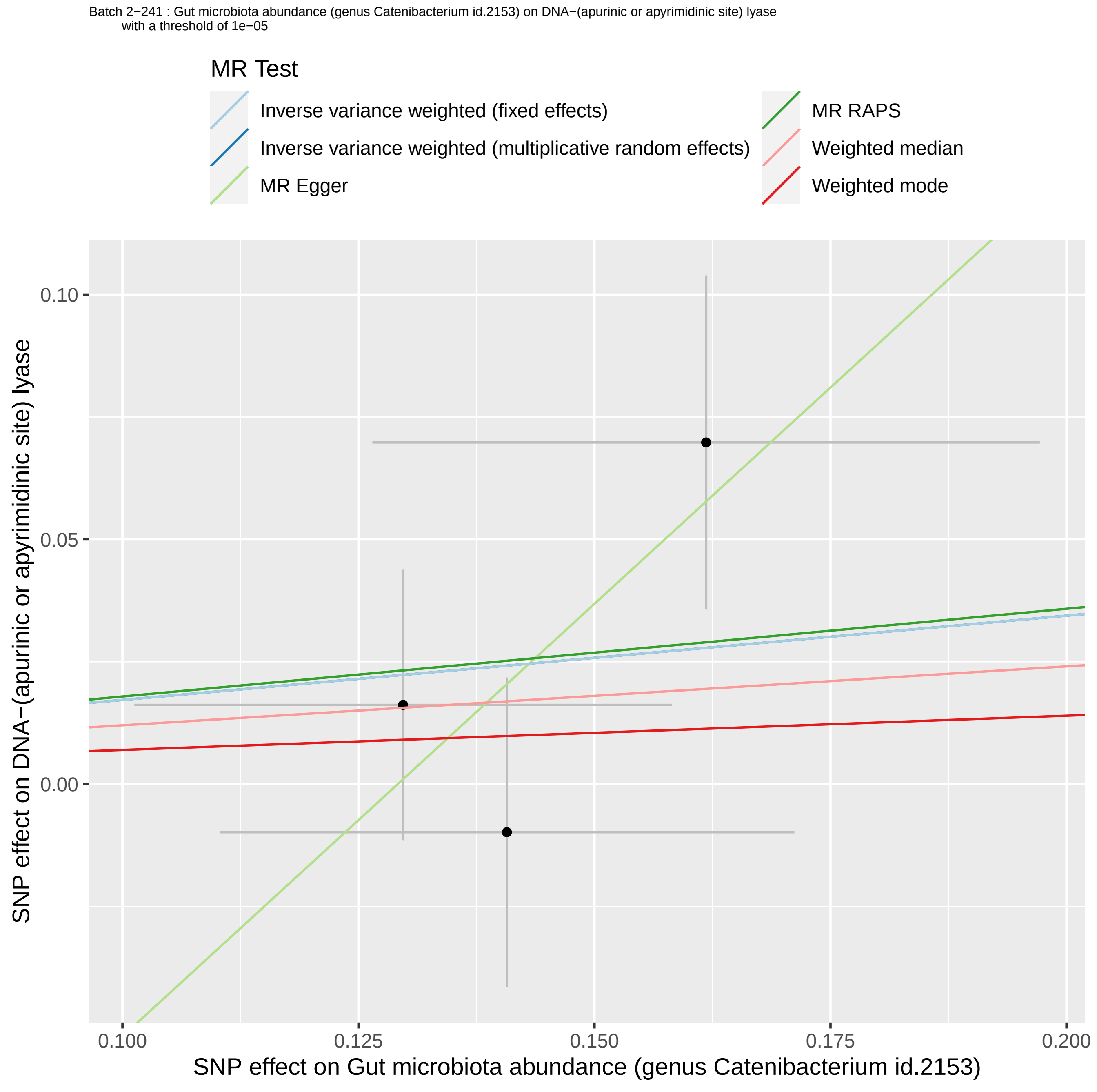

Batch 2−241 : Gut microbiota abundance (genus Catenibacterium id.2153) on DNA−(apurinic or apyrimidinic site) lyase
with a threshold of 1e−05
MR Test
Inverse variance weighted (fixed effects)
Inverse variance weighted (multiplicative random effects)
MR Egger
MR RAPS
Weighted median
Weighted mode
0.10
0.05
0.00
0.100
0.125
0.150
0.175
0.200
SNP effect on DNA−(apurinic or apyrimidinic site) lyase
SNP effect on Gut microbiota abundance (genus Catenibacterium id.2153)

Batch 2−242 : Gut microbiota abundance (genus Parasutterella id.2892) on DNA−(apurinic or apyrimidinic site) lyase
with a threshold of 1e−05

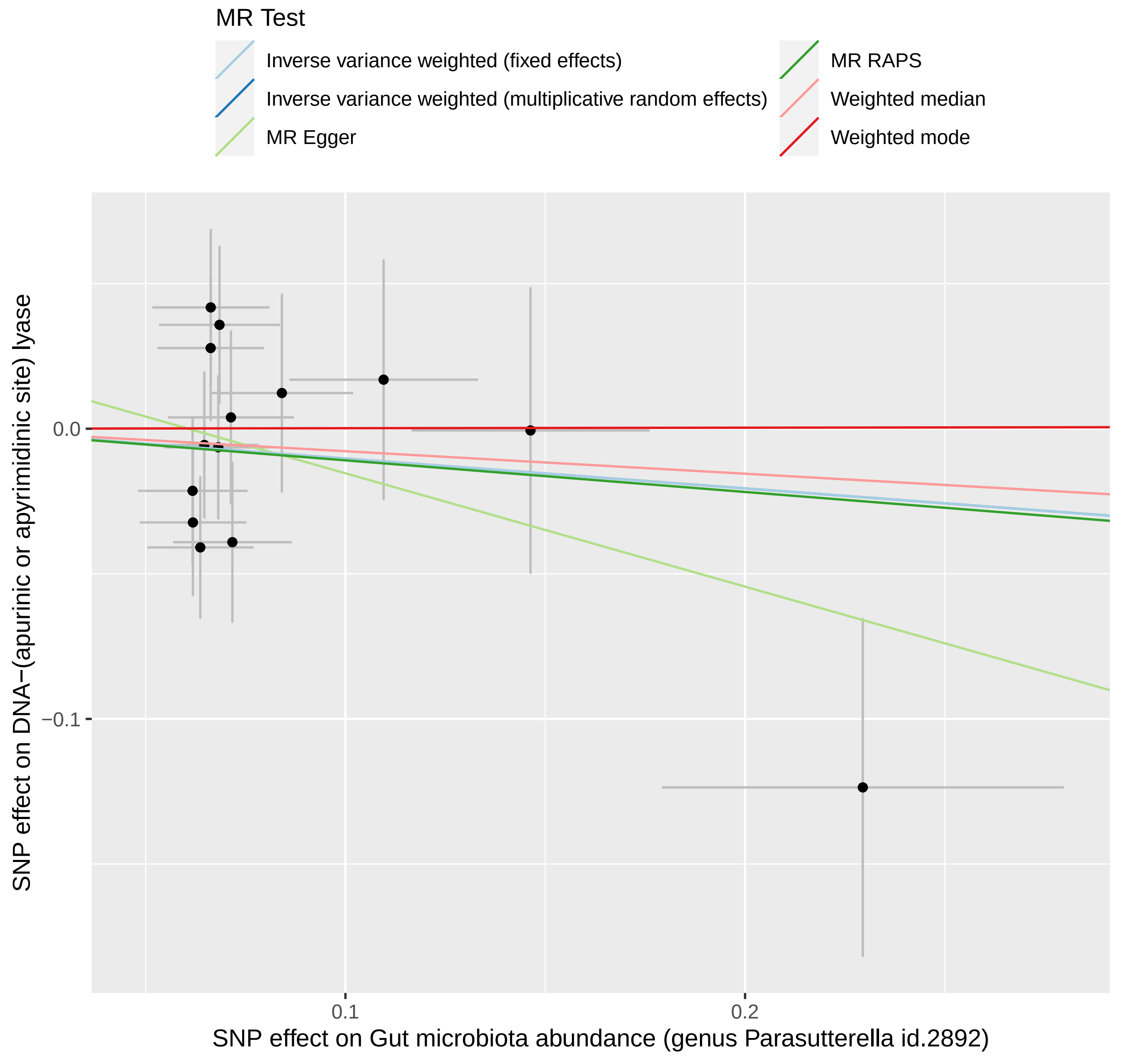

Batch 2−243 : Gut microbiota abundance (genus Catenibacterium id.2153) on Amyloid−like protein 2
with a threshold of 1e−05

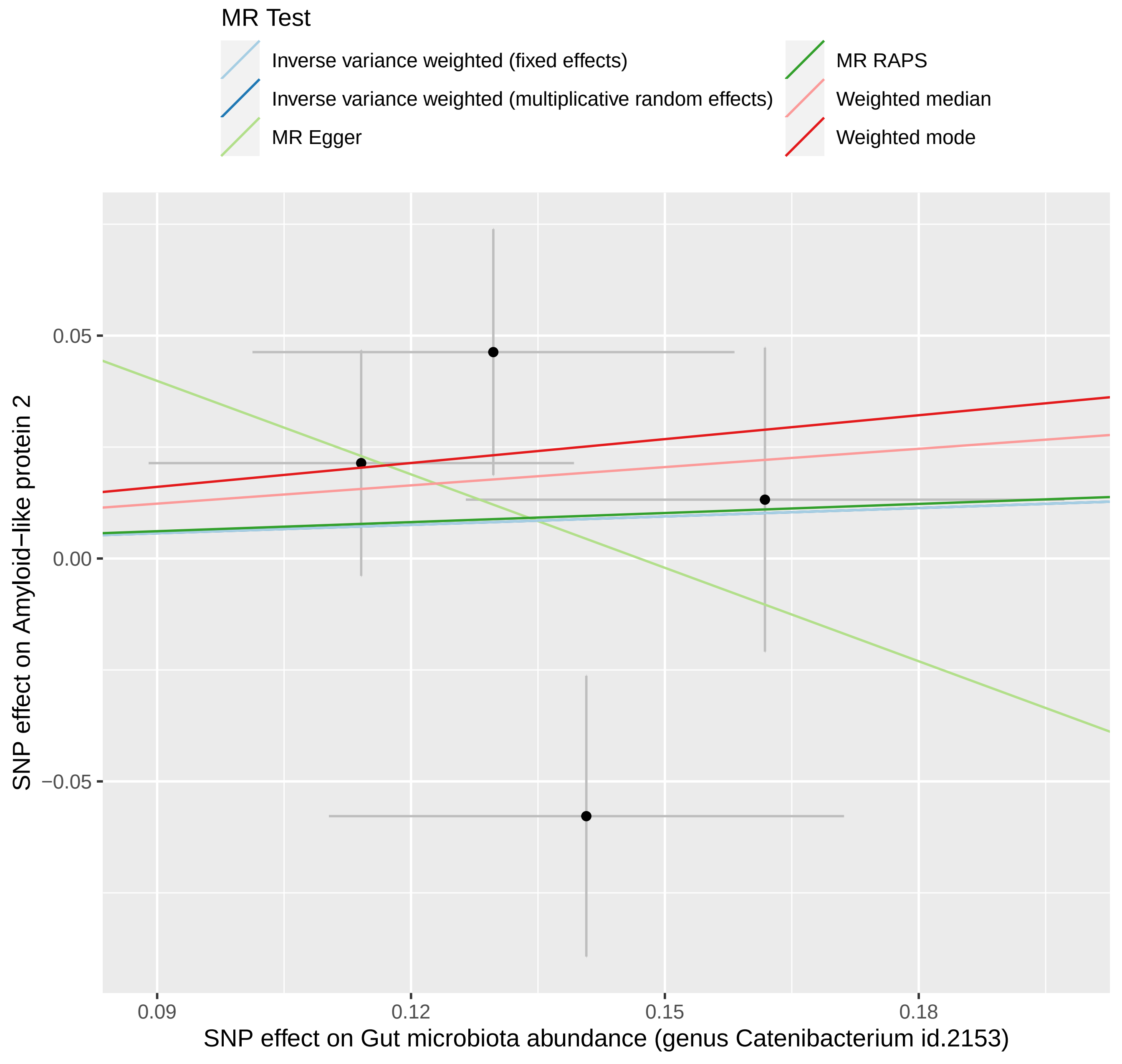

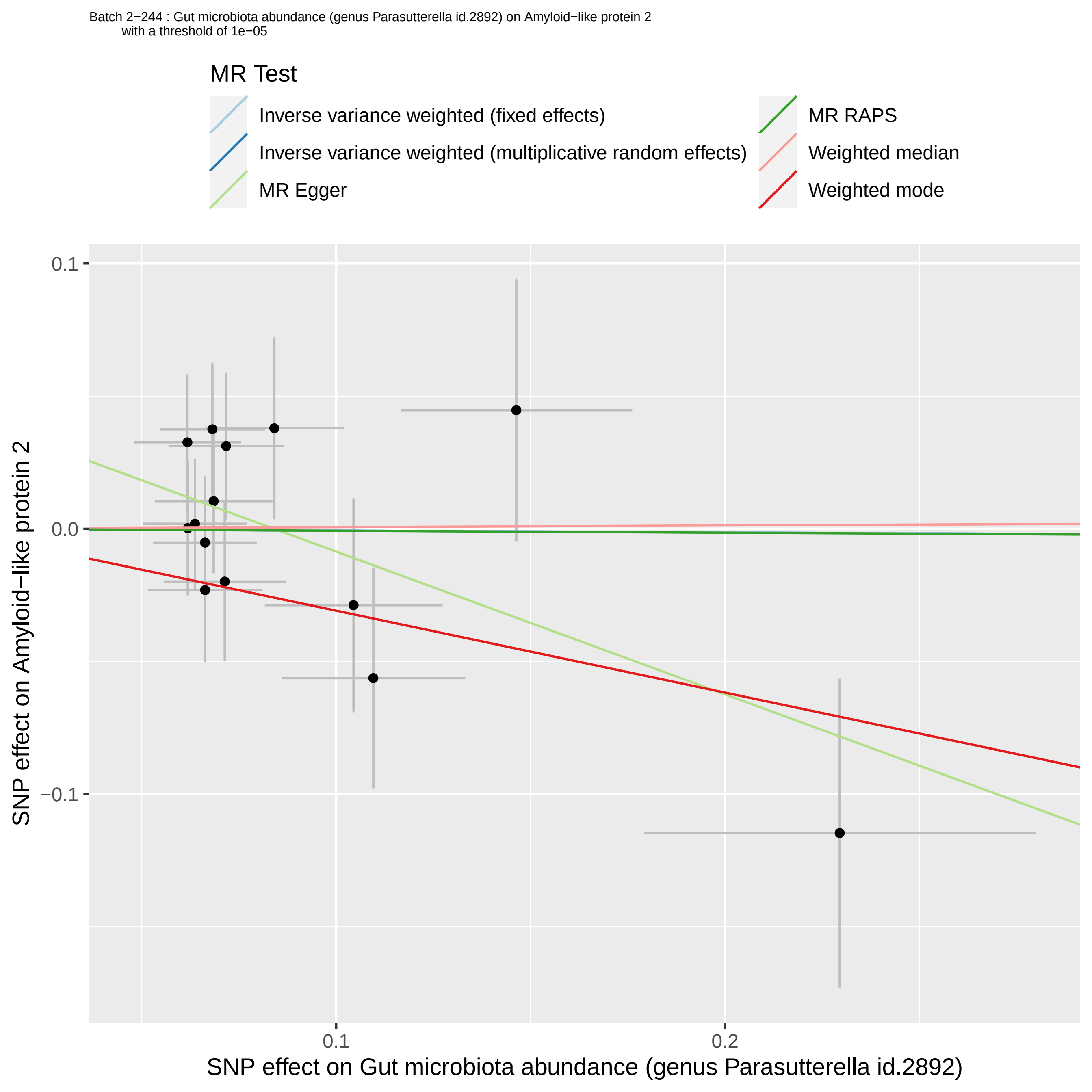

Batch 2−244 : Gut microbiota abundance (genus Parasutterella id.2892) on Amyloid−like protein 2
with a threshold of 1e−05
MR Test
Inverse variance weighted (fixed effects)
Inverse variance weighted (multiplicative random effects)
MR Egger
MR RAPS
Weighted median
Weighted mode
0.1
0.0
−0.1
0.1
0.2
SNP effect on Amyloid−like protein 2
SNP effect on Gut microbiota abundance (genus Parasutterella id.2892)

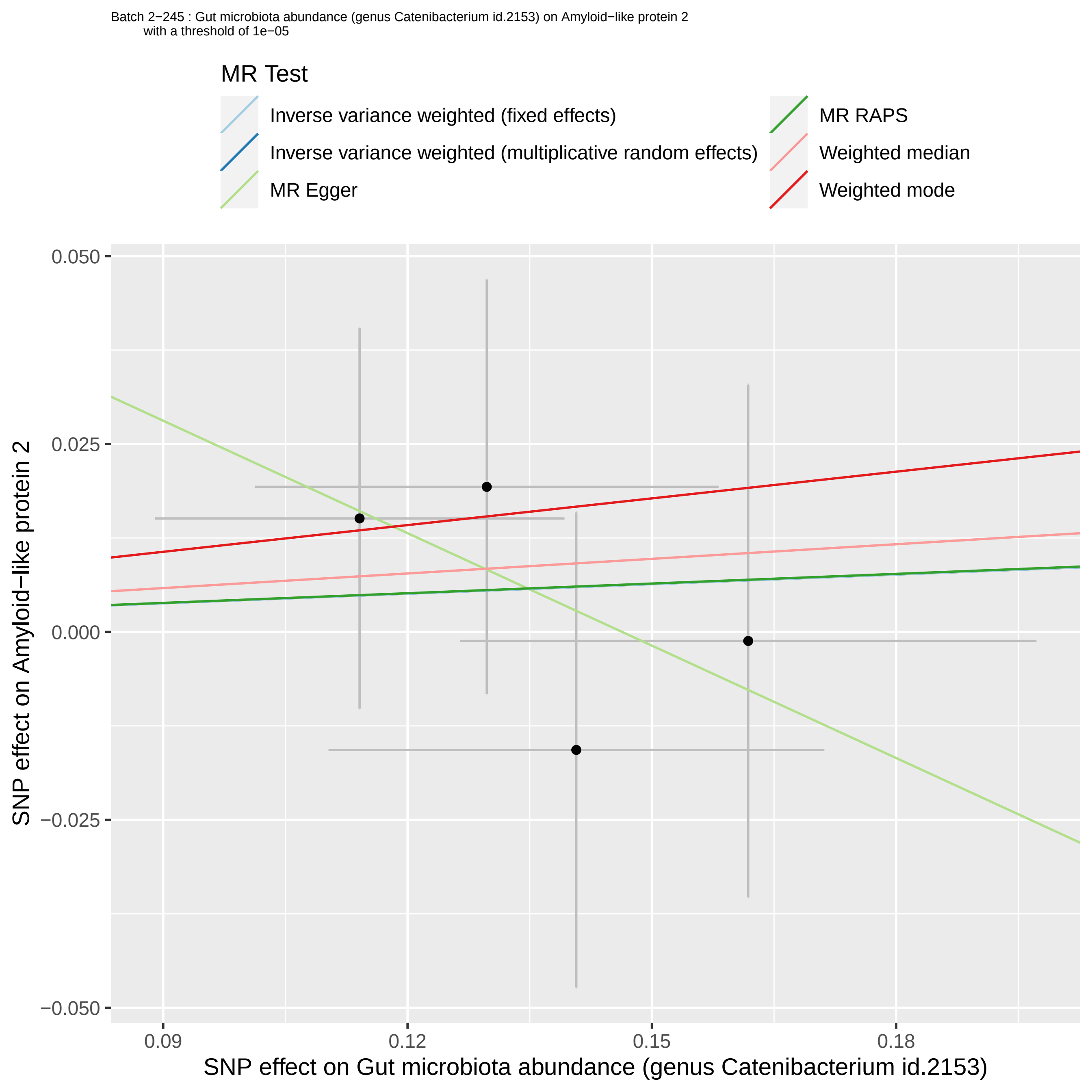
Batch 2−245 : Gut microbiota abundance (genus Catenibacterium id.2153) on Amyloid−like protein 2
with a threshold of 1e−05
MR Test
Inverse variance weighted (fixed effects)
Inverse variance weighted (multiplicative random effects)
MR Egger
MR RAPS
Weighted median
Weighted mode
0.050
0.025
0.000
−0.025
−0.050
SNP effect on Amyloid−like protein 2
0.09
0.12
0.15
0.18
SNP effect on Gut microbiota abundance (genus Catenibacterium id.2153)

Batch 2−246 : Gut microbiota abundance (genus Parasutterella id.2892) on Amyloid−like protein 2
with a threshold of 1e−05

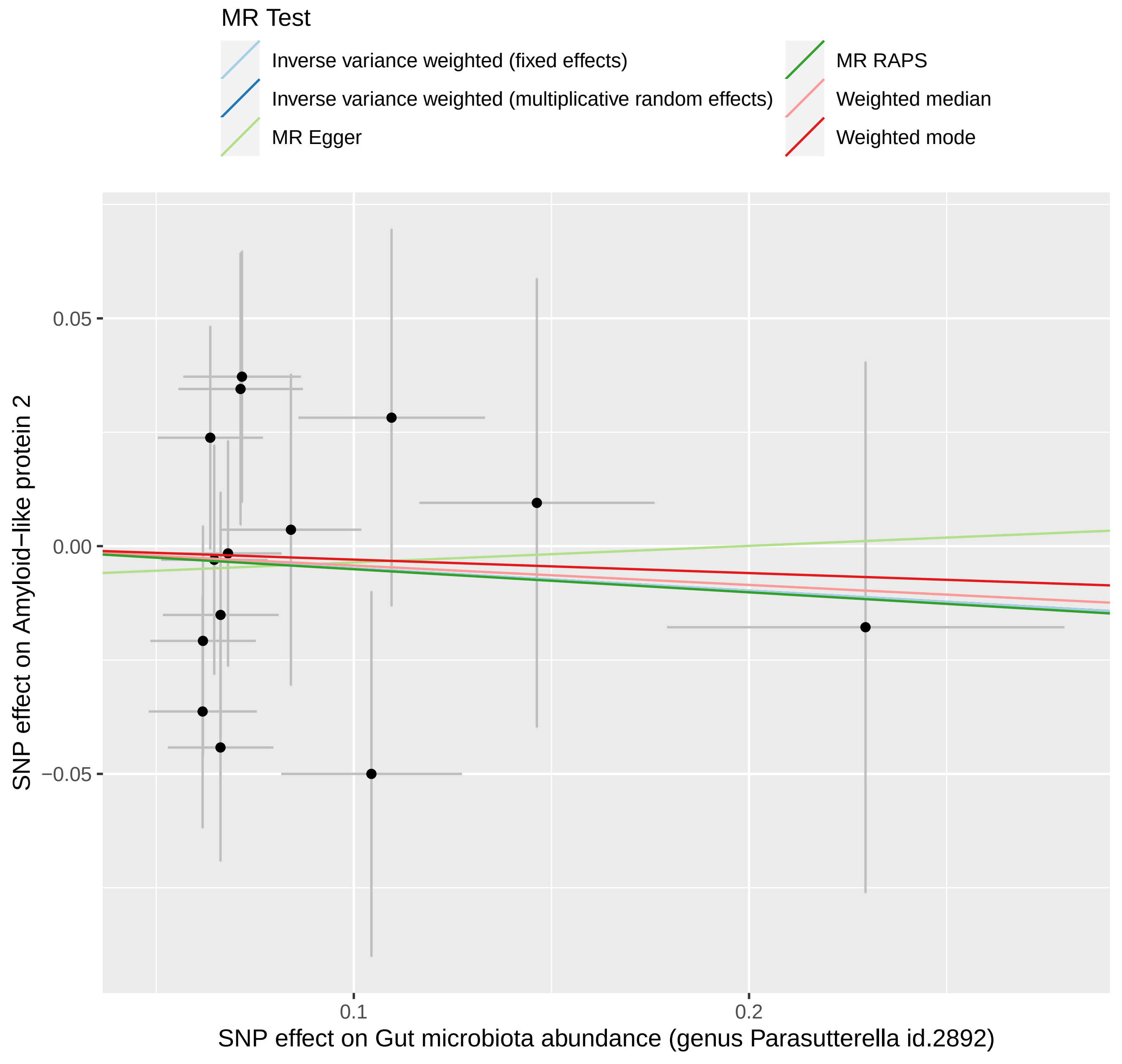

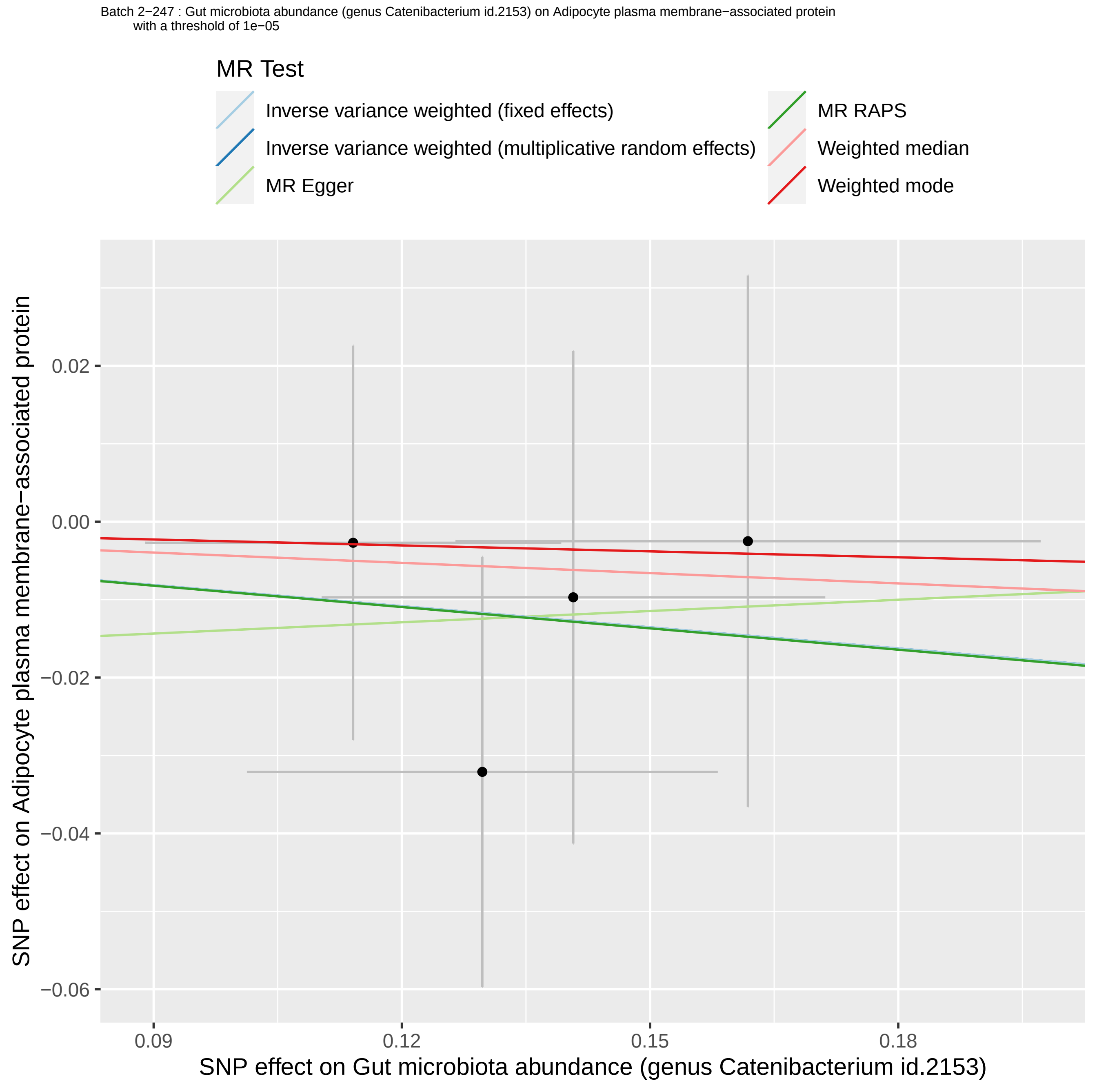

Batch 2−247 : Gut microbiota abundance (genus Catenibacterium id.2153) on Adipocyte plasma membrane−associated protein
with a threshold of 1e−05
MR Test
Inverse variance weighted (fixed effects)
Inverse variance weighted (multiplicative random effects)
MR Egger
MR RAPS
Weighted median
Weighted mode
SNP effect on Adipocyte plasma membrane−associated protein
0.02
0.00
−0.02
−0.04
−0.06
0.09
0.12
0.15
0.18
SNP effect on Gut microbiota abundance (genus Catenibacterium id.2153)

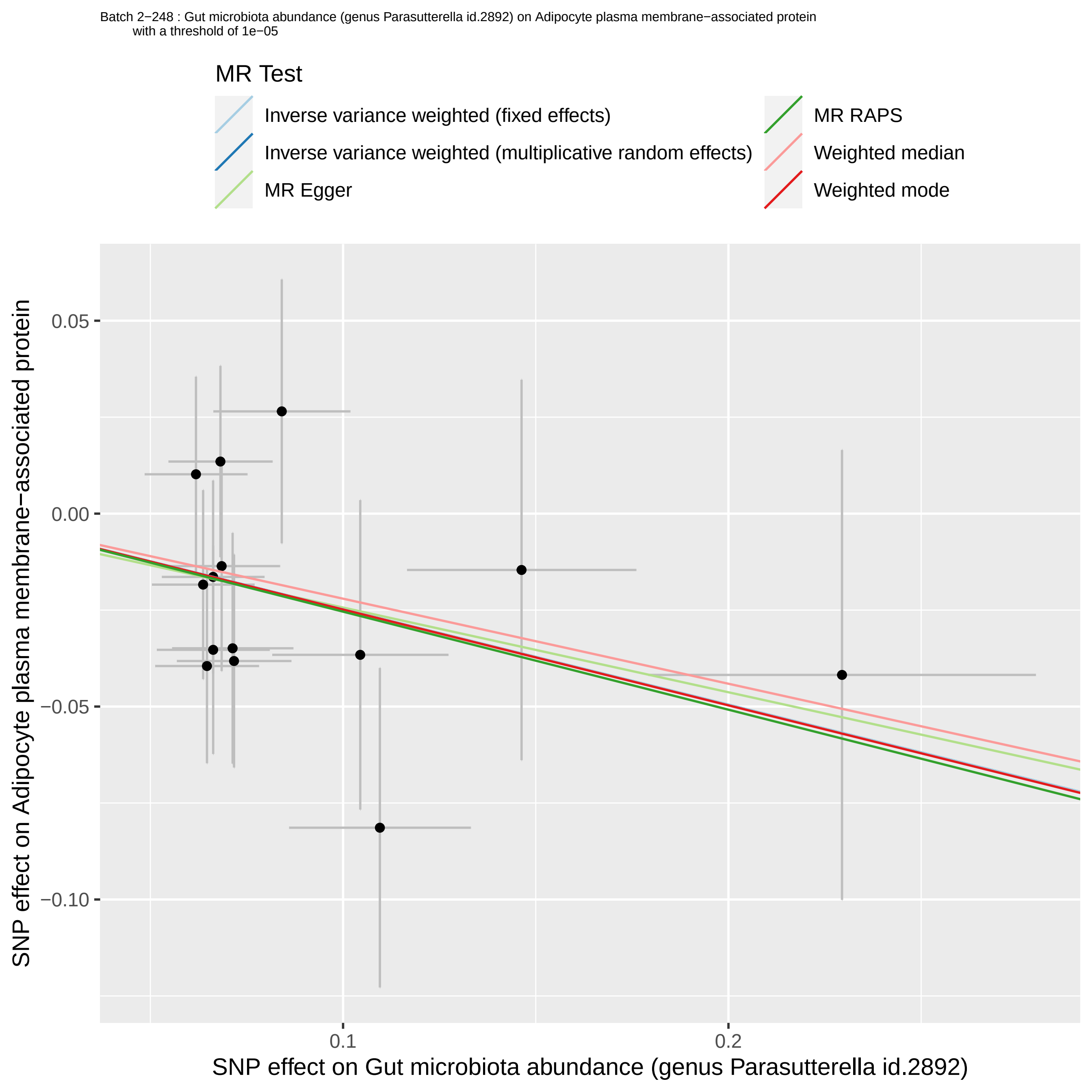

Batch 2−248 : Gut microbiota abundance (genus Parasutterella id.2892) on Adipocyte plasma membrane−associated protein
with a threshold of 1e−05
MR Test
Inverse variance weighted (fixed effects)
Inverse variance weighted (multiplicative random effects)
MR Egger
MR RAPS
Weighted median
Weighted mode
0.05
0.00
−0.05
−0.10
0.1
0.2
SNP effect on Adipocyte plasma membrane−associated protein
SNP effect on Gut microbiota abundance (genus Parasutterella id.2892)

Batch 2−249 : Gut microbiota abundance (genus Catenibacterium id.2153) on Apolipoprotein A−V
with a threshold of 1e−05

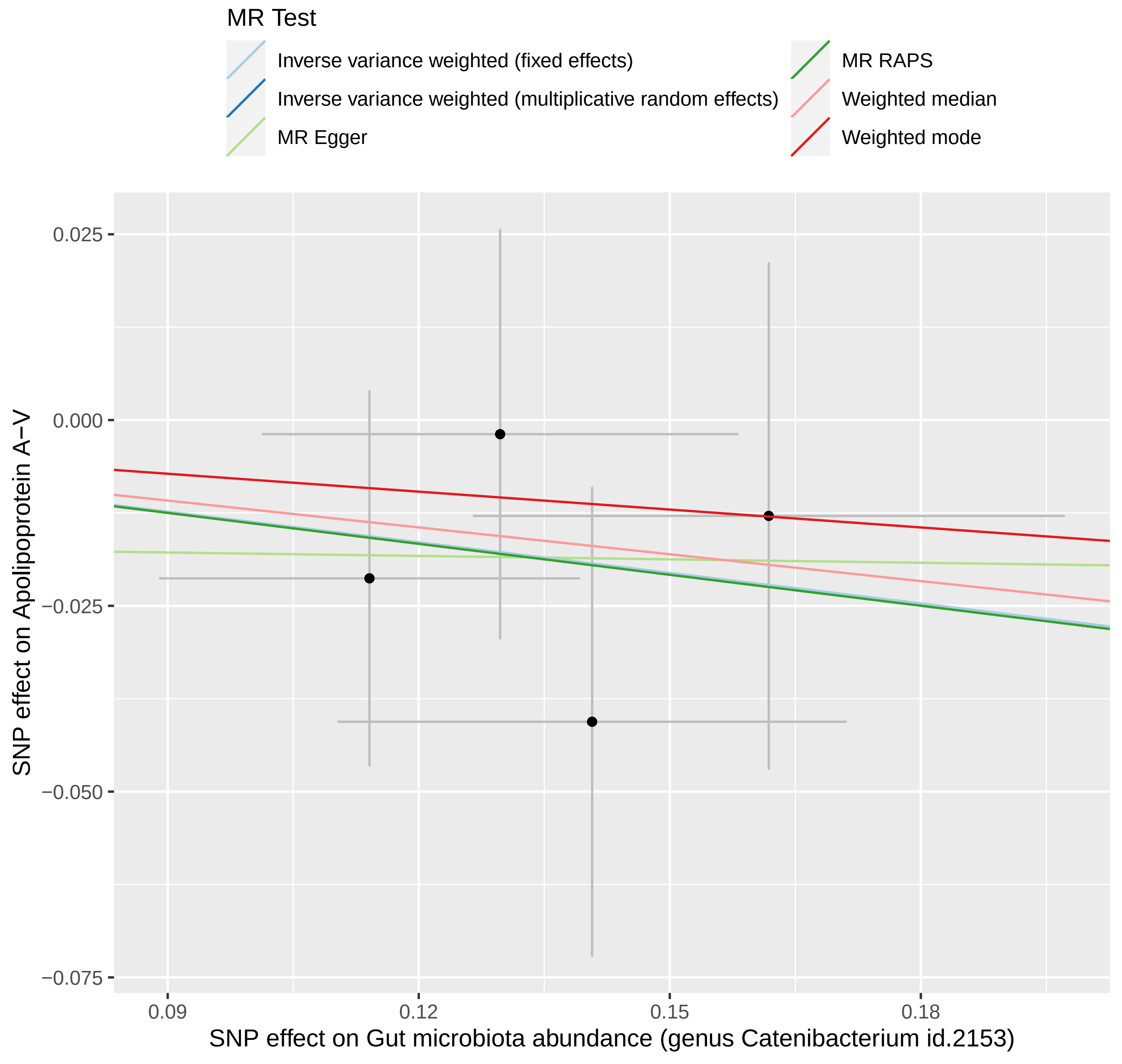

Batch 2−250 : Gut microbiota abundance (genus Parasutterella id.2892) on Apolipoprotein A−V
with a threshold of 1e−05

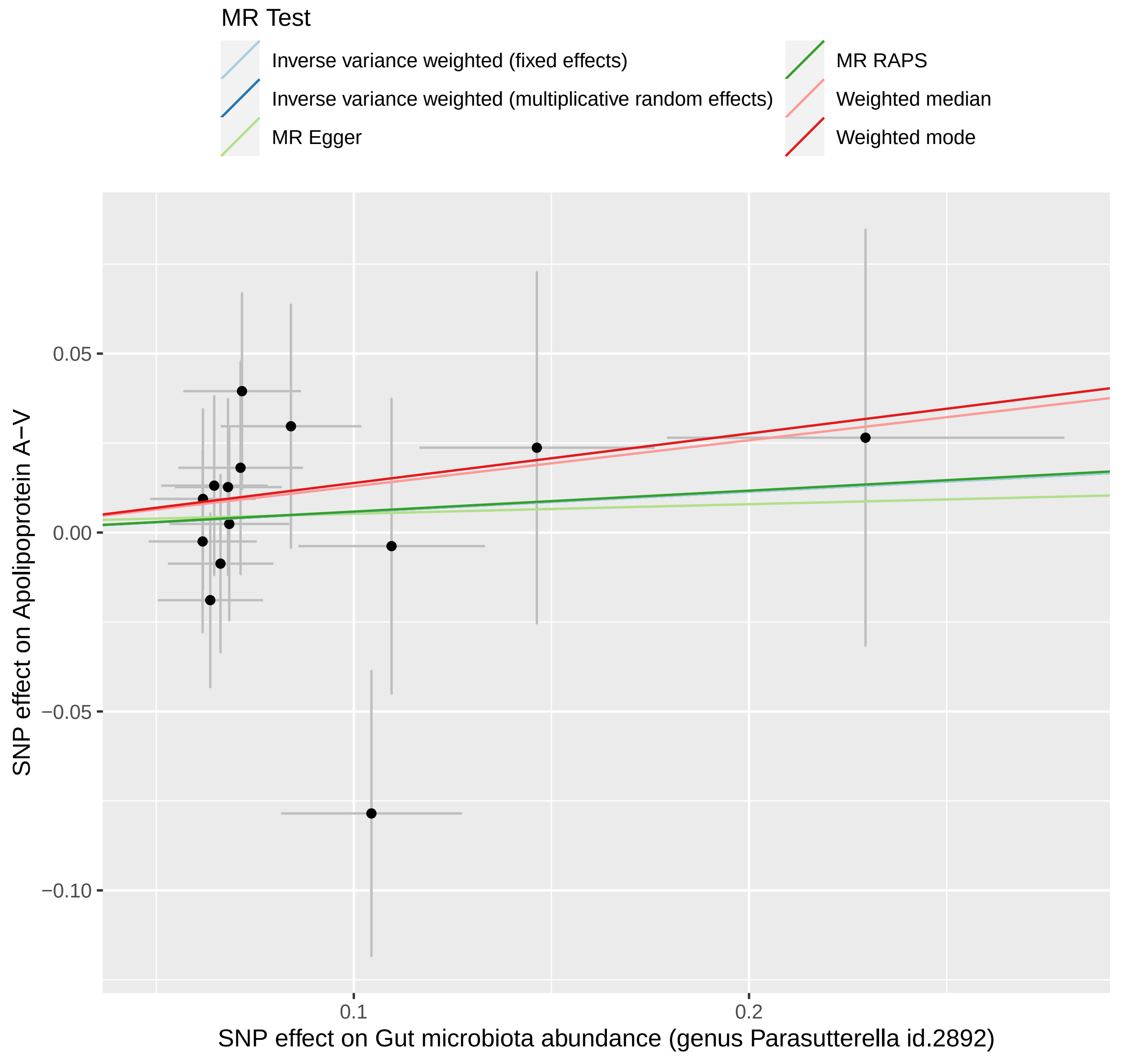

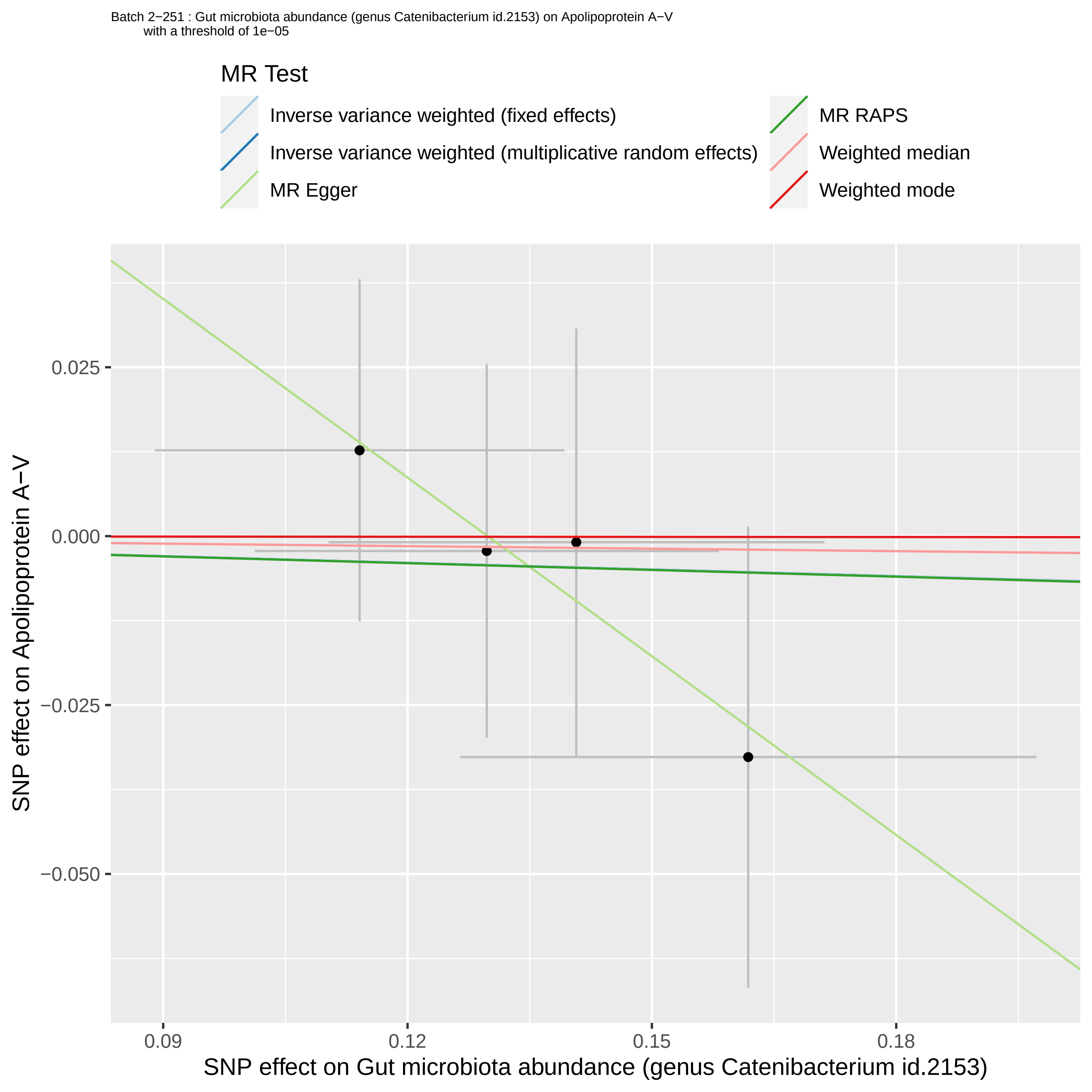

Batch 2−251 : Gut microbiota abundance (genus Catenibacterium id.2153) on Apolipoprotein A−V
with a threshold of 1e−05
MR Test
Inverse variance weighted (fixed effects)
Inverse variance weighted (multiplicative random effects)
MR Egger
MR RAPS
Weighted median
Weighted mode
0.025
0.000
−0.025
−0.050
0.09
0.12
0.15
0.18
SNP effect on Apolipoprotein A−V
SNP effect on Gut microbiota abundance (genus Catenibacterium id.2153)

Batch 2−252 : Gut microbiota abundance (genus Parasutterella id.2892) on Apolipoprotein A−V
with a threshold of 1e−05

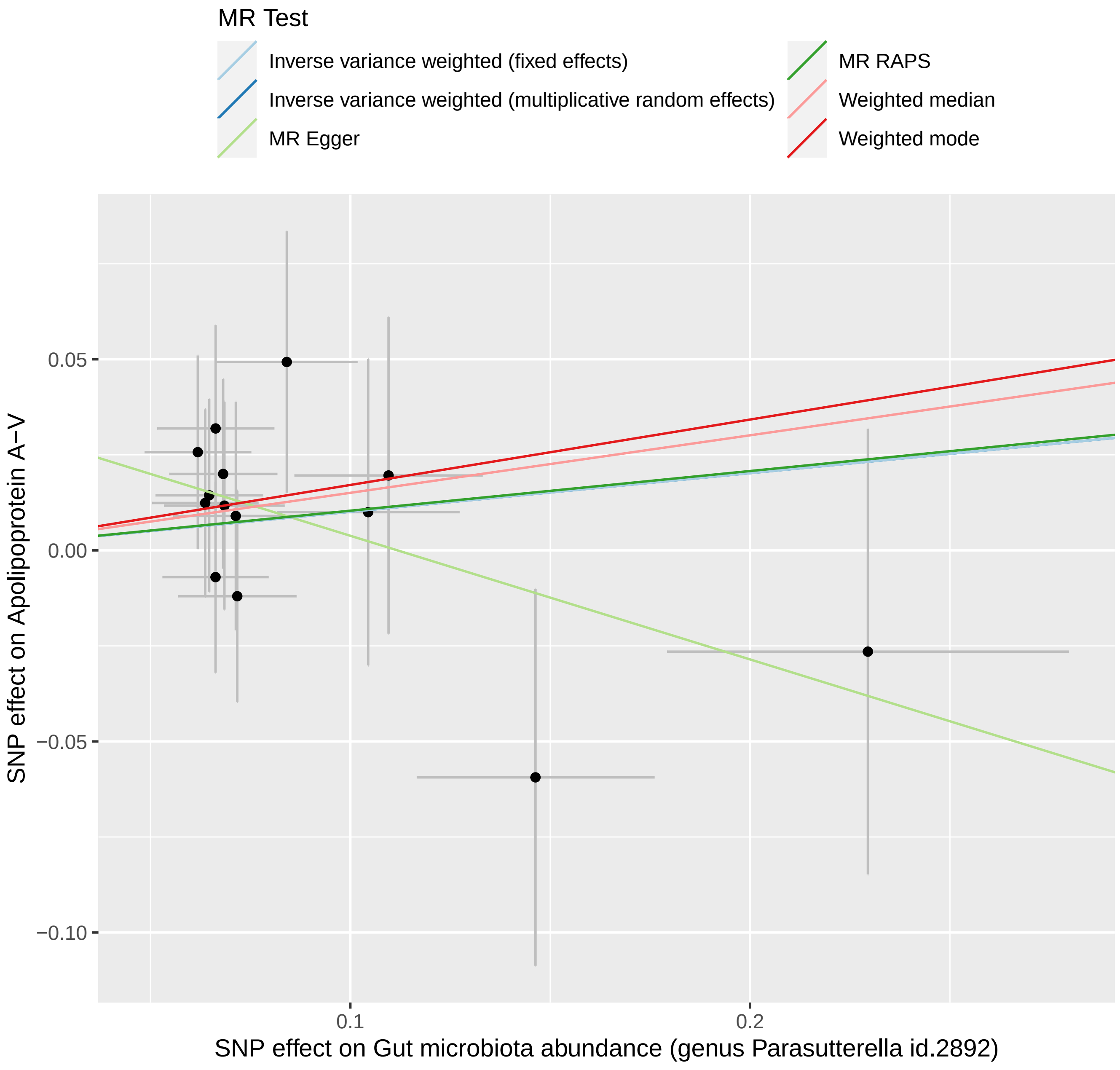

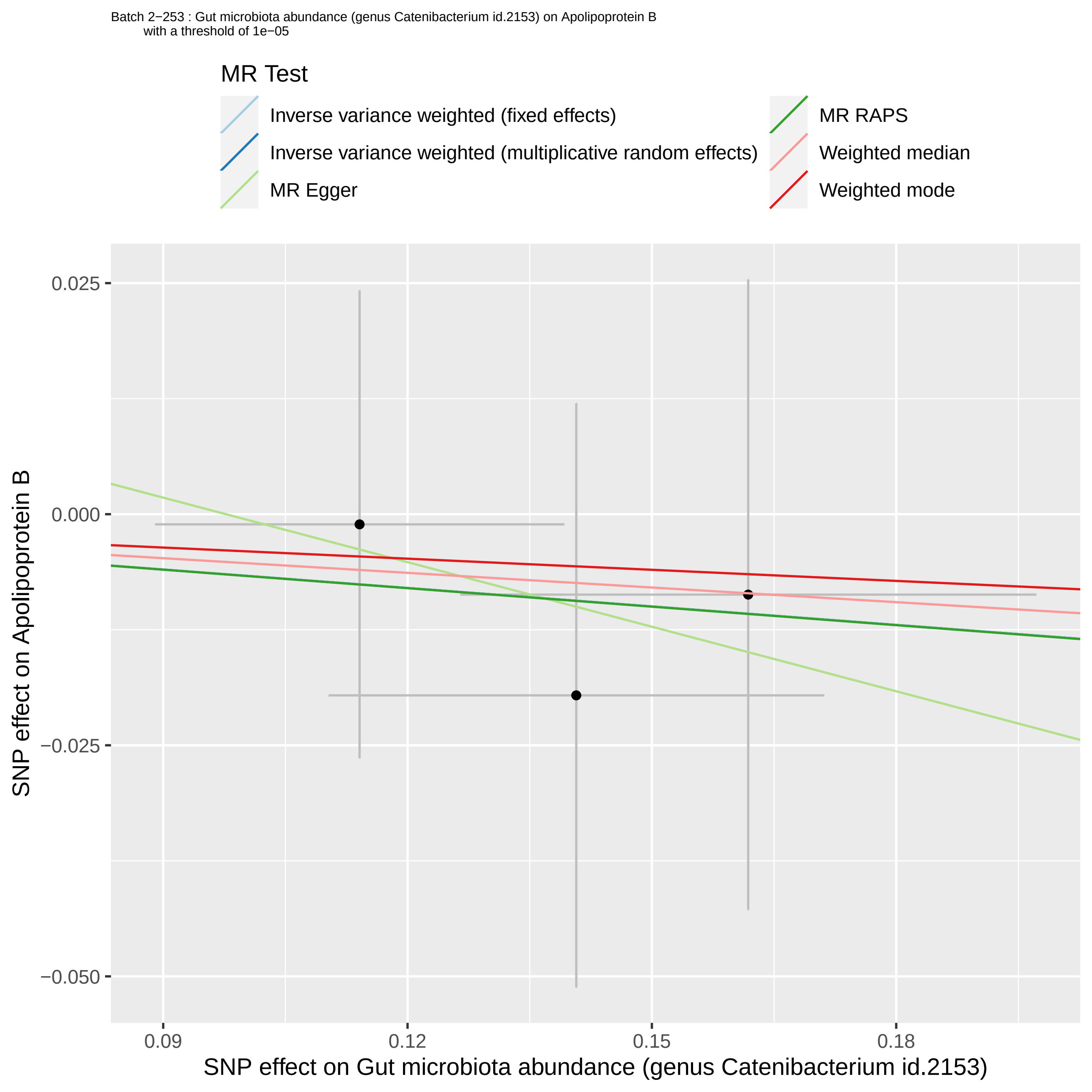

Batch 2−253 : Gut microbiota abundance (genus Catenibacterium id.2153) on Apolipoprotein B
with a threshold of 1e−05
MR Test
Inverse variance weighted (fixed effects)
Inverse variance weighted (multiplicative random effects)
MR Egger
MR RAPS
Weighted median
Weighted mode
0.025
0.000
−0.025
−0.050
0.09
0.12
0.15
0.18
SNP effect on Apolipoprotein B
SNP effect on Gut microbiota abundance (genus Catenibacterium id.2153)

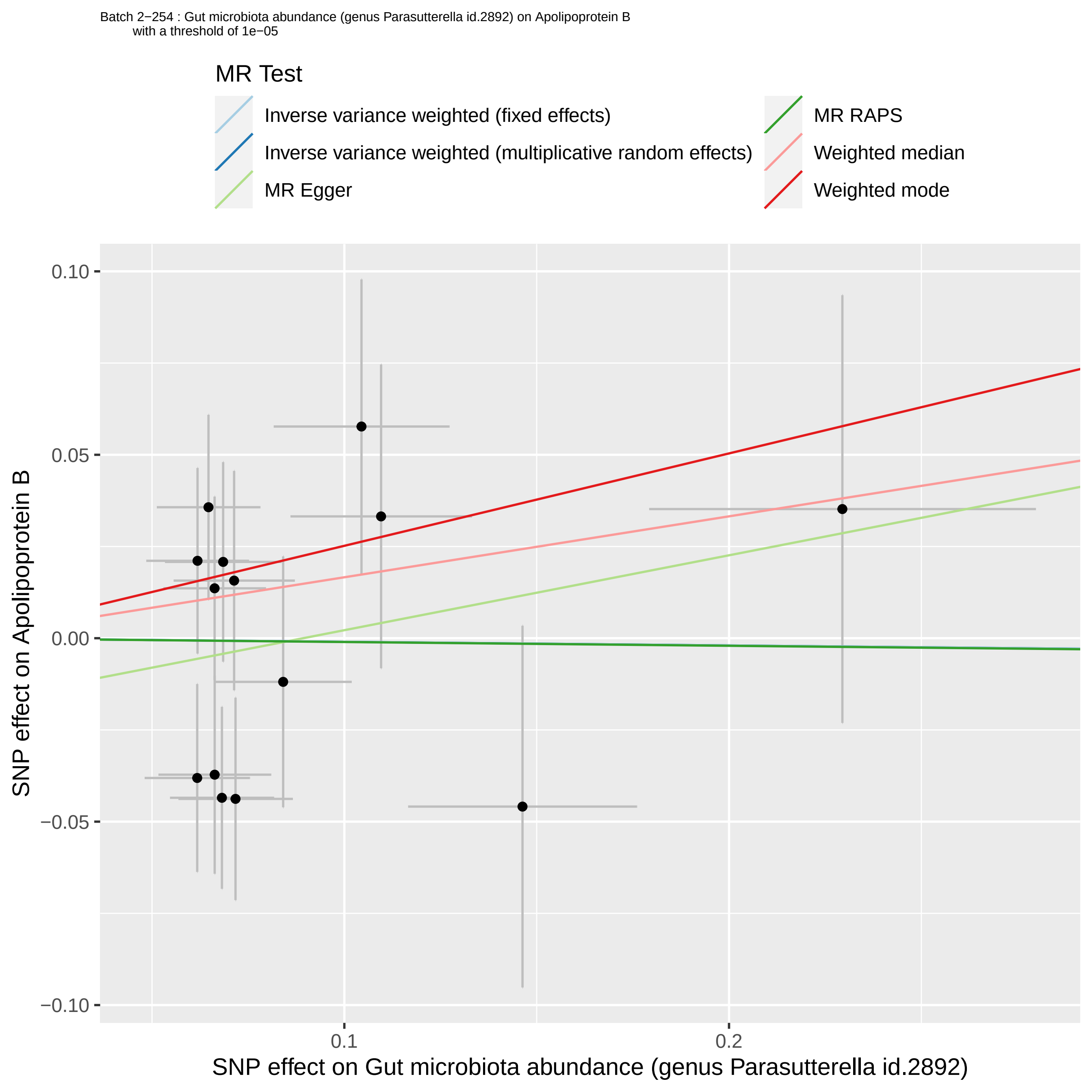
Batch 2−254 : Gut microbiota abundance (genus Parasutterella id.2892) on Apolipoprotein B
with a threshold of 1e−05
MR Test
Inverse variance weighted (fixed effects)
Inverse variance weighted (multiplicative random effects)
MR Egger
MR RAPS
Weighted median
Weighted mode
0.10
0.05
0.00
−0.05
−0.10
0.1
0.2
SNP effect on Apolipoprotein B
SNP effect on Gut microbiota abundance (genus Parasutterella id.2892)

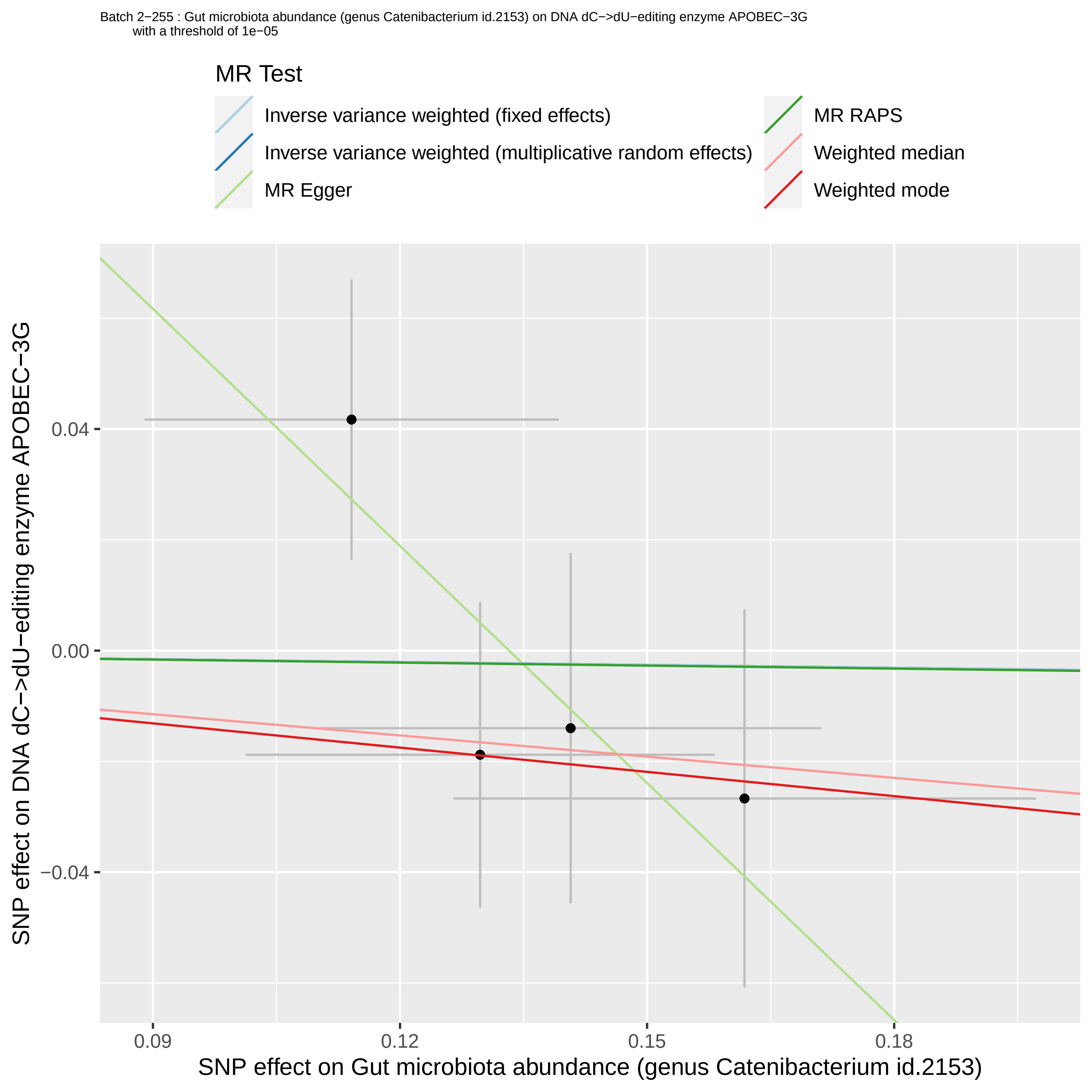
Batch 2−255 : Gut microbiota abundance (genus Catenibacterium id.2153) on DNA dC−>dU−editing enzyme APOBEC−3G
with a threshold of 1e−05
MR Test
Inverse variance weighted (fixed effects)
Inverse variance weighted (multiplicative random effects)
MR Egger
MR RAPS
Weighted median
Weighted mode
0.04
0.00
−0.04
0.09
0.12
0.15
0.18
SNP effect on DNA dC−>dU−editing enzyme APOBEC−3G
SNP effect on Gut microbiota abundance (genus Catenibacterium id.2153)

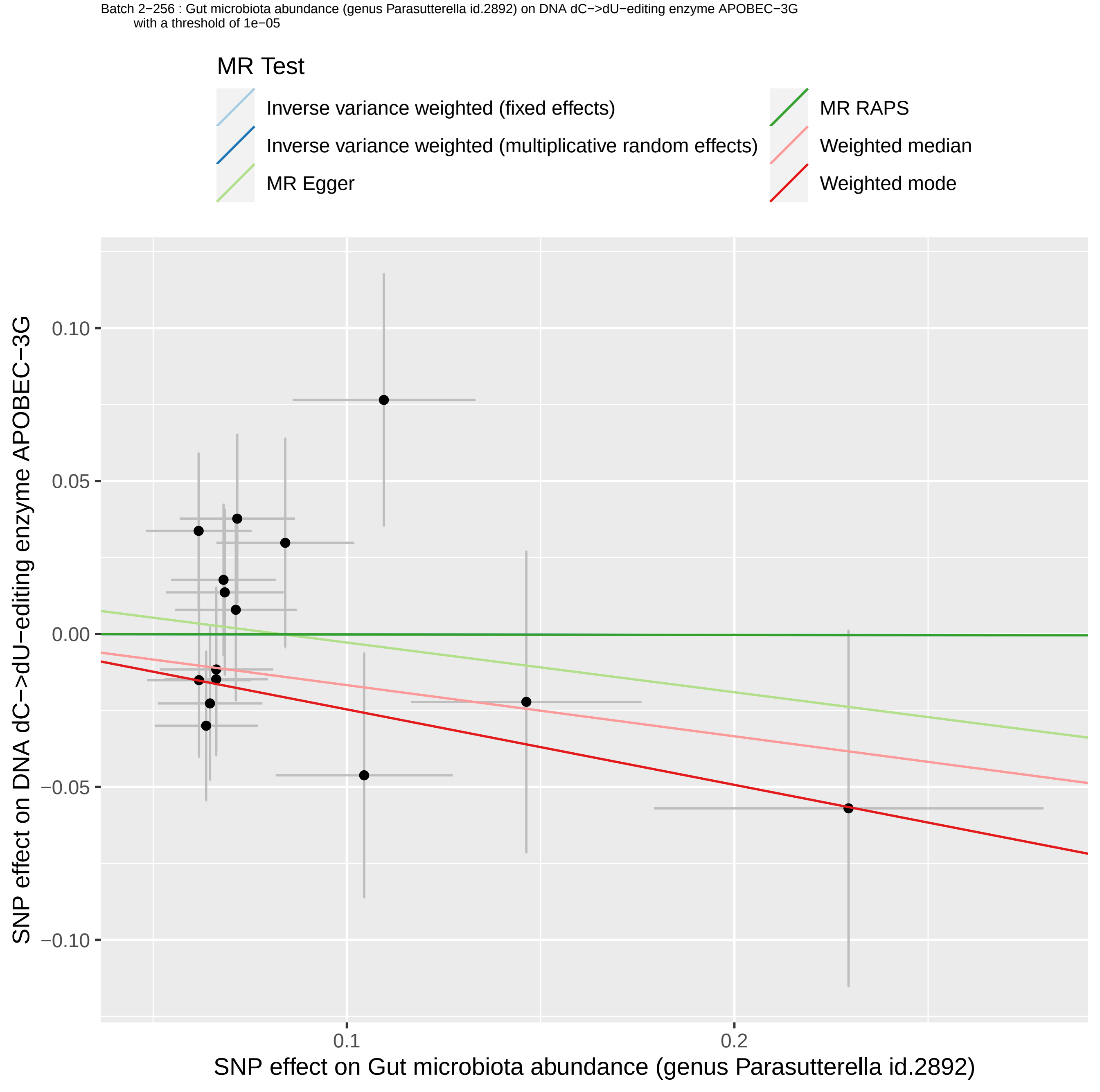
Batch 2−256 : Gut microbiota abundance (genus Parasutterella id.2892) on DNA dC−>dU−editing enzyme APOBEC−3G
with a threshold of 1e−05
MR Test
Inverse variance weighted (fixed effects)
Inverse variance weighted (multiplicative random effects)
MR Egger
MR RAPS
Weighted median
Weighted mode
0.10
0.05
0.00
−0.05
−0.10
0.1
0.2
SNP effect on DNA dC−>dU−editing enzyme APOBEC−3G
SNP effect on Gut microbiota abundance (genus Parasutterella id.2892)

Batch 2−257 : Gut microbiota abundance (genus Catenibacterium id.2153) on Apolipoprotein D
with a threshold of 1e−05

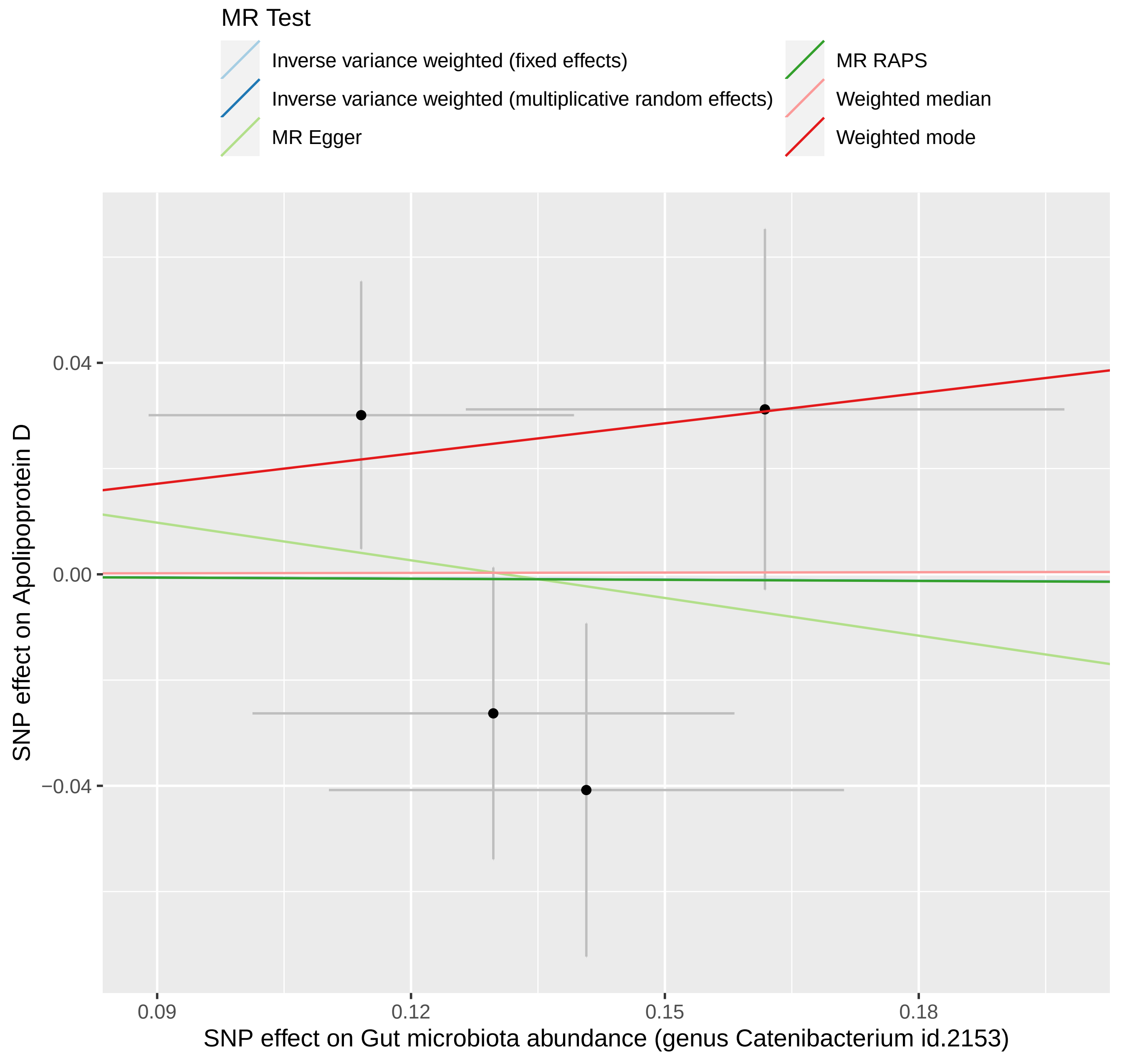

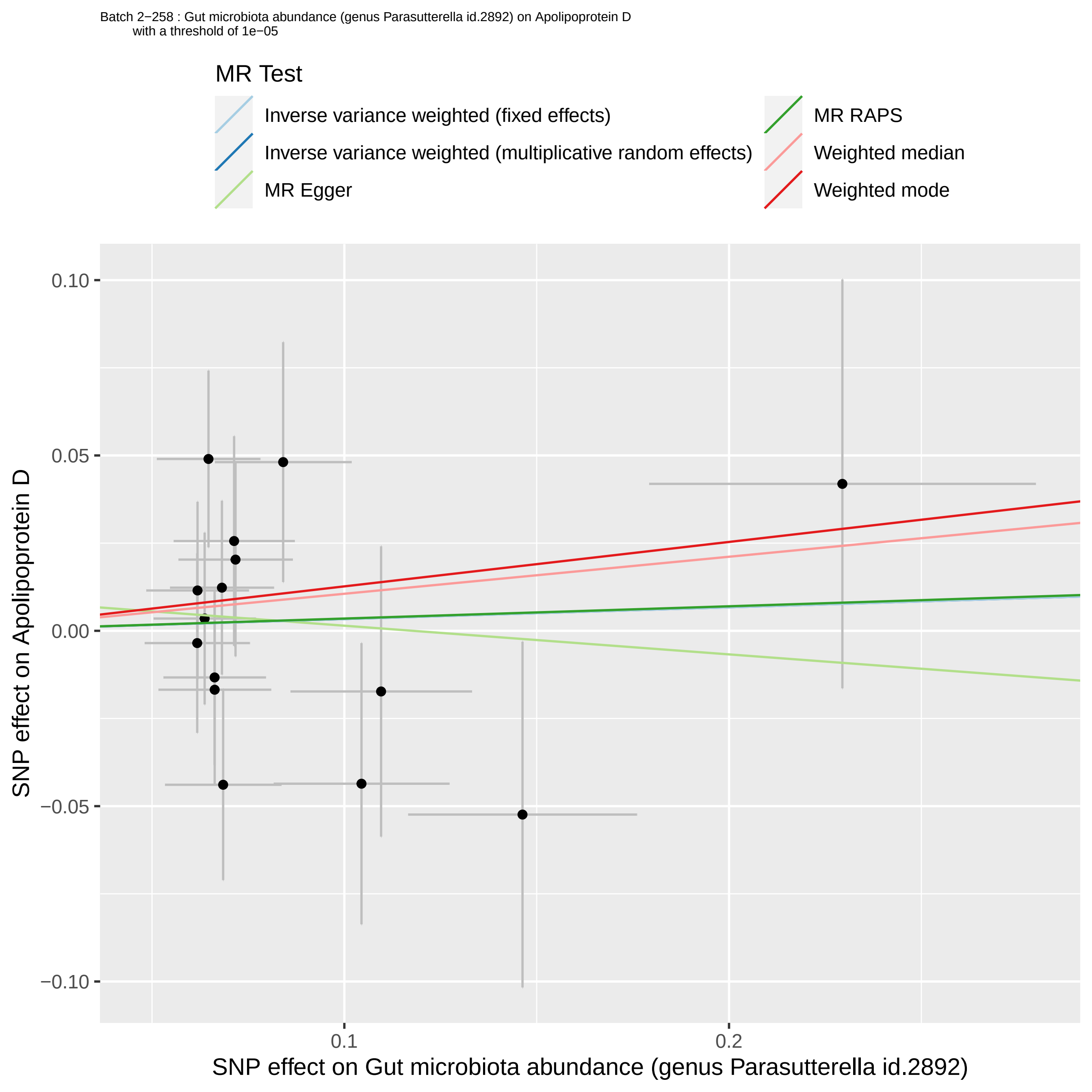

Batch 2−258 : Gut microbiota abundance (genus Parasutterella id.2892) on Apolipoprotein D
with a threshold of 1e−05
MR Test
Inverse variance weighted (fixed effects)
Inverse variance weighted (multiplicative random effects)
MR Egger
MR RAPS
Weighted median
Weighted mode
0.10
0.05
0.00
−0.05
−0.10
0.1
0.2
SNP effect on Apolipoprotein D
SNP effect on Gut microbiota abundance (genus Parasutterella id.2892)

Batch 2−259 : Gut microbiota abundance (genus Catenibacterium id.2153) on Apolipoprotein D
with a threshold of 1e−05

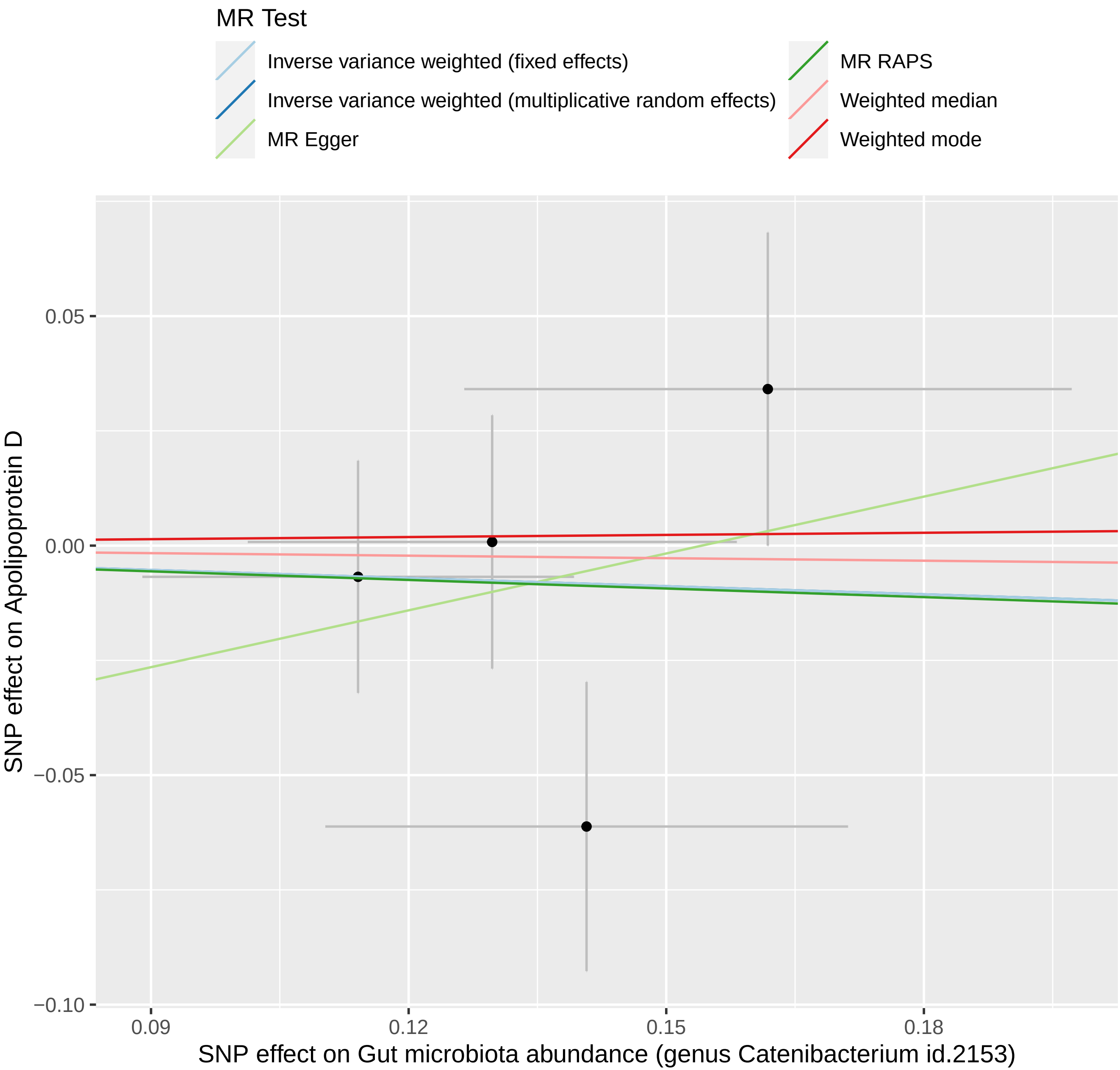

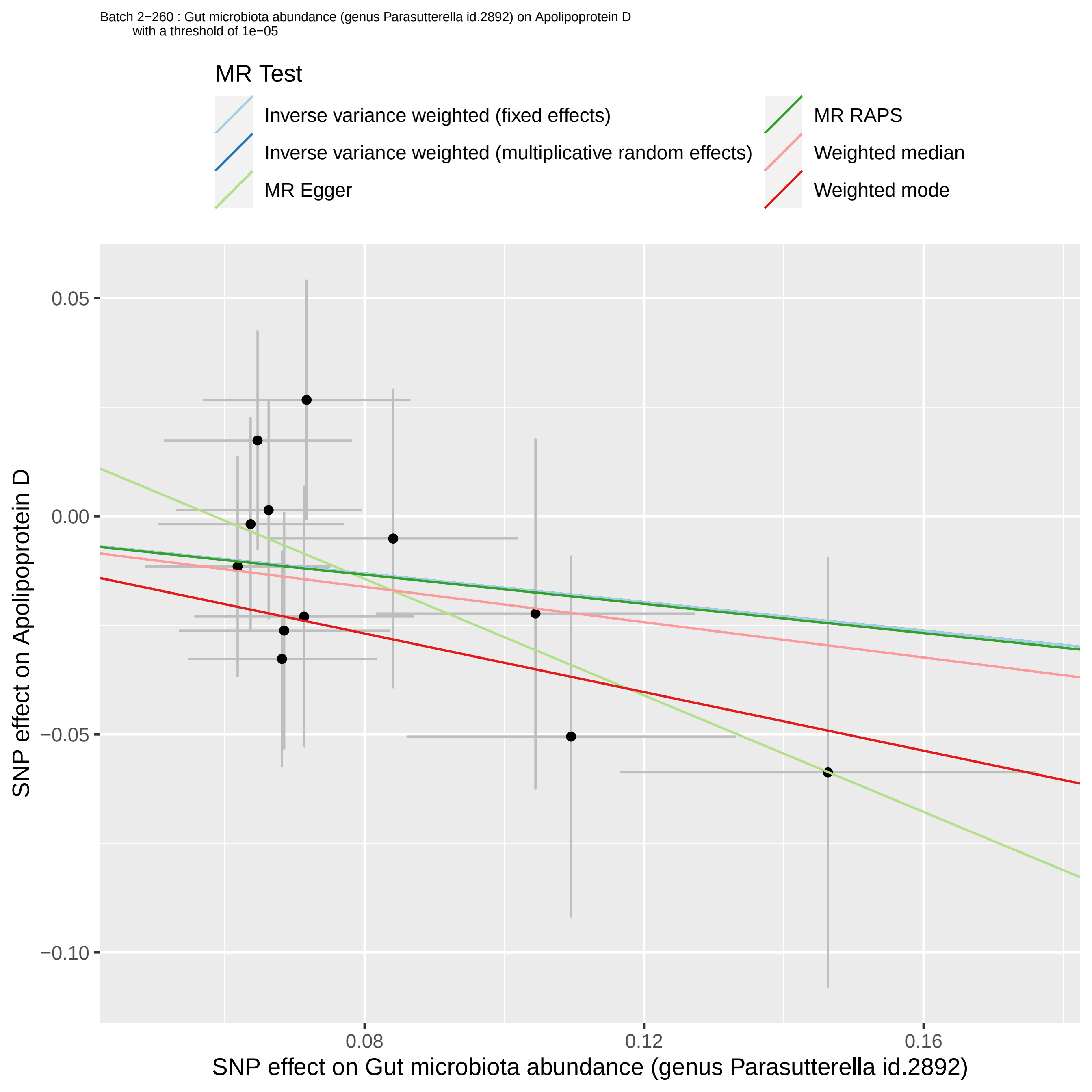
Batch 2−260 : Gut microbiota abundance (genus Parasutterella id.2892) on Apolipoprotein D
with a threshold of 1e−05
MR Test
Inverse variance weighted (fixed effects)
Inverse variance weighted (multiplicative random effects)
MR Egger
MR RAPS
Weighted median
Weighted mode
0.05
0.00
−0.05
−0.10
0.08
0.12
0.16
SNP effect on Apolipoprotein D
SNP effect on Gut microbiota abundance (genus Parasutterella id.2892)

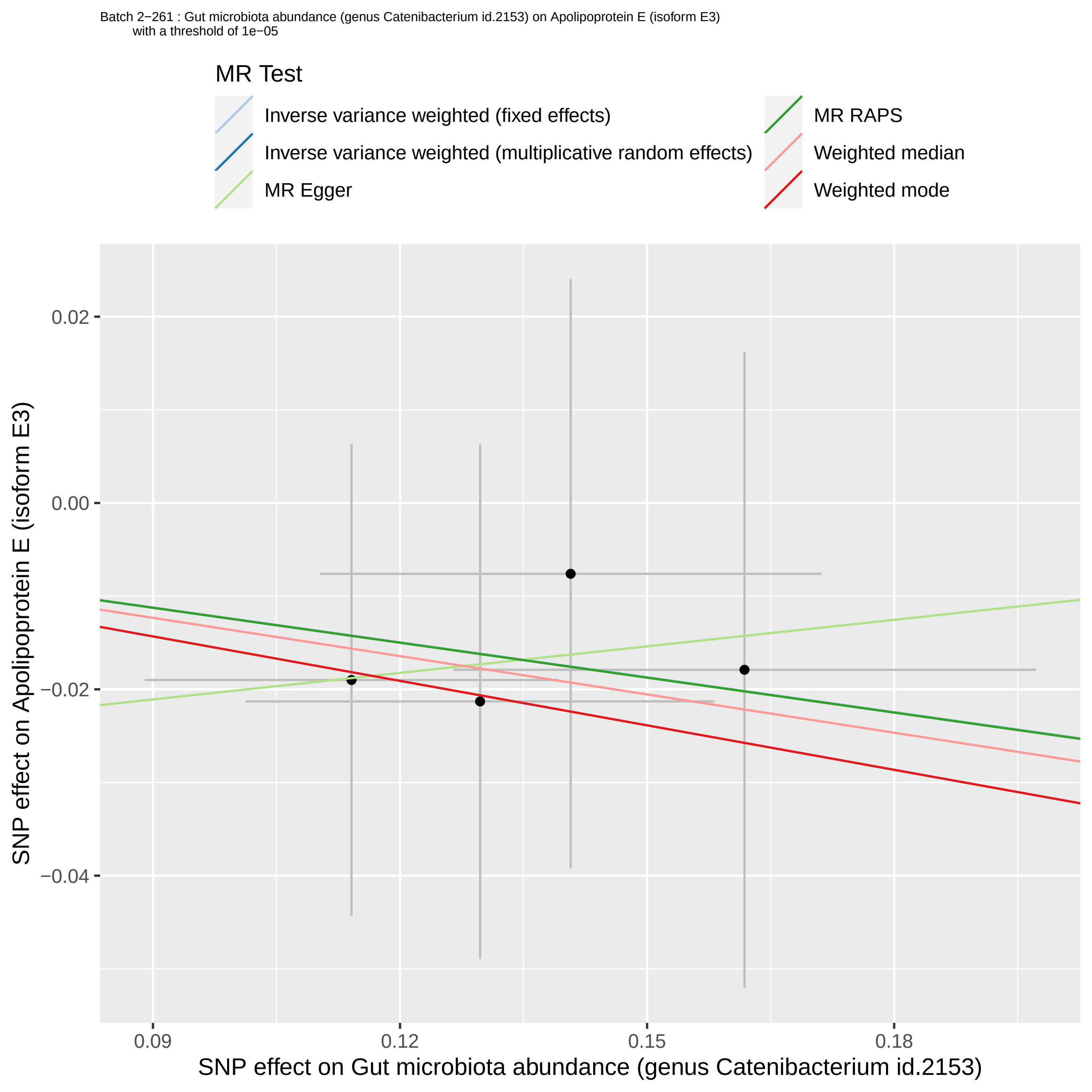
Batch 2−261 : Gut microbiota abundance (genus Catenibacterium id.2153) on Apolipoprotein E (isoform E3)
with a threshold of 1e−05
MR Test
Inverse variance weighted (fixed effects)
Inverse variance weighted (multiplicative random effects)
MR Egger
MR RAPS
Weighted median
Weighted mode
0.02
0.00
−0.02
−0.04
0.09
0.12
0.15
0.18
SNP effect on Apolipoprotein E (isoform E3)
SNP effect on Gut microbiota abundance (genus Catenibacterium id.2153)

Batch 2−262 : Gut microbiota abundance (genus Parasutterella id.2892) on Apolipoprotein E (isoform E3) with a threshold of 1e−05

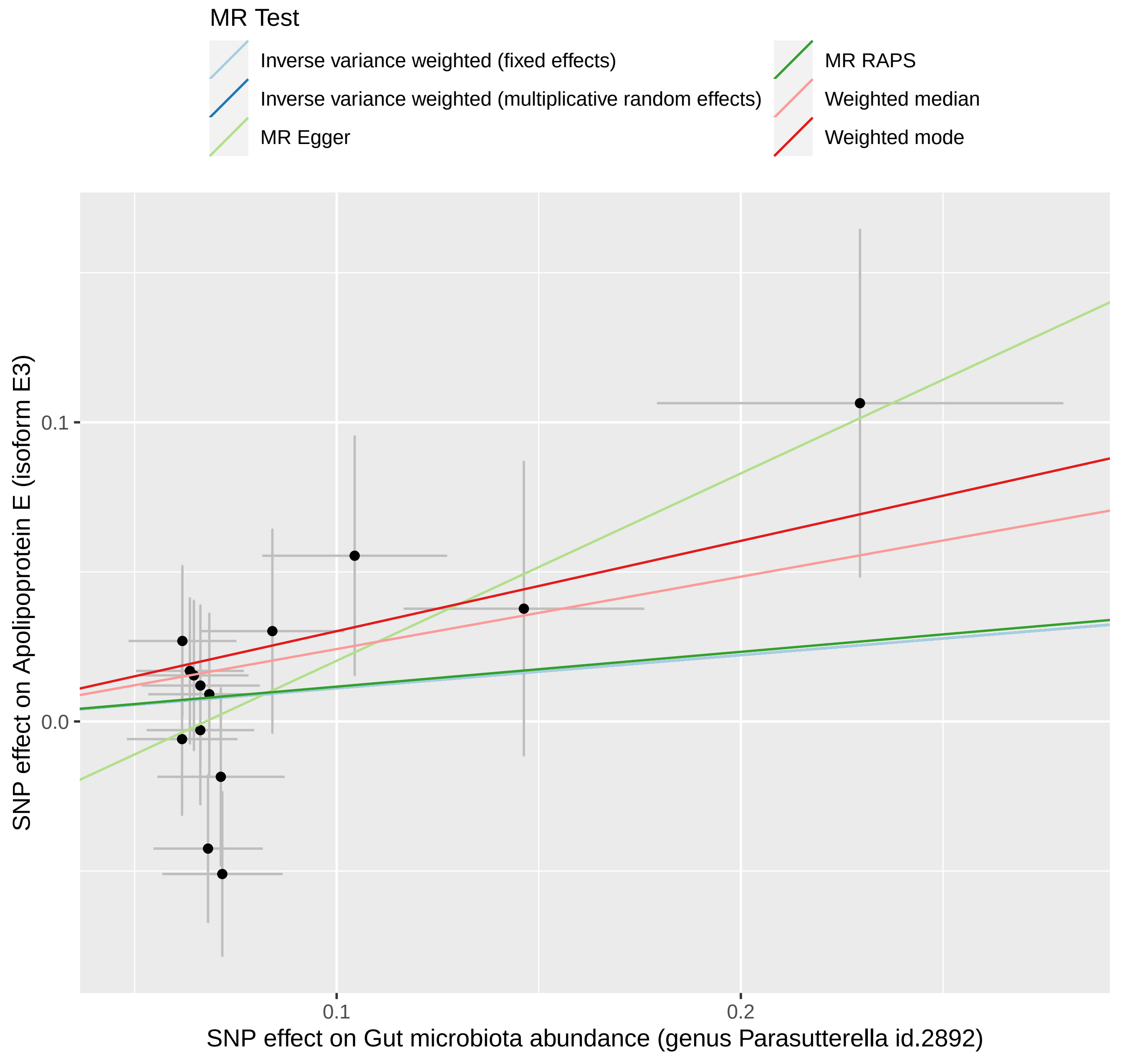

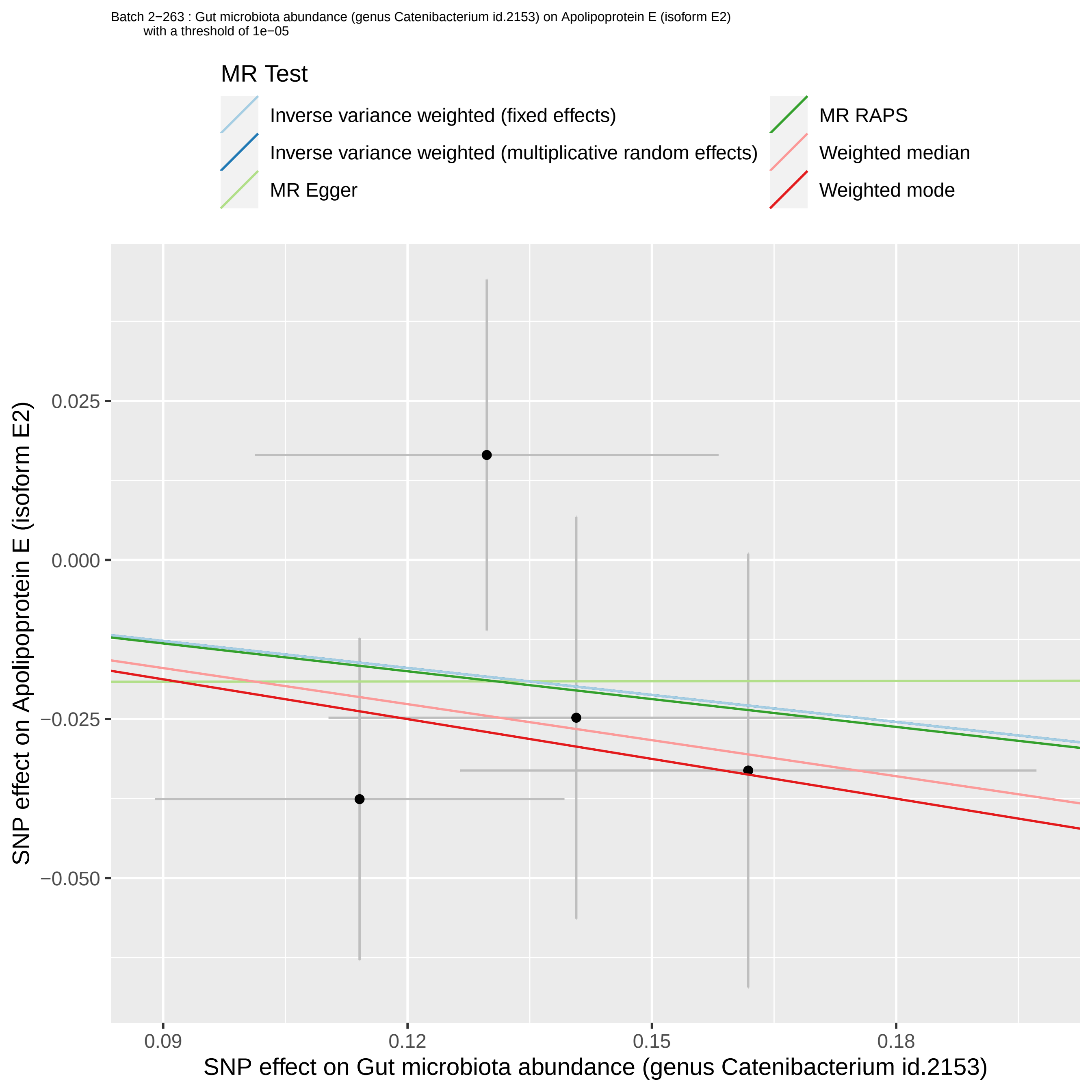

Batch 2−263 : Gut microbiota abundance (genus Catenibacterium id.2153) on Apolipoprotein E (isoform E2)
with a threshold of 1e−05
MR Test
Inverse variance weighted (fixed effects)
Inverse variance weighted (multiplicative random effects)
MR Egger
MR RAPS
Weighted median
Weighted mode
0.025
0.000
−0.025
−0.050
0.09
0.12
0.15
0.18
SNP effect on Apolipoprotein E (isoform E2)
SNP effect on Gut microbiota abundance (genus Catenibacterium id.2153)

Batch 2−264 : Gut microbiota abundance (genus Parasutterella id.2892) on Apolipoprotein E (isoform E2)
with a threshold of 1e−05

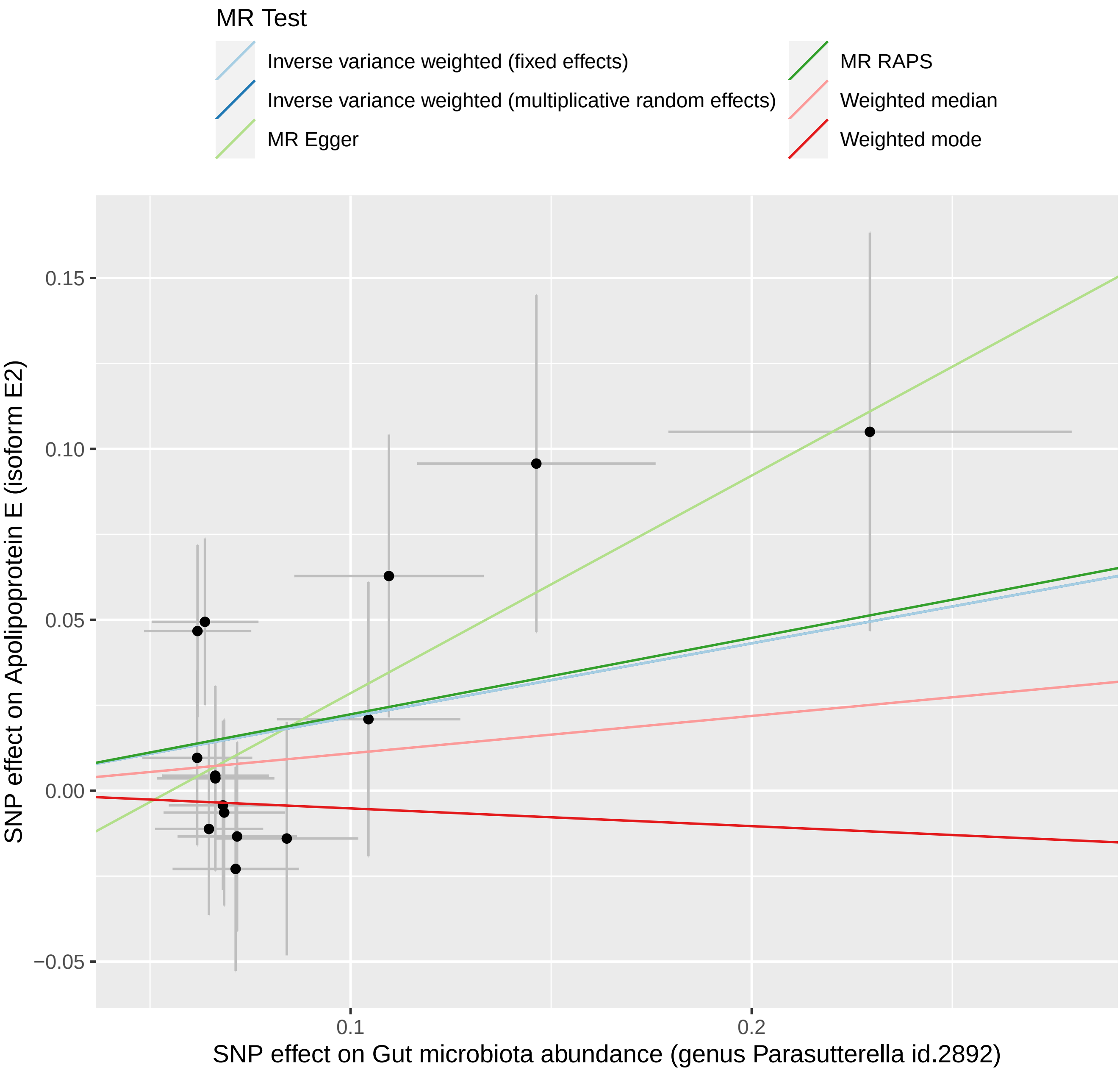

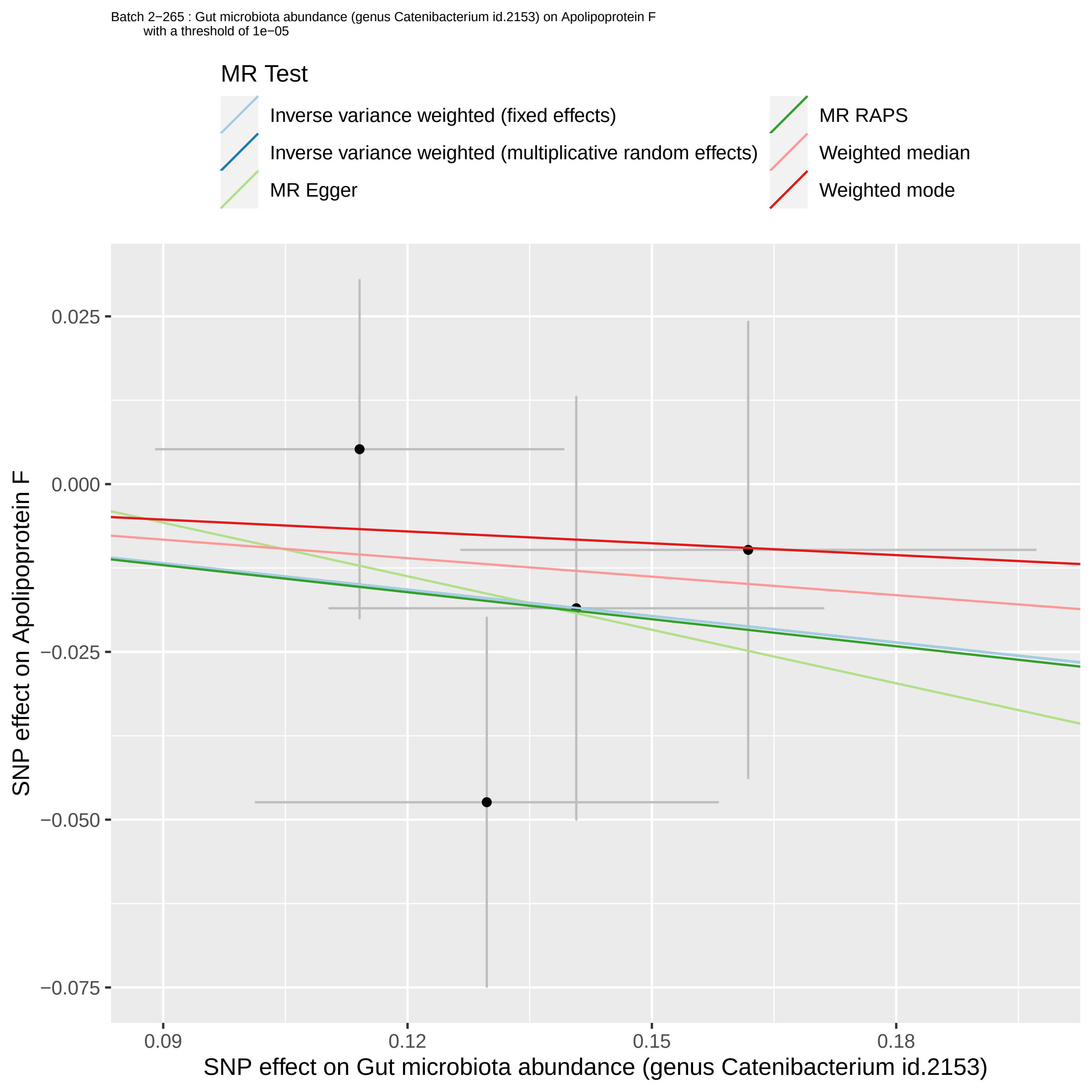
Batch 2−265 : Gut microbiota abundance (genus Catenibacterium id.2153) on Apolipoprotein F
with a threshold of 1e−05
MR Test
Inverse variance weighted (fixed effects)
Inverse variance weighted (multiplicative random effects)
MR Egger
MR RAPS
Weighted median
Weighted mode
0.025
0.000
−0.025
−0.050
−0.075
0.09
0.12
0.15
0.18
SNP effect on Apolipoprotein F
SNP effect on Gut microbiota abundance (genus Catenibacterium id.2153)

Batch 2−266 : Gut microbiota abundance (genus Parasutterella id.2892) on Apolipoprotein F
with a threshold of 1e−05

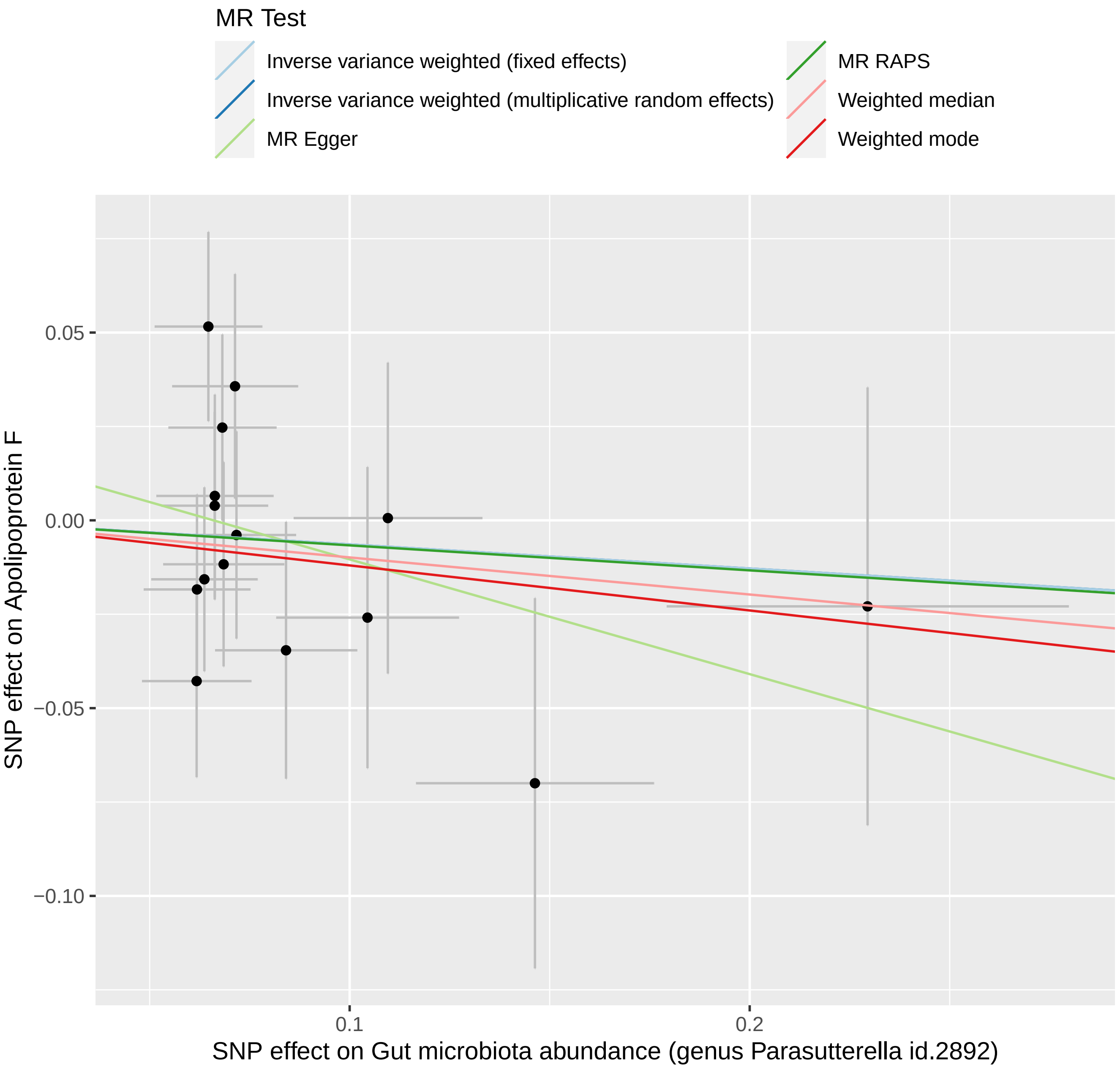

Batch 2−267 : Gut microbiota abundance (genus Catenibacterium id.2153) on Apolipoprotein L1
with a threshold of 1e−05

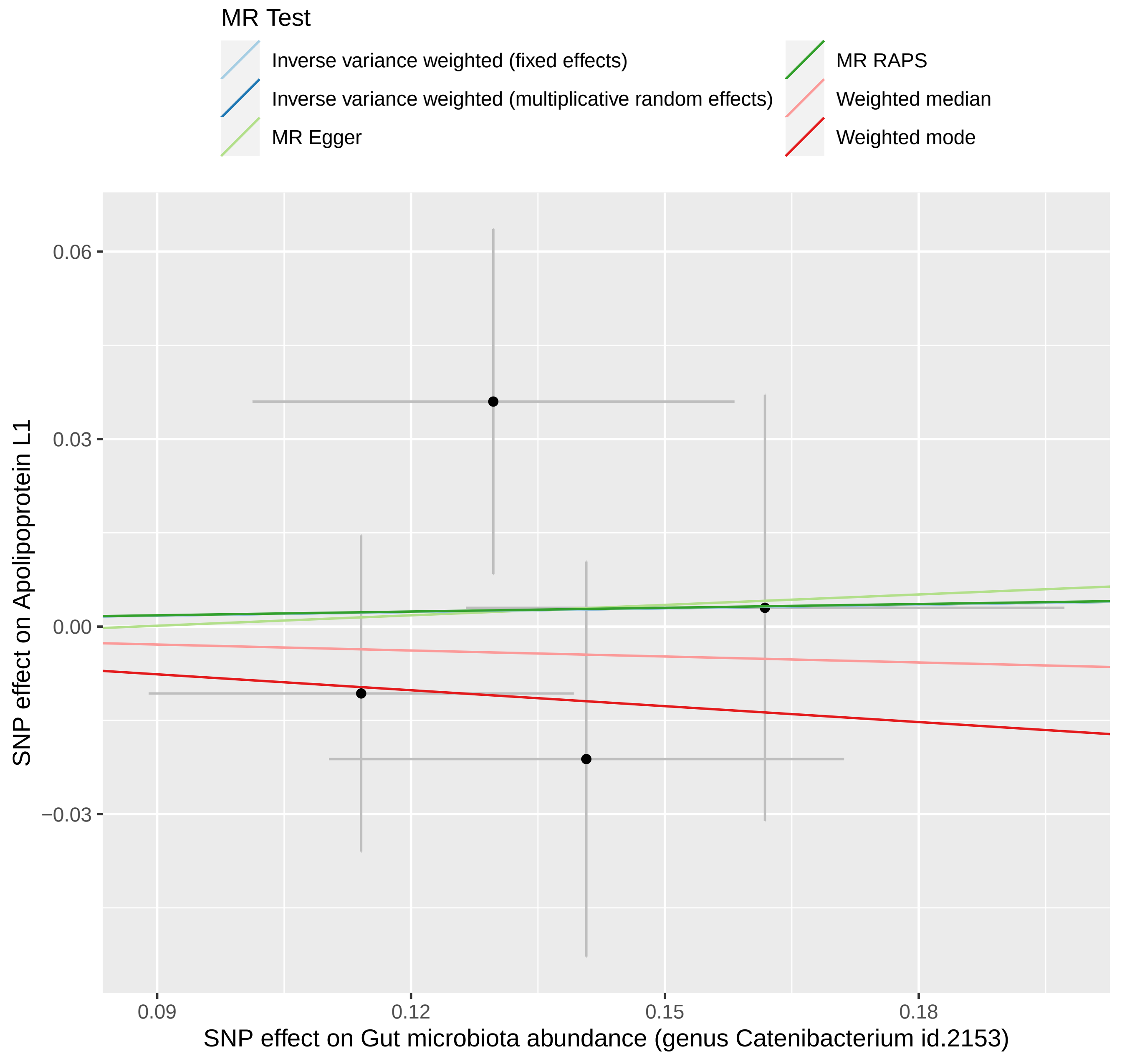

Batch 2−268 : Gut microbiota abundance (genus Parasutterella id.2892) on Apolipoprotein L1
with a threshold of 1e−05

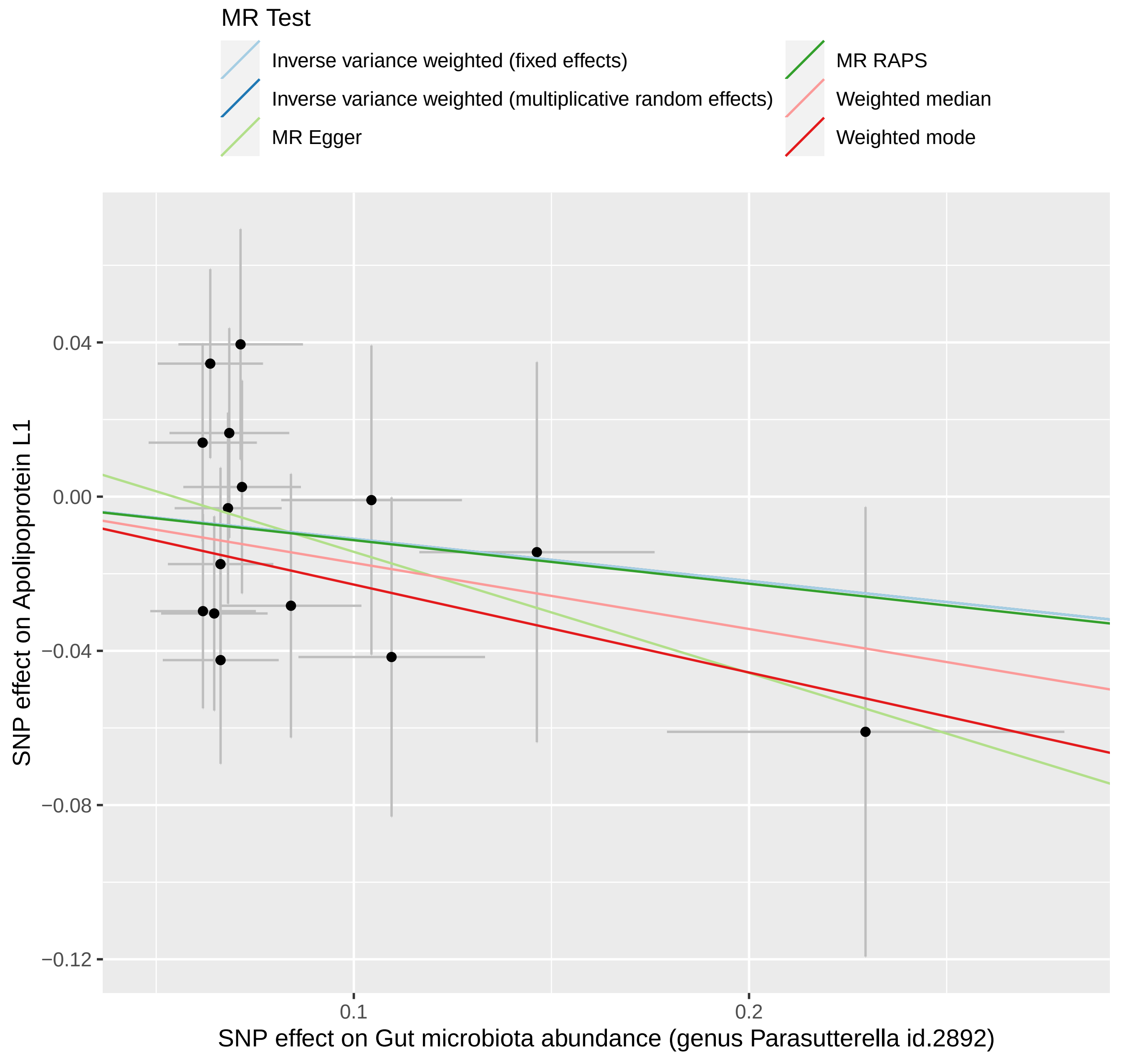

Batch 2−269 : Gut microbiota abundance (genus Catenibacterium id.2153) on Apolipoprotein L1
with a threshold of 1e−05

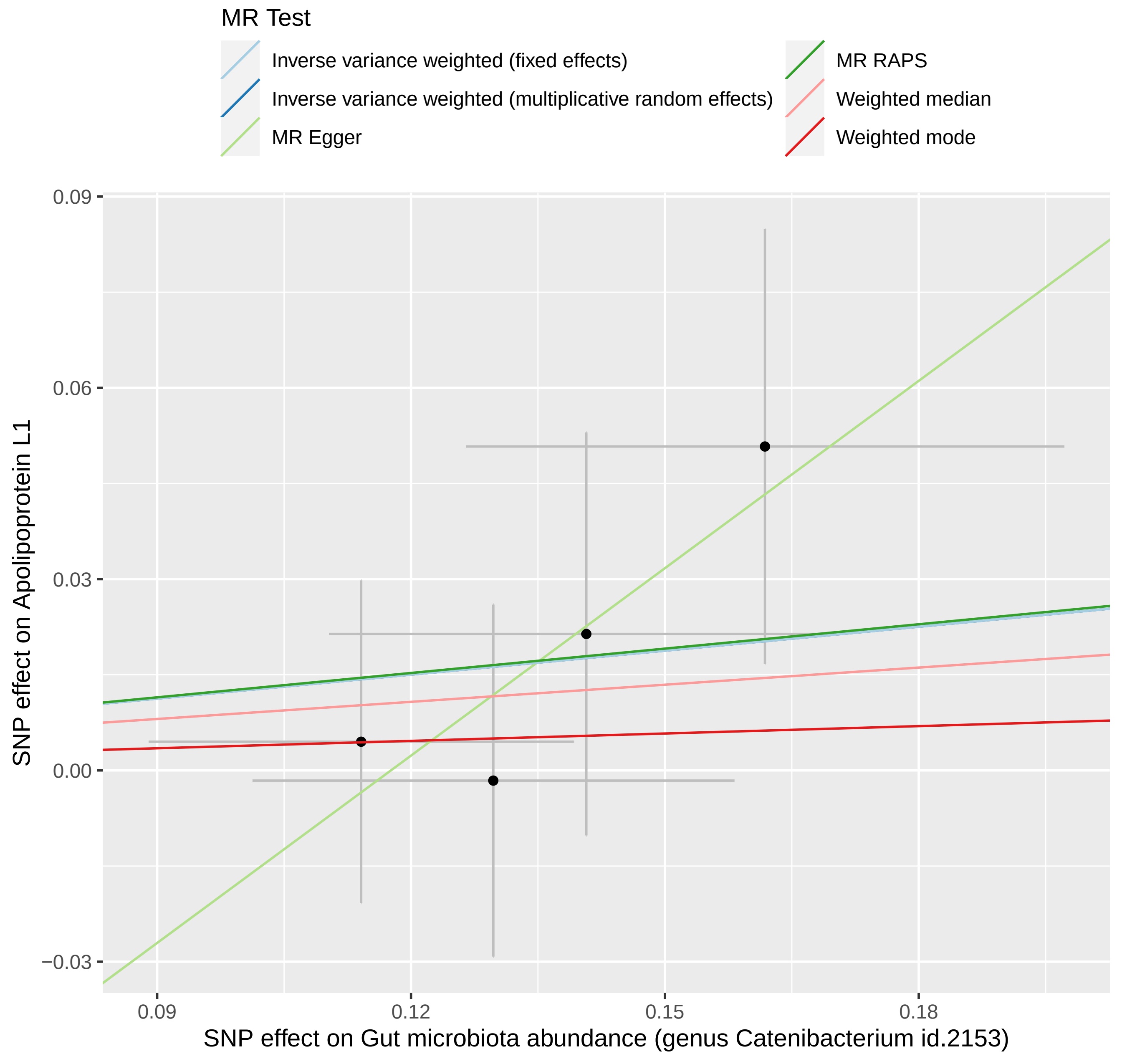

Batch 2−270 : Gut microbiota abundance (genus Parasutterella id.2892) on Apolipoprotein L1
with a threshold of 1e−05

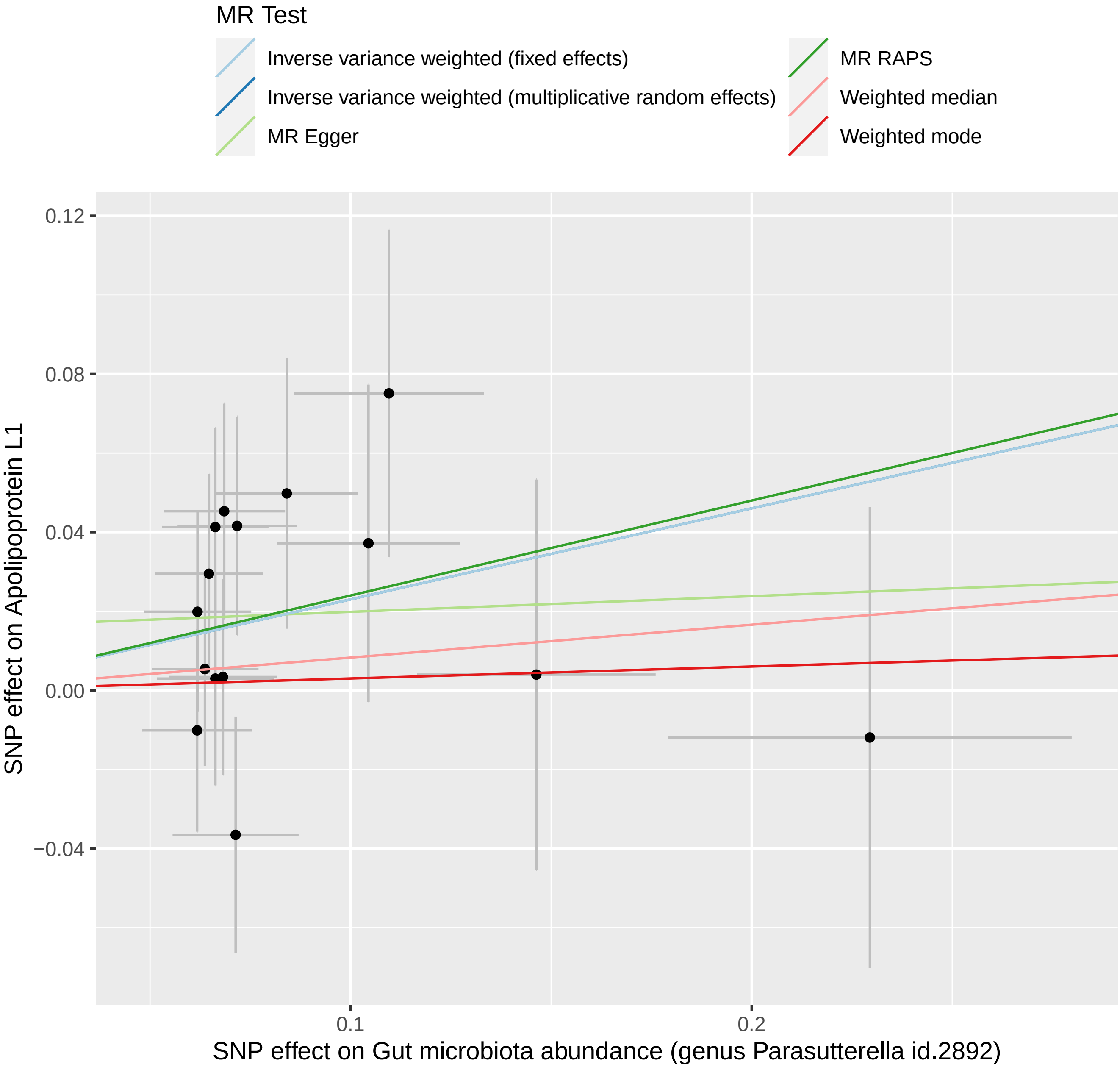

Batch 2−271 : Gut microbiota abundance (genus Catenibacterium id.2153) on Apolipoprotein M
with a threshold of 1e−05

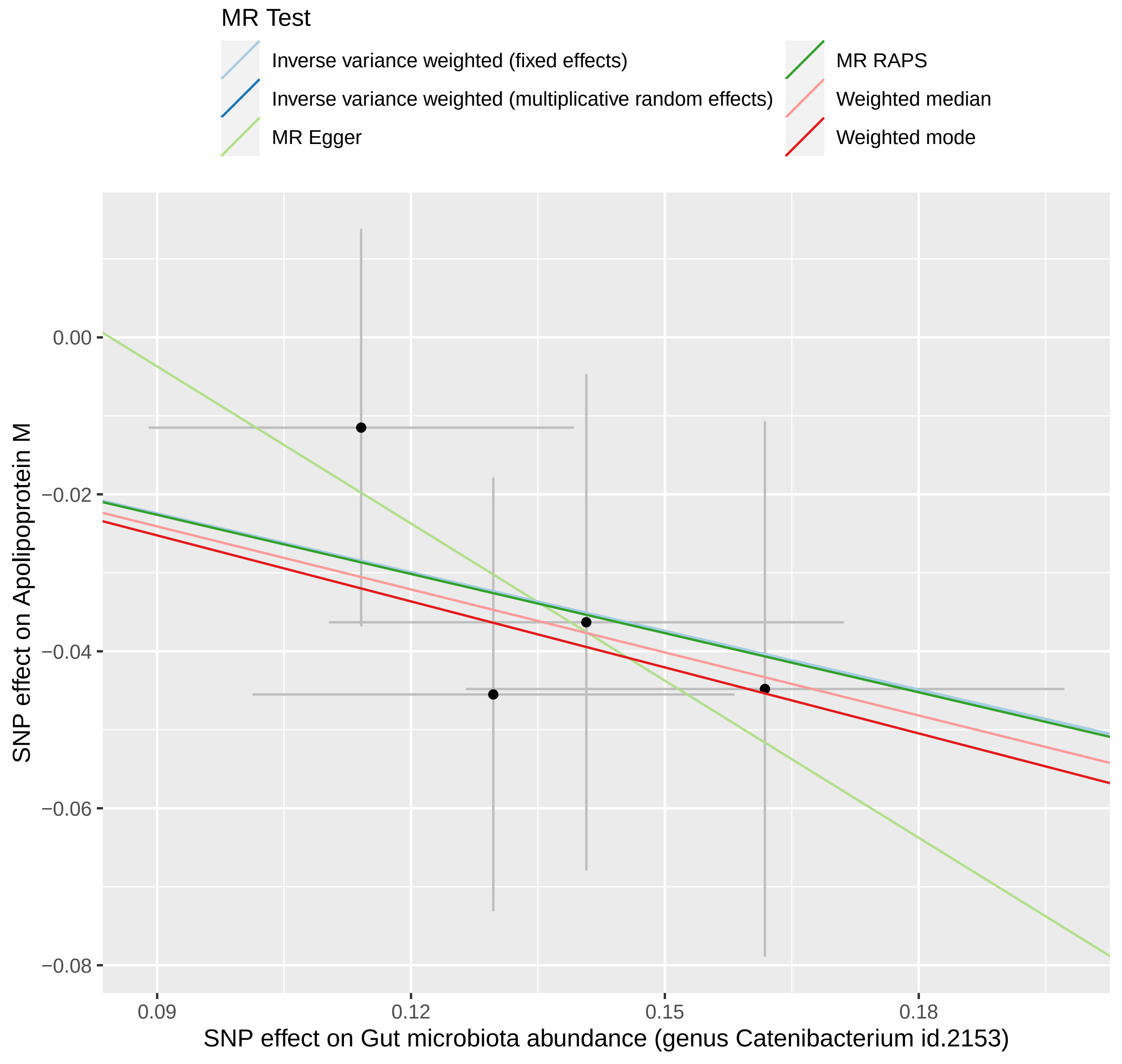

Batch 2−272 : Gut microbiota abundance (genus Parasutterella id.2892) on Apolipoprotein M
with a threshold of 1e−05

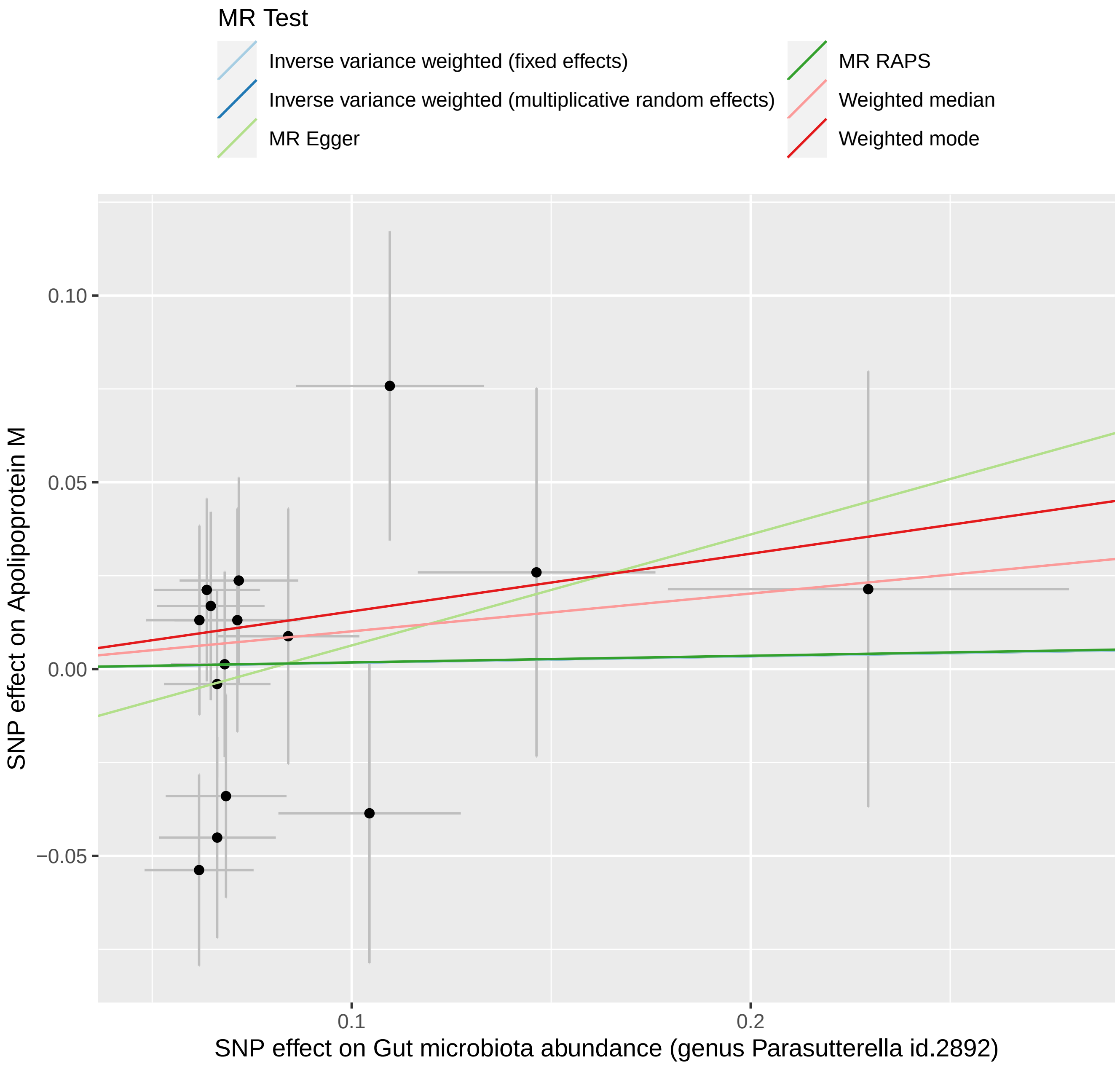

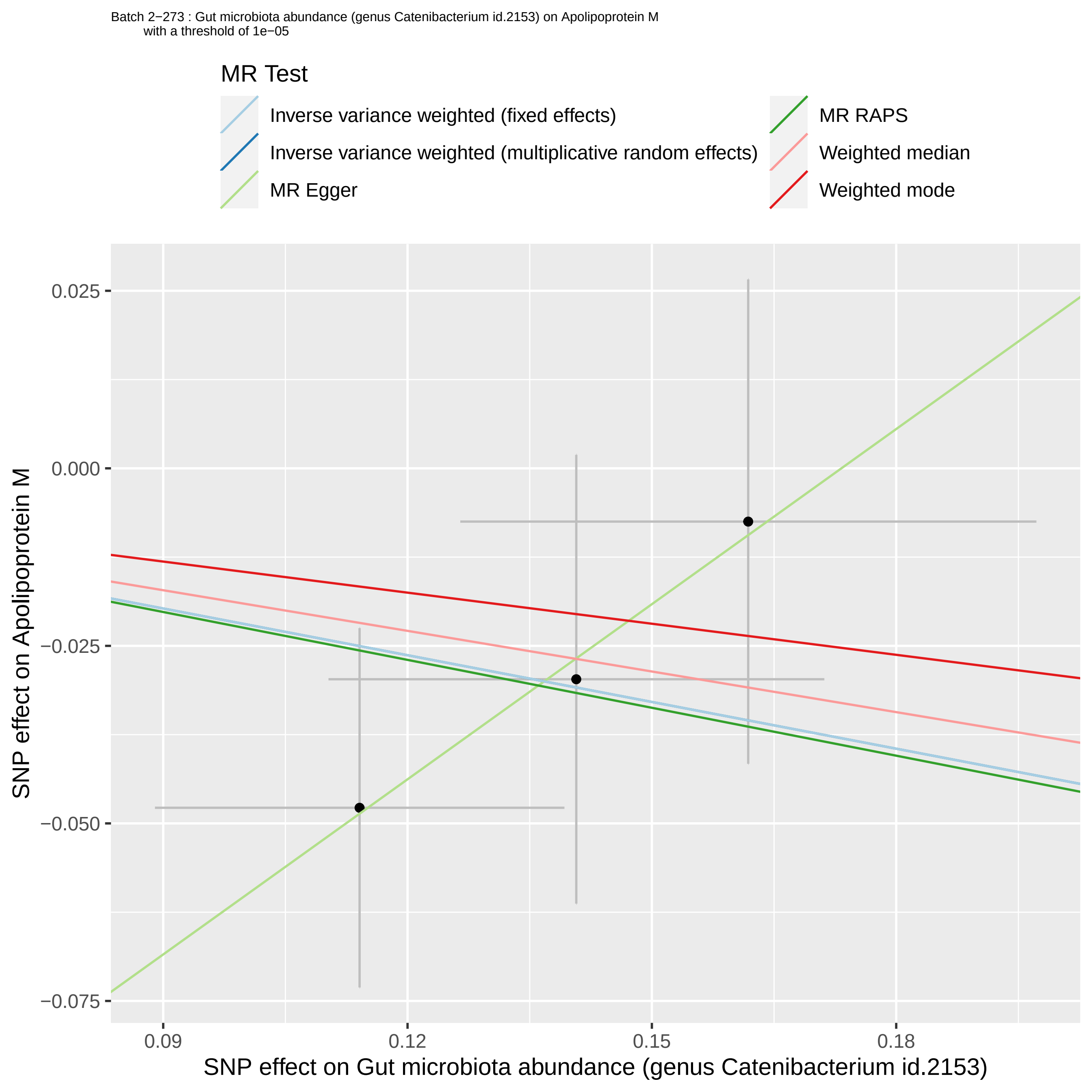

Batch 2−273 : Gut microbiota abundance (genus Catenibacterium id.2153) on Apolipoprotein M
with a threshold of 1e−05
MR Test
Inverse variance weighted (fixed effects)
Inverse variance weighted (multiplicative random effects)
MR Egger
MR RAPS
Weighted median
Weighted mode
0.025
0.000
−0.025
−0.050
−0.075
0.09
0.12
0.15
0.18
SNP effect on Apolipoprotein M
SNP effect on Gut microbiota abundance (genus Catenibacterium id.2153)

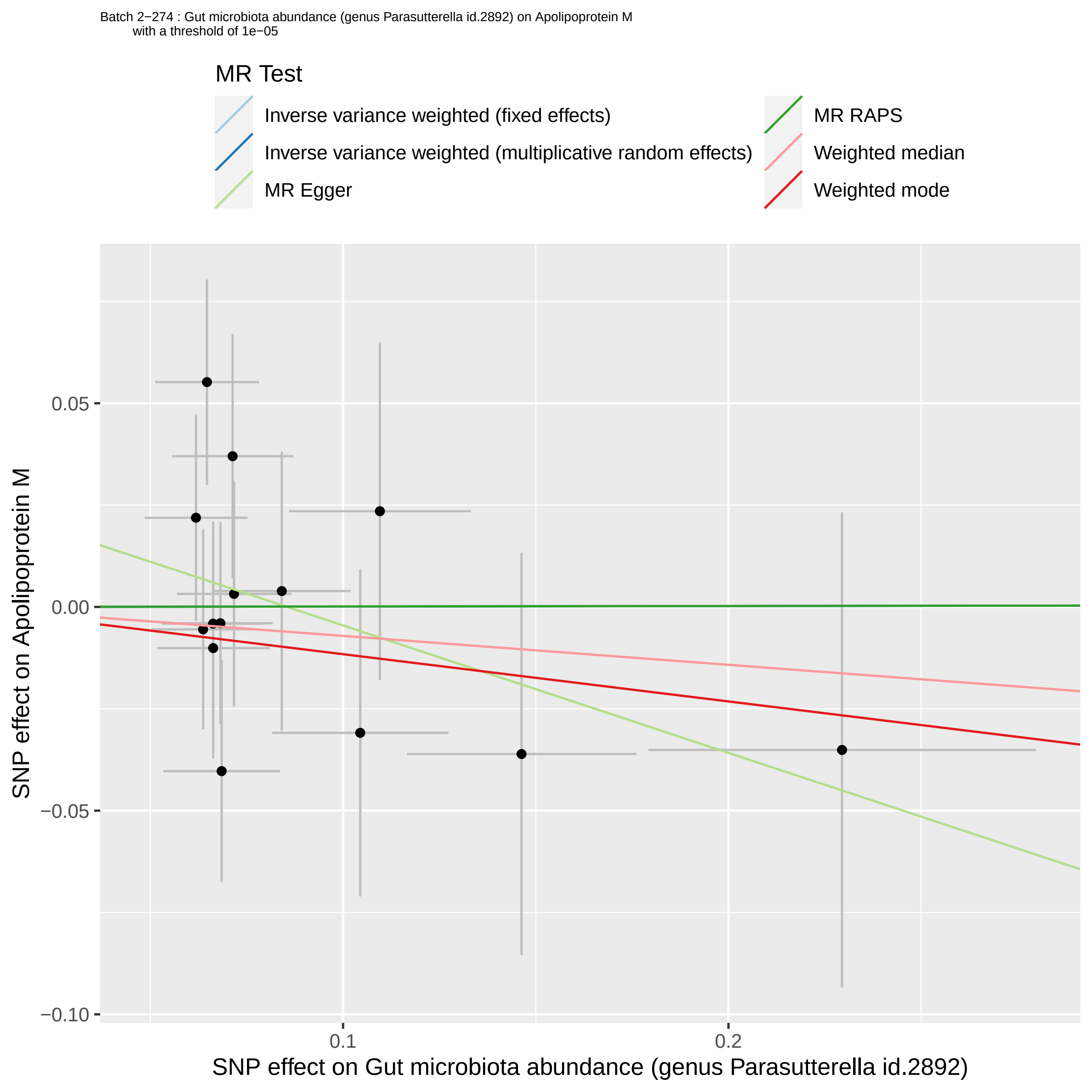

Batch 2−274 : Gut microbiota abundance (genus Parasutterella id.2892) on Apolipoprotein M
with a threshold of 1e−05
MR Test
Inverse variance weighted (fixed effects)
Inverse variance weighted (multiplicative random effects)
MR Egger
MR RAPS
Weighted median
Weighted mode
0.05
0.00
−0.05
−0.10
0.1
0.2
SNP effect on Apolipoprotein M
SNP effect on Gut microbiota abundance (genus Parasutterella id.2892)

Batch 2−275 : Gut microbiota abundance (genus Catenibacterium id.2153) on MICOS complex subunit MIC26
with a threshold of 1e−05

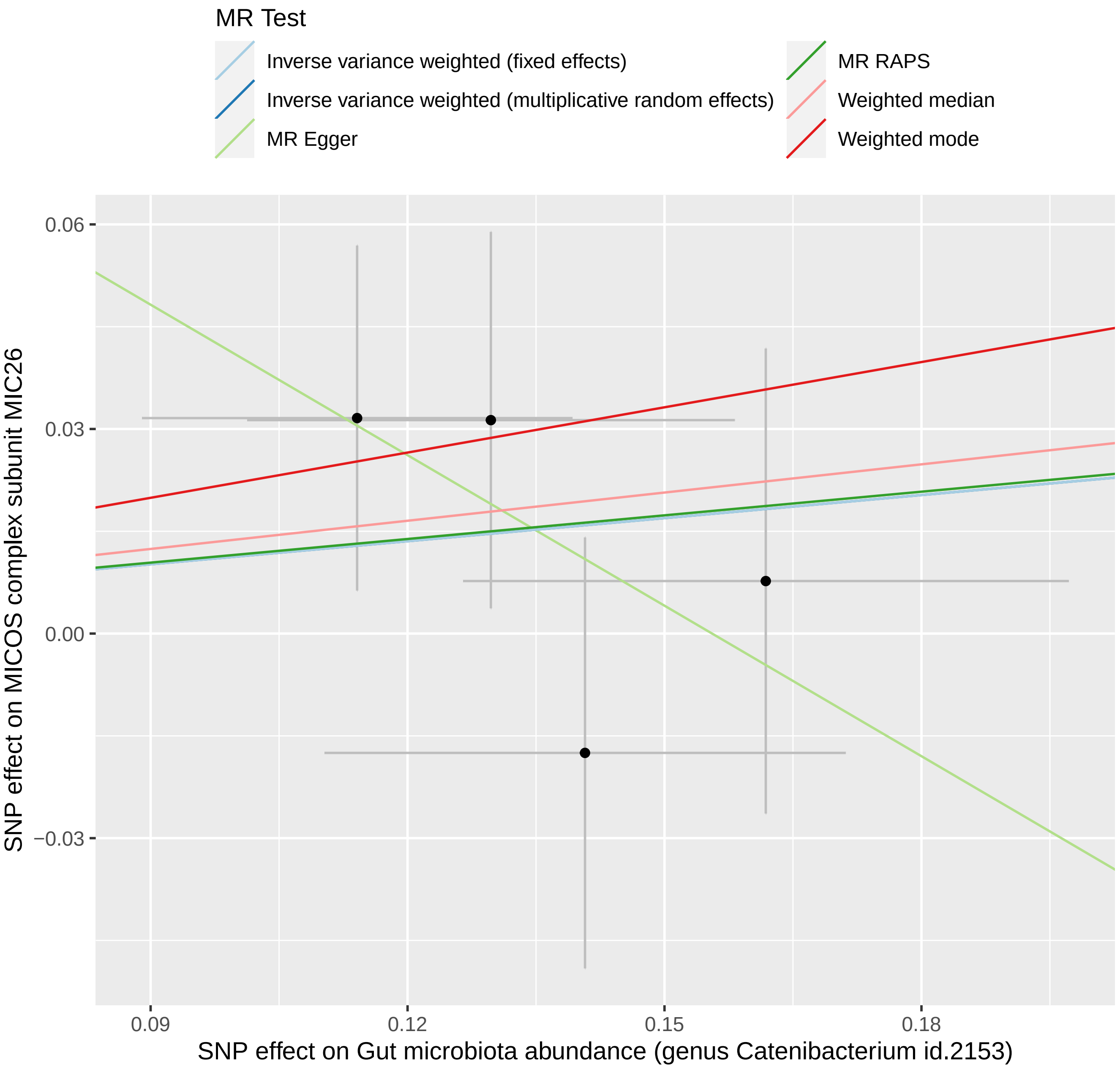

Batch 2−276 : Gut microbiota abundance (genus Parasutterella id.2892) on MICOS complex subunit MIC26
with a threshold of 1e−05

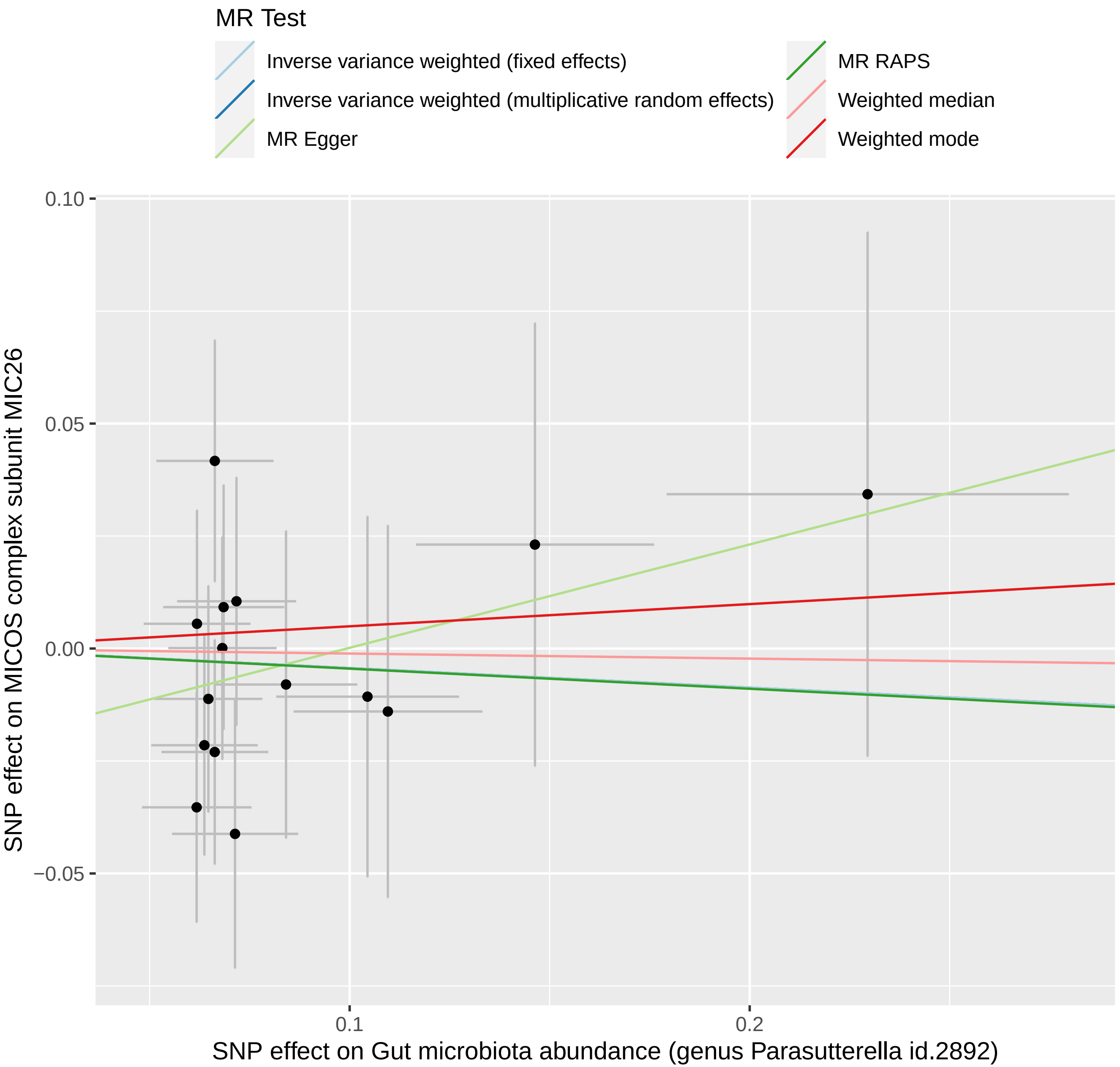

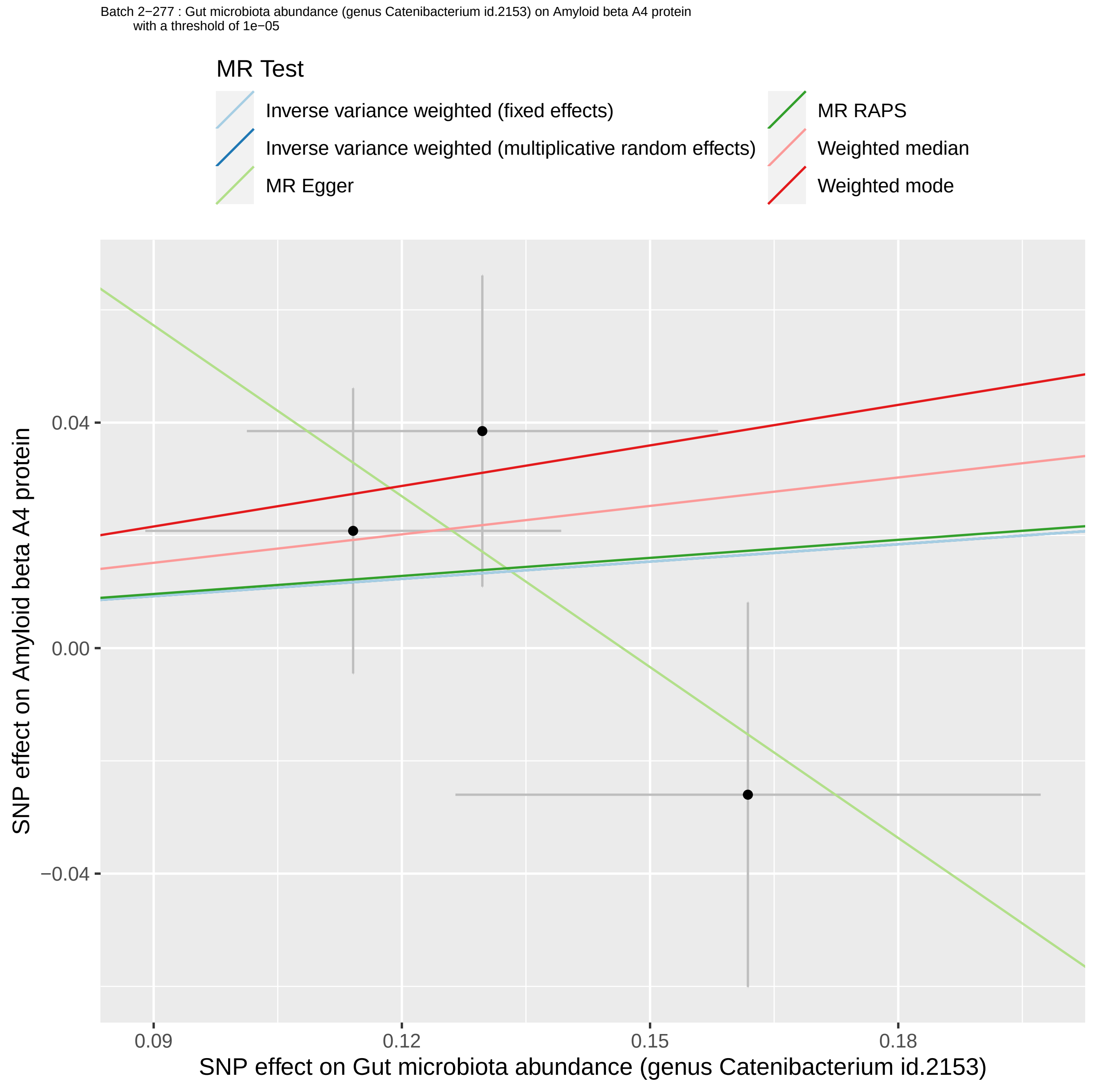

Batch 2−277 : Gut microbiota abundance (genus Catenibacterium id.2153) on Amyloid beta A4 protein
with a threshold of 1e−05
MR Test
Inverse variance weighted (fixed effects)
Inverse variance weighted (multiplicative random effects)
MR Egger
MR RAPS
Weighted median
Weighted mode
0.04
0.00
−0.04
0.09
0.12
0.15
0.18
SNP effect on Amyloid beta A4 protein
SNP effect on Gut microbiota abundance (genus Catenibacterium id.2153)

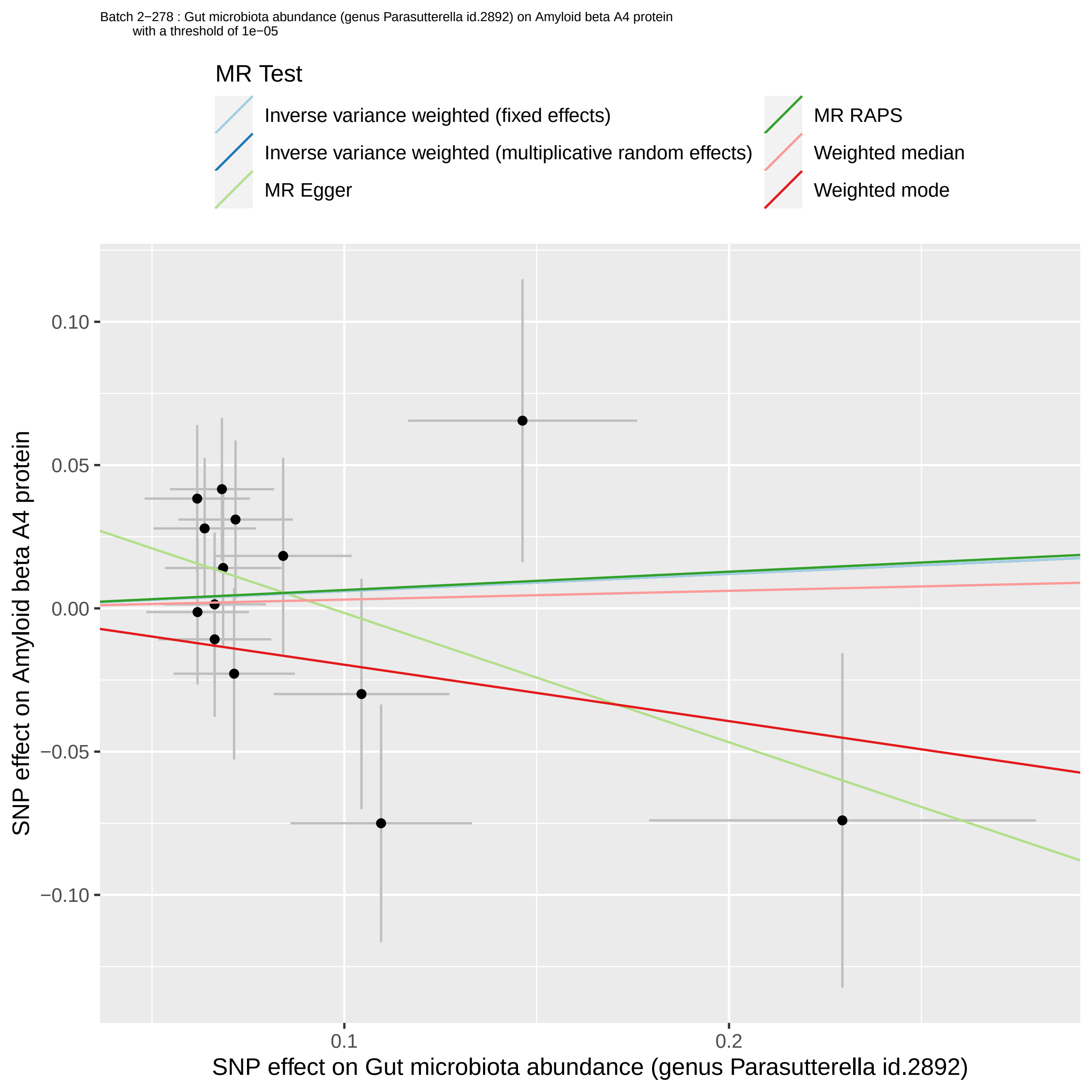
Batch 2−278 : Gut microbiota abundance (genus Parasutterella id.2892) on Amyloid beta A4 protein
with a threshold of 1e−05
MR Test
Inverse variance weighted (fixed effects)
Inverse variance weighted (multiplicative random effects)
MR Egger
MR RAPS
Weighted median
Weighted mode
0.10
0.05
0.00
−0.05
−0.10
0.1
0.2
SNP effect on Amyloid beta A4 protein
SNP effect on Gut microbiota abundance (genus Parasutterella id.2892)

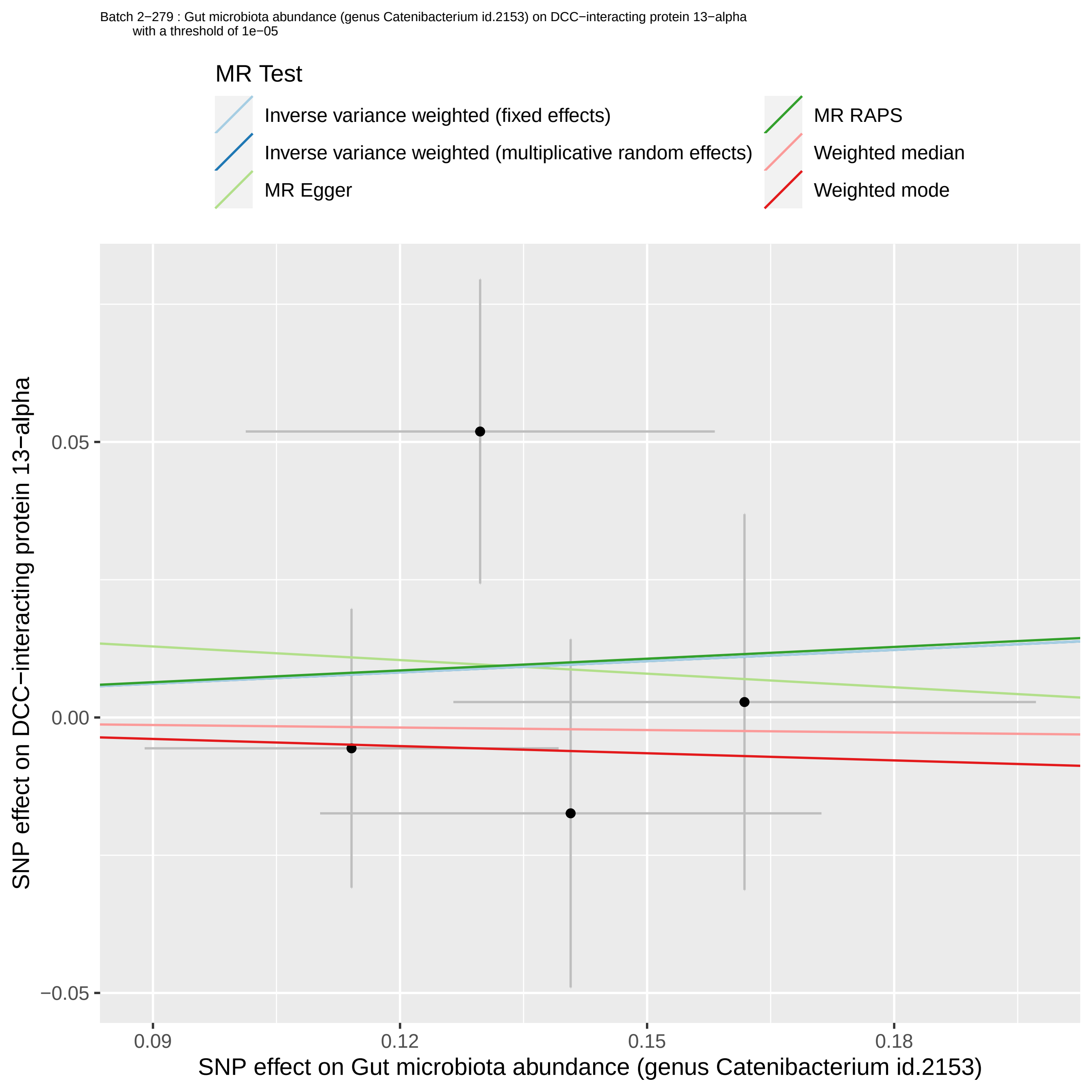

Batch 2−279 : Gut microbiota abundance (genus Catenibacterium id.2153) on DCC−interacting protein 13−alpha
with a threshold of 1e−05
MR Test
Inverse variance weighted (fixed effects)
Inverse variance weighted (multiplicative random effects)
MR Egger
MR RAPS
Weighted median
Weighted mode
0.05
0.00
−0.05
0.09
0.12
0.15
0.18
SNP effect on DCC−interacting protein 13−alpha
SNP effect on Gut microbiota abundance (genus Catenibacterium id.2153)

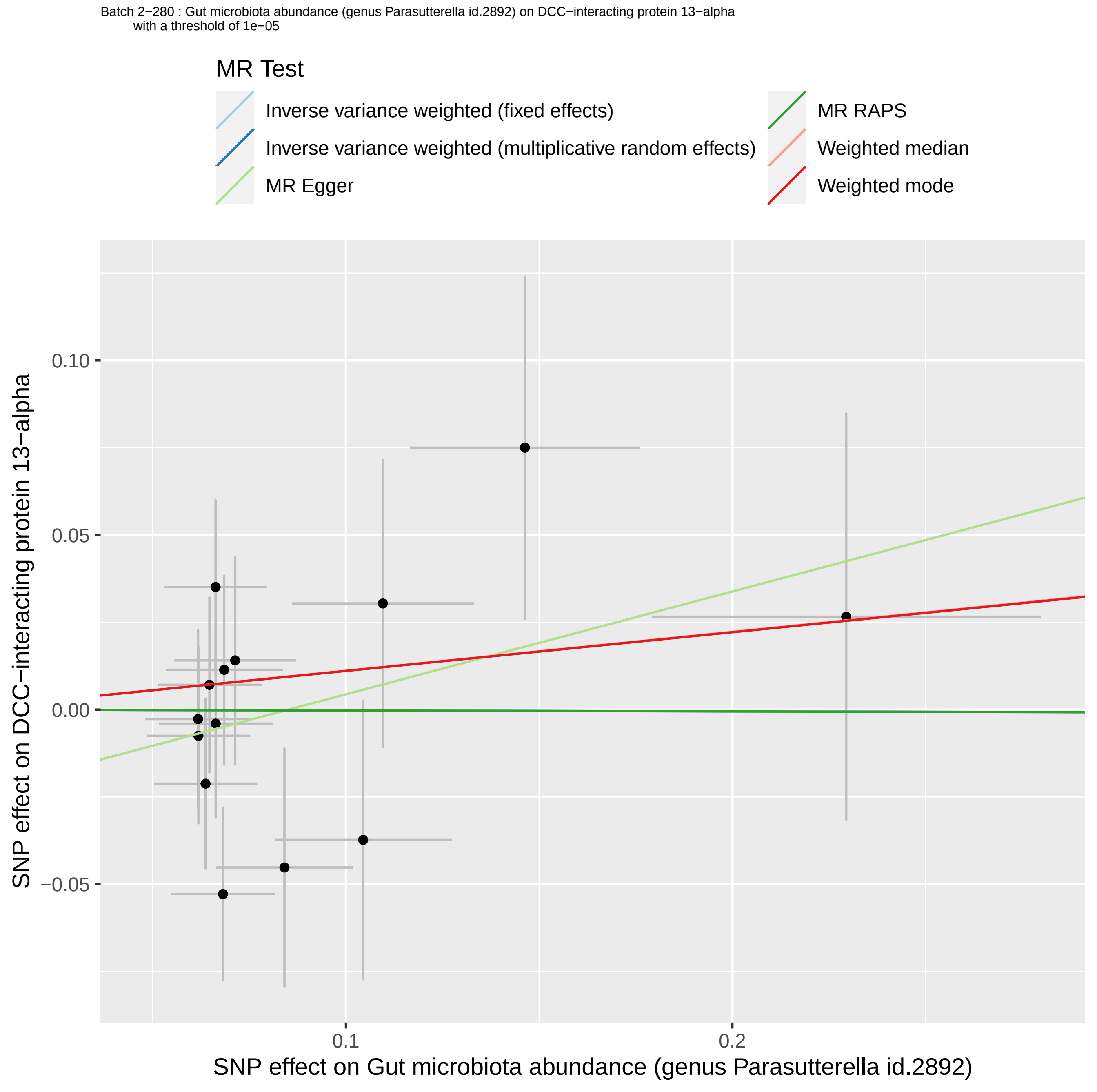

Batch 2−280 : Gut microbiota abundance (genus Parasutterella id.2892) on DCC−interacting protein 13−alpha
with a threshold of 1e−05
MR Test
Inverse variance weighted (fixed effects)
Inverse variance weighted (multiplicative random effects)
MR Egger
MR RAPS
Weighted median
Weighted mode
0.10
0.05
0.00
−0.05
0.1
0.2
SNP effect on DCC−interacting protein 13−alpha
SNP effect on Gut microbiota abundance (genus Parasutterella id.2892)

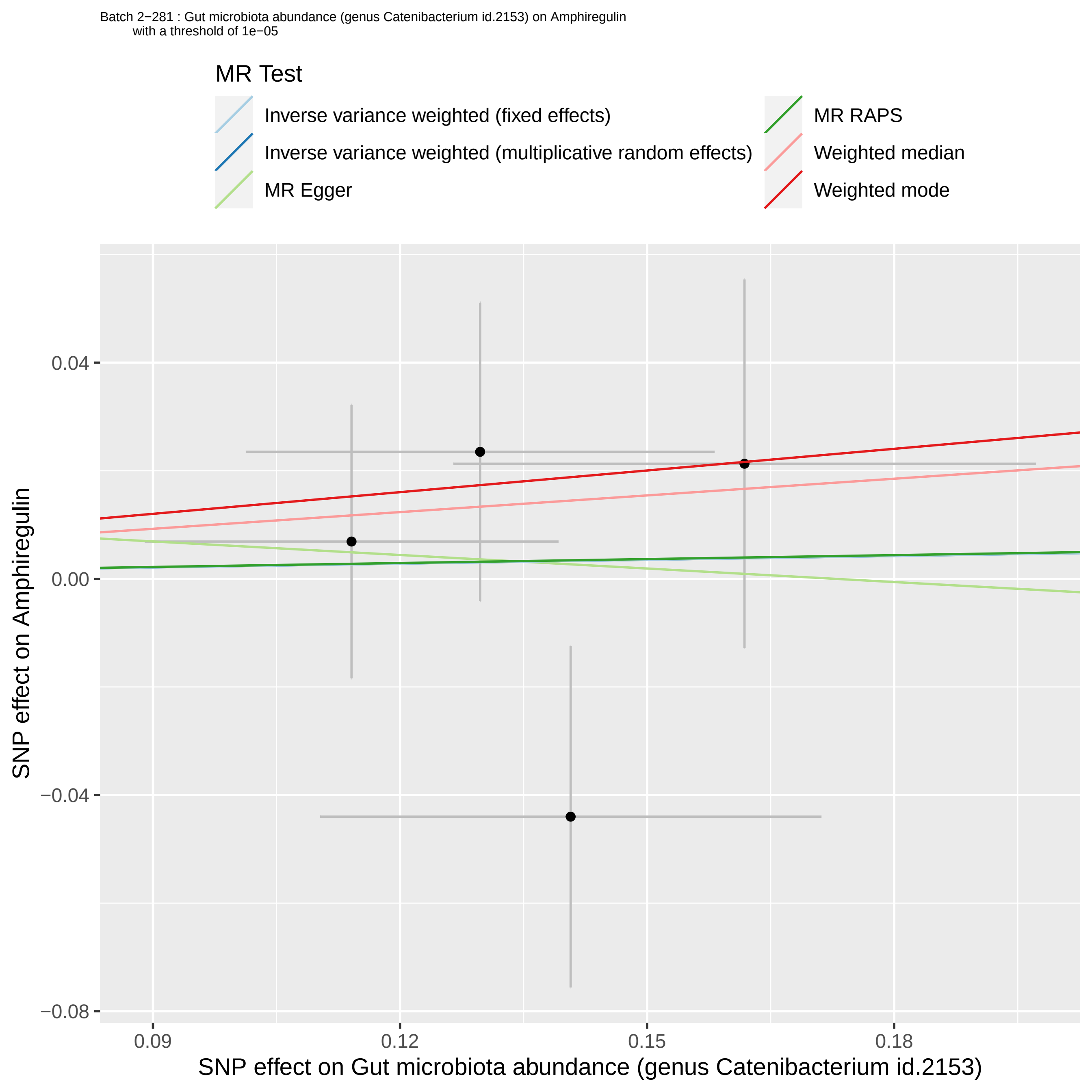
Batch 2−281 : Gut microbiota abundance (genus Catenibacterium id.2153) on Amphiregulin
with a threshold of 1e−05
MR Test
Inverse variance weighted (fixed effects)
Inverse variance weighted (multiplicative random effects)
MR Egger
MR RAPS
Weighted median
Weighted mode
0.04
0.00
−0.04
−0.08
0.09
0.12
0.15
0.18
SNP effect on Amphiregulin
SNP effect on Gut microbiota abundance (genus Catenibacterium id.2153)

Batch 2−282 : Gut microbiota abundance (genus Parasutterella id.2892) on Amphiregulin
with a threshold of 1e−05

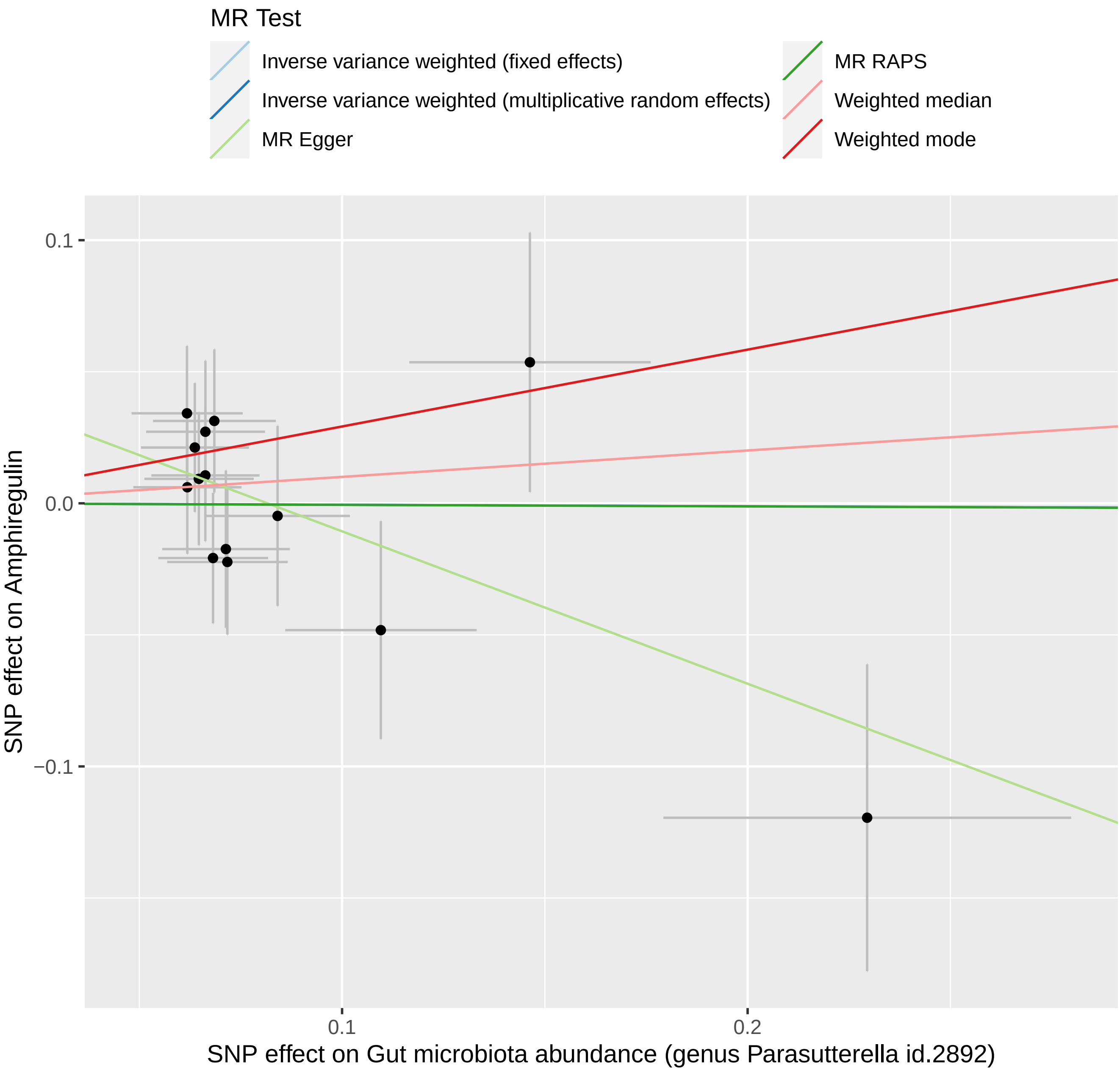

Batch 2−283 : Gut microbiota abundance (genus Catenibacterium id.2153) on ADP−ribosylation factor 3
with a threshold of 1e−05

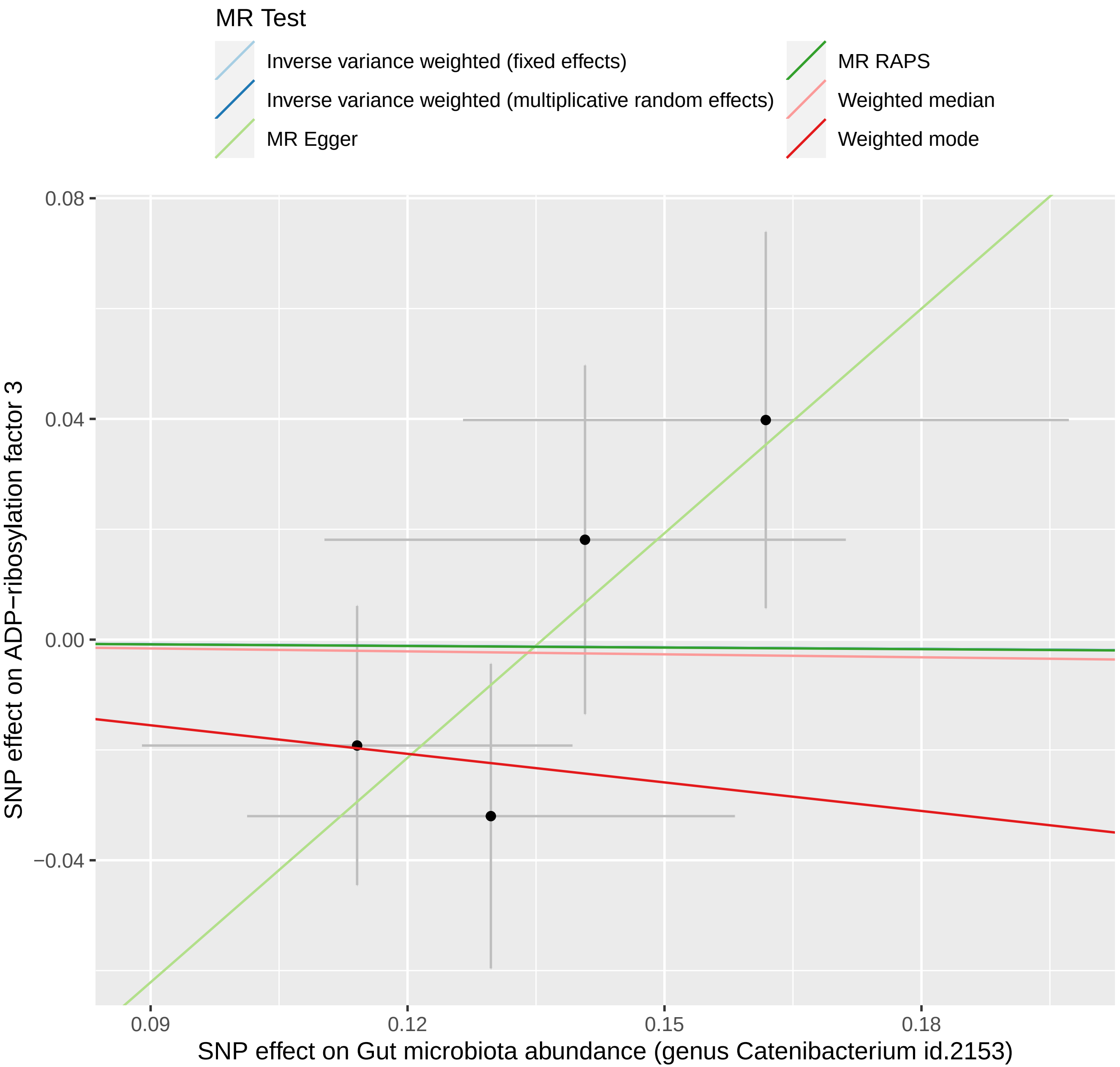

Batch 2−284 : Gut microbiota abundance (genus Parasutterella id.2892) on ADP−ribosylation factor 3
with a threshold of 1e−05

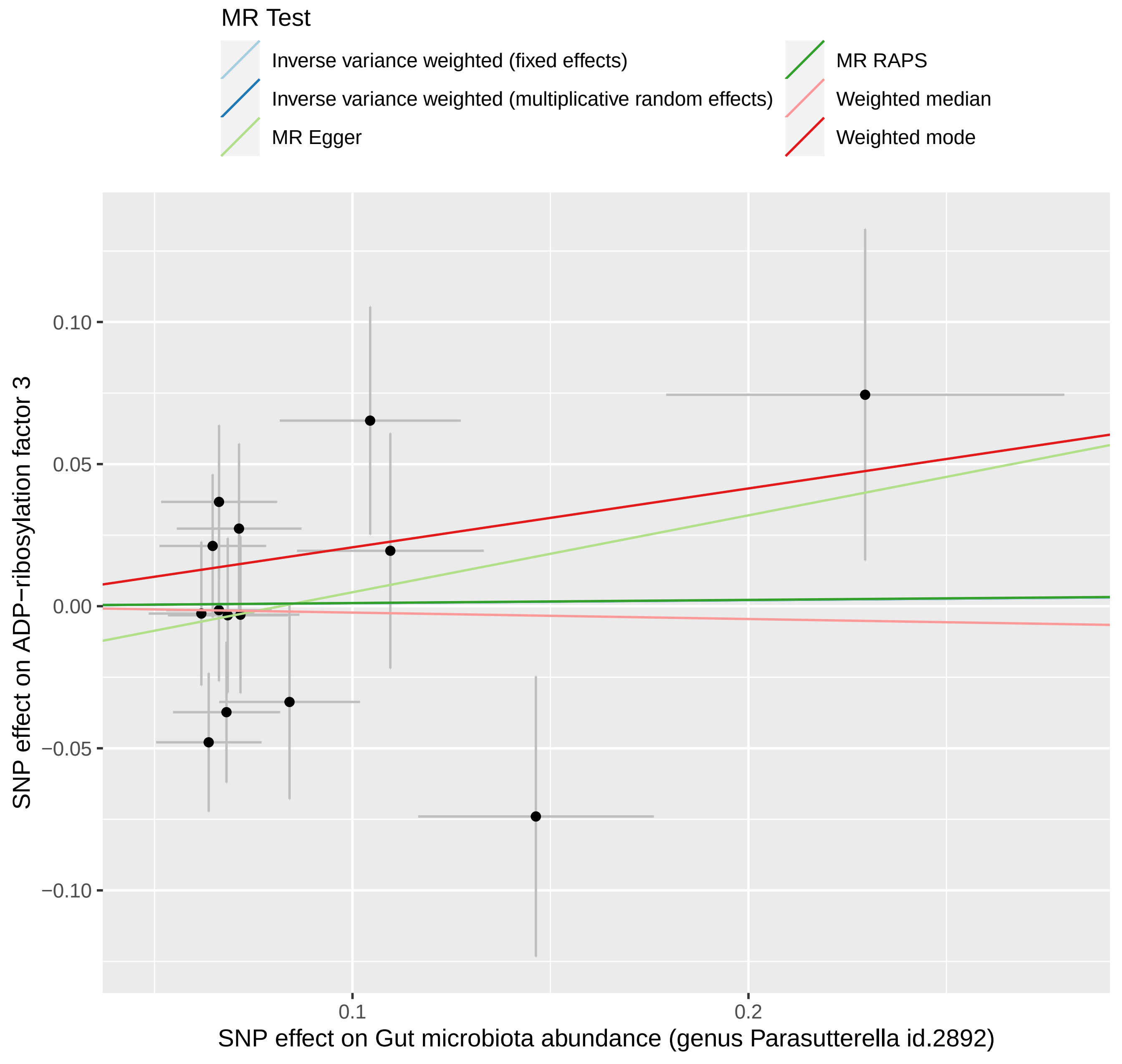

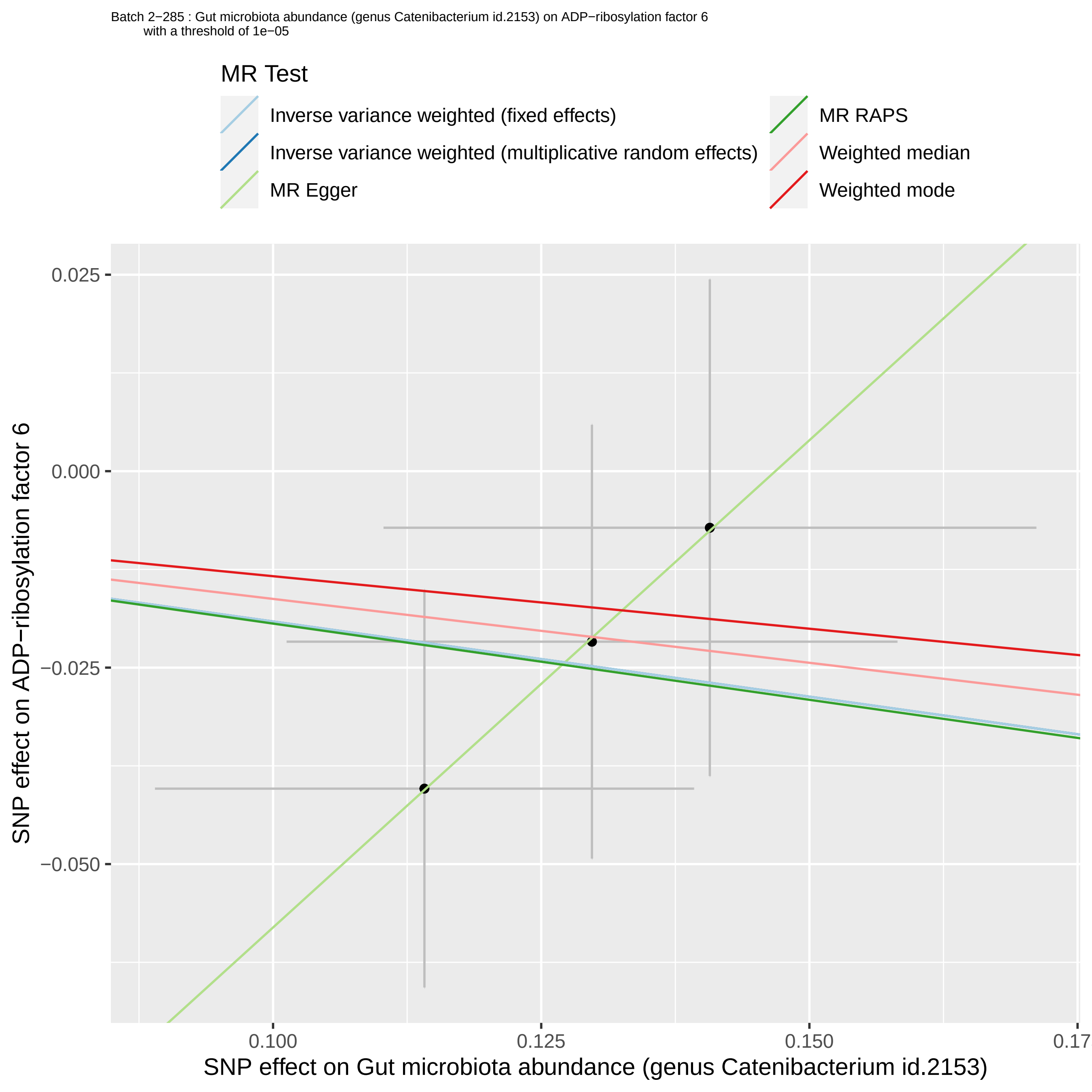

Batch 2−285 : Gut microbiota abundance (genus Catenibacterium id.2153) on ADP−ribosylation factor 6
with a threshold of 1e−05
MR Test
Inverse variance weighted (fixed effects)
Inverse variance weighted (multiplicative random effects)
MR Egger
MR RAPS
Weighted median
Weighted mode
0.025
0.000
−0.025
−0.050
SNP effect on ADP−ribosylation factor 6
0.100
0.125
0.150
0.17
SNP effect on Gut microbiota abundance (genus Catenibacterium id.2153)

Batch 2−286 : Gut microbiota abundance (genus Parasutterella id.2892) on ADP−ribosylation factor 6
with a threshold of 1e−05

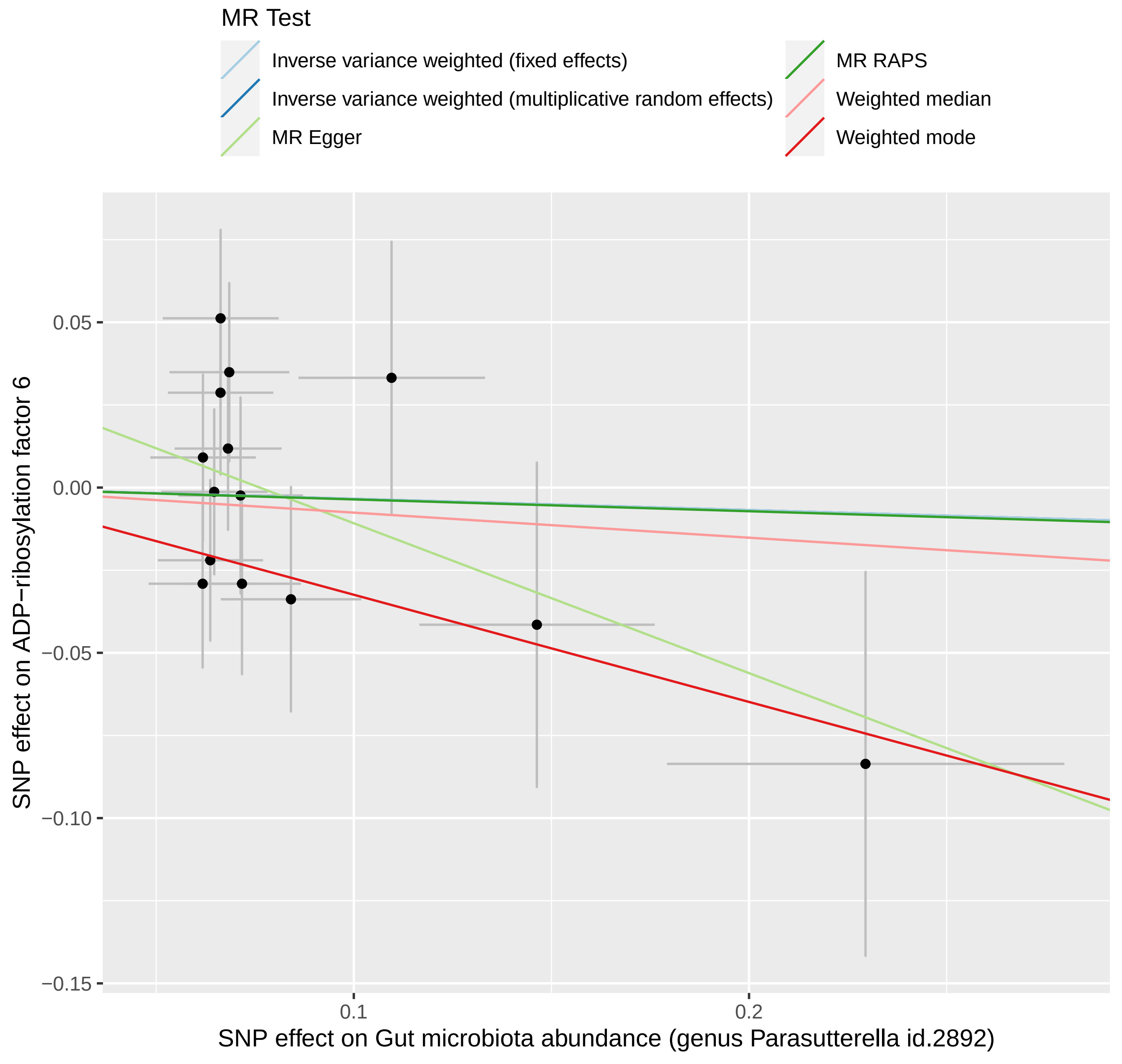

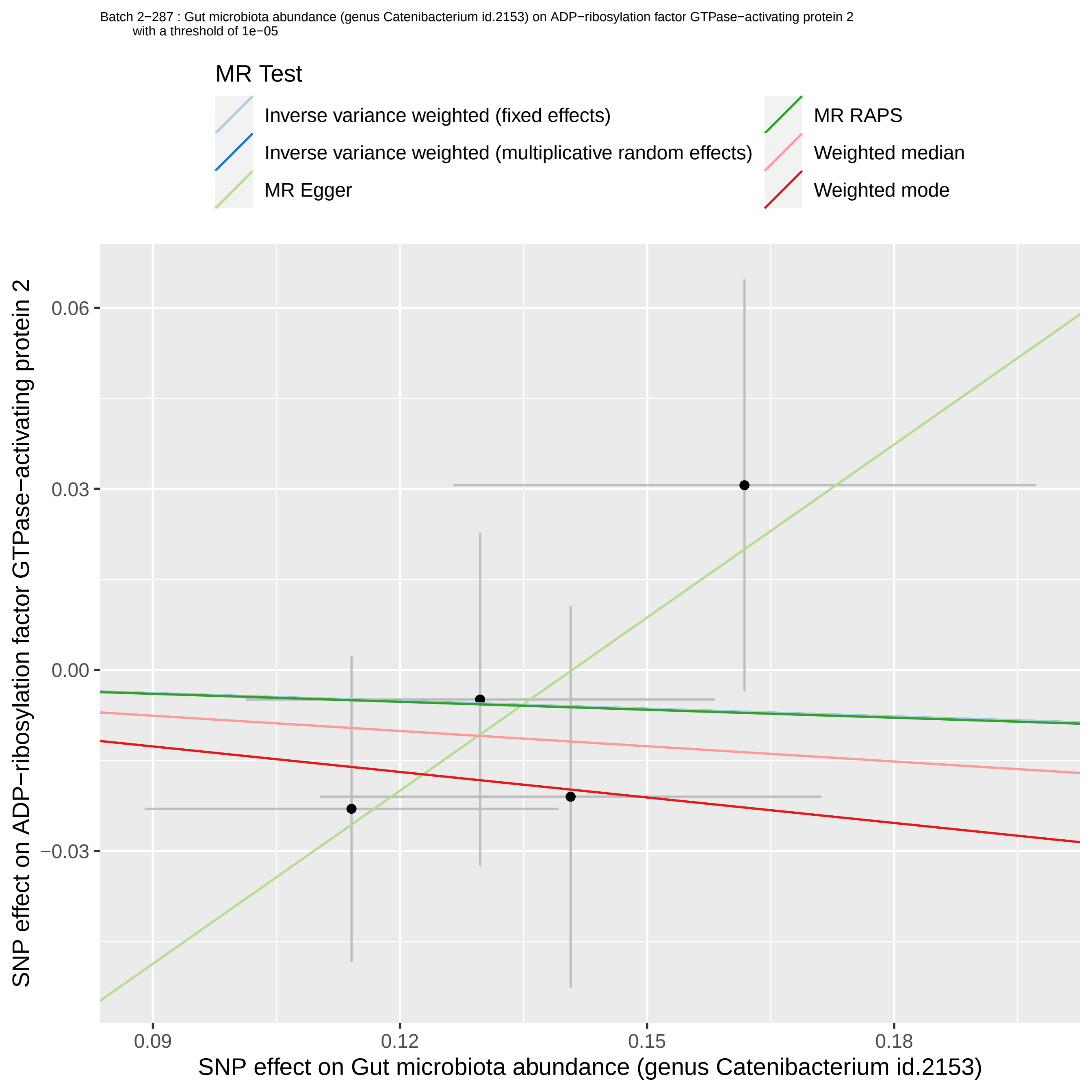

Batch 2−287 : Gut microbiota abundance (genus Catenibacterium id.2153) on ADP−ribosylation factor GTPase−activating protein 2
with a threshold of 1e−05
MR Test
Inverse variance weighted (fixed effects)
Inverse variance weighted (multiplicative random effects)
MR Egger
MR RAPS
Weighted median
Weighted mode
0.06
0.03
0.00
−0.03
0.09
0.12
0.15
0.18
SNP effect on ADP−ribosylation factor GTPase−activating protein 2
SNP effect on Gut microbiota abundance (genus Catenibacterium id.2153)

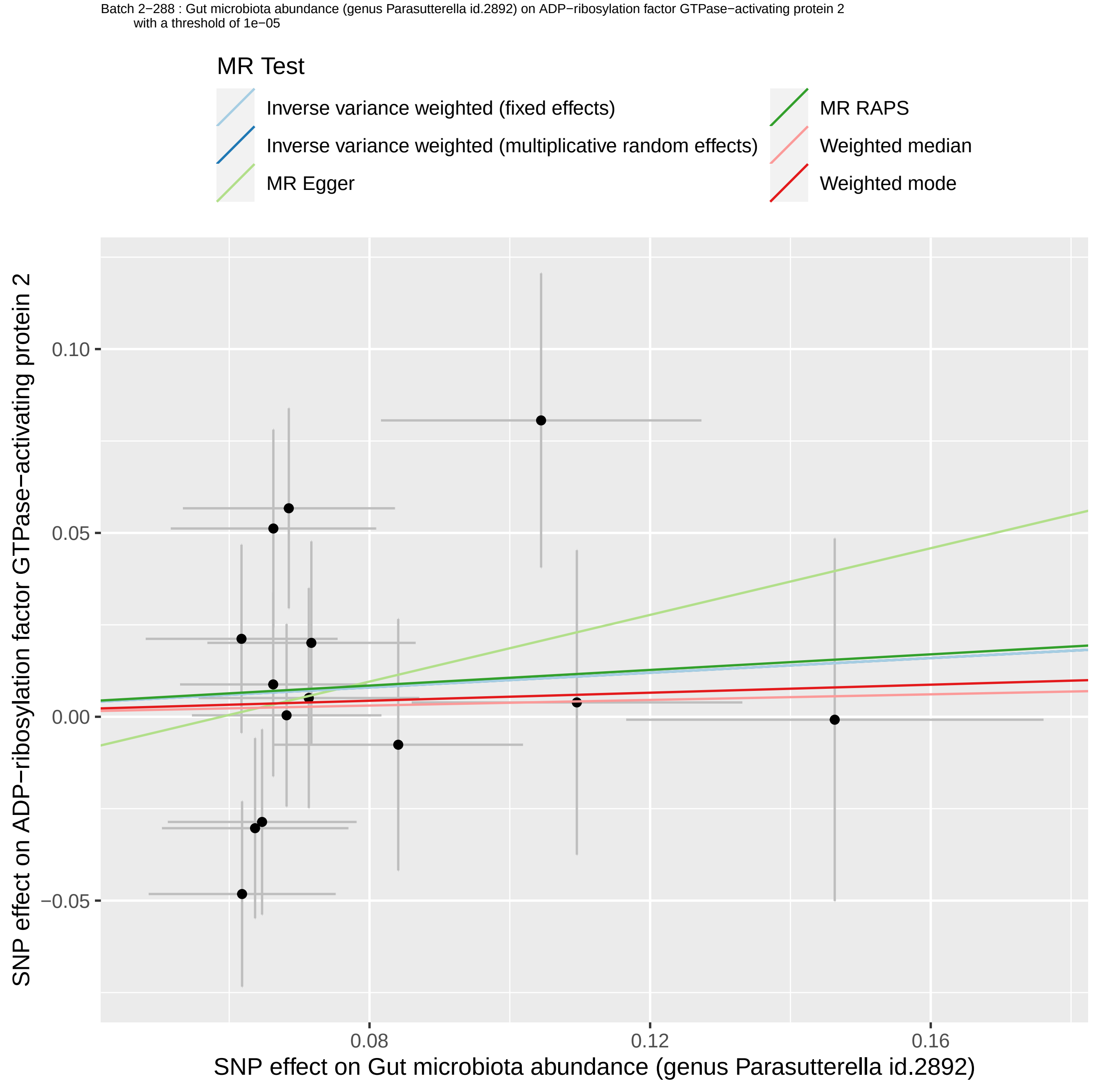

Batch 2−288 : Gut microbiota abundance (genus Parasutterella id.2892) on ADP−ribosylation factor GTPase−activating protein 2
with a threshold of 1e−05
MR Test
Inverse variance weighted (fixed effects)
Inverse variance weighted (multiplicative random effects)
MR Egger
MR RAPS
Weighted median
Weighted mode
0.10
0.05
0.00
−0.05
0.08
0.12
0.16
SNP effect on ADP−ribosylation factor GTPase−activating protein 2
SNP effect on Gut microbiota abundance (genus Parasutterella id.2892)

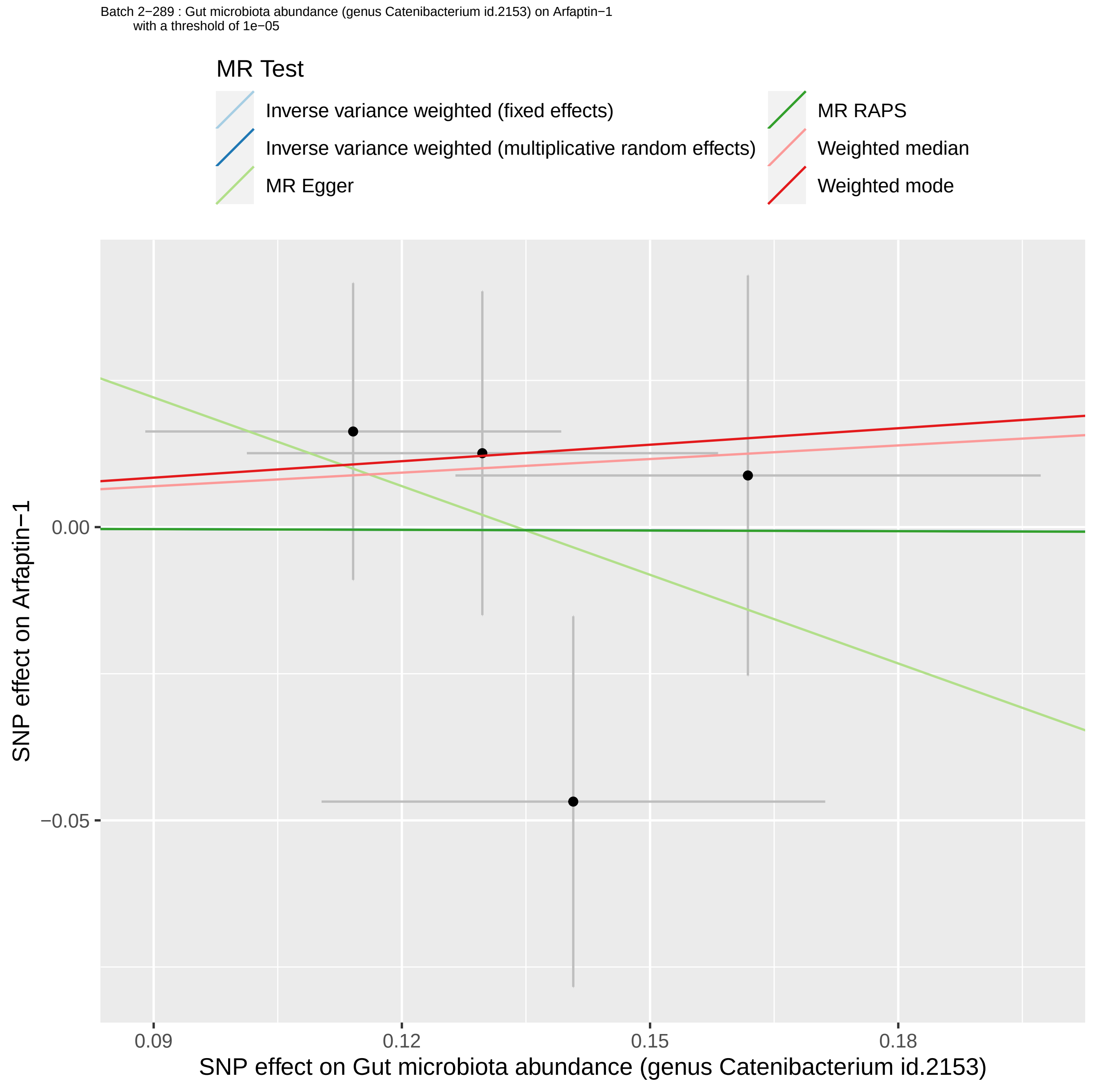
Batch 2−289 : Gut microbiota abundance (genus Catenibacterium id.2153) on Arfaptin−1
with a threshold of 1e−05
MR Test
Inverse variance weighted (fixed effects)
Inverse variance weighted (multiplicative random effects)
MR Egger
MR RAPS
Weighted median
Weighted mode
0.00
−0.05
0.09
0.12
0.15
0.18
SNP effect on Arfaptin−1
SNP effect on Gut microbiota abundance (genus Catenibacterium id.2153)

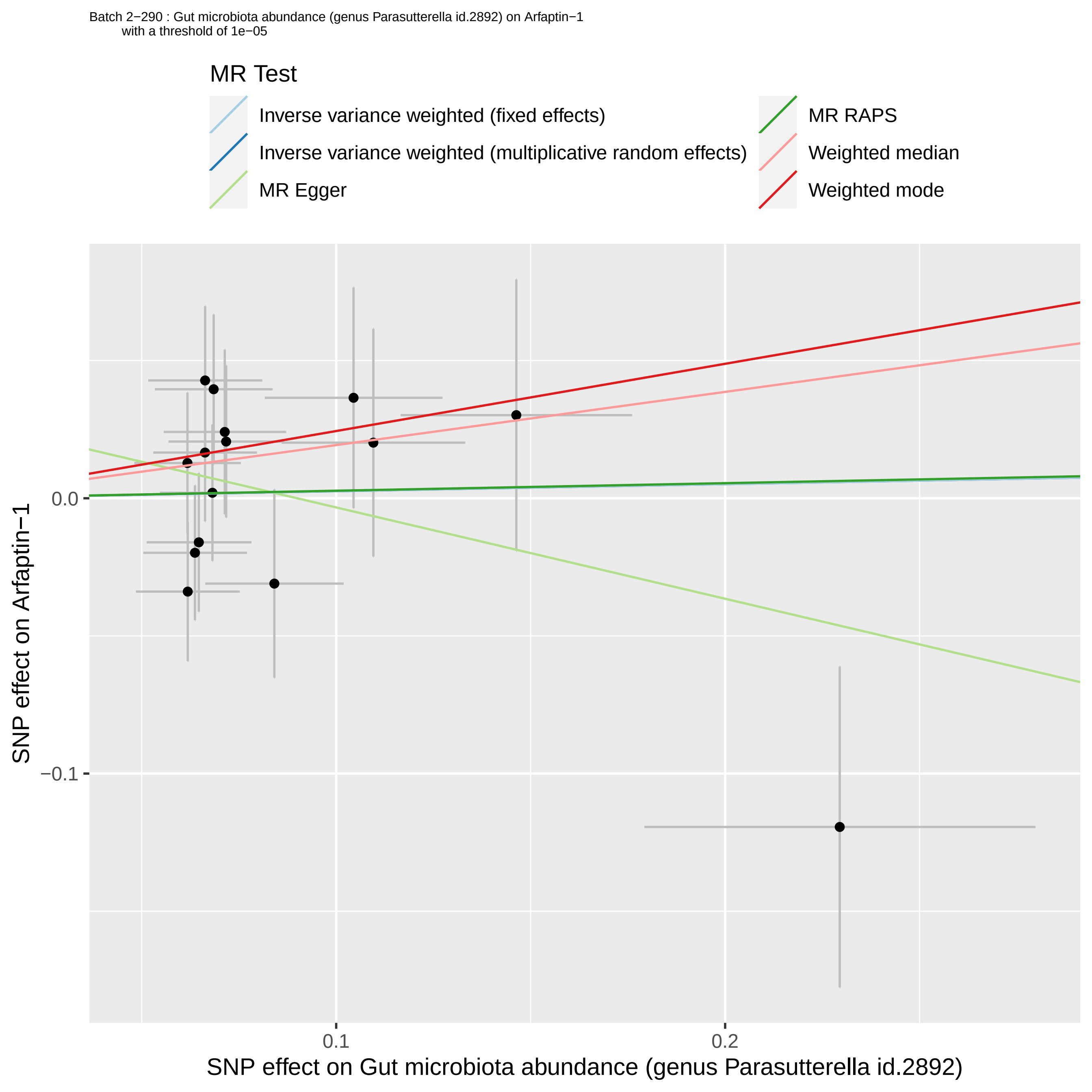
Batch 2−290 : Gut microbiota abundance (genus Parasutterella id.2892) on Arfaptin−1
with a threshold of 1e−05
MR Test
Inverse variance weighted (fixed effects)
Inverse variance weighted (multiplicative random effects)
MR Egger
MR RAPS
Weighted median
Weighted mode
0.0
−0.1
0.1
0.2
SNP effect on Arfaptin−1
SNP effect on Gut microbiota abundance (genus Parasutterella id.2892)

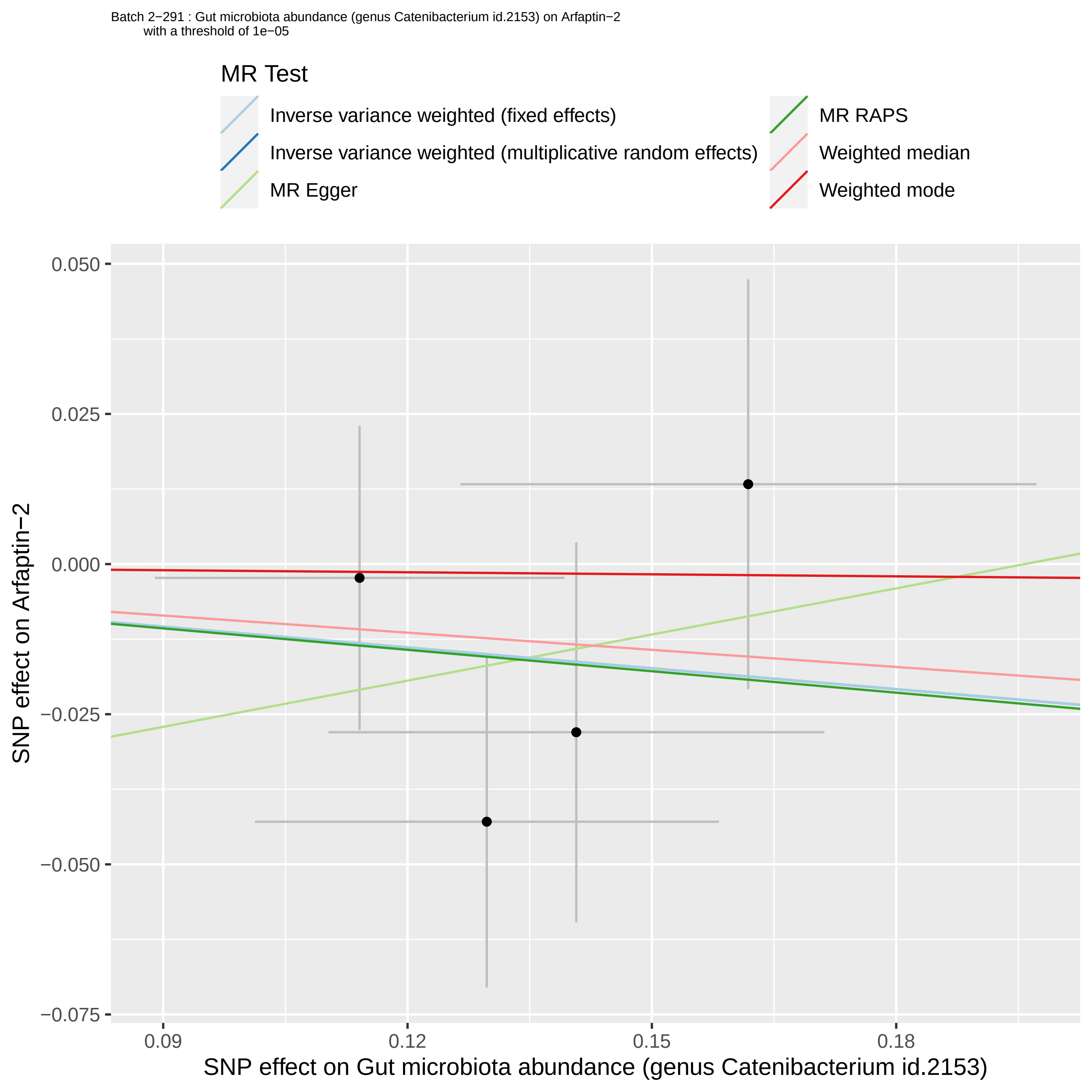
Batch 2−291 : Gut microbiota abundance (genus Catenibacterium id.2153) on Arfaptin−2
with a threshold of 1e−05
MR Test
Inverse variance weighted (fixed effects)
Inverse variance weighted (multiplicative random effects)
MR Egger
MR RAPS
Weighted median
Weighted mode
0.050
0.025
0.000
−0.025
−0.050
−0.075
0.09
0.12
0.15
0.18
SNP effect on Arfaptin−2
SNP effect on Gut microbiota abundance (genus Catenibacterium id.2153)

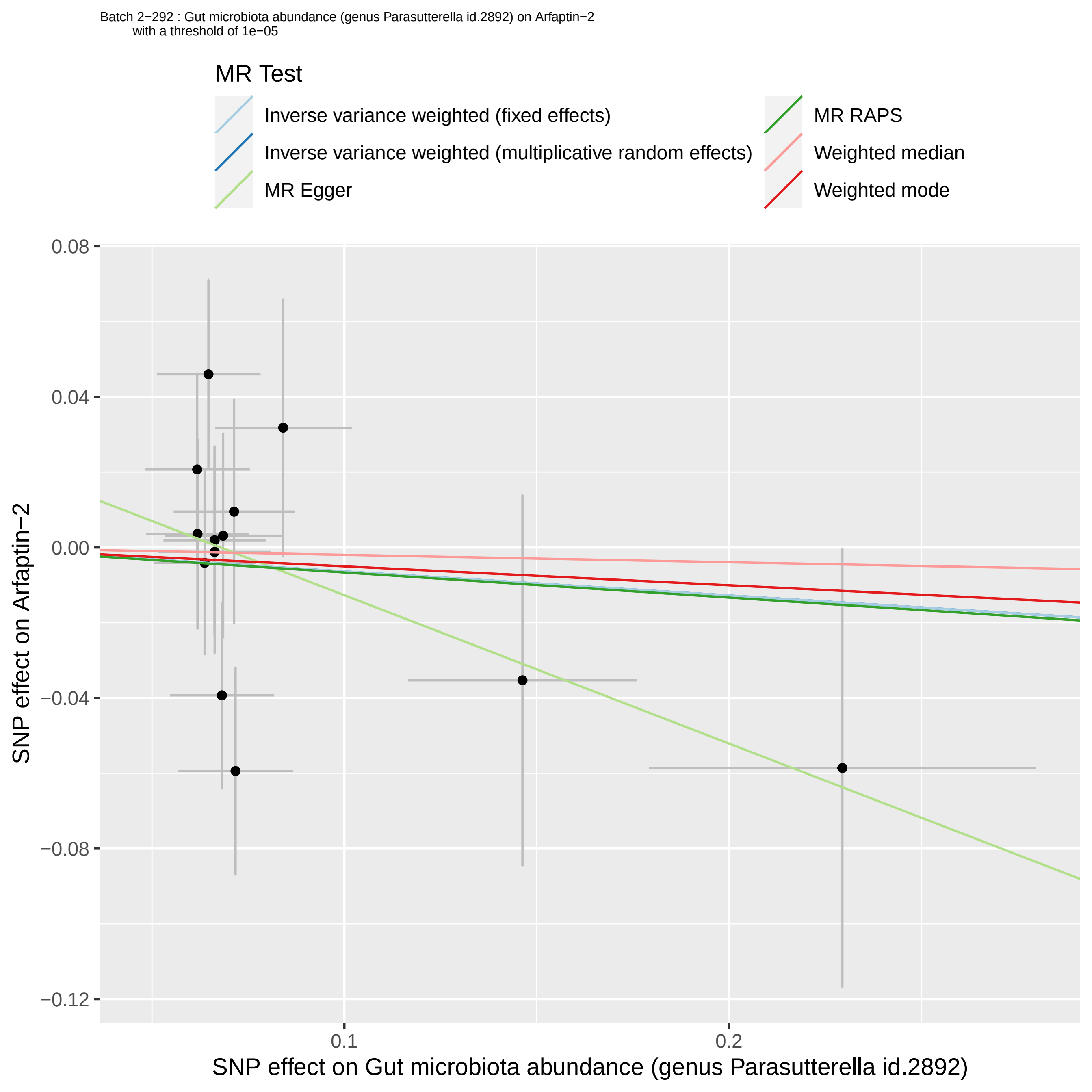
Batch 2−292 : Gut microbiota abundance (genus Parasutterella id.2892) on Arfaptin−2
with a threshold of 1e−05
MR Test
Inverse variance weighted (fixed effects)
Inverse variance weighted (multiplicative random effects)
MR Egger
MR RAPS
Weighted median
Weighted mode
0.08
0.04
0.00
−0.04
−0.08
−0.12
0.1
0.2
SNP effect on Arfaptin−2
SNP effect on Gut microbiota abundance (genus Parasutterella id.2892)

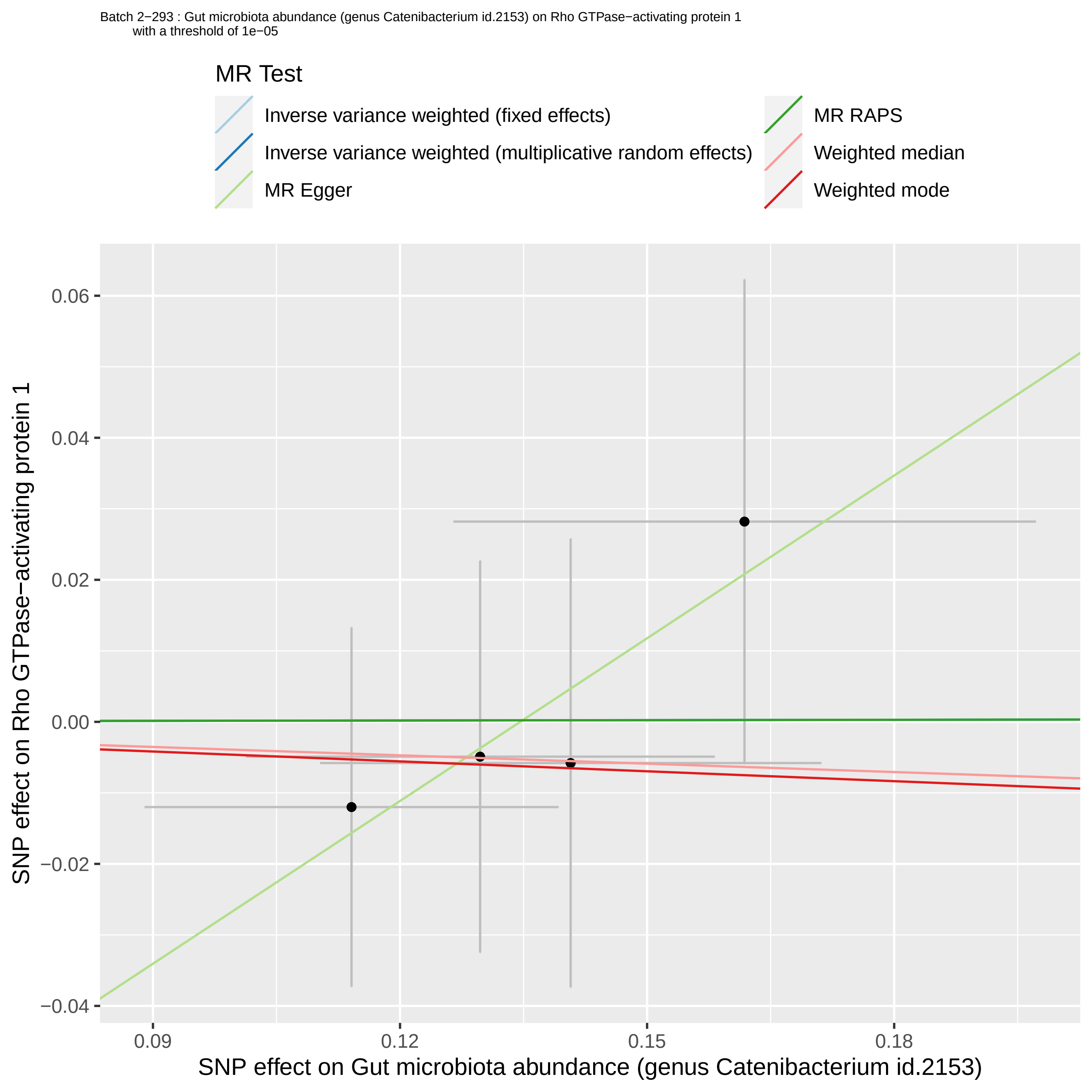
Batch 2−293 : Gut microbiota abundance (genus Catenibacterium id.2153) on Rho GTPase−activating protein 1
with a threshold of 1e−05
MR Test
Inverse variance weighted (fixed effects)
Inverse variance weighted (multiplicative random effects)
MR Egger
MR RAPS
Weighted median
Weighted mode
0.06
0.04
0.02
0.00
−0.02
−0.04
0.09
0.12
0.15
0.18
SNP effect on Rho GTPase−activating protein 1
SNP effect on Gut microbiota abundance (genus Catenibacterium id.2153)

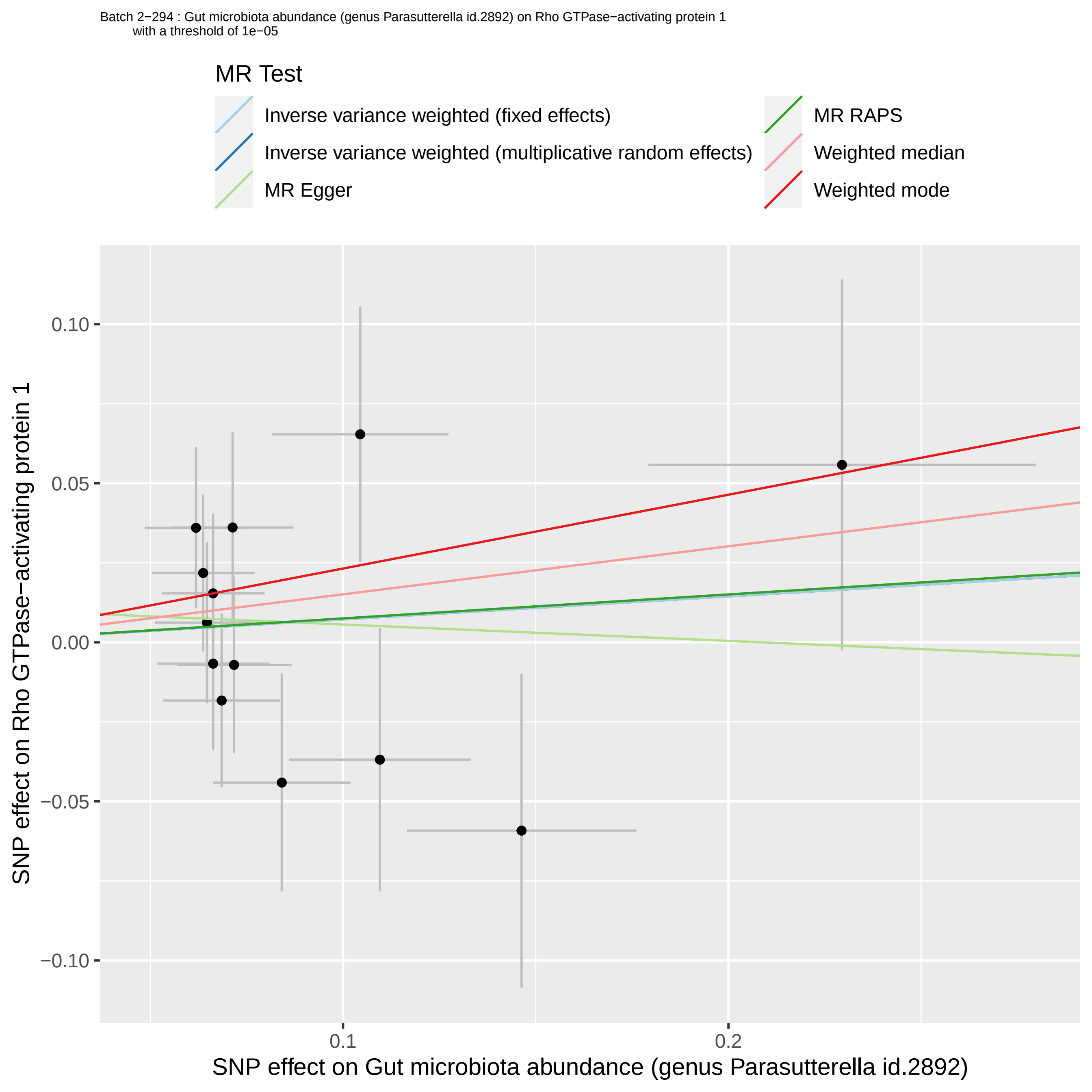

Batch 2−294 : Gut microbiota abundance (genus Parasutterella id.2892) on Rho GTPase−activating protein 1
with a threshold of 1e−05
MR Test
Inverse variance weighted (fixed effects)
Inverse variance weighted (multiplicative random effects)
MR Egger
MR RAPS
Weighted median
Weighted mode
0.10
0.05
0.00
−0.05
−0.10
0.1
0.2
SNP effect on Rho GTPase−activating protein 1
SNP effect on Gut microbiota abundance (genus Parasutterella id.2892)

Batch 2−295 : Gut microbiota abundance (genus Catenibacterium id.2153) on Rho GTPase−activating protein 25
with a threshold of 1e−05

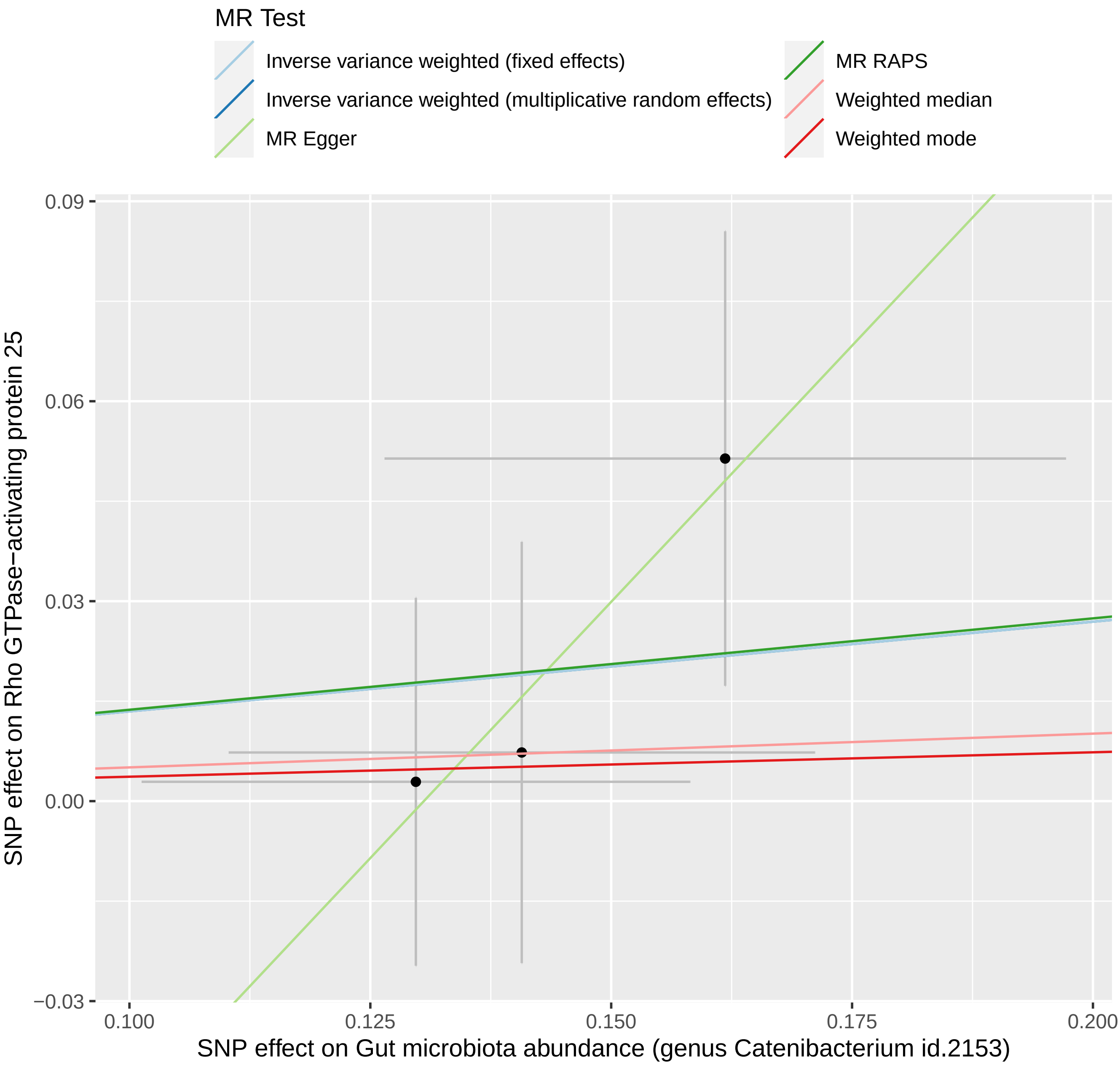

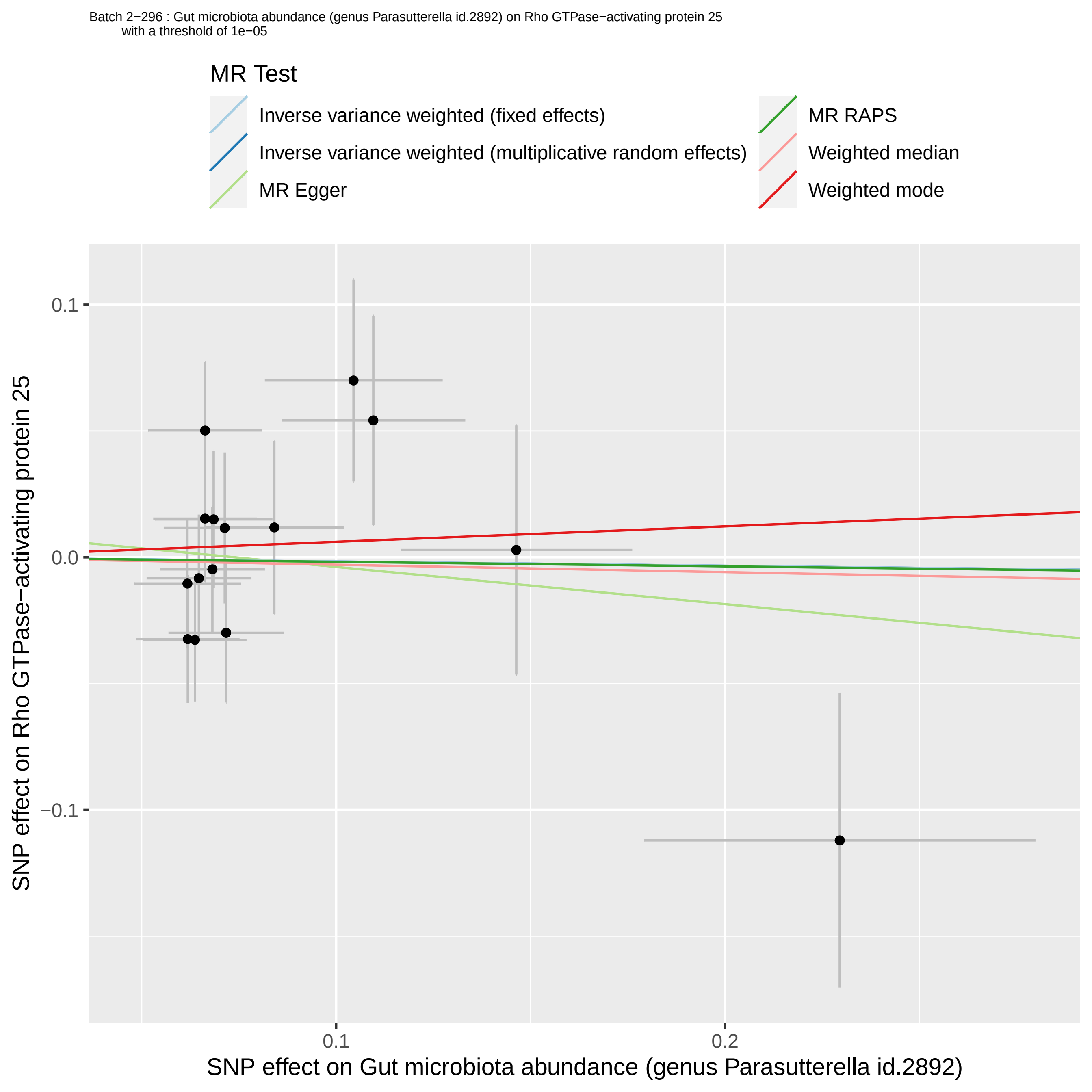

Batch 2−296 : Gut microbiota abundance (genus Parasutterella id.2892) on Rho GTPase−activating protein 25
with a threshold of 1e−05
MR Test
Inverse variance weighted (fixed effects)
Inverse variance weighted (multiplicative random effects)
MR Egger
MR RAPS
Weighted median
Weighted mode
0.1
0.0
−0.1
SNP effect on Rho GTPase−activating protein 25
0.1
0.2
SNP effect on Gut microbiota abundance (genus Parasutterella id.2892)

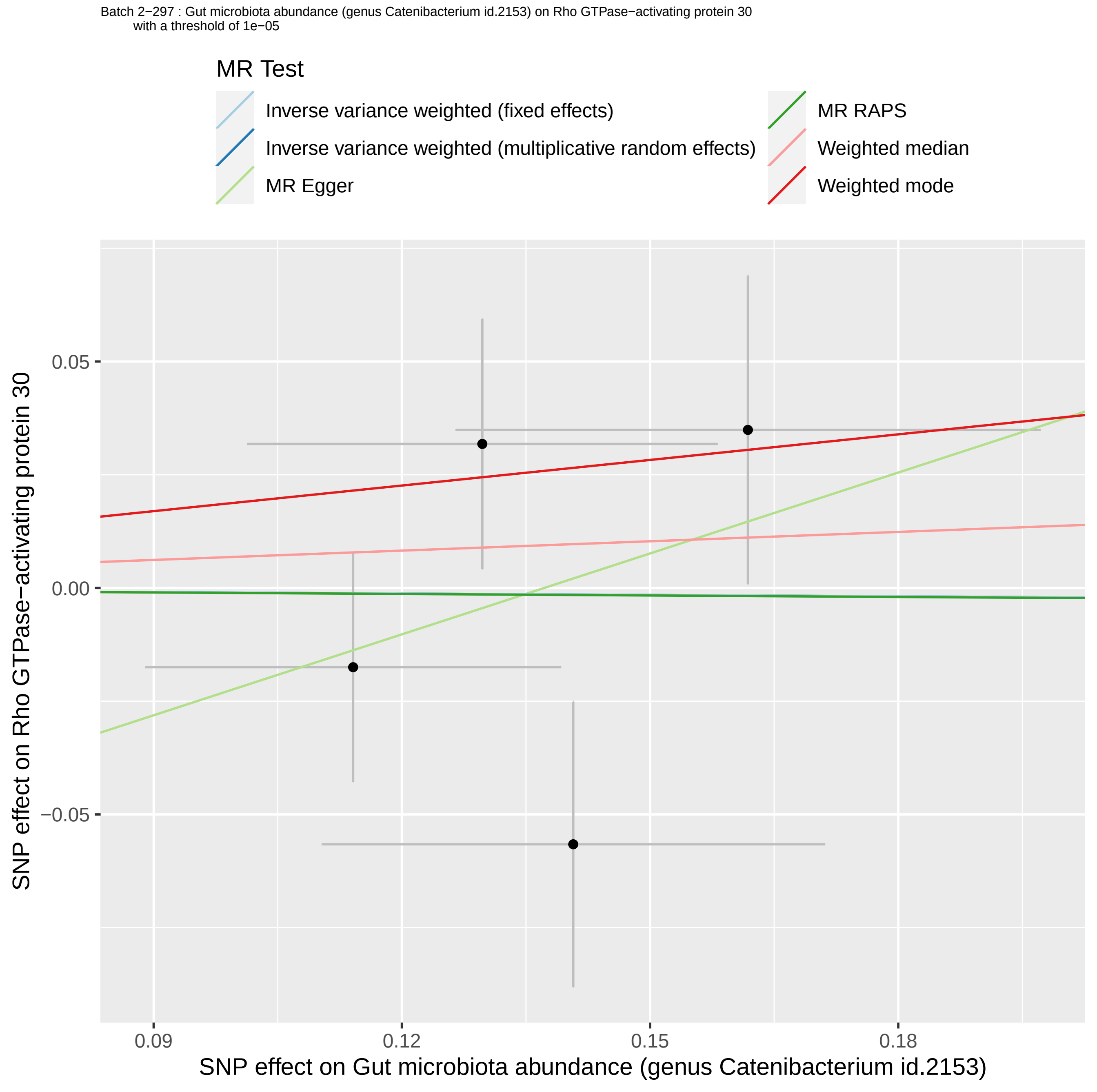

Batch 2−297 : Gut microbiota abundance (genus Catenibacterium id.2153) on Rho GTPase−activating protein 30
with a threshold of 1e−05
MR Test
Inverse variance weighted (fixed effects)
Inverse variance weighted (multiplicative random effects)
MR Egger
MR RAPS
Weighted median
Weighted mode
0.05
0.00
−0.05
0.09
0.12
0.15
0.18
SNP effect on Rho GTPase−activating protein 30
SNP effect on Gut microbiota abundance (genus Catenibacterium id.2153)

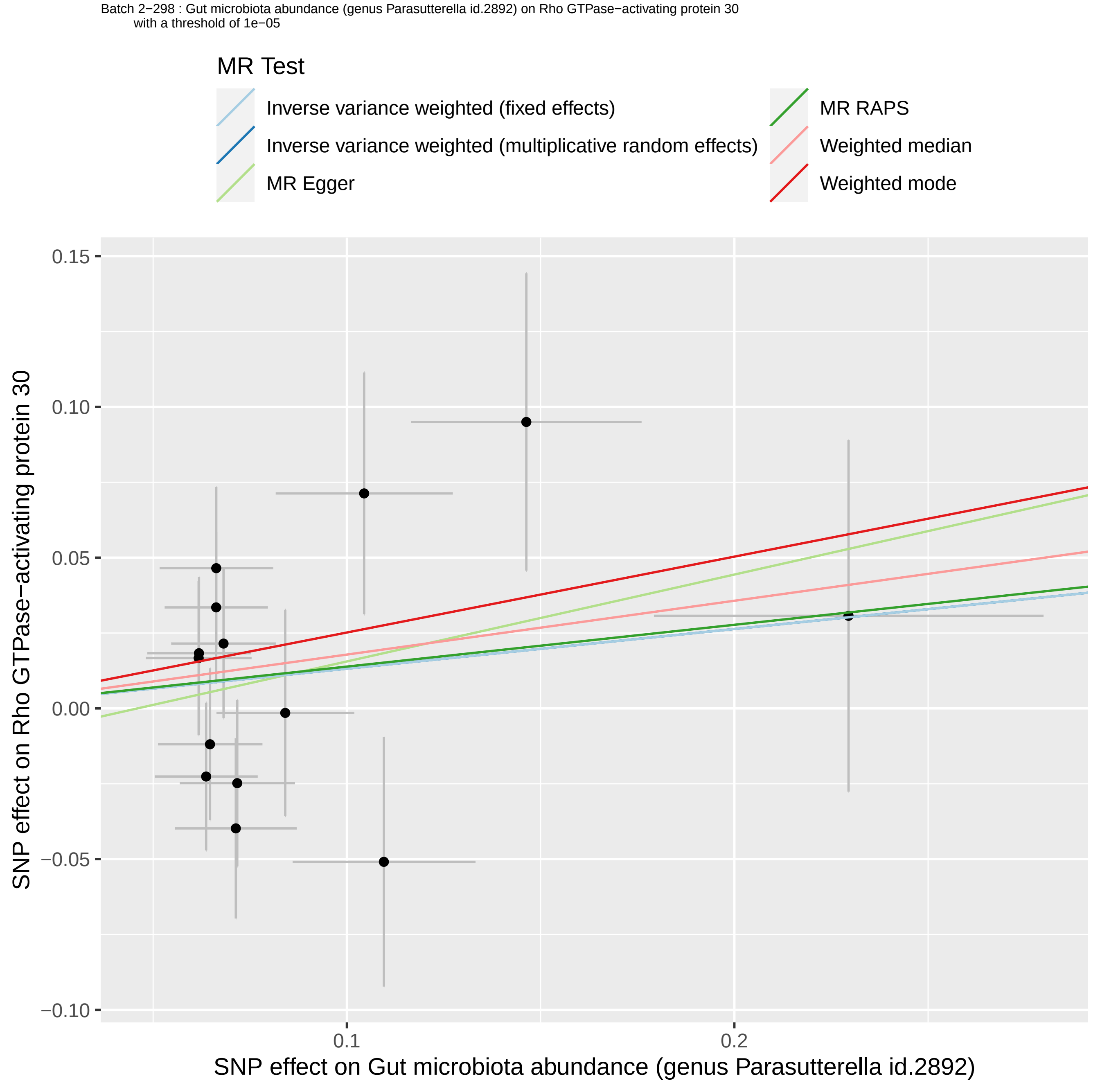
Batch 2−298 : Gut microbiota abundance (genus Parasutterella id.2892) on Rho GTPase−activating protein 30
with a threshold of 1e−05
MR Test
Inverse variance weighted (fixed effects)
Inverse variance weighted (multiplicative random effects)
MR Egger
MR RAPS
Weighted median
Weighted mode
0.15
0.10
0.05
0.00
−0.05
−0.10
0.1
0.2
SNP effect on Rho GTPase−activating protein 30
SNP effect on Gut microbiota abundance (genus Parasutterella id.2892)

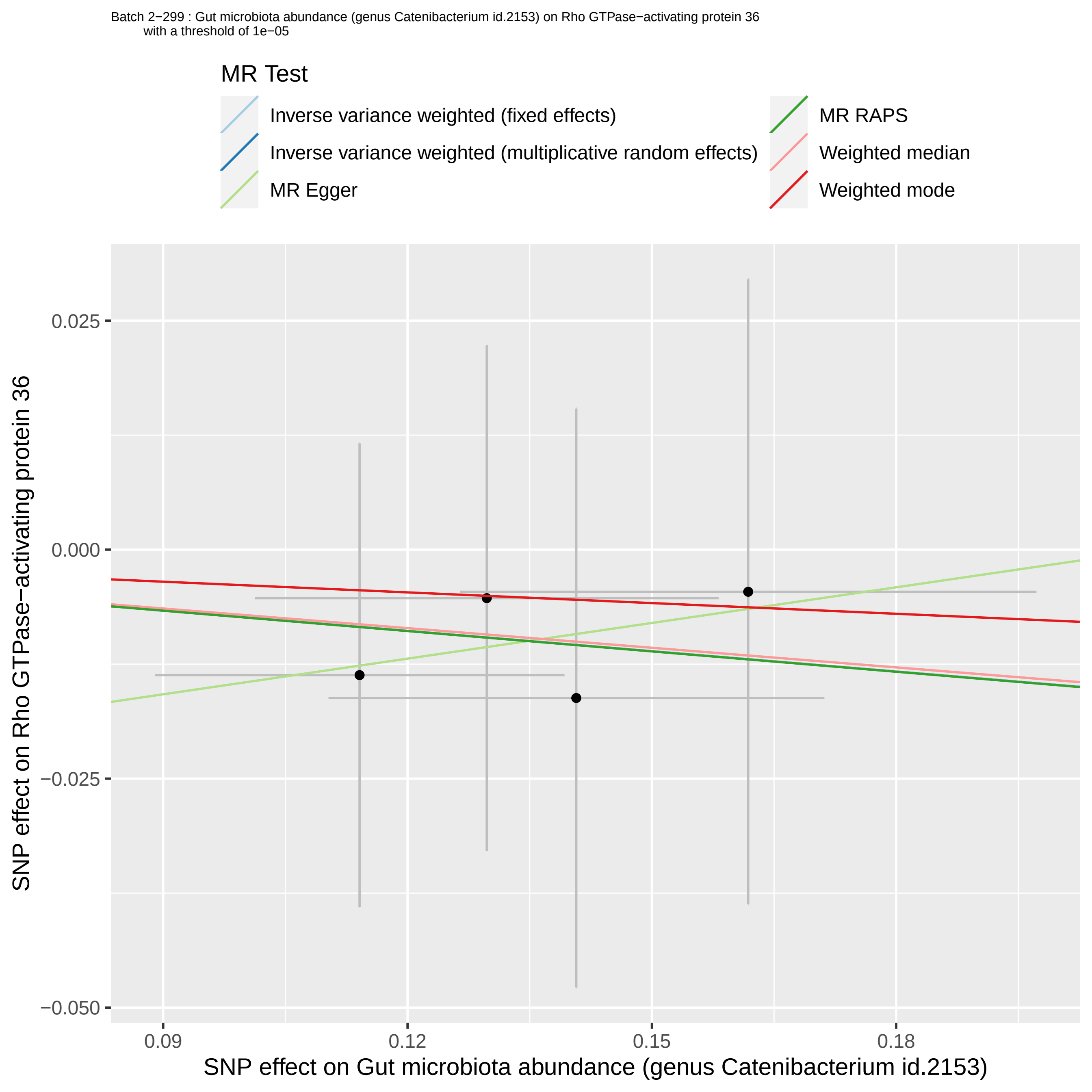

Batch 2−299 : Gut microbiota abundance (genus Catenibacterium id.2153) on Rho GTPase−activating protein 36
with a threshold of 1e−05
MR Test
Inverse variance weighted (fixed effects)
Inverse variance weighted (multiplicative random effects)
MR Egger
MR RAPS
Weighted median
Weighted mode
0.025
0.000
−0.025
−0.050
0.09
0.12
0.15
0.18
SNP effect on Rho GTPase−activating protein 36
SNP effect on Gut microbiota abundance (genus Catenibacterium id.2153)

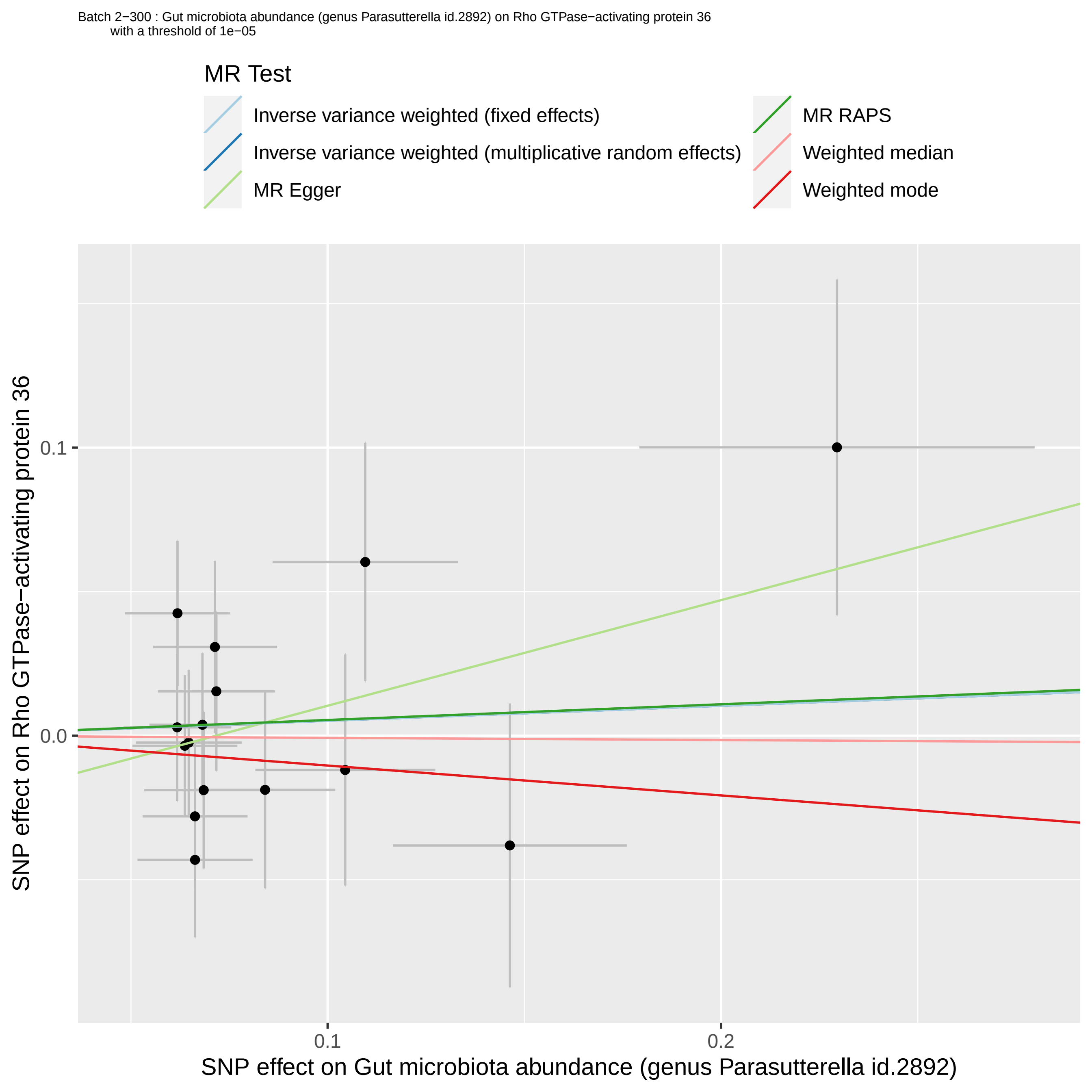

Batch 2−300 : Gut microbiota abundance (genus Parasutterella id.2892) on Rho GTPase−activating protein 36
with a threshold of 1e−05
MR Test
Inverse variance weighted (fixed effects)
Inverse variance weighted (multiplicative random effects)
MR Egger
MR RAPS
Weighted median
Weighted mode
0.1
0.0
0.1
0.2
SNP effect on Rho GTPase−activating protein 36
SNP effect on Gut microbiota abundance (genus Parasutterella id.2892)

Batch 2−301 : Gut microbiota abundance (genus Catenibacterium id.2153) on Rho GTPase−activating protein 5
with a threshold of 1e−05

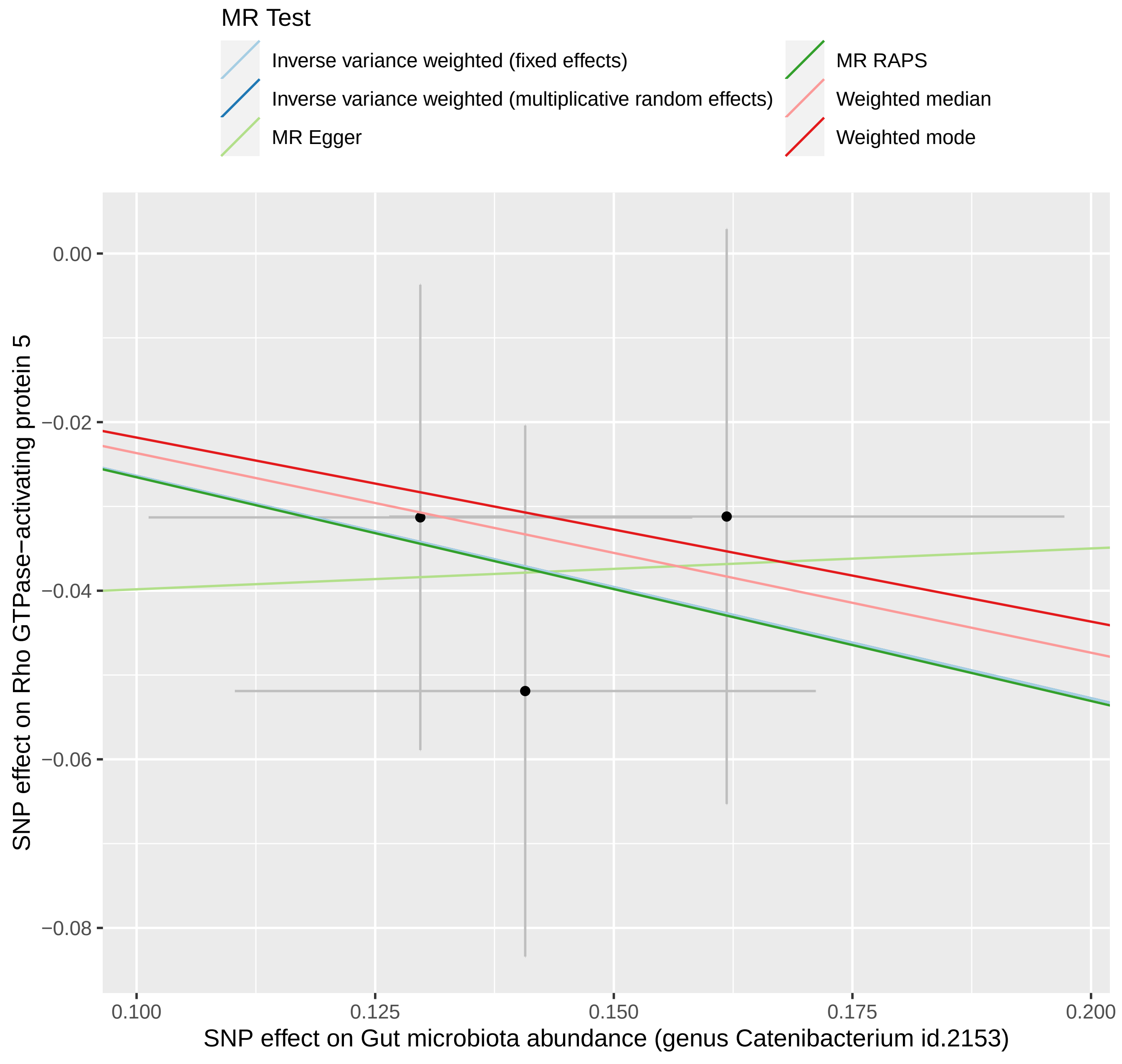

Batch 2−302 : Gut microbiota abundance (genus Parasutterella id.2892) on Rho GTPase−activating protein 5
with a threshold of 1e−05

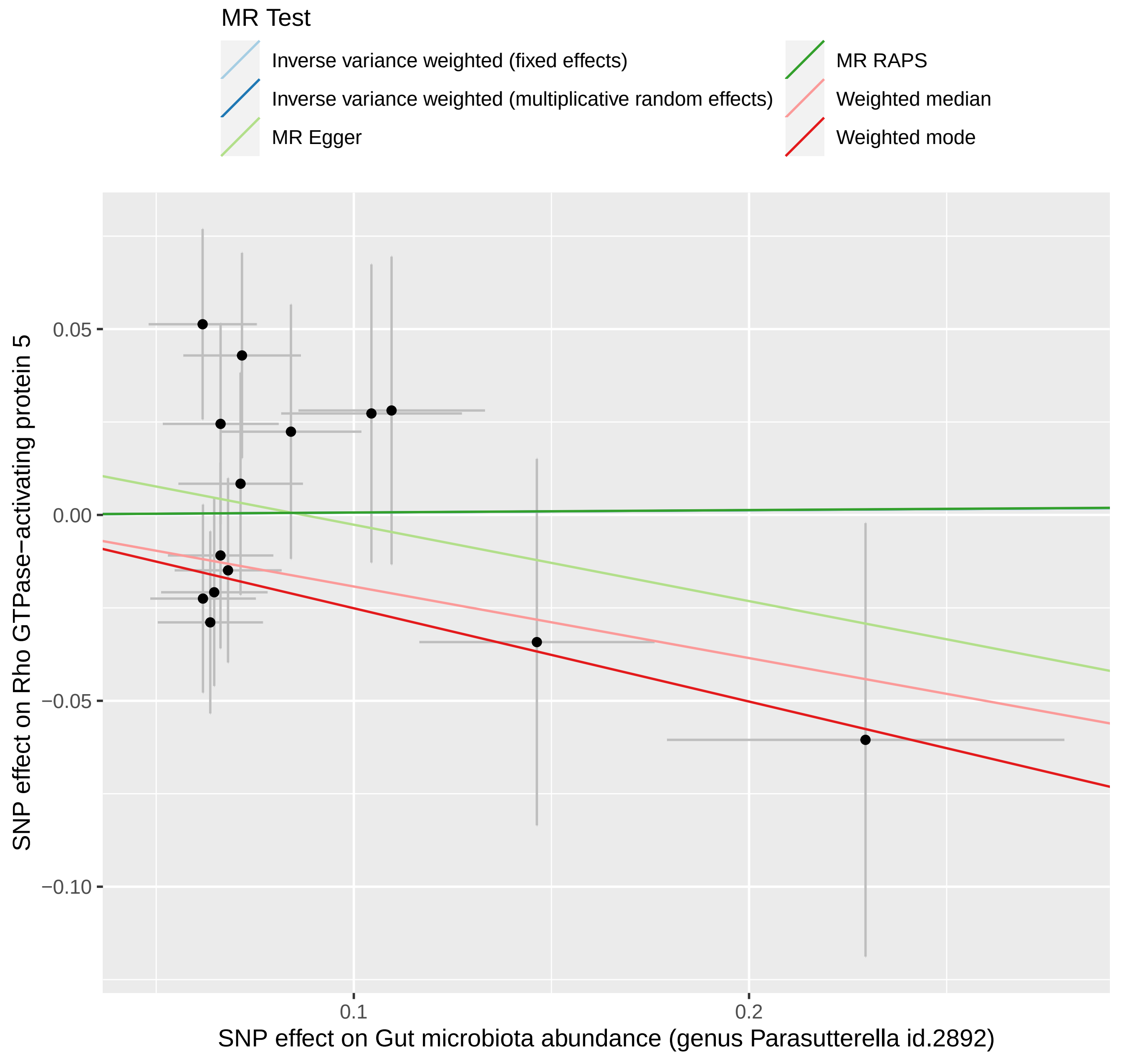

Batch 2−303 : Gut microbiota abundance (genus Catenibacterium id.2153) on Rho GDP−dissociation inhibitor 1
with a threshold of 1e−05

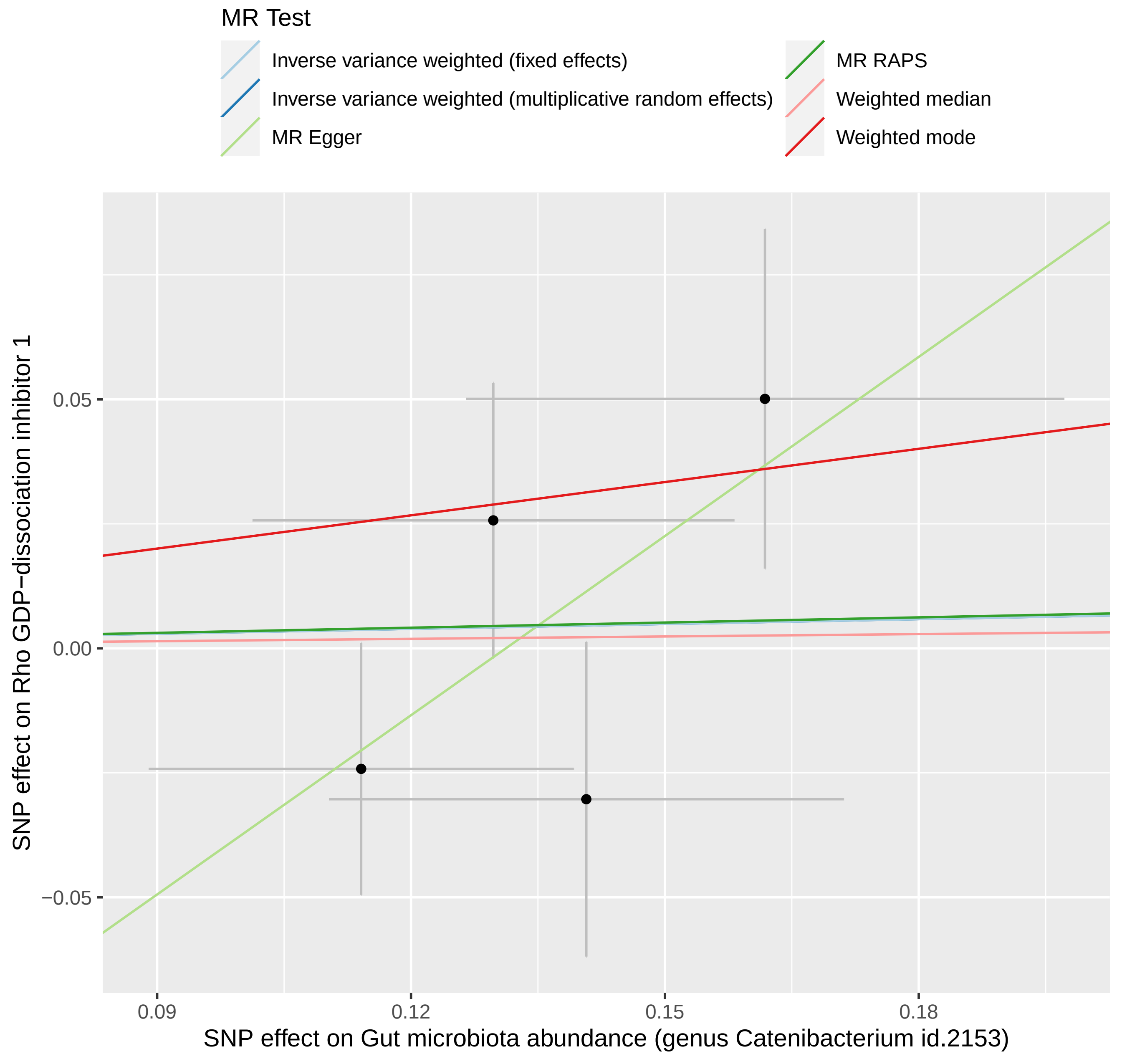

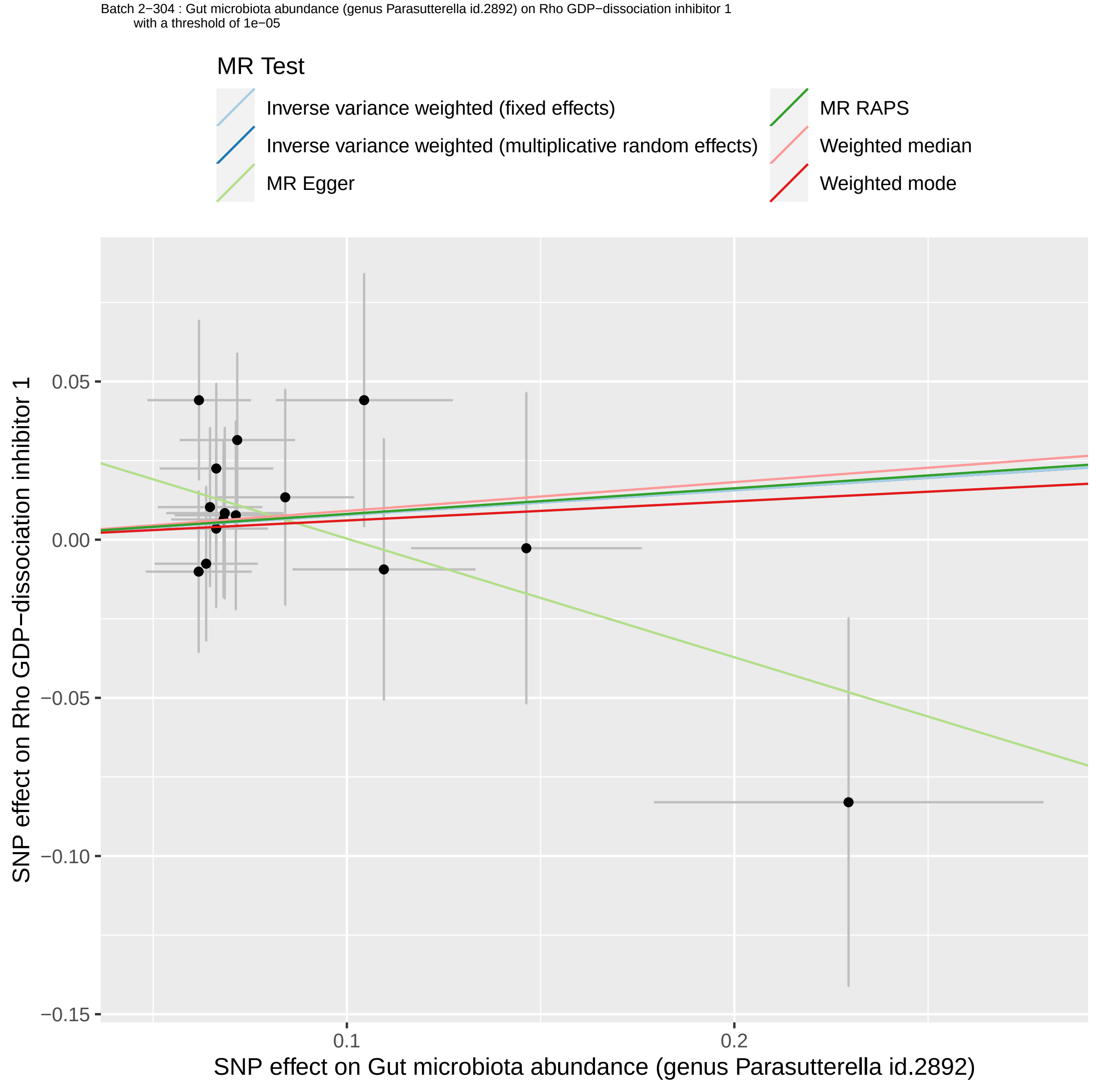

Batch 2−304 : Gut microbiota abundance (genus Parasutterella id.2892) on Rho GDP−dissociation inhibitor 1
with a threshold of 1e−05
MR Test
Inverse variance weighted (fixed effects)
Inverse variance weighted (multiplicative random effects)
MR Egger
MR RAPS
Weighted median
Weighted mode
0.05
0.00
−0.05
−0.10
−0.15
0.1
0.2
SNP effect on Rho GDP−dissociation inhibitor 1
SNP effect on Gut microbiota abundance (genus Parasutterella id.2892)

Batch 2−305 : Gut microbiota abundance (genus Catenibacterium id.2153) on Rho GDP−dissociation inhibitor 2 with a threshold of 1e−05

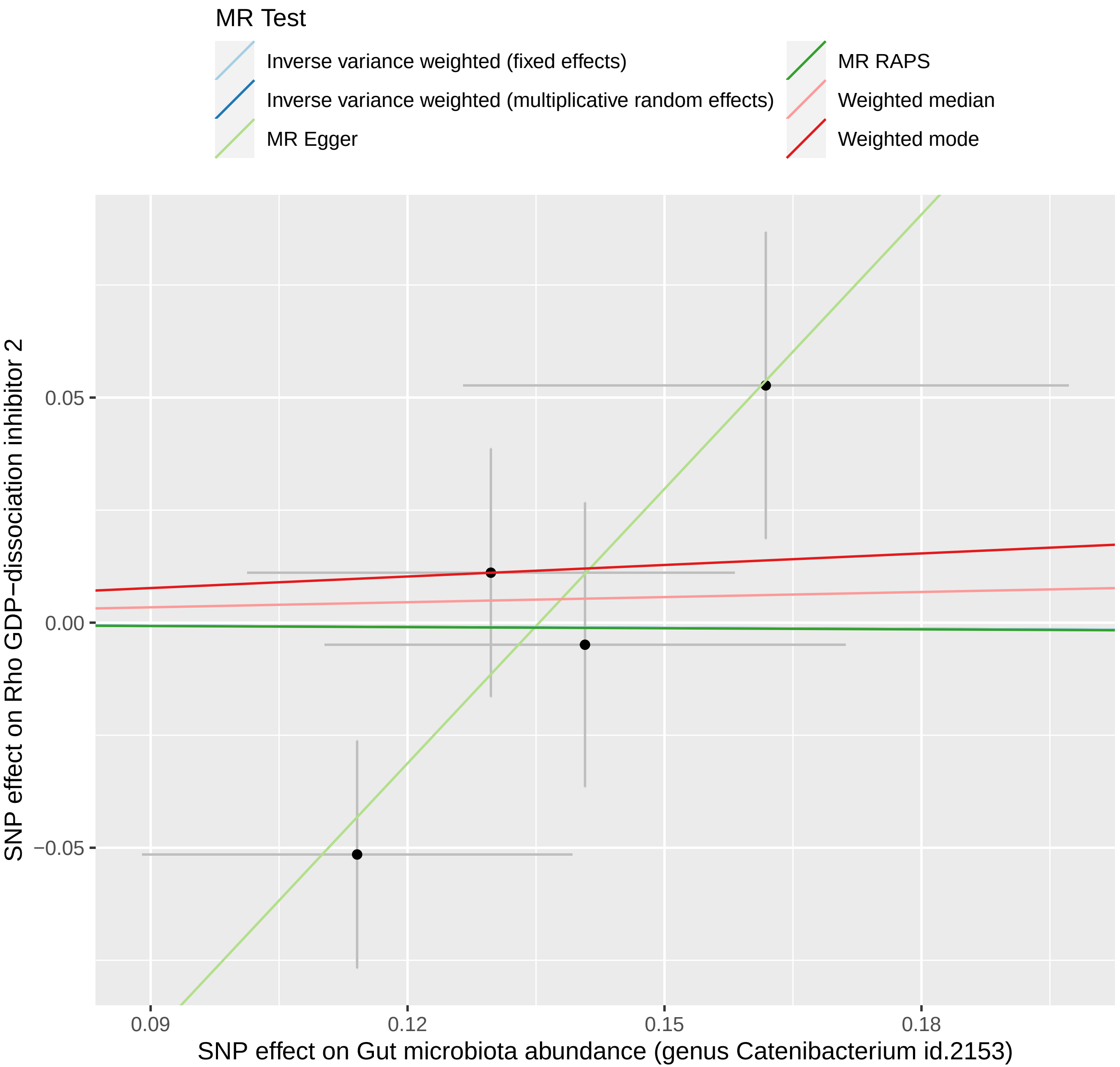

Batch 2−306 : Gut microbiota abundance (genus Parasutterella id.2892) on Rho GDP−dissociation inhibitor 2
with a threshold of 1e−05

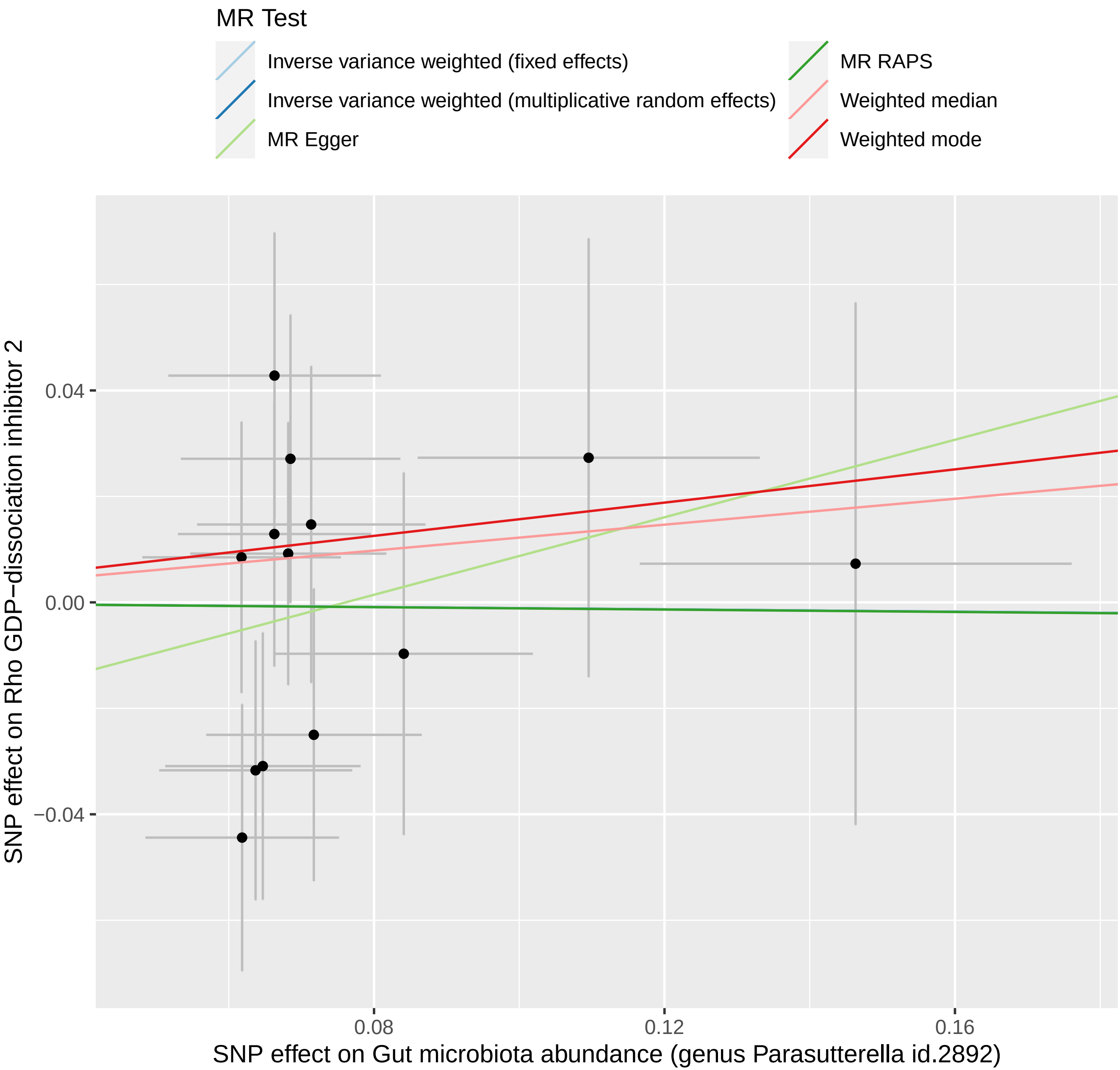

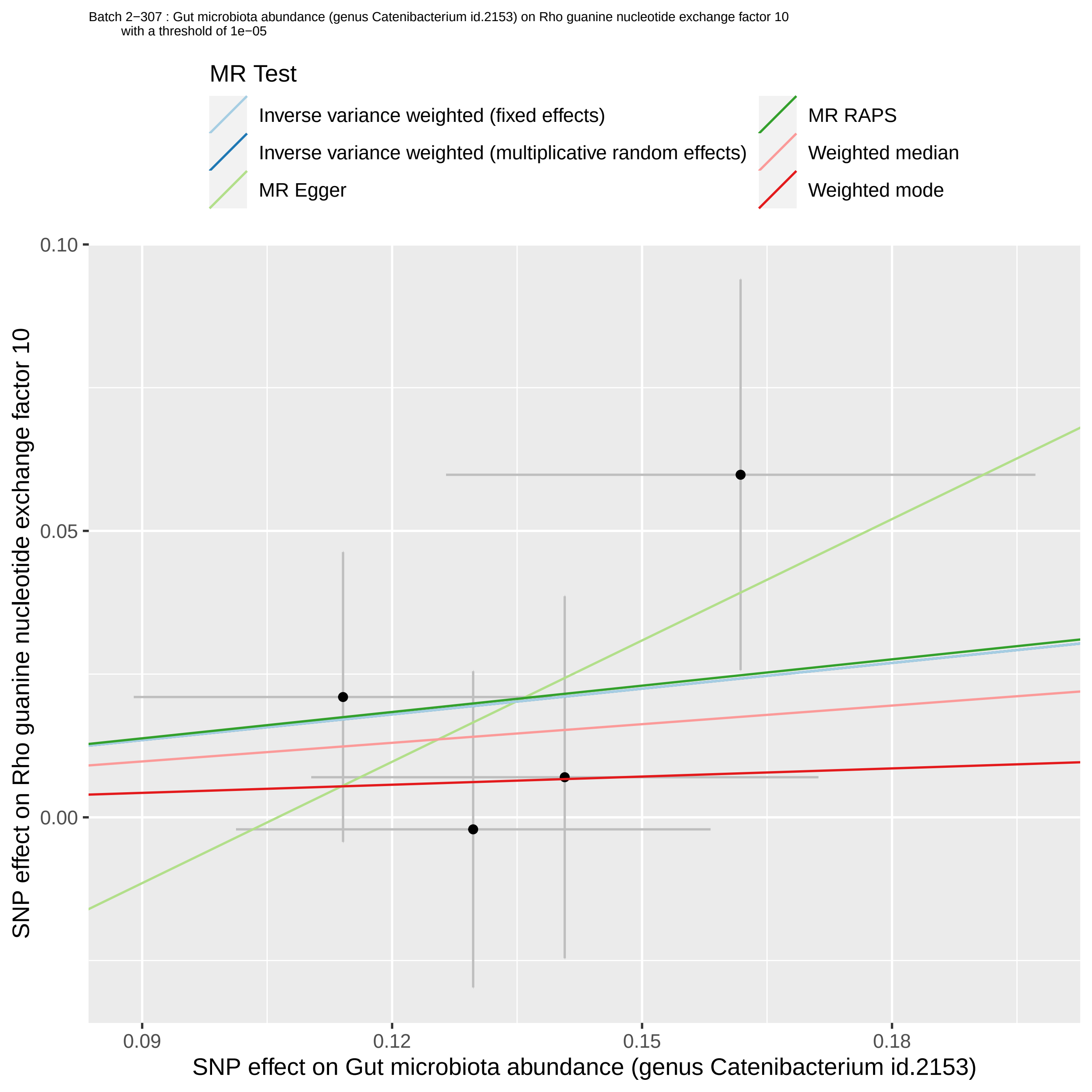
Batch 2−307 : Gut microbiota abundance (genus Catenibacterium id.2153) on Rho guanine nucleotide exchange factor 10
with a threshold of 1e−05
MR Test
Inverse variance weighted (fixed effects)
Inverse variance weighted (multiplicative random effects)
MR Egger
MR RAPS
Weighted median
Weighted mode
0.10
0.05
0.00
0.09
0.12
0.15
0.18
SNP effect on Rho guanine nucleotide exchange factor 10
SNP effect on Gut microbiota abundance (genus Catenibacterium id.2153)

Batch 2−308 : Gut microbiota abundance (genus Parasutterella id.2892) on Rho guanine nucleotide exchange factor 10
with a threshold of 1e−05

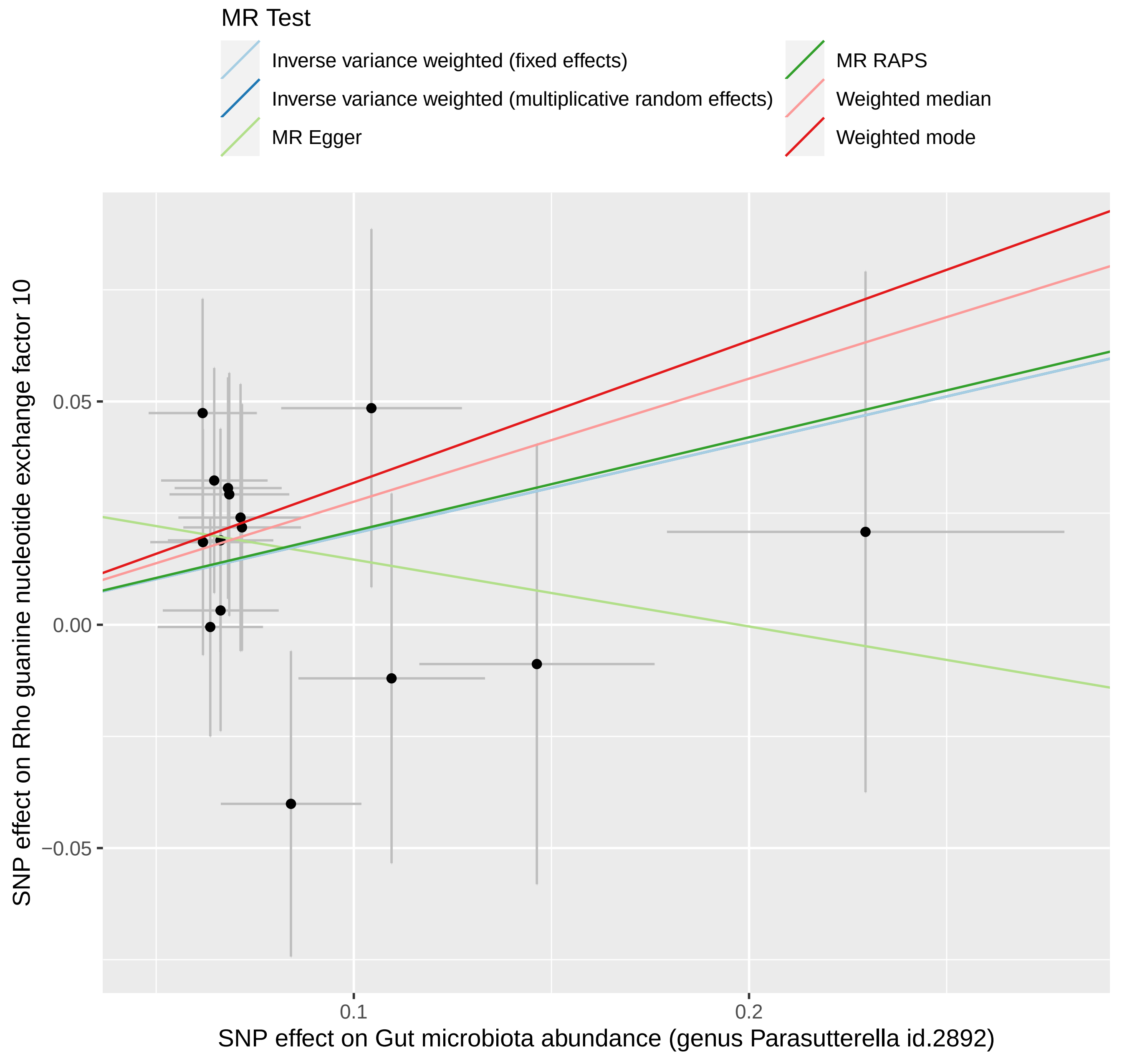

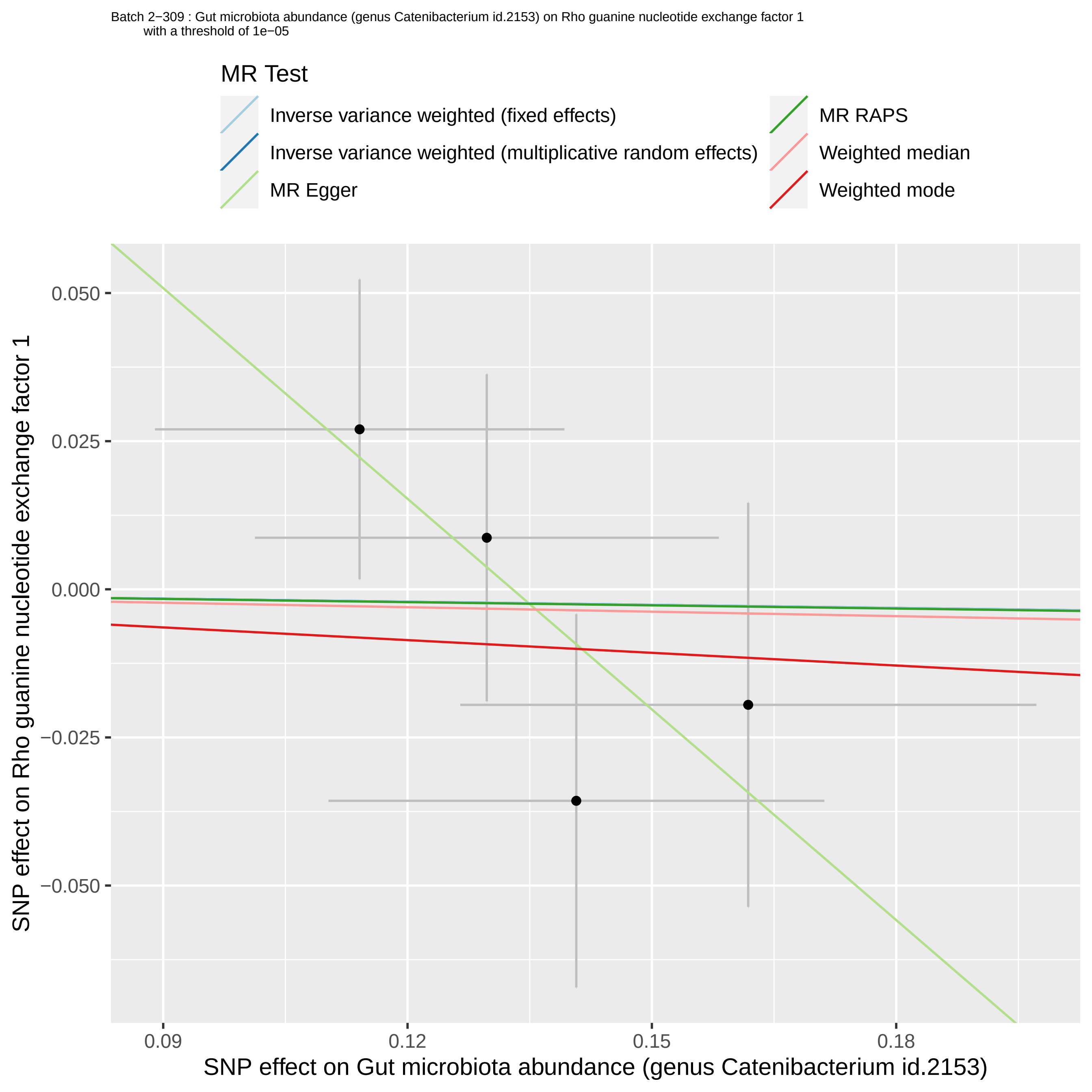

Batch 2−309 : Gut microbiota abundance (genus Catenibacterium id.2153) on Rho guanine nucleotide exchange factor 1
with a threshold of 1e−05
MR Test
Inverse variance weighted (fixed effects)
Inverse variance weighted (multiplicative random effects)
MR Egger
MR RAPS
Weighted median
Weighted mode
0.050
0.025
0.000
−0.025
−0.050
0.09
0.12
0.15
0.18
SNP effect on Rho guanine nucleotide exchange factor 1
SNP effect on Gut microbiota abundance (genus Catenibacterium id.2153)

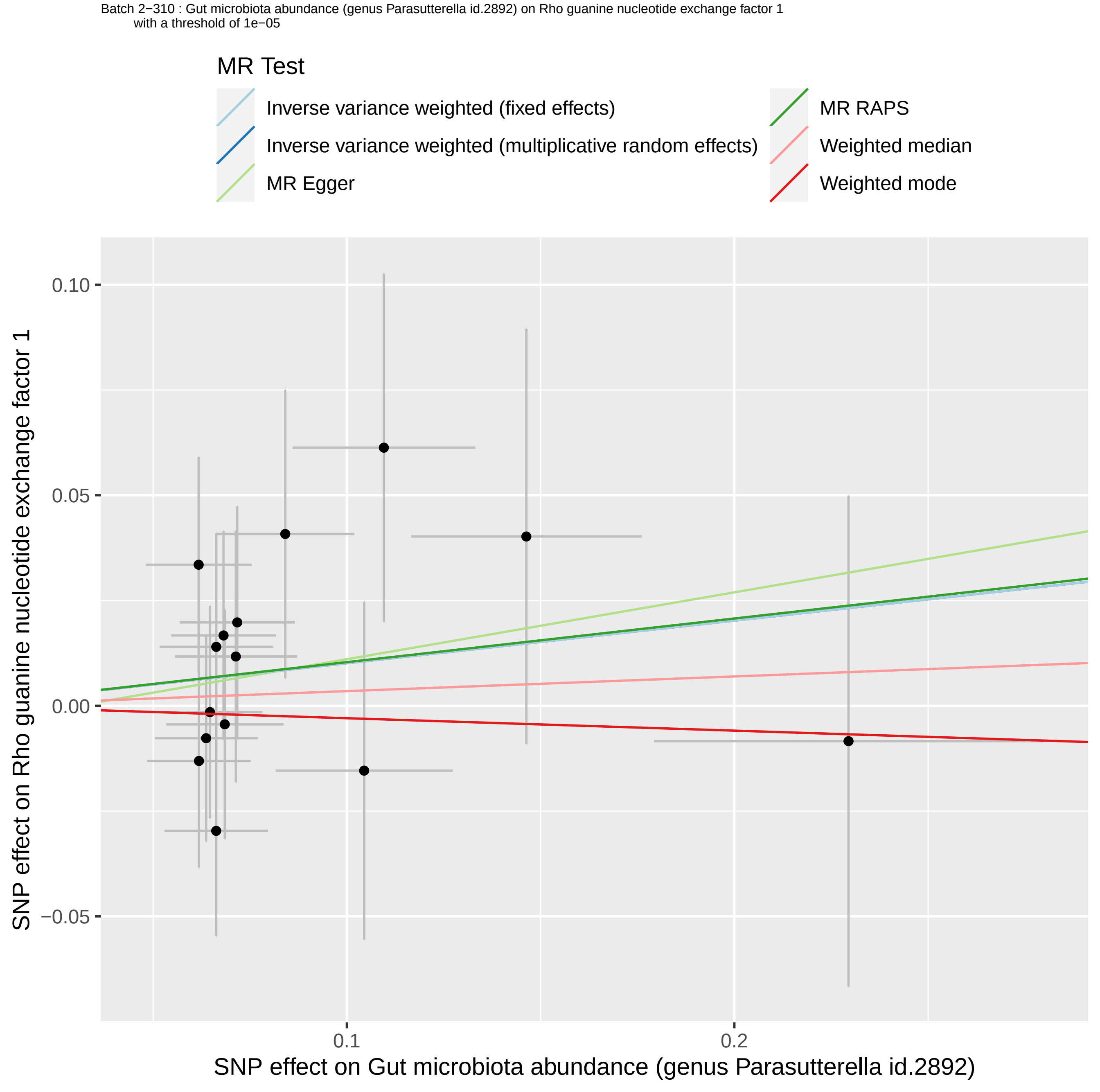

Batch 2−310 : Gut microbiota abundance (genus Parasutterella id.2892) on Rho guanine nucleotide exchange factor 1
with a threshold of 1e−05
MR Test
Inverse variance weighted (fixed effects)
Inverse variance weighted (multiplicative random effects)
MR Egger
MR RAPS
Weighted median
Weighted mode
0.10
0.05
0.00
−0.05
0.1
0.2
SNP effect on Rho guanine nucleotide exchange factor 1
SNP effect on Gut microbiota abundance (genus Parasutterella id.2892)

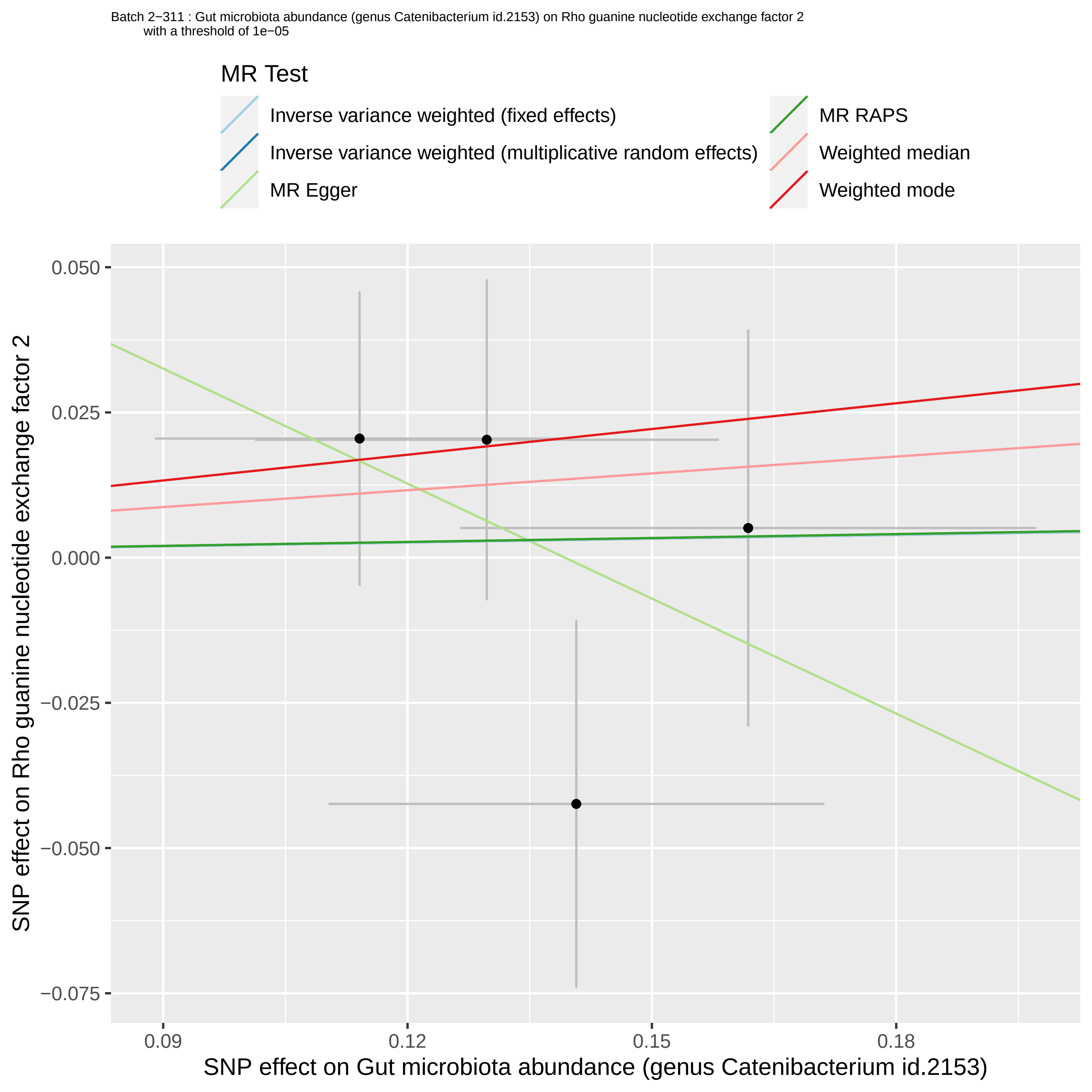

Batch 2−311 : Gut microbiota abundance (genus Catenibacterium id.2153) on Rho guanine nucleotide exchange factor 2 with a threshold of 1e−05
MR Test
Inverse variance weighted (fixed effects)
Inverse variance weighted (multiplicative random effects)
MR Egger
MR RAPS
Weighted median
Weighted mode
0.050
0.025
0.000
−0.025
−0.050
−0.075
0.09
0.12
0.15
0.18
SNP effect on Rho guanine nucleotide exchange factor 2
SNP effect on Gut microbiota abundance (genus Catenibacterium id.2153)

Batch 2−312 : Gut microbiota abundance (genus Parasutterella id.2892) on Rho guanine nucleotide exchange factor 2
with a threshold of 1e−05

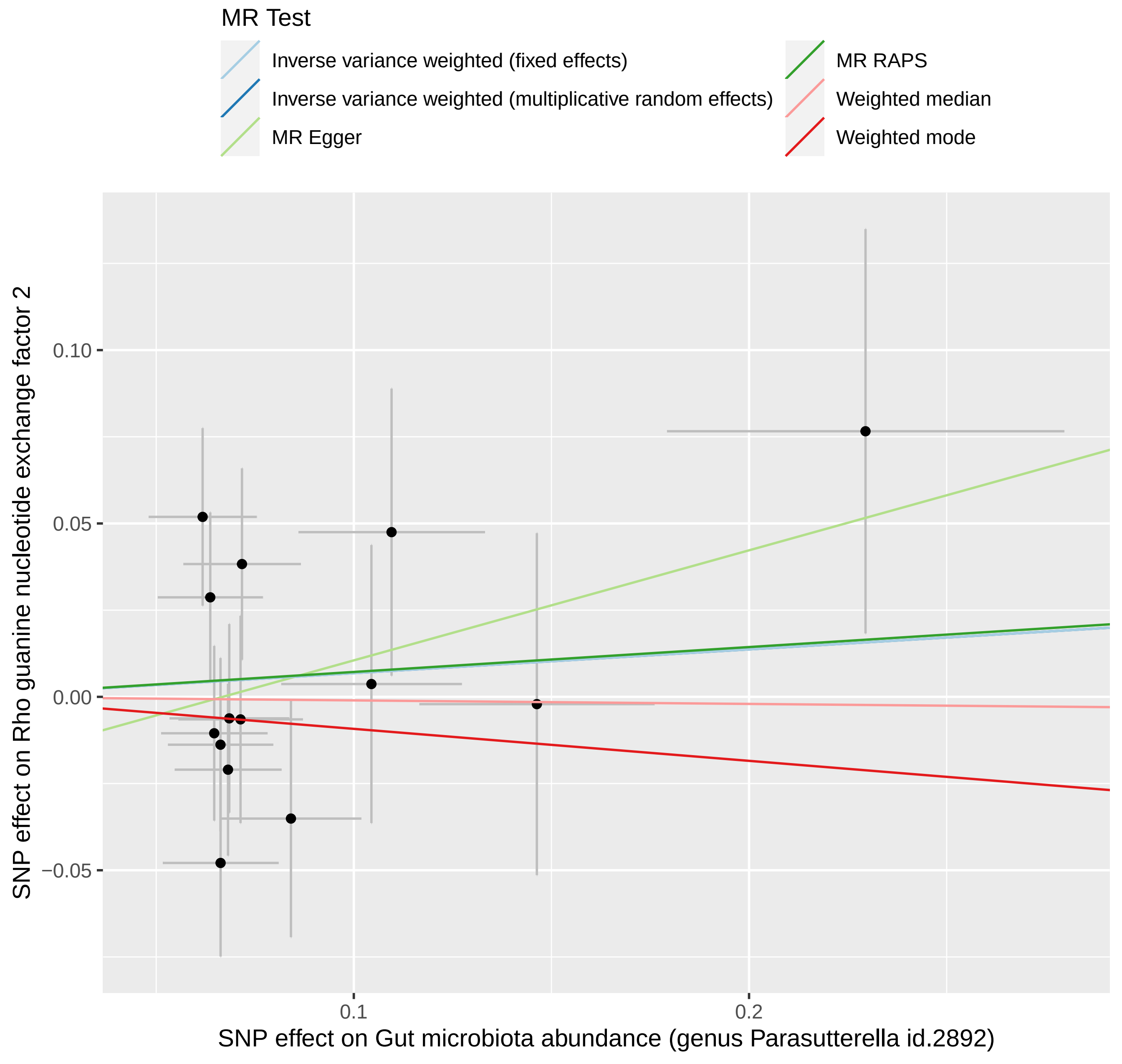

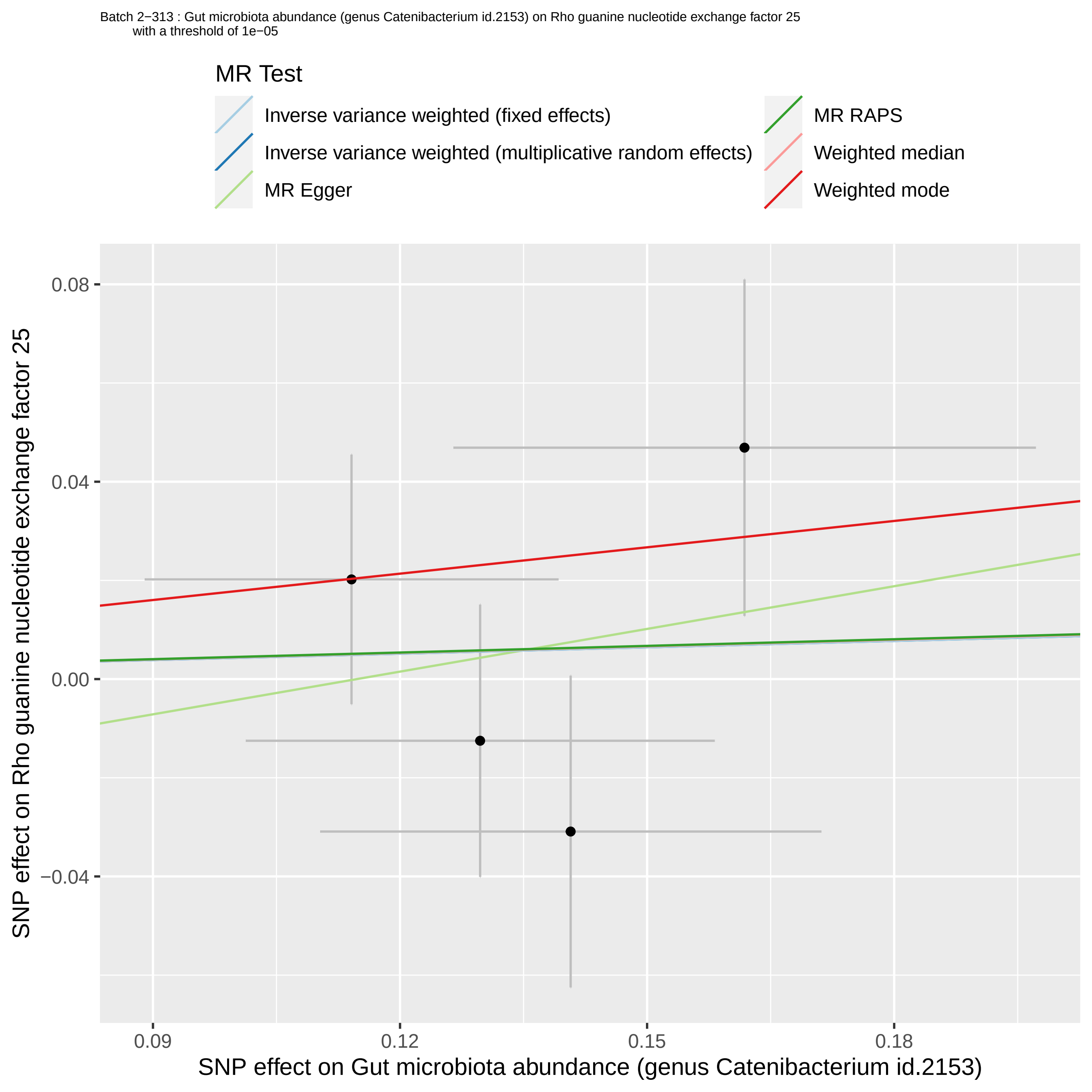

Batch 2−313 : Gut microbiota abundance (genus Catenibacterium id.2153) on Rho guanine nucleotide exchange factor 25
with a threshold of 1e−05
MR Test
Inverse variance weighted (fixed effects)
Inverse variance weighted (multiplicative random effects)
MR Egger
MR RAPS
Weighted median
Weighted mode
0.08
0.04
0.00
−0.04
0.09
0.12
0.15
0.18
SNP effect on Rho guanine nucleotide exchange factor 25
SNP effect on Gut microbiota abundance (genus Catenibacterium id.2153)

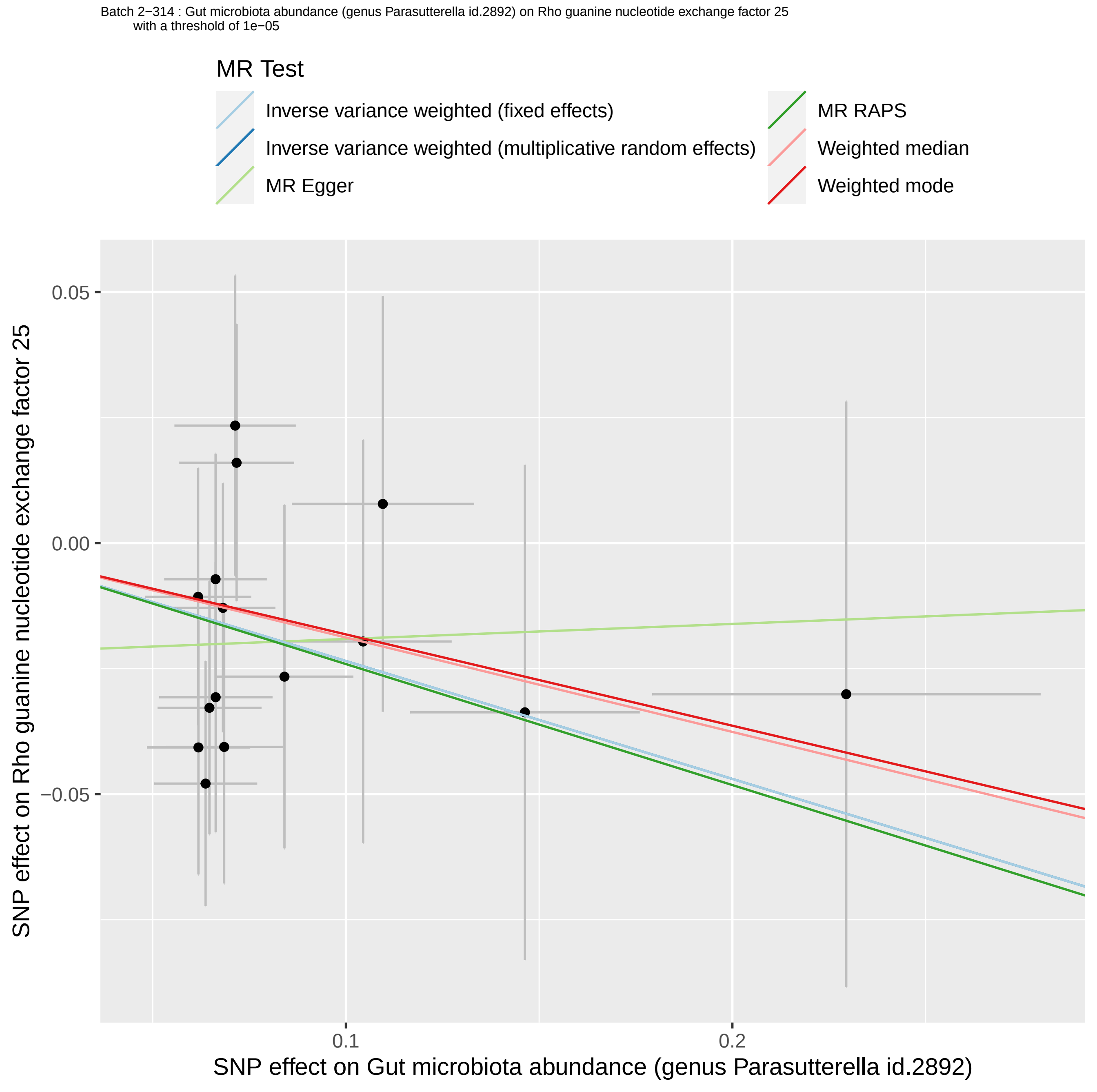

Batch 2−314 : Gut microbiota abundance (genus Parasutterella id.2892) on Rho guanine nucleotide exchange factor 25
with a threshold of 1e−05
MR Test
Inverse variance weighted (fixed effects)
Inverse variance weighted (multiplicative random effects)
MR Egger
MR RAPS
Weighted median
Weighted mode
0.05
0.00
−0.05
0.1
0.2
SNP effect on Rho guanine nucleotide exchange factor 25
SNP effect on Gut microbiota abundance (genus Parasutterella id.2892)

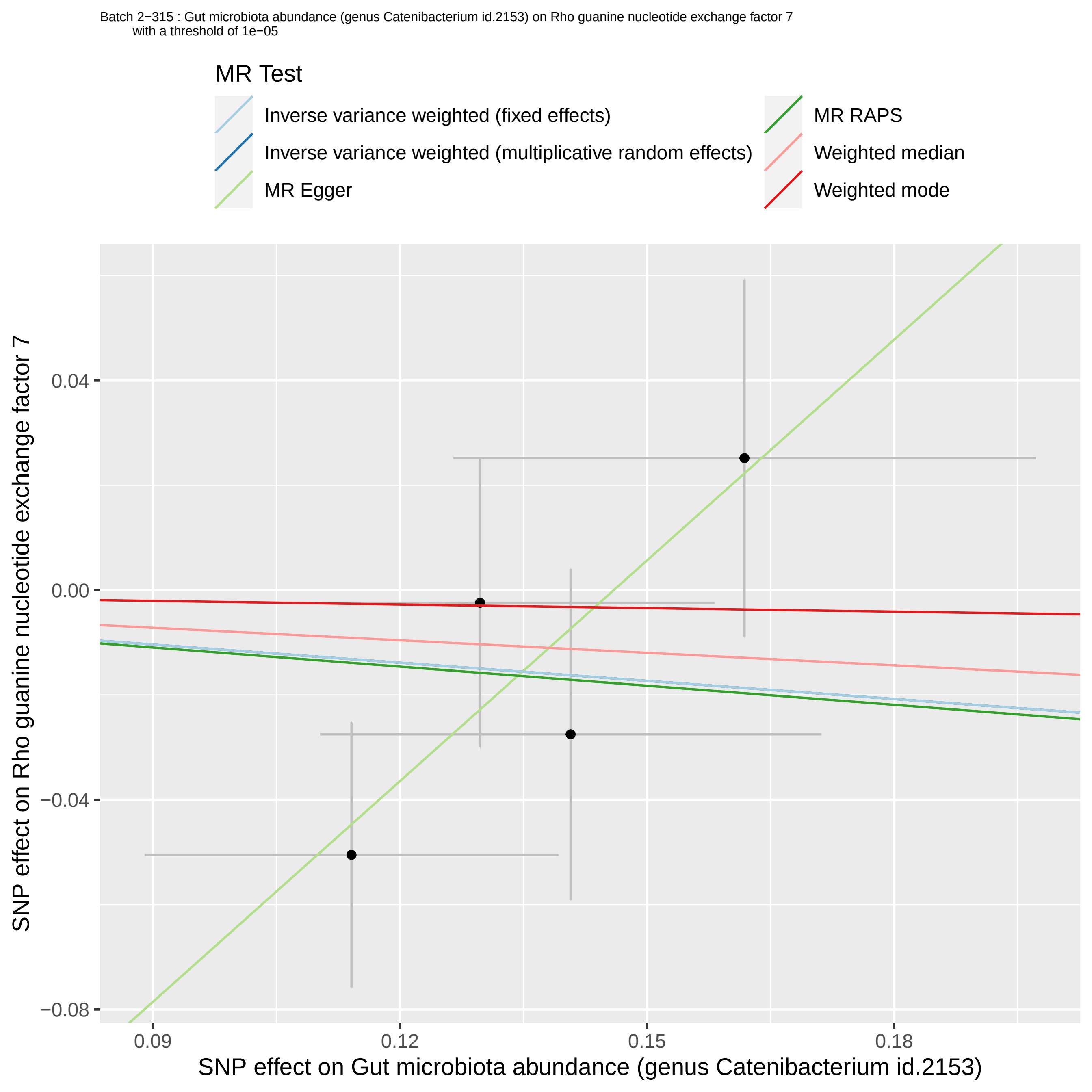

Batch 2−315 : Gut microbiota abundance (genus Catenibacterium id.2153) on Rho guanine nucleotide exchange factor 7
with a threshold of 1e−05
MR Test
Inverse variance weighted (fixed effects)
Inverse variance weighted (multiplicative random effects)
MR Egger
MR RAPS
Weighted median
Weighted mode
0.04
0.00
−0.04
−0.08
0.09
0.12
0.15
0.18
SNP effect on Rho guanine nucleotide exchange factor 7
SNP effect on Gut microbiota abundance (genus Catenibacterium id.2153)

Batch 2−316 : Gut microbiota abundance (genus Parasutterella id.2892) on Rho guanine nucleotide exchange factor 7
with a threshold of 1e−05

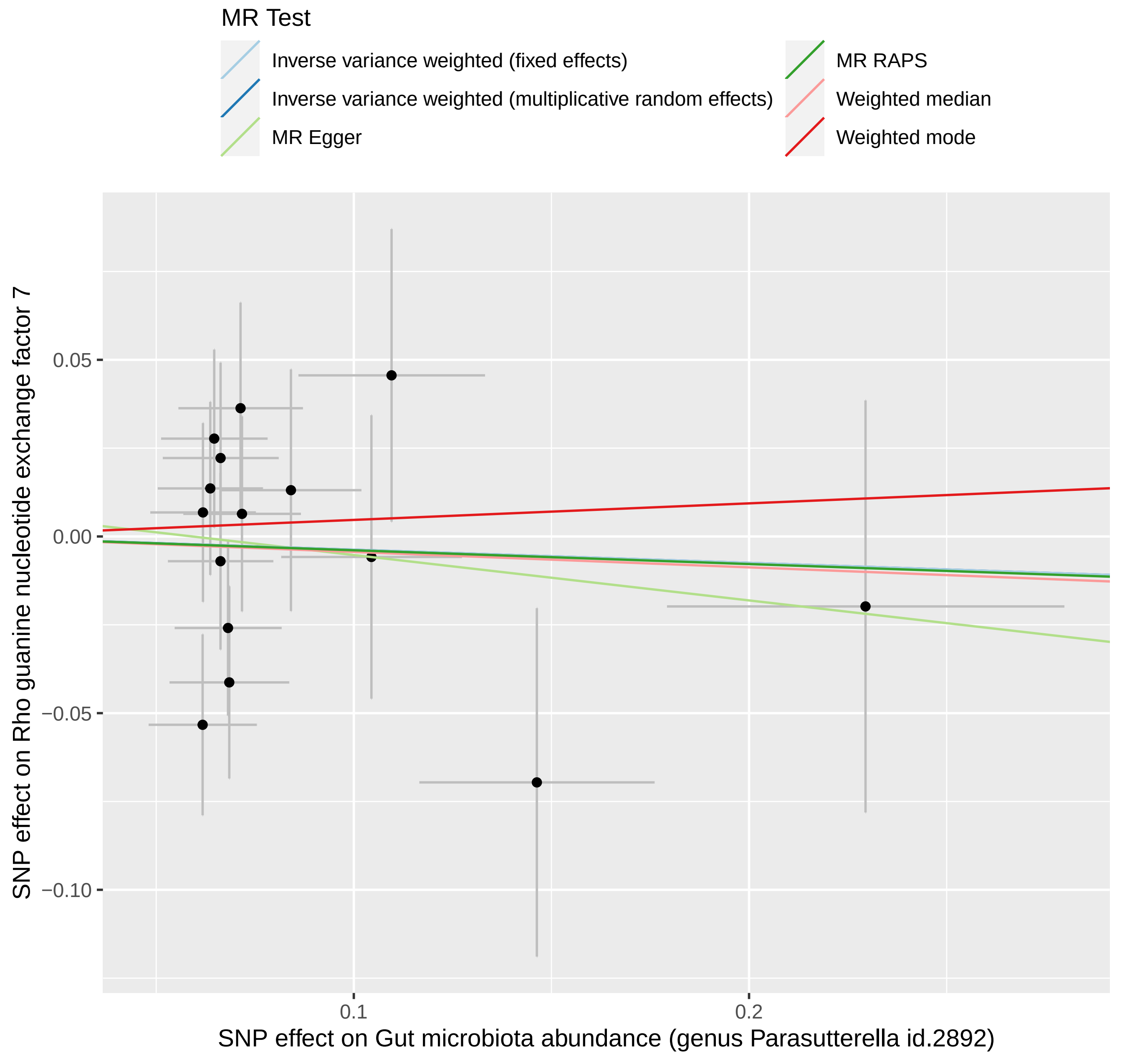

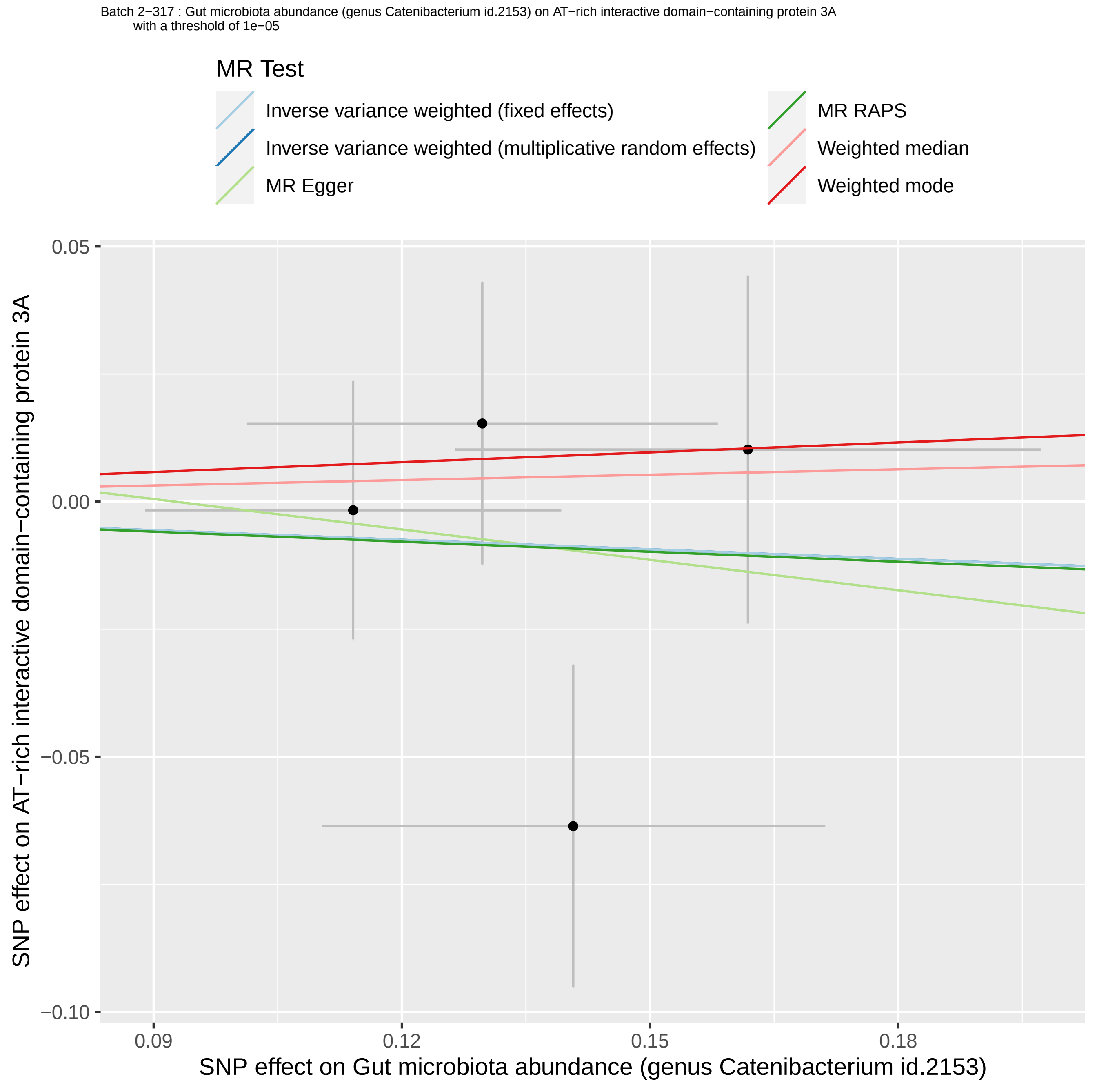

Batch 2−317 : Gut microbiota abundance (genus Catenibacterium id.2153) on AT−rich interactive domain−containing protein 3A
with a threshold of 1e−05
MR Test
Inverse variance weighted (fixed effects)
Inverse variance weighted (multiplicative random effects)
MR Egger
MR RAPS
Weighted median
Weighted mode
0.05
0.00
−0.05
−0.10
0.09
0.12
0.15
0.18
SNP effect on AT−rich interactive domain−containing protein 3A
SNP effect on Gut microbiota abundance (genus Catenibacterium id.2153)

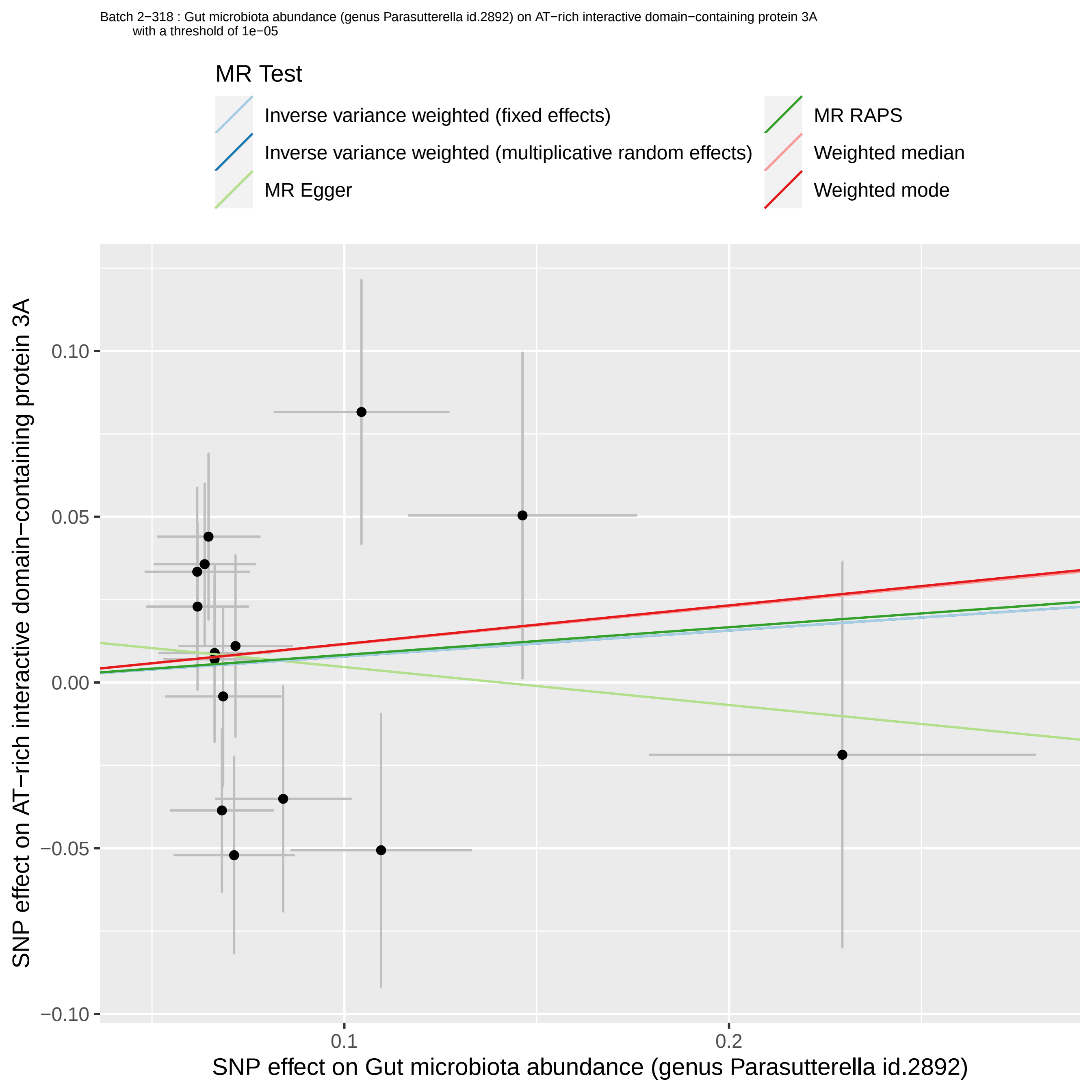

Batch 2−318 : Gut microbiota abundance (genus Parasutterella id.2892) on AT−rich interactive domain−containing protein 3A
with a threshold of 1e−05
MR Test
Inverse variance weighted (fixed effects)
Inverse variance weighted (multiplicative random effects)
MR Egger
MR RAPS
Weighted median
Weighted mode
0.10
0.05
0.00
−0.05
−0.10
0.1
0.2
SNP effect on AT−rich interactive domain−containing protein 3A
SNP effect on Gut microbiota abundance (genus Parasutterella id.2892)

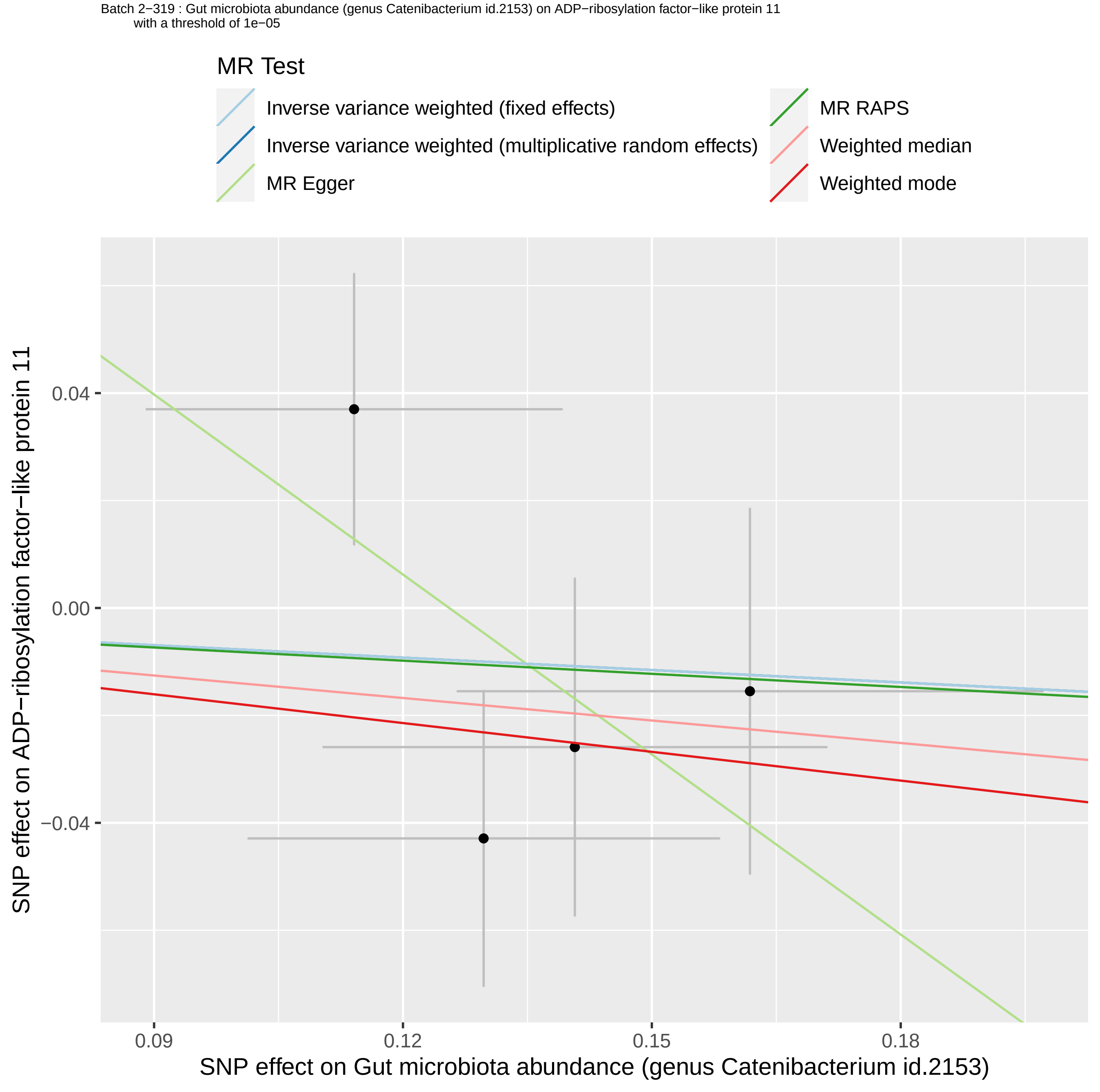

Batch 2−319 : Gut microbiota abundance (genus Catenibacterium id.2153) on ADP−ribosylation factor−like protein 11
with a threshold of 1e−05
MR Test
Inverse variance weighted (fixed effects)
Inverse variance weighted (multiplicative random effects)
MR Egger
MR RAPS
Weighted median
Weighted mode
0.04
0.00
−0.04
0.09
0.12
0.15
0.18
SNP effect on Gut microbiota abundance (genus Catenibacterium id.2153)
SNP effect on ADP−ribosylation factor−like protein 11

Batch 2−320 : Gut microbiota abundance (genus Parasutterella id.2892) on ADP−ribosylation factor−like protein 11
with a threshold of 1e−05

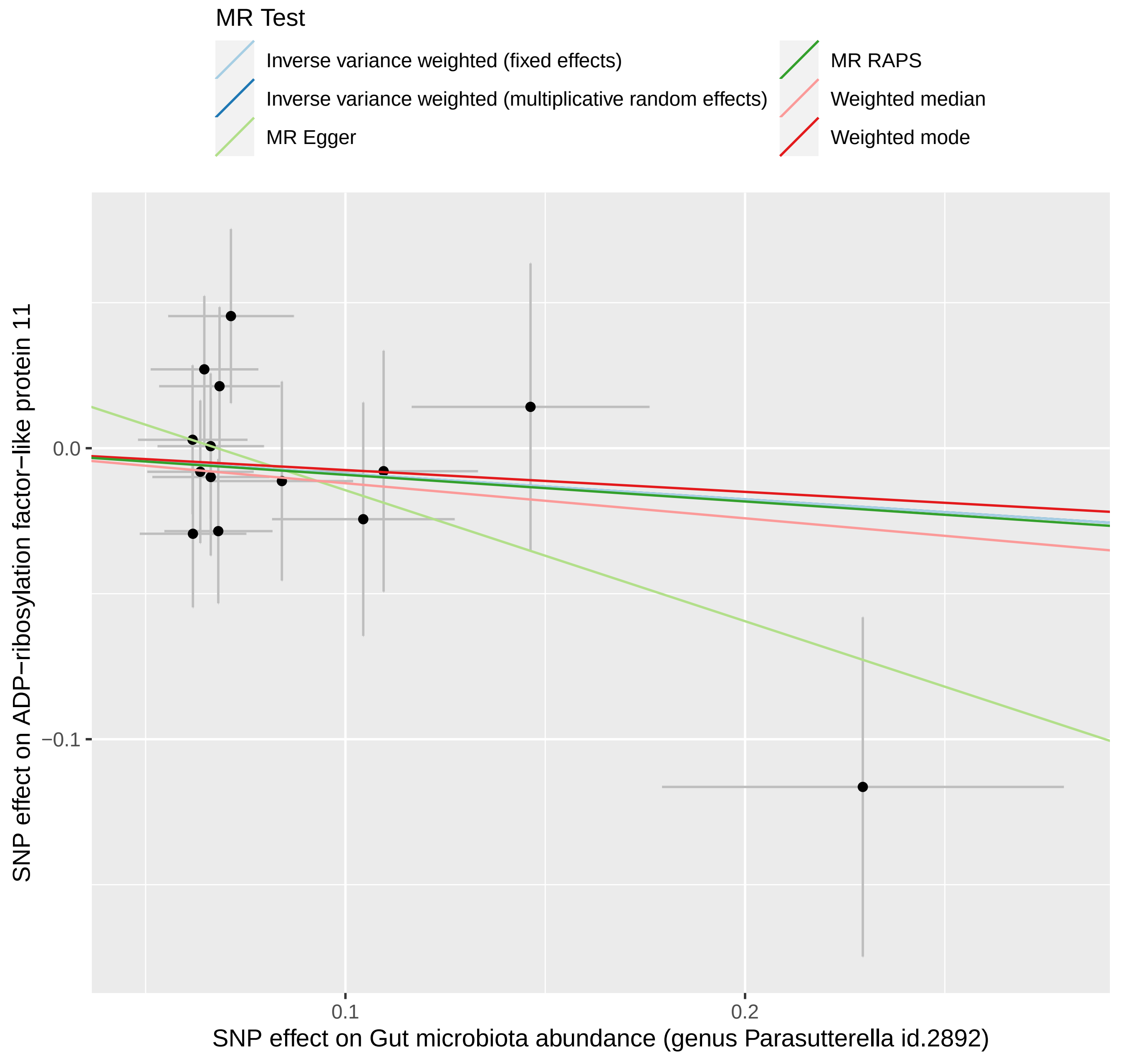

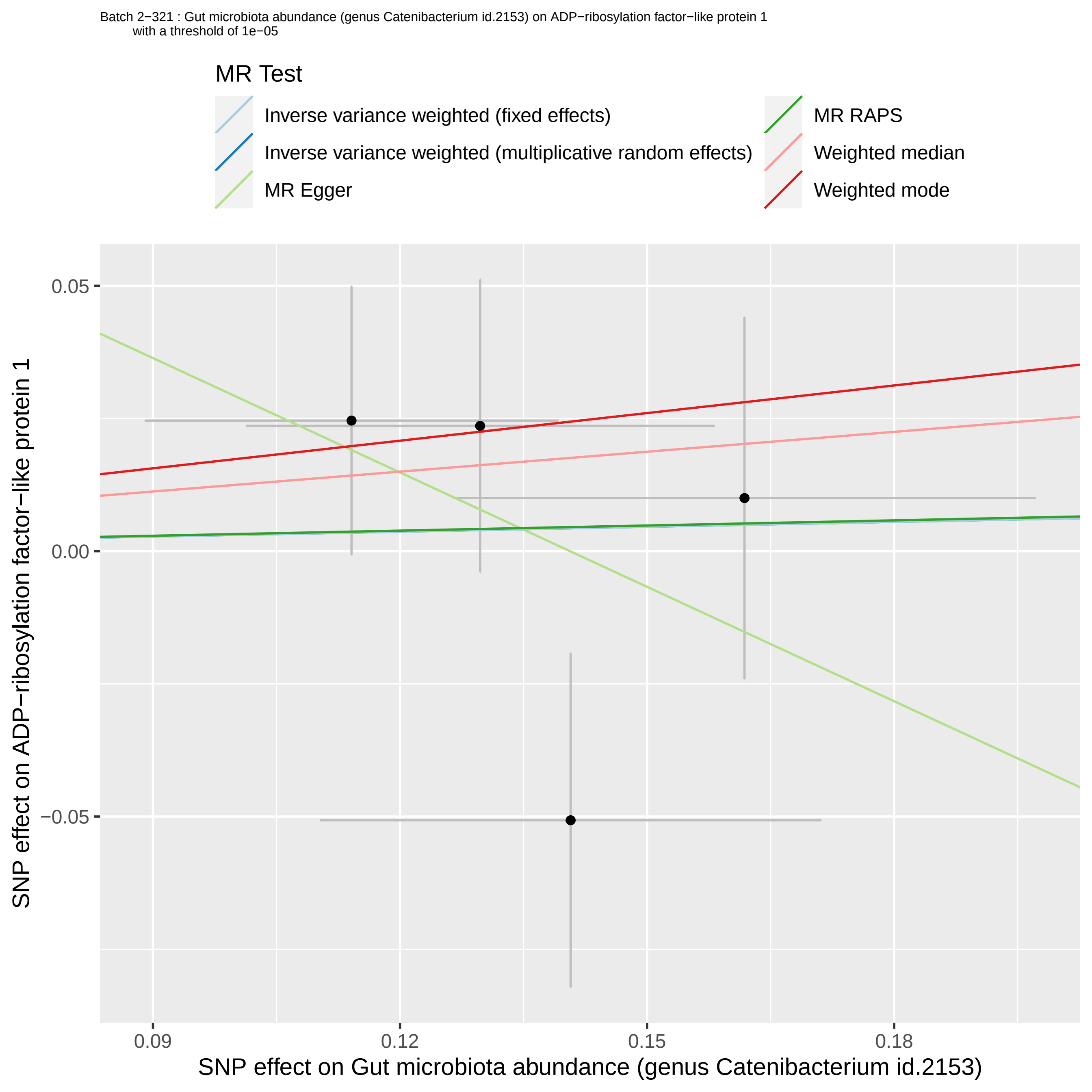

Batch 2−321 : Gut microbiota abundance (genus Catenibacterium id.2153) on ADP−ribosylation factor−like protein 1
with a threshold of 1e−05
MR Test
Inverse variance weighted (fixed effects)
Inverse variance weighted (multiplicative random effects)
MR Egger
MR RAPS
Weighted median
Weighted mode
0.05
0.00
−0.05
0.09
0.12
0.15
0.18
SNP effect on ADP−ribosylation factor−like protein 1
SNP effect on Gut microbiota abundance (genus Catenibacterium id.2153)

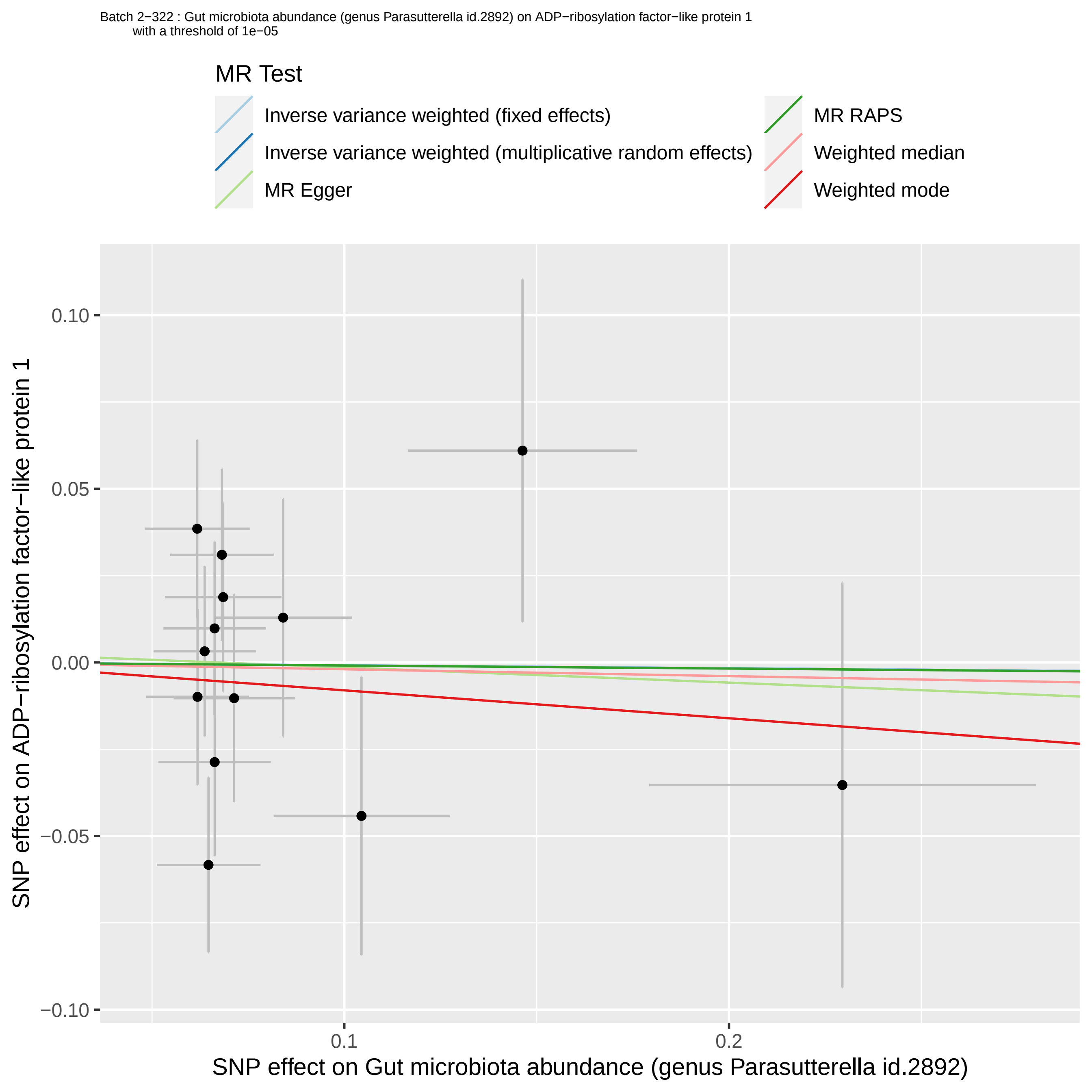
Batch 2−322 : Gut microbiota abundance (genus Parasutterella id.2892) on ADP−ribosylation factor−like protein 1
with a threshold of 1e−05
MR Test
Inverse variance weighted (fixed effects)
Inverse variance weighted (multiplicative random effects)
MR Egger
MR RAPS
Weighted median
Weighted mode
SNP effect on ADP−ribosylation factor−like protein 1
0.10
0.05
0.00
−0.05
−0.10
0.1
0.2
SNP effect on Gut microbiota abundance (genus Parasutterella id.2892)

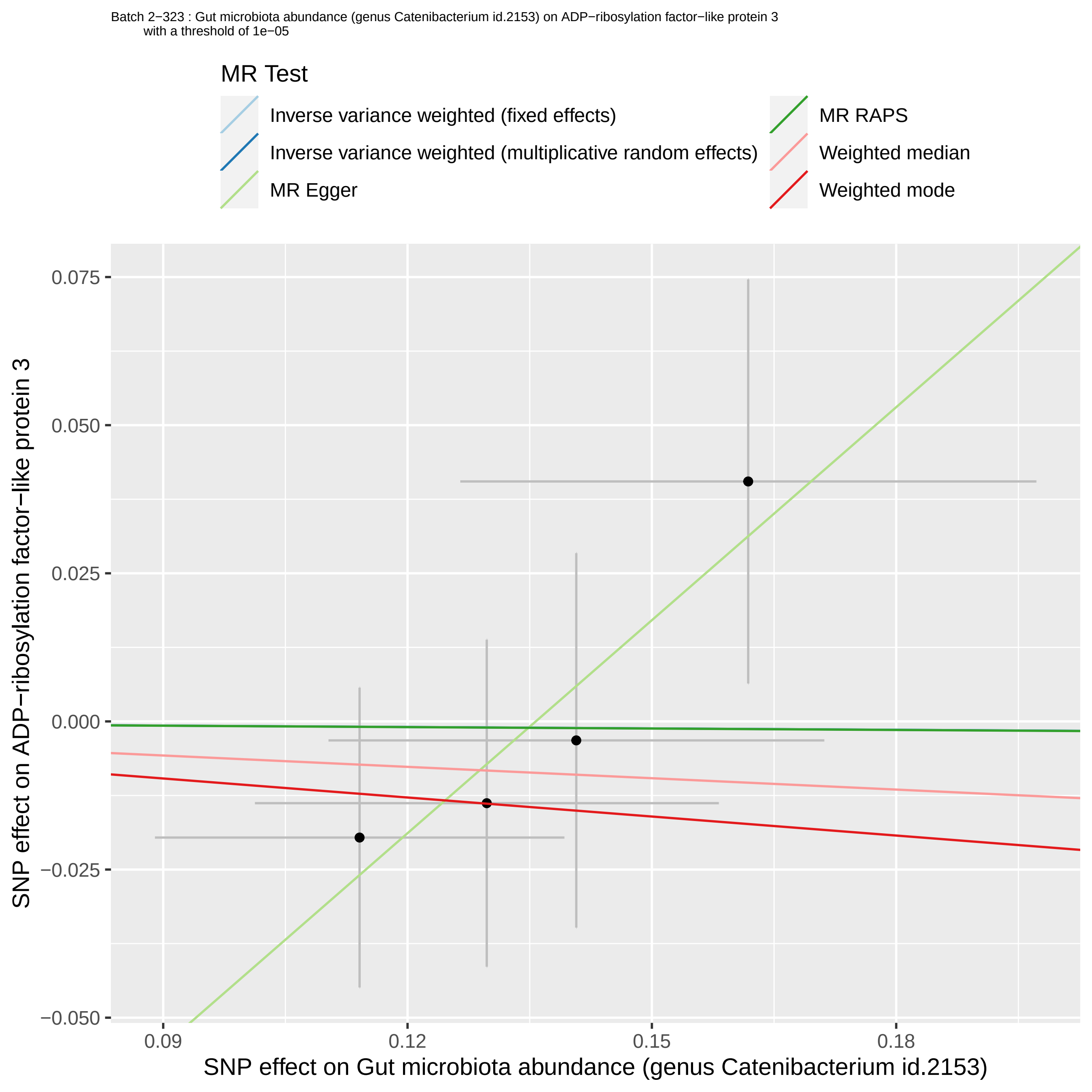

Batch 2−323 : Gut microbiota abundance (genus Catenibacterium id.2153) on ADP−ribosylation factor−like protein 3
with a threshold of 1e−05
MR Test
Inverse variance weighted (fixed effects)
Inverse variance weighted (multiplicative random effects)
MR Egger
MR RAPS
Weighted median
Weighted mode
0.075
0.050
0.025
0.000
−0.025
−0.050
0.09
0.12
0.15
0.18
SNP effect on ADP−ribosylation factor−like protein 3
SNP effect on Gut microbiota abundance (genus Catenibacterium id.2153)

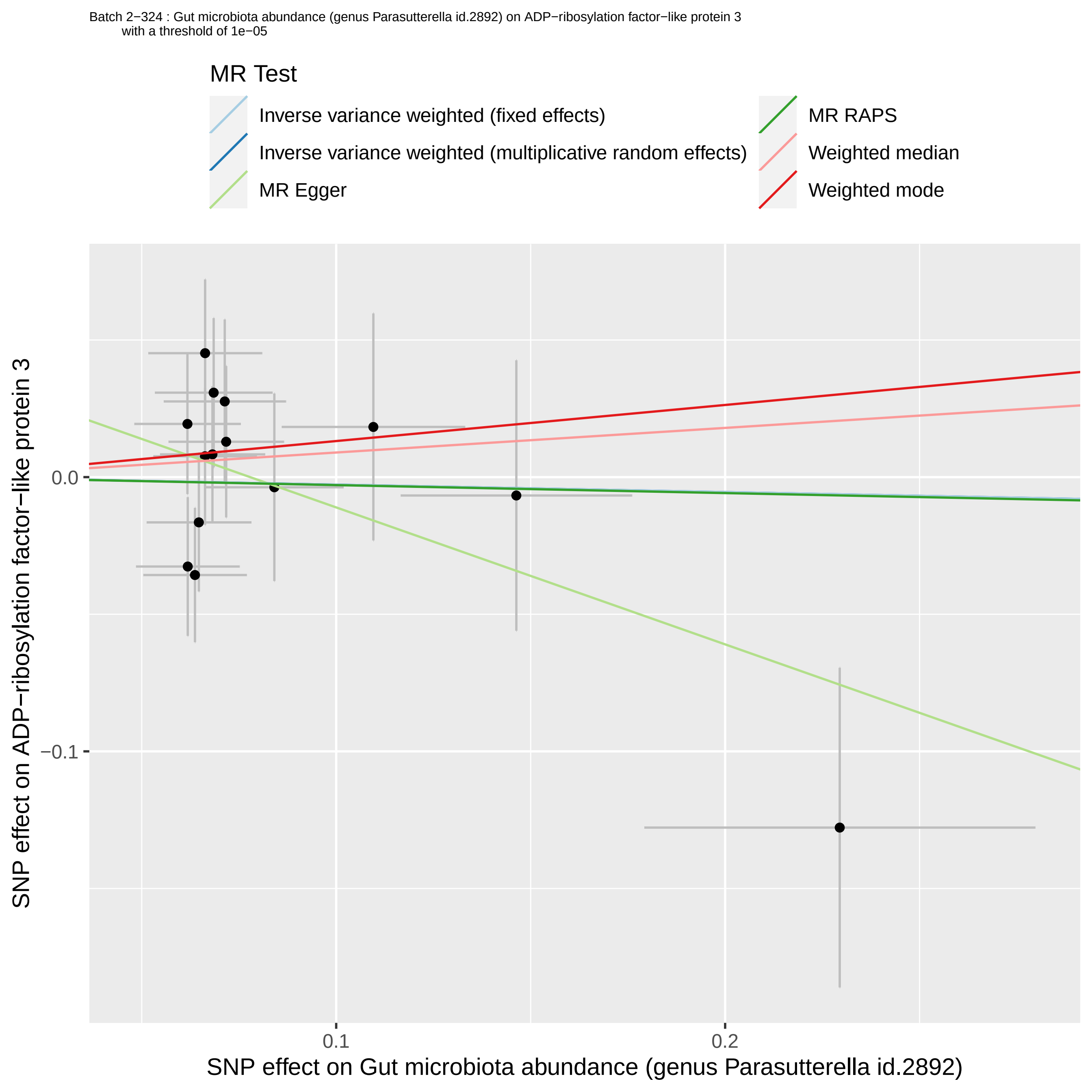

Batch 2−324 : Gut microbiota abundance (genus Parasutterella id.2892) on ADP−ribosylation factor−like protein 3
with a threshold of 1e−05
MR Test
Inverse variance weighted (fixed effects)
Inverse variance weighted (multiplicative random effects)
MR Egger
MR RAPS
Weighted median
Weighted mode
0.0
−0.1
0.1
0.2
SNP effect on ADP−ribosylation factor−like protein 3
SNP effect on Gut microbiota abundance (genus Parasutterella id.2892)

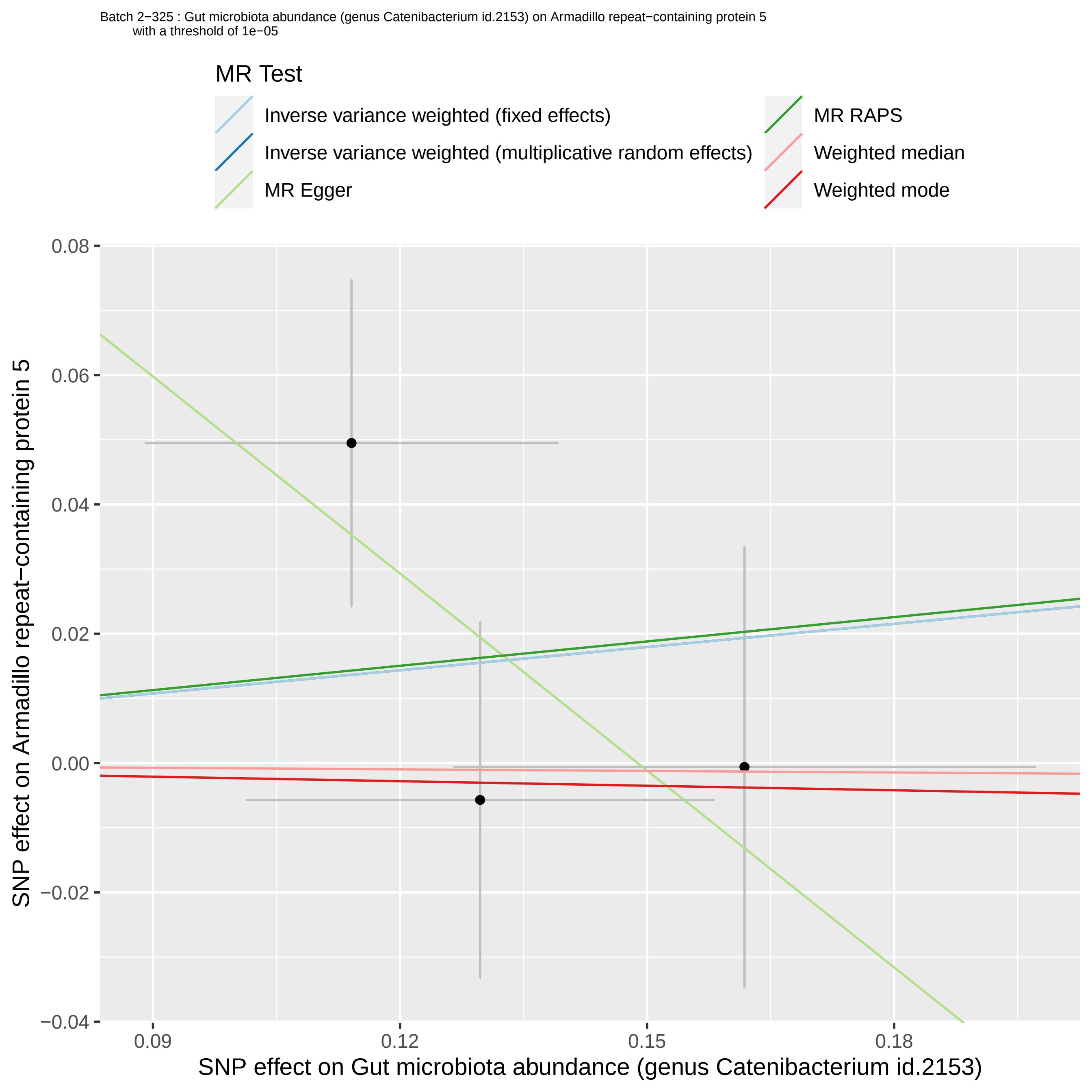
Batch 2−325 : Gut microbiota abundance (genus Catenibacterium id.2153) on Armadillo repeat−containing protein 5
with a threshold of 1e−05
MR Test
Inverse variance weighted (fixed effects)
Inverse variance weighted (multiplicative random effects)
MR Egger
MR RAPS
Weighted median
Weighted mode
0.08
0.06
0.04
0.02
0.00
−0.02
−0.04
0.09
0.12
0.15
0.18
SNP effect on Armadillo repeat−containing protein 5
SNP effect on Gut microbiota abundance (genus Catenibacterium id.2153)

Batch 2−326 : Gut microbiota abundance (genus Parasutterella id.2892) on Armadillo repeat−containing protein 5
with a threshold of 1e−05

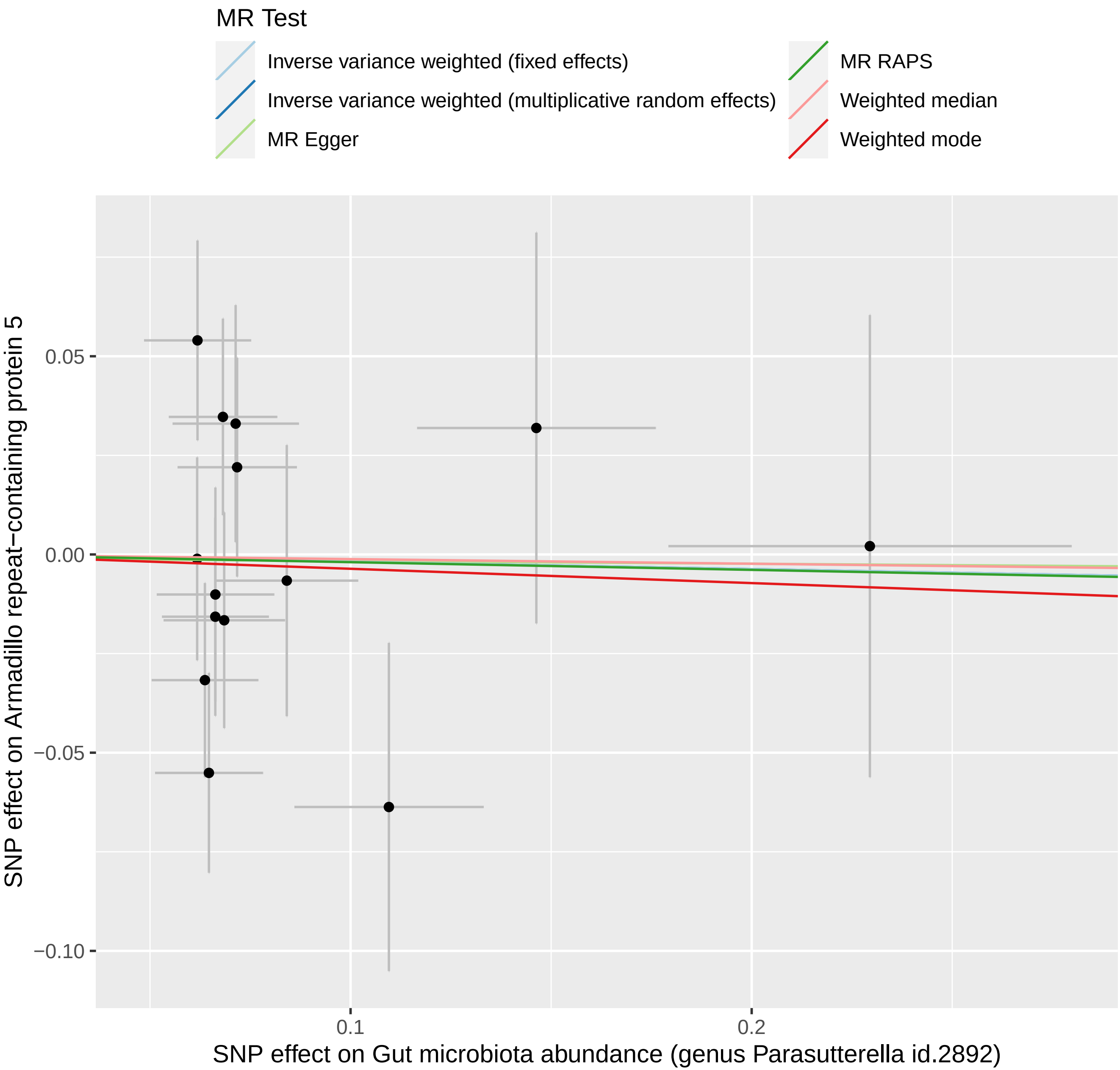

Batch 2−327 : Gut microbiota abundance (genus Catenibacterium id.2153) on Armadillo repeat−containing protein 5
with a threshold of 1e−05

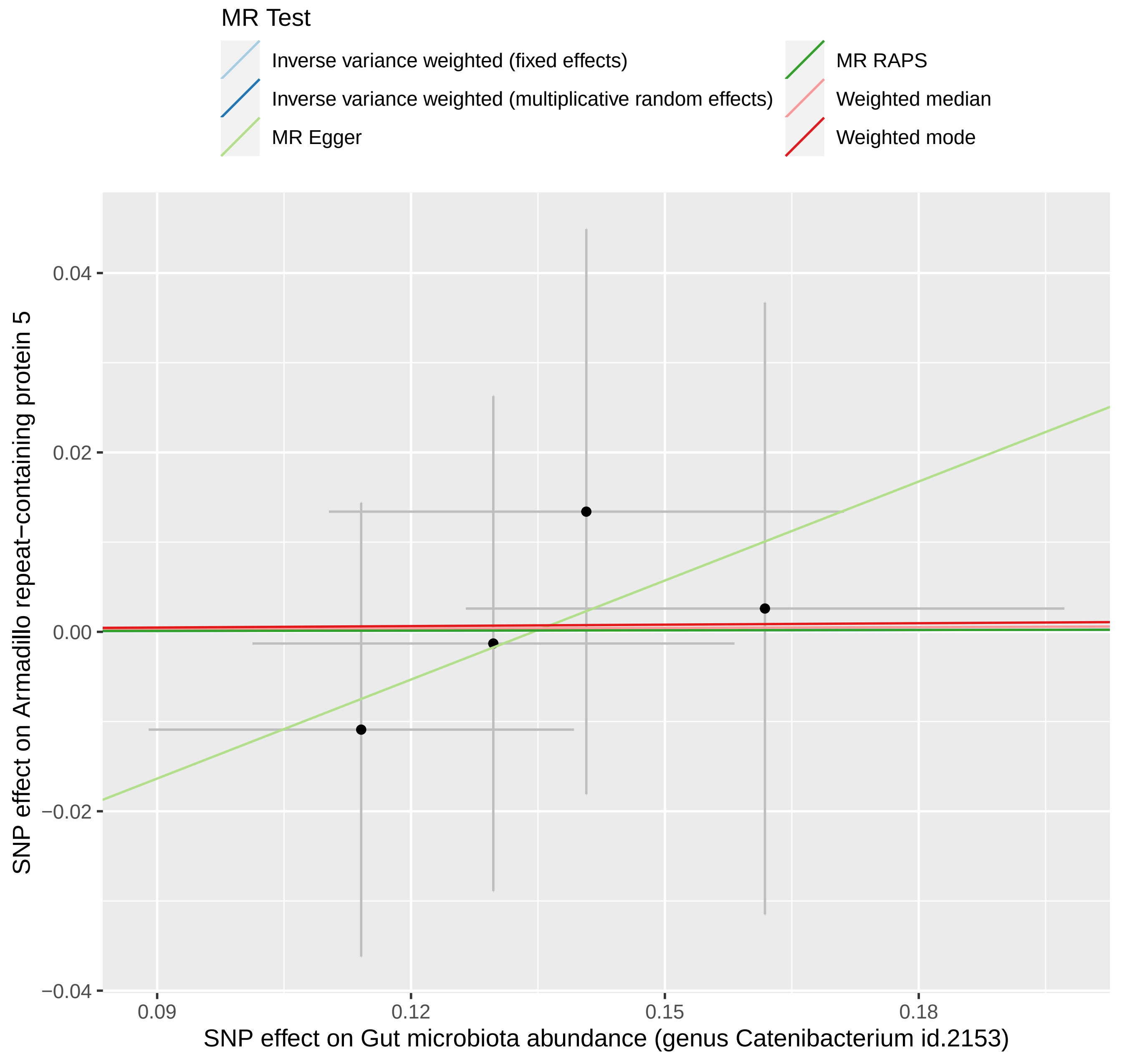

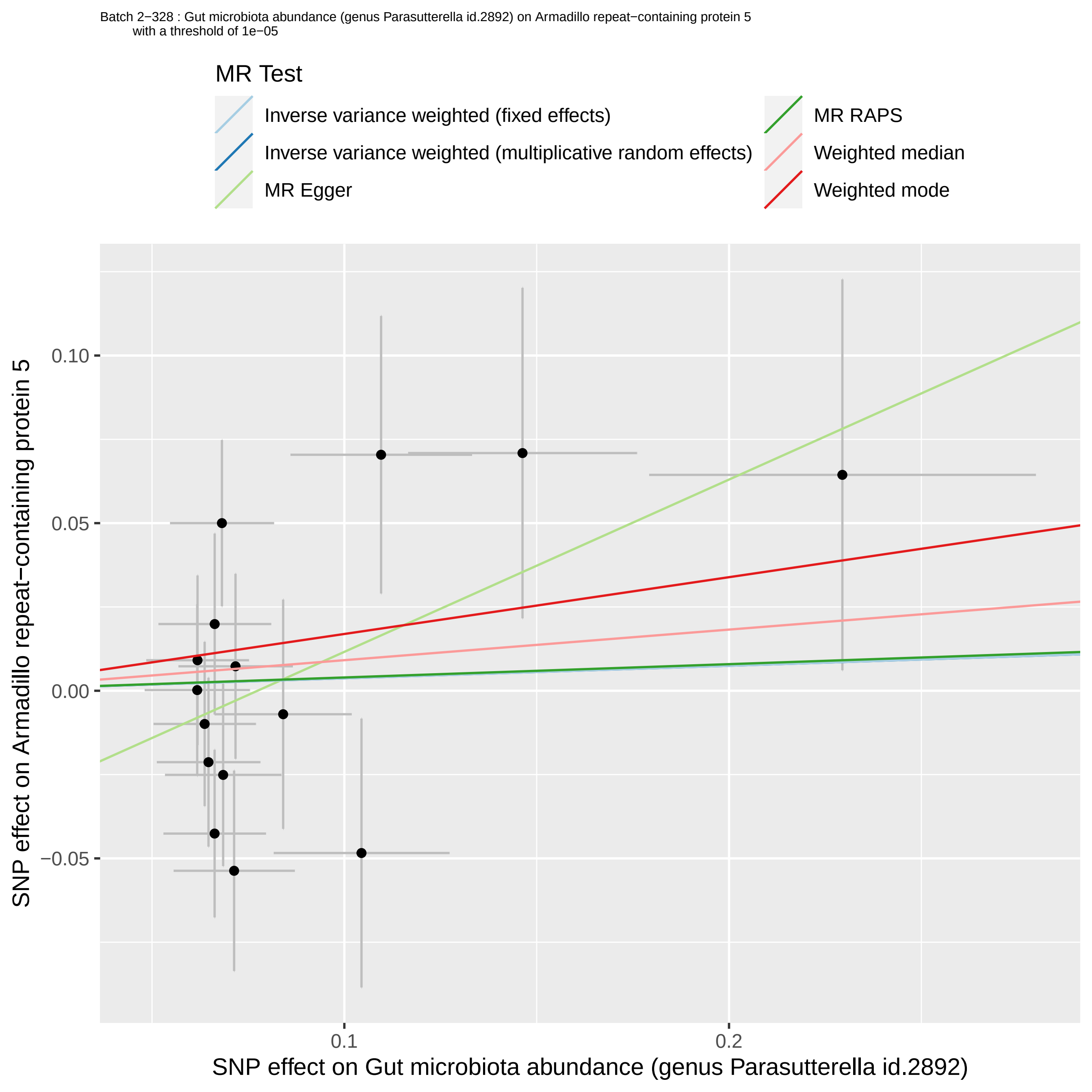

Batch 2−328 : Gut microbiota abundance (genus Parasutterella id.2892) on Armadillo repeat−containing protein 5
with a threshold of 1e−05
MR Test
Inverse variance weighted (fixed effects)
Inverse variance weighted (multiplicative random effects)
MR Egger
MR RAPS
Weighted median
Weighted mode
0.10
0.05
0.00
−0.05
0.1
0.2
SNP effect on Armadillo repeat−containing protein 5
SNP effect on Gut microbiota abundance (genus Parasutterella id.2892)

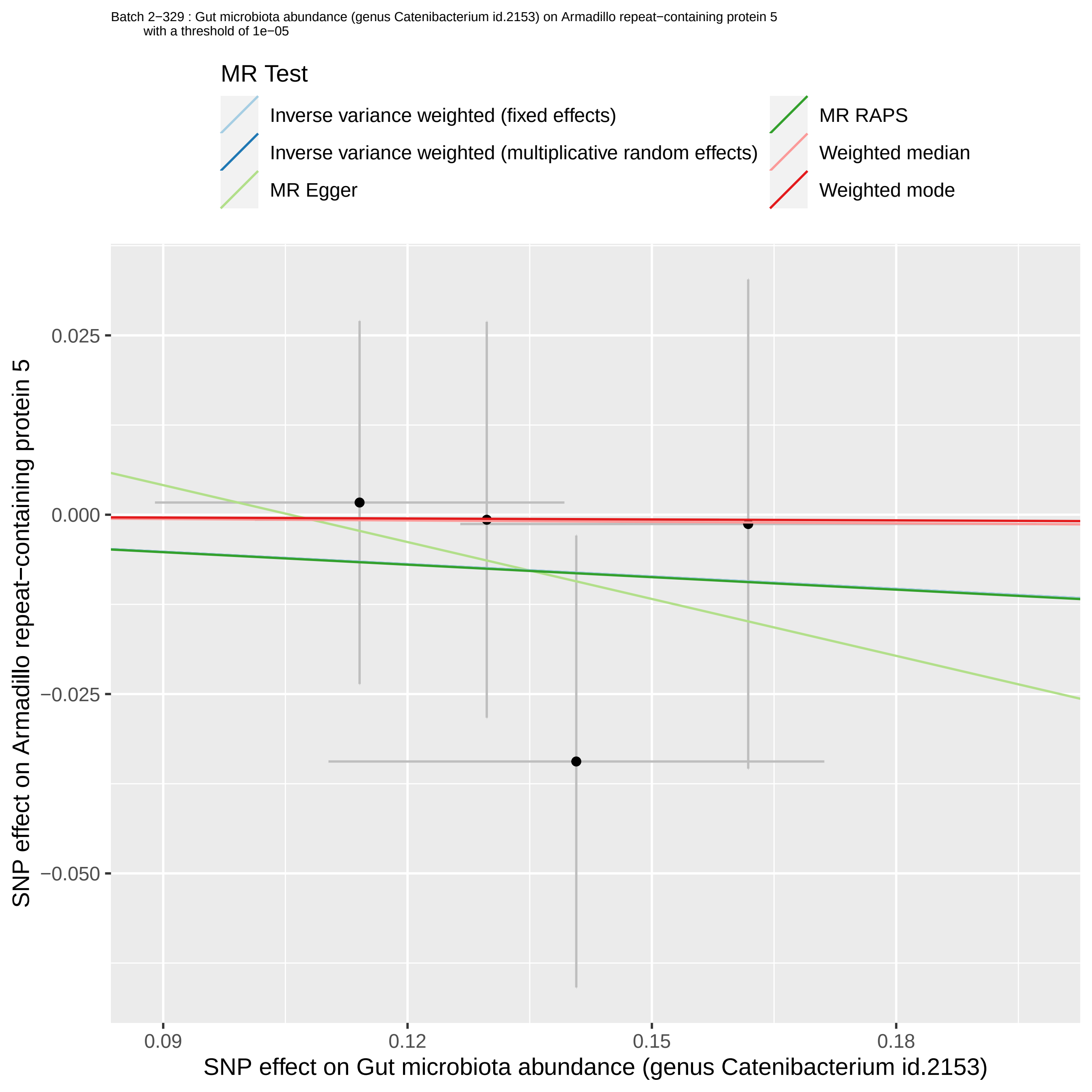

Batch 2−329 : Gut microbiota abundance (genus Catenibacterium id.2153) on Armadillo repeat−containing protein 5
with a threshold of 1e−05
MR Test
Inverse variance weighted (fixed effects)
Inverse variance weighted (multiplicative random effects)
MR Egger
MR RAPS
Weighted median
Weighted mode
0.025
0.000
−0.025
−0.050
0.09
0.12
0.15
0.18
SNP effect on Armadillo repeat−containing protein 5
SNP effect on Gut microbiota abundance (genus Catenibacterium id.2153)

Batch 2−330 : Gut microbiota abundance (genus Parasutterella id.2892) on Armadillo repeat−containing protein 5
with a threshold of 1e−05

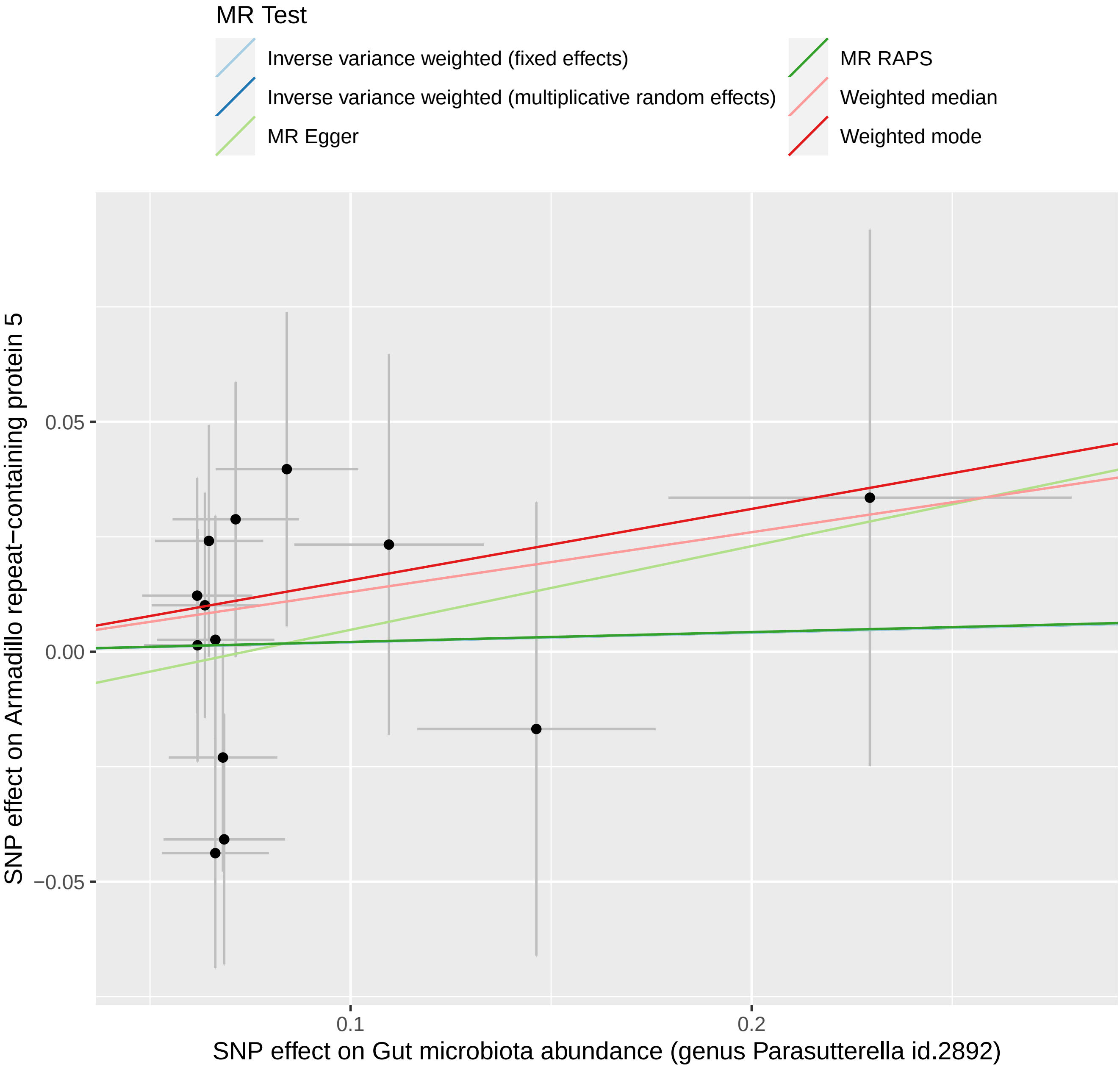

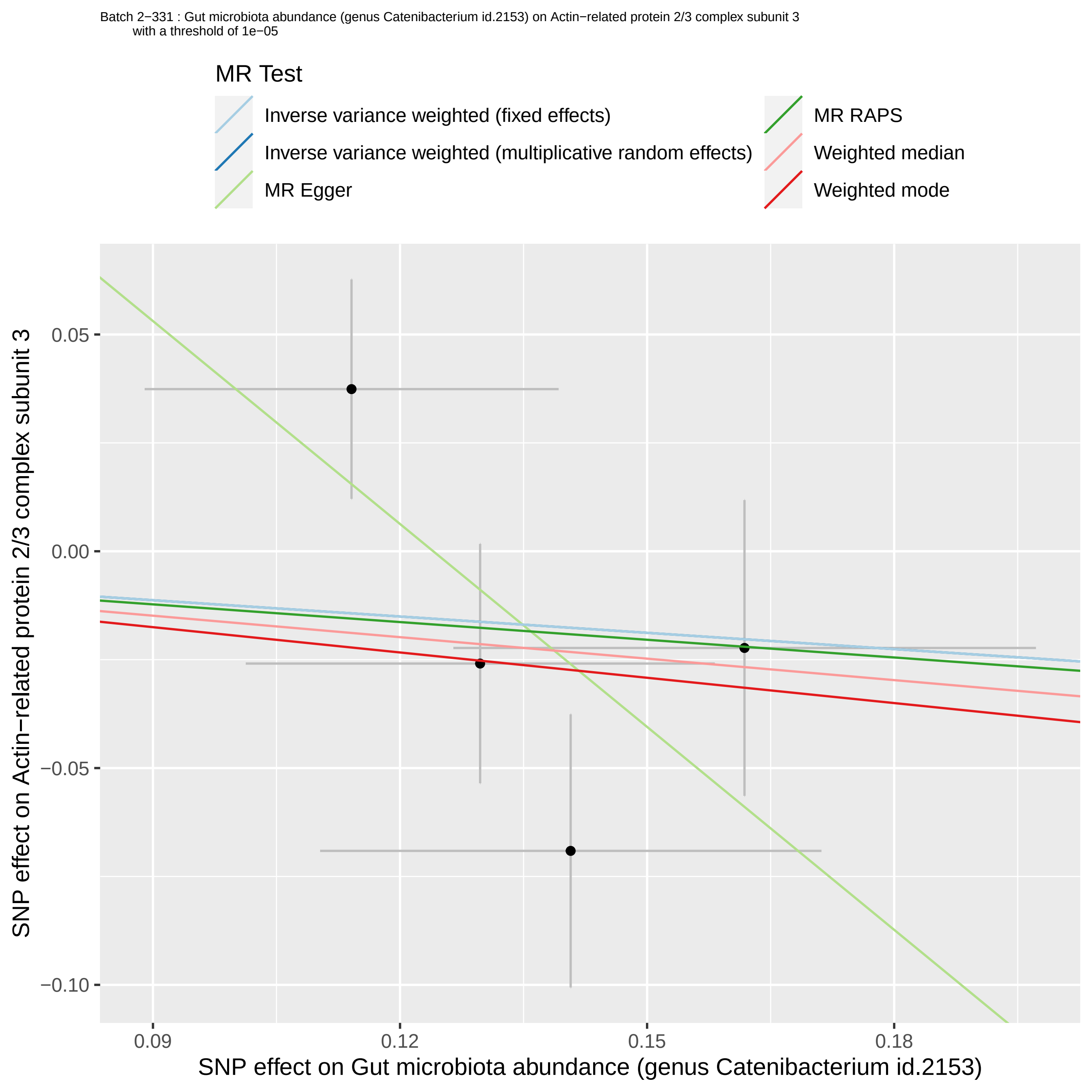

Batch 2−331 : Gut microbiota abundance (genus Catenibacterium id.2153) on Actin−related protein 2/3 complex subunit 3
with a threshold of 1e−05
MR Test
Inverse variance weighted (fixed effects)
Inverse variance weighted (multiplicative random effects)
MR Egger
MR RAPS
Weighted median
Weighted mode
0.05
0.00
−0.05
−0.10
0.09
0.12
0.15
0.18
SNP effect on Actin−related protein 2/3 complex subunit 3
SNP effect on Gut microbiota abundance (genus Catenibacterium id.2153)

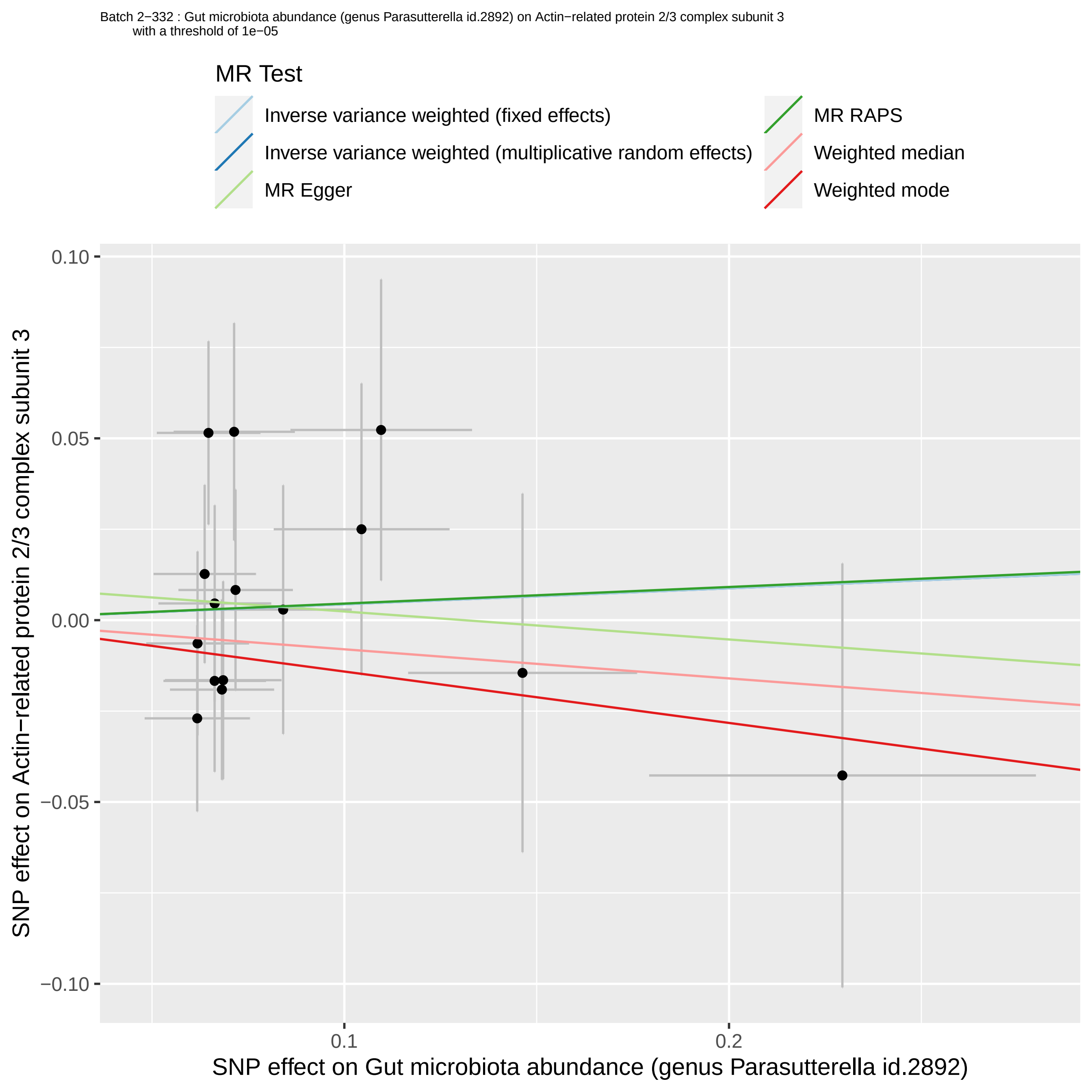

Batch 2−332 : Gut microbiota abundance (genus Parasutterella id.2892) on Actin−related protein 2/3 complex subunit 3
with a threshold of 1e−05
MR Test
Inverse variance weighted (fixed effects)
Inverse variance weighted (multiplicative random effects)
MR Egger
MR RAPS
Weighted median
Weighted mode
0.10
0.05
0.00
−0.05
−0.10
0.1
0.2
SNP effect on Actin−related protein 2/3 complex subunit 3
SNP effect on Gut microbiota abundance (genus Parasutterella id.2892)

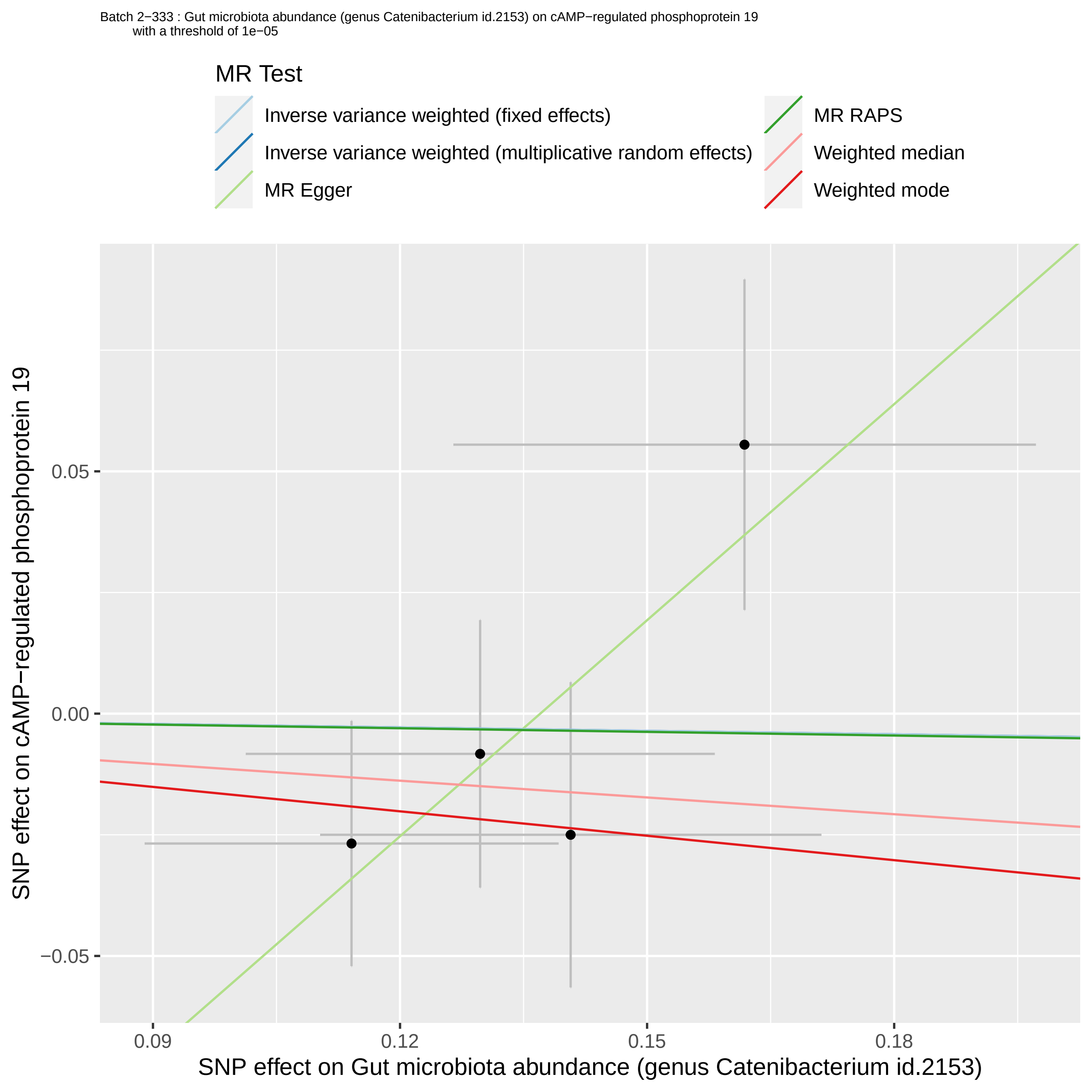

Batch 2−333 : Gut microbiota abundance (genus Catenibacterium id.2153) on cAMP−regulated phosphoprotein 19
with a threshold of 1e−05
MR Test
Inverse variance weighted (fixed effects)
Inverse variance weighted (multiplicative random effects)
MR Egger
MR RAPS
Weighted median
Weighted mode
0.05
0.00
−0.05
0.09
0.12
0.15
0.18
SNP effect on cAMP−regulated phosphoprotein 19
SNP effect on Gut microbiota abundance (genus Catenibacterium id.2153)

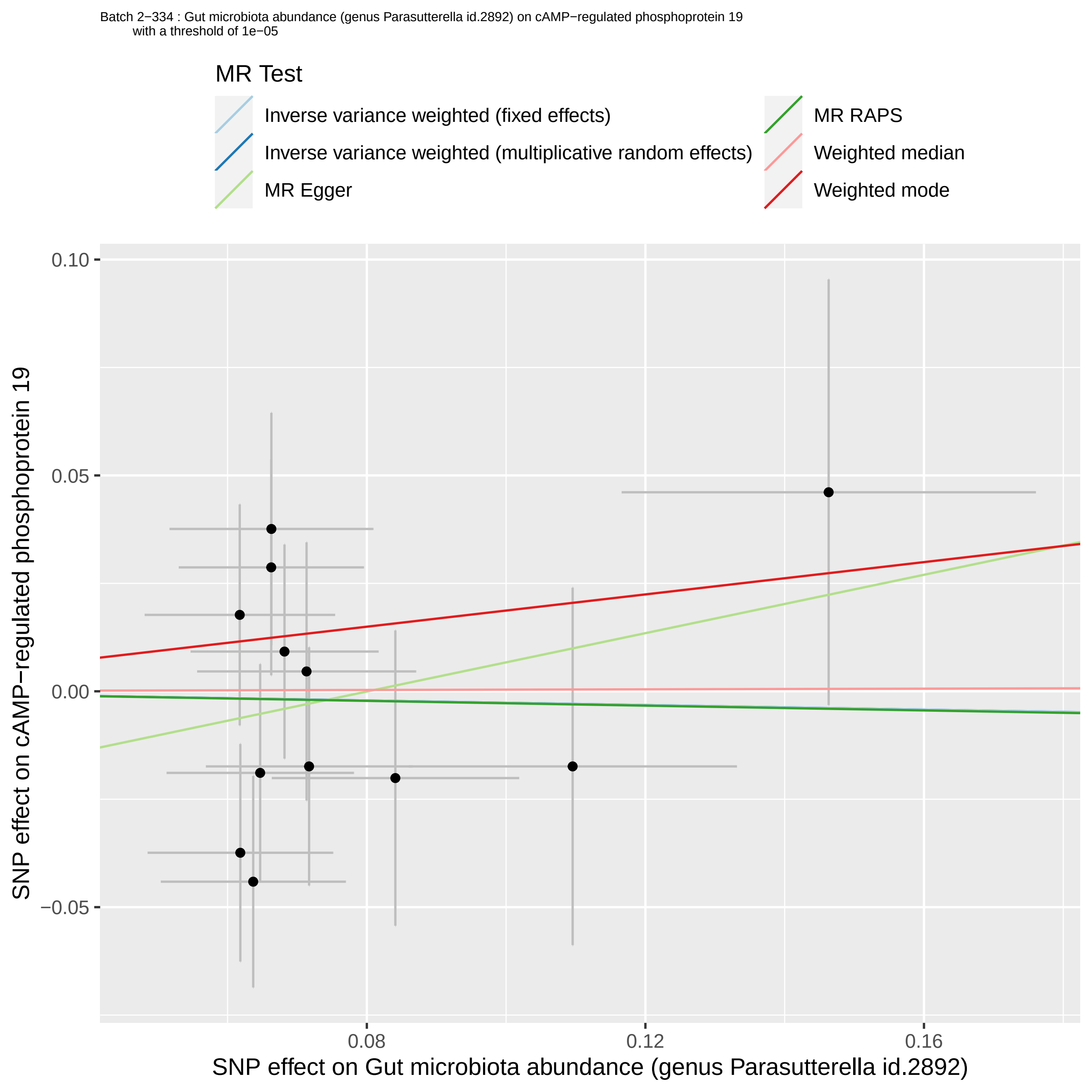
Batch 2−334 : Gut microbiota abundance (genus Parasutterella id.2892) on cAMP−regulated phosphoprotein 19
with a threshold of 1e−05
MR Test
Inverse variance weighted (fixed effects)
Inverse variance weighted (multiplicative random effects)
MR Egger
MR RAPS
Weighted median
Weighted mode
0.10
0.05
0.00
−0.05
0.08
0.12
0.16
SNP effect on cAMP−regulated phosphoprotein 19
SNP effect on Gut microbiota abundance (genus Parasutterella id.2892)

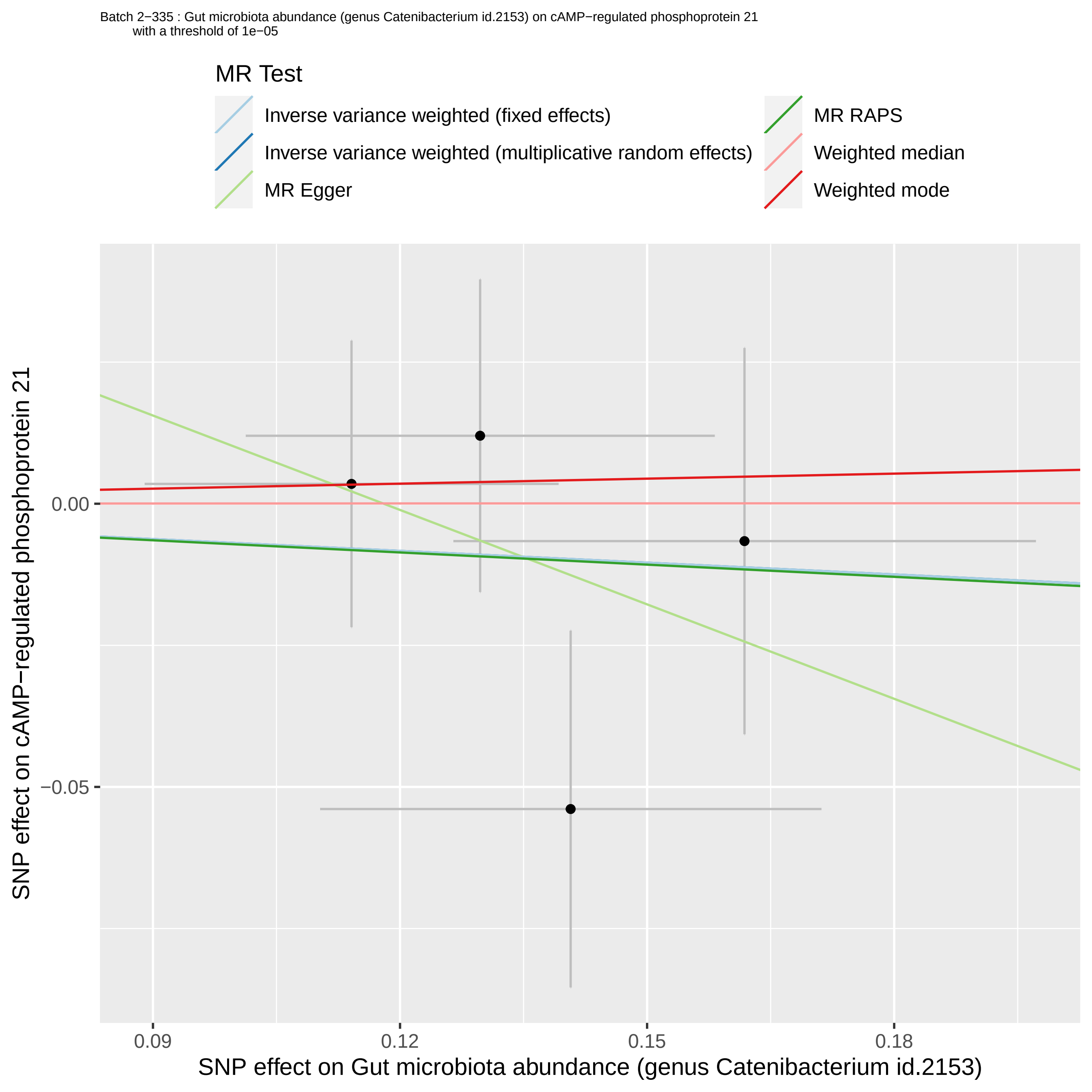
Batch 2−335 : Gut microbiota abundance (genus Catenibacterium id.2153) on cAMP−regulated phosphoprotein 21
with a threshold of 1e−05
MR Test
Inverse variance weighted (fixed effects)
Inverse variance weighted (multiplicative random effects)
MR Egger
MR RAPS
Weighted median
Weighted mode
0.00
−0.05
0.09
0.12
0.15
0.18
SNP effect on cAMP−regulated phosphoprotein 21
SNP effect on Gut microbiota abundance (genus Catenibacterium id.2153)

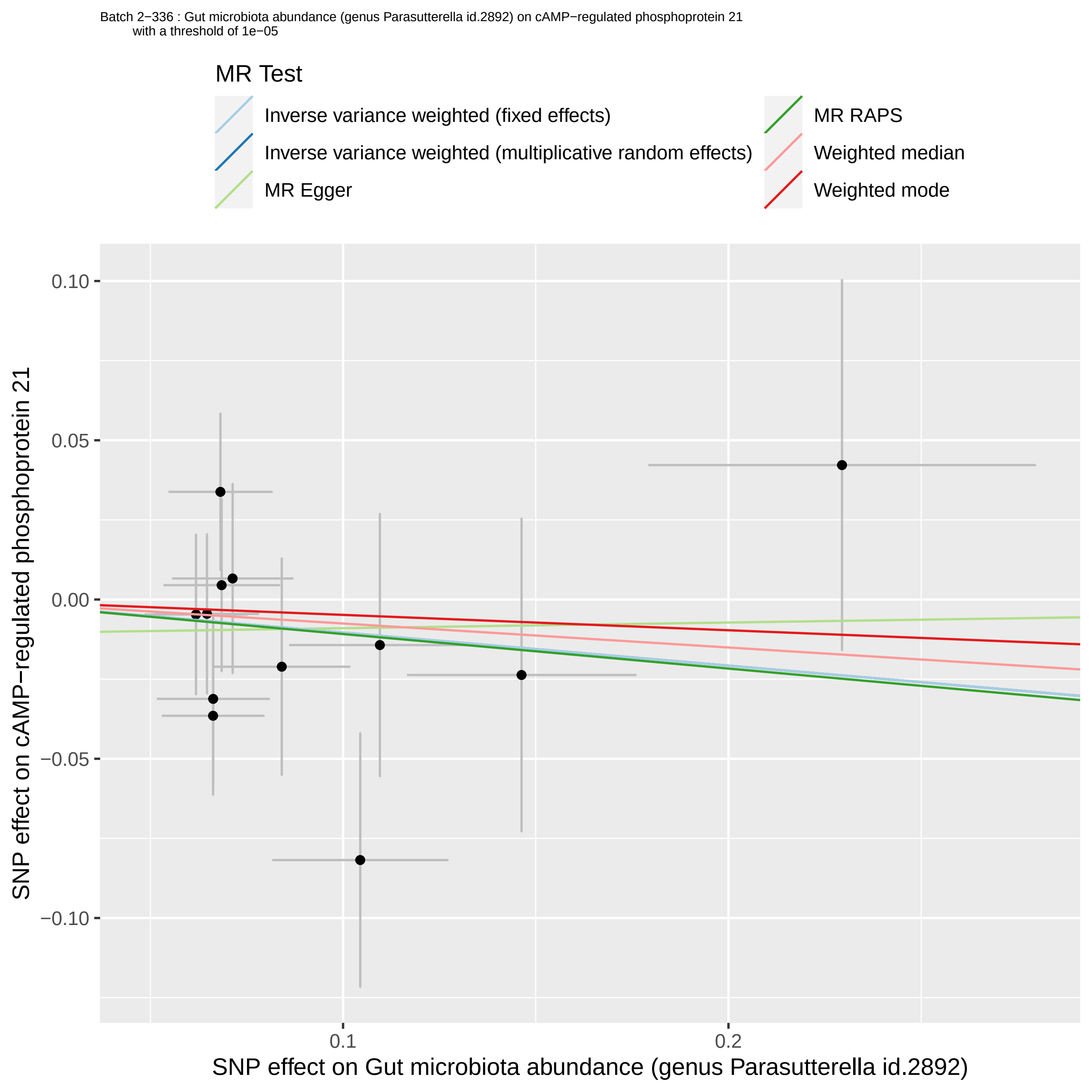
Batch 2−336 : Gut microbiota abundance (genus Parasutterella id.2892) on cAMP−regulated phosphoprotein 21
with a threshold of 1e−05
MR Test
Inverse variance weighted (fixed effects)
Inverse variance weighted (multiplicative random effects)
MR Egger
MR RAPS
Weighted median
Weighted mode
SNP effect on cAMP−regulated phosphoprotein 21
0.10
0.05
0.00
−0.05
−0.10
0.1
0.2
SNP effect on Gut microbiota abundance (genus Parasutterella id.2892)

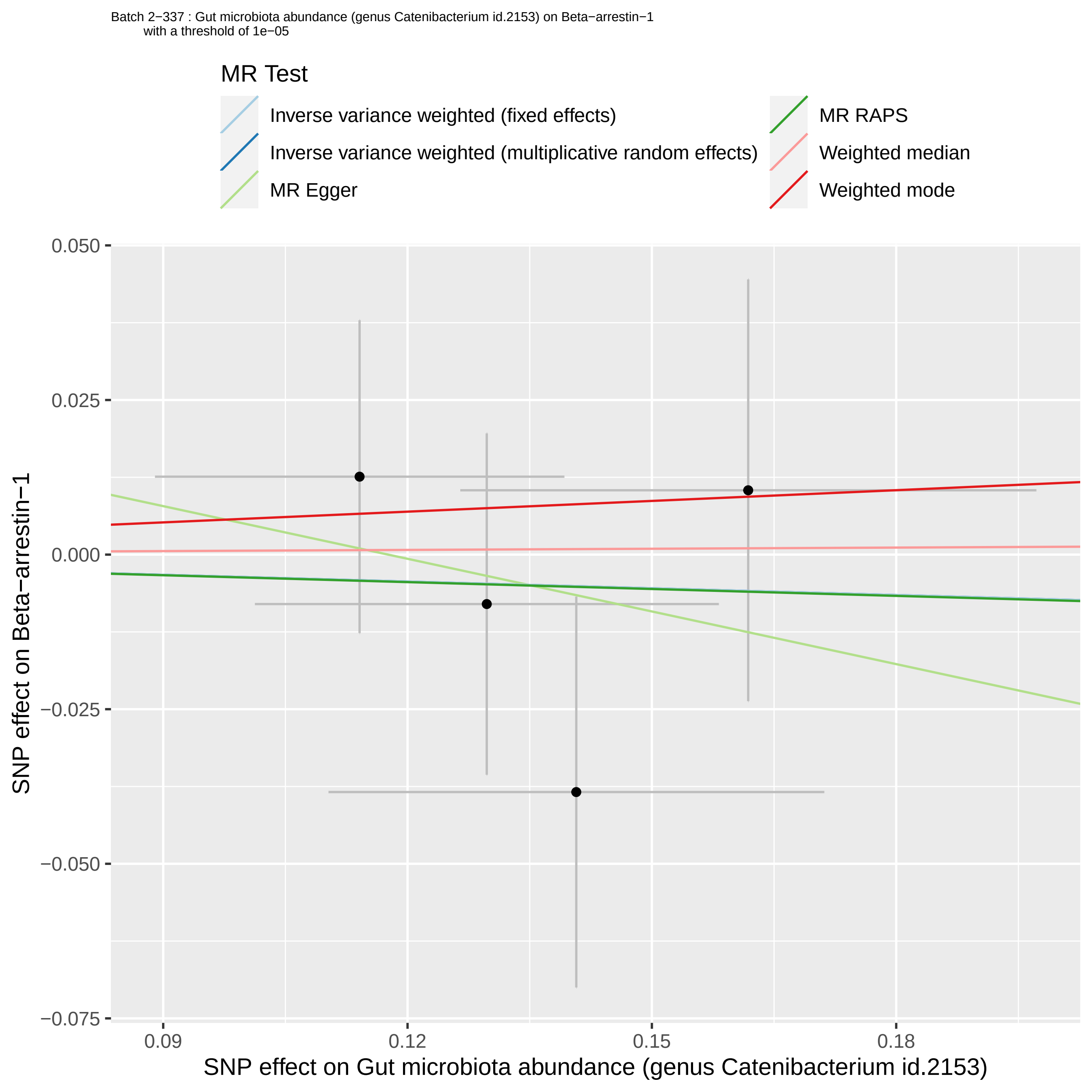
Batch 2−337 : Gut microbiota abundance (genus Catenibacterium id.2153) on Beta−arrestin−1
with a threshold of 1e−05
MR Test
Inverse variance weighted (fixed effects)
Inverse variance weighted (multiplicative random effects)
MR Egger
MR RAPS
Weighted median
Weighted mode
0.050
0.025
0.000
−0.025
−0.050
−0.075
0.09
0.12
0.15
0.18
SNP effect on Beta−arrestin−1
SNP effect on Gut microbiota abundance (genus Catenibacterium id.2153)

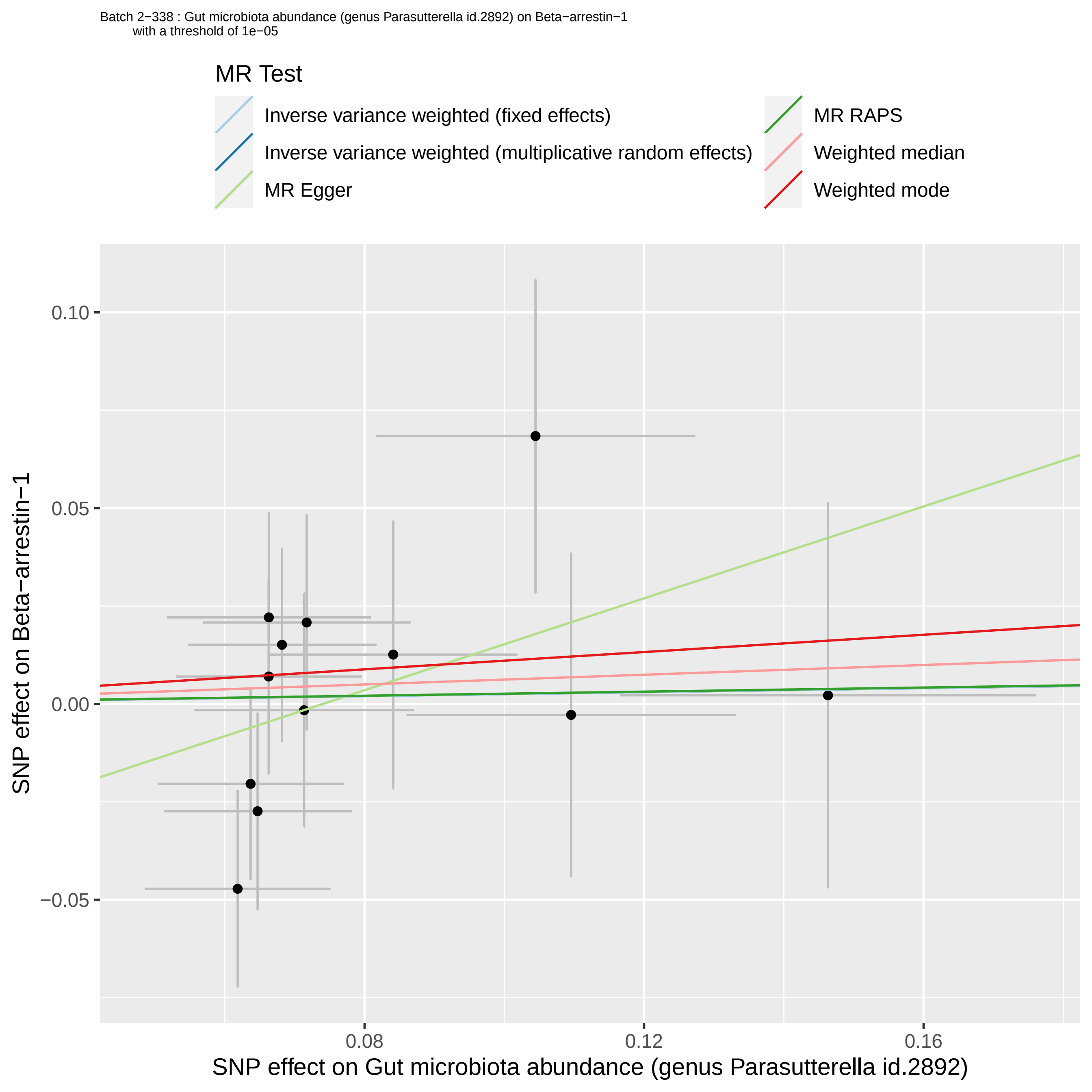

Batch 2−338 : Gut microbiota abundance (genus Parasutterella id.2892) on Beta−arrestin−1
with a threshold of 1e−05
MR Test
Inverse variance weighted (fixed effects)
Inverse variance weighted (multiplicative random effects)
MR Egger
MR RAPS
Weighted median
Weighted mode
0.10
0.05
0.00
−0.05
0.08
0.12
0.16
SNP effect on Beta−arrestin−1
SNP effect on Gut microbiota abundance (genus Parasutterella id.2892)

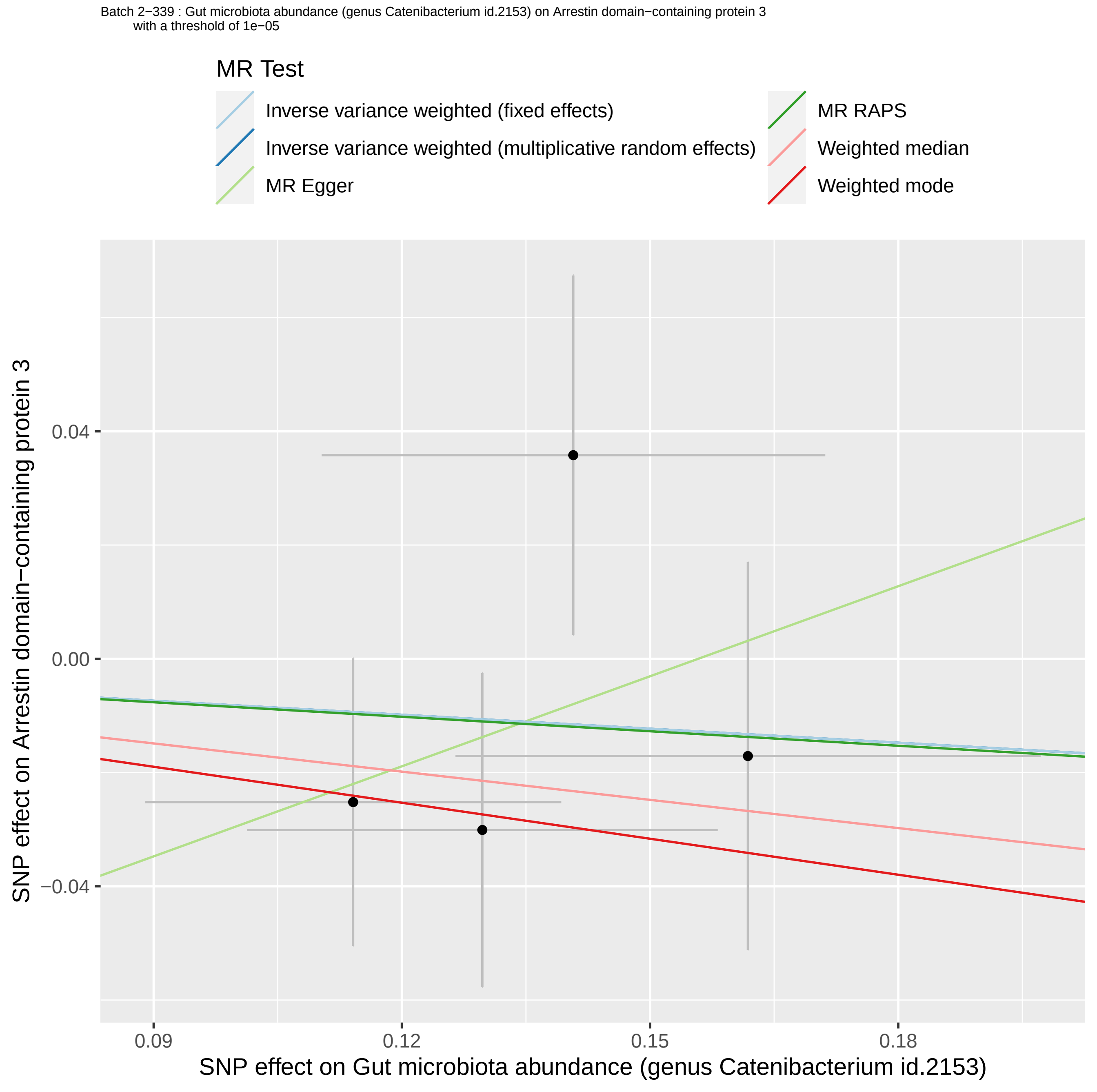

Batch 2−339 : Gut microbiota abundance (genus Catenibacterium id.2153) on Arrestin domain−containing protein 3
with a threshold of 1e−05
MR Test
Inverse variance weighted (fixed effects)
Inverse variance weighted (multiplicative random effects)
MR Egger
MR RAPS
Weighted median
Weighted mode
0.04
0.00
−0.04
0.09
0.12
0.15
0.18
SNP effect on Arrestin domain−containing protein 3
SNP effect on Gut microbiota abundance (genus Catenibacterium id.2153)

Batch 2−340 : Gut microbiota abundance (genus Parasutterella id.2892) on Arrestin domain−containing protein 3
with a threshold of 1e−05

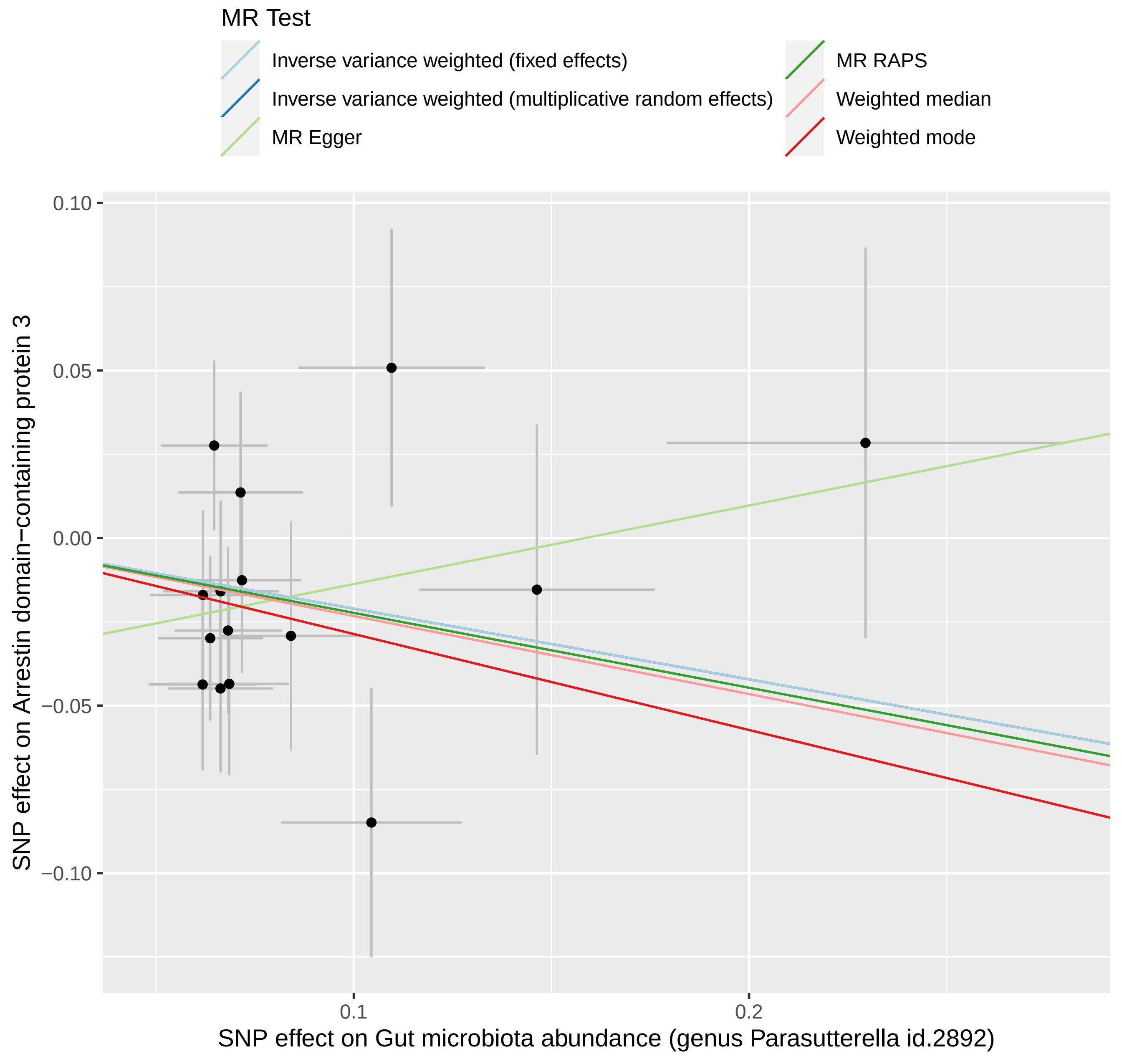

Batch 2−341 : Gut microbiota abundance (genus Catenibacterium id.2153) on Arylsulfatase A
with a threshold of 1e−05

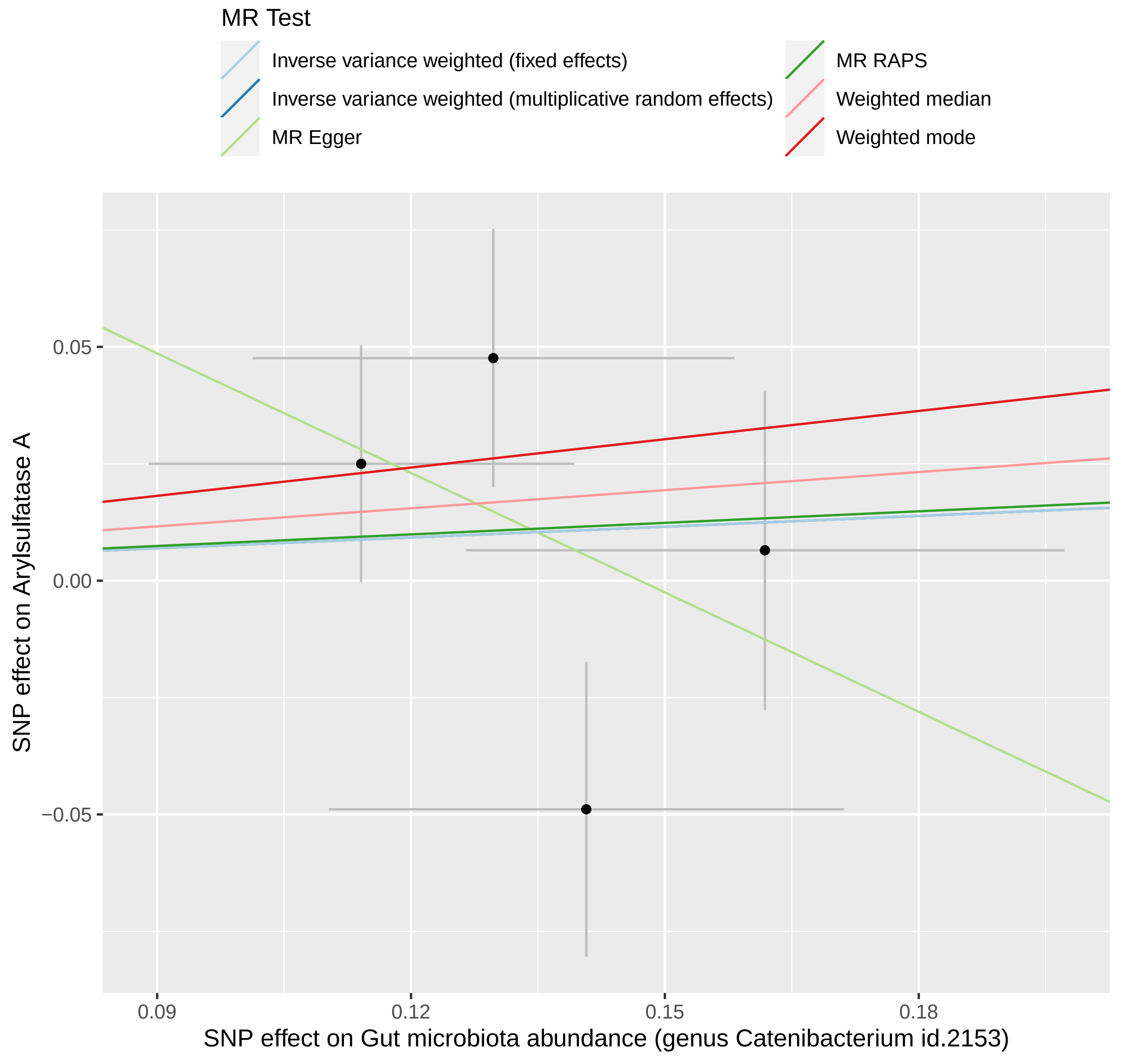

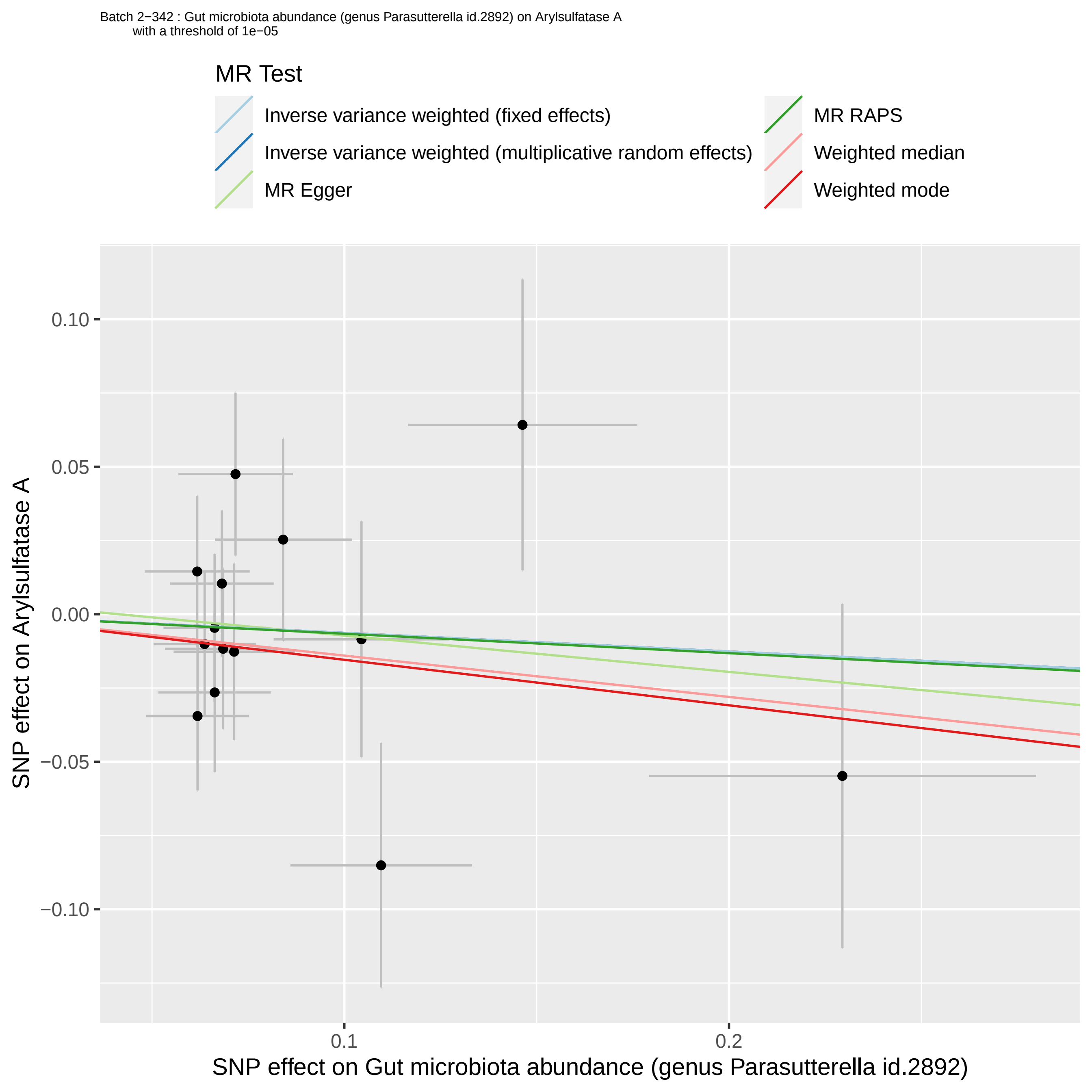

Batch 2−342 : Gut microbiota abundance (genus Parasutterella id.2892) on Arylsulfatase A
with a threshold of 1e−05
MR Test
Inverse variance weighted (fixed effects)
Inverse variance weighted (multiplicative random effects)
MR Egger
MR RAPS
Weighted median
Weighted mode
SNP effect on Arylsulfatase A
0.10
0.05
0.00
−0.05
−0.10
0.1
0.2
SNP effect on Gut microbiota abundance (genus Parasutterella id.2892)

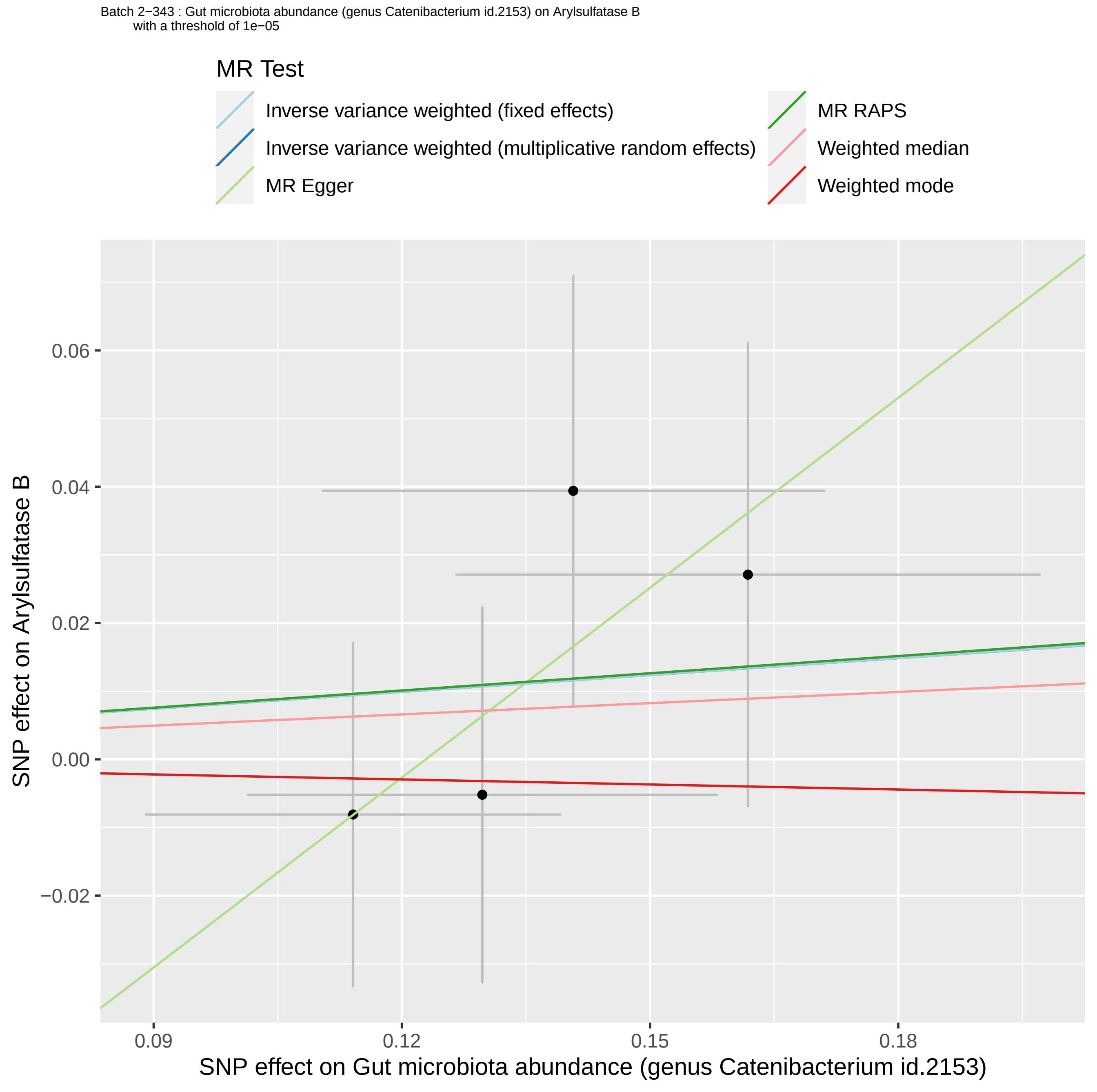

Batch 2−343 : Gut microbiota abundance (genus Catenibacterium id.2153) on Arylsulfatase B
with a threshold of 1e−05
MR Test
Inverse variance weighted (fixed effects)
Inverse variance weighted (multiplicative random effects)
MR Egger
MR RAPS
Weighted median
Weighted mode
0.06
0.04
0.02
0.00
−0.02
SNP effect on Arylsulfatase B
0.09
0.12
0.15
0.18
SNP effect on Gut microbiota abundance (genus Catenibacterium id.2153)

Batch 2−344 : Gut microbiota abundance (genus Parasutterella id.2892) on Arylsulfatase B
with a threshold of 1e−05

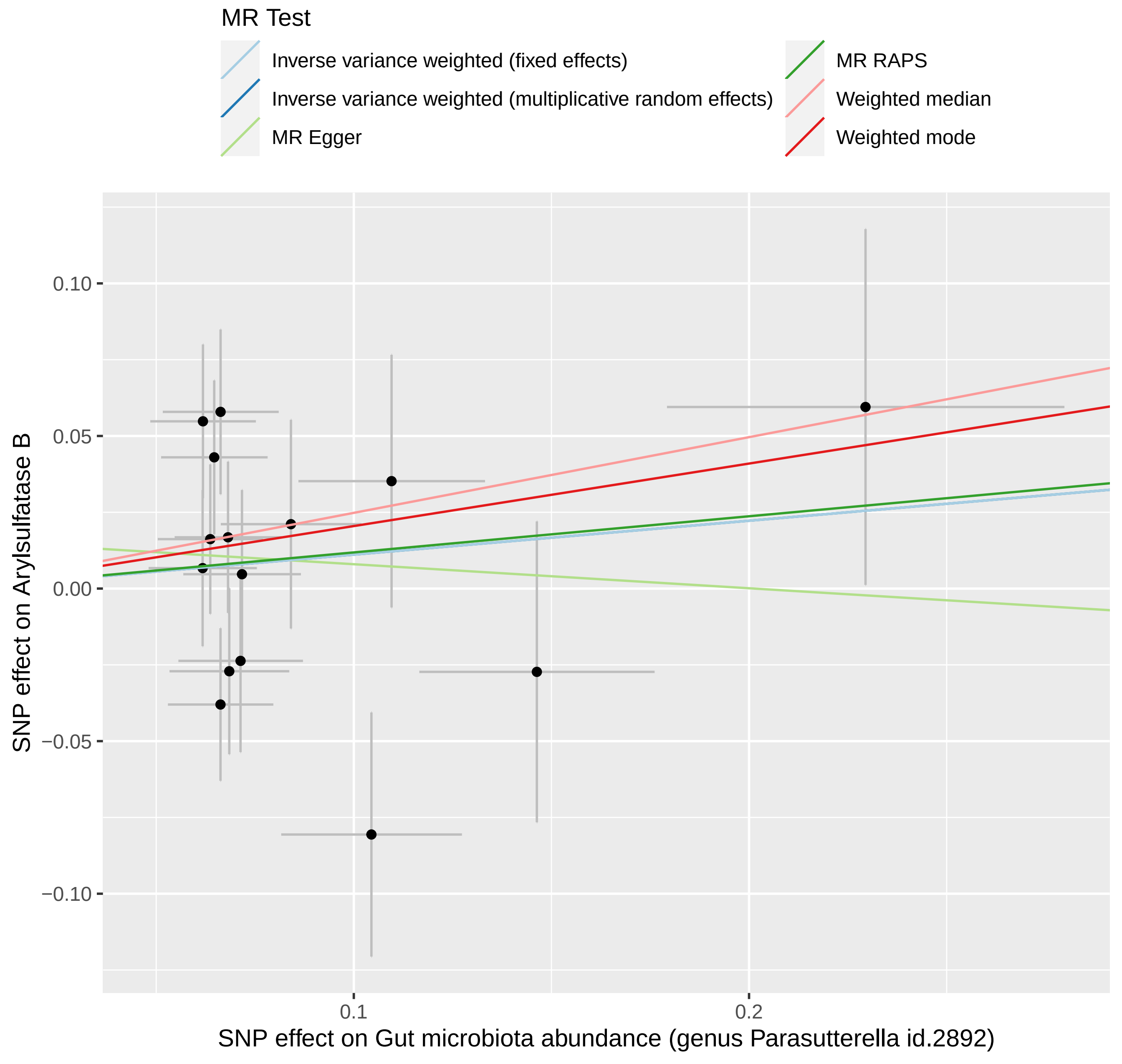

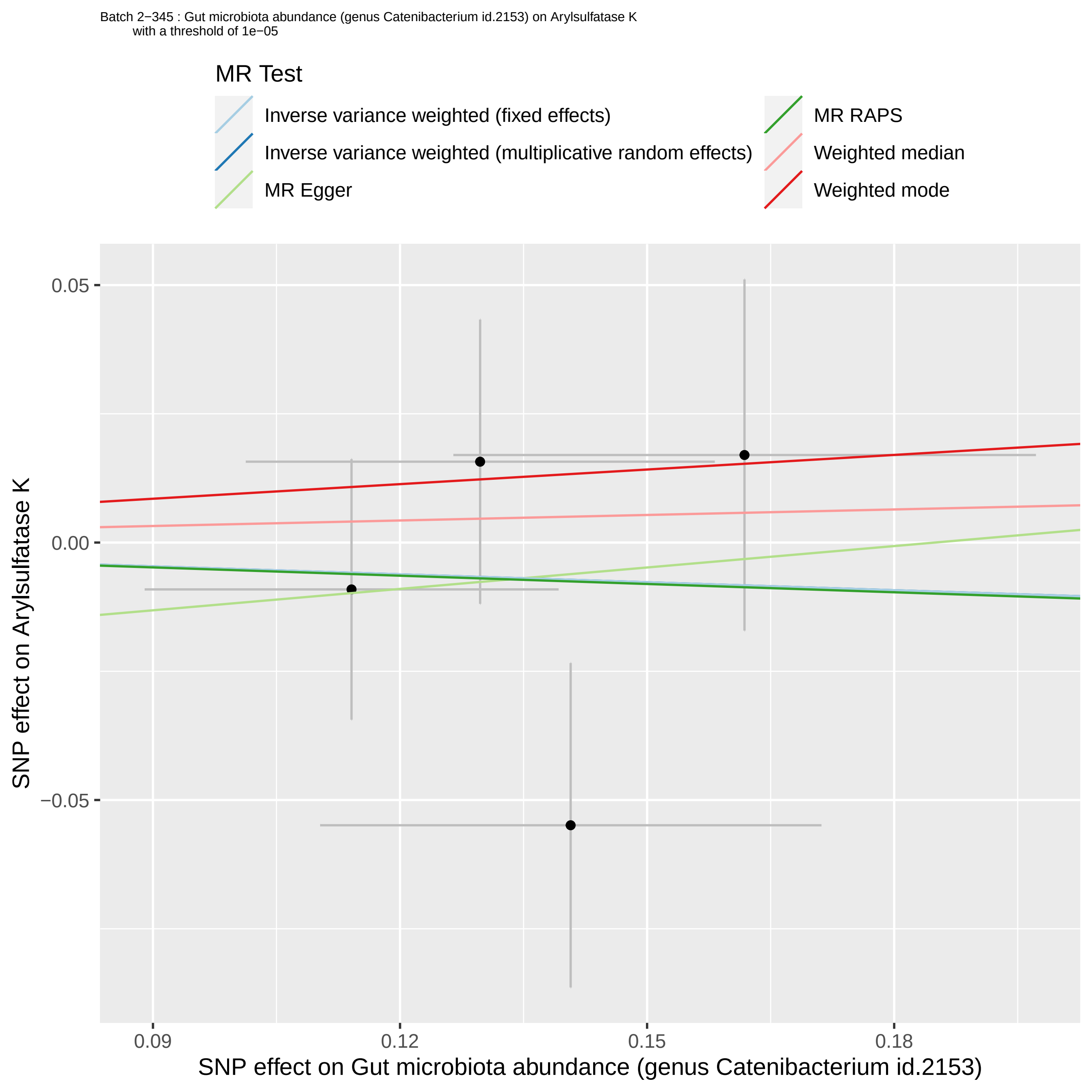
Batch 2−345 : Gut microbiota abundance (genus Catenibacterium id.2153) on Arylsulfatase K
with a threshold of 1e−05
MR Test
Inverse variance weighted (fixed effects)
Inverse variance weighted (multiplicative random effects)
MR Egger
MR RAPS
Weighted median
Weighted mode
0.05
0.00
−0.05
0.09
0.12
0.15
0.18
SNP effect on Arylsulfatase K
SNP effect on Gut microbiota abundance (genus Catenibacterium id.2153)

Batch 2−346 : Gut microbiota abundance (genus Parasutterella id.2892) on Arylsulfatase K
with a threshold of 1e−05

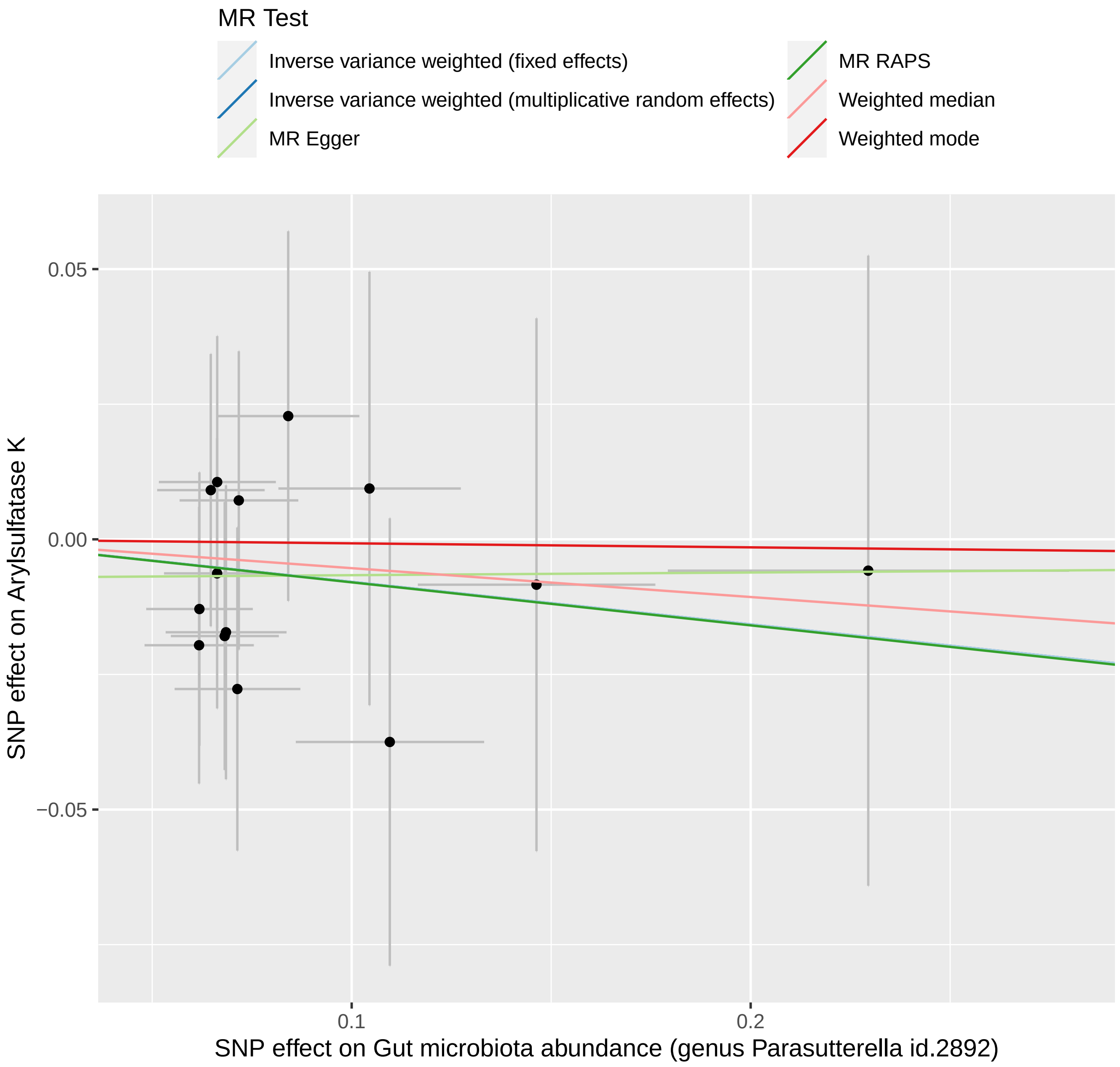

Batch 2−347 : Gut microbiota abundance (genus Catenibacterium id.2153) on Ecto−ADP−ribosyltransferase 3
with a threshold of 1e−05

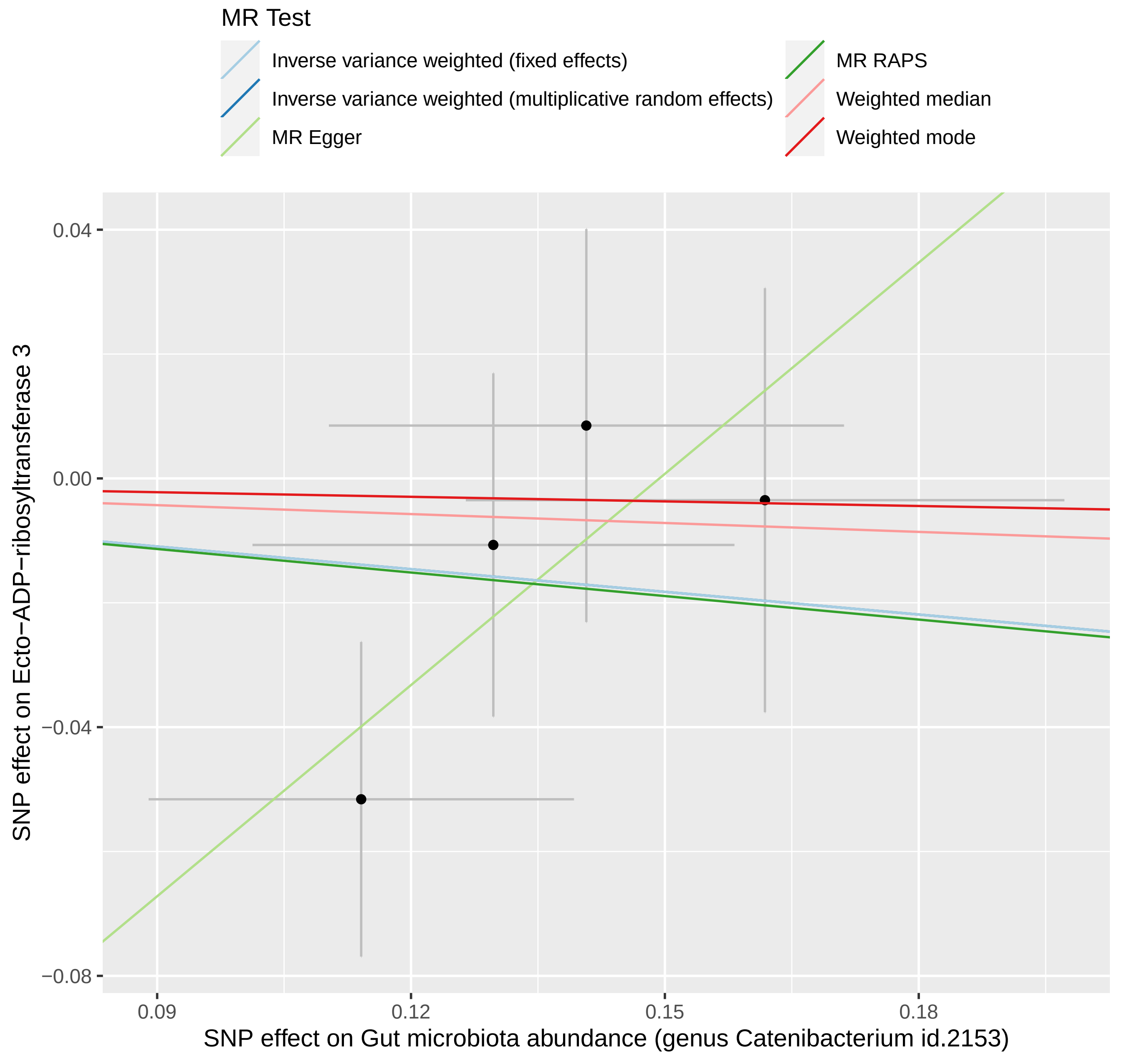

Batch 2−348 : Gut microbiota abundance (genus Parasutterella id.2892) on Ecto−ADP−ribosyltransferase 3
with a threshold of 1e−05

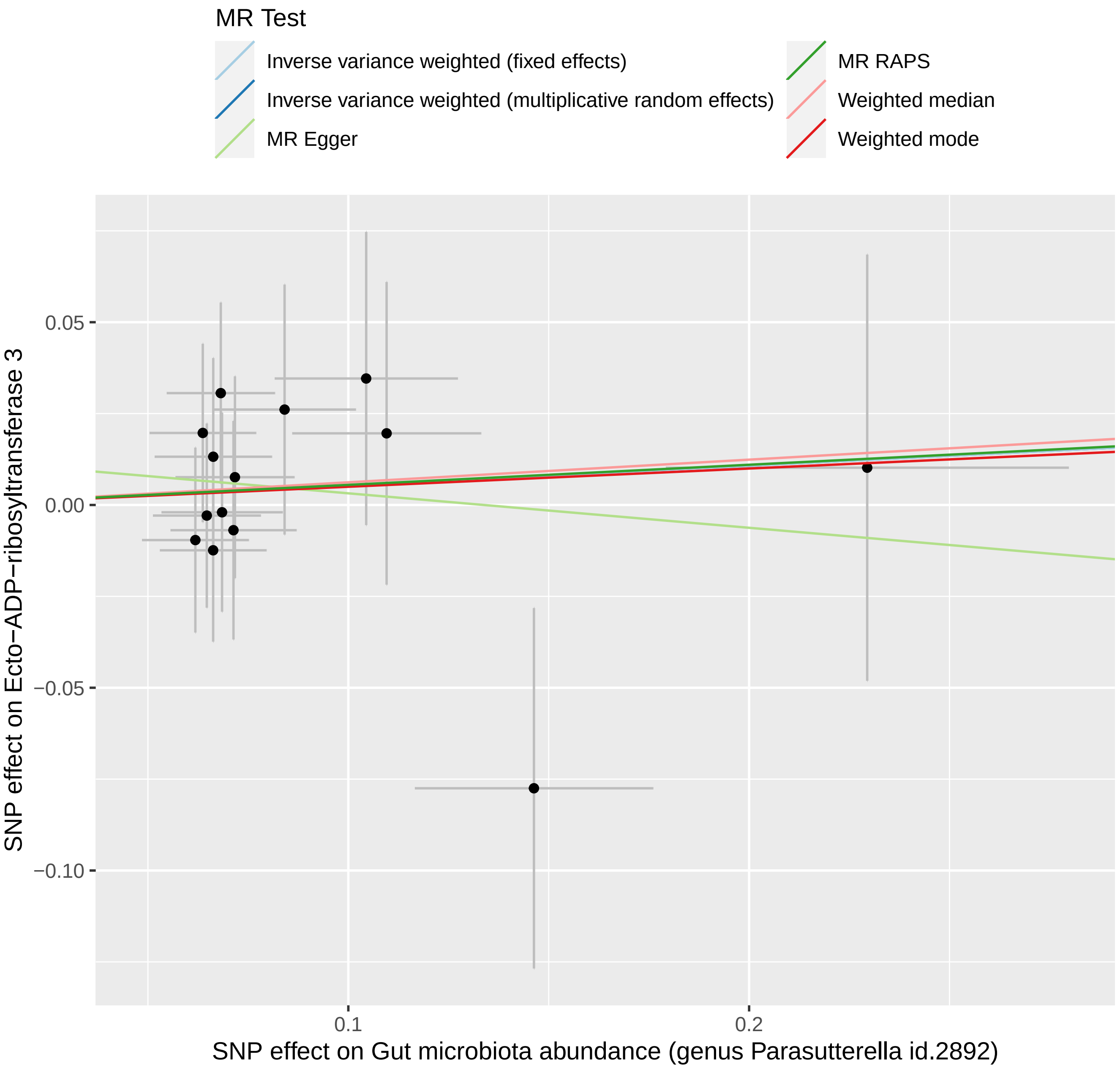

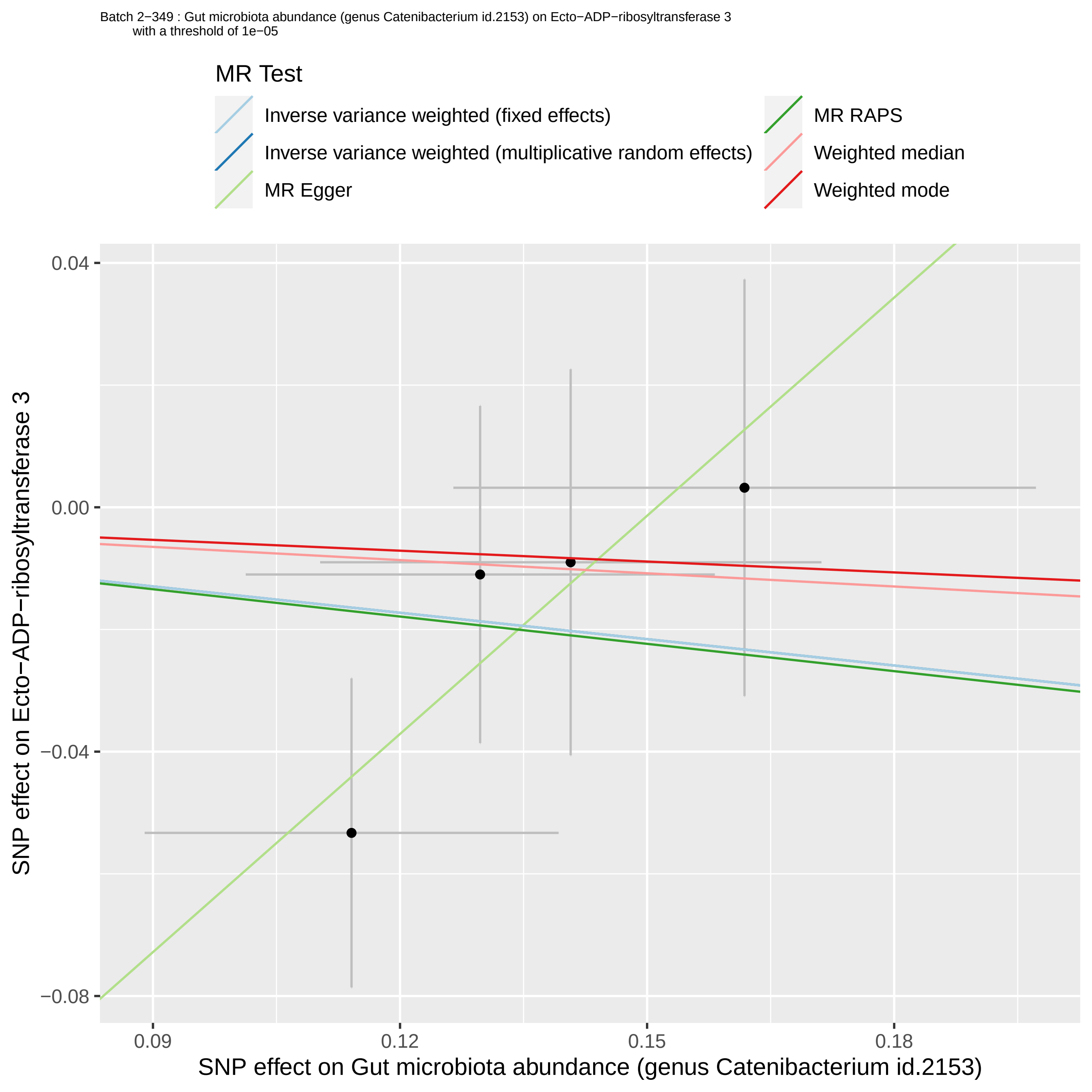

Batch 2−349 : Gut microbiota abundance (genus Catenibacterium id.2153) on Ecto−ADP−ribosyltransferase 3
with a threshold of 1e−05
MR Test
Inverse variance weighted (fixed effects)
Inverse variance weighted (multiplicative random effects)
MR Egger
MR RAPS
Weighted median
Weighted mode
0.04
0.00
−0.04
−0.08
0.09
0.12
0.15
0.18
SNP effect on Ecto−ADP−ribosyltransferase 3
SNP effect on Gut microbiota abundance (genus Catenibacterium id.2153)

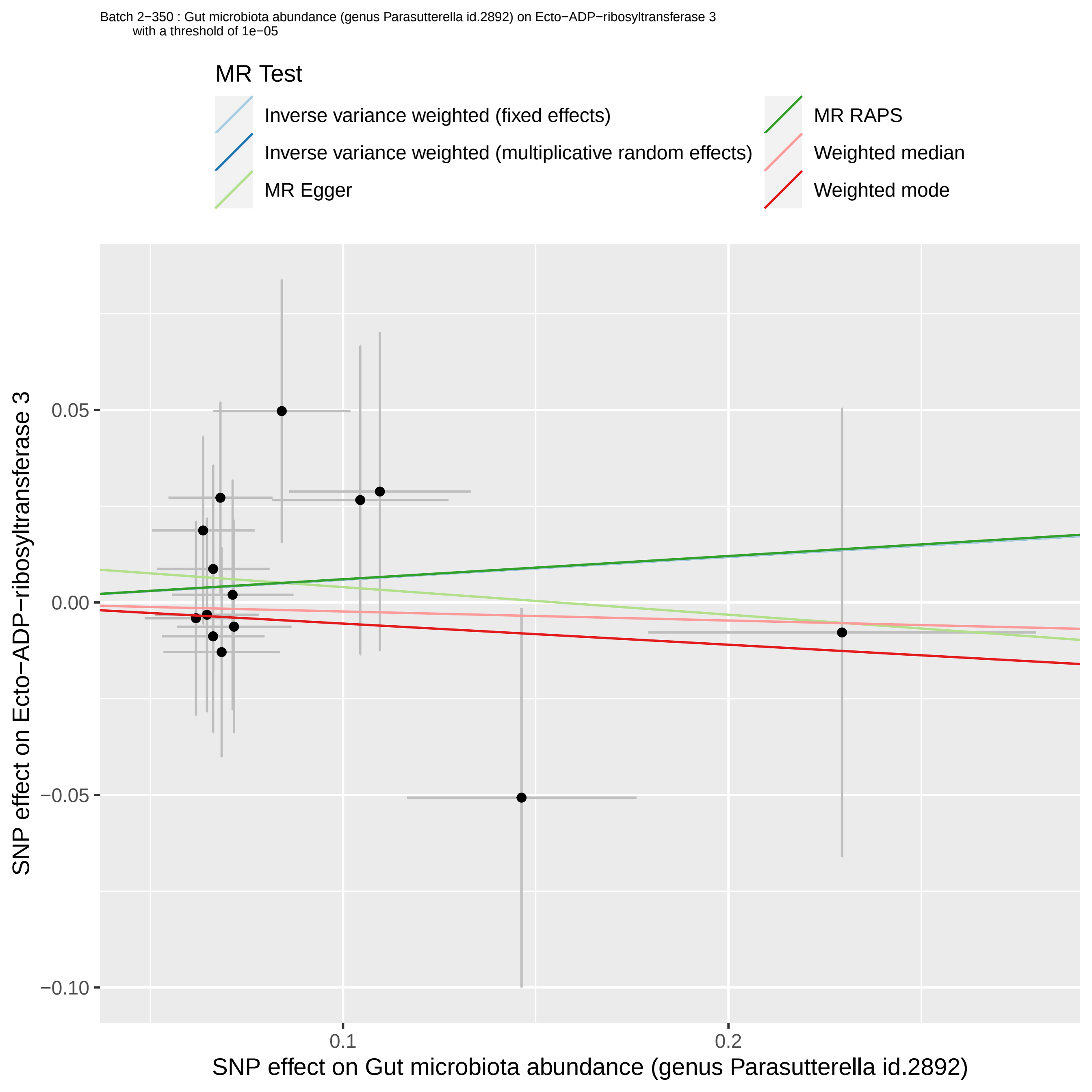
Batch 2−350 : Gut microbiota abundance (genus Parasutterella id.2892) on Ecto−ADP−ribosyltransferase 3
with a threshold of 1e−05
MR Test
Inverse variance weighted (fixed effects)
Inverse variance weighted (multiplicative random effects)
MR Egger
MR RAPS
Weighted median
Weighted mode
0.05
0.00
−0.05
−0.10
0.1
0.2
SNP effect on Ecto−ADP−ribosyltransferase 3
SNP effect on Gut microbiota abundance (genus Parasutterella id.2892)

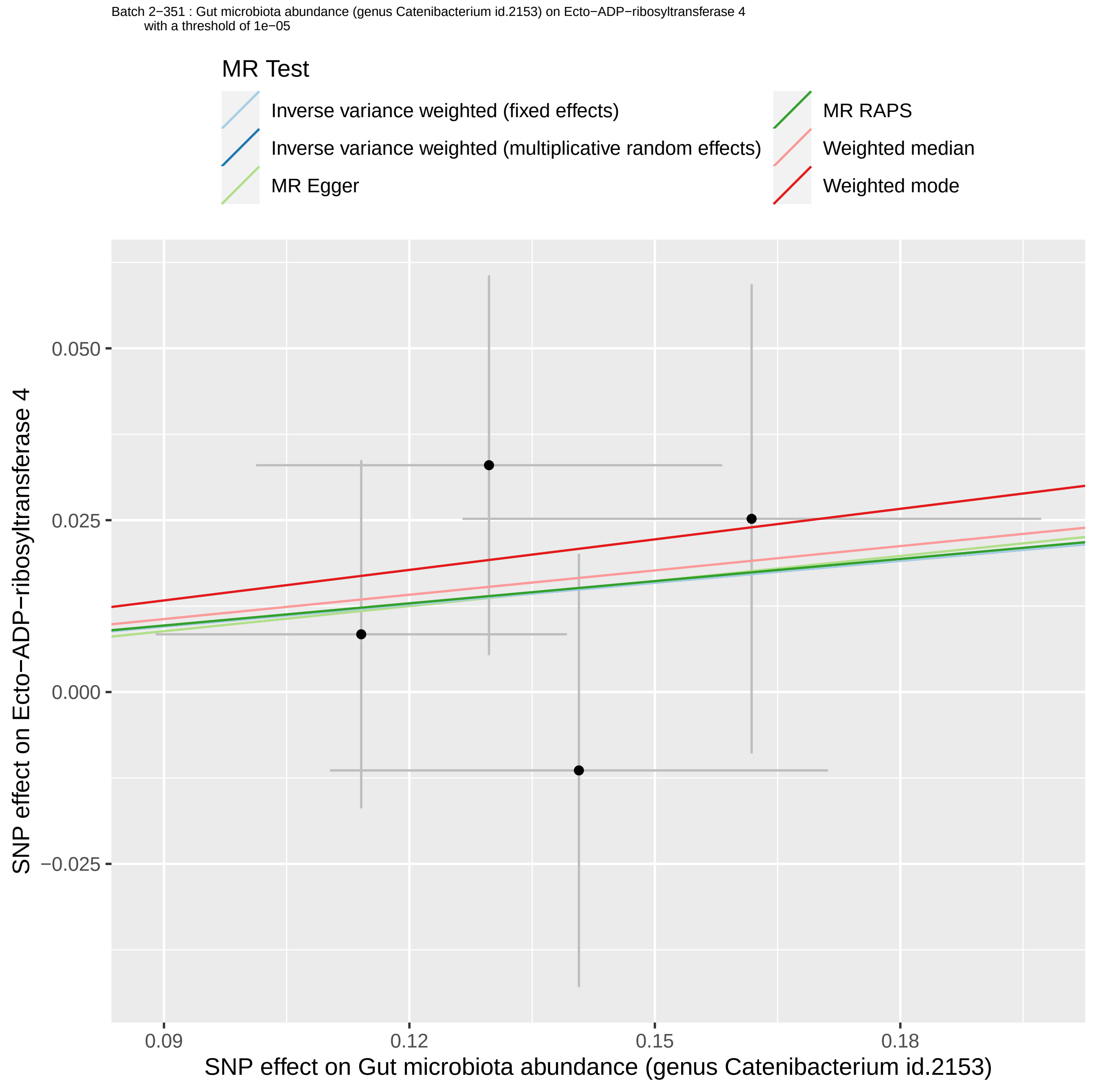
Batch 2−351 : Gut microbiota abundance (genus Catenibacterium id.2153) on Ecto−ADP−ribosyltransferase 4
with a threshold of 1e−05
MR Test
Inverse variance weighted (fixed effects)
Inverse variance weighted (multiplicative random effects)
MR Egger
MR RAPS
Weighted median
Weighted mode
0.050
0.025
0.000
−0.025
0.09
0.12
0.15
0.18
SNP effect on Ecto−ADP−ribosyltransferase 4
SNP effect on Gut microbiota abundance (genus Catenibacterium id.2153)

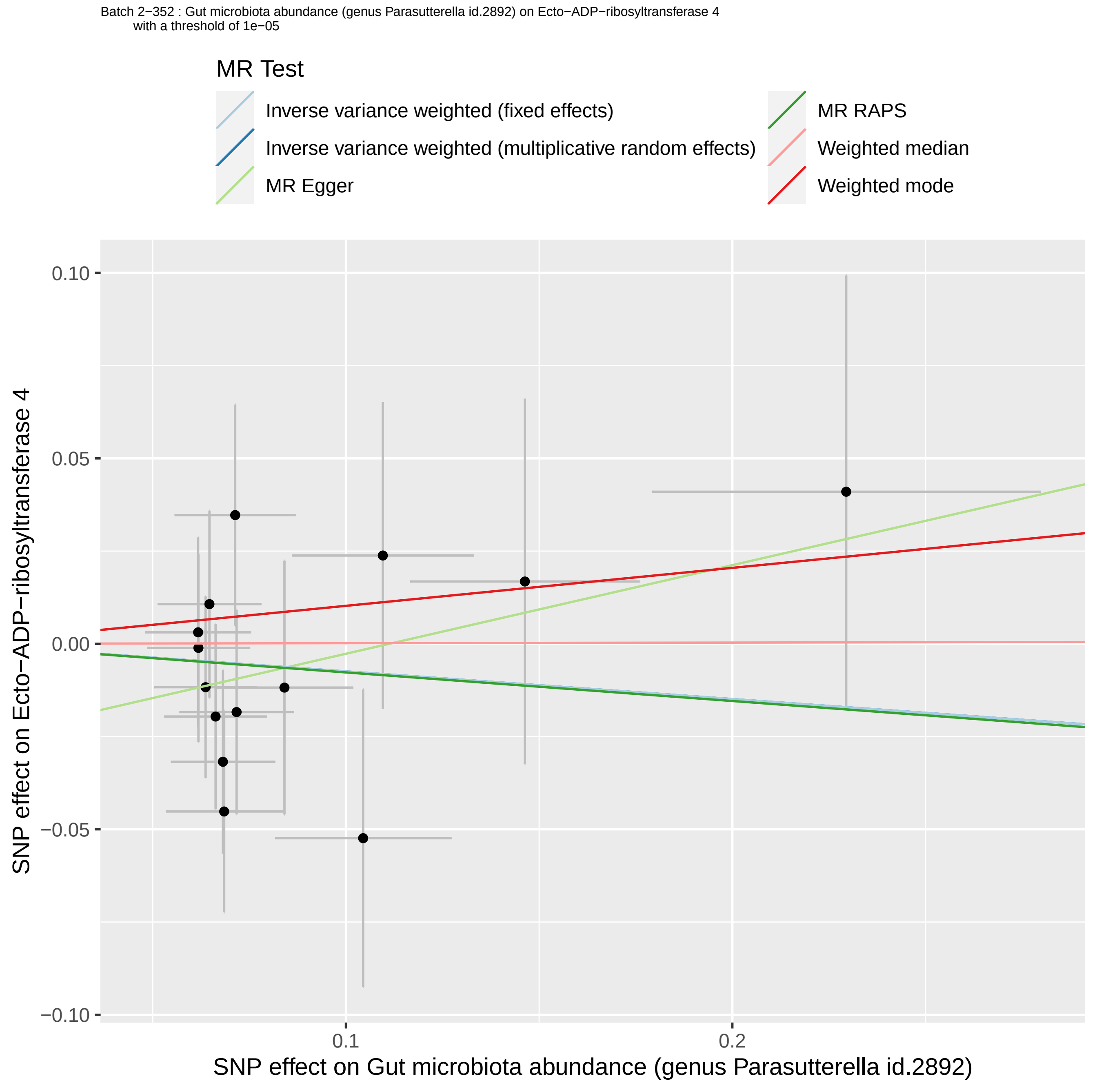

Batch 2−352 : Gut microbiota abundance (genus Parasutterella id.2892) on Ecto−ADP−ribosyltransferase 4
with a threshold of 1e−05
MR Test
Inverse variance weighted (fixed effects)
Inverse variance weighted (multiplicative random effects)
MR Egger
MR RAPS
Weighted median
Weighted mode
0.10
0.05
0.00
−0.05
−0.10
0.1
0.2
SNP effect on Ecto−ADP−ribosyltransferase 4
SNP effect on Gut microbiota abundance (genus Parasutterella id.2892)

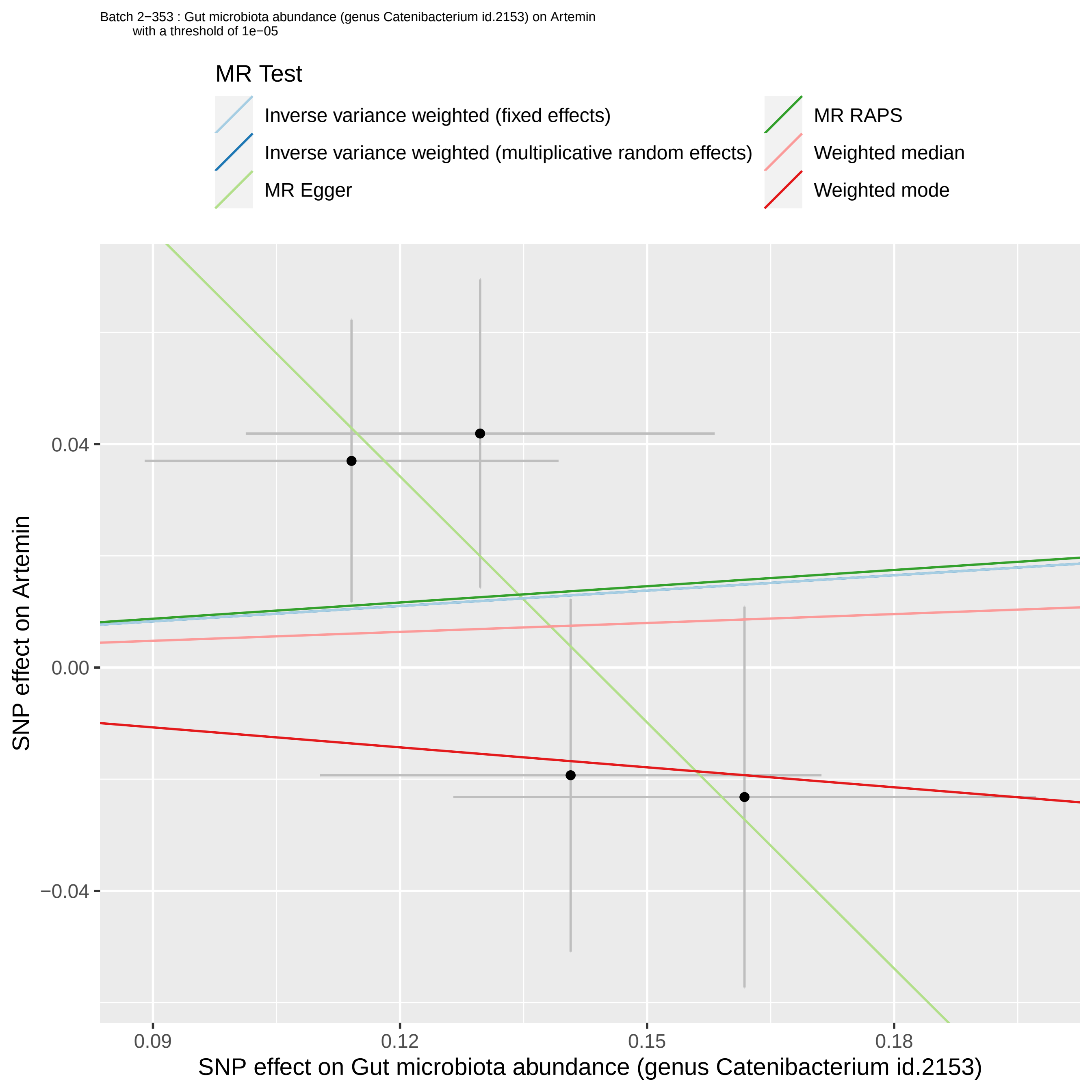
Batch 2−353 : Gut microbiota abundance (genus Catenibacterium id.2153) on Artemin
with a threshold of 1e−05
MR Test
Inverse variance weighted (fixed effects)
Inverse variance weighted (multiplicative random effects)
MR Egger
MR RAPS
Weighted median
Weighted mode
0.04
0.00
−0.04
0.09
0.12
0.15
0.18
SNP effect on Artemin
SNP effect on Gut microbiota abundance (genus Catenibacterium id.2153)

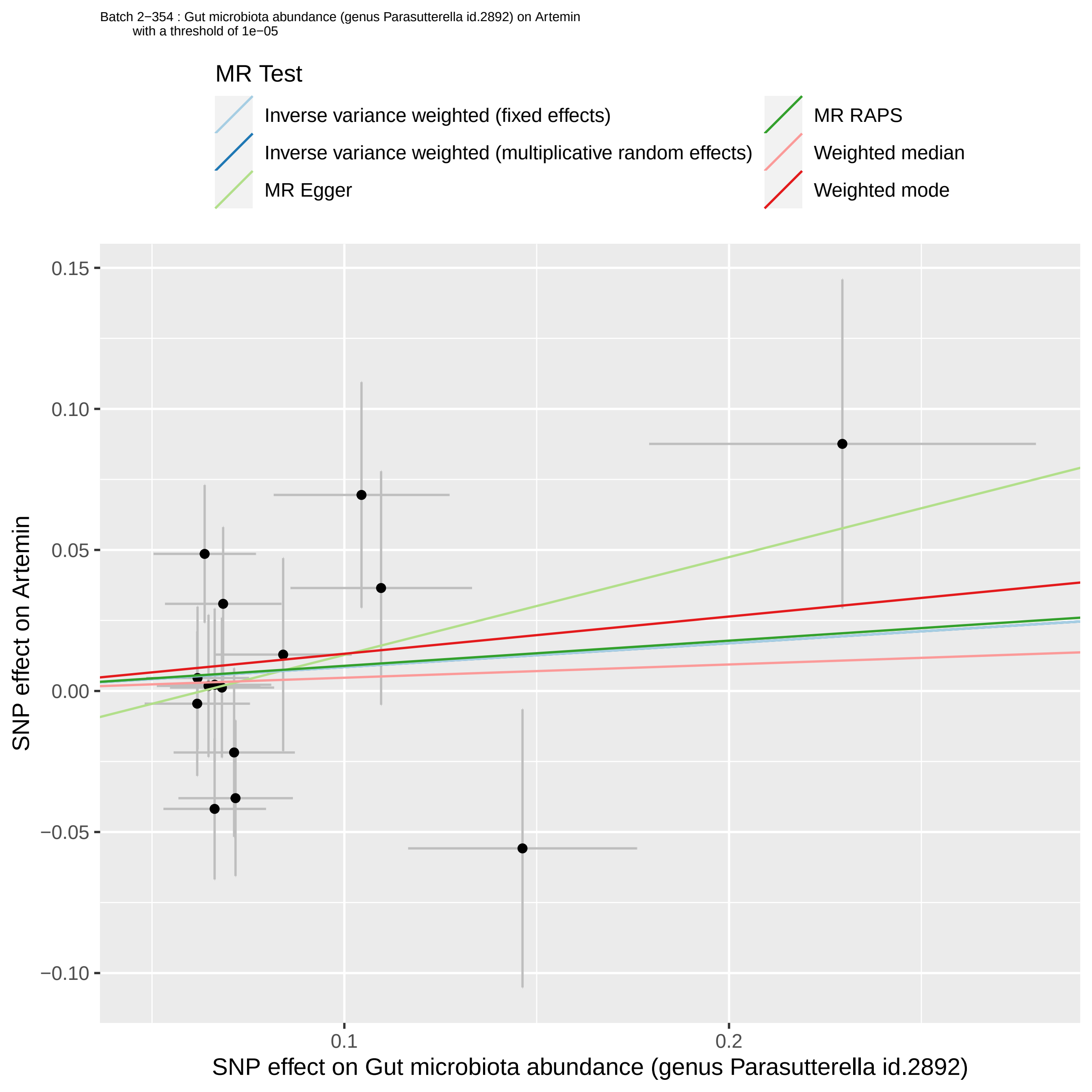
Batch 2−354 : Gut microbiota abundance (genus Parasutterella id.2892) on Artemin
with a threshold of 1e−05
MR Test
Inverse variance weighted (fixed effects)
Inverse variance weighted (multiplicative random effects)
MR Egger
MR RAPS
Weighted median
Weighted mode
0.15
0.10
0.05
0.00
−0.05
−0.10
0.1
0.2
SNP effect on Artemin
SNP effect on Gut microbiota abundance (genus Parasutterella id.2892)

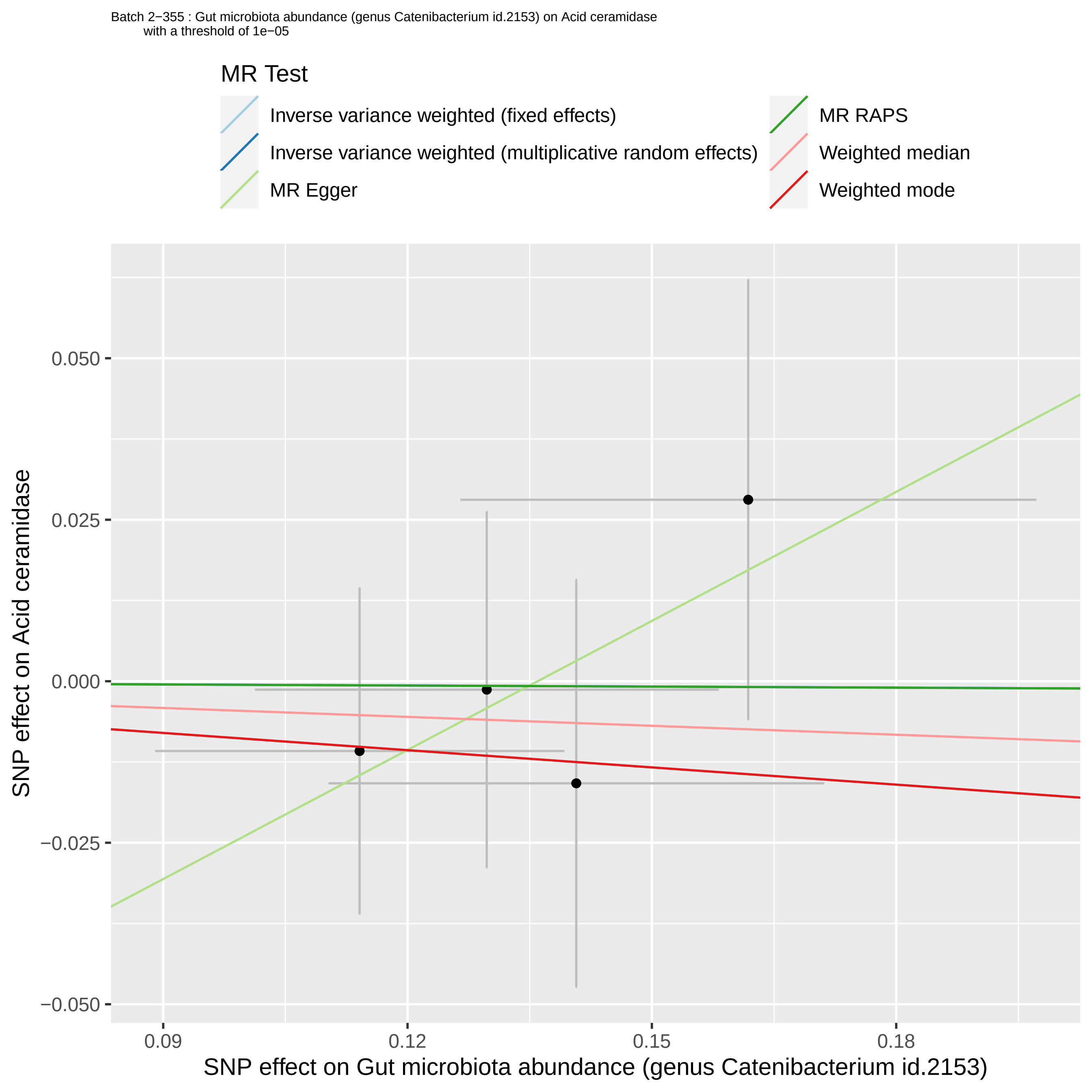
Batch 2−355 : Gut microbiota abundance (genus Catenibacterium id.2153) on Acid ceramidase
with a threshold of 1e−05
MR Test
Inverse variance weighted (fixed effects)
Inverse variance weighted (multiplicative random effects)
MR Egger
MR RAPS
Weighted median
Weighted mode
0.050
0.025
0.000
−0.025
−0.050
0.09
0.12
0.15
0.18
SNP effect on Acid ceramidase
SNP effect on Gut microbiota abundance (genus Catenibacterium id.2153)

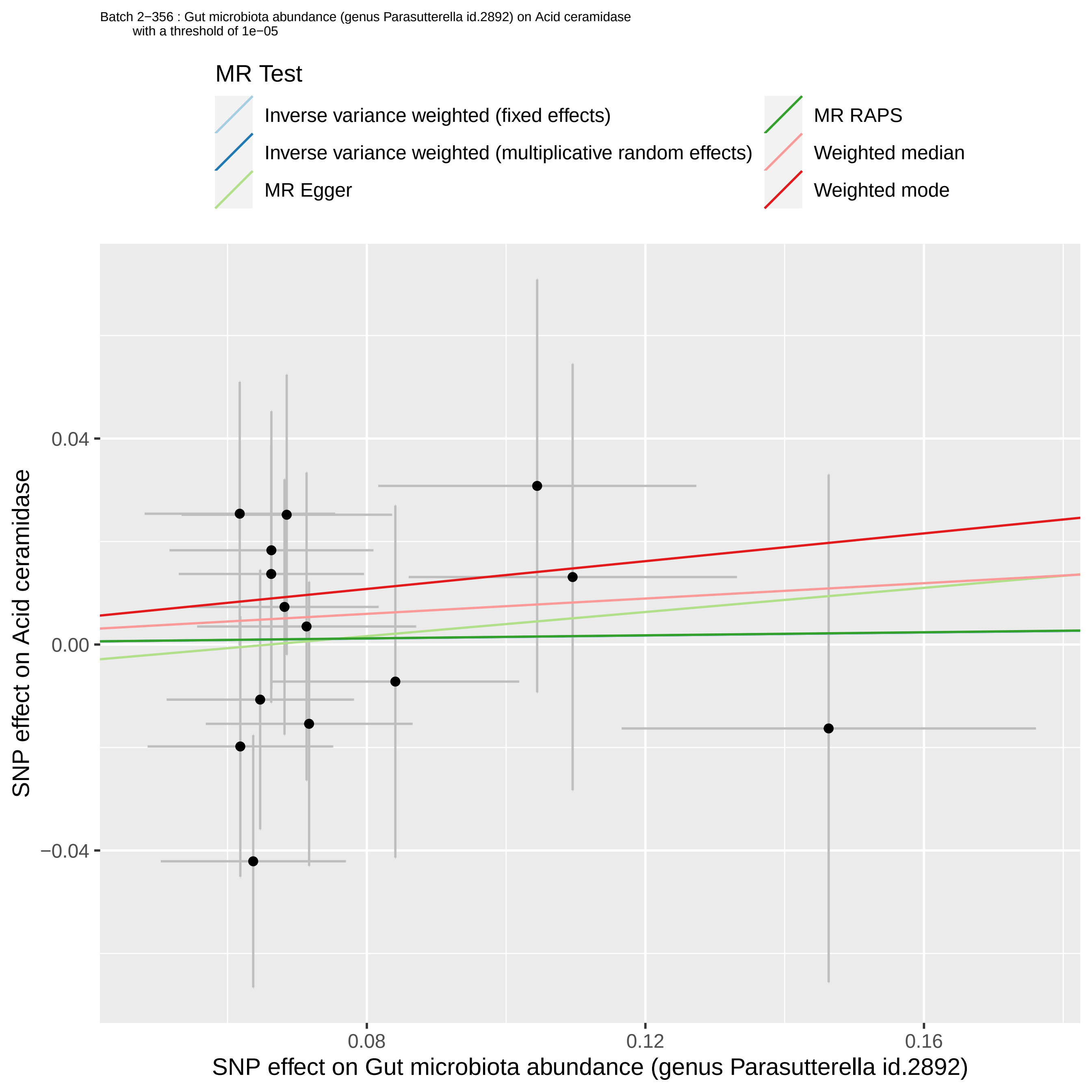
Batch 2−356 : Gut microbiota abundance (genus Parasutterella id.2892) on Acid ceramidase
with a threshold of 1e−05
MR Test
Inverse variance weighted (fixed effects)
Inverse variance weighted (multiplicative random effects)
MR Egger
MR RAPS
Weighted median
Weighted mode
SNP effect on Acid ceramidase
0.04
0.00
−0.04
0.08
0.12
0.16
SNP effect on Gut microbiota abundance (genus Parasutterella id.2892)

Batch 2−357 : Gut microbiota abundance (genus Catenibacterium id.2153) on Neutral ceramidase
with a threshold of 1e−05

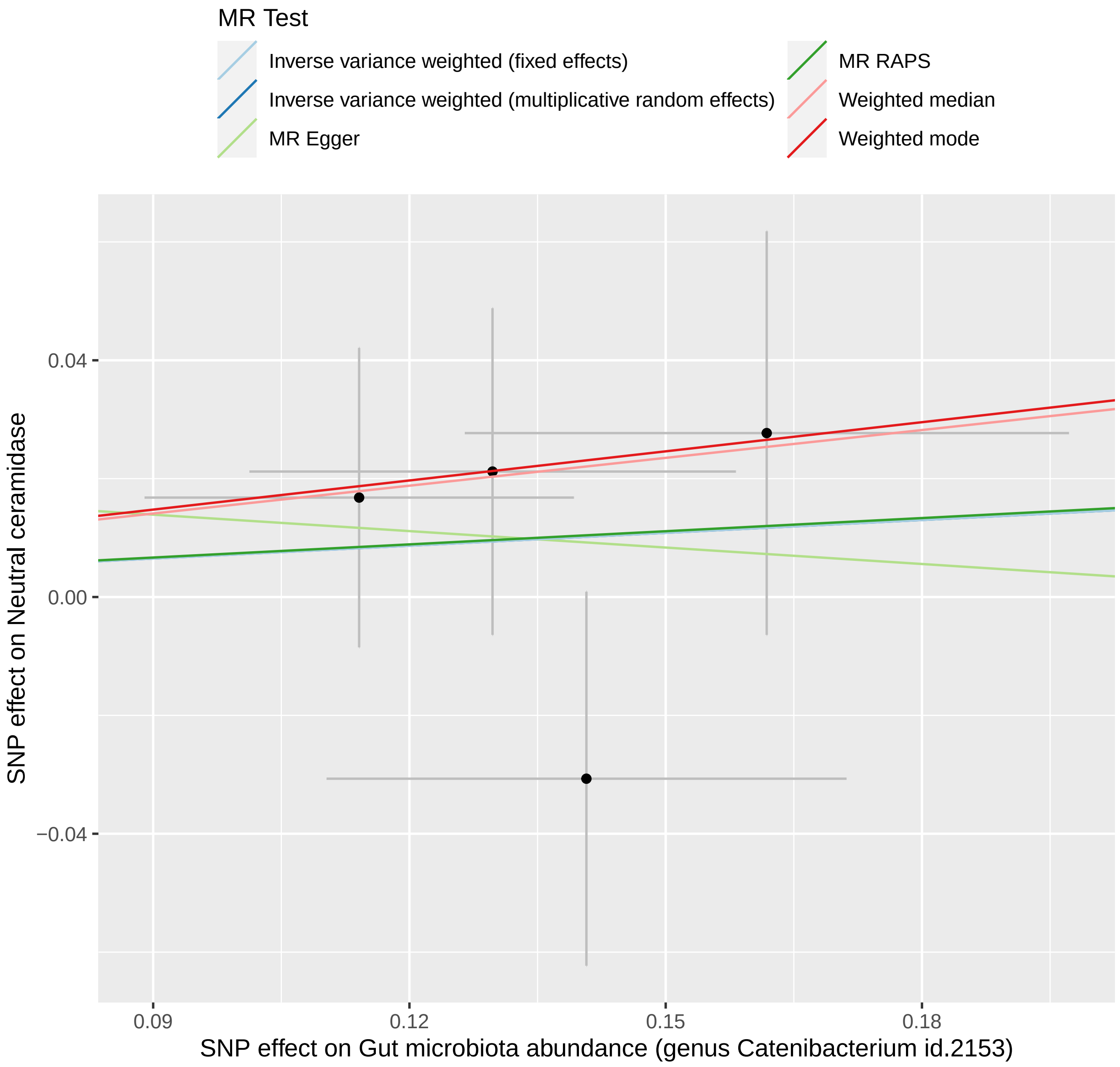

Batch 2−358 : Gut microbiota abundance (genus Parasutterella id.2892) on Neutral ceramidase
with a threshold of 1e−05

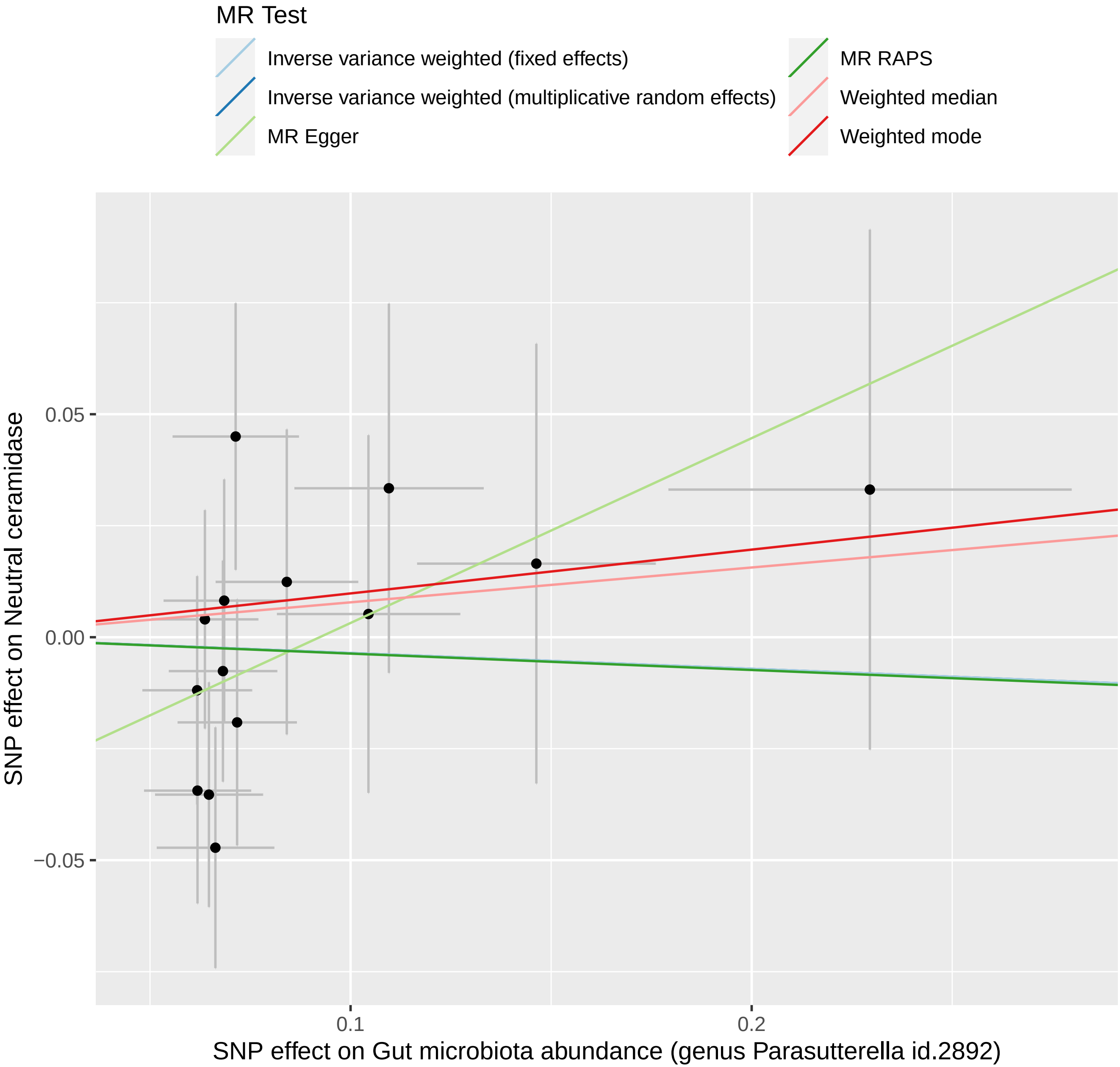

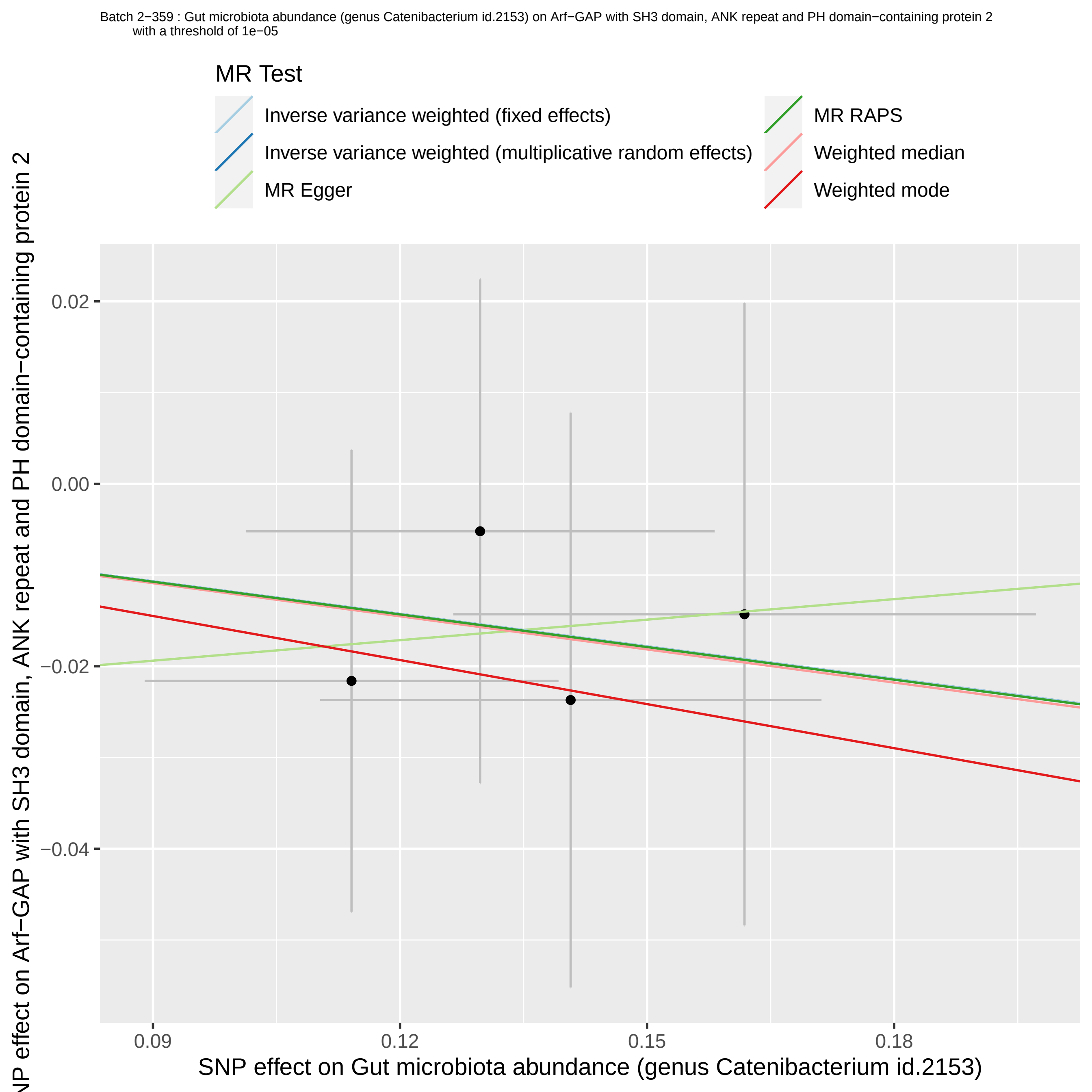
Batch 2−359 : Gut microbiota abundance (genus Catenibacterium id.2153) on Arf−GAP with SH3 domain, ANK repeat and PH domain−containing protein 2 with a threshold of 1e−05
MR Test
Inverse variance weighted (fixed effects)
Inverse variance weighted (multiplicative random effects)
MR Egger
MR RAPS
Weighted median
Weighted mode
0.02
0.00
−0.02
−0.04
0.09
0.12
0.15
0.18
SNP effect on Gut microbiota abundance (genus Catenibacterium id.2153)
NP effect on Arf−GAP with SH3 domain, ANK repeat and PH domain−containing protein 2

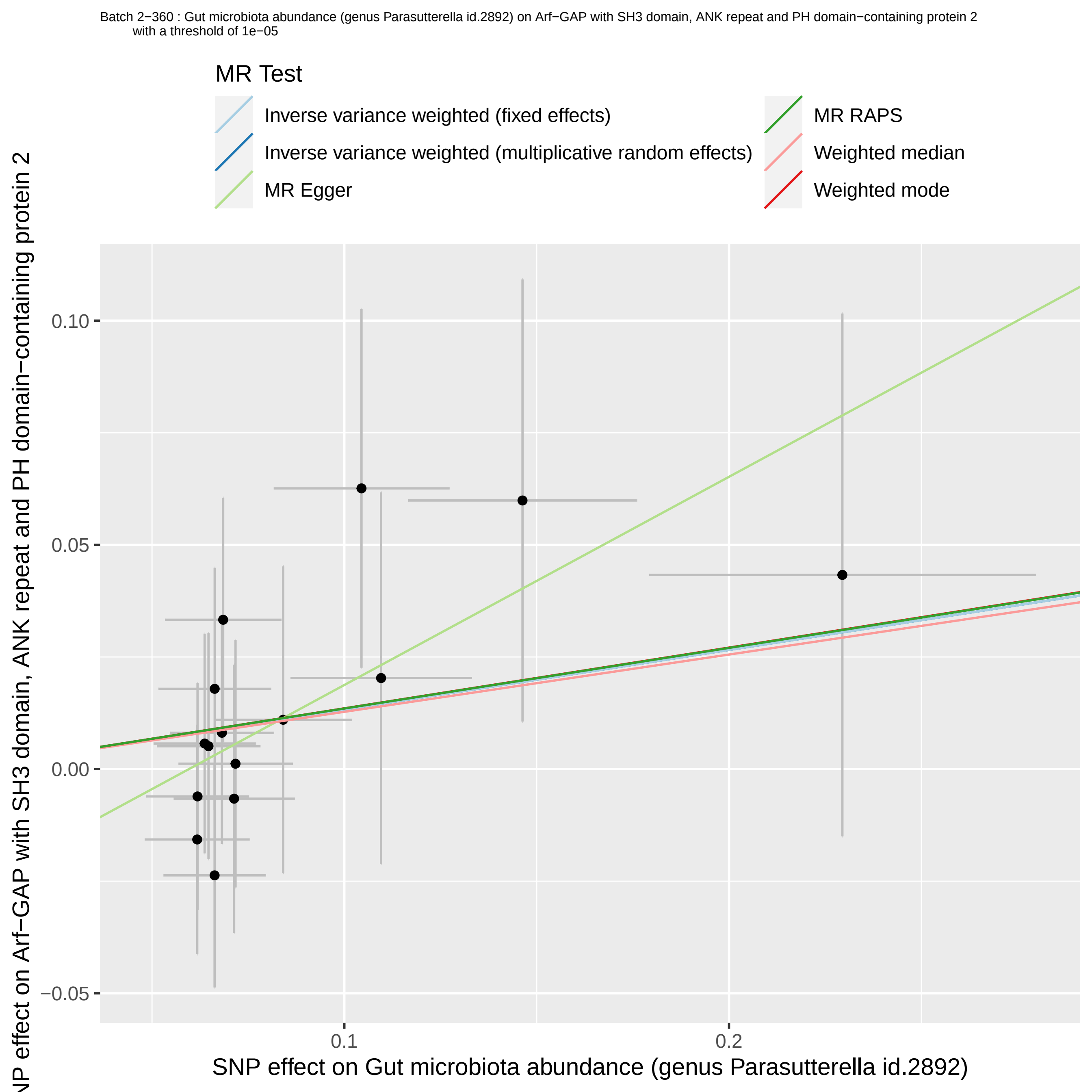

Batch 2−360 : Gut microbiota abundance (genus Parasutterella id.2892) on Arf−GAP with SH3 domain, ANK repeat and PH domain−containing protein 2 with a threshold of 1e−05
MR Test
Inverse variance weighted (fixed effects)
Inverse variance weighted (multiplicative random effects)
MR Egger
MR RAPS
Weighted median
Weighted mode
0.10
0.05
0.00
−0.05
0.1
0.2
SNP effect on Gut microbiota abundance (genus Parasutterella id.2892)
SNP effect on Arf−GAP with SH3 domain, ANK repeat and PH domain−containing protein 2

Batch 2−361 : Gut microbiota abundance (genus Catenibacterium id.2153) on Activating signal cointegrator 1 complex subunit 1 with a threshold of 1e−05

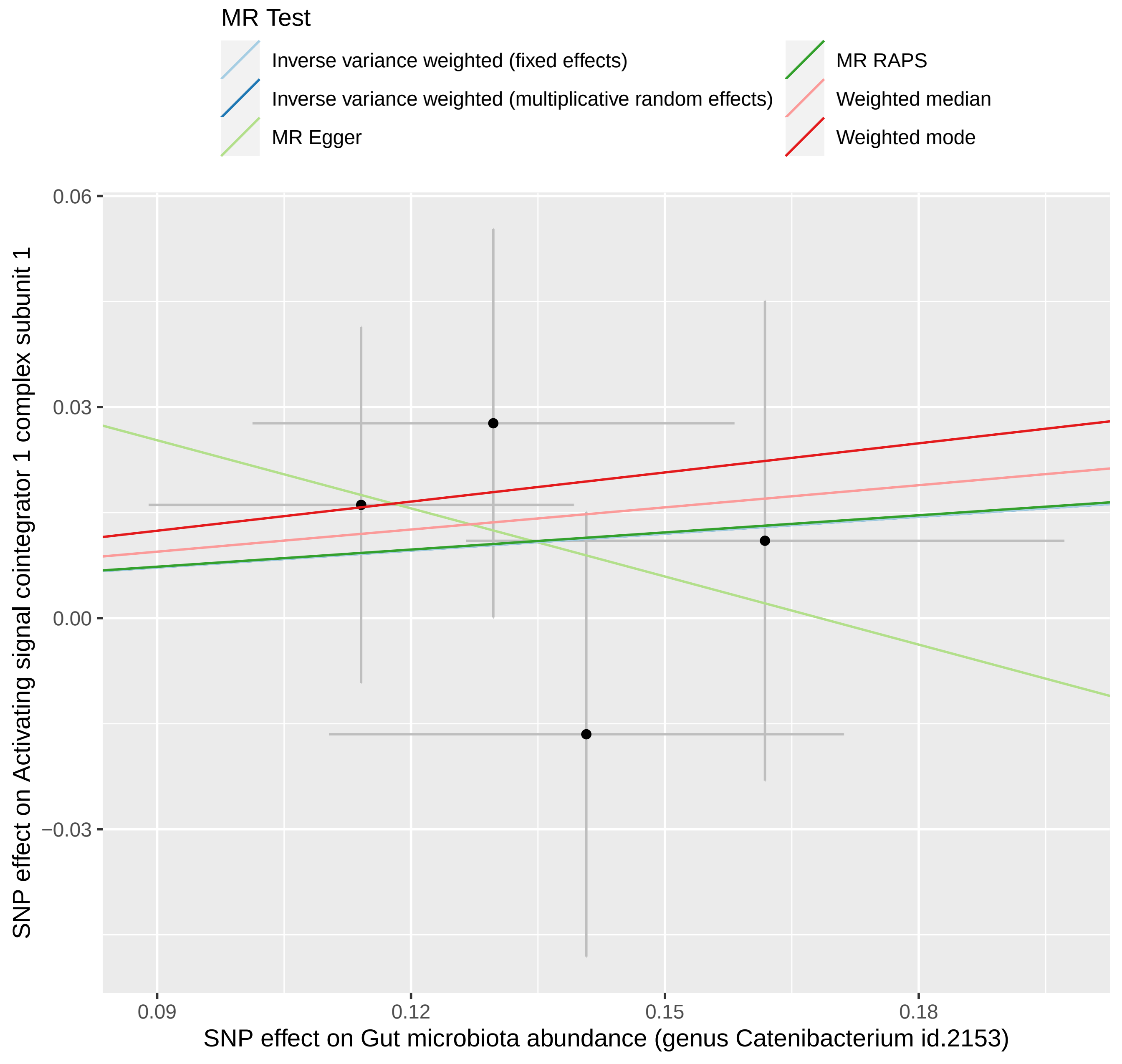

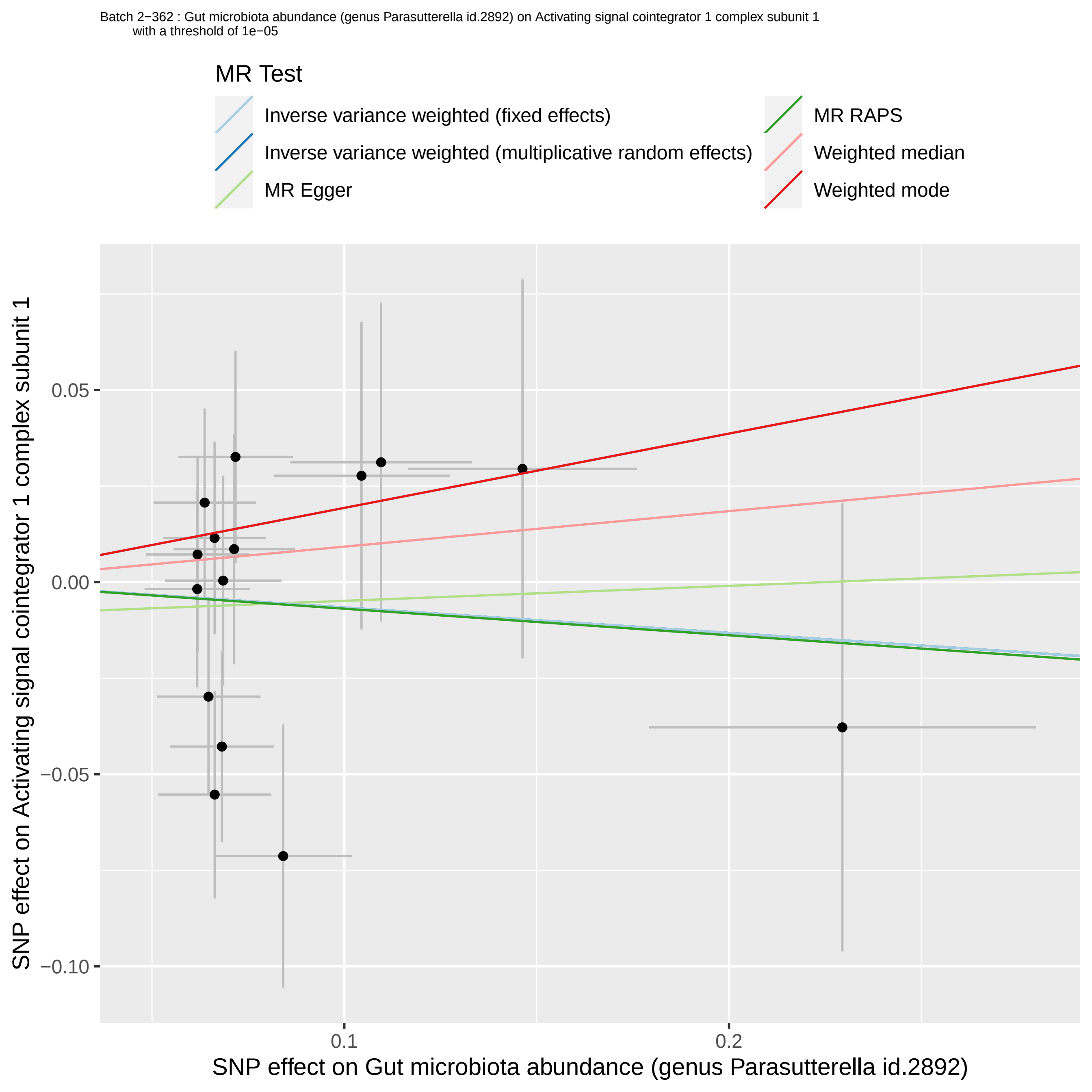
Batch 2−362 : Gut microbiota abundance (genus Parasutterella id.2892) on Activating signal cointegrator 1 complex subunit 1
with a threshold of 1e−05
MR Test
Inverse variance weighted (fixed effects)
Inverse variance weighted (multiplicative random effects)
MR Egger
MR RAPS
Weighted median
Weighted mode
0.05
0.00
−0.05
−0.10
0.1
0.2
SNP effect on Activating signal cointegrator 1 complex subunit 1
SNP effect on Gut microbiota abundance (genus Parasutterella id.2892)

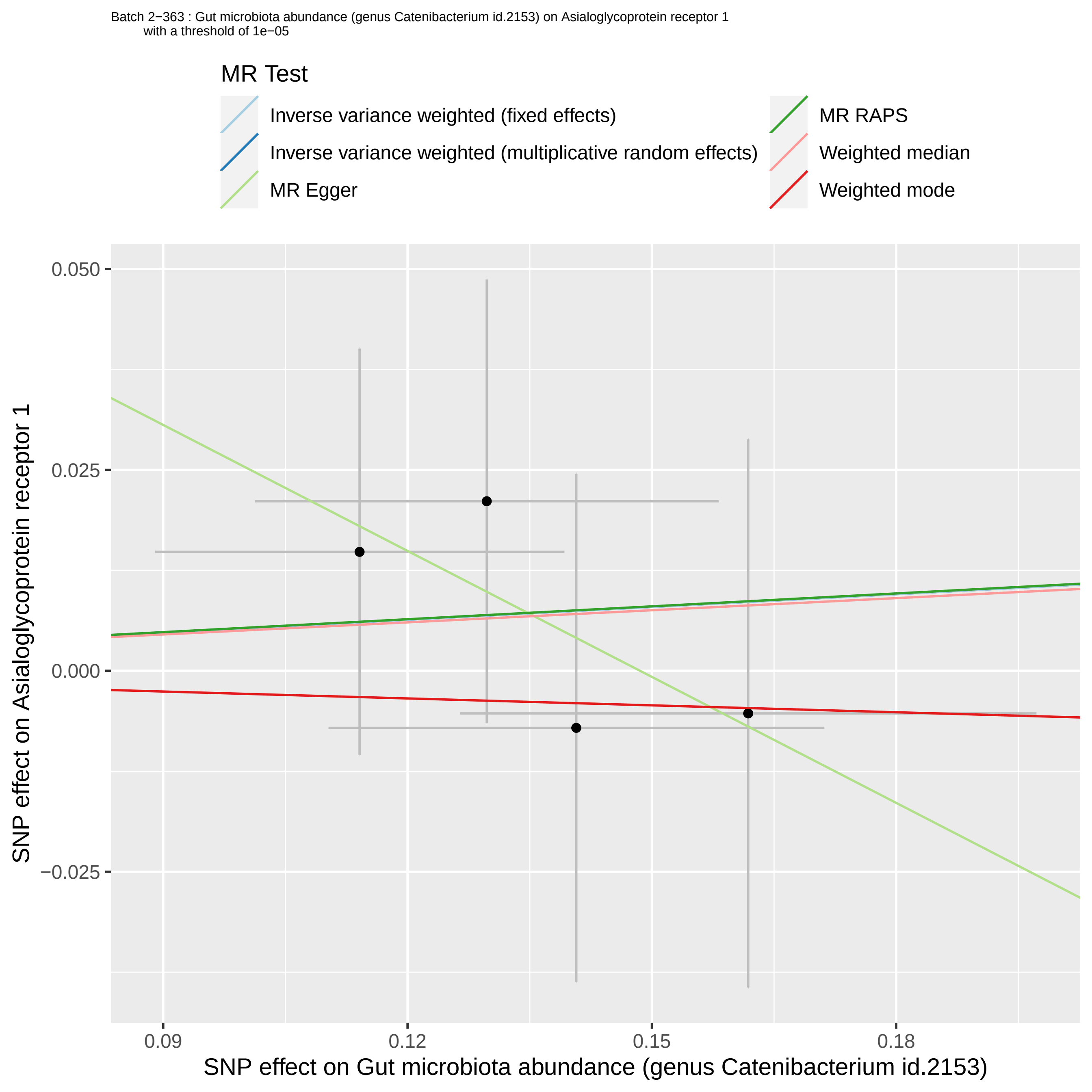

Batch 2−363 : Gut microbiota abundance (genus Catenibacterium id.2153) on Asialoglycoprotein receptor 1
with a threshold of 1e−05
MR Test
Inverse variance weighted (fixed effects)
Inverse variance weighted (multiplicative random effects)
MR Egger
MR RAPS
Weighted median
Weighted mode
0.050
0.025
0.000
−0.025
0.09
0.12
0.15
0.18
SNP effect on Asialoglycoprotein receptor 1
SNP effect on Gut microbiota abundance (genus Catenibacterium id.2153)

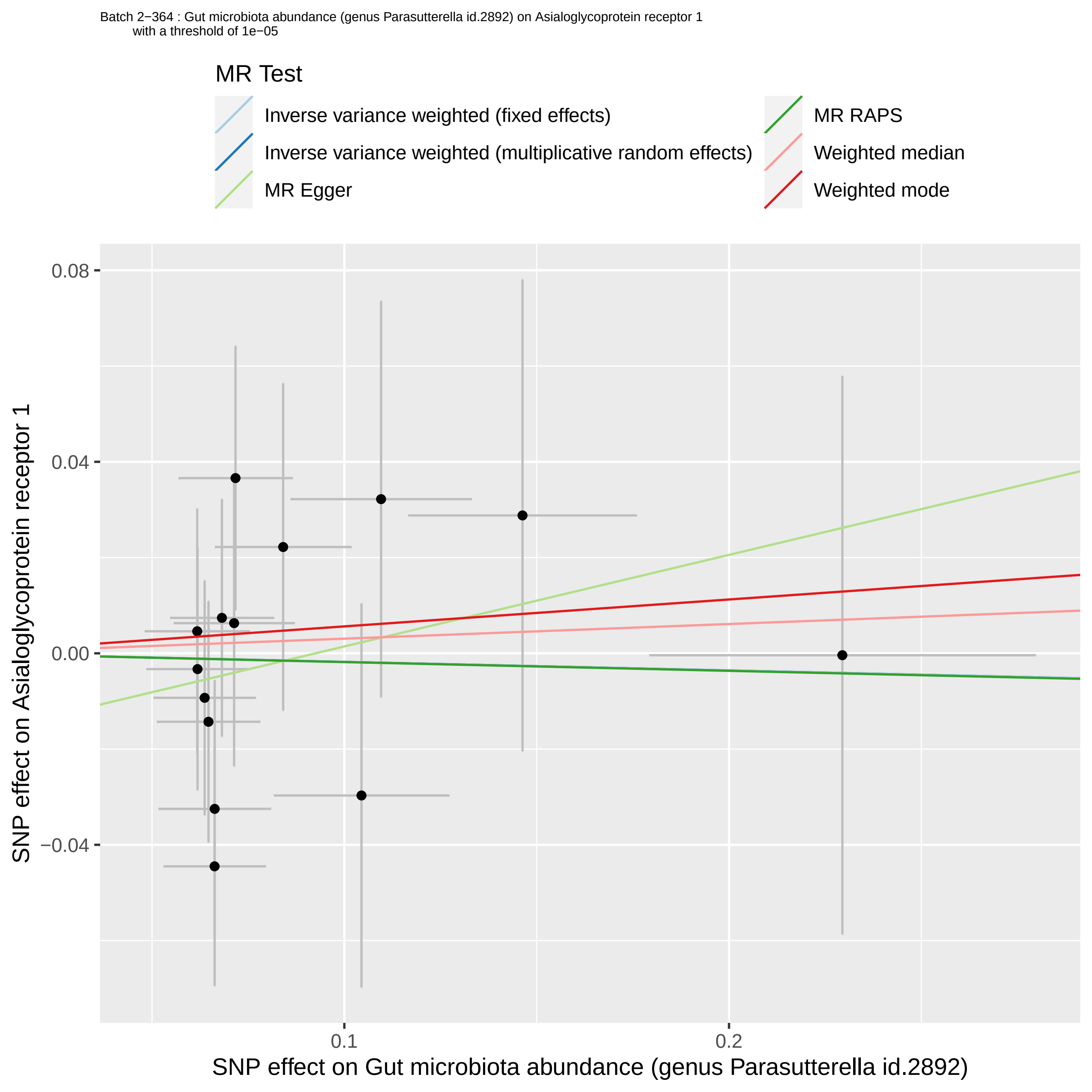

Batch 2−364 : Gut microbiota abundance (genus Parasutterella id.2892) on Asialoglycoprotein receptor 1
with a threshold of 1e−05
MR Test
Inverse variance weighted (fixed effects)
Inverse variance weighted (multiplicative random effects)
MR Egger
MR RAPS
Weighted median
Weighted mode
0.08
0.04
0.00
−0.04
0.1
0.2
SNP effect on Asialoglycoprotein receptor 1
SNP effect on Gut microbiota abundance (genus Parasutterella id.2892)

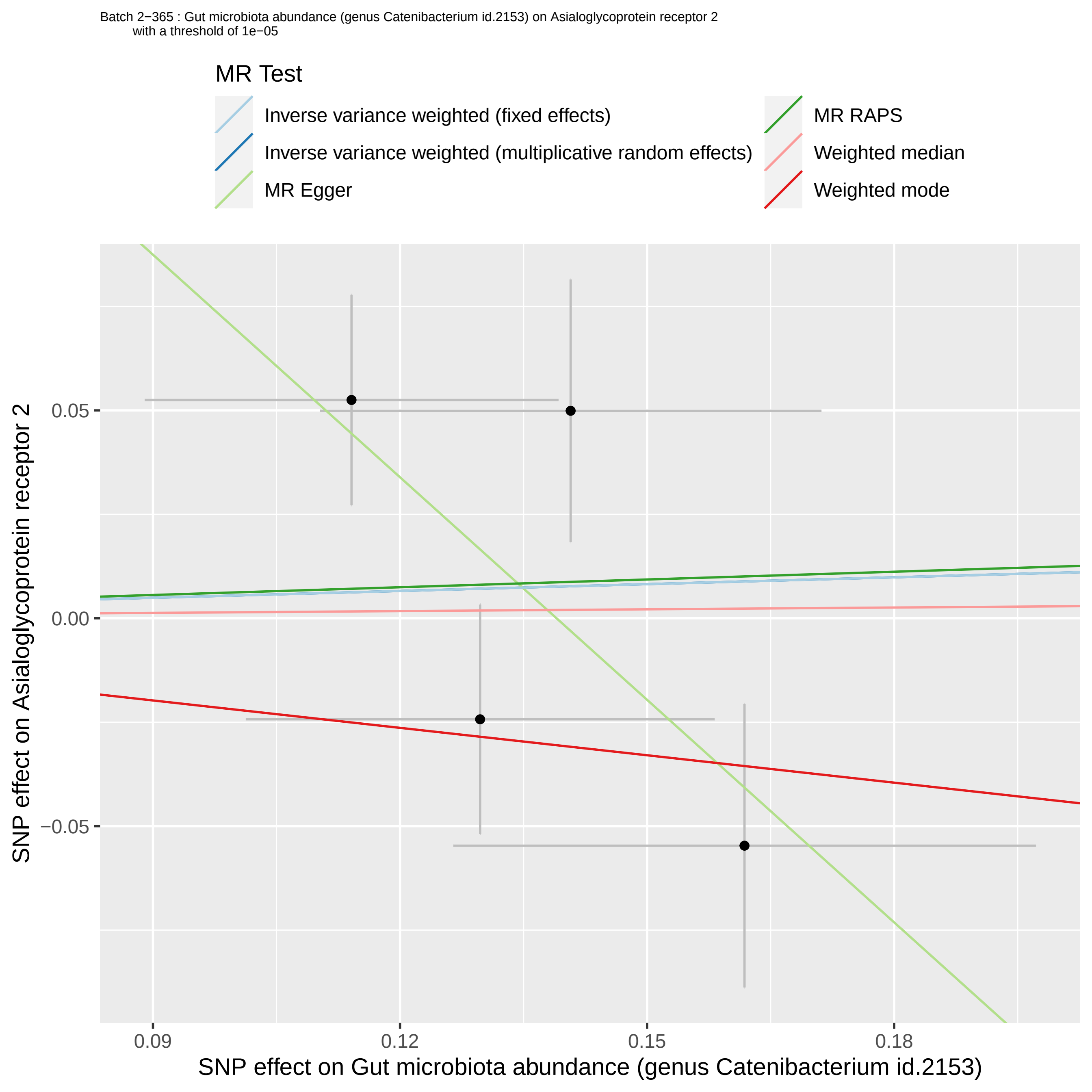

Batch 2−365 : Gut microbiota abundance (genus Catenibacterium id.2153) on Asialoglycoprotein receptor 2
with a threshold of 1e−05
MR Test
Inverse variance weighted (fixed effects)
Inverse variance weighted (multiplicative random effects)
MR Egger
MR RAPS
Weighted median
Weighted mode
0.05
0.00
−0.05
0.09
0.12
0.15
0.18
SNP effect on Asialoglycoprotein receptor 2
SNP effect on Gut microbiota abundance (genus Catenibacterium id.2153)

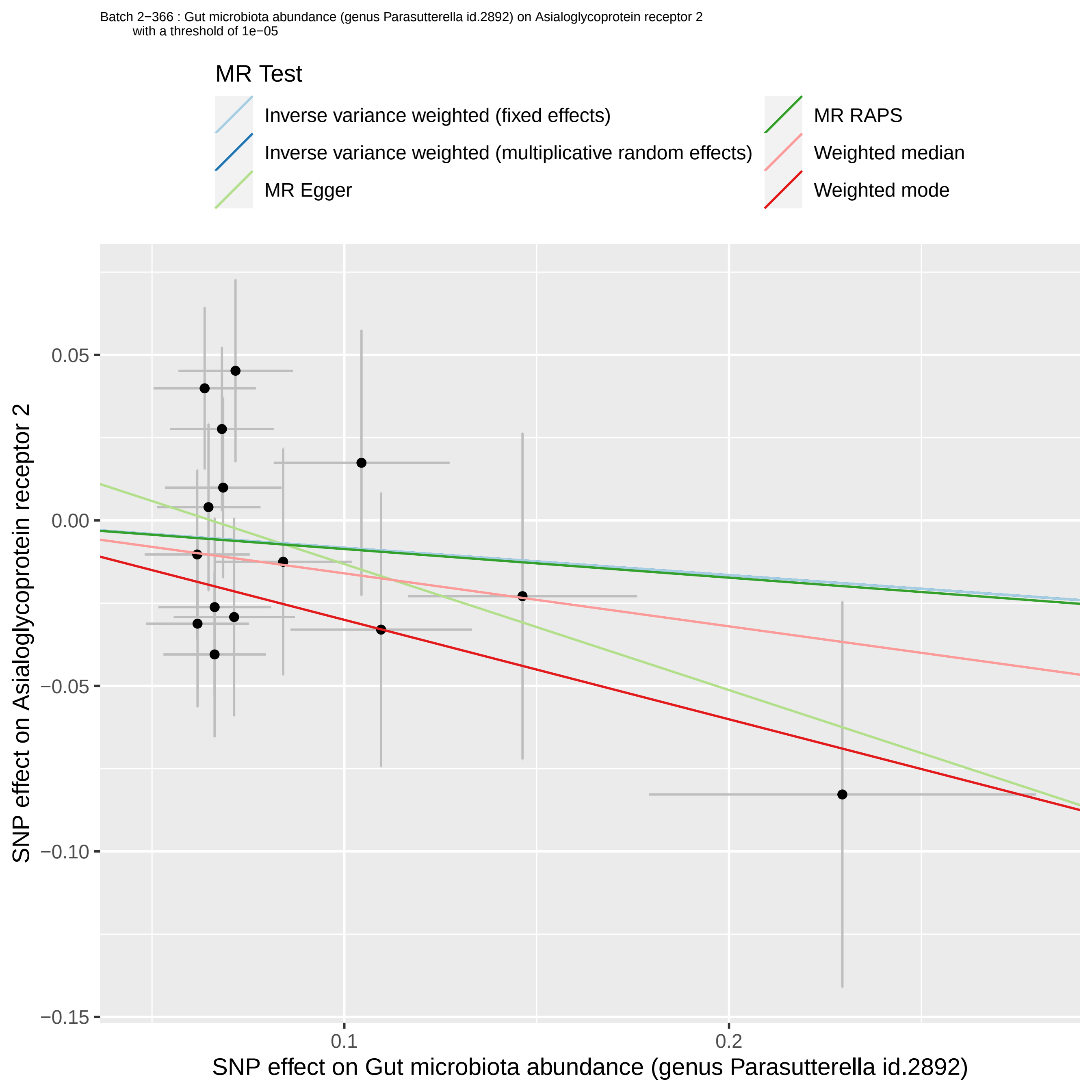
Batch 2−366 : Gut microbiota abundance (genus Parasutterella id.2892) on Asialoglycoprotein receptor 2
with a threshold of 1e−05
MR Test
Inverse variance weighted (fixed effects)
Inverse variance weighted (multiplicative random effects)
MR Egger
MR RAPS
Weighted median
Weighted mode
0.05
0.00
−0.05
−0.10
−0.15
0.1
0.2
SNP effect on Asialoglycoprotein receptor 2
SNP effect on Gut microbiota abundance (genus Parasutterella id.2892)

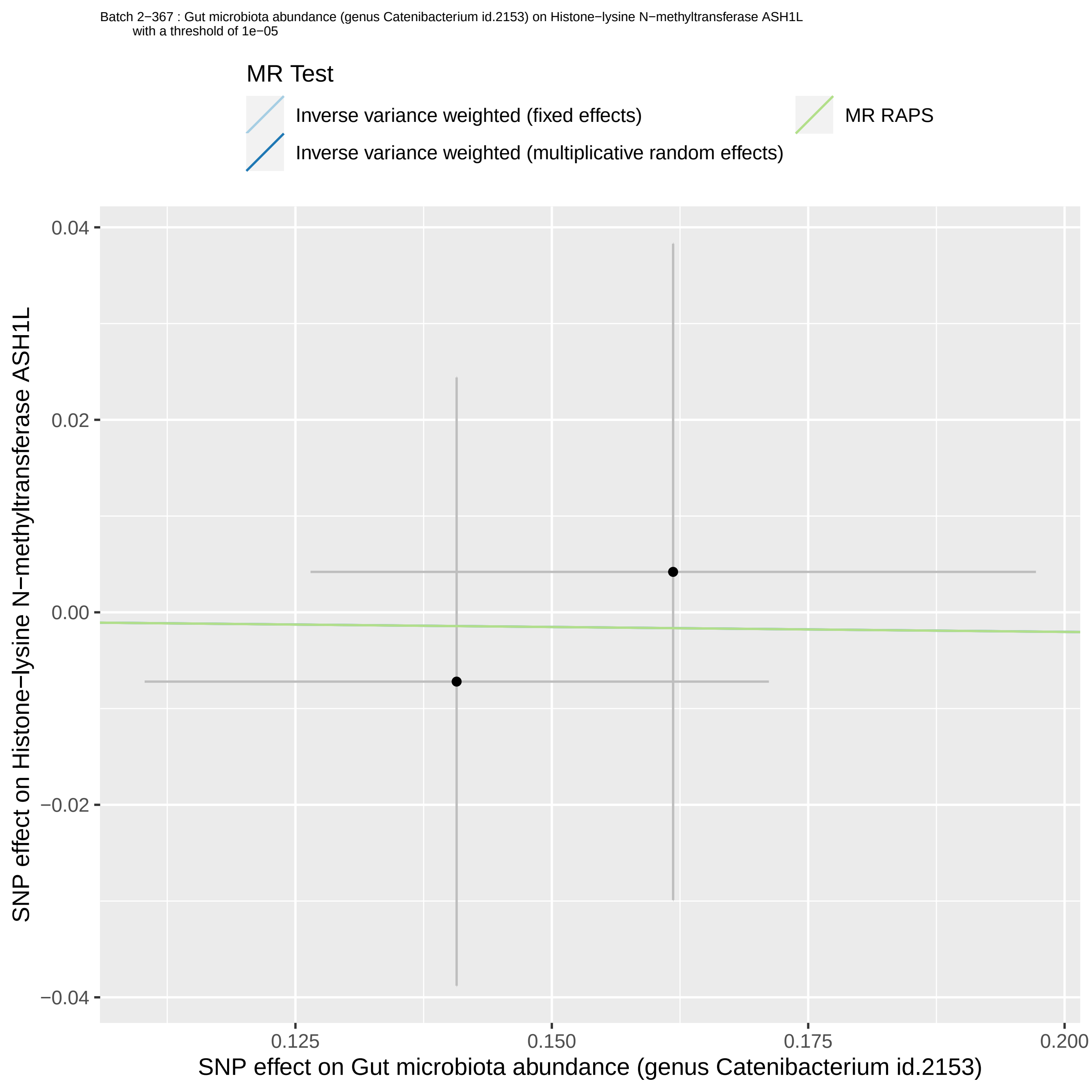
Batch 2−367 : Gut microbiota abundance (genus Catenibacterium id.2153) on Histone−lysine N−methyltransferase ASH1L
with a threshold of 1e−05
MR Test
Inverse variance weighted (fixed effects)
Inverse variance weighted (multiplicative random effects)
MR RAPS
0.04
0.02
0.00
−0.02
−0.04
0.125
0.150
0.175
0.200
SNP effect on Histone−lysine N−methyltransferase ASH1L
SNP effect on Gut microbiota abundance (genus Catenibacterium id.2153)

Batch 2−368 : Gut microbiota abundance (genus Parasutterella id.2892) on Histone−lysine N−methyltransferase ASH1L
with a threshold of 1e−05

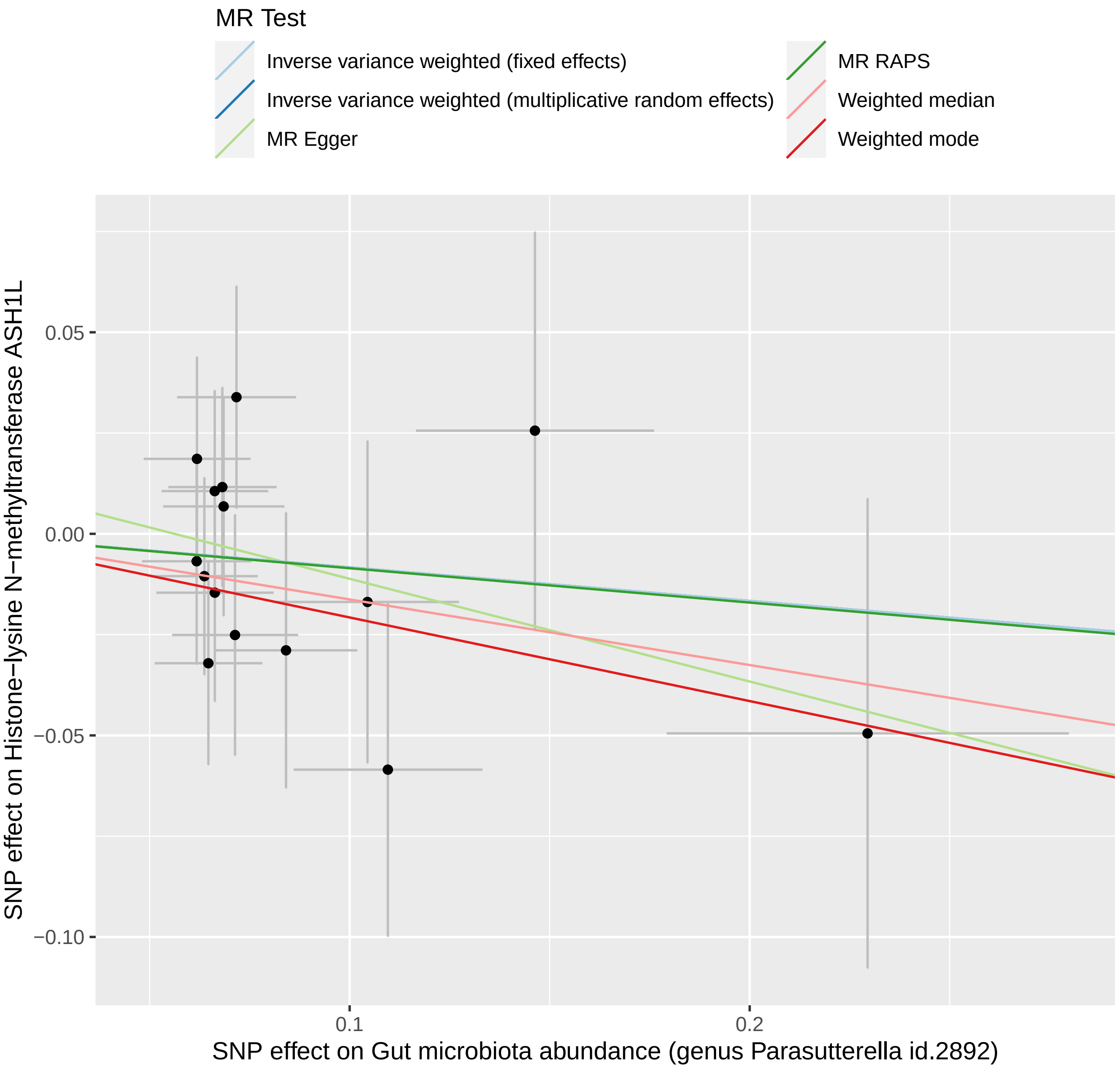

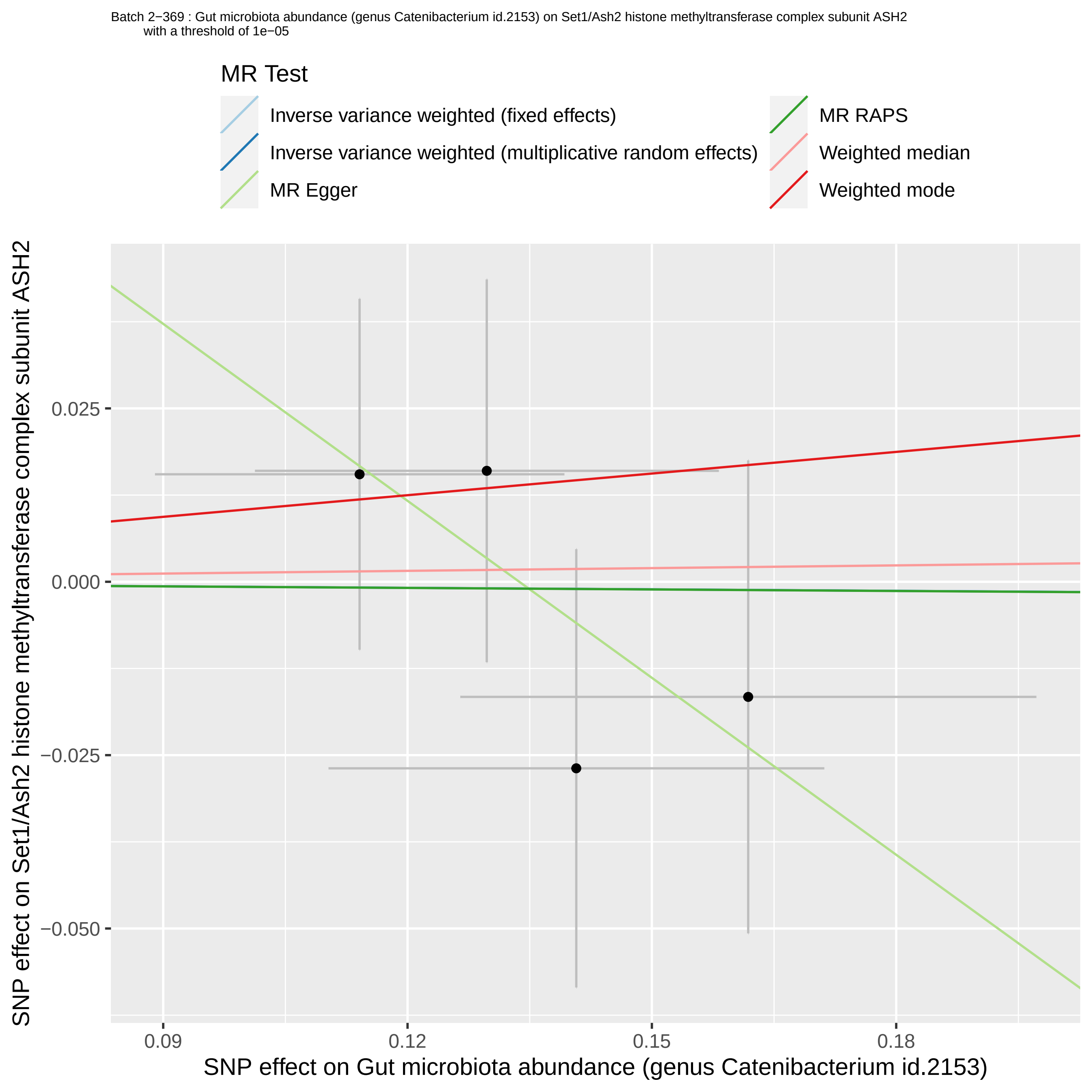
Batch 2−369 : Gut microbiota abundance (genus Catenibacterium id.2153) on Set1/Ash2 histone methyltransferase complex subunit ASH2
with a threshold of 1e−05
MR Test
Inverse variance weighted (fixed effects)
Inverse variance weighted (multiplicative random effects)
MR Egger
MR RAPS
Weighted median
Weighted mode
0.025
0.000
−0.025
−0.050
0.09
0.12
0.15
0.18
SNP effect on Set1/Ash2 histone methyltransferase complex subunit ASH2
SNP effect on Gut microbiota abundance (genus Catenibacterium id.2153)

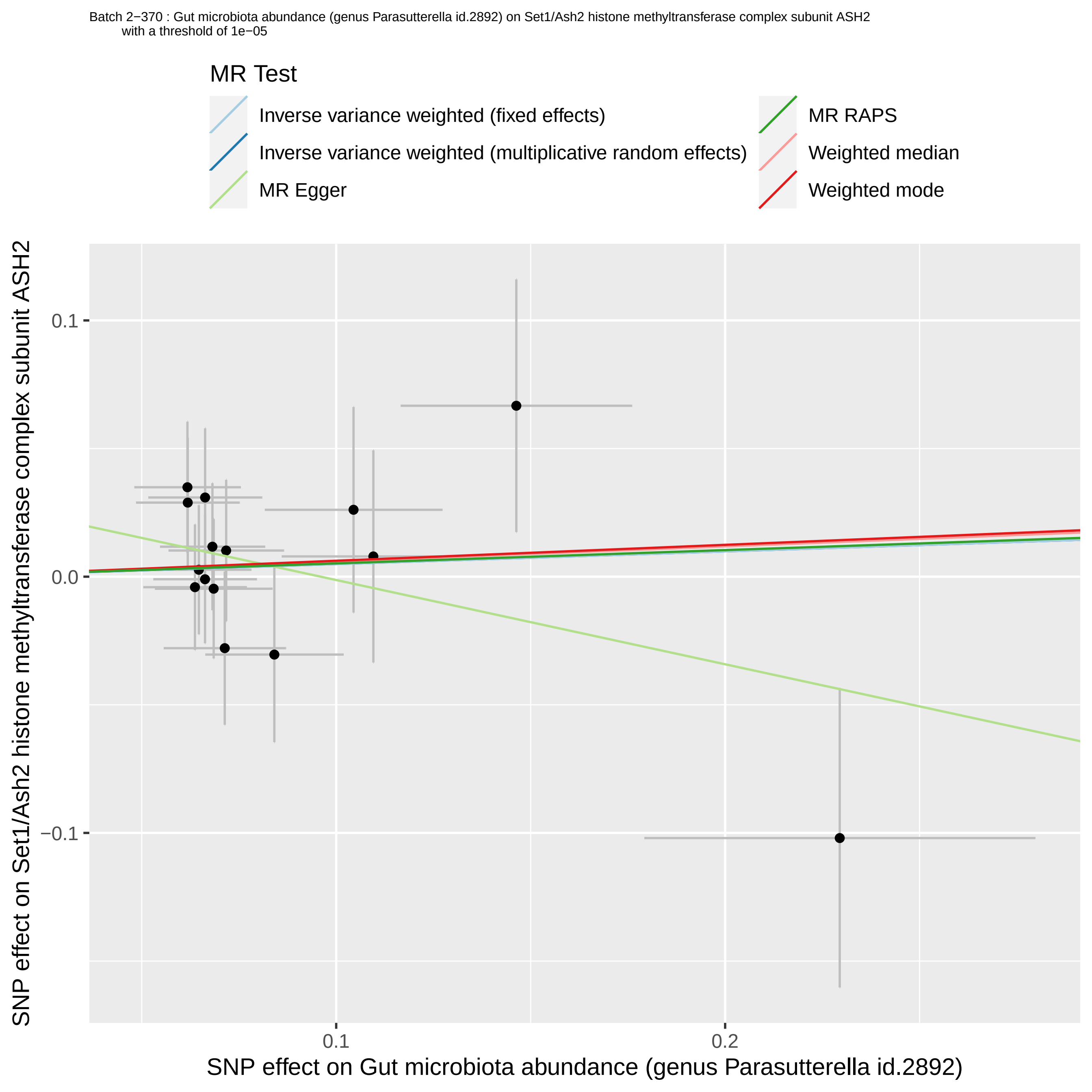

Batch 2−370 : Gut microbiota abundance (genus Parasutterella id.2892) on Set1/Ash2 histone methyltransferase complex subunit ASH2
with a threshold of 1e−05
MR Test
Inverse variance weighted (fixed effects)
Inverse variance weighted (multiplicative random effects)
MR Egger
MR RAPS
Weighted median
Weighted mode
0.1
0.0
−0.1
0.1
0.2
SNP effect on Set1/Ash2 histone methyltransferase complex subunit ASH2
SNP effect on Gut microbiota abundance (genus Parasutterella id.2892)

Batch 2−371 : Gut microbiota abundance (genus Catenibacterium id.2153) on Acid−sensing ion channel 4
with a threshold of 1e−05

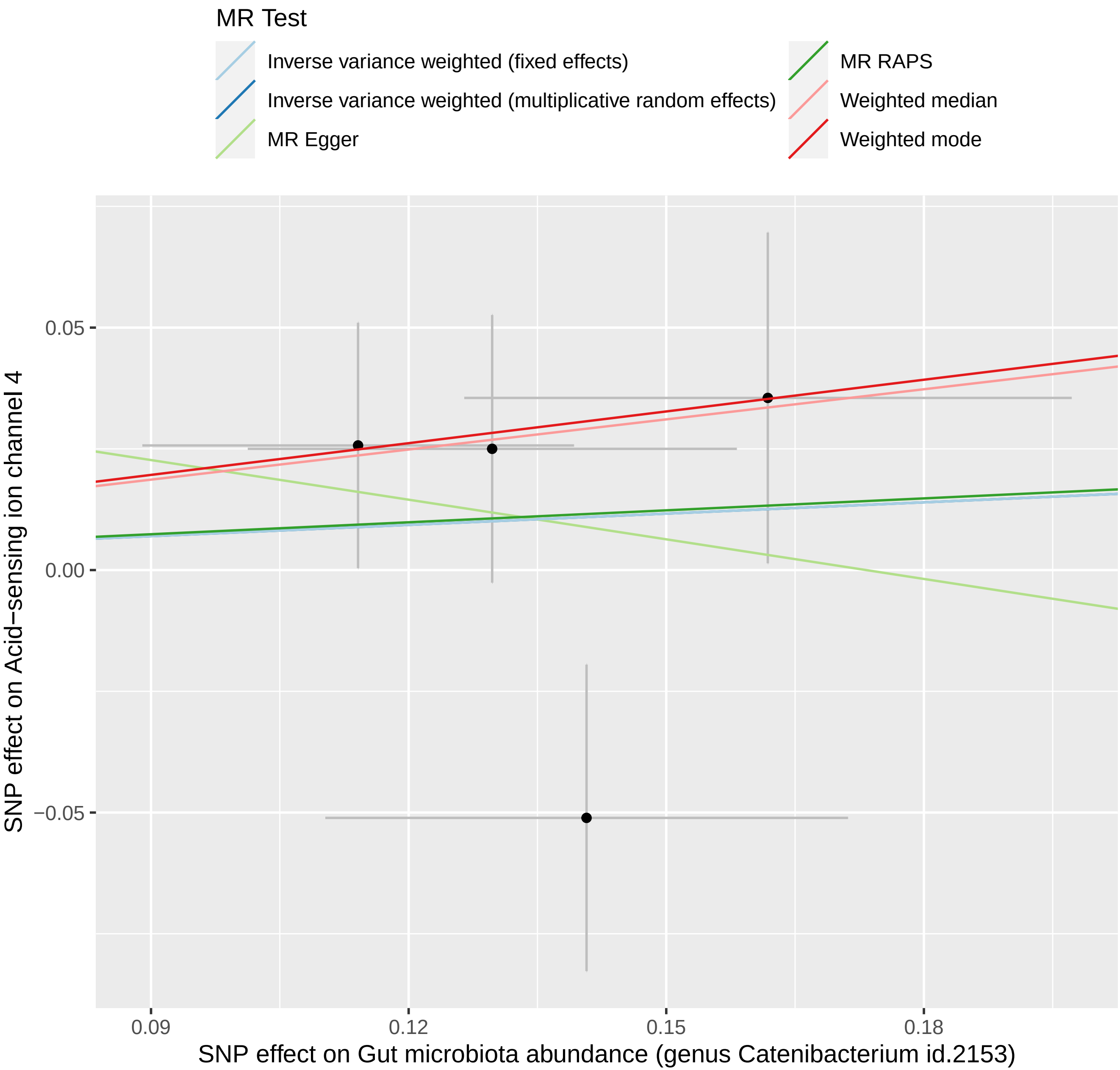

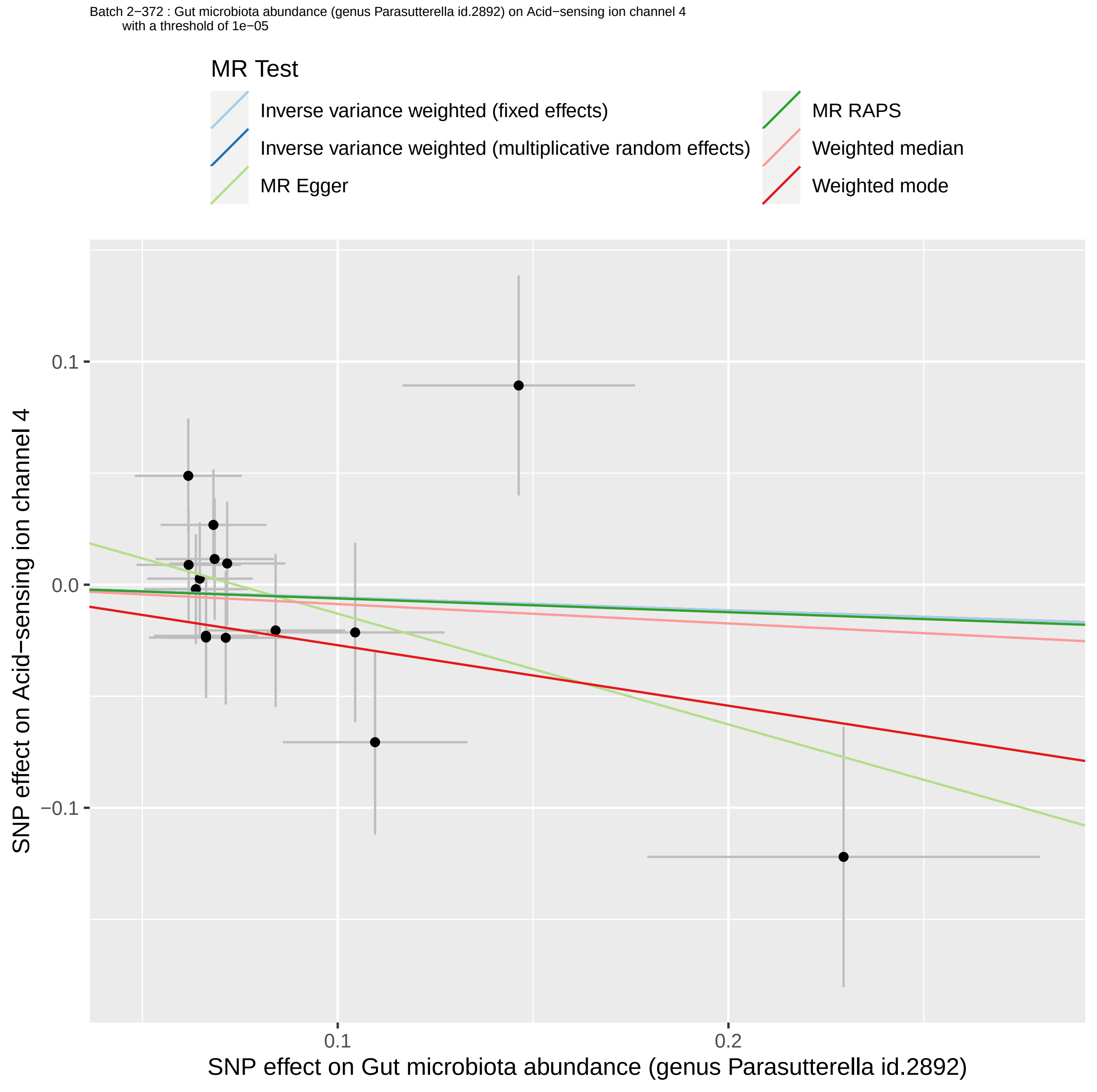

Batch 2−372 : Gut microbiota abundance (genus Parasutterella id.2892) on Acid−sensing ion channel 4
with a threshold of 1e−05
MR Test
Inverse variance weighted (fixed effects)
Inverse variance weighted (multiplicative random effects)
MR Egger
MR RAPS
Weighted median
Weighted mode
0.1
0.0
−0.1
0.1
0.2
SNP effect on Acid−sensing ion channel 4
SNP effect on Gut microbiota abundance (genus Parasutterella id.2892)

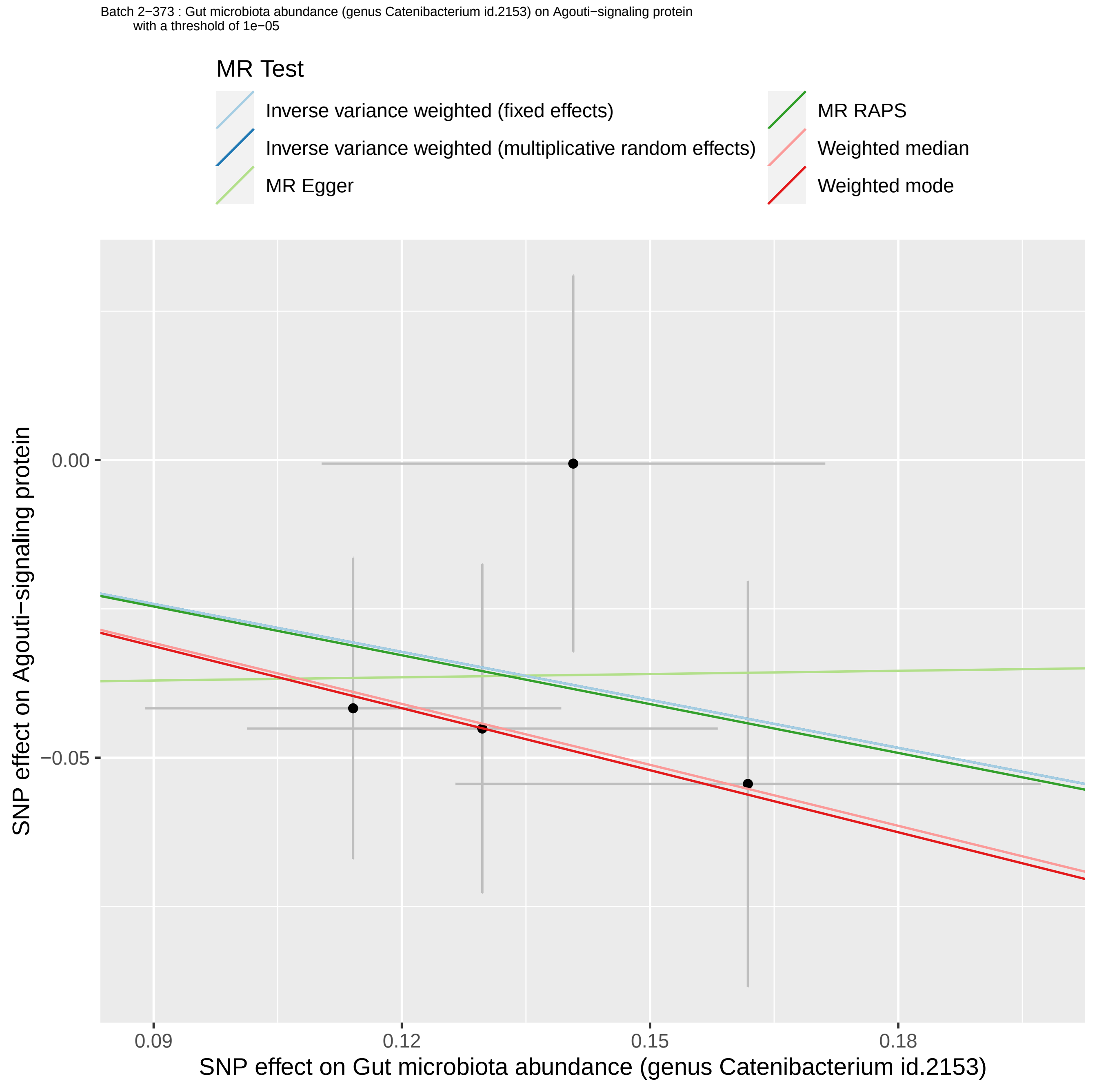
Batch 2−373 : Gut microbiota abundance (genus Catenibacterium id.2153) on Agouti−signaling protein
with a threshold of 1e−05
MR Test
Inverse variance weighted (fixed effects)
Inverse variance weighted (multiplicative random effects)
MR Egger
MR RAPS
Weighted median
Weighted mode
0.00
−0.05
0.09
0.12
0.15
0.18
SNP effect on Agouti−signaling protein
SNP effect on Gut microbiota abundance (genus Catenibacterium id.2153)

Batch 2−374 : Gut microbiota abundance (genus Parasutterella id.2892) on Agouti−signaling protein
with a threshold of 1e−05

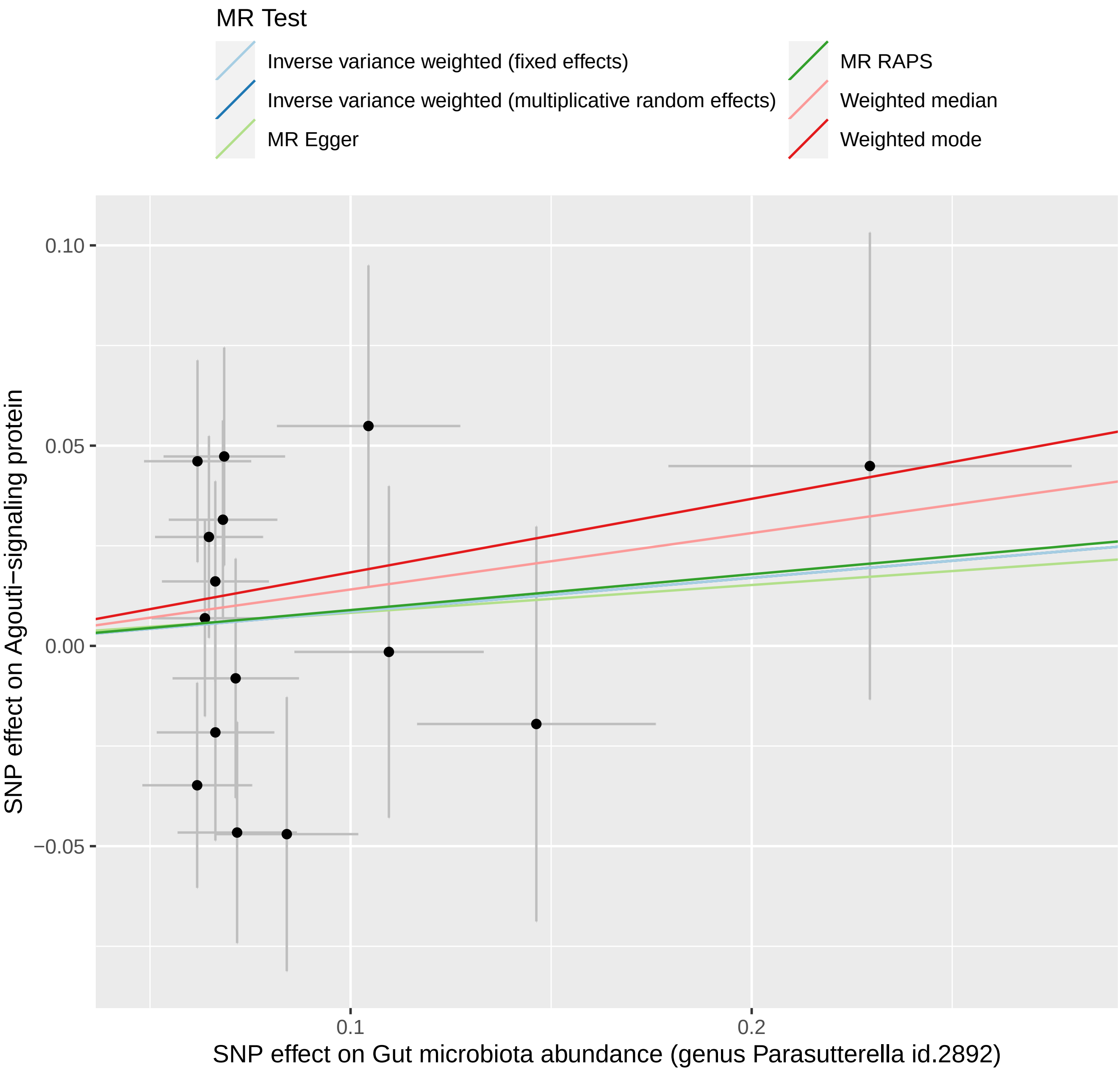

Batch 2−375 : Gut microbiota abundance (genus Catenibacterium id.2153) on Argininosuccinate lyase
with a threshold of 1e−05

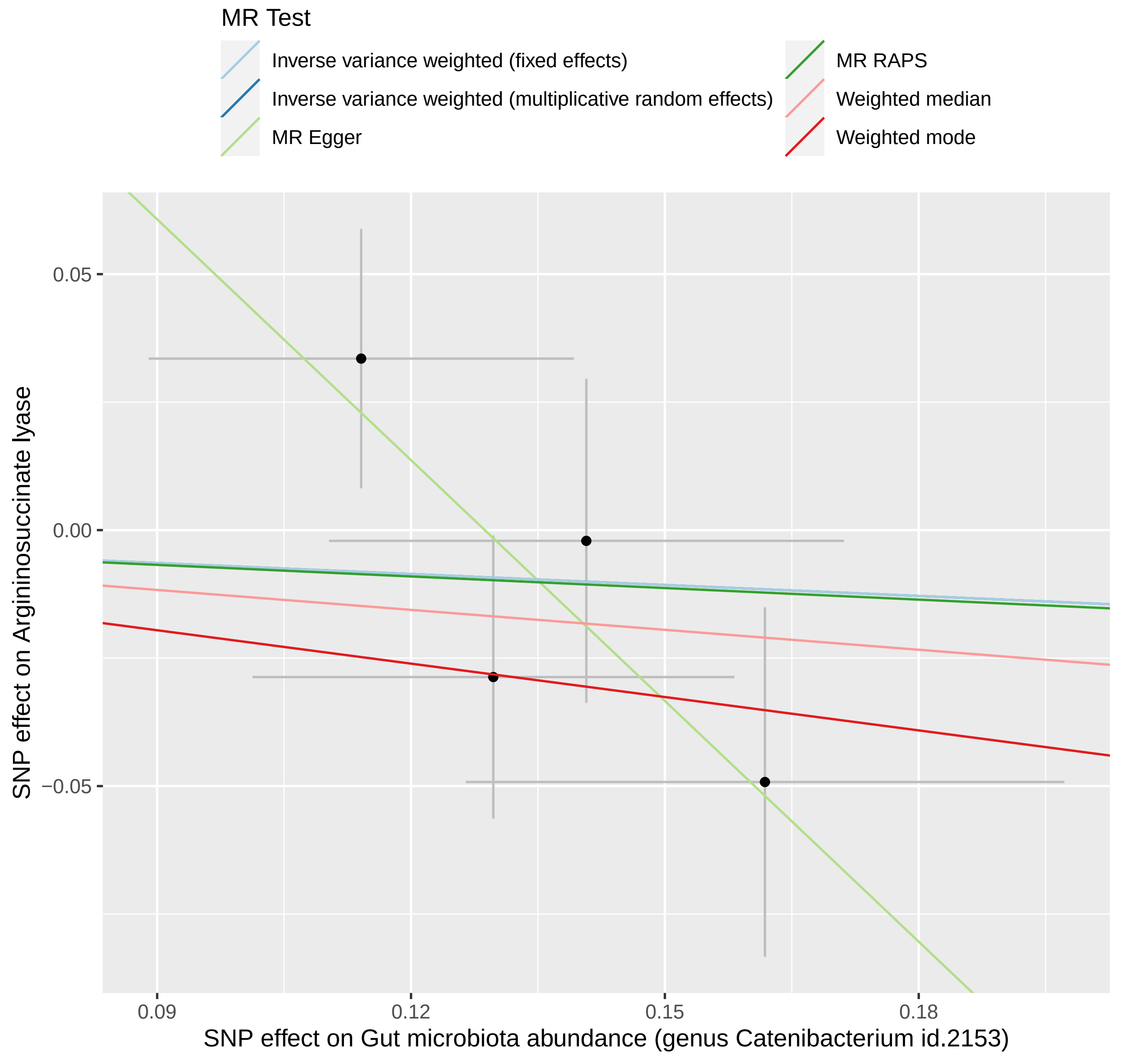

Batch 2−376 : Gut microbiota abundance (genus Parasutterella id.2892) on Argininosuccinate lyase
with a threshold of 1e−05

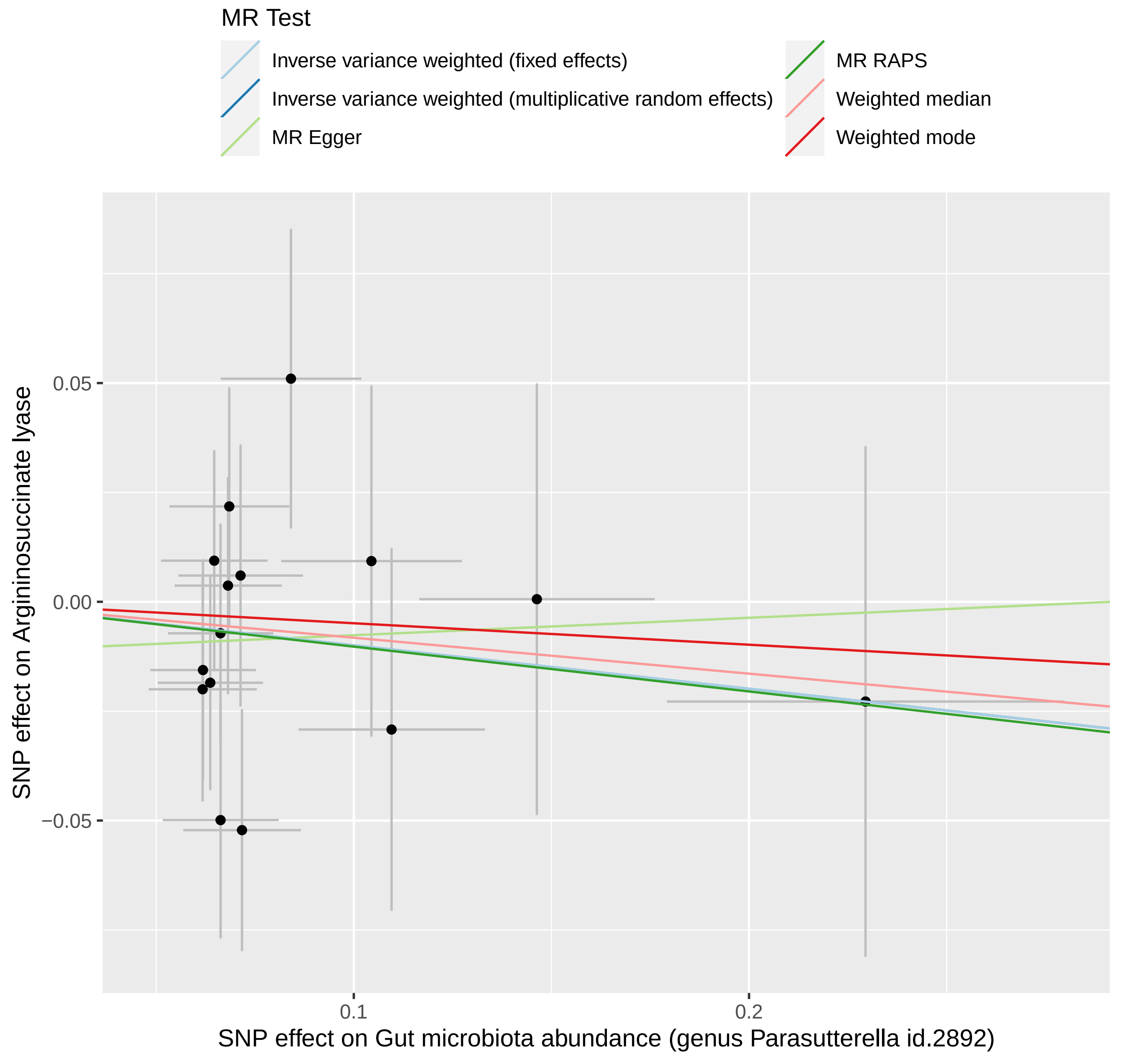

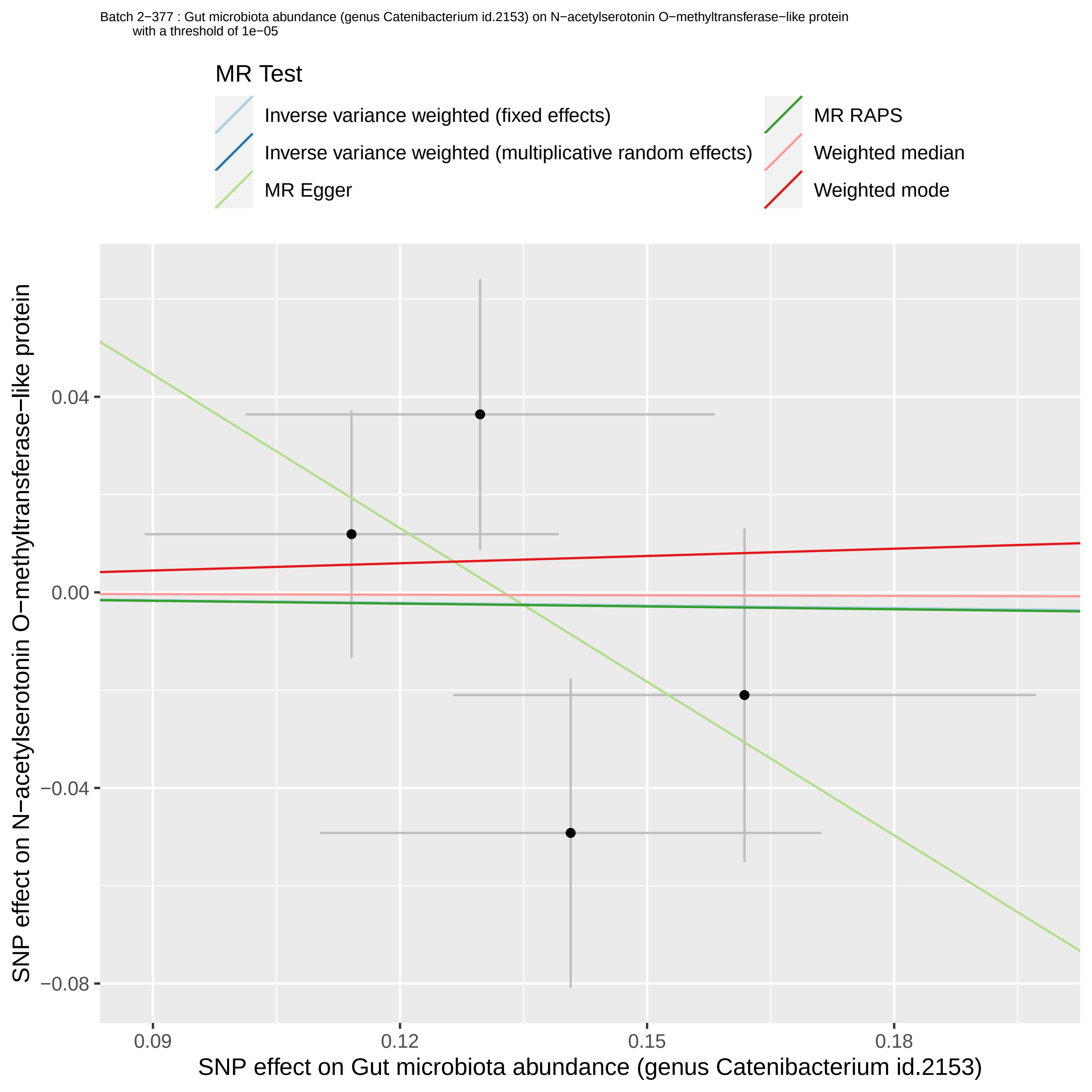
Batch 2−377 : Gut microbiota abundance (genus Catenibacterium id.2153) on N−acetylserotonin O−methyltransferase−like protein
with a threshold of 1e−05
MR Test
Inverse variance weighted (fixed effects)
Inverse variance weighted (multiplicative random effects)
MR Egger
MR RAPS
Weighted median
Weighted mode
SNP effect on N−acetylserotonin O−methyltransferase−like protein
0.04
0.00
−0.04
−0.08
0.09
0.12
0.15
0.18
SNP effect on Gut microbiota abundance (genus Catenibacterium id.2153)

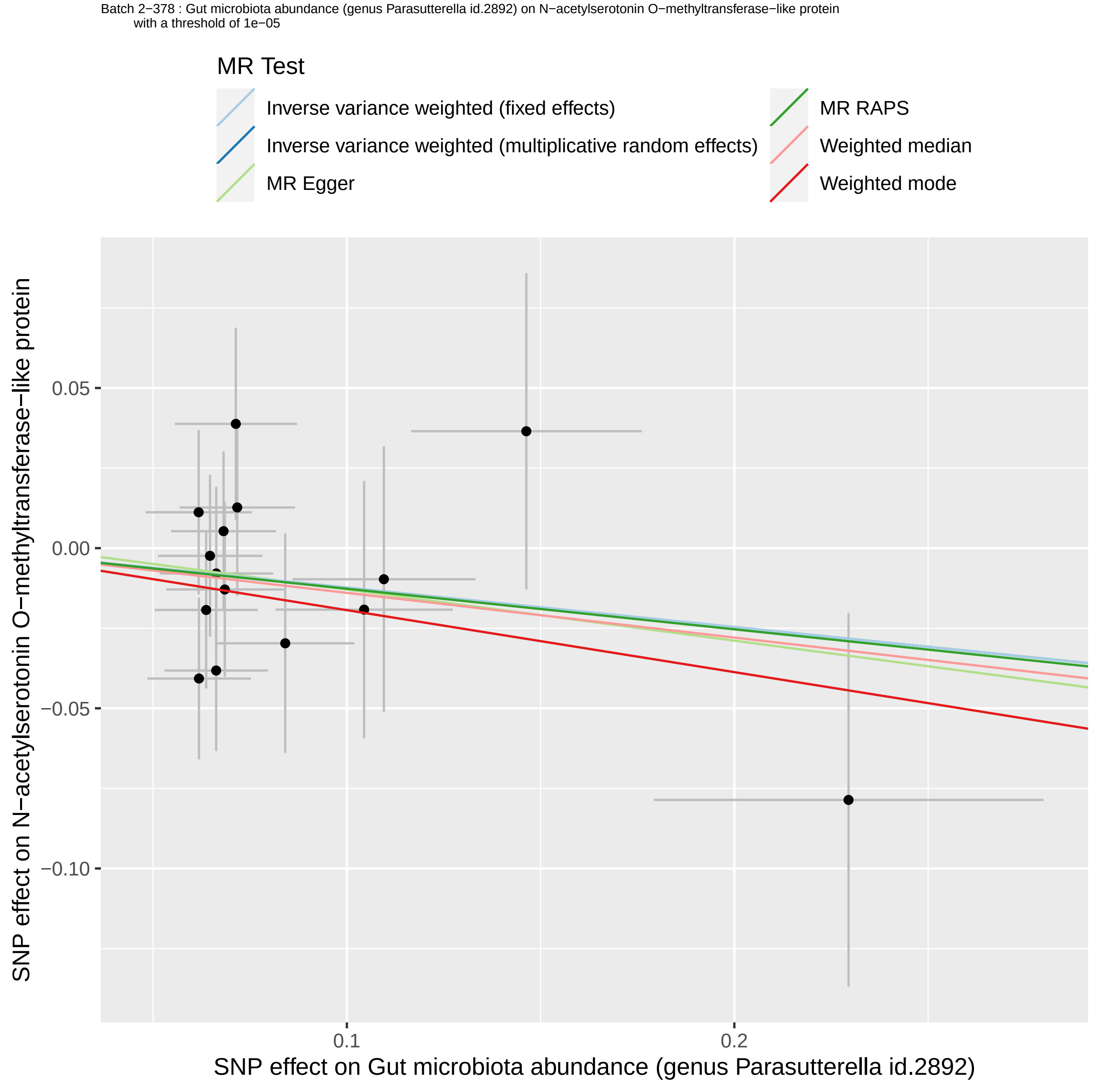

Batch 2−378 : Gut microbiota abundance (genus Parasutterella id.2892) on N−acetylserotonin O−methyltransferase−like protein
with a threshold of 1e−05
MR Test
Inverse variance weighted (fixed effects)
Inverse variance weighted (multiplicative random effects)
MR Egger
MR RAPS
Weighted median
Weighted mode
0.05
0.00
−0.05
−0.10
0.1
0.2
SNP effect on N−acetylserotonin O−methyltransferase−like protein
SNP effect on Gut microbiota abundance (genus Parasutterella id.2892)

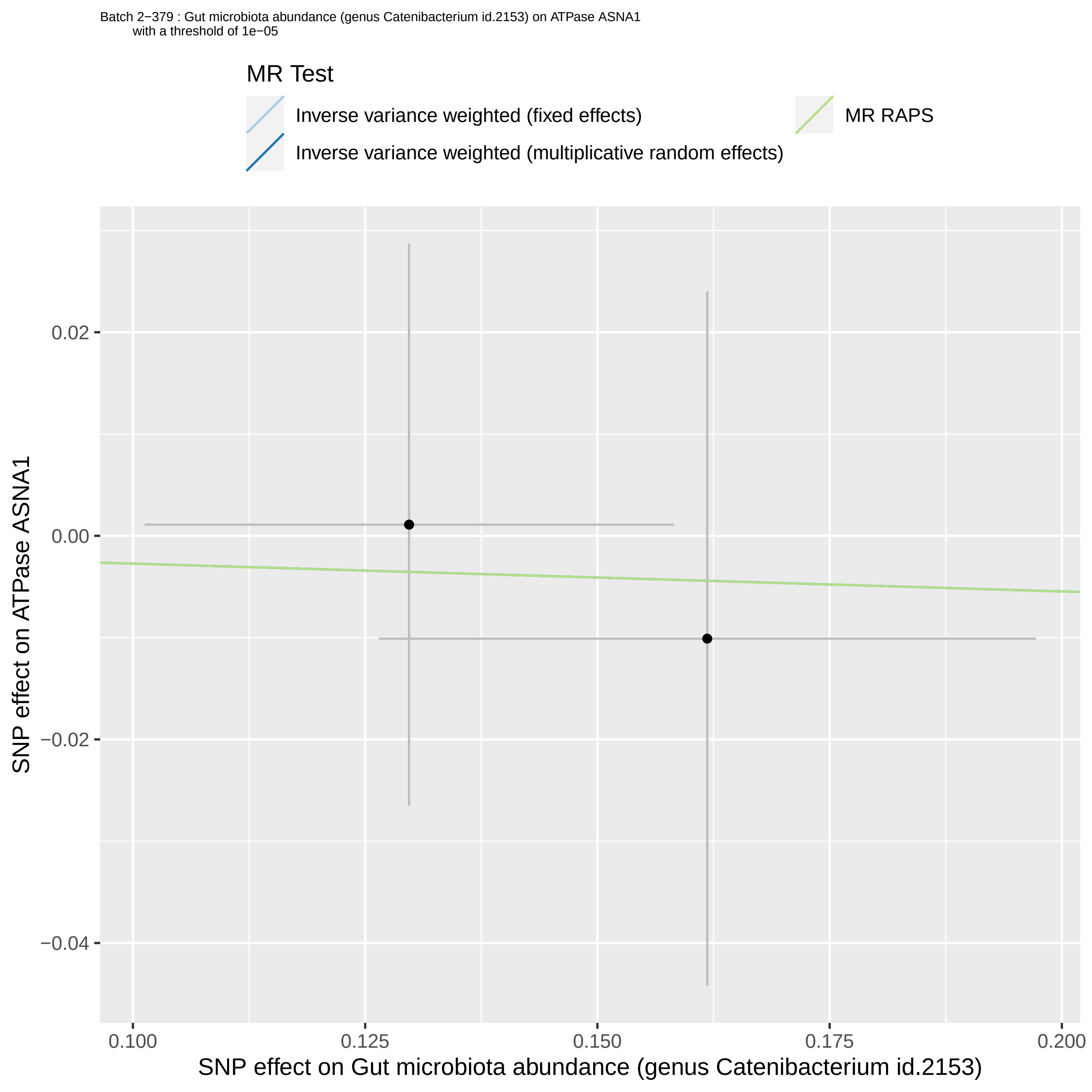

Batch 2−379 : Gut microbiota abundance (genus Catenibacterium id.2153) on ATPase ASNA1
with a threshold of 1e−05
MR Test
Inverse variance weighted (fixed effects)
Inverse variance weighted (multiplicative random effects)
MR RAPS
0.02
0.00
−0.02
−0.04
SNP effect on ATPase ASNA1
0.100
0.125
0.150
0.175
0.200
SNP effect on Gut microbiota abundance (genus Catenibacterium id.2153)

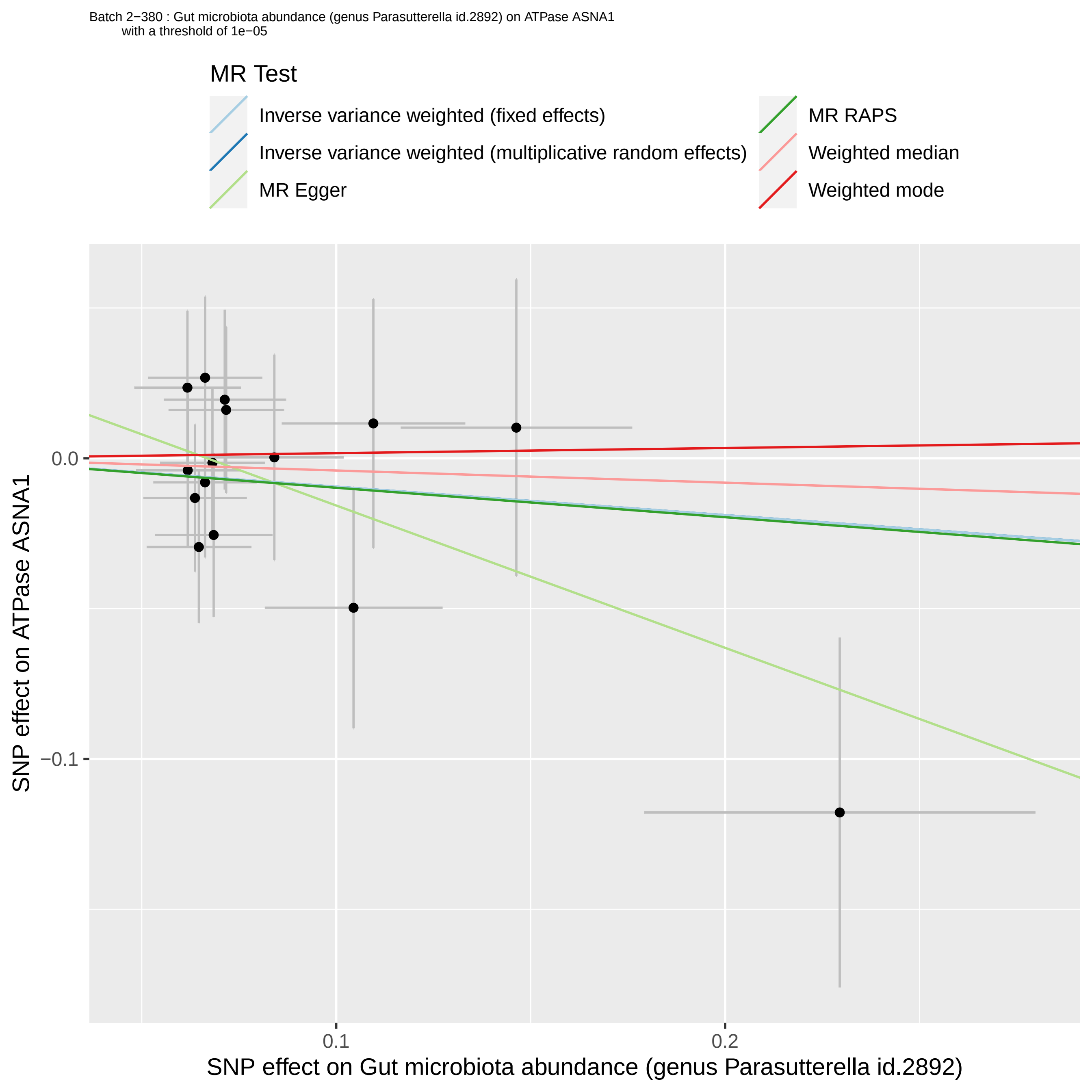

Batch 2−380 : Gut microbiota abundance (genus Parasutterella id.2892) on ATPase ASNA1
with a threshold of 1e−05
MR Test
Inverse variance weighted (fixed effects)
Inverse variance weighted (multiplicative random effects)
MR Egger
MR RAPS
Weighted median
Weighted mode
0.0
−0.1
0.1
0.2
SNP effect on ATPase ASNA1
SNP effect on Gut microbiota abundance (genus Parasutterella id.2892)

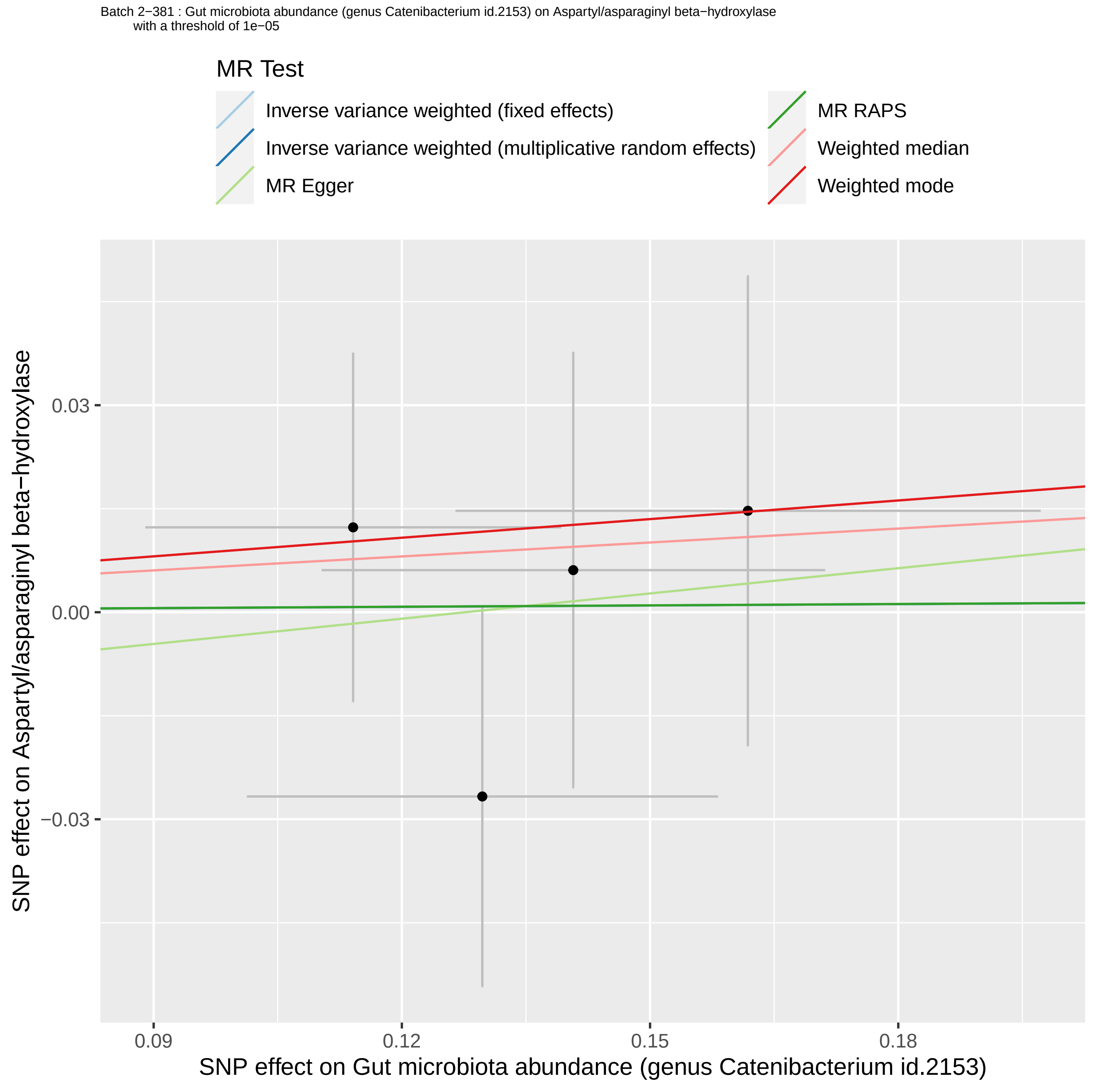
Batch 2−381 : Gut microbiota abundance (genus Catenibacterium id.2153) on Aspartyl/asparaginyl beta−hydroxylase
with a threshold of 1e−05
MR Test
Inverse variance weighted (fixed effects)
Inverse variance weighted (multiplicative random effects)
MR Egger
MR RAPS
Weighted median
Weighted mode
0.03
0.00
−0.03
0.09
0.12
0.15
0.18
SNP effect on Aspartyl/asparaginyl beta−hydroxylase
SNP effect on Gut microbiota abundance (genus Catenibacterium id.2153)

Batch 2−382 : Gut microbiota abundance (genus Parasutterella id.2892) on Aspartyl/asparaginyl beta−hydroxylase
with a threshold of 1e−05

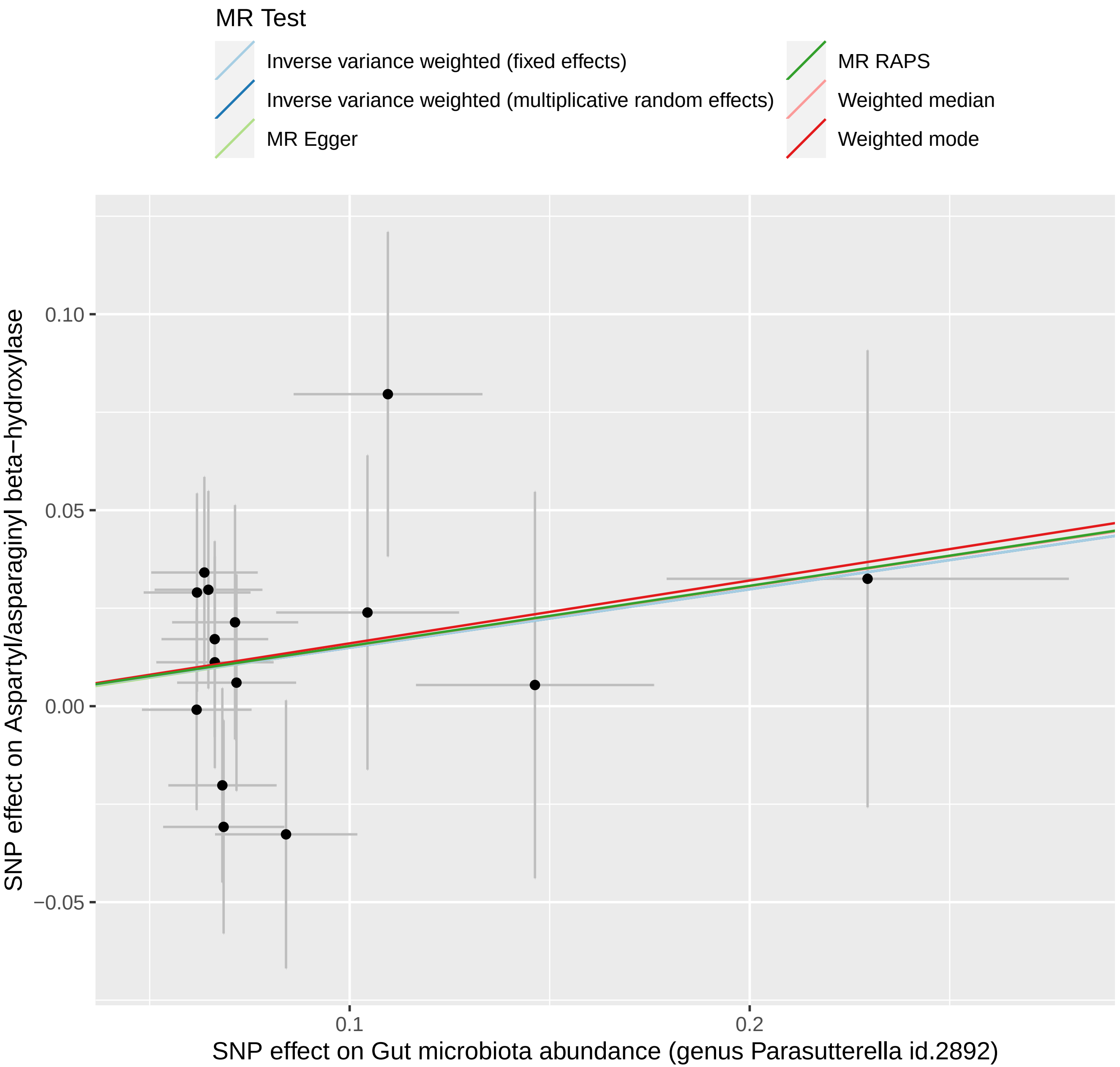

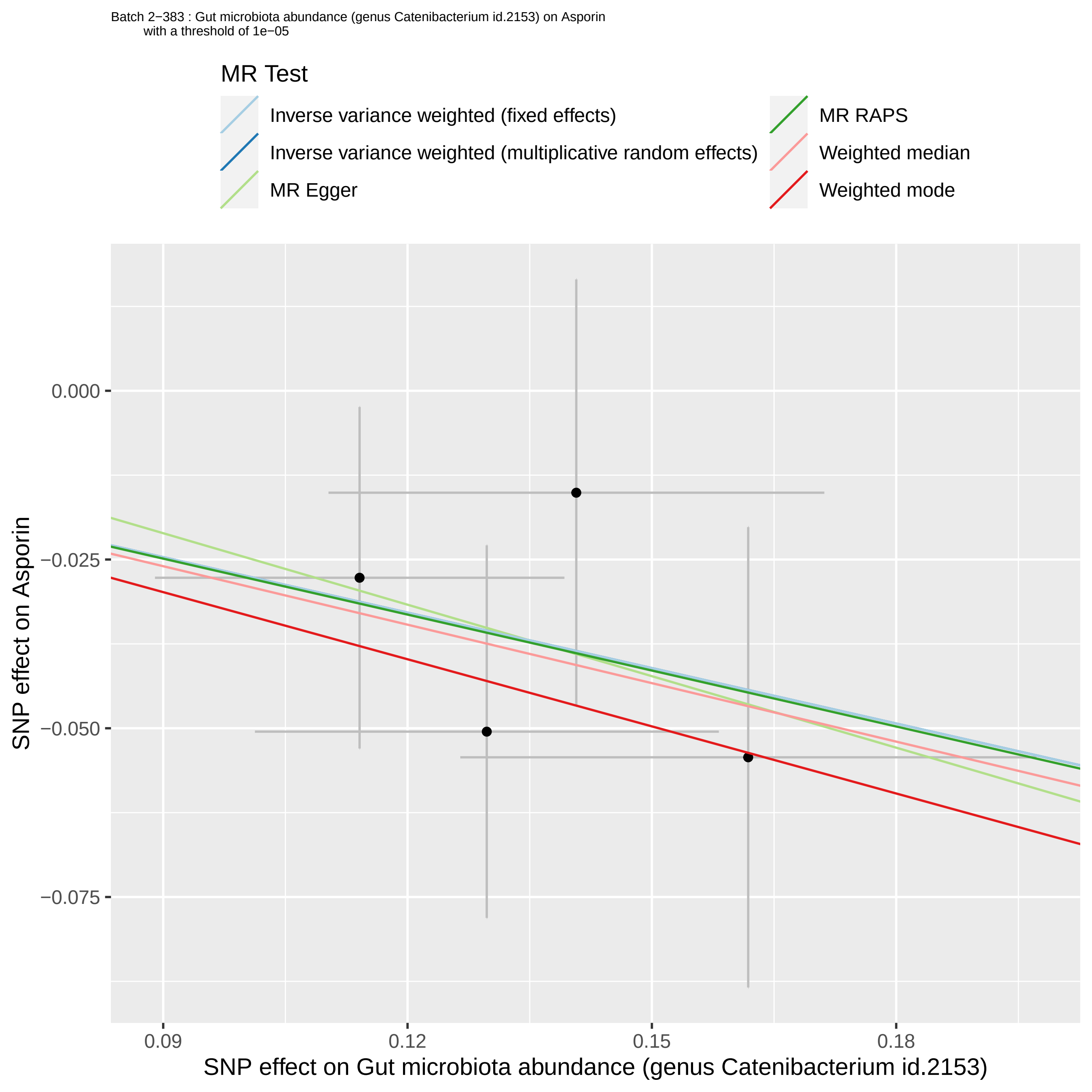
Batch 2−383 : Gut microbiota abundance (genus Catenibacterium id.2153) on Asporin
with a threshold of 1e−05
MR Test
Inverse variance weighted (fixed effects)
Inverse variance weighted (multiplicative random effects)
MR Egger
MR RAPS
Weighted median
Weighted mode
0.000
−0.025
−0.050
−0.075
0.09
0.12
0.15
0.18
SNP effect on Asporin
SNP effect on Gut microbiota abundance (genus Catenibacterium id.2153)

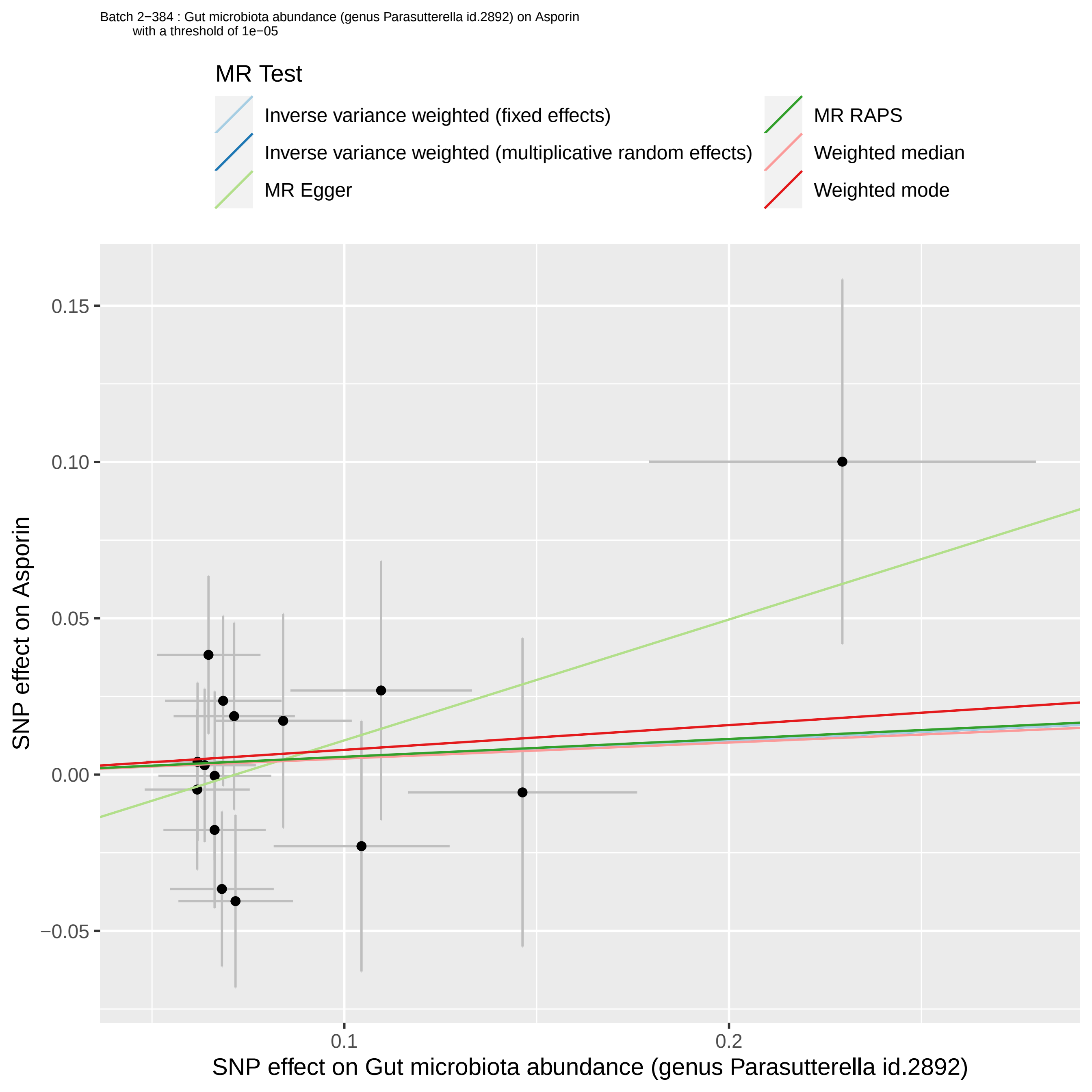

Batch 2−384 : Gut microbiota abundance (genus Parasutterella id.2892) on Asporin
with a threshold of 1e−05
MR Test
Inverse variance weighted (fixed effects)
Inverse variance weighted (multiplicative random effects)
MR Egger
MR RAPS
Weighted median
Weighted mode
0.15
0.10
0.05
0.00
−0.05
0.1
0.2
SNP effect on Asporin
SNP effect on Gut microbiota abundance (genus Parasutterella id.2892)

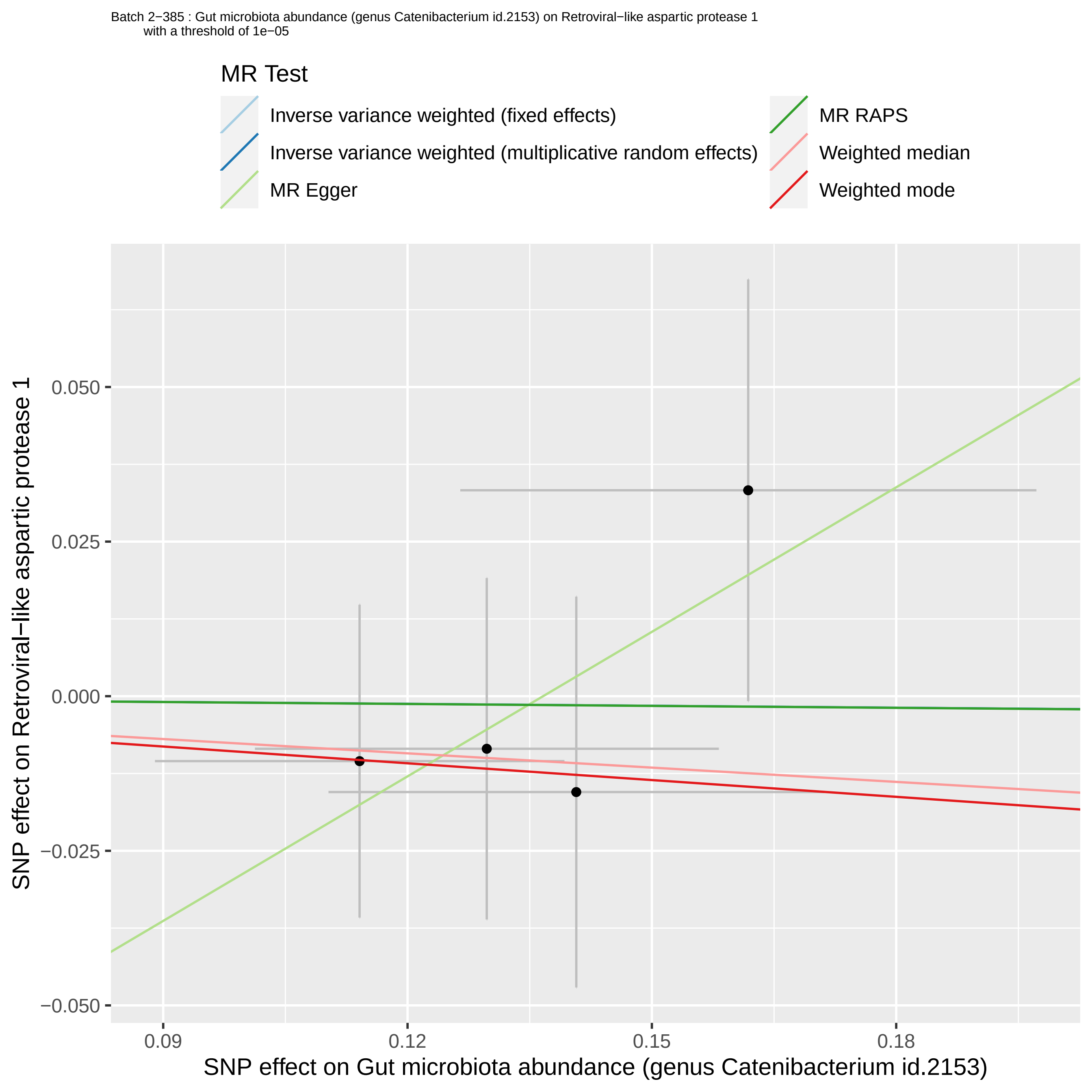
Batch 2−385 : Gut microbiota abundance (genus Catenibacterium id.2153) on Retroviral−like aspartic protease 1
with a threshold of 1e−05
MR Test
Inverse variance weighted (fixed effects)
Inverse variance weighted (multiplicative random effects)
MR Egger
MR RAPS
Weighted median
Weighted mode
0.050
0.025
0.000
−0.025
−0.050
0.09
0.12
0.15
0.18
SNP effect on Retroviral−like aspartic protease 1
SNP effect on Gut microbiota abundance (genus Catenibacterium id.2153)

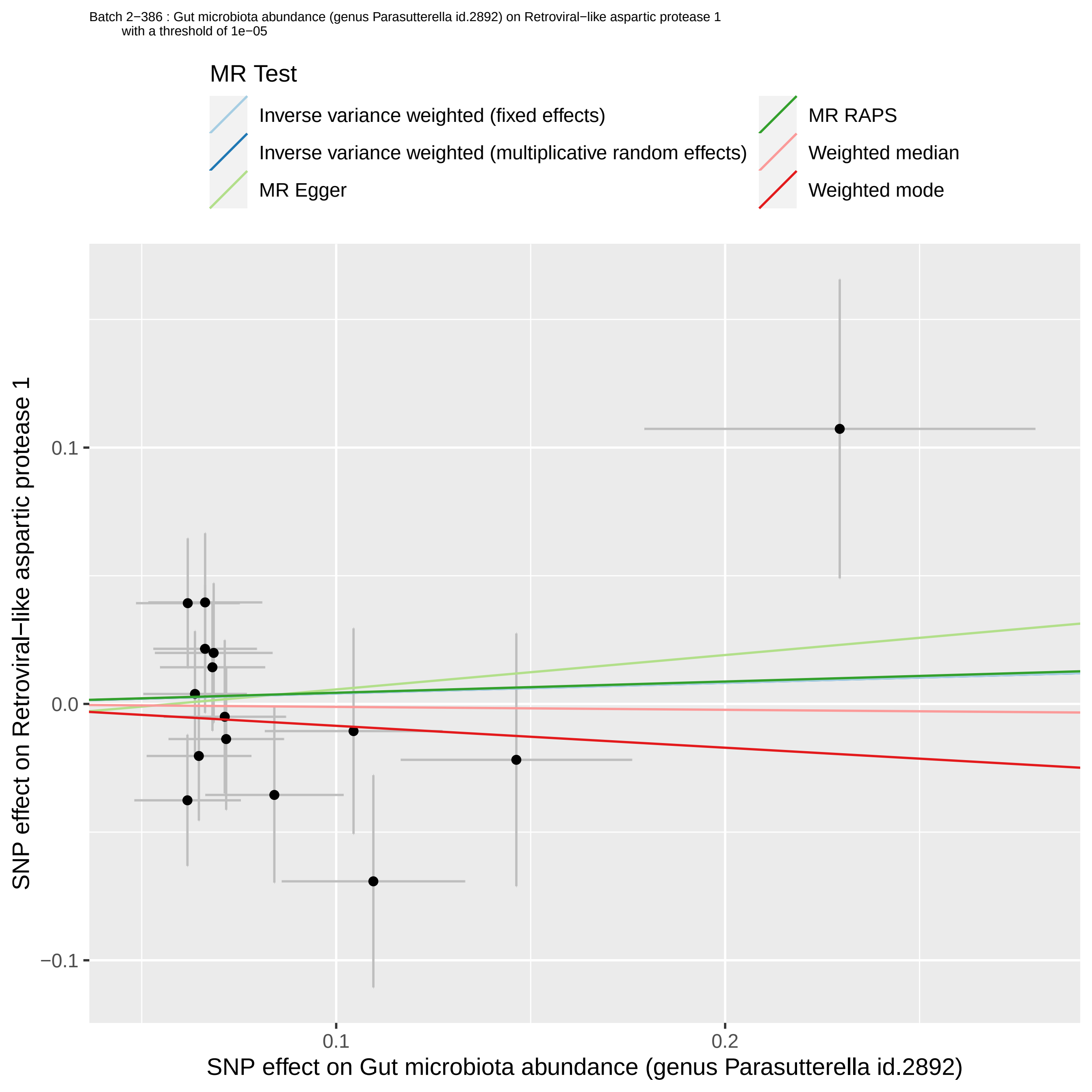

Batch 2−386 : Gut microbiota abundance (genus Parasutterella id.2892) on Retroviral−like aspartic protease 1
with a threshold of 1e−05
MR Test
Inverse variance weighted (fixed effects)
Inverse variance weighted (multiplicative random effects)
MR Egger
MR RAPS
Weighted median
Weighted mode
0.1
0.0
−0.1
0.1
0.2
SNP effect on Retroviral−like aspartic protease 1
SNP effect on Gut microbiota abundance (genus Parasutterella id.2892)

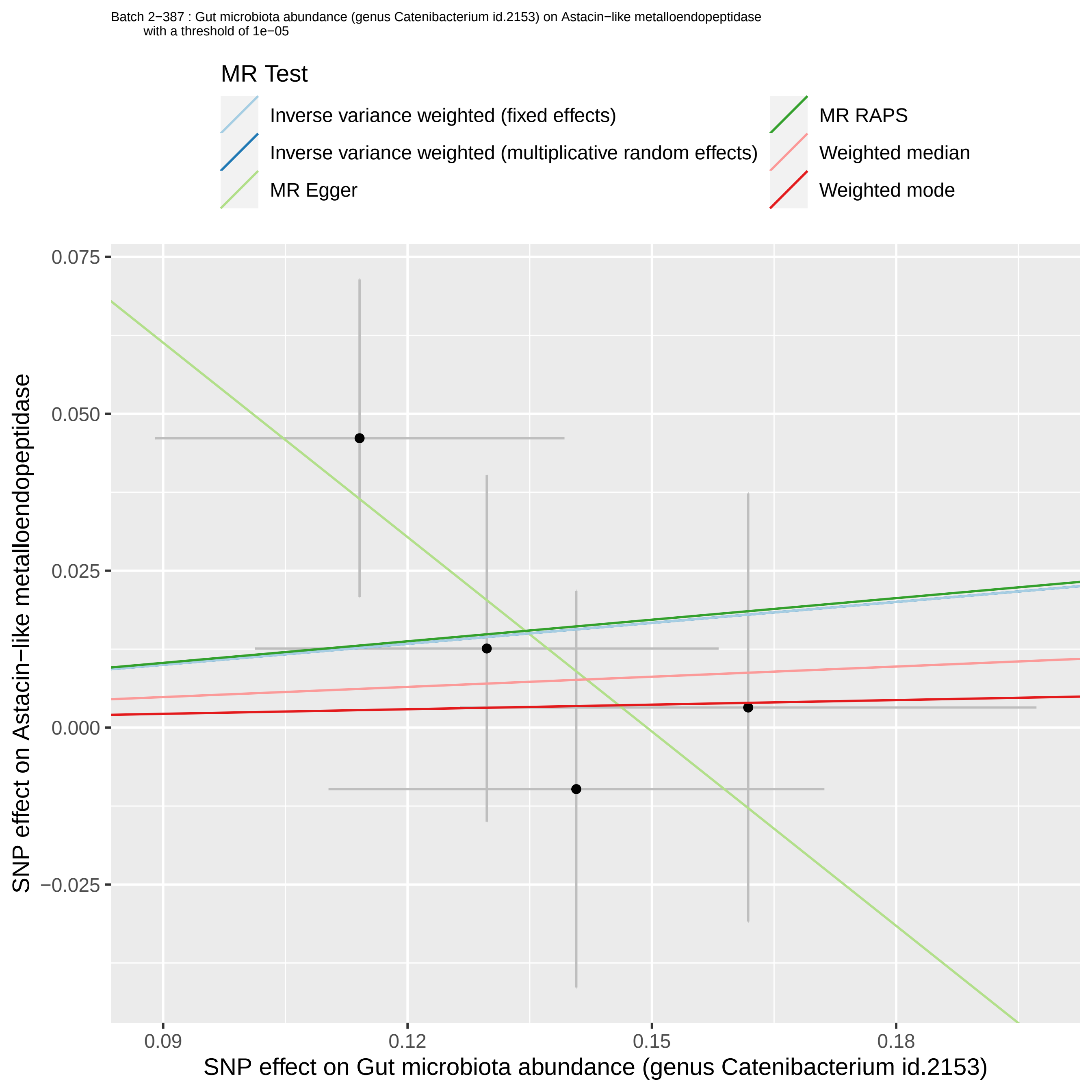

Batch 2−387 : Gut microbiota abundance (genus Catenibacterium id.2153) on Astacin−like metalloendopeptidase
with a threshold of 1e−05
MR Test
Inverse variance weighted (fixed effects)
Inverse variance weighted (multiplicative random effects)
MR Egger
MR RAPS
Weighted median
Weighted mode
0.075
0.050
0.025
0.000
−0.025
0.09
0.12
0.15
0.18
SNP effect on Astacin−like metalloendopeptidase
SNP effect on Gut microbiota abundance (genus Catenibacterium id.2153)

Batch 2−388 : Gut microbiota abundance (genus Parasutterella id.2892) on Astacin−like metalloendopeptidase
with a threshold of 1e−05

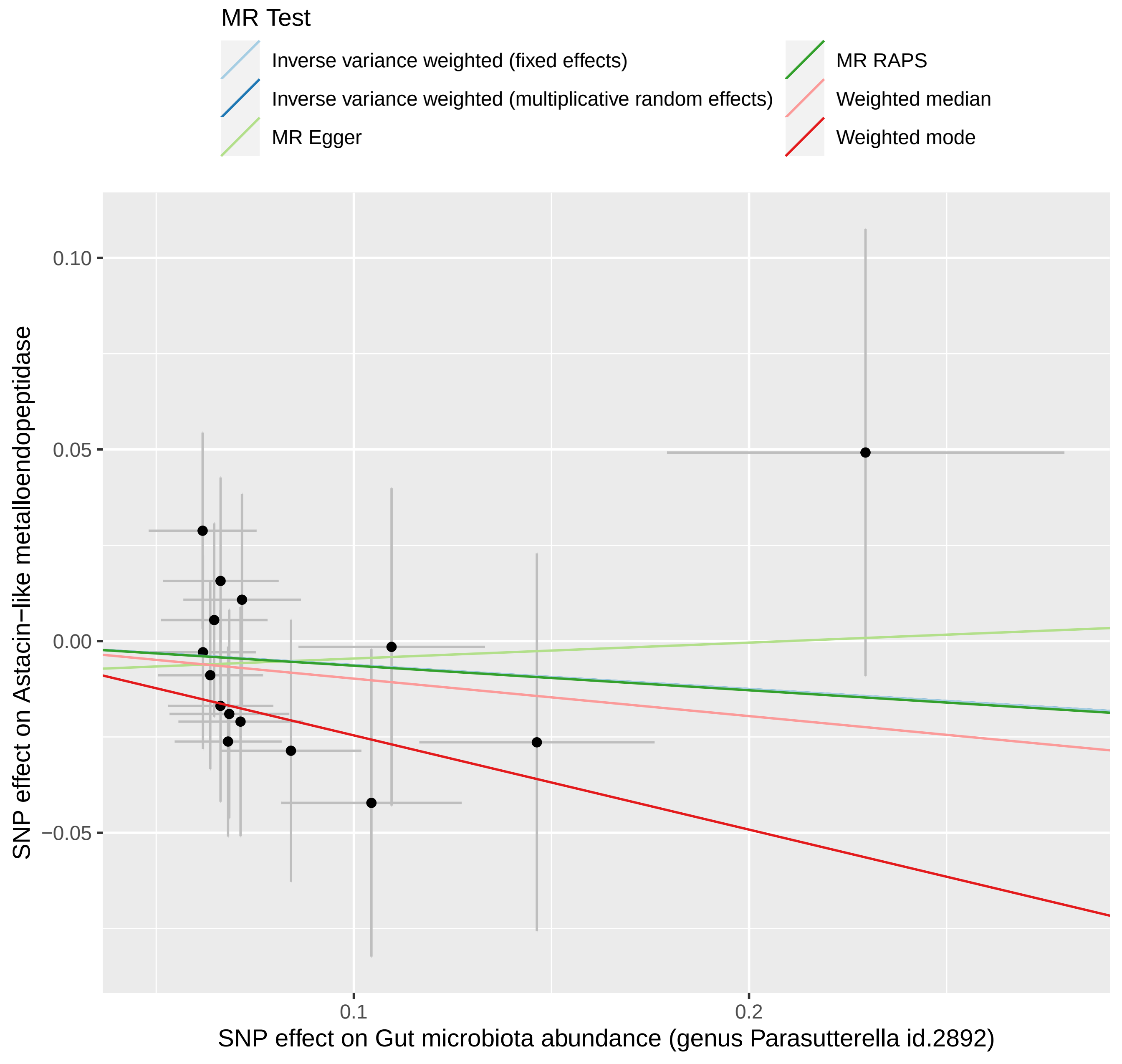

Batch 2−389 : Gut microbiota abundance (genus Catenibacterium id.2153) on ATPase family AAA domain−containing protein 1
with a threshold of 1e−05

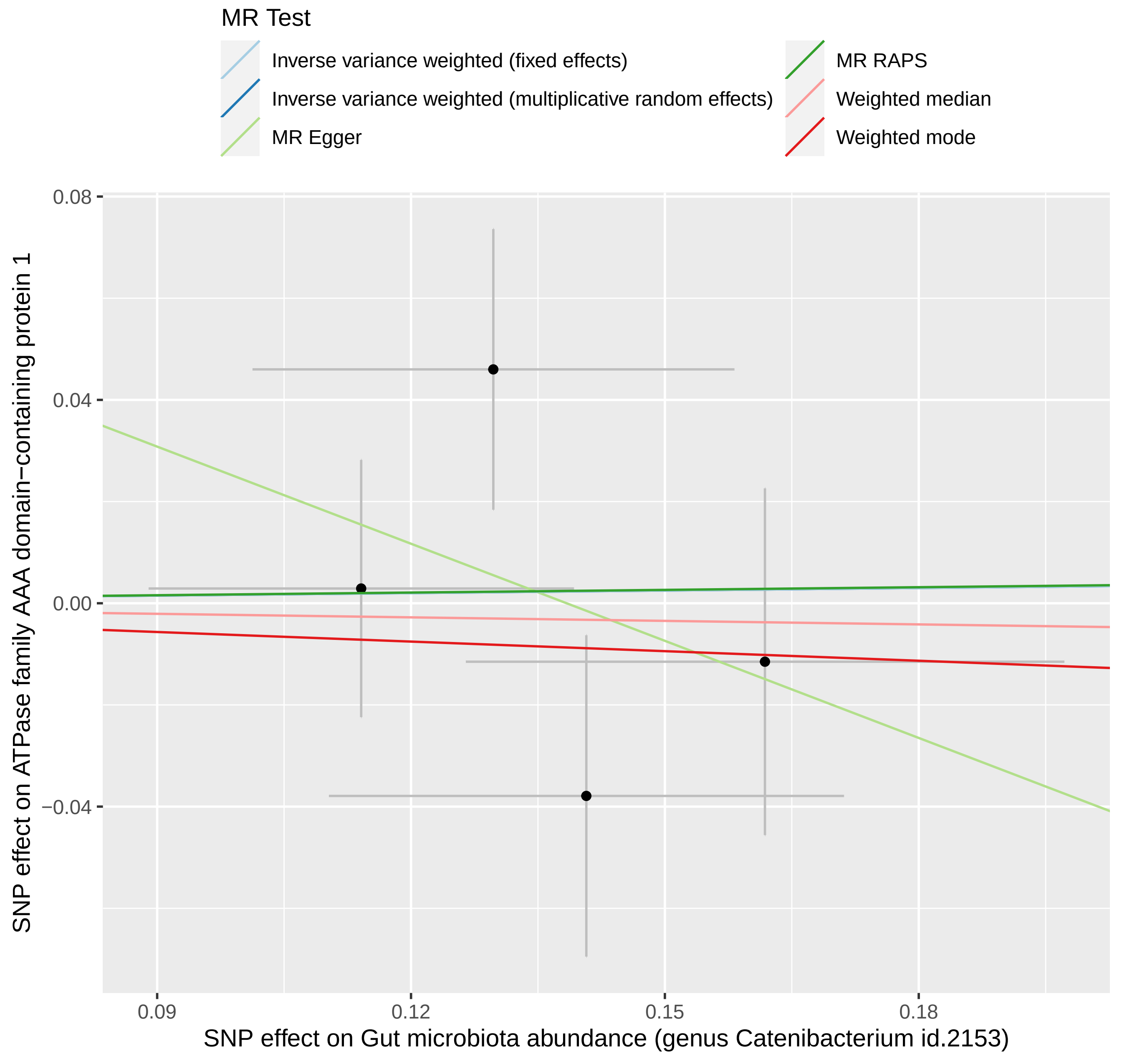

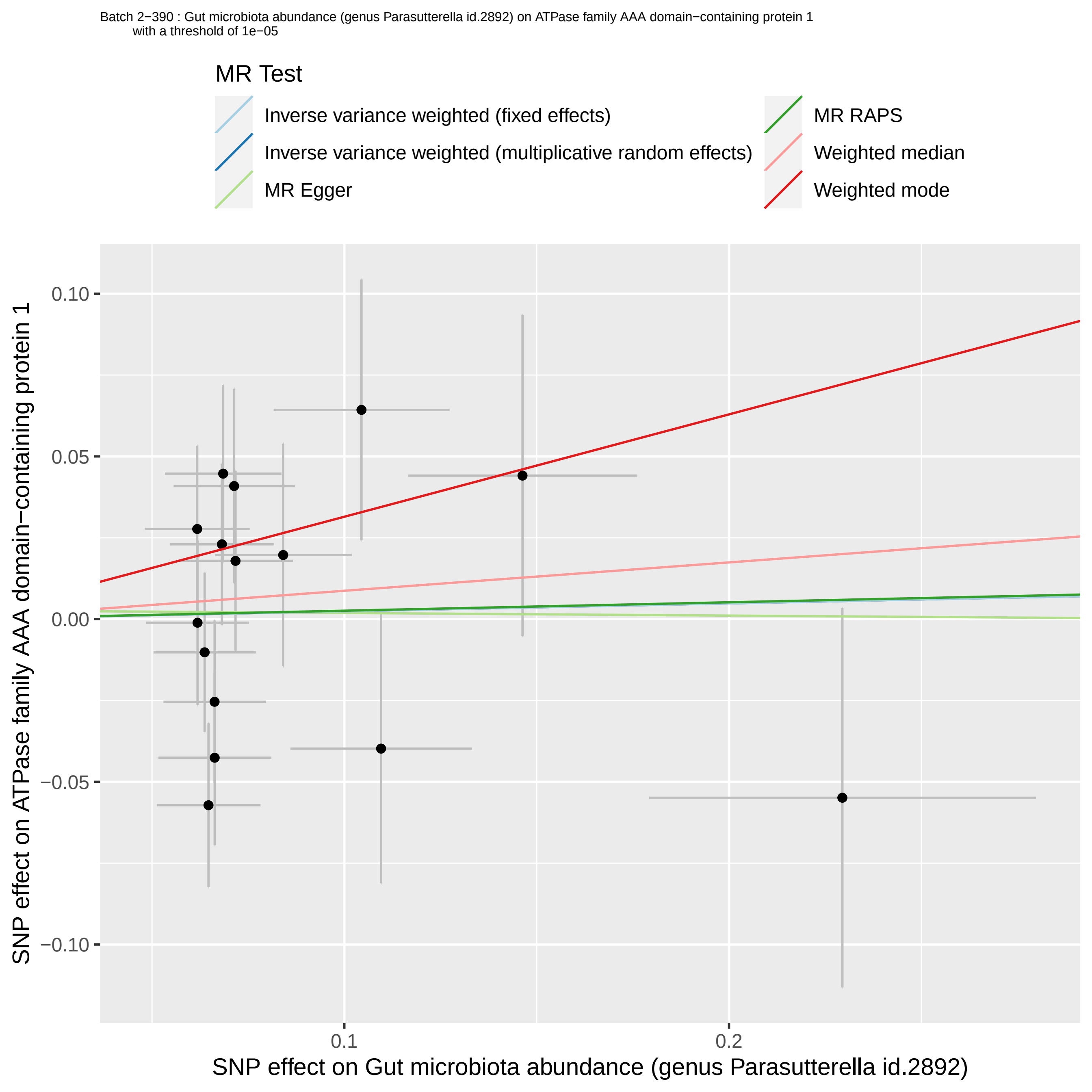

Batch 2−390 : Gut microbiota abundance (genus Parasutterella id.2892) on ATPase family AAA domain−containing protein 1
with a threshold of 1e−05
MR Test
Inverse variance weighted (fixed effects)
Inverse variance weighted (multiplicative random effects)
MR Egger
MR RAPS
Weighted median
Weighted mode
0.10
0.05
0.00
−0.05
−0.10
0.1
0.2
SNP effect on ATPase family AAA domain−containing protein 1
SNP effect on Gut microbiota abundance (genus Parasutterella id.2892)

Batch 2−391 : Gut microbiota abundance (genus Catenibacterium id.2153) on ATPase family AAA domain−containing protein 2 with a threshold of 1e−05

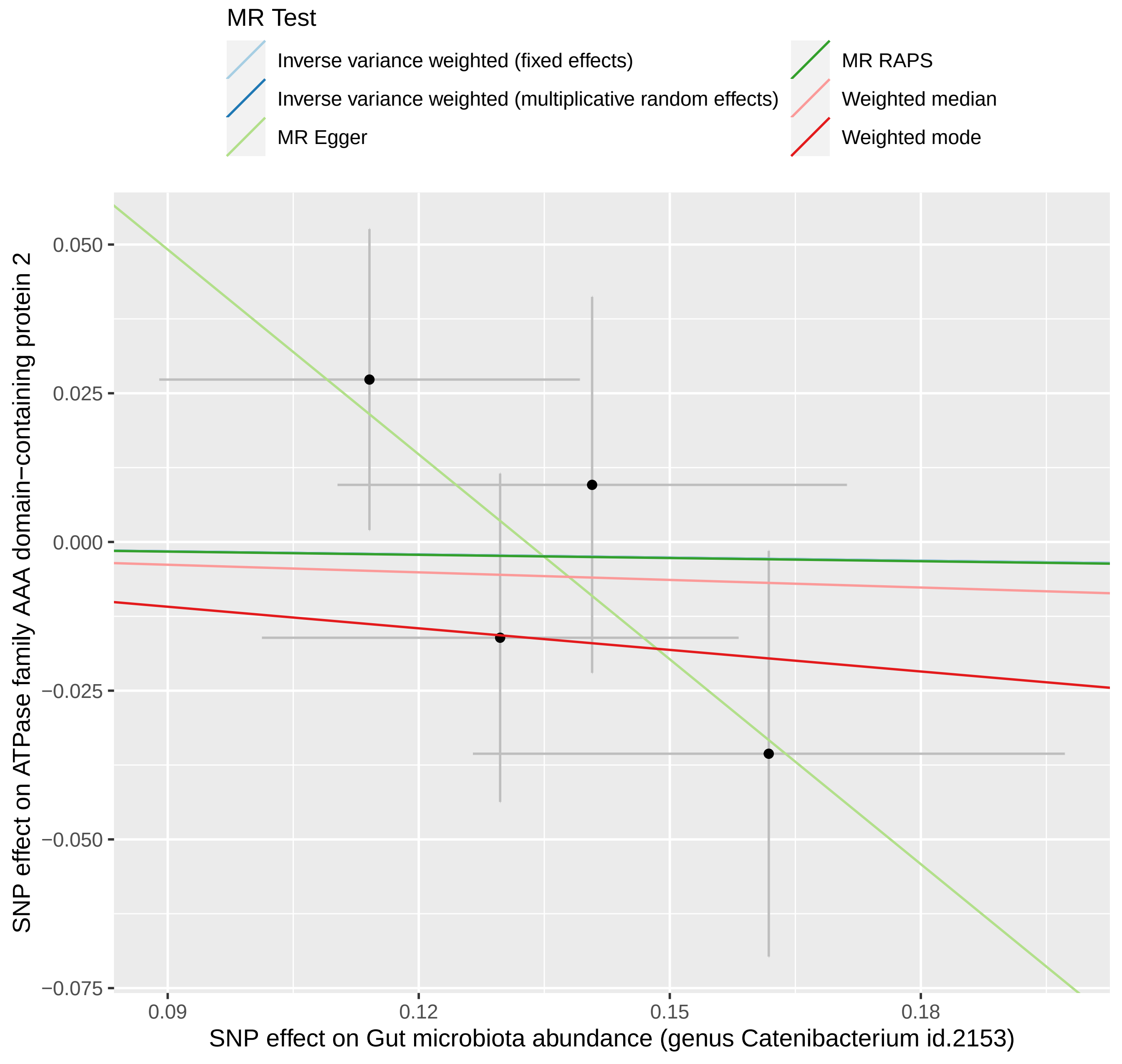

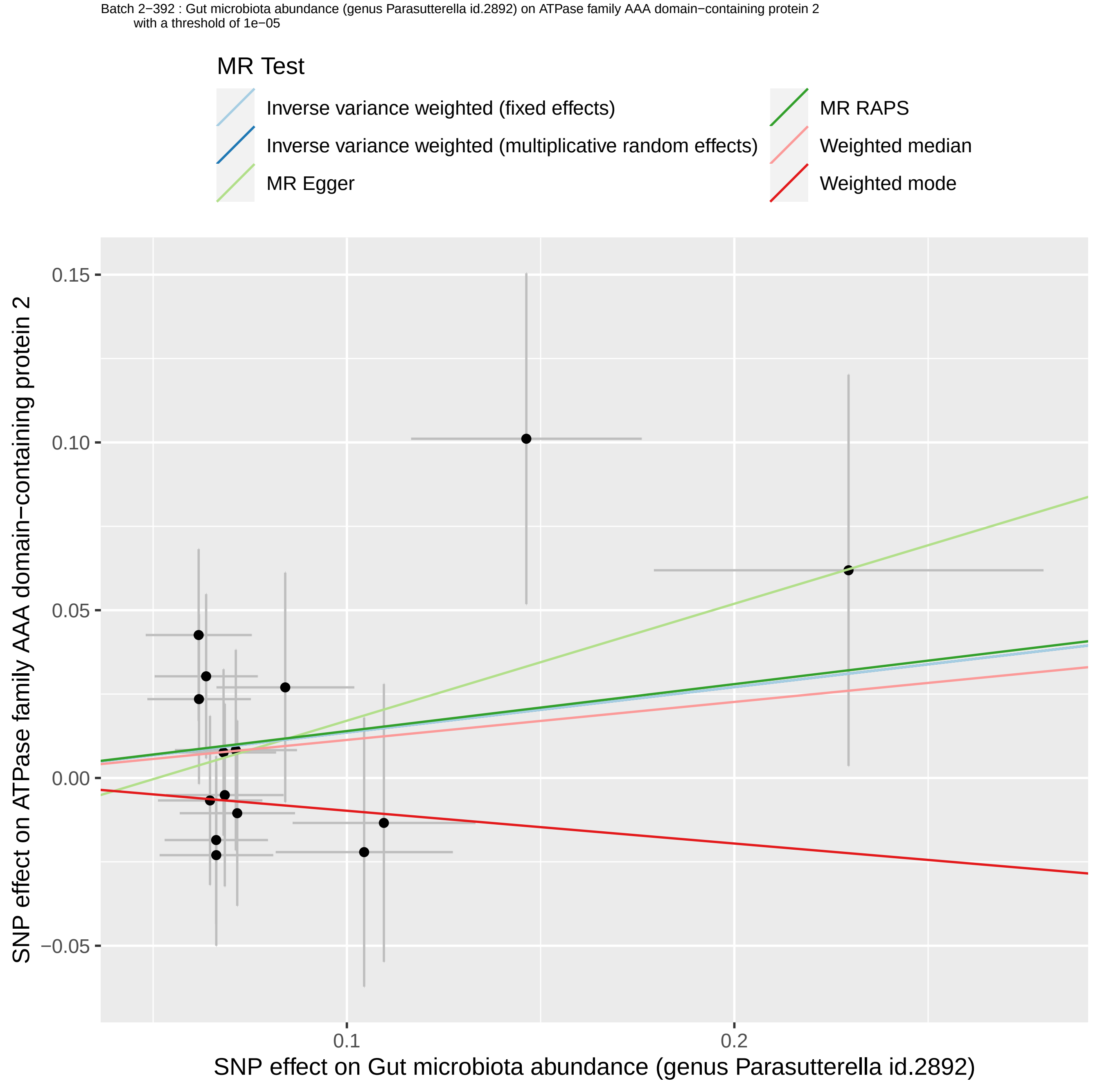

Batch 2−392 : Gut microbiota abundance (genus Parasutterella id.2892) on ATPase family AAA domain−containing protein 2
with a threshold of 1e−05
MR Test
Inverse variance weighted (fixed effects)
Inverse variance weighted (multiplicative random effects)
MR Egger
MR RAPS
Weighted median
Weighted mode
0.15
0.10
0.05
0.00
−0.05
0.1
0.2
SNP effect on ATPase family AAA domain−containing protein 2
SNP effect on Gut microbiota abundance (genus Parasutterella id.2892)

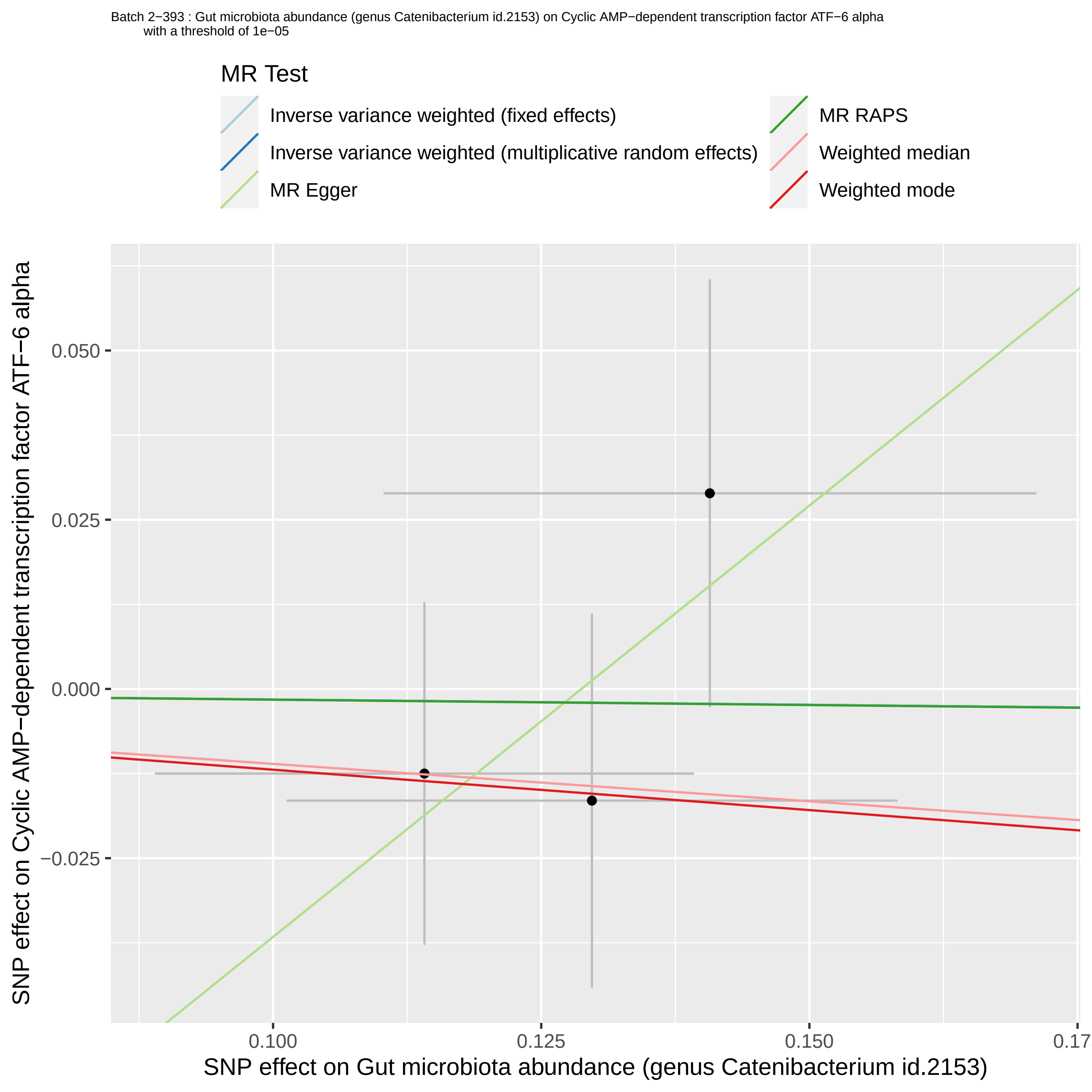

Batch 2−393 : Gut microbiota abundance (genus Catenibacterium id.2153) on Cyclic AMP−dependent transcription factor ATF−6 alpha
with a threshold of 1e−05
MR Test
Inverse variance weighted (fixed effects)
Inverse variance weighted (multiplicative random effects)
MR Egger
MR RAPS
Weighted median
Weighted mode
0.050
0.025
0.000
−0.025
0.100
0.125
0.150
0.17
SNP effect on Cyclic AMP−dependent transcription factor ATF−6 alpha
SNP effect on Gut microbiota abundance (genus Catenibacterium id.2153)

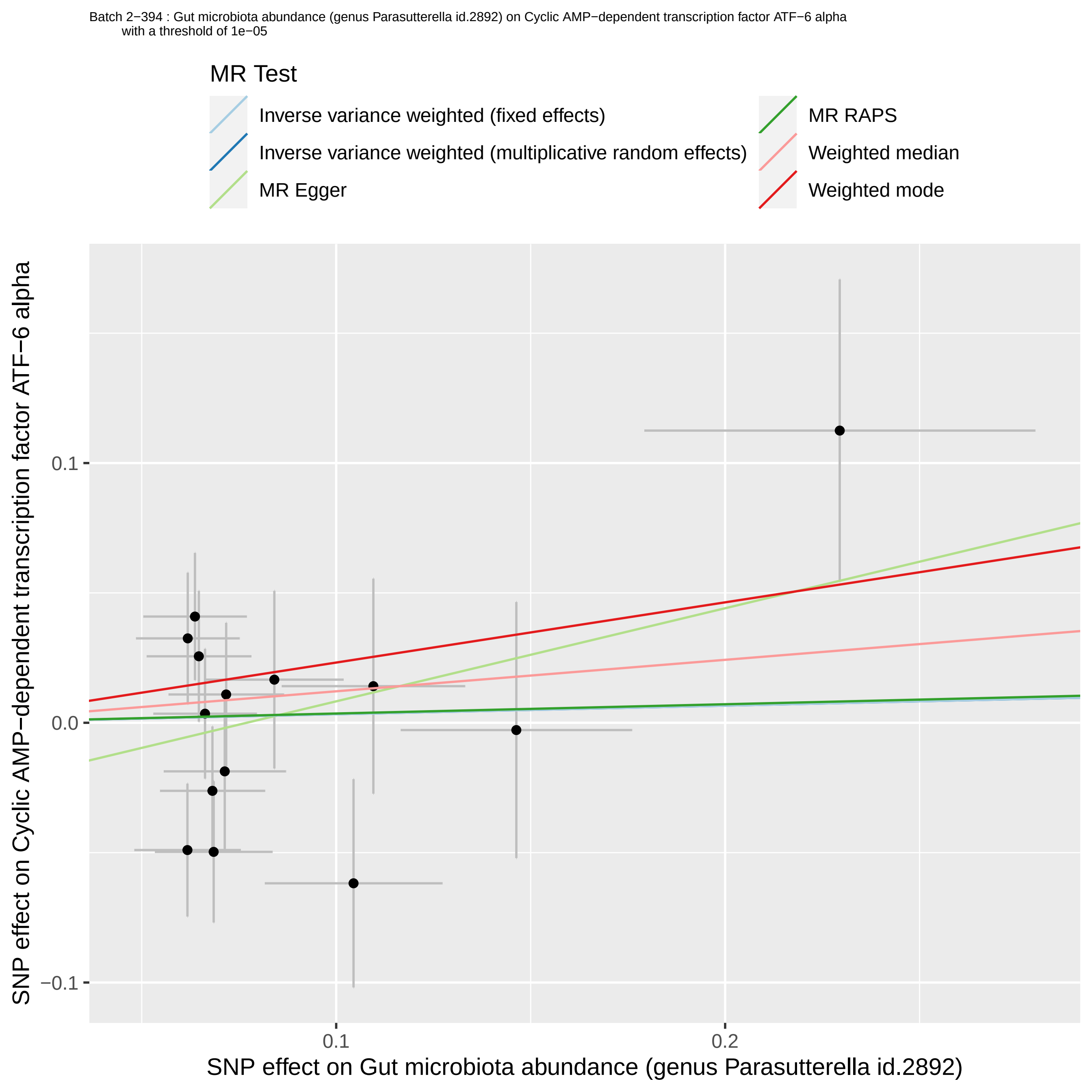

Batch 2−394 : Gut microbiota abundance (genus Parasutterella id.2892) on Cyclic AMP−dependent transcription factor ATF−6 alpha
with a threshold of 1e−05
MR Test
Inverse variance weighted (fixed effects)
Inverse variance weighted (multiplicative random effects)
MR Egger
MR RAPS
Weighted median
Weighted mode
SNP effect on Cyclic AMP−dependent transcription factor ATF−6 alpha
0.1
0.0
−0.1
0.1
0.2
SNP effect on Gut microbiota abundance (genus Parasutterella id.2892)

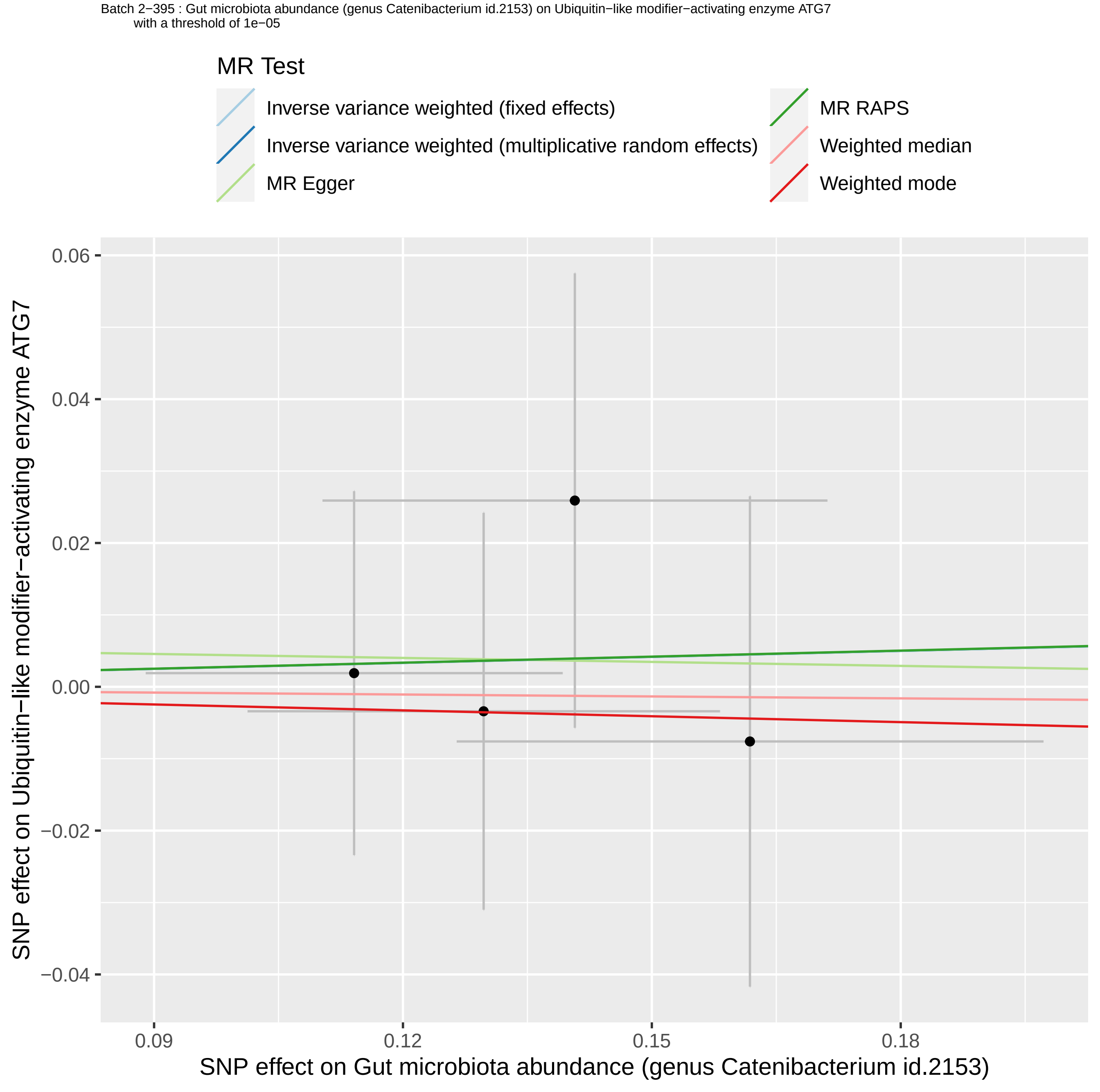

Batch 2−395 : Gut microbiota abundance (genus Catenibacterium id.2153) on Ubiquitin−like modifier−activating enzyme ATG7
with a threshold of 1e−05
MR Test
Inverse variance weighted (fixed effects)
Inverse variance weighted (multiplicative random effects)
MR Egger
MR RAPS
Weighted median
Weighted mode
SNP effect on Ubiquitin−like modifier−activating enzyme ATG7
0.06
0.04
0.02
0.00
−0.02
−0.04
0.09
0.12
0.15
0.18
SNP effect on Gut microbiota abundance (genus Catenibacterium id.2153)

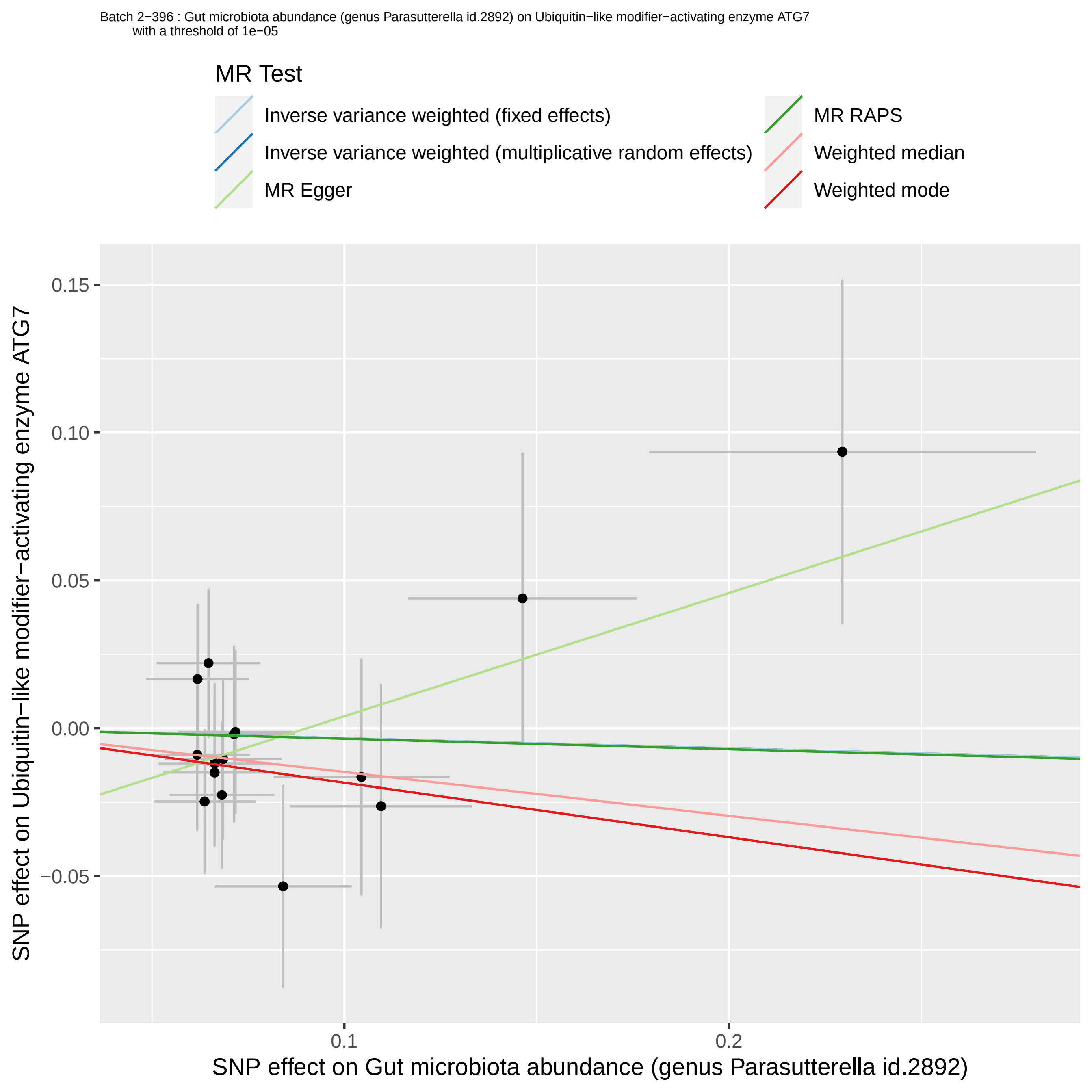

Batch 2−396 : Gut microbiota abundance (genus Parasutterella id.2892) on Ubiquitin−like modifier−activating enzyme ATG7
with a threshold of 1e−05
MR Test
Inverse variance weighted (fixed effects)
Inverse variance weighted (multiplicative random effects)
MR Egger
MR RAPS
Weighted median
Weighted mode
0.15
0.10
0.05
0.00
−0.05
0.1
0.2
SNP effect on Ubiquitin−like modifier−activating enzyme ATG7
SNP effect on Gut microbiota abundance (genus Parasutterella id.2892)

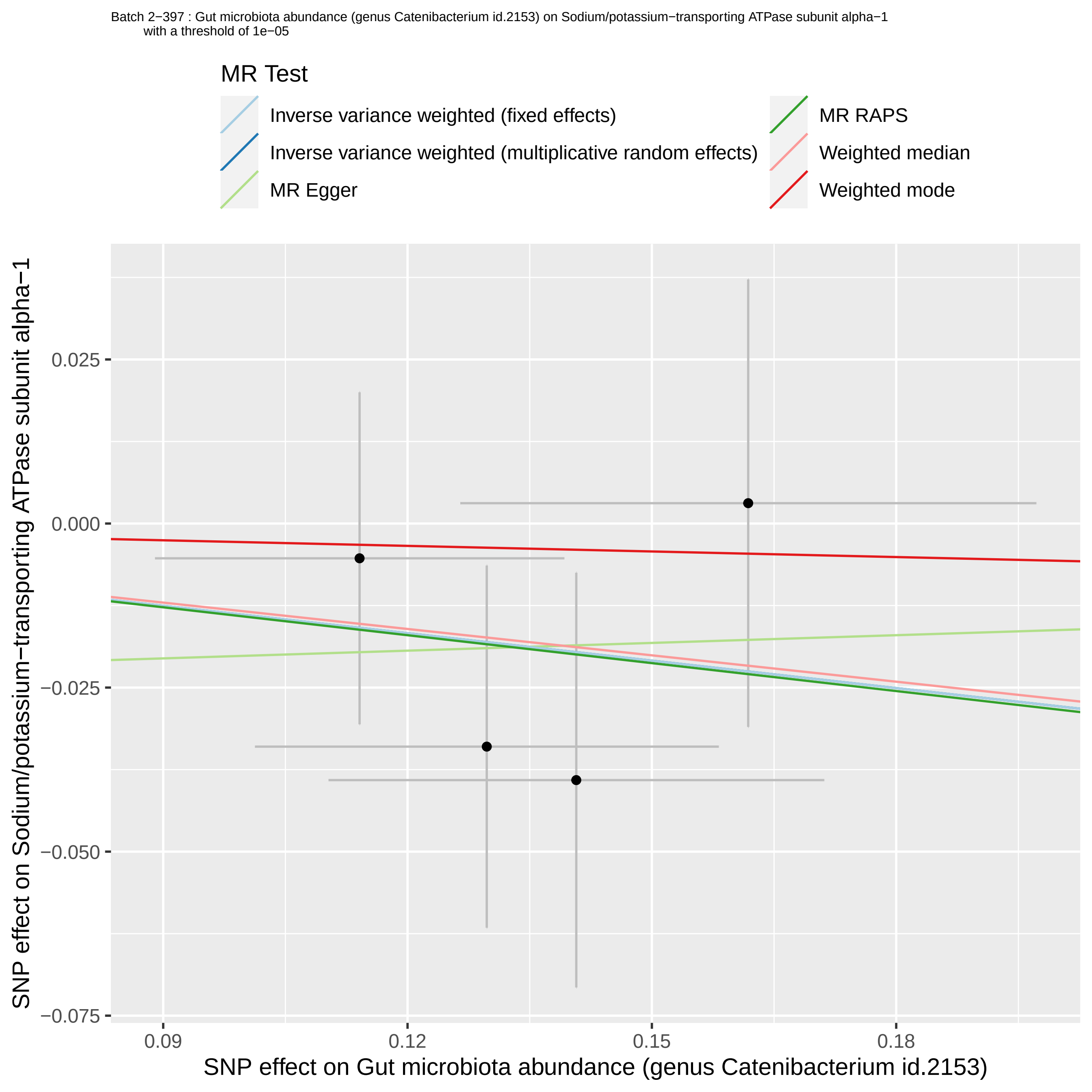

Batch 2−397 : Gut microbiota abundance (genus Catenibacterium id.2153) on Sodium/potassium−transporting ATPase subunit alpha−1
with a threshold of 1e−05
MR Test
Inverse variance weighted (fixed effects)
Inverse variance weighted (multiplicative random effects)
MR Egger
MR RAPS
Weighted median
Weighted mode
SNP effect on Sodium/potassium−transporting ATPase subunit alpha−1
0.025
0.000
−0.025
−0.050
−0.075
0.09
0.12
0.15
0.18
SNP effect on Gut microbiota abundance (genus Catenibacterium id.2153)

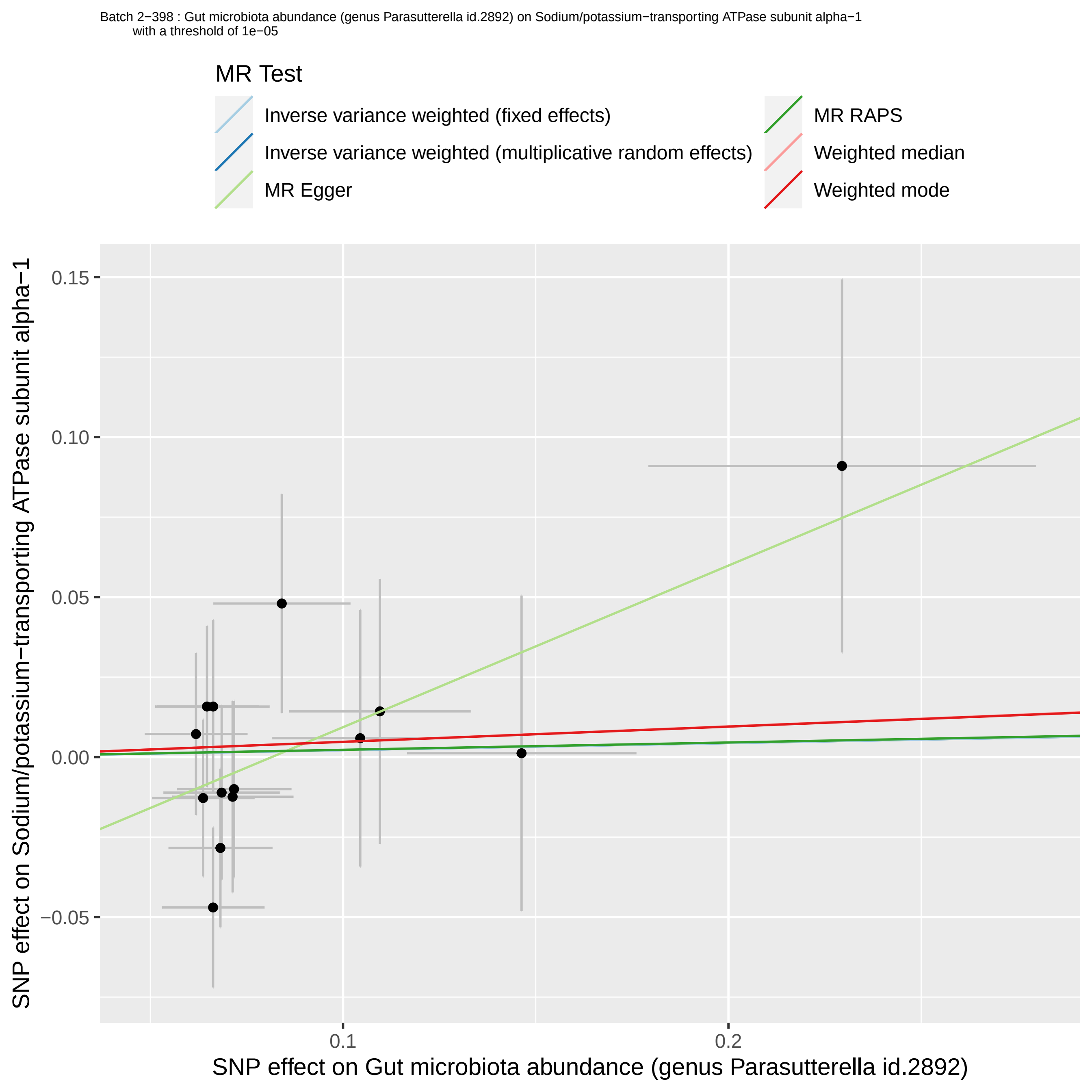
Batch 2−398 : Gut microbiota abundance (genus Parasutterella id.2892) on Sodium/potassium−transporting ATPase subunit alpha−1
with a threshold of 1e−05
MR Test
Inverse variance weighted (fixed effects)
Inverse variance weighted (multiplicative random effects)
MR Egger
MR RAPS
Weighted median
Weighted mode
0.15
0.10
0.05
0.00
−0.05
0.1
0.2
SNP effect on Sodium/potassium−transporting ATPase subunit alpha−1
SNP effect on Gut microbiota abundance (genus Parasutterella id.2892)

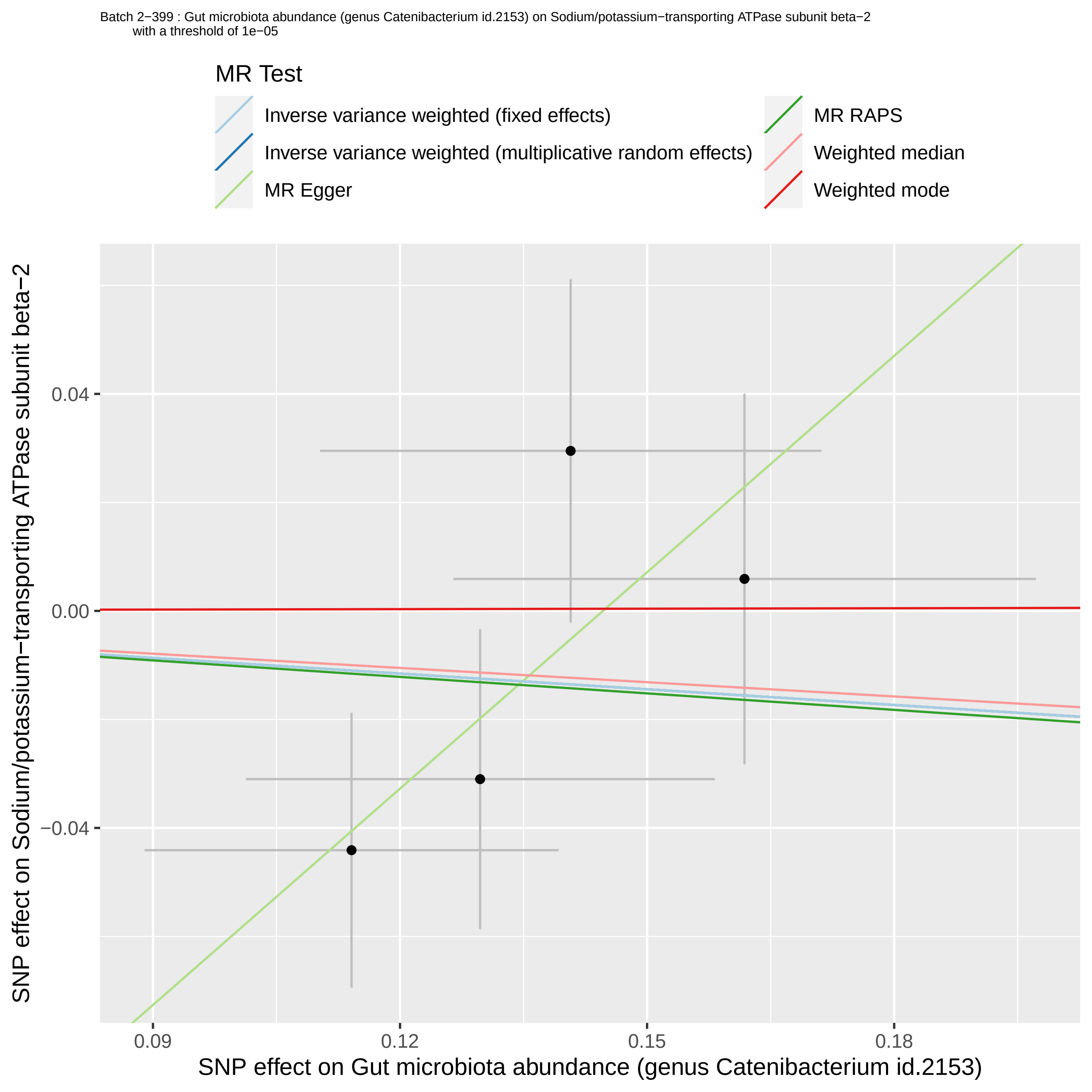
Batch 2−399 : Gut microbiota abundance (genus Catenibacterium id.2153) on Sodium/potassium−transporting ATPase subunit beta−2
with a threshold of 1e−05
MR Test
Inverse variance weighted (fixed effects)
Inverse variance weighted (multiplicative random effects)
MR Egger
MR RAPS
Weighted median
Weighted mode
0.04
0.00
−0.04
0.09
0.12
0.15
0.18
SNP effect on Sodium/potassium−transporting ATPase subunit beta−2
SNP effect on Gut microbiota abundance (genus Catenibacterium id.2153)

Batch 2−400 : Gut microbiota abundance (genus Parasutterella id.2892) on Sodium/potassium−transporting ATPase subunit beta−2
with a threshold of 1e−05

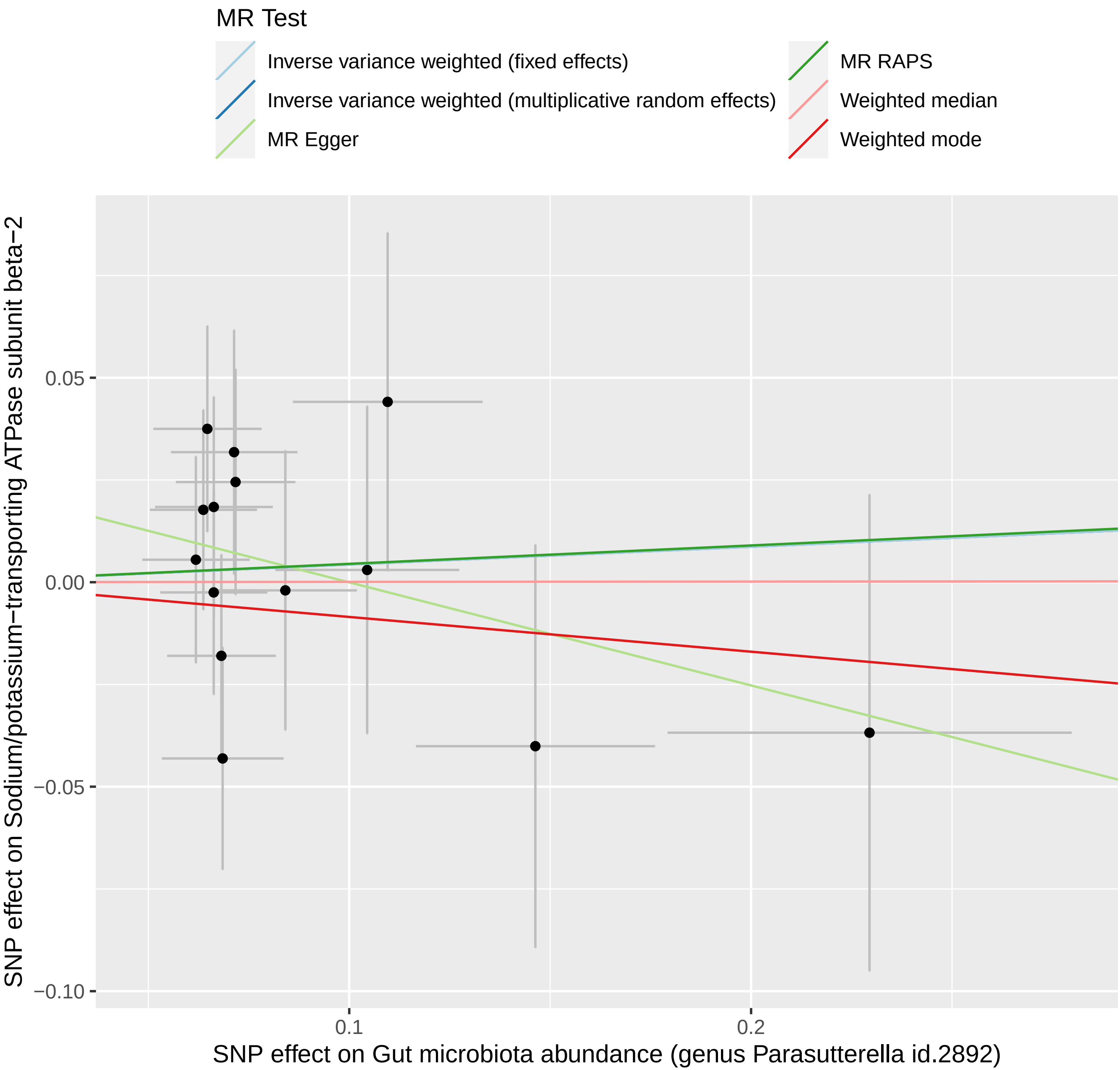

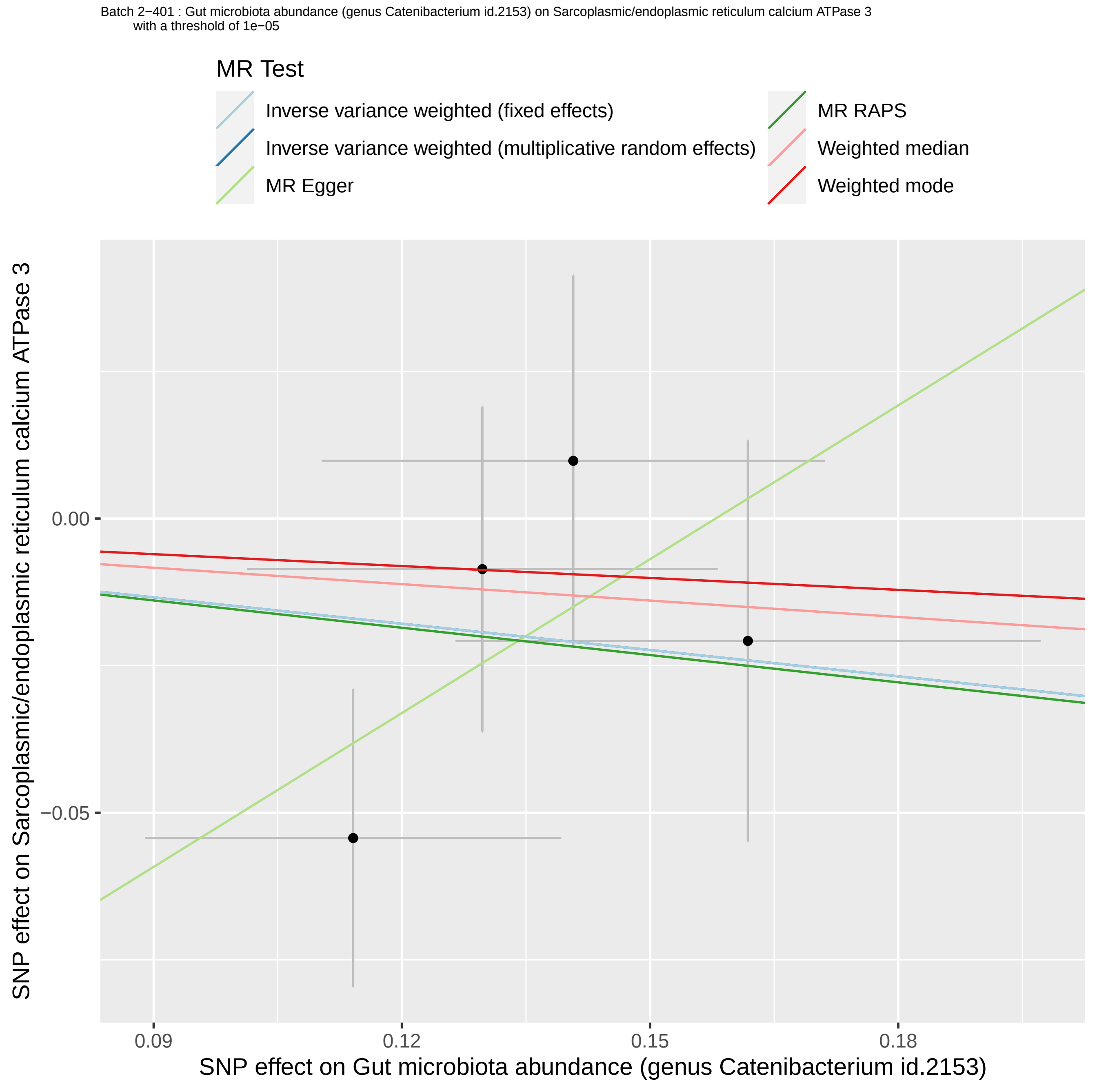

Batch 2−401 : Gut microbiota abundance (genus Catenibacterium id.2153) on Sarcoplasmic/endoplasmic reticulum calcium ATPase 3
with a threshold of 1e−05
MR Test
Inverse variance weighted (fixed effects)
Inverse variance weighted (multiplicative random effects)
MR Egger
MR RAPS
Weighted median
Weighted mode
0.00
−0.05
0.09
0.12
0.15
0.18
SNP effect on Gut microbiota abundance (genus Catenibacterium id.2153)
SNP effect on Sarcoplasmic/endoplasmic reticulum calcium ATPase 3

Batch 2−402 : Gut microbiota abundance (genus Parasutterella id.2892) on Sarcoplasmic/endoplasmic reticulum calcium ATPase 3
with a threshold of 1e−05

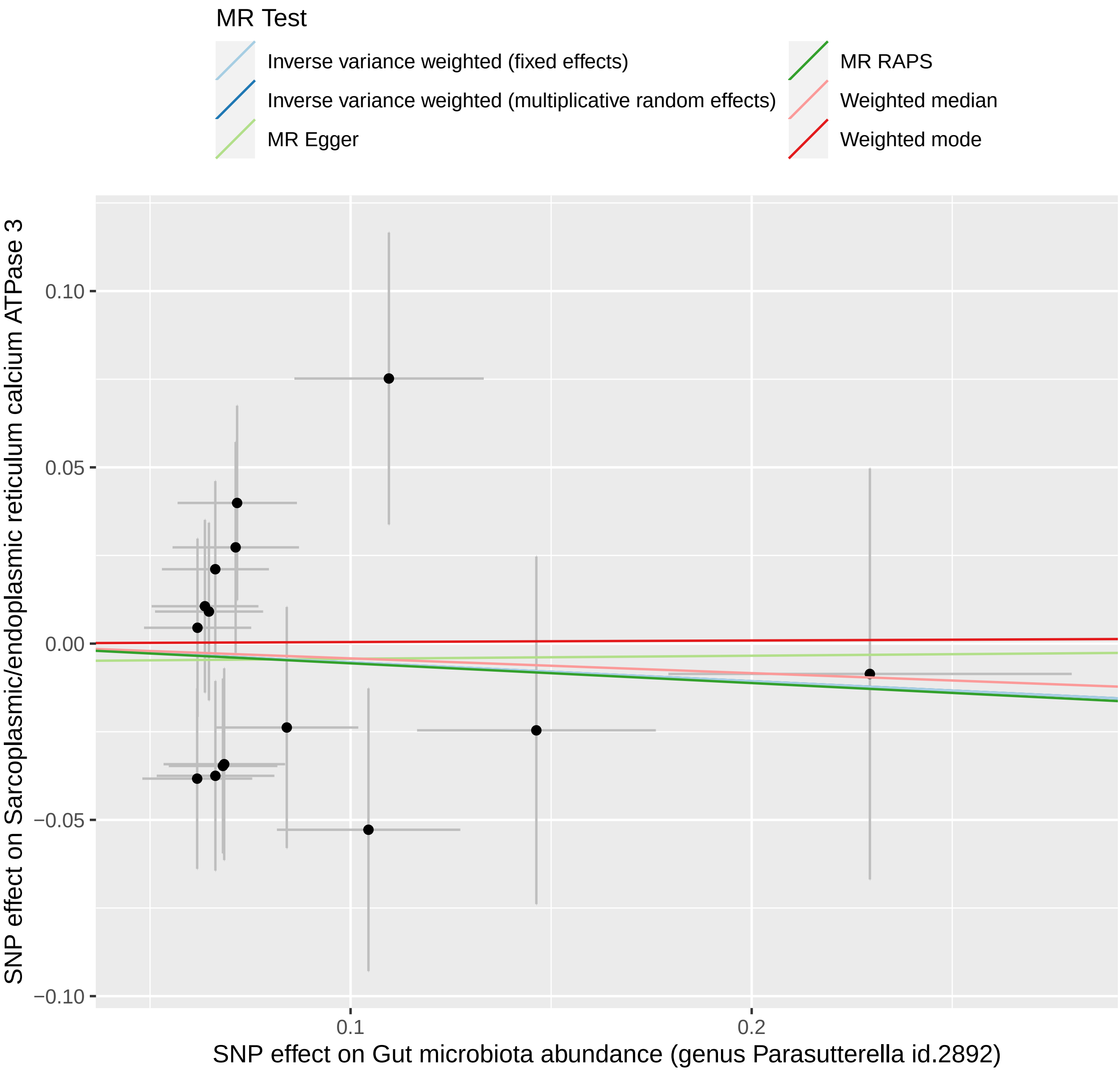

Batch 2−403 : Gut microbiota abundance (genus Catenibacterium id.2153) on Potassium−transporting ATPase subunit beta
with a threshold of 1e−05

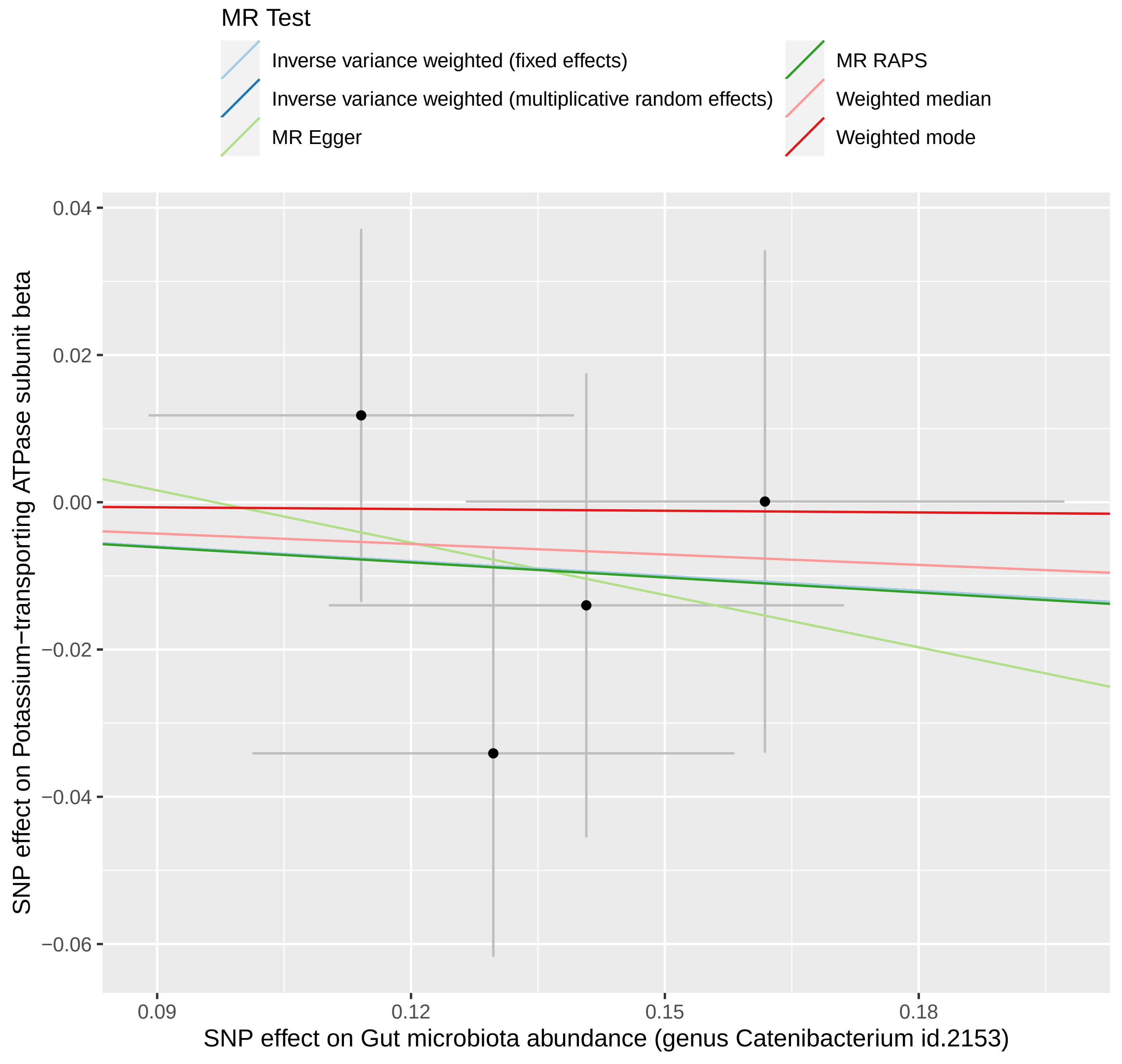

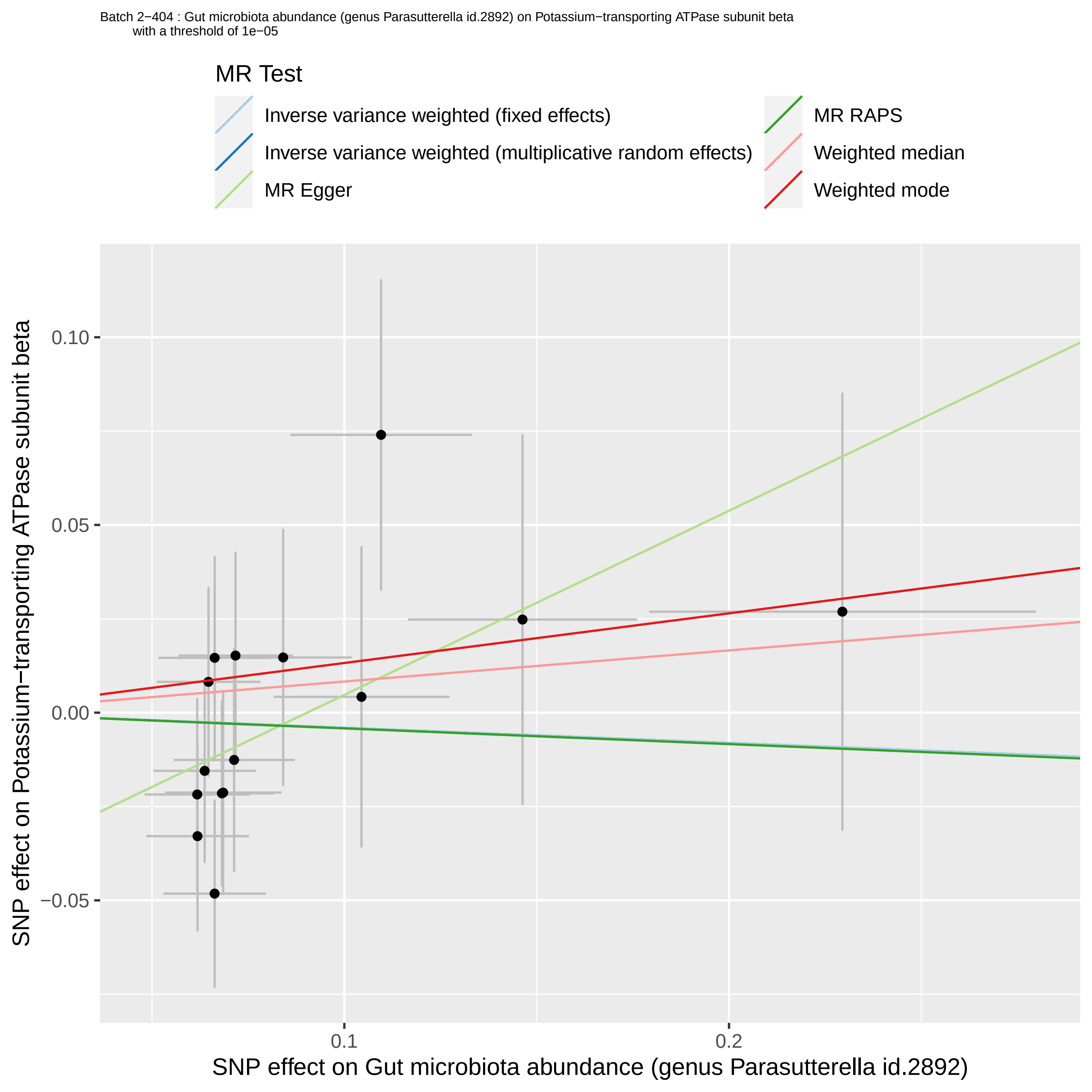

Batch 2−404 : Gut microbiota abundance (genus Parasutterella id.2892) on Potassium−transporting ATPase subunit beta
with a threshold of 1e−05
MR Test
Inverse variance weighted (fixed effects)
Inverse variance weighted (multiplicative random effects)
MR Egger
MR RAPS
Weighted median
Weighted mode
0.10
0.05
0.00
−0.05
0.1
0.2
SNP effect on Potassium−transporting ATPase subunit beta
SNP effect on Gut microbiota abundance (genus Parasutterella id.2892)

Batch 2−405 : Gut microbiota abundance (genus Catenibacterium id.2153) on ATP synthase subunit beta, mitochondrial
with a threshold of 1e−05

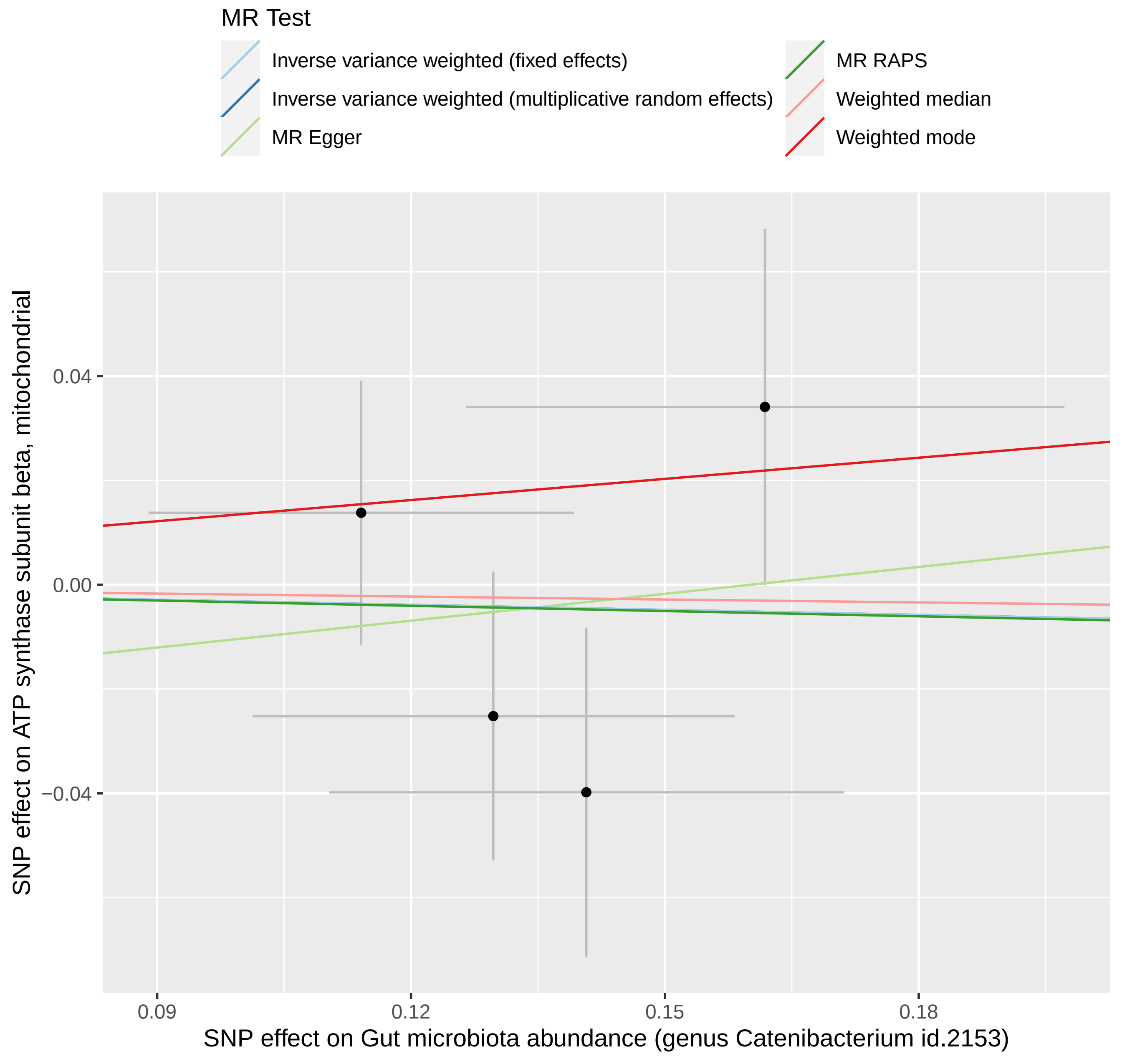

Batch 2−406 : Gut microbiota abundance (genus Parasutterella id.2892) on ATP synthase subunit beta, mitochondrial
with a threshold of 1e−05

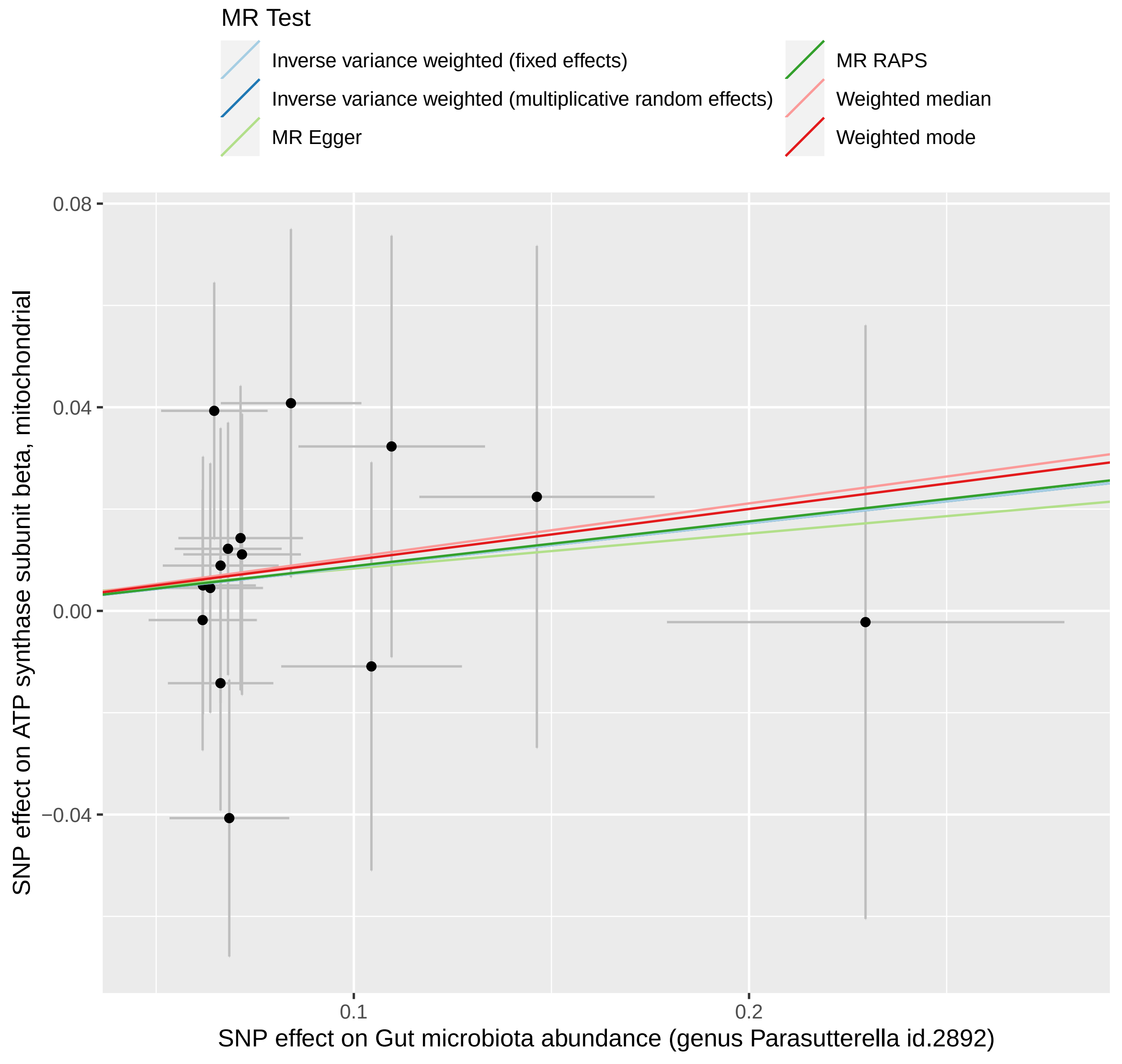

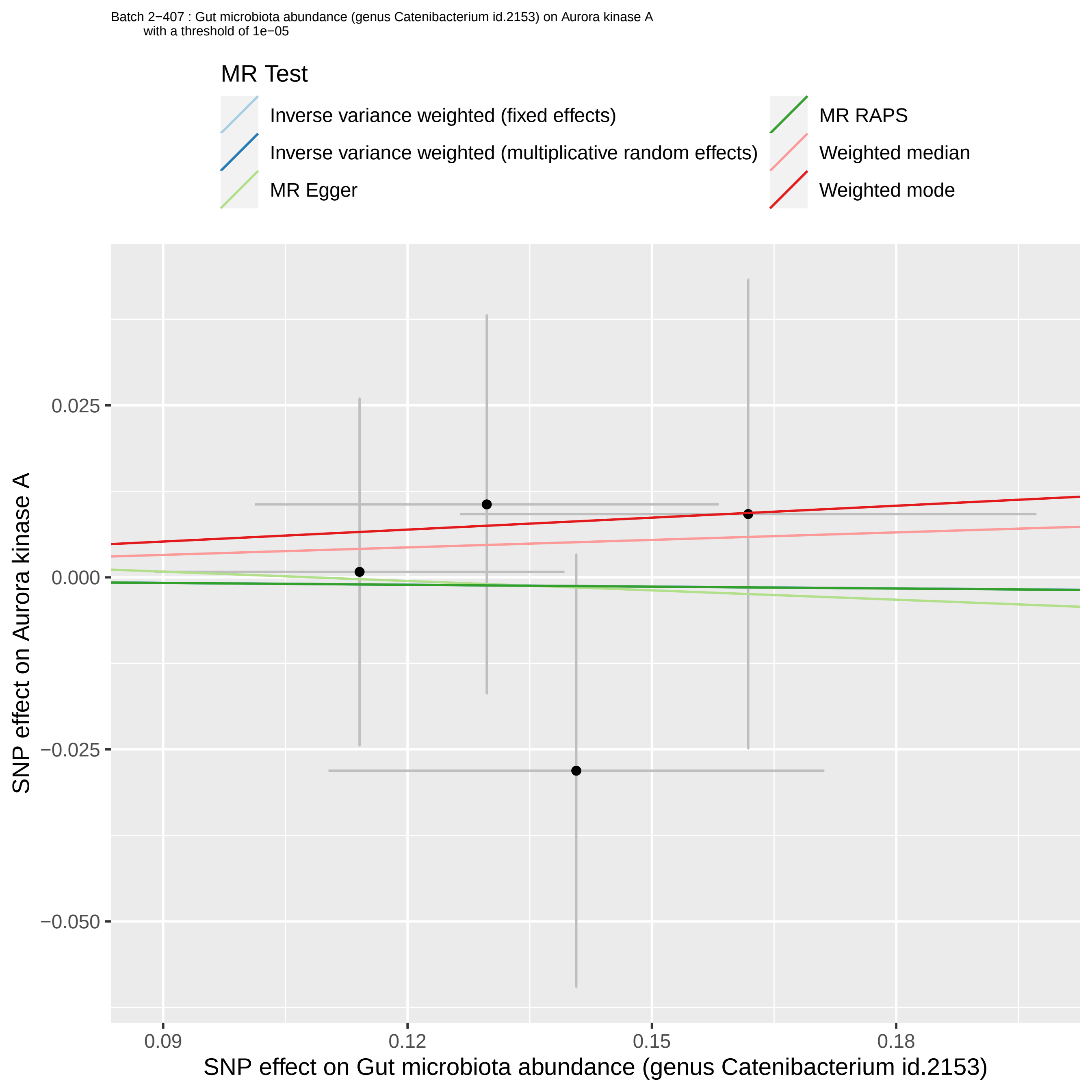
Batch 2−407 : Gut microbiota abundance (genus Catenibacterium id.2153) on Aurora kinase A
with a threshold of 1e−05
MR Test
Inverse variance weighted (fixed effects)
Inverse variance weighted (multiplicative random effects)
MR Egger
MR RAPS
Weighted median
Weighted mode
0.025
0.000
−0.025
−0.050
0.09
0.12
0.15
0.18
SNP effect on Aurora kinase A
SNP effect on Gut microbiota abundance (genus Catenibacterium id.2153)

Batch 2−408 : Gut microbiota abundance (genus Parasutterella id.2892) on Aurora kinase A
with a threshold of 1e−05

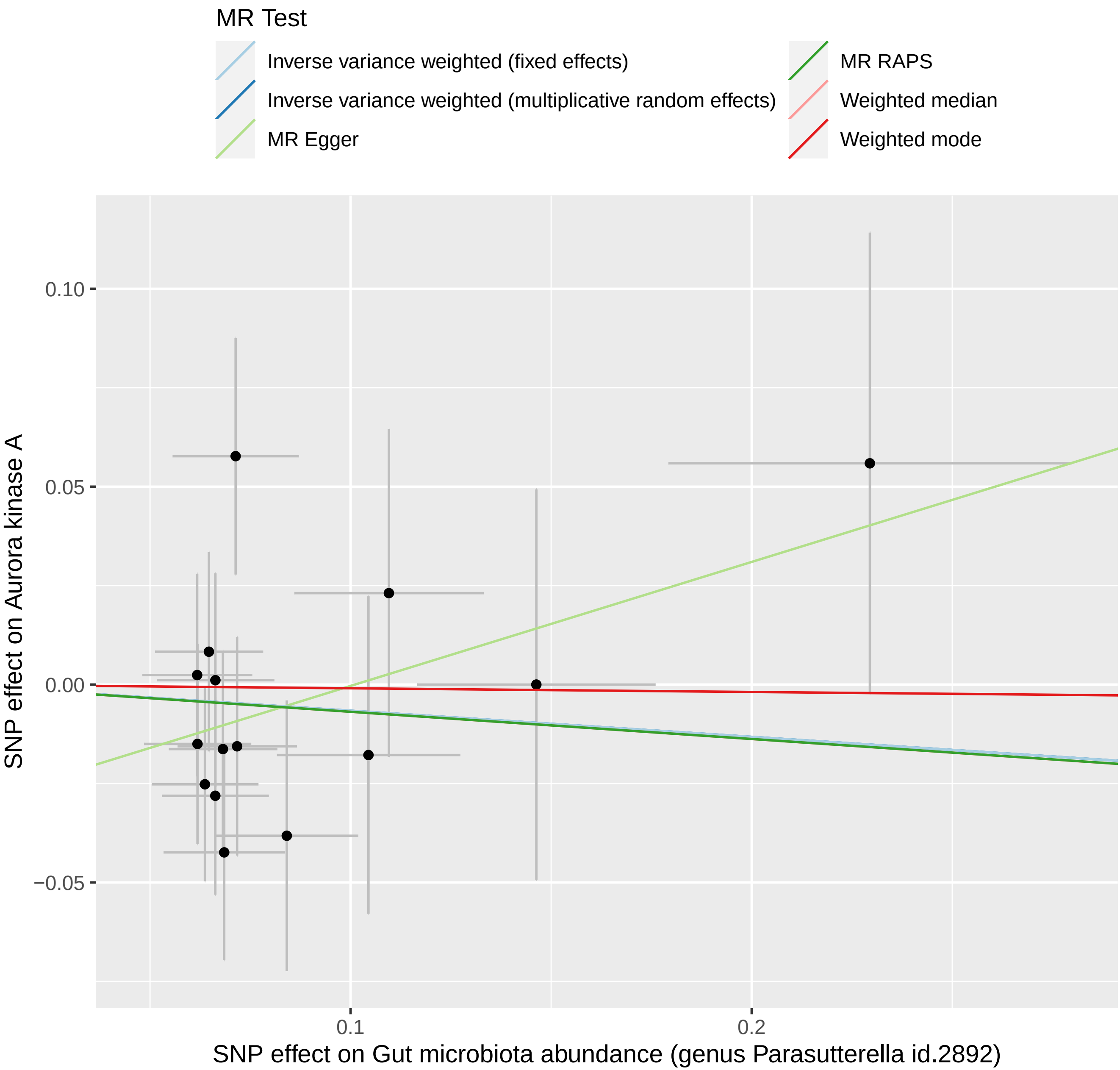

Batch 2−409 : Gut microbiota abundance (genus Catenibacterium id.2153) on Aurora kinase B
with a threshold of 1e−05

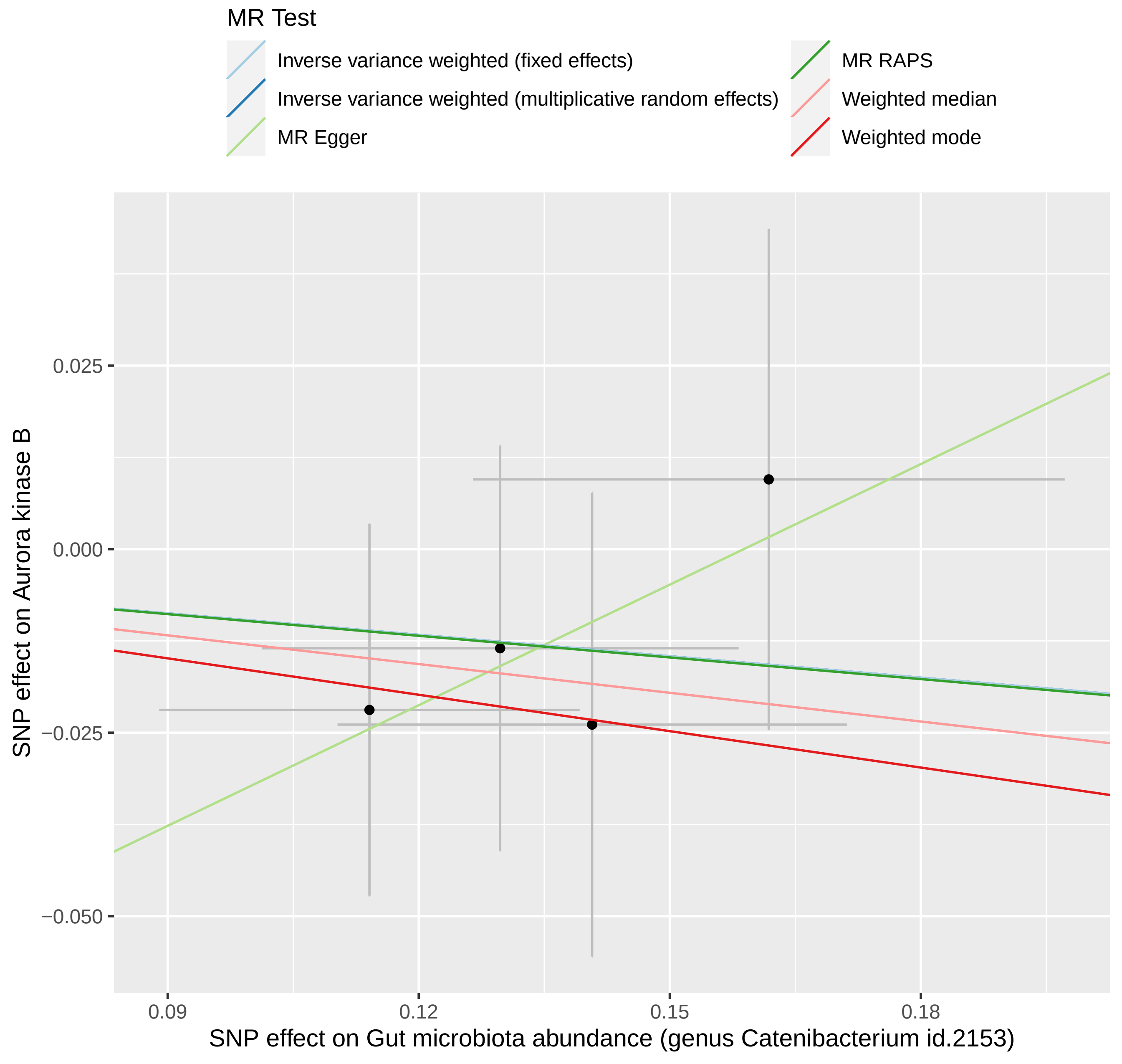

Batch 2−410 : Gut microbiota abundance (genus Parasutterella id.2892) on Aurora kinase B
with a threshold of 1e−05

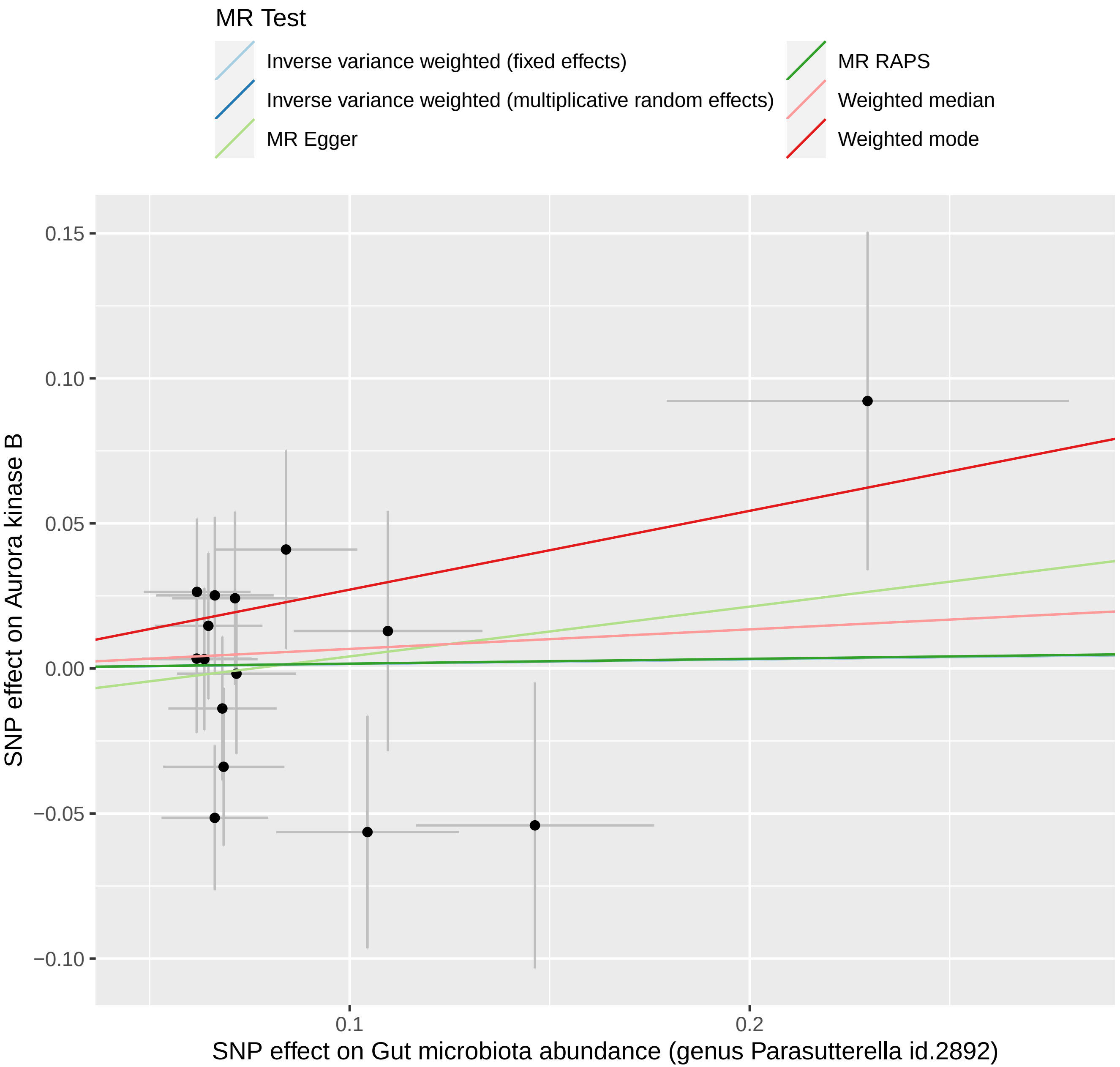

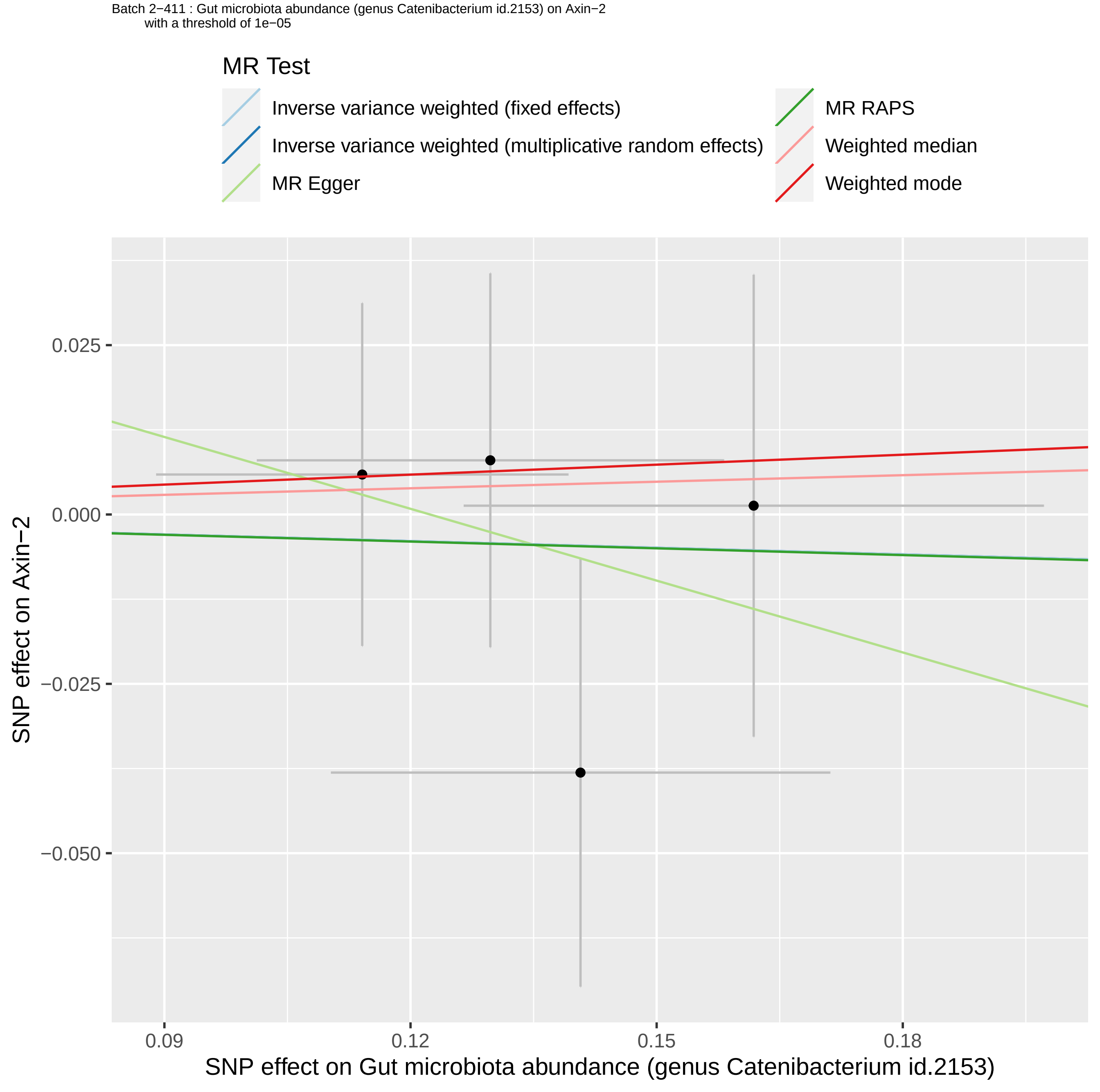

Batch 2−411 : Gut microbiota abundance (genus Catenibacterium id.2153) on Axin−2
with a threshold of 1e−05
MR Test
Inverse variance weighted (fixed effects)
Inverse variance weighted (multiplicative random effects)
MR Egger
MR RAPS
Weighted median
Weighted mode
0.025
0.000
−0.025
−0.050
0.09
0.12
0.15
0.18
SNP effect on Axin−2
SNP effect on Gut microbiota abundance (genus Catenibacterium id.2153)

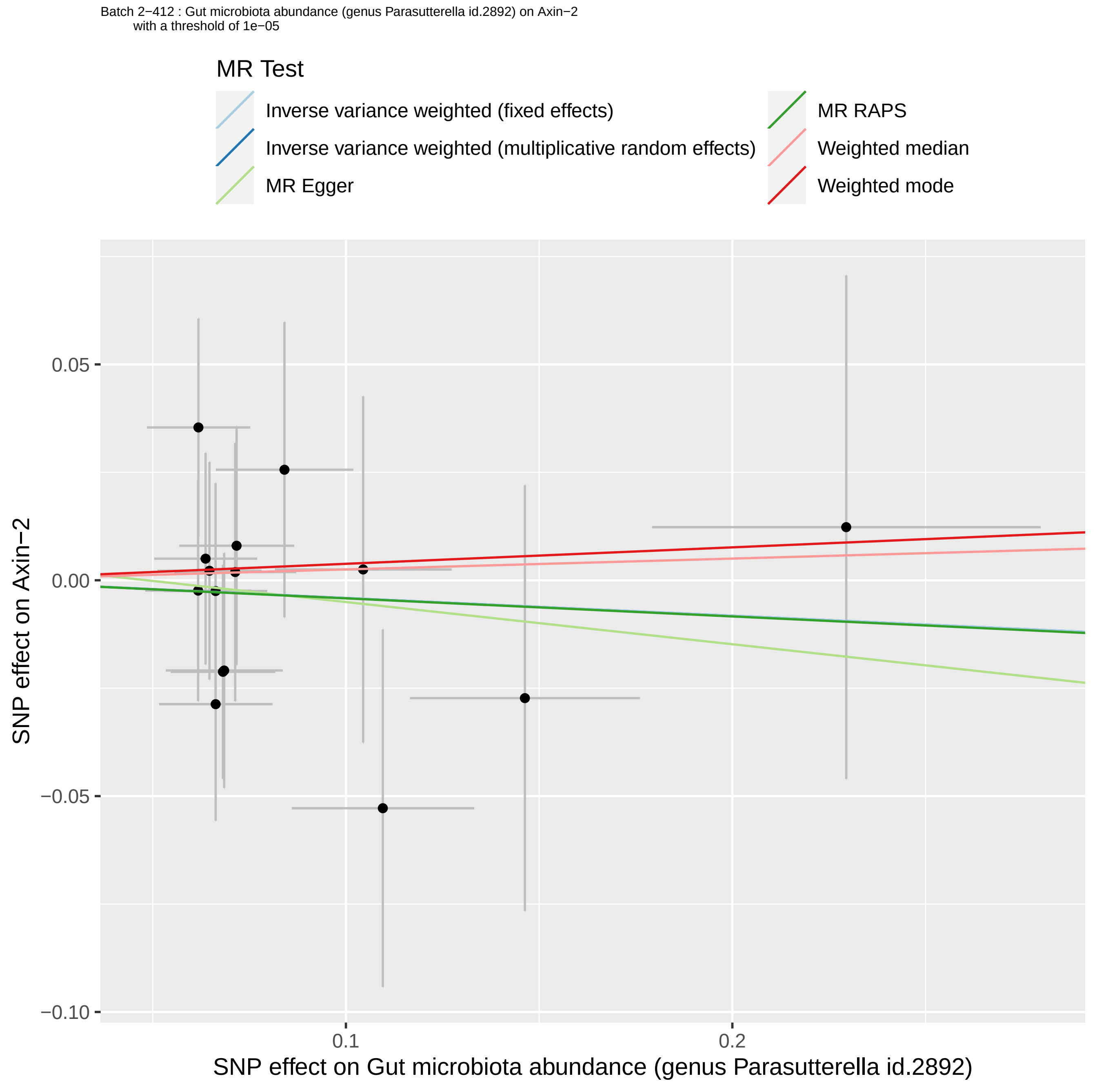

Batch 2−412 : Gut microbiota abundance (genus Parasutterella id.2892) on Axin−2
with a threshold of 1e−05
MR Test
Inverse variance weighted (fixed effects)
Inverse variance weighted (multiplicative random effects)
MR Egger
MR RAPS
Weighted median
Weighted mode
0.05
0.00
−0.05
−0.10
0.1
0.2
SNP effect on Axin−2
SNP effect on Gut microbiota abundance (genus Parasutterella id.2892)

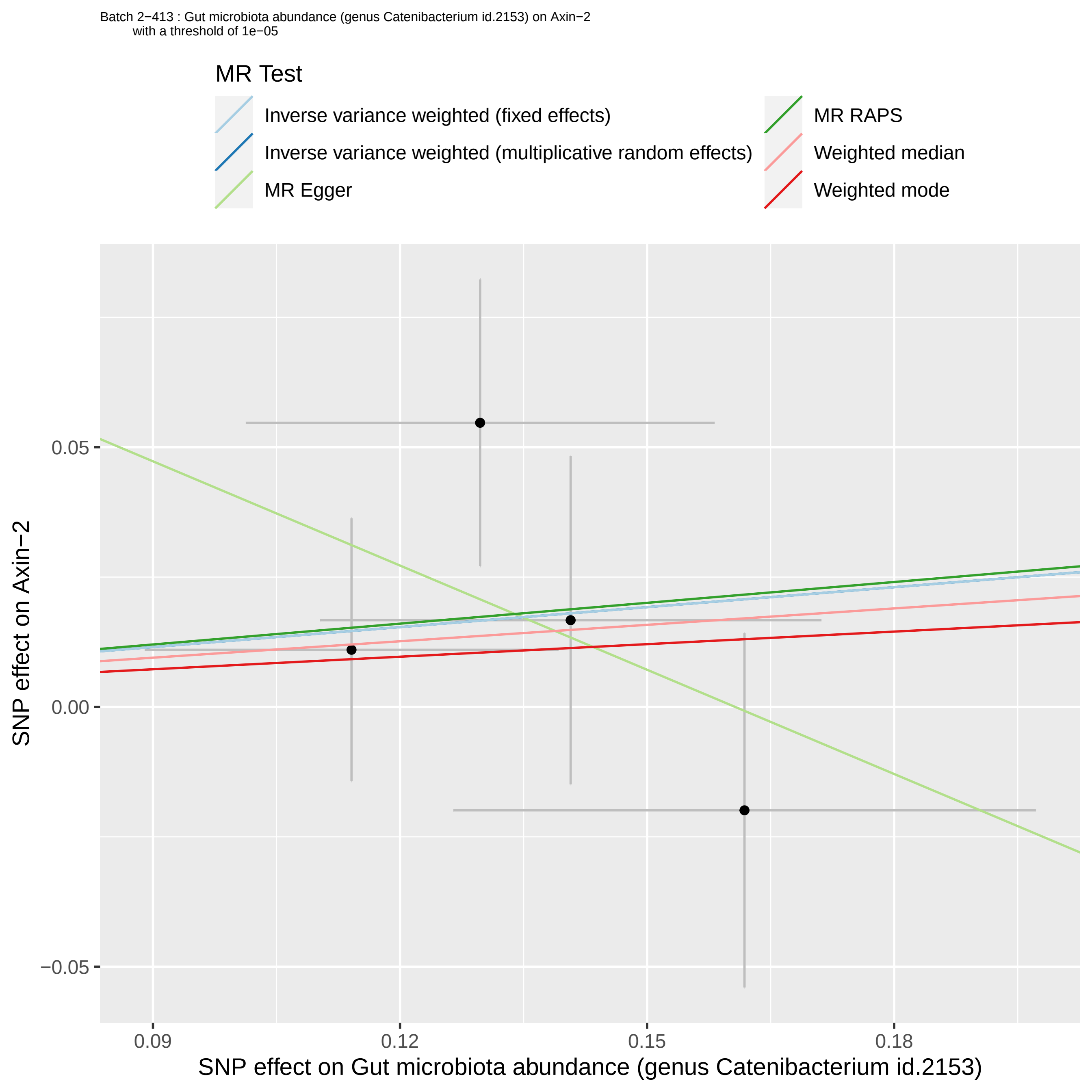
Batch 2−413 : Gut microbiota abundance (genus Catenibacterium id.2153) on Axin−2
with a threshold of 1e−05
MR Test
Inverse variance weighted (fixed effects)
Inverse variance weighted (multiplicative random effects)
MR Egger
MR RAPS
Weighted median
Weighted mode
0.05
0.00
−0.05
SNP effect on Axin−2
0.09
0.12
0.15
0.18
SNP effect on Gut microbiota abundance (genus Catenibacterium id.2153)

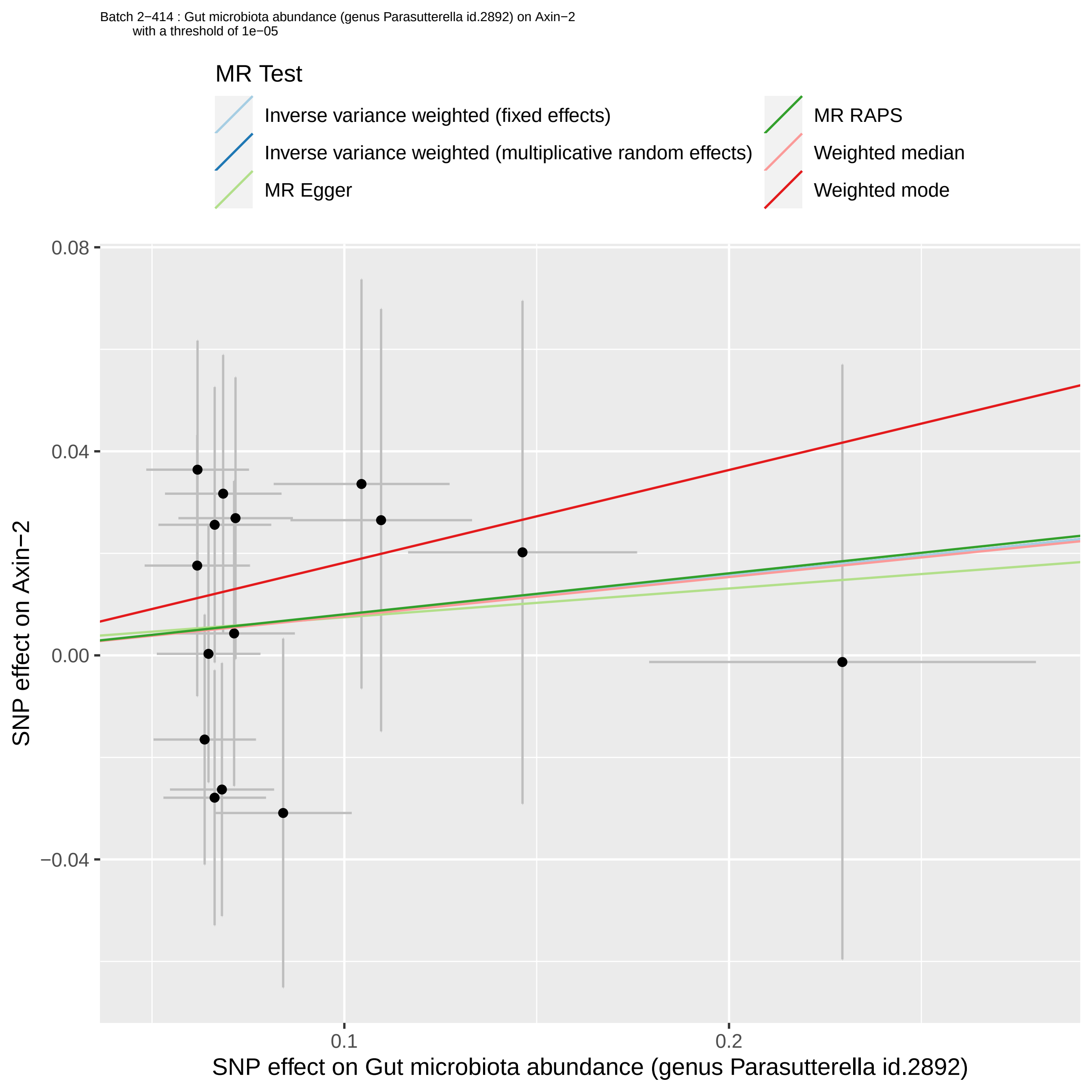

Batch 2−414 : Gut microbiota abundance (genus Parasutterella id.2892) on Axin−2
with a threshold of 1e−05
MR Test
Inverse variance weighted (fixed effects)
Inverse variance weighted (multiplicative random effects)
MR Egger
MR RAPS
Weighted median
Weighted mode
0.08
0.04
0.00
−0.04
0.1
0.2
SNP effect on Axin−2
SNP effect on Gut microbiota abundance (genus Parasutterella id.2892)

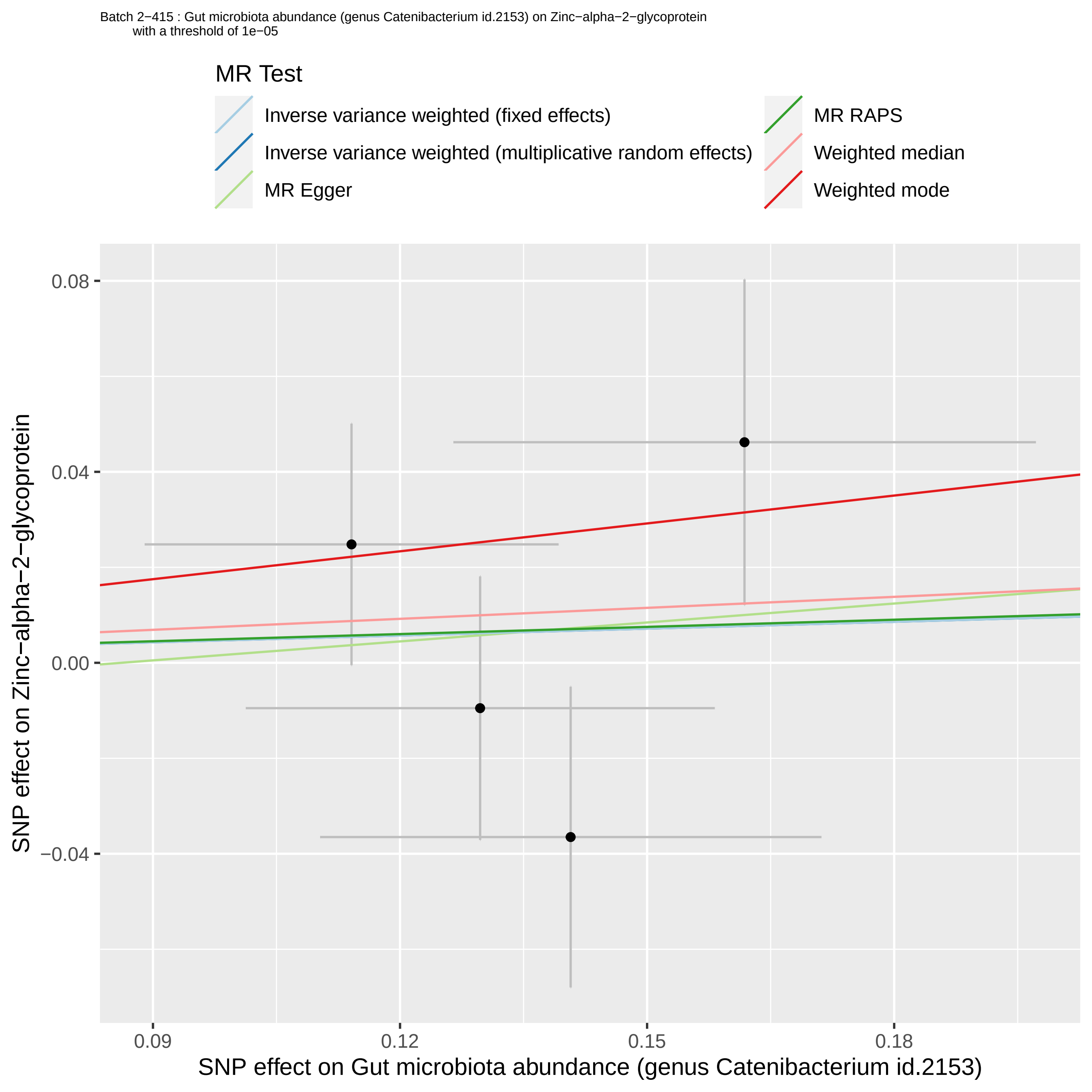

Batch 2−415 : Gut microbiota abundance (genus Catenibacterium id.2153) on Zinc−alpha−2−glycoprotein
with a threshold of 1e−05
MR Test
Inverse variance weighted (fixed effects)
Inverse variance weighted (multiplicative random effects)
MR Egger
MR RAPS
Weighted median
Weighted mode
0.08
0.04
0.00
−0.04
0.09
0.12
0.15
0.18
SNP effect on Zinc−alpha−2−glycoprotein
SNP effect on Gut microbiota abundance (genus Catenibacterium id.2153)

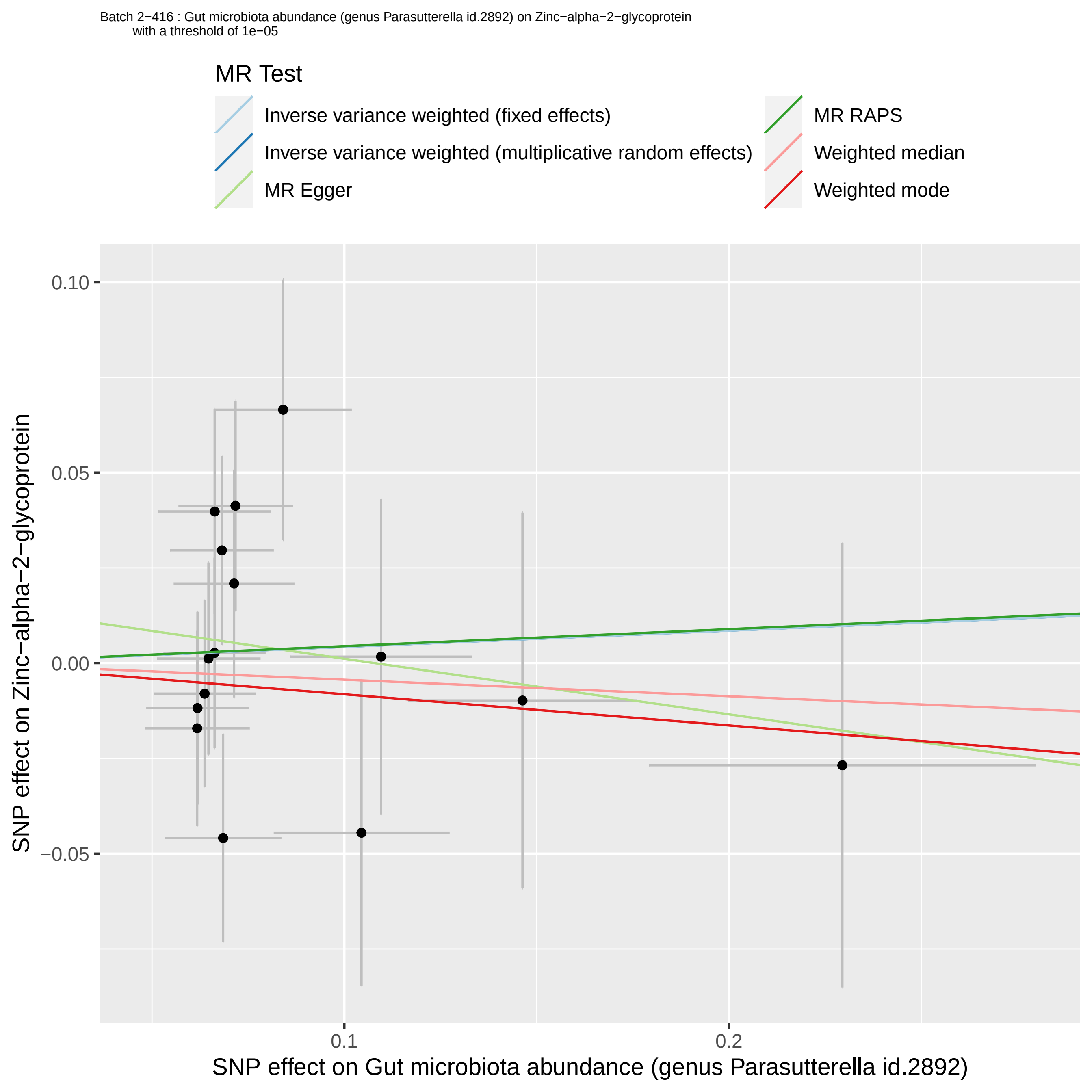

Batch 2−416 : Gut microbiota abundance (genus Parasutterella id.2892) on Zinc−alpha−2−glycoprotein
with a threshold of 1e−05
MR Test
Inverse variance weighted (fixed effects)
Inverse variance weighted (multiplicative random effects)
MR Egger
MR RAPS
Weighted median
Weighted mode
0.10
0.05
0.00
−0.05
0.1
0.2
SNP effect on Zinc−alpha−2−glycoprotein
SNP effect on Gut microbiota abundance (genus Parasutterella id.2892)

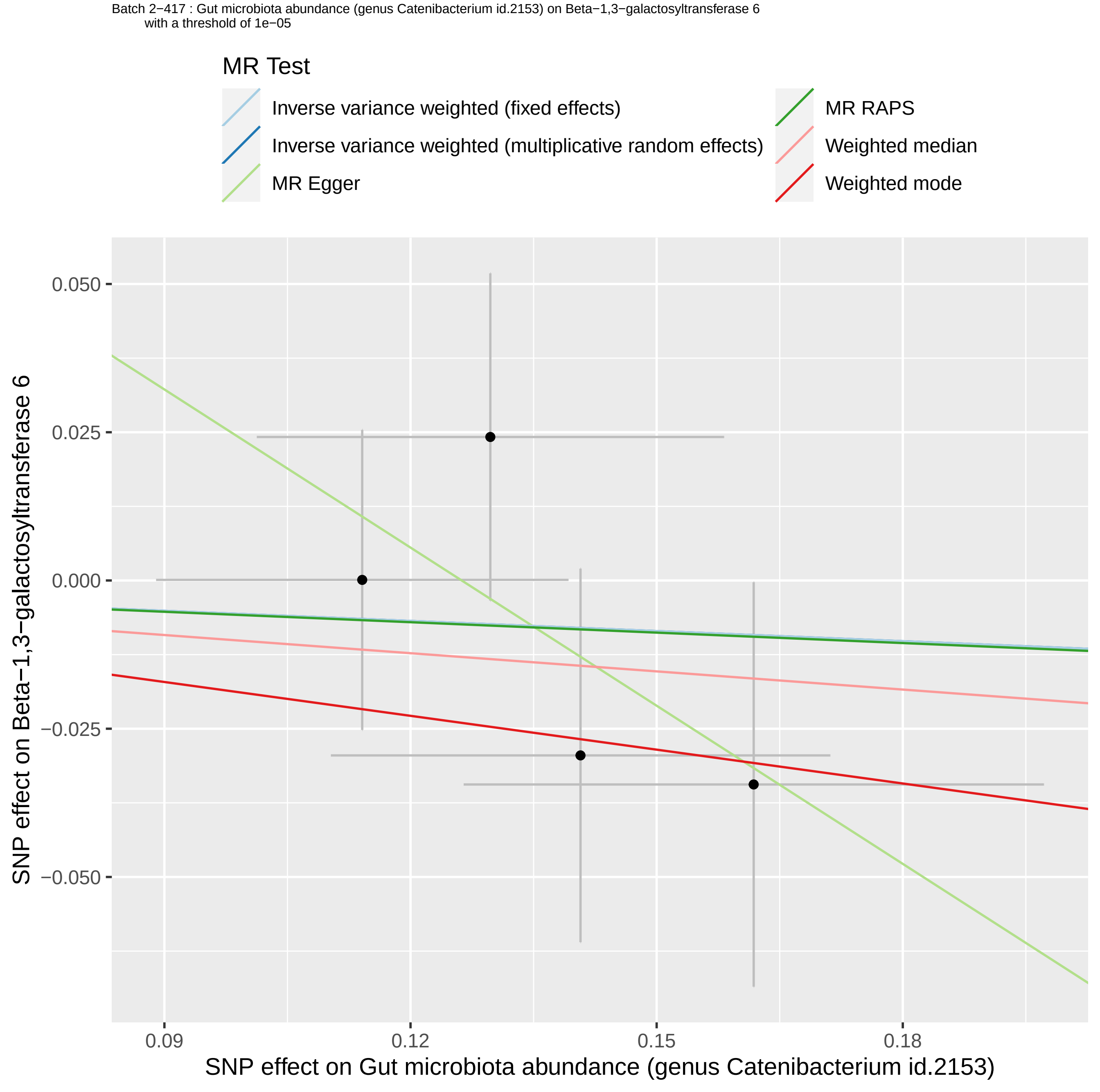
Batch 2−417 : Gut microbiota abundance (genus Catenibacterium id.2153) on Beta−1,3−galactosyltransferase 6
with a threshold of 1e−05
MR Test
Inverse variance weighted (fixed effects)
Inverse variance weighted (multiplicative random effects)
MR Egger
MR RAPS
Weighted median
Weighted mode
0.050
0.025
0.000
−0.025
−0.050
0.09
0.12
0.15
0.18
SNP effect on Beta−1,3−galactosyltransferase 6
SNP effect on Gut microbiota abundance (genus Catenibacterium id.2153)

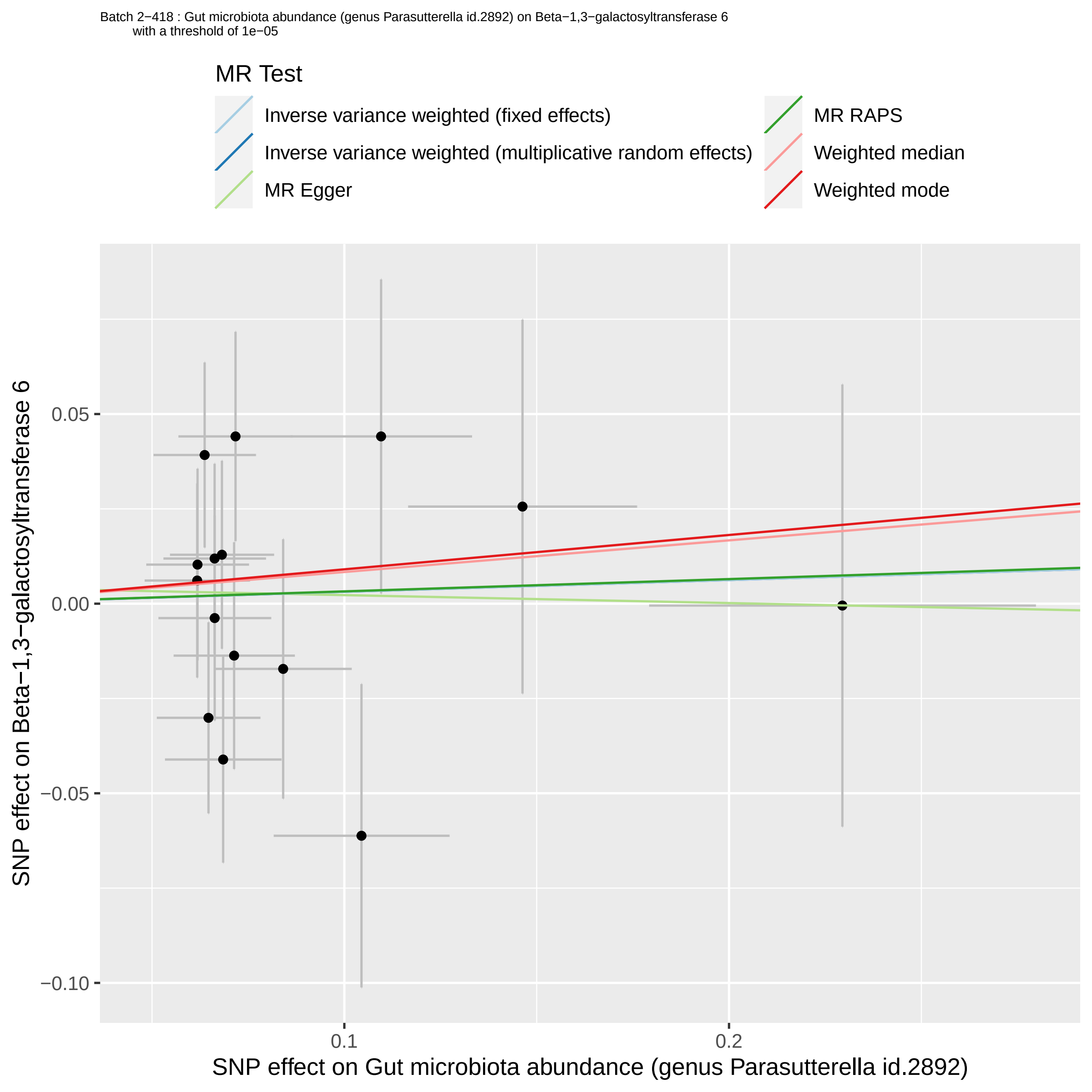
Batch 2−418 : Gut microbiota abundance (genus Parasutterella id.2892) on Beta−1,3−galactosyltransferase 6
with a threshold of 1e−05
MR Test
Inverse variance weighted (fixed effects)
Inverse variance weighted (multiplicative random effects)
MR Egger
MR RAPS
Weighted median
Weighted mode
0.05
0.00
−0.05
−0.10
0.1
0.2
SNP effect on Beta−1,3−galactosyltransferase 6
SNP effect on Gut microbiota abundance (genus Parasutterella id.2892)

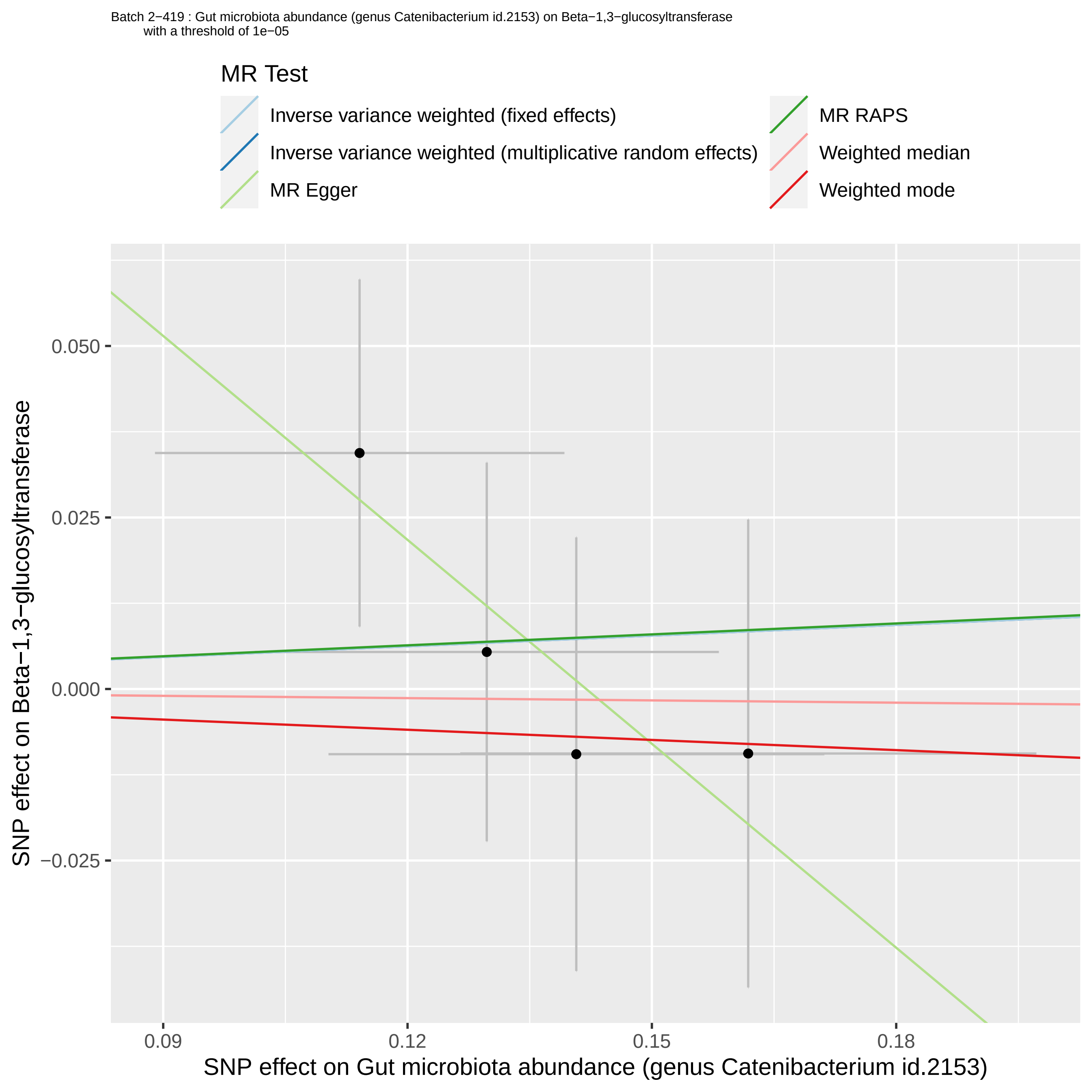
Batch 2−419 : Gut microbiota abundance (genus Catenibacterium id.2153) on Beta−1,3−glucosyltransferase
with a threshold of 1e−05
MR Test
Inverse variance weighted (fixed effects)
Inverse variance weighted (multiplicative random effects)
MR Egger
MR RAPS
Weighted median
Weighted mode
SNP effect on Beta−1,3−glucosyltransferase
0.050
0.025
0.000
−0.025
0.09
0.12
0.15
0.18
SNP effect on Gut microbiota abundance (genus Catenibacterium id.2153)

Batch 2−420 : Gut microbiota abundance (genus Parasutterella id.2892) on Beta−1,3−glucosyltransferase
with a threshold of 1e−05

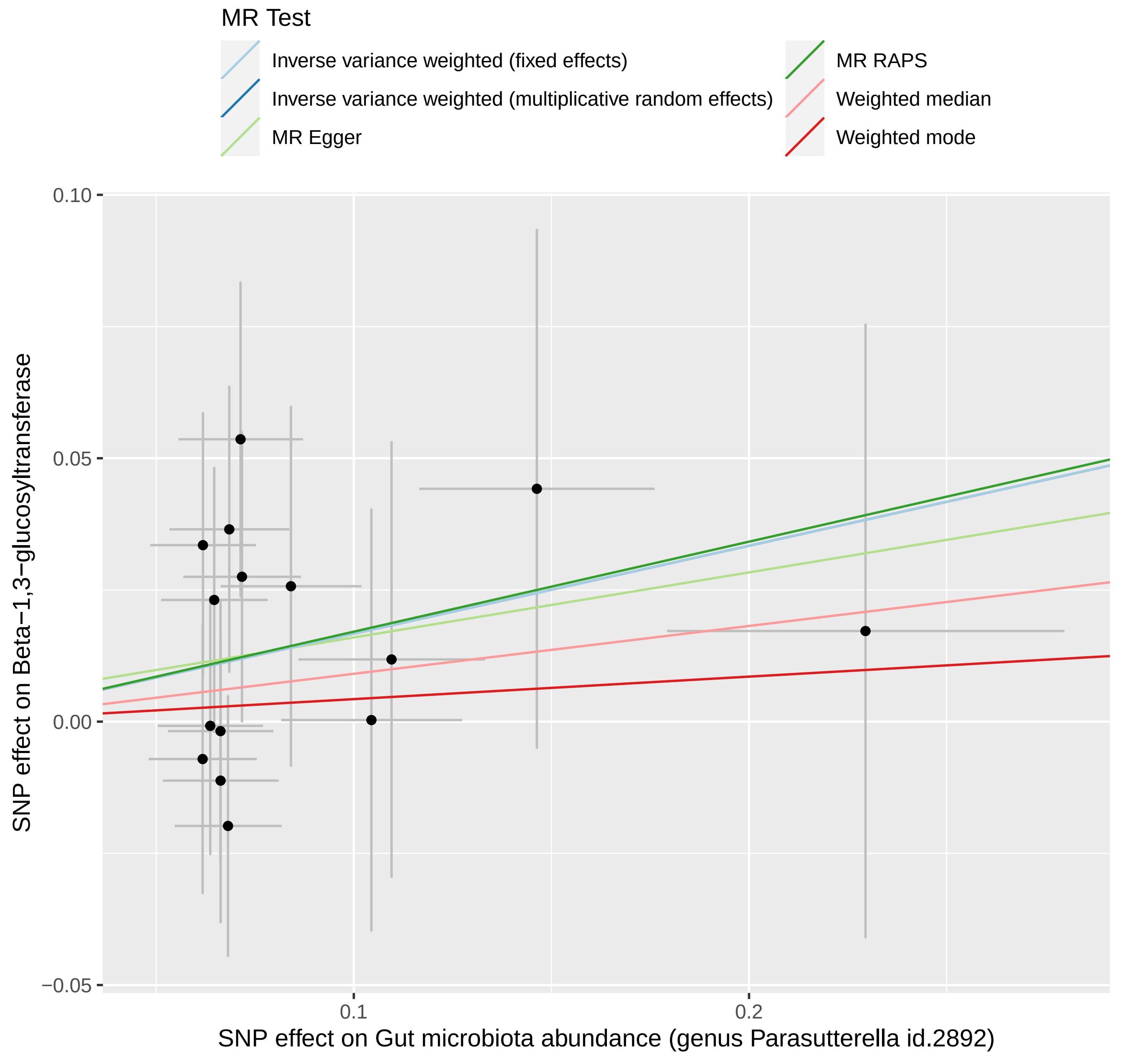

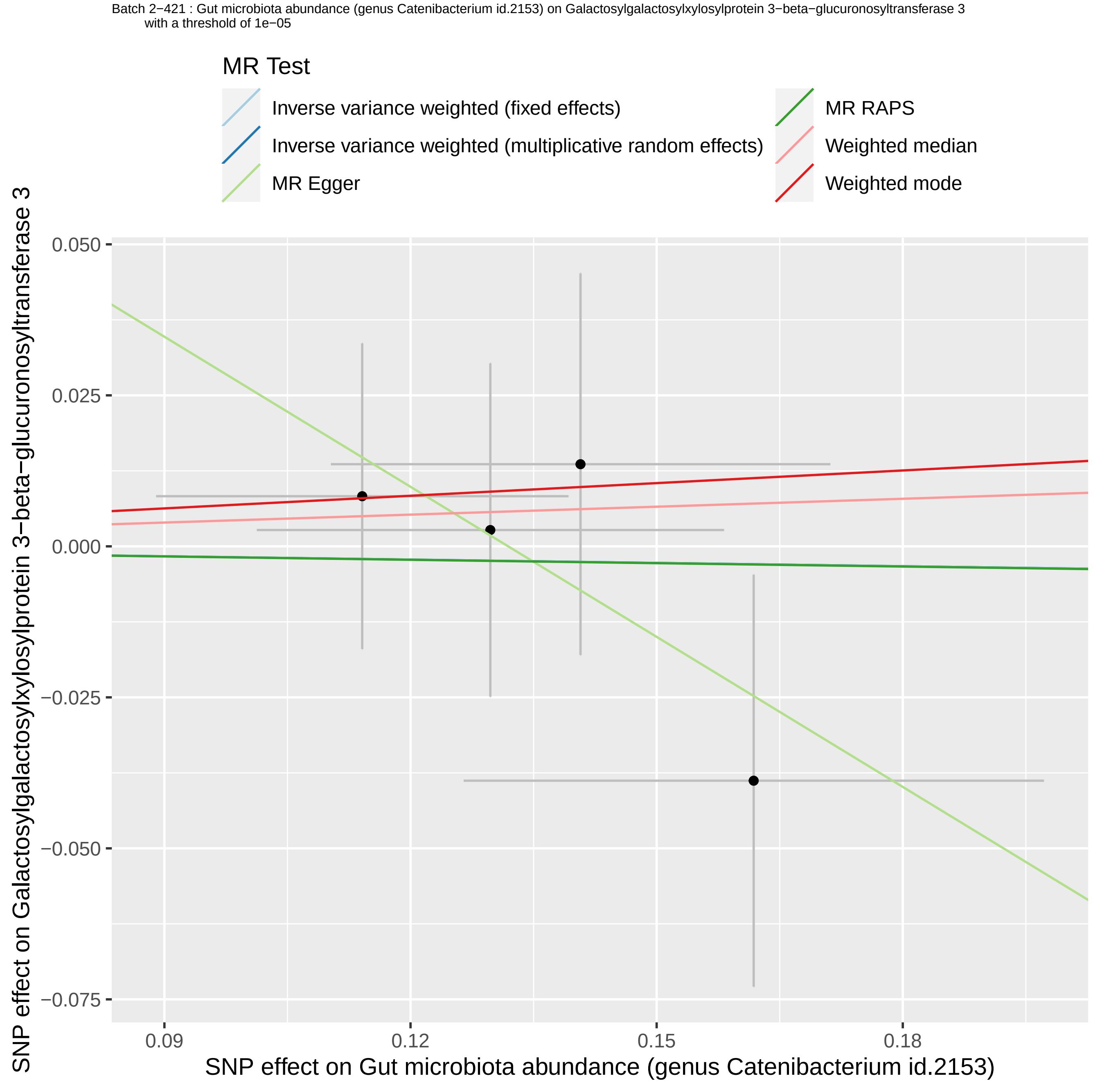

Batch 2−421 : Gut microbiota abundance (genus Catenibacterium id.2153) on Galactosylgalactosylxylosylprotein 3−beta−glucuronosyltransferase 3 with a threshold of 1e−05
MR Test
Inverse variance weighted (fixed effects)
Inverse variance weighted (multiplicative random effects)
MR Egger
MR RAPS
Weighted median
Weighted mode
0.050
0.025
0.000
−0.025
−0.050
−0.075
0.09
0.12
0.15
0.18
SNP effect on Galactosylgalactosylxylosylprotein 3−beta−glucuronosyltransferase 3
SNP effect on Gut microbiota abundance (genus Catenibacterium id.2153)

Batch 2−422 : Gut microbiota abundance (genus Parasutterella id.2892) on Galactosylgalactosylxylosylprotein 3−beta−glucuronosyltransferase 3
with a threshold of 1e−05

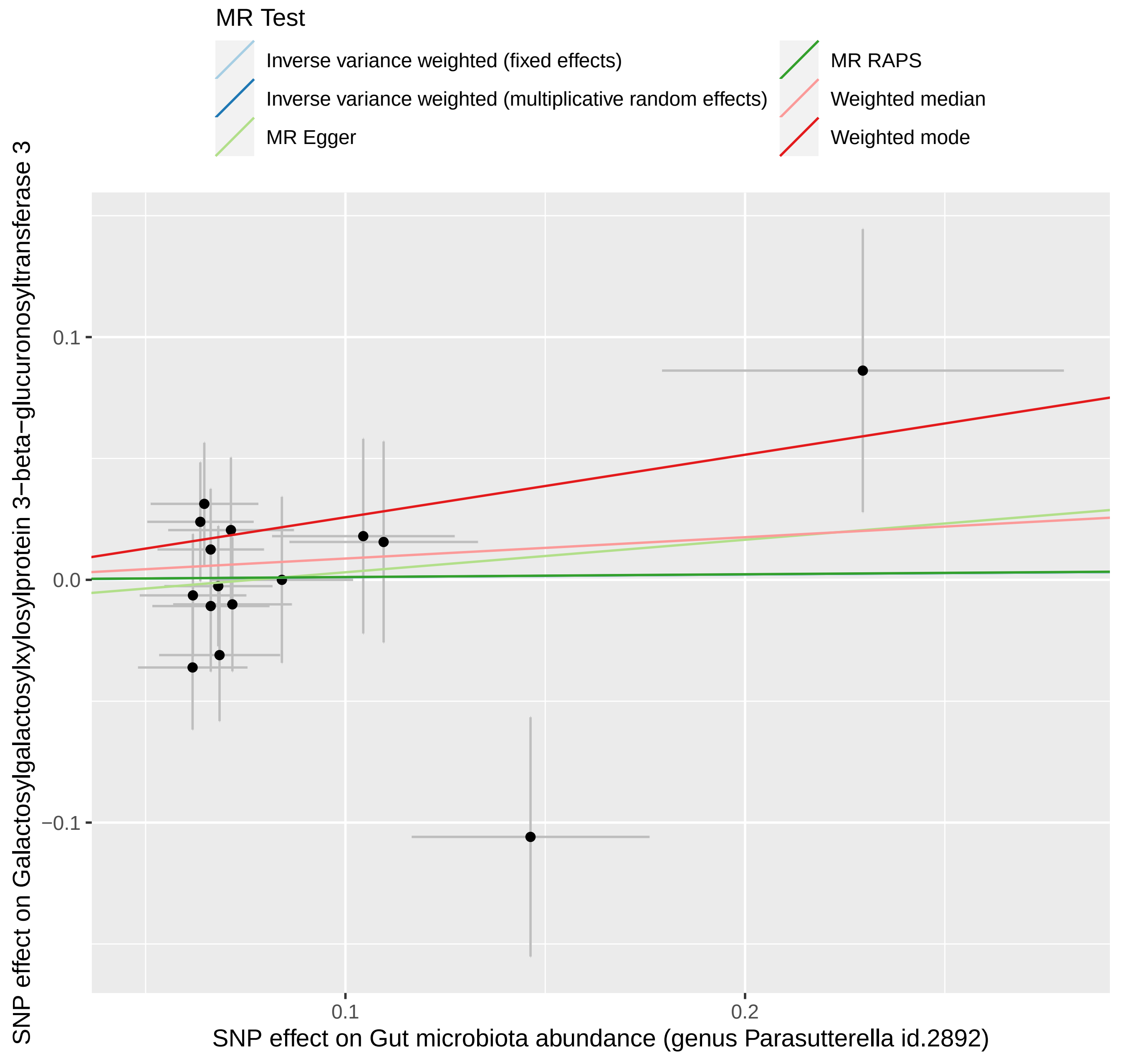

Batch 2−423 : Gut microbiota abundance (genus Catenibacterium id.2153) on Beta−1,4−glucuronyltransferase 1
with a threshold of 1e−05

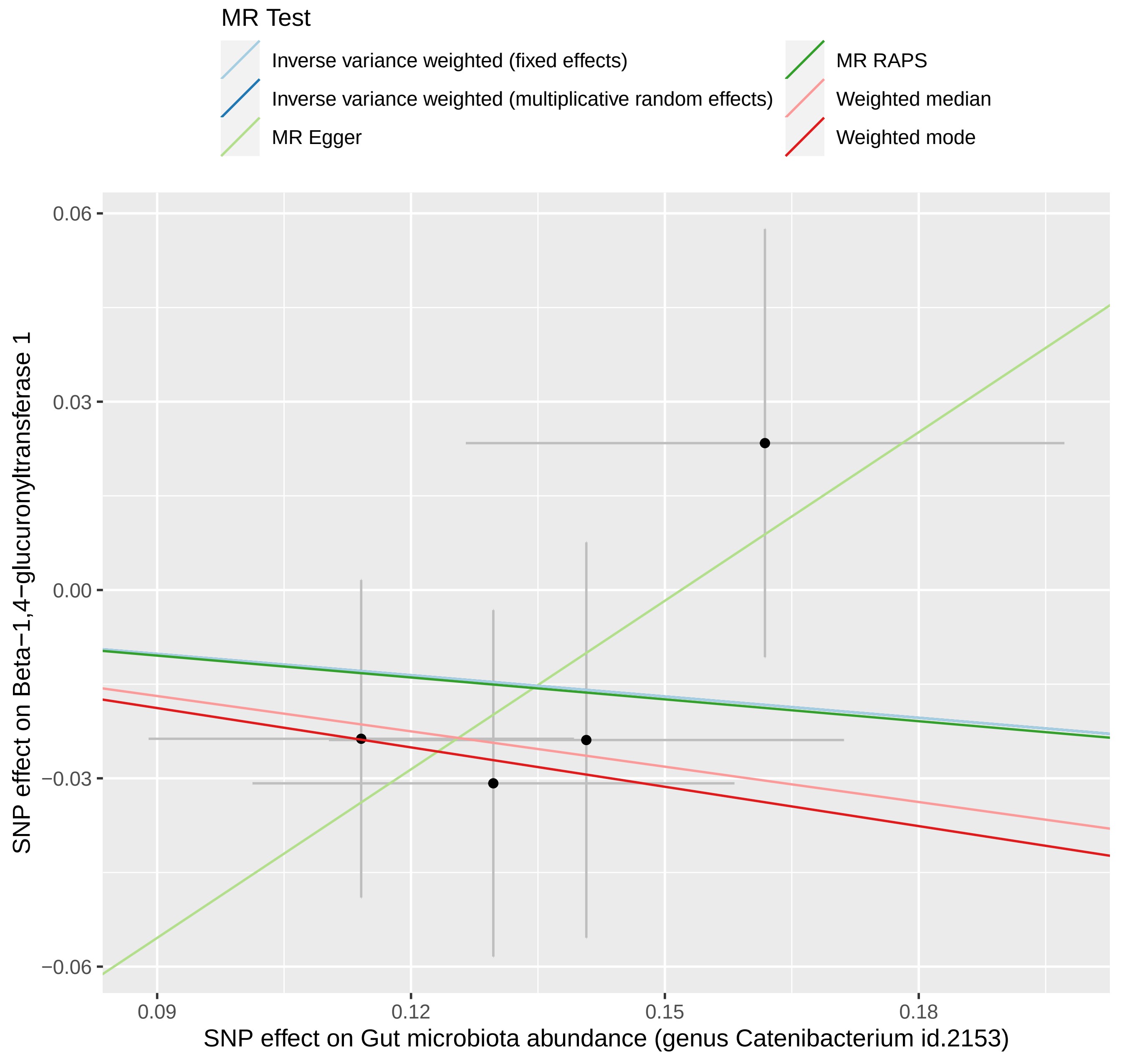

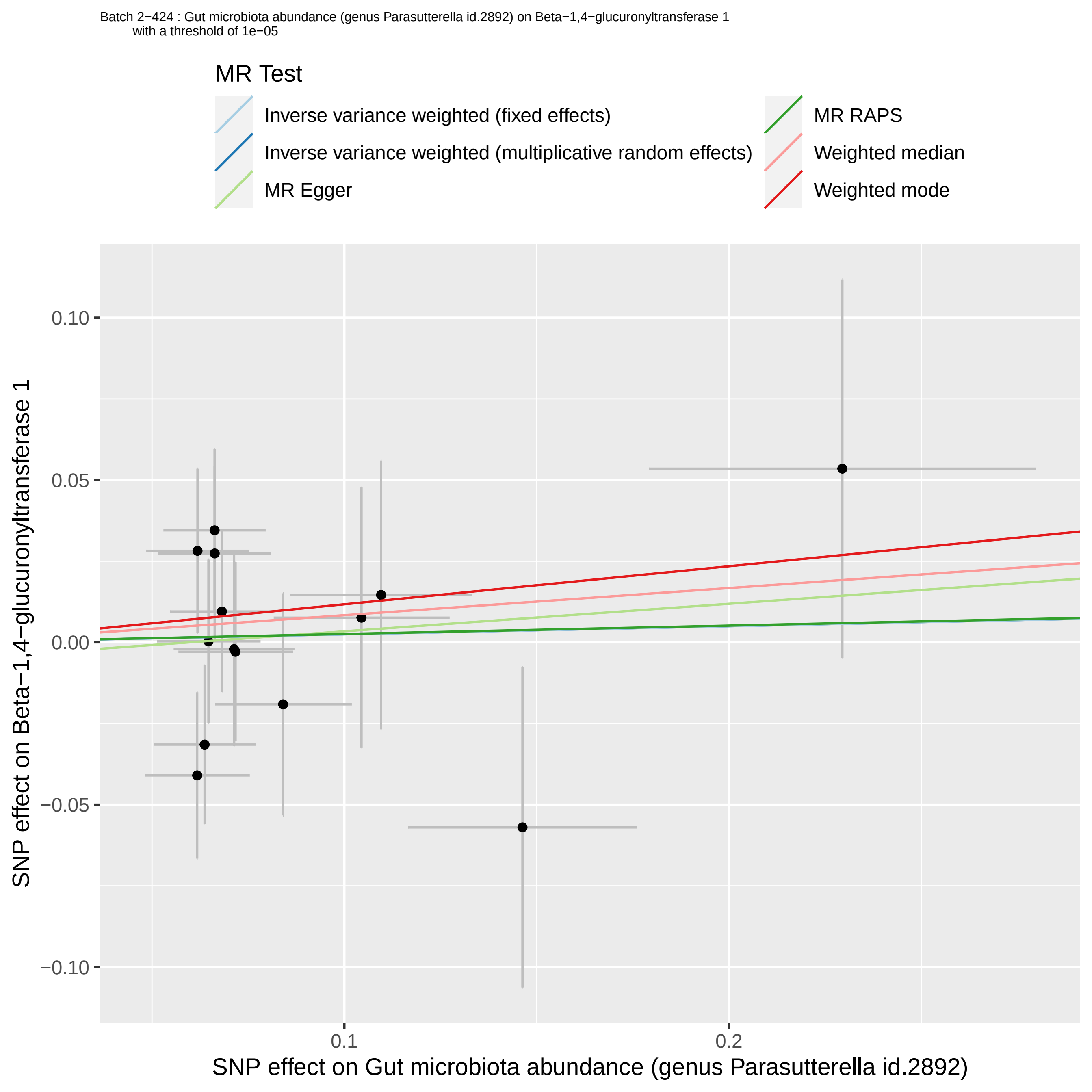

Batch 2−424 : Gut microbiota abundance (genus Parasutterella id.2892) on Beta−1,4−glucuronyltransferase 1
with a threshold of 1e−05
MR Test
Inverse variance weighted (fixed effects)
Inverse variance weighted (multiplicative random effects)
MR Egger
MR RAPS
Weighted median
Weighted mode
0.10
0.05
0.00
−0.05
−0.10
0.1
0.2
SNP effect on Beta−1,4−glucuronyltransferase 1
SNP effect on Gut microbiota abundance (genus Parasutterella id.2892)

Batch 2−425 : Gut microbiota abundance (genus Catenibacterium id.2153) on N−acetyllactosaminide beta−1,3−N−acetylglucosaminyltransferase 2 with a threshold of 1e−05

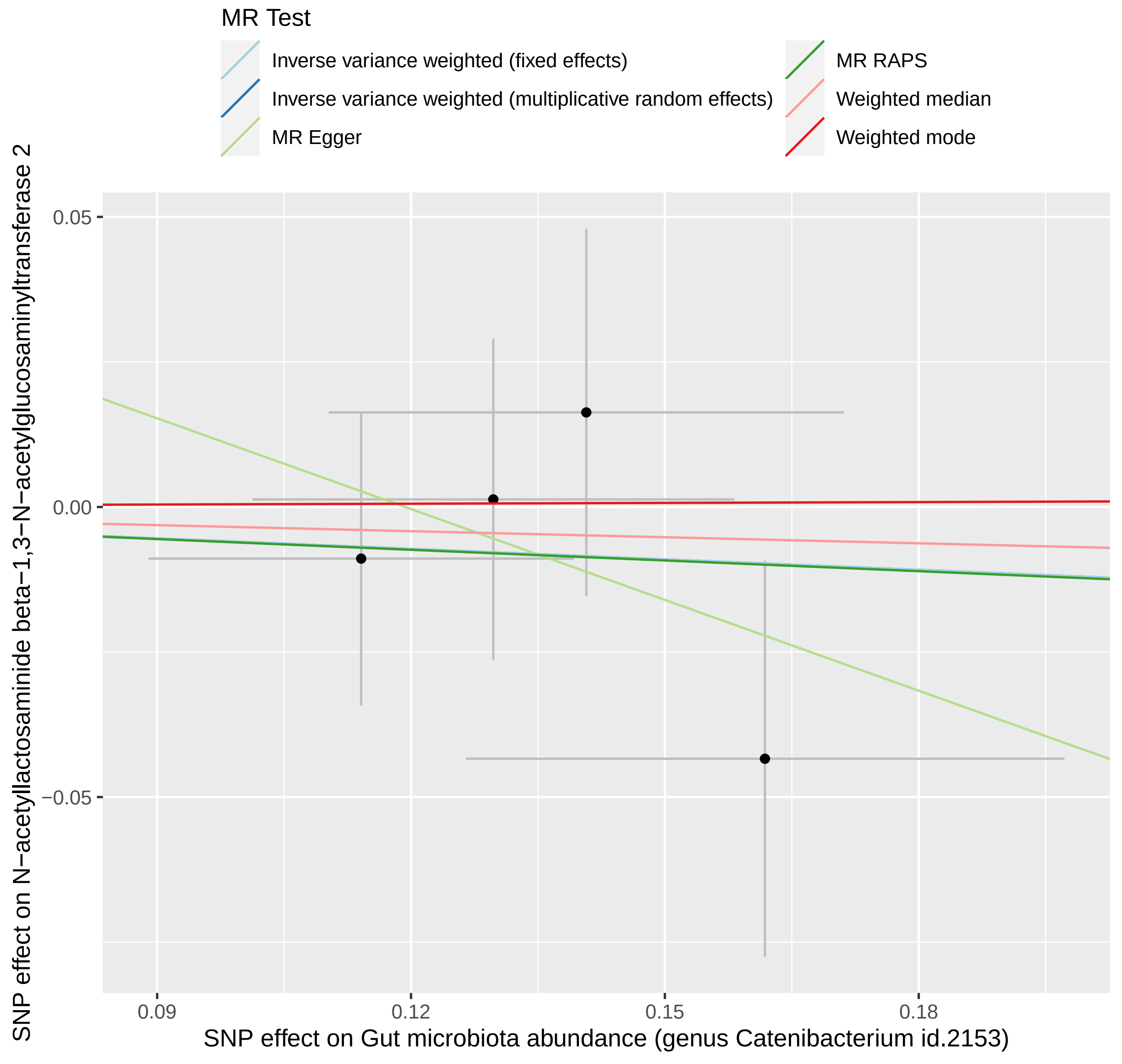

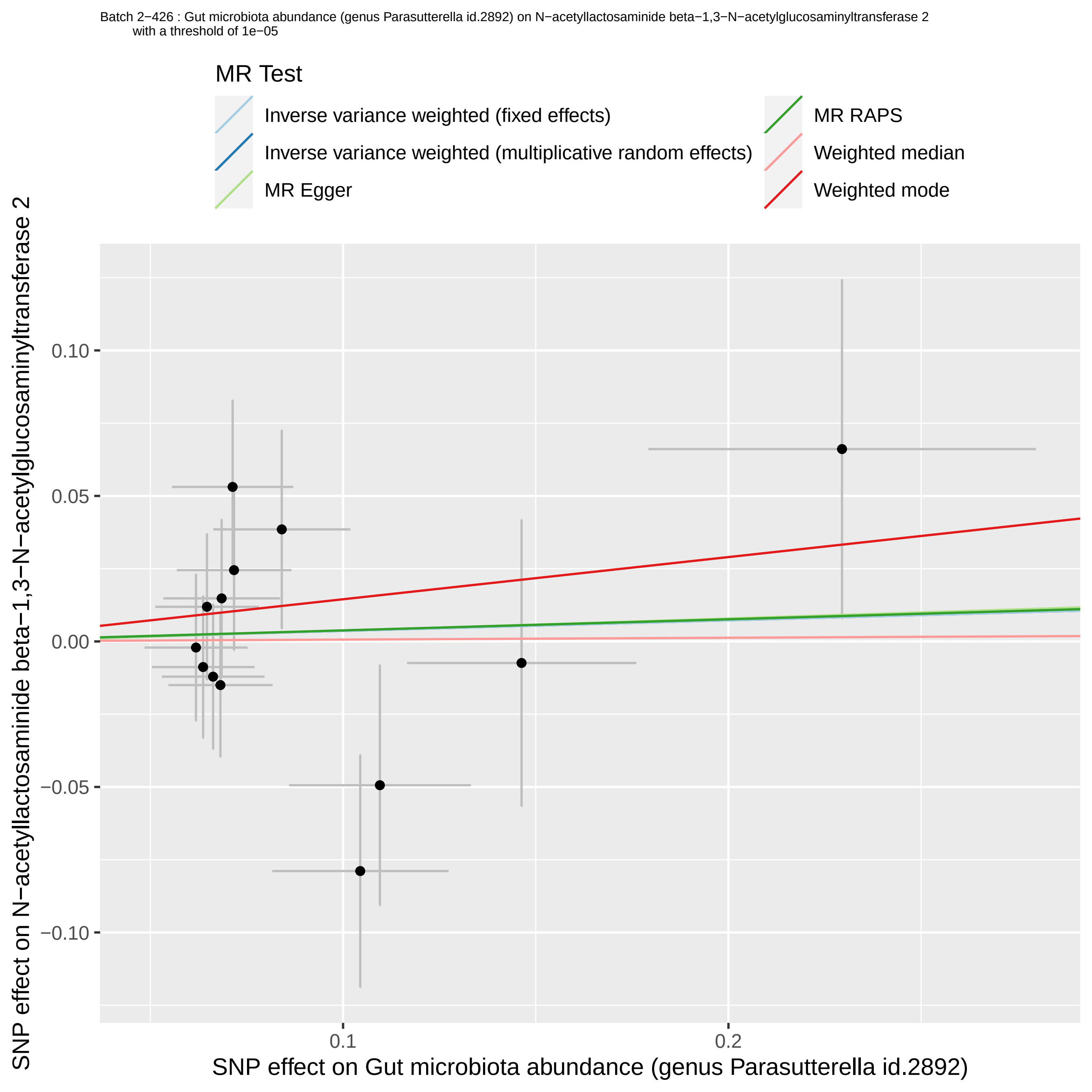

Batch 2−426 : Gut microbiota abundance (genus Parasutterella id.2892) on N−acetyllactosaminide beta−1,3−N−acetylglucosaminyltransferase 2 with a threshold of 1e−05
MR Test
Inverse variance weighted (fixed effects)
Inverse variance weighted (multiplicative random effects)
MR Egger
MR RAPS
Weighted median
Weighted mode
0.10
0.05
0.00
−0.05
−0.10
0.1
0.2
SNP effect on N−acetyllactosaminide beta−1,3−N−acetylglucosaminyltransferase 2
SNP effect on Gut microbiota abundance (genus Parasutterella id.2892)

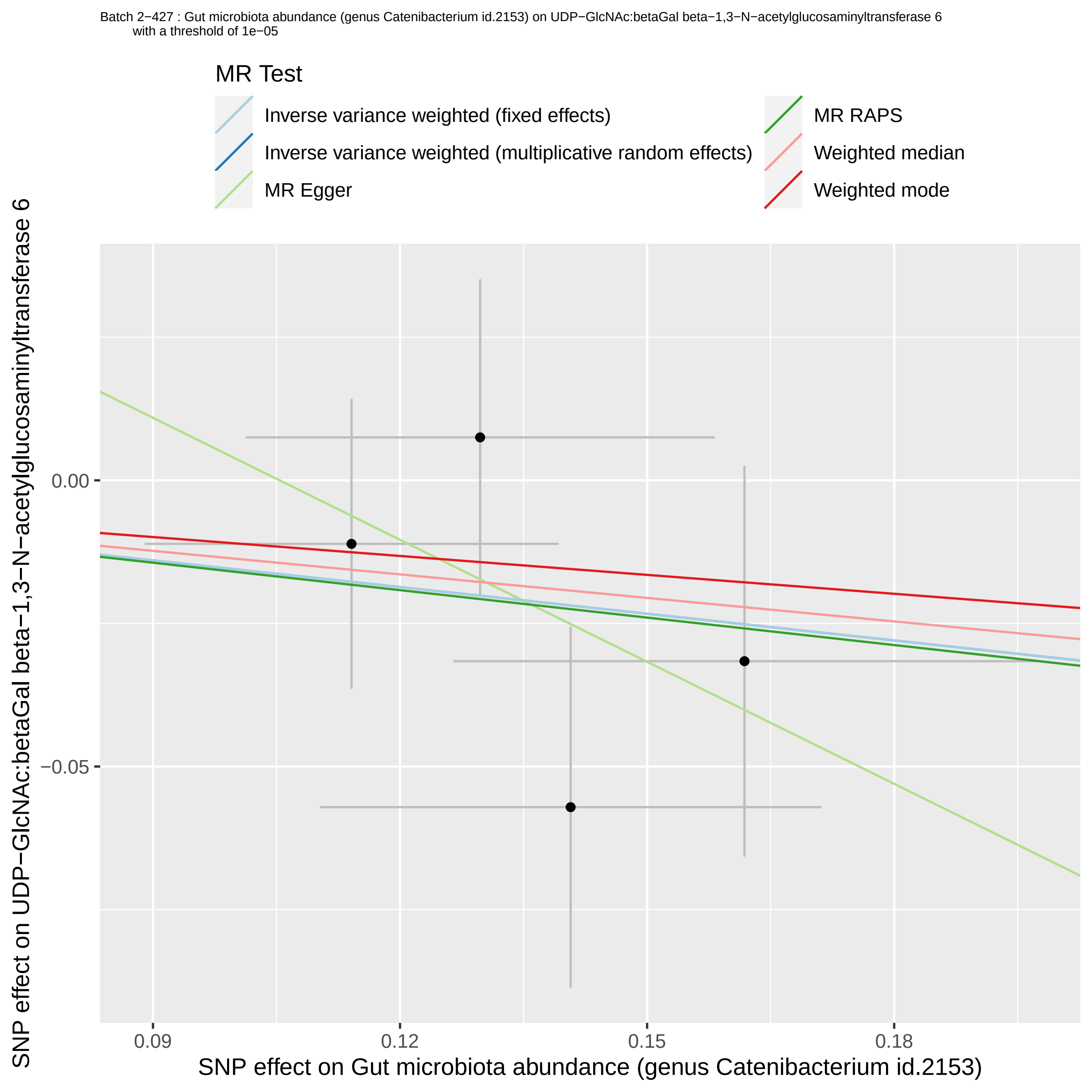

Batch 2−427 : Gut microbiota abundance (genus Catenibacterium id.2153) on UDP−GlcNAc:betaGal beta−1,3−N−acetylglucosaminyltransferase 6
with a threshold of 1e−05
MR Test
Inverse variance weighted (fixed effects)
Inverse variance weighted (multiplicative random effects)
MR Egger
MR RAPS
Weighted median
Weighted mode
0.00
−0.05
0.09
0.12
0.15
0.18
SNP effect on UDP−GlcNAc:betaGal beta−1,3−N−acetylglucosaminyltransferase 6
SNP effect on Gut microbiota abundance (genus Catenibacterium id.2153)

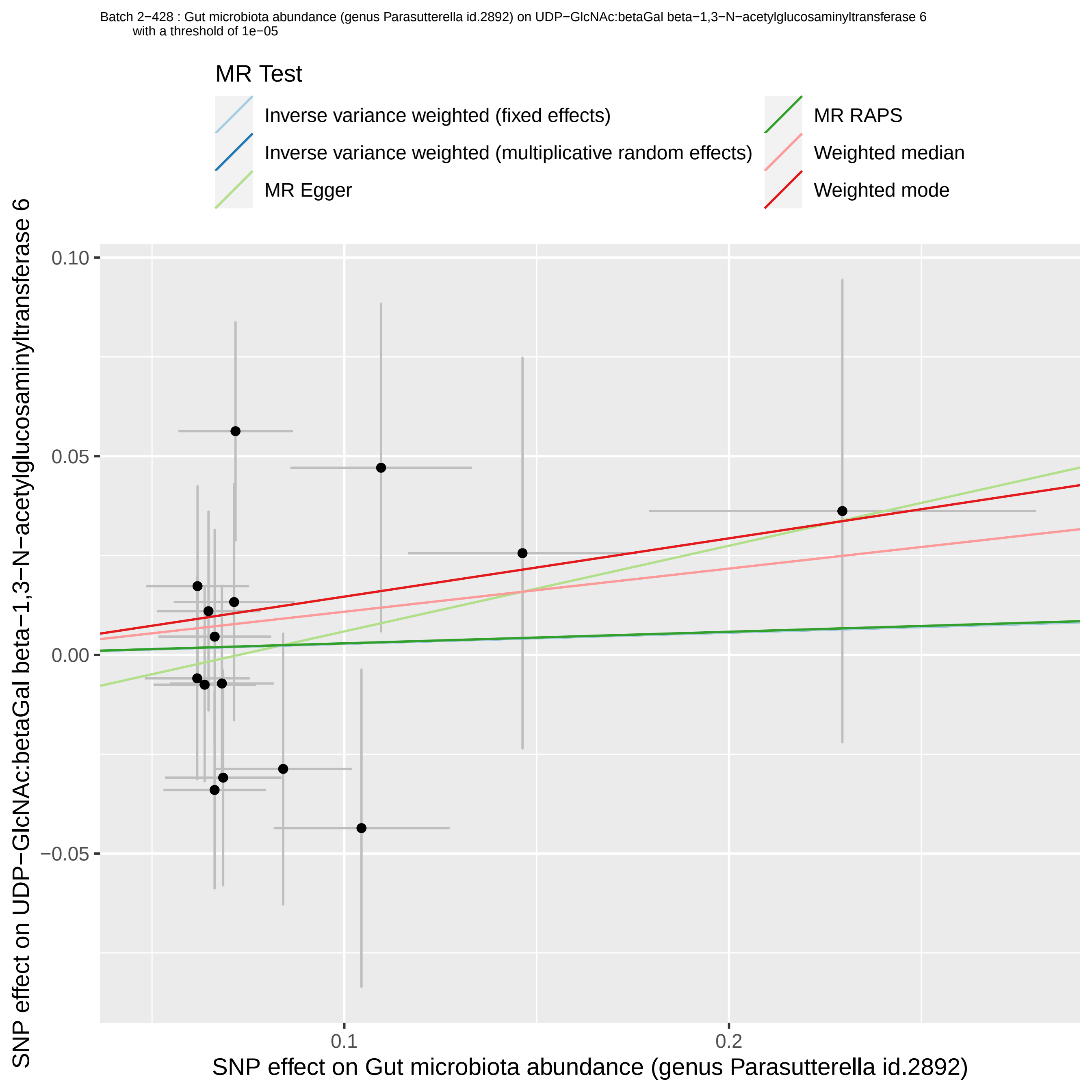
Batch 2−428 : Gut microbiota abundance (genus Parasutterella id.2892) on UDP−GlcNAc:betaGal beta−1,3−N−acetylglucosaminyltransferase 6
with a threshold of 1e−05
MR Test
Inverse variance weighted (fixed effects)
Inverse variance weighted (multiplicative random effects)
MR Egger
MR RAPS
Weighted median
Weighted mode
0.10
0.05
0.00
−0.05
0.1
0.2
SNP effect on UDP−GlcNAc:betaGal beta−1,3−N−acetylglucosaminyltransferase 6
SNP effect on Gut microbiota abundance (genus Parasutterella id.2892)

Batch 2−429 : Gut microbiota abundance (genus Catenibacterium id.2153) on UDP−GlcNAc:betaGal beta−1,3−N−acetylglucosaminyltransferase 8
with a threshold of 1e−05

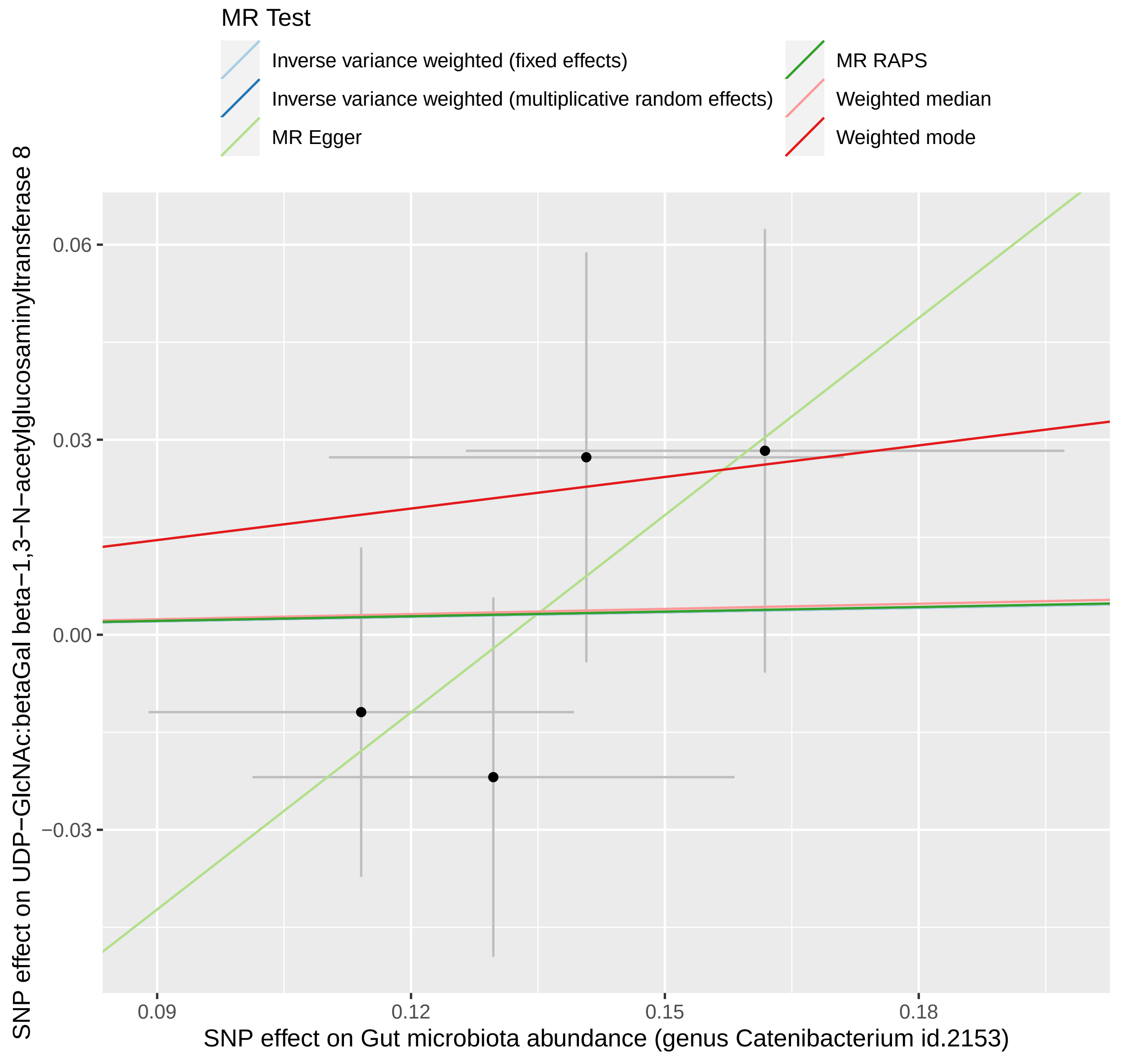

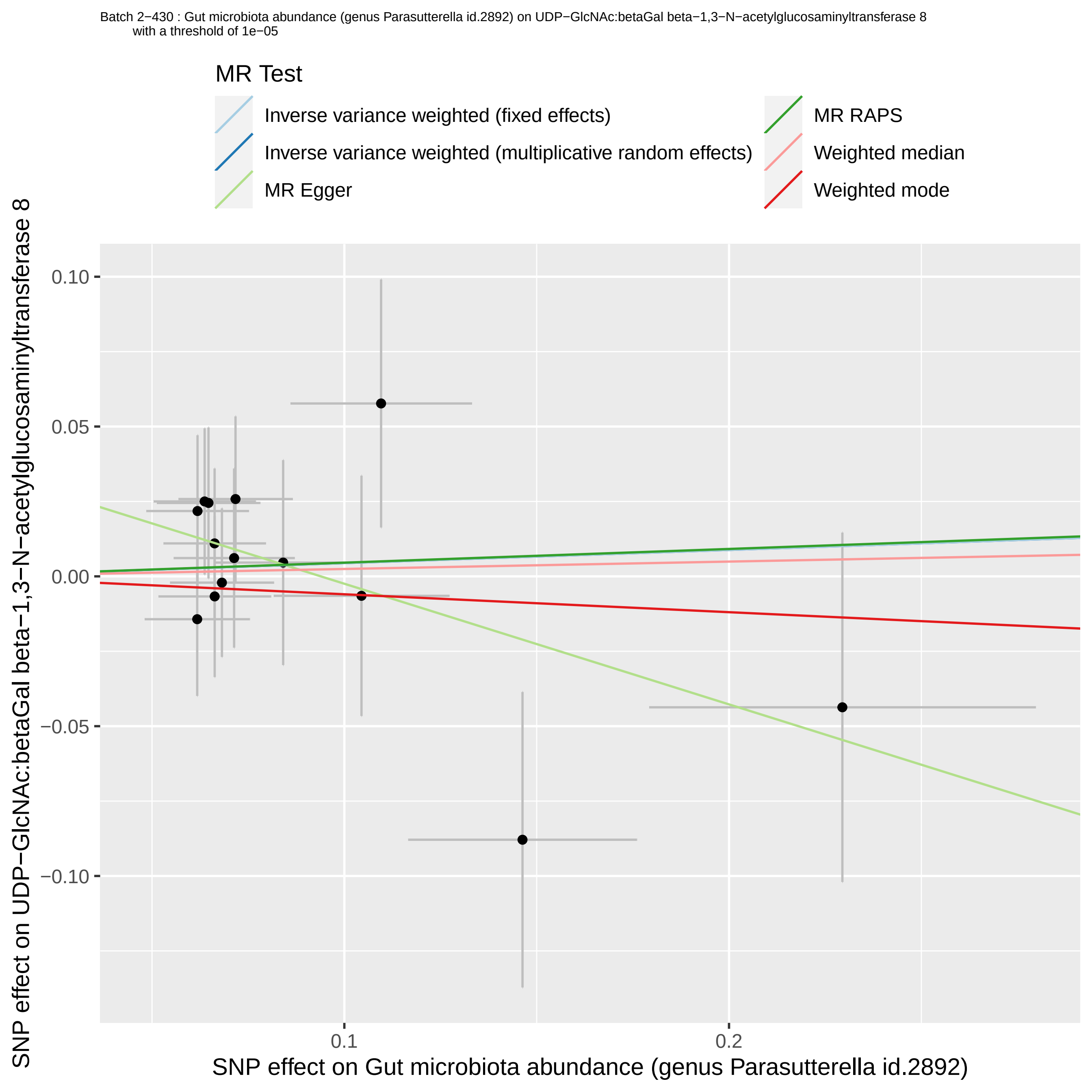
Batch 2−430 : Gut microbiota abundance (genus Parasutterella id.2892) on UDP−GlcNAc:betaGal beta−1,3−N−acetylglucosaminyltransferase 8
with a threshold of 1e−05
MR Test
Inverse variance weighted (fixed effects)
Inverse variance weighted (multiplicative random effects)
MR Egger
MR RAPS
Weighted median
Weighted mode
0.10
0.05
0.00
−0.05
−0.10
0.1
0.2
SNP effect on UDP−GlcNAc:betaGal beta−1,3−N−acetylglucosaminyltransferase 8
SNP effect on Gut microbiota abundance (genus Parasutterella id.2892)

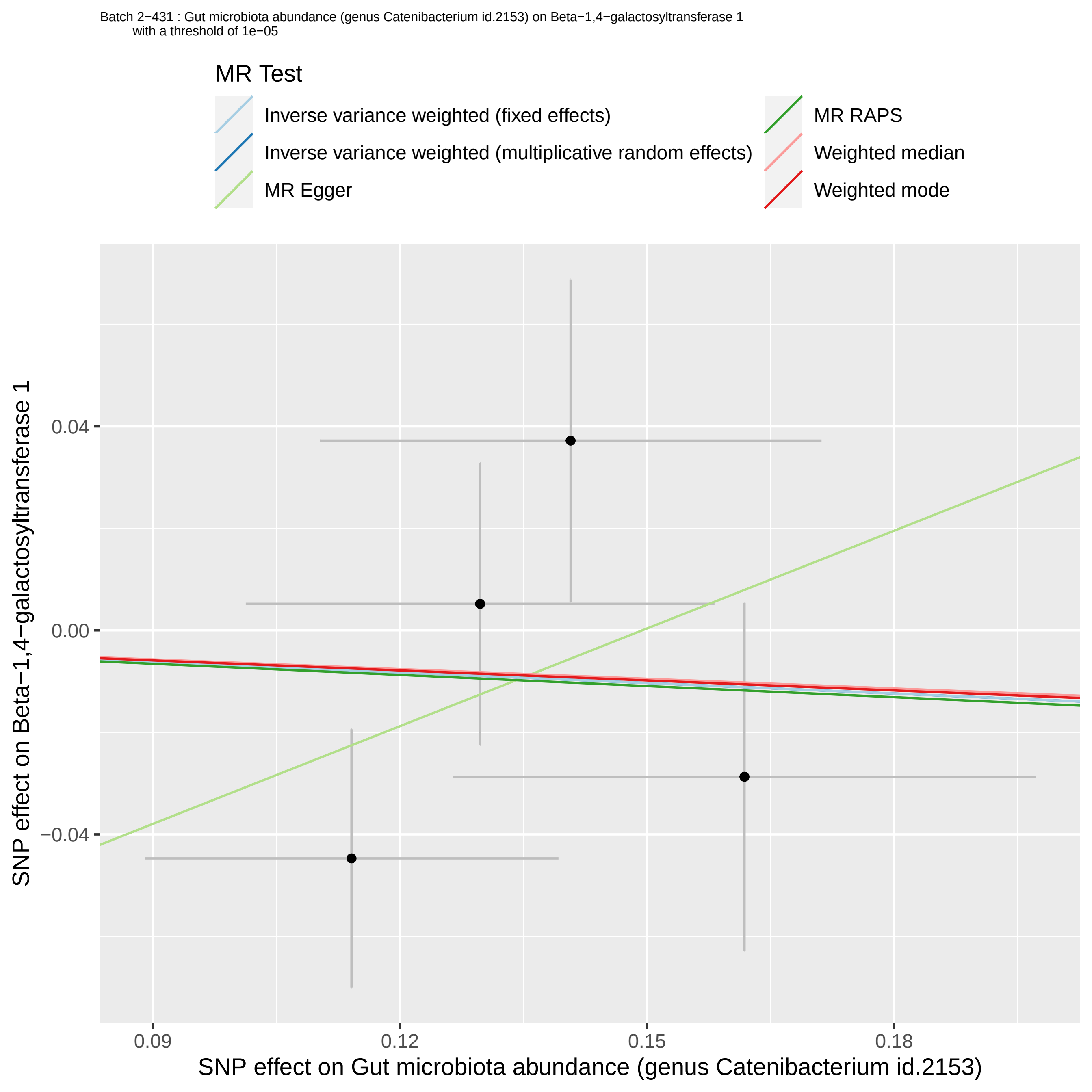
Batch 2−431 : Gut microbiota abundance (genus Catenibacterium id.2153) on Beta−1,4−galactosyltransferase 1
with a threshold of 1e−05
MR Test
Inverse variance weighted (fixed effects)
Inverse variance weighted (multiplicative random effects)
MR Egger
MR RAPS
Weighted median
Weighted mode
0.04
0.00
−0.04
0.09
0.12
0.15
0.18
SNP effect on Beta−1,4−galactosyltransferase 1
SNP effect on Gut microbiota abundance (genus Catenibacterium id.2153)

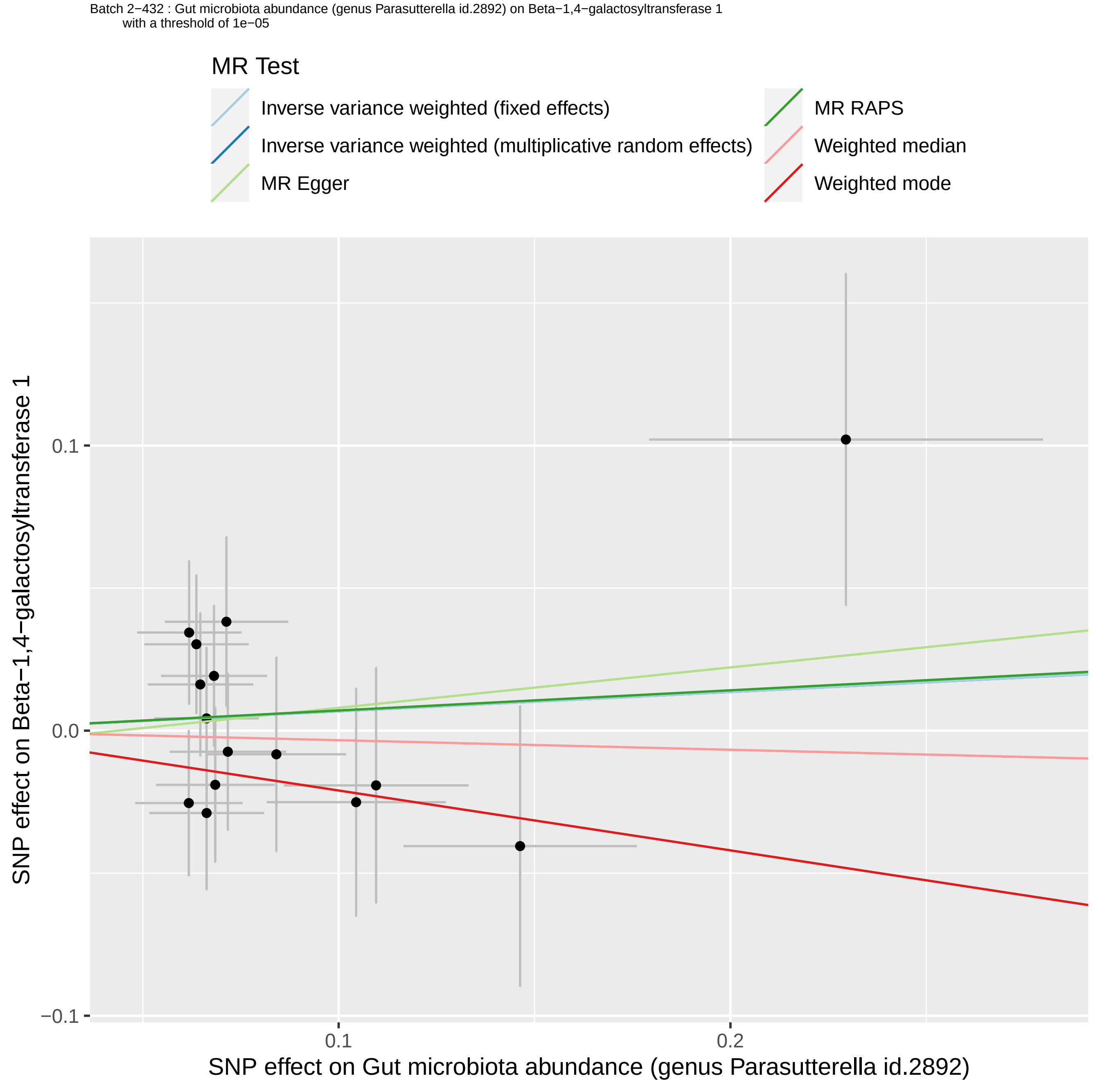

Batch 2−432 : Gut microbiota abundance (genus Parasutterella id.2892) on Beta−1,4−galactosyltransferase 1
with a threshold of 1e−05
MR Test
Inverse variance weighted (fixed effects)
Inverse variance weighted (multiplicative random effects)
MR Egger
MR RAPS
Weighted median
Weighted mode
SNP effect on Beta−1,4−galactosyltransferase 1
0.1
0.0
−0.1
0.1
0.2
SNP effect on Gut microbiota abundance (genus Parasutterella id.2892)

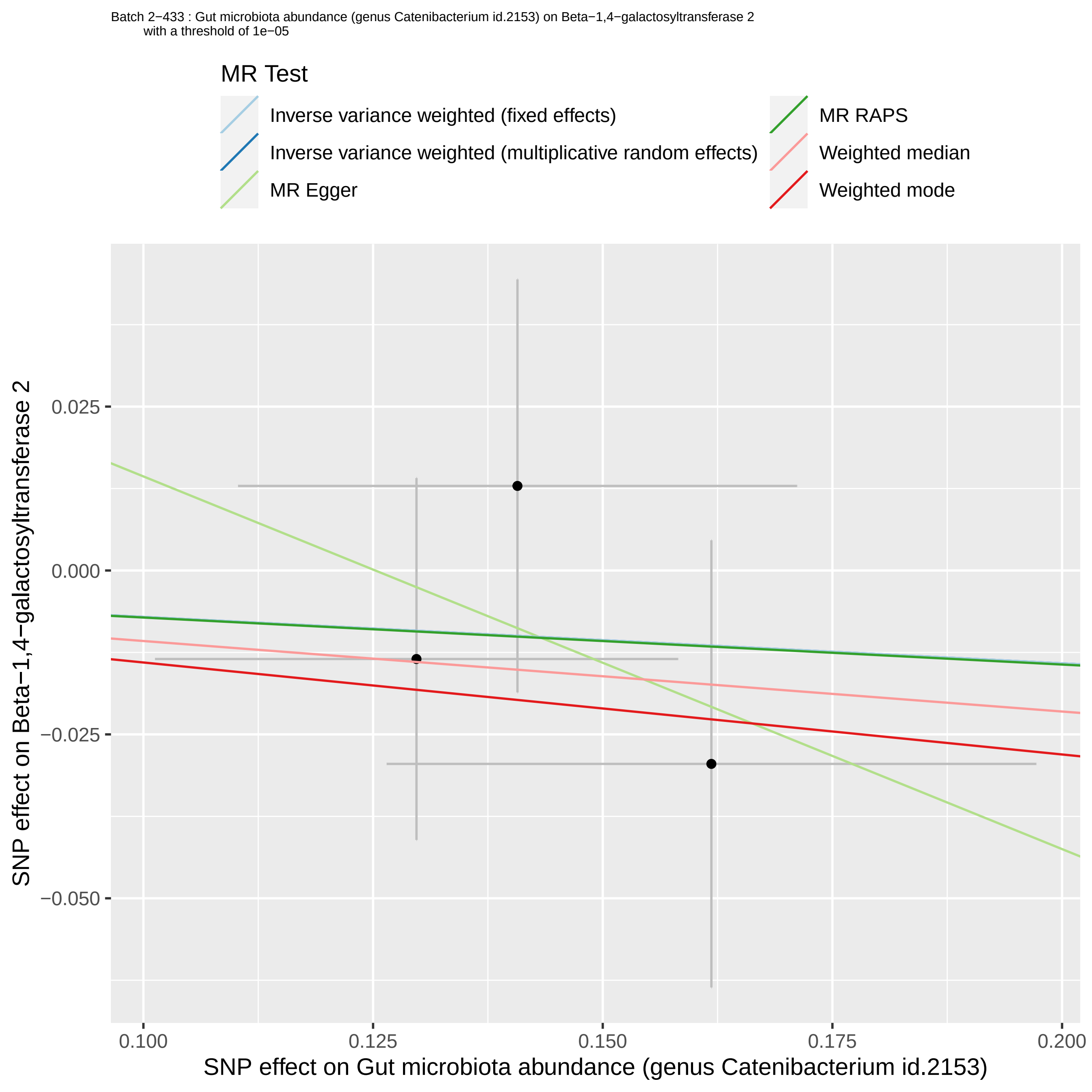
Batch 2−433 : Gut microbiota abundance (genus Catenibacterium id.2153) on Beta−1,4−galactosyltransferase 2
with a threshold of 1e−05
MR Test
Inverse variance weighted (fixed effects)
Inverse variance weighted (multiplicative random effects)
MR Egger
MR RAPS
Weighted median
Weighted mode
SNP effect on Beta−1,4−galactosyltransferase 2
0.025
0.000
−0.025
−0.050
0.100
0.125
0.150
0.175
0.200
SNP effect on Gut microbiota abundance (genus Catenibacterium id.2153)

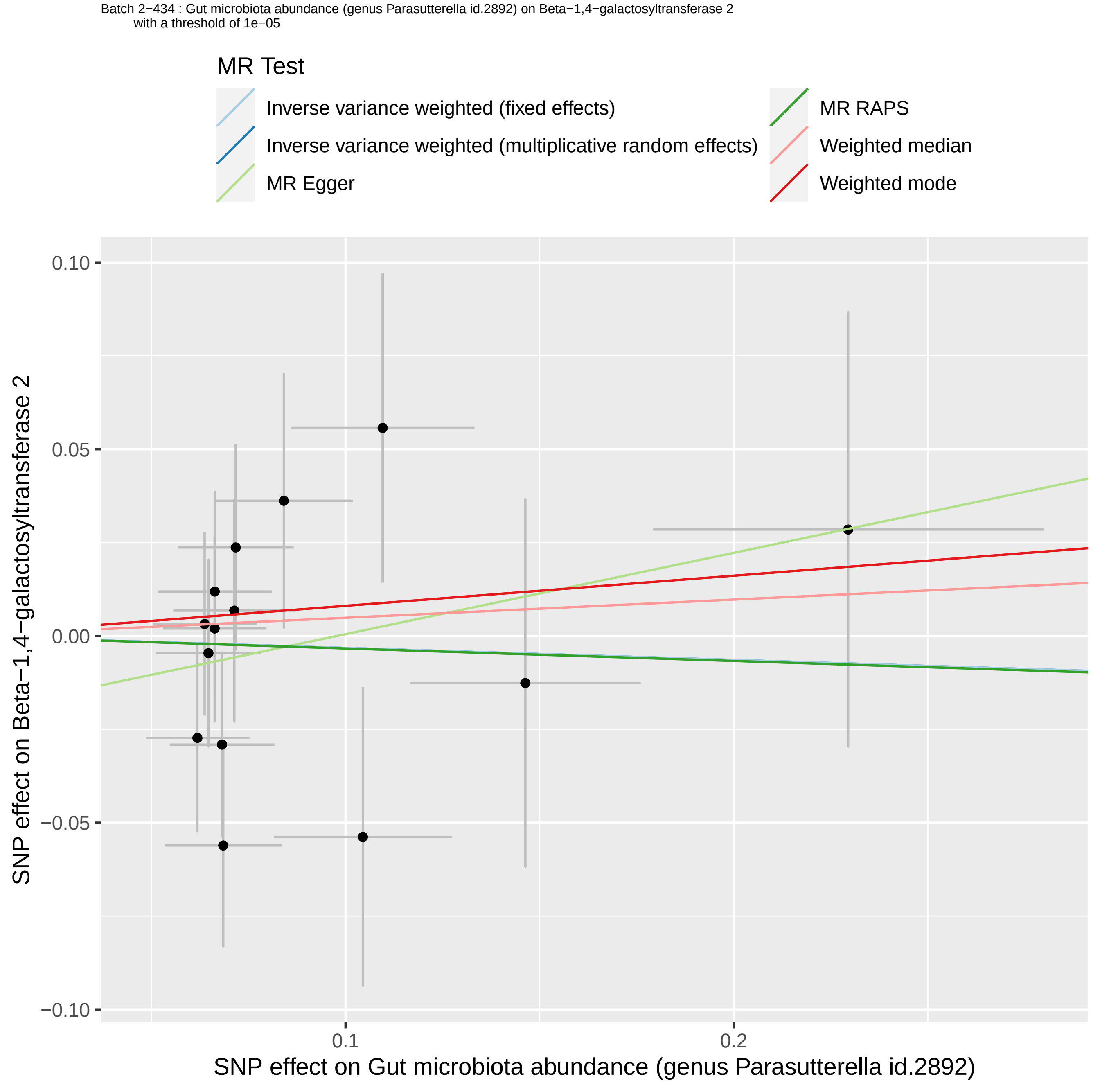

Batch 2−434 : Gut microbiota abundance (genus Parasutterella id.2892) on Beta−1,4−galactosyltransferase 2
with a threshold of 1e−05
MR Test
Inverse variance weighted (fixed effects)
Inverse variance weighted (multiplicative random effects)
MR Egger
MR RAPS
Weighted median
Weighted mode
0.10
0.05
0.00
−0.05
−0.10
0.1
0.2
SNP effect on Beta−1,4−galactosyltransferase 2
SNP effect on Gut microbiota abundance (genus Parasutterella id.2892)

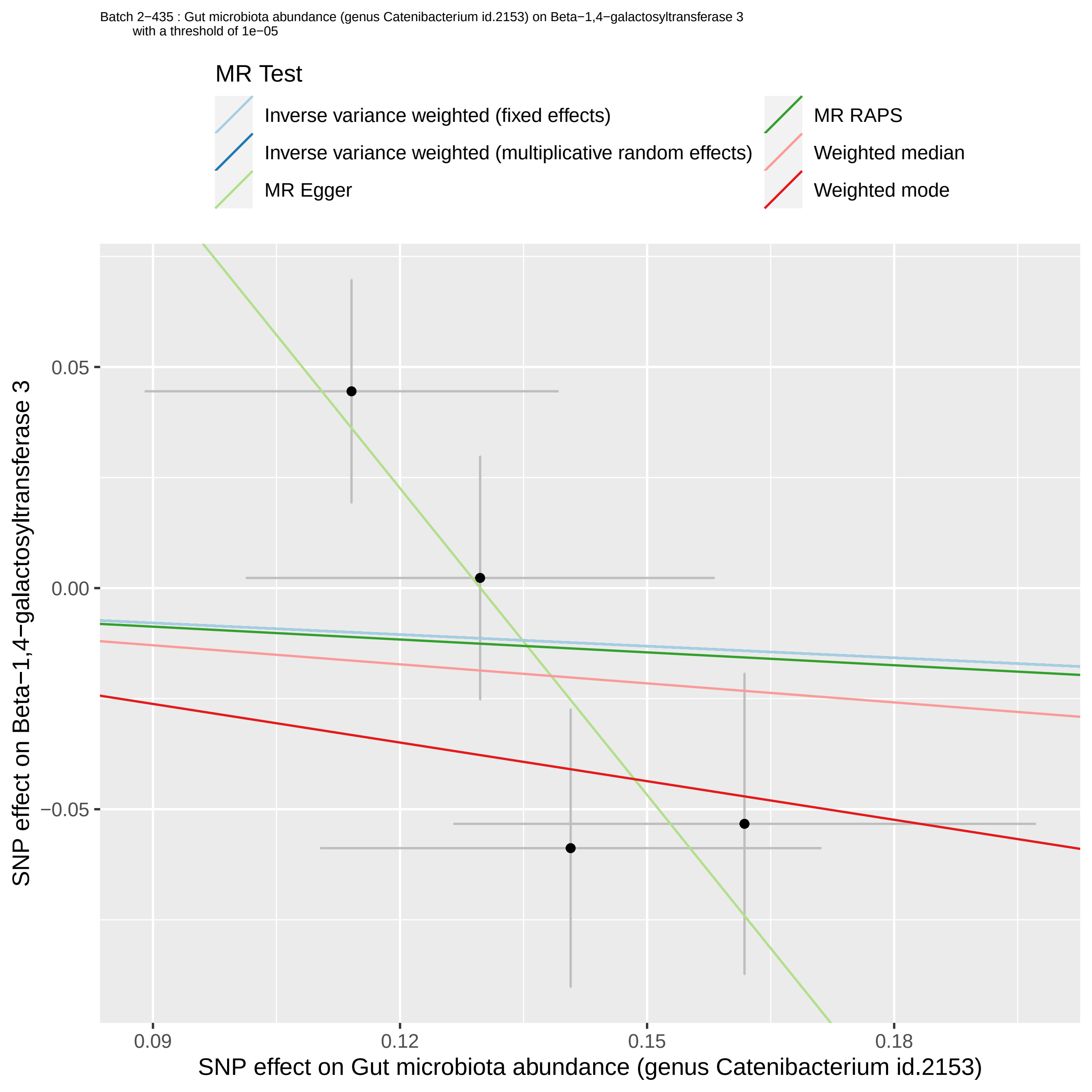
Batch 2−435 : Gut microbiota abundance (genus Catenibacterium id.2153) on Beta−1,4−galactosyltransferase 3
with a threshold of 1e−05
MR Test
Inverse variance weighted (fixed effects)
Inverse variance weighted (multiplicative random effects)
MR Egger
MR RAPS
Weighted median
Weighted mode
0.05
0.00
−0.05
0.09
0.12
0.15
0.18
SNP effect on Beta−1,4−galactosyltransferase 3
SNP effect on Gut microbiota abundance (genus Catenibacterium id.2153)

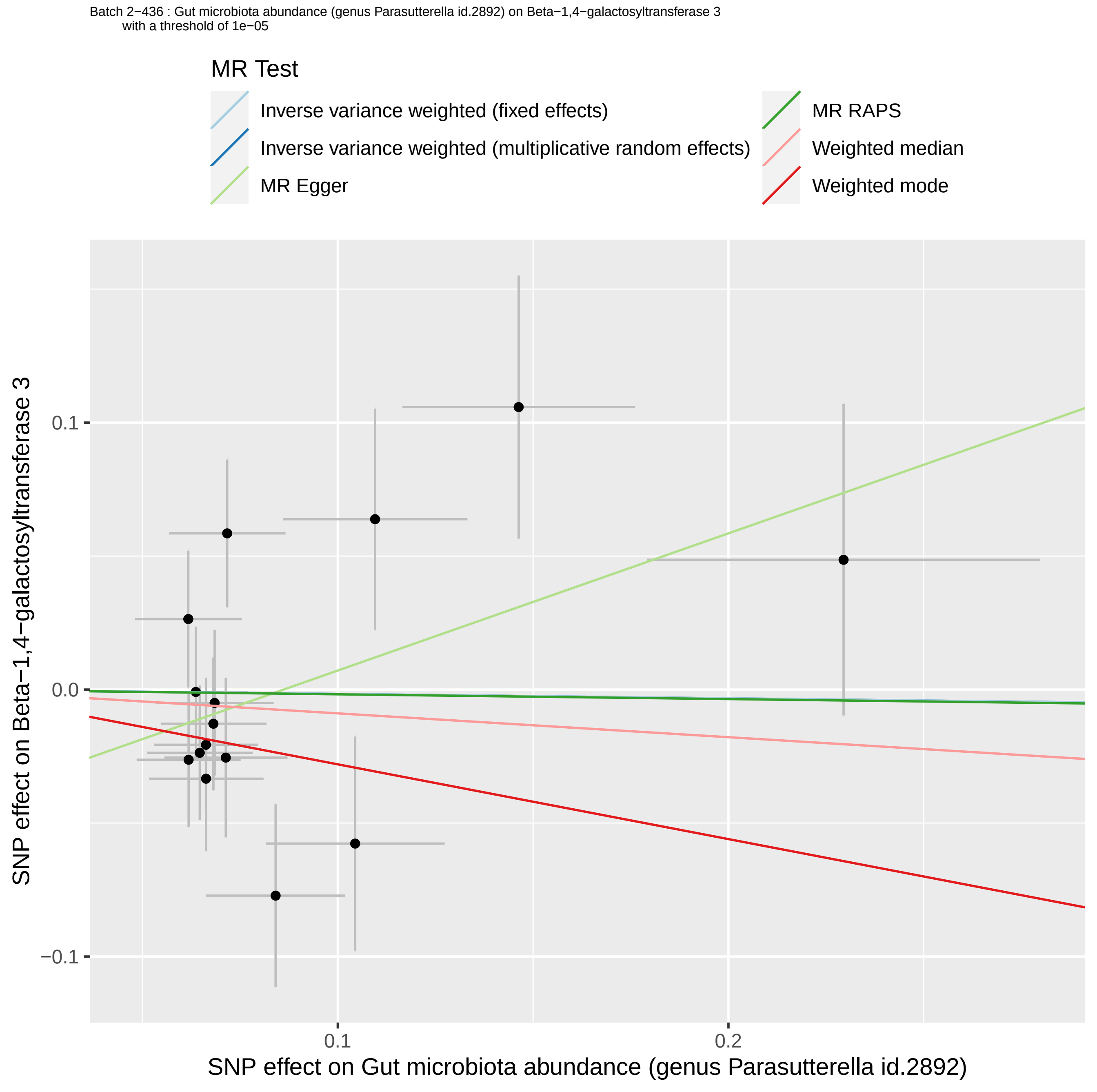
Batch 2−436 : Gut microbiota abundance (genus Parasutterella id.2892) on Beta−1,4−galactosyltransferase 3
with a threshold of 1e−05
MR Test
Inverse variance weighted (fixed effects)
Inverse variance weighted (multiplicative random effects)
MR Egger
MR RAPS
Weighted median
Weighted mode
SNP effect on Beta−1,4−galactosyltransferase 3
0.1
0.0
−0.1
0.1
0.2
SNP effect on Gut microbiota abundance (genus Parasutterella id.2892)

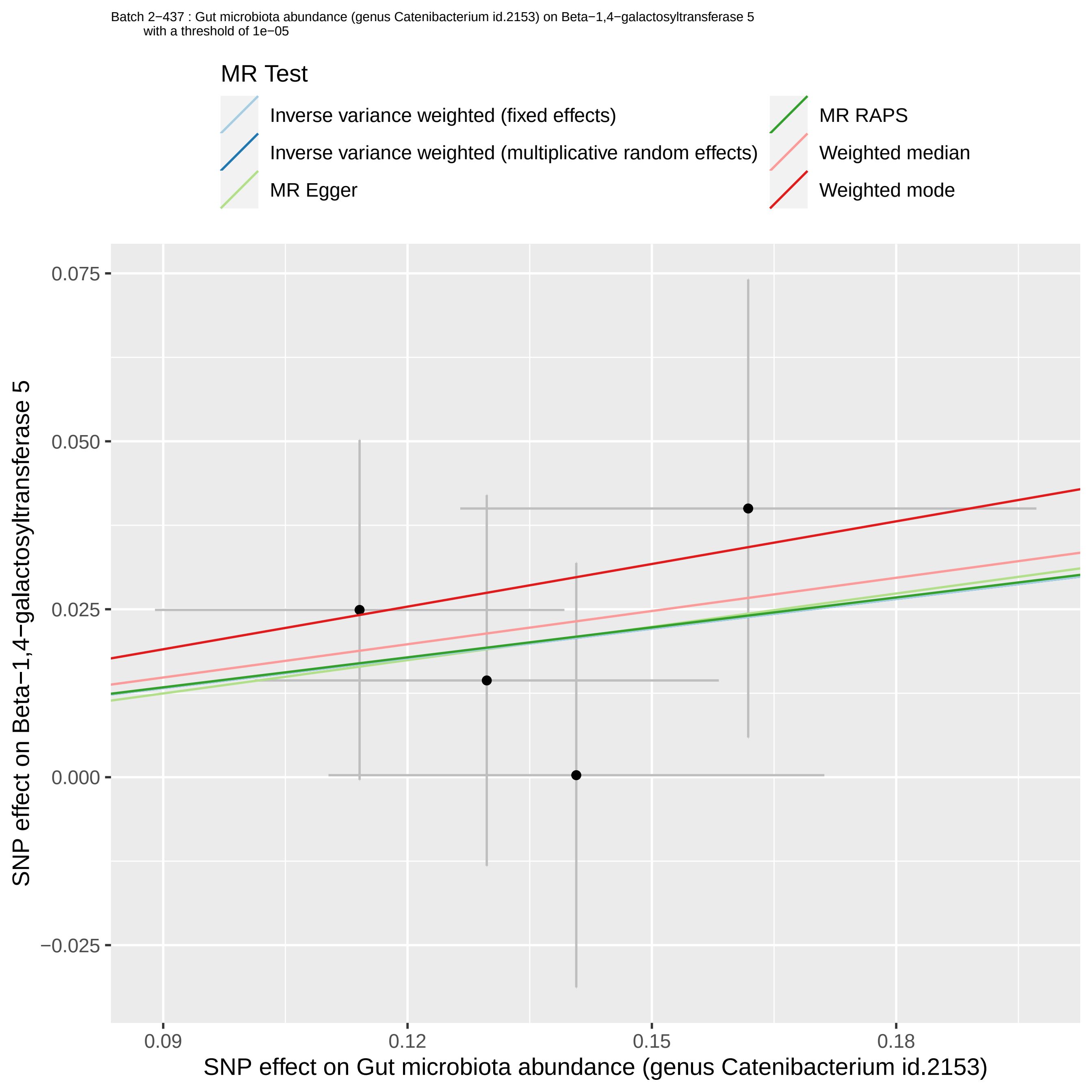

Batch 2−437 : Gut microbiota abundance (genus Catenibacterium id.2153) on Beta−1,4−galactosyltransferase 5
with a threshold of 1e−05
MR Test
Inverse variance weighted (fixed effects)
Inverse variance weighted (multiplicative random effects)
MR Egger
MR RAPS
Weighted median
Weighted mode
0.075
0.050
0.025
0.000
−0.025
0.09
0.12
0.15
0.18
SNP effect on Beta−1,4−galactosyltransferase 5
SNP effect on Gut microbiota abundance (genus Catenibacterium id.2153)

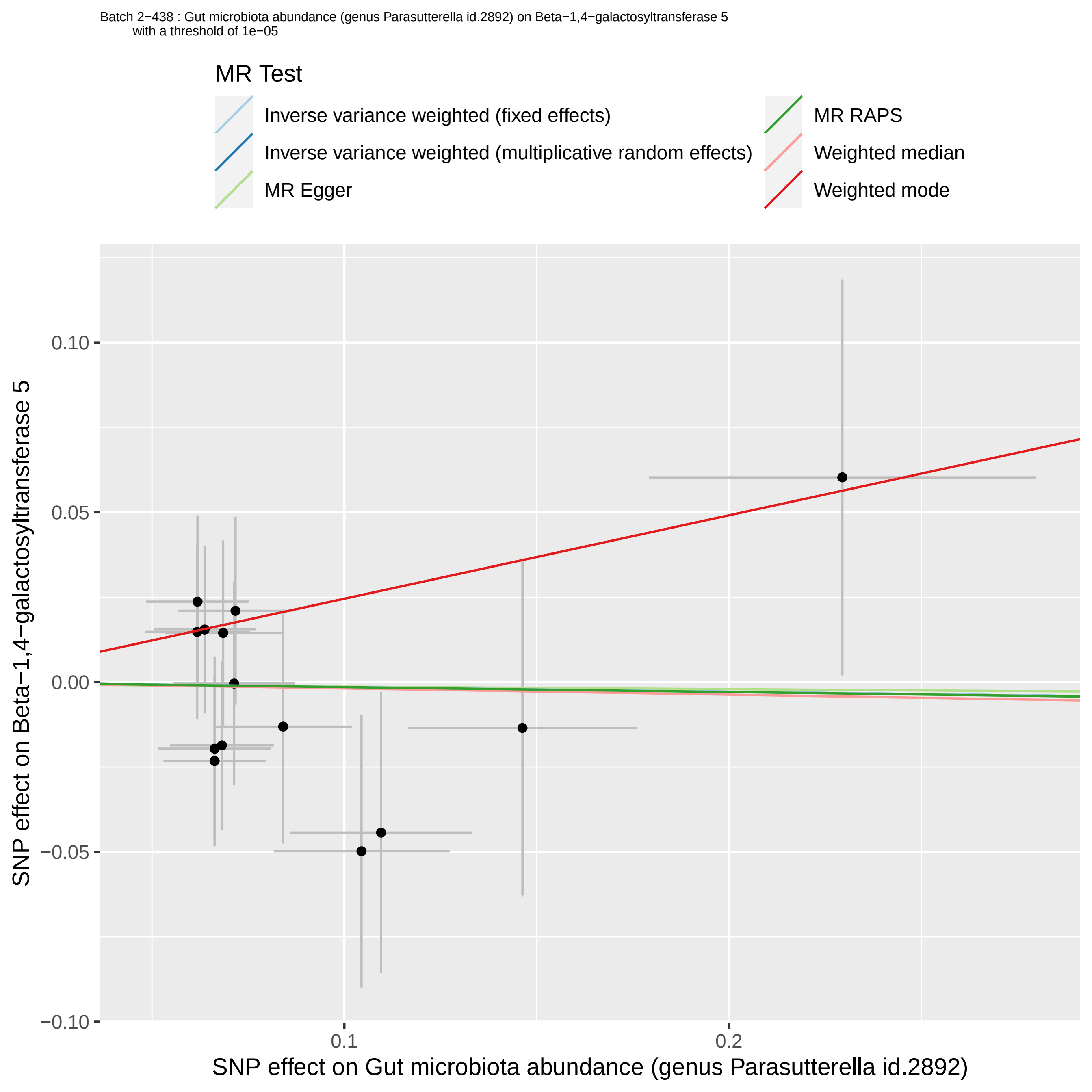

Batch 2−438 : Gut microbiota abundance (genus Parasutterella id.2892) on Beta−1,4−galactosyltransferase 5
with a threshold of 1e−05
MR Test
Inverse variance weighted (fixed effects)
Inverse variance weighted (multiplicative random effects)
MR Egger
MR RAPS
Weighted median
Weighted mode
0.10
0.05
0.00
−0.05
−0.10
0.1
0.2
SNP effect on Beta−1,4−galactosyltransferase 5
SNP effect on Gut microbiota abundance (genus Parasutterella id.2892)

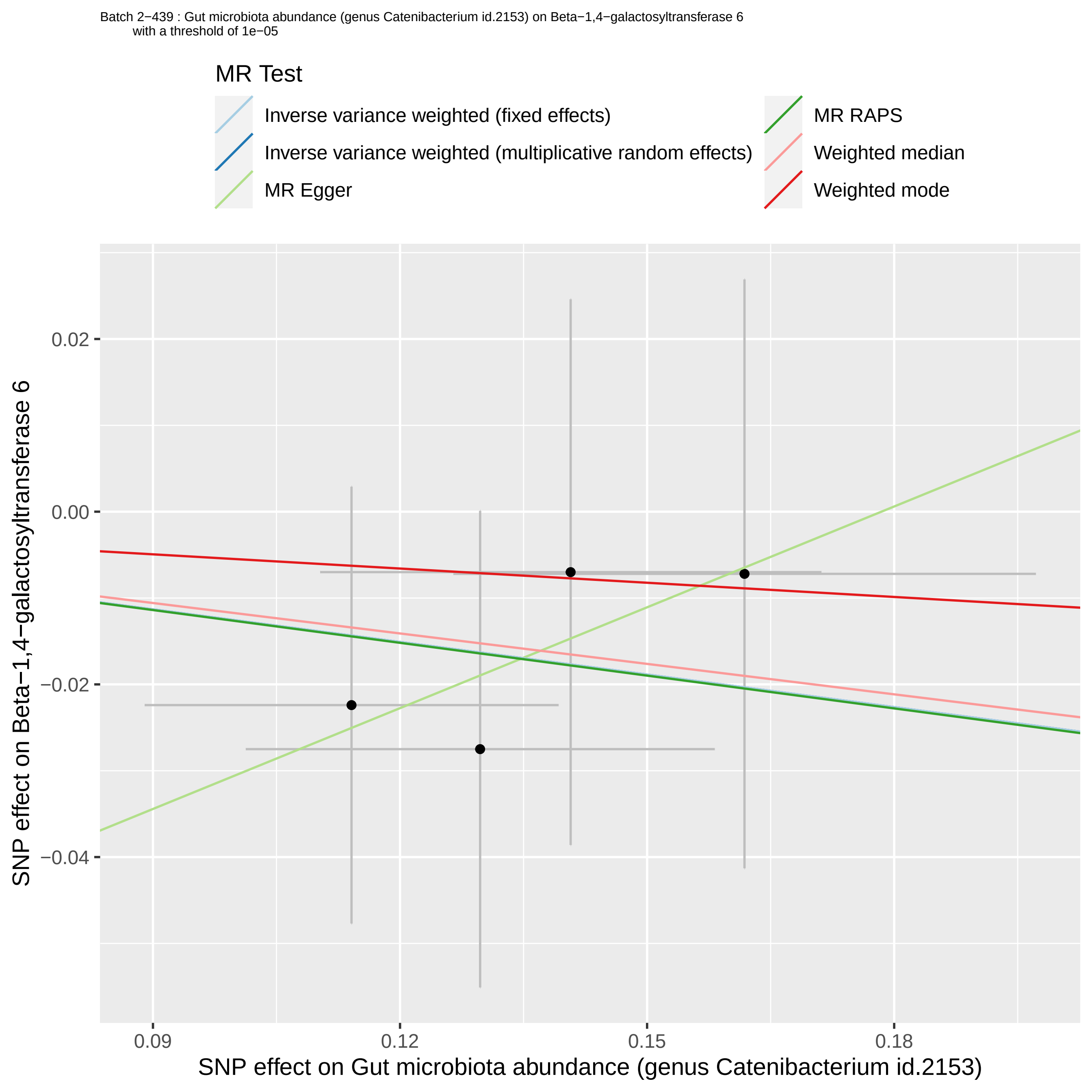
Batch 2−439 : Gut microbiota abundance (genus Catenibacterium id.2153) on Beta−1,4−galactosyltransferase 6
with a threshold of 1e−05
MR Test
Inverse variance weighted (fixed effects)
Inverse variance weighted (multiplicative random effects)
MR Egger
MR RAPS
Weighted median
Weighted mode
0.02
0.00
−0.02
−0.04
SNP effect on Beta−1,4−galactosyltransferase 6
0.09
0.12
0.15
0.18
SNP effect on Gut microbiota abundance (genus Catenibacterium id.2153)

Batch 2−440 : Gut microbiota abundance (genus Parasutterella id.2892) on Beta−1,4−galactosyltransferase 6
with a threshold of 1e−05

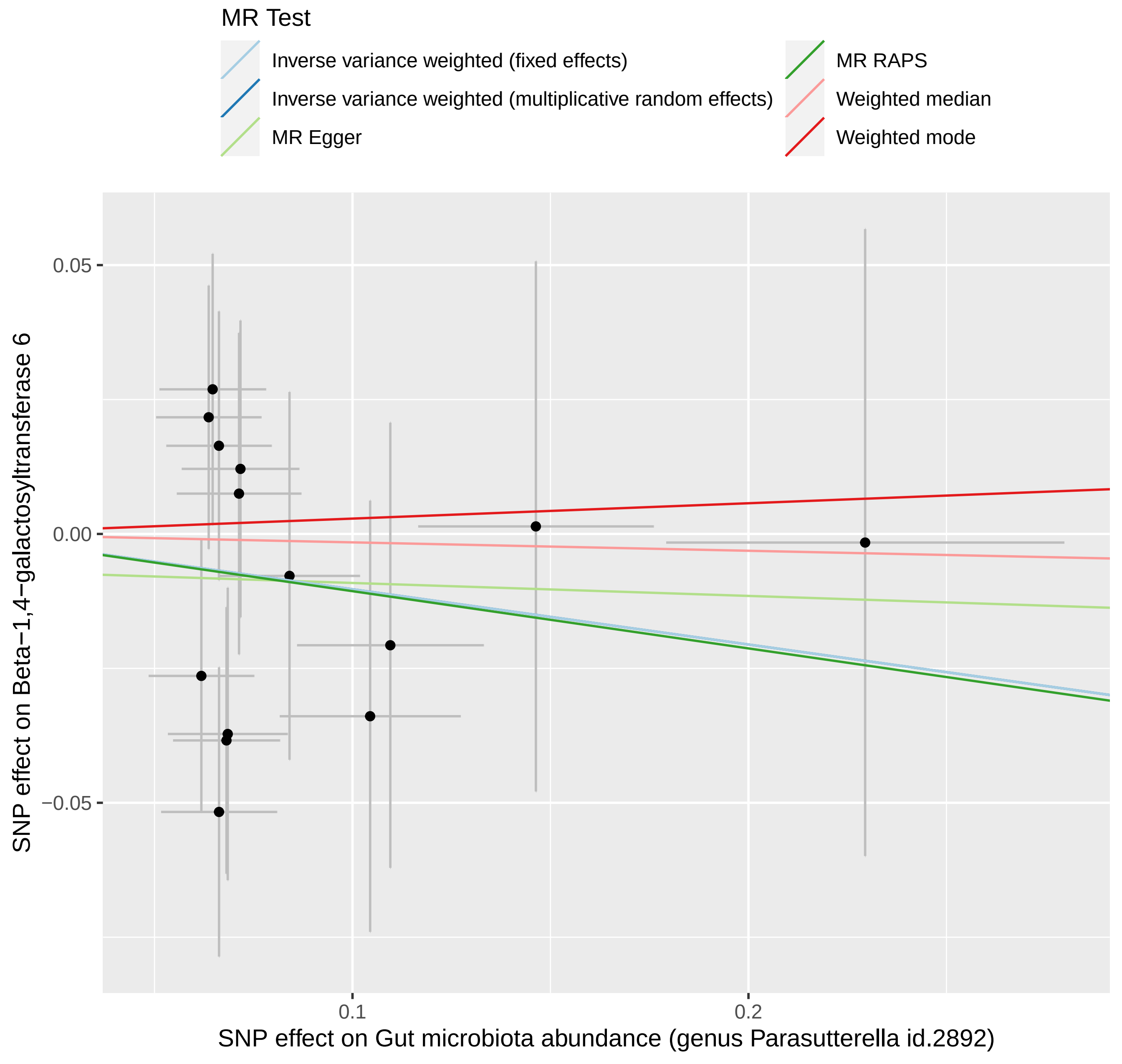

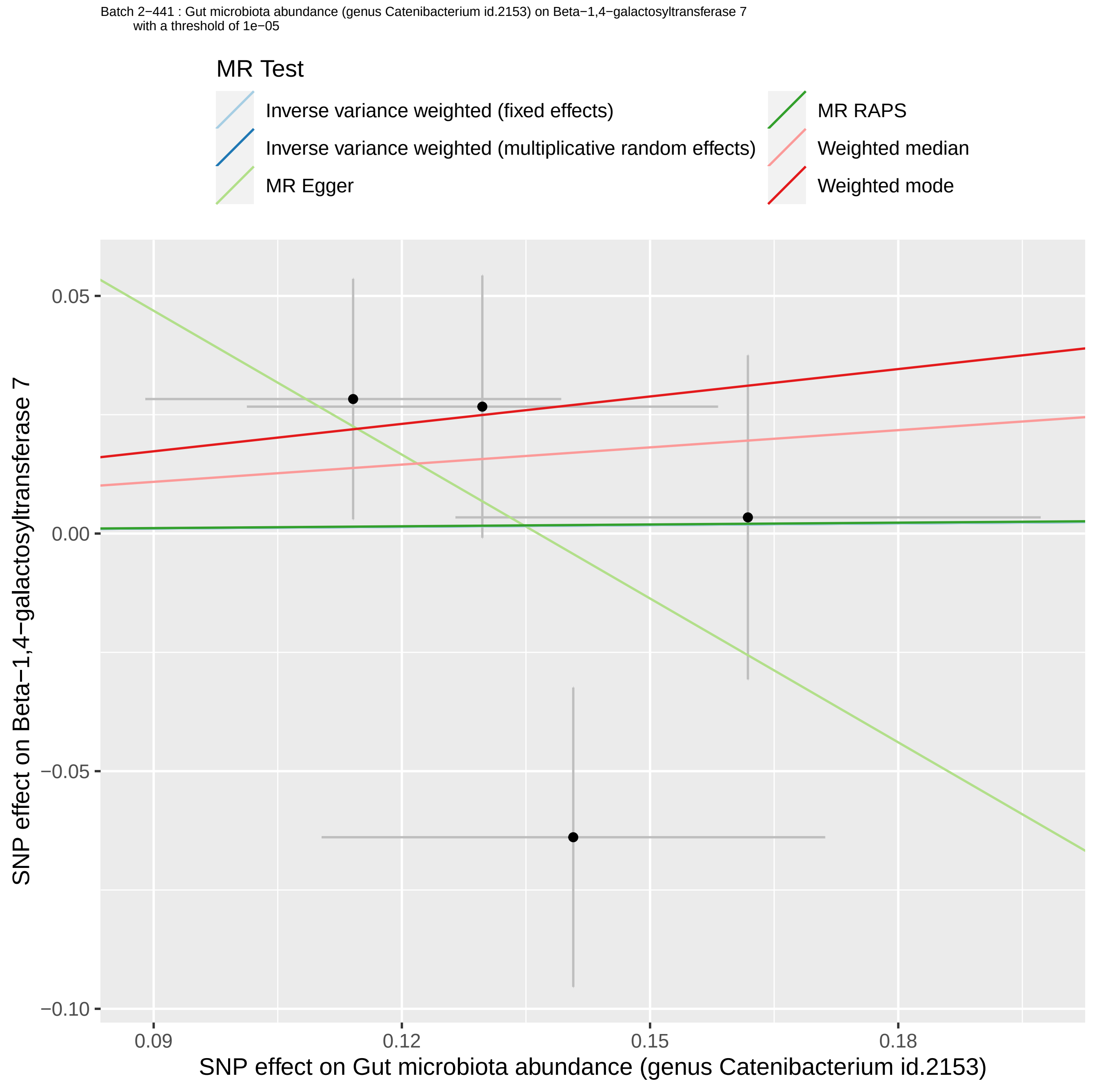

Batch 2−441 : Gut microbiota abundance (genus Catenibacterium id.2153) on Beta−1,4−galactosyltransferase 7
with a threshold of 1e−05
MR Test
Inverse variance weighted (fixed effects)
Inverse variance weighted (multiplicative random effects)
MR Egger
MR RAPS
Weighted median
Weighted mode
0.05
0.00
−0.05
−0.10
0.09
0.12
0.15
0.18
SNP effect on Beta−1,4−galactosyltransferase 7
SNP effect on Gut microbiota abundance (genus Catenibacterium id.2153)

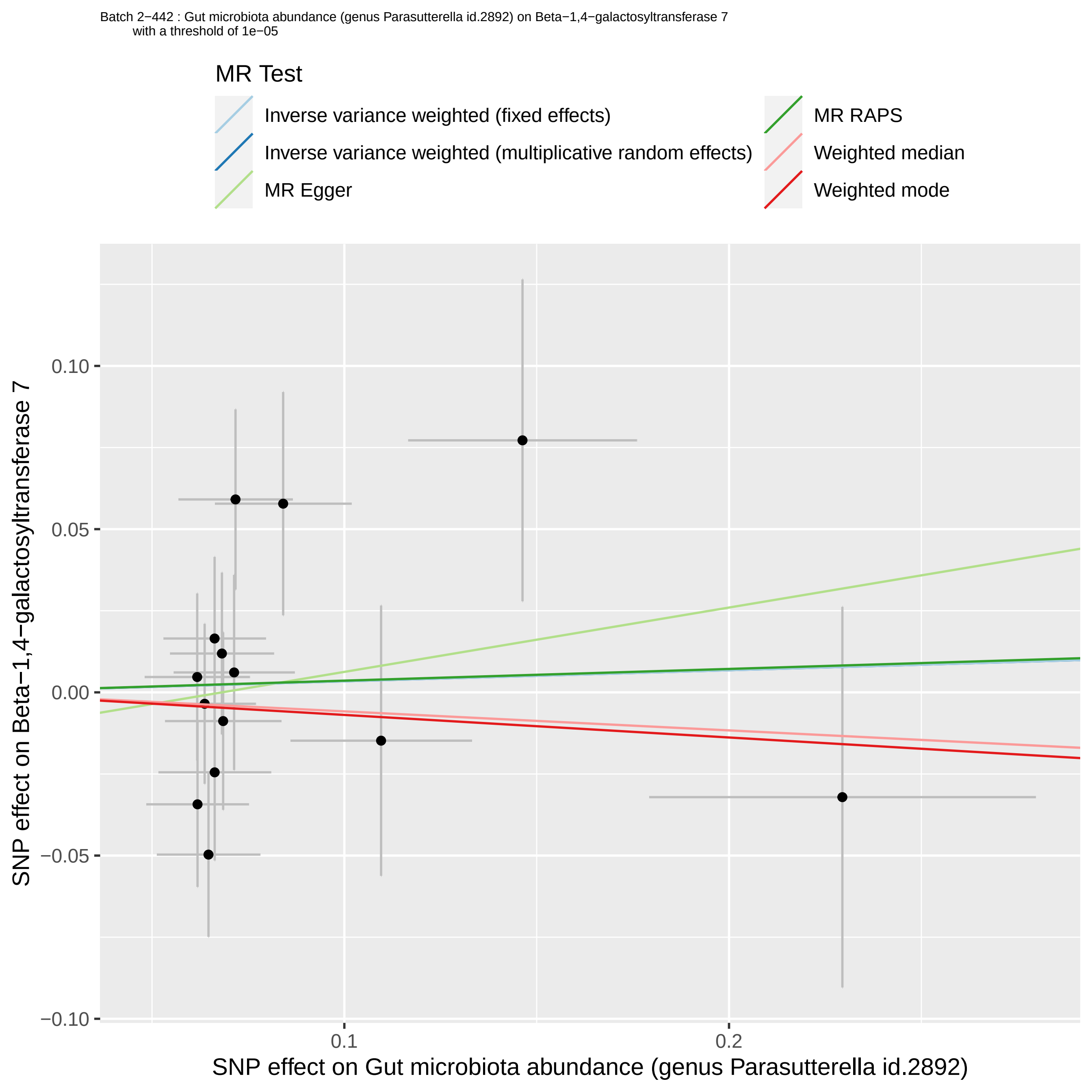
Batch 2−442 : Gut microbiota abundance (genus Parasutterella id.2892) on Beta−1,4−galactosyltransferase 7
with a threshold of 1e−05
MR Test
Inverse variance weighted (fixed effects)
Inverse variance weighted (multiplicative random effects)
MR Egger
MR RAPS
Weighted median
Weighted mode
SNP effect on Beta−1,4−galactosyltransferase 7
0.10
0.05
0.00
−0.05
−0.10
0.1
0.2
SNP effect on Gut microbiota abundance (genus Parasutterella id.2892)

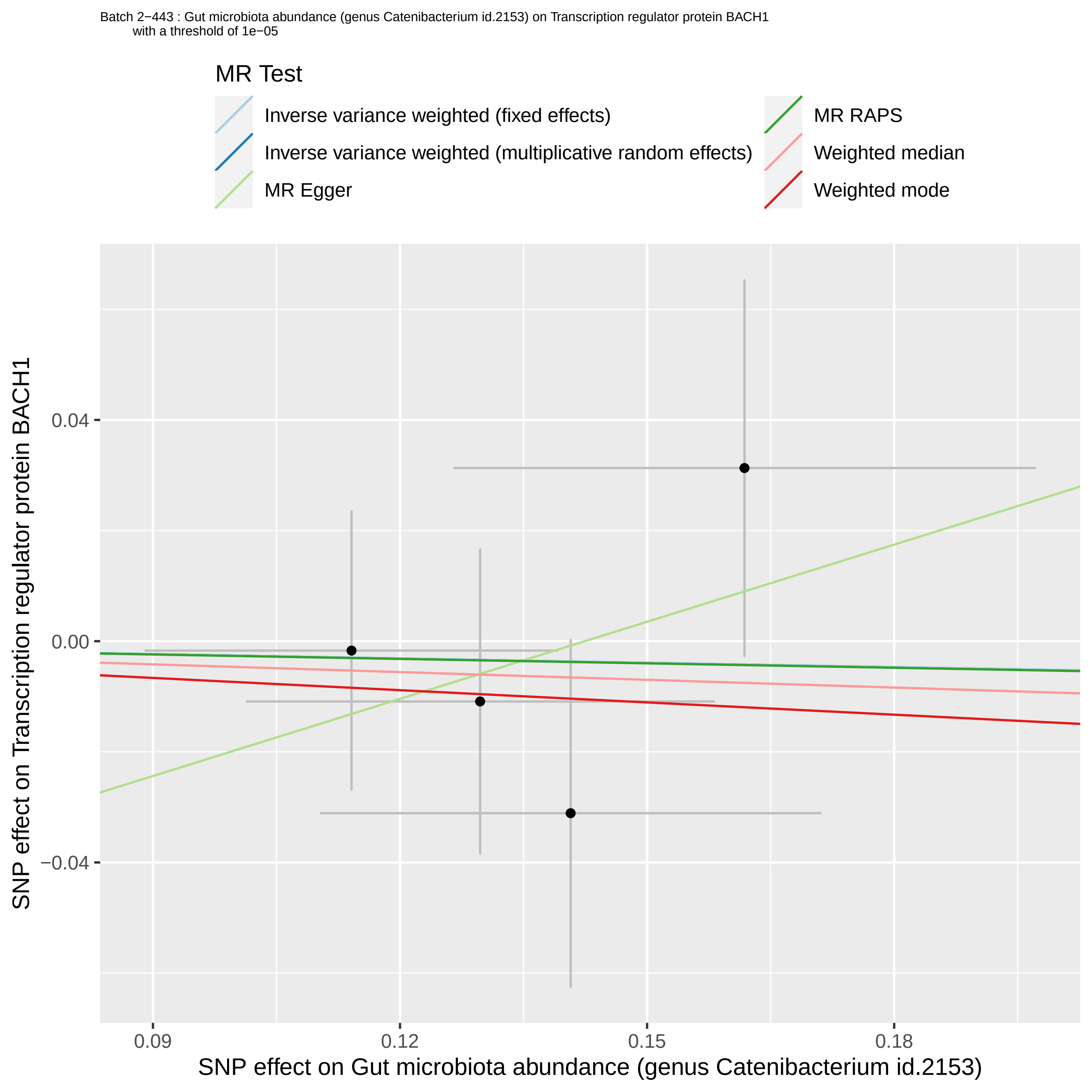

Batch 2−443 : Gut microbiota abundance (genus Catenibacterium id.2153) on Transcription regulator protein BACH1
with a threshold of 1e−05
MR Test
Inverse variance weighted (fixed effects)
Inverse variance weighted (multiplicative random effects)
MR Egger
MR RAPS
Weighted median
Weighted mode
0.04
0.00
−0.04
0.09
0.12
0.15
0.18
SNP effect on Transcription regulator protein BACH1
SNP effect on Gut microbiota abundance (genus Catenibacterium id.2153)

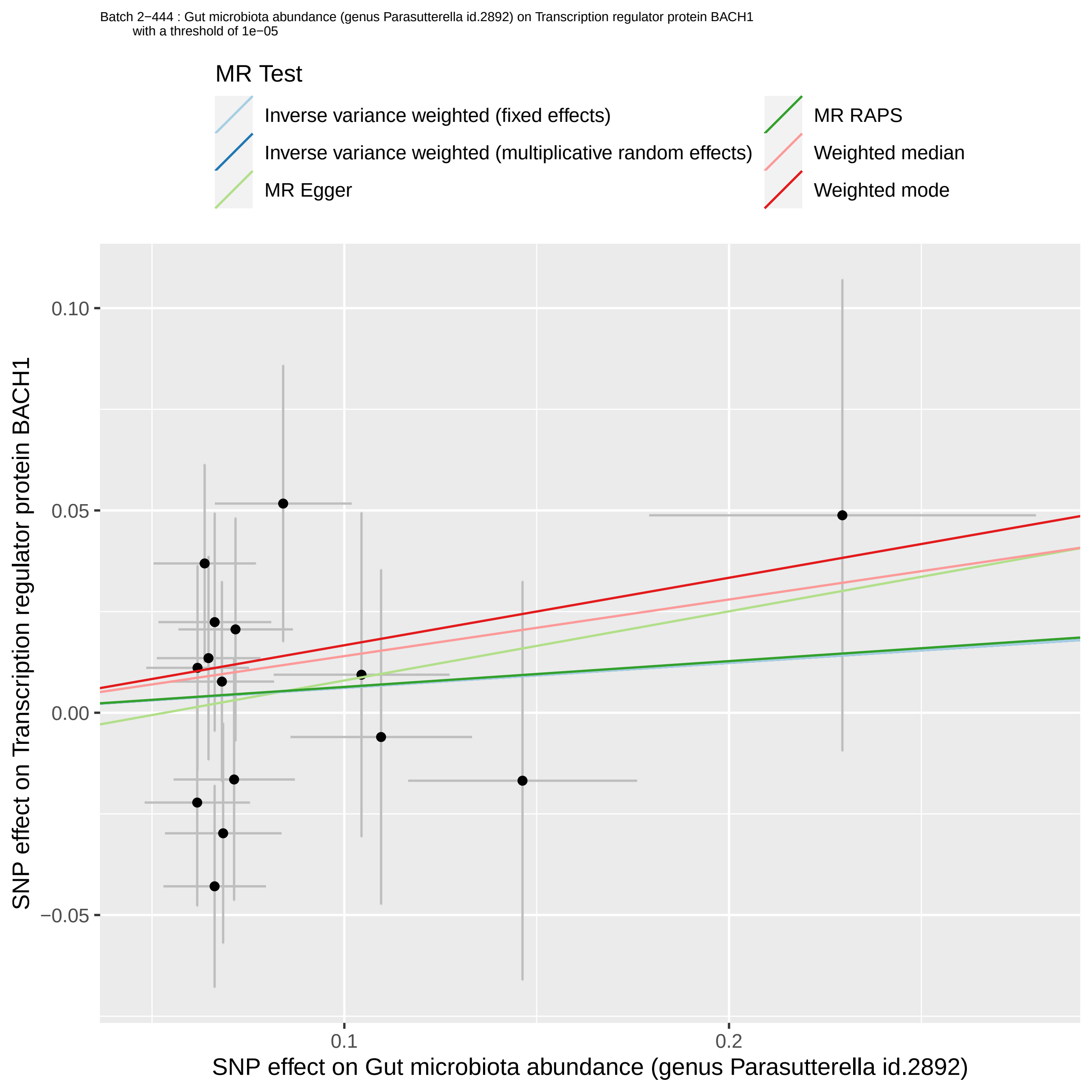
Batch 2−444 : Gut microbiota abundance (genus Parasutterella id.2892) on Transcription regulator protein BACH1
with a threshold of 1e−05
MR Test
Inverse variance weighted (fixed effects)
Inverse variance weighted (multiplicative random effects)
MR Egger
MR RAPS
Weighted median
Weighted mode
0.10
0.05
0.00
−0.05
0.1
0.2
SNP effect on Transcription regulator protein BACH1
SNP effect on Gut microbiota abundance (genus Parasutterella id.2892)

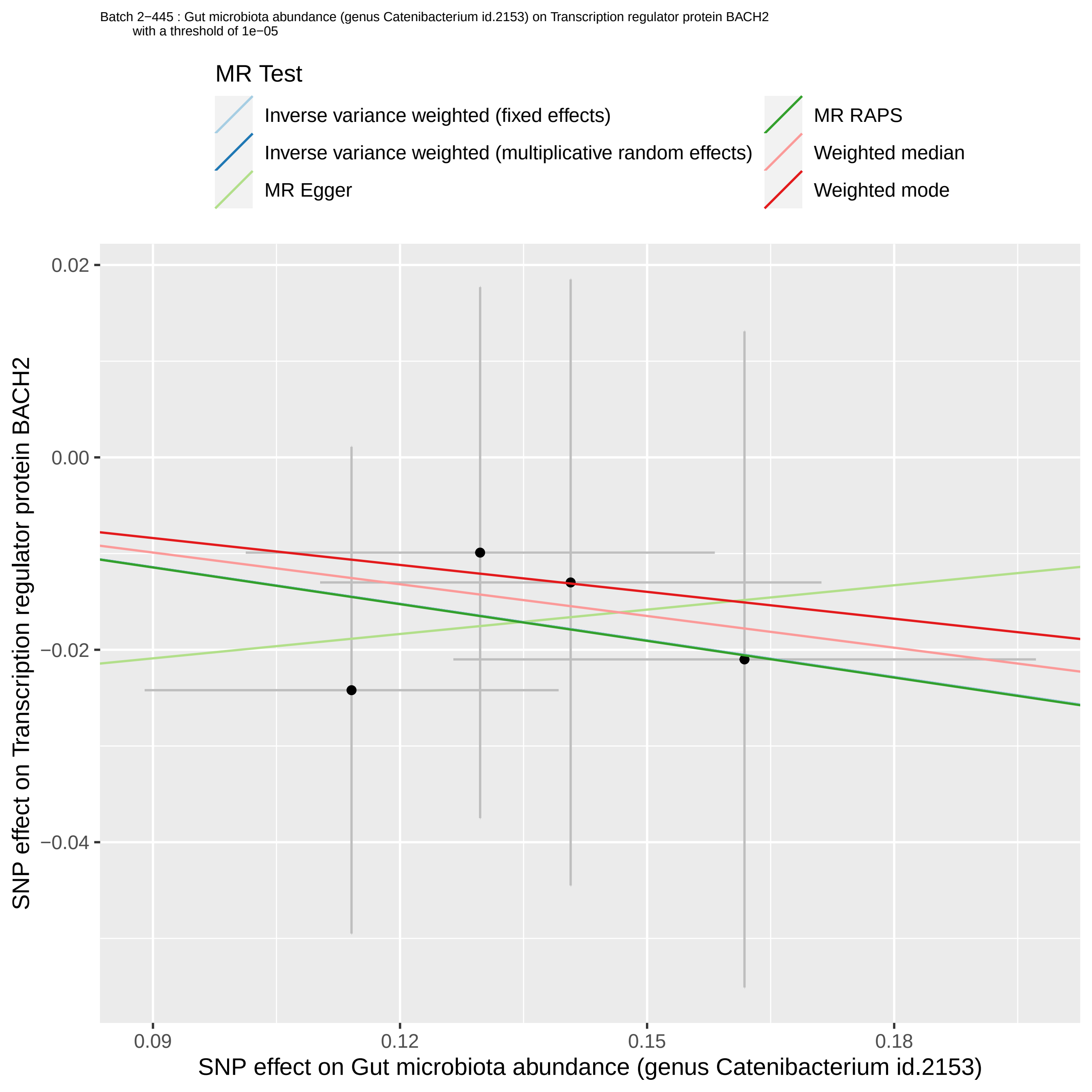

Batch 2−445 : Gut microbiota abundance (genus Catenibacterium id.2153) on Transcription regulator protein BACH2
with a threshold of 1e−05
MR Test
Inverse variance weighted (fixed effects)
Inverse variance weighted (multiplicative random effects)
MR Egger
MR RAPS
Weighted median
Weighted mode
0.02
0.00
−0.02
−0.04
0.09
0.12
0.15
0.18
SNP effect on Transcription regulator protein BACH2
SNP effect on Gut microbiota abundance (genus Catenibacterium id.2153)

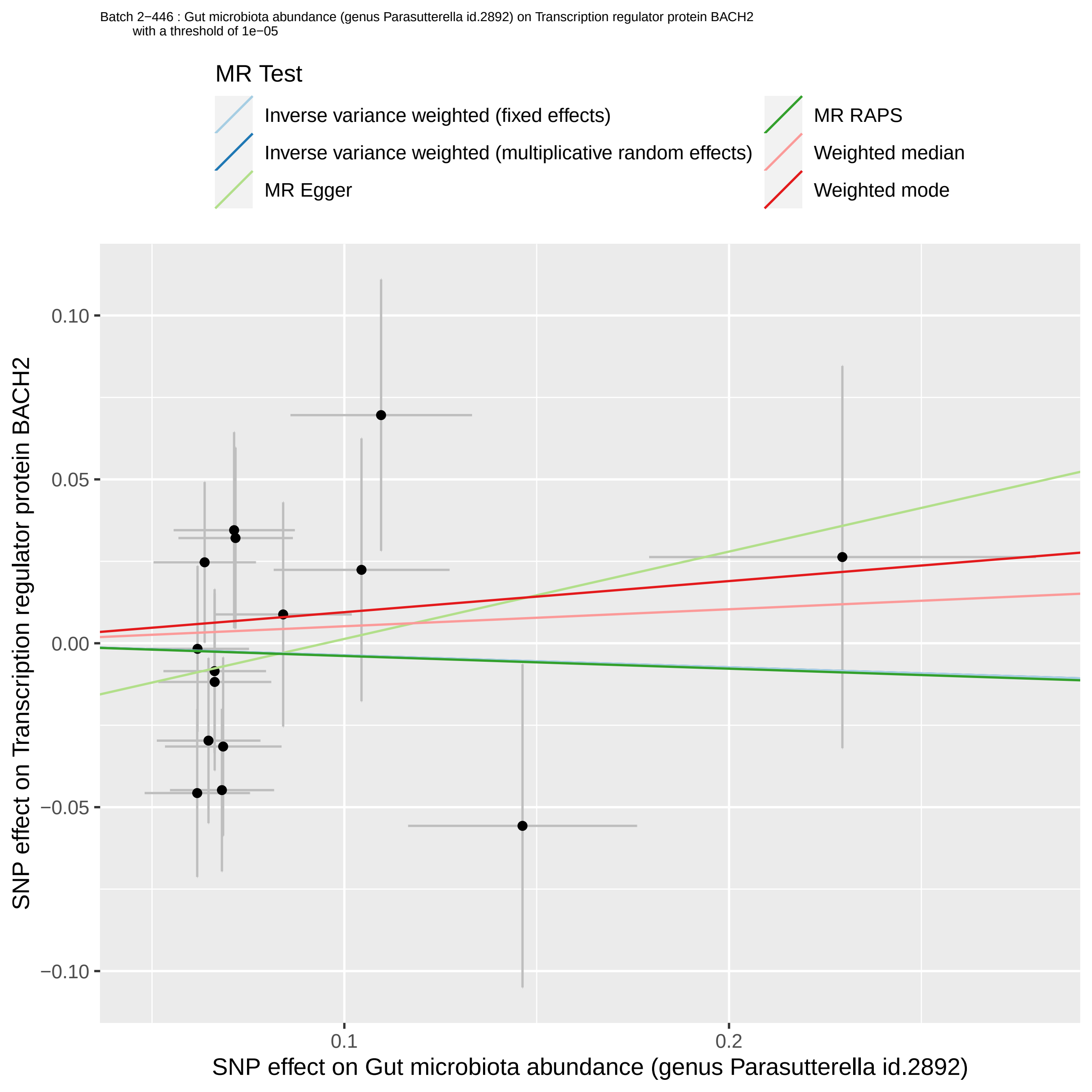

Batch 2−446 : Gut microbiota abundance (genus Parasutterella id.2892) on Transcription regulator protein BACH2
with a threshold of 1e−05
MR Test
Inverse variance weighted (fixed effects)
Inverse variance weighted (multiplicative random effects)
MR Egger
MR RAPS
Weighted median
Weighted mode
0.10
0.05
0.00
−0.05
−0.10
0.1
0.2
SNP effect on Transcription regulator protein BACH2
SNP effect on Gut microbiota abundance (genus Parasutterella id.2892)

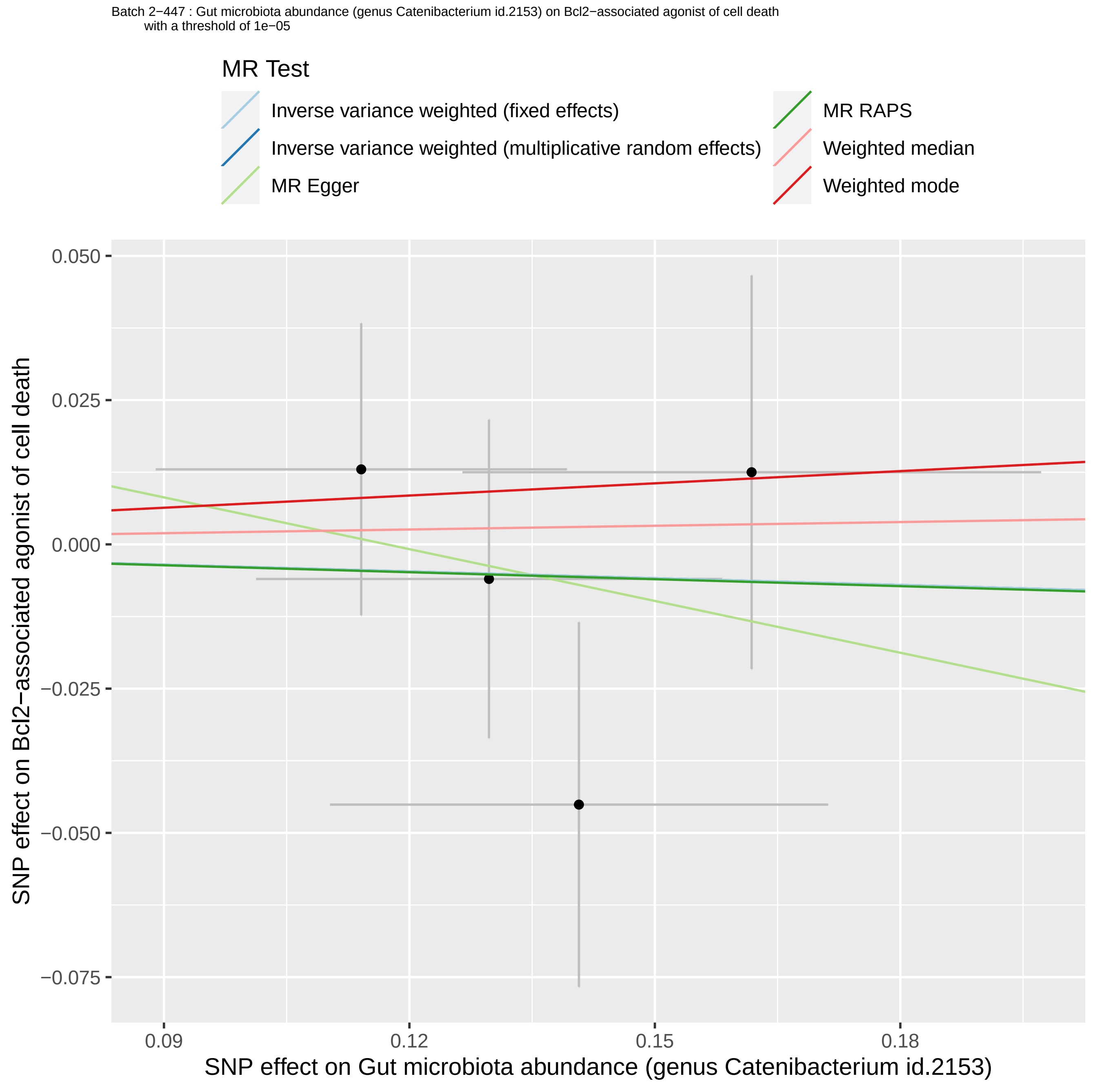

Batch 2−447 : Gut microbiota abundance (genus Catenibacterium id.2153) on Bcl2−associated agonist of cell death
with a threshold of 1e−05
MR Test
Inverse variance weighted (fixed effects)
Inverse variance weighted (multiplicative random effects)
MR Egger
MR RAPS
Weighted median
Weighted mode
0.050
0.025
0.000
−0.025
−0.050
−0.075
0.09
0.12
0.15
0.18
SNP effect on Bcl2−associated agonist of cell death
SNP effect on Gut microbiota abundance (genus Catenibacterium id.2153)

Batch 2−448 : Gut microbiota abundance (genus Parasutterella id.2892) on Bcl2−associated agonist of cell death
with a threshold of 1e−05

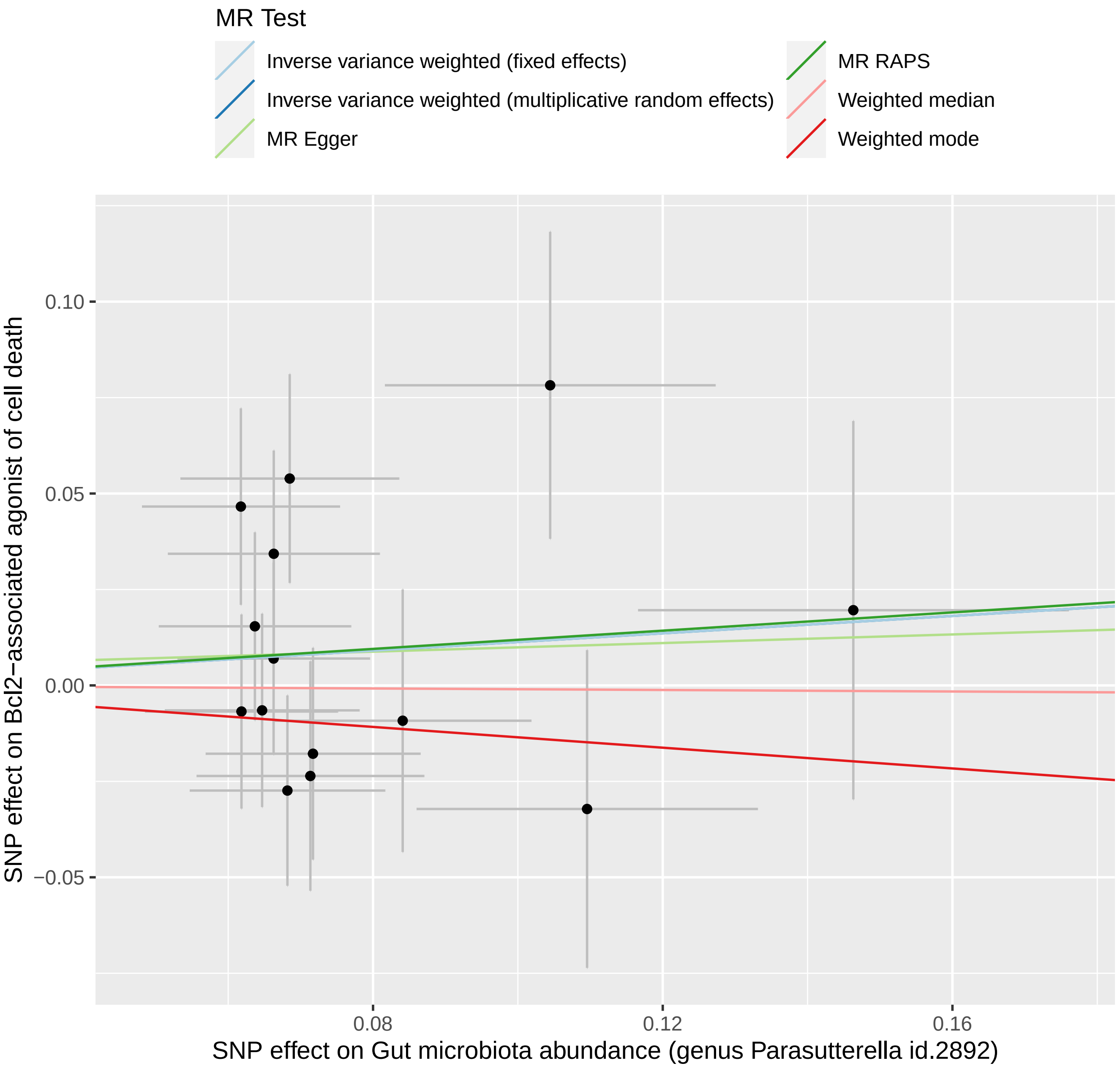

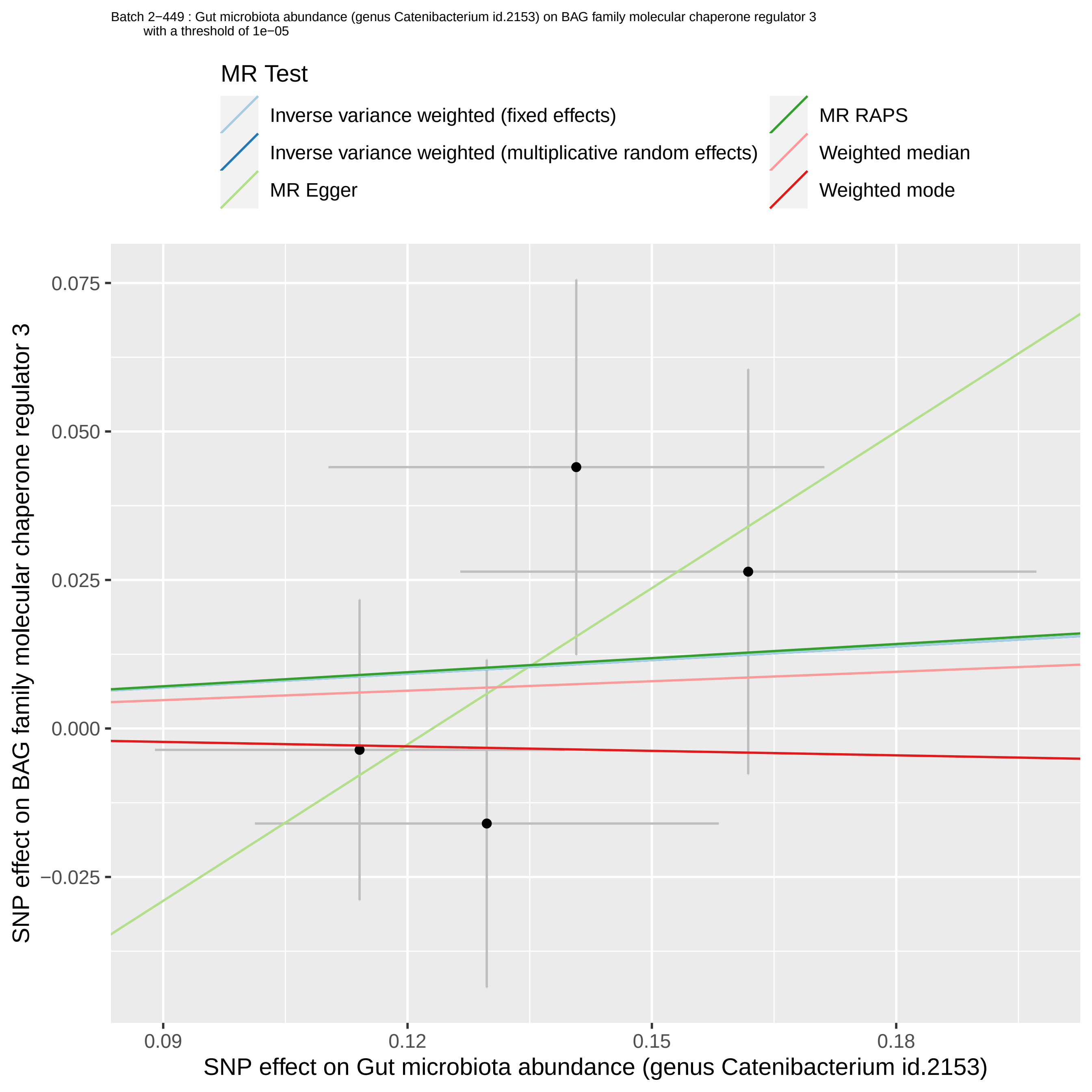
Batch 2−449 : Gut microbiota abundance (genus Catenibacterium id.2153) on BAG family molecular chaperone regulator 3 with a threshold of 1e−05
MR Test
Inverse variance weighted (fixed effects)
Inverse variance weighted (multiplicative random effects)
MR Egger
MR RAPS
Weighted median
Weighted mode
0.075
0.050
0.025
0.000
−0.025
0.09
0.12
0.15
0.18
SNP effect on BAG family molecular chaperone regulator 3
SNP effect on Gut microbiota abundance (genus Catenibacterium id.2153)

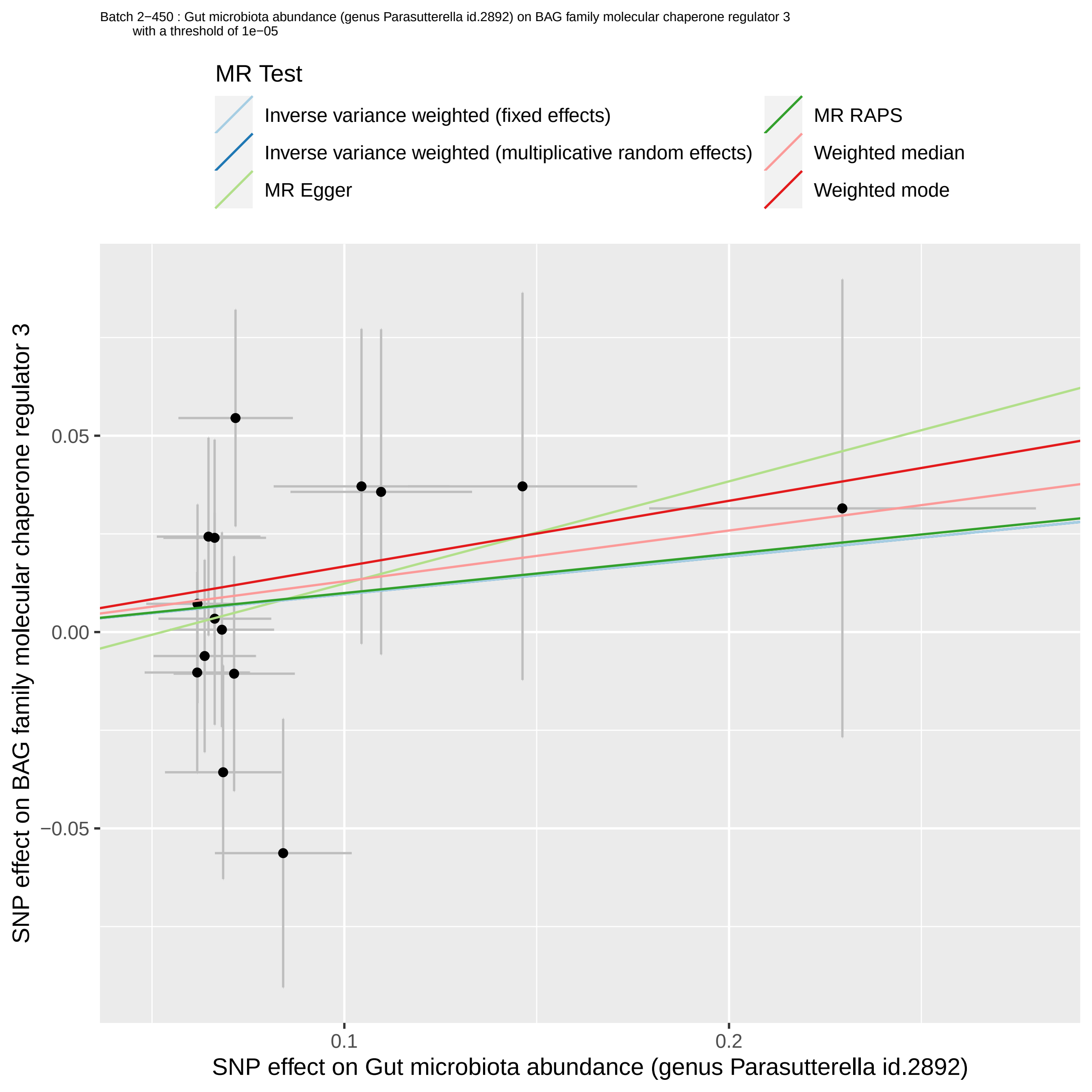
Batch 2−450 : Gut microbiota abundance (genus Parasutterella id.2892) on BAG family molecular chaperone regulator 3
with a threshold of 1e−05
MR Test
Inverse variance weighted (fixed effects)
Inverse variance weighted (multiplicative random effects)
MR Egger
MR RAPS
Weighted median
Weighted mode
0.05
0.00
−0.05
0.1
0.2
SNP effect on BAG family molecular chaperone regulator 3
SNP effect on Gut microbiota abundance (genus Parasutterella id.2892)

Batch 2−451 : Gut microbiota abundance (genus Catenibacterium id.2153) on BAG family molecular chaperone regulator 4
with a threshold of 1e−05

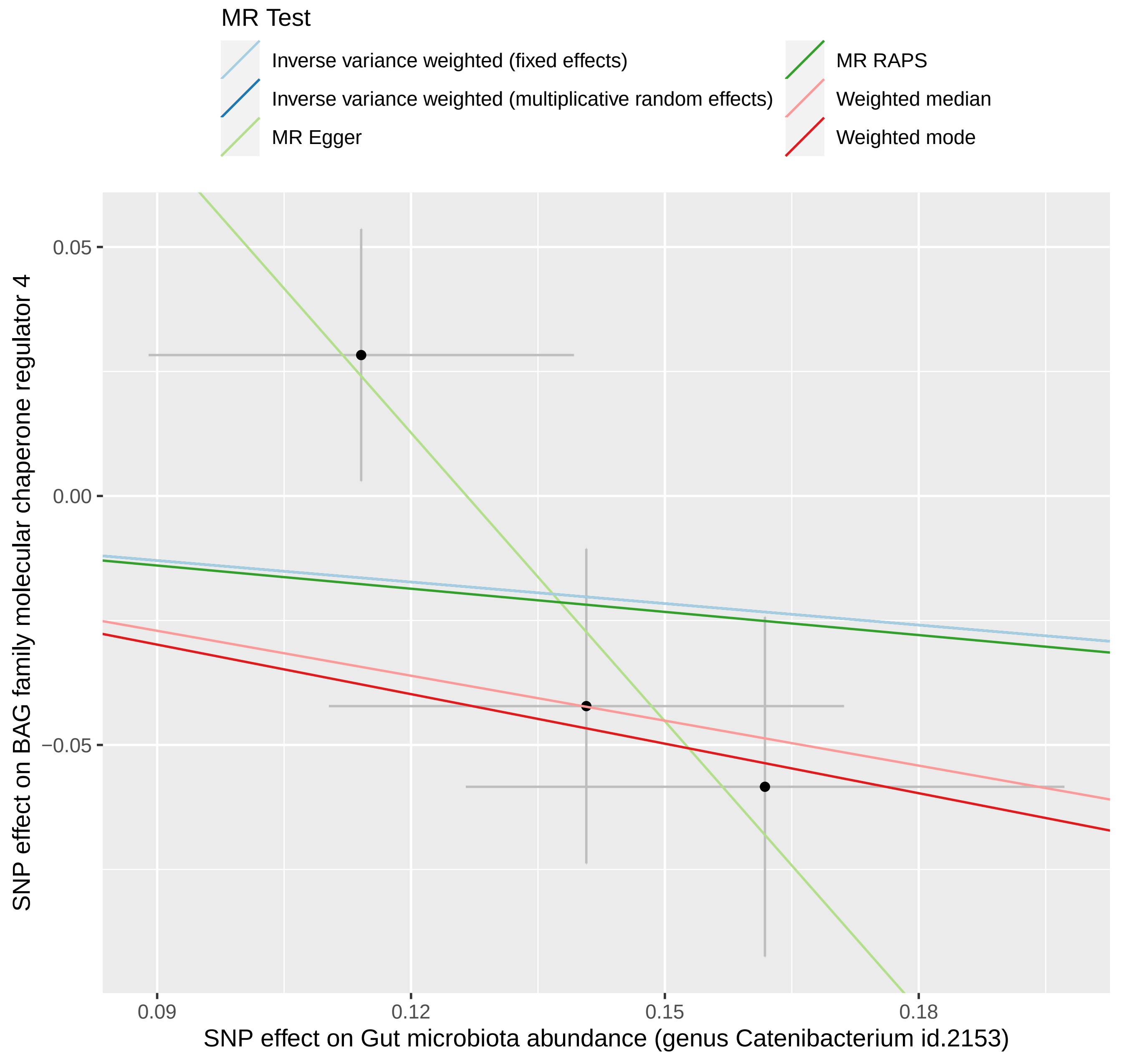

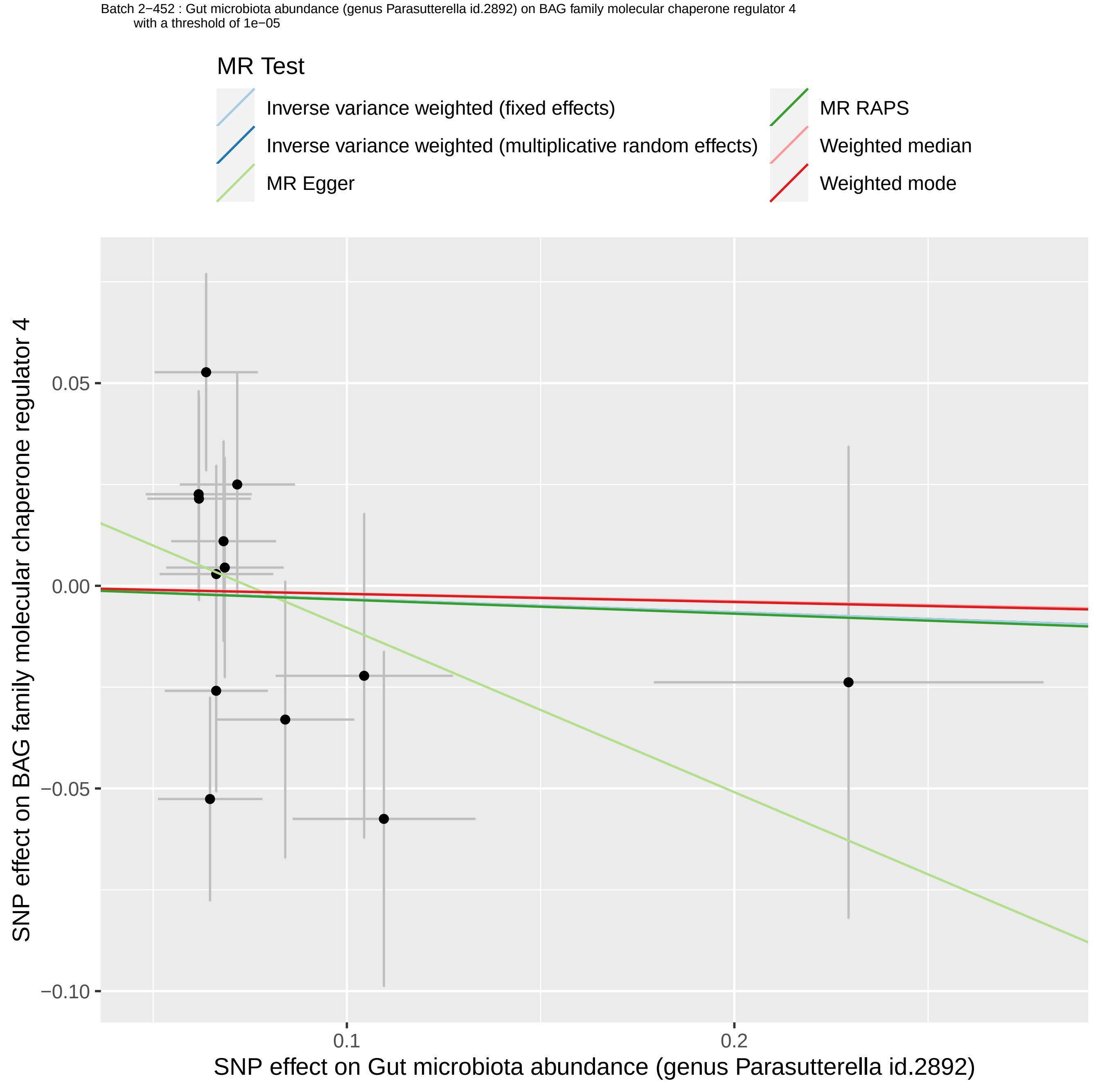

Batch 2−452 : Gut microbiota abundance (genus Parasutterella id.2892) on BAG family molecular chaperone regulator 4
with a threshold of 1e−05
MR Test
Inverse variance weighted (fixed effects)
Inverse variance weighted (multiplicative random effects)
MR Egger
MR RAPS
Weighted median
Weighted mode
0.05
0.00
−0.05
−0.10
0.1
0.2
SNP effect on BAG family molecular chaperone regulator 4
SNP effect on Gut microbiota abundance (genus Parasutterella id.2892)

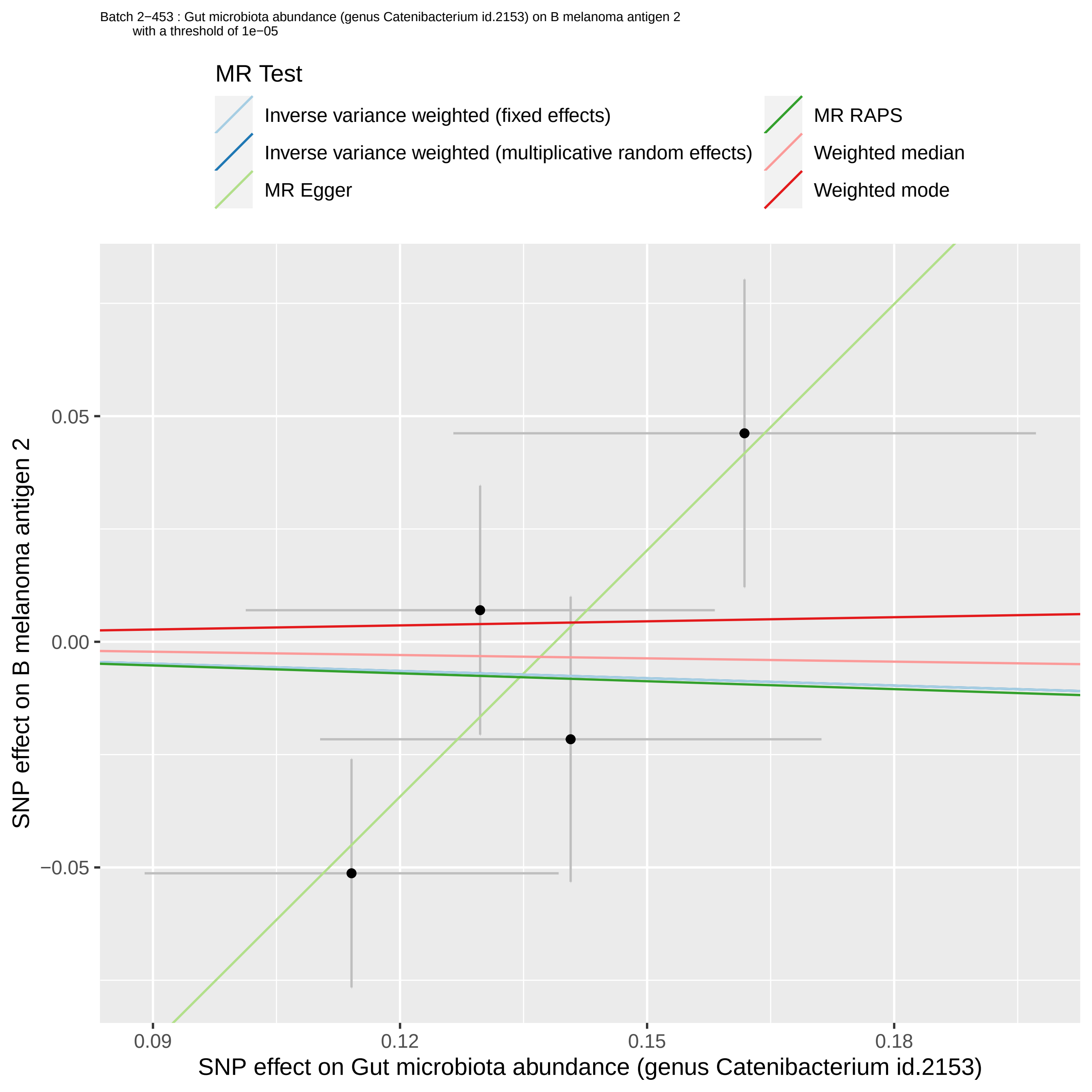
Batch 2−453 : Gut microbiota abundance (genus Catenibacterium id.2153) on B melanoma antigen 2
with a threshold of 1e−05
MR Test
Inverse variance weighted (fixed effects)
Inverse variance weighted (multiplicative random effects)
MR Egger
MR RAPS
Weighted median
Weighted mode
0.05
0.00
−0.05
0.09
0.12
0.15
0.18
SNP effect on B melanoma antigen 2
SNP effect on Gut microbiota abundance (genus Catenibacterium id.2153)

Batch 2−454 : Gut microbiota abundance (genus Parasutterella id.2892) on B melanoma antigen 2
with a threshold of 1e−05

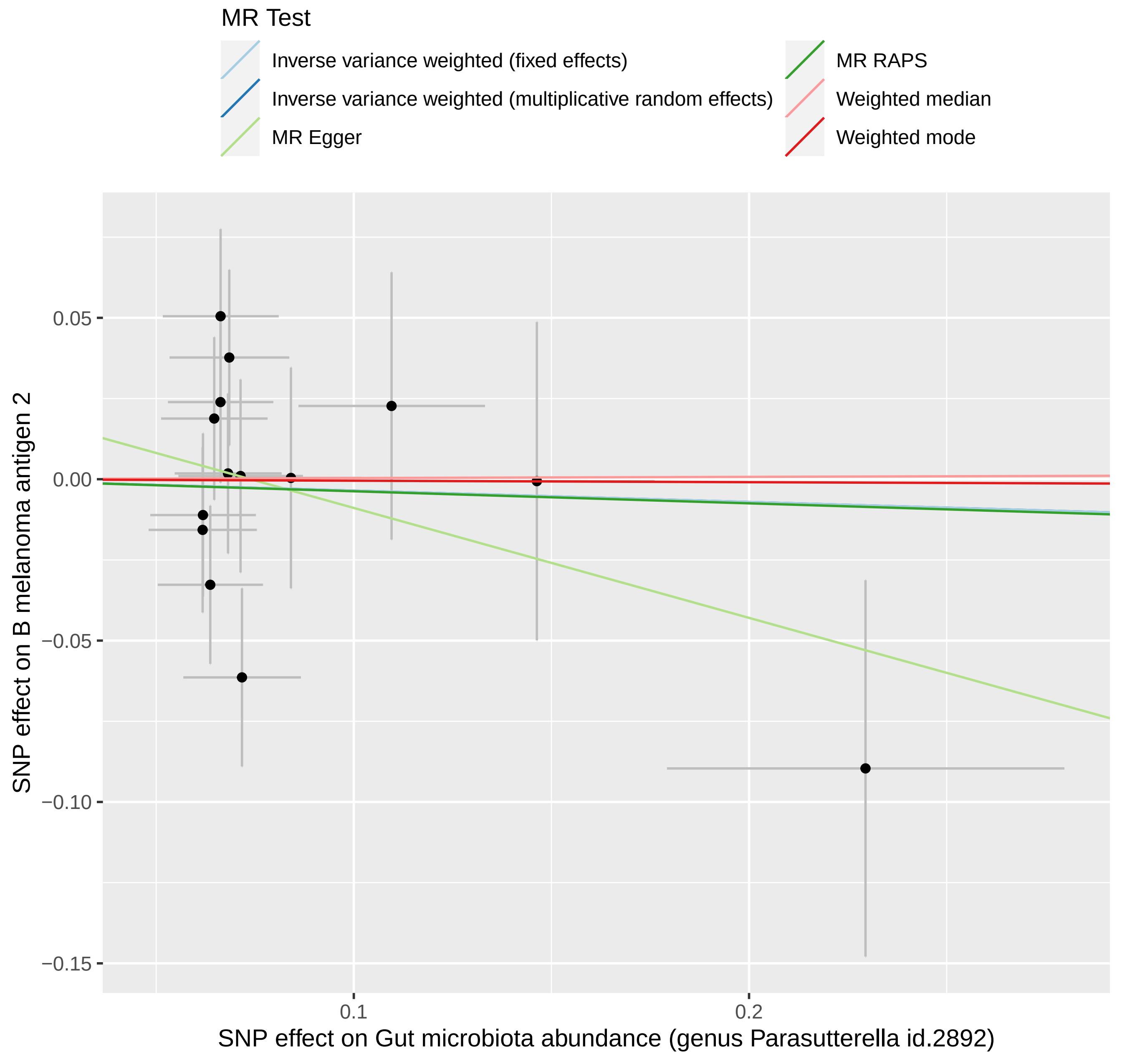

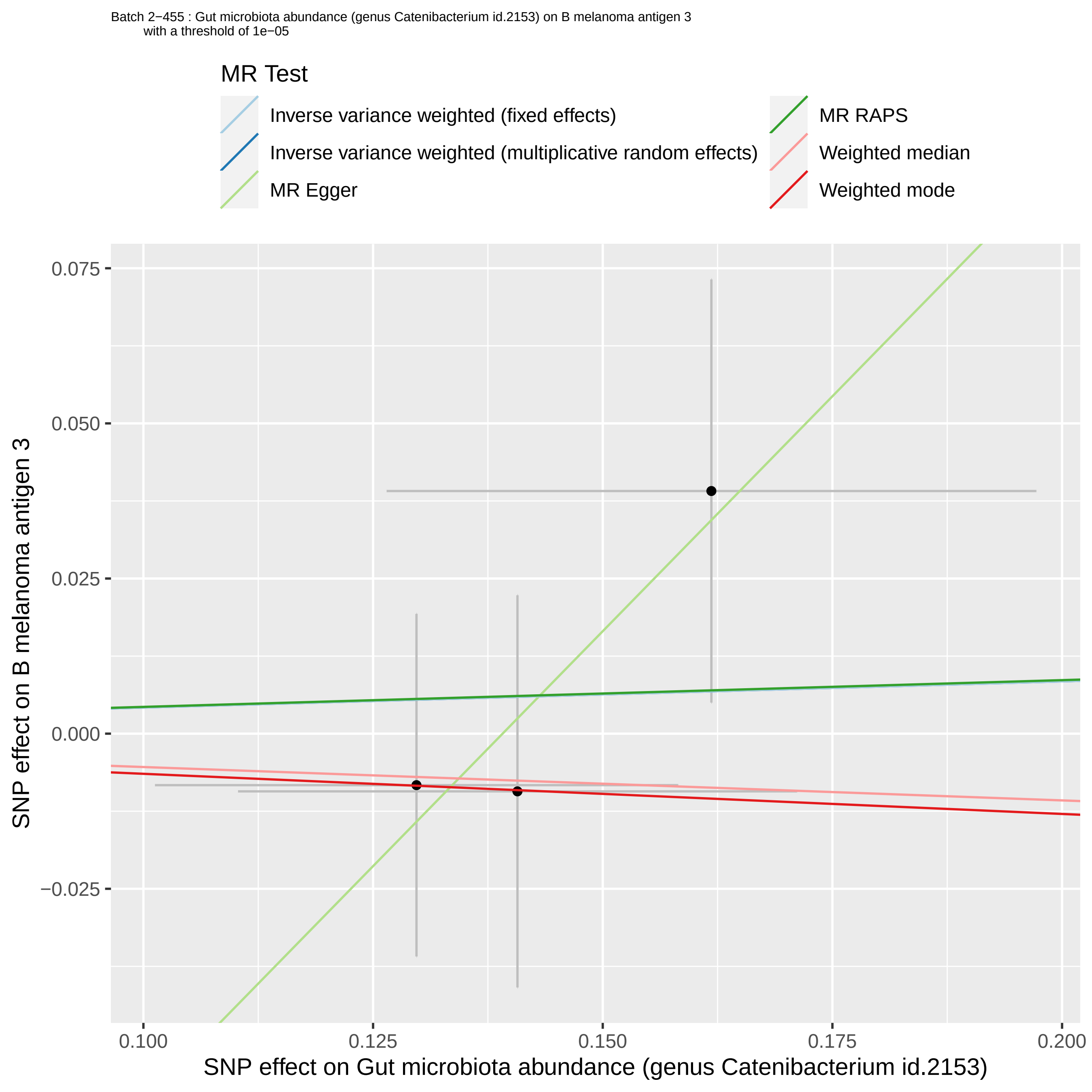
Batch 2−455 : Gut microbiota abundance (genus Catenibacterium id.2153) on B melanoma antigen 3
with a threshold of 1e−05
MR Test
Inverse variance weighted (fixed effects)
Inverse variance weighted (multiplicative random effects)
MR Egger
MR RAPS
Weighted median
Weighted mode
SNP effect on B melanoma antigen 3
0.075
0.050
0.025
0.000
−0.025
0.100
0.125
0.150
0.175
0.200
SNP effect on Gut microbiota abundance (genus Catenibacterium id.2153)

Batch 2−456 : Gut microbiota abundance (genus Parasutterella id.2892) on B melanoma antigen 3
with a threshold of 1e−05

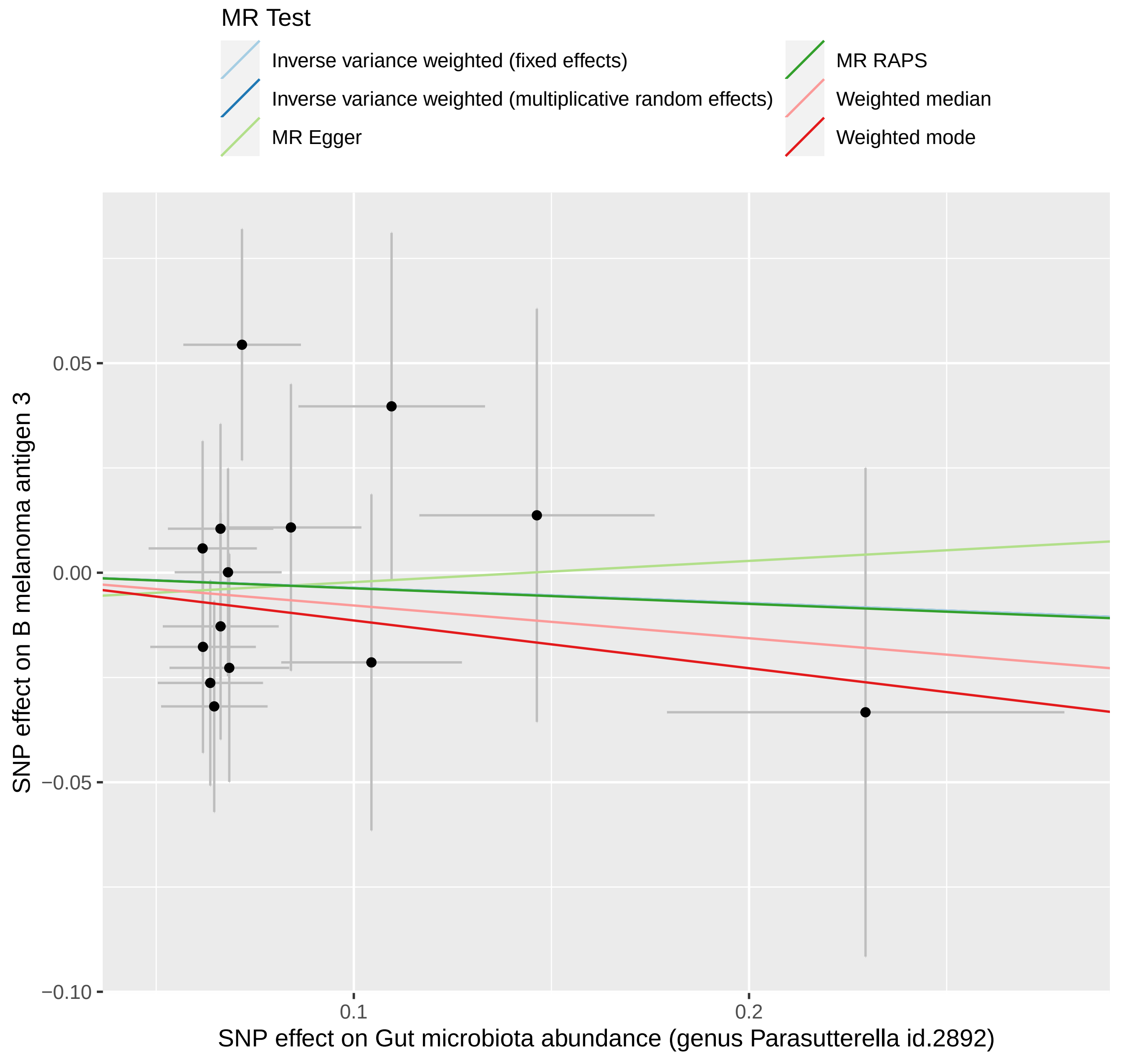

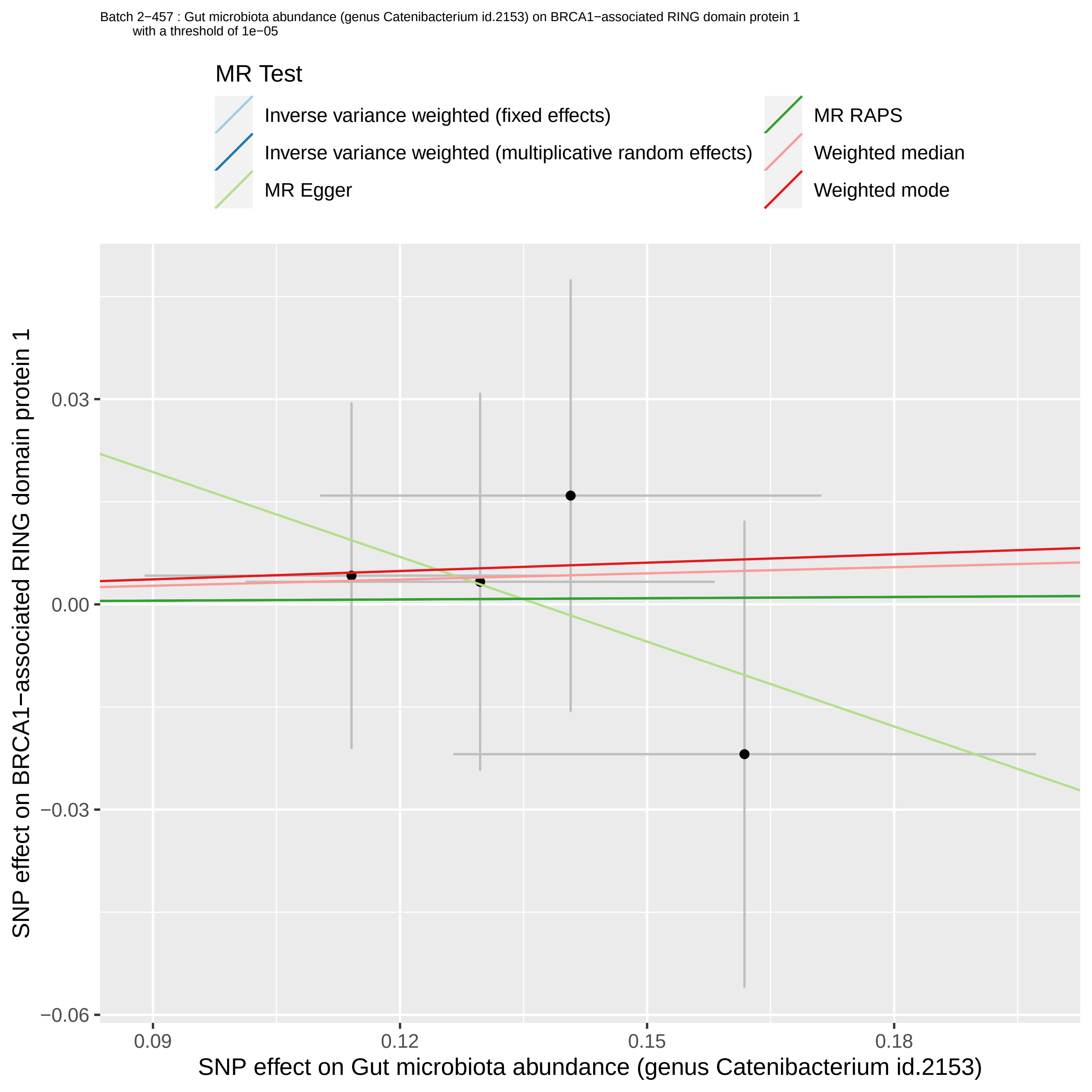
Batch 2−457 : Gut microbiota abundance (genus Catenibacterium id.2153) on BRCA1−associated RING domain protein 1
with a threshold of 1e−05
MR Test
Inverse variance weighted (fixed effects)
Inverse variance weighted (multiplicative random effects)
MR Egger
MR RAPS
Weighted median
Weighted mode
0.03
0.00
−0.03
−0.06
0.09
0.12
0.15
0.18
SNP effect on BRCA1−associated RING domain protein 1
SNP effect on Gut microbiota abundance (genus Catenibacterium id.2153)

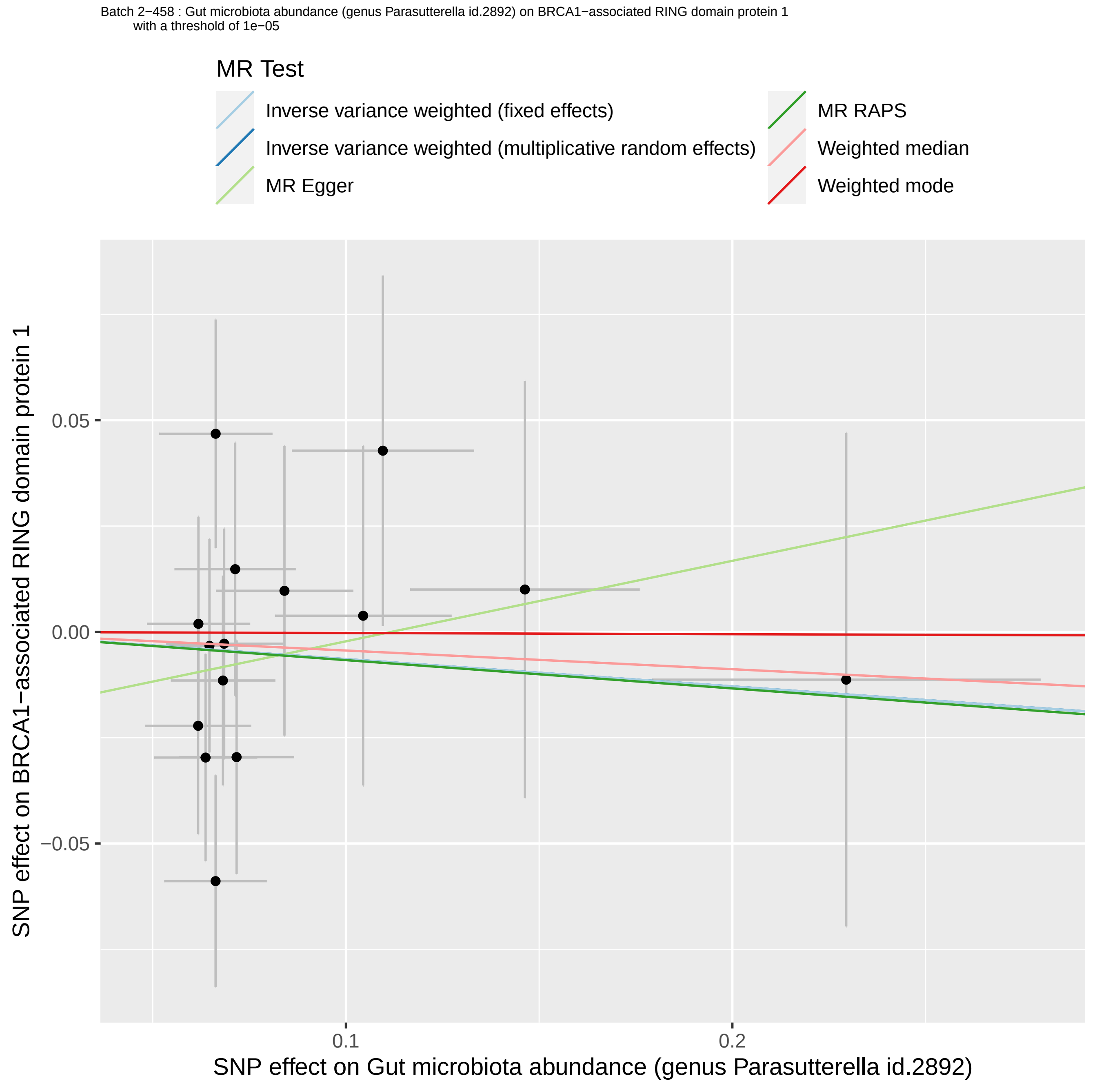
Batch 2−458 : Gut microbiota abundance (genus Parasutterella id.2892) on BRCA1−associated RING domain protein 1
with a threshold of 1e−05
MR Test
Inverse variance weighted (fixed effects)
Inverse variance weighted (multiplicative random effects)
MR Egger
MR RAPS
Weighted median
Weighted mode
0.05
0.00
−0.05
0.1
0.2
SNP effect on BRCA1−associated RING domain protein 1
SNP effect on Gut microbiota abundance (genus Parasutterella id.2892)

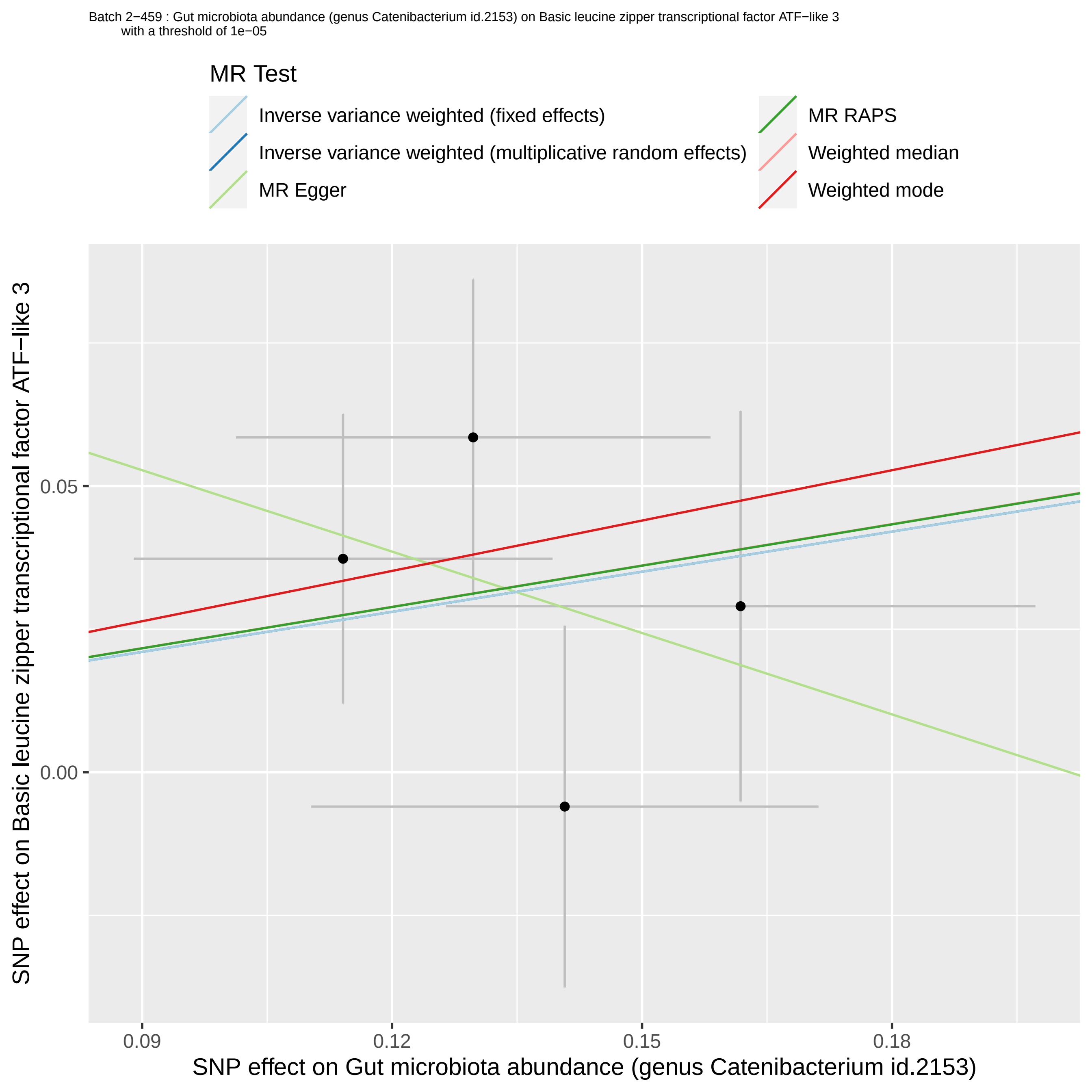

Batch 2−459 : Gut microbiota abundance (genus Catenibacterium id.2153) on Basic leucine zipper transcriptional factor ATF−like 3
with a threshold of 1e−05
MR Test
Inverse variance weighted (fixed effects)
Inverse variance weighted (multiplicative random effects)
MR Egger
MR RAPS
Weighted median
Weighted mode
SNP effect on Basic leucine zipper transcriptional factor ATF−like 3
0.05
0.00
0.09
0.12
0.15
0.18
SNP effect on Gut microbiota abundance (genus Catenibacterium id.2153)

Batch 2−460 : Gut microbiota abundance (genus Parasutterella id.2892) on Basic leucine zipper transcriptional factor ATF−like 3
with a threshold of 1e−05

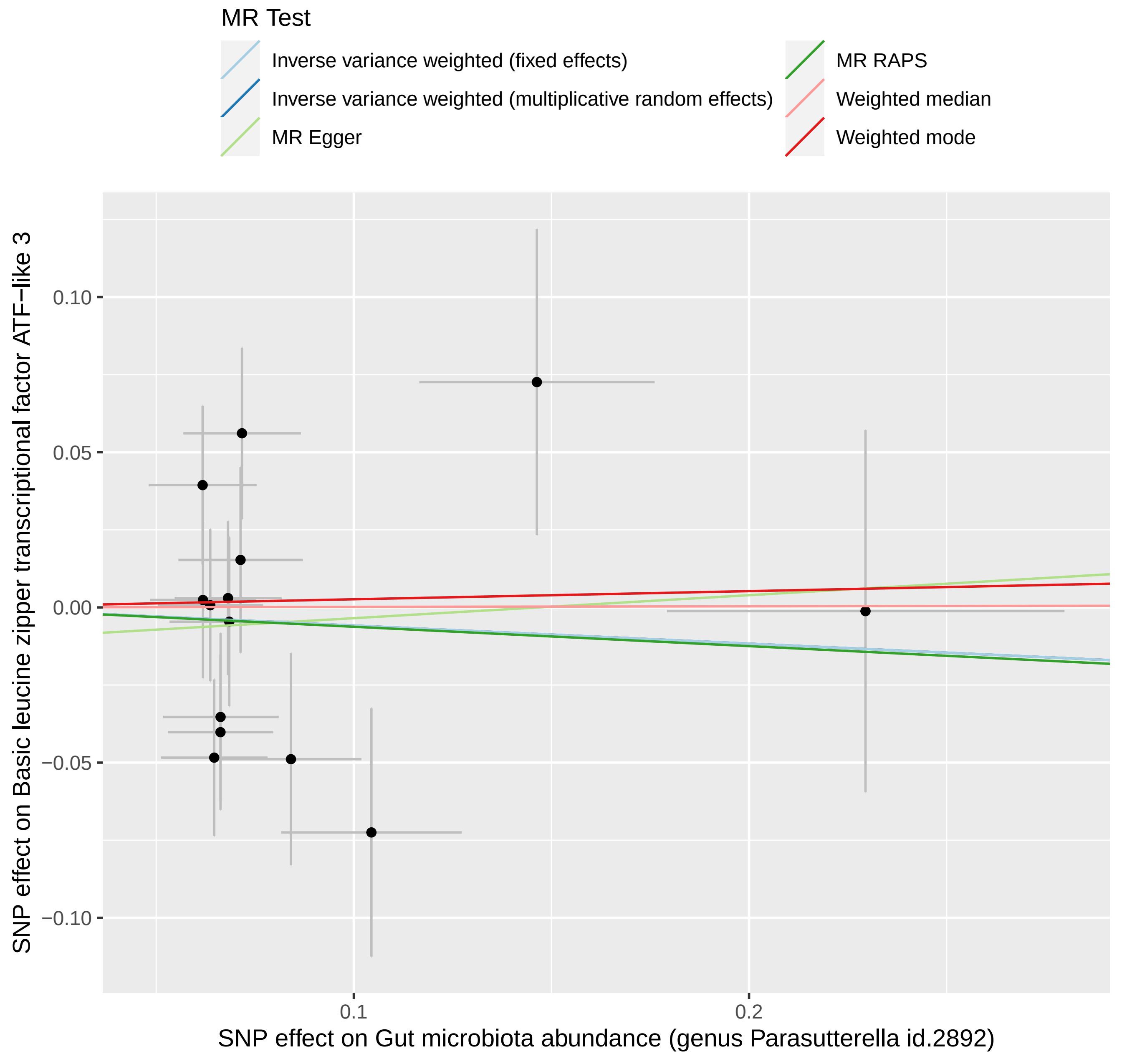

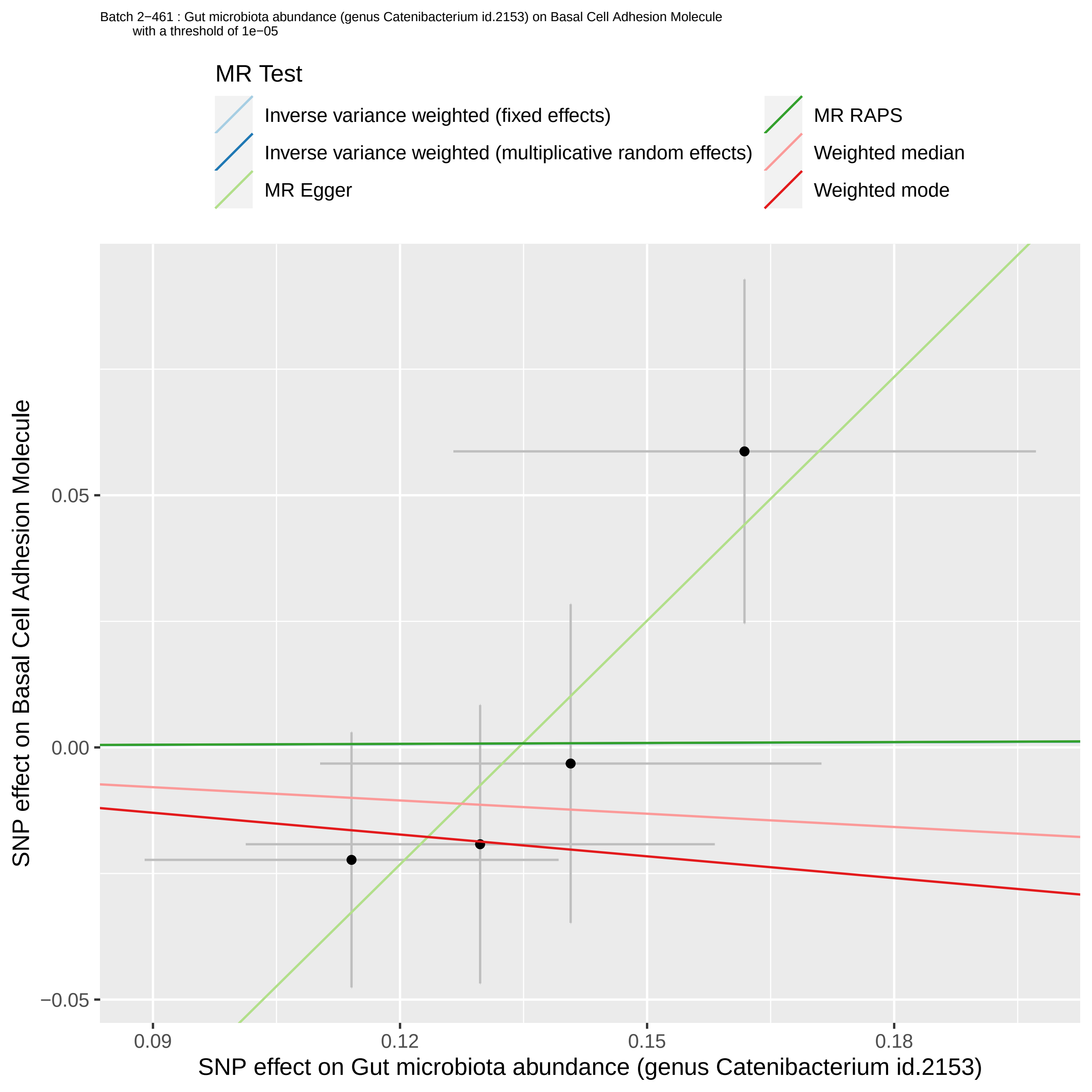

Batch 2−461 : Gut microbiota abundance (genus Catenibacterium id.2153) on Basal Cell Adhesion Molecule
with a threshold of 1e−05
MR Test
Inverse variance weighted (fixed effects)
Inverse variance weighted (multiplicative random effects)
MR Egger
MR RAPS
Weighted median
Weighted mode
0.05
0.00
−0.05
0.09
0.12
0.15
0.18
SNP effect on Basal Cell Adhesion Molecule
SNP effect on Gut microbiota abundance (genus Catenibacterium id.2153)

Batch 2−462 : Gut microbiota abundance (genus Parasutterella id.2892) on Basal Cell Adhesion Molecule
with a threshold of 1e−05

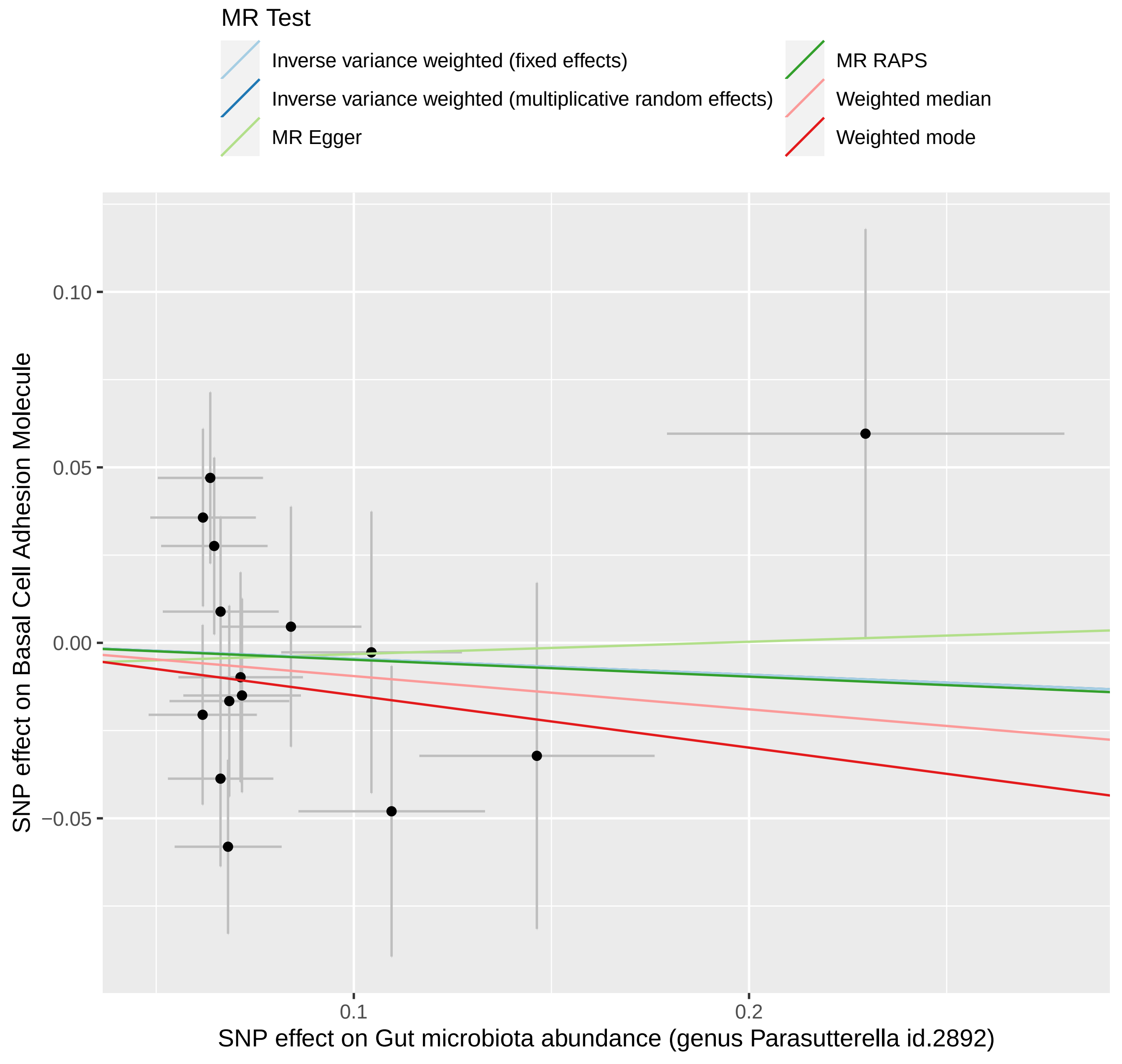

Batch 2−463 : Gut microbiota abundance (genus Catenibacterium id.2153) on Brevican core protein
with a threshold of 1e−05

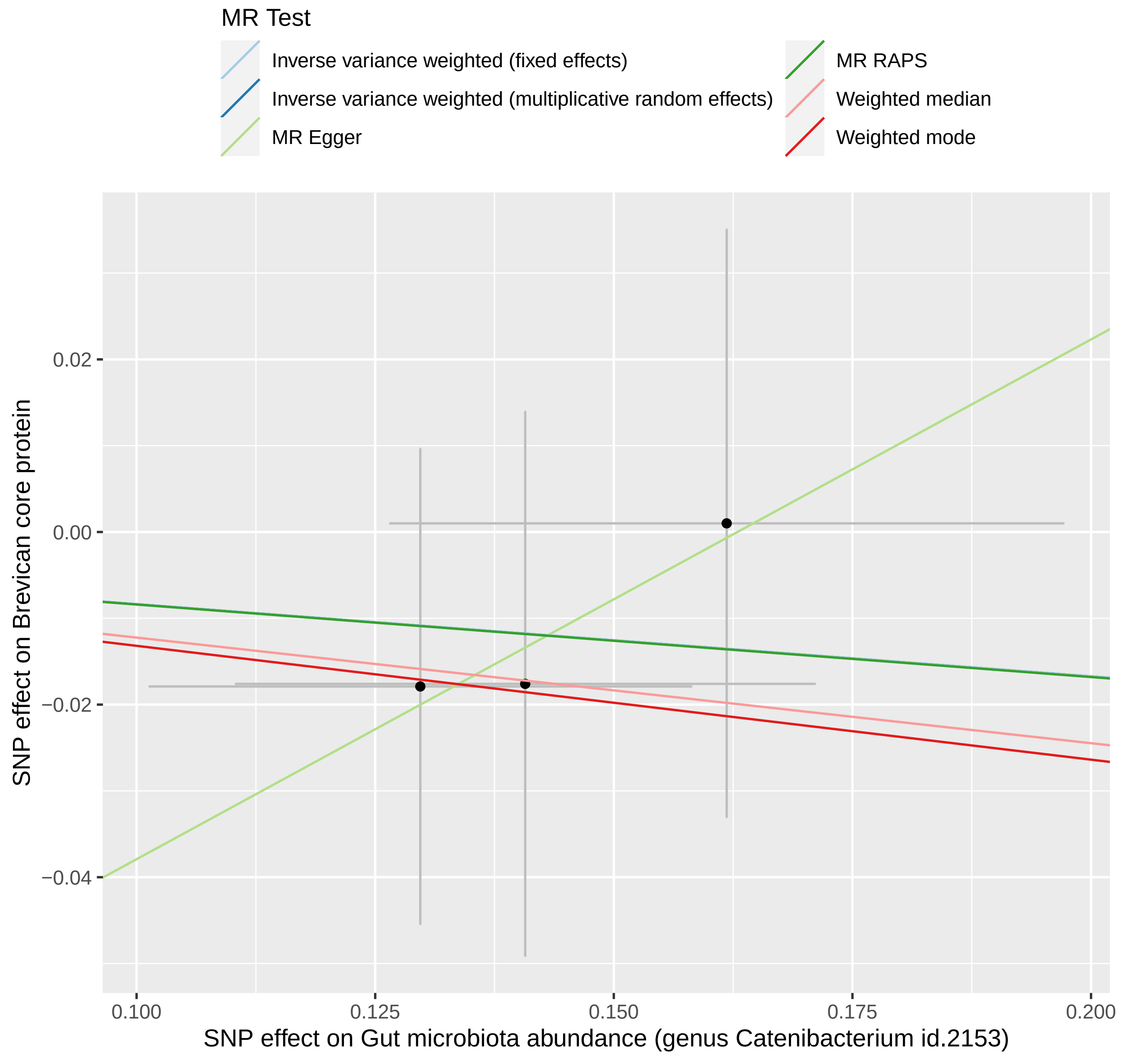

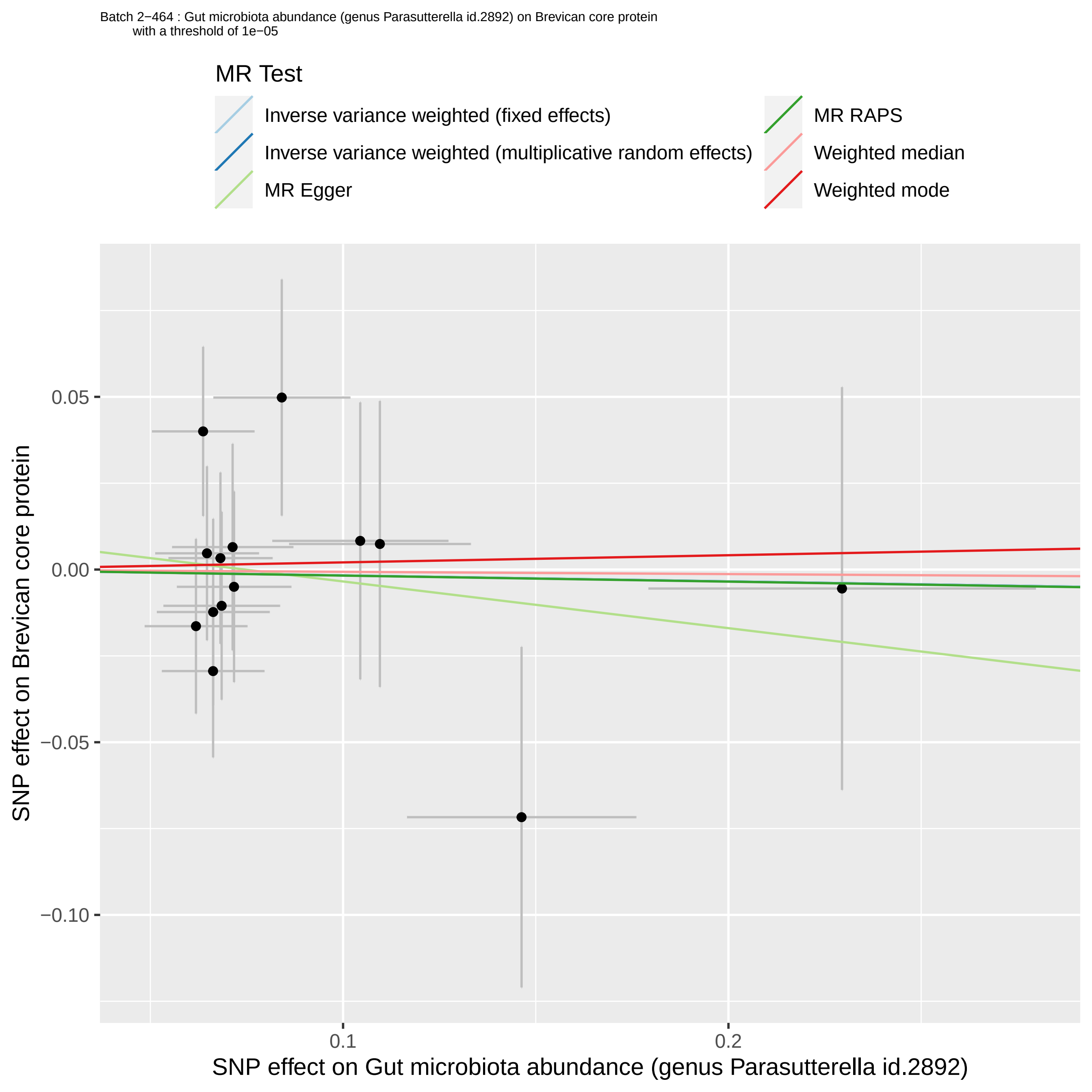

Batch 2−464 : Gut microbiota abundance (genus Parasutterella id.2892) on Brevican core protein
with a threshold of 1e−05
MR Test
Inverse variance weighted (fixed effects)
Inverse variance weighted (multiplicative random effects)
MR Egger
MR RAPS
Weighted median
Weighted mode
0.05
0.00
−0.05
−0.10
0.1
0.2
SNP effect on Brevican core protein
SNP effect on Gut microbiota abundance (genus Parasutterella id.2892)

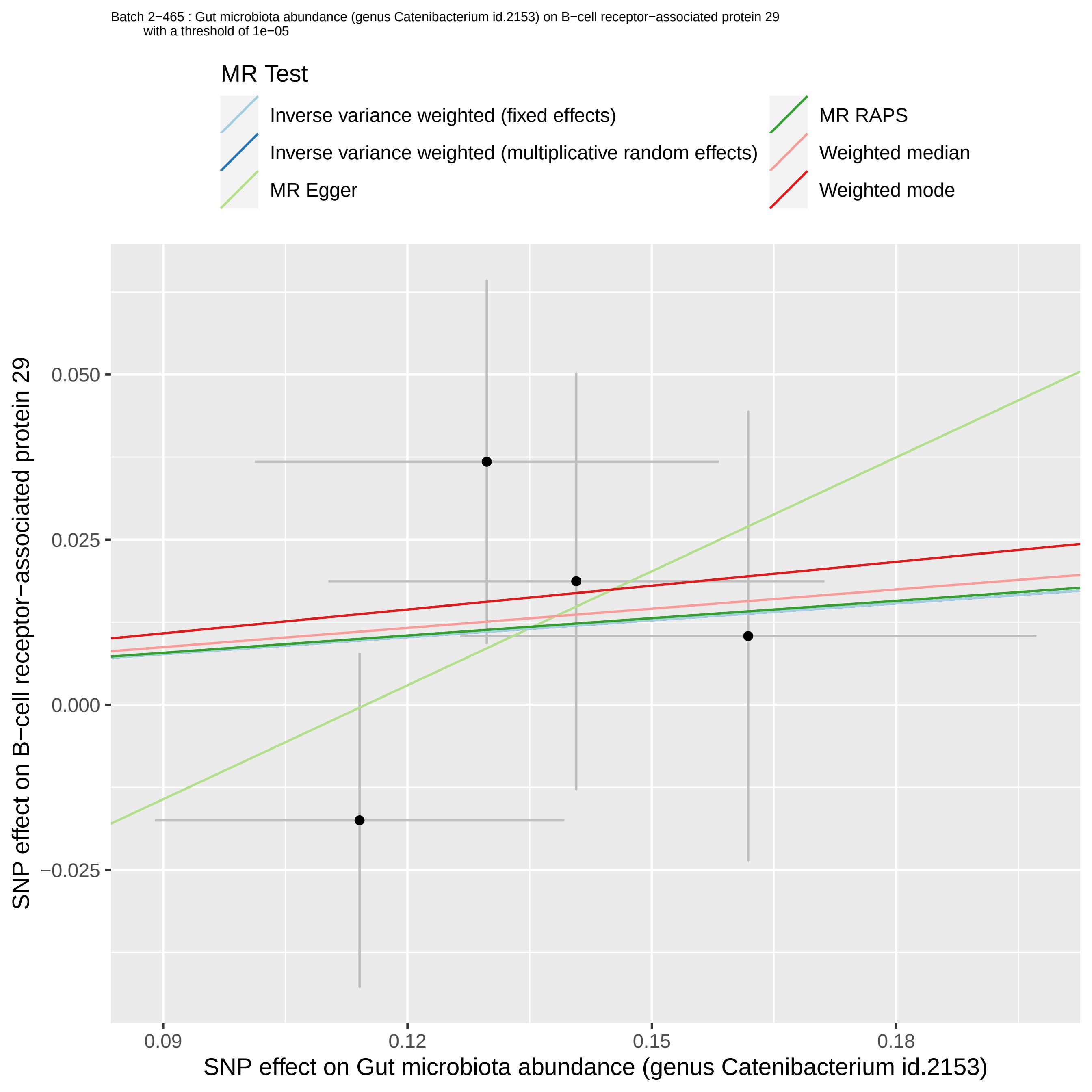

Batch 2−465 : Gut microbiota abundance (genus Catenibacterium id.2153) on B−cell receptor−associated protein 29
with a threshold of 1e−05
MR Test
Inverse variance weighted (fixed effects)
Inverse variance weighted (multiplicative random effects)
MR Egger
MR RAPS
Weighted median
Weighted mode
0.050
0.025
0.000
−0.025
0.09
0.12
0.15
0.18
SNP effect on B−cell receptor−associated protein 29
SNP effect on Gut microbiota abundance (genus Catenibacterium id.2153)

Batch 2−466 : Gut microbiota abundance (genus Parasutterella id.2892) on B−cell receptor−associated protein 29
with a threshold of 1e−05

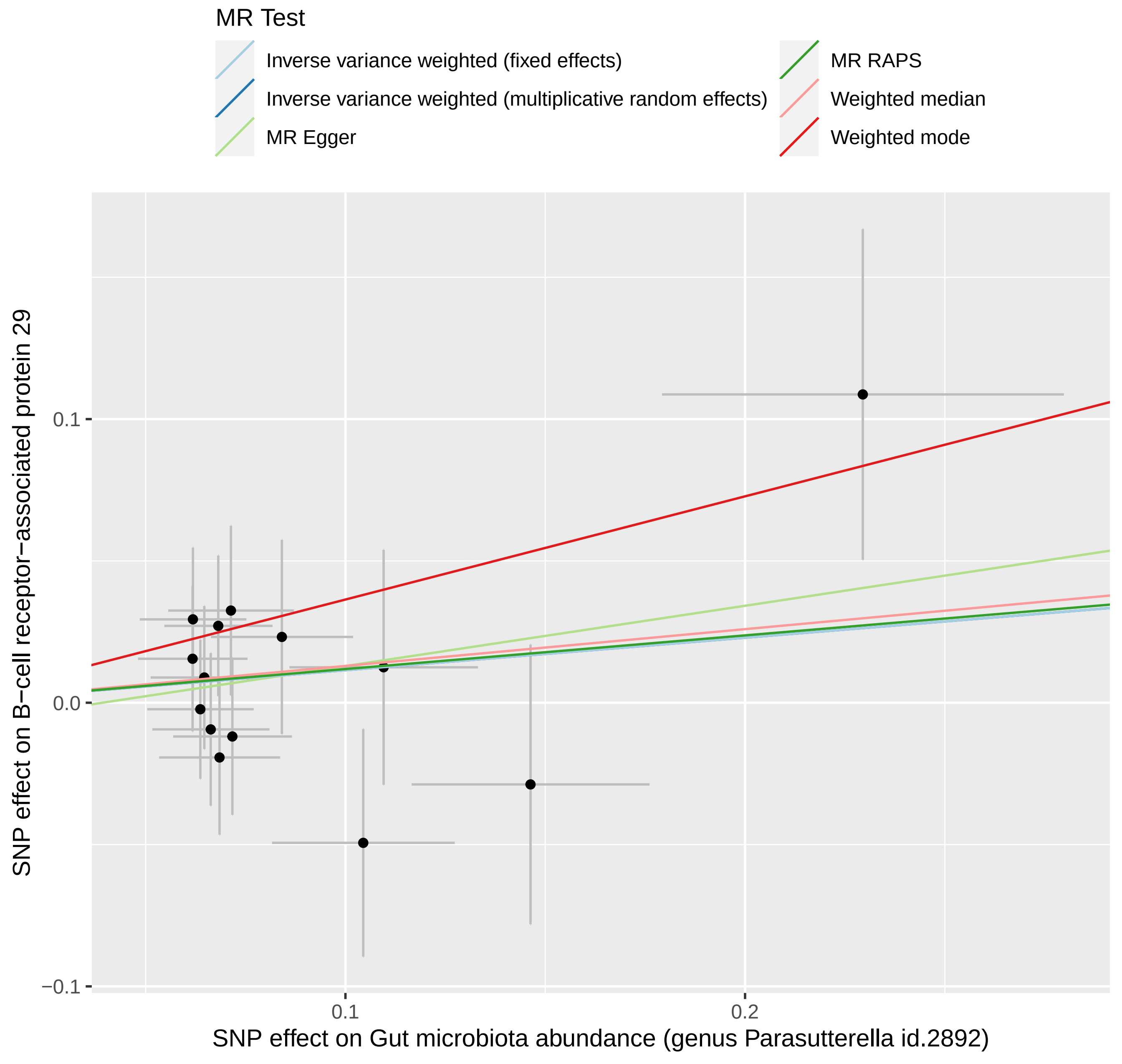

Batch 2−467 : Gut microbiota abundance (genus Catenibacterium id.2153) on Breast cancer anti−estrogen resistance protein 3
with a threshold of 1e−05

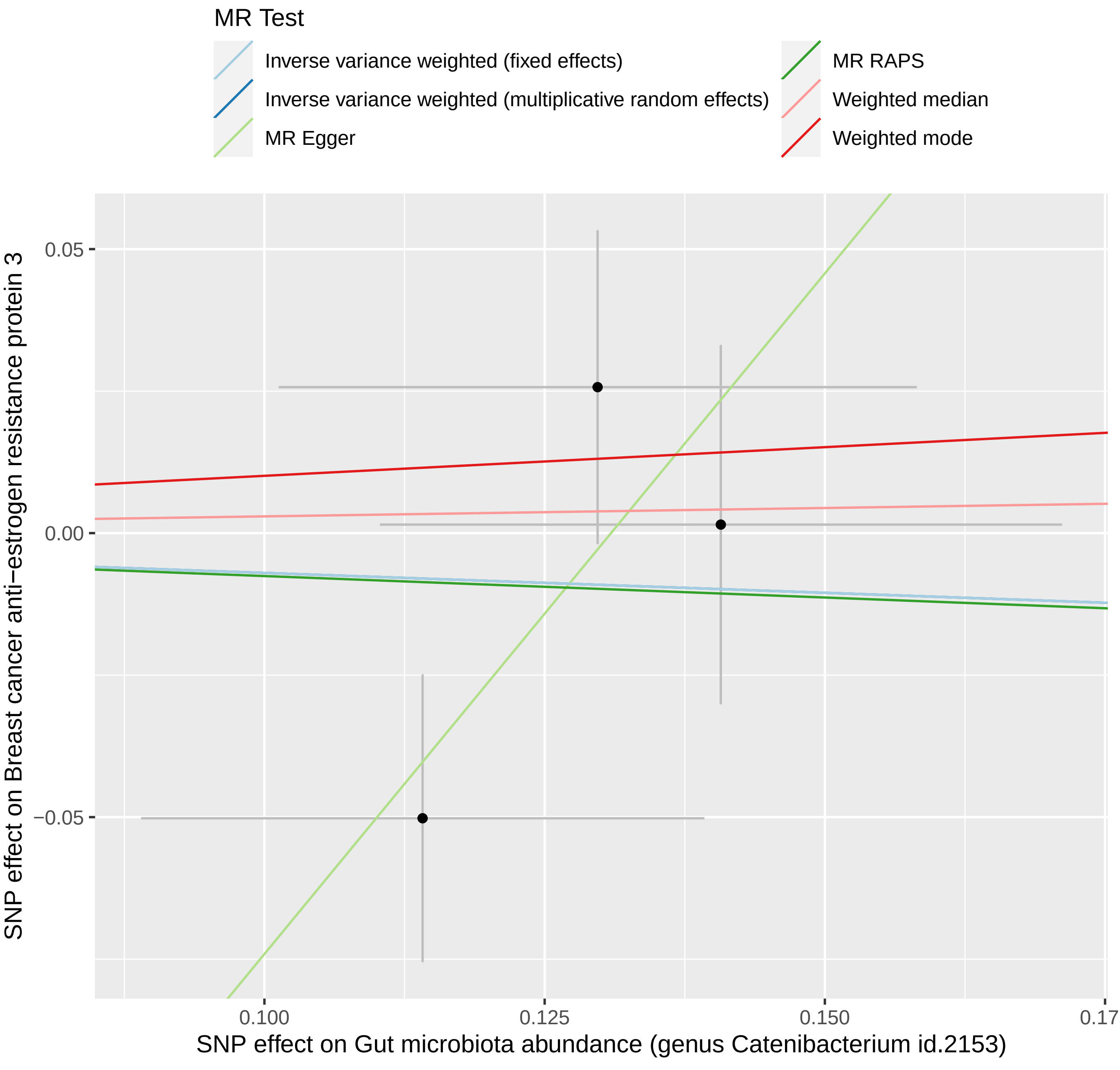

Batch 2−468 : Gut microbiota abundance (genus Parasutterella id.2892) on Breast cancer anti−estrogen resistance protein 3 with a threshold of 1e−05

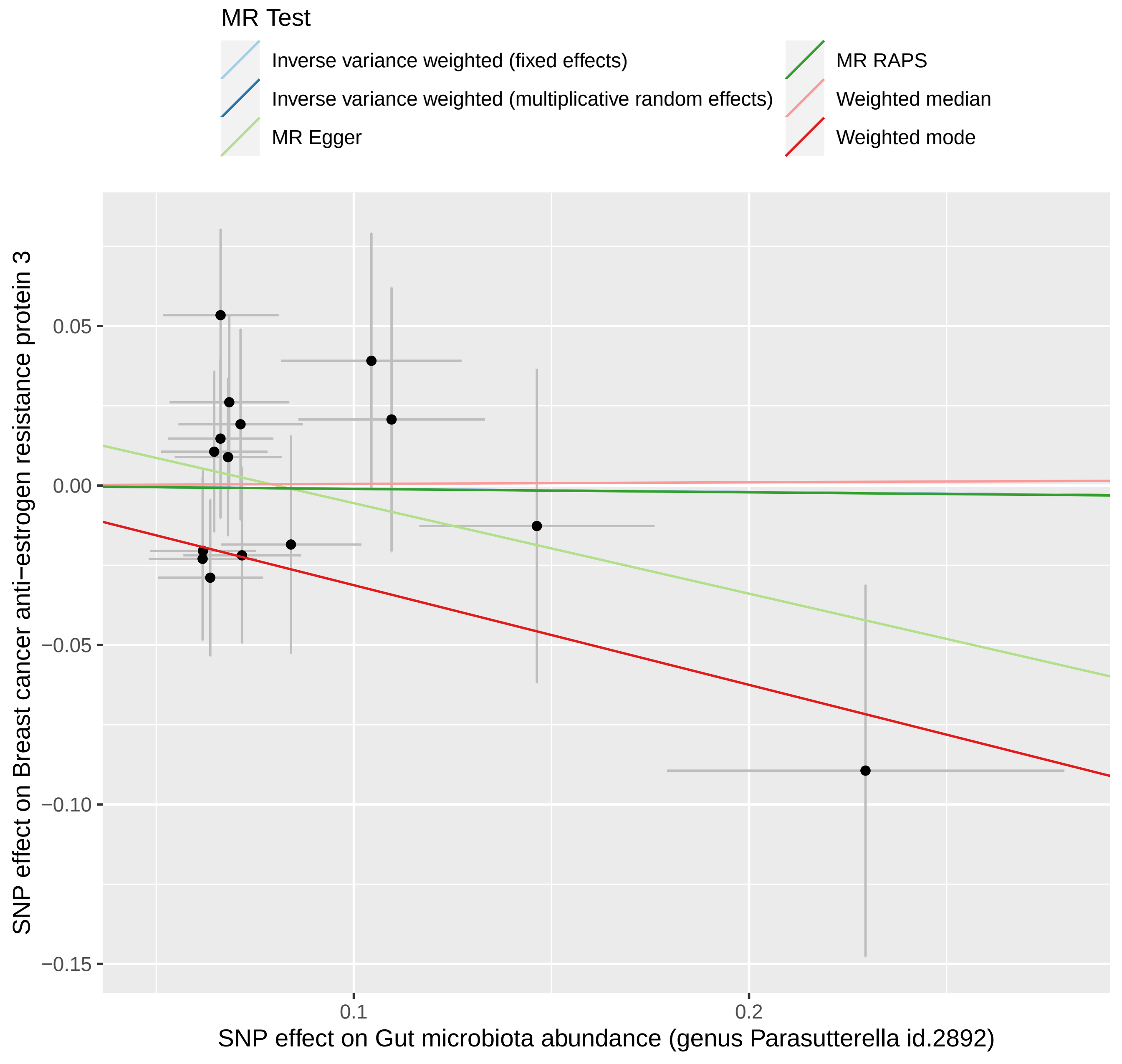

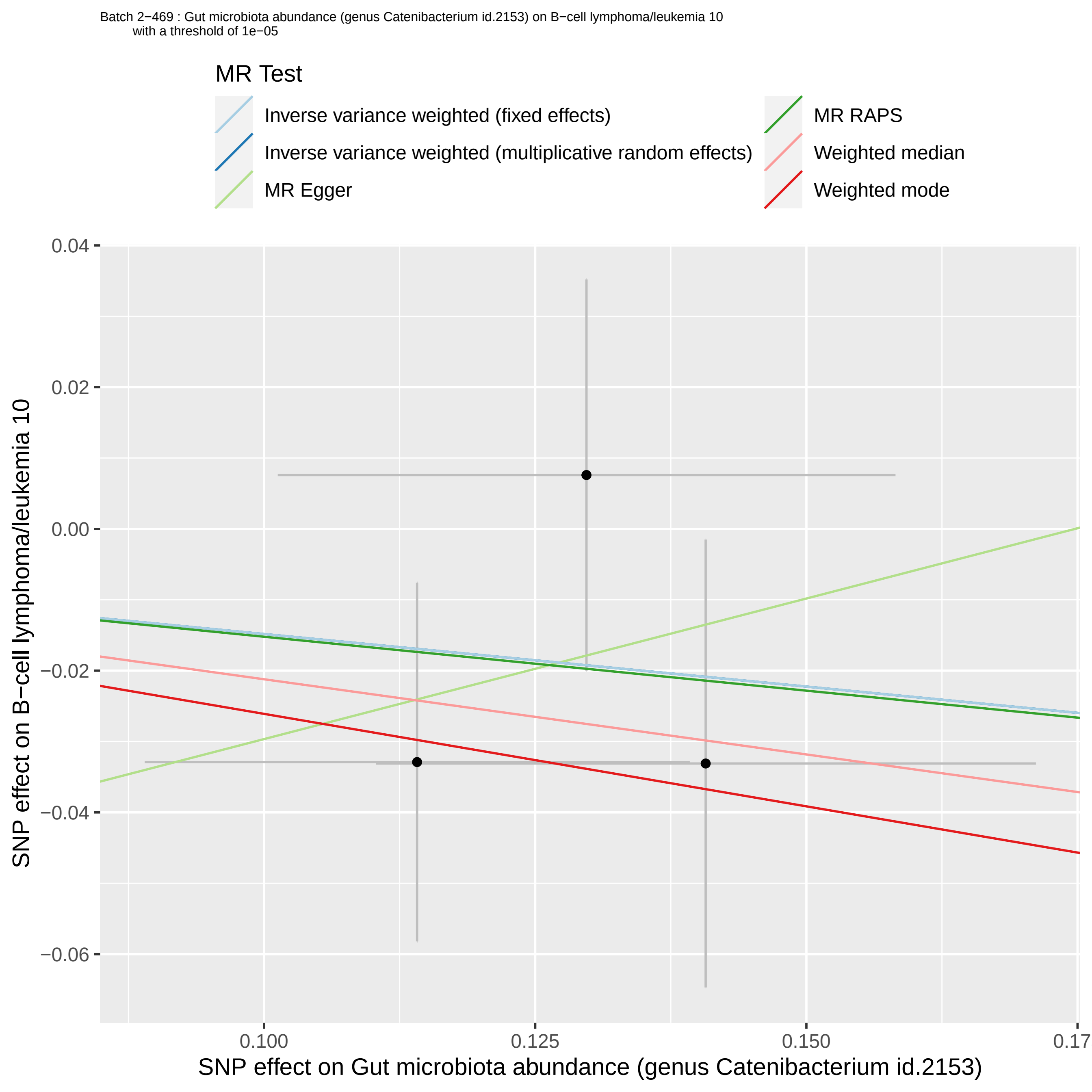

Batch 2−469 : Gut microbiota abundance (genus Catenibacterium id.2153) on B−cell lymphoma/leukemia 10
with a threshold of 1e−05
MR Test
Inverse variance weighted (fixed effects)
Inverse variance weighted (multiplicative random effects)
MR Egger
MR RAPS
Weighted median
Weighted mode
0.04
0.02
0.00
−0.02
−0.04
−0.06
0.100
0.125
0.150
0.17
SNP effect on B−cell lymphoma/leukemia 10
SNP effect on Gut microbiota abundance (genus Catenibacterium id.2153)

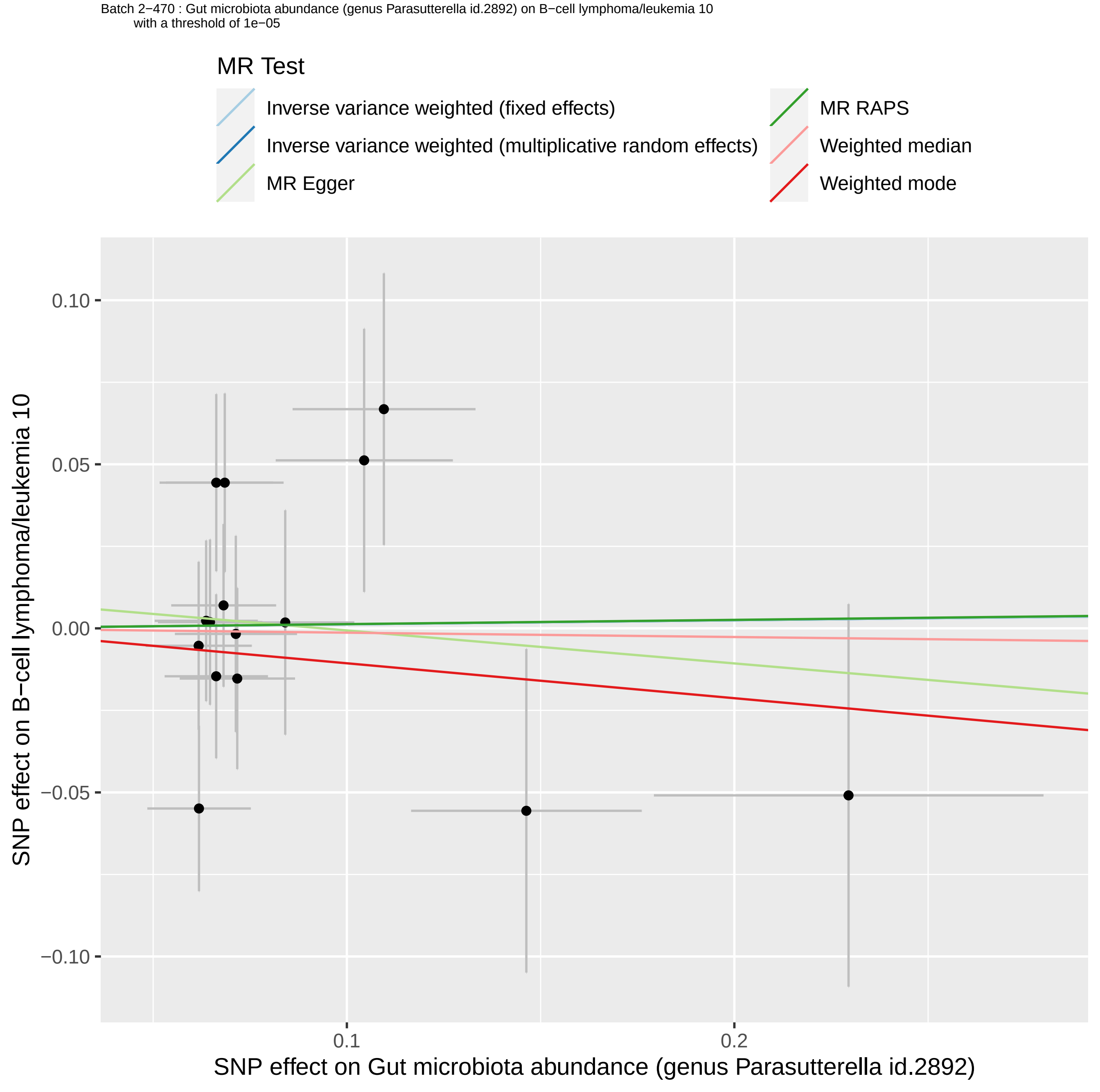
Batch 2−470 : Gut microbiota abundance (genus Parasutterella id.2892) on B−cell lymphoma/leukemia 10
with a threshold of 1e−05
MR Test
Inverse variance weighted (fixed effects)
Inverse variance weighted (multiplicative random effects)
MR Egger
MR RAPS
Weighted median
Weighted mode
0.10
0.05
0.00
−0.05
−0.10
0.1
0.2
SNP effect on B−cell lymphoma/leukemia 10
SNP effect on Gut microbiota abundance (genus Parasutterella id.2892)

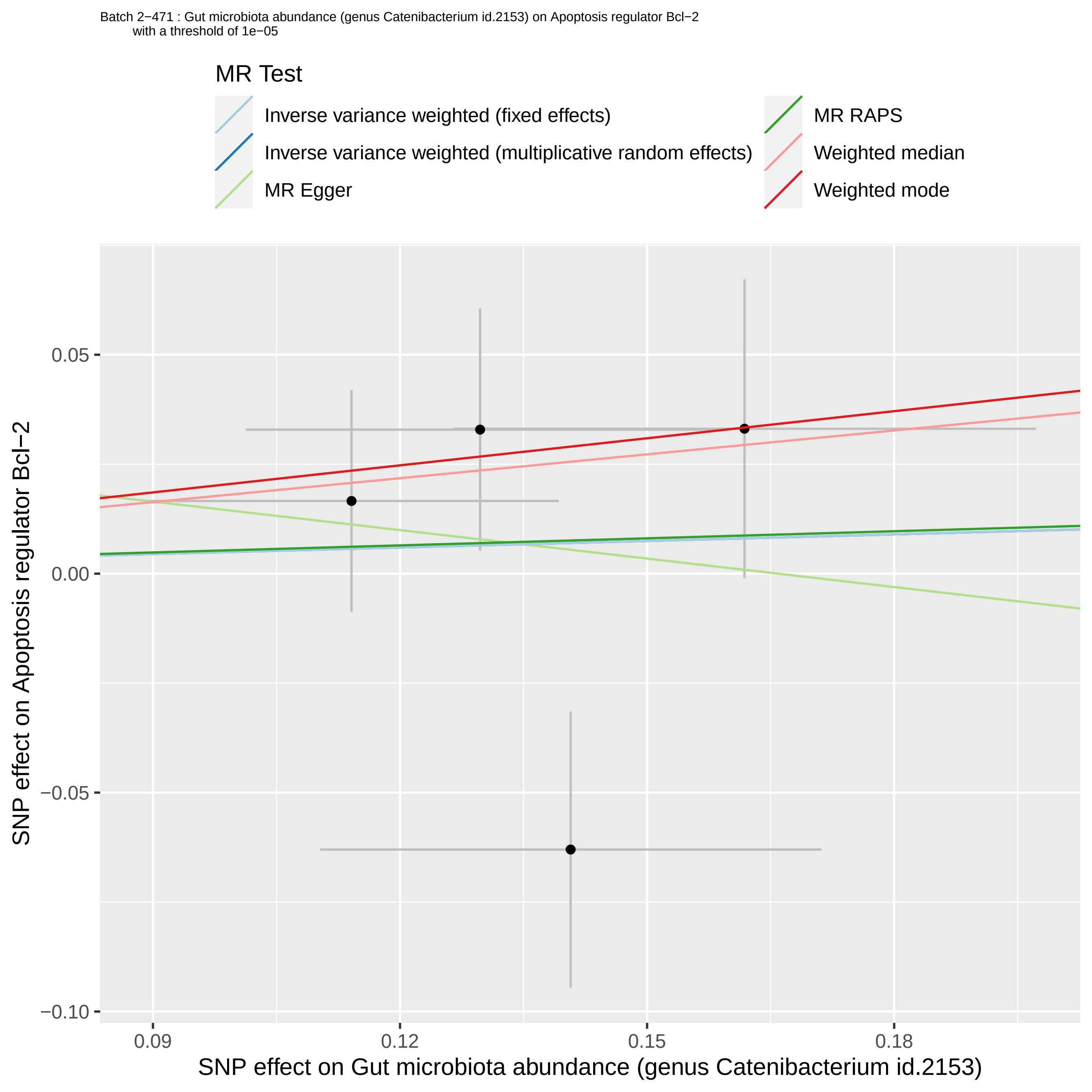

Batch 2−471 : Gut microbiota abundance (genus Catenibacterium id.2153) on Apoptosis regulator Bcl−2
with a threshold of 1e−05
MR Test
Inverse variance weighted (fixed effects)
Inverse variance weighted (multiplicative random effects)
MR Egger
MR RAPS
Weighted median
Weighted mode
0.05
0.00
−0.05
−0.10
0.09
0.12
0.15
0.18
SNP effect on Apoptosis regulator Bcl−2
SNP effect on Gut microbiota abundance (genus Catenibacterium id.2153)

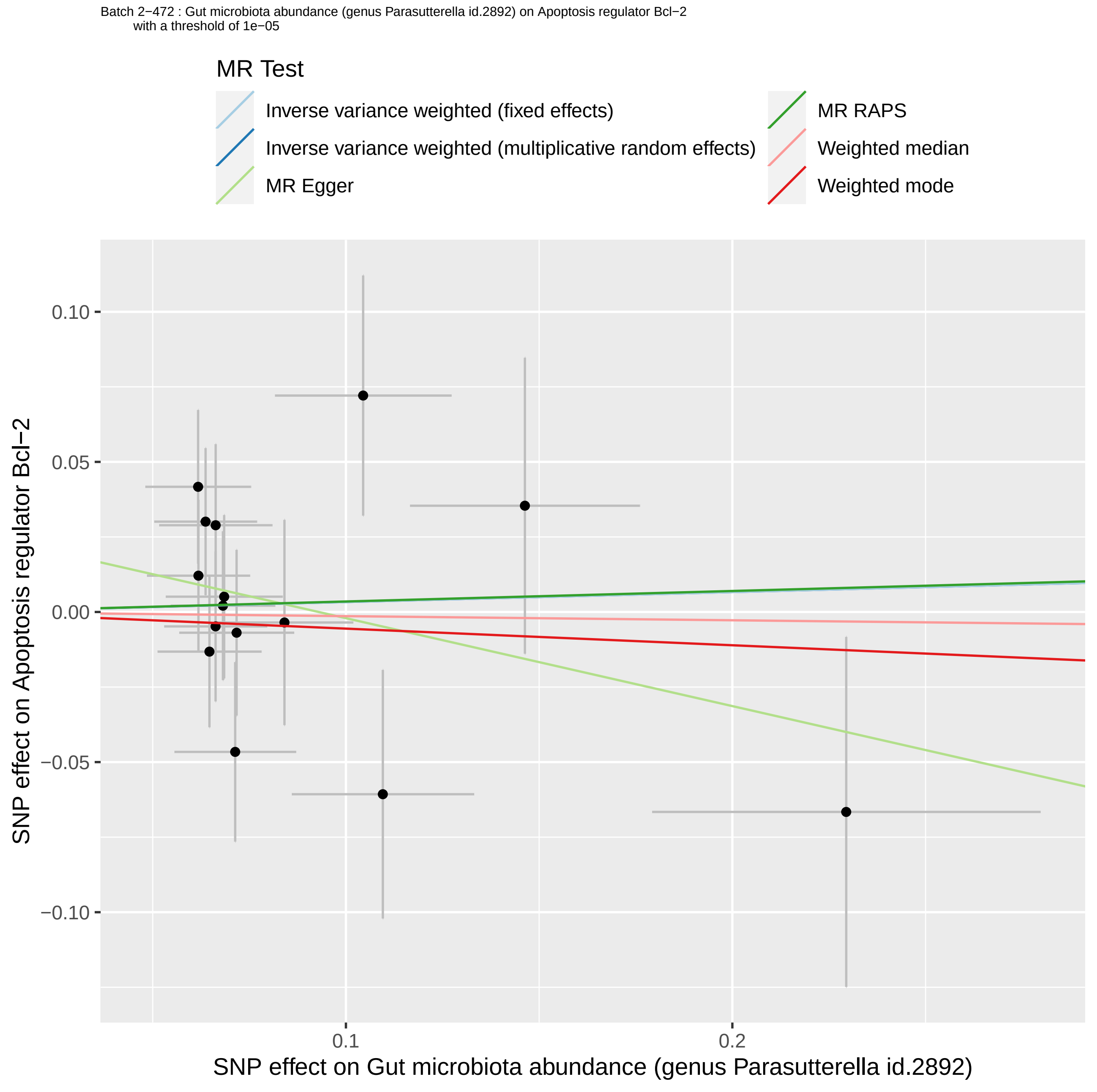

Batch 2−472 : Gut microbiota abundance (genus Parasutterella id.2892) on Apoptosis regulator Bcl−2
with a threshold of 1e−05
MR Test
Inverse variance weighted (fixed effects)
Inverse variance weighted (multiplicative random effects)
MR Egger
MR RAPS
Weighted median
Weighted mode
0.10
0.05
0.00
−0.05
−0.10
0.1
0.2
SNP effect on Apoptosis regulator Bcl−2
SNP effect on Gut microbiota abundance (genus Parasutterella id.2892)

Batch 2−473 : Gut microbiota abundance (genus Catenibacterium id.2153) on Bcl−2−related protein A1
with a threshold of 1e−05

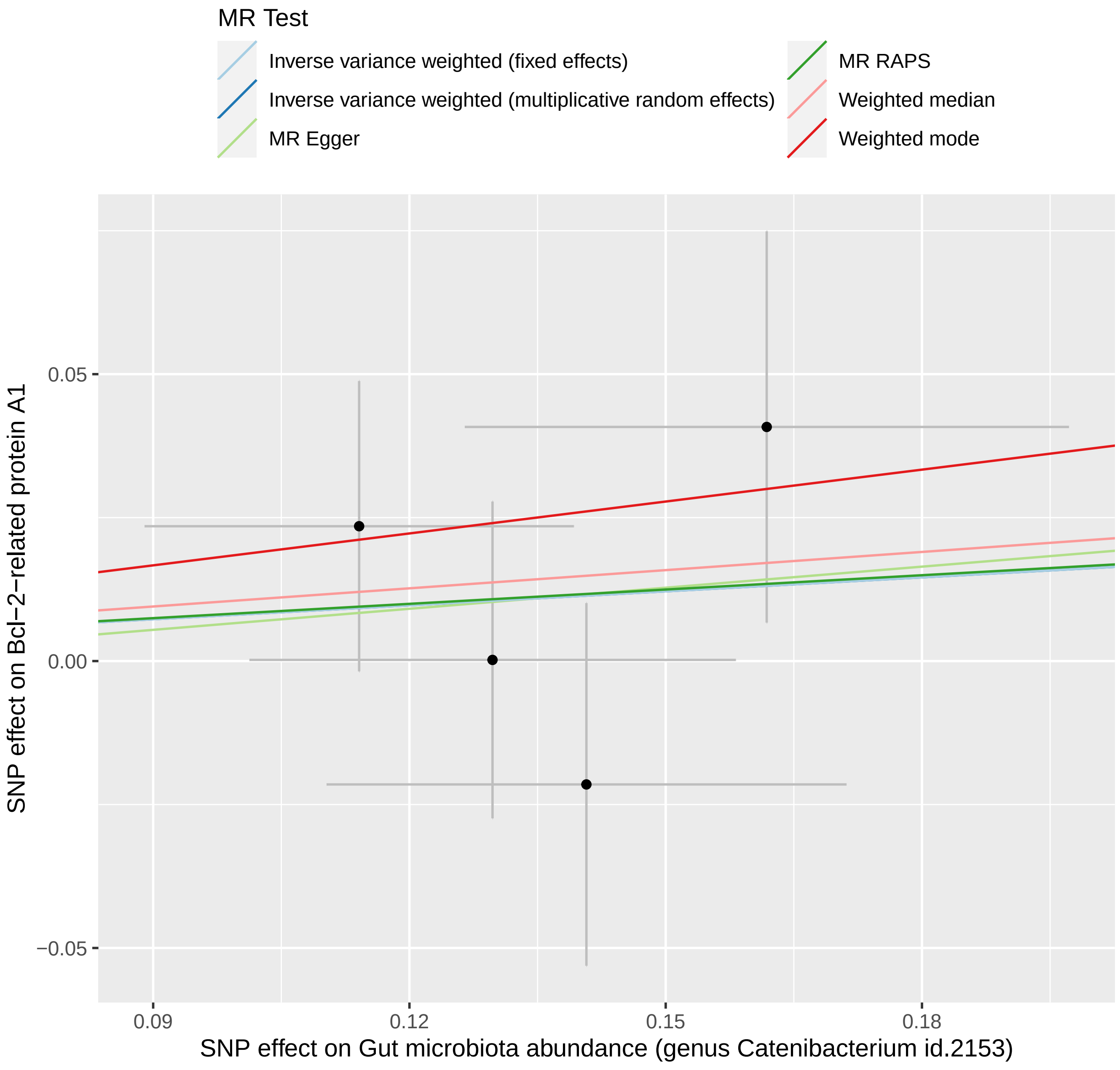

Batch 2−474 : Gut microbiota abundance (genus Parasutterella id.2892) on Bcl−2−related protein A1
with a threshold of 1e−05

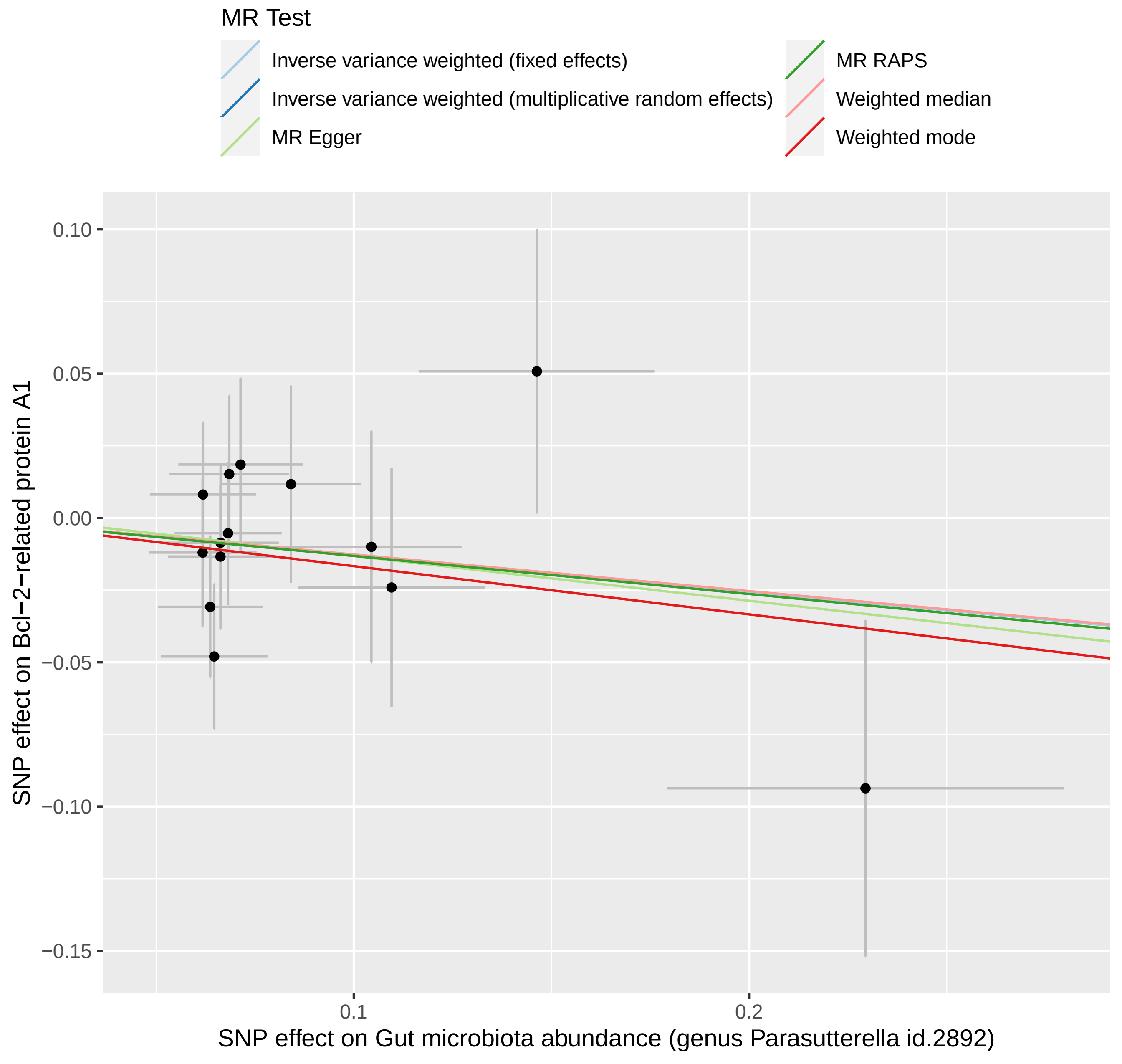

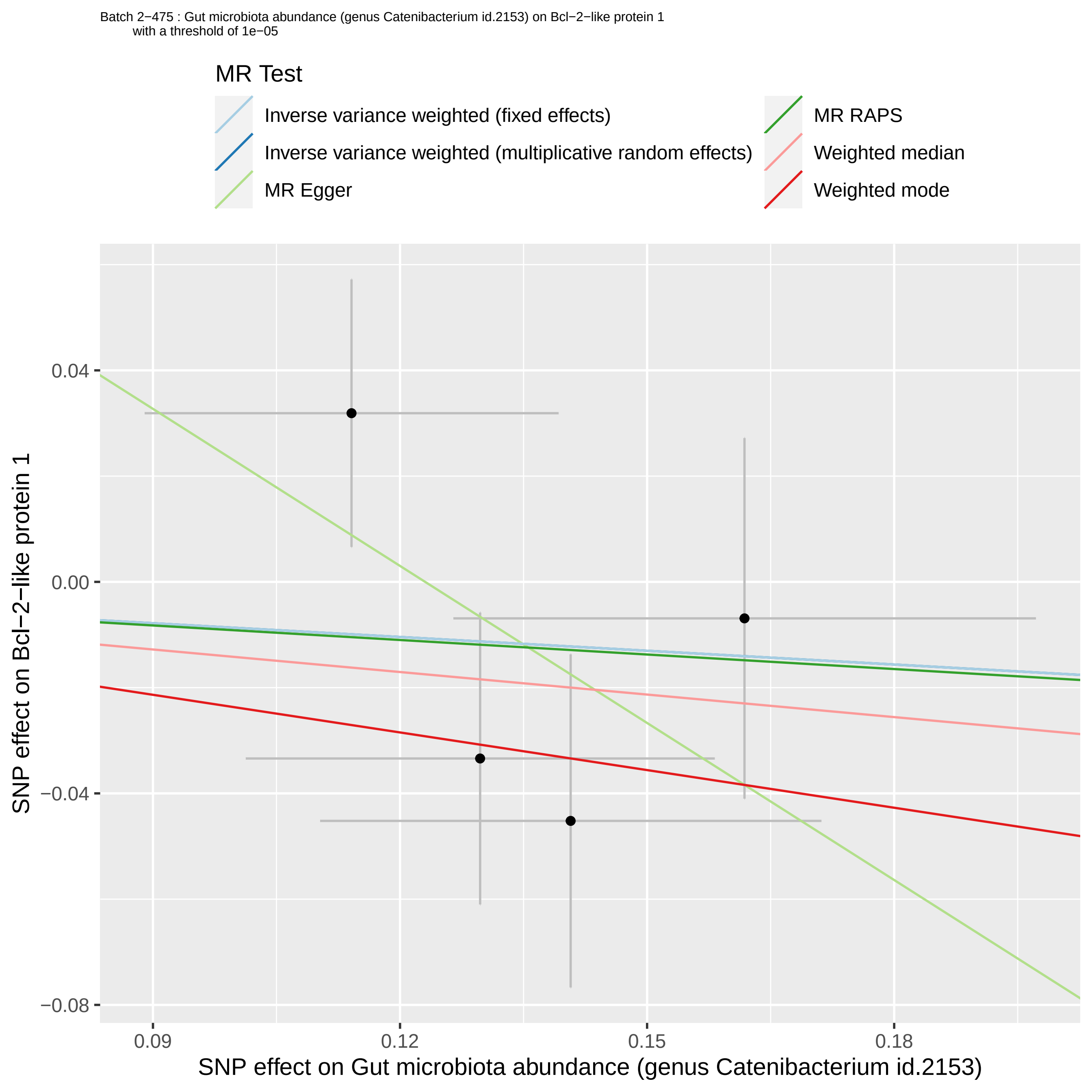
Batch 2−475 : Gut microbiota abundance (genus Catenibacterium id.2153) on Bcl−2−like protein 1
with a threshold of 1e−05
MR Test
Inverse variance weighted (fixed effects)
Inverse variance weighted (multiplicative random effects)
MR Egger
MR RAPS
Weighted median
Weighted mode
0.04
0.00
−0.04
−0.08
0.09
0.12
0.15
0.18
SNP effect on Bcl−2−like protein 1
SNP effect on Gut microbiota abundance (genus Catenibacterium id.2153)

Batch 2−476 : Gut microbiota abundance (genus Parasutterella id.2892) on Bcl−2−like protein 1
with a threshold of 1e−05

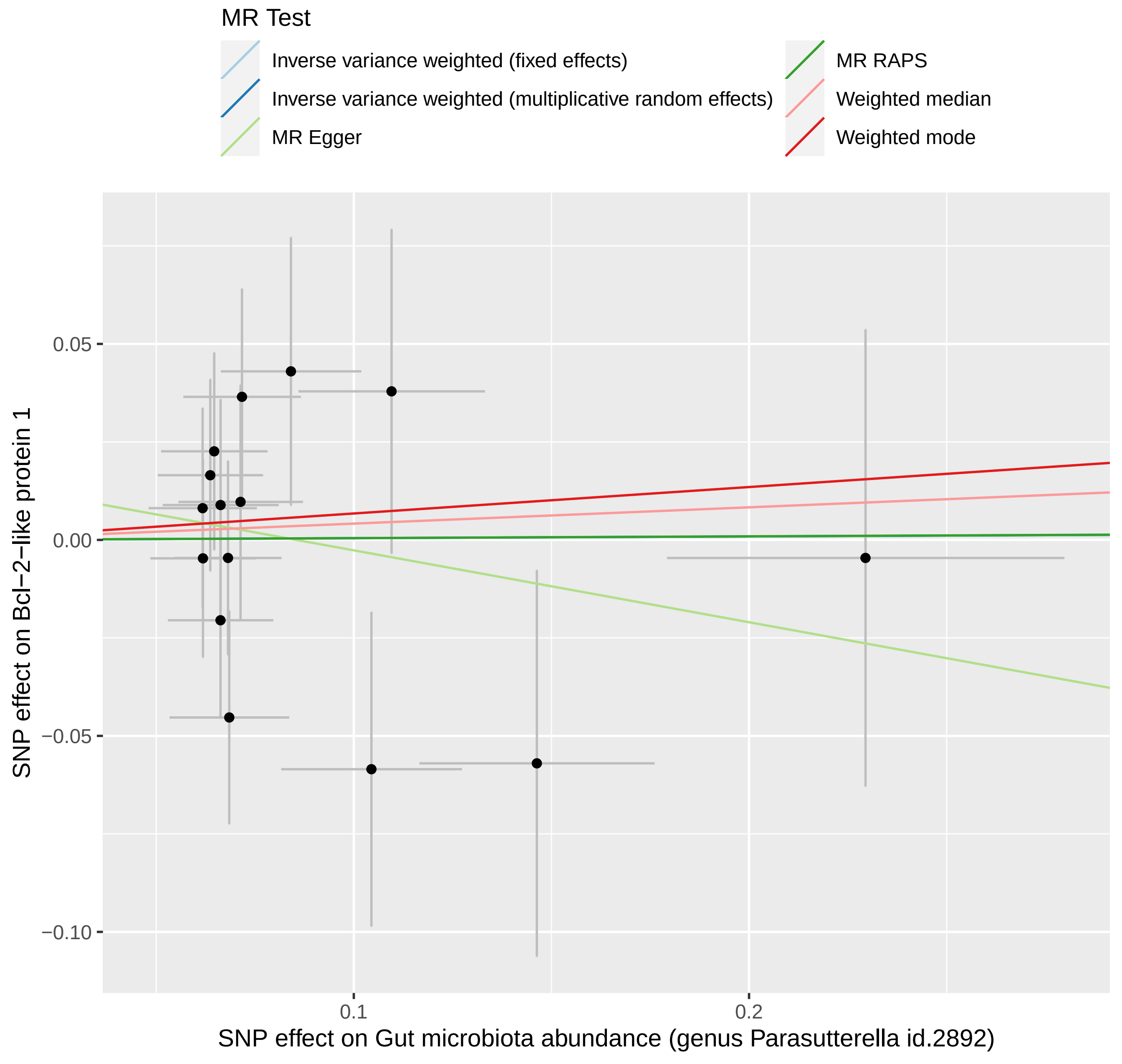

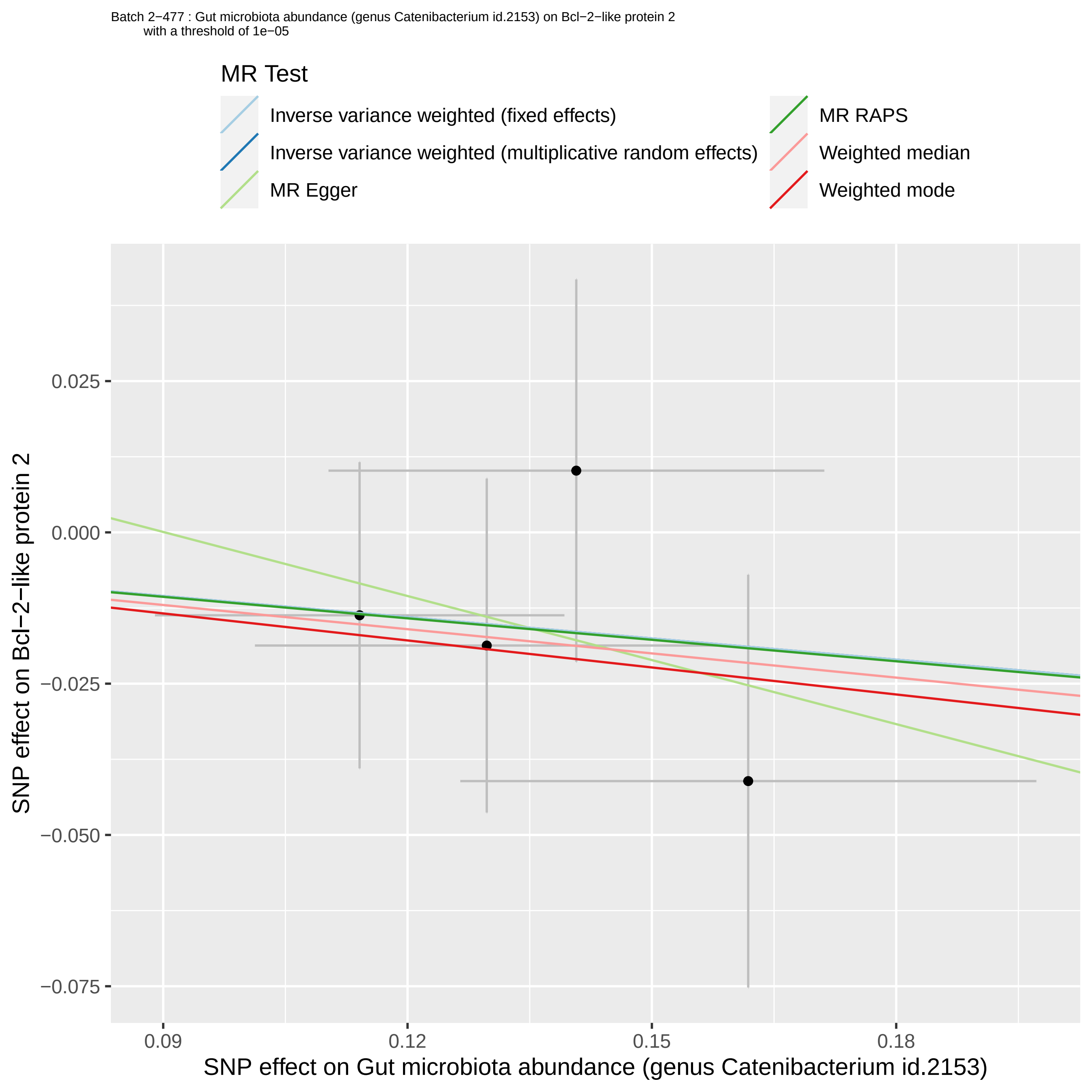

Batch 2−477 : Gut microbiota abundance (genus Catenibacterium id.2153) on Bcl−2−like protein 2
with a threshold of 1e−05
MR Test
Inverse variance weighted (fixed effects)
Inverse variance weighted (multiplicative random effects)
MR Egger
MR RAPS
Weighted median
Weighted mode
0.025
0.000
−0.025
−0.050
−0.075
0.09
0.12
0.15
0.18
SNP effect on Bcl−2−like protein 2
SNP effect on Gut microbiota abundance (genus Catenibacterium id.2153)

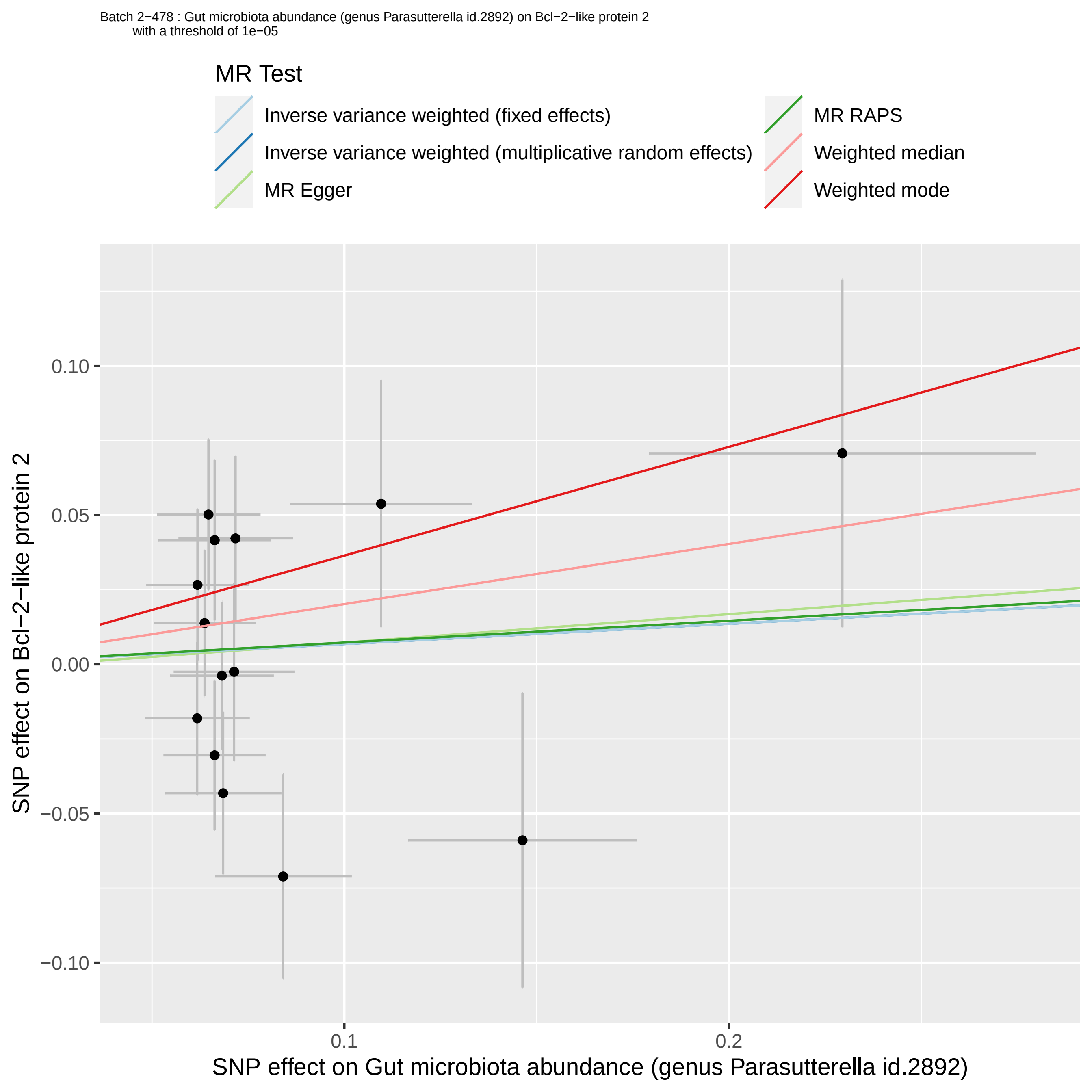

Batch 2−478 : Gut microbiota abundance (genus Parasutterella id.2892) on Bcl−2−like protein 2
with a threshold of 1e−05
MR Test
Inverse variance weighted (fixed effects)
Inverse variance weighted (multiplicative random effects)
MR Egger
MR RAPS
Weighted median
Weighted mode
SNP effect on Bcl−2−like protein 2
0.10
0.05
0.00
−0.05
−0.10
0.1
0.2
SNP effect on Gut microbiota abundance (genus Parasutterella id.2892)

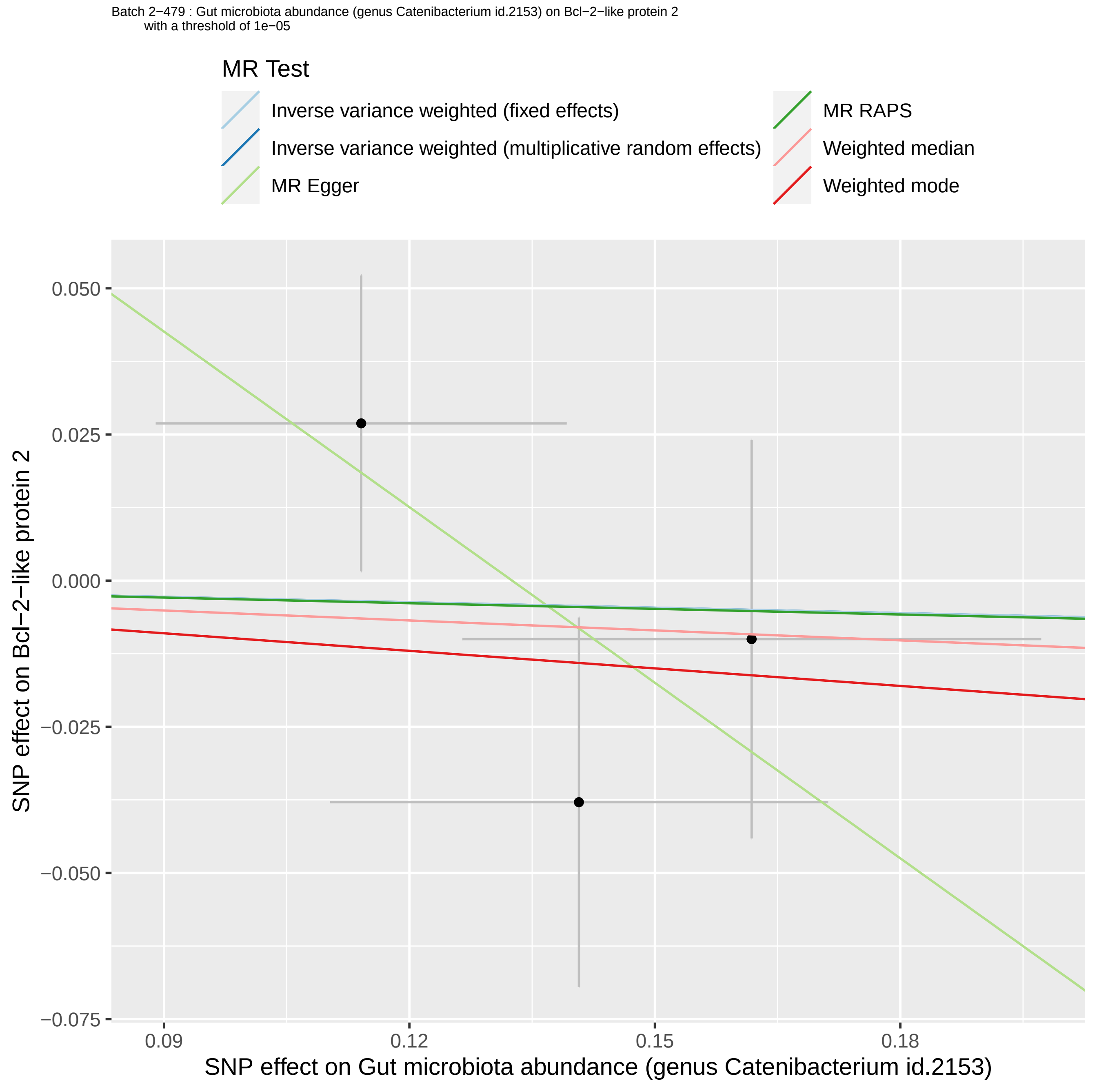

Batch 2−479 : Gut microbiota abundance (genus Catenibacterium id.2153) on Bcl−2−like protein 2
with a threshold of 1e−05
MR Test
Inverse variance weighted (fixed effects)
Inverse variance weighted (multiplicative random effects)
MR Egger
MR RAPS
Weighted median
Weighted mode
0.050
0.025
0.000
−0.025
−0.050
−0.075
0.09
0.12
0.15
0.18
SNP effect on Bcl−2−like protein 2
SNP effect on Gut microbiota abundance (genus Catenibacterium id.2153)

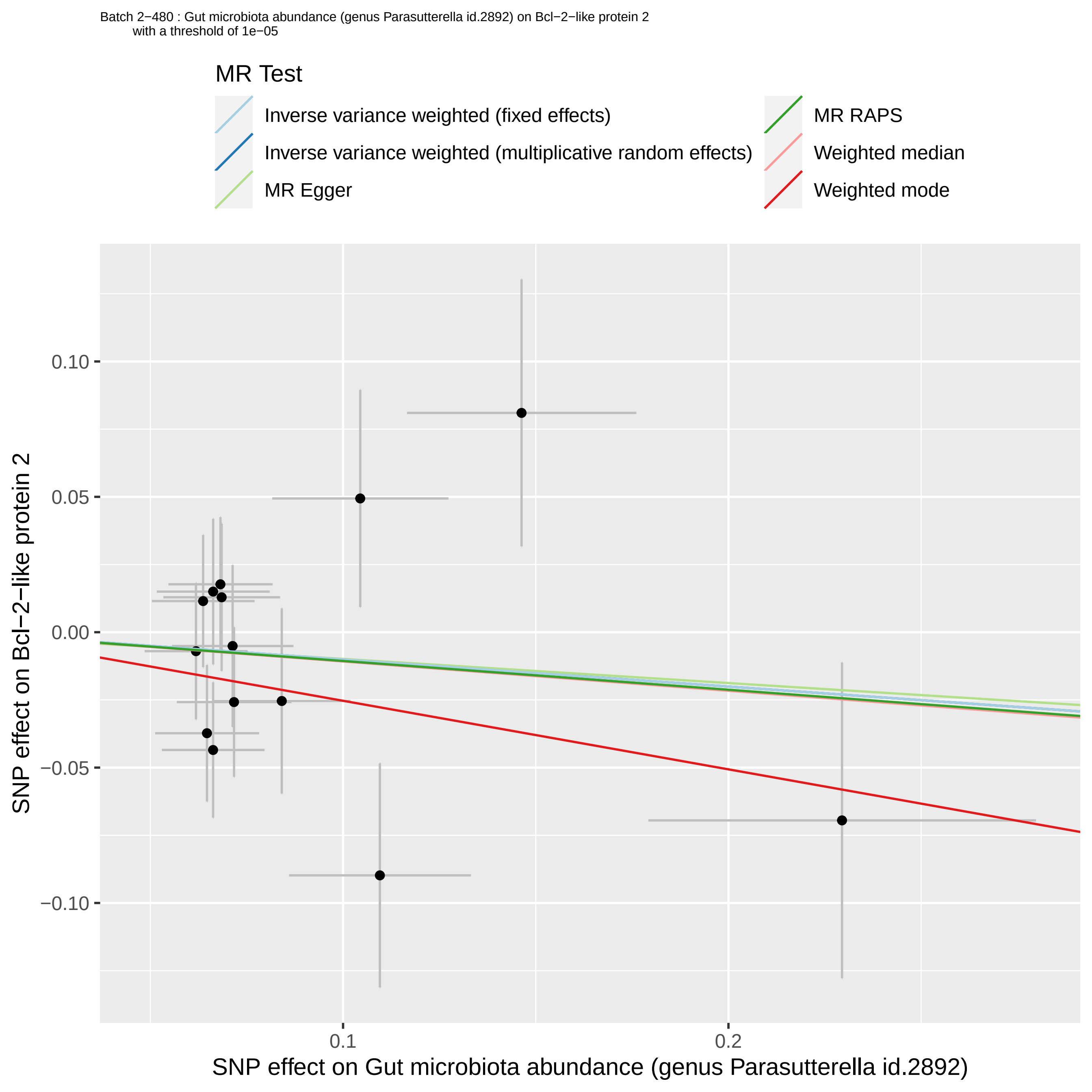
Batch 2−480 : Gut microbiota abundance (genus Parasutterella id.2892) on Bcl−2−like protein 2
with a threshold of 1e−05
MR Test
Inverse variance weighted (fixed effects)
Inverse variance weighted (multiplicative random effects)
MR Egger
MR RAPS
Weighted median
Weighted mode
0.10
0.05
0.00
−0.05
−0.10
0.1
0.2
SNP effect on Bcl−2−like protein 2
SNP effect on Gut microbiota abundance (genus Parasutterella id.2892)

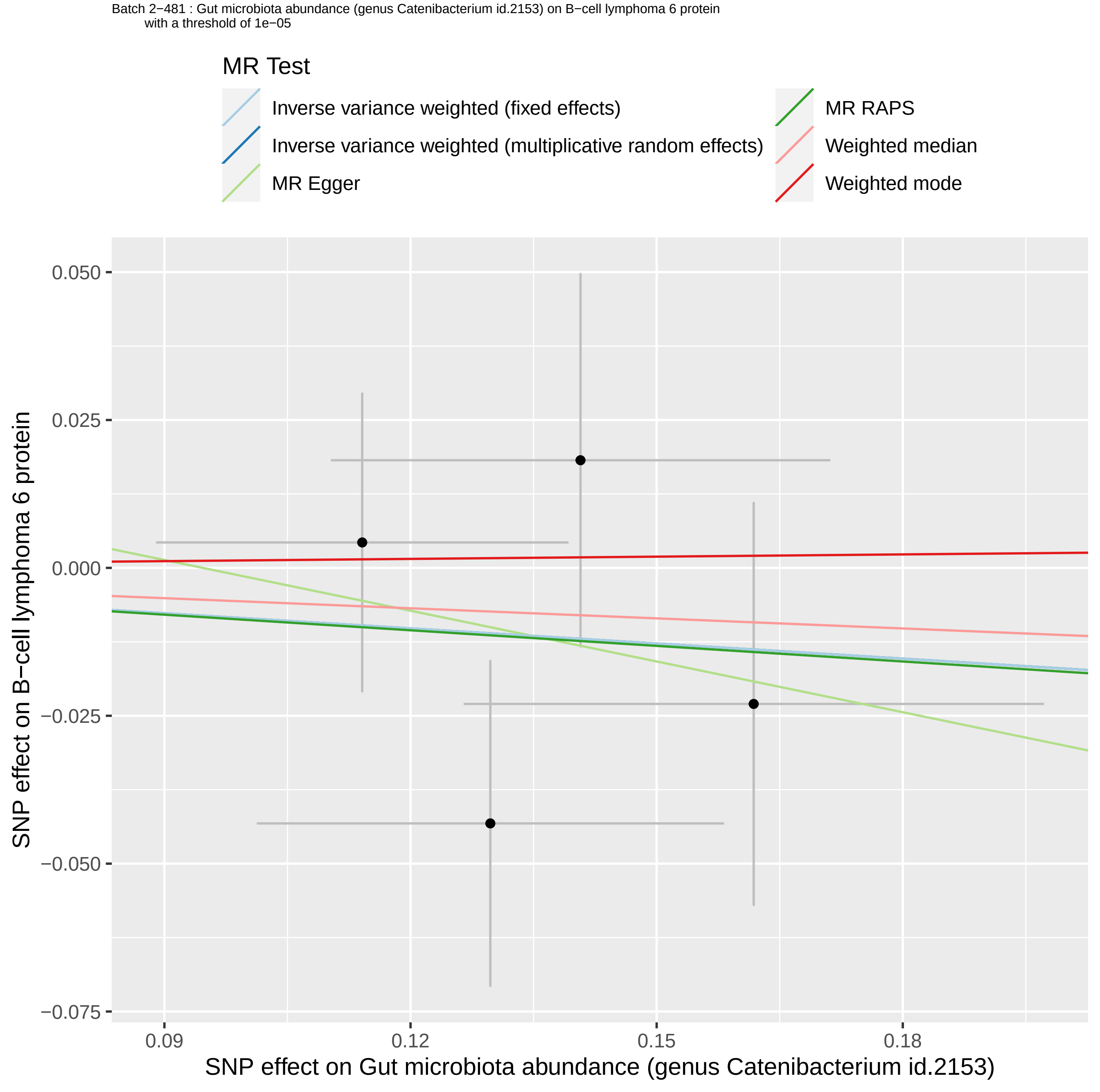
Batch 2−481 : Gut microbiota abundance (genus Catenibacterium id.2153) on B−cell lymphoma 6 protein
with a threshold of 1e−05
MR Test
Inverse variance weighted (fixed effects)
Inverse variance weighted (multiplicative random effects)
MR Egger
MR RAPS
Weighted median
Weighted mode
0.050
0.025
0.000
−0.025
−0.050
−0.075
0.09
0.12
0.15
0.18
SNP effect on B−cell lymphoma 6 protein
SNP effect on Gut microbiota abundance (genus Catenibacterium id.2153)

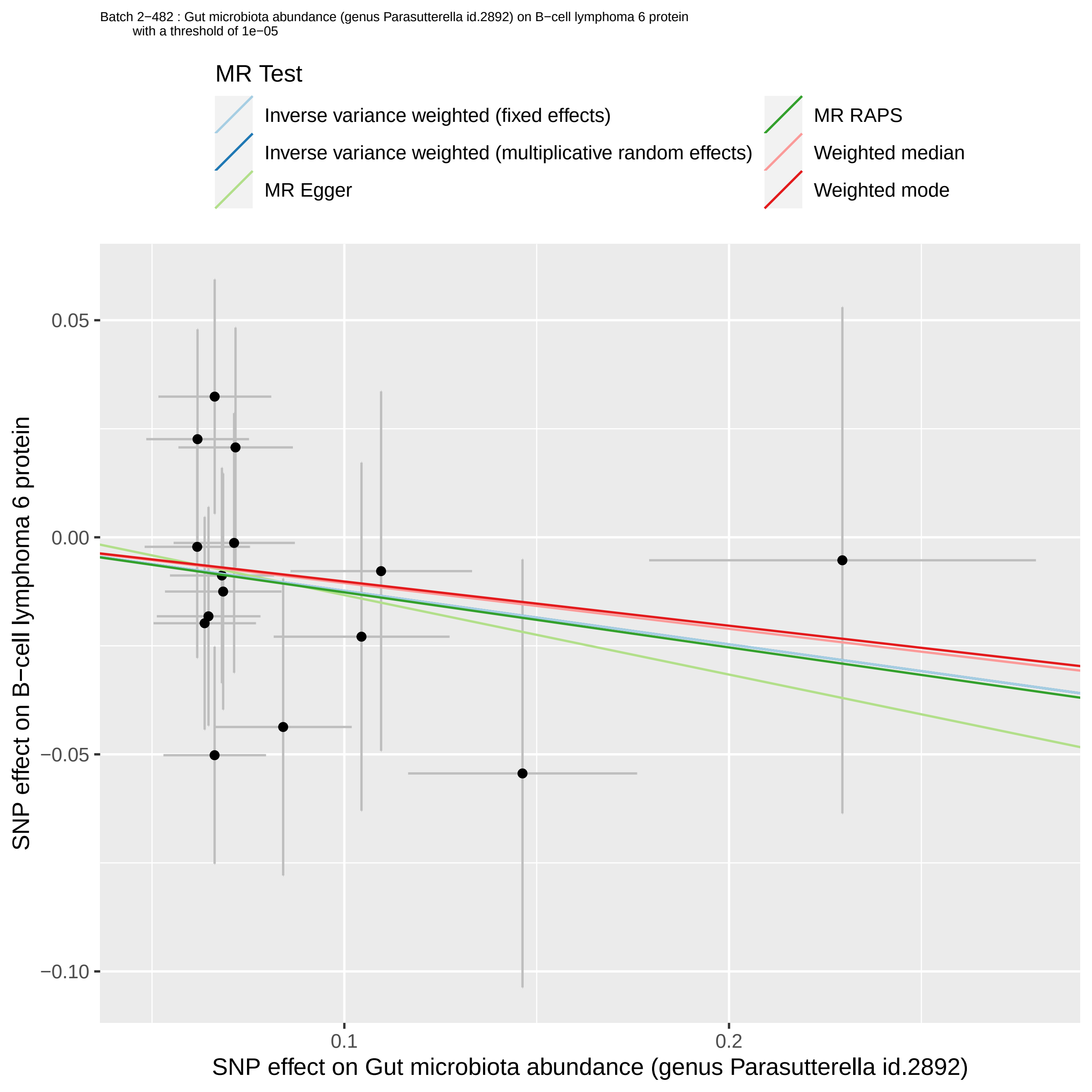
Batch 2−482 : Gut microbiota abundance (genus Parasutterella id.2892) on B−cell lymphoma 6 protein
with a threshold of 1e−05
MR Test
Inverse variance weighted (fixed effects)
Inverse variance weighted (multiplicative random effects)
MR Egger
MR RAPS
Weighted median
Weighted mode
0.05
0.00
−0.05
−0.10
0.1
0.2
SNP effect on B−cell lymphoma 6 protein
SNP effect on Gut microbiota abundance (genus Parasutterella id.2892)

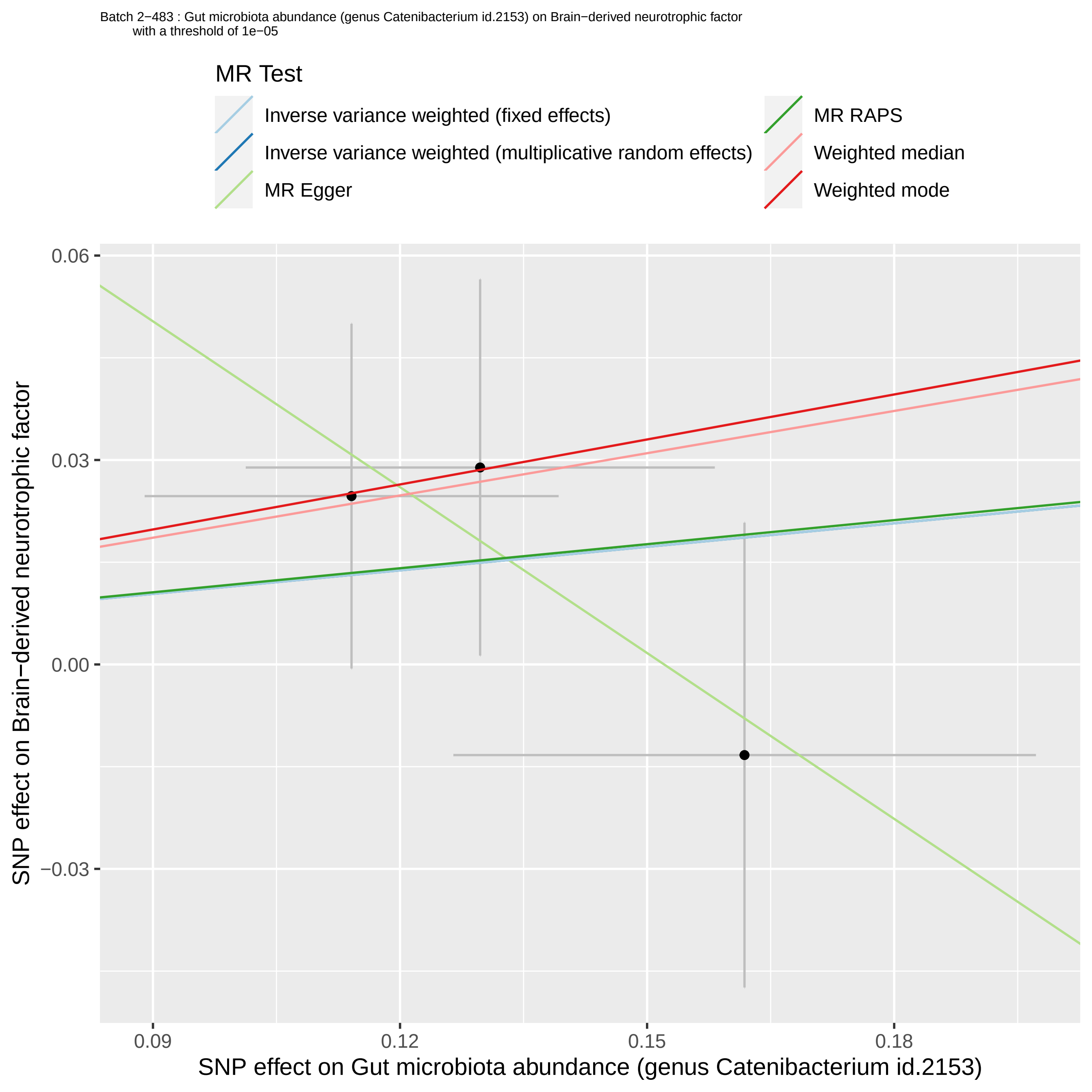

Batch 2−483 : Gut microbiota abundance (genus Catenibacterium id.2153) on Brain−derived neurotrophic factor
with a threshold of 1e−05
MR Test
Inverse variance weighted (fixed effects)
Inverse variance weighted (multiplicative random effects)
MR Egger
MR RAPS
Weighted median
Weighted mode
0.06
0.03
0.00
−0.03
0.09
0.12
0.15
0.18
SNP effect on Brain−derived neurotrophic factor
SNP effect on Gut microbiota abundance (genus Catenibacterium id.2153)

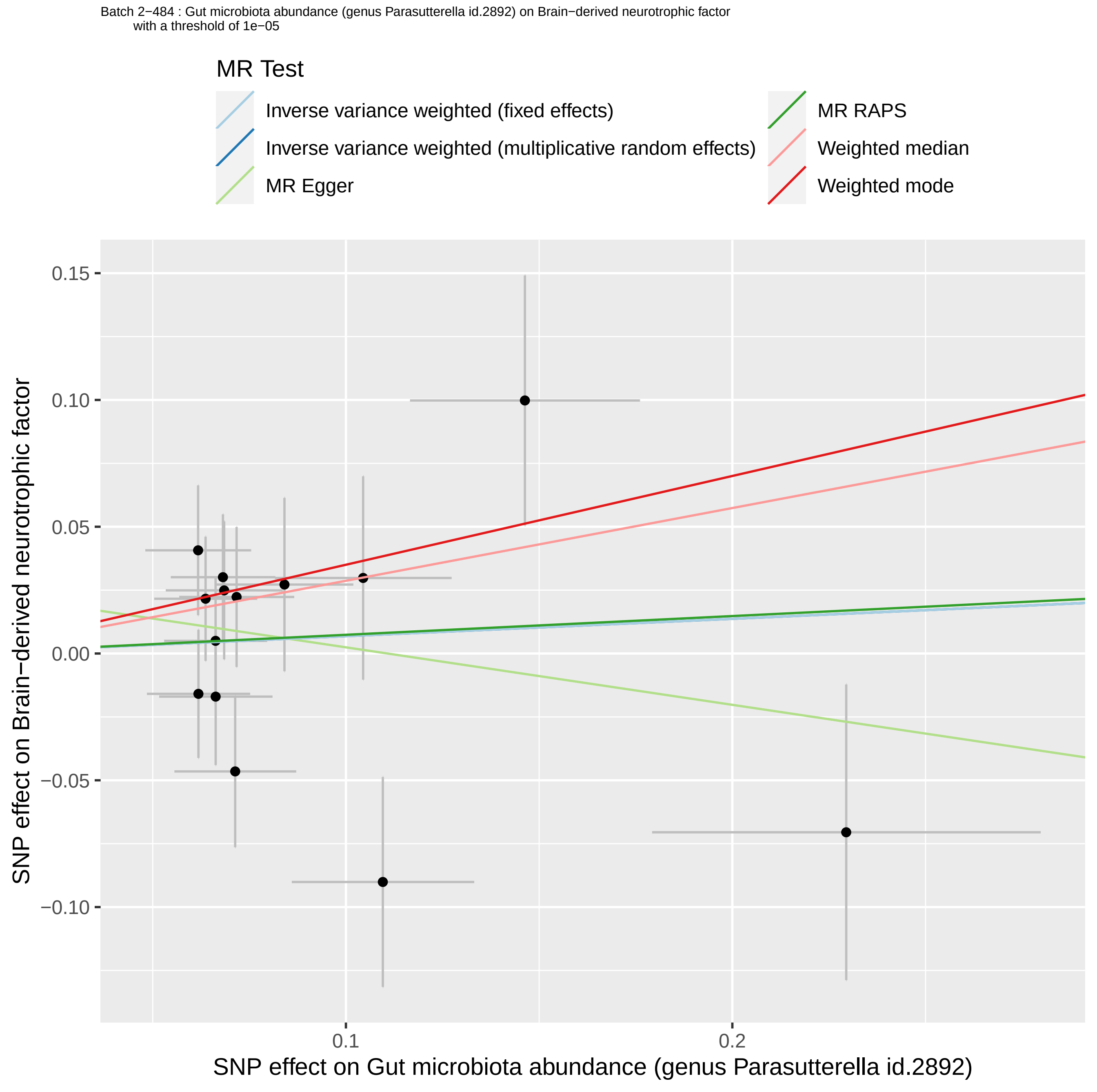

Batch 2−484 : Gut microbiota abundance (genus Parasutterella id.2892) on Brain−derived neurotrophic factor
with a threshold of 1e−05
MR Test
Inverse variance weighted (fixed effects)
Inverse variance weighted (multiplicative random effects)
MR Egger
MR RAPS
Weighted median
Weighted mode
0.15
0.10
0.05
0.00
−0.05
−0.10
0.1
0.2
SNP effect on Brain−derived neurotrophic factor
SNP effect on Gut microbiota abundance (genus Parasutterella id.2892)

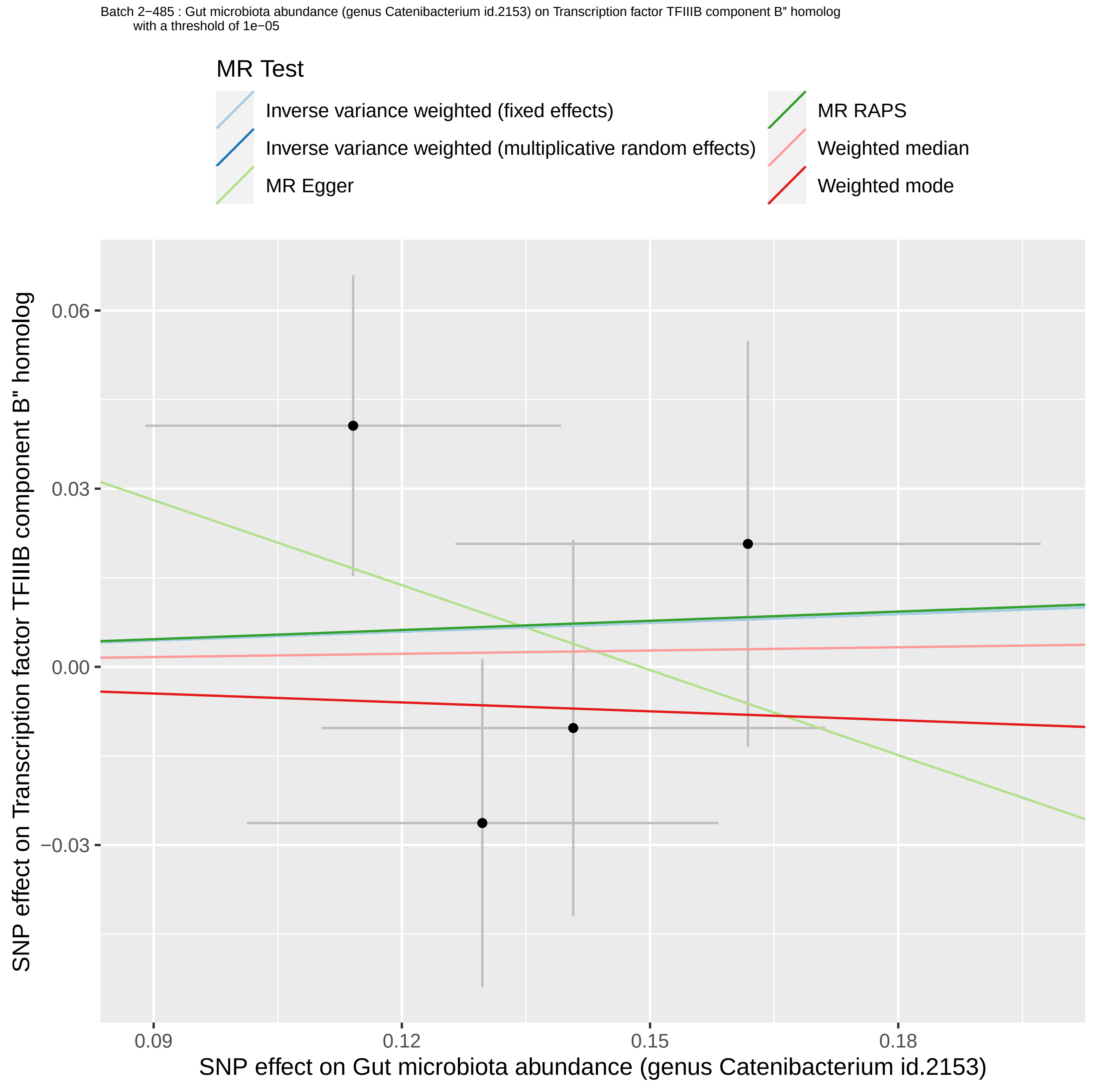

Batch 2−485 : Gut microbiota abundance (genus Catenibacterium id.2153) on Transcription factor TFIIIB component B" homolog
with a threshold of 1e−05
MR Test
Inverse variance weighted (fixed effects)
Inverse variance weighted (multiplicative random effects)
MR Egger
MR RAPS
Weighted median
Weighted mode
0.06
0.03
0.00
−0.03
0.09
0.12
0.15
0.18
SNP effect on Transcription factor TFIIIB component B" homolog
SNP effect on Gut microbiota abundance (genus Catenibacterium id.2153)

Batch 2−486 : Gut microbiota abundance (genus Parasutterella id.2892) on Transcription factor TFIIIB component B" homolog
with a threshold of 1e−05

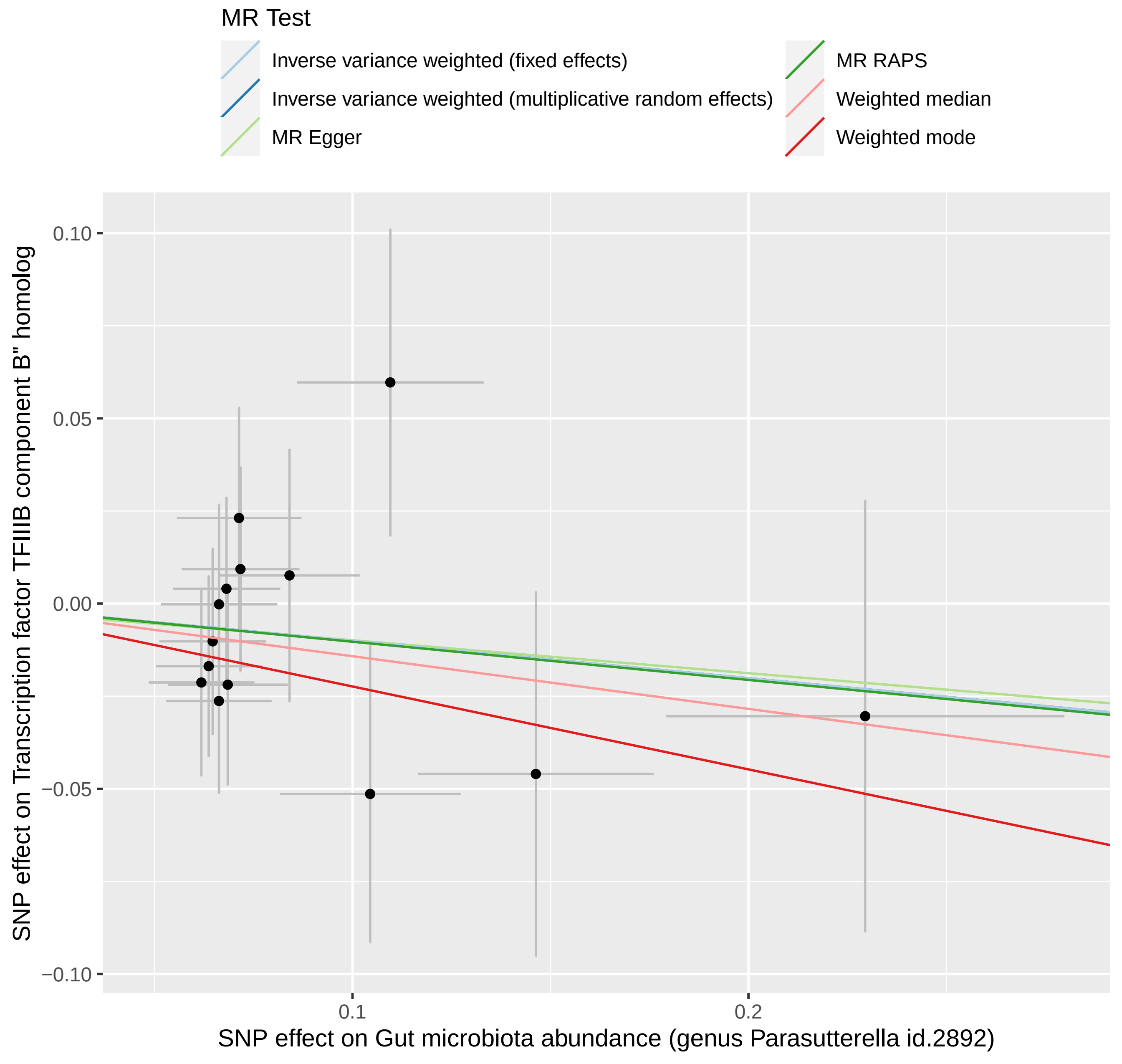

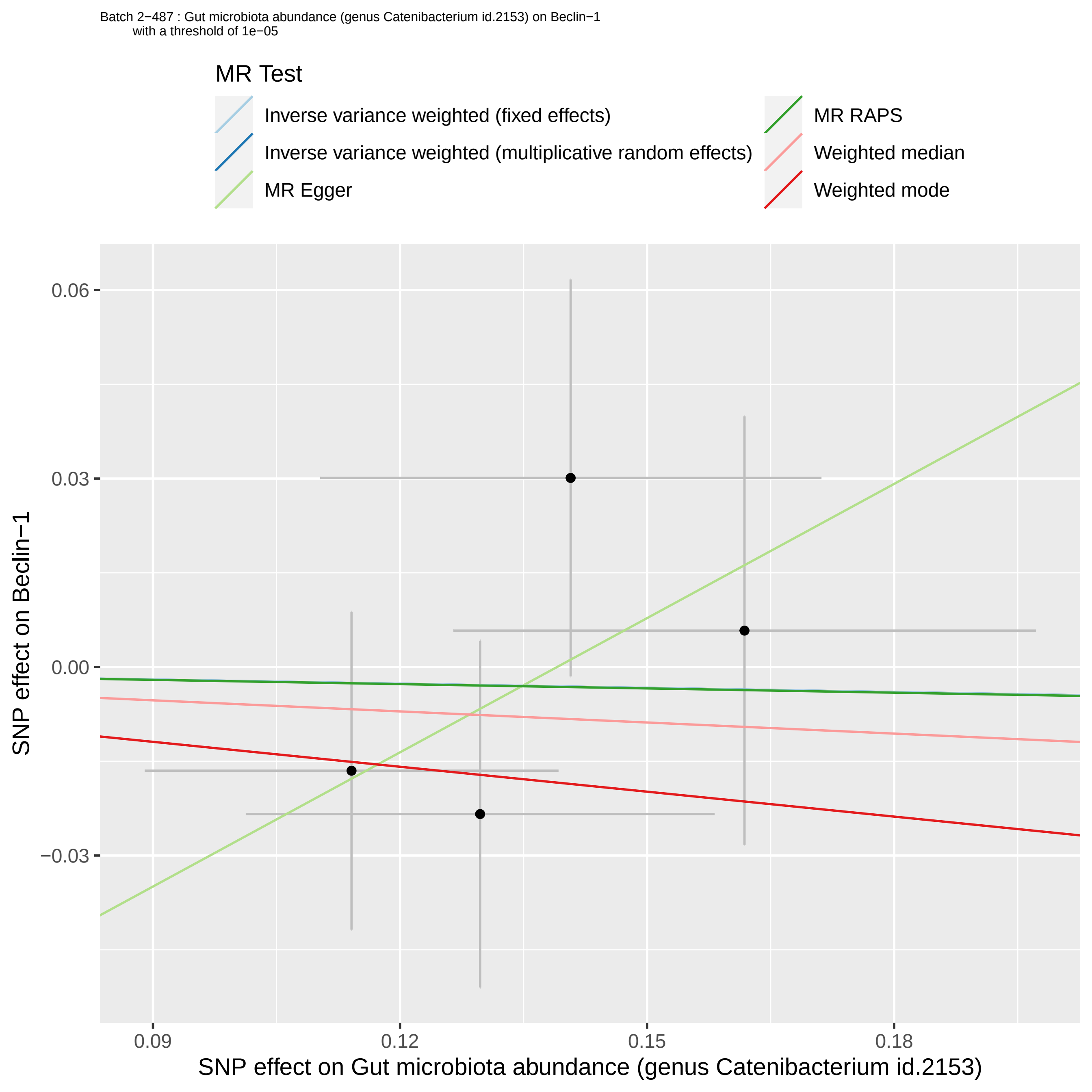
Batch 2−487 : Gut microbiota abundance (genus Catenibacterium id.2153) on Beclin−1
with a threshold of 1e−05
MR Test
Inverse variance weighted (fixed effects)
Inverse variance weighted (multiplicative random effects)
MR Egger
MR RAPS
Weighted median
Weighted mode
0.06
0.03
0.00
−0.03
0.09
0.12
0.15
0.18
SNP effect on Beclin−1
SNP effect on Gut microbiota abundance (genus Catenibacterium id.2153)

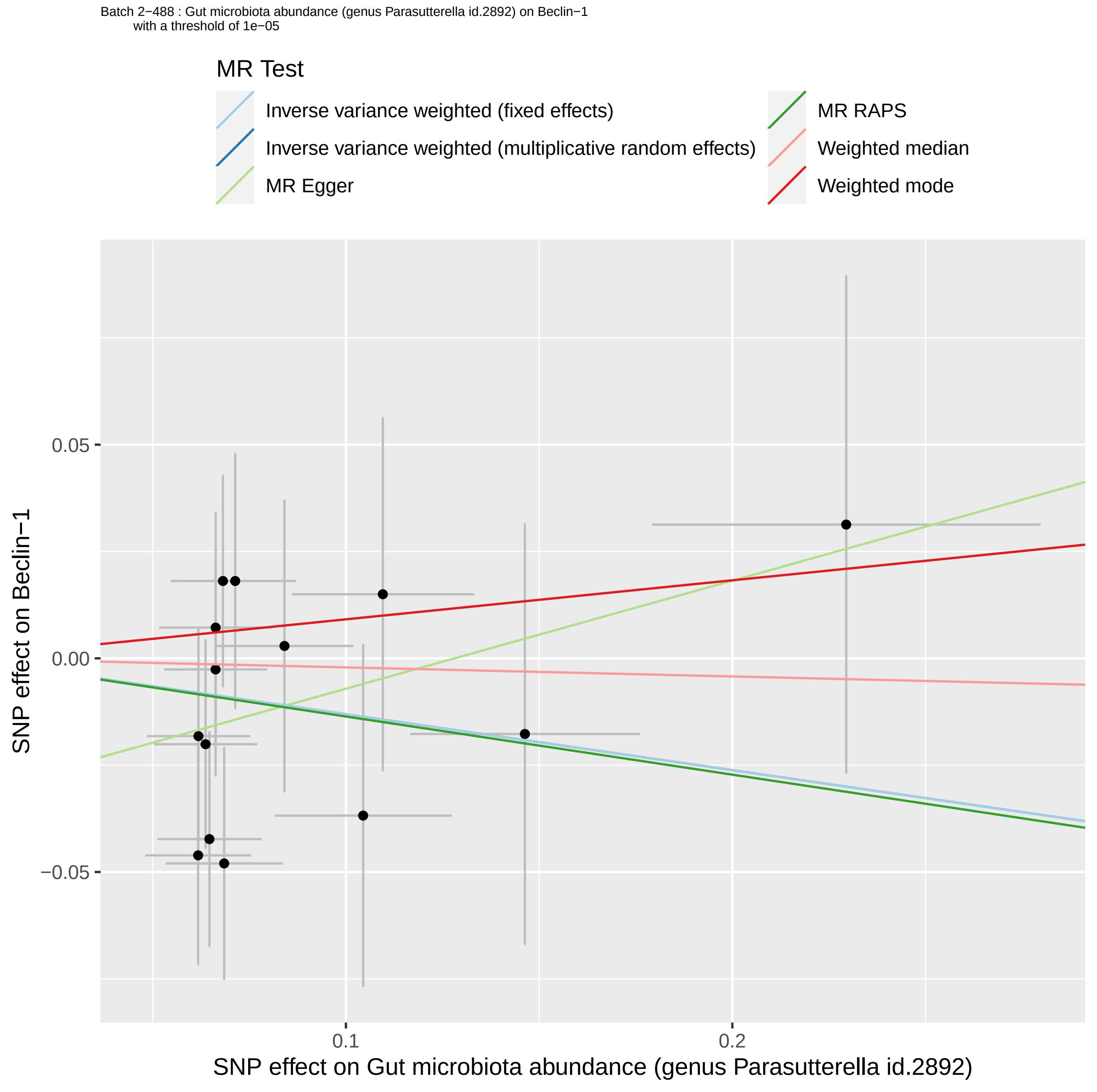
Batch 2−488 : Gut microbiota abundance (genus Parasutterella id.2892) on Beclin−1
with a threshold of 1e−05
MR Test
Inverse variance weighted (fixed effects)
Inverse variance weighted (multiplicative random effects)
MR Egger
MR RAPS
Weighted median
Weighted mode
0.05
0.00
−0.05
0.1
0.2
SNP effect on Beclin−1
SNP effect on Gut microbiota abundance (genus Parasutterella id.2892)

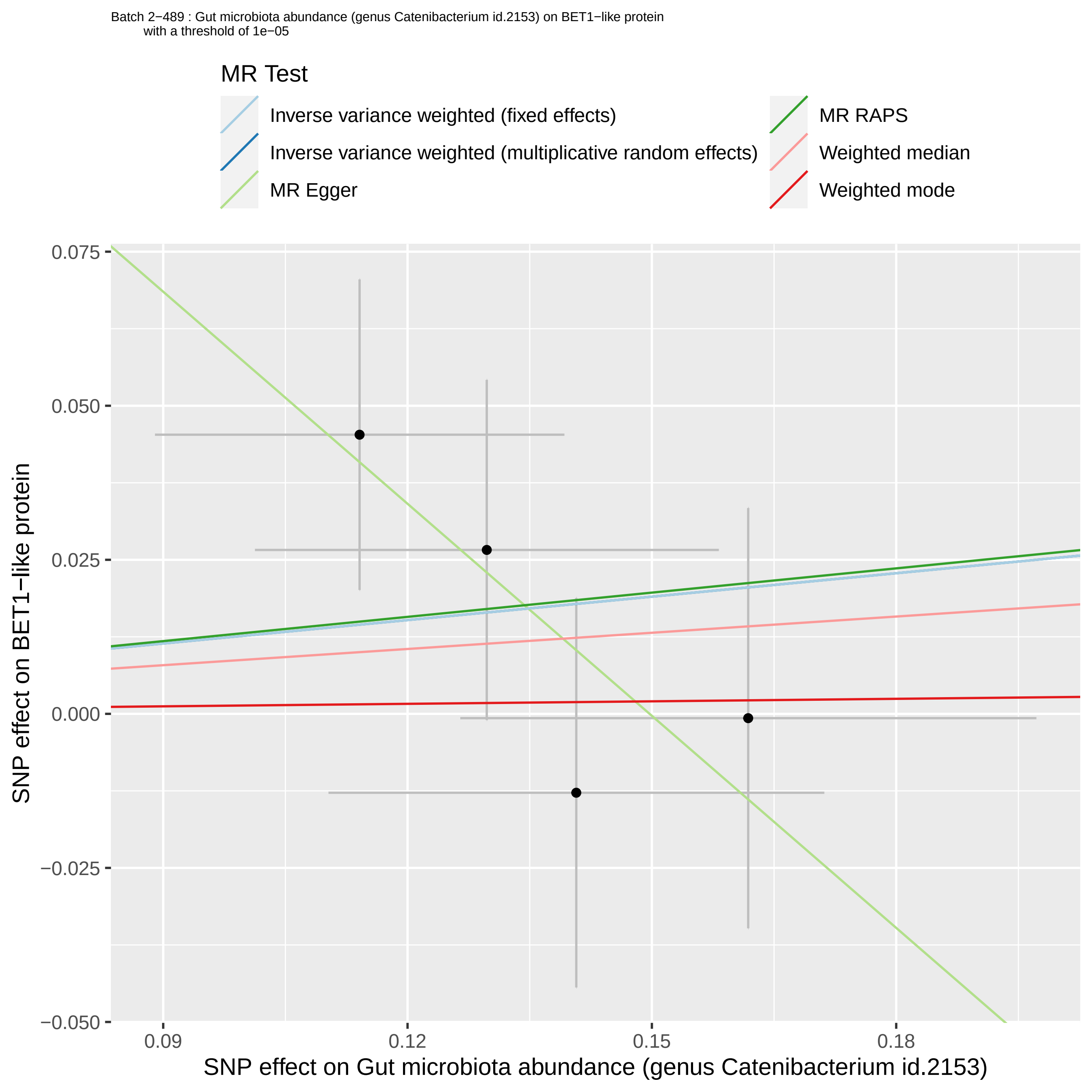
Batch 2−489 : Gut microbiota abundance (genus Catenibacterium id.2153) on BET1−like protein
with a threshold of 1e−05
MR Test
Inverse variance weighted (fixed effects)
Inverse variance weighted (multiplicative random effects)
MR Egger
MR RAPS
Weighted median
Weighted mode
0.075
0.050
0.025
0.000
−0.025
−0.050
0.09
0.12
0.15
0.18
SNP effect on BET1−like protein
SNP effect on Gut microbiota abundance (genus Catenibacterium id.2153)

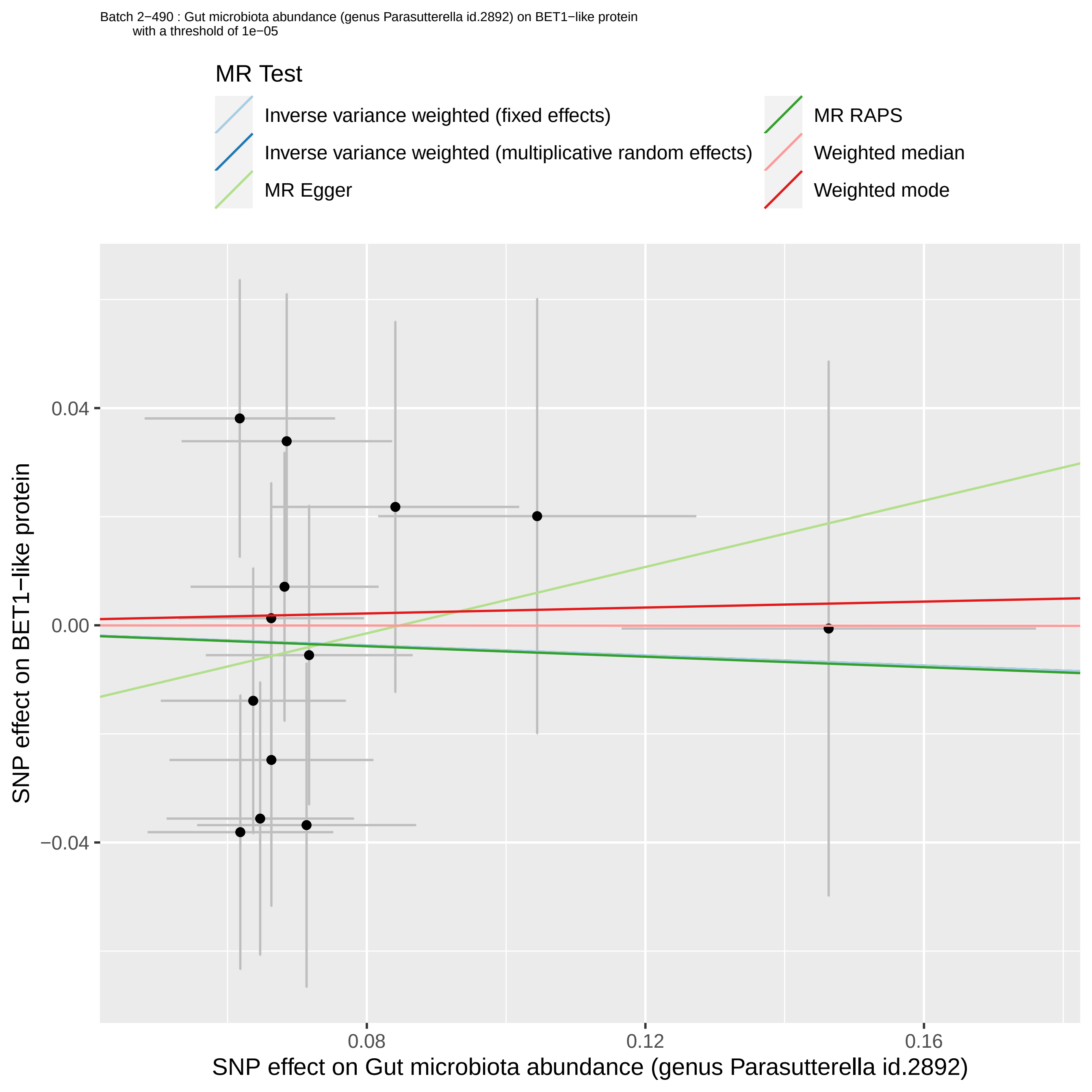
Batch 2−490 : Gut microbiota abundance (genus Parasutterella id.2892) on BET1−like protein
with a threshold of 1e−05
MR Test
Inverse variance weighted (fixed effects)
Inverse variance weighted (multiplicative random effects)
MR Egger
MR RAPS
Weighted median
Weighted mode
0.04
0.00
−0.04
0.08
0.12
0.16
SNP effect on BET1−like protein
SNP effect on Gut microbiota abundance (genus Parasutterella id.2892)

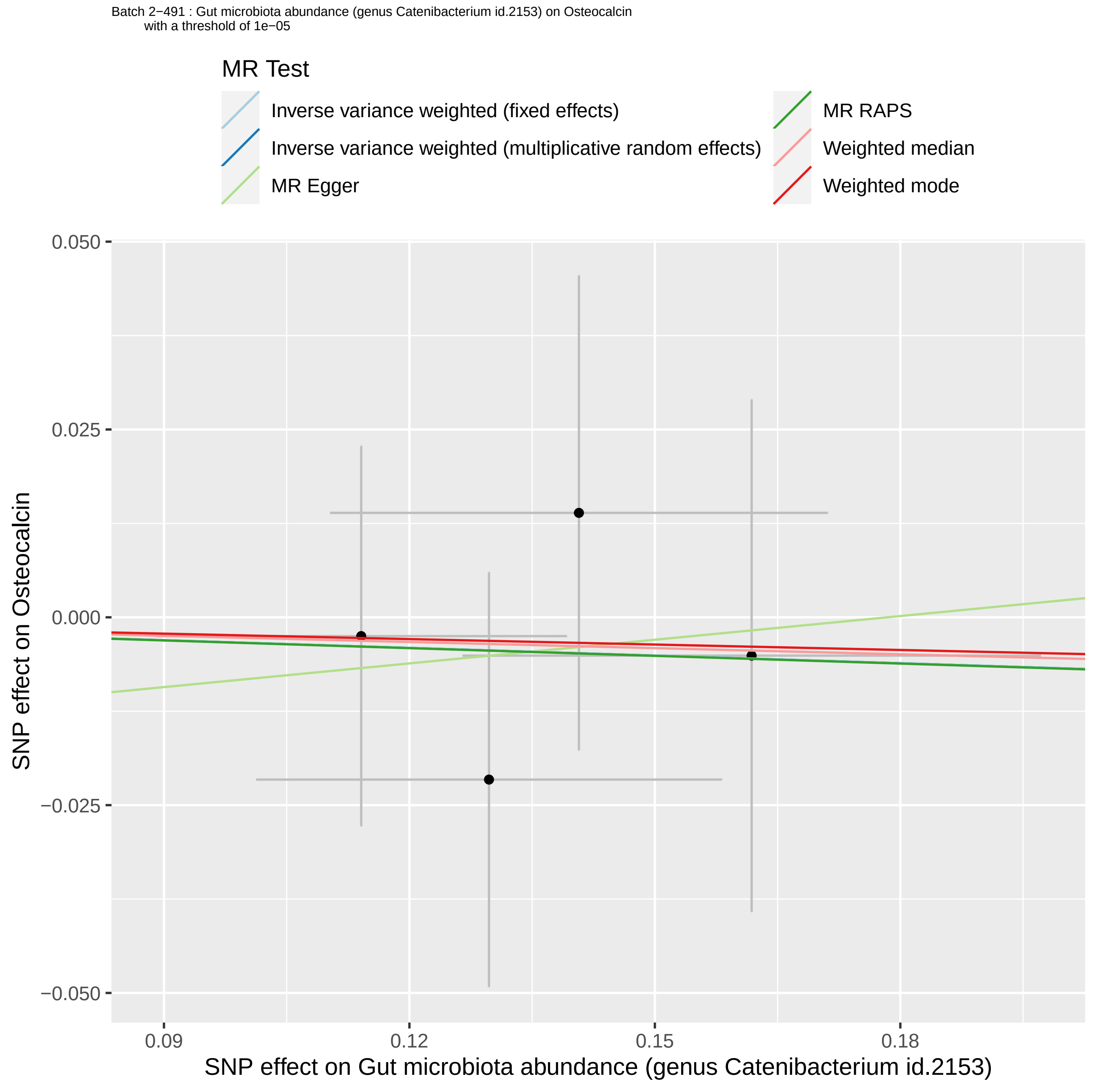
Batch 2−491 : Gut microbiota abundance (genus Catenibacterium id.2153) on Osteocalcin
with a threshold of 1e−05
MR Test
Inverse variance weighted (fixed effects)
Inverse variance weighted (multiplicative random effects)
MR Egger
MR RAPS
Weighted median
Weighted mode
0.050
0.025
0.000
−0.025
−0.050
0.09
0.12
0.15
0.18
SNP effect on Osteocalcin
SNP effect on Gut microbiota abundance (genus Catenibacterium id.2153)

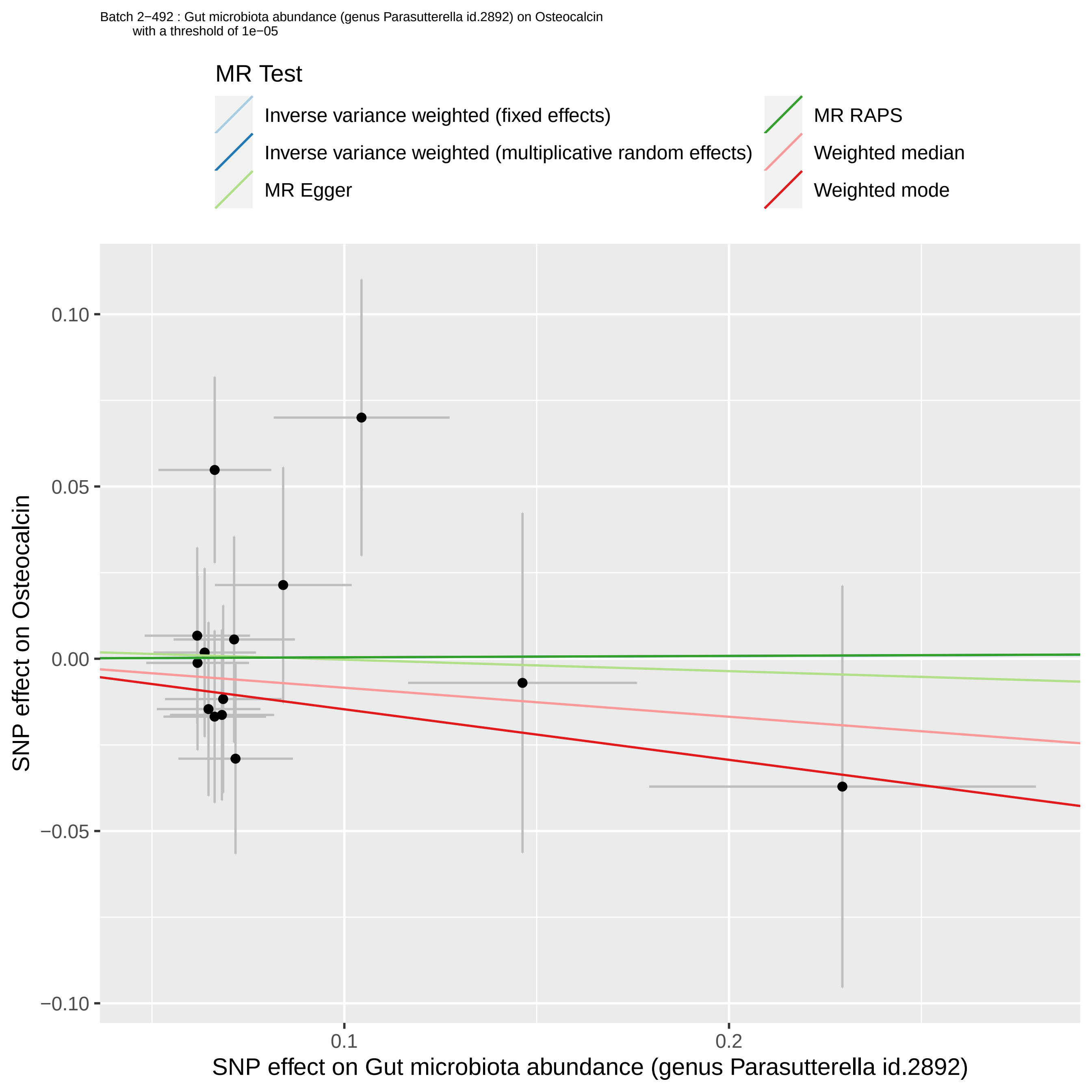

Batch 2−492 : Gut microbiota abundance (genus Parasutterella id.2892) on Osteocalcin
with a threshold of 1e−05
MR Test
Inverse variance weighted (fixed effects)
Inverse variance weighted (multiplicative random effects)
MR Egger
MR RAPS
Weighted median
Weighted mode
0.10
0.05
0.00
−0.05
−0.10
0.1
0.2
SNP effect on Osteocalcin
SNP effect on Gut microbiota abundance (genus Parasutterella id.2892)

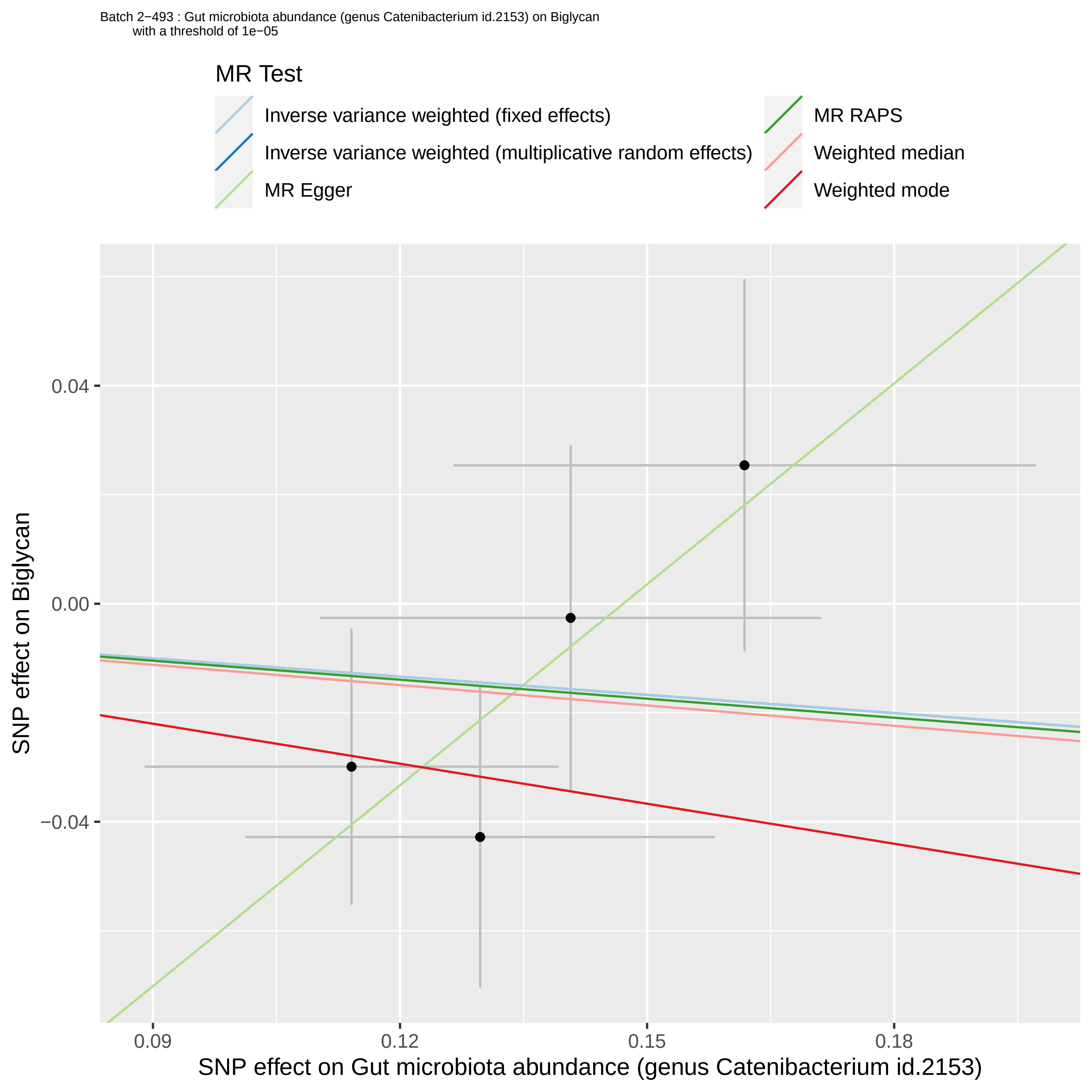
Batch 2−493 : Gut microbiota abundance (genus Catenibacterium id.2153) on Biglycan
with a threshold of 1e−05
MR Test
Inverse variance weighted (fixed effects)
Inverse variance weighted (multiplicative random effects)
MR Egger
MR RAPS
Weighted median
Weighted mode
0.04
0.00
−0.04
0.09
0.12
0.15
0.18
SNP effect on Biglycan
SNP effect on Gut microbiota abundance (genus Catenibacterium id.2153)

Batch 2−494 : Gut microbiota abundance (genus Parasutterella id.2892) on Biglycan
with a threshold of 1e−05

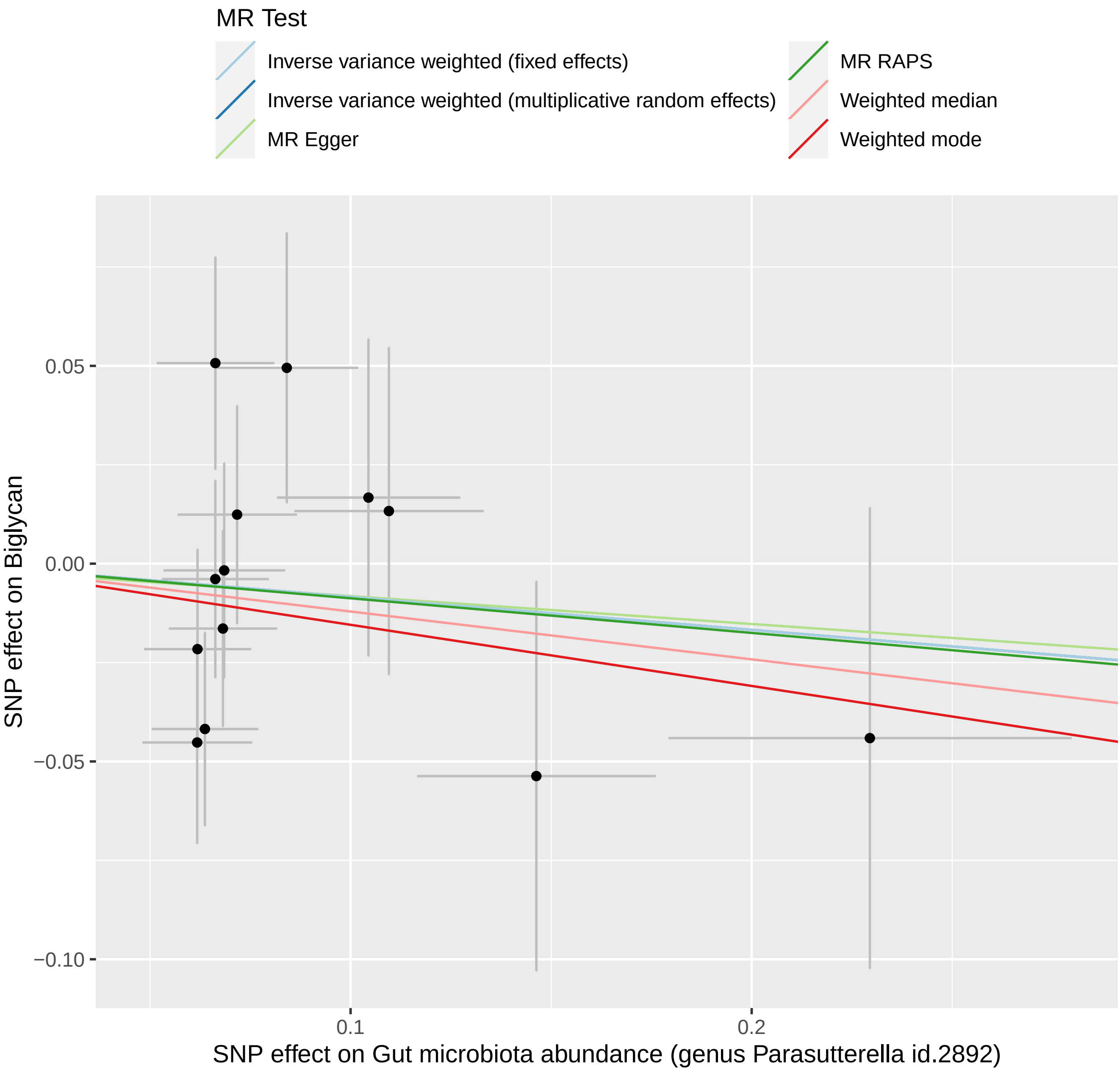

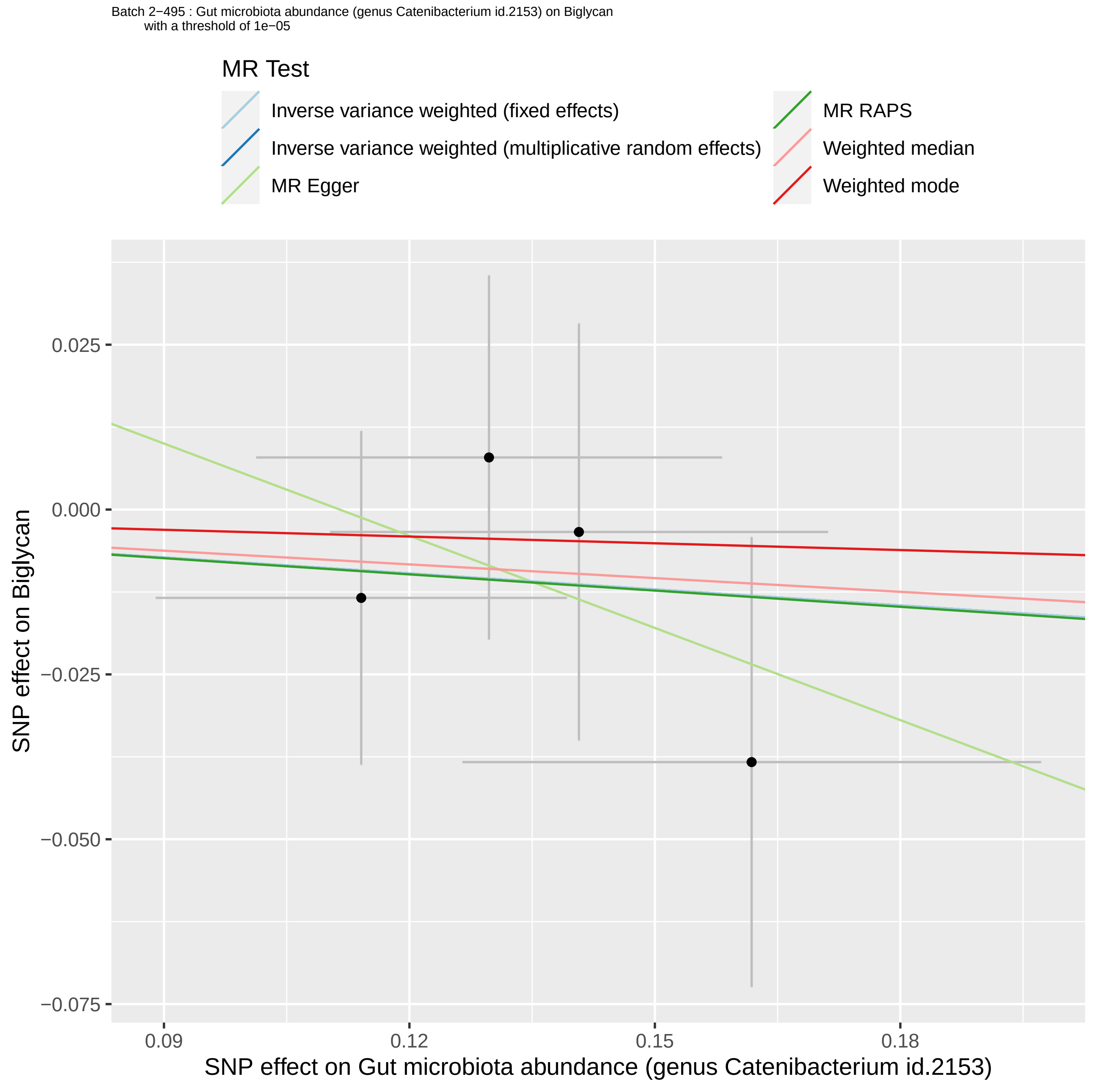

Batch 2−495 : Gut microbiota abundance (genus Catenibacterium id.2153) on Biglycan
with a threshold of 1e−05
MR Test
Inverse variance weighted (fixed effects)
Inverse variance weighted (multiplicative random effects)
MR Egger
MR RAPS
Weighted median
Weighted mode
0.025
0.000
−0.025
−0.050
−0.075
SNP effect on Biglycan
0.09
0.12
0.15
0.18
SNP effect on Gut microbiota abundance (genus Catenibacterium id.2153)

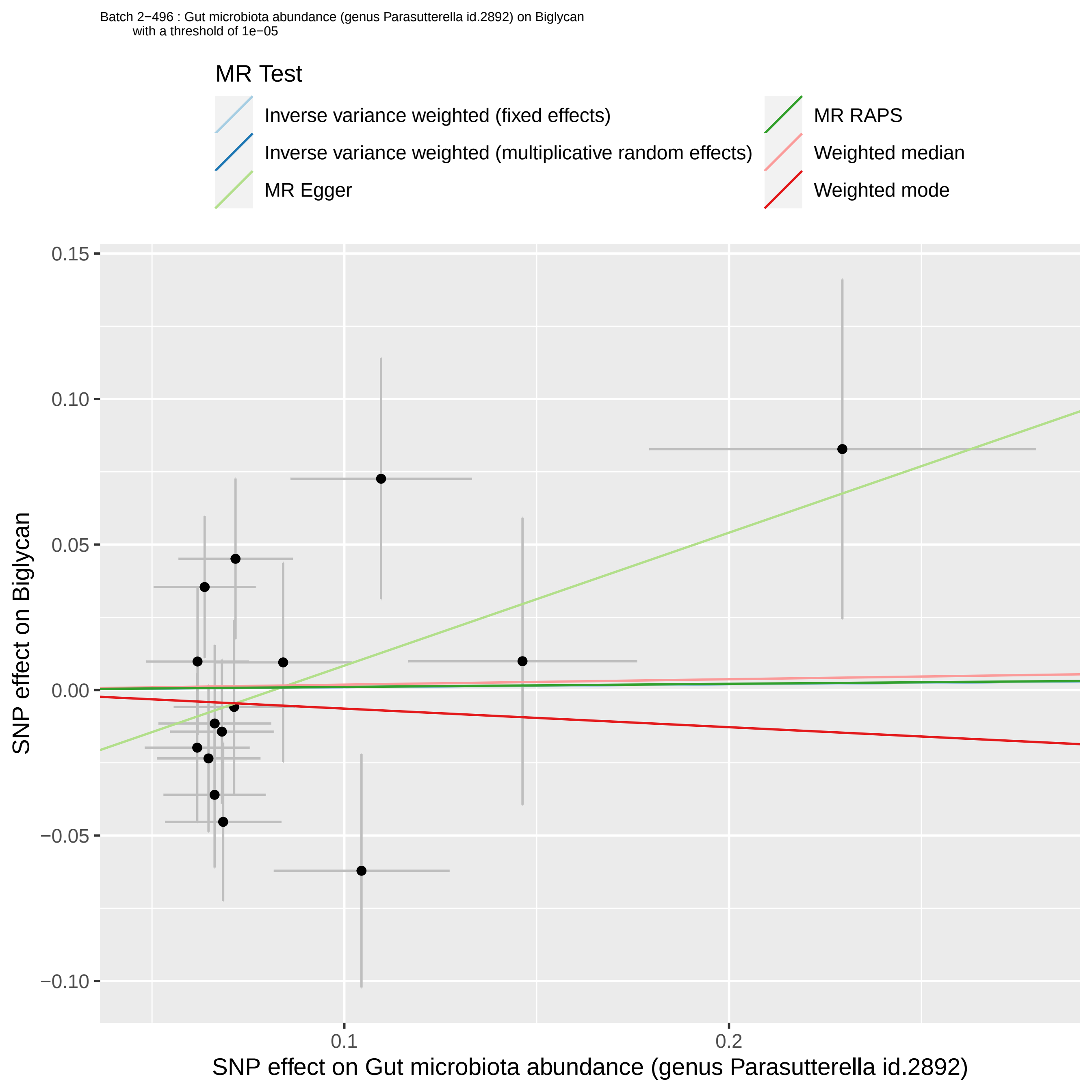
Batch 2−496 : Gut microbiota abundance (genus Parasutterella id.2892) on Biglycan
with a threshold of 1e−05
MR Test
Inverse variance weighted (fixed effects)
Inverse variance weighted (multiplicative random effects)
MR Egger
MR RAPS
Weighted median
Weighted mode
0.15
0.10
0.05
0.00
−0.05
−0.10
0.1
0.2
SNP effect on Biglycan
SNP effect on Gut microbiota abundance (genus Parasutterella id.2892)

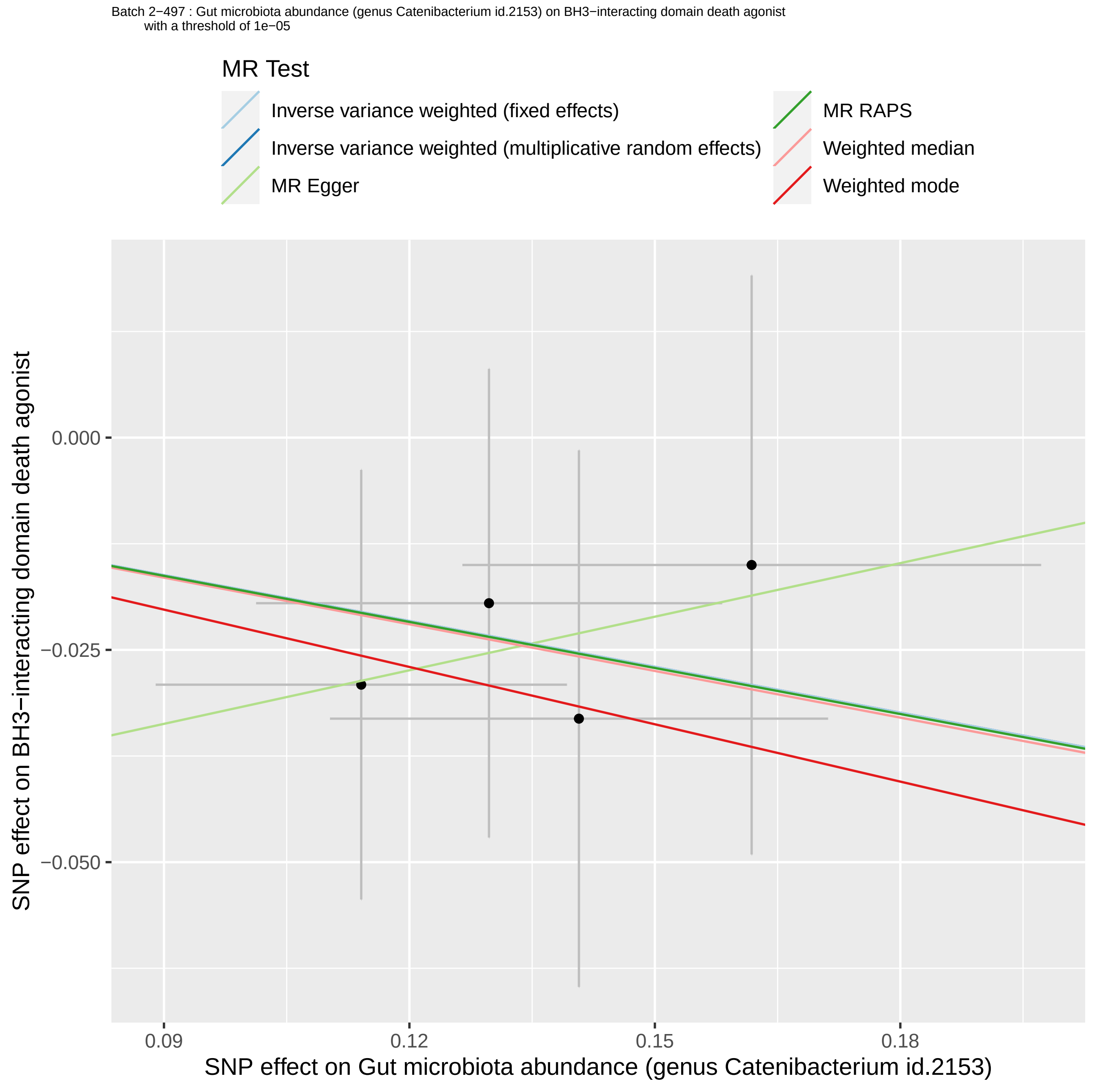

Batch 2−497 : Gut microbiota abundance (genus Catenibacterium id.2153) on BH3−interacting domain death agonist
with a threshold of 1e−05
MR Test
Inverse variance weighted (fixed effects)
Inverse variance weighted (multiplicative random effects)
MR Egger
MR RAPS
Weighted median
Weighted mode
0.000
−0.025
−0.050
0.09
0.12
0.15
0.18
SNP effect on BH3−interacting domain death agonist
SNP effect on Gut microbiota abundance (genus Catenibacterium id.2153)

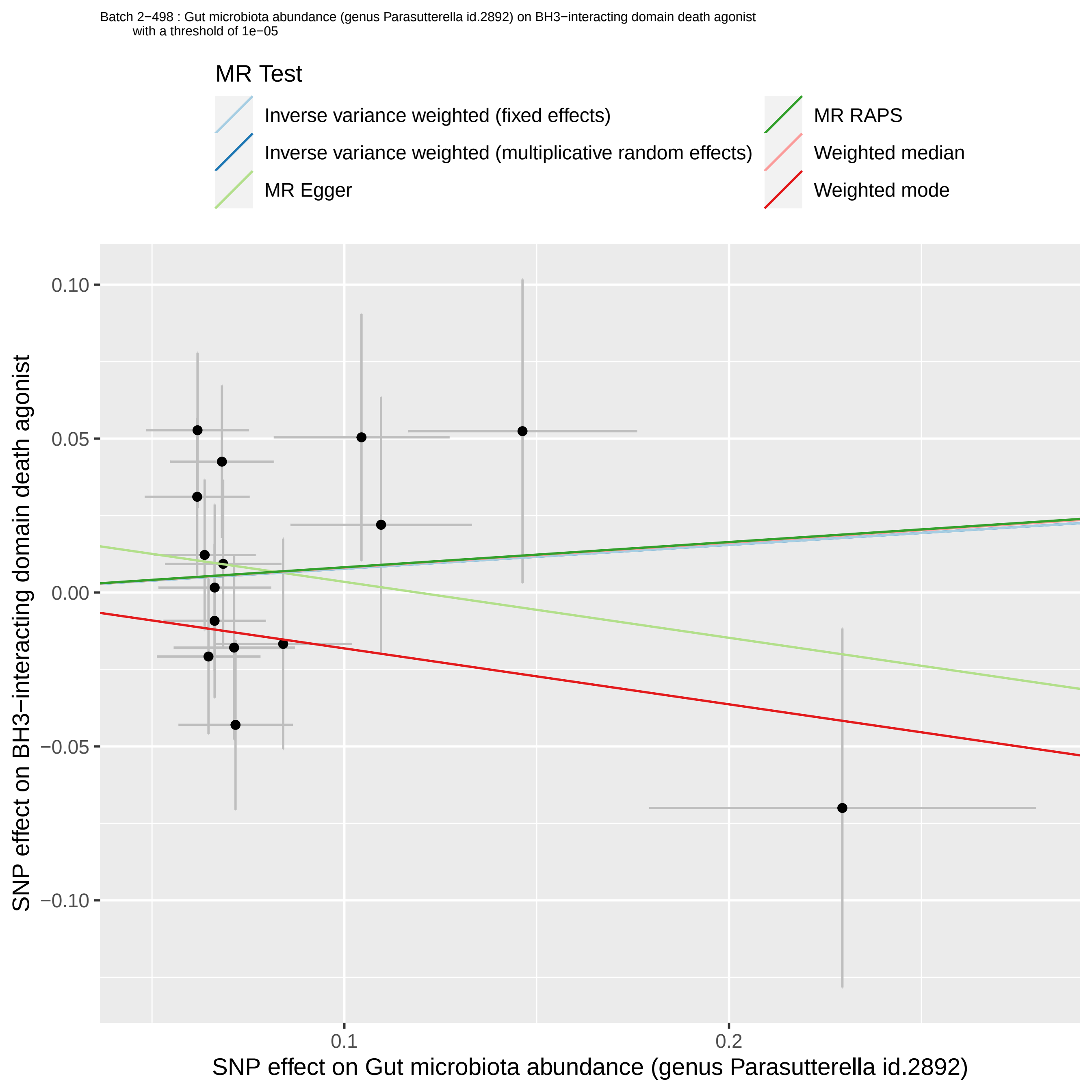

Batch 2−498 : Gut microbiota abundance (genus Parasutterella id.2892) on BH3−interacting domain death agonist
with a threshold of 1e−05
MR Test
Inverse variance weighted (fixed effects)
Inverse variance weighted (multiplicative random effects)
MR Egger
MR RAPS
Weighted median
Weighted mode
0.10
0.05
0.00
−0.05
−0.10
0.1
0.2
SNP effect on BH3−interacting domain death agonist
SNP effect on Gut microbiota abundance (genus Parasutterella id.2892)

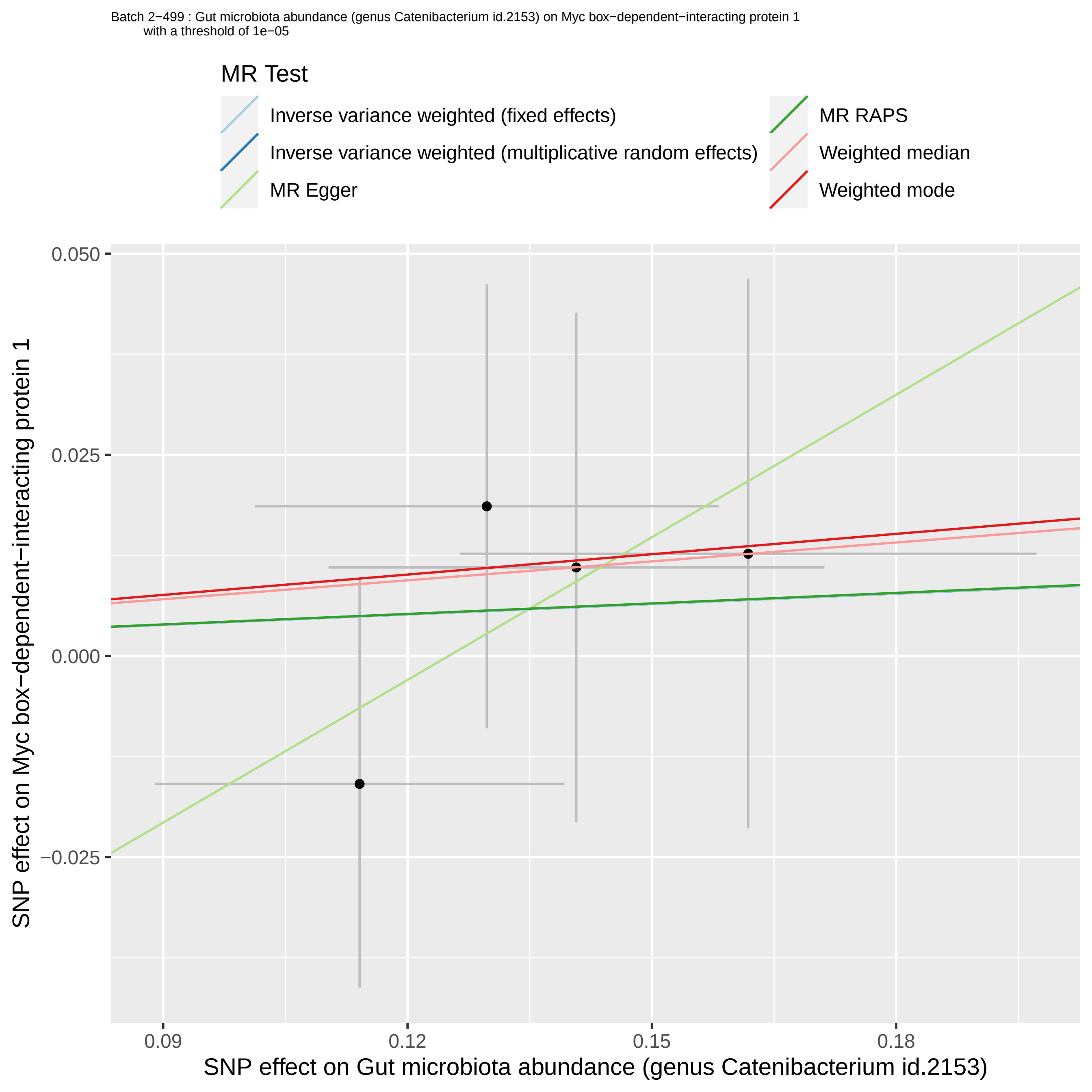
Batch 2−499 : Gut microbiota abundance (genus Catenibacterium id.2153) on Myc box−dependent−interacting protein 1
with a threshold of 1e−05
MR Test
Inverse variance weighted (fixed effects)
Inverse variance weighted (multiplicative random effects)
MR Egger
MR RAPS
Weighted median
Weighted mode
0.050
0.025
0.000
−0.025
0.09
0.12
0.15
0.18
SNP effect on Myc box−dependent−interacting protein 1
SNP effect on Gut microbiota abundance (genus Catenibacterium id.2153)

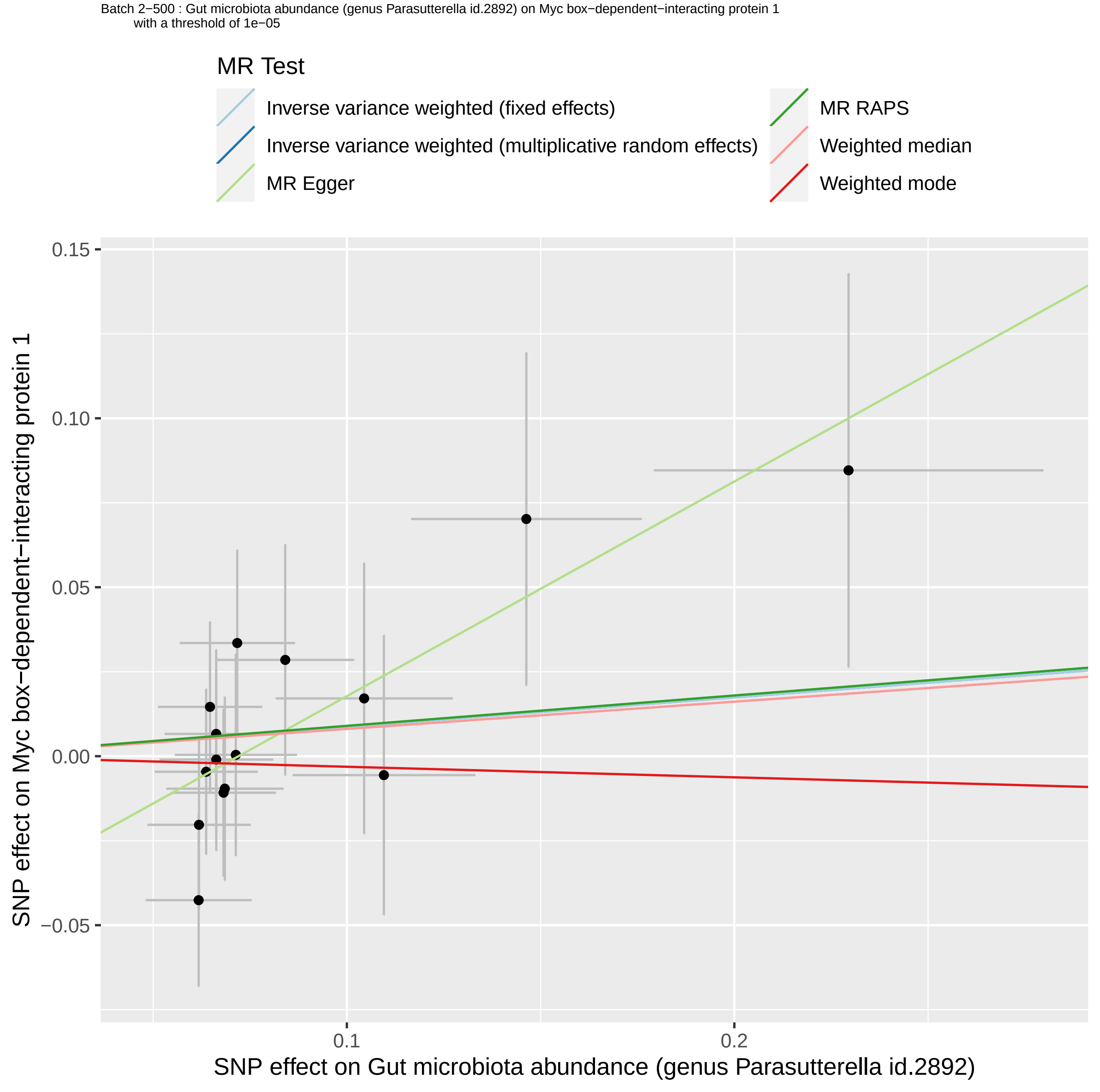
Batch 2−500 : Gut microbiota abundance (genus Parasutterella id.2892) on Myc box−dependent−interacting protein 1
with a threshold of 1e−05
MR Test
Inverse variance weighted (fixed effects)
Inverse variance weighted (multiplicative random effects)
MR Egger
MR RAPS
Weighted median
Weighted mode
0.15
0.10
0.05
0.00
−0.05
0.1
0.2
SNP effect on Myc box−dependent−interacting protein 1
SNP effect on Gut microbiota abundance (genus Parasutterella id.2892)

Batch 2−501 : Gut microbiota abundance (genus Catenibacterium id.2153) on Baculoviral IAP repeat−containing protein 3
with a threshold of 1e−05

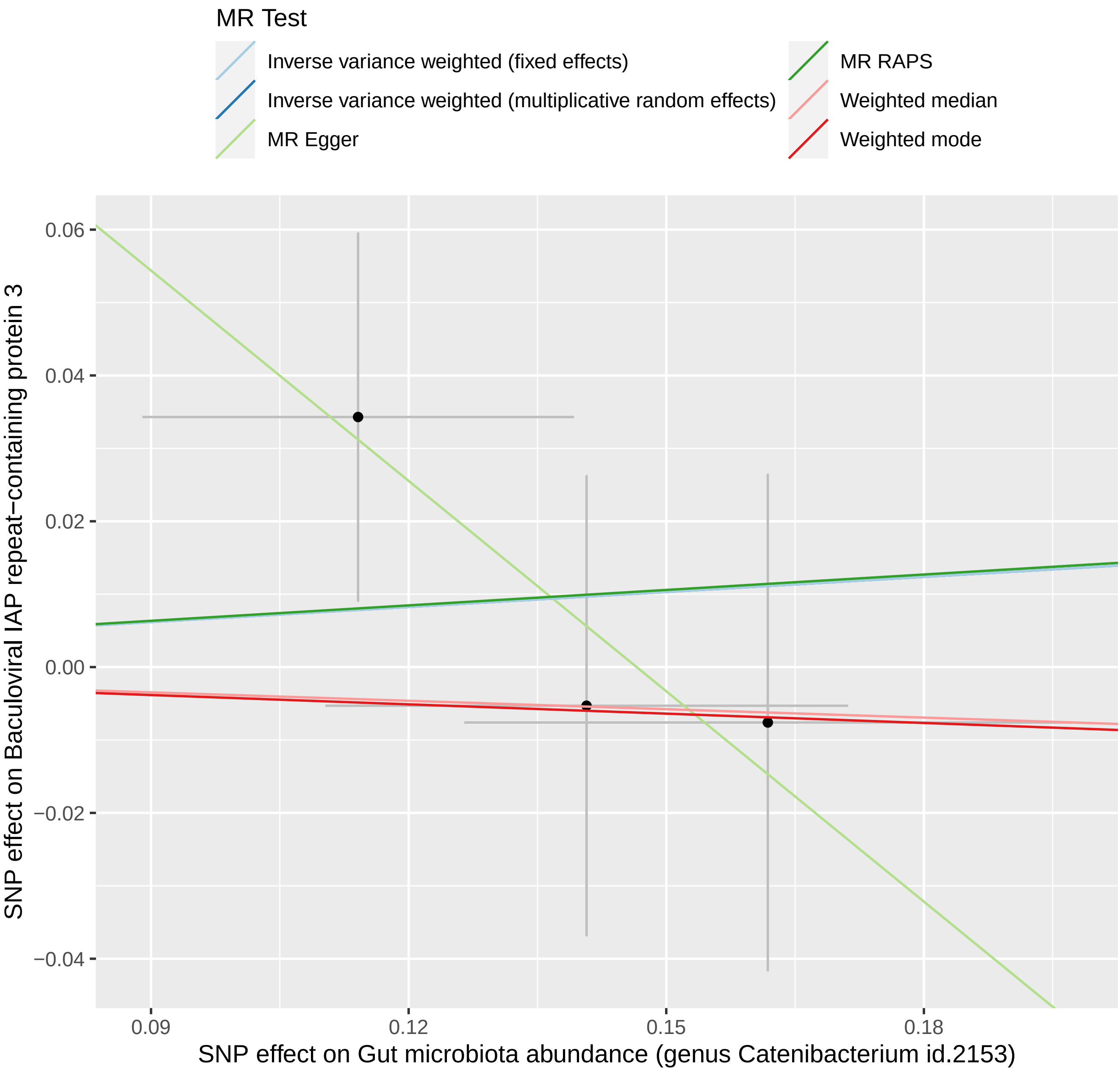

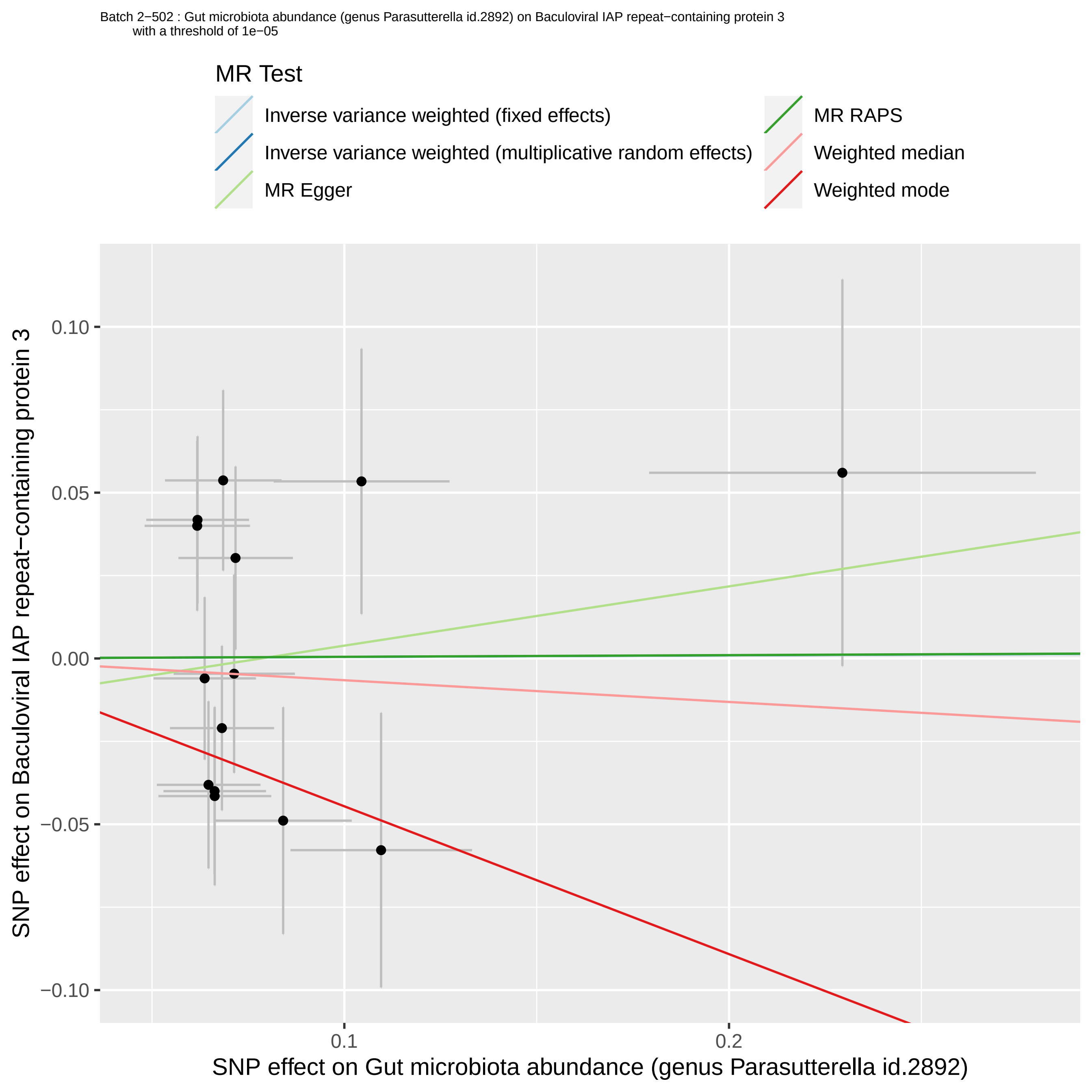

Batch 2−502 : Gut microbiota abundance (genus Parasutterella id.2892) on Baculoviral IAP repeat−containing protein 3
with a threshold of 1e−05
MR Test
Inverse variance weighted (fixed effects)
Inverse variance weighted (multiplicative random effects)
MR Egger
MR RAPS
Weighted median
Weighted mode
0.10
0.05
0.00
−0.05
−0.10
0.1
0.2
SNP effect on Baculoviral IAP repeat−containing protein 3
SNP effect on Gut microbiota abundance (genus Parasutterella id.2892)

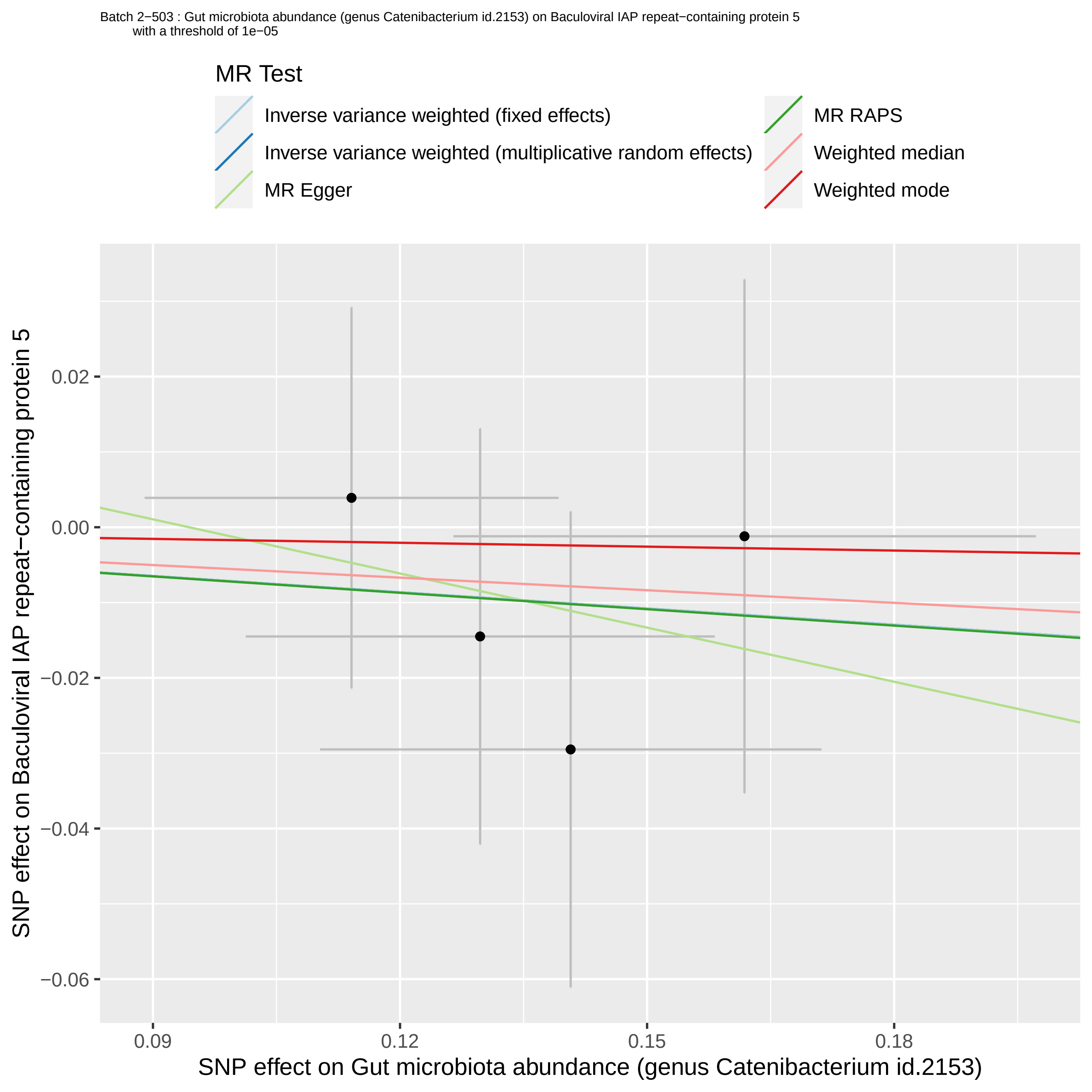
Batch 2−503 : Gut microbiota abundance (genus Catenibacterium id.2153) on Baculoviral IAP repeat−containing protein 5
with a threshold of 1e−05
MR Test
Inverse variance weighted (fixed effects)
Inverse variance weighted (multiplicative random effects)
MR Egger
MR RAPS
Weighted median
Weighted mode
0.02
0.00
−0.02
−0.04
−0.06
0.09
0.12
0.15
0.18
SNP effect on Baculoviral IAP repeat−containing protein 5
SNP effect on Gut microbiota abundance (genus Catenibacterium id.2153)

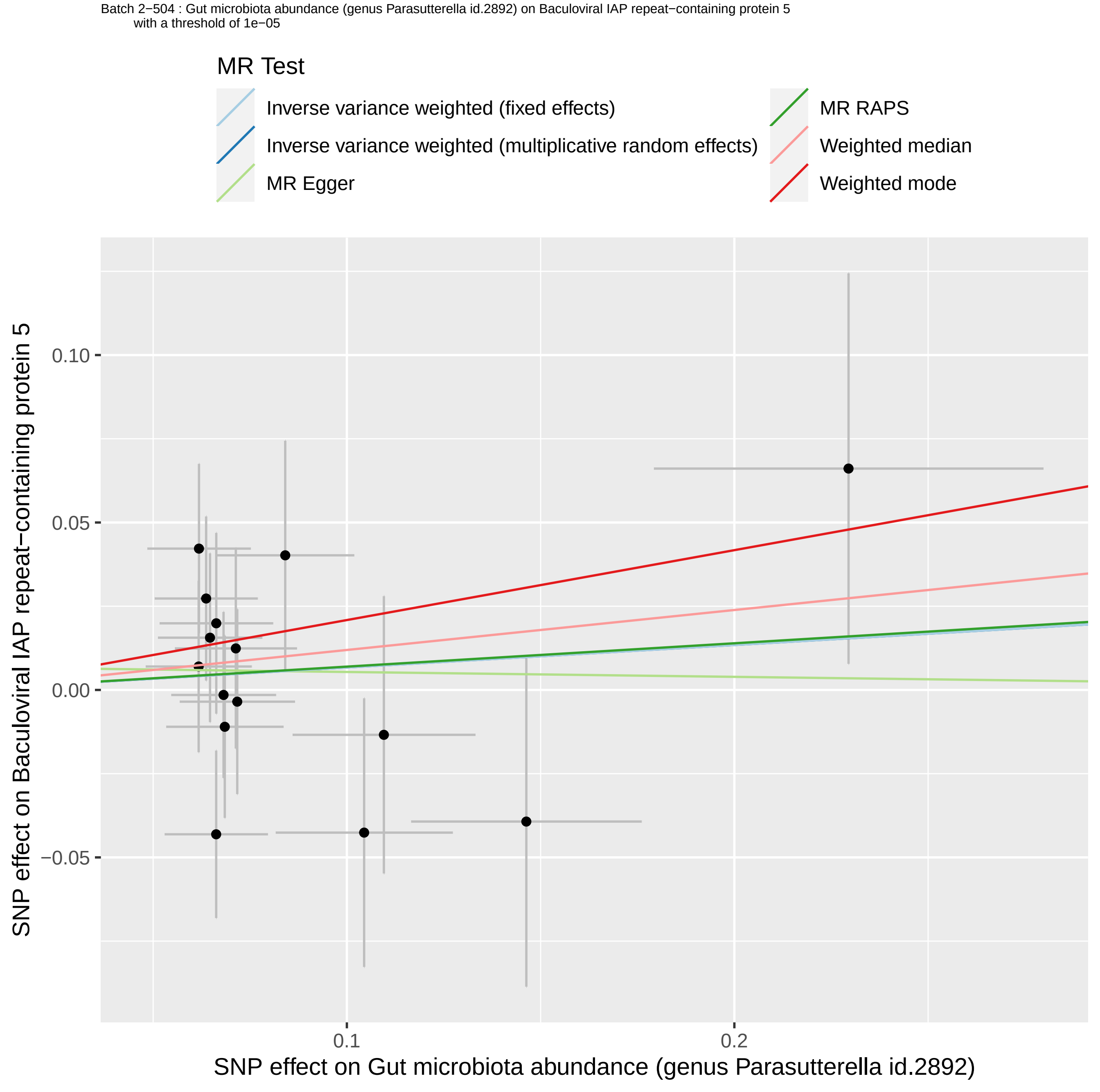
Batch 2−504 : Gut microbiota abundance (genus Parasutterella id.2892) on Baculoviral IAP repeat−containing protein 5
with a threshold of 1e−05
MR Test
Inverse variance weighted (fixed effects)
Inverse variance weighted (multiplicative random effects)
MR Egger
MR RAPS
Weighted median
Weighted mode
0.10
0.05
0.00
−0.05
0.1
0.2
SNP effect on Baculoviral IAP repeat−containing protein 5
SNP effect on Gut microbiota abundance (genus Parasutterella id.2892)

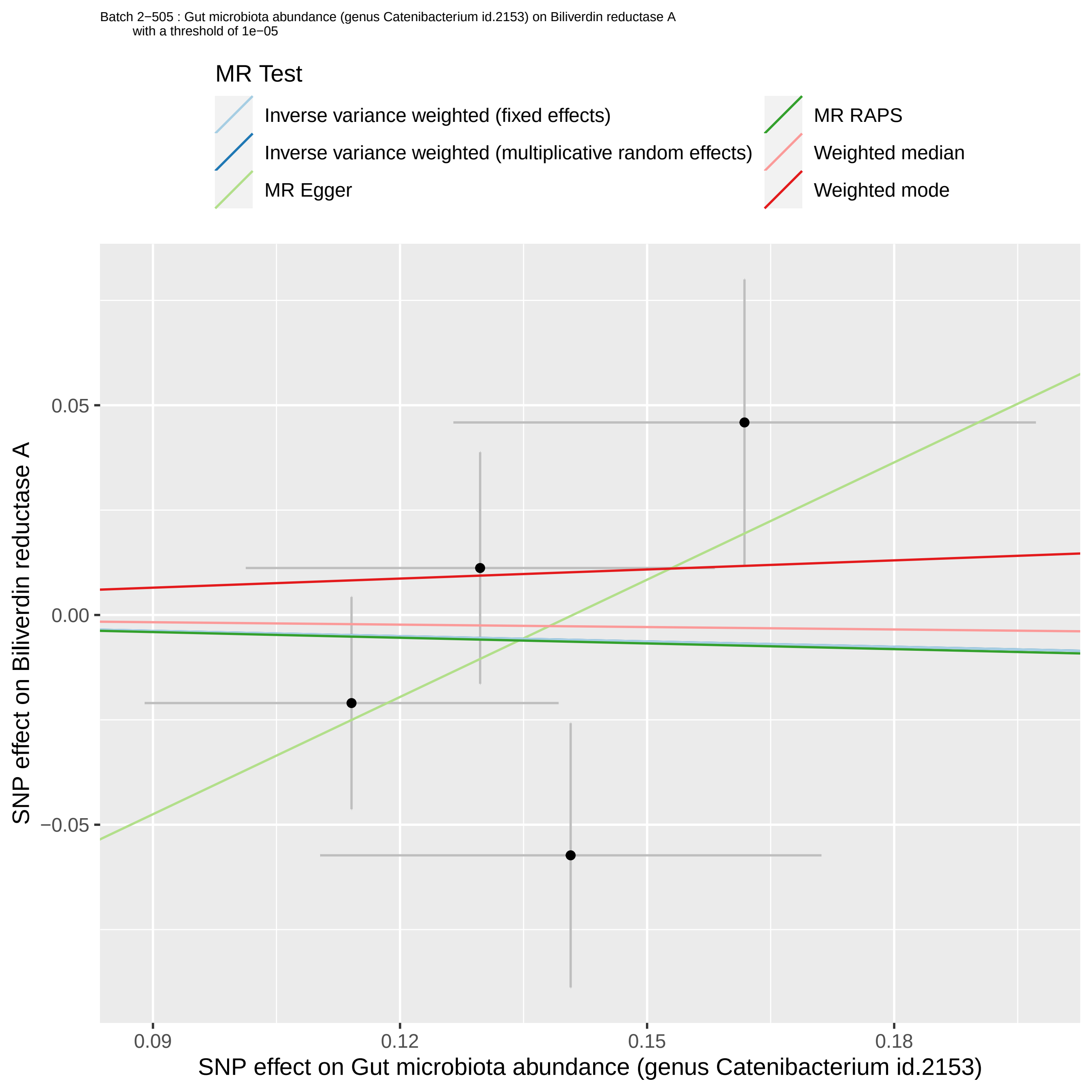
Batch 2−505 : Gut microbiota abundance (genus Catenibacterium id.2153) on Biliverdin reductase A
with a threshold of 1e−05
MR Test
Inverse variance weighted (fixed effects)
Inverse variance weighted (multiplicative random effects)
MR Egger
MR RAPS
Weighted median
Weighted mode
0.05
0.00
−0.05
0.09
0.12
0.15
0.18
SNP effect on Biliverdin reductase A
SNP effect on Gut microbiota abundance (genus Catenibacterium id.2153)

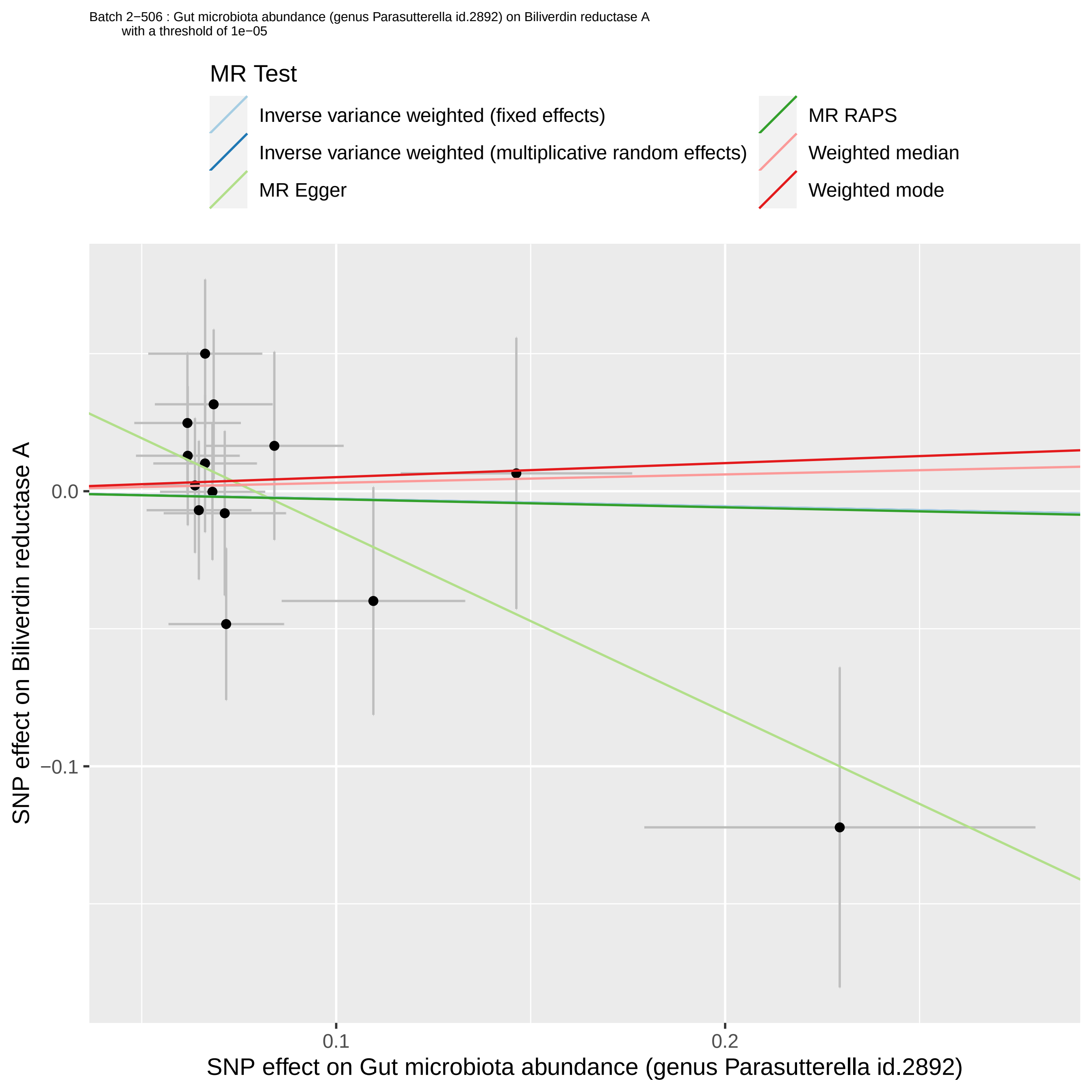

Batch 2−506 : Gut microbiota abundance (genus Parasutterella id.2892) on Biliverdin reductase A
with a threshold of 1e−05
MR Test
Inverse variance weighted (fixed effects)
Inverse variance weighted (multiplicative random effects)
MR Egger
MR RAPS
Weighted median
Weighted mode
0.0
−0.1
0.1
0.2
SNP effect on Biliverdin reductase A
SNP effect on Gut microbiota abundance (genus Parasutterella id.2892)

Batch 2−507 : Gut microbiota abundance (genus Catenibacterium id.2153) on Bone morphogenetic protein 10
with a threshold of 1e−05

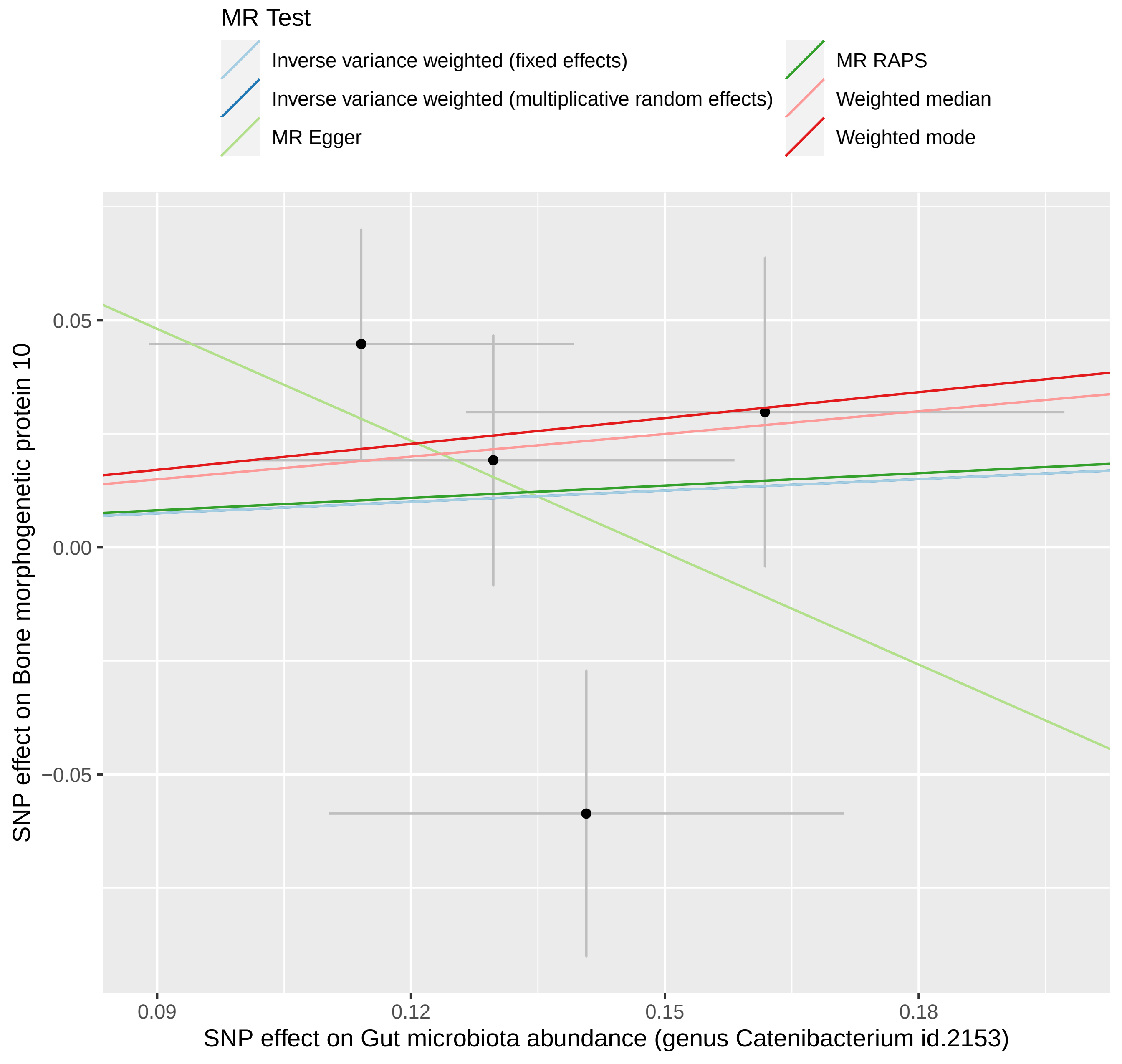

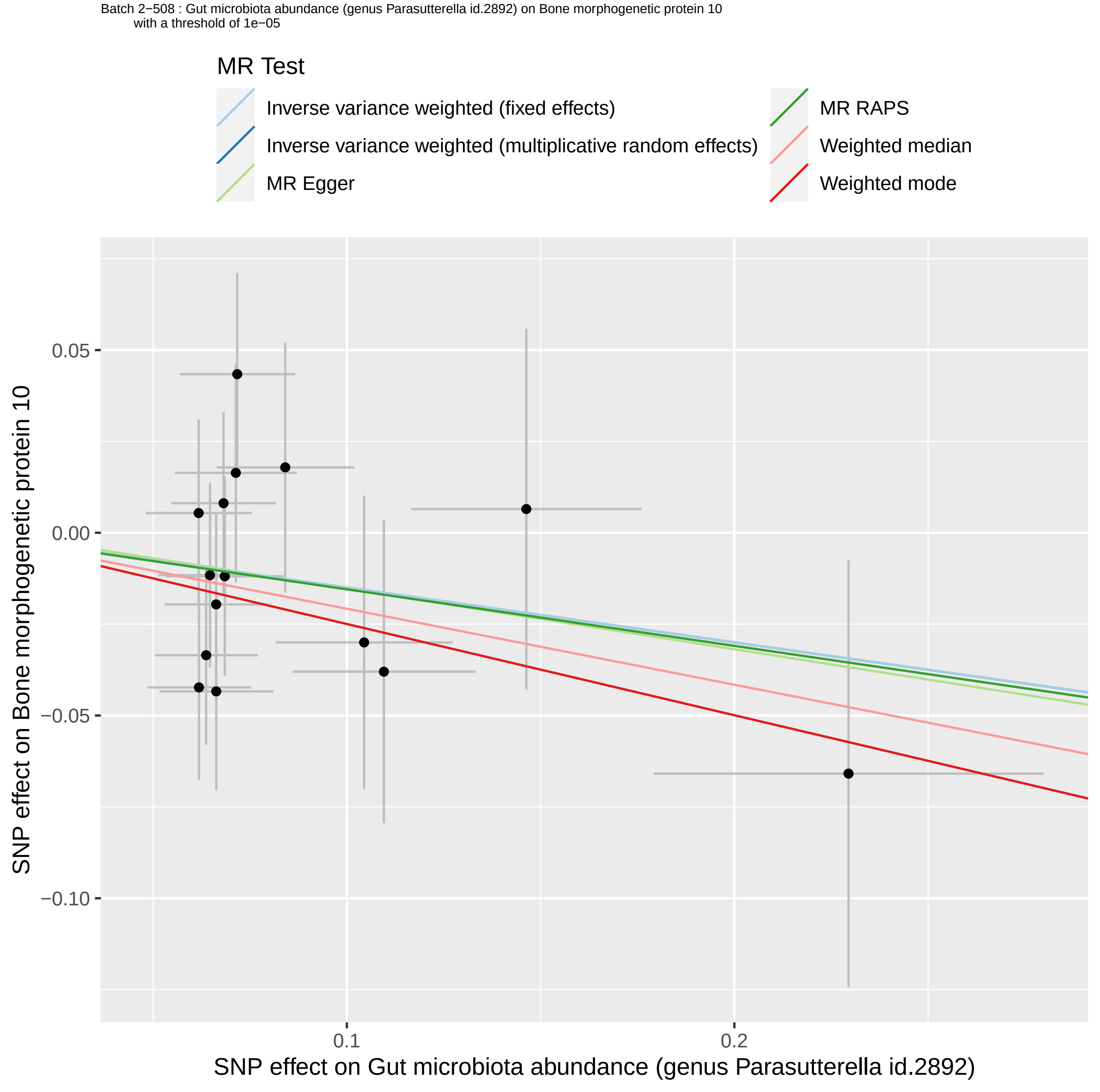
Batch 2−508 : Gut microbiota abundance (genus Parasutterella id.2892) on Bone morphogenetic protein 10
with a threshold of 1e−05
MR Test
Inverse variance weighted (fixed effects)
Inverse variance weighted (multiplicative random effects)
MR Egger
MR RAPS
Weighted median
Weighted mode
0.05
0.00
−0.05
−0.10
0.1
0.2
SNP effect on Bone morphogenetic protein 10
SNP effect on Gut microbiota abundance (genus Parasutterella id.2892)

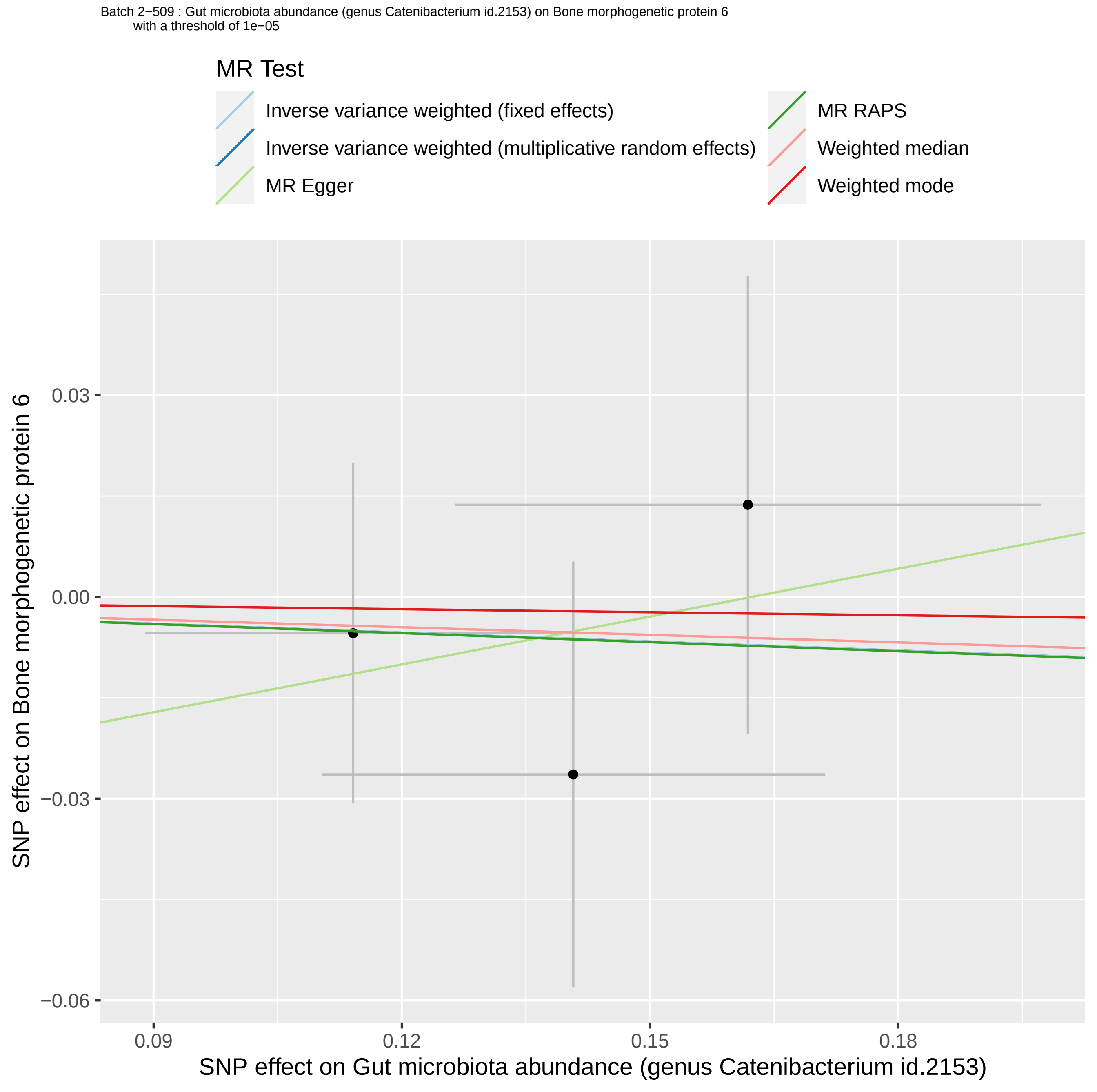

Batch 2−509 : Gut microbiota abundance (genus Catenibacterium id.2153) on Bone morphogenetic protein 6
with a threshold of 1e−05
MR Test
Inverse variance weighted (fixed effects)
Inverse variance weighted (multiplicative random effects)
MR Egger
MR RAPS
Weighted median
Weighted mode
0.03
0.00
−0.03
−0.06
0.09
0.12
0.15
0.18
SNP effect on Bone morphogenetic protein 6
SNP effect on Gut microbiota abundance (genus Catenibacterium id.2153)

Batch 2−510 : Gut microbiota abundance (genus Parasutterella id.2892) on Bone morphogenetic protein 6
with a threshold of 1e−05

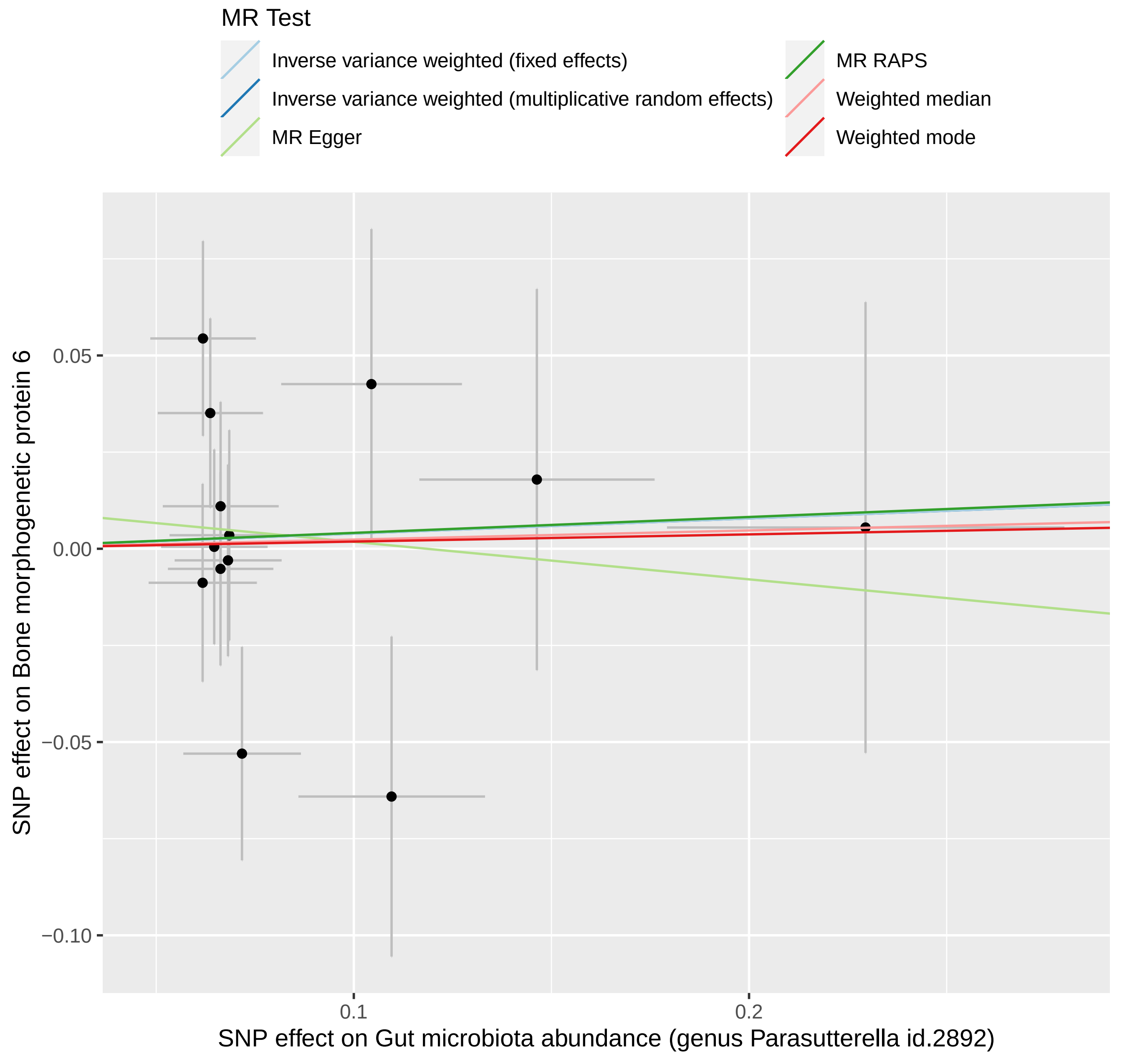

Batch 2−511 : Gut microbiota abundance (genus Catenibacterium id.2153) on Bone morphogenetic protein 6
with a threshold of 1e−05

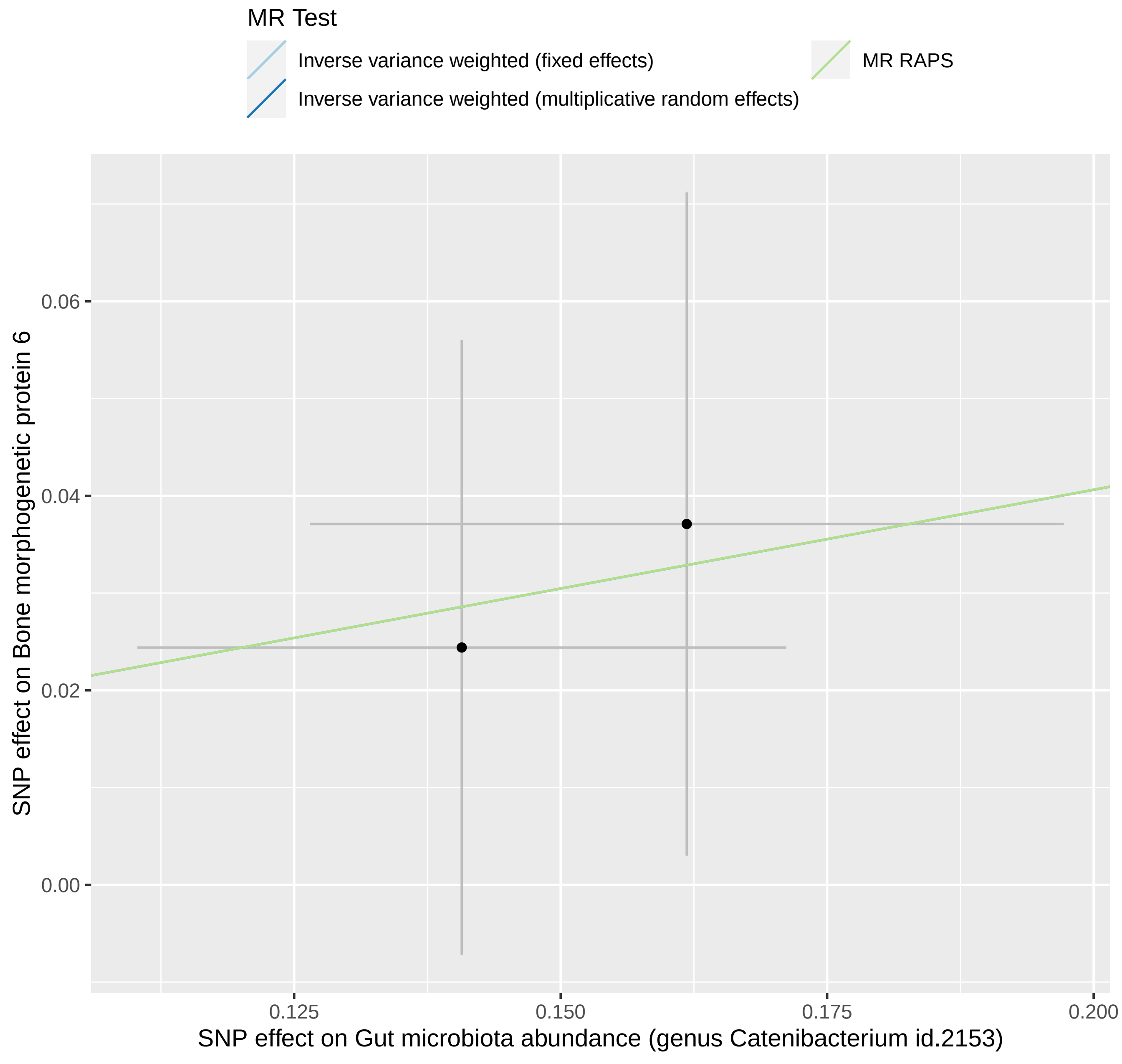

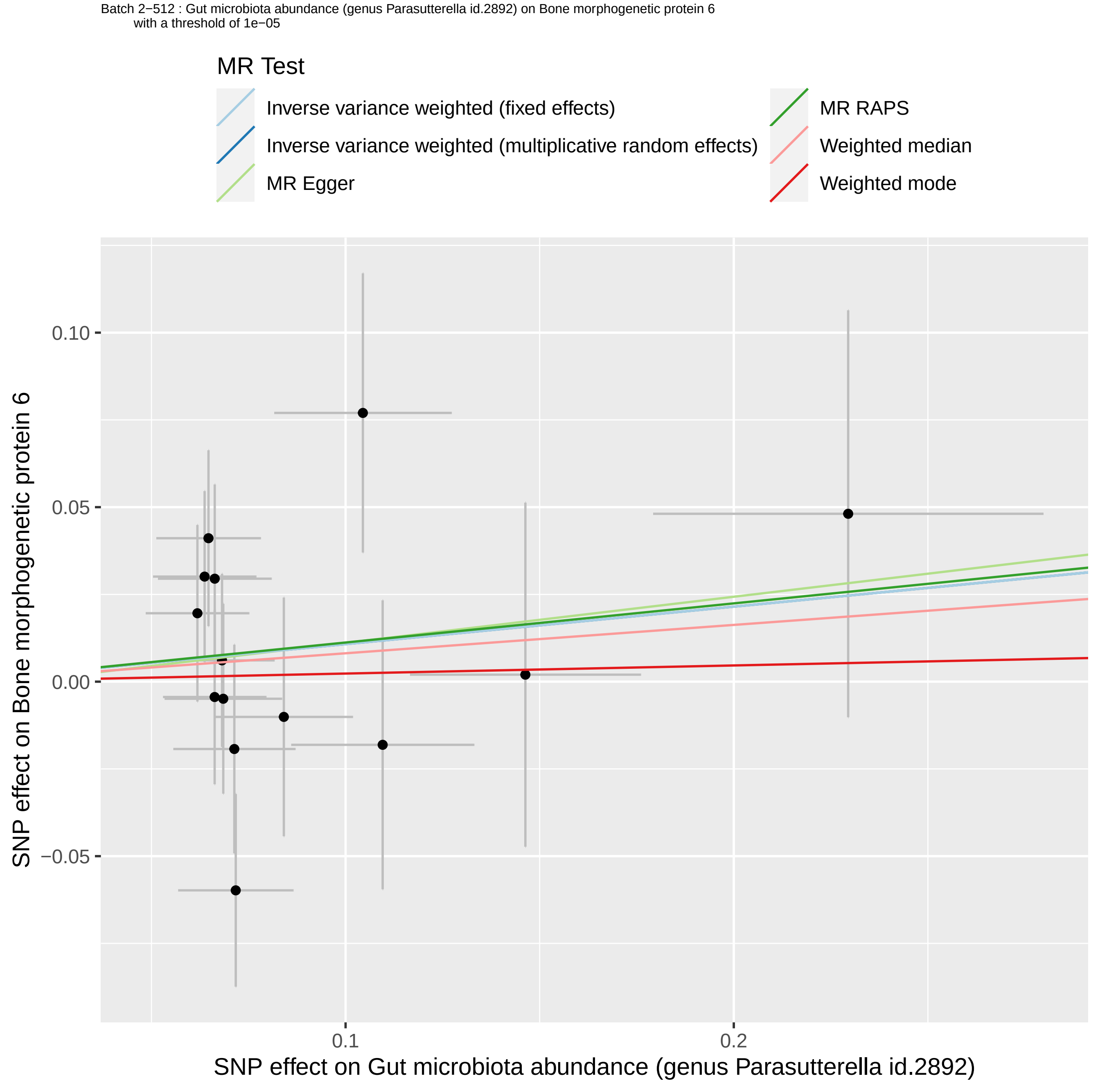
Batch 2−512 : Gut microbiota abundance (genus Parasutterella id.2892) on Bone morphogenetic protein 6
with a threshold of 1e−05
MR Test
Inverse variance weighted (fixed effects)
Inverse variance weighted (multiplicative random effects)
MR Egger
MR RAPS
Weighted median
Weighted mode
0.10
0.05
0.00
−0.05
0.1
0.2
SNP effect on Bone morphogenetic protein 6
SNP effect on Gut microbiota abundance (genus Parasutterella id.2892)

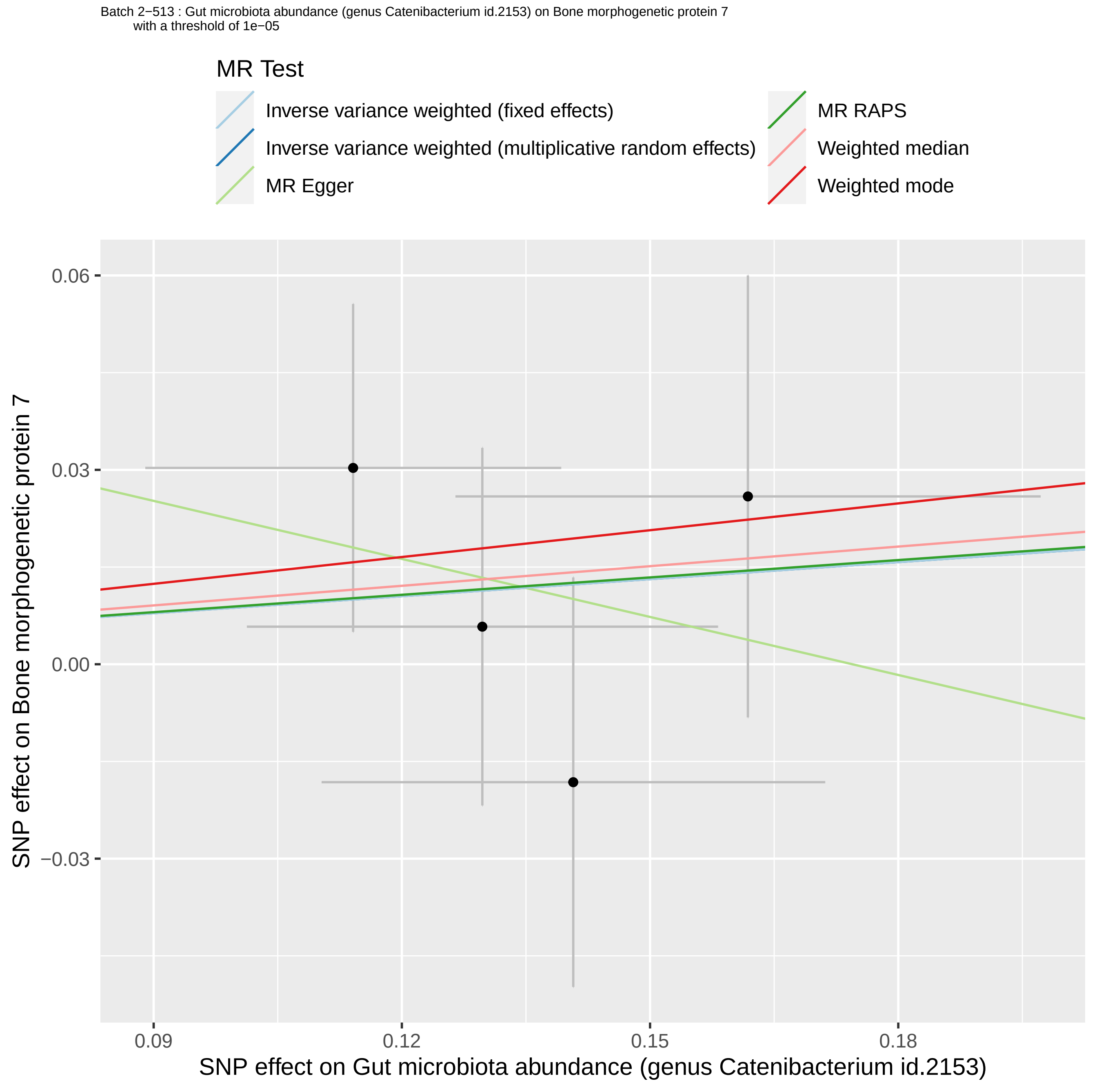

Batch 2−513 : Gut microbiota abundance (genus Catenibacterium id.2153) on Bone morphogenetic protein 7
with a threshold of 1e−05
MR Test
Inverse variance weighted (fixed effects)
Inverse variance weighted (multiplicative random effects)
MR Egger
MR RAPS
Weighted median
Weighted mode
0.06
0.03
0.00
−0.03
0.09
0.12
0.15
0.18
SNP effect on Bone morphogenetic protein 7
SNP effect on Gut microbiota abundance (genus Catenibacterium id.2153)

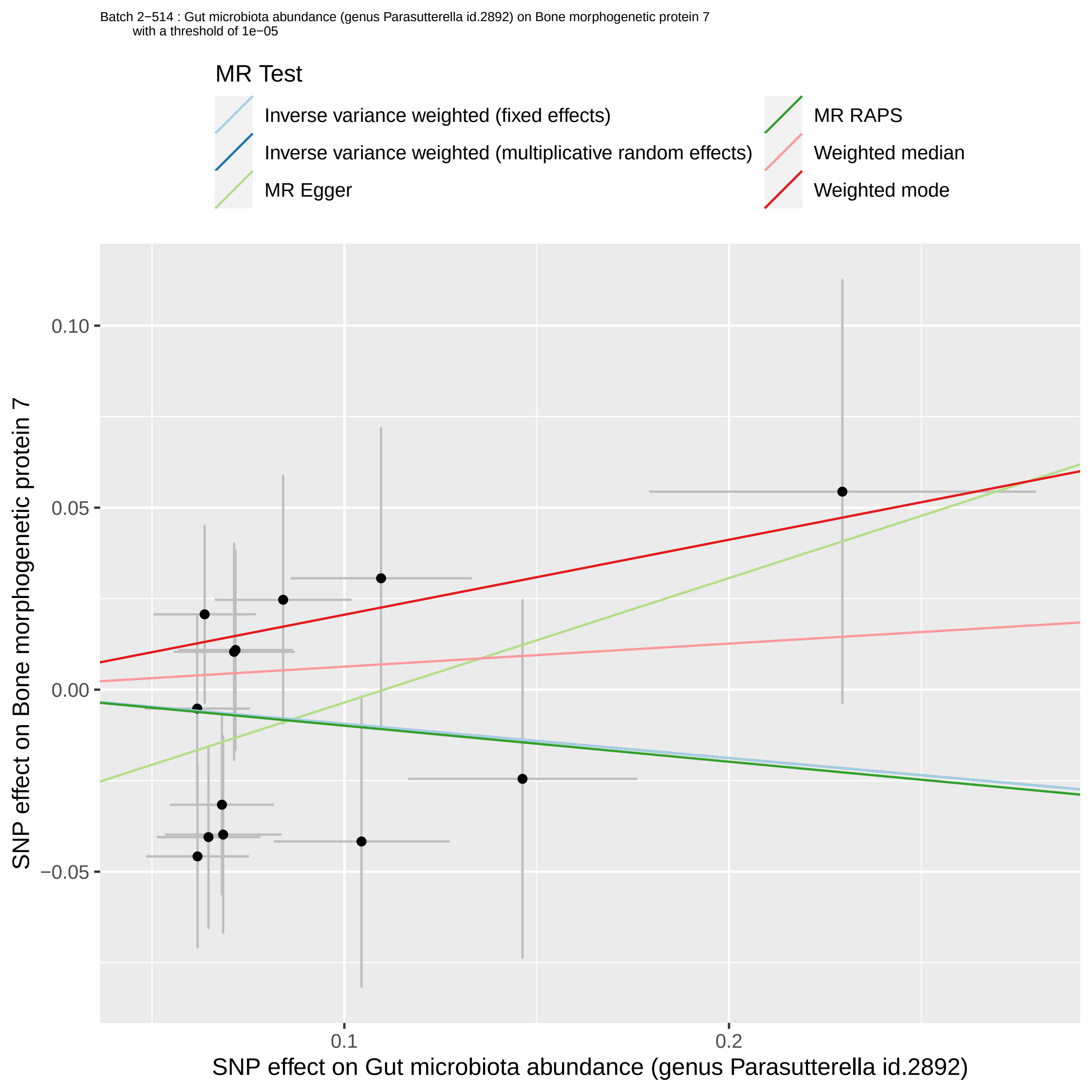
Batch 2−514 : Gut microbiota abundance (genus Parasutterella id.2892) on Bone morphogenetic protein 7
with a threshold of 1e−05
MR Test
Inverse variance weighted (fixed effects)
Inverse variance weighted (multiplicative random effects)
MR Egger
MR RAPS
Weighted median
Weighted mode
0.10
0.05
0.00
−0.05
0.1
0.2
SNP effect on Bone morphogenetic protein 7
SNP effect on Gut microbiota abundance (genus Parasutterella id.2892)

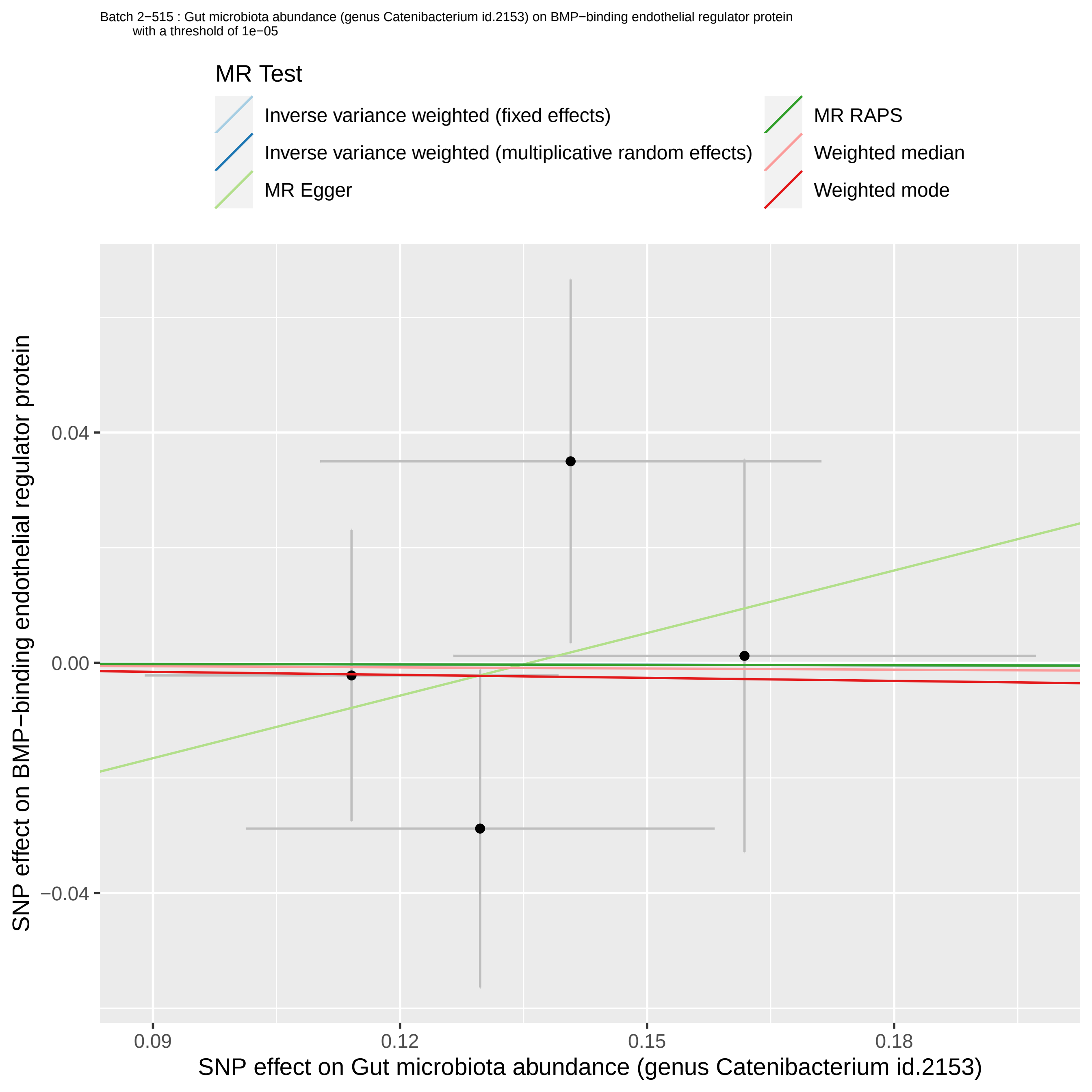
Batch 2−515 : Gut microbiota abundance (genus Catenibacterium id.2153) on BMP−binding endothelial regulator protein
with a threshold of 1e−05
MR Test
Inverse variance weighted (fixed effects)
Inverse variance weighted (multiplicative random effects)
MR Egger
MR RAPS
Weighted median
Weighted mode
0.04
0.00
−0.04
0.09
0.12
0.15
0.18
SNP effect on BMP−binding endothelial regulator protein
SNP effect on Gut microbiota abundance (genus Catenibacterium id.2153)

Batch 2−516 : Gut microbiota abundance (genus Parasutterella id.2892) on BMP−binding endothelial regulator protein
with a threshold of 1e−05

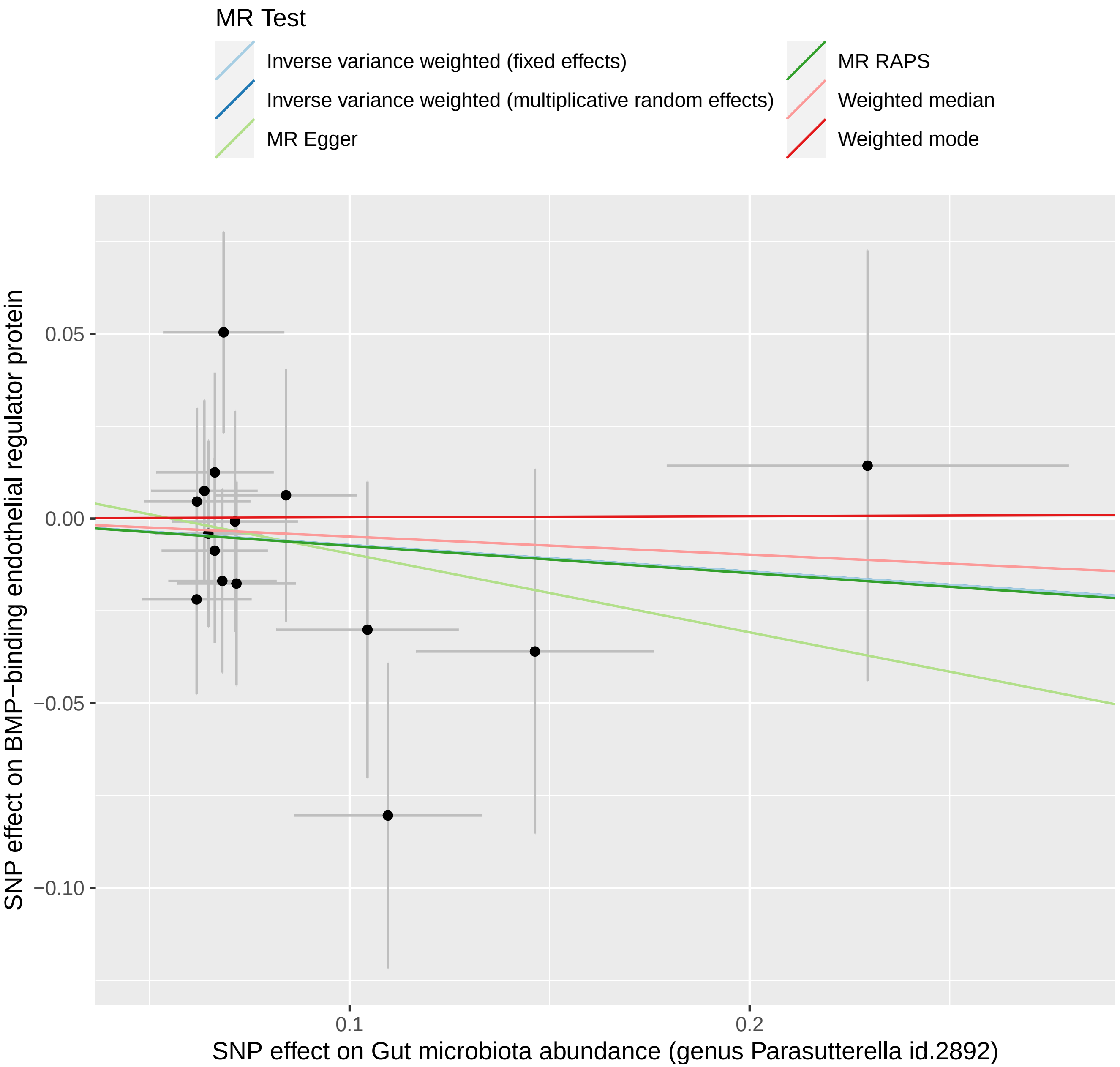

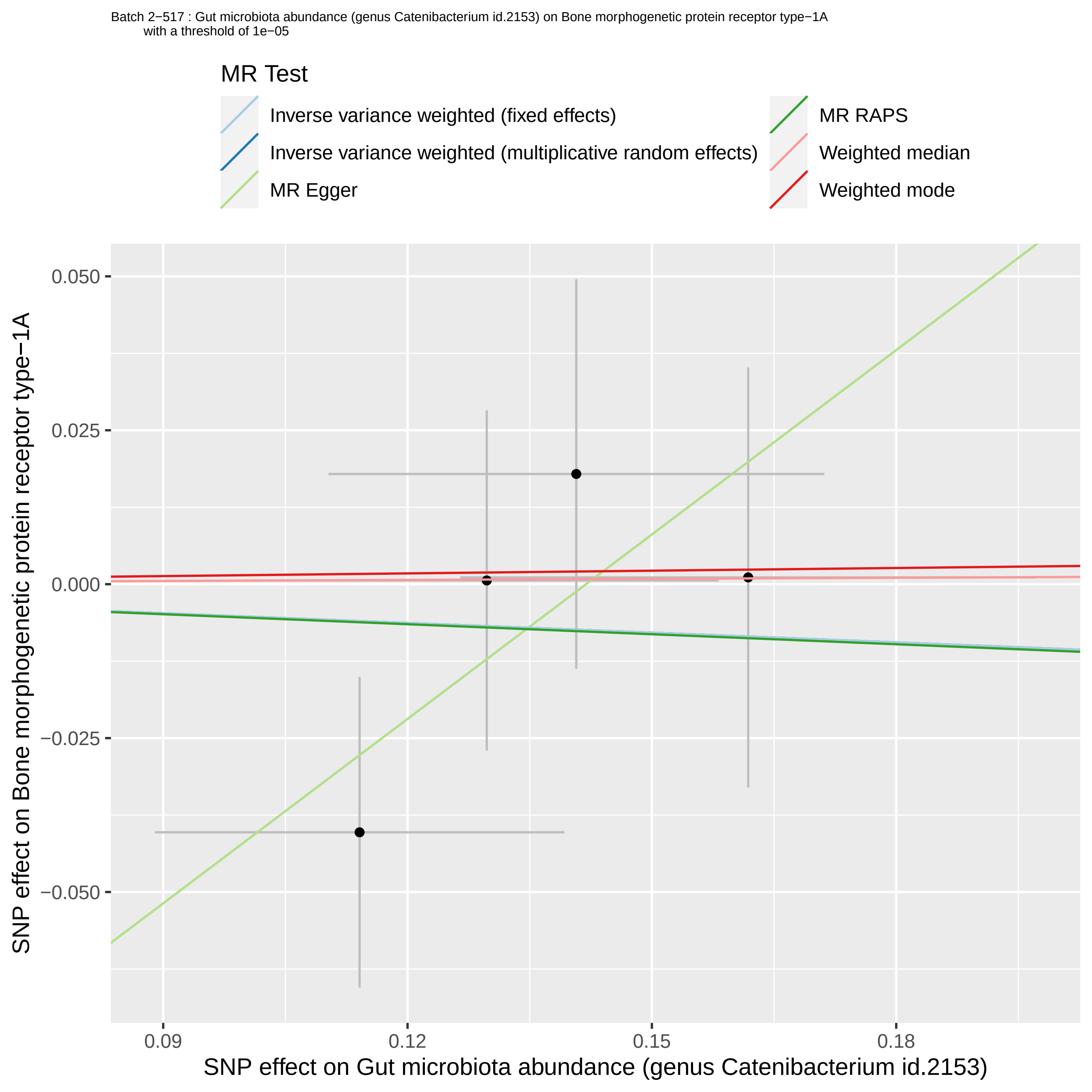

Batch 2−517 : Gut microbiota abundance (genus Catenibacterium id.2153) on Bone morphogenetic protein receptor type−1A
with a threshold of 1e−05
MR Test
Inverse variance weighted (fixed effects)
Inverse variance weighted (multiplicative random effects)
MR Egger
MR RAPS
Weighted median
Weighted mode
0.050
0.025
0.000
−0.025
−0.050
0.09
0.12
0.15
0.18
SNP effect on Bone morphogenetic protein receptor type−1A
SNP effect on Gut microbiota abundance (genus Catenibacterium id.2153)

Batch 2−518 : Gut microbiota abundance (genus Parasutterella id.2892) on Bone morphogenetic protein receptor type−1A
with a threshold of 1e−05

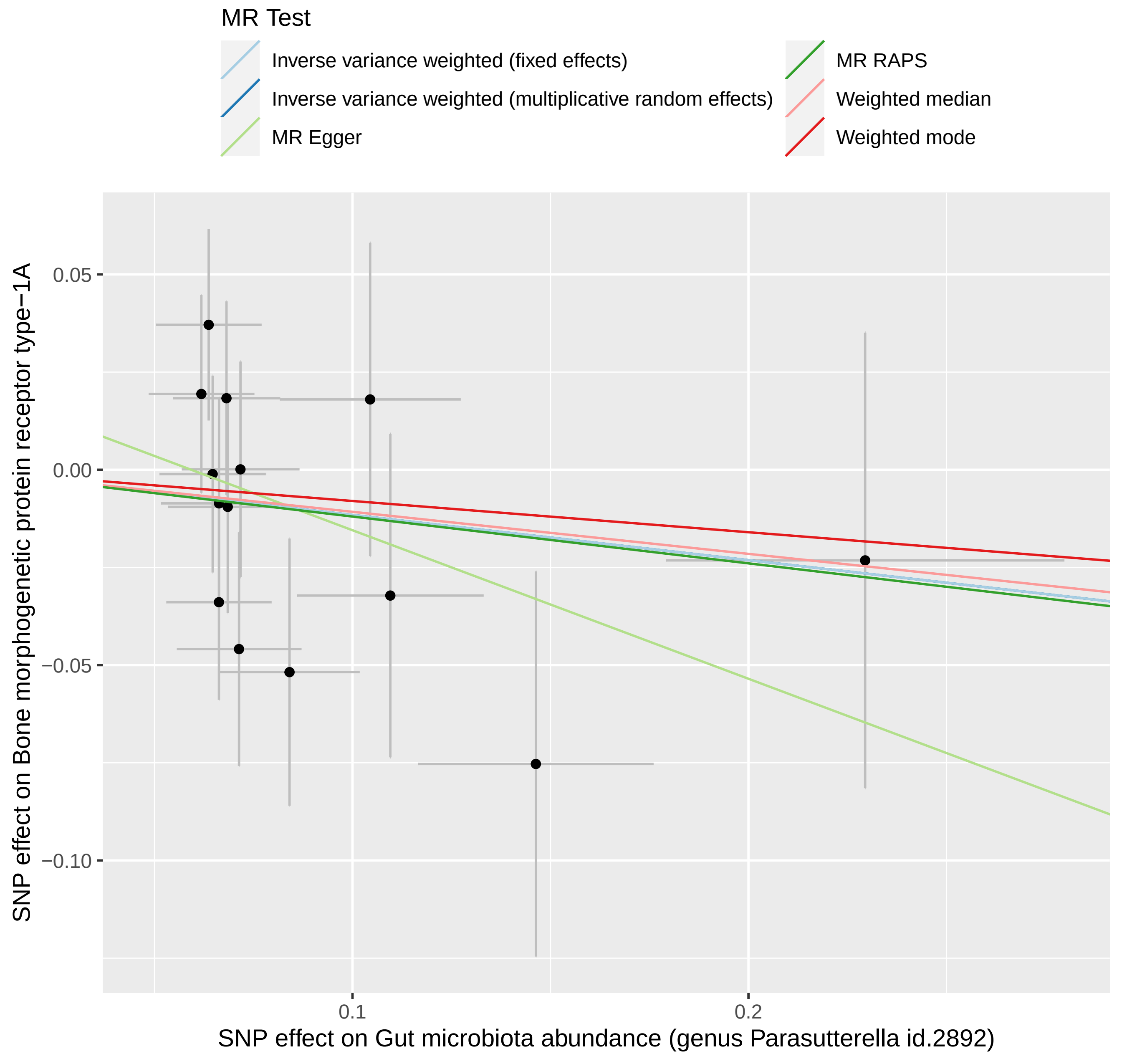

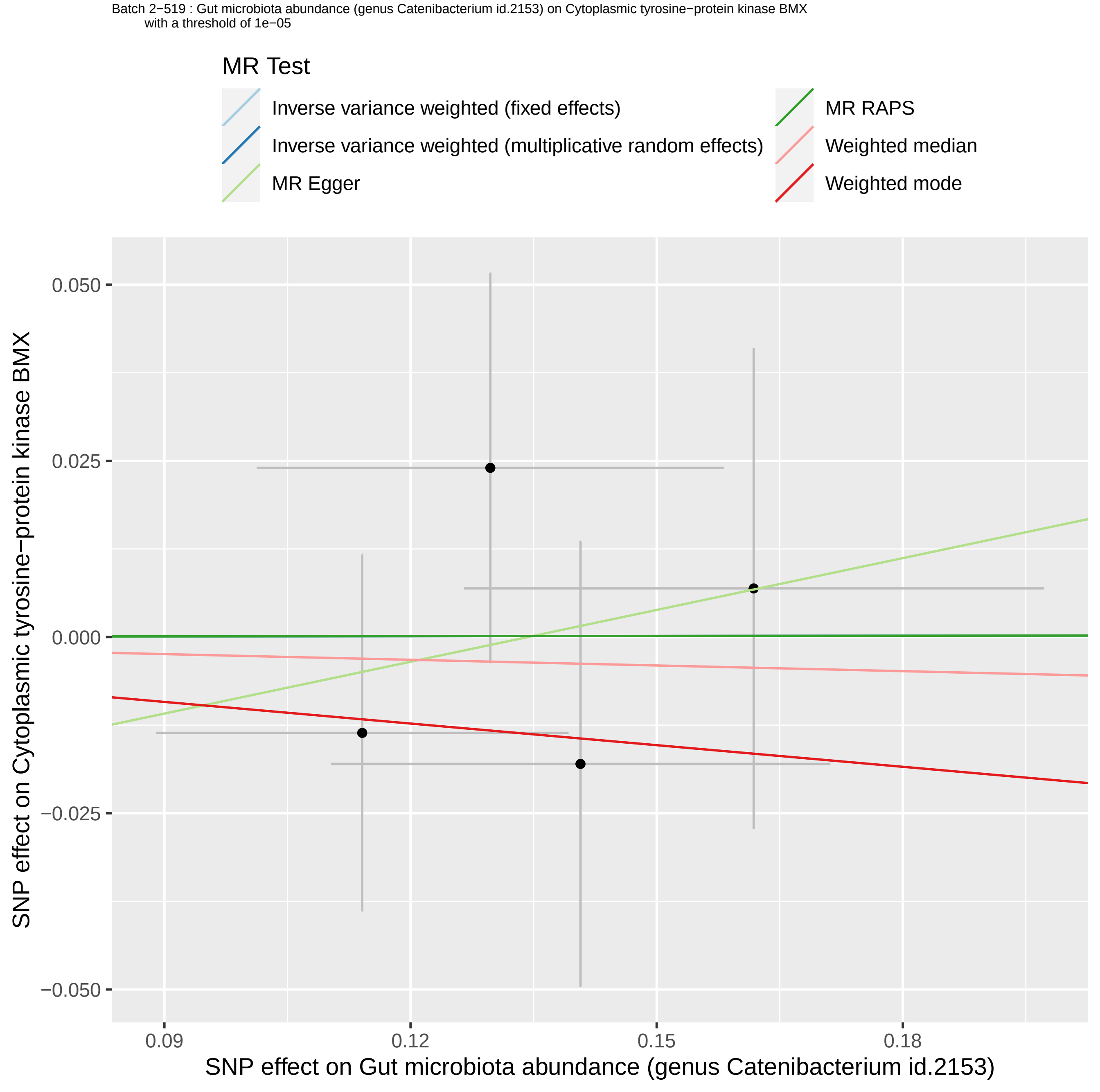
Batch 2−519 : Gut microbiota abundance (genus Catenibacterium id.2153) on Cytoplasmic tyrosine−protein kinase BMX
with a threshold of 1e−05
MR Test
Inverse variance weighted (fixed effects)
Inverse variance weighted (multiplicative random effects)
MR Egger
MR RAPS
Weighted median
Weighted mode
0.050
0.025
0.000
−0.025
−0.050
0.09
0.12
0.15
0.18
SNP effect on Cytoplasmic tyrosine−protein kinase BMX
SNP effect on Gut microbiota abundance (genus Catenibacterium id.2153)

Batch 2−520 : Gut microbiota abundance (genus Parasutterella id.2892) on Cytoplasmic tyrosine−protein kinase BMX
with a threshold of 1e−05

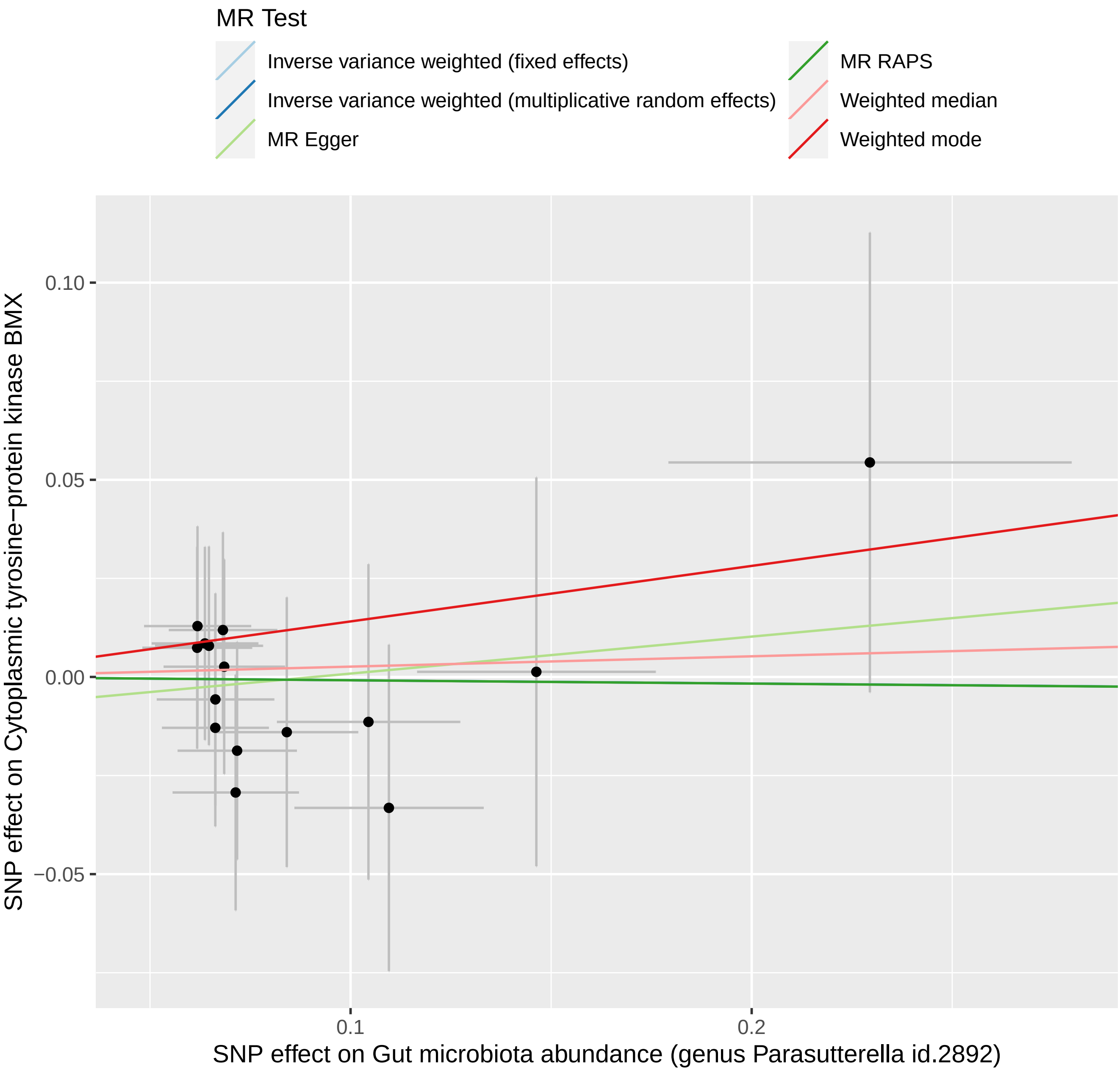

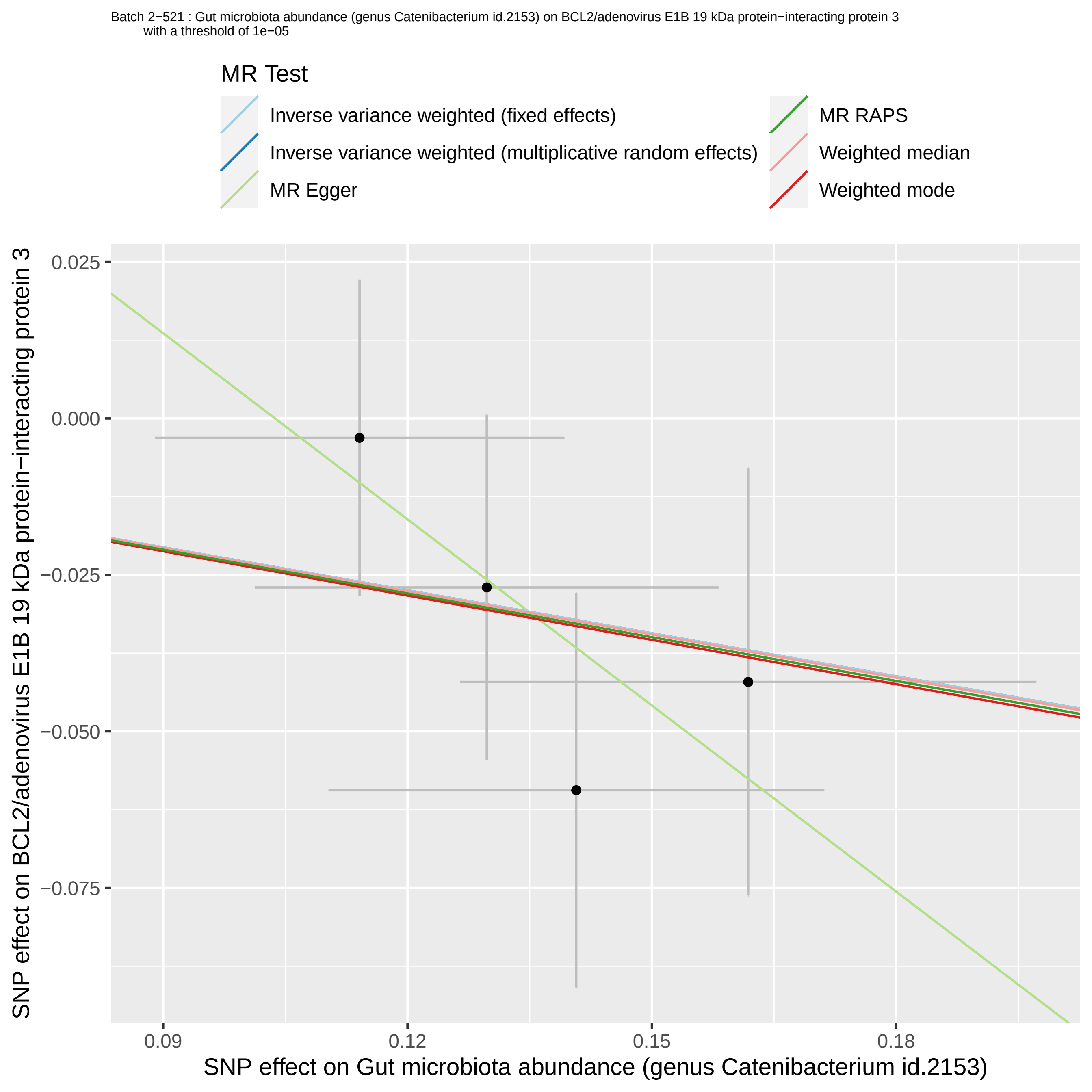

Batch 2−521 : Gut microbiota abundance (genus Catenibacterium id.2153) on BCL2/adenovirus E1B 19 kDa protein−interacting protein 3
with a threshold of 1e−05
MR Test
Inverse variance weighted (fixed effects)
Inverse variance weighted (multiplicative random effects)
MR Egger
MR RAPS
Weighted median
Weighted mode
0.025
0.000
−0.025
−0.050
−0.075
0.09
0.12
0.15
0.18
SNP effect on BCL2/adenovirus E1B 19 kDa protein−interacting protein 3
SNP effect on Gut microbiota abundance (genus Catenibacterium id.2153)

Batch 2−522 : Gut microbiota abundance (genus Parasutterella id.2892) on BCL2/adenovirus E1B 19 kDa protein−interacting protein 3
with a threshold of 1e−05

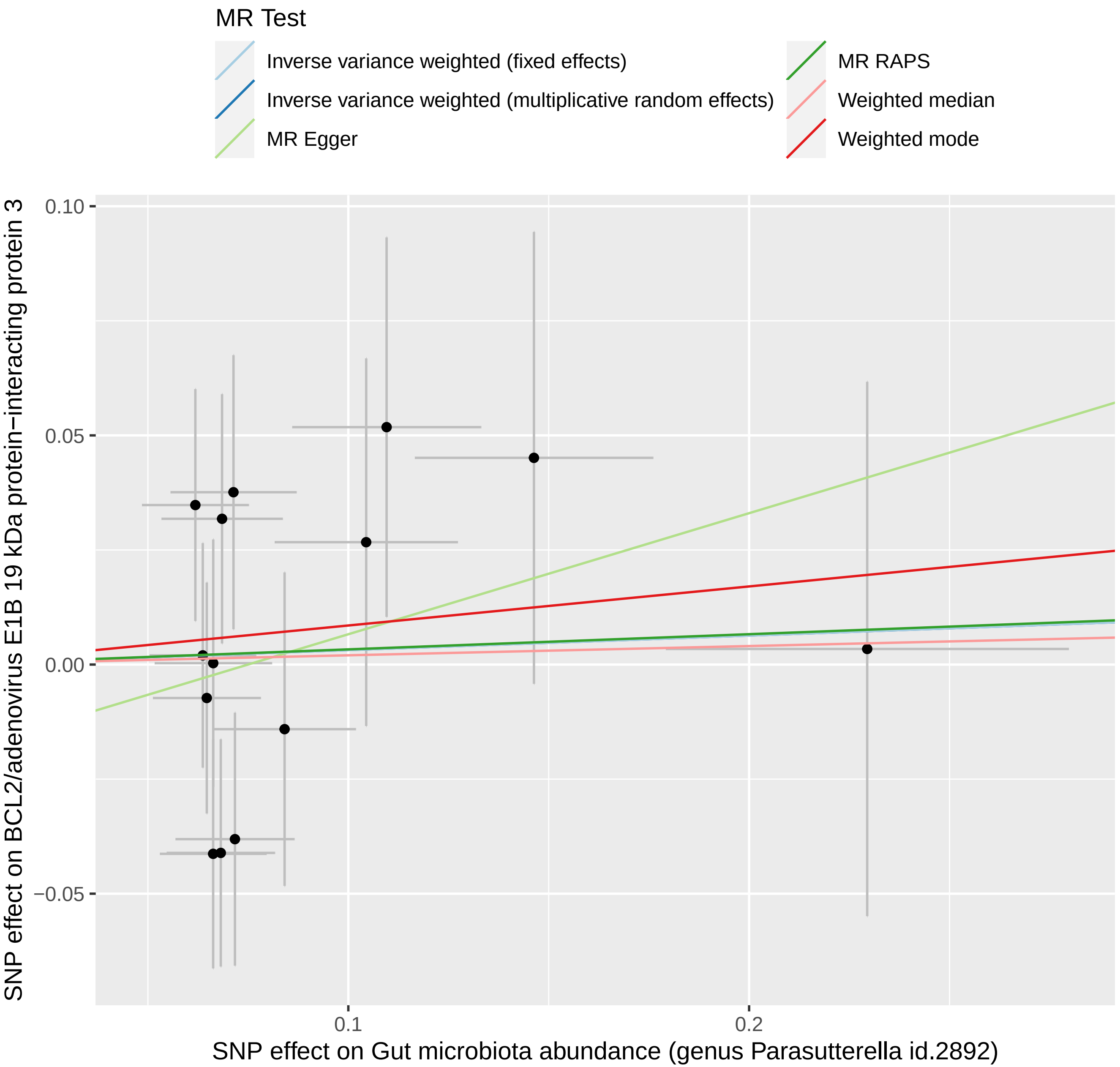

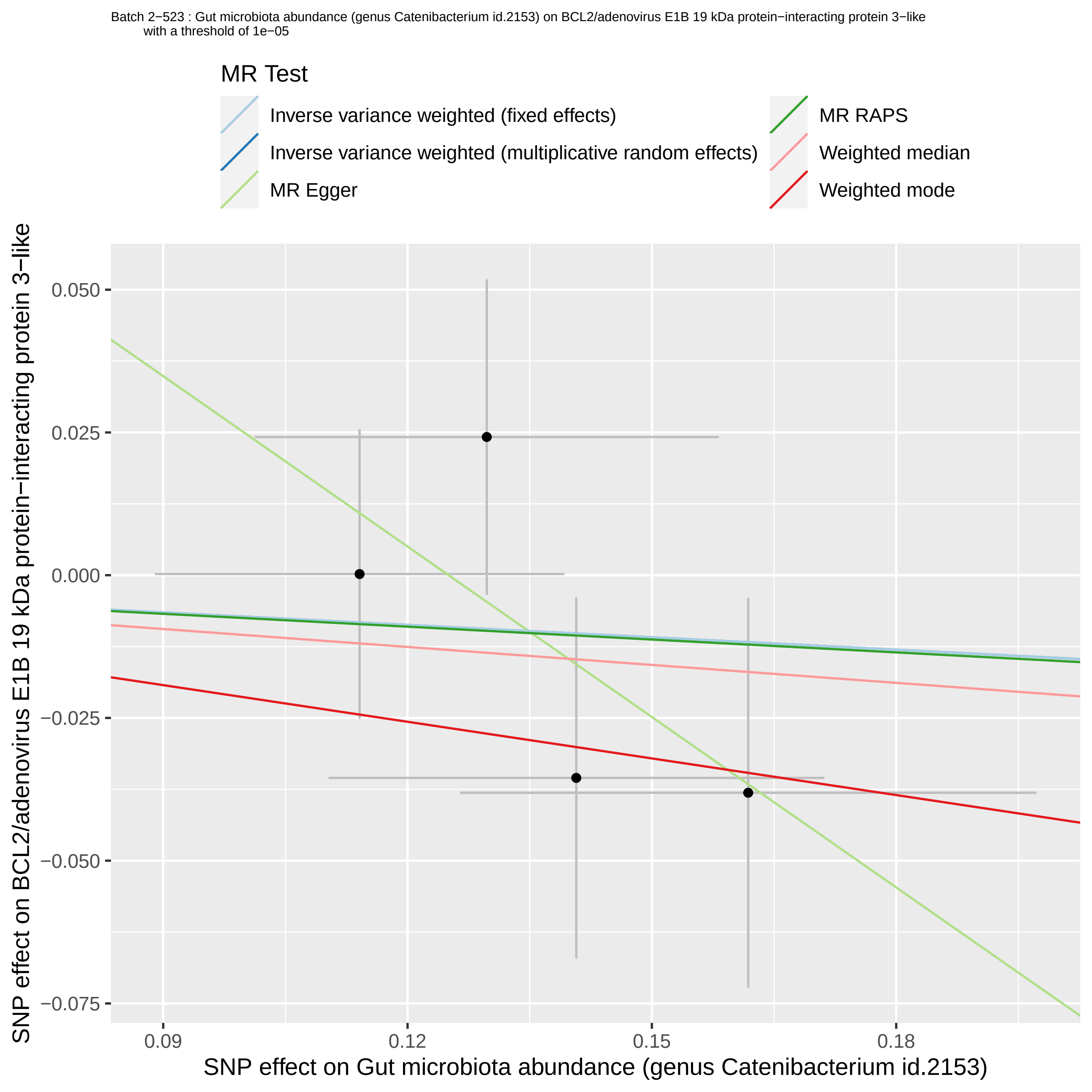

Batch 2−523 : Gut microbiota abundance (genus Catenibacterium id.2153) on BCL2/adenovirus E1B 19 kDa protein−interacting protein 3−like
with a threshold of 1e−05
MR Test
Inverse variance weighted (fixed effects)
Inverse variance weighted (multiplicative random effects)
MR Egger
MR RAPS
Weighted median
Weighted mode
0.050
0.025
0.000
−0.025
−0.050
−0.075
0.09
0.12
0.15
0.18
SNP effect on BCL2/adenovirus E1B 19 kDa protein−interacting protein 3−like
SNP effect on Gut microbiota abundance (genus Catenibacterium id.2153)

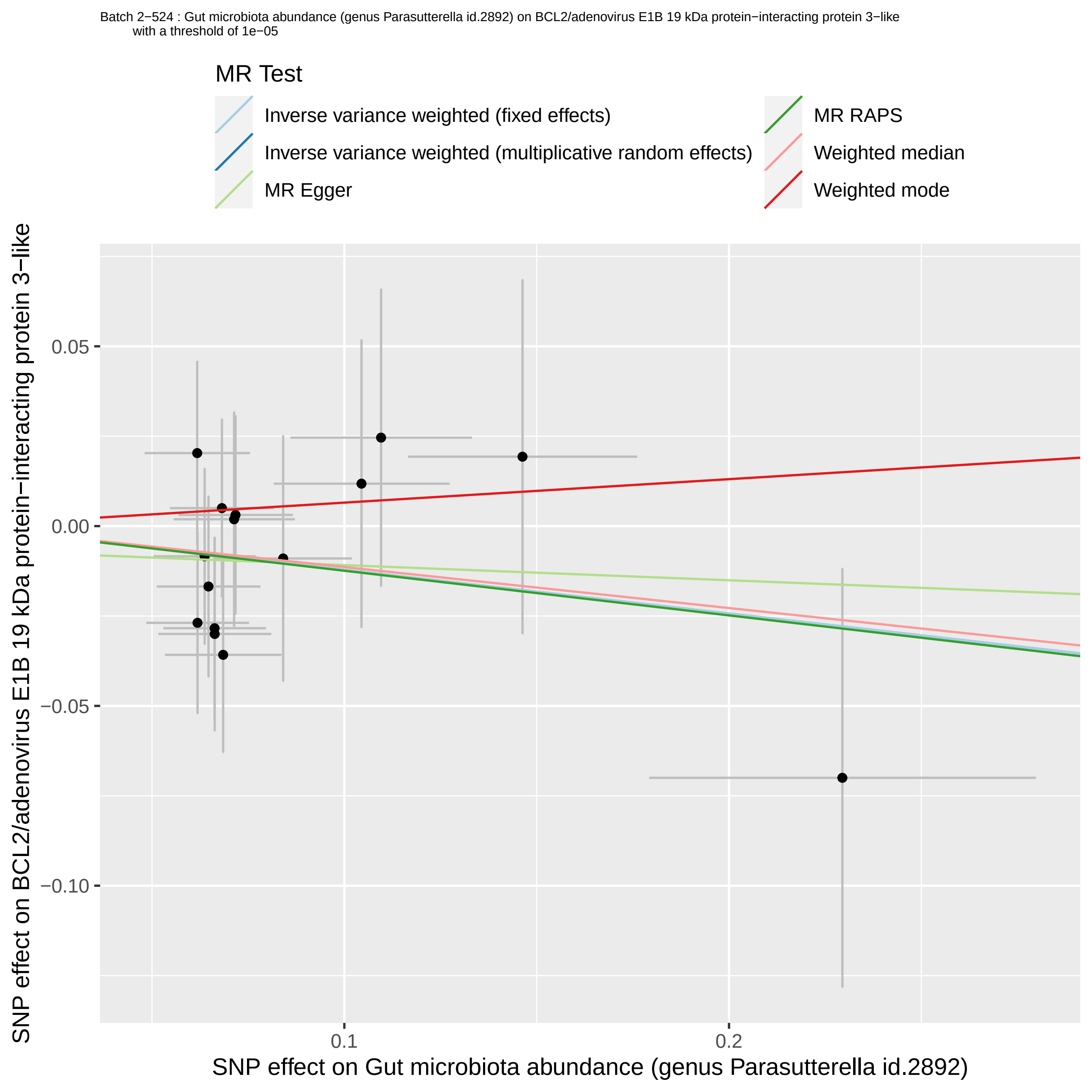

Batch 2−524 : Gut microbiota abundance (genus Parasutterella id.2892) on BCL2/adenovirus E1B 19 kDa protein−interacting protein 3−like
with a threshold of 1e−05
MR Test
Inverse variance weighted (fixed effects)
Inverse variance weighted (multiplicative random effects)
MR Egger
MR RAPS
Weighted median
Weighted mode
SNP effect on BCL2/adenovirus E1B 19 kDa protein−interacting protein 3−like
0.05
0.00
−0.05
−0.10
0.1
0.2
SNP effect on Gut microbiota abundance (genus Parasutterella id.2892)

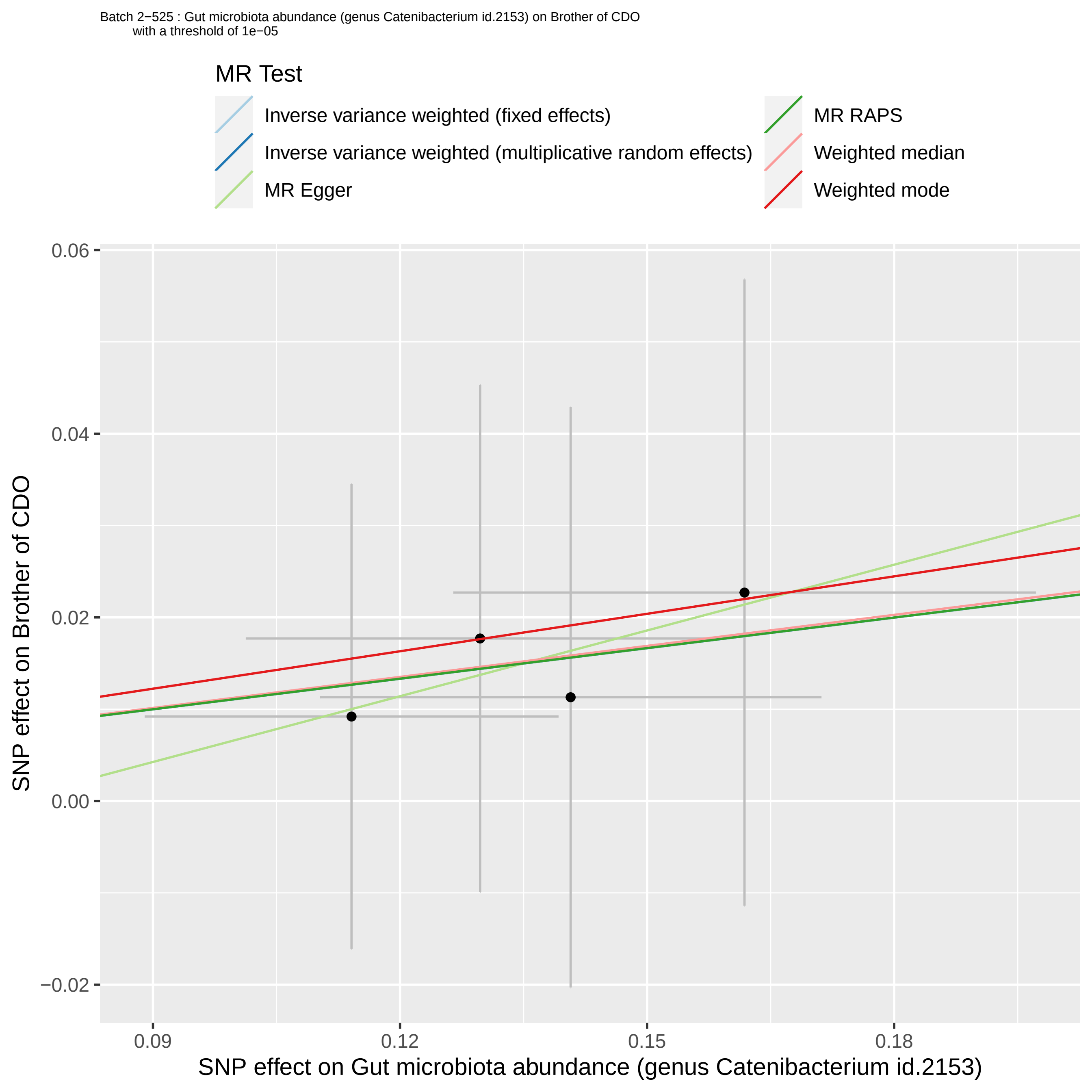

Batch 2−525 : Gut microbiota abundance (genus Catenibacterium id.2153) on Brother of CDO
with a threshold of 1e−05
MR Test
Inverse variance weighted (fixed effects)
Inverse variance weighted (multiplicative random effects)
MR Egger
MR RAPS
Weighted median
Weighted mode
0.06
0.04
0.02
0.00
−0.02
SNP effect on Brother of CDO
0.09
0.12
0.15
0.18
SNP effect on Gut microbiota abundance (genus Catenibacterium id.2153)

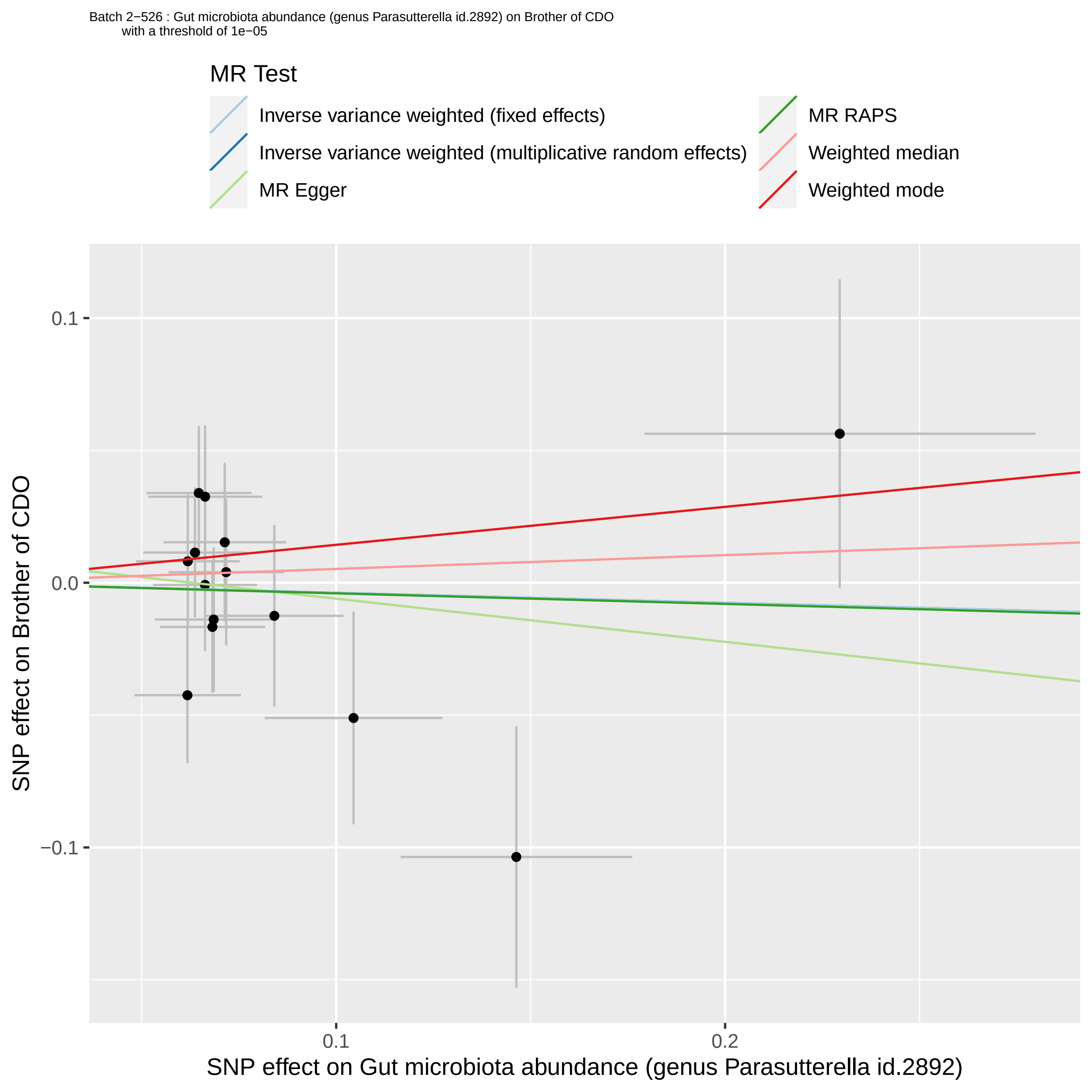

Batch 2−526 : Gut microbiota abundance (genus Parasutterella id.2892) on Brother of CDO
with a threshold of 1e−05
MR Test
Inverse variance weighted (fixed effects)
Inverse variance weighted (multiplicative random effects)
MR Egger
MR RAPS
Weighted median
Weighted mode
0.1
0.0
−0.1
0.1
0.2
SNP effect on Brother of CDO
SNP effect on Gut microbiota abundance (genus Parasutterella id.2892)

Batch 2−527 : Gut microbiota abundance (genus Catenibacterium id.2153) on BolA−like protein 2
with a threshold of 1e−05

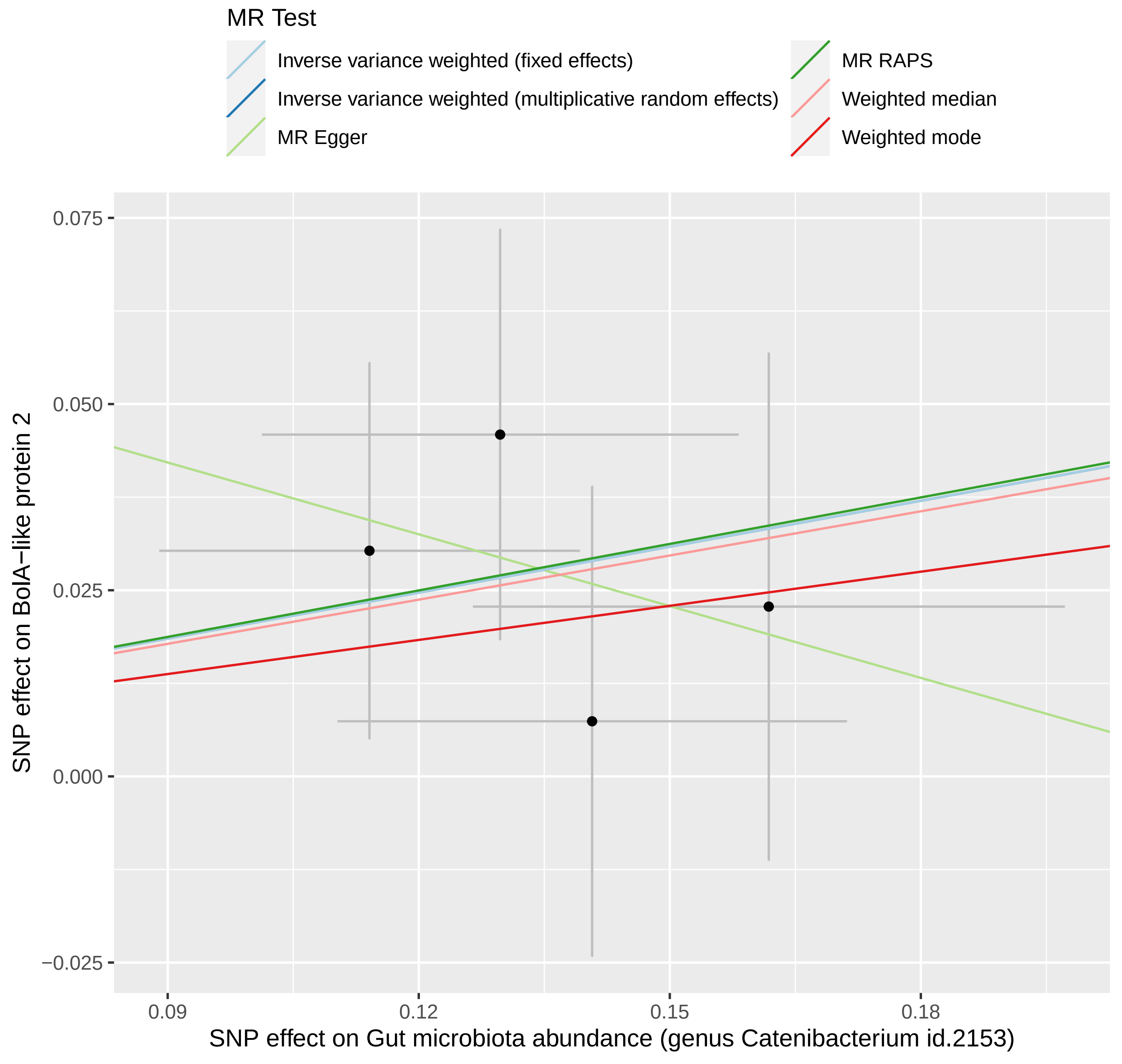

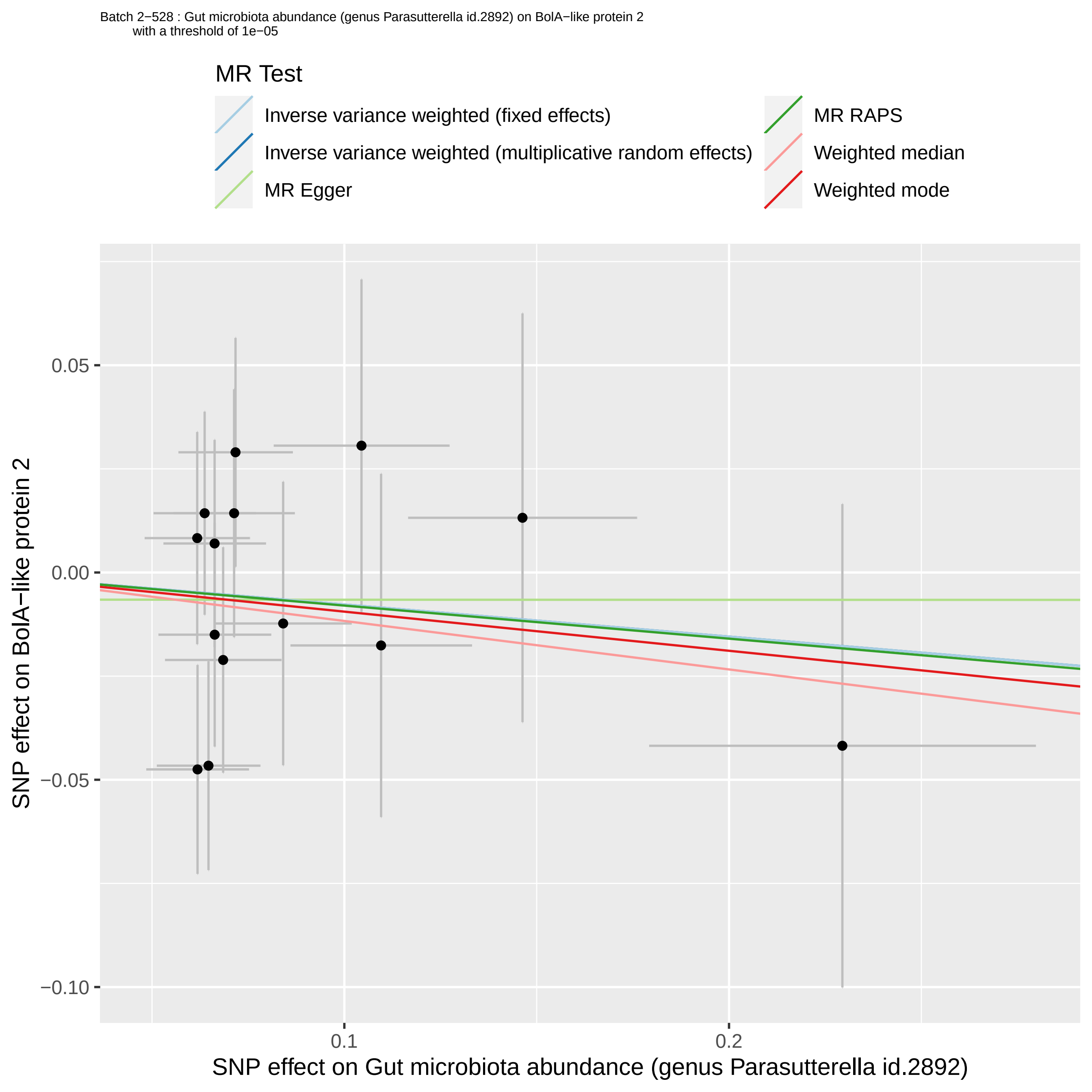
Batch 2−528 : Gut microbiota abundance (genus Parasutterella id.2892) on BolA−like protein 2
with a threshold of 1e−05
MR Test
Inverse variance weighted (fixed effects)
Inverse variance weighted (multiplicative random effects)
MR Egger
MR RAPS
Weighted median
Weighted mode
0.05
0.00
−0.05
−0.10
0.1
0.2
SNP effect on BolA−like protein 2
SNP effect on Gut microbiota abundance (genus Parasutterella id.2892)

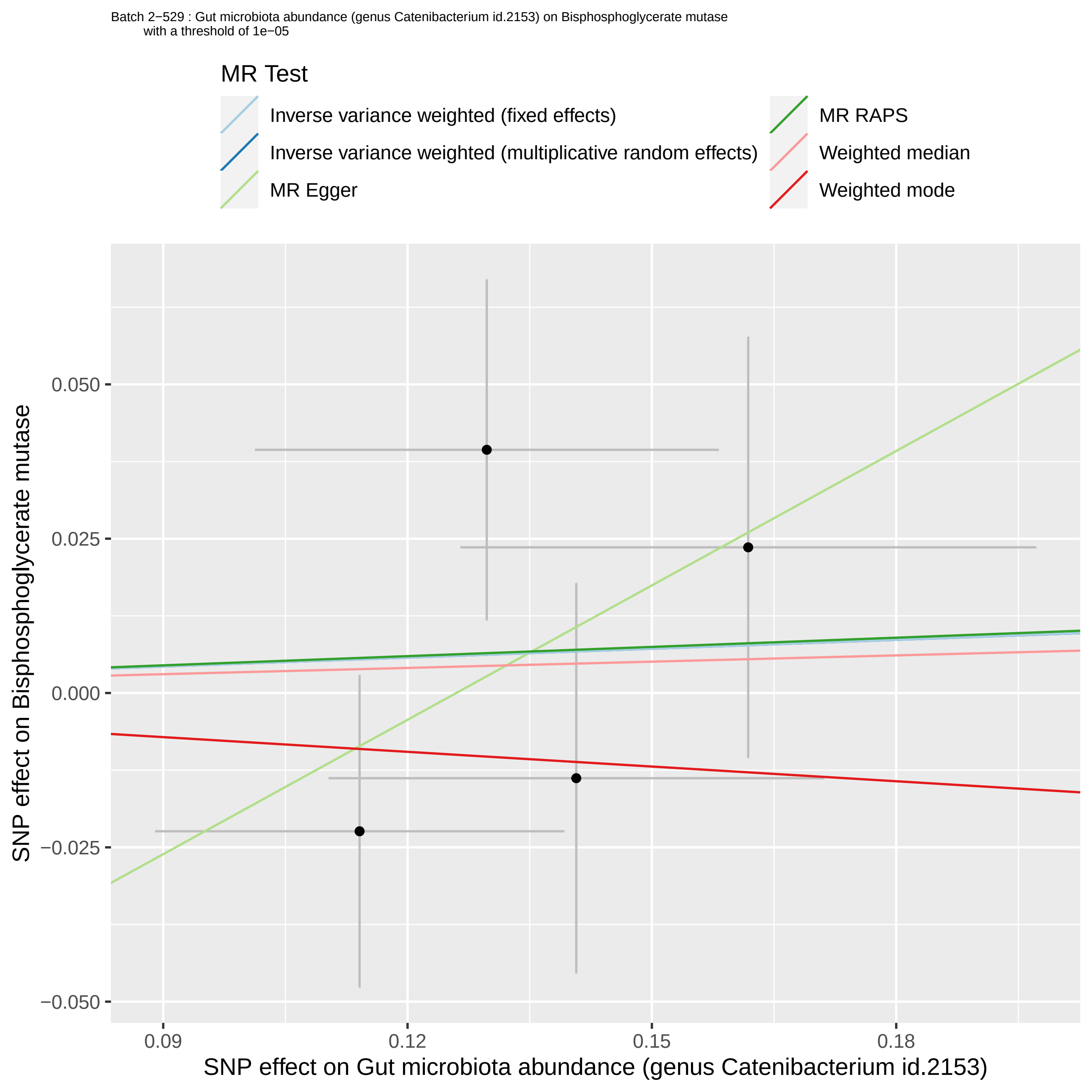

Batch 2−529 : Gut microbiota abundance (genus Catenibacterium id.2153) on Bisphosphoglycerate mutase
with a threshold of 1e−05
MR Test
Inverse variance weighted (fixed effects)
Inverse variance weighted (multiplicative random effects)
MR Egger
MR RAPS
Weighted median
Weighted mode
0.050
0.025
0.000
−0.025
−0.050
0.09
0.12
0.15
0.18
SNP effect on Bisphosphoglycerate mutase
SNP effect on Gut microbiota abundance (genus Catenibacterium id.2153)

Batch 2−530 : Gut microbiota abundance (genus Parasutterella id.2892) on Bisphosphoglycerate mutase with a threshold of 1e−05

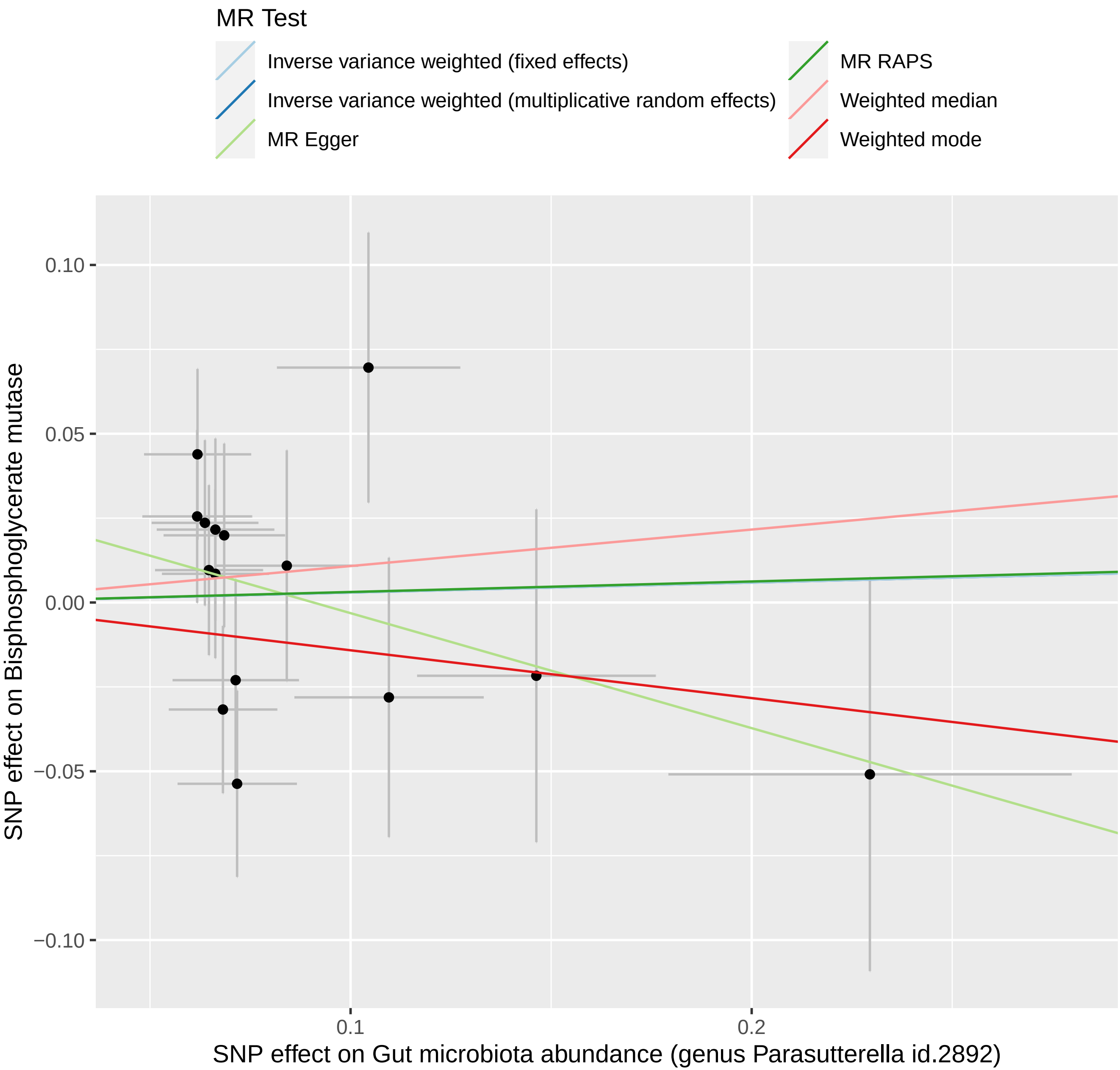

Batch 2−531 : Gut microbiota abundance (genus Catenibacterium id.2153) on Bactericidal permeability−increasing protein
with a threshold of 1e−05

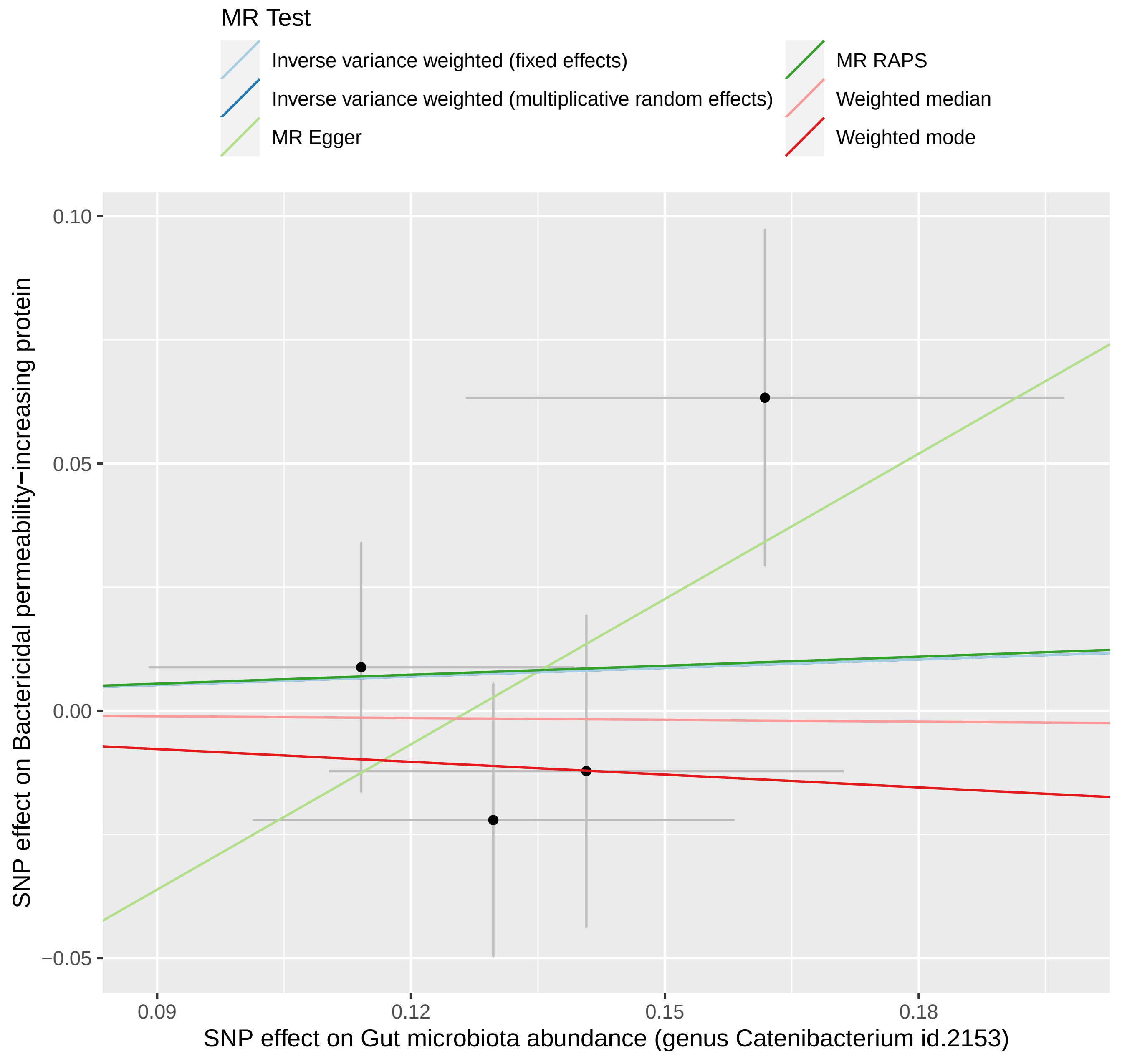

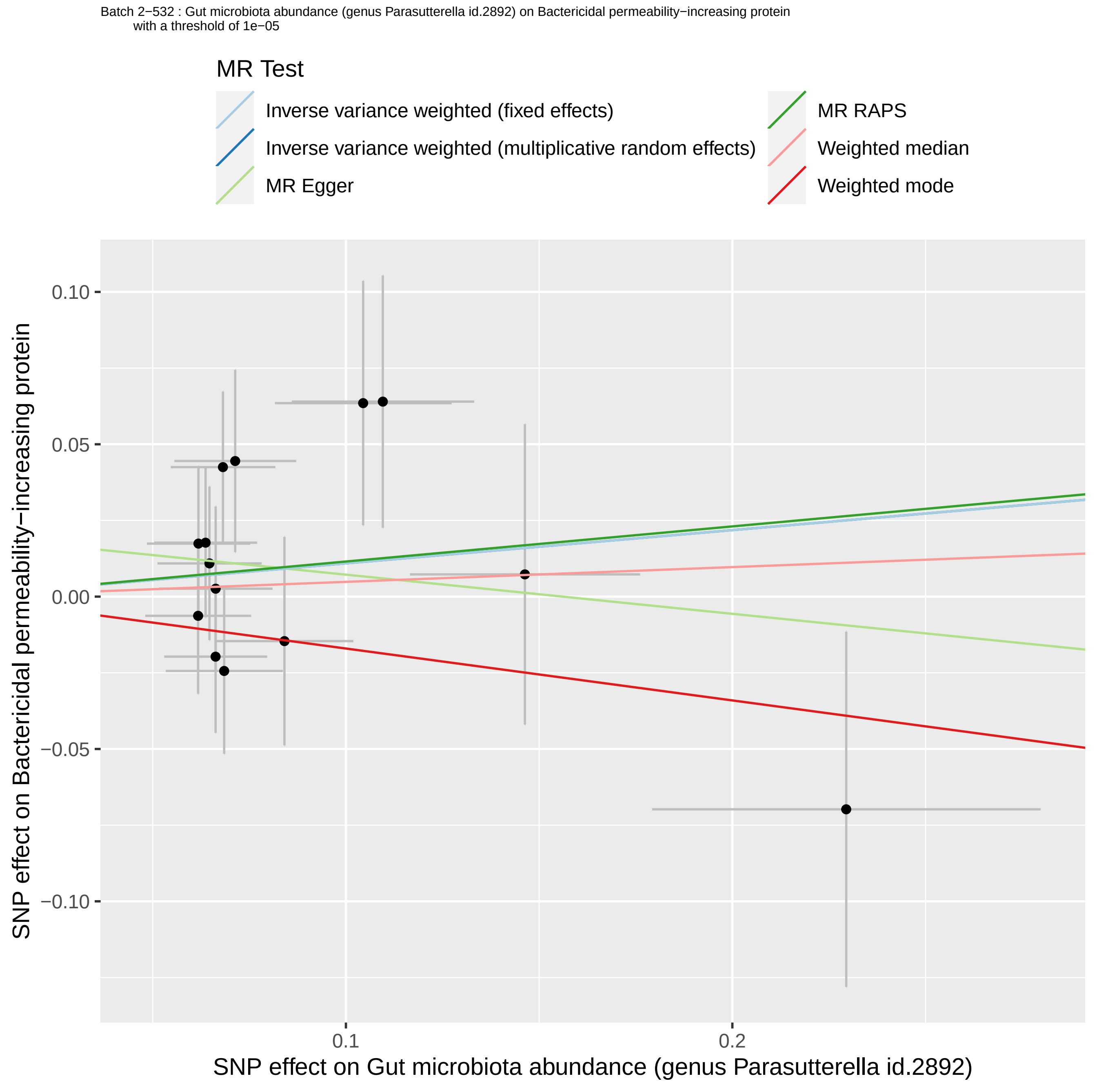
Batch 2−532 : Gut microbiota abundance (genus Parasutterella id.2892) on Bactericidal permeability−increasing protein
with a threshold of 1e−05
MR Test
Inverse variance weighted (fixed effects)
Inverse variance weighted (multiplicative random effects)
MR Egger
MR RAPS
Weighted median
Weighted mode
0.10
0.05
0.00
−0.05
−0.10
0.1
0.2
SNP effect on Bactericidal permeability−increasing protein
SNP effect on Gut microbiota abundance (genus Parasutterella id.2892)

Batch 2−533 : Gut microbiota abundance (genus Catenibacterium id.2153) on BPI fold−containing family A member 1
with a threshold of 1e−05

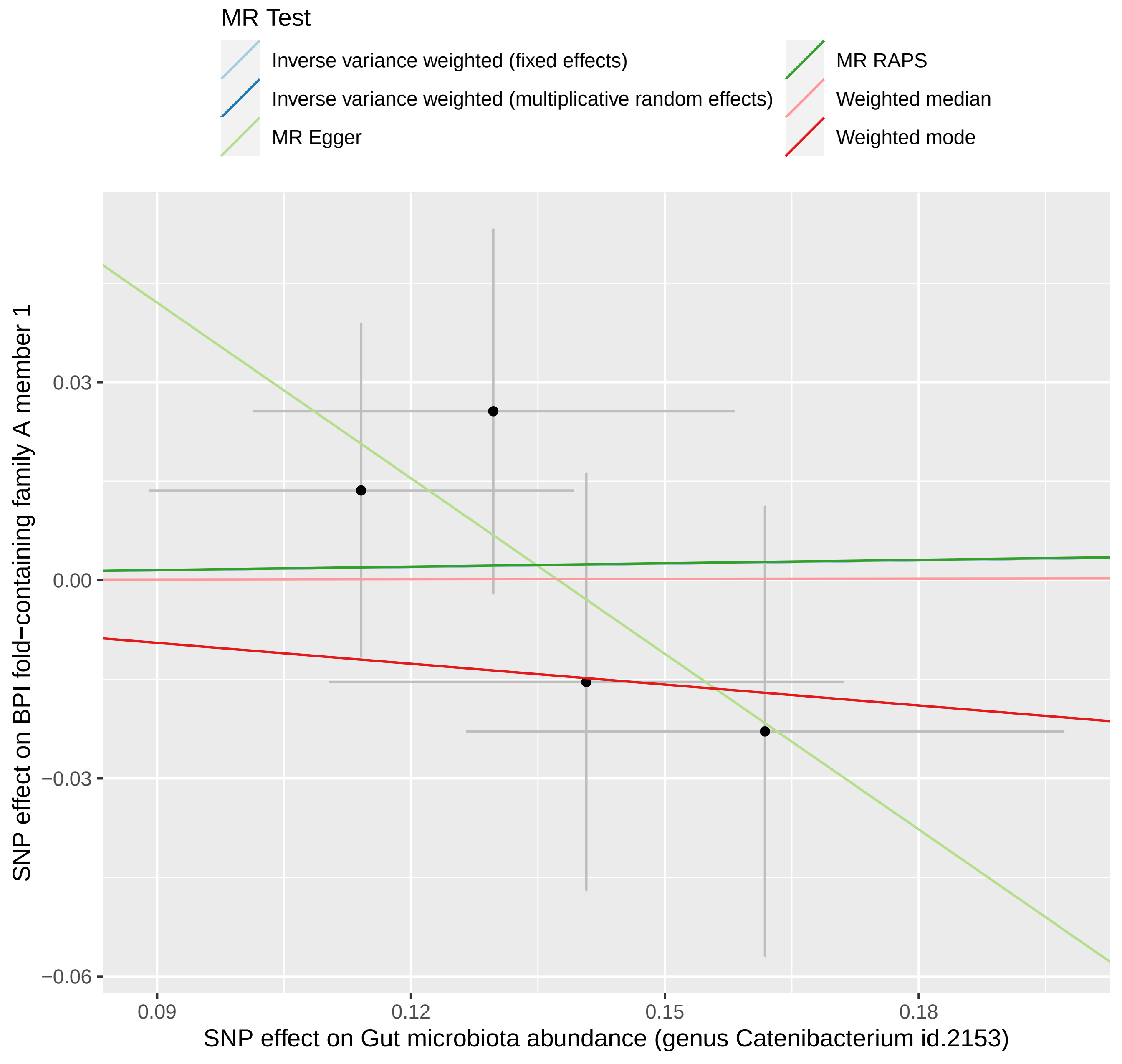

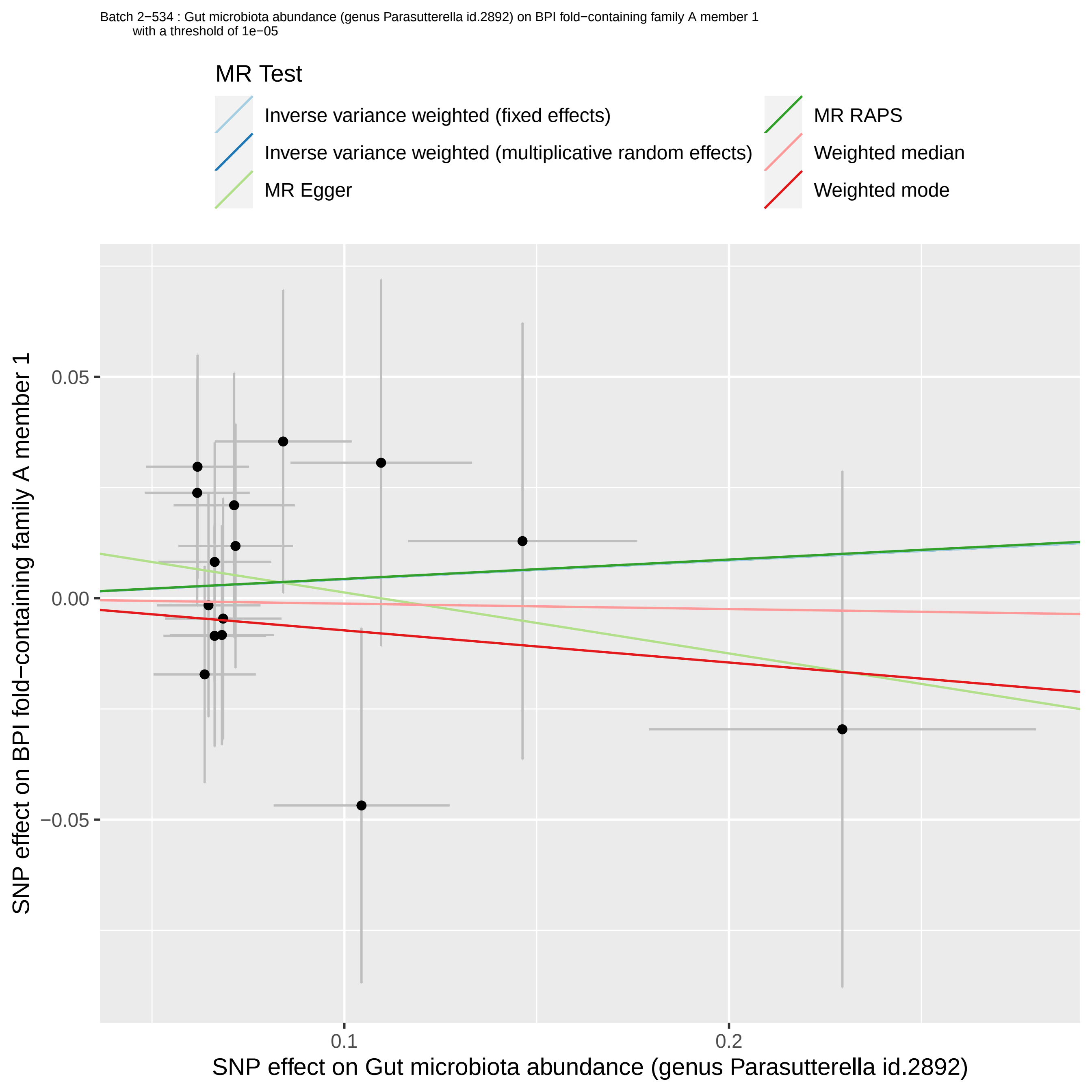
Batch 2−534 : Gut microbiota abundance (genus Parasutterella id.2892) on BPI fold−containing family A member 1
with a threshold of 1e−05
MR Test
Inverse variance weighted (fixed effects)
Inverse variance weighted (multiplicative random effects)
MR Egger
MR RAPS
Weighted median
Weighted mode
0.05
0.00
−0.05
0.1
0.2
SNP effect on BPI fold−containing family A member 1
SNP effect on Gut microbiota abundance (genus Parasutterella id.2892)

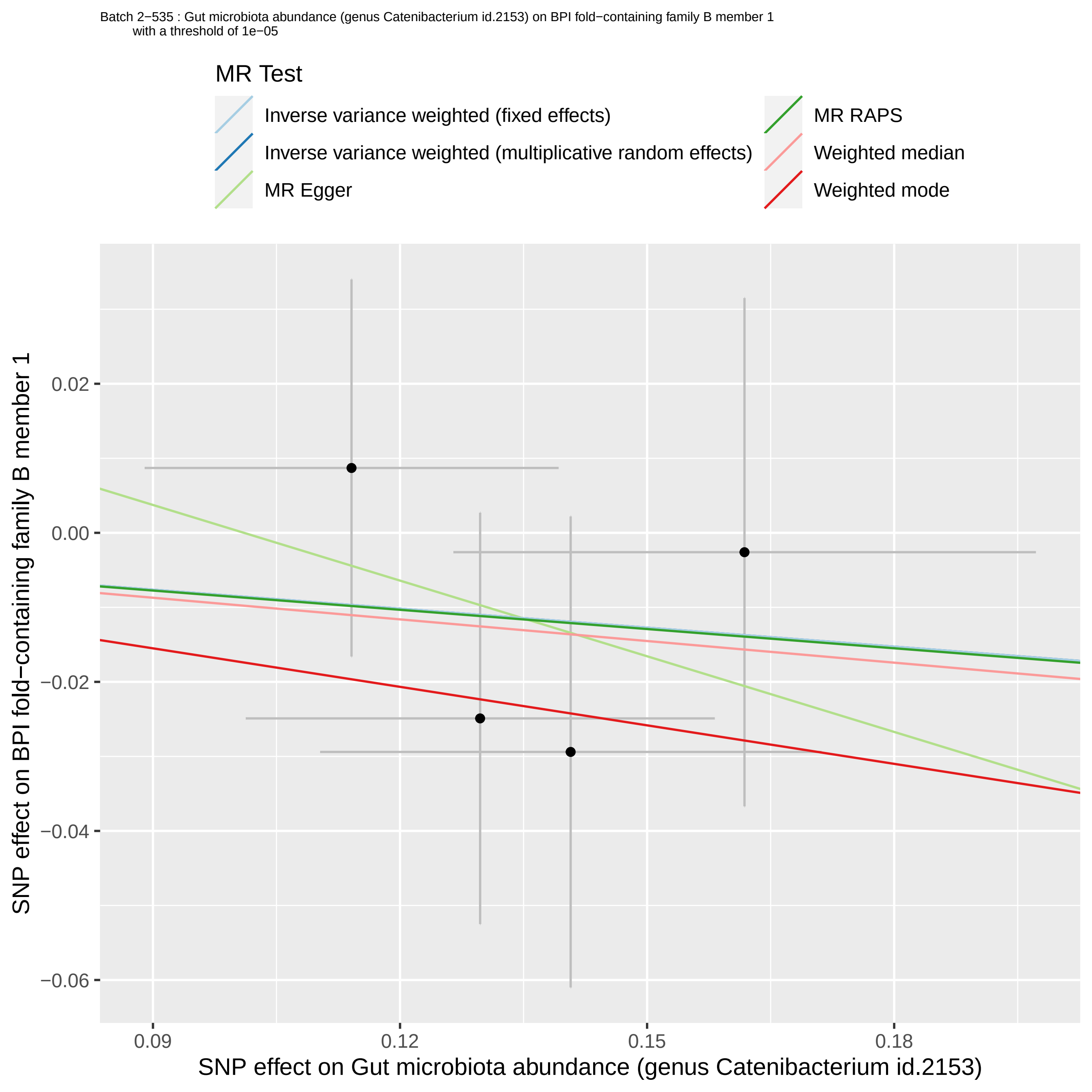
Batch 2−535 : Gut microbiota abundance (genus Catenibacterium id.2153) on BPI fold−containing family B member 1
with a threshold of 1e−05
MR Test
Inverse variance weighted (fixed effects)
Inverse variance weighted (multiplicative random effects)
MR Egger
MR RAPS
Weighted median
Weighted mode
0.02
0.00
−0.02
−0.04
−0.06
SNP effect on BPI fold−containing family B member 1
0.09
0.12
0.15
0.18
SNP effect on Gut microbiota abundance (genus Catenibacterium id.2153)

Batch 2−536 : Gut microbiota abundance (genus Parasutterella id.2892) on BPI fold−containing family B member 1
with a threshold of 1e−05

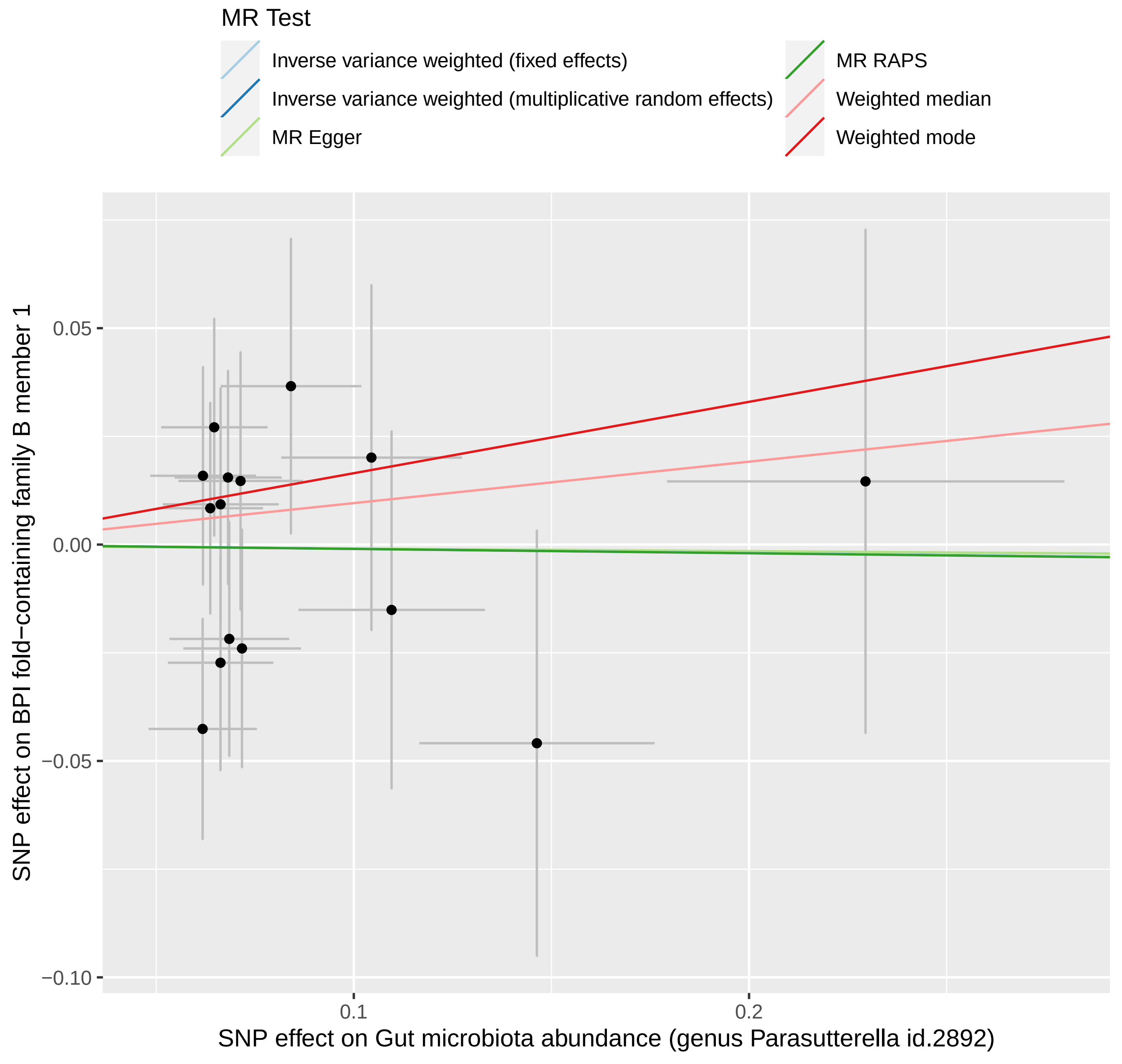

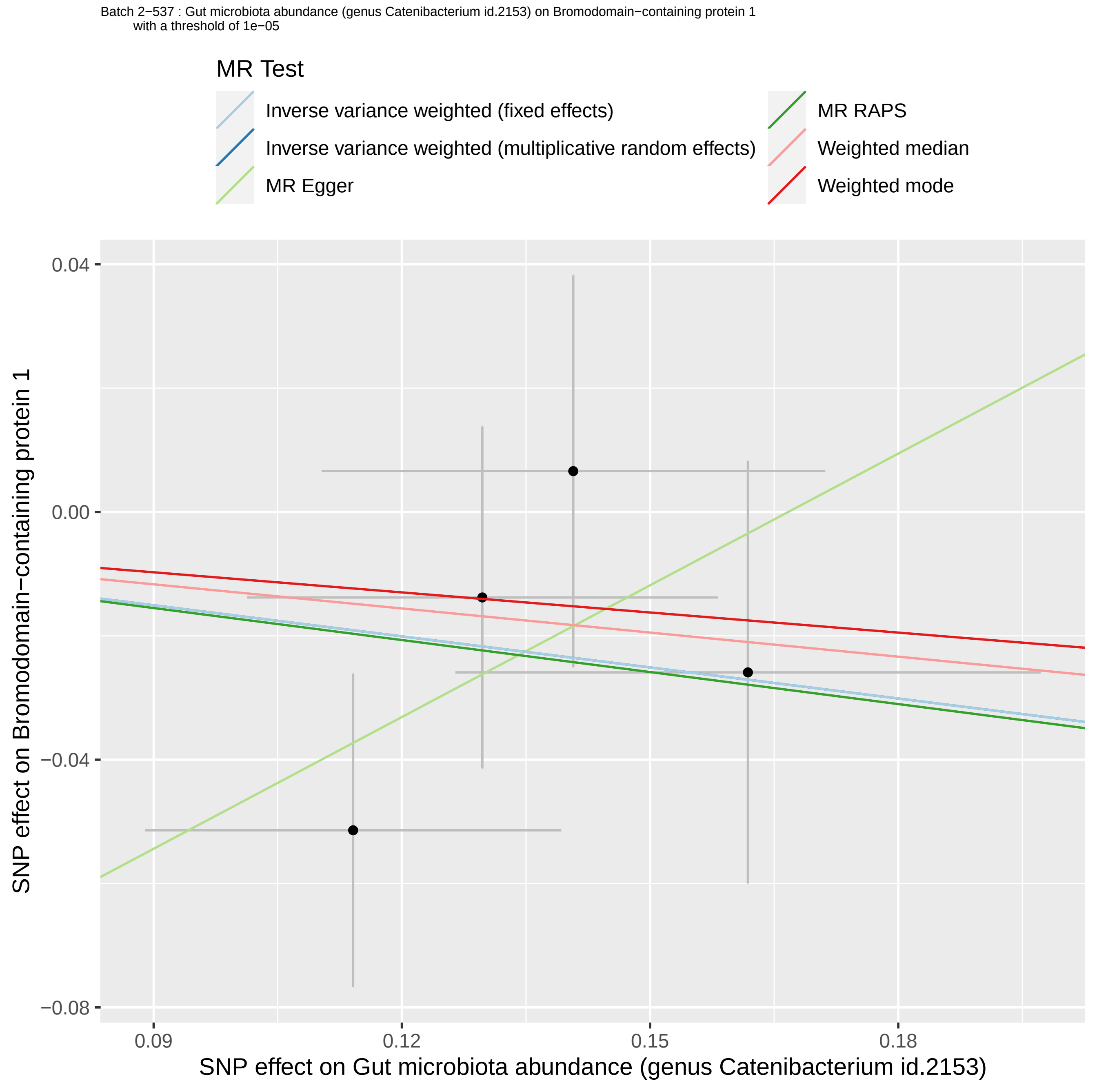
Batch 2−537 : Gut microbiota abundance (genus Catenibacterium id.2153) on Bromodomain−containing protein 1
with a threshold of 1e−05
MR Test
Inverse variance weighted (fixed effects)
Inverse variance weighted (multiplicative random effects)
MR Egger
MR RAPS
Weighted median
Weighted mode
0.04
0.00
−0.04
−0.08
0.09
0.12
0.15
0.18
SNP effect on Bromodomain−containing protein 1
SNP effect on Gut microbiota abundance (genus Catenibacterium id.2153)

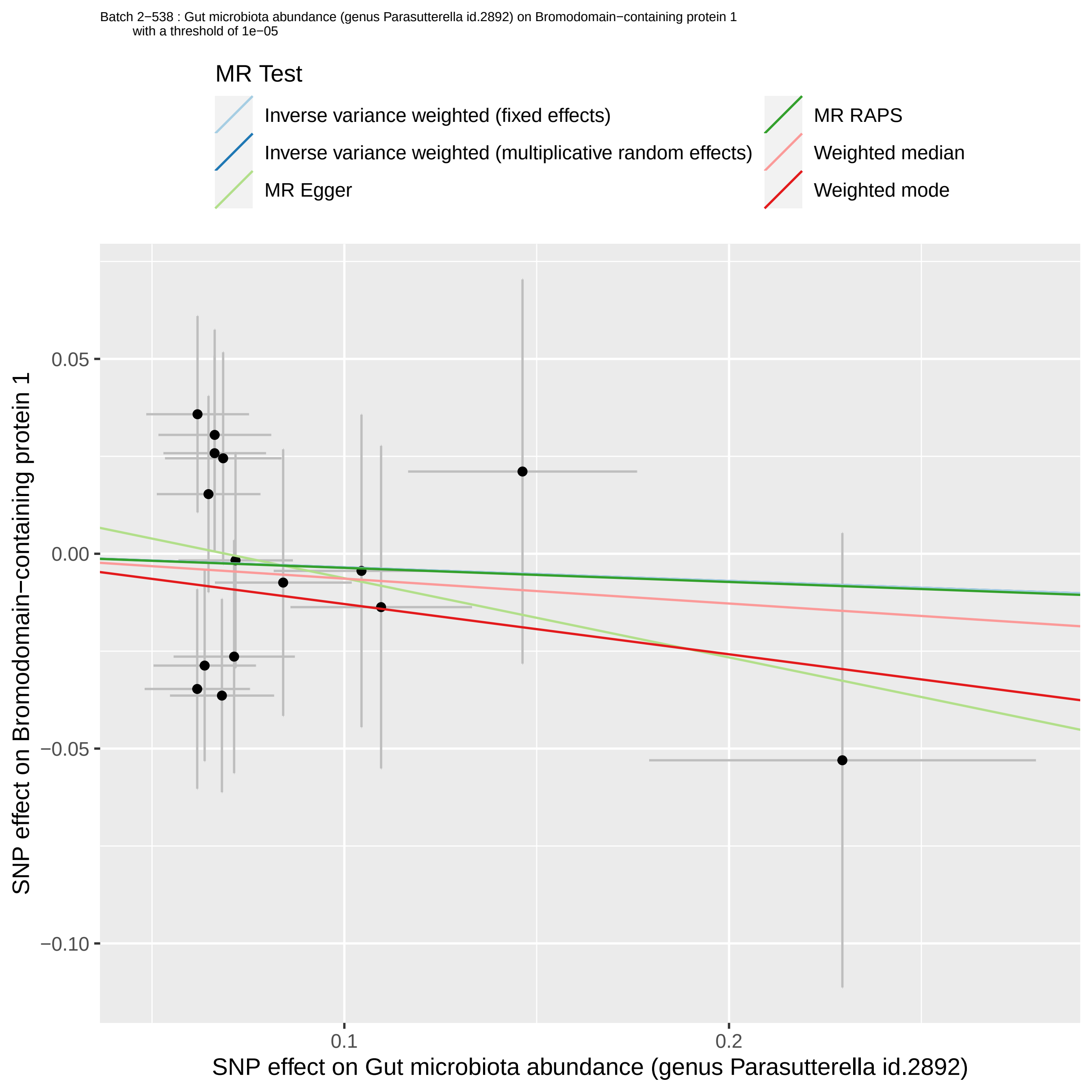

Batch 2−538 : Gut microbiota abundance (genus Parasutterella id.2892) on Bromodomain−containing protein 1
with a threshold of 1e−05
MR Test
Inverse variance weighted (fixed effects)
Inverse variance weighted (multiplicative random effects)
MR Egger
MR RAPS
Weighted median
Weighted mode
0.05
0.00
−0.05
−0.10
0.1
0.2
SNP effect on Bromodomain−containing protein 1
SNP effect on Gut microbiota abundance (genus Parasutterella id.2892)

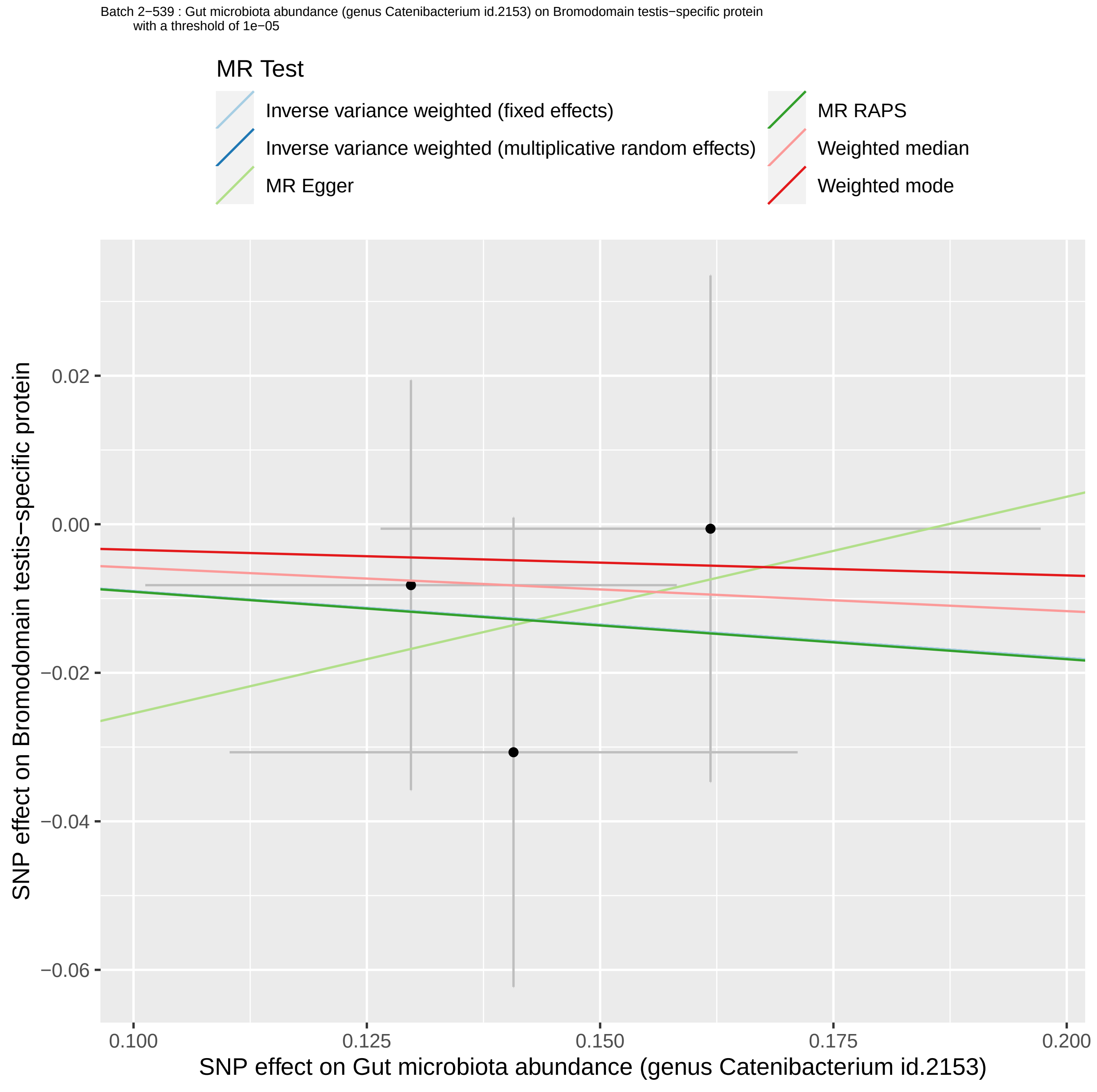

Batch 2−539 : Gut microbiota abundance (genus Catenibacterium id.2153) on Bromodomain testis−specific protein
with a threshold of 1e−05
MR Test
Inverse variance weighted (fixed effects)
Inverse variance weighted (multiplicative random effects)
MR Egger
MR RAPS
Weighted median
Weighted mode
0.02
0.00
−0.02
−0.04
−0.06
0.100
0.125
0.150
0.175
0.200
SNP effect on Bromodomain testis−specific protein
SNP effect on Gut microbiota abundance (genus Catenibacterium id.2153)

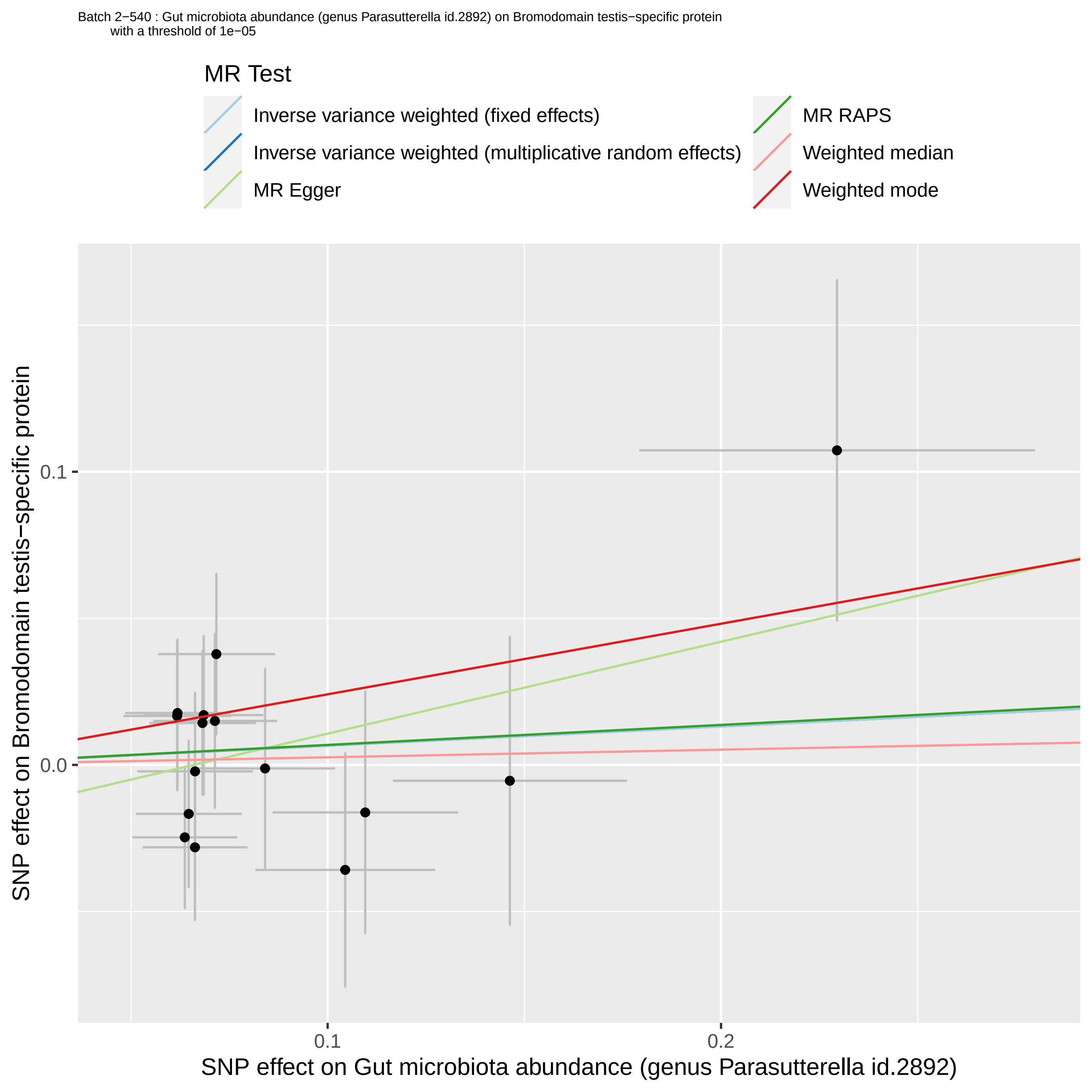
Batch 2−540 : Gut microbiota abundance (genus Parasutterella id.2892) on Bromodomain testis−specific protein
with a threshold of 1e−05
MR Test
Inverse variance weighted (fixed effects)
Inverse variance weighted (multiplicative random effects)
MR Egger
MR RAPS
Weighted median
Weighted mode
0.1
0.0
0.1
0.2
SNP effect on Bromodomain testis−specific protein
SNP effect on Gut microbiota abundance (genus Parasutterella id.2892)

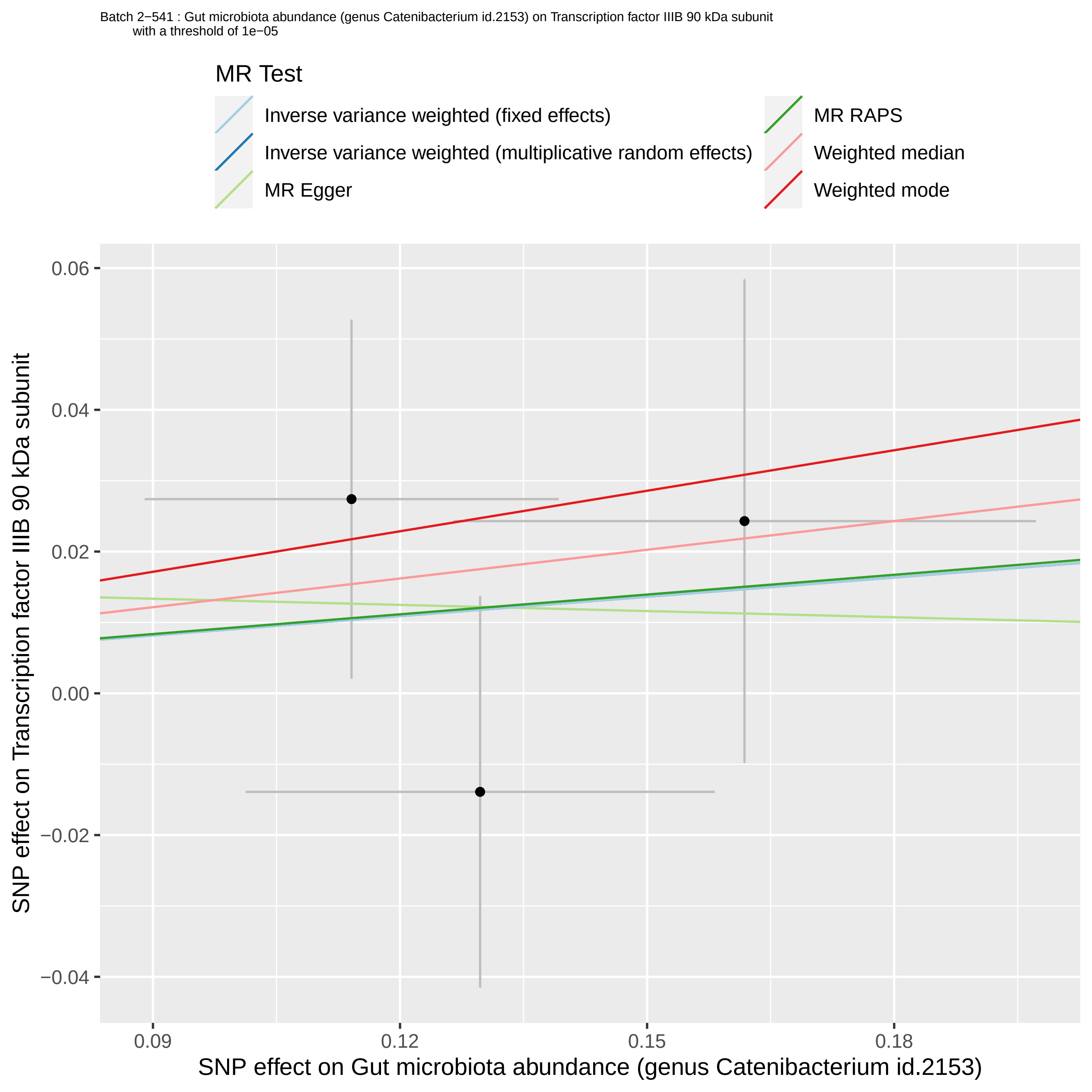

Batch 2−541 : Gut microbiota abundance (genus Catenibacterium id.2153) on Transcription factor IIIB 90 kDa subunit
with a threshold of 1e−05
MR Test
Inverse variance weighted (fixed effects)
Inverse variance weighted (multiplicative random effects)
MR Egger
MR RAPS
Weighted median
Weighted mode
SNP effect on Transcription factor IIIB 90 kDa subunit
0.06
0.04
0.02
0.00
−0.02
−0.04
0.09
0.12
0.15
0.18
SNP effect on Gut microbiota abundance (genus Catenibacterium id.2153)

Batch 2−542 : Gut microbiota abundance (genus Parasutterella id.2892) on Transcription factor IIIB 90 kDa subunit
with a threshold of 1e−05

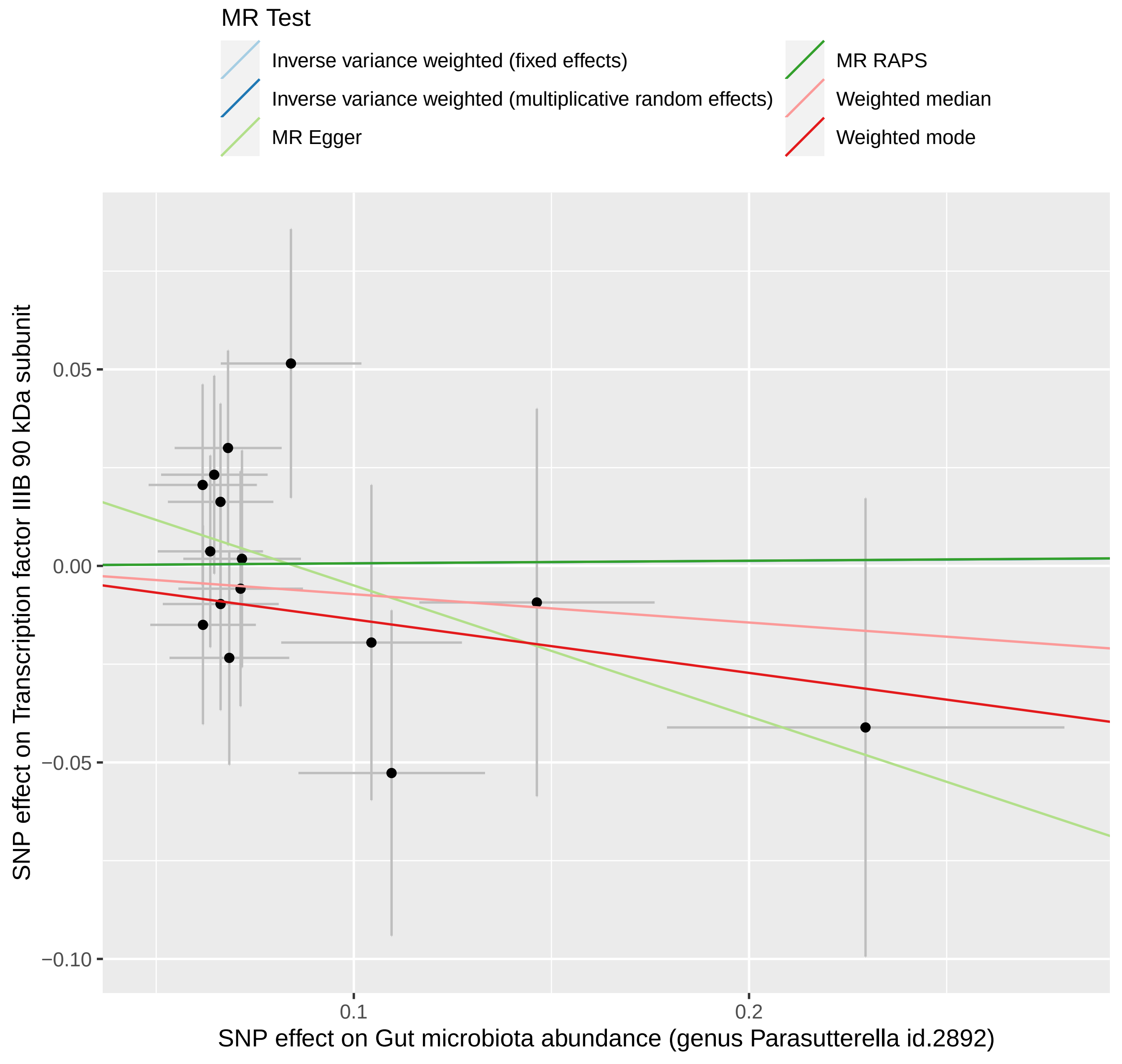

Batch 2−543 : Gut microbiota abundance (genus Catenibacterium id.2153) on BRICHOS domain−containing protein 5
with a threshold of 1e−05

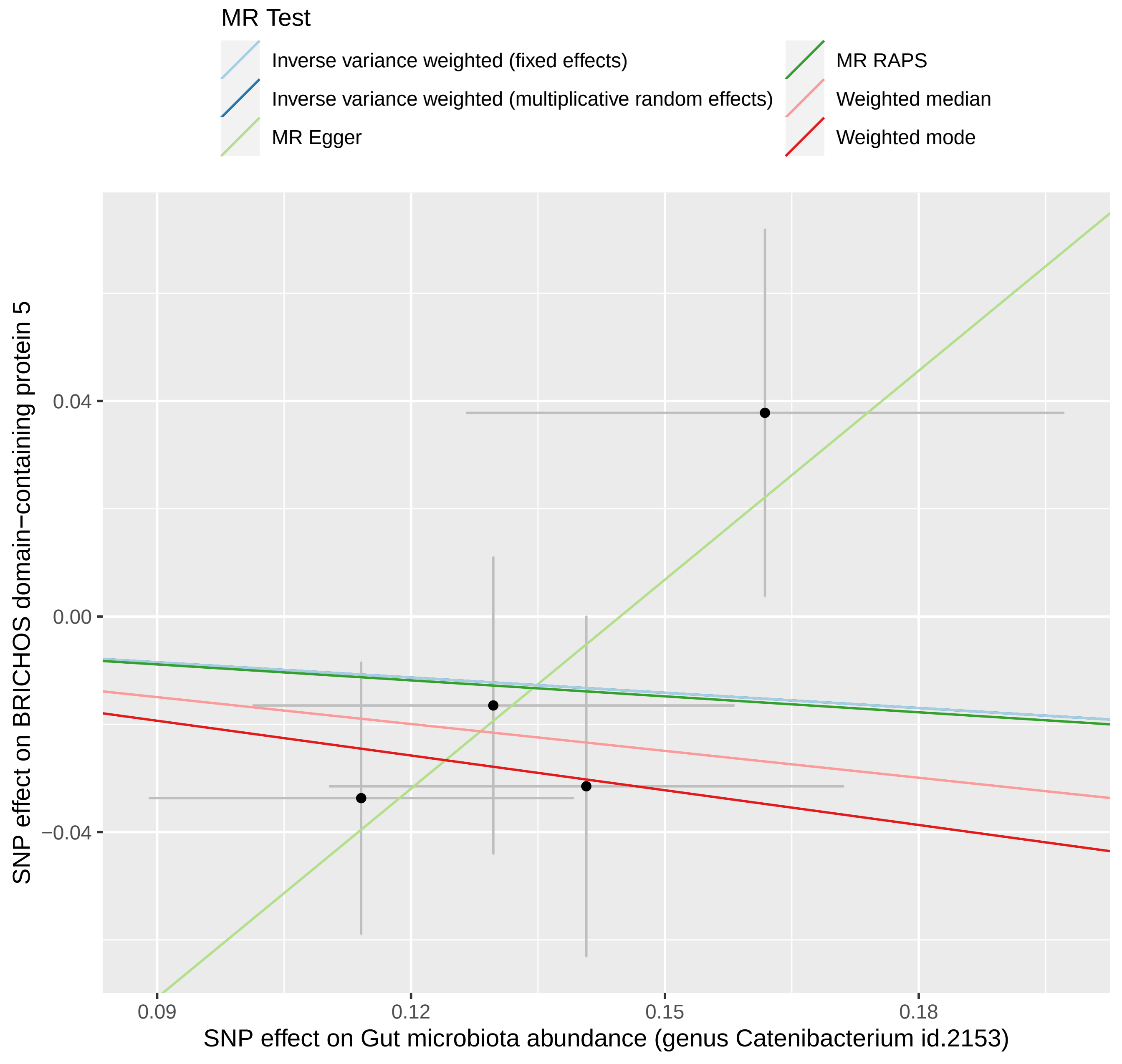

Batch 2−544 : Gut microbiota abundance (genus Parasutterella id.2892) on BRICHOS domain−containing protein 5
with a threshold of 1e−05

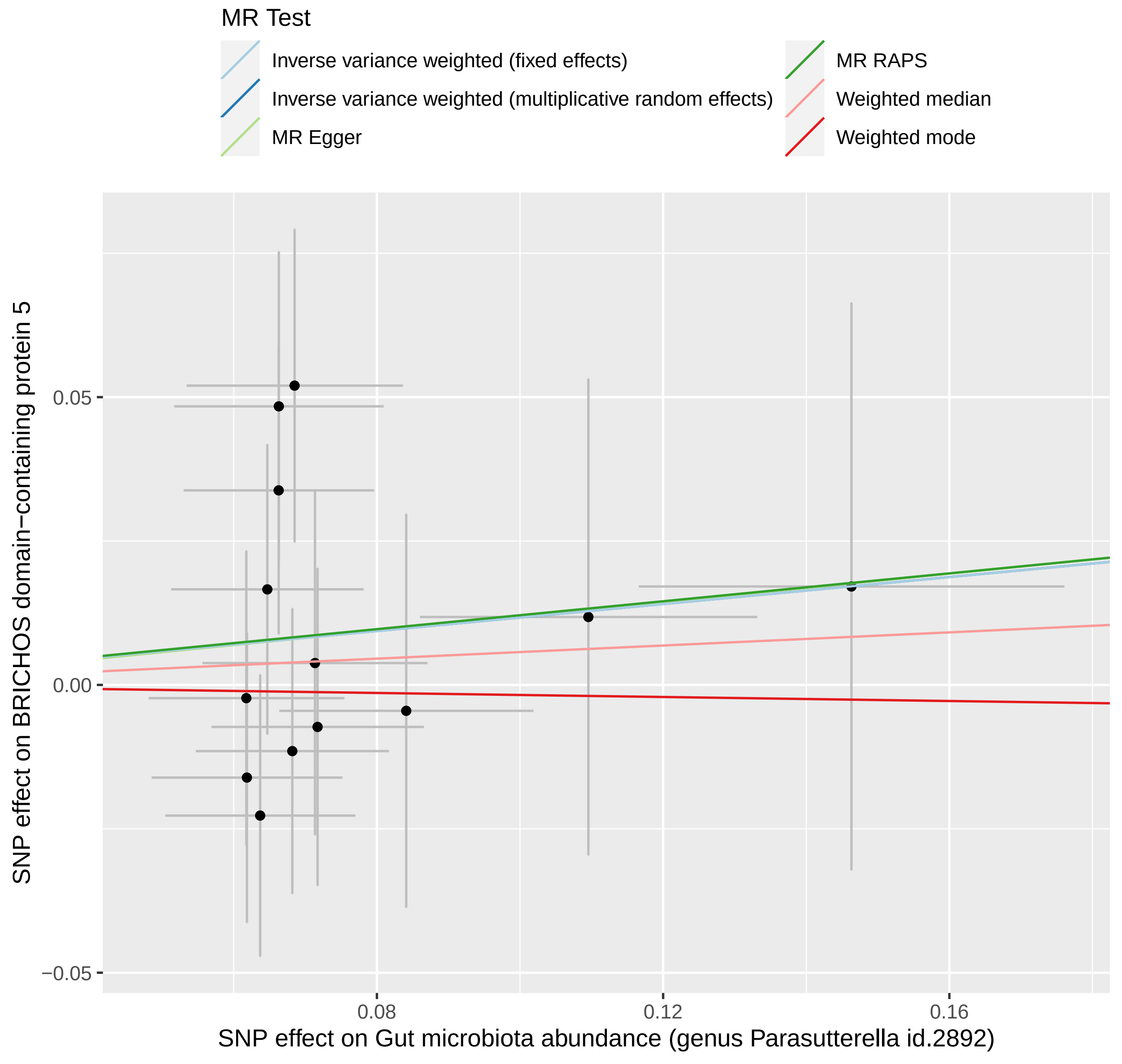

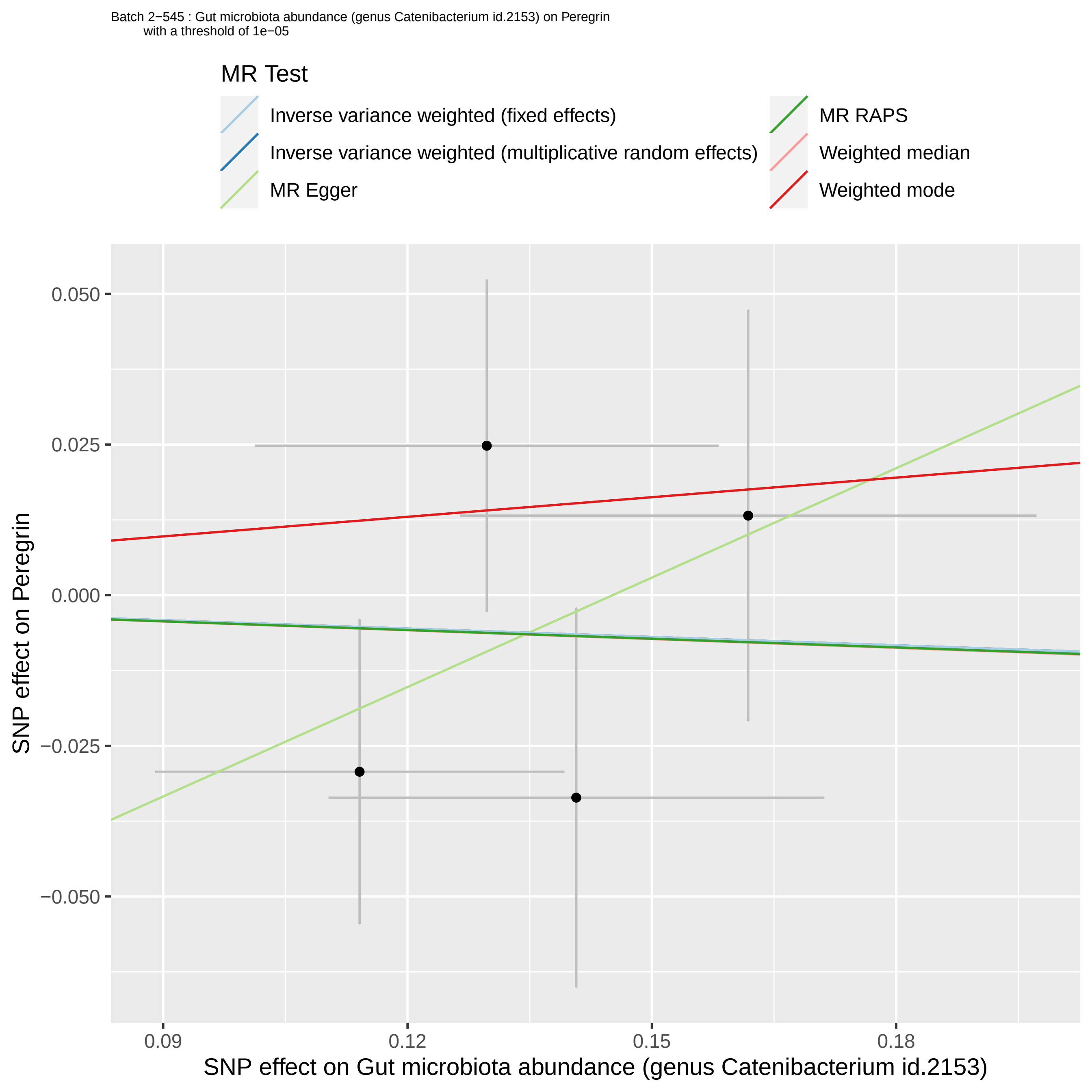
Batch 2−545 : Gut microbiota abundance (genus Catenibacterium id.2153) on Peregrin
with a threshold of 1e−05
MR Test
Inverse variance weighted (fixed effects)
Inverse variance weighted (multiplicative random effects)
MR Egger
MR RAPS
Weighted median
Weighted mode
0.050
0.025
0.000
−0.025
−0.050
SNP effect on Peregrin
0.09
0.12
0.15
0.18
SNP effect on Gut microbiota abundance (genus Catenibacterium id.2153)

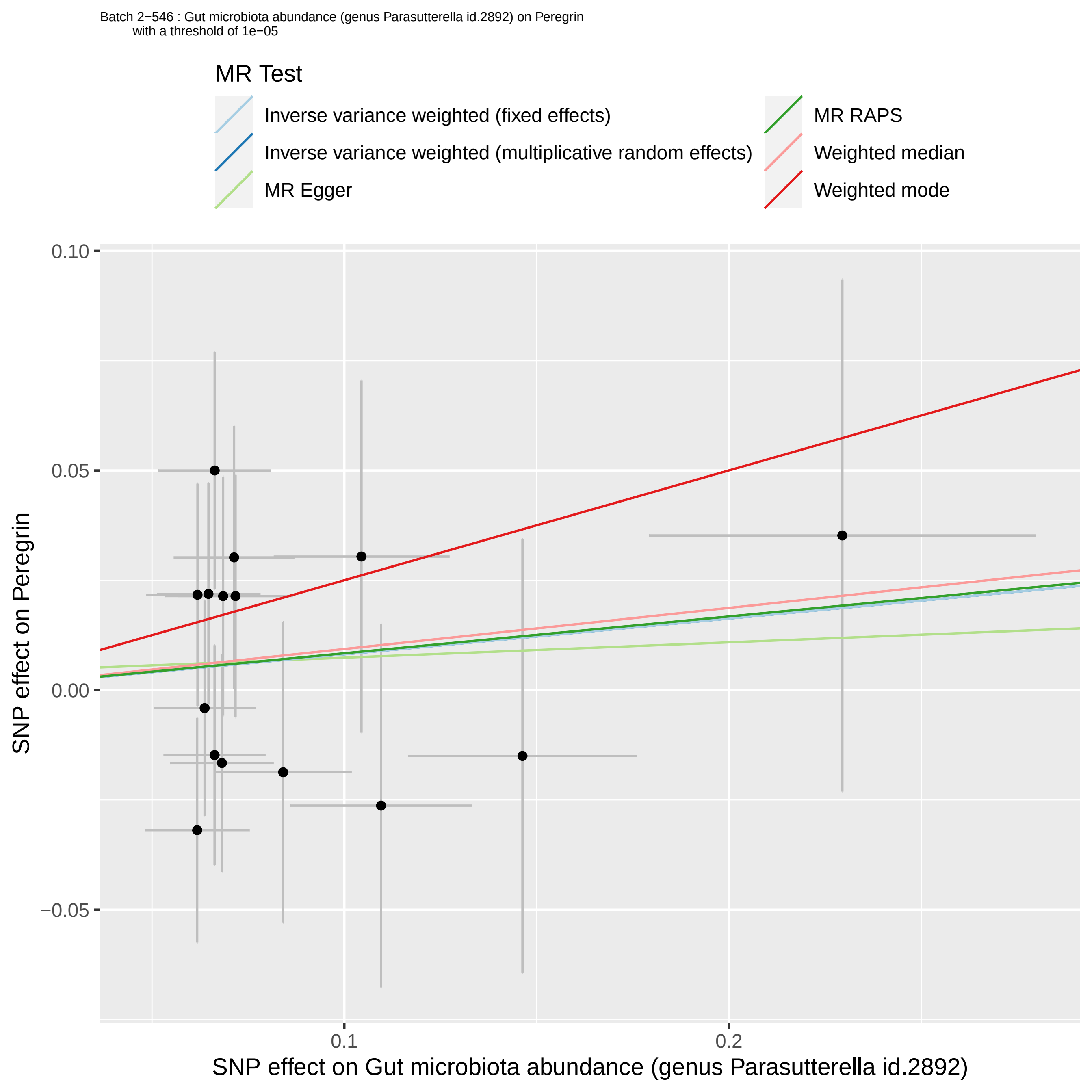
Batch 2−546 : Gut microbiota abundance (genus Parasutterella id.2892) on Peregrin
with a threshold of 1e−05
MR Test
Inverse variance weighted (fixed effects)
Inverse variance weighted (multiplicative random effects)
MR Egger
MR RAPS
Weighted median
Weighted mode
0.10
0.05
0.00
−0.05
0.1
0.2
SNP effect on Peregrin
SNP effect on Gut microbiota abundance (genus Parasutterella id.2892)

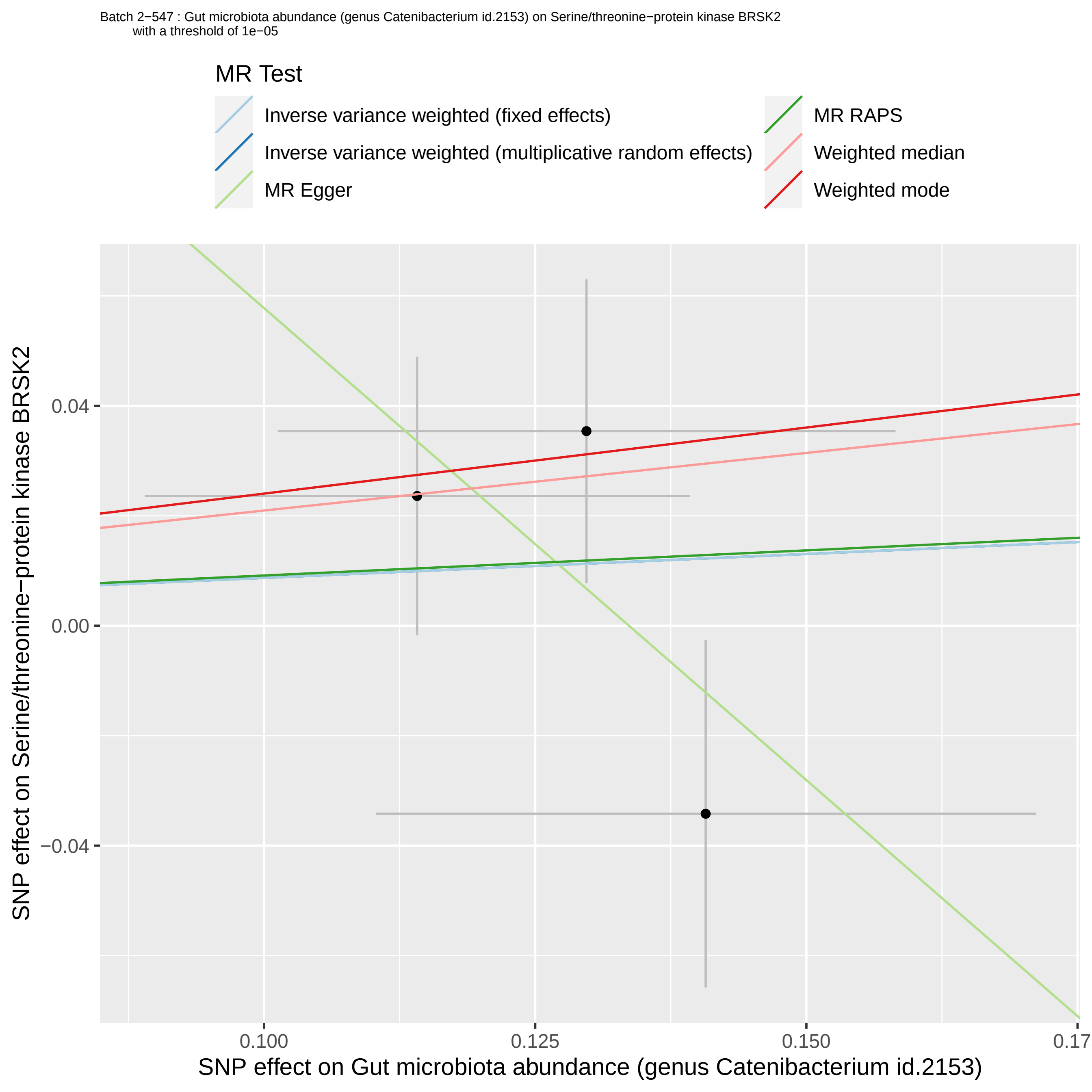
Batch 2−547 : Gut microbiota abundance (genus Catenibacterium id.2153) on Serine/threonine−protein kinase BRSK2
with a threshold of 1e−05
MR Test
Inverse variance weighted (fixed effects)
Inverse variance weighted (multiplicative random effects)
MR Egger
MR RAPS
Weighted median
Weighted mode
0.04
0.00
−0.04
0.100
0.125
0.150
0.17
SNP effect on Serine/threonine−protein kinase BRSK2
SNP effect on Gut microbiota abundance (genus Catenibacterium id.2153)

Batch 2−548 : Gut microbiota abundance (genus Parasutterella id.2892) on Serine/threonine−protein kinase BRSK2
with a threshold of 1e−05

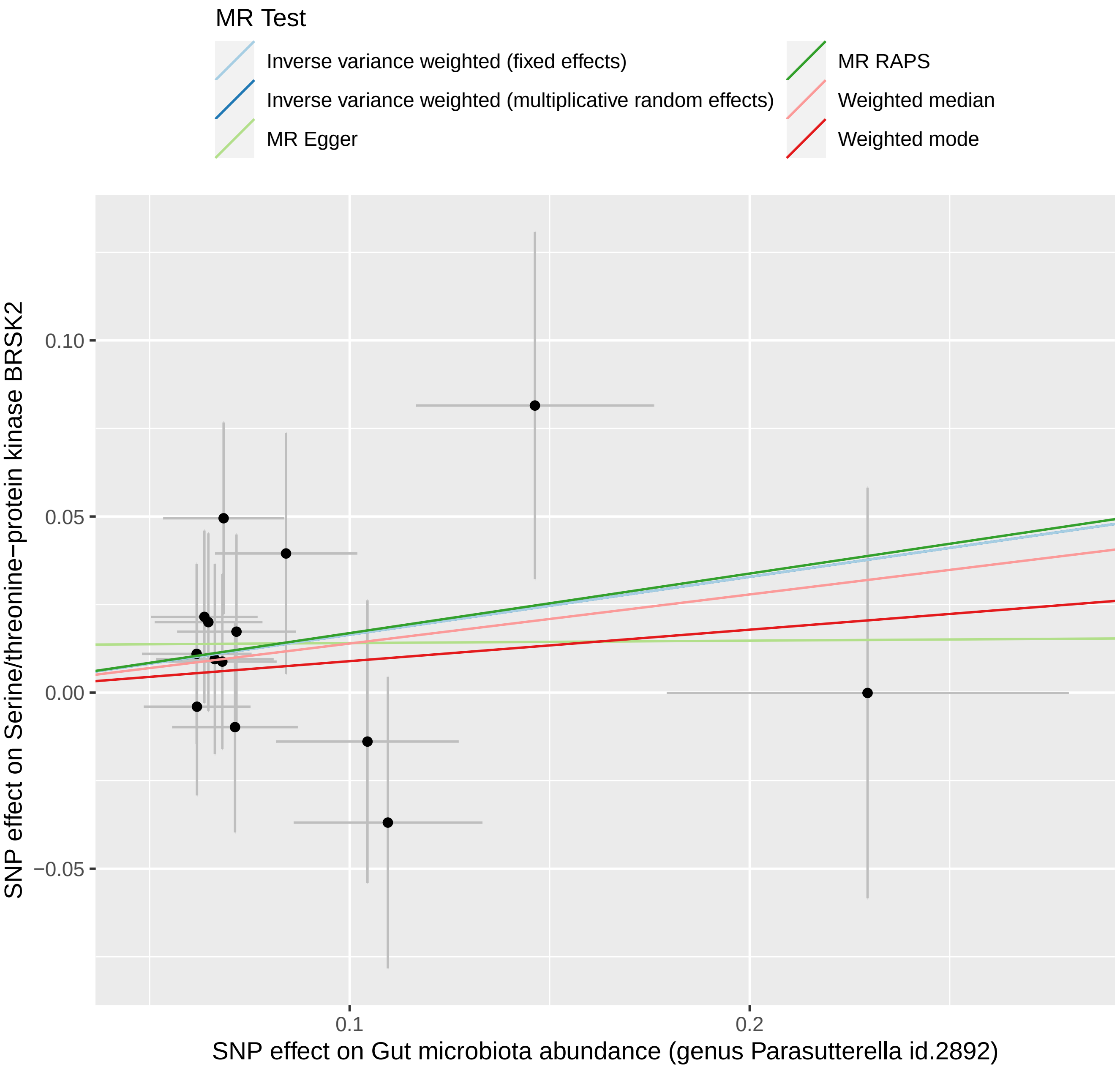

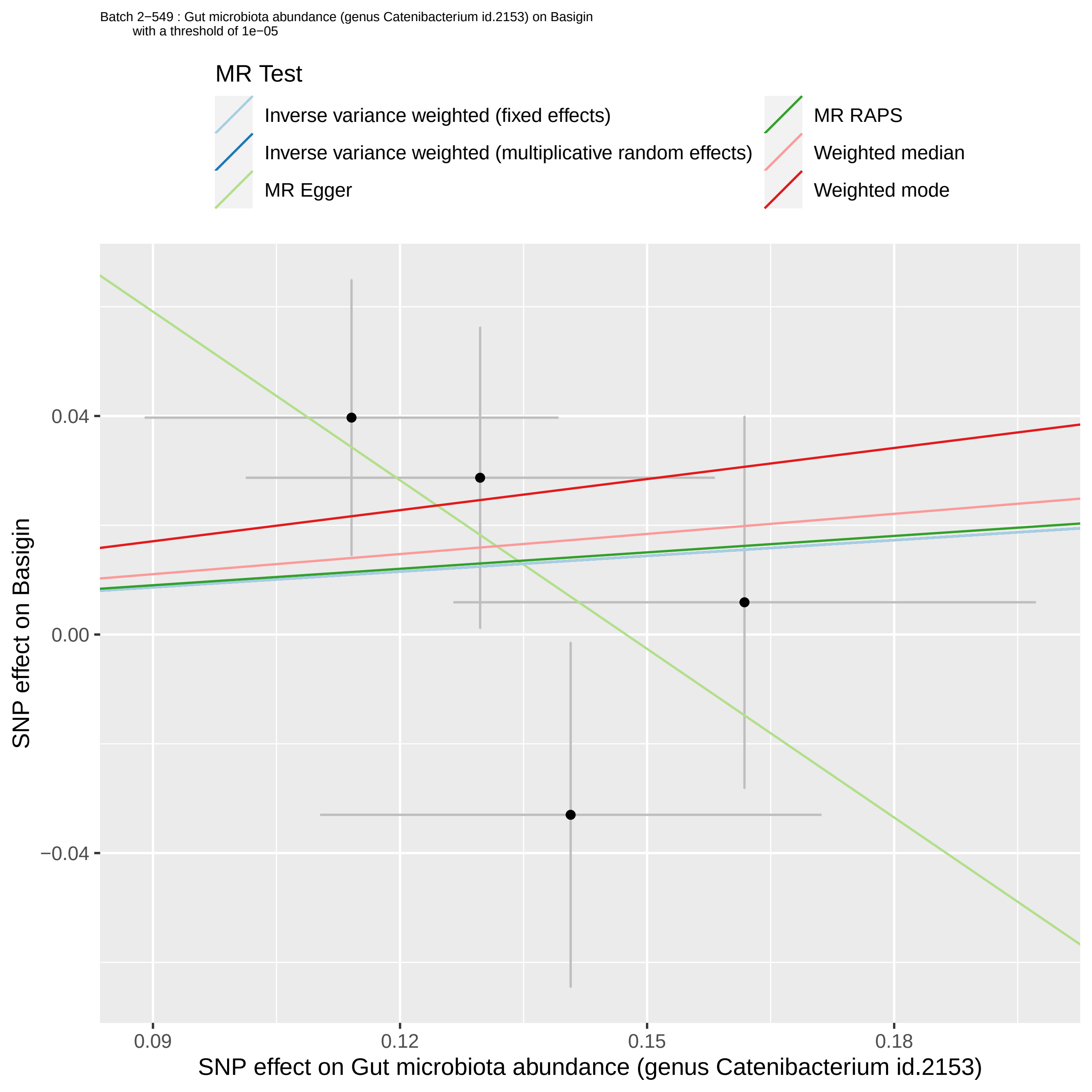

Batch 2−549 : Gut microbiota abundance (genus Catenibacterium id.2153) on Basigin
with a threshold of 1e−05
MR Test
Inverse variance weighted (fixed effects)
Inverse variance weighted (multiplicative random effects)
MR Egger
MR RAPS
Weighted median
Weighted mode
0.04
0.00
−0.04
0.09
0.12
0.15
0.18
SNP effect on Basigin
SNP effect on Gut microbiota abundance (genus Catenibacterium id.2153)

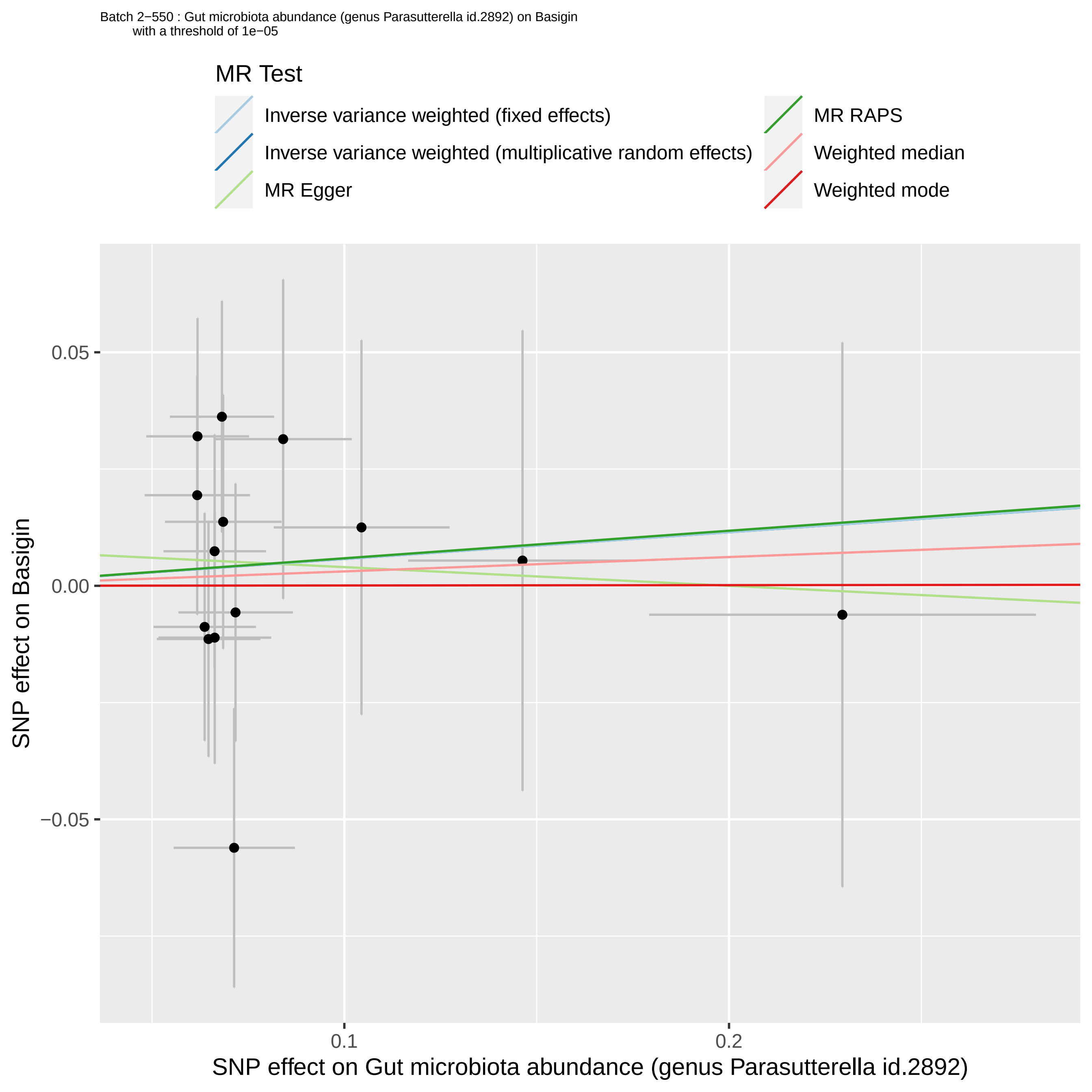
Batch 2−550 : Gut microbiota abundance (genus Parasutterella id.2892) on Basigin
with a threshold of 1e−05
MR Test
Inverse variance weighted (fixed effects)
Inverse variance weighted (multiplicative random effects)
MR Egger
MR RAPS
Weighted median
Weighted mode
0.05
0.00
−0.05
0.1
0.2
SNP effect on Basigin
SNP effect on Gut microbiota abundance (genus Parasutterella id.2892)

Batch 2−551 : Gut microbiota abundance (genus Catenibacterium id.2153) on ADP−ribosyl cyclase/cyclic ADP−ribose hydrolase 2
with a threshold of 1e−05

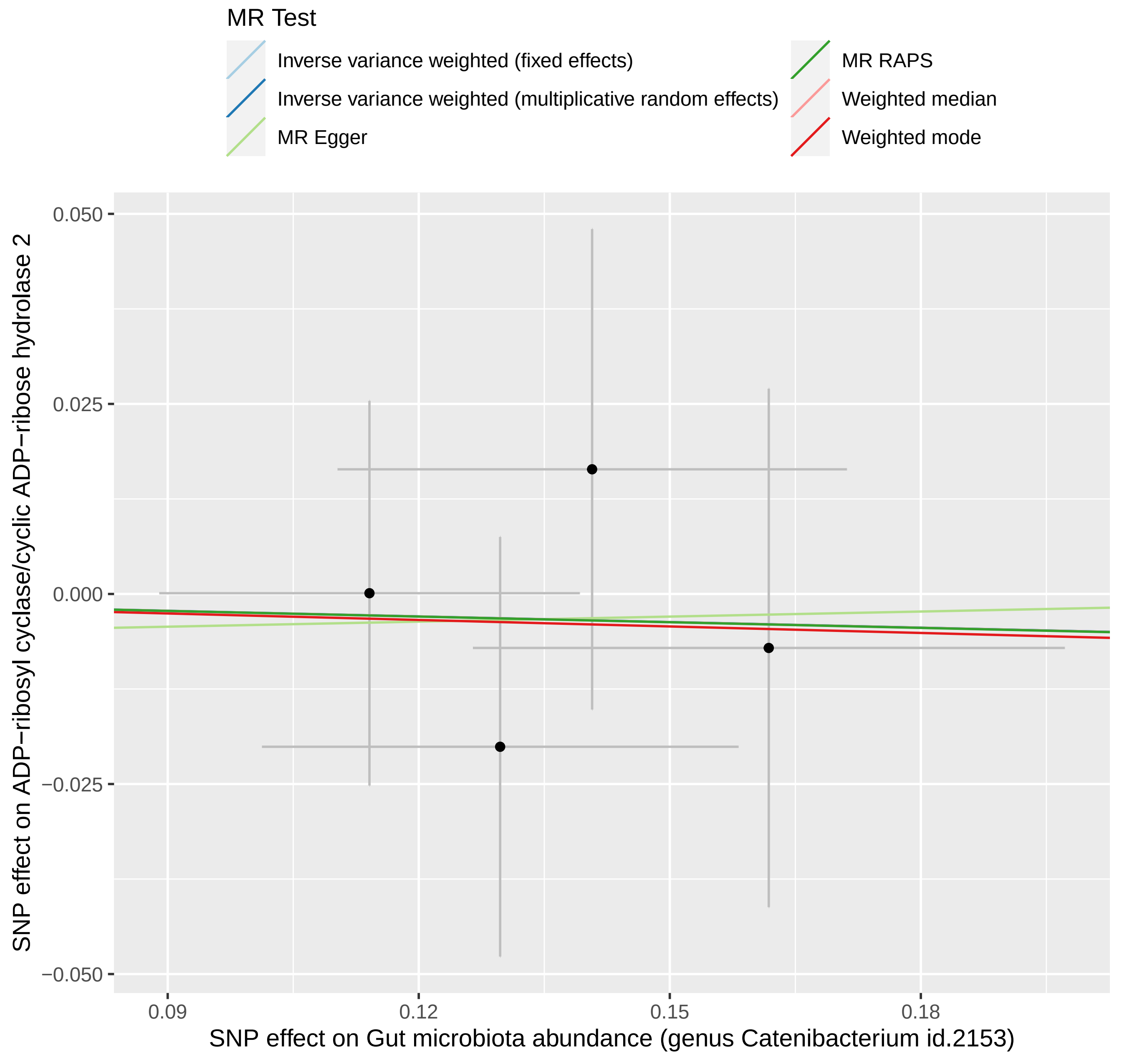

Batch 2−552 : Gut microbiota abundance (genus Parasutterella id.2892) on ADP−ribosyl cyclase/cyclic ADP−ribose hydrolase 2
with a threshold of 1e−05

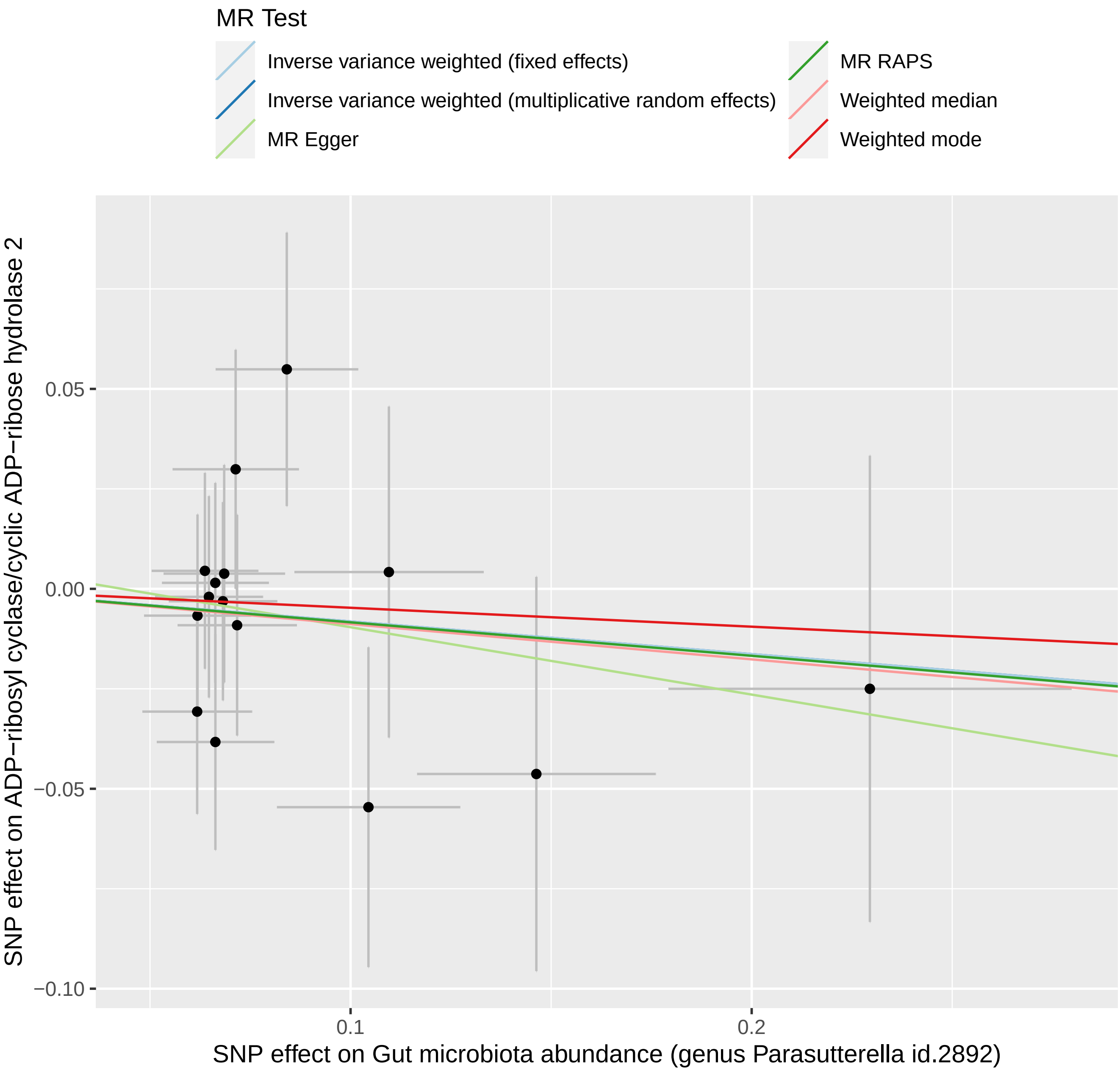

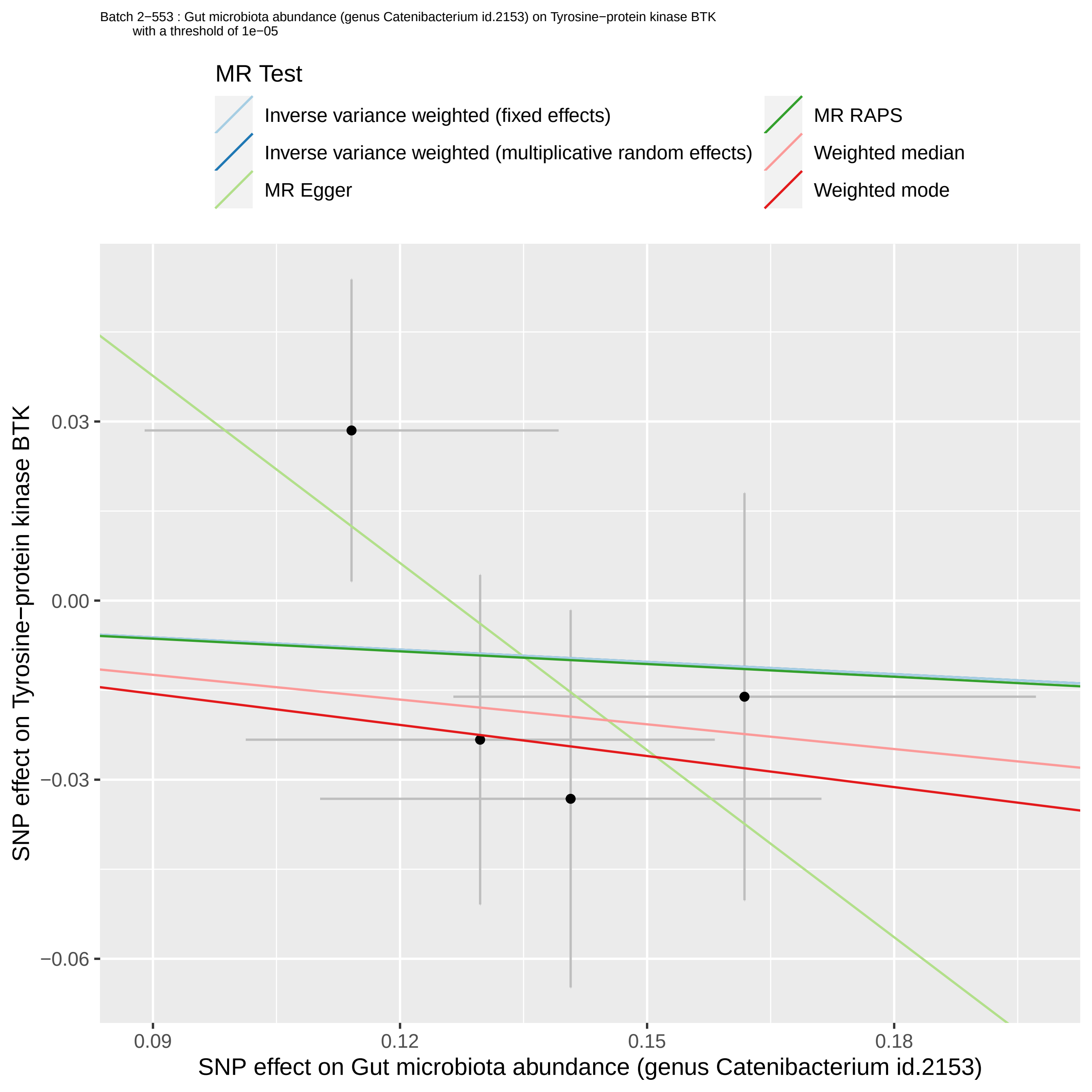
Batch 2−553 : Gut microbiota abundance (genus Catenibacterium id.2153) on Tyrosine−protein kinase BTK
with a threshold of 1e−05
MR Test
Inverse variance weighted (fixed effects)
Inverse variance weighted (multiplicative random effects)
MR Egger
MR RAPS
Weighted median
Weighted mode
SNP effect on Tyrosine−protein kinase BTK
0.03
0.00
−0.03
−0.06
0.09
0.12
0.15
0.18
SNP effect on Gut microbiota abundance (genus Catenibacterium id.2153)

Batch 2−554 : Gut microbiota abundance (genus Parasutterella id.2892) on Tyrosine−protein kinase BTK
with a threshold of 1e−05

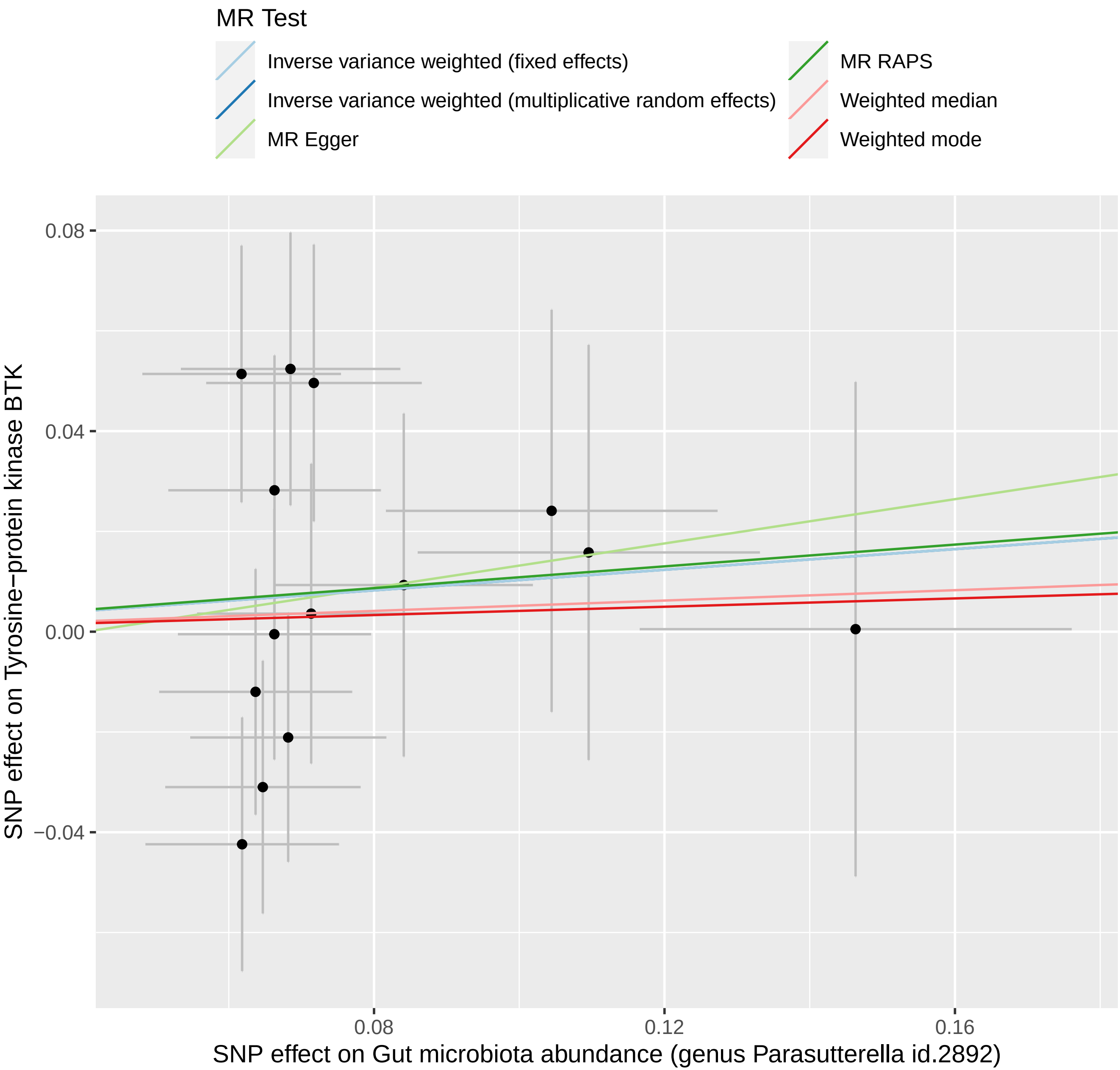

Batch 2−555 : Gut microbiota abundance (genus Catenibacterium id.2153) on Butyrophilin−like protein 9
with a threshold of 1e−05

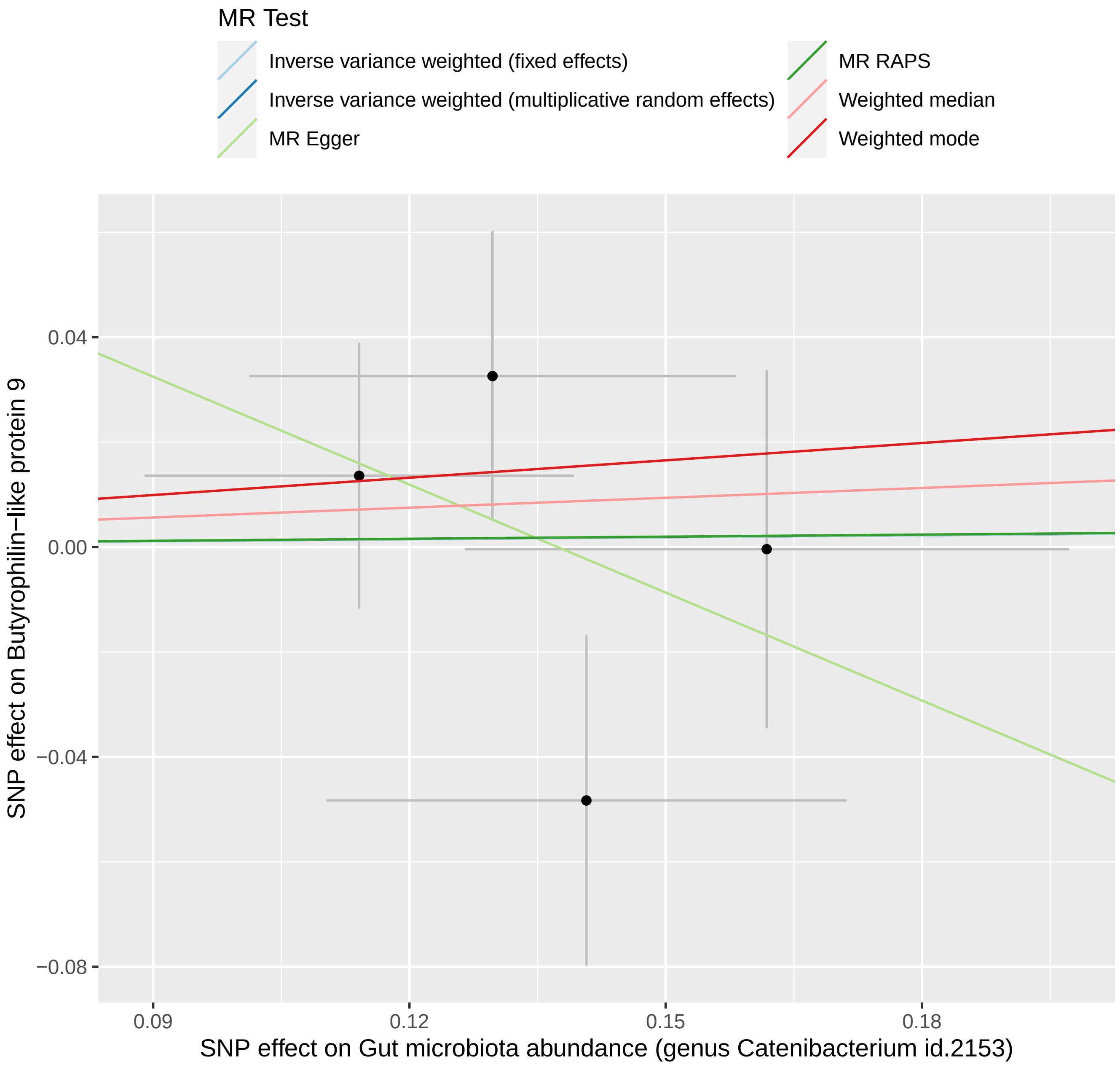

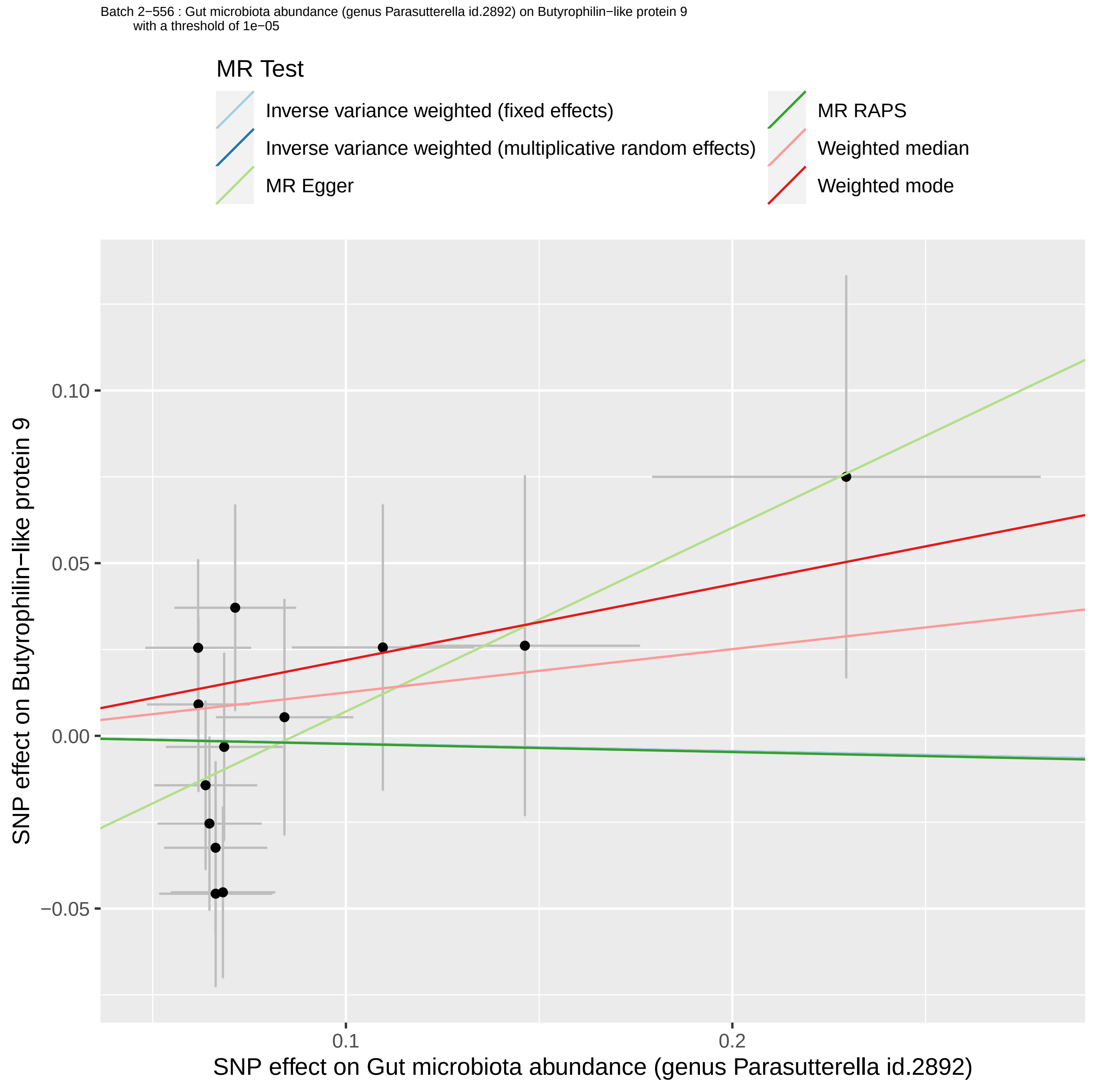

Batch 2−556 : Gut microbiota abundance (genus Parasutterella id.2892) on Butyrophilin−like protein 9
with a threshold of 1e−05
MR Test
Inverse variance weighted (fixed effects)
Inverse variance weighted (multiplicative random effects)
MR Egger
MR RAPS
Weighted median
Weighted mode
0.10
0.05
0.00
−0.05
0.1
0.2
SNP effect on Butyrophilin−like protein 9
SNP effect on Gut microbiota abundance (genus Parasutterella id.2892)

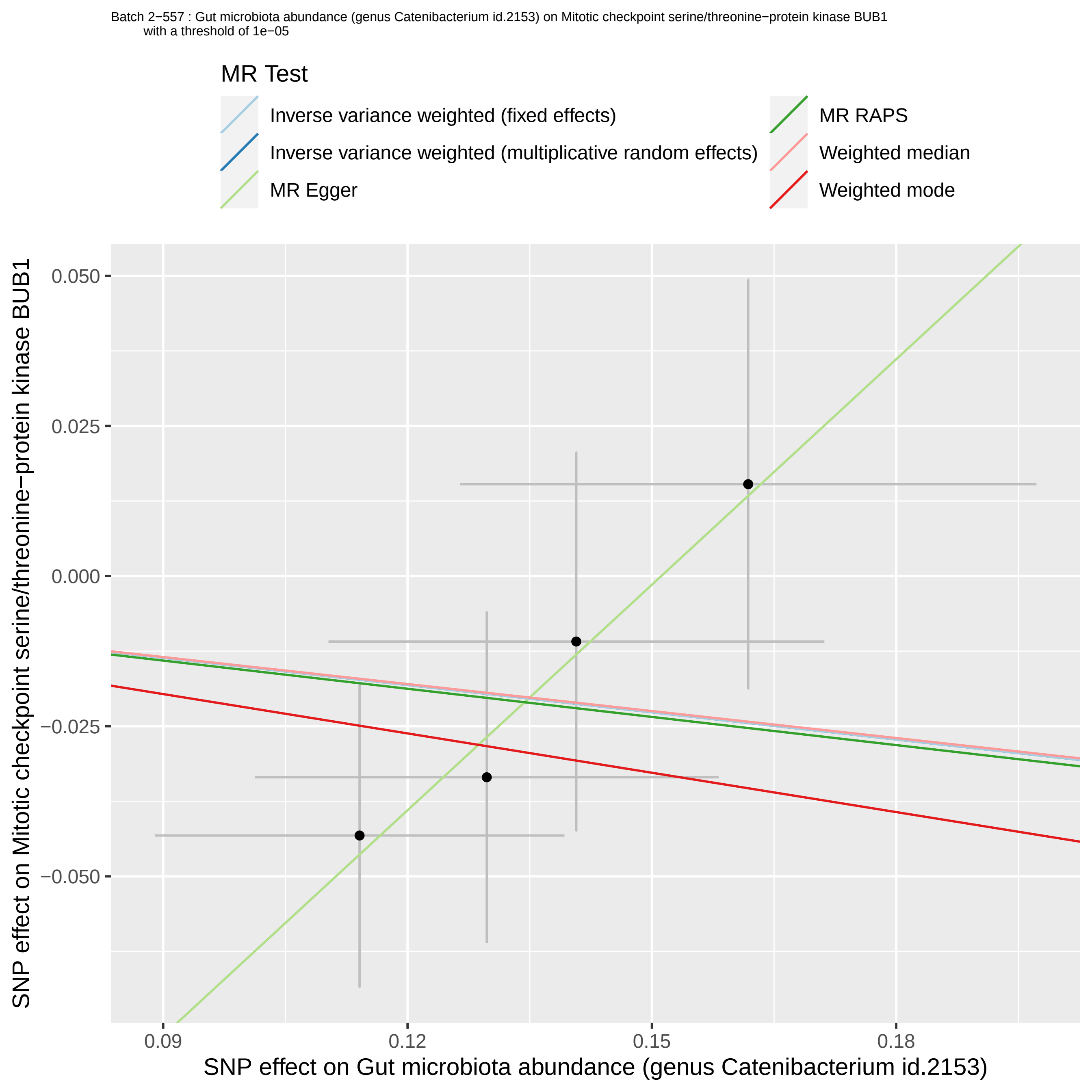

Batch 2−557 : Gut microbiota abundance (genus Catenibacterium id.2153) on Mitotic checkpoint serine/threonine−protein kinase BUB1
with a threshold of 1e−05
MR Test
Inverse variance weighted (fixed effects)
Inverse variance weighted (multiplicative random effects)
MR Egger
MR RAPS
Weighted median
Weighted mode
SNP effect on Mitotic checkpoint serine/threonine−protein kinase BUB1
0.050
0.025
0.000
−0.025
−0.050
0.09
0.12
0.15
0.18
SNP effect on Gut microbiota abundance (genus Catenibacterium id.2153)

Batch 2−558 : Gut microbiota abundance (genus Parasutterella id.2892) on Mitotic checkpoint serine/threonine−protein kinase BUB1
with a threshold of 1e−05

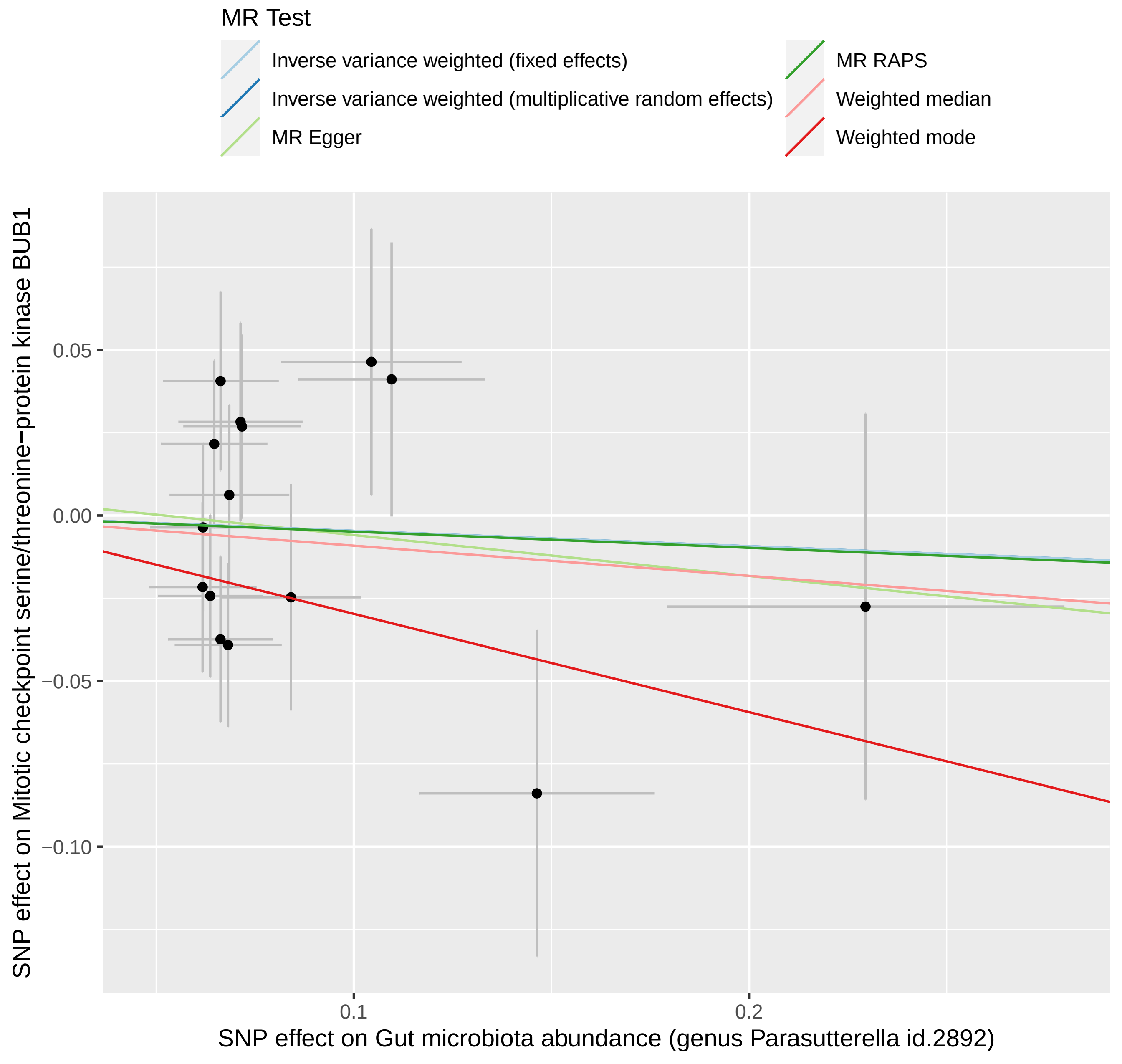

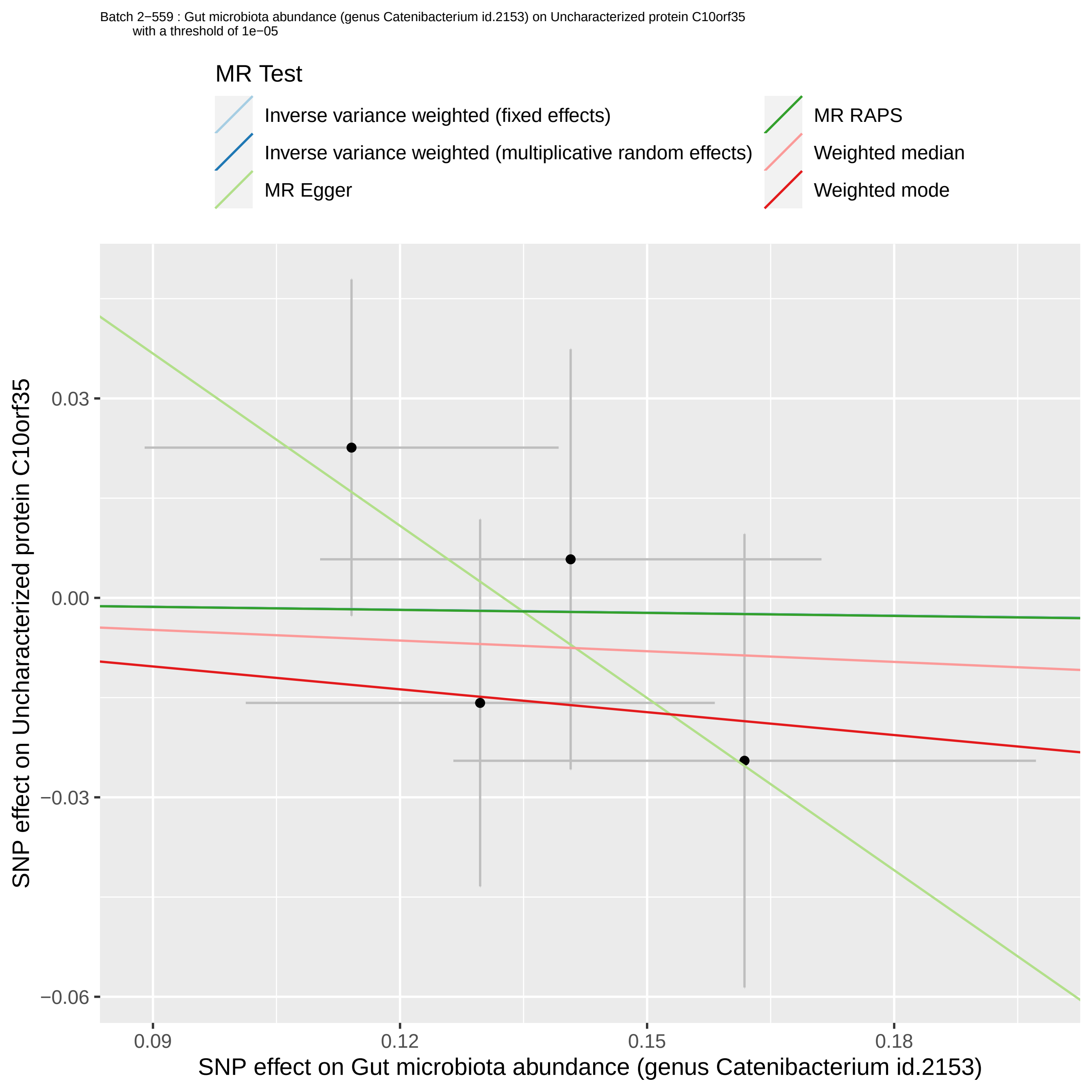

Batch 2−559 : Gut microbiota abundance (genus Catenibacterium id.2153) on Uncharacterized protein C10orf35
with a threshold of 1e−05
MR Test
Inverse variance weighted (fixed effects)
Inverse variance weighted (multiplicative random effects)
MR Egger
MR RAPS
Weighted median
Weighted mode
0.03
0.00
−0.03
−0.06
0.09
0.12
0.15
0.18
SNP effect on Uncharacterized protein C10orf35
SNP effect on Gut microbiota abundance (genus Catenibacterium id.2153)

Batch 2−560 : Gut microbiota abundance (genus Parasutterella id.2892) on Uncharacterized protein C10orf35
with a threshold of 1e−05

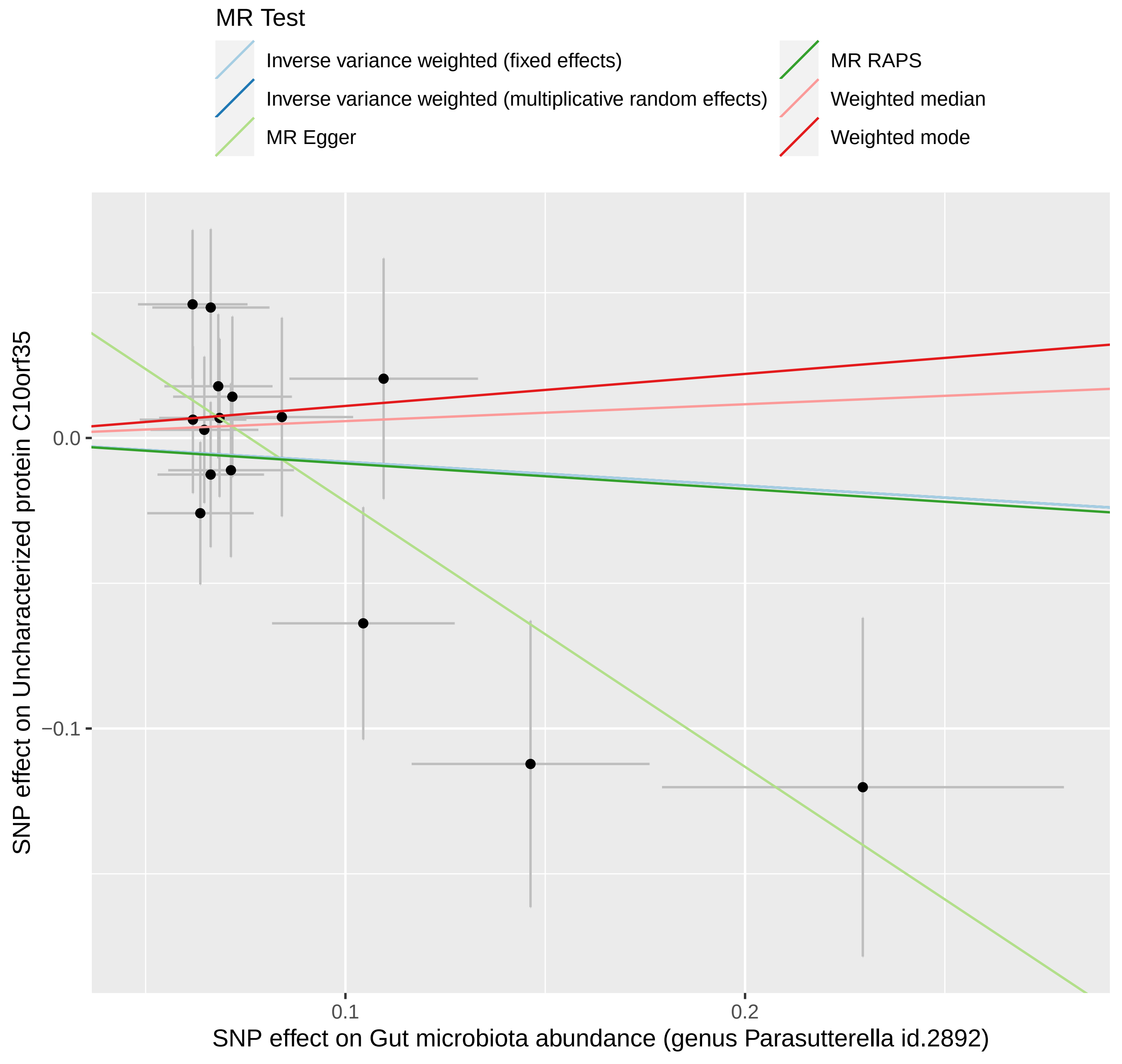

Batch 2−561 : Gut microbiota abundance (genus Catenibacterium id.2153) on Platelet receptor Gi24
with a threshold of 1e−05

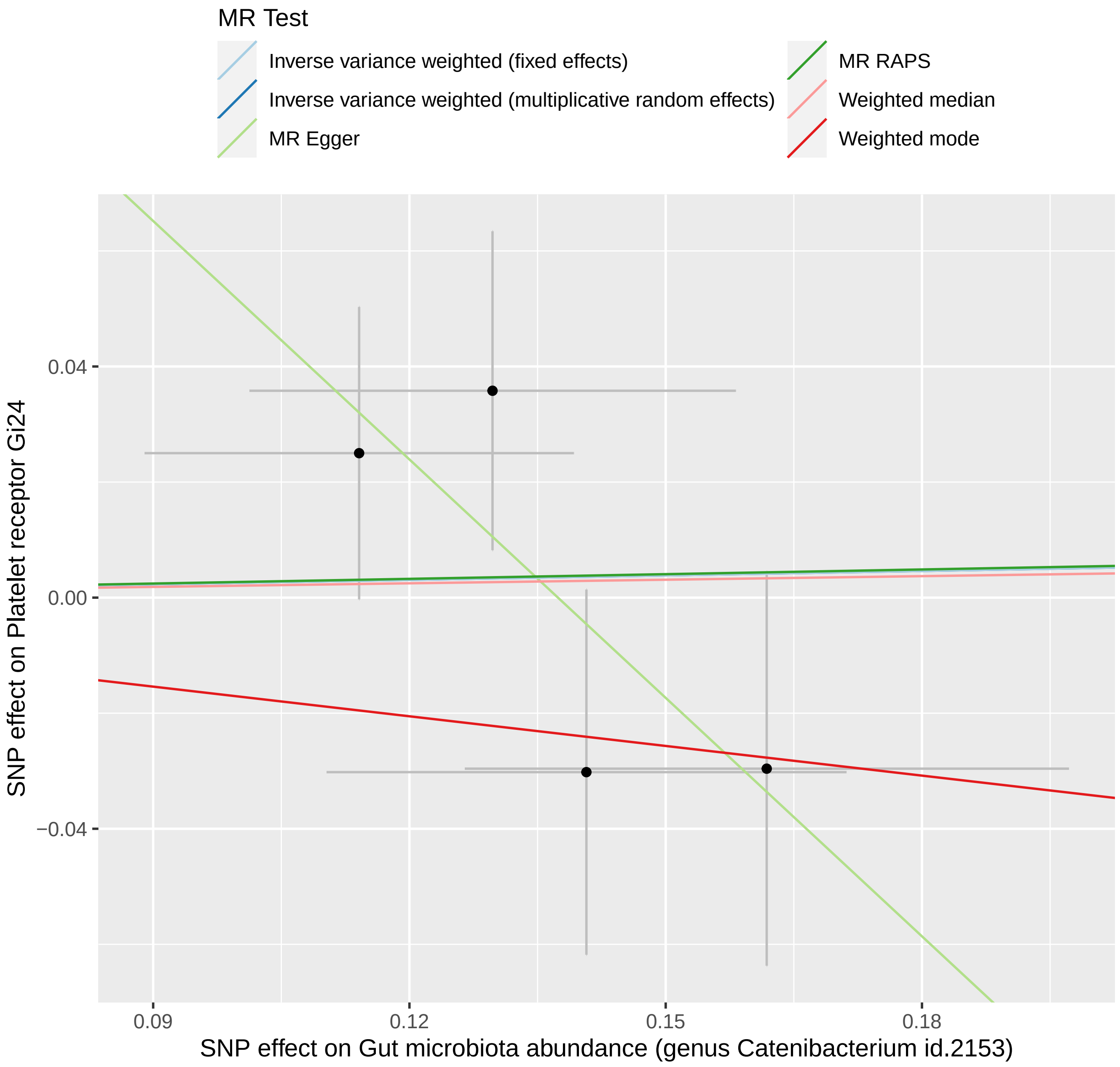

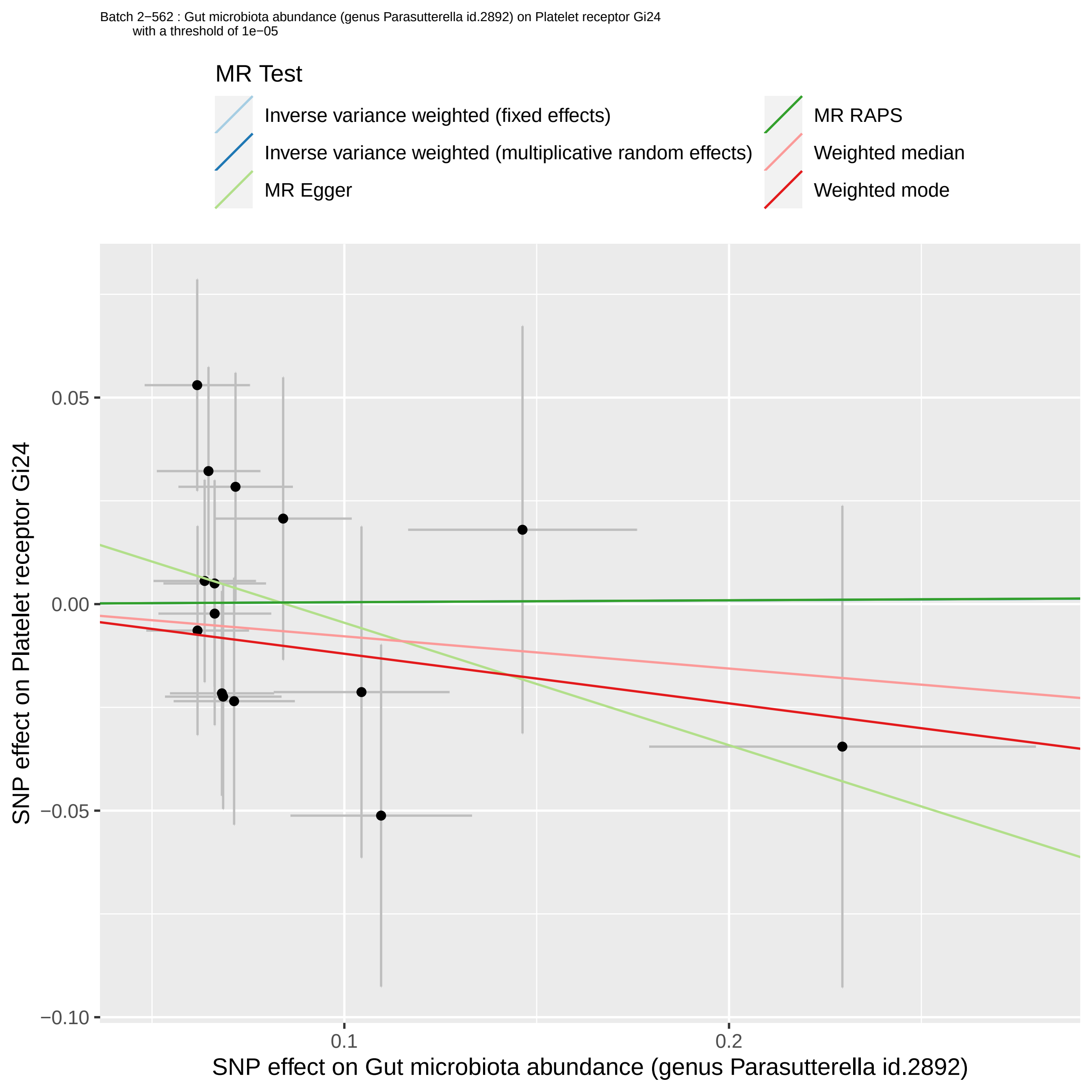
Batch 2−562 : Gut microbiota abundance (genus Parasutterella id.2892) on Platelet receptor Gi24
with a threshold of 1e−05
MR Test
Inverse variance weighted (fixed effects)
Inverse variance weighted (multiplicative random effects)
MR Egger
MR RAPS
Weighted median
Weighted mode
0.05
0.00
−0.05
−0.10
0.1
0.2
SNP effect on Platelet receptor Gi24
SNP effect on Gut microbiota abundance (genus Parasutterella id.2892)

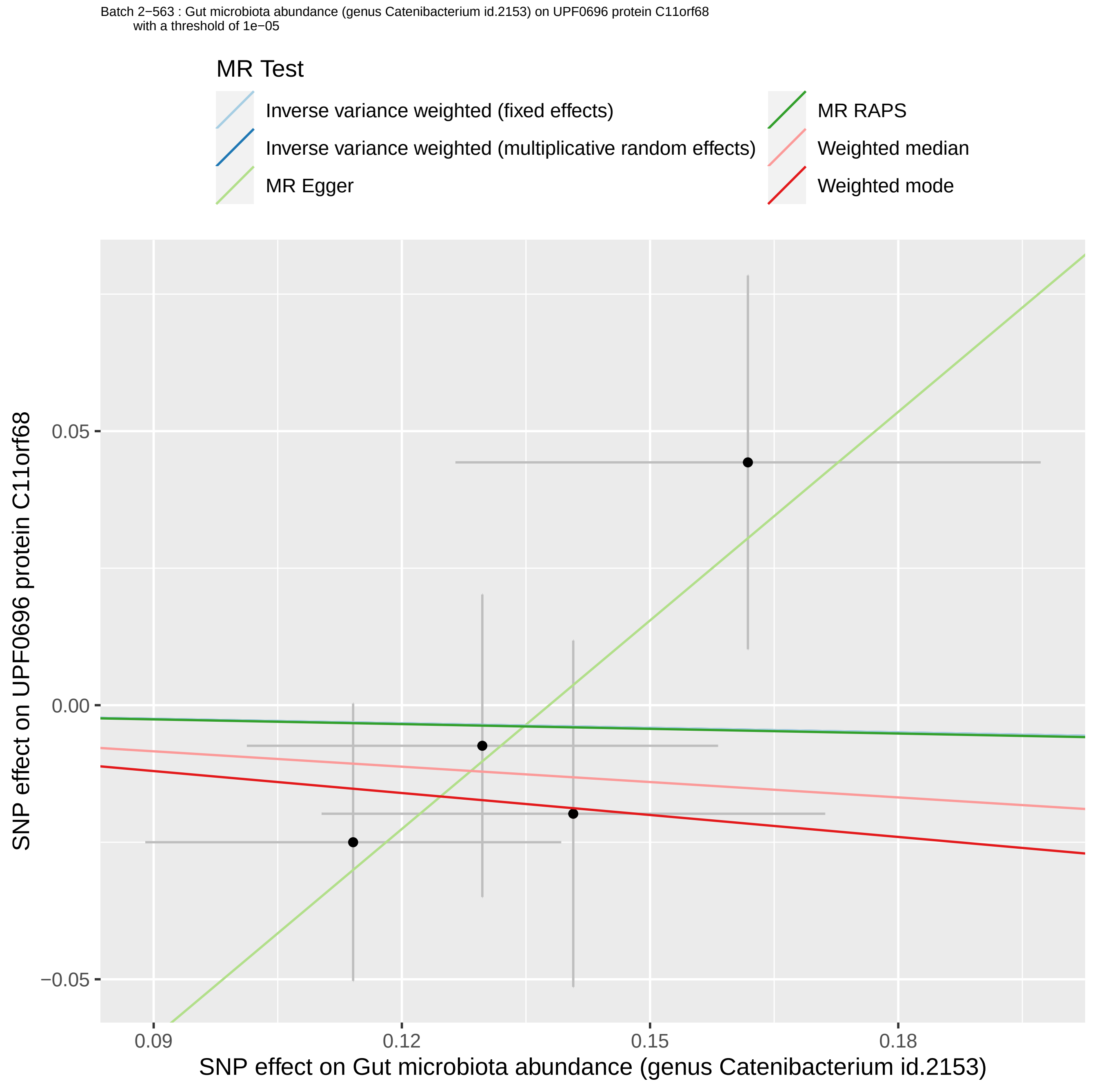

Batch 2−563 : Gut microbiota abundance (genus Catenibacterium id.2153) on UPF0696 protein C11orf68
with a threshold of 1e−05
MR Test
Inverse variance weighted (fixed effects)
Inverse variance weighted (multiplicative random effects)
MR Egger
MR RAPS
Weighted median
Weighted mode
0.05
0.00
−0.05
0.09
0.12
0.15
0.18
SNP effect on UPF0696 protein C11orf68
SNP effect on Gut microbiota abundance (genus Catenibacterium id.2153)

Batch 2−564 : Gut microbiota abundance (genus Parasutterella id.2892) on UPF0696 protein C11orf68
with a threshold of 1e−05

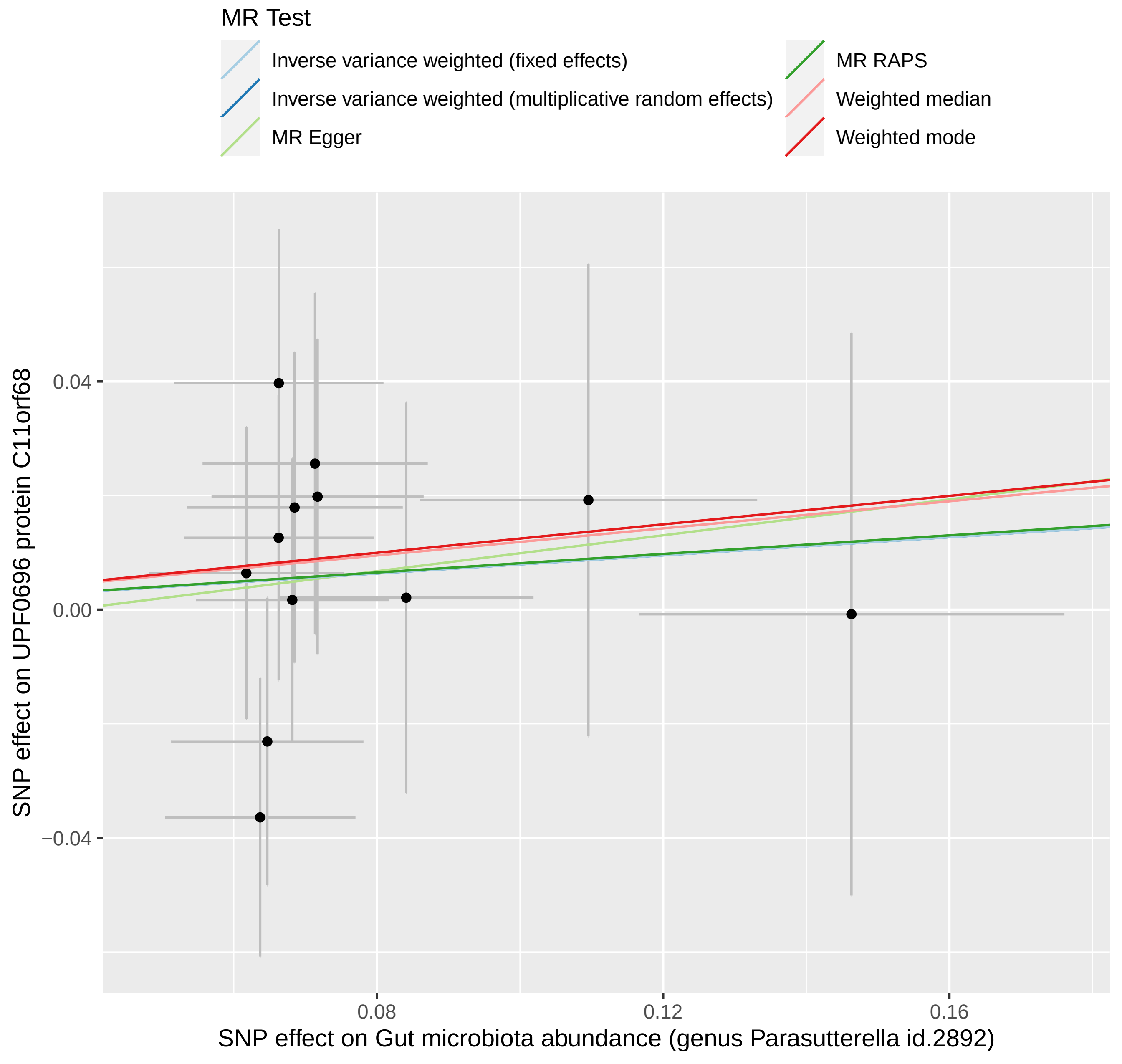

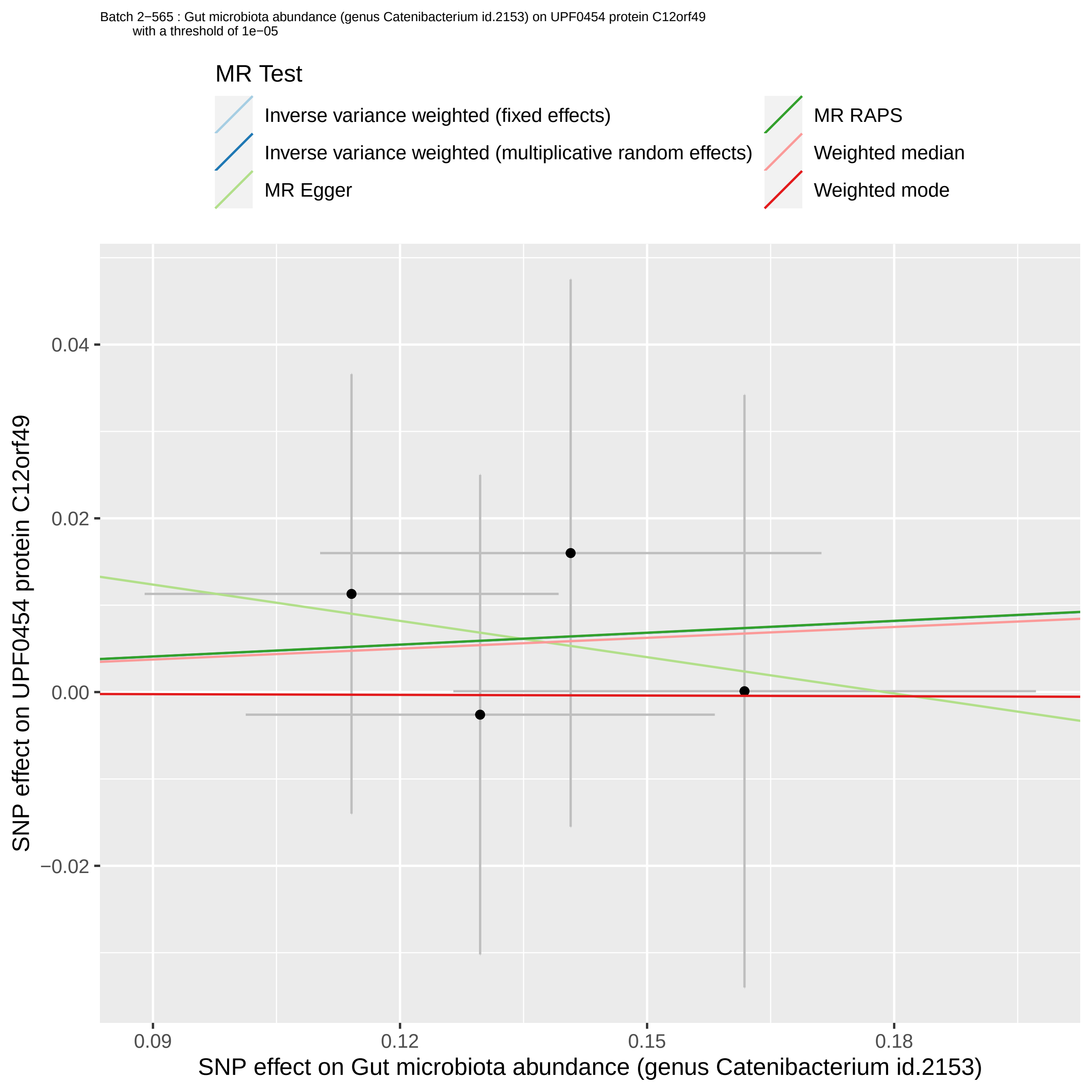

Batch 2−565 : Gut microbiota abundance (genus Catenibacterium id.2153) on UPF0454 protein C12orf49
with a threshold of 1e−05
MR Test
Inverse variance weighted (fixed effects)
Inverse variance weighted (multiplicative random effects)
MR Egger
MR RAPS
Weighted median
Weighted mode
SNP effect on UPF0454 protein C12orf49
0.04
0.02
0.00
−0.02
0.09
0.12
0.15
0.18
SNP effect on Gut microbiota abundance (genus Catenibacterium id.2153)

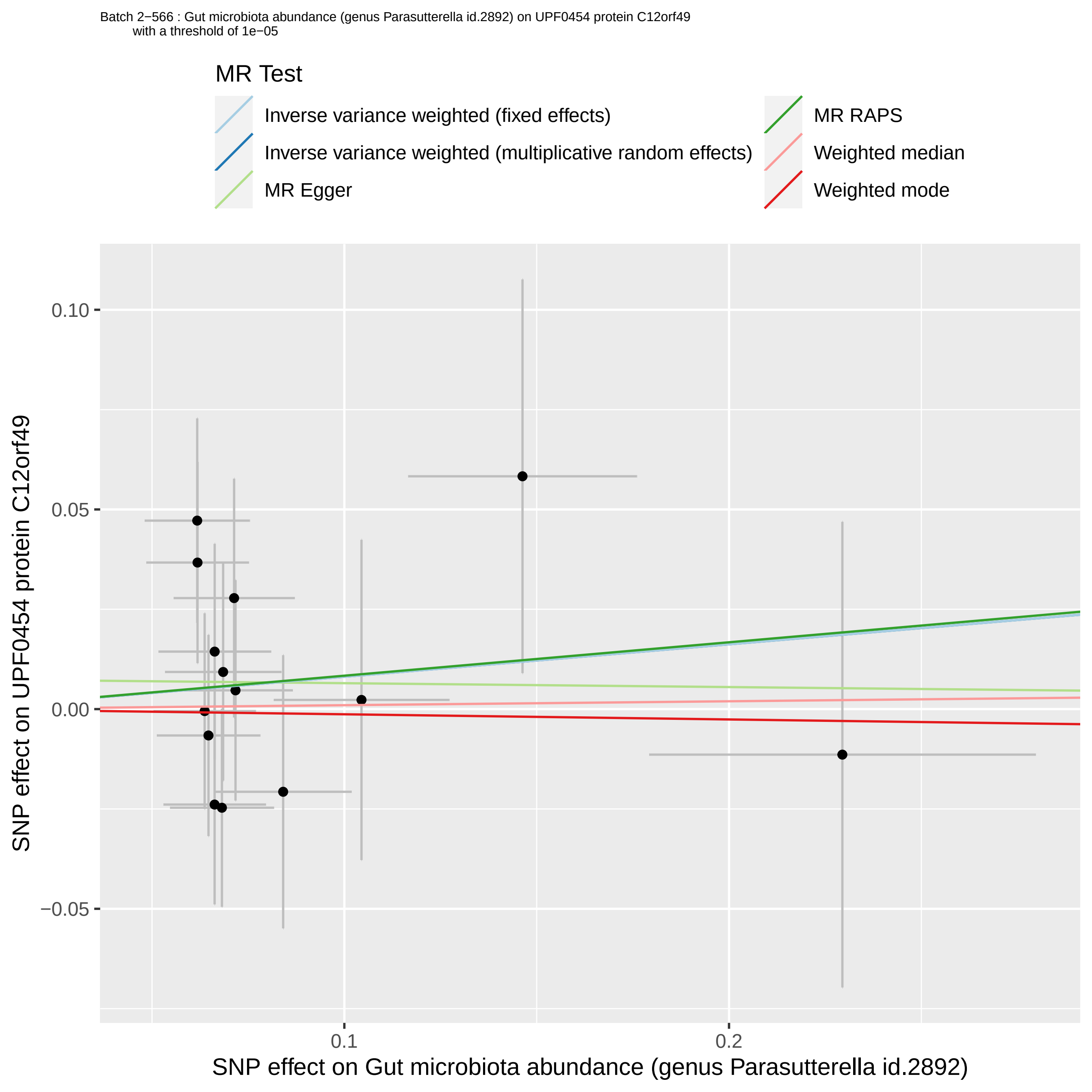

Batch 2−566 : Gut microbiota abundance (genus Parasutterella id.2892) on UPF0454 protein C12orf49
with a threshold of 1e−05
MR Test
Inverse variance weighted (fixed effects)
Inverse variance weighted (multiplicative random effects)
MR Egger
MR RAPS
Weighted median
Weighted mode
0.10
0.05
0.00
−0.05
0.1
0.2
SNP effect on UPF0454 protein C12orf49
SNP effect on Gut microbiota abundance (genus Parasutterella id.2892)

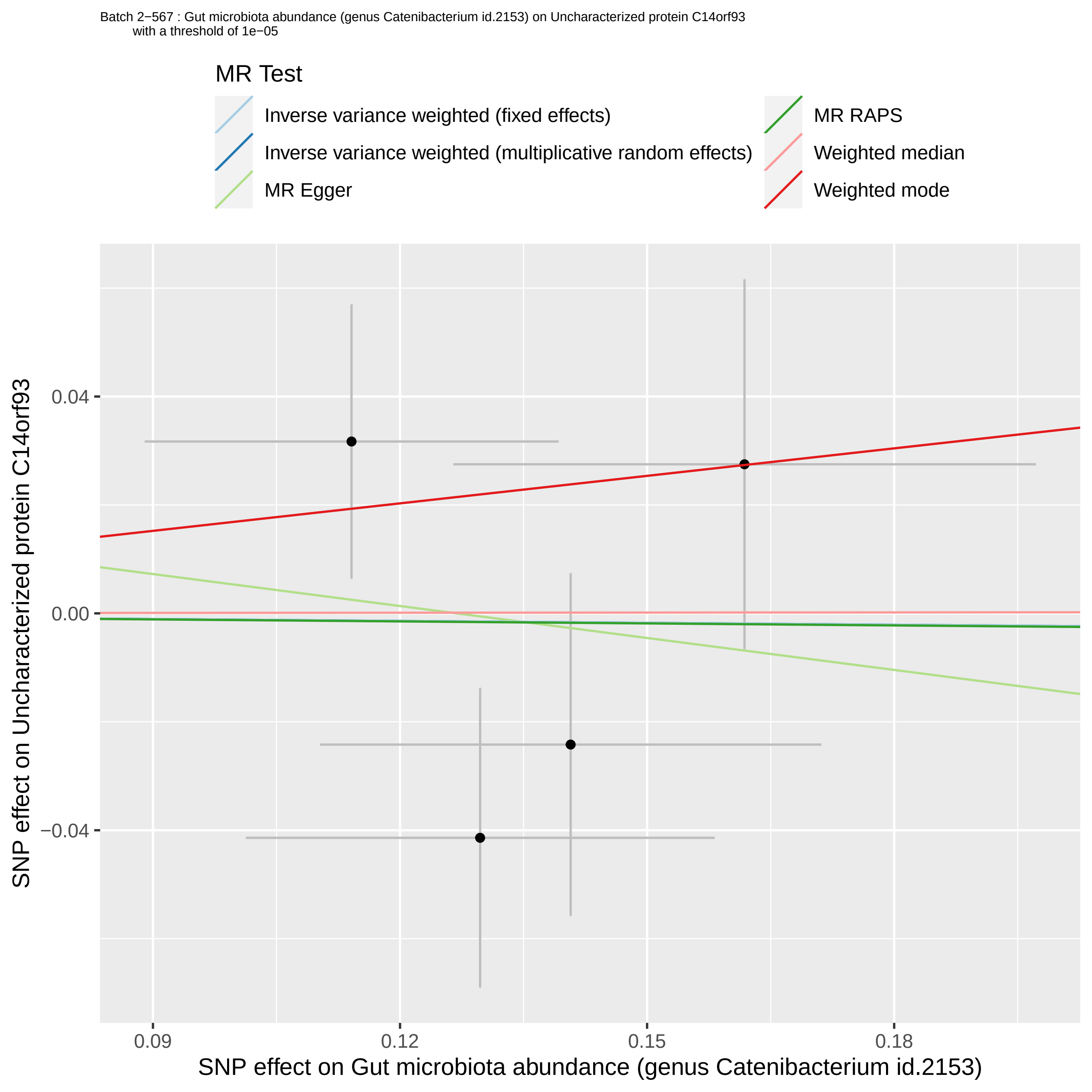
Batch 2−567 : Gut microbiota abundance (genus Catenibacterium id.2153) on Uncharacterized protein C14orf93
with a threshold of 1e−05
MR Test
Inverse variance weighted (fixed effects)
Inverse variance weighted (multiplicative random effects)
MR Egger
MR RAPS
Weighted median
Weighted mode
0.04
0.00
−0.04
0.09
0.12
0.15
0.18
SNP effect on Uncharacterized protein C14orf93
SNP effect on Gut microbiota abundance (genus Catenibacterium id.2153)

Batch 2−568 : Gut microbiota abundance (genus Parasutterella id.2892) on Uncharacterized protein C14orf93
with a threshold of 1e−05

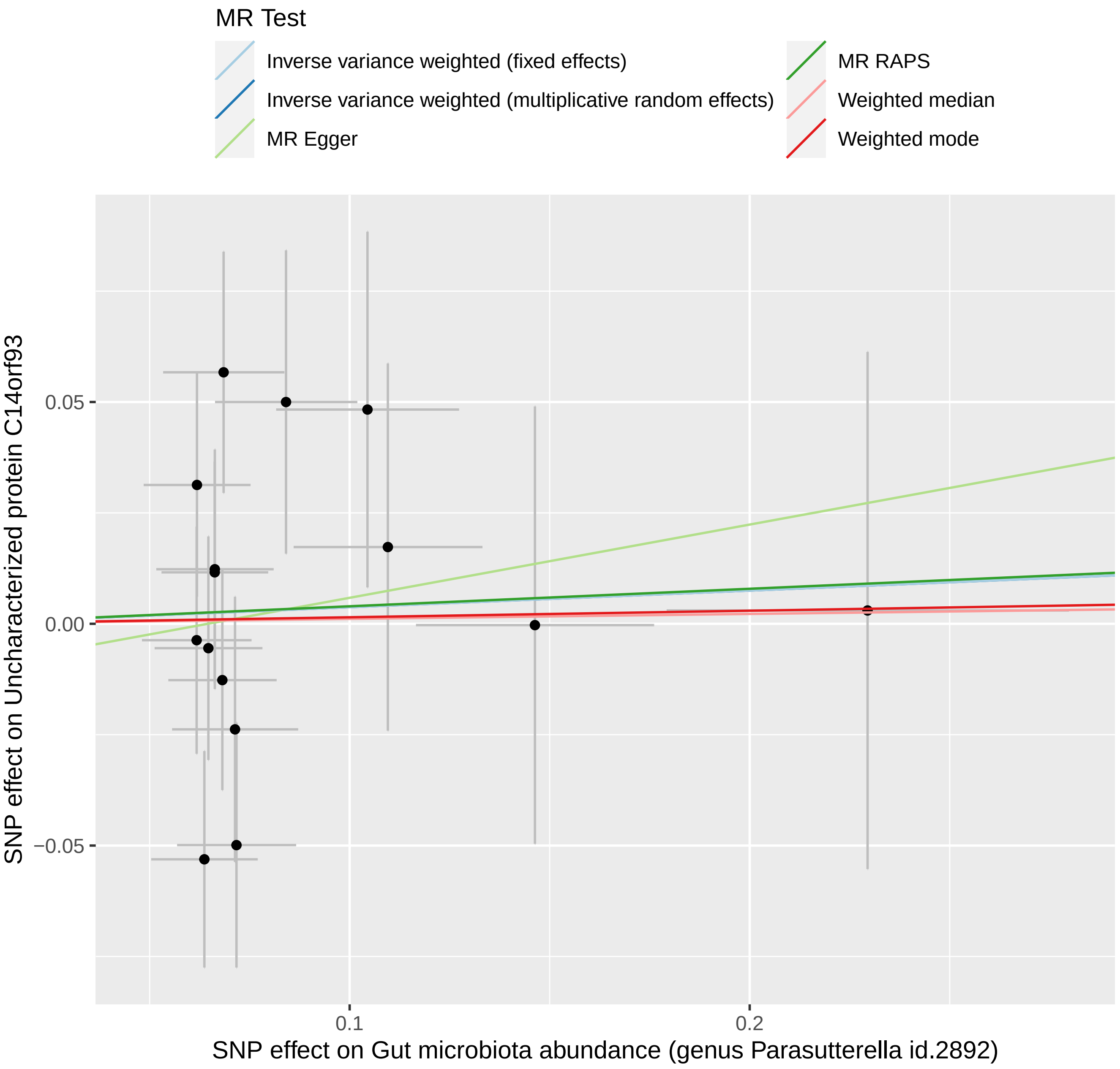

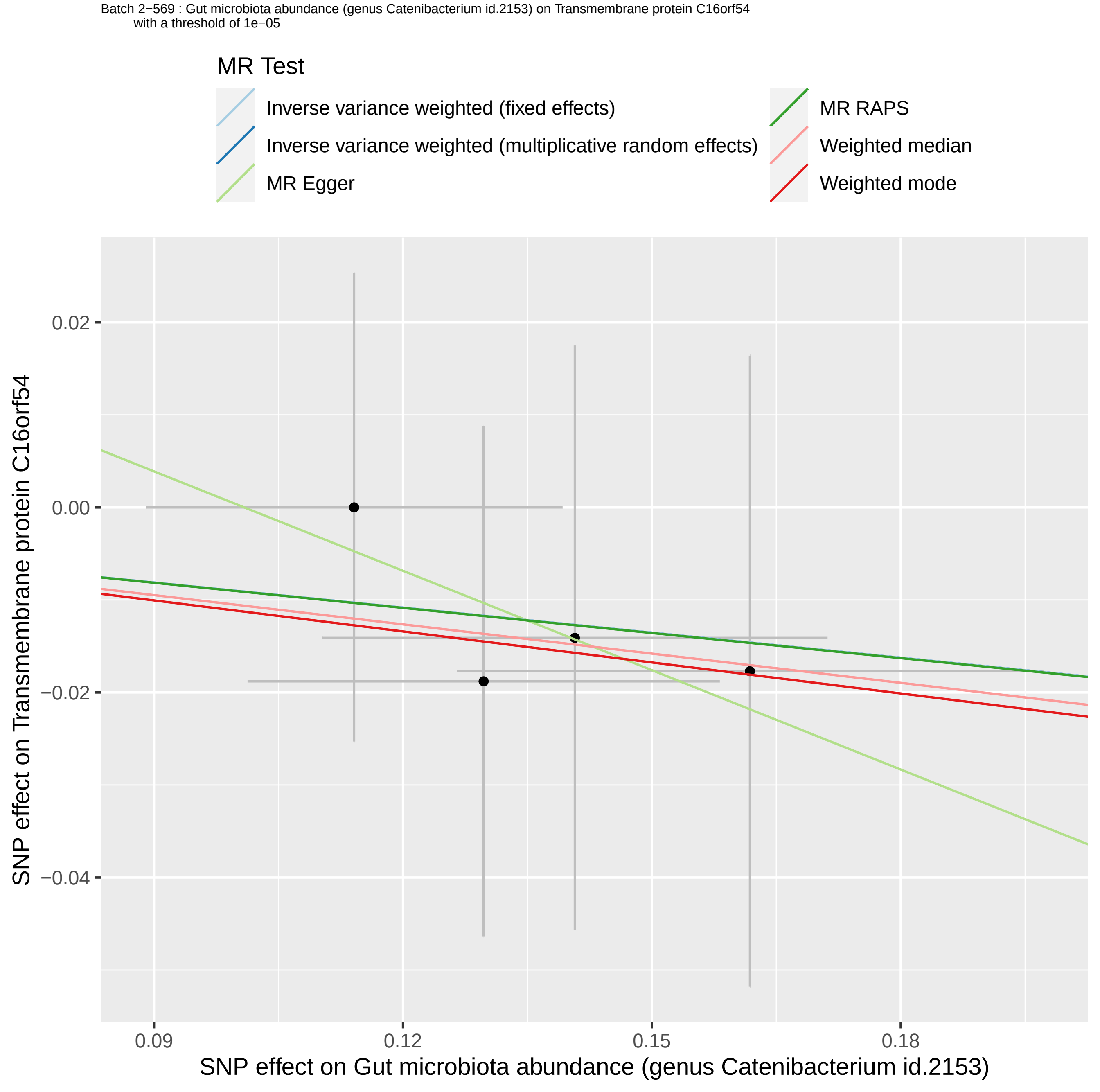
Batch 2−569 : Gut microbiota abundance (genus Catenibacterium id.2153) on Transmembrane protein C16orf54
with a threshold of 1e−05
MR Test
Inverse variance weighted (fixed effects)
Inverse variance weighted (multiplicative random effects)
MR Egger
MR RAPS
Weighted median
Weighted mode
0.02
0.00
−0.02
−0.04
0.09
0.12
0.15
0.18
SNP effect on Transmembrane protein C16orf54
SNP effect on Gut microbiota abundance (genus Catenibacterium id.2153)

Batch 2−570 : Gut microbiota abundance (genus Parasutterella id.2892) on Transmembrane protein C16orf54
with a threshold of 1e−05

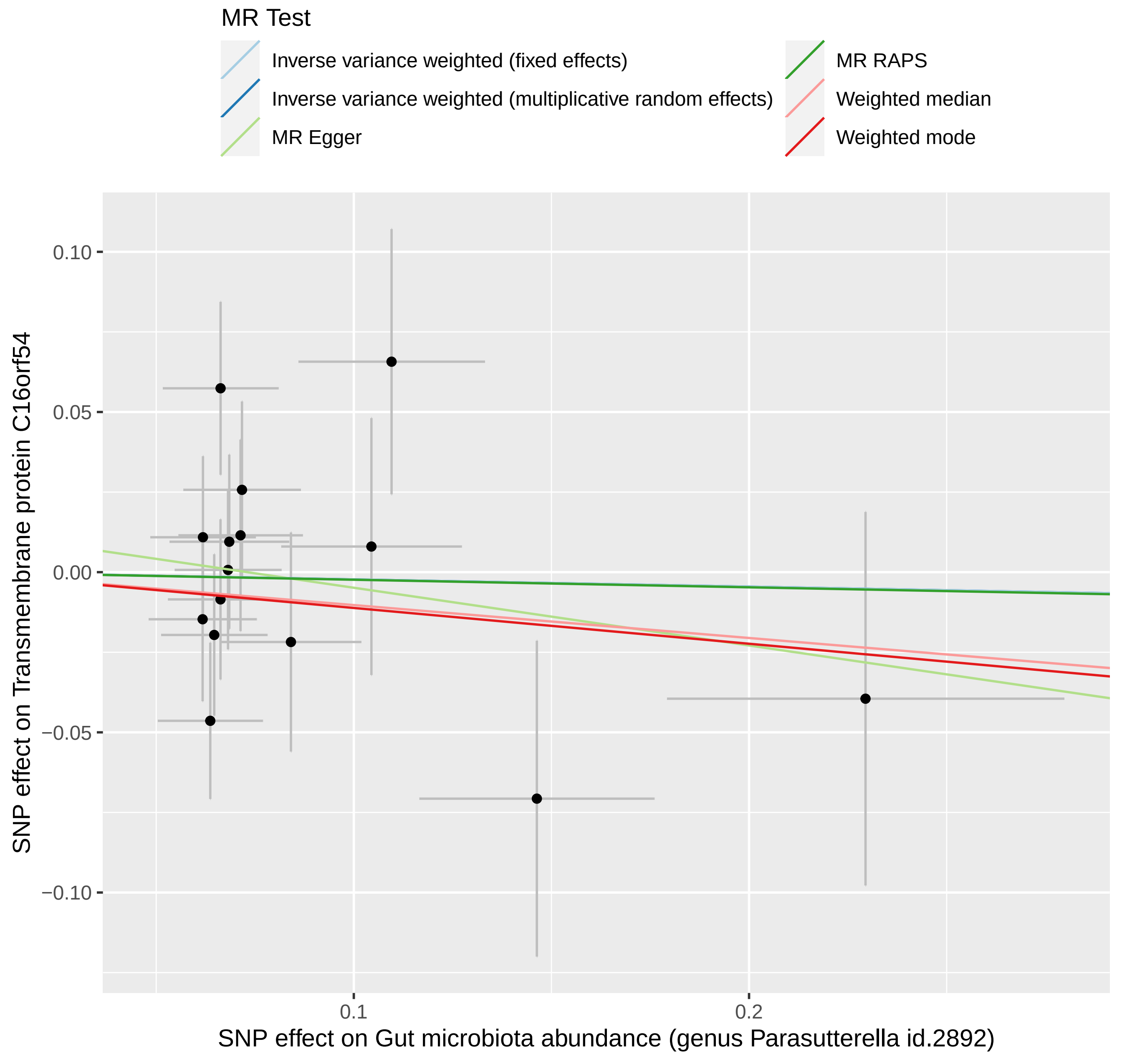

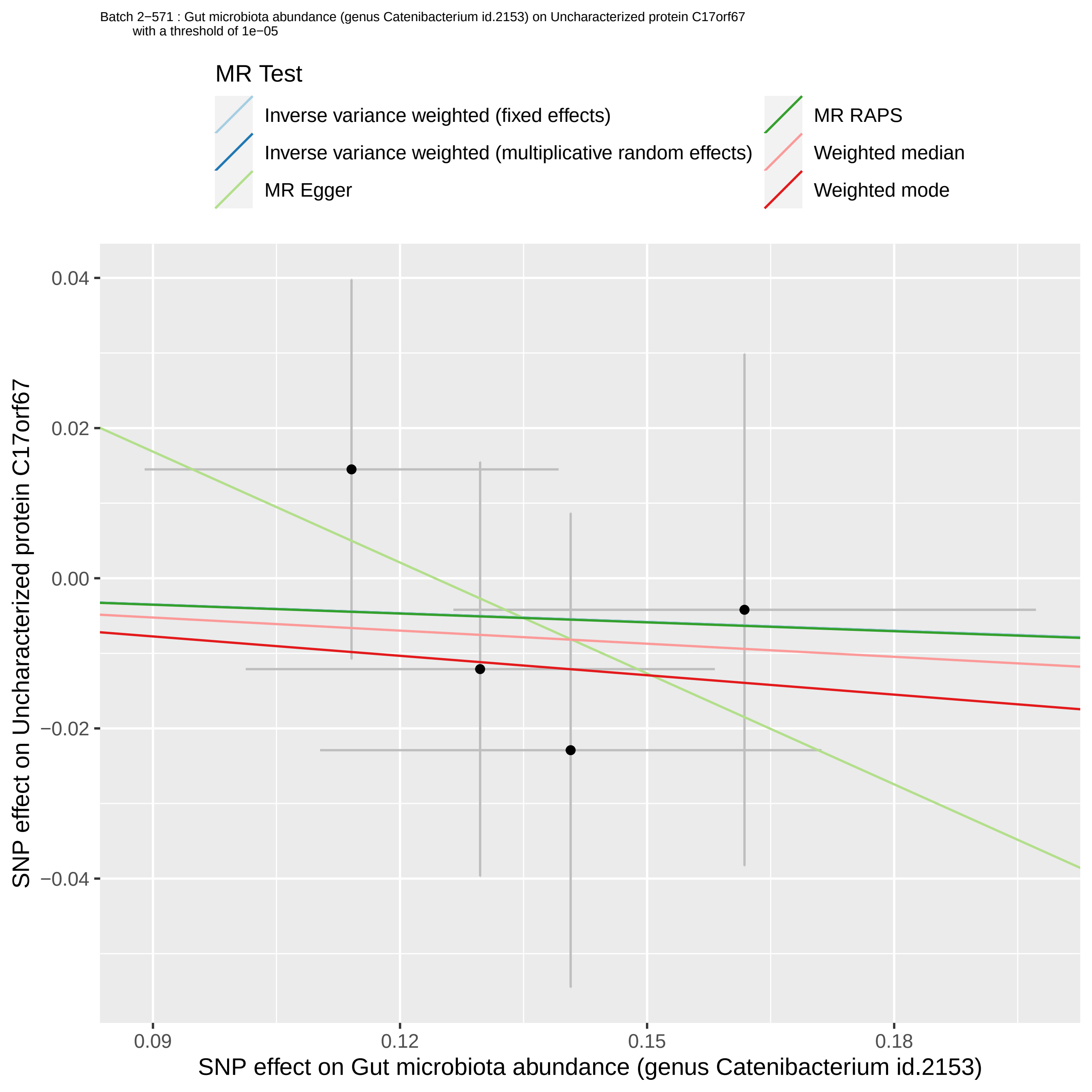
Batch 2−571 : Gut microbiota abundance (genus Catenibacterium id.2153) on Uncharacterized protein C17orf67
with a threshold of 1e−05
MR Test
Inverse variance weighted (fixed effects)
Inverse variance weighted (multiplicative random effects)
MR Egger
MR RAPS
Weighted median
Weighted mode
0.04
0.02
0.00
−0.02
−0.04
0.09
0.12
0.15
0.18
SNP effect on Uncharacterized protein C17orf67
SNP effect on Gut microbiota abundance (genus Catenibacterium id.2153)

Batch 2−572 : Gut microbiota abundance (genus Parasutterella id.2892) on Uncharacterized protein C17orf67
with a threshold of 1e−05

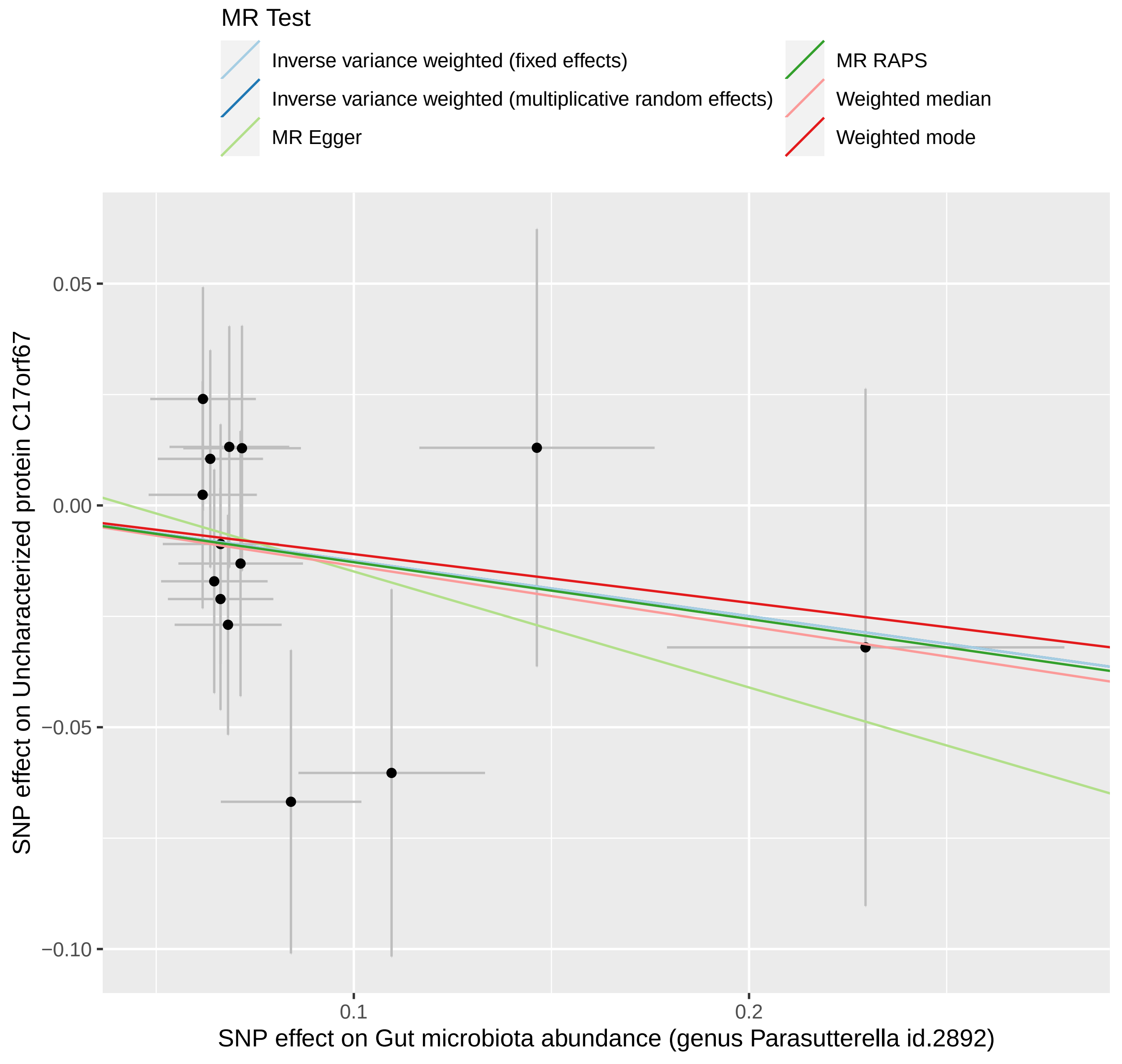

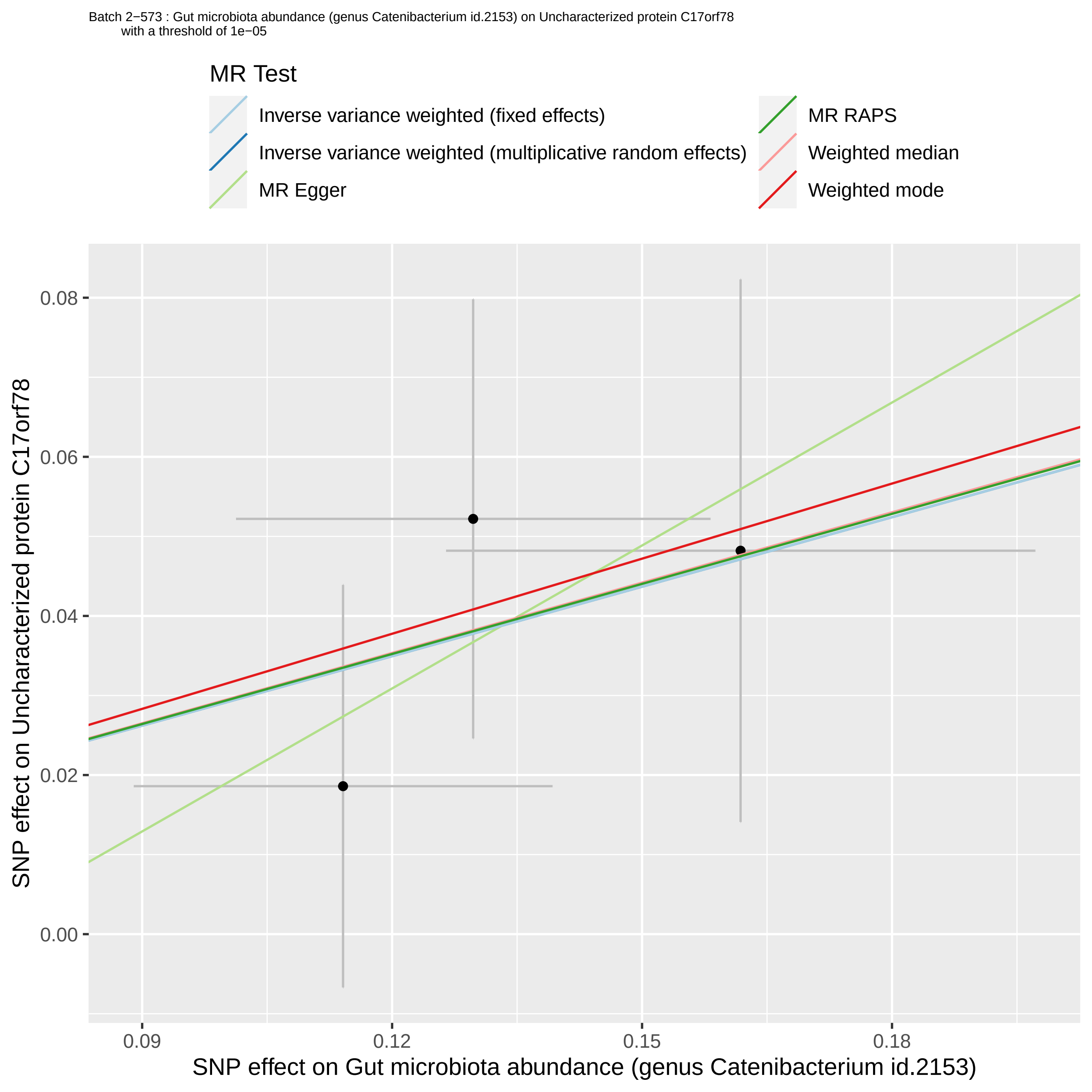
Batch 2−573 : Gut microbiota abundance (genus Catenibacterium id.2153) on Uncharacterized protein C17orf78
with a threshold of 1e−05
MR Test
Inverse variance weighted (fixed effects)
Inverse variance weighted (multiplicative random effects)
MR Egger
MR RAPS
Weighted median
Weighted mode
SNP effect on Uncharacterized protein C17orf78
0.08
0.06
0.04
0.02
0.00
0.09
0.12
0.15
0.18
SNP effect on Gut microbiota abundance (genus Catenibacterium id.2153)

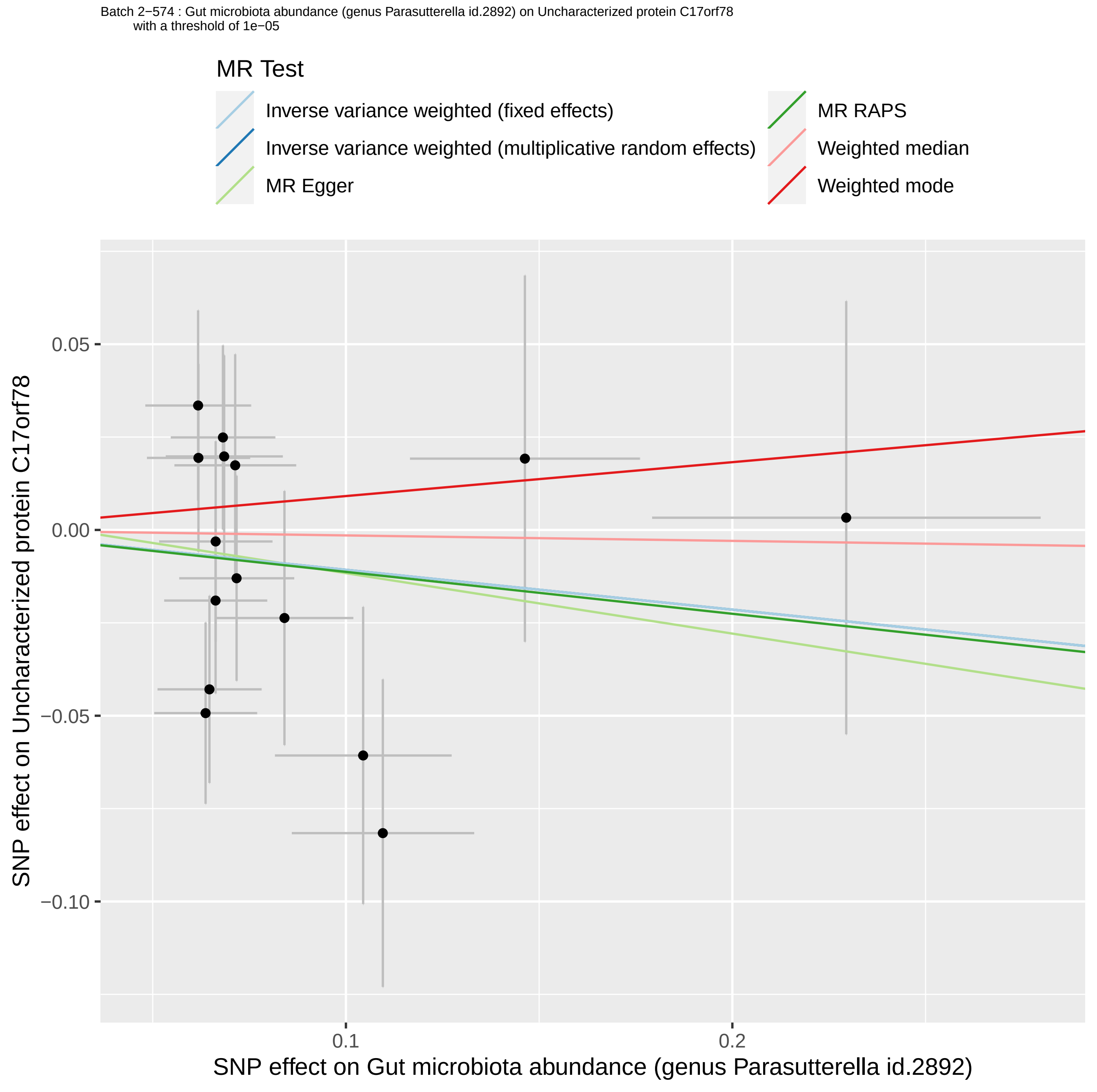
Batch 2−574 : Gut microbiota abundance (genus Parasutterella id.2892) on Uncharacterized protein C17orf78
with a threshold of 1e−05
MR Test
Inverse variance weighted (fixed effects)
Inverse variance weighted (multiplicative random effects)
MR Egger
MR RAPS
Weighted median
Weighted mode
0.05
0.00
−0.05
−0.10
0.1
0.2
SNP effect on Uncharacterized protein C17orf78
SNP effect on Gut microbiota abundance (genus Parasutterella id.2892)

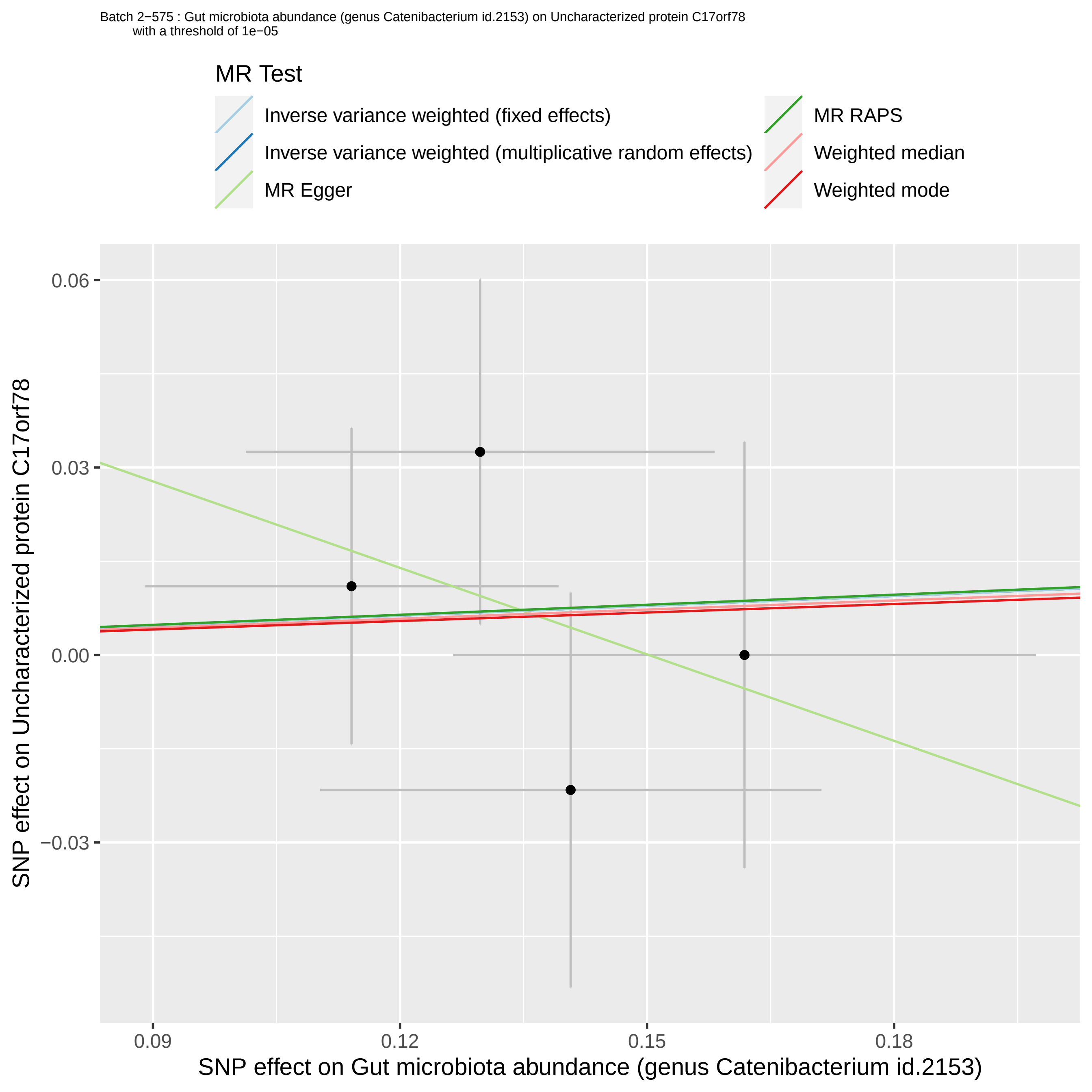

Batch 2−575 : Gut microbiota abundance (genus Catenibacterium id.2153) on Uncharacterized protein C17orf78
with a threshold of 1e−05
MR Test
Inverse variance weighted (fixed effects)
Inverse variance weighted (multiplicative random effects)
MR Egger
MR RAPS
Weighted median
Weighted mode
0.06
0.03
0.00
−0.03
0.09
0.12
0.15
0.18
SNP effect on Uncharacterized protein C17orf78
SNP effect on Gut microbiota abundance (genus Catenibacterium id.2153)

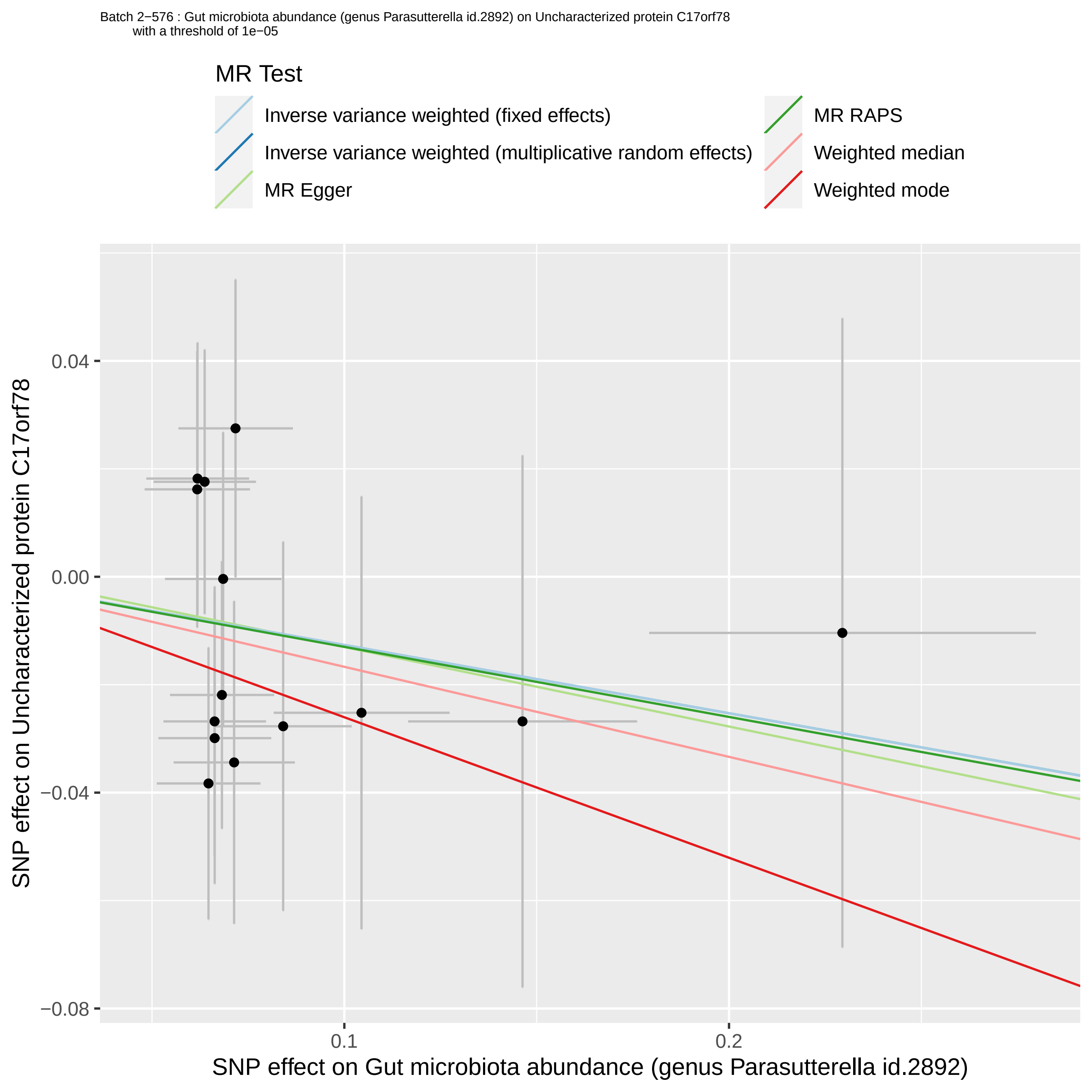
Batch 2−576 : Gut microbiota abundance (genus Parasutterella id.2892) on Uncharacterized protein C17orf78
with a threshold of 1e−05
MR Test
Inverse variance weighted (fixed effects)
Inverse variance weighted (multiplicative random effects)
MR Egger
MR RAPS
Weighted median
Weighted mode
0.04
0.00
−0.04
−0.08
0.1
0.2
SNP effect on Uncharacterized protein C17orf78
SNP effect on Gut microbiota abundance (genus Parasutterella id.2892)

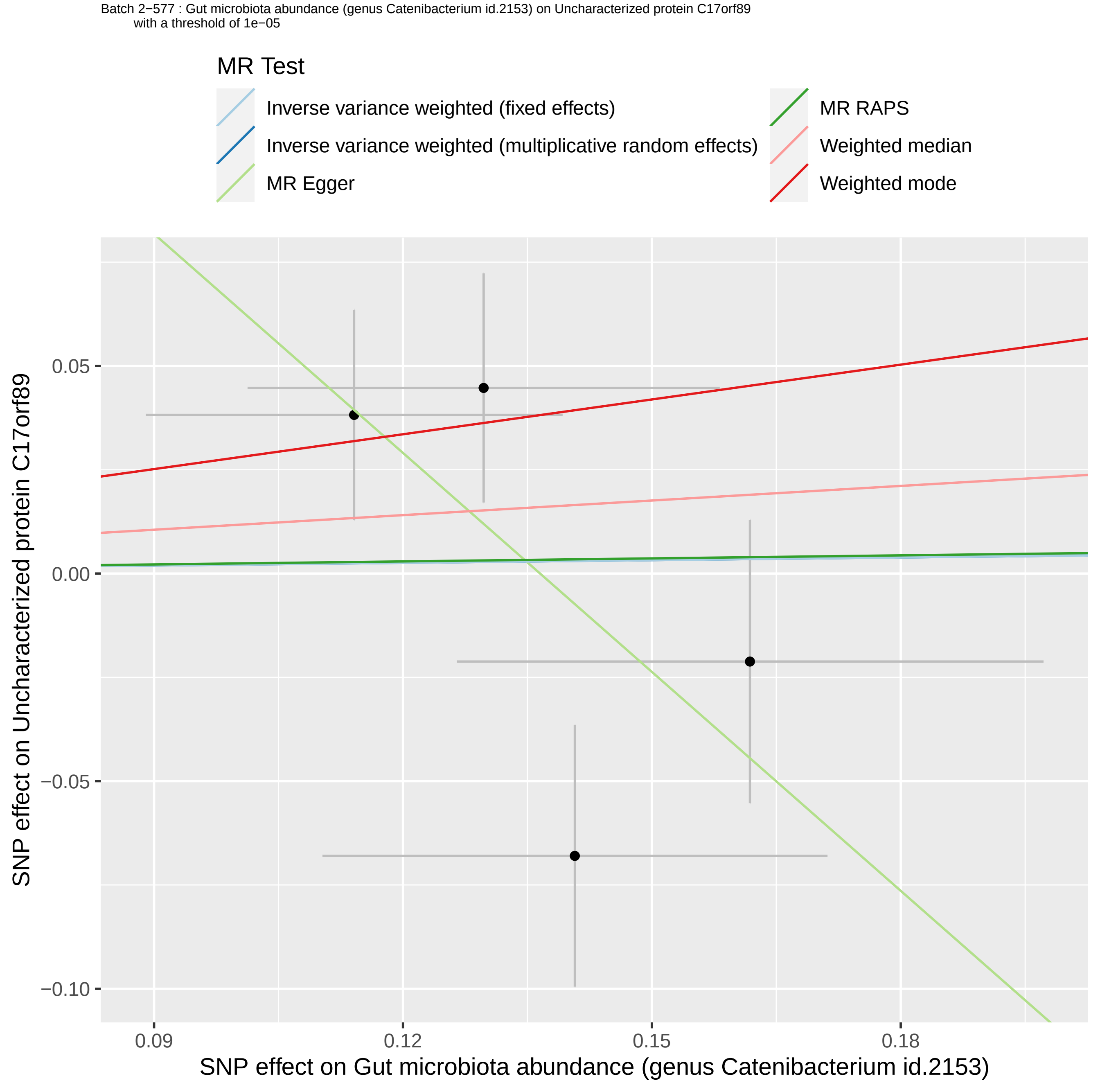

Batch 2−577 : Gut microbiota abundance (genus Catenibacterium id.2153) on Uncharacterized protein C17orf89
with a threshold of 1e−05
MR Test
Inverse variance weighted (fixed effects)
Inverse variance weighted (multiplicative random effects)
MR Egger
MR RAPS
Weighted median
Weighted mode
0.05
0.00
−0.05
−0.10
0.09
0.12
0.15
0.18
SNP effect on Uncharacterized protein C17orf89
SNP effect on Gut microbiota abundance (genus Catenibacterium id.2153)

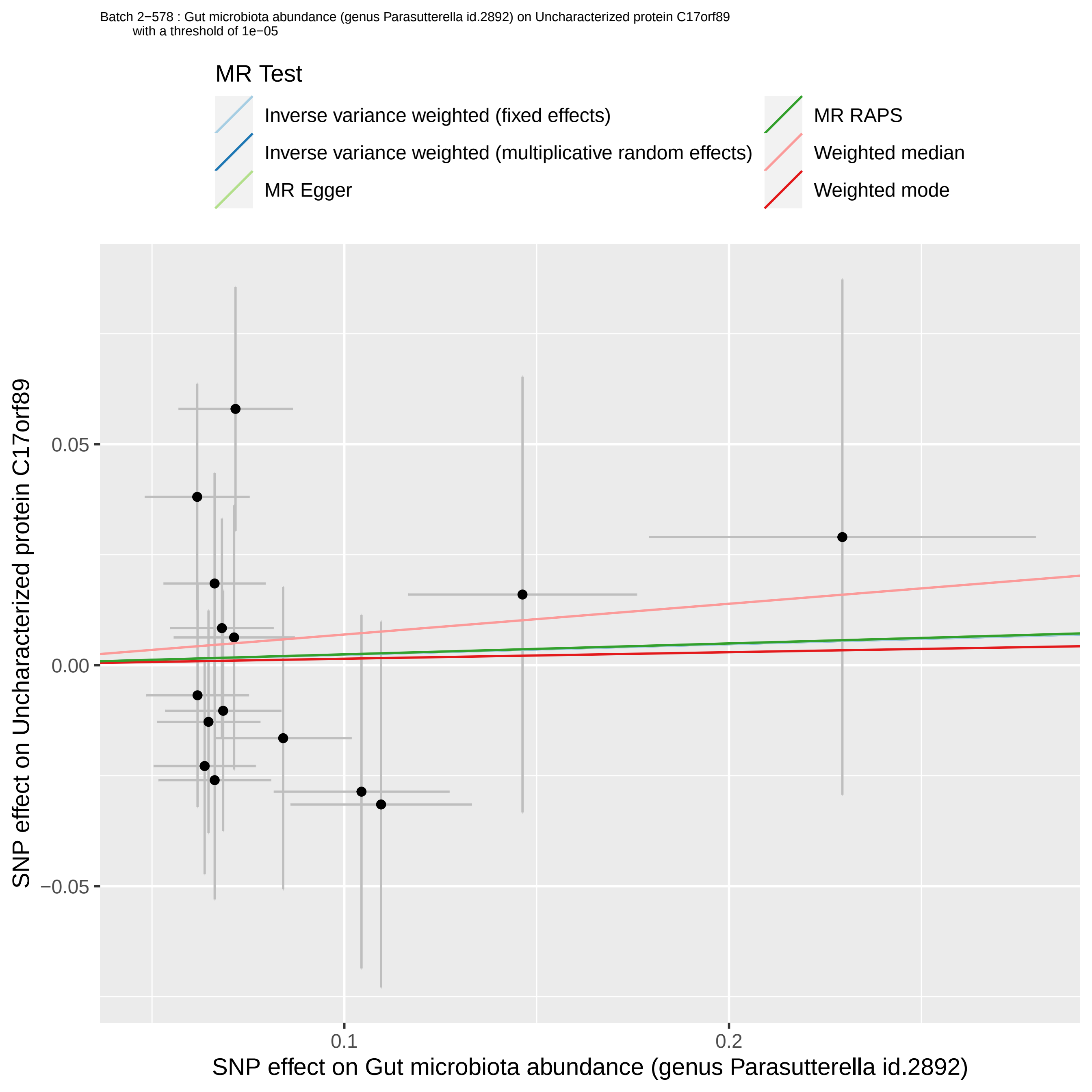
Batch 2−578 : Gut microbiota abundance (genus Parasutterella id.2892) on Uncharacterized protein C17orf89
with a threshold of 1e−05
MR Test
Inverse variance weighted (fixed effects)
Inverse variance weighted (multiplicative random effects)
MR Egger
MR RAPS
Weighted median
Weighted mode
SNP effect on Uncharacterized protein C17orf89
0.05
0.00
−0.05
0.1
0.2
SNP effect on Gut microbiota abundance (genus Parasutterella id.2892)

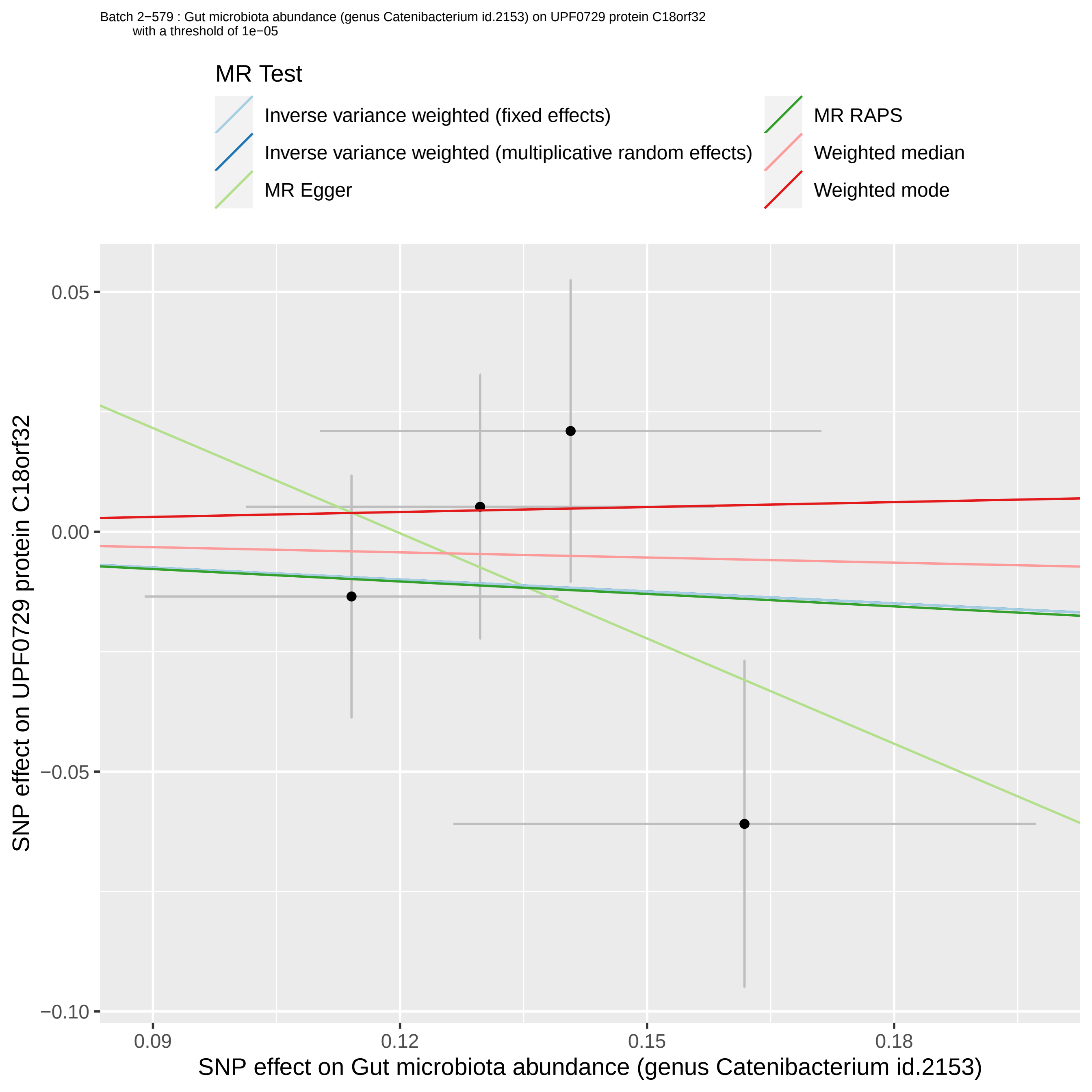
Batch 2−579 : Gut microbiota abundance (genus Catenibacterium id.2153) on UPF0729 protein C18orf32
with a threshold of 1e−05
MR Test
Inverse variance weighted (fixed effects)
Inverse variance weighted (multiplicative random effects)
MR Egger
MR RAPS
Weighted median
Weighted mode
SNP effect on UPF0729 protein C18orf32
SNP effect on Gut microbiota abundance (genus Catenibacterium id.2153)
0.05
0.00
−0.05
−0.10
0.09
0.12
0.15
0.18

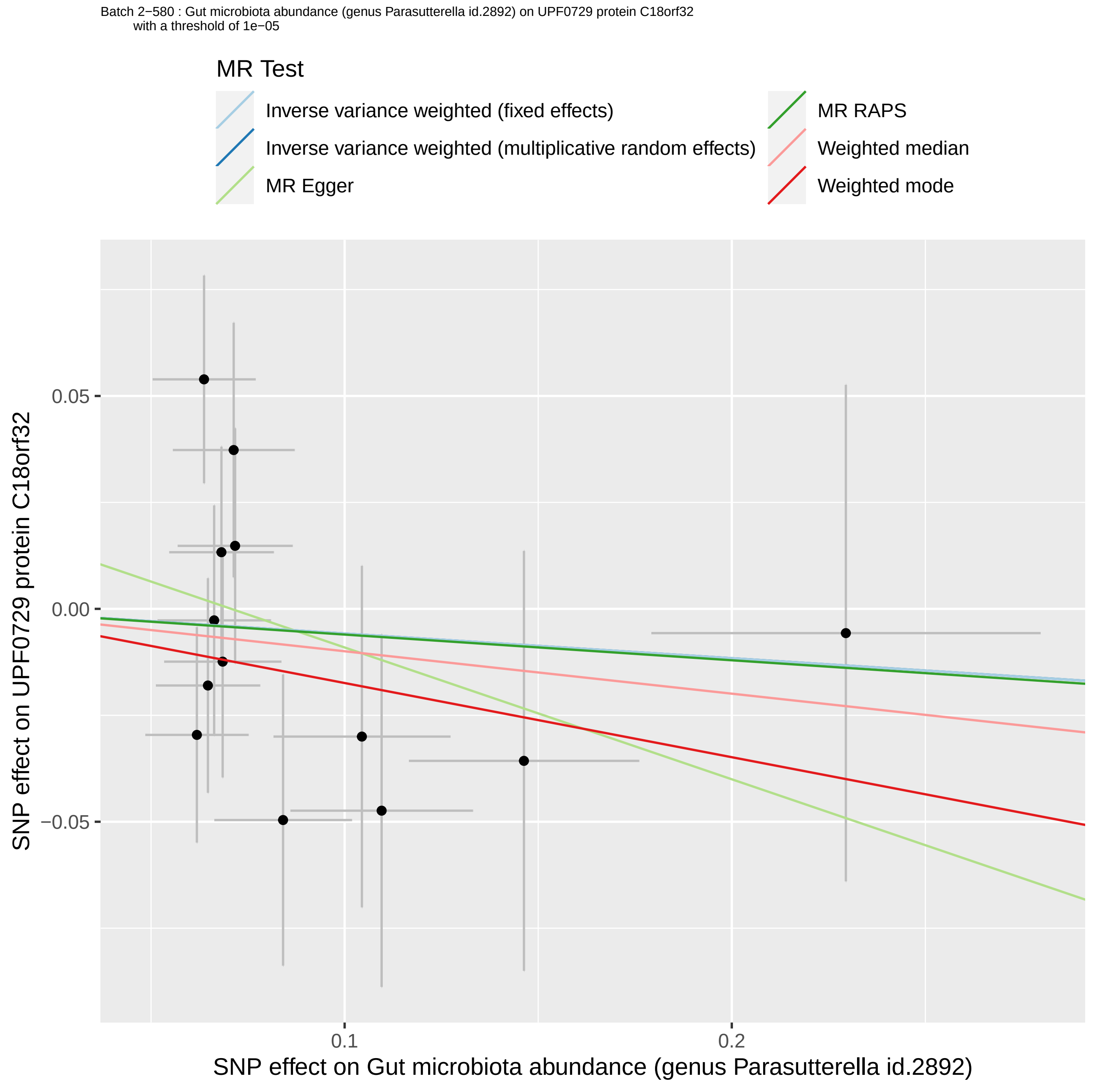

Batch 2−580 : Gut microbiota abundance (genus Parasutterella id.2892) on UPF0729 protein C18orf32
with a threshold of 1e−05
MR Test
Inverse variance weighted (fixed effects)
Inverse variance weighted (multiplicative random effects)
MR Egger
MR RAPS
Weighted median
Weighted mode
0.05
0.00
−0.05
0.1
0.2
SNP effect on UPF0729 protein C18orf32
SNP effect on Gut microbiota abundance (genus Parasutterella id.2892)

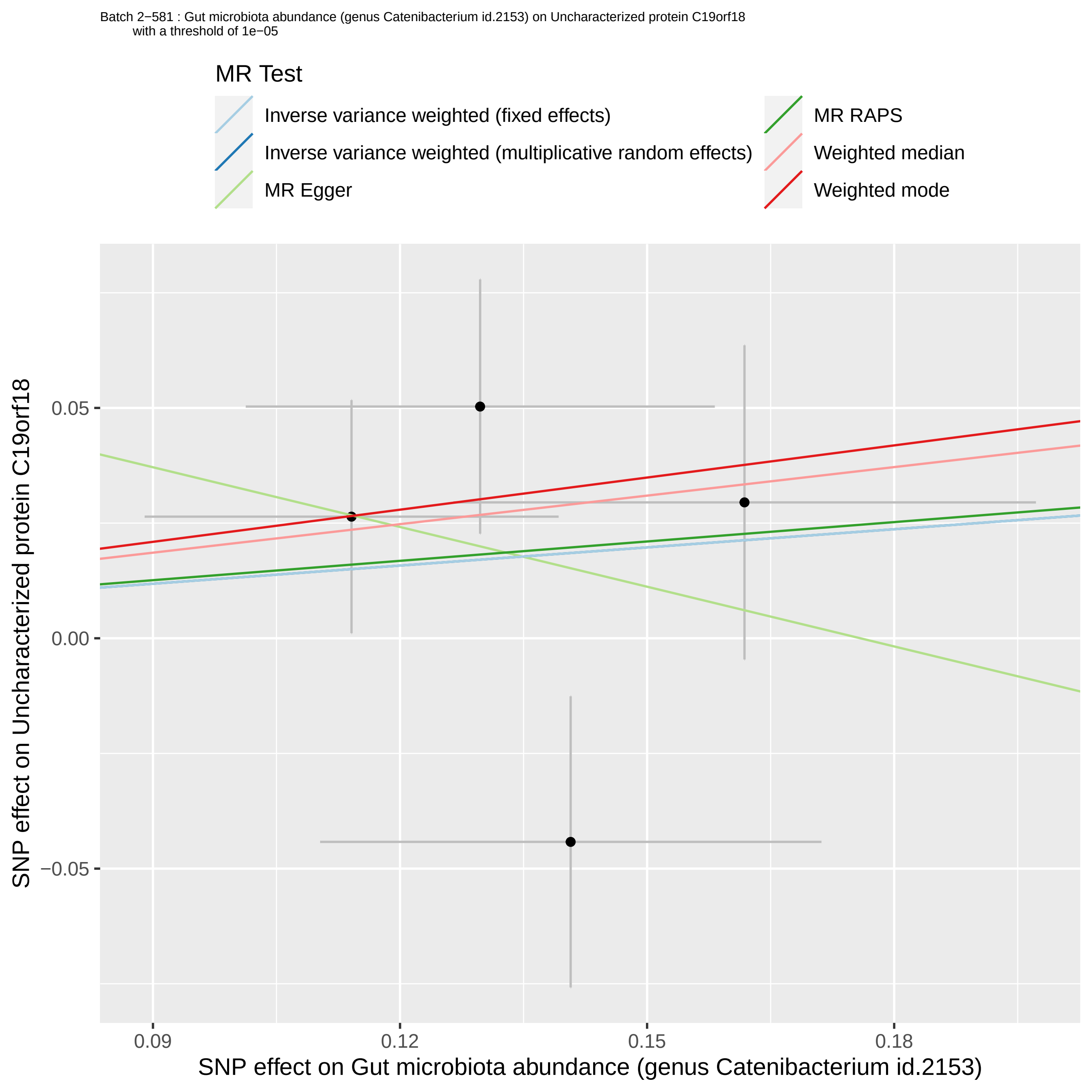
Batch 2−581 : Gut microbiota abundance (genus Catenibacterium id.2153) on Uncharacterized protein C19orf18
with a threshold of 1e−05
MR Test
Inverse variance weighted (fixed effects)
Inverse variance weighted (multiplicative random effects)
MR Egger
MR RAPS
Weighted median
Weighted mode
0.05
0.00
−0.05
0.09
0.12
0.15
0.18
SNP effect on Uncharacterized protein C19orf18
SNP effect on Gut microbiota abundance (genus Catenibacterium id.2153)

Batch 2−582 : Gut microbiota abundance (genus Parasutterella id.2892) on Uncharacterized protein C19orf18
with a threshold of 1e−05

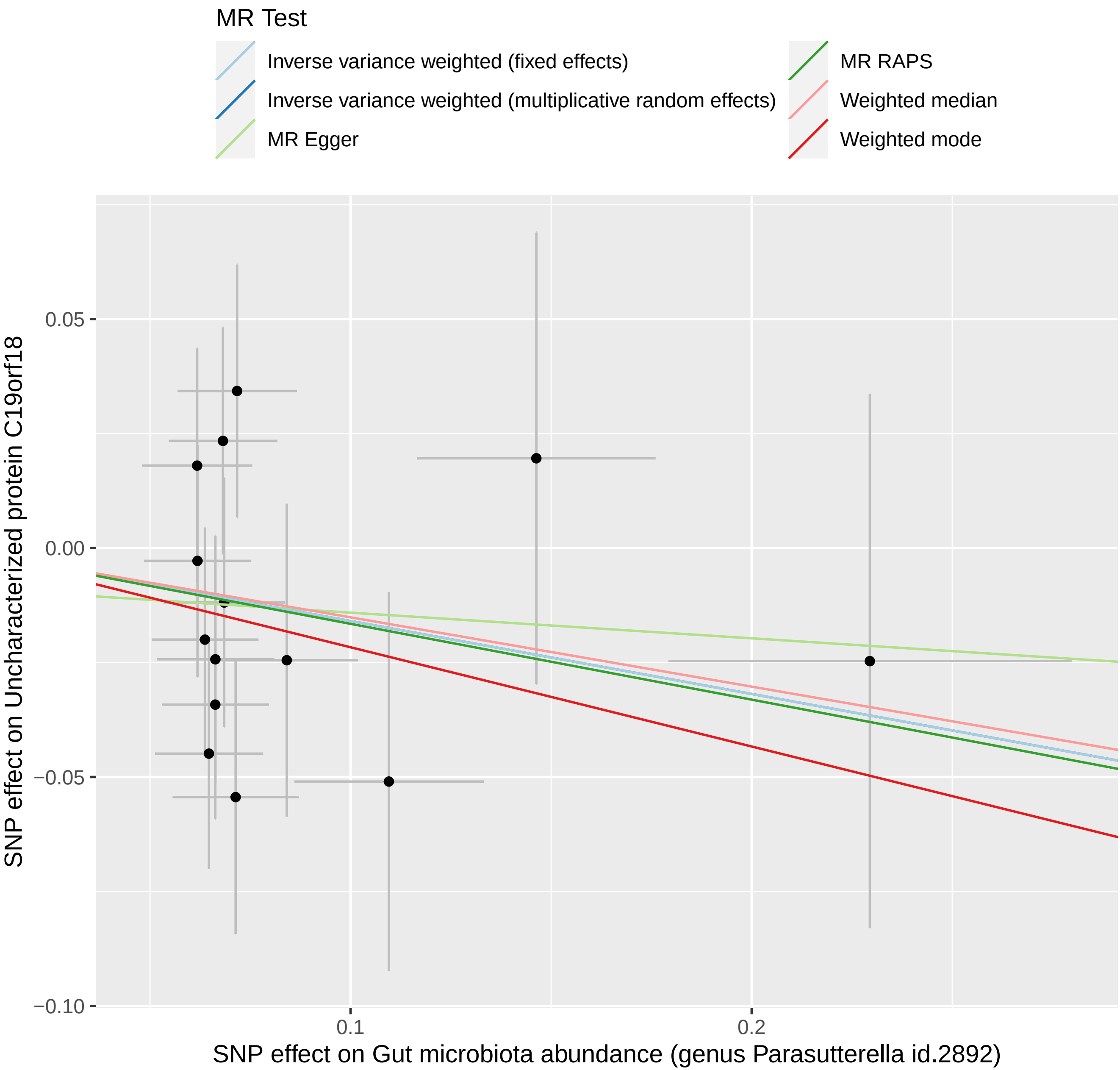

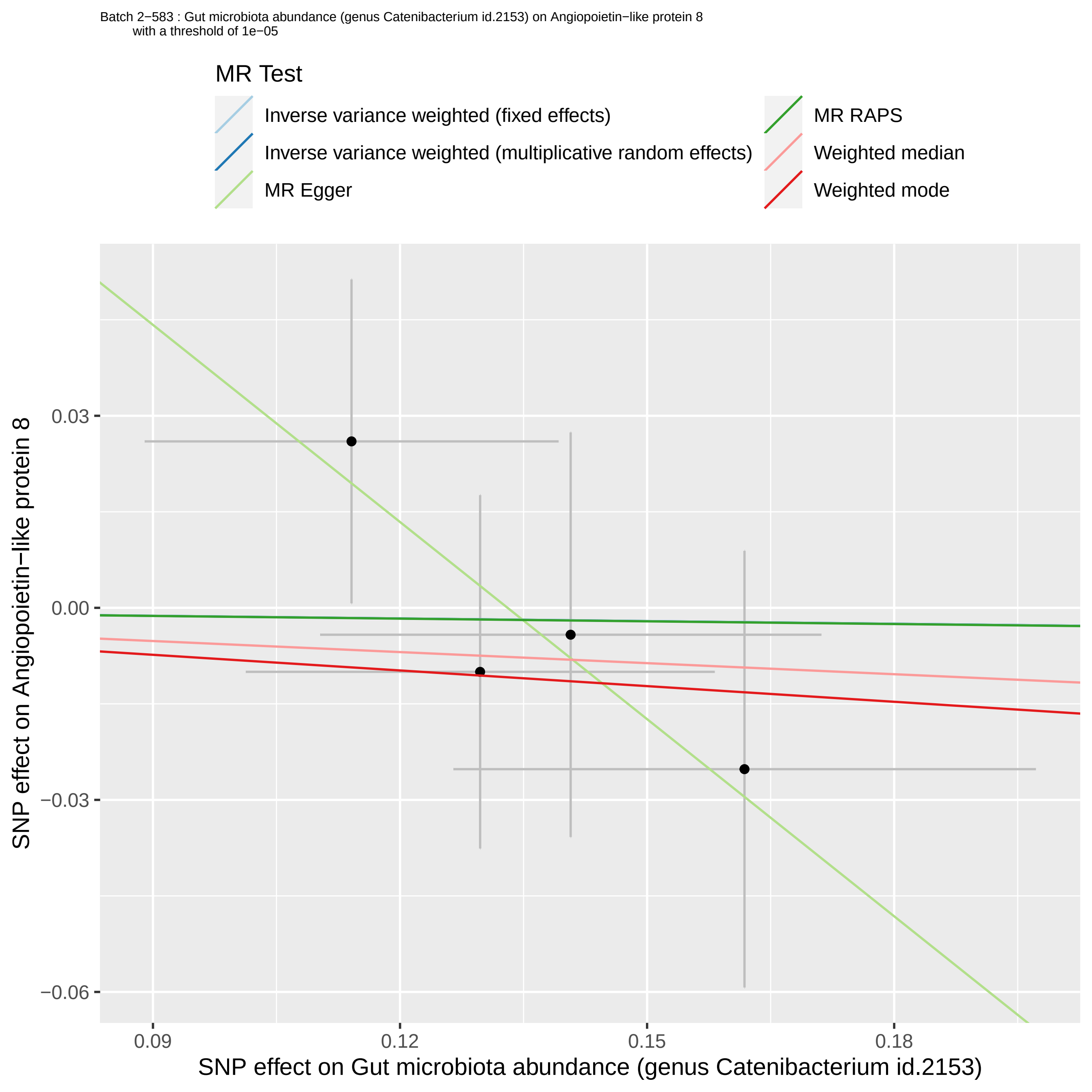
Batch 2−583 : Gut microbiota abundance (genus Catenibacterium id.2153) on Angiopoietin−like protein 8
with a threshold of 1e−05
MR Test
Inverse variance weighted (fixed effects)
Inverse variance weighted (multiplicative random effects)
MR Egger
MR RAPS
Weighted median
Weighted mode
0.03
0.00
−0.03
−0.06
0.09
0.12
0.15
0.18
SNP effect on Angiopoietin−like protein 8
SNP effect on Gut microbiota abundance (genus Catenibacterium id.2153)

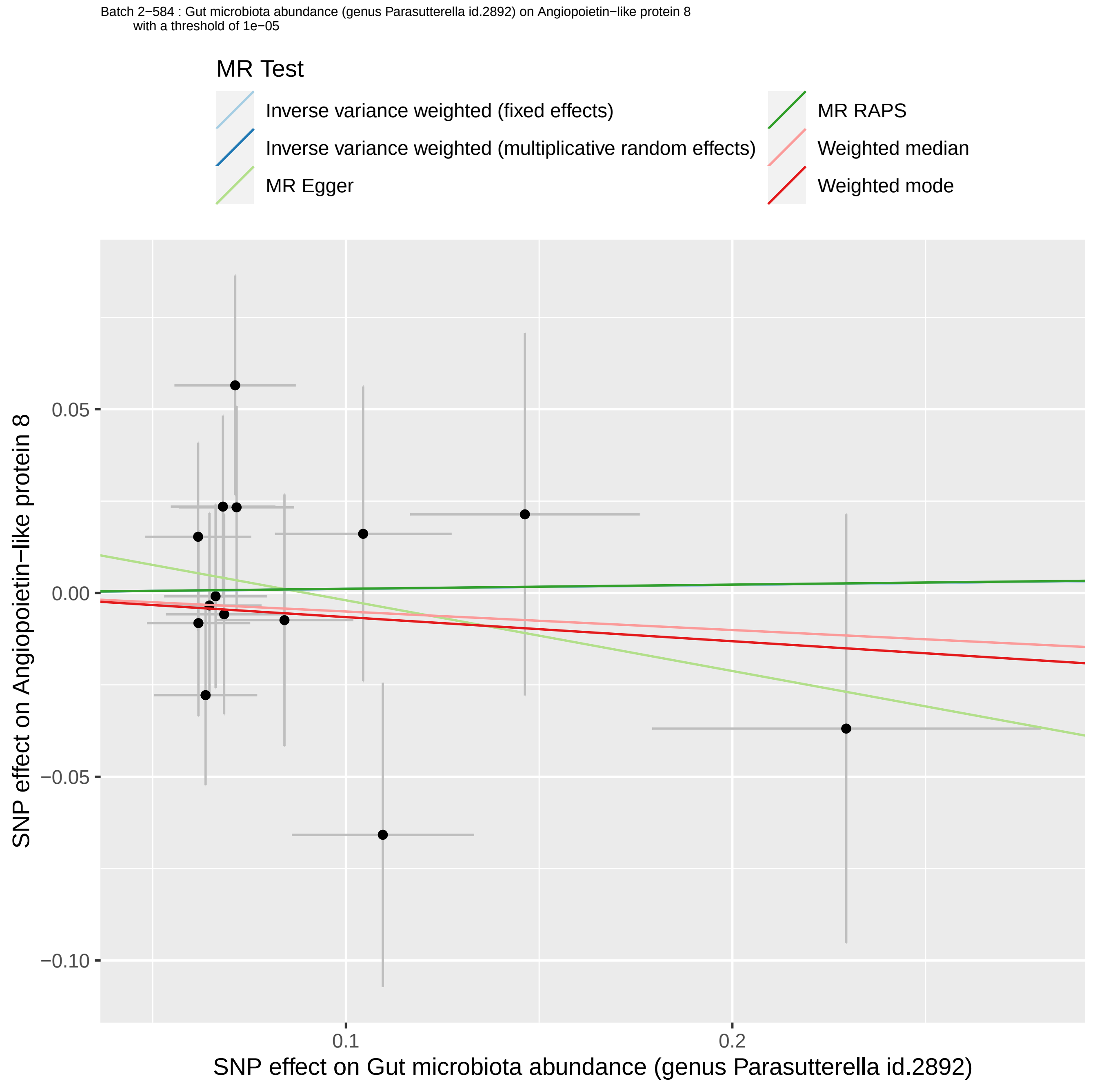

Batch 2−584 : Gut microbiota abundance (genus Parasutterella id.2892) on Angiopoietin−like protein 8
with a threshold of 1e−05
MR Test
Inverse variance weighted (fixed effects)
Inverse variance weighted (multiplicative random effects)
MR Egger
MR RAPS
Weighted median
Weighted mode
0.05
0.00
−0.05
−0.10
0.1
0.2
SNP effect on Angiopoietin−like protein 8
SNP effect on Gut microbiota abundance (genus Parasutterella id.2892)

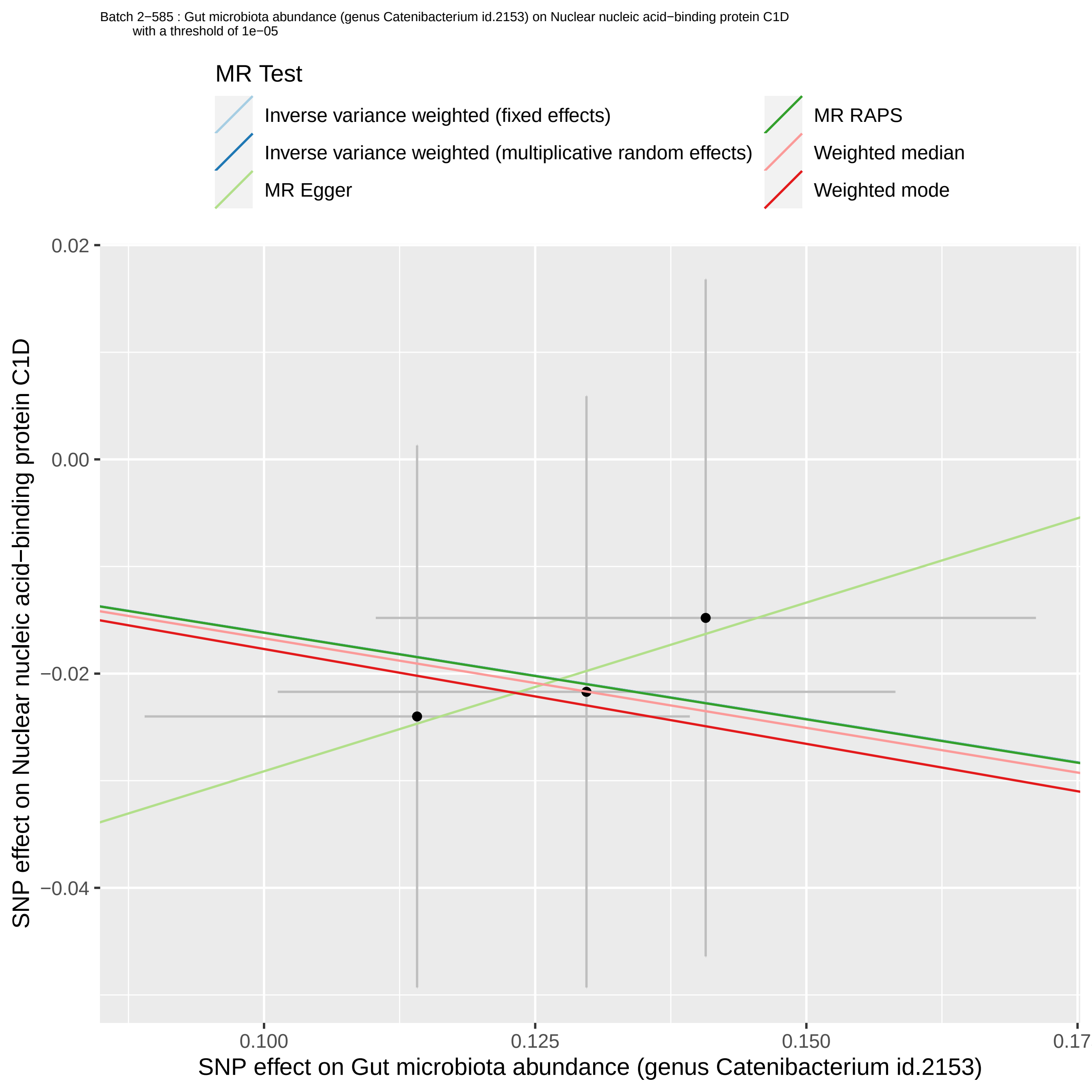
Batch 2−585 : Gut microbiota abundance (genus Catenibacterium id.2153) on Nuclear nucleic acid−binding protein C1D
with a threshold of 1e−05
MR Test
Inverse variance weighted (fixed effects)
Inverse variance weighted (multiplicative random effects)
MR Egger
MR RAPS
Weighted median
Weighted mode
0.02
0.00
−0.02
−0.04
0.100
0.125
0.150
0.17
SNP effect on Nuclear nucleic acid−binding protein C1D
SNP effect on Gut microbiota abundance (genus Catenibacterium id.2153)

Batch 2−586 : Gut microbiota abundance (genus Parasutterella id.2892) on Nuclear nucleic acid−binding protein C1D
with a threshold of 1e−05

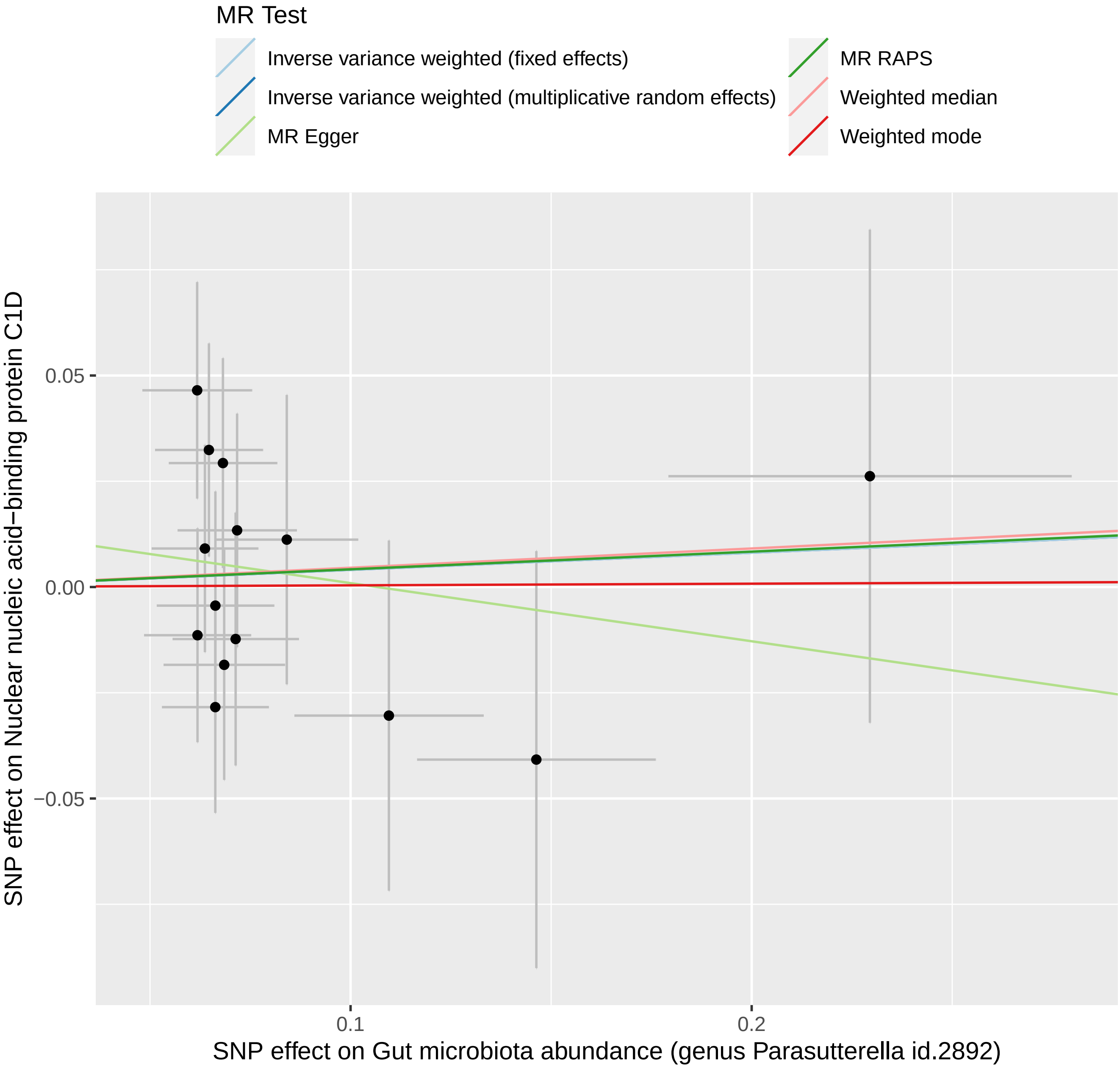

Batch 2−587 : Gut microbiota abundance (genus Catenibacterium id.2153) on C1GALT1−specific chaperone 1
with a threshold of 1e−05

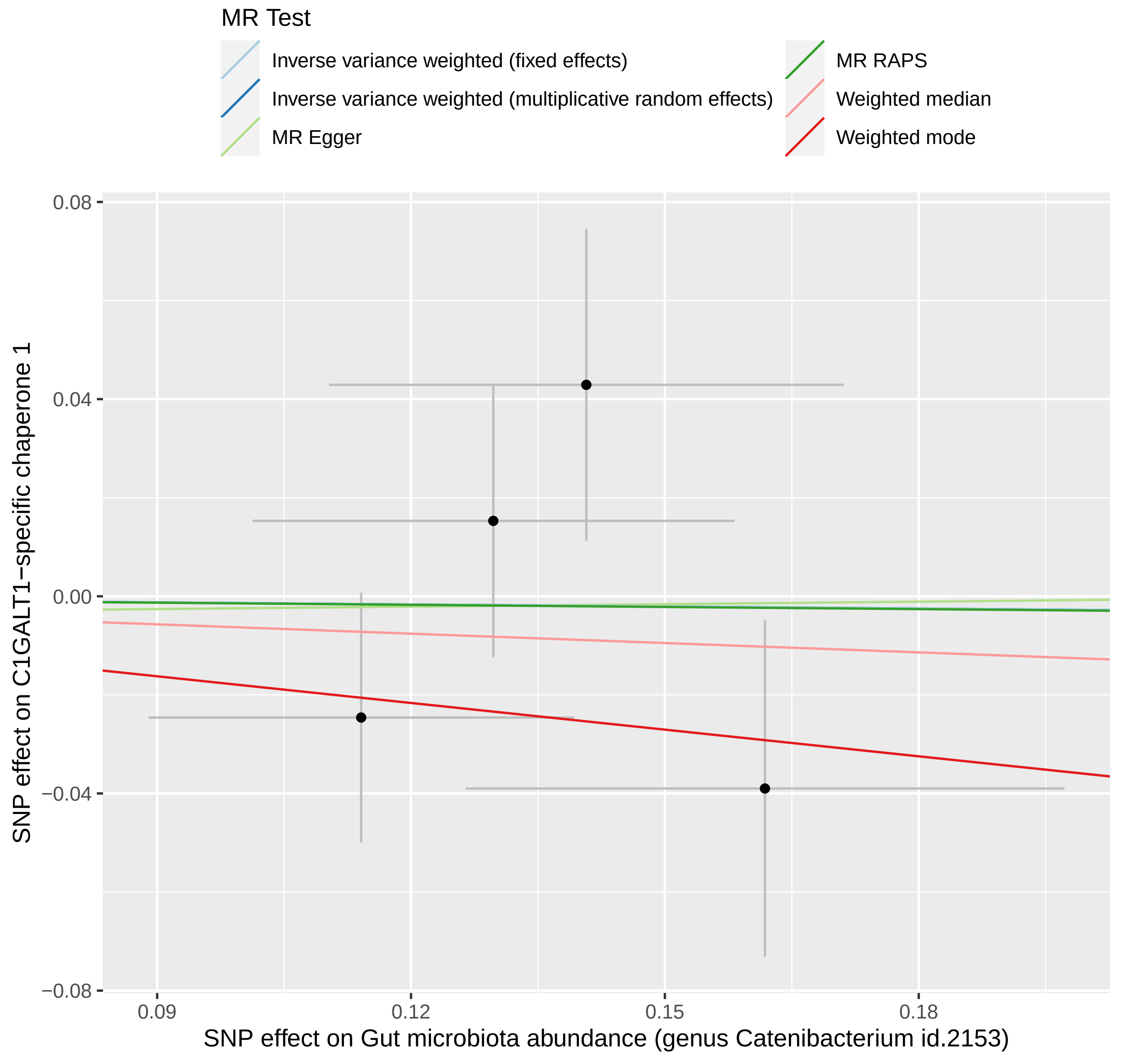

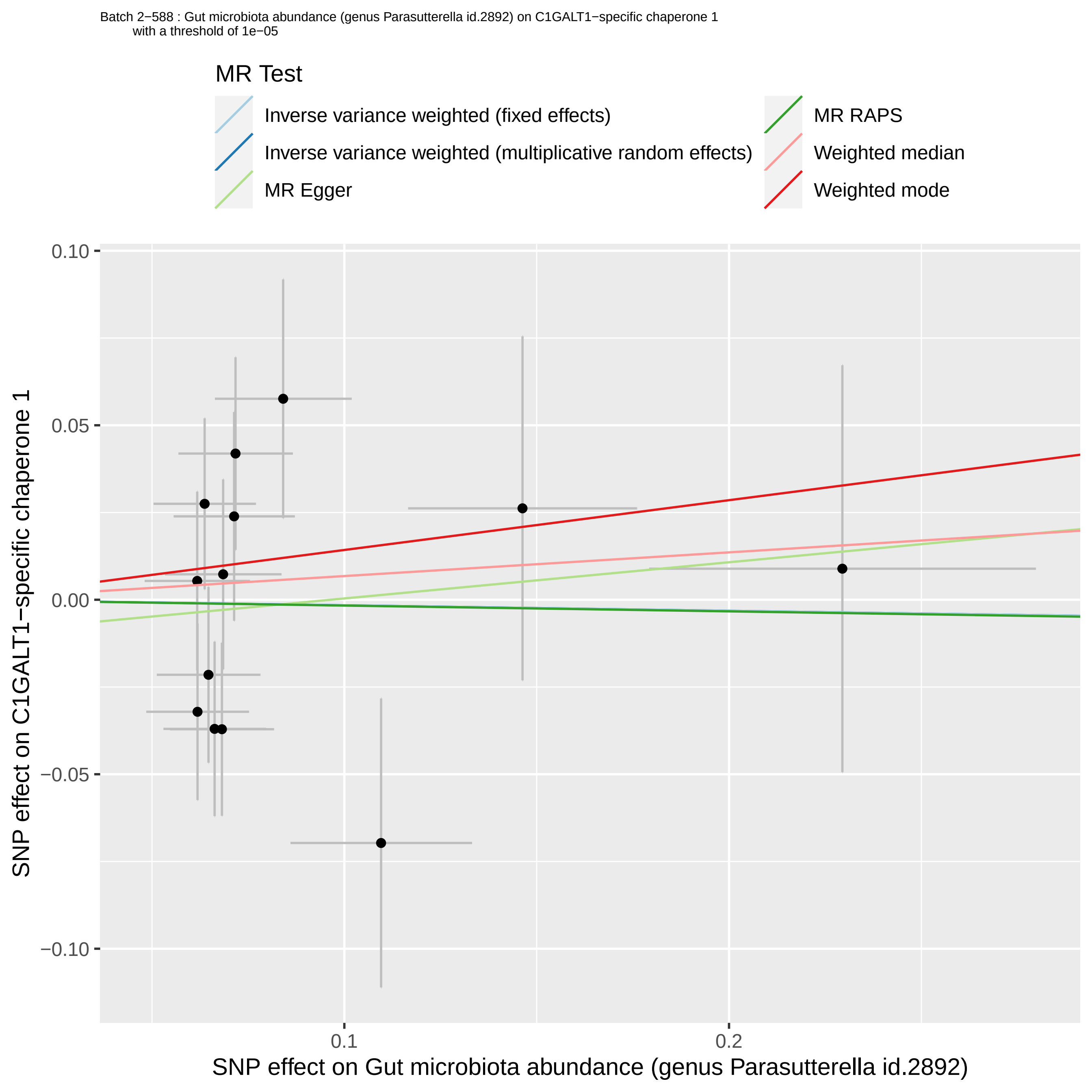

Batch 2−588 : Gut microbiota abundance (genus Parasutterella id.2892) on C1GALT1−specific chaperone 1
with a threshold of 1e−05
MR Test
Inverse variance weighted (fixed effects)
Inverse variance weighted (multiplicative random effects)
MR Egger
MR RAPS
Weighted median
Weighted mode
0.10
0.05
0.00
−0.05
−0.10
0.1
0.2
SNP effect on C1GALT1−specific chaperone 1
SNP effect on Gut microbiota abundance (genus Parasutterella id.2892)

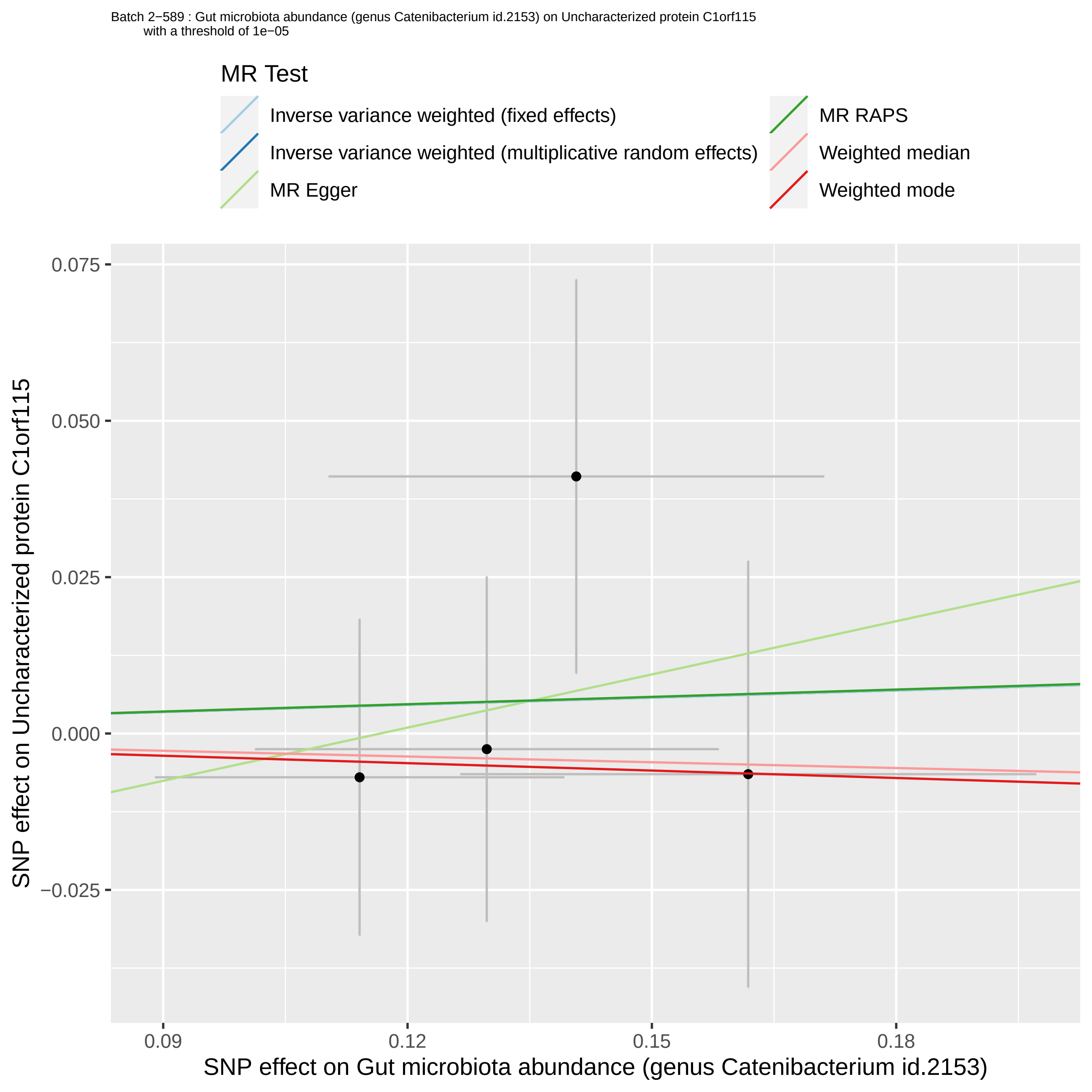
Batch 2−589 : Gut microbiota abundance (genus Catenibacterium id.2153) on Uncharacterized protein C1orf115
with a threshold of 1e−05
MR Test
Inverse variance weighted (fixed effects)
Inverse variance weighted (multiplicative random effects)
MR Egger
MR RAPS
Weighted median
Weighted mode
0.075
0.050
0.025
0.000
−0.025
0.09
0.12
0.15
0.18
SNP effect on Uncharacterized protein C1orf115
SNP effect on Gut microbiota abundance (genus Catenibacterium id.2153)

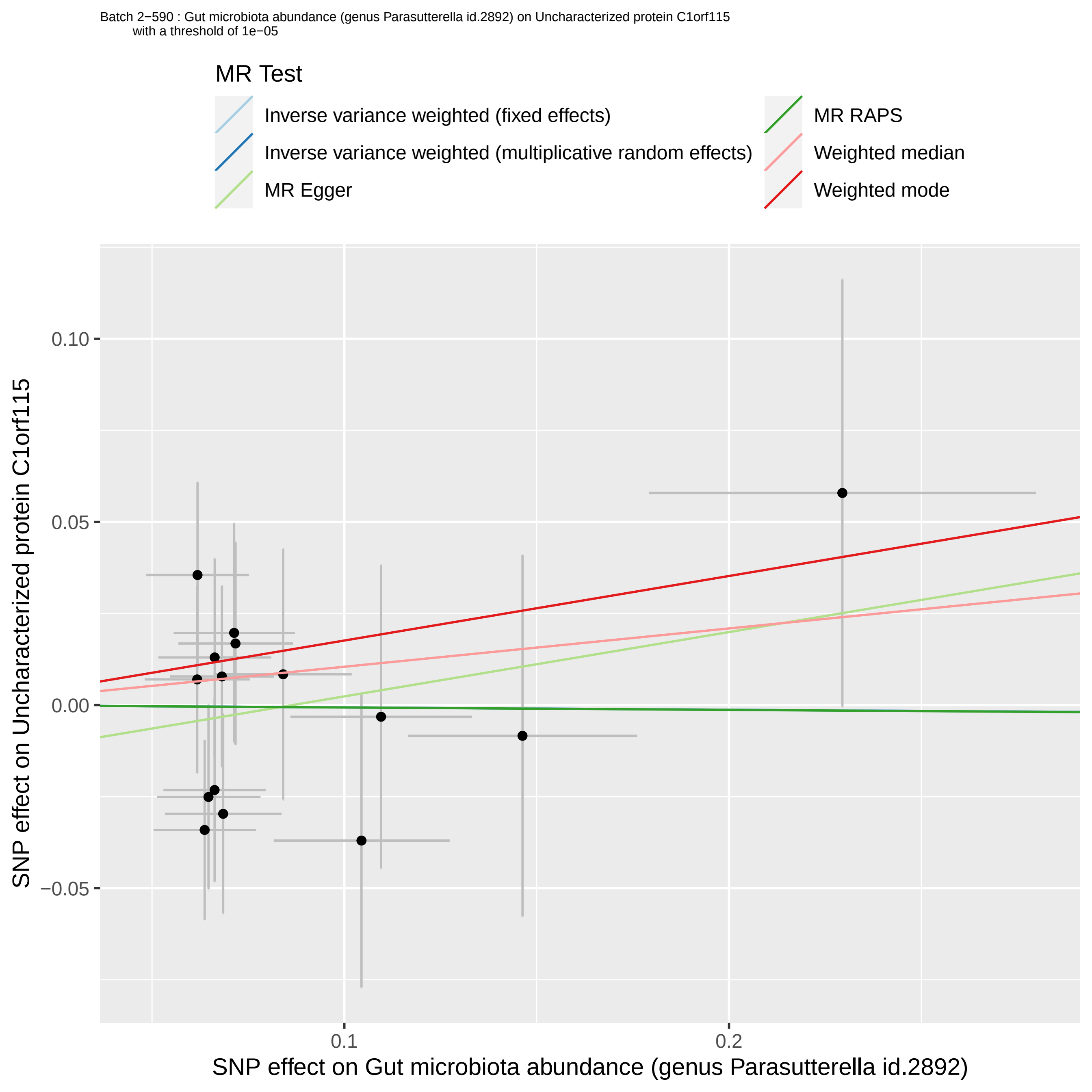

Batch 2−590 : Gut microbiota abundance (genus Parasutterella id.2892) on Uncharacterized protein C1orf115
with a threshold of 1e−05
MR Test
Inverse variance weighted (fixed effects)
Inverse variance weighted (multiplicative random effects)
MR Egger
MR RAPS
Weighted median
Weighted mode
0.10
0.05
0.00
−0.05
0.1
0.2
SNP effect on Uncharacterized protein C1orf115
SNP effect on Gut microbiota abundance (genus Parasutterella id.2892)

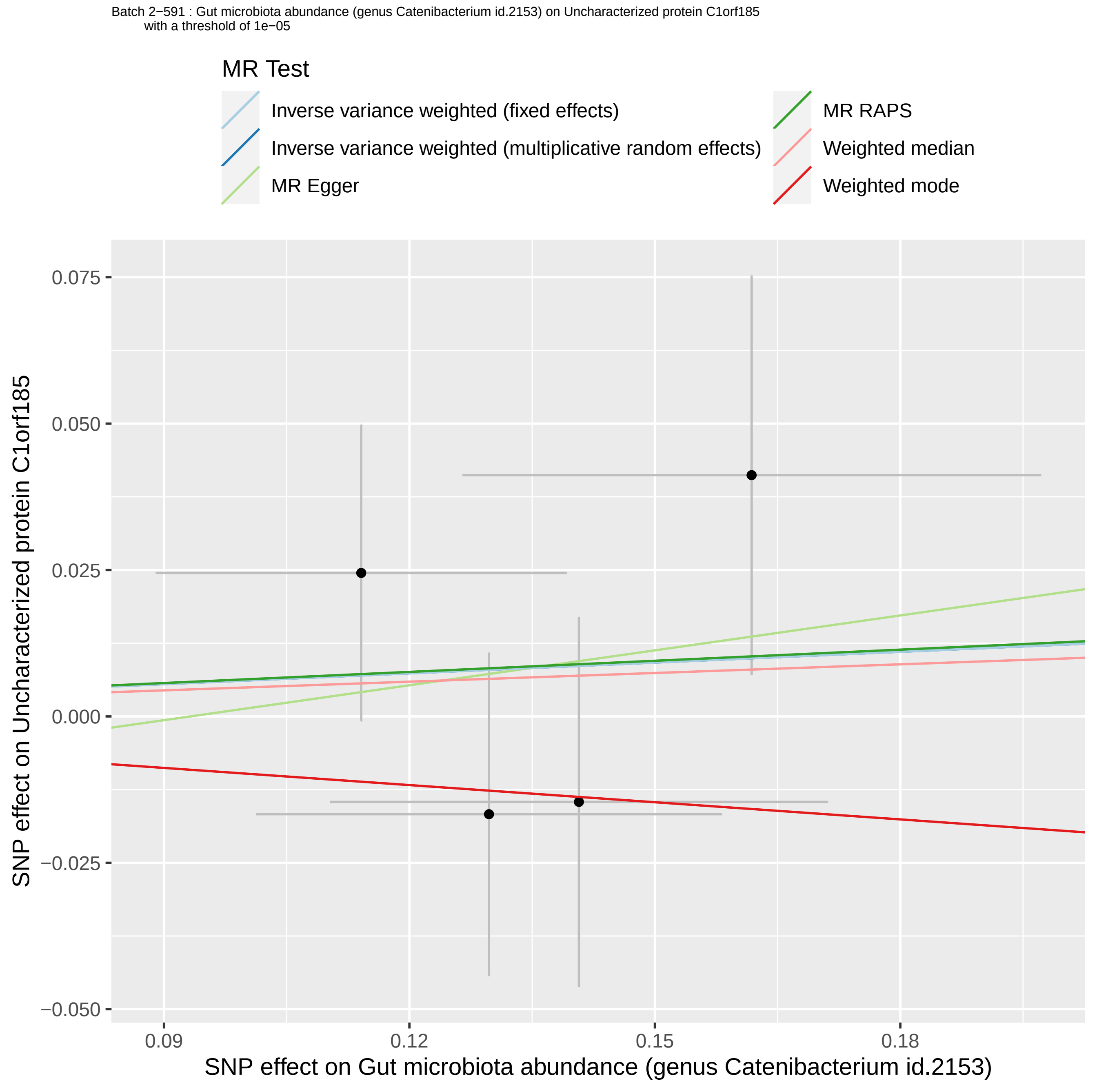
Batch 2−591 : Gut microbiota abundance (genus Catenibacterium id.2153) on Uncharacterized protein C1orf185
with a threshold of 1e−05
MR Test
Inverse variance weighted (fixed effects)
Inverse variance weighted (multiplicative random effects)
MR Egger
MR RAPS
Weighted median
Weighted mode
0.075
0.050
0.025
0.000
−0.025
−0.050
0.09
0.12
0.15
0.18
SNP effect on Uncharacterized protein C1orf185
SNP effect on Gut microbiota abundance (genus Catenibacterium id.2153)

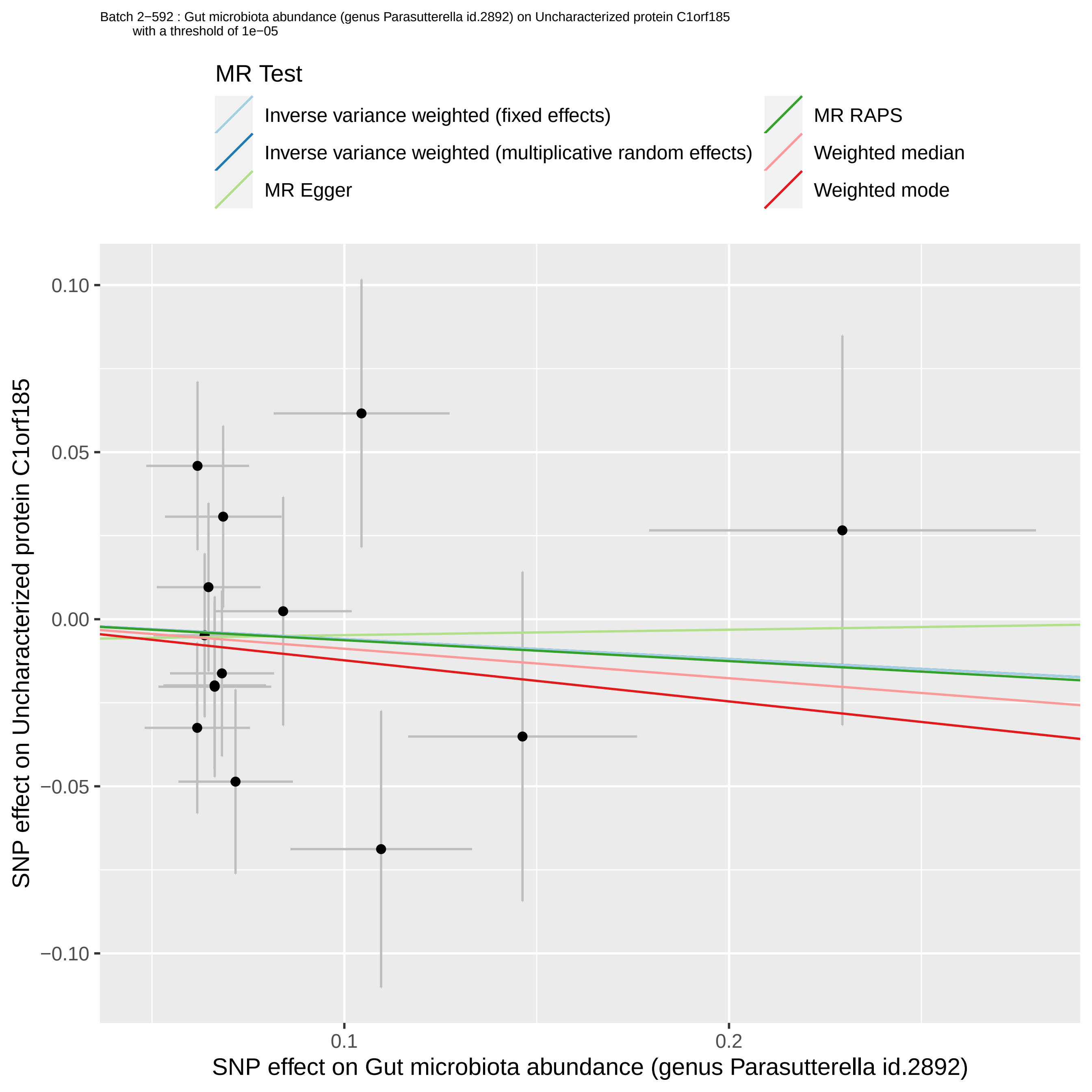
Batch 2−592 : Gut microbiota abundance (genus Parasutterella id.2892) on Uncharacterized protein C1orf185
with a threshold of 1e−05
MR Test
Inverse variance weighted (fixed effects)
Inverse variance weighted (multiplicative random effects)
MR Egger
MR RAPS
Weighted median
Weighted mode
0.10
0.05
0.00
−0.05
−0.10
0.1
0.2
SNP effect on Uncharacterized protein C1orf185
SNP effect on Gut microbiota abundance (genus Parasutterella id.2892)

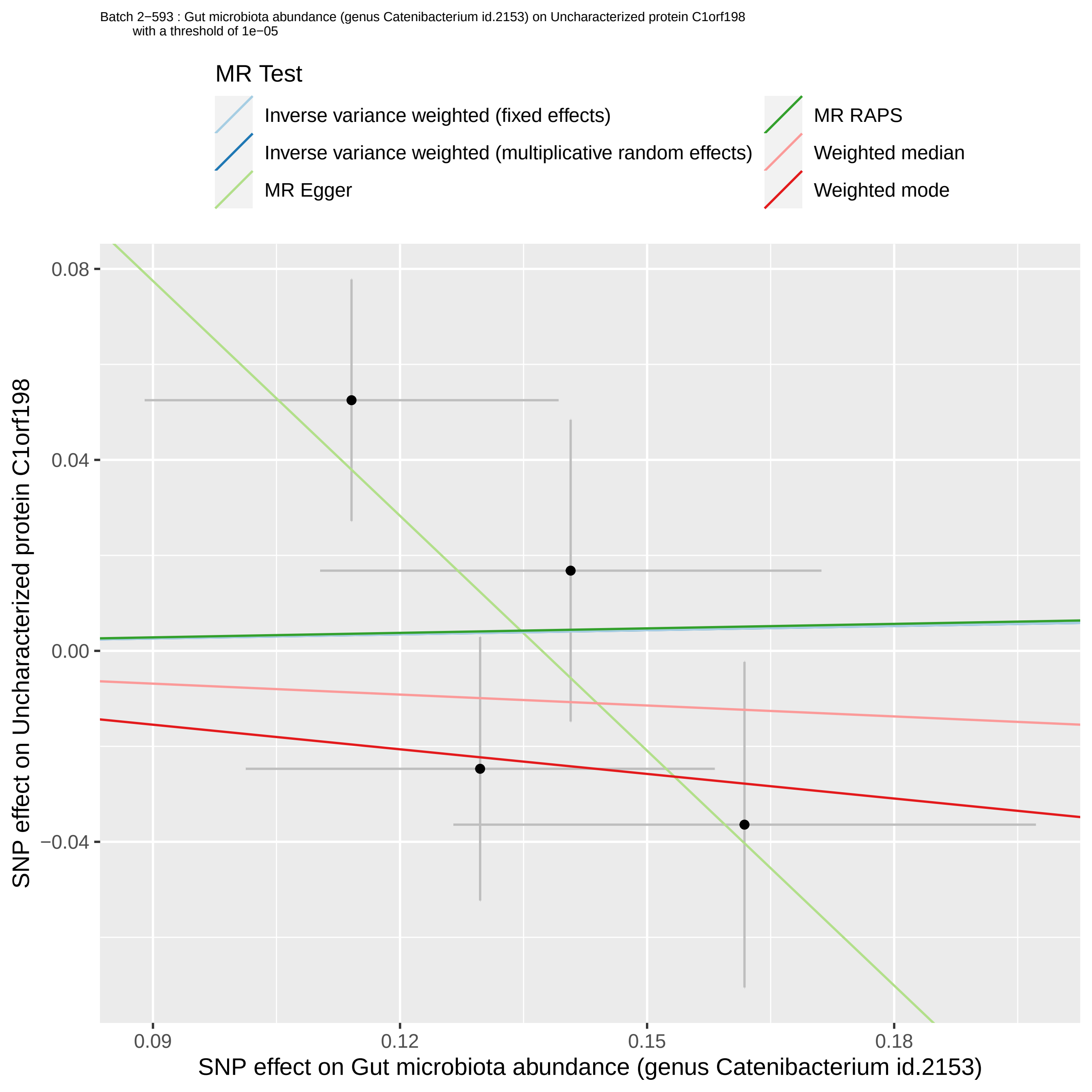
Batch 2−593 : Gut microbiota abundance (genus Catenibacterium id.2153) on Uncharacterized protein C1orf198
with a threshold of 1e−05
MR Test
Inverse variance weighted (fixed effects)
Inverse variance weighted (multiplicative random effects)
MR Egger
MR RAPS
Weighted median
Weighted mode
0.08
0.04
0.00
−0.04
0.09
0.12
0.15
0.18
SNP effect on Uncharacterized protein C1orf198
SNP effect on Gut microbiota abundance (genus Catenibacterium id.2153)

Batch 2−594 : Gut microbiota abundance (genus Parasutterella id.2892) on Uncharacterized protein C1orf198
with a threshold of 1e−05

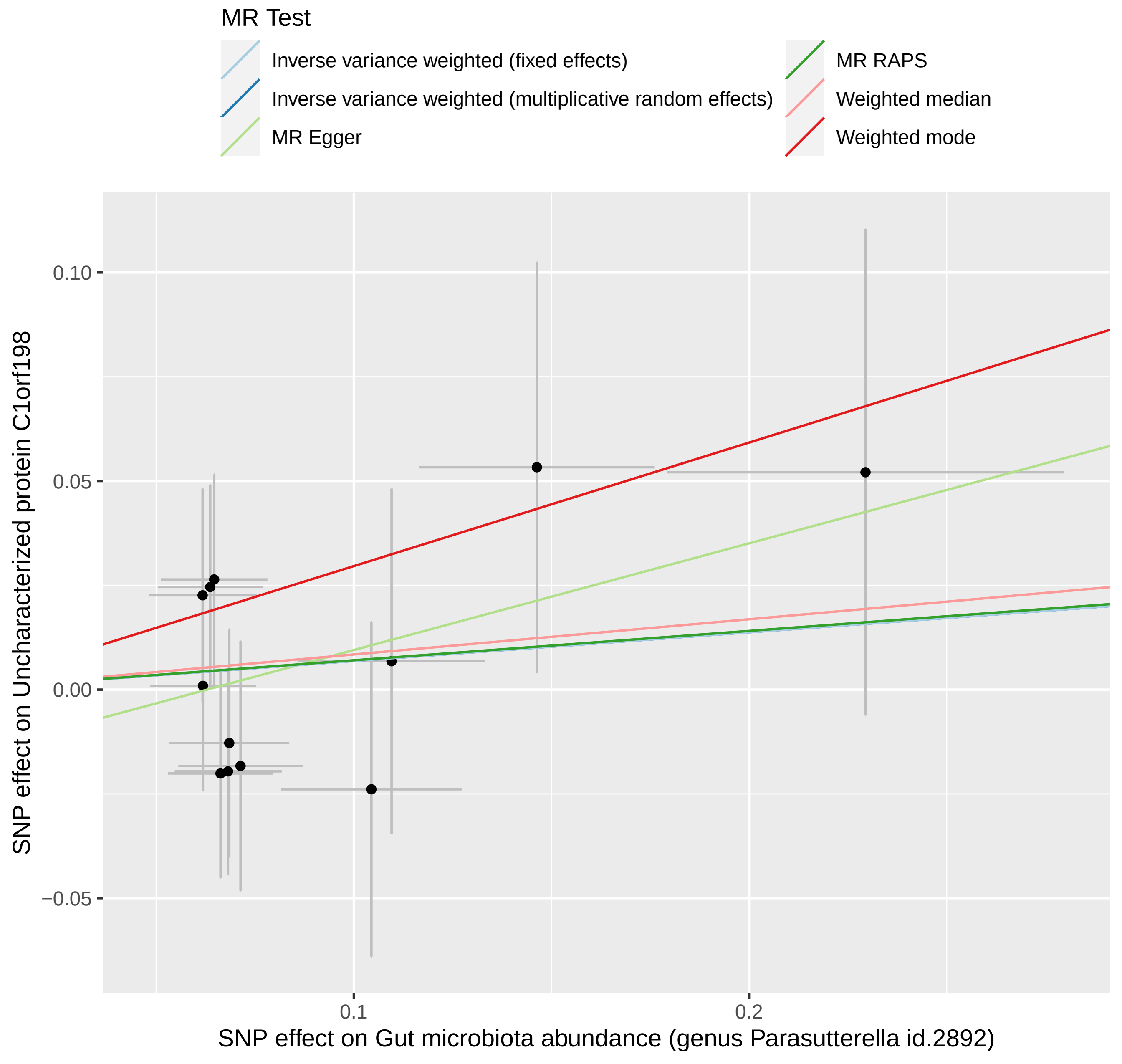

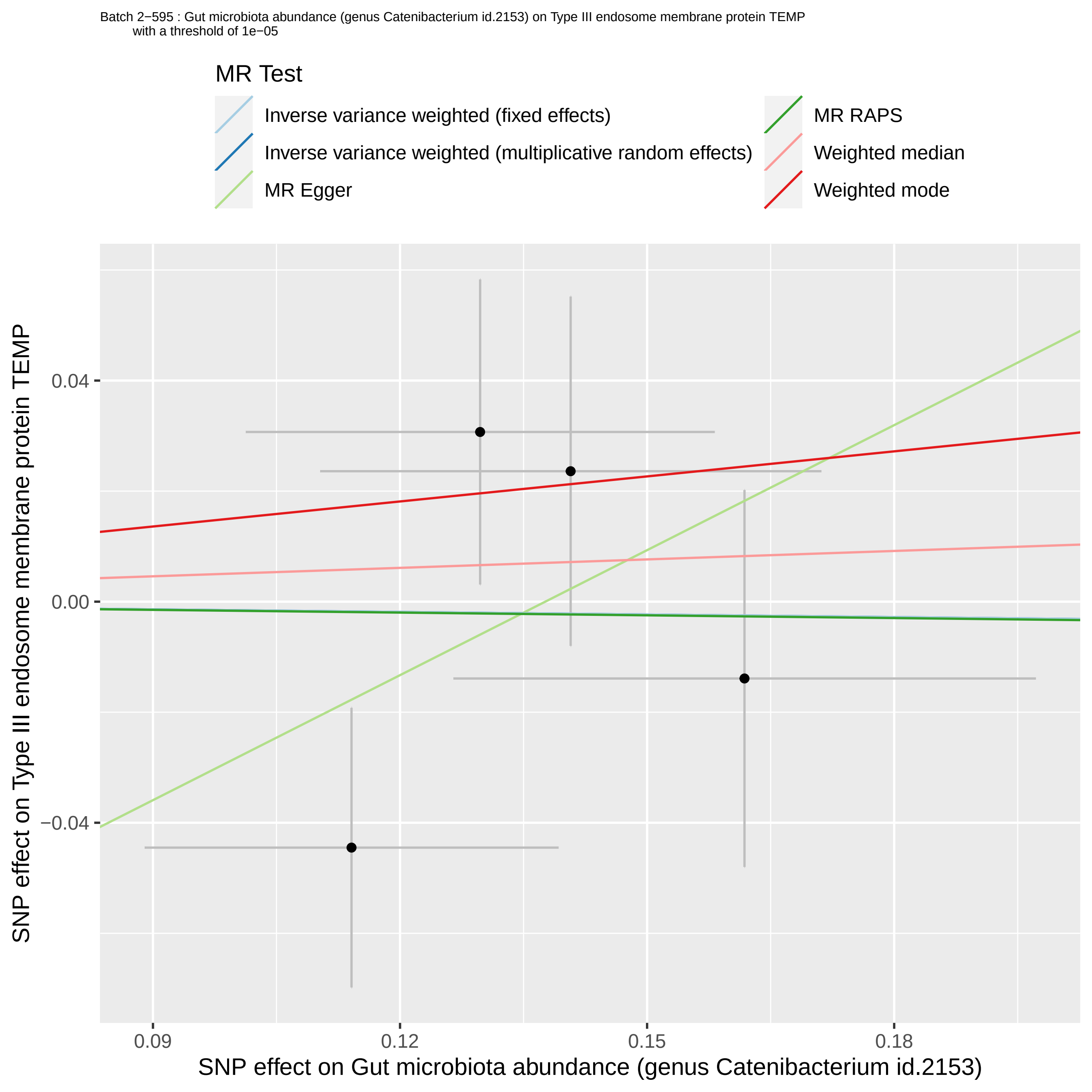
Batch 2−595 : Gut microbiota abundance (genus Catenibacterium id.2153) on Type III endosome membrane protein TEMP
with a threshold of 1e−05
MR Test
Inverse variance weighted (fixed effects)
Inverse variance weighted (multiplicative random effects)
MR Egger
MR RAPS
Weighted median
Weighted mode
0.04
0.00
−0.04
0.09
0.12
0.15
0.18
SNP effect on Type III endosome membrane protein TEMP
SNP effect on Gut microbiota abundance (genus Catenibacterium id.2153)

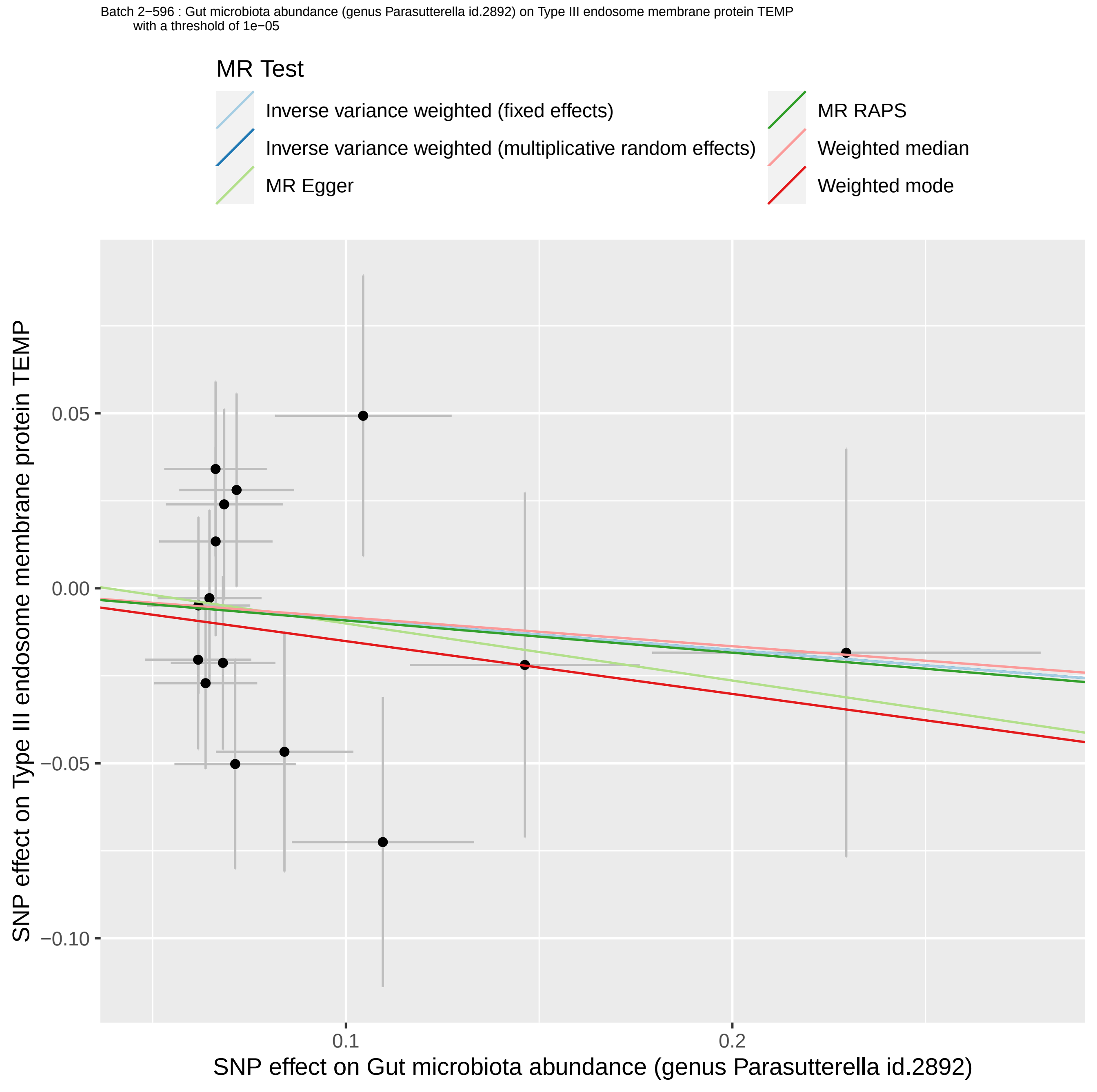

Batch 2−596 : Gut microbiota abundance (genus Parasutterella id.2892) on Type III endosome membrane protein TEMP
with a threshold of 1e−05
MR Test
Inverse variance weighted (fixed effects)
Inverse variance weighted (multiplicative random effects)
MR Egger
MR RAPS
Weighted median
Weighted mode
0.05
0.00
−0.05
−0.10
0.1
0.2
SNP effect on Type III endosome membrane protein TEMP
SNP effect on Gut microbiota abundance (genus Parasutterella id.2892)

Batch 2−597 : Gut microbiota abundance (genus Catenibacterium id.2153) on Uncharacterized protein C1orf226 with a threshold of 1e−05

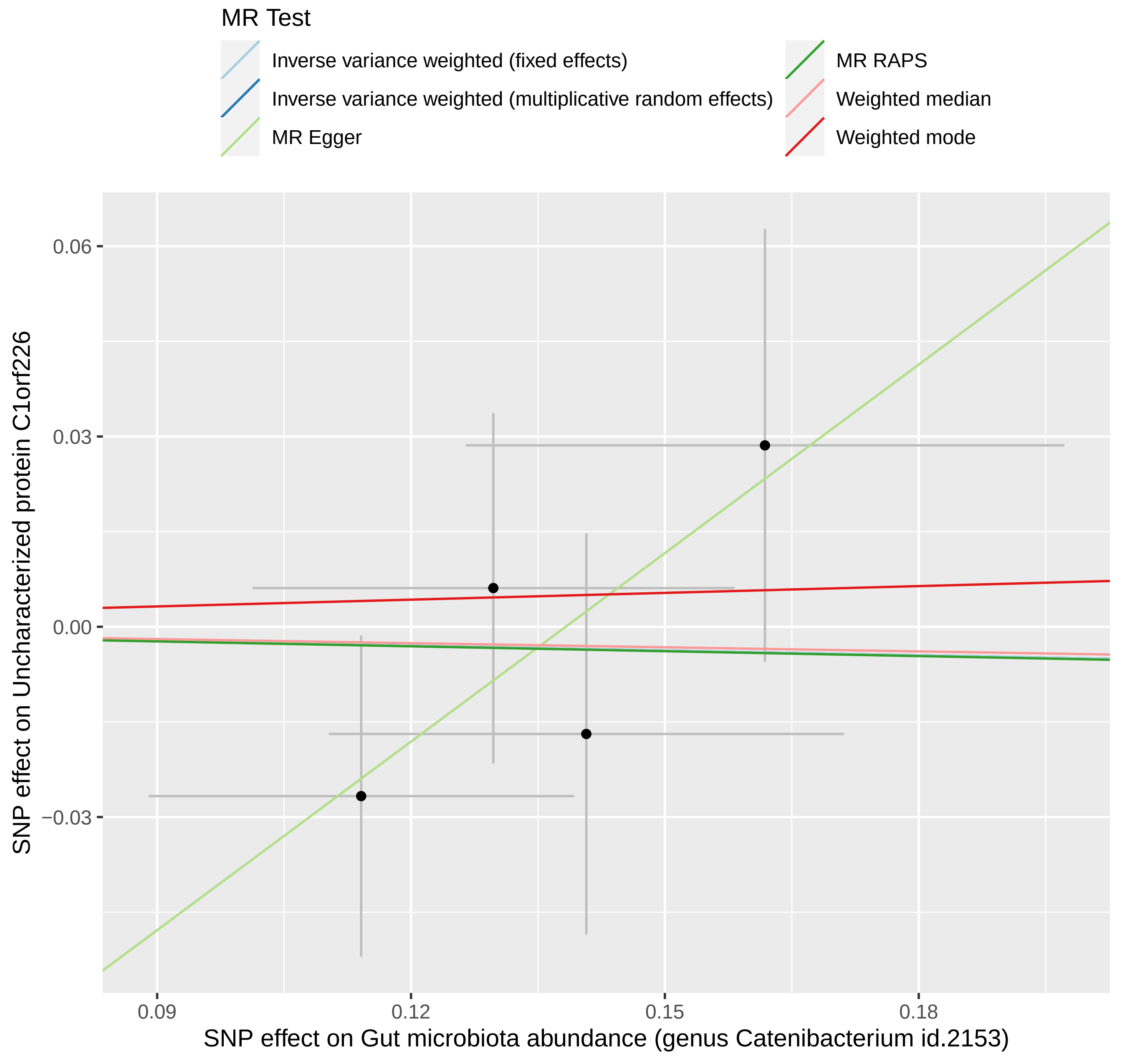

Batch 2−598 : Gut microbiota abundance (genus Parasutterella id.2892) on Uncharacterized protein C1orf226
with a threshold of 1e−05

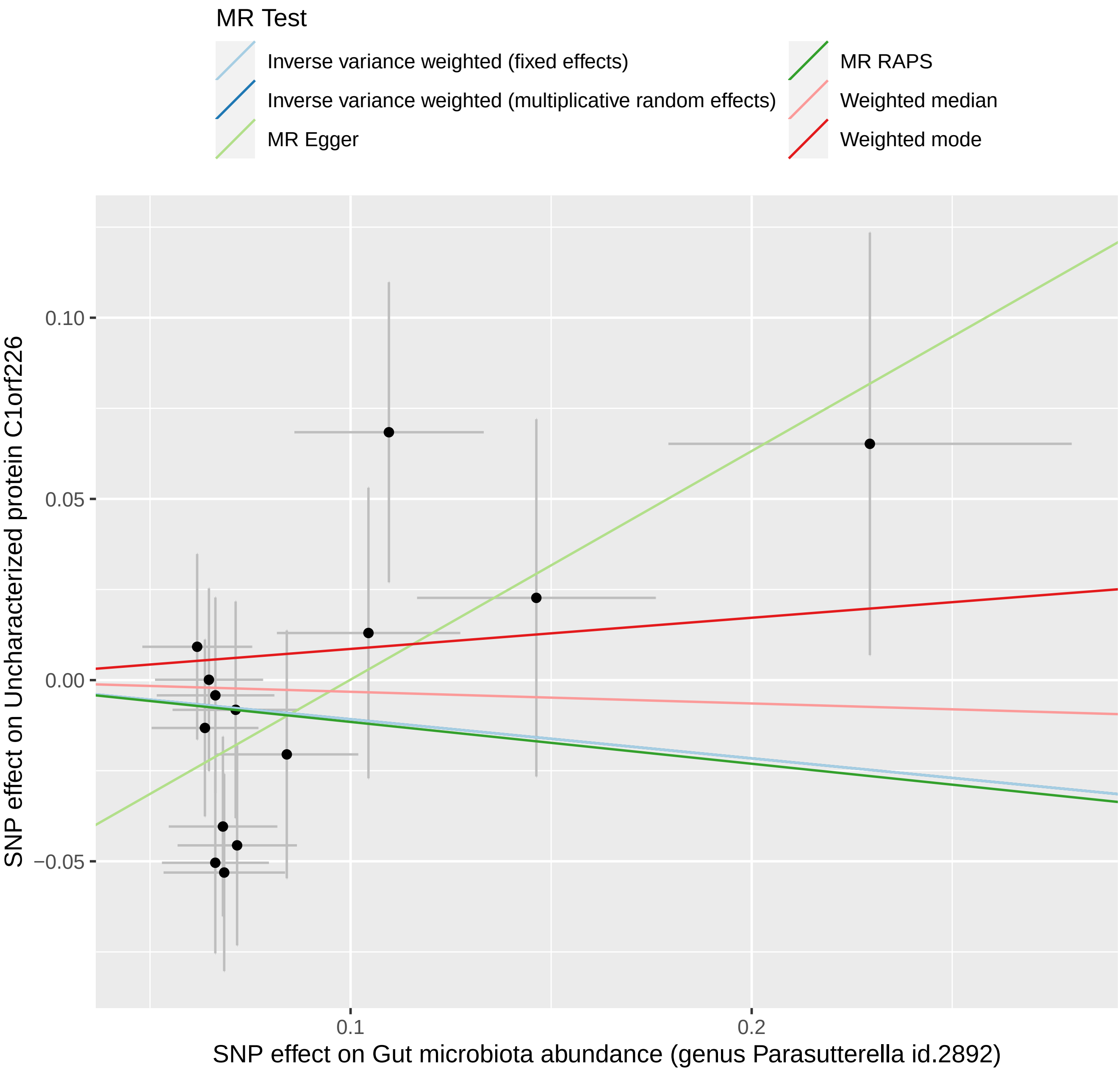

Batch 2−599 : Gut microbiota abundance (genus Catenibacterium id.2153) on Complement component 1 Q subcomponent−binding protein, mitochondrial with a threshold of 1e−05

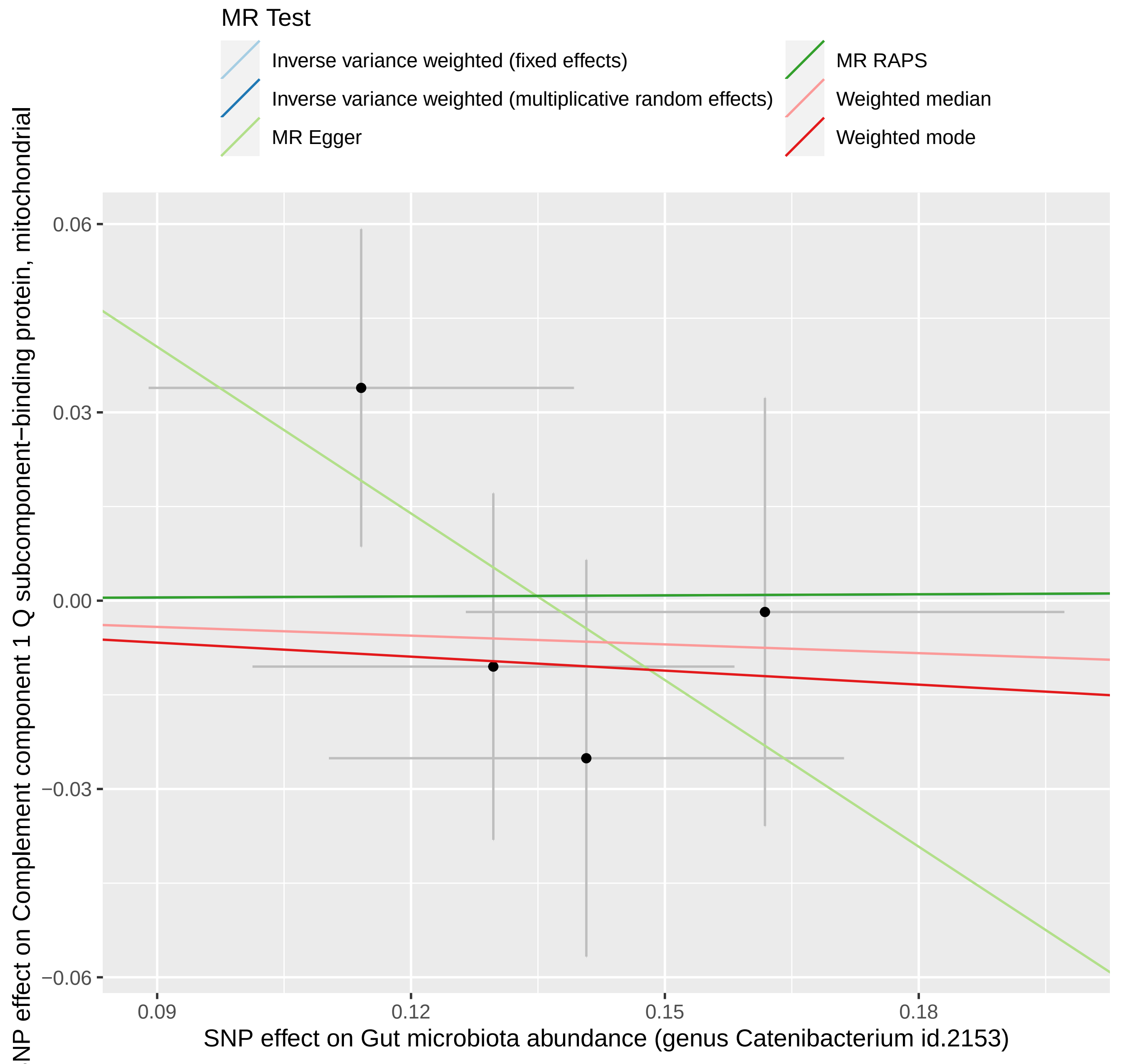

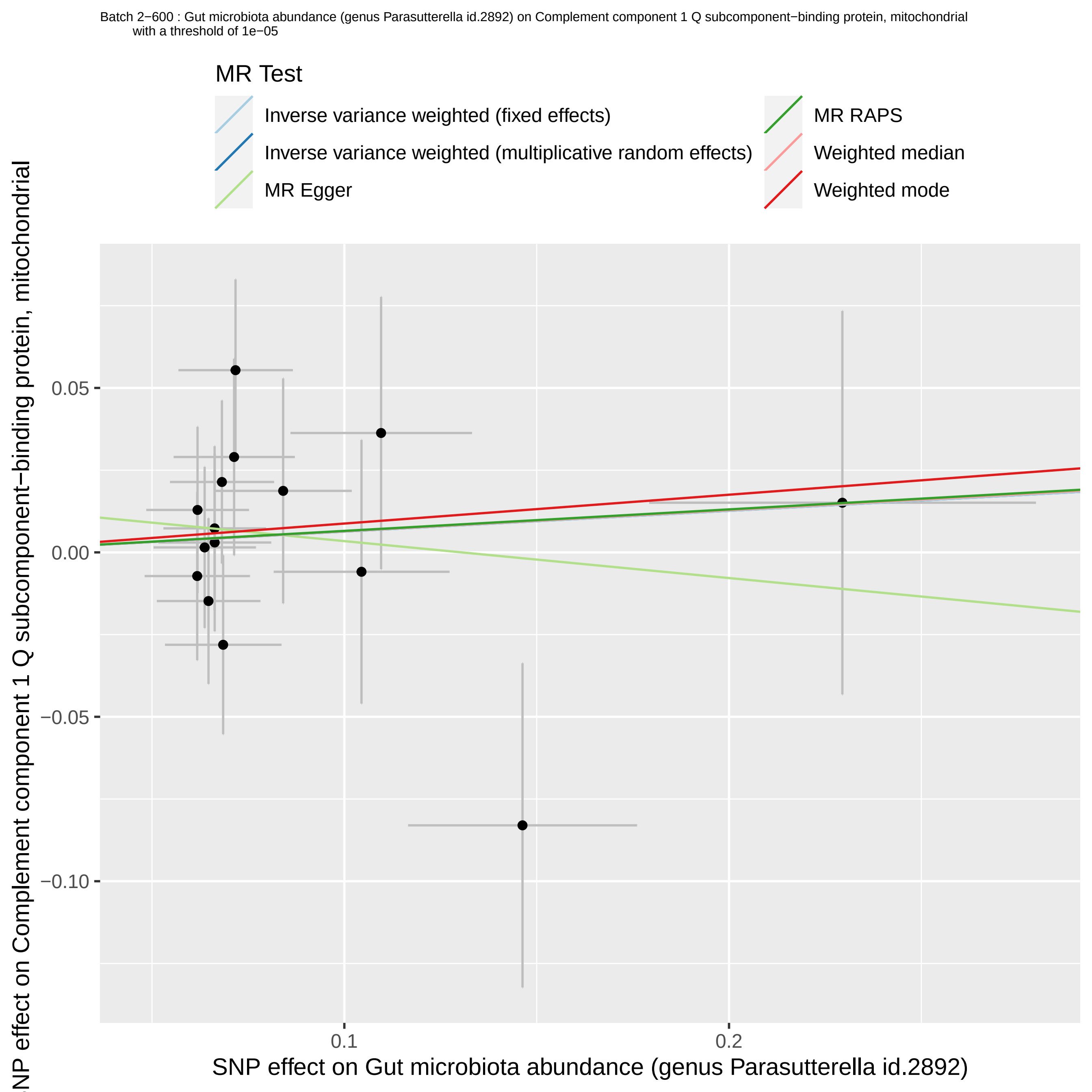

Batch 2−600 : Gut microbiota abundance (genus Parasutterella id.2892) on Complement component 1 Q subcomponent−binding protein, mitochondrial with a threshold of 1e−05
MR Test
Inverse variance weighted (fixed effects)
Inverse variance weighted (multiplicative random effects)
MR Egger
MR RAPS
Weighted median
Weighted mode
0.05
0.00
−0.05
−0.10
0.1
0.2
SNP effect on Gut microbiota abundance (genus Parasutterella id.2892)
SNP effect on Complement component 1 Q subcomponent−binding protein, mitochondrial

Batch 2−601 : Gut microbiota abundance (genus Catenibacterium id.2153) on Complement C1q subcomponent subunit C
with a threshold of 1e−05

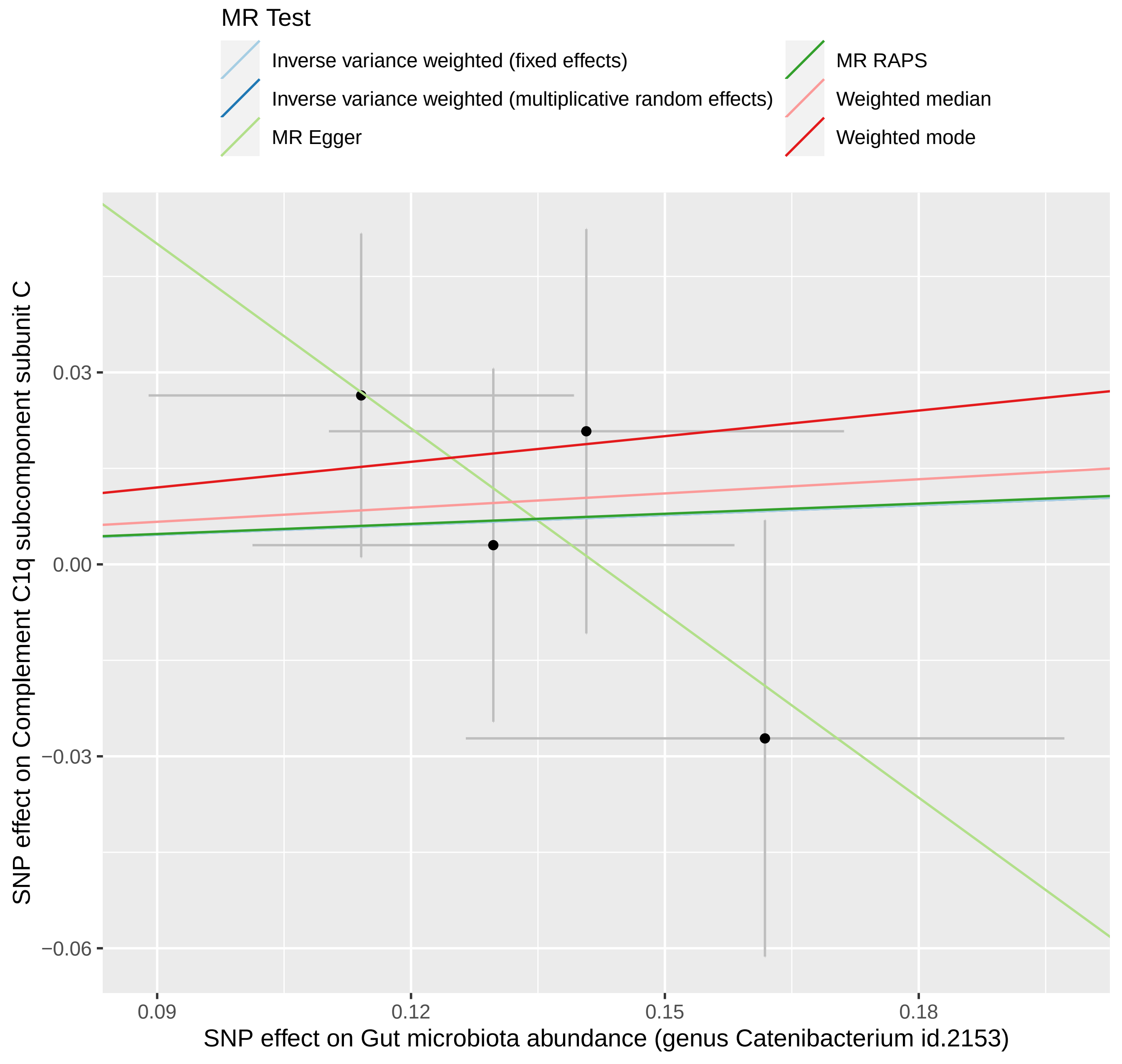

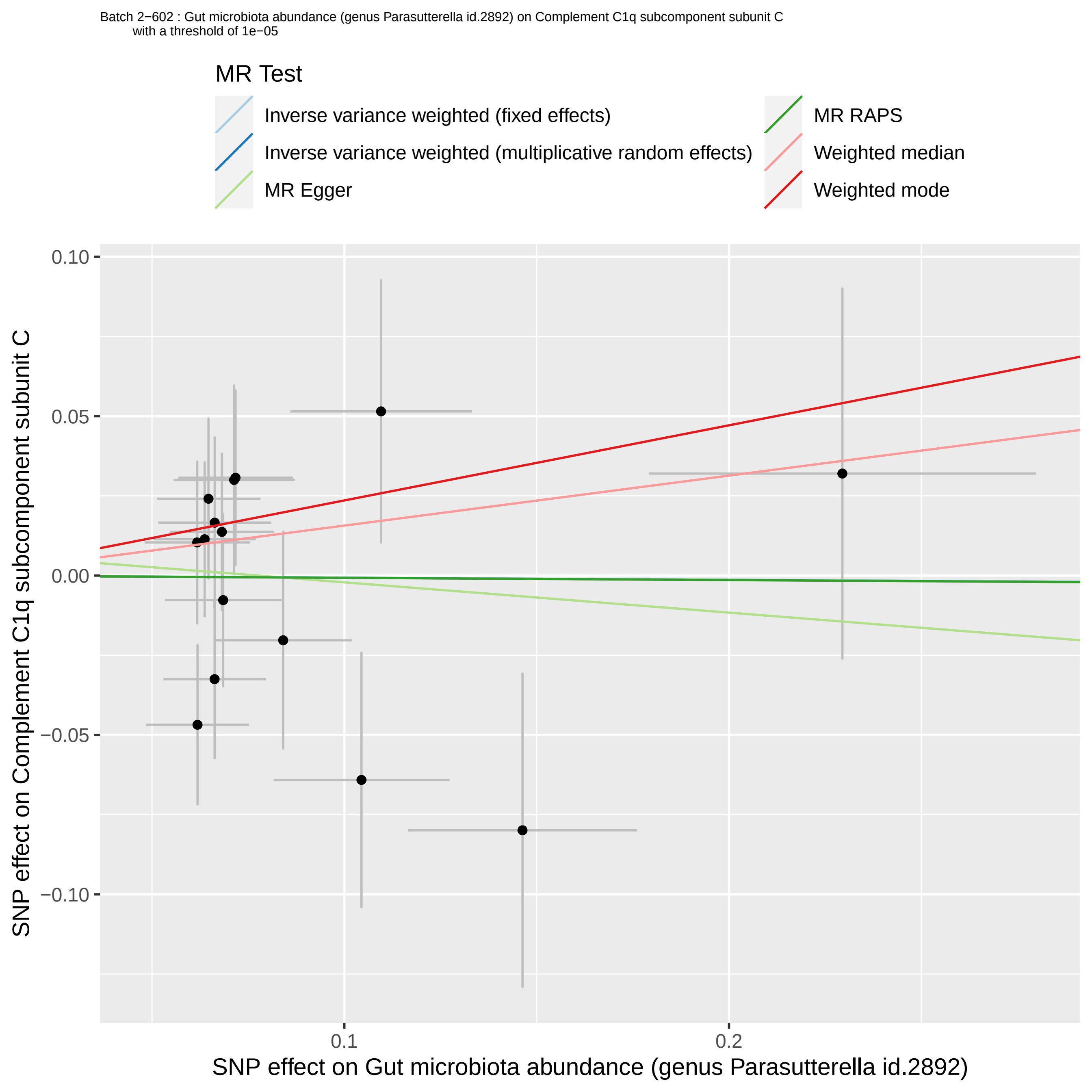

Batch 2−602 : Gut microbiota abundance (genus Parasutterella id.2892) on Complement C1q subcomponent subunit C
with a threshold of 1e−05
MR Test
Inverse variance weighted (fixed effects)
Inverse variance weighted (multiplicative random effects)
MR Egger
MR RAPS
Weighted median
Weighted mode
0.10
0.05
0.00
−0.05
−0.10
0.1
0.2
SNP effect on Complement C1q subcomponent subunit C
SNP effect on Gut microbiota abundance (genus Parasutterella id.2892)

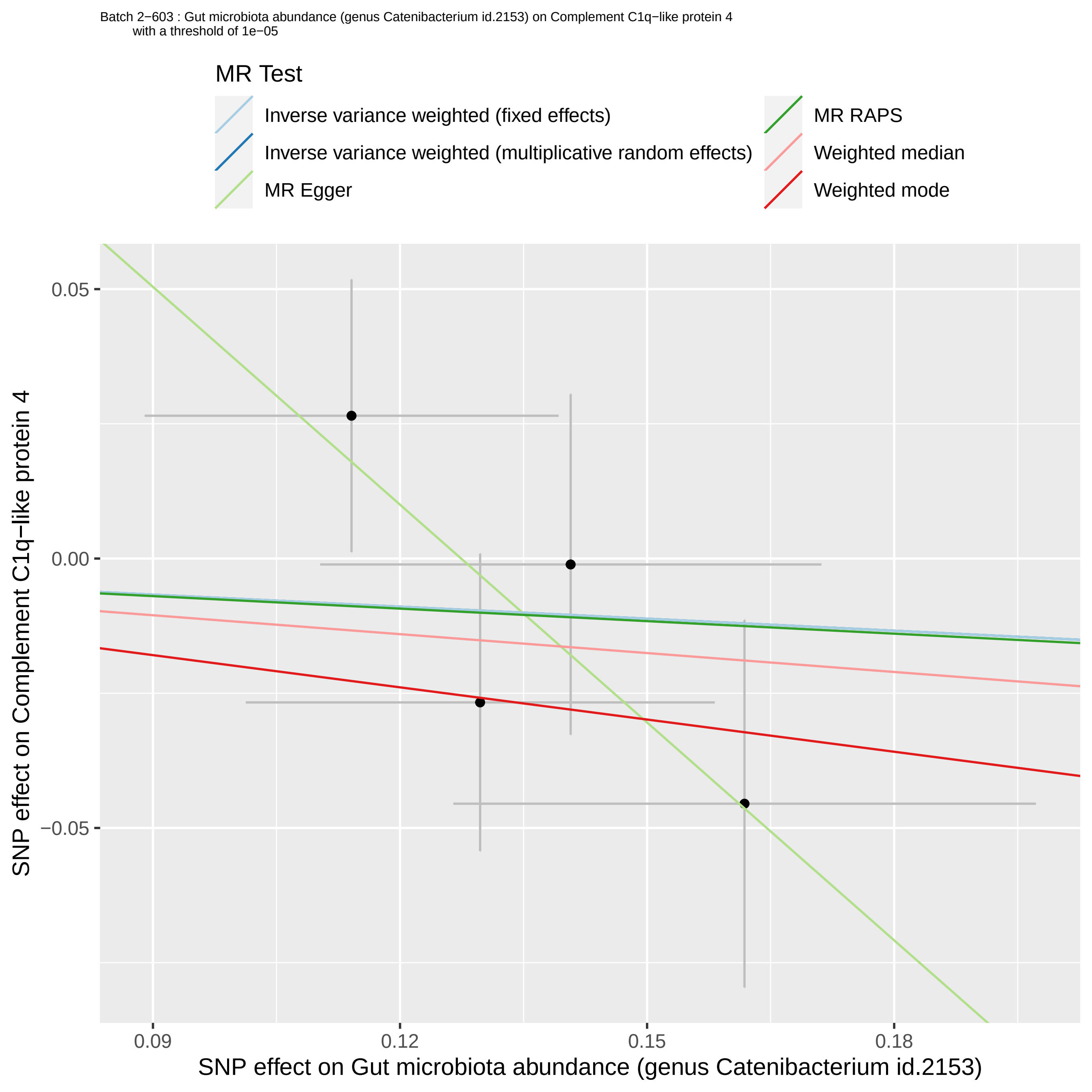
Batch 2−603 : Gut microbiota abundance (genus Catenibacterium id.2153) on Complement C1q−like protein 4
with a threshold of 1e−05
MR Test
Inverse variance weighted (fixed effects)
Inverse variance weighted (multiplicative random effects)
MR Egger
MR RAPS
Weighted median
Weighted mode
0.05
0.00
−0.05
0.09
0.12
0.15
0.18
SNP effect on Complement C1q−like protein 4
SNP effect on Gut microbiota abundance (genus Catenibacterium id.2153)

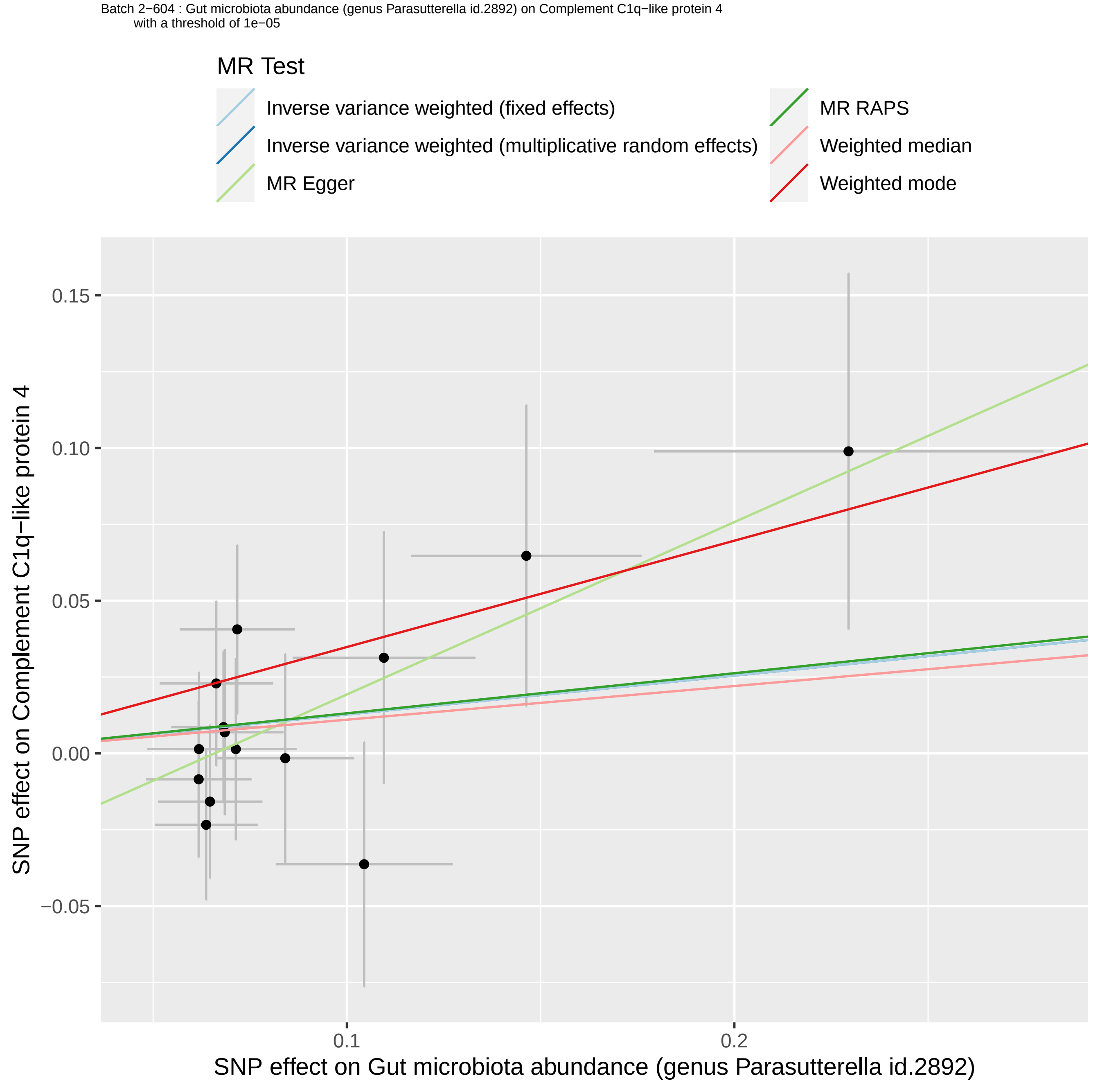

Batch 2−604 : Gut microbiota abundance (genus Parasutterella id.2892) on Complement C1q−like protein 4
with a threshold of 1e−05
MR Test
Inverse variance weighted (fixed effects)
Inverse variance weighted (multiplicative random effects)
MR Egger
MR RAPS
Weighted median
Weighted mode
0.15
0.10
0.05
0.00
−0.05
0.1
0.2
SNP effect on Complement C1q−like protein 4
SNP effect on Gut microbiota abundance (genus Parasutterella id.2892)

Batch 2−605 : Gut microbiota abundance (genus Catenibacterium id.2153) on Complement C1q tumor necrosis factor−related protein 1
with a threshold of 1e−05

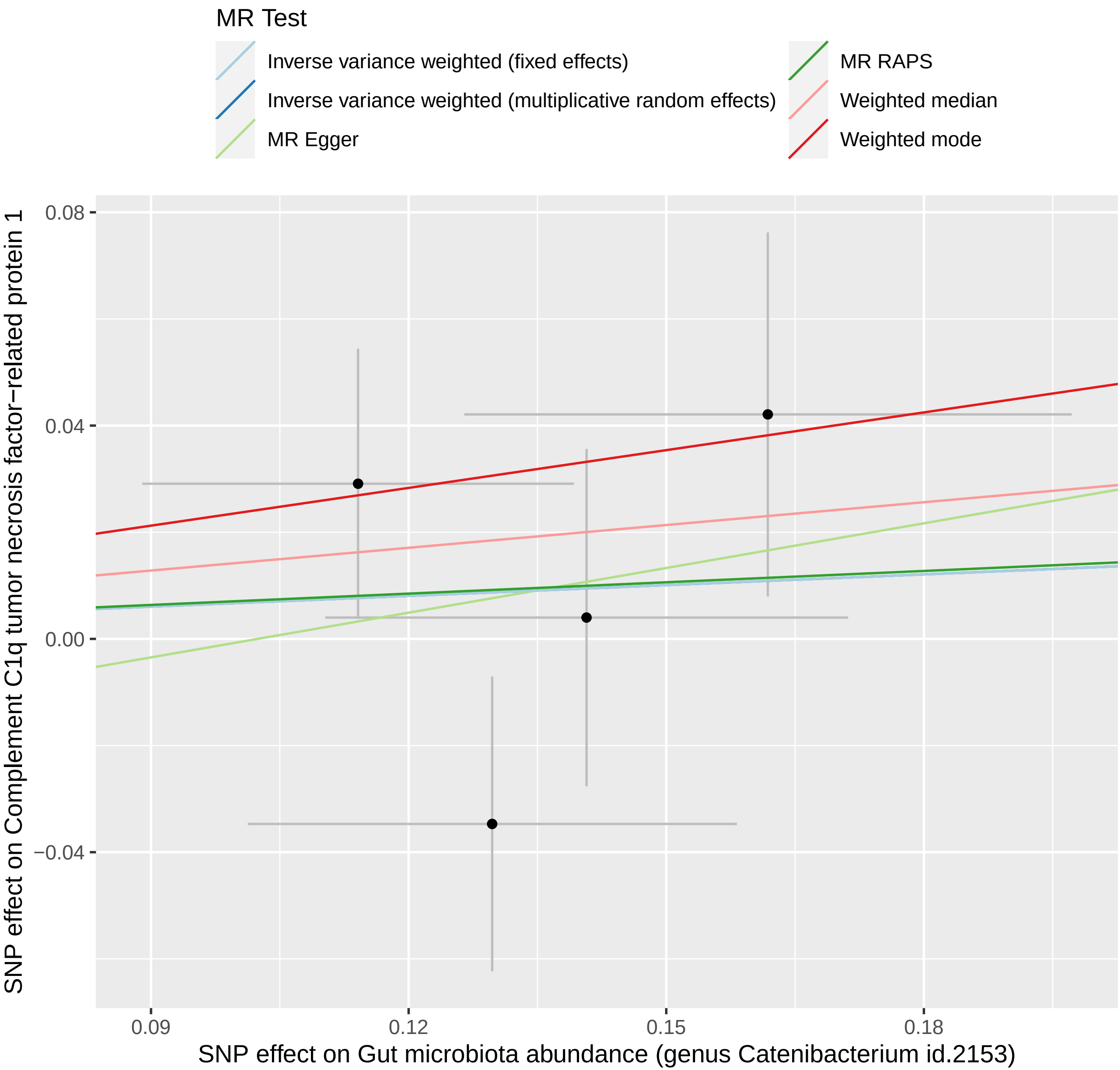

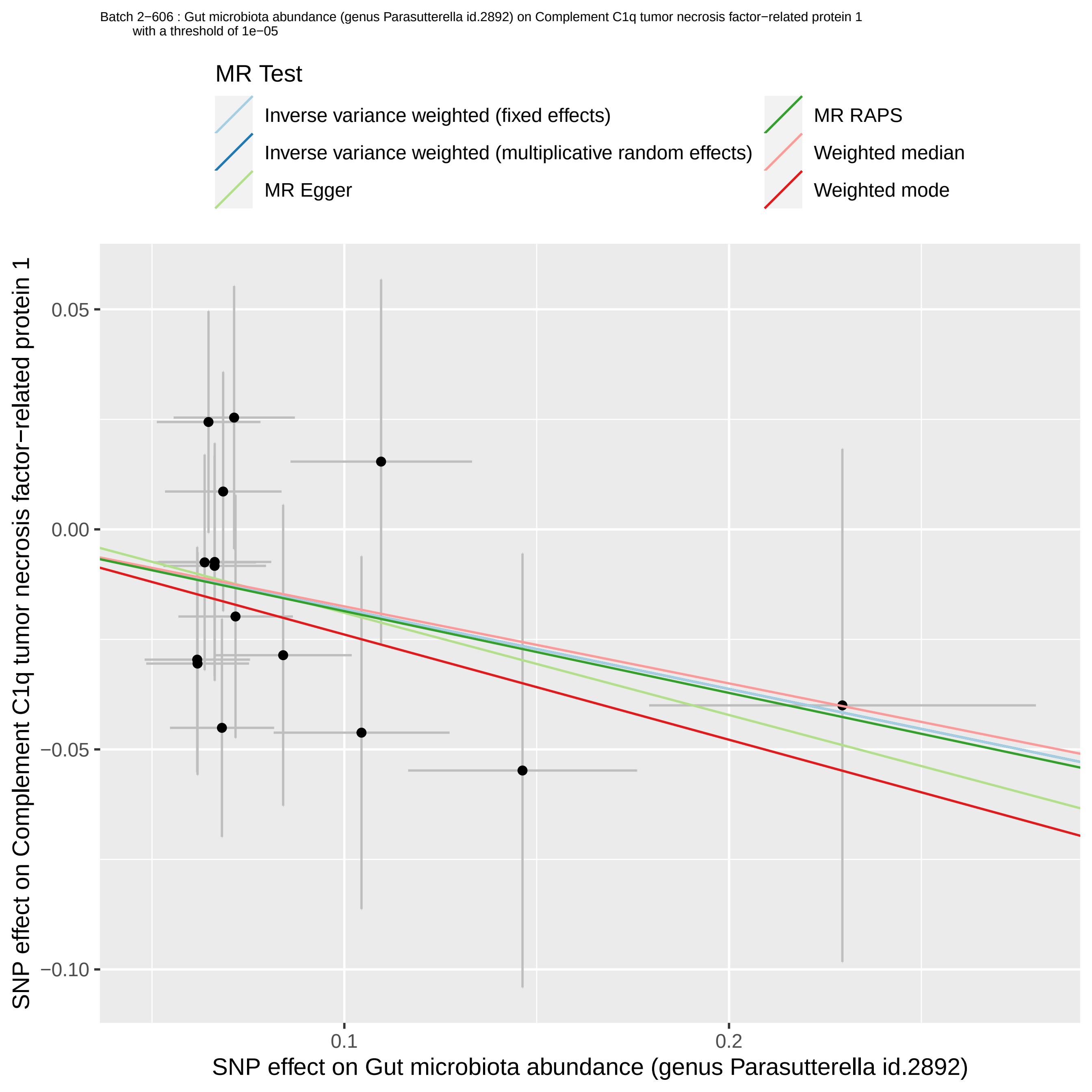
Batch 2−606 : Gut microbiota abundance (genus Parasutterella id.2892) on Complement C1q tumor necrosis factor−related protein 1
with a threshold of 1e−05
MR Test
Inverse variance weighted (fixed effects)
Inverse variance weighted (multiplicative random effects)
MR Egger
MR RAPS
Weighted median
Weighted mode
0.05
0.00
−0.05
−0.10
0.1
0.2
SNP effect on Complement C1q tumor necrosis factor−related protein 1
SNP effect on Gut microbiota abundance (genus Parasutterella id.2892)

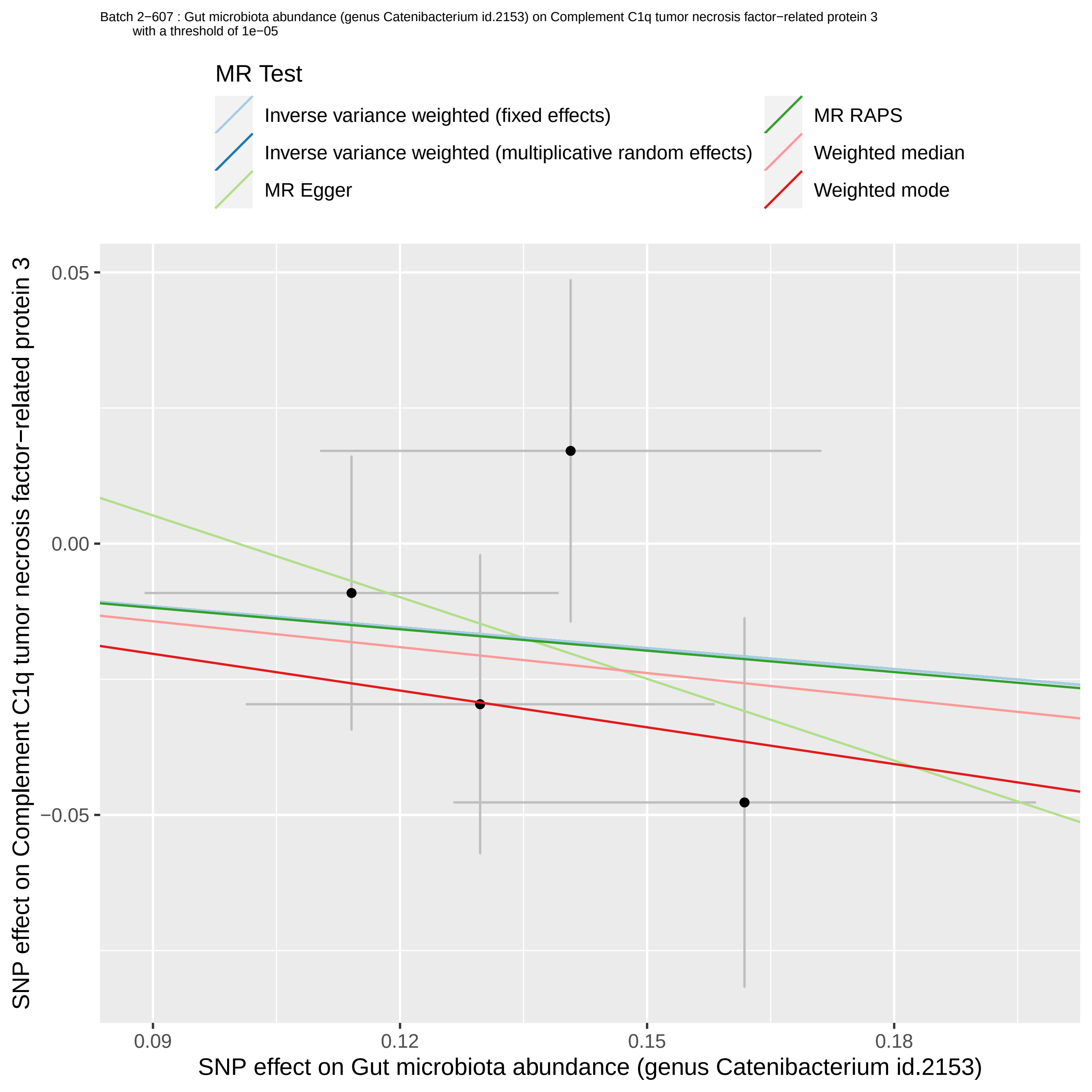

Batch 2−607 : Gut microbiota abundance (genus Catenibacterium id.2153) on Complement C1q tumor necrosis factor−related protein 3
with a threshold of 1e−05
MR Test
Inverse variance weighted (fixed effects)
Inverse variance weighted (multiplicative random effects)
MR Egger
MR RAPS
Weighted median
Weighted mode
0.05
0.00
−0.05
0.09
0.12
0.15
0.18
SNP effect on Complement C1q tumor necrosis factor−related protein 3
SNP effect on Gut microbiota abundance (genus Catenibacterium id.2153)

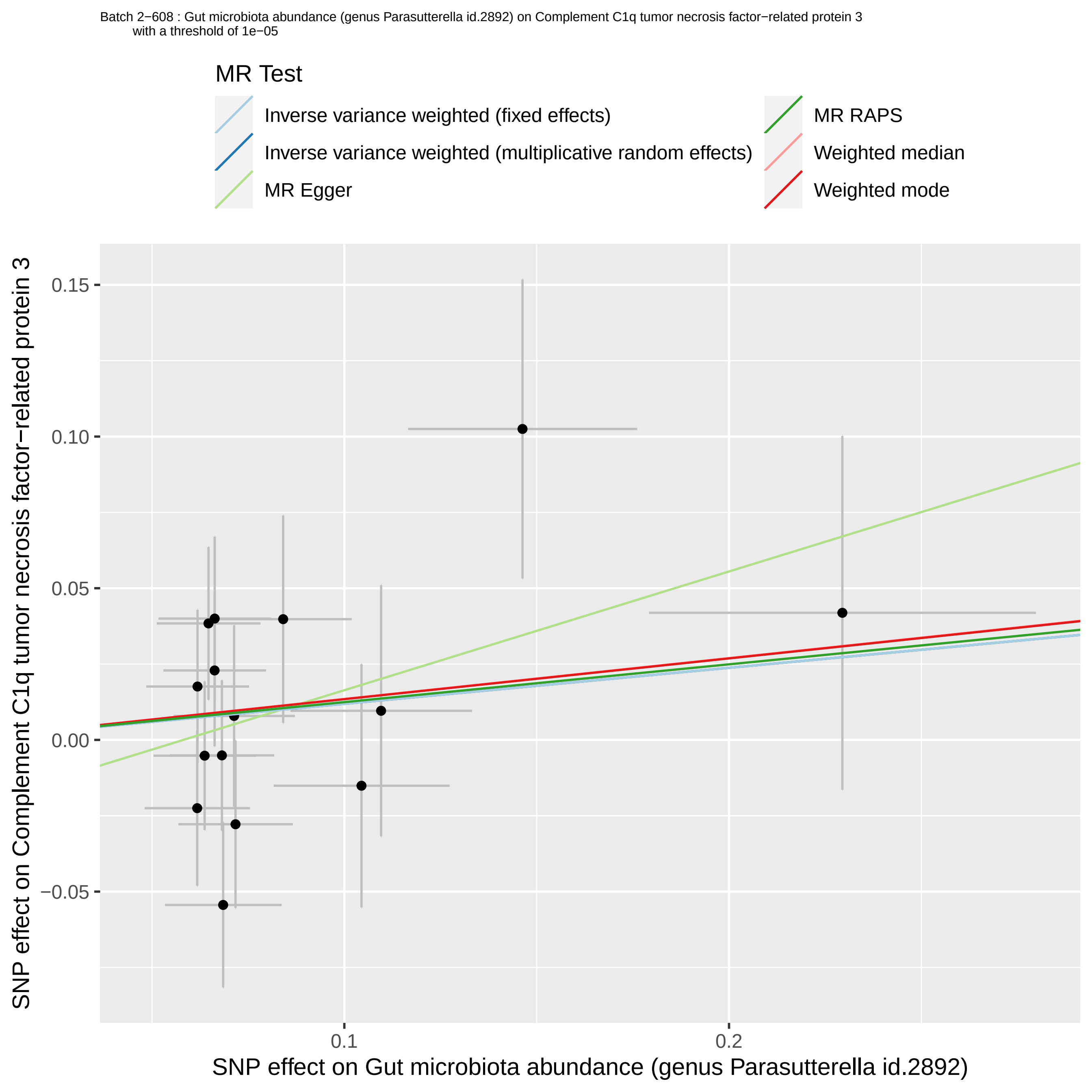
Batch 2−608 : Gut microbiota abundance (genus Parasutterella id.2892) on Complement C1q tumor necrosis factor−related protein 3
with a threshold of 1e−05
MR Test
Inverse variance weighted (fixed effects)
Inverse variance weighted (multiplicative random effects)
MR Egger
MR RAPS
Weighted median
Weighted mode
SNP effect on Complement C1q tumor necrosis factor−related protein 3
0.15
0.10
0.05
0.00
−0.05
0.1
0.2
SNP effect on Gut microbiota abundance (genus Parasutterella id.2892)

Batch 2−609 : Gut microbiota abundance (genus Catenibacterium id.2153) on Complement C1q tumor necrosis factor−related protein 5
with a threshold of 1e−05

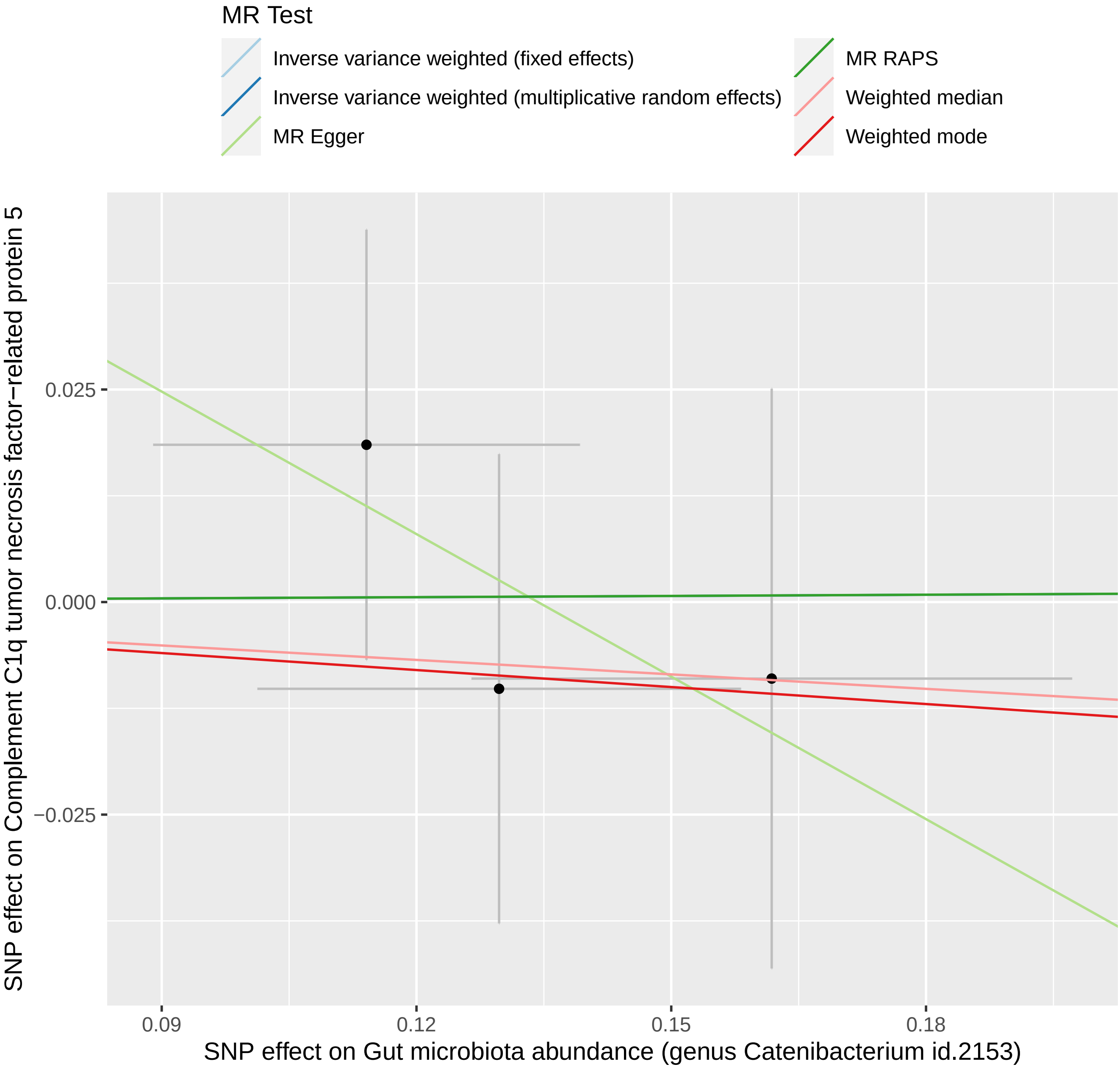

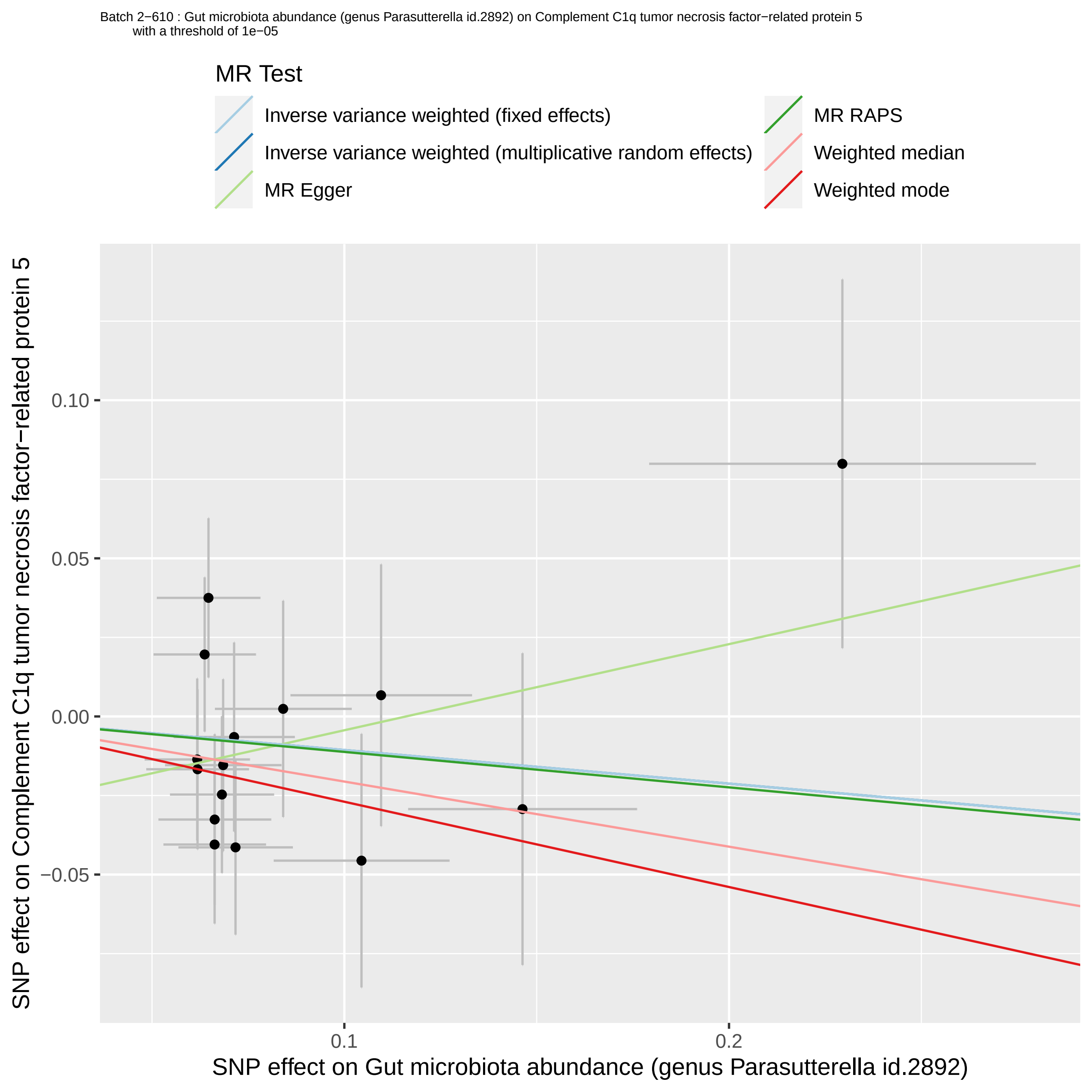

Batch 2−610 : Gut microbiota abundance (genus Parasutterella id.2892) on Complement C1q tumor necrosis factor−related protein 5
with a threshold of 1e−05
MR Test
Inverse variance weighted (fixed effects)
Inverse variance weighted (multiplicative random effects)
MR Egger
MR RAPS
Weighted median
Weighted mode
0.10
0.05
0.00
−0.05
0.1
0.2
SNP effect on Complement C1q tumor necrosis factor−related protein 5
SNP effect on Gut microbiota abundance (genus Parasutterella id.2892)

Batch 2−611 : Gut microbiota abundance (genus Catenibacterium id.2153) on Complement C1q and tumor necrosis factor−related protein 9A
with a threshold of 1e−05

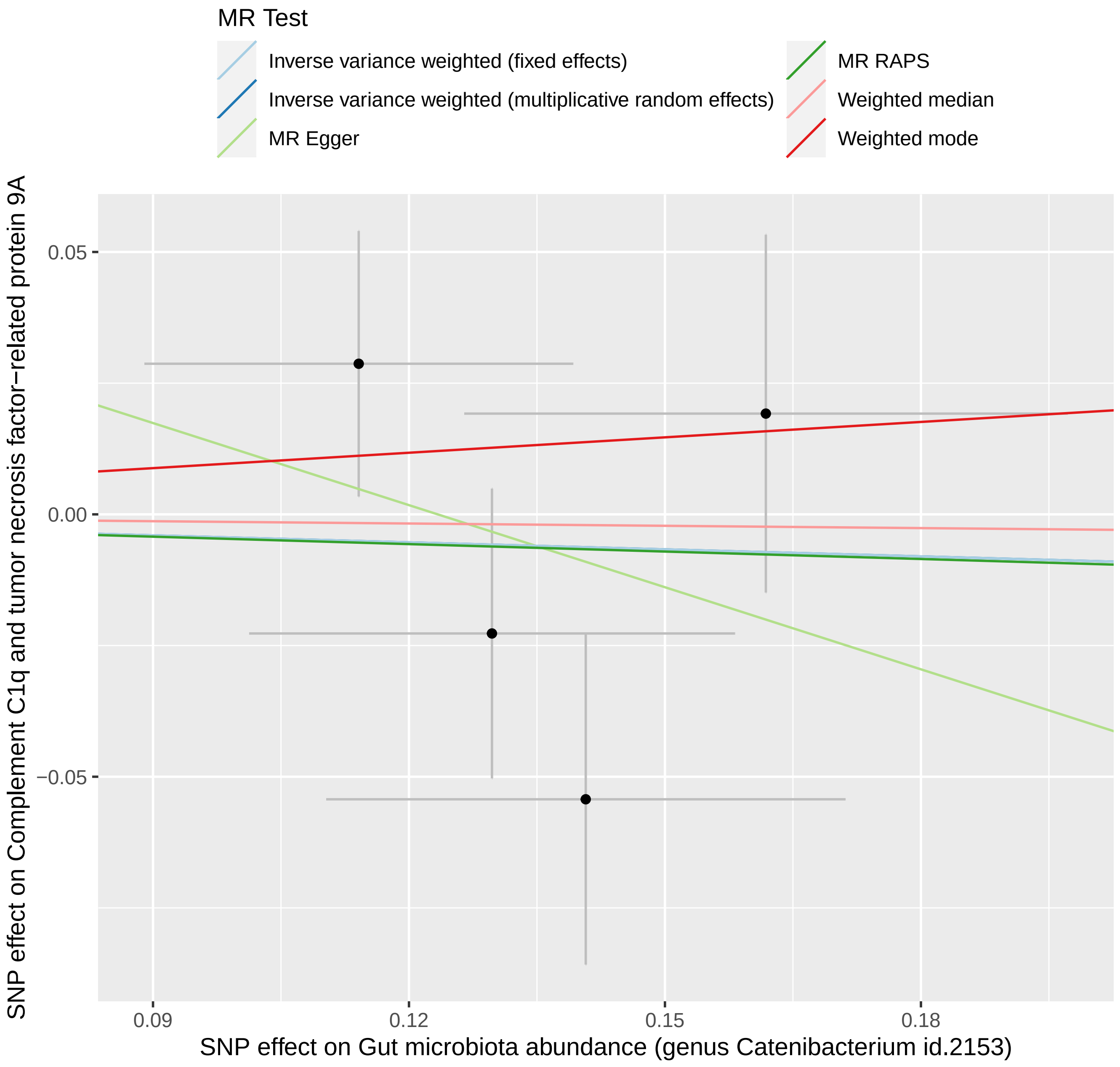

Batch 2−612 : Gut microbiota abundance (genus Parasutterella id.2892) on Complement C1q and tumor necrosis factor−related protein 9A
with a threshold of 1e−05

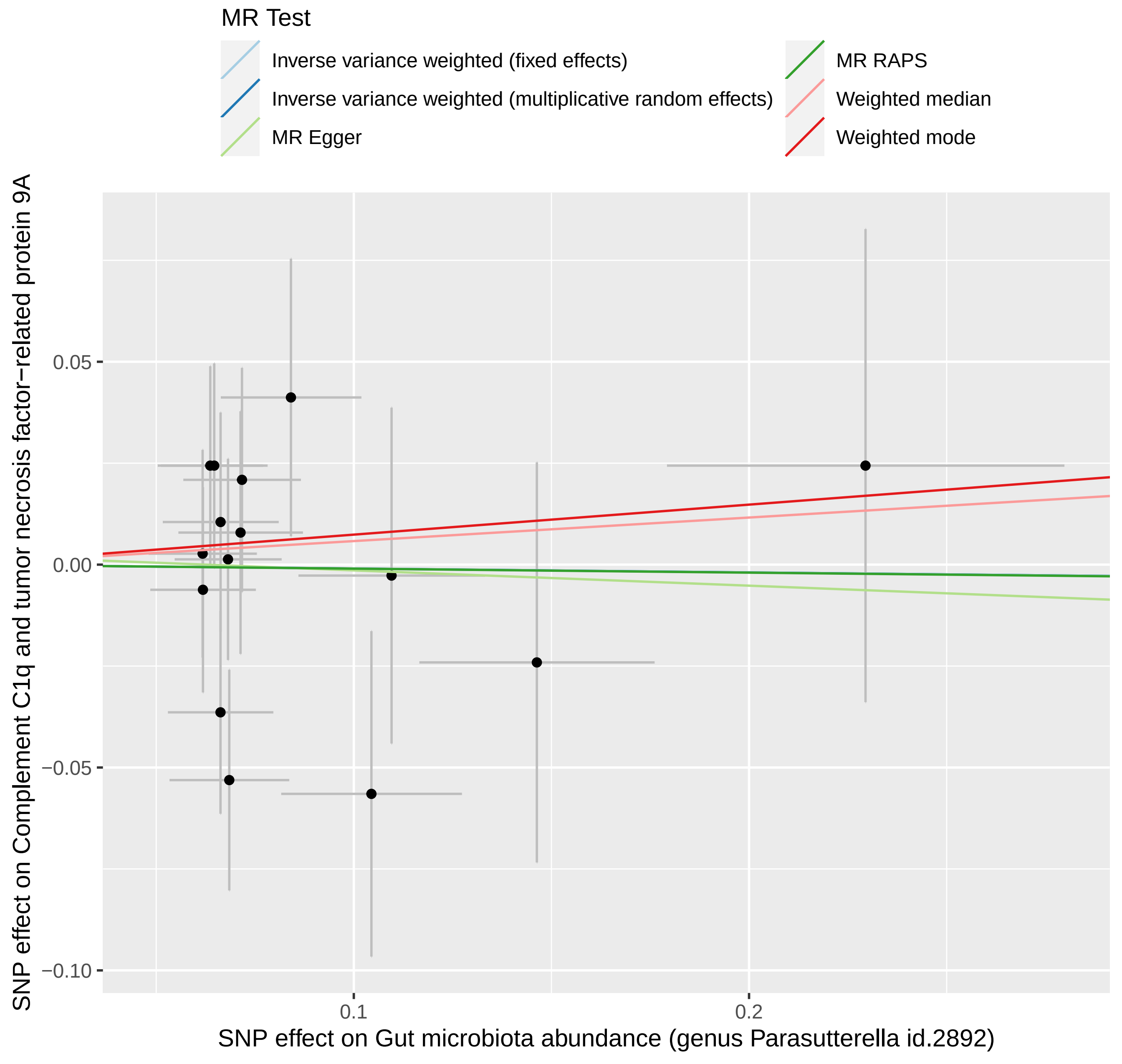

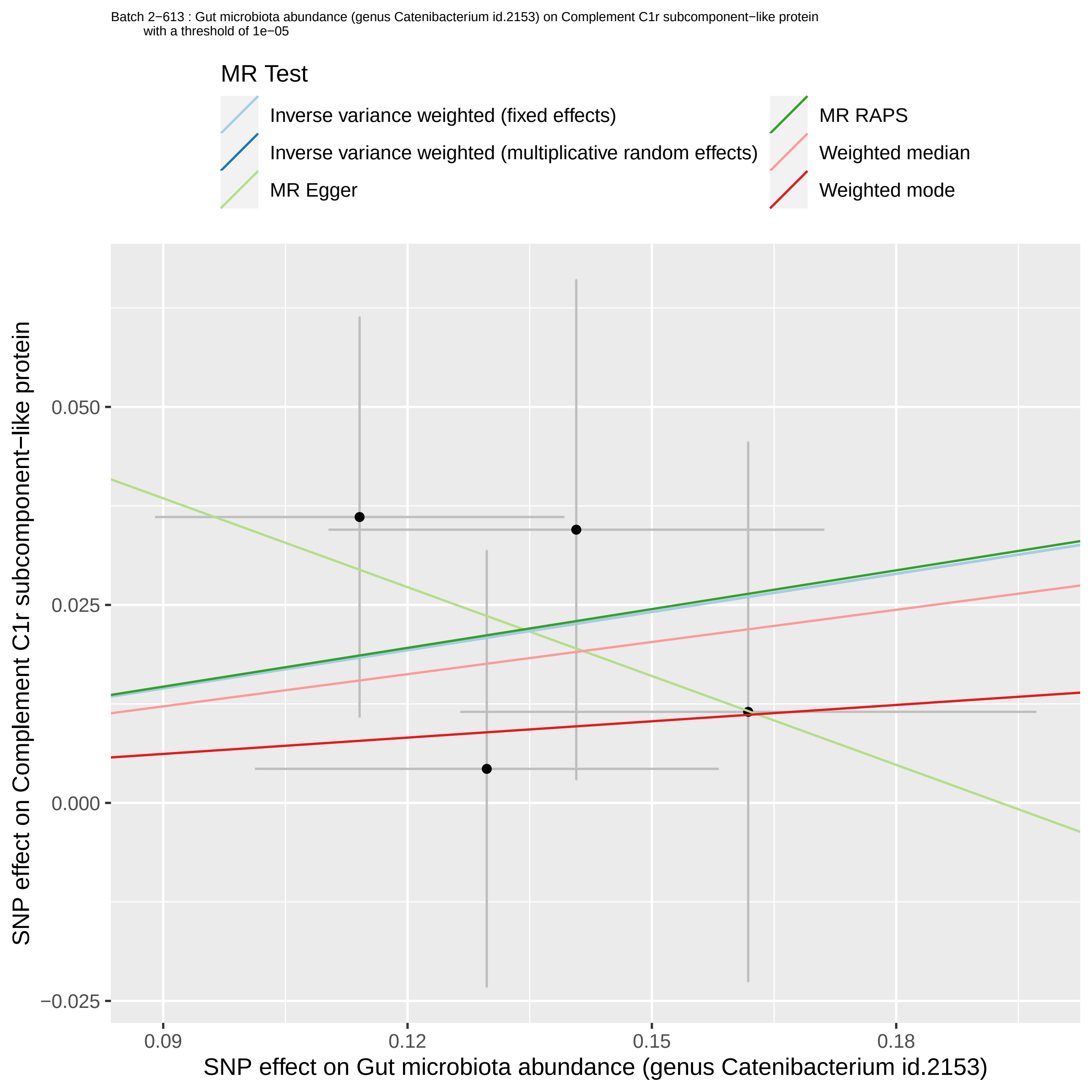
Batch 2−613 : Gut microbiota abundance (genus Catenibacterium id.2153) on Complement C1r subcomponent−like protein
with a threshold of 1e−05
MR Test
Inverse variance weighted (fixed effects)
Inverse variance weighted (multiplicative random effects)
MR Egger
MR RAPS
Weighted median
Weighted mode
0.050
0.025
0.000
−0.025
0.09
0.12
0.15
0.18
SNP effect on Complement C1r subcomponent−like protein
SNP effect on Gut microbiota abundance (genus Catenibacterium id.2153)

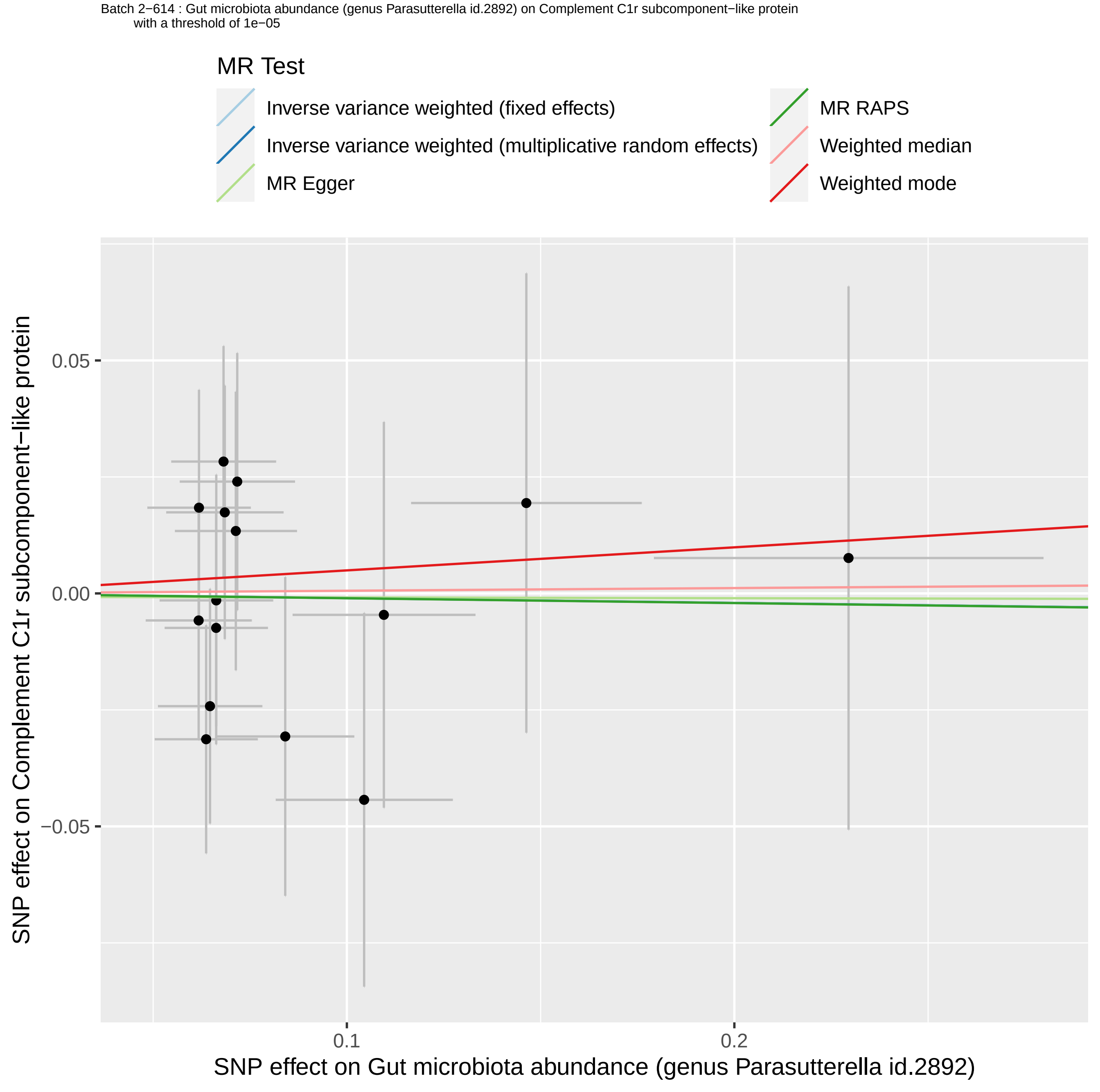
Batch 2−614 : Gut microbiota abundance (genus Parasutterella id.2892) on Complement C1r subcomponent−like protein
with a threshold of 1e−05
MR Test
Inverse variance weighted (fixed effects)
Inverse variance weighted (multiplicative random effects)
MR Egger
MR RAPS
Weighted median
Weighted mode
0.05
0.00
−0.05
0.1
0.2
SNP effect on Complement C1r subcomponent−like protein
SNP effect on Gut microbiota abundance (genus Parasutterella id.2892)

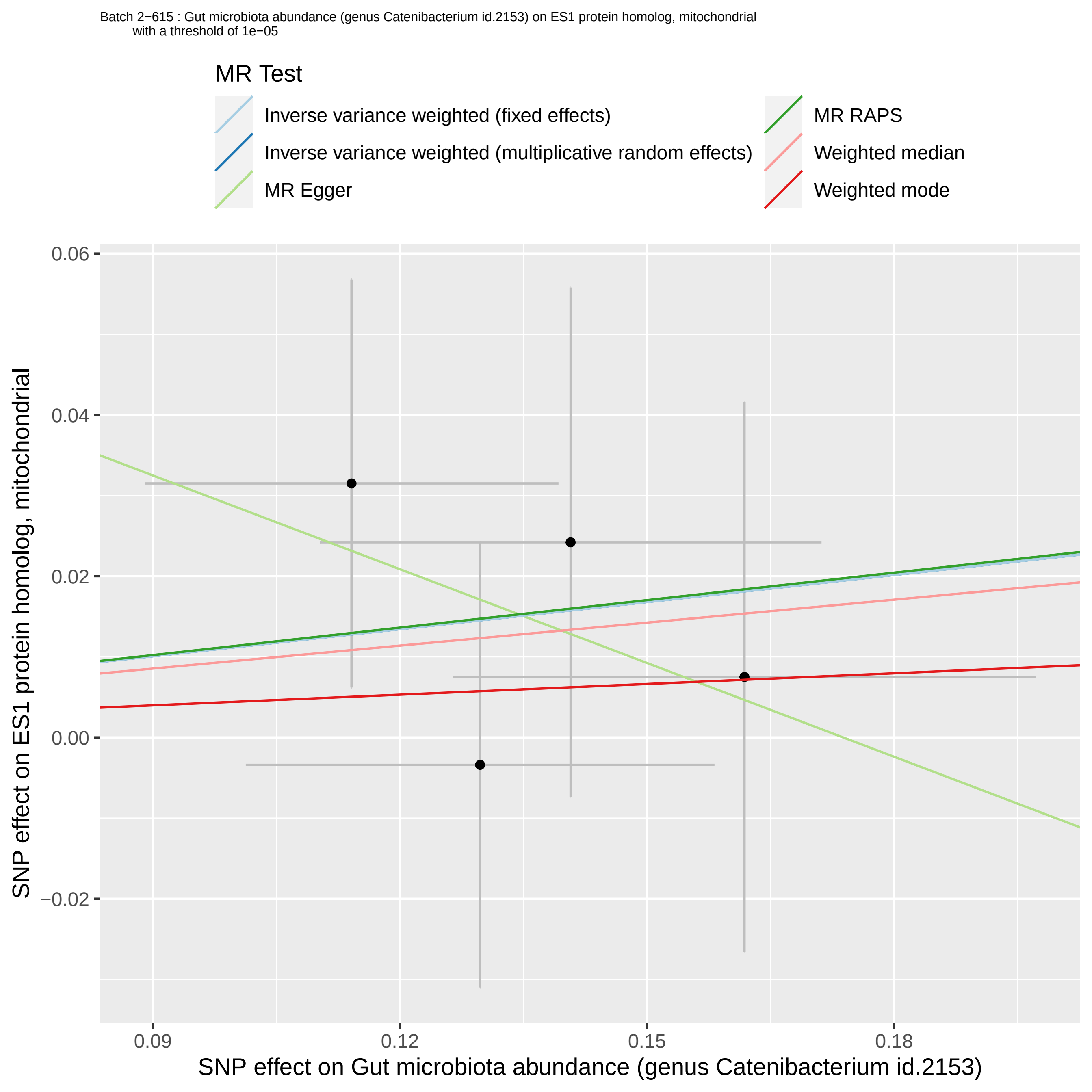

Batch 2−615 : Gut microbiota abundance (genus Catenibacterium id.2153) on ES1 protein homolog, mitochondrial
with a threshold of 1e−05
MR Test
Inverse variance weighted (fixed effects)
Inverse variance weighted (multiplicative random effects)
MR Egger
MR RAPS
Weighted median
Weighted mode
0.06
0.04
0.02
0.00
−0.02
0.09
0.12
0.15
0.18
SNP effect on ES1 protein homolog, mitochondrial
SNP effect on Gut microbiota abundance (genus Catenibacterium id.2153)

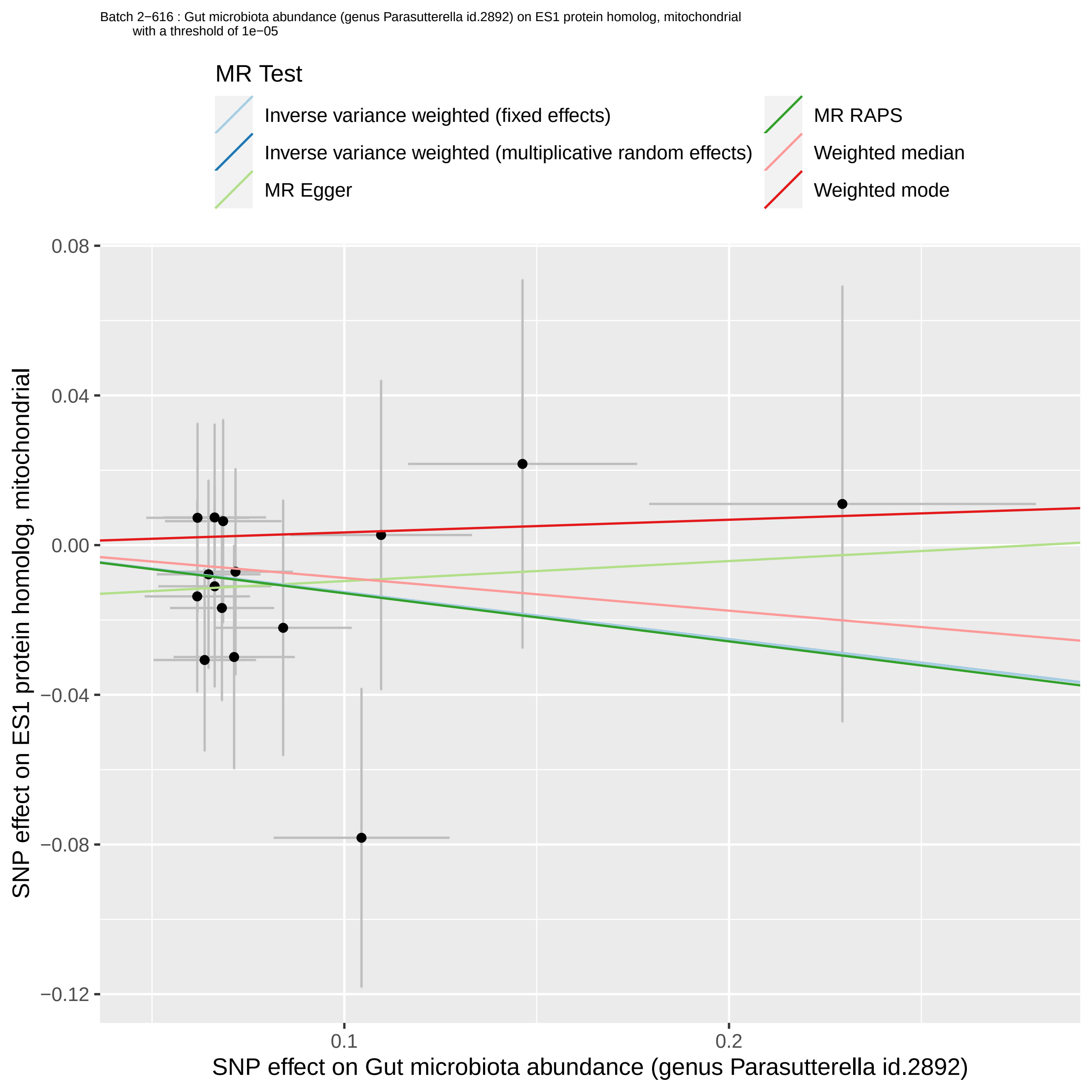
Batch 2−616 : Gut microbiota abundance (genus Parasutterella id.2892) on ES1 protein homolog, mitochondrial
with a threshold of 1e−05
MR Test
Inverse variance weighted (fixed effects)
Inverse variance weighted (multiplicative random effects)
MR Egger
MR RAPS
Weighted median
Weighted mode
0.08
0.04
0.00
−0.04
−0.08
−0.12
0.1
0.2
SNP effect on ES1 protein homolog, mitochondrial
SNP effect on Gut microbiota abundance (genus Parasutterella id.2892)

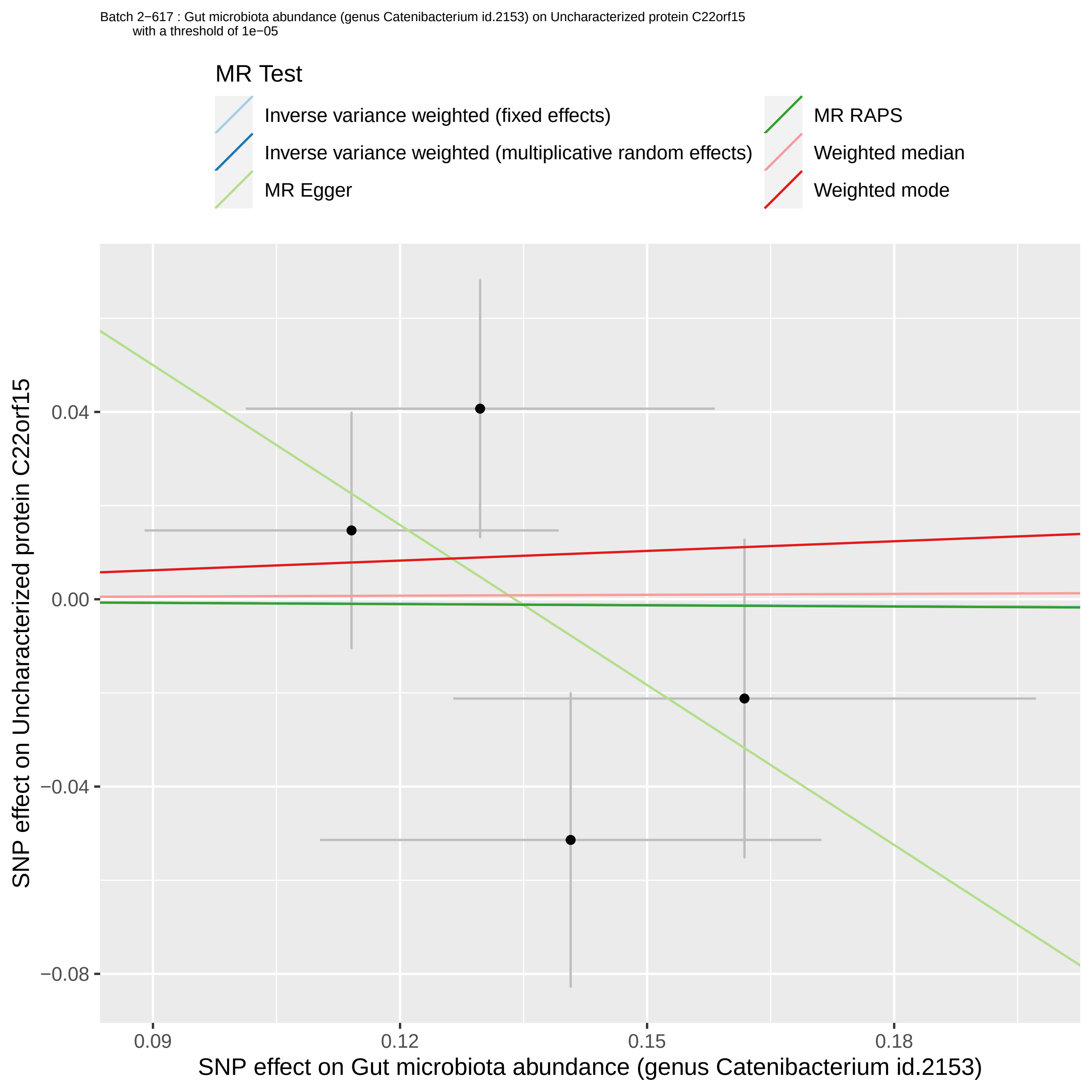
Batch 2−617 : Gut microbiota abundance (genus Catenibacterium id.2153) on Uncharacterized protein C22orf15
with a threshold of 1e−05
MR Test
Inverse variance weighted (fixed effects)
Inverse variance weighted (multiplicative random effects)
MR Egger
MR RAPS
Weighted median
Weighted mode
0.04
0.00
−0.04
−0.08
0.09
0.12
0.15
0.18
SNP effect on Uncharacterized protein C22orf15
SNP effect on Gut microbiota abundance (genus Catenibacterium id.2153)

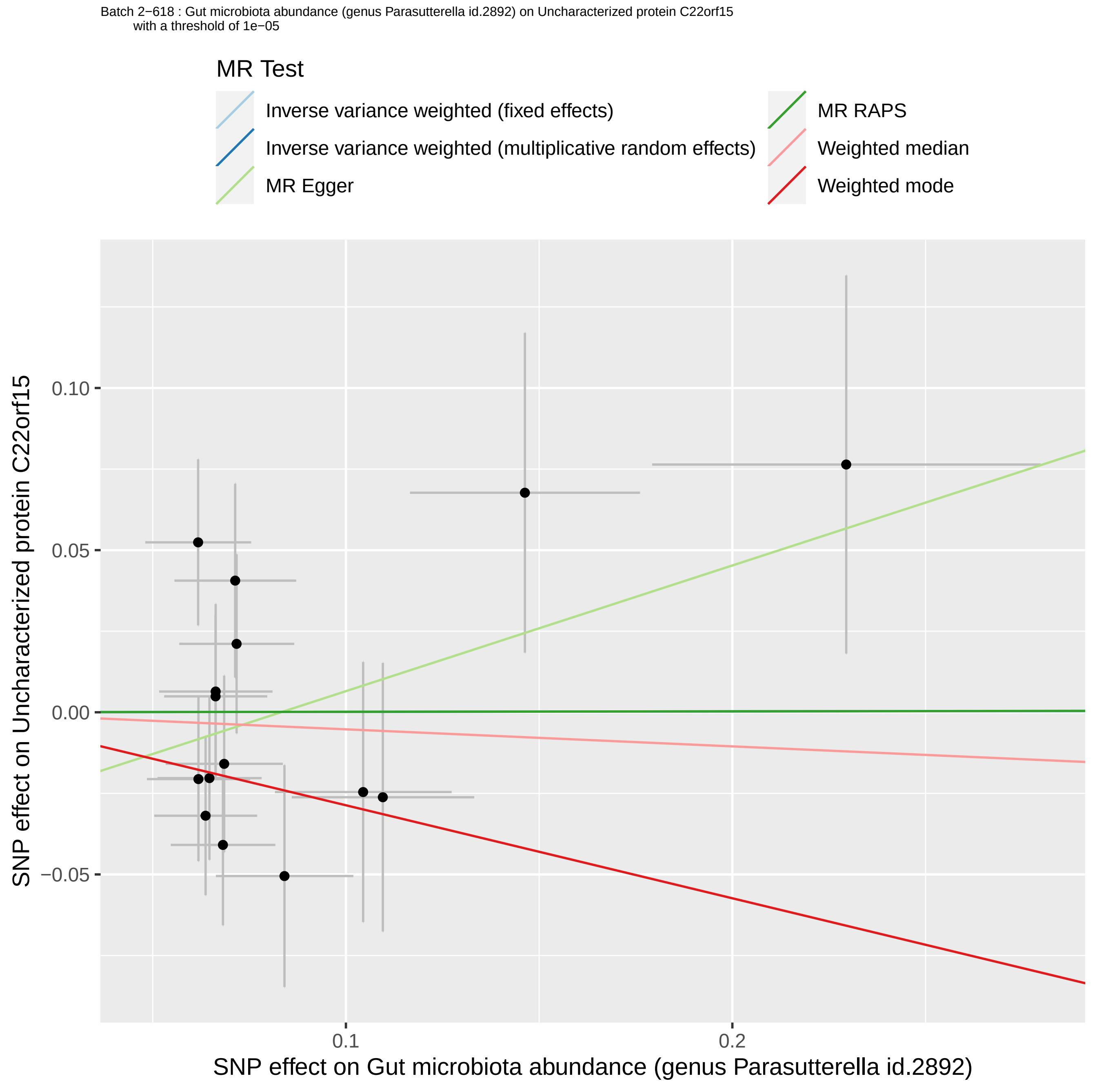

Batch 2−618 : Gut microbiota abundance (genus Parasutterella id.2892) on Uncharacterized protein C22orf15
with a threshold of 1e−05
MR Test
Inverse variance weighted (fixed effects)
Inverse variance weighted (multiplicative random effects)
MR Egger
MR RAPS
Weighted median
Weighted mode
0.10
0.05
0.00
−0.05
0.1
0.2
SNP effect on Uncharacterized protein C22orf15
SNP effect on Gut microbiota abundance (genus Parasutterella id.2892)

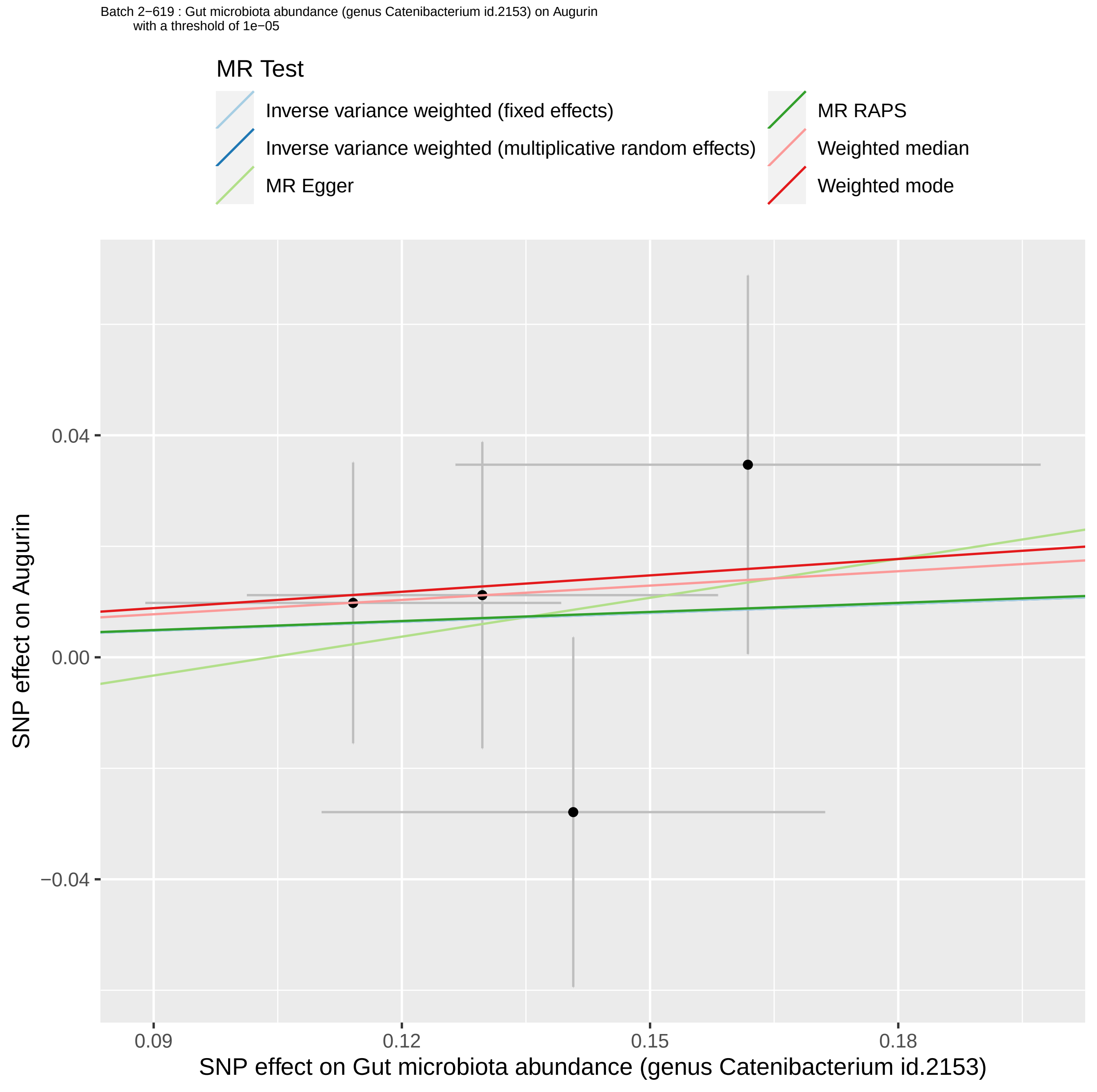

Batch 2−619 : Gut microbiota abundance (genus Catenibacterium id.2153) on Augurin
with a threshold of 1e−05
MR Test
Inverse variance weighted (fixed effects)
Inverse variance weighted (multiplicative random effects)
MR Egger
MR RAPS
Weighted median
Weighted mode
0.04
0.00
−0.04
0.09
0.12
0.15
0.18
SNP effect on Augurin
SNP effect on Gut microbiota abundance (genus Catenibacterium id.2153)

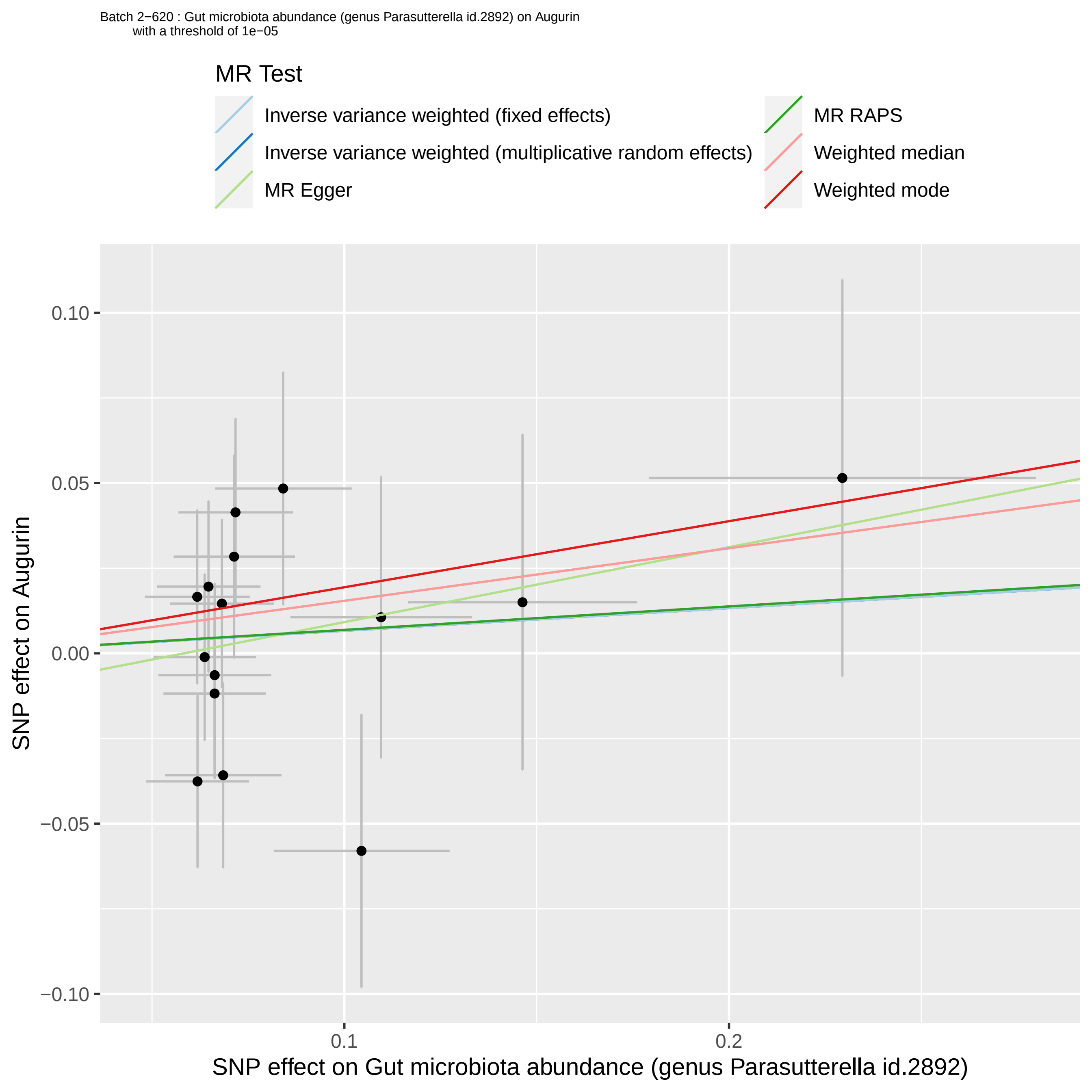
Batch 2−620 : Gut microbiota abundance (genus Parasutterella id.2892) on Augurin
with a threshold of 1e−05
MR Test
Inverse variance weighted (fixed effects)
Inverse variance weighted (multiplicative random effects)
MR Egger
MR RAPS
Weighted median
Weighted mode
0.10
0.05
0.00
−0.05
−0.10
0.1
0.2
SNP effect on Augurin
SNP effect on Gut microbiota abundance (genus Parasutterella id.2892)

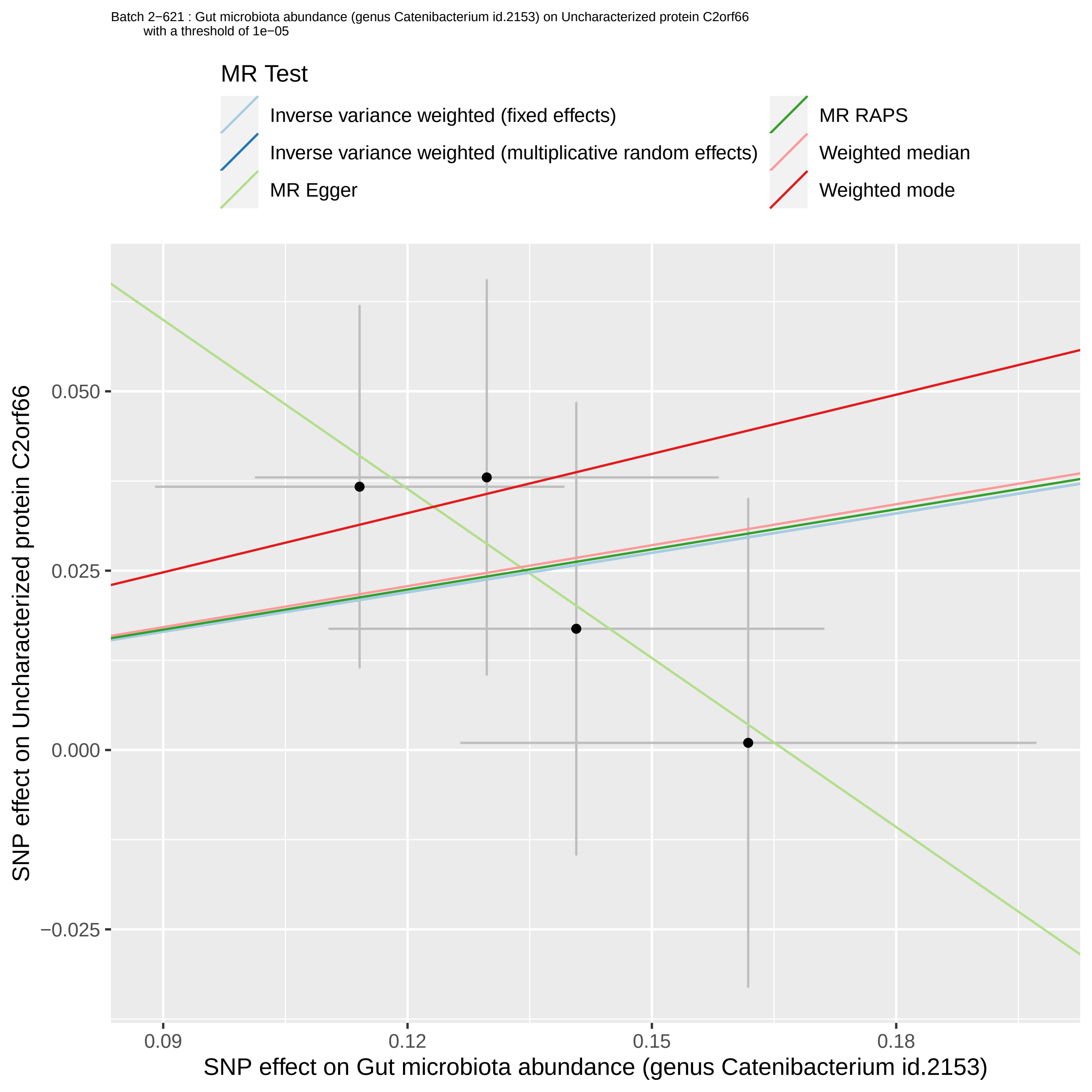
Batch 2−621 : Gut microbiota abundance (genus Catenibacterium id.2153) on Uncharacterized protein C2orf66
with a threshold of 1e−05
MR Test
Inverse variance weighted (fixed effects)
Inverse variance weighted (multiplicative random effects)
MR Egger
MR RAPS
Weighted median
Weighted mode
SNP effect on Uncharacterized protein C2orf66
0.050
0.025
0.000
−0.025
0.09
0.12
0.15
0.18
SNP effect on Gut microbiota abundance (genus Catenibacterium id.2153)

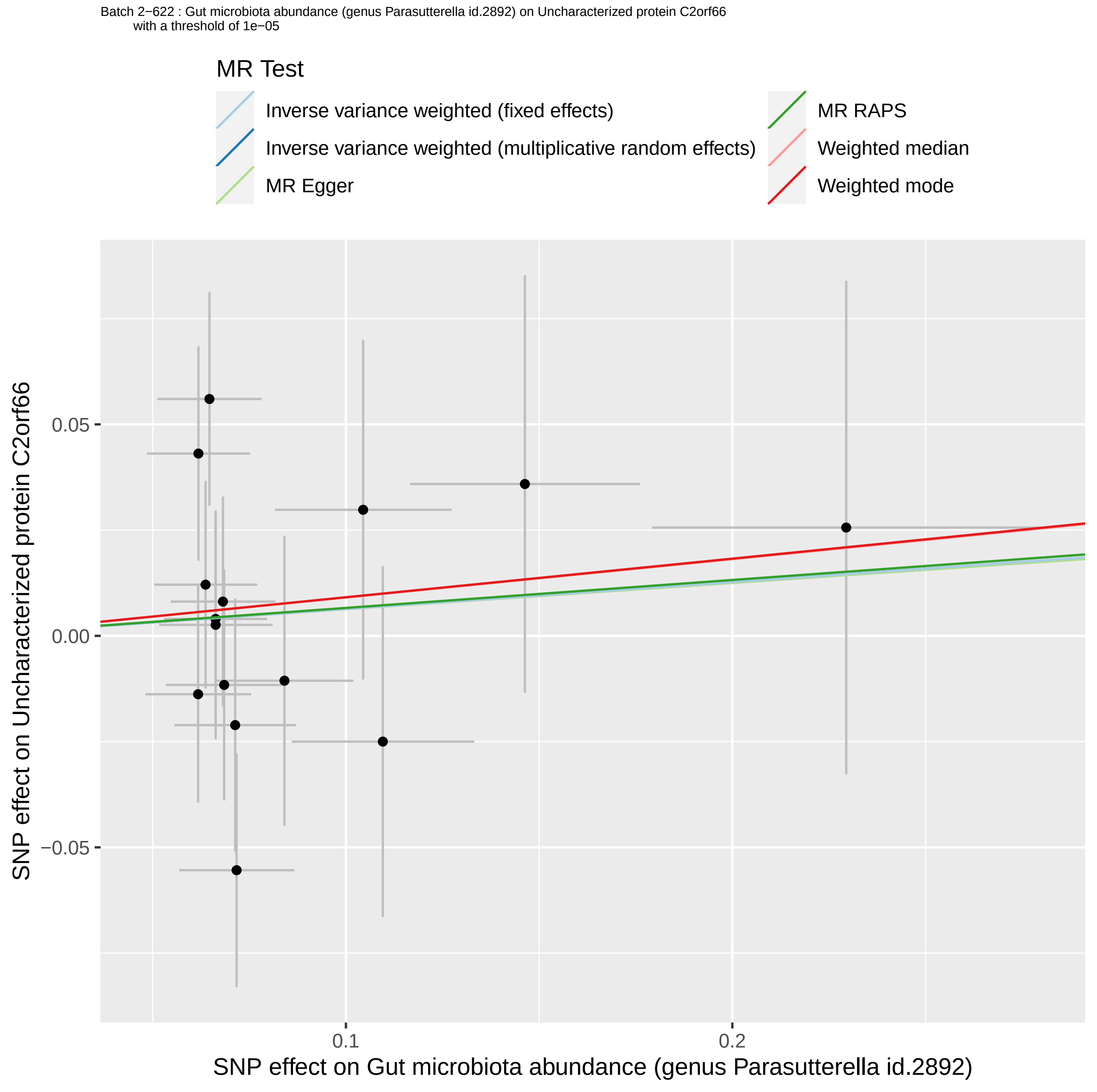
Batch 2−622 : Gut microbiota abundance (genus Parasutterella id.2892) on Uncharacterized protein C2orf66
with a threshold of 1e−05
MR Test
Inverse variance weighted (fixed effects)
Inverse variance weighted (multiplicative random effects)
MR Egger
MR RAPS
Weighted median
Weighted mode
0.05
0.00
−0.05
0.1
0.2
SNP effect on Uncharacterized protein C2orf66
SNP effect on Gut microbiota abundance (genus Parasutterella id.2892)

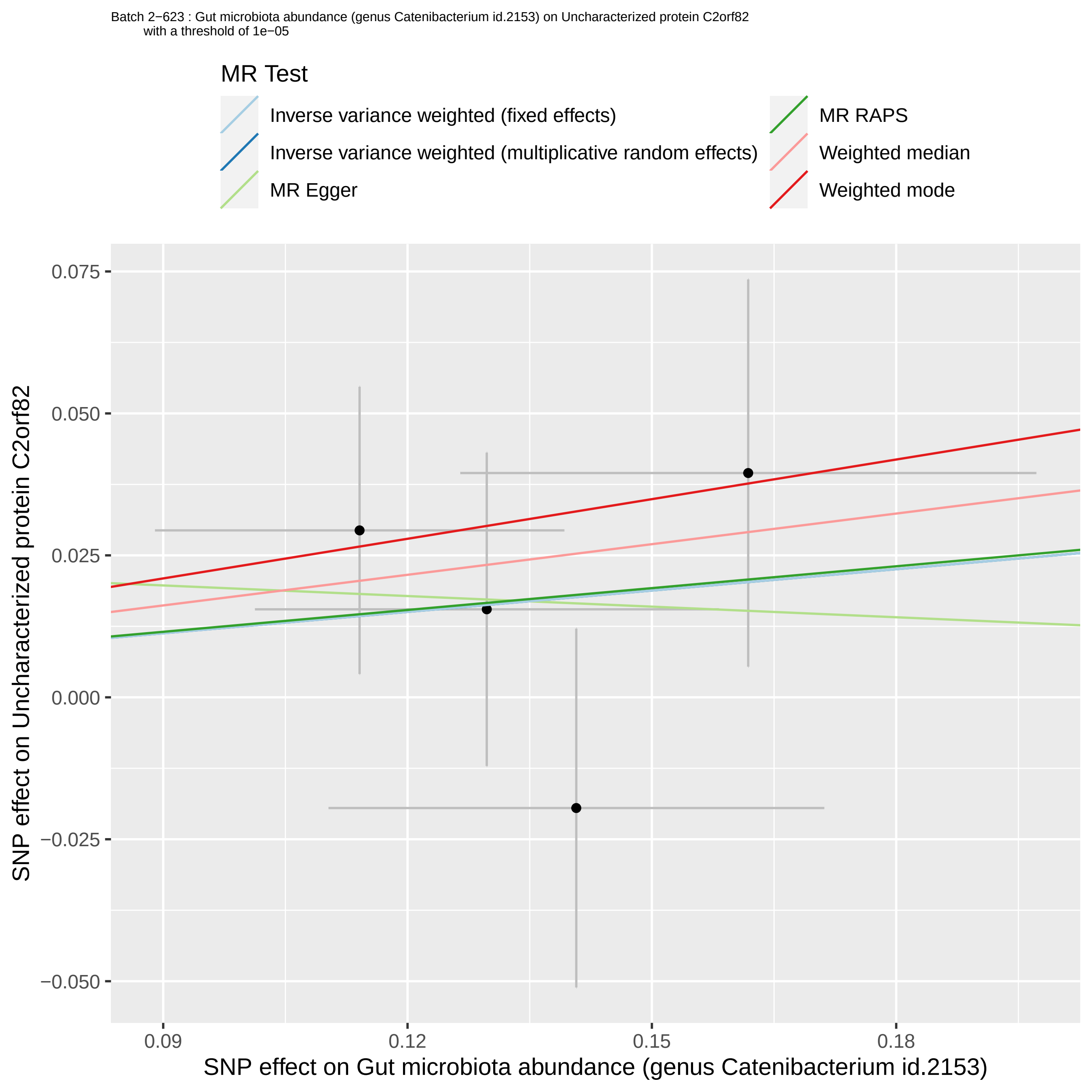
Batch 2−623 : Gut microbiota abundance (genus Catenibacterium id.2153) on Uncharacterized protein C2orf82
with a threshold of 1e−05
MR Test
Inverse variance weighted (fixed effects)
Inverse variance weighted (multiplicative random effects)
MR Egger
MR RAPS
Weighted median
Weighted mode
SNP effect on Uncharacterized protein C2orf82
0.075
0.050
0.025
0.000
−0.025
−0.050
0.09
0.12
0.15
0.18
SNP effect on Gut microbiota abundance (genus Catenibacterium id.2153)

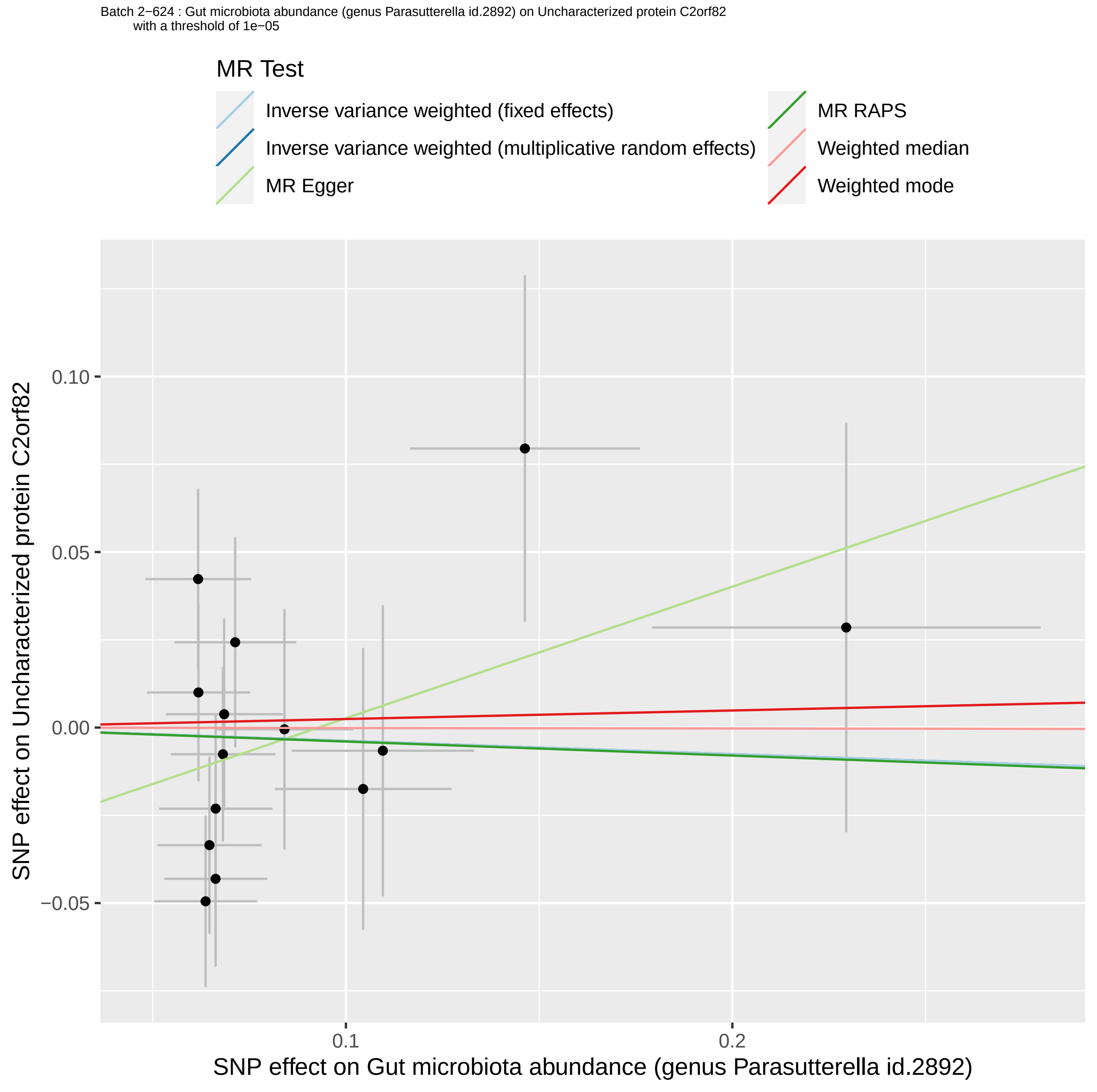

Batch 2−624 : Gut microbiota abundance (genus Parasutterella id.2892) on Uncharacterized protein C2orf82
with a threshold of 1e−05
MR Test
Inverse variance weighted (fixed effects)
Inverse variance weighted (multiplicative random effects)
MR Egger
MR RAPS
Weighted median
Weighted mode
0.10
0.05
0.00
−0.05
0.1
0.2
SNP effect on Uncharacterized protein C2orf82
SNP effect on Gut microbiota abundance (genus Parasutterella id.2892)

Batch 2−625 : Gut microbiota abundance (genus Catenibacterium id.2153) on C3a anaphylatoxin des Arginine
with a threshold of 1e−05

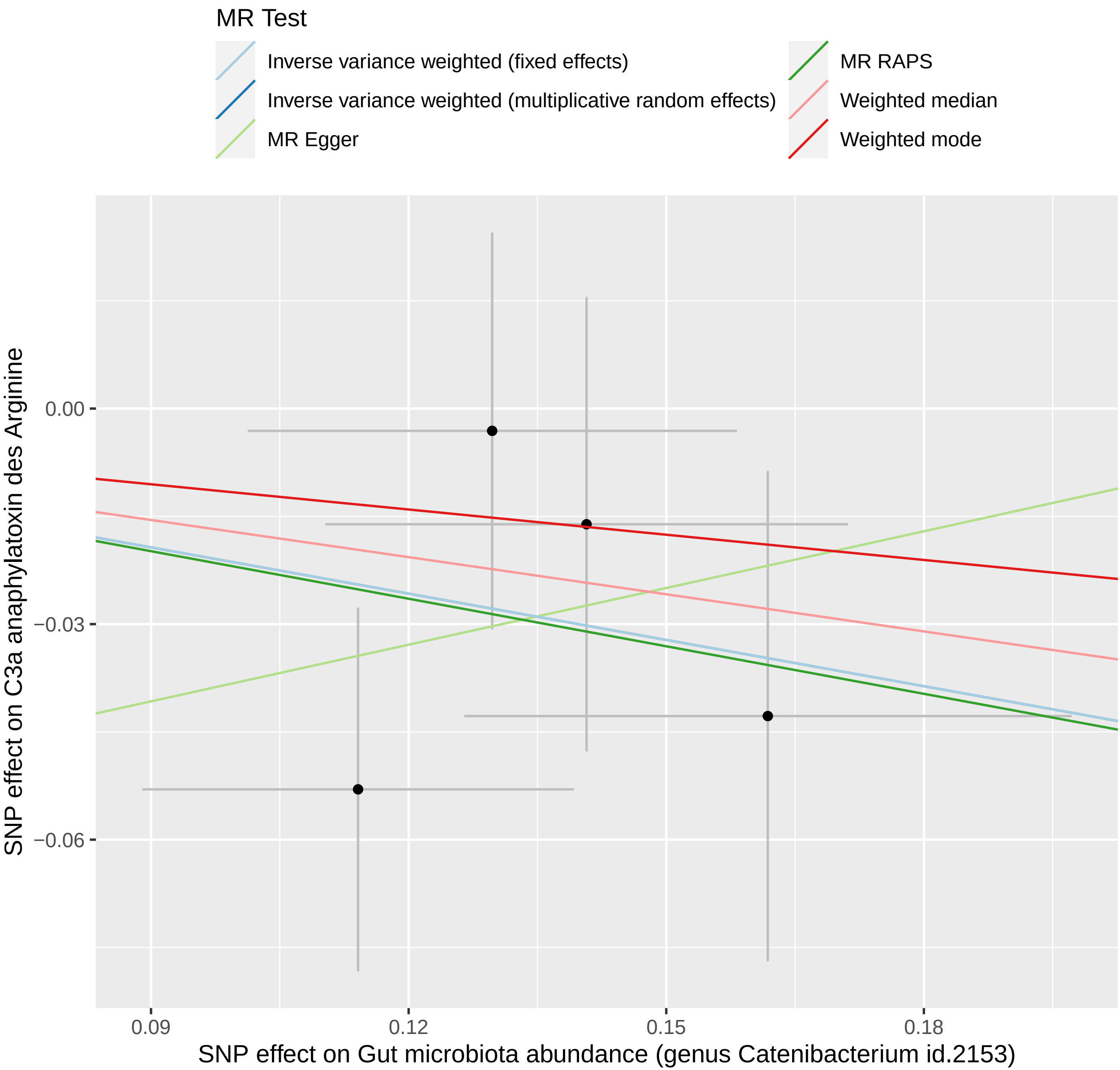

Batch 2−626 : Gut microbiota abundance (genus Parasutterella id.2892) on C3a anaphylatoxin des Arginine
with a threshold of 1e−05

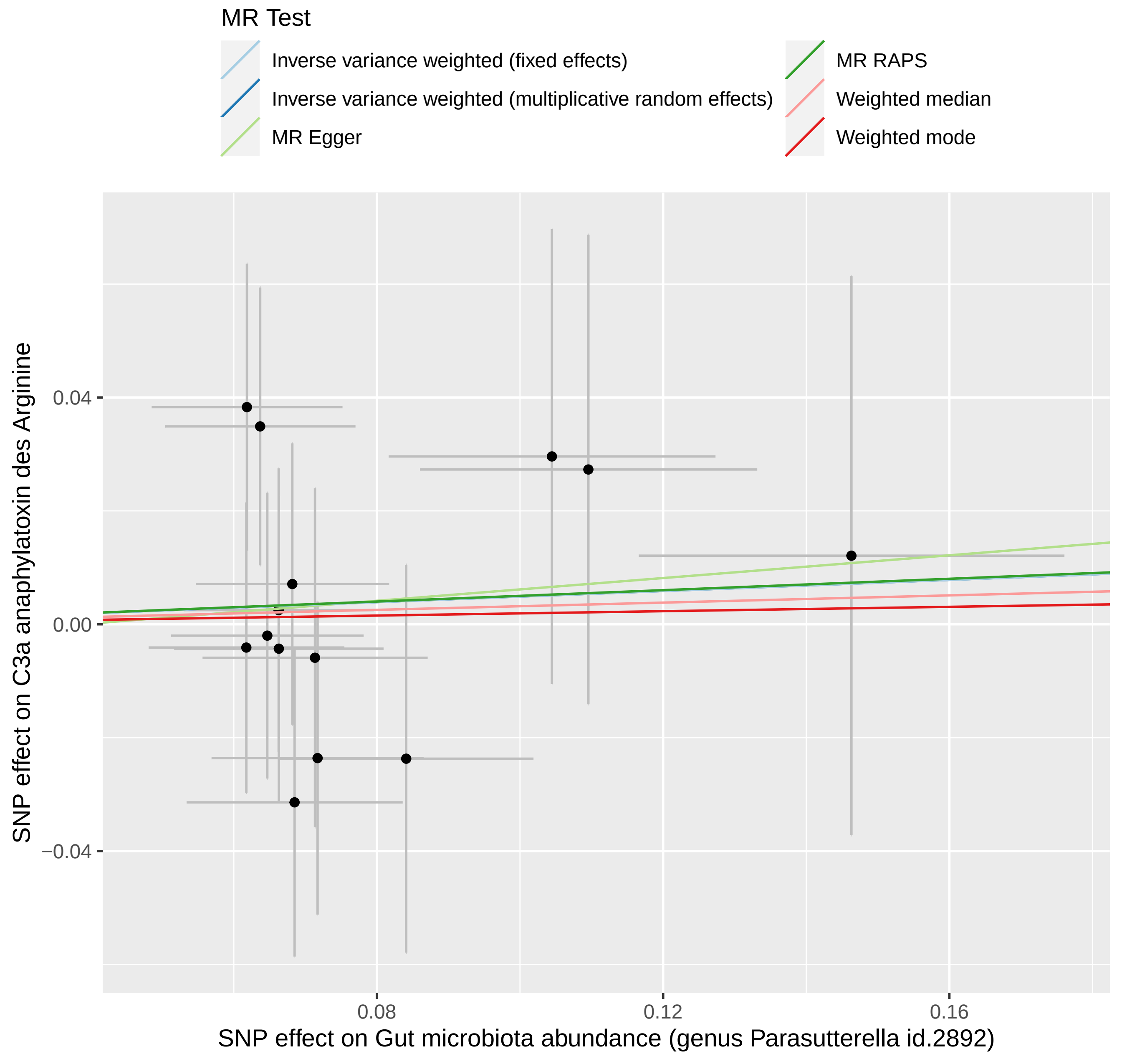

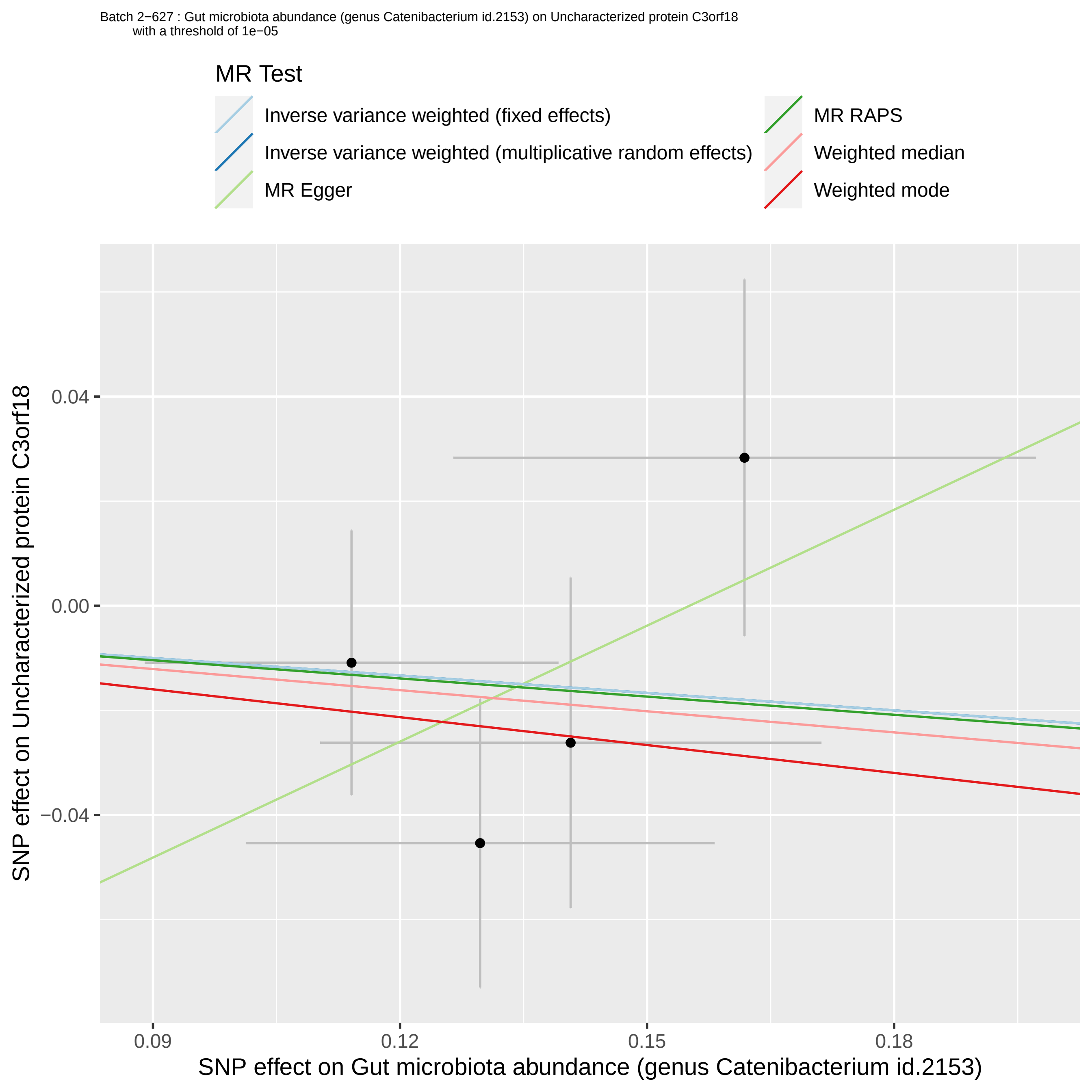

Batch 2−627 : Gut microbiota abundance (genus Catenibacterium id.2153) on Uncharacterized protein C3orf18
with a threshold of 1e−05
MR Test
Inverse variance weighted (fixed effects)
Inverse variance weighted (multiplicative random effects)
MR Egger
MR RAPS
Weighted median
Weighted mode
0.04
0.00
−0.04
0.09
0.12
0.15
0.18
SNP effect on Uncharacterized protein C3orf18
SNP effect on Gut microbiota abundance (genus Catenibacterium id.2153)

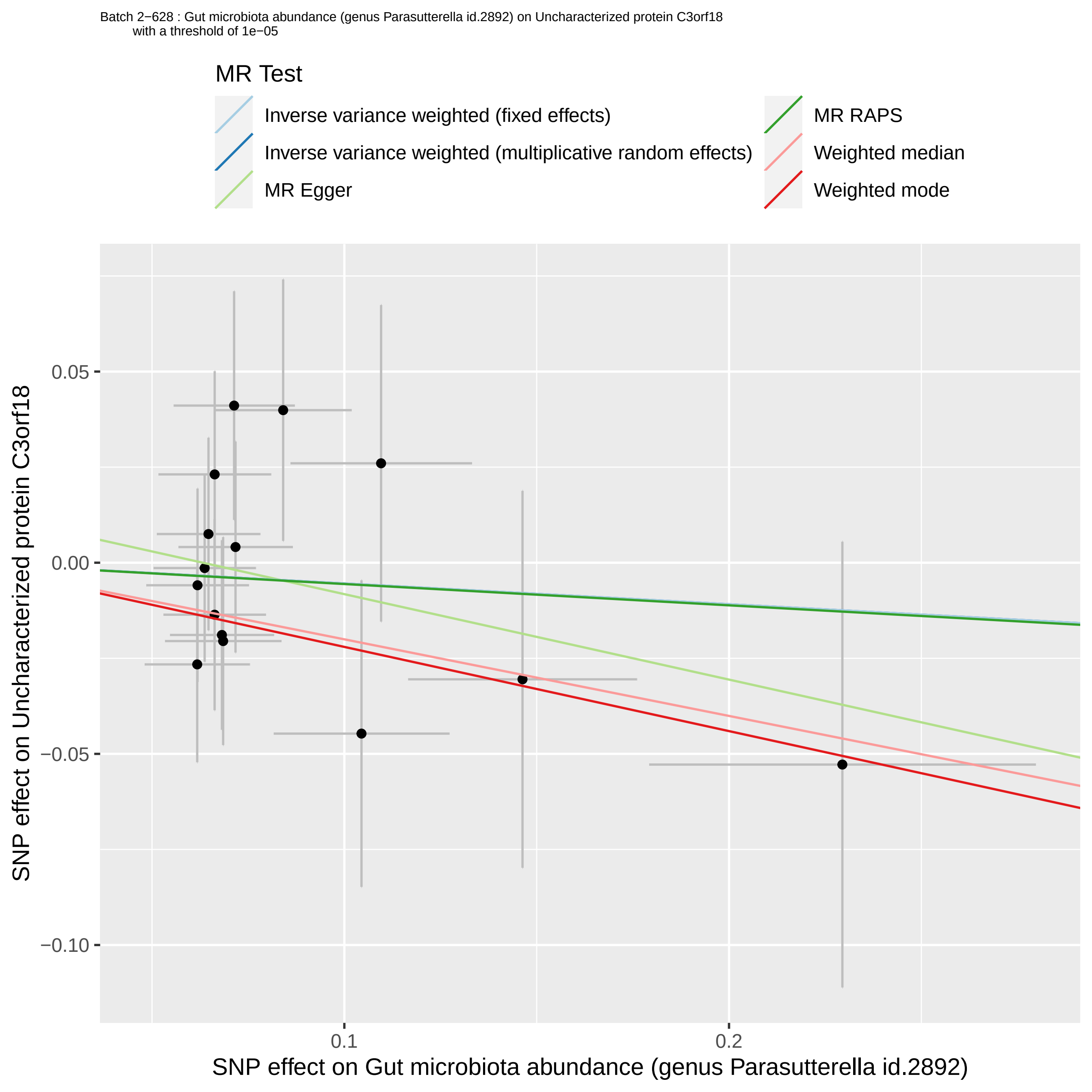
Batch 2−628 : Gut microbiota abundance (genus Parasutterella id.2892) on Uncharacterized protein C3orf18
with a threshold of 1e−05
MR Test
Inverse variance weighted (fixed effects)
Inverse variance weighted (multiplicative random effects)
MR Egger
MR RAPS
Weighted median
Weighted mode
0.05
0.00
−0.05
−0.10
0.1
0.2
SNP effect on Uncharacterized protein C3orf18
SNP effect on Gut microbiota abundance (genus Parasutterella id.2892)

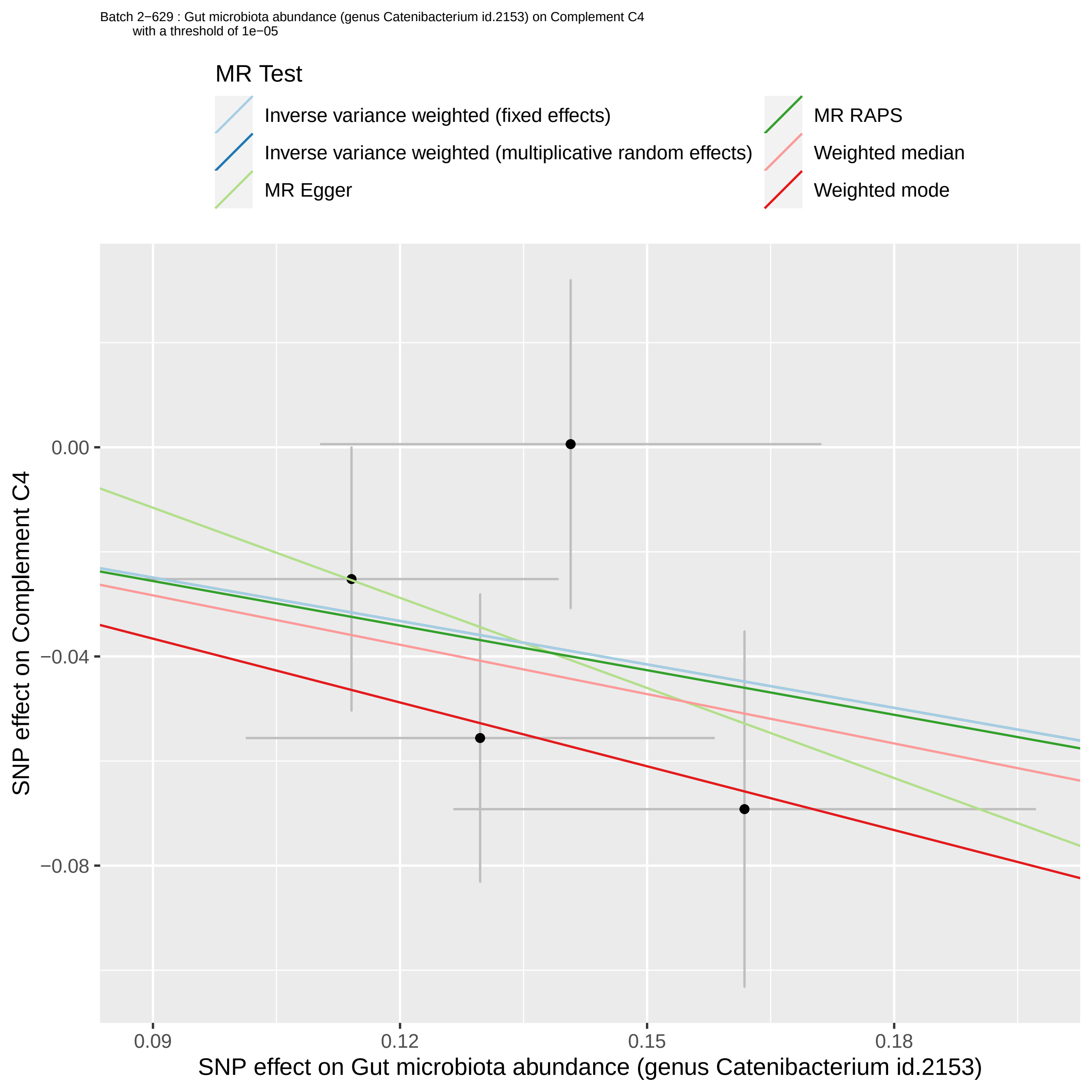
Batch 2−629 : Gut microbiota abundance (genus Catenibacterium id.2153) on Complement C4
with a threshold of 1e−05
MR Test
Inverse variance weighted (fixed effects)
Inverse variance weighted (multiplicative random effects)
MR Egger
MR RAPS
Weighted median
Weighted mode
SNP effect on Complement C4
0.00
−0.04
−0.08
0.09
0.12
0.15
0.18
SNP effect on Gut microbiota abundance (genus Catenibacterium id.2153)

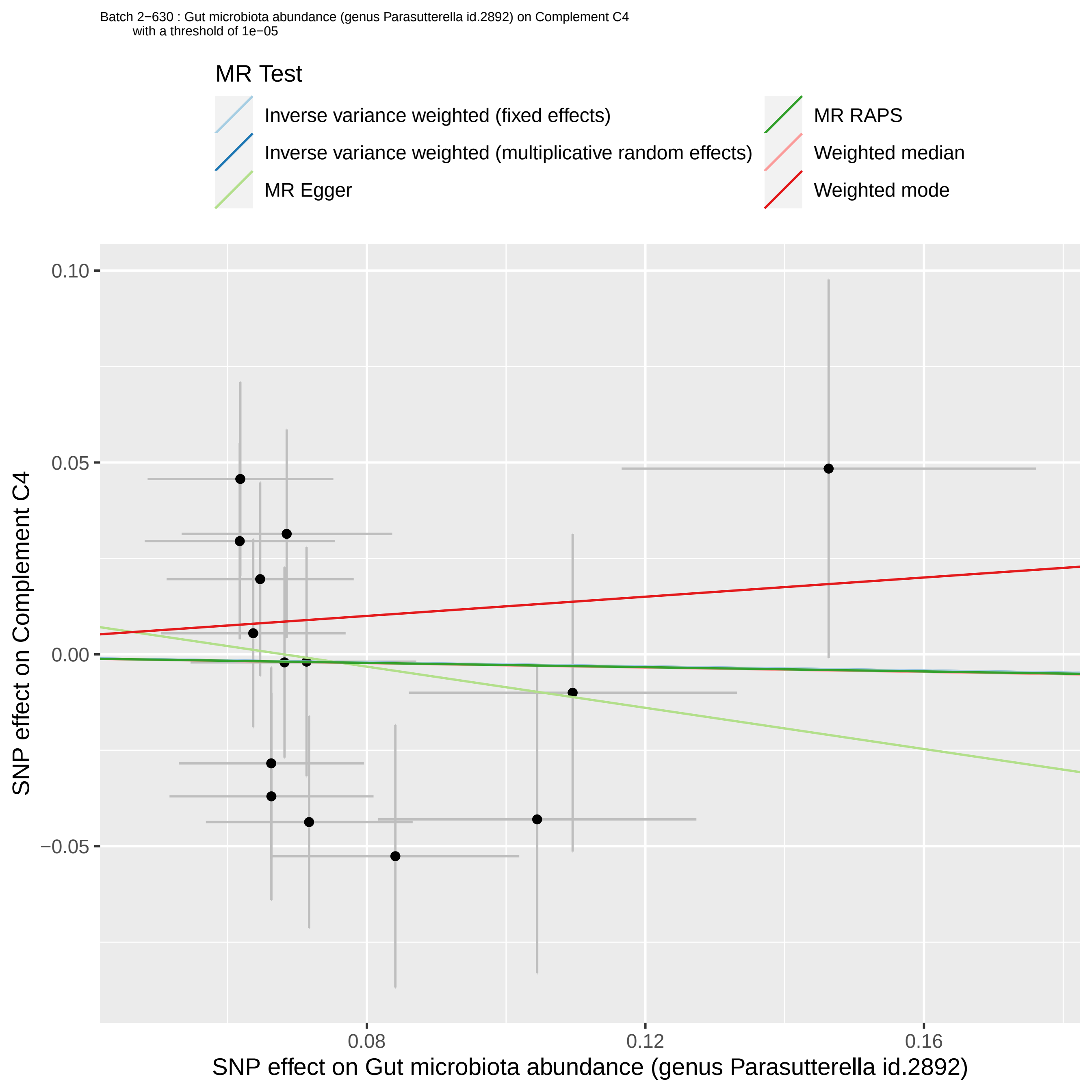

Batch 2−630 : Gut microbiota abundance (genus Parasutterella id.2892) on Complement C4
with a threshold of 1e−05
MR Test
Inverse variance weighted (fixed effects)
Inverse variance weighted (multiplicative random effects)
MR Egger
MR RAPS
Weighted median
Weighted mode
0.10
0.05
0.00
−0.05
0.08
0.12
0.16
SNP effect on Complement C4
SNP effect on Gut microbiota abundance (genus Parasutterella id.2892)

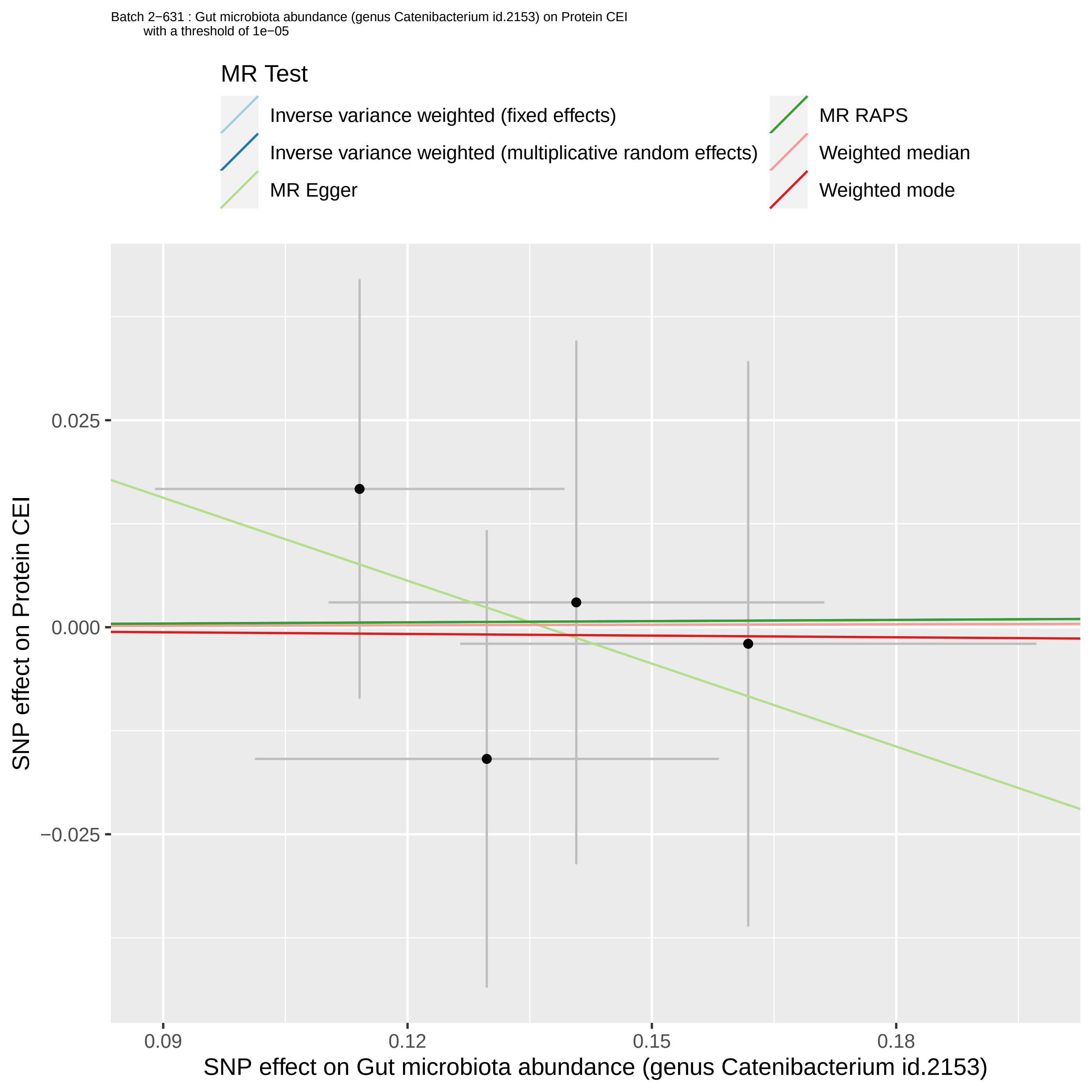
Batch 2−631 : Gut microbiota abundance (genus Catenibacterium id.2153) on Protein CEI
with a threshold of 1e−05
MR Test
Inverse variance weighted (fixed effects)
Inverse variance weighted (multiplicative random effects)
MR Egger
MR RAPS
Weighted median
Weighted mode
0.025
0.000
−0.025
0.09
0.12
0.15
0.18
SNP effect on Protein CEI
SNP effect on Gut microbiota abundance (genus Catenibacterium id.2153)

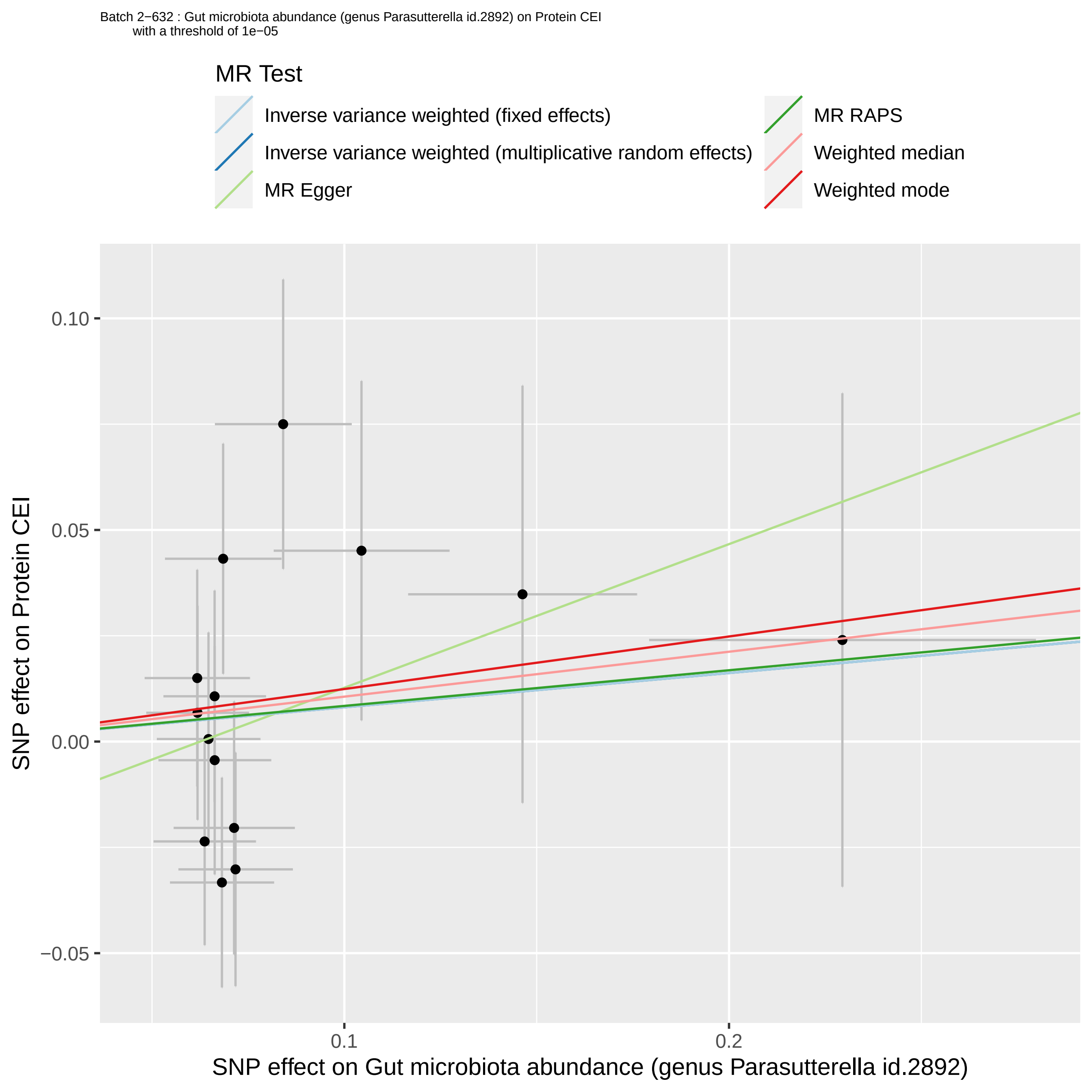
Batch 2−632 : Gut microbiota abundance (genus Parasutterella id.2892) on Protein CEI
with a threshold of 1e−05
MR Test
Inverse variance weighted (fixed effects)
Inverse variance weighted (multiplicative random effects)
MR Egger
MR RAPS
Weighted median
Weighted mode
0.10
0.05
0.00
−0.05
0.1
0.2
SNP effect on Protein CEI
SNP effect on Gut microbiota abundance (genus Parasutterella id.2892)

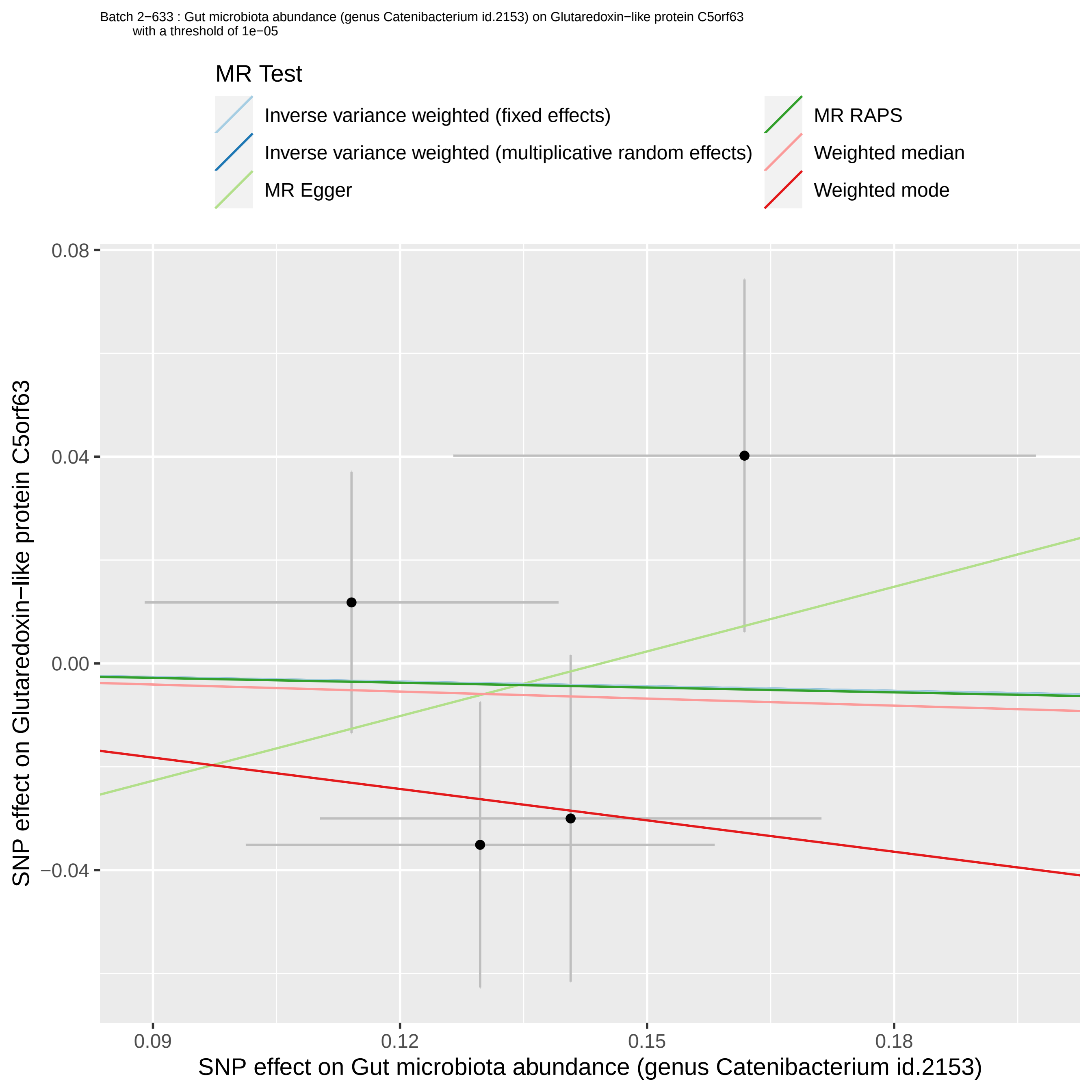
Batch 2−633 : Gut microbiota abundance (genus Catenibacterium id.2153) on Glutaredoxin−like protein C5orf63
with a threshold of 1e−05
MR Test
Inverse variance weighted (fixed effects)
Inverse variance weighted (multiplicative random effects)
MR Egger
MR RAPS
Weighted median
Weighted mode
0.08
0.04
0.00
−0.04
0.09
0.12
0.15
0.18
SNP effect on Glutaredoxin−like protein C5orf63
SNP effect on Gut microbiota abundance (genus Catenibacterium id.2153)

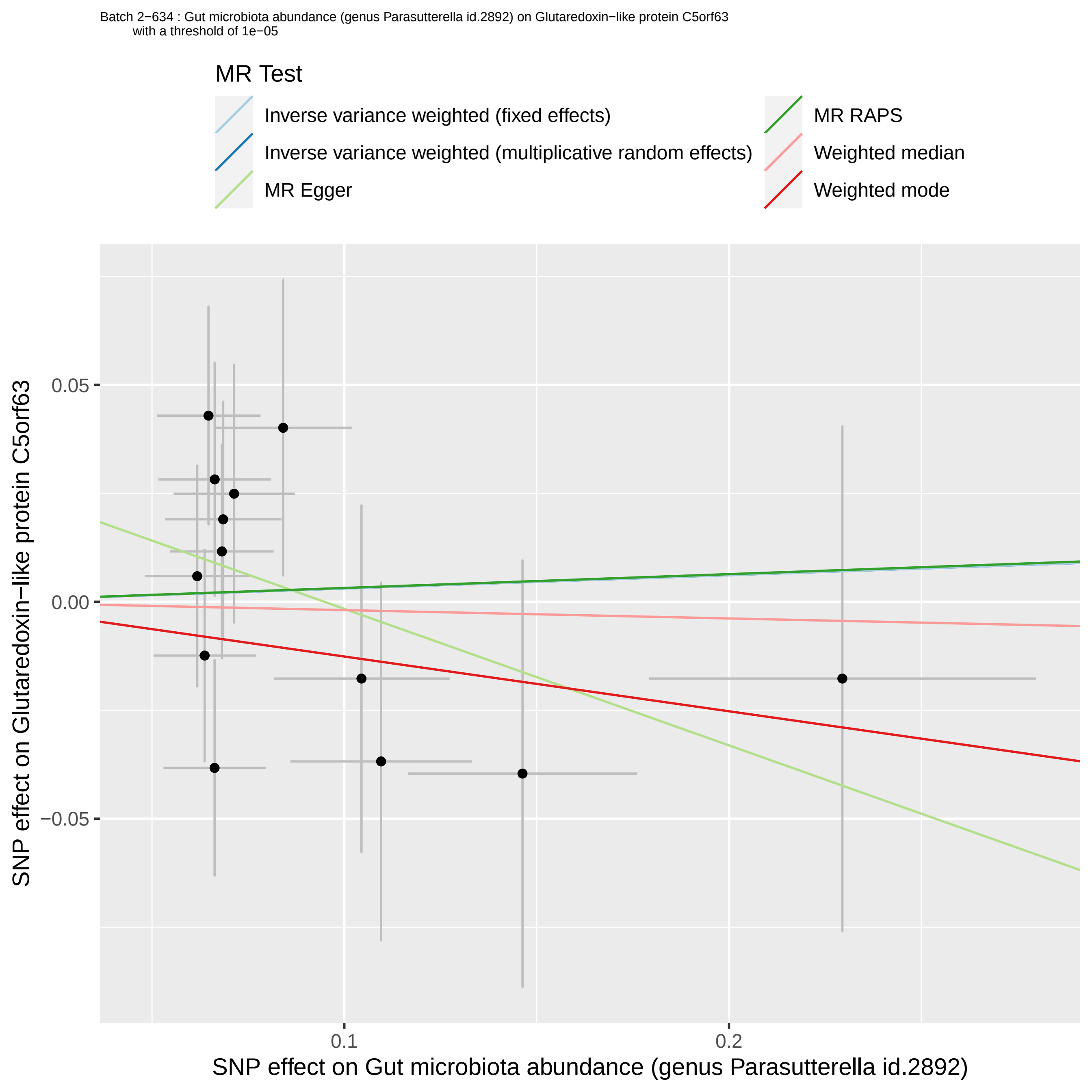
Batch 2−634 : Gut microbiota abundance (genus Parasutterella id.2892) on Glutaredoxin−like protein C5orf63
with a threshold of 1e−05
MR Test
Inverse variance weighted (fixed effects)
Inverse variance weighted (multiplicative random effects)
MR Egger
MR RAPS
Weighted median
Weighted mode
0.05
0.00
−0.05
0.1
0.2
SNP effect on Glutaredoxin−like protein C5orf63
SNP effect on Gut microbiota abundance (genus Parasutterella id.2892)

Batch 2−635 : Gut microbiota abundance (genus Catenibacterium id.2153) on Complement component C6
with a threshold of 1e−05

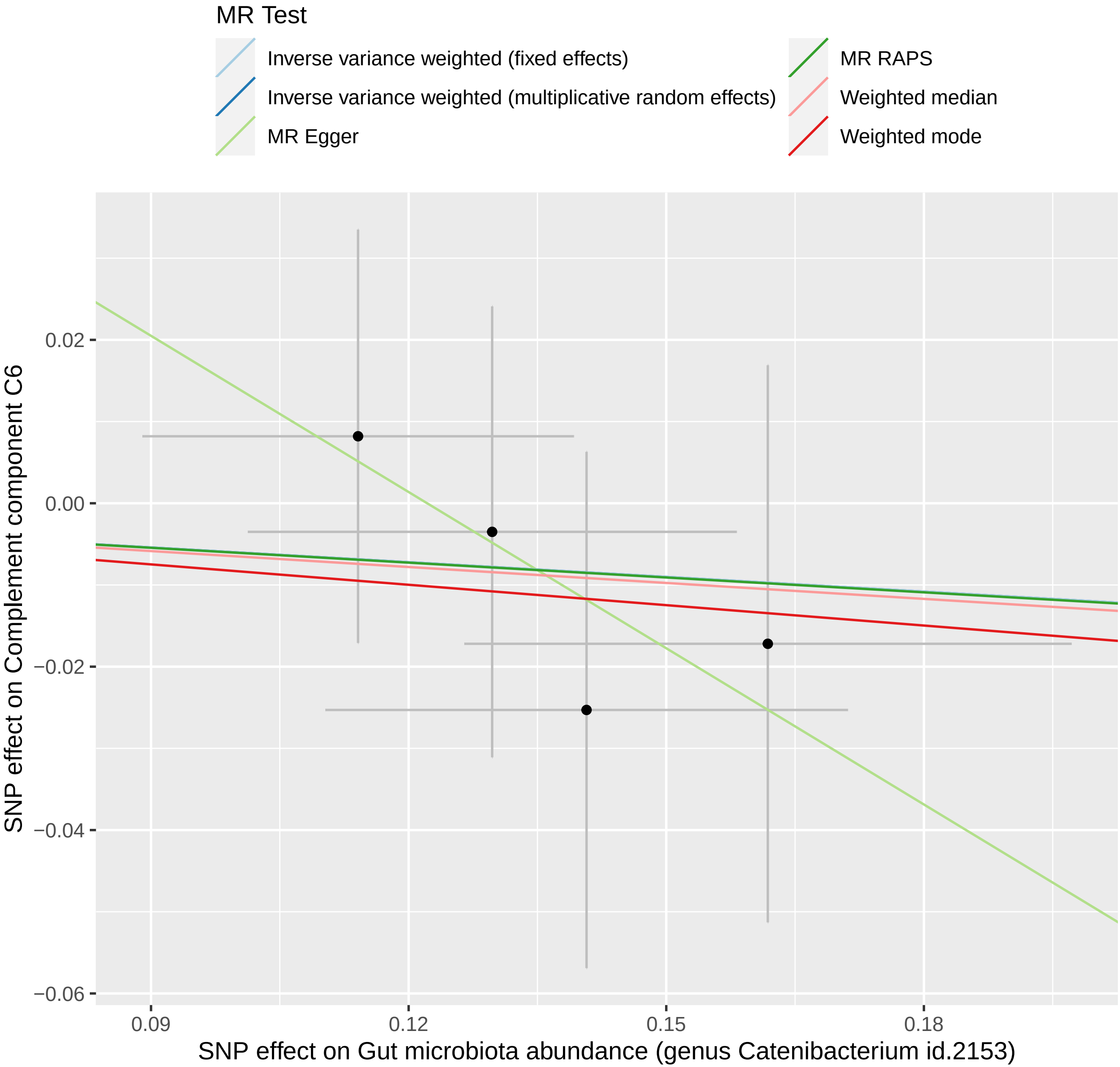

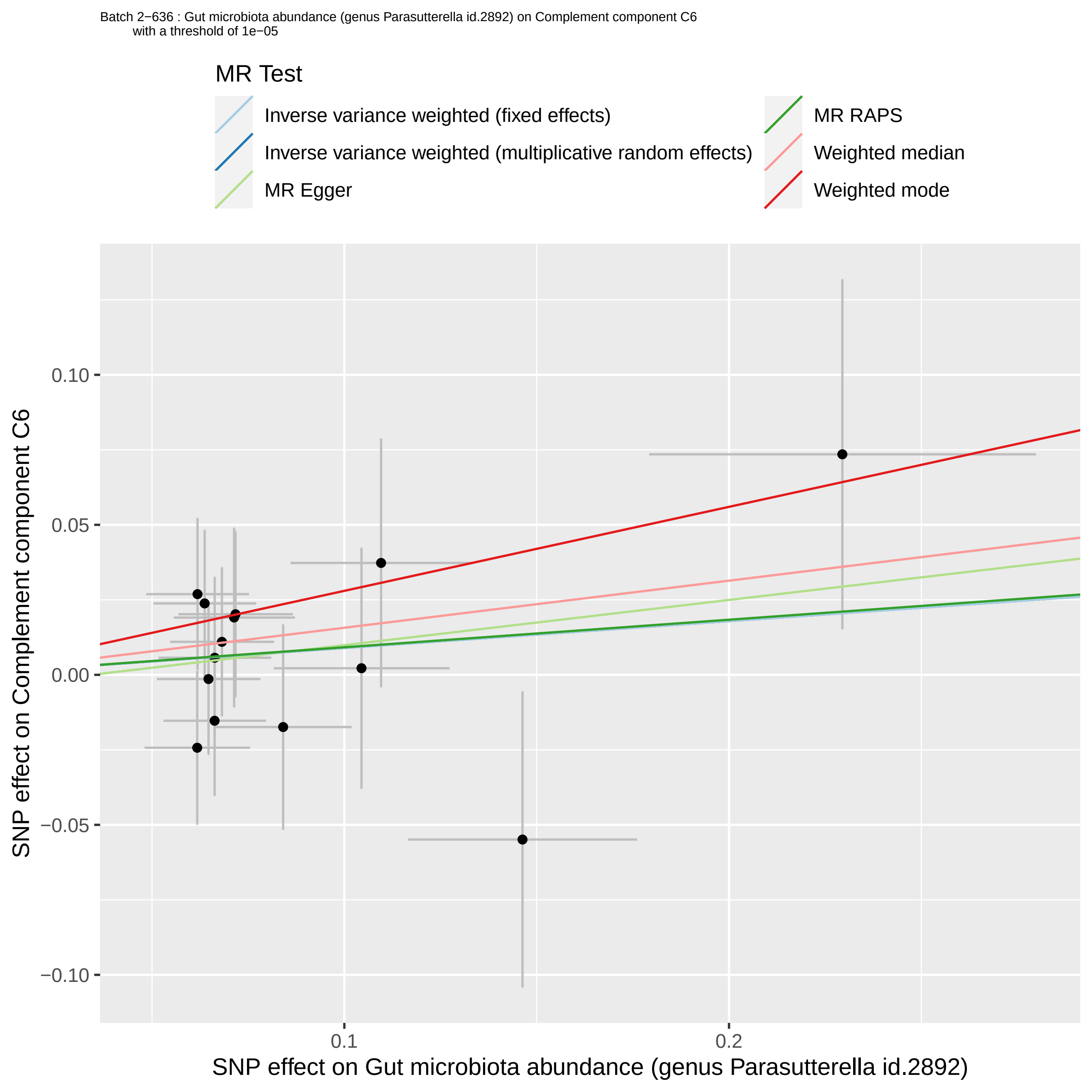

Batch 2−636 : Gut microbiota abundance (genus Parasutterella id.2892) on Complement component C6
with a threshold of 1e−05
MR Test
Inverse variance weighted (fixed effects)
Inverse variance weighted (multiplicative random effects)
MR Egger
MR RAPS
Weighted median
Weighted mode
0.10
0.05
0.00
−0.05
−0.10
0.1
0.2
SNP effect on Complement component C6
SNP effect on Gut microbiota abundance (genus Parasutterella id.2892)

Batch 2−637 : Gut microbiota abundance (genus Catenibacterium id.2153) on Uncharacterized protein C6orf226
with a threshold of 1e−05

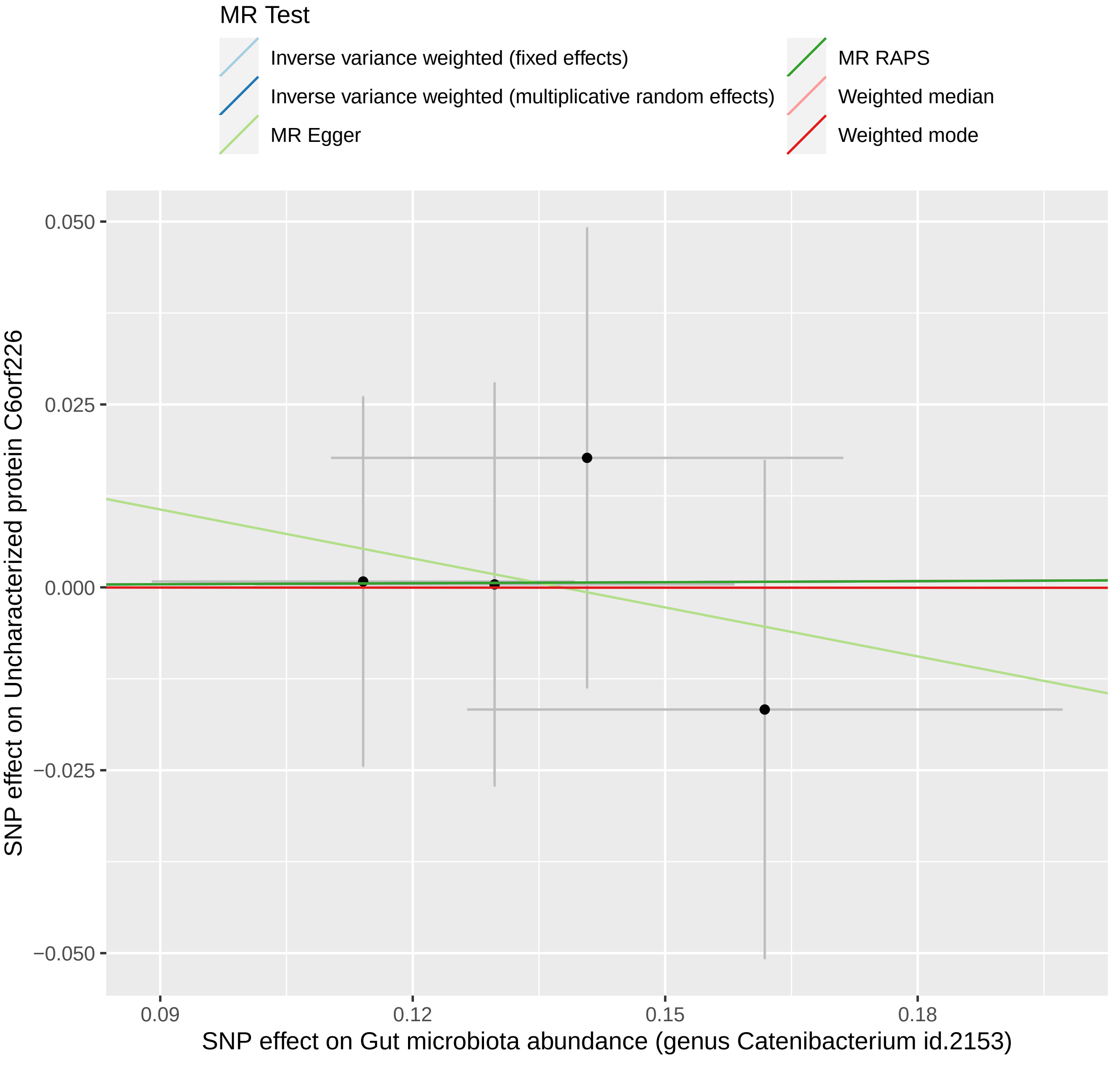

Batch 2−638 : Gut microbiota abundance (genus Parasutterella id.2892) on Uncharacterized protein C6orf226
with a threshold of 1e−05

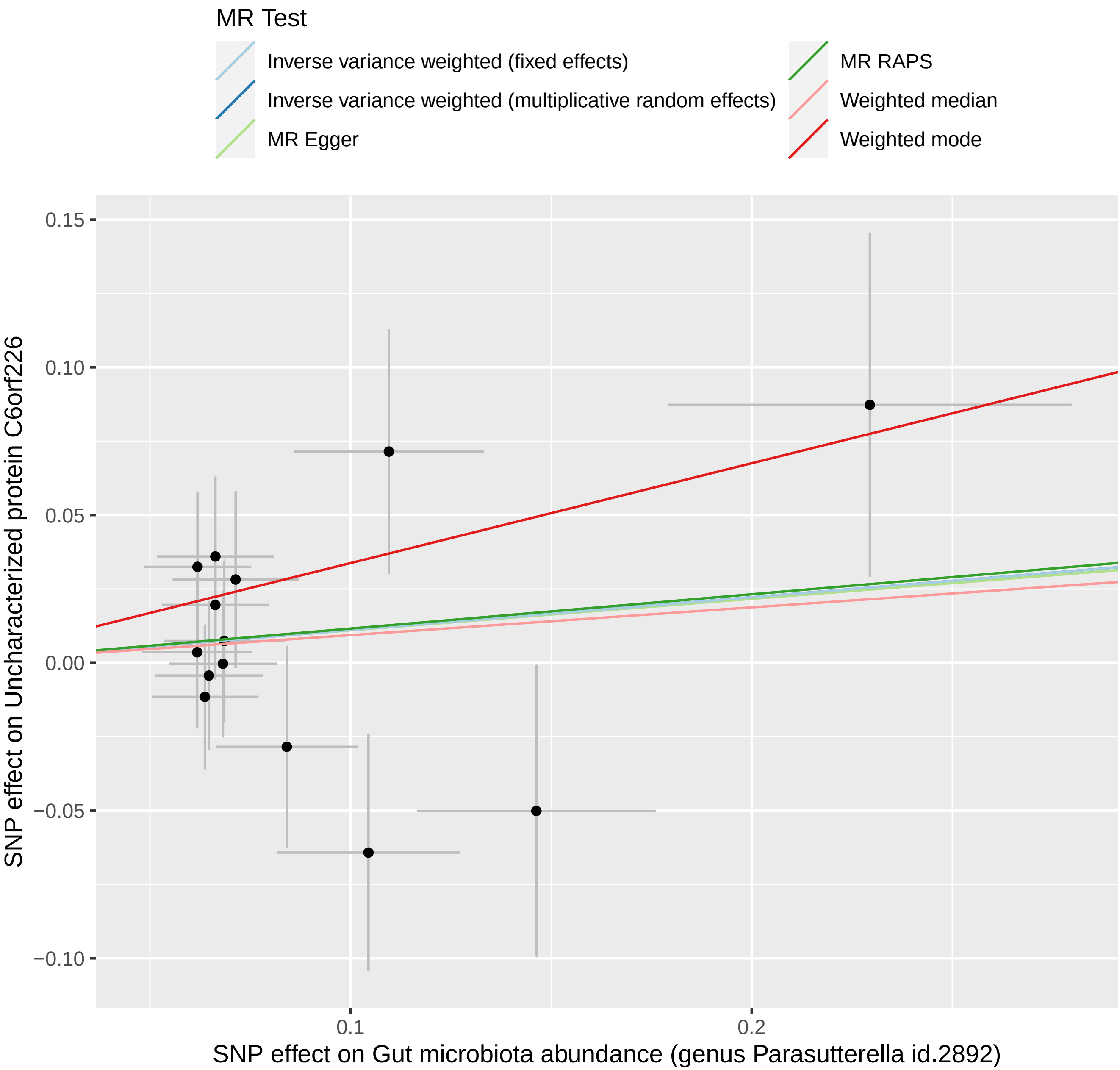

Batch 2−639 : Gut microbiota abundance (genus Catenibacterium id.2153) on Bombesin receptor−activated protein C6orf89
with a threshold of 1e−05

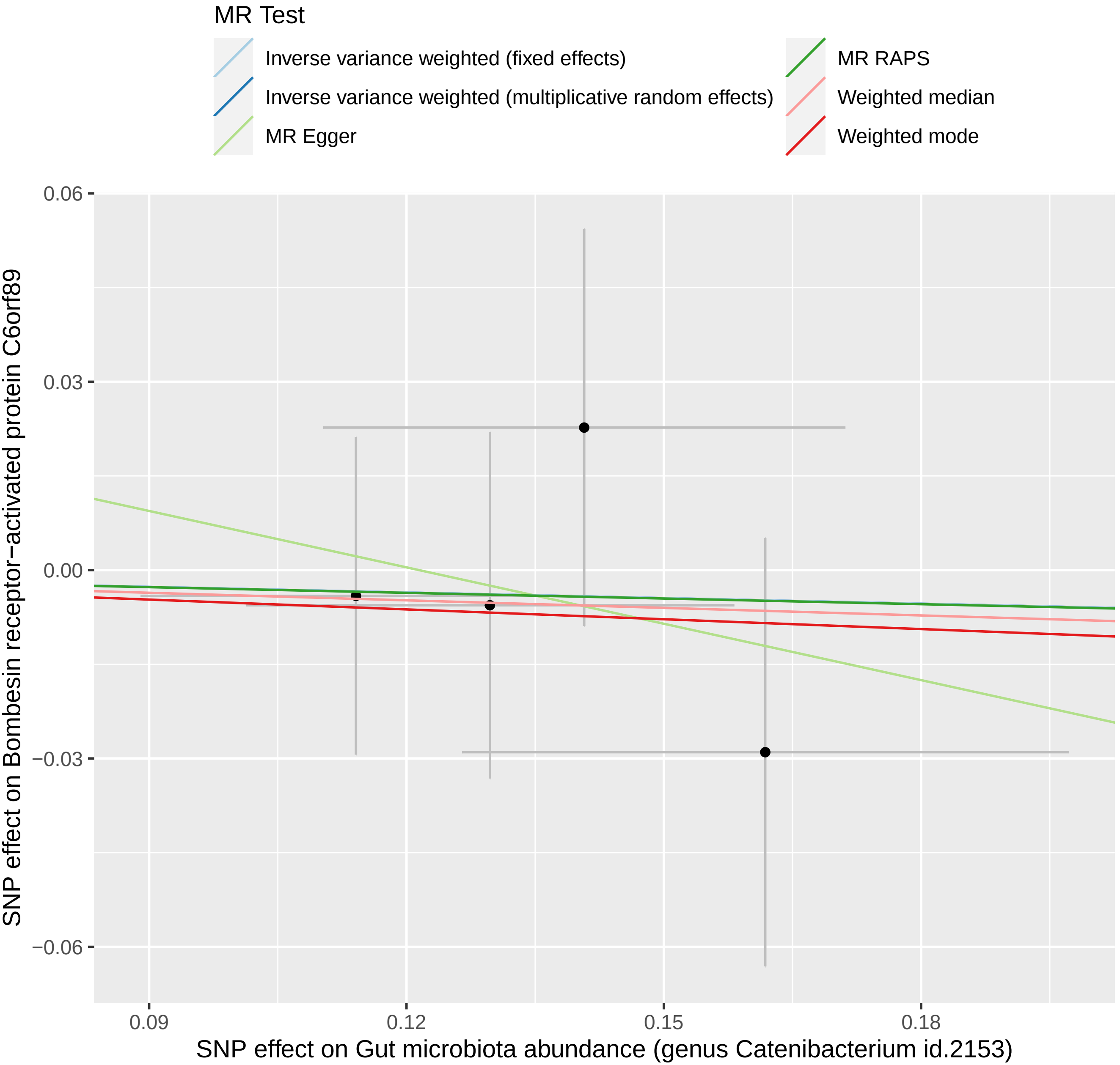

Batch 2−640 : Gut microbiota abundance (genus Parasutterella id.2892) on Bombesin receptor−activated protein C6orf89 with a threshold of 1e−05

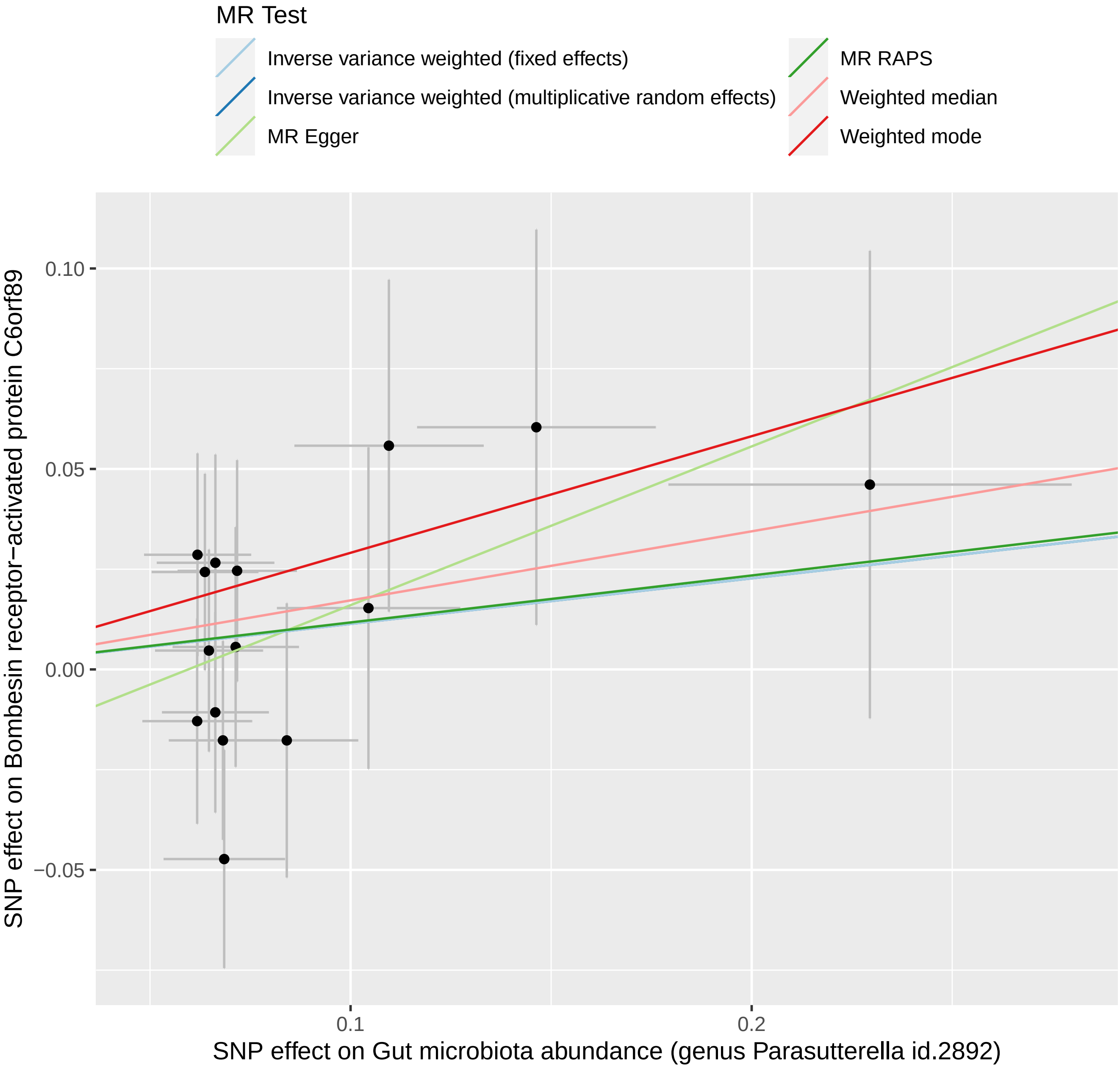

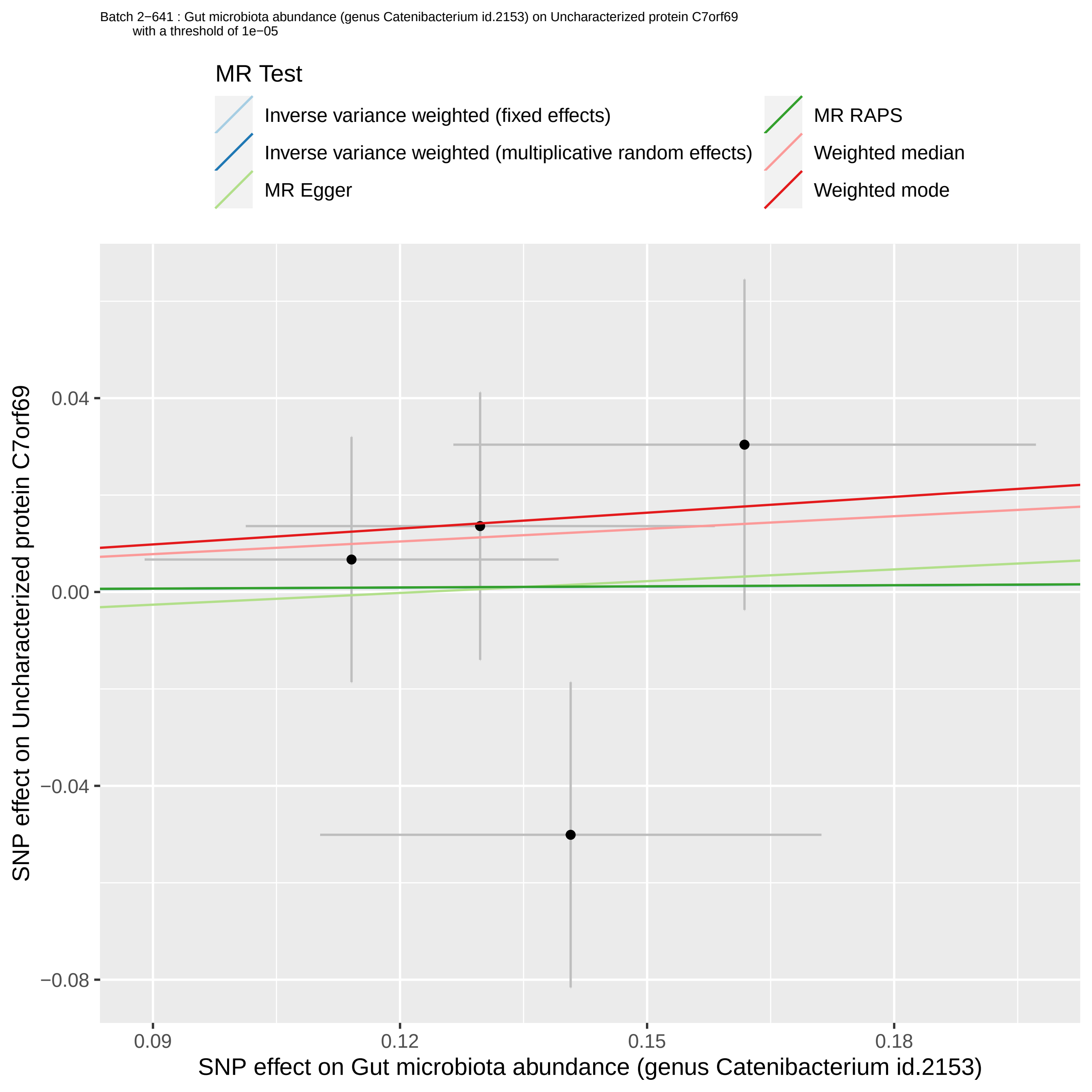

Batch 2−641 : Gut microbiota abundance (genus Catenibacterium id.2153) on Uncharacterized protein C7orf69
with a threshold of 1e−05
MR Test
Inverse variance weighted (fixed effects)
Inverse variance weighted (multiplicative random effects)
MR Egger
MR RAPS
Weighted median
Weighted mode
0.04
0.00
−0.04
−0.08
0.09
0.12
0.15
0.18
SNP effect on Uncharacterized protein C7orf69
SNP effect on Gut microbiota abundance (genus Catenibacterium id.2153)

Batch 2−642 : Gut microbiota abundance (genus Parasutterella id.2892) on Uncharacterized protein C7orf69
with a threshold of 1e−05

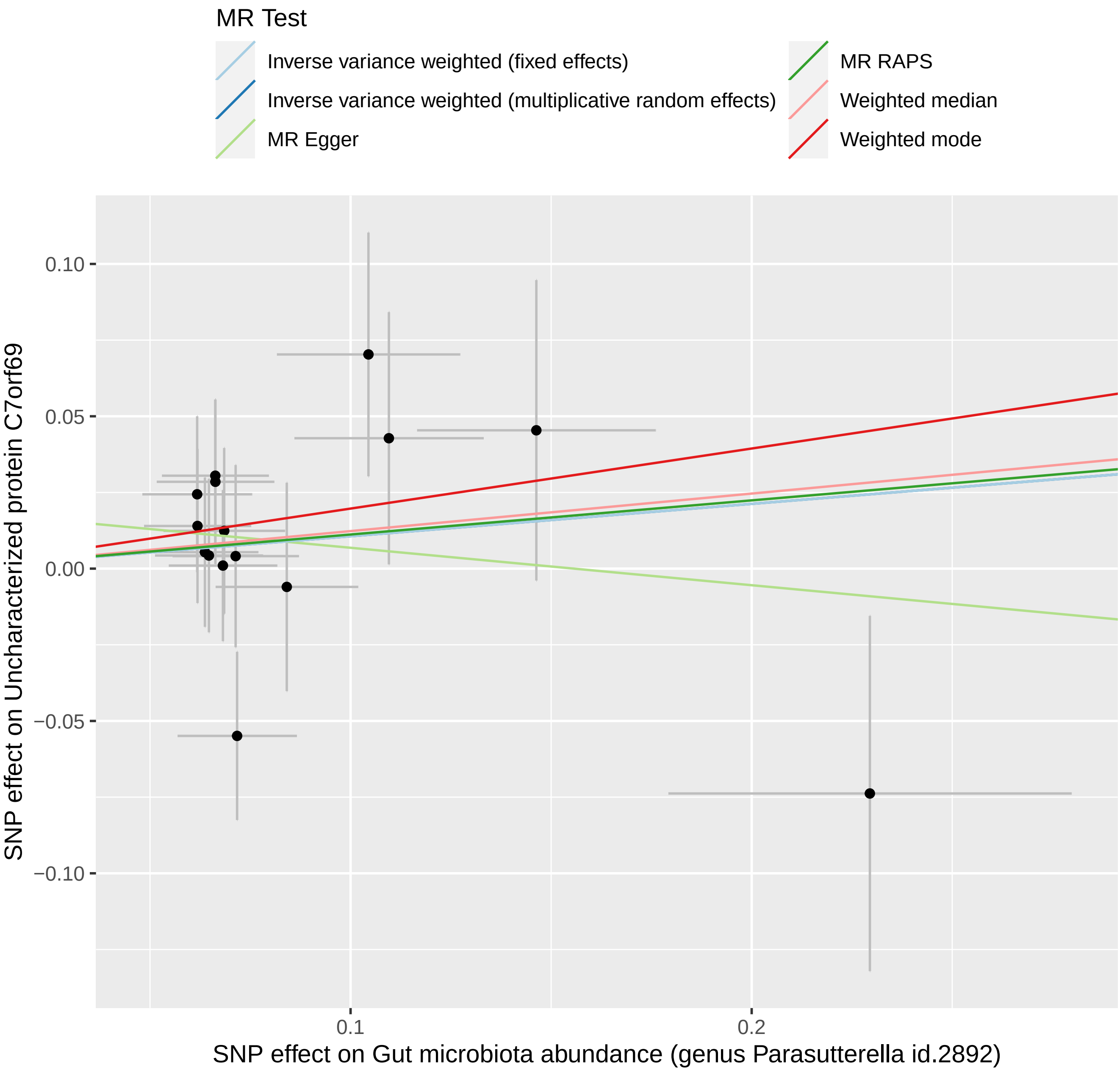

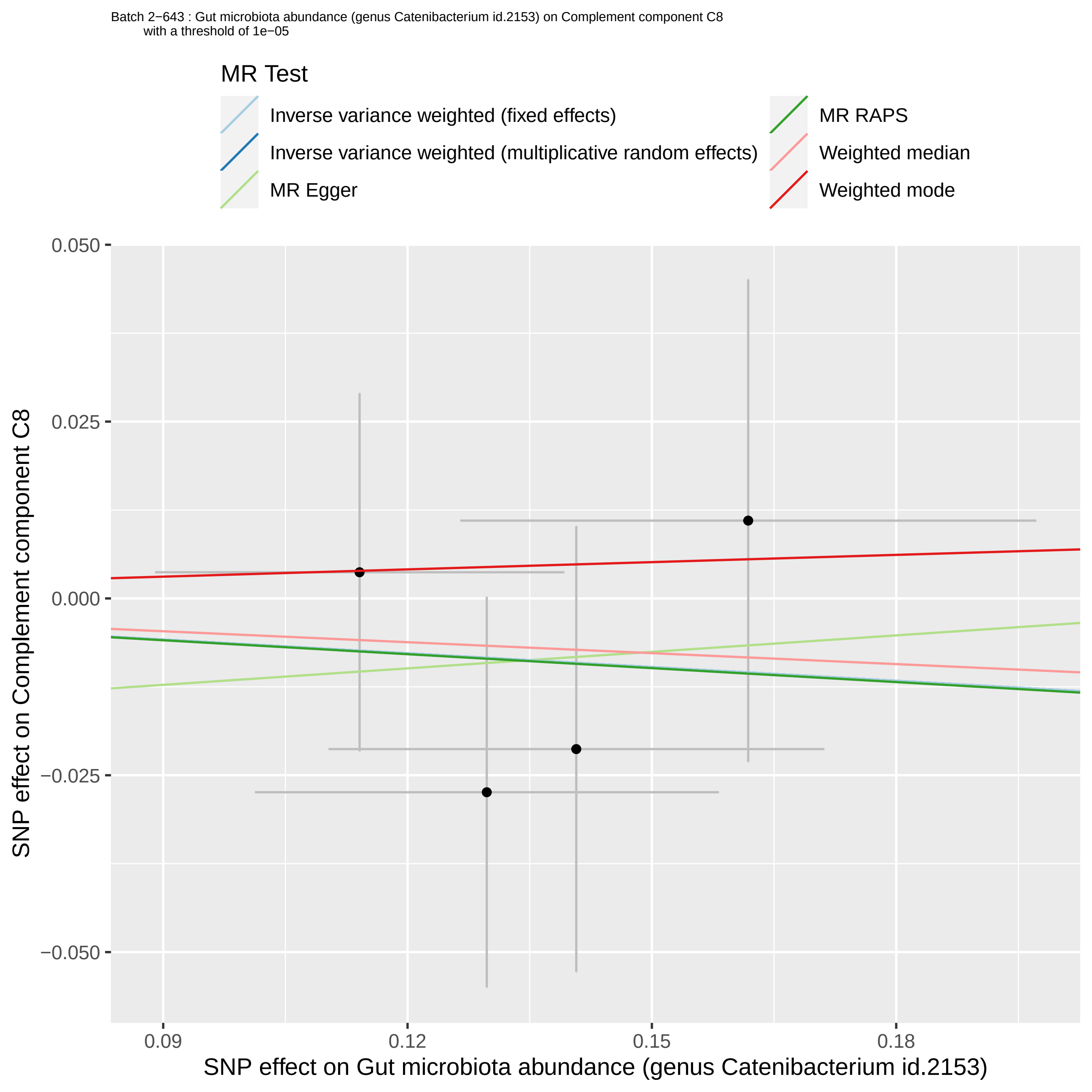
Batch 2−643 : Gut microbiota abundance (genus Catenibacterium id.2153) on Complement component C8
with a threshold of 1e−05
MR Test
Inverse variance weighted (fixed effects)
Inverse variance weighted (multiplicative random effects)
MR Egger
MR RAPS
Weighted median
Weighted mode
0.050
0.025
0.000
−0.025
−0.050
0.09
0.12
0.15
0.18
SNP effect on Complement component C8
SNP effect on Gut microbiota abundance (genus Catenibacterium id.2153)

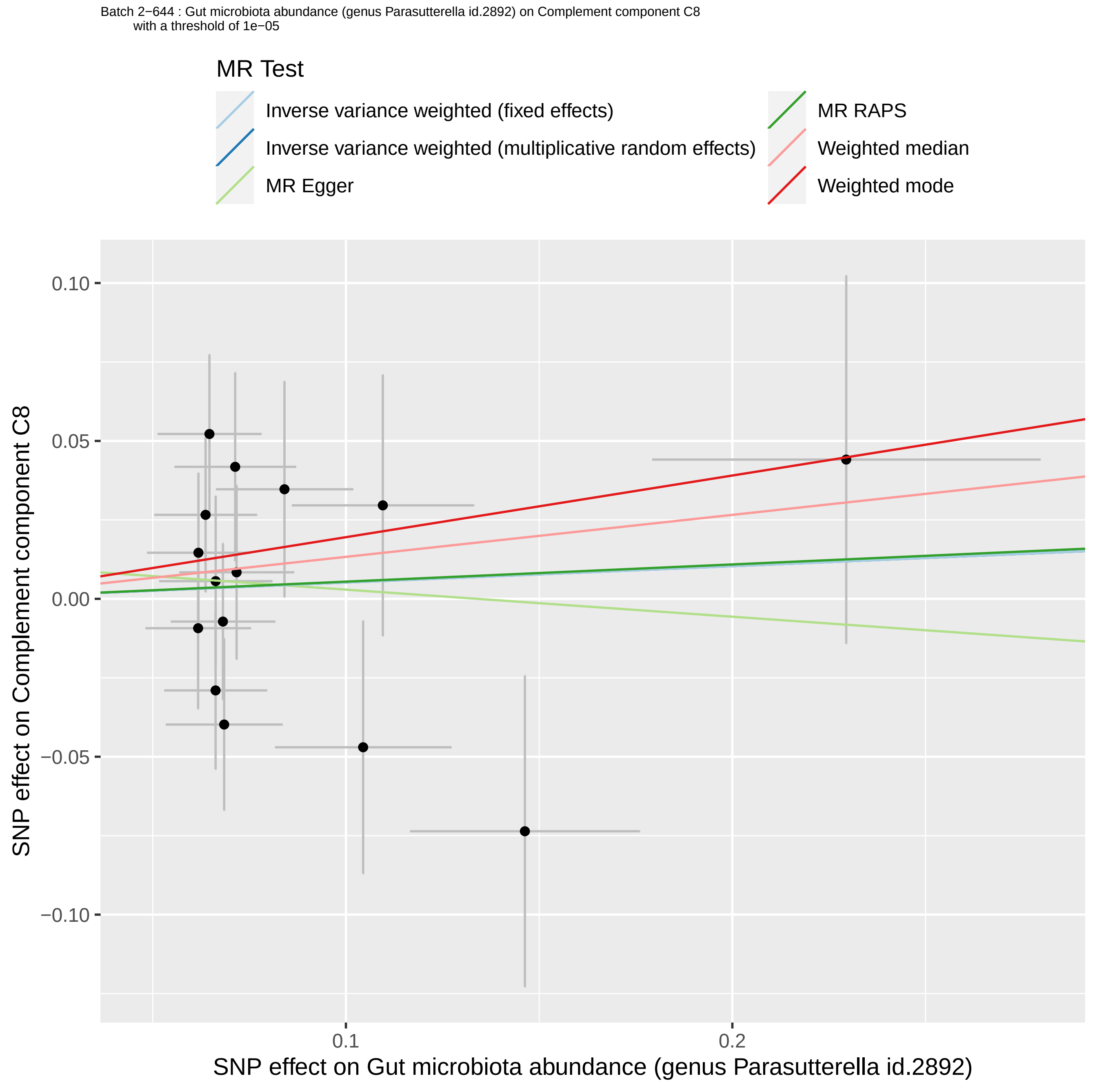
Batch 2−644 : Gut microbiota abundance (genus Parasutterella id.2892) on Complement component C8
with a threshold of 1e−05
MR Test
Inverse variance weighted (fixed effects)
Inverse variance weighted (multiplicative random effects)
MR Egger
MR RAPS
Weighted median
Weighted mode
0.10
0.05
0.00
−0.05
−0.10
0.1
0.2
SNP effect on Complement component C8
SNP effect on Gut microbiota abundance (genus Parasutterella id.2892)

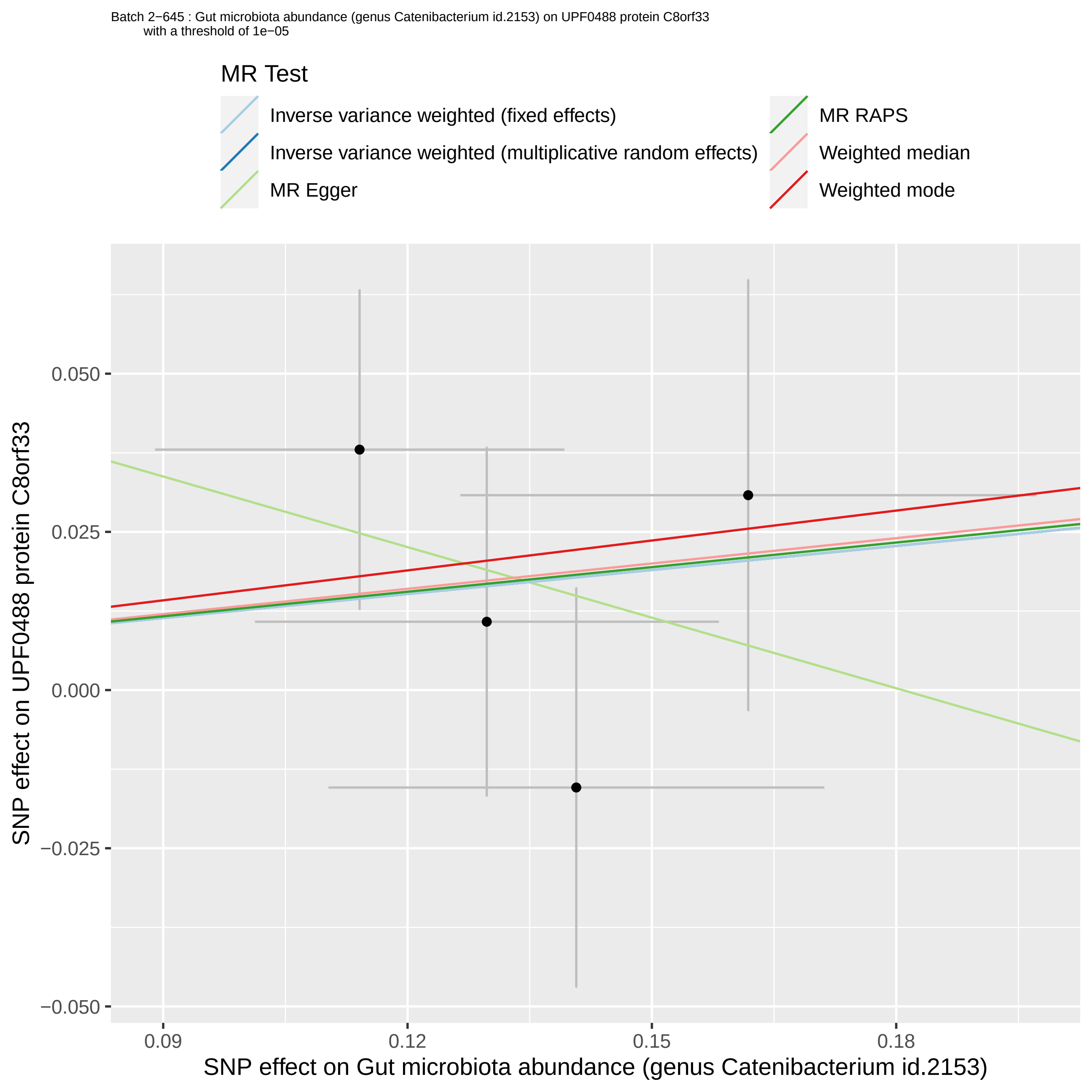

Batch 2−645 : Gut microbiota abundance (genus Catenibacterium id.2153) on UPF0488 protein C8orf33
with a threshold of 1e−05
MR Test
Inverse variance weighted (fixed effects)
Inverse variance weighted (multiplicative random effects)
MR Egger
MR RAPS
Weighted median
Weighted mode
0.050
0.025
0.000
−0.025
−0.050
0.09
0.12
0.15
0.18
SNP effect on UPF0488 protein C8orf33
SNP effect on Gut microbiota abundance (genus Catenibacterium id.2153)

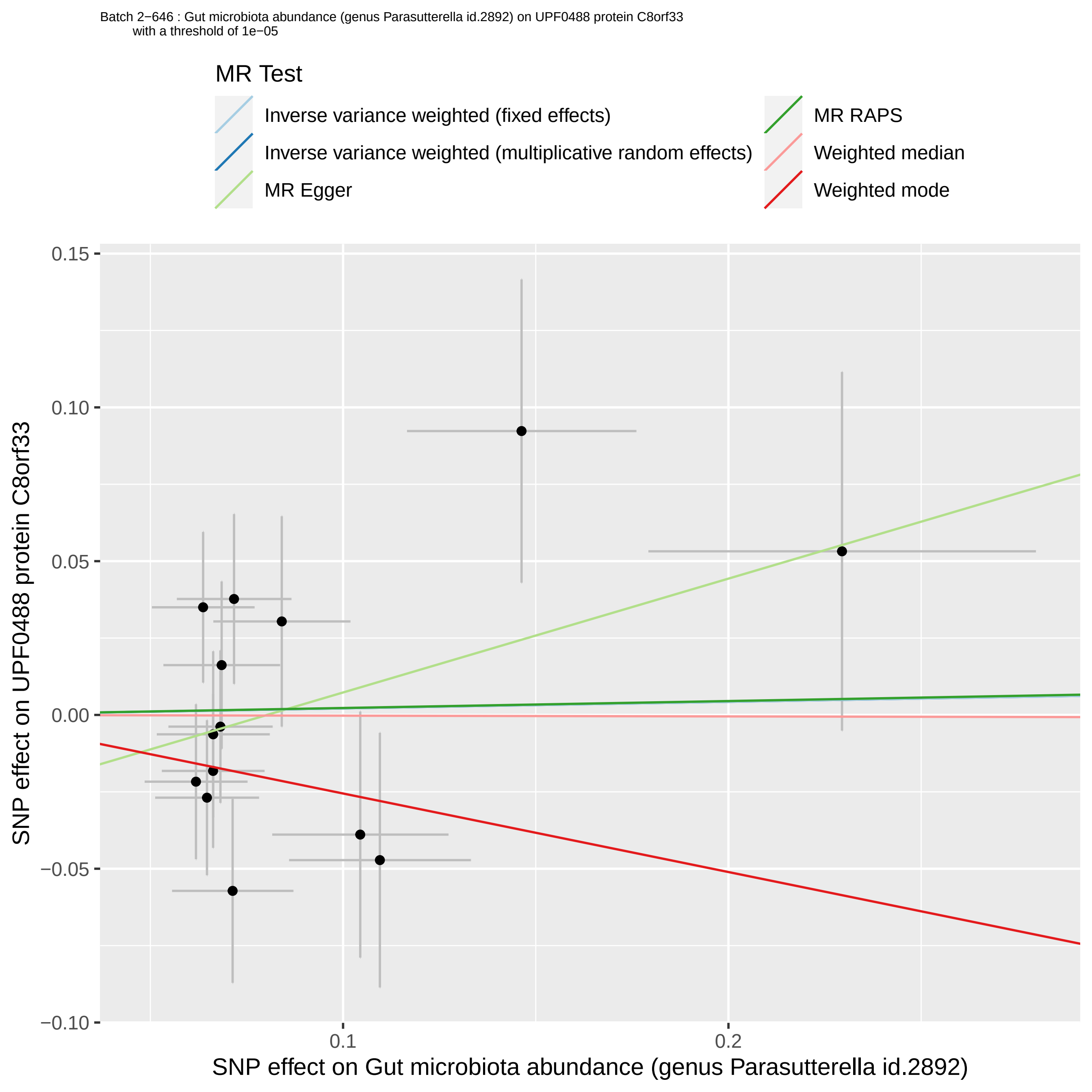

Batch 2−646 : Gut microbiota abundance (genus Parasutterella id.2892) on UPF0488 protein C8orf33
with a threshold of 1e−05
MR Test
Inverse variance weighted (fixed effects)
Inverse variance weighted (multiplicative random effects)
MR Egger
MR RAPS
Weighted median
Weighted mode
0.15
0.10
0.05
0.00
−0.05
−0.10
0.1
0.2
SNP effect on UPF0488 protein C8orf33
SNP effect on Gut microbiota abundance (genus Parasutterella id.2892)

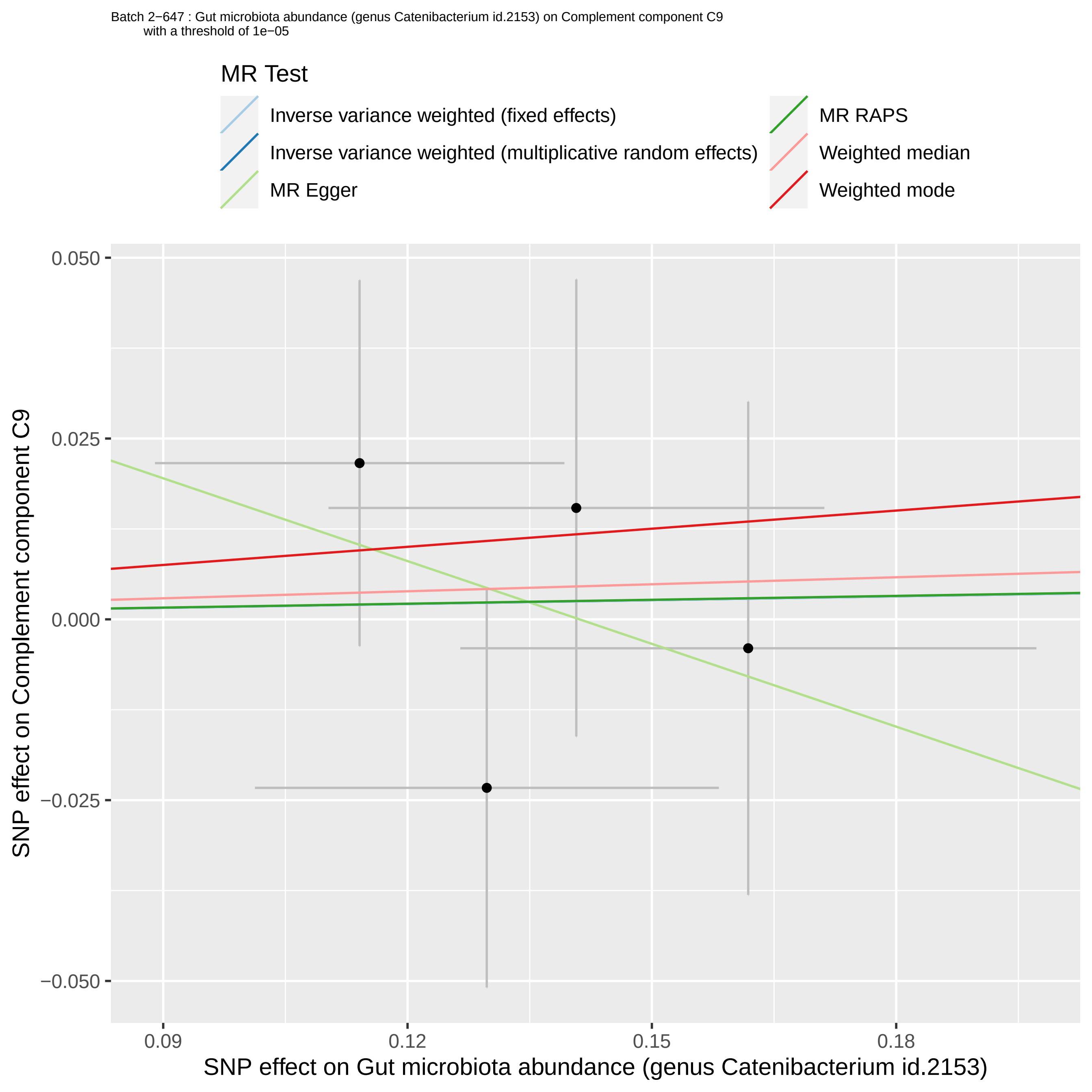
Batch 2−647 : Gut microbiota abundance (genus Catenibacterium id.2153) on Complement component C9
with a threshold of 1e−05
MR Test
Inverse variance weighted (fixed effects)
Inverse variance weighted (multiplicative random effects)
MR Egger
MR RAPS
Weighted median
Weighted mode
0.050
0.025
0.000
−0.025
−0.050
0.09
0.12
0.15
0.18
SNP effect on Complement component C9
SNP effect on Gut microbiota abundance (genus Catenibacterium id.2153)

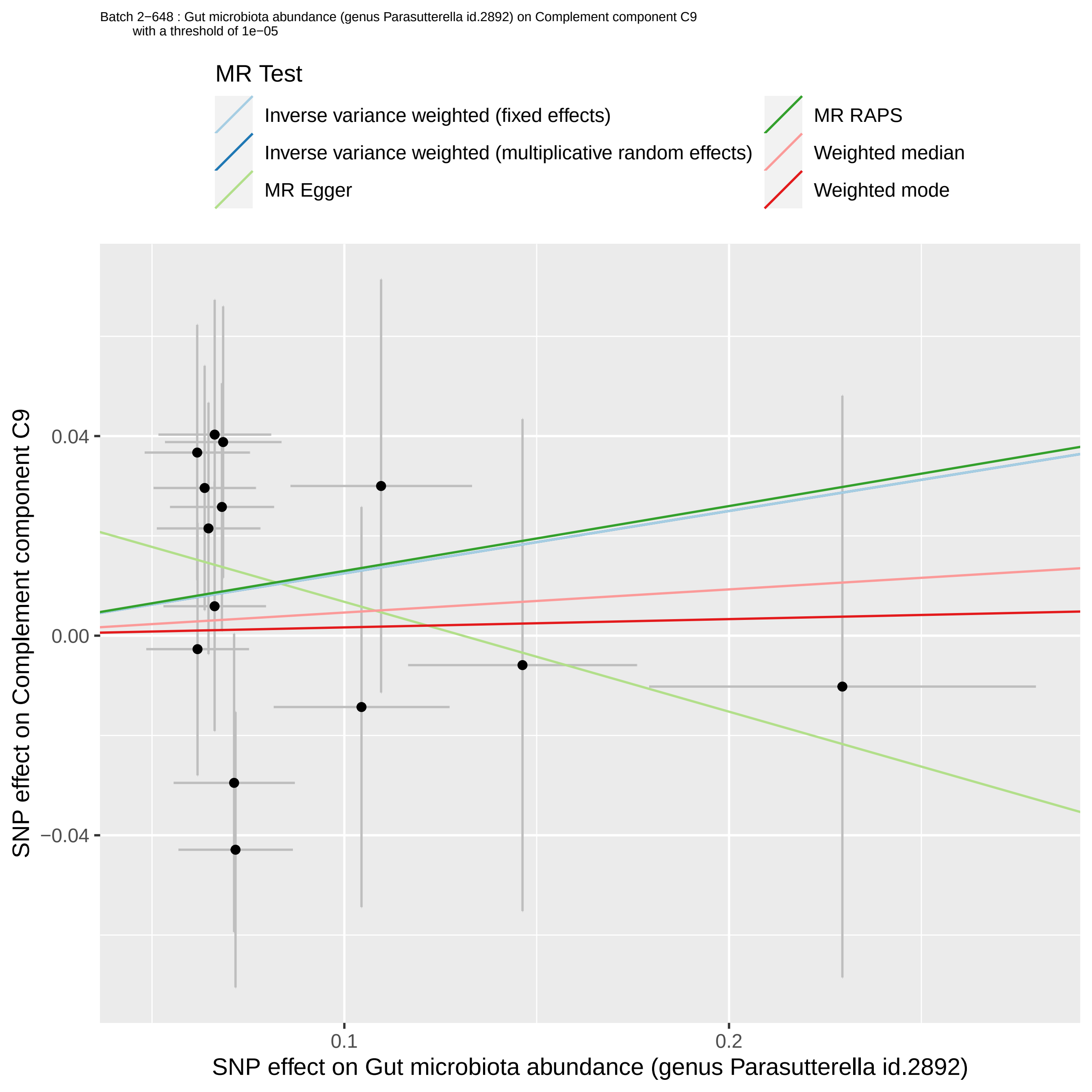
Batch 2−648 : Gut microbiota abundance (genus Parasutterella id.2892) on Complement component C9
with a threshold of 1e−05
MR Test
Inverse variance weighted (fixed effects)
Inverse variance weighted (multiplicative random effects)
MR Egger
MR RAPS
Weighted median
Weighted mode
0.04
0.00
−0.04
0.1
0.2
SNP effect on Complement component C9
SNP effect on Gut microbiota abundance (genus Parasutterella id.2892)

Batch 2−649 : Gut microbiota abundance (genus Catenibacterium id.2153) on Bcl10−interacting CARD protein
with a threshold of 1e−05

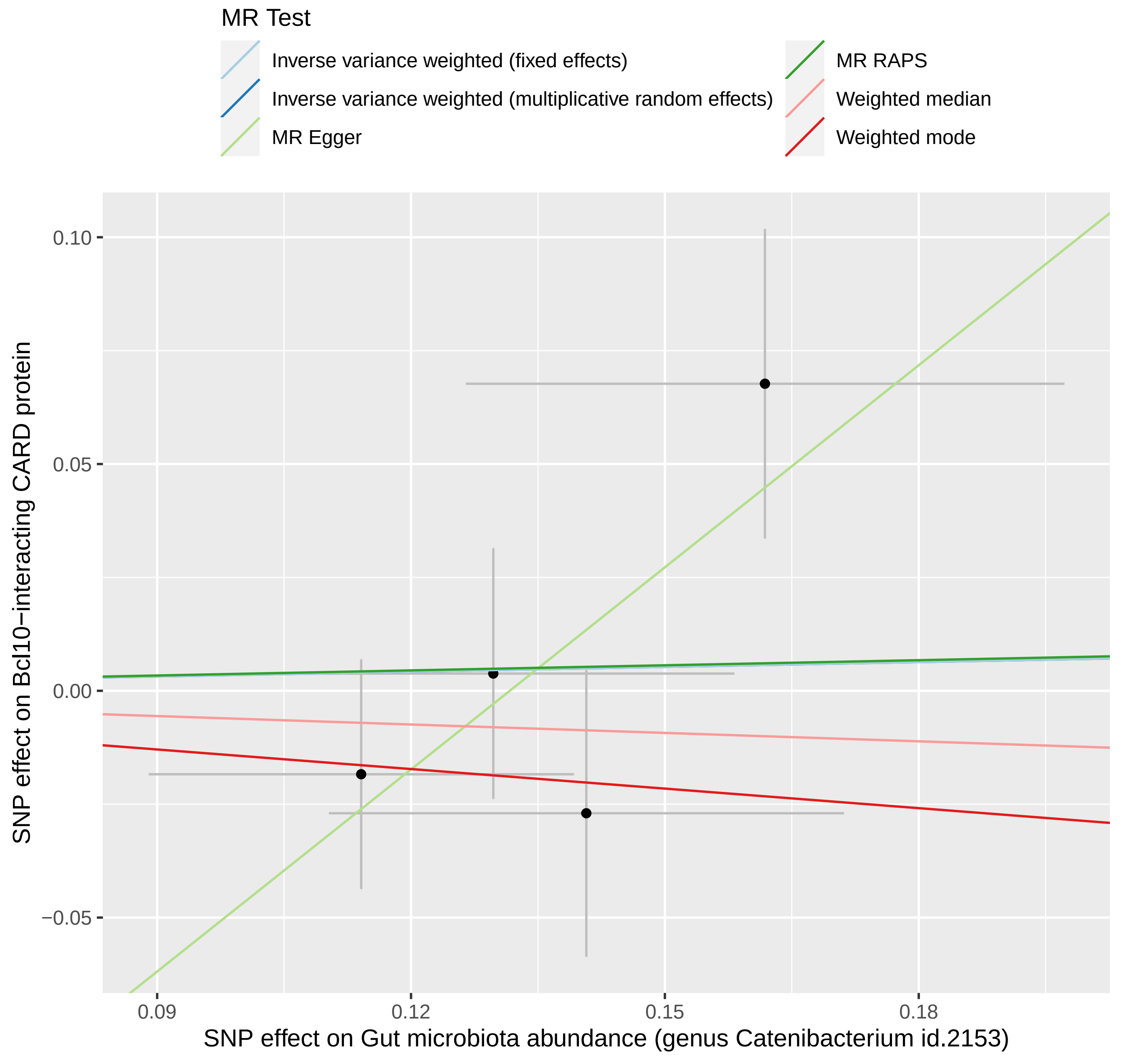

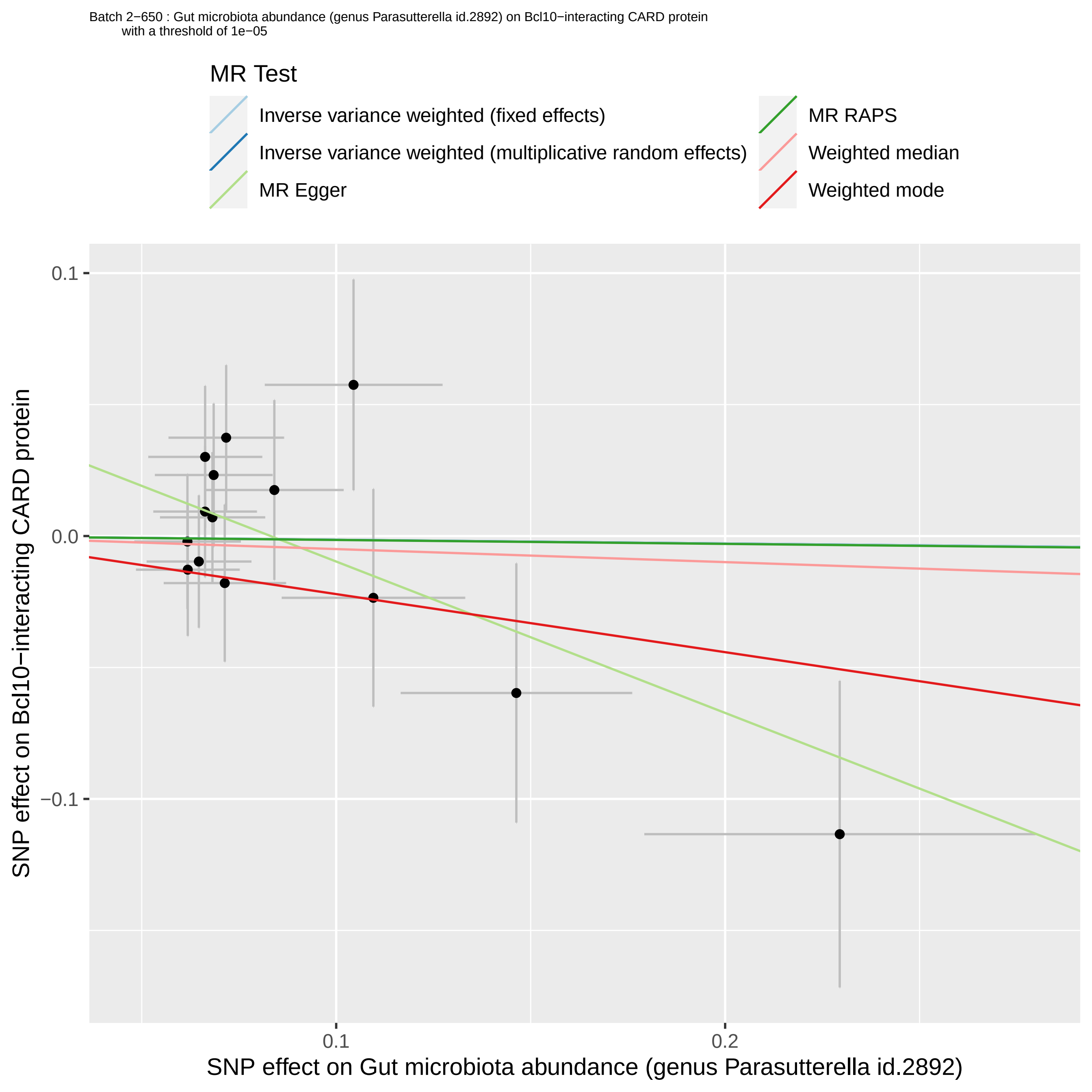

Batch 2−650 : Gut microbiota abundance (genus Parasutterella id.2892) on Bcl10−interacting CARD protein
with a threshold of 1e−05
MR Test
Inverse variance weighted (fixed effects)
Inverse variance weighted (multiplicative random effects)
MR Egger
MR RAPS
Weighted median
Weighted mode
0.1
0.0
−0.1
0.1
0.2
SNP effect on Bcl10−interacting CARD protein
SNP effect on Gut microbiota abundance (genus Parasutterella id.2892)

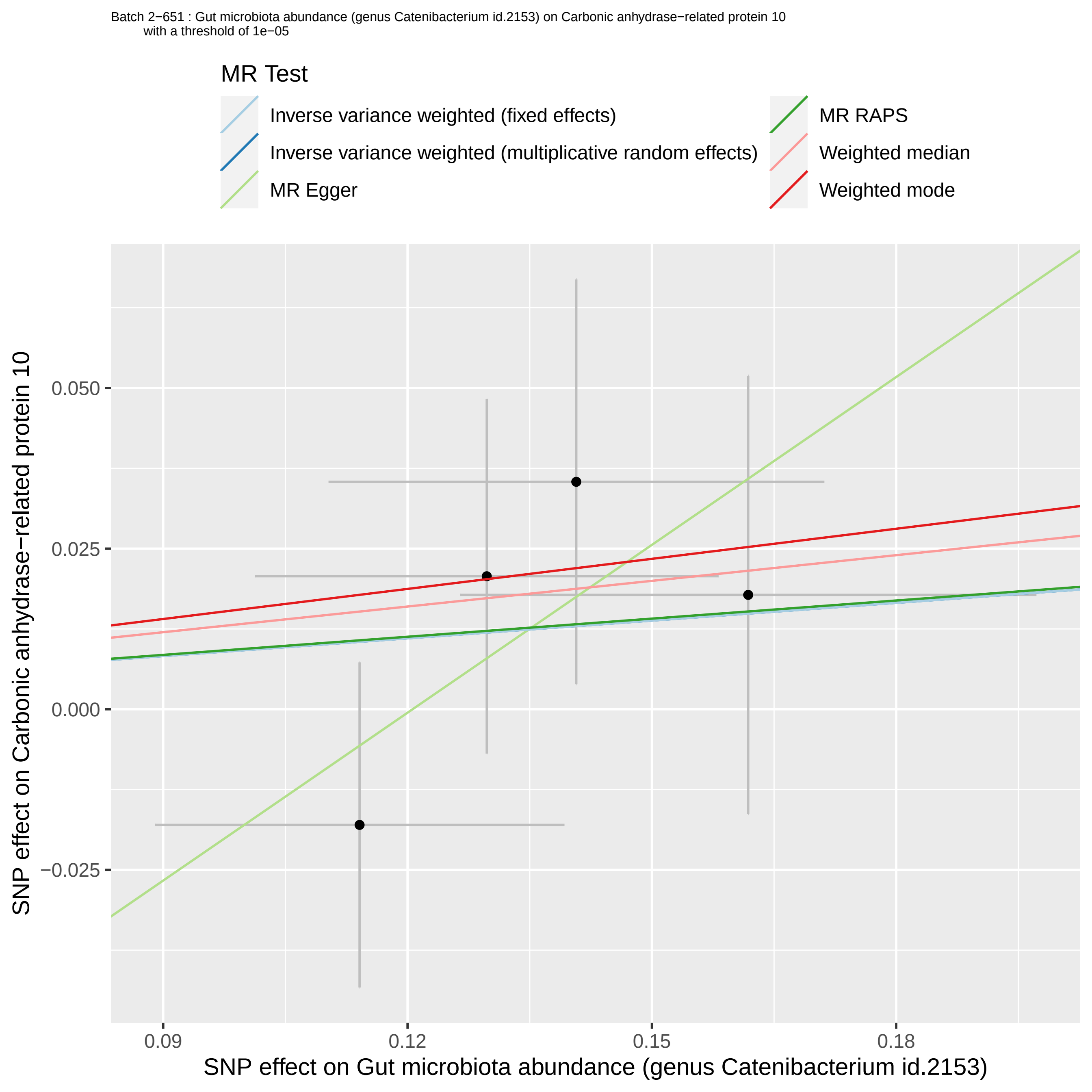

Batch 2−651 : Gut microbiota abundance (genus Catenibacterium id.2153) on Carbonic anhydrase−related protein 10
with a threshold of 1e−05
MR Test
Inverse variance weighted (fixed effects)
Inverse variance weighted (multiplicative random effects)
MR Egger
MR RAPS
Weighted median
Weighted mode
0.050
0.025
0.000
−0.025
0.09
0.12
0.15
0.18
SNP effect on Carbonic anhydrase−related protein 10
SNP effect on Gut microbiota abundance (genus Catenibacterium id.2153)

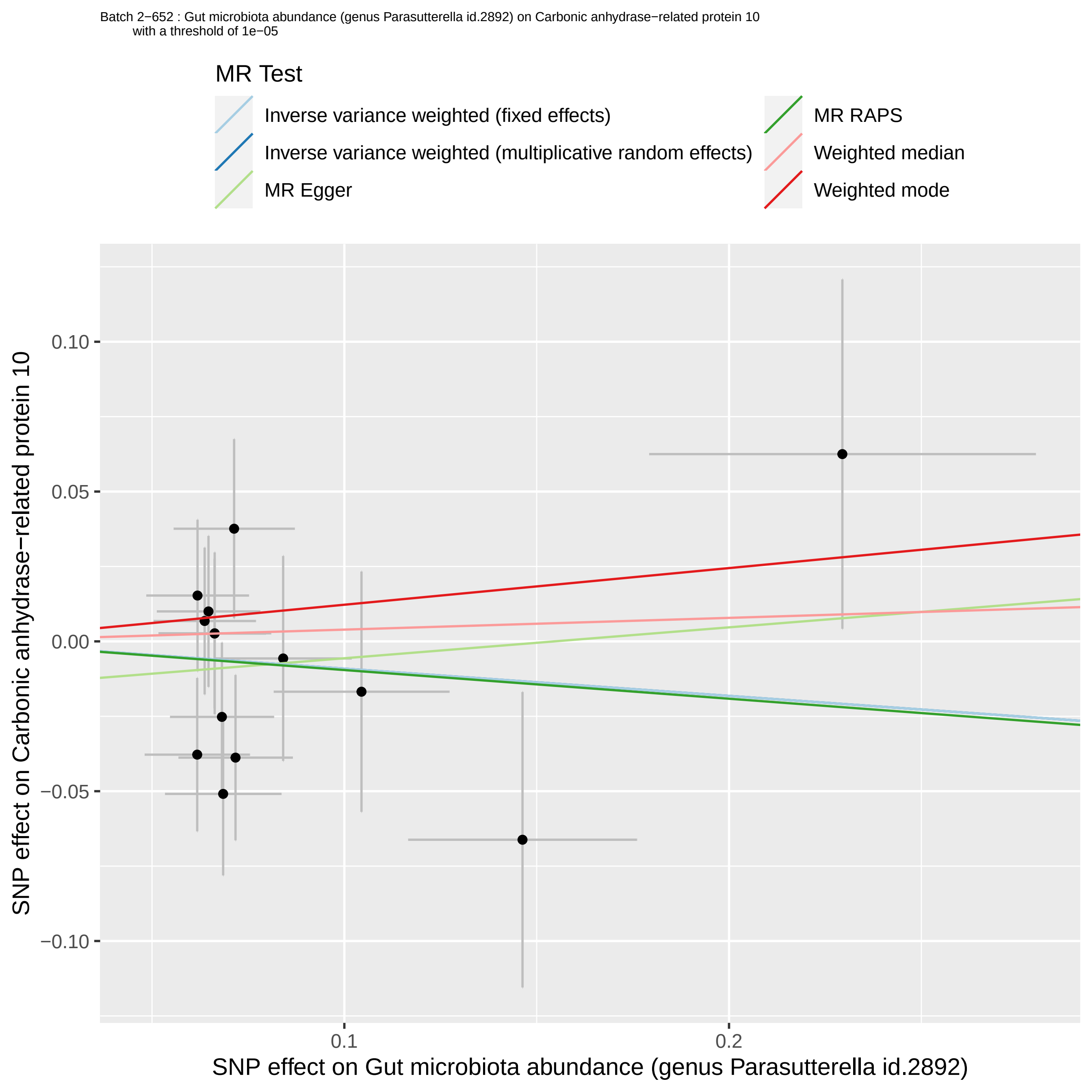
Batch 2−652 : Gut microbiota abundance (genus Parasutterella id.2892) on Carbonic anhydrase−related protein 10
with a threshold of 1e−05
MR Test
Inverse variance weighted (fixed effects)
Inverse variance weighted (multiplicative random effects)
MR Egger
MR RAPS
Weighted median
Weighted mode
0.10
0.05
0.00
−0.05
−0.10
0.1
0.2
SNP effect on Carbonic anhydrase−related protein 10
SNP effect on Gut microbiota abundance (genus Parasutterella id.2892)

Batch 2−653 : Gut microbiota abundance (genus Catenibacterium id.2153) on Carbonic anhydrase−related protein 10
with a threshold of 1e−05

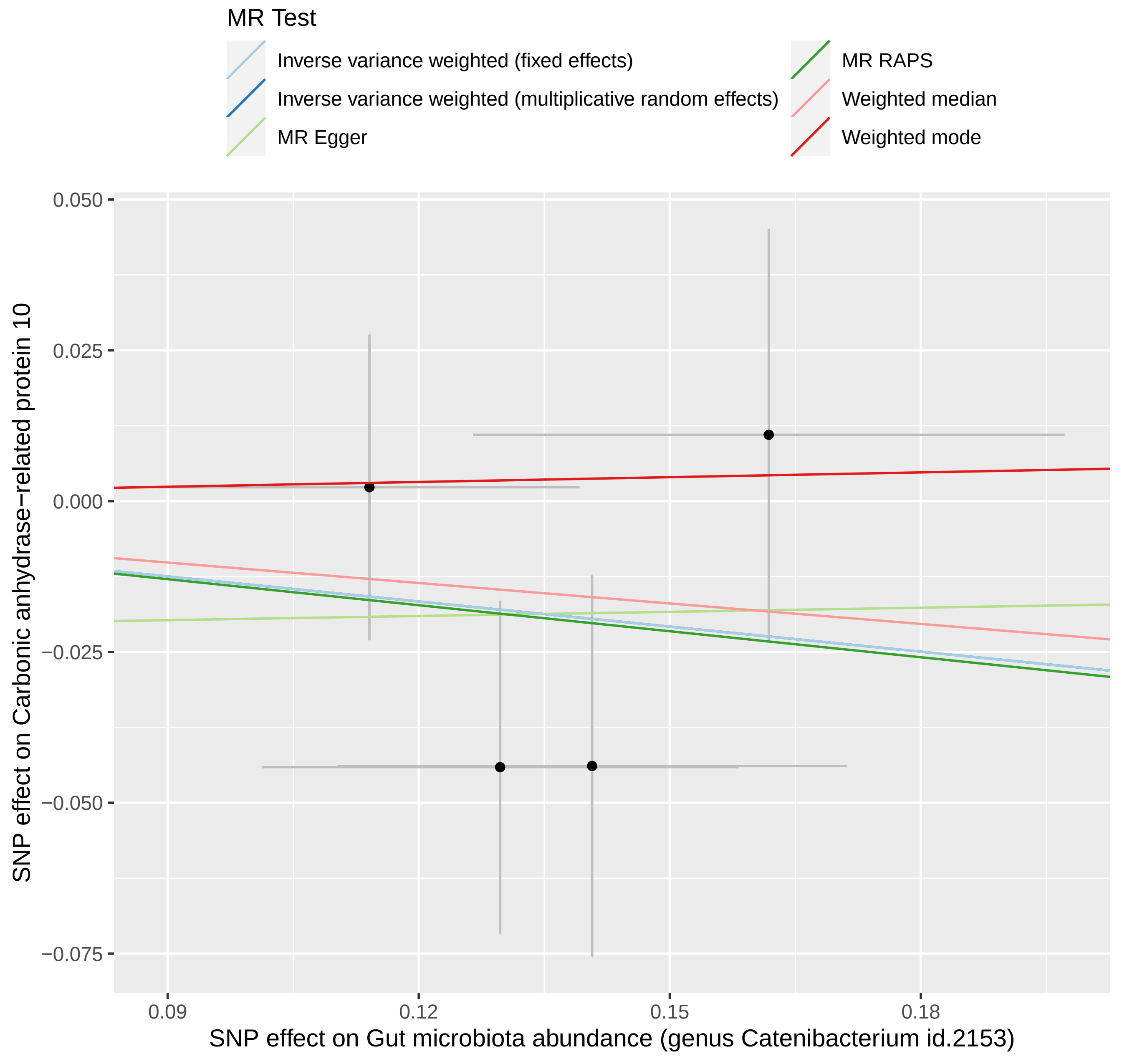

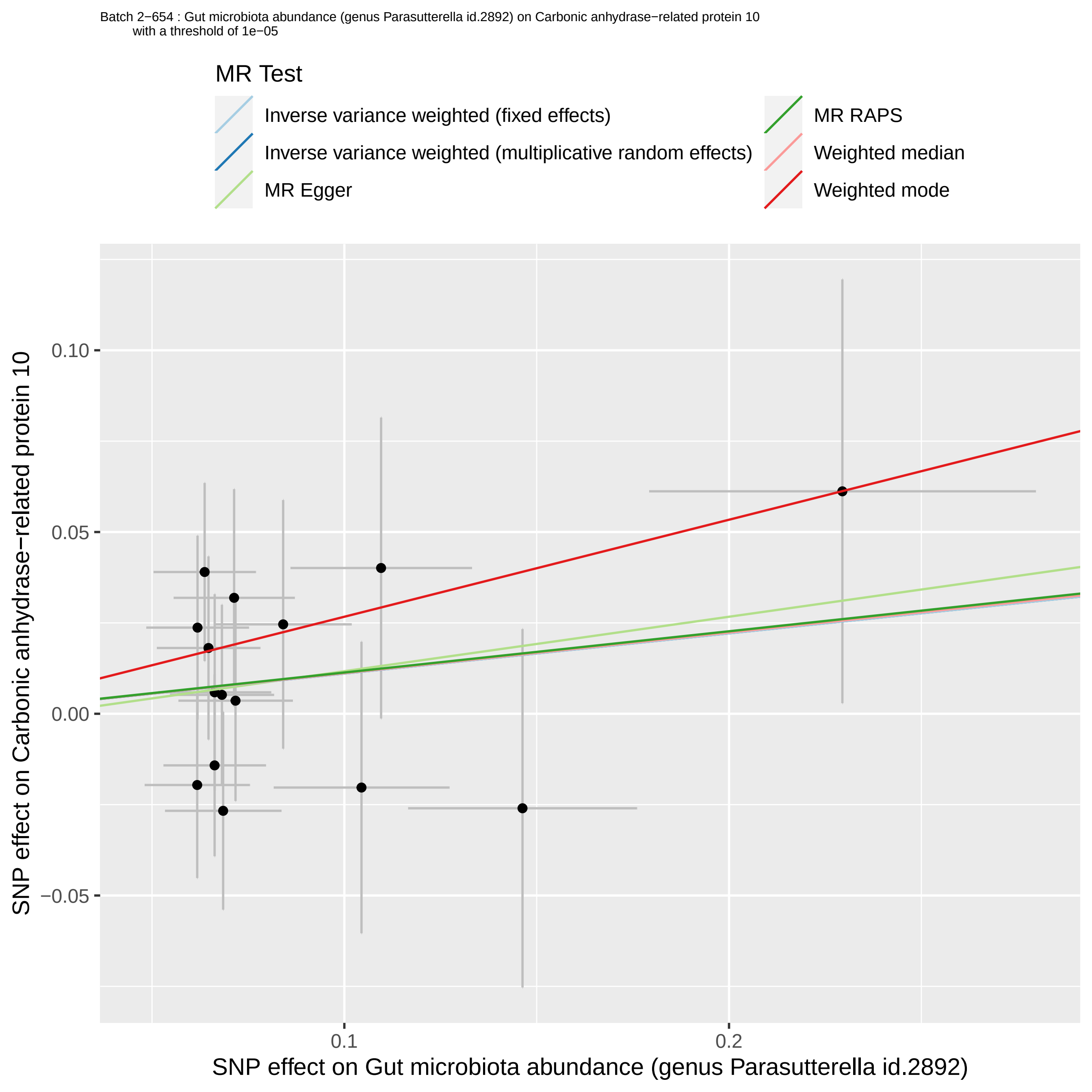

Batch 2−654 : Gut microbiota abundance (genus Parasutterella id.2892) on Carbonic anhydrase−related protein 10
with a threshold of 1e−05
MR Test
Inverse variance weighted (fixed effects)
Inverse variance weighted (multiplicative random effects)
MR Egger
MR RAPS
Weighted median
Weighted mode
0.10
0.05
0.00
−0.05
0.1
0.2
SNP effect on Carbonic anhydrase−related protein 10
SNP effect on Gut microbiota abundance (genus Parasutterella id.2892)

Batch 2−655 : Gut microbiota abundance (genus Catenibacterium id.2153) on Carbonic anhydrase 1
with a threshold of 1e−05

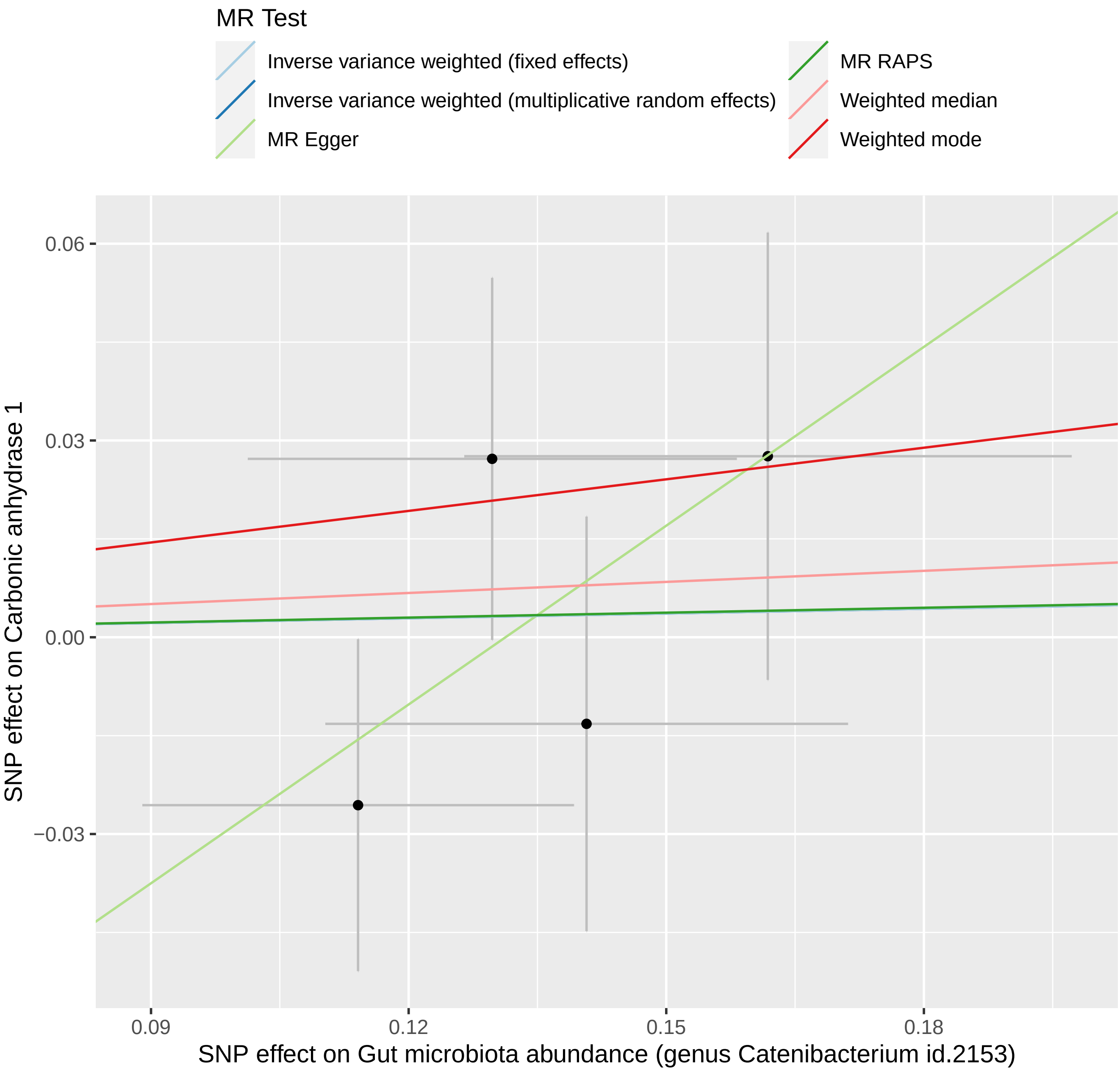

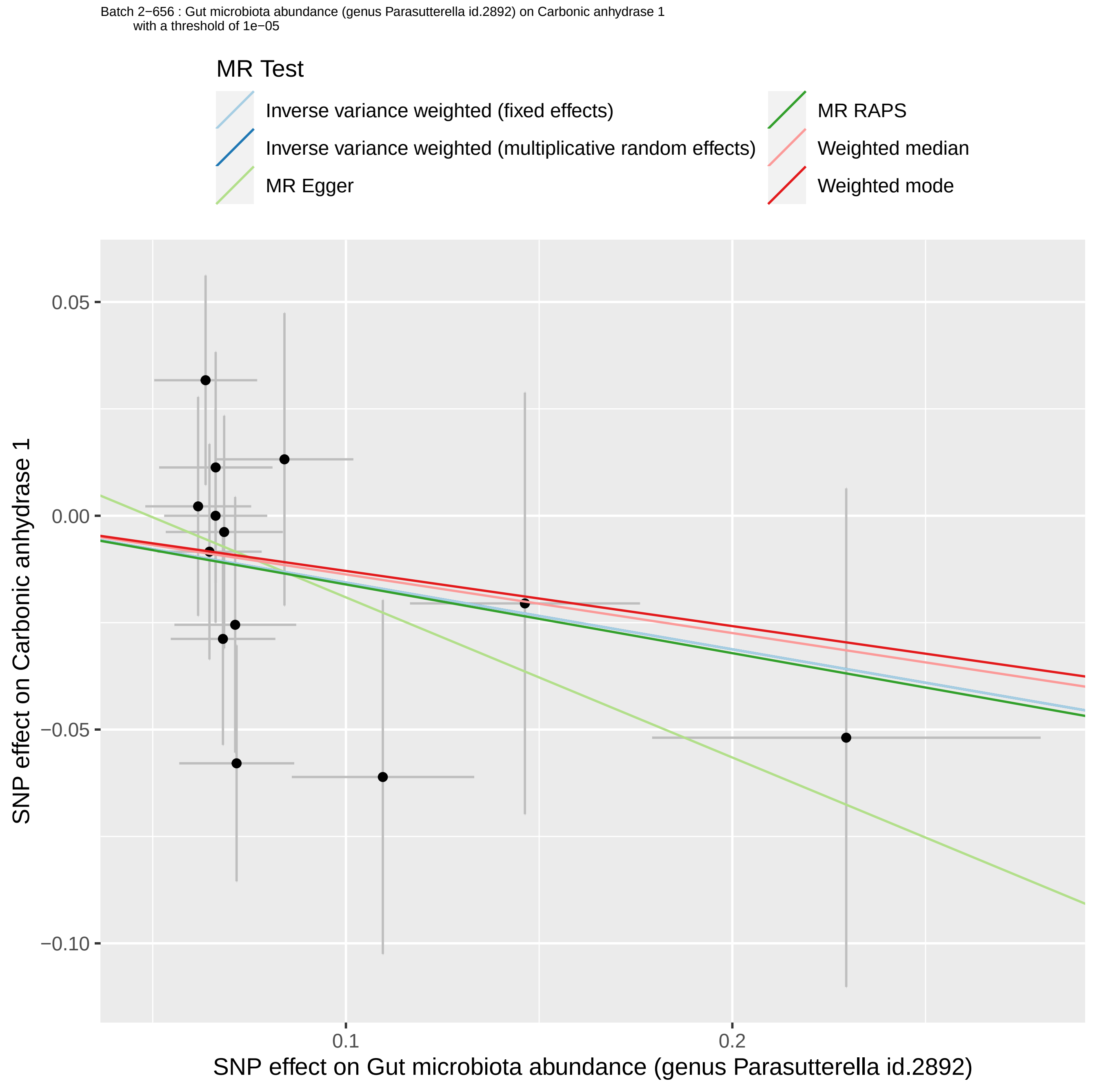
Batch 2−656 : Gut microbiota abundance (genus Parasutterella id.2892) on Carbonic anhydrase 1
with a threshold of 1e−05
MR Test
Inverse variance weighted (fixed effects)
Inverse variance weighted (multiplicative random effects)
MR Egger
MR RAPS
Weighted median
Weighted mode
0.05
0.00
−0.05
−0.10
0.1
0.2
SNP effect on Carbonic anhydrase 1
SNP effect on Gut microbiota abundance (genus Parasutterella id.2892)

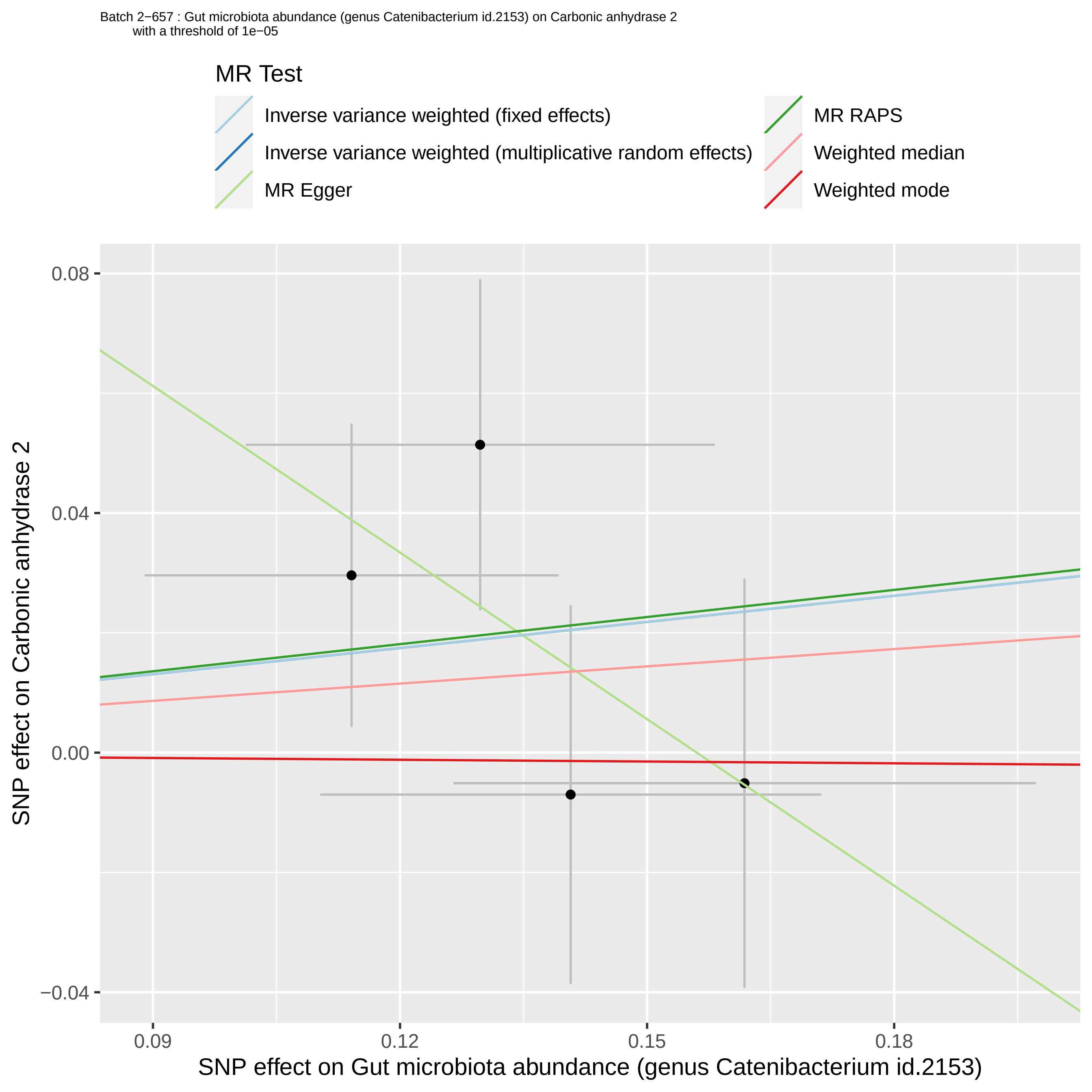

Batch 2−657 : Gut microbiota abundance (genus Catenibacterium id.2153) on Carbonic anhydrase 2
with a threshold of 1e−05
MR Test
Inverse variance weighted (fixed effects)
Inverse variance weighted (multiplicative random effects)
MR Egger
MR RAPS
Weighted median
Weighted mode
0.08
0.04
0.00
−0.04
0.09
0.12
0.15
0.18
SNP effect on Carbonic anhydrase 2
SNP effect on Gut microbiota abundance (genus Catenibacterium id.2153)

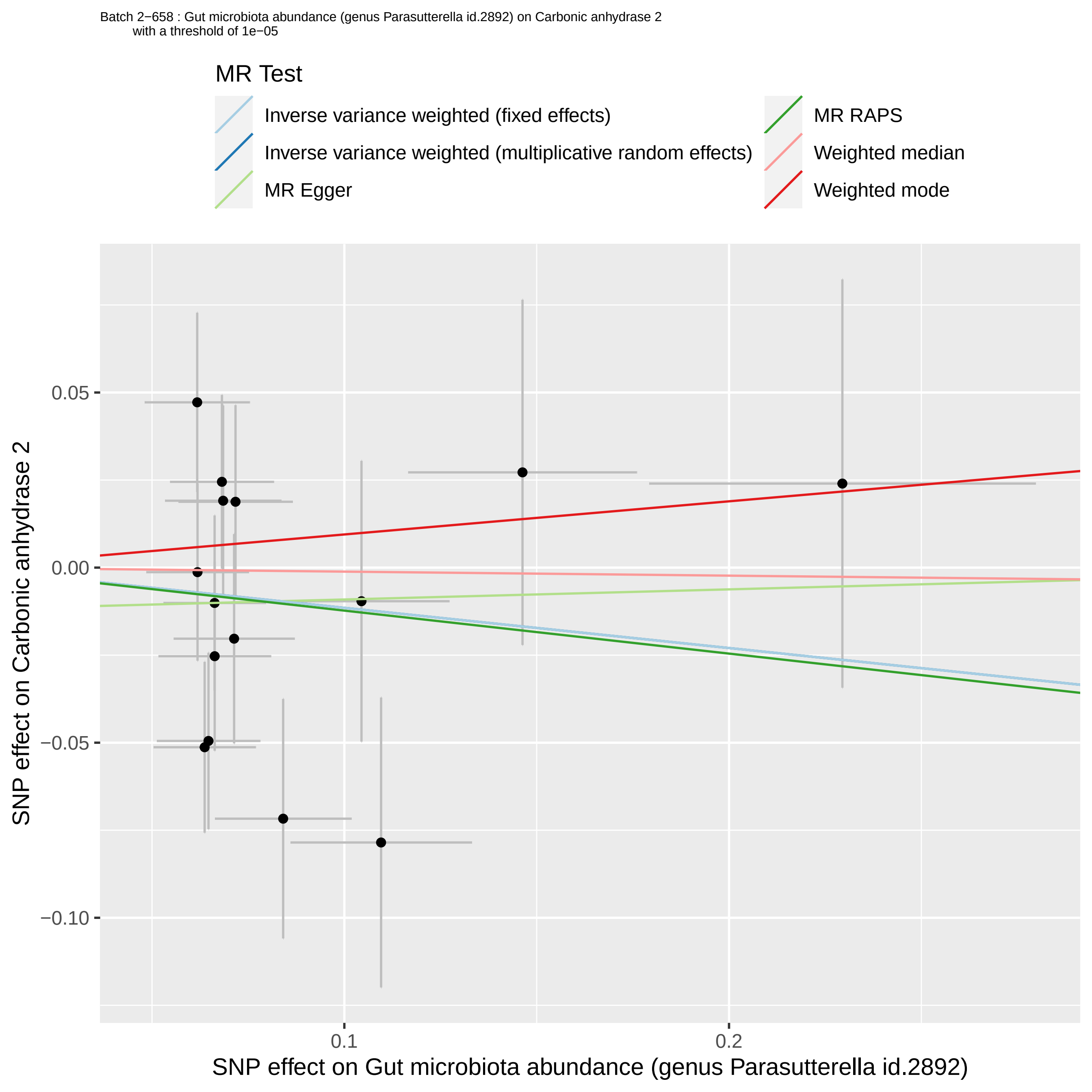

Batch 2−658 : Gut microbiota abundance (genus Parasutterella id.2892) on Carbonic anhydrase 2
with a threshold of 1e−05
MR Test
Inverse variance weighted (fixed effects)
Inverse variance weighted (multiplicative random effects)
MR Egger
MR RAPS
Weighted median
Weighted mode
0.05
0.00
−0.05
−0.10
0.1
0.2
SNP effect on Carbonic anhydrase 2
SNP effect on Gut microbiota abundance (genus Parasutterella id.2892)

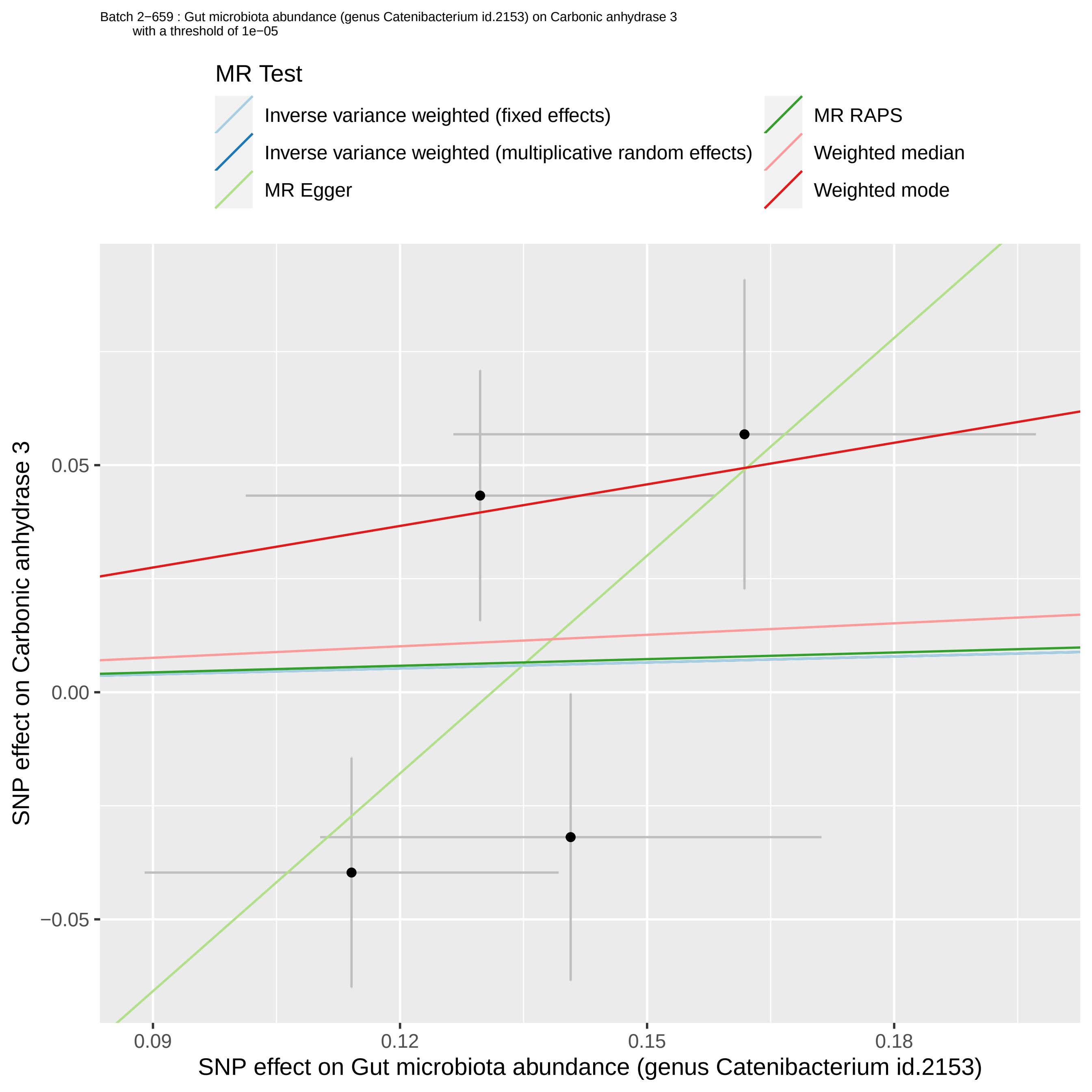

Batch 2−659 : Gut microbiota abundance (genus Catenibacterium id.2153) on Carbonic anhydrase 3
with a threshold of 1e−05
MR Test
Inverse variance weighted (fixed effects)
Inverse variance weighted (multiplicative random effects)
MR Egger
MR RAPS
Weighted median
Weighted mode
0.05
0.00
−0.05
0.09
0.12
0.15
0.18
SNP effect on Carbonic anhydrase 3
SNP effect on Gut microbiota abundance (genus Catenibacterium id.2153)

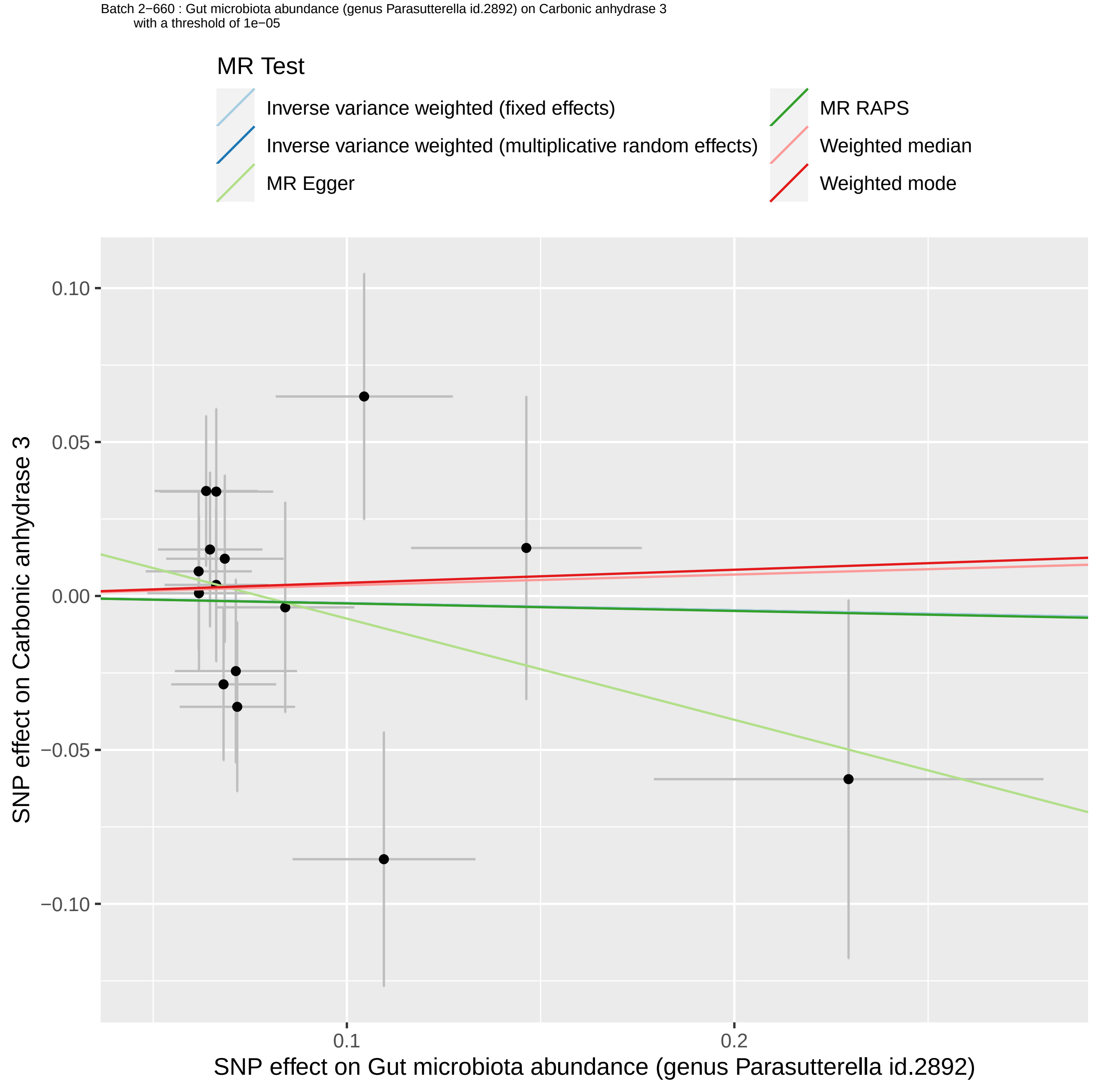

Batch 2−660 : Gut microbiota abundance (genus Parasutterella id.2892) on Carbonic anhydrase 3
with a threshold of 1e−05
MR Test
Inverse variance weighted (fixed effects)
Inverse variance weighted (multiplicative random effects)
MR Egger
MR RAPS
Weighted median
Weighted mode
0.10
0.05
0.00
−0.05
−0.10
0.1
0.2
SNP effect on Carbonic anhydrase 3
SNP effect on Gut microbiota abundance (genus Parasutterella id.2892)

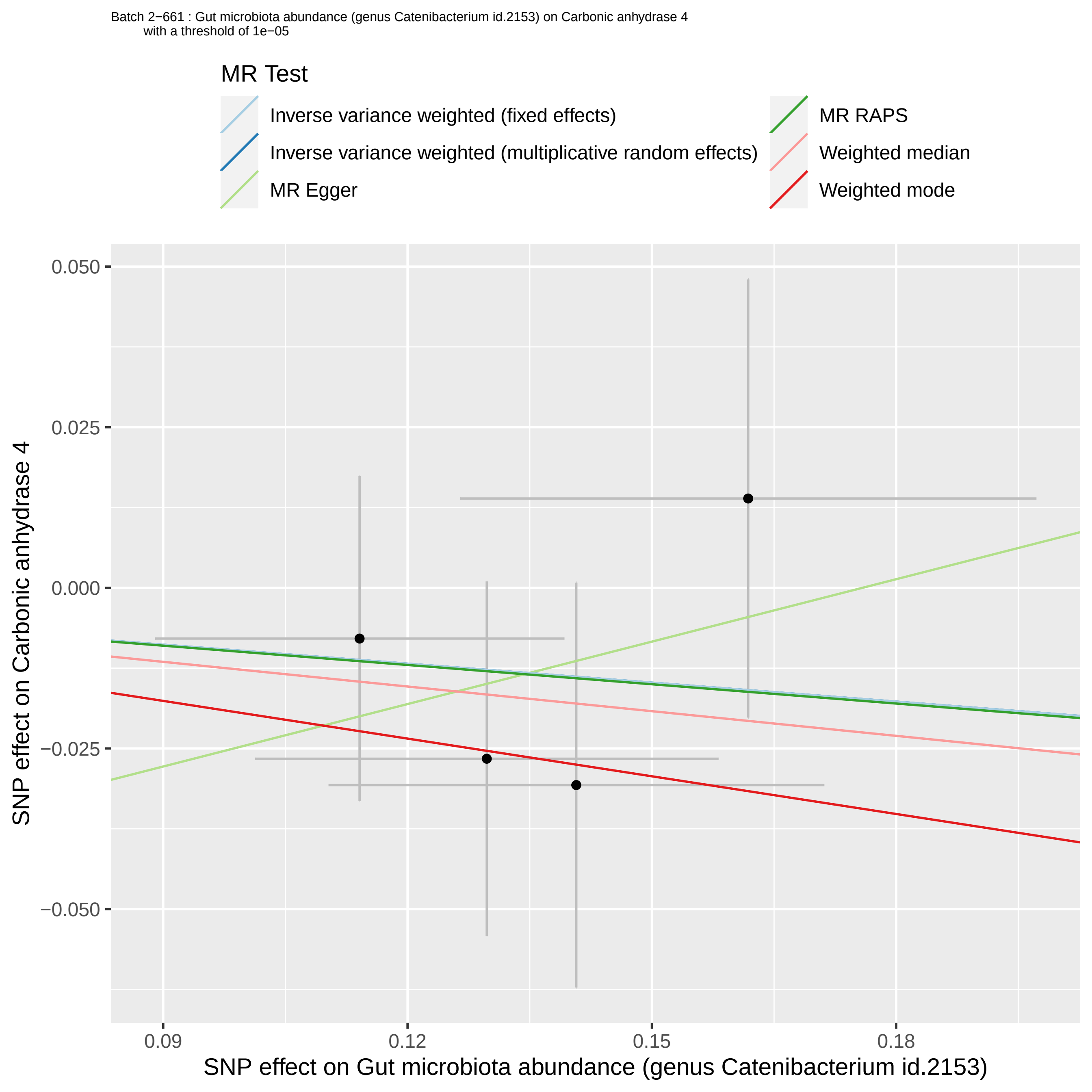

Batch 2−661 : Gut microbiota abundance (genus Catenibacterium id.2153) on Carbonic anhydrase 4
with a threshold of 1e−05
MR Test
Inverse variance weighted (fixed effects)
Inverse variance weighted (multiplicative random effects)
MR Egger
MR RAPS
Weighted median
Weighted mode
0.050
0.025
0.000
−0.025
−0.050
0.09
0.12
0.15
0.18
SNP effect on Carbonic anhydrase 4
SNP effect on Gut microbiota abundance (genus Catenibacterium id.2153)

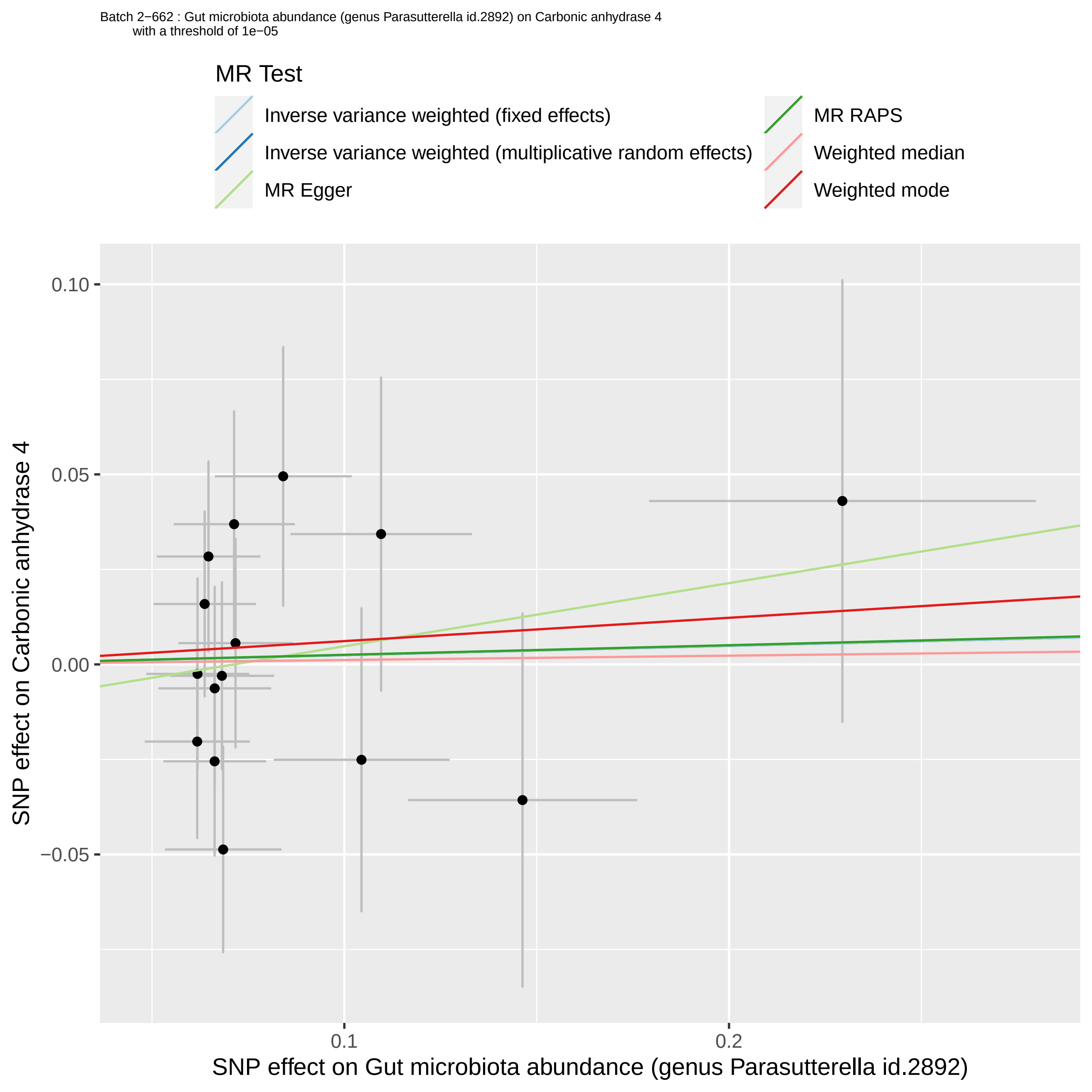

Batch 2−662 : Gut microbiota abundance (genus Parasutterella id.2892) on Carbonic anhydrase 4
with a threshold of 1e−05
MR Test
Inverse variance weighted (fixed effects)
Inverse variance weighted (multiplicative random effects)
MR Egger
MR RAPS
Weighted median
Weighted mode
0.10
0.05
0.00
−0.05
0.1
0.2
SNP effect on Carbonic anhydrase 4
SNP effect on Gut microbiota abundance (genus Parasutterella id.2892)

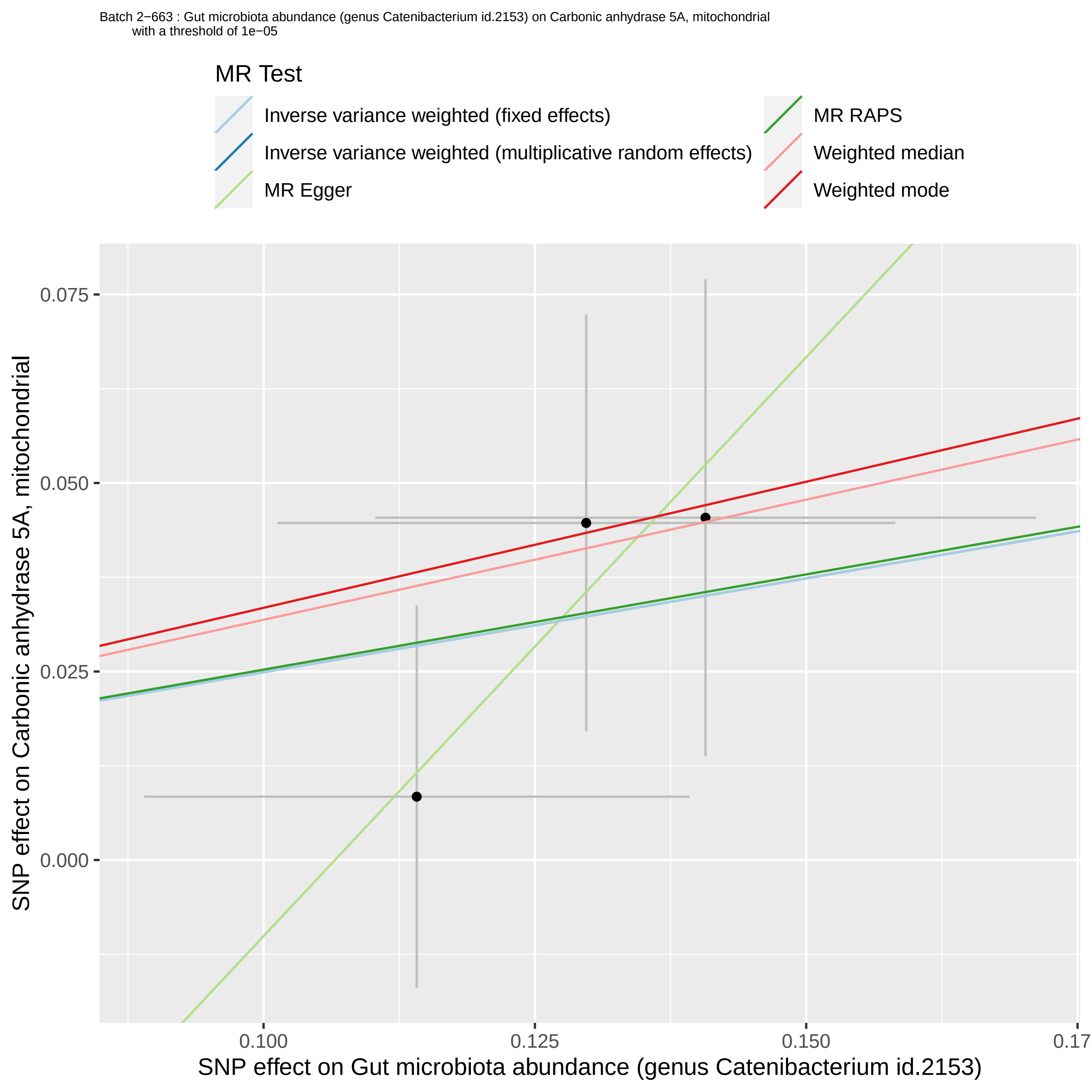
Batch 2−663 : Gut microbiota abundance (genus Catenibacterium id.2153) on Carbonic anhydrase 5A, mitochondrial
with a threshold of 1e−05
MR Test
Inverse variance weighted (fixed effects)
Inverse variance weighted (multiplicative random effects)
MR Egger
MR RAPS
Weighted median
Weighted mode
0.075
0.050
0.025
0.000
0.100
0.125
0.150
0.17
SNP effect on Carbonic anhydrase 5A, mitochondrial
SNP effect on Gut microbiota abundance (genus Catenibacterium id.2153)

Batch 2−664 : Gut microbiota abundance (genus Parasutterella id.2892) on Carbonic anhydrase 5A, mitochondrial
with a threshold of 1e−05

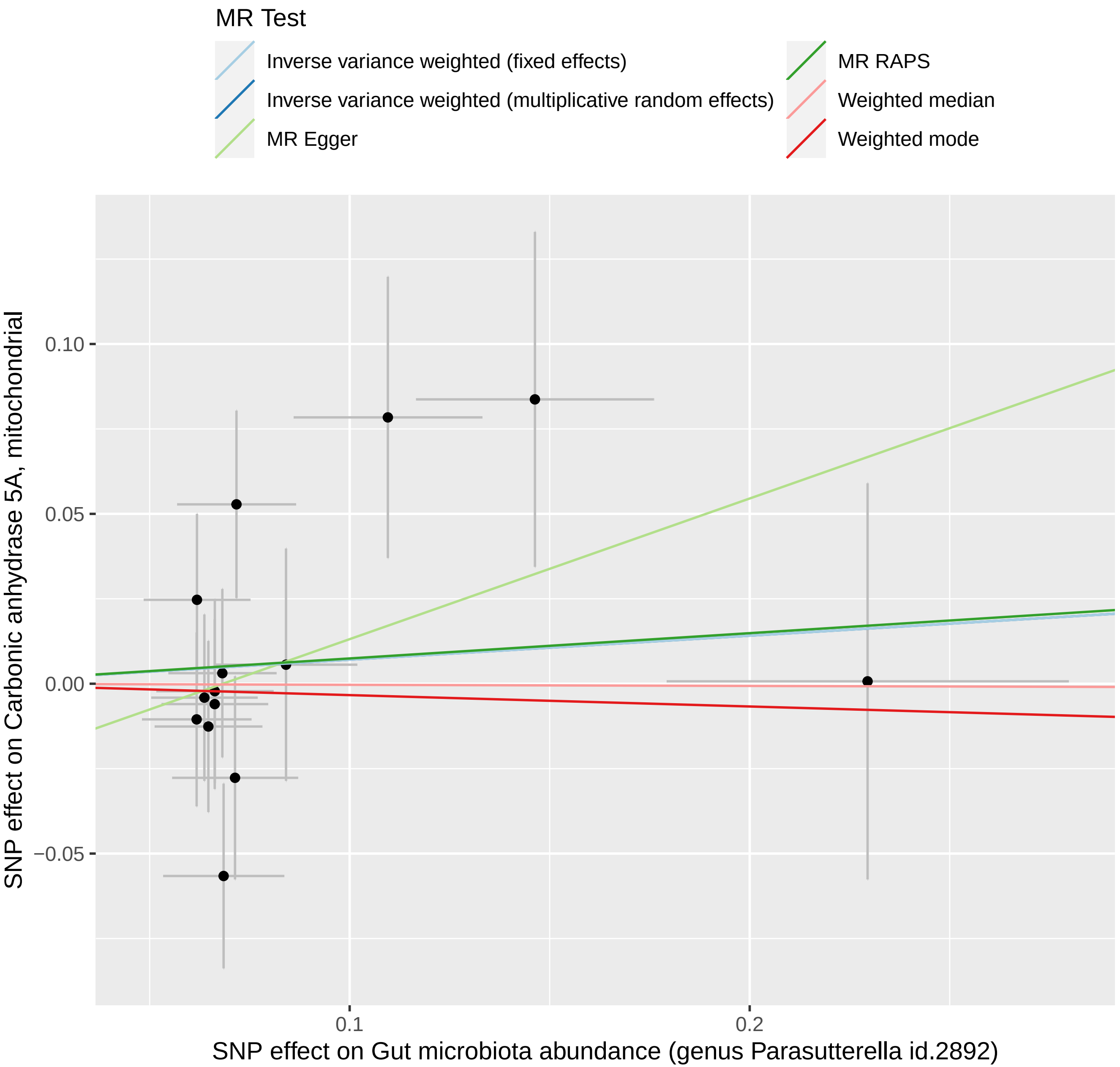

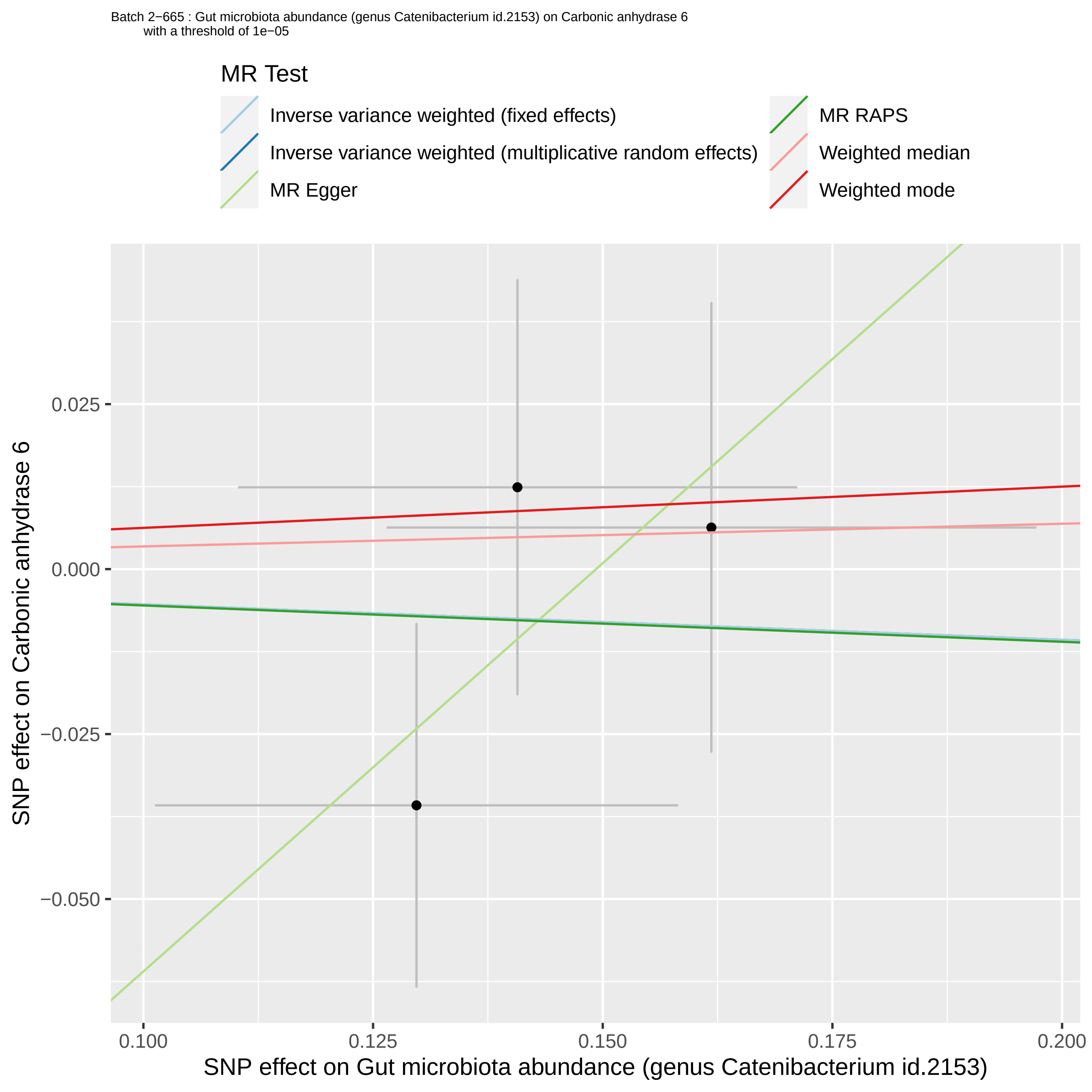
Batch 2−665 : Gut microbiota abundance (genus Catenibacterium id.2153) on Carbonic anhydrase 6
with a threshold of 1e−05
MR Test
Inverse variance weighted (fixed effects)
Inverse variance weighted (multiplicative random effects)
MR Egger
MR RAPS
Weighted median
Weighted mode
0.025
0.000
−0.025
−0.050
0.100
0.125
0.150
0.175
0.200
SNP effect on Carbonic anhydrase 6
SNP effect on Gut microbiota abundance (genus Catenibacterium id.2153)

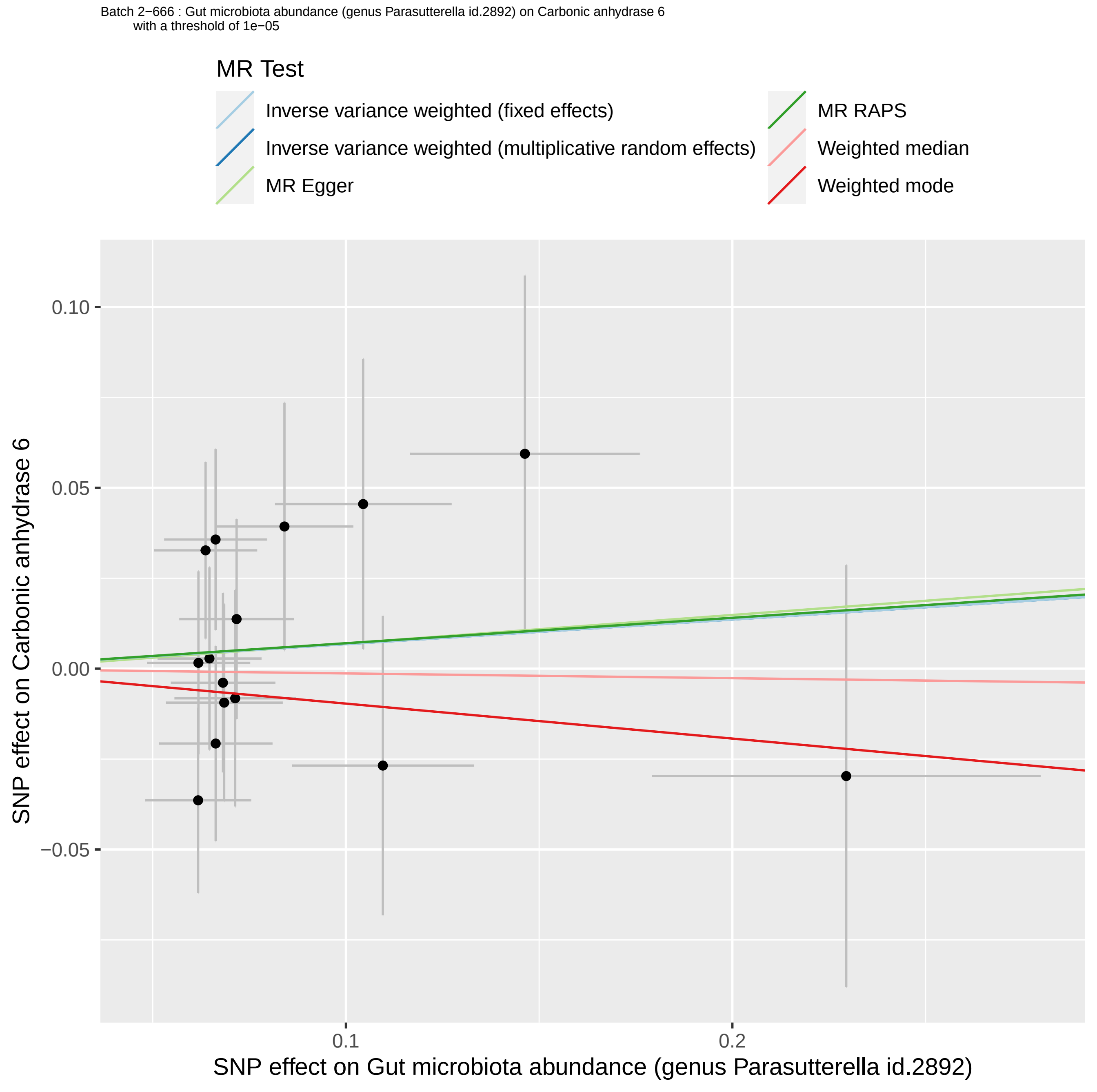

Batch 2−666 : Gut microbiota abundance (genus Parasutterella id.2892) on Carbonic anhydrase 6
with a threshold of 1e−05
MR Test
Inverse variance weighted (fixed effects)
Inverse variance weighted (multiplicative random effects)
MR Egger
MR RAPS
Weighted median
Weighted mode
0.10
0.05
0.00
−0.05
0.1
0.2
SNP effect on Carbonic anhydrase 6
SNP effect on Gut microbiota abundance (genus Parasutterella id.2892)

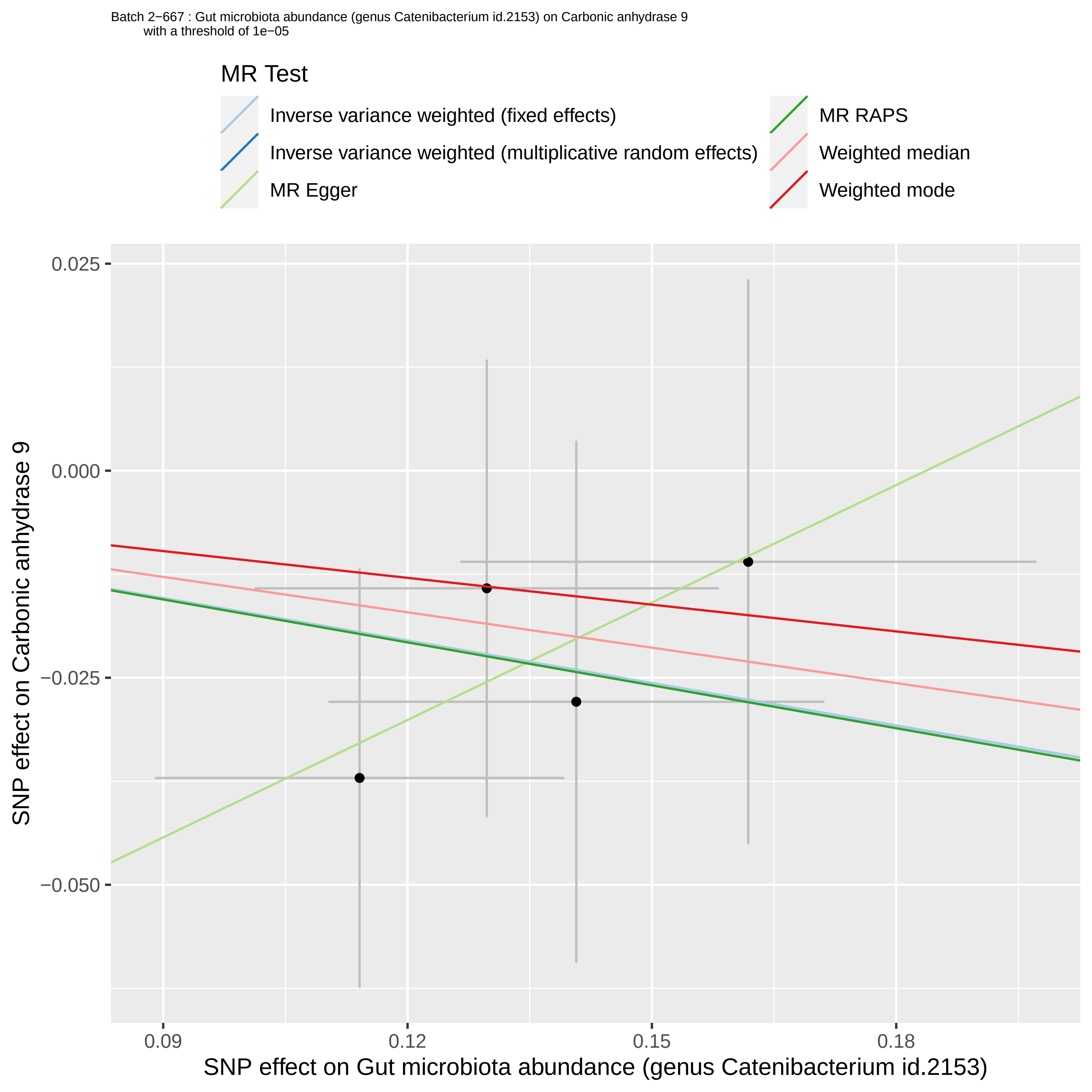

Batch 2−667 : Gut microbiota abundance (genus Catenibacterium id.2153) on Carbonic anhydrase 9
with a threshold of 1e−05
MR Test
Inverse variance weighted (fixed effects)
Inverse variance weighted (multiplicative random effects)
MR Egger
MR RAPS
Weighted median
Weighted mode
0.025
0.000
−0.025
−0.050
0.09
0.12
0.15
0.18
SNP effect on Carbonic anhydrase 9
SNP effect on Gut microbiota abundance (genus Catenibacterium id.2153)

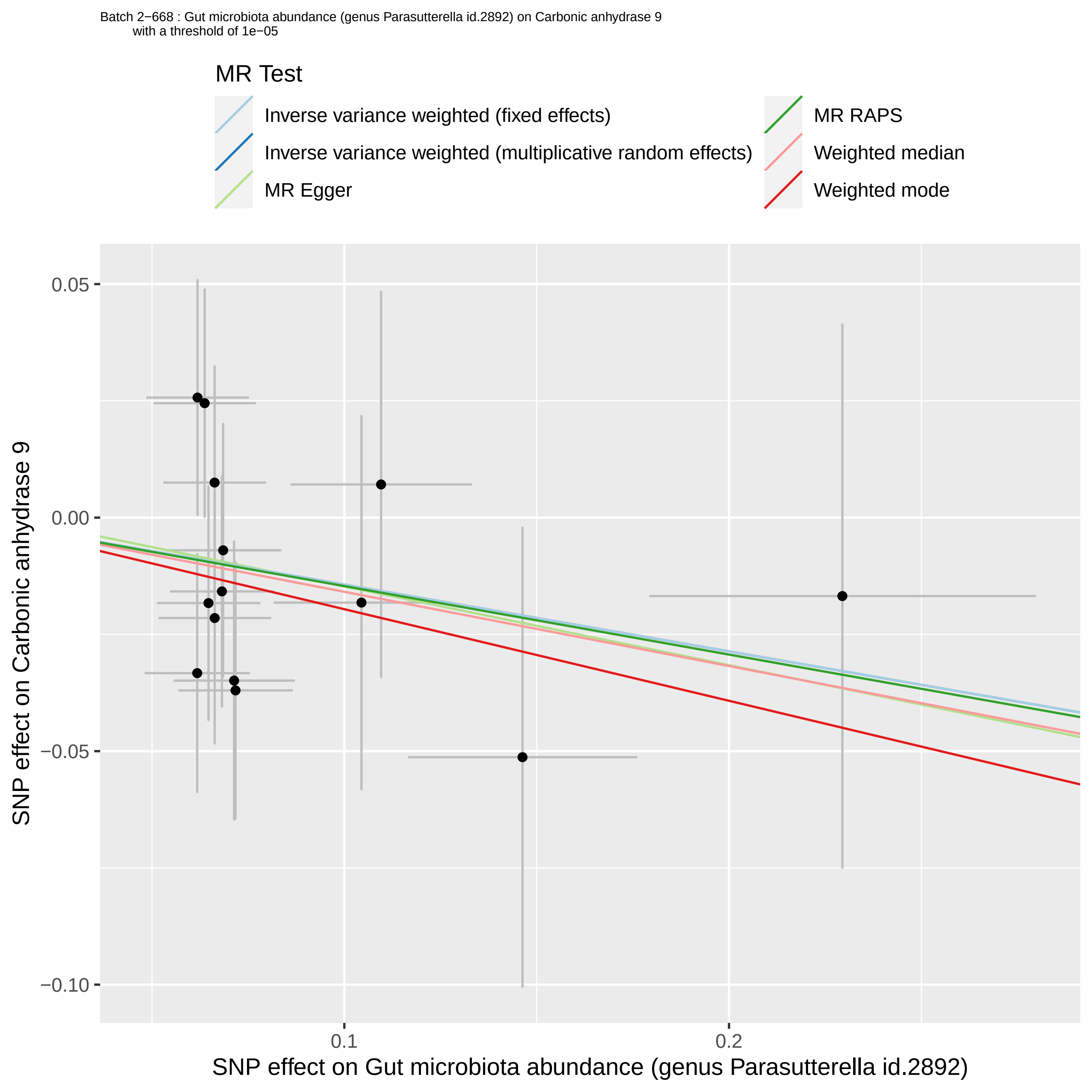
Batch 2−668 : Gut microbiota abundance (genus Parasutterella id.2892) on Carbonic anhydrase 9
with a threshold of 1e−05
MR Test
Inverse variance weighted (fixed effects)
Inverse variance weighted (multiplicative random effects)
MR Egger
MR RAPS
Weighted median
Weighted mode
0.05
0.00
−0.05
−0.10
0.1
0.2
SNP effect on Carbonic anhydrase 9
SNP effect on Gut microbiota abundance (genus Parasutterella id.2892)

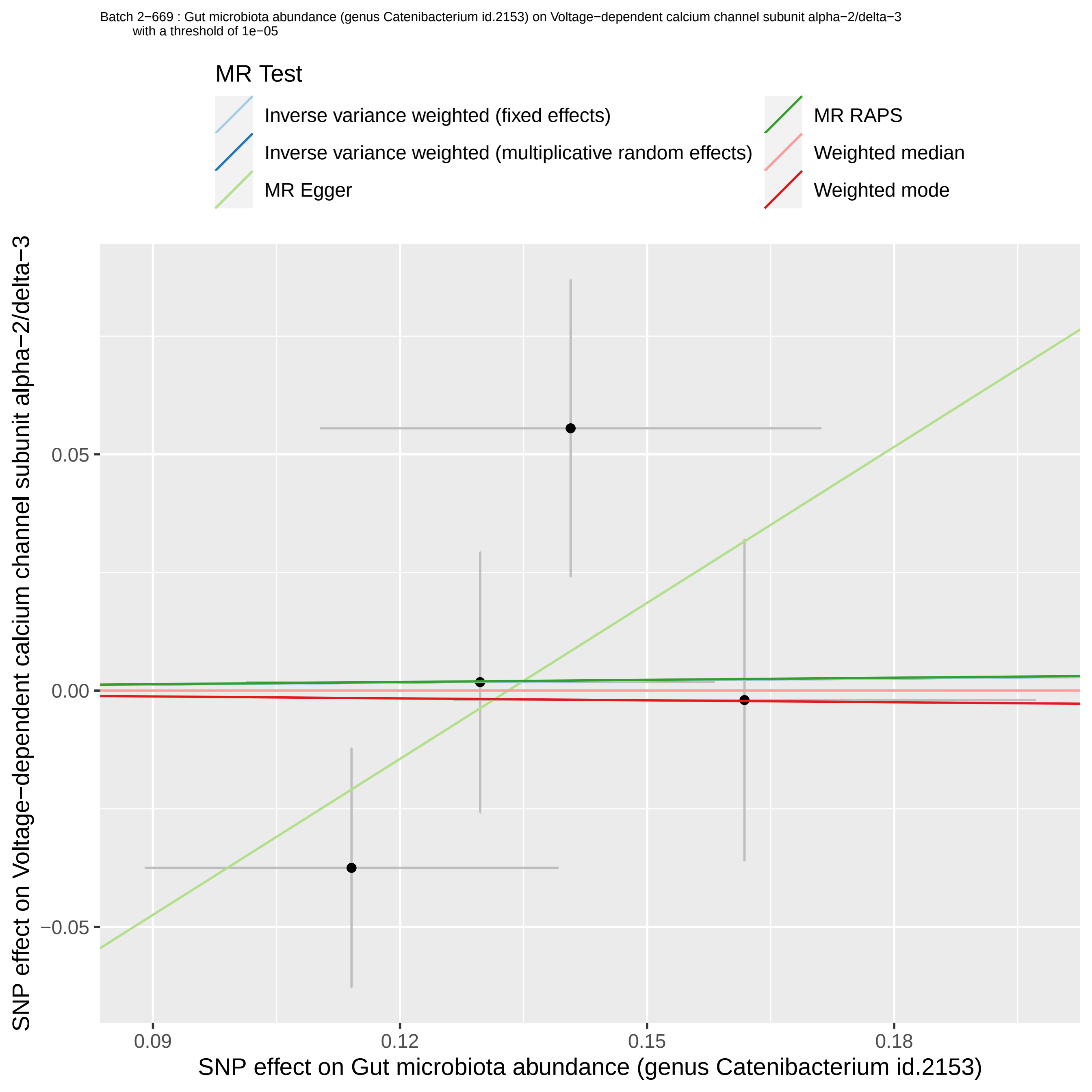

Batch 2−669 : Gut microbiota abundance (genus Catenibacterium id.2153) on Voltage−dependent calcium channel subunit alpha−2/delta−3
with a threshold of 1e−05
MR Test
Inverse variance weighted (fixed effects)
Inverse variance weighted (multiplicative random effects)
MR Egger
MR RAPS
Weighted median
Weighted mode
SNP effect on Voltage−dependent calcium channel subunit alpha−2/delta−3
0.05
0.00
−0.05
0.09
0.12
0.15
0.18
SNP effect on Gut microbiota abundance (genus Catenibacterium id.2153)

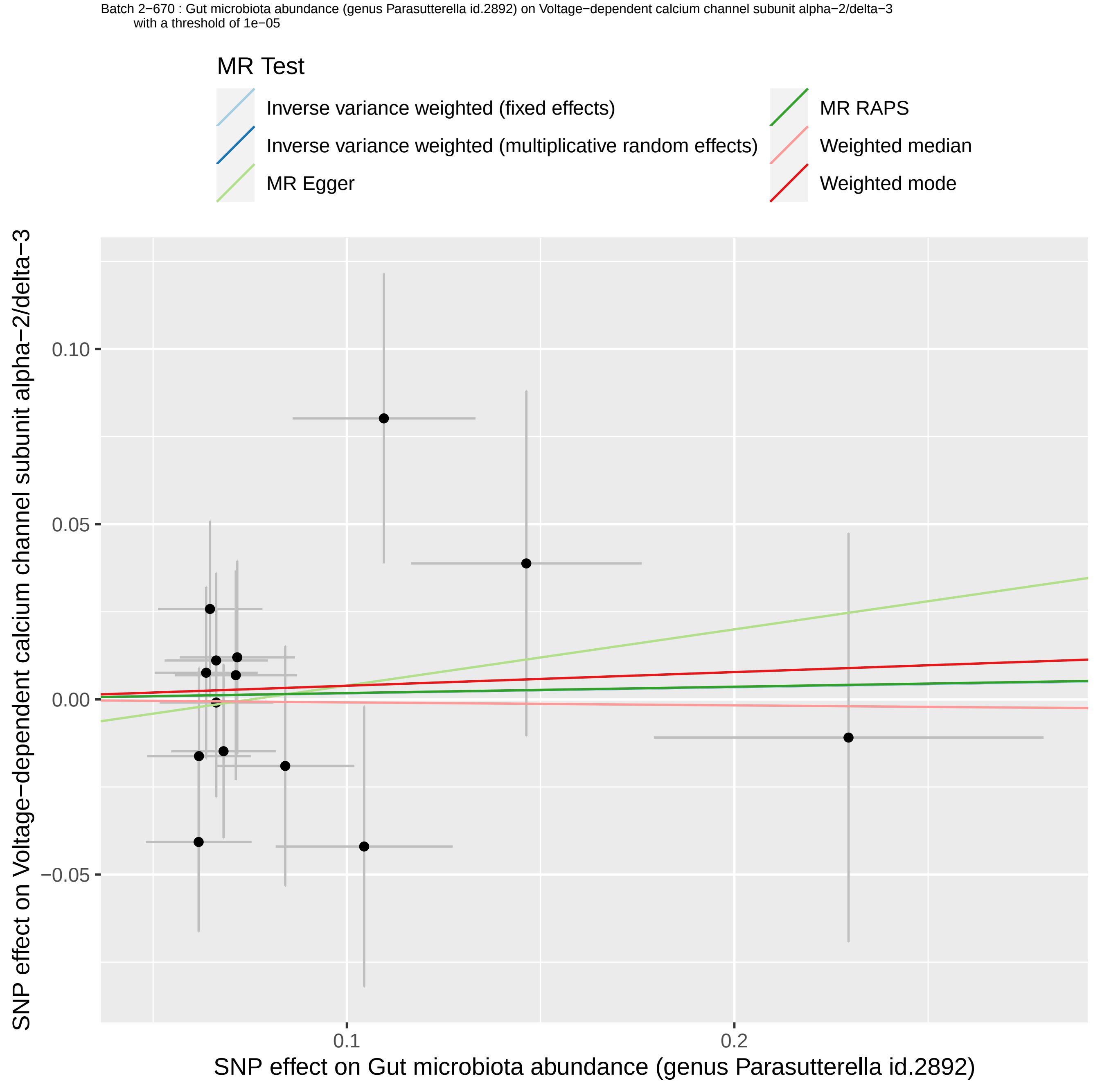

Batch 2−670 : Gut microbiota abundance (genus Parasutterella id.2892) on Voltage−dependent calcium channel subunit alpha−2/delta−3
with a threshold of 1e−05
MR Test
Inverse variance weighted (fixed effects)
Inverse variance weighted (multiplicative random effects)
MR Egger
MR RAPS
Weighted median
Weighted mode
SNP effect on Voltage−dependent calcium channel subunit alpha−2/delta−3
0.10
0.05
0.00
−0.05
0.1
0.2
SNP effect on Gut microbiota abundance (genus Parasutterella id.2892)

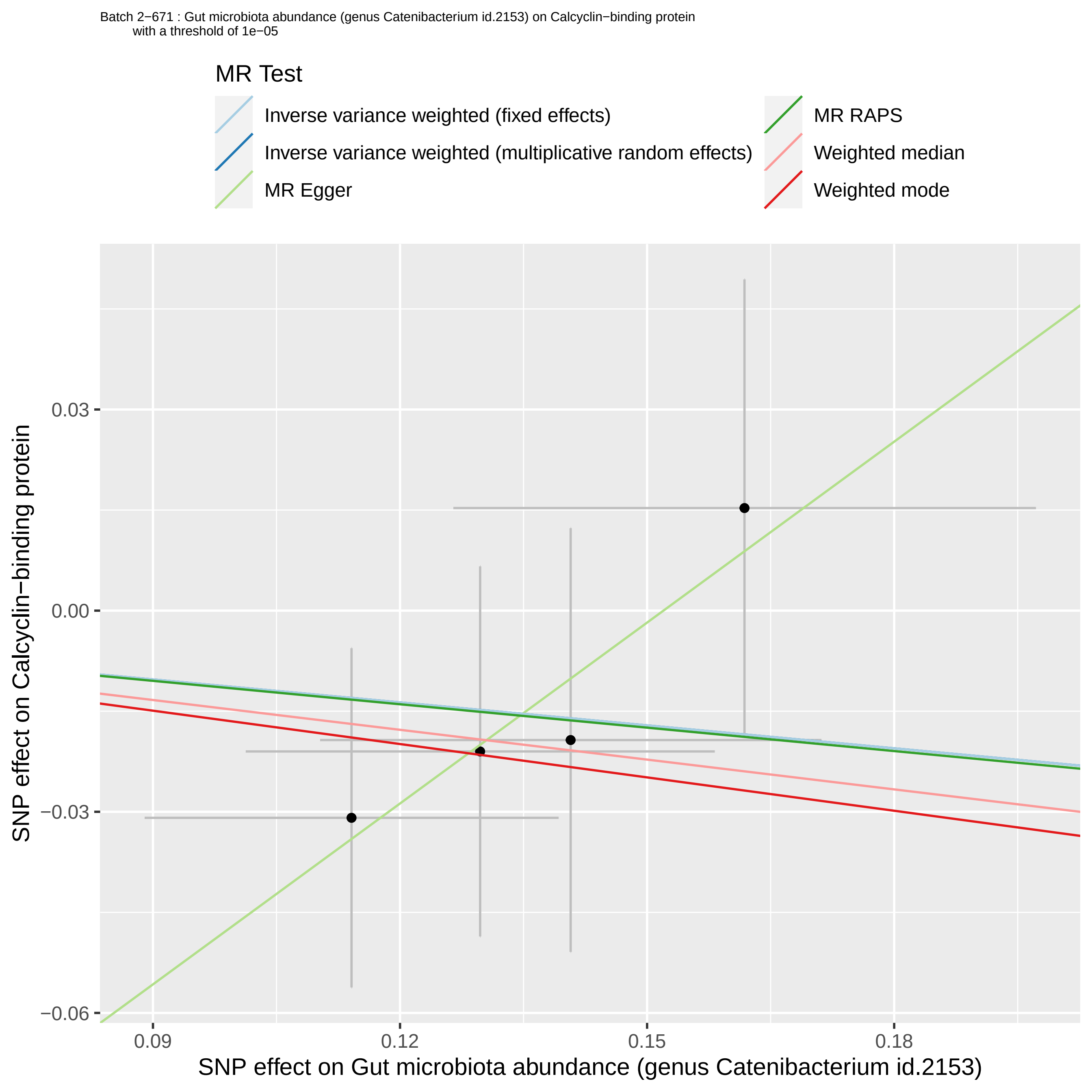

Batch 2−671 : Gut microbiota abundance (genus Catenibacterium id.2153) on Calcyclin−binding protein
with a threshold of 1e−05
MR Test
Inverse variance weighted (fixed effects)
Inverse variance weighted (multiplicative random effects)
MR Egger
MR RAPS
Weighted median
Weighted mode
0.03
0.00
−0.03
−0.06
0.09
0.12
0.15
0.18
SNP effect on Calcyclin−binding protein
SNP effect on Gut microbiota abundance (genus Catenibacterium id.2153)

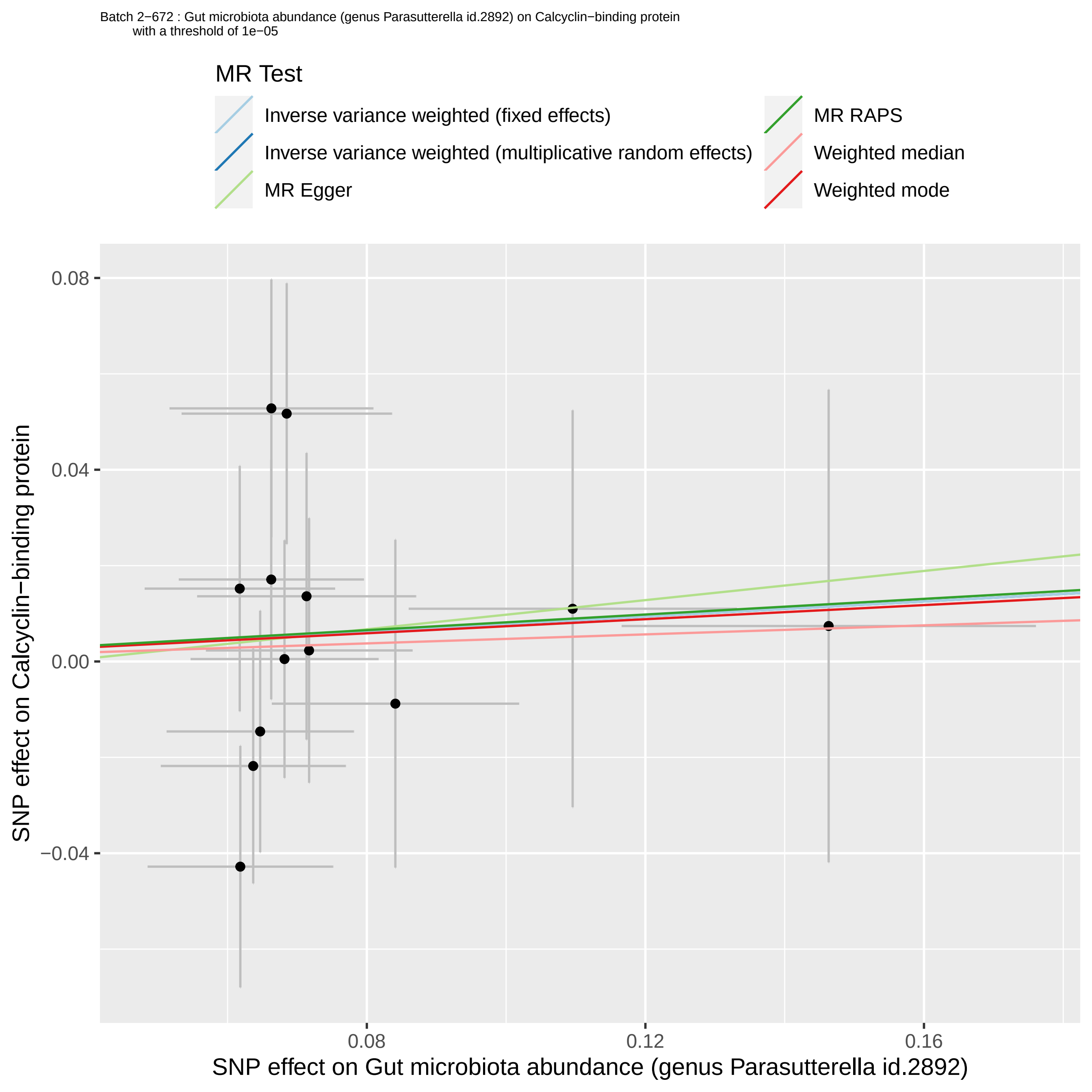

Batch 2−672 : Gut microbiota abundance (genus Parasutterella id.2892) on Calcyclin−binding protein
with a threshold of 1e−05
MR Test
Inverse variance weighted (fixed effects)
Inverse variance weighted (multiplicative random effects)
MR Egger
MR RAPS
Weighted median
Weighted mode
0.08
0.04
0.00
−0.04
0.08
0.12
0.16
SNP effect on Calcyclin−binding protein
SNP effect on Gut microbiota abundance (genus Parasutterella id.2892)

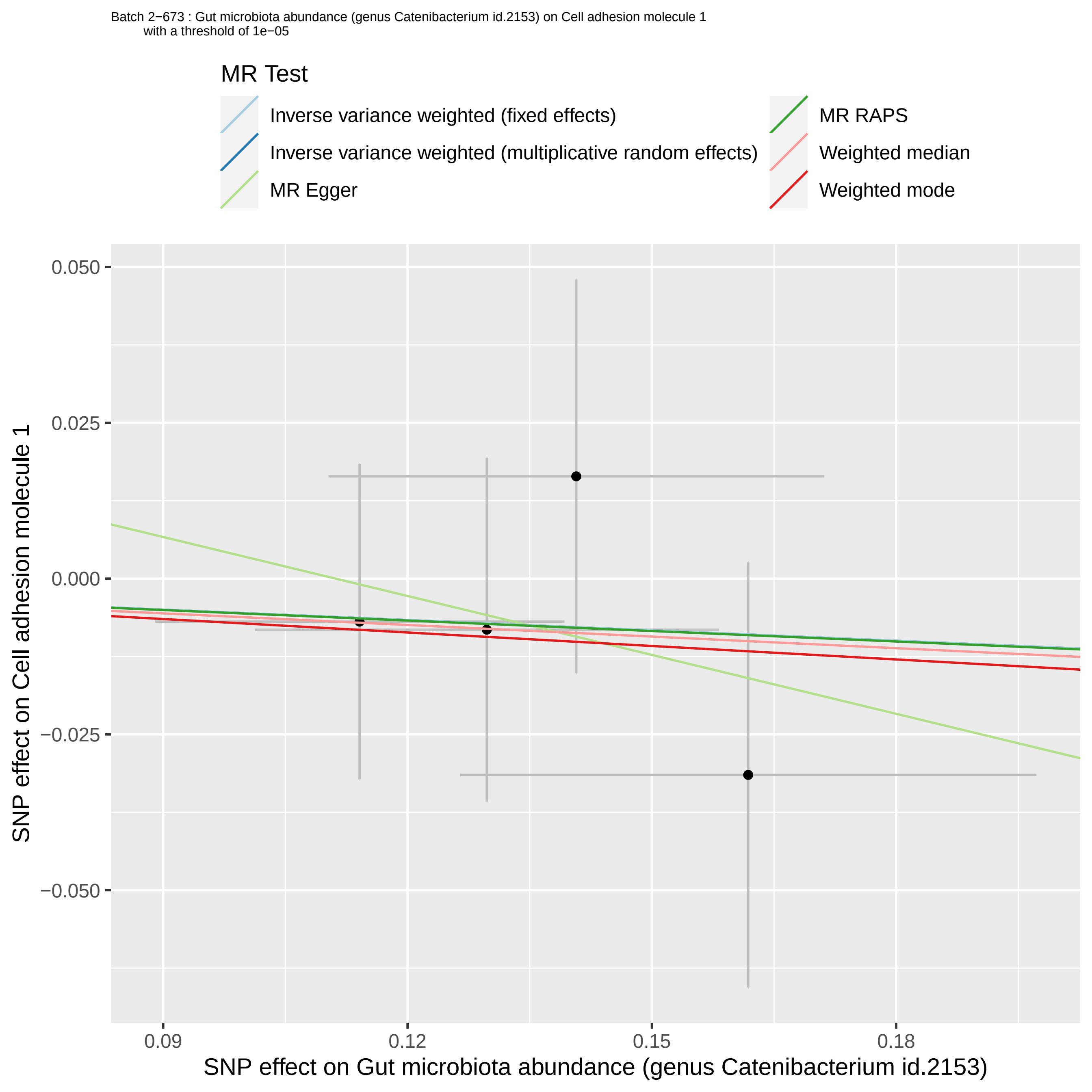
Batch 2−673 : Gut microbiota abundance (genus Catenibacterium id.2153) on Cell adhesion molecule 1
with a threshold of 1e−05
MR Test
Inverse variance weighted (fixed effects)
Inverse variance weighted (multiplicative random effects)
MR Egger
MR RAPS
Weighted median
Weighted mode
0.050
0.025
0.000
−0.025
−0.050
0.09
0.12
0.15
0.18
SNP effect on Cell adhesion molecule 1
SNP effect on Gut microbiota abundance (genus Catenibacterium id.2153)

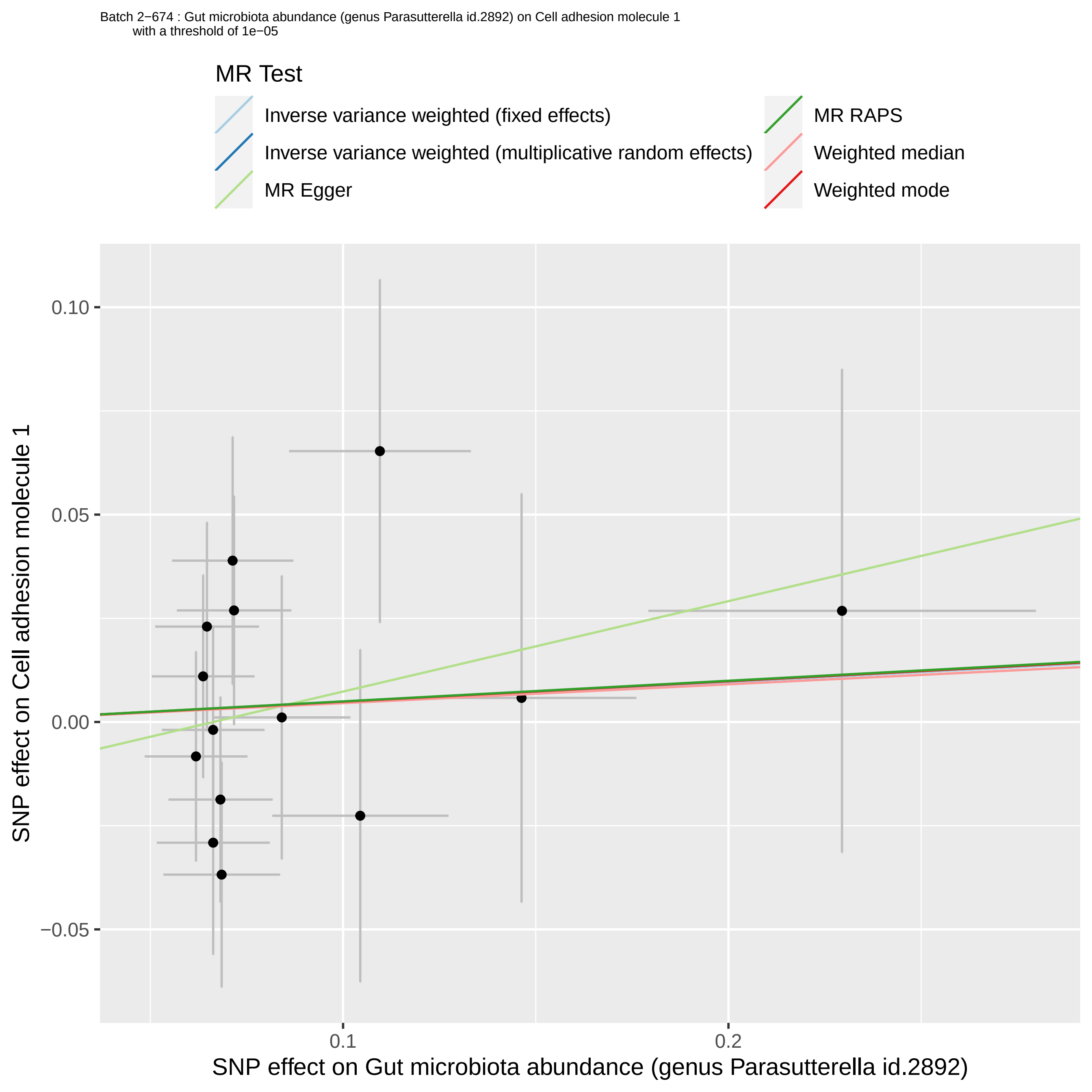
Batch 2−674 : Gut microbiota abundance (genus Parasutterella id.2892) on Cell adhesion molecule 1
with a threshold of 1e−05
MR Test
Inverse variance weighted (fixed effects)
Inverse variance weighted (multiplicative random effects)
MR Egger
MR RAPS
Weighted median
Weighted mode
0.10
0.05
0.00
−0.05
0.1
0.2
SNP effect on Cell adhesion molecule 1
SNP effect on Gut microbiota abundance (genus Parasutterella id.2892)

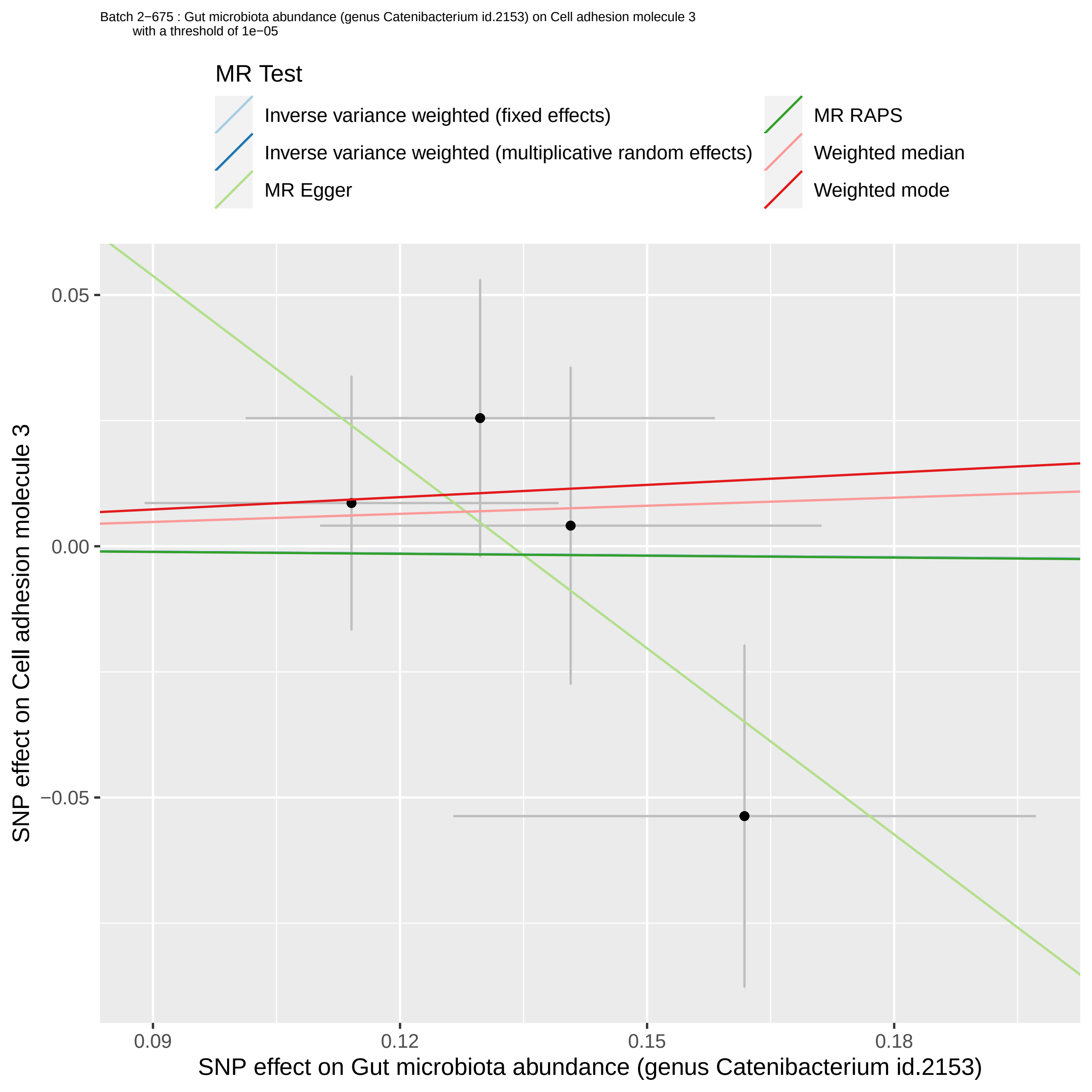
Batch 2−675 : Gut microbiota abundance (genus Catenibacterium id.2153) on Cell adhesion molecule 3
with a threshold of 1e−05
MR Test
Inverse variance weighted (fixed effects)
Inverse variance weighted (multiplicative random effects)
MR Egger
MR RAPS
Weighted median
Weighted mode
0.05
0.00
−0.05
0.09
0.12
0.15
0.18
SNP effect on Cell adhesion molecule 3
SNP effect on Gut microbiota abundance (genus Catenibacterium id.2153)

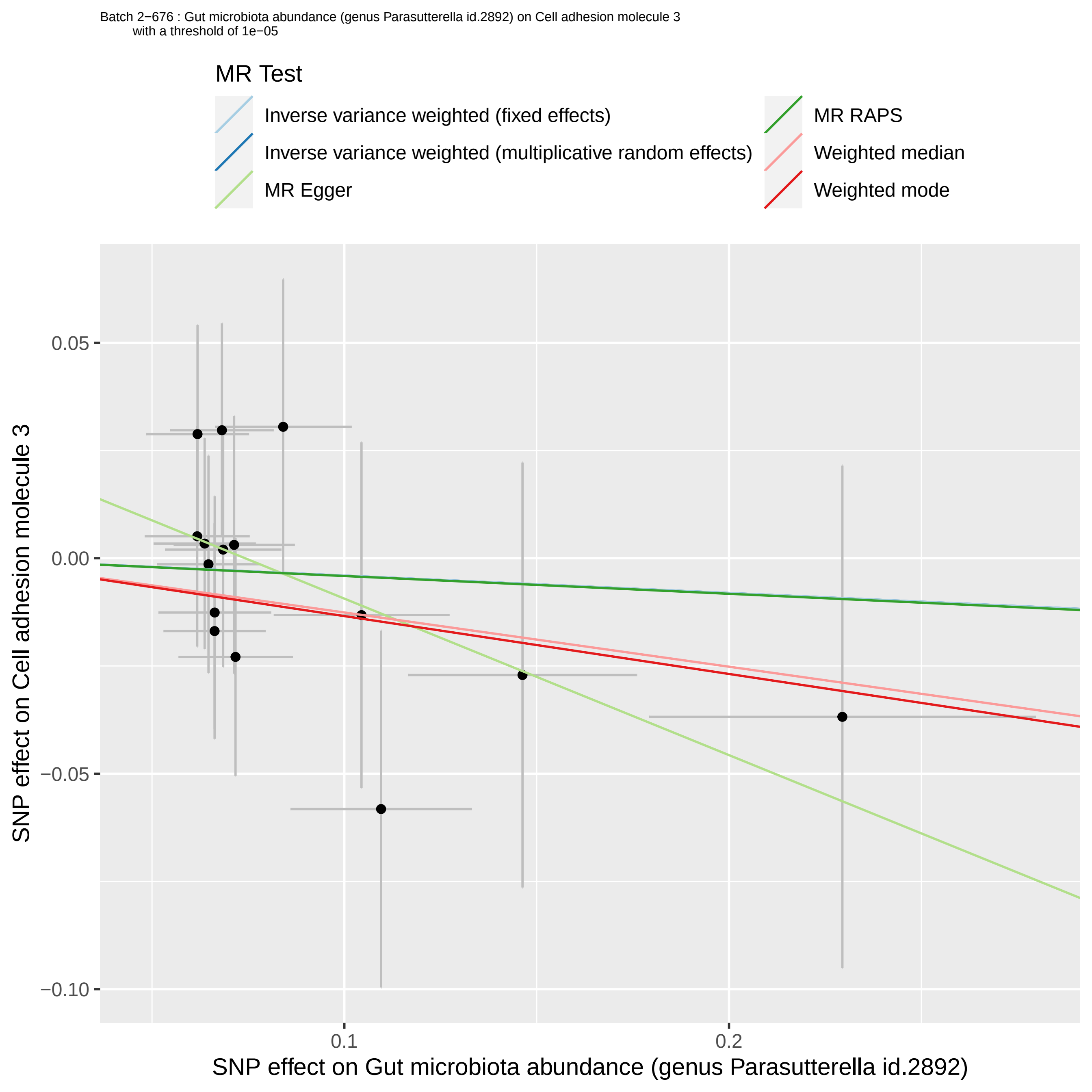

Batch 2−676 : Gut microbiota abundance (genus Parasutterella id.2892) on Cell adhesion molecule 3
with a threshold of 1e−05
MR Test
Inverse variance weighted (fixed effects)
Inverse variance weighted (multiplicative random effects)
MR Egger
MR RAPS
Weighted median
Weighted mode
0.05
0.00
−0.05
−0.10
0.1
0.2
SNP effect on Cell adhesion molecule 3
SNP effect on Gut microbiota abundance (genus Parasutterella id.2892)

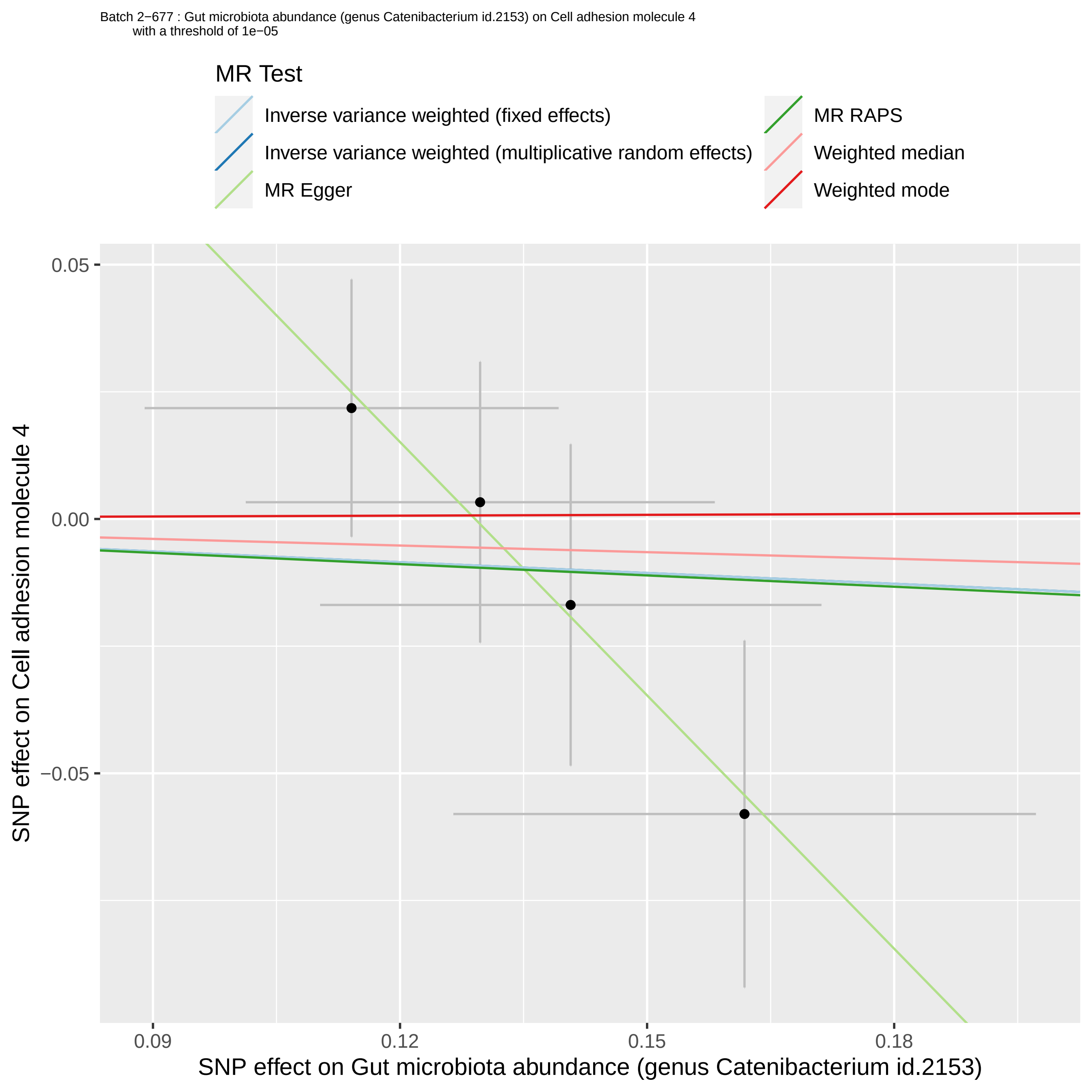
Batch 2−677 : Gut microbiota abundance (genus Catenibacterium id.2153) on Cell adhesion molecule 4
with a threshold of 1e−05
MR Test
Inverse variance weighted (fixed effects)
Inverse variance weighted (multiplicative random effects)
MR Egger
MR RAPS
Weighted median
Weighted mode
0.05
0.00
−0.05
0.09
0.12
0.15
0.18
SNP effect on Cell adhesion molecule 4
SNP effect on Gut microbiota abundance (genus Catenibacterium id.2153)

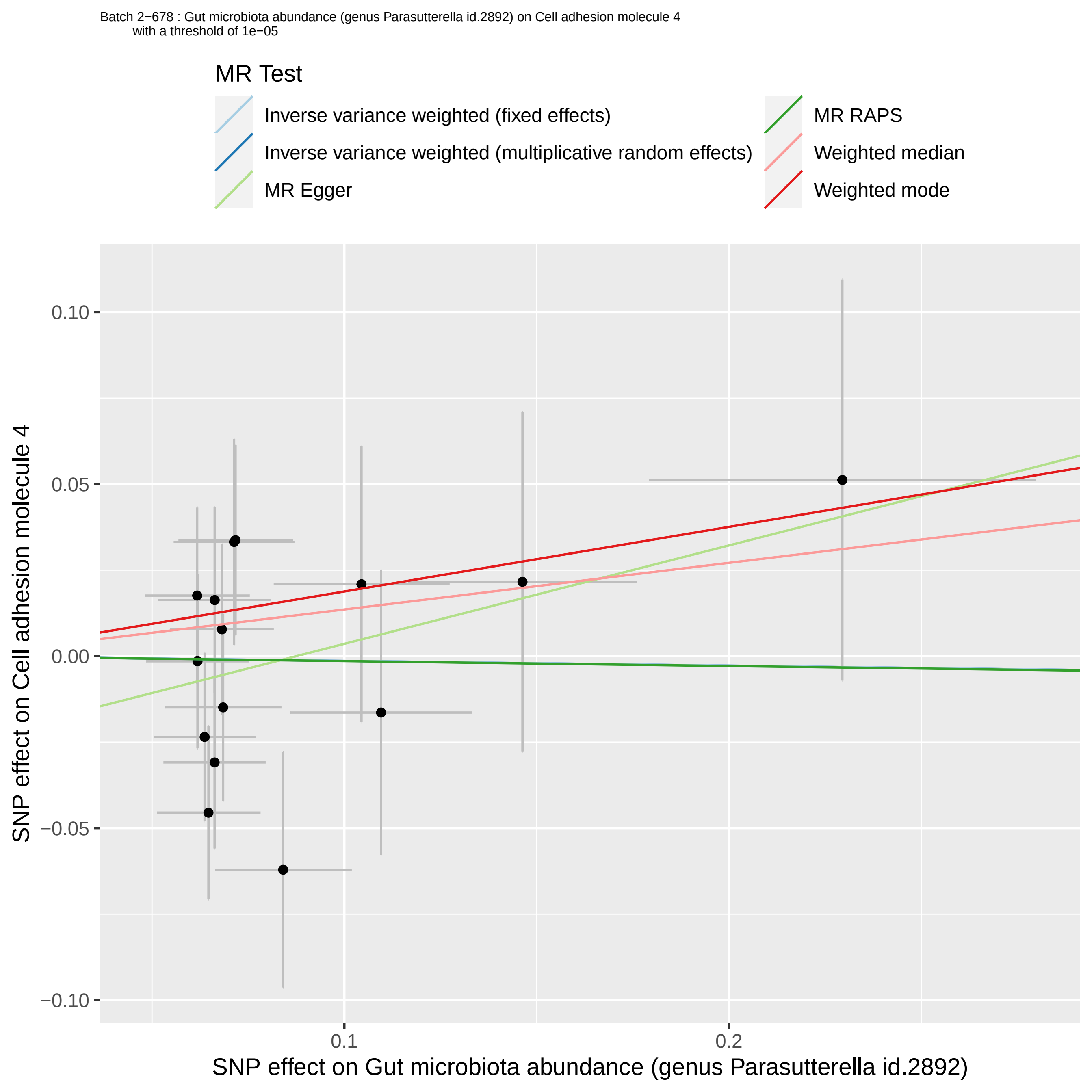

Batch 2−678 : Gut microbiota abundance (genus Parasutterella id.2892) on Cell adhesion molecule 4
with a threshold of 1e−05
MR Test
Inverse variance weighted (fixed effects)
Inverse variance weighted (multiplicative random effects)
MR Egger
MR RAPS
Weighted median
Weighted mode
0.10
0.05
0.00
−0.05
−0.10
0.1
0.2
SNP effect on Cell adhesion molecule 4
SNP effect on Gut microbiota abundance (genus Parasutterella id.2892)

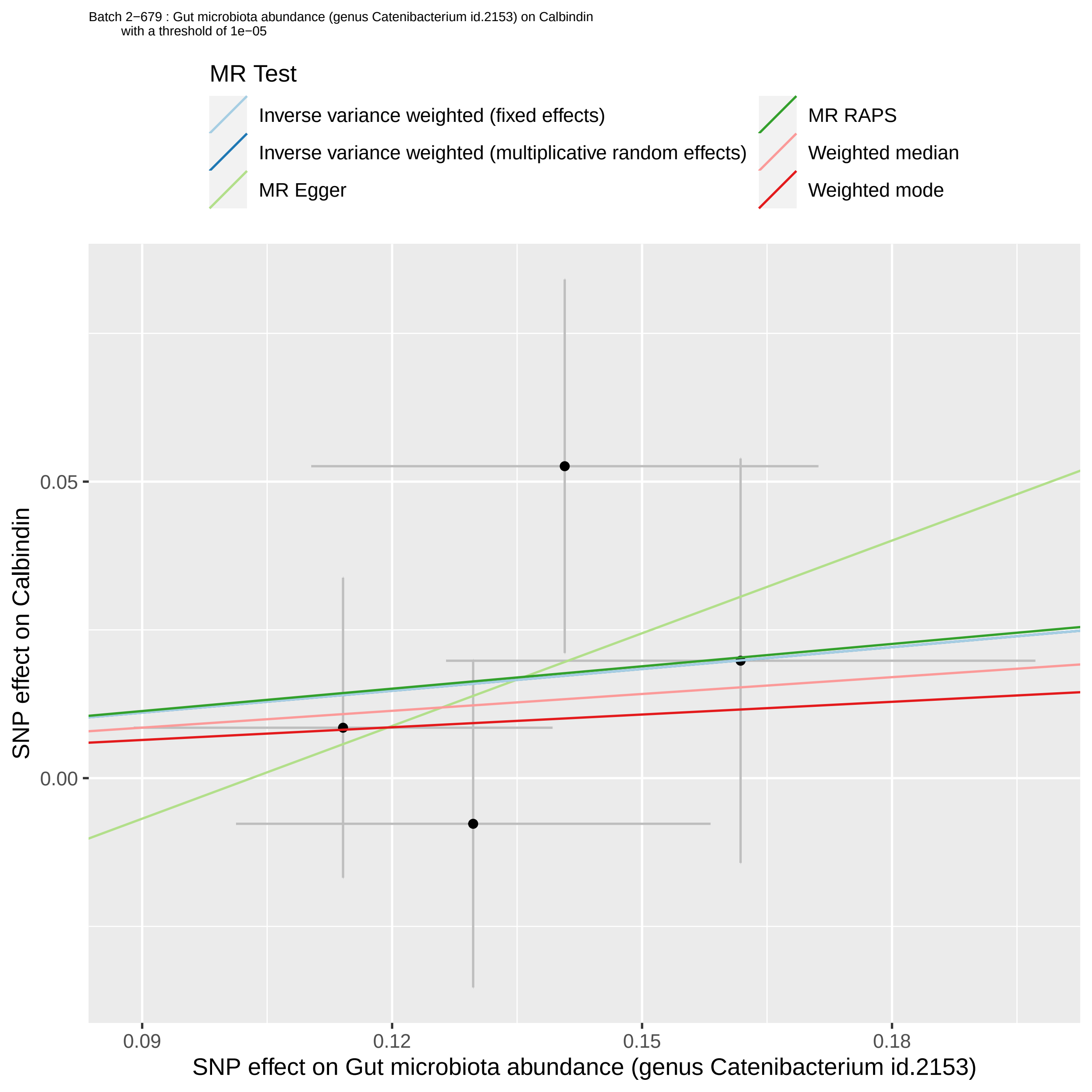

Batch 2−679 : Gut microbiota abundance (genus Catenibacterium id.2153) on Calbindin
with a threshold of 1e−05
MR Test
Inverse variance weighted (fixed effects)
Inverse variance weighted (multiplicative random effects)
MR Egger
MR RAPS
Weighted median
Weighted mode
SNP effect on Calbindin
0.05
0.00
0.09
0.12
0.15
0.18
SNP effect on Gut microbiota abundance (genus Catenibacterium id.2153)

Batch 2−680 : Gut microbiota abundance (genus Parasutterella id.2892) on Calbindin
with a threshold of 1e−05

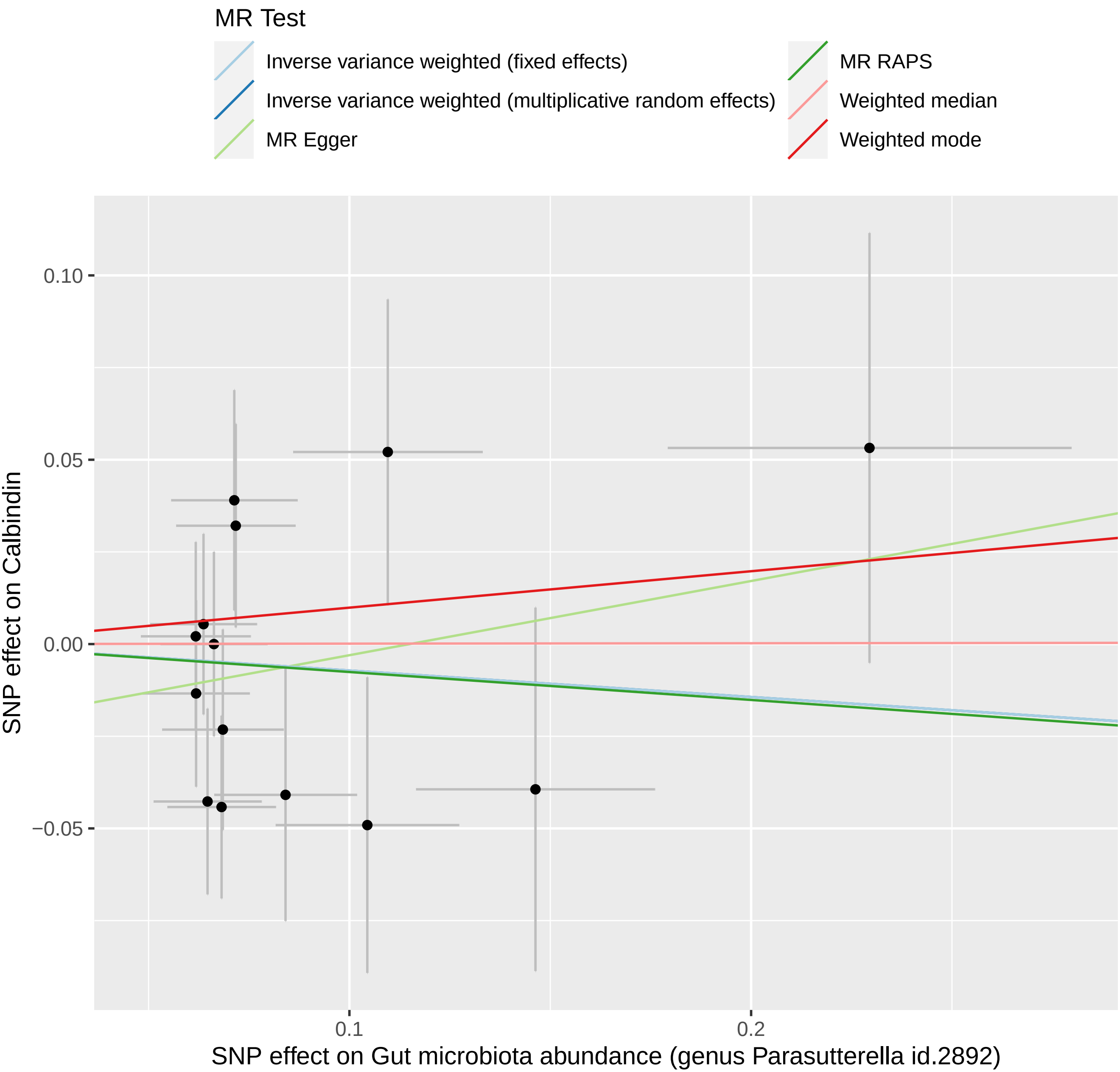

Batch 2−681 : Gut microbiota abundance (genus Catenibacterium id.2153) on Procalcitonin
with a threshold of 1e−05

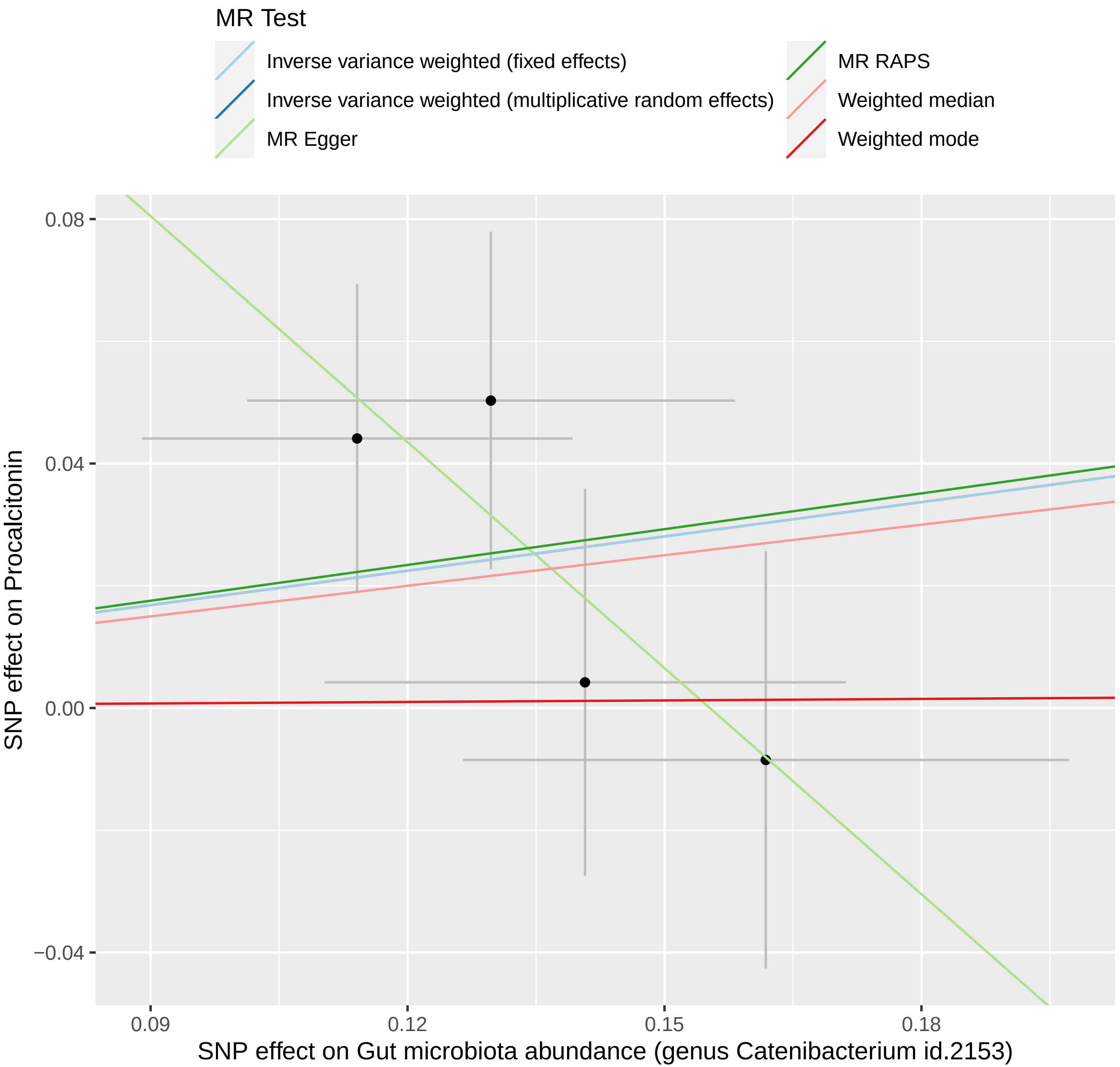

Batch 2−682 : Gut microbiota abundance (genus Parasutterella id.2892) on Procalcitonin
with a threshold of 1e−05

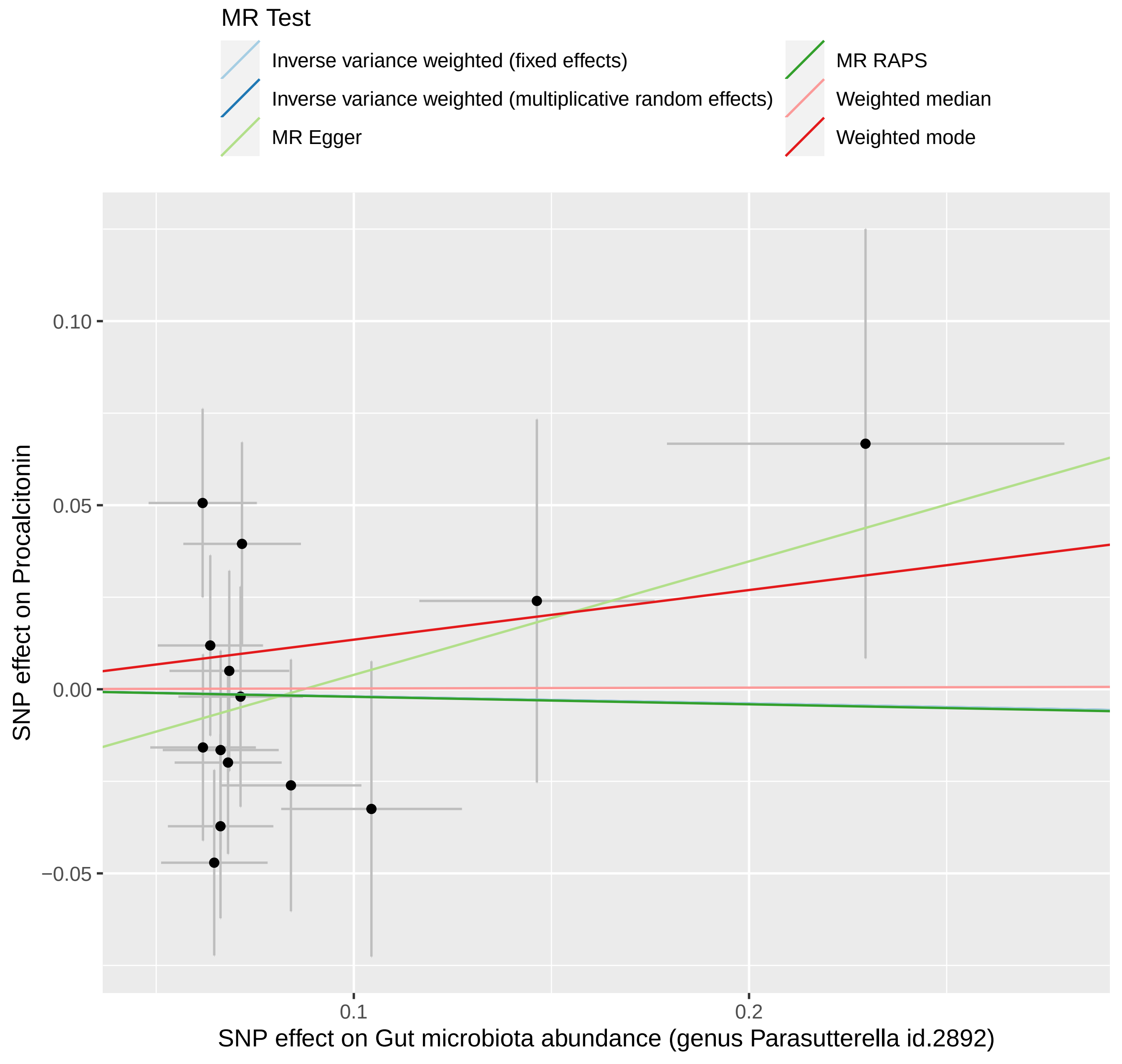

Batch 2−683 : Gut microbiota abundance (genus Catenibacterium id.2153) on Calcium−binding and coiled−coil domain−containing protein 2
with a threshold of 1e−05

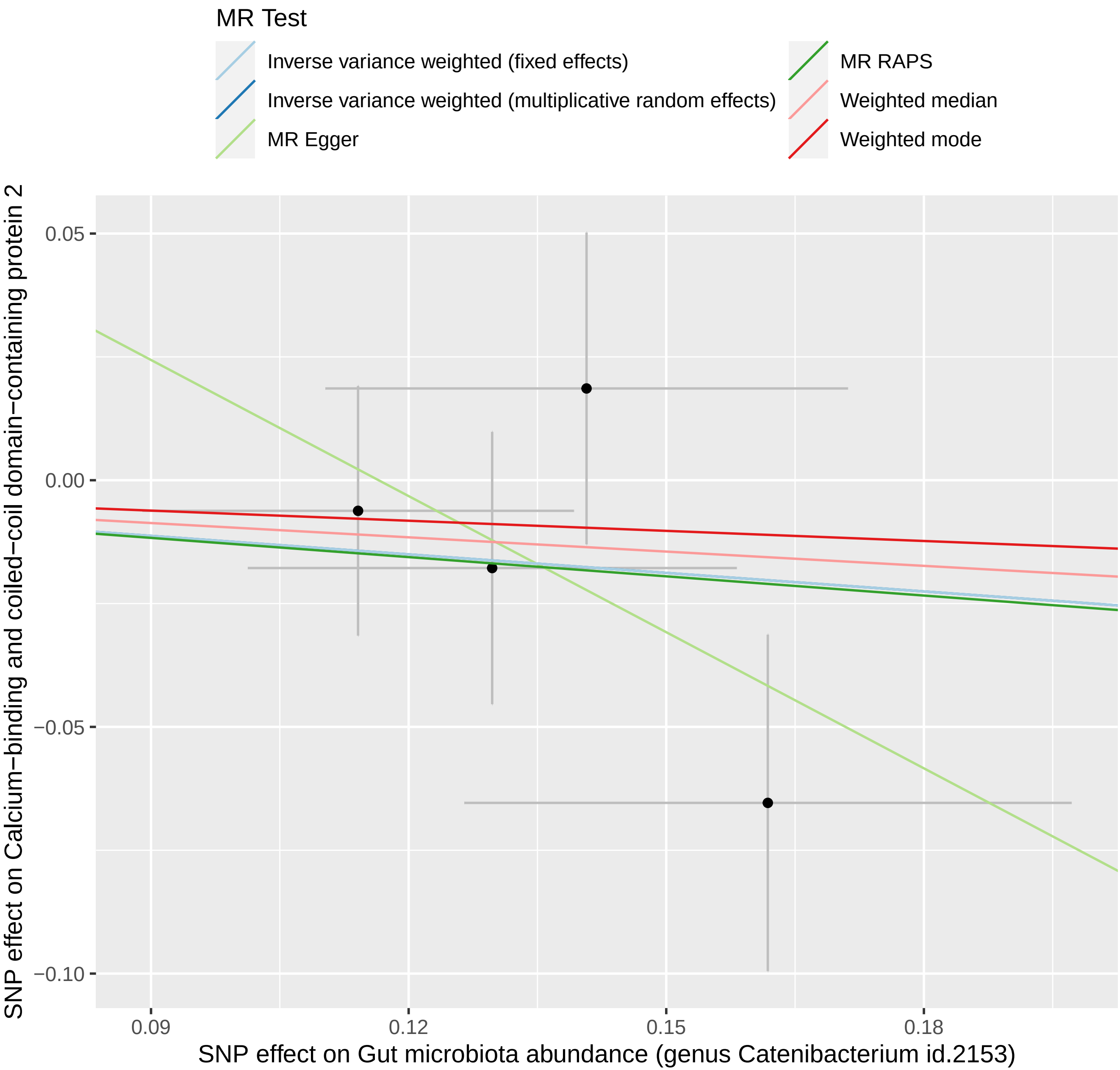

Batch 2−684 : Gut microbiota abundance (genus Parasutterella id.2892) on Calcium−binding and coiled−coil domain−containing protein 2
with a threshold of 1e−05

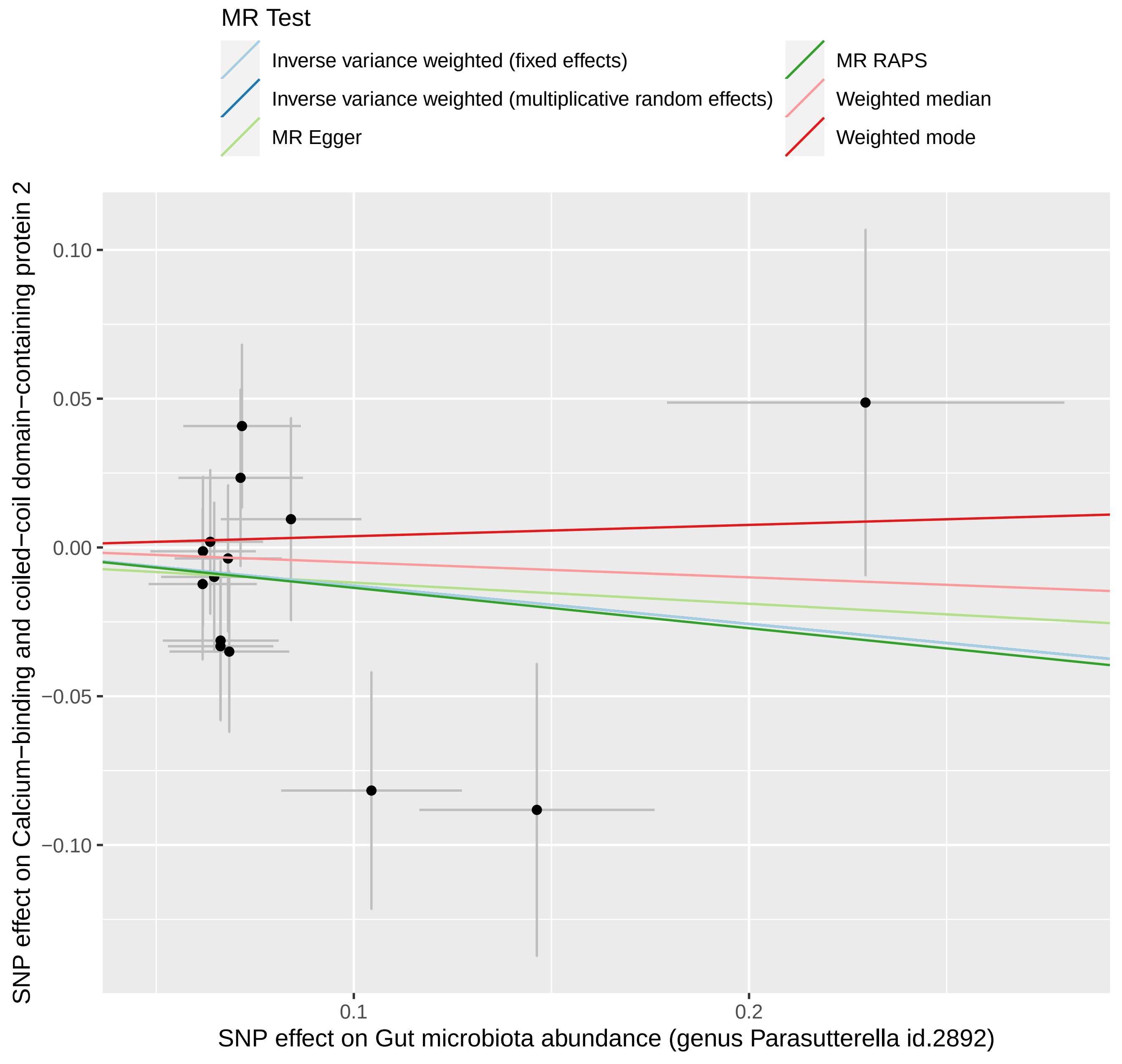

Batch 2−685 : Gut microbiota abundance (genus Catenibacterium id.2153) on Calcium−binding protein 8
with a threshold of 1e−05

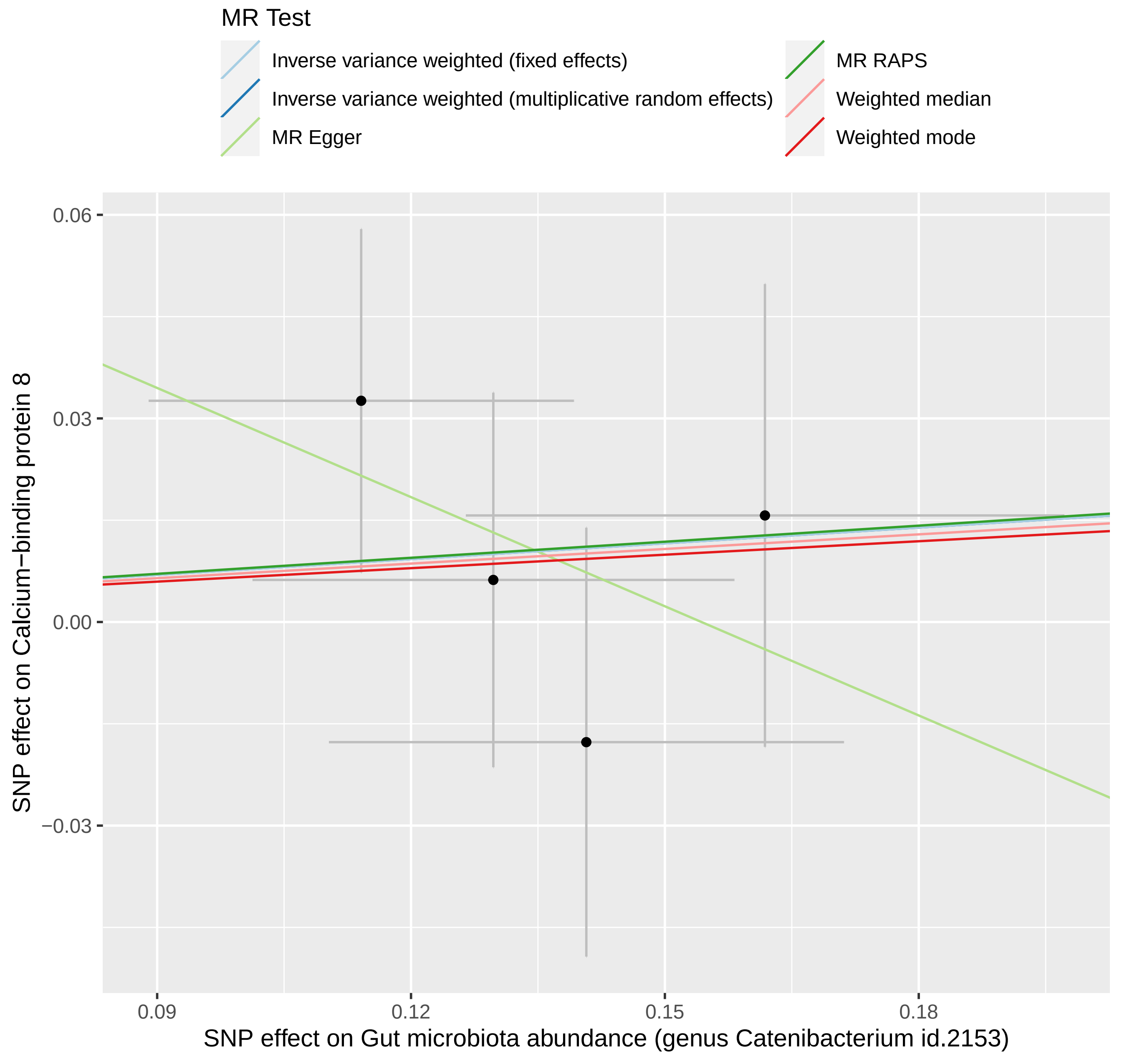

Batch 2−686 : Gut microbiota abundance (genus Parasutterella id.2892) on Calcium−binding protein 8
with a threshold of 1e−05

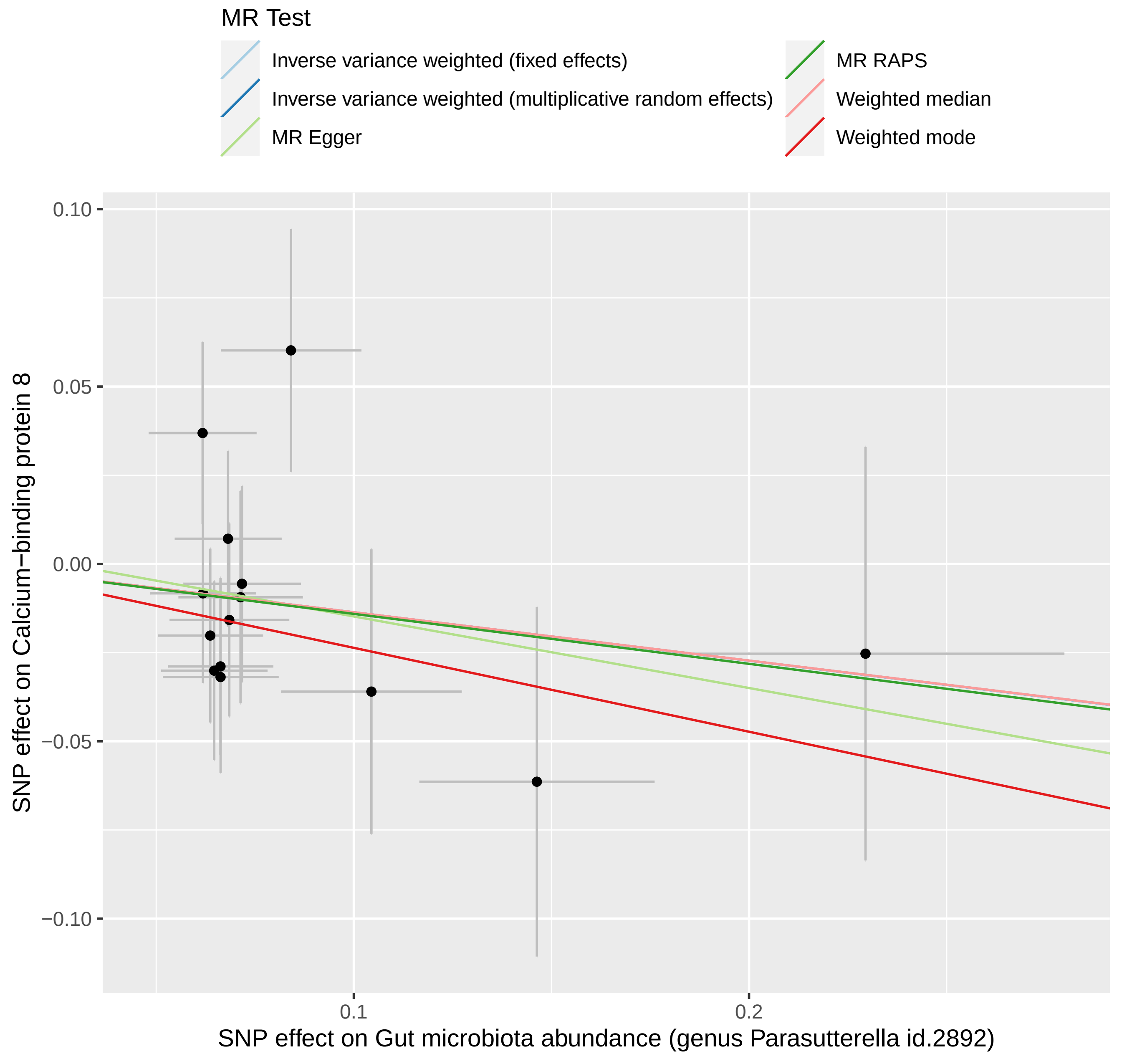

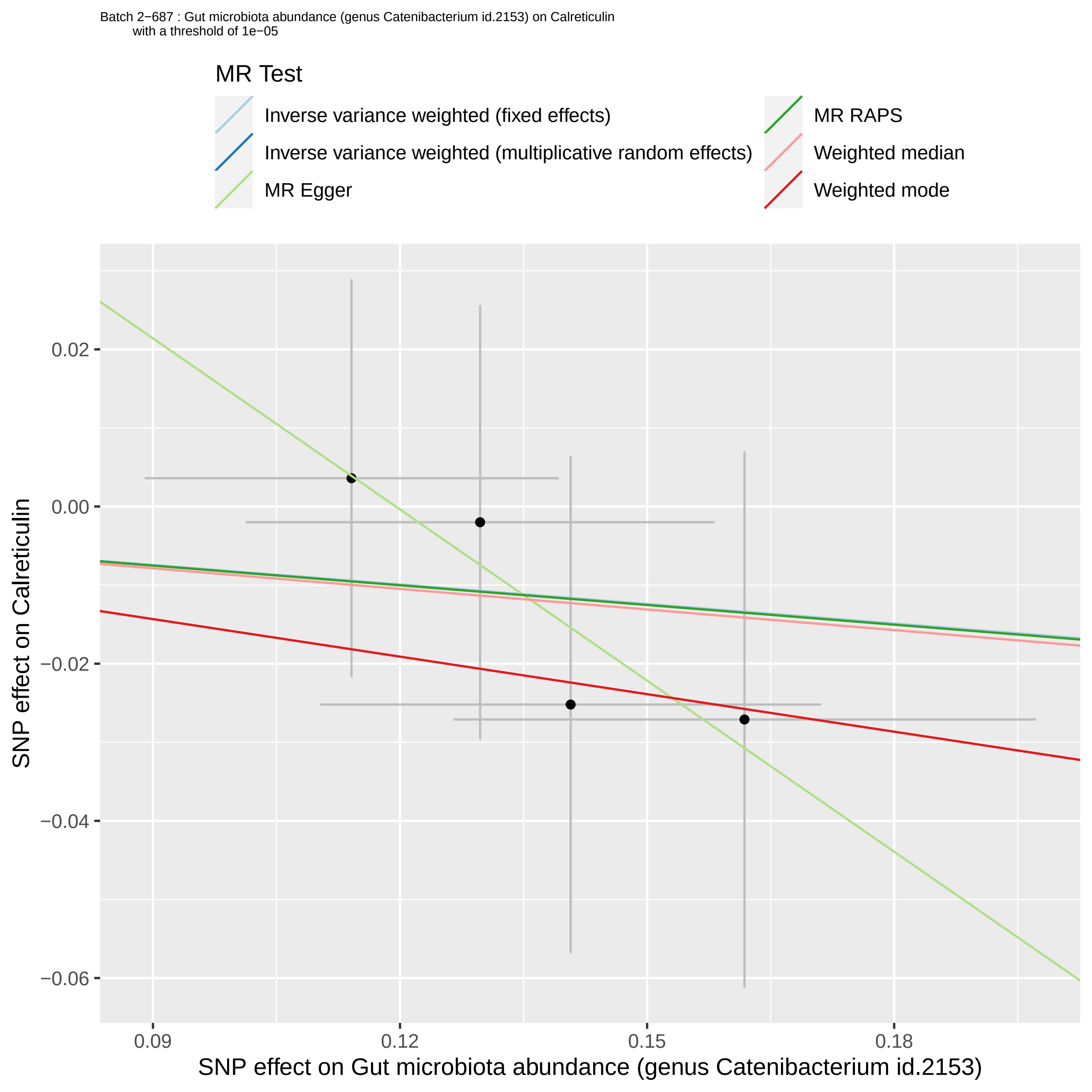

Batch 2−687 : Gut microbiota abundance (genus Catenibacterium id.2153) on Calreticulin
with a threshold of 1e−05
MR Test
Inverse variance weighted (fixed effects)
Inverse variance weighted (multiplicative random effects)
MR Egger
MR RAPS
Weighted median
Weighted mode
0.02
0.00
−0.02
−0.04
−0.06
0.09
0.12
0.15
0.18
SNP effect on Calreticulin
SNP effect on Gut microbiota abundance (genus Catenibacterium id.2153)

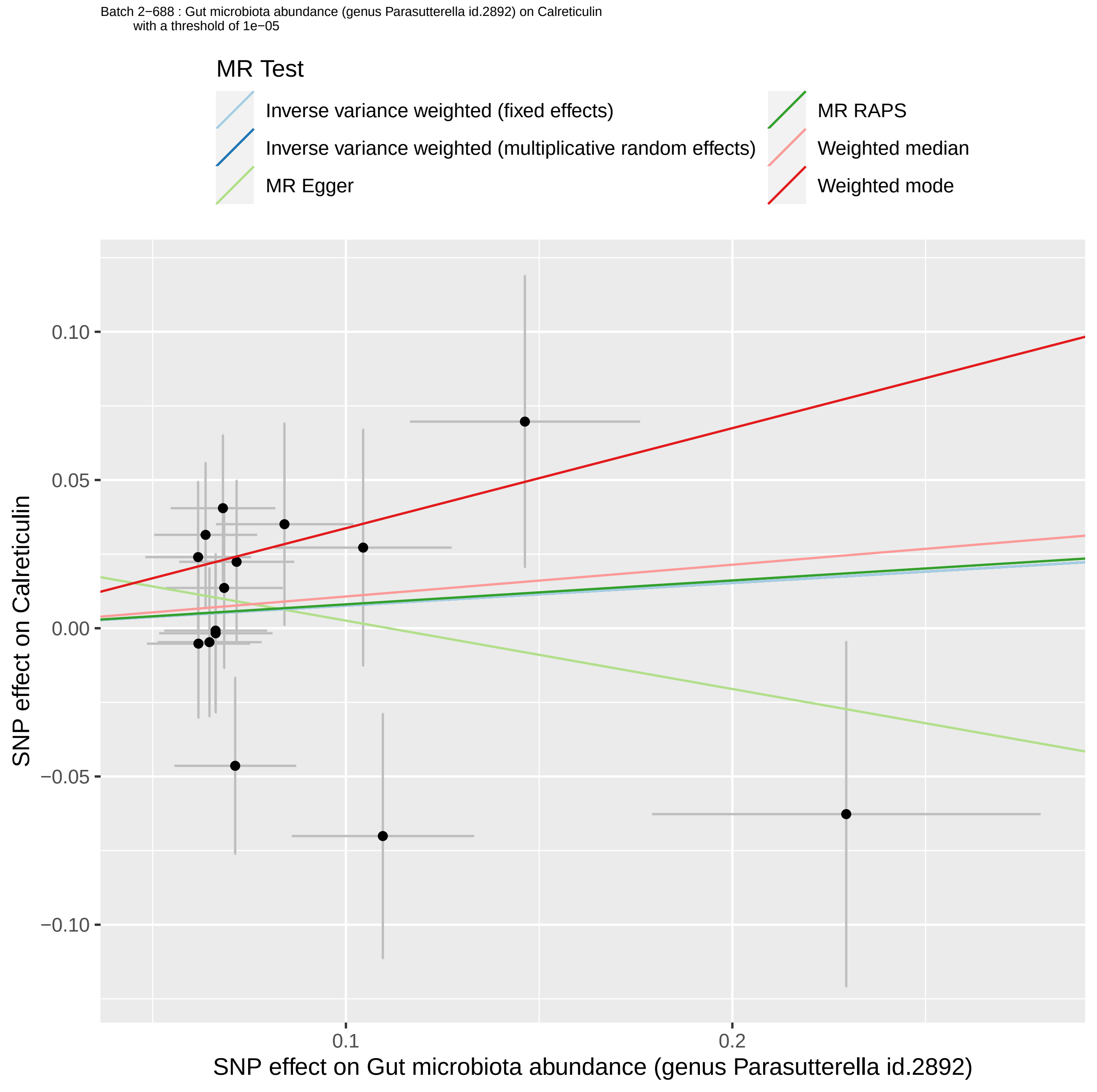

Batch 2−688 : Gut microbiota abundance (genus Parasutterella id.2892) on Calreticulin
with a threshold of 1e−05
MR Test
Inverse variance weighted (fixed effects)
Inverse variance weighted (multiplicative random effects)
MR Egger
MR RAPS
Weighted median
Weighted mode
0.10
0.05
0.00
−0.05
−0.10
SNP effect on Calreticulin
0.1
0.2
SNP effect on Gut microbiota abundance (genus Parasutterella id.2892)

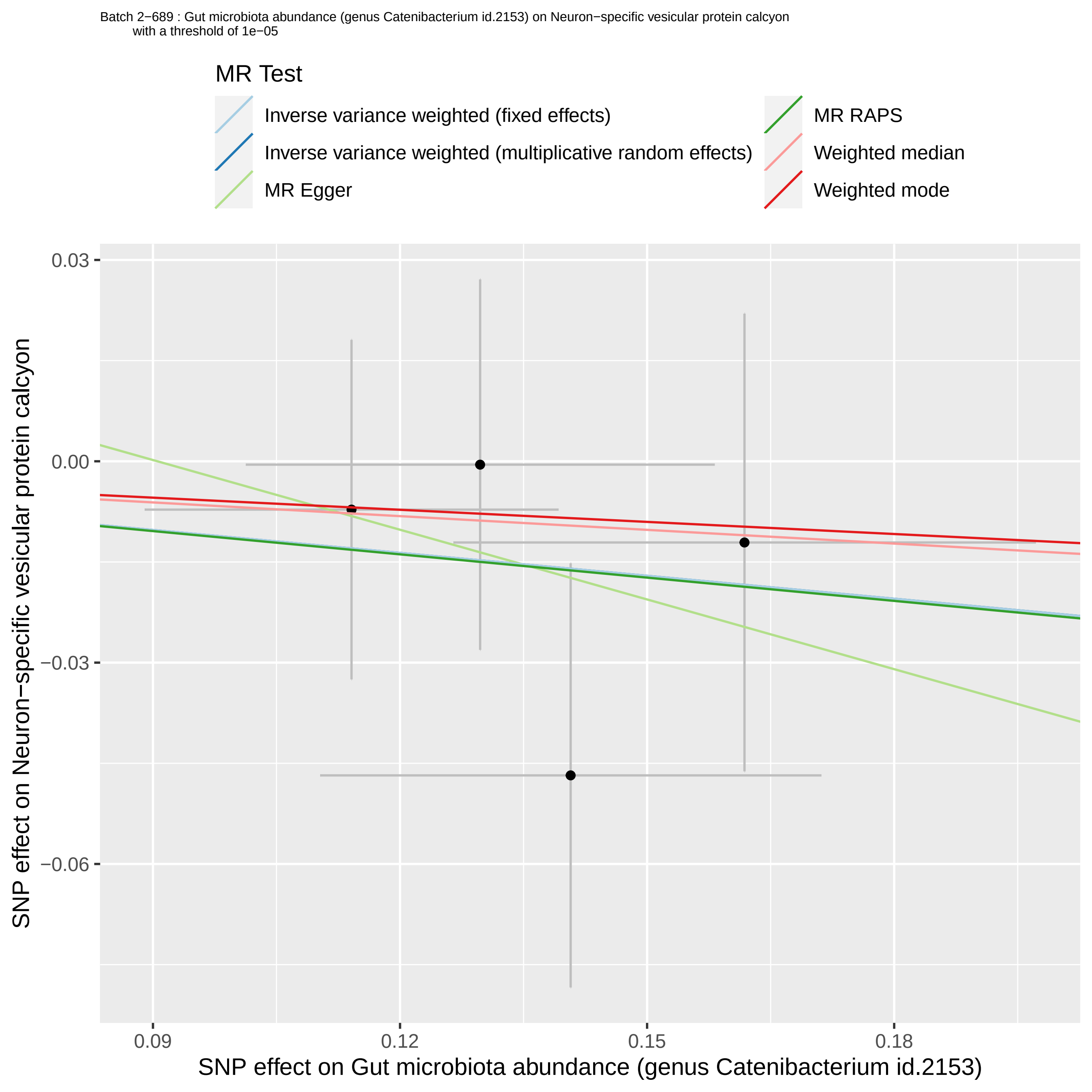
Batch 2−689 : Gut microbiota abundance (genus Catenibacterium id.2153) on Neuron−specific vesicular protein calcyon
with a threshold of 1e−05
MR Test
Inverse variance weighted (fixed effects)
Inverse variance weighted (multiplicative random effects)
MR Egger
MR RAPS
Weighted median
Weighted mode
0.03
0.00
−0.03
−0.06
0.09
0.12
0.15
0.18
SNP effect on Neuron−specific vesicular protein calcyon
SNP effect on Gut microbiota abundance (genus Catenibacterium id.2153)

Batch 2−690 : Gut microbiota abundance (genus Parasutterella id.2892) on Neuron−specific vesicular protein calcyon
with a threshold of 1e−05

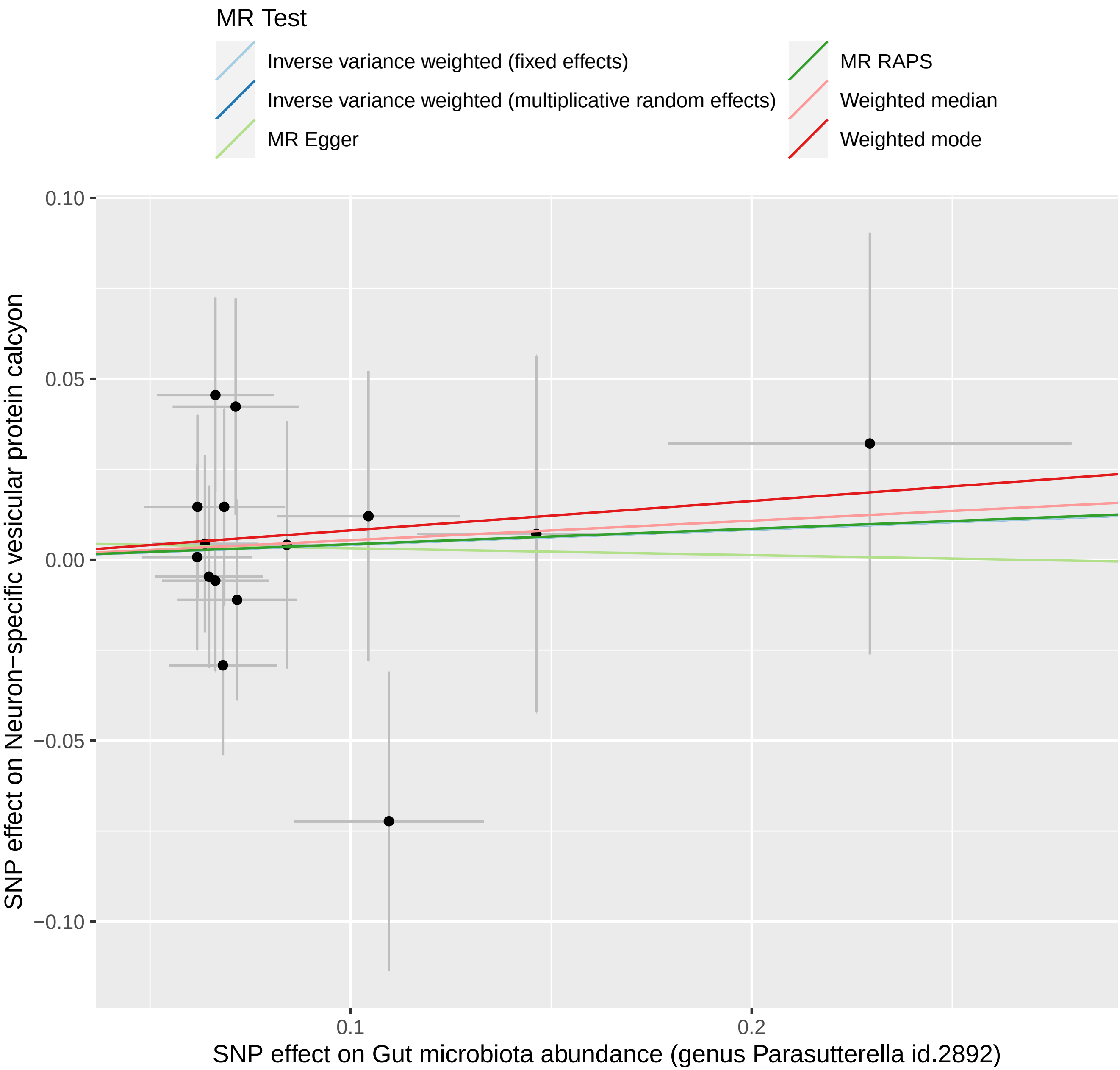

Batch 2−691 : Gut microbiota abundance (genus Catenibacterium id.2153) on Calcium/calmodulin−dependent protein kinase type 1
with a threshold of 1e−05

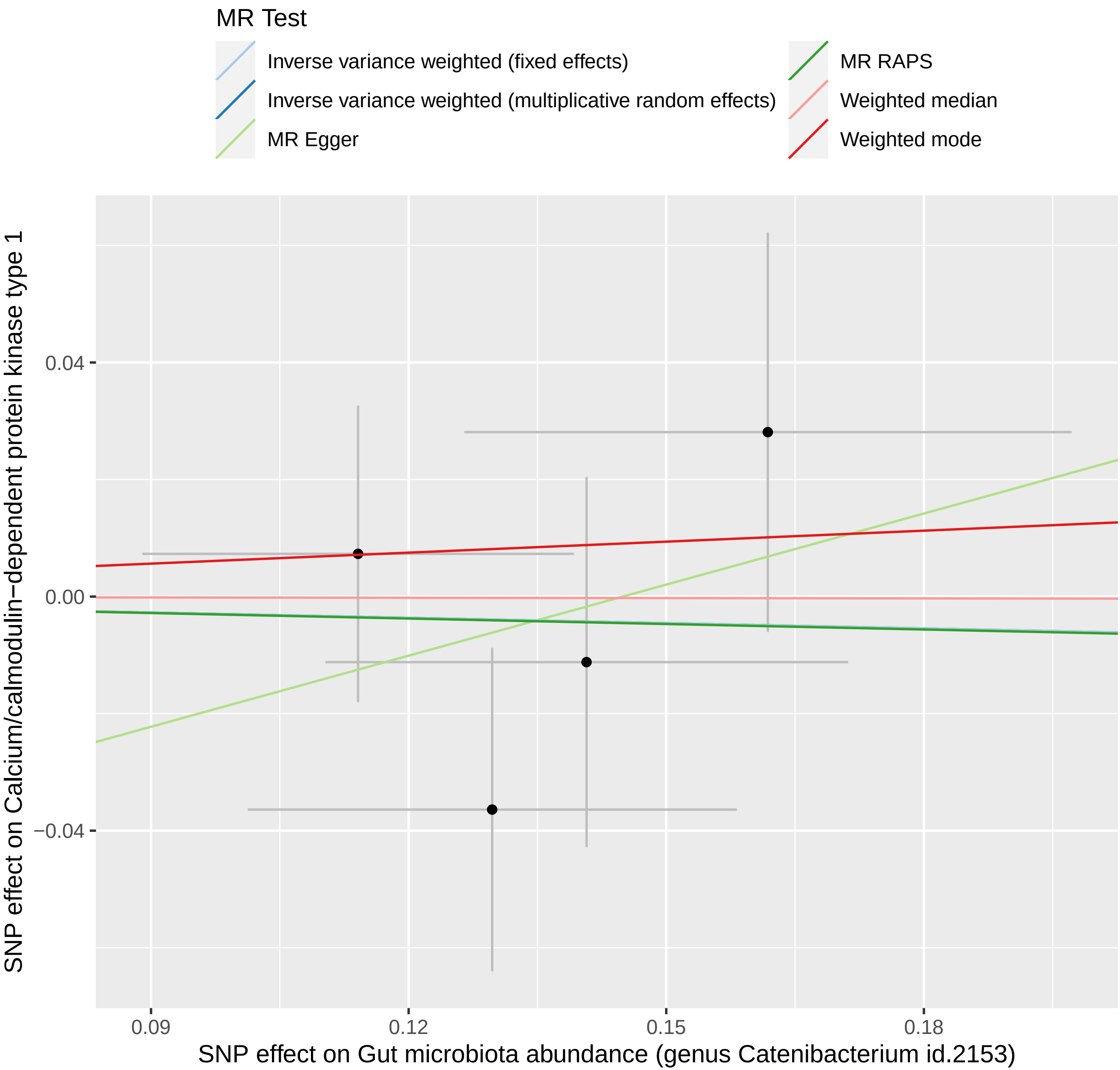

Batch 2−692 : Gut microbiota abundance (genus Parasutterella id.2892) on Calcium/calmodulin−dependent protein kinase type 1
with a threshold of 1e−05

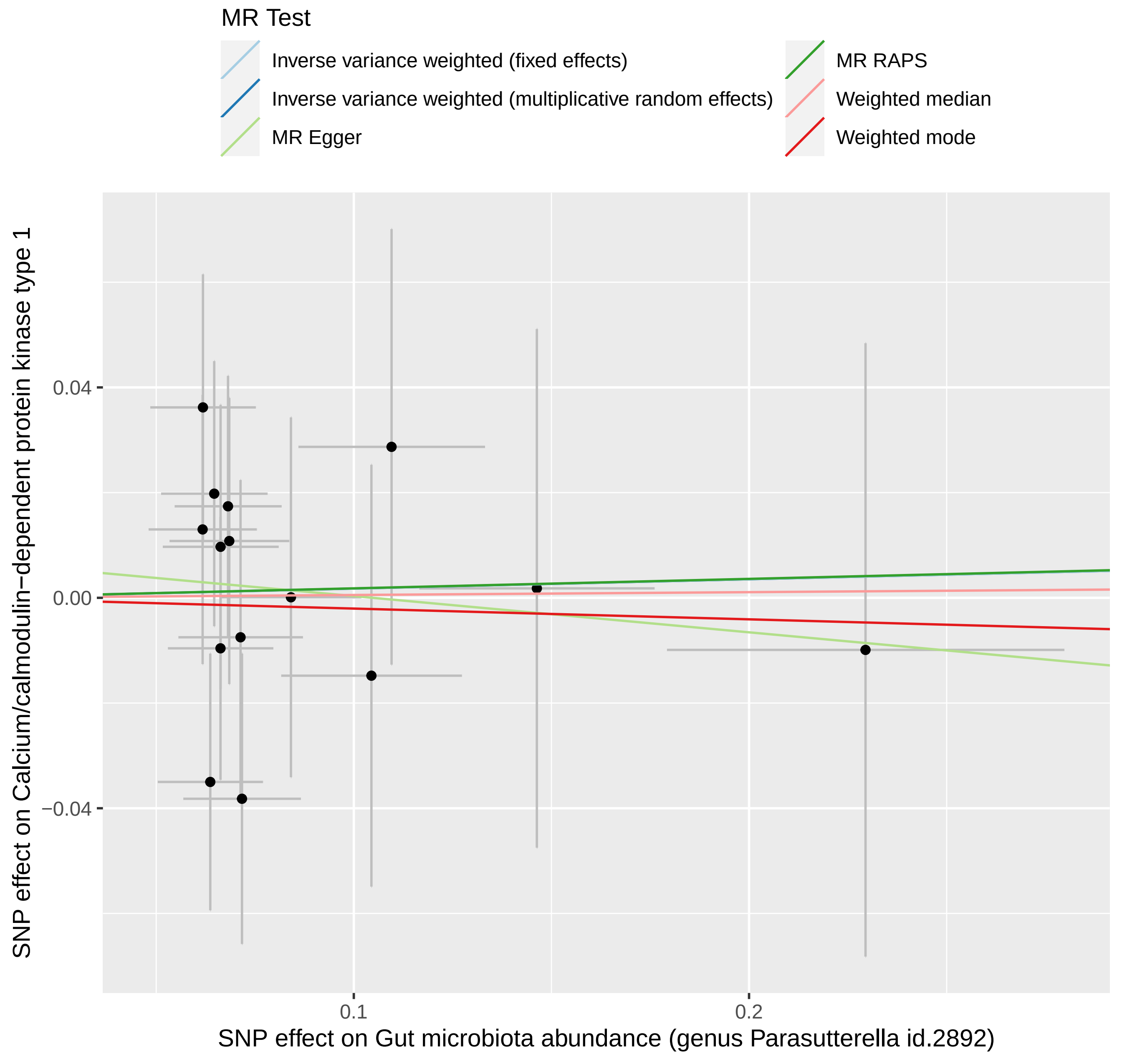

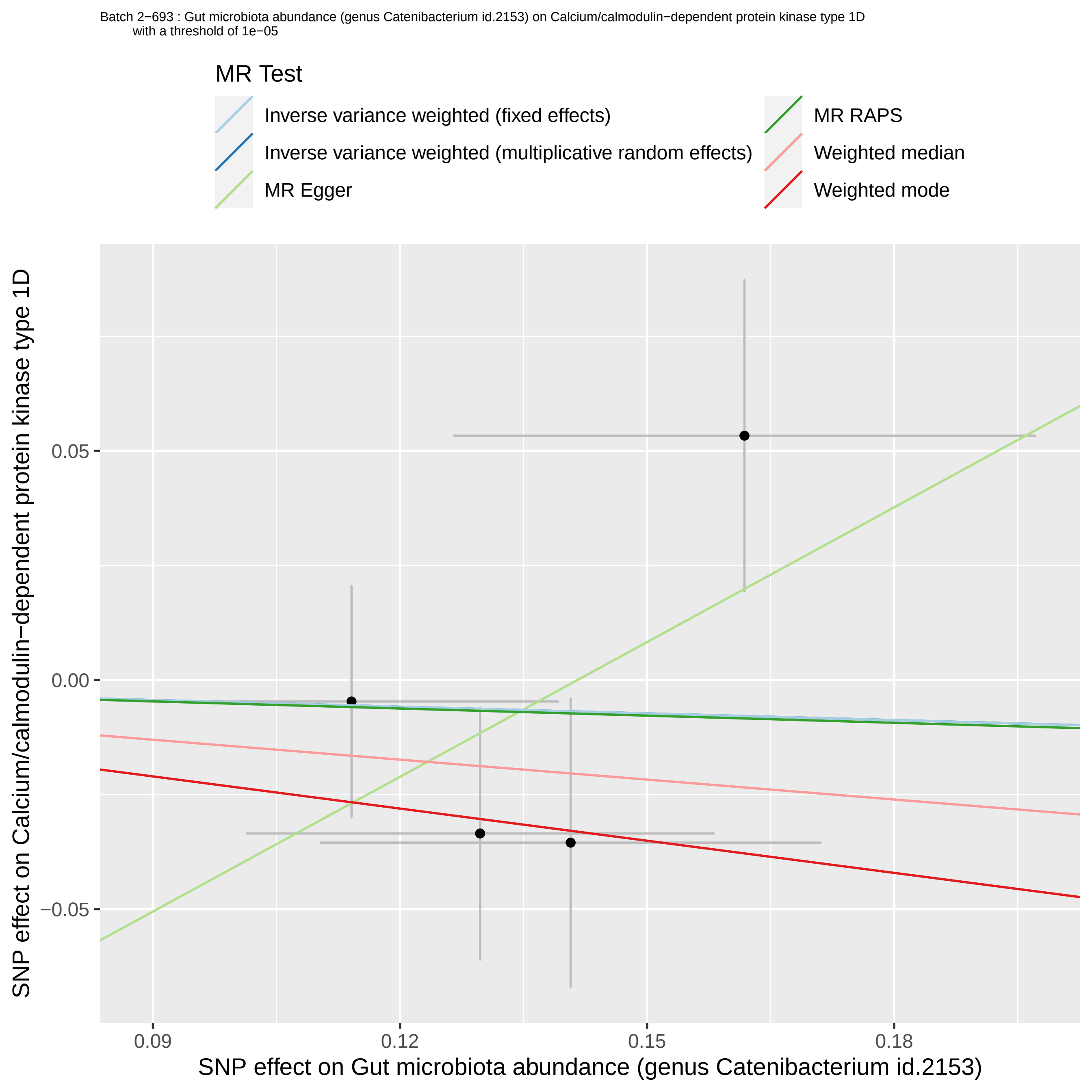

Batch 2−693 : Gut microbiota abundance (genus Catenibacterium id.2153) on Calcium/calmodulin−dependent protein kinase type 1D
with a threshold of 1e−05
MR Test
Inverse variance weighted (fixed effects)
Inverse variance weighted (multiplicative random effects)
MR Egger
MR RAPS
Weighted median
Weighted mode
SNP effect on Calcium/calmodulin−dependent protein kinase type 1D
0.05
0.00
−0.05
0.09
0.12
0.15
0.18
SNP effect on Gut microbiota abundance (genus Catenibacterium id.2153)

Batch 2−694 : Gut microbiota abundance (genus Parasutterella id.2892) on Calcium/calmodulin−dependent protein kinase type 1D with a threshold of 1e−05

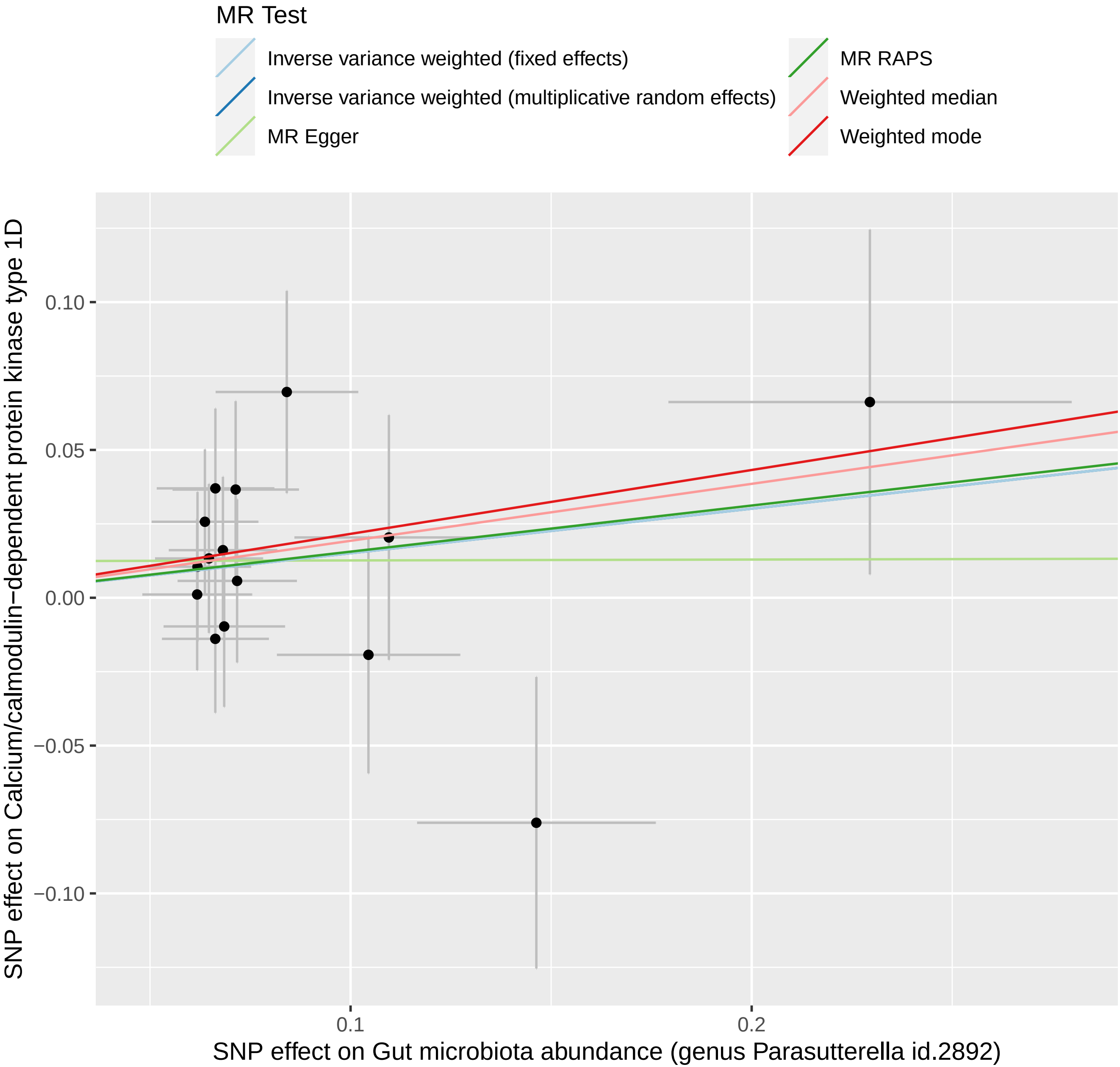

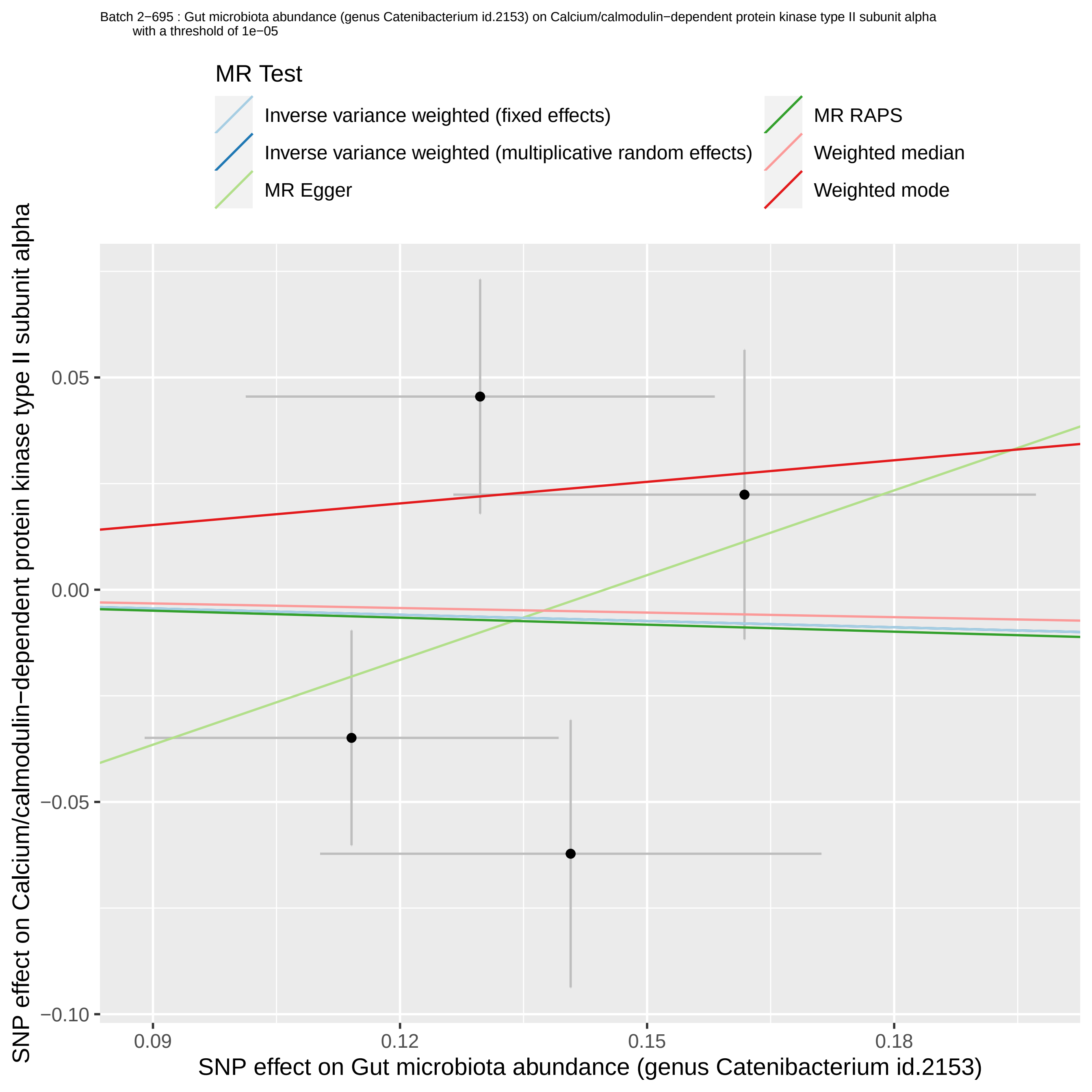
Batch 2−695 : Gut microbiota abundance (genus Catenibacterium id.2153) on Calcium/calmodulin−dependent protein kinase type II subunit alpha with a threshold of 1e−05
MR Test
Inverse variance weighted (fixed effects)
Inverse variance weighted (multiplicative random effects)
MR Egger
MR RAPS
Weighted median
Weighted mode
0.05
0.00
−0.05
−0.10
0.09
0.12
0.15
0.18
SNP effect on Calcium/calmodulin−dependent protein kinase type II subunit alpha
SNP effect on Gut microbiota abundance (genus Catenibacterium id.2153)

Batch 2−696 : Gut microbiota abundance (genus Parasutterella id.2892) on Calcium/calmodulin−dependent protein kinase type II subunit alpha with a threshold of 1e−05

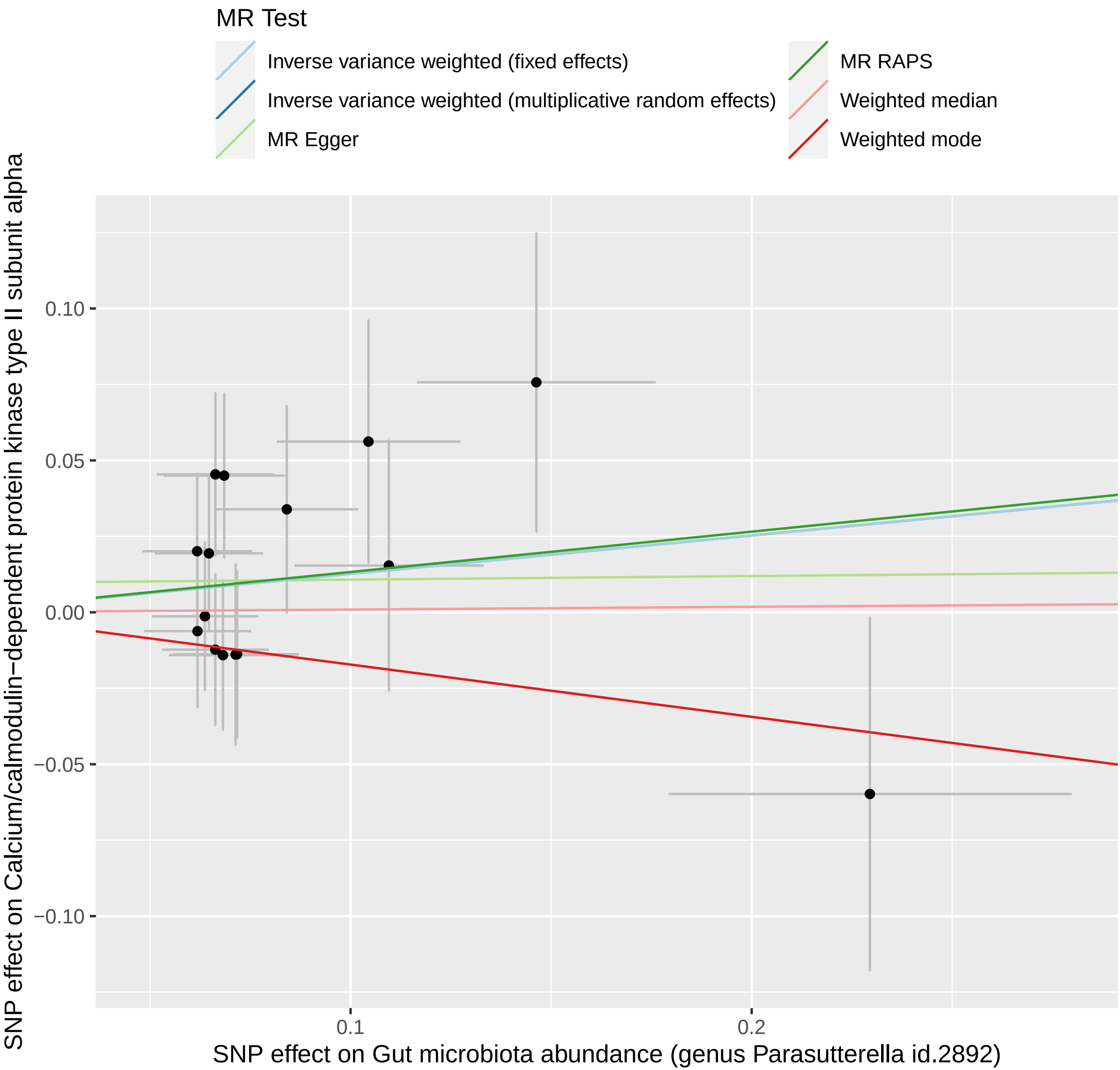

Batch 2−697 : Gut microbiota abundance (genus Catenibacterium id.2153) on Calcium/calmodulin−dependent protein kinase type II subunit beta
with a threshold of 1e−05

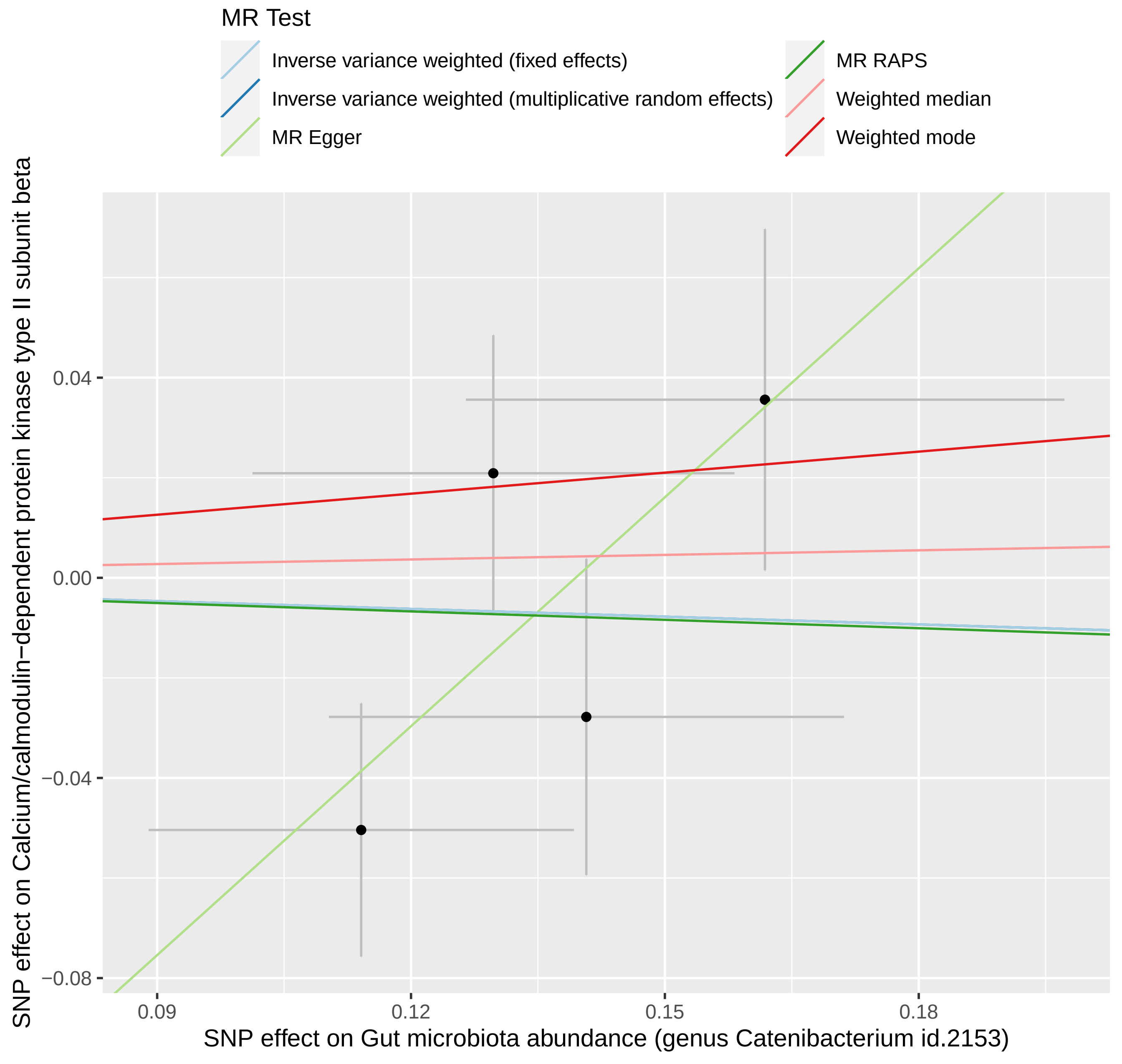

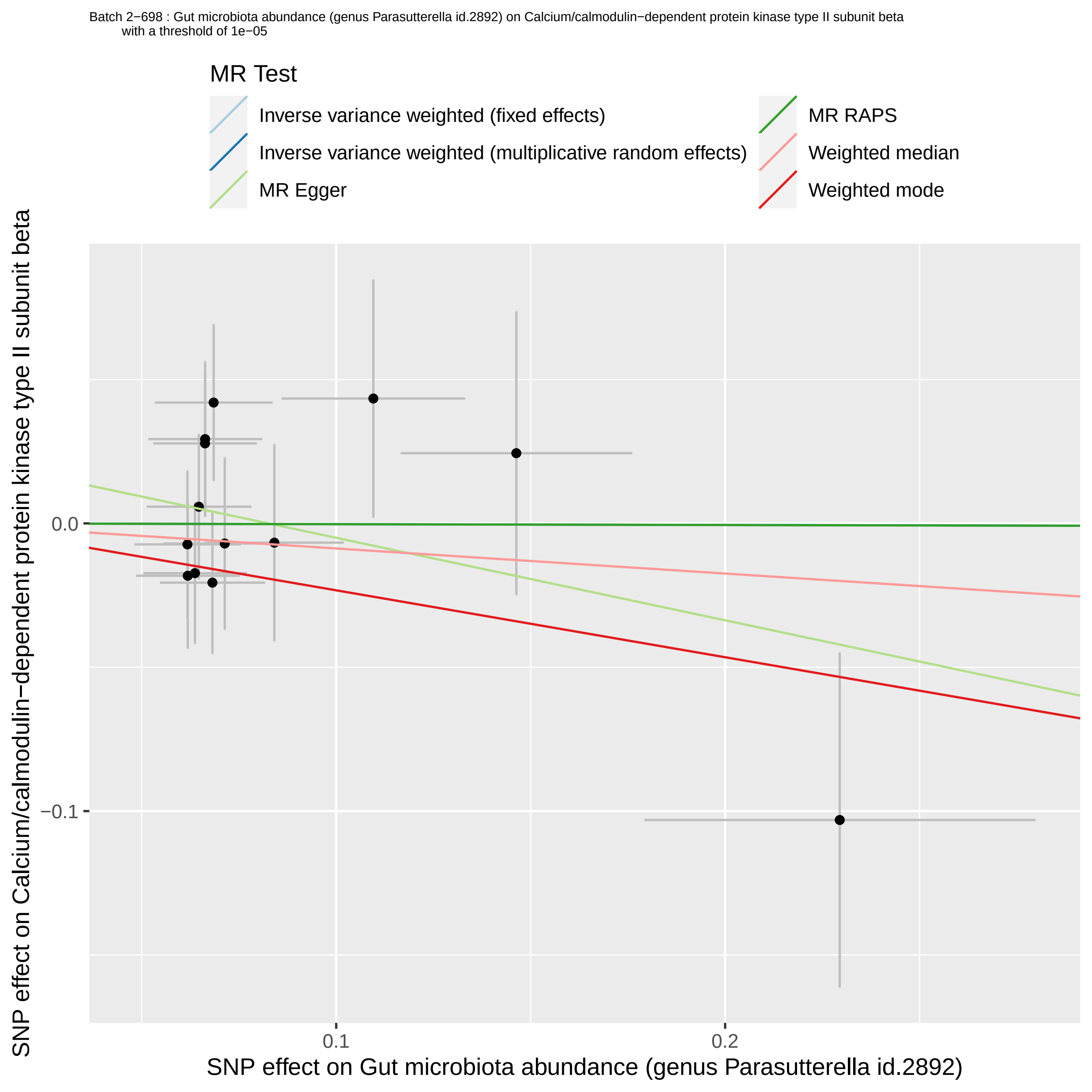
Batch 2−698 : Gut microbiota abundance (genus Parasutterella id.2892) on Calcium/calmodulin−dependent protein kinase type II subunit beta
with a threshold of 1e−05
MR Test
Inverse variance weighted (fixed effects)
Inverse variance weighted (multiplicative random effects)
MR Egger
MR RAPS
Weighted median
Weighted mode
SNP effect on Calcium/calmodulin−dependent protein kinase type II subunit beta
0.0
−0.1
0.1
0.2
SNP effect on Gut microbiota abundance (genus Parasutterella id.2892)

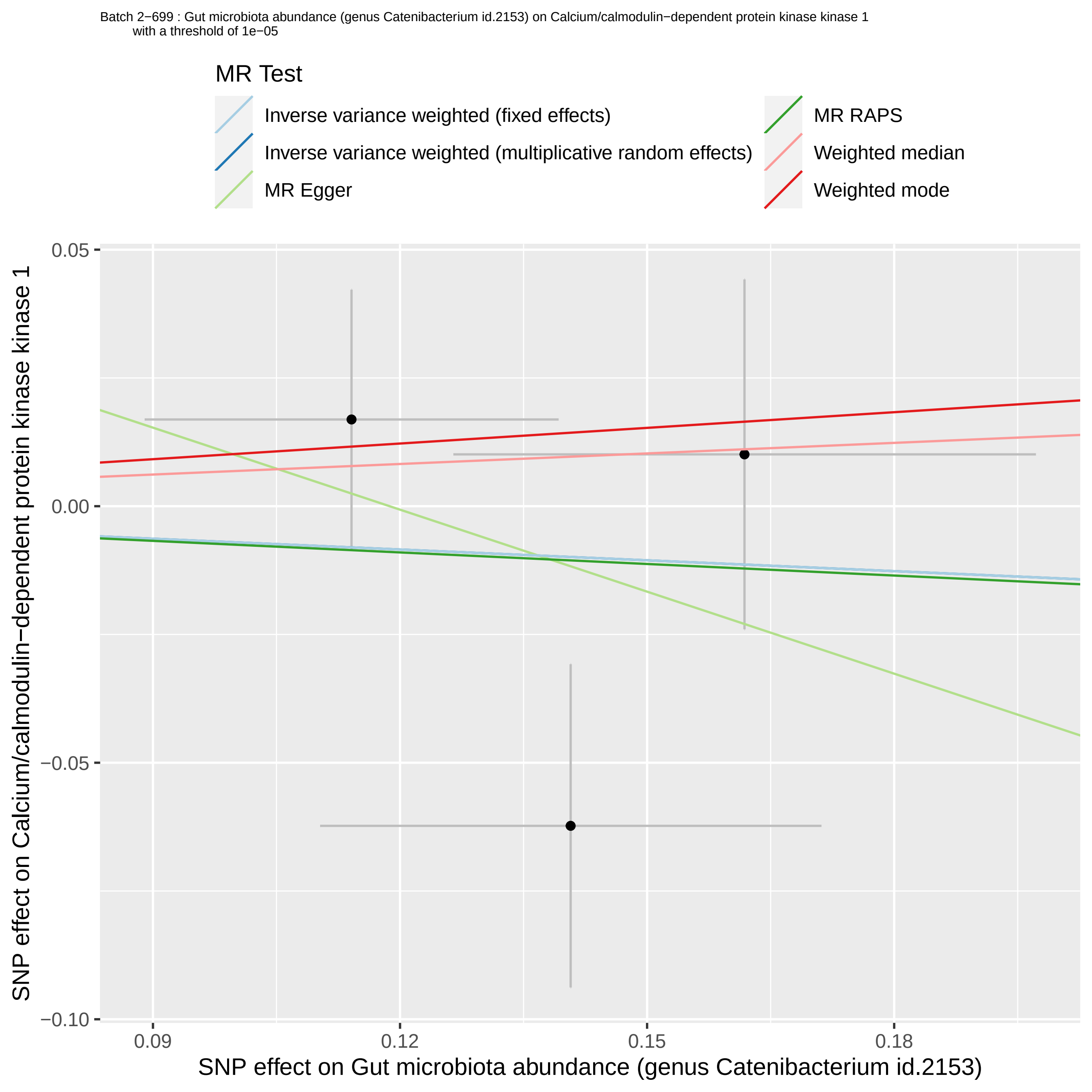

Batch 2−699 : Gut microbiota abundance (genus Catenibacterium id.2153) on Calcium/calmodulin−dependent protein kinase kinase 1
with a threshold of 1e−05
MR Test
Inverse variance weighted (fixed effects)
Inverse variance weighted (multiplicative random effects)
MR Egger
MR RAPS
Weighted median
Weighted mode
0.05
0.00
−0.05
−0.10
0.09
0.12
0.15
0.18
SNP effect on Calcium/calmodulin−dependent protein kinase kinase 1
SNP effect on Gut microbiota abundance (genus Catenibacterium id.2153)

Batch 2−700 : Gut microbiota abundance (genus Parasutterella id.2892) on Calcium/calmodulin−dependent protein kinase kinase 1
with a threshold of 1e−05

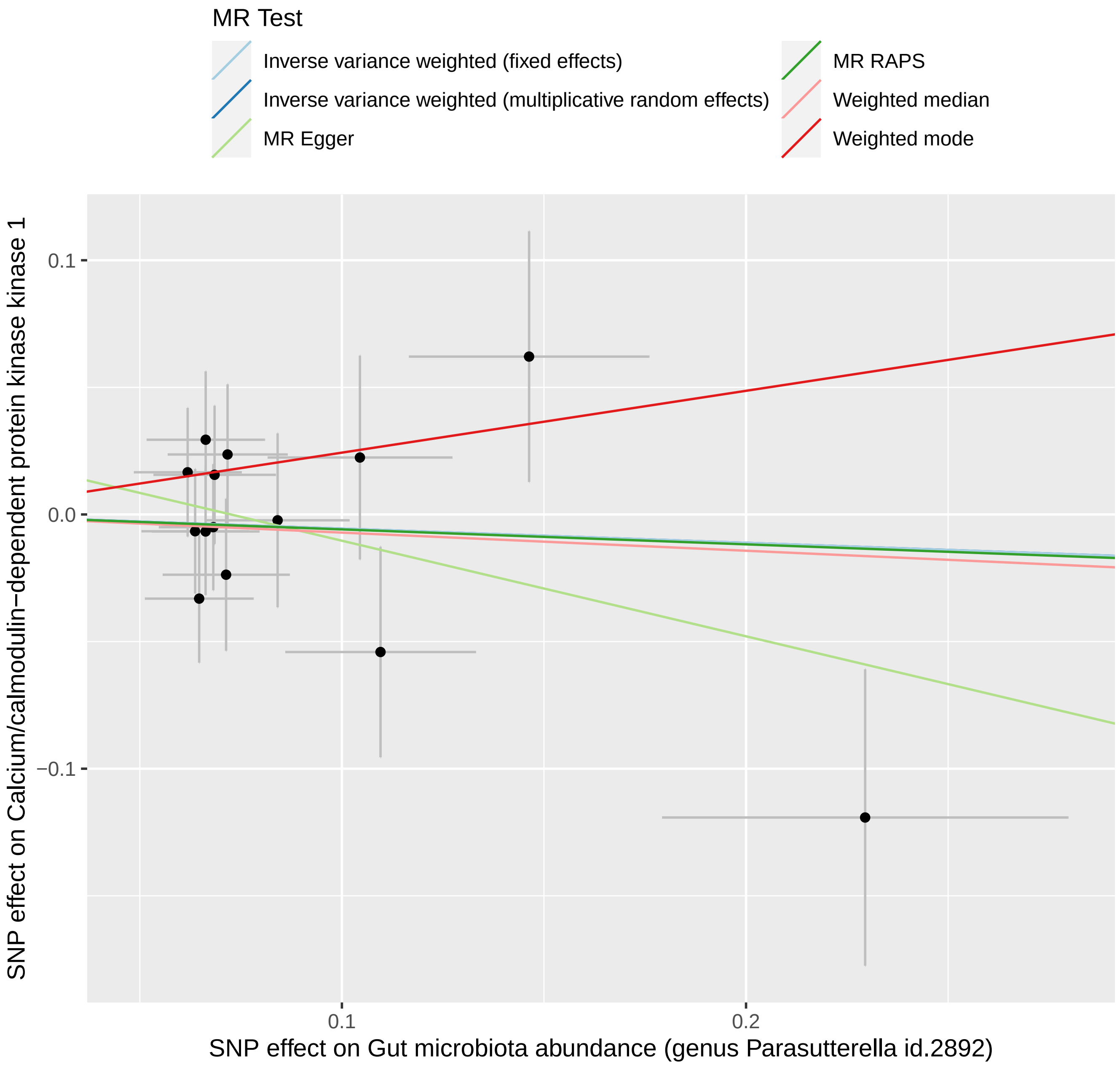

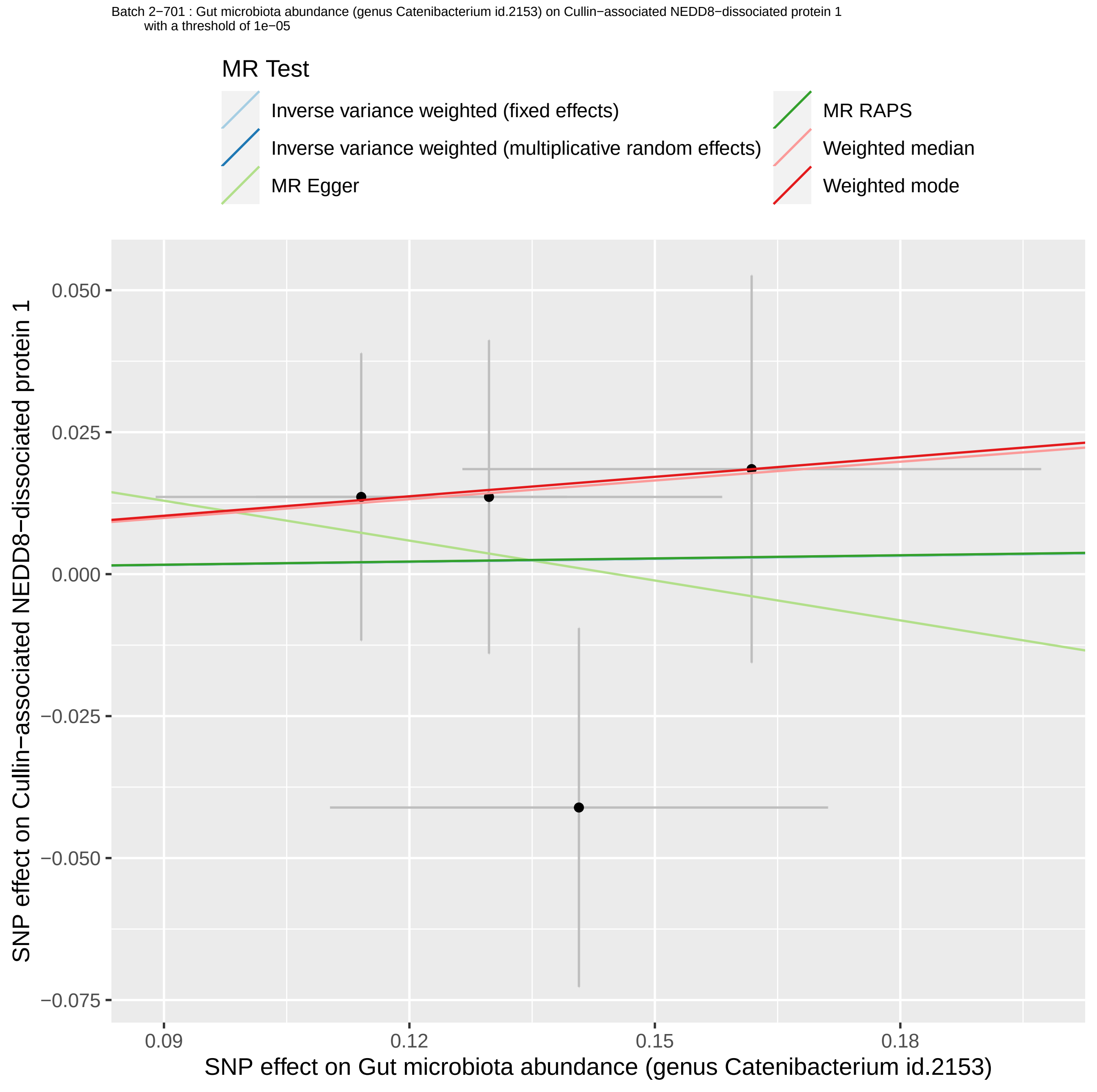

Batch 2−701 : Gut microbiota abundance (genus Catenibacterium id.2153) on Cullin−associated NEDD8−dissociated protein 1
with a threshold of 1e−05
MR Test
Inverse variance weighted (fixed effects)
Inverse variance weighted (multiplicative random effects)
MR Egger
MR RAPS
Weighted median
Weighted mode
0.050
0.025
0.000
−0.025
−0.050
−0.075
0.09
0.12
0.15
0.18
SNP effect on Cullin−associated NEDD8−dissociated protein 1
SNP effect on Gut microbiota abundance (genus Catenibacterium id.2153)

Batch 2−702 : Gut microbiota abundance (genus Parasutterella id.2892) on Cullin−associated NEDD8−dissociated protein 1
with a threshold of 1e−05

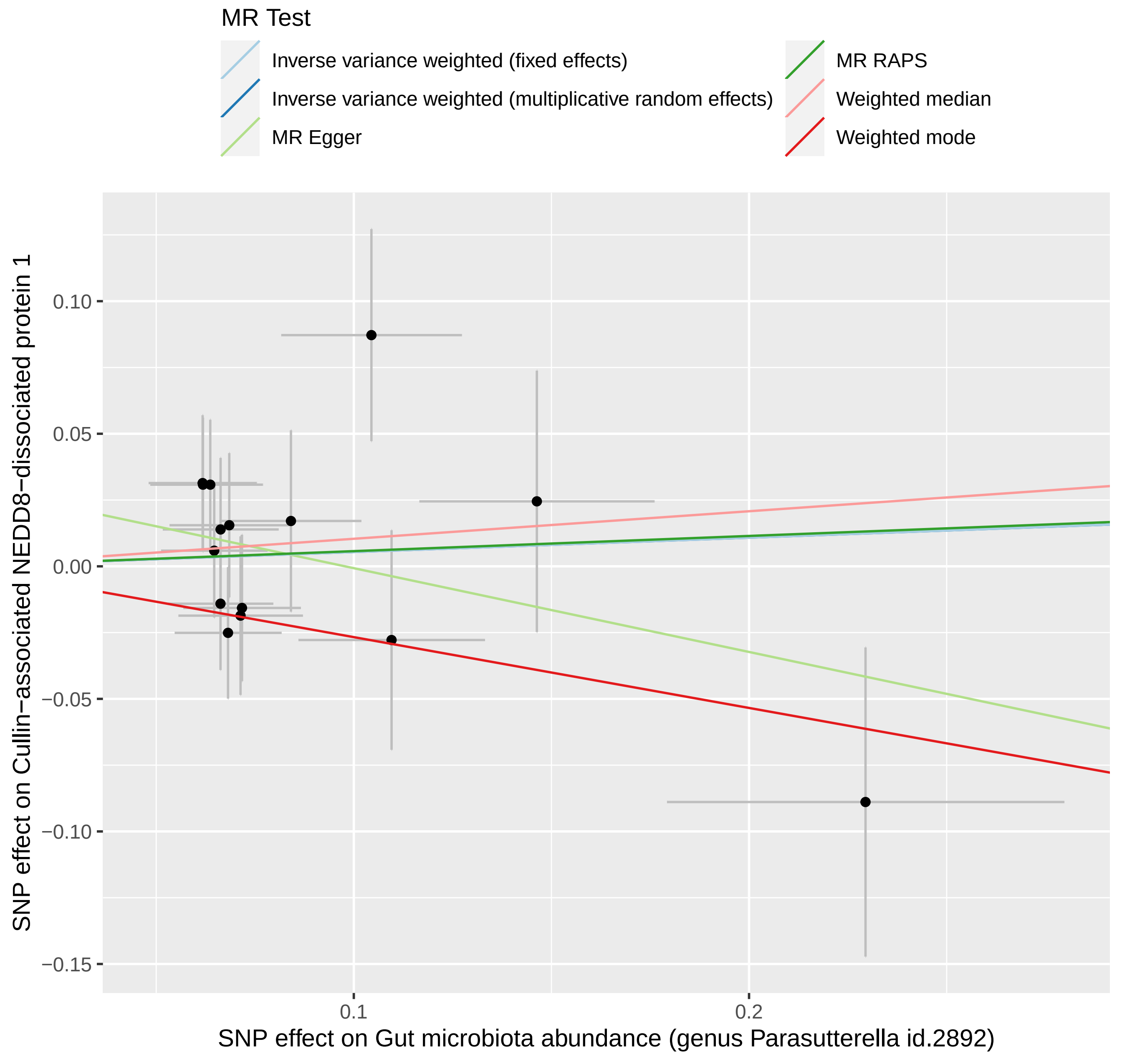

Batch 2−703 : Gut microbiota abundance (genus Catenibacterium id.2153) on Soluble calcium−activated nucleotidase 1 with a threshold of 1e−05

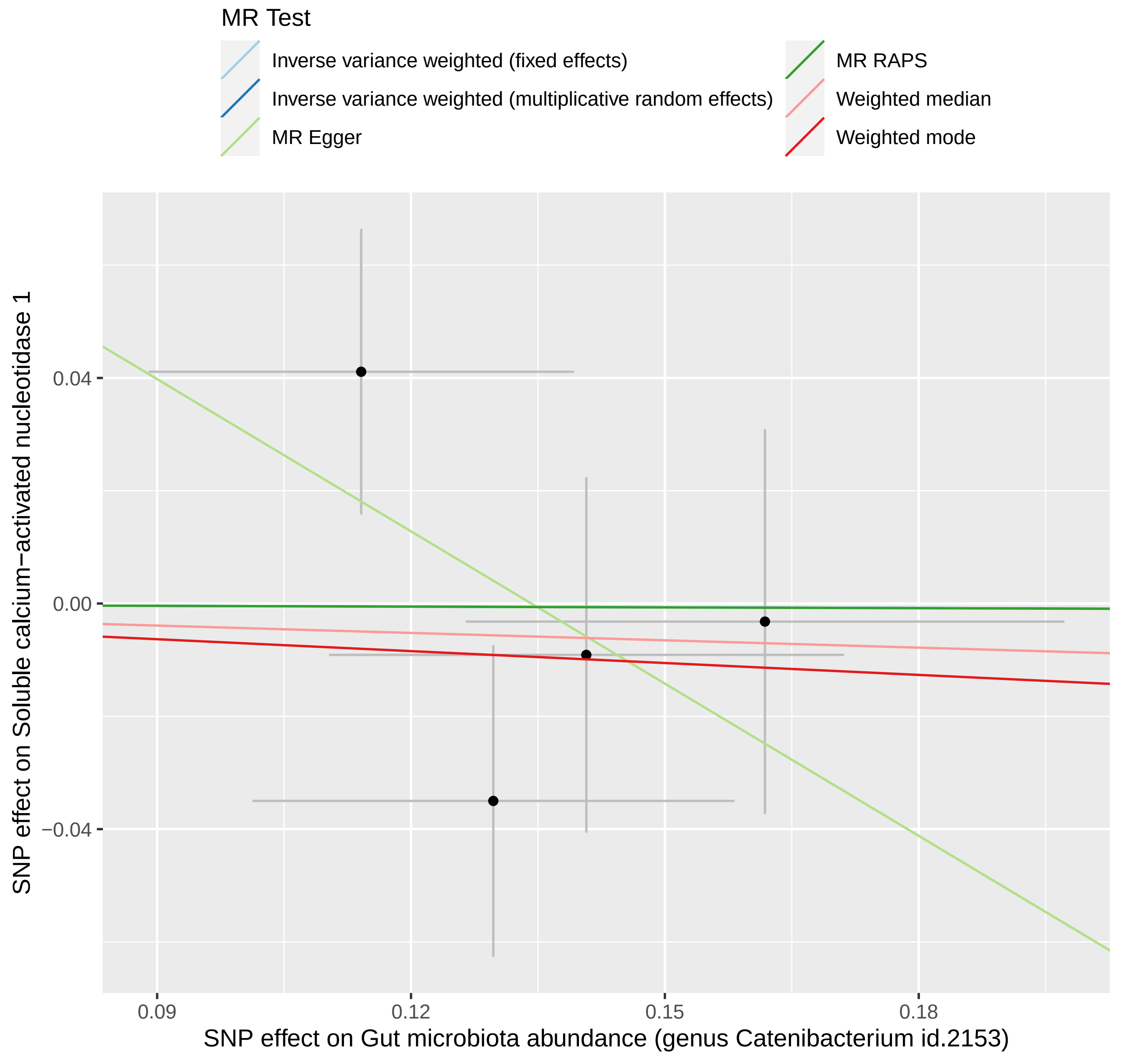

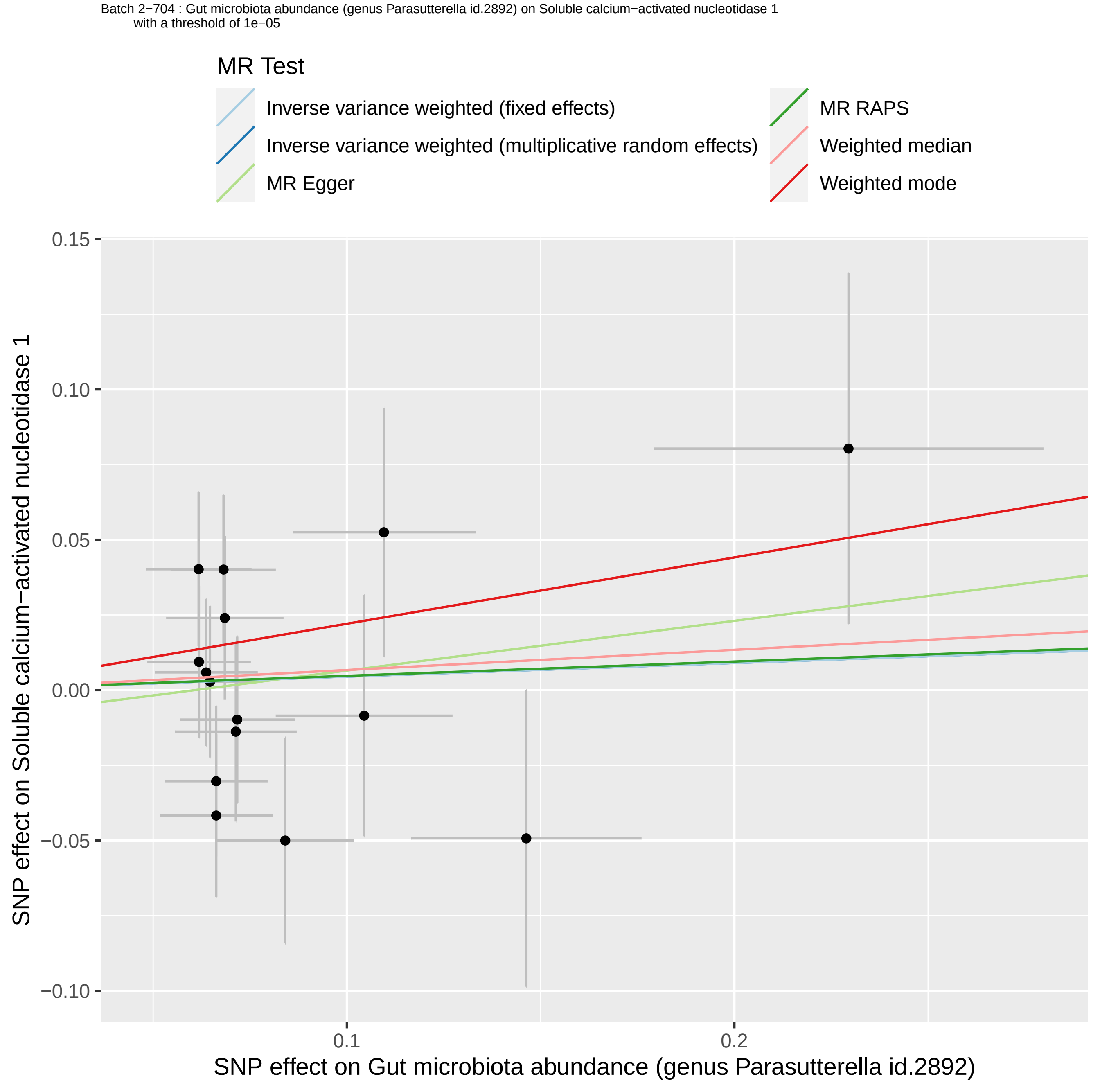

Batch 2−704 : Gut microbiota abundance (genus Parasutterella id.2892) on Soluble calcium−activated nucleotidase 1
with a threshold of 1e−05
MR Test
Inverse variance weighted (fixed effects)
Inverse variance weighted (multiplicative random effects)
MR Egger
MR RAPS
Weighted median
Weighted mode
0.15
0.10
0.05
0.00
−0.05
−0.10
0.1
0.2
SNP effect on Soluble calcium−activated nucleotidase 1
SNP effect on Gut microbiota abundance (genus Parasutterella id.2892)

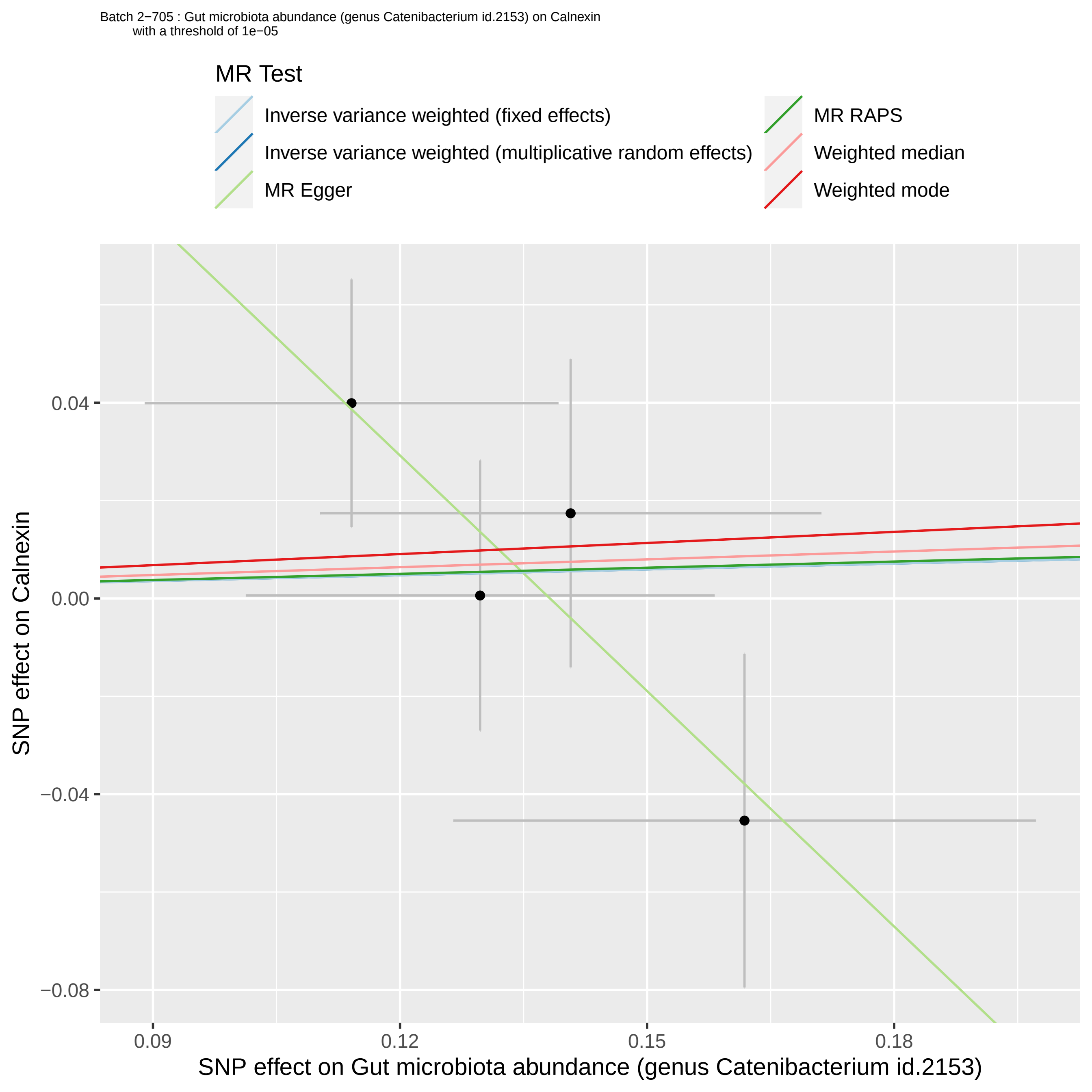

Batch 2−705 : Gut microbiota abundance (genus Catenibacterium id.2153) on Calnexin
with a threshold of 1e−05
MR Test
Inverse variance weighted (fixed effects)
Inverse variance weighted (multiplicative random effects)
MR Egger
MR RAPS
Weighted median
Weighted mode
0.04
0.00
−0.04
−0.08
SNP effect on Calnexin
0.09
0.12
0.15
0.18
SNP effect on Gut microbiota abundance (genus Catenibacterium id.2153)

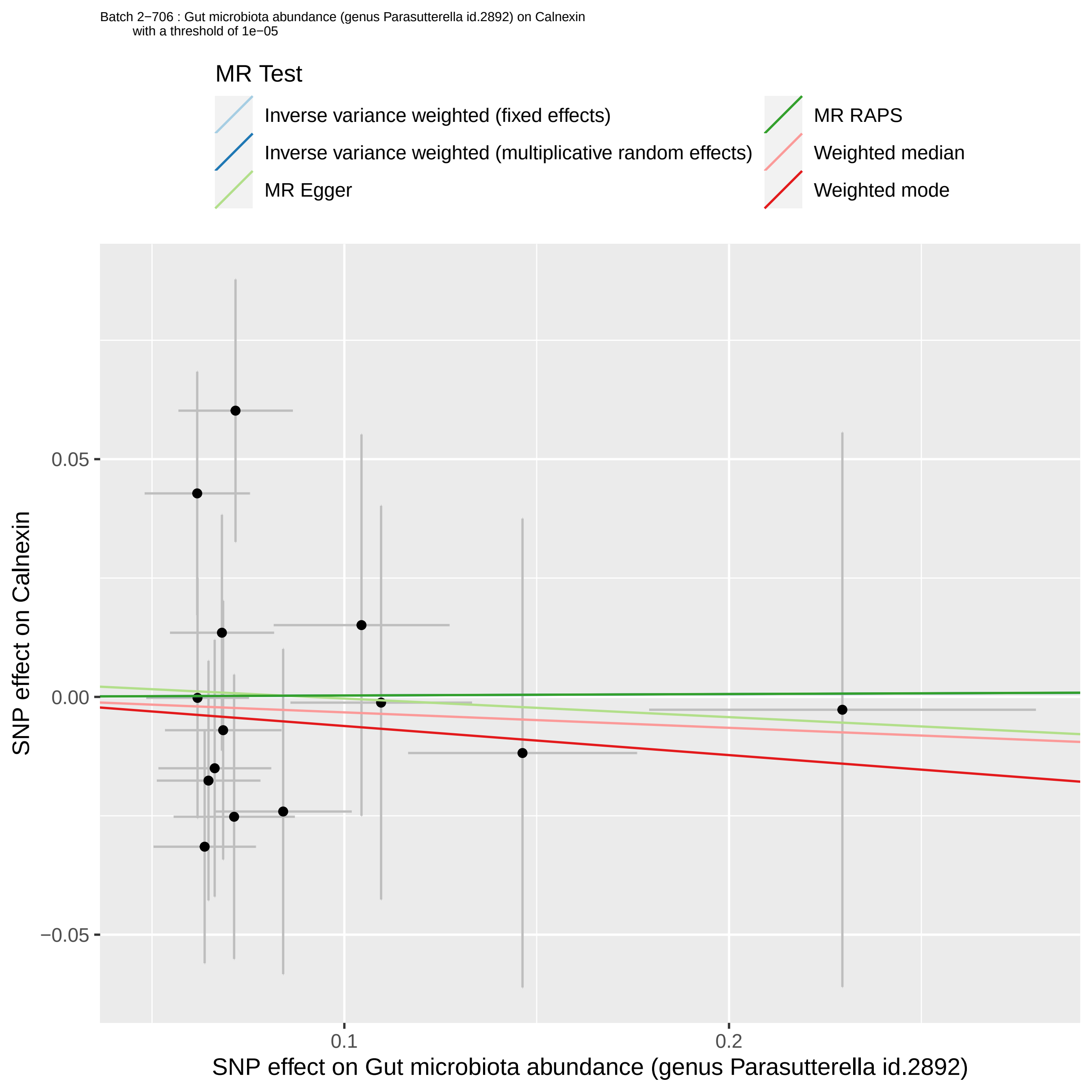
Batch 2−706 : Gut microbiota abundance (genus Parasutterella id.2892) on Calnexin
with a threshold of 1e−05
MR Test
Inverse variance weighted (fixed effects)
Inverse variance weighted (multiplicative random effects)
MR Egger
MR RAPS
Weighted median
Weighted mode
0.05
0.00
−0.05
0.1
0.2
SNP effect on Calnexin
SNP effect on Gut microbiota abundance (genus Parasutterella id.2892)

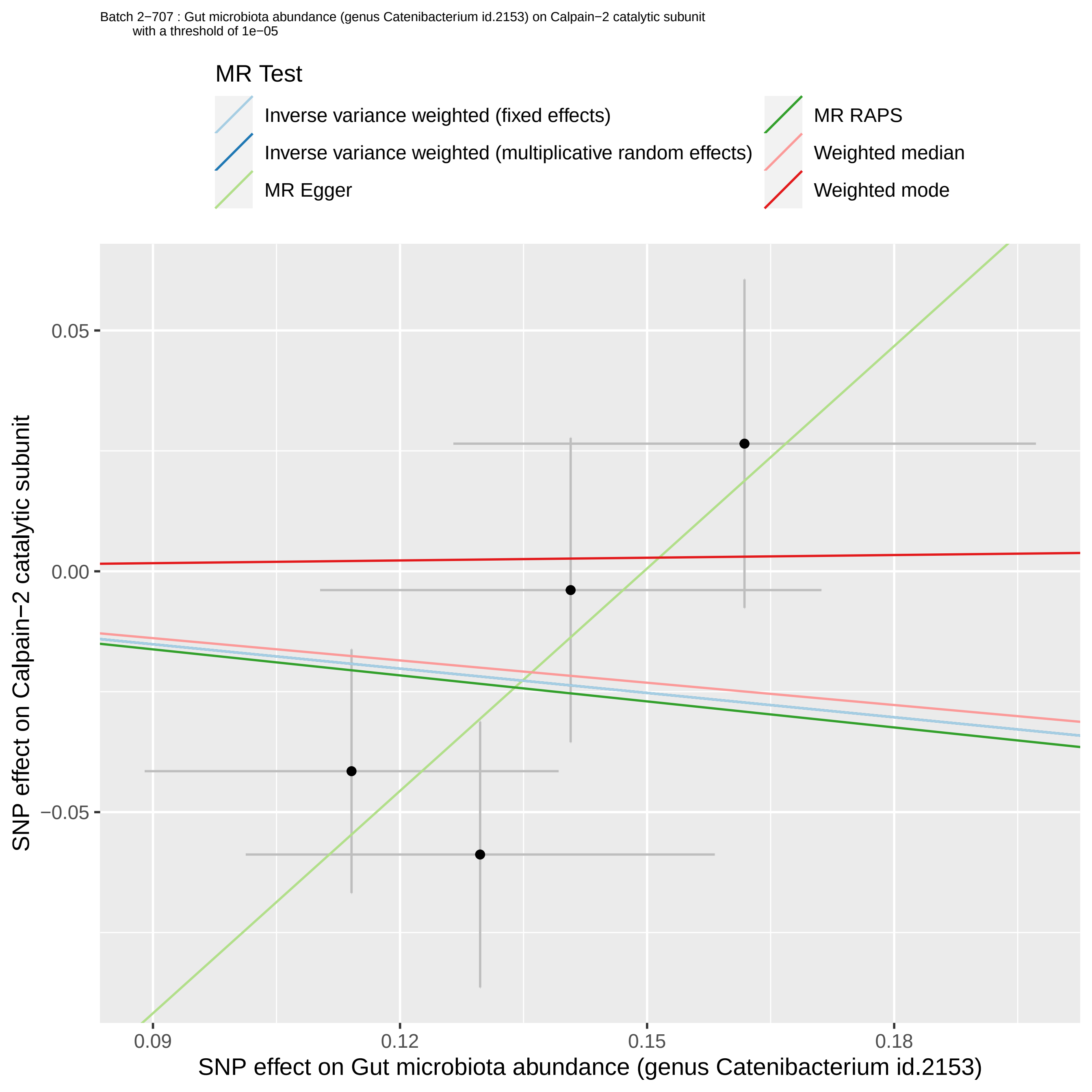
Batch 2−707 : Gut microbiota abundance (genus Catenibacterium id.2153) on Calpain−2 catalytic subunit
with a threshold of 1e−05
MR Test
Inverse variance weighted (fixed effects)
Inverse variance weighted (multiplicative random effects)
MR Egger
MR RAPS
Weighted median
Weighted mode
0.05
0.00
−0.05
0.09
0.12
0.15
0.18
SNP effect on Calpain−2 catalytic subunit
SNP effect on Gut microbiota abundance (genus Catenibacterium id.2153)

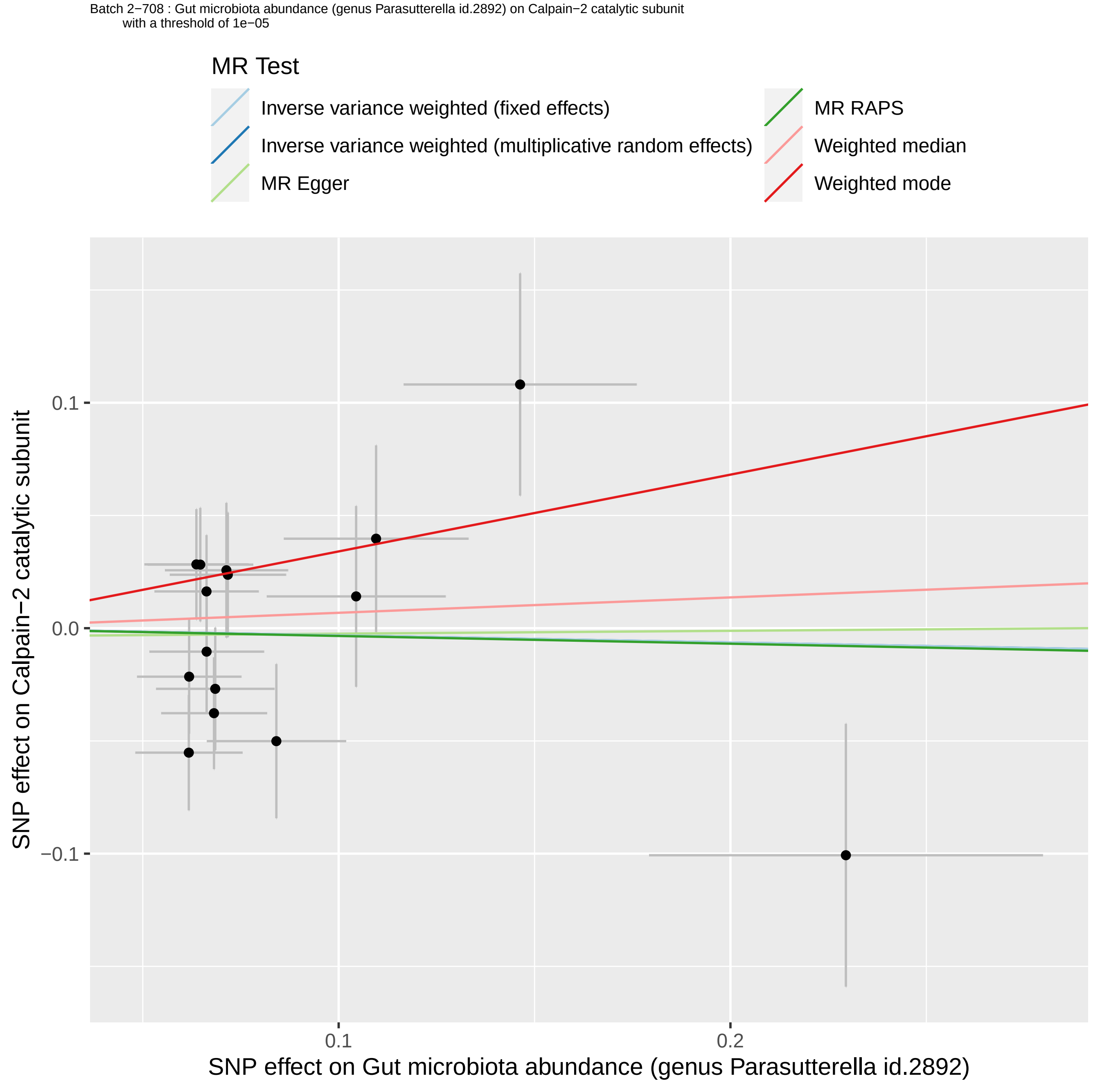

Batch 2−708 : Gut microbiota abundance (genus Parasutterella id.2892) on Calpain−2 catalytic subunit
with a threshold of 1e−05
MR Test
Inverse variance weighted (fixed effects)
Inverse variance weighted (multiplicative random effects)
MR Egger
MR RAPS
Weighted median
Weighted mode
0.1
0.0
−0.1
0.1
0.2
SNP effect on Calpain−2 catalytic subunit
SNP effect on Gut microbiota abundance (genus Parasutterella id.2892)

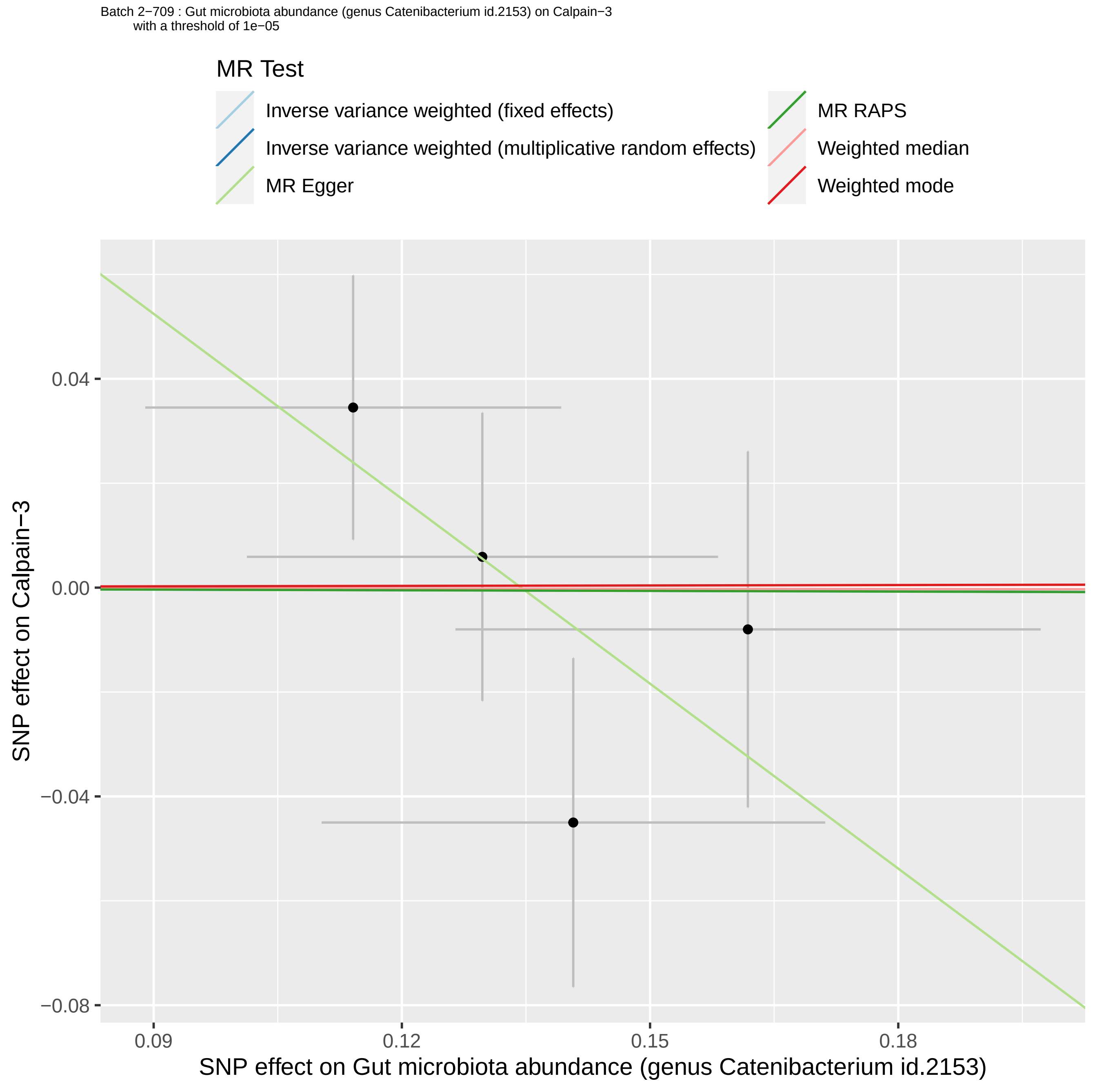

Batch 2−709 : Gut microbiota abundance (genus Catenibacterium id.2153) on Calpain−3
with a threshold of 1e−05
MR Test
Inverse variance weighted (fixed effects)
Inverse variance weighted (multiplicative random effects)
MR Egger
MR RAPS
Weighted median
Weighted mode
0.04
0.00
−0.04
−0.08
0.09
0.12
0.15
0.18
SNP effect on Calpain−3
SNP effect on Gut microbiota abundance (genus Catenibacterium id.2153)

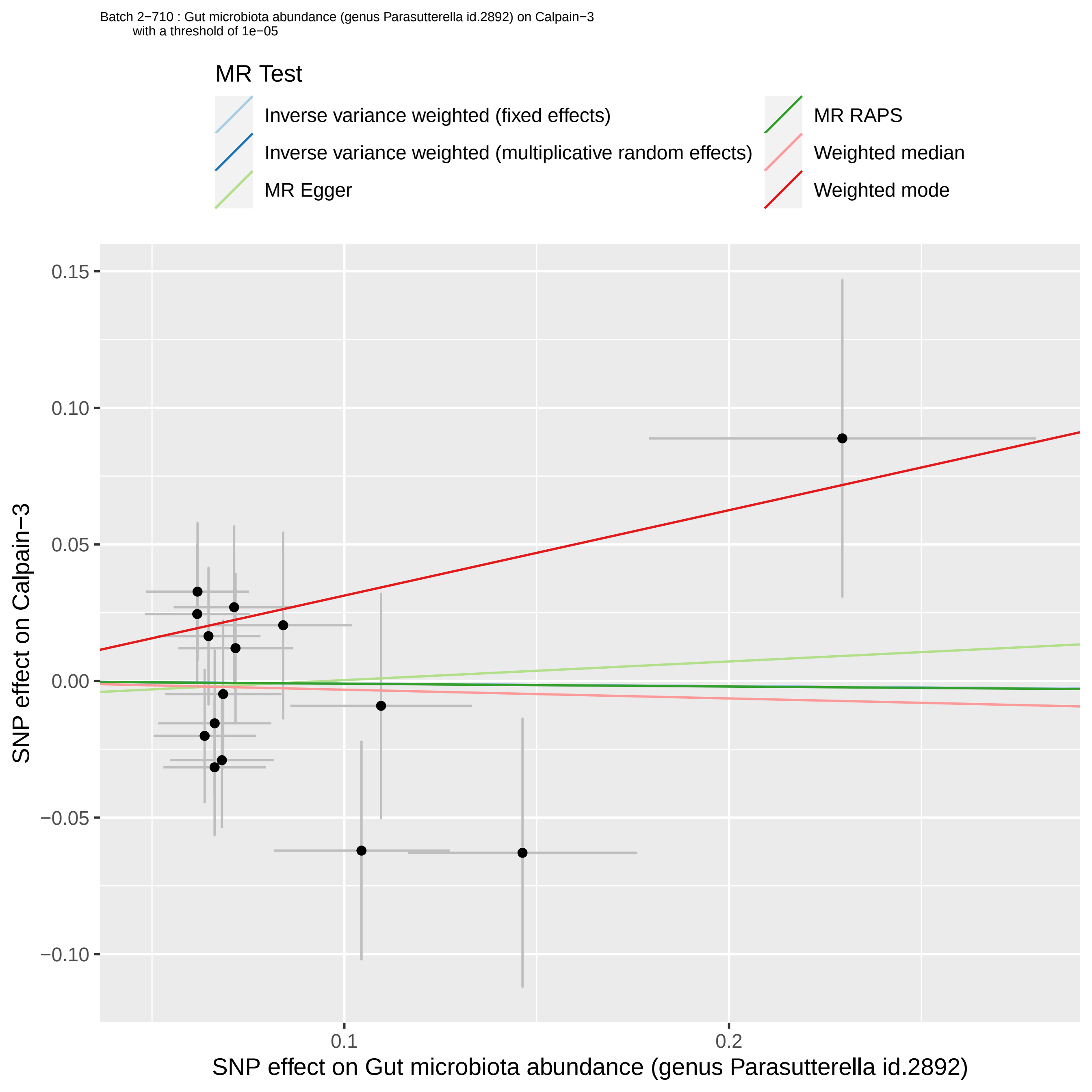
Batch 2−710 : Gut microbiota abundance (genus Parasutterella id.2892) on Calpain−3
with a threshold of 1e−05
MR Test
Inverse variance weighted (fixed effects)
Inverse variance weighted (multiplicative random effects)
MR Egger
MR RAPS
Weighted median
Weighted mode
0.15
0.10
0.05
0.00
−0.05
−0.10
SNP effect on Calpain−3
0.1
0.2
SNP effect on Gut microbiota abundance (genus Parasutterella id.2892)

Batch 2−711 : Gut microbiota abundance (genus Catenibacterium id.2153) on Calcyphosin−like protein
with a threshold of 1e−05

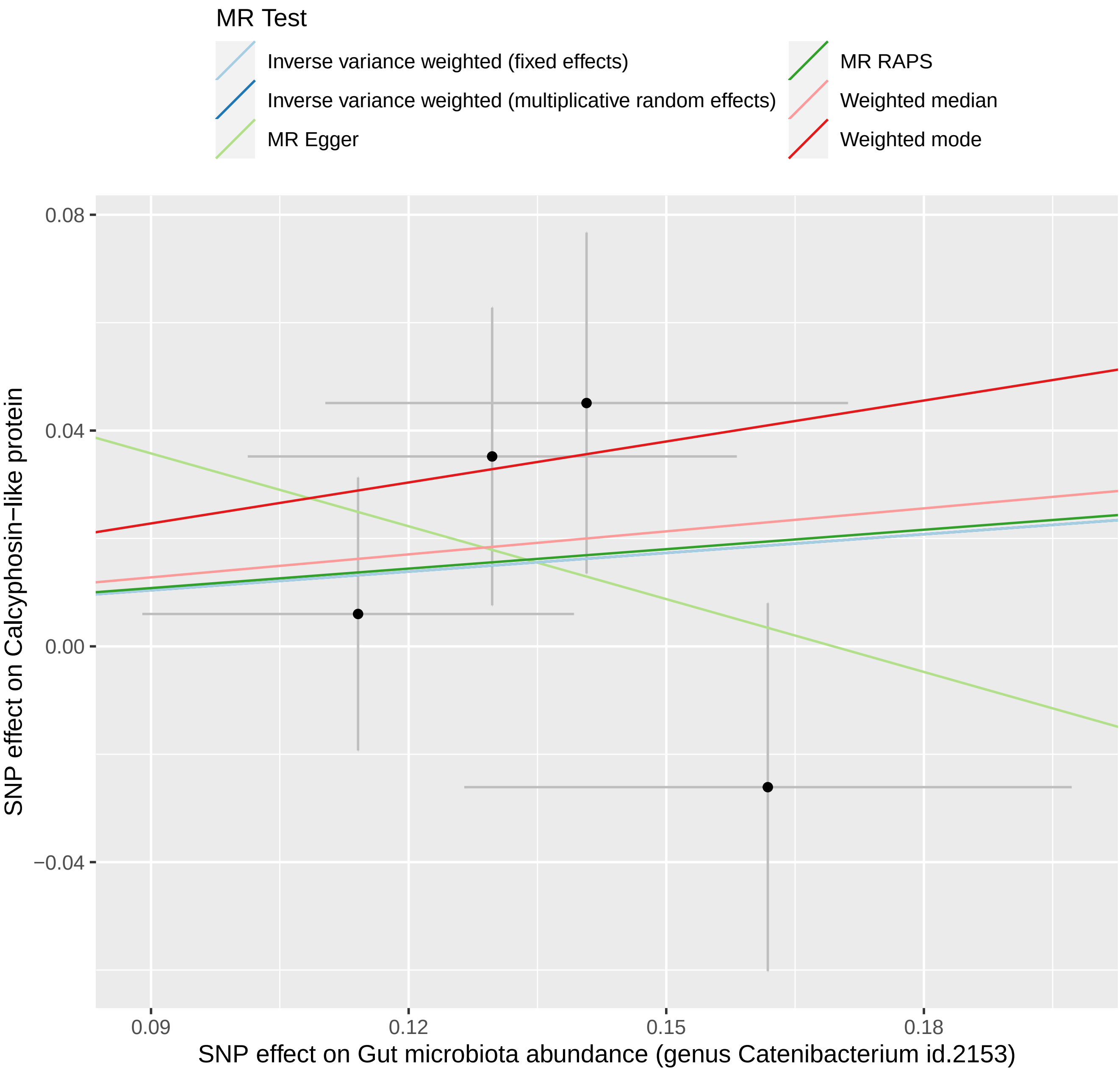

Batch 2−712 : Gut microbiota abundance (genus Parasutterella id.2892) on Calcyphosin−like protein
with a threshold of 1e−05

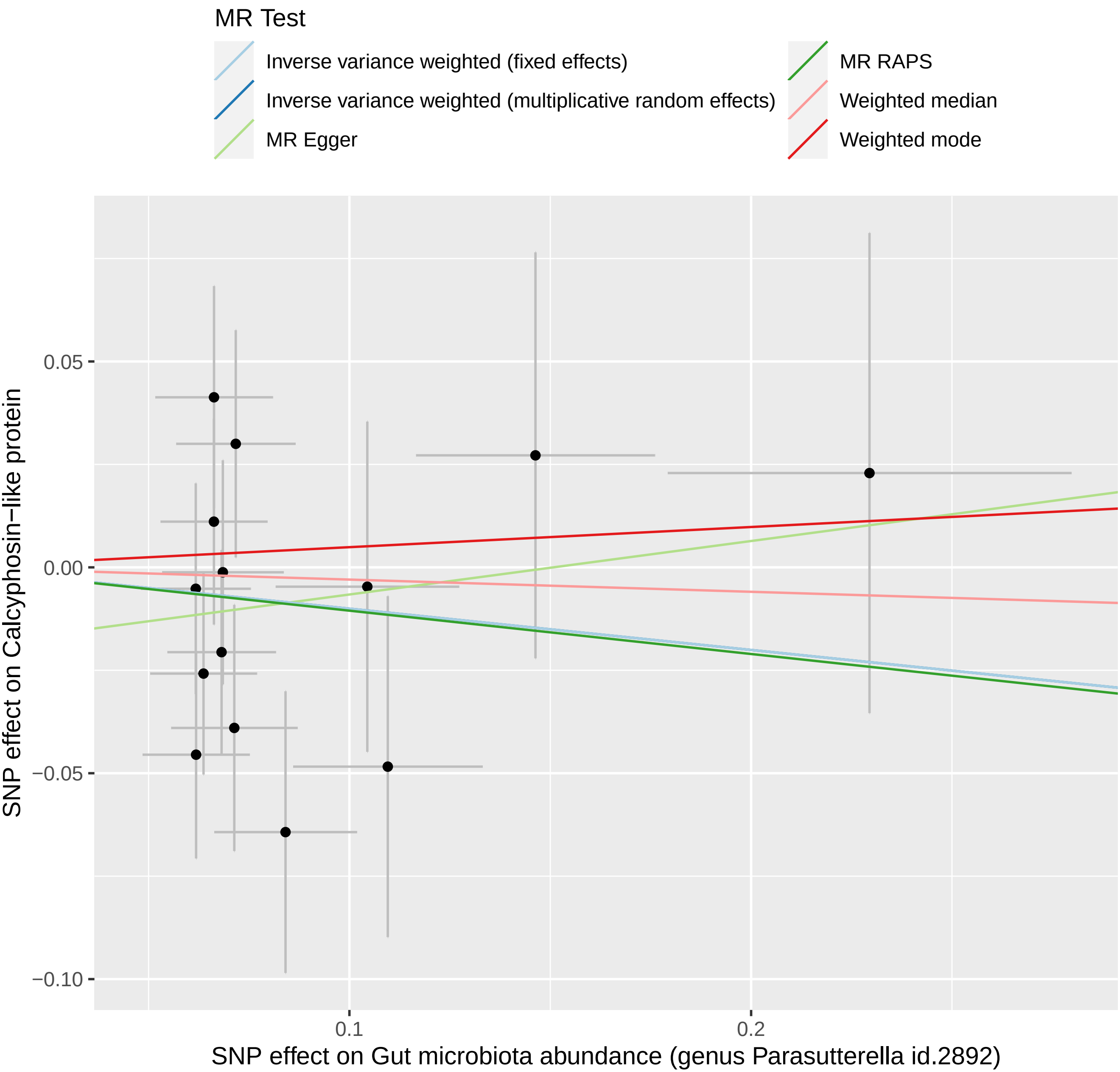

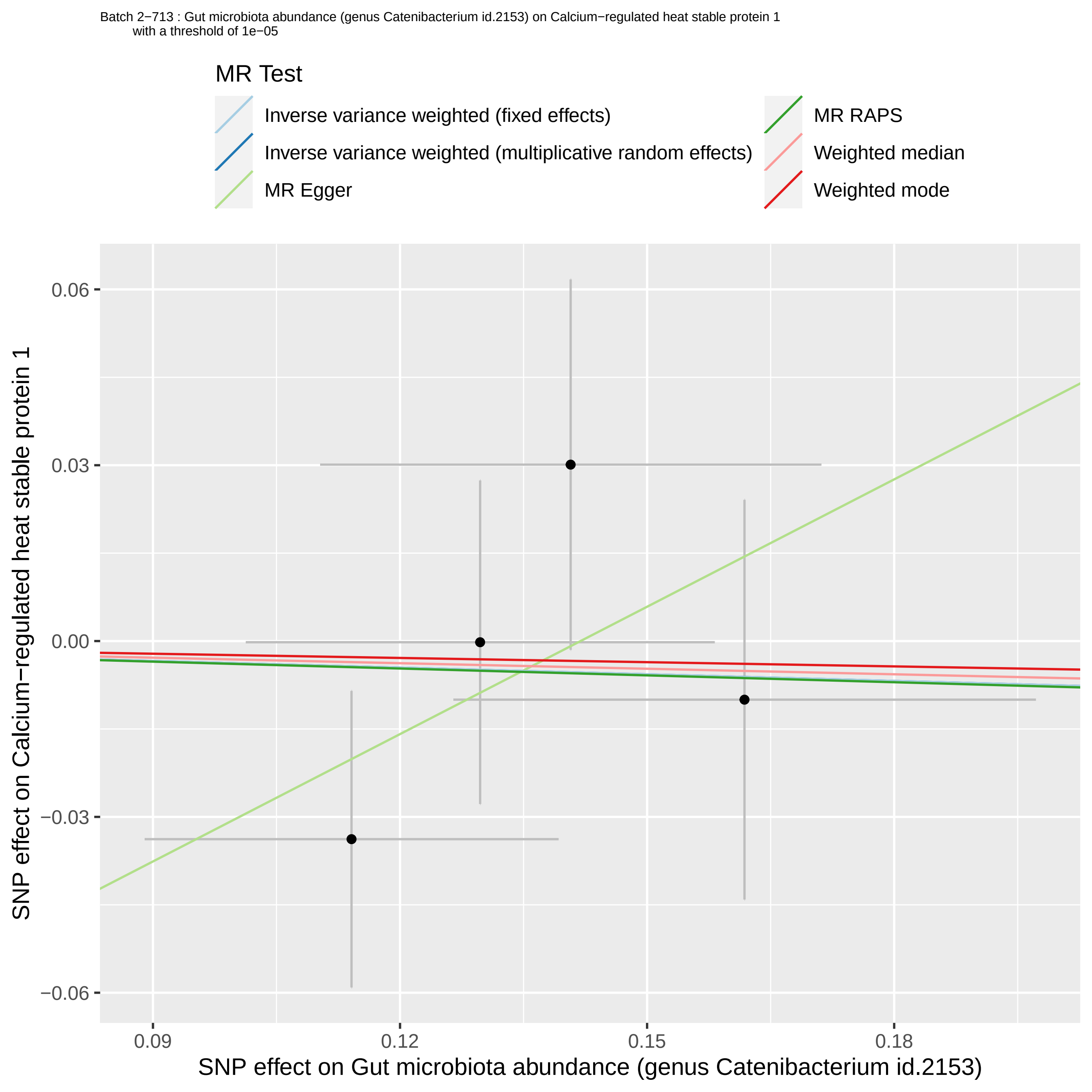
Batch 2−713 : Gut microbiota abundance (genus Catenibacterium id.2153) on Calcium−regulated heat stable protein 1
with a threshold of 1e−05
MR Test
Inverse variance weighted (fixed effects)
Inverse variance weighted (multiplicative random effects)
MR Egger
MR RAPS
Weighted median
Weighted mode
0.06
0.03
0.00
−0.03
−0.06
0.09
0.12
0.15
0.18
SNP effect on Calcium−regulated heat stable protein 1
SNP effect on Gut microbiota abundance (genus Catenibacterium id.2153)

Batch 2−714 : Gut microbiota abundance (genus Parasutterella id.2892) on Calcium−regulated heat stable protein 1
with a threshold of 1e−05

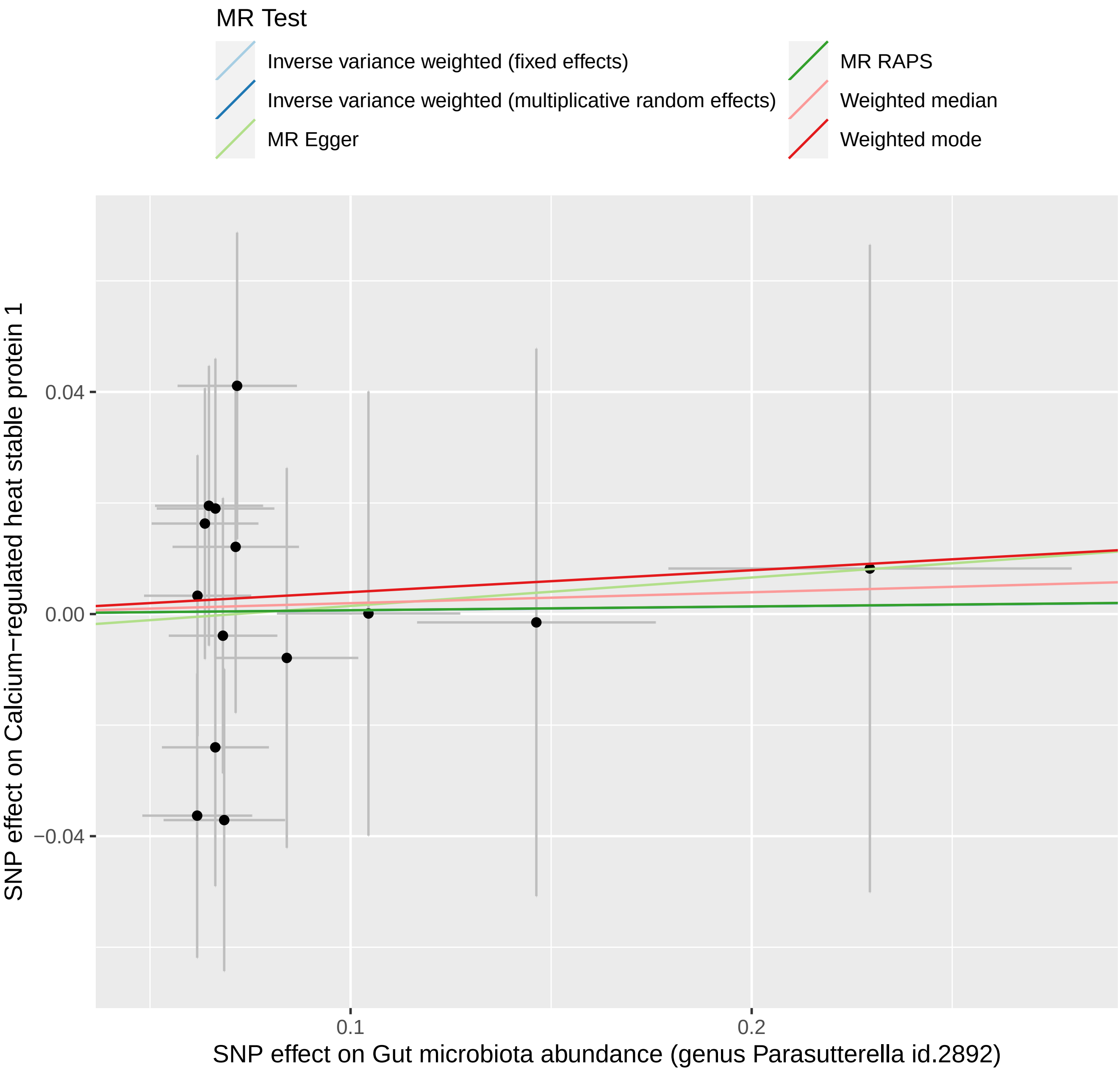

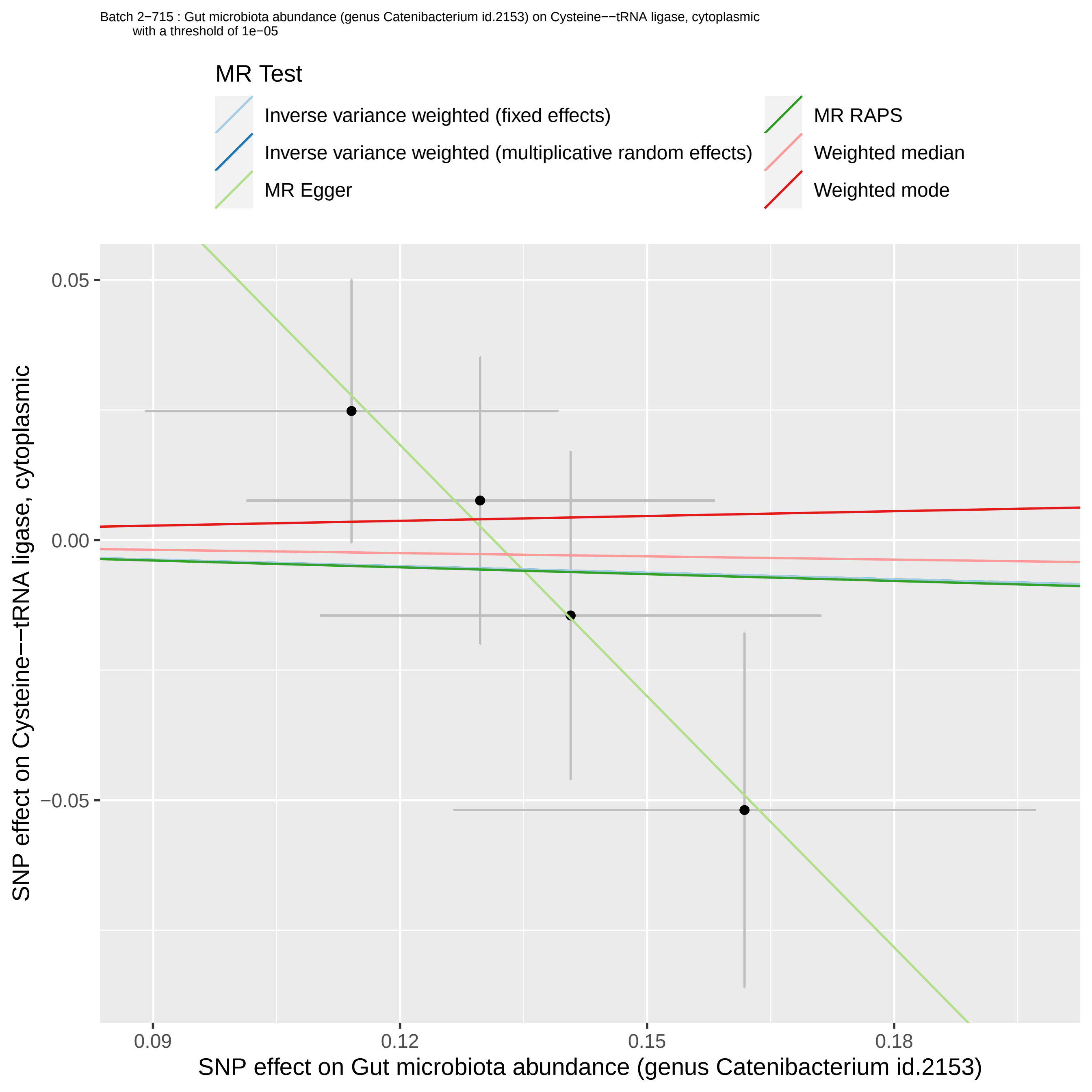

Batch 2−715 : Gut microbiota abundance (genus Catenibacterium id.2153) on Cysteine−−tRNA ligase, cytoplasmic
with a threshold of 1e−05
MR Test
Inverse variance weighted (fixed effects)
Inverse variance weighted (multiplicative random effects)
MR Egger
MR RAPS
Weighted median
Weighted mode
0.05
0.00
−0.05
0.09
0.12
0.15
0.18
SNP effect on Cysteine−−tRNA ligase, cytoplasmic
SNP effect on Gut microbiota abundance (genus Catenibacterium id.2153)

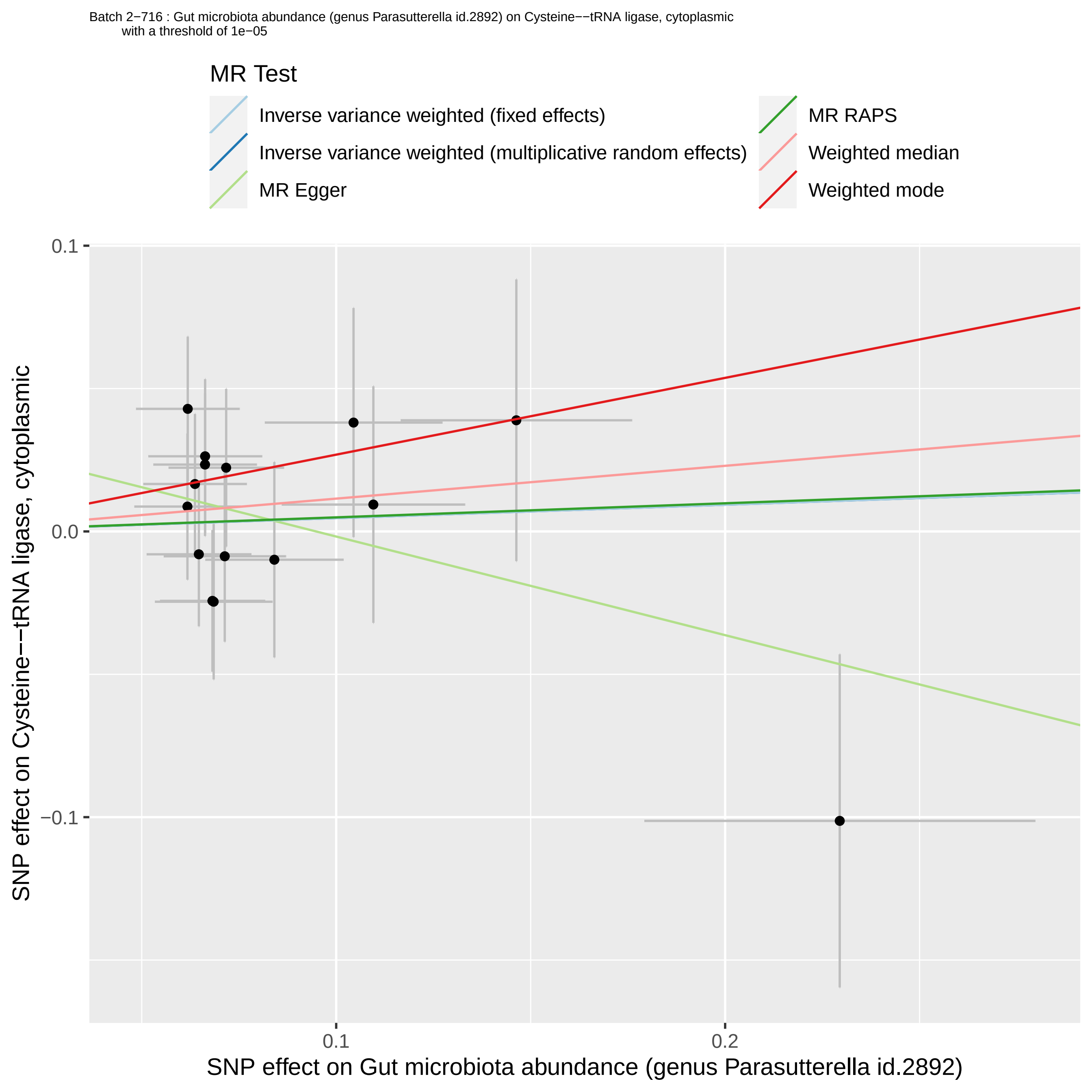

Batch 2−716 : Gut microbiota abundance (genus Parasutterella id.2892) on Cysteine−−tRNA ligase, cytoplasmic
with a threshold of 1e−05
MR Test
Inverse variance weighted (fixed effects)
Inverse variance weighted (multiplicative random effects)
MR Egger
MR RAPS
Weighted median
Weighted mode
0.1
0.0
−0.1
0.1
0.2
SNP effect on Cysteine−−tRNA ligase, cytoplasmic
SNP effect on Gut microbiota abundance (genus Parasutterella id.2892)

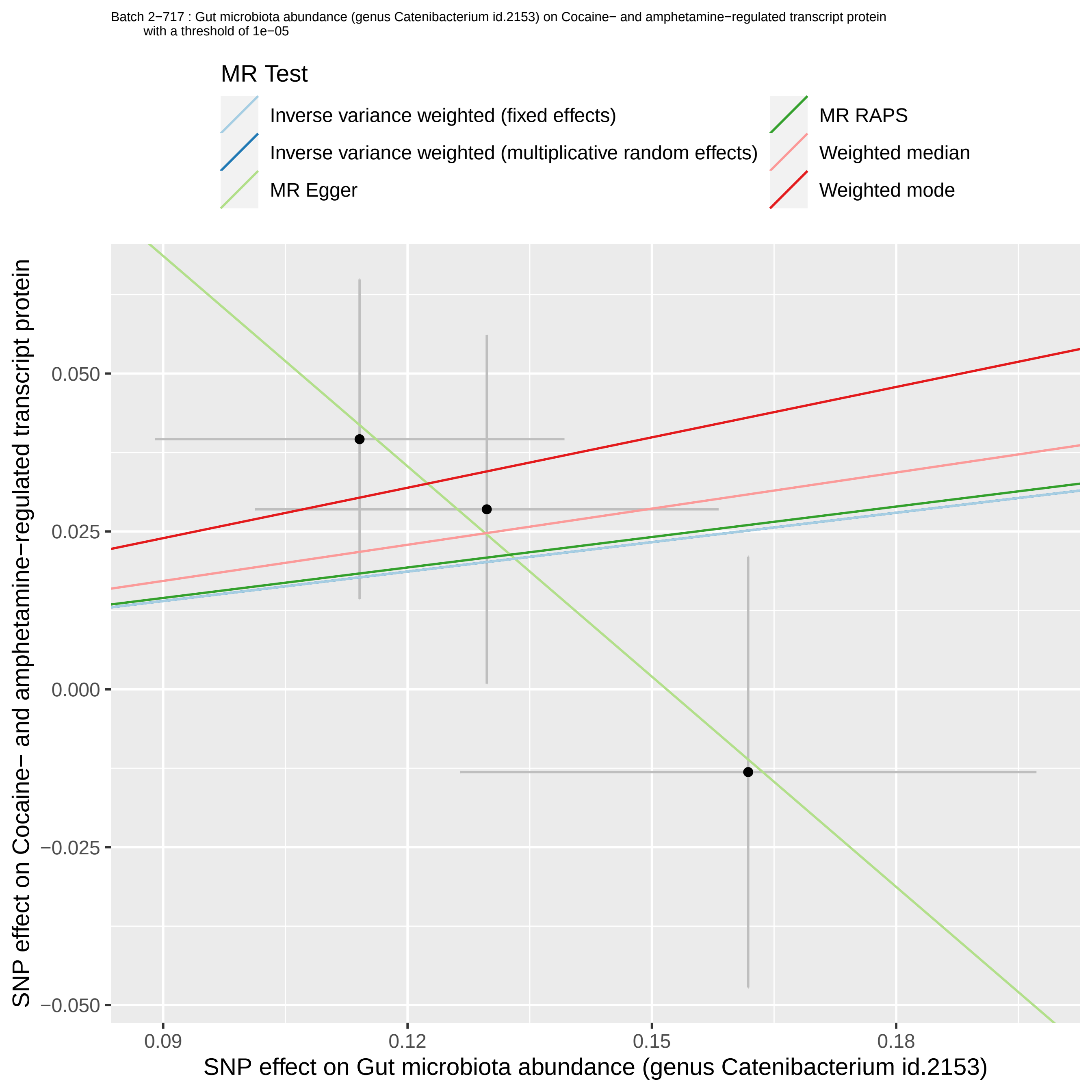

Batch 2−717 : Gut microbiota abundance (genus Catenibacterium id.2153) on Cocaine− and amphetamine−regulated transcript protein
with a threshold of 1e−05
MR Test
Inverse variance weighted (fixed effects)
Inverse variance weighted (multiplicative random effects)
MR Egger
MR RAPS
Weighted median
Weighted mode
SNP effect on Cocaine− and amphetamine−regulated transcript protein
0.050
0.025
0.000
−0.025
−0.050
0.09
0.12
0.15
0.18
SNP effect on Gut microbiota abundance (genus Catenibacterium id.2153)

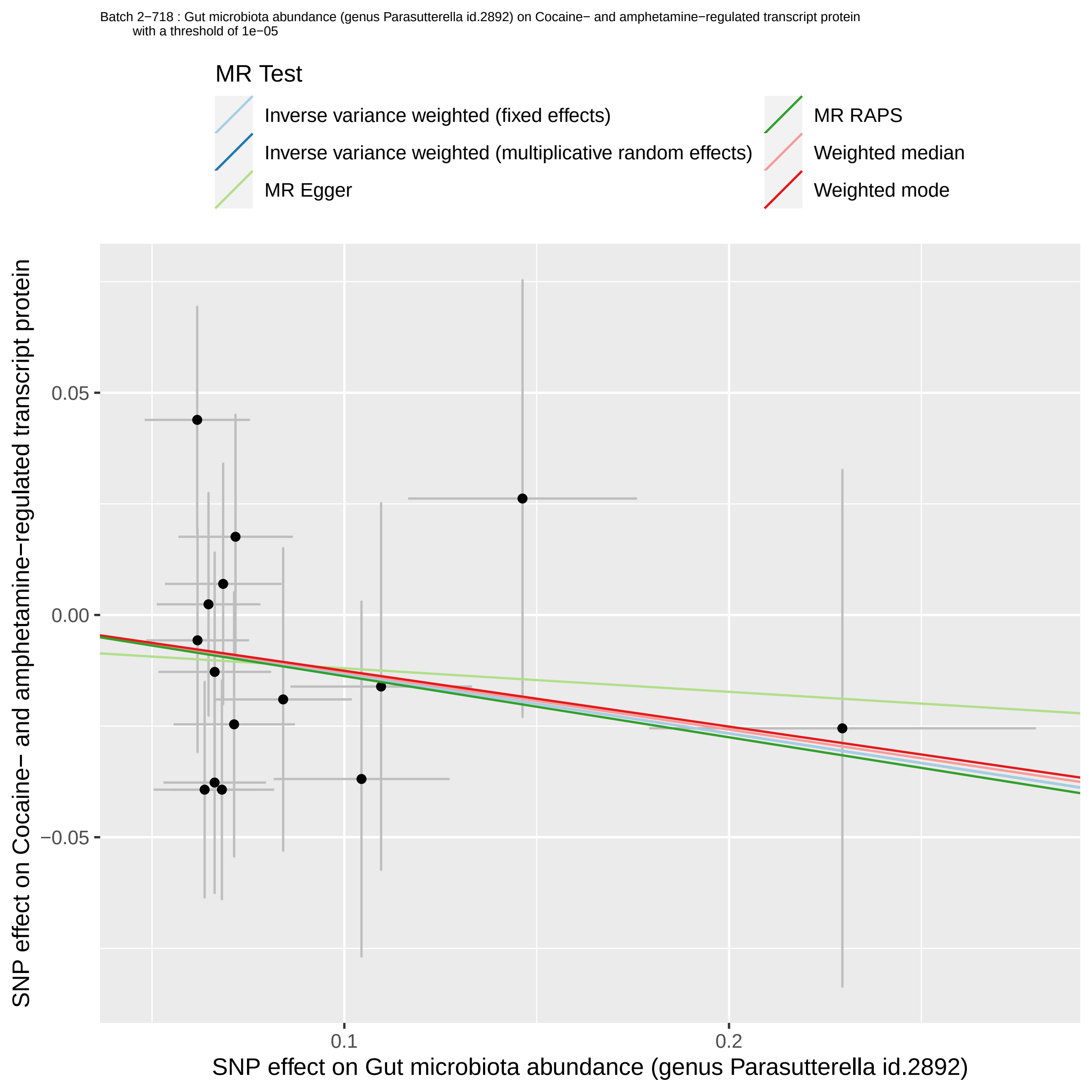
Batch 2−718 : Gut microbiota abundance (genus Parasutterella id.2892) on Cocaine− and amphetamine−regulated transcript protein
with a threshold of 1e−05
MR Test
Inverse variance weighted (fixed effects)
Inverse variance weighted (multiplicative random effects)
MR Egger
MR RAPS
Weighted median
Weighted mode
0.05
0.00
−0.05
0.1
0.2
SNP effect on Cocaine− and amphetamine−regulated transcript protein
SNP effect on Gut microbiota abundance (genus Parasutterella id.2892)

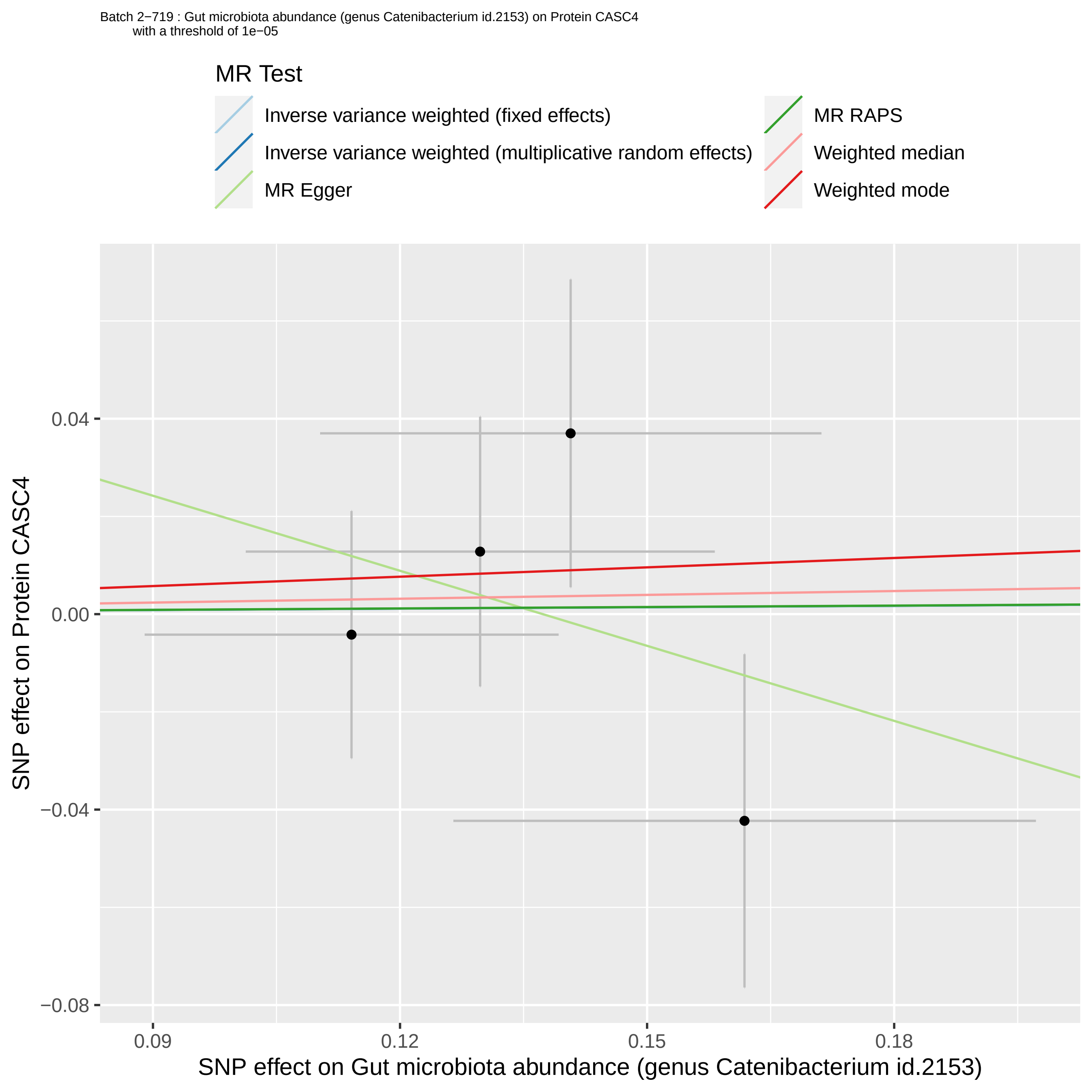
Batch 2−719 : Gut microbiota abundance (genus Catenibacterium id.2153) on Protein CASC4
with a threshold of 1e−05
MR Test
Inverse variance weighted (fixed effects)
Inverse variance weighted (multiplicative random effects)
MR Egger
MR RAPS
Weighted median
Weighted mode
SNP effect on Protein CASC4
0.04
0.00
−0.04
−0.08
0.09
0.12
0.15
0.18
SNP effect on Gut microbiota abundance (genus Catenibacterium id.2153)

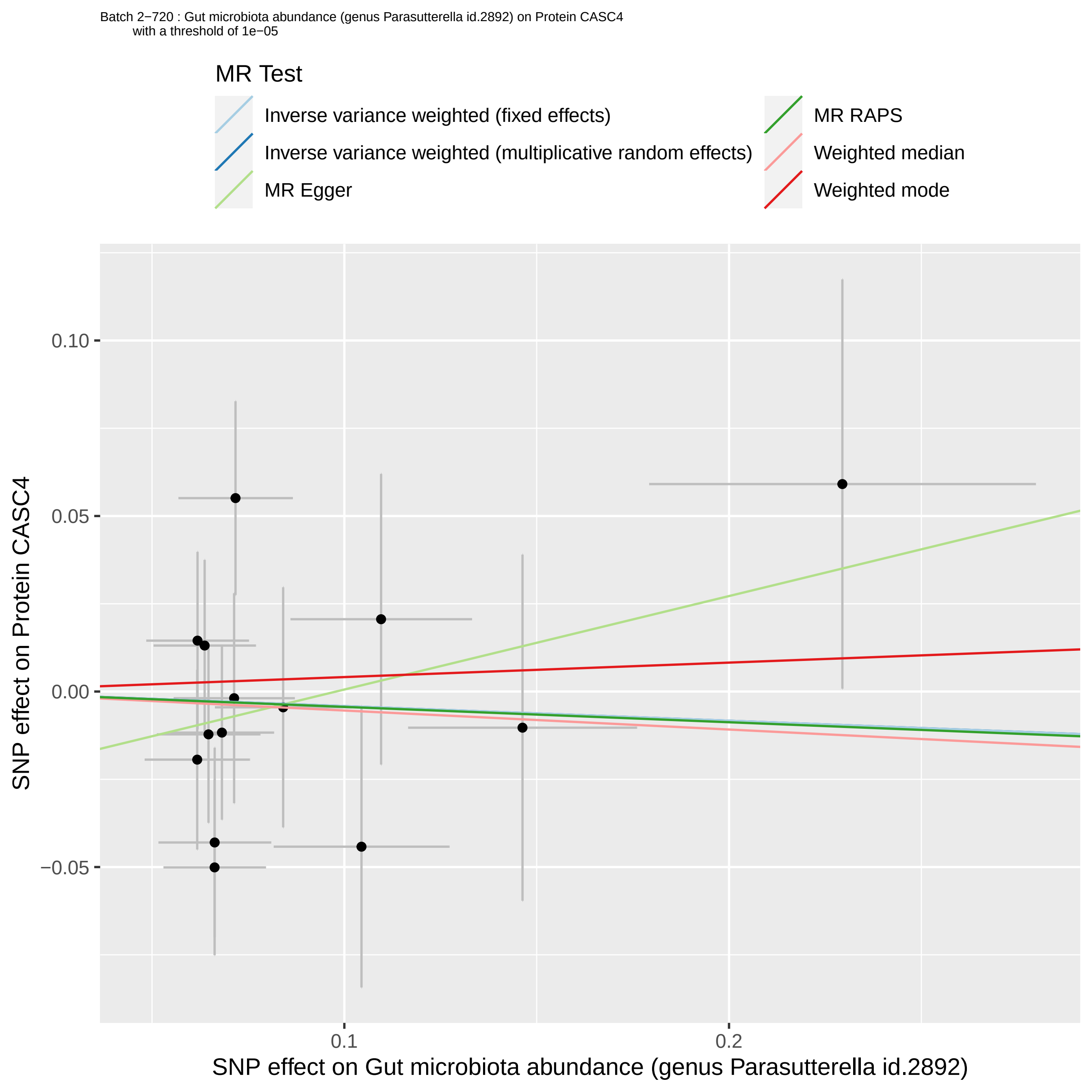

Batch 2−720 : Gut microbiota abundance (genus Parasutterella id.2892) on Protein CASC4
with a threshold of 1e−05
MR Test
Inverse variance weighted (fixed effects)
Inverse variance weighted (multiplicative random effects)
MR Egger
MR RAPS
Weighted median
Weighted mode
0.10
0.05
0.00
−0.05
0.1
0.2
SNP effect on Protein CASC4
SNP effect on Gut microbiota abundance (genus Parasutterella id.2892)

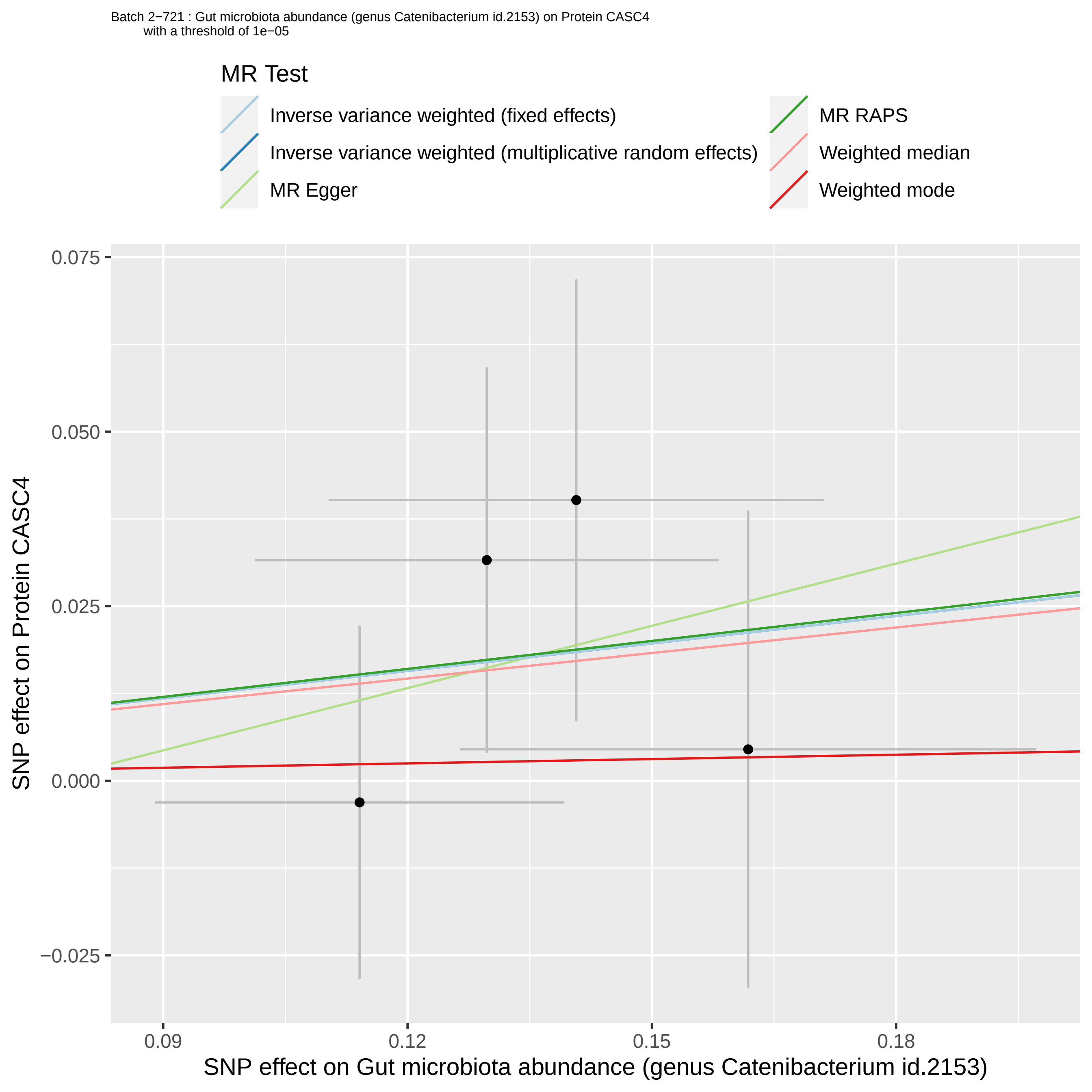
Batch 2−721 : Gut microbiota abundance (genus Catenibacterium id.2153) on Protein CASC4
with a threshold of 1e−05
MR Test
Inverse variance weighted (fixed effects)
Inverse variance weighted (multiplicative random effects)
MR Egger
MR RAPS
Weighted median
Weighted mode
0.075
0.050
0.025
0.000
−0.025
0.09
0.12
0.15
0.18
SNP effect on Protein CASC4
SNP effect on Gut microbiota abundance (genus Catenibacterium id.2153)

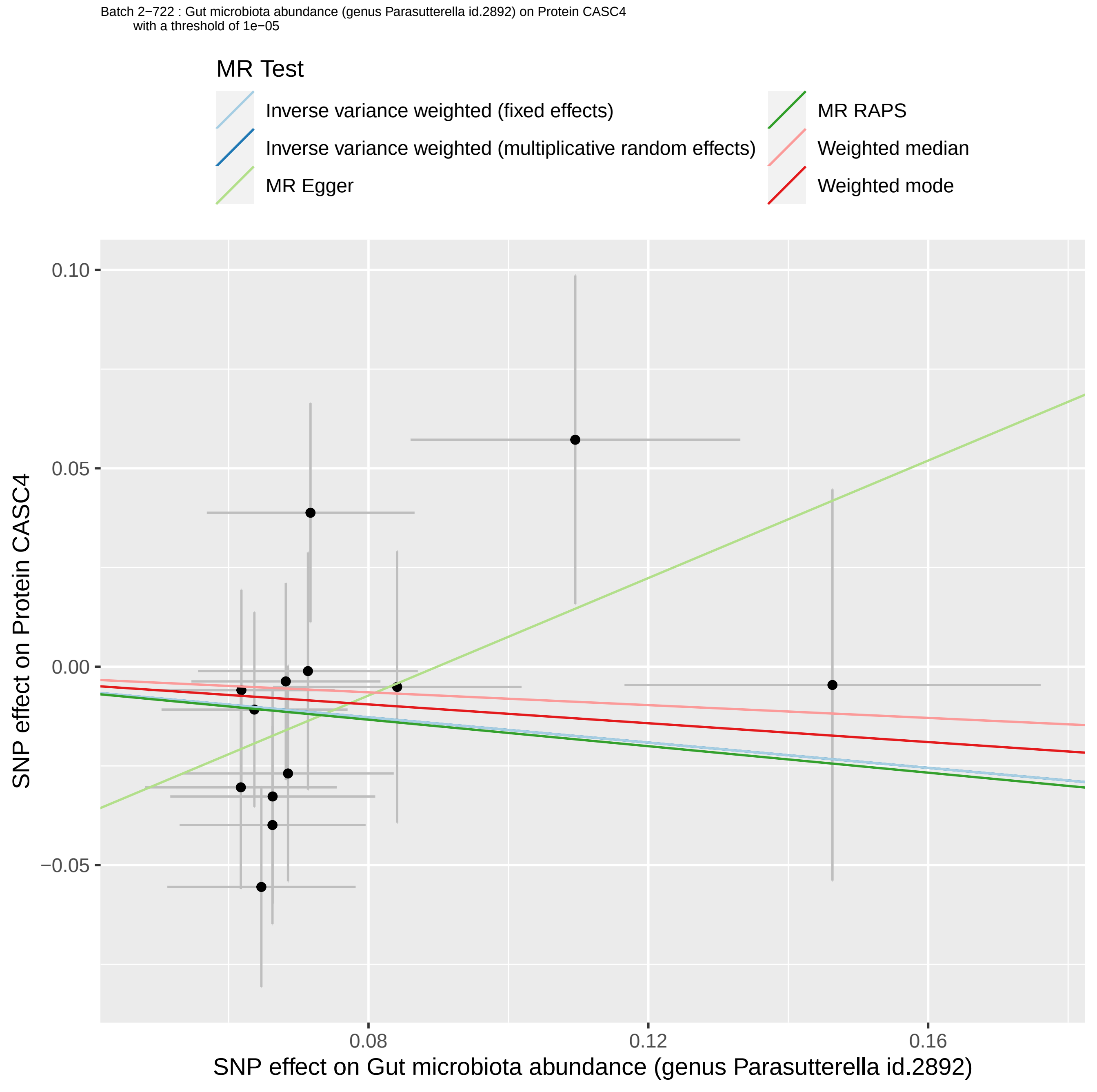
Batch 2−722 : Gut microbiota abundance (genus Parasutterella id.2892) on Protein CASC4
with a threshold of 1e−05
MR Test
Inverse variance weighted (fixed effects)
Inverse variance weighted (multiplicative random effects)
MR Egger
MR RAPS
Weighted median
Weighted mode
SNP effect on Protein CASC4
0.10
0.05
0.00
−0.05
0.08
0.12
0.16
SNP effect on Gut microbiota abundance (genus Parasutterella id.2892)

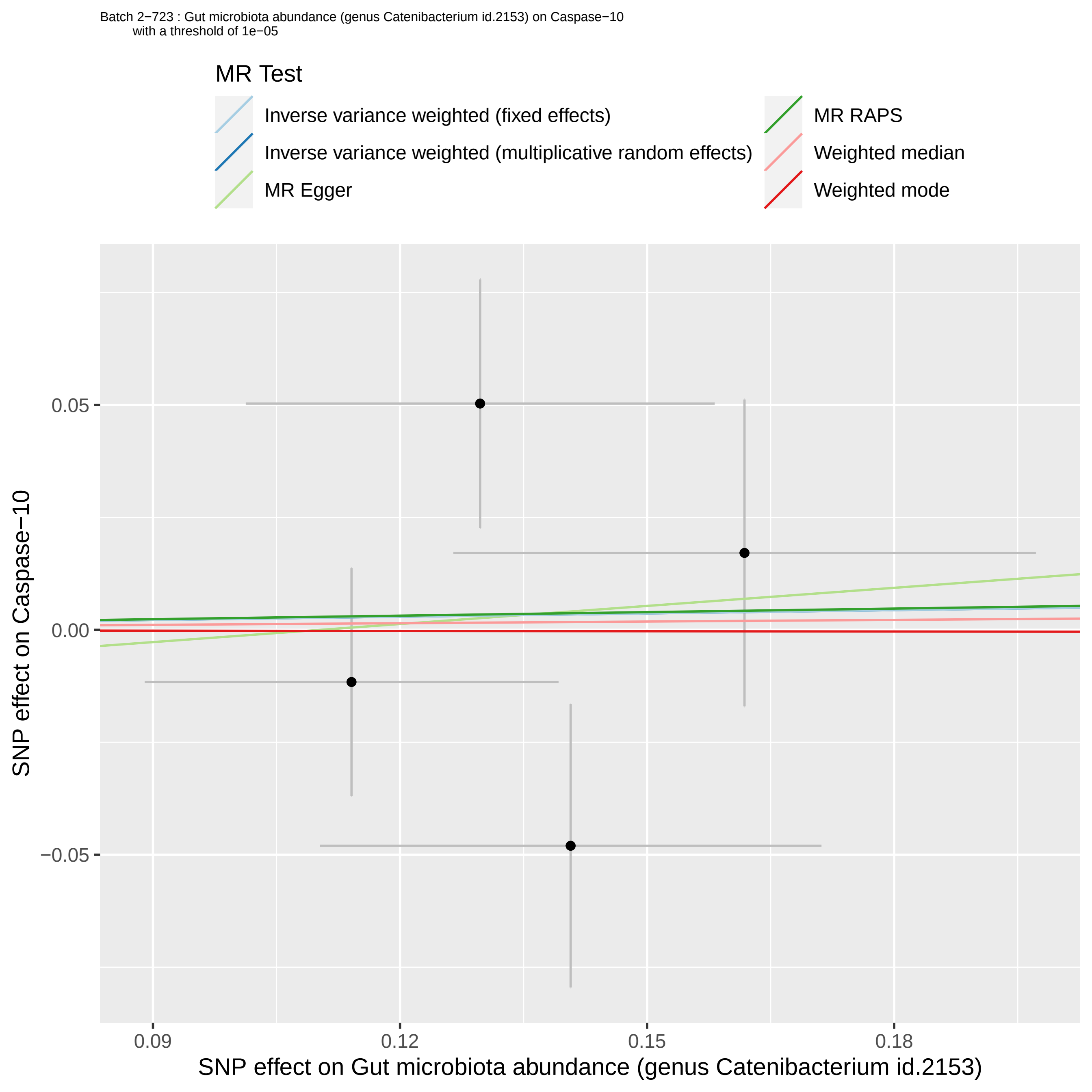

Batch 2−723 : Gut microbiota abundance (genus Catenibacterium id.2153) on Caspase−10
with a threshold of 1e−05
MR Test
Inverse variance weighted (fixed effects)
Inverse variance weighted (multiplicative random effects)
MR Egger
MR RAPS
Weighted median
Weighted mode
0.05
0.00
−0.05
0.09
0.12
0.15
0.18
SNP effect on Caspase−10
SNP effect on Gut microbiota abundance (genus Catenibacterium id.2153)

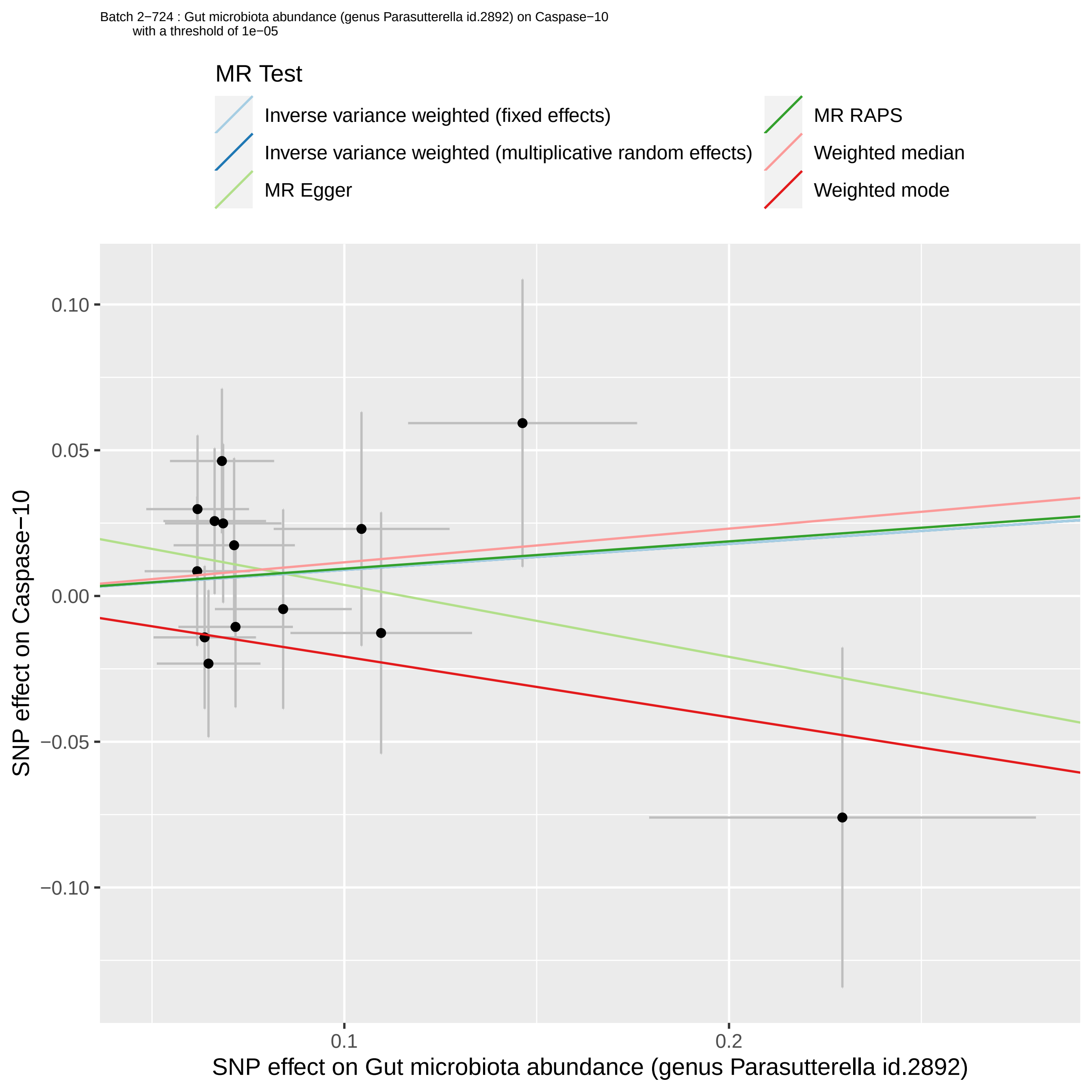

Batch 2−724 : Gut microbiota abundance (genus Parasutterella id.2892) on Caspase−10
with a threshold of 1e−05
MR Test
Inverse variance weighted (fixed effects)
Inverse variance weighted (multiplicative random effects)
MR Egger
MR RAPS
Weighted median
Weighted mode
0.10
0.05
0.00
−0.05
−0.10
SNP effect on Caspase−10
0.1
0.2
SNP effect on Gut microbiota abundance (genus Parasutterella id.2892)

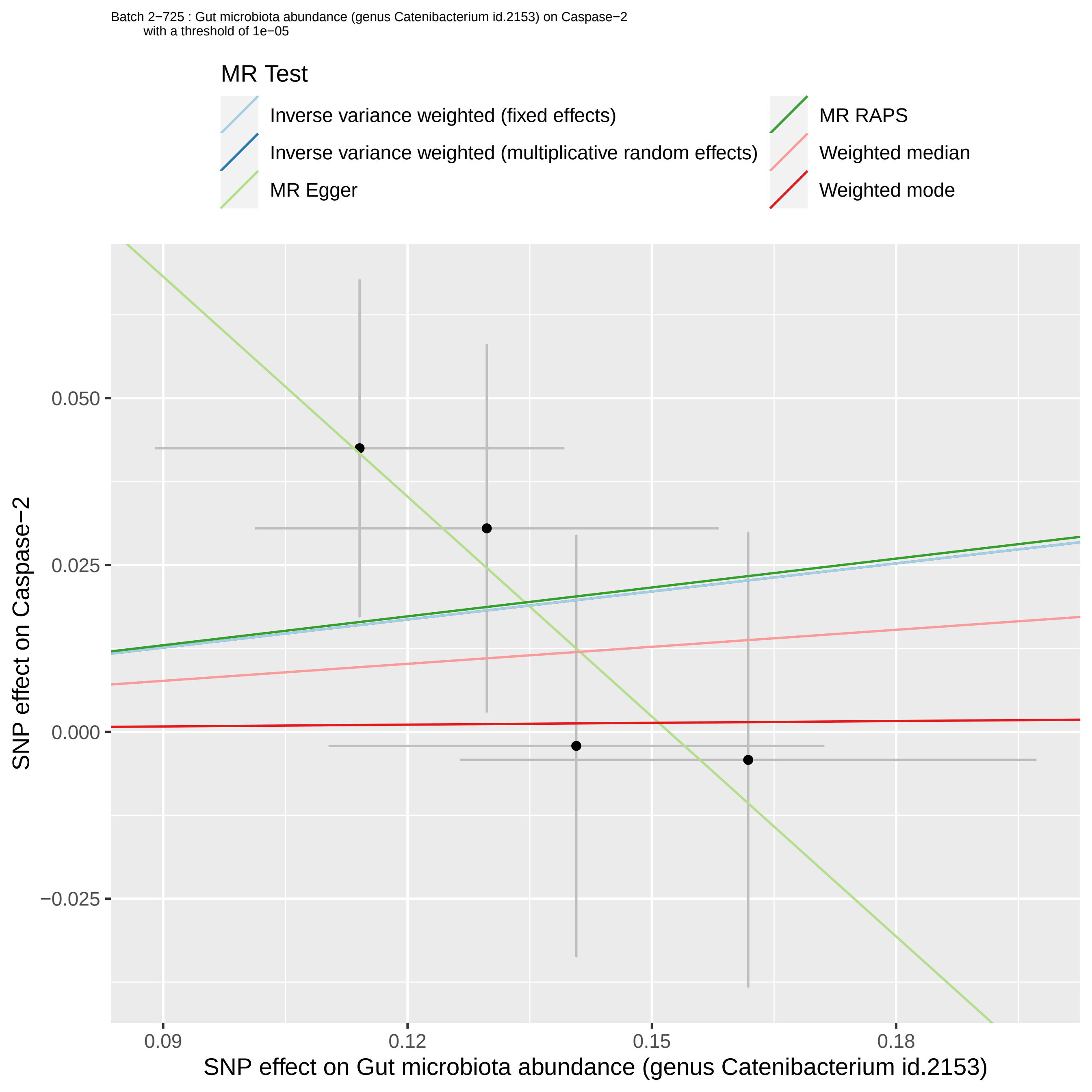

Batch 2−725 : Gut microbiota abundance (genus Catenibacterium id.2153) on Caspase−2
with a threshold of 1e−05
MR Test
Inverse variance weighted (fixed effects)
Inverse variance weighted (multiplicative random effects)
MR Egger
MR RAPS
Weighted median
Weighted mode
SNP effect on Caspase−2
0.050
0.025
0.000
−0.025
0.09
0.12
0.15
0.18
SNP effect on Gut microbiota abundance (genus Catenibacterium id.2153)

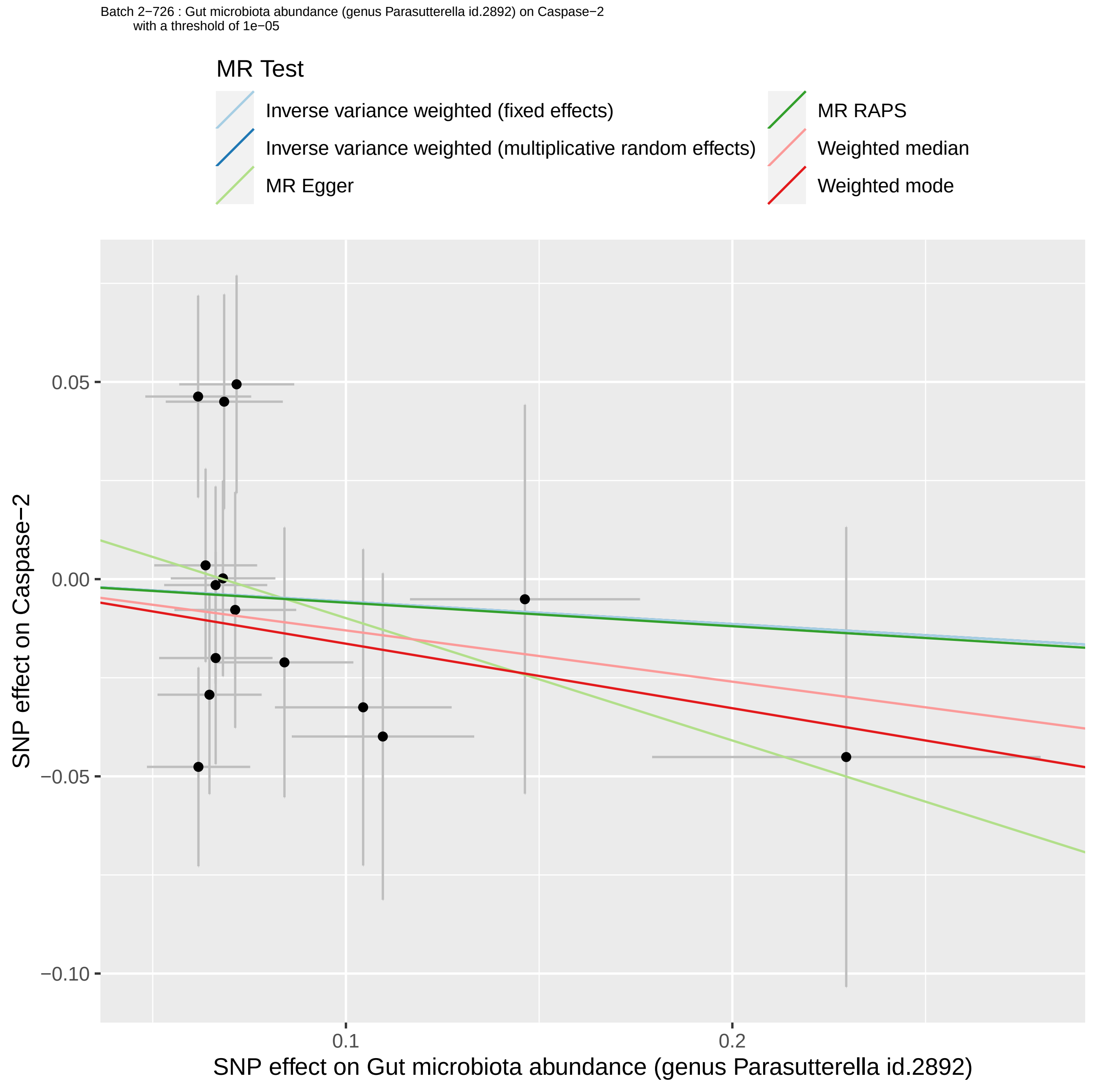
Batch 2−726 : Gut microbiota abundance (genus Parasutterella id.2892) on Caspase−2
with a threshold of 1e−05
MR Test
Inverse variance weighted (fixed effects)
Inverse variance weighted (multiplicative random effects)
MR Egger
MR RAPS
Weighted median
Weighted mode
0.05
0.00
−0.05
−0.10
0.1
0.2
SNP effect on Caspase−2
SNP effect on Gut microbiota abundance (genus Parasutterella id.2892)

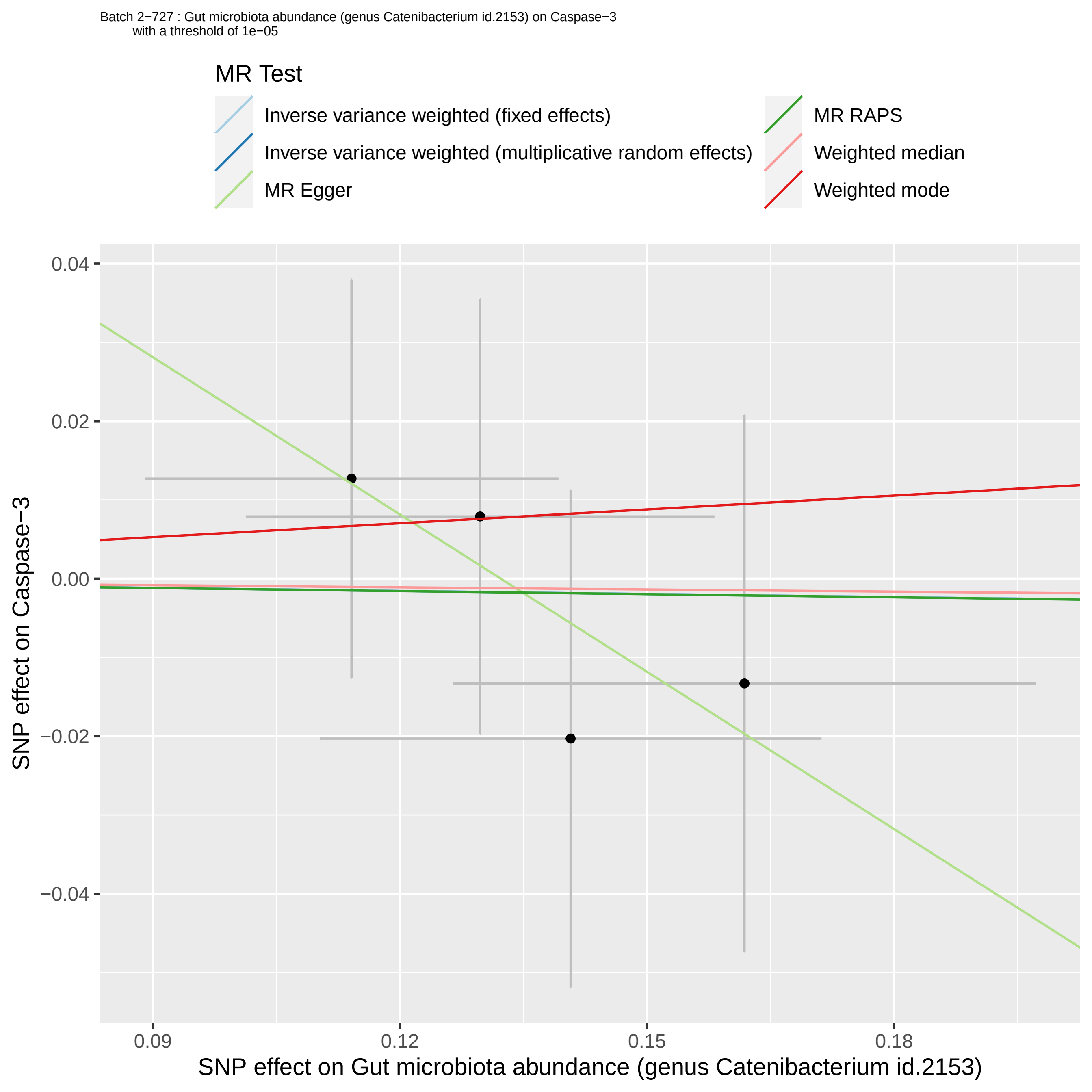
Batch 2−727 : Gut microbiota abundance (genus Catenibacterium id.2153) on Caspase−3
with a threshold of 1e−05
MR Test
Inverse variance weighted (fixed effects)
Inverse variance weighted (multiplicative random effects)
MR Egger
MR RAPS
Weighted median
Weighted mode
0.04
0.02
0.00
−0.02
−0.04
SNP effect on Caspase−3
0.09
0.12
0.15
0.18
SNP effect on Gut microbiota abundance (genus Catenibacterium id.2153)

Batch 2−728 : Gut microbiota abundance (genus Parasutterella id.2892) on Caspase−3
with a threshold of 1e−05

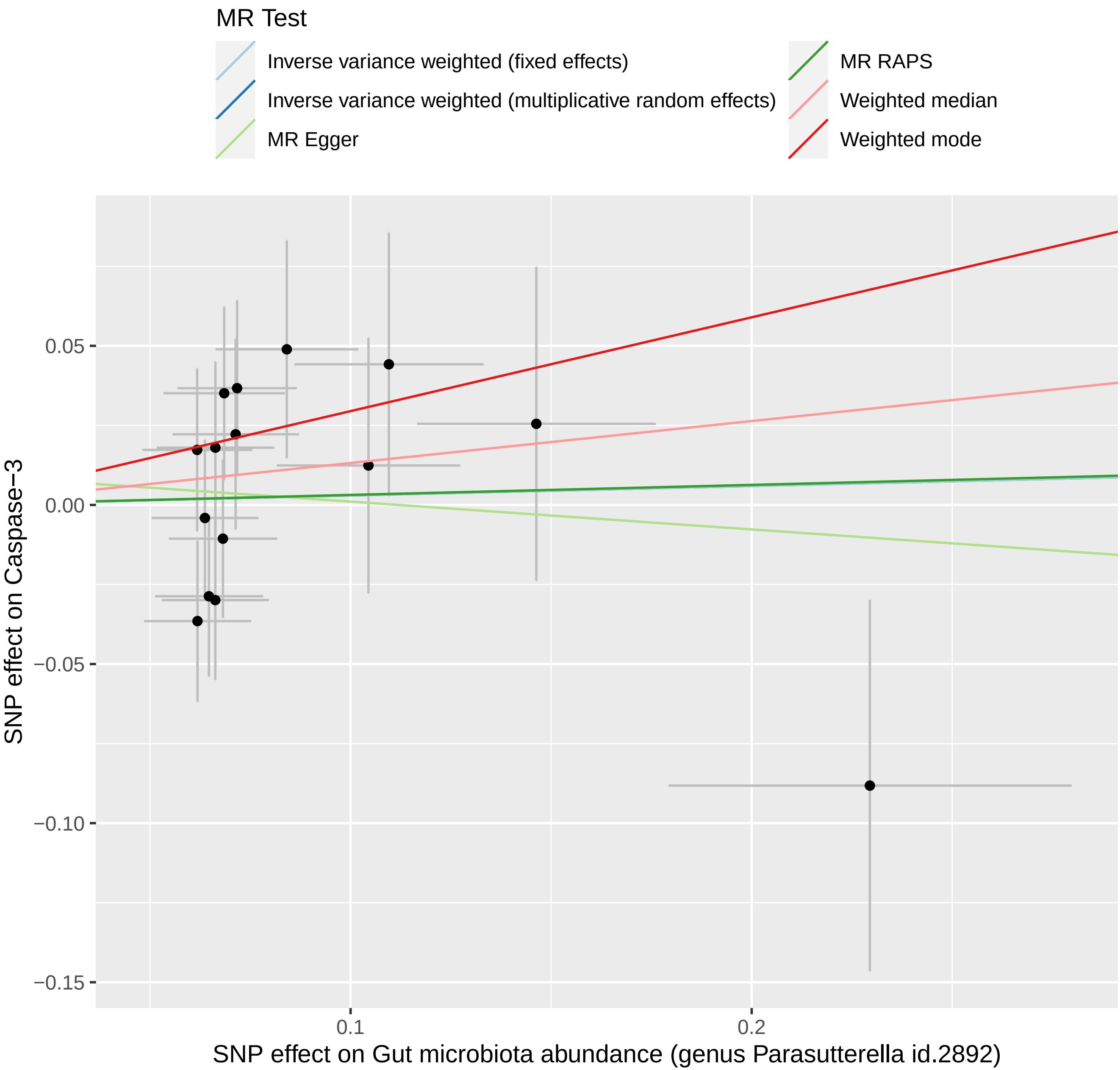

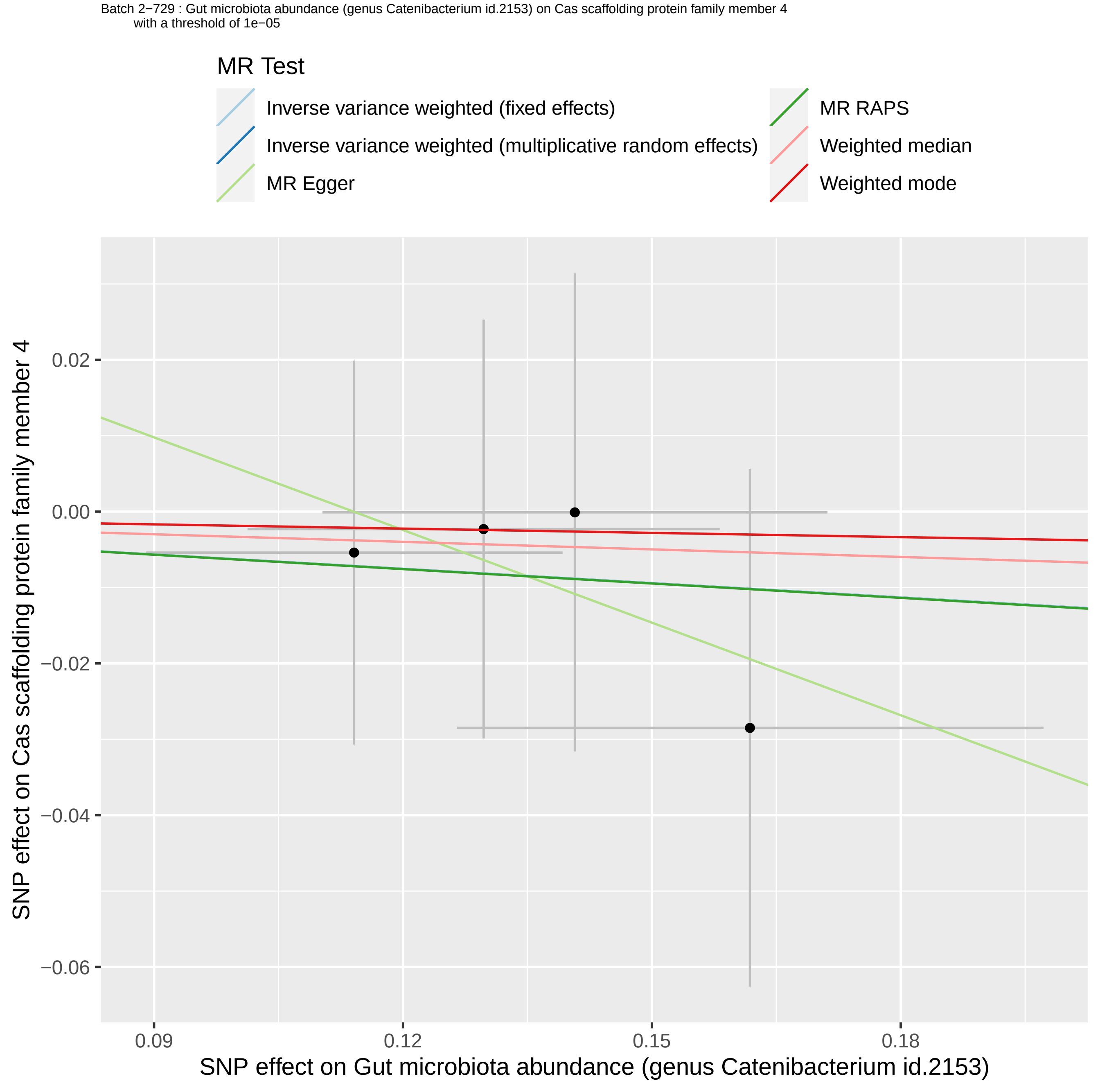

Batch 2−729 : Gut microbiota abundance (genus Catenibacterium id.2153) on Cas scaffolding protein family member 4
with a threshold of 1e−05
MR Test
Inverse variance weighted (fixed effects)
Inverse variance weighted (multiplicative random effects)
MR Egger
MR RAPS
Weighted median
Weighted mode
0.02
0.00
−0.02
−0.04
−0.06
0.09
0.12
0.15
0.18
SNP effect on Cas scaffolding protein family member 4
SNP effect on Gut microbiota abundance (genus Catenibacterium id.2153)

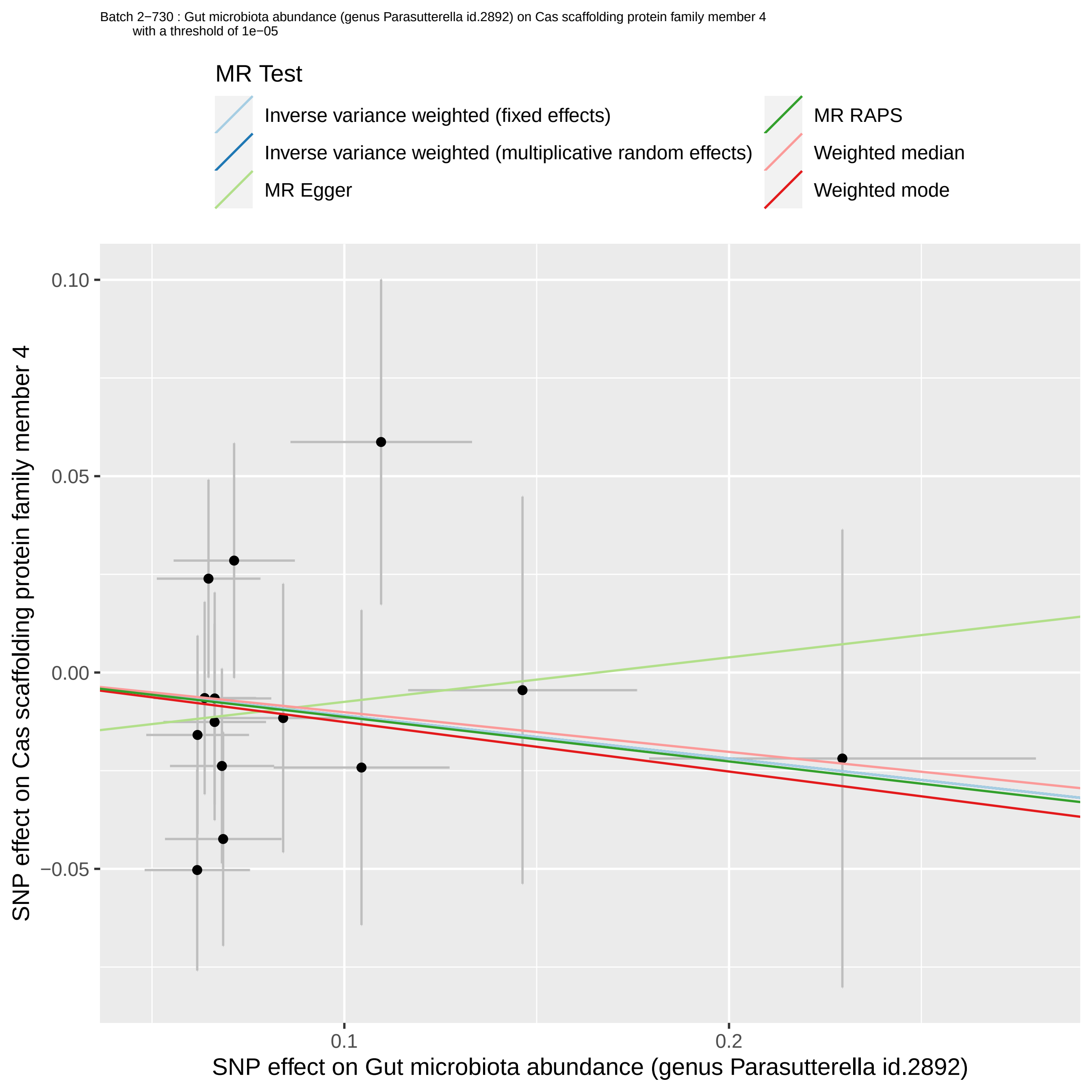

Batch 2−730 : Gut microbiota abundance (genus Parasutterella id.2892) on Cas scaffolding protein family member 4
with a threshold of 1e−05
MR Test
Inverse variance weighted (fixed effects)
Inverse variance weighted (multiplicative random effects)
MR Egger
MR RAPS
Weighted median
Weighted mode
0.10
0.05
0.00
−0.05
0.1
0.2
SNP effect on Cas scaffolding protein family member 4
SNP effect on Gut microbiota abundance (genus Parasutterella id.2892)

Batch 2−731 : Gut microbiota abundance (genus Catenibacterium id.2153) on Calpastatin
with a threshold of 1e−05

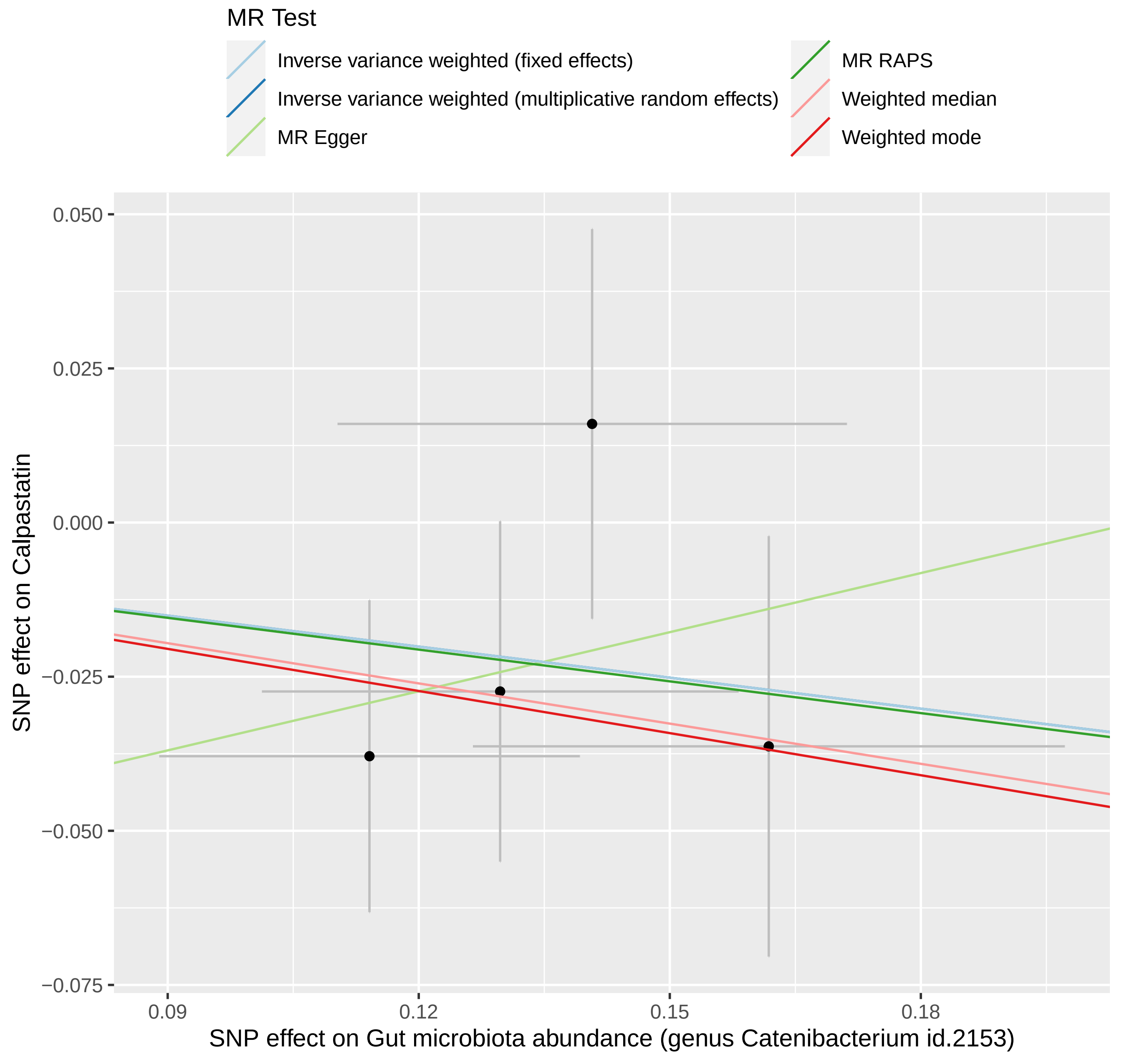

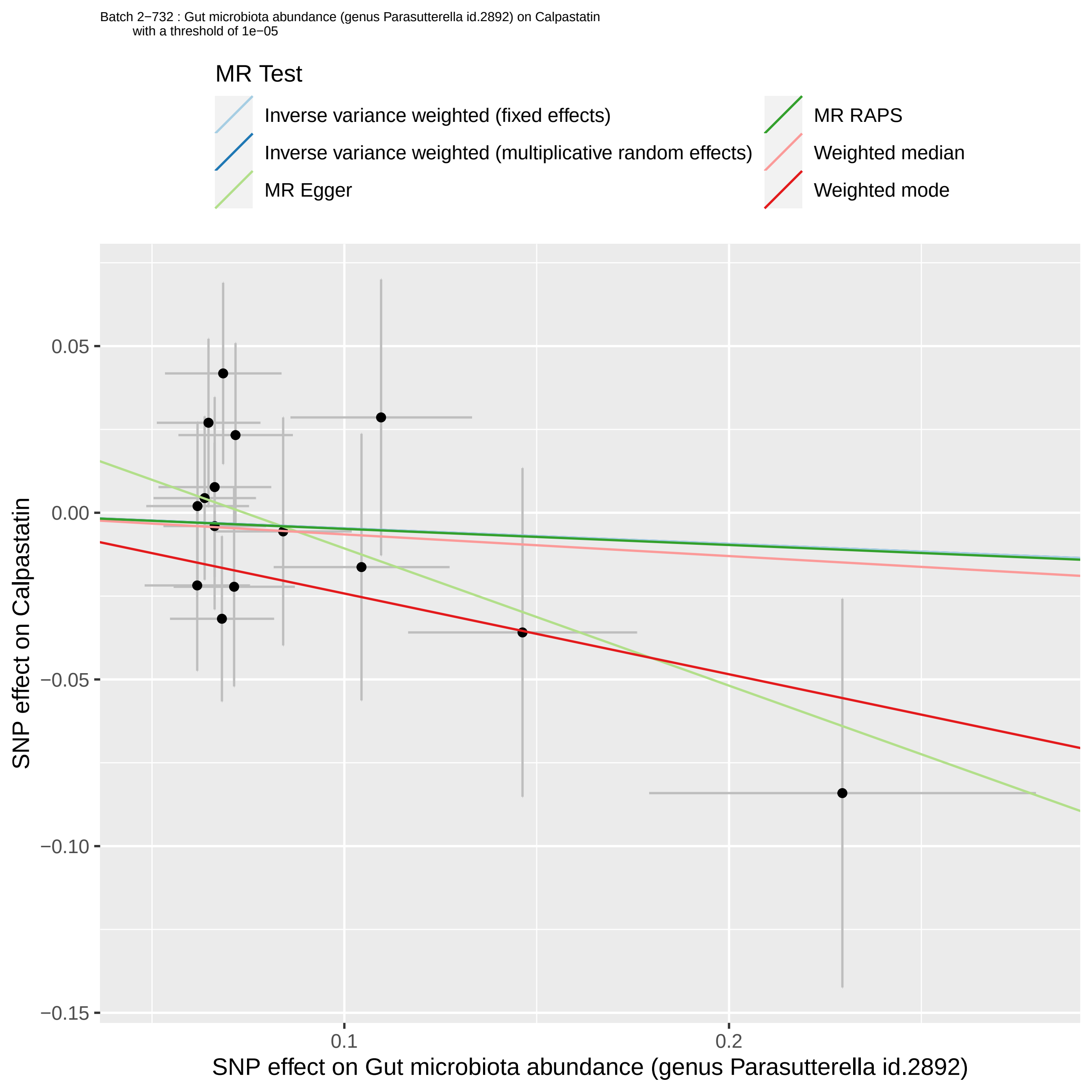

Batch 2−732 : Gut microbiota abundance (genus Parasutterella id.2892) on Calpastatin
with a threshold of 1e−05
MR Test
Inverse variance weighted (fixed effects)
Inverse variance weighted (multiplicative random effects)
MR Egger
MR RAPS
Weighted median
Weighted mode
0.05
0.00
−0.05
−0.10
−0.15
0.1
0.2
SNP effect on Calpastatin
SNP effect on Gut microbiota abundance (genus Parasutterella id.2892)

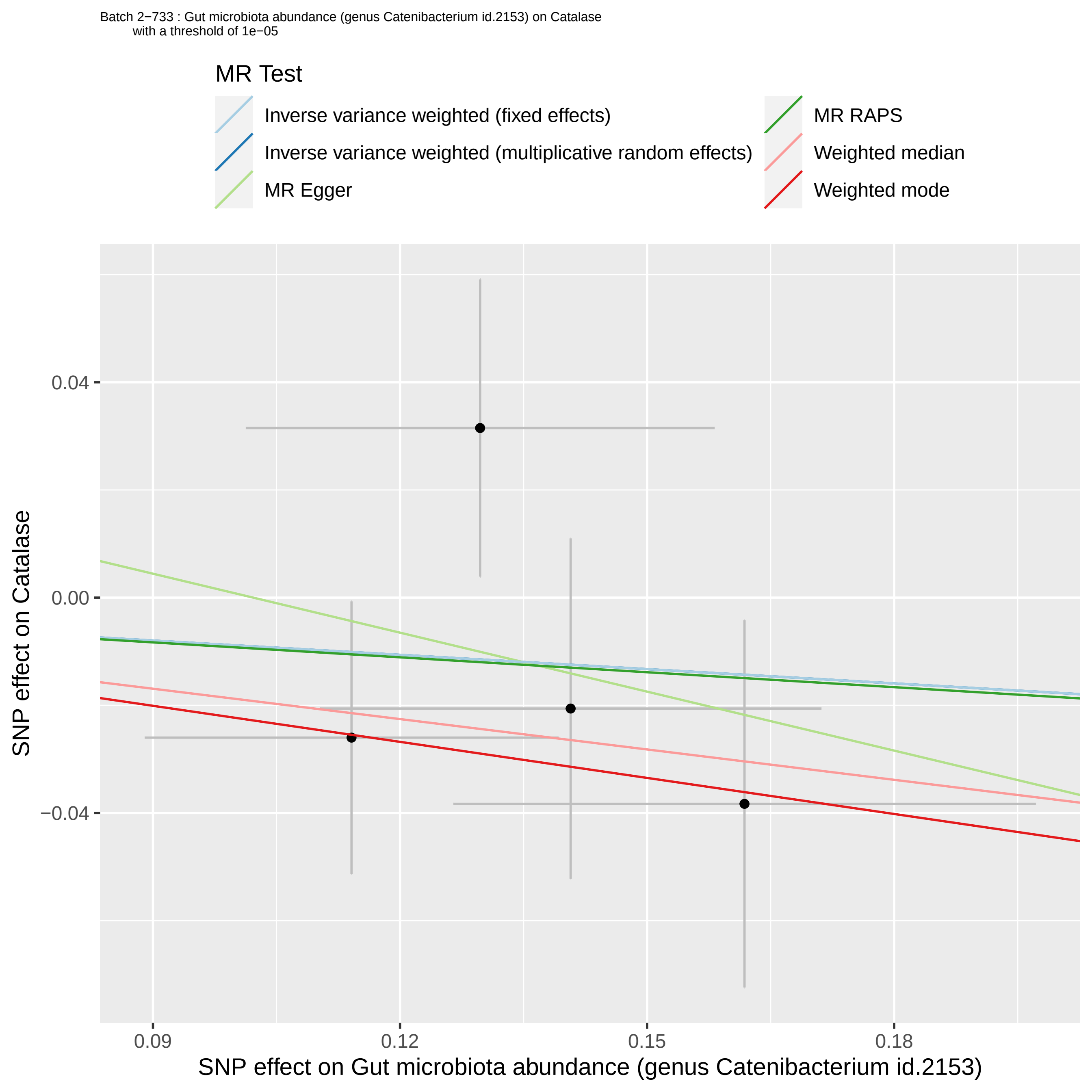
Batch 2−733 : Gut microbiota abundance (genus Catenibacterium id.2153) on Catalase
with a threshold of 1e−05
MR Test
Inverse variance weighted (fixed effects)
Inverse variance weighted (multiplicative random effects)
MR Egger
MR RAPS
Weighted median
Weighted mode
0.04
0.00
−0.04
0.09
0.12
0.15
0.18
SNP effect on Catalase
SNP effect on Gut microbiota abundance (genus Catenibacterium id.2153)

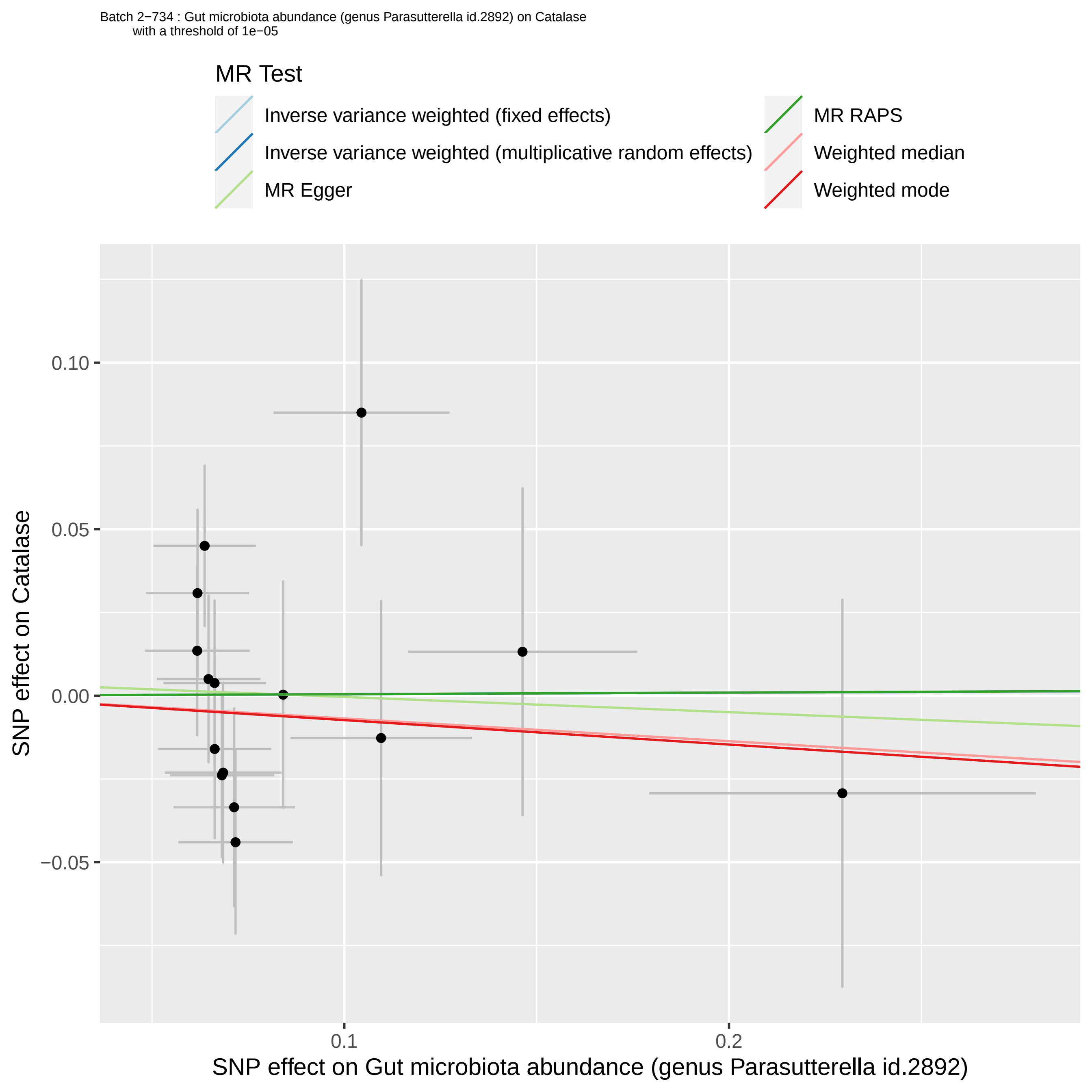

Batch 2−734 : Gut microbiota abundance (genus Parasutterella id.2892) on Catalase
with a threshold of 1e−05
MR Test
Inverse variance weighted (fixed effects)
Inverse variance weighted (multiplicative random effects)
MR Egger
MR RAPS
Weighted median
Weighted mode
0.10
0.05
0.00
−0.05
0.1
0.2
SNP effect on Catalase
SNP effect on Gut microbiota abundance (genus Parasutterella id.2892)

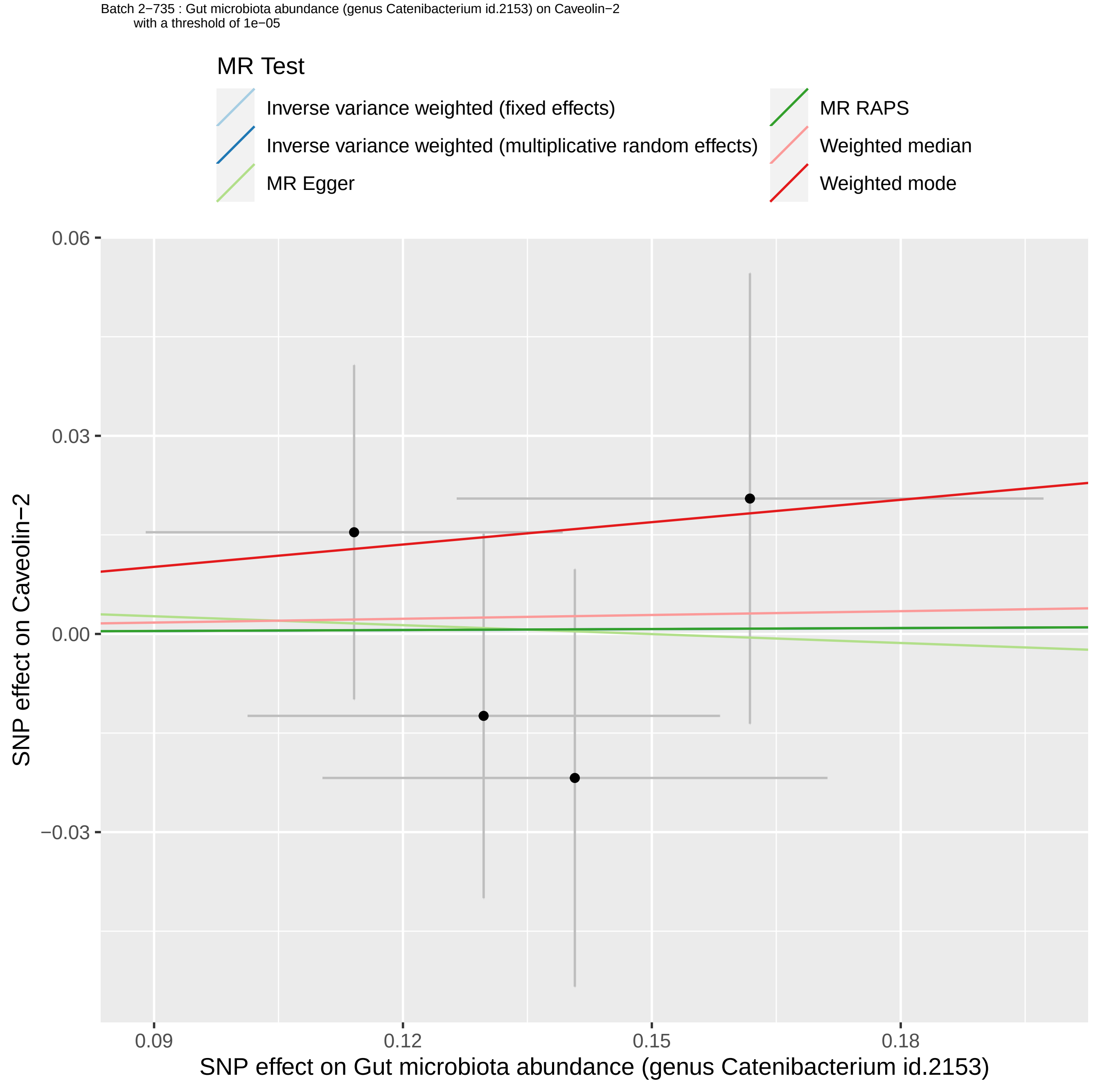
Batch 2−735 : Gut microbiota abundance (genus Catenibacterium id.2153) on Caveolin−2
with a threshold of 1e−05
MR Test
Inverse variance weighted (fixed effects)
Inverse variance weighted (multiplicative random effects)
MR Egger
MR RAPS
Weighted median
Weighted mode
0.06
0.03
0.00
−0.03
0.09
0.12
0.15
0.18
SNP effect on Caveolin−2
SNP effect on Gut microbiota abundance (genus Catenibacterium id.2153)

Batch 2−736 : Gut microbiota abundance (genus Parasutterella id.2892) on Caveolin−2
with a threshold of 1e−05

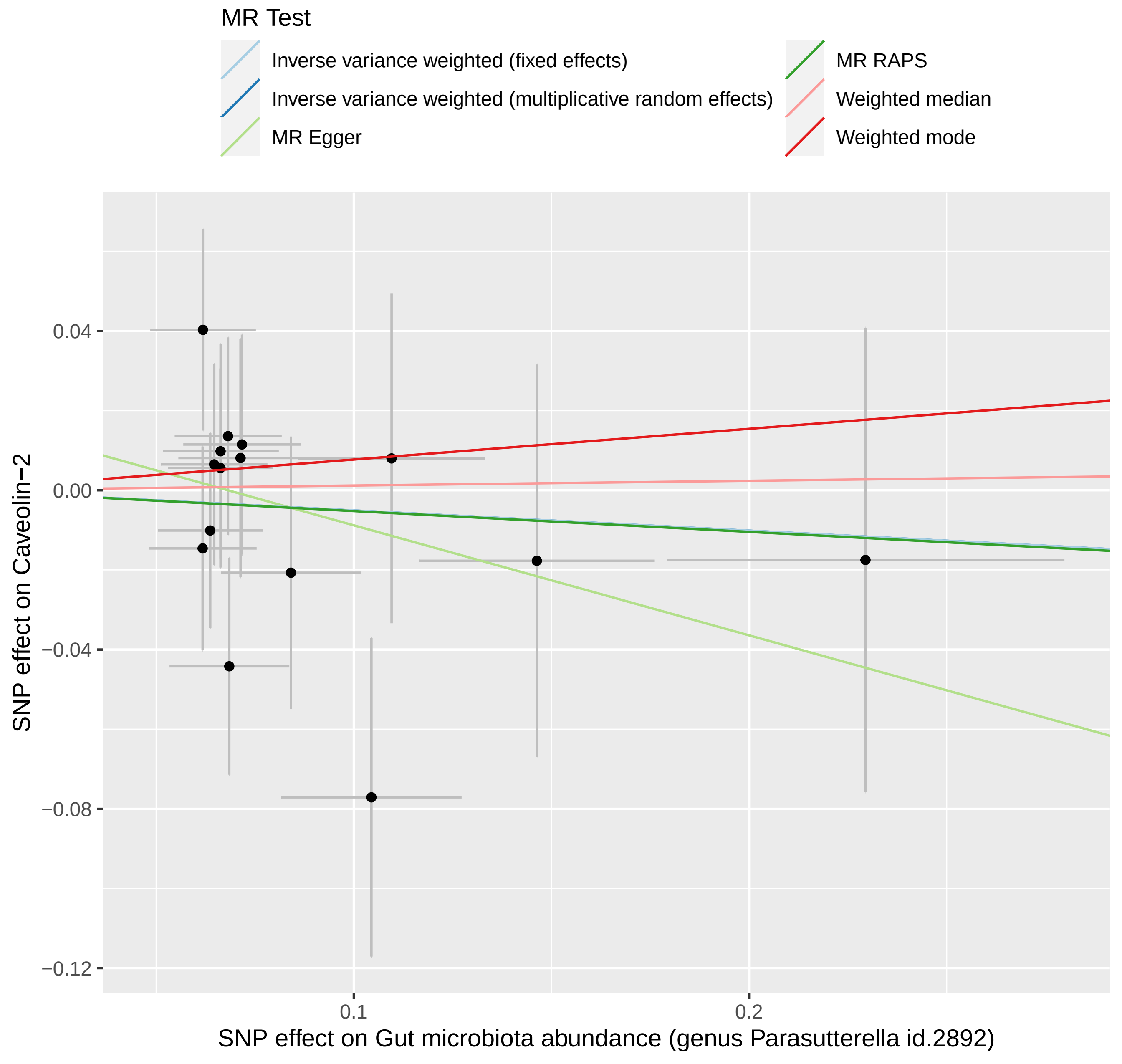

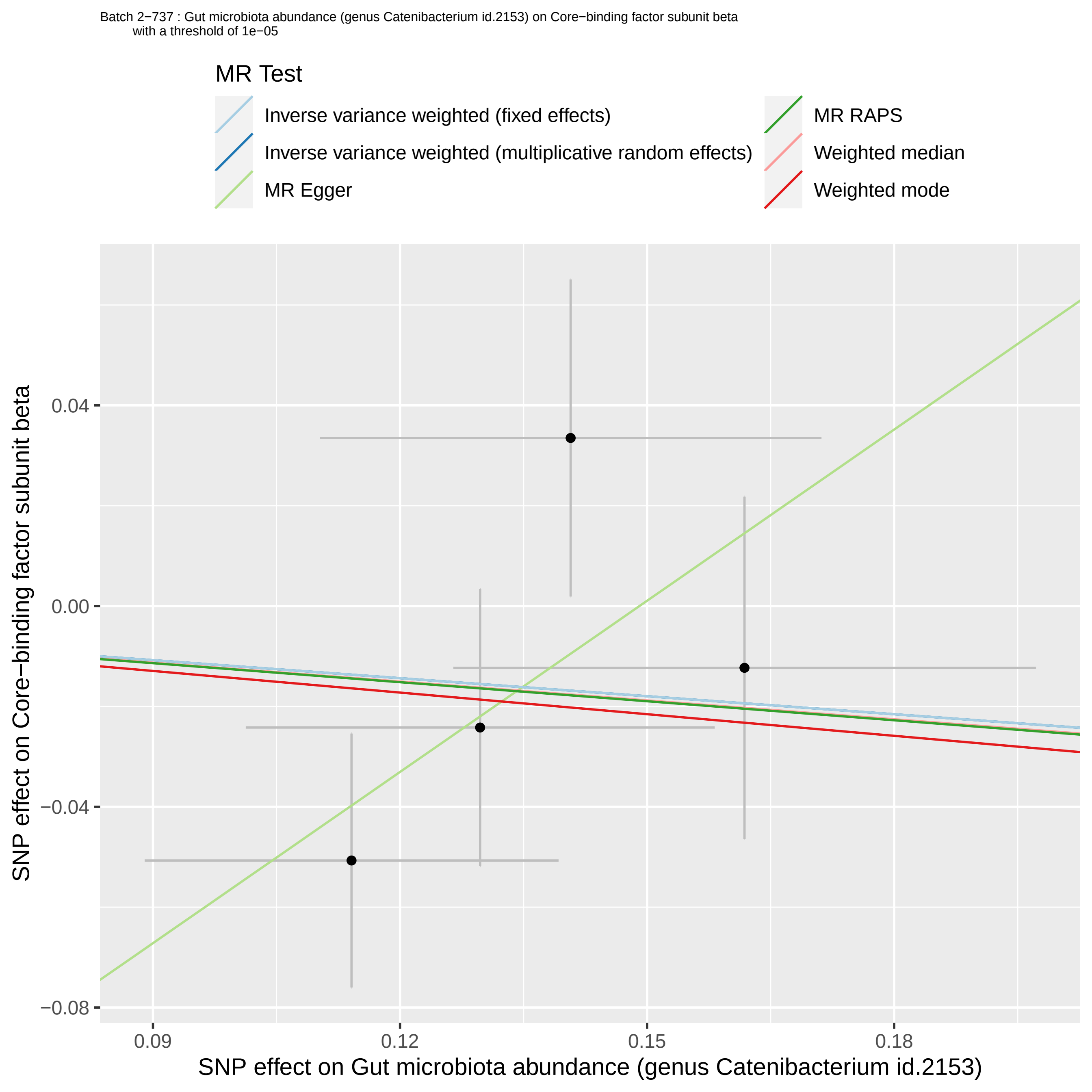

Batch 2−737 : Gut microbiota abundance (genus Catenibacterium id.2153) on Core−binding factor subunit beta
with a threshold of 1e−05
MR Test
Inverse variance weighted (fixed effects)
Inverse variance weighted (multiplicative random effects)
MR Egger
MR RAPS
Weighted median
Weighted mode
SNP effect on Core−binding factor subunit beta
0.04
0.00
−0.04
−0.08
0.09
0.12
0.15
0.18
SNP effect on Gut microbiota abundance (genus Catenibacterium id.2153)

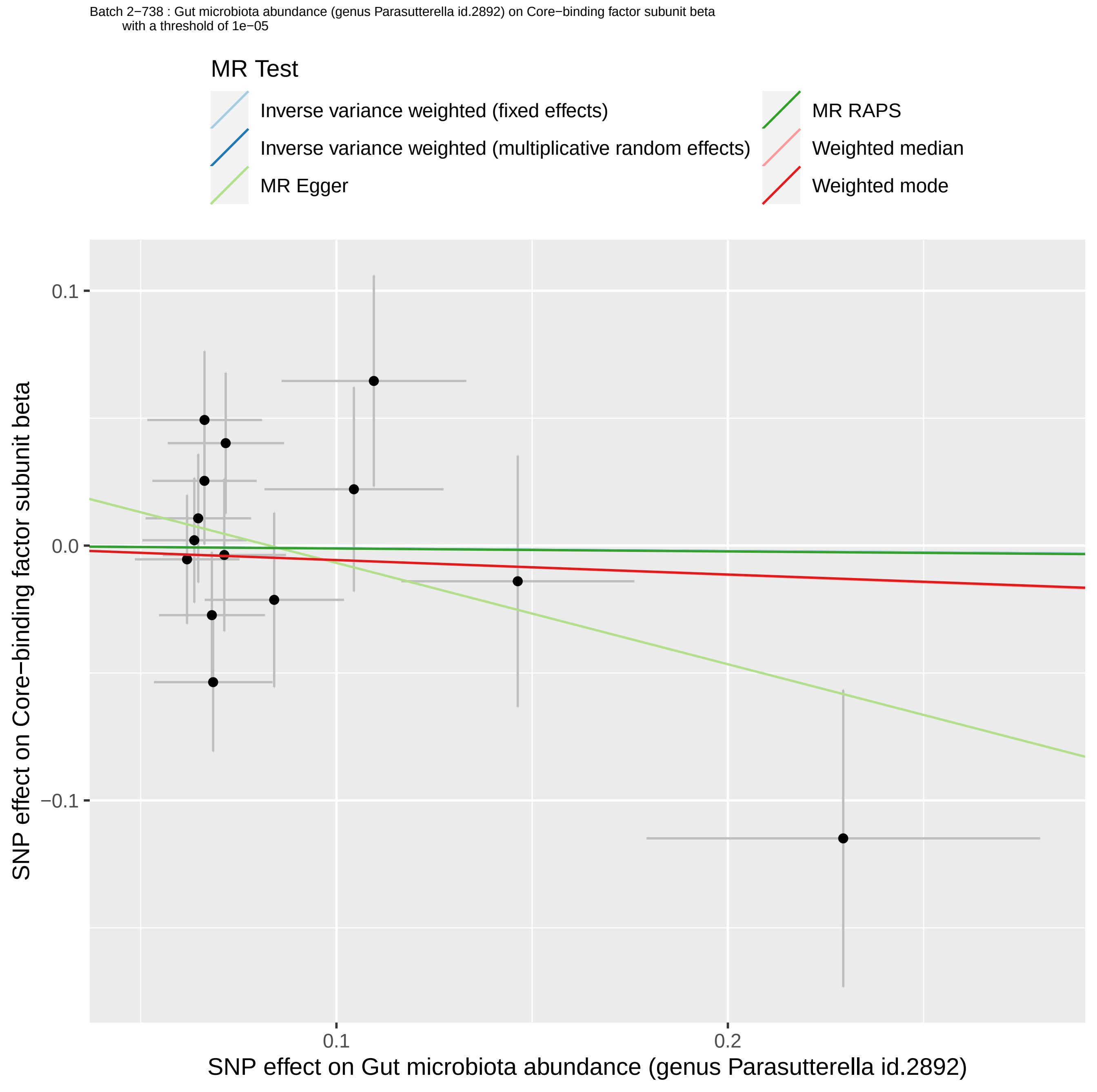
Batch 2−738 : Gut microbiota abundance (genus Parasutterella id.2892) on Core−binding factor subunit beta
with a threshold of 1e−05
MR Test
Inverse variance weighted (fixed effects)
Inverse variance weighted (multiplicative random effects)
MR Egger
MR RAPS
Weighted median
Weighted mode
0.1
0.0
−0.1
0.1
0.2
SNP effect on Core−binding factor subunit beta
SNP effect on Gut microbiota abundance (genus Parasutterella id.2892)

Batch 2−739 : Gut microbiota abundance (genus Catenibacterium id.2153) on E3 ubiquitin−protein ligase CBL
with a threshold of 1e−05

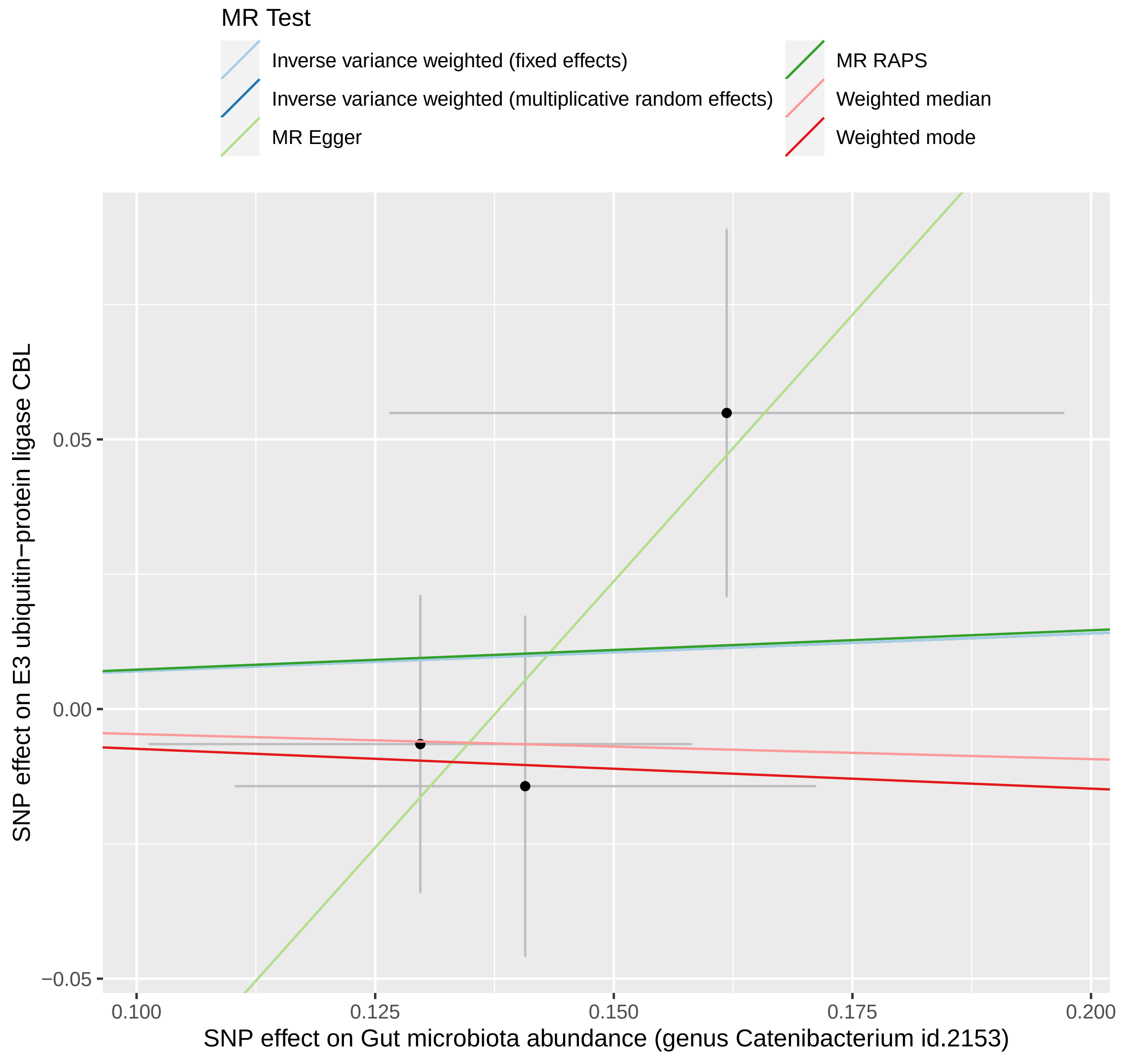

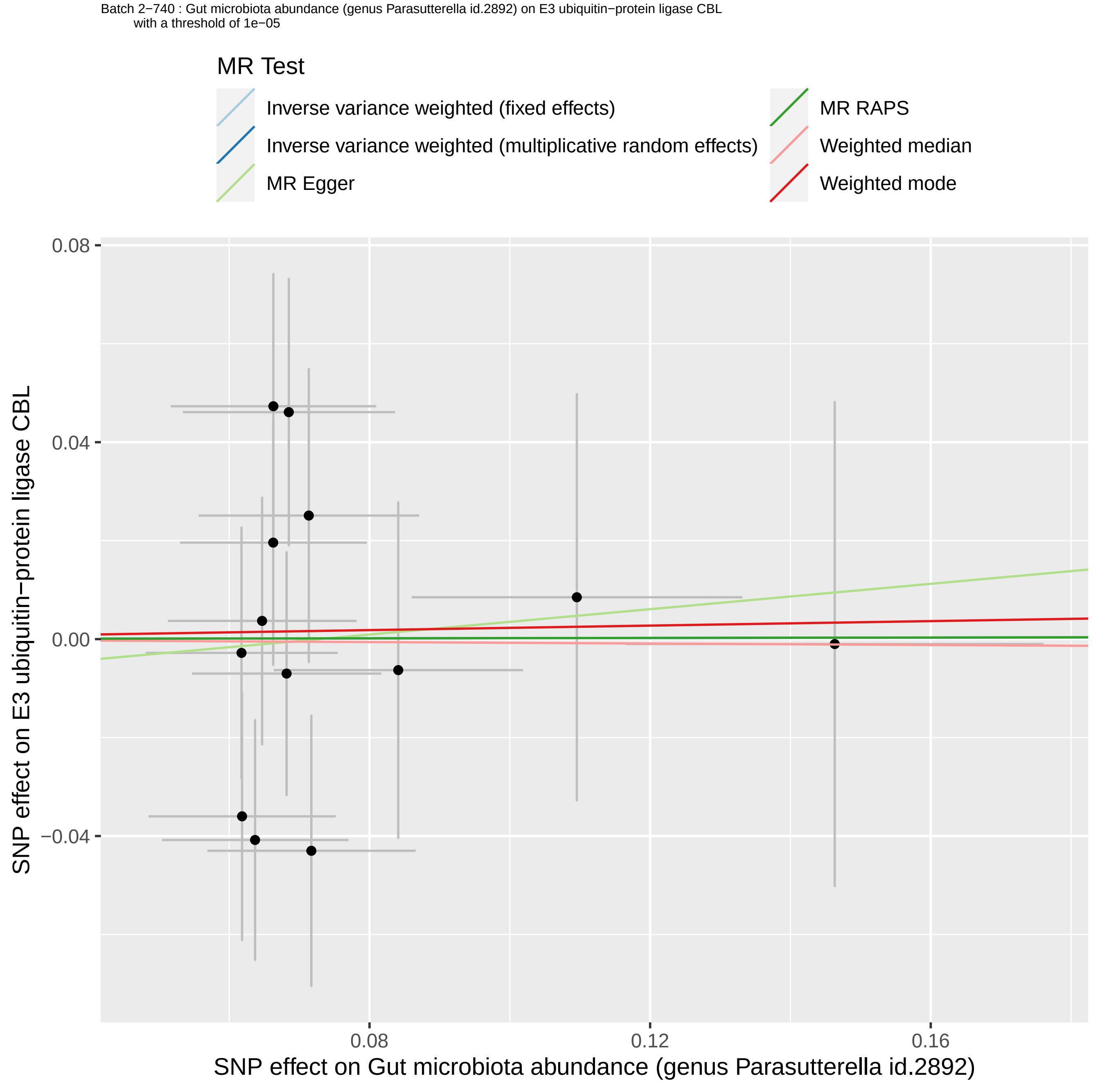
Batch 2−740 : Gut microbiota abundance (genus Parasutterella id.2892) on E3 ubiquitin−protein ligase CBL
with a threshold of 1e−05
MR Test
Inverse variance weighted (fixed effects)
Inverse variance weighted (multiplicative random effects)
MR Egger
MR RAPS
Weighted median
Weighted mode
0.08
0.04
0.00
−0.04
0.08
0.12
0.16
SNP effect on E3 ubiquitin−protein ligase CBL
SNP effect on Gut microbiota abundance (genus Parasutterella id.2892)

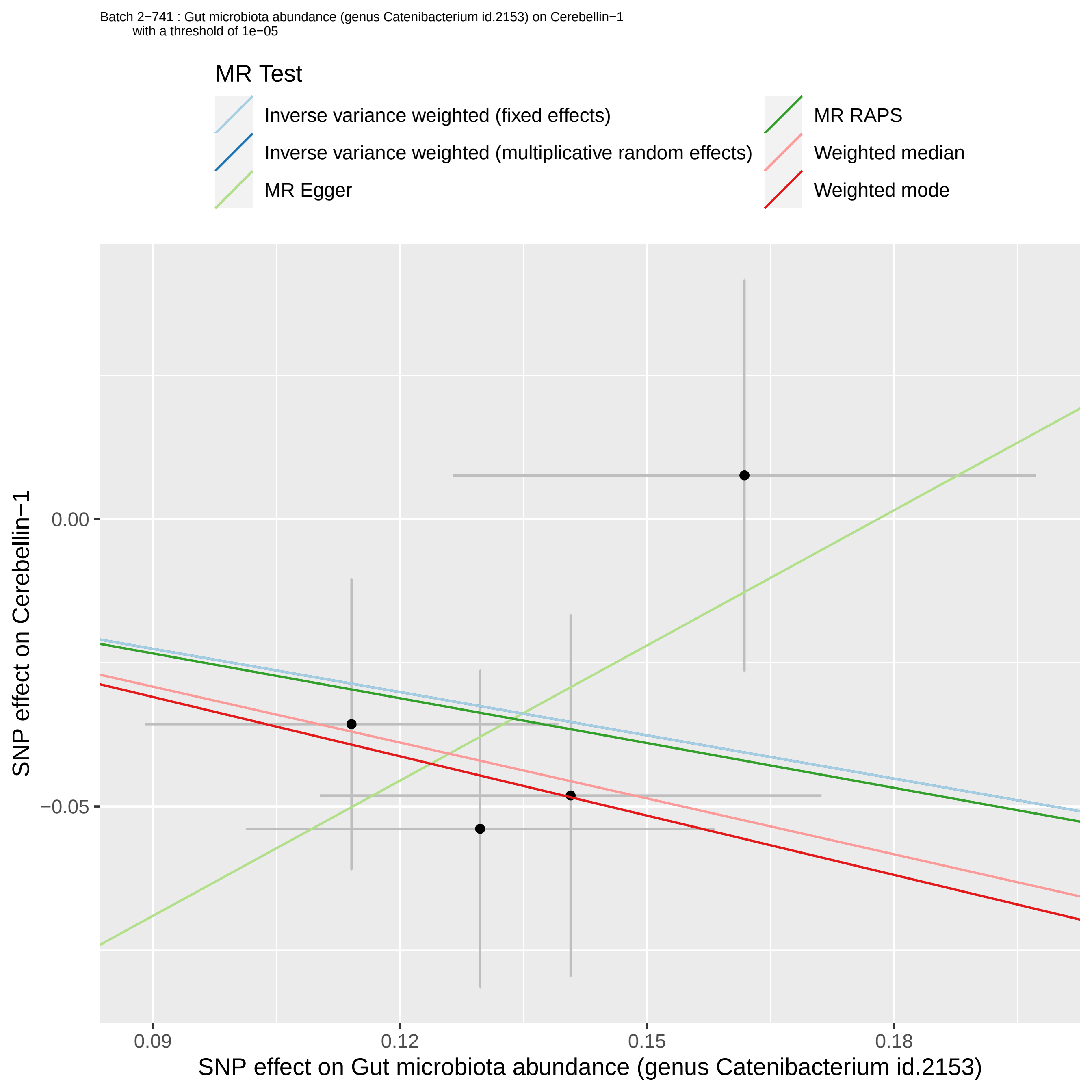

Batch 2−741 : Gut microbiota abundance (genus Catenibacterium id.2153) on Cerebellin−1
with a threshold of 1e−05
MR Test
Inverse variance weighted (fixed effects)
Inverse variance weighted (multiplicative random effects)
MR Egger
MR RAPS
Weighted median
Weighted mode
0.00
−0.05
0.09
0.12
0.15
0.18
SNP effect on Cerebellin−1
SNP effect on Gut microbiota abundance (genus Catenibacterium id.2153)

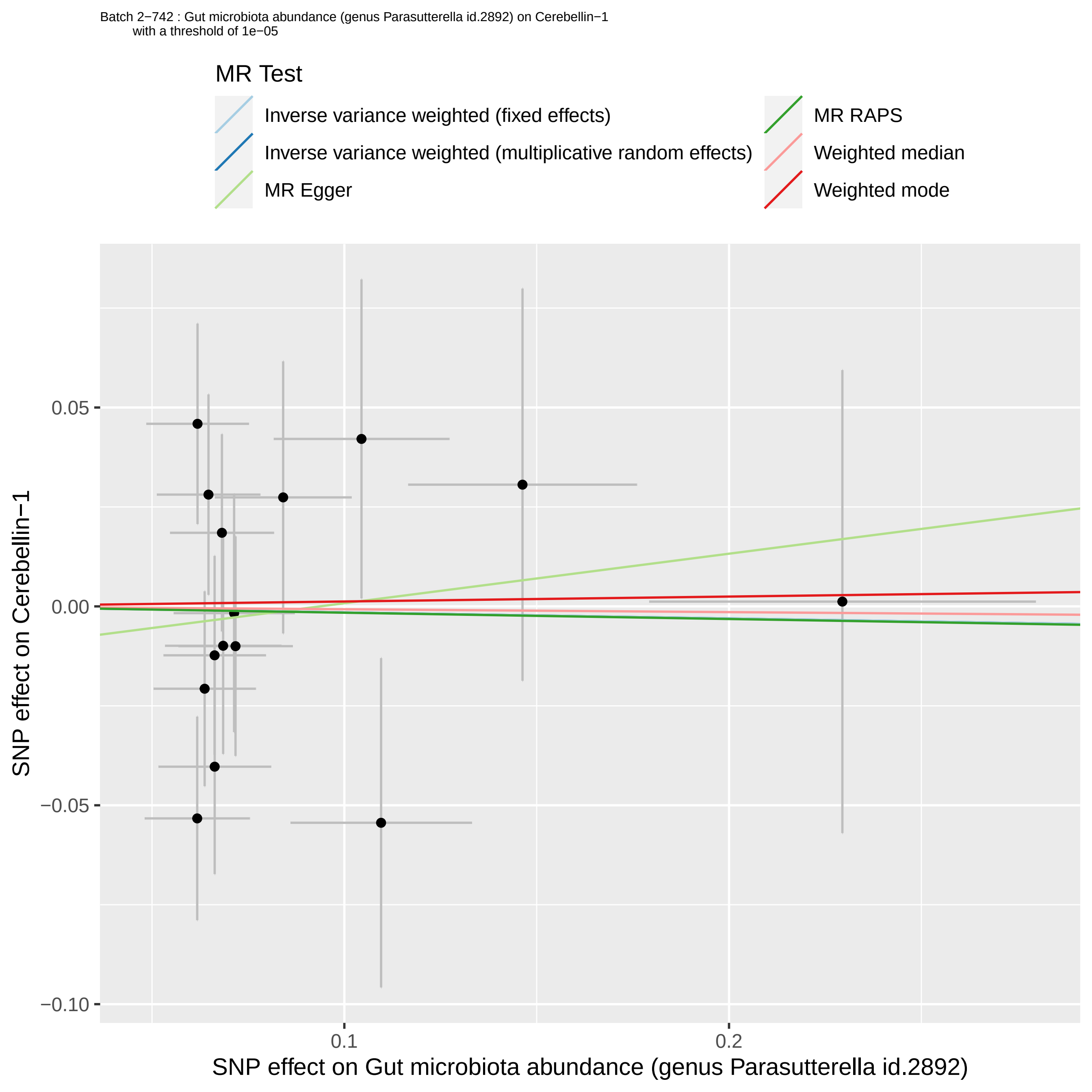

Batch 2−742 : Gut microbiota abundance (genus Parasutterella id.2892) on Cerebellin−1
with a threshold of 1e−05
MR Test
Inverse variance weighted (fixed effects)
Inverse variance weighted (multiplicative random effects)
MR Egger
MR RAPS
Weighted median
Weighted mode
0.05
0.00
−0.05
−0.10
0.1
0.2
SNP effect on Cerebellin−1
SNP effect on Gut microbiota abundance (genus Parasutterella id.2892)

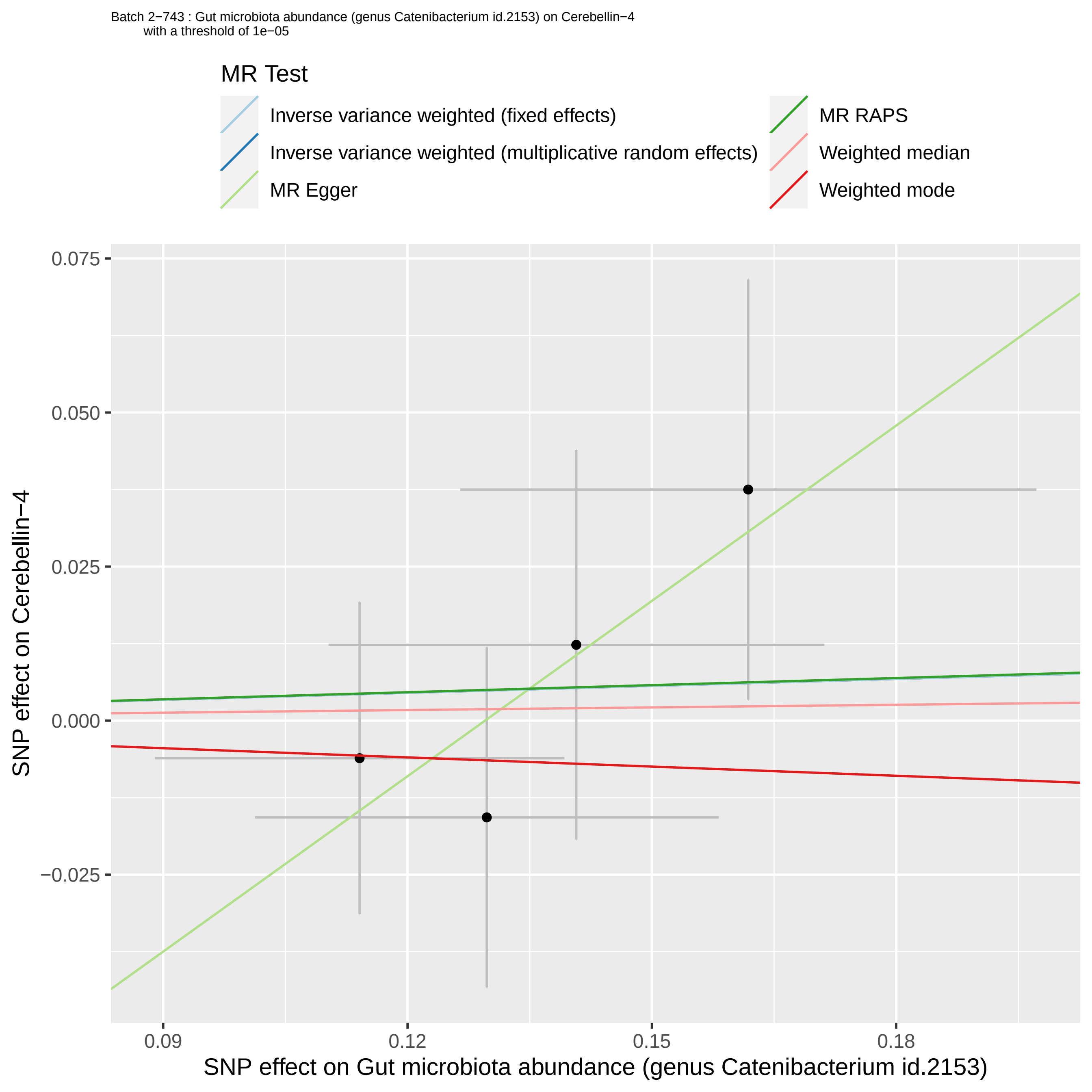

Batch 2−743 : Gut microbiota abundance (genus Catenibacterium id.2153) on Cerebellin−4
with a threshold of 1e−05
MR Test
Inverse variance weighted (fixed effects)
Inverse variance weighted (multiplicative random effects)
MR Egger
MR RAPS
Weighted median
Weighted mode
0.075
0.050
0.025
0.000
−0.025
0.09
0.12
0.15
0.18
SNP effect on Cerebellin−4
SNP effect on Gut microbiota abundance (genus Catenibacterium id.2153)

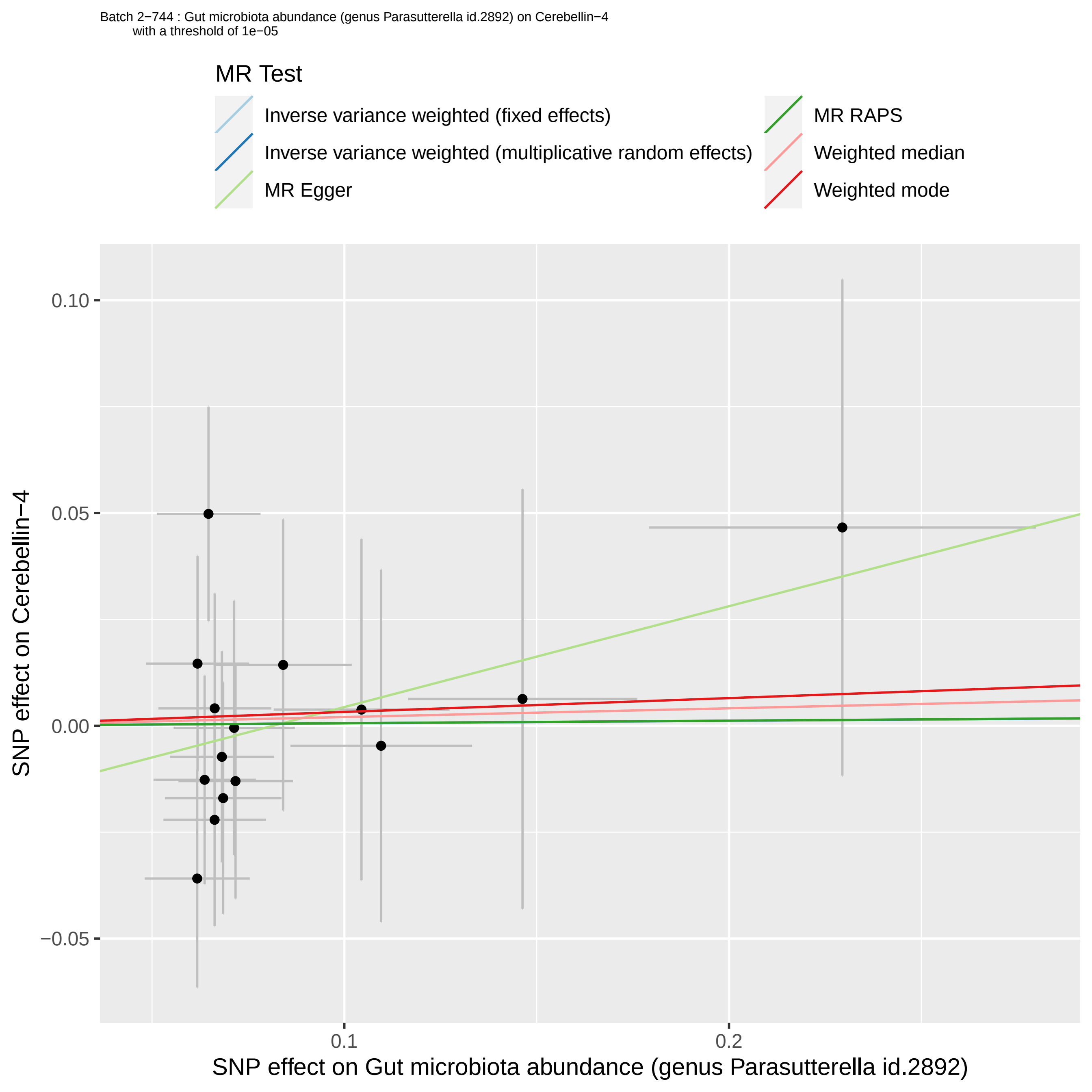
Batch 2−744 : Gut microbiota abundance (genus Parasutterella id.2892) on Cerebellin−4
with a threshold of 1e−05
MR Test
Inverse variance weighted (fixed effects)
Inverse variance weighted (multiplicative random effects)
MR Egger
MR RAPS
Weighted median
Weighted mode
0.10
0.05
0.00
−0.05
0.1
0.2
SNP effect on Cerebellin−4
SNP effect on Gut microbiota abundance (genus Parasutterella id.2892)

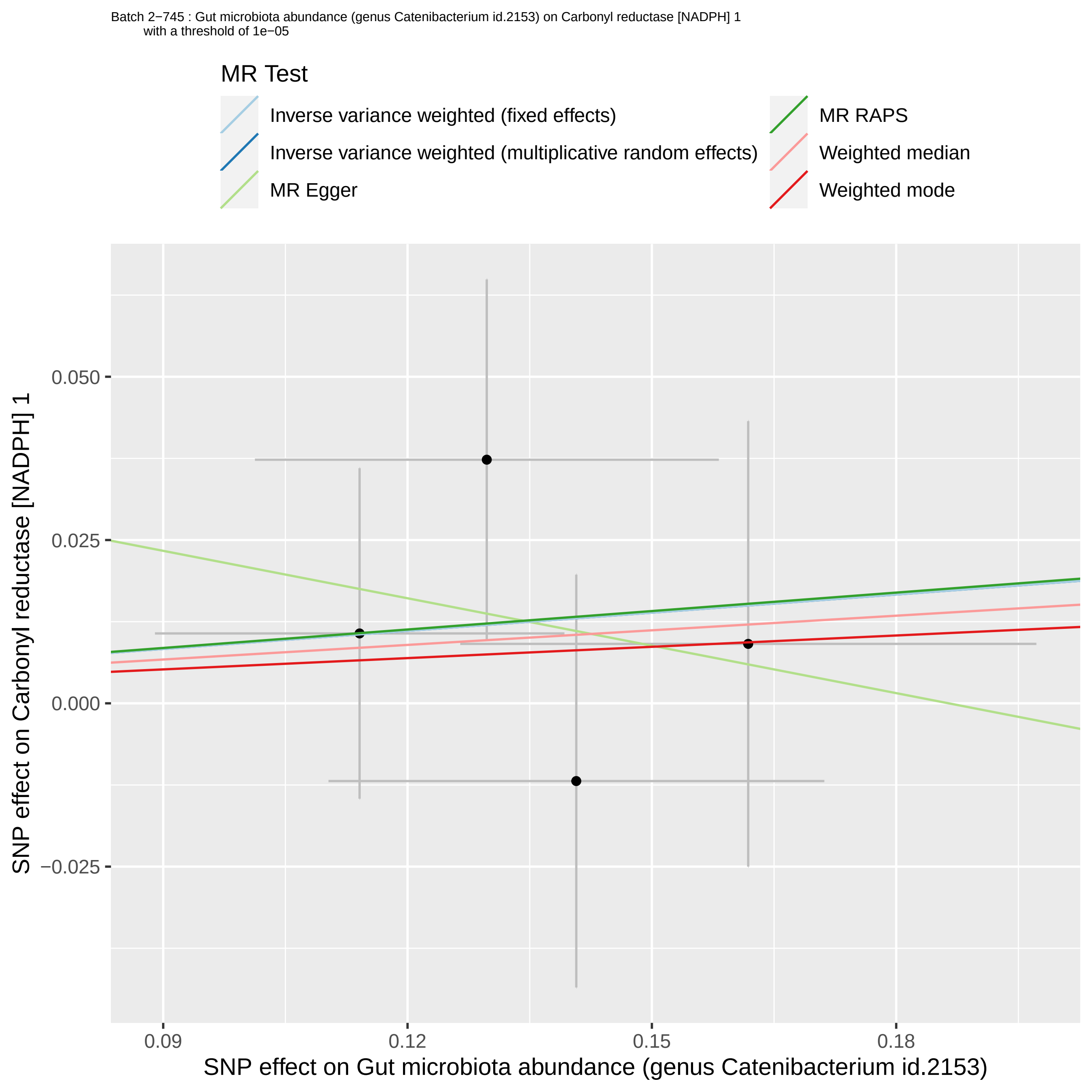

Batch 2−745 : Gut microbiota abundance (genus Catenibacterium id.2153) on Carbonyl reductase [NADPH] 1
with a threshold of 1e−05
MR Test
Inverse variance weighted (fixed effects)
Inverse variance weighted (multiplicative random effects)
MR Egger
MR RAPS
Weighted median
Weighted mode
0.050
0.025
0.000
−0.025
0.09
0.12
0.15
0.18
SNP effect on Carbonyl reductase [NADPH] 1
SNP effect on Gut microbiota abundance (genus Catenibacterium id.2153)

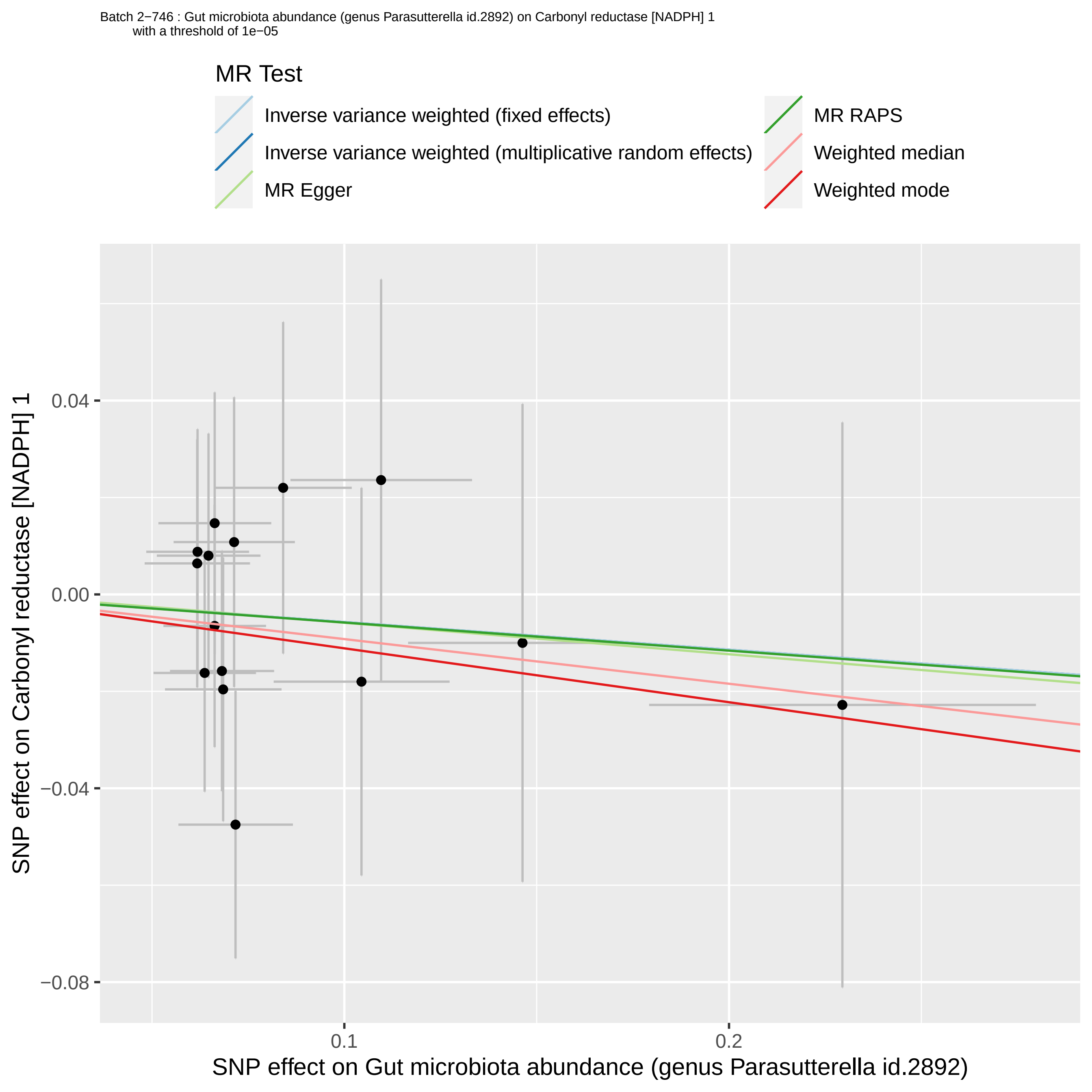

Batch 2−746 : Gut microbiota abundance (genus Parasutterella id.2892) on Carbonyl reductase [NADPH] 1
with a threshold of 1e−05
MR Test
Inverse variance weighted (fixed effects)
Inverse variance weighted (multiplicative random effects)
MR Egger
MR RAPS
Weighted median
Weighted mode
0.04
0.00
−0.04
−0.08
0.1
0.2
SNP effect on Carbonyl reductase [NADPH] 1
SNP effect on Gut microbiota abundance (genus Parasutterella id.2892)

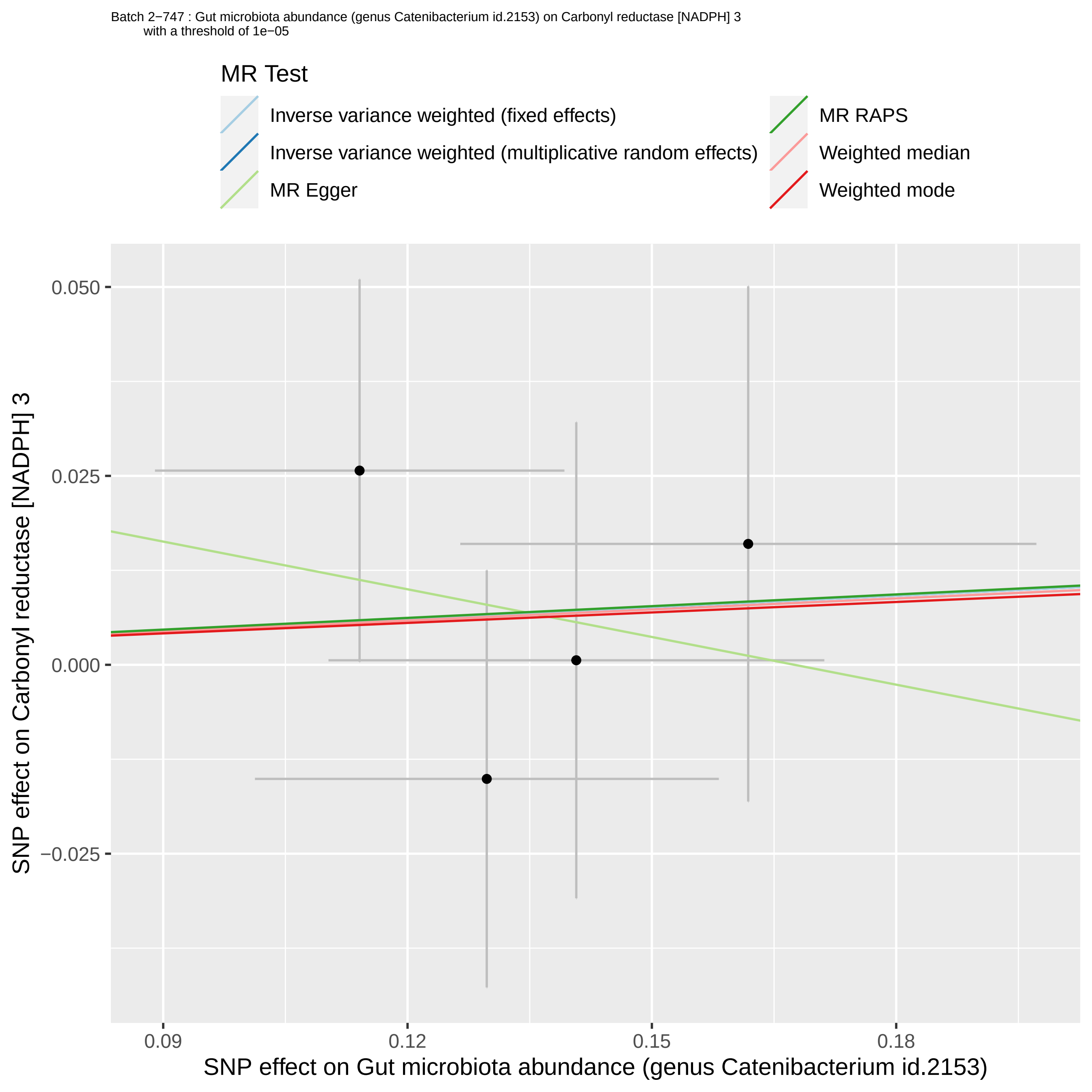

Batch 2−747 : Gut microbiota abundance (genus Catenibacterium id.2153) on Carbonyl reductase [NADPH] 3
with a threshold of 1e−05
MR Test
Inverse variance weighted (fixed effects)
Inverse variance weighted (multiplicative random effects)
MR Egger
MR RAPS
Weighted median
Weighted mode
0.050
0.025
0.000
−0.025
0.09
0.12
0.15
0.18
SNP effect on Carbonyl reductase [NADPH] 3
SNP effect on Gut microbiota abundance (genus Catenibacterium id.2153)

Batch 2−748 : Gut microbiota abundance (genus Parasutterella id.2892) on Carbonyl reductase [NADPH] 3
with a threshold of 1e−05

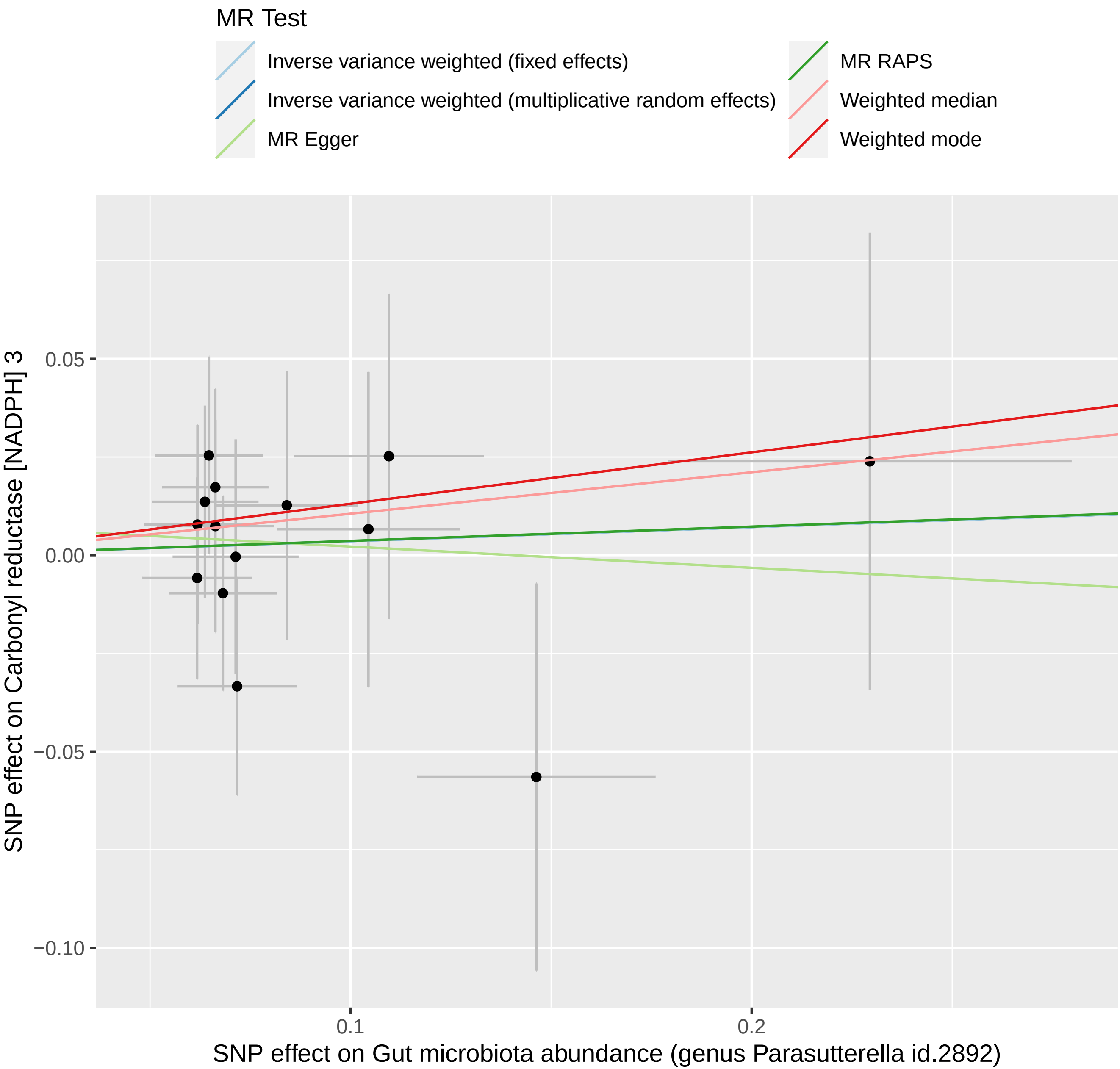

Batch 2−749 : Gut microbiota abundance (genus Catenibacterium id.2153) on Cystathionine beta−synthase
with a threshold of 1e−05

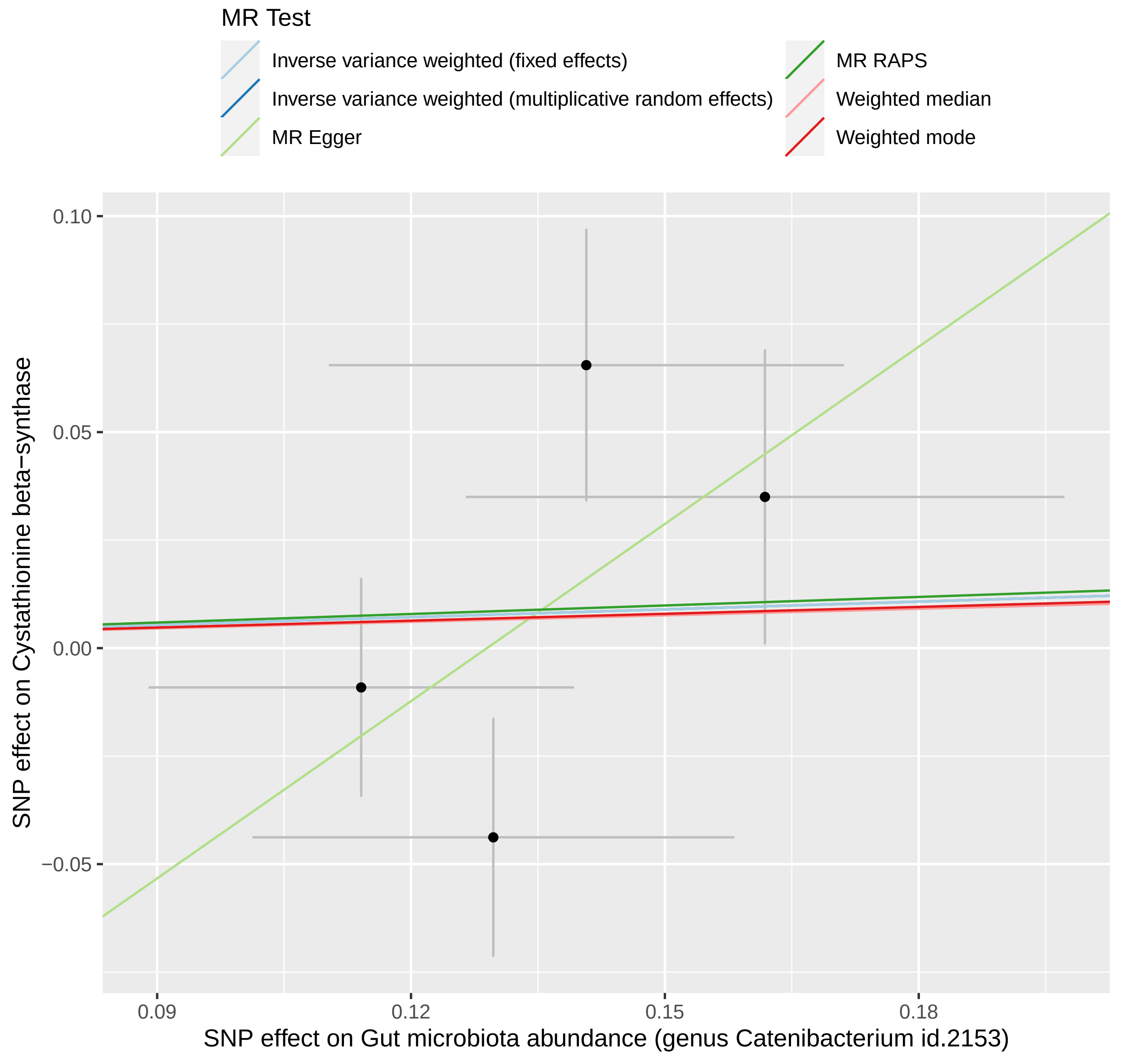

Batch 2−750 : Gut microbiota abundance (genus Parasutterella id.2892) on Cystathionine beta−synthase
with a threshold of 1e−05

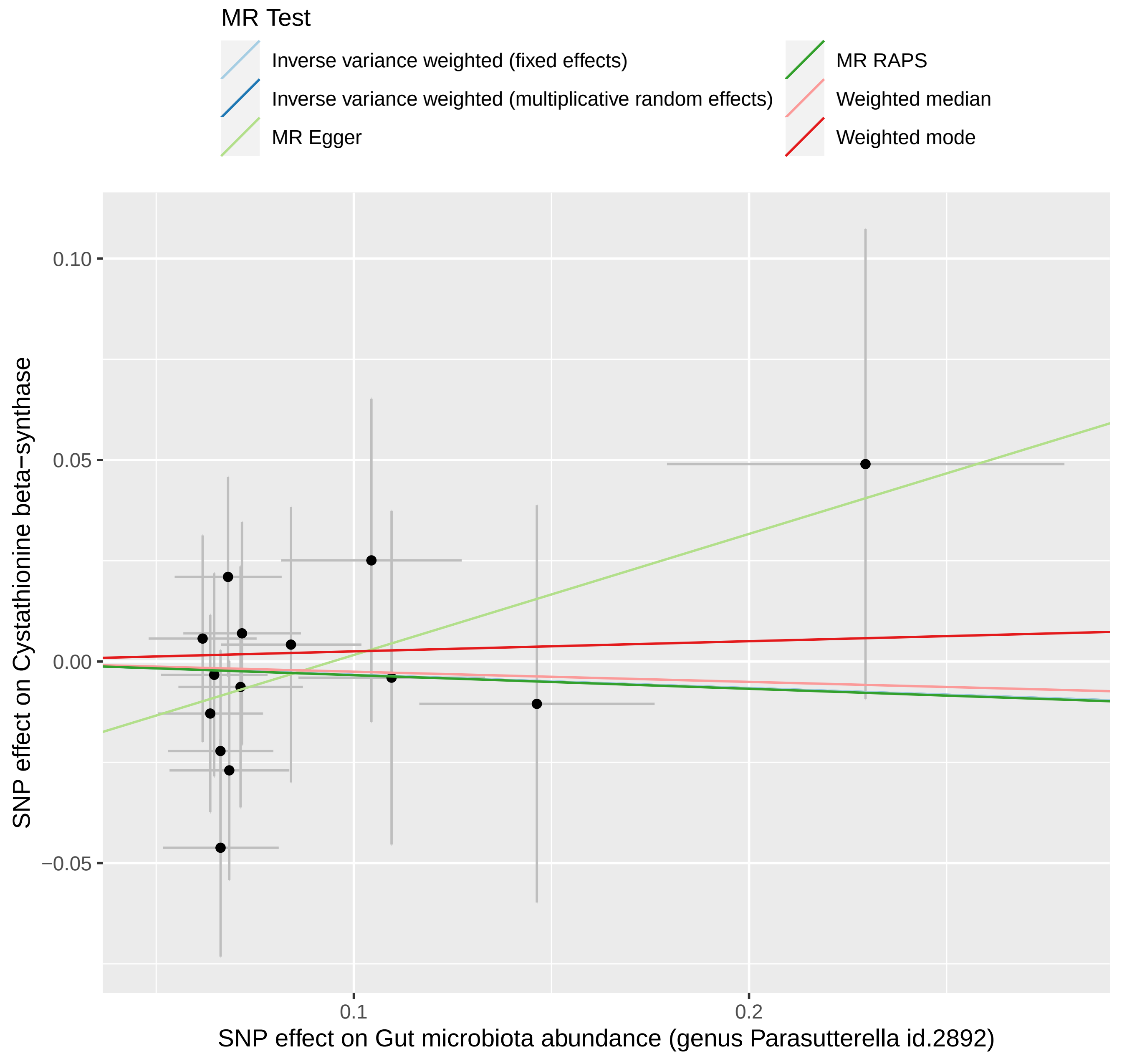

Batch 2−751 : Gut microbiota abundance (genus Catenibacterium id.2153) on Chromobox protein homolog 5
with a threshold of 1e−05

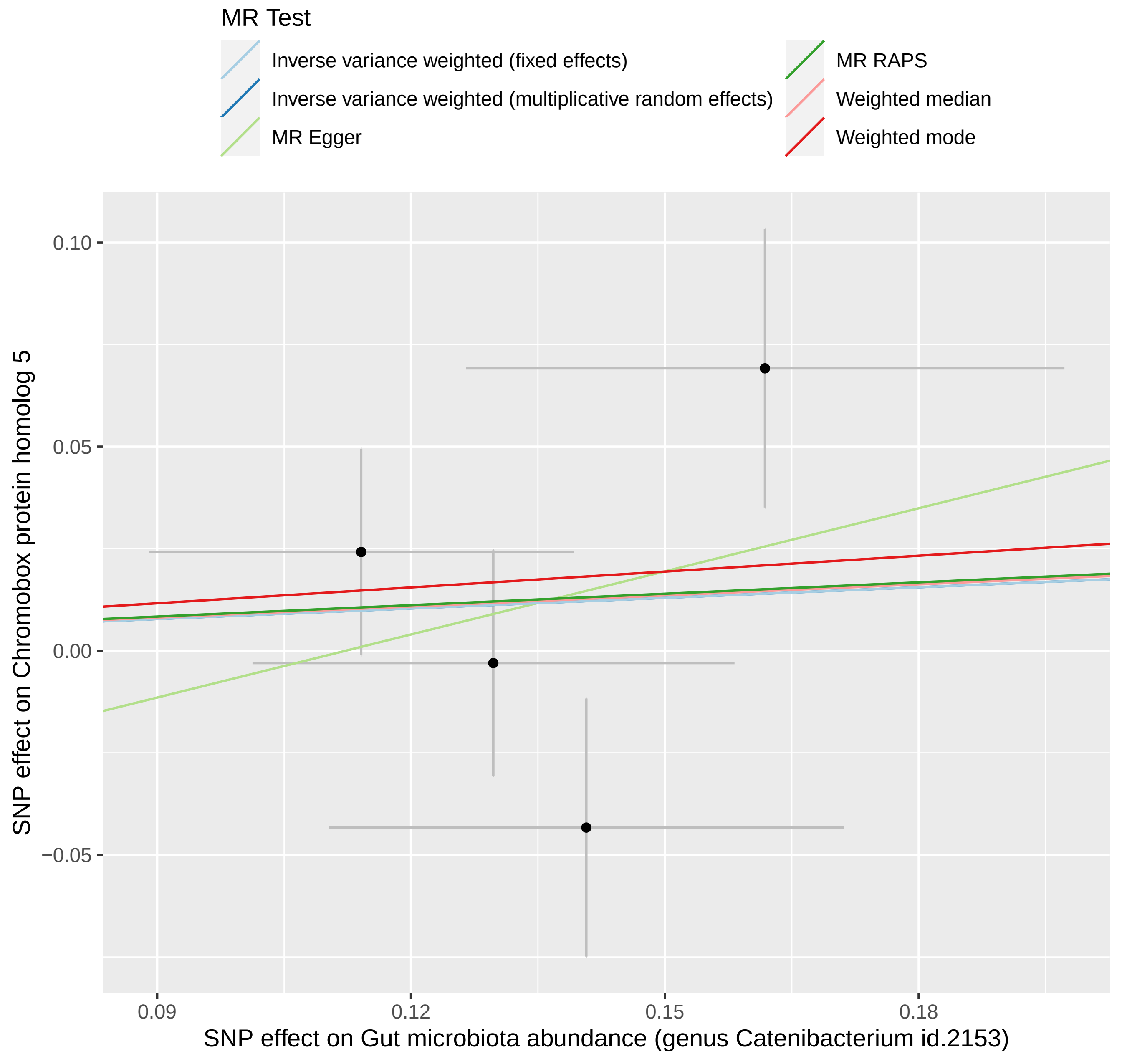

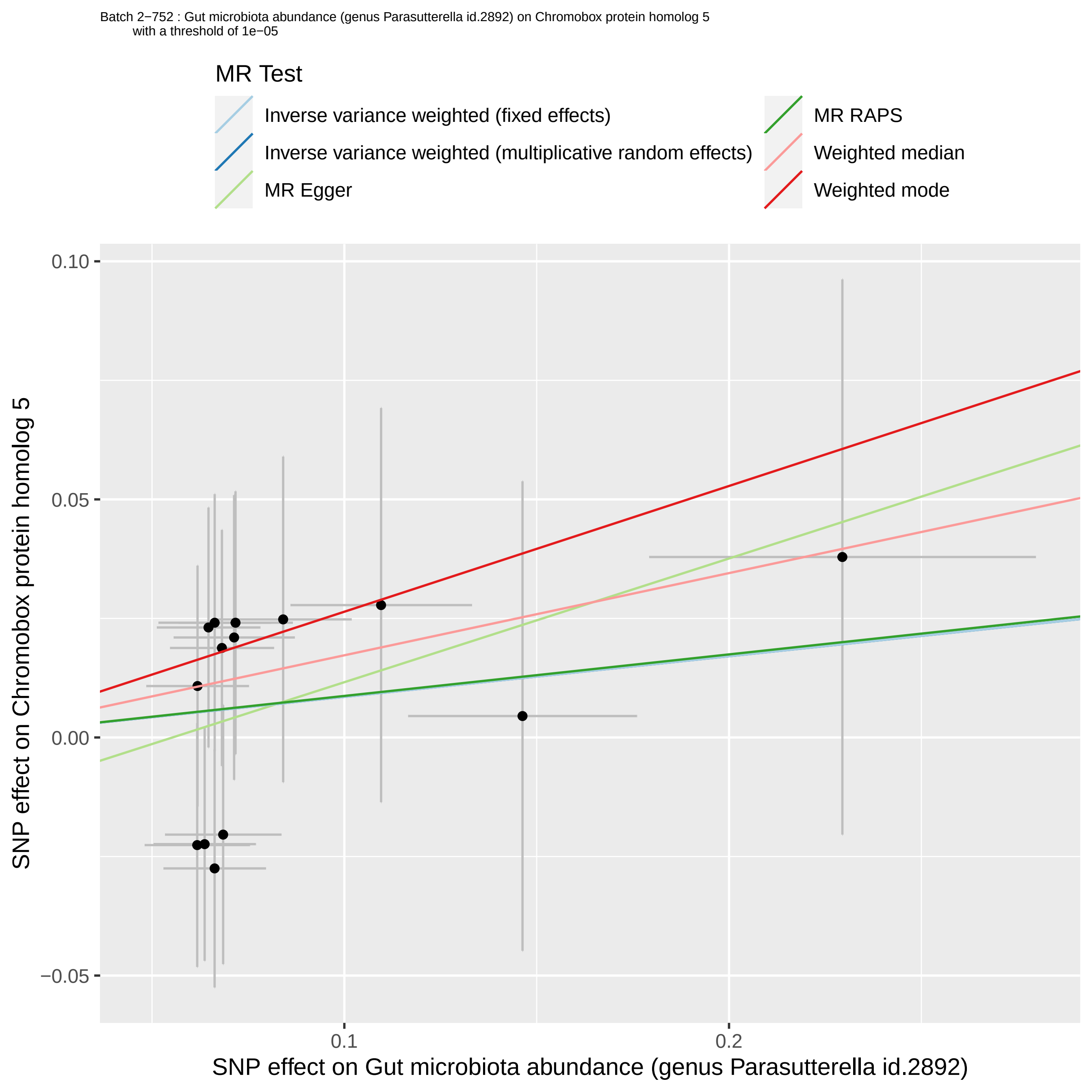
Batch 2−752 : Gut microbiota abundance (genus Parasutterella id.2892) on Chromobox protein homolog 5
with a threshold of 1e−05
MR Test
Inverse variance weighted (fixed effects)
Inverse variance weighted (multiplicative random effects)
MR Egger
MR RAPS
Weighted median
Weighted mode
0.10
0.05
0.00
−0.05
0.1
0.2
SNP effect on Chromobox protein homolog 5
SNP effect on Gut microbiota abundance (genus Parasutterella id.2892)

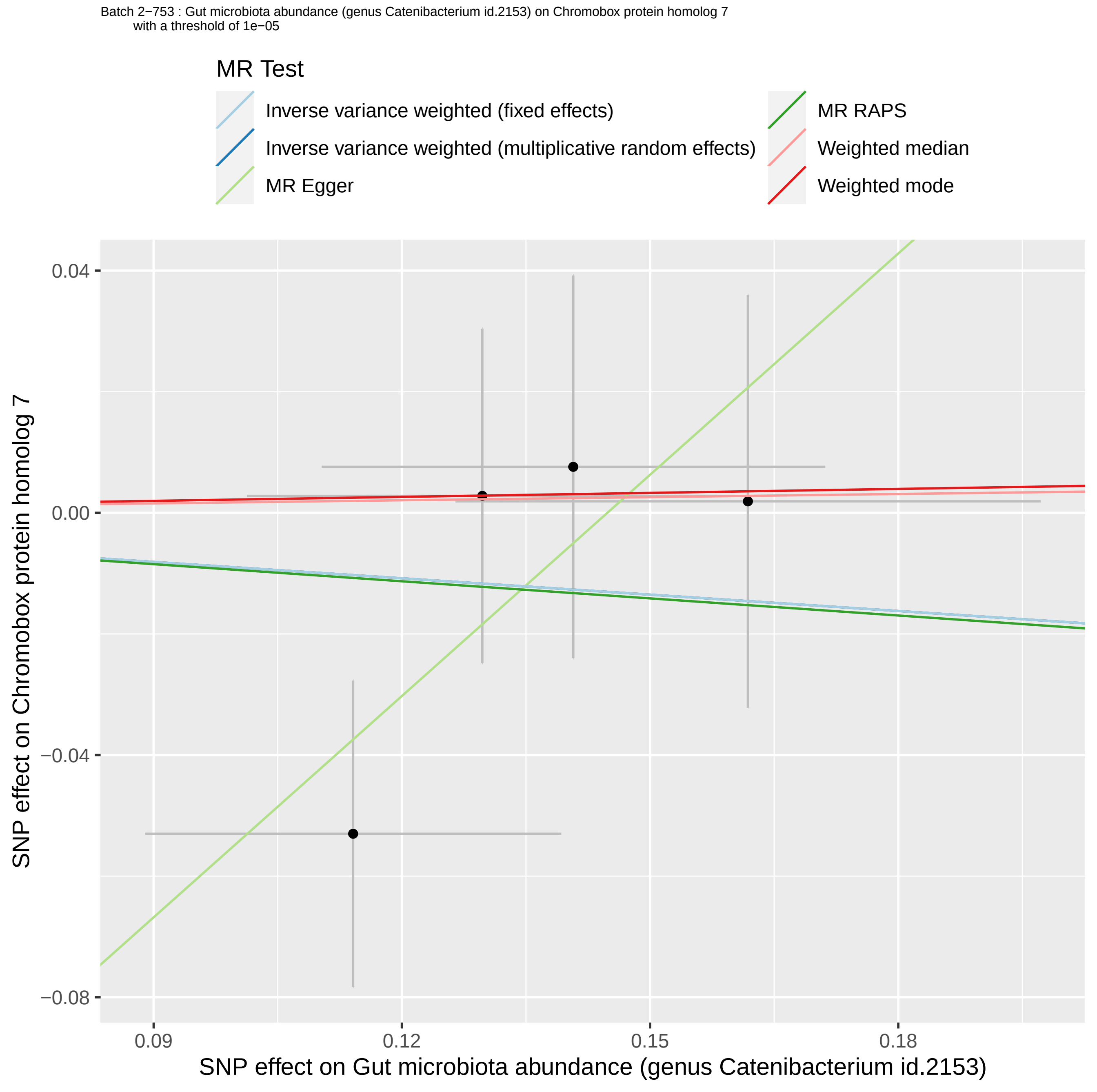

Batch 2−753 : Gut microbiota abundance (genus Catenibacterium id.2153) on Chromobox protein homolog 7
with a threshold of 1e−05
MR Test
Inverse variance weighted (fixed effects)
Inverse variance weighted (multiplicative random effects)
MR Egger
MR RAPS
Weighted median
Weighted mode
0.04
0.00
−0.04
−0.08
0.09
0.12
0.15
0.18
SNP effect on Chromobox protein homolog 7
SNP effect on Gut microbiota abundance (genus Catenibacterium id.2153)

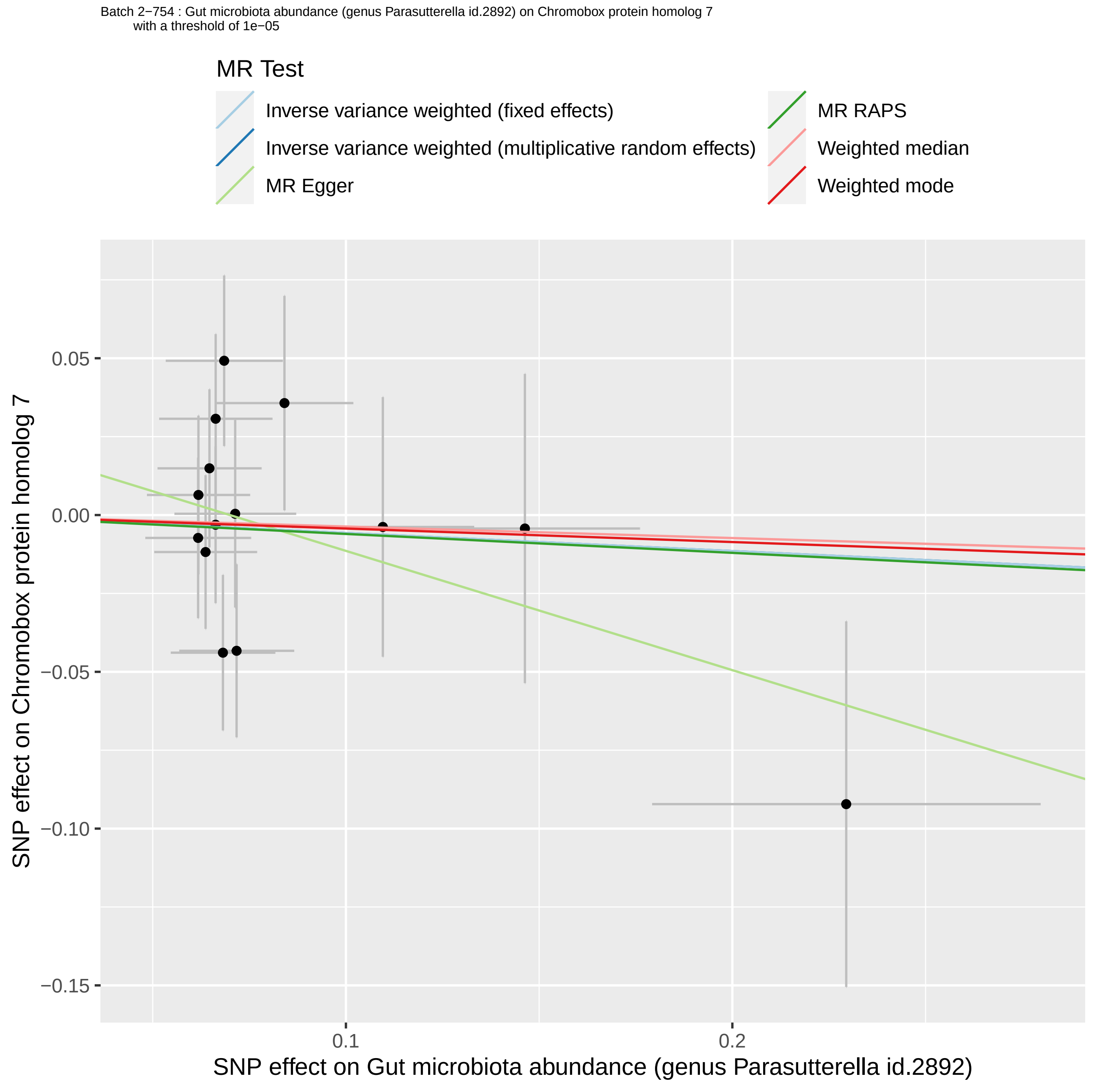

Batch 2−754 : Gut microbiota abundance (genus Parasutterella id.2892) on Chromobox protein homolog 7
with a threshold of 1e−05
MR Test
Inverse variance weighted (fixed effects)
Inverse variance weighted (multiplicative random effects)
MR Egger
MR RAPS
Weighted median
Weighted mode
0.05
0.00
−0.05
−0.10
−0.15
0.1
0.2
SNP effect on Chromobox protein homolog 7
SNP effect on Gut microbiota abundance (genus Parasutterella id.2892)

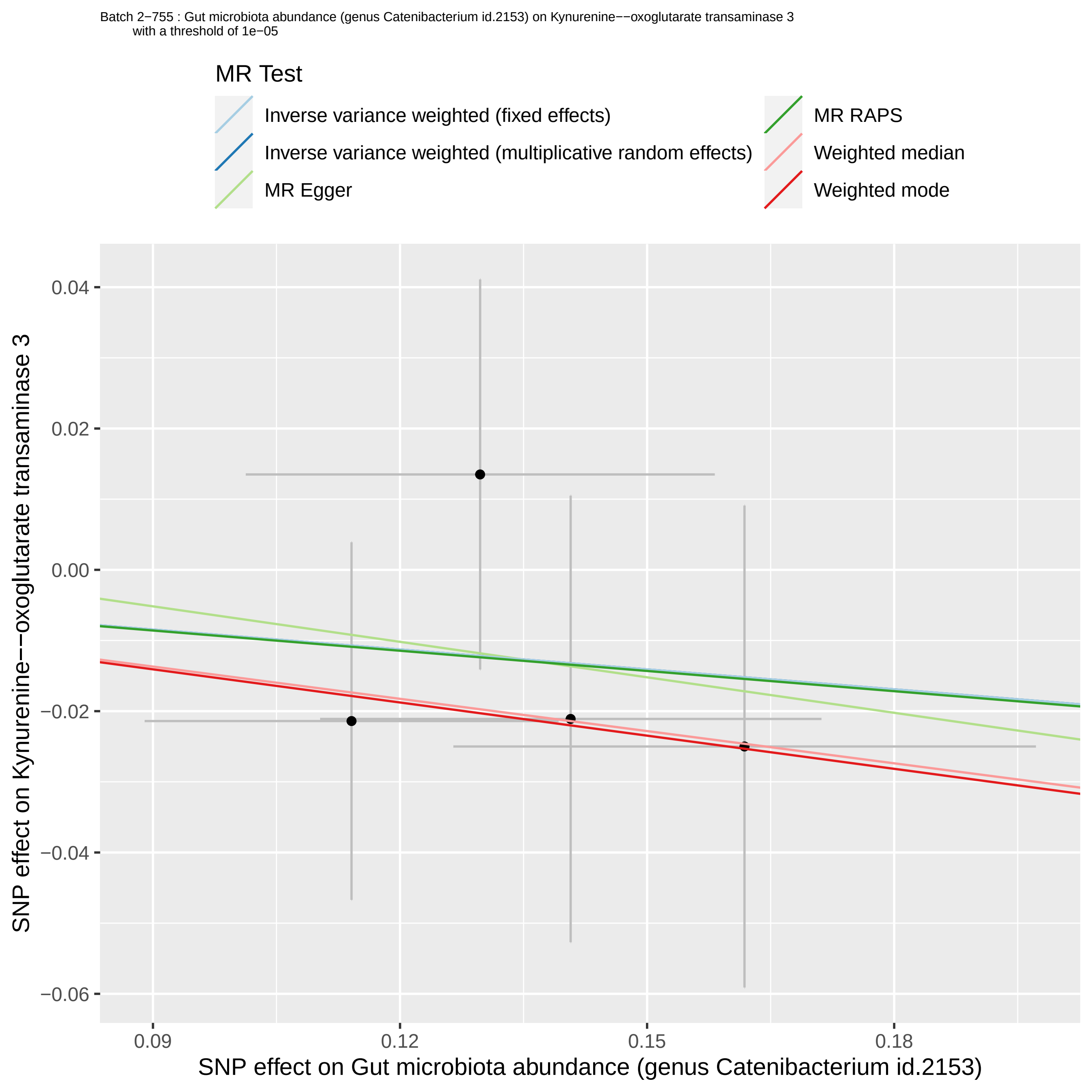
Batch 2−755 : Gut microbiota abundance (genus Catenibacterium id.2153) on Kynurenine−−oxoglutarate transaminase 3
with a threshold of 1e−05
MR Test
Inverse variance weighted (fixed effects)
Inverse variance weighted (multiplicative random effects)
MR Egger
MR RAPS
Weighted median
Weighted mode
0.04
0.02
0.00
−0.02
−0.04
−0.06
0.09
0.12
0.15
0.18
SNP effect on Kynurenine−−oxoglutarate transaminase 3
SNP effect on Gut microbiota abundance (genus Catenibacterium id.2153)

Batch 2−756 : Gut microbiota abundance (genus Parasutterella id.2892) on Kynurenine−−oxoglutarate transaminase 3
with a threshold of 1e−05

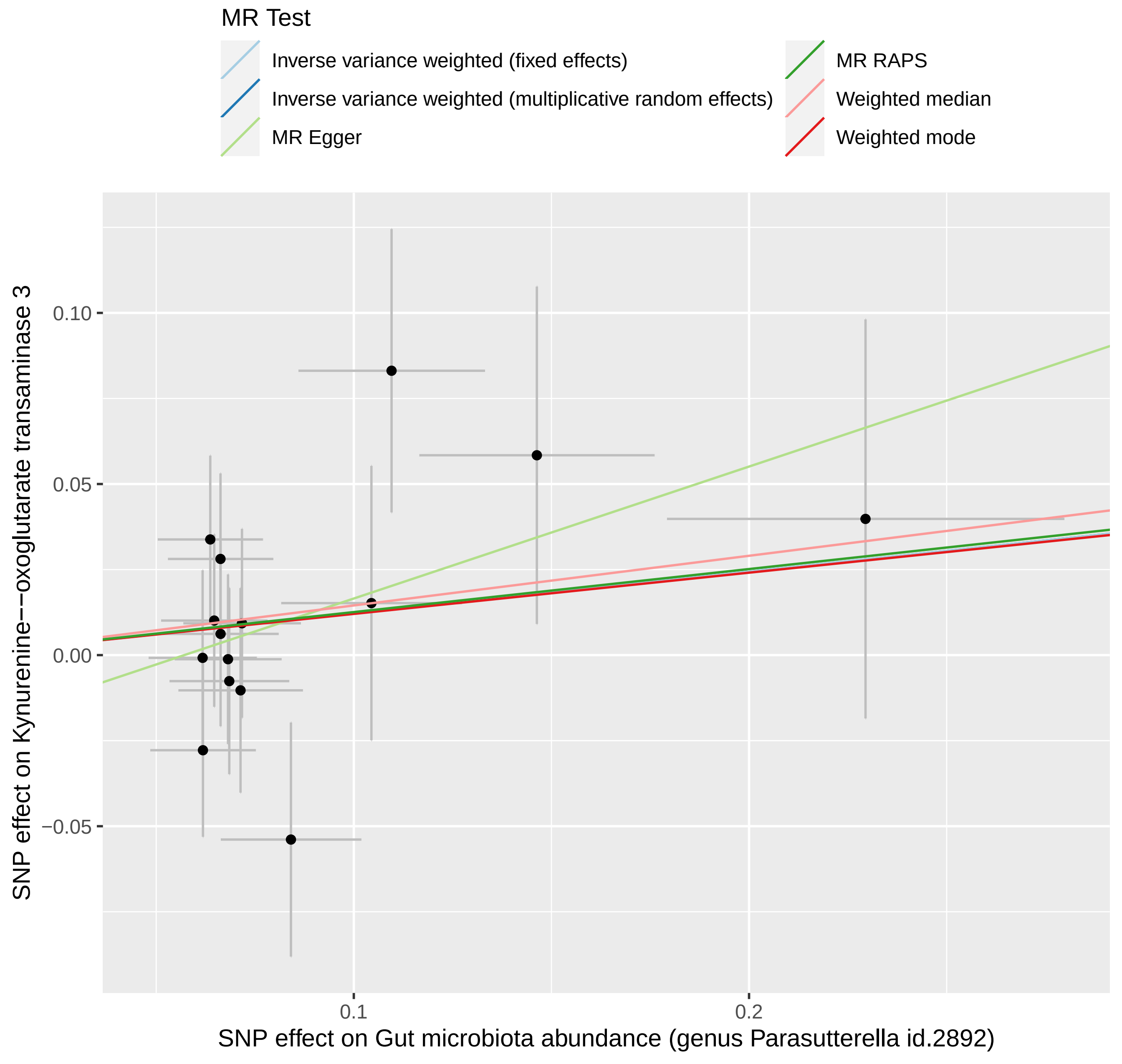

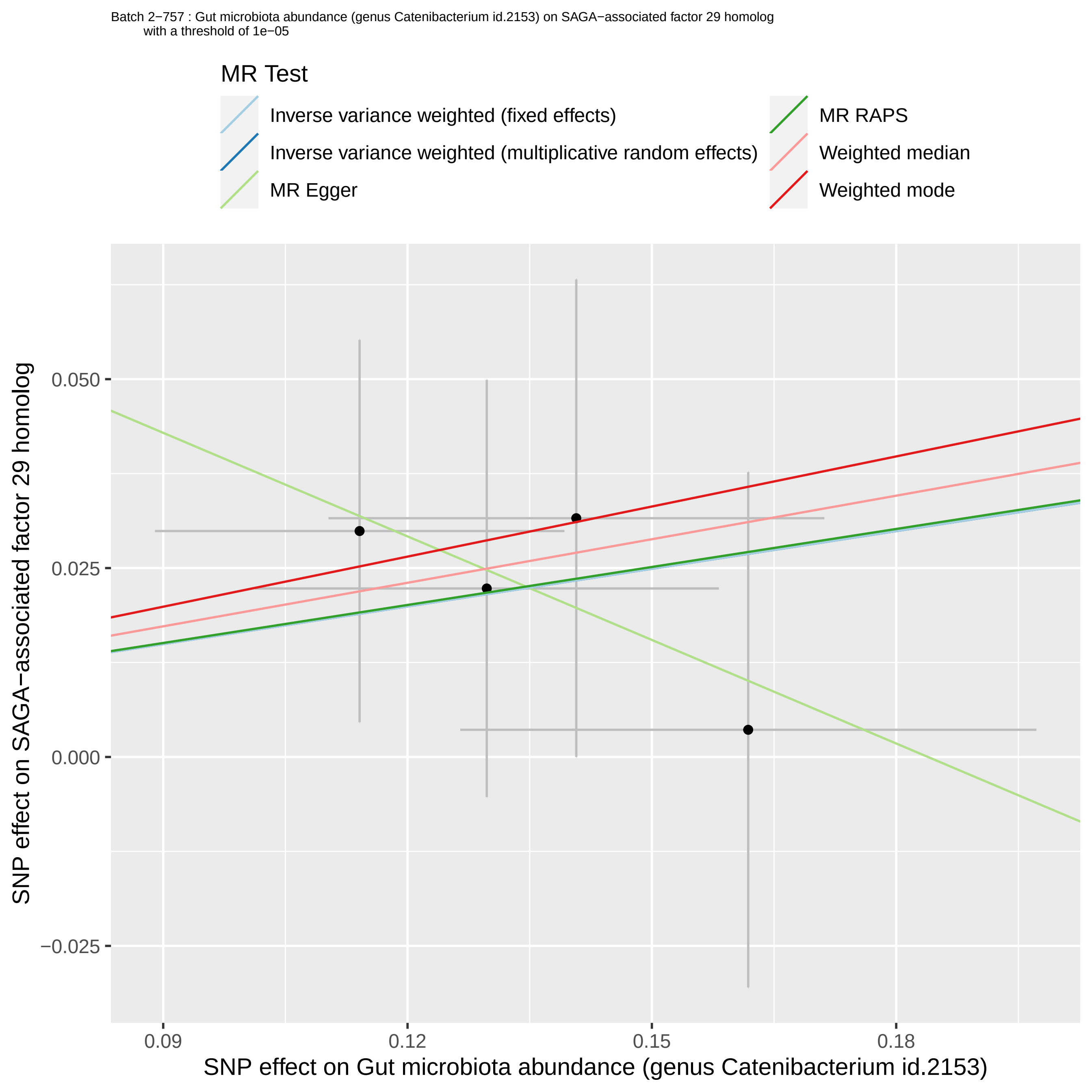
Batch 2−757 : Gut microbiota abundance (genus Catenibacterium id.2153) on SAGA−associated factor 29 homolog
with a threshold of 1e−05
MR Test
Inverse variance weighted (fixed effects)
Inverse variance weighted (multiplicative random effects)
MR Egger
MR RAPS
Weighted median
Weighted mode
SNP effect on SAGA−associated factor 29 homolog
0.050
0.025
0.000
−0.025
0.09
0.12
0.15
0.18
SNP effect on Gut microbiota abundance (genus Catenibacterium id.2153)

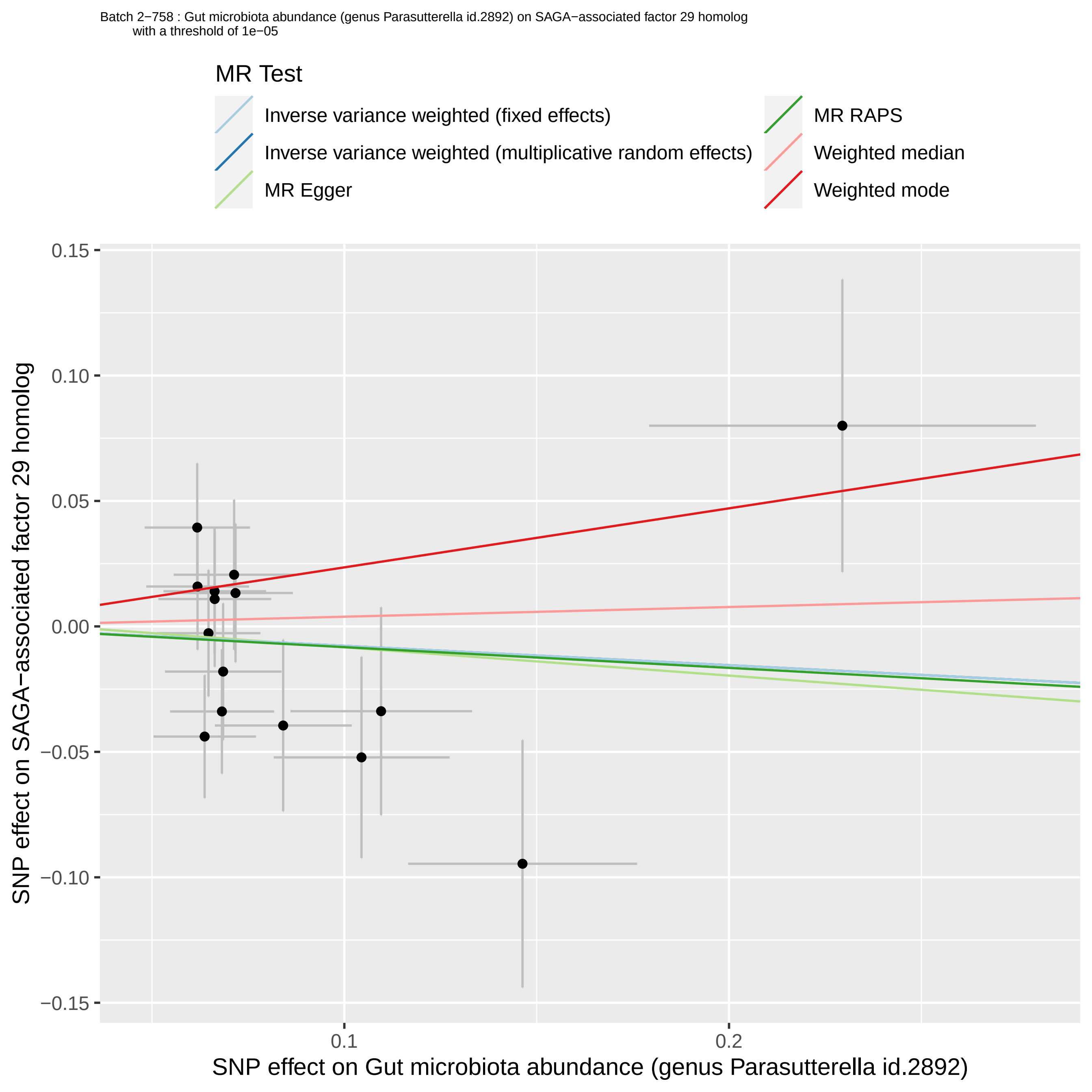

Batch 2−758 : Gut microbiota abundance (genus Parasutterella id.2892) on SAGA−associated factor 29 homolog
with a threshold of 1e−05
MR Test
Inverse variance weighted (fixed effects)
Inverse variance weighted (multiplicative random effects)
MR Egger
MR RAPS
Weighted median
Weighted mode
0.15
0.10
0.05
0.00
−0.05
−0.10
−0.15
0.1
0.2
SNP effect on SAGA−associated factor 29 homolog
SNP effect on Gut microbiota abundance (genus Parasutterella id.2892)

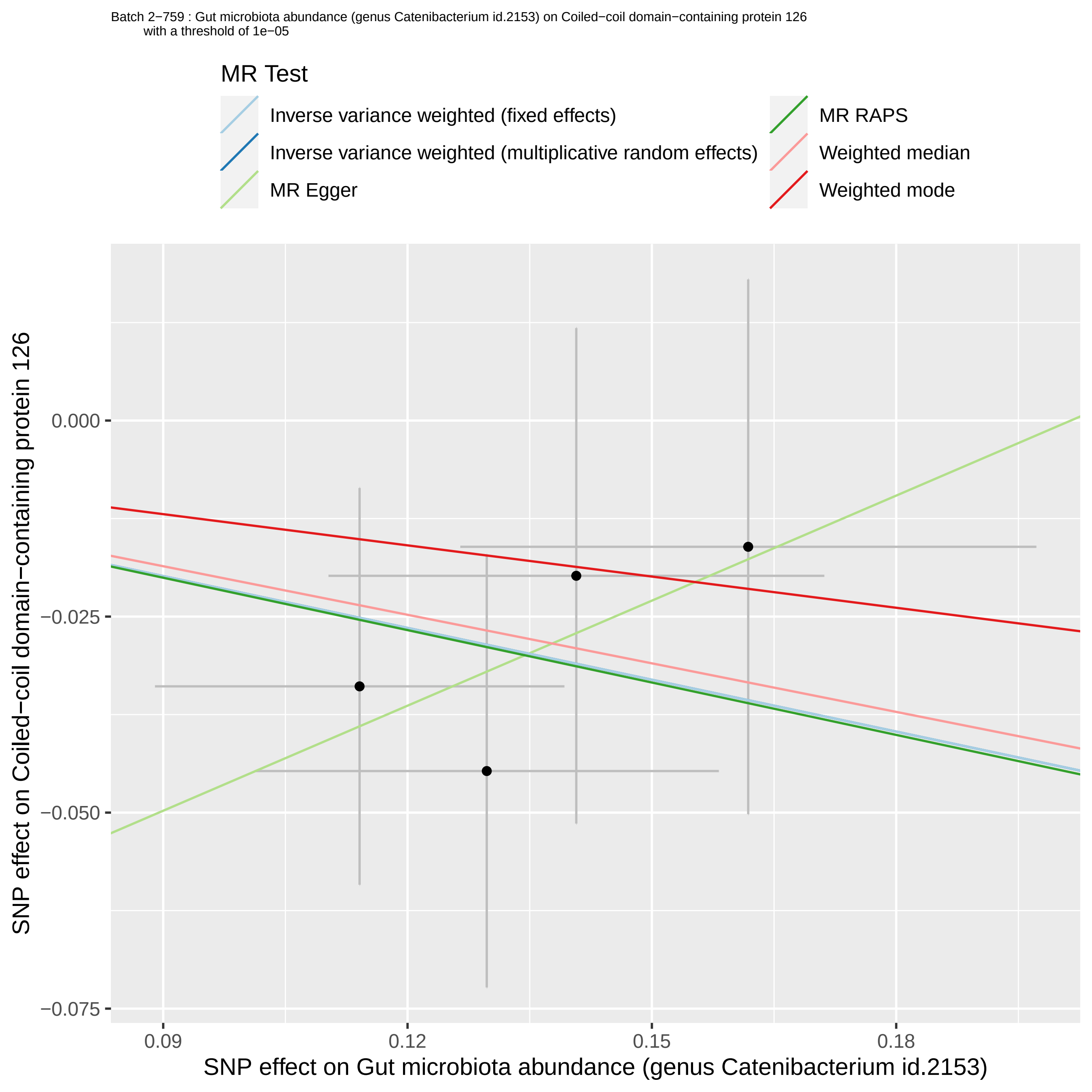

Batch 2−759 : Gut microbiota abundance (genus Catenibacterium id.2153) on Coiled−coil domain−containing protein 126 with a threshold of 1e−05
MR Test
Inverse variance weighted (fixed effects)
Inverse variance weighted (multiplicative random effects)
MR Egger
MR RAPS
Weighted median
Weighted mode
SNP effect on Coiled−coil domain−containing protein 126
0.000
−0.025
−0.050
−0.075
0.09
0.12
0.15
0.18
SNP effect on Gut microbiota abundance (genus Catenibacterium id.2153)

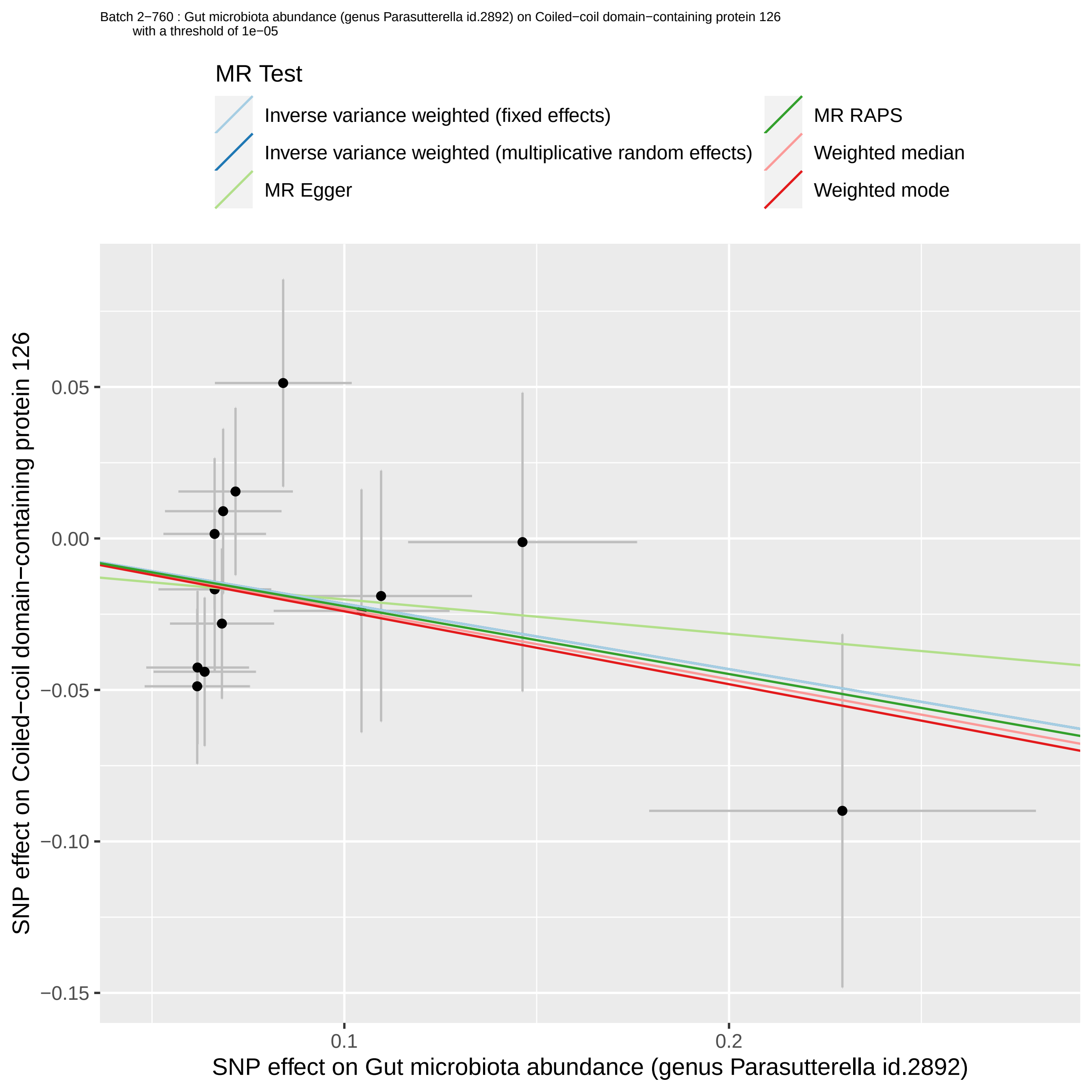

Batch 2−760 : Gut microbiota abundance (genus Parasutterella id.2892) on Coiled−coil domain−containing protein 126
with a threshold of 1e−05
MR Test
Inverse variance weighted (fixed effects)
Inverse variance weighted (multiplicative random effects)
MR Egger
MR RAPS
Weighted median
Weighted mode
0.05
0.00
−0.05
−0.10
−0.15
0.1
0.2
SNP effect on Coiled−coil domain−containing protein 126
SNP effect on Gut microbiota abundance (genus Parasutterella id.2892)

Batch 2−761 : Gut microbiota abundance (genus Catenibacterium id.2153) on Coiled−coil domain−containing protein 134
with a threshold of 1e−05

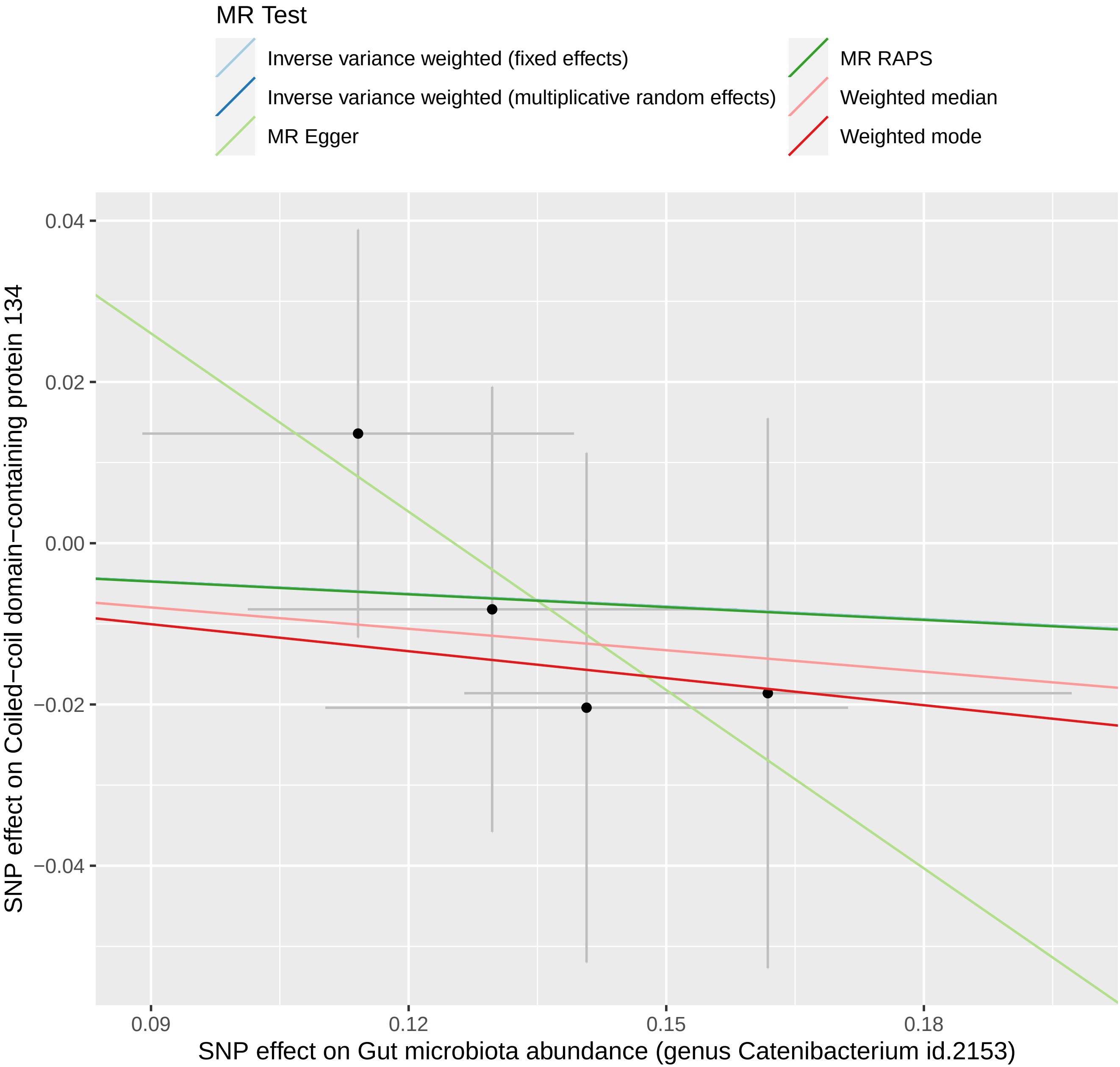

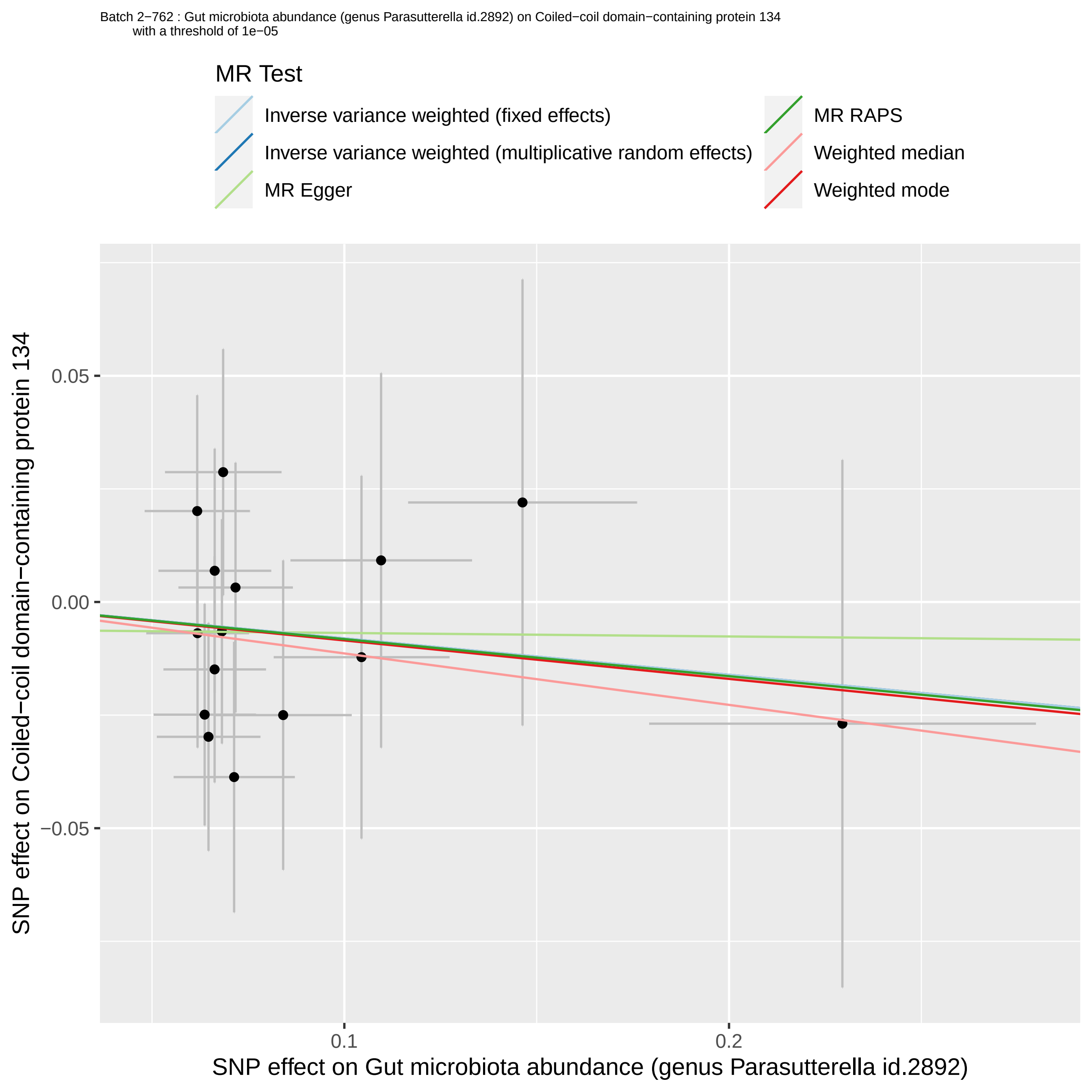

Batch 2−762 : Gut microbiota abundance (genus Parasutterella id.2892) on Coiled−coil domain−containing protein 134
with a threshold of 1e−05
MR Test
Inverse variance weighted (fixed effects)
Inverse variance weighted (multiplicative random effects)
MR Egger
MR RAPS
Weighted median
Weighted mode
0.05
0.00
−0.05
0.1
0.2
SNP effect on Coiled−coil domain−containing protein 134
SNP effect on Gut microbiota abundance (genus Parasutterella id.2892)

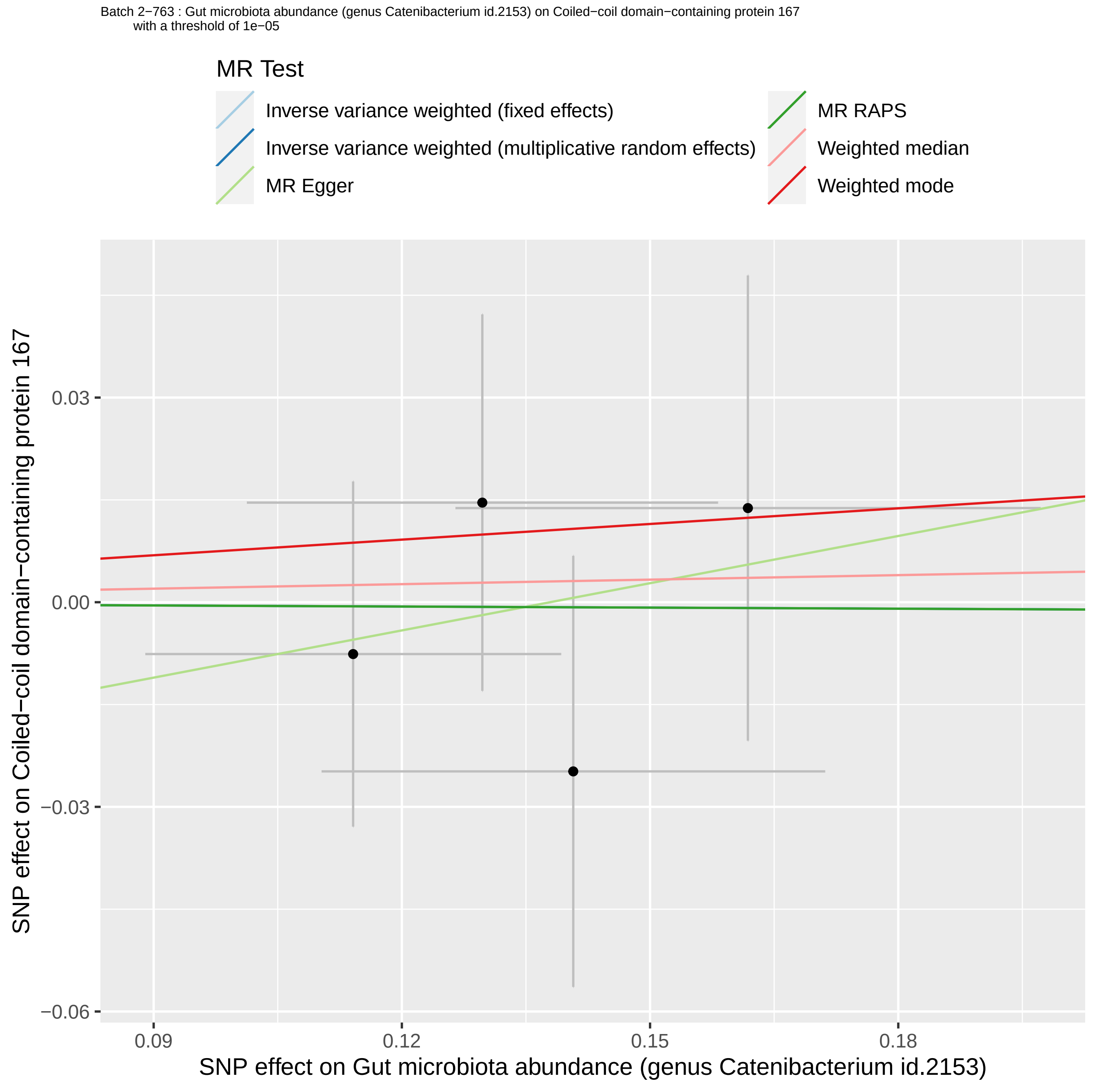

Batch 2−763 : Gut microbiota abundance (genus Catenibacterium id.2153) on Coiled−coil domain−containing protein 167
with a threshold of 1e−05
MR Test
Inverse variance weighted (fixed effects)
Inverse variance weighted (multiplicative random effects)
MR Egger
MR RAPS
Weighted median
Weighted mode
0.03
0.00
−0.03
−0.06
0.09
0.12
0.15
0.18
SNP effect on Coiled−coil domain−containing protein 167
SNP effect on Gut microbiota abundance (genus Catenibacterium id.2153)

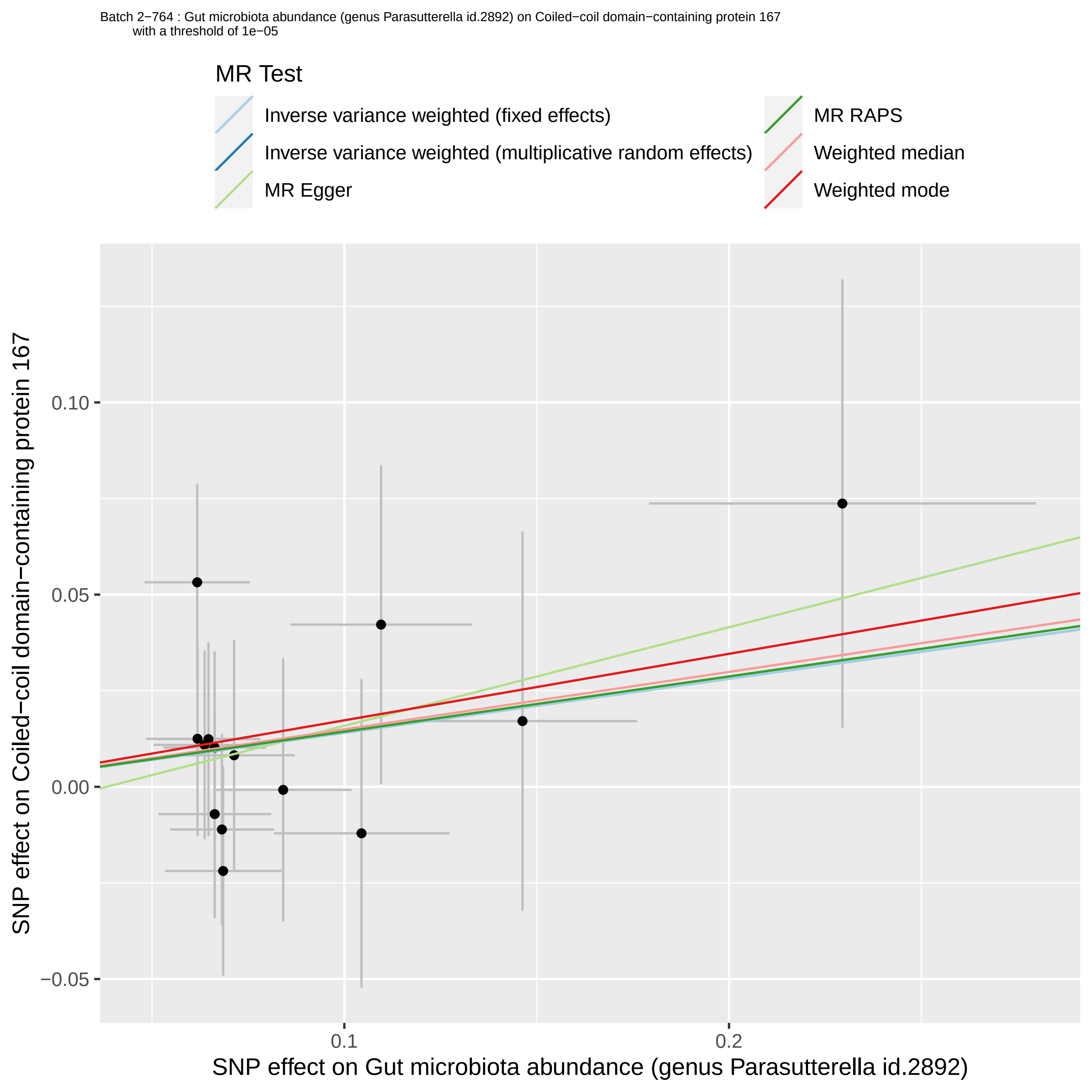
Batch 2−764 : Gut microbiota abundance (genus Parasutterella id.2892) on Coiled−coil domain−containing protein 167
with a threshold of 1e−05
MR Test
Inverse variance weighted (fixed effects)
Inverse variance weighted (multiplicative random effects)
MR Egger
MR RAPS
Weighted median
Weighted mode
SNP effect on Coiled−coil domain−containing protein 167
0.10
0.05
0.00
−0.05
0.1
0.2
SNP effect on Gut microbiota abundance (genus Parasutterella id.2892)

Batch 2−765 : Gut microbiota abundance (genus Catenibacterium id.2153) on Coiled−coil domain−containing protein 51 with a threshold of 1e−05

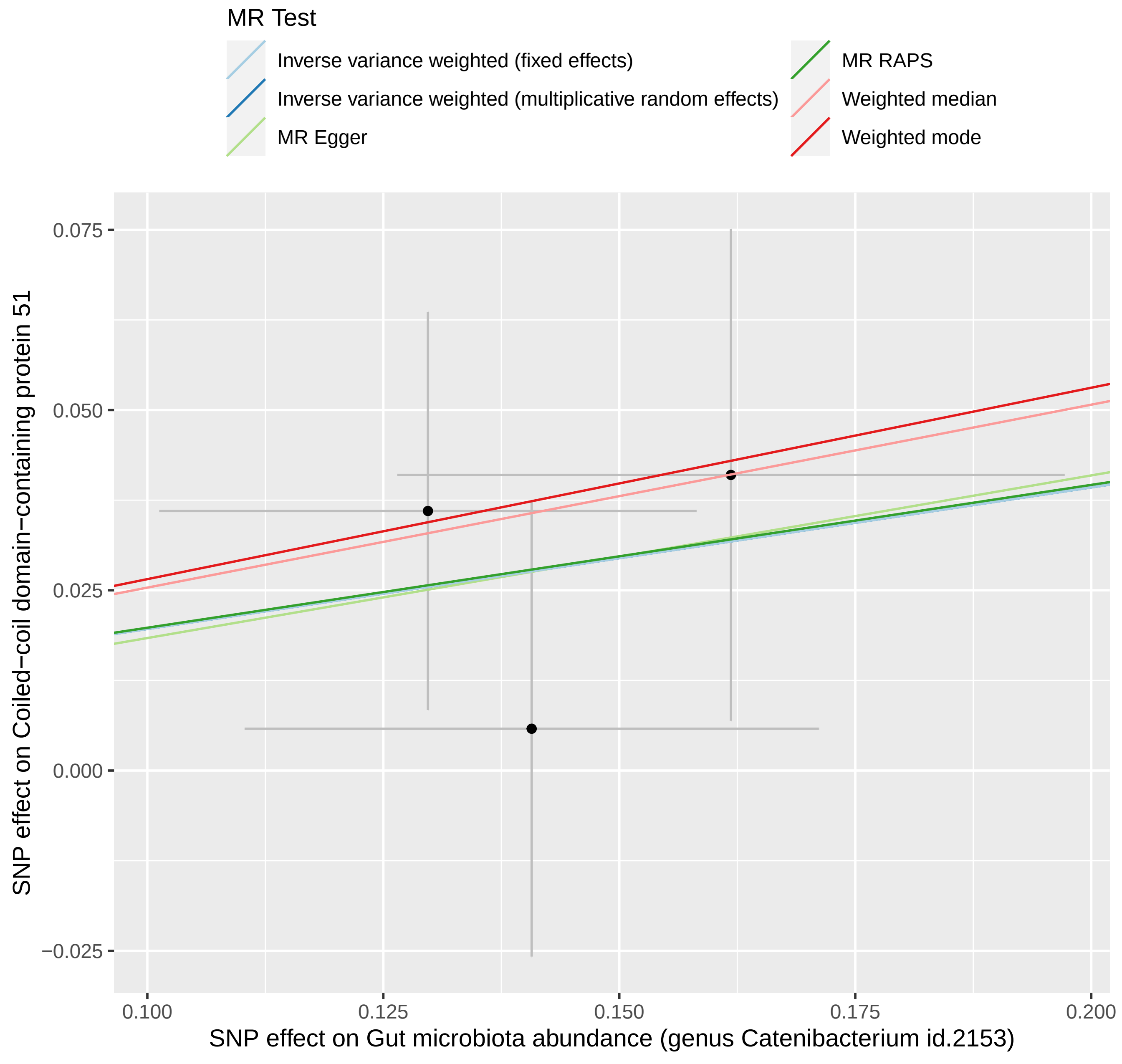

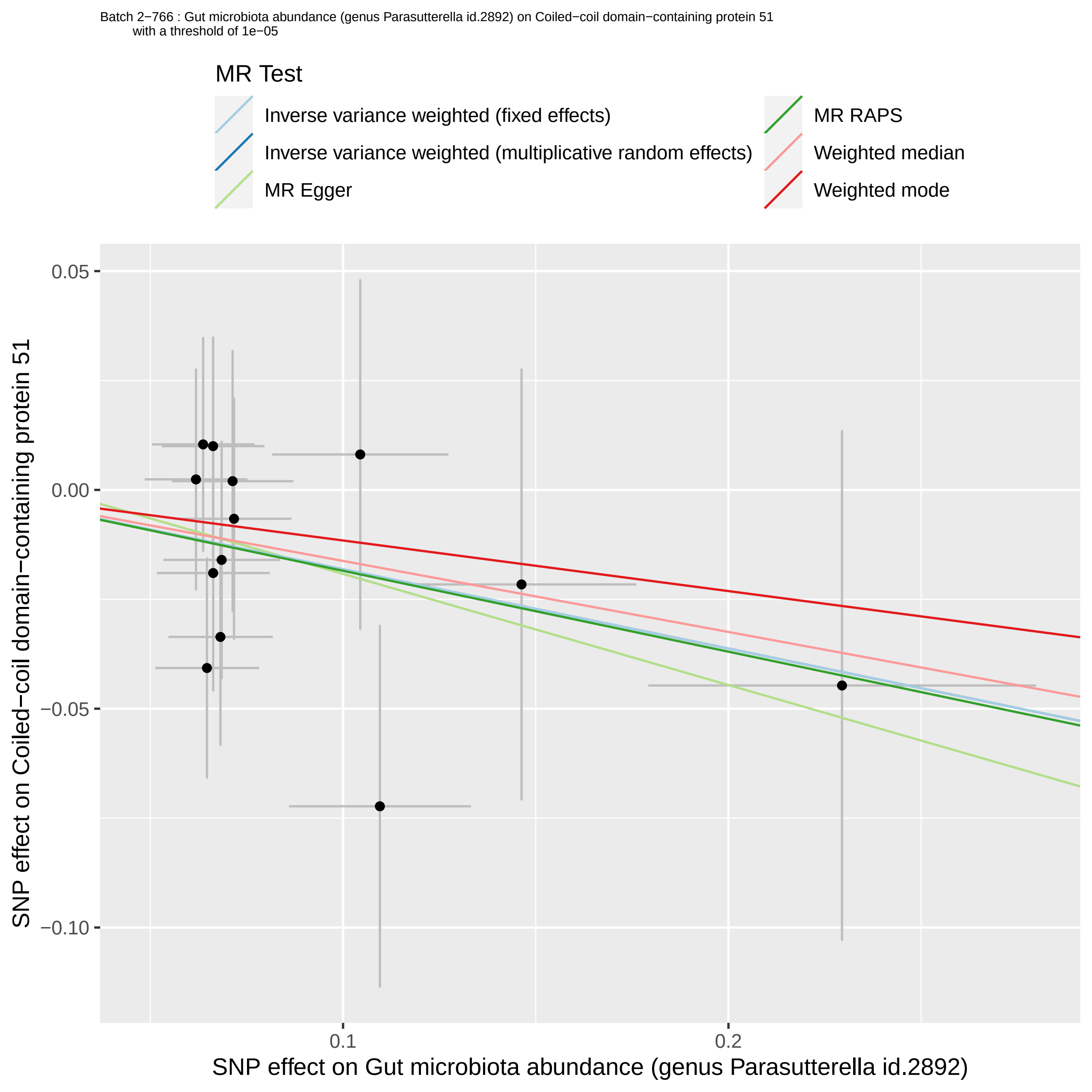
Batch 2−766 : Gut microbiota abundance (genus Parasutterella id.2892) on Coiled−coil domain−containing protein 51
with a threshold of 1e−05
MR Test
Inverse variance weighted (fixed effects)
Inverse variance weighted (multiplicative random effects)
MR Egger
MR RAPS
Weighted median
Weighted mode
0.05
0.00
−0.05
−0.10
0.1
0.2
SNP effect on Coiled−coil domain−containing protein 51
SNP effect on Gut microbiota abundance (genus Parasutterella id.2892)

Batch 2−767 : Gut microbiota abundance (genus Catenibacterium id.2153) on Coiled−coil domain−containing protein 80
with a threshold of 1e−05

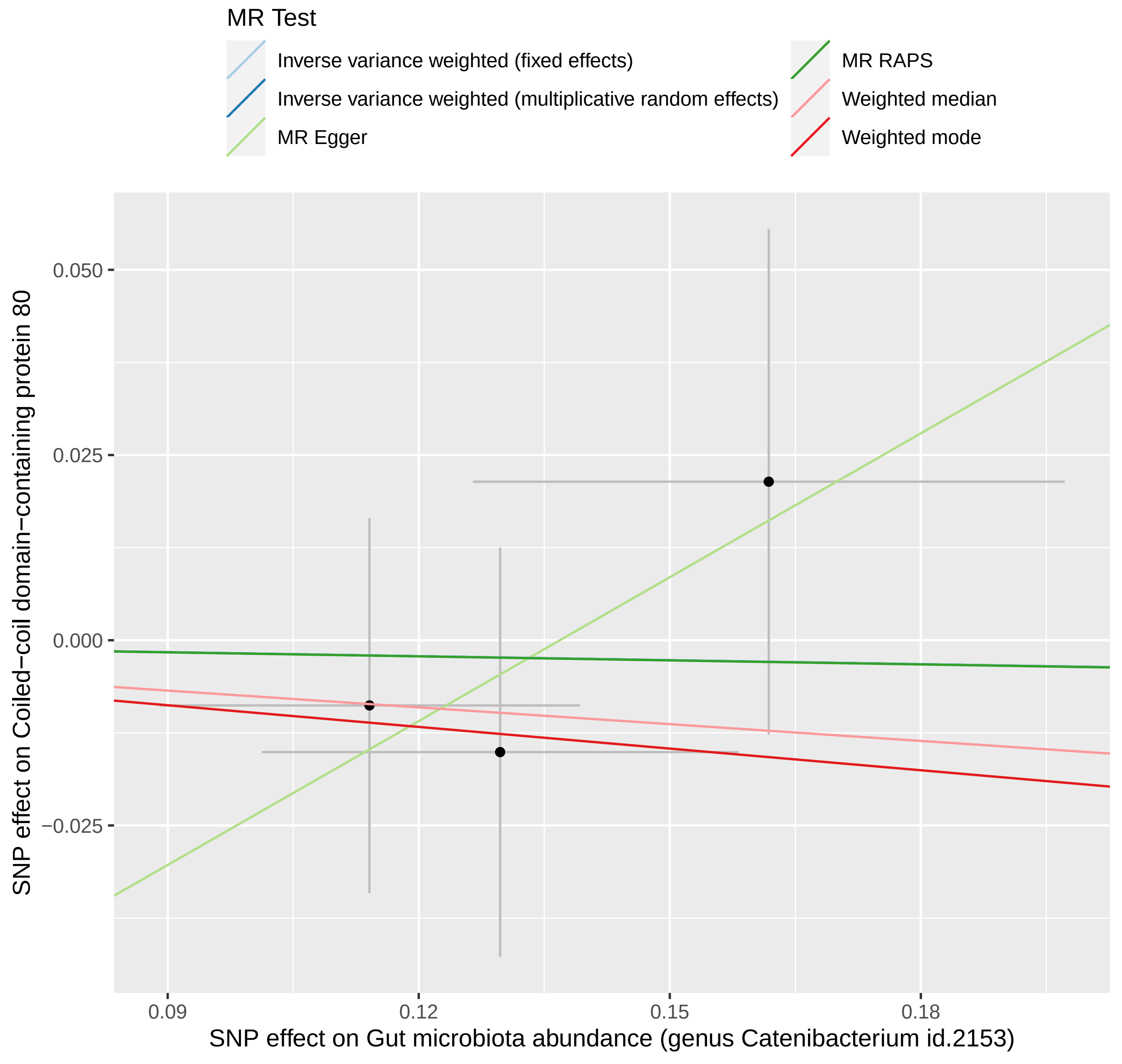

Batch 2−768 : Gut microbiota abundance (genus Parasutterella id.2892) on Coiled−coil domain−containing protein 80
with a threshold of 1e−05

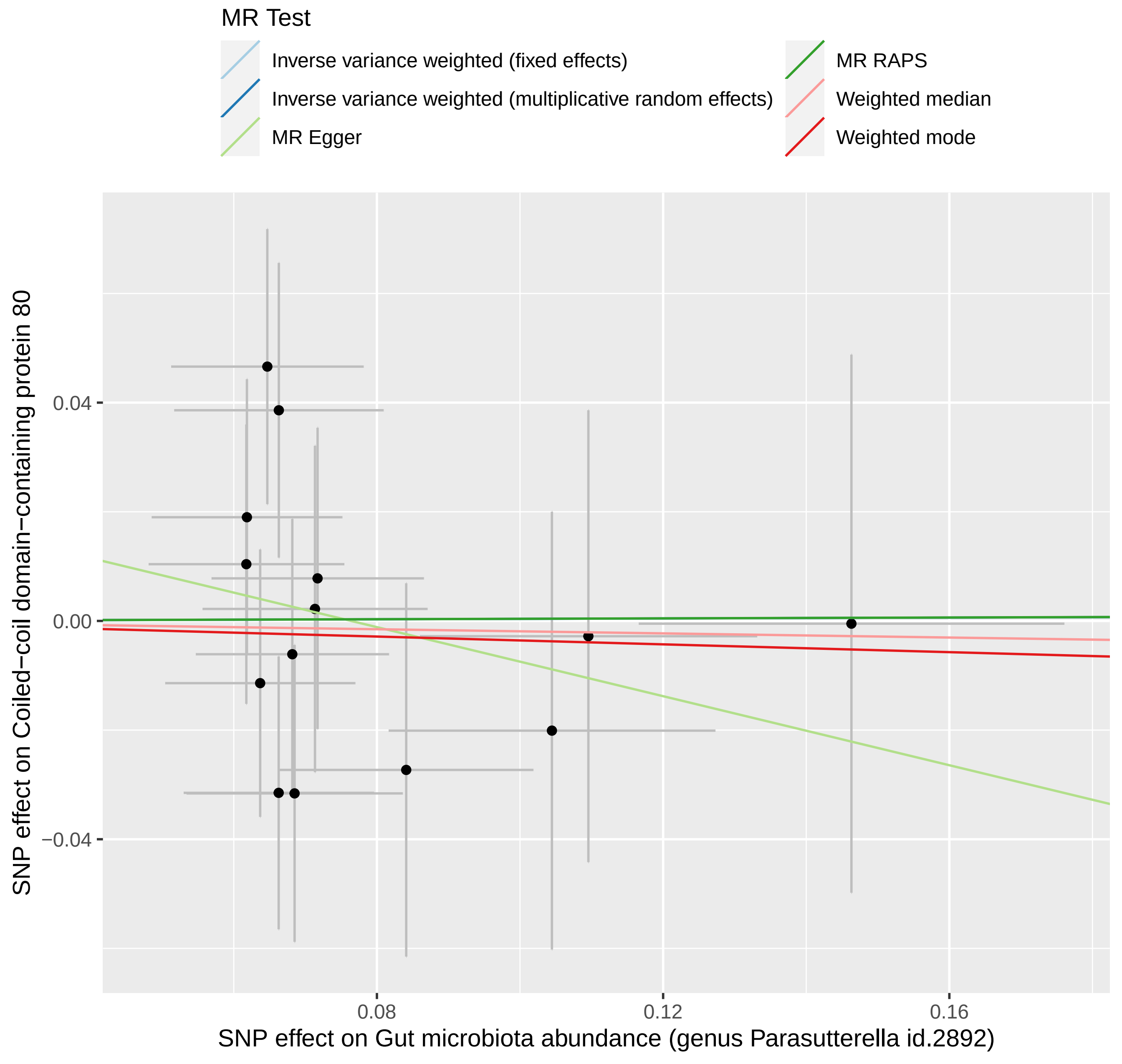

Batch 2−769 : Gut microbiota abundance (genus Catenibacterium id.2153) on Coiled−coil domain−containing protein 90B, mitochondrial
with a threshold of 1e−05

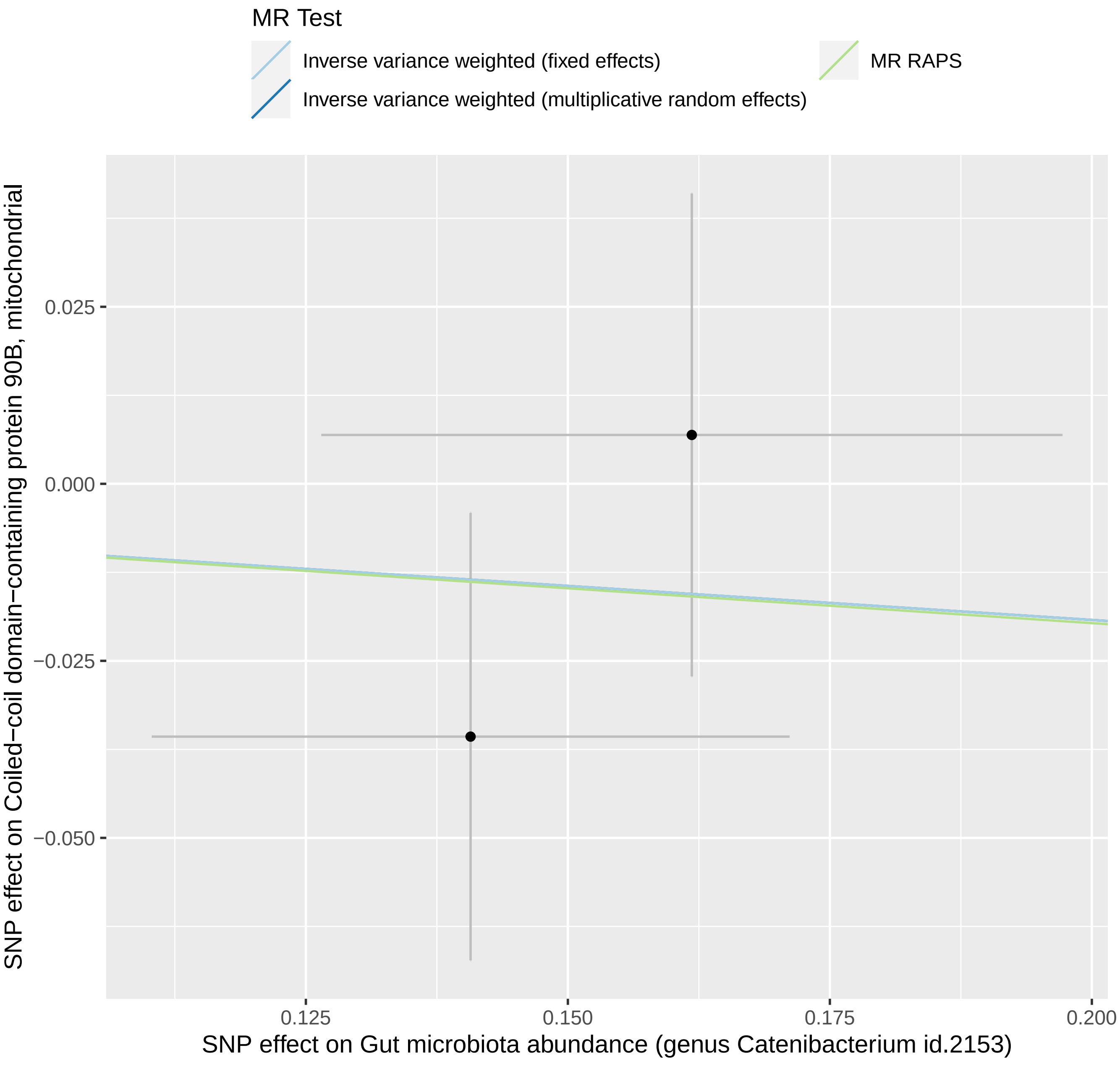

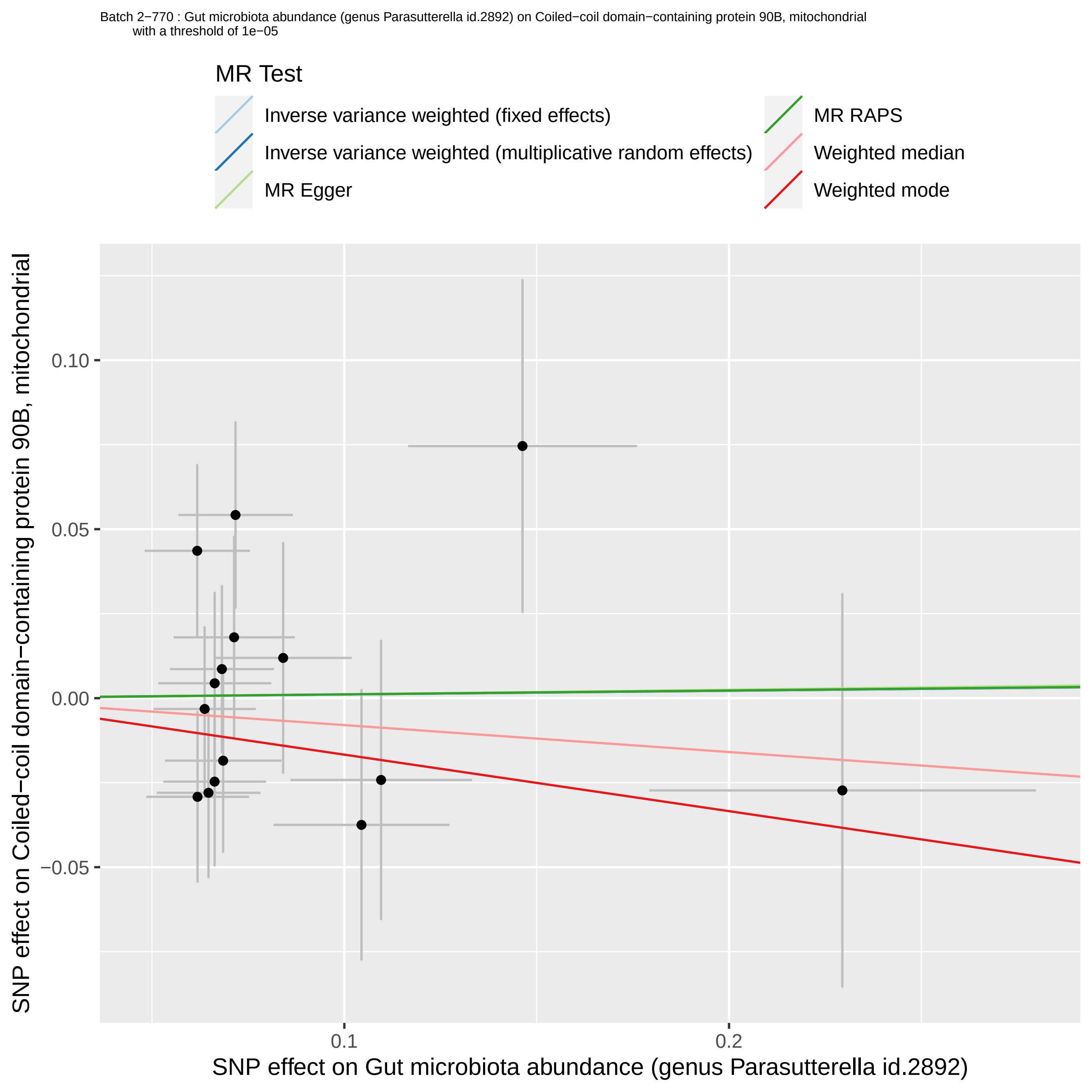

Batch 2−770 : Gut microbiota abundance (genus Parasutterella id.2892) on Coiled−coil domain−containing protein 90B, mitochondrial
with a threshold of 1e−05
MR Test
Inverse variance weighted (fixed effects)
Inverse variance weighted (multiplicative random effects)
MR Egger
MR RAPS
Weighted median
Weighted mode
0.10
0.05
0.00
−0.05
0.1
0.2
SNP effect on Coiled−coil domain−containing protein 90B, mitochondrial
SNP effect on Gut microbiota abundance (genus Parasutterella id.2892)

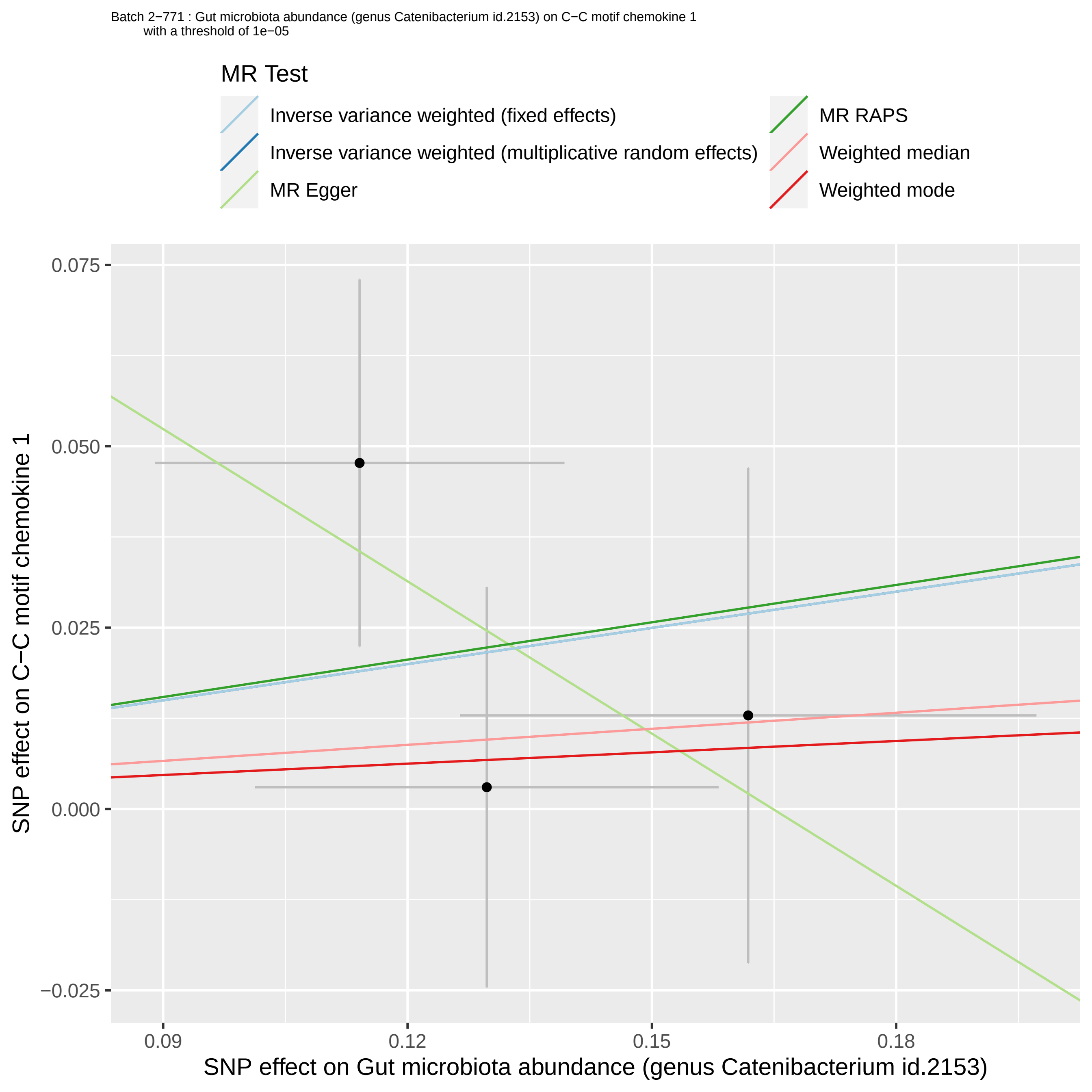

Batch 2−771 : Gut microbiota abundance (genus Catenibacterium id.2153) on C−C motif chemokine 1
with a threshold of 1e−05
MR Test
Inverse variance weighted (fixed effects)
Inverse variance weighted (multiplicative random effects)
MR Egger
MR RAPS
Weighted median
Weighted mode
0.075
0.050
0.025
0.000
−0.025
0.09
0.12
0.15
0.18
SNP effect on C−C motif chemokine 1
SNP effect on Gut microbiota abundance (genus Catenibacterium id.2153)

Batch 2−772 : Gut microbiota abundance (genus Parasutterella id.2892) on C−C motif chemokine 1
with a threshold of 1e−05

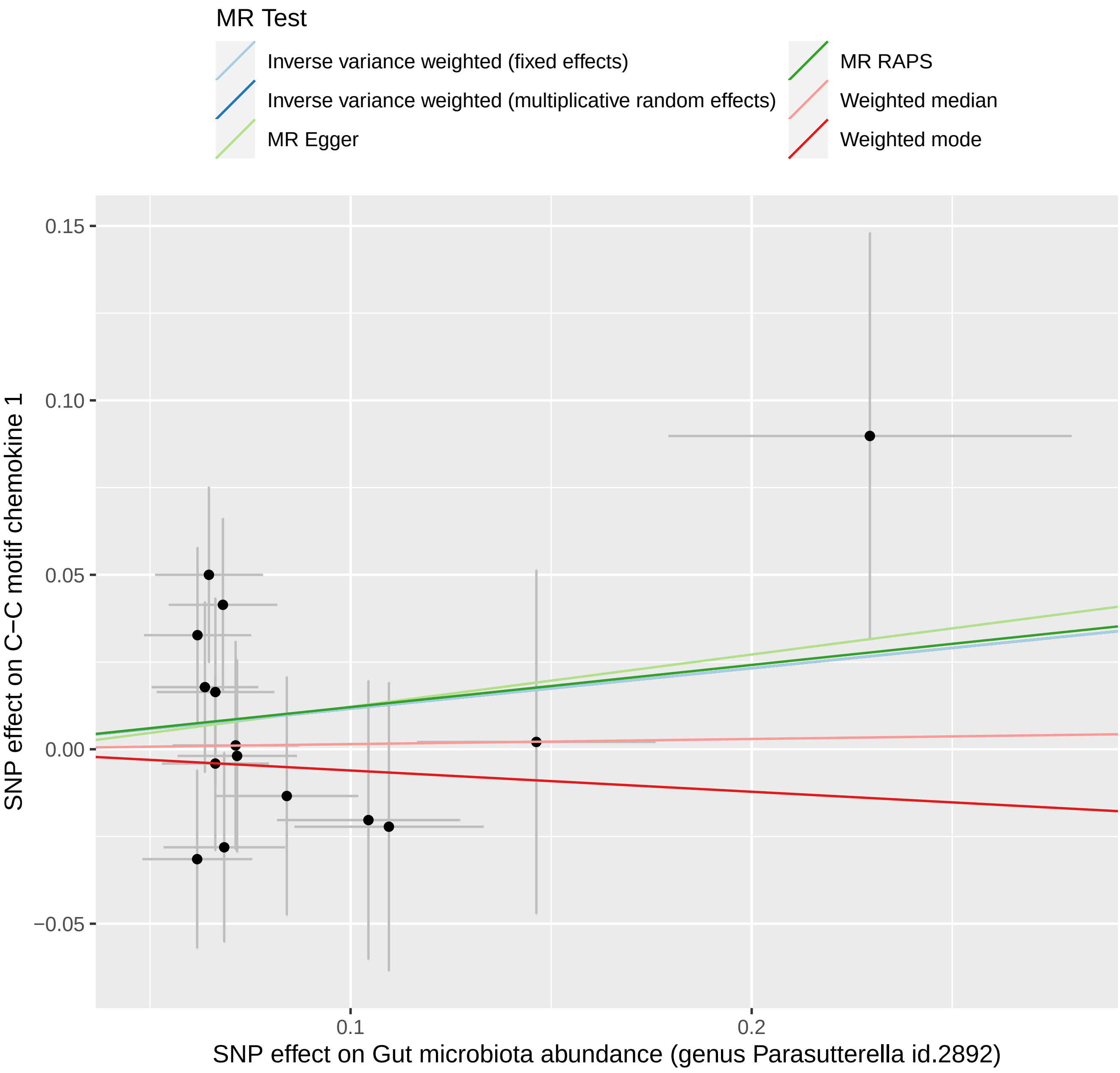

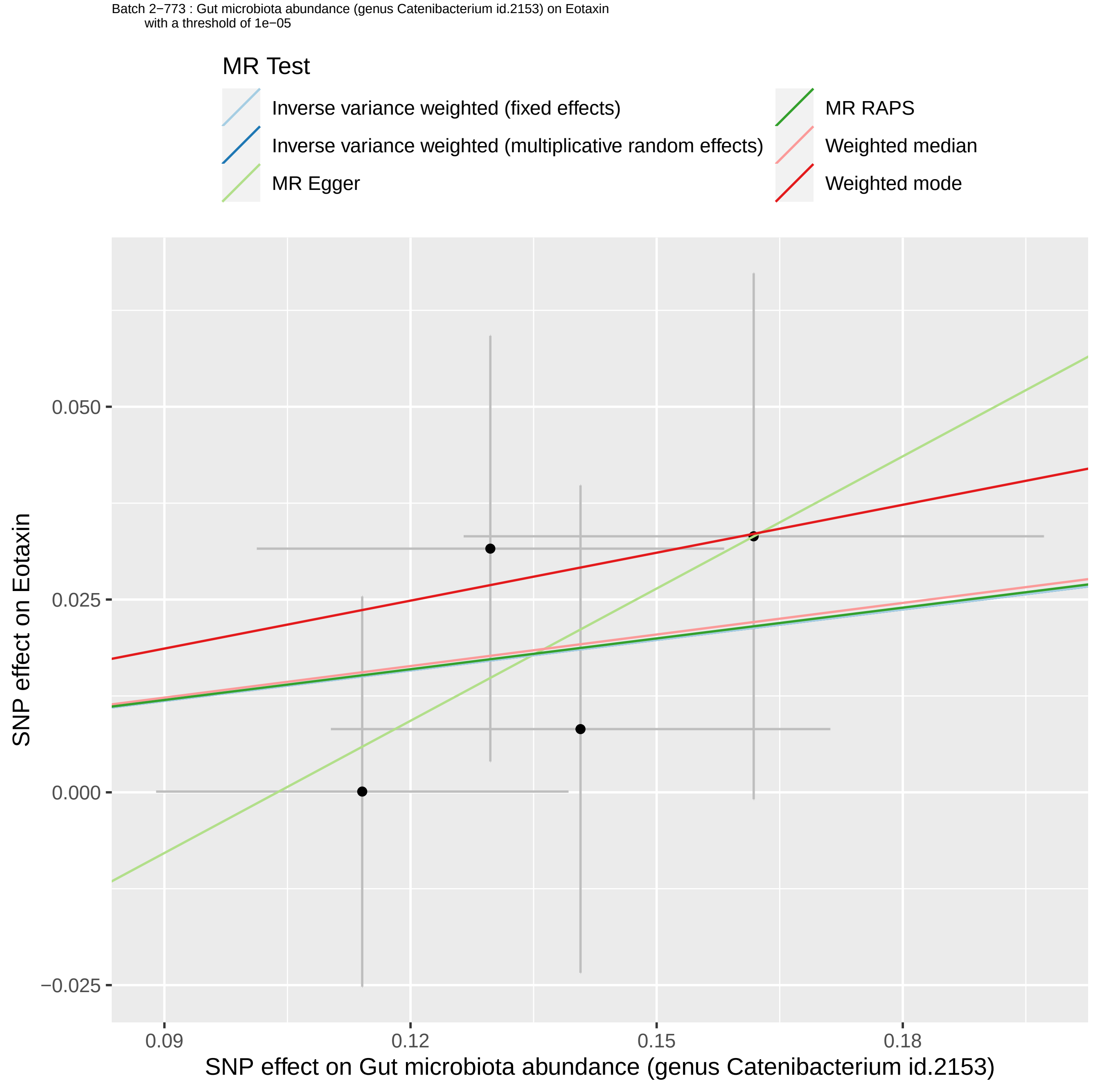
Batch 2−773 : Gut microbiota abundance (genus Catenibacterium id.2153) on Eotaxin
with a threshold of 1e−05
MR Test
Inverse variance weighted (fixed effects)
Inverse variance weighted (multiplicative random effects)
MR Egger
MR RAPS
Weighted median
Weighted mode
0.050
0.025
0.000
−0.025
0.09
0.12
0.15
0.18
SNP effect on Eotaxin
SNP effect on Gut microbiota abundance (genus Catenibacterium id.2153)

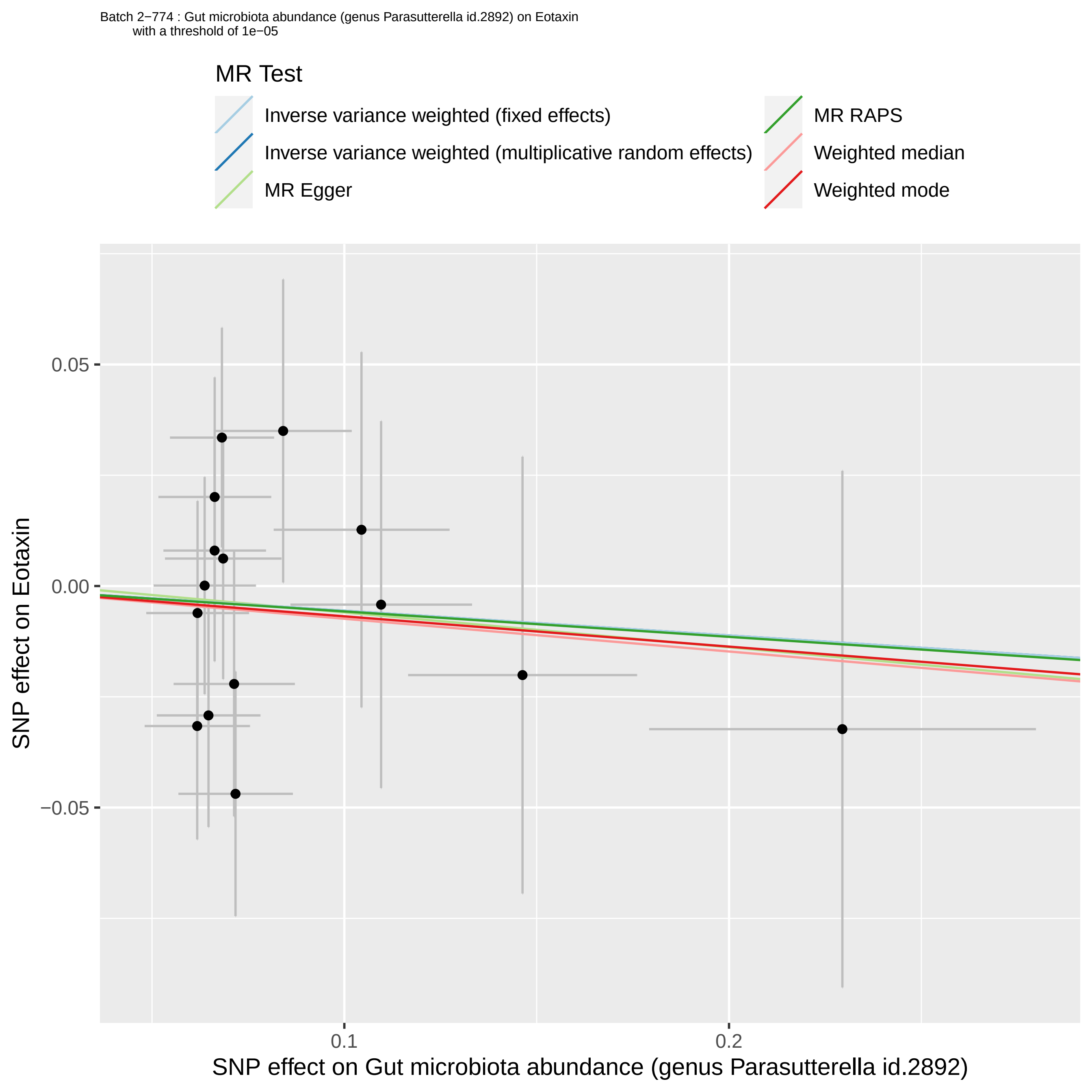

Batch 2−774 : Gut microbiota abundance (genus Parasutterella id.2892) on Eotaxin
with a threshold of 1e−05
MR Test
Inverse variance weighted (fixed effects)
Inverse variance weighted (multiplicative random effects)
MR Egger
MR RAPS
Weighted median
Weighted mode
0.05
0.00
−0.05
0.1
0.2
SNP effect on Eotaxin
SNP effect on Gut microbiota abundance (genus Parasutterella id.2892)

Batch 2−775 : Gut microbiota abundance (genus Catenibacterium id.2153) on C−C motif chemokine 1
with a threshold of 1e−05

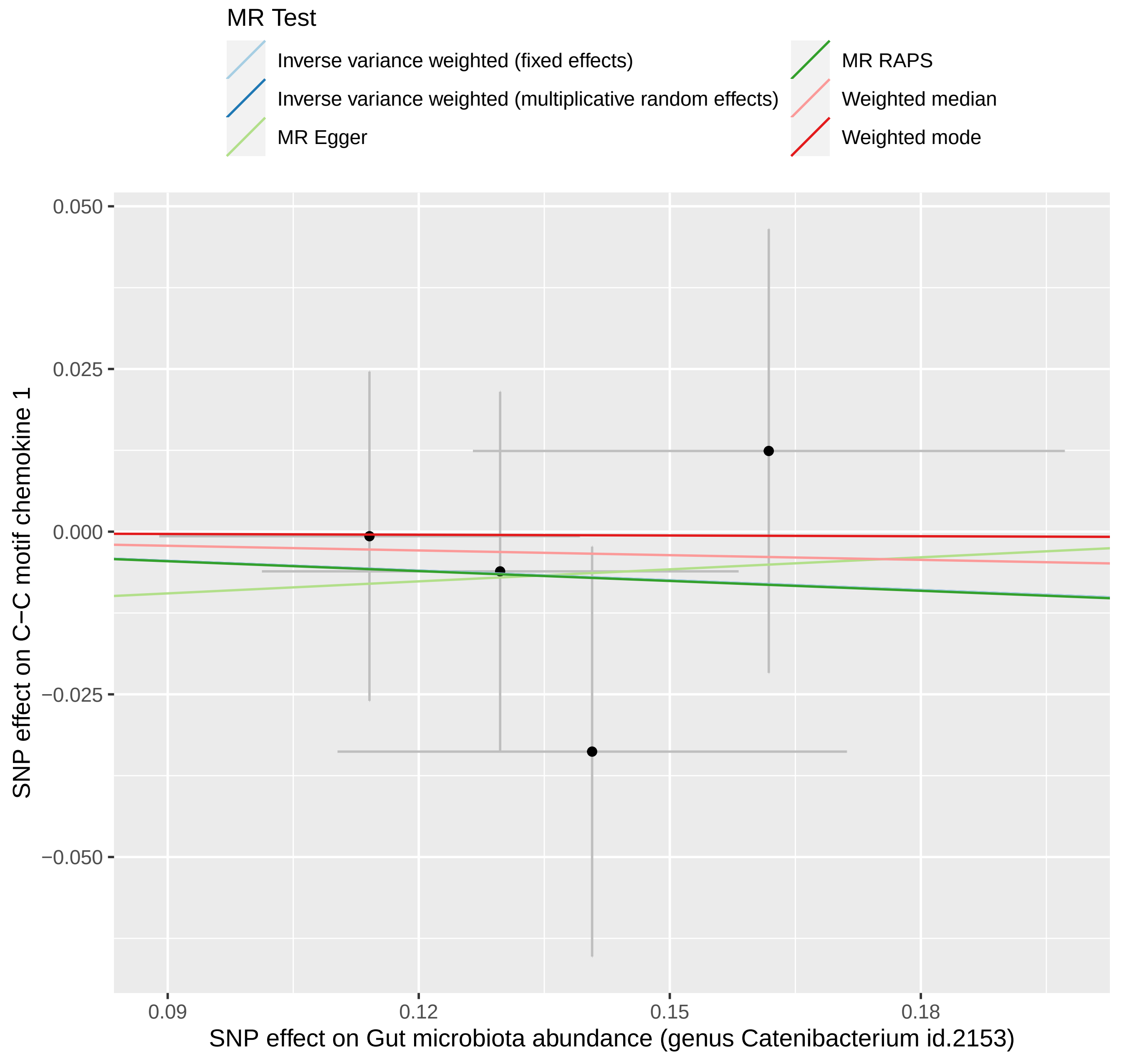

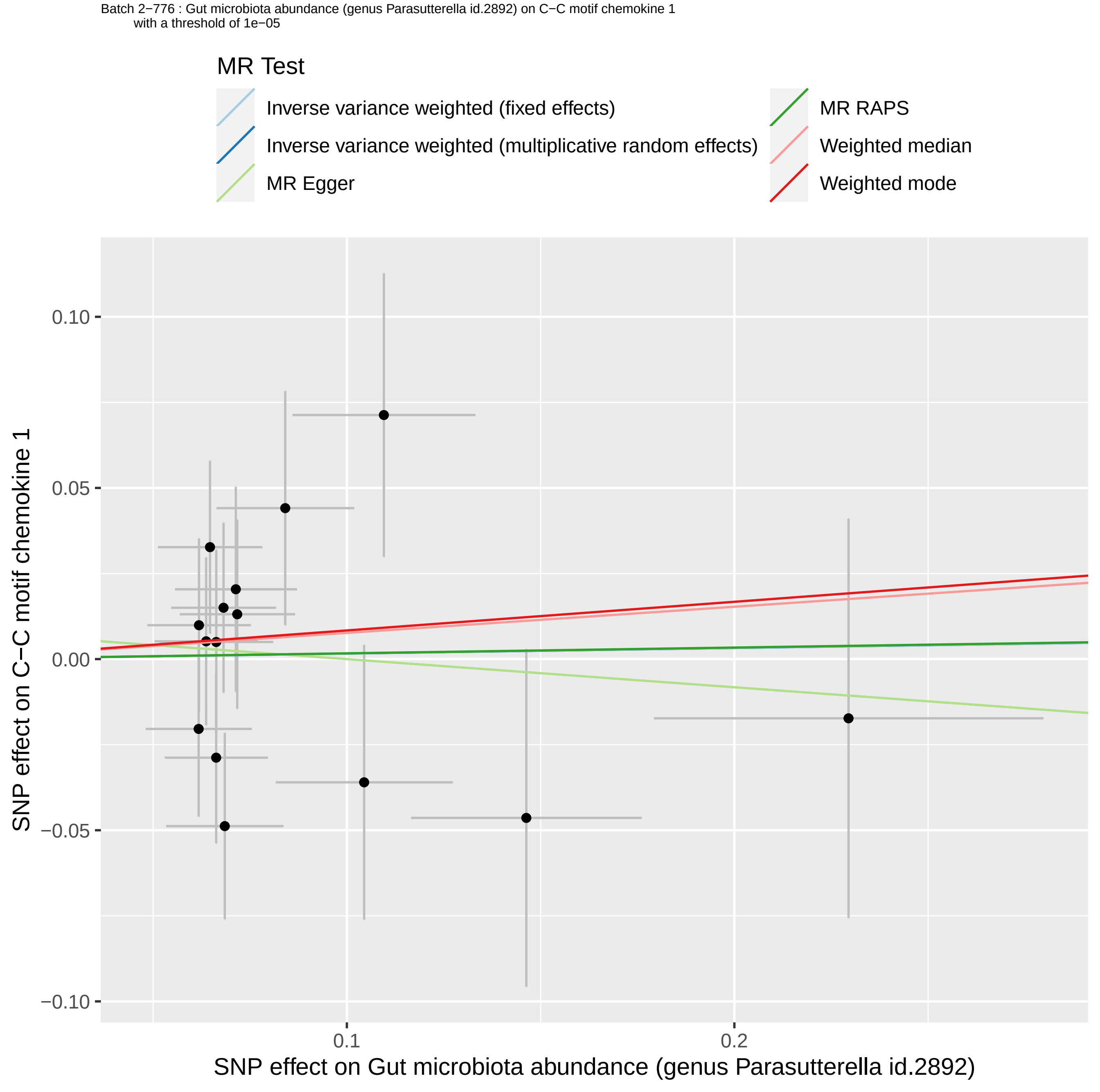

Batch 2−776 : Gut microbiota abundance (genus Parasutterella id.2892) on C−C motif chemokine 1
with a threshold of 1e−05
MR Test
Inverse variance weighted (fixed effects)
Inverse variance weighted (multiplicative random effects)
MR Egger
MR RAPS
Weighted median
Weighted mode
0.10
0.05
0.00
−0.05
−0.10
0.1
0.2
SNP effect on C−C motif chemokine 1
SNP effect on Gut microbiota abundance (genus Parasutterella id.2892)

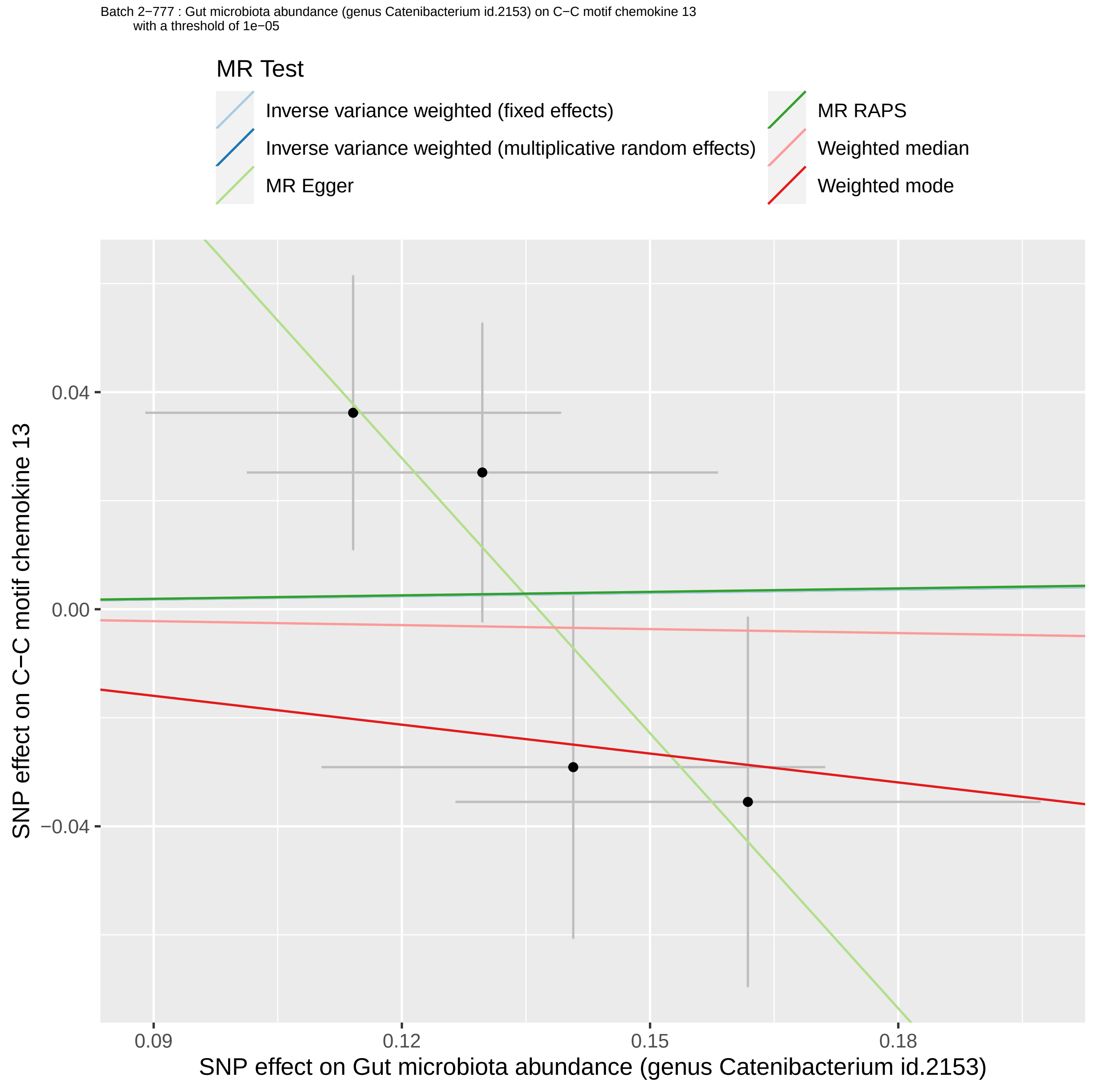

Batch 2−777 : Gut microbiota abundance (genus Catenibacterium id.2153) on C−C motif chemokine 13
with a threshold of 1e−05
MR Test
Inverse variance weighted (fixed effects)
Inverse variance weighted (multiplicative random effects)
MR Egger
MR RAPS
Weighted median
Weighted mode
0.04
0.00
−0.04
0.09
0.12
0.15
0.18
SNP effect on C−C motif chemokine 13
SNP effect on Gut microbiota abundance (genus Catenibacterium id.2153)

Batch 2−778 : Gut microbiota abundance (genus Parasutterella id.2892) on C−C motif chemokine 13
with a threshold of 1e−05

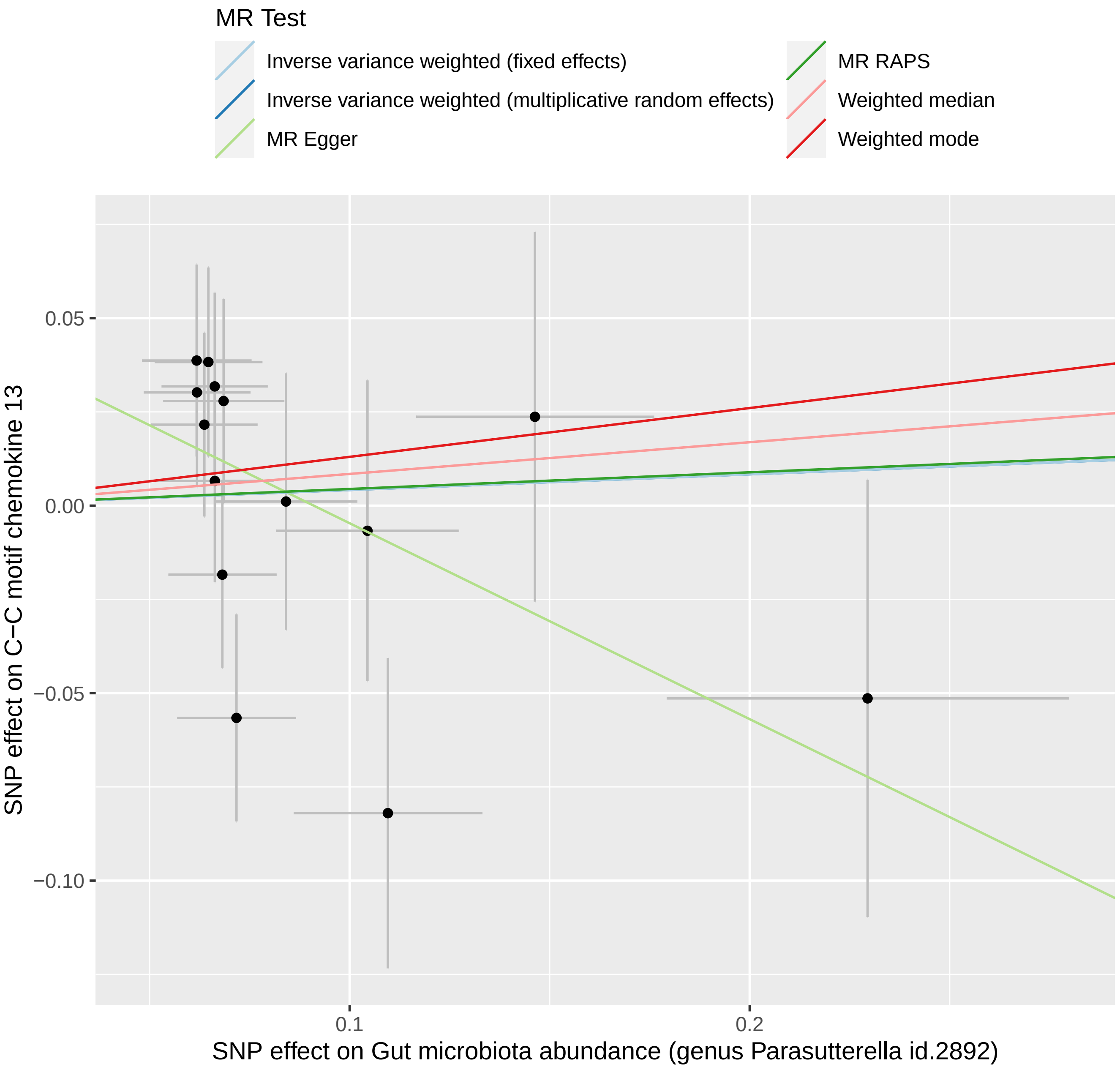

Batch 2−779 : Gut microbiota abundance (genus Catenibacterium id.2153) on C−C motif chemokine 14
with a threshold of 1e−05

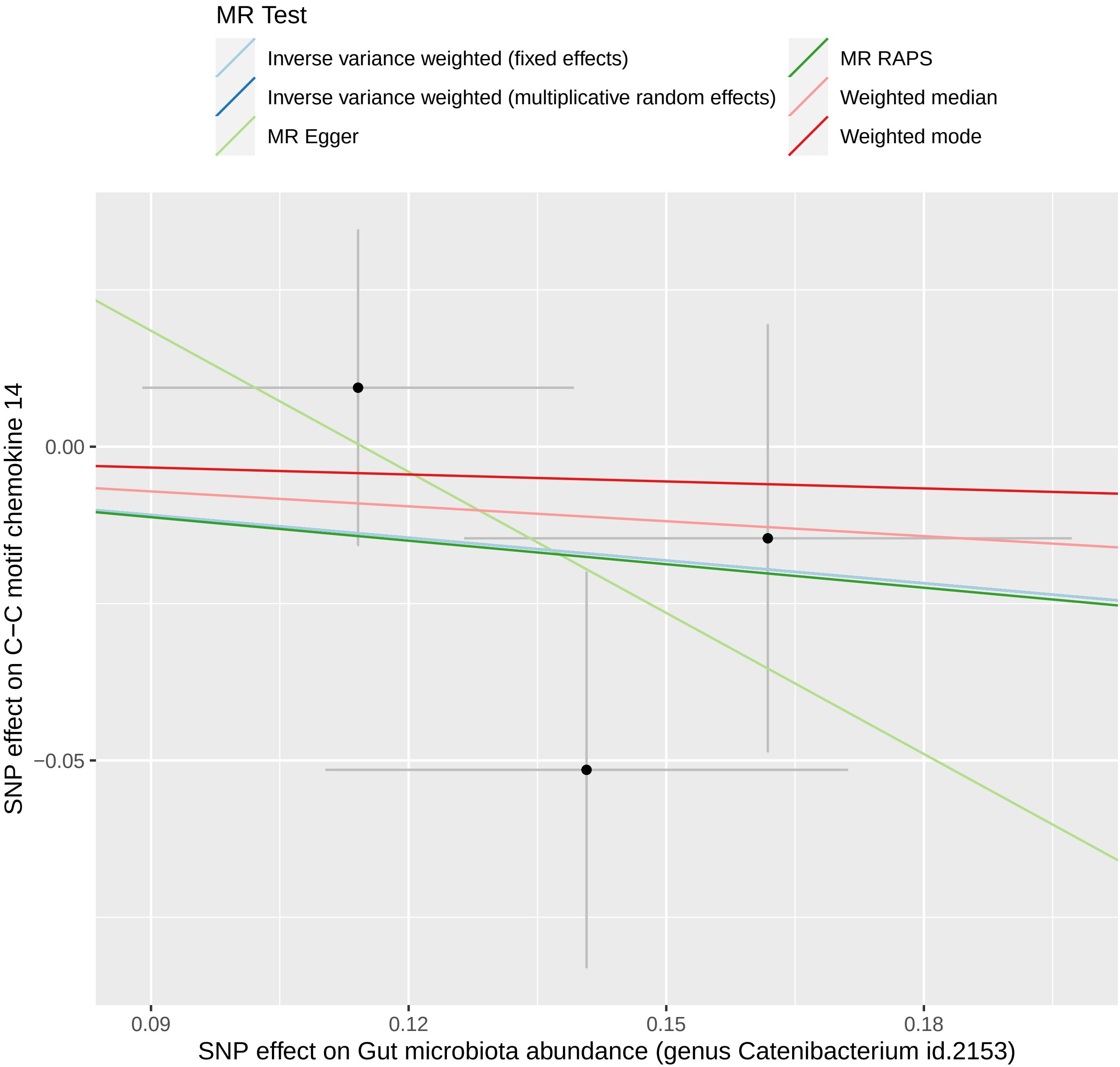

Batch 2−780 : Gut microbiota abundance (genus Parasutterella id.2892) on C−C motif chemokine 14
with a threshold of 1e−05

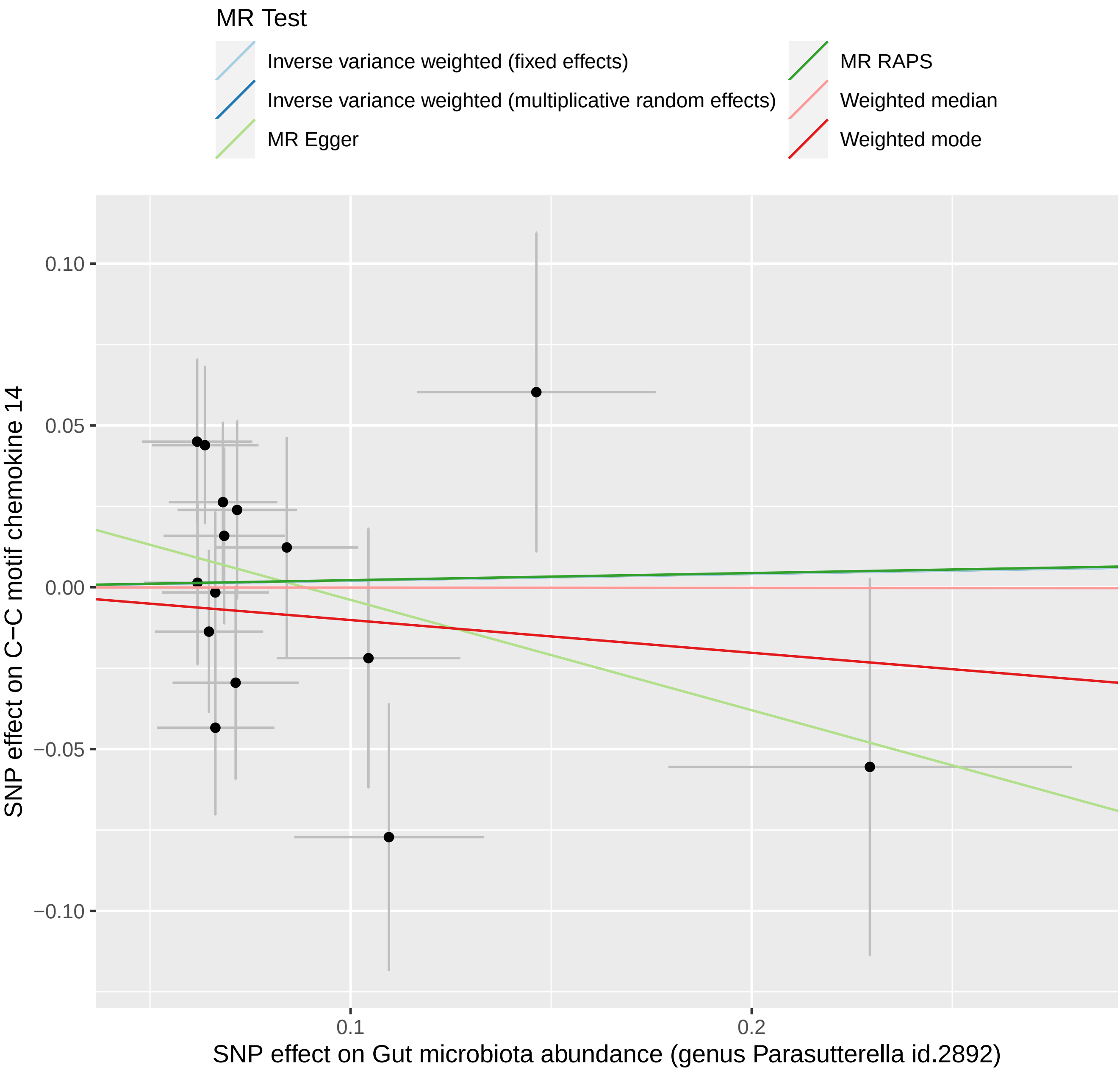

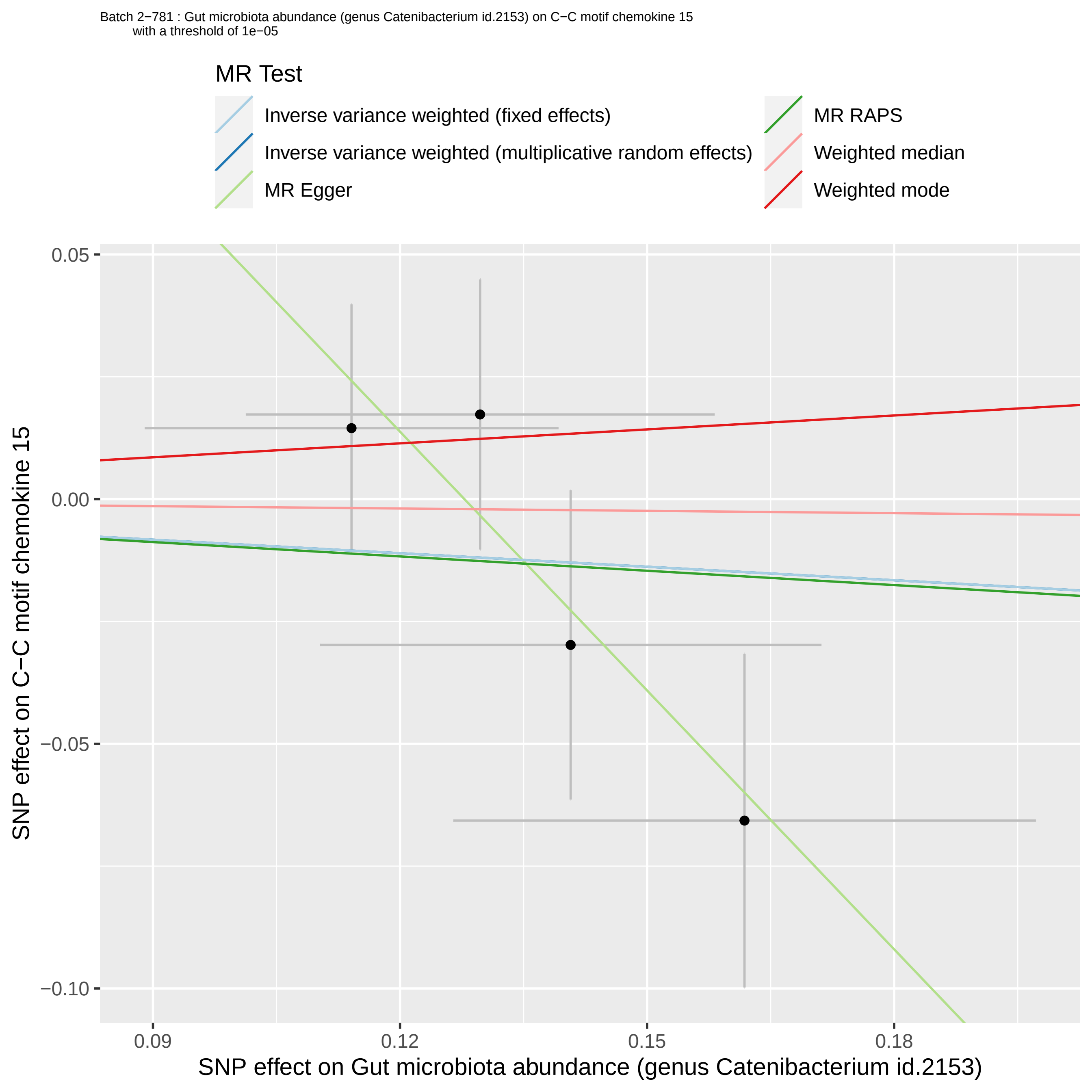
Batch 2−781 : Gut microbiota abundance (genus Catenibacterium id.2153) on C−C motif chemokine 15
with a threshold of 1e−05
MR Test
Inverse variance weighted (fixed effects)
Inverse variance weighted (multiplicative random effects)
MR Egger
MR RAPS
Weighted median
Weighted mode
0.05
0.00
−0.05
−0.10
0.09
0.12
0.15
0.18
SNP effect on C−C motif chemokine 15
SNP effect on Gut microbiota abundance (genus Catenibacterium id.2153)

Batch 2−782 : Gut microbiota abundance (genus Parasutterella id.2892) on C−C motif chemokine 15
with a threshold of 1e−05

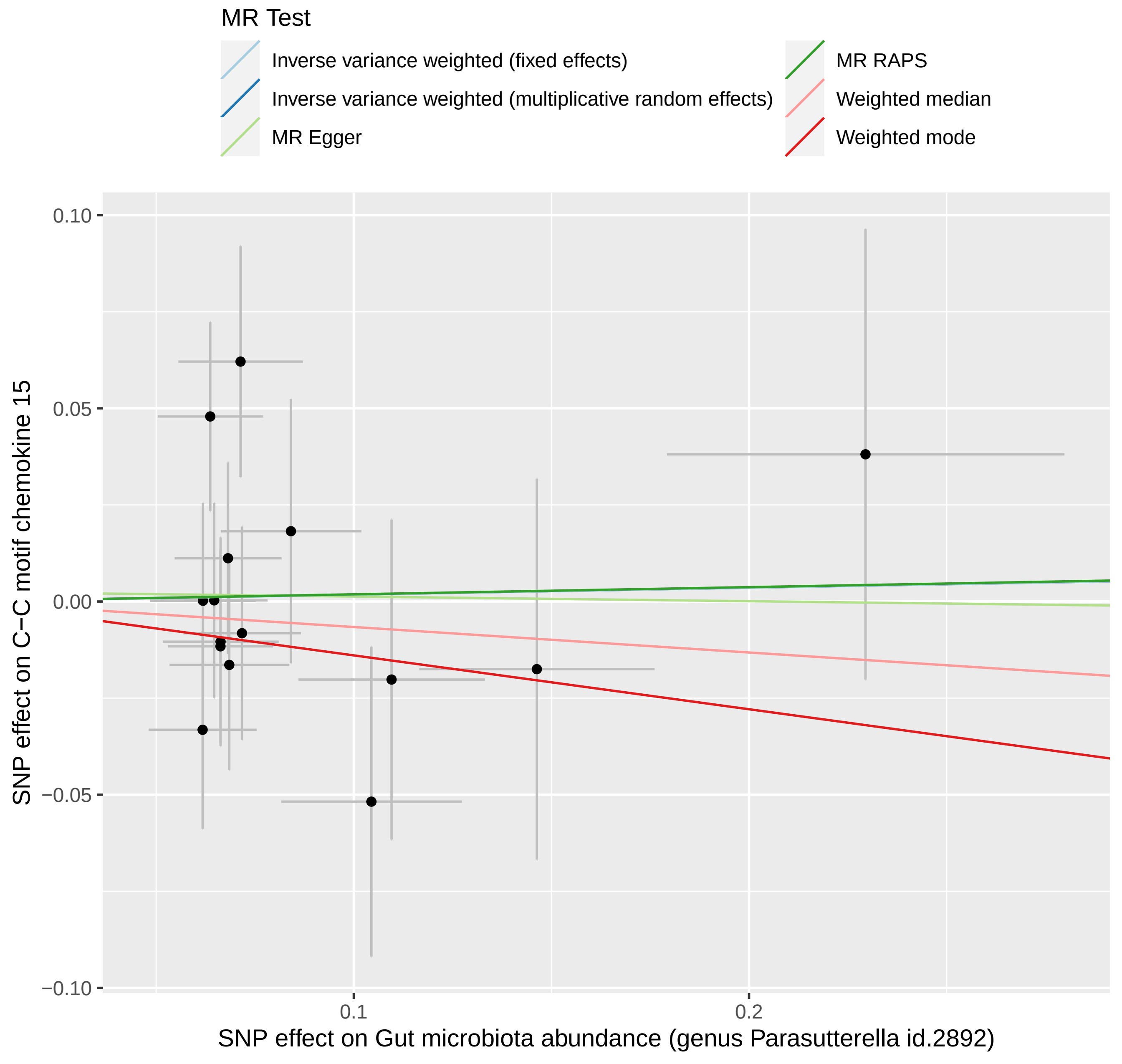

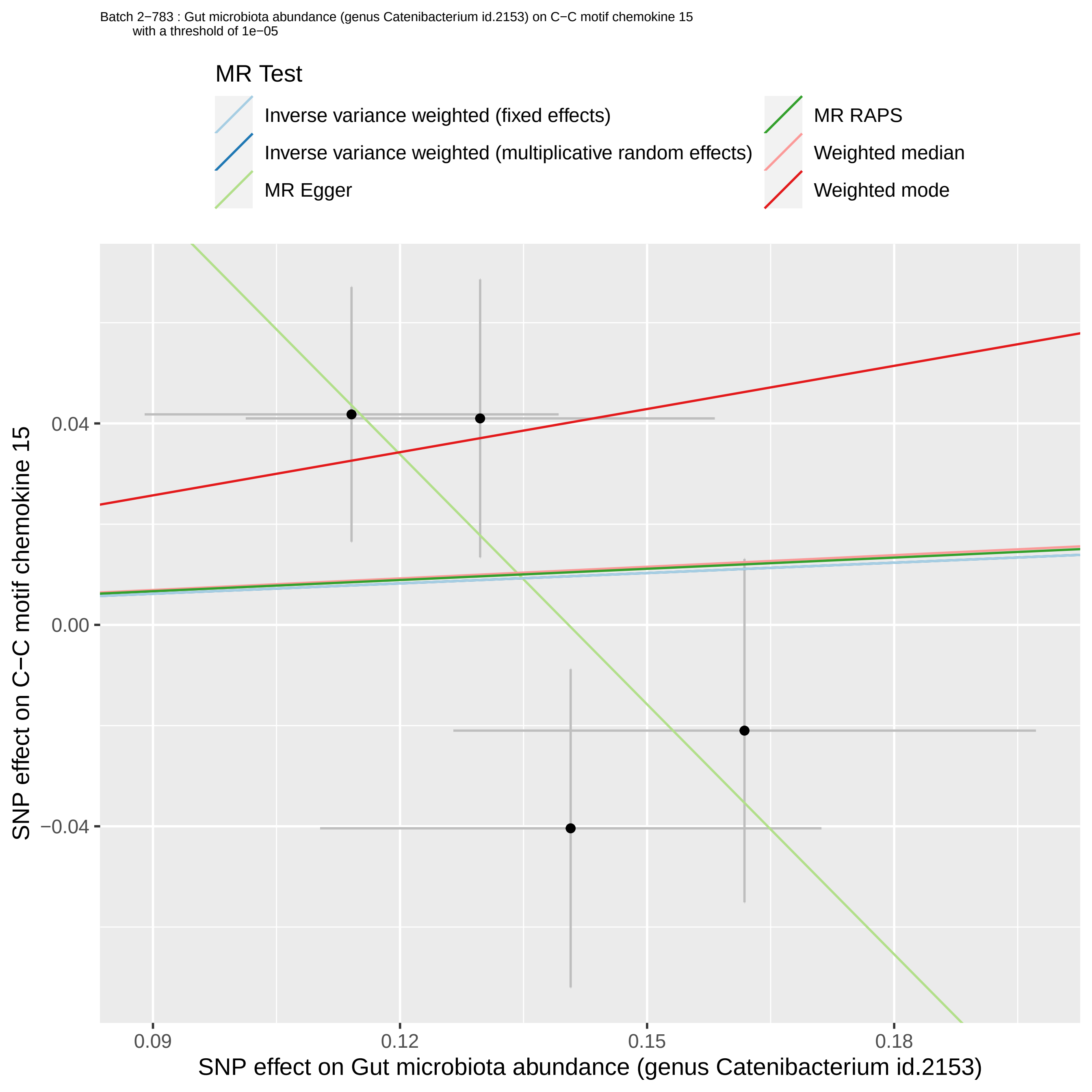

Batch 2−783 : Gut microbiota abundance (genus Catenibacterium id.2153) on C−C motif chemokine 15
with a threshold of 1e−05
MR Test
Inverse variance weighted (fixed effects)
Inverse variance weighted (multiplicative random effects)
MR Egger
MR RAPS
Weighted median
Weighted mode
SNP effect on C−C motif chemokine 15
0.04
0.00
−0.04
0.09
0.12
0.15
0.18
SNP effect on Gut microbiota abundance (genus Catenibacterium id.2153)

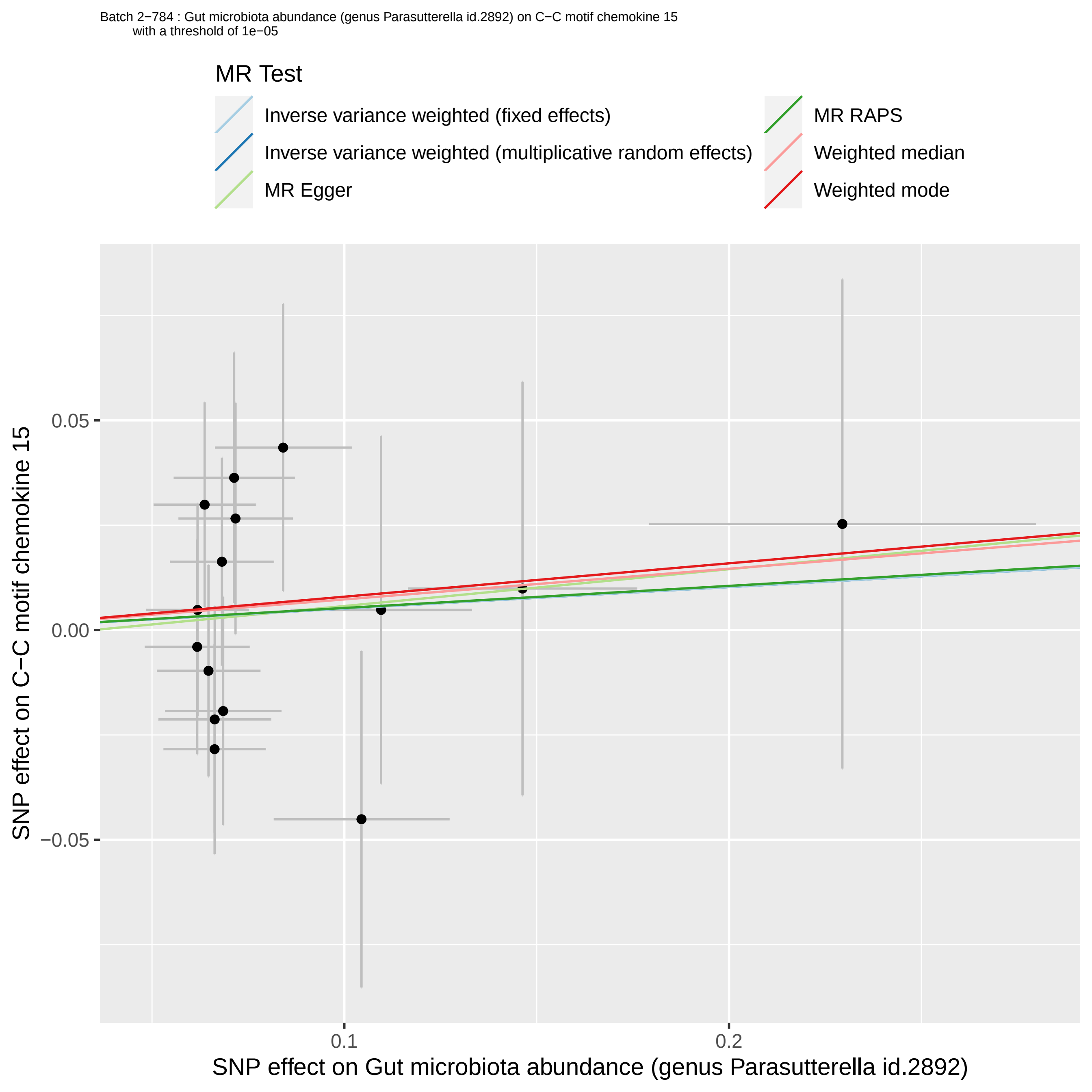

Batch 2−784 : Gut microbiota abundance (genus Parasutterella id.2892) on C−C motif chemokine 15
with a threshold of 1e−05
MR Test
Inverse variance weighted (fixed effects)
Inverse variance weighted (multiplicative random effects)
MR Egger
MR RAPS
Weighted median
Weighted mode
0.05
0.00
−0.05
0.1
0.2
SNP effect on C−C motif chemokine 15
SNP effect on Gut microbiota abundance (genus Parasutterella id.2892)

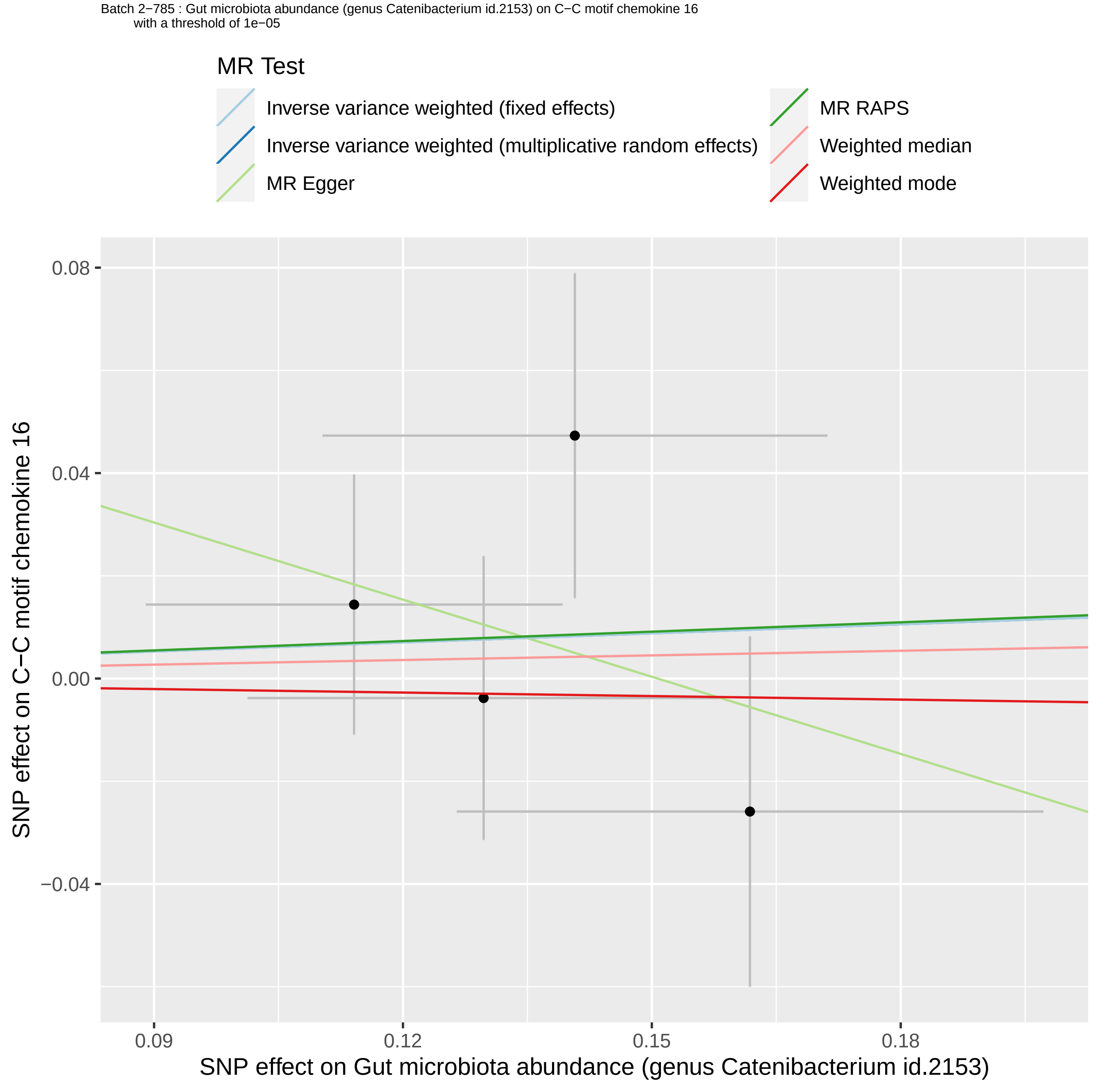

Batch 2−785 : Gut microbiota abundance (genus Catenibacterium id.2153) on C−C motif chemokine 16
with a threshold of 1e−05
MR Test
Inverse variance weighted (fixed effects)
Inverse variance weighted (multiplicative random effects)
MR Egger
MR RAPS
Weighted median
Weighted mode
0.08
0.04
0.00
−0.04
0.09
0.12
0.15
0.18
SNP effect on C−C motif chemokine 16
SNP effect on Gut microbiota abundance (genus Catenibacterium id.2153)

Batch 2−786 : Gut microbiota abundance (genus Parasutterella id.2892) on C−C motif chemokine 16
with a threshold of 1e−05

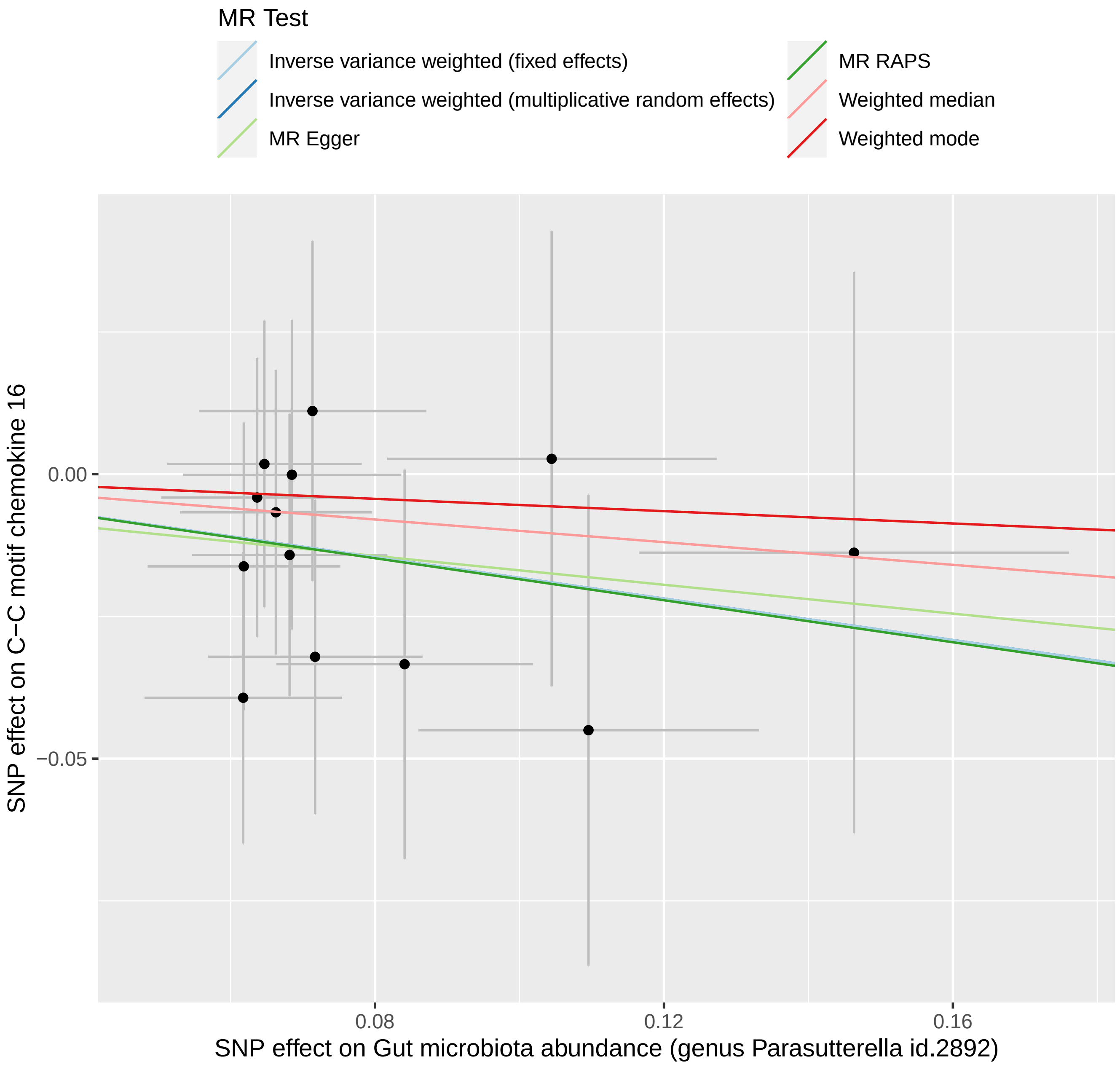

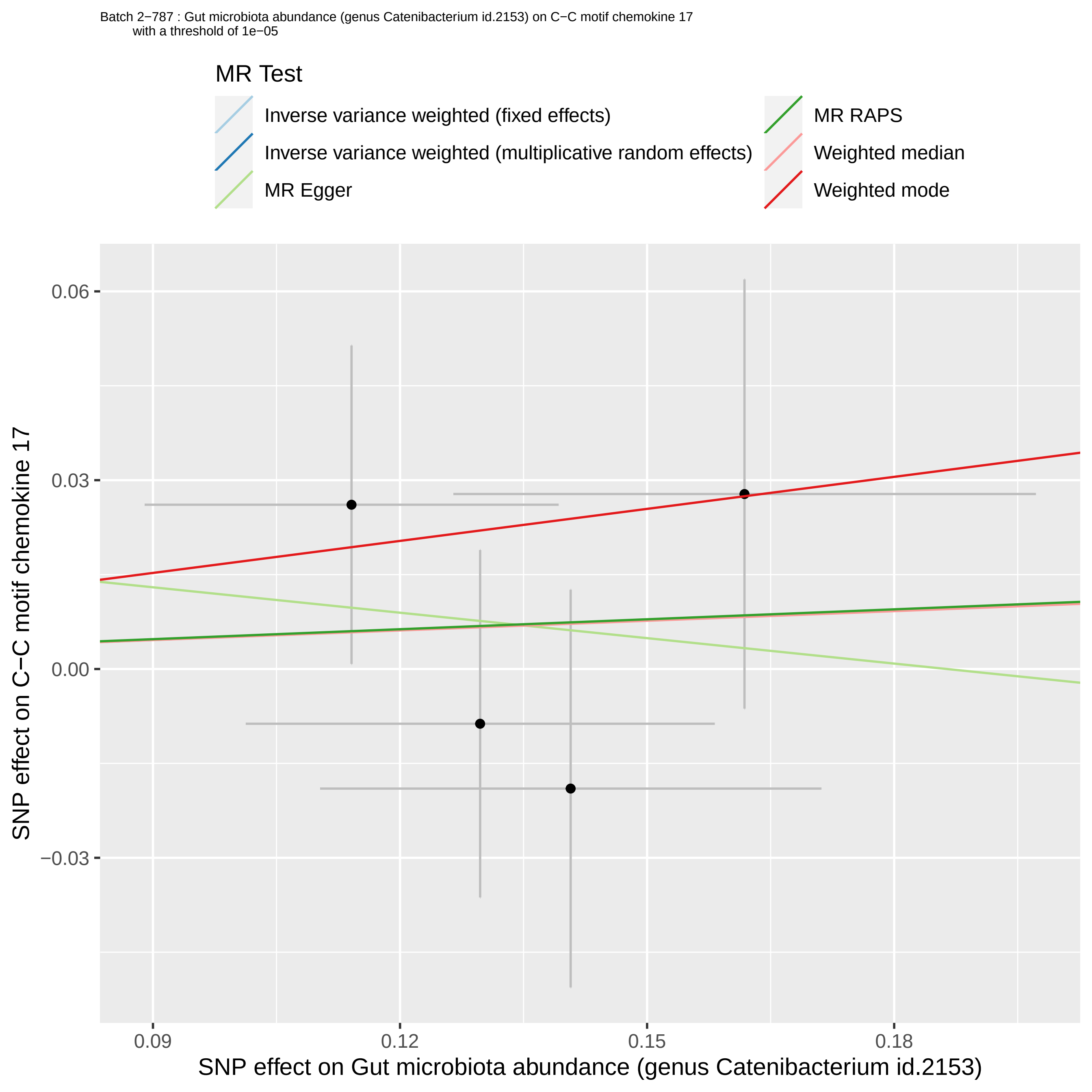
Batch 2−787 : Gut microbiota abundance (genus Catenibacterium id.2153) on C−C motif chemokine 17
with a threshold of 1e−05
MR Test
Inverse variance weighted (fixed effects)
Inverse variance weighted (multiplicative random effects)
MR Egger
MR RAPS
Weighted median
Weighted mode
0.06
0.03
0.00
−0.03
0.09
0.12
0.15
0.18
SNP effect on C−C motif chemokine 17
SNP effect on Gut microbiota abundance (genus Catenibacterium id.2153)

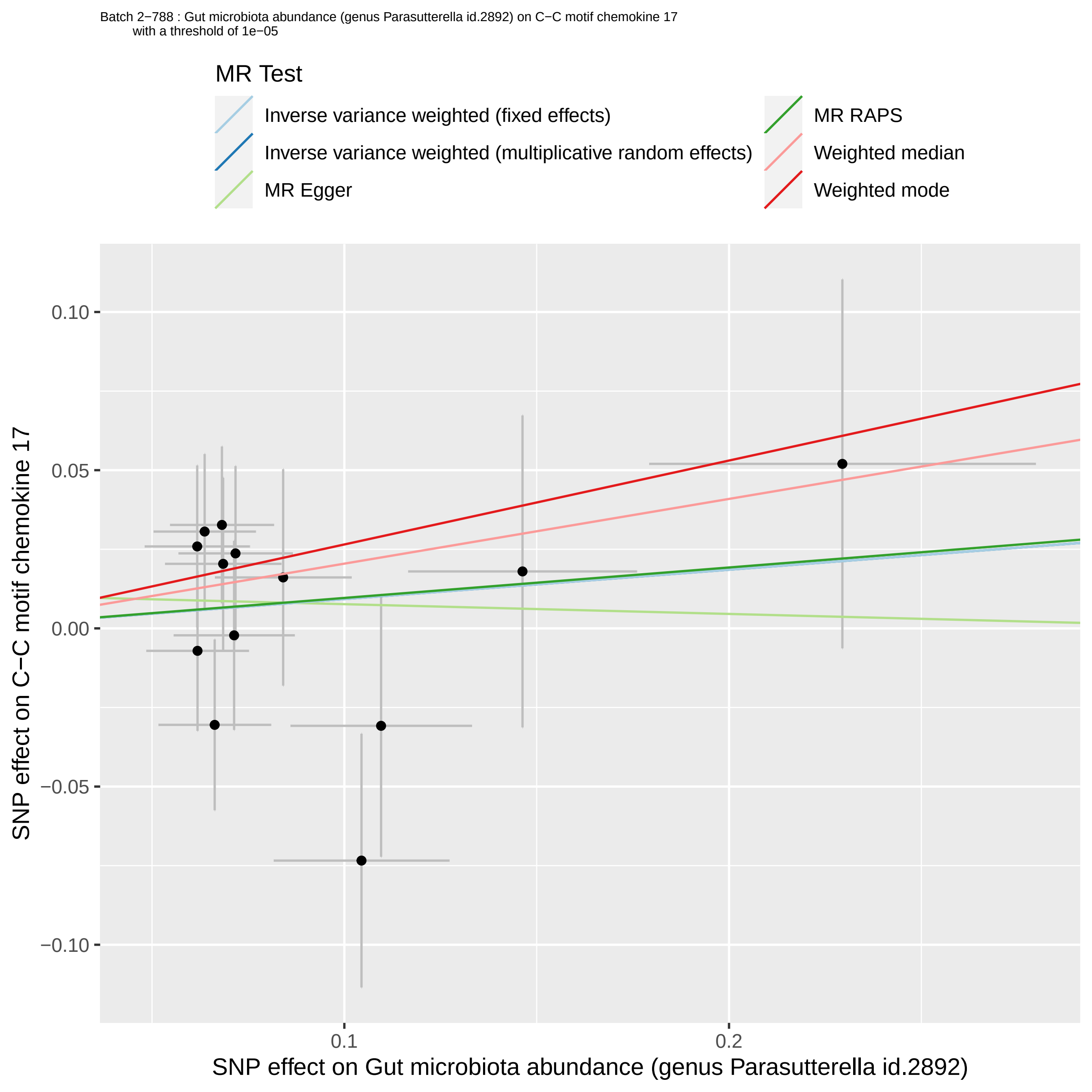

Batch 2−788 : Gut microbiota abundance (genus Parasutterella id.2892) on C−C motif chemokine 17
with a threshold of 1e−05
MR Test
Inverse variance weighted (fixed effects)
Inverse variance weighted (multiplicative random effects)
MR Egger
MR RAPS
Weighted median
Weighted mode
SNP effect on C−C motif chemokine 17
0.10
0.05
0.00
−0.05
−0.10
0.1
0.2
SNP effect on Gut microbiota abundance (genus Parasutterella id.2892)

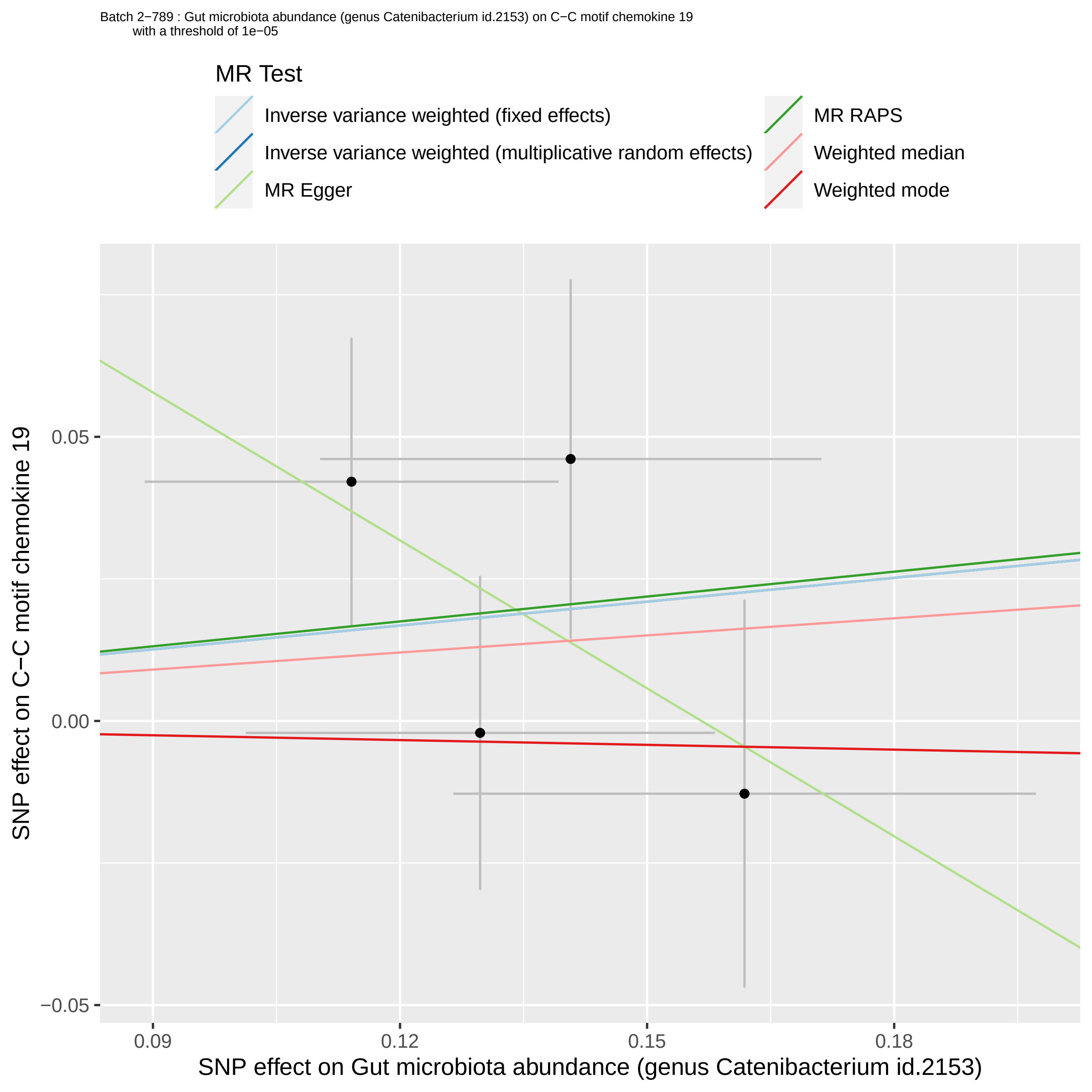
Batch 2−789 : Gut microbiota abundance (genus Catenibacterium id.2153) on C−C motif chemokine 19
with a threshold of 1e−05
MR Test
Inverse variance weighted (fixed effects)
Inverse variance weighted (multiplicative random effects)
MR Egger
MR RAPS
Weighted median
Weighted mode
0.05
0.00
−0.05
0.09
0.12
0.15
0.18
SNP effect on C−C motif chemokine 19
SNP effect on Gut microbiota abundance (genus Catenibacterium id.2153)

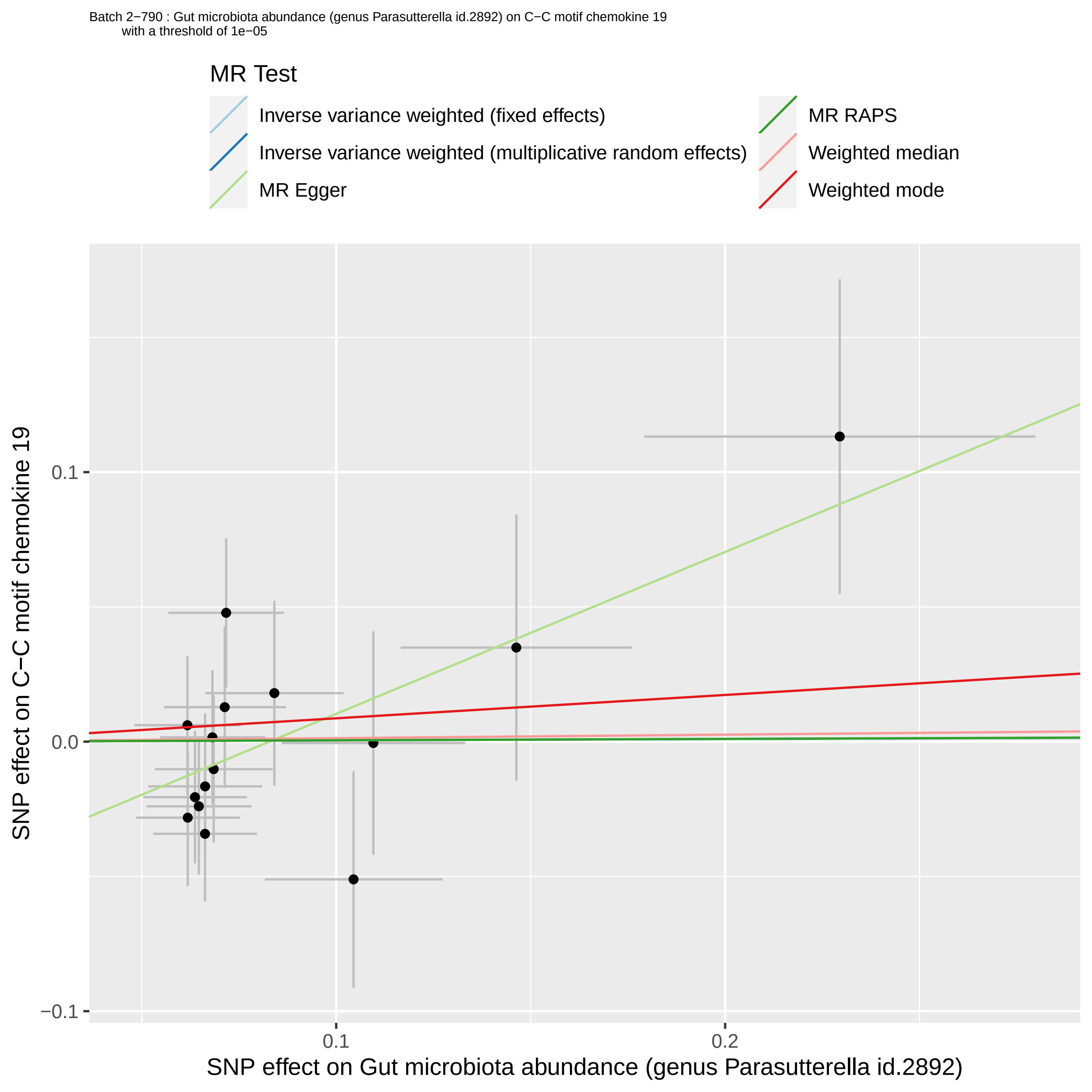
Batch 2−790 : Gut microbiota abundance (genus Parasutterella id.2892) on C−C motif chemokine 19
with a threshold of 1e−05
MR Test
Inverse variance weighted (fixed effects)
Inverse variance weighted (multiplicative random effects)
MR Egger
MR RAPS
Weighted median
Weighted mode
0.1
0.0
−0.1
0.1
0.2
SNP effect on C−C motif chemokine 19
SNP effect on Gut microbiota abundance (genus Parasutterella id.2892)

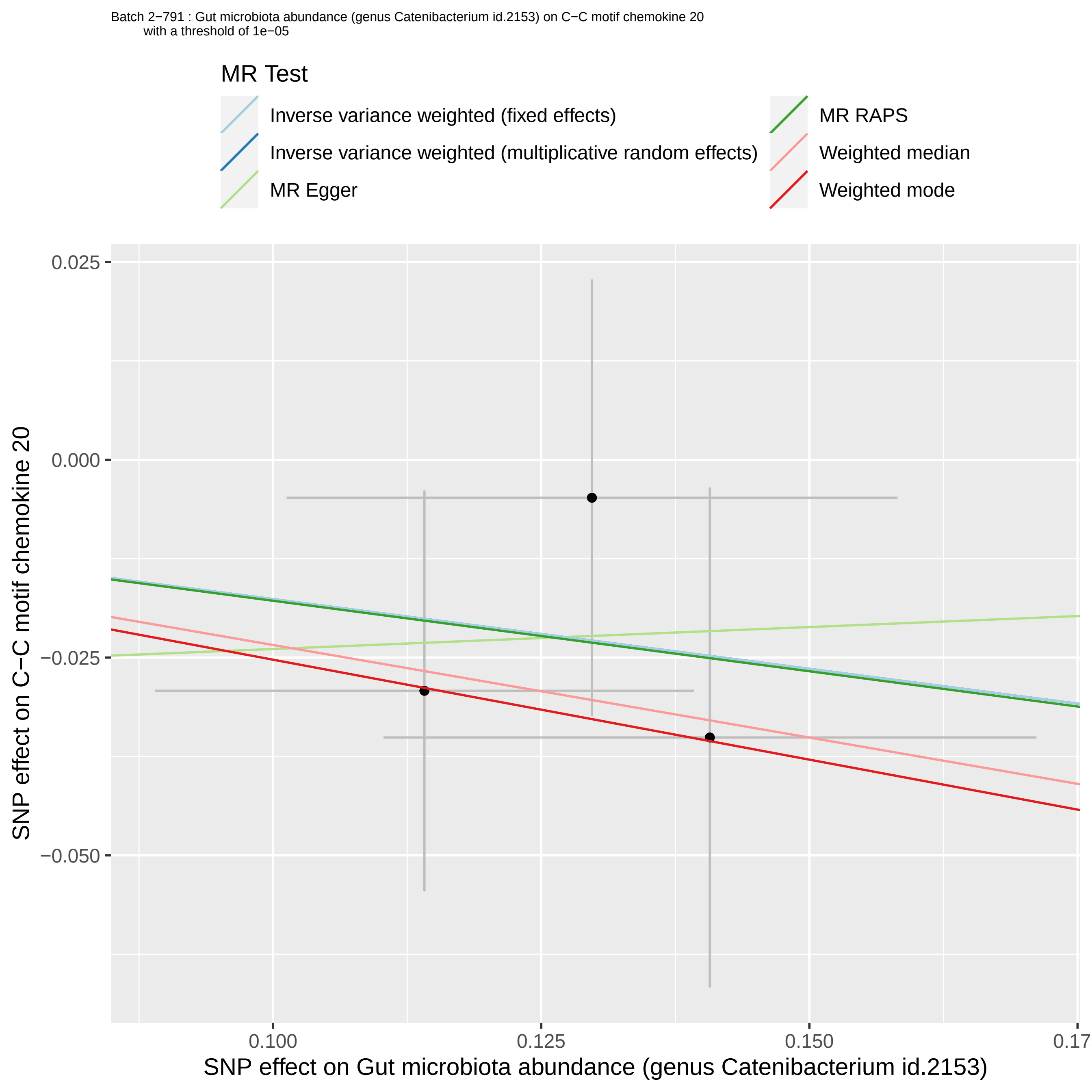

Batch 2−791 : Gut microbiota abundance (genus Catenibacterium id.2153) on C−C motif chemokine 20
with a threshold of 1e−05
MR Test
Inverse variance weighted (fixed effects)
Inverse variance weighted (multiplicative random effects)
MR Egger
MR RAPS
Weighted median
Weighted mode
0.025
0.000
−0.025
−0.050
0.100
0.125
0.150
0.17
SNP effect on C−C motif chemokine 20
SNP effect on Gut microbiota abundance (genus Catenibacterium id.2153)

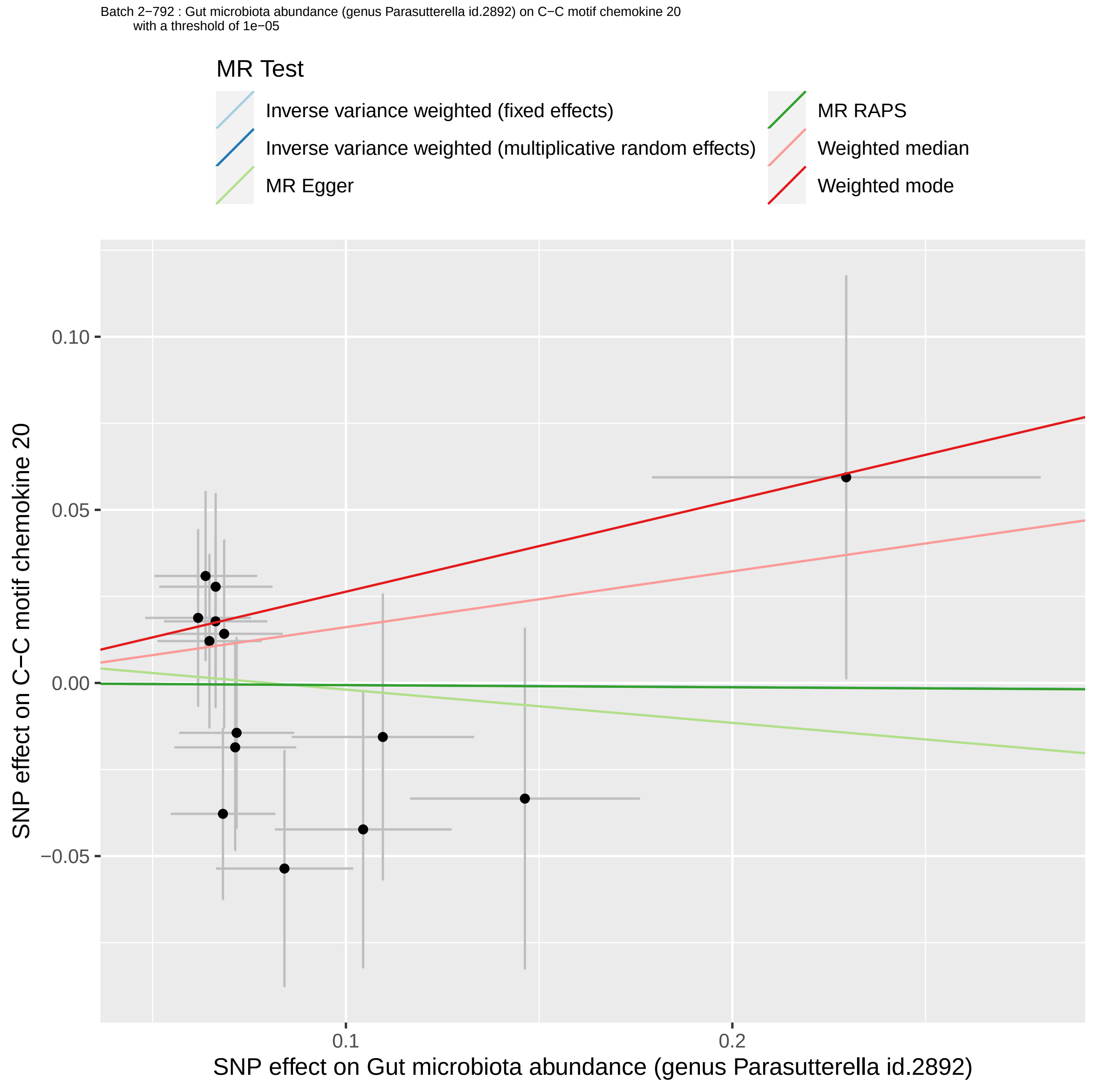

Batch 2−792 : Gut microbiota abundance (genus Parasutterella id.2892) on C−C motif chemokine 20
with a threshold of 1e−05
MR Test
Inverse variance weighted (fixed effects)
Inverse variance weighted (multiplicative random effects)
MR Egger
MR RAPS
Weighted median
Weighted mode
0.10
0.05
0.00
−0.05
0.1
0.2
SNP effect on C−C motif chemokine 20
SNP effect on Gut microbiota abundance (genus Parasutterella id.2892)

Batch 2−793 : Gut microbiota abundance (genus Catenibacterium id.2153) on C−C motif chemokine 21
with a threshold of 1e−05

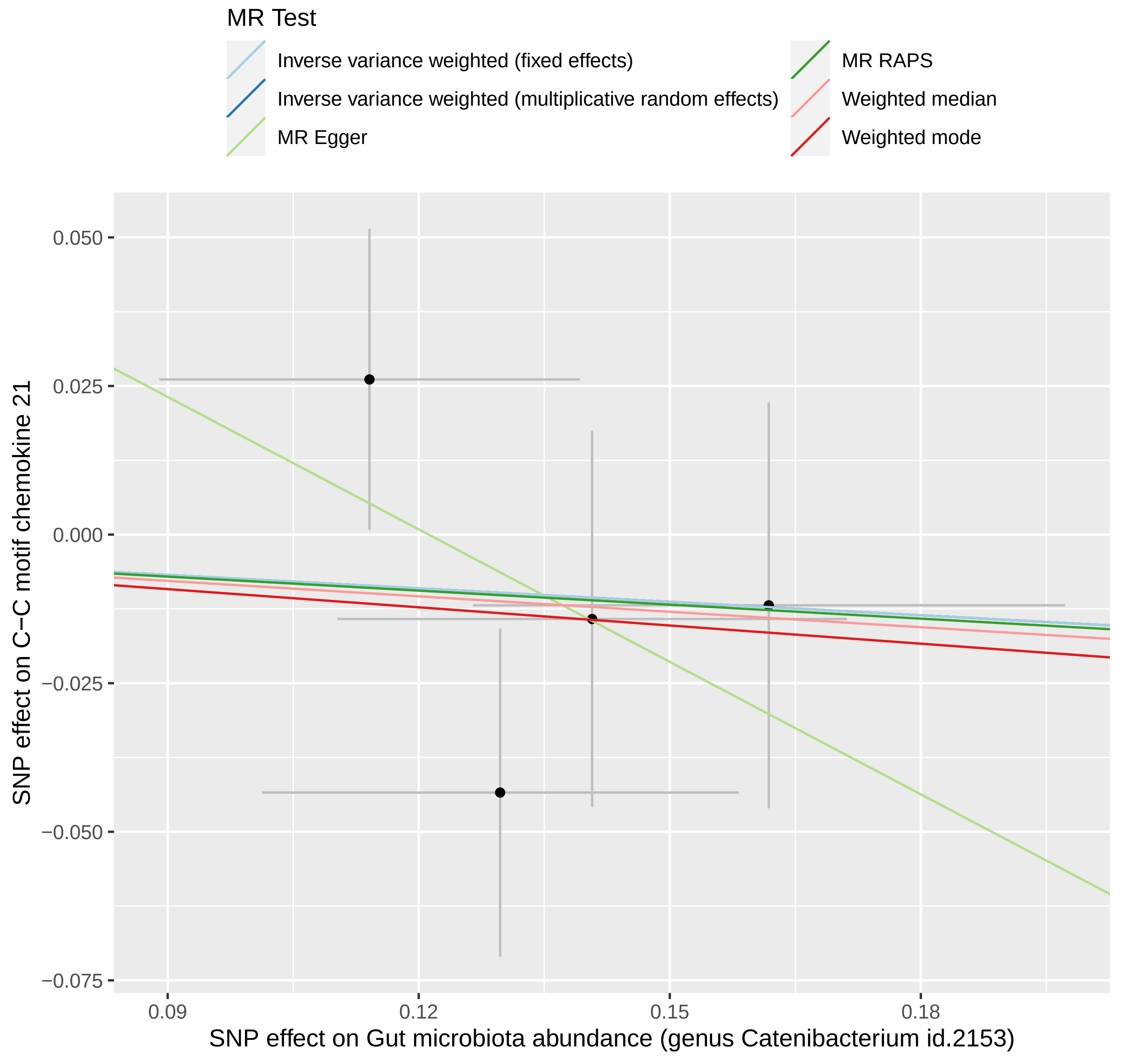

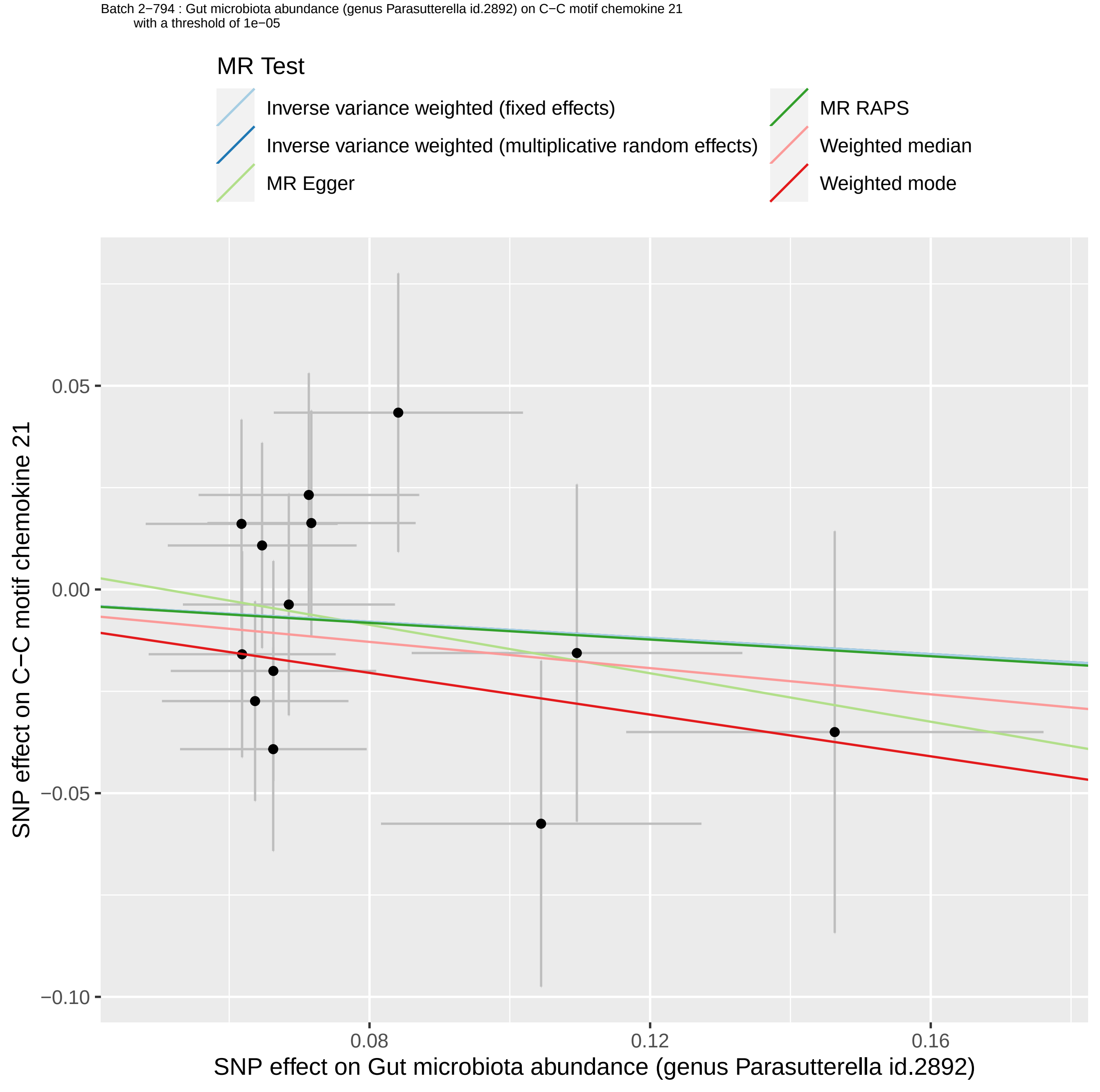

Batch 2−794 : Gut microbiota abundance (genus Parasutterella id.2892) on C−C motif chemokine 21
with a threshold of 1e−05
MR Test
Inverse variance weighted (fixed effects)
Inverse variance weighted (multiplicative random effects)
MR Egger
MR RAPS
Weighted median
Weighted mode
0.05
0.00
−0.05
−0.10
0.08
0.12
0.16
SNP effect on C−C motif chemokine 21
SNP effect on Gut microbiota abundance (genus Parasutterella id.2892)

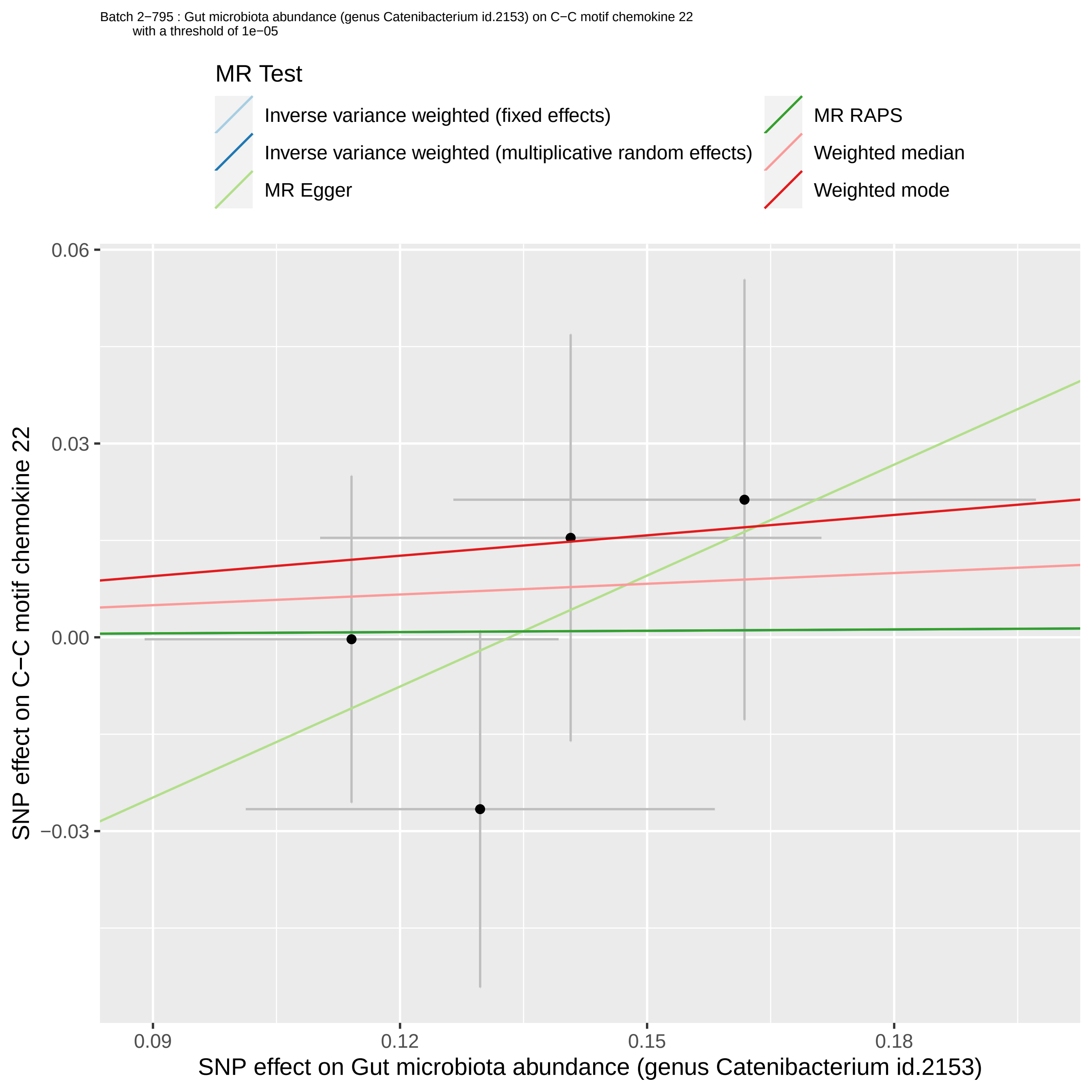

Batch 2−795 : Gut microbiota abundance (genus Catenibacterium id.2153) on C−C motif chemokine 22
with a threshold of 1e−05
MR Test
Inverse variance weighted (fixed effects)
Inverse variance weighted (multiplicative random effects)
MR Egger
MR RAPS
Weighted median
Weighted mode
0.06
0.03
0.00
−0.03
0.09
0.12
0.15
0.18
SNP effect on C−C motif chemokine 22
SNP effect on Gut microbiota abundance (genus Catenibacterium id.2153)

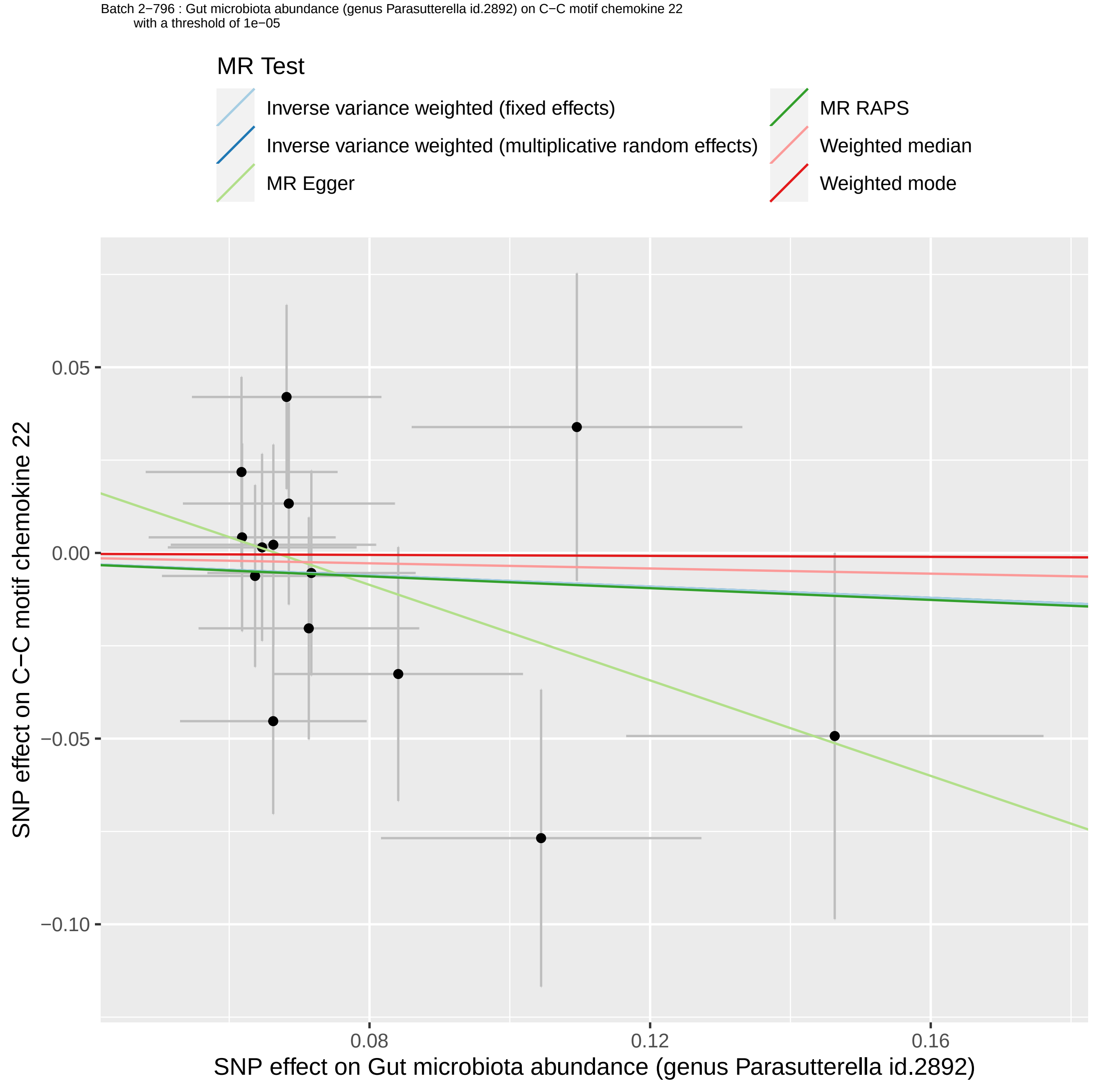

Batch 2−796 : Gut microbiota abundance (genus Parasutterella id.2892) on C−C motif chemokine 22
with a threshold of 1e−05
MR Test
Inverse variance weighted (fixed effects)
Inverse variance weighted (multiplicative random effects)
MR Egger
MR RAPS
Weighted median
Weighted mode
0.05
0.00
−0.05
−0.10
0.08
0.12
0.16
SNP effect on C−C motif chemokine 22
SNP effect on Gut microbiota abundance (genus Parasutterella id.2892)

Batch 2−797 : Gut microbiota abundance (genus Catenibacterium id.2153) on C−C motif chemokine 23
with a threshold of 1e−05

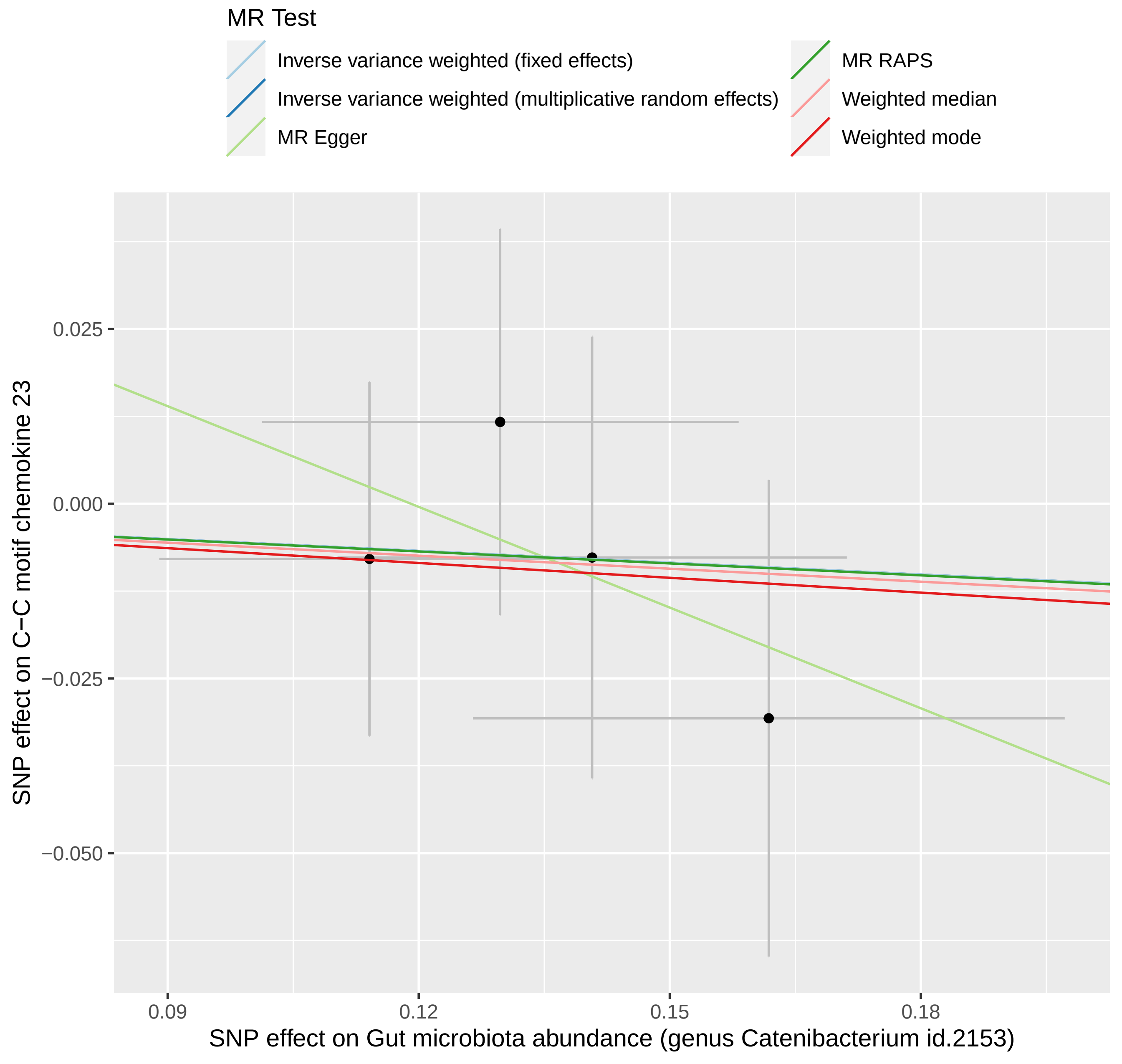

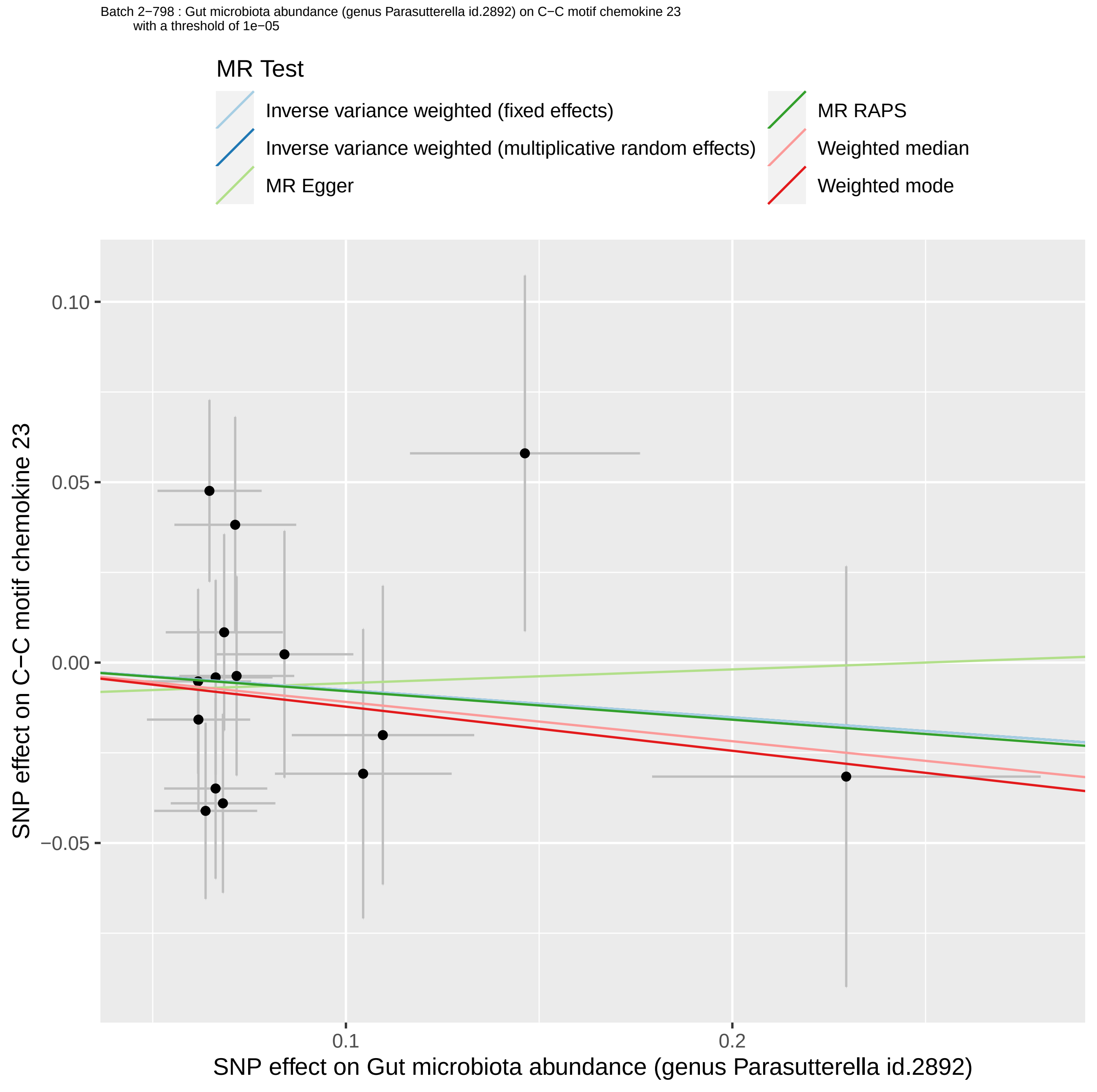
Batch 2−798 : Gut microbiota abundance (genus Parasutterella id.2892) on C−C motif chemokine 23
with a threshold of 1e−05
MR Test
Inverse variance weighted (fixed effects)
Inverse variance weighted (multiplicative random effects)
MR Egger
MR RAPS
Weighted median
Weighted mode
0.10
0.05
0.00
−0.05
0.1
0.2
SNP effect on C−C motif chemokine 23
SNP effect on Gut microbiota abundance (genus Parasutterella id.2892)

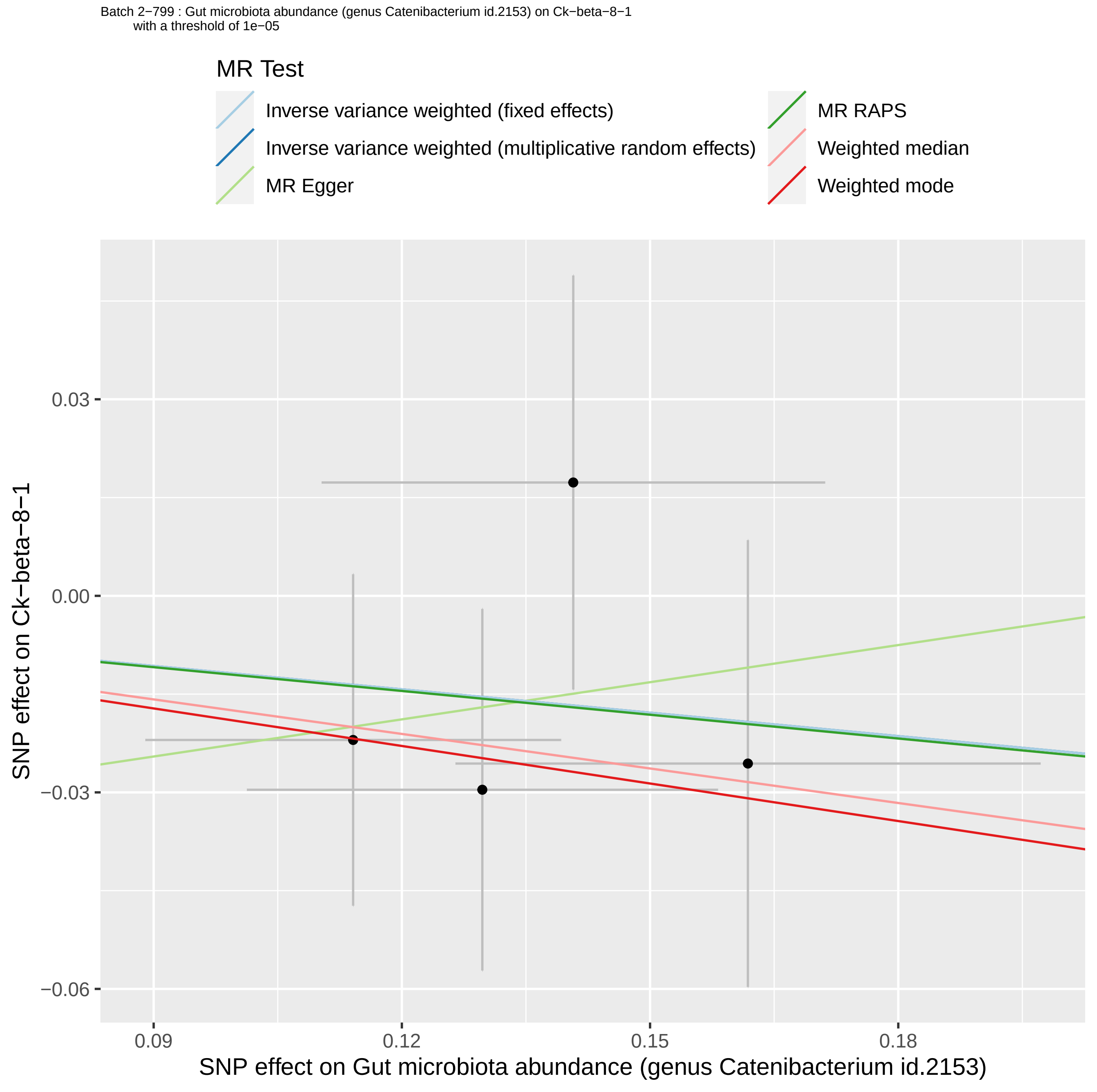
Batch 2−799 : Gut microbiota abundance (genus Catenibacterium id.2153) on Ck−beta−8−1
with a threshold of 1e−05
MR Test
Inverse variance weighted (fixed effects)
Inverse variance weighted (multiplicative random effects)
MR Egger
MR RAPS
Weighted median
Weighted mode
0.03
0.00
−0.03
−0.06
0.09
0.12
0.15
0.18
SNP effect on Ck−beta−8−1
SNP effect on Gut microbiota abundance (genus Catenibacterium id.2153)

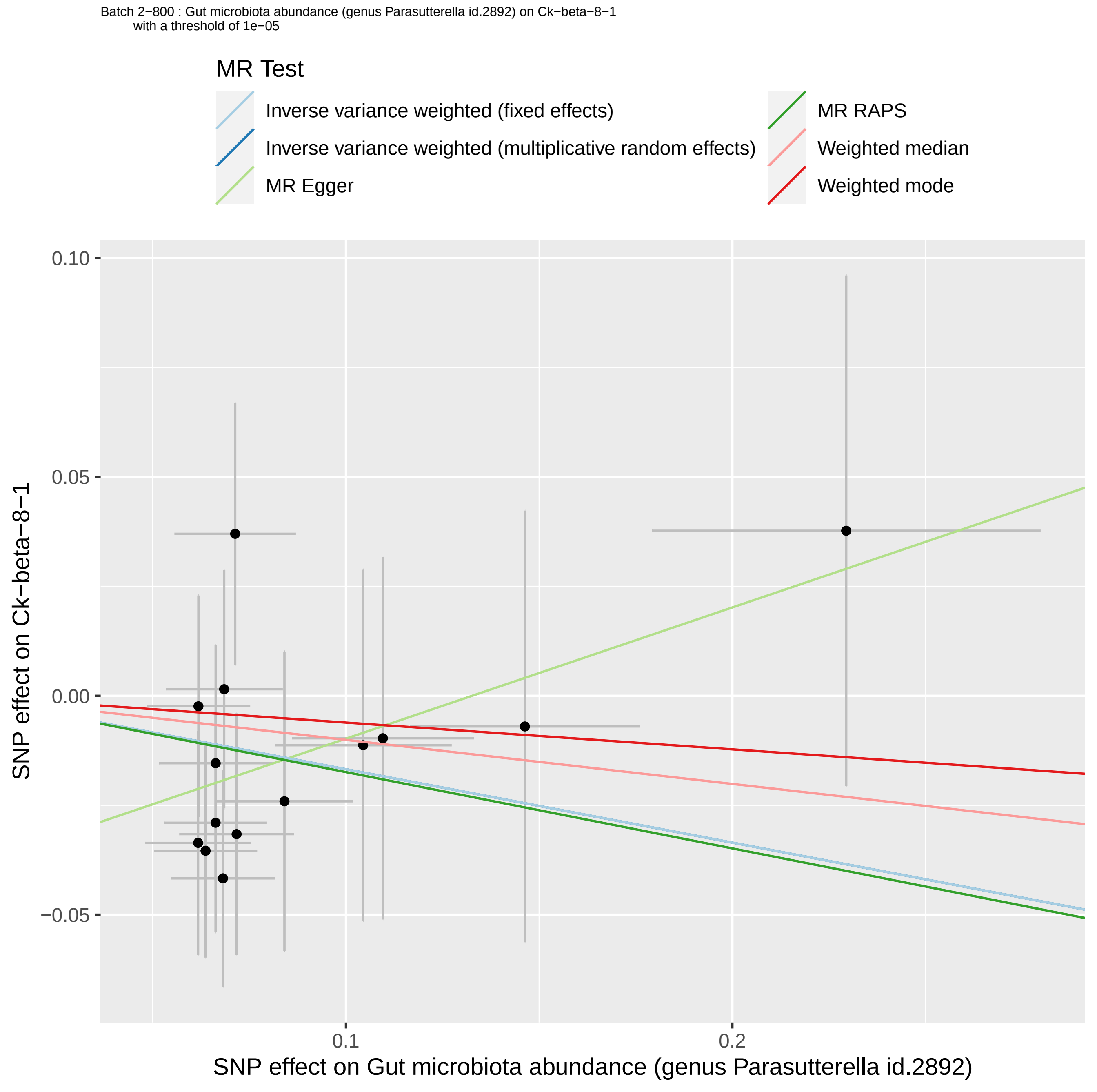
Batch 2−800 : Gut microbiota abundance (genus Parasutterella id.2892) on Ck−beta−8−1
with a threshold of 1e−05
MR Test
Inverse variance weighted (fixed effects)
Inverse variance weighted (multiplicative random effects)
MR Egger
MR RAPS
Weighted median
Weighted mode
0.10
0.05
0.00
−0.05
0.1
0.2
SNP effect on Ck−beta−8−1
SNP effect on Gut microbiota abundance (genus Parasutterella id.2892)

Batch 2−801 : Gut microbiota abundance (genus Catenibacterium id.2153) on C−C motif chemokine 24
with a threshold of 1e−05

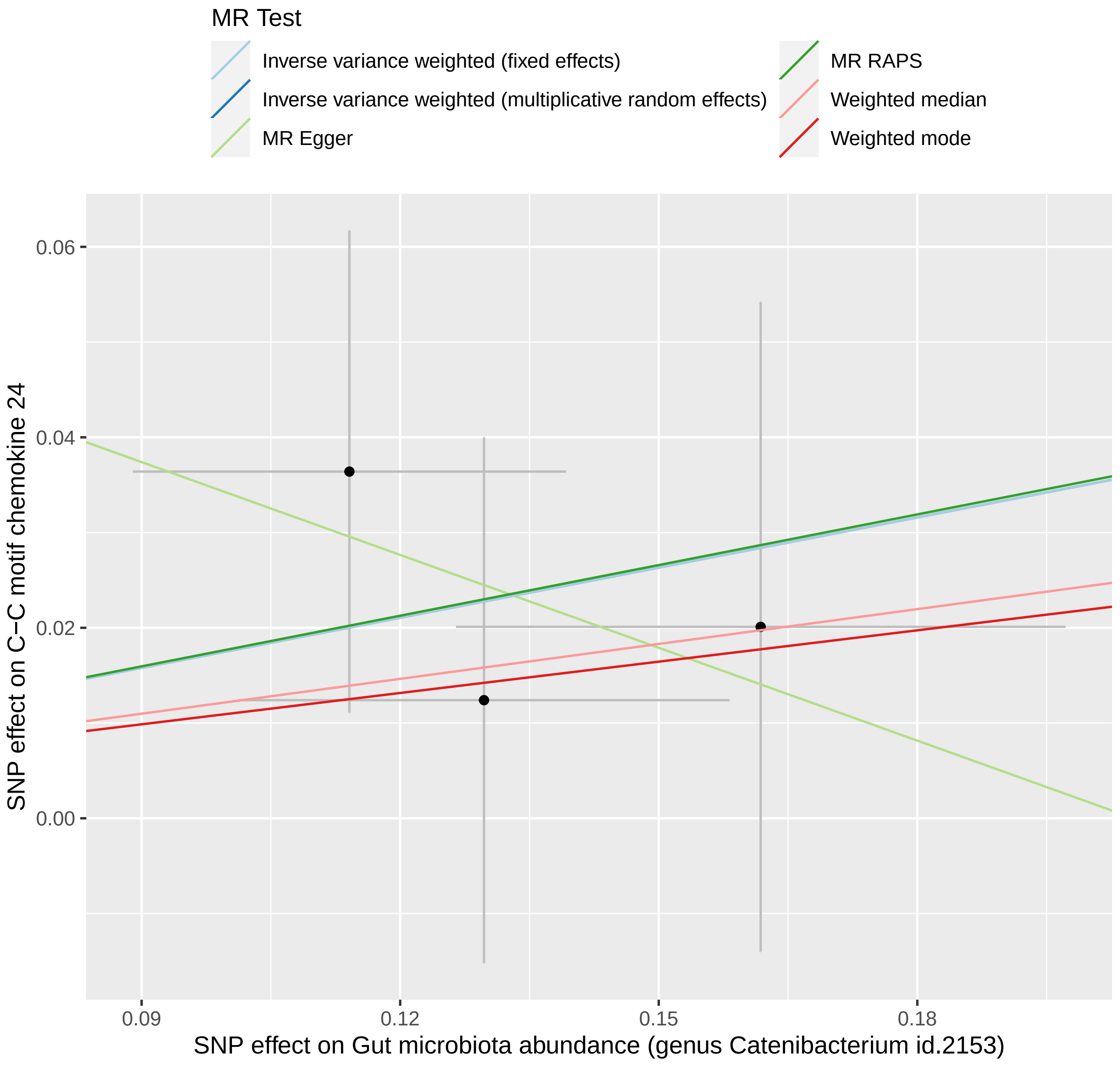

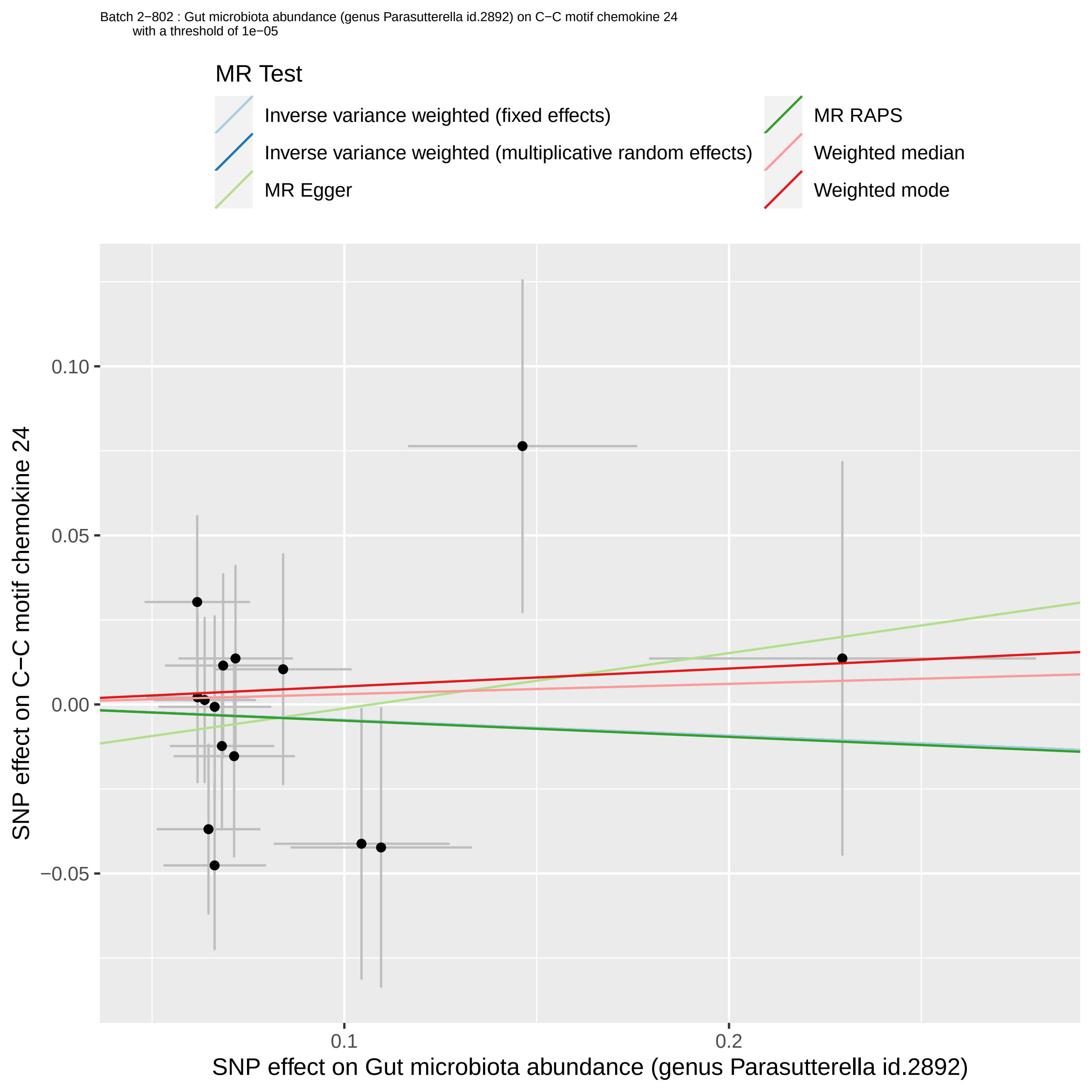
Batch 2−802 : Gut microbiota abundance (genus Parasutterella id.2892) on C−C motif chemokine 24
with a threshold of 1e−05
MR Test
Inverse variance weighted (fixed effects)
Inverse variance weighted (multiplicative random effects)
MR Egger
MR RAPS
Weighted median
Weighted mode
0.10
0.05
0.00
−0.05
0.1
0.2
SNP effect on C−C motif chemokine 24
SNP effect on Gut microbiota abundance (genus Parasutterella id.2892)

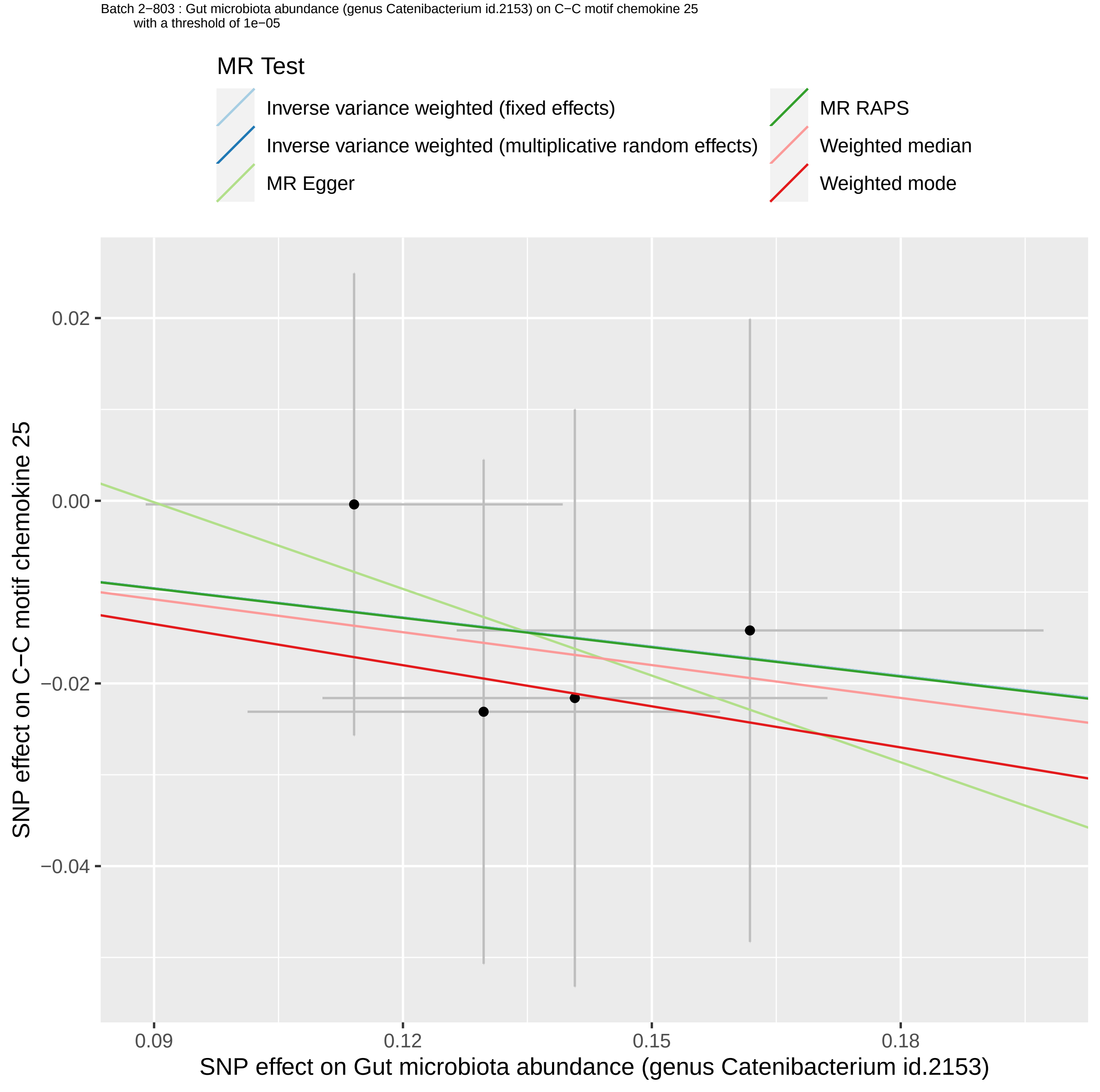

Batch 2−803 : Gut microbiota abundance (genus Catenibacterium id.2153) on C−C motif chemokine 25
with a threshold of 1e−05
MR Test
Inverse variance weighted (fixed effects)
Inverse variance weighted (multiplicative random effects)
MR Egger
MR RAPS
Weighted median
Weighted mode
0.02
0.00
−0.02
−0.04
0.09
0.12
0.15
0.18
SNP effect on C−C motif chemokine 25
SNP effect on Gut microbiota abundance (genus Catenibacterium id.2153)

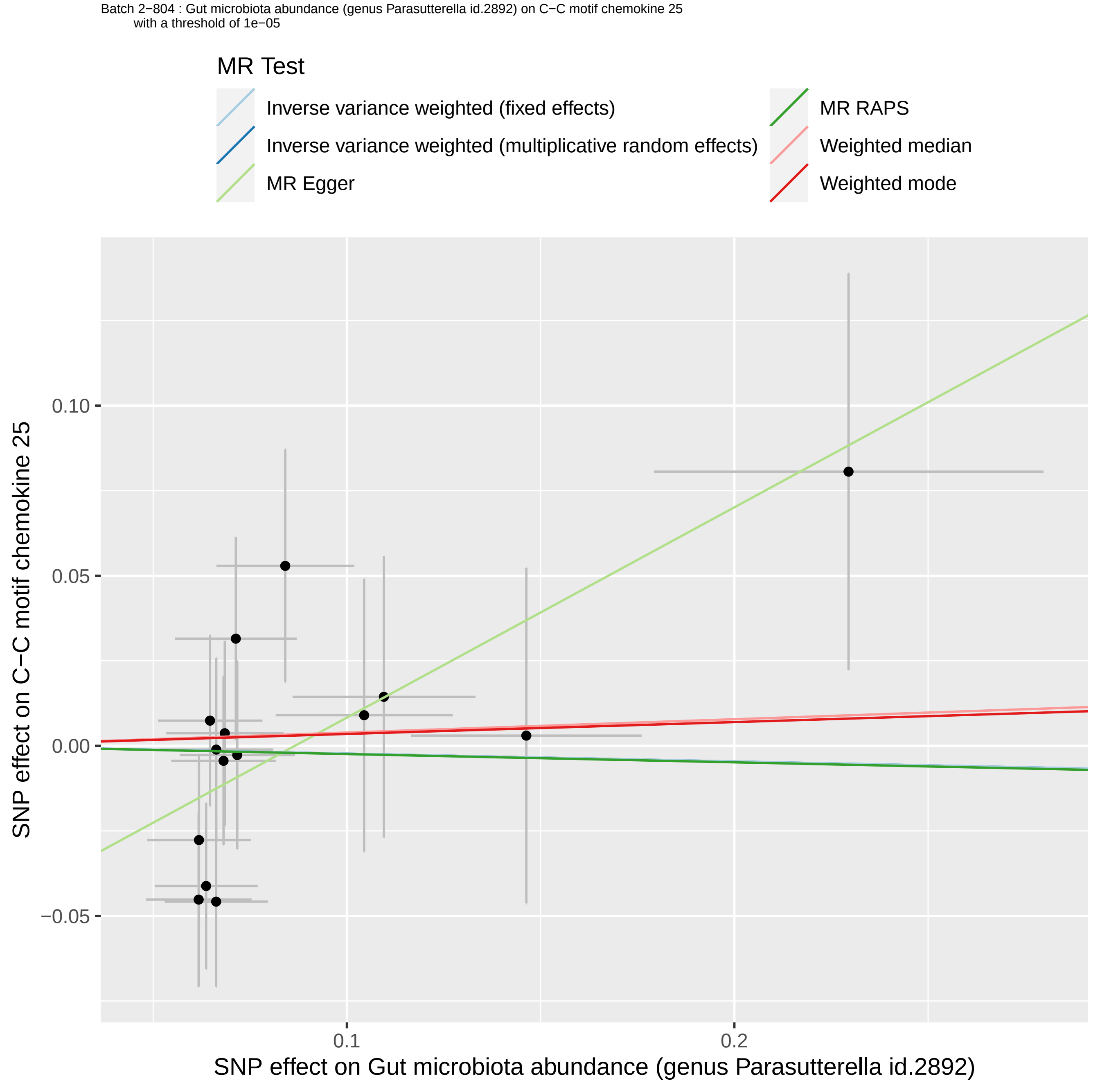

Batch 2−804 : Gut microbiota abundance (genus Parasutterella id.2892) on C−C motif chemokine 25
with a threshold of 1e−05
MR Test
Inverse variance weighted (fixed effects)
Inverse variance weighted (multiplicative random effects)
MR Egger
MR RAPS
Weighted median
Weighted mode
0.10
0.05
0.00
−0.05
0.1
0.2
SNP effect on C−C motif chemokine 25
SNP effect on Gut microbiota abundance (genus Parasutterella id.2892)

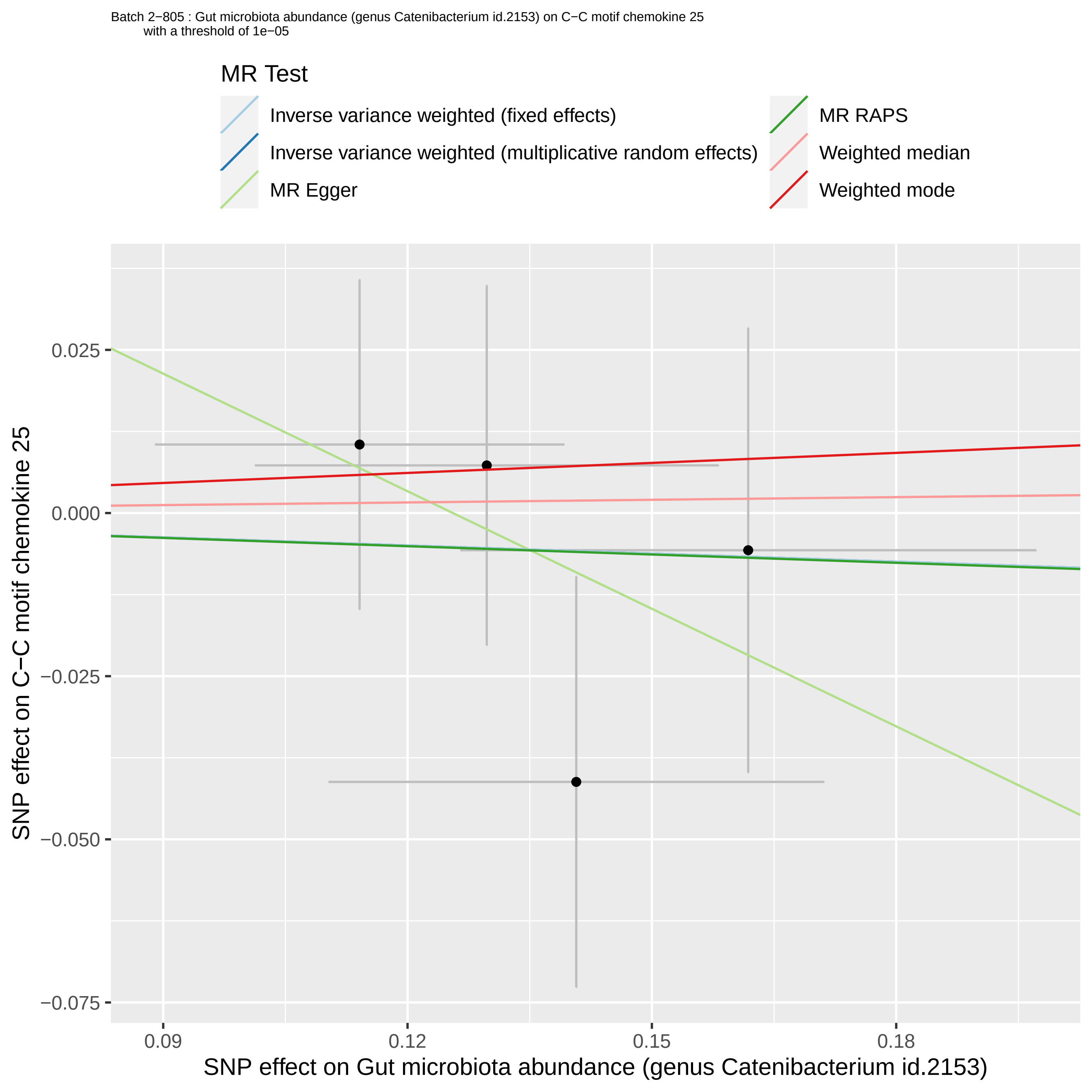

Batch 2−805 : Gut microbiota abundance (genus Catenibacterium id.2153) on C−C motif chemokine 25
with a threshold of 1e−05
MR Test
Inverse variance weighted (fixed effects)
Inverse variance weighted (multiplicative random effects)
MR Egger
MR RAPS
Weighted median
Weighted mode
0.025
0.000
−0.025
−0.050
−0.075
0.09
0.12
0.15
0.18
SNP effect on C−C motif chemokine 25
SNP effect on Gut microbiota abundance (genus Catenibacterium id.2153)

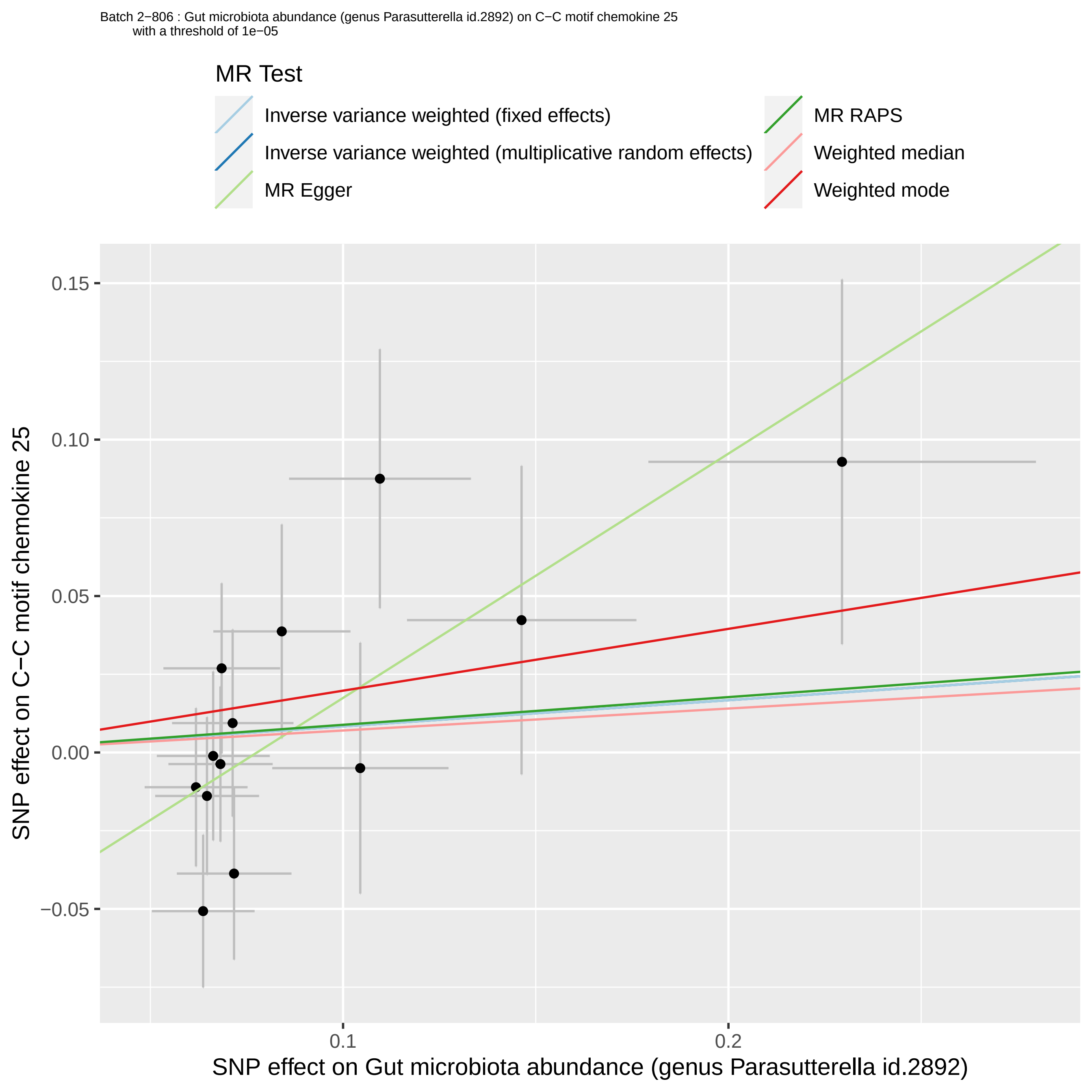
Batch 2−806 : Gut microbiota abundance (genus Parasutterella id.2892) on C−C motif chemokine 25
with a threshold of 1e−05
MR Test
Inverse variance weighted (fixed effects)
Inverse variance weighted (multiplicative random effects)
MR Egger
MR RAPS
Weighted median
Weighted mode
0.15
0.10
0.05
0.00
−0.05
0.1
0.2
SNP effect on C−C motif chemokine 25
SNP effect on Gut microbiota abundance (genus Parasutterella id.2892)

Batch 2−807 : Gut microbiota abundance (genus Catenibacterium id.2153) on C−C motif chemokine 26
with a threshold of 1e−05

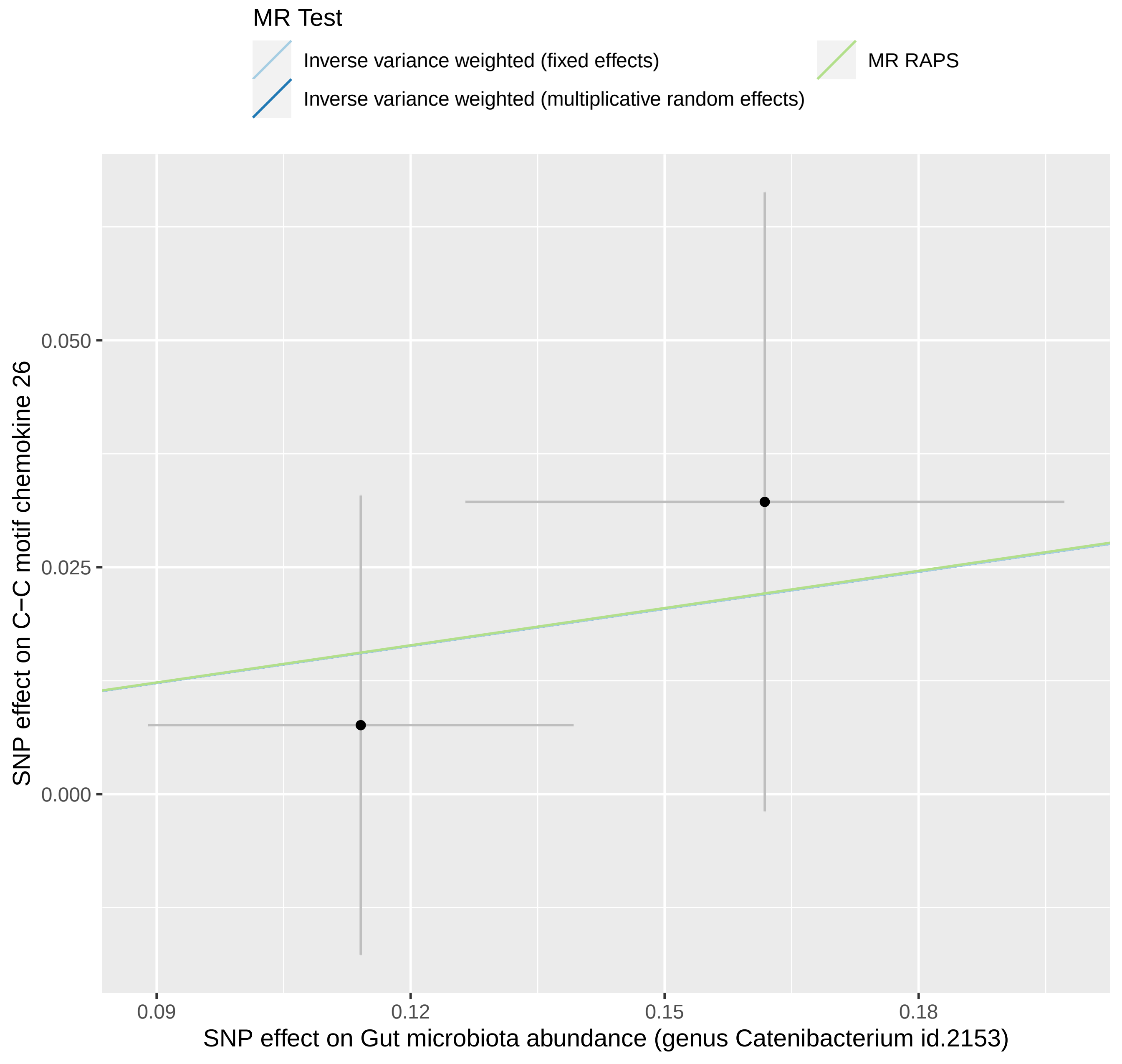

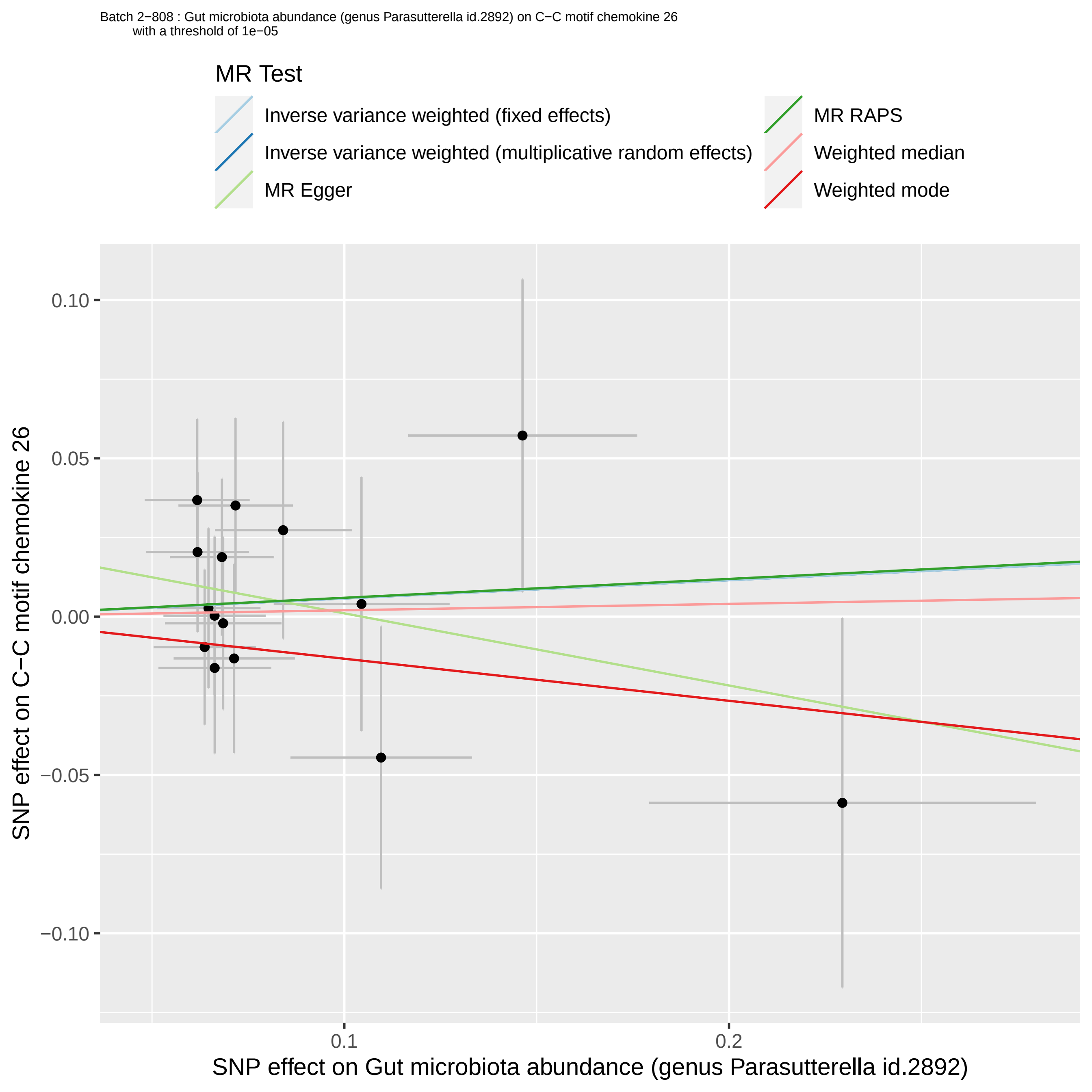

Batch 2−808 : Gut microbiota abundance (genus Parasutterella id.2892) on C−C motif chemokine 26
with a threshold of 1e−05
MR Test
Inverse variance weighted (fixed effects)
Inverse variance weighted (multiplicative random effects)
MR Egger
MR RAPS
Weighted median
Weighted mode
0.10
0.05
0.00
−0.05
−0.10
0.1
0.2
SNP effect on C−C motif chemokine 26
SNP effect on Gut microbiota abundance (genus Parasutterella id.2892)

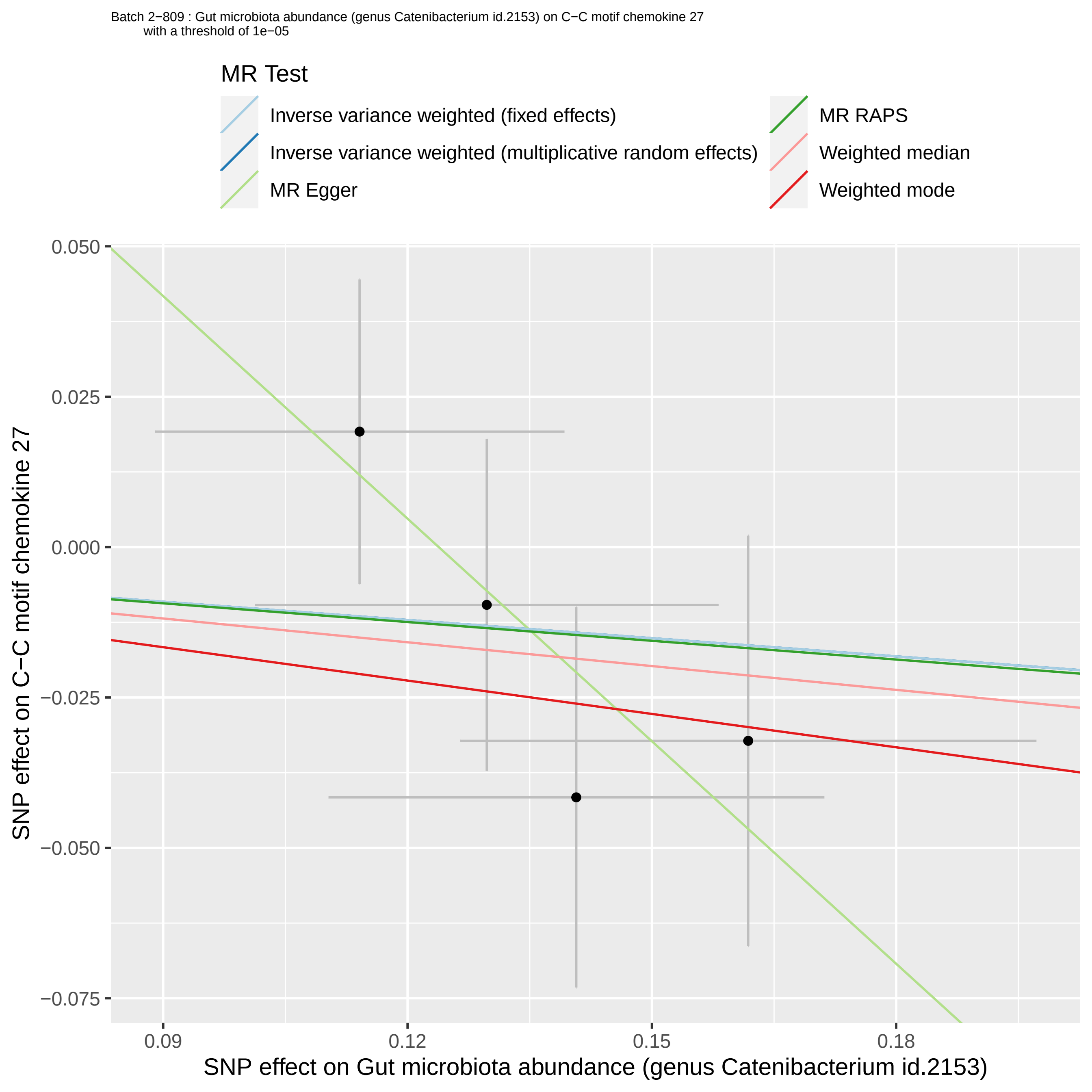

Batch 2−809 : Gut microbiota abundance (genus Catenibacterium id.2153) on C−C motif chemokine 27
with a threshold of 1e−05
MR Test
Inverse variance weighted (fixed effects)
Inverse variance weighted (multiplicative random effects)
MR Egger
MR RAPS
Weighted median
Weighted mode
0.050
0.025
0.000
−0.025
−0.050
−0.075
0.09
0.12
0.15
0.18
SNP effect on C−C motif chemokine 27
SNP effect on Gut microbiota abundance (genus Catenibacterium id.2153)

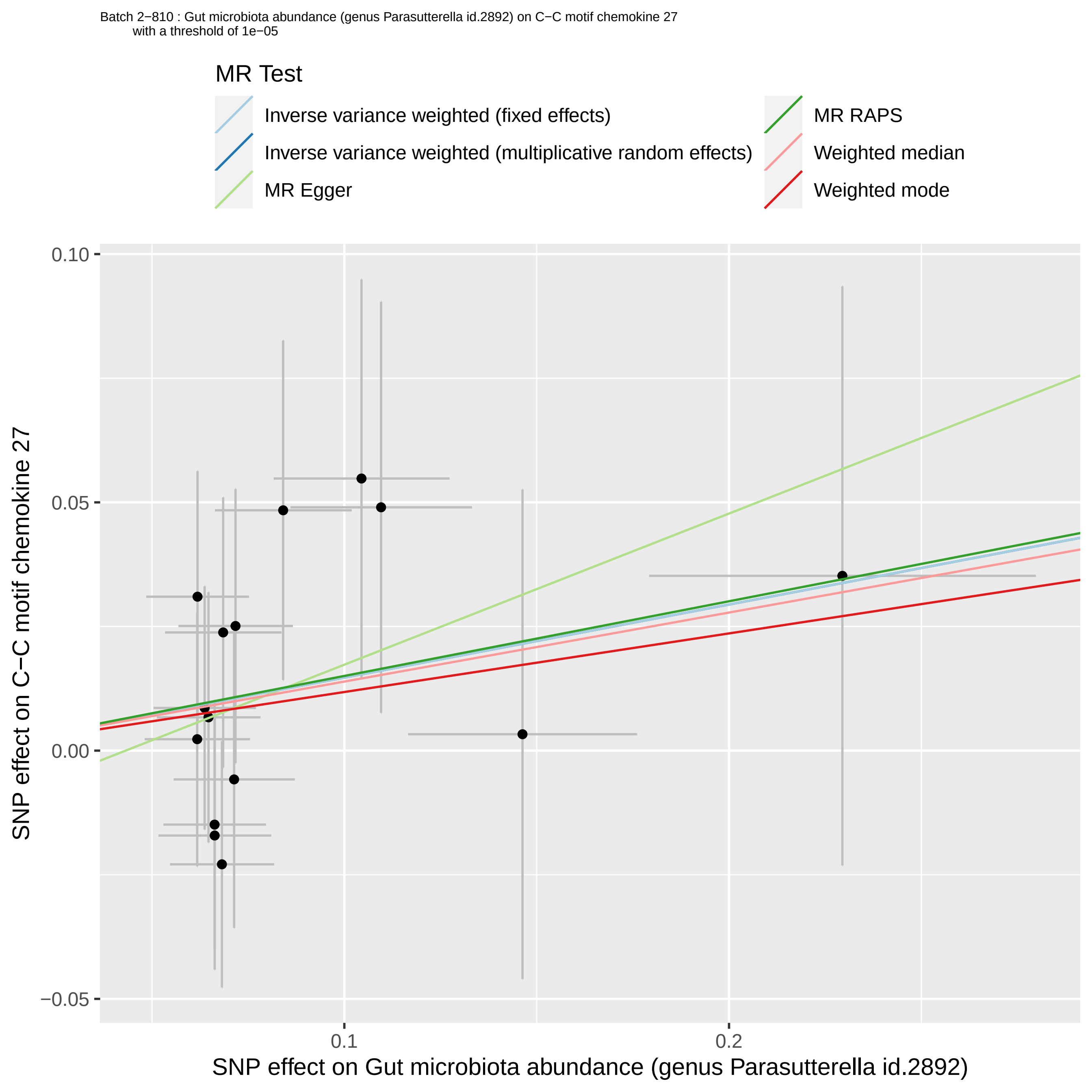

Batch 2−810 : Gut microbiota abundance (genus Parasutterella id.2892) on C−C motif chemokine 27
with a threshold of 1e−05
MR Test
Inverse variance weighted (fixed effects)
Inverse variance weighted (multiplicative random effects)
MR Egger
MR RAPS
Weighted median
Weighted mode
0.10
0.05
0.00
−0.05
0.1
0.2
SNP effect on C−C motif chemokine 27
SNP effect on Gut microbiota abundance (genus Parasutterella id.2892)

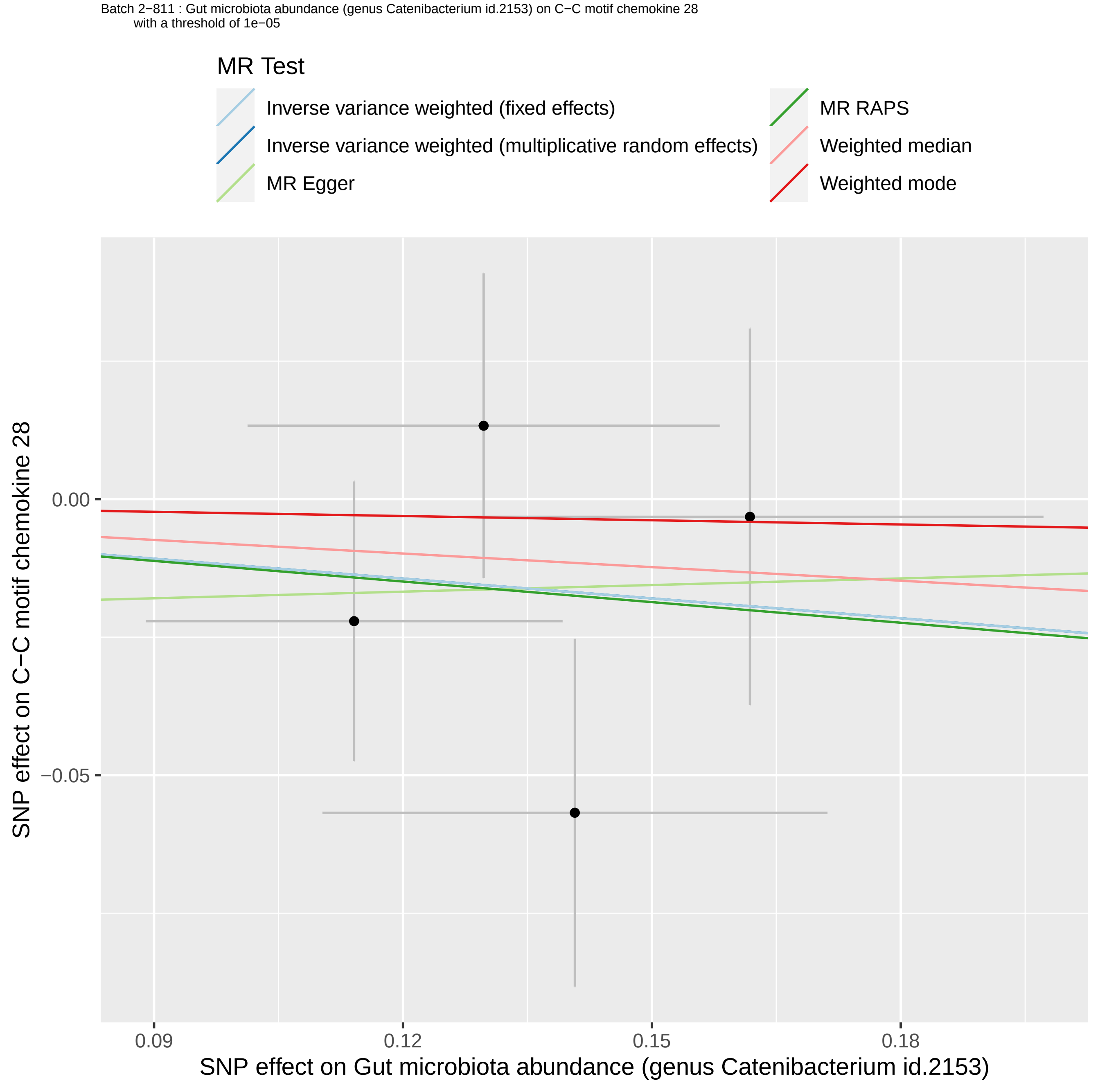
Batch 2−811 : Gut microbiota abundance (genus Catenibacterium id.2153) on C−C motif chemokine 28
with a threshold of 1e−05
MR Test
Inverse variance weighted (fixed effects)
Inverse variance weighted (multiplicative random effects)
MR Egger
MR RAPS
Weighted median
Weighted mode
0.00
−0.05
0.09
0.12
0.15
0.18
SNP effect on Gut microbiota abundance (genus Catenibacterium id.2153)
SNP effect on C−C motif chemokine 28

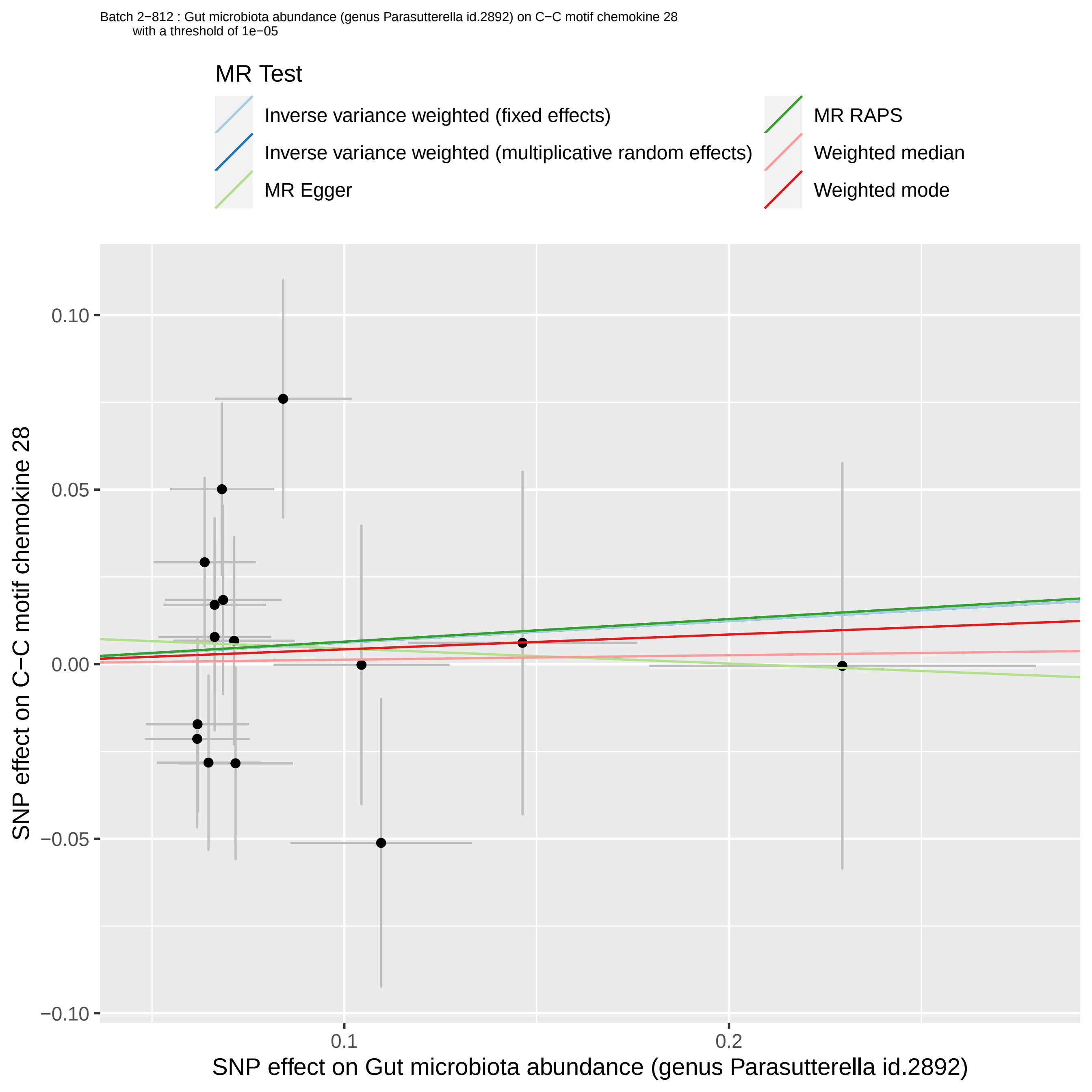

Batch 2−812 : Gut microbiota abundance (genus Parasutterella id.2892) on C−C motif chemokine 28
with a threshold of 1e−05
MR Test
Inverse variance weighted (fixed effects)
Inverse variance weighted (multiplicative random effects)
MR Egger
MR RAPS
Weighted median
Weighted mode
0.10
0.05
0.00
−0.05
−0.10
0.1
0.2
SNP effect on C−C motif chemokine 28
SNP effect on Gut microbiota abundance (genus Parasutterella id.2892)

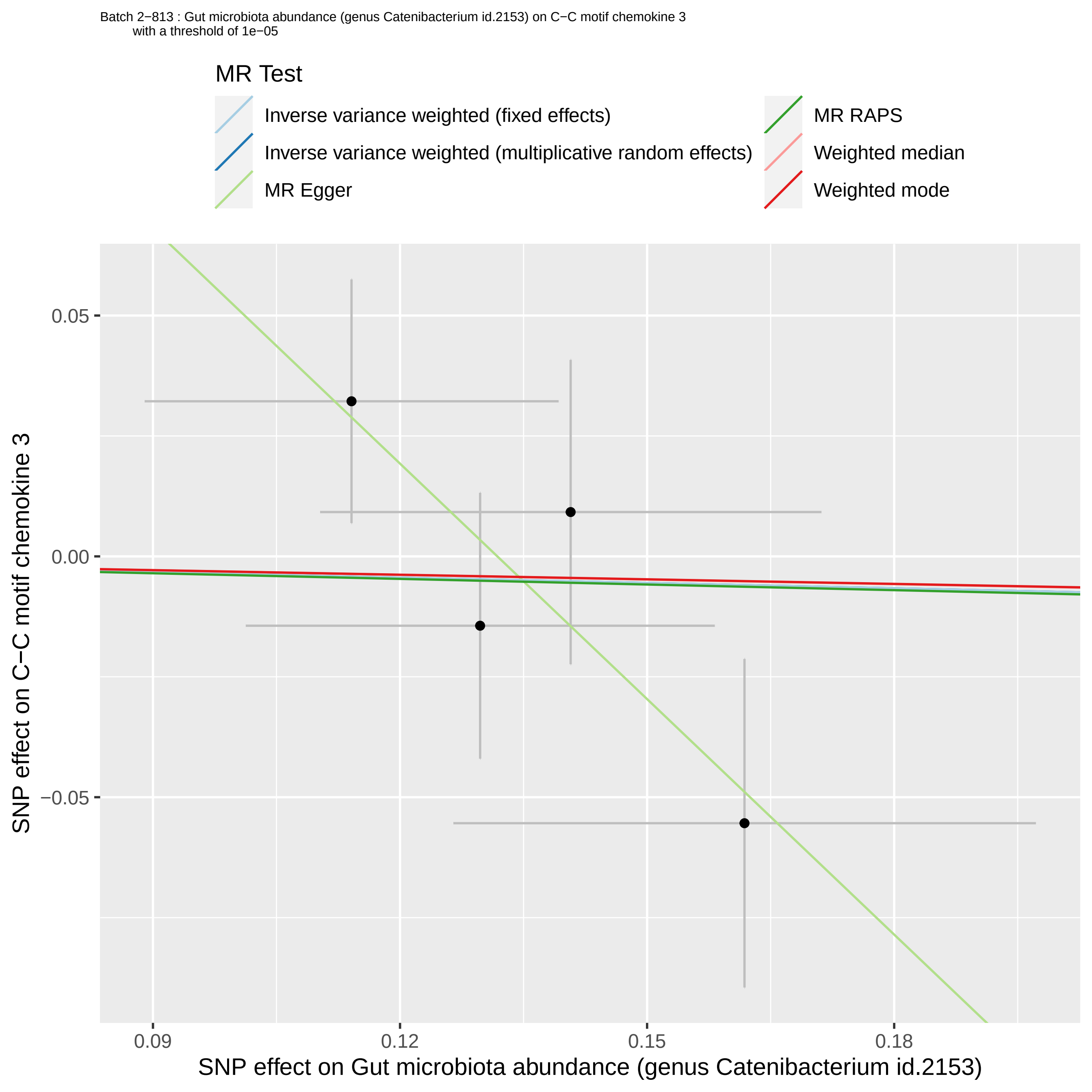
Batch 2−813 : Gut microbiota abundance (genus Catenibacterium id.2153) on C−C motif chemokine 3
with a threshold of 1e−05
MR Test
Inverse variance weighted (fixed effects)
Inverse variance weighted (multiplicative random effects)
MR Egger
MR RAPS
Weighted median
Weighted mode
0.05
0.00
−0.05
0.09
0.12
0.15
0.18
SNP effect on C−C motif chemokine 3
SNP effect on Gut microbiota abundance (genus Catenibacterium id.2153)

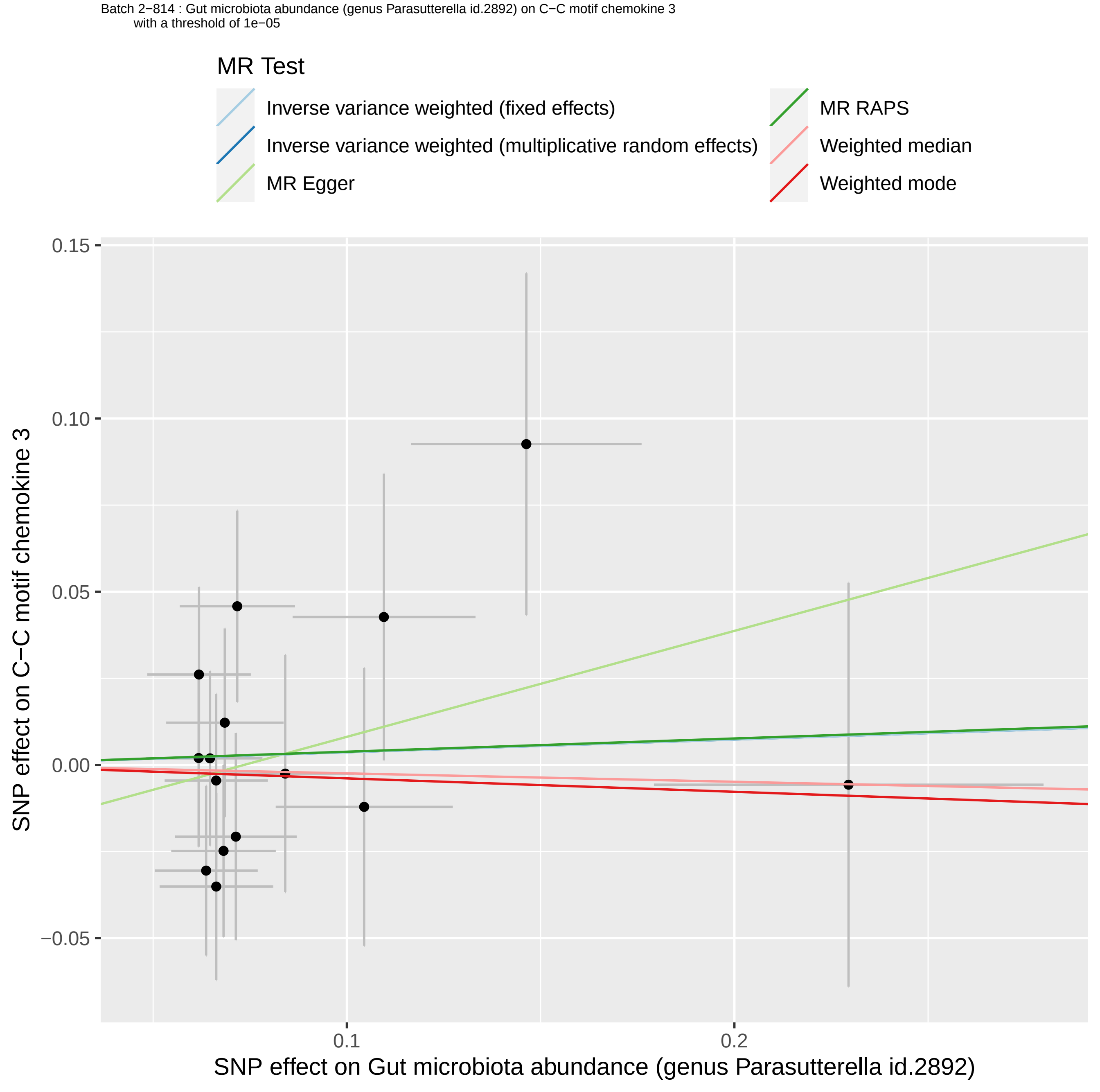
Batch 2−814 : Gut microbiota abundance (genus Parasutterella id.2892) on C−C motif chemokine 3
with a threshold of 1e−05
MR Test
Inverse variance weighted (fixed effects)
Inverse variance weighted (multiplicative random effects)
MR Egger
MR RAPS
Weighted median
Weighted mode
0.15
0.10
0.05
0.00
−0.05
0.1
0.2
SNP effect on C−C motif chemokine 3
SNP effect on Gut microbiota abundance (genus Parasutterella id.2892)

Batch 2−815 : Gut microbiota abundance (genus Catenibacterium id.2153) on C−C motif chemokine 3−like 1
with a threshold of 1e−05

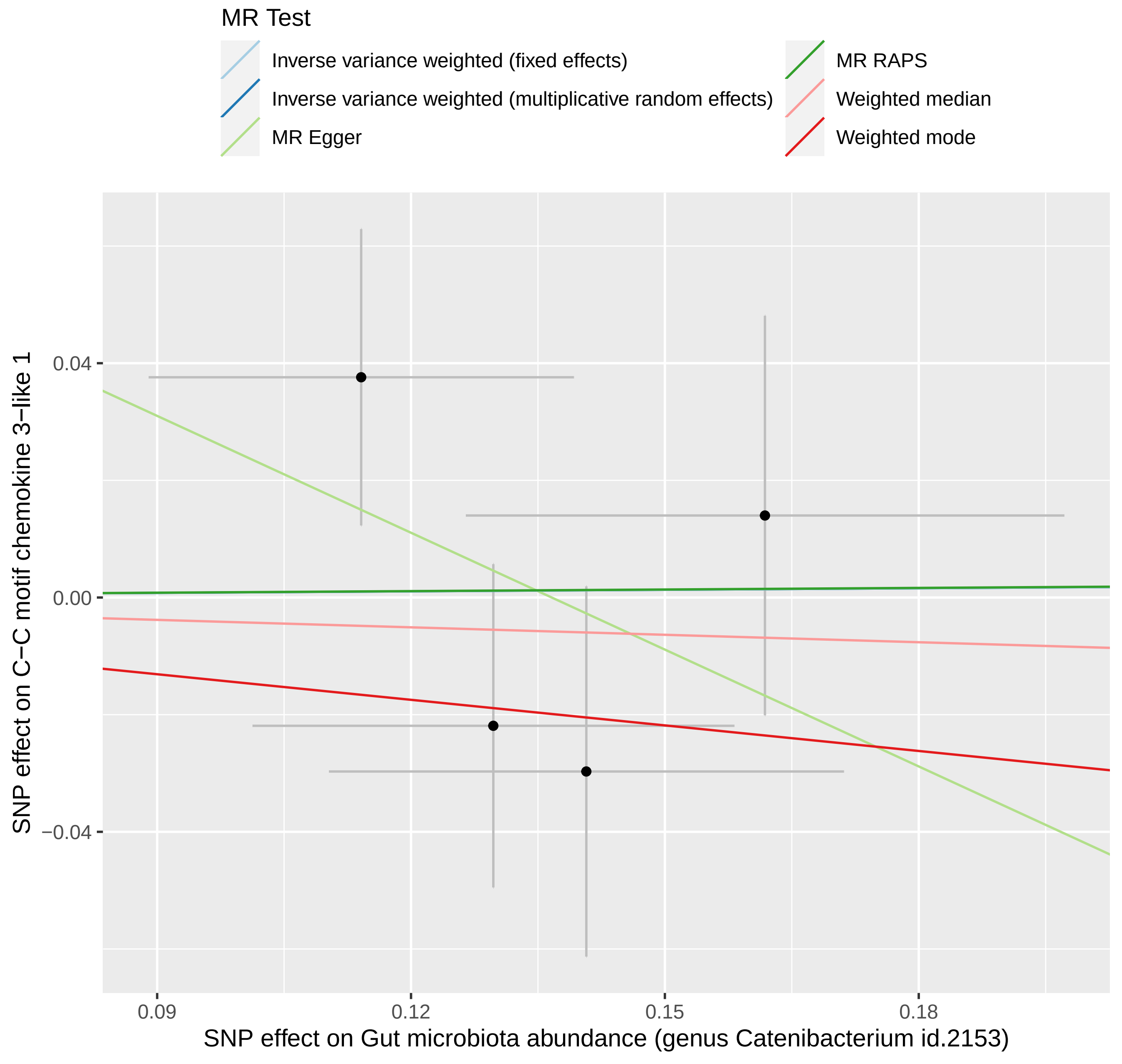

Batch 2−816 : Gut microbiota abundance (genus Parasutterella id.2892) on C−C motif chemokine 3−like 1
with a threshold of 1e−05

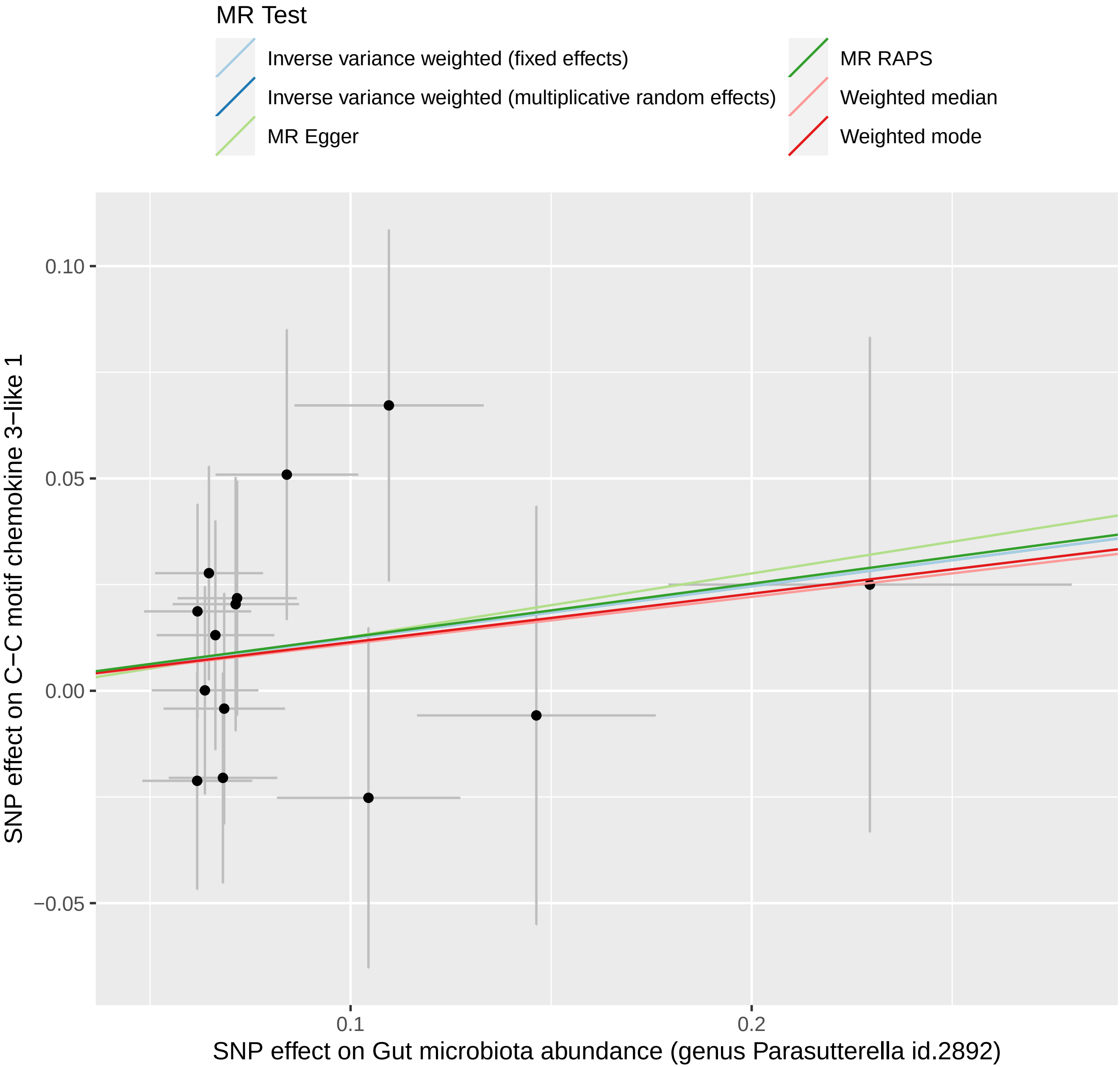

Batch 2−817 : Gut microbiota abundance (genus Catenibacterium id.2153) on C−C motif chemokine 5
with a threshold of 1e−05

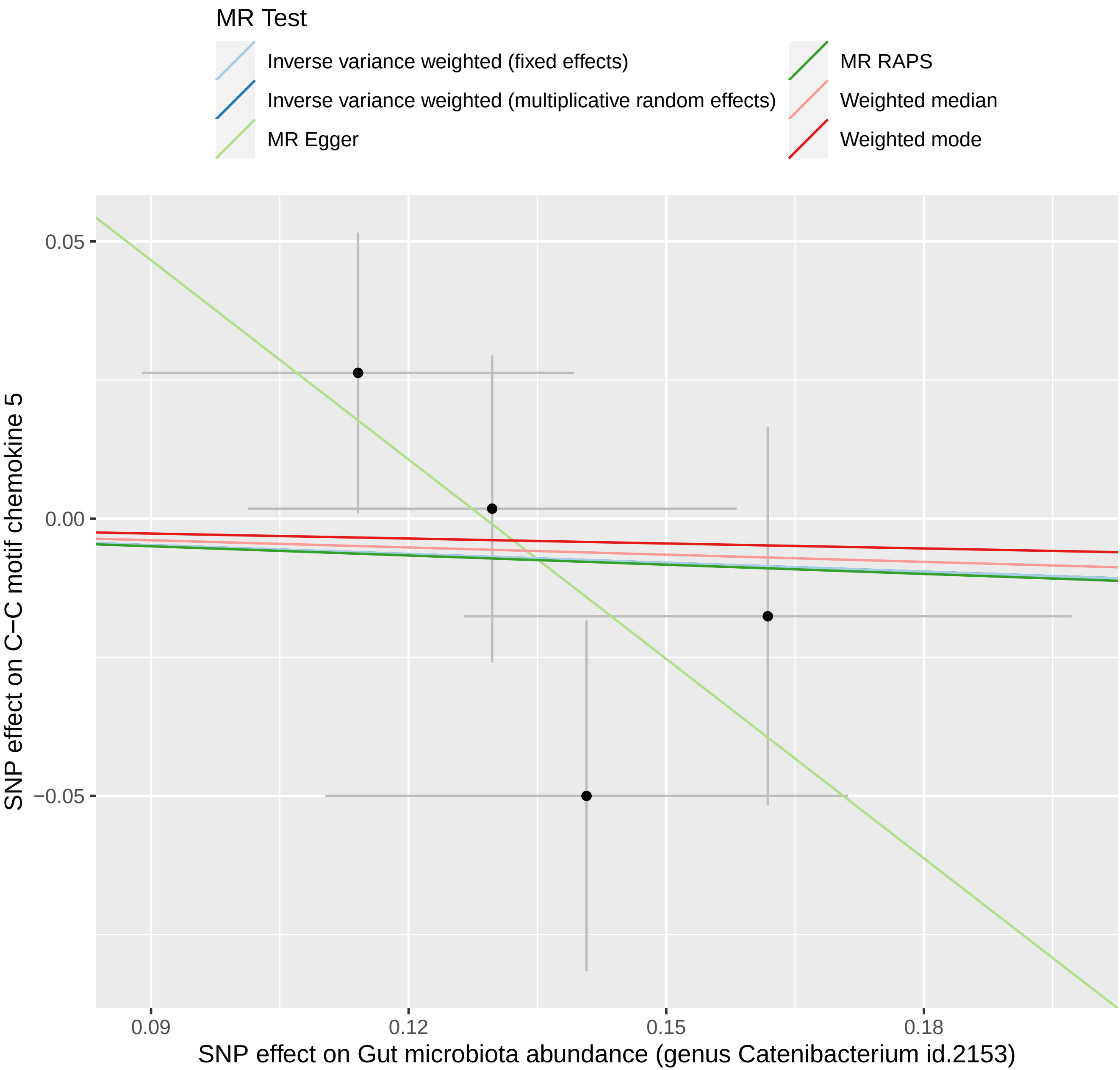

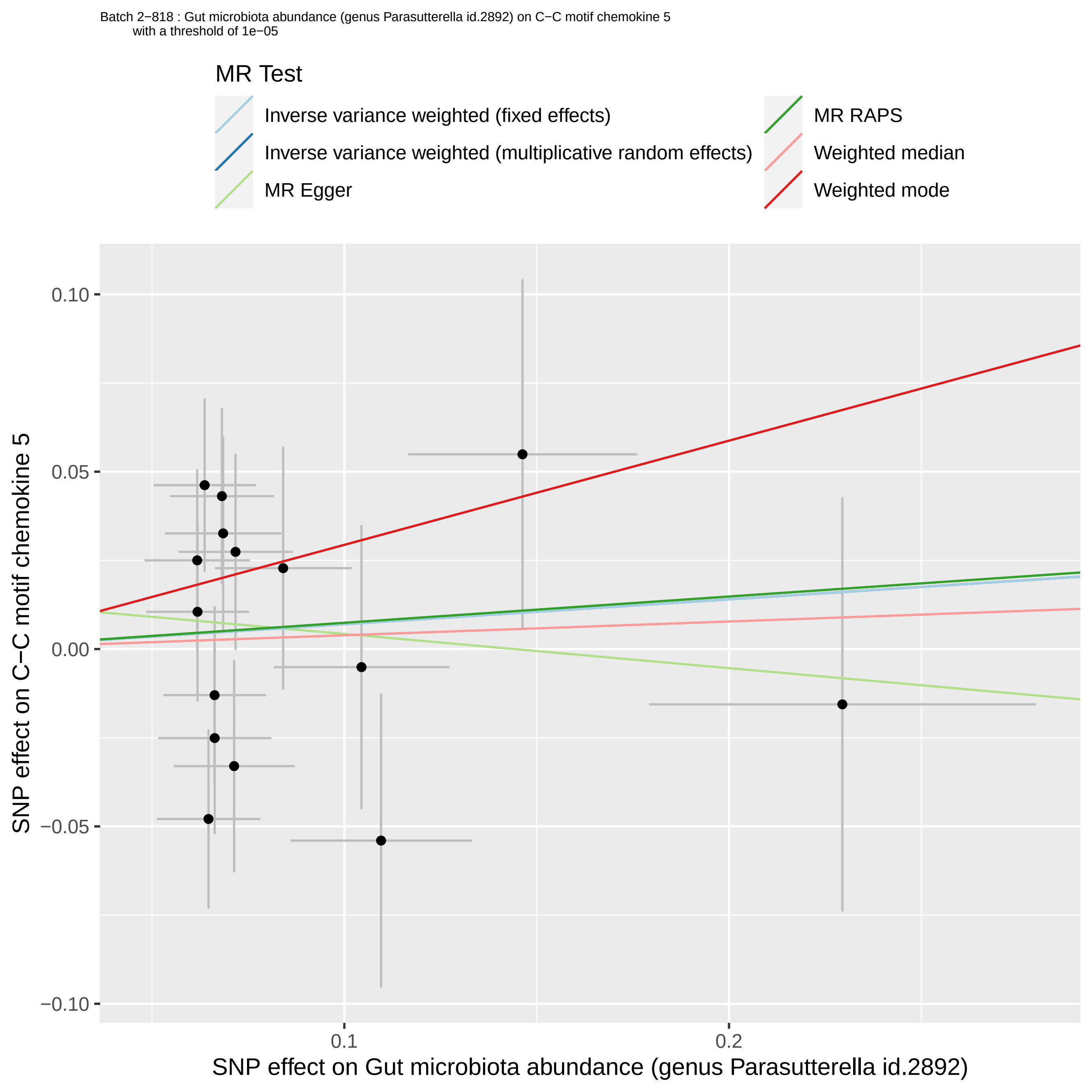
Batch 2−818 : Gut microbiota abundance (genus Parasutterella id.2892) on C−C motif chemokine 5
with a threshold of 1e−05
MR Test
Inverse variance weighted (fixed effects)
Inverse variance weighted (multiplicative random effects)
MR Egger
MR RAPS
Weighted median
Weighted mode
0.10
0.05
0.00
−0.05
−0.10
0.1
0.2
SNP effect on C−C motif chemokine 5
SNP effect on Gut microbiota abundance (genus Parasutterella id.2892)

Batch 2−819 : Gut microbiota abundance (genus Catenibacterium id.2153) on C−C motif chemokine 7
with a threshold of 1e−05

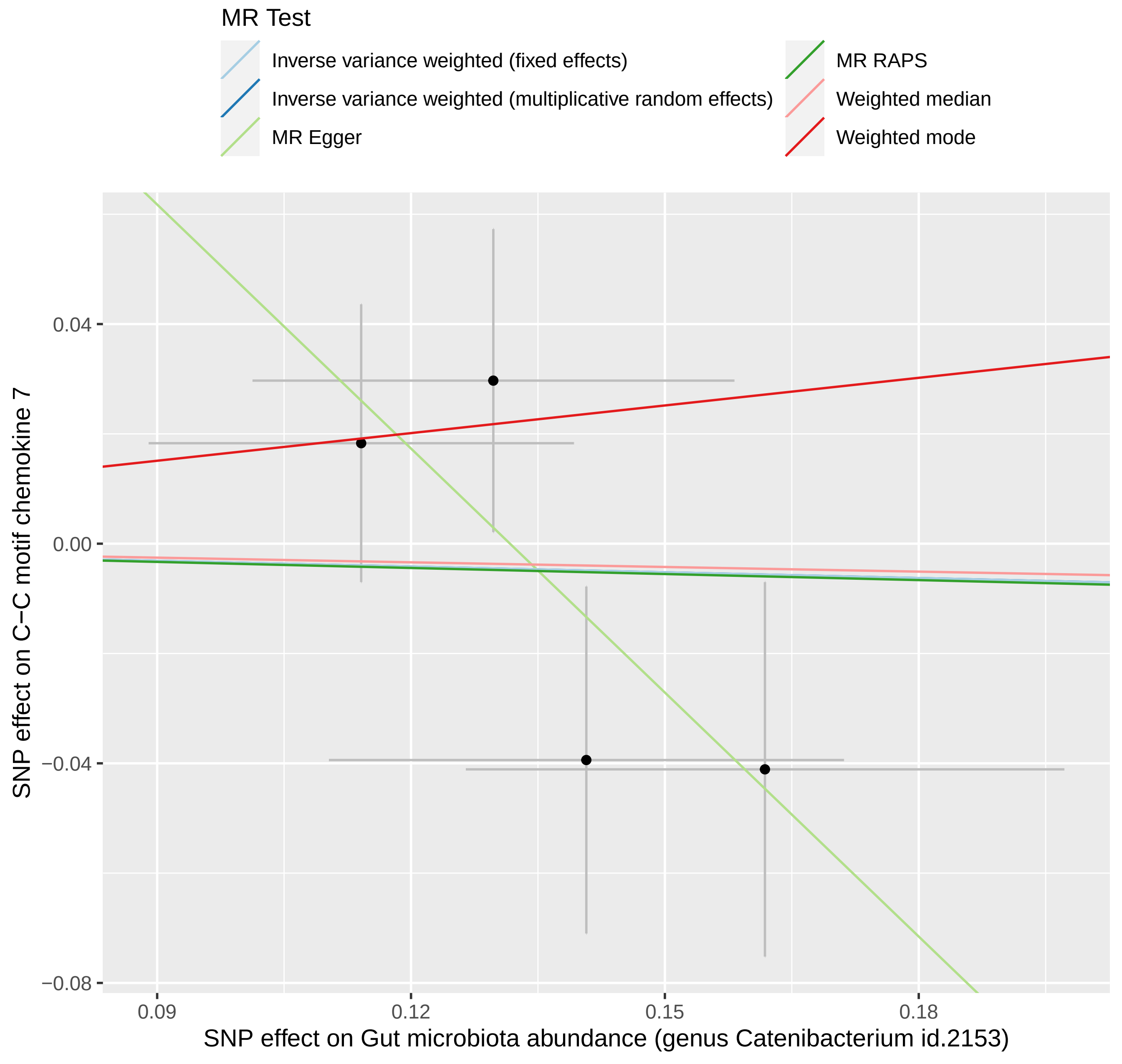

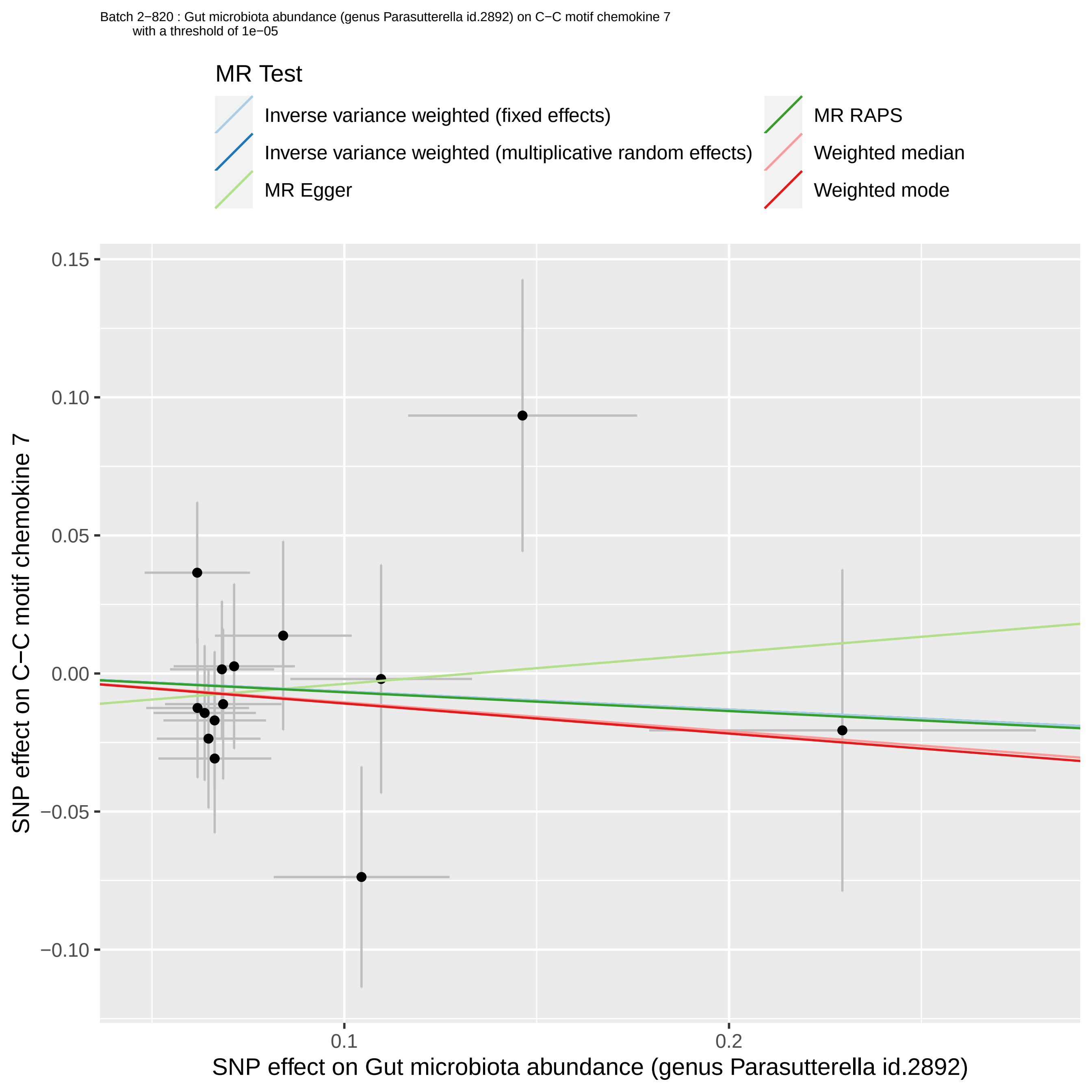
Batch 2−820 : Gut microbiota abundance (genus Parasutterella id.2892) on C−C motif chemokine 7
with a threshold of 1e−05
MR Test
Inverse variance weighted (fixed effects)
Inverse variance weighted (multiplicative random effects)
MR Egger
MR RAPS
Weighted median
Weighted mode
0.15
0.10
0.05
0.00
−0.05
−0.10
SNP effect on C−C motif chemokine 7
0.1
0.2
SNP effect on Gut microbiota abundance (genus Parasutterella id.2892)

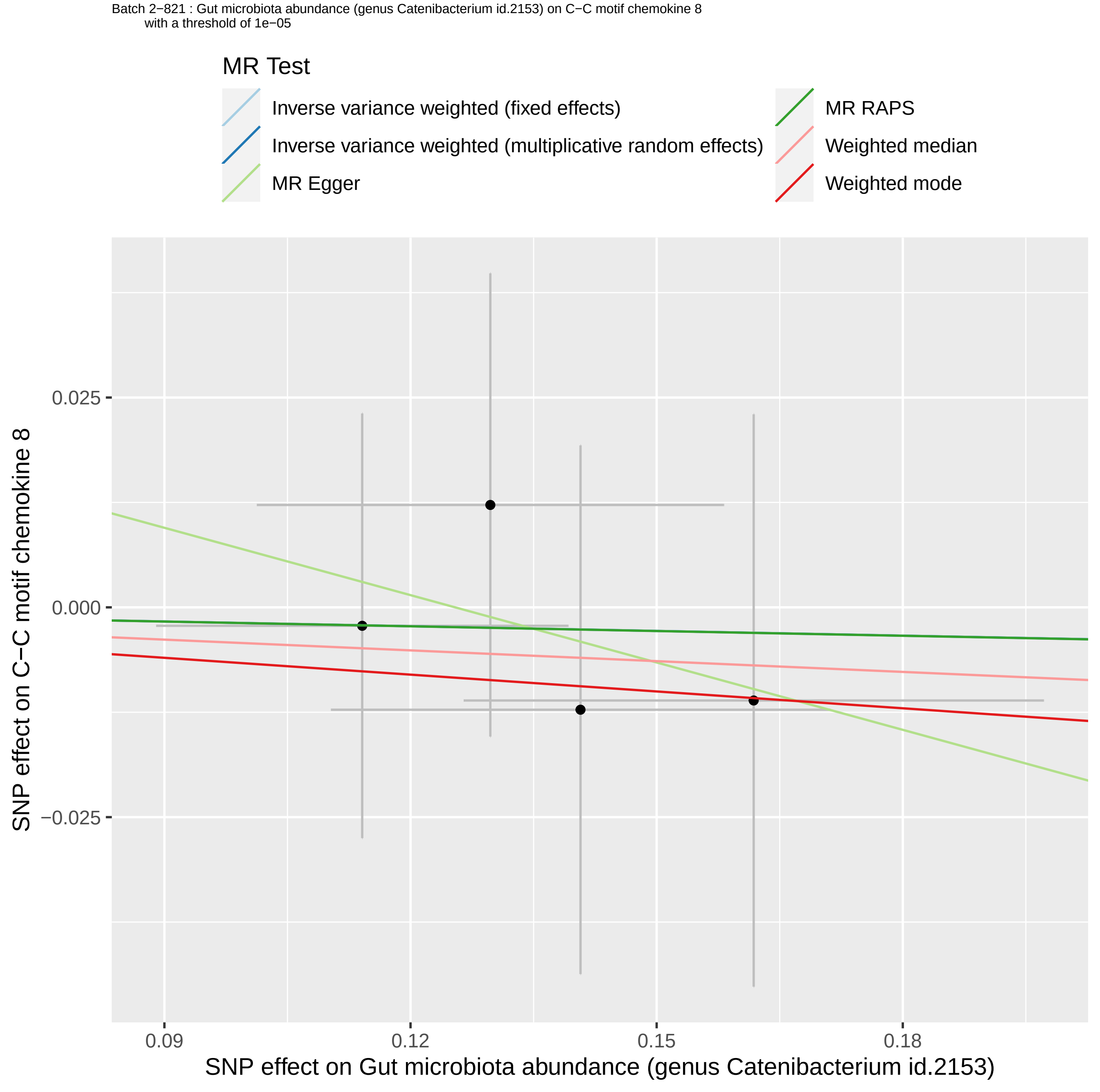
Batch 2−821 : Gut microbiota abundance (genus Catenibacterium id.2153) on C−C motif chemokine 8
with a threshold of 1e−05
MR Test
Inverse variance weighted (fixed effects)
Inverse variance weighted (multiplicative random effects)
MR Egger
MR RAPS
Weighted median
Weighted mode
0.025
0.000
−0.025
0.09
0.12
0.15
0.18
SNP effect on C−C motif chemokine 8
SNP effect on Gut microbiota abundance (genus Catenibacterium id.2153)

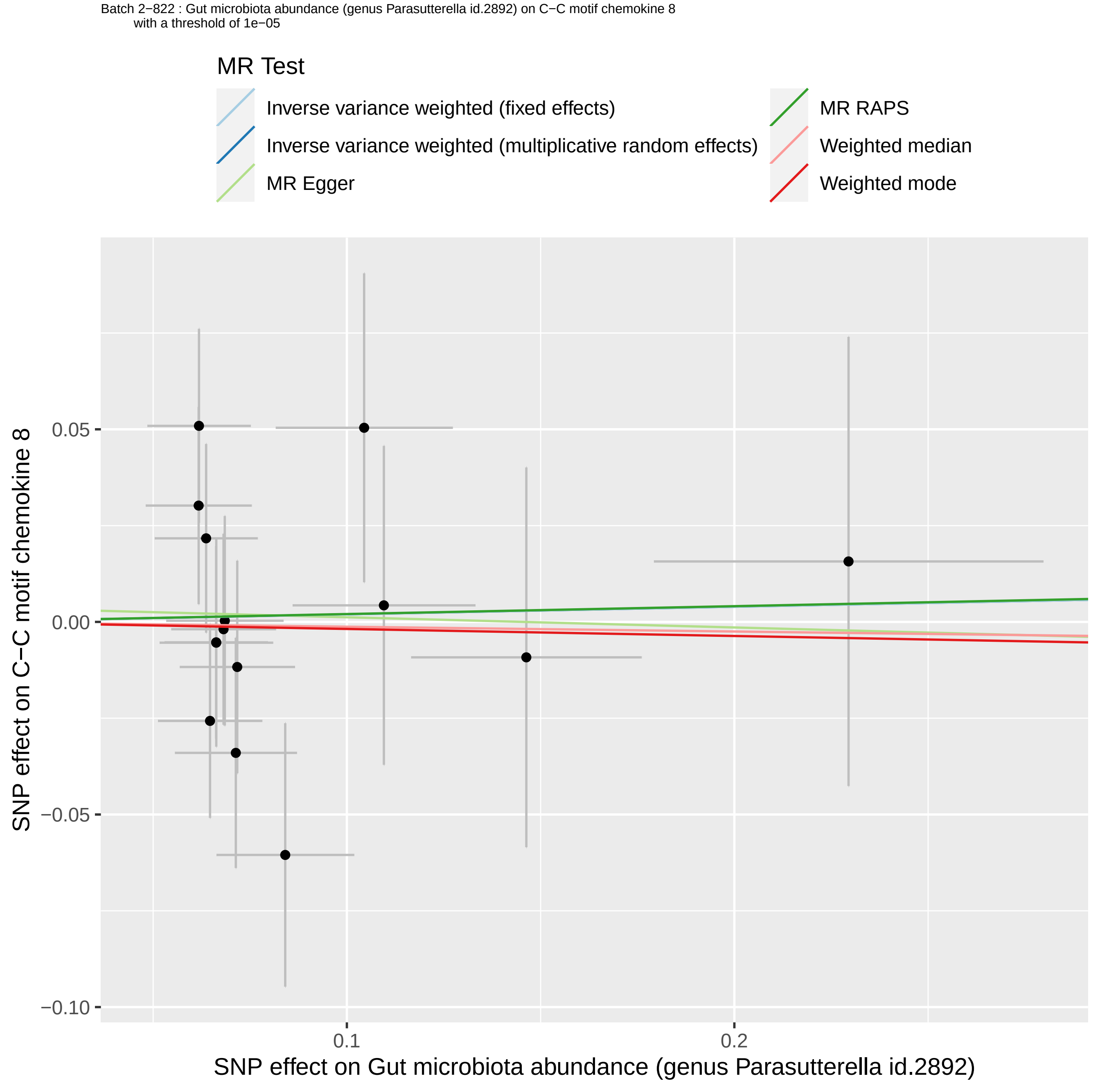

Batch 2−822 : Gut microbiota abundance (genus Parasutterella id.2892) on C−C motif chemokine 8
with a threshold of 1e−05
MR Test
Inverse variance weighted (fixed effects)
Inverse variance weighted (multiplicative random effects)
MR Egger
MR RAPS
Weighted median
Weighted mode
SNP effect on C−C motif chemokine 8
0.05
0.00
−0.05
−0.10
0.1
0.2
SNP effect on Gut microbiota abundance (genus Parasutterella id.2892)

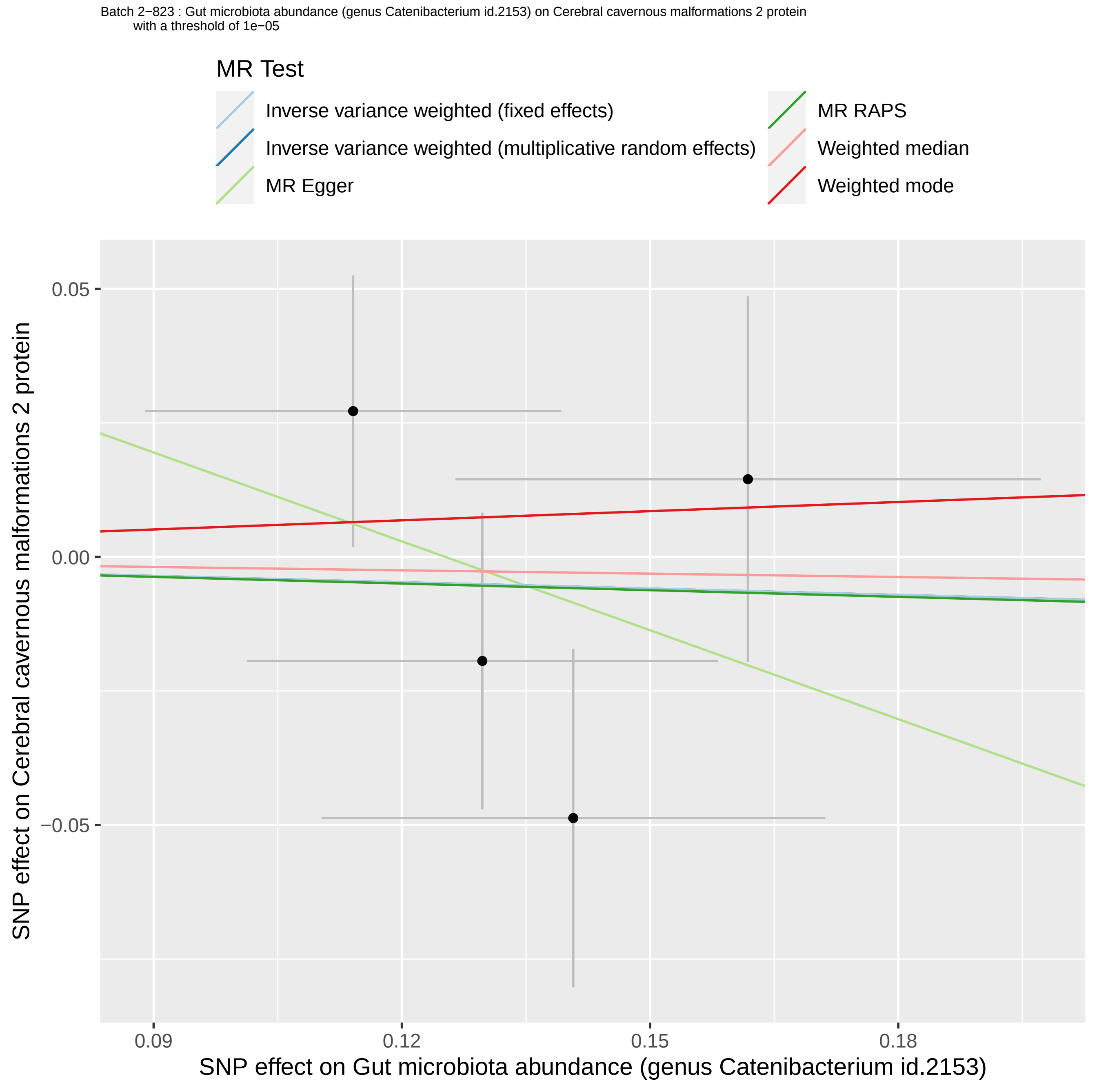

Batch 2−823 : Gut microbiota abundance (genus Catenibacterium id.2153) on Cerebral cavernous malformations 2 protein
with a threshold of 1e−05
MR Test
Inverse variance weighted (fixed effects)
Inverse variance weighted (multiplicative random effects)
MR Egger
MR RAPS
Weighted median
Weighted mode
0.05
0.00
−0.05
0.09
0.12
0.15
0.18
SNP effect on Cerebral cavernous malformations 2 protein
SNP effect on Gut microbiota abundance (genus Catenibacterium id.2153)

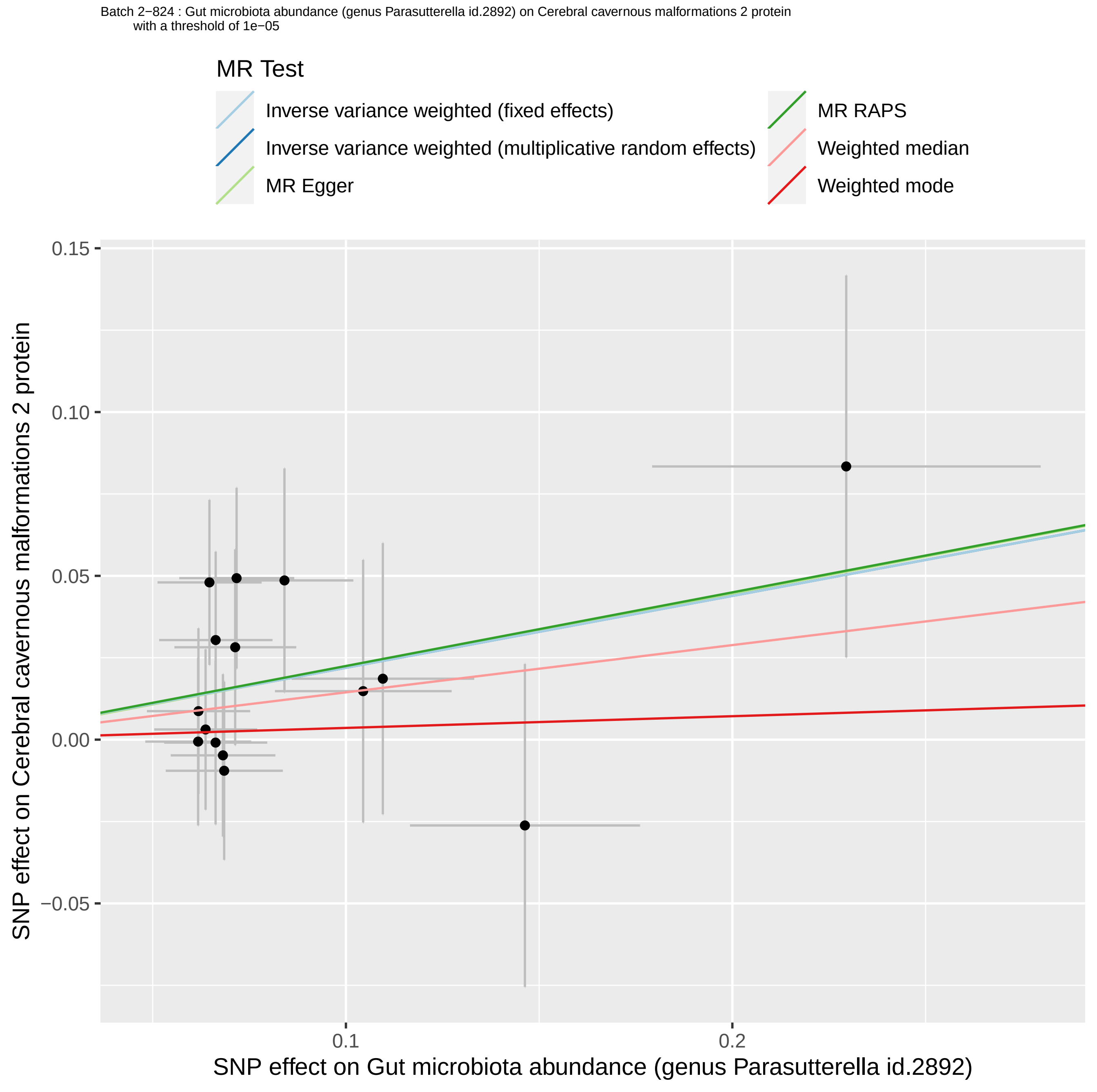

Batch 2−824 : Gut microbiota abundance (genus Parasutterella id.2892) on Cerebral cavernous malformations 2 protein
with a threshold of 1e−05
MR Test
Inverse variance weighted (fixed effects)
Inverse variance weighted (multiplicative random effects)
MR Egger
MR RAPS
Weighted median
Weighted mode
0.15
0.10
0.05
0.00
−0.05
0.1
0.2
SNP effect on Cerebral cavernous malformations 2 protein
SNP effect on Gut microbiota abundance (genus Parasutterella id.2892)

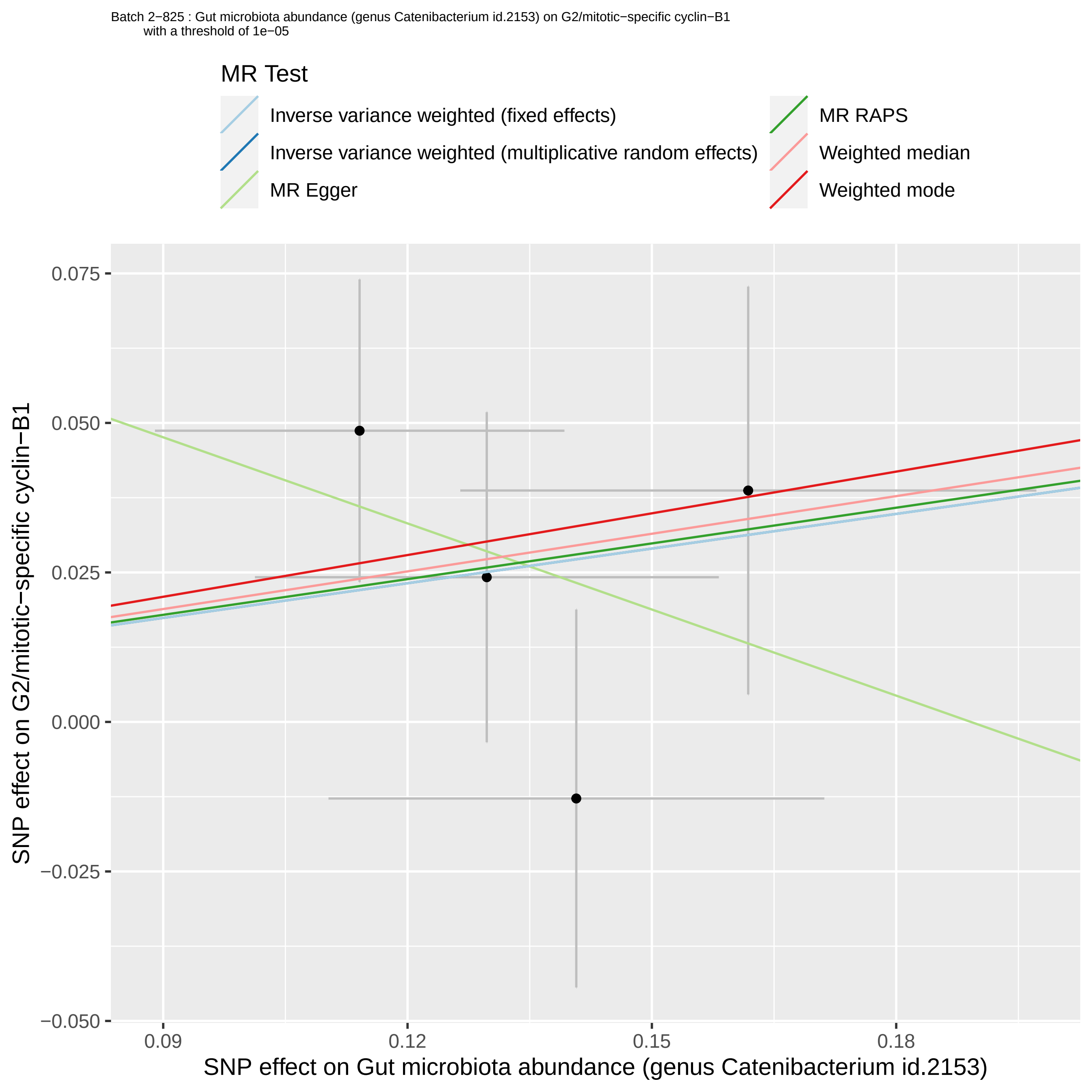

Batch 2−825 : Gut microbiota abundance (genus Catenibacterium id.2153) on G2/mitotic−specific cyclin−B1
with a threshold of 1e−05
MR Test
Inverse variance weighted (fixed effects)
Inverse variance weighted (multiplicative random effects)
MR Egger
MR RAPS
Weighted median
Weighted mode
0.075
0.050
0.025
0.000
−0.025
−0.050
0.09
0.12
0.15
0.18
SNP effect on G2/mitotic−specific cyclin−B1
SNP effect on Gut microbiota abundance (genus Catenibacterium id.2153)

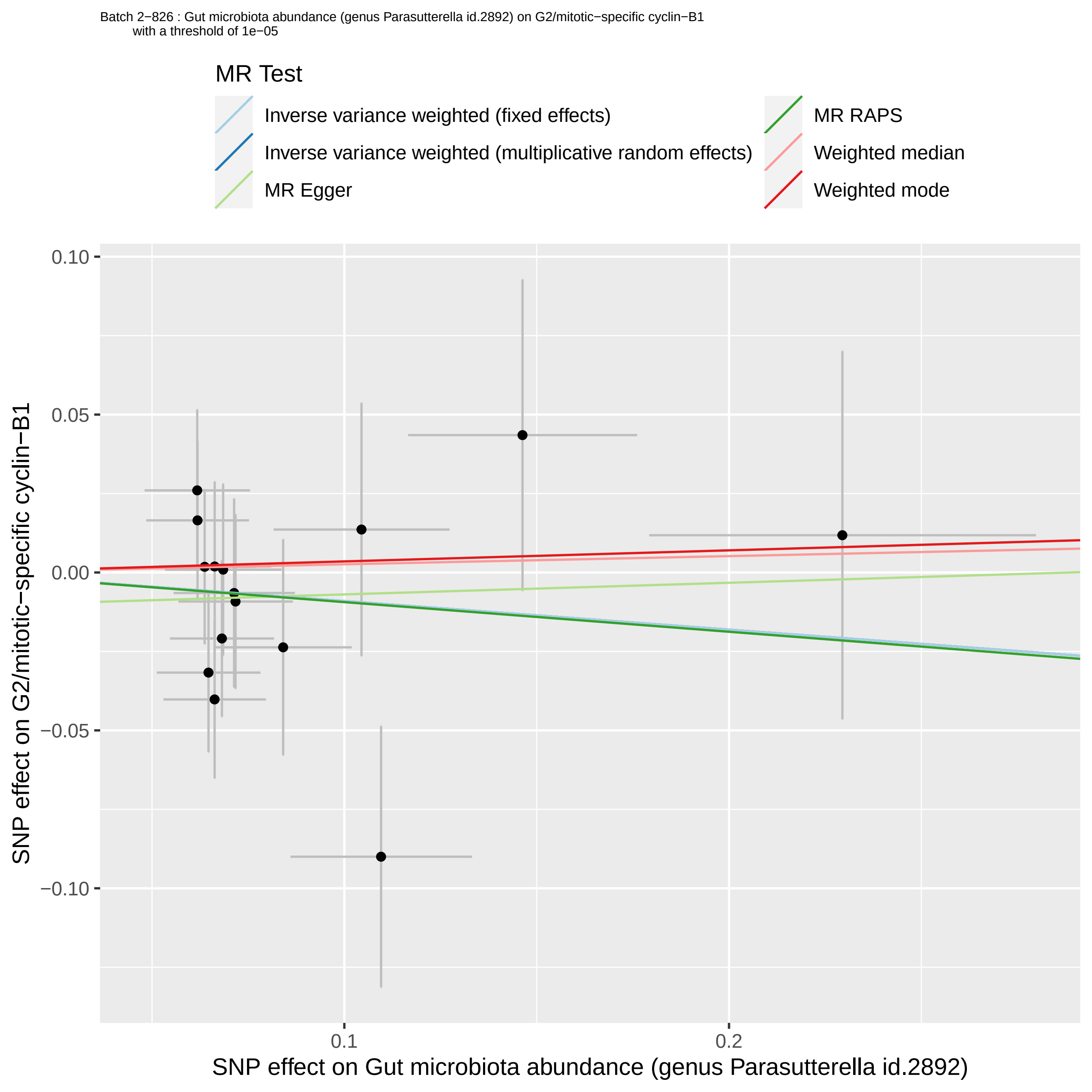

Batch 2−826 : Gut microbiota abundance (genus Parasutterella id.2892) on G2/mitotic−specific cyclin−B1
with a threshold of 1e−05
MR Test
Inverse variance weighted (fixed effects)
Inverse variance weighted (multiplicative random effects)
MR Egger
MR RAPS
Weighted median
Weighted mode
0.10
0.05
0.00
−0.05
−0.10
0.1
0.2
SNP effect on G2/mitotic−specific cyclin−B1
SNP effect on Gut microbiota abundance (genus Parasutterella id.2892)

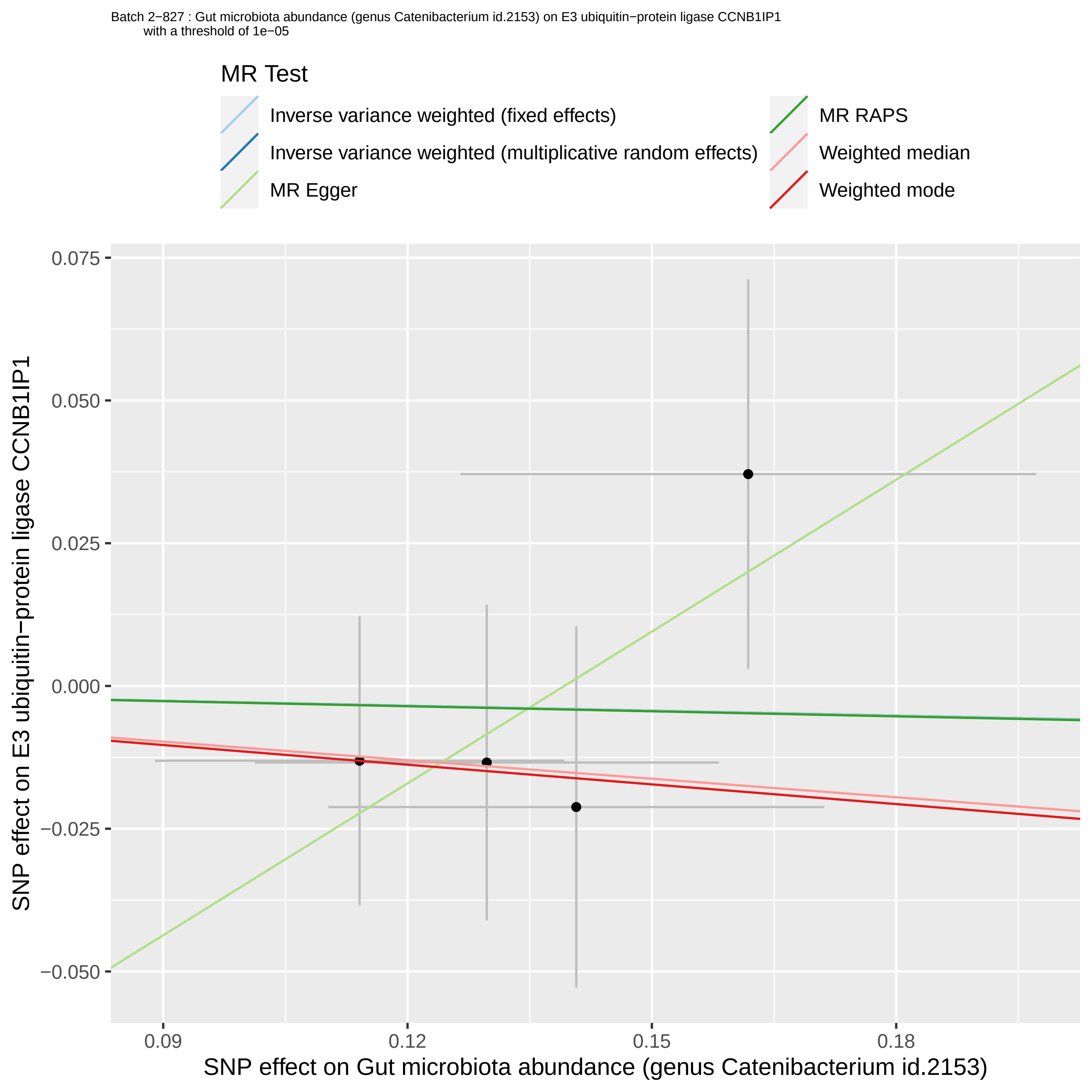

Batch 2−827 : Gut microbiota abundance (genus Catenibacterium id.2153) on E3 ubiquitin−protein ligase CCNB1IP1
with a threshold of 1e−05
MR Test
Inverse variance weighted (fixed effects)
Inverse variance weighted (multiplicative random effects)
MR Egger
MR RAPS
Weighted median
Weighted mode
0.075
0.050
0.025
0.000
−0.025
−0.050
0.09
0.12
0.15
0.18
SNP effect on E3 ubiquitin−protein ligase CCNB1IP1
SNP effect on Gut microbiota abundance (genus Catenibacterium id.2153)

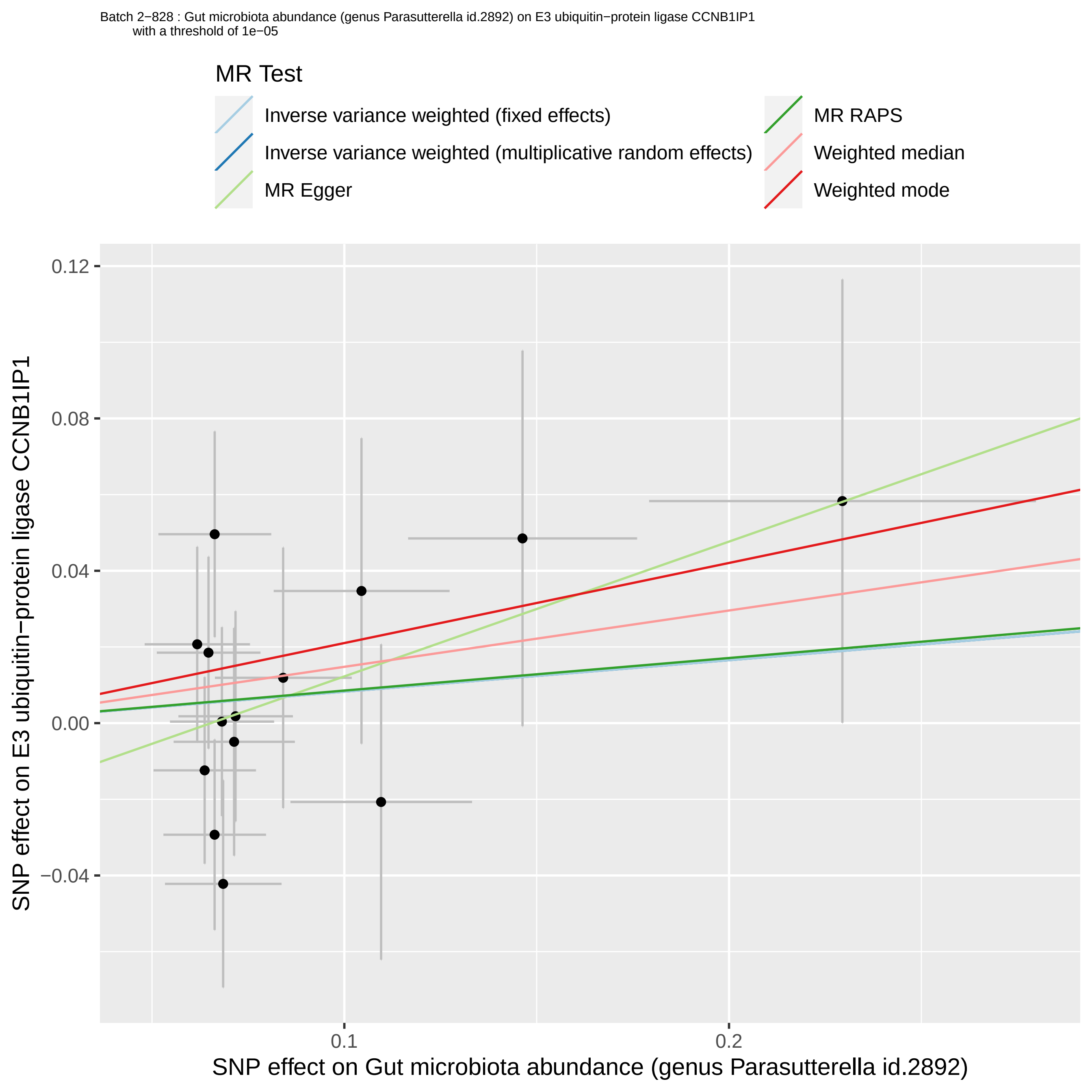

Batch 2−828 : Gut microbiota abundance (genus Parasutterella id.2892) on E3 ubiquitin−protein ligase CCNB1IP1
with a threshold of 1e−05
MR Test
Inverse variance weighted (fixed effects)
Inverse variance weighted (multiplicative random effects)
MR Egger
MR RAPS
Weighted median
Weighted mode
0.12
0.08
0.04
0.00
−0.04
0.1
0.2
SNP effect on E3 ubiquitin−protein ligase CCNB1IP1
SNP effect on Gut microbiota abundance (genus Parasutterella id.2892)

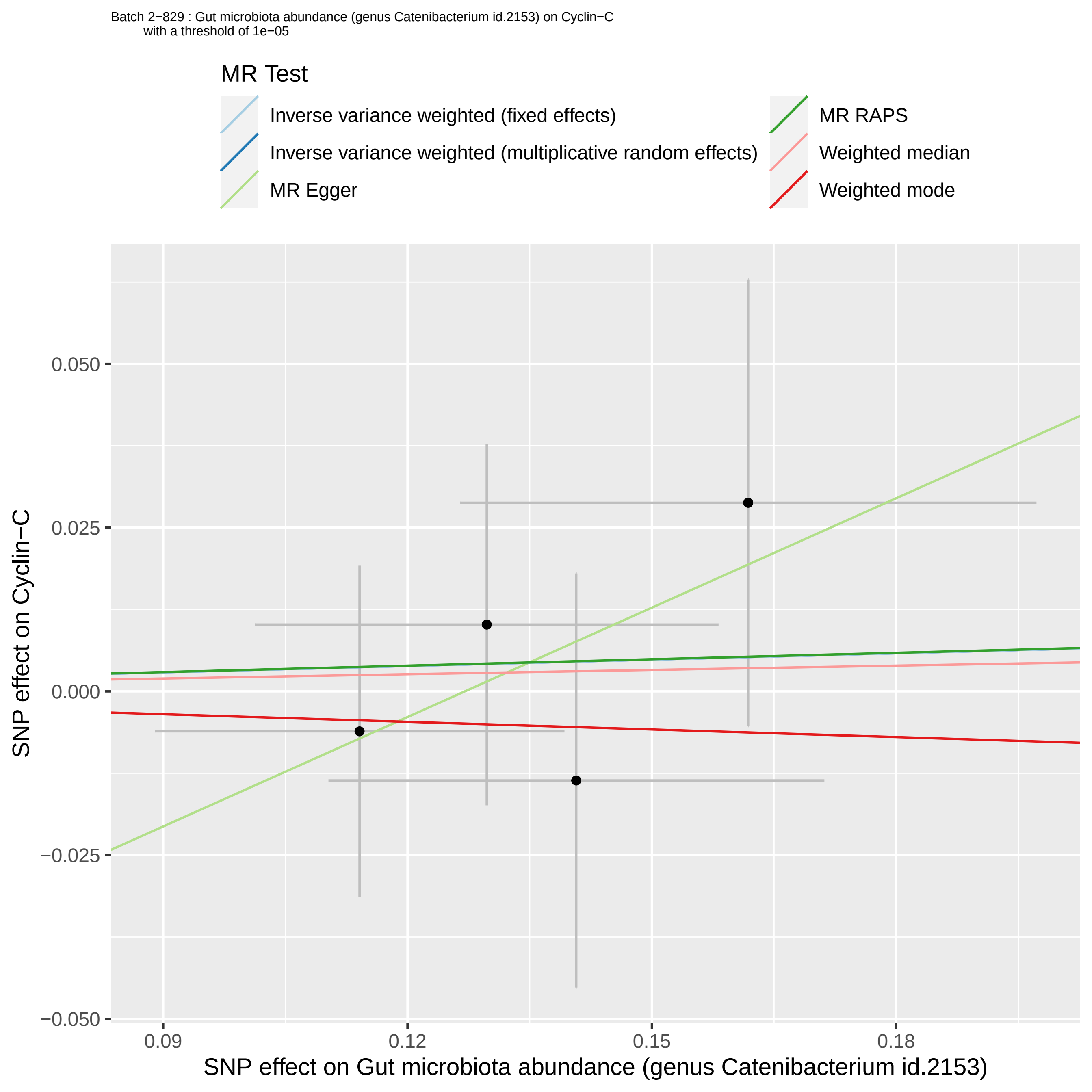
Batch 2−829 : Gut microbiota abundance (genus Catenibacterium id.2153) on Cyclin−C
with a threshold of 1e−05
MR Test
Inverse variance weighted (fixed effects)
Inverse variance weighted (multiplicative random effects)
MR Egger
MR RAPS
Weighted median
Weighted mode
0.050
0.025
0.000
−0.025
−0.050
0.09
0.12
0.15
0.18
SNP effect on Cyclin−C
SNP effect on Gut microbiota abundance (genus Catenibacterium id.2153)

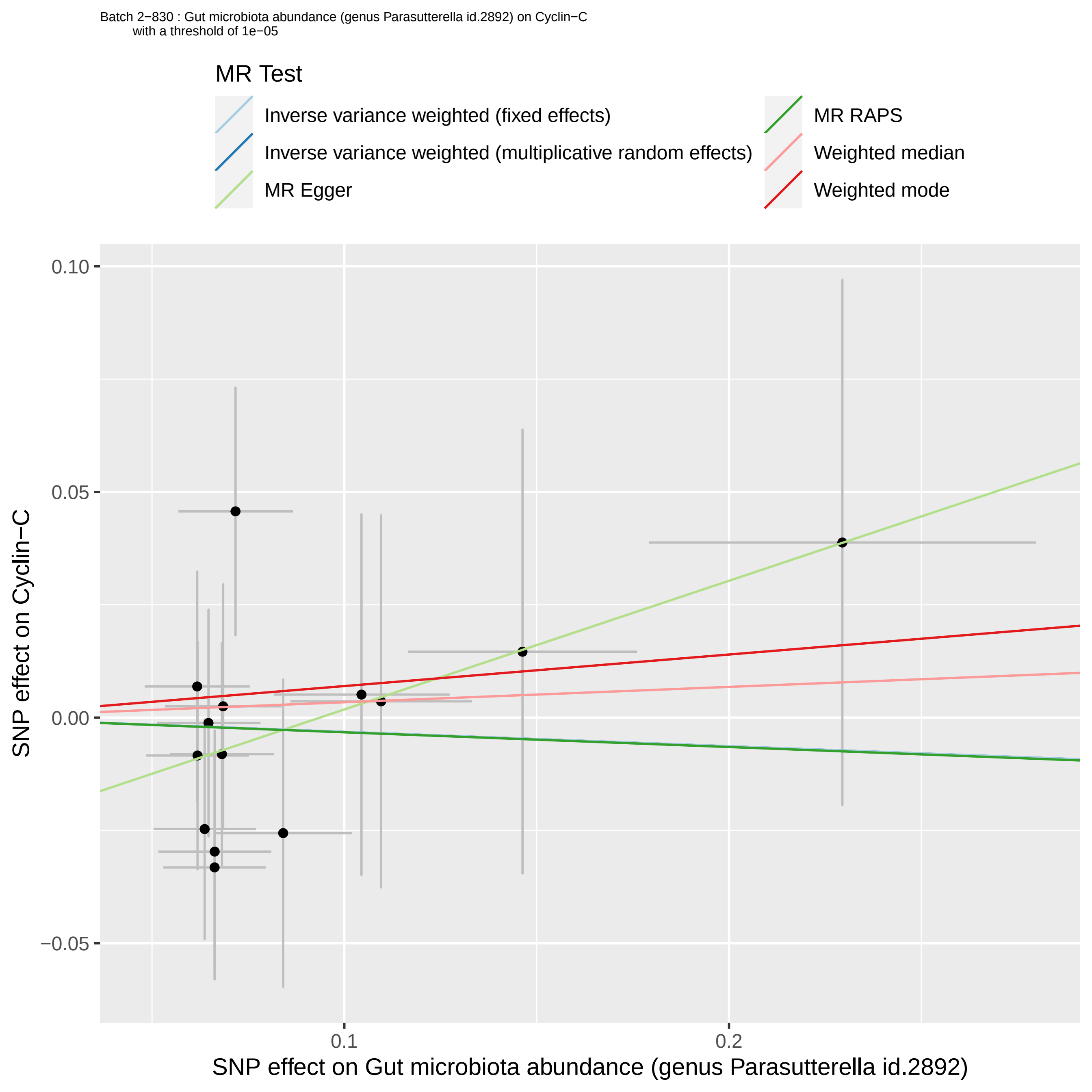

Batch 2−830 : Gut microbiota abundance (genus Parasutterella id.2892) on Cyclin−C
with a threshold of 1e−05
MR Test
Inverse variance weighted (fixed effects)
Inverse variance weighted (multiplicative random effects)
MR Egger
MR RAPS
Weighted median
Weighted mode
SNP effect on Cyclin−C
0.10
0.05
0.00
−0.05
0.1
0.2
SNP effect on Gut microbiota abundance (genus Parasutterella id.2892)

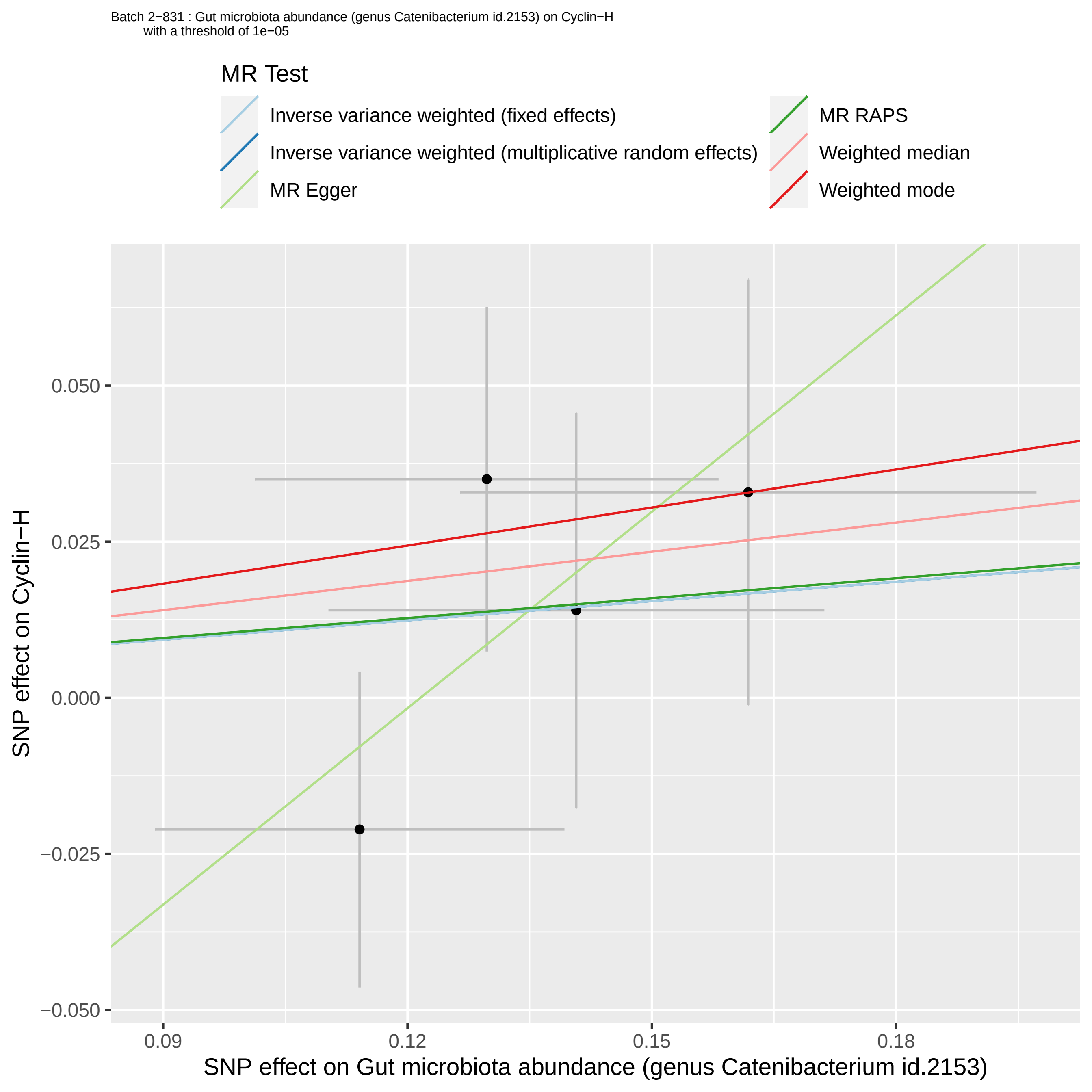

Batch 2−831 : Gut microbiota abundance (genus Catenibacterium id.2153) on Cyclin−H
with a threshold of 1e−05
MR Test
Inverse variance weighted (fixed effects)
Inverse variance weighted (multiplicative random effects)
MR Egger
MR RAPS
Weighted median
Weighted mode
0.050
0.025
0.000
−0.025
−0.050
SNP effect on Cyclin−H
0.09
0.12
0.15
0.18
SNP effect on Gut microbiota abundance (genus Catenibacterium id.2153)

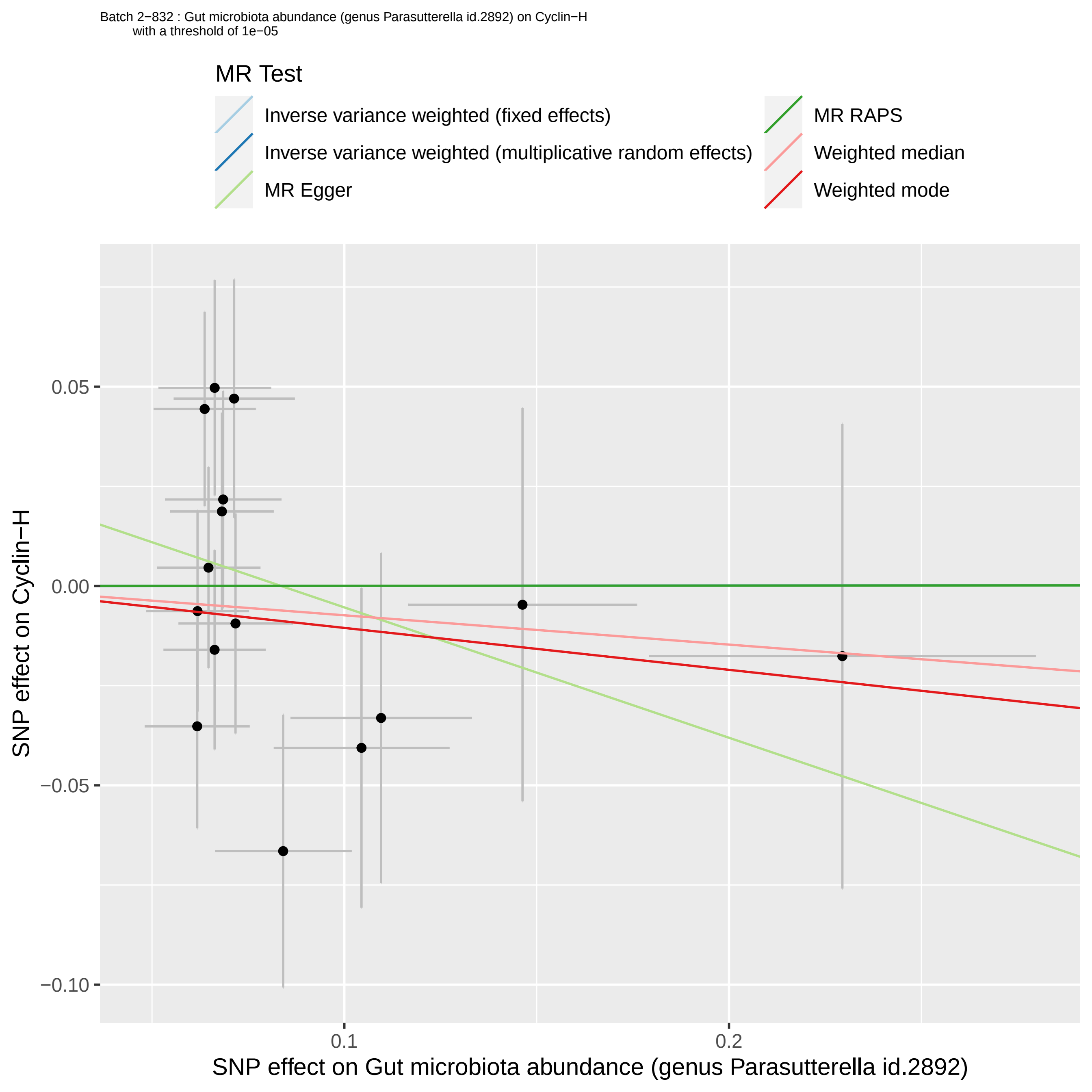
Batch 2−832 : Gut microbiota abundance (genus Parasutterella id.2892) on Cyclin−H
with a threshold of 1e−05
MR Test
Inverse variance weighted (fixed effects)
Inverse variance weighted (multiplicative random effects)
MR Egger
MR RAPS
Weighted median
Weighted mode
SNP effect on Cyclin−H
0.05
0.00
−0.05
−0.10
0.1
0.2
SNP effect on Gut microbiota abundance (genus Parasutterella id.2892)

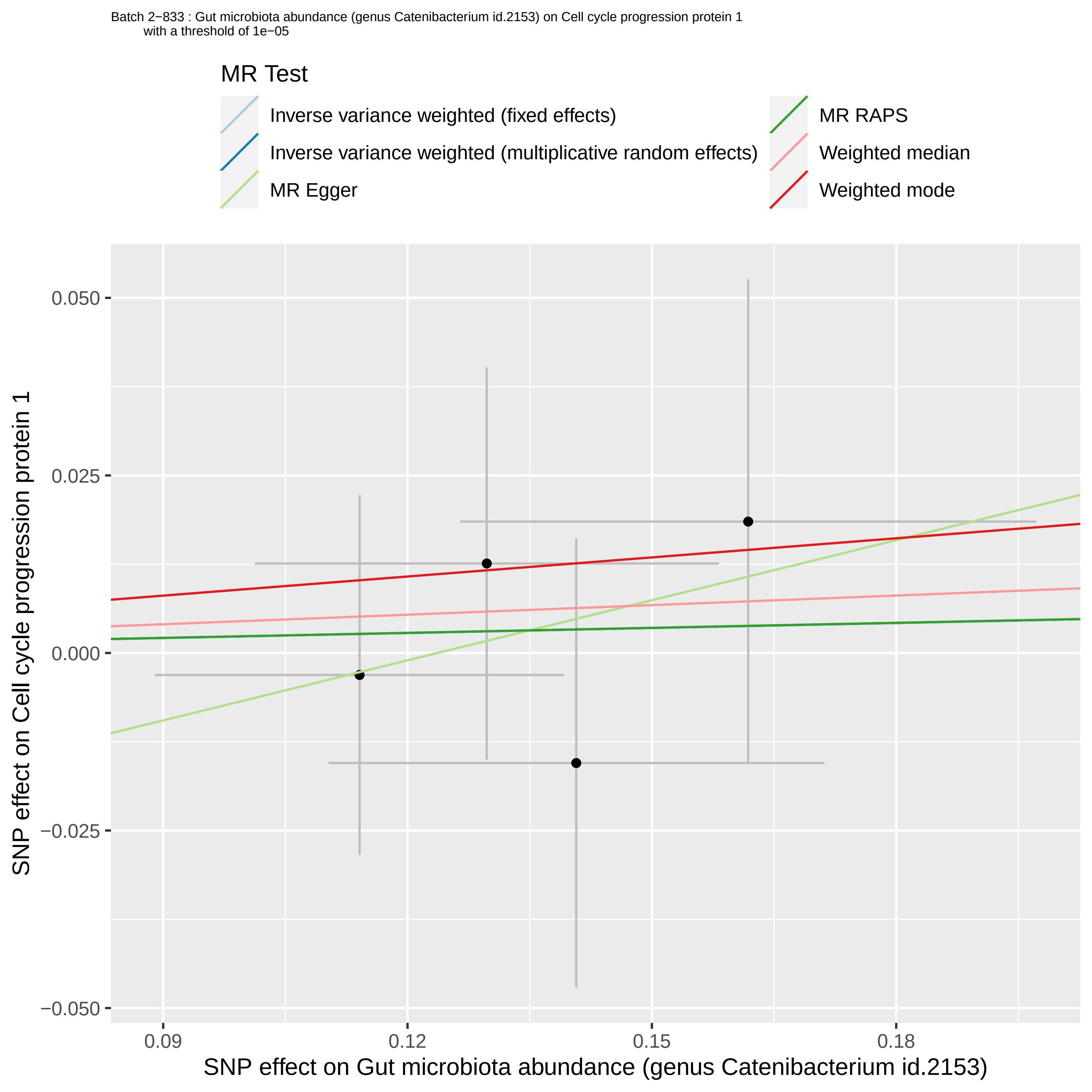
Batch 2−833 : Gut microbiota abundance (genus Catenibacterium id.2153) on Cell cycle progression protein 1
with a threshold of 1e−05
MR Test
Inverse variance weighted (fixed effects)
Inverse variance weighted (multiplicative random effects)
MR Egger
MR RAPS
Weighted median
Weighted mode
0.050
0.025
0.000
−0.025
−0.050
0.09
0.12
0.15
0.18
SNP effect on Cell cycle progression protein 1
SNP effect on Gut microbiota abundance (genus Catenibacterium id.2153)

Batch 2−834 : Gut microbiota abundance (genus Parasutterella id.2892) on Cell cycle progression protein 1
with a threshold of 1e−05

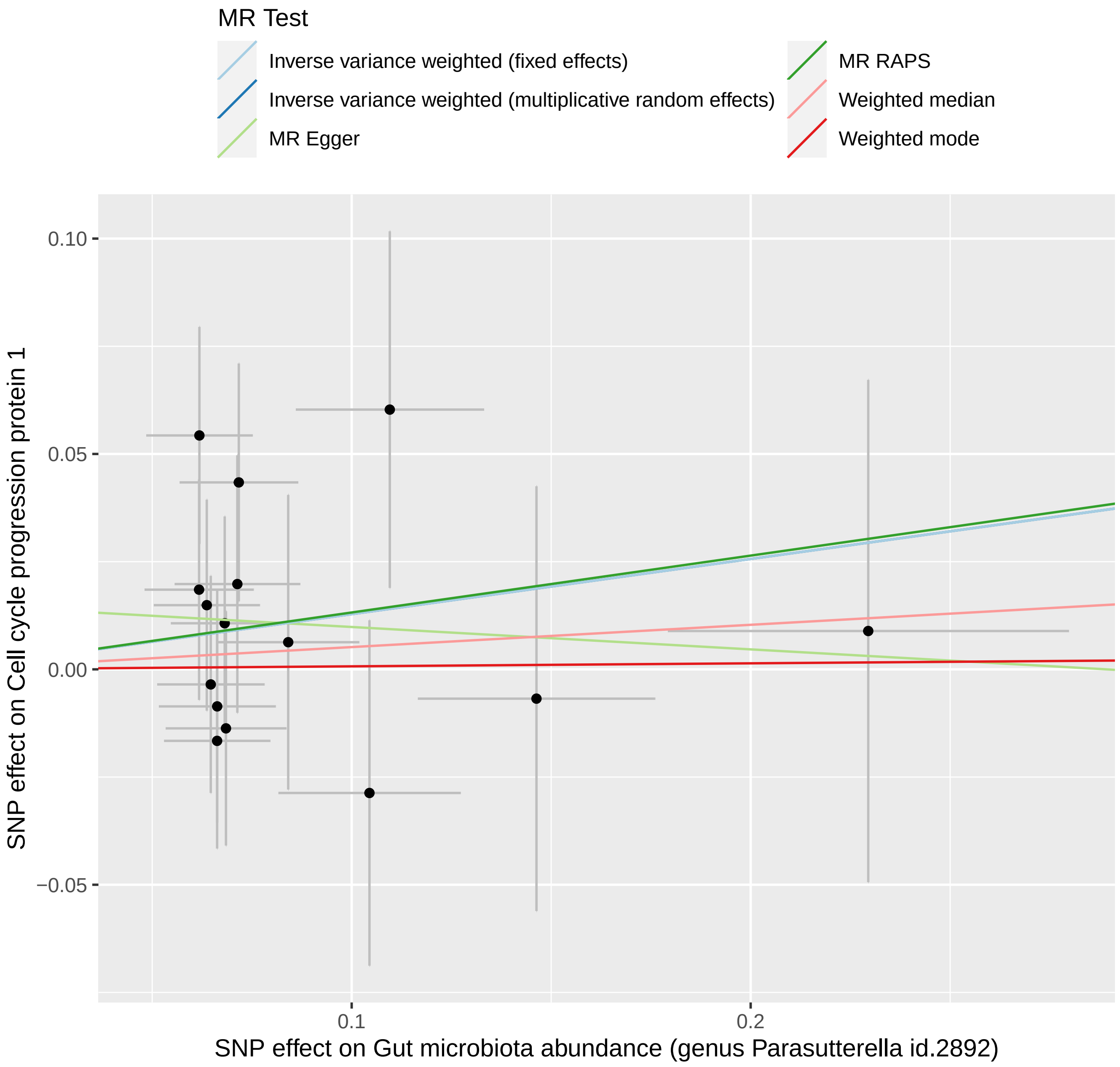

Batch 2−835 : Gut microbiota abundance (genus Catenibacterium id.2153) on CD109 antigen
with a threshold of 1e−05

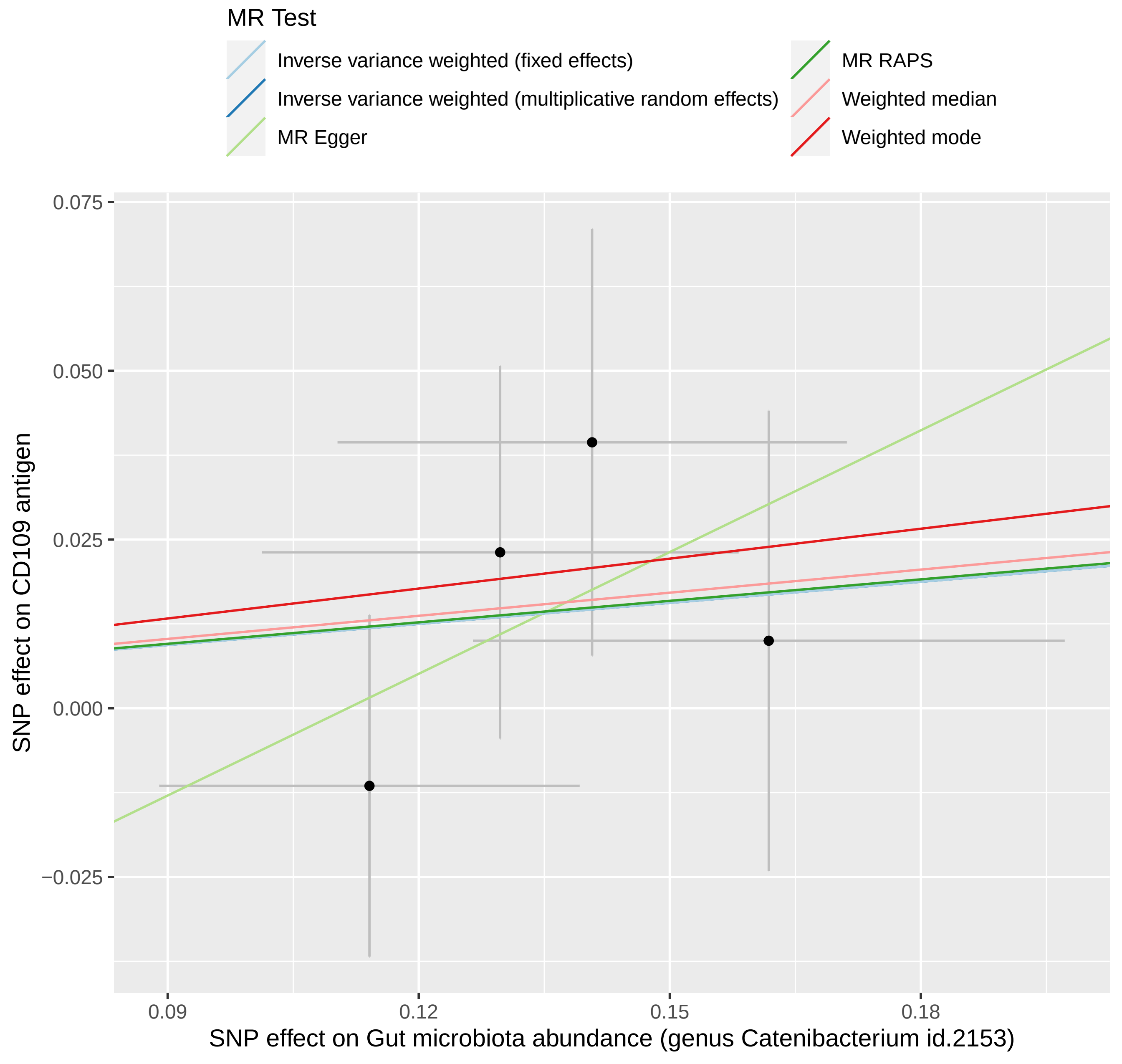

Batch 2−836 : Gut microbiota abundance (genus Parasutterella id.2892) on CD109 antigen
with a threshold of 1e−05

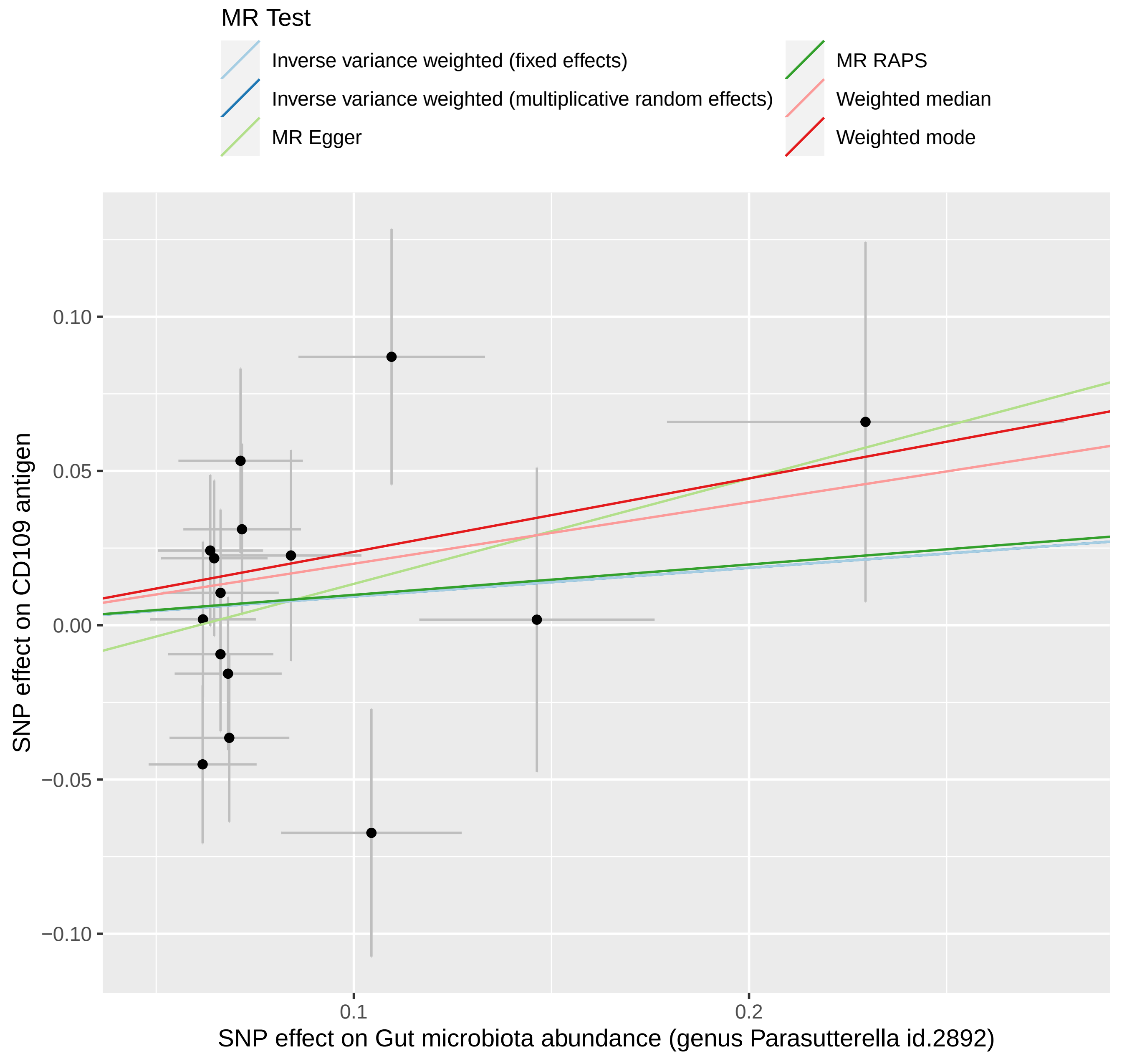

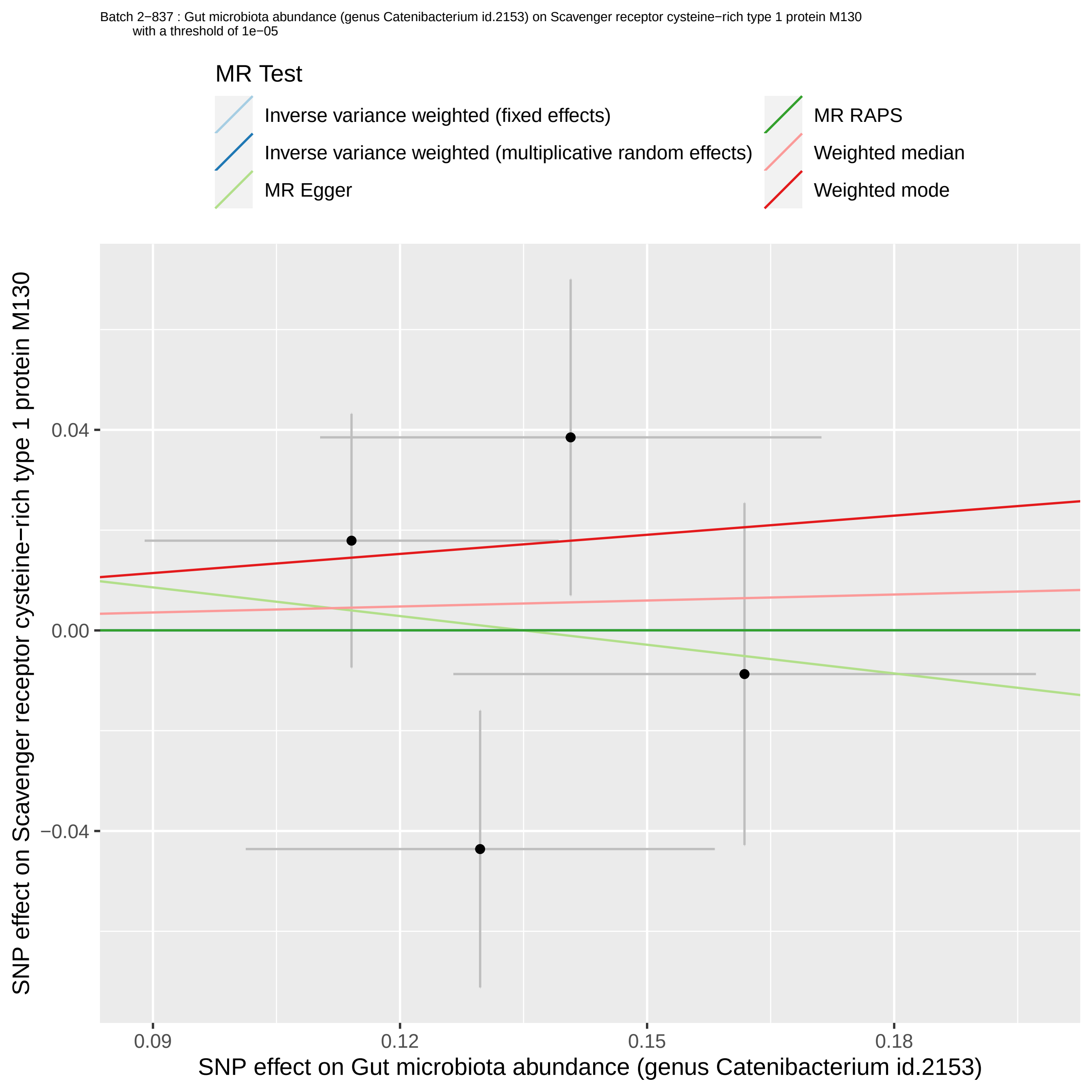

Batch 2−837 : Gut microbiota abundance (genus Catenibacterium id.2153) on Scavenger receptor cysteine−rich type 1 protein M130
with a threshold of 1e−05
MR Test
Inverse variance weighted (fixed effects)
Inverse variance weighted (multiplicative random effects)
MR Egger
MR RAPS
Weighted median
Weighted mode
0.04
0.00
−0.04
0.09
0.12
0.15
0.18
SNP effect on Scavenger receptor cysteine−rich type 1 protein M130
SNP effect on Gut microbiota abundance (genus Catenibacterium id.2153)

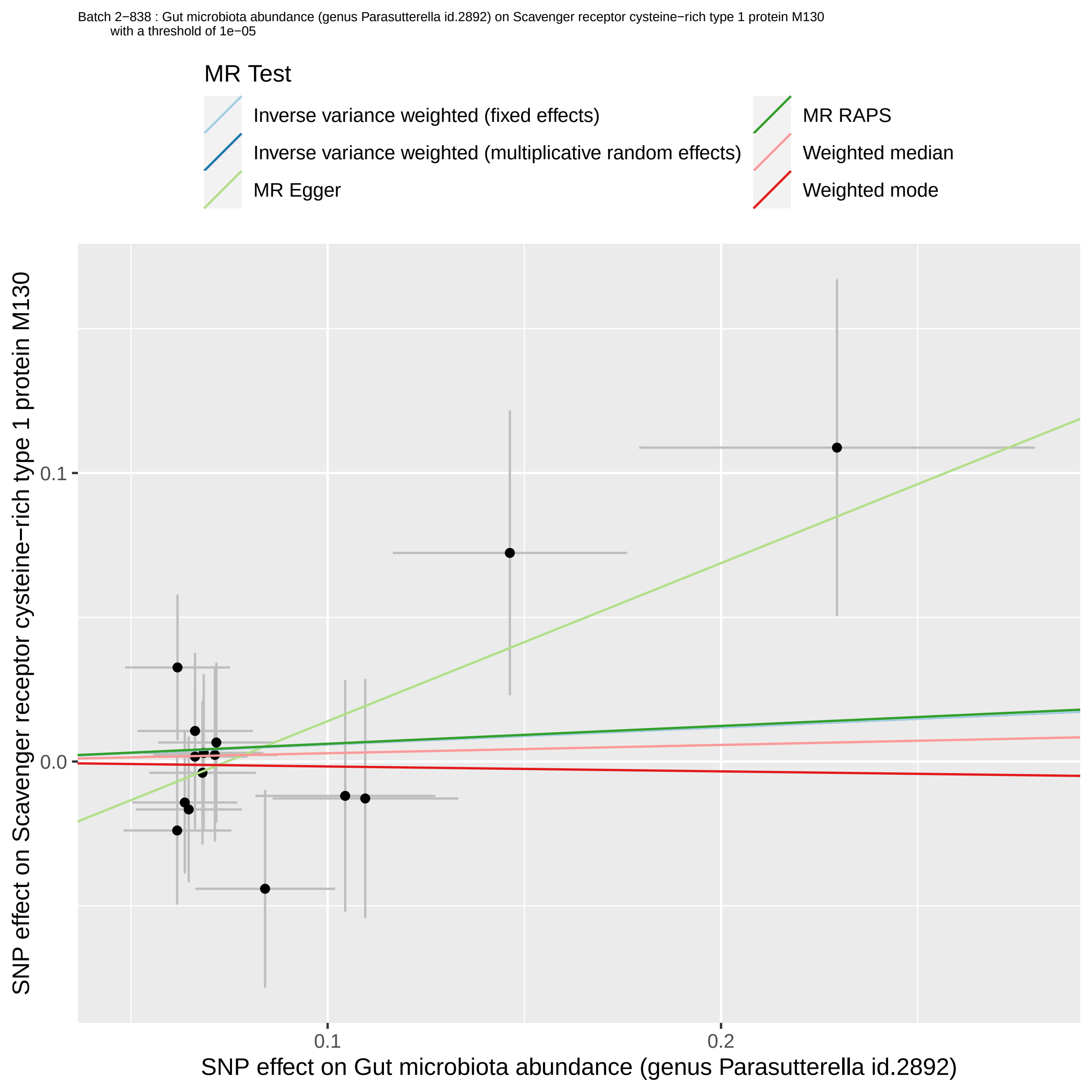

Batch 2−838 : Gut microbiota abundance (genus Parasutterella id.2892) on Scavenger receptor cysteine−rich type 1 protein M130
with a threshold of 1e−05
MR Test
Inverse variance weighted (fixed effects)
Inverse variance weighted (multiplicative random effects)
MR Egger
MR RAPS
Weighted median
Weighted mode
SNP effect on Scavenger receptor cysteine−rich type 1 protein M130
0.1
0.0
0.1
0.2
SNP effect on Gut microbiota abundance (genus Parasutterella id.2892)

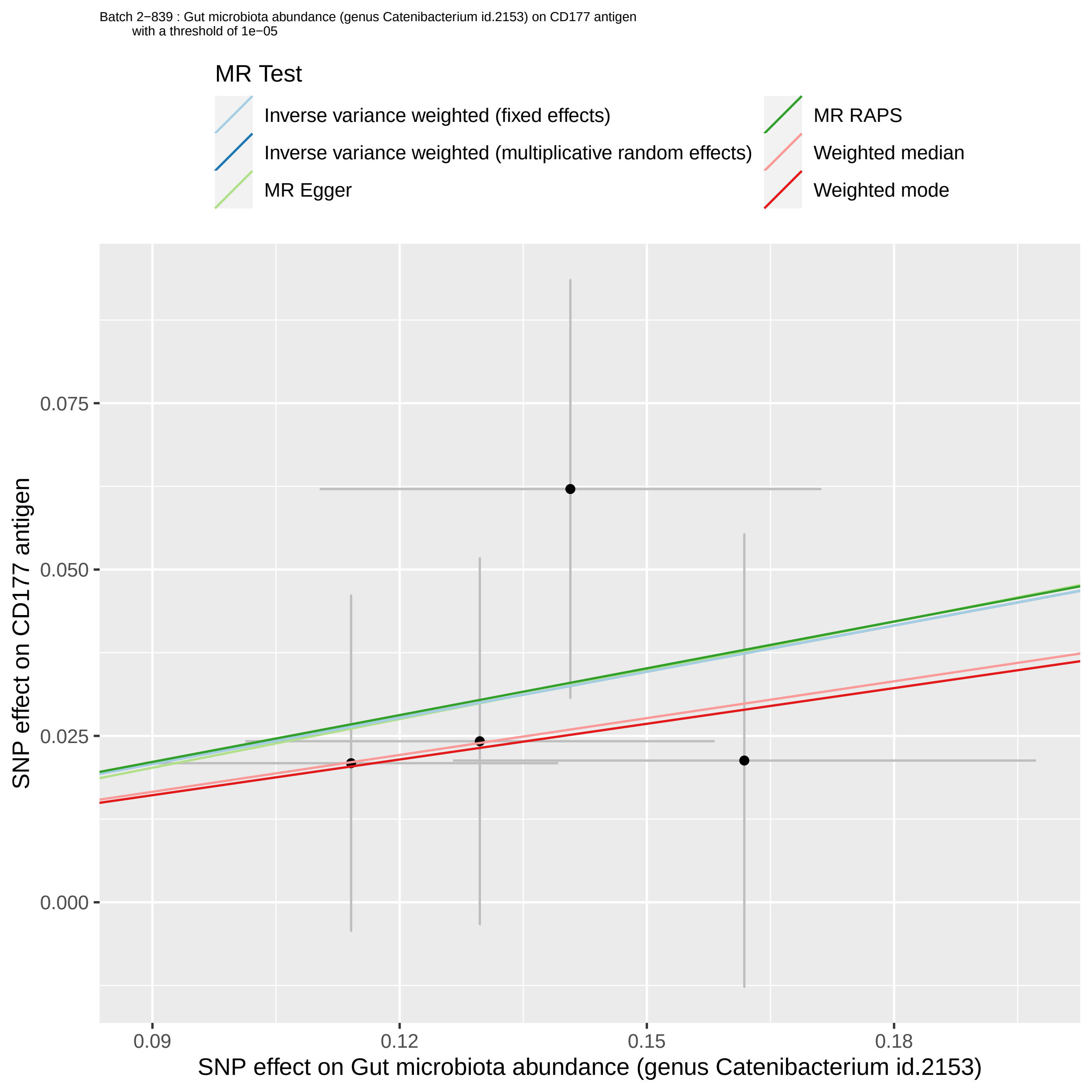
Batch 2−839 : Gut microbiota abundance (genus Catenibacterium id.2153) on CD177 antigen
with a threshold of 1e−05
MR Test
Inverse variance weighted (fixed effects)
Inverse variance weighted (multiplicative random effects)
MR Egger
MR RAPS
Weighted median
Weighted mode
0.075
0.050
0.025
0.000
0.09
0.12
0.15
0.18
SNP effect on CD177 antigen
SNP effect on Gut microbiota abundance (genus Catenibacterium id.2153)

Batch 2−840 : Gut microbiota abundance (genus Parasutterella id.2892) on CD177 antigen
with a threshold of 1e−05

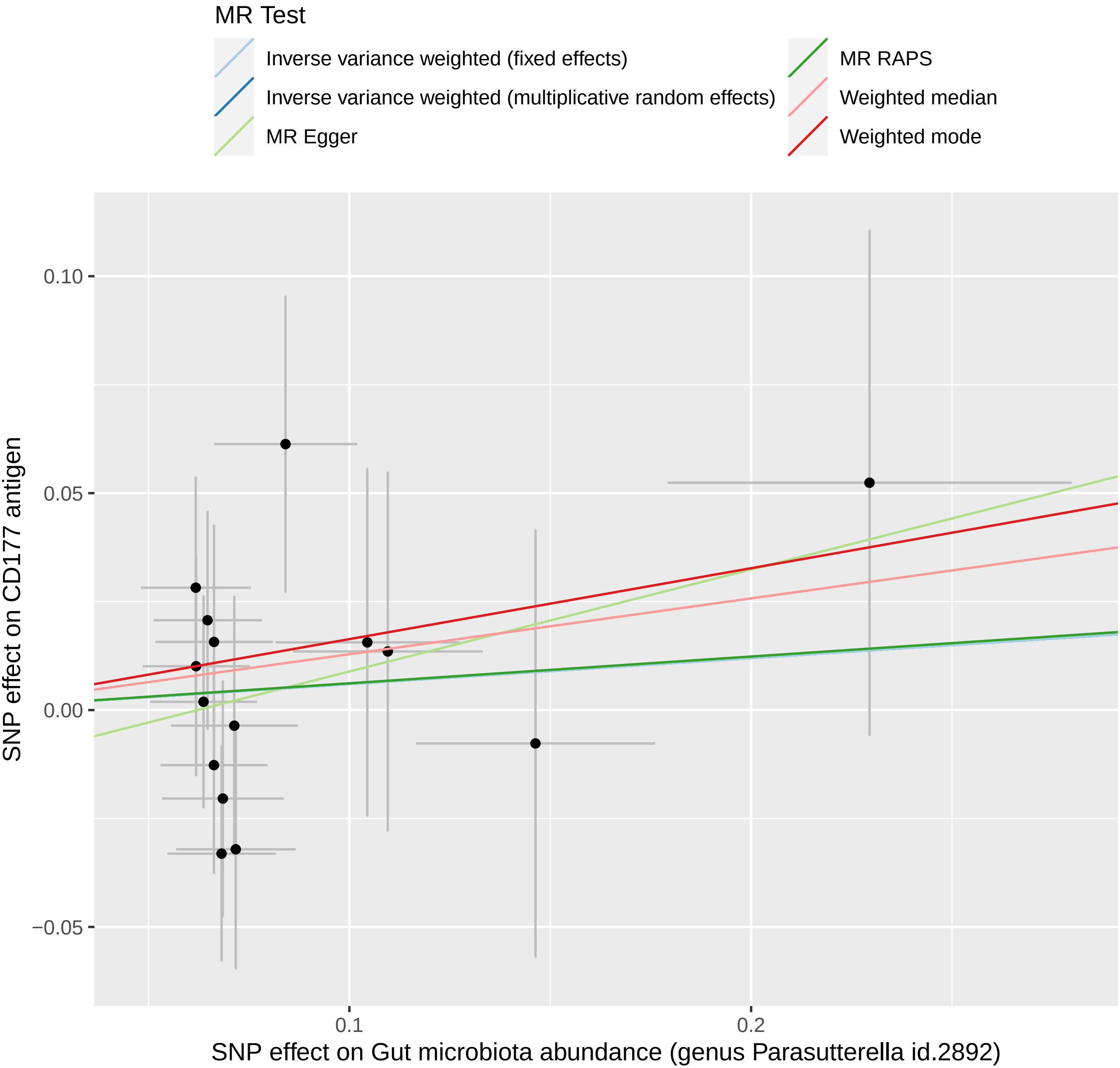

Batch 2−841 : Gut microbiota abundance (genus Catenibacterium id.2153) on Antigen−presenting glycoprotein CD1d
with a threshold of 1e−05

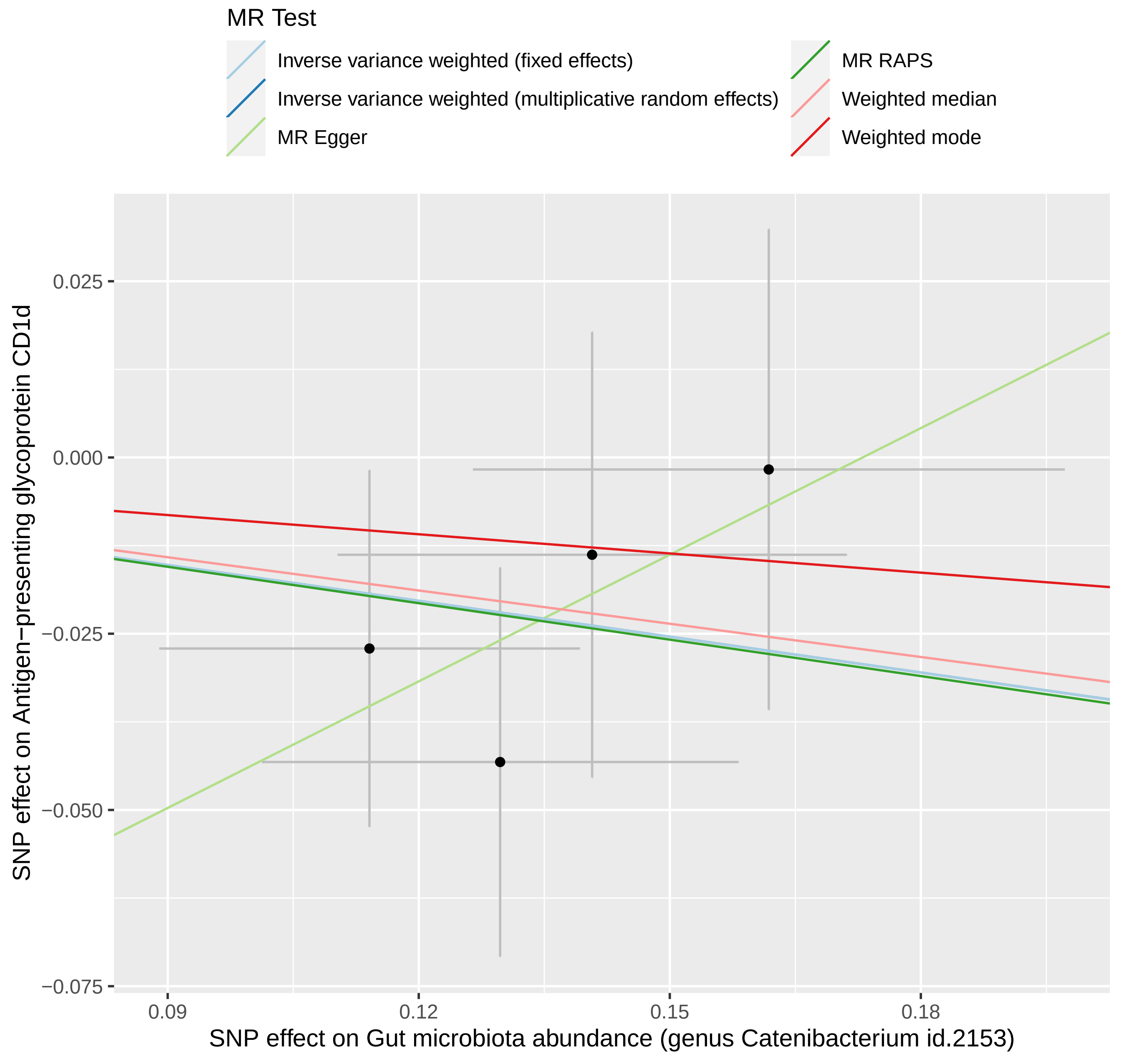

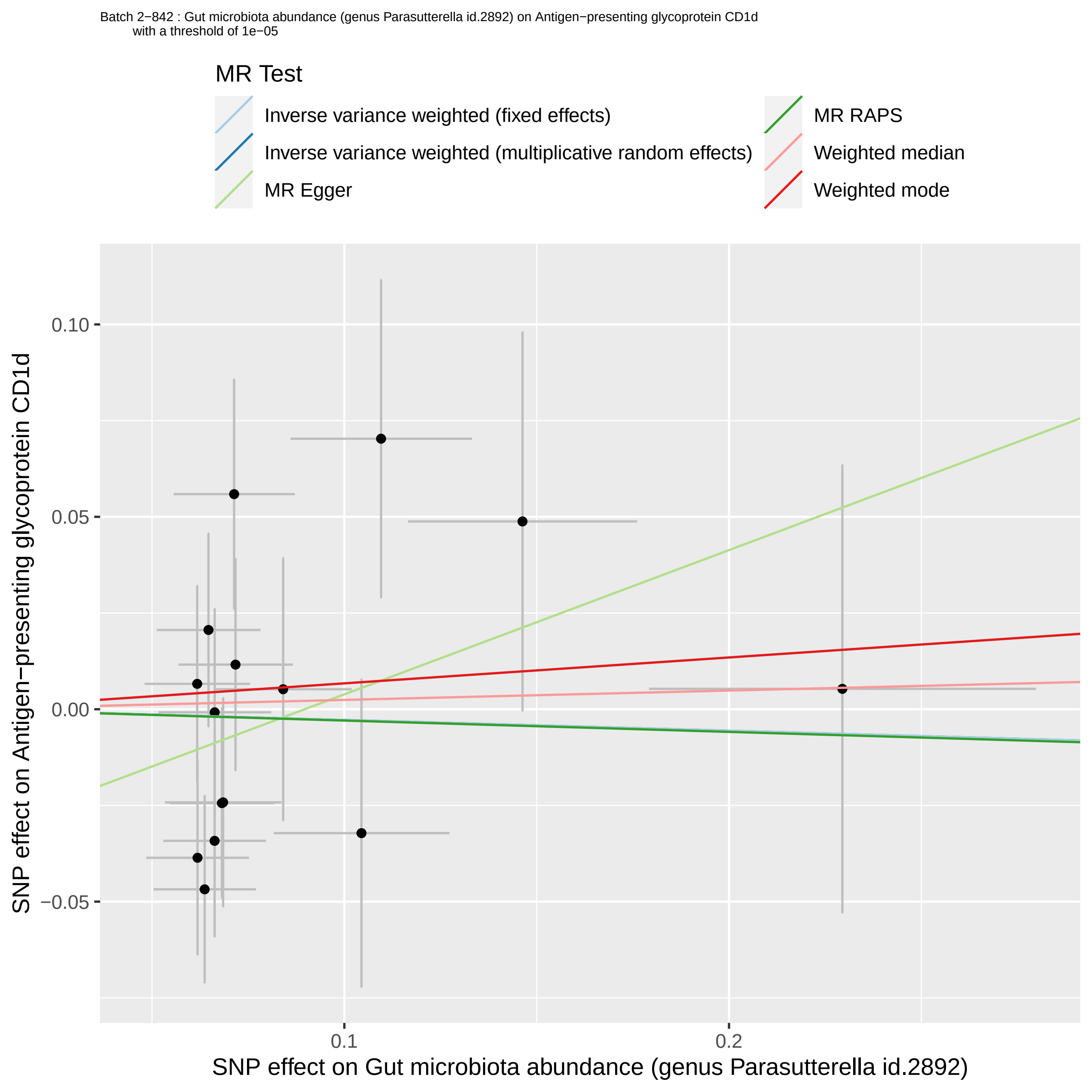

Batch 2−842 : Gut microbiota abundance (genus Parasutterella id.2892) on Antigen−presenting glycoprotein CD1d
with a threshold of 1e−05
MR Test
Inverse variance weighted (fixed effects)
Inverse variance weighted (multiplicative random effects)
MR Egger
MR RAPS
Weighted median
Weighted mode
0.10
0.05
0.00
−0.05
0.1
0.2
SNP effect on Antigen−presenting glycoprotein CD1d
SNP effect on Gut microbiota abundance (genus Parasutterella id.2892)

Batch 2−843 : Gut microbiota abundance (genus Catenibacterium id.2153) on OX−2 membrane glycoprotein
with a threshold of 1e−05

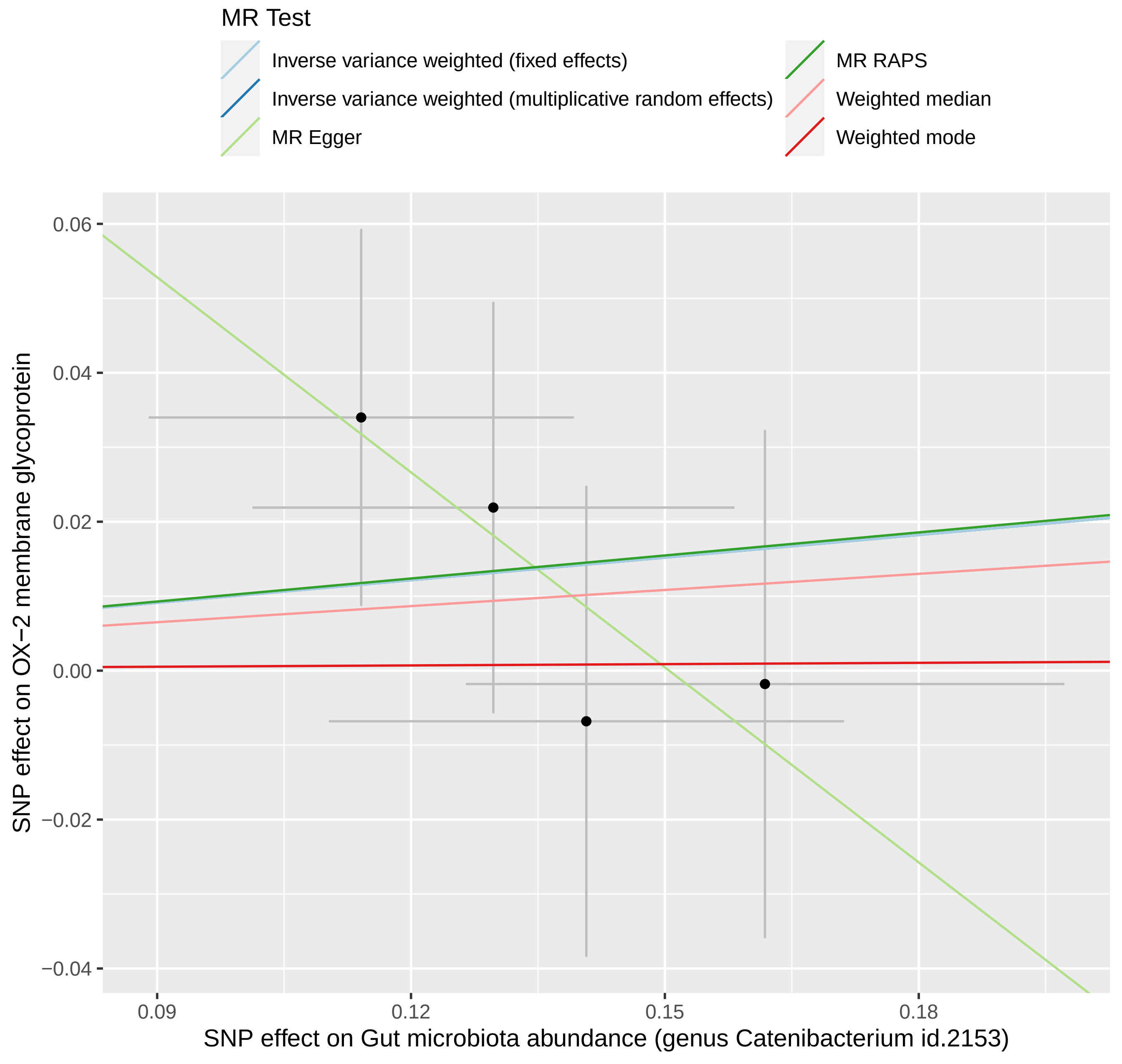

Batch 2−844 : Gut microbiota abundance (genus Parasutterella id.2892) on OX−2 membrane glycoprotein
with a threshold of 1e−05

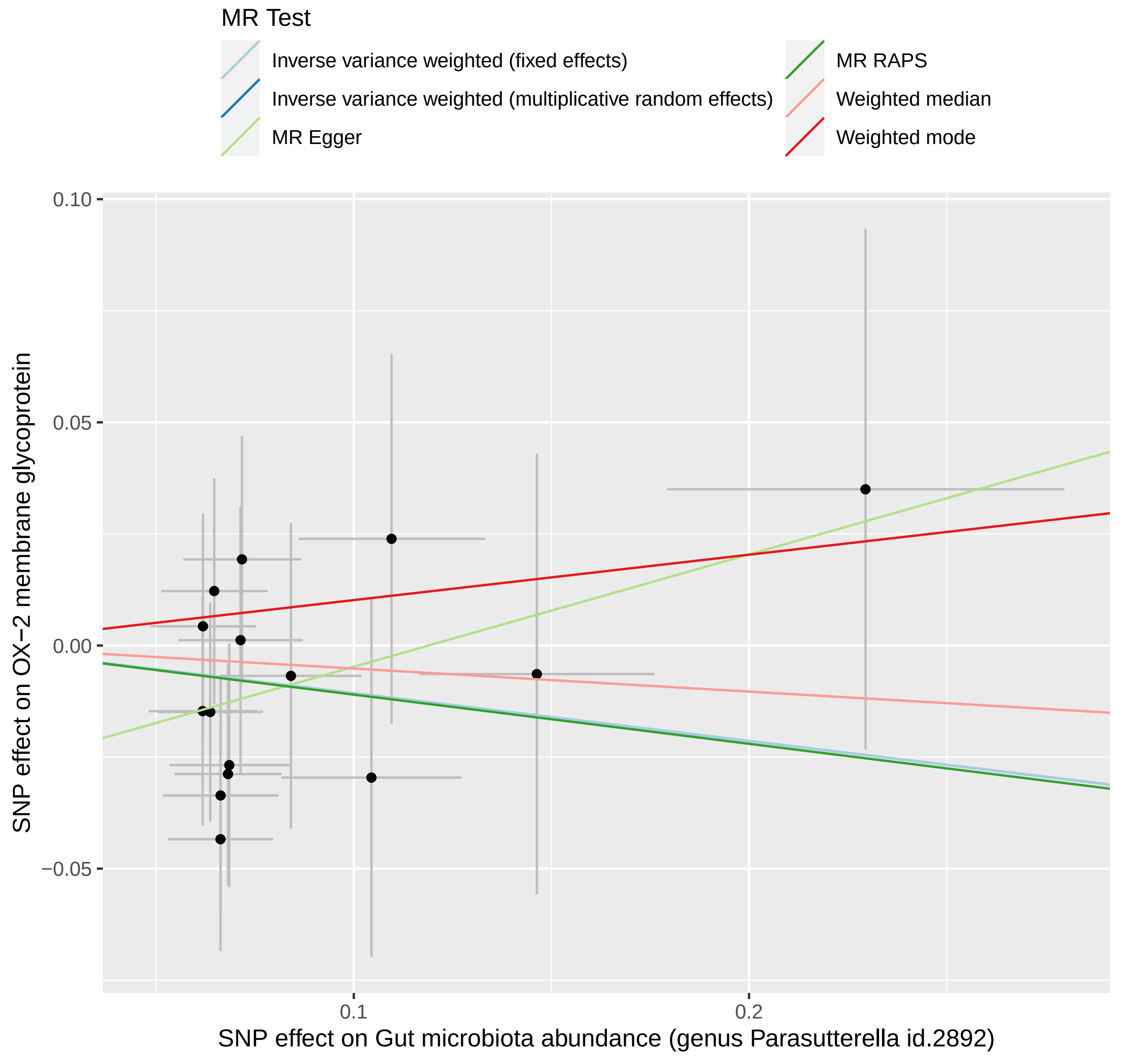

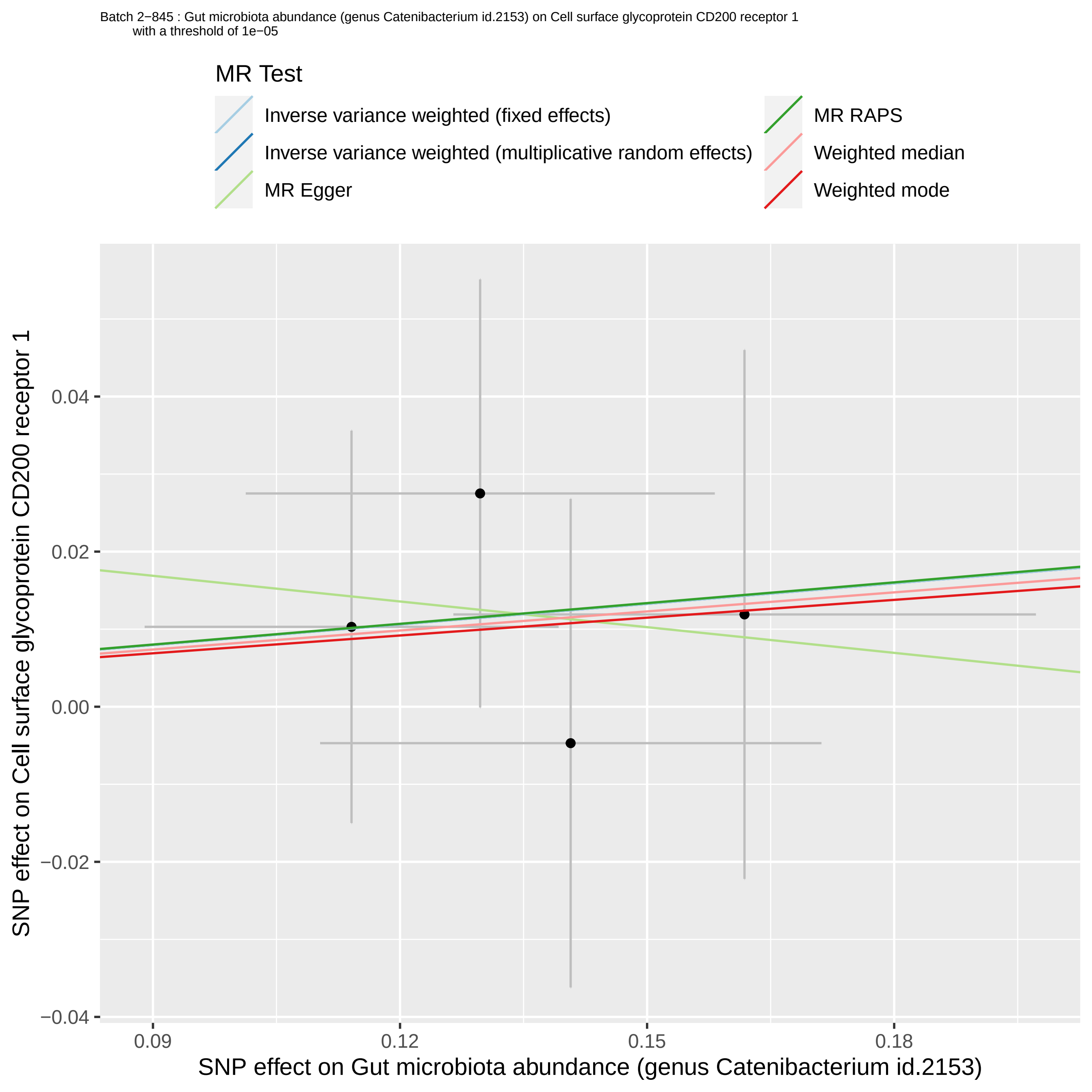

Batch 2−845 : Gut microbiota abundance (genus Catenibacterium id.2153) on Cell surface glycoprotein CD200 receptor 1
with a threshold of 1e−05
MR Test
Inverse variance weighted (fixed effects)
Inverse variance weighted (multiplicative random effects)
MR Egger
MR RAPS
Weighted median
Weighted mode
0.04
0.02
0.00
−0.02
−0.04
0.09
0.12
0.15
0.18
SNP effect on Cell surface glycoprotein CD200 receptor 1
SNP effect on Gut microbiota abundance (genus Catenibacterium id.2153)

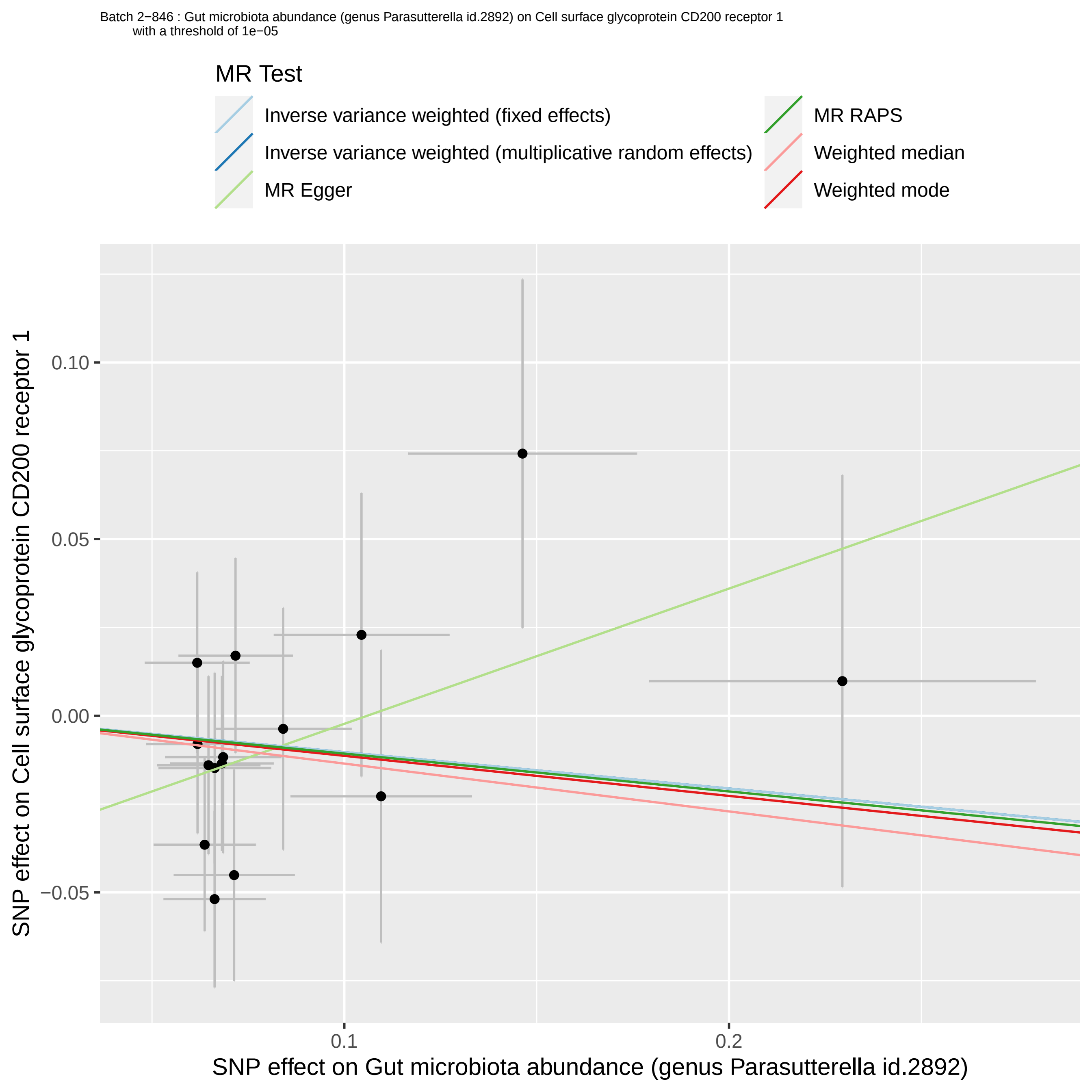

Batch 2−846 : Gut microbiota abundance (genus Parasutterella id.2892) on Cell surface glycoprotein CD200 receptor 1
with a threshold of 1e−05
MR Test
Inverse variance weighted (fixed effects)
Inverse variance weighted (multiplicative random effects)
MR Egger
MR RAPS
Weighted median
Weighted mode
0.10
0.05
0.00
−0.05
0.1
0.2
SNP effect on Cell surface glycoprotein CD200 receptor 1
SNP effect on Gut microbiota abundance (genus Parasutterella id.2892)

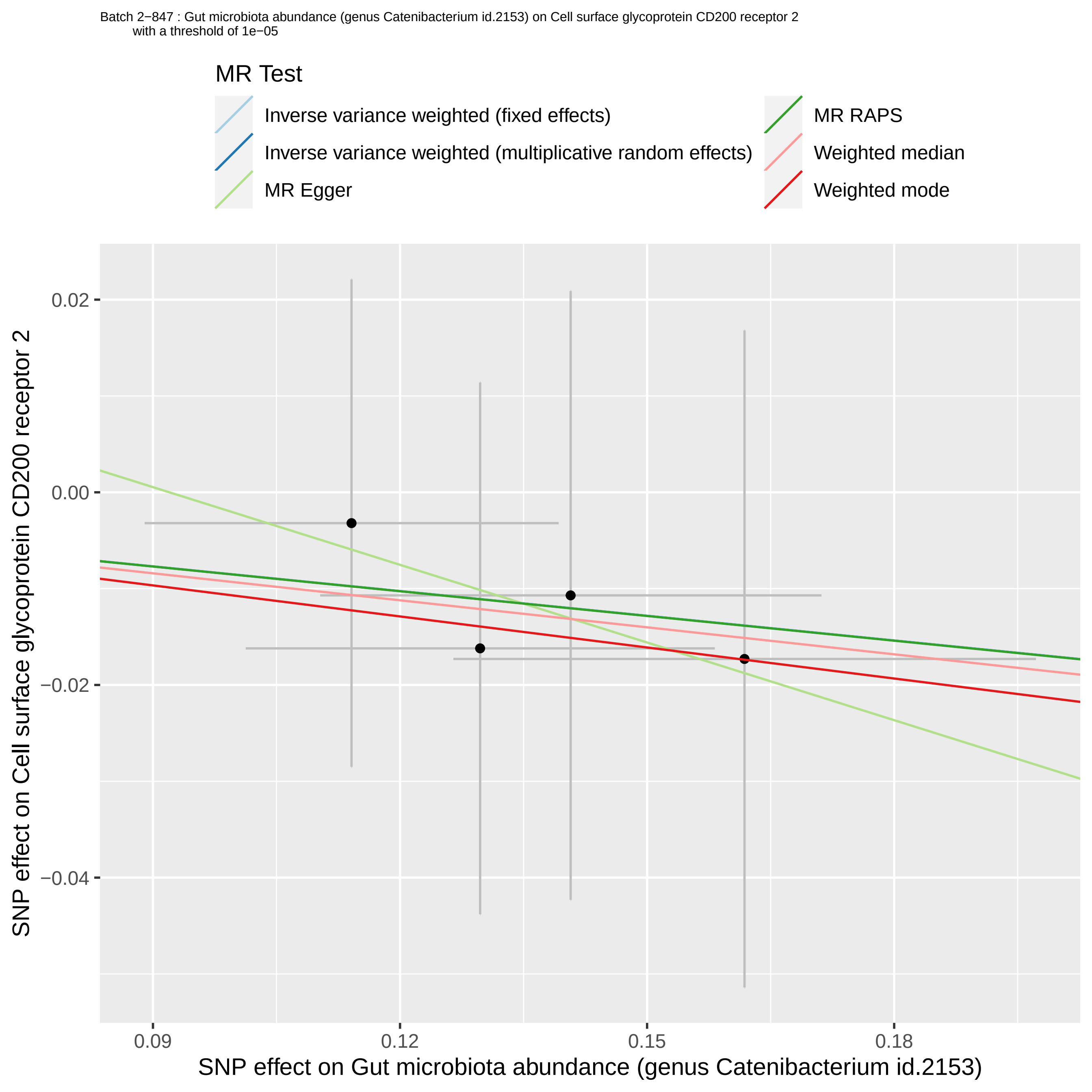

Batch 2−847 : Gut microbiota abundance (genus Catenibacterium id.2153) on Cell surface glycoprotein CD200 receptor 2
with a threshold of 1e−05
MR Test
Inverse variance weighted (fixed effects)
Inverse variance weighted (multiplicative random effects)
MR Egger
MR RAPS
Weighted median
Weighted mode
0.02
0.00
−0.02
−0.04
0.09
0.12
0.15
0.18
SNP effect on Cell surface glycoprotein CD200 receptor 2
SNP effect on Gut microbiota abundance (genus Catenibacterium id.2153)

Batch 2−848 : Gut microbiota abundance (genus Parasutterella id.2892) on Cell surface glycoprotein CD200 receptor 2
with a threshold of 1e−05

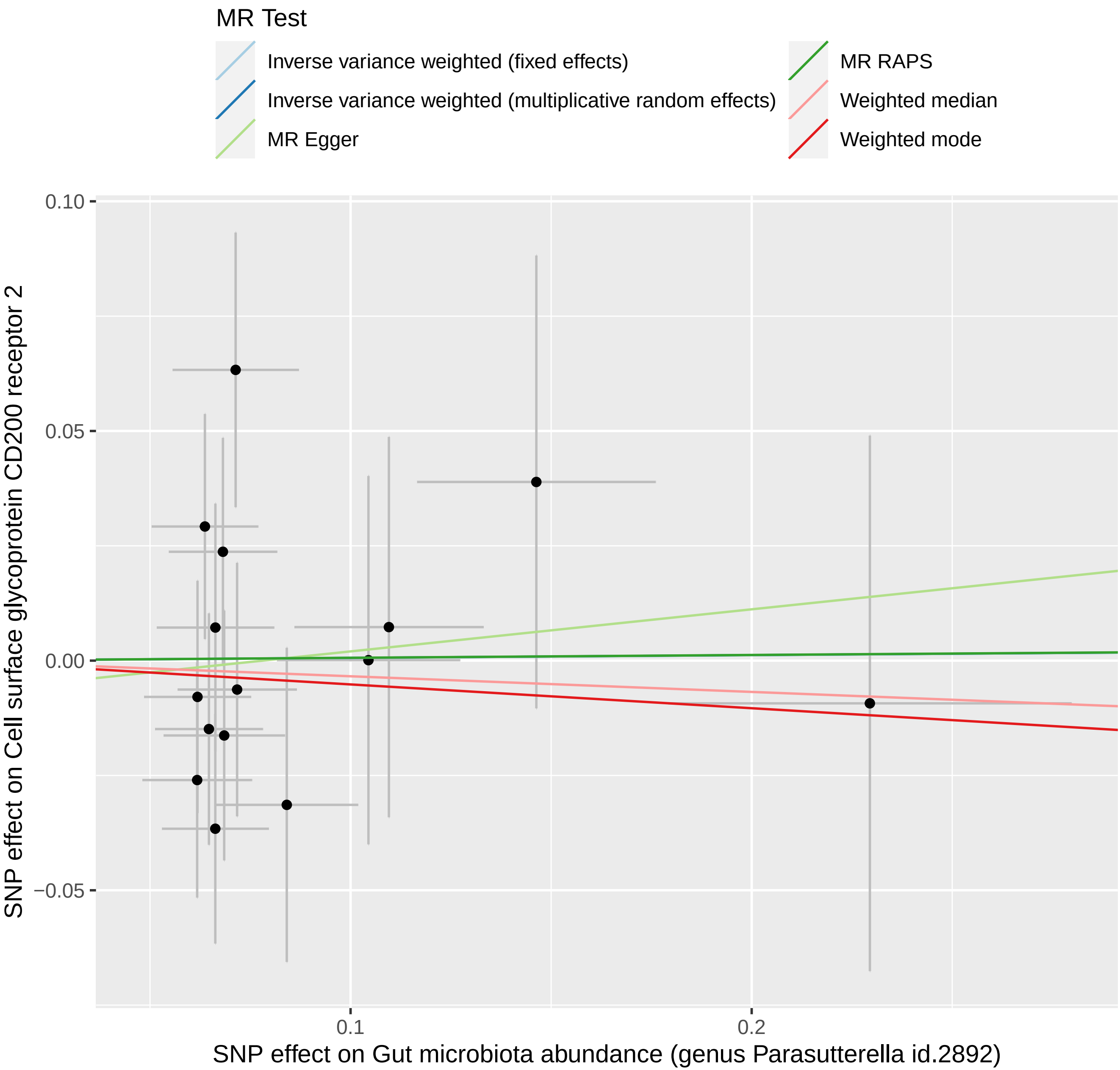

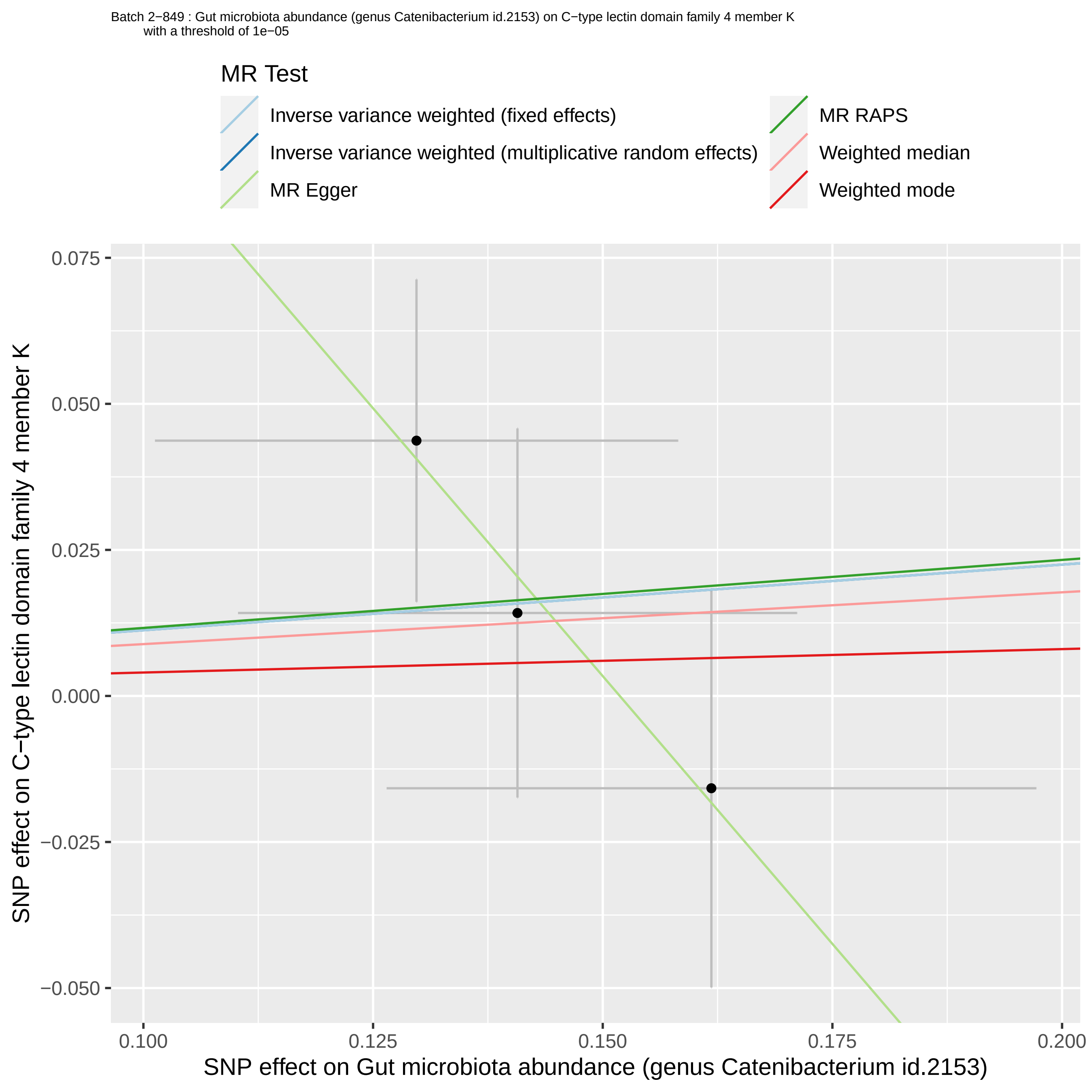
Batch 2−849 : Gut microbiota abundance (genus Catenibacterium id.2153) on C−type lectin domain family 4 member K
with a threshold of 1e−05
MR Test
Inverse variance weighted (fixed effects)
Inverse variance weighted (multiplicative random effects)
MR Egger
MR RAPS
Weighted median
Weighted mode
0.075
0.050
0.025
0.000
−0.025
−0.050
SNP effect on C−type lectin domain family 4 member K
0.100
0.125
0.150
0.175
0.200
SNP effect on Gut microbiota abundance (genus Catenibacterium id.2153)

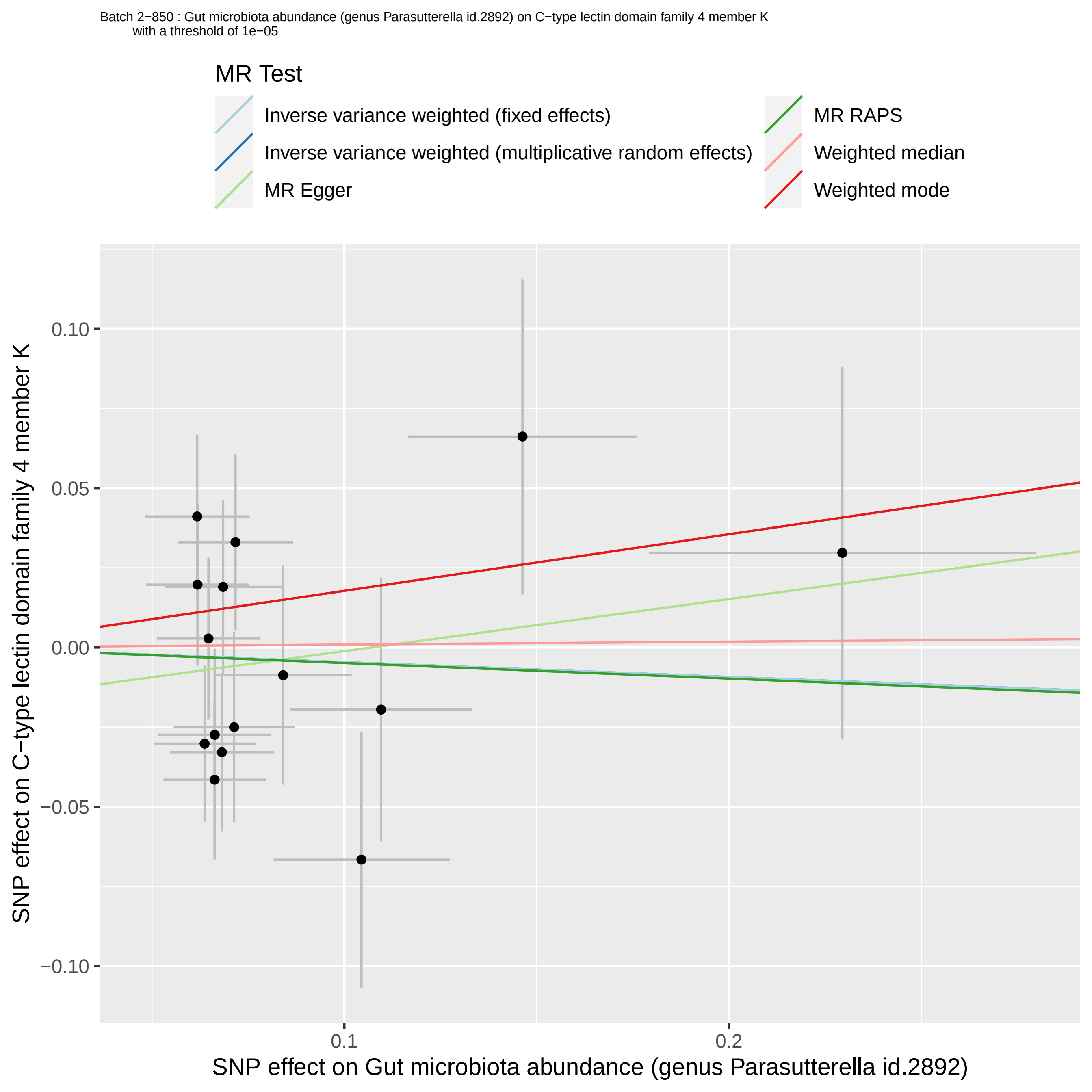
Batch 2−850 : Gut microbiota abundance (genus Parasutterella id.2892) on C−type lectin domain family 4 member K
with a threshold of 1e−05
MR Test
Inverse variance weighted (fixed effects)
Inverse variance weighted (multiplicative random effects)
MR Egger
MR RAPS
Weighted median
Weighted mode
0.10
0.05
0.00
−0.05
−0.10
0.1
0.2
SNP effect on C−type lectin domain family 4 member K
SNP effect on Gut microbiota abundance (genus Parasutterella id.2892)

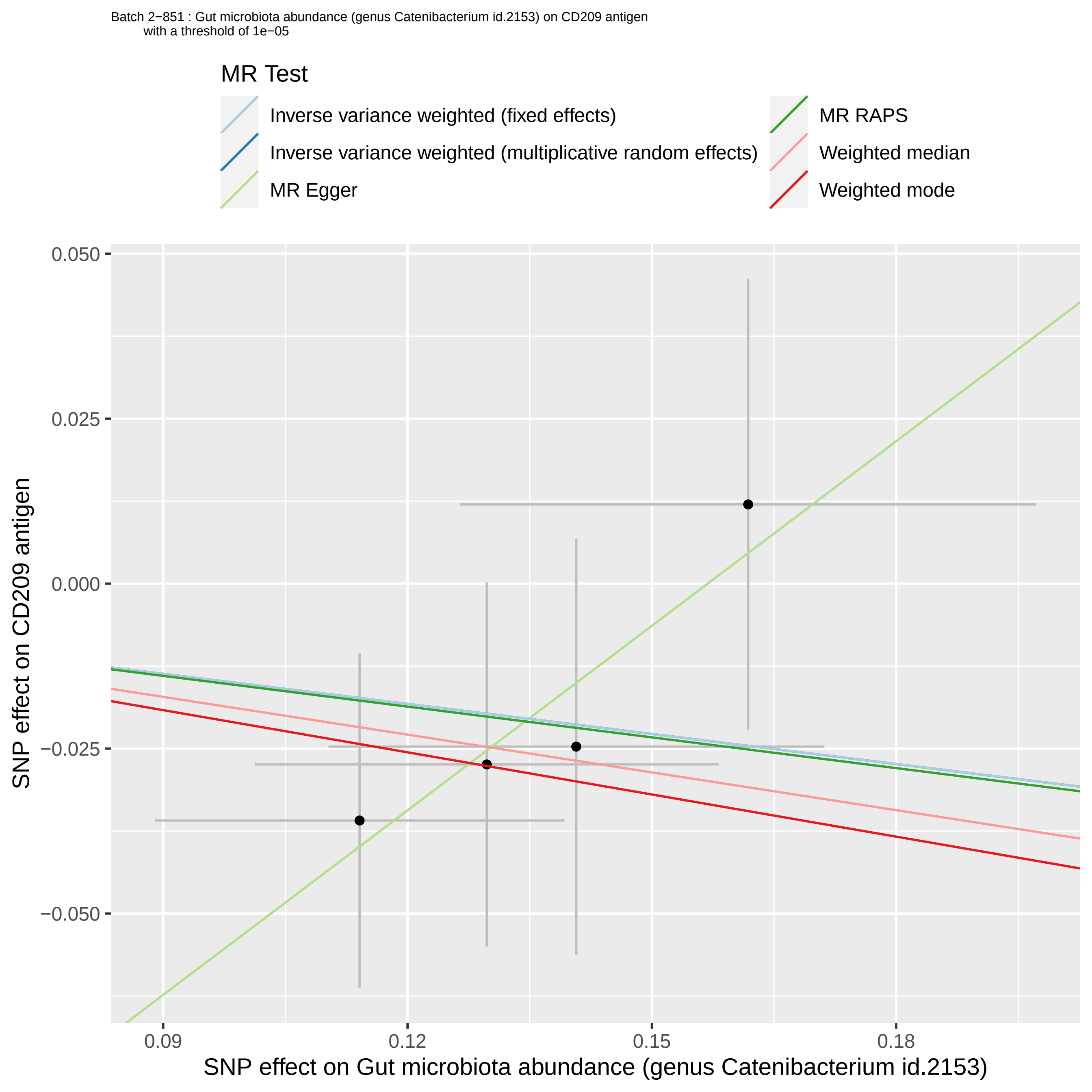
Batch 2−851 : Gut microbiota abundance (genus Catenibacterium id.2153) on CD209 antigen
with a threshold of 1e−05
MR Test
Inverse variance weighted (fixed effects)
Inverse variance weighted (multiplicative random effects)
MR Egger
MR RAPS
Weighted median
Weighted mode
0.050
0.025
0.000
−0.025
−0.050
0.09
0.12
0.15
0.18
SNP effect on CD209 antigen
SNP effect on Gut microbiota abundance (genus Catenibacterium id.2153)

Batch 2−852 : Gut microbiota abundance (genus Parasutterella id.2892) on CD209 antigen
with a threshold of 1e−05

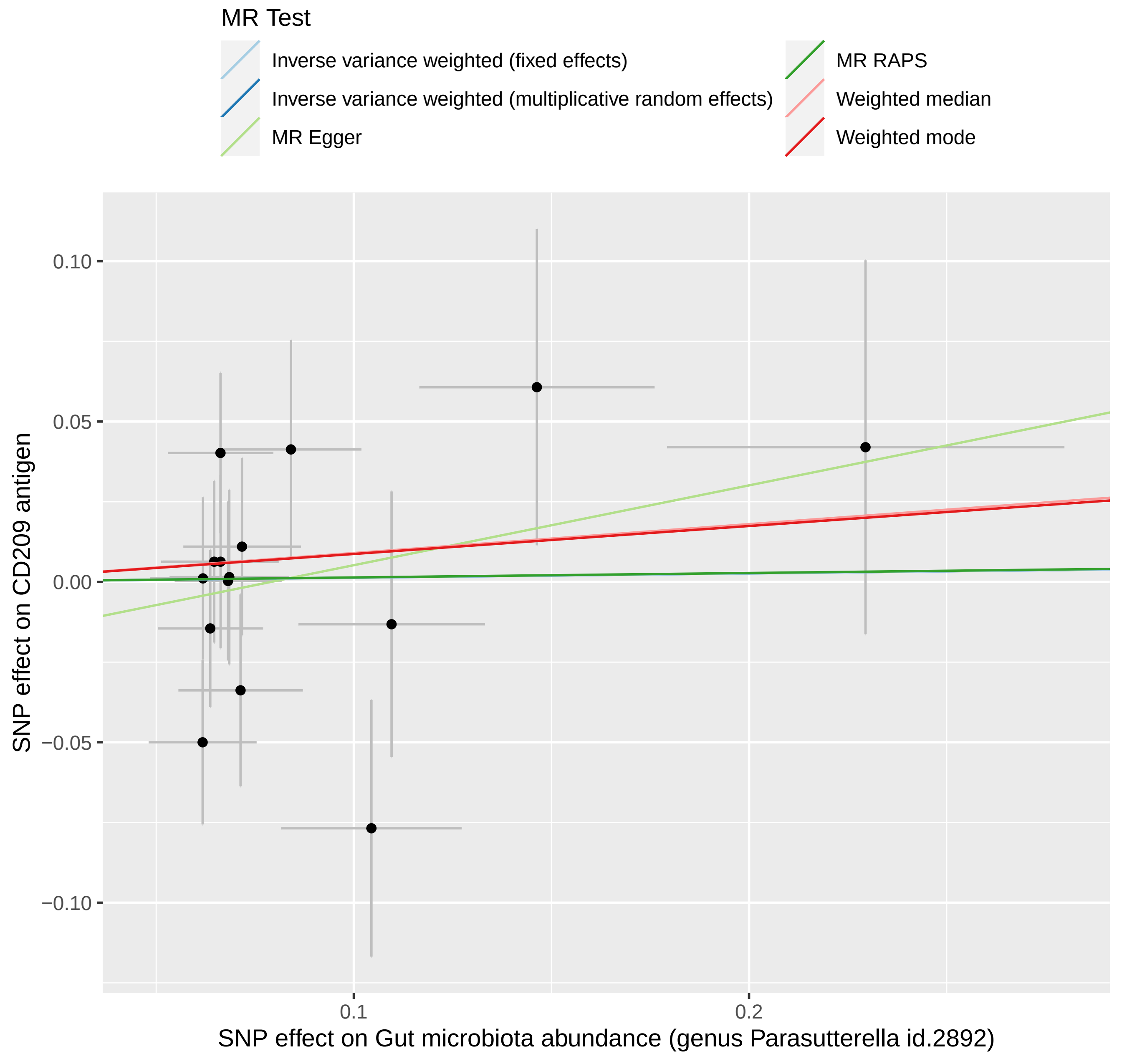

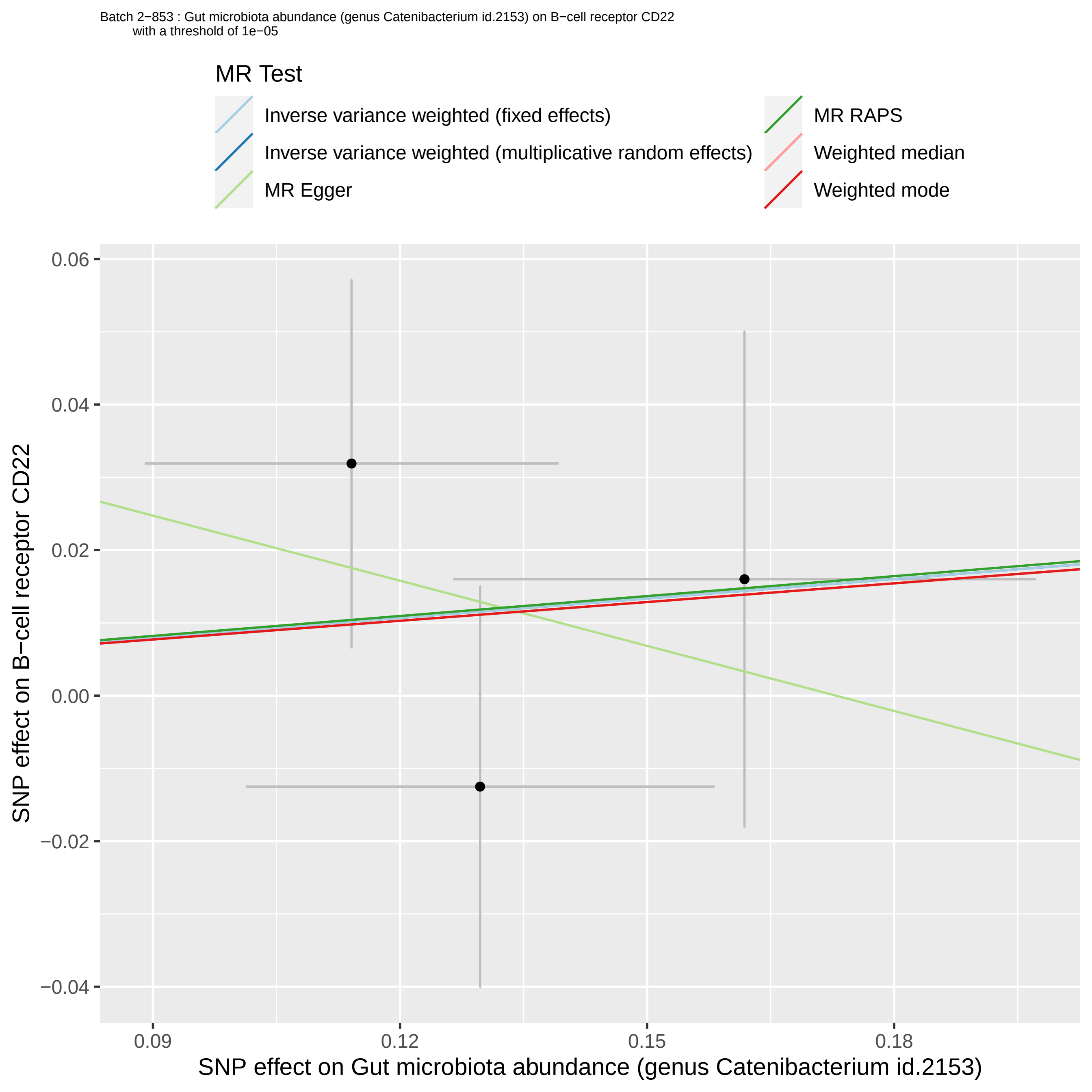

Batch 2−853 : Gut microbiota abundance (genus Catenibacterium id.2153) on B−cell receptor CD22
with a threshold of 1e−05
MR Test
Inverse variance weighted (fixed effects)
Inverse variance weighted (multiplicative random effects)
MR Egger
MR RAPS
Weighted median
Weighted mode
0.06
0.04
0.02
0.00
−0.02
−0.04
0.09
0.12
0.15
0.18
SNP effect on B−cell receptor CD22
SNP effect on Gut microbiota abundance (genus Catenibacterium id.2153)

Batch 2−854 : Gut microbiota abundance (genus Parasutterella id.2892) on B−cell receptor CD22
with a threshold of 1e−05

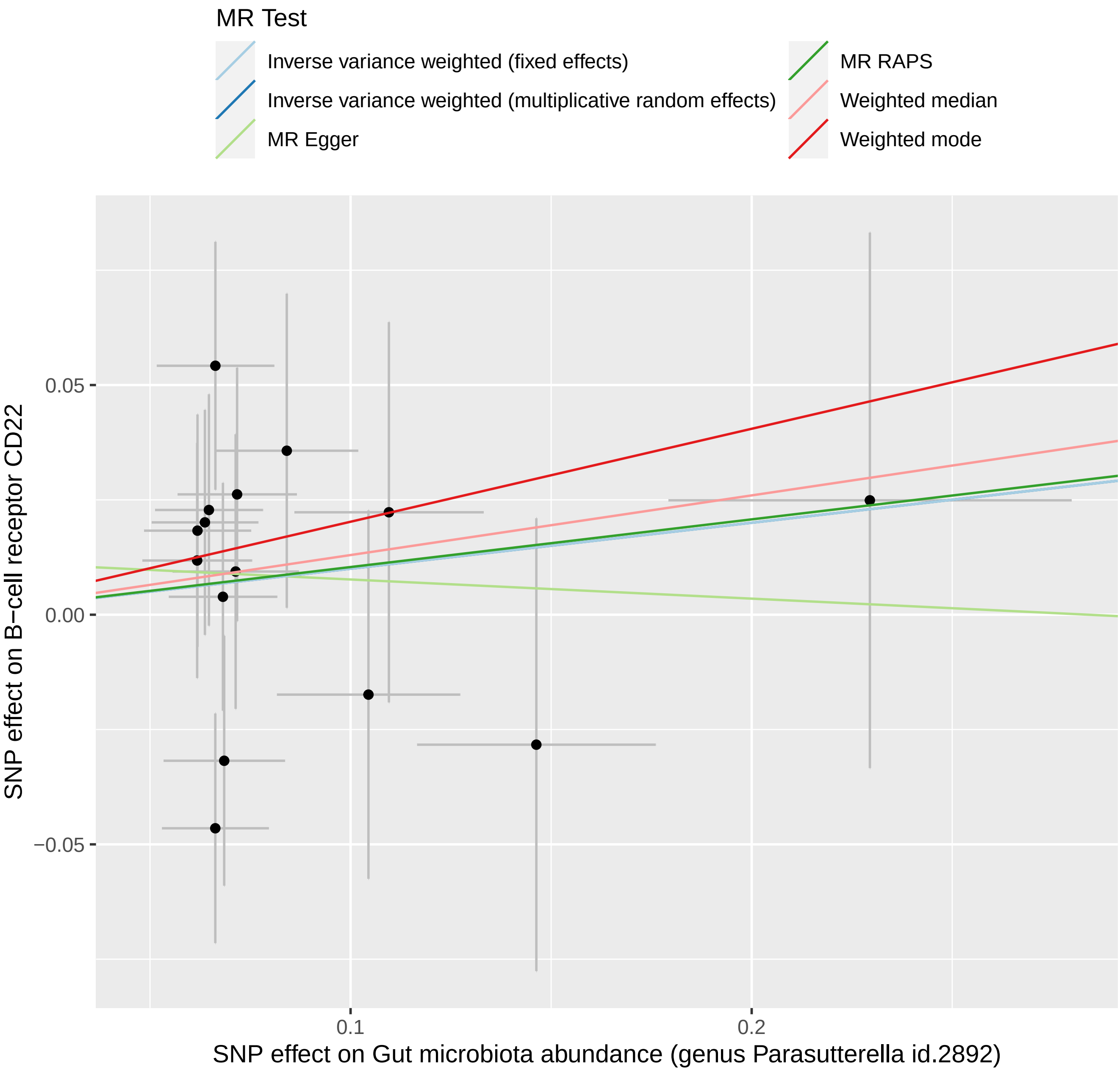

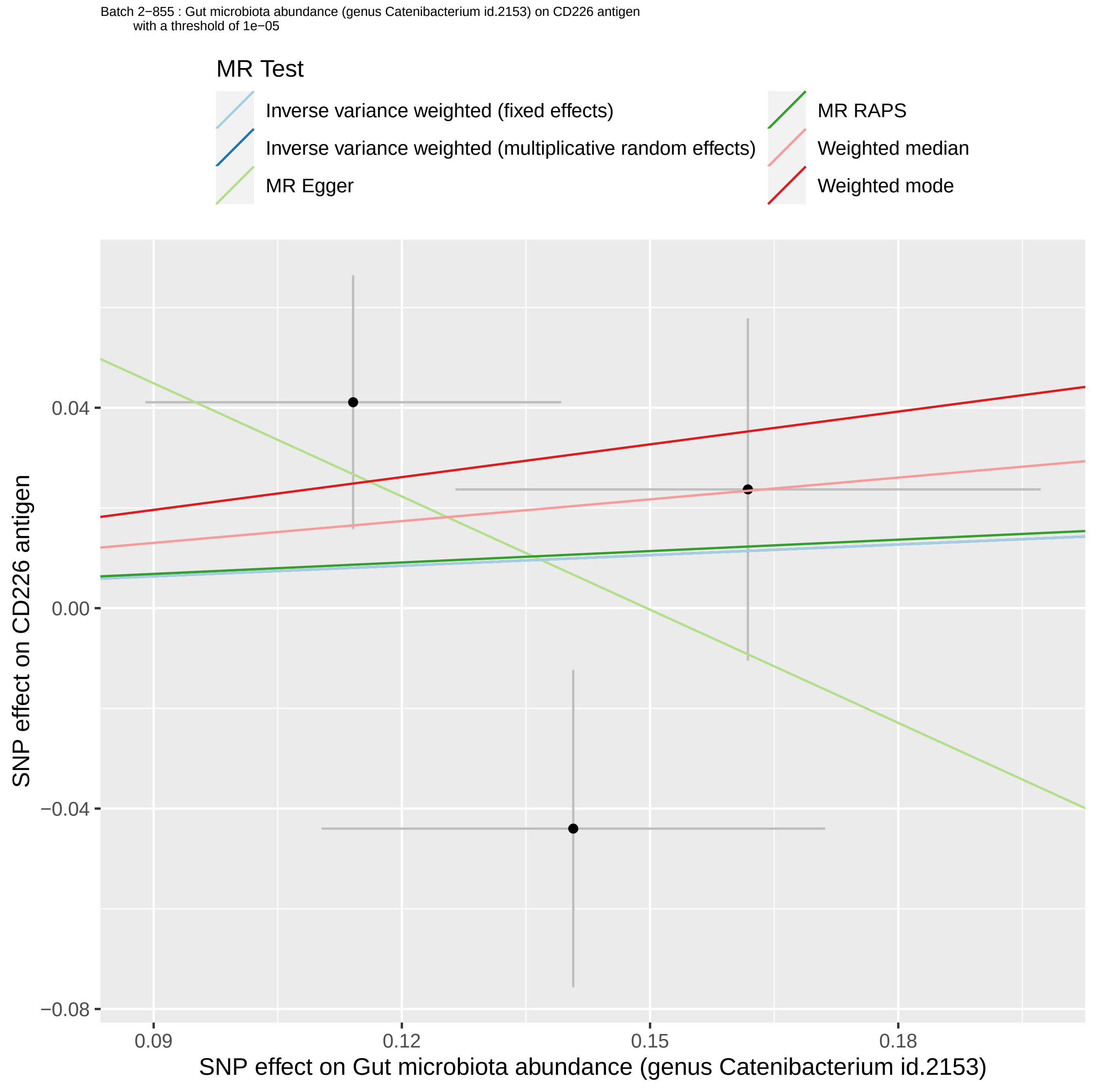

Batch 2−855 : Gut microbiota abundance (genus Catenibacterium id.2153) on CD226 antigen
with a threshold of 1e−05
MR Test
Inverse variance weighted (fixed effects)
Inverse variance weighted (multiplicative random effects)
MR Egger
MR RAPS
Weighted median
Weighted mode
0.04
0.00
−0.04
−0.08
0.09
0.12
0.15
0.18
SNP effect on CD226 antigen
SNP effect on Gut microbiota abundance (genus Catenibacterium id.2153)

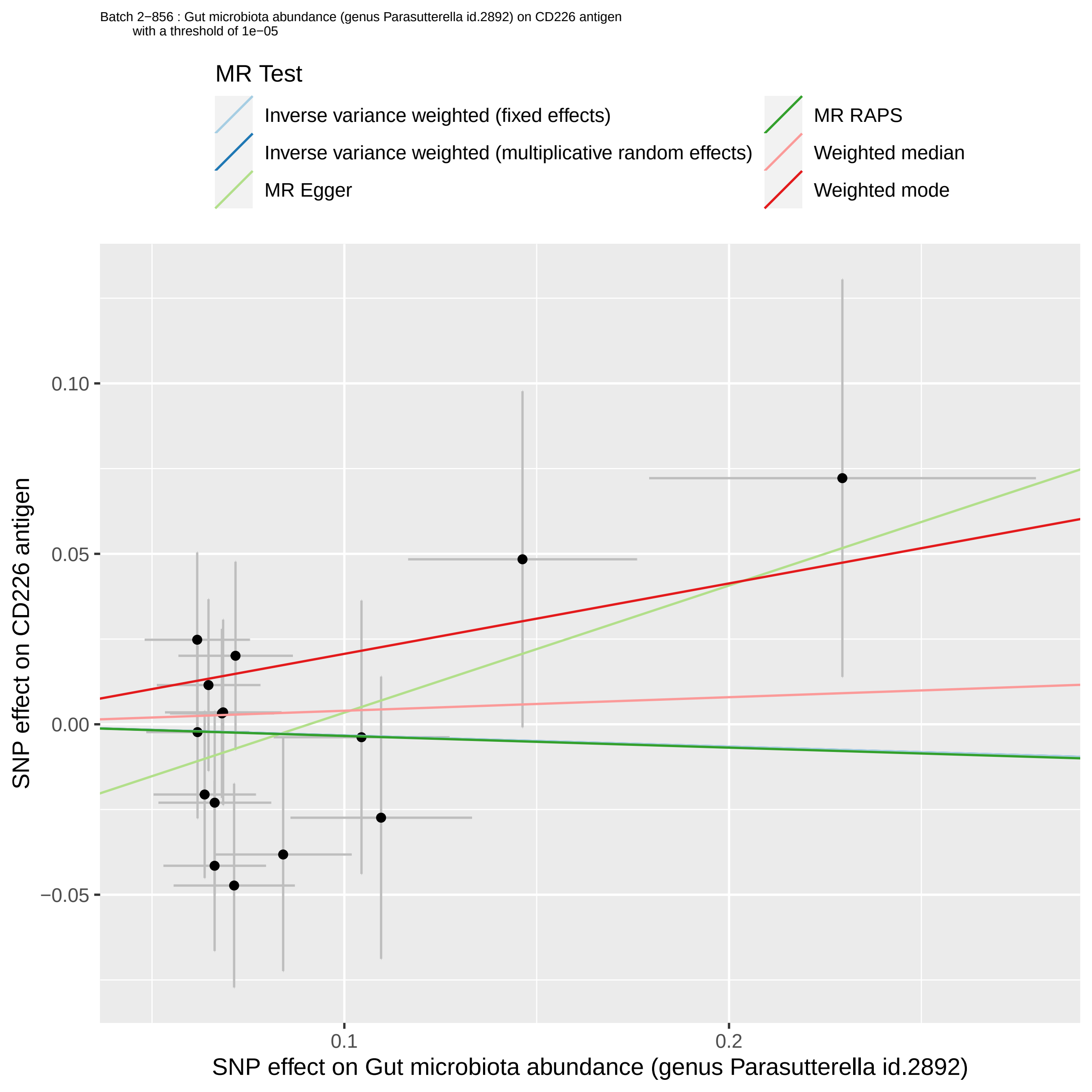
Batch 2−856 : Gut microbiota abundance (genus Parasutterella id.2892) on CD226 antigen
with a threshold of 1e−05
MR Test
Inverse variance weighted (fixed effects)
Inverse variance weighted (multiplicative random effects)
MR Egger
MR RAPS
Weighted median
Weighted mode
0.10
0.05
0.00
−0.05
0.1
0.2
SNP effect on CD226 antigen
SNP effect on Gut microbiota abundance (genus Parasutterella id.2892)

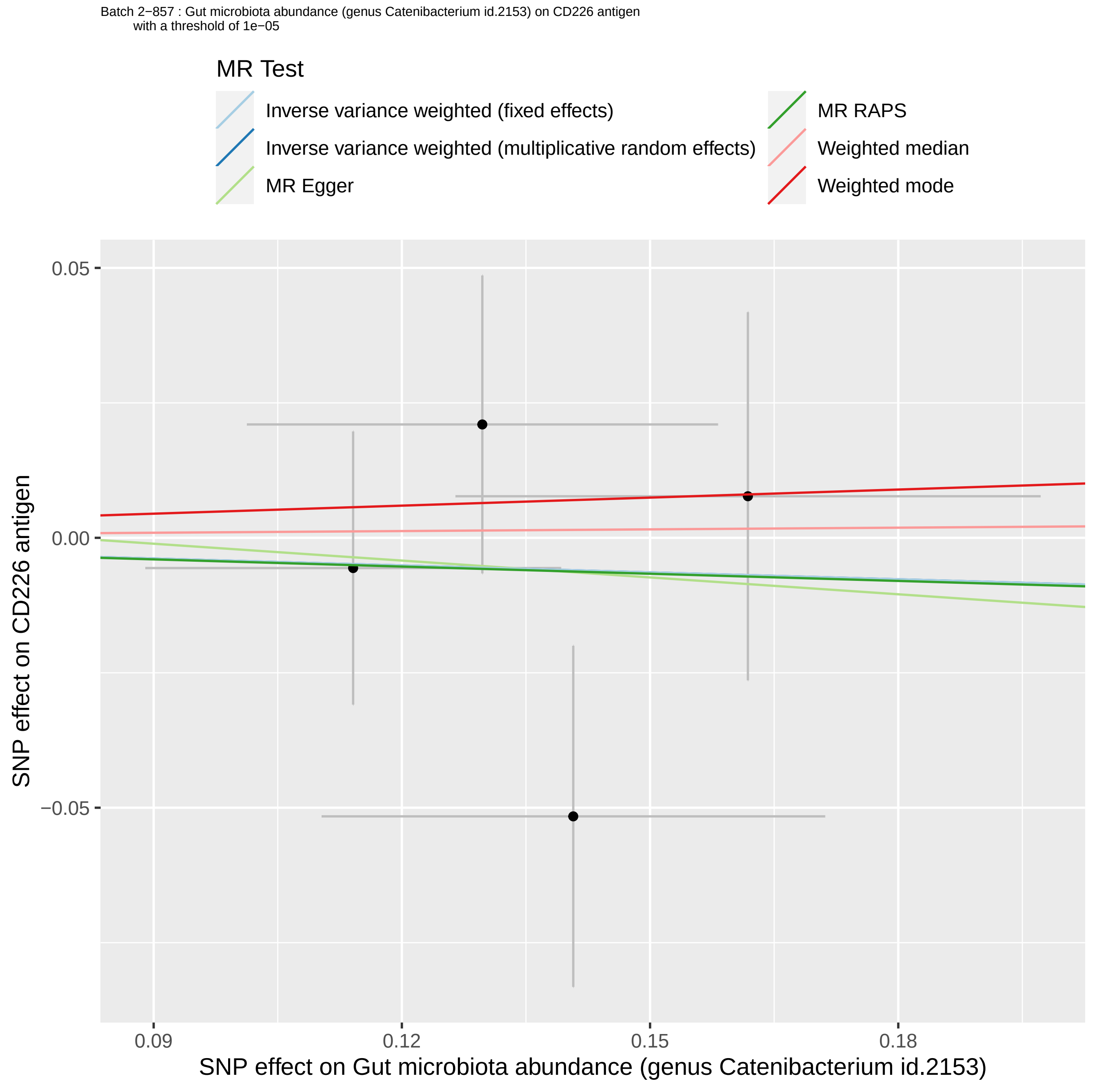
Batch 2−857 : Gut microbiota abundance (genus Catenibacterium id.2153) on CD226 antigen
with a threshold of 1e−05
MR Test
Inverse variance weighted (fixed effects)
Inverse variance weighted (multiplicative random effects)
MR Egger
MR RAPS
Weighted median
Weighted mode
0.05
0.00
−0.05
0.09
0.12
0.15
0.18
SNP effect on CD226 antigen
SNP effect on Gut microbiota abundance (genus Catenibacterium id.2153)

Batch 2−858 : Gut microbiota abundance (genus Parasutterella id.2892) on CD226 antigen
with a threshold of 1e−05

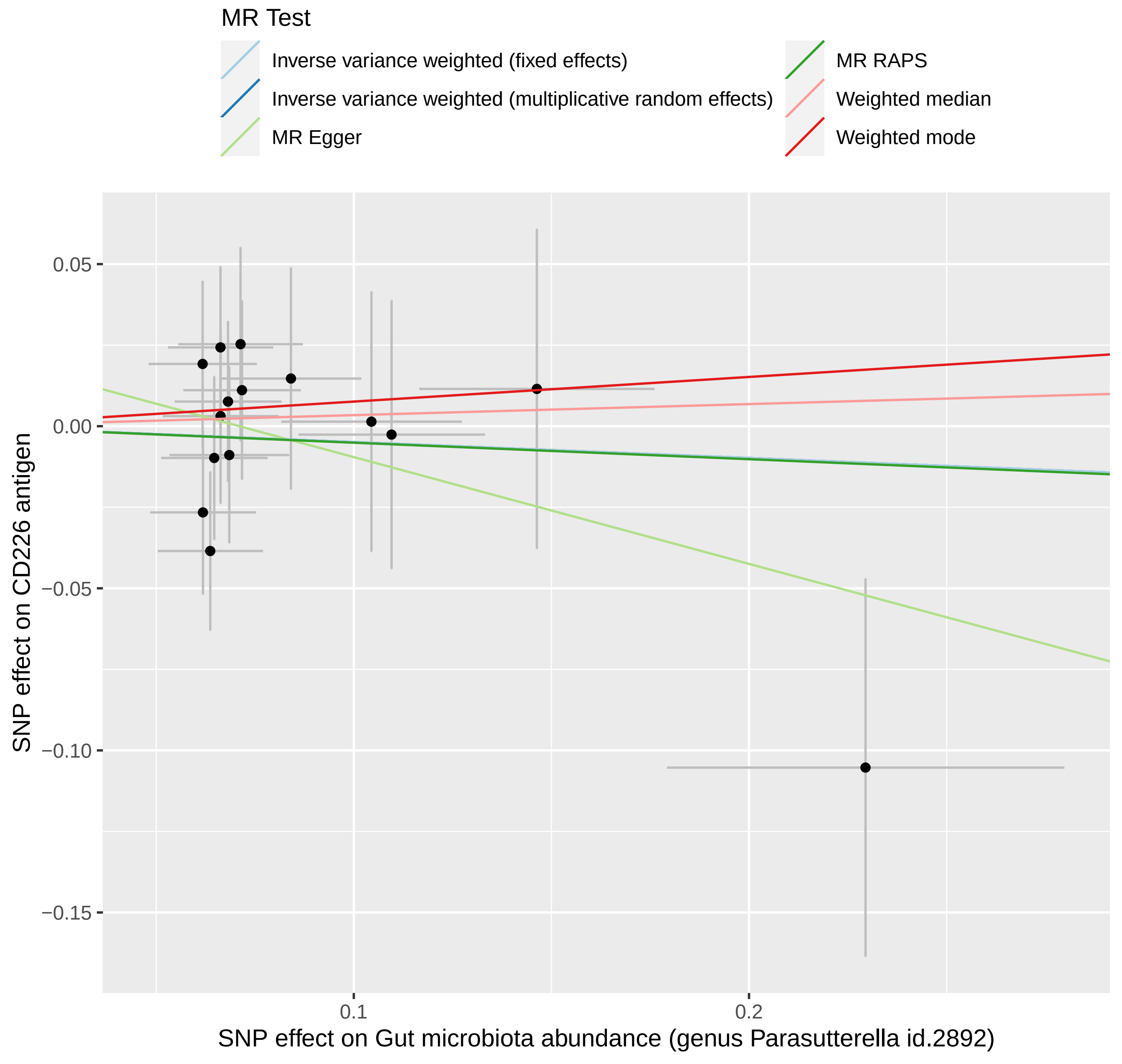

Batch 2−859 : Gut microbiota abundance (genus Catenibacterium id.2153) on T−cell surface antigen CD2
with a threshold of 1e−05

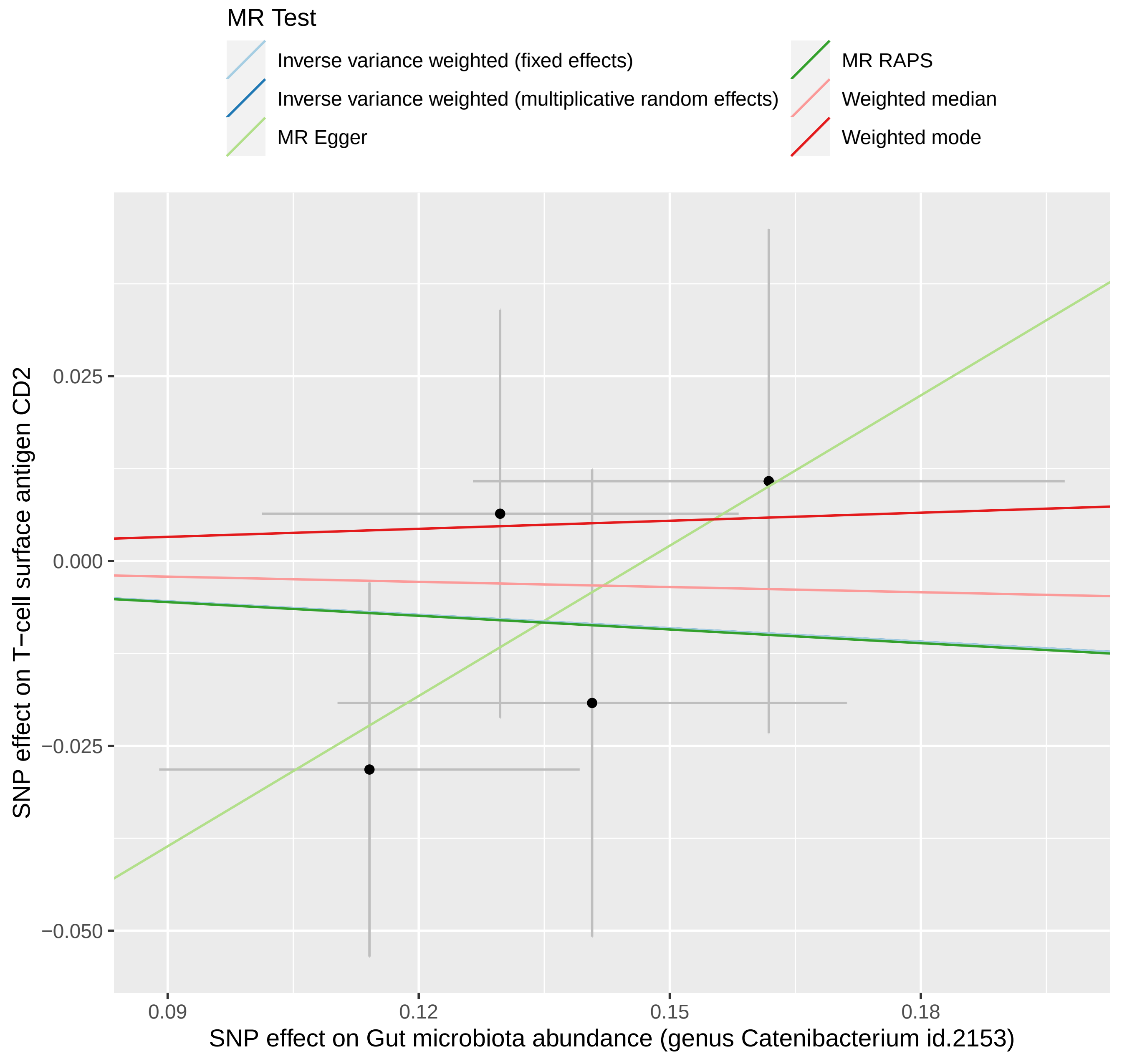

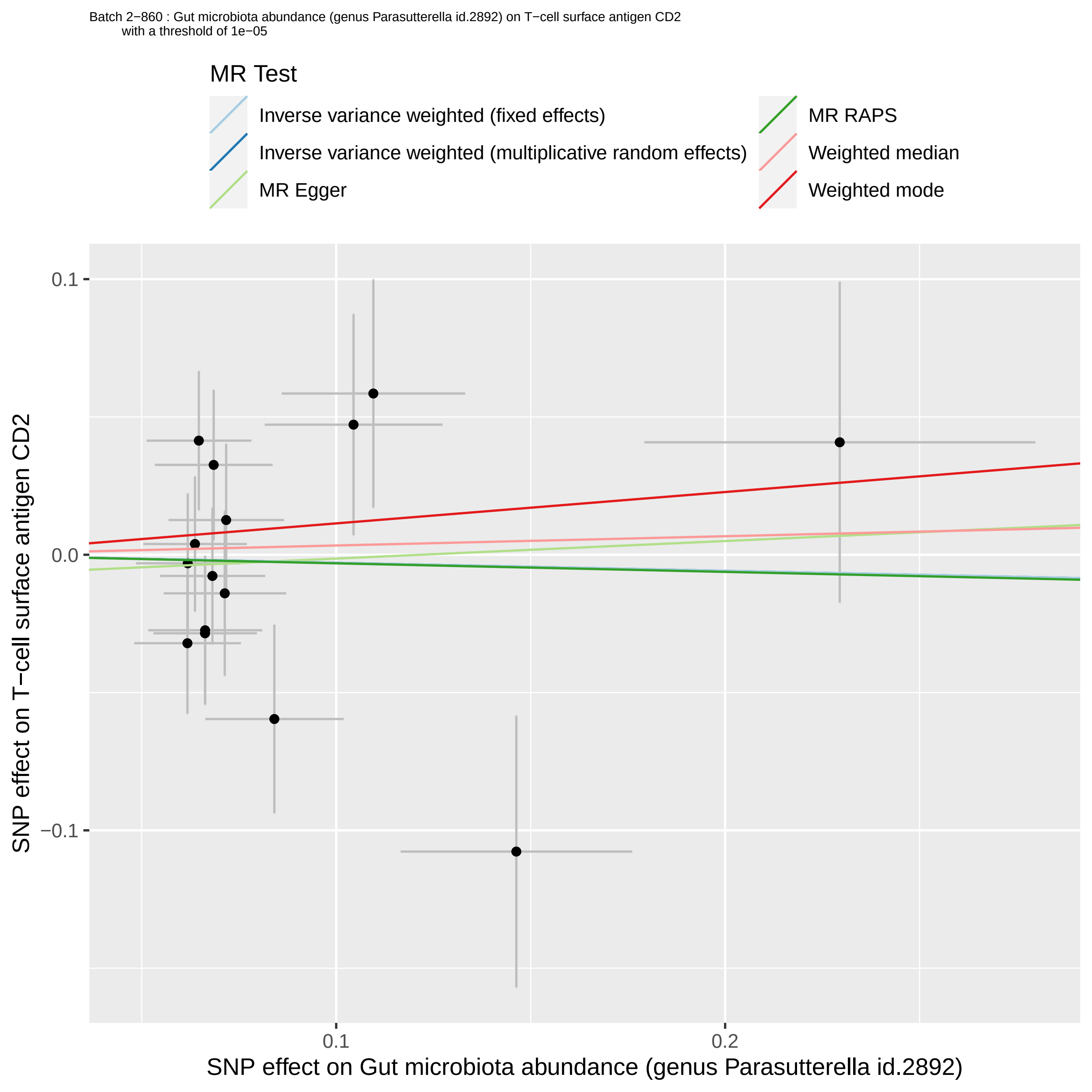

Batch 2−860 : Gut microbiota abundance (genus Parasutterella id.2892) on T−cell surface antigen CD2
with a threshold of 1e−05
MR Test
Inverse variance weighted (fixed effects)
Inverse variance weighted (multiplicative random effects)
MR Egger
MR RAPS
Weighted median
Weighted mode
0.1
0.0
−0.1
0.1
0.2
SNP effect on T−cell surface antigen CD2
SNP effect on Gut microbiota abundance (genus Parasutterella id.2892)

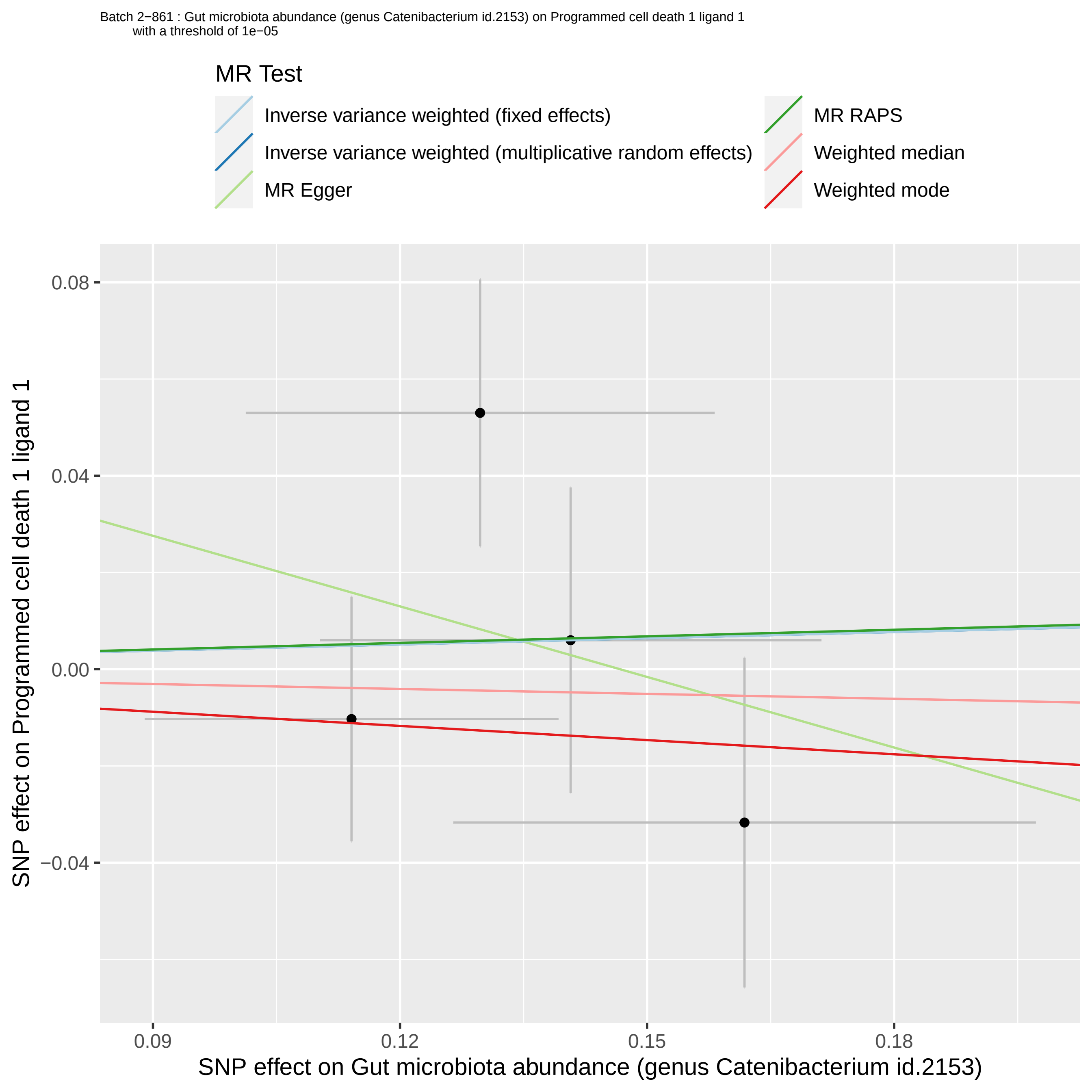
Batch 2−861 : Gut microbiota abundance (genus Catenibacterium id.2153) on Programmed cell death 1 ligand 1
with a threshold of 1e−05
MR Test
Inverse variance weighted (fixed effects)
Inverse variance weighted (multiplicative random effects)
MR Egger
MR RAPS
Weighted median
Weighted mode
0.08
0.04
0.00
−0.04
0.09
0.12
0.15
0.18
SNP effect on Programmed cell death 1 ligand 1
SNP effect on Gut microbiota abundance (genus Catenibacterium id.2153)

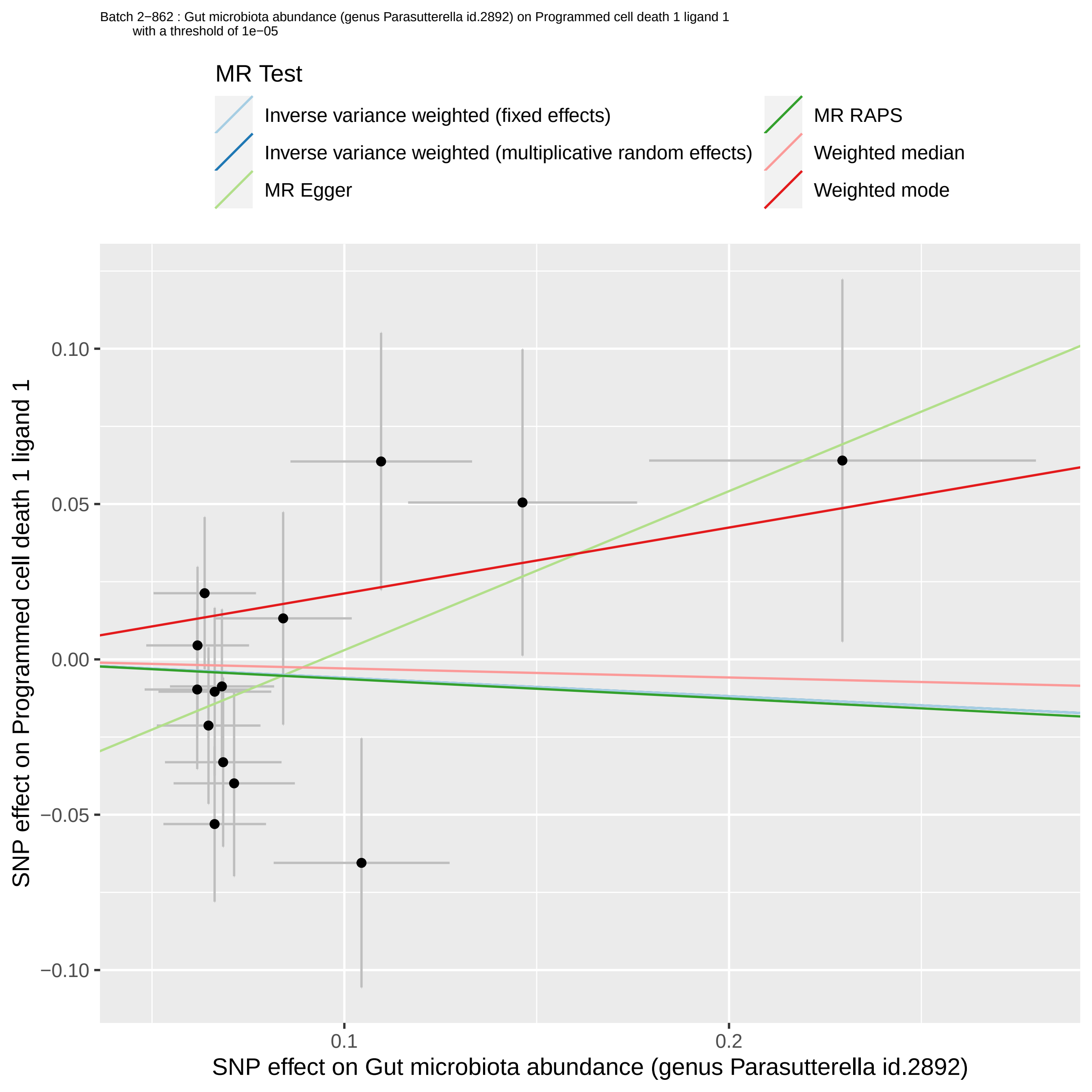
Batch 2−862 : Gut microbiota abundance (genus Parasutterella id.2892) on Programmed cell death 1 ligand 1
with a threshold of 1e−05
MR Test
Inverse variance weighted (fixed effects)
Inverse variance weighted (multiplicative random effects)
MR Egger
MR RAPS
Weighted median
Weighted mode
0.10
0.05
0.00
−0.05
−0.10
0.1
0.2
SNP effect on Programmed cell death 1 ligand 1
SNP effect on Gut microbiota abundance (genus Parasutterella id.2892)

Batch 2−863 : Gut microbiota abundance (genus Catenibacterium id.2153) on CMRF35−like molecule 8
with a threshold of 1e−05

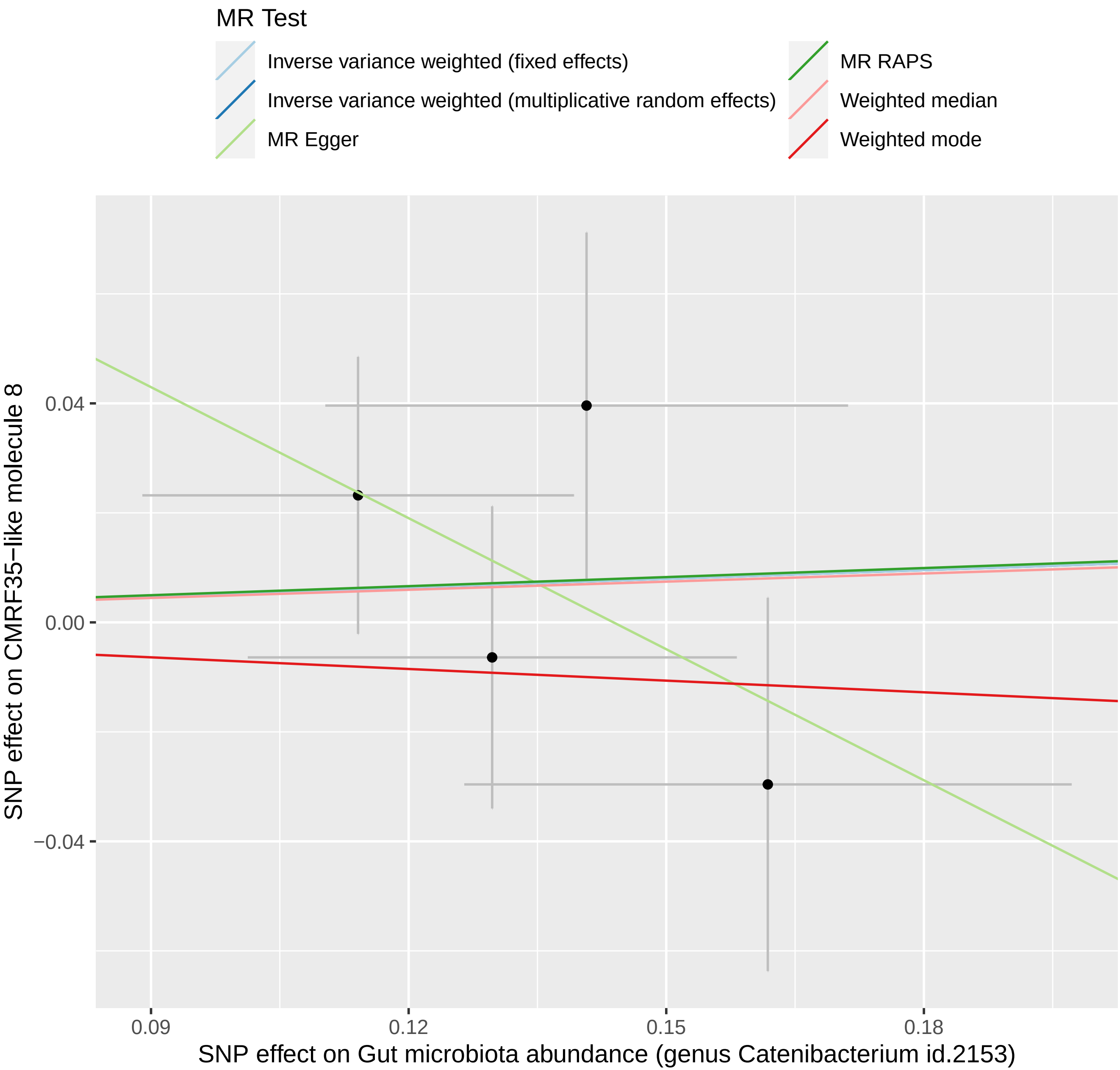

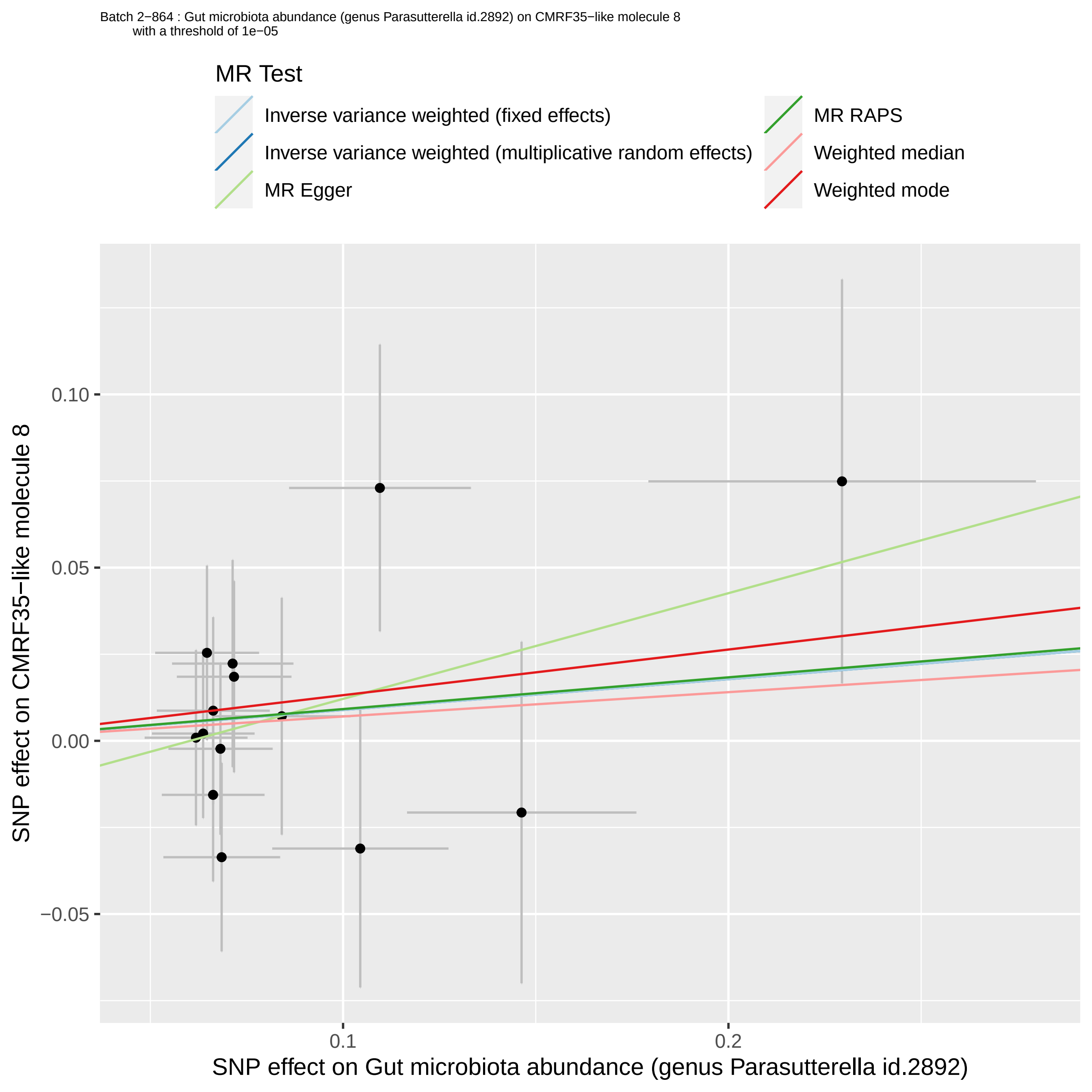

Batch 2−864 : Gut microbiota abundance (genus Parasutterella id.2892) on CMRF35−like molecule 8
with a threshold of 1e−05
MR Test
Inverse variance weighted (fixed effects)
Inverse variance weighted (multiplicative random effects)
MR Egger
MR RAPS
Weighted median
Weighted mode
0.10
0.05
0.00
−0.05
0.1
0.2
SNP effect on CMRF35−like molecule 8
SNP effect on Gut microbiota abundance (genus Parasutterella id.2892)

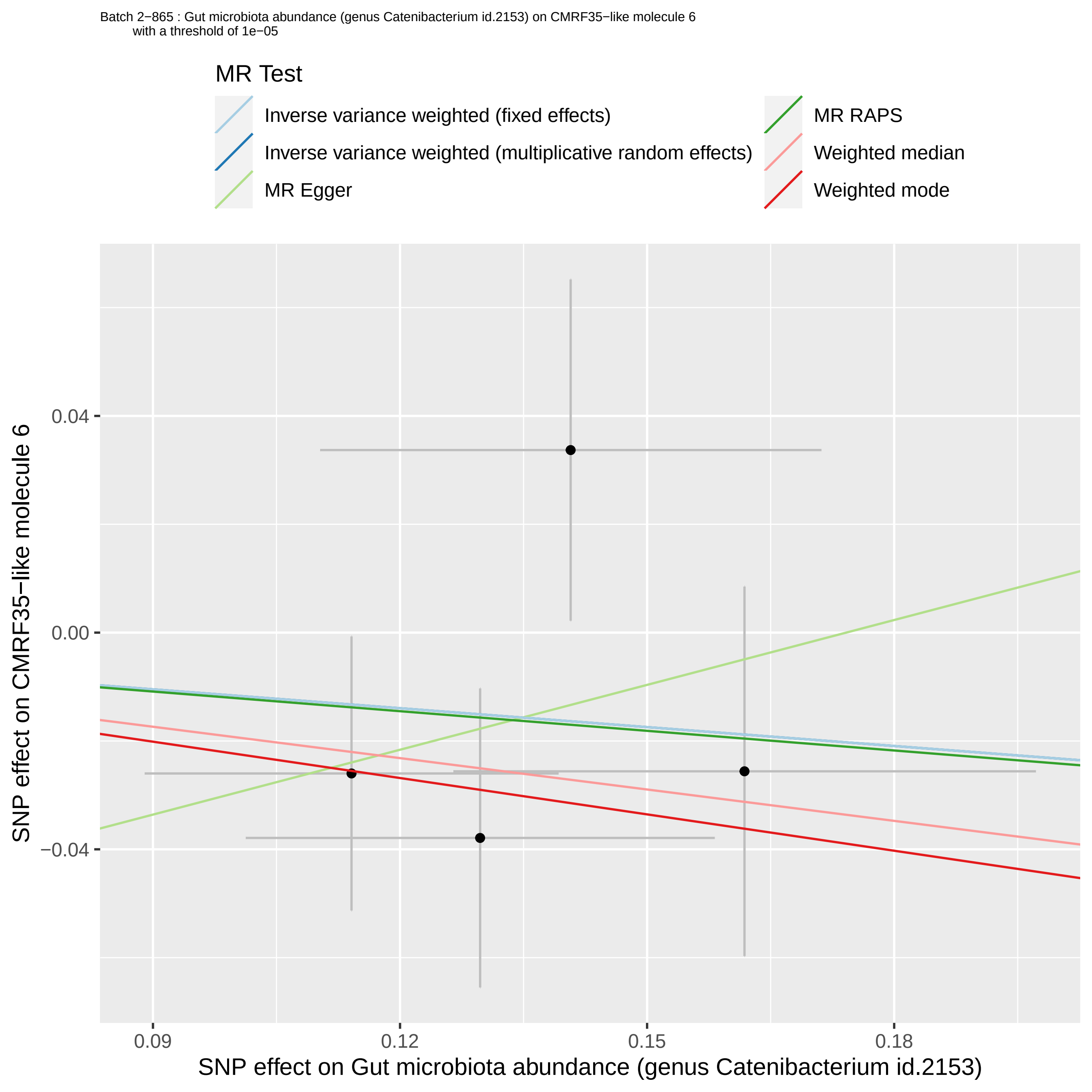

Batch 2−865 : Gut microbiota abundance (genus Catenibacterium id.2153) on CMRF35−like molecule 6
with a threshold of 1e−05
MR Test
Inverse variance weighted (fixed effects)
Inverse variance weighted (multiplicative random effects)
MR Egger
MR RAPS
Weighted median
Weighted mode
0.04
0.00
−0.04
0.09
0.12
0.15
0.18
SNP effect on CMRF35−like molecule 6
SNP effect on Gut microbiota abundance (genus Catenibacterium id.2153)

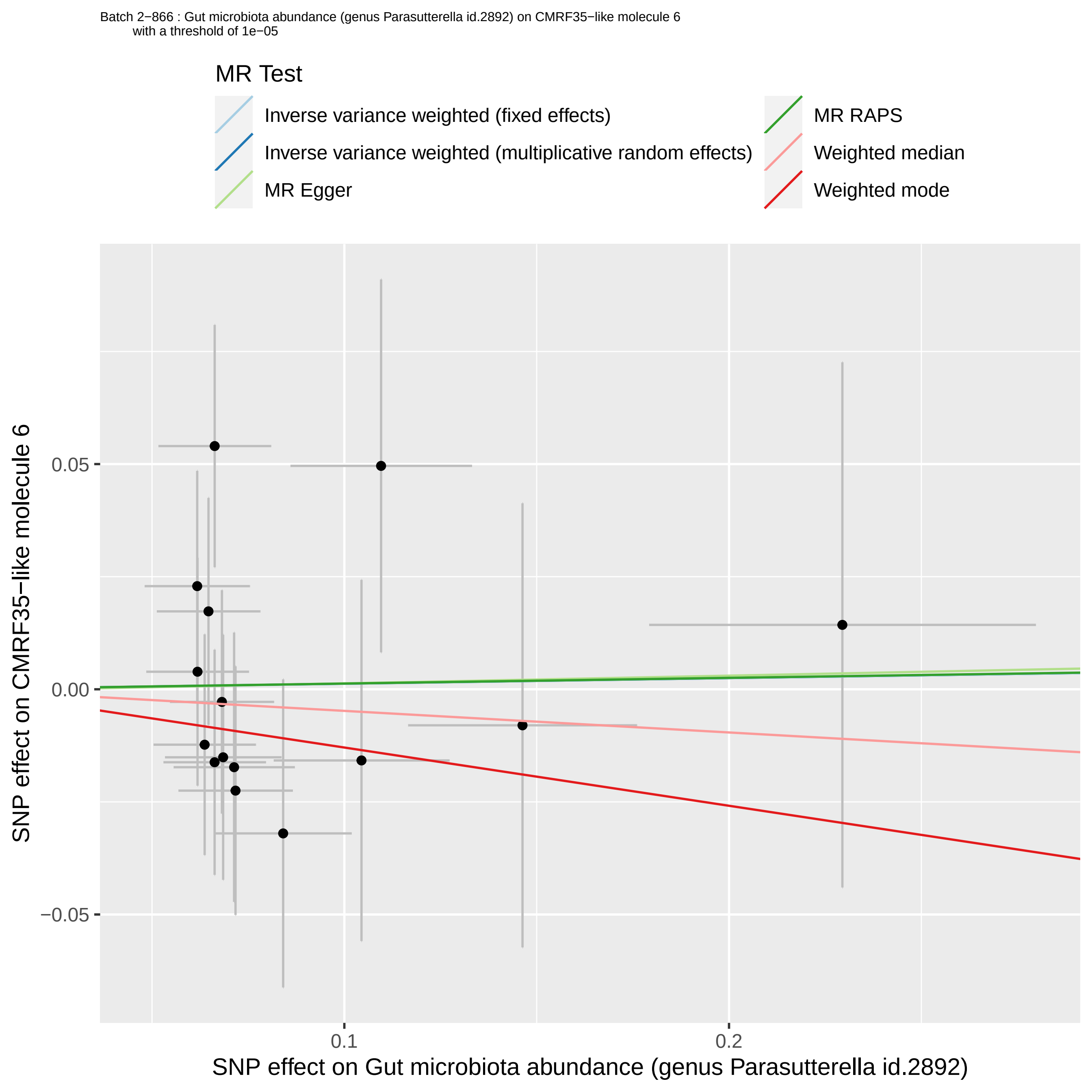

Batch 2−866 : Gut microbiota abundance (genus Parasutterella id.2892) on CMRF35−like molecule 6
with a threshold of 1e−05
MR Test
Inverse variance weighted (fixed effects)
Inverse variance weighted (multiplicative random effects)
MR Egger
MR RAPS
Weighted median
Weighted mode
0.05
0.00
−0.05
0.1
0.2
SNP effect on CMRF35−like molecule 6
SNP effect on Gut microbiota abundance (genus Parasutterella id.2892)

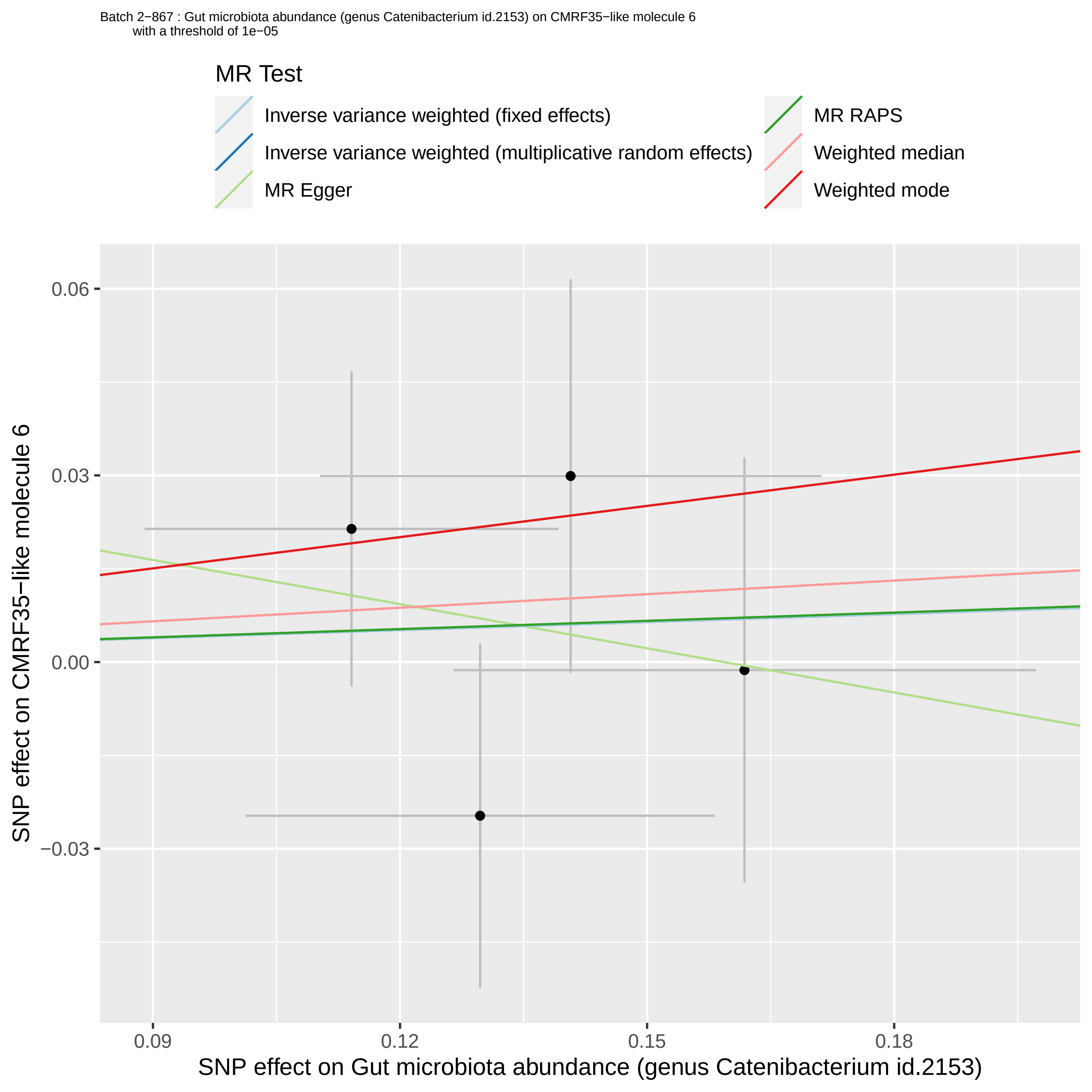

Batch 2−867 : Gut microbiota abundance (genus Catenibacterium id.2153) on CMRF35−like molecule 6
with a threshold of 1e−05
MR Test
Inverse variance weighted (fixed effects)
Inverse variance weighted (multiplicative random effects)
MR Egger
MR RAPS
Weighted median
Weighted mode
0.06
0.03
0.00
−0.03
0.09
0.12
0.15
0.18
SNP effect on CMRF35−like molecule 6
SNP effect on Gut microbiota abundance (genus Catenibacterium id.2153)

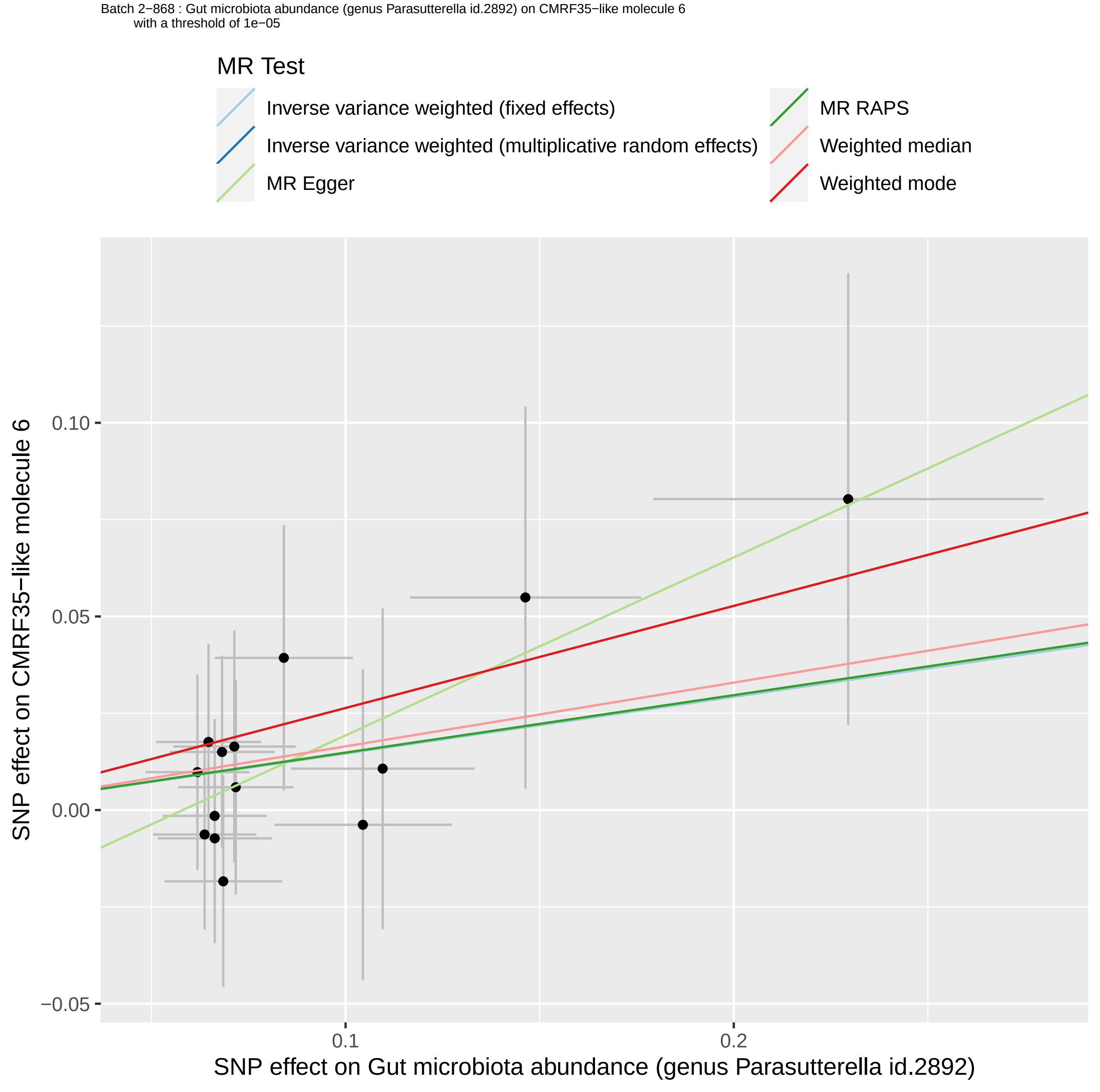
Batch 2−868 : Gut microbiota abundance (genus Parasutterella id.2892) on CMRF35−like molecule 6
with a threshold of 1e−05
MR Test
Inverse variance weighted (fixed effects)
Inverse variance weighted (multiplicative random effects)
MR Egger
MR RAPS
Weighted median
Weighted mode
SNP effect on CMRF35−like molecule 6
0.10
0.05
0.00
−0.05
0.1
0.2
SNP effect on Gut microbiota abundance (genus Parasutterella id.2892)

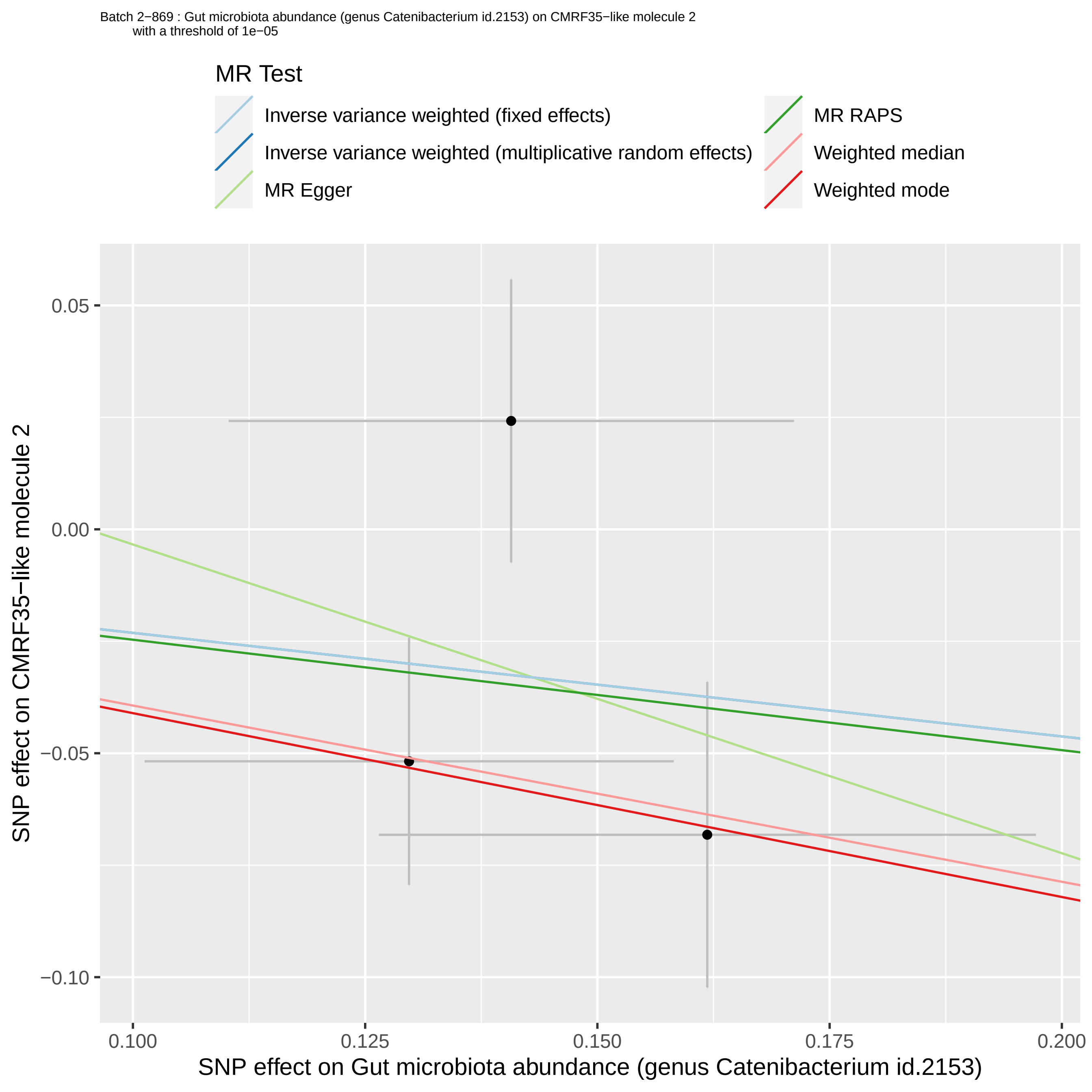

Batch 2−869 : Gut microbiota abundance (genus Catenibacterium id.2153) on CMRF35−like molecule 2
with a threshold of 1e−05
MR Test
Inverse variance weighted (fixed effects)
Inverse variance weighted (multiplicative random effects)
MR Egger
MR RAPS
Weighted median
Weighted mode
0.05
0.00
−0.05
−0.10
0.100
0.125
0.150
0.175
0.200
SNP effect on CMRF35−like molecule 2
SNP effect on Gut microbiota abundance (genus Catenibacterium id.2153)

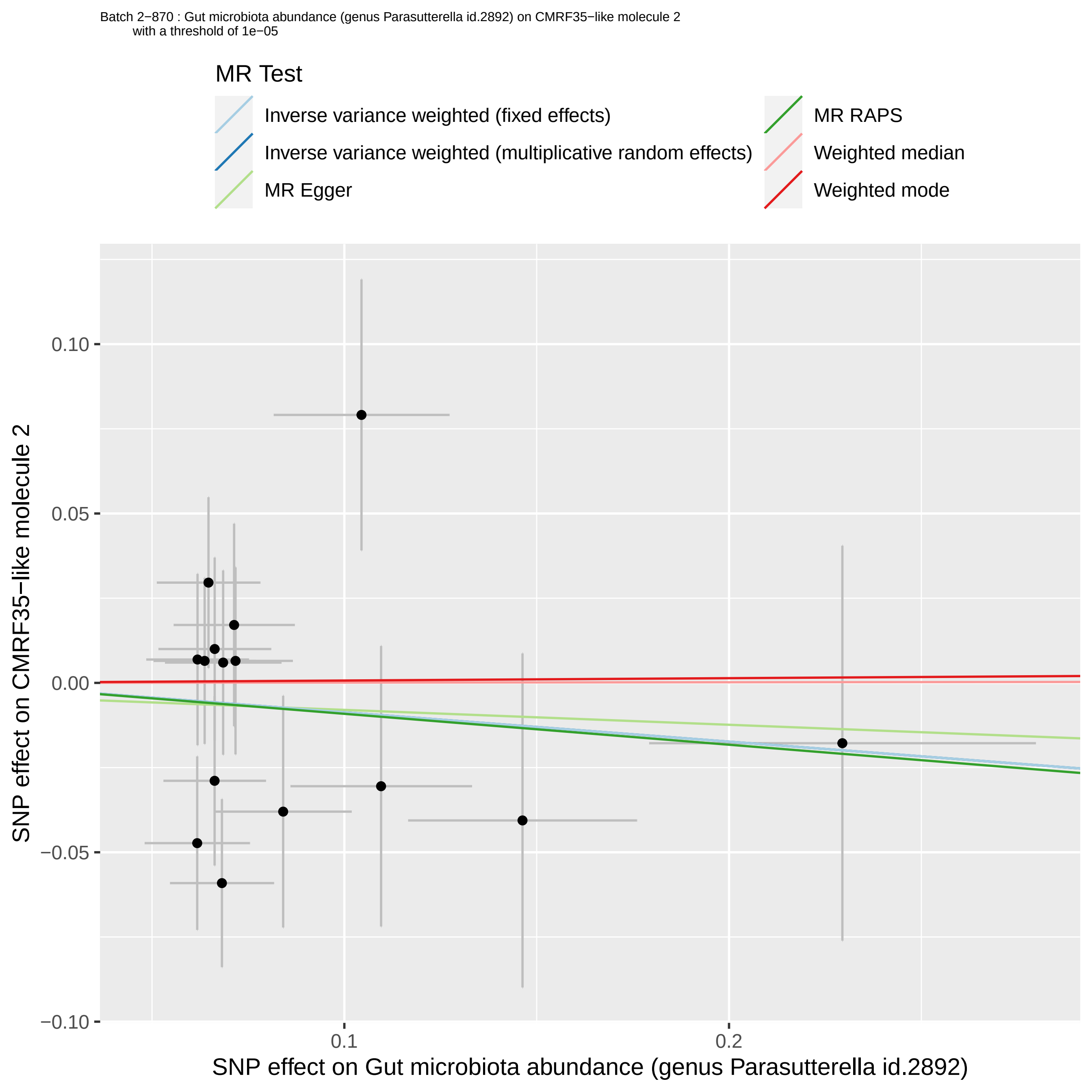
Batch 2−870 : Gut microbiota abundance (genus Parasutterella id.2892) on CMRF35−like molecule 2
with a threshold of 1e−05
MR Test
Inverse variance weighted (fixed effects)
Inverse variance weighted (multiplicative random effects)
MR Egger
MR RAPS
Weighted median
Weighted mode
0.10
0.05
0.00
−0.05
−0.10
0.1
0.2
SNP effect on CMRF35−like molecule 2
SNP effect on Gut microbiota abundance (genus Parasutterella id.2892)

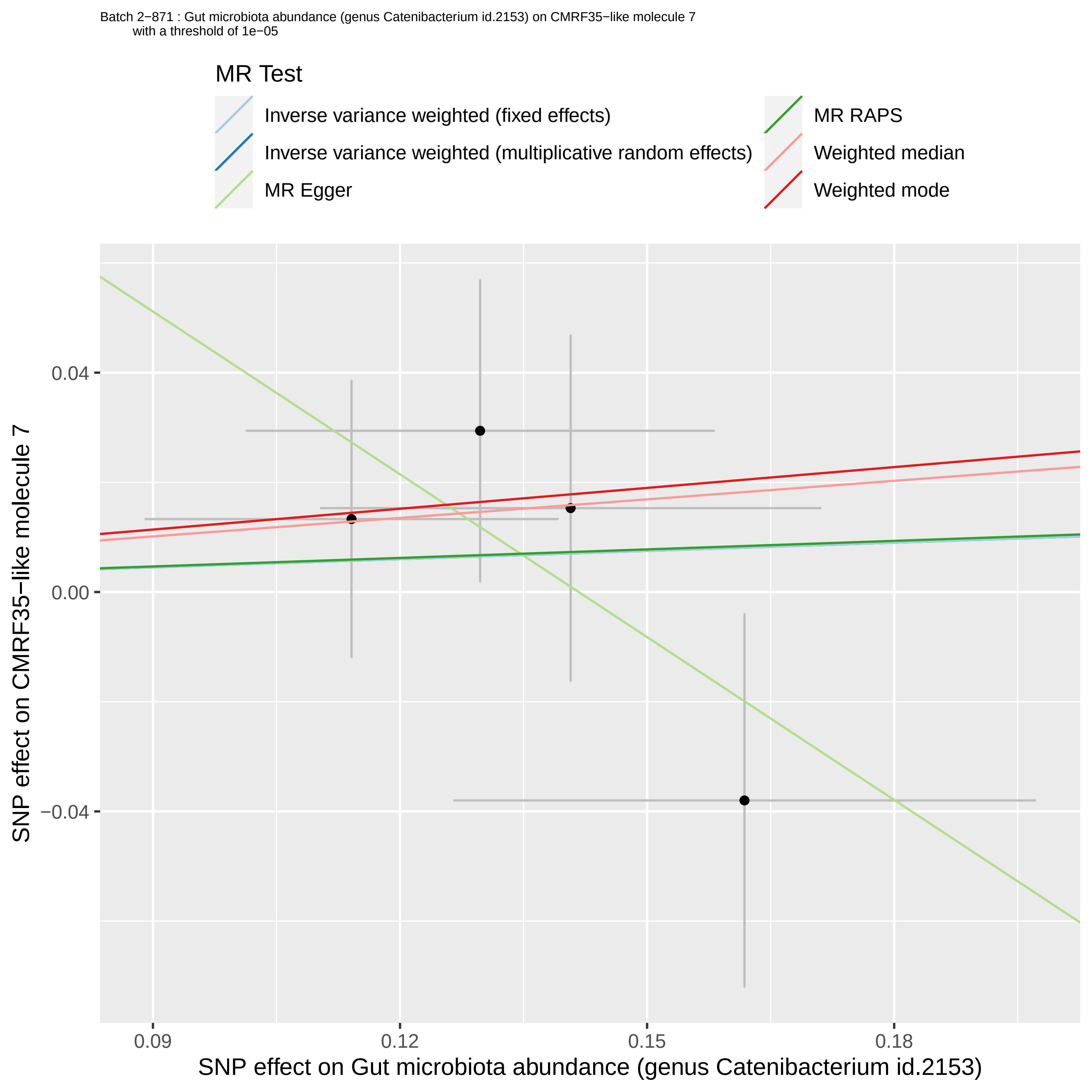
Batch 2−871 : Gut microbiota abundance (genus Catenibacterium id.2153) on CMRF35−like molecule 7
with a threshold of 1e−05
MR Test
Inverse variance weighted (fixed effects)
Inverse variance weighted (multiplicative random effects)
MR Egger
MR RAPS
Weighted median
Weighted mode
0.04
0.00
−0.04
0.09
0.12
0.15
0.18
SNP effect on CMRF35−like molecule 7
SNP effect on Gut microbiota abundance (genus Catenibacterium id.2153)

Batch 2−872 : Gut microbiota abundance (genus Parasutterella id.2892) on CMRF35−like molecule 7
with a threshold of 1e−05

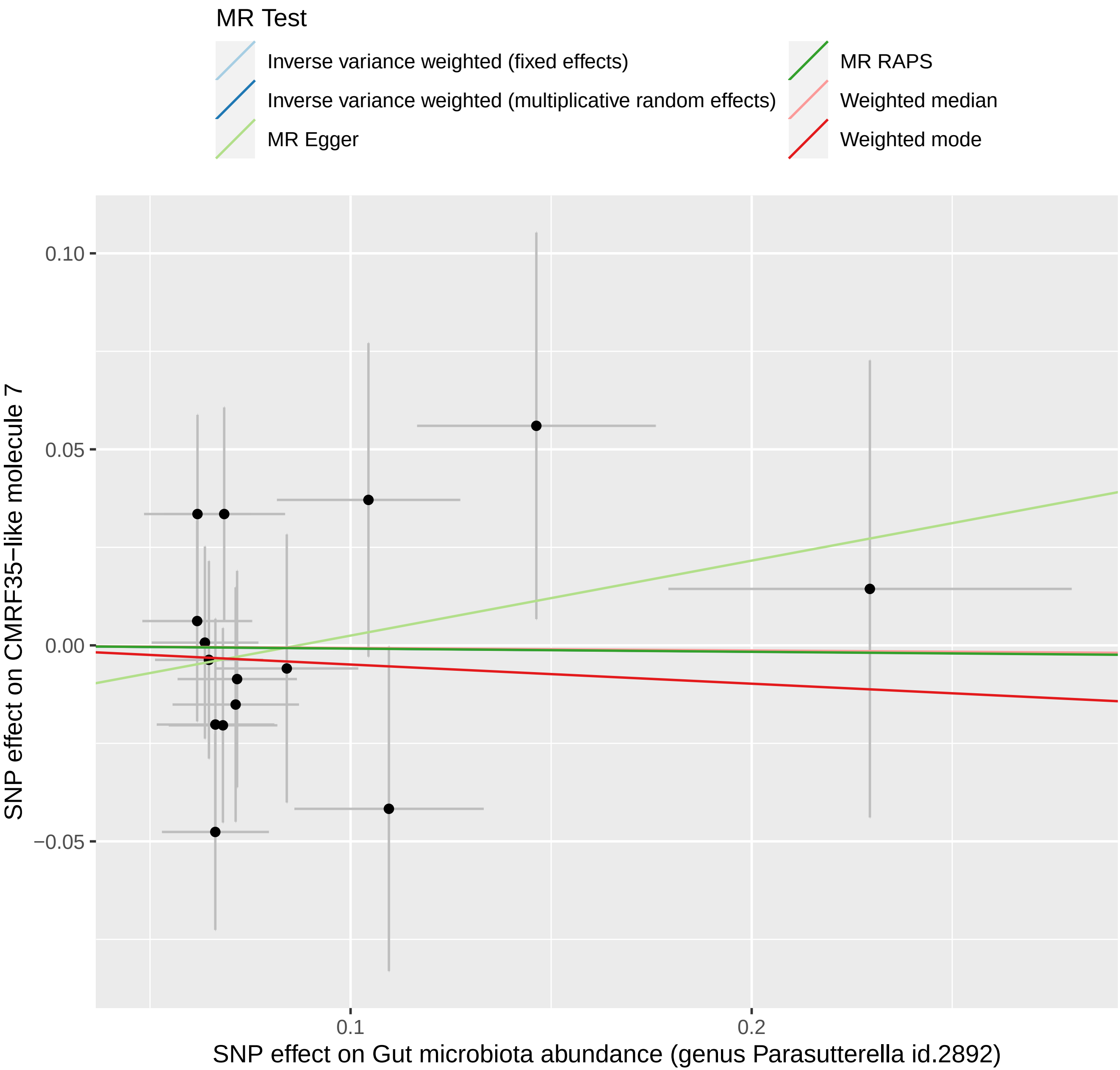

Batch 2−873 : Gut microbiota abundance (genus Catenibacterium id.2153) on CMRF35−like molecule 1
with a threshold of 1e−05

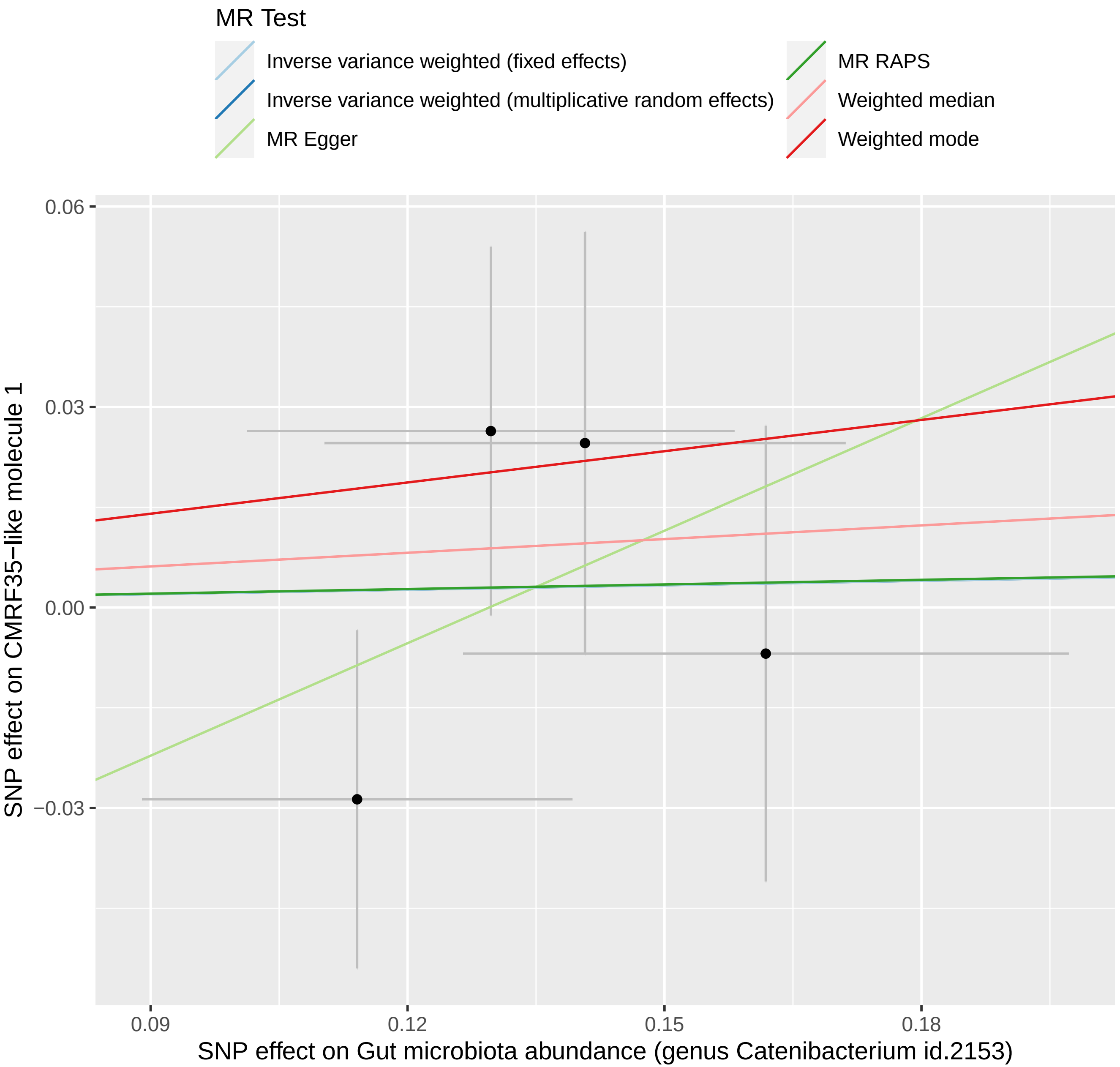

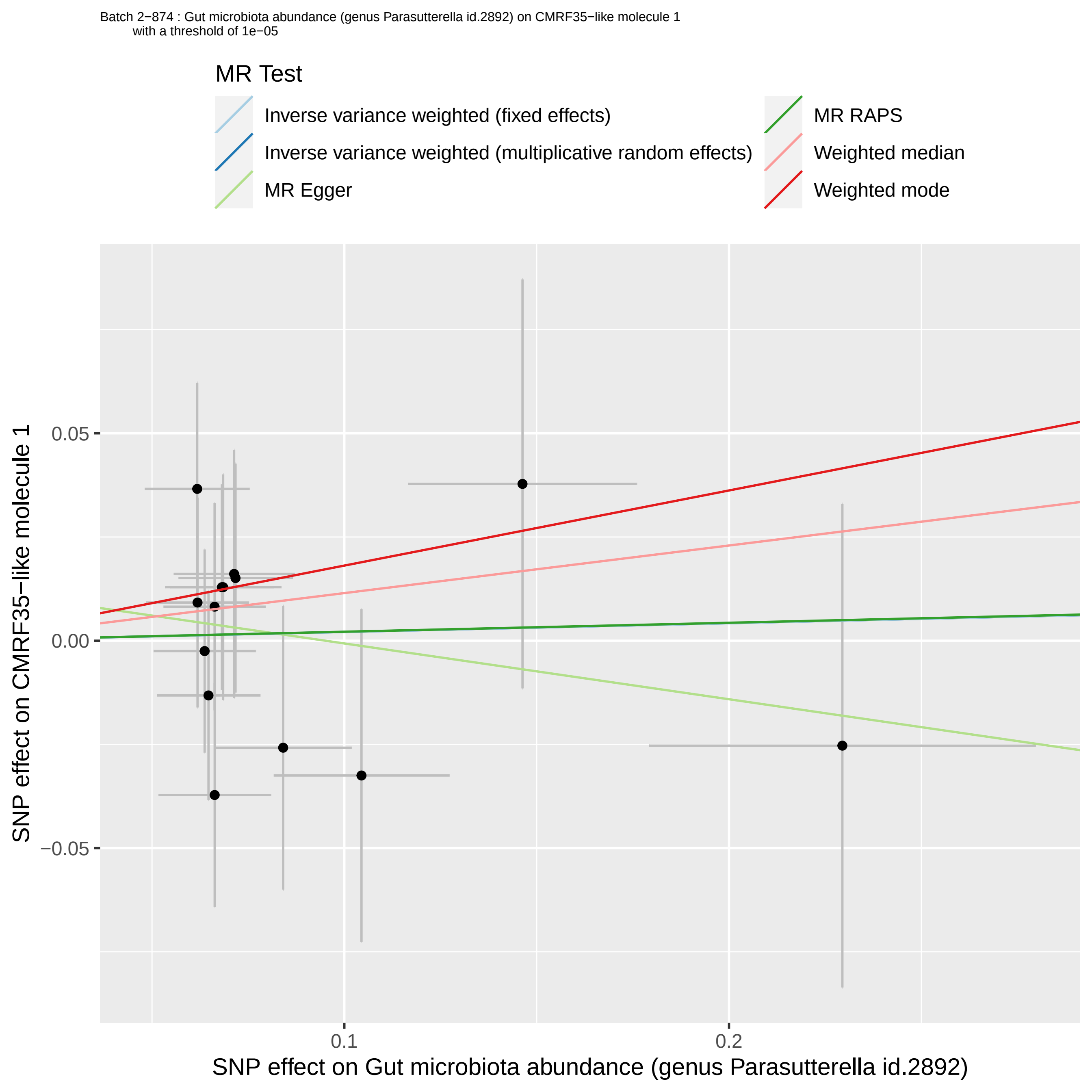
Batch 2−874 : Gut microbiota abundance (genus Parasutterella id.2892) on CMRF35−like molecule 1
with a threshold of 1e−05
MR Test
Inverse variance weighted (fixed effects)
Inverse variance weighted (multiplicative random effects)
MR Egger
MR RAPS
Weighted median
Weighted mode
SNP effect on CMRF35−like molecule 1
0.05
0.00
−0.05
0.1
0.2
SNP effect on Gut microbiota abundance (genus Parasutterella id.2892)

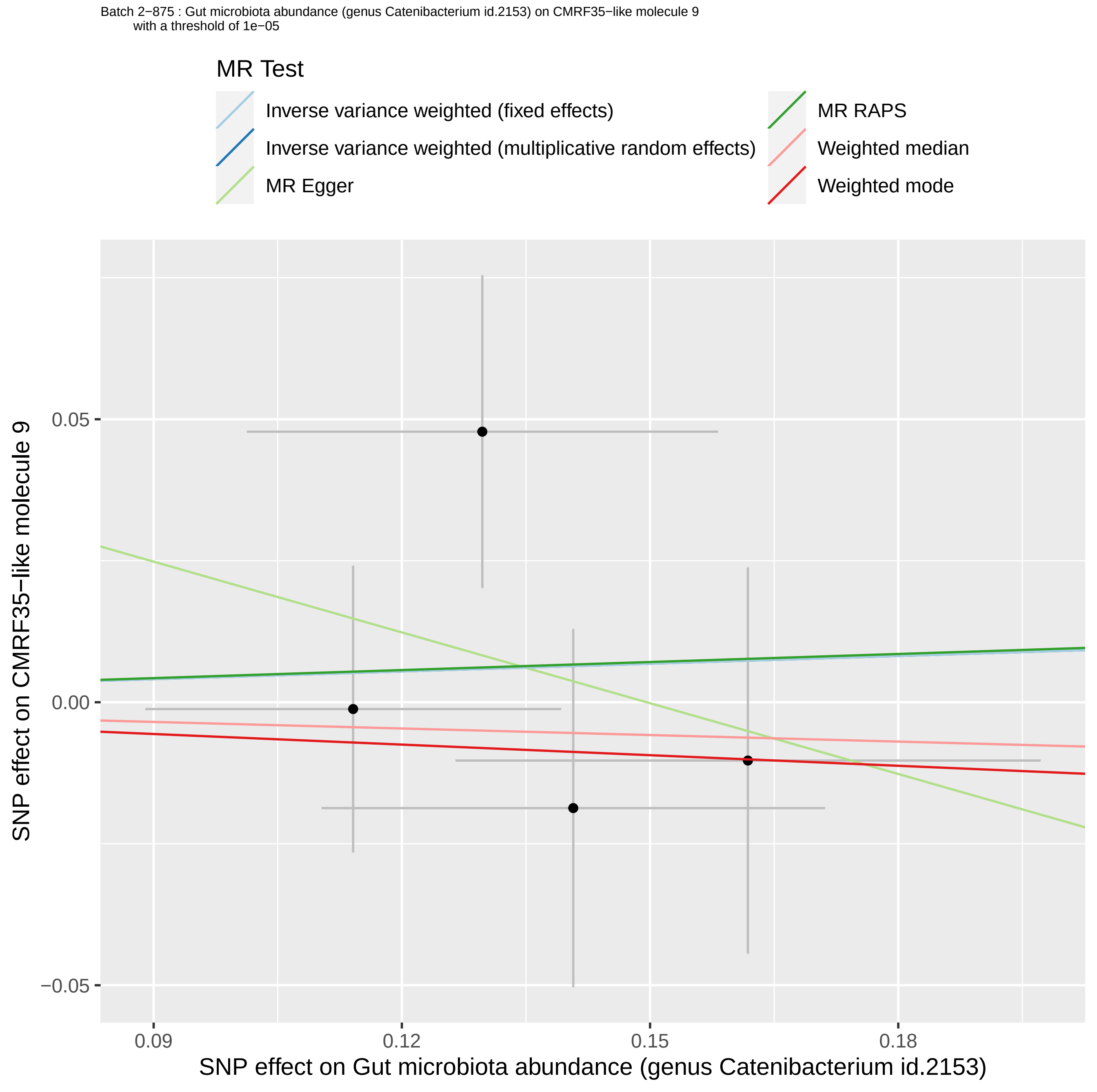

Batch 2−875 : Gut microbiota abundance (genus Catenibacterium id.2153) on CMRF35−like molecule 9
with a threshold of 1e−05
MR Test
Inverse variance weighted (fixed effects)
Inverse variance weighted (multiplicative random effects)
MR Egger
MR RAPS
Weighted median
Weighted mode
SNP effect on CMRF35−like molecule 9
0.05
0.00
−0.05
0.09
0.12
0.15
0.18
SNP effect on Gut microbiota abundance (genus Catenibacterium id.2153)

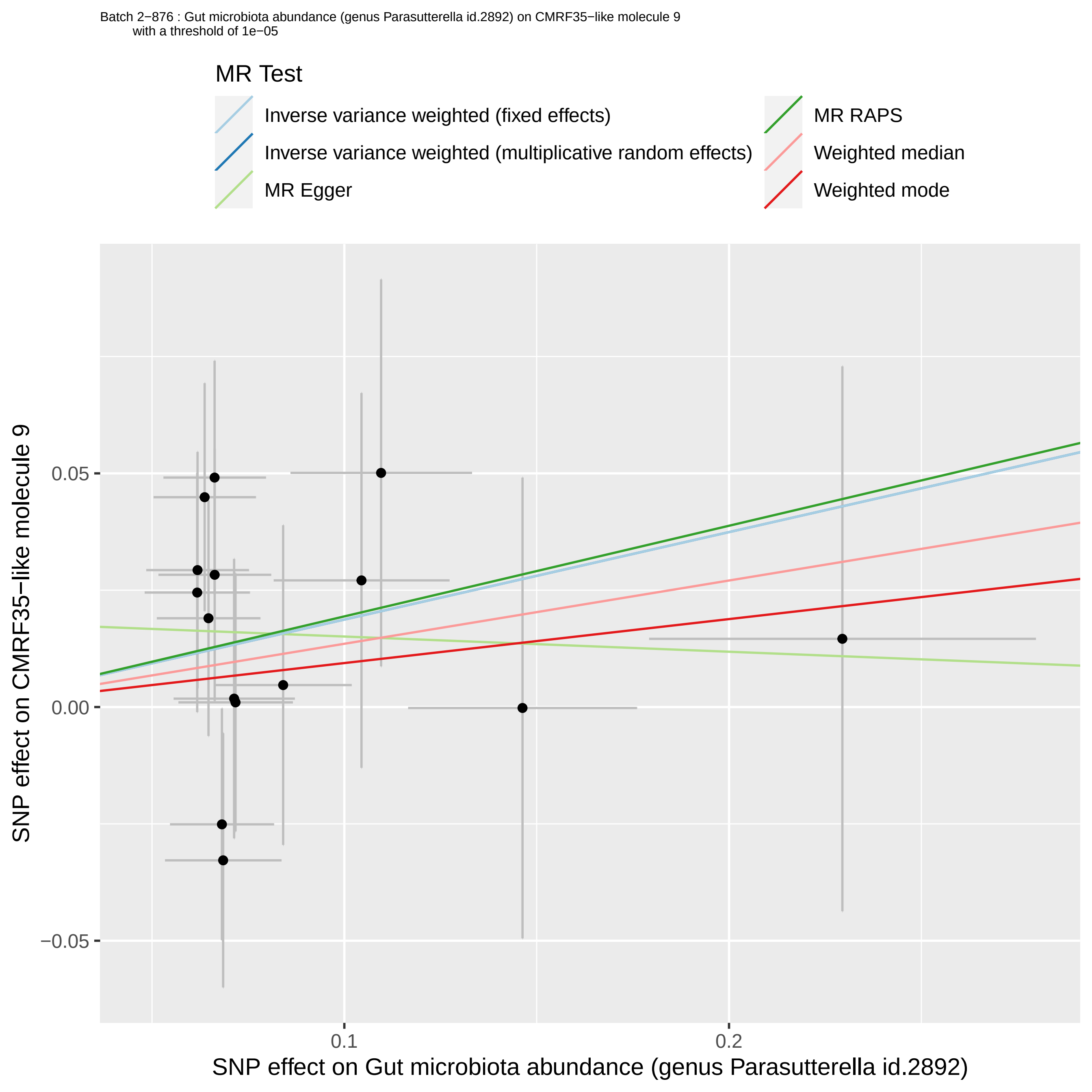

Batch 2−876 : Gut microbiota abundance (genus Parasutterella id.2892) on CMRF35−like molecule 9
with a threshold of 1e−05
MR Test
Inverse variance weighted (fixed effects)
Inverse variance weighted (multiplicative random effects)
MR Egger
MR RAPS
Weighted median
Weighted mode
0.05
0.00
−0.05
0.1
0.2
SNP effect on CMRF35−like molecule 9
SNP effect on Gut microbiota abundance (genus Parasutterella id.2892)

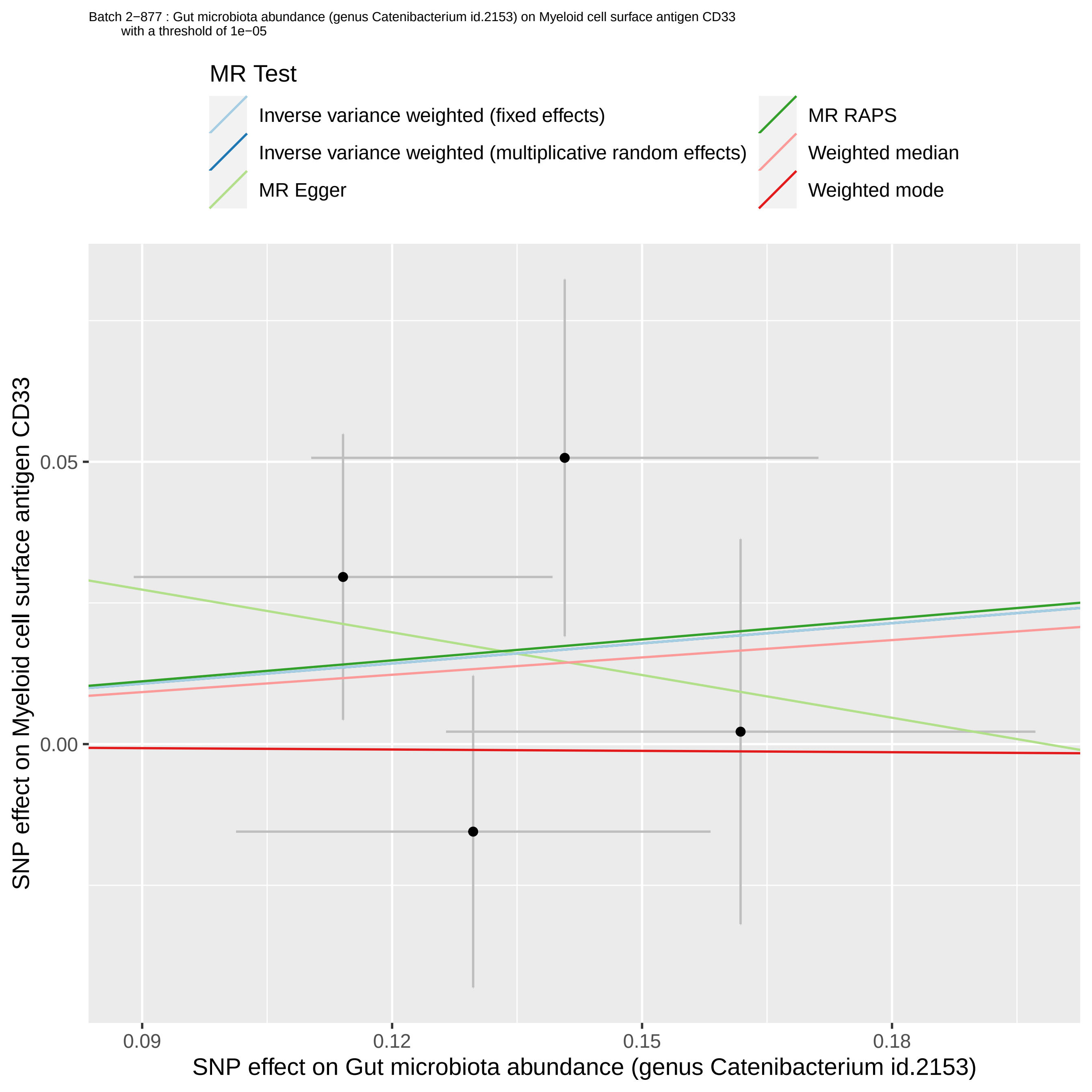

Batch 2−877 : Gut microbiota abundance (genus Catenibacterium id.2153) on Myeloid cell surface antigen CD33
with a threshold of 1e−05
MR Test
Inverse variance weighted (fixed effects)
Inverse variance weighted (multiplicative random effects)
MR Egger
MR RAPS
Weighted median
Weighted mode
SNP effect on Myeloid cell surface antigen CD33
0.05
0.00
0.09
0.12
0.15
0.18
SNP effect on Gut microbiota abundance (genus Catenibacterium id.2153)

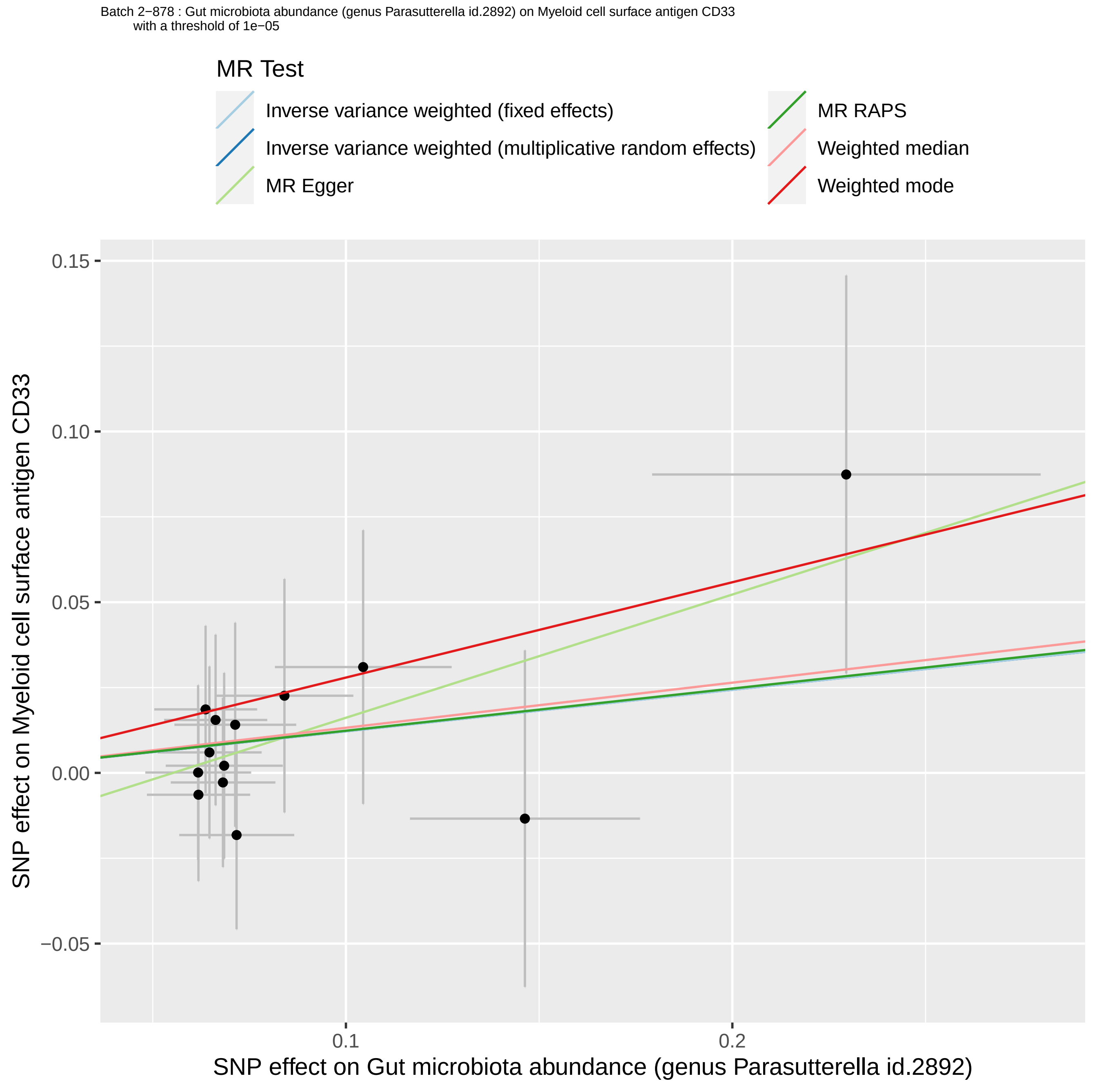

Batch 2−878 : Gut microbiota abundance (genus Parasutterella id.2892) on Myeloid cell surface antigen CD33
with a threshold of 1e−05
MR Test
Inverse variance weighted (fixed effects)
Inverse variance weighted (multiplicative random effects)
MR Egger
MR RAPS
Weighted median
Weighted mode
0.15
0.10
0.05
0.00
−0.05
0.1
0.2
SNP effect on Myeloid cell surface antigen CD33
SNP effect on Gut microbiota abundance (genus Parasutterella id.2892)

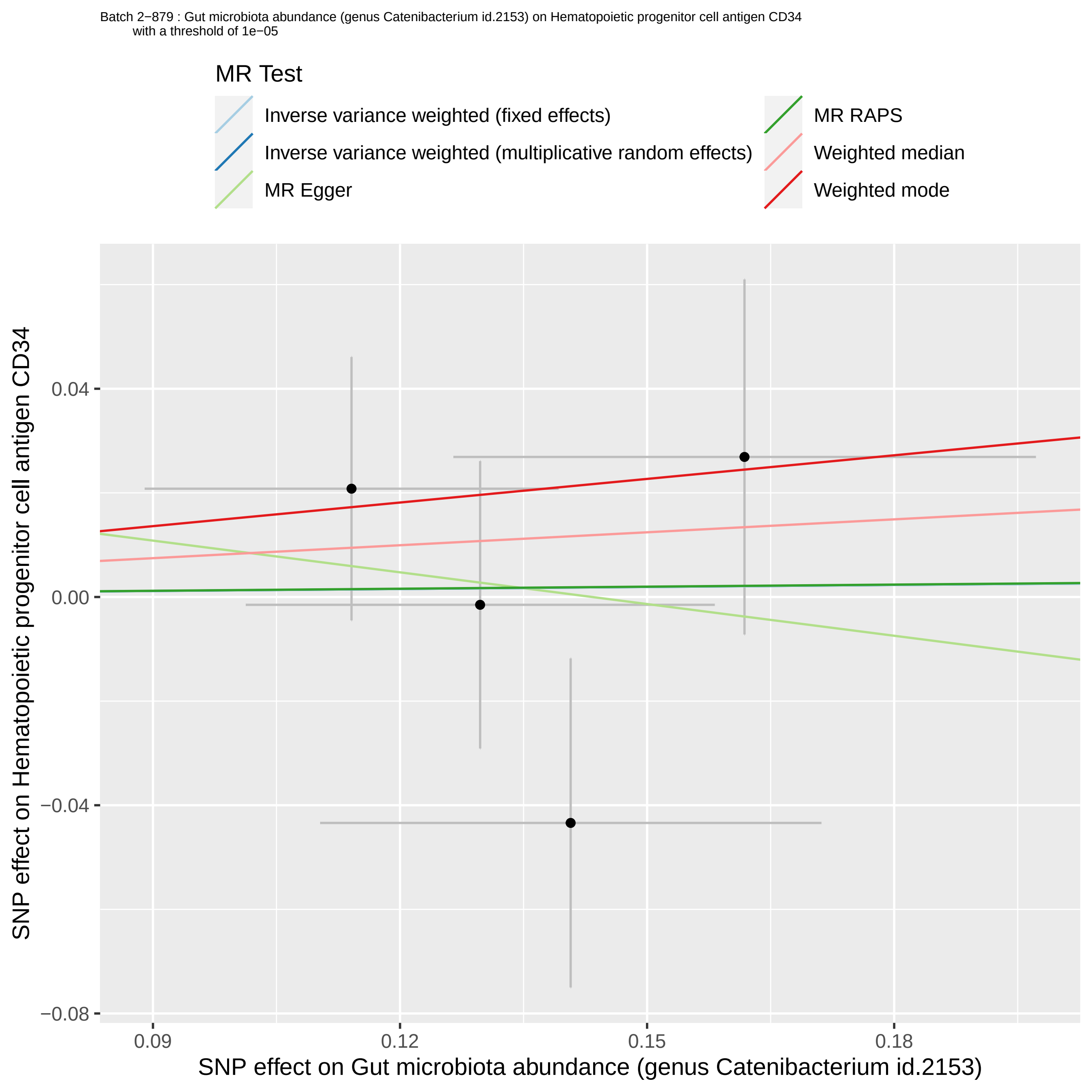

Batch 2−879 : Gut microbiota abundance (genus Catenibacterium id.2153) on Hematopoietic progenitor cell antigen CD34
with a threshold of 1e−05
MR Test
Inverse variance weighted (fixed effects)
Inverse variance weighted (multiplicative random effects)
MR Egger
MR RAPS
Weighted median
Weighted mode
0.04
0.00
−0.04
−0.08
0.09
0.12
0.15
0.18
SNP effect on Hematopoietic progenitor cell antigen CD34
SNP effect on Gut microbiota abundance (genus Catenibacterium id.2153)

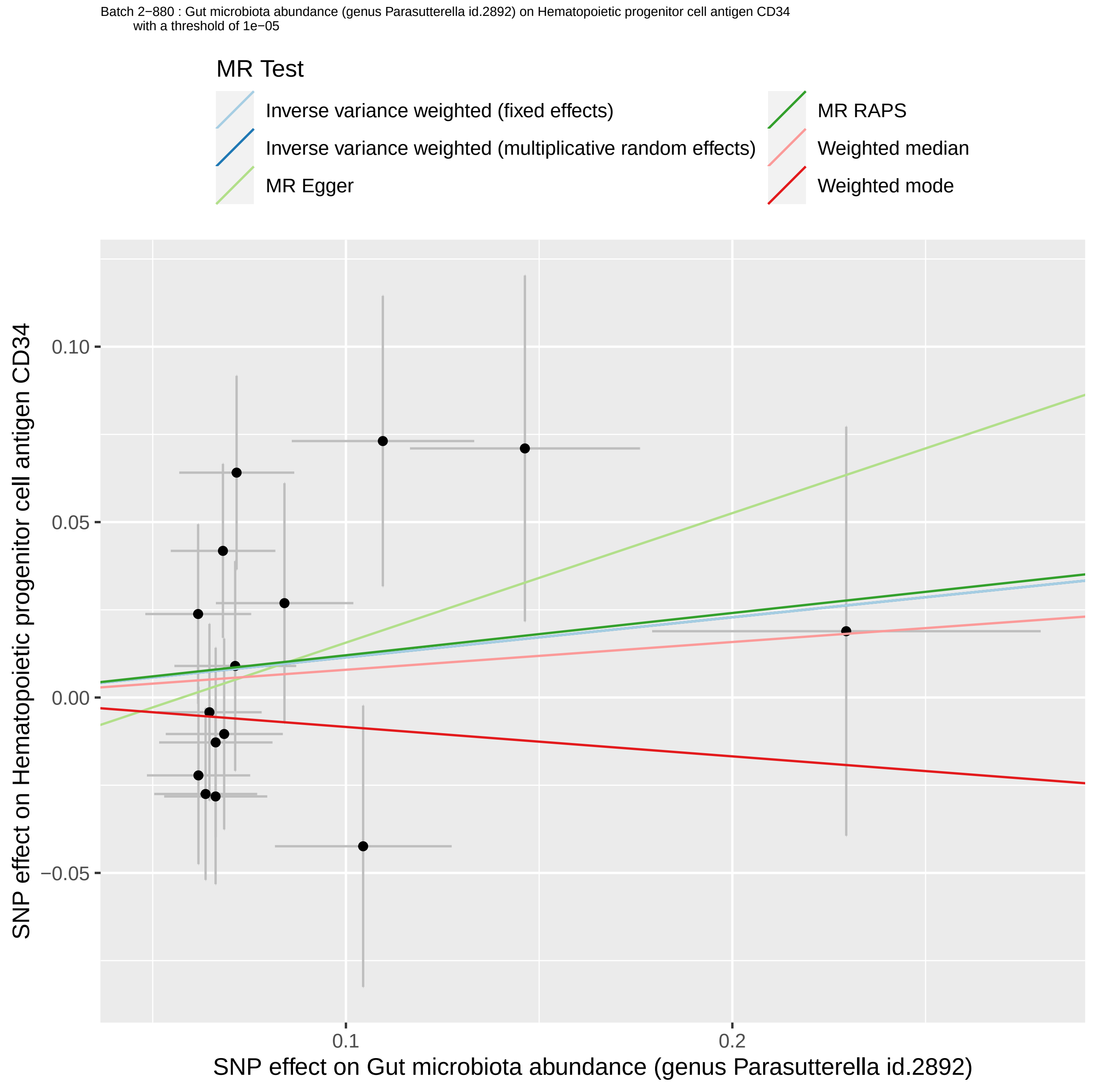
Batch 2−880 : Gut microbiota abundance (genus Parasutterella id.2892) on Hematopoietic progenitor cell antigen CD34
with a threshold of 1e−05
MR Test
Inverse variance weighted (fixed effects)
Inverse variance weighted (multiplicative random effects)
MR Egger
MR RAPS
Weighted median
Weighted mode
0.10
0.05
0.00
−0.05
0.1
0.2
SNP effect on Hematopoietic progenitor cell antigen CD34
SNP effect on Gut microbiota abundance (genus Parasutterella id.2892)

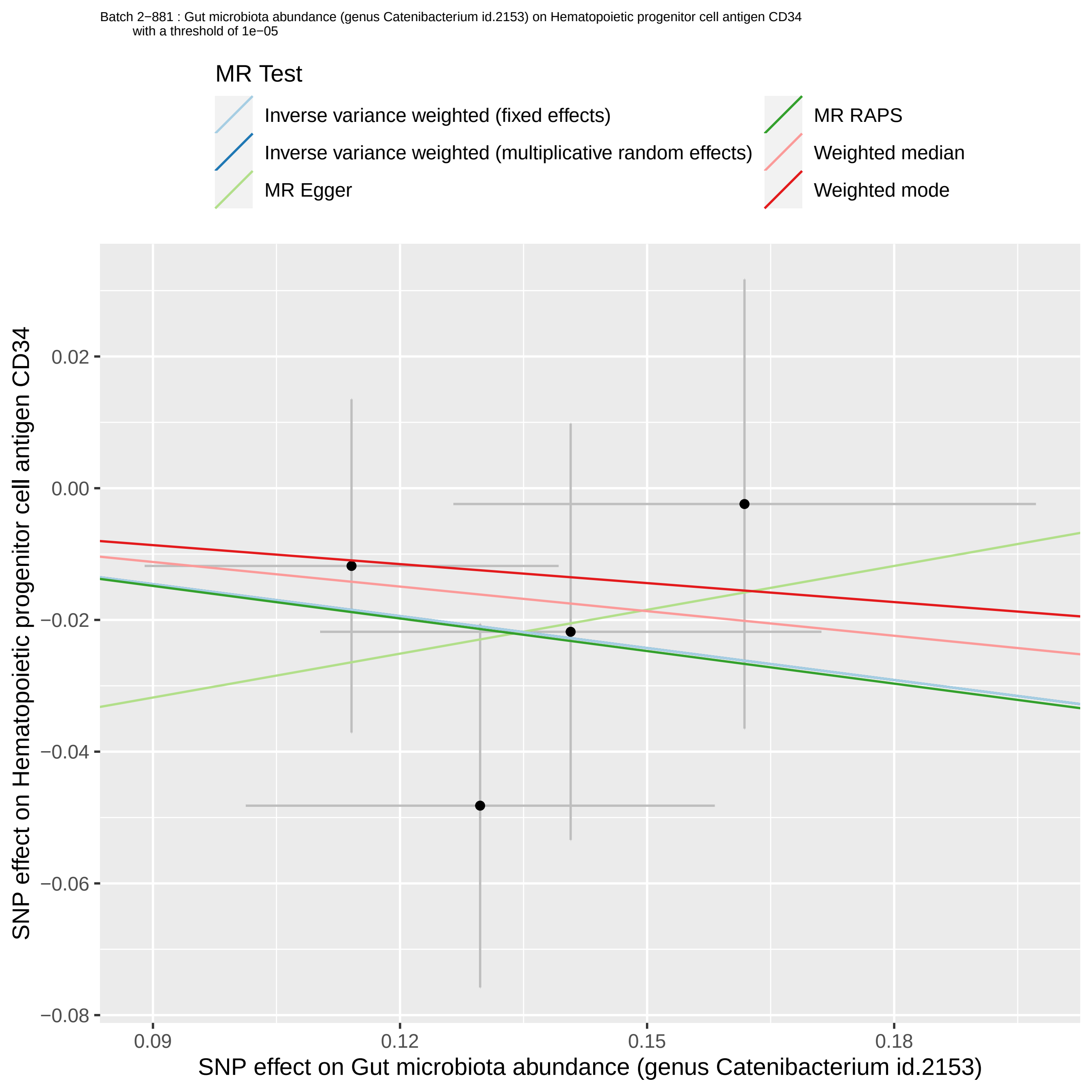
Batch 2−881 : Gut microbiota abundance (genus Catenibacterium id.2153) on Hematopoietic progenitor cell antigen CD34
with a threshold of 1e−05
MR Test
Inverse variance weighted (fixed effects)
Inverse variance weighted (multiplicative random effects)
MR Egger
MR RAPS
Weighted median
Weighted mode
0.02
0.00
−0.02
−0.04
−0.06
−0.08
0.09
0.12
0.15
0.18
SNP effect on Hematopoietic progenitor cell antigen CD34
SNP effect on Gut microbiota abundance (genus Catenibacterium id.2153)

Batch 2−882 : Gut microbiota abundance (genus Parasutterella id.2892) on Hematopoietic progenitor cell antigen CD34
with a threshold of 1e−05

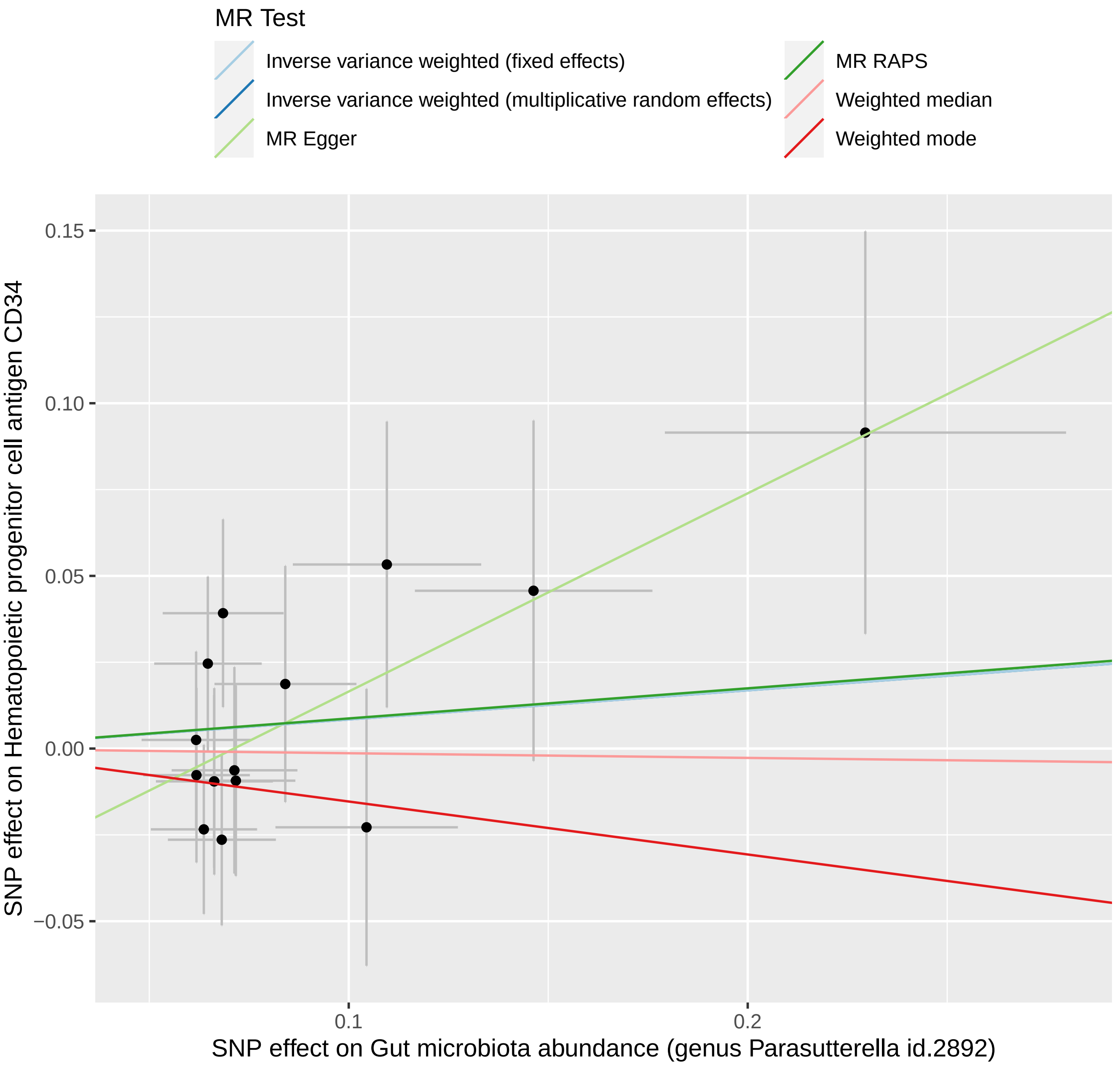

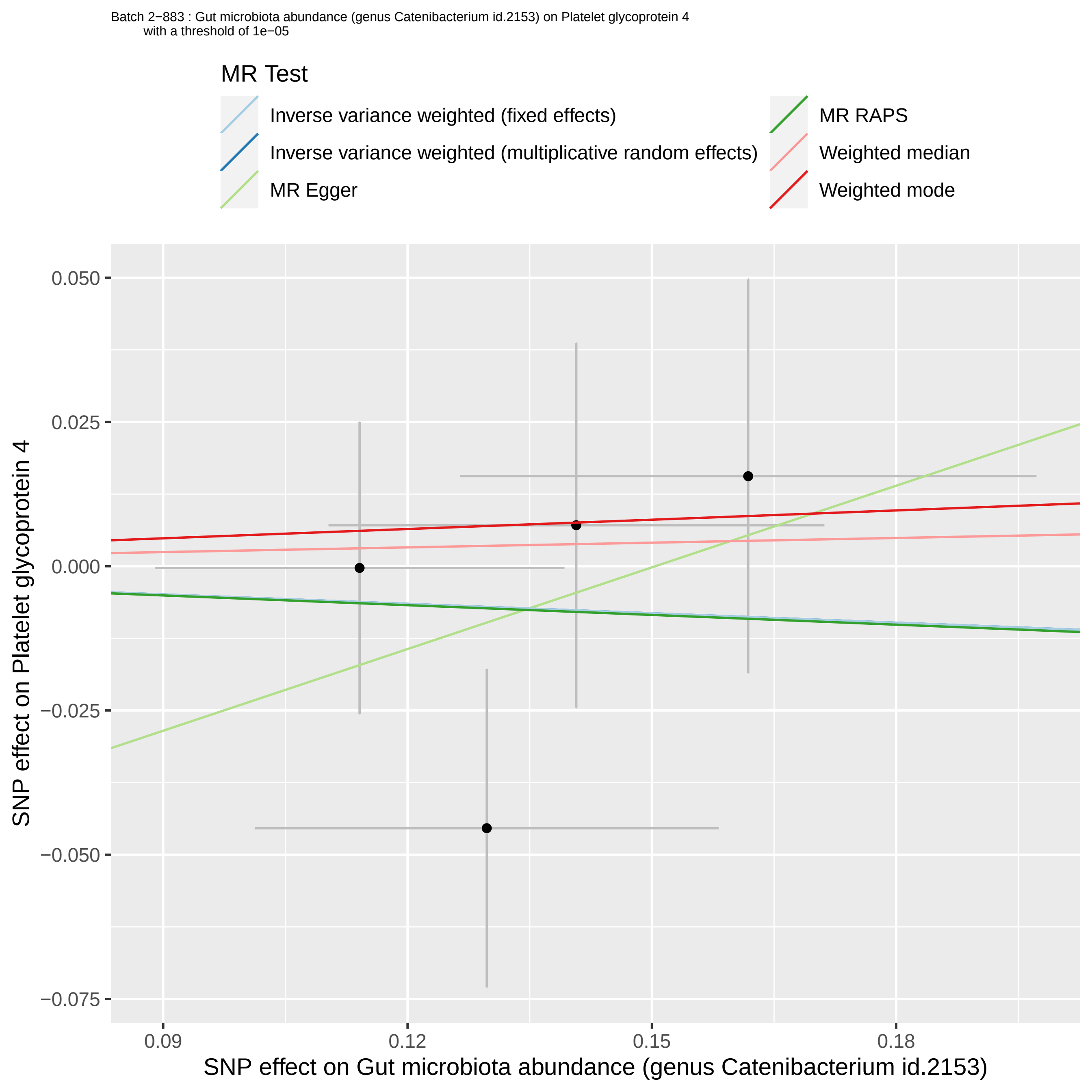
Batch 2−883 : Gut microbiota abundance (genus Catenibacterium id.2153) on Platelet glycoprotein 4
with a threshold of 1e−05
MR Test
Inverse variance weighted (fixed effects)
Inverse variance weighted (multiplicative random effects)
MR Egger
MR RAPS
Weighted median
Weighted mode
0.050
0.025
0.000
−0.025
−0.050
−0.075
SNP effect on Platelet glycoprotein 4
0.09
0.12
0.15
0.18
SNP effect on Gut microbiota abundance (genus Catenibacterium id.2153)

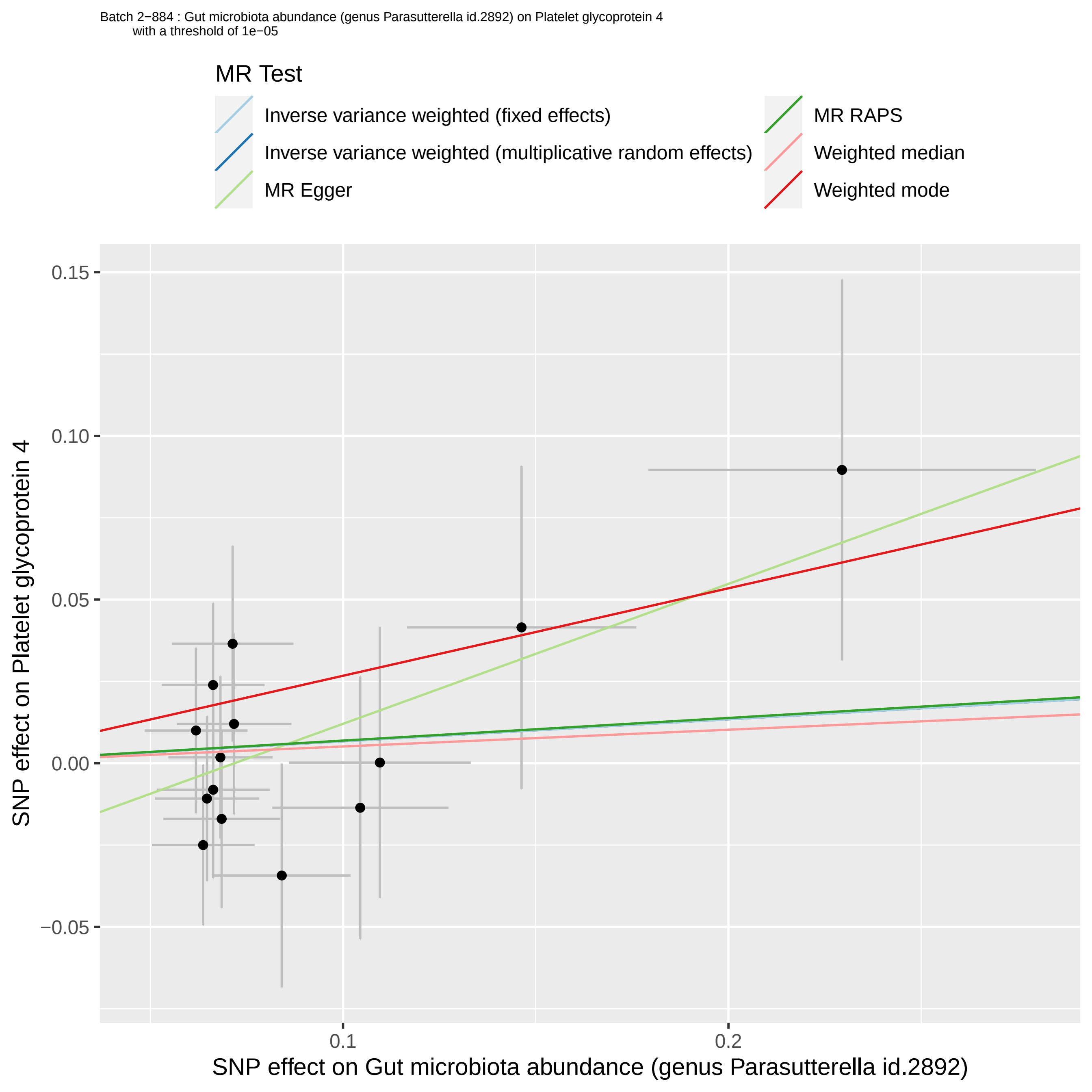
Batch 2−884 : Gut microbiota abundance (genus Parasutterella id.2892) on Platelet glycoprotein 4
with a threshold of 1e−05
MR Test
Inverse variance weighted (fixed effects)
Inverse variance weighted (multiplicative random effects)
MR Egger
MR RAPS
Weighted median
Weighted mode
0.15
0.10
0.05
0.00
−0.05
0.1
0.2
SNP effect on Platelet glycoprotein 4
SNP effect on Gut microbiota abundance (genus Parasutterella id.2892)

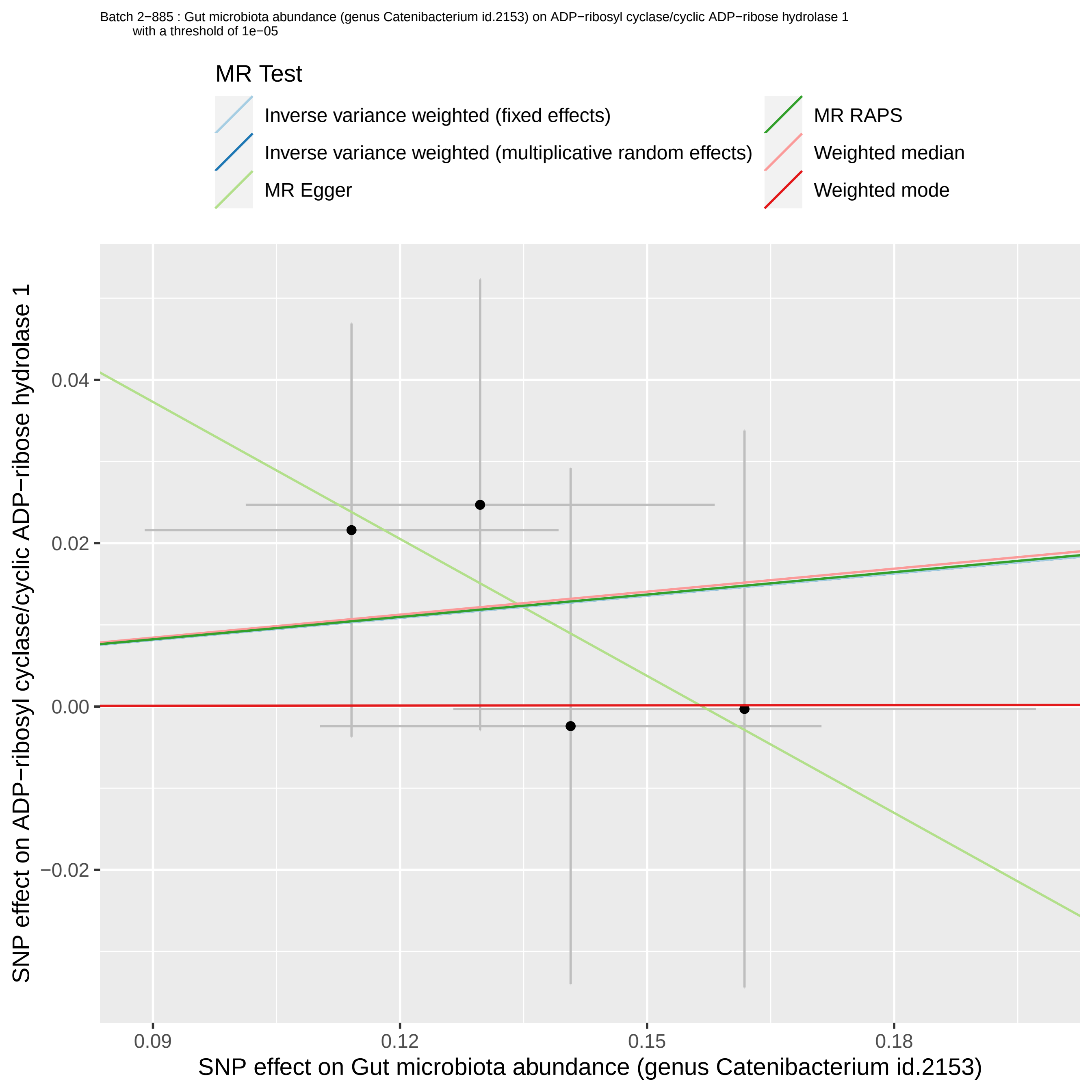

Batch 2−885 : Gut microbiota abundance (genus Catenibacterium id.2153) on ADP−ribosyl cyclase/cyclic ADP−ribose hydrolase 1
with a threshold of 1e−05
MR Test
Inverse variance weighted (fixed effects)
Inverse variance weighted (multiplicative random effects)
MR Egger
MR RAPS
Weighted median
Weighted mode
SNP effect on ADP−ribosyl cyclase/cyclic ADP−ribose hydrolase 1
0.04
0.02
0.00
−0.02
0.09
0.12
0.15
0.18
SNP effect on Gut microbiota abundance (genus Catenibacterium id.2153)

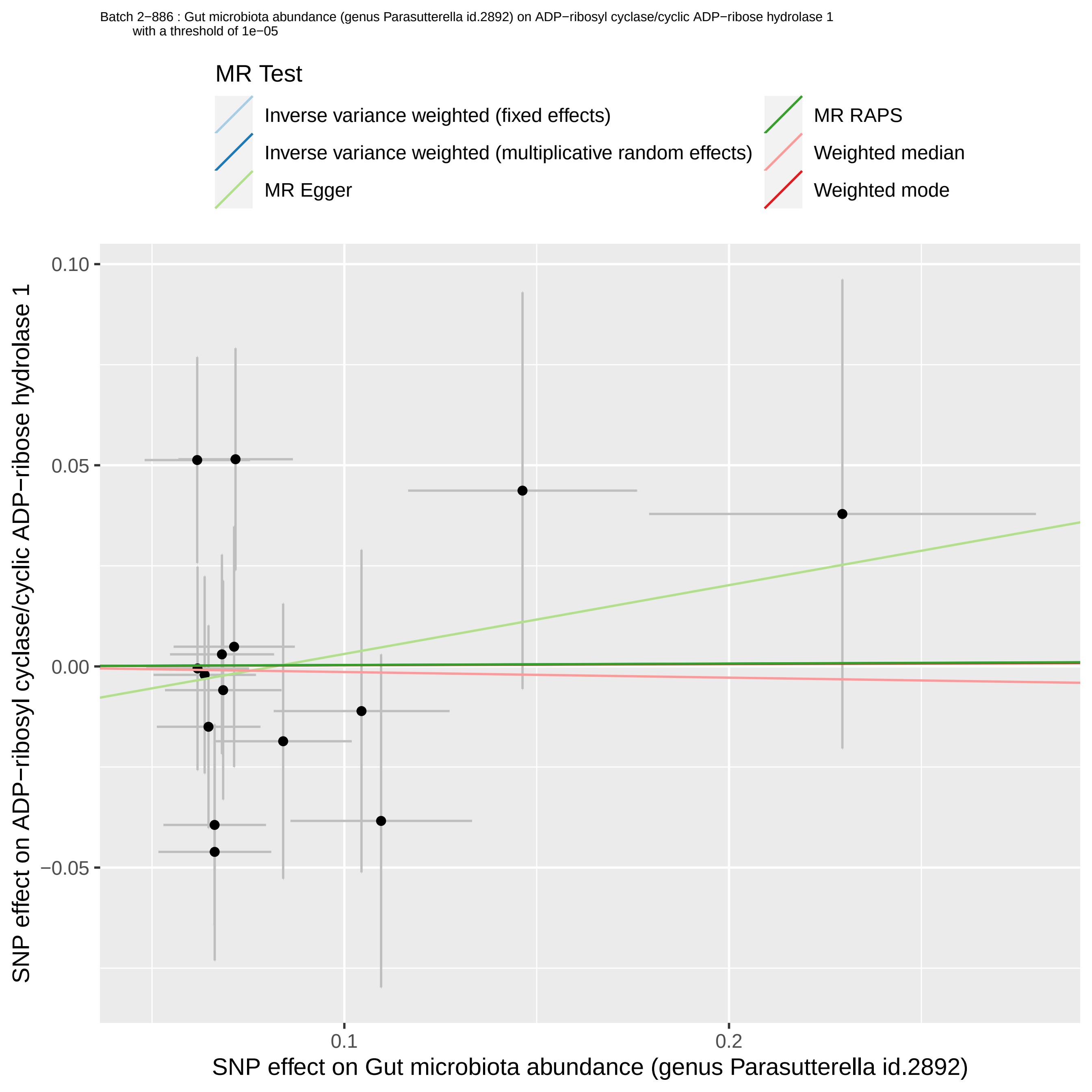
Batch 2−886 : Gut microbiota abundance (genus Parasutterella id.2892) on ADP−ribosyl cyclase/cyclic ADP−ribose hydrolase 1
with a threshold of 1e−05
MR Test
Inverse variance weighted (fixed effects)
Inverse variance weighted (multiplicative random effects)
MR Egger
MR RAPS
Weighted median
Weighted mode
0.10
0.05
0.00
−0.05
0.1
0.2
SNP effect on ADP−ribosyl cyclase/cyclic ADP−ribose hydrolase 1
SNP effect on Gut microbiota abundance (genus Parasutterella id.2892)

Batch 2−887 : Gut microbiota abundance (genus Catenibacterium id.2153) on ADP−ribosyl cyclase/cyclic ADP−ribose hydrolase 1 with a threshold of 1e−05

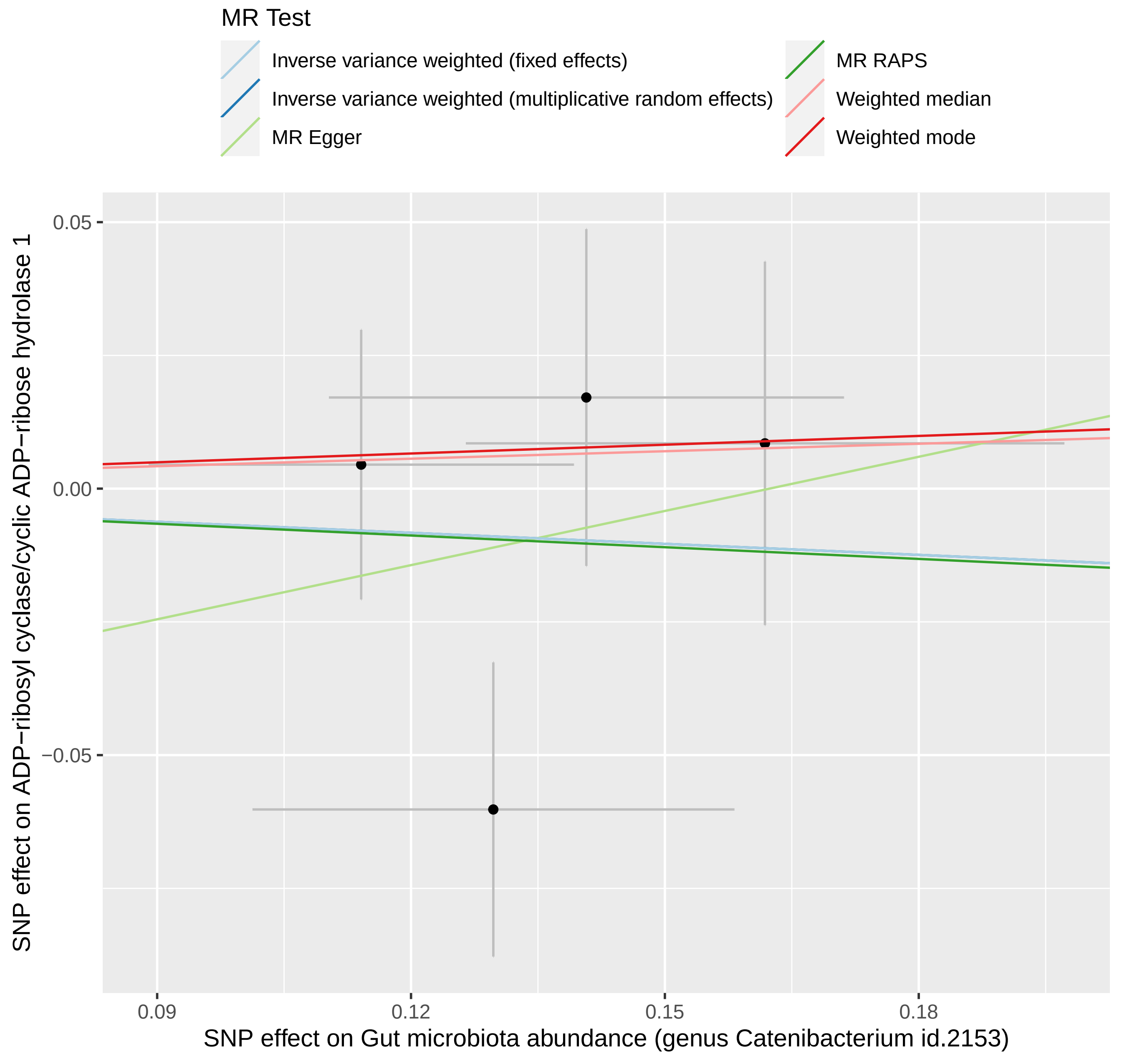

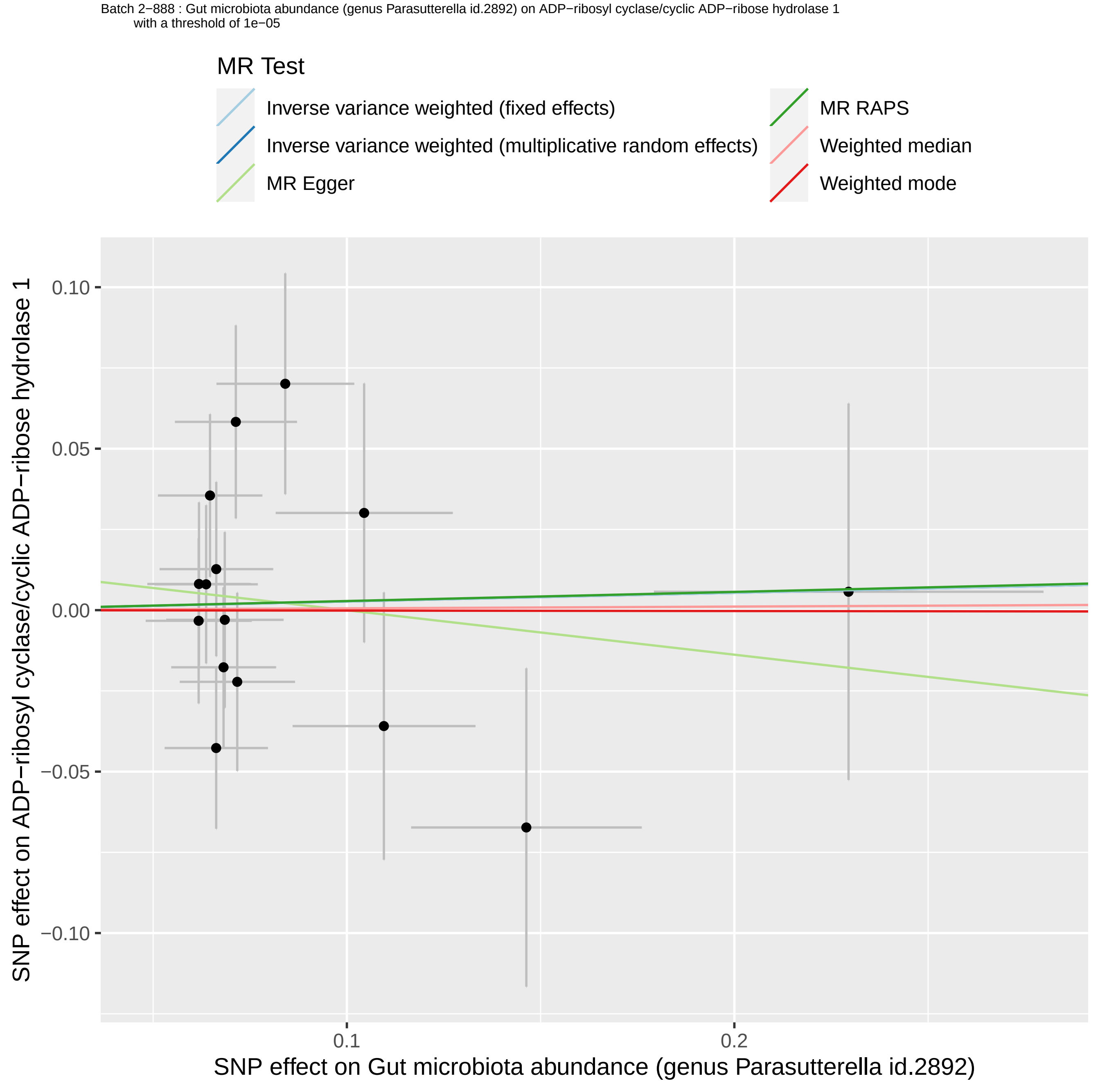

Batch 2−888 : Gut microbiota abundance (genus Parasutterella id.2892) on ADP−ribosyl cyclase/cyclic ADP−ribose hydrolase 1
with a threshold of 1e−05
MR Test
Inverse variance weighted (fixed effects)
Inverse variance weighted (multiplicative random effects)
MR Egger
MR RAPS
Weighted median
Weighted mode
SNP effect on ADP−ribosyl cyclase/cyclic ADP−ribose hydrolase 1
0.10
0.05
0.00
−0.05
−0.10
0.1
0.2
SNP effect on Gut microbiota abundance (genus Parasutterella id.2892)

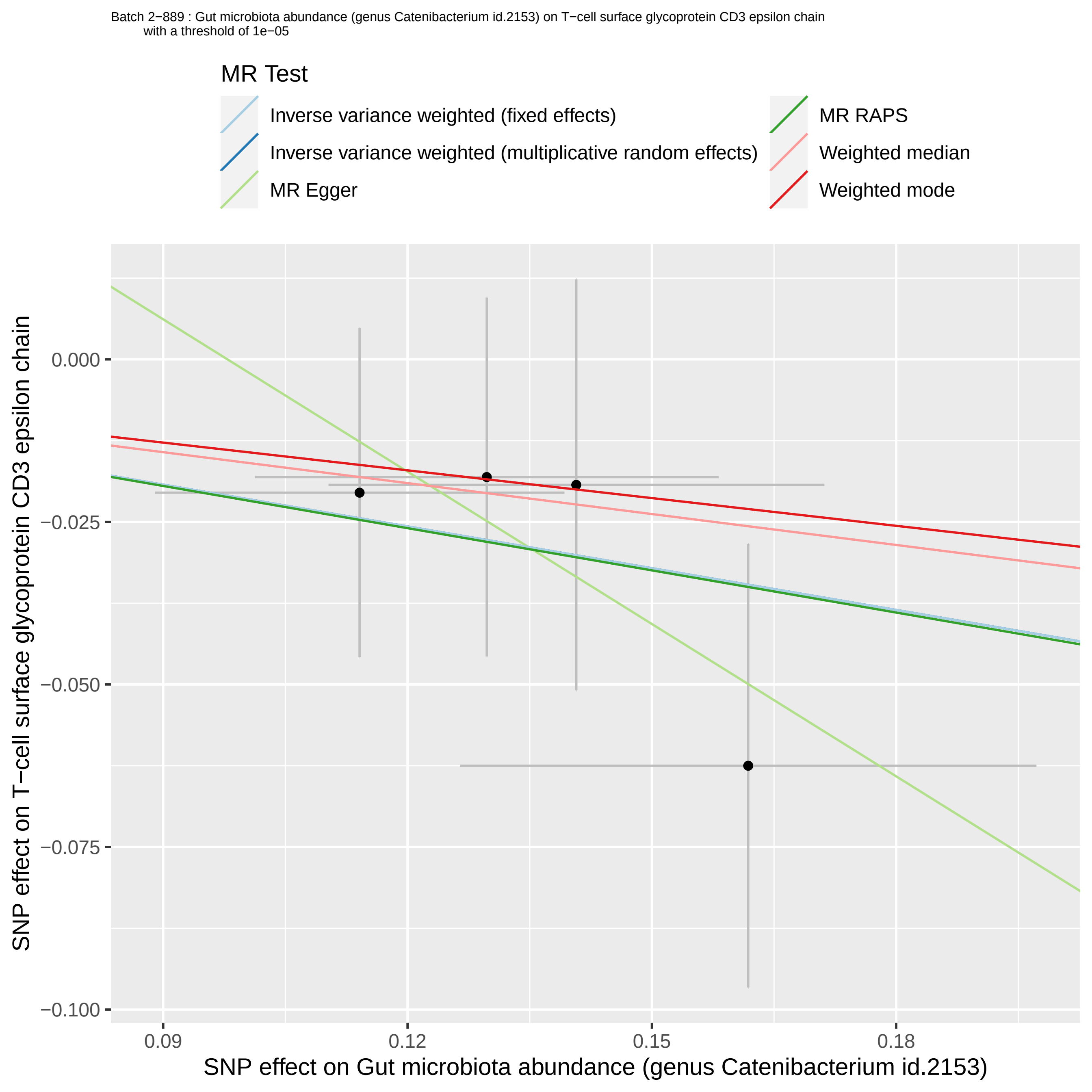
Batch 2−889 : Gut microbiota abundance (genus Catenibacterium id.2153) on T−cell surface glycoprotein CD3 epsilon chain
with a threshold of 1e−05
MR Test
Inverse variance weighted (fixed effects)
Inverse variance weighted (multiplicative random effects)
MR Egger
MR RAPS
Weighted median
Weighted mode
0.000
−0.025
−0.050
−0.075
−0.100
SNP effect on T−cell surface glycoprotein CD3 epsilon chain
0.09
0.12
0.15
0.18
SNP effect on Gut microbiota abundance (genus Catenibacterium id.2153)

Batch 2−890 : Gut microbiota abundance (genus Parasutterella id.2892) on T−cell surface glycoprotein CD3 epsilon chain
with a threshold of 1e−05

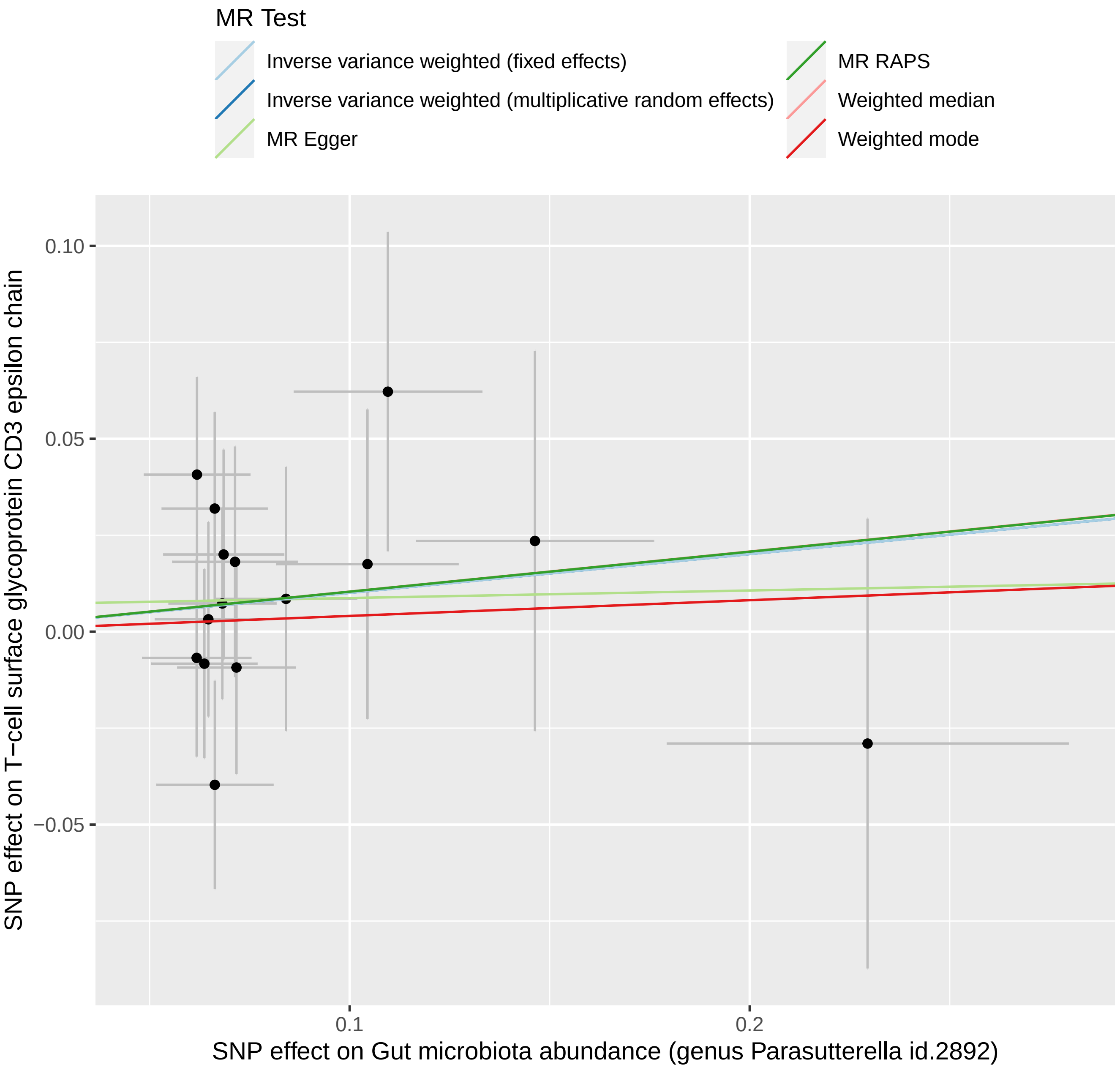

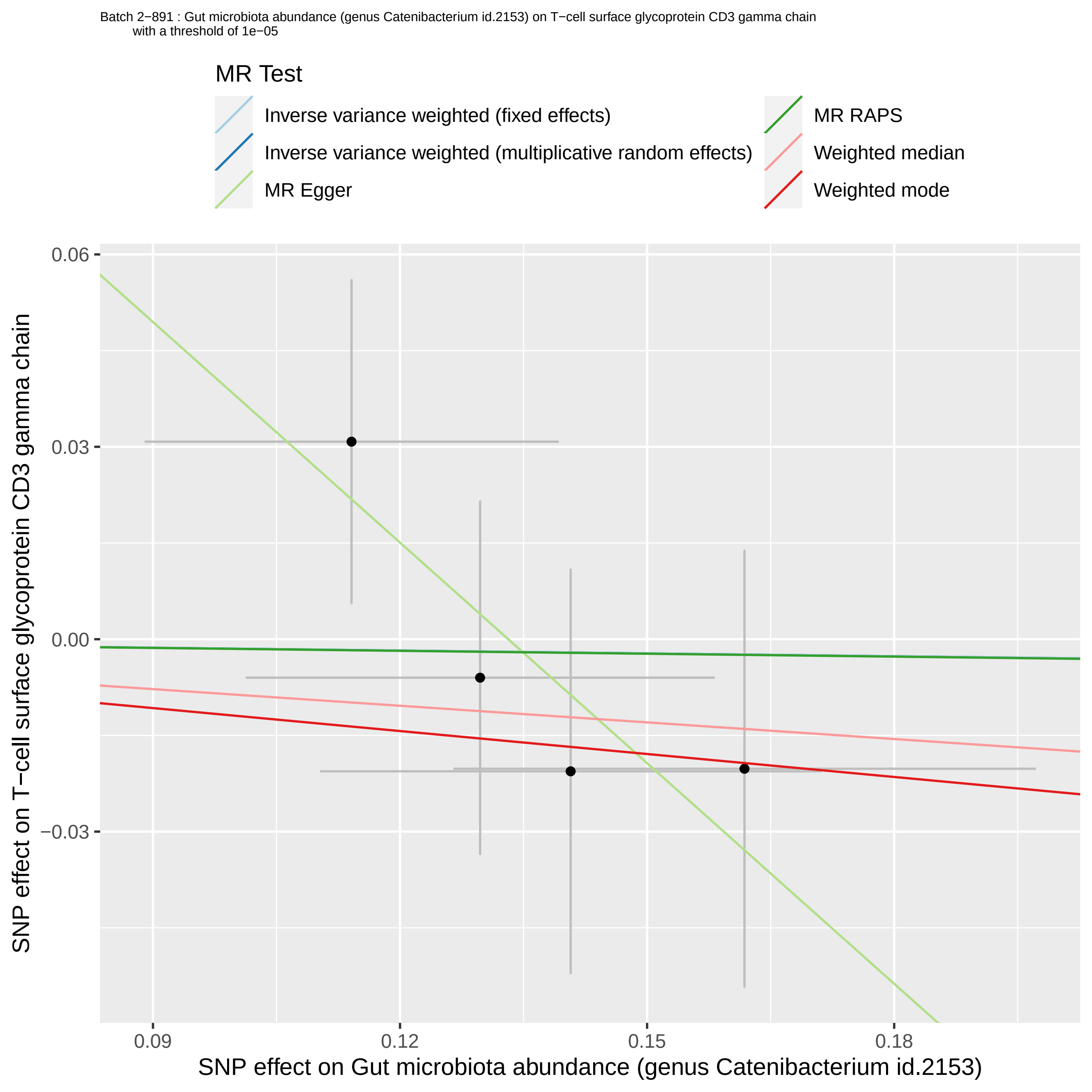
Batch 2−891 : Gut microbiota abundance (genus Catenibacterium id.2153) on T−cell surface glycoprotein CD3 gamma chain
with a threshold of 1e−05
MR Test
Inverse variance weighted (fixed effects)
Inverse variance weighted (multiplicative random effects)
MR Egger
MR RAPS
Weighted median
Weighted mode
0.06
0.03
0.00
−0.03
0.09
0.12
0.15
0.18
SNP effect on T−cell surface glycoprotein CD3 gamma chain
SNP effect on Gut microbiota abundance (genus Catenibacterium id.2153)

Batch 2−892 : Gut microbiota abundance (genus Parasutterella id.2892) on T−cell surface glycoprotein CD3 gamma chain
with a threshold of 1e−05

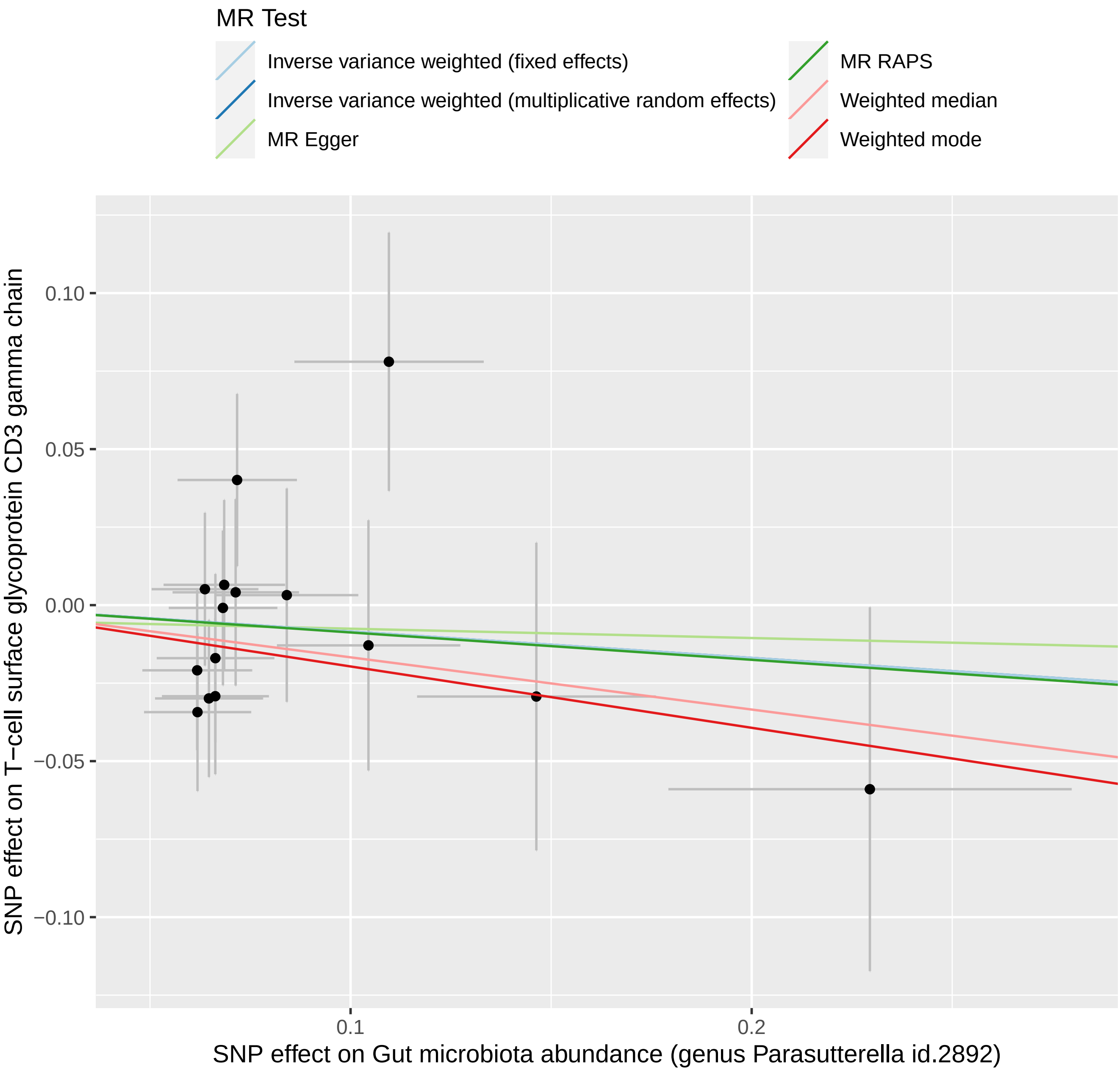

Batch 2−893 : Gut microbiota abundance (genus Catenibacterium id.2153) on CD40 ligand with a threshold of 1e−05

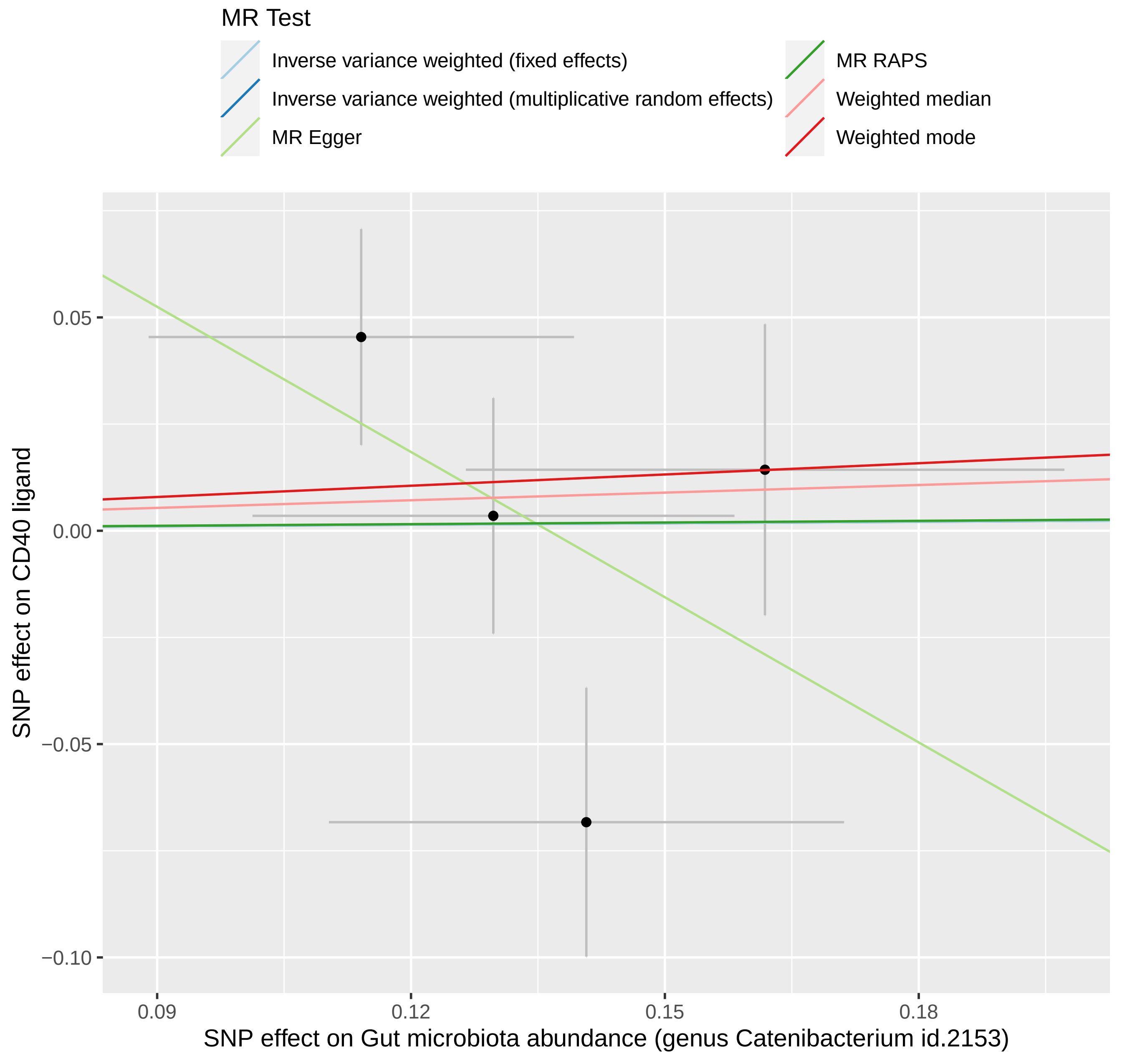

Batch 2−894 : Gut microbiota abundance (genus Parasutterella id.2892) on CD40 ligand
with a threshold of 1e−05

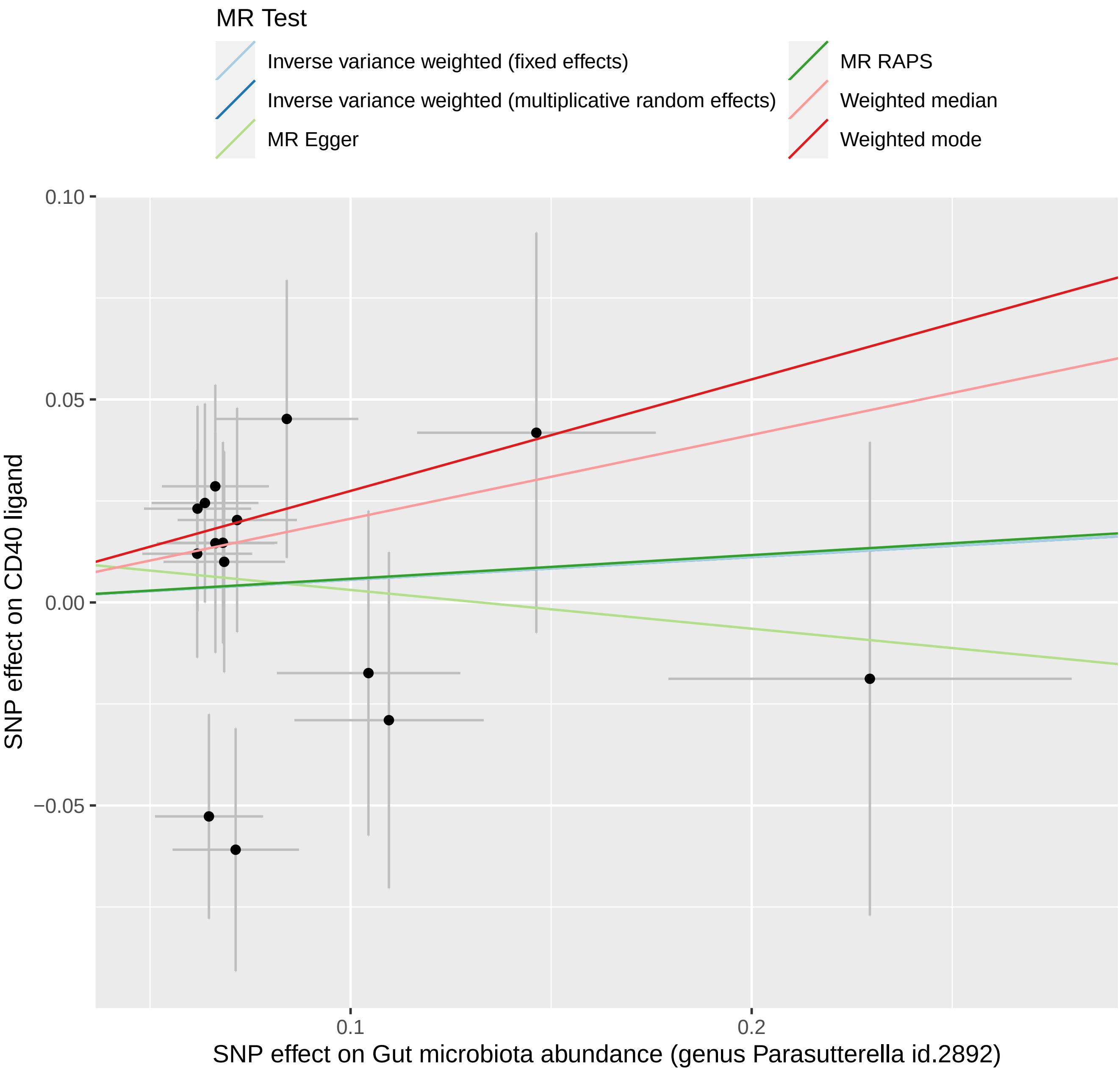

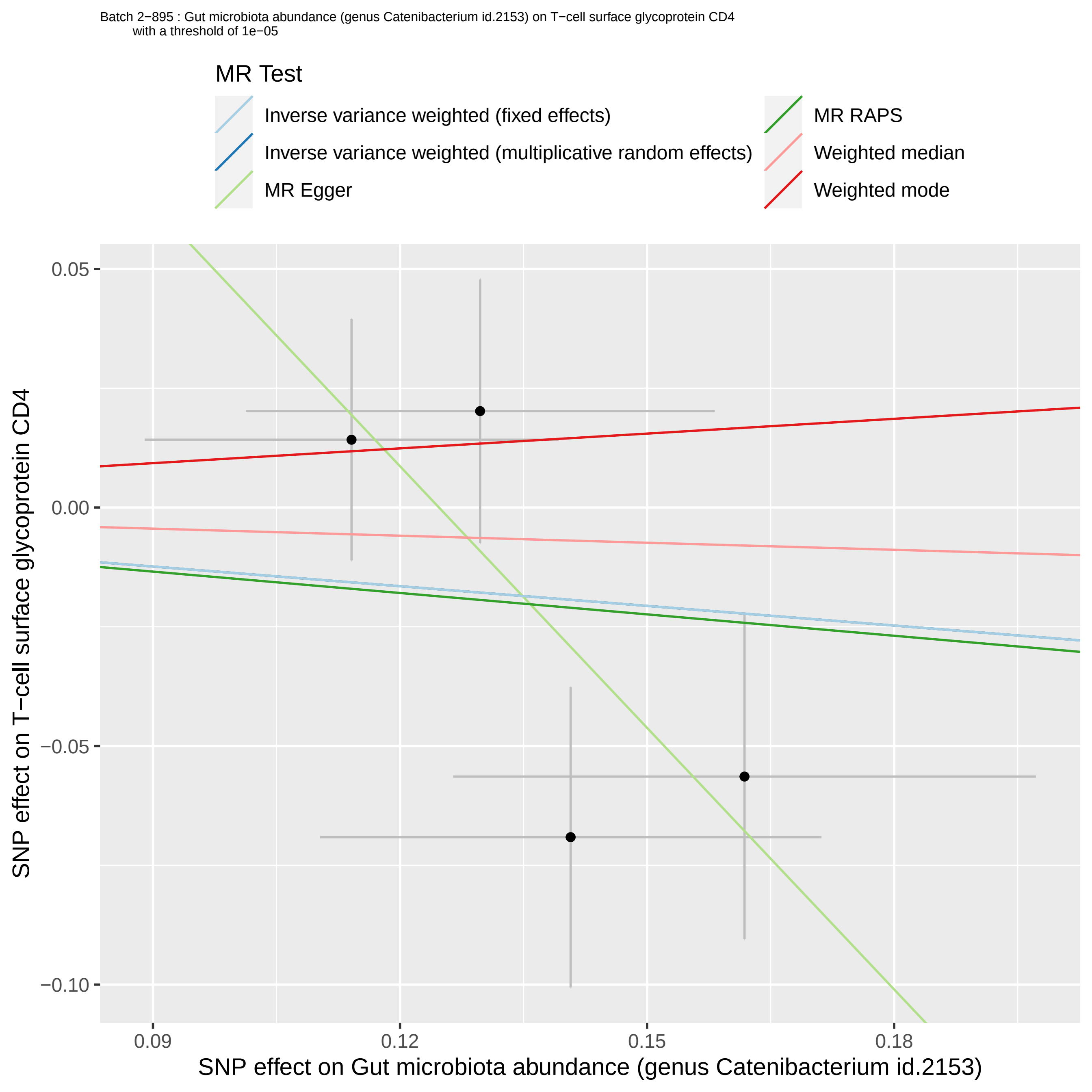
Batch 2−895 : Gut microbiota abundance (genus Catenibacterium id.2153) on T−cell surface glycoprotein CD4
with a threshold of 1e−05
MR Test
Inverse variance weighted (fixed effects)
Inverse variance weighted (multiplicative random effects)
MR Egger
MR RAPS
Weighted median
Weighted mode
0.05
0.00
−0.05
−0.10
0.09
0.12
0.15
0.18
SNP effect on T−cell surface glycoprotein CD4
SNP effect on Gut microbiota abundance (genus Catenibacterium id.2153)

Batch 2−896 : Gut microbiota abundance (genus Parasutterella id.2892) on T−cell surface glycoprotein CD4
with a threshold of 1e−05

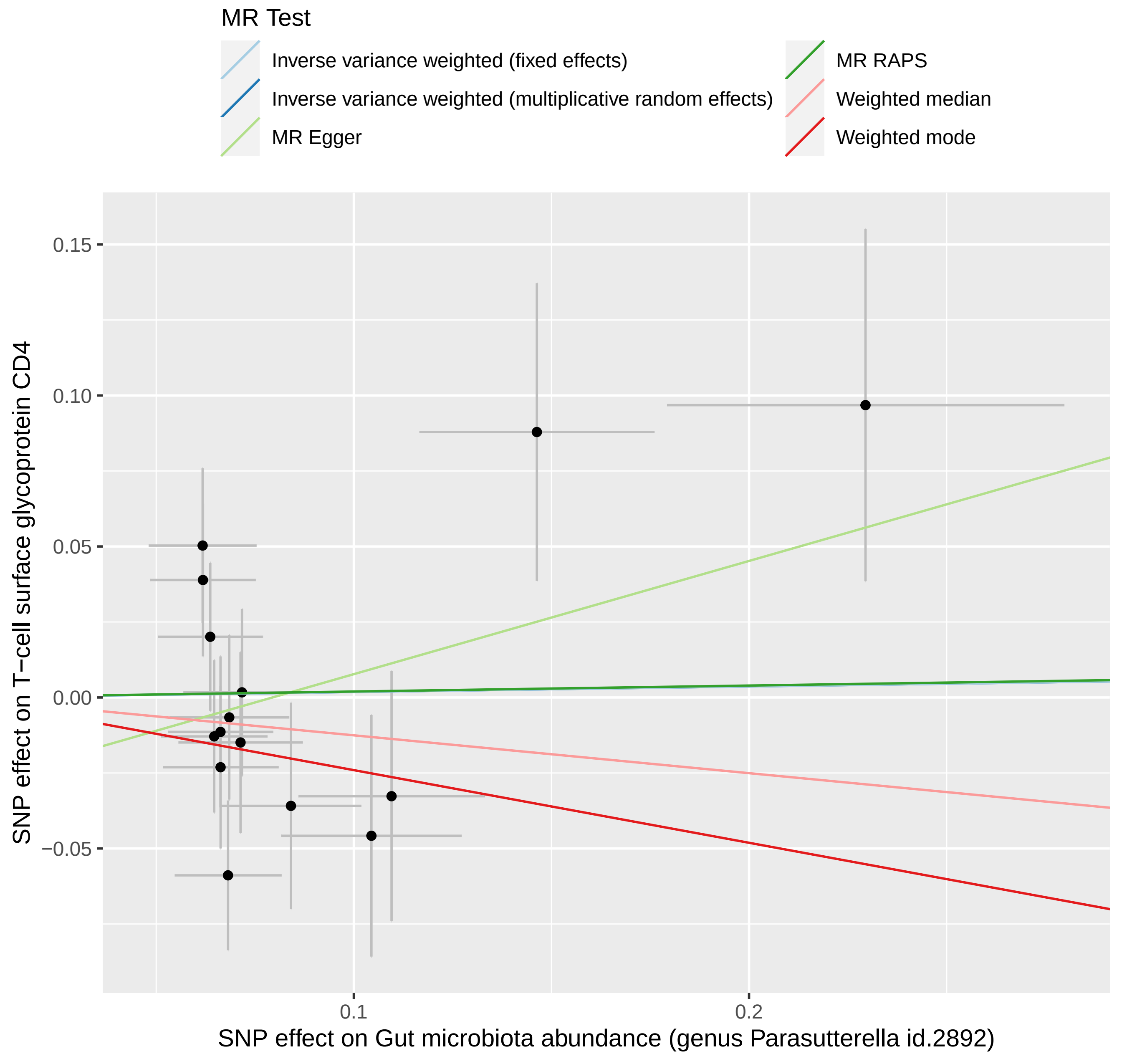

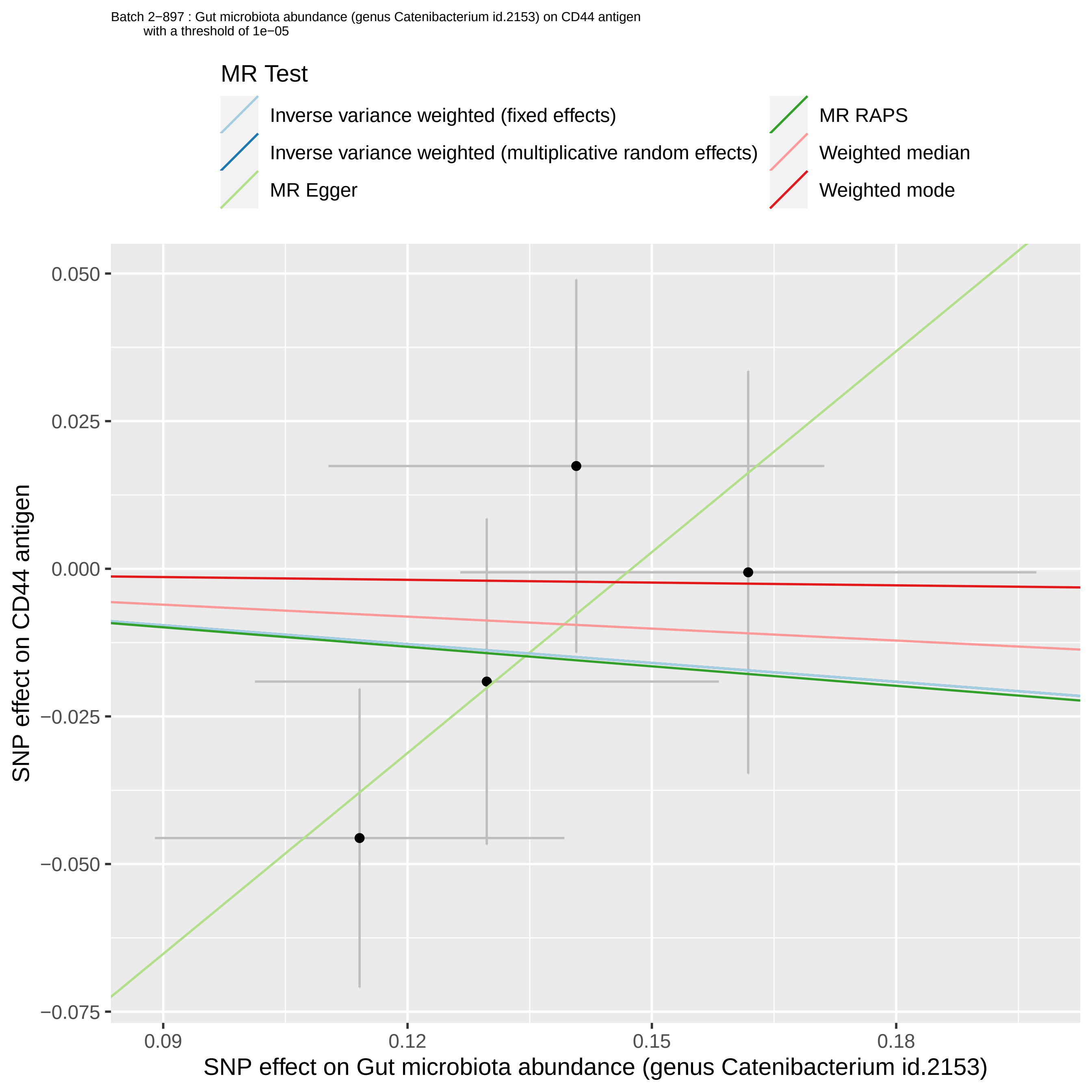
Batch 2−897 : Gut microbiota abundance (genus Catenibacterium id.2153) on CD44 antigen
with a threshold of 1e−05
MR Test
Inverse variance weighted (fixed effects)
Inverse variance weighted (multiplicative random effects)
MR Egger
MR RAPS
Weighted median
Weighted mode
0.050
0.025
0.000
−0.025
−0.050
−0.075
0.09
0.12
0.15
0.18
SNP effect on CD44 antigen
SNP effect on Gut microbiota abundance (genus Catenibacterium id.2153)

Batch 2−898 : Gut microbiota abundance (genus Parasutterella id.2892) on CD44 antigen
with a threshold of 1e−05

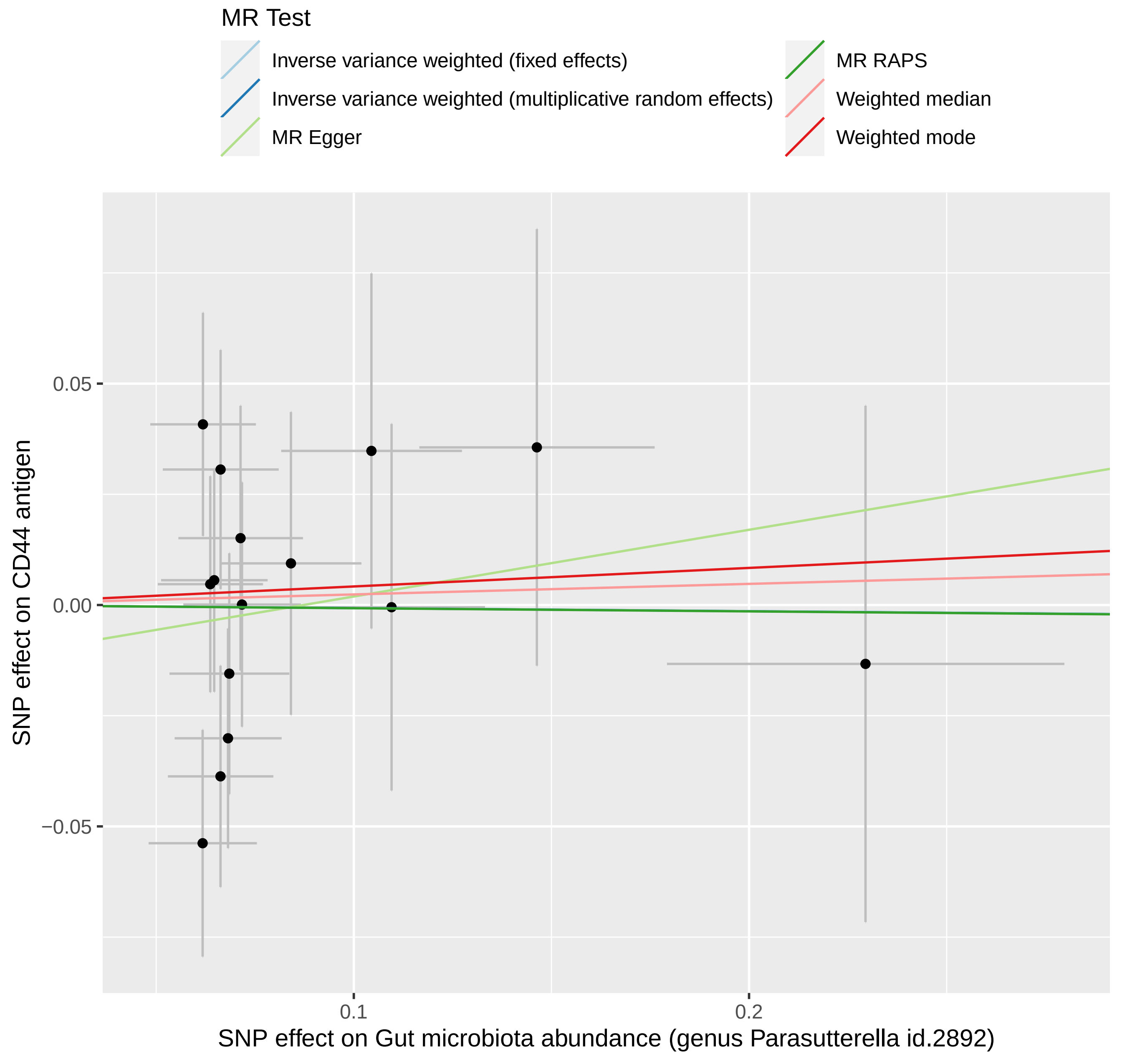

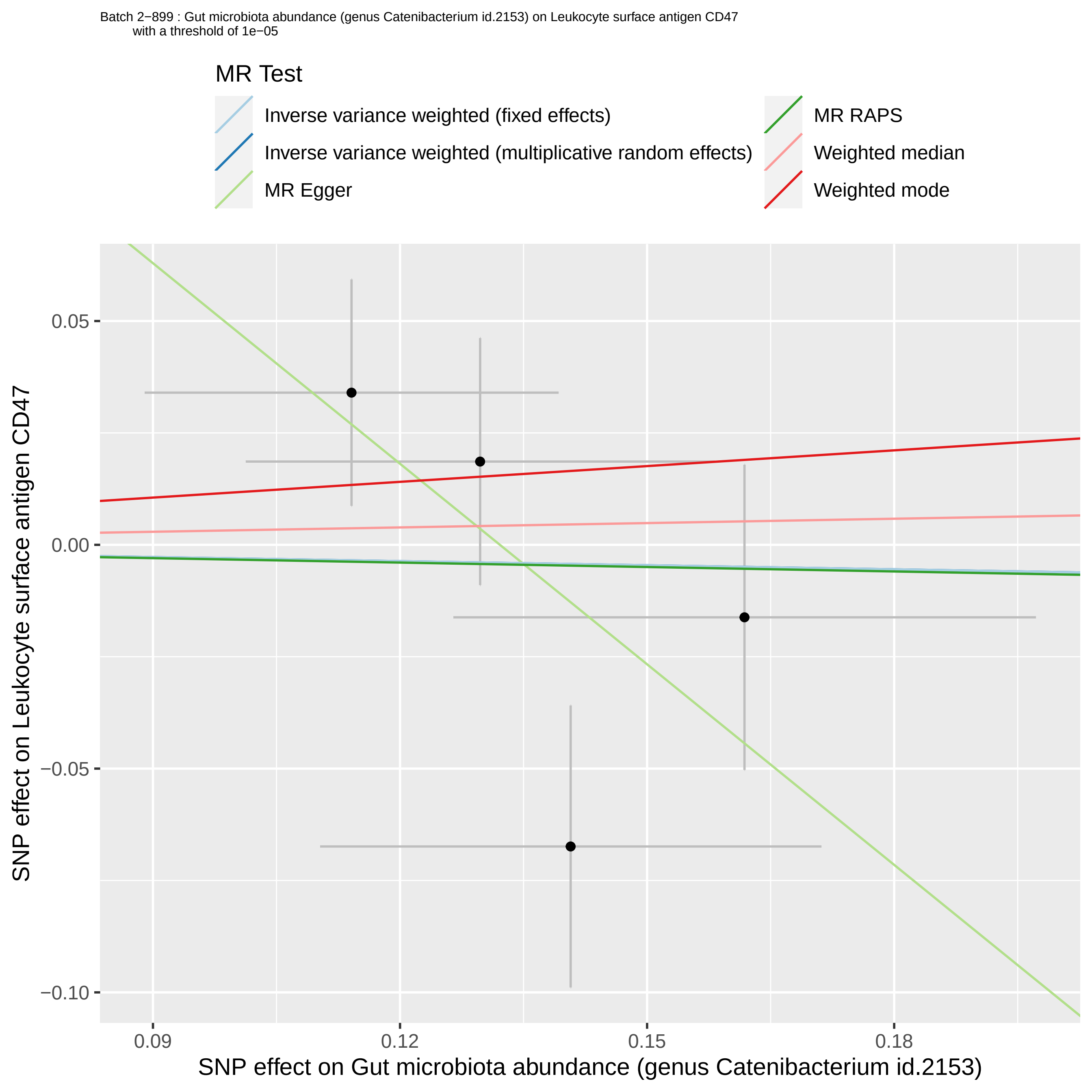
Batch 2−899 : Gut microbiota abundance (genus Catenibacterium id.2153) on Leukocyte surface antigen CD47
with a threshold of 1e−05
MR Test
Inverse variance weighted (fixed effects)
Inverse variance weighted (multiplicative random effects)
MR Egger
MR RAPS
Weighted median
Weighted mode
0.05
0.00
−0.05
−0.10
0.09
0.12
0.15
0.18
SNP effect on Leukocyte surface antigen CD47
SNP effect on Gut microbiota abundance (genus Catenibacterium id.2153)

Batch 2−900 : Gut microbiota abundance (genus Parasutterella id.2892) on Leukocyte surface antigen CD47
with a threshold of 1e−05

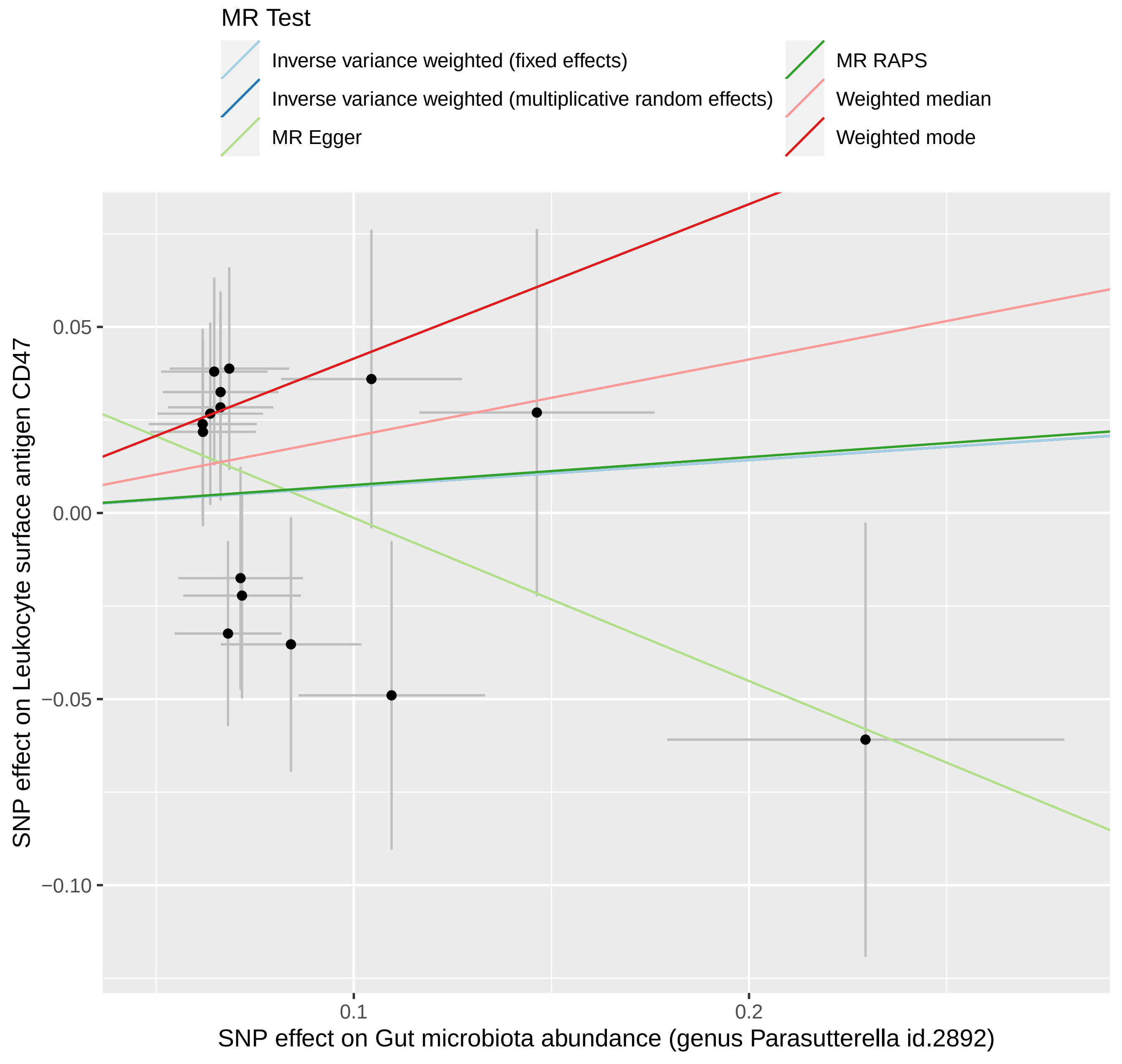

Batch 2−901 : Gut microbiota abundance (genus Catenibacterium id.2153) on CD48 antigen
with a threshold of 1e−05

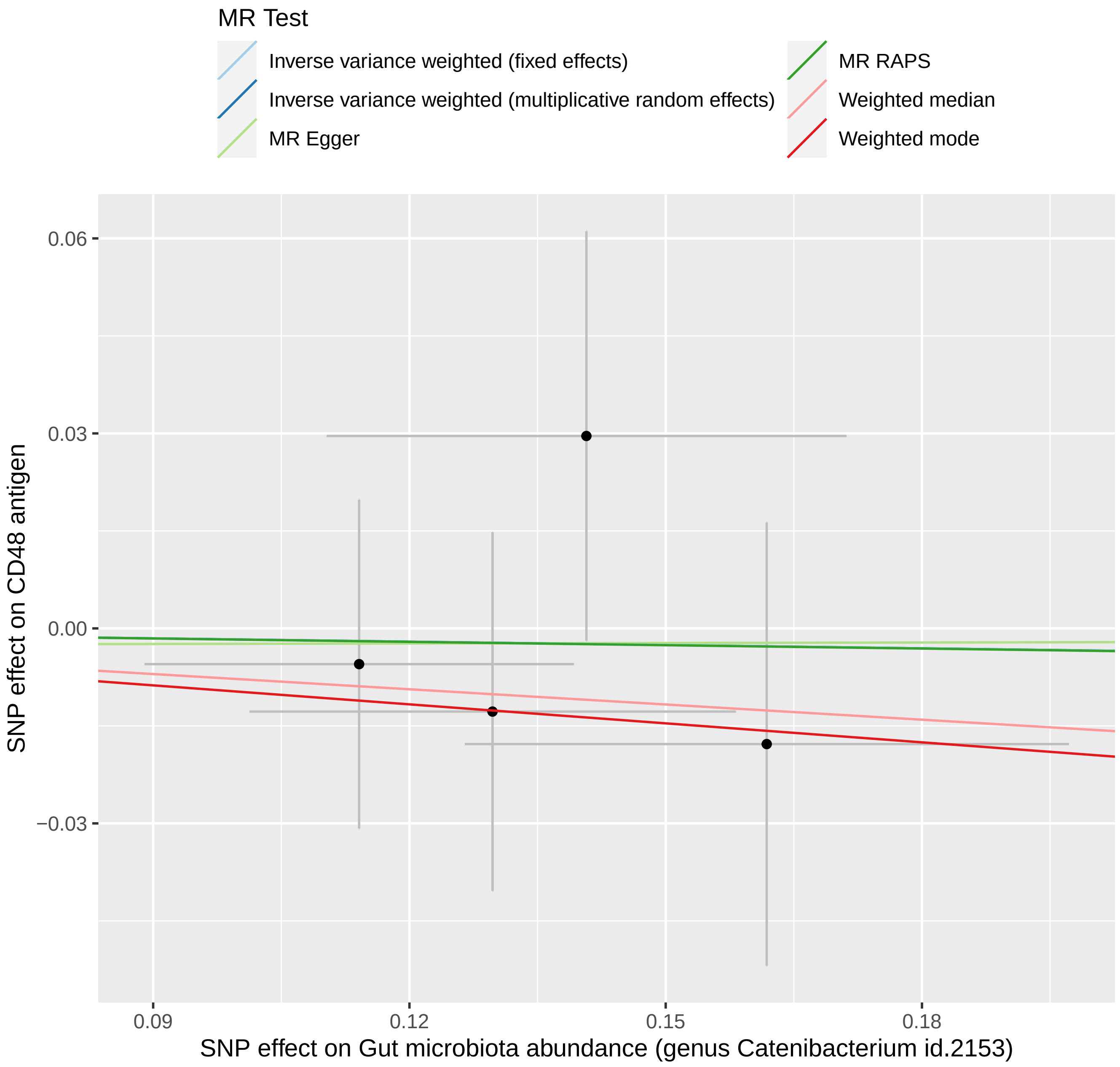

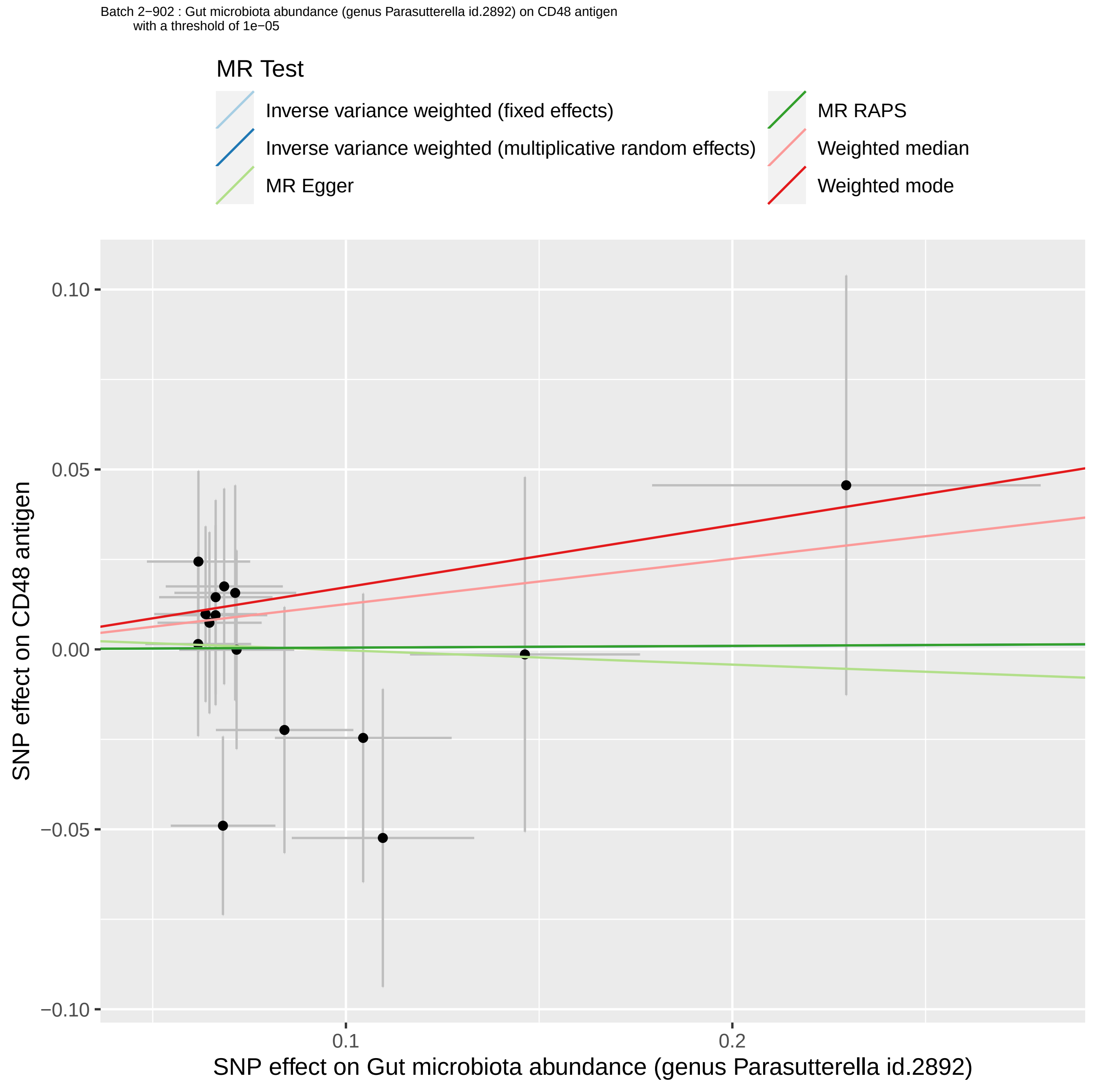

Batch 2−902 : Gut microbiota abundance (genus Parasutterella id.2892) on CD48 antigen
with a threshold of 1e−05
MR Test
Inverse variance weighted (fixed effects)
Inverse variance weighted (multiplicative random effects)
MR Egger
MR RAPS
Weighted median
Weighted mode
0.10
0.05
0.00
−0.05
−0.10
0.1
0.2
SNP effect on CD48 antigen
SNP effect on Gut microbiota abundance (genus Parasutterella id.2892)

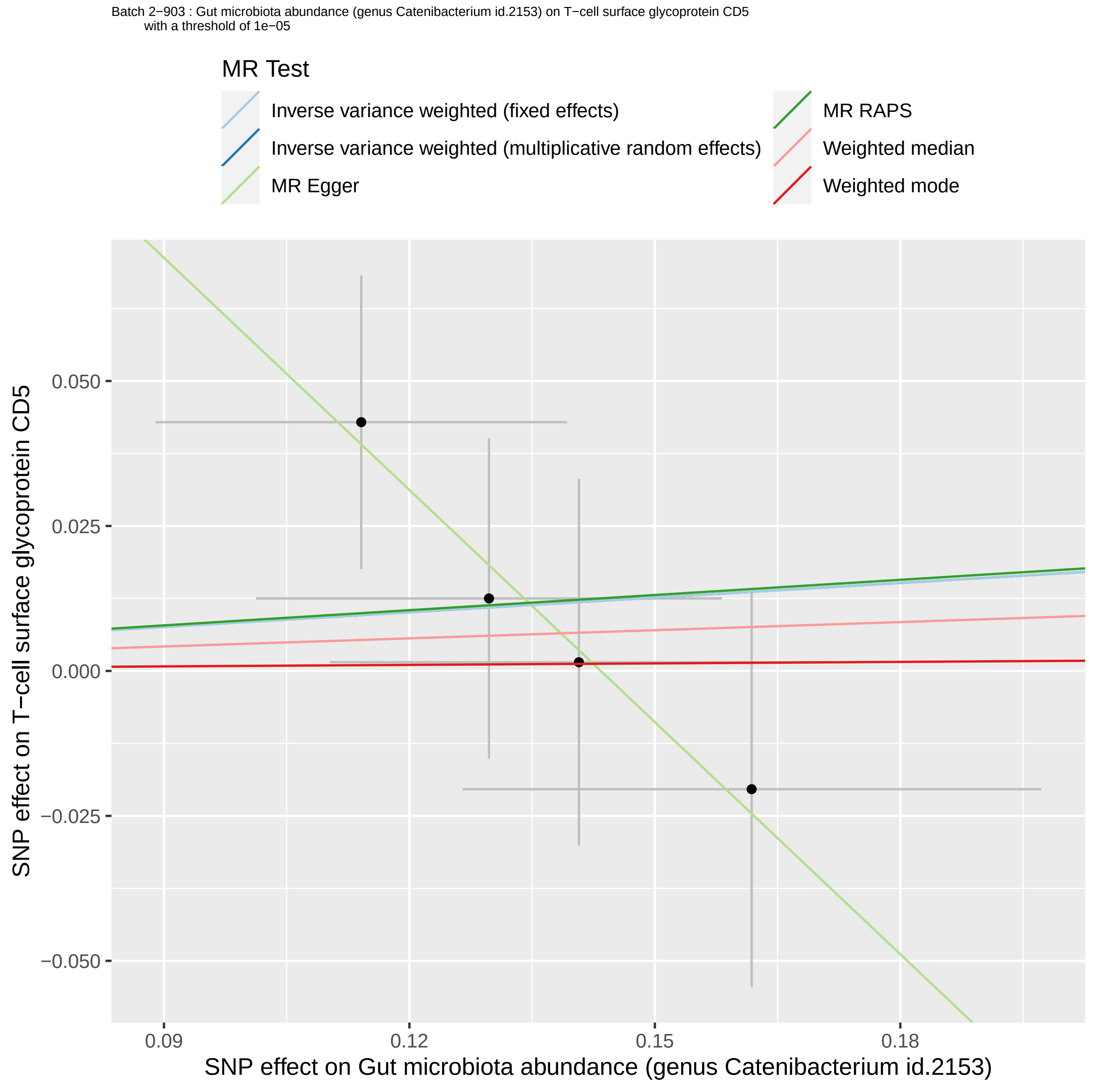
Batch 2−903 : Gut microbiota abundance (genus Catenibacterium id.2153) on T−cell surface glycoprotein CD5
with a threshold of 1e−05
MR Test
Inverse variance weighted (fixed effects)
Inverse variance weighted (multiplicative random effects)
MR Egger
MR RAPS
Weighted median
Weighted mode
0.050
0.025
0.000
−0.025
−0.050
0.09
0.12
0.15
0.18
SNP effect on T−cell surface glycoprotein CD5
SNP effect on Gut microbiota abundance (genus Catenibacterium id.2153)

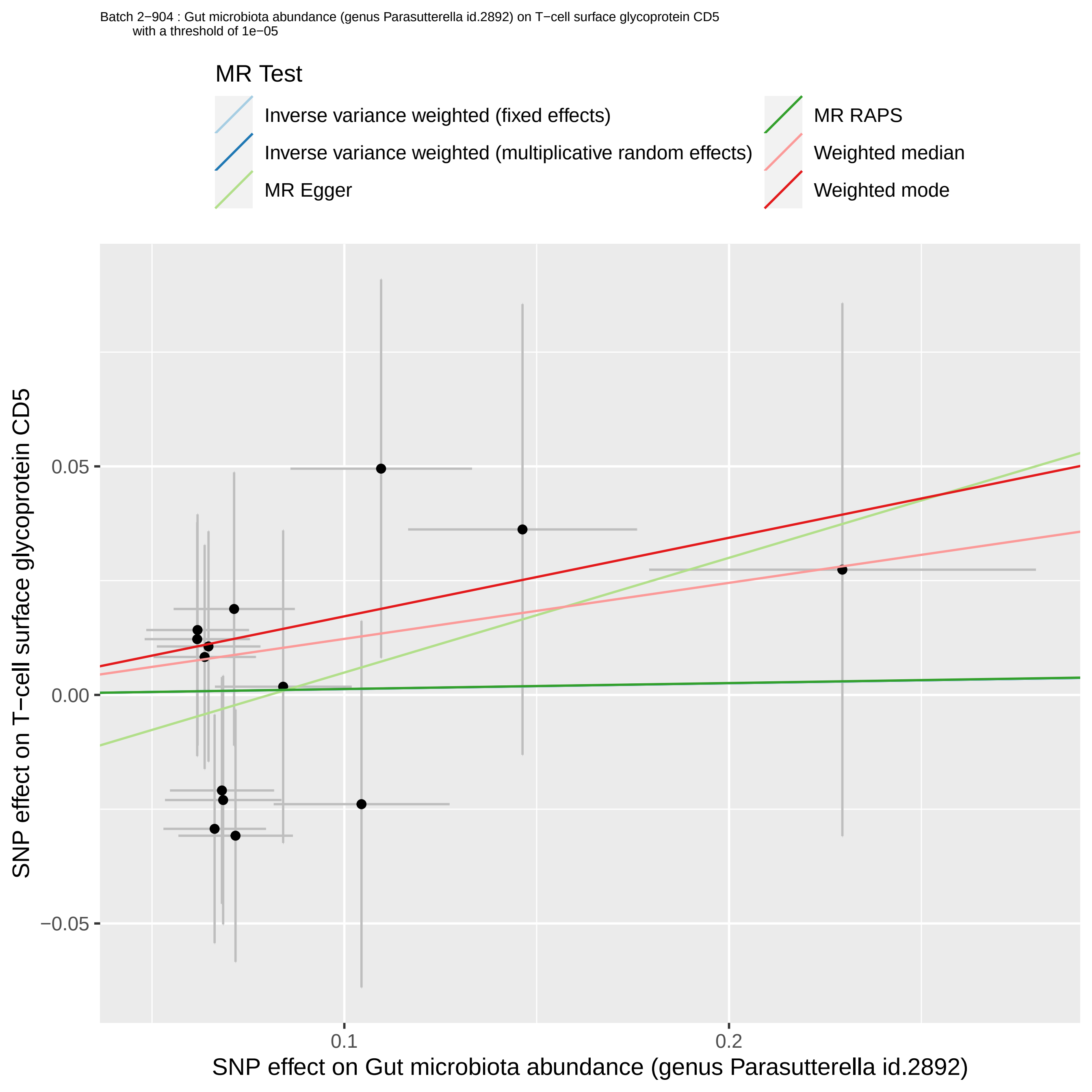
Batch 2−904 : Gut microbiota abundance (genus Parasutterella id.2892) on T−cell surface glycoprotein CD5
with a threshold of 1e−05
MR Test
Inverse variance weighted (fixed effects)
Inverse variance weighted (multiplicative random effects)
MR Egger
MR RAPS
Weighted median
Weighted mode
SNP effect on T−cell surface glycoprotein CD5
0.05
0.00
−0.05
0.1
0.2
SNP effect on Gut microbiota abundance (genus Parasutterella id.2892)

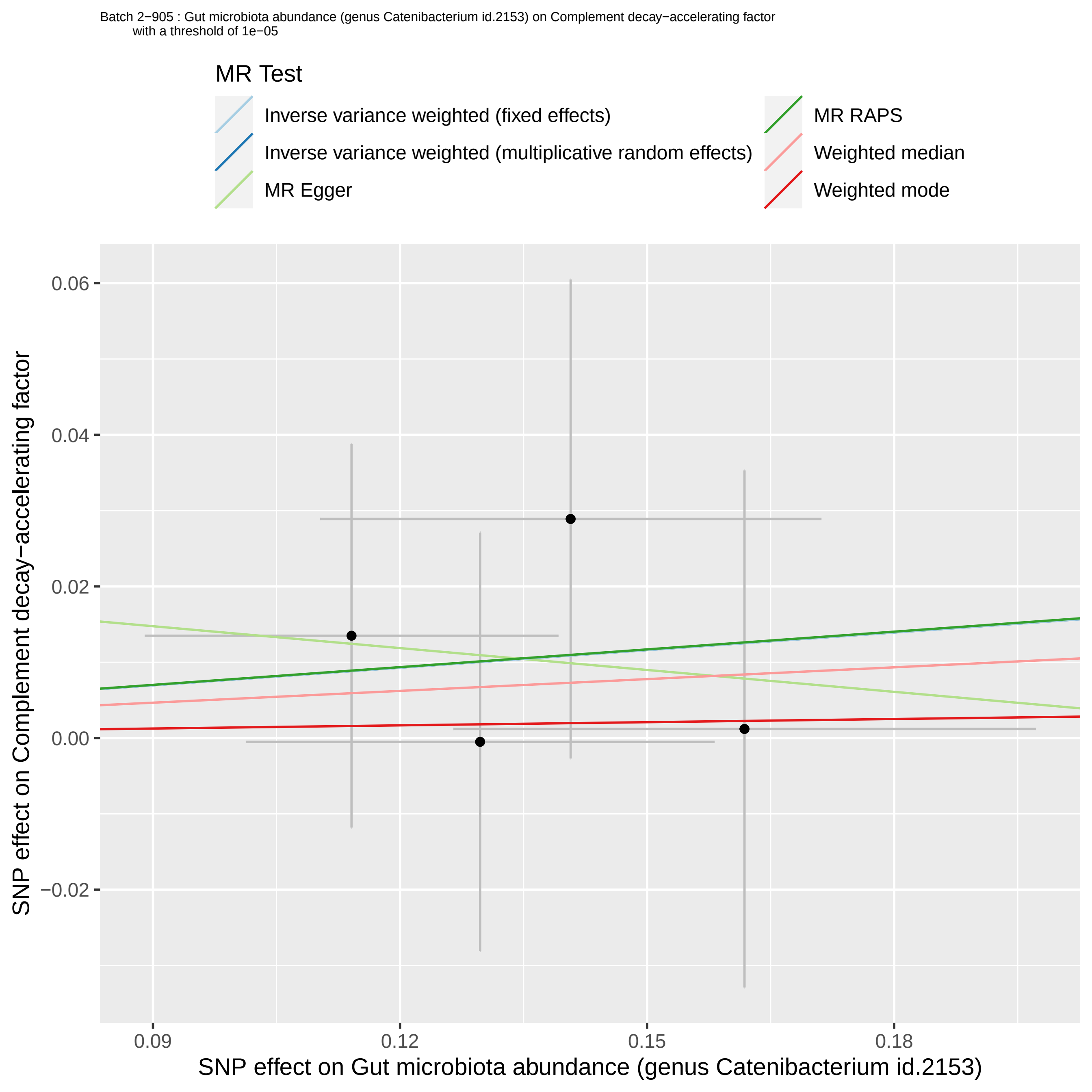
Batch 2−905 : Gut microbiota abundance (genus Catenibacterium id.2153) on Complement decay−accelerating factor
with a threshold of 1e−05
MR Test
Inverse variance weighted (fixed effects)
Inverse variance weighted (multiplicative random effects)
MR Egger
MR RAPS
Weighted median
Weighted mode
0.06
0.04
0.02
0.00
−0.02
0.09
0.12
0.15
0.18
SNP effect on Complement decay−accelerating factor
SNP effect on Gut microbiota abundance (genus Catenibacterium id.2153)

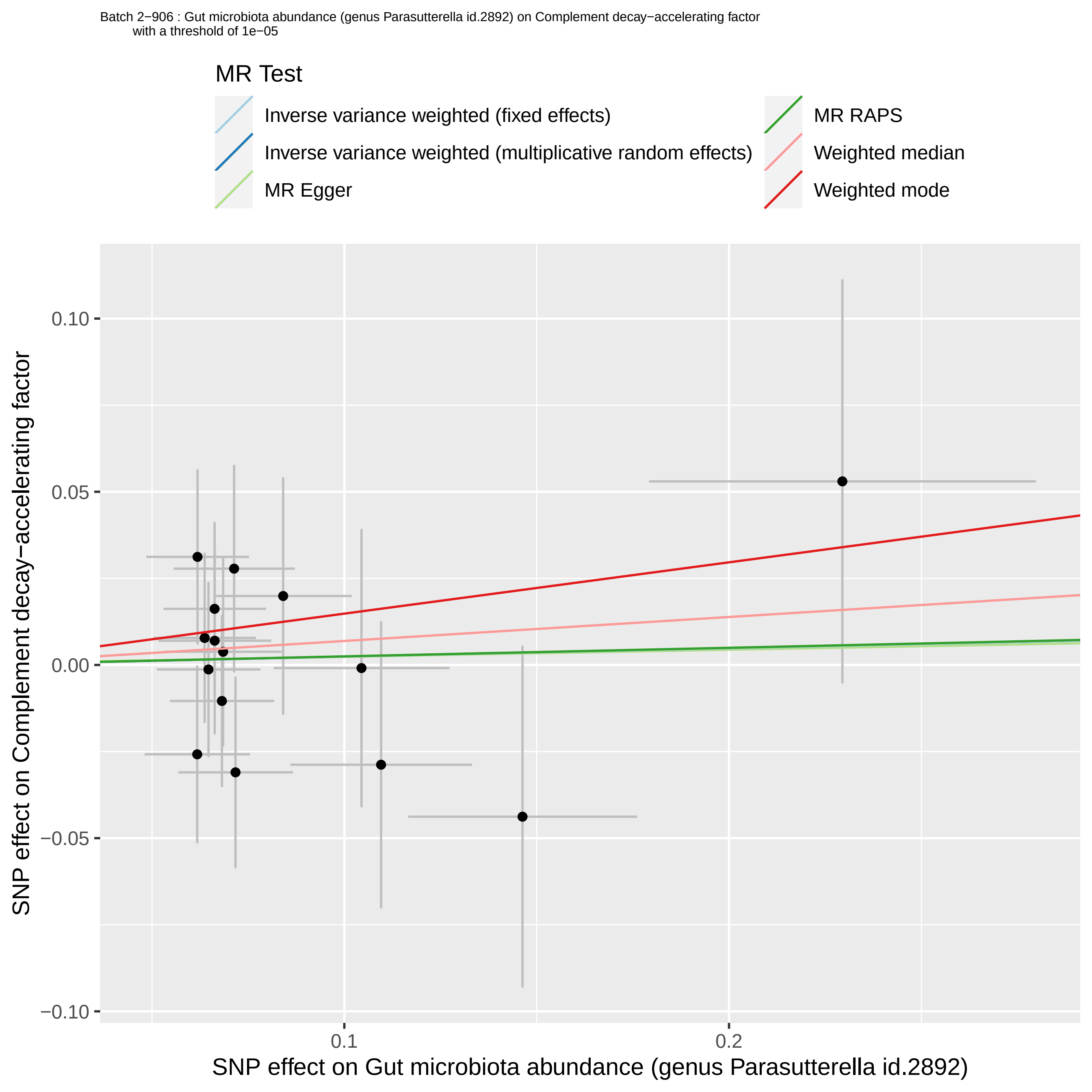

Batch 2−906 : Gut microbiota abundance (genus Parasutterella id.2892) on Complement decay−accelerating factor
with a threshold of 1e−05
MR Test
Inverse variance weighted (fixed effects)
Inverse variance weighted (multiplicative random effects)
MR Egger
MR RAPS
Weighted median
Weighted mode
0.10
0.05
0.00
−0.05
−0.10
0.1
0.2
SNP effect on Complement decay−accelerating factor
SNP effect on Gut microbiota abundance (genus Parasutterella id.2892)

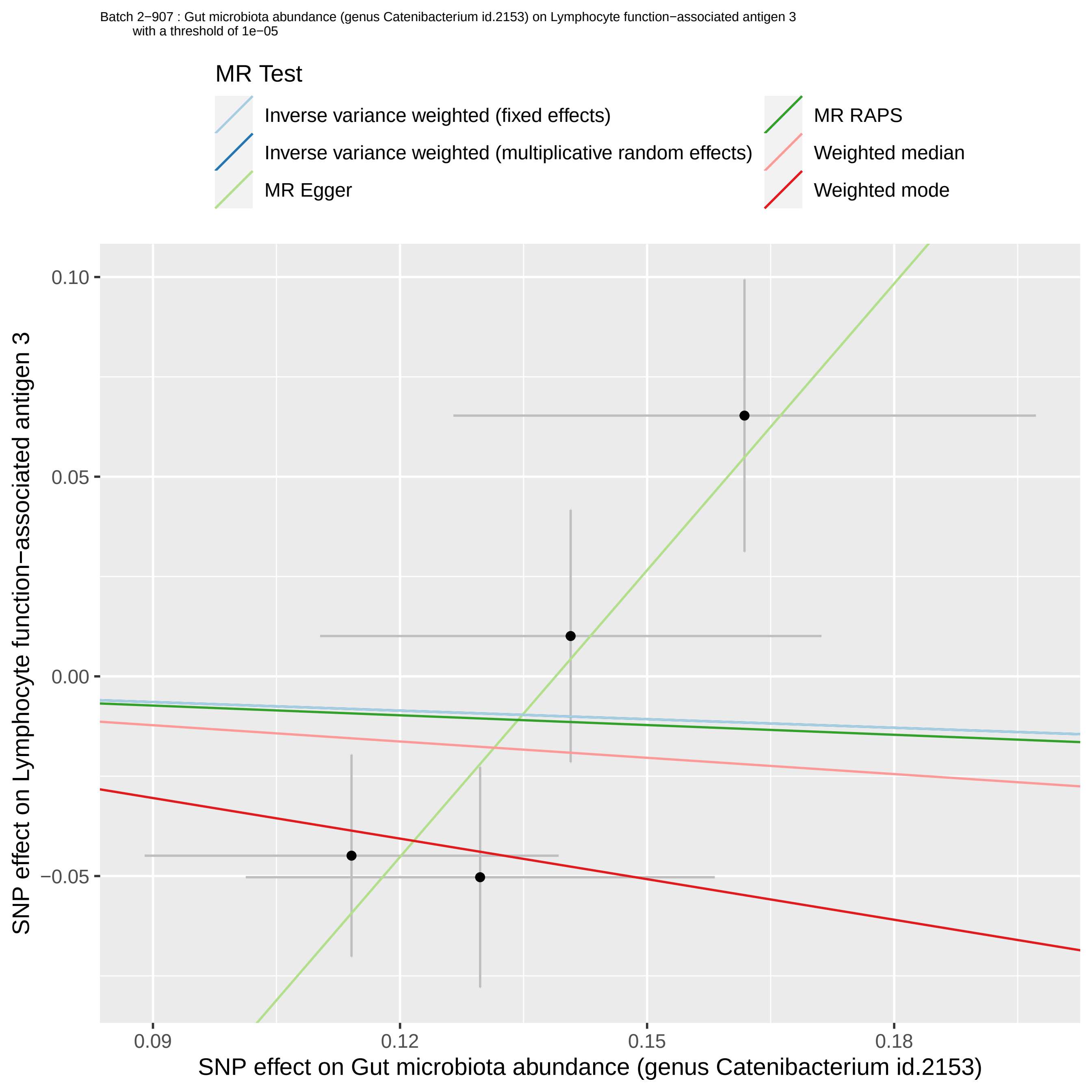

Batch 2−907 : Gut microbiota abundance (genus Catenibacterium id.2153) on Lymphocyte function−associated antigen 3
with a threshold of 1e−05
MR Test
Inverse variance weighted (fixed effects)
Inverse variance weighted (multiplicative random effects)
MR Egger
MR RAPS
Weighted median
Weighted mode
0.10
0.05
0.00
−0.05
0.09
0.12
0.15
0.18
SNP effect on Lymphocyte function−associated antigen 3
SNP effect on Gut microbiota abundance (genus Catenibacterium id.2153)

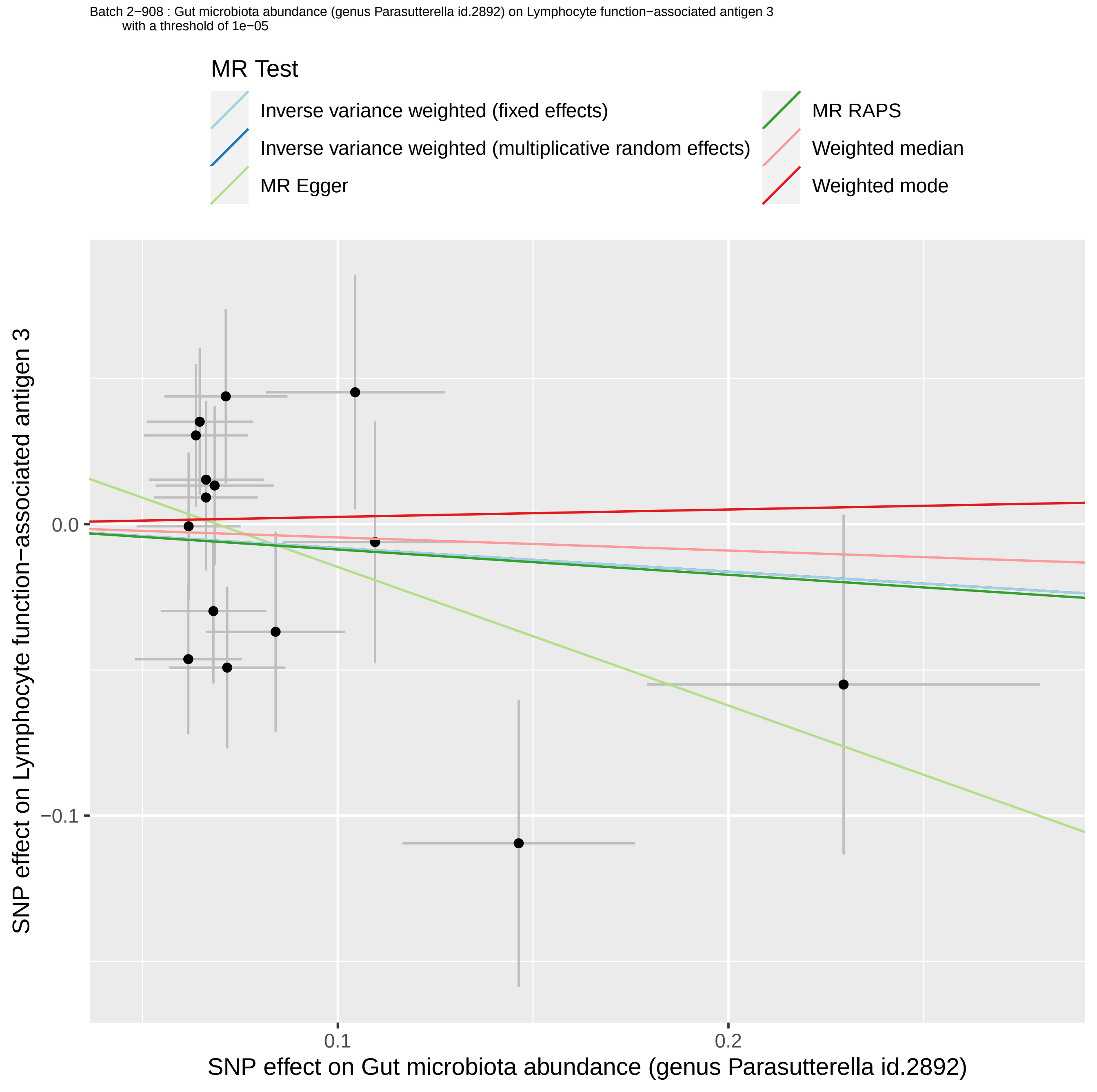

Batch 2−908 : Gut microbiota abundance (genus Parasutterella id.2892) on Lymphocyte function−associated antigen 3
with a threshold of 1e−05
MR Test
Inverse variance weighted (fixed effects)
Inverse variance weighted (multiplicative random effects)
MR Egger
MR RAPS
Weighted median
Weighted mode
0.0
−0.1
0.1
0.2
SNP effect on Lymphocyte function−associated antigen 3
SNP effect on Gut microbiota abundance (genus Parasutterella id.2892)

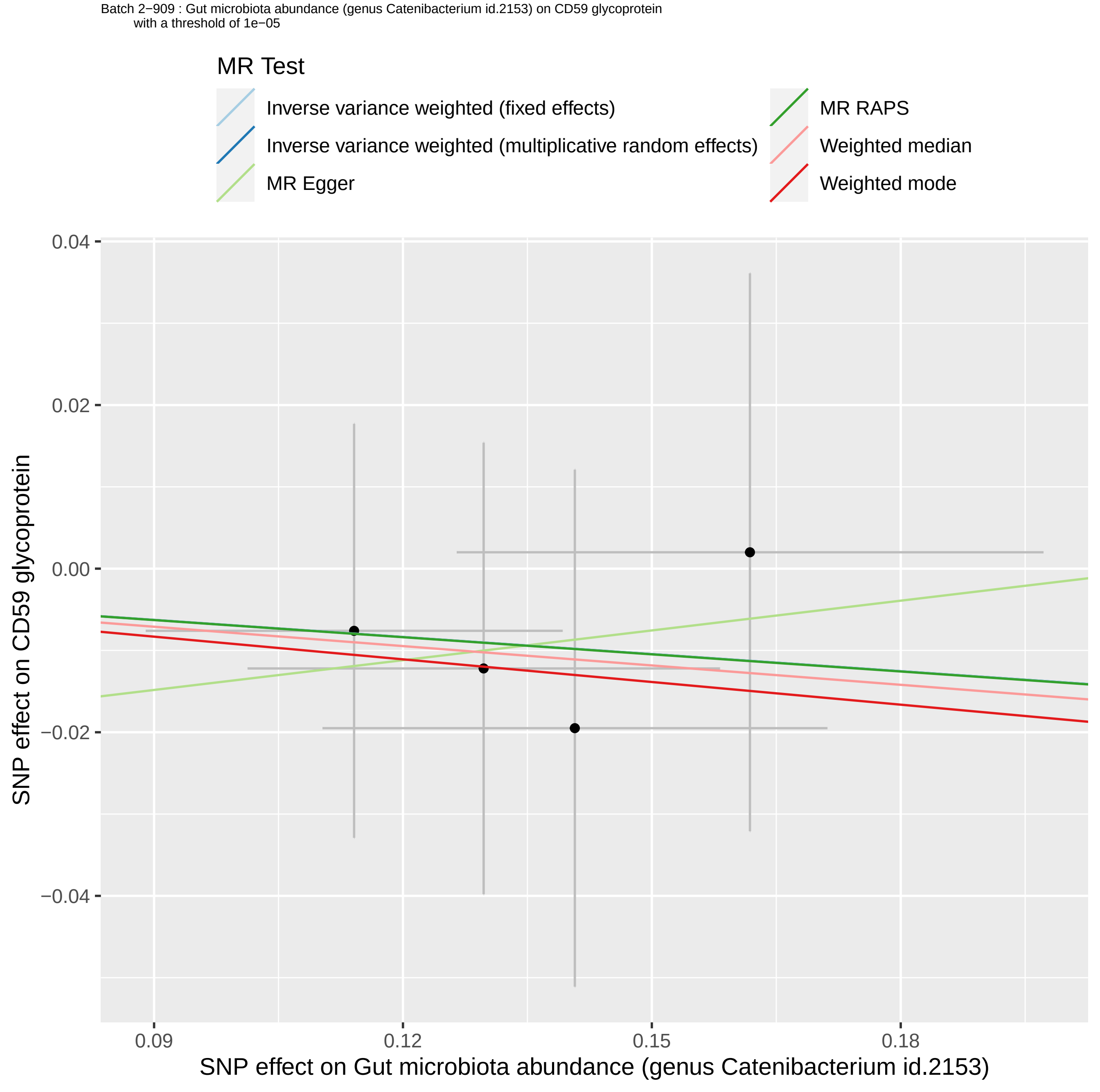
Batch 2−909 : Gut microbiota abundance (genus Catenibacterium id.2153) on CD59 glycoprotein
with a threshold of 1e−05
MR Test
Inverse variance weighted (fixed effects)
Inverse variance weighted (multiplicative random effects)
MR Egger
MR RAPS
Weighted median
Weighted mode
0.04
0.02
0.00
−0.02
−0.04
0.09
0.12
0.15
0.18
SNP effect on CD59 glycoprotein
SNP effect on Gut microbiota abundance (genus Catenibacterium id.2153)

Batch 2−910 : Gut microbiota abundance (genus Parasutterella id.2892) on CD59 glycoprotein
with a threshold of 1e−05

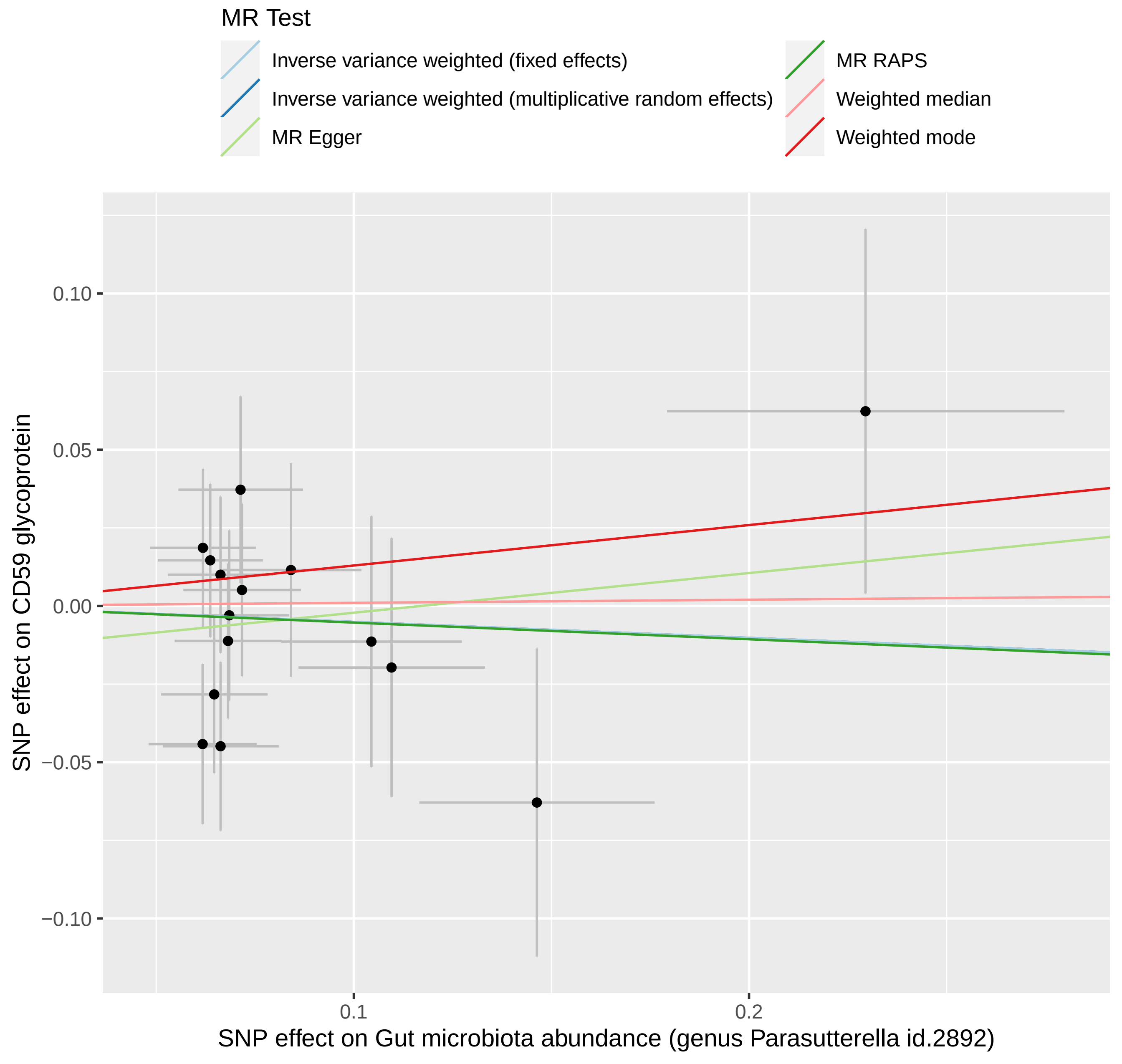

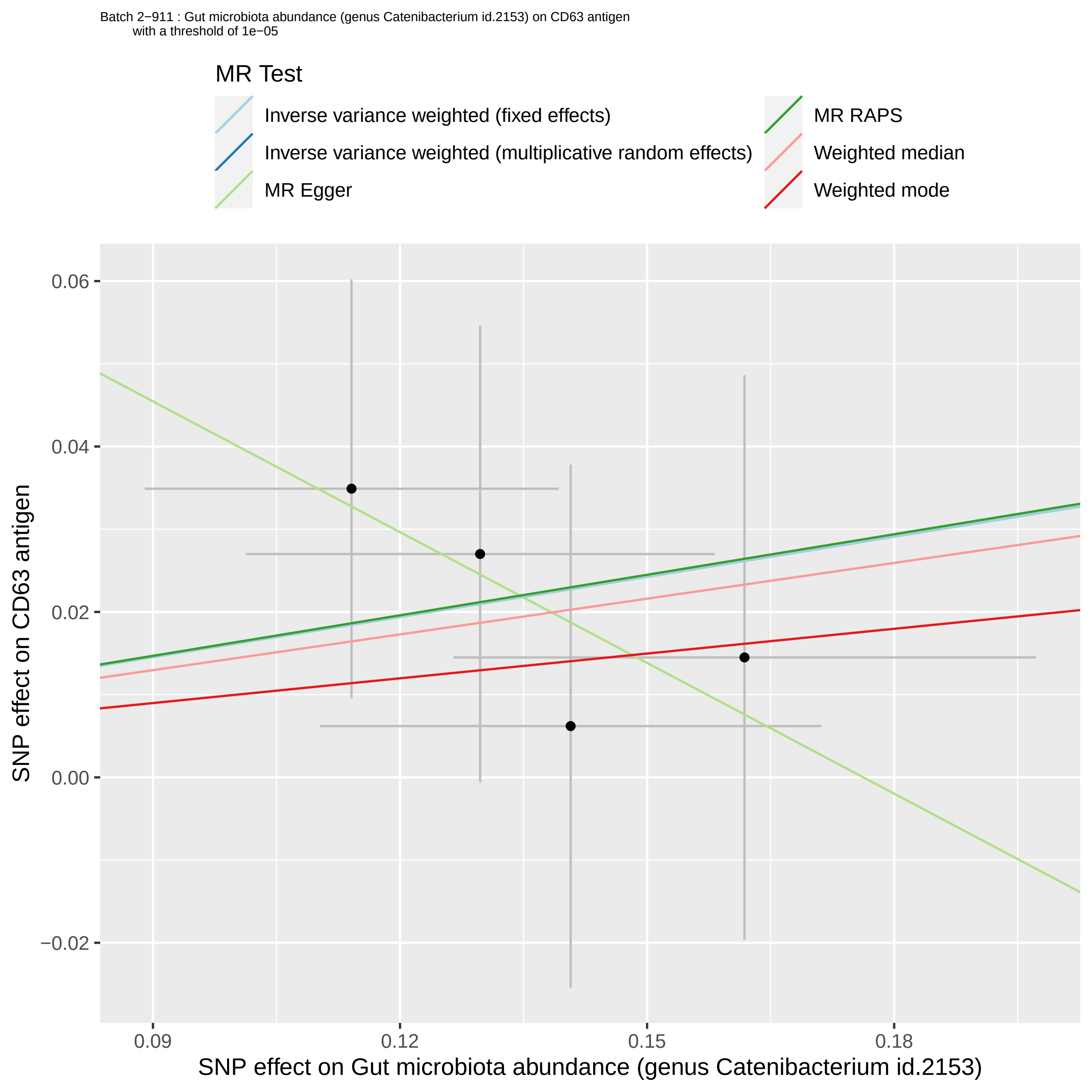
Batch 2−911 : Gut microbiota abundance (genus Catenibacterium id.2153) on CD63 antigen
with a threshold of 1e−05
MR Test
Inverse variance weighted (fixed effects)
Inverse variance weighted (multiplicative random effects)
MR Egger
MR RAPS
Weighted median
Weighted mode
0.06
0.04
0.02
0.00
−0.02
0.09
0.12
0.15
0.18
SNP effect on CD63 antigen
SNP effect on Gut microbiota abundance (genus Catenibacterium id.2153)

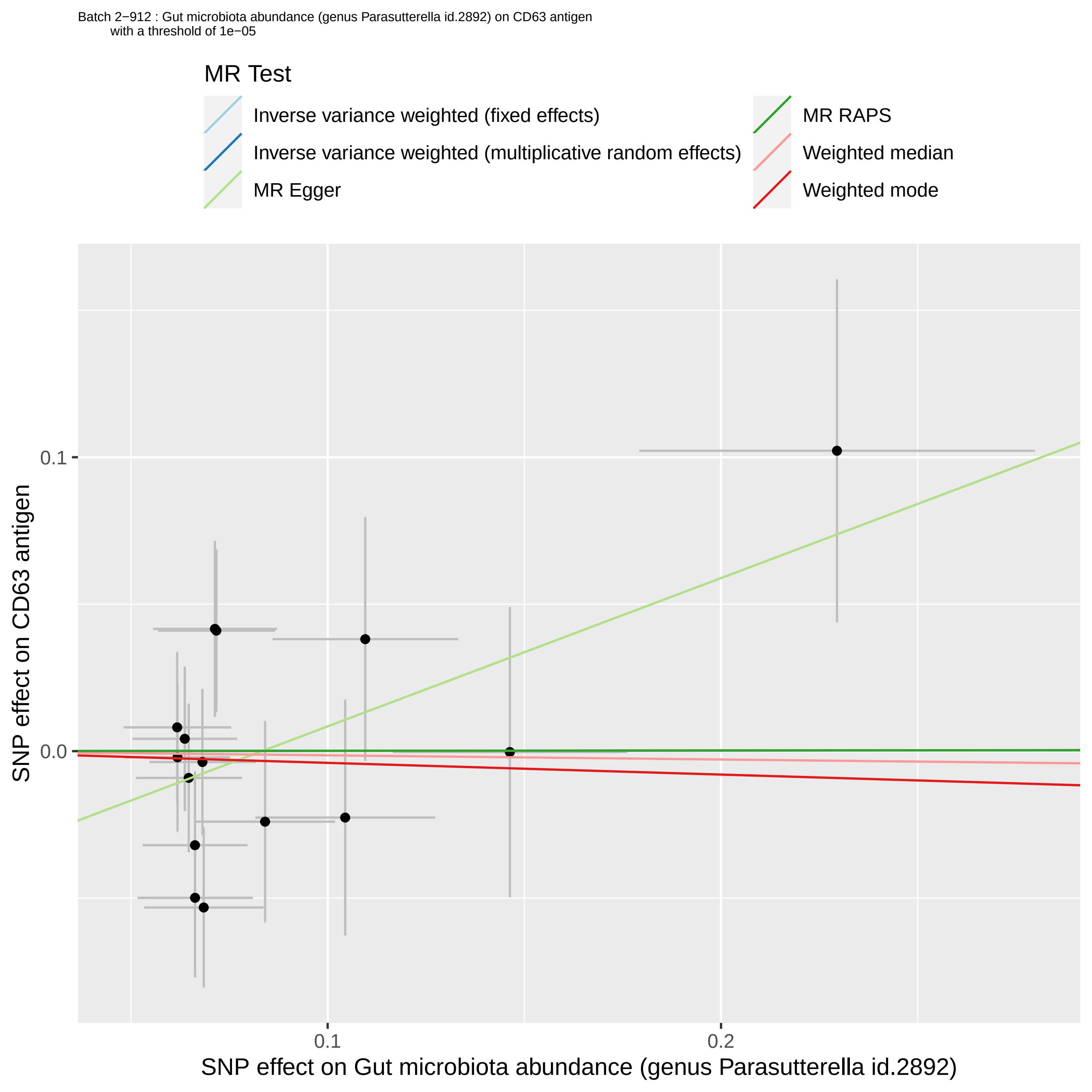

Batch 2−912 : Gut microbiota abundance (genus Parasutterella id.2892) on CD63 antigen
with a threshold of 1e−05
MR Test
Inverse variance weighted (fixed effects)
Inverse variance weighted (multiplicative random effects)
MR Egger
MR RAPS
Weighted median
Weighted mode
0.1
0.0
0.1
0.2
SNP effect on CD63 antigen
SNP effect on Gut microbiota abundance (genus Parasutterella id.2892)

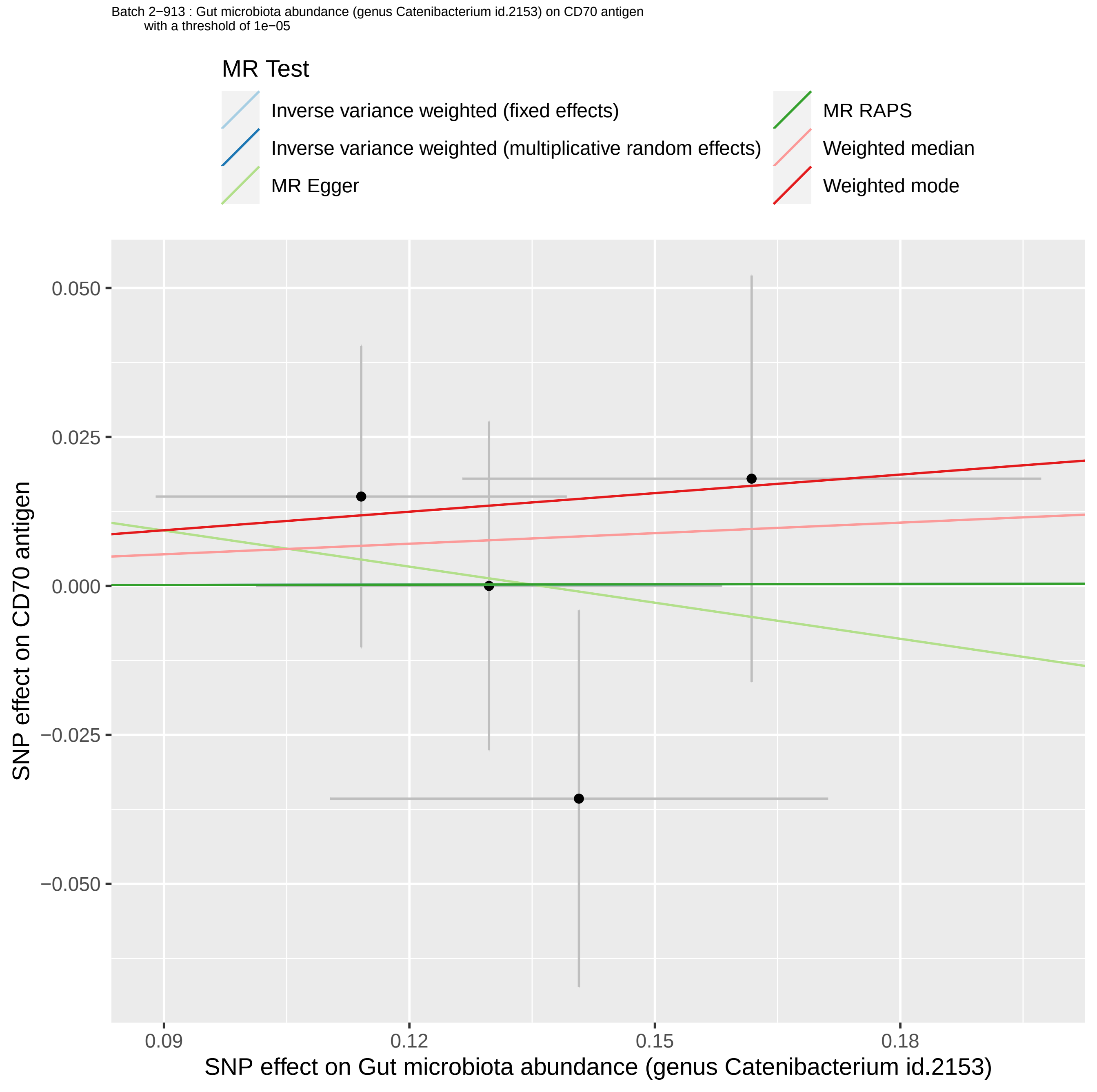
Batch 2−913 : Gut microbiota abundance (genus Catenibacterium id.2153) on CD70 antigen
with a threshold of 1e−05
MR Test
Inverse variance weighted (fixed effects)
Inverse variance weighted (multiplicative random effects)
MR Egger
MR RAPS
Weighted median
Weighted mode
SNP effect on CD70 antigen
0.050
0.025
0.000
−0.025
−0.050
0.09
0.12
0.15
0.18
SNP effect on Gut microbiota abundance (genus Catenibacterium id.2153)

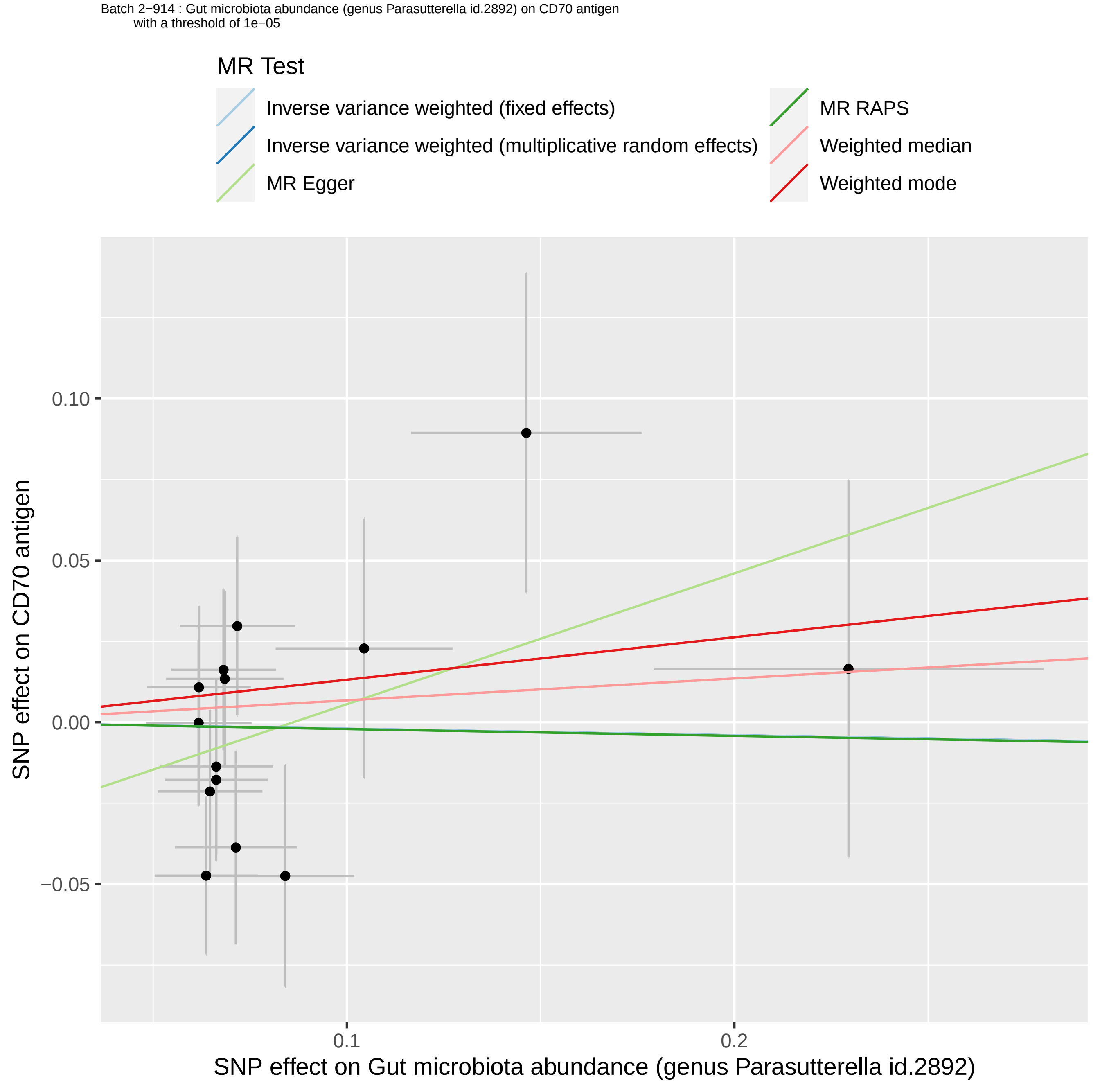

Batch 2−914 : Gut microbiota abundance (genus Parasutterella id.2892) on CD70 antigen
with a threshold of 1e−05
MR Test
Inverse variance weighted (fixed effects)
Inverse variance weighted (multiplicative random effects)
MR Egger
MR RAPS
Weighted median
Weighted mode
0.10
0.05
0.00
−0.05
0.1
0.2
SNP effect on CD70 antigen
SNP effect on Gut microbiota abundance (genus Parasutterella id.2892)

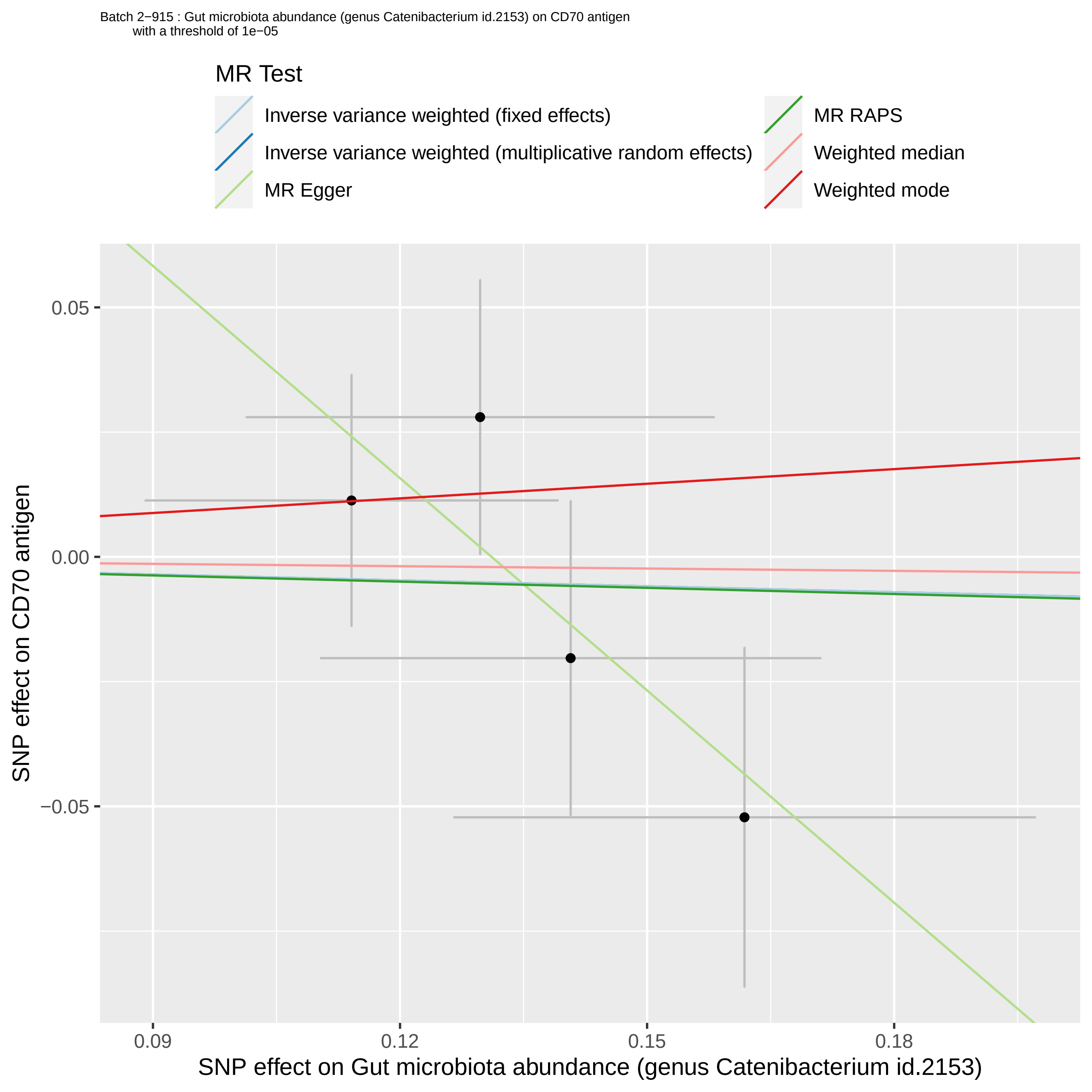

Batch 2−915 : Gut microbiota abundance (genus Catenibacterium id.2153) on CD70 antigen
with a threshold of 1e−05
MR Test
Inverse variance weighted (fixed effects)
Inverse variance weighted (multiplicative random effects)
MR Egger
MR RAPS
Weighted median
Weighted mode
0.05
0.00
−0.05
0.09
0.12
0.15
0.18
SNP effect on CD70 antigen
SNP effect on Gut microbiota abundance (genus Catenibacterium id.2153)

Batch 2−916 : Gut microbiota abundance (genus Parasutterella id.2892) on CD70 antigen
with a threshold of 1e−05

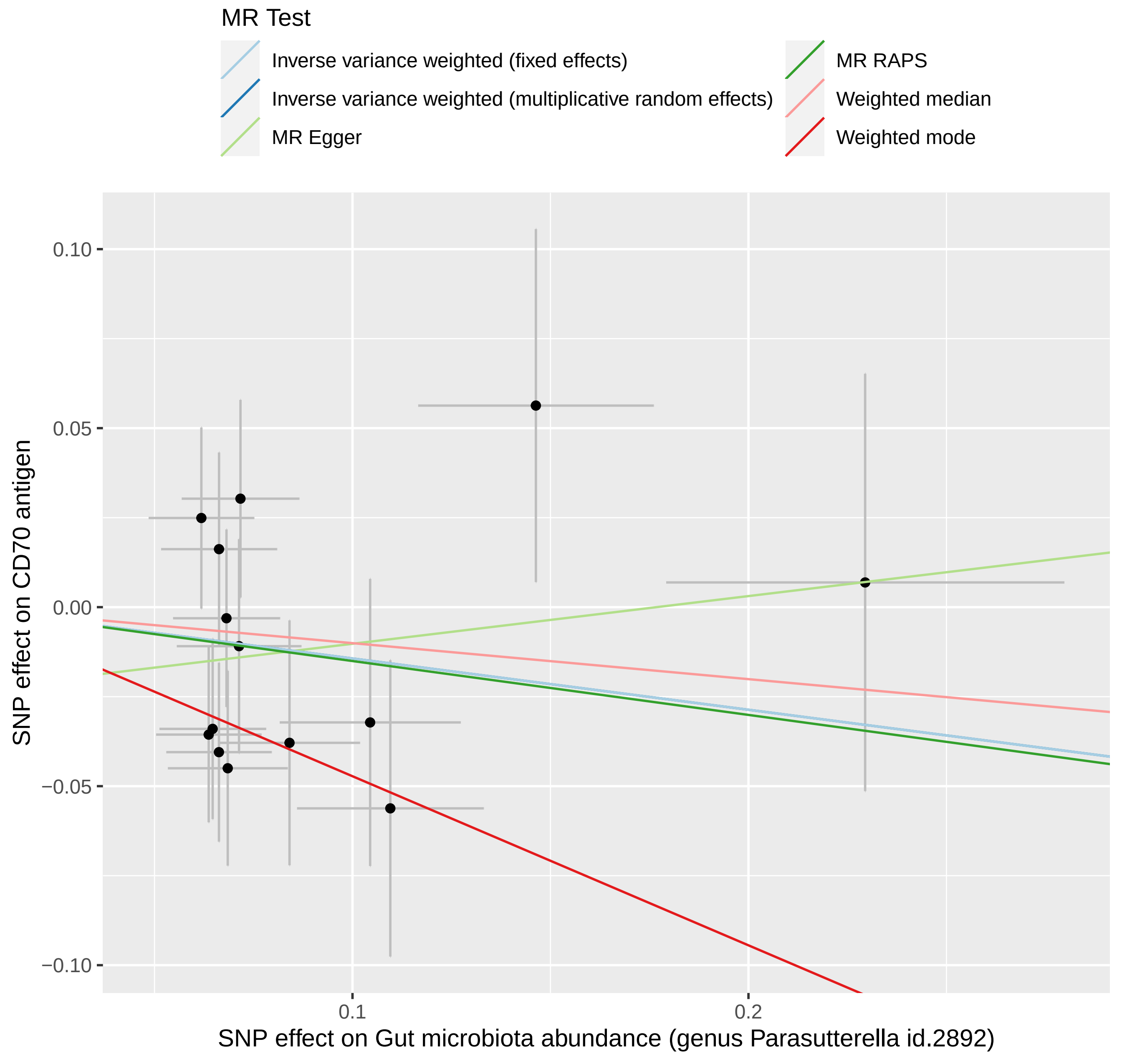

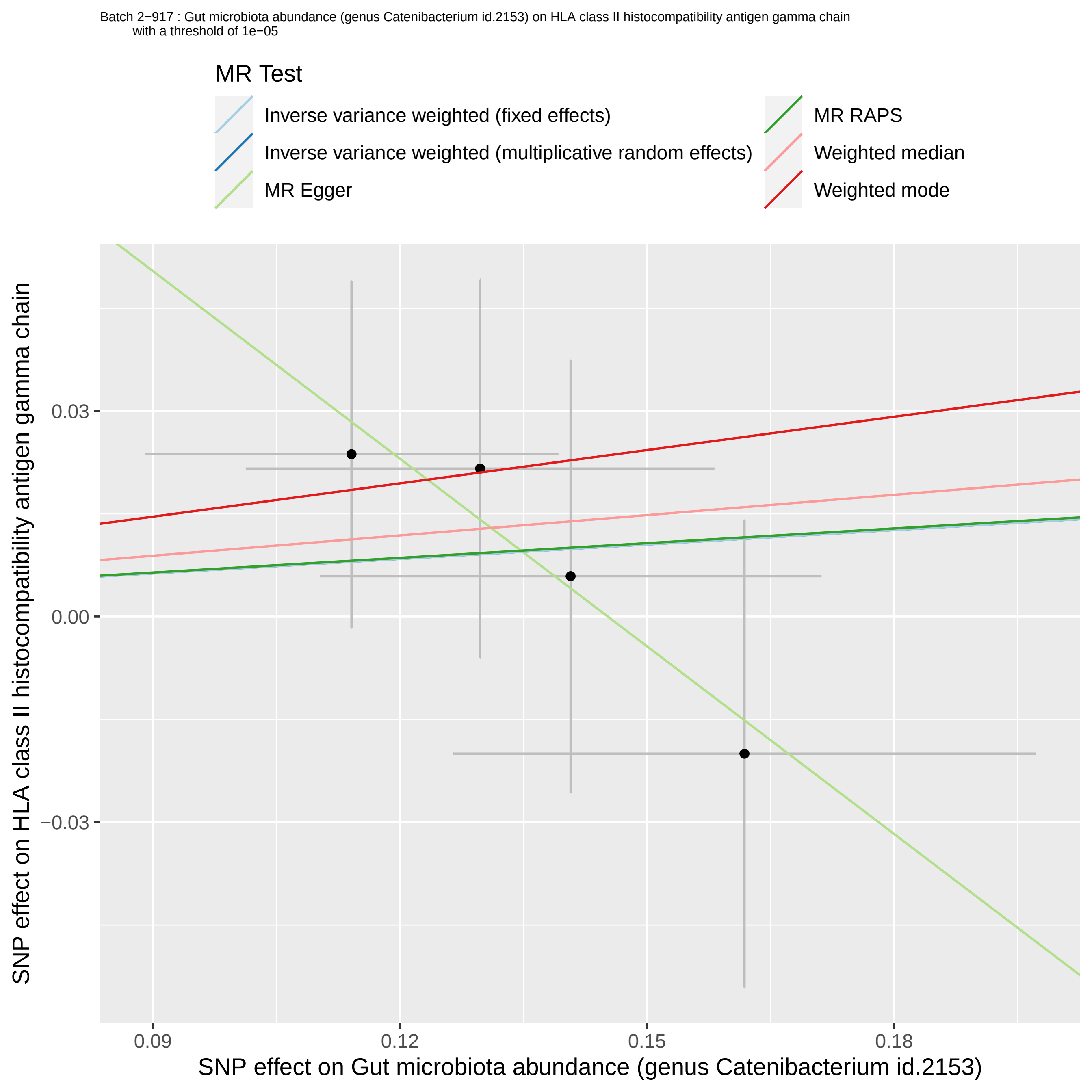

Batch 2−917 : Gut microbiota abundance (genus Catenibacterium id.2153) on HLA class II histocompatibility antigen gamma chain
with a threshold of 1e−05
MR Test
Inverse variance weighted (fixed effects)
Inverse variance weighted (multiplicative random effects)
MR Egger
MR RAPS
Weighted median
Weighted mode
SNP effect on HLA class II histocompatibility antigen gamma chain
0.03
0.00
−0.03
0.09
0.12
0.15
0.18
SNP effect on Gut microbiota abundance (genus Catenibacterium id.2153)

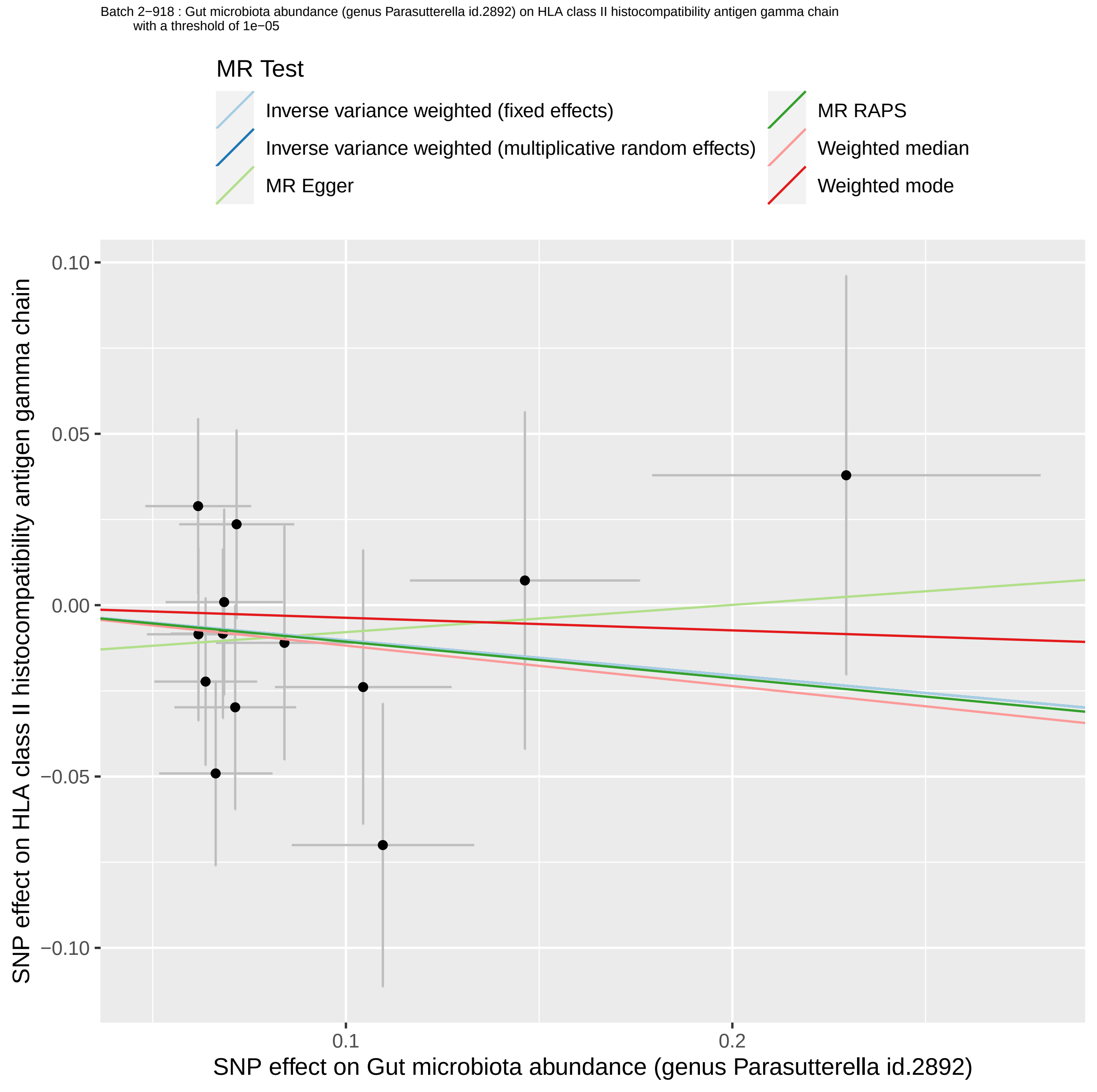

Batch 2−918 : Gut microbiota abundance (genus Parasutterella id.2892) on HLA class II histocompatibility antigen gamma chain
with a threshold of 1e−05
MR Test
Inverse variance weighted (fixed effects)
Inverse variance weighted (multiplicative random effects)
MR Egger
MR RAPS
Weighted median
Weighted mode
0.10
0.05
0.00
−0.05
−0.10
0.1
0.2
SNP effect on HLA class II histocompatibility antigen gamma chain
SNP effect on Gut microbiota abundance (genus Parasutterella id.2892)

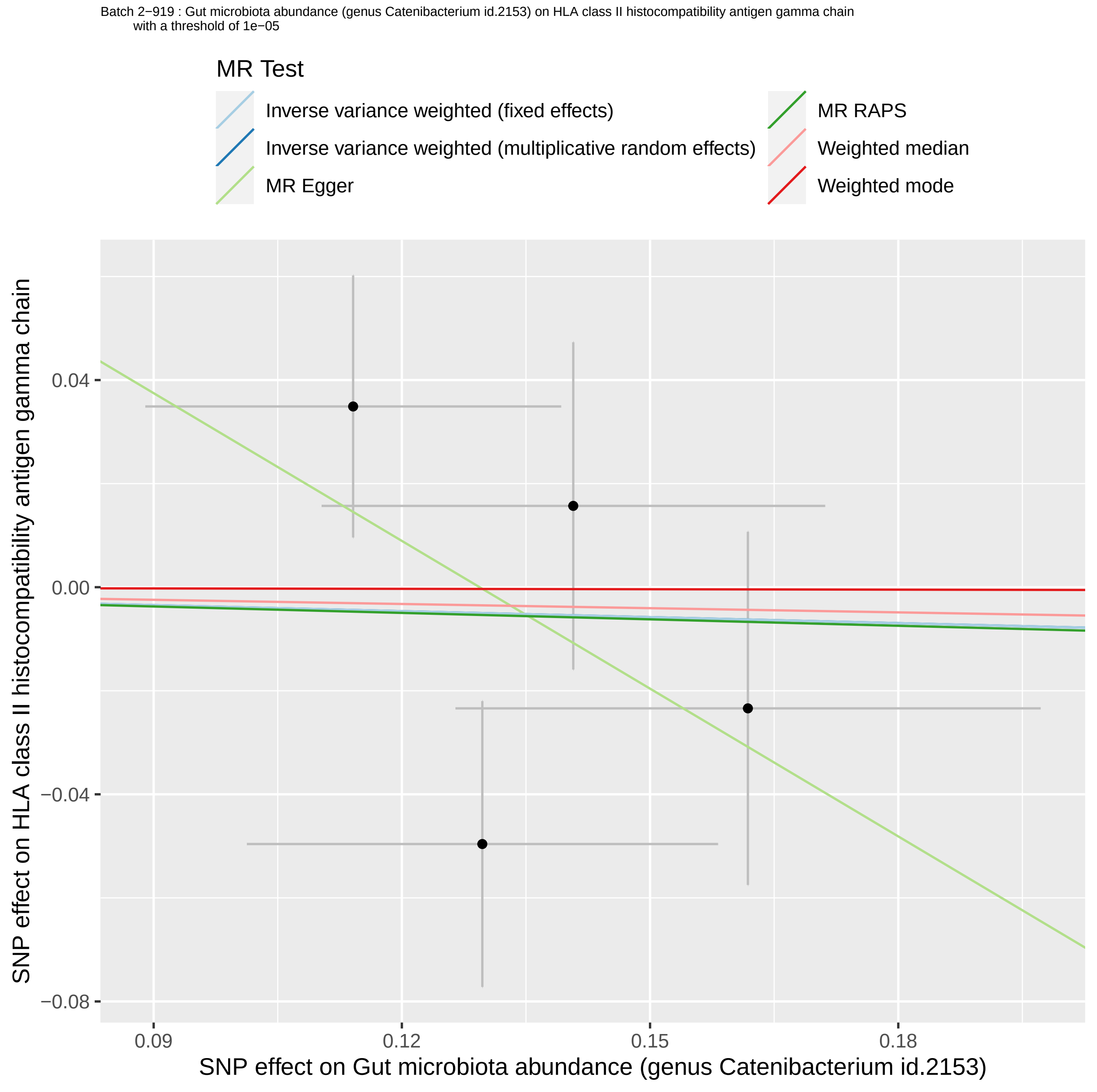

Batch 2−919 : Gut microbiota abundance (genus Catenibacterium id.2153) on HLA class II histocompatibility antigen gamma chain
with a threshold of 1e−05
MR Test
Inverse variance weighted (fixed effects)
Inverse variance weighted (multiplicative random effects)
MR Egger
MR RAPS
Weighted median
Weighted mode
SNP effect on HLA class II histocompatibility antigen gamma chain
0.04
0.00
−0.04
−0.08
0.09
0.12
0.15
0.18
SNP effect on Gut microbiota abundance (genus Catenibacterium id.2153)

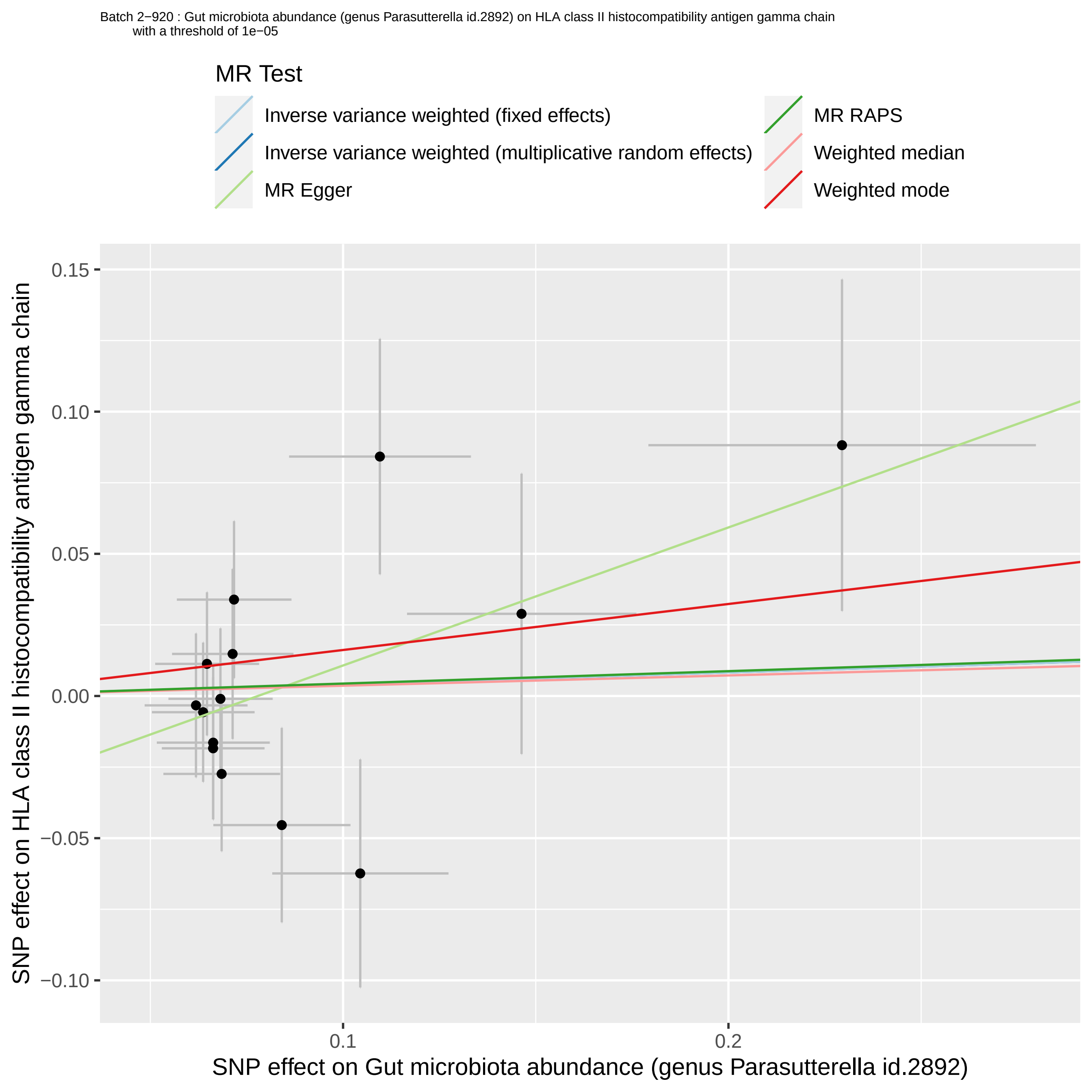

Batch 2−920 : Gut microbiota abundance (genus Parasutterella id.2892) on HLA class II histocompatibility antigen gamma chain
with a threshold of 1e−05
MR Test
Inverse variance weighted (fixed effects)
Inverse variance weighted (multiplicative random effects)
MR Egger
MR RAPS
Weighted median
Weighted mode
SNP effect on HLA class II histocompatibility antigen gamma chain
0.15
0.10
0.05
0.00
−0.05
−0.10
0.1
0.2
SNP effect on Gut microbiota abundance (genus Parasutterella id.2892)

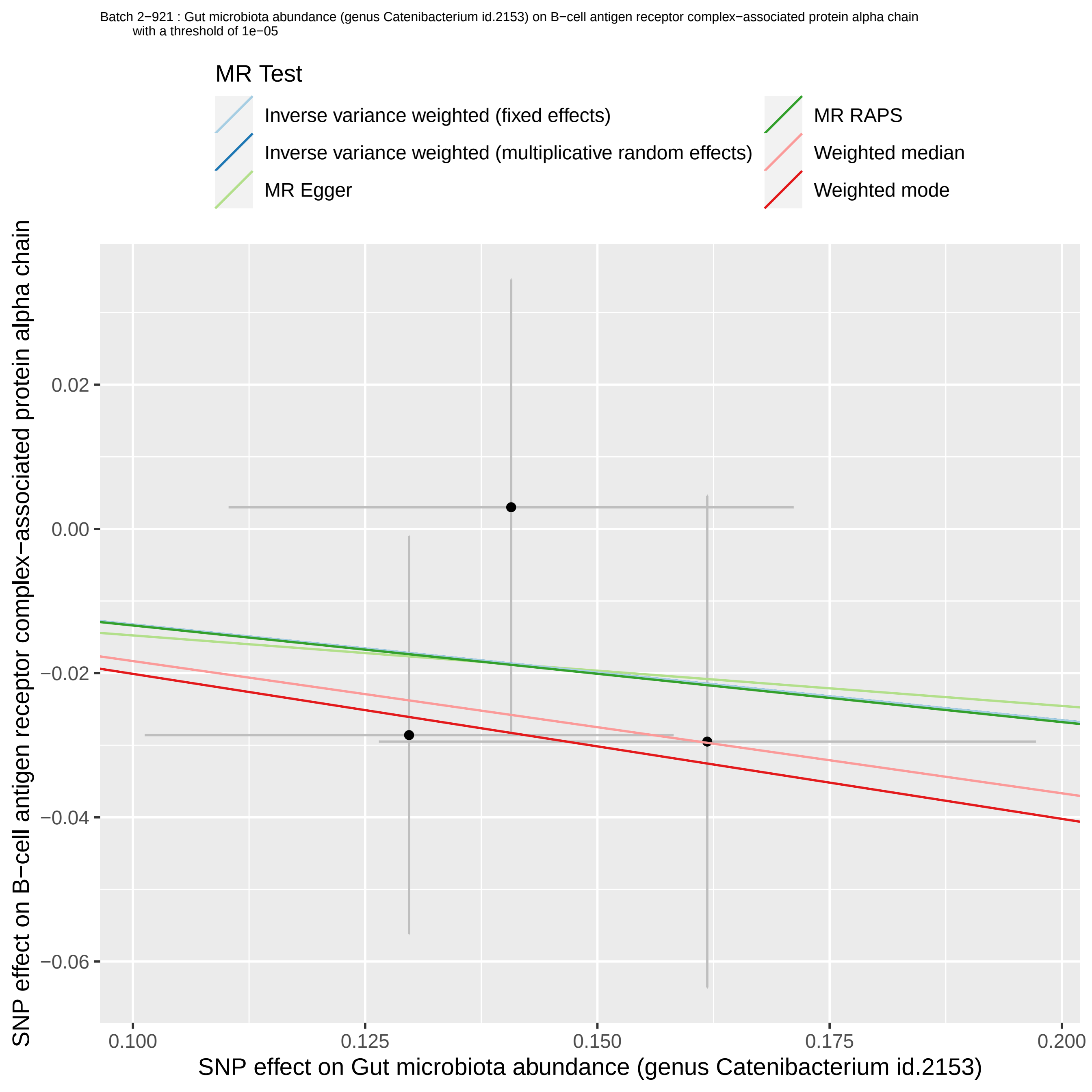
Batch 2−921 : Gut microbiota abundance (genus Catenibacterium id.2153) on B−cell antigen receptor complex−associated protein alpha chain
with a threshold of 1e−05
MR Test
Inverse variance weighted (fixed effects)
Inverse variance weighted (multiplicative random effects)
MR Egger
MR RAPS
Weighted median
Weighted mode
SNP effect on B−cell antigen receptor complex−associated protein alpha chain
0.02
0.00
−0.02
−0.04
−0.06
0.100
0.125
0.150
0.175
0.200
SNP effect on Gut microbiota abundance (genus Catenibacterium id.2153)

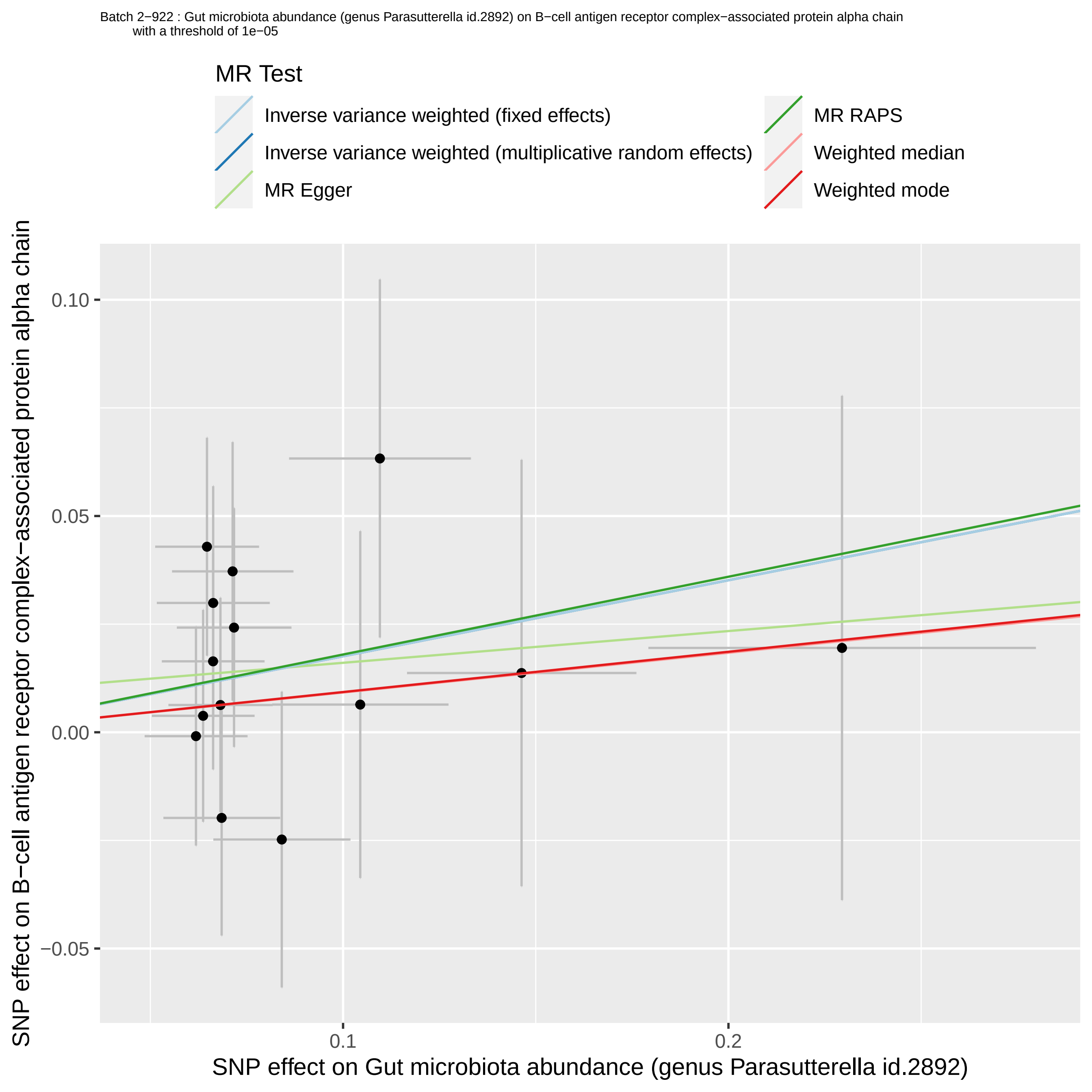
Batch 2−922 : Gut microbiota abundance (genus Parasutterella id.2892) on B−cell antigen receptor complex−associated protein alpha chain
with a threshold of 1e−05
MR Test
Inverse variance weighted (fixed effects)
Inverse variance weighted (multiplicative random effects)
MR Egger
MR RAPS
Weighted median
Weighted mode
0.10
0.05
0.00
−0.05
0.1
0.2
SNP effect on B−cell antigen receptor complex−associated protein alpha chain
SNP effect on Gut microbiota abundance (genus Parasutterella id.2892)

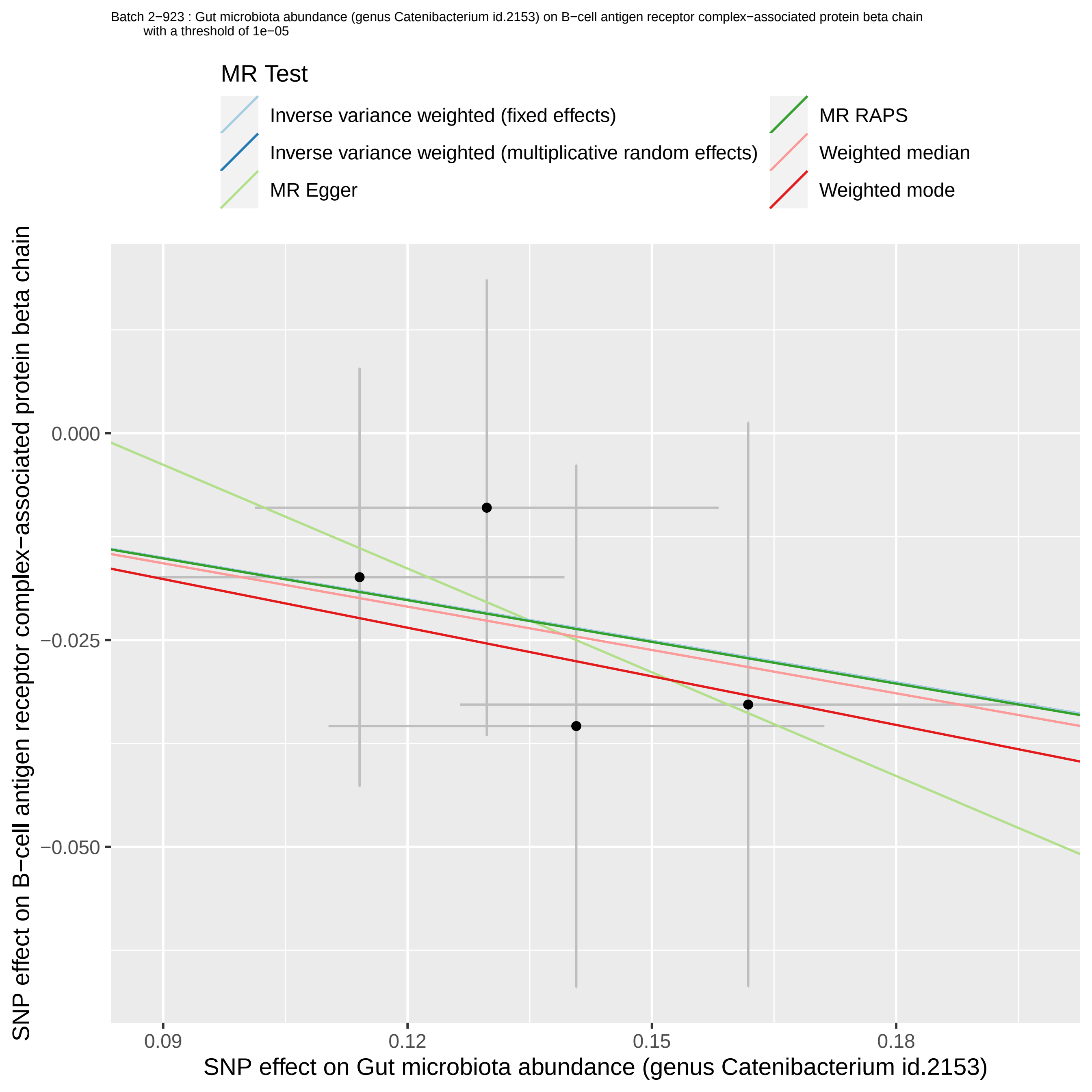

Batch 2−923 : Gut microbiota abundance (genus Catenibacterium id.2153) on B−cell antigen receptor complex−associated protein beta chain
with a threshold of 1e−05
MR Test
Inverse variance weighted (fixed effects)
Inverse variance weighted (multiplicative random effects)
MR Egger
MR RAPS
Weighted median
Weighted mode
0.000
−0.025
−0.050
0.09
0.12
0.15
0.18
SNP effect on B−cell antigen receptor complex−associated protein beta chain
SNP effect on Gut microbiota abundance (genus Catenibacterium id.2153)

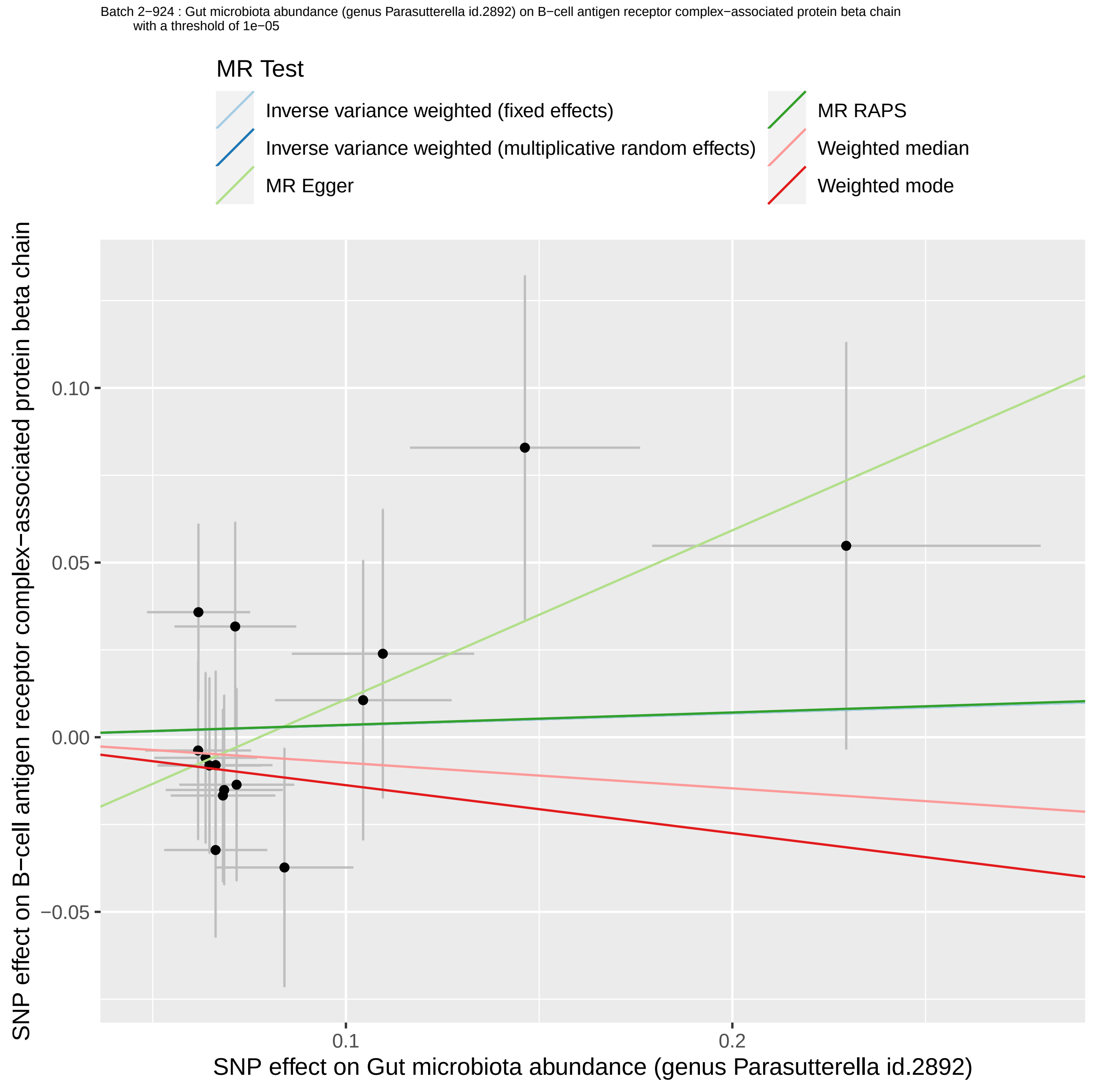

Batch 2−924 : Gut microbiota abundance (genus Parasutterella id.2892) on B−cell antigen receptor complex−associated protein beta chain
with a threshold of 1e−05
MR Test
Inverse variance weighted (fixed effects)
Inverse variance weighted (multiplicative random effects)
MR Egger
MR RAPS
Weighted median
Weighted mode
SNP effect on B−cell antigen receptor complex−associated protein beta chain
0.10
0.05
0.00
−0.05
0.1
0.2
SNP effect on Gut microbiota abundance (genus Parasutterella id.2892)

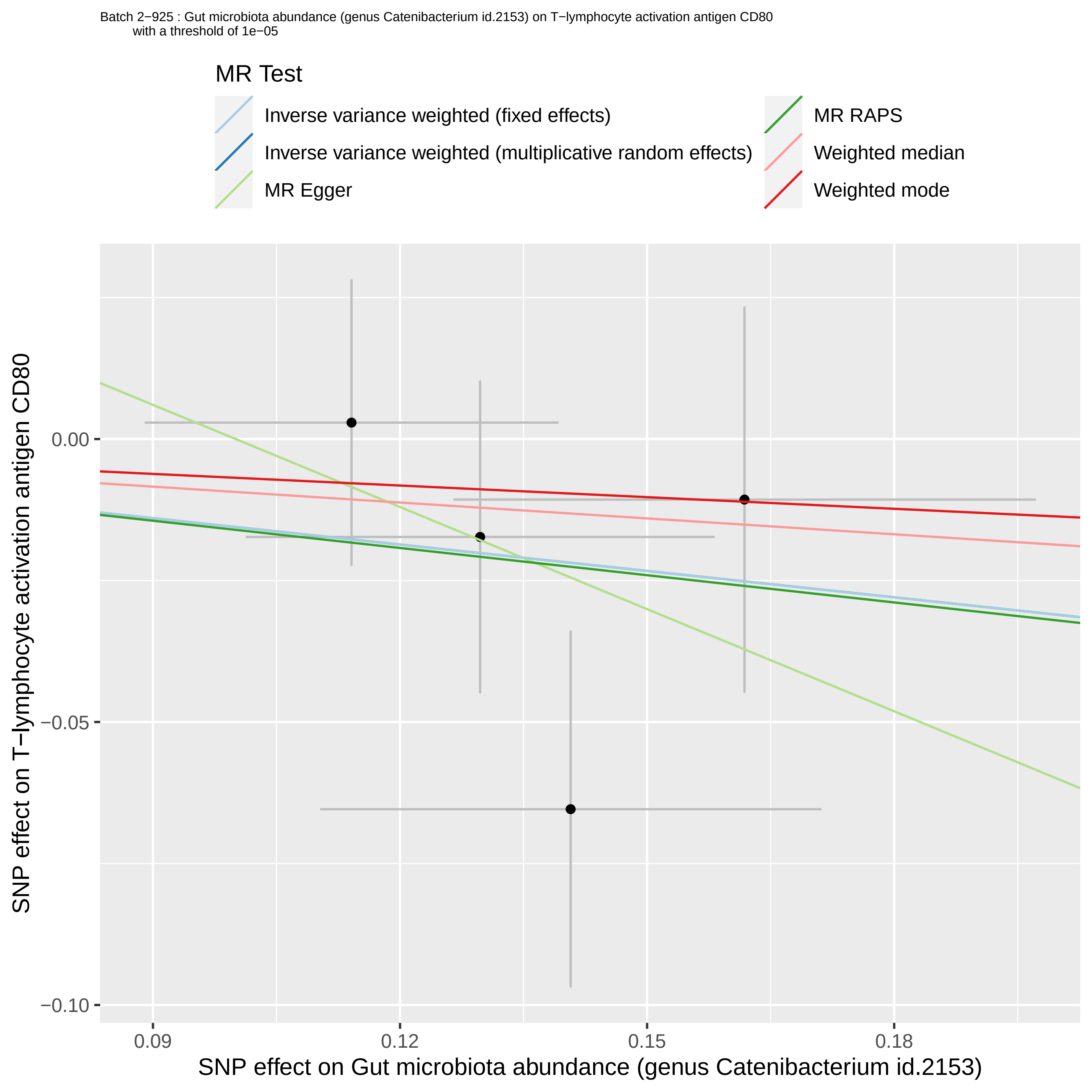
Batch 2−925 : Gut microbiota abundance (genus Catenibacterium id.2153) on T−lymphocyte activation antigen CD80
with a threshold of 1e−05
MR Test
Inverse variance weighted (fixed effects)
Inverse variance weighted (multiplicative random effects)
MR Egger
MR RAPS
Weighted median
Weighted mode
0.00
−0.05
−0.10
0.09
0.12
0.15
0.18
SNP effect on T−lymphocyte activation antigen CD80
SNP effect on Gut microbiota abundance (genus Catenibacterium id.2153)

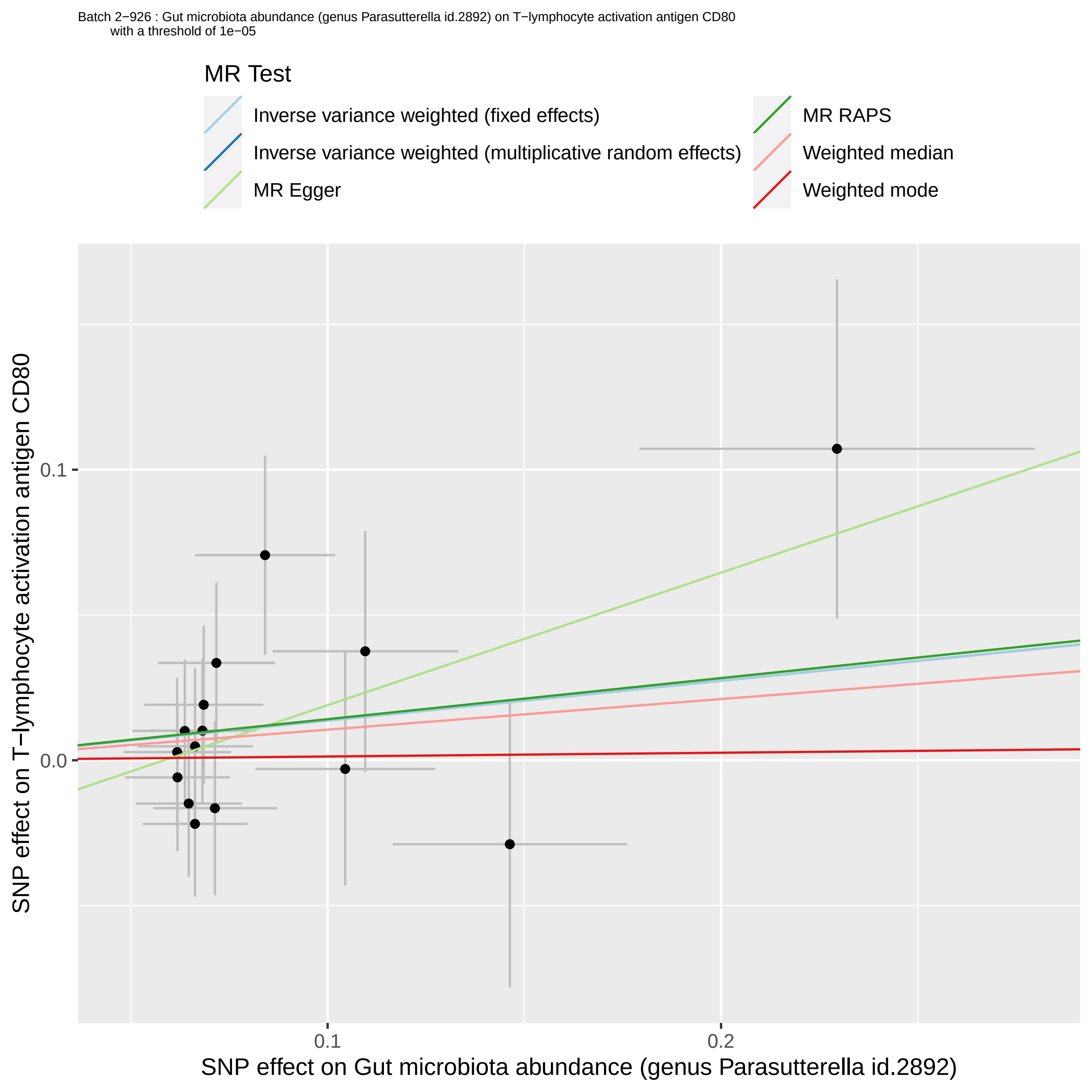
Batch 2−926 : Gut microbiota abundance (genus Parasutterella id.2892) on T−lymphocyte activation antigen CD80
with a threshold of 1e−05
MR Test
Inverse variance weighted (fixed effects)
Inverse variance weighted (multiplicative random effects)
MR Egger
MR RAPS
Weighted median
Weighted mode
SNP effect on T−lymphocyte activation antigen CD80
0.1
0.0
0.1
0.2
SNP effect on Gut microbiota abundance (genus Parasutterella id.2892)

Batch 2−927 : Gut microbiota abundance (genus Catenibacterium id.2153) on SLAM family member 5
with a threshold of 1e−05

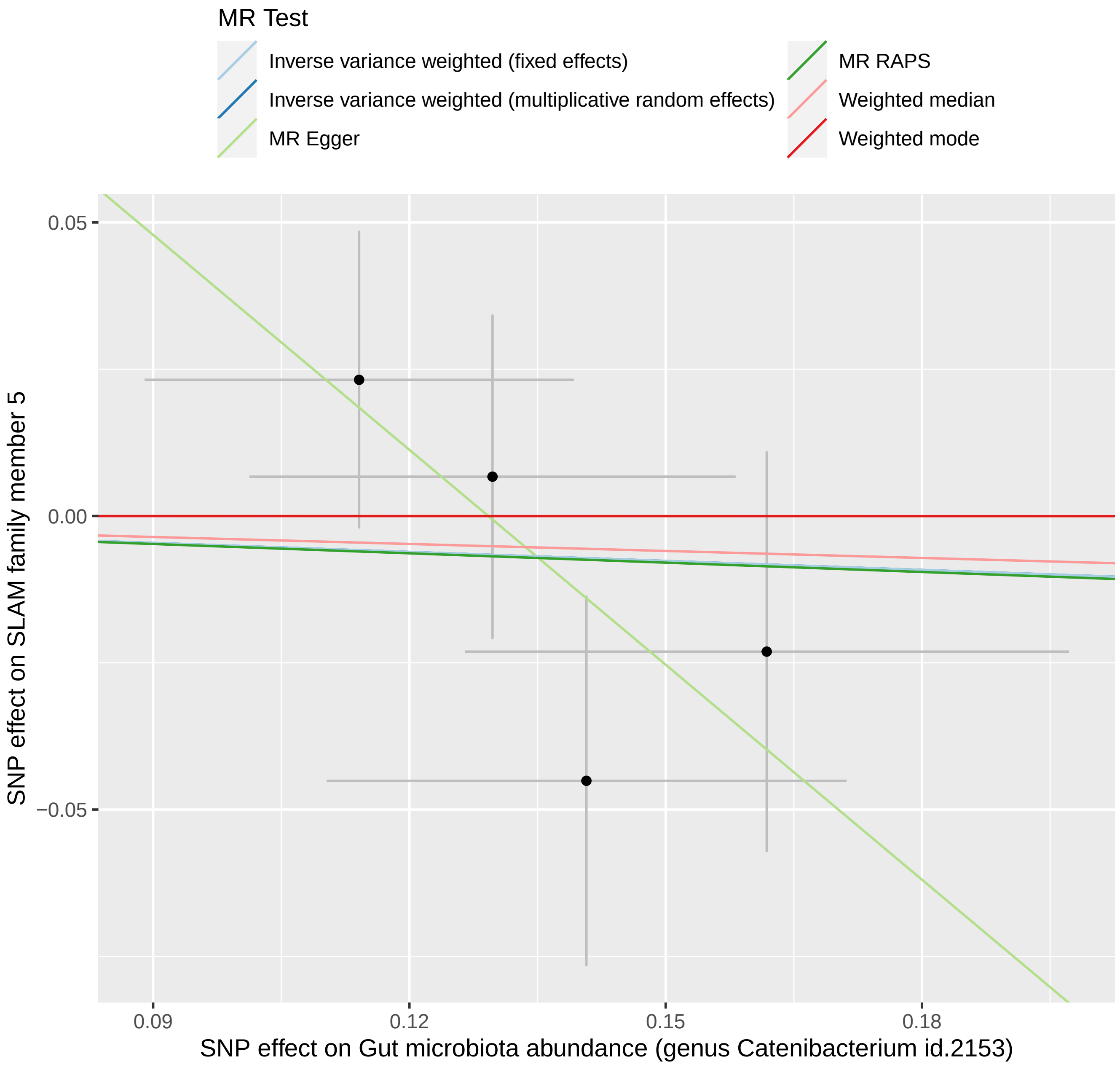

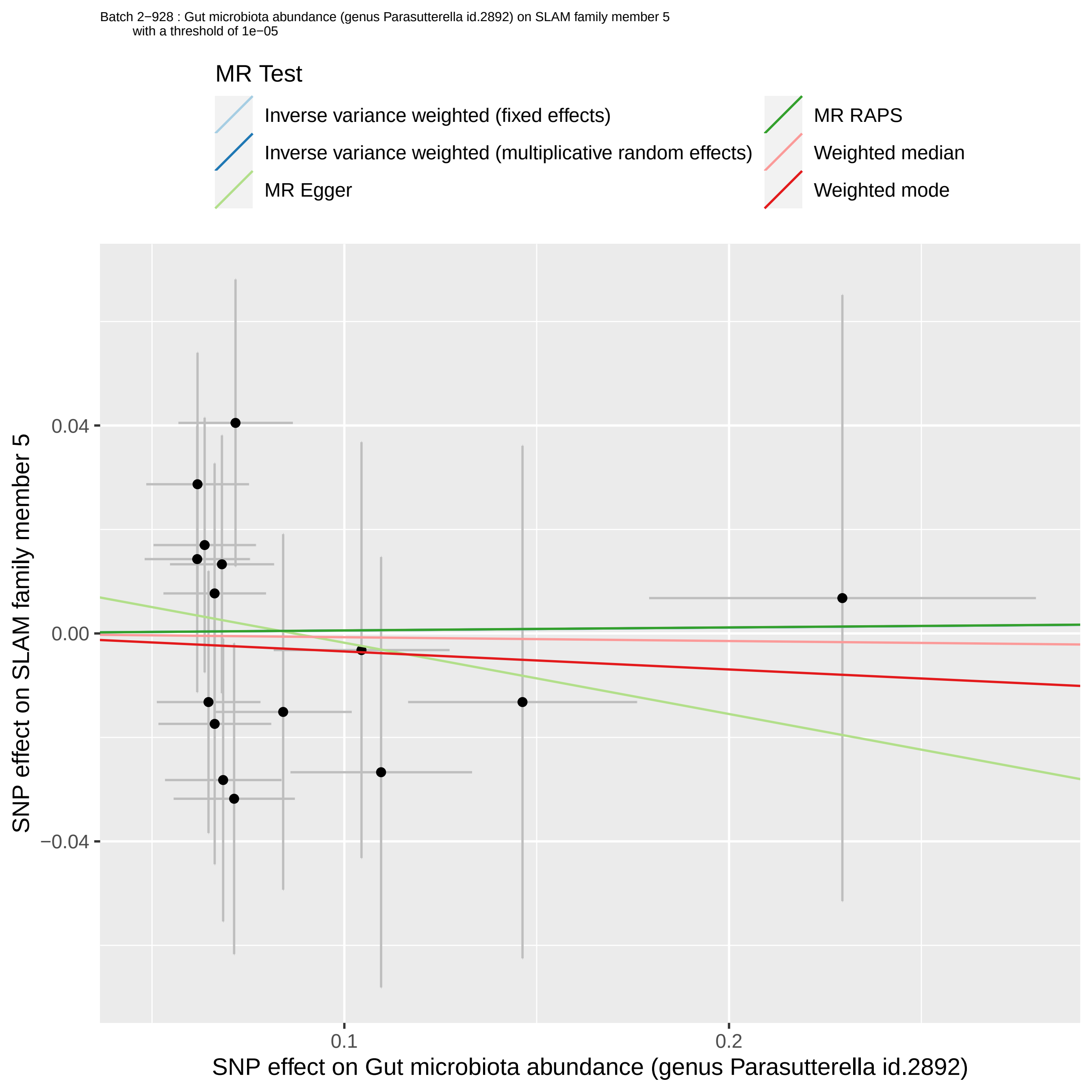
Batch 2−928 : Gut microbiota abundance (genus Parasutterella id.2892) on SLAM family member 5
with a threshold of 1e−05
MR Test
Inverse variance weighted (fixed effects)
Inverse variance weighted (multiplicative random effects)
MR Egger
MR RAPS
Weighted median
Weighted mode
0.04
0.00
−0.04
0.1
0.2
SNP effect on SLAM family member 5
SNP effect on Gut microbiota abundance (genus Parasutterella id.2892)

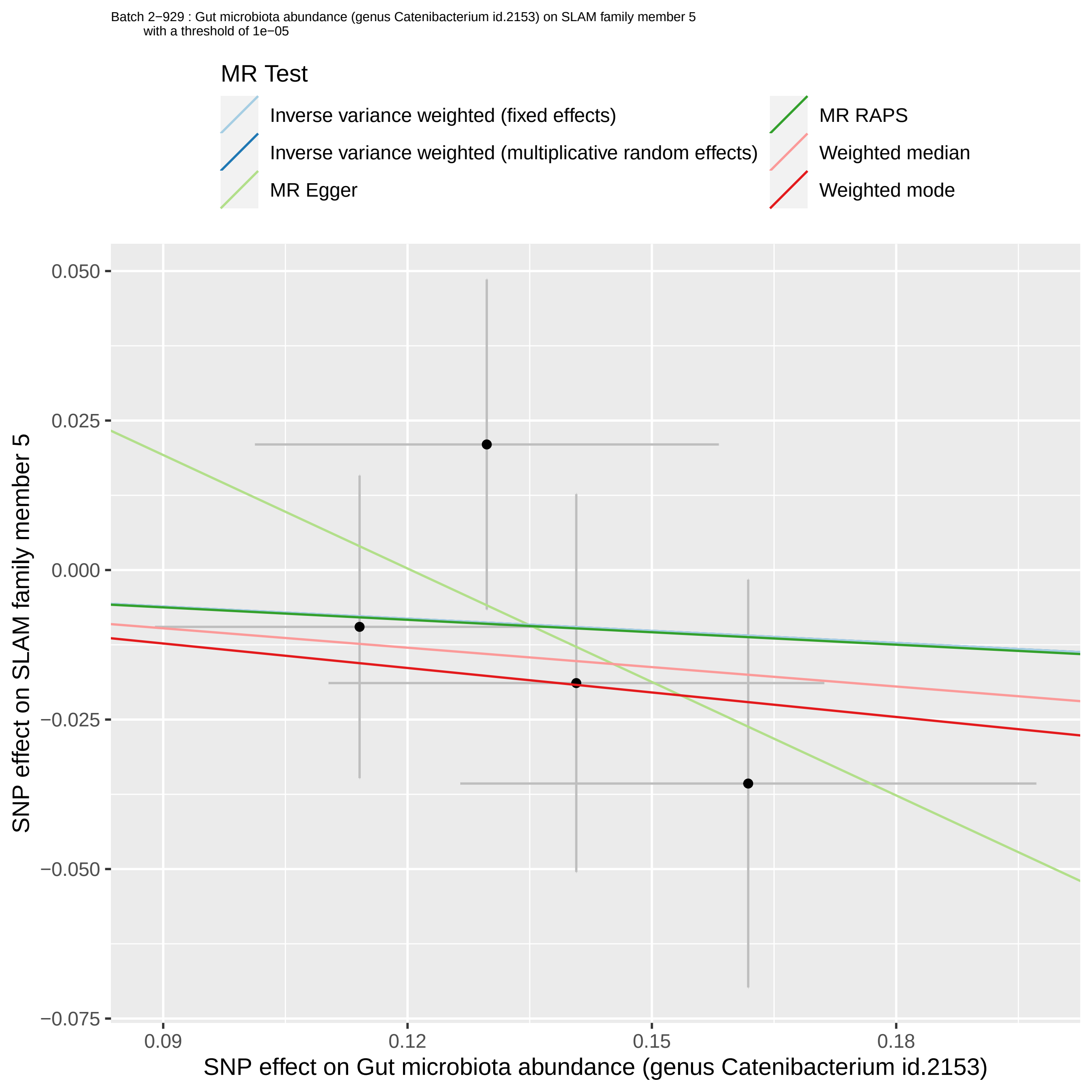

Batch 2−929 : Gut microbiota abundance (genus Catenibacterium id.2153) on SLAM family member 5
with a threshold of 1e−05
MR Test
Inverse variance weighted (fixed effects)
Inverse variance weighted (multiplicative random effects)
MR Egger
MR RAPS
Weighted median
Weighted mode
0.050
0.025
0.000
−0.025
−0.050
−0.075
0.09
0.12
0.15
0.18
SNP effect on SLAM family member 5
SNP effect on Gut microbiota abundance (genus Catenibacterium id.2153)

Batch 2−930 : Gut microbiota abundance (genus Parasutterella id.2892) on SLAM family member 5
with a threshold of 1e−05

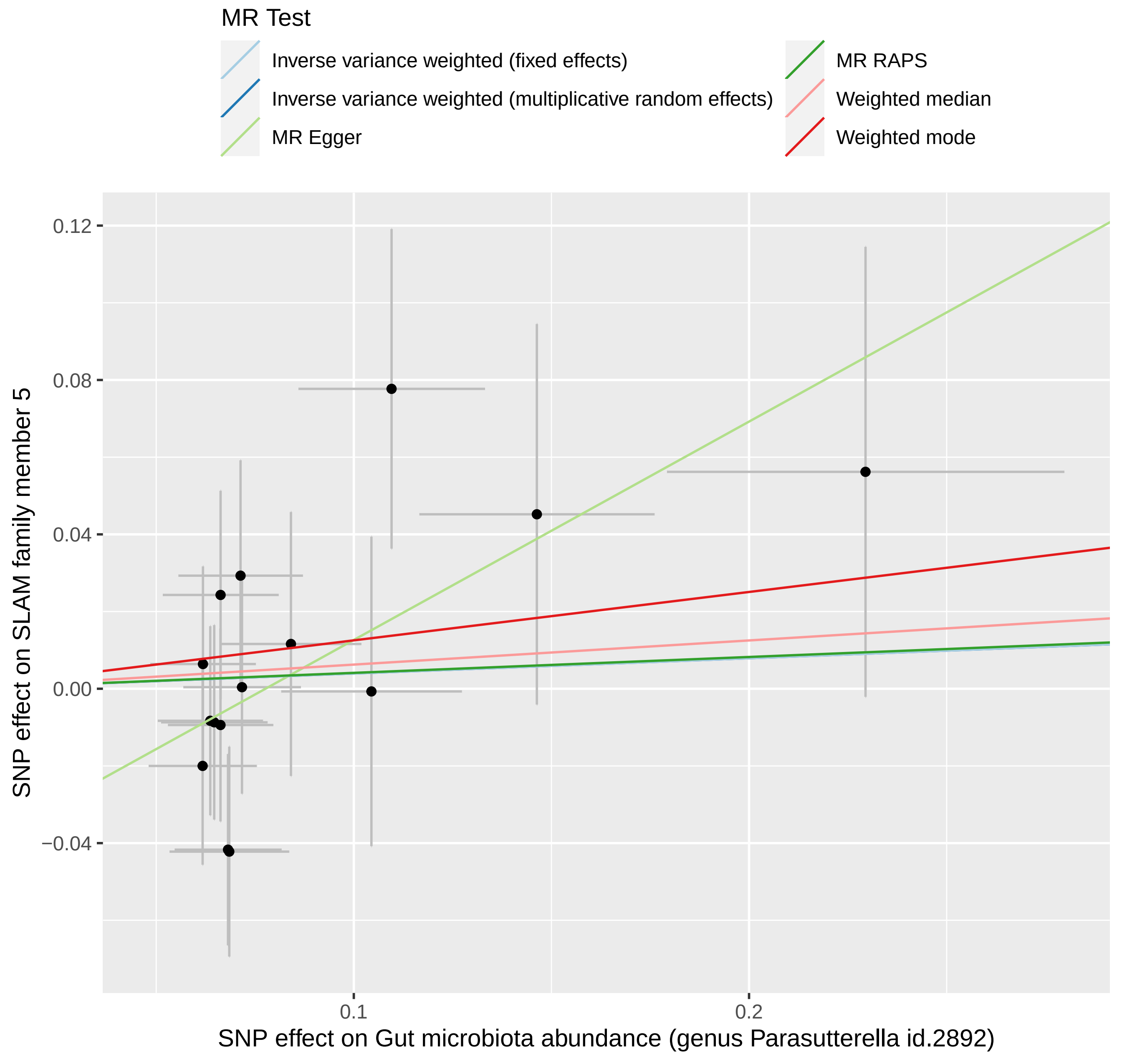

Batch 2−931 : Gut microbiota abundance (genus Catenibacterium id.2153) on T−lymphocyte activation antigen CD86
with a threshold of 1e−05

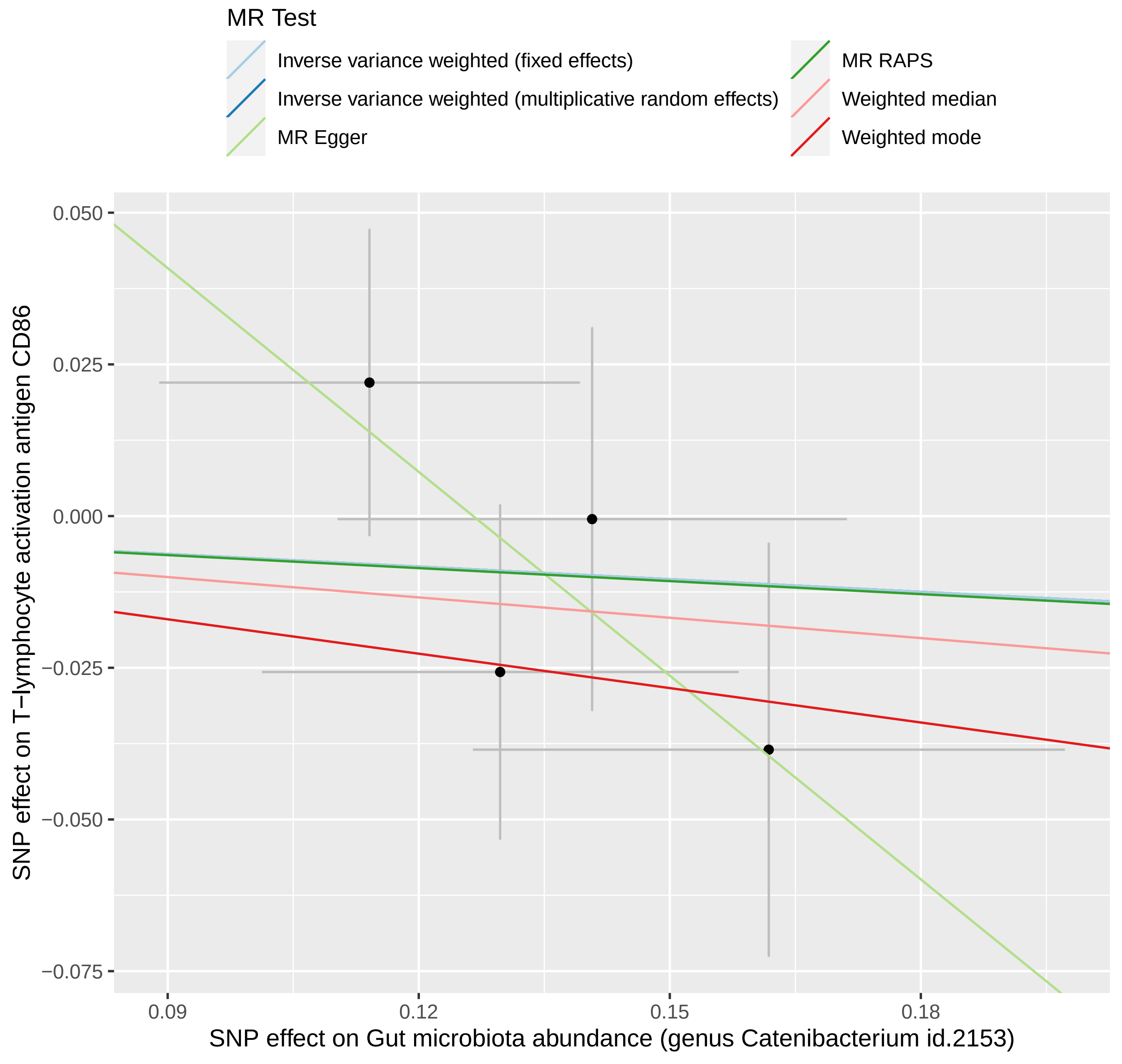

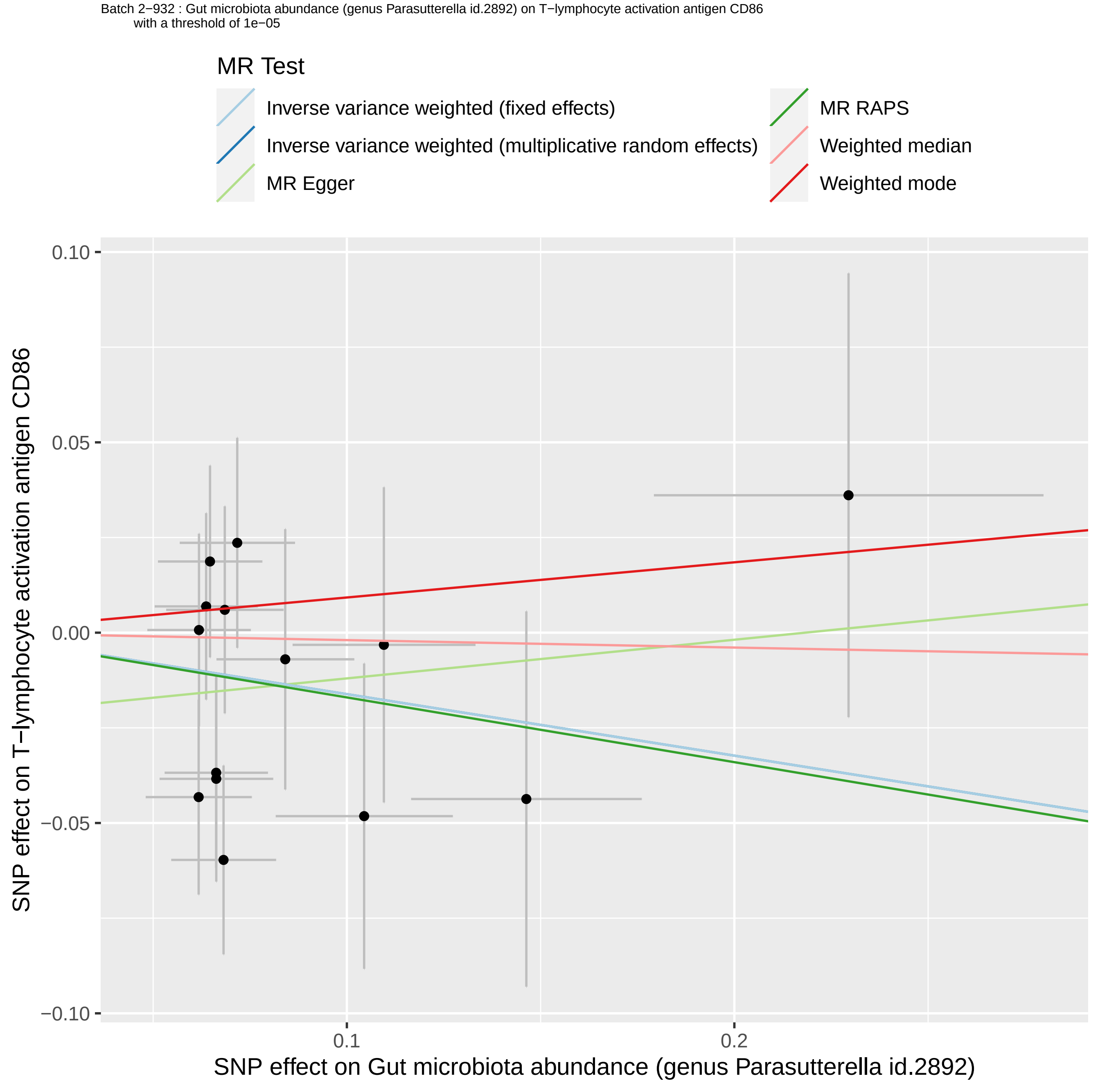

Batch 2−932 : Gut microbiota abundance (genus Parasutterella id.2892) on T−lymphocyte activation antigen CD86
with a threshold of 1e−05
MR Test
Inverse variance weighted (fixed effects)
Inverse variance weighted (multiplicative random effects)
MR Egger
MR RAPS
Weighted median
Weighted mode
0.10
0.05
0.00
−0.05
−0.10
0.1
0.2
SNP effect on T−lymphocyte activation antigen CD86
SNP effect on Gut microbiota abundance (genus Parasutterella id.2892)

Batch 2−933 : Gut microbiota abundance (genus Catenibacterium id.2153) on T−cell surface glycoprotein CD8 beta chain with a threshold of 1e−05

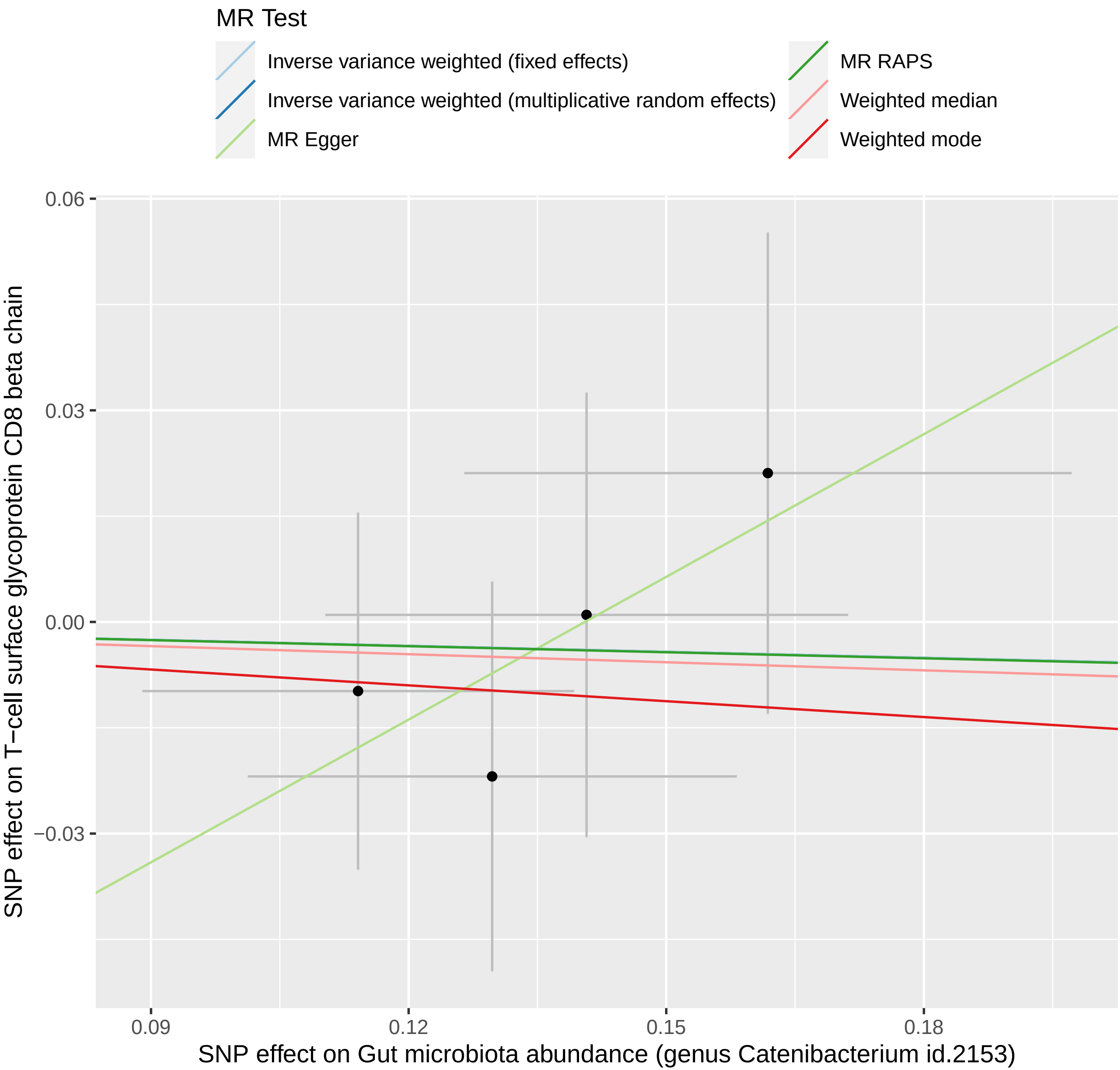

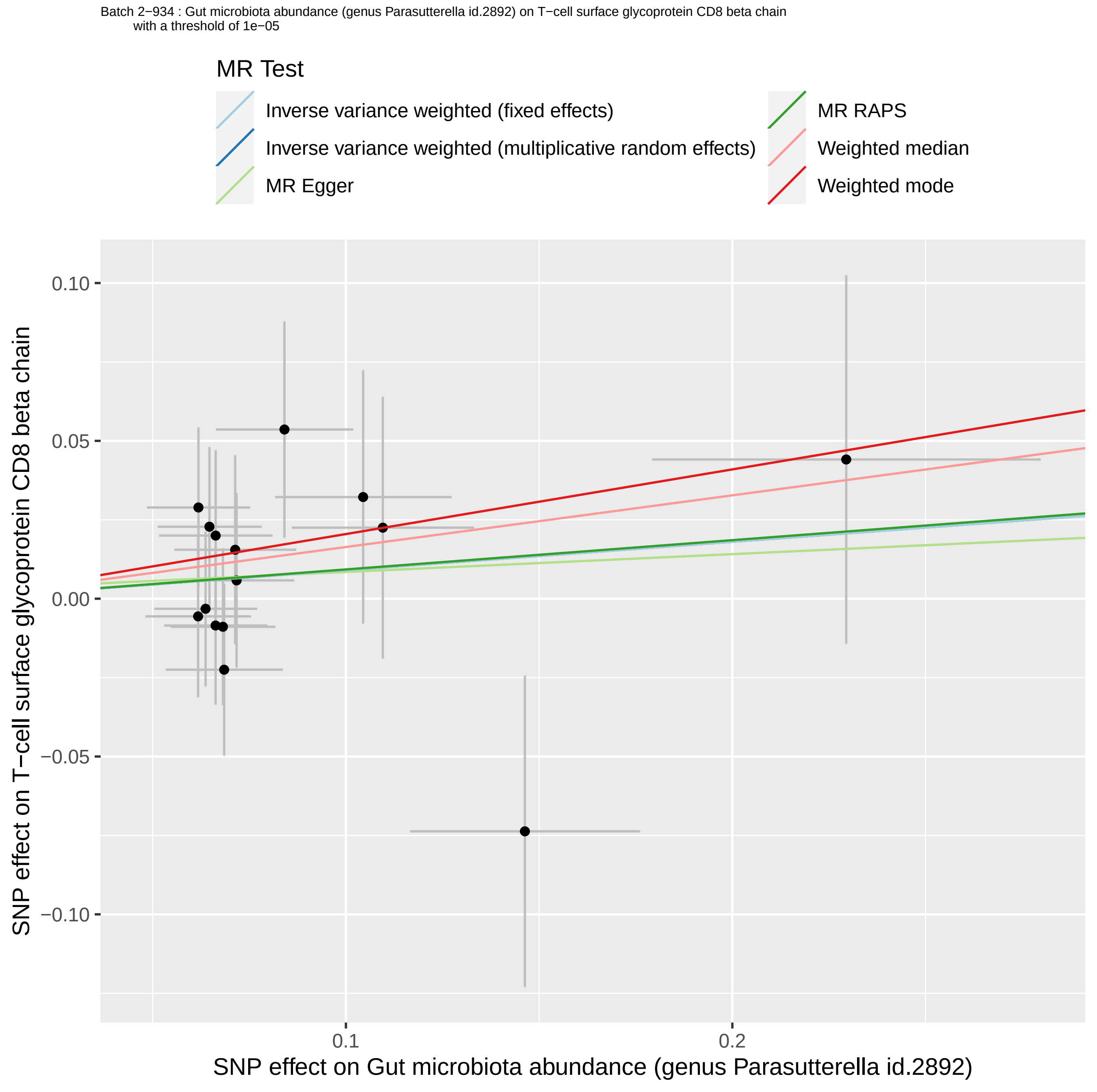
Batch 2−934 : Gut microbiota abundance (genus Parasutterella id.2892) on T−cell surface glycoprotein CD8 beta chain
with a threshold of 1e−05
MR Test
Inverse variance weighted (fixed effects)
Inverse variance weighted (multiplicative random effects)
MR Egger
MR RAPS
Weighted median
Weighted mode
0.10
0.05
0.00
−0.05
−0.10
0.1
0.2
SNP effect on T−cell surface glycoprotein CD8 beta chain
SNP effect on Gut microbiota abundance (genus Parasutterella id.2892)

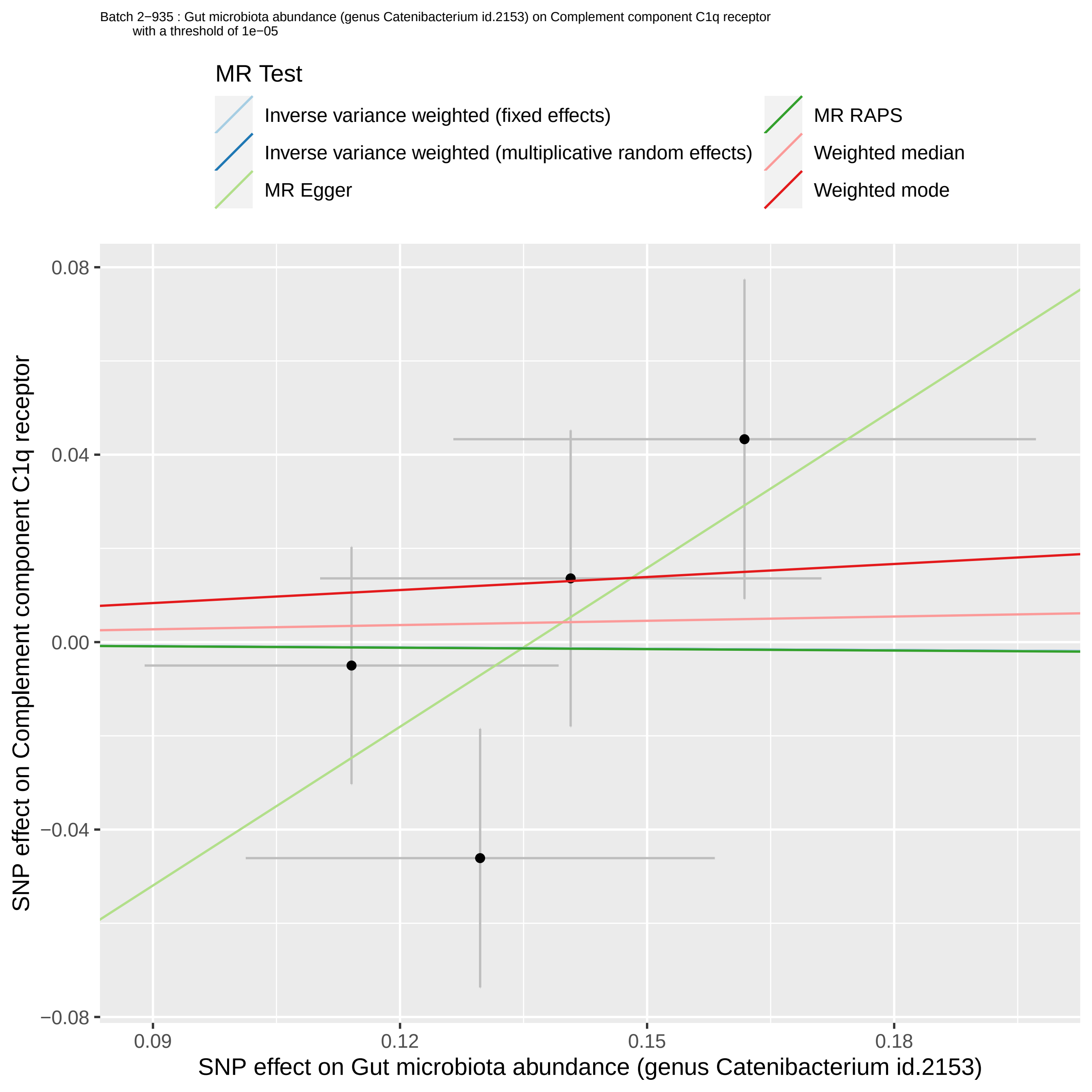
Batch 2−935 : Gut microbiota abundance (genus Catenibacterium id.2153) on Complement component C1q receptor
with a threshold of 1e−05
MR Test
Inverse variance weighted (fixed effects)
Inverse variance weighted (multiplicative random effects)
MR Egger
MR RAPS
Weighted median
Weighted mode
0.08
0.04
0.00
−0.04
−0.08
0.09
0.12
0.15
0.18
SNP effect on Complement component C1q receptor
SNP effect on Gut microbiota abundance (genus Catenibacterium id.2153)

Batch 2−936 : Gut microbiota abundance (genus Parasutterella id.2892) on Complement component C1q receptor
with a threshold of 1e−05

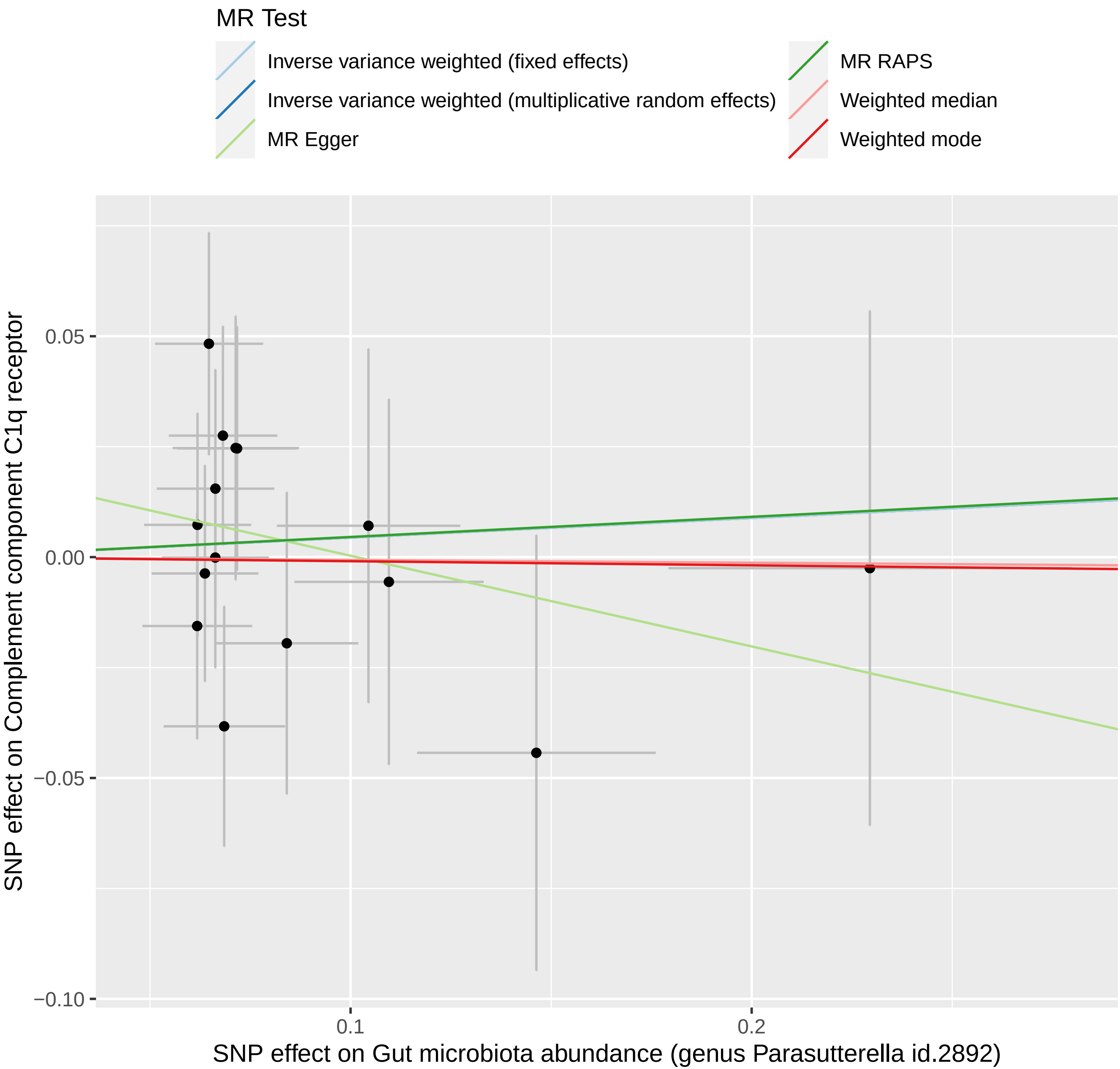

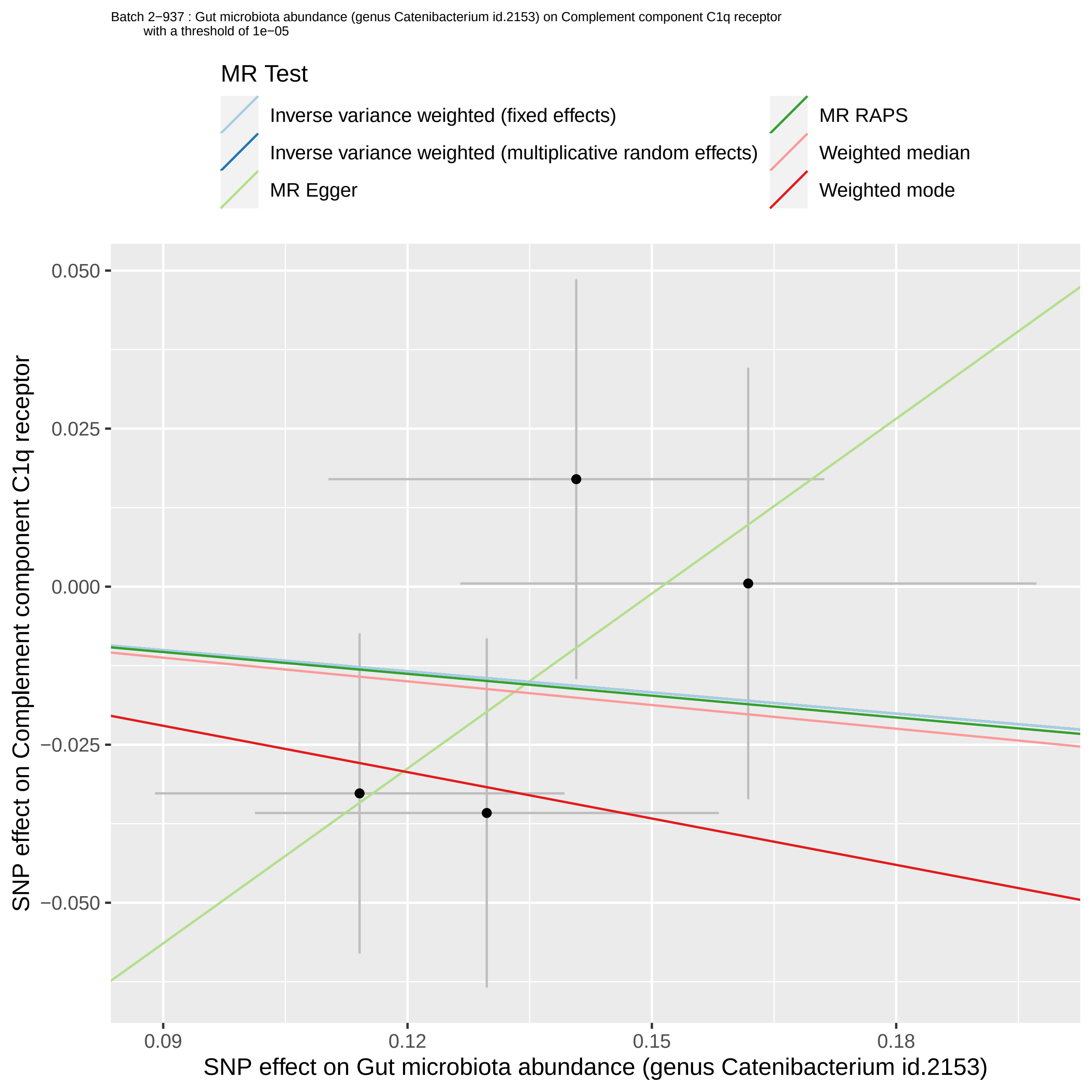
Batch 2−937 : Gut microbiota abundance (genus Catenibacterium id.2153) on Complement component C1q receptor
with a threshold of 1e−05
MR Test
Inverse variance weighted (fixed effects)
Inverse variance weighted (multiplicative random effects)
MR Egger
MR RAPS
Weighted median
Weighted mode
0.050
0.025
0.000
−0.025
−0.050
0.09
0.12
0.15
0.18
SNP effect on Complement component C1q receptor
SNP effect on Gut microbiota abundance (genus Catenibacterium id.2153)

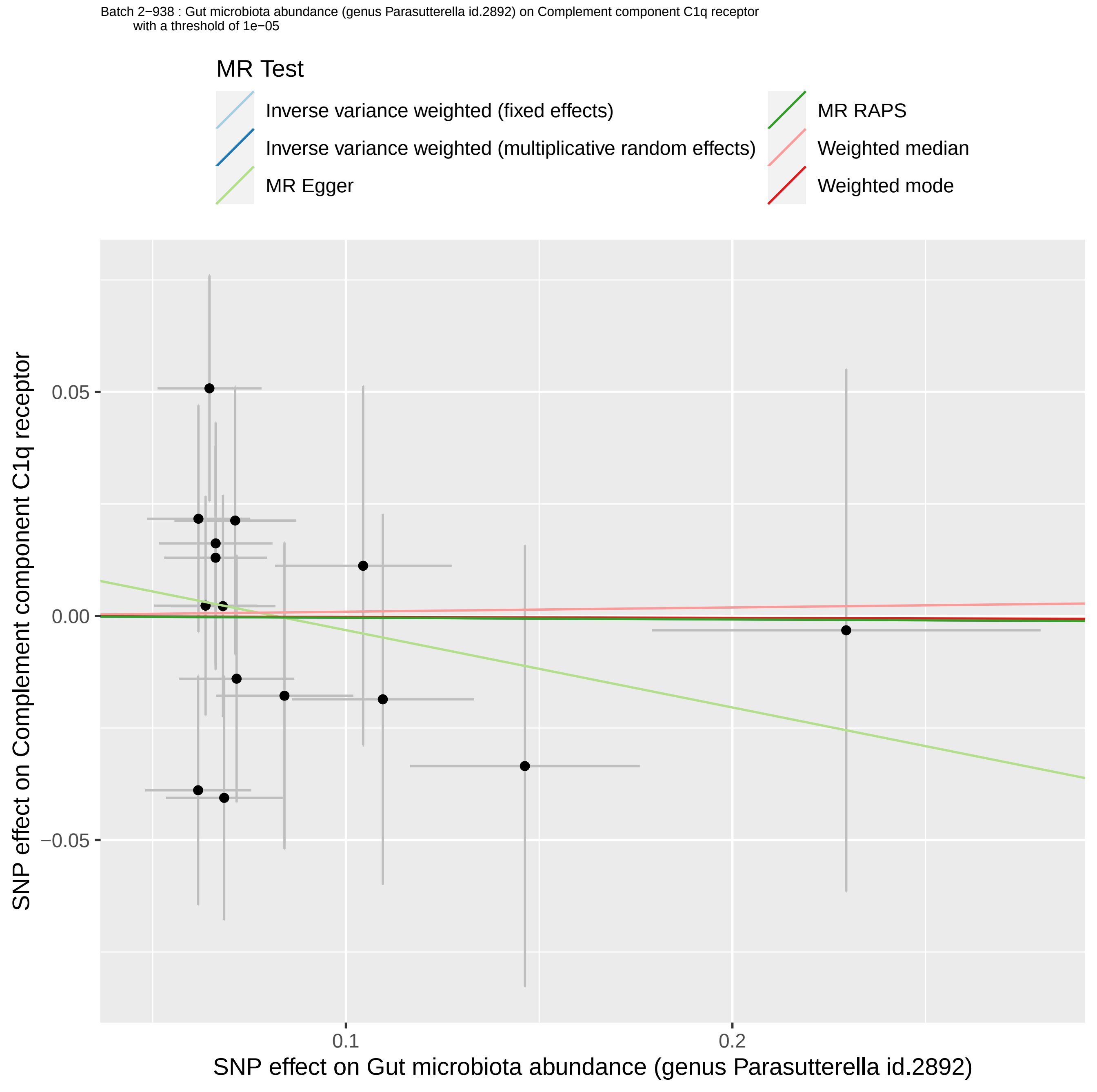

Batch 2−938 : Gut microbiota abundance (genus Parasutterella id.2892) on Complement component C1q receptor
with a threshold of 1e−05
MR Test
Inverse variance weighted (fixed effects)
Inverse variance weighted (multiplicative random effects)
MR Egger
MR RAPS
Weighted median
Weighted mode
0.05
0.00
−0.05
0.1
0.2
SNP effect on Complement component C1q receptor
SNP effect on Gut microbiota abundance (genus Parasutterella id.2892)

Batch 2−939 : Gut microbiota abundance (genus Catenibacterium id.2153) on T−cell surface protein tactile
with a threshold of 1e−05

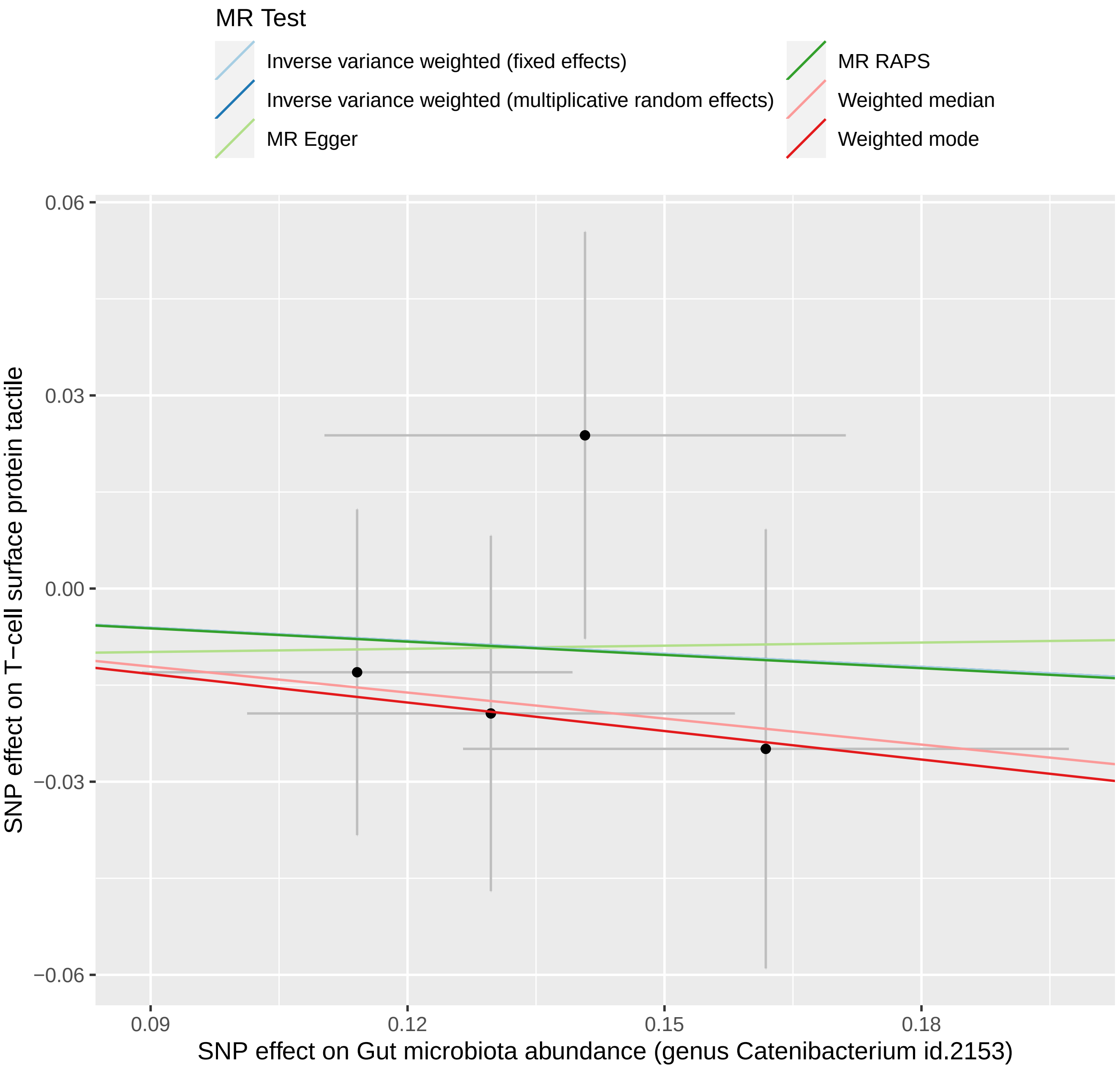

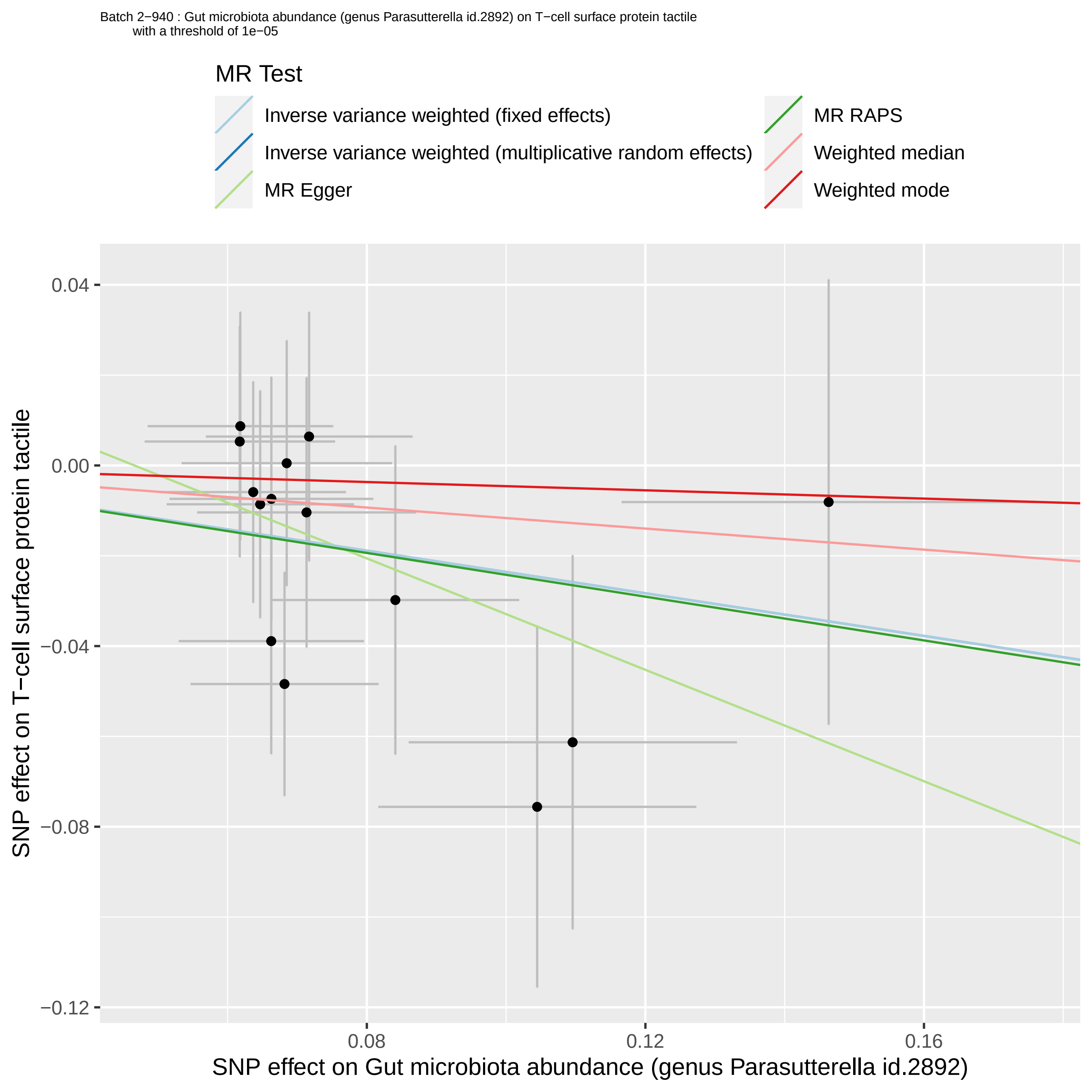

Batch 2−940 : Gut microbiota abundance (genus Parasutterella id.2892) on T−cell surface protein tactile
with a threshold of 1e−05
MR Test
Inverse variance weighted (fixed effects)
Inverse variance weighted (multiplicative random effects)
MR Egger
MR RAPS
Weighted median
Weighted mode
0.04
0.00
−0.04
−0.08
−0.12
0.08
0.12
0.16
SNP effect on T−cell surface protein tactile
SNP effect on Gut microbiota abundance (genus Parasutterella id.2892)

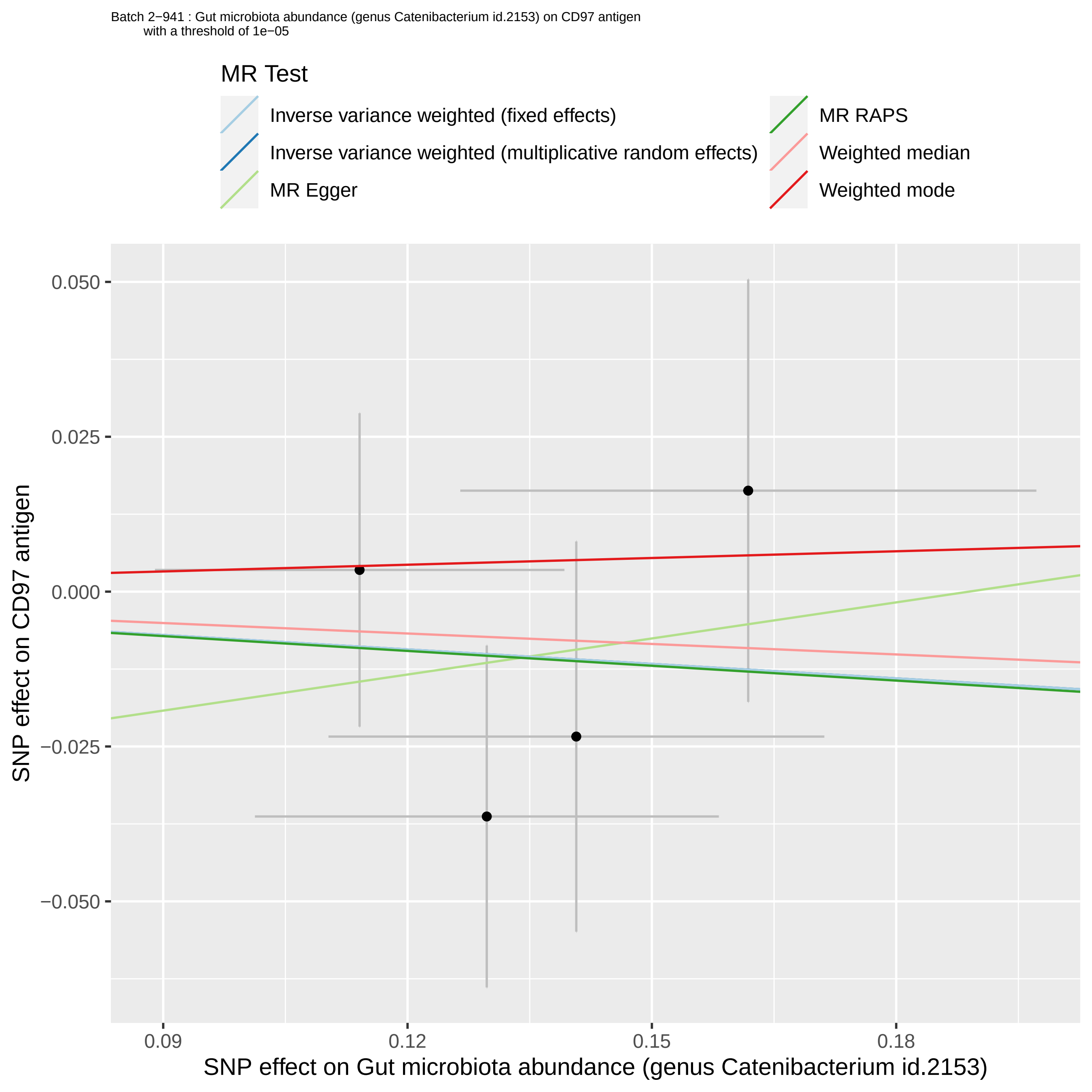
Batch 2−941 : Gut microbiota abundance (genus Catenibacterium id.2153) on CD97 antigen
with a threshold of 1e−05
MR Test
Inverse variance weighted (fixed effects)
Inverse variance weighted (multiplicative random effects)
MR Egger
MR RAPS
Weighted median
Weighted mode
0.050
0.025
0.000
−0.025
−0.050
0.09
0.12
0.15
0.18
SNP effect on CD97 antigen
SNP effect on Gut microbiota abundance (genus Catenibacterium id.2153)

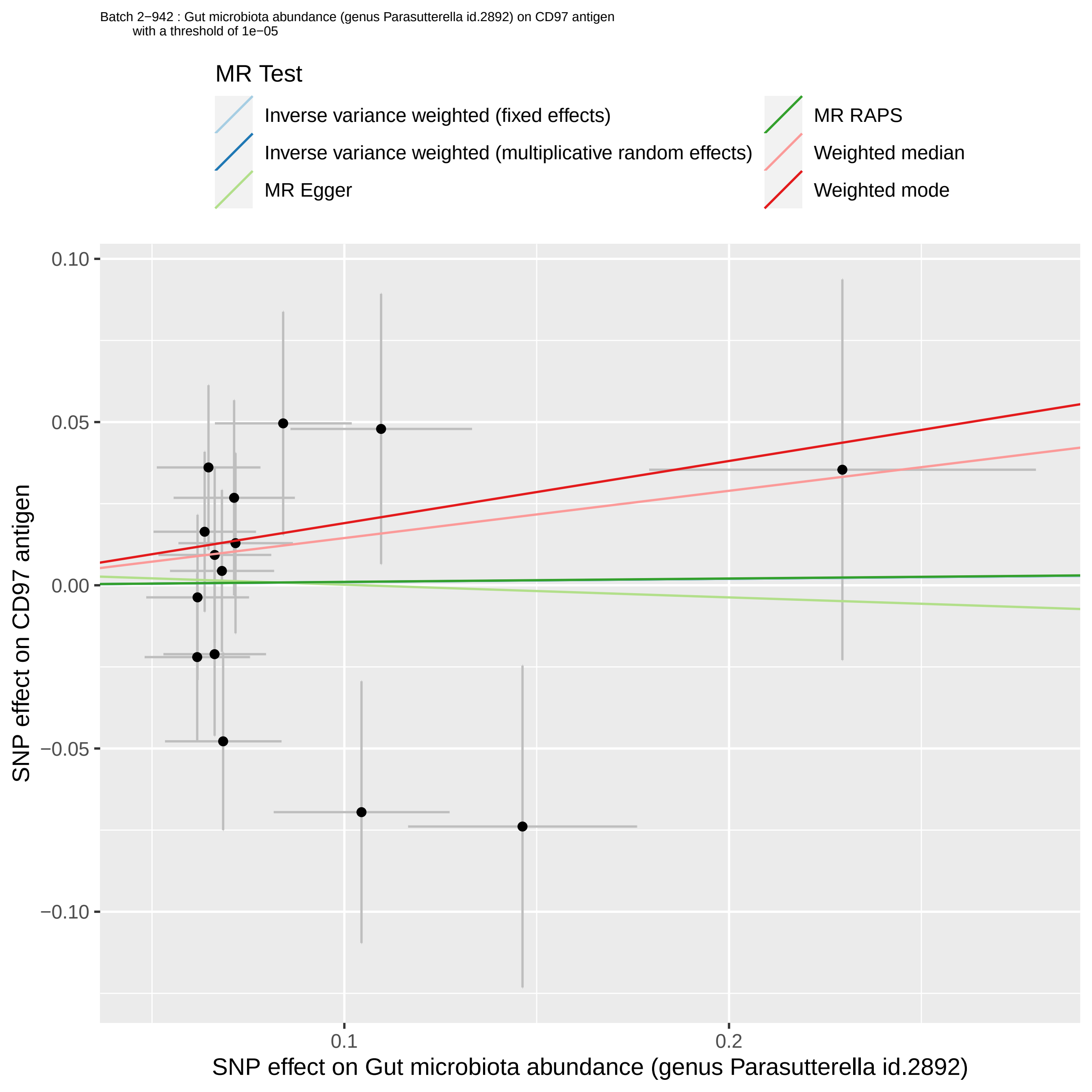
Batch 2−942 : Gut microbiota abundance (genus Parasutterella id.2892) on CD97 antigen
with a threshold of 1e−05
MR Test
Inverse variance weighted (fixed effects)
Inverse variance weighted (multiplicative random effects)
MR Egger
MR RAPS
Weighted median
Weighted mode
0.10
0.05
0.00
−0.05
−0.10
0.1
0.2
SNP effect on CD97 antigen
SNP effect on Gut microbiota abundance (genus Parasutterella id.2892)

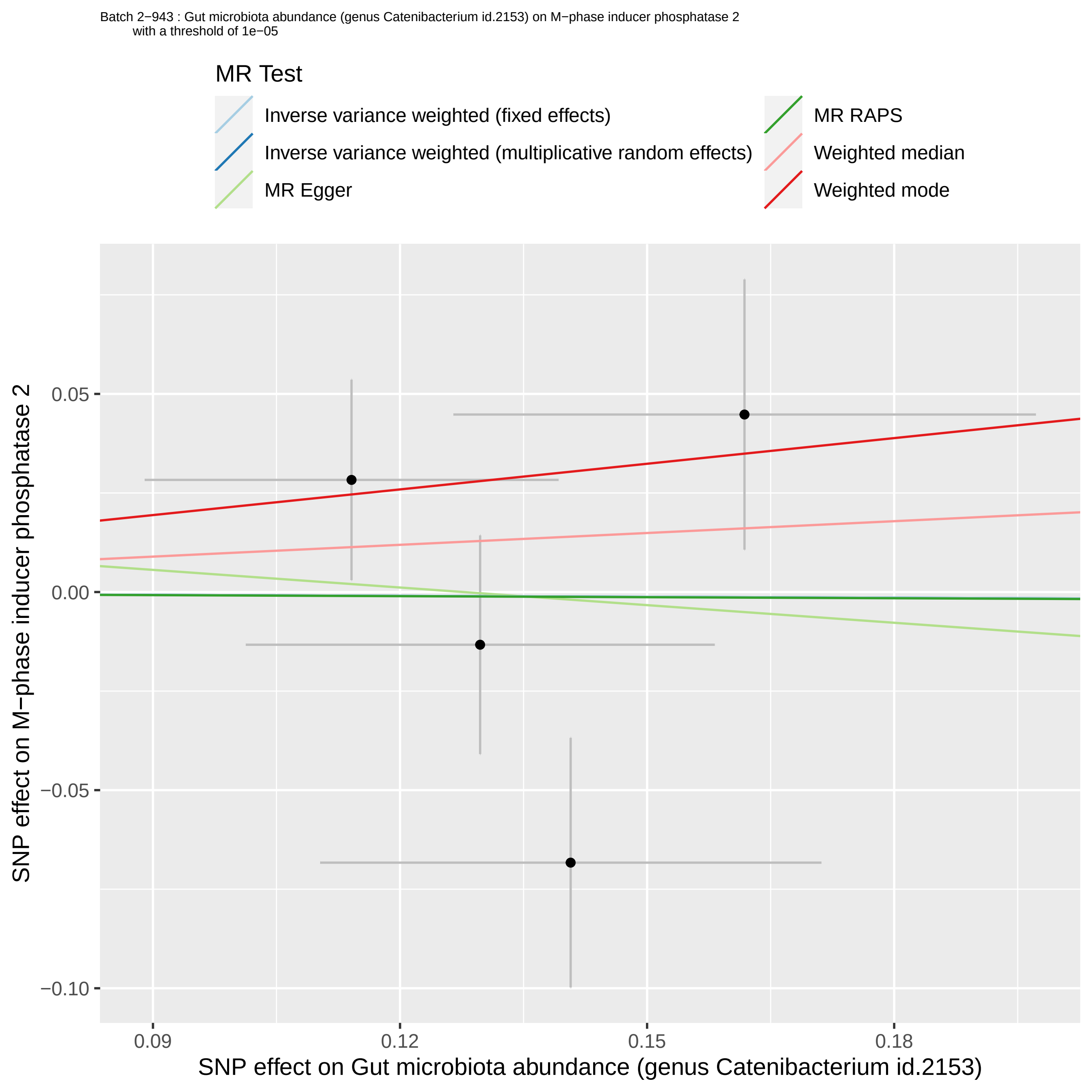
Batch 2−943 : Gut microbiota abundance (genus Catenibacterium id.2153) on M−phase inducer phosphatase 2
with a threshold of 1e−05
MR Test
Inverse variance weighted (fixed effects)
Inverse variance weighted (multiplicative random effects)
MR Egger
MR RAPS
Weighted median
Weighted mode
SNP effect on M−phase inducer phosphatase 2
0.05
0.00
−0.05
−0.10
0.09
0.12
0.15
0.18
SNP effect on Gut microbiota abundance (genus Catenibacterium id.2153)

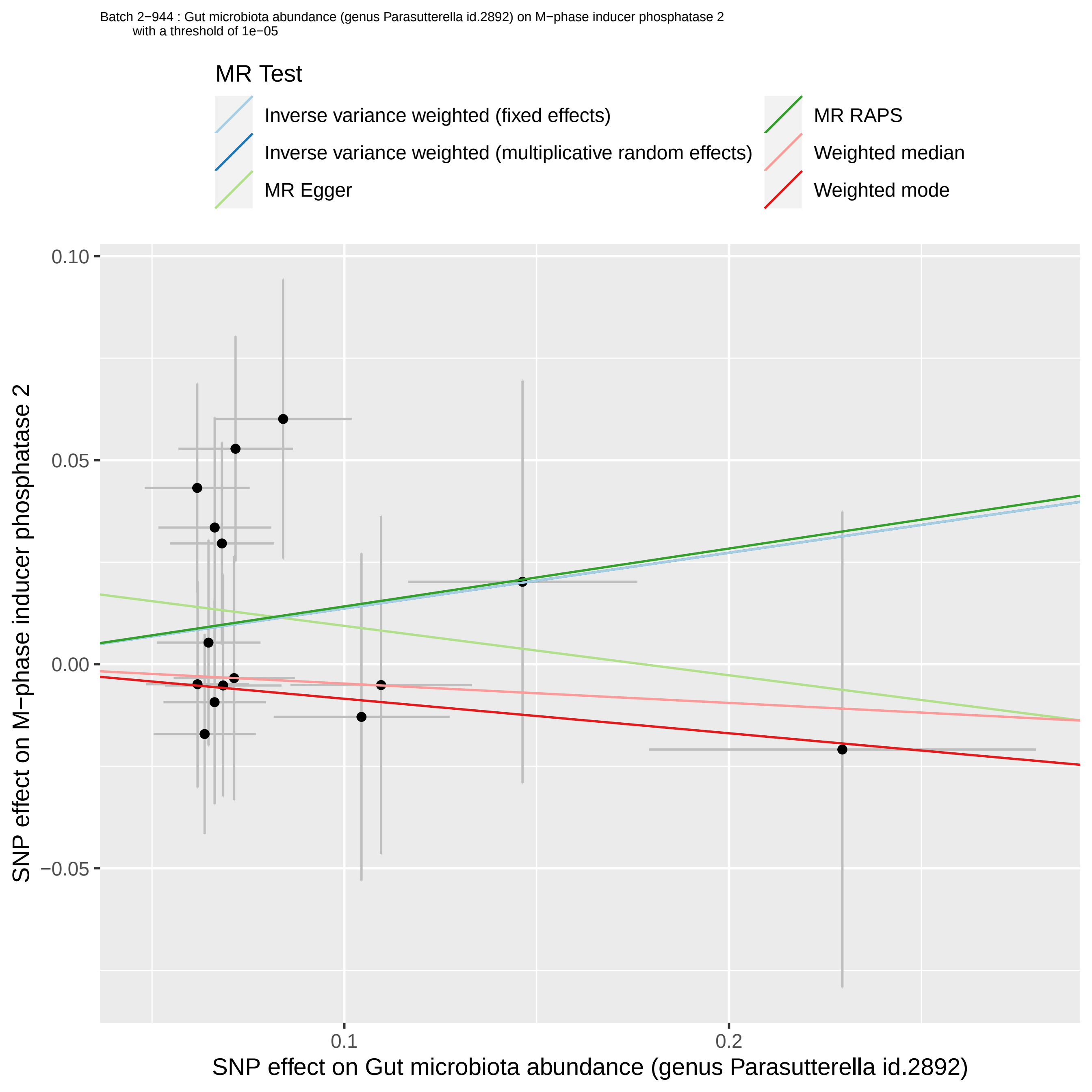
Batch 2−944 : Gut microbiota abundance (genus Parasutterella id.2892) on M−phase inducer phosphatase 2
with a threshold of 1e−05
MR Test
Inverse variance weighted (fixed effects)
Inverse variance weighted (multiplicative random effects)
MR Egger
MR RAPS
Weighted median
Weighted mode
0.10
0.05
0.00
−0.05
0.1
0.2
SNP effect on M−phase inducer phosphatase 2
SNP effect on Gut microbiota abundance (genus Parasutterella id.2892)

Batch 2−945 : Gut microbiota abundance (genus Catenibacterium id.2153) on Cyclin−dependent kinase 1:G2/mitotic−specific cyclin−B1 complex
with a threshold of 1e−05

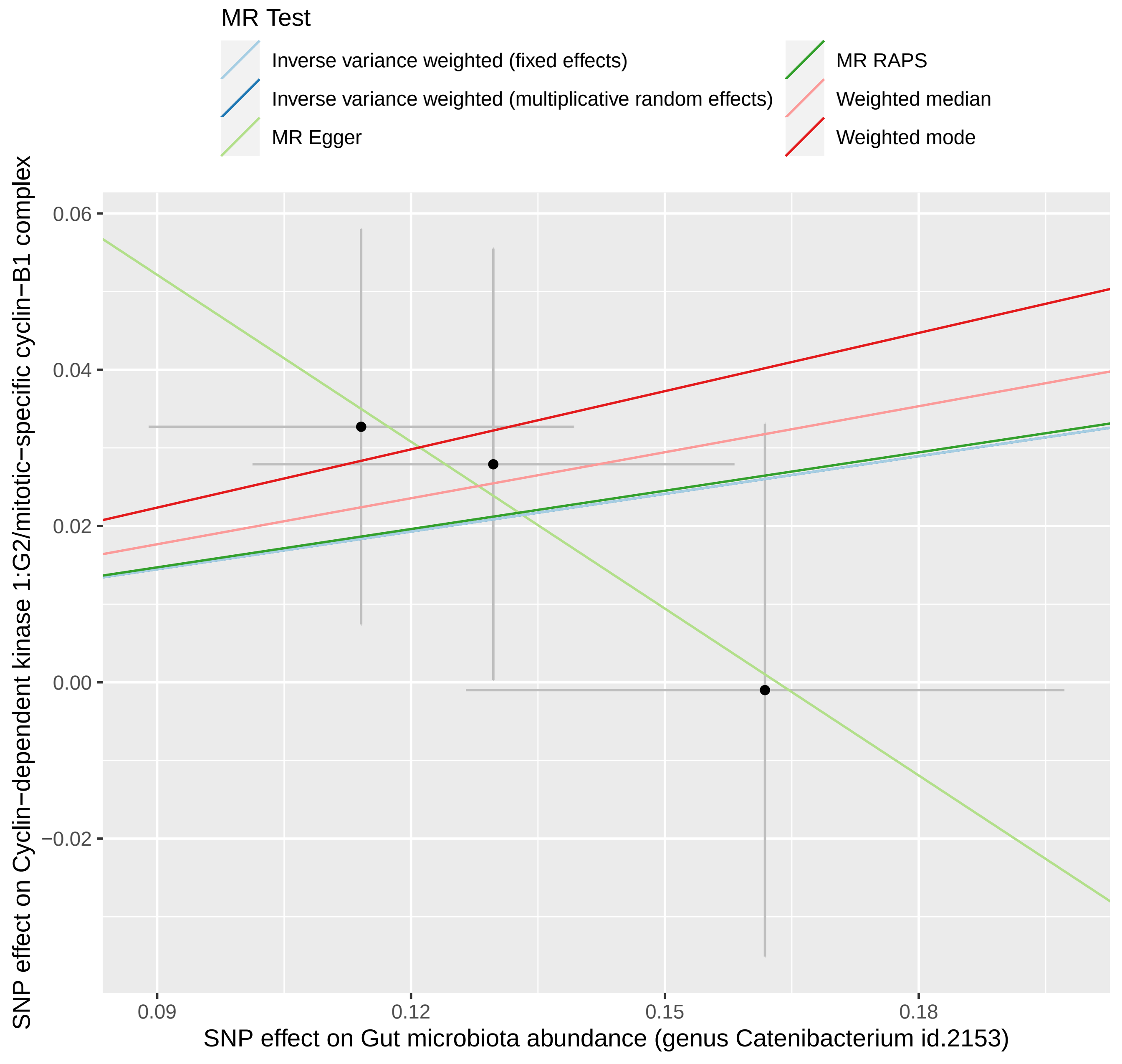

Batch 2−946 : Gut microbiota abundance (genus Parasutterella id.2892) on Cyclin−dependent kinase 1:G2/mitotic−specific cyclin−B1 complex with a threshold of 1e−05

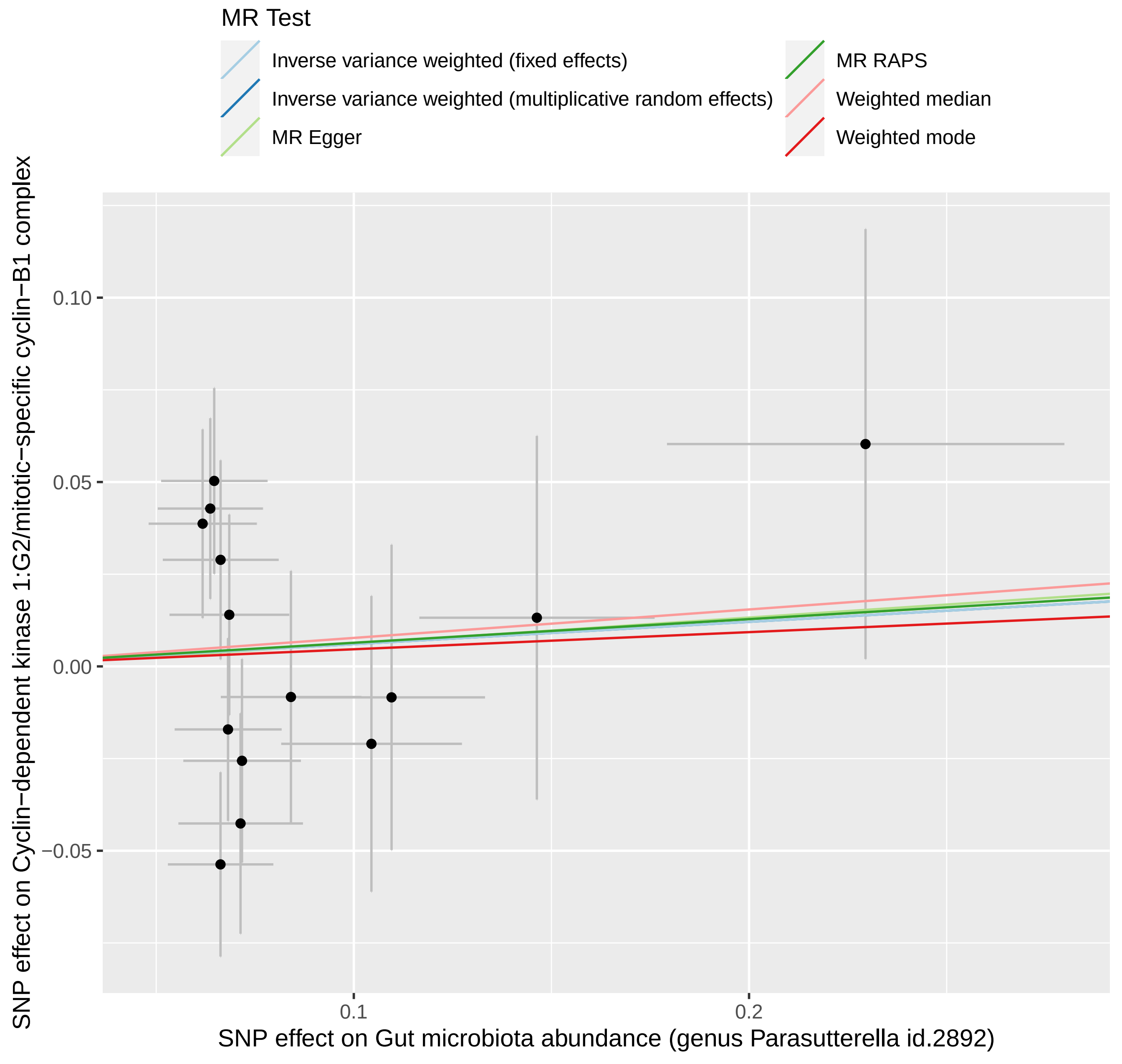

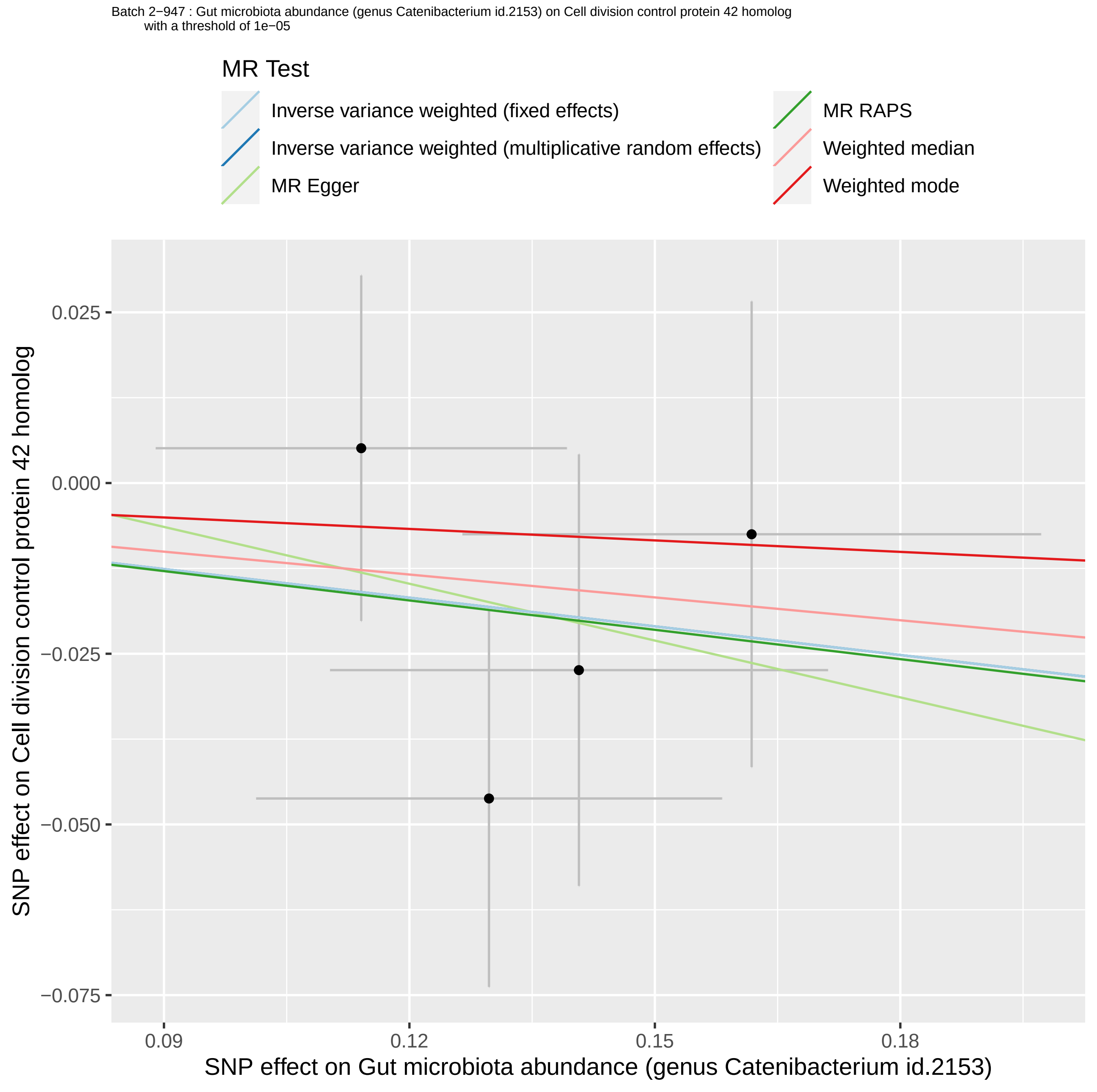

Batch 2−947 : Gut microbiota abundance (genus Catenibacterium id.2153) on Cell division control protein 42 homolog
with a threshold of 1e−05
MR Test
Inverse variance weighted (fixed effects)
Inverse variance weighted (multiplicative random effects)
MR Egger
MR RAPS
Weighted median
Weighted mode
0.025
0.000
−0.025
−0.050
−0.075
0.09
0.12
0.15
0.18
SNP effect on Cell division control protein 42 homolog
SNP effect on Gut microbiota abundance (genus Catenibacterium id.2153)

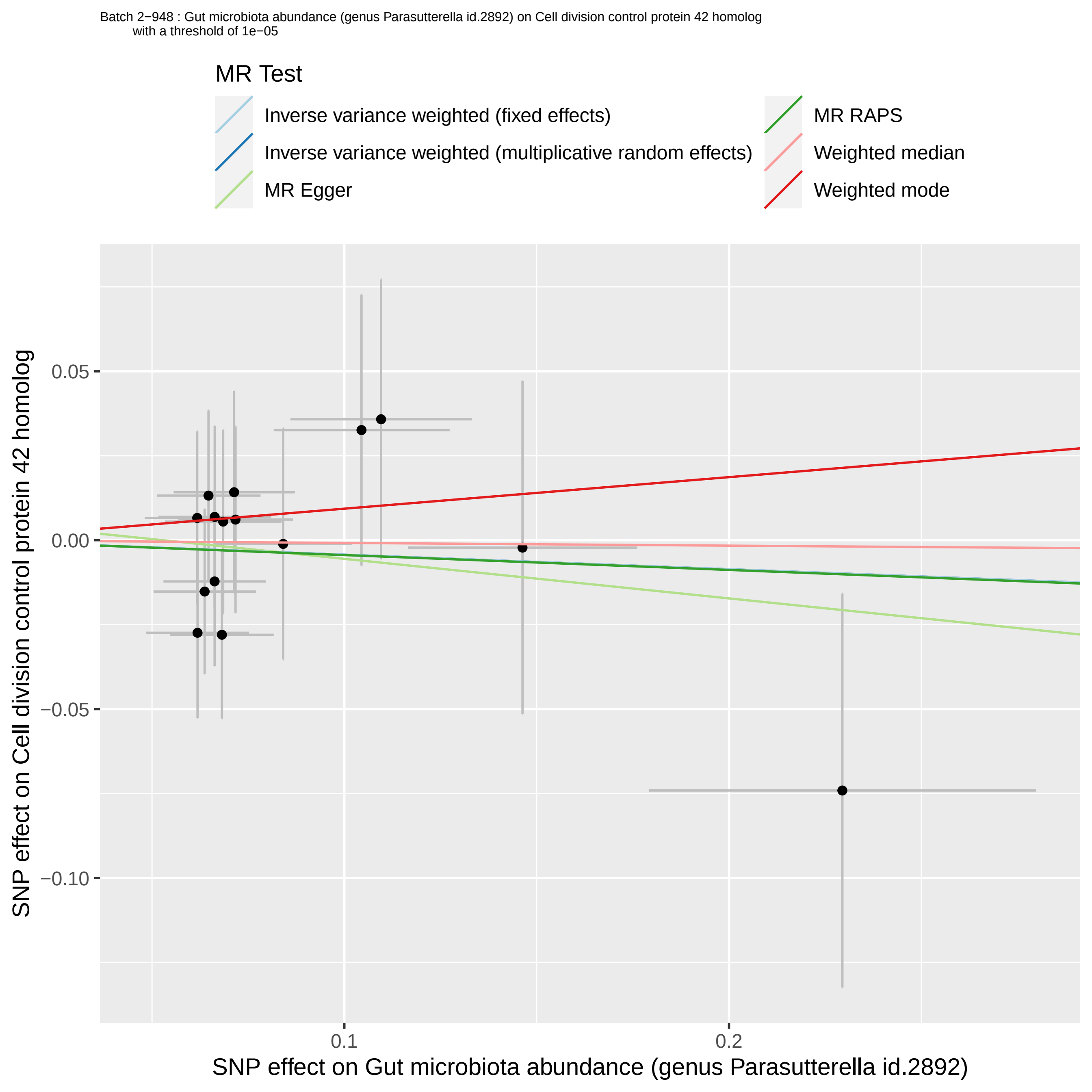
Batch 2−948 : Gut microbiota abundance (genus Parasutterella id.2892) on Cell division control protein 42 homolog
with a threshold of 1e−05
MR Test
Inverse variance weighted (fixed effects)
Inverse variance weighted (multiplicative random effects)
MR Egger
MR RAPS
Weighted median
Weighted mode
0.05
0.00
−0.05
−0.10
0.1
0.2
SNP effect on Cell division control protein 42 homolog
SNP effect on Gut microbiota abundance (genus Parasutterella id.2892)

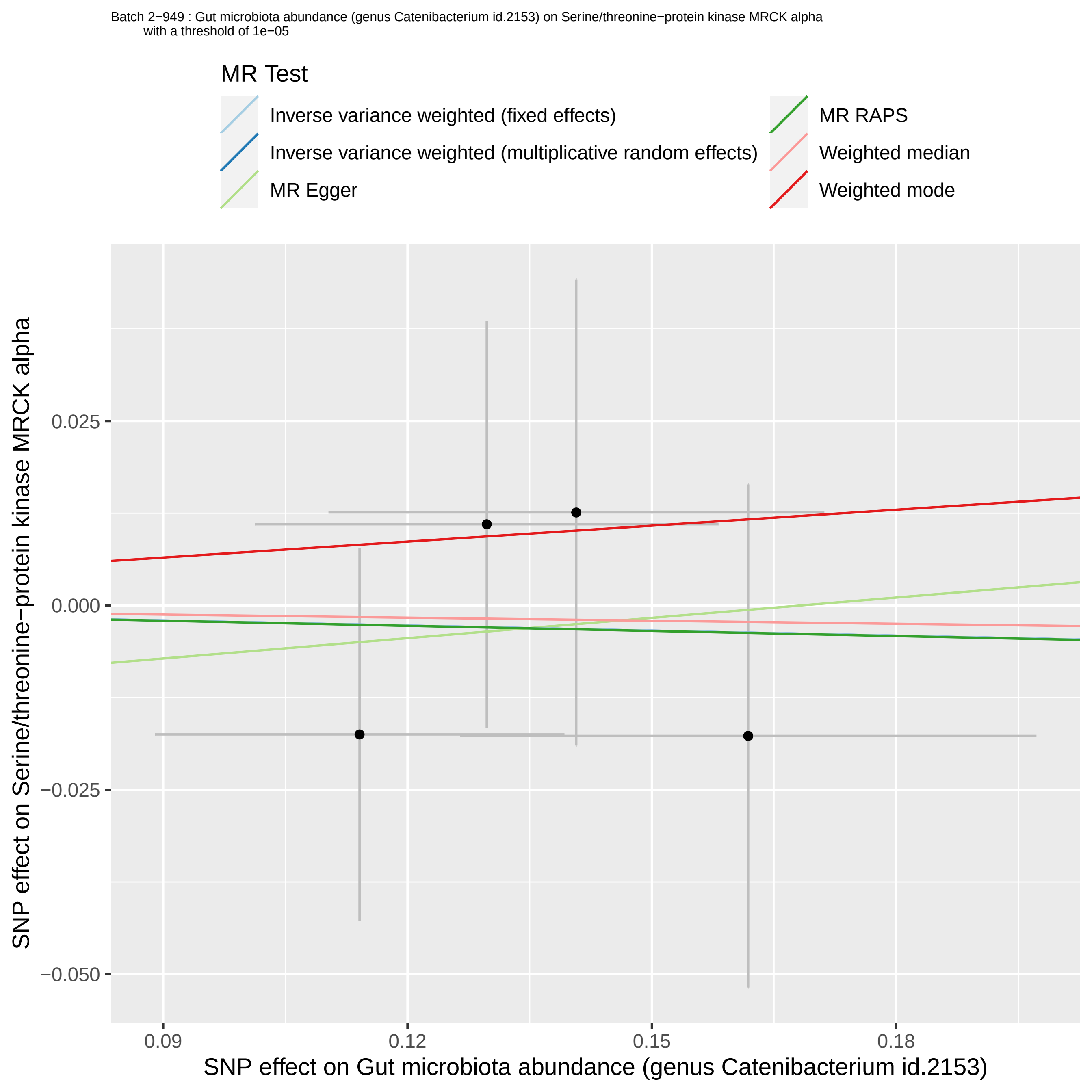
Batch 2−949 : Gut microbiota abundance (genus Catenibacterium id.2153) on Serine/threonine−protein kinase MRCK alpha
with a threshold of 1e−05
MR Test
Inverse variance weighted (fixed effects)
Inverse variance weighted (multiplicative random effects)
MR Egger
MR RAPS
Weighted median
Weighted mode
0.025
0.000
−0.025
−0.050
0.09
0.12
0.15
0.18
SNP effect on Serine/threonine−protein kinase MRCK alpha
SNP effect on Gut microbiota abundance (genus Catenibacterium id.2153)

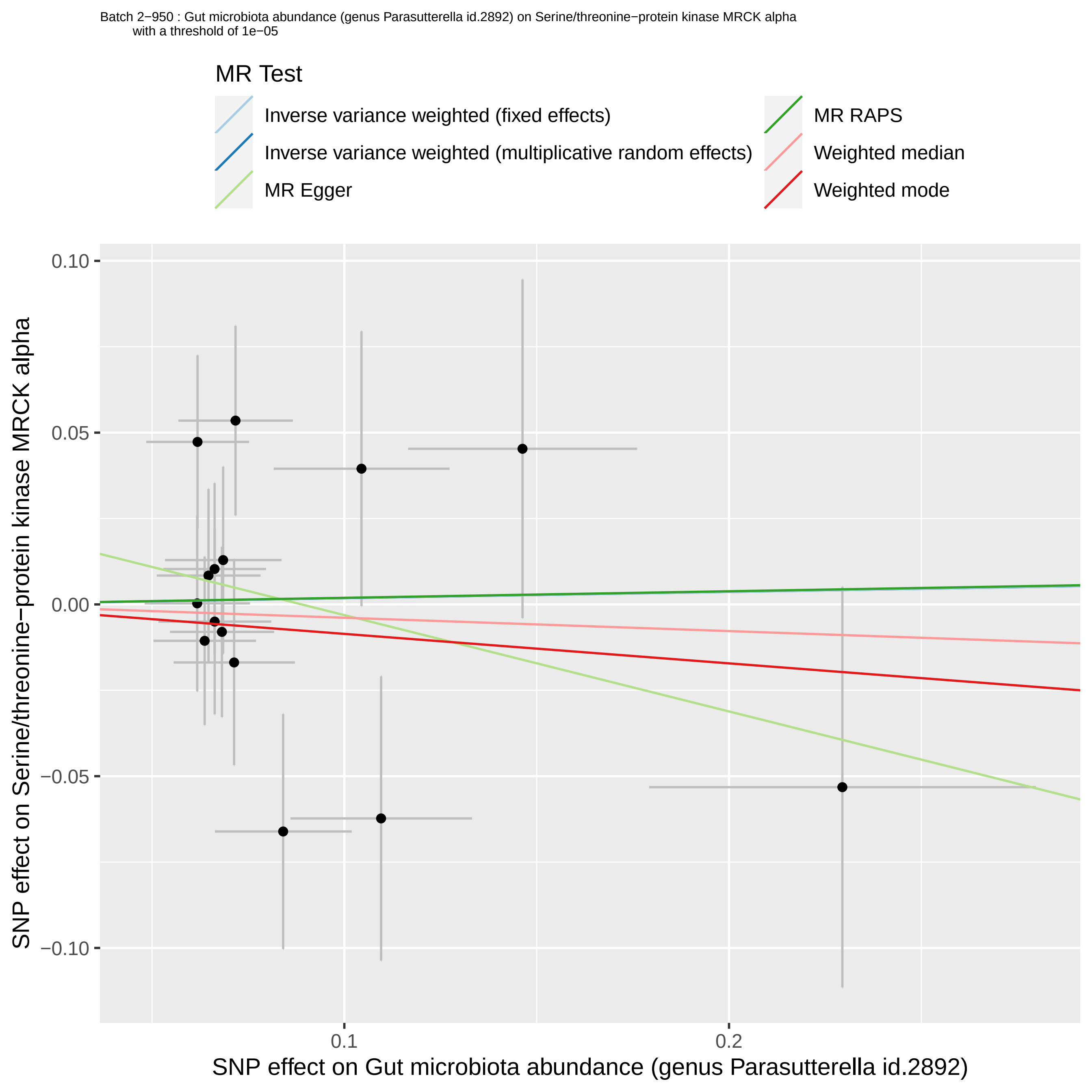
Batch 2−950 : Gut microbiota abundance (genus Parasutterella id.2892) on Serine/threonine−protein kinase MRCK alpha
with a threshold of 1e−05
MR Test
Inverse variance weighted (fixed effects)
Inverse variance weighted (multiplicative random effects)
MR Egger
MR RAPS
Weighted median
Weighted mode
0.10
0.05
0.00
−0.05
−0.10
0.1
0.2
SNP effect on Serine/threonine−protein kinase MRCK alpha
SNP effect on Gut microbiota abundance (genus Parasutterella id.2892)

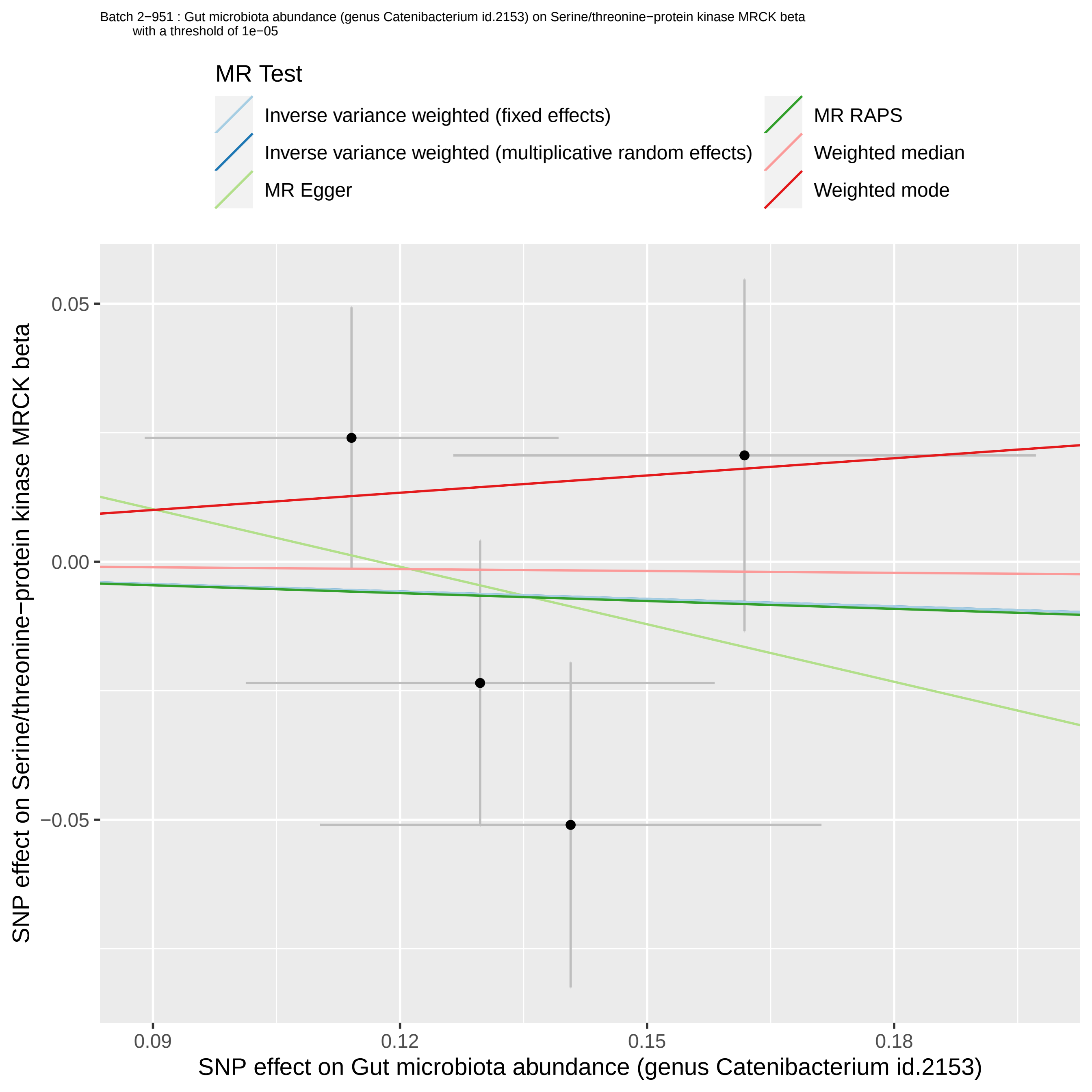
Batch 2−951 : Gut microbiota abundance (genus Catenibacterium id.2153) on Serine/threonine−protein kinase MRCK beta
with a threshold of 1e−05
MR Test
Inverse variance weighted (fixed effects)
Inverse variance weighted (multiplicative random effects)
MR Egger
MR RAPS
Weighted median
Weighted mode
0.05
0.00
−0.05
0.09
0.12
0.15
0.18
SNP effect on Serine/threonine−protein kinase MRCK beta
SNP effect on Gut microbiota abundance (genus Catenibacterium id.2153)

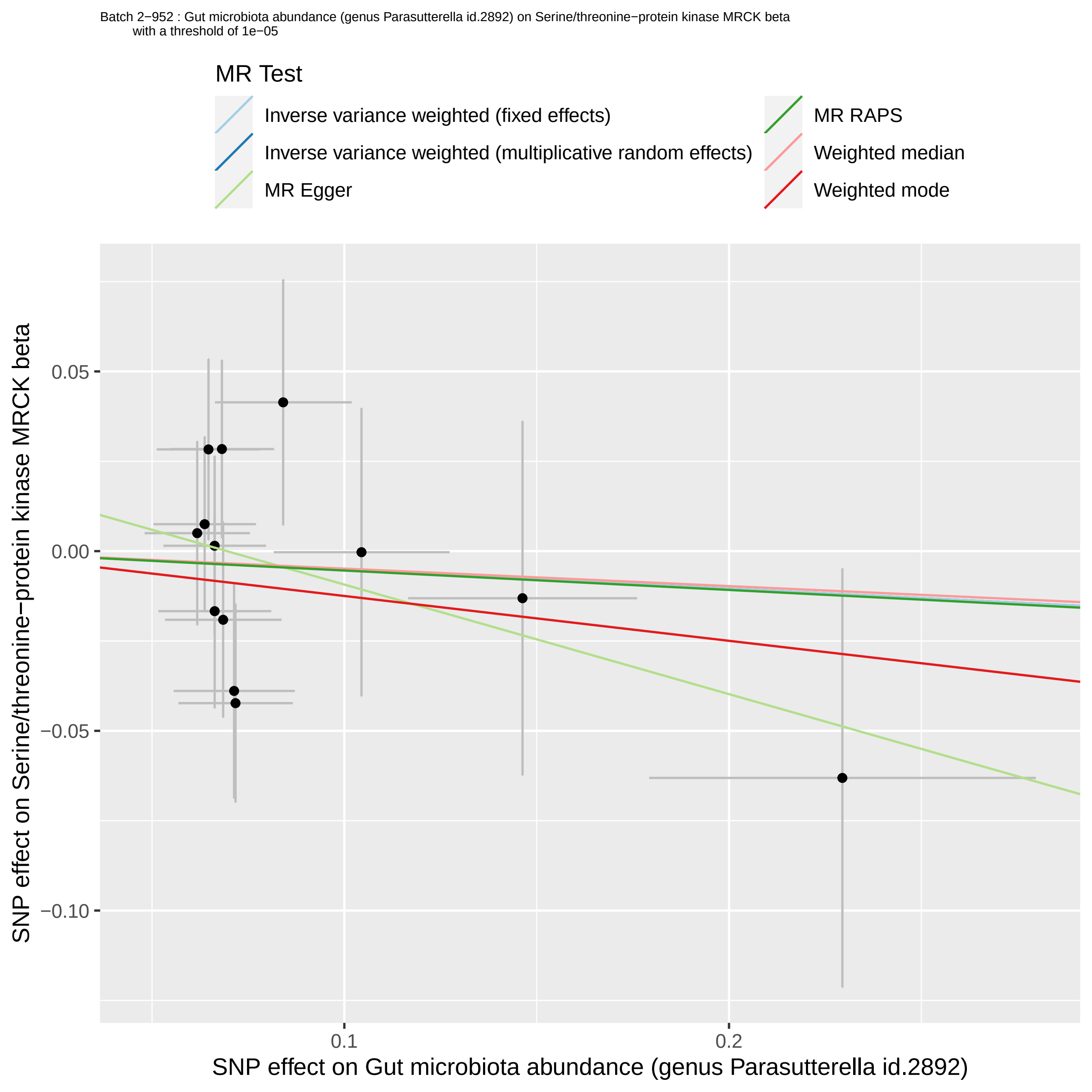
Batch 2−952 : Gut microbiota abundance (genus Parasutterella id.2892) on Serine/threonine−protein kinase MRCK beta
with a threshold of 1e−05
MR Test
Inverse variance weighted (fixed effects)
Inverse variance weighted (multiplicative random effects)
MR Egger
MR RAPS
Weighted median
Weighted mode
0.05
0.00
−0.05
−0.10
0.1
0.2
SNP effect on Serine/threonine−protein kinase MRCK beta
SNP effect on Gut microbiota abundance (genus Parasutterella id.2892)

Batch 2−953 : Gut microbiota abundance (genus Catenibacterium id.2153) on CUB domain−containing protein 1
with a threshold of 1e−05

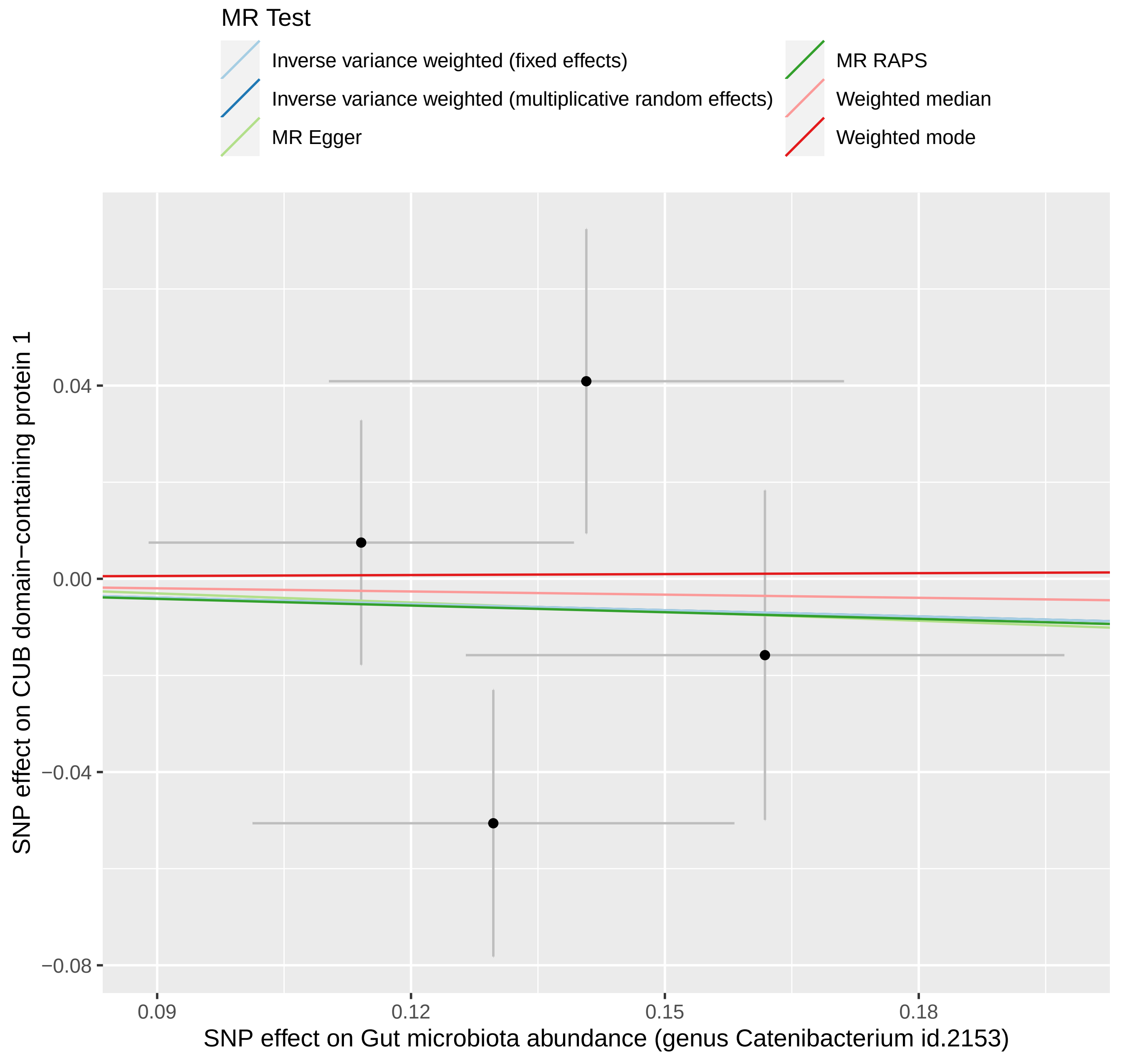

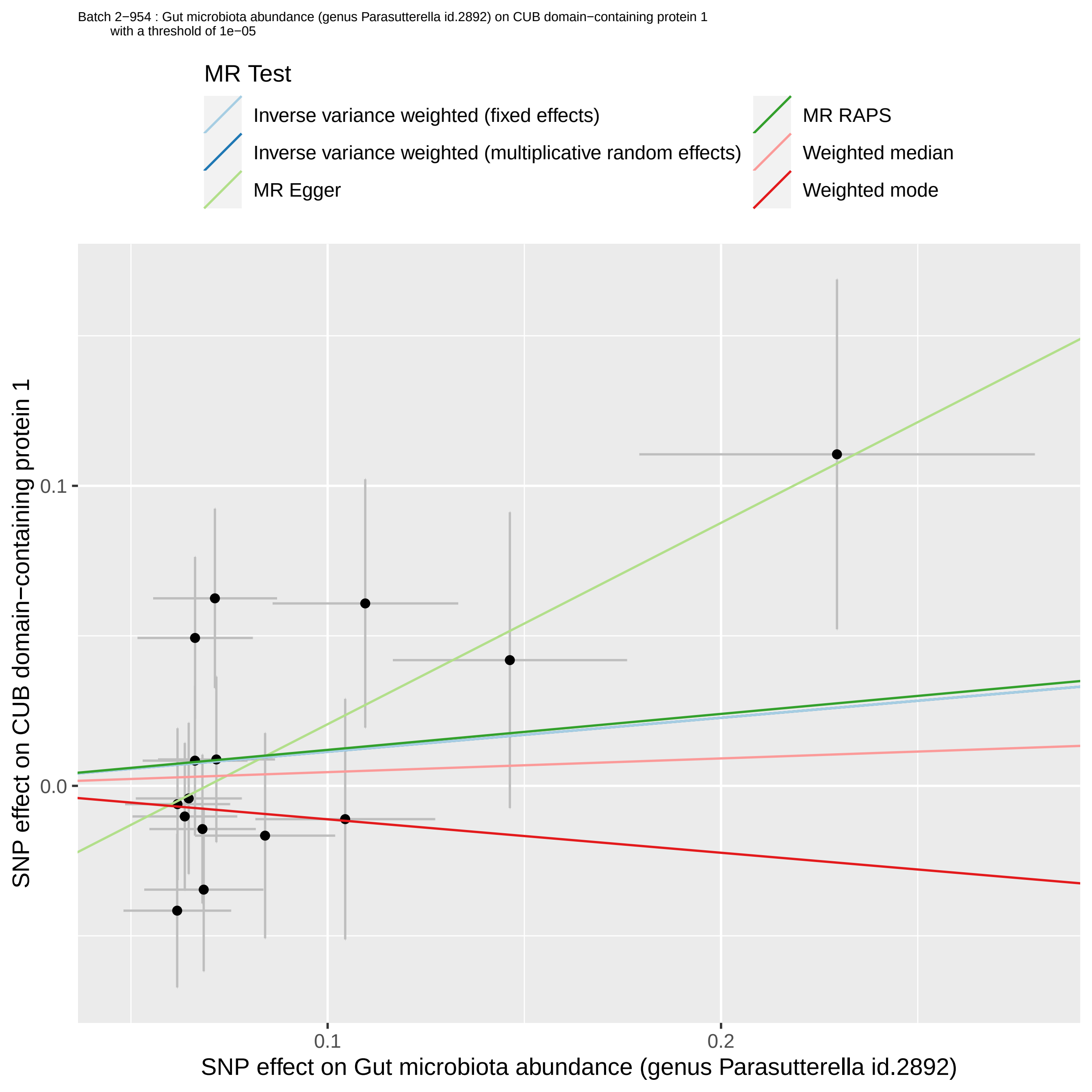

Batch 2−954 : Gut microbiota abundance (genus Parasutterella id.2892) on CUB domain−containing protein 1
with a threshold of 1e−05
MR Test
Inverse variance weighted (fixed effects)
Inverse variance weighted (multiplicative random effects)
MR Egger
MR RAPS
Weighted median
Weighted mode
SNP effect on CUB domain−containing protein 1
0.1
0.0
0.1
0.2
SNP effect on Gut microbiota abundance (genus Parasutterella id.2892)

Batch 2−955 : Gut microbiota abundance (genus Catenibacterium id.2153) on Cadherin−12
with a threshold of 1e−05

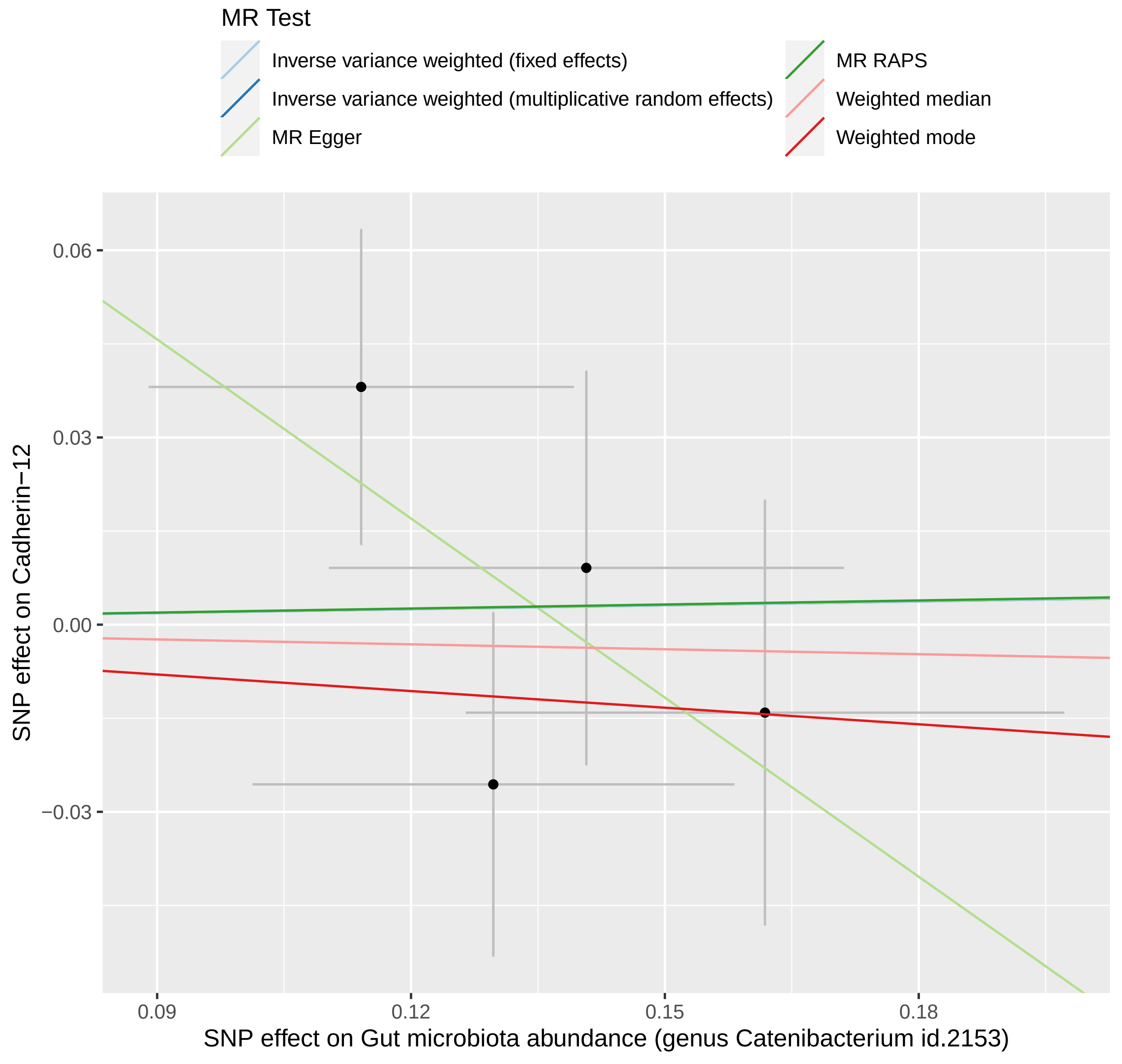

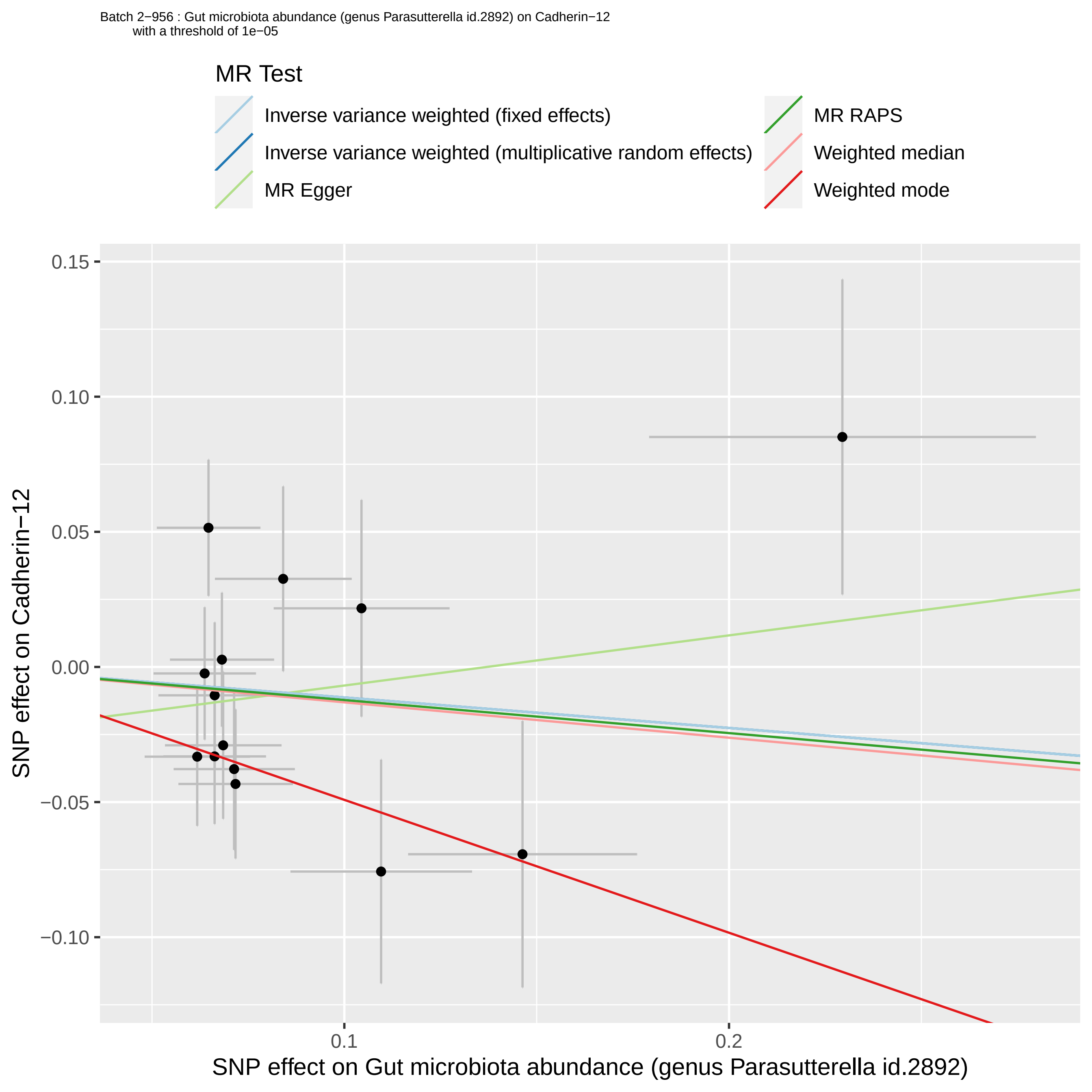

Batch 2−956 : Gut microbiota abundance (genus Parasutterella id.2892) on Cadherin−12
with a threshold of 1e−05
MR Test
Inverse variance weighted (fixed effects)
Inverse variance weighted (multiplicative random effects)
MR Egger
MR RAPS
Weighted median
Weighted mode
0.15
0.10
0.05
0.00
−0.05
−0.10
0.1
0.2
SNP effect on Cadherin−12
SNP effect on Gut microbiota abundance (genus Parasutterella id.2892)

Batch 2−957 : Gut microbiota abundance (genus Catenibacterium id.2153) on Cadherin−12
with a threshold of 1e−05

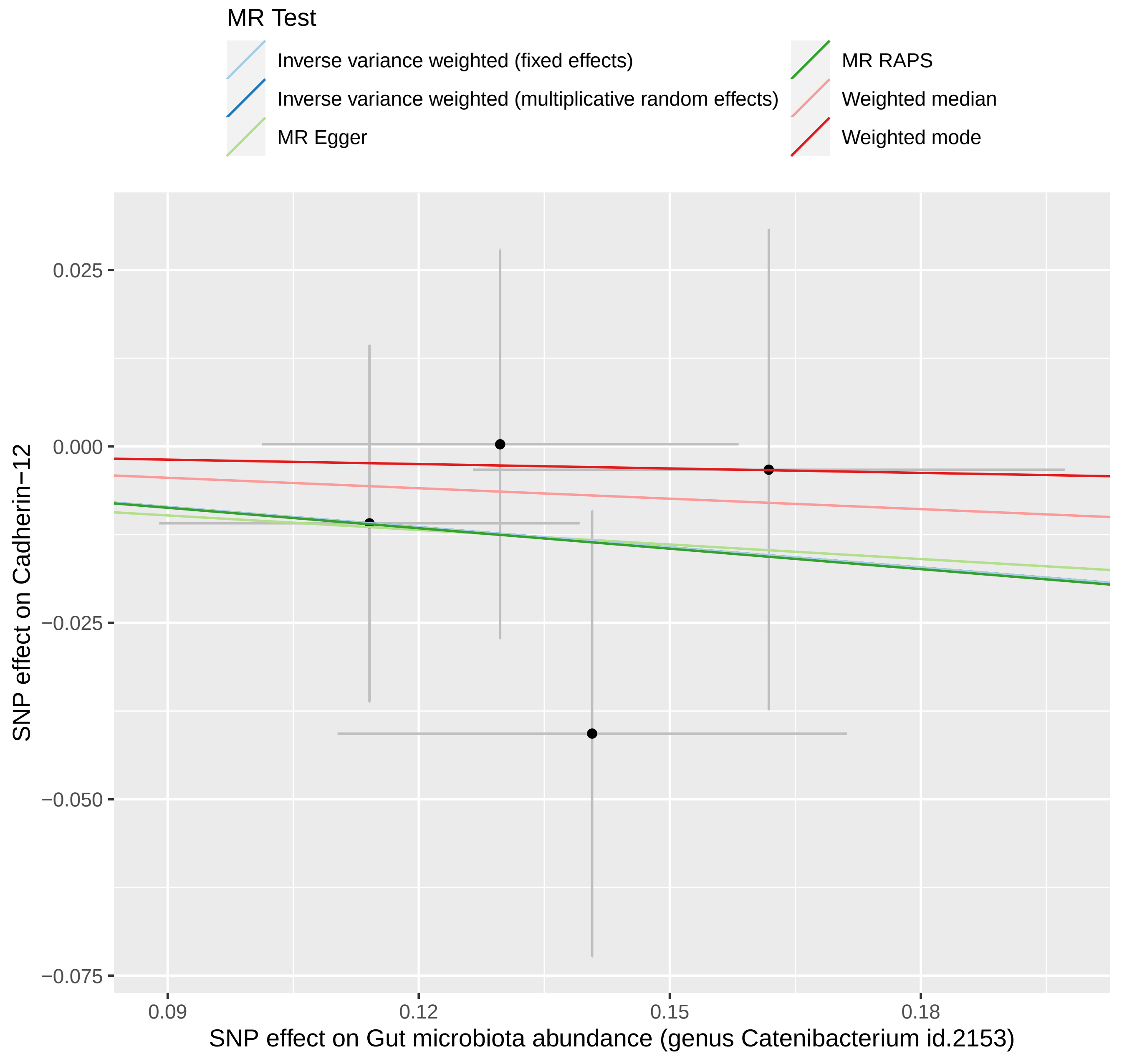

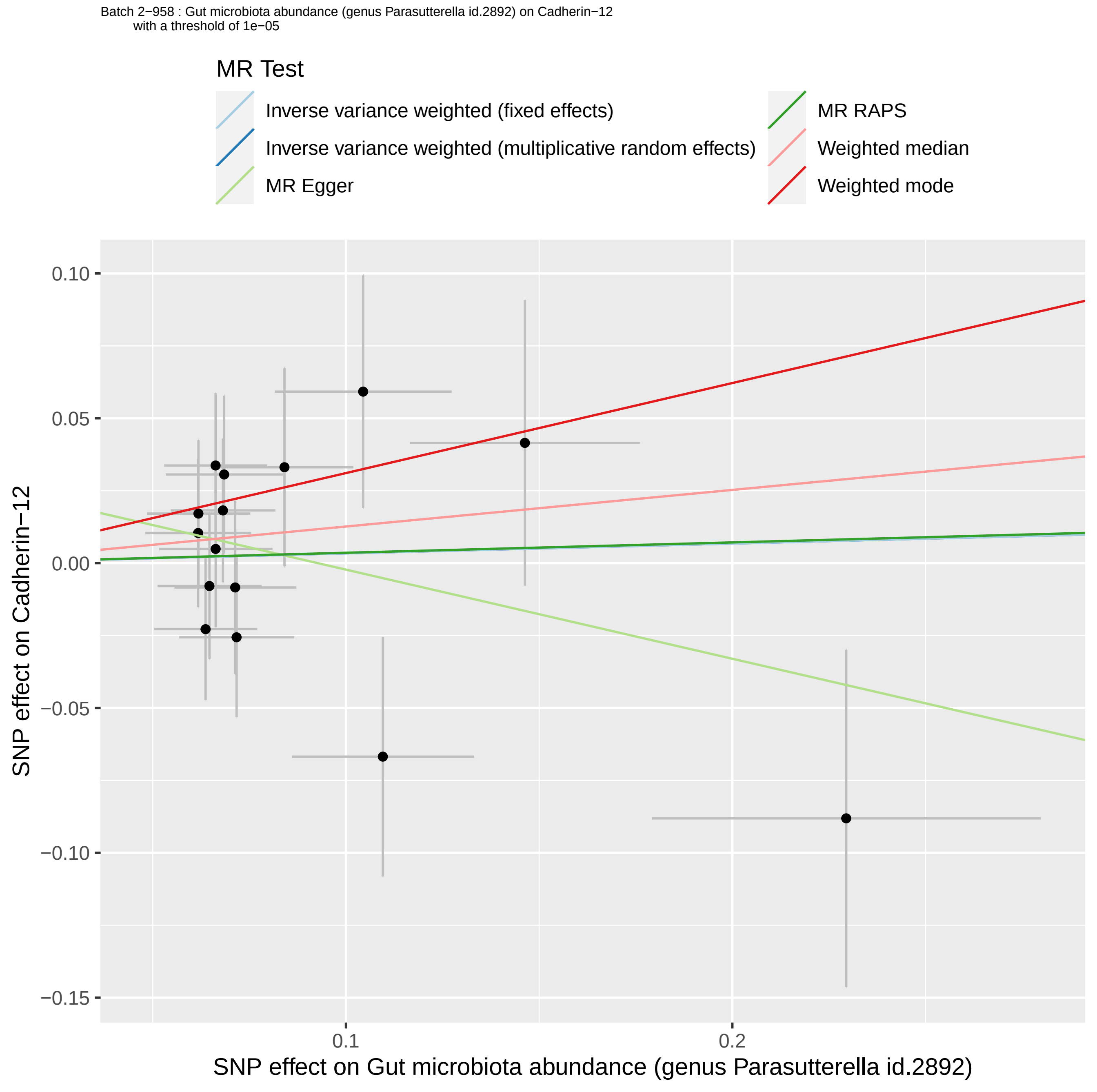
Batch 2−958 : Gut microbiota abundance (genus Parasutterella id.2892) on Cadherin−12
with a threshold of 1e−05
MR Test
Inverse variance weighted (fixed effects)
Inverse variance weighted (multiplicative random effects)
MR Egger
MR RAPS
Weighted median
Weighted mode
0.10
0.05
0.00
−0.05
−0.10
−0.15
0.1
0.2
SNP effect on Cadherin−12
SNP effect on Gut microbiota abundance (genus Parasutterella id.2892)

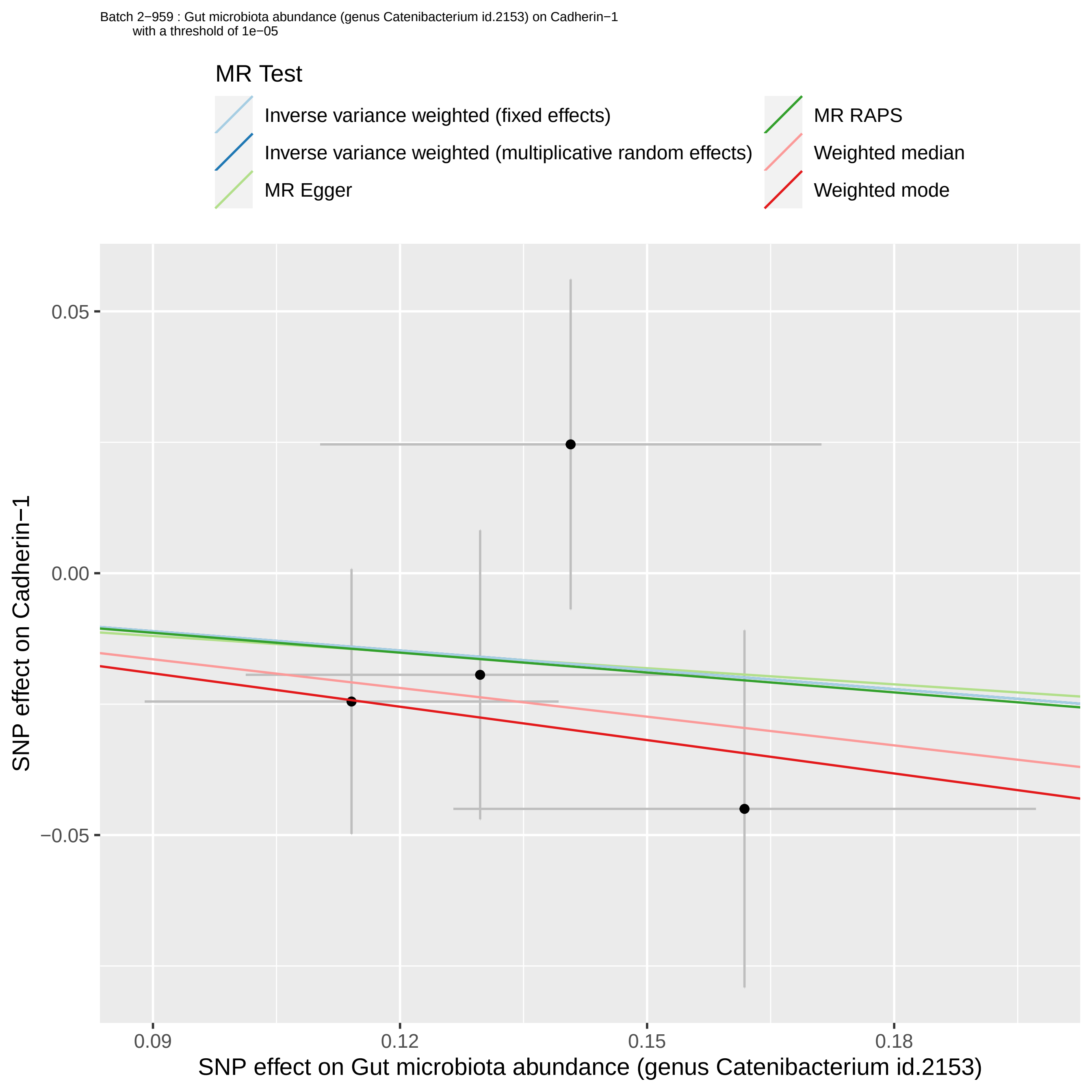

Batch 2−959 : Gut microbiota abundance (genus Catenibacterium id.2153) on Cadherin−1
with a threshold of 1e−05
MR Test
Inverse variance weighted (fixed effects)
Inverse variance weighted (multiplicative random effects)
MR Egger
MR RAPS
Weighted median
Weighted mode
0.05
0.00
−0.05
0.09
0.12
0.15
0.18
SNP effect on Cadherin−1
SNP effect on Gut microbiota abundance (genus Catenibacterium id.2153)

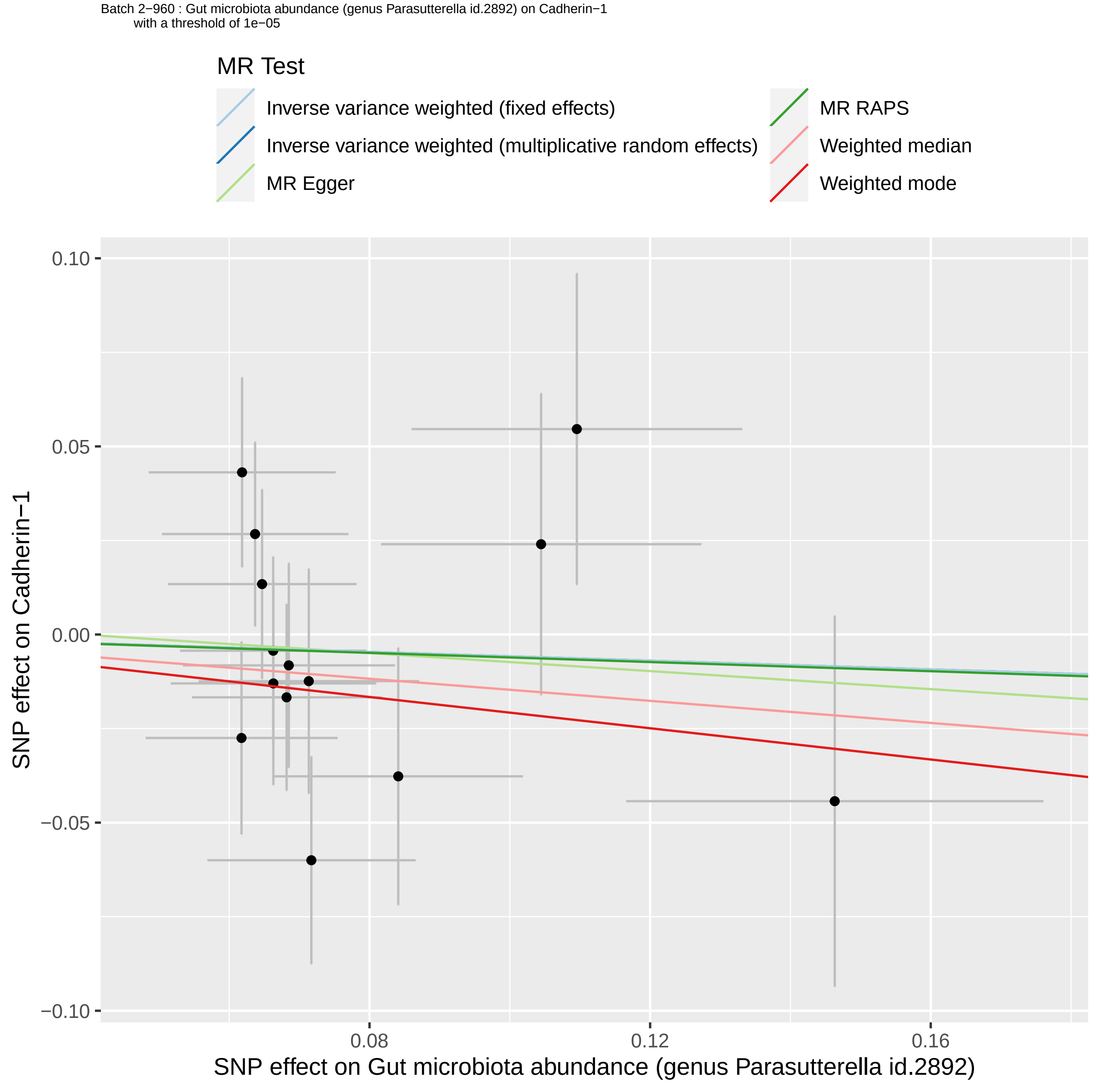

Batch 2−960 : Gut microbiota abundance (genus Parasutterella id.2892) on Cadherin−1
with a threshold of 1e−05
MR Test
Inverse variance weighted (fixed effects)
Inverse variance weighted (multiplicative random effects)
MR Egger
MR RAPS
Weighted median
Weighted mode
0.10
0.05
0.00
−0.05
−0.10
0.08
0.12
0.16
SNP effect on Cadherin−1
SNP effect on Gut microbiota abundance (genus Parasutterella id.2892)

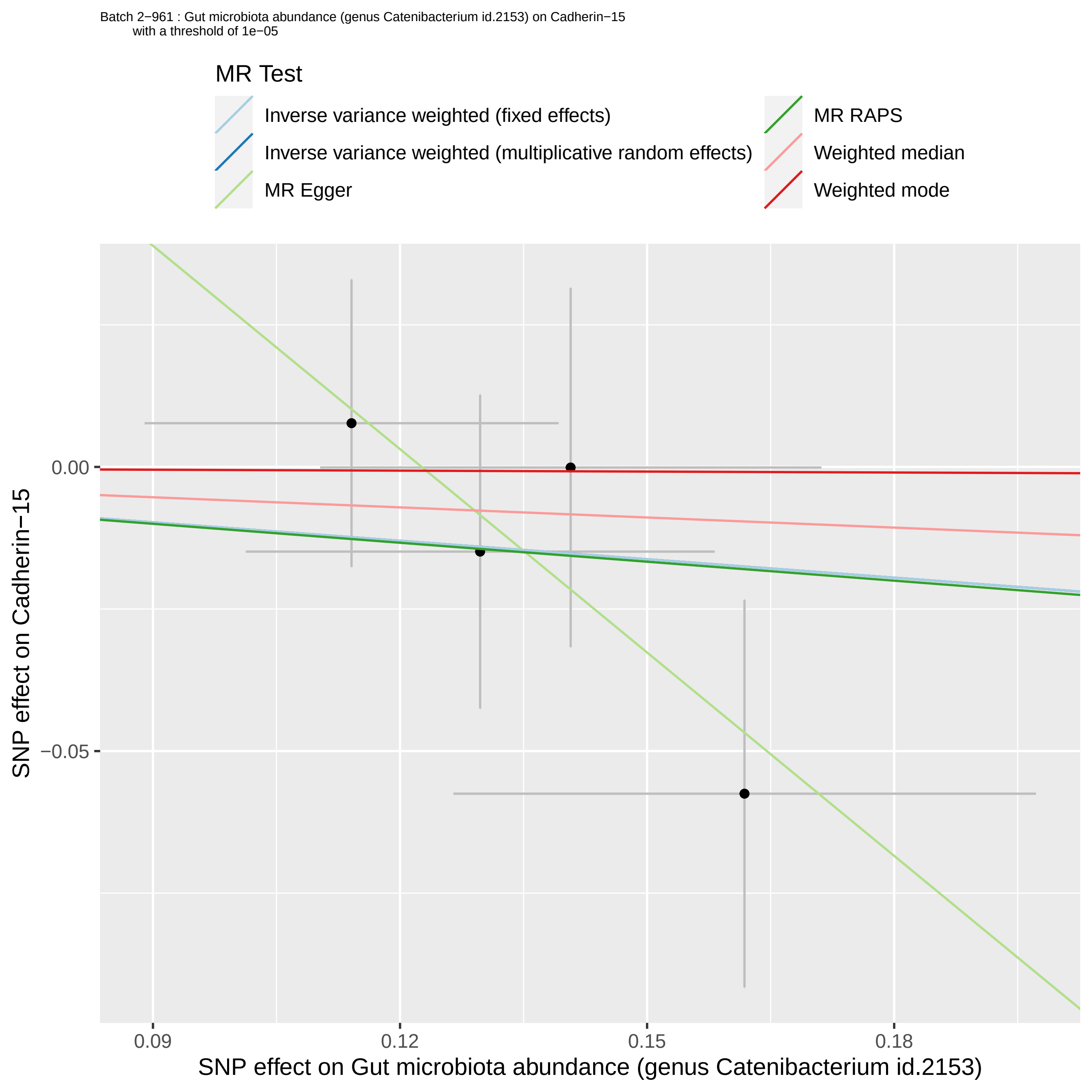
Batch 2−961 : Gut microbiota abundance (genus Catenibacterium id.2153) on Cadherin−15
with a threshold of 1e−05
MR Test
Inverse variance weighted (fixed effects)
Inverse variance weighted (multiplicative random effects)
MR Egger
MR RAPS
Weighted median
Weighted mode
0.00
−0.05
SNP effect on Cadherin−15
0.09
0.12
0.15
0.18
SNP effect on Gut microbiota abundance (genus Catenibacterium id.2153)

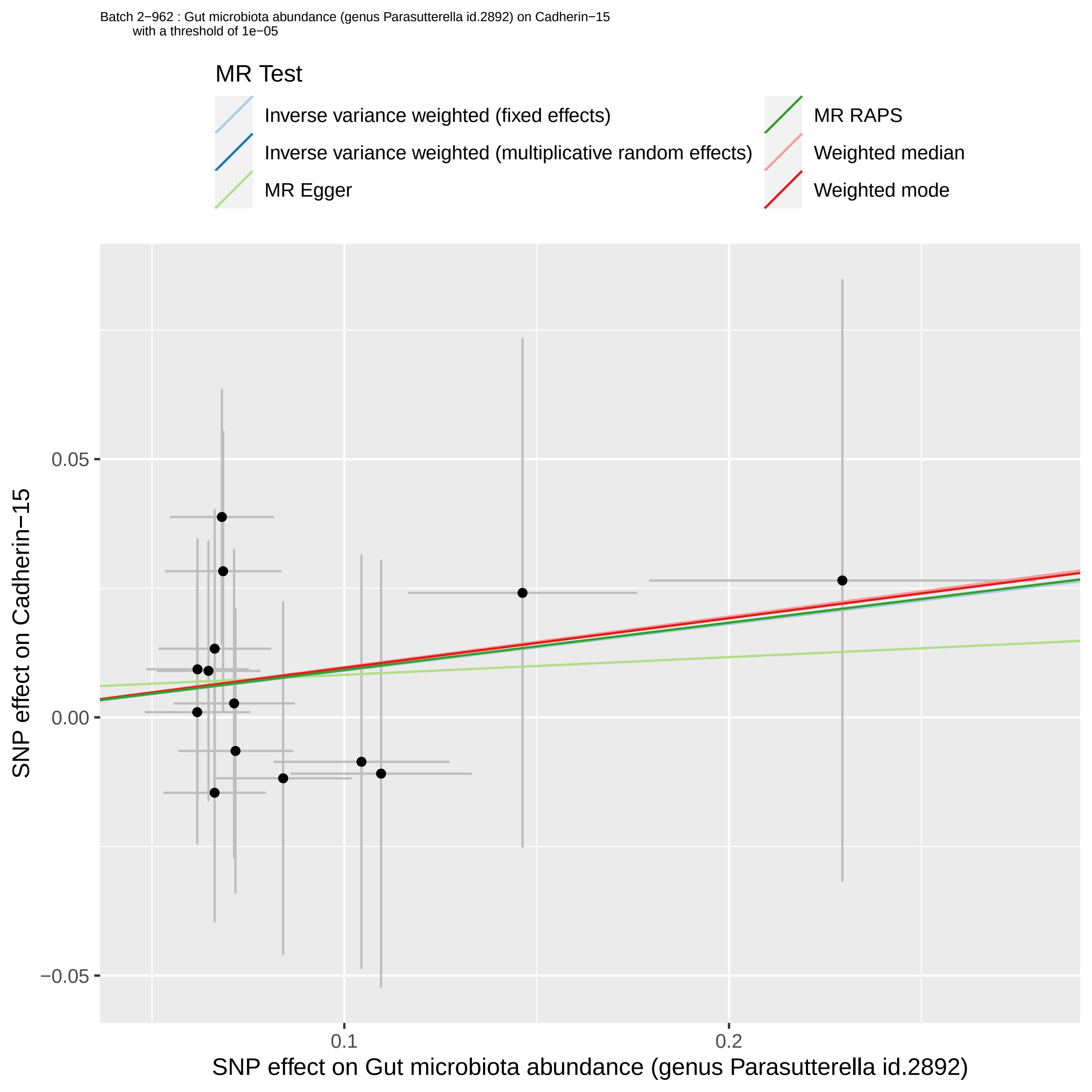
Batch 2−962 : Gut microbiota abundance (genus Parasutterella id.2892) on Cadherin−15
with a threshold of 1e−05
MR Test
Inverse variance weighted (fixed effects)
Inverse variance weighted (multiplicative random effects)
MR Egger
MR RAPS
Weighted median
Weighted mode
SNP effect on Cadherin−15
0.05
0.00
−0.05
0.1
0.2
SNP effect on Gut microbiota abundance (genus Parasutterella id.2892)

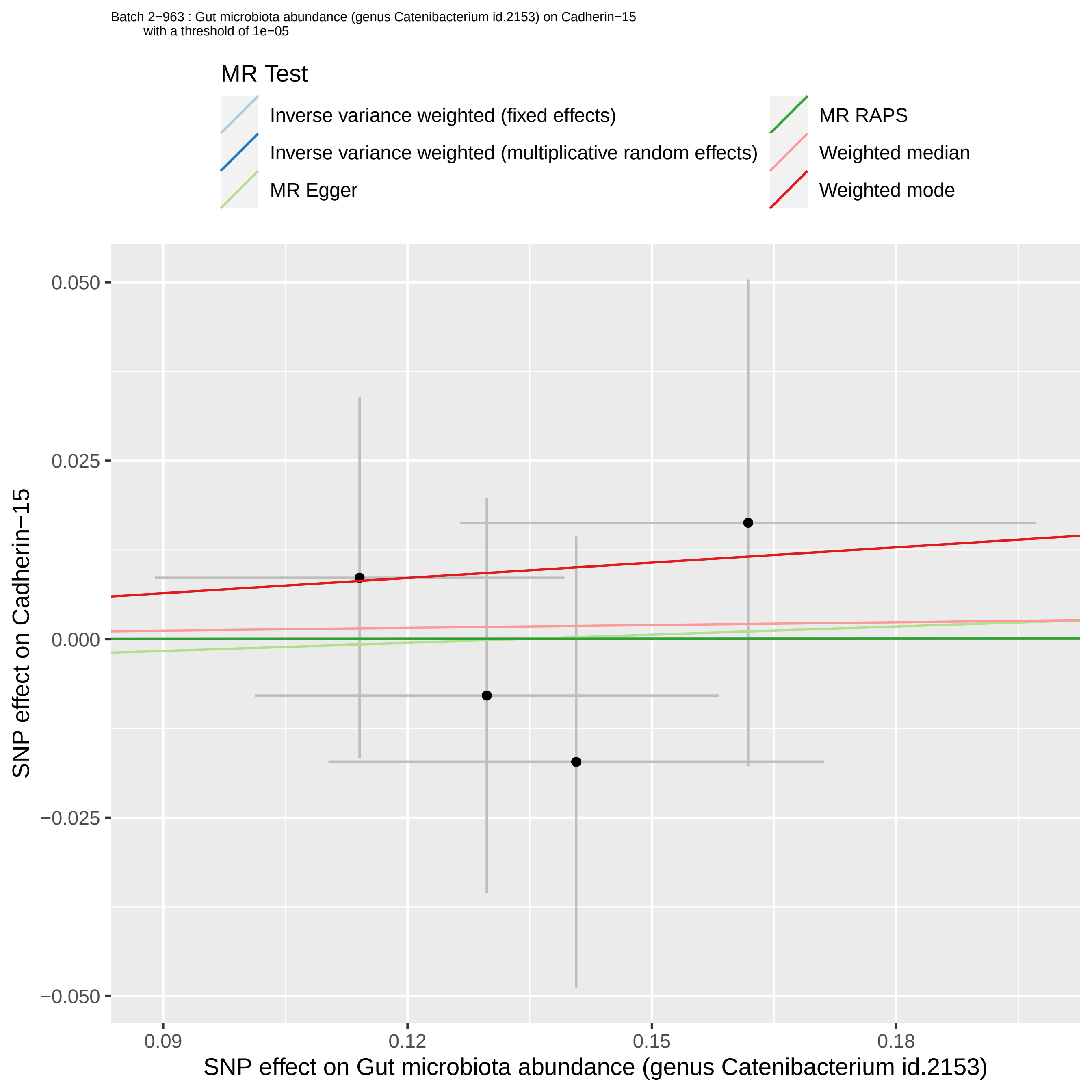
Batch 2−963 : Gut microbiota abundance (genus Catenibacterium id.2153) on Cadherin−15
with a threshold of 1e−05
MR Test
Inverse variance weighted (fixed effects)
Inverse variance weighted (multiplicative random effects)
MR Egger
MR RAPS
Weighted median
Weighted mode
0.050
0.025
0.000
−0.025
−0.050
SNP effect on Cadherin−15
0.09
0.12
0.15
0.18
SNP effect on Gut microbiota abundance (genus Catenibacterium id.2153)

Batch 2−964 : Gut microbiota abundance (genus Parasutterella id.2892) on Cadherin−15
with a threshold of 1e−05

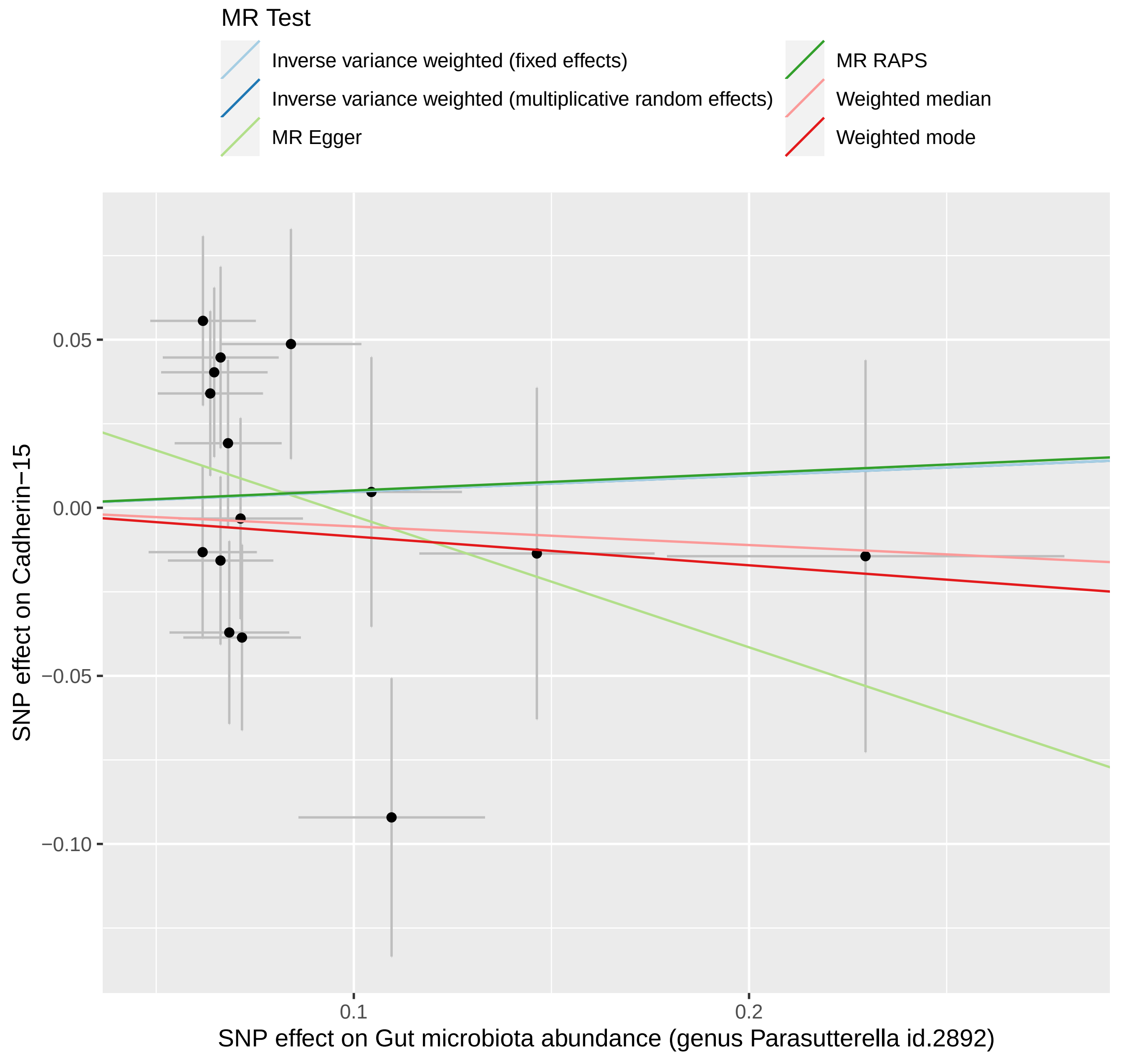

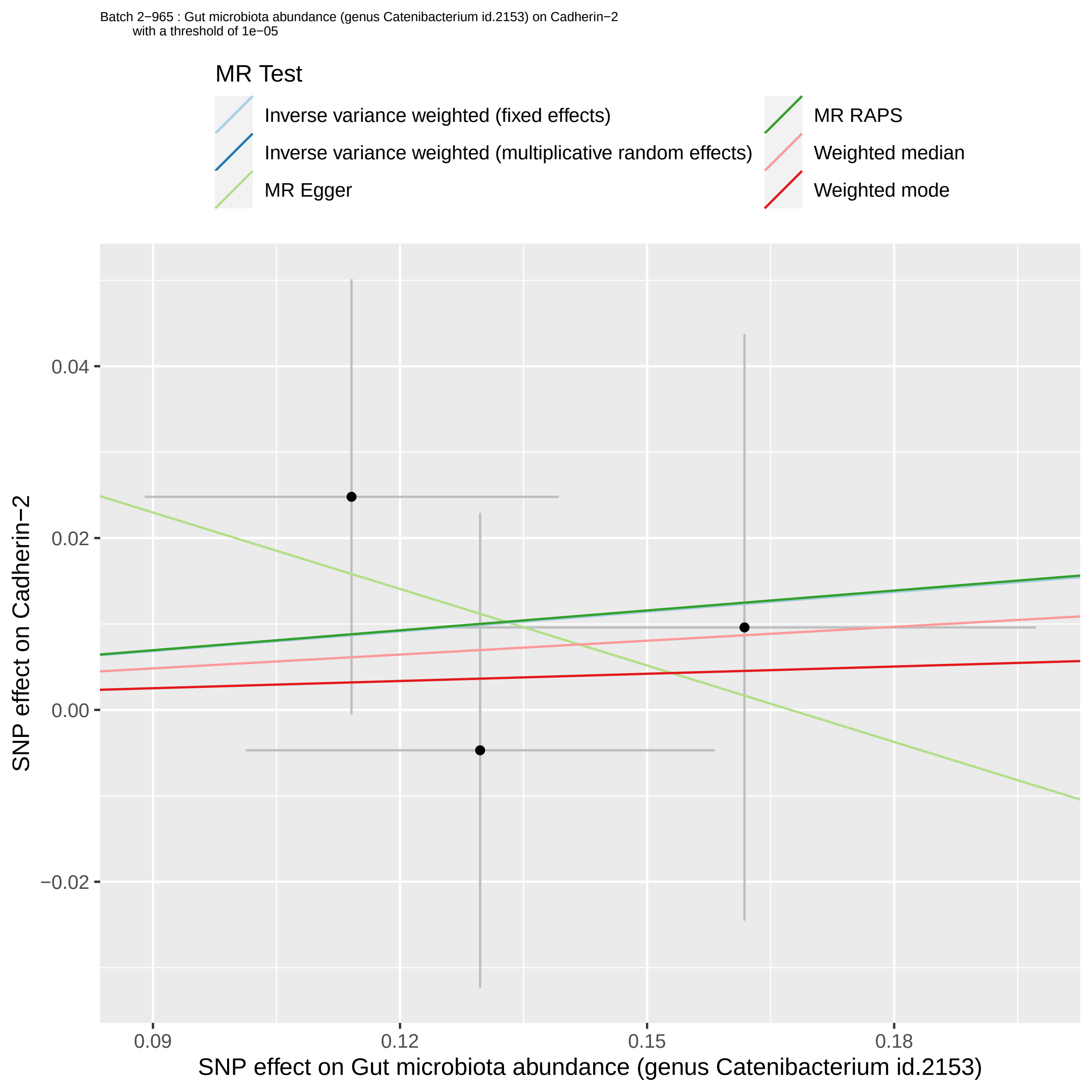
Batch 2−965 : Gut microbiota abundance (genus Catenibacterium id.2153) on Cadherin−2
with a threshold of 1e−05
MR Test
Inverse variance weighted (fixed effects)
Inverse variance weighted (multiplicative random effects)
MR Egger
MR RAPS
Weighted median
Weighted mode
0.04
0.02
0.00
−0.02
0.09
0.12
0.15
0.18
SNP effect on Cadherin−2
SNP effect on Gut microbiota abundance (genus Catenibacterium id.2153)

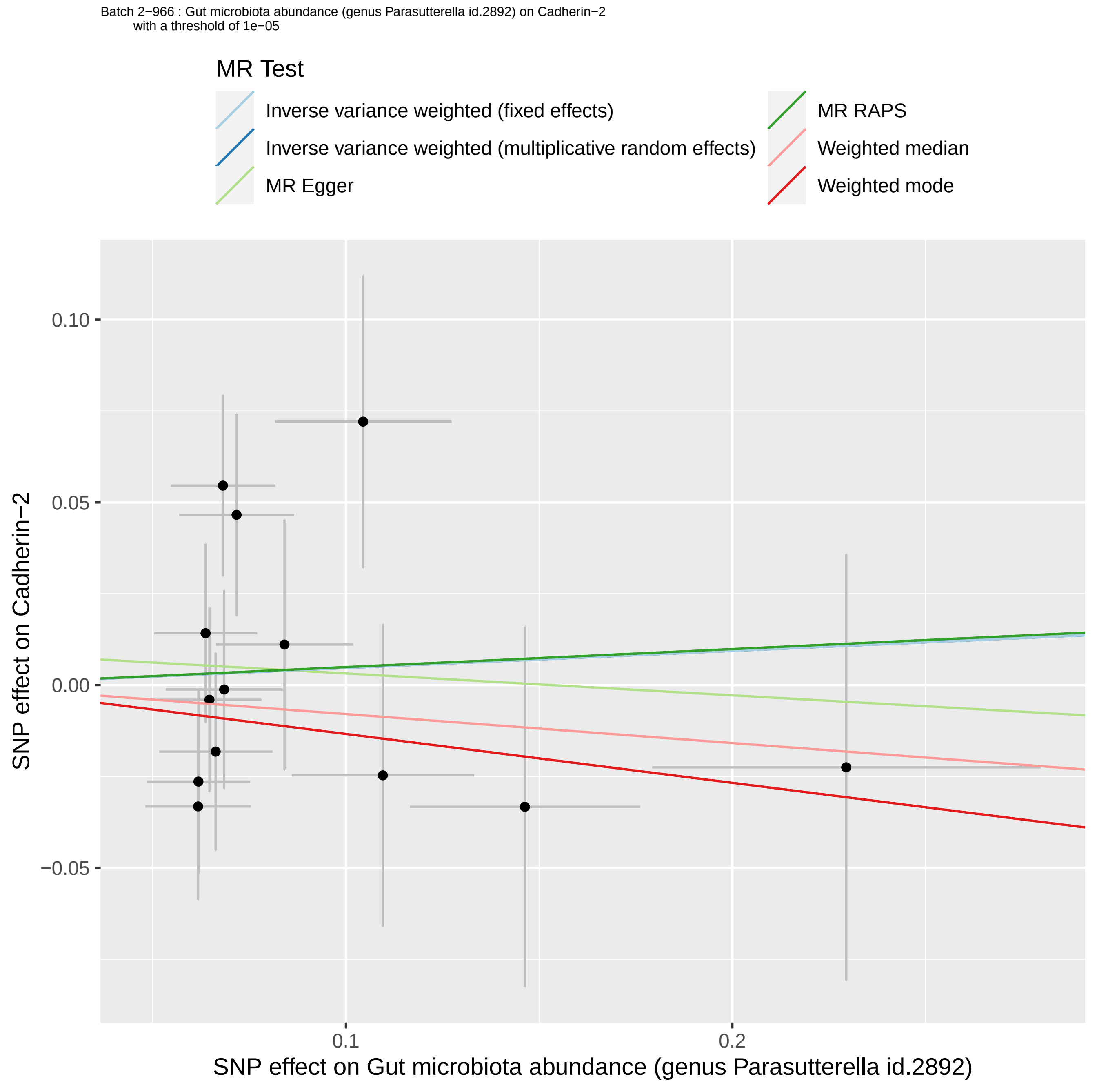
Batch 2−966 : Gut microbiota abundance (genus Parasutterella id.2892) on Cadherin−2
with a threshold of 1e−05
MR Test
Inverse variance weighted (fixed effects)
Inverse variance weighted (multiplicative random effects)
MR Egger
MR RAPS
Weighted median
Weighted mode
0.10
0.05
0.00
−0.05
0.1
0.2
SNP effect on Cadherin−2
SNP effect on Gut microbiota abundance (genus Parasutterella id.2892)

Batch 2−967 : Gut microbiota abundance (genus Catenibacterium id.2153) on Cadherin−3
with a threshold of 1e−05

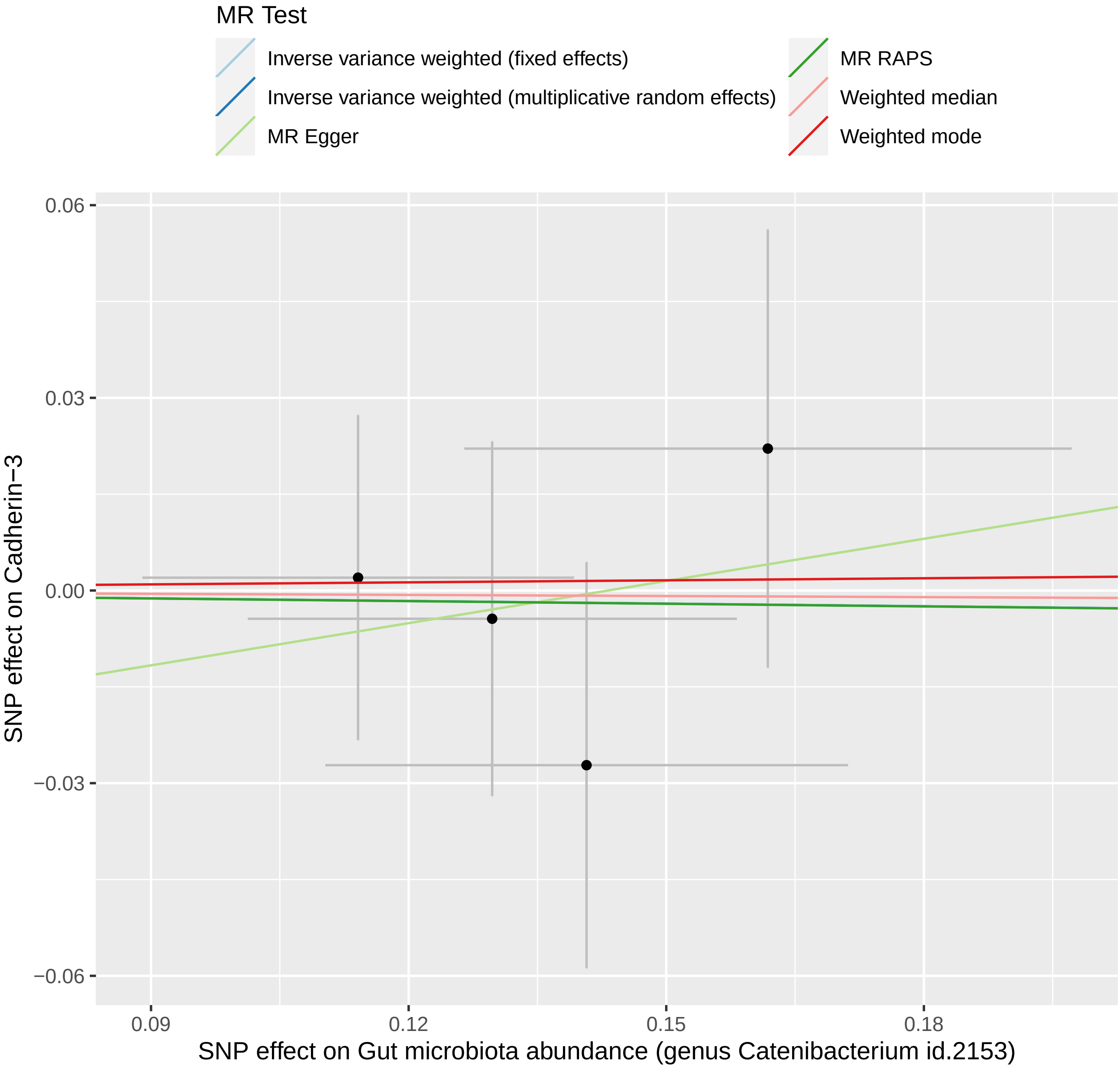

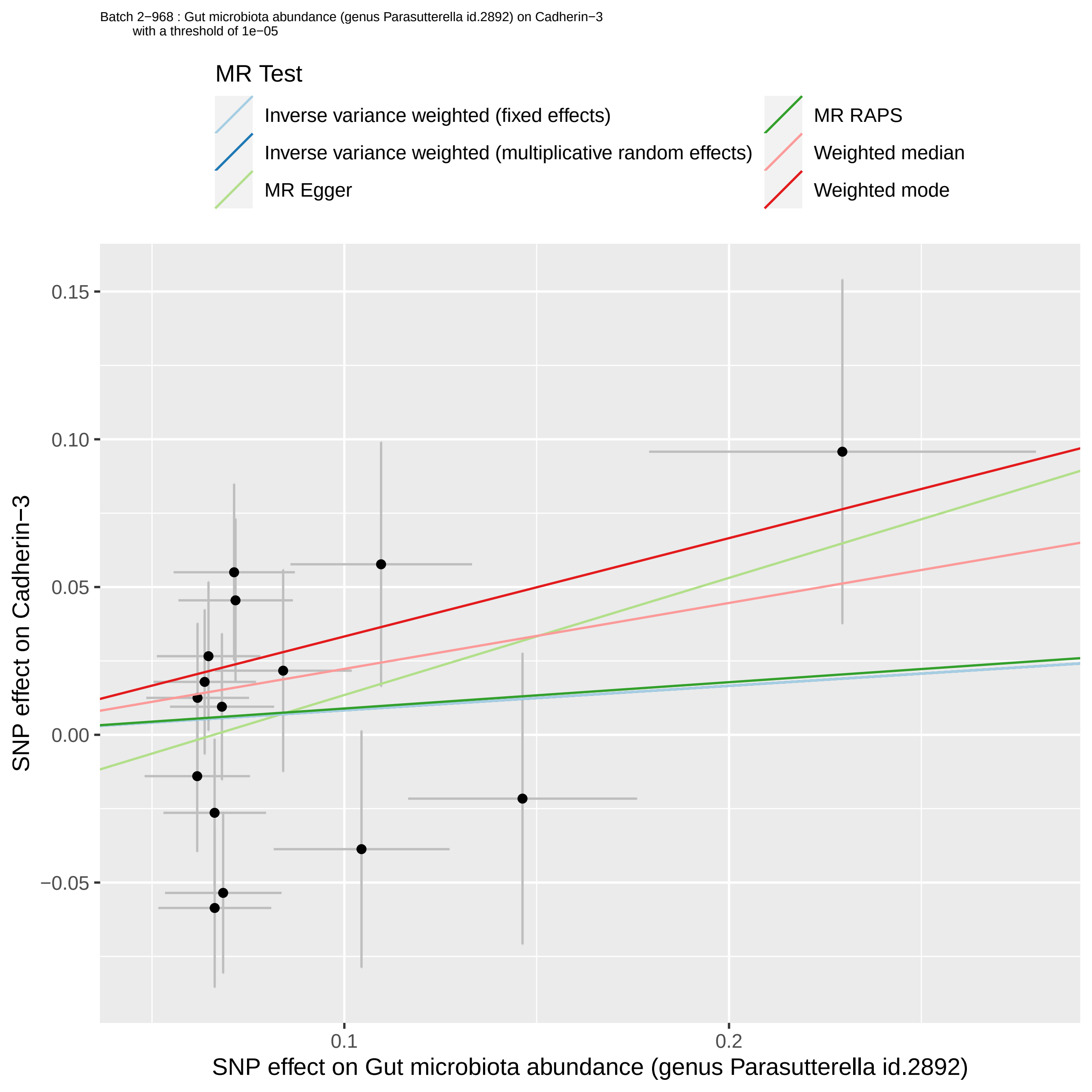
Batch 2−968 : Gut microbiota abundance (genus Parasutterella id.2892) on Cadherin−3
with a threshold of 1e−05
MR Test
Inverse variance weighted (fixed effects)
Inverse variance weighted (multiplicative random effects)
MR Egger
MR RAPS
Weighted median
Weighted mode
0.15
0.10
0.05
0.00
−0.05
0.1
0.2
SNP effect on Cadherin−3
SNP effect on Gut microbiota abundance (genus Parasutterella id.2892)

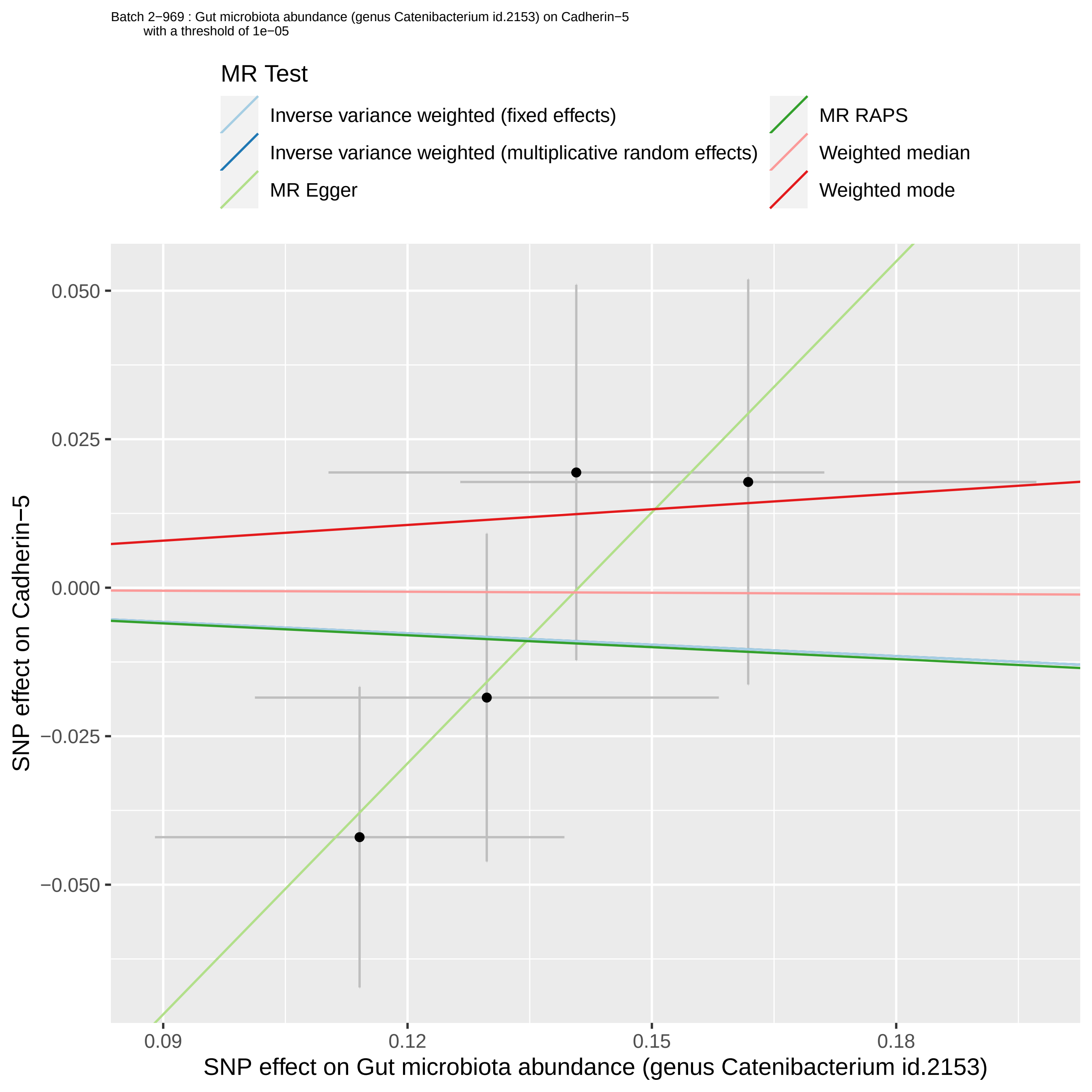
Batch 2−969 : Gut microbiota abundance (genus Catenibacterium id.2153) on Cadherin−5
with a threshold of 1e−05
MR Test
Inverse variance weighted (fixed effects)
Inverse variance weighted (multiplicative random effects)
MR Egger
MR RAPS
Weighted median
Weighted mode
0.050
0.025
0.000
−0.025
−0.050
SNP effect on Cadherin−5
0.09
0.12
0.15
0.18
SNP effect on Gut microbiota abundance (genus Catenibacterium id.2153)

Batch 2−970 : Gut microbiota abundance (genus Parasutterella id.2892) on Cadherin−5
with a threshold of 1e−05

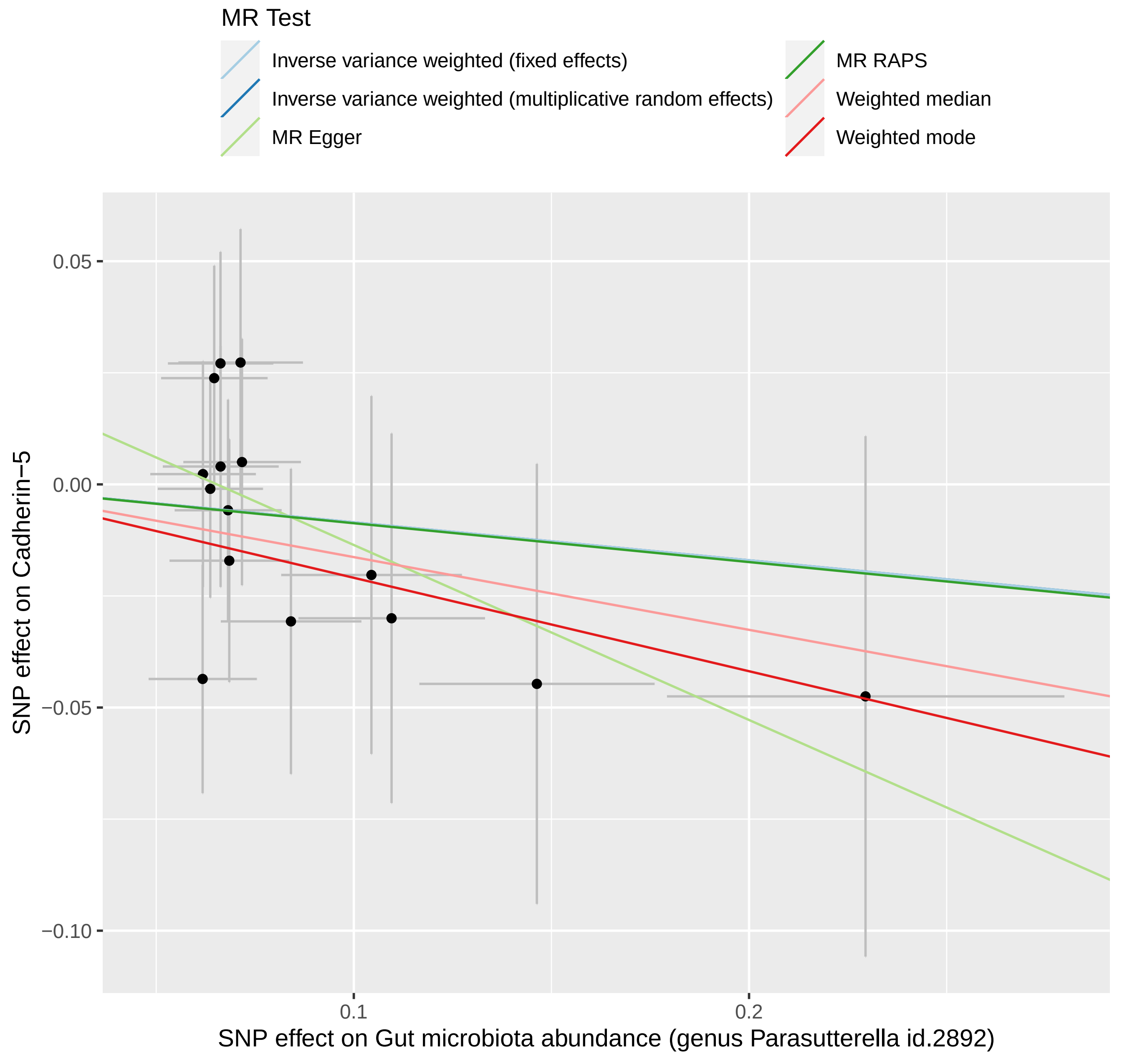

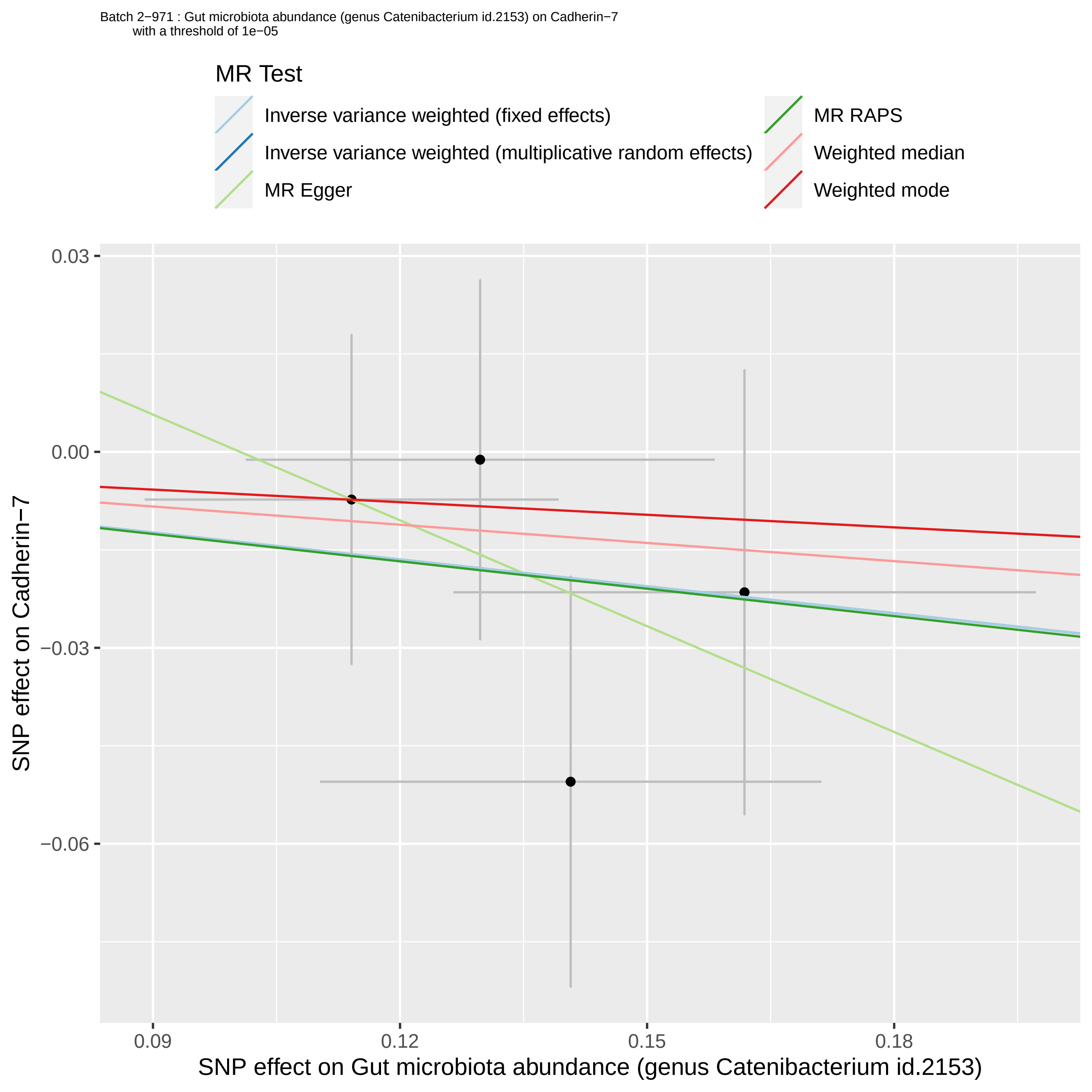

Batch 2−971 : Gut microbiota abundance (genus Catenibacterium id.2153) on Cadherin−7
with a threshold of 1e−05
MR Test
Inverse variance weighted (fixed effects)
Inverse variance weighted (multiplicative random effects)
MR Egger
MR RAPS
Weighted median
Weighted mode
0.03
0.00
−0.03
−0.06
0.09
0.12
0.15
0.18
SNP effect on Cadherin−7
SNP effect on Gut microbiota abundance (genus Catenibacterium id.2153)

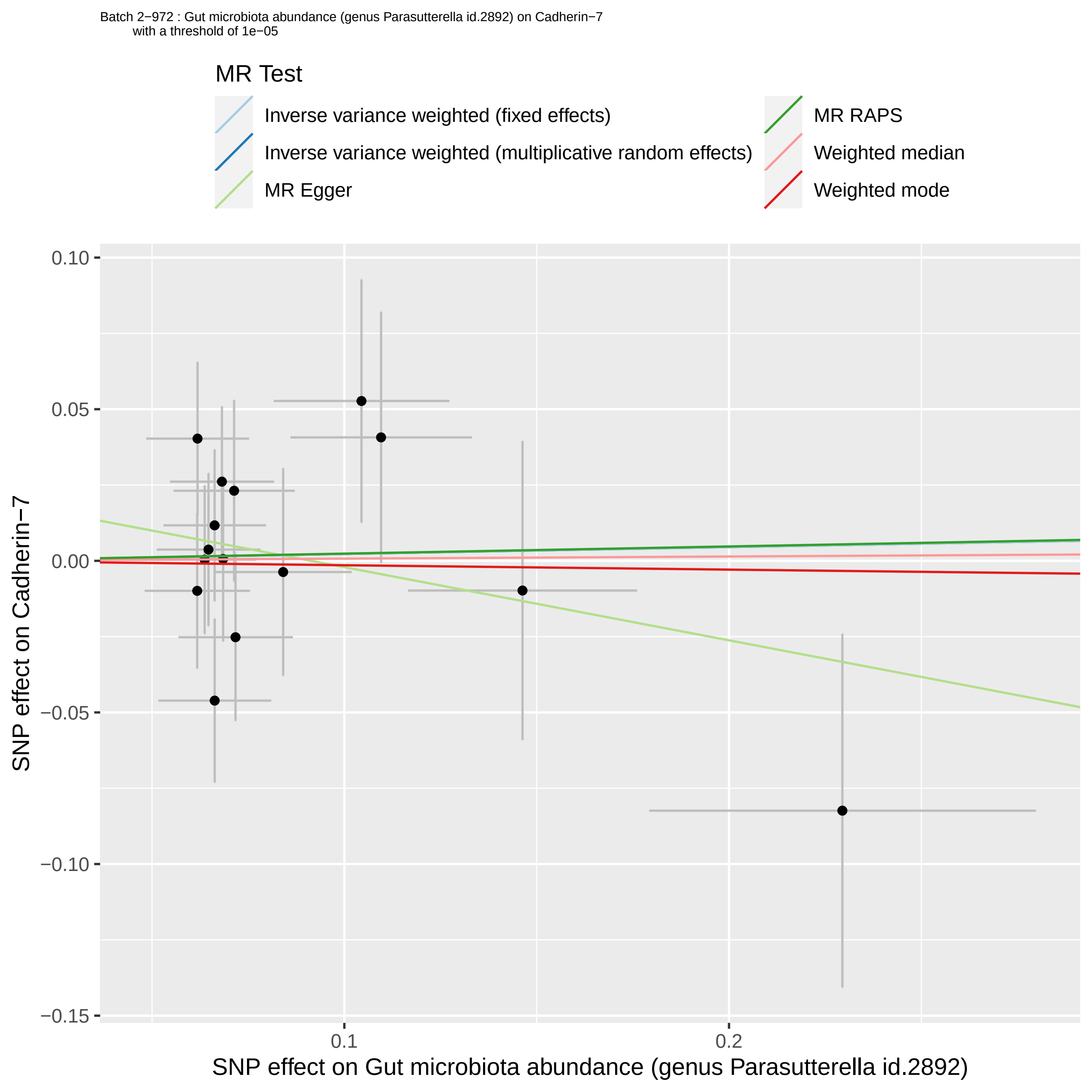

Batch 2−972 : Gut microbiota abundance (genus Parasutterella id.2892) on Cadherin−7
with a threshold of 1e−05
MR Test
Inverse variance weighted (fixed effects)
Inverse variance weighted (multiplicative random effects)
MR Egger
MR RAPS
Weighted median
Weighted mode
0.10
0.05
0.00
−0.05
−0.10
−0.15
0.1
0.2
SNP effect on Cadherin−7
SNP effect on Gut microbiota abundance (genus Parasutterella id.2892)

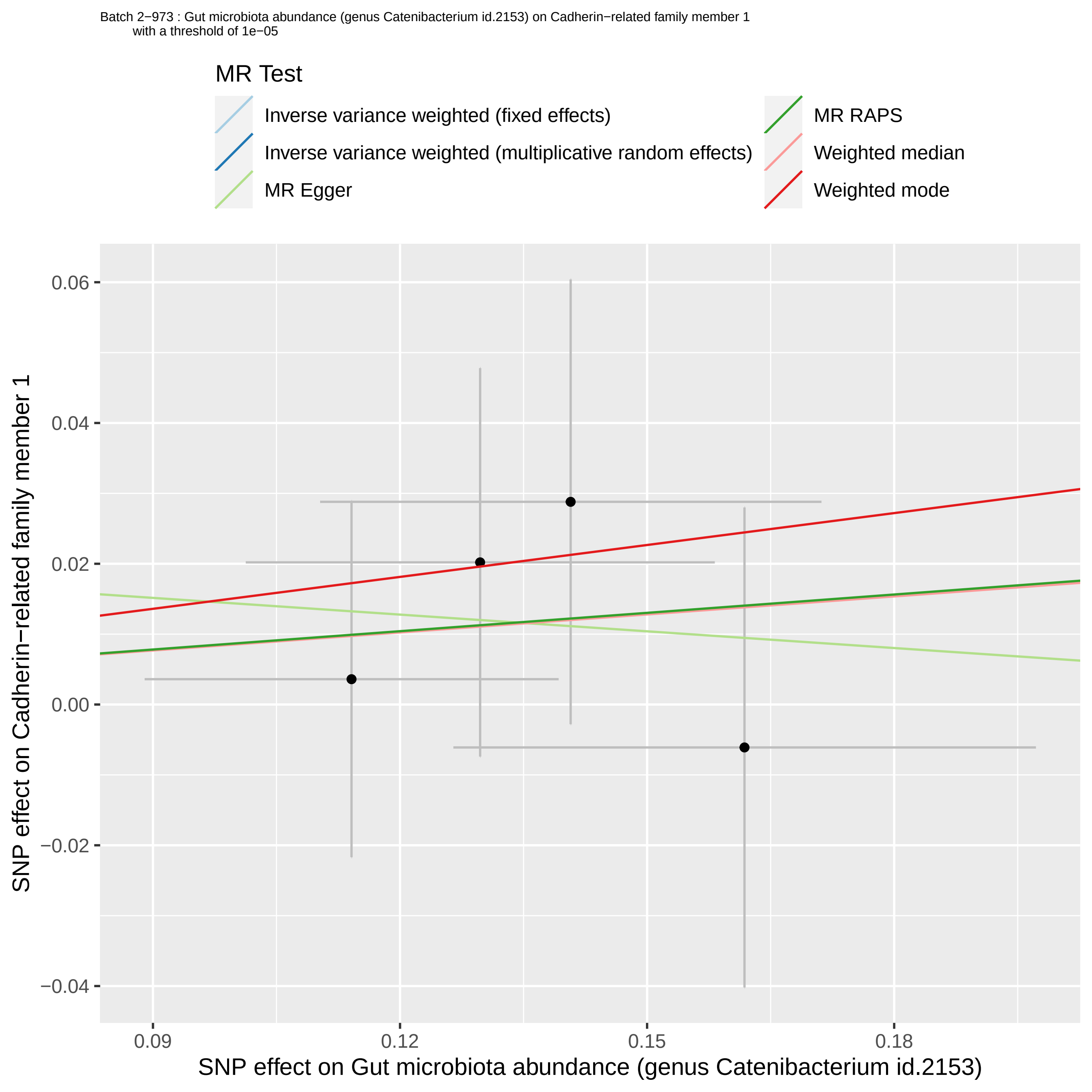

Batch 2−973 : Gut microbiota abundance (genus Catenibacterium id.2153) on Cadherin−related family member 1
with a threshold of 1e−05
MR Test
Inverse variance weighted (fixed effects)
Inverse variance weighted (multiplicative random effects)
MR Egger
MR RAPS
Weighted median
Weighted mode
0.06
0.04
0.02
0.00
−0.02
−0.04
0.09
0.12
0.15
0.18
SNP effect on Cadherin−related family member 1
SNP effect on Gut microbiota abundance (genus Catenibacterium id.2153)

Batch 2−974 : Gut microbiota abundance (genus Parasutterella id.2892) on Cadherin−related family member 1
with a threshold of 1e−05

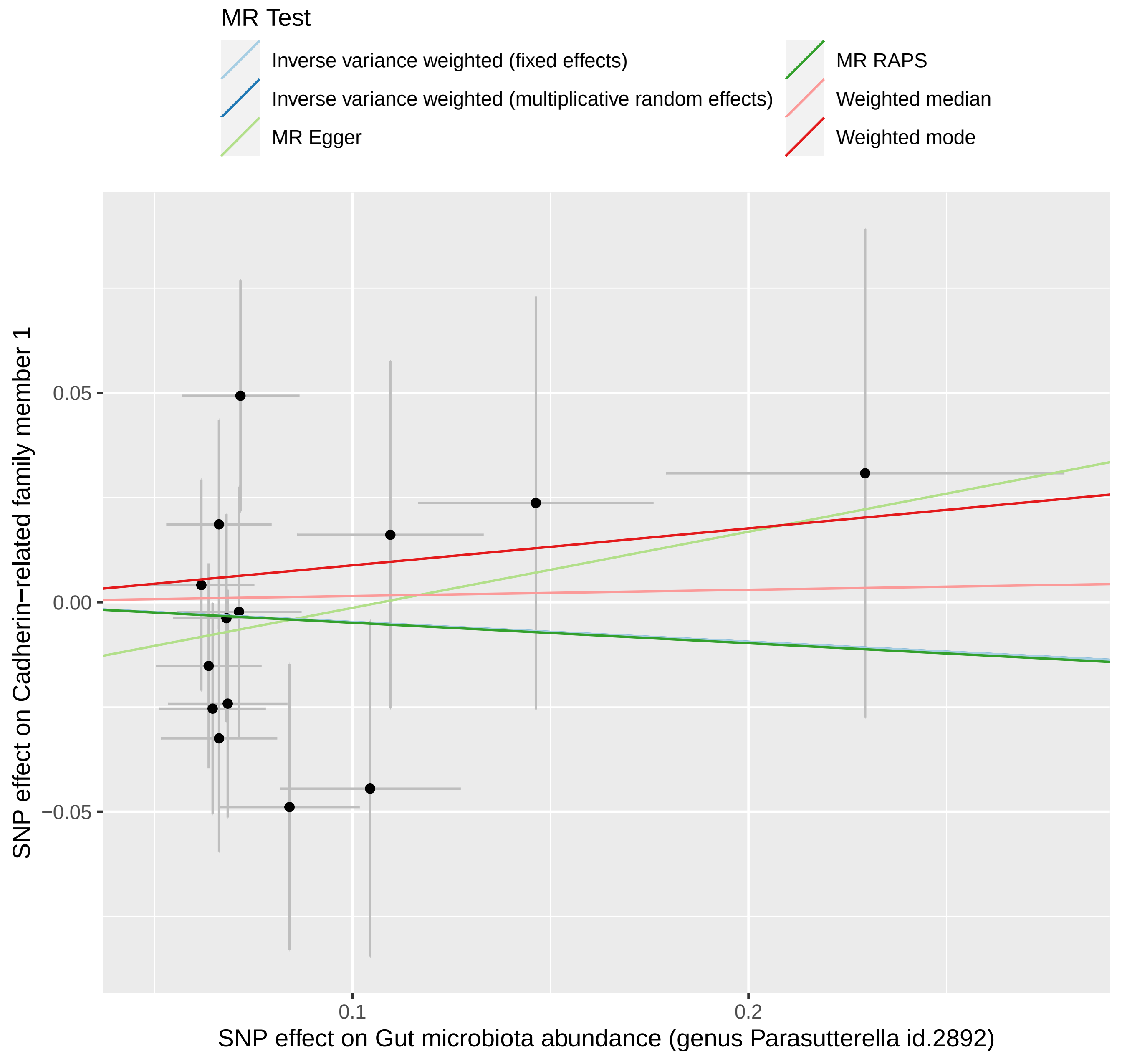

Batch 2−975 : Gut microbiota abundance (genus Catenibacterium id.2153) on Cadherin−related family member 3
with a threshold of 1e−05

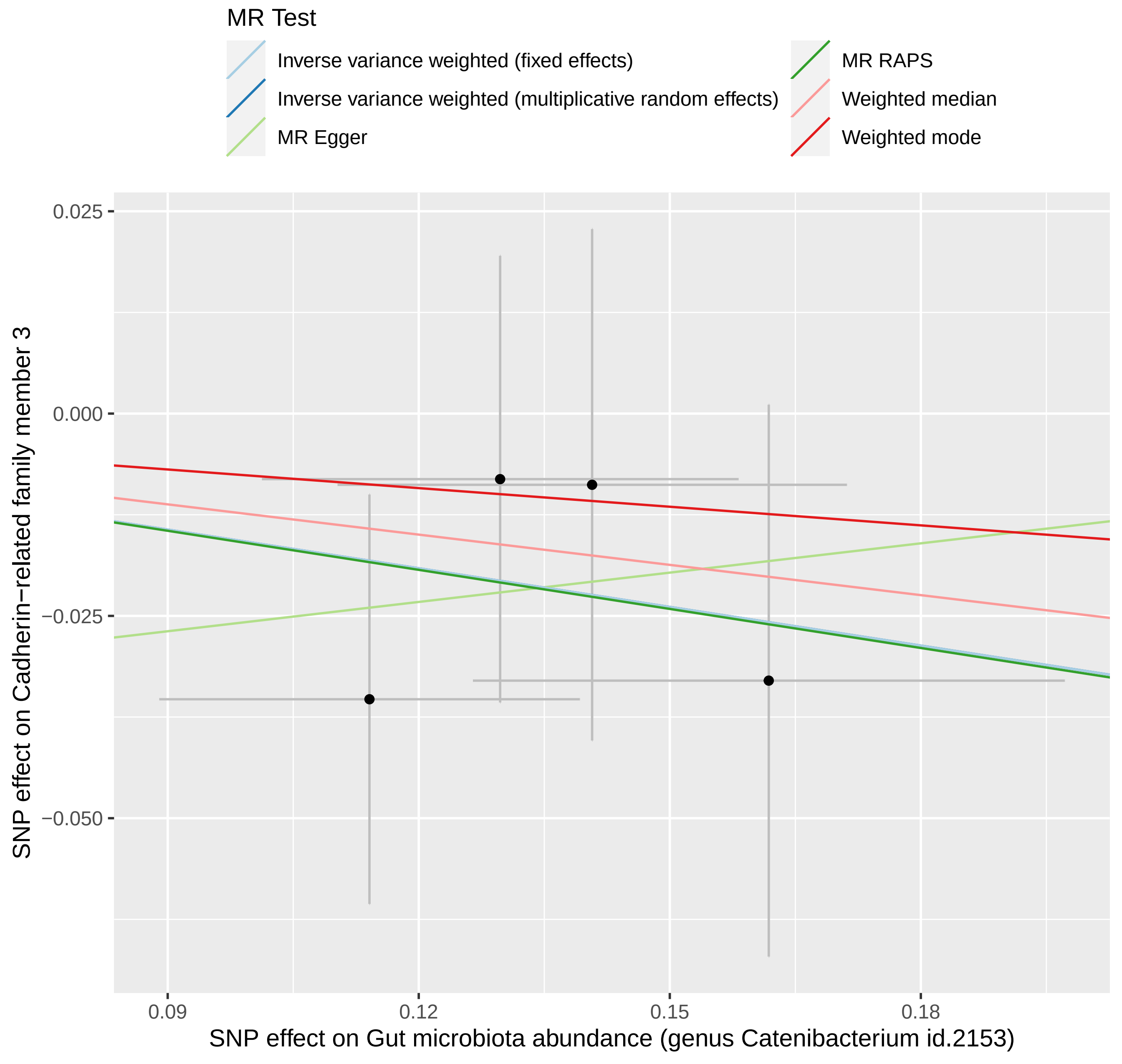

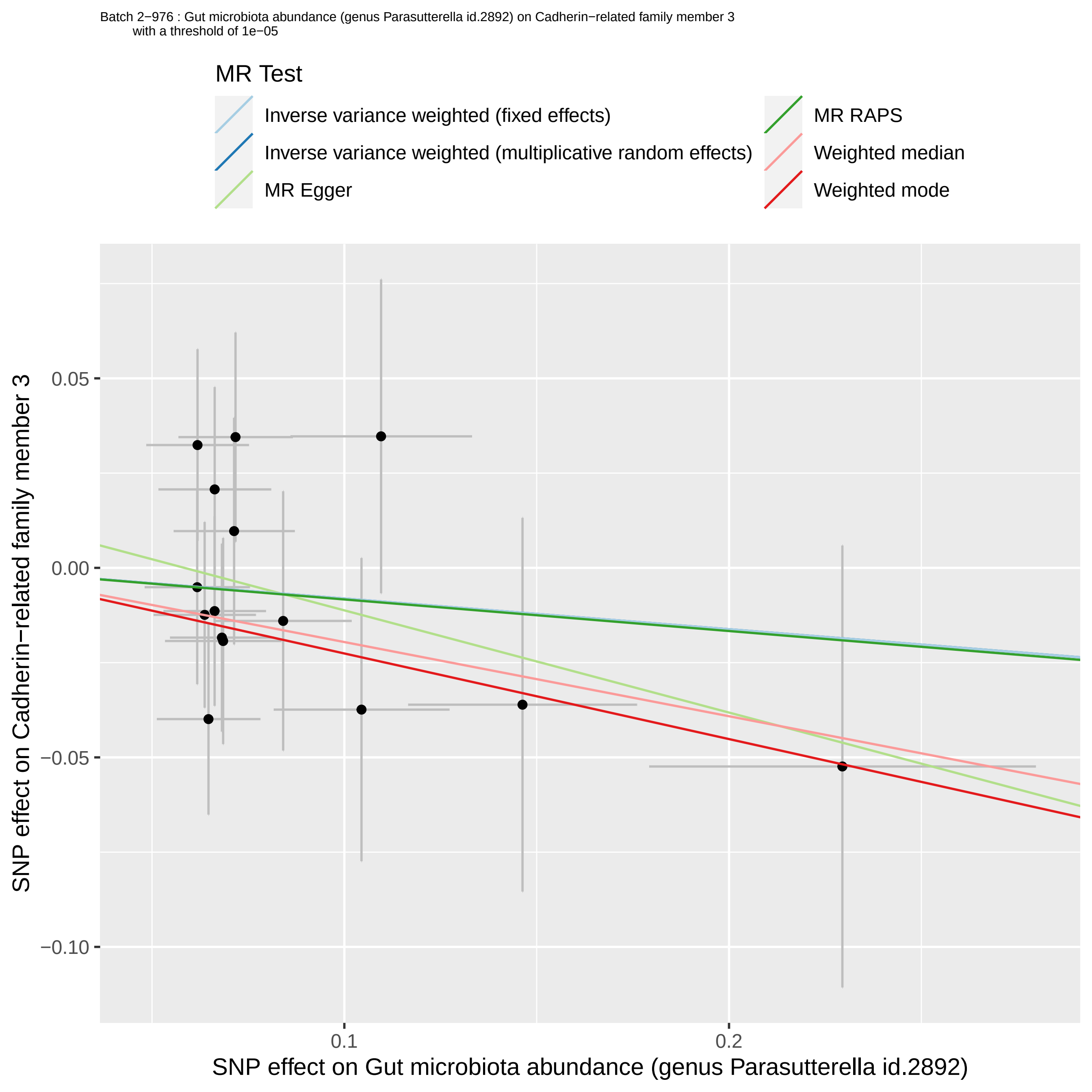

Batch 2−976 : Gut microbiota abundance (genus Parasutterella id.2892) on Cadherin−related family member 3
with a threshold of 1e−05
MR Test
Inverse variance weighted (fixed effects)
Inverse variance weighted (multiplicative random effects)
MR Egger
MR RAPS
Weighted median
Weighted mode
SNP effect on Cadherin−related family member 3
0.05
0.00
−0.05
−0.10
0.1
0.2
SNP effect on Gut microbiota abundance (genus Parasutterella id.2892)

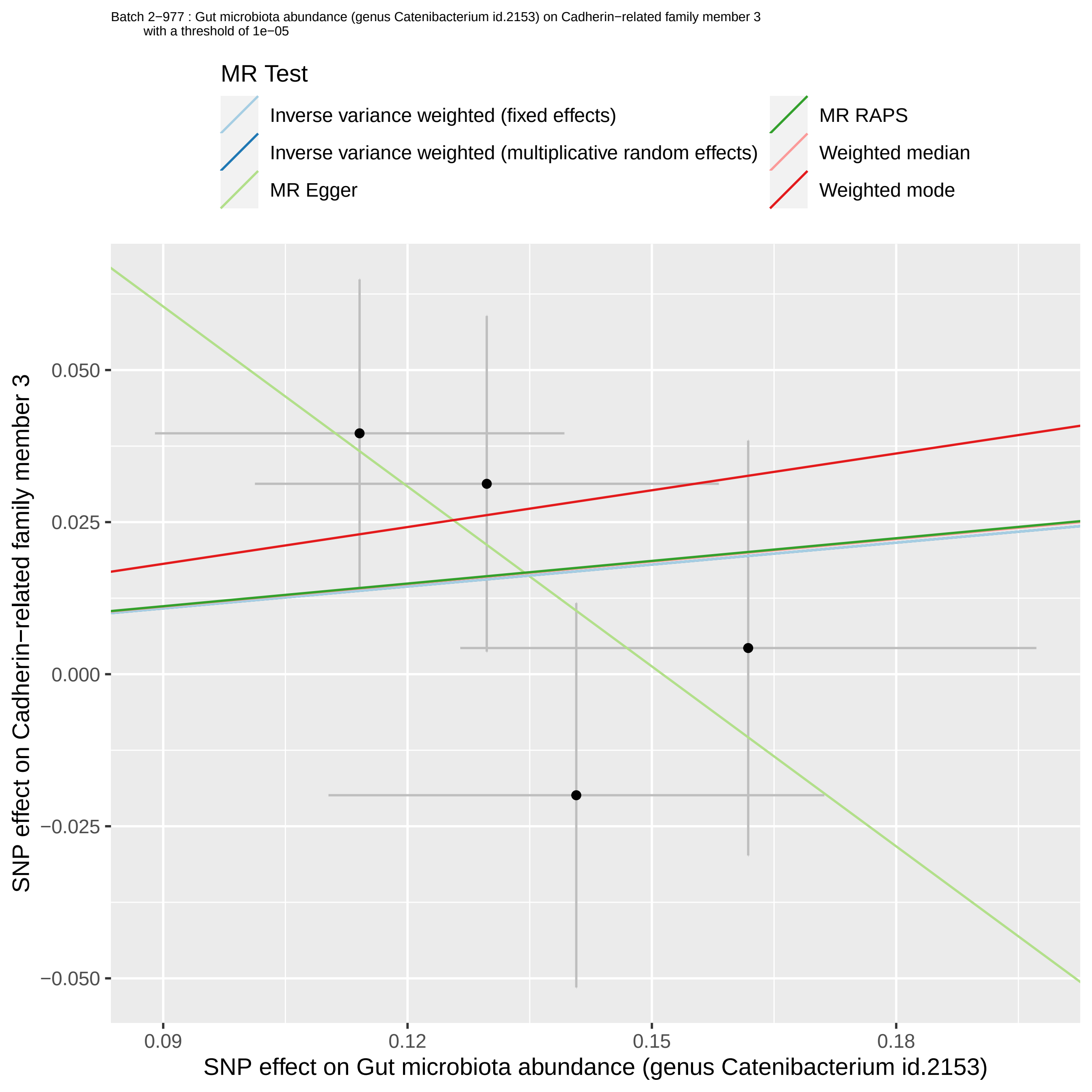
Batch 2−977 : Gut microbiota abundance (genus Catenibacterium id.2153) on Cadherin−related family member 3
with a threshold of 1e−05
MR Test
Inverse variance weighted (fixed effects)
Inverse variance weighted (multiplicative random effects)
MR Egger
MR RAPS
Weighted median
Weighted mode
0.050
0.025
0.000
−0.025
−0.050
0.09
0.12
0.15
0.18
SNP effect on Cadherin−related family member 3
SNP effect on Gut microbiota abundance (genus Catenibacterium id.2153)

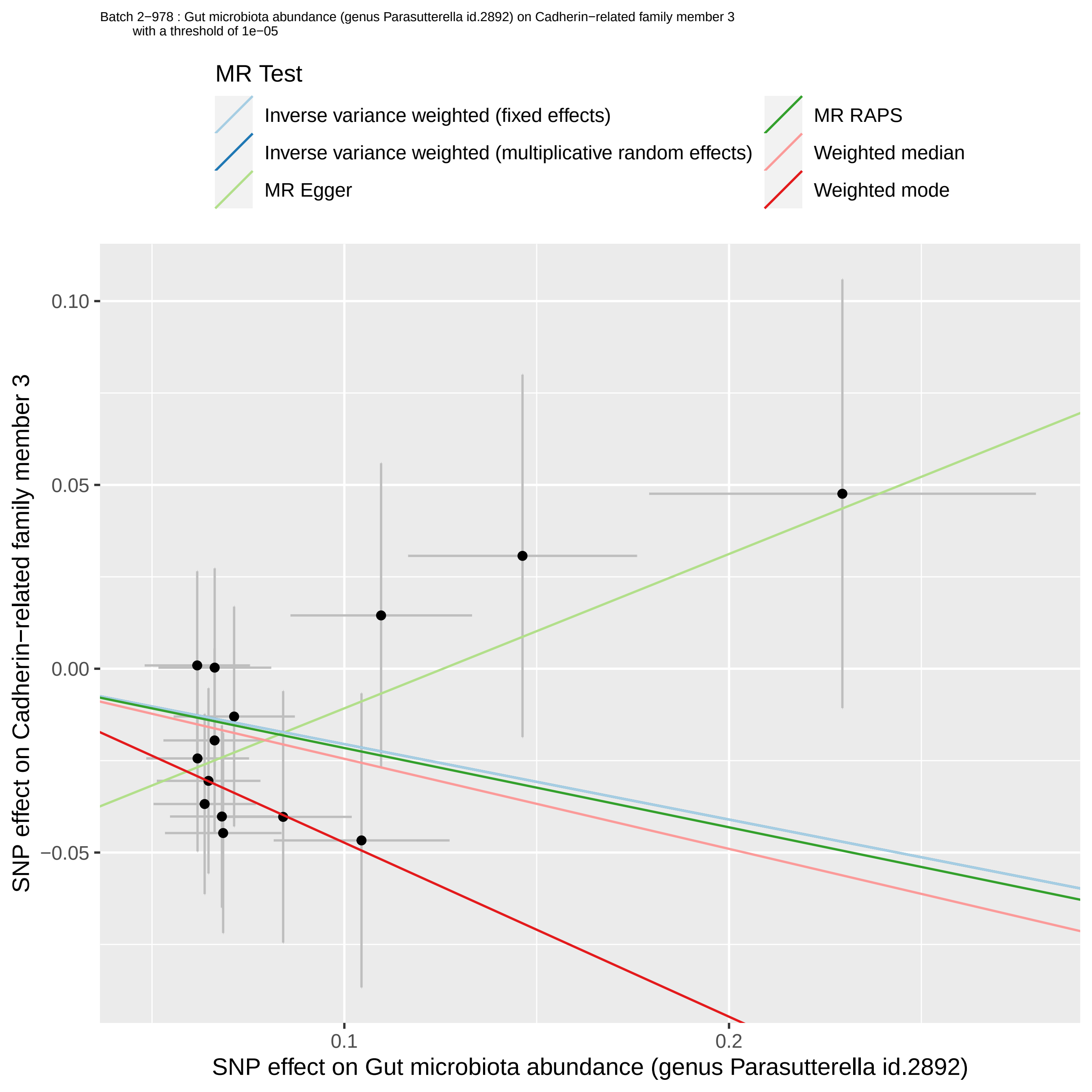
Batch 2−978 : Gut microbiota abundance (genus Parasutterella id.2892) on Cadherin−related family member 3
with a threshold of 1e−05
MR Test
Inverse variance weighted (fixed effects)
Inverse variance weighted (multiplicative random effects)
MR Egger
MR RAPS
Weighted median
Weighted mode
0.10
0.05
0.00
−0.05
0.1
0.2
SNP effect on Cadherin−related family member 3
SNP effect on Gut microbiota abundance (genus Parasutterella id.2892)

Batch 2−979 : Gut microbiota abundance (genus Catenibacterium id.2153) on Cadherin−related family member 5
with a threshold of 1e−05

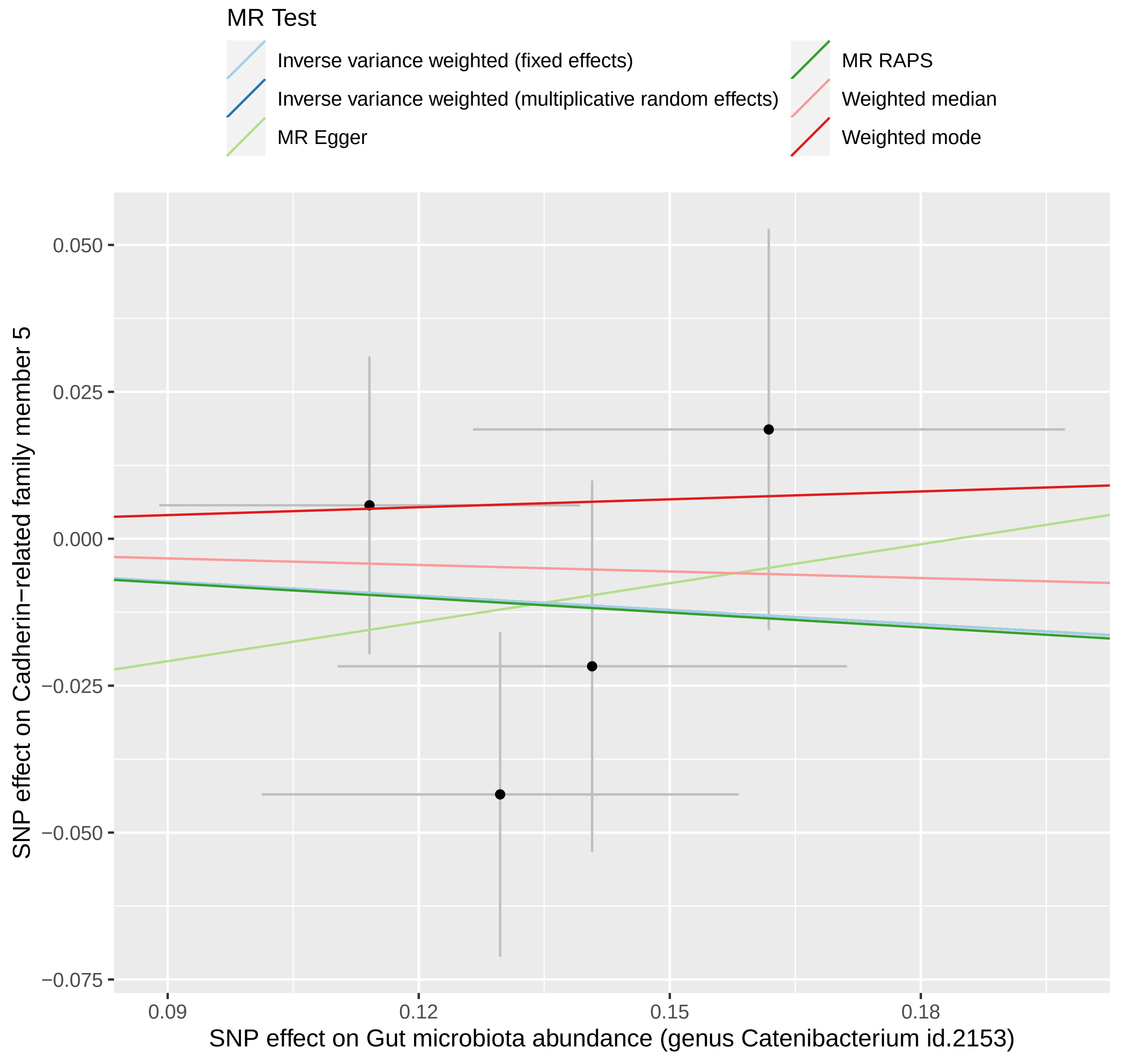

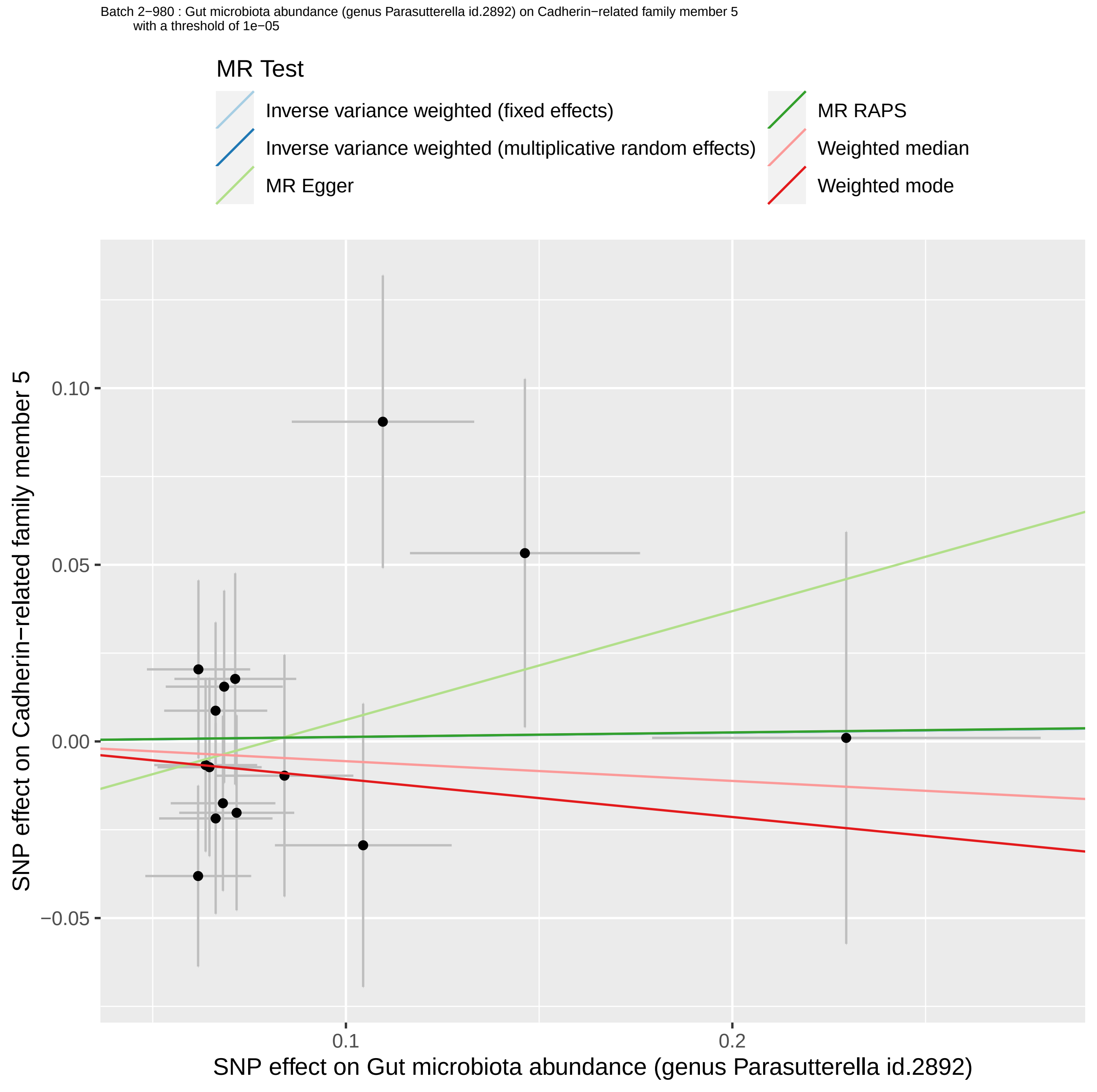
Batch 2−980 : Gut microbiota abundance (genus Parasutterella id.2892) on Cadherin−related family member 5
with a threshold of 1e−05
MR Test
Inverse variance weighted (fixed effects)
Inverse variance weighted (multiplicative random effects)
MR Egger
MR RAPS
Weighted median
Weighted mode
SNP effect on Cadherin−related family member 5
0.10
0.05
0.00
−0.05
0.1
0.2
SNP effect on Gut microbiota abundance (genus Parasutterella id.2892)

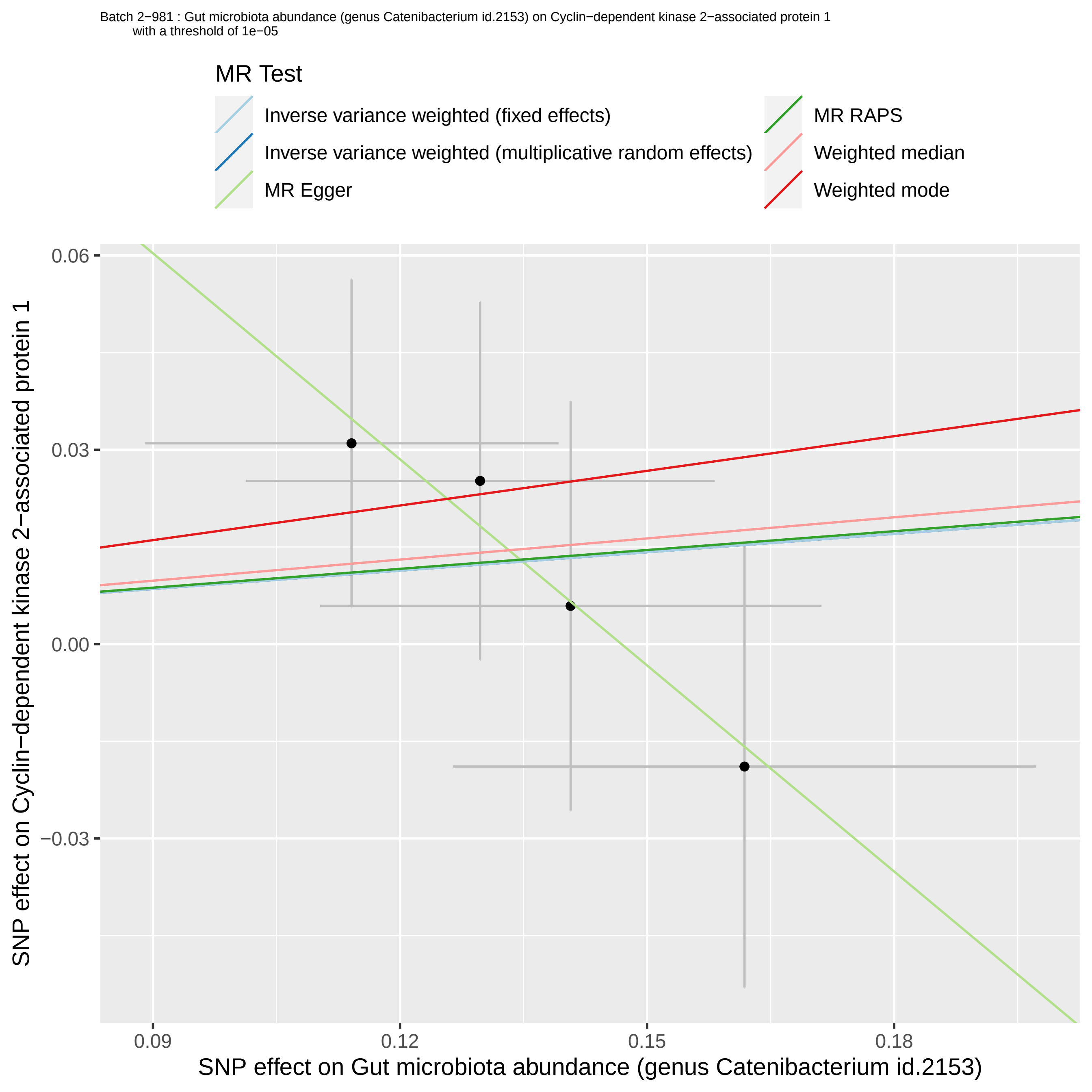
Batch 2−981 : Gut microbiota abundance (genus Catenibacterium id.2153) on Cyclin−dependent kinase 2−associated protein 1
with a threshold of 1e−05
MR Test
Inverse variance weighted (fixed effects)
Inverse variance weighted (multiplicative random effects)
MR Egger
MR RAPS
Weighted median
Weighted mode
0.06
0.03
0.00
−0.03
0.09
0.12
0.15
0.18
SNP effect on Gut microbiota abundance (genus Catenibacterium id.2153)
SNP effect on Cyclin−dependent kinase 2−associated protein 1

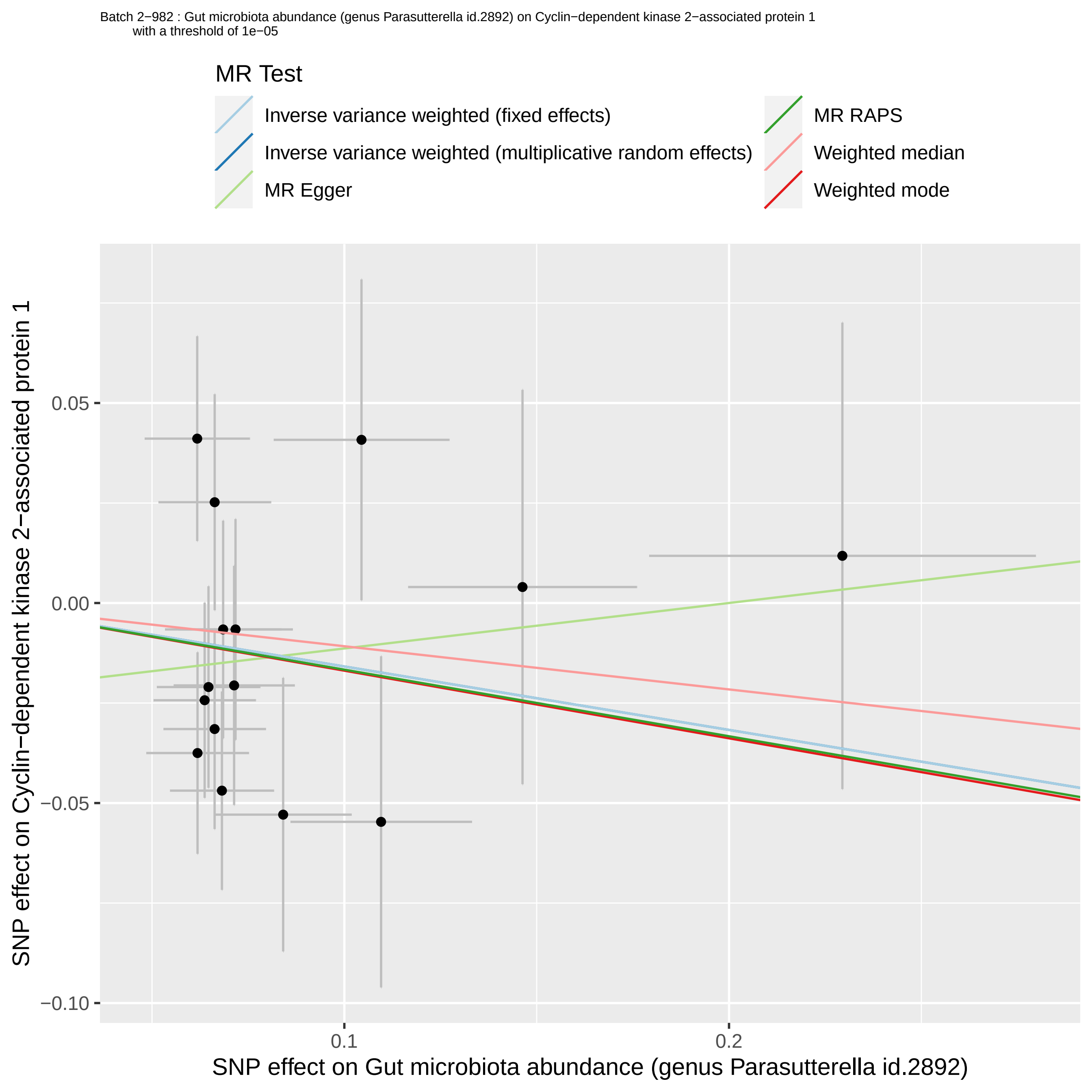
Batch 2−982 : Gut microbiota abundance (genus Parasutterella id.2892) on Cyclin−dependent kinase 2−associated protein 1
with a threshold of 1e−05
MR Test
Inverse variance weighted (fixed effects)
Inverse variance weighted (multiplicative random effects)
MR Egger
MR RAPS
Weighted median
Weighted mode
0.05
0.00
−0.05
−0.10
0.1
0.2
SNP effect on Cyclin−dependent kinase 2−associated protein 1
SNP effect on Gut microbiota abundance (genus Parasutterella id.2892)

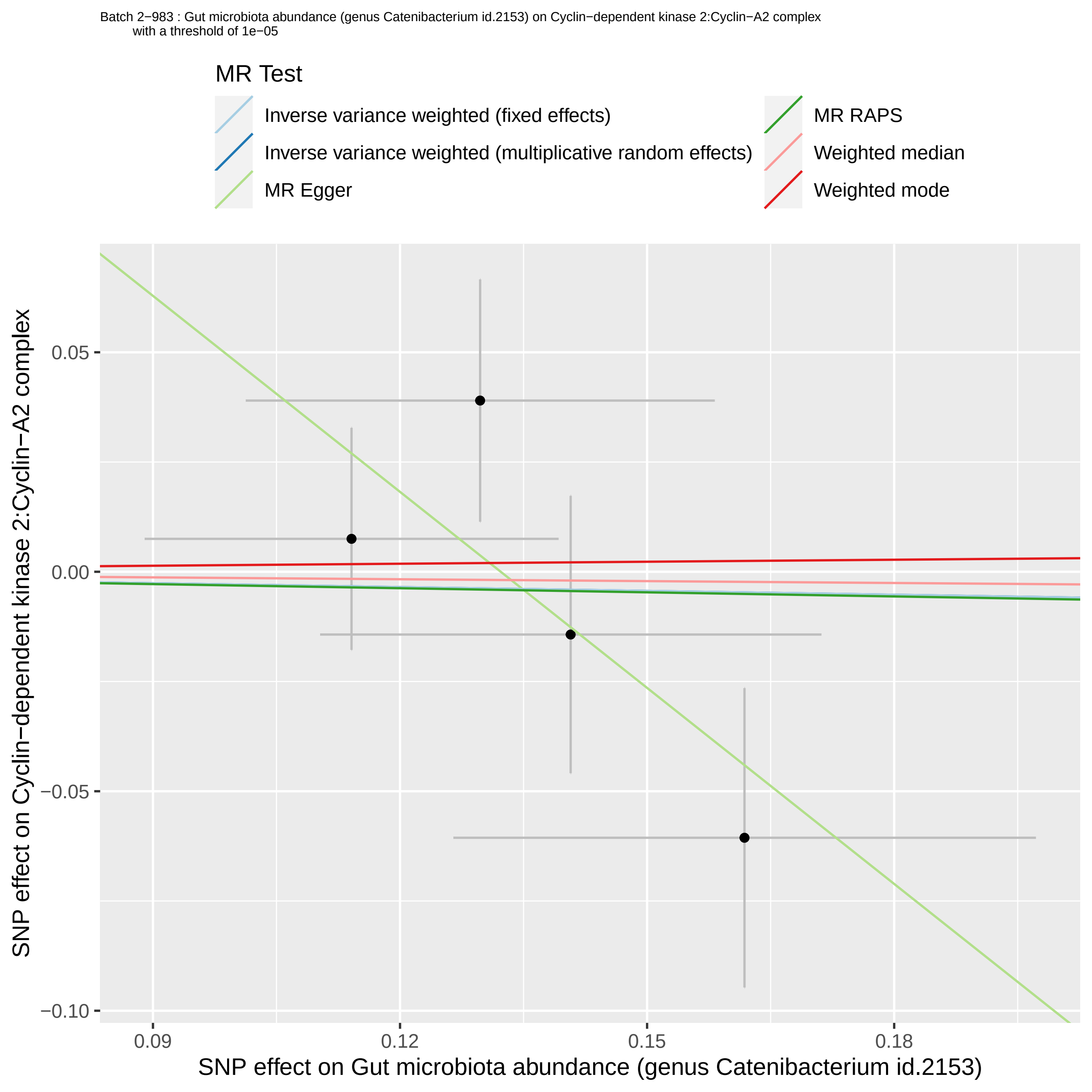
Batch 2−983 : Gut microbiota abundance (genus Catenibacterium id.2153) on Cyclin−dependent kinase 2:Cyclin−A2 complex
with a threshold of 1e−05
MR Test
Inverse variance weighted (fixed effects)
Inverse variance weighted (multiplicative random effects)
MR Egger
MR RAPS
Weighted median
Weighted mode
0.05
0.00
−0.05
−0.10
0.09
0.12
0.15
0.18
SNP effect on Cyclin−dependent kinase 2:Cyclin−A2 complex
SNP effect on Gut microbiota abundance (genus Catenibacterium id.2153)

Batch 2−984 : Gut microbiota abundance (genus Parasutterella id.2892) on Cyclin−dependent kinase 2:Cyclin−A2 complex
with a threshold of 1e−05

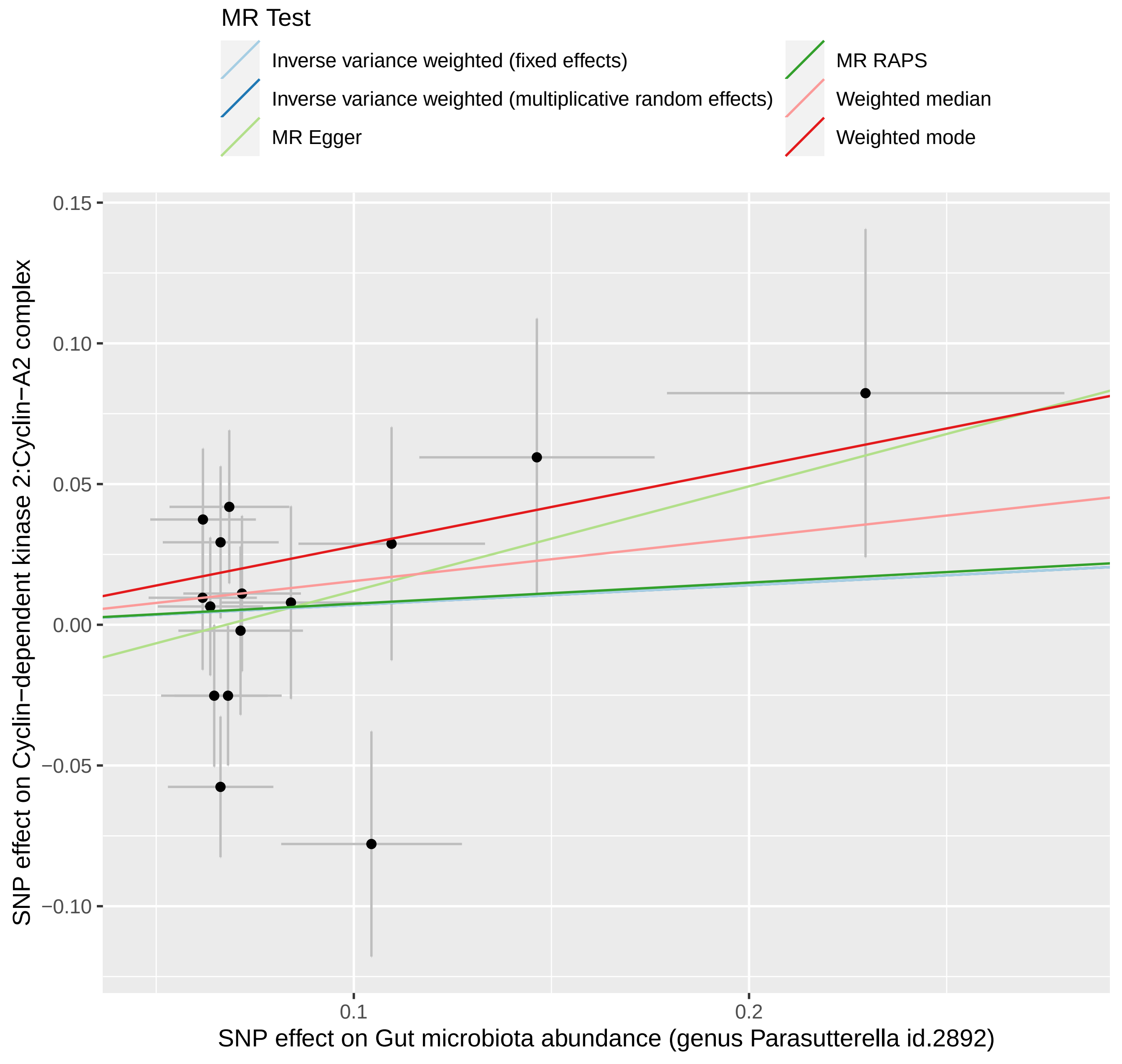

Batch 2−985 : Gut microbiota abundance (genus Catenibacterium id.2153) on Cyclin−dependent kinase 5:Cyclin−dependent kinase 5 activator 1 complex
with a threshold of 1e−05

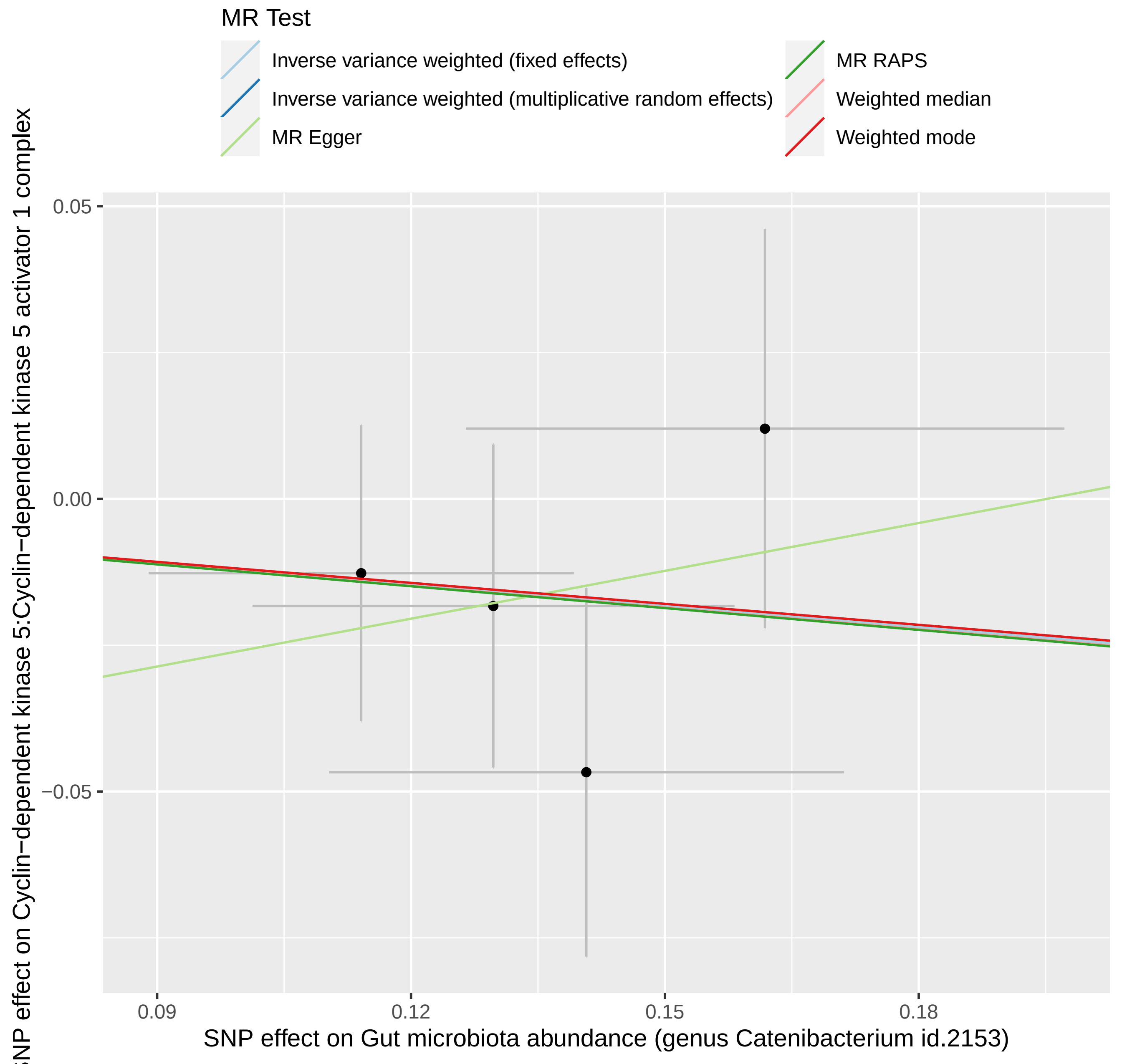

Batch 2−986 : Gut microbiota abundance (genus Parasutterella id.2892) on Cyclin−dependent kinase 5:Cyclin−dependent kinase 5 activator 1 complex with a threshold of 1e−05

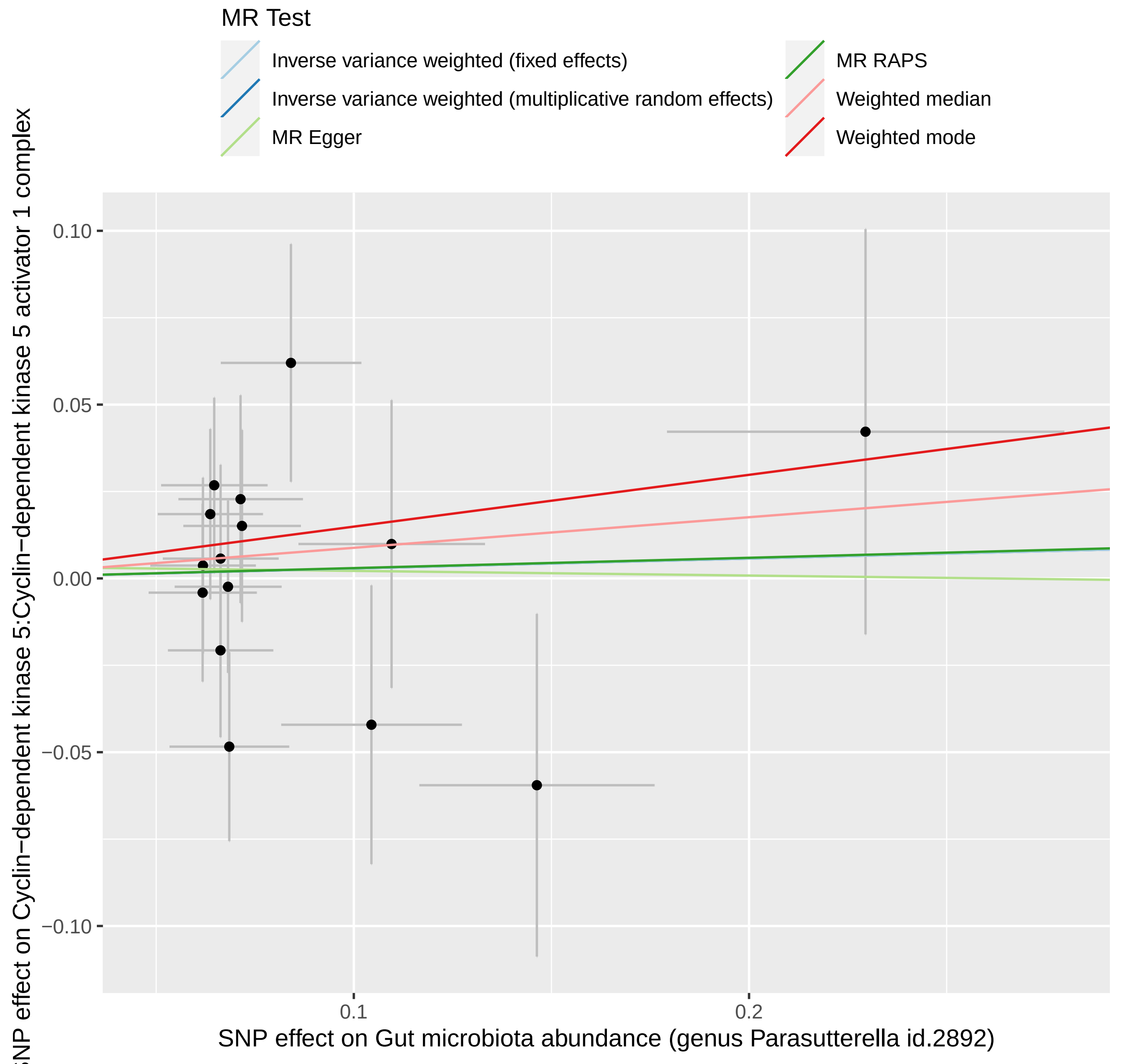

Batch 2−987 : Gut microbiota abundance (genus Catenibacterium id.2153) on Cyclin−dependent kinase 8:Cyclin−C complex
with a threshold of 1e−05

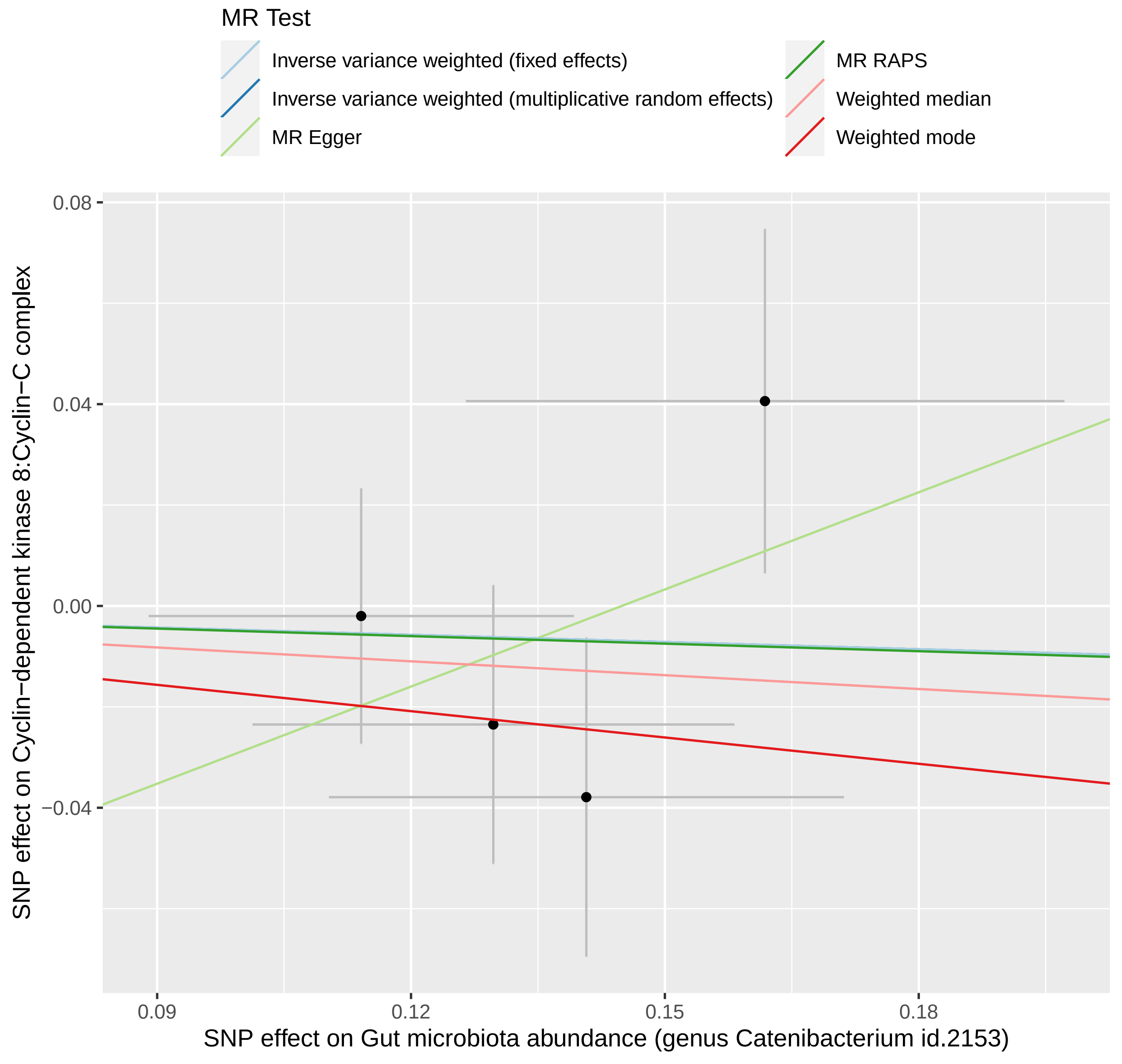

Batch 2−988 : Gut microbiota abundance (genus Parasutterella id.2892) on Cyclin−dependent kinase 8:Cyclin−C complex
with a threshold of 1e−05

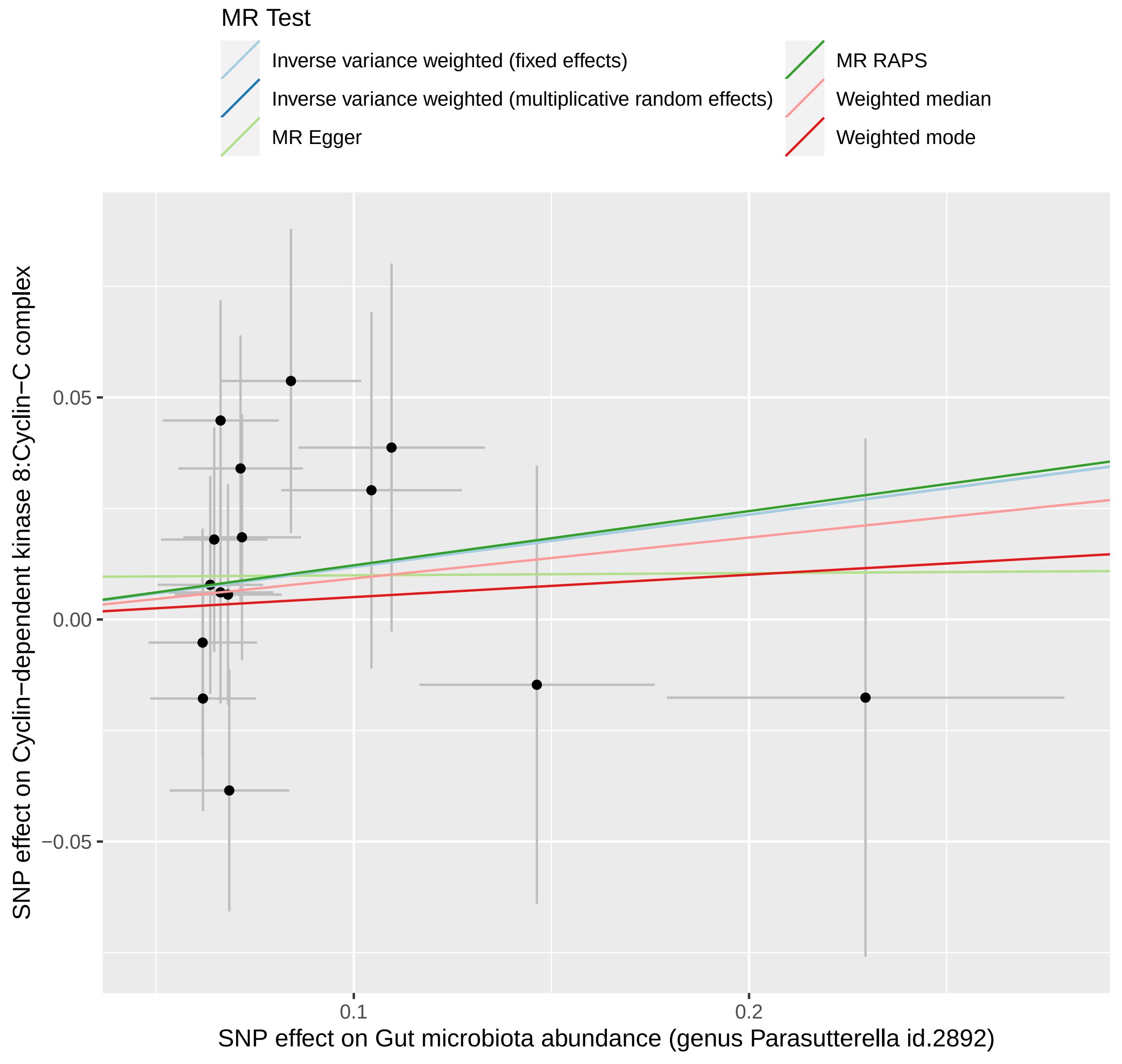

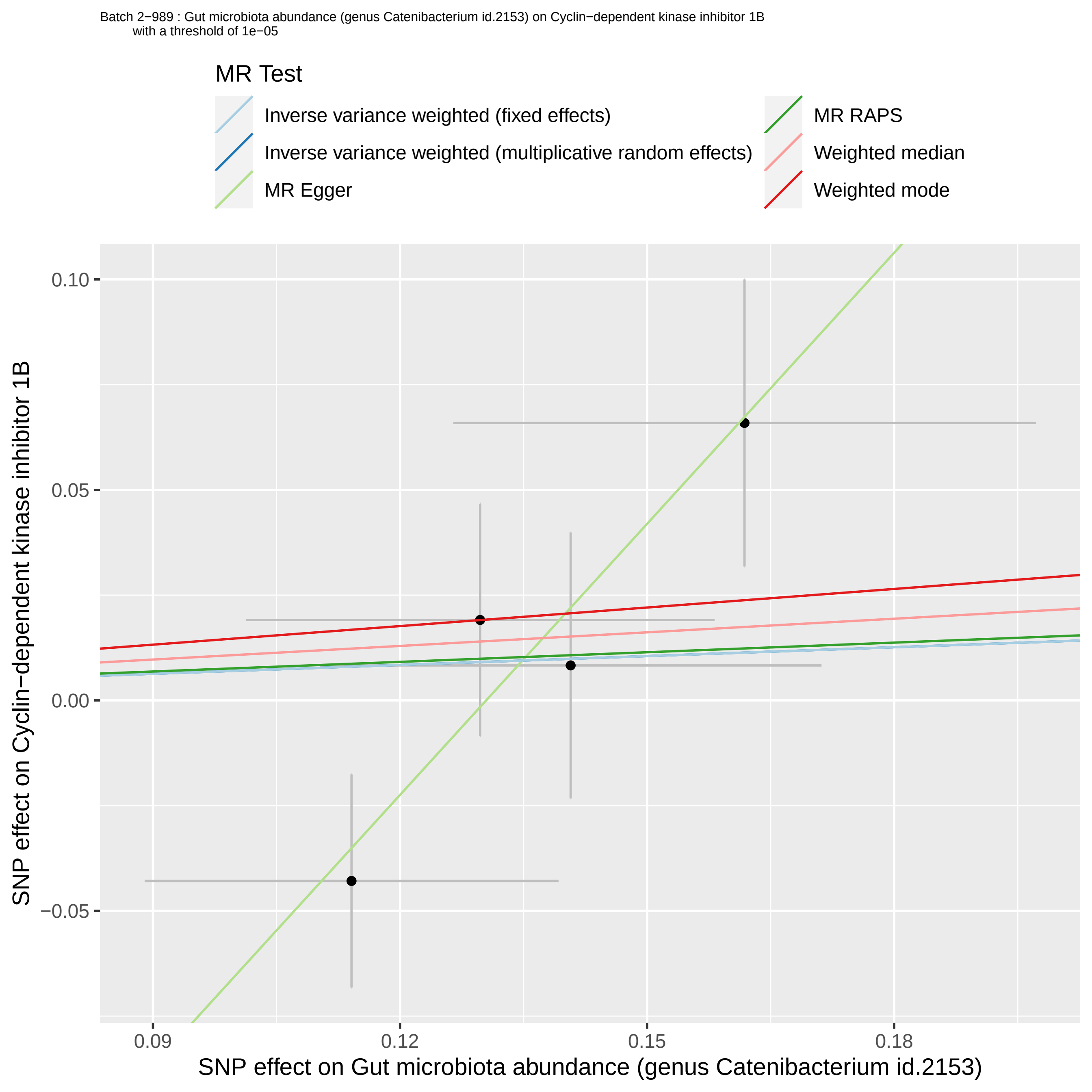

Batch 2−989 : Gut microbiota abundance (genus Catenibacterium id.2153) on Cyclin−dependent kinase inhibitor 1B
with a threshold of 1e−05
MR Test
Inverse variance weighted (fixed effects)
Inverse variance weighted (multiplicative random effects)
MR Egger
MR RAPS
Weighted median
Weighted mode
0.10
0.05
0.00
−0.05
0.09
0.12
0.15
0.18
SNP effect on Cyclin−dependent kinase inhibitor 1B
SNP effect on Gut microbiota abundance (genus Catenibacterium id.2153)

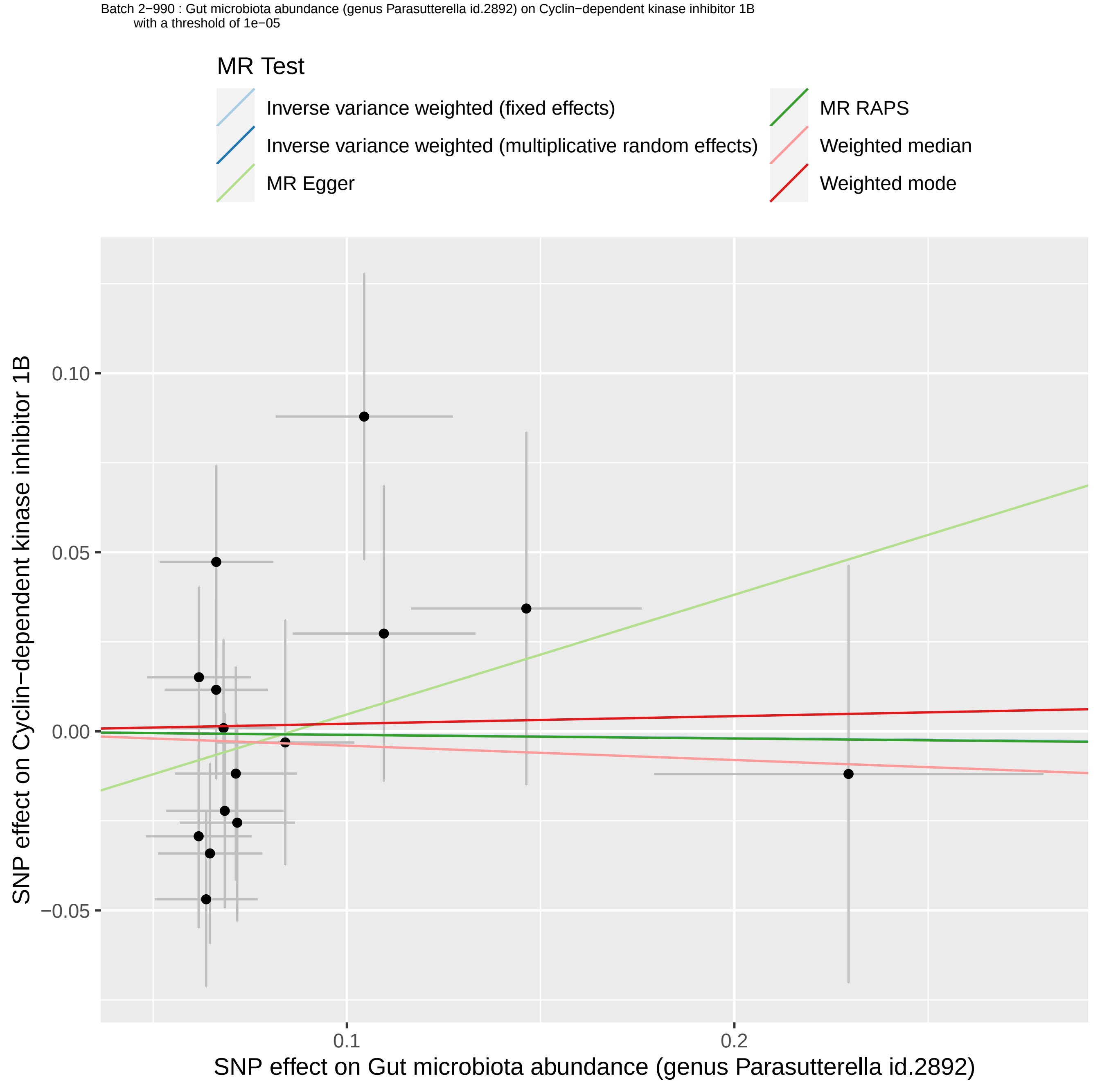

Batch 2−990 : Gut microbiota abundance (genus Parasutterella id.2892) on Cyclin−dependent kinase inhibitor 1B
with a threshold of 1e−05
MR Test
Inverse variance weighted (fixed effects)
Inverse variance weighted (multiplicative random effects)
MR Egger
MR RAPS
Weighted median
Weighted mode
SNP effect on Cyclin−dependent kinase inhibitor 1B
0.10
0.05
0.00
−0.05
0.1
0.2
SNP effect on Gut microbiota abundance (genus Parasutterella id.2892)

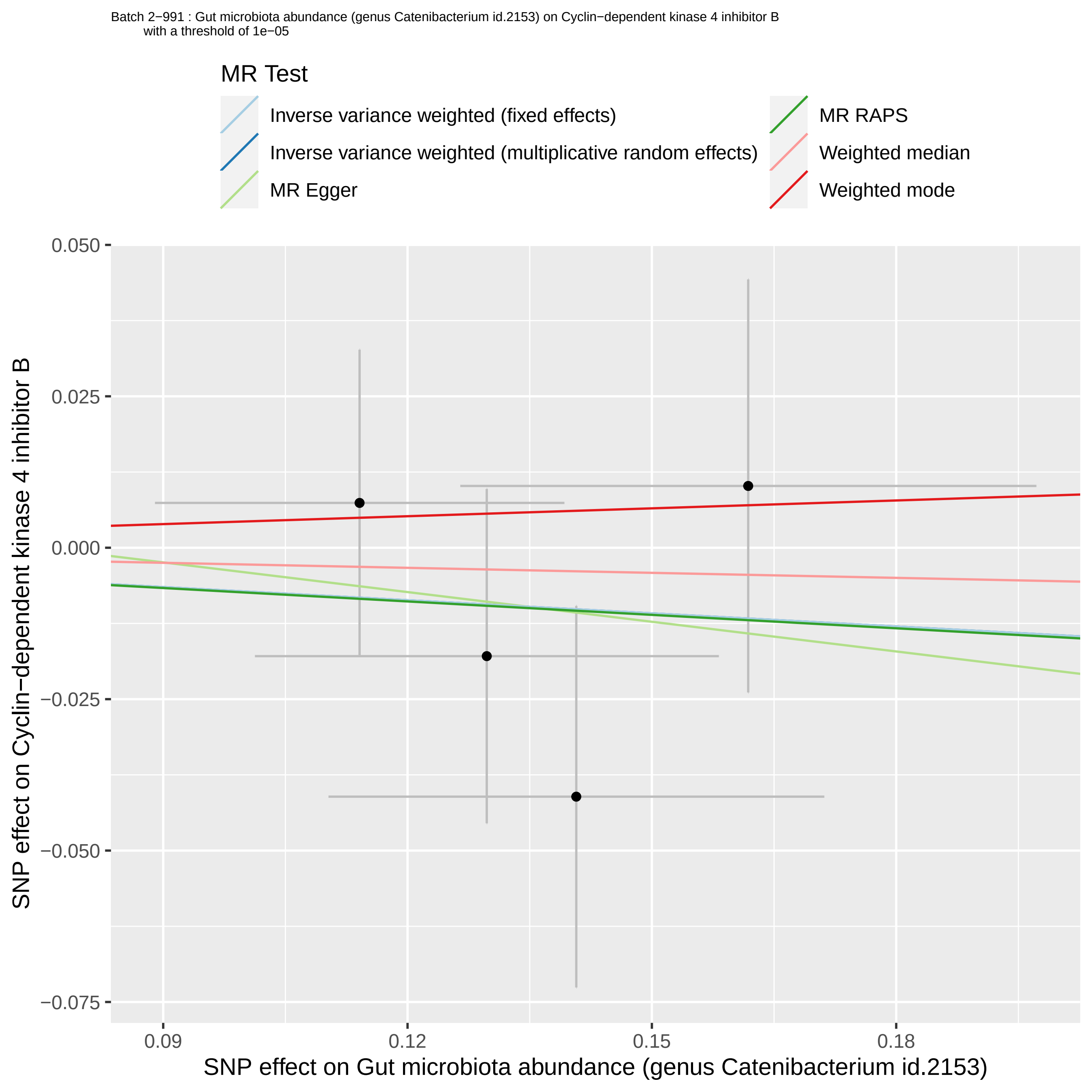

Batch 2−991 : Gut microbiota abundance (genus Catenibacterium id.2153) on Cyclin−dependent kinase 4 inhibitor B
with a threshold of 1e−05
MR Test
Inverse variance weighted (fixed effects)
Inverse variance weighted (multiplicative random effects)
MR Egger
MR RAPS
Weighted median
Weighted mode
0.050
0.025
0.000
−0.025
−0.050
−0.075
SNP effect on Cyclin−dependent kinase 4 inhibitor B
0.09
0.12
0.15
0.18
SNP effect on Gut microbiota abundance (genus Catenibacterium id.2153)

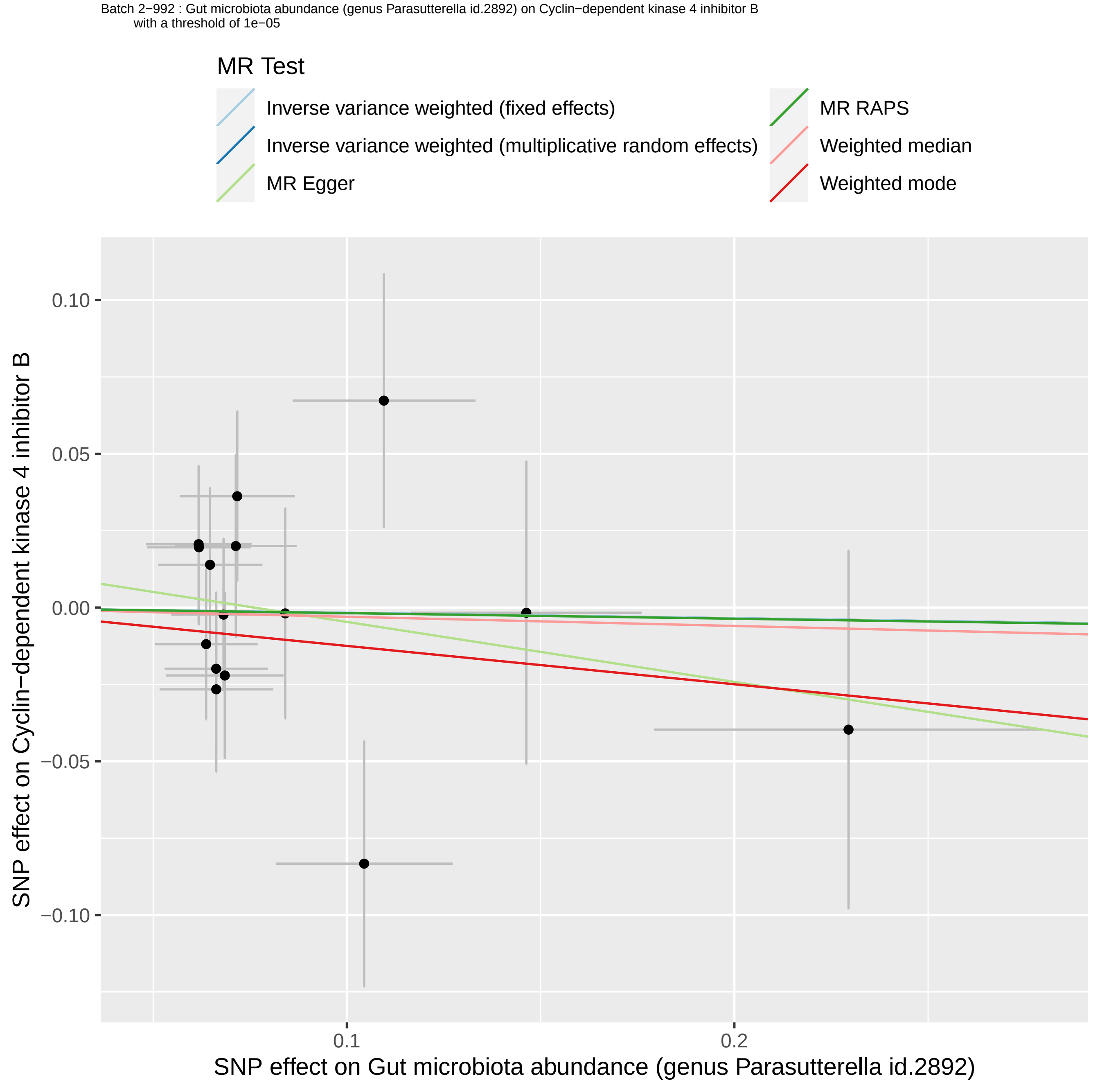
Batch 2−992 : Gut microbiota abundance (genus Parasutterella id.2892) on Cyclin−dependent kinase 4 inhibitor B
with a threshold of 1e−05
MR Test
Inverse variance weighted (fixed effects)
Inverse variance weighted (multiplicative random effects)
MR Egger
MR RAPS
Weighted median
Weighted mode
0.10
0.05
0.00
−0.05
−0.10
0.1
0.2
SNP effect on Cyclin−dependent kinase 4 inhibitor B
SNP effect on Gut microbiota abundance (genus Parasutterella id.2892)

Batch 2−993 : Gut microbiota abundance (genus Catenibacterium id.2153) on Cyclin−dependent kinase inhibitor 3
with a threshold of 1e−05

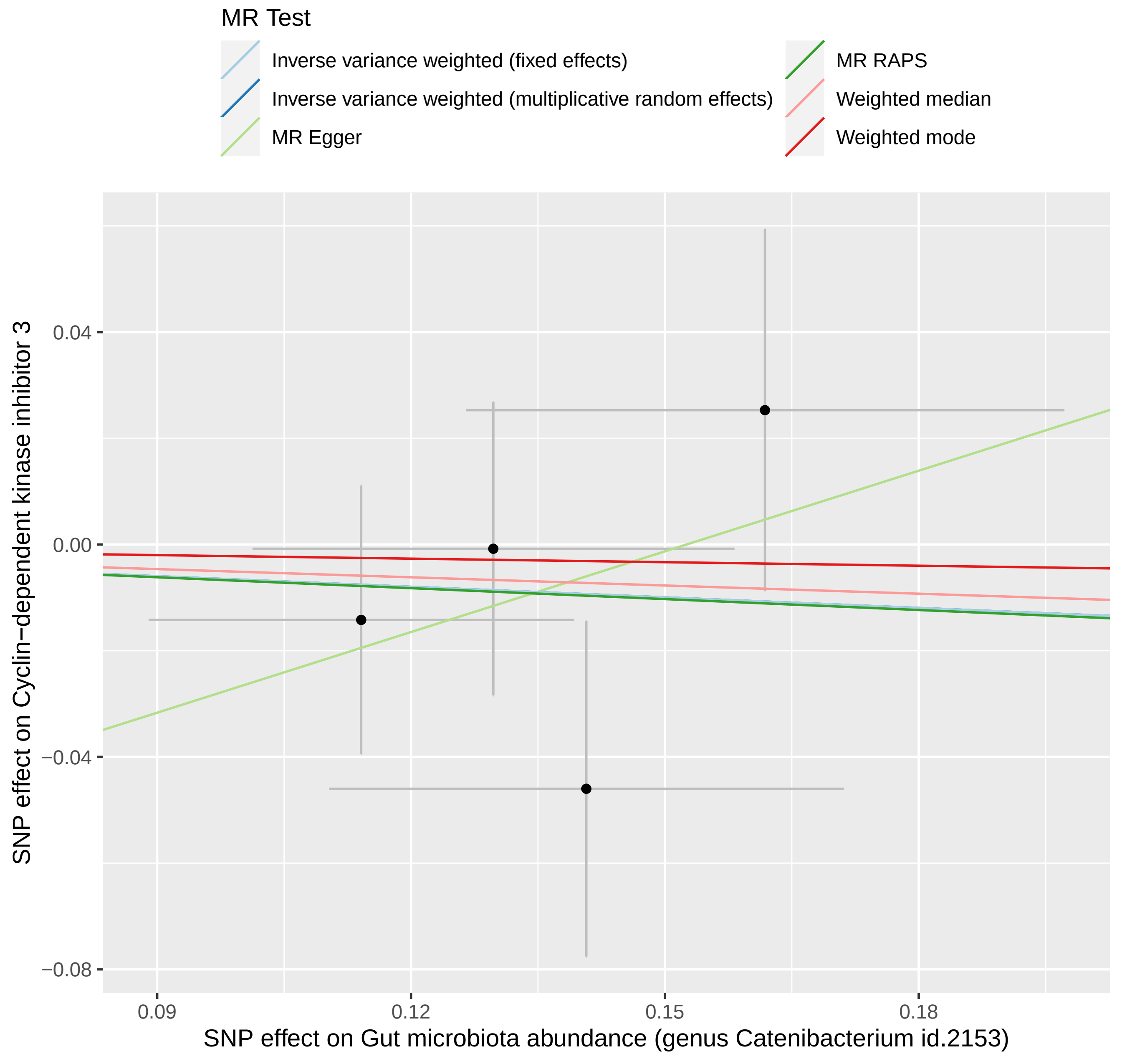

Batch 2−994 : Gut microbiota abundance (genus Parasutterella id.2892) on Cyclin−dependent kinase inhibitor 3
with a threshold of 1e−05

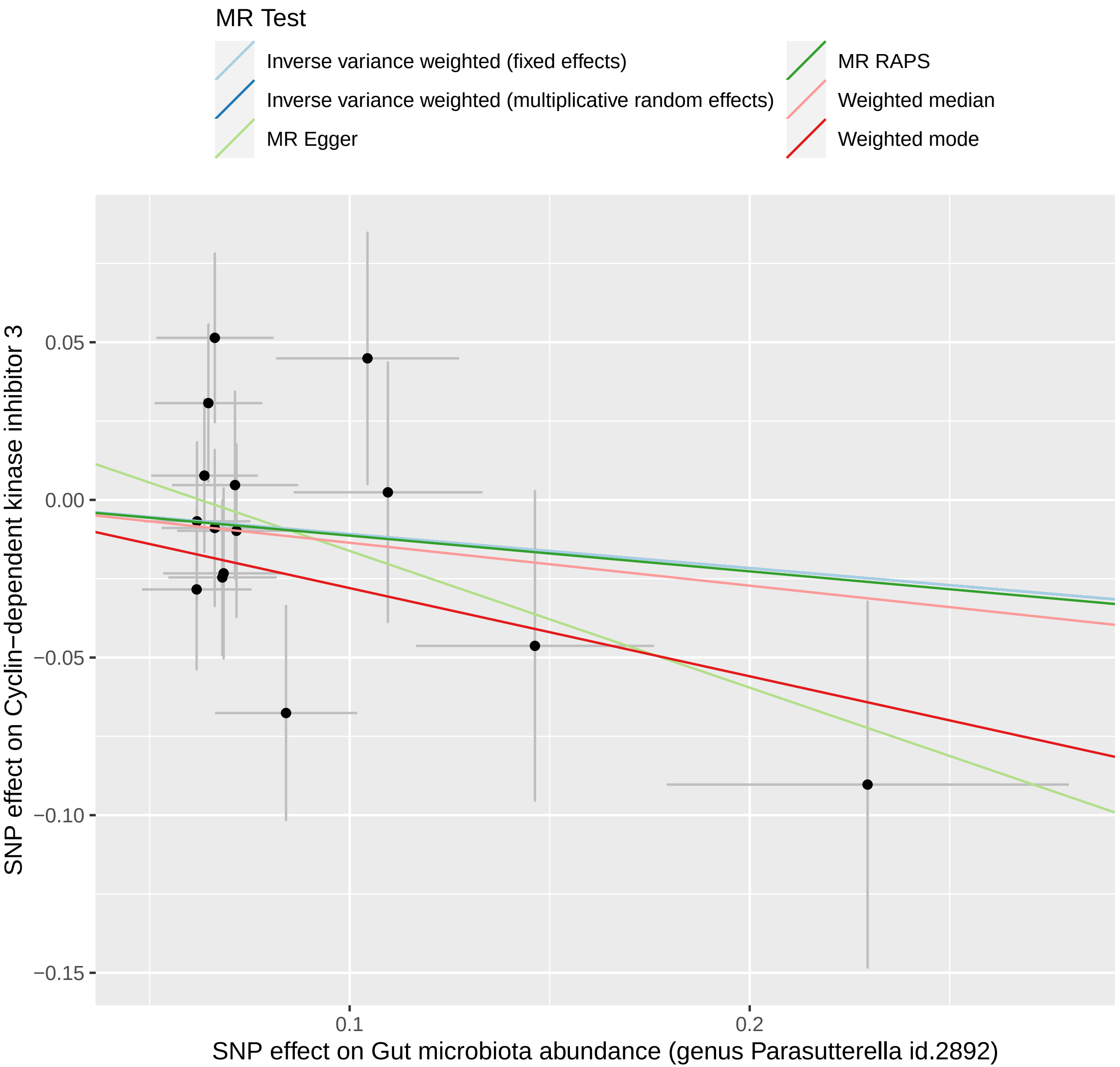

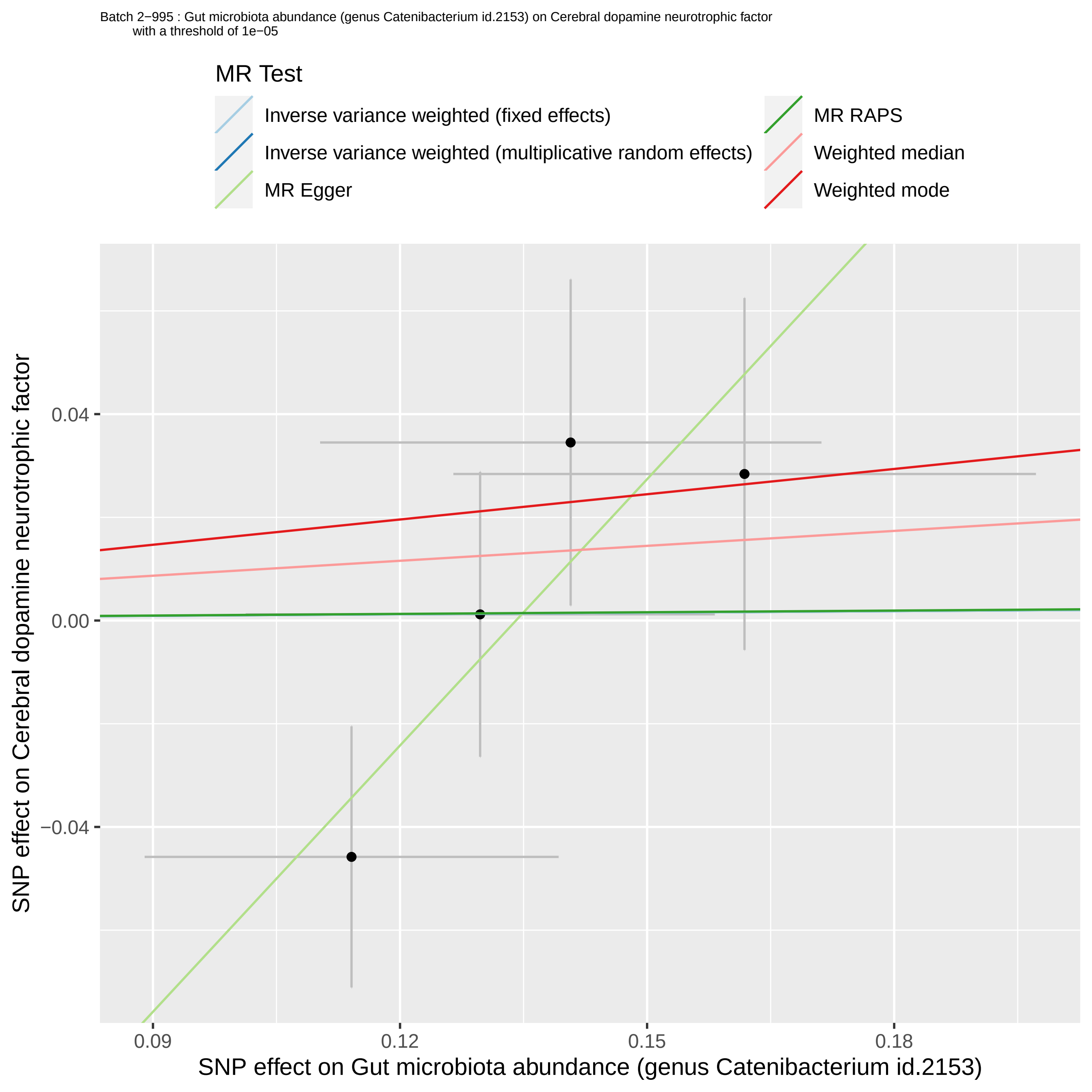

Batch 2−995 : Gut microbiota abundance (genus Catenibacterium id.2153) on Cerebral dopamine neurotrophic factor
with a threshold of 1e−05
MR Test
Inverse variance weighted (fixed effects)
Inverse variance weighted (multiplicative random effects)
MR Egger
MR RAPS
Weighted median
Weighted mode
0.04
0.00
−0.04
0.09
0.12
0.15
0.18
SNP effect on Cerebral dopamine neurotrophic factor
SNP effect on Gut microbiota abundance (genus Catenibacterium id.2153)

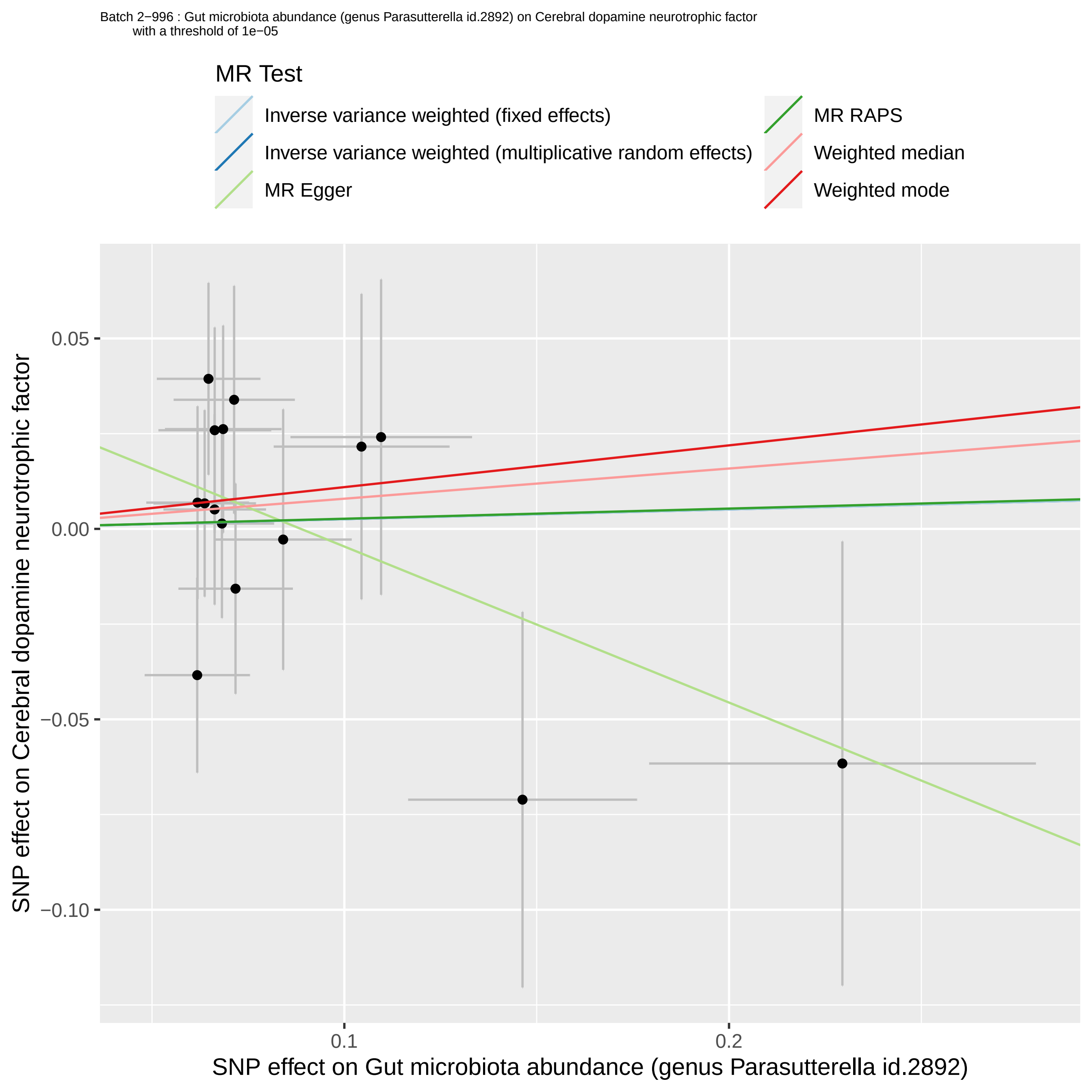

Batch 2−996 : Gut microbiota abundance (genus Parasutterella id.2892) on Cerebral dopamine neurotrophic factor
with a threshold of 1e−05
MR Test
Inverse variance weighted (fixed effects)
Inverse variance weighted (multiplicative random effects)
MR Egger
MR RAPS
Weighted median
Weighted mode
0.05
0.00
−0.05
−0.10
0.1
0.2
SNP effect on Cerebral dopamine neurotrophic factor
SNP effect on Gut microbiota abundance (genus Parasutterella id.2892)

Batch 2−997 : Gut microbiota abundance (genus Catenibacterium id.2153) on Cell adhesion molecule−related/down−regulated by oncogenes
with a threshold of 1e−05

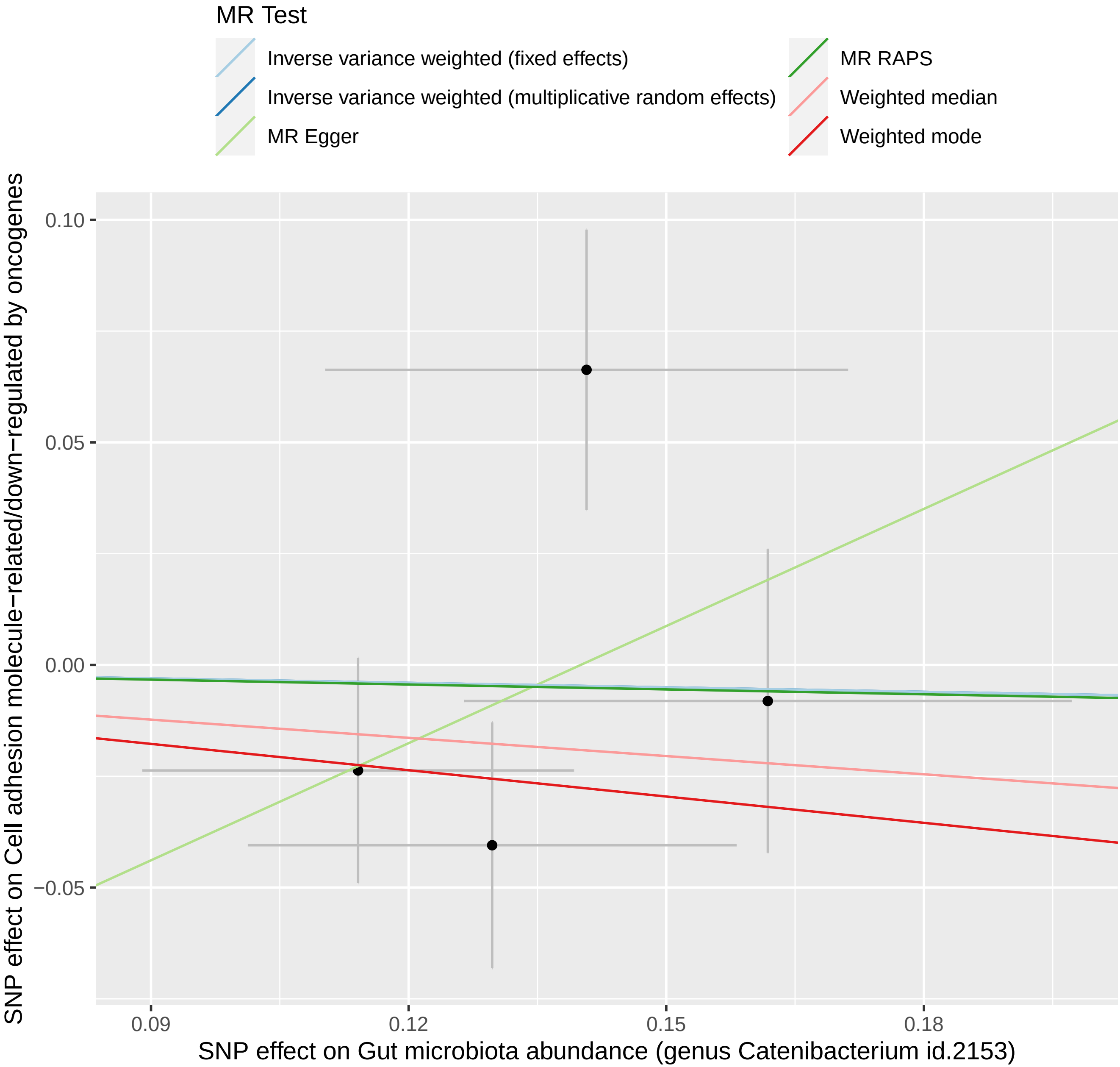

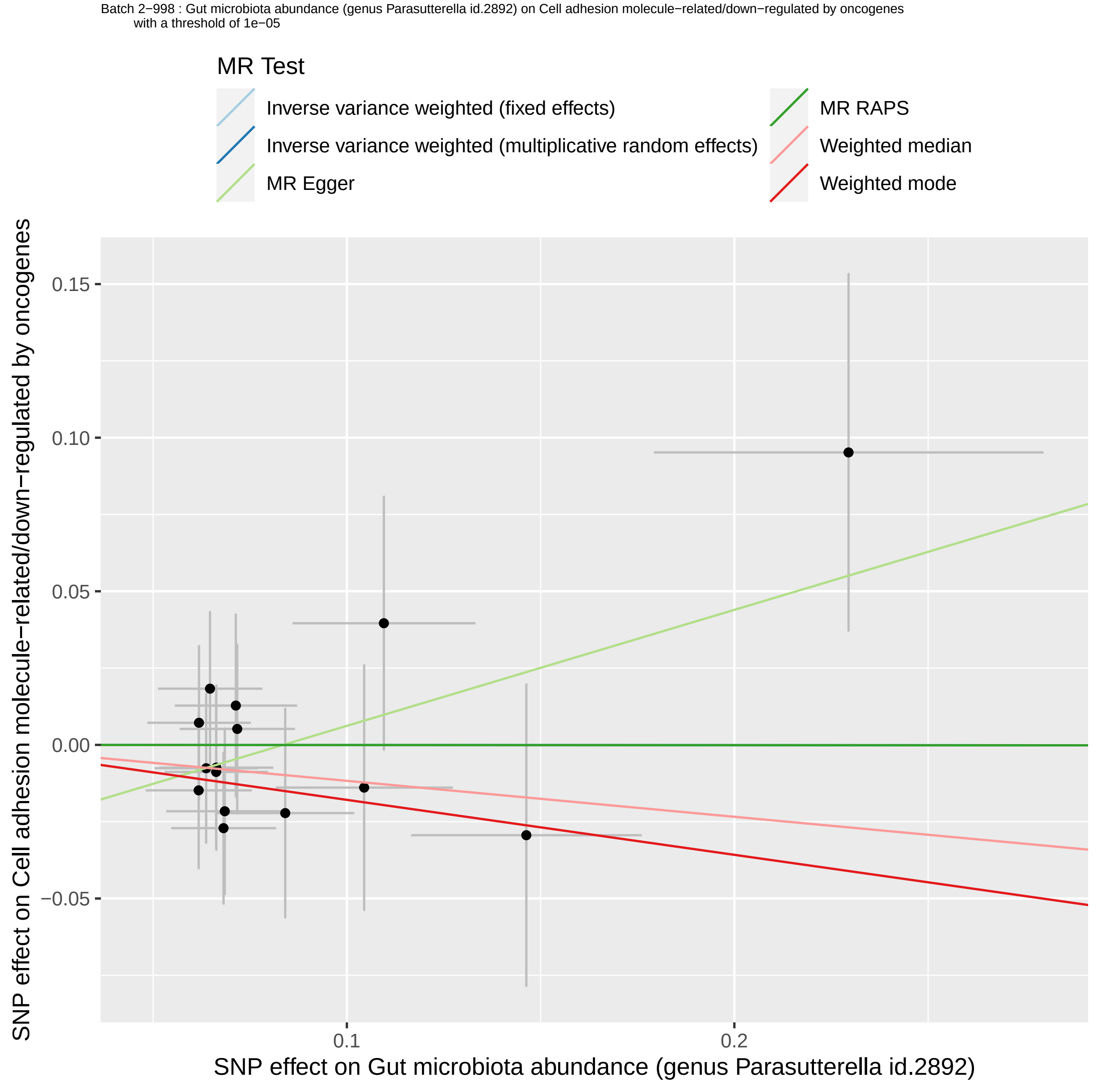

Batch 2−998 : Gut microbiota abundance (genus Parasutterella id.2892) on Cell adhesion molecule−related/down−regulated by oncogenes with a threshold of 1e−05
MR Test
Inverse variance weighted (fixed effects)
Inverse variance weighted (multiplicative random effects)
MR Egger
MR RAPS
Weighted median
Weighted mode
SNP effect on Cell adhesion molecule−related/down−regulated by oncogenes
0.15
0.10
0.05
0.00
−0.05
0.1
0.2
SNP effect on Gut microbiota abundance (genus Parasutterella id.2892)

Batch 2−999 : Gut microbiota abundance (genus Catenibacterium id.2153) on Corneodesmosin
with a threshold of 1e−05

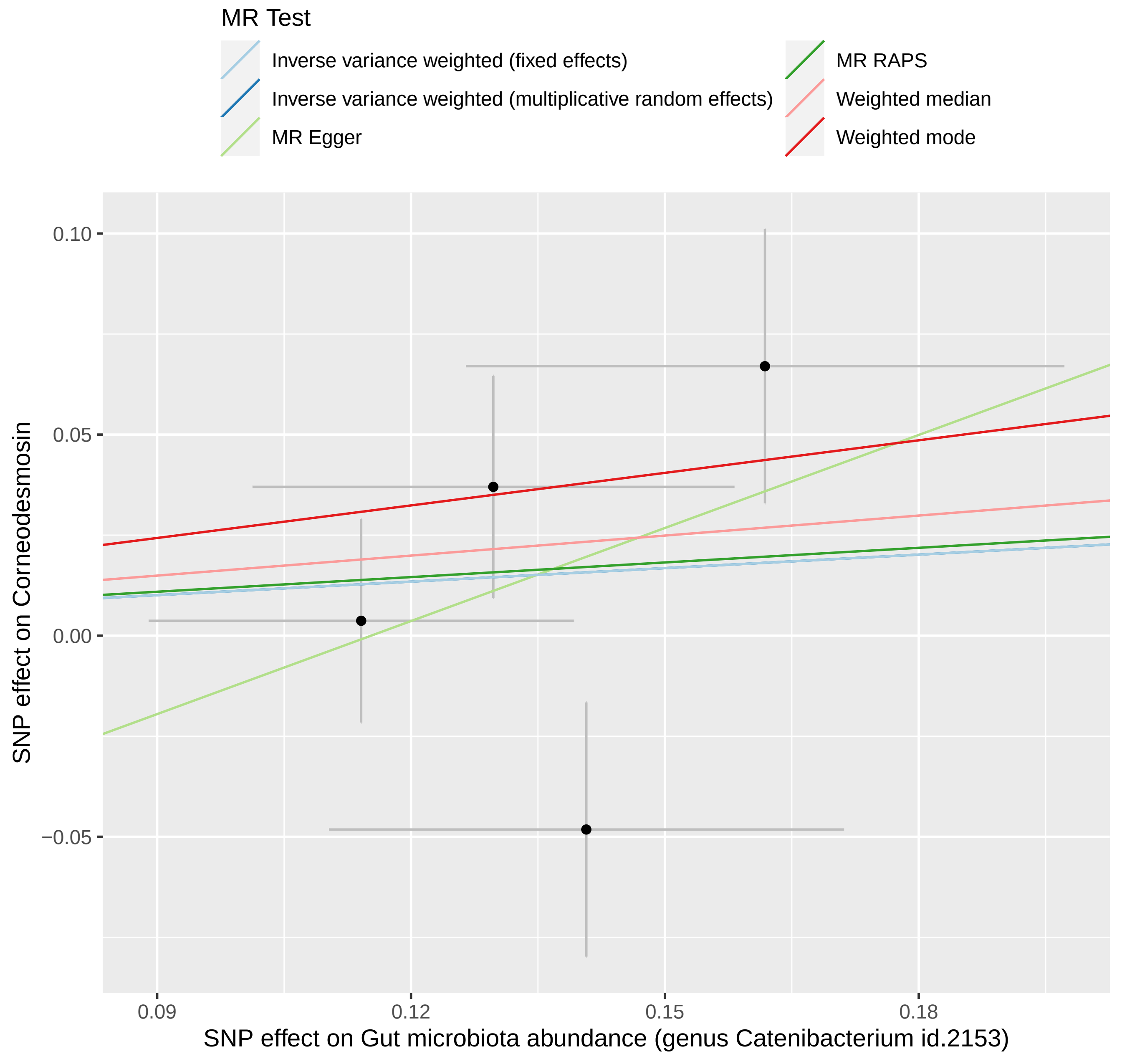

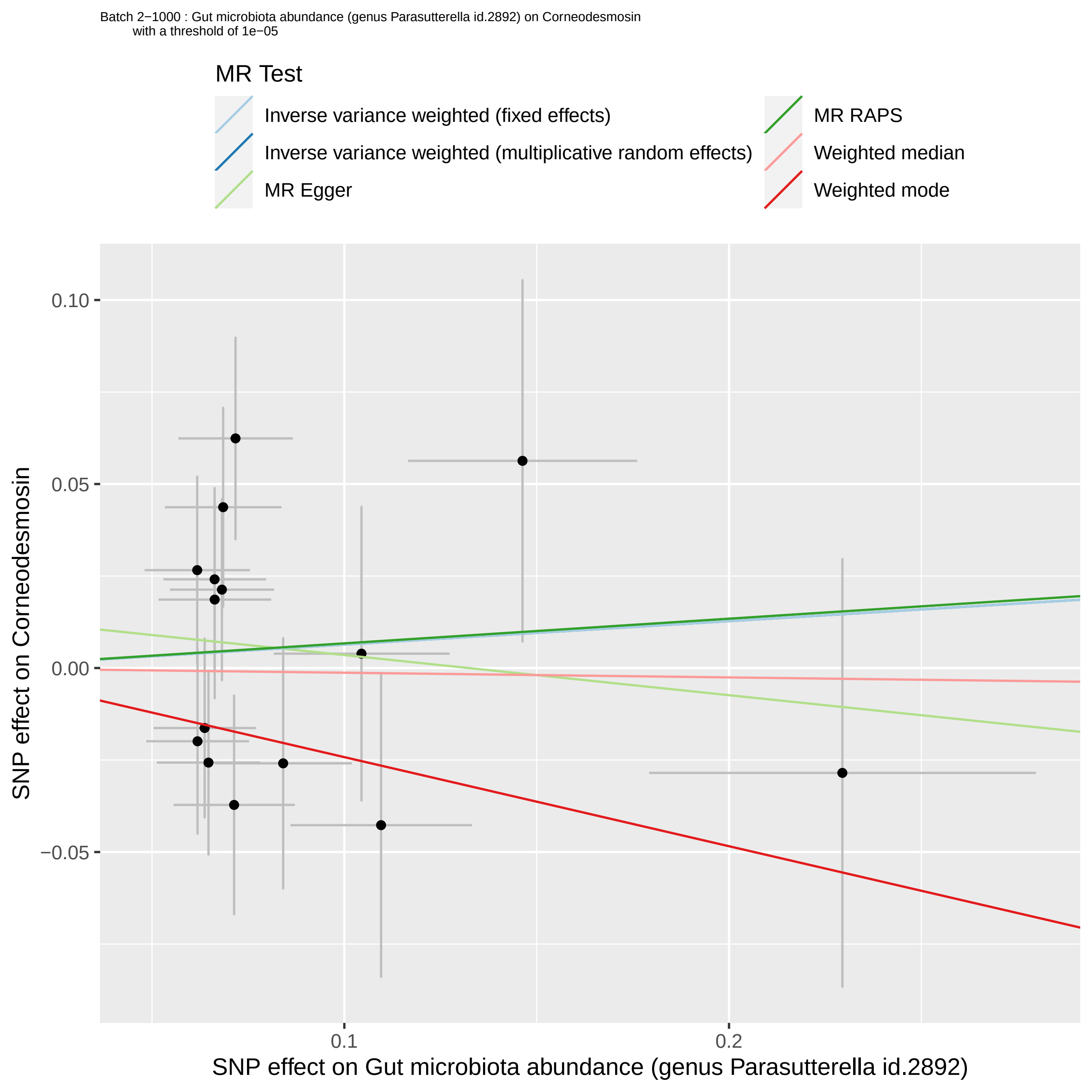
Batch 2−1000 : Gut microbiota abundance (genus Parasutterella id.2892) on Corneodesmosin
with a threshold of 1e−05
MR Test
Inverse variance weighted (fixed effects)
Inverse variance weighted (multiplicative random effects)
MR Egger
MR RAPS
Weighted median
Weighted mode
0.10
0.05
0.00
−0.05
0.1
0.2
SNP effect on Corneodesmosin
SNP effect on Gut microbiota abundance (genus Parasutterella id.2892)

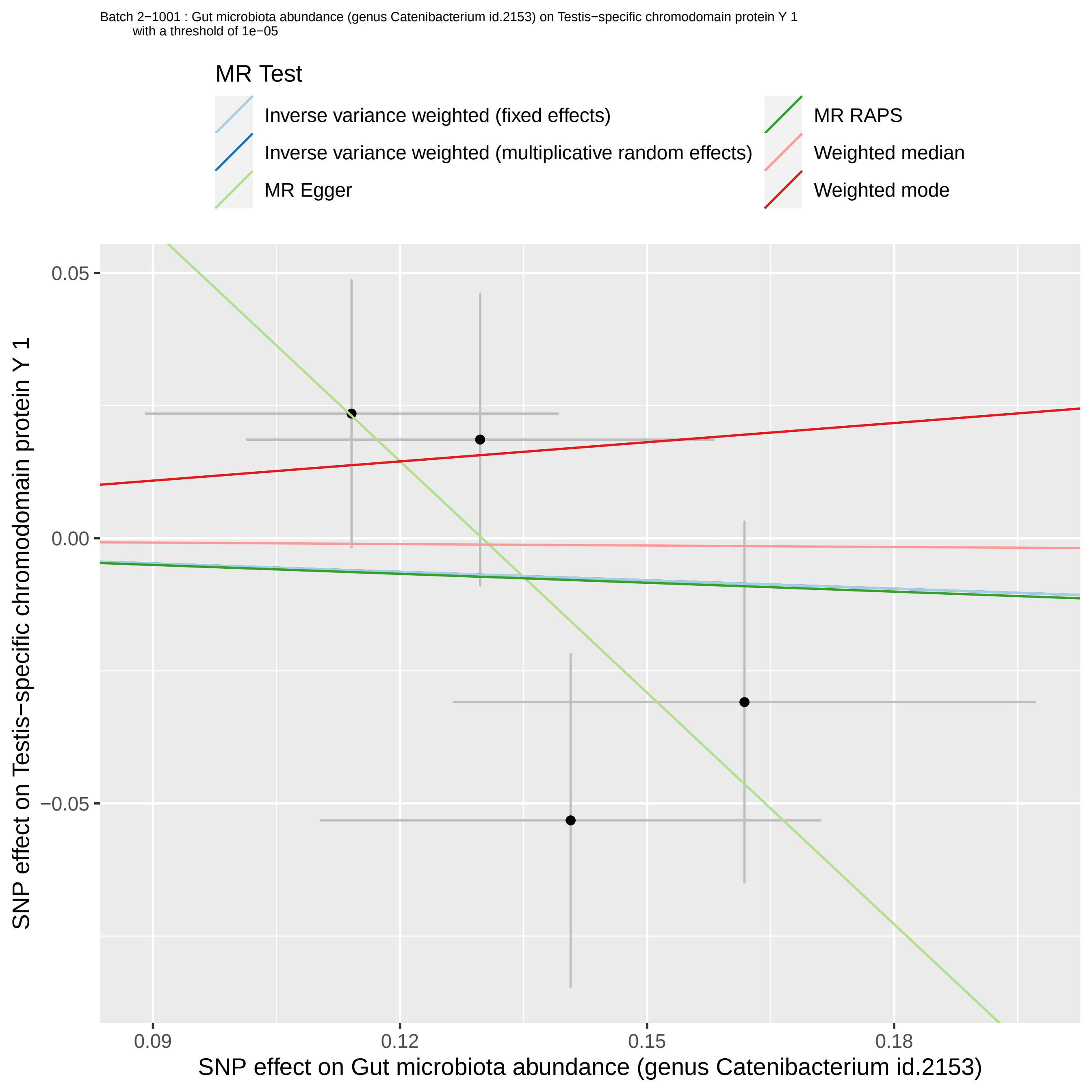
Batch 2−1001 : Gut microbiota abundance (genus Catenibacterium id.2153) on Testis−specific chromodomain protein Y 1
with a threshold of 1e−05
MR Test
Inverse variance weighted (fixed effects)
Inverse variance weighted (multiplicative random effects)
MR Egger
MR RAPS
Weighted median
Weighted mode
0.05
0.00
−0.05
0.09
0.12
0.15
0.18
SNP effect on Testis−specific chromodomain protein Y 1
SNP effect on Gut microbiota abundance (genus Catenibacterium id.2153)

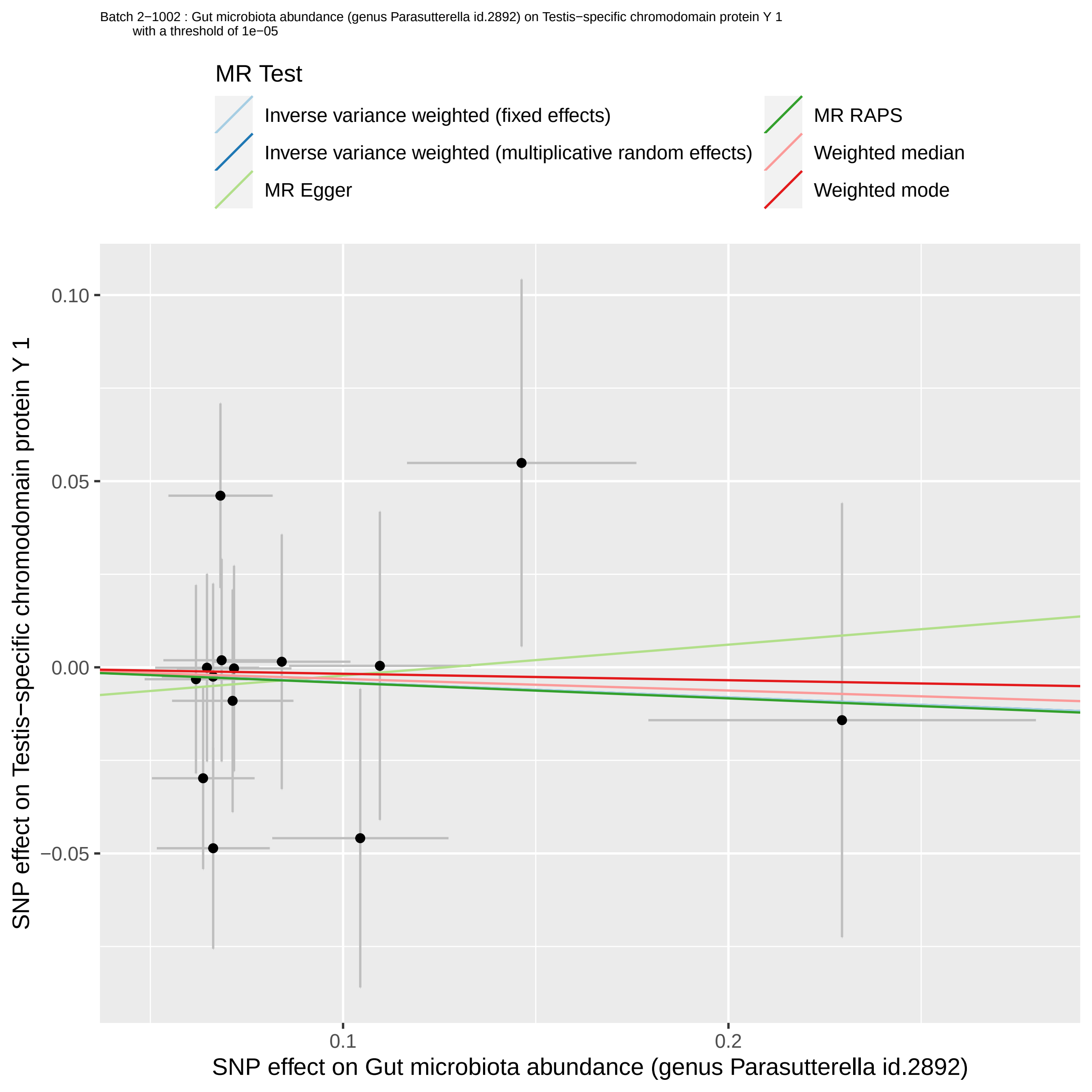
Batch 2−1002 : Gut microbiota abundance (genus Parasutterella id.2892) on Testis−specific chromodomain protein Y 1
with a threshold of 1e−05
MR Test
Inverse variance weighted (fixed effects)
Inverse variance weighted (multiplicative random effects)
MR Egger
MR RAPS
Weighted median
Weighted mode
0.10
0.05
0.00
−0.05
0.1
0.2
SNP effect on Testis−specific chromodomain protein Y 1
SNP effect on Gut microbiota abundance (genus Parasutterella id.2892)

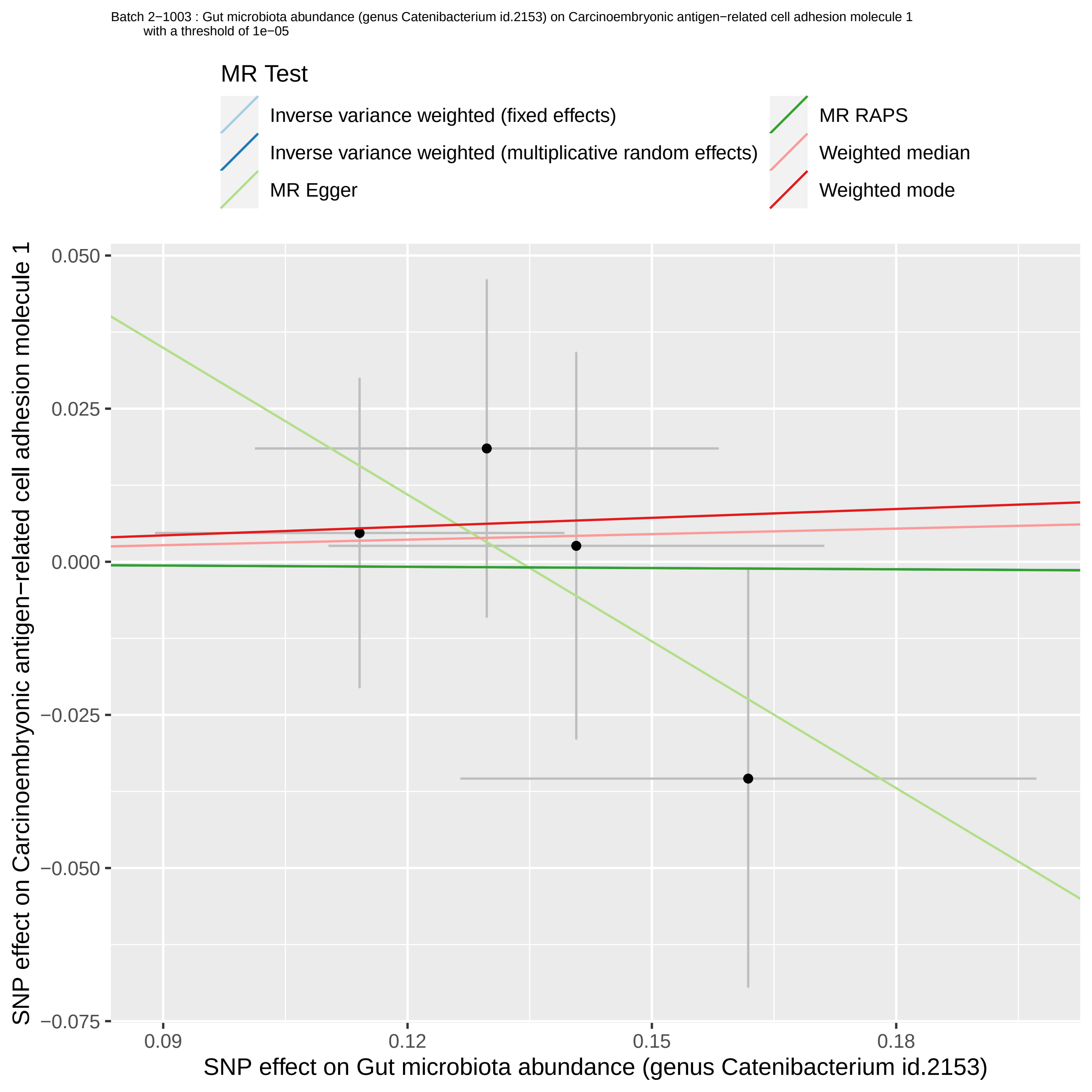
Batch 2−1003 : Gut microbiota abundance (genus Catenibacterium id.2153) on Carcinoembryonic antigen−related cell adhesion molecule 1
with a threshold of 1e−05
MR Test
Inverse variance weighted (fixed effects)
Inverse variance weighted (multiplicative random effects)
MR Egger
MR RAPS
Weighted median
Weighted mode
SNP effect on Carcinoembryonic antigen−related cell adhesion molecule 1
0.050
0.025
0.000
−0.025
−0.050
−0.075
0.09
0.12
0.15
0.18
SNP effect on Gut microbiota abundance (genus Catenibacterium id.2153)

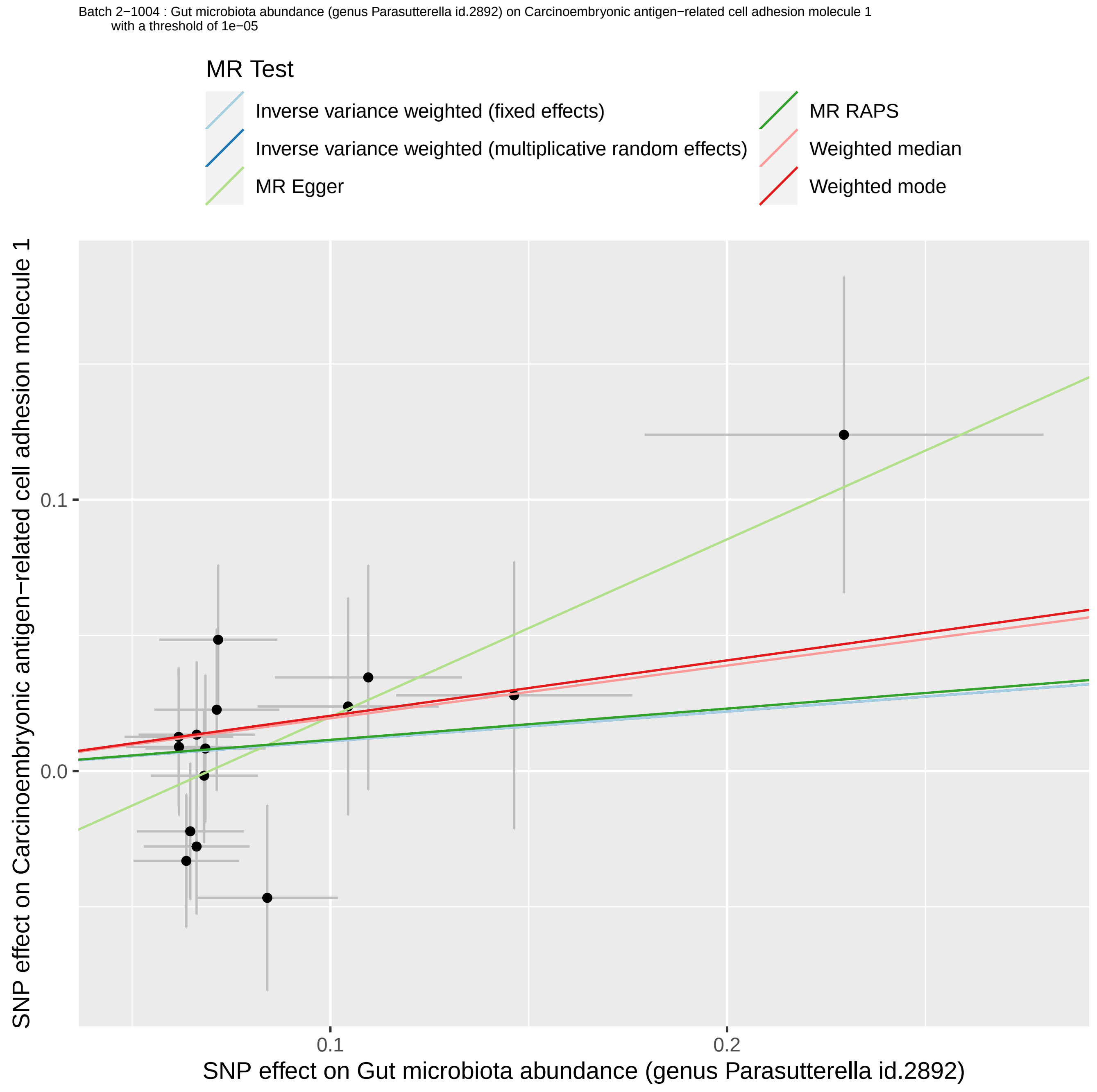

Batch 2−1004 : Gut microbiota abundance (genus Parasutterella id.2892) on Carcinoembryonic antigen−related cell adhesion molecule 1
with a threshold of 1e−05
MR Test
Inverse variance weighted (fixed effects)
Inverse variance weighted (multiplicative random effects)
MR Egger
MR RAPS
Weighted median
Weighted mode
0.1
0.0
0.1
0.2
SNP effect on Carcinoembryonic antigen−related cell adhesion molecule 1
SNP effect on Gut microbiota abundance (genus Parasutterella id.2892)

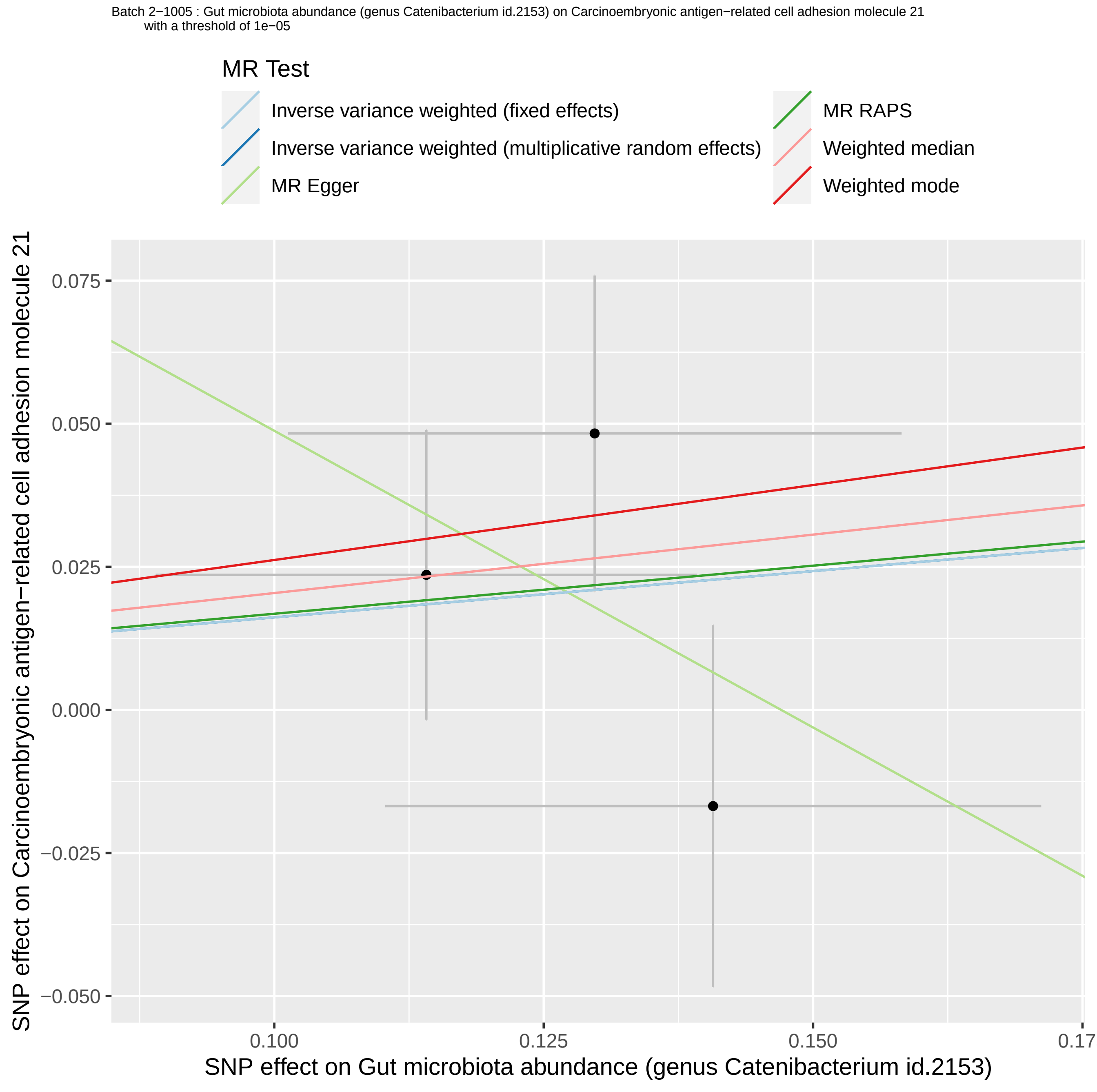
Batch 2−1005 : Gut microbiota abundance (genus Catenibacterium id.2153) on Carcinoembryonic antigen−related cell adhesion molecule 21
with a threshold of 1e−05
MR Test
Inverse variance weighted (fixed effects)
Inverse variance weighted (multiplicative random effects)
MR Egger
MR RAPS
Weighted median
Weighted mode
0.075
0.050
0.025
0.000
−0.025
−0.050
0.100
0.125
0.150
0.17
SNP effect on Carcinoembryonic antigen−related cell adhesion molecule 21
SNP effect on Gut microbiota abundance (genus Catenibacterium id.2153)

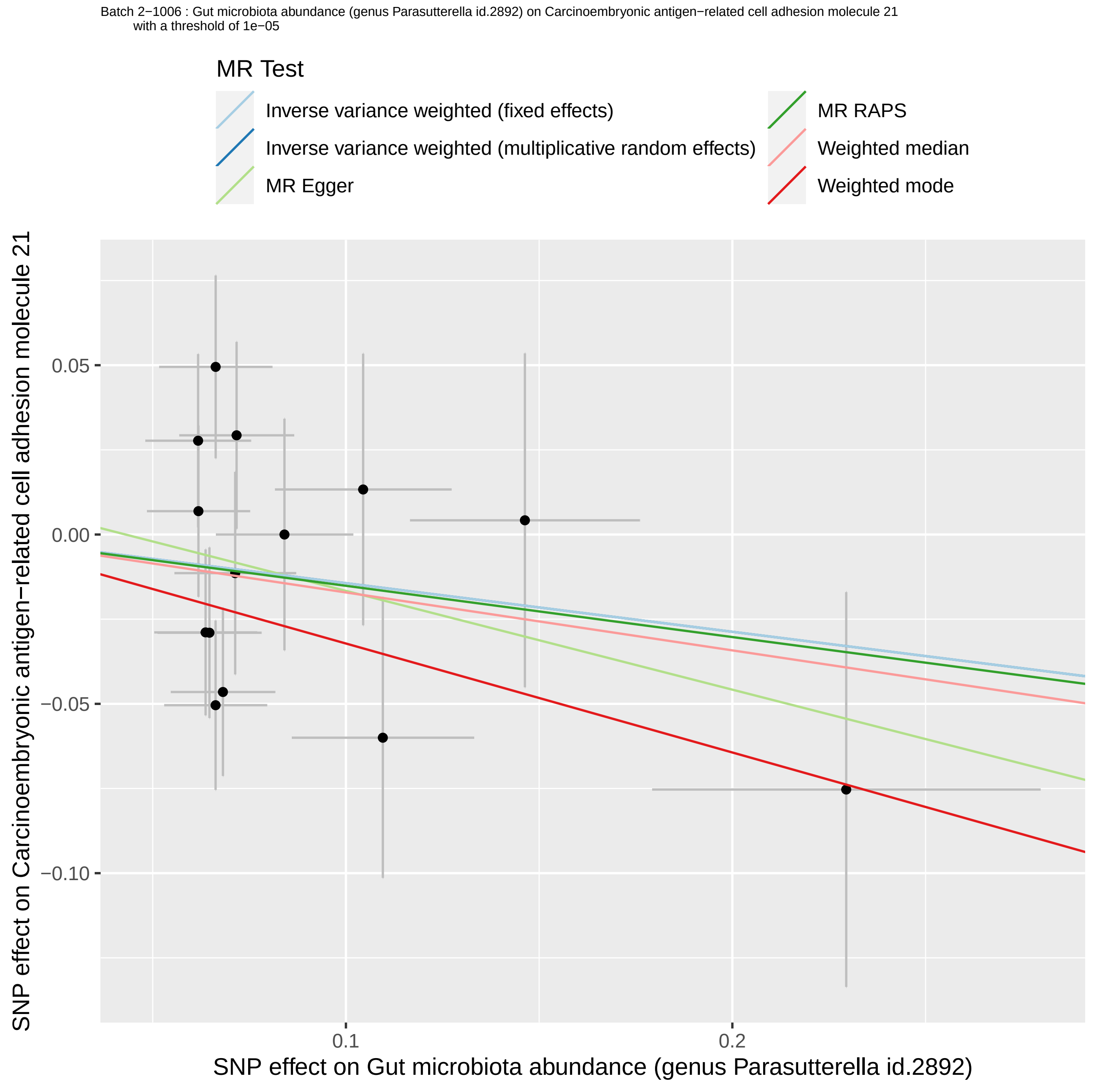

Batch 2−1006 : Gut microbiota abundance (genus Parasutterella id.2892) on Carcinoembryonic antigen−related cell adhesion molecule 21
with a threshold of 1e−05
MR Test
Inverse variance weighted (fixed effects)
Inverse variance weighted (multiplicative random effects)
MR Egger
MR RAPS
Weighted median
Weighted mode
SNP effect on Carcinoembryonic antigen−related cell adhesion molecule 21
0.05
0.00
−0.05
−0.10
0.1
0.2
SNP effect on Gut microbiota abundance (genus Parasutterella id.2892)

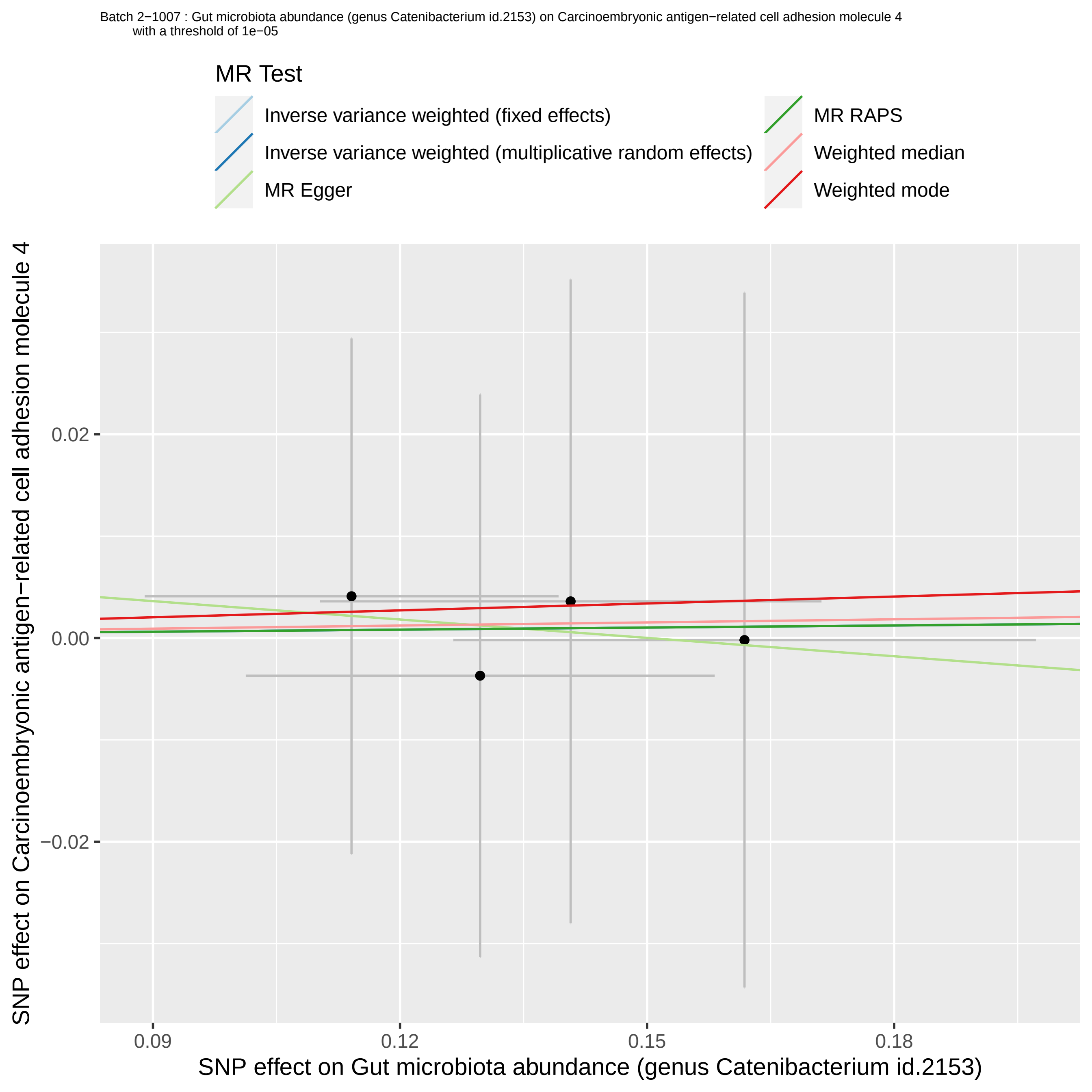

Batch 2−1007 : Gut microbiota abundance (genus Catenibacterium id.2153) on Carcinoembryonic antigen−related cell adhesion molecule 4
with a threshold of 1e−05
MR Test
Inverse variance weighted (fixed effects)
Inverse variance weighted (multiplicative random effects)
MR Egger
MR RAPS
Weighted median
Weighted mode
SNP effect on Carcinoembryonic antigen−related cell adhesion molecule 4
0.02
0.00
−0.02
0.09
0.12
0.15
0.18
SNP effect on Gut microbiota abundance (genus Catenibacterium id.2153)

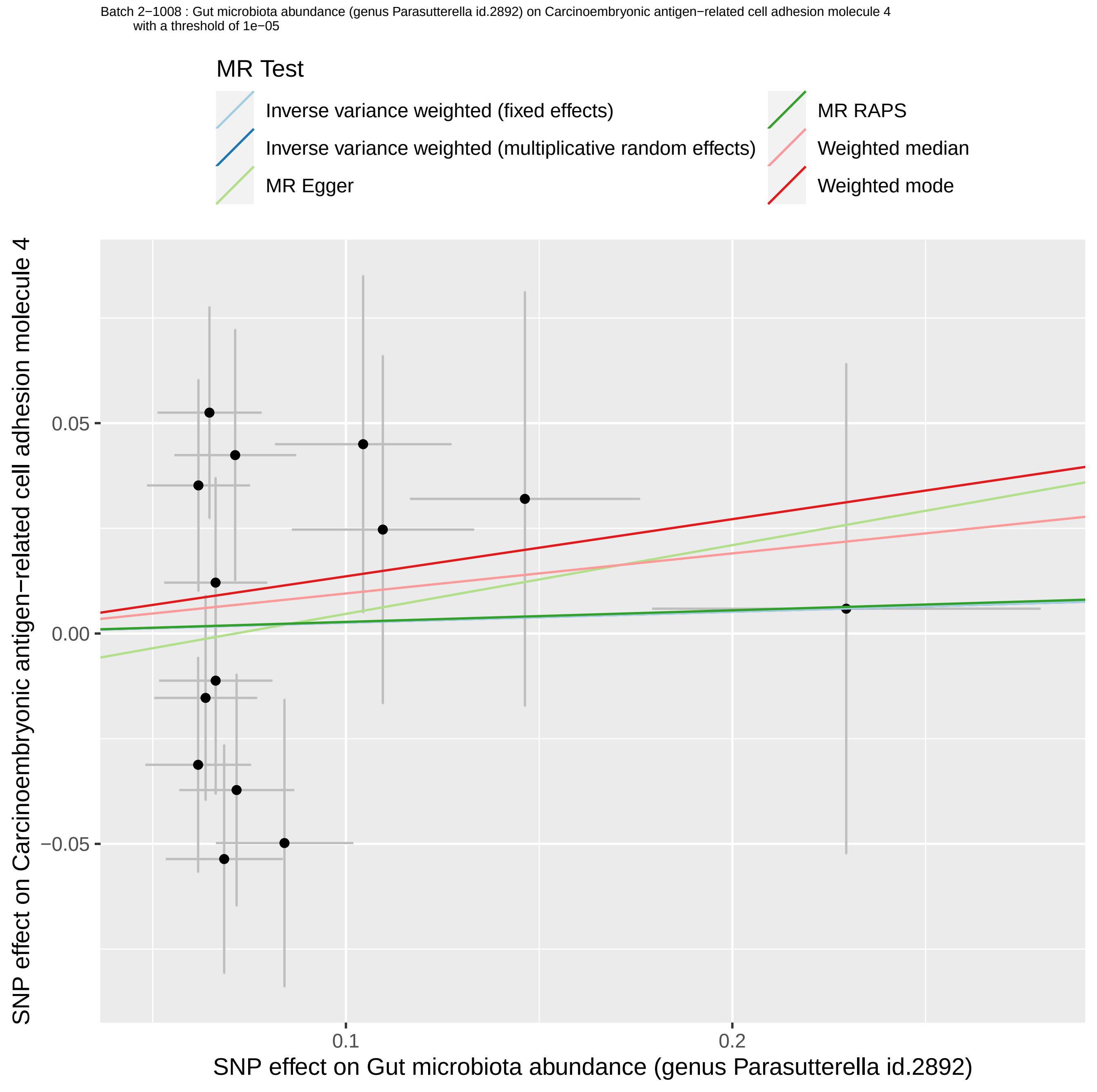
Batch 2−1008 : Gut microbiota abundance (genus Parasutterella id.2892) on Carcinoembryonic antigen−related cell adhesion molecule 4
with a threshold of 1e−05
MR Test
Inverse variance weighted (fixed effects)
Inverse variance weighted (multiplicative random effects)
MR Egger
MR RAPS
Weighted median
Weighted mode
0.05
0.00
−0.05
0.1
0.2
SNP effect on Carcinoembryonic antigen−related cell adhesion molecule 4
SNP effect on Gut microbiota abundance (genus Parasutterella id.2892)

Batch 2−1009 : Gut microbiota abundance (genus Catenibacterium id.2153) on CCAAT/enhancer−binding protein beta
with a threshold of 1e−05

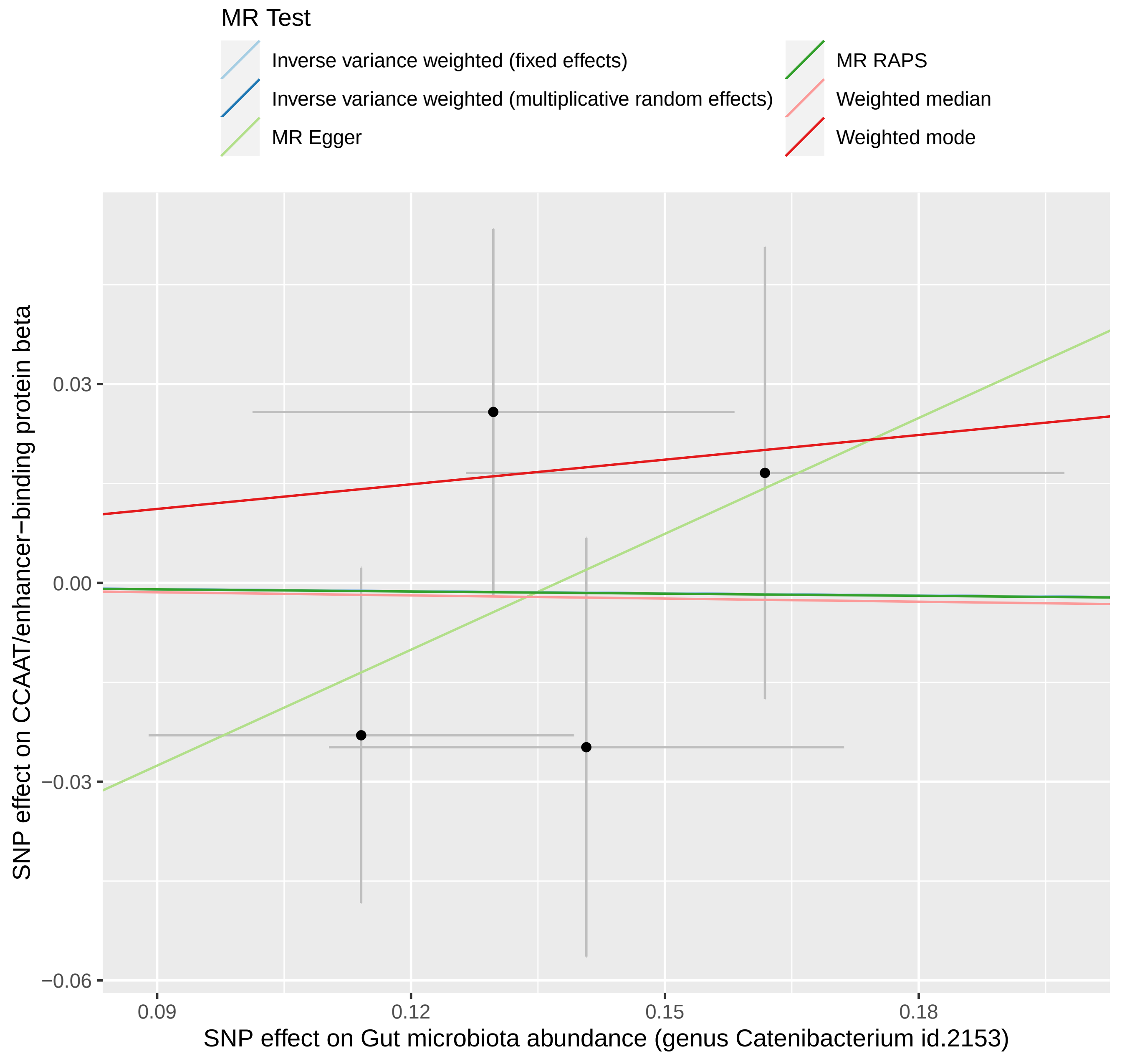

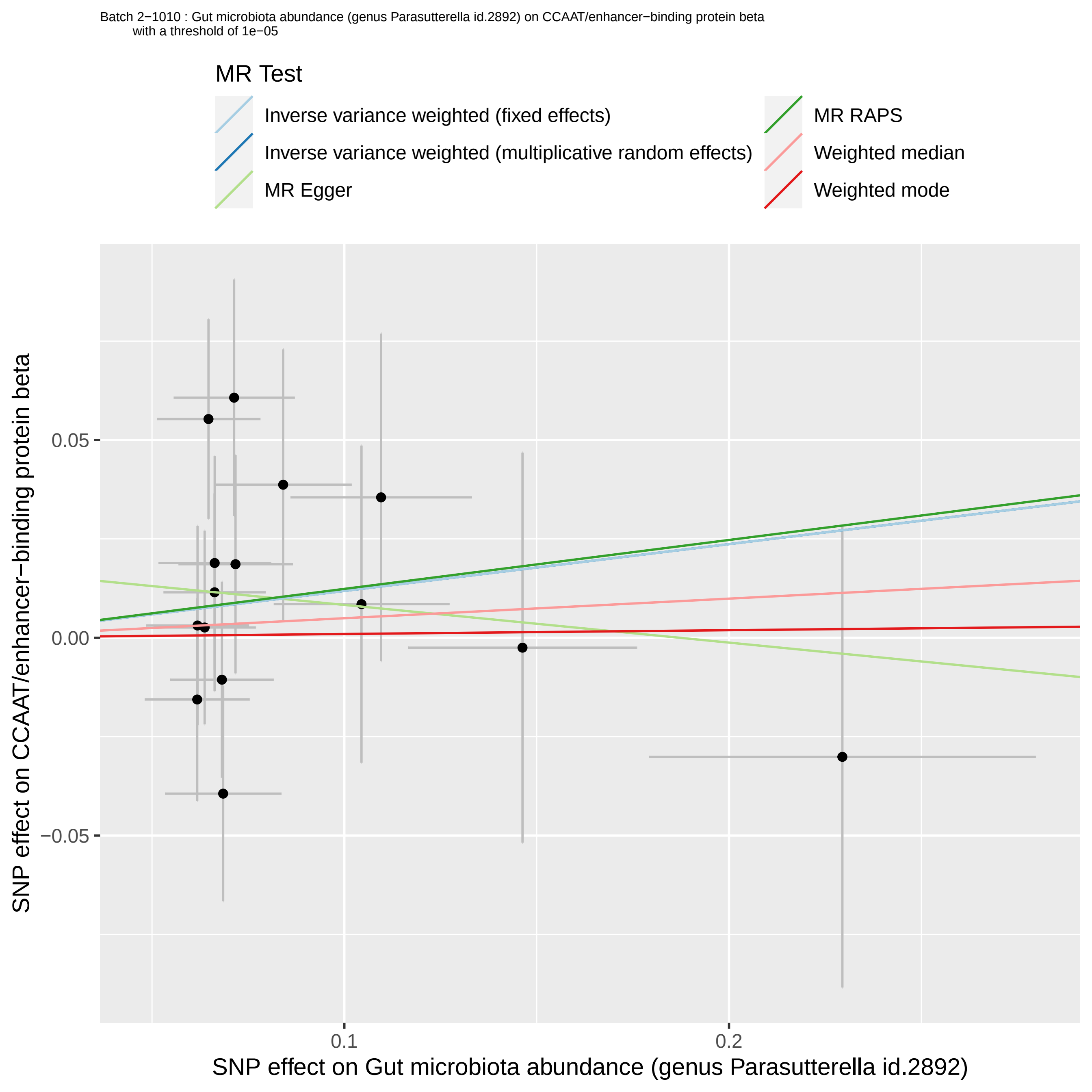
Batch 2−1010 : Gut microbiota abundance (genus Parasutterella id.2892) on CCAAT/enhancer−binding protein beta
with a threshold of 1e−05
MR Test
Inverse variance weighted (fixed effects)
Inverse variance weighted (multiplicative random effects)
MR Egger
MR RAPS
Weighted median
Weighted mode
0.05
0.00
−0.05
0.1
0.2
SNP effect on CCAAT/enhancer−binding protein beta
SNP effect on Gut microbiota abundance (genus Parasutterella id.2892)

Batch 2−1011 : Gut microbiota abundance (genus Catenibacterium id.2153) on Adenosine deaminase CECR1
with a threshold of 1e−05

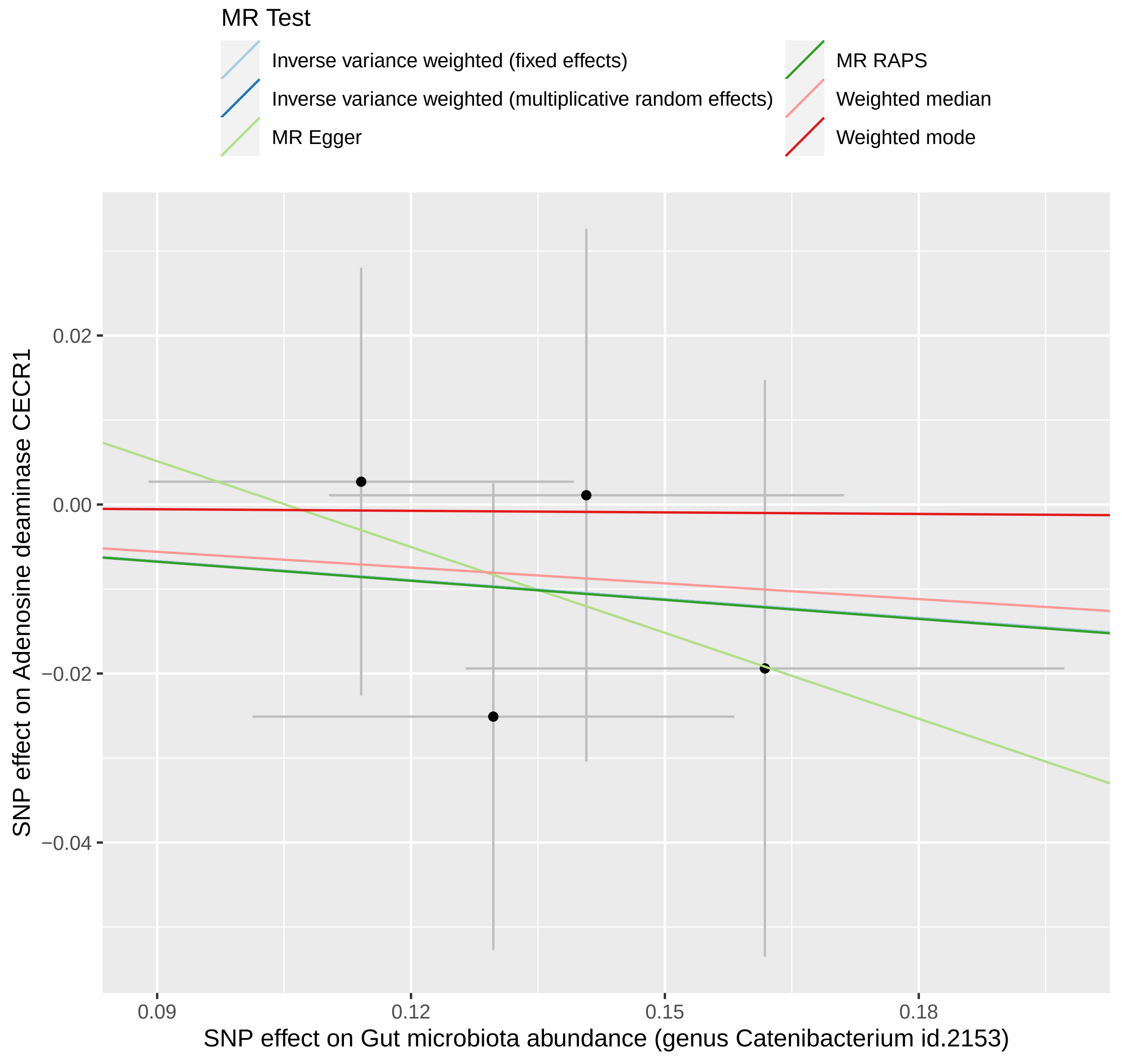

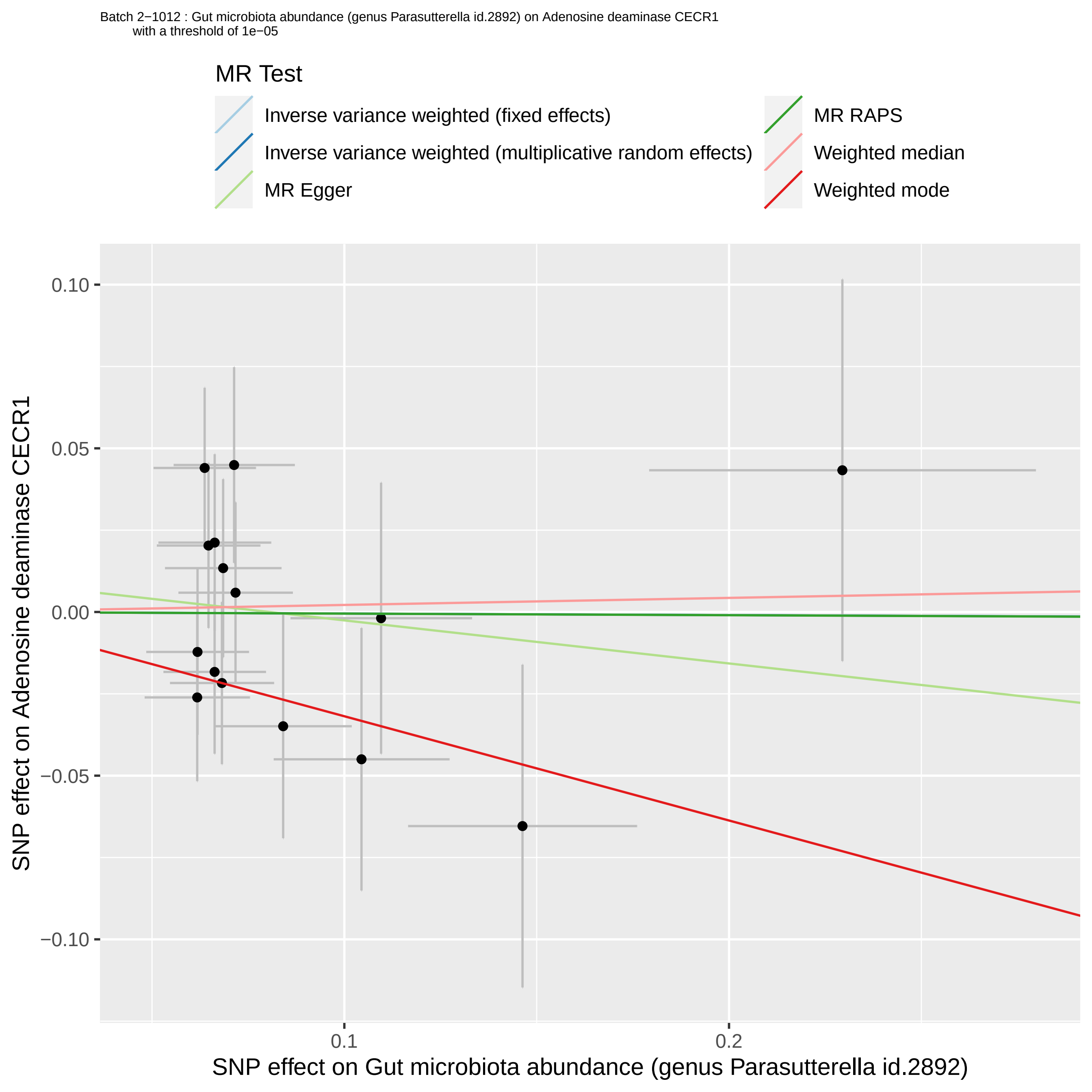

Batch 2−1012 : Gut microbiota abundance (genus Parasutterella id.2892) on Adenosine deaminase CECR1
with a threshold of 1e−05
MR Test
Inverse variance weighted (fixed effects)
Inverse variance weighted (multiplicative random effects)
MR Egger
MR RAPS
Weighted median
Weighted mode
0.10
0.05
0.00
−0.05
−0.10
0.1
0.2
SNP effect on Adenosine deaminase CECR1
SNP effect on Gut microbiota abundance (genus Parasutterella id.2892)

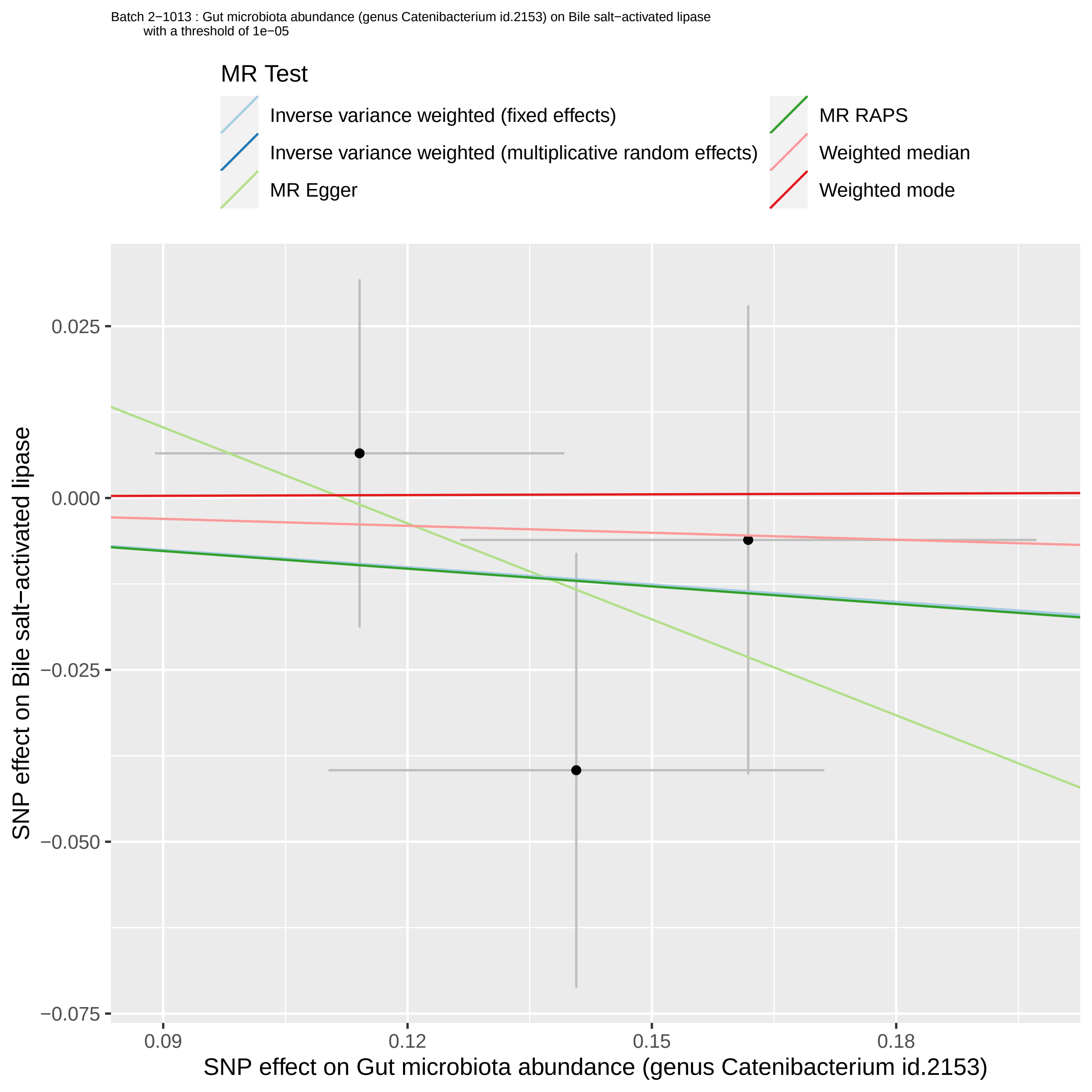

Batch 2−1013 : Gut microbiota abundance (genus Catenibacterium id.2153) on Bile salt−activated lipase
with a threshold of 1e−05
MR Test
Inverse variance weighted (fixed effects)
Inverse variance weighted (multiplicative random effects)
MR Egger
MR RAPS
Weighted median
Weighted mode
0.025
0.000
−0.025
−0.050
−0.075
0.09
0.12
0.15
0.18
SNP effect on Bile salt−activated lipase
SNP effect on Gut microbiota abundance (genus Catenibacterium id.2153)

Batch 2−1014 : Gut microbiota abundance (genus Parasutterella id.2892) on Bile salt−activated lipase
with a threshold of 1e−05

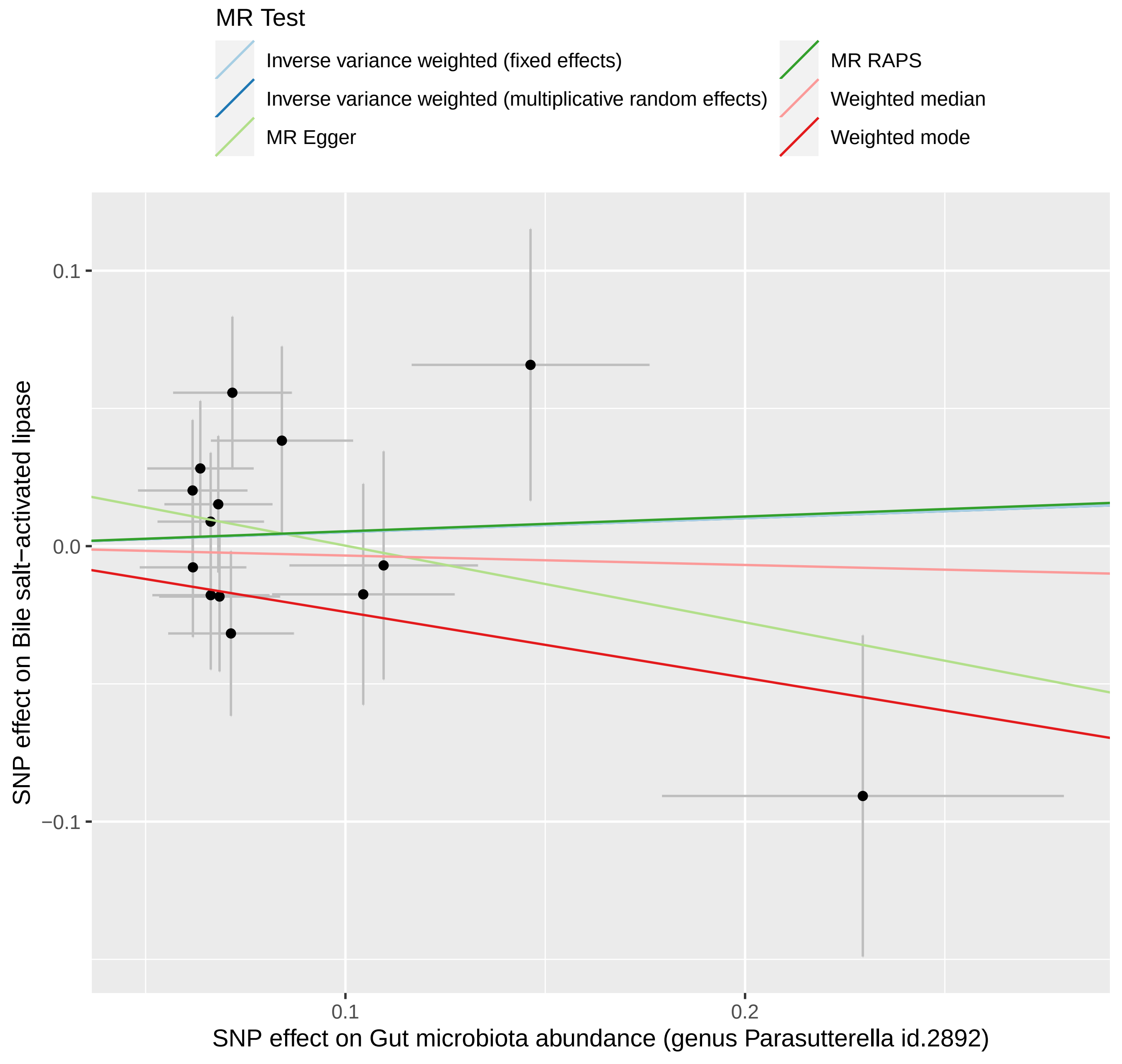

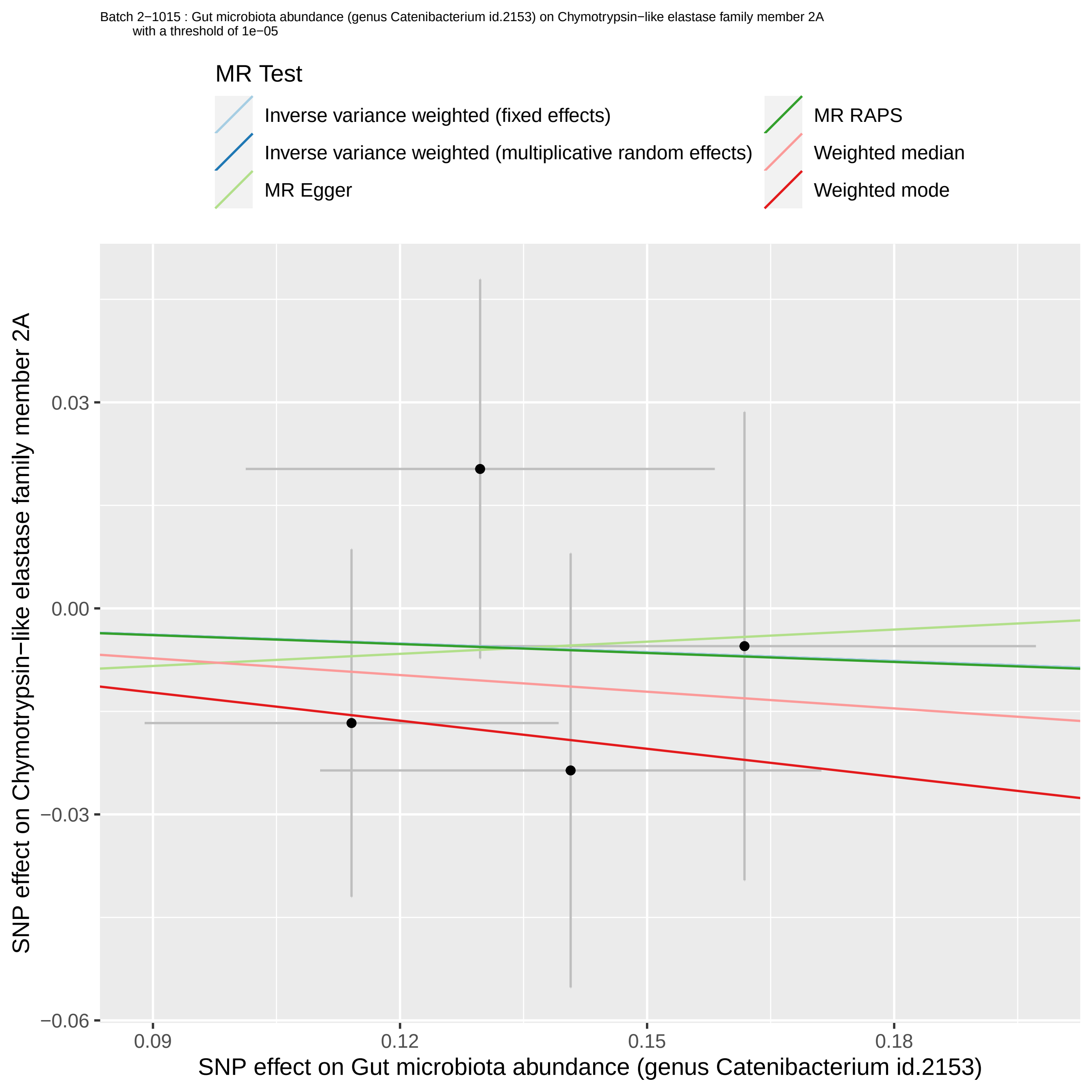
Batch 2−1015 : Gut microbiota abundance (genus Catenibacterium id.2153) on Chymotrypsin−like elastase family member 2A
with a threshold of 1e−05
MR Test
Inverse variance weighted (fixed effects)
Inverse variance weighted (multiplicative random effects)
MR Egger
MR RAPS
Weighted median
Weighted mode
0.03
0.00
−0.03
−0.06
0.09
0.12
0.15
0.18
SNP effect on Chymotrypsin−like elastase family member 2A
SNP effect on Gut microbiota abundance (genus Catenibacterium id.2153)

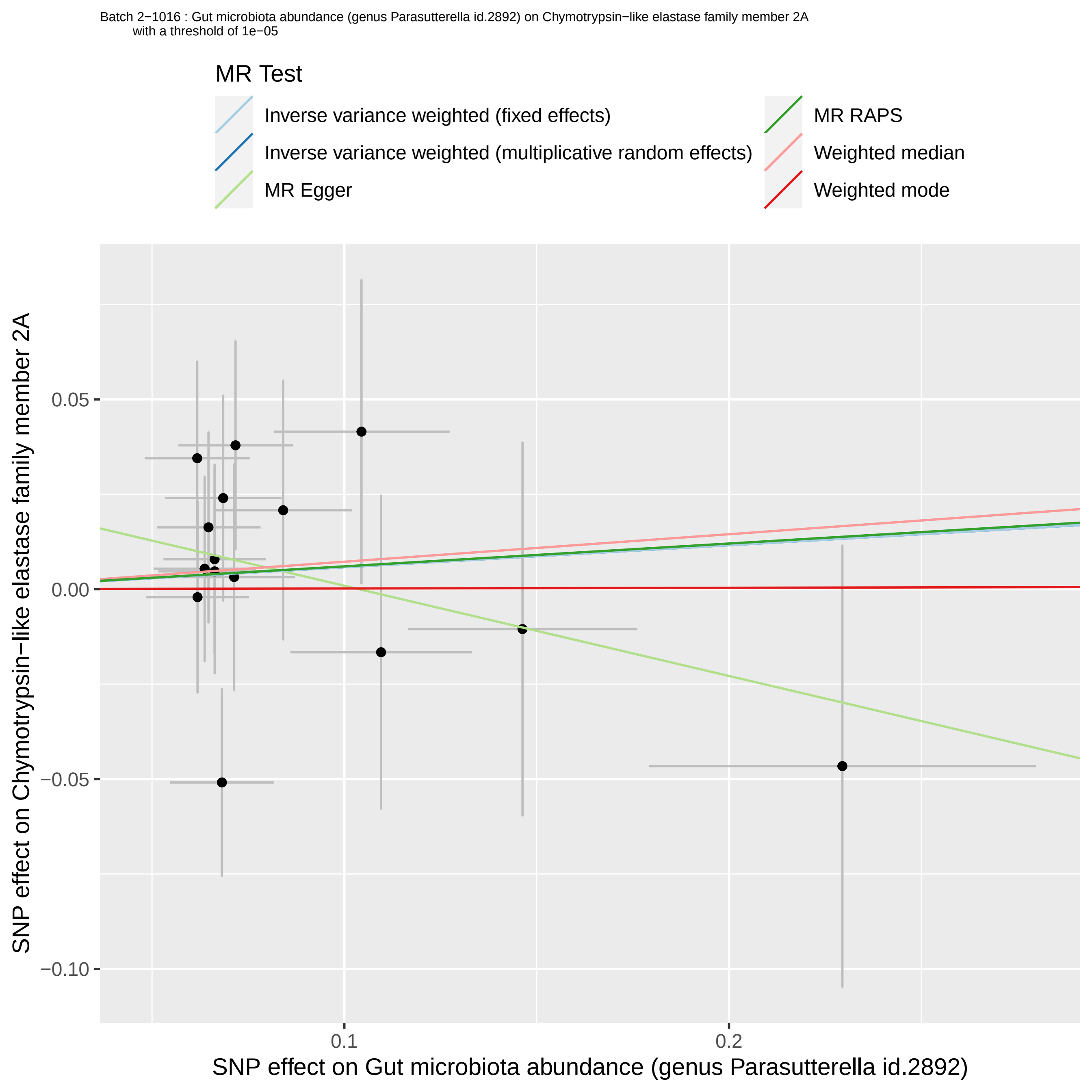
Batch 2−1016 : Gut microbiota abundance (genus Parasutterella id.2892) on Chymotrypsin−like elastase family member 2A
with a threshold of 1e−05
MR Test
Inverse variance weighted (fixed effects)
Inverse variance weighted (multiplicative random effects)
MR Egger
MR RAPS
Weighted median
Weighted mode
0.05
0.00
−0.05
−0.10
0.1
0.2
SNP effect on Chymotrypsin−like elastase family member 2A
SNP effect on Gut microbiota abundance (genus Parasutterella id.2892)

Batch 2−1017 : Gut microbiota abundance (genus Catenibacterium id.2153) on Chymotrypsin−like elastase family member 3B
with a threshold of 1e−05

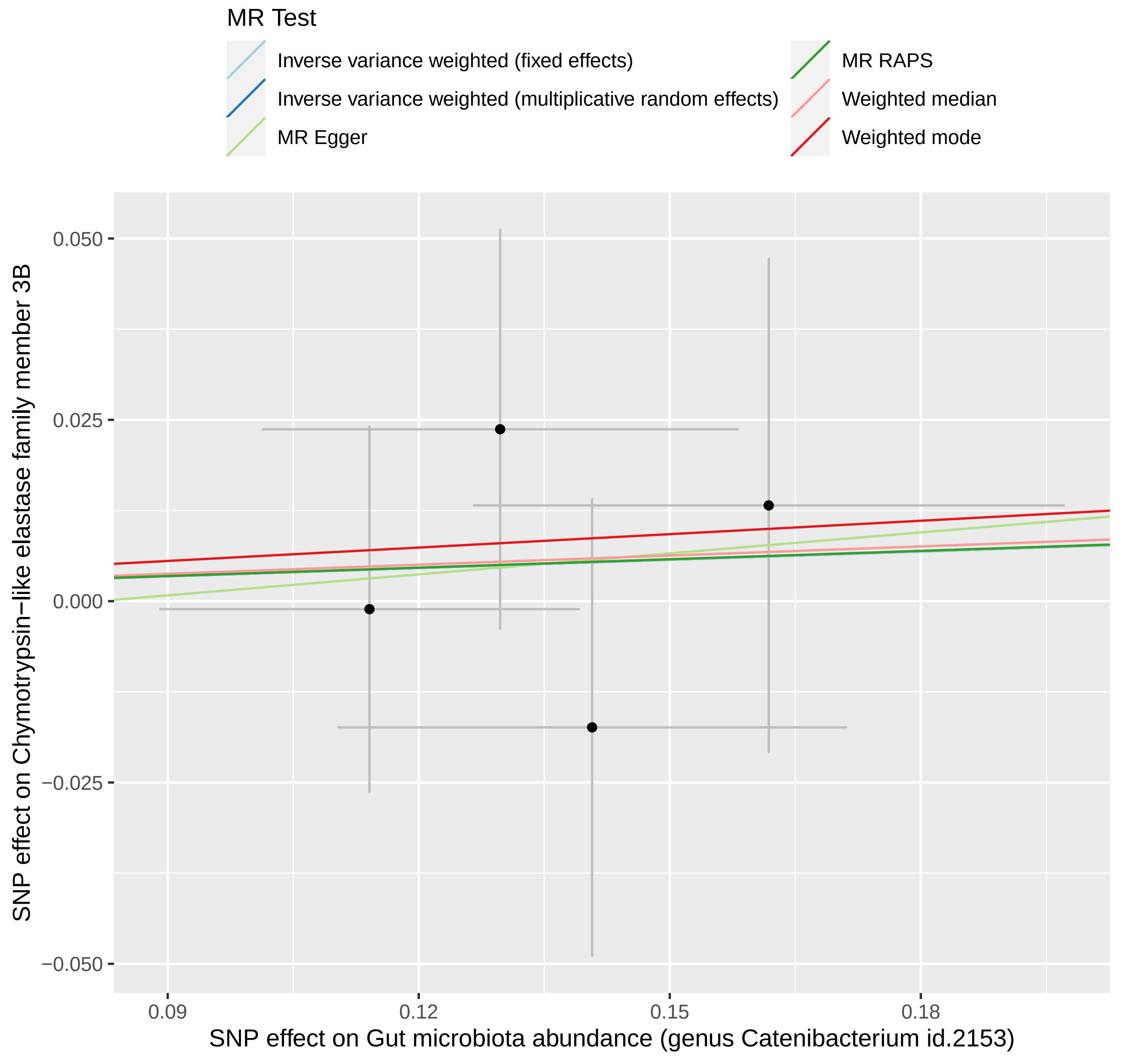

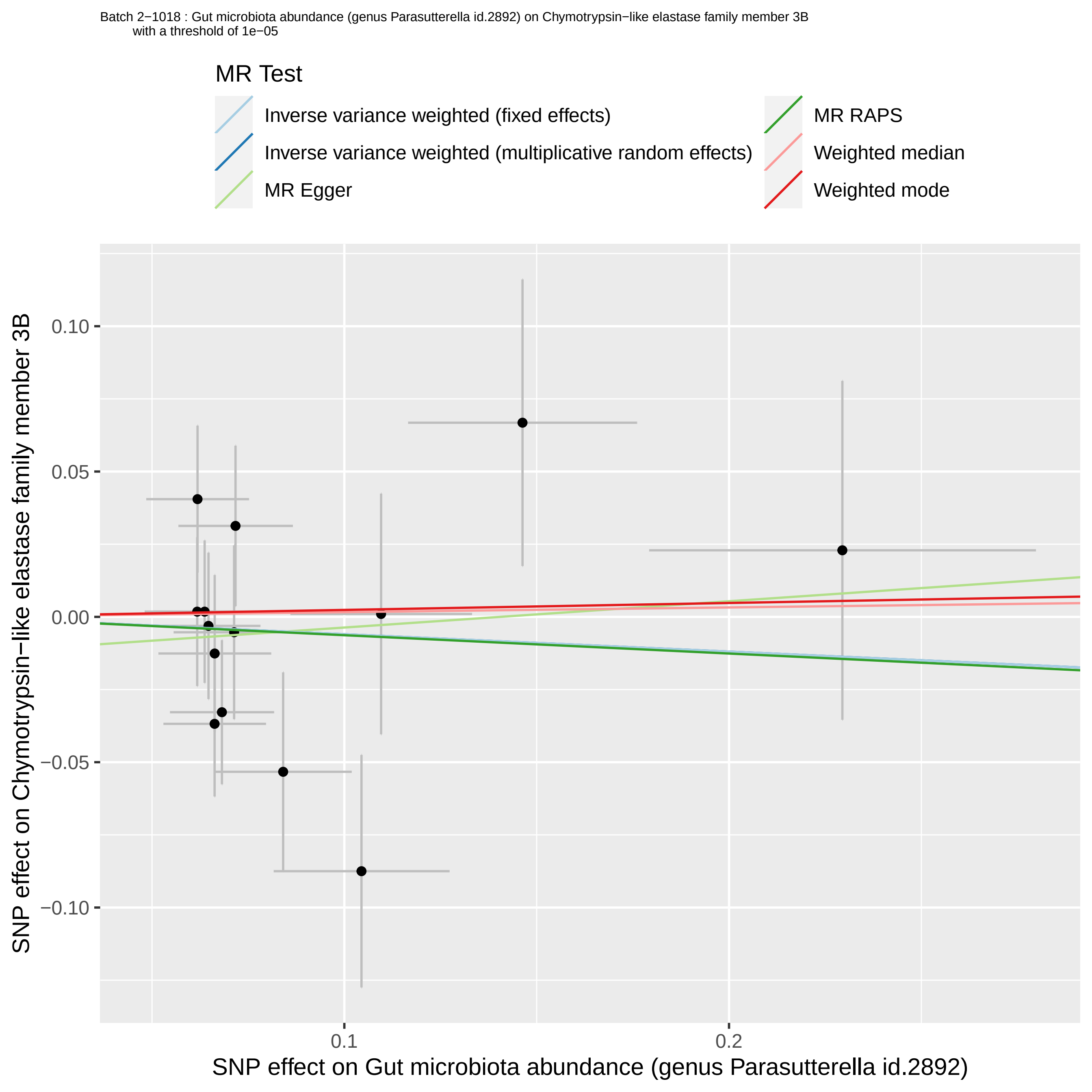
Batch 2−1018 : Gut microbiota abundance (genus Parasutterella id.2892) on Chymotrypsin−like elastase family member 3B
with a threshold of 1e−05
MR Test
Inverse variance weighted (fixed effects)
Inverse variance weighted (multiplicative random effects)
MR Egger
MR RAPS
Weighted median
Weighted mode
0.10
0.05
0.00
−0.05
−0.10
0.1
0.2
SNP effect on Chymotrypsin−like elastase family member 3B
SNP effect on Gut microbiota abundance (genus Parasutterella id.2892)

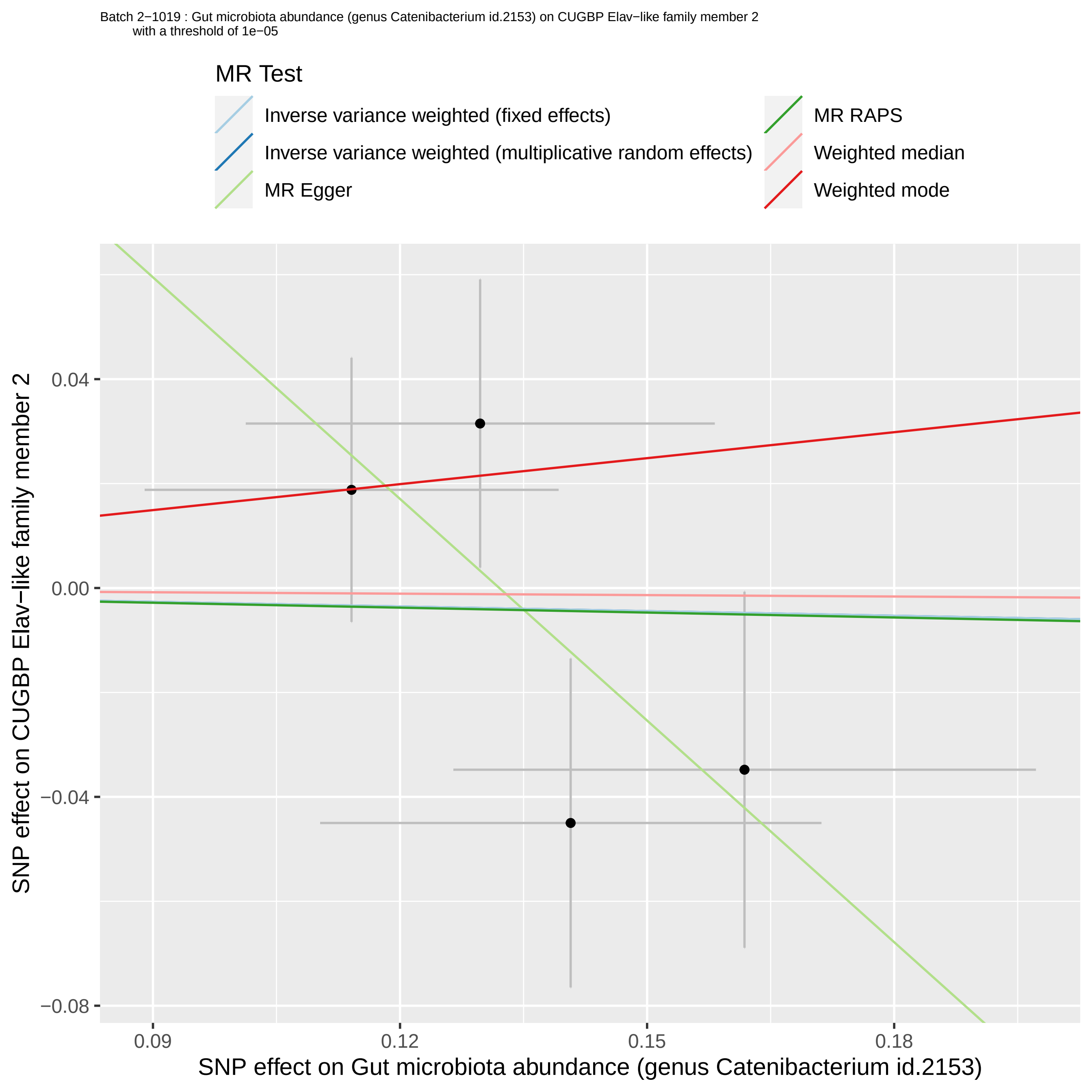

Batch 2−1019 : Gut microbiota abundance (genus Catenibacterium id.2153) on CUGBP Elav−like family member 2
with a threshold of 1e−05
MR Test
Inverse variance weighted (fixed effects)
Inverse variance weighted (multiplicative random effects)
MR Egger
MR RAPS
Weighted median
Weighted mode
SNP effect on CUGBP Elav−like family member 2
0.04
0.00
−0.04
−0.08
0.09
0.12
0.15
0.18
SNP effect on Gut microbiota abundance (genus Catenibacterium id.2153)

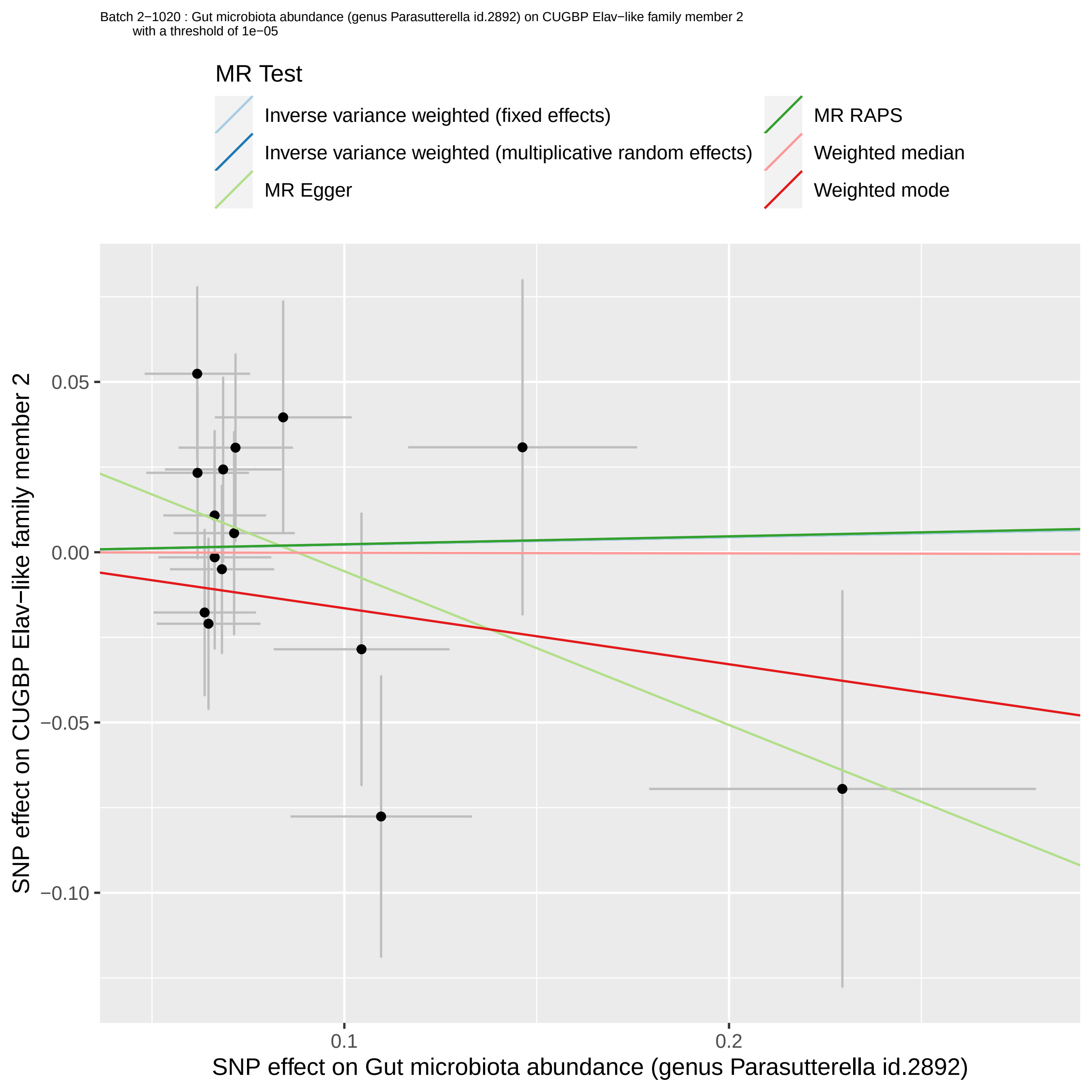
Batch 2−1020 : Gut microbiota abundance (genus Parasutterella id.2892) on CUGBP Elav−like family member 2
with a threshold of 1e−05
MR Test
Inverse variance weighted (fixed effects)
Inverse variance weighted (multiplicative random effects)
MR Egger
MR RAPS
Weighted median
Weighted mode
0.05
0.00
−0.05
−0.10
0.1
0.2
SNP effect on CUGBP Elav−like family member 2
SNP effect on Gut microbiota abundance (genus Parasutterella id.2892)

Batch 2−1021 : Gut microbiota abundance (genus Catenibacterium id.2153) on Centromere protein W
with a threshold of 1e−05

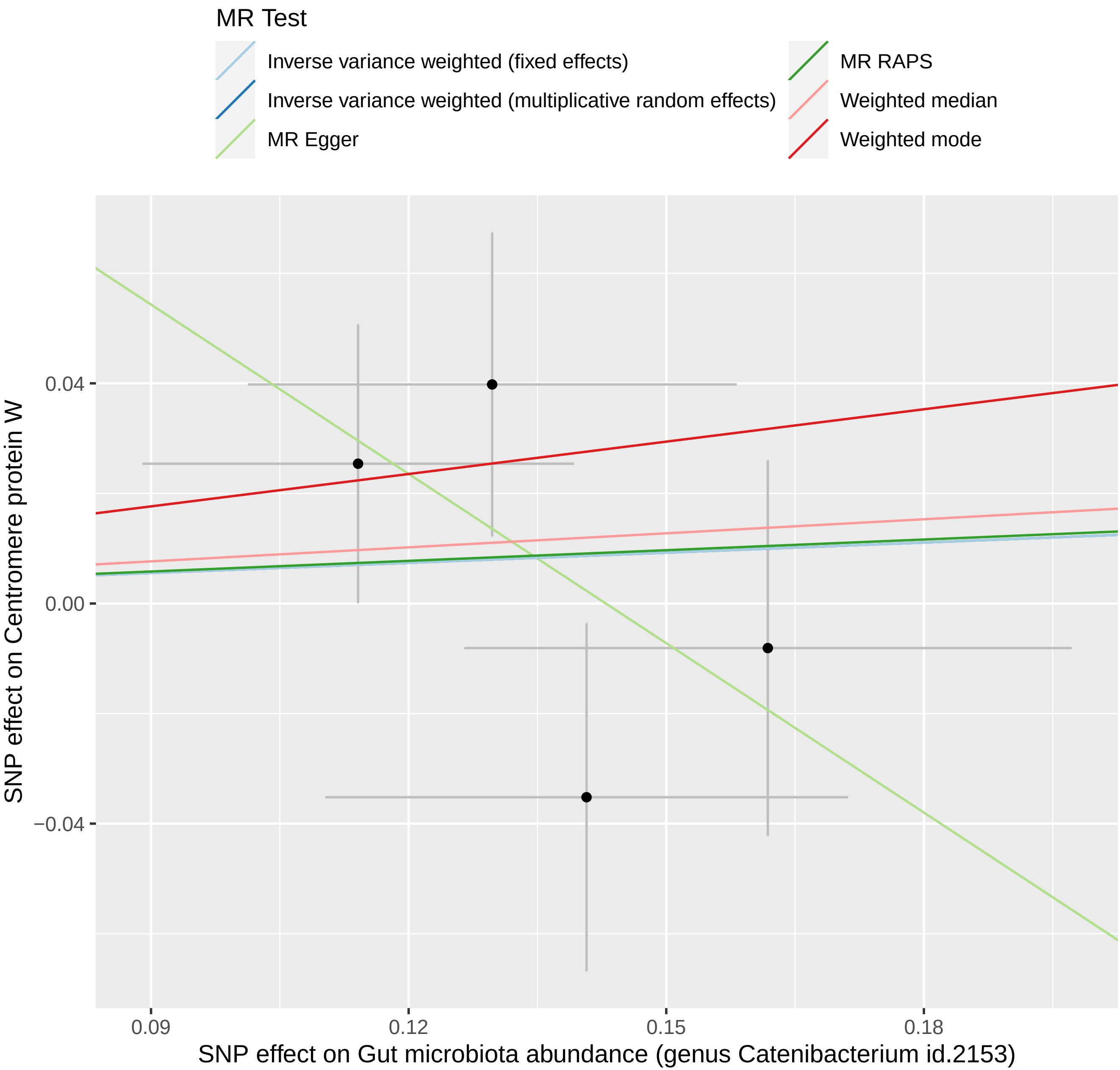

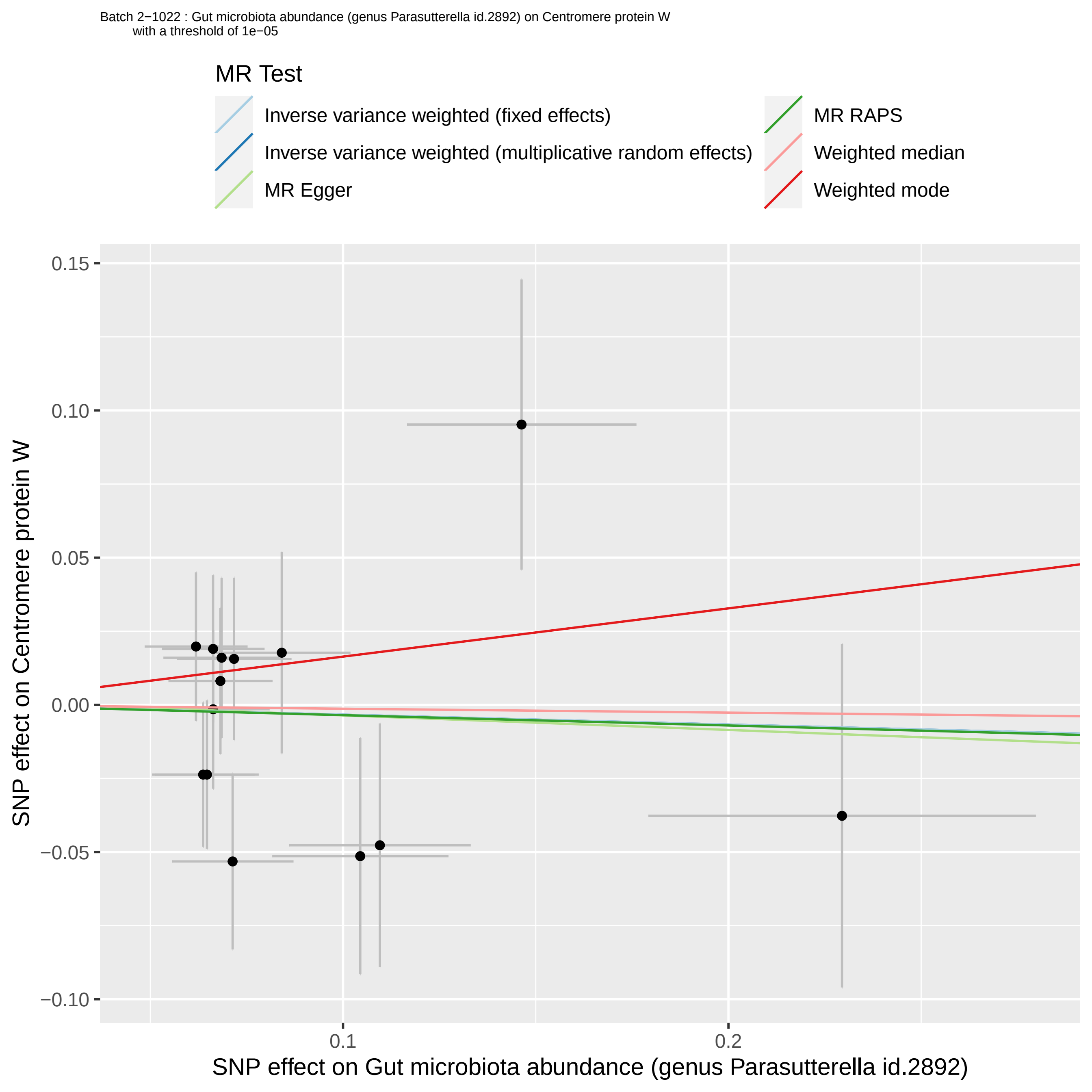

Batch 2−1022 : Gut microbiota abundance (genus Parasutterella id.2892) on Centromere protein W
with a threshold of 1e−05
MR Test
Inverse variance weighted (fixed effects)
Inverse variance weighted (multiplicative random effects)
MR Egger
MR RAPS
Weighted median
Weighted mode
0.15
0.10
0.05
0.00
−0.05
−0.10
0.1
0.2
SNP effect on Centromere protein W
SNP effect on Gut microbiota abundance (genus Parasutterella id.2892)

Batch 2−1023 : Gut microbiota abundance (genus Catenibacterium id.2153) on Centrosomal protein of 57 kDa
with a threshold of 1e−05

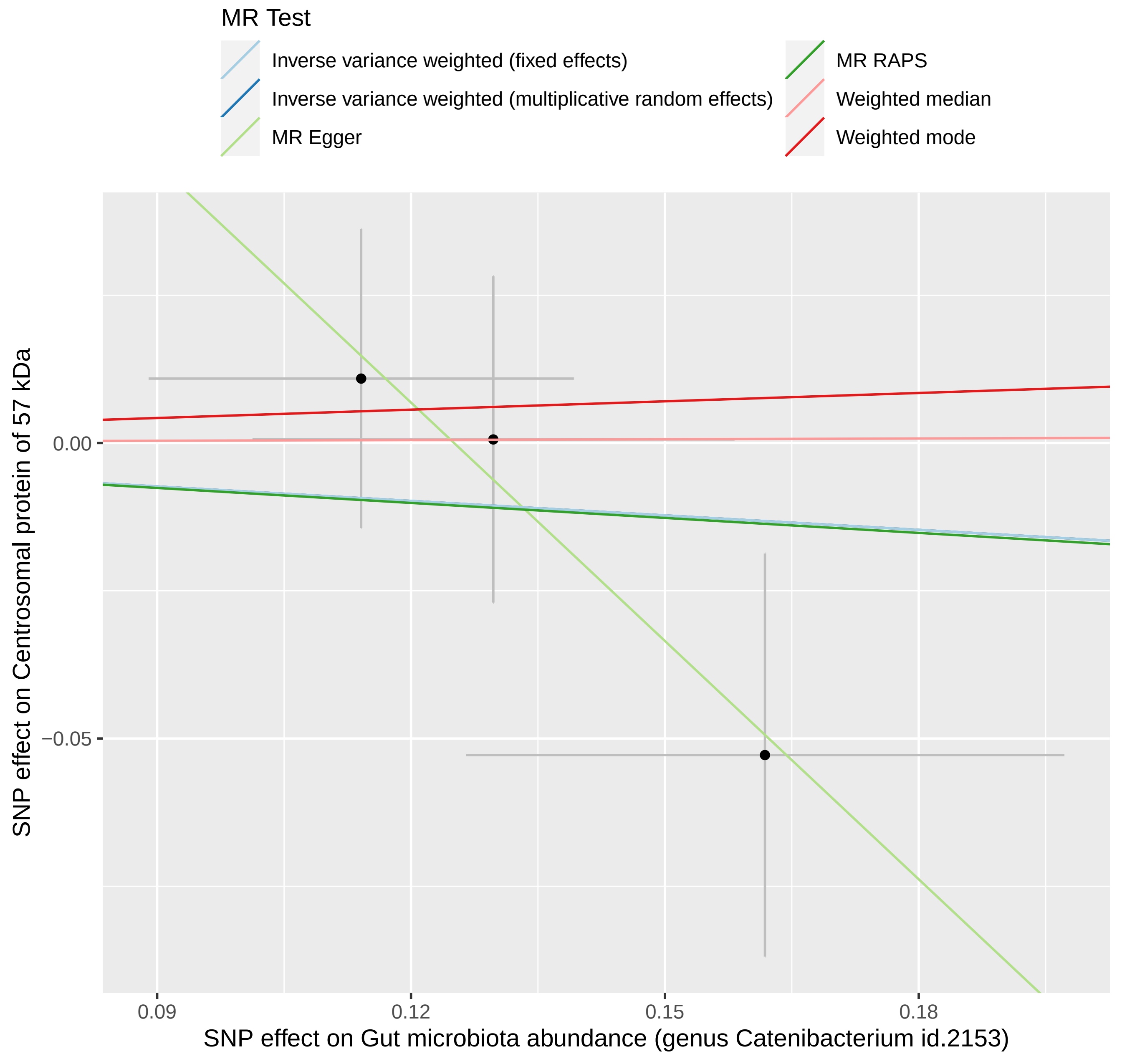

Batch 2−1024 : Gut microbiota abundance (genus Parasutterella id.2892) on Centrosomal protein of 57 kDa
with a threshold of 1e−05

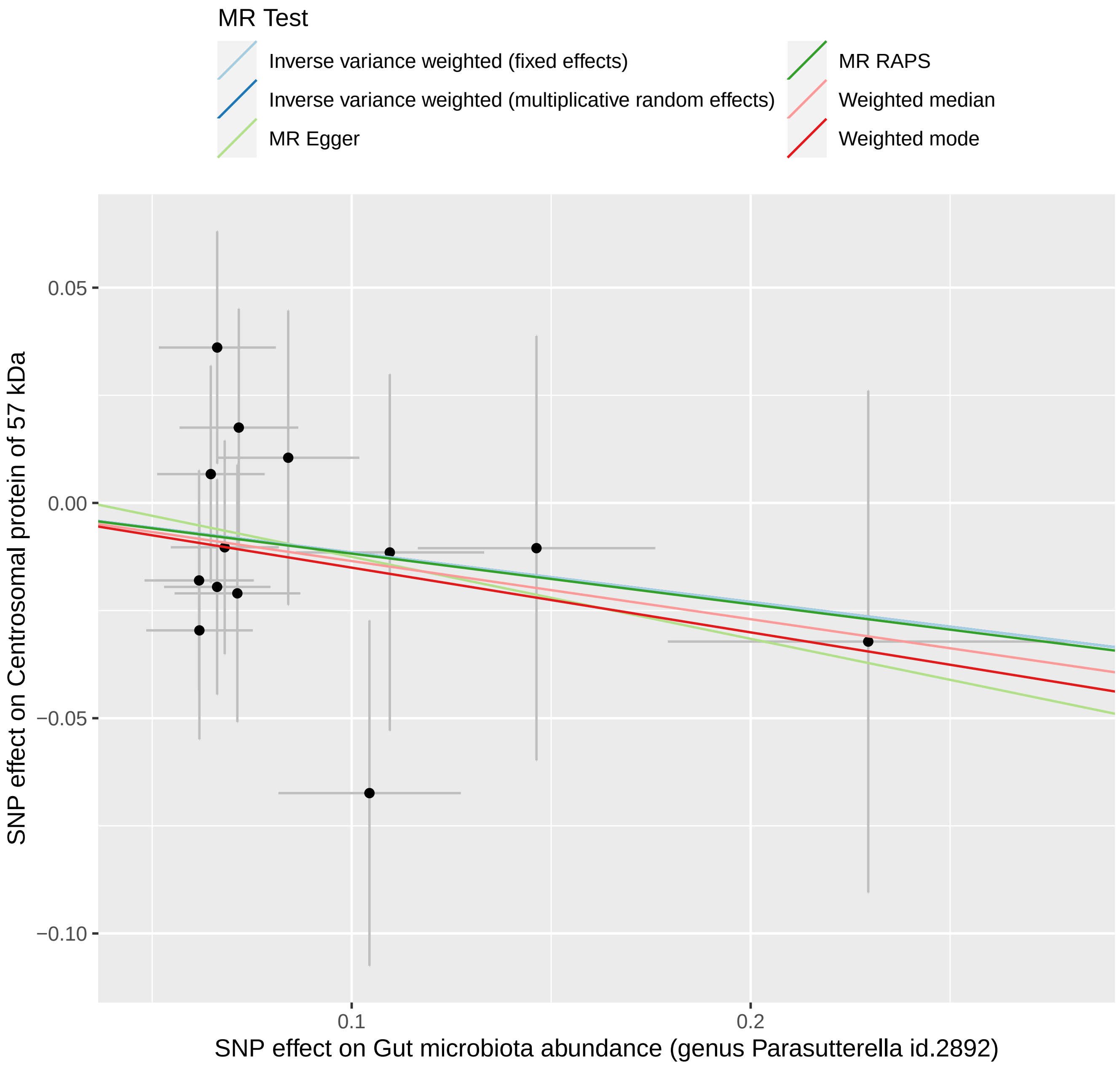

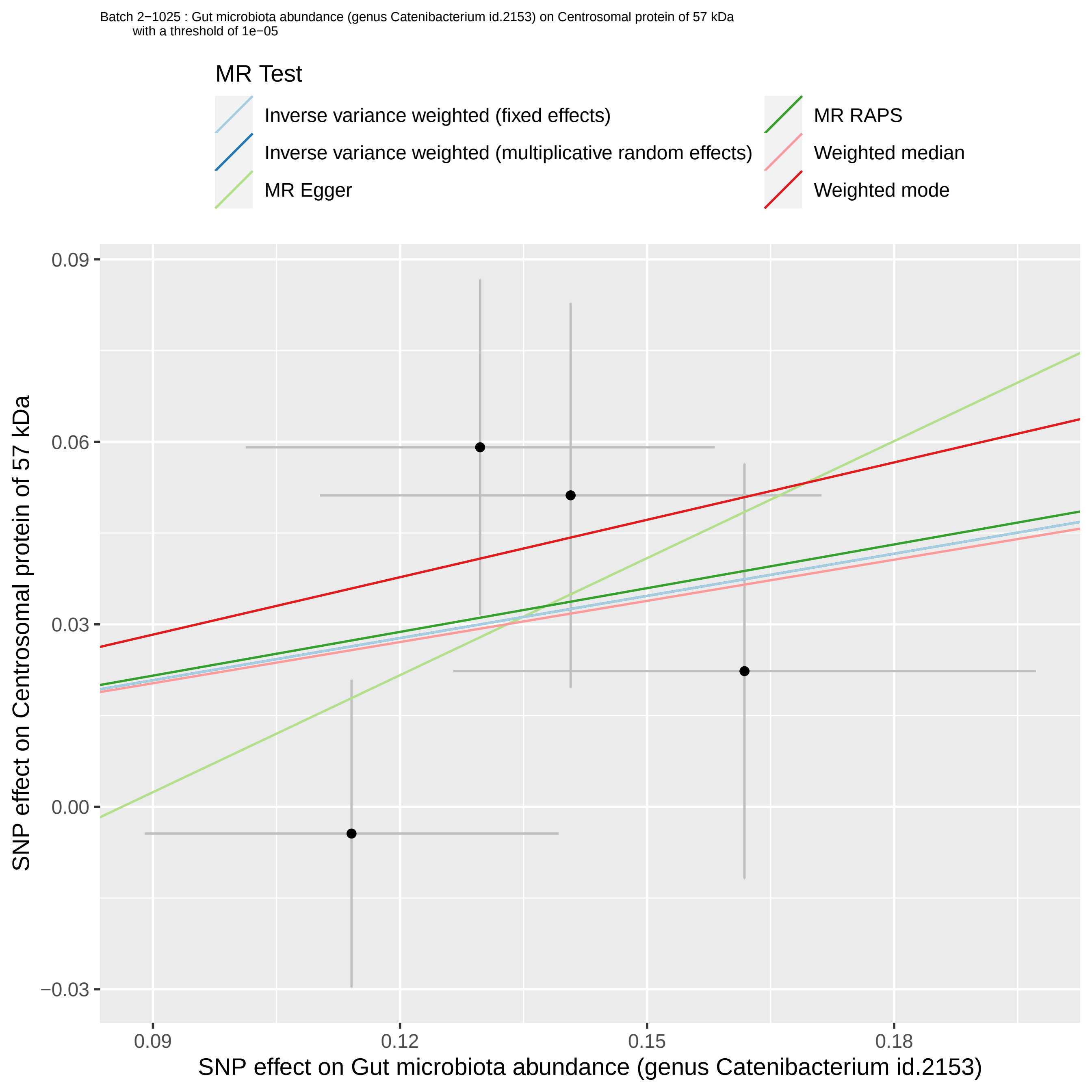

Batch 2−1025 : Gut microbiota abundance (genus Catenibacterium id.2153) on Centrosomal protein of 57 kDa
with a threshold of 1e−05
MR Test
Inverse variance weighted (fixed effects)
Inverse variance weighted (multiplicative random effects)
MR Egger
MR RAPS
Weighted median
Weighted mode
0.09
0.06
0.03
0.00
−0.03
0.09
0.12
0.15
0.18
SNP effect on Centrosomal protein of 57 kDa
SNP effect on Gut microbiota abundance (genus Catenibacterium id.2153)

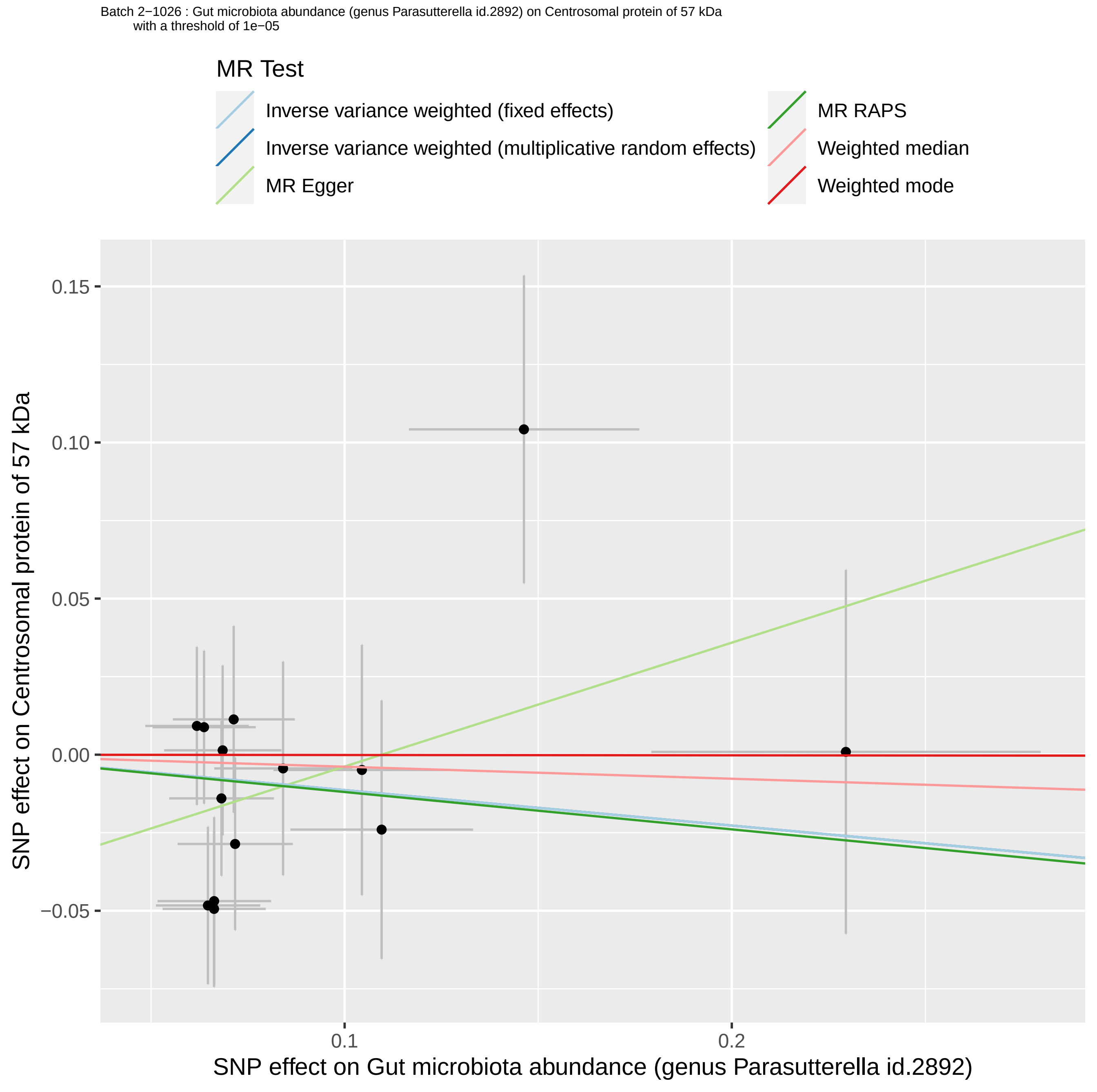

Batch 2−1026 : Gut microbiota abundance (genus Parasutterella id.2892) on Centrosomal protein of 57 kDa
with a threshold of 1e−05
MR Test
Inverse variance weighted (fixed effects)
Inverse variance weighted (multiplicative random effects)
MR Egger
MR RAPS
Weighted median
Weighted mode
0.15
0.10
0.05
0.00
−0.05
0.1
0.2
SNP effect on Centrosomal protein of 57 kDa
SNP effect on Gut microbiota abundance (genus Parasutterella id.2892)

Batch 2−1027 : Gut microbiota abundance (genus Catenibacterium id.2153) on Centrosomal protein of 57 kDa
with a threshold of 1e−05

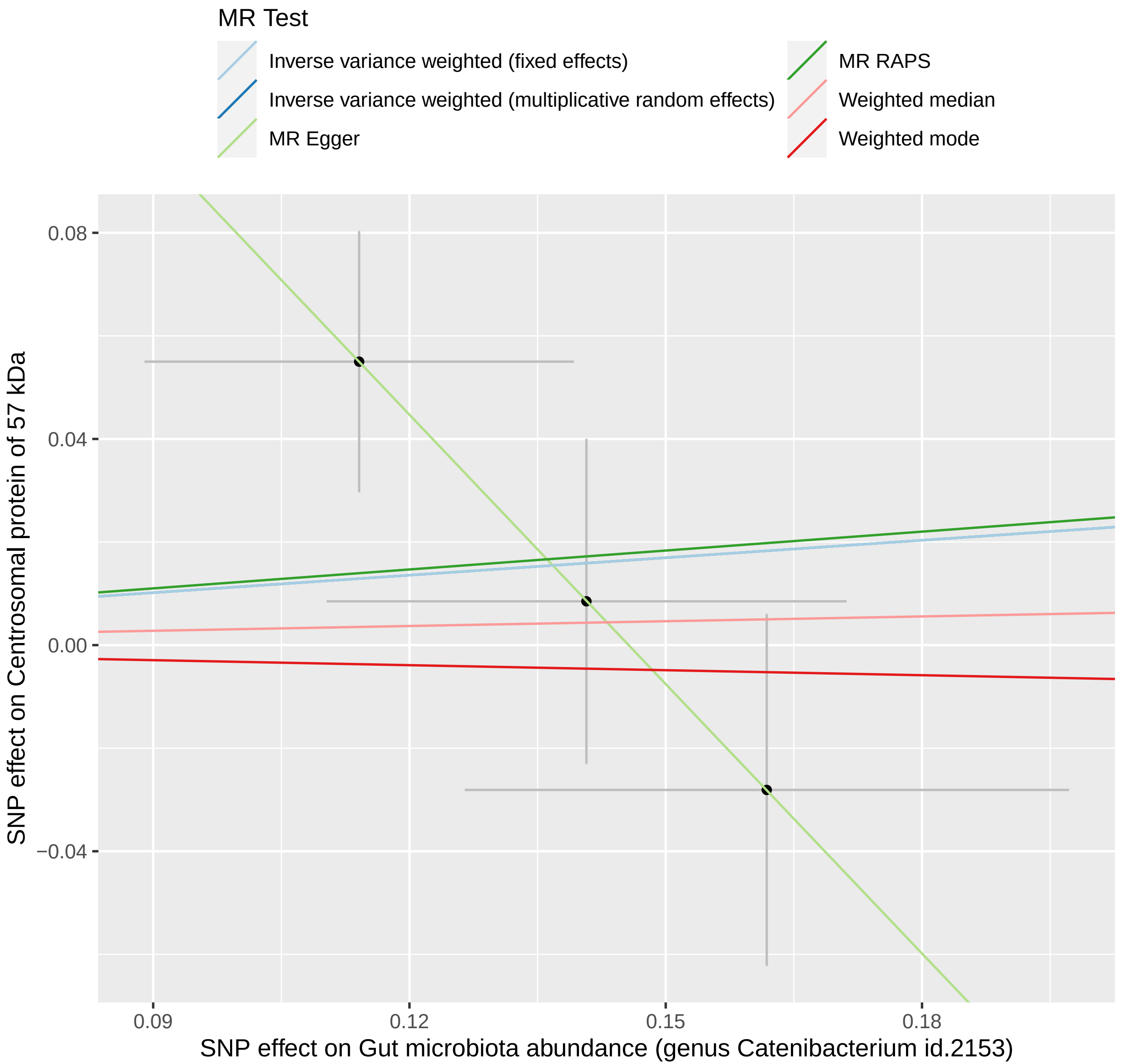

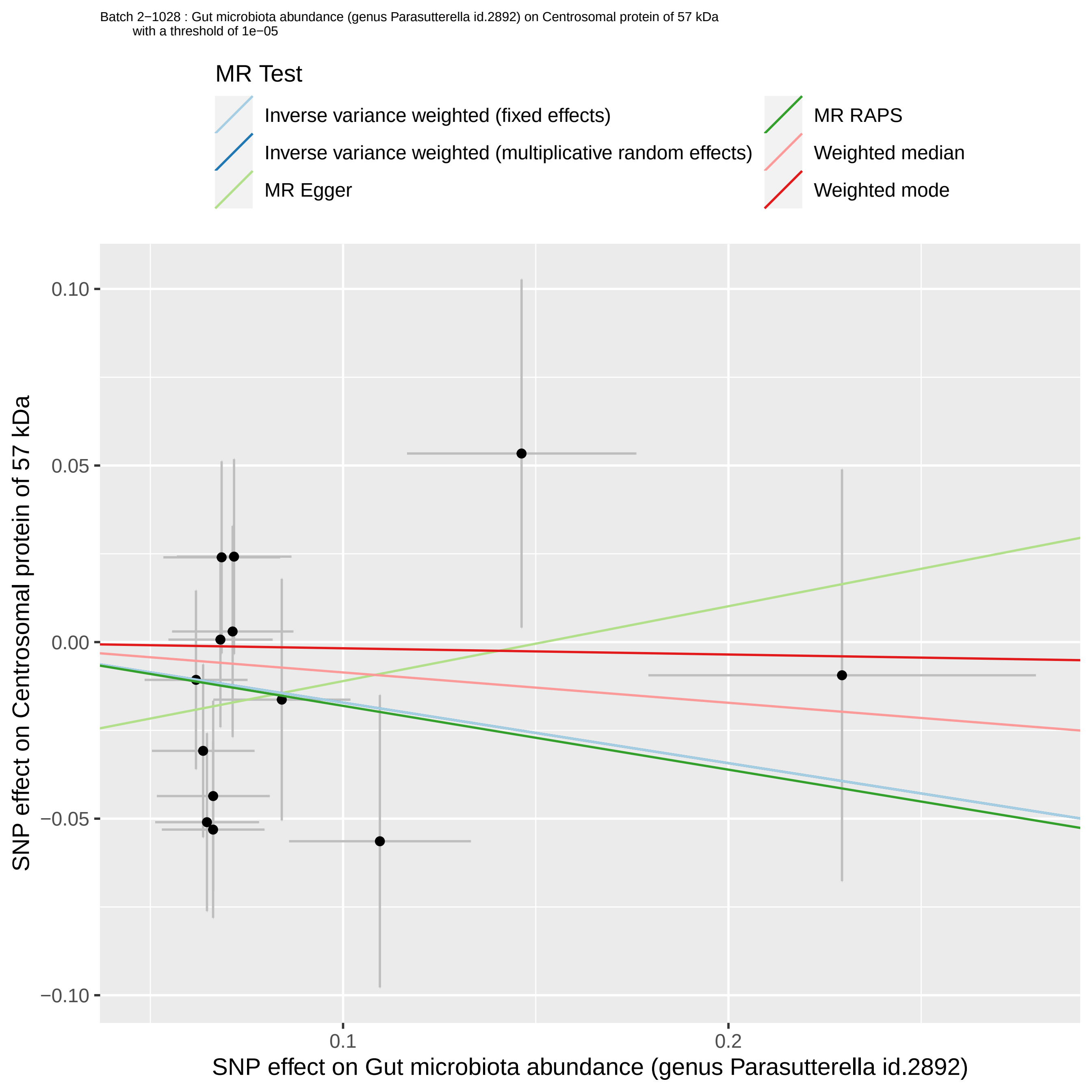

Batch 2−1028 : Gut microbiota abundance (genus Parasutterella id.2892) on Centrosomal protein of 57 kDa
with a threshold of 1e−05
MR Test
Inverse variance weighted (fixed effects)
Inverse variance weighted (multiplicative random effects)
MR Egger
MR RAPS
Weighted median
Weighted mode
SNP effect on Centrosomal protein of 57 kDa
0.10
0.05
0.00
−0.05
−0.10
0.1
0.2
SNP effect on Gut microbiota abundance (genus Parasutterella id.2892)

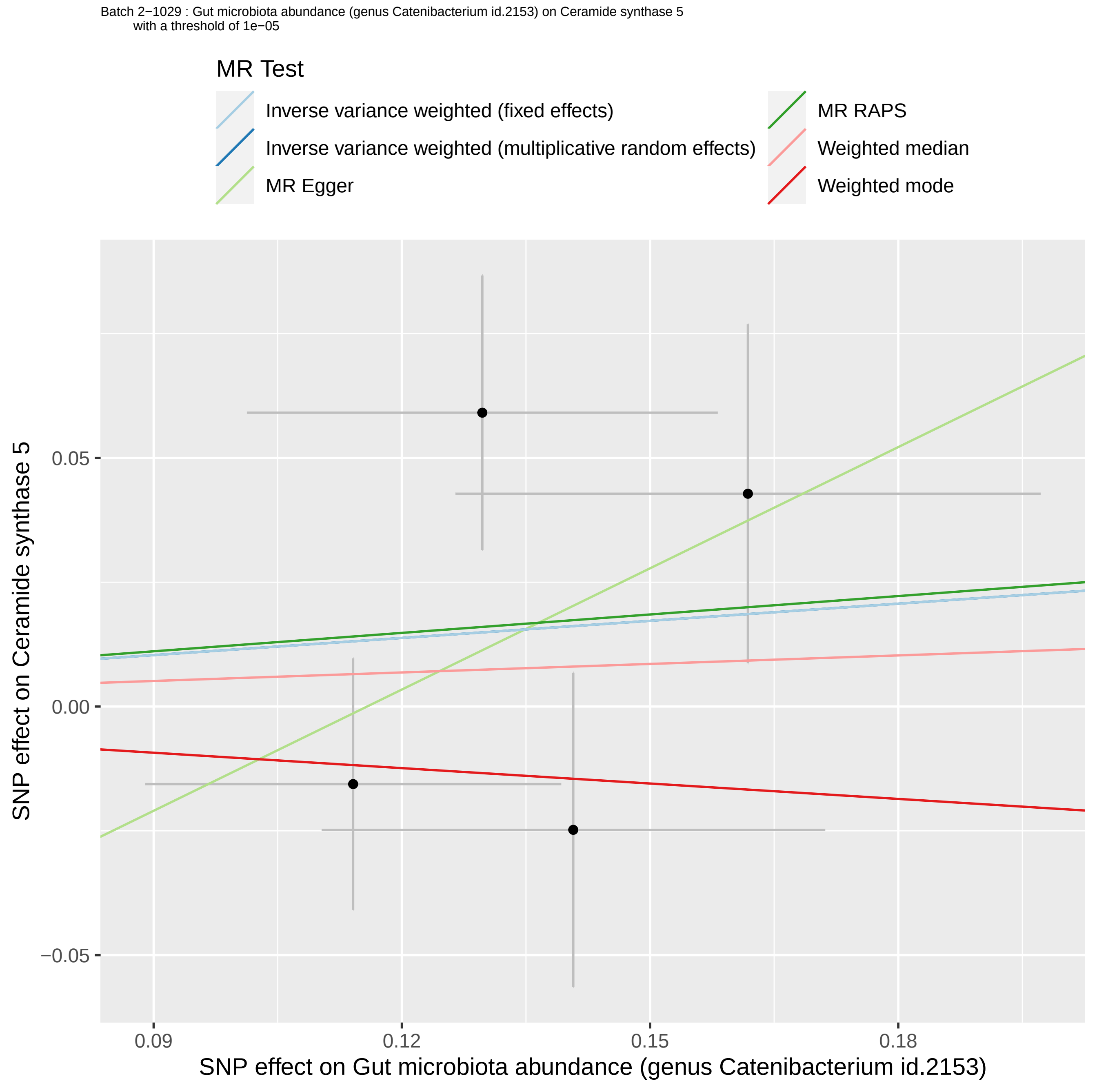

Batch 2−1029 : Gut microbiota abundance (genus Catenibacterium id.2153) on Ceramide synthase 5
with a threshold of 1e−05
MR Test
Inverse variance weighted (fixed effects)
Inverse variance weighted (multiplicative random effects)
MR Egger
MR RAPS
Weighted median
Weighted mode
SNP effect on Ceramide synthase 5
0.05
0.00
−0.05
0.09
0.12
0.15
0.18
SNP effect on Gut microbiota abundance (genus Catenibacterium id.2153)

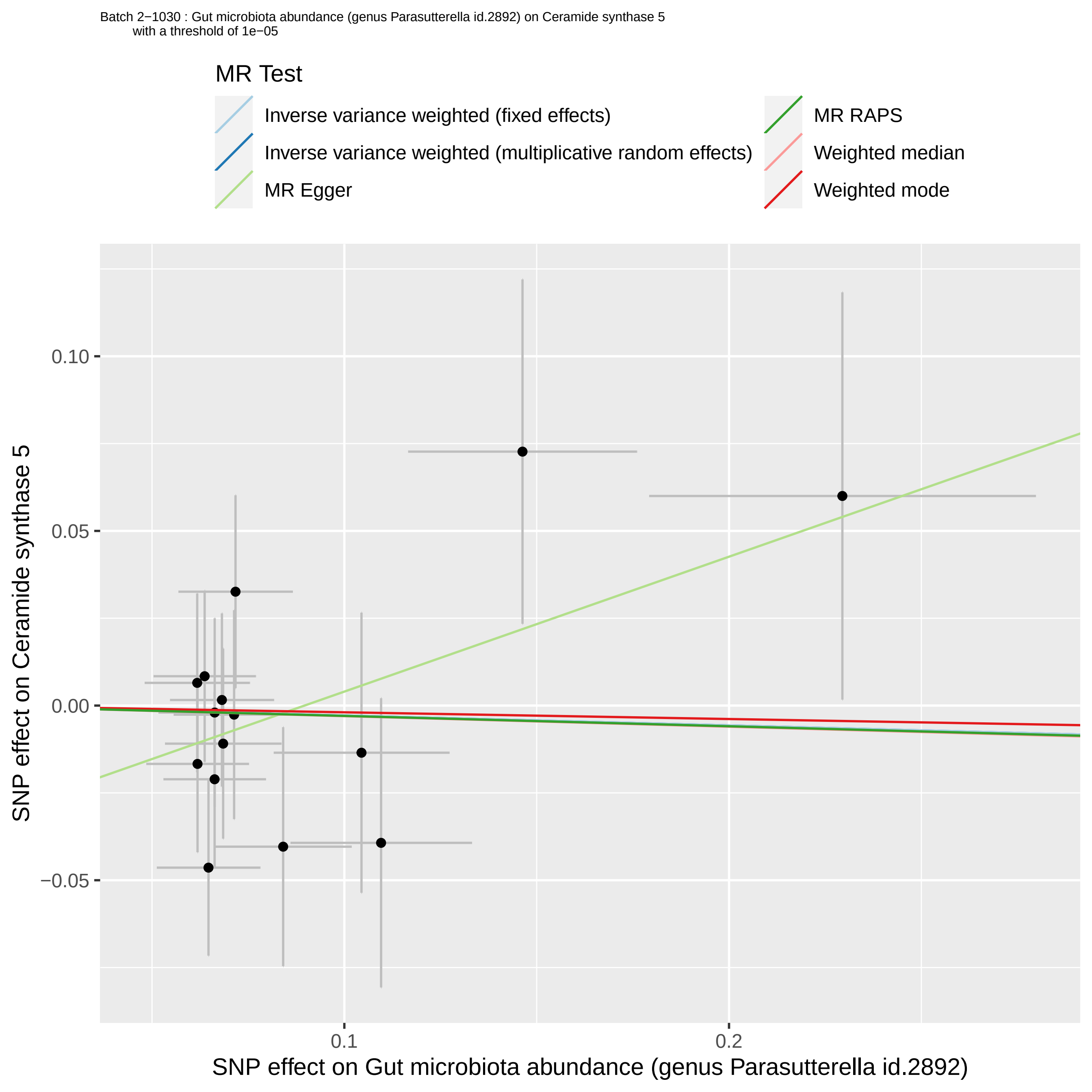
Batch 2−1030 : Gut microbiota abundance (genus Parasutterella id.2892) on Ceramide synthase 5
with a threshold of 1e−05
MR Test
Inverse variance weighted (fixed effects)
Inverse variance weighted (multiplicative random effects)
MR Egger
MR RAPS
Weighted median
Weighted mode
0.10
0.05
0.00
−0.05
0.1
0.2
SNP effect on Ceramide synthase 5
SNP effect on Gut microbiota abundance (genus Parasutterella id.2892)

Batch 2−1031 : Gut microbiota abundance (genus Catenibacterium id.2153) on Centrin−2
with a threshold of 1e−05

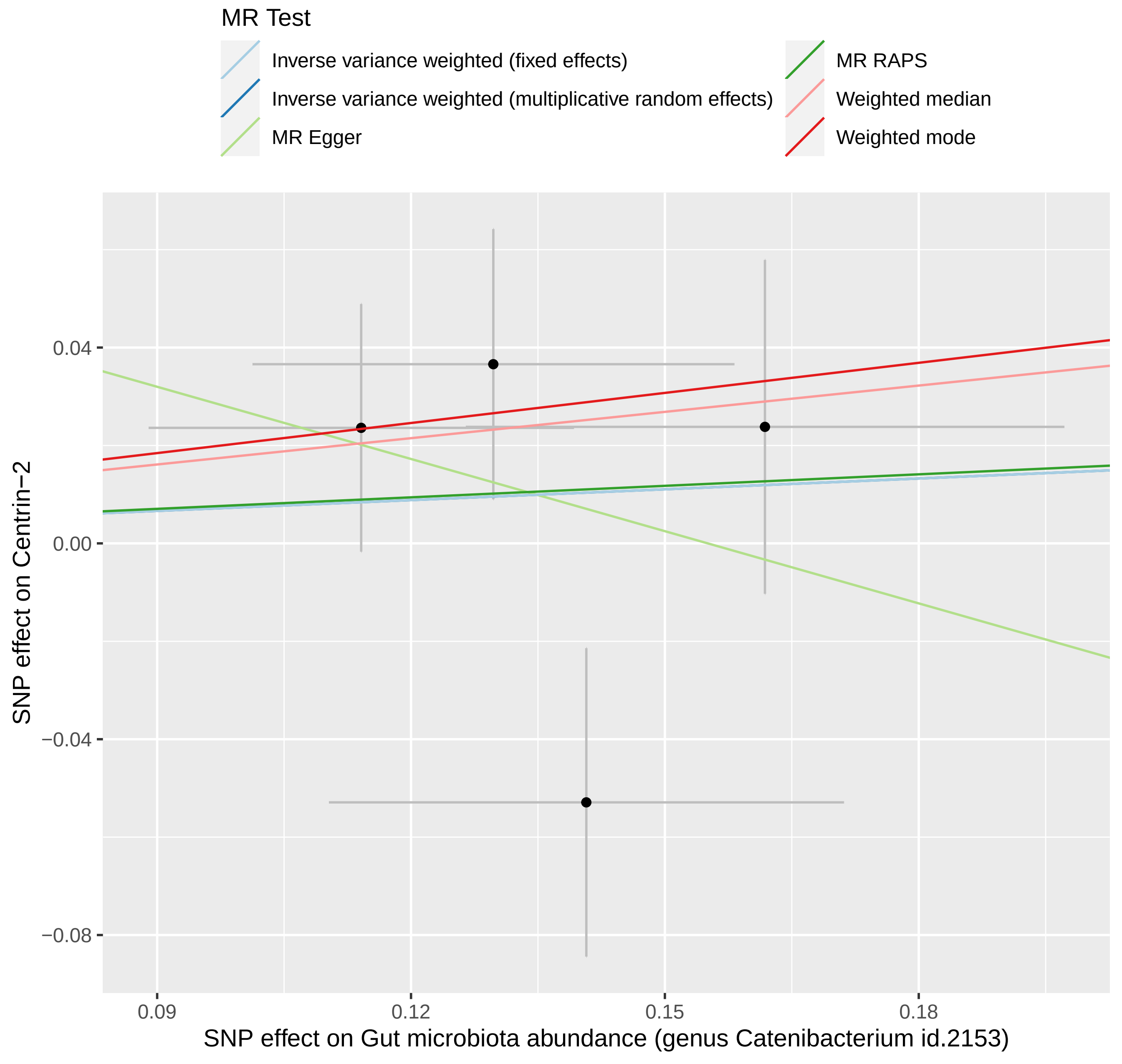

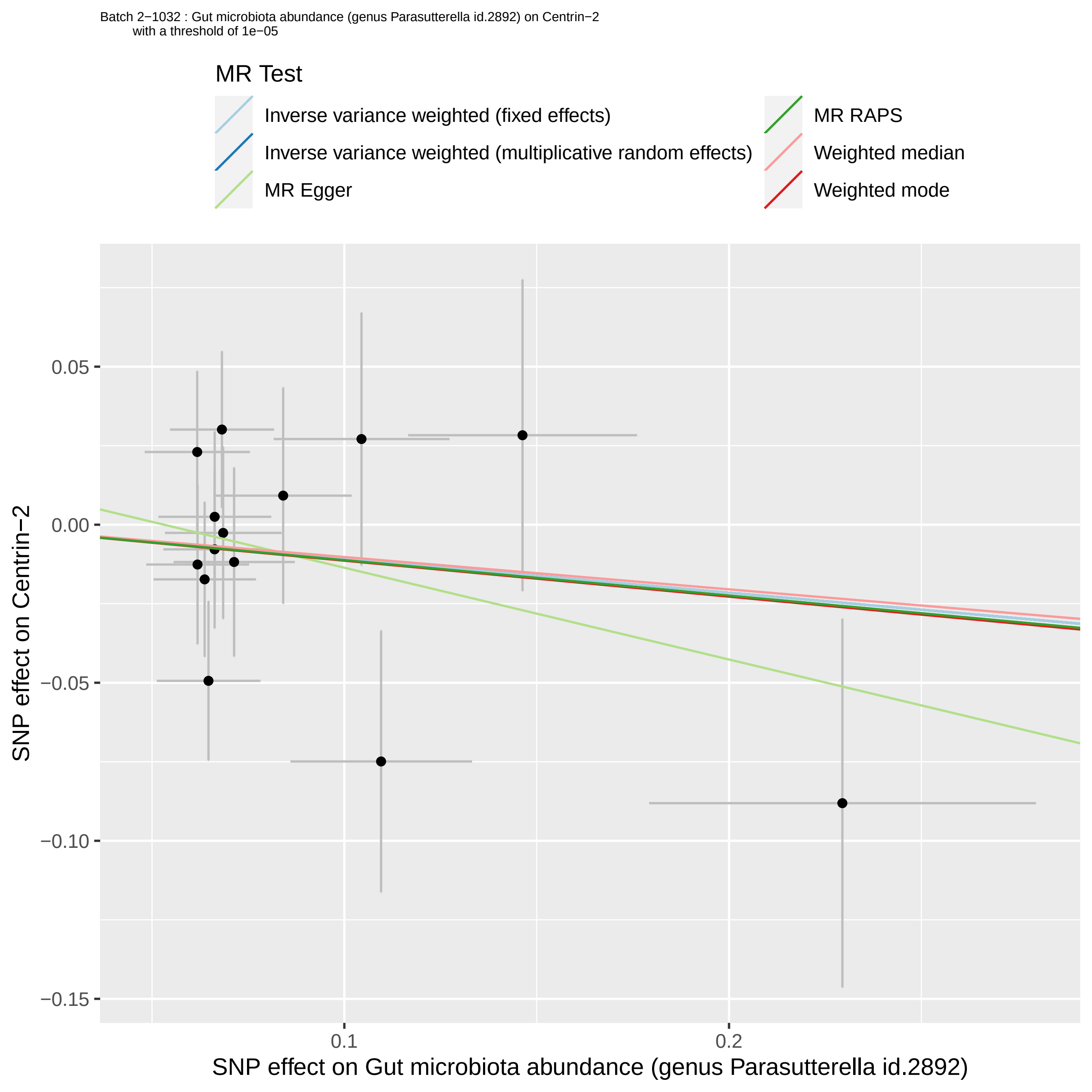
Batch 2−1032 : Gut microbiota abundance (genus Parasutterella id.2892) on Centrin−2
with a threshold of 1e−05
MR Test
Inverse variance weighted (fixed effects)
Inverse variance weighted (multiplicative random effects)
MR Egger
MR RAPS
Weighted median
Weighted mode
0.05
0.00
−0.05
−0.10
−0.15
0.1
0.2
SNP effect on Centrin−2
SNP effect on Gut microbiota abundance (genus Parasutterella id.2892)

Batch 2−1033 : Gut microbiota abundance (genus Catenibacterium id.2153) on Cilia− and flagella−associated protein 45
with a threshold of 1e−05

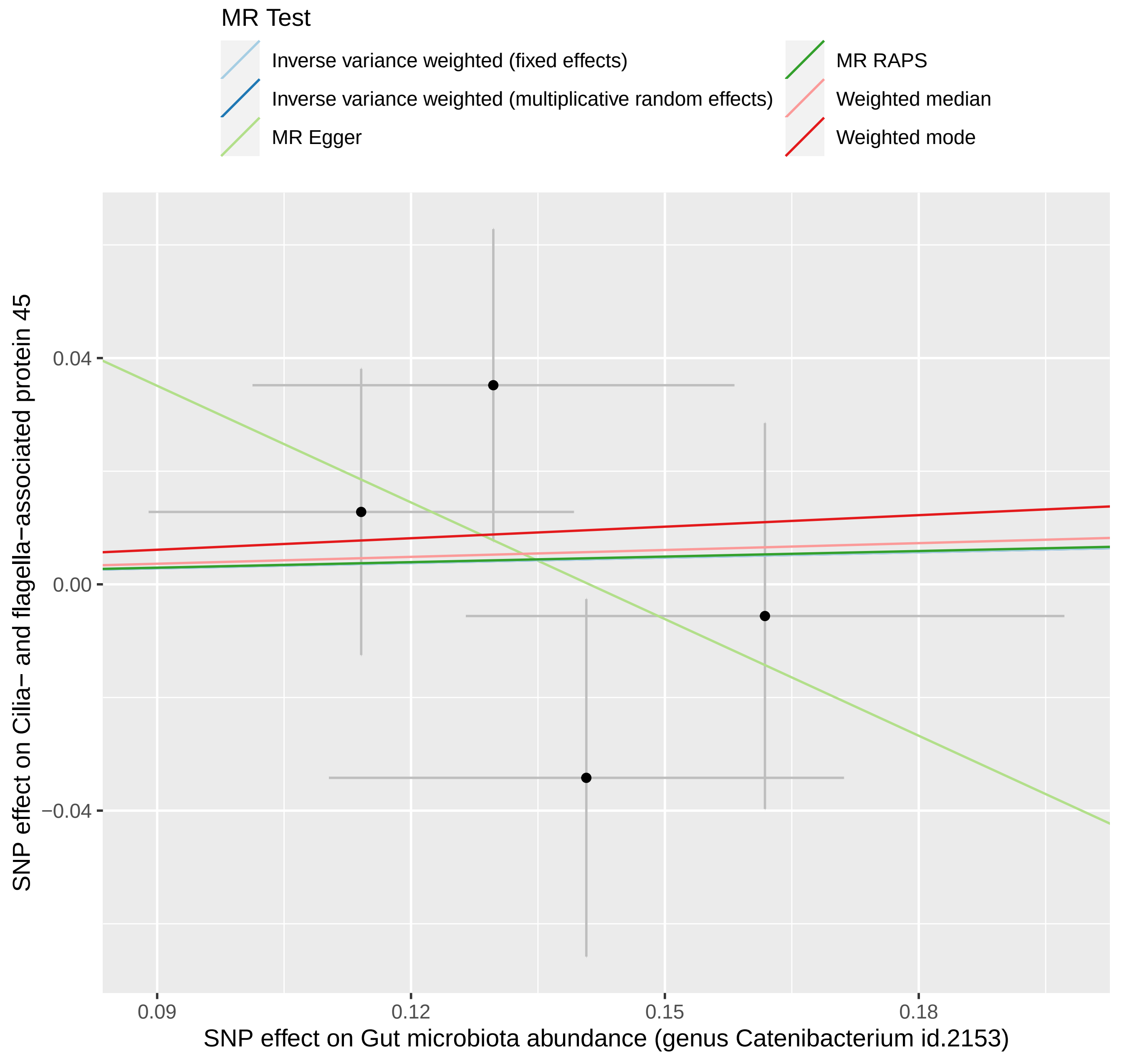

Batch 2−1034 : Gut microbiota abundance (genus Parasutterella id.2892) on Cilia− and flagella−associated protein 45
with a threshold of 1e−05

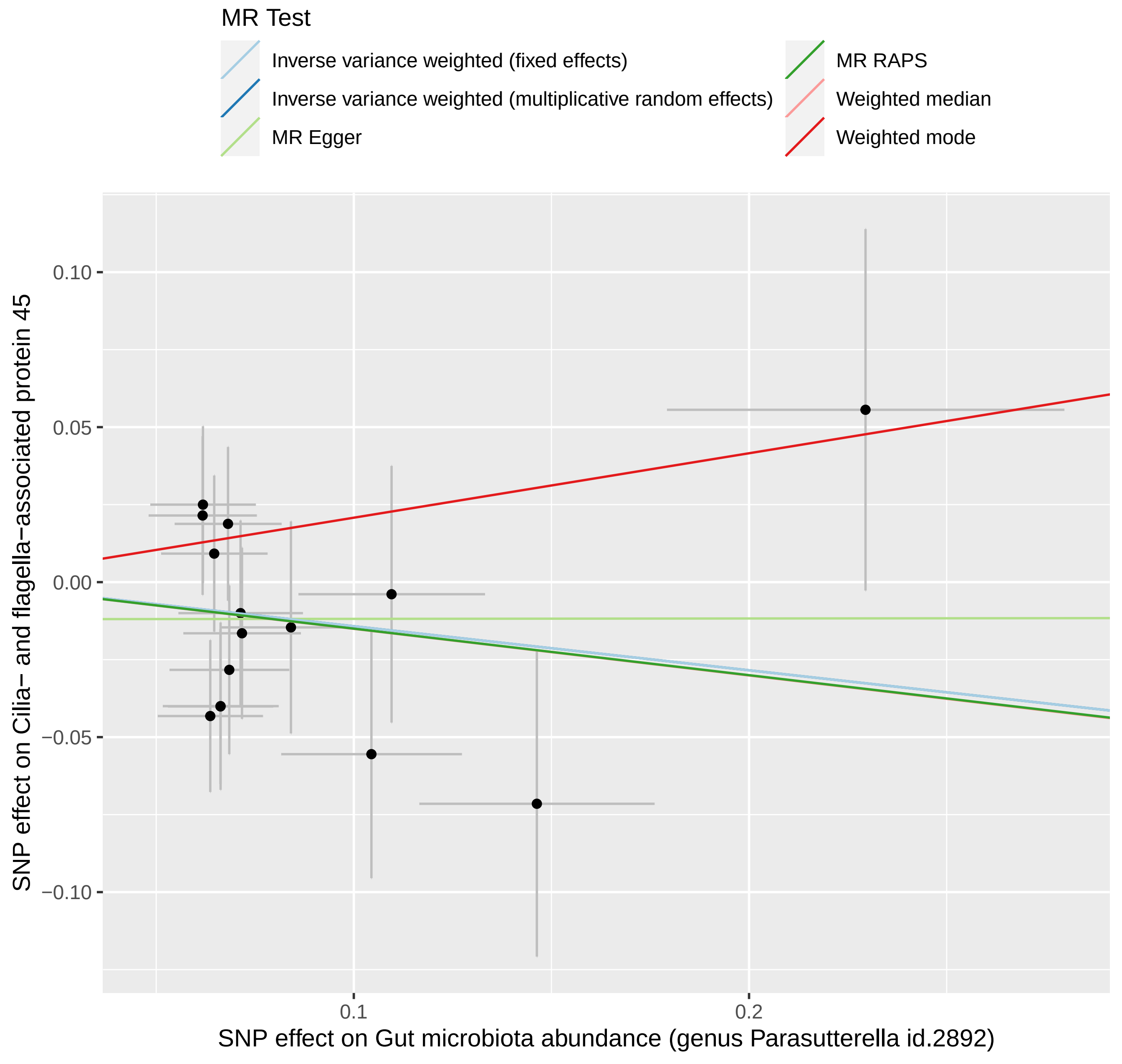

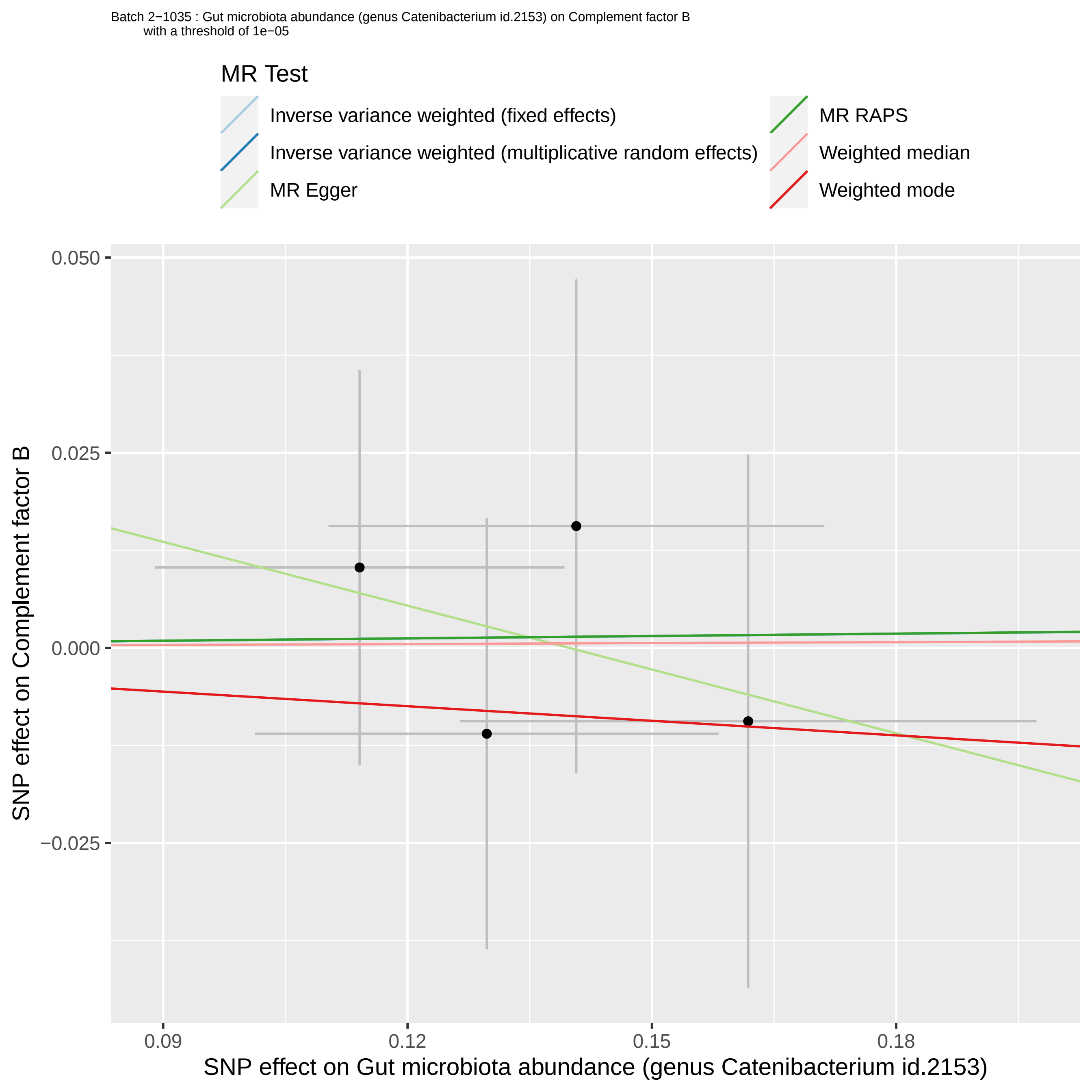

Batch 2−1035 : Gut microbiota abundance (genus Catenibacterium id.2153) on Complement factor B
with a threshold of 1e−05
MR Test
Inverse variance weighted (fixed effects)
Inverse variance weighted (multiplicative random effects)
MR Egger
MR RAPS
Weighted median
Weighted mode
0.050
0.025
0.000
−0.025
SNP effect on Complement factor B
0.09
0.12
0.15
0.18
SNP effect on Gut microbiota abundance (genus Catenibacterium id.2153)

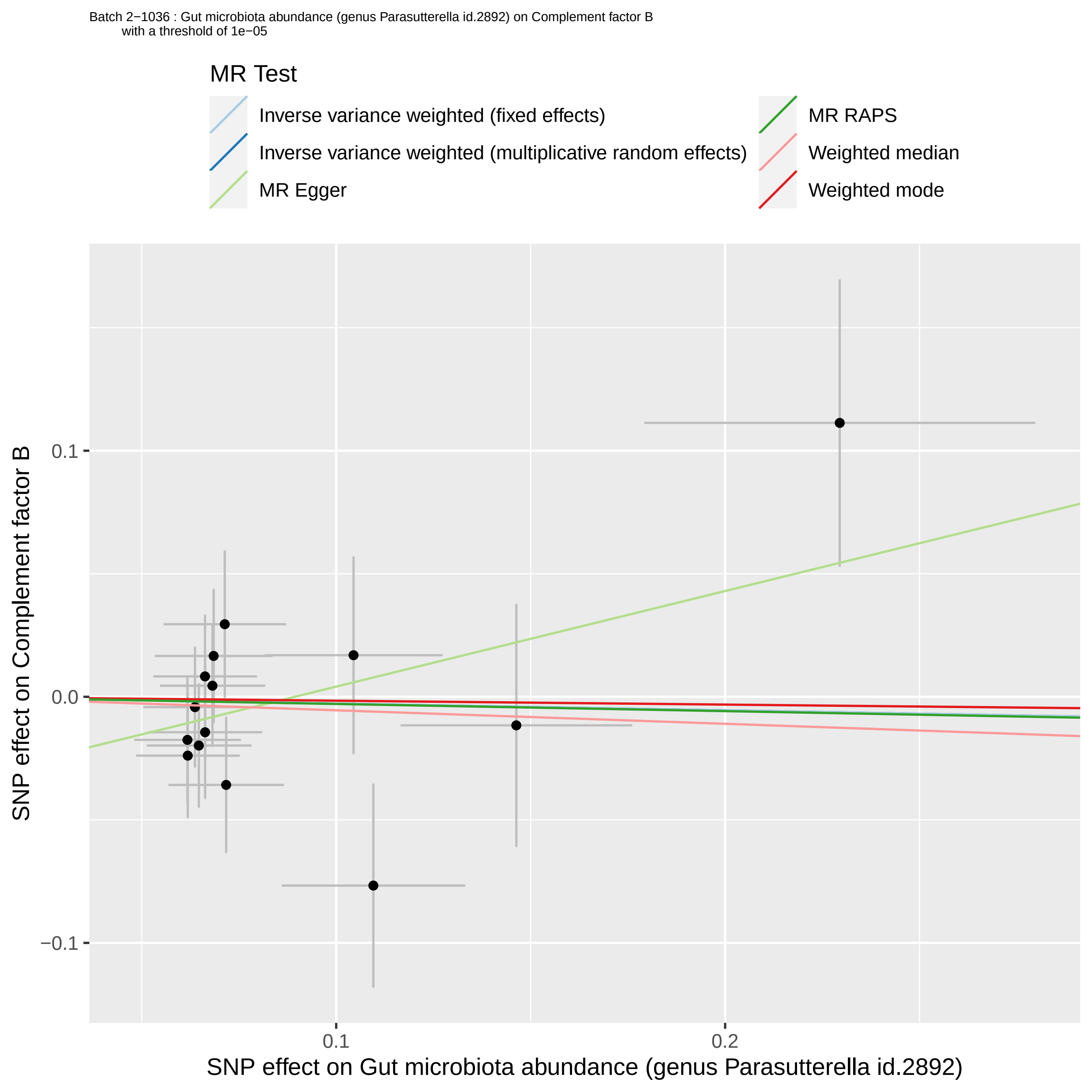
Batch 2−1036 : Gut microbiota abundance (genus Parasutterella id.2892) on Complement factor B
with a threshold of 1e−05
MR Test
Inverse variance weighted (fixed effects)
Inverse variance weighted (multiplicative random effects)
MR Egger
MR RAPS
Weighted median
Weighted mode
0.1
0.0
−0.1
0.1
0.2
SNP effect on Complement factor B
SNP effect on Gut microbiota abundance (genus Parasutterella id.2892)

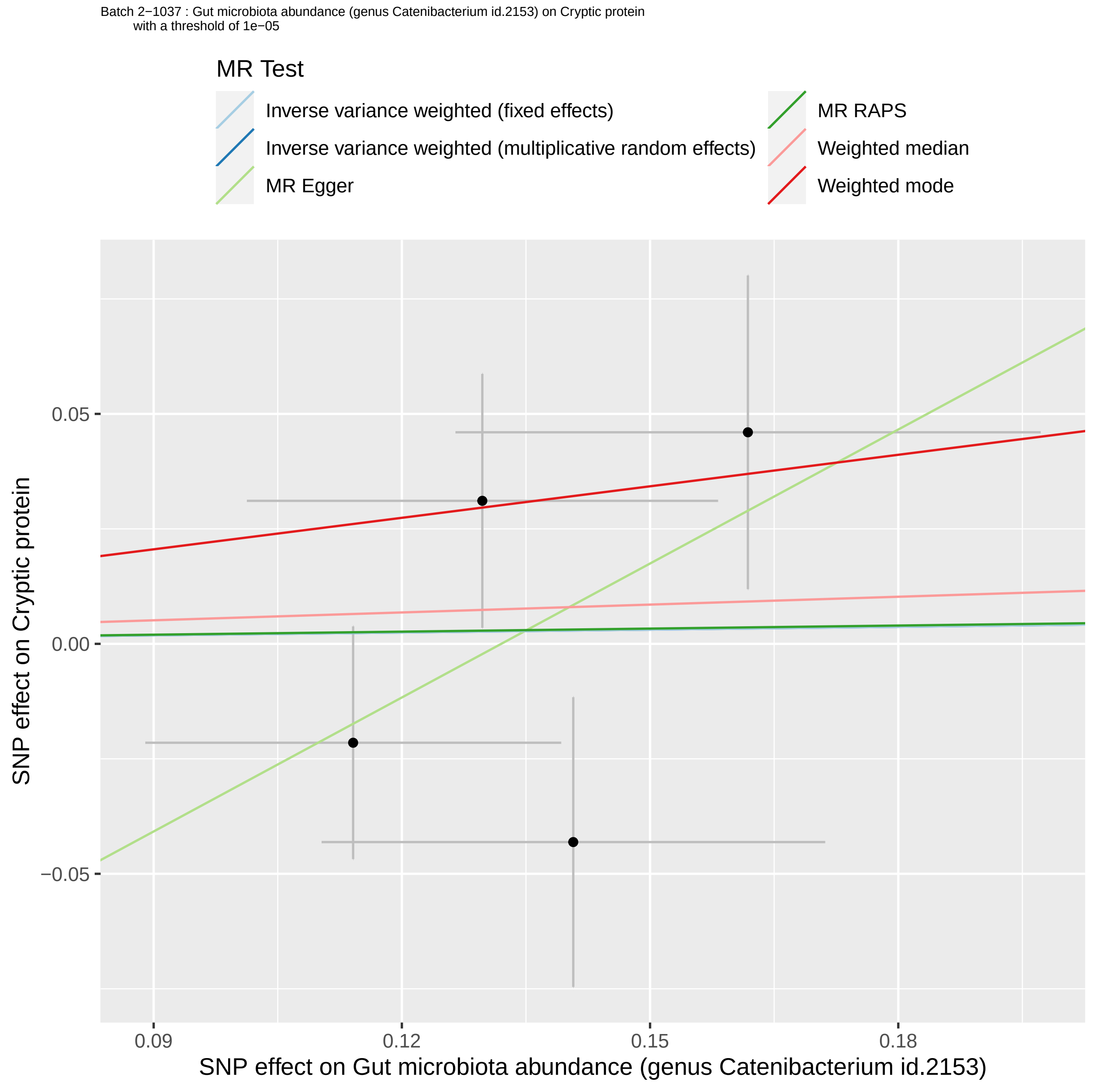
Batch 2−1037 : Gut microbiota abundance (genus Catenibacterium id.2153) on Cryptic protein
with a threshold of 1e−05
MR Test
Inverse variance weighted (fixed effects)
Inverse variance weighted (multiplicative random effects)
MR Egger
MR RAPS
Weighted median
Weighted mode
0.05
0.00
−0.05
0.09
0.12
0.15
0.18
SNP effect on Cryptic protein
SNP effect on Gut microbiota abundance (genus Catenibacterium id.2153)

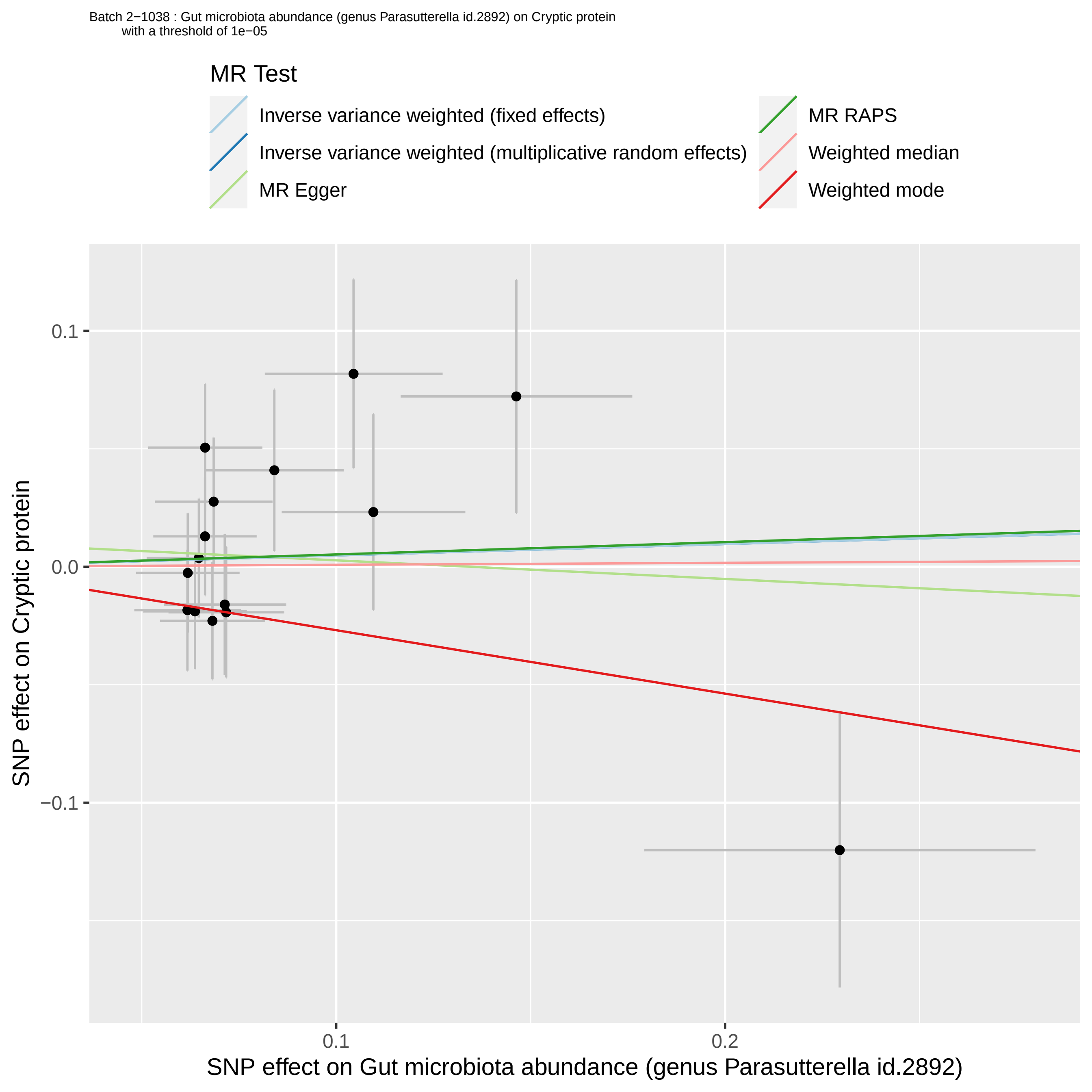
Batch 2−1038 : Gut microbiota abundance (genus Parasutterella id.2892) on Cryptic protein
with a threshold of 1e−05
MR Test
Inverse variance weighted (fixed effects)
Inverse variance weighted (multiplicative random effects)
MR Egger
MR RAPS
Weighted median
Weighted mode
0.1
0.0
−0.1
0.1
0.2
SNP effect on Cryptic protein
SNP effect on Gut microbiota abundance (genus Parasutterella id.2892)

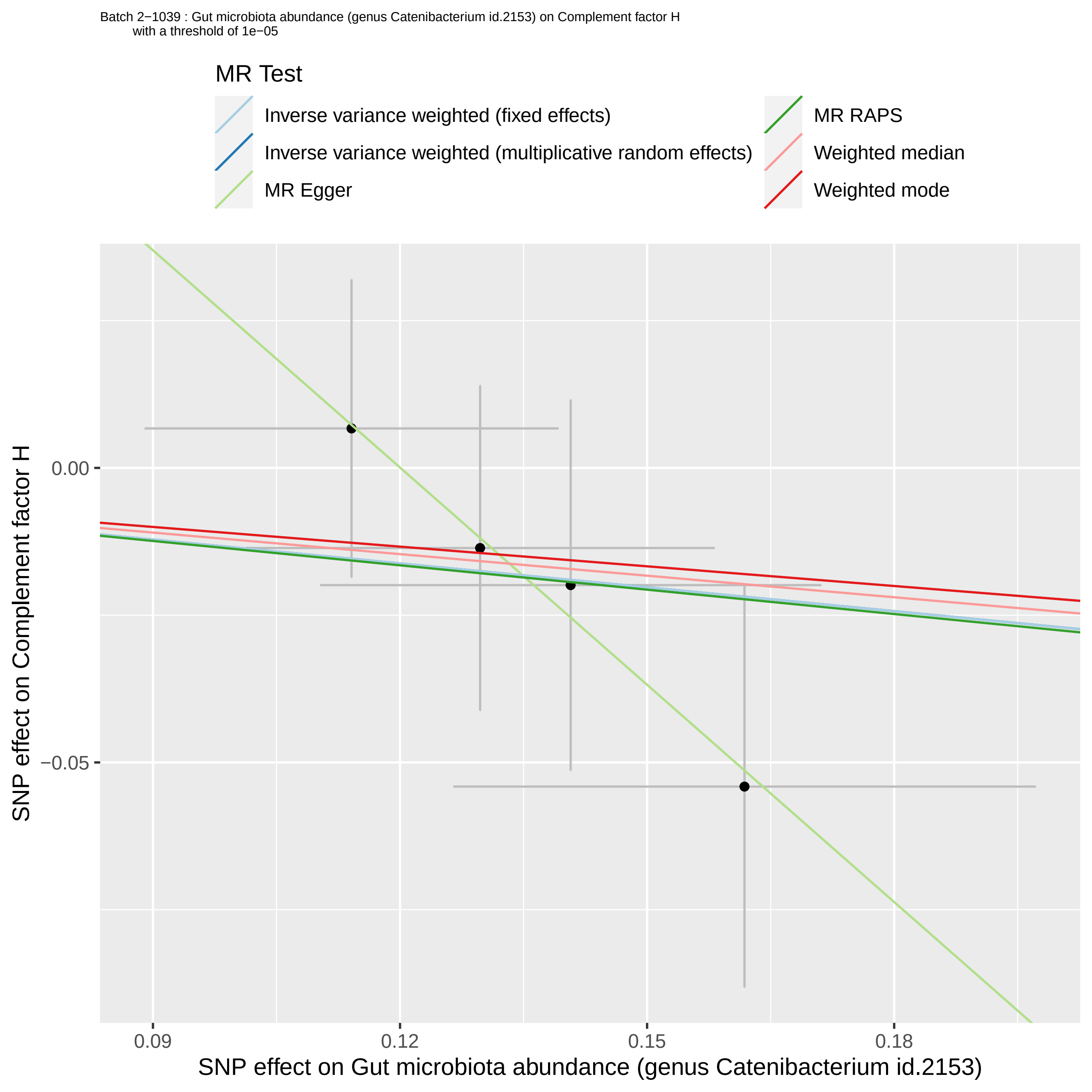
Batch 2−1039 : Gut microbiota abundance (genus Catenibacterium id.2153) on Complement factor H
with a threshold of 1e−05
MR Test
Inverse variance weighted (fixed effects)
Inverse variance weighted (multiplicative random effects)
MR Egger
MR RAPS
Weighted median
Weighted mode
SNP effect on Complement factor H
0.00
−0.05
0.09
0.12
0.15
0.18
SNP effect on Gut microbiota abundance (genus Catenibacterium id.2153)

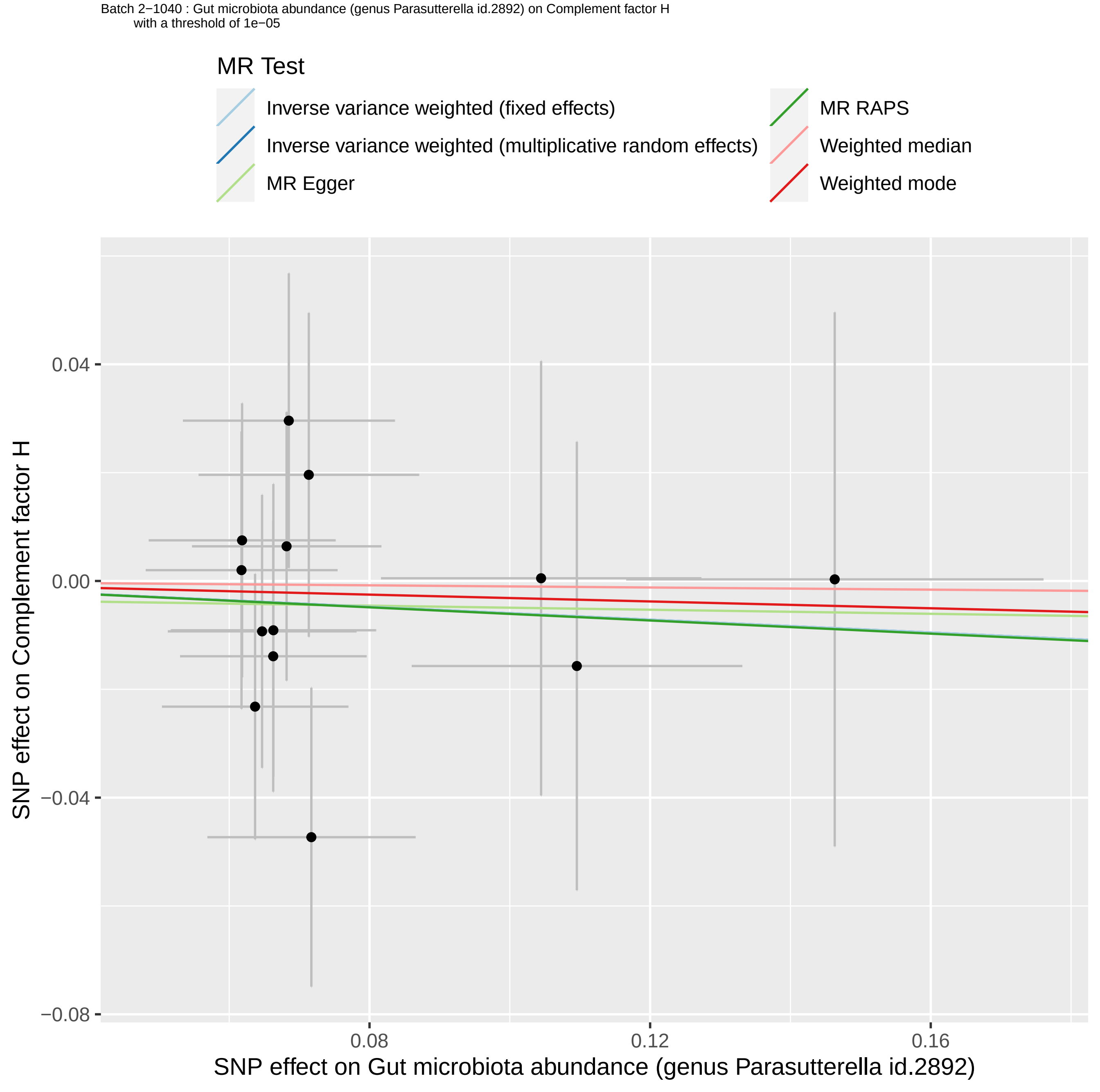

Batch 2−1040 : Gut microbiota abundance (genus Parasutterella id.2892) on Complement factor H
with a threshold of 1e−05
MR Test
Inverse variance weighted (fixed effects)
Inverse variance weighted (multiplicative random effects)
MR Egger
MR RAPS
Weighted median
Weighted mode
0.04
0.00
−0.04
−0.08
0.08
0.12
0.16
SNP effect on Complement factor H
SNP effect on Gut microbiota abundance (genus Parasutterella id.2892)

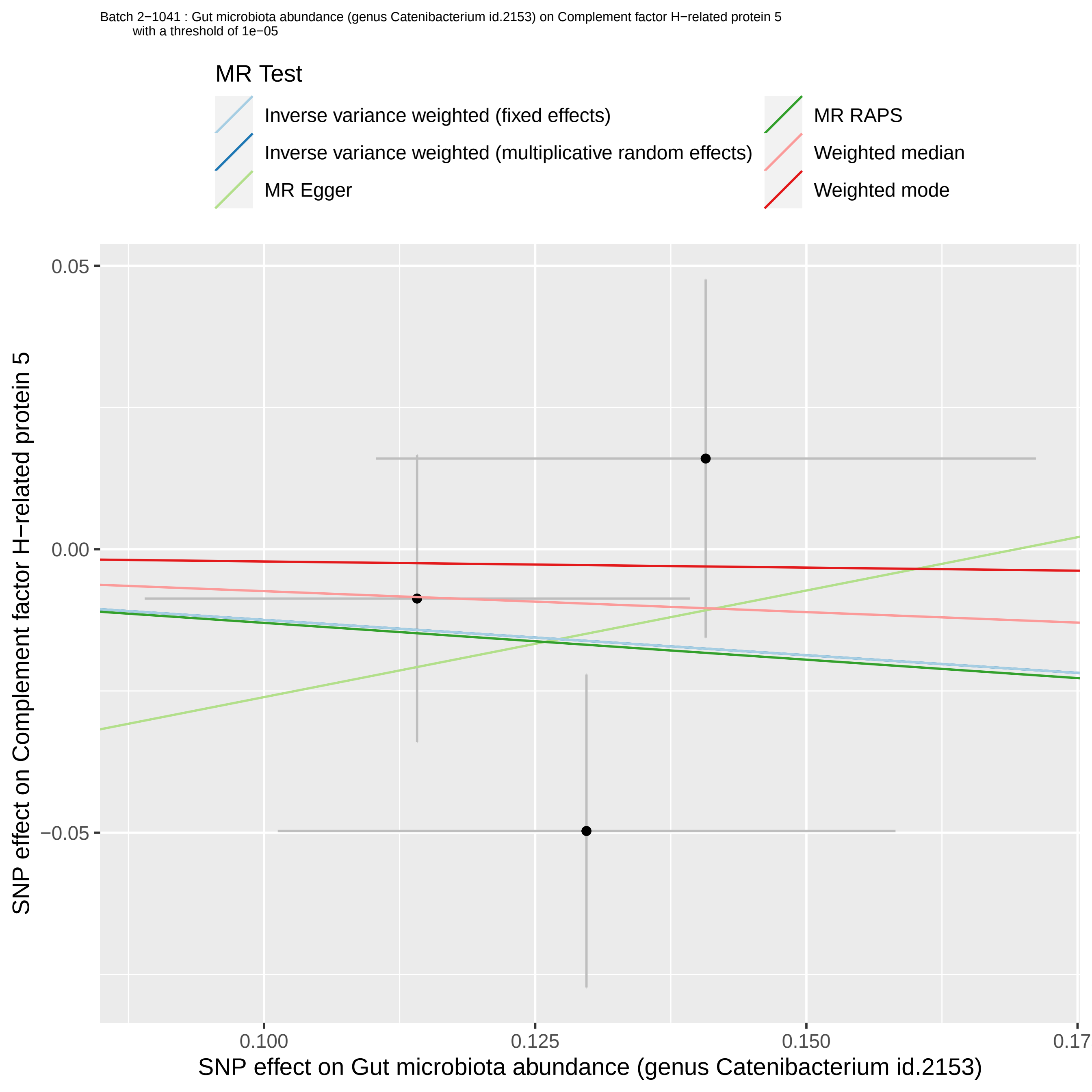

Batch 2−1041 : Gut microbiota abundance (genus Catenibacterium id.2153) on Complement factor H−related protein 5
with a threshold of 1e−05
MR Test
Inverse variance weighted (fixed effects)
Inverse variance weighted (multiplicative random effects)
MR Egger
MR RAPS
Weighted median
Weighted mode
0.05
0.00
−0.05
0.100
0.125
0.150
0.17
SNP effect on Complement factor H−related protein 5
SNP effect on Gut microbiota abundance (genus Catenibacterium id.2153)

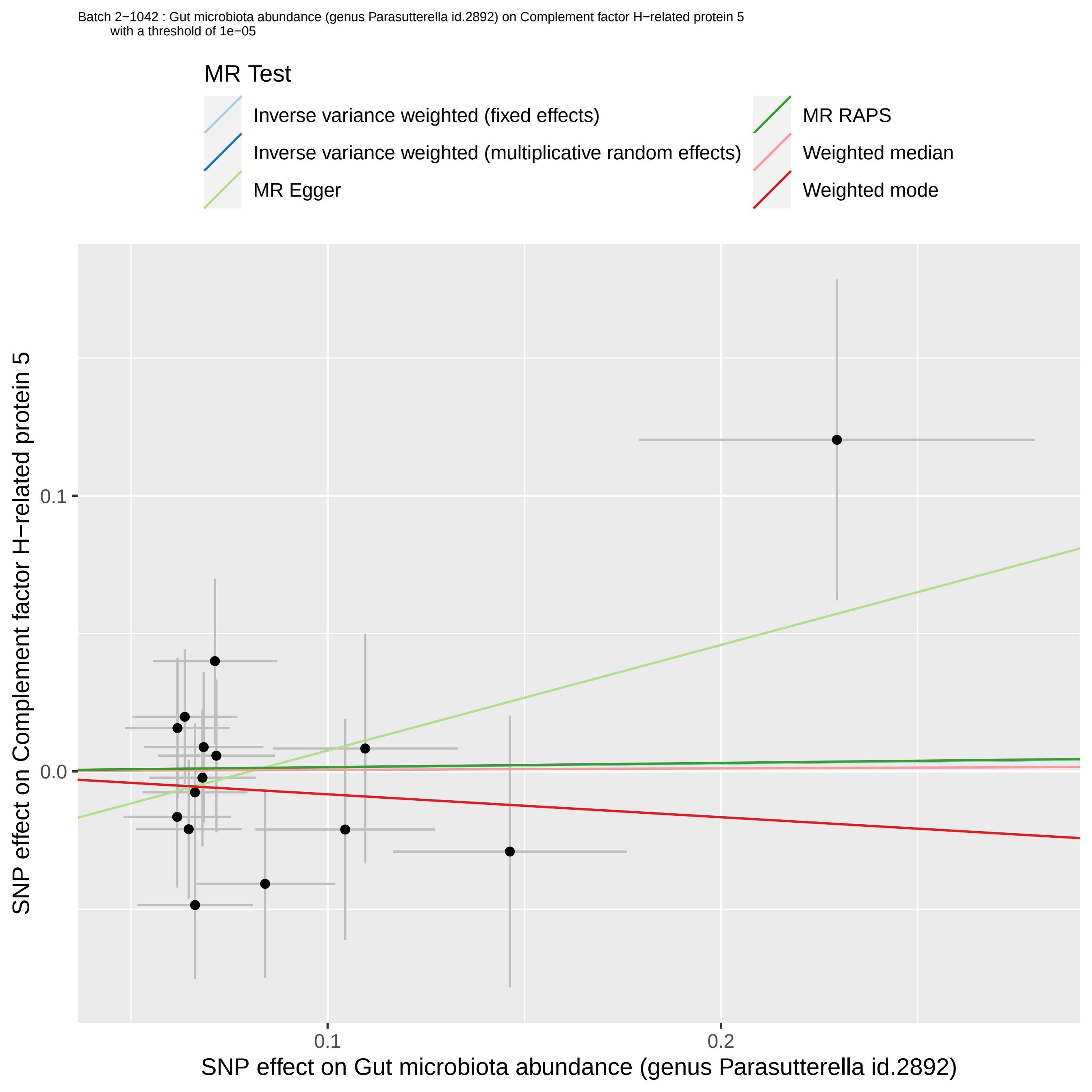
Batch 2−1042 : Gut microbiota abundance (genus Parasutterella id.2892) on Complement factor H−related protein 5
with a threshold of 1e−05
MR Test
Inverse variance weighted (fixed effects)
Inverse variance weighted (multiplicative random effects)
MR Egger
MR RAPS
Weighted median
Weighted mode
SNP effect on Complement factor H−related protein 5
0.1
0.0
0.1
0.2
SNP effect on Gut microbiota abundance (genus Parasutterella id.2892)

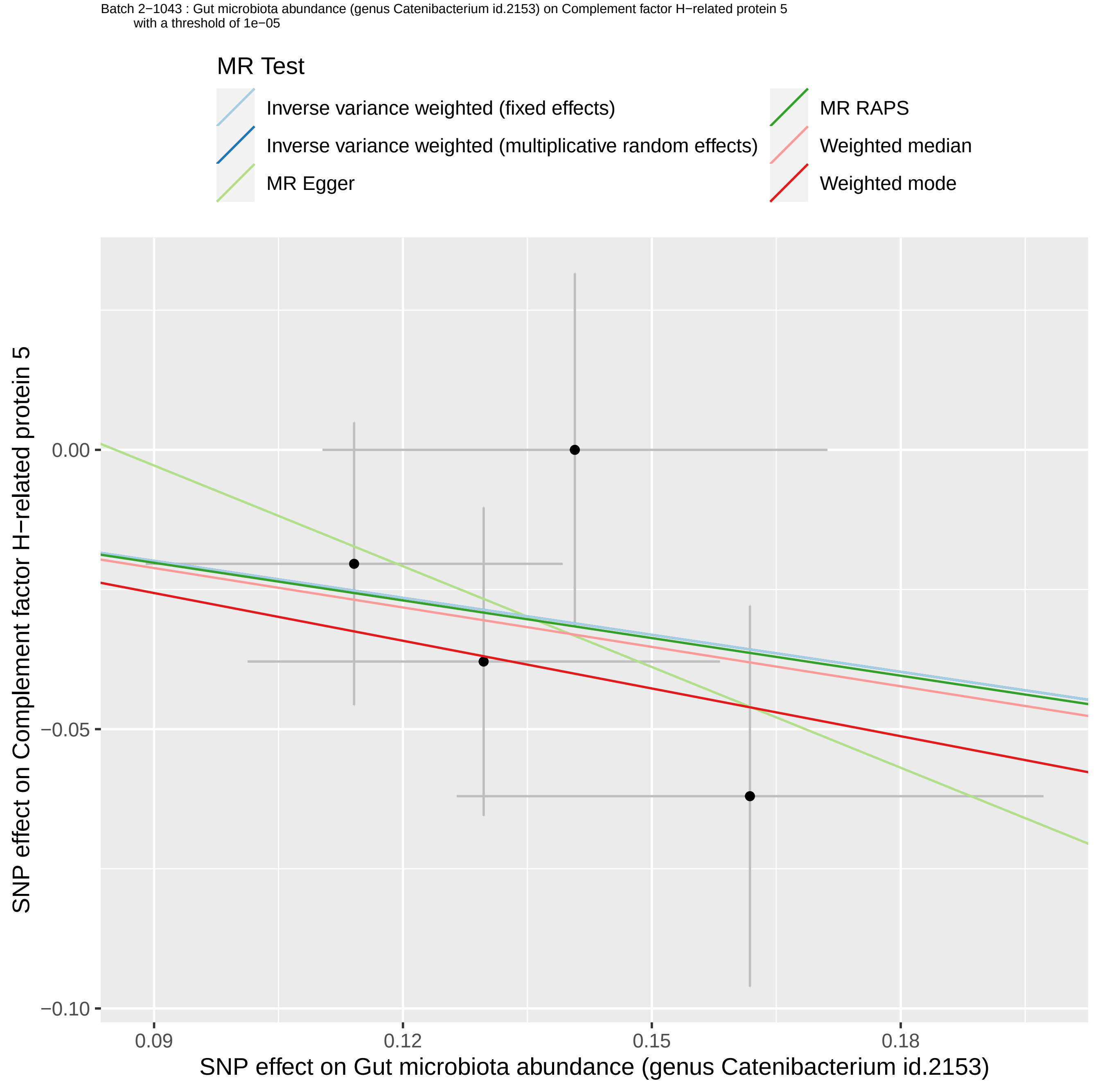

Batch 2−1043 : Gut microbiota abundance (genus Catenibacterium id.2153) on Complement factor H−related protein 5
with a threshold of 1e−05
MR Test
Inverse variance weighted (fixed effects)
Inverse variance weighted (multiplicative random effects)
MR Egger
MR RAPS
Weighted median
Weighted mode
SNP effect on Complement factor H−related protein 5
0.00
−0.05
−0.10
0.09
0.12
0.15
0.18
SNP effect on Gut microbiota abundance (genus Catenibacterium id.2153)

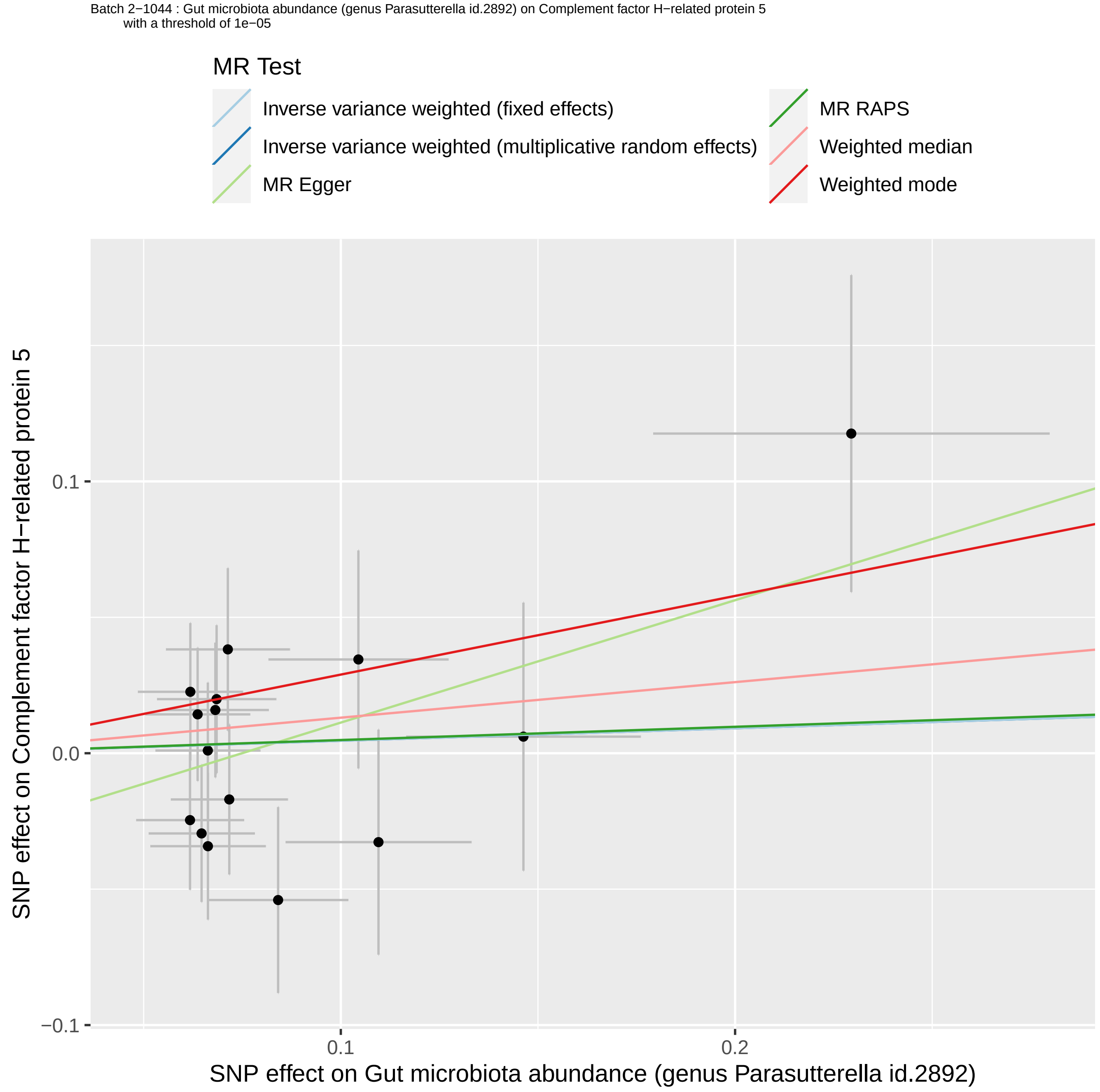
Batch 2−1044 : Gut microbiota abundance (genus Parasutterella id.2892) on Complement factor H−related protein 5
with a threshold of 1e−05
MR Test
Inverse variance weighted (fixed effects)
Inverse variance weighted (multiplicative random effects)
MR Egger
MR RAPS
Weighted median
Weighted mode
SNP effect on Complement factor H−related protein 5
0.1
0.0
−0.1
0.1
0.2
SNP effect on Gut microbiota abundance (genus Parasutterella id.2892)

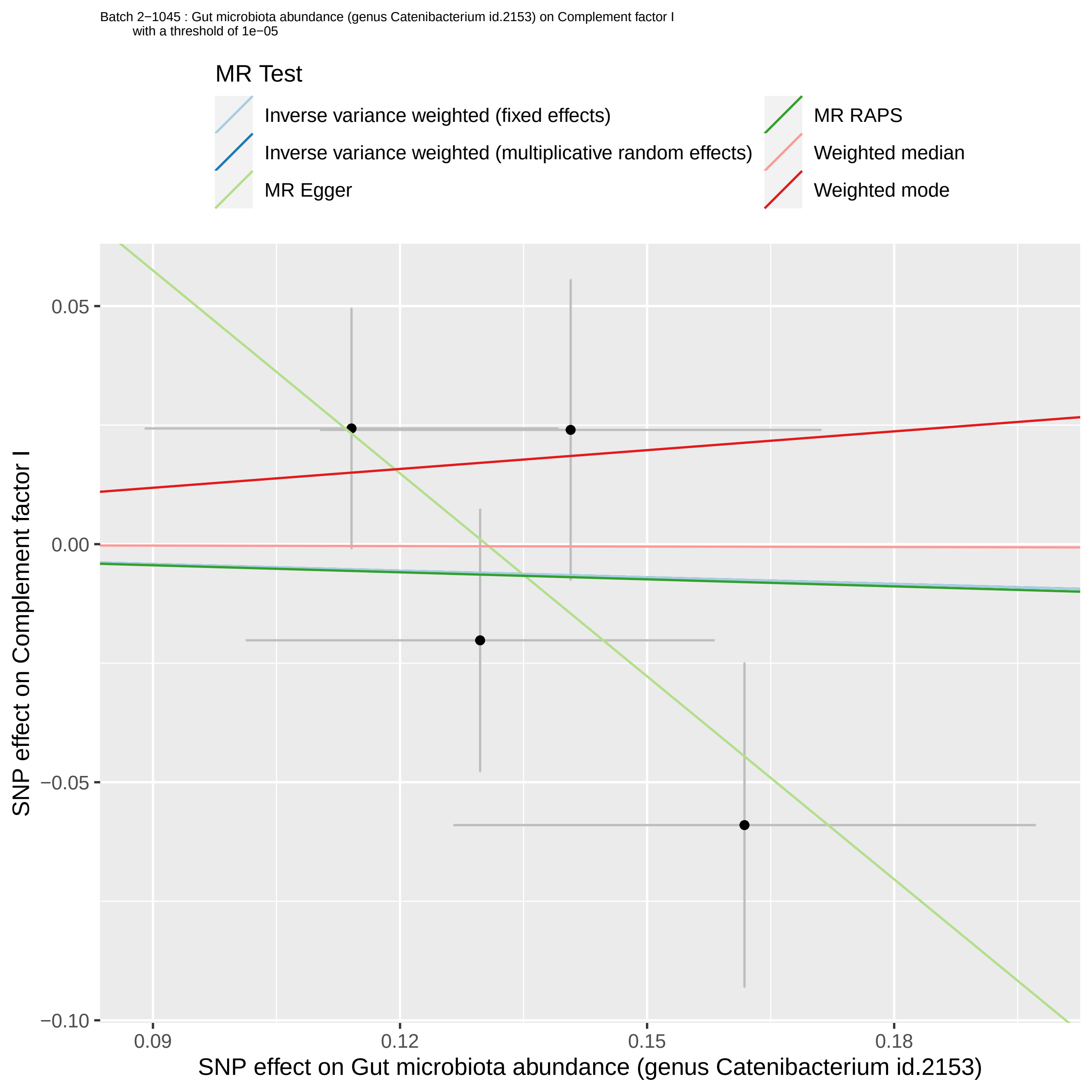
Batch 2−1045 : Gut microbiota abundance (genus Catenibacterium id.2153) on Complement factor I
with a threshold of 1e−05
MR Test
Inverse variance weighted (fixed effects)
Inverse variance weighted (multiplicative random effects)
MR Egger
MR RAPS
Weighted median
Weighted mode
0.05
0.00
−0.05
−0.10
0.09
0.12
0.15
0.18
SNP effect on Complement factor I
SNP effect on Gut microbiota abundance (genus Catenibacterium id.2153)

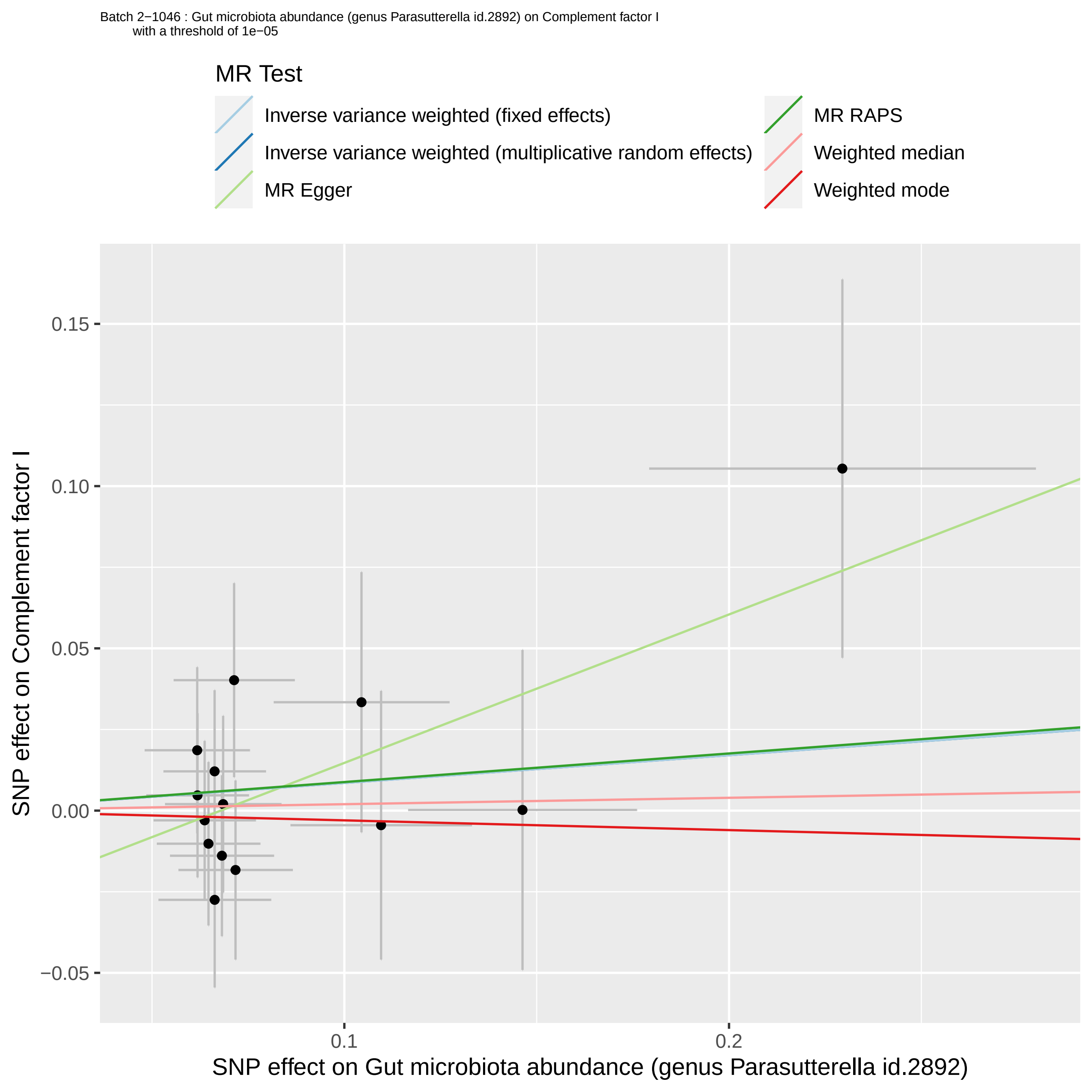

Batch 2−1046 : Gut microbiota abundance (genus Parasutterella id.2892) on Complement factor I
with a threshold of 1e−05
MR Test
Inverse variance weighted (fixed effects)
Inverse variance weighted (multiplicative random effects)
MR Egger
MR RAPS
Weighted median
Weighted mode
0.15
0.10
0.05
0.00
−0.05
0.1
0.2
SNP effect on Complement factor I
SNP effect on Gut microbiota abundance (genus Parasutterella id.2892)

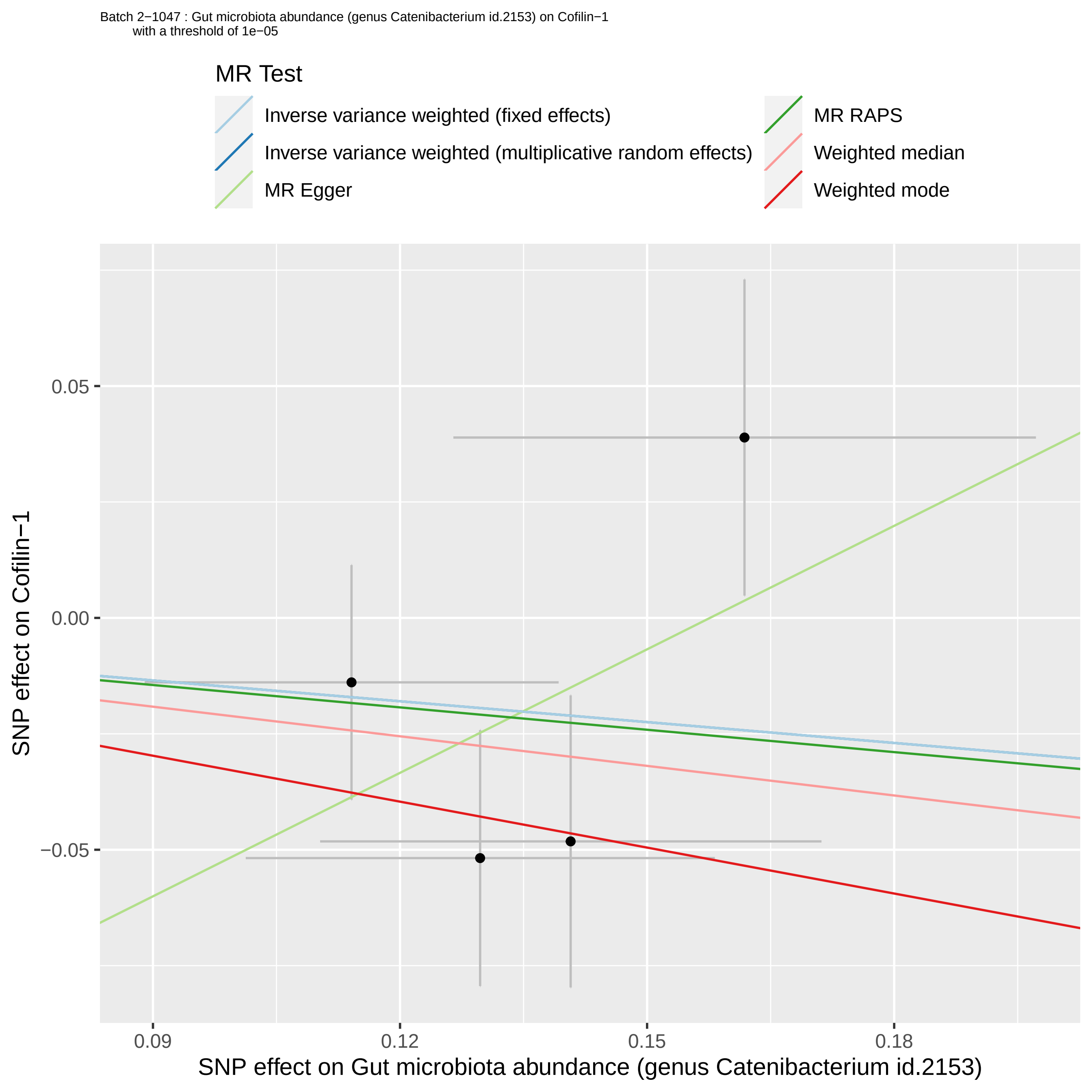

Batch 2−1047 : Gut microbiota abundance (genus Catenibacterium id.2153) on Cofilin−1
with a threshold of 1e−05
MR Test
Inverse variance weighted (fixed effects)
Inverse variance weighted (multiplicative random effects)
MR Egger
MR RAPS
Weighted median
Weighted mode
0.05
0.00
−0.05
0.09
0.12
0.15
0.18
SNP effect on Cofilin−1
SNP effect on Gut microbiota abundance (genus Catenibacterium id.2153)

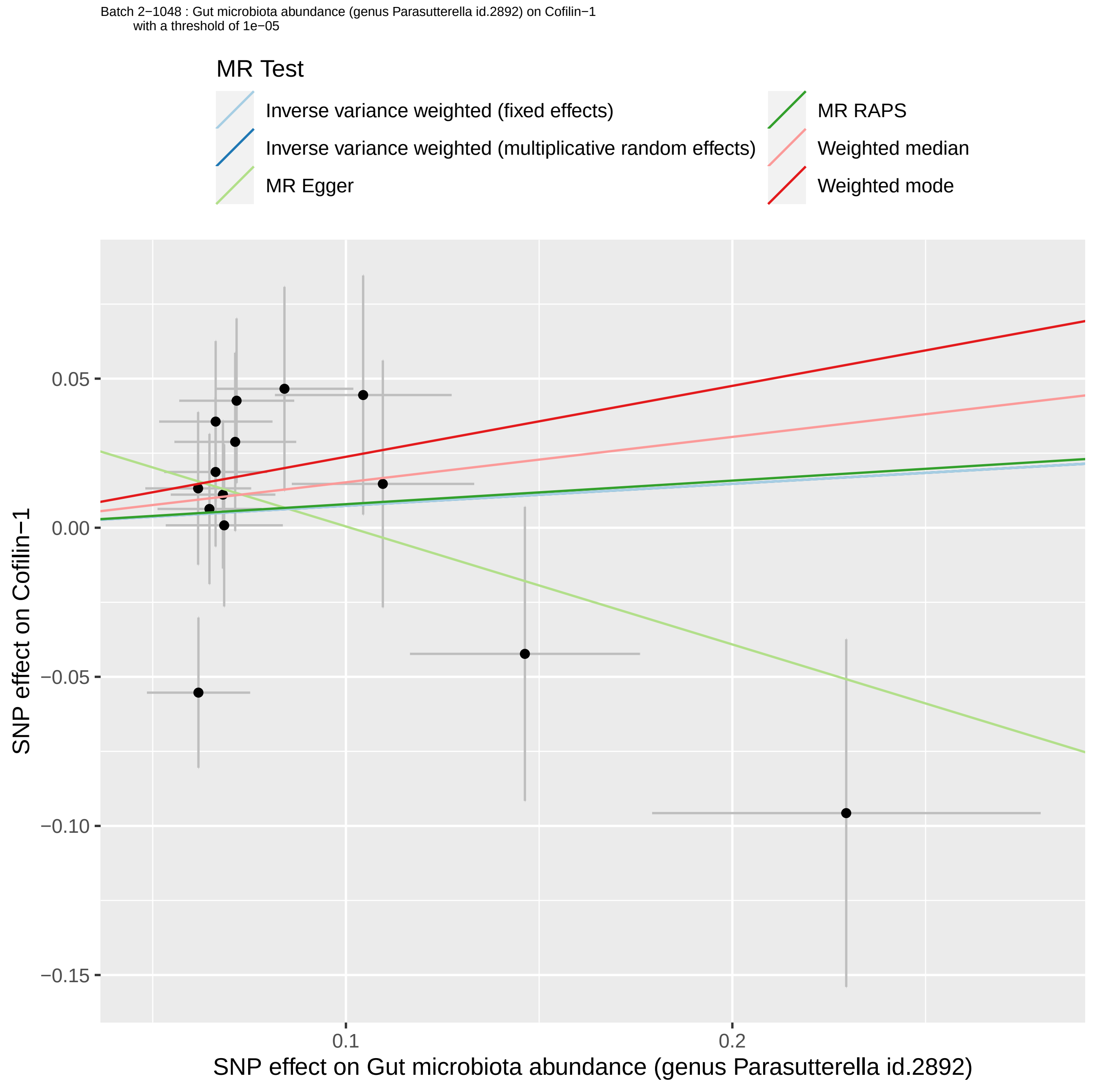
Batch 2−1048 : Gut microbiota abundance (genus Parasutterella id.2892) on Cofilin−1
with a threshold of 1e−05
MR Test
Inverse variance weighted (fixed effects)
Inverse variance weighted (multiplicative random effects)
MR Egger
MR RAPS
Weighted median
Weighted mode
0.05
0.00
−0.05
−0.10
−0.15
0.1
0.2
SNP effect on Cofilin−1
SNP effect on Gut microbiota abundance (genus Parasutterella id.2892)

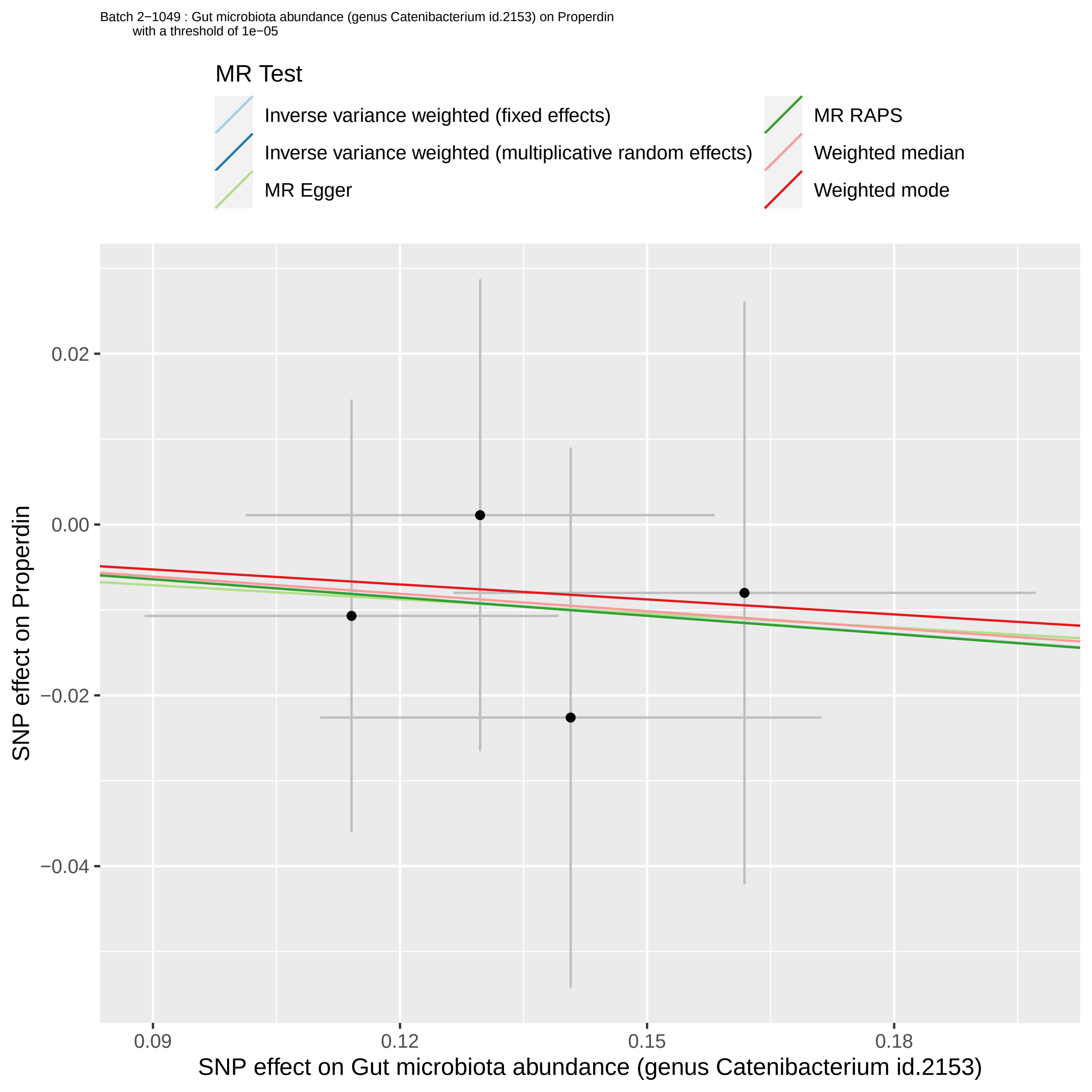

Batch 2−1049 : Gut microbiota abundance (genus Catenibacterium id.2153) on Properdin
with a threshold of 1e−05
MR Test
Inverse variance weighted (fixed effects)
Inverse variance weighted (multiplicative random effects)
MR Egger
MR RAPS
Weighted median
Weighted mode
0.02
0.00
−0.02
−0.04
0.09
0.12
0.15
0.18
SNP effect on Properdin
SNP effect on Gut microbiota abundance (genus Catenibacterium id.2153)

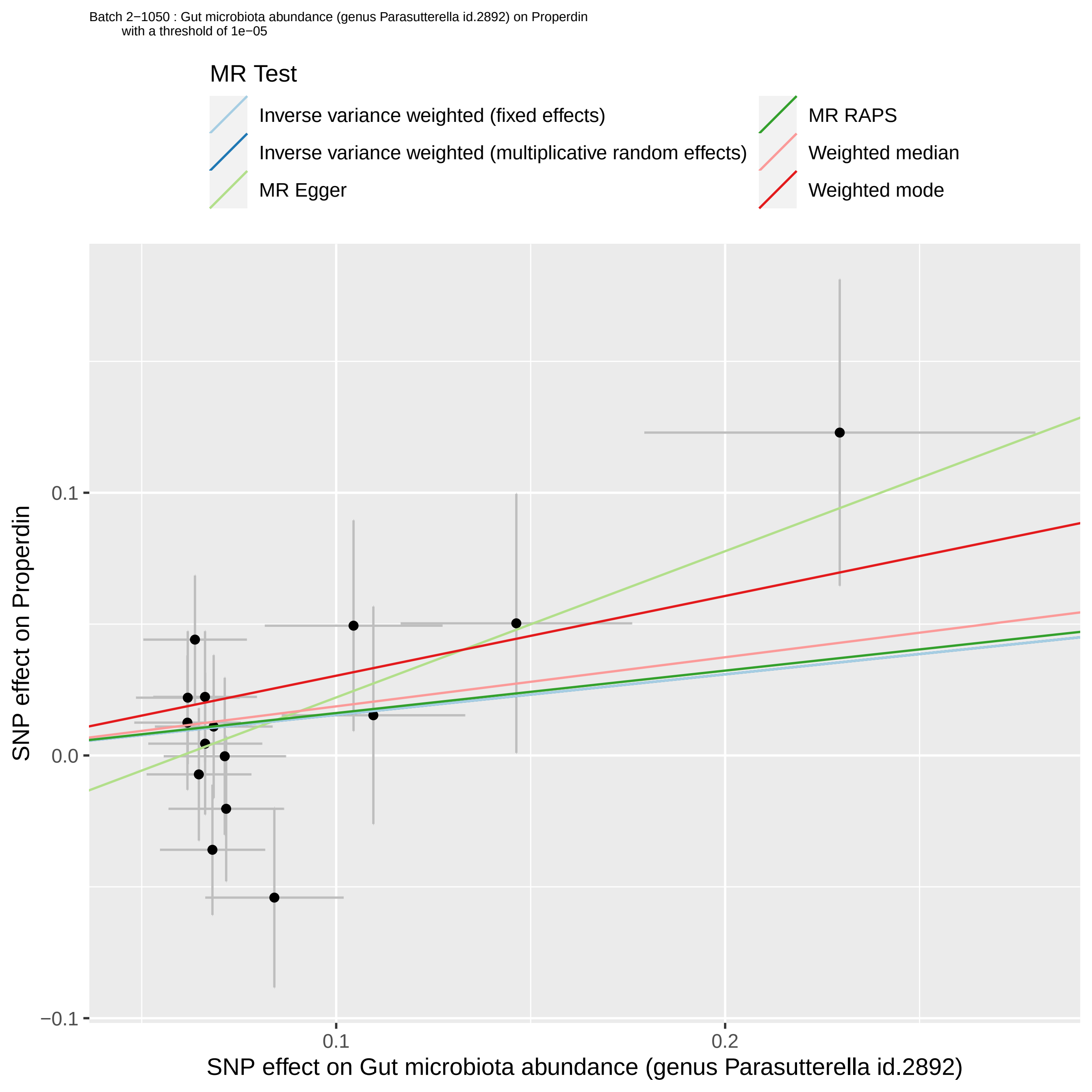
Batch 2−1050 : Gut microbiota abundance (genus Parasutterella id.2892) on Properdin
with a threshold of 1e−05
MR Test
Inverse variance weighted (fixed effects)
Inverse variance weighted (multiplicative random effects)
MR Egger
MR RAPS
Weighted median
Weighted mode
SNP effect on Properdin
0.1
0.0
−0.1
0.1
0.2
SNP effect on Gut microbiota abundance (genus Parasutterella id.2892)

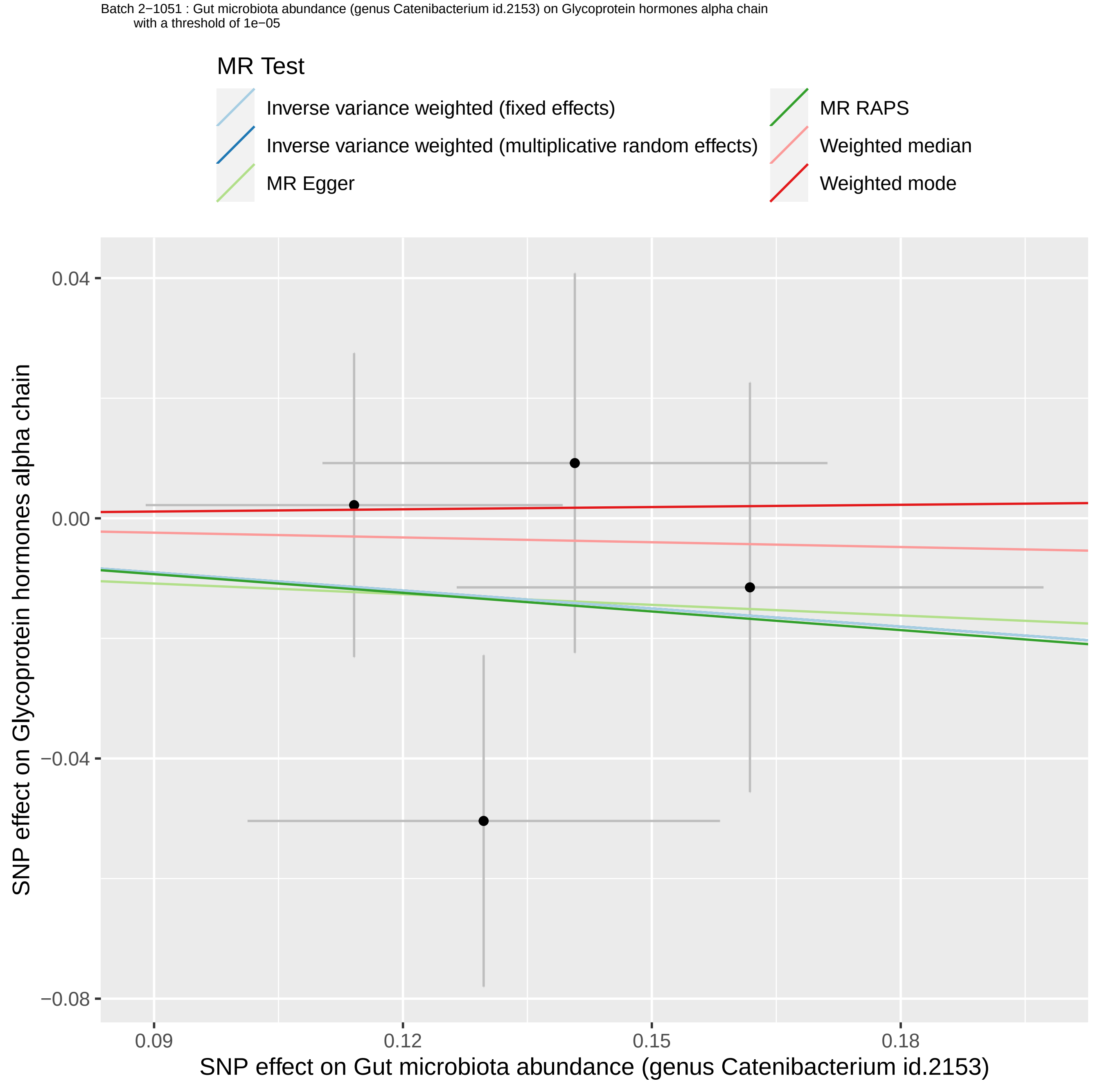
Batch 2−1051 : Gut microbiota abundance (genus Catenibacterium id.2153) on Glycoprotein hormones alpha chain
with a threshold of 1e−05
MR Test
Inverse variance weighted (fixed effects)
Inverse variance weighted (multiplicative random effects)
MR Egger
MR RAPS
Weighted median
Weighted mode
0.04
0.00
−0.04
−0.08
0.09
0.12
0.15
0.18
SNP effect on Glycoprotein hormones alpha chain
SNP effect on Gut microbiota abundance (genus Catenibacterium id.2153)

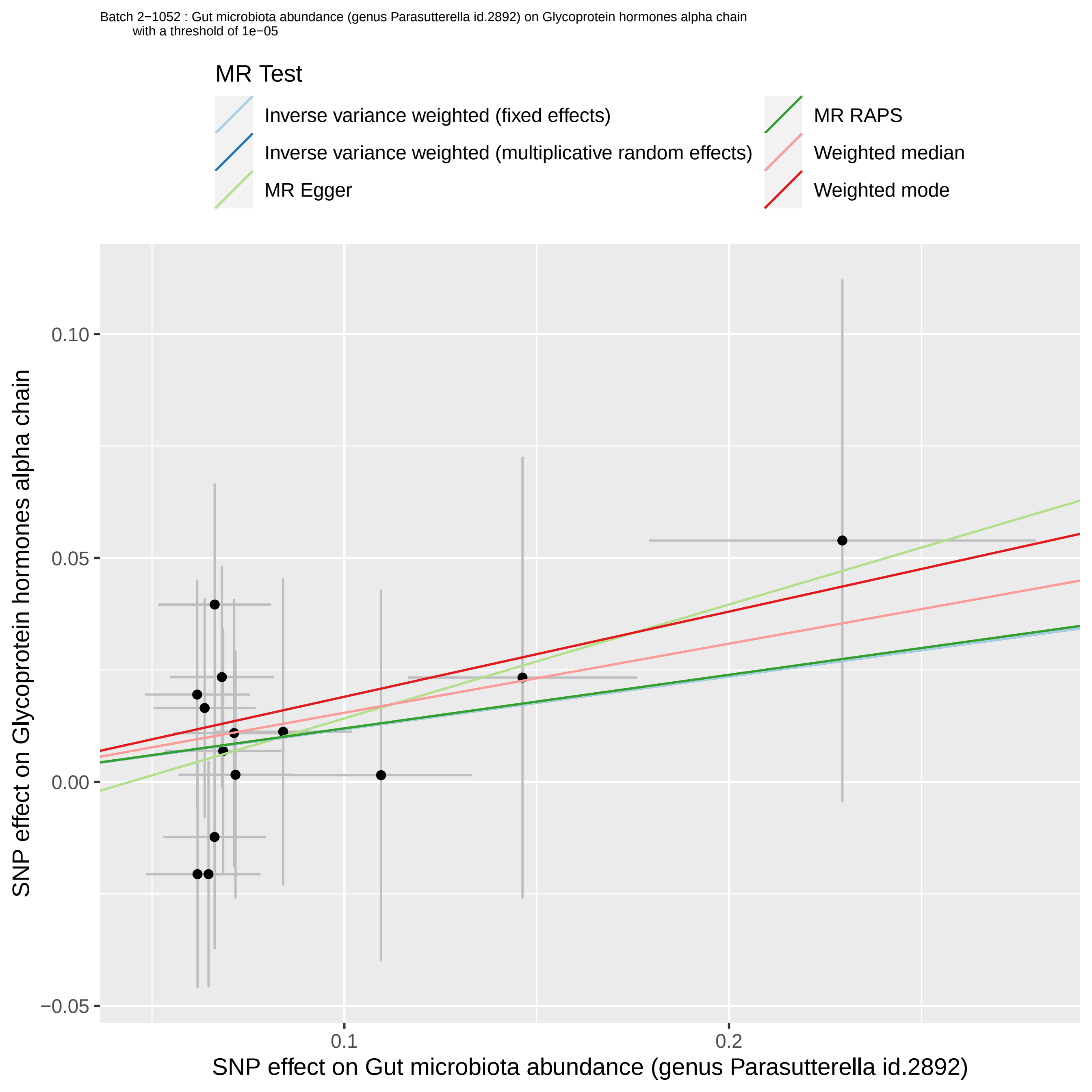
Batch 2−1052 : Gut microbiota abundance (genus Parasutterella id.2892) on Glycoprotein hormones alpha chain
with a threshold of 1e−05
MR Test
Inverse variance weighted (fixed effects)
Inverse variance weighted (multiplicative random effects)
MR Egger
MR RAPS
Weighted median
Weighted mode
0.10
0.05
0.00
−0.05
0.1
0.2
SNP effect on Glycoprotein hormones alpha chain
SNP effect on Gut microbiota abundance (genus Parasutterella id.2892)

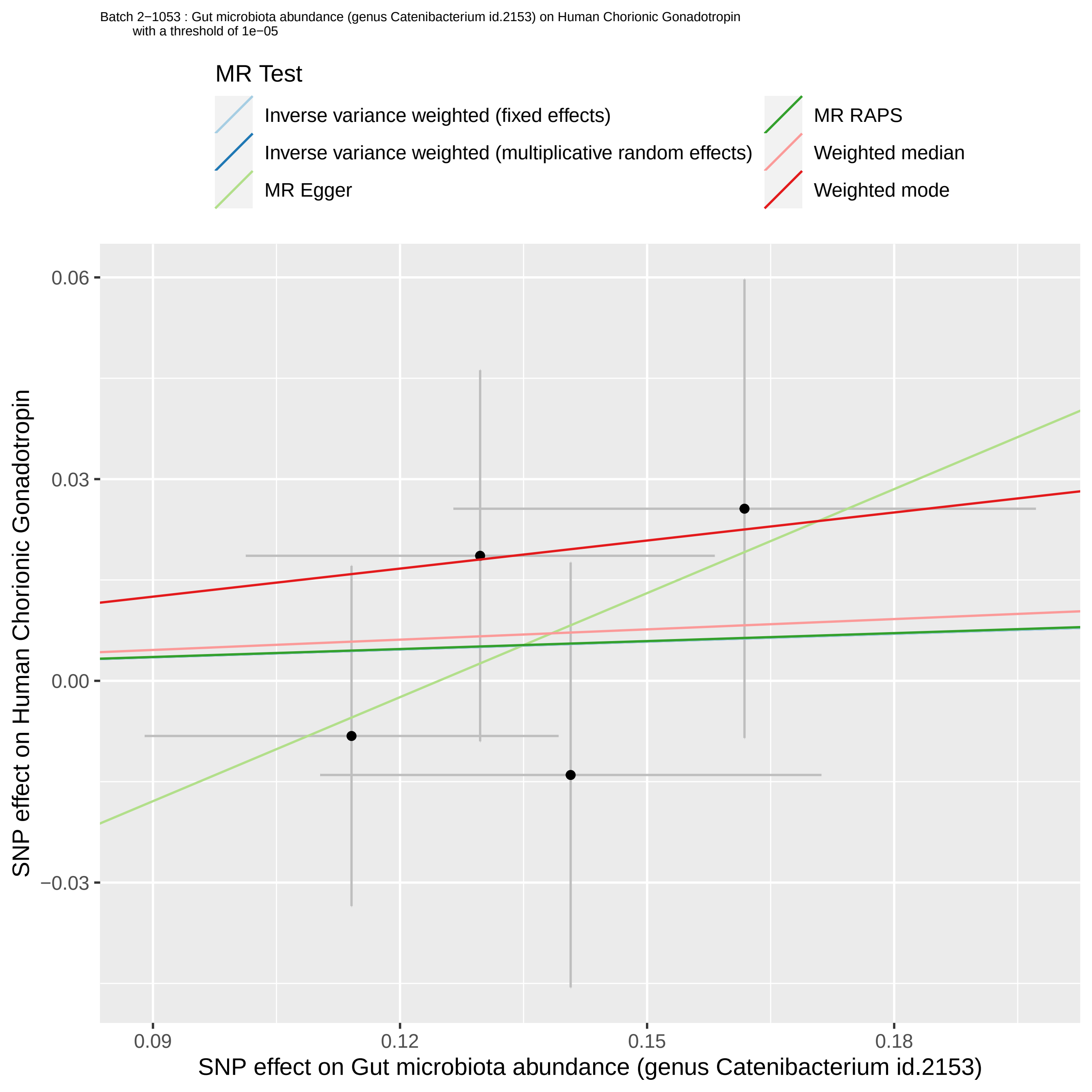
Batch 2−1053 : Gut microbiota abundance (genus Catenibacterium id.2153) on Human Chorionic Gonadotropin
with a threshold of 1e−05
MR Test
Inverse variance weighted (fixed effects)
Inverse variance weighted (multiplicative random effects)
MR Egger
MR RAPS
Weighted median
Weighted mode
0.06
0.03
0.00
−0.03
0.09
0.12
0.15
0.18
SNP effect on Human Chorionic Gonadotropin
SNP effect on Gut microbiota abundance (genus Catenibacterium id.2153)

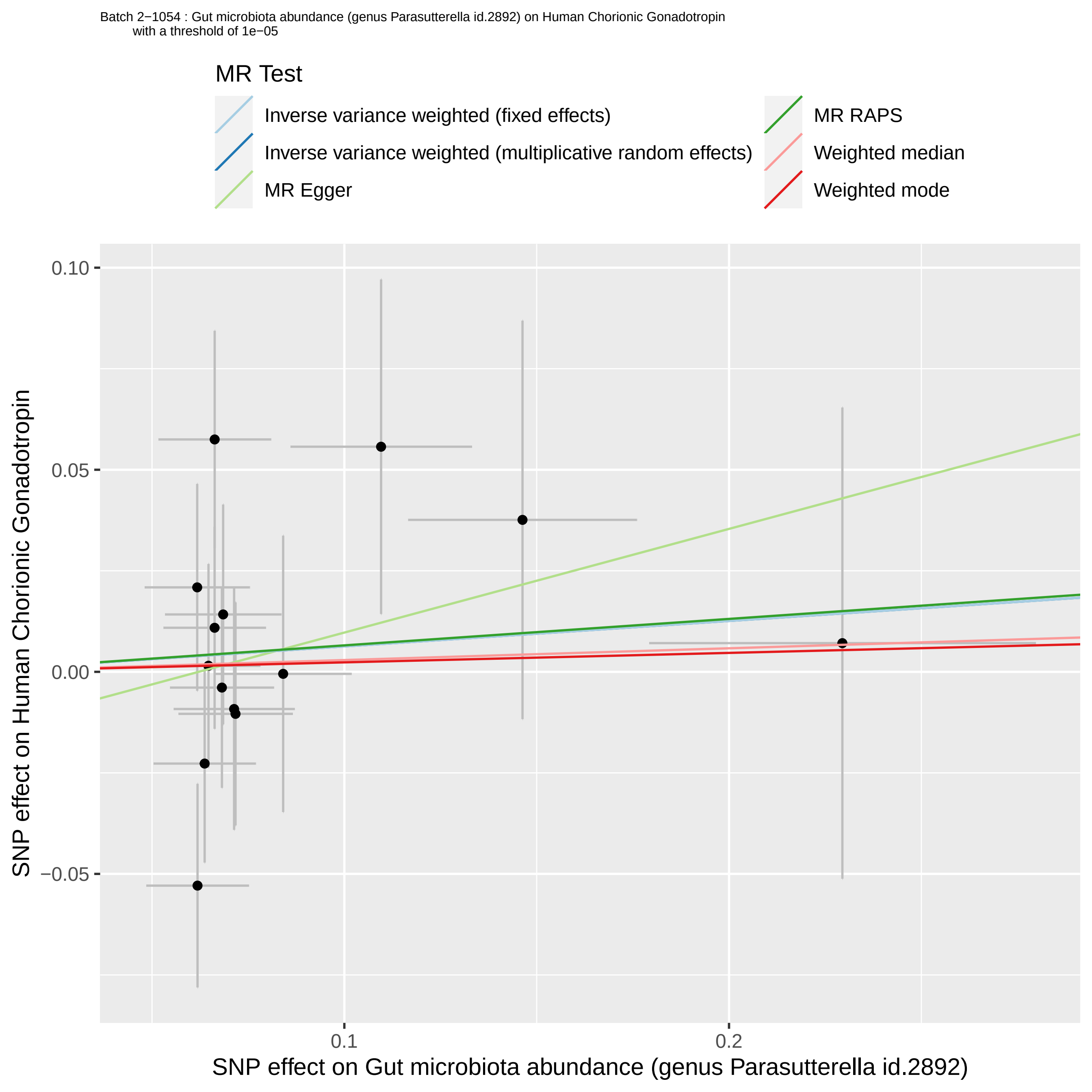
Batch 2−1054 : Gut microbiota abundance (genus Parasutterella id.2892) on Human Chorionic Gonadotropin
with a threshold of 1e−05
MR Test
Inverse variance weighted (fixed effects)
Inverse variance weighted (multiplicative random effects)
MR Egger
MR RAPS
Weighted median
Weighted mode
0.10
0.05
0.00
−0.05
0.1
0.2
SNP effect on Human Chorionic Gonadotropin
SNP effect on Gut microbiota abundance (genus Parasutterella id.2892)

Batch 2−1055 : Gut microbiota abundance (genus Catenibacterium id.2153) on Follicle stimulating hormone
with a threshold of 1e−05

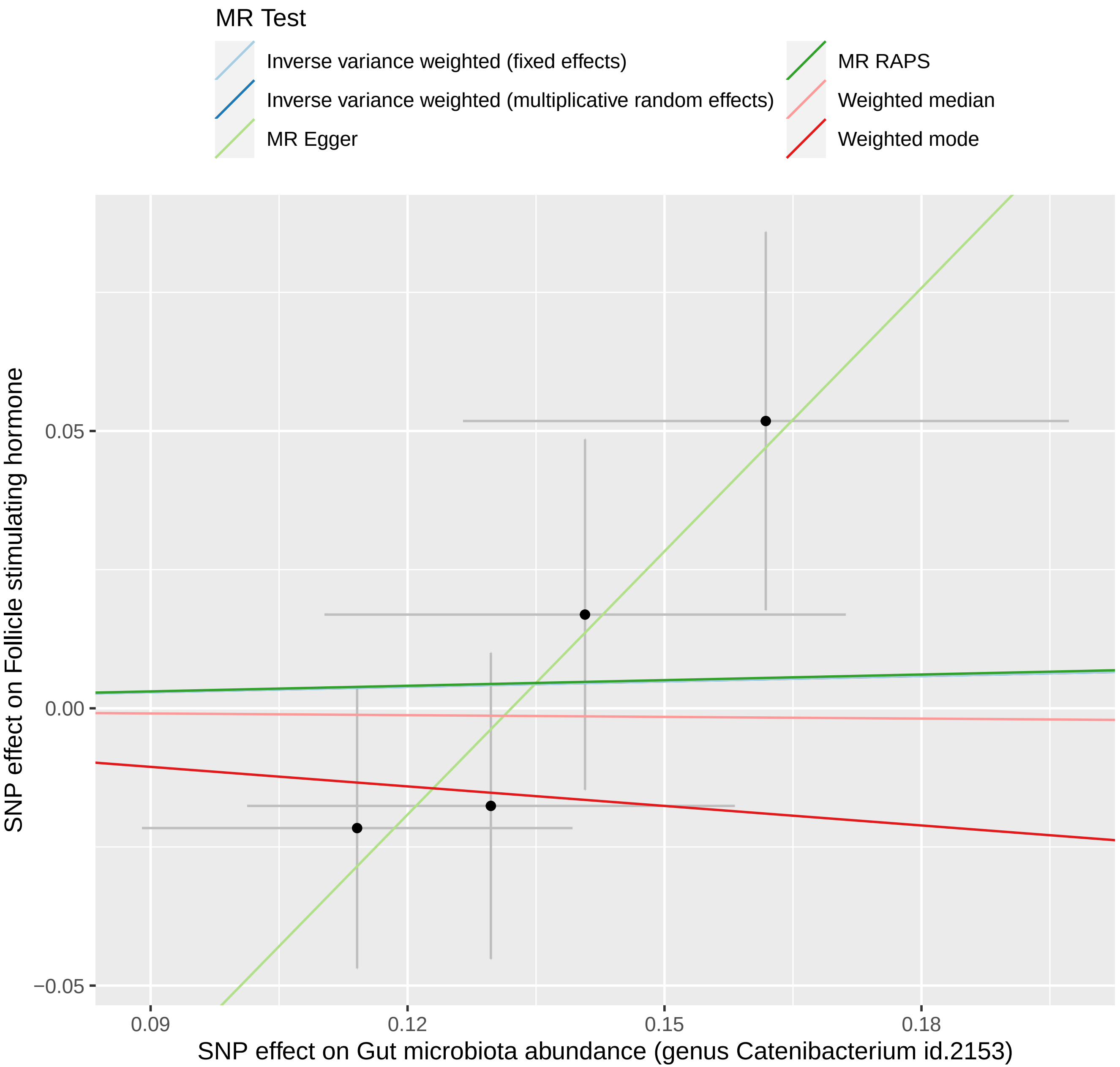

Batch 2−1056 : Gut microbiota abundance (genus Parasutterella id.2892) on Follicle stimulating hormone
with a threshold of 1e−05

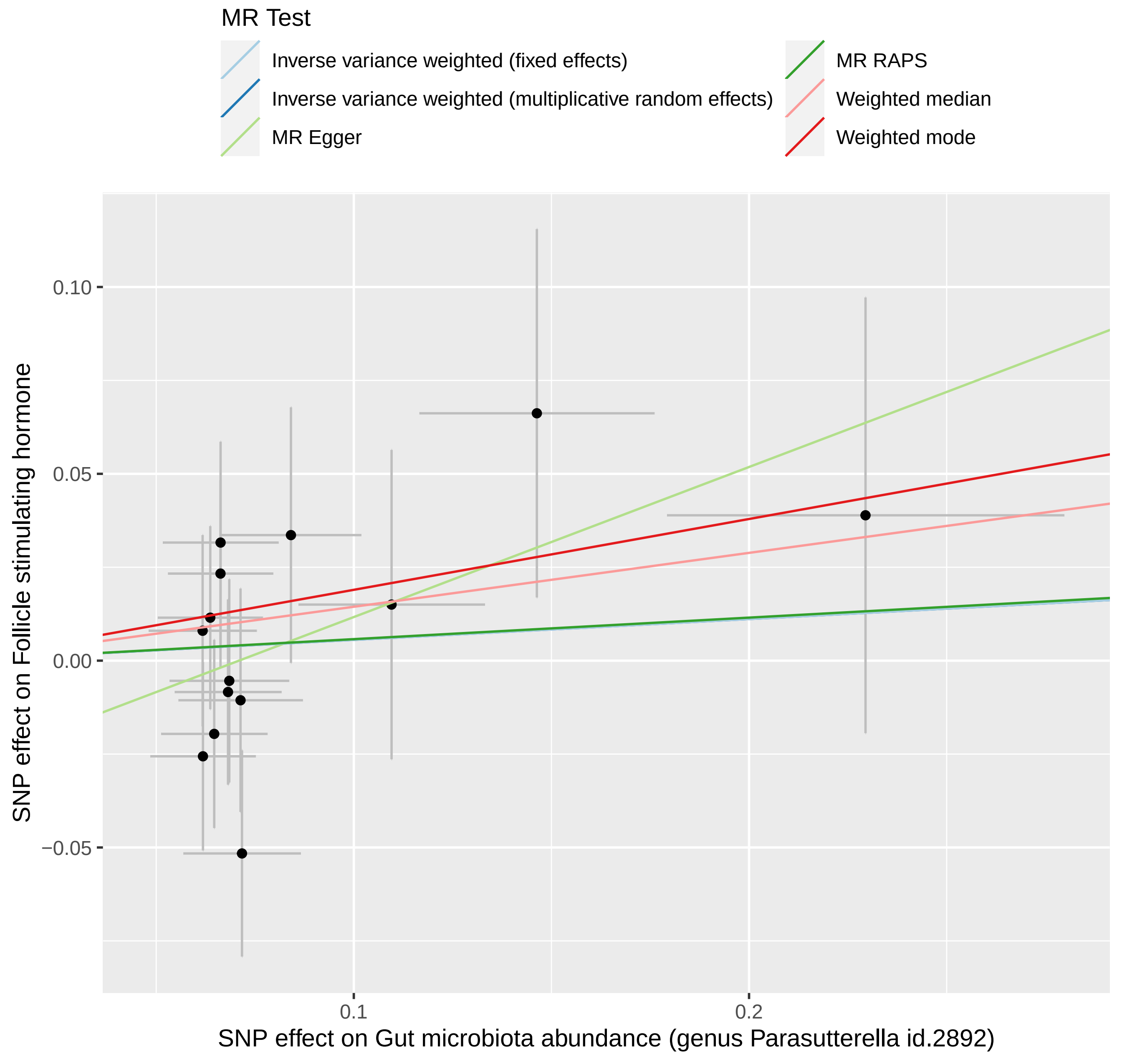

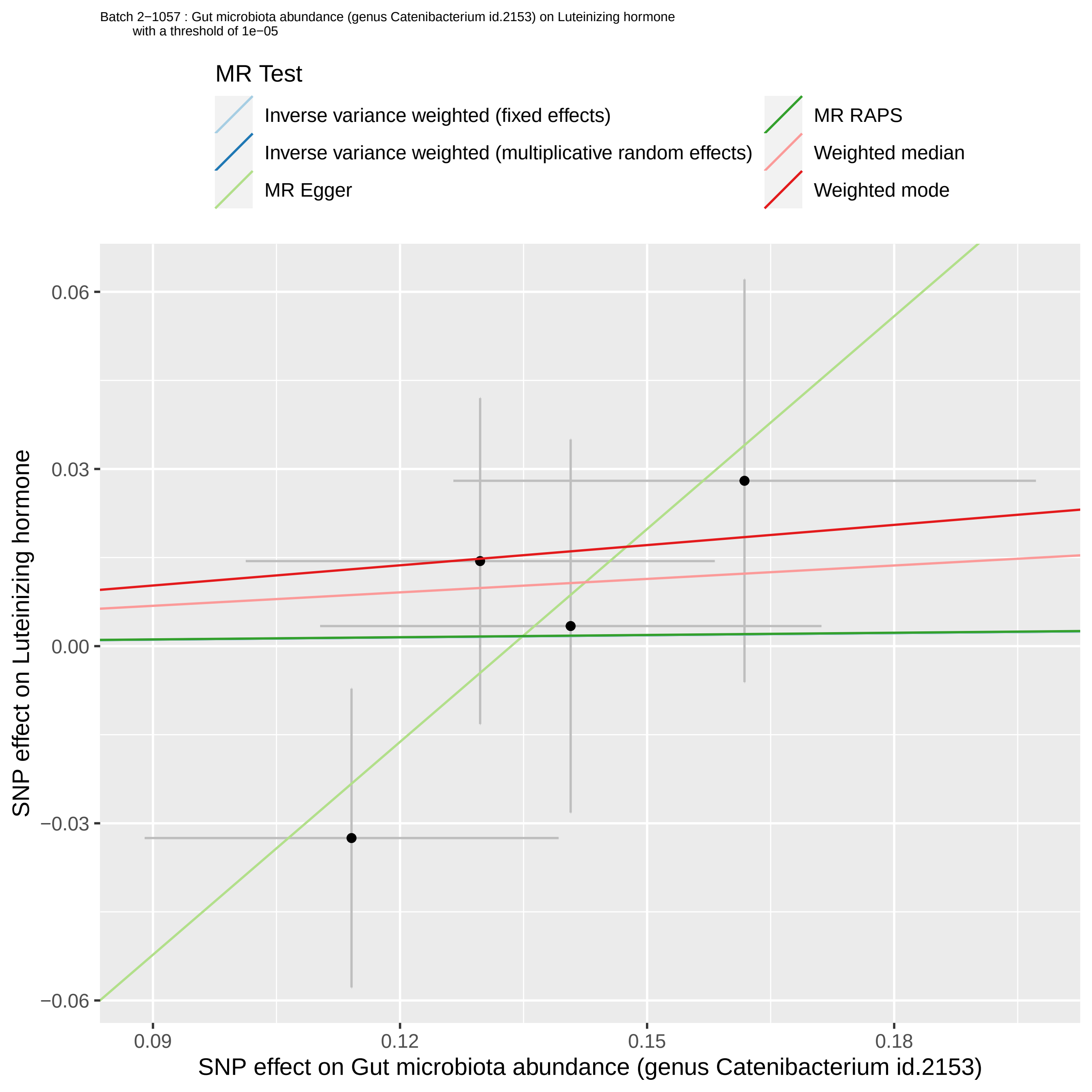
Batch 2−1057 : Gut microbiota abundance (genus Catenibacterium id.2153) on Luteinizing hormone
with a threshold of 1e−05
MR Test
Inverse variance weighted (fixed effects)
Inverse variance weighted (multiplicative random effects)
MR Egger
MR RAPS
Weighted median
Weighted mode
0.06
0.03
0.00
−0.03
−0.06
0.09
0.12
0.15
0.18
SNP effect on Luteinizing hormone
SNP effect on Gut microbiota abundance (genus Catenibacterium id.2153)

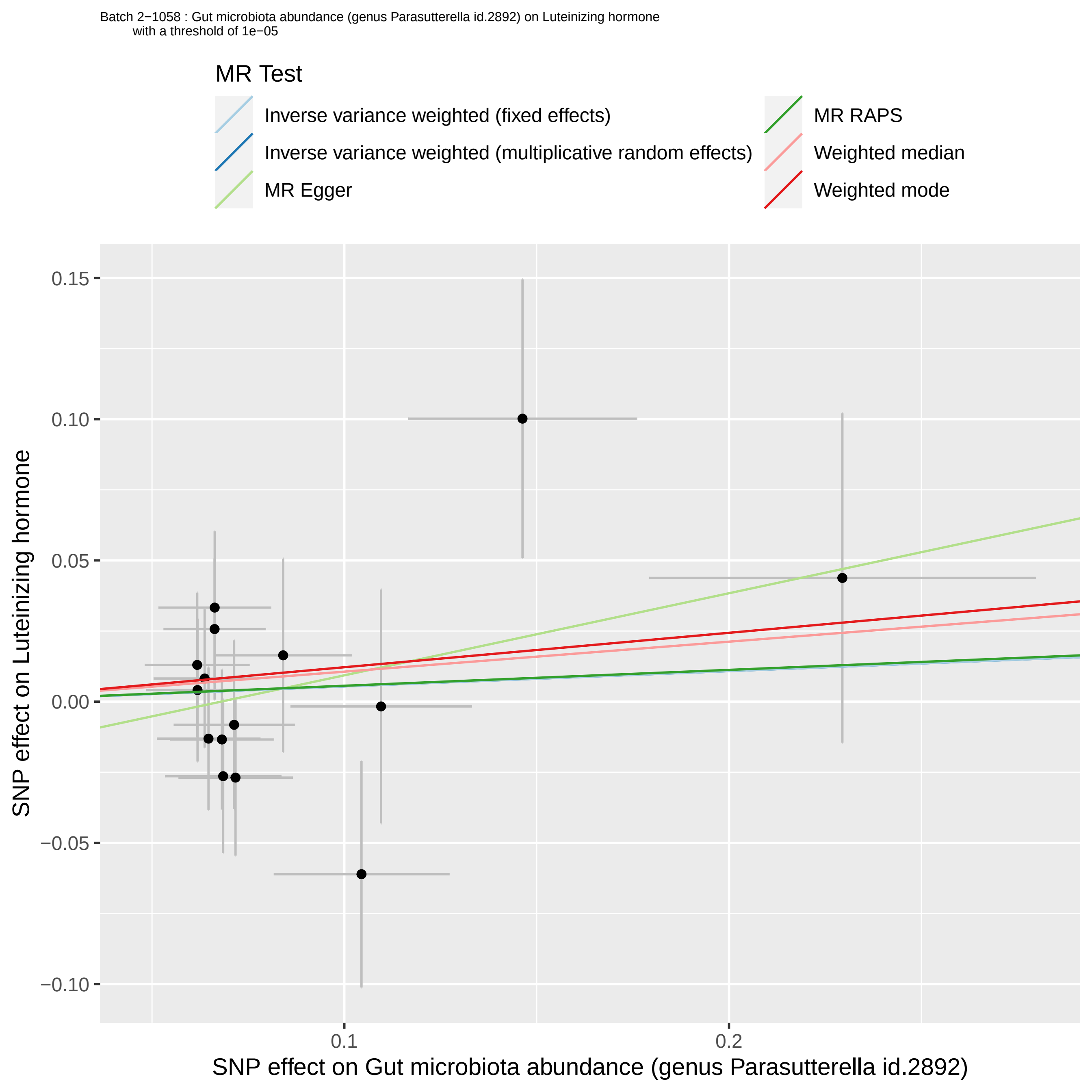
Batch 2−1058 : Gut microbiota abundance (genus Parasutterella id.2892) on Luteinizing hormone
with a threshold of 1e−05
MR Test
Inverse variance weighted (fixed effects)
Inverse variance weighted (multiplicative random effects)
MR Egger
MR RAPS
Weighted median
Weighted mode
SNP effect on Luteinizing hormone
0.15
0.10
0.05
0.00
−0.05
−0.10
0.1
0.2
SNP effect on Gut microbiota abundance (genus Parasutterella id.2892)

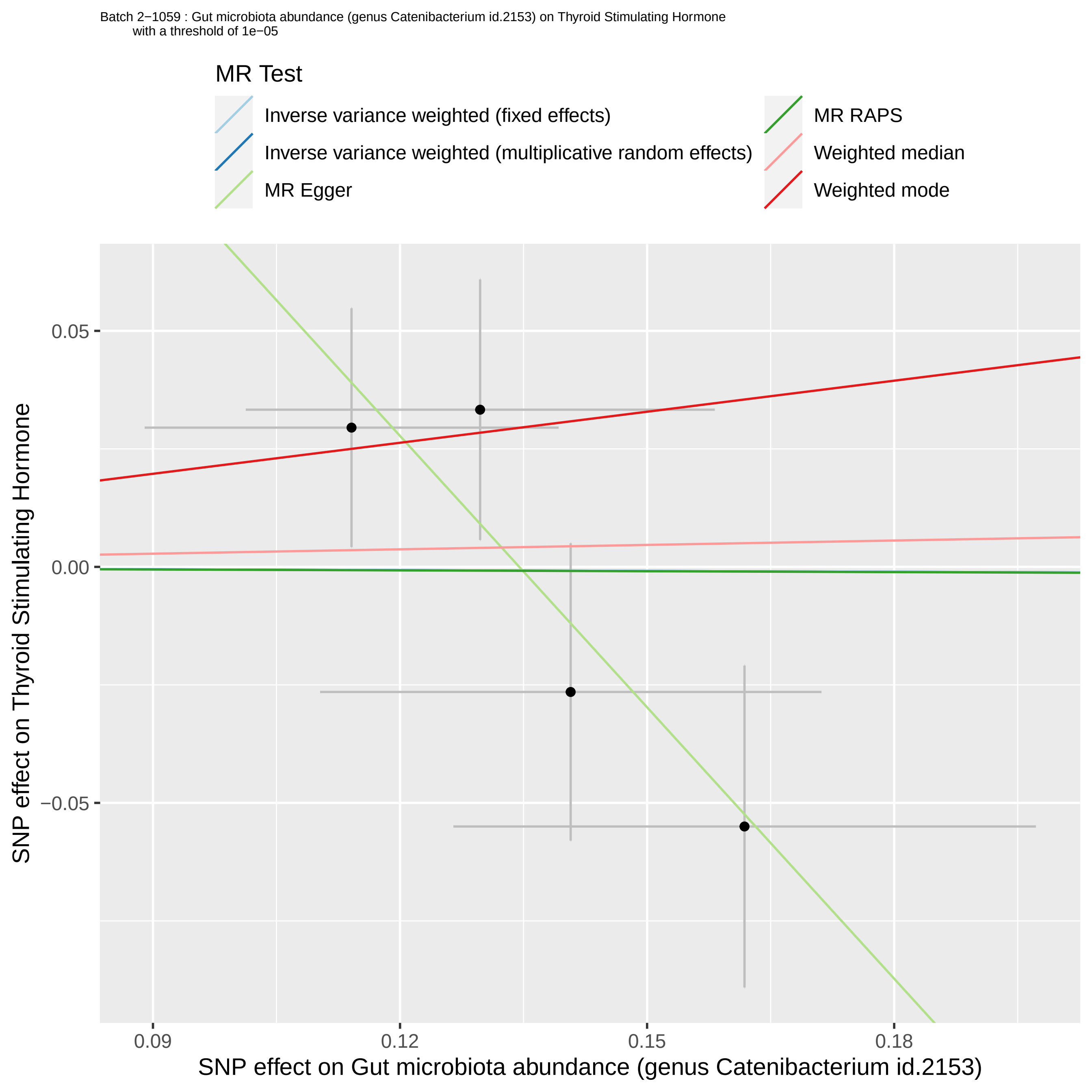
Batch 2−1059 : Gut microbiota abundance (genus Catenibacterium id.2153) on Thyroid Stimulating Hormone
with a threshold of 1e−05
MR Test
Inverse variance weighted (fixed effects)
Inverse variance weighted (multiplicative random effects)
MR Egger
MR RAPS
Weighted median
Weighted mode
0.05
0.00
−0.05
0.09
0.12
0.15
0.18
SNP effect on Thyroid Stimulating Hormone
SNP effect on Gut microbiota abundance (genus Catenibacterium id.2153)

Batch 2−1060 : Gut microbiota abundance (genus Parasutterella id.2892) on Thyroid Stimulating Hormone
with a threshold of 1e−05

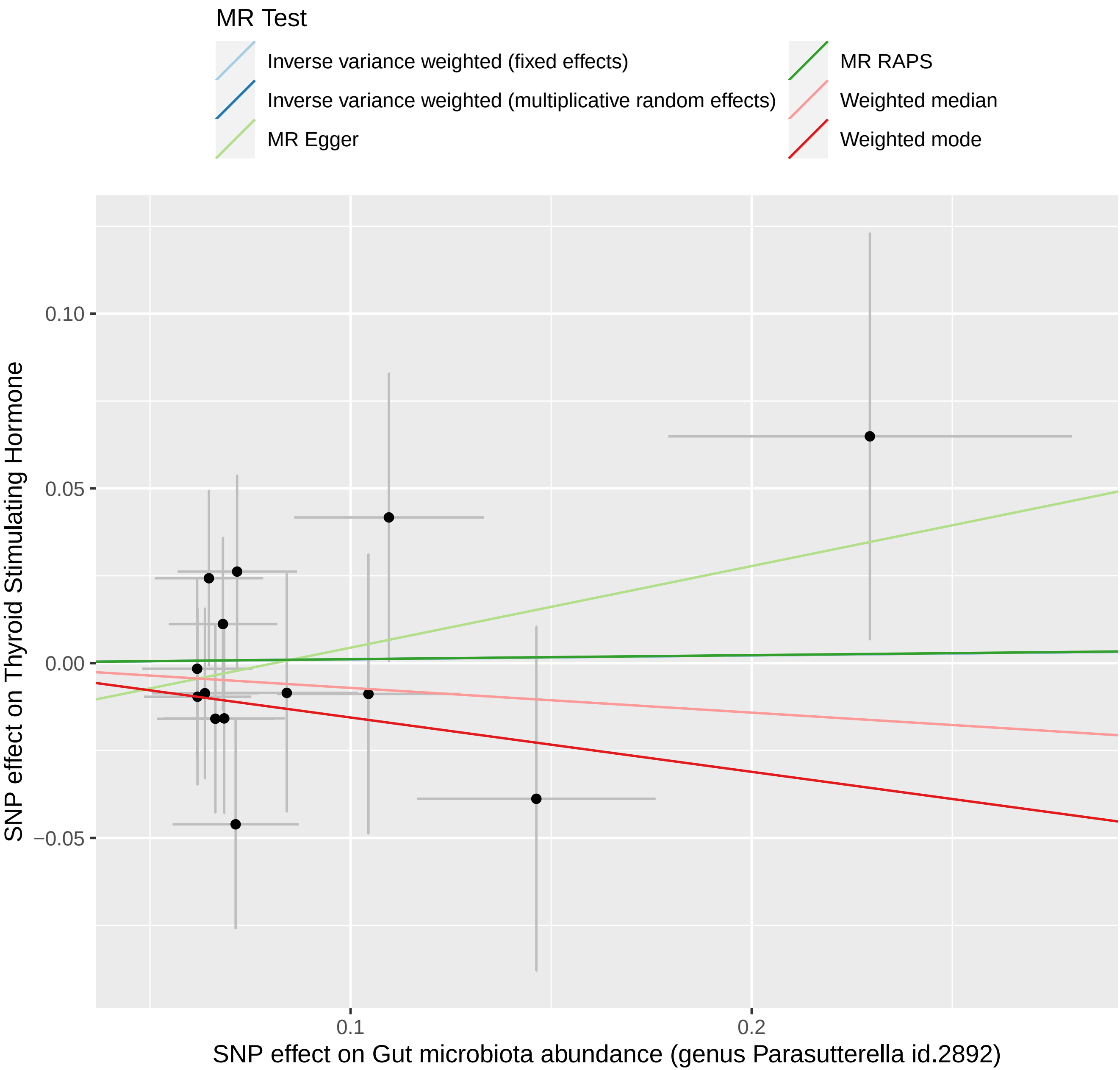

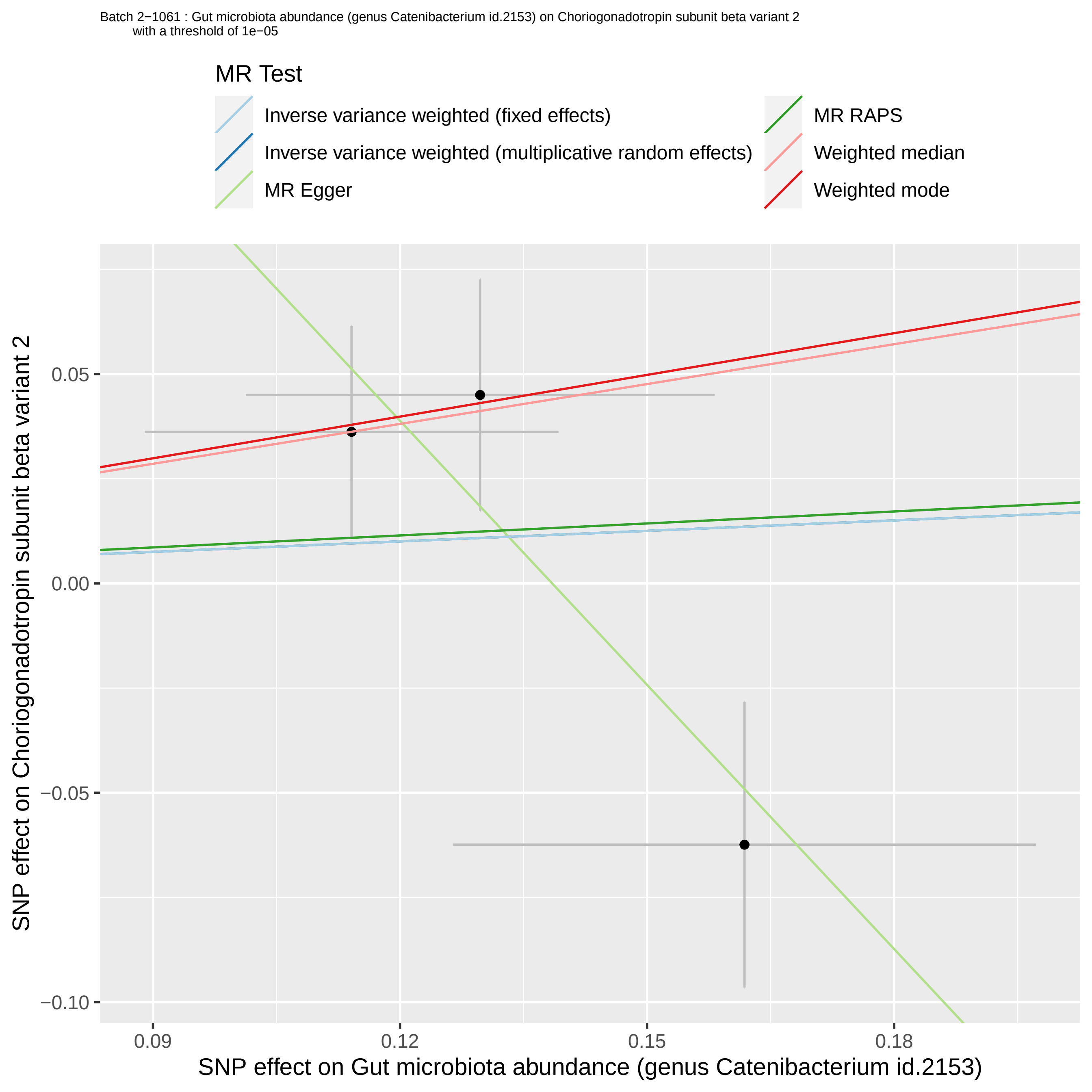
Batch 2−1061 : Gut microbiota abundance (genus Catenibacterium id.2153) on Choriogonadotropin subunit beta variant 2
with a threshold of 1e−05
MR Test
Inverse variance weighted (fixed effects)
Inverse variance weighted (multiplicative random effects)
MR Egger
MR RAPS
Weighted median
Weighted mode
0.05
0.00
−0.05
−0.10
0.09
0.12
0.15
0.18
SNP effect on Choriogonadotropin subunit beta variant 2
SNP effect on Gut microbiota abundance (genus Catenibacterium id.2153)

Batch 2−1062 : Gut microbiota abundance (genus Parasutterella id.2892) on Choriogonadotropin subunit beta variant 2
with a threshold of 1e−05

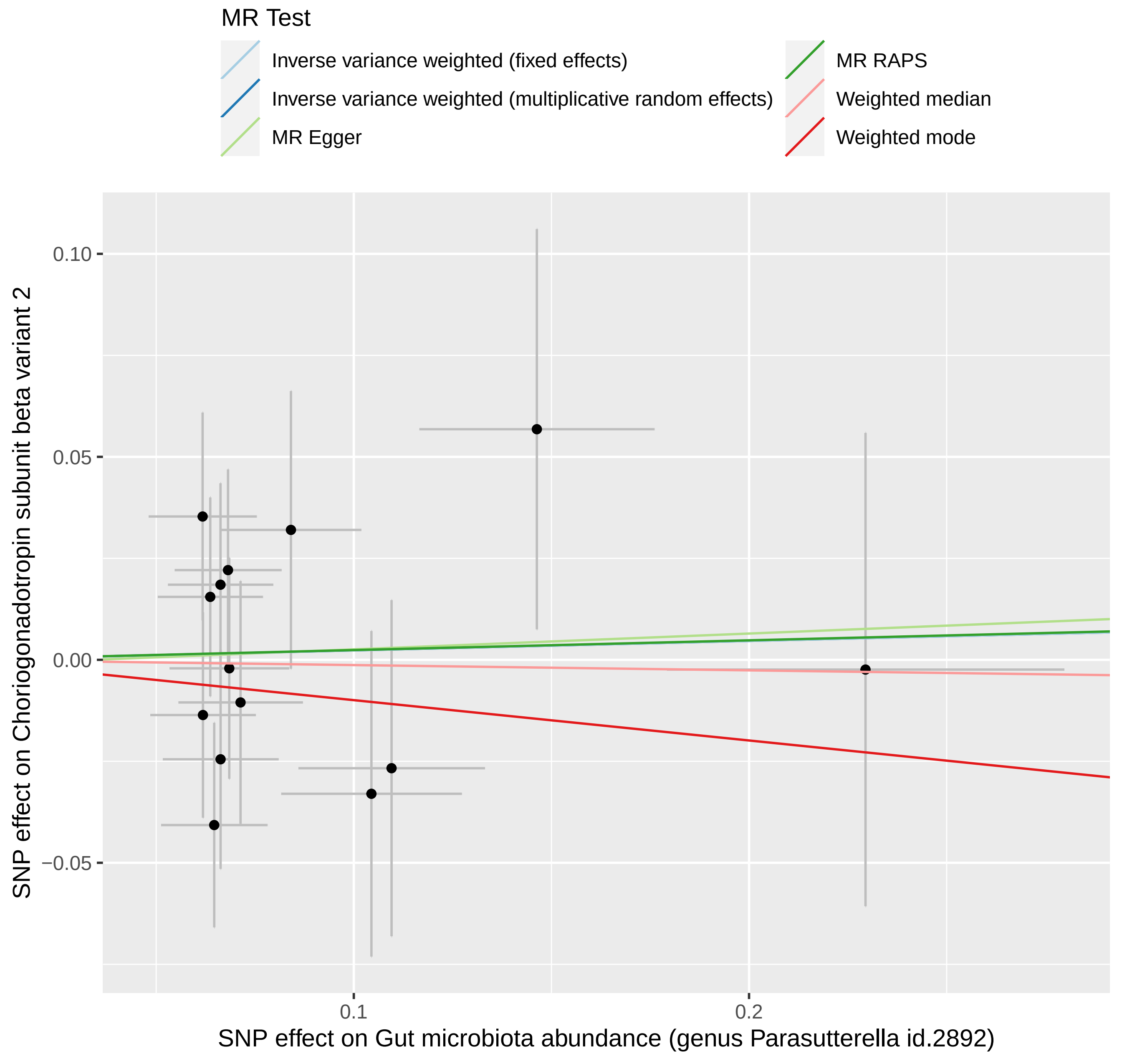

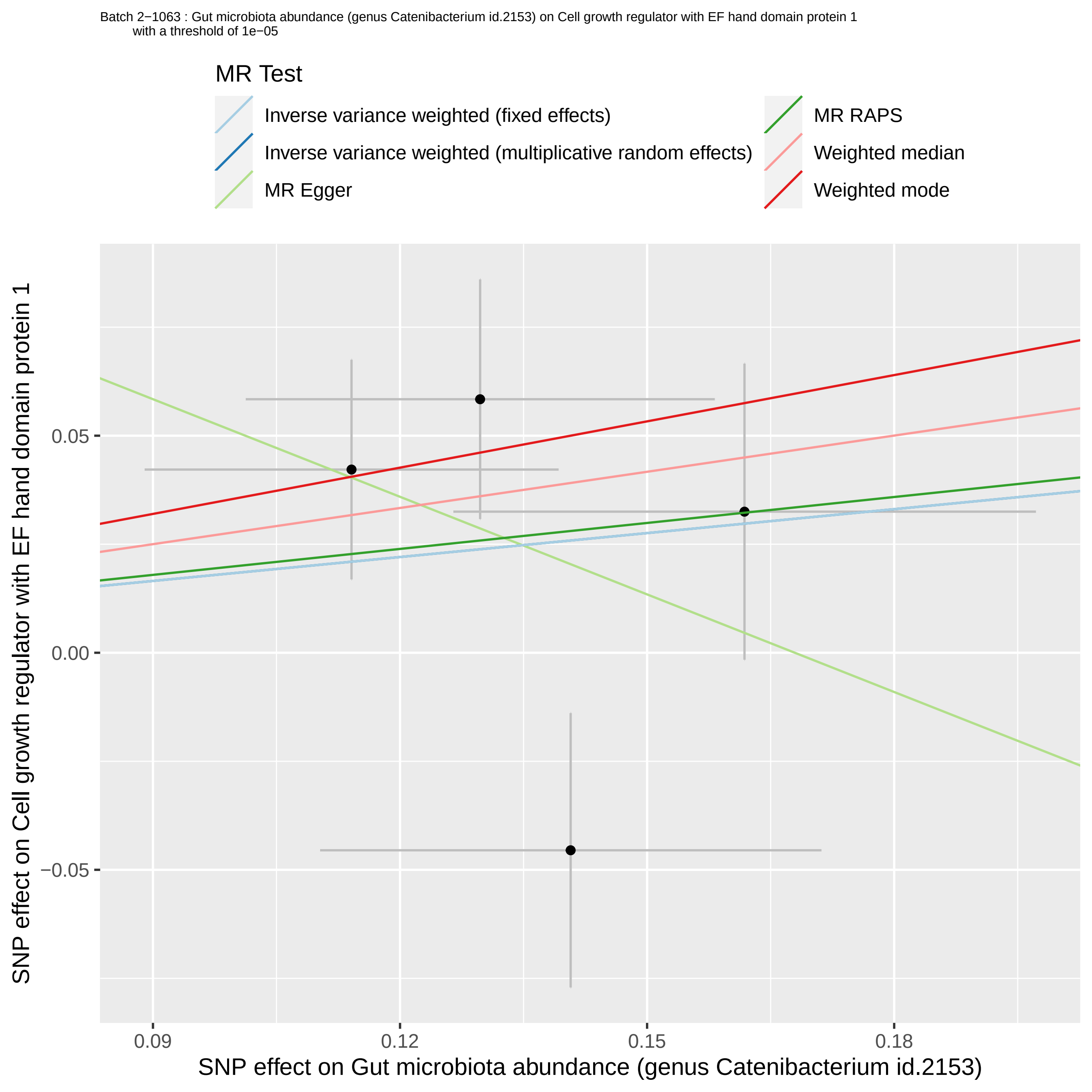
Batch 2−1063 : Gut microbiota abundance (genus Catenibacterium id.2153) on Cell growth regulator with EF hand domain protein 1
with a threshold of 1e−05
MR Test
Inverse variance weighted (fixed effects)
Inverse variance weighted (multiplicative random effects)
MR Egger
MR RAPS
Weighted median
Weighted mode
0.05
0.00
−0.05
0.09
0.12
0.15
0.18
SNP effect on Cell growth regulator with EF hand domain protein 1
SNP effect on Gut microbiota abundance (genus Catenibacterium id.2153)

Batch 2−1064 : Gut microbiota abundance (genus Parasutterella id.2892) on Cell growth regulator with EF hand domain protein 1
with a threshold of 1e−05

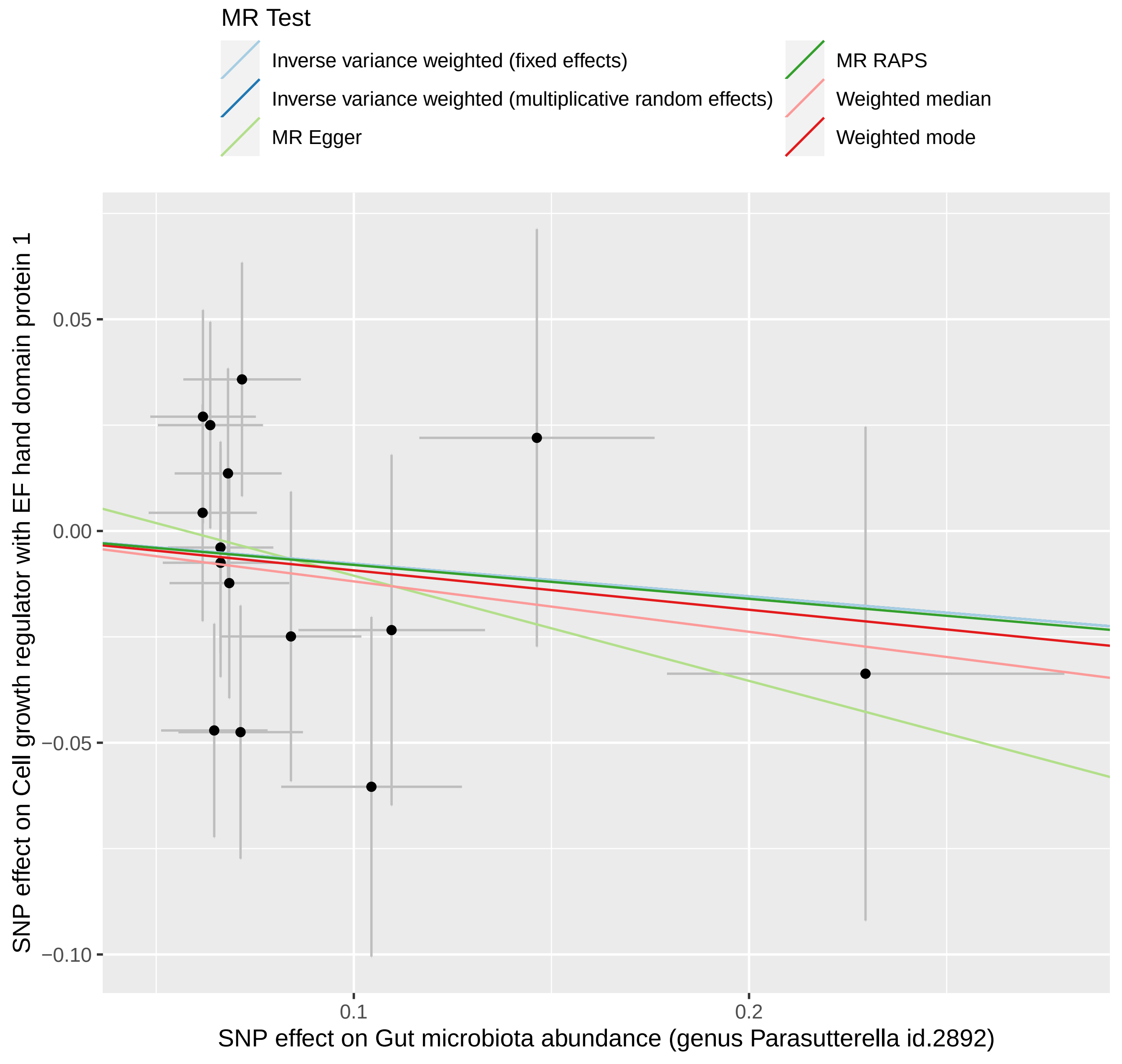

Batch 2−1065 : Gut microbiota abundance (genus Catenibacterium id.2153) on Chondroadherin
with a threshold of 1e−05

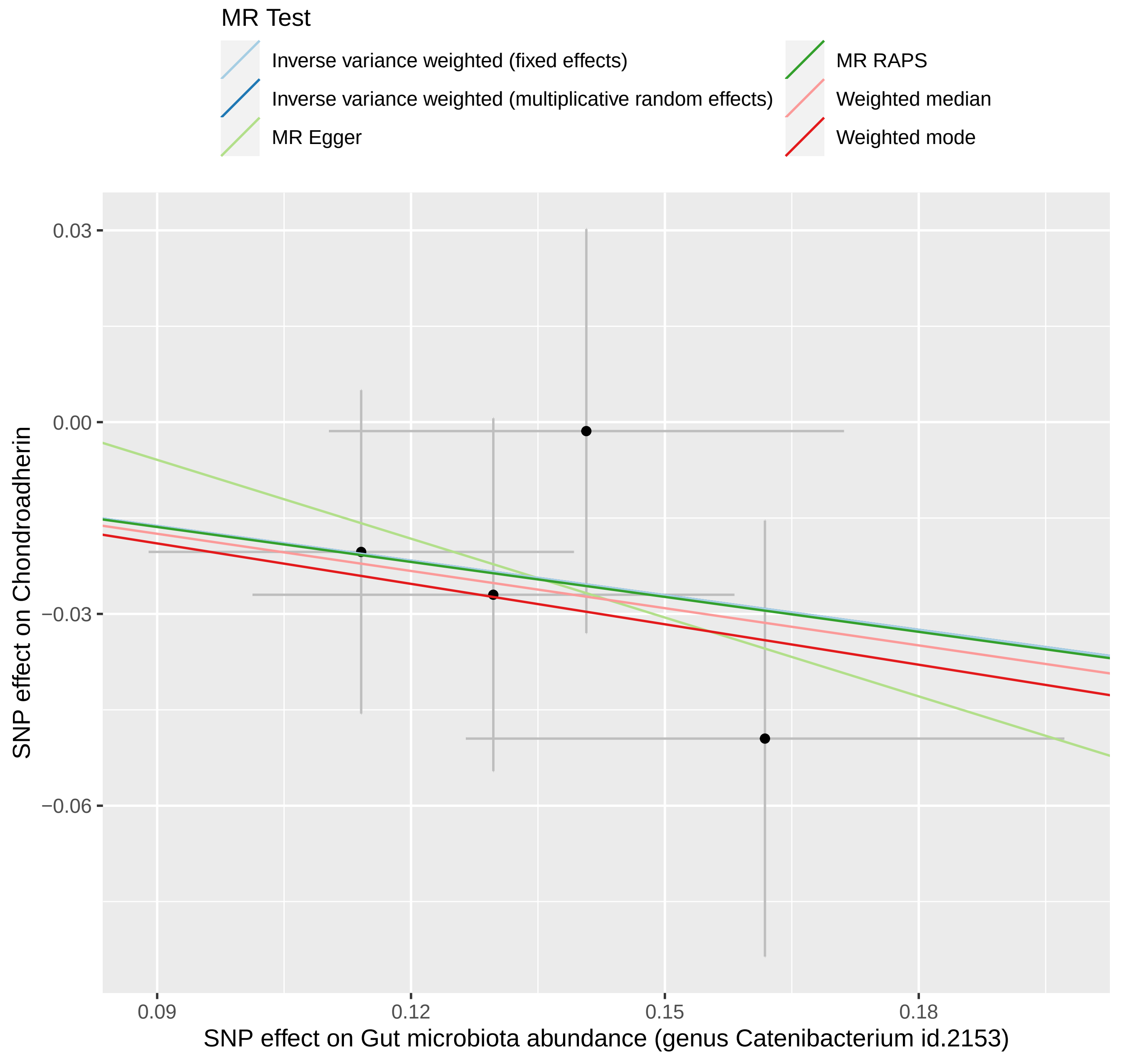

Batch 2−1066 : Gut microbiota abundance (genus Parasutterella id.2892) on Chondroadherin
with a threshold of 1e−05

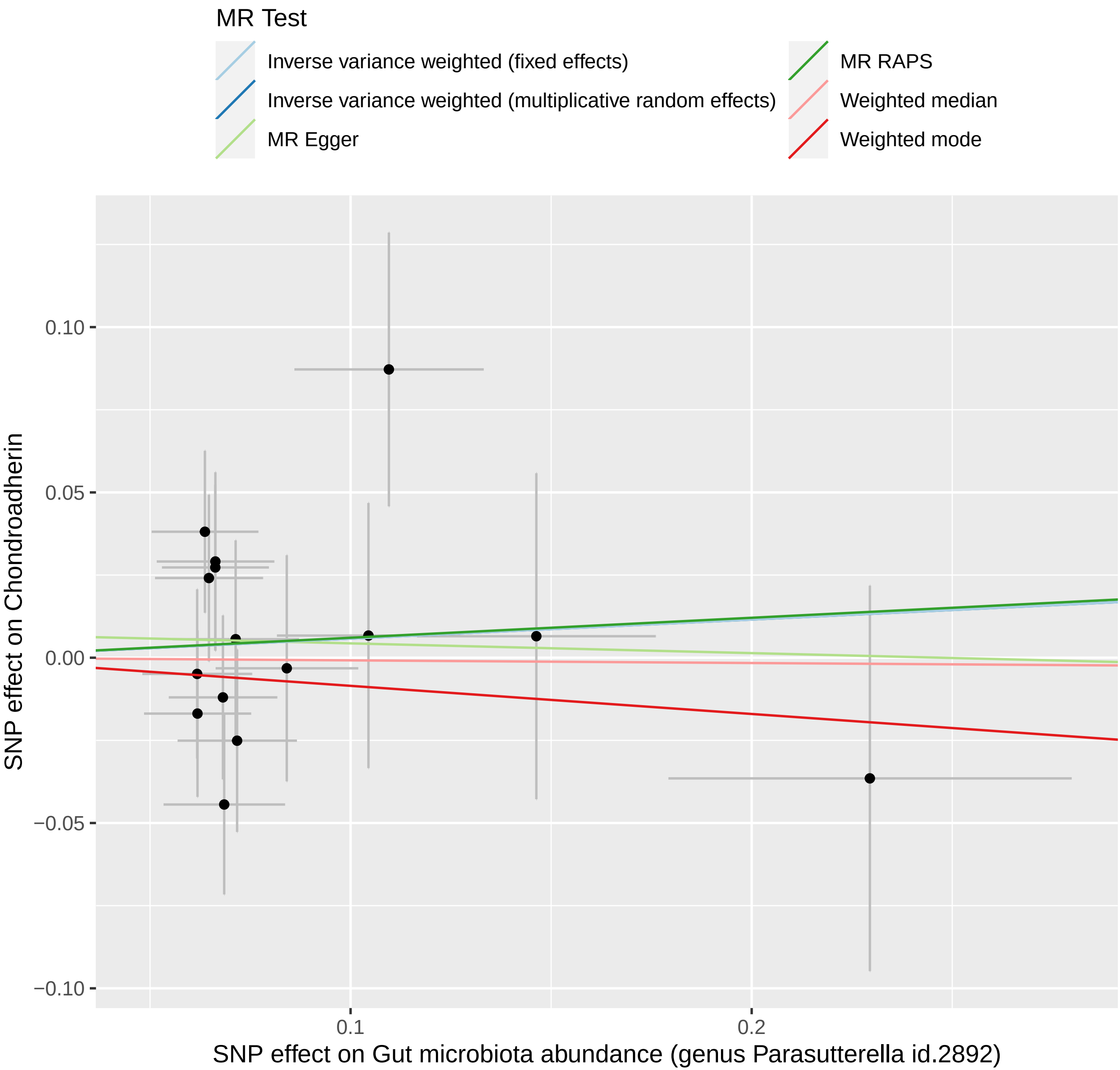

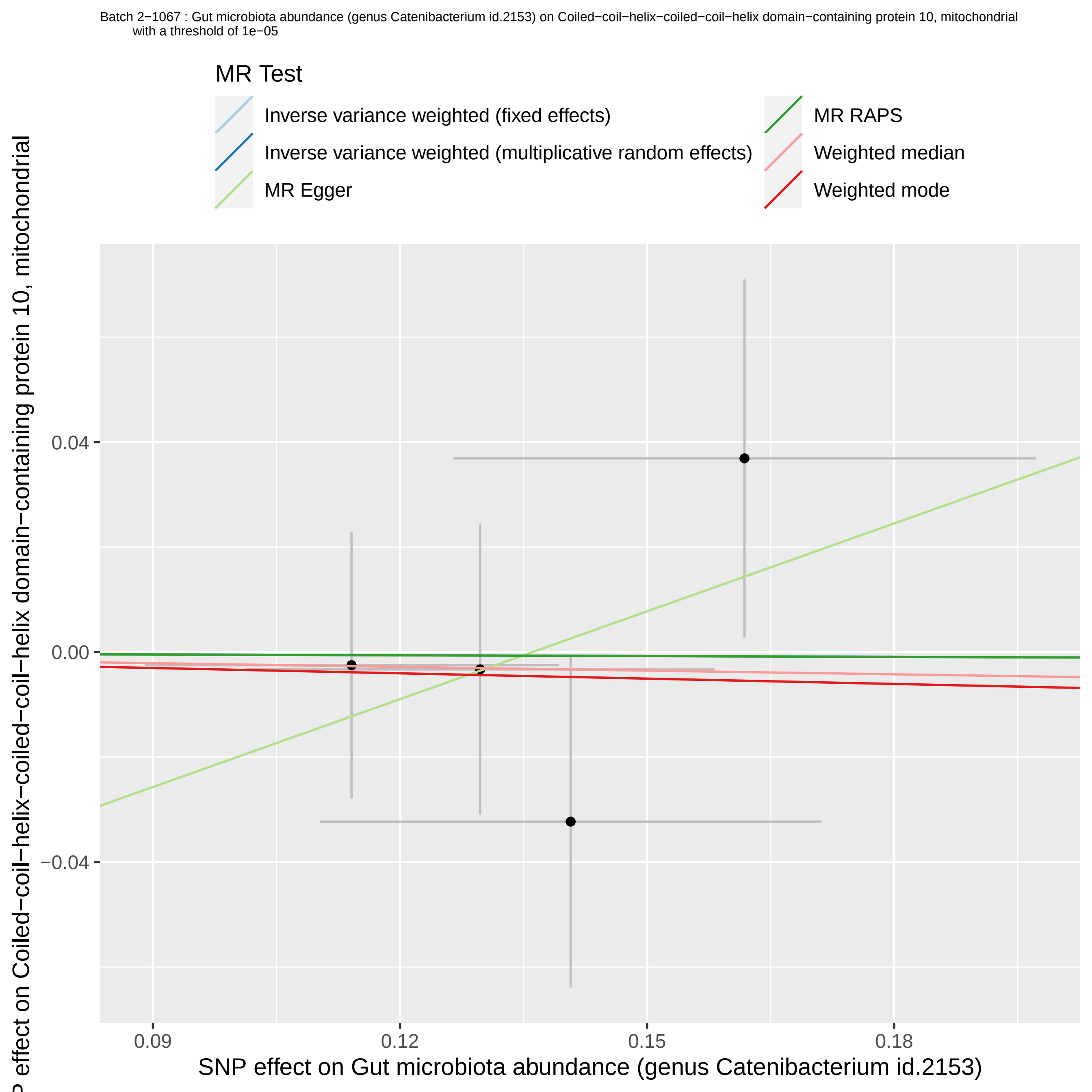

Batch 2−1067 : Gut microbiota abundance (genus Catenibacterium id.2153) on Coiled−coil−helix−coiled−coil−helix domain−containing protein 10, mitochondrial with a threshold of 1e−05
MR Test
Inverse variance weighted (fixed effects)
Inverse variance weighted (multiplicative random effects)
MR Egger
MR RAPS
Weighted median
Weighted mode
0.04
0.00
−0.04
0.09
0.12
0.15
0.18
SNP effect on Gut microbiota abundance (genus Catenibacterium id.2153)
P effect on Coiled−coil−helix−coiled−coil−helix domain−containing protein 10, mitochondrial

Batch 2−1068 : Gut microbiota abundance (genus Parasutterella id.2892) on Coiled−coil−helix−coiled−coil−helix domain−containing protein 10, mitochondrial with a threshold of 1e−05

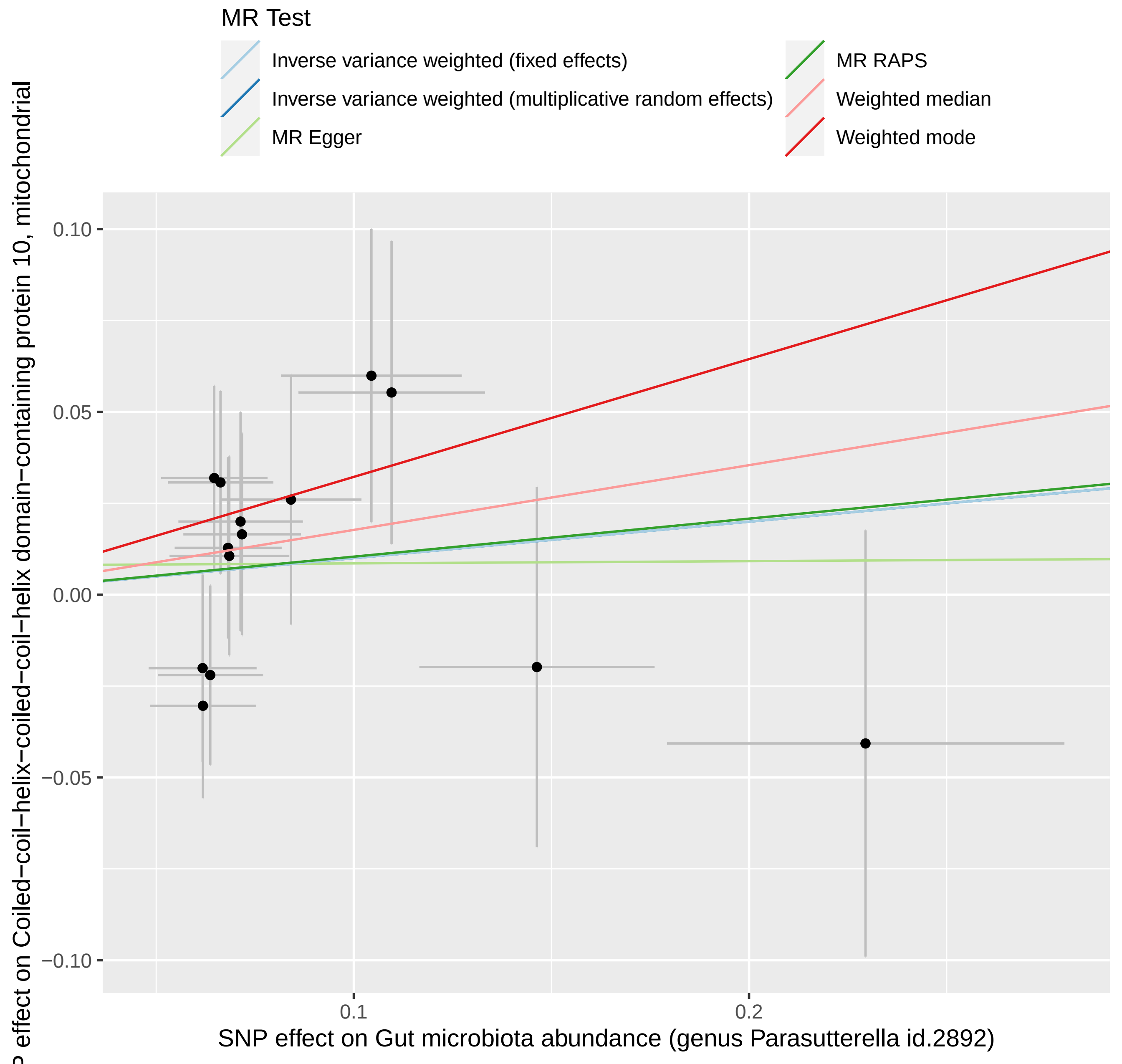

Batch 2−1069 : Gut microbiota abundance (genus Catenibacterium id.2153) on Coiled−coil−helix−coiled−coil−helix domain−containing protein 2
with a threshold of 1e−05

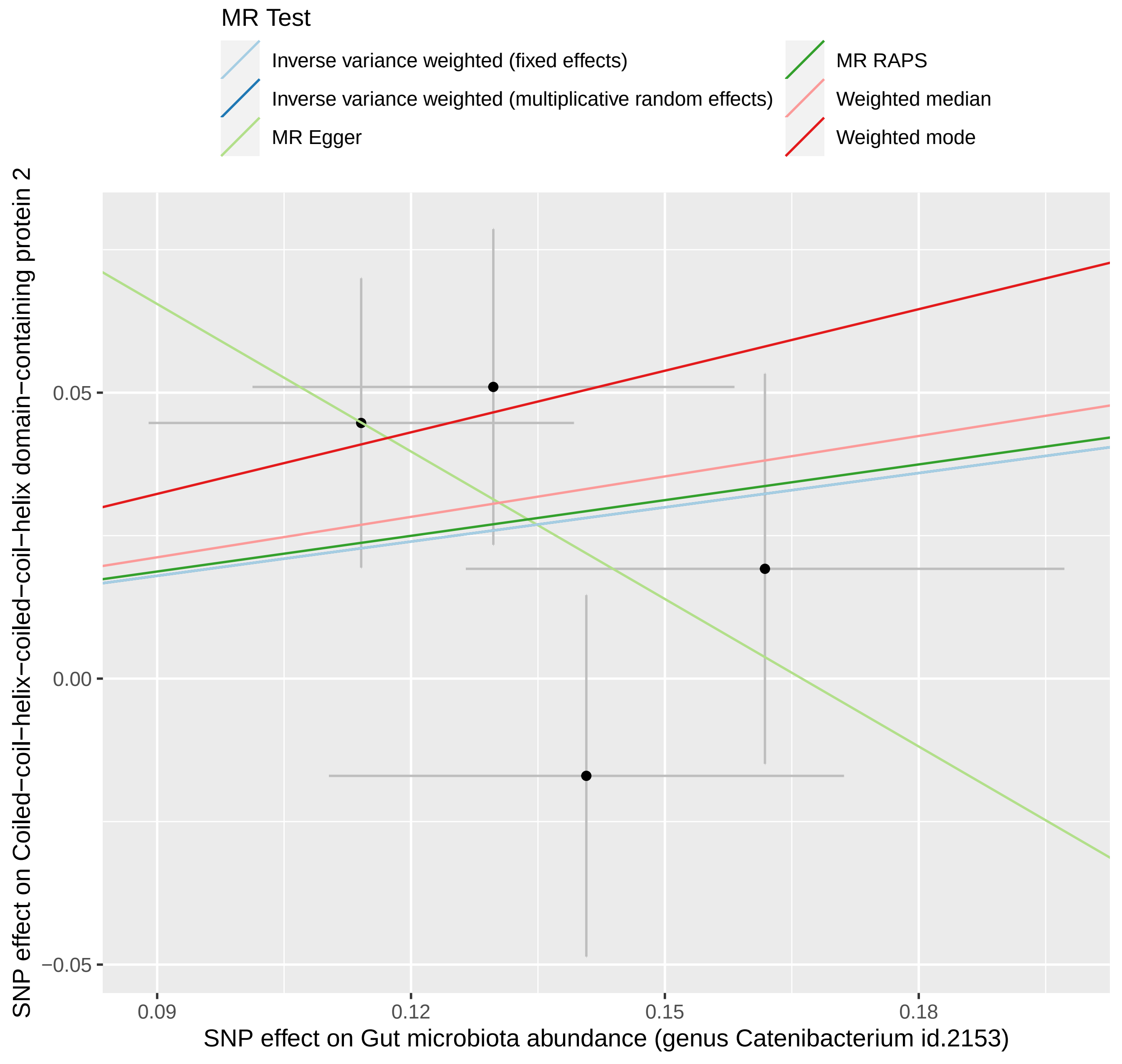

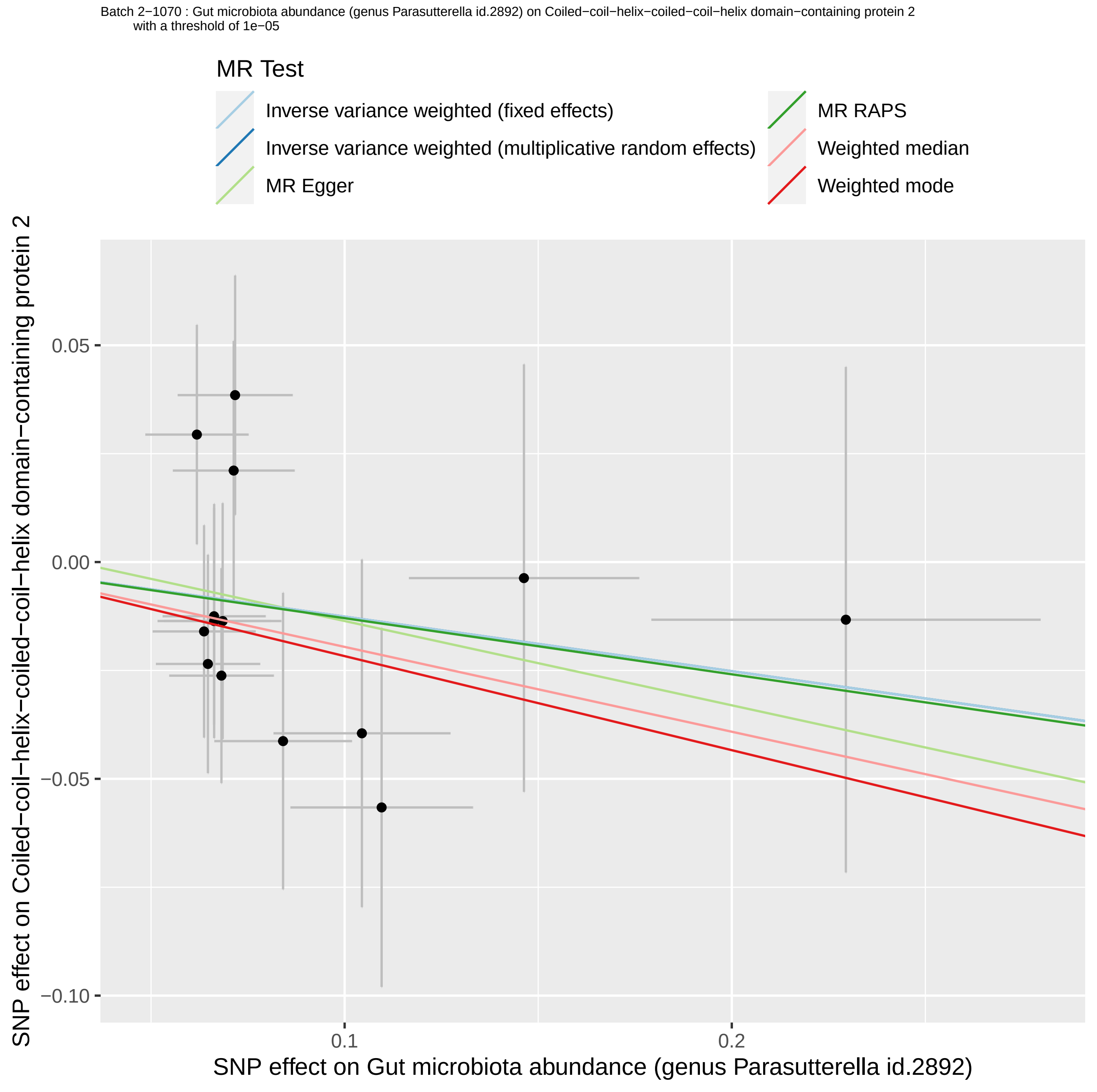

Batch 2−1070 : Gut microbiota abundance (genus Parasutterella id.2892) on Coiled−coil−helix−coiled−coil−helix domain−containing protein 2
with a threshold of 1e−05
MR Test
Inverse variance weighted (fixed effects)
Inverse variance weighted (multiplicative random effects)
MR Egger
MR RAPS
Weighted median
Weighted mode
0.05
0.00
−0.05
−0.10
0.1
0.2
SNP effect on Coiled−coil−helix−coiled−coil−helix domain−containing protein 2
SNP effect on Gut microbiota abundance (genus Parasutterella id.2892)

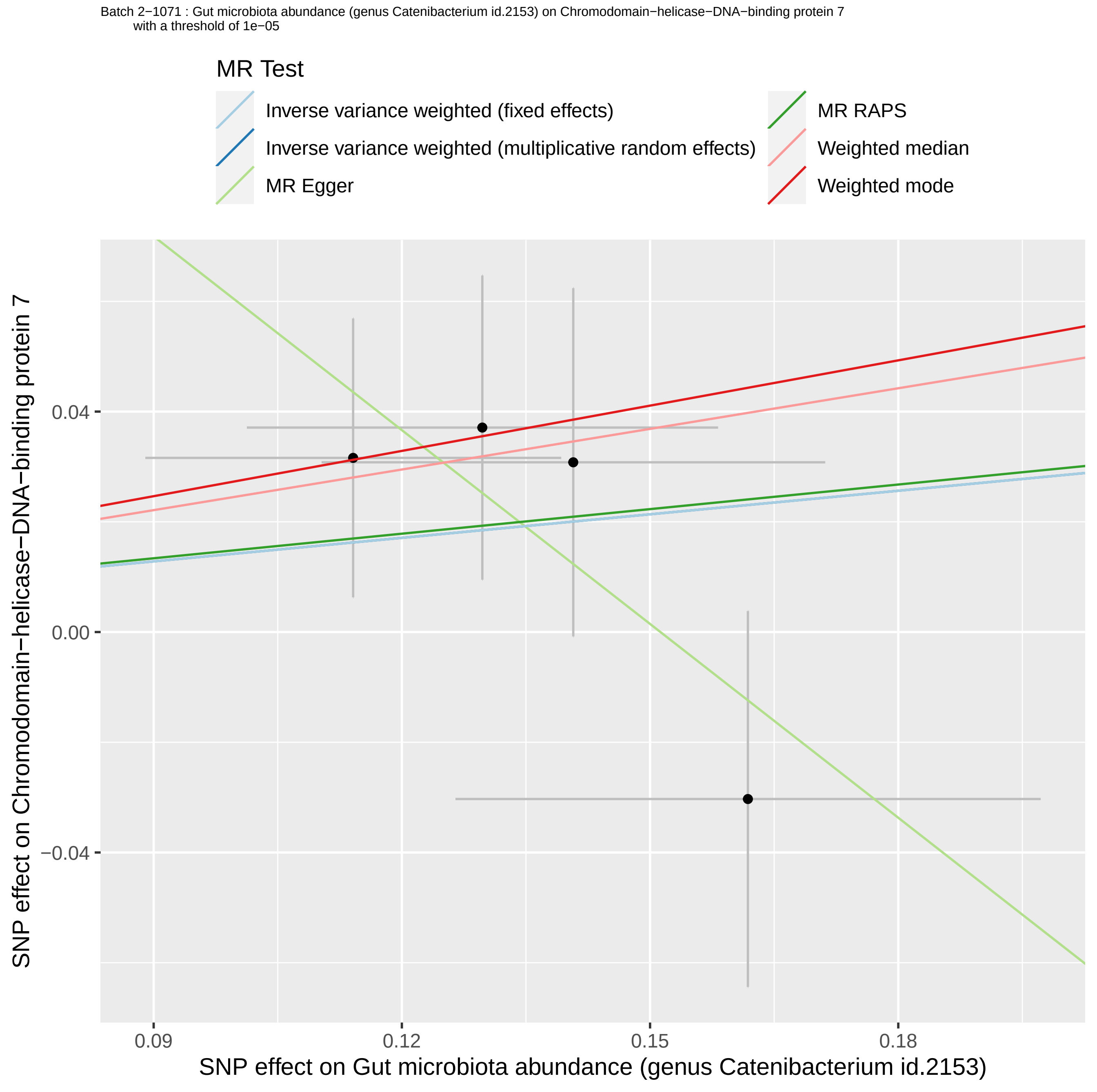
Batch 2−1071 : Gut microbiota abundance (genus Catenibacterium id.2153) on Chromodomain−helicase−DNA−binding protein 7
with a threshold of 1e−05
MR Test
Inverse variance weighted (fixed effects)
Inverse variance weighted (multiplicative random effects)
MR Egger
MR RAPS
Weighted median
Weighted mode
0.04
0.00
−0.04
0.09
0.12
0.15
0.18
SNP effect on Chromodomain−helicase−DNA−binding protein 7
SNP effect on Gut microbiota abundance (genus Catenibacterium id.2153)

Batch 2−1072 : Gut microbiota abundance (genus Parasutterella id.2892) on Chromodomain−helicase−DNA−binding protein 7
with a threshold of 1e−05

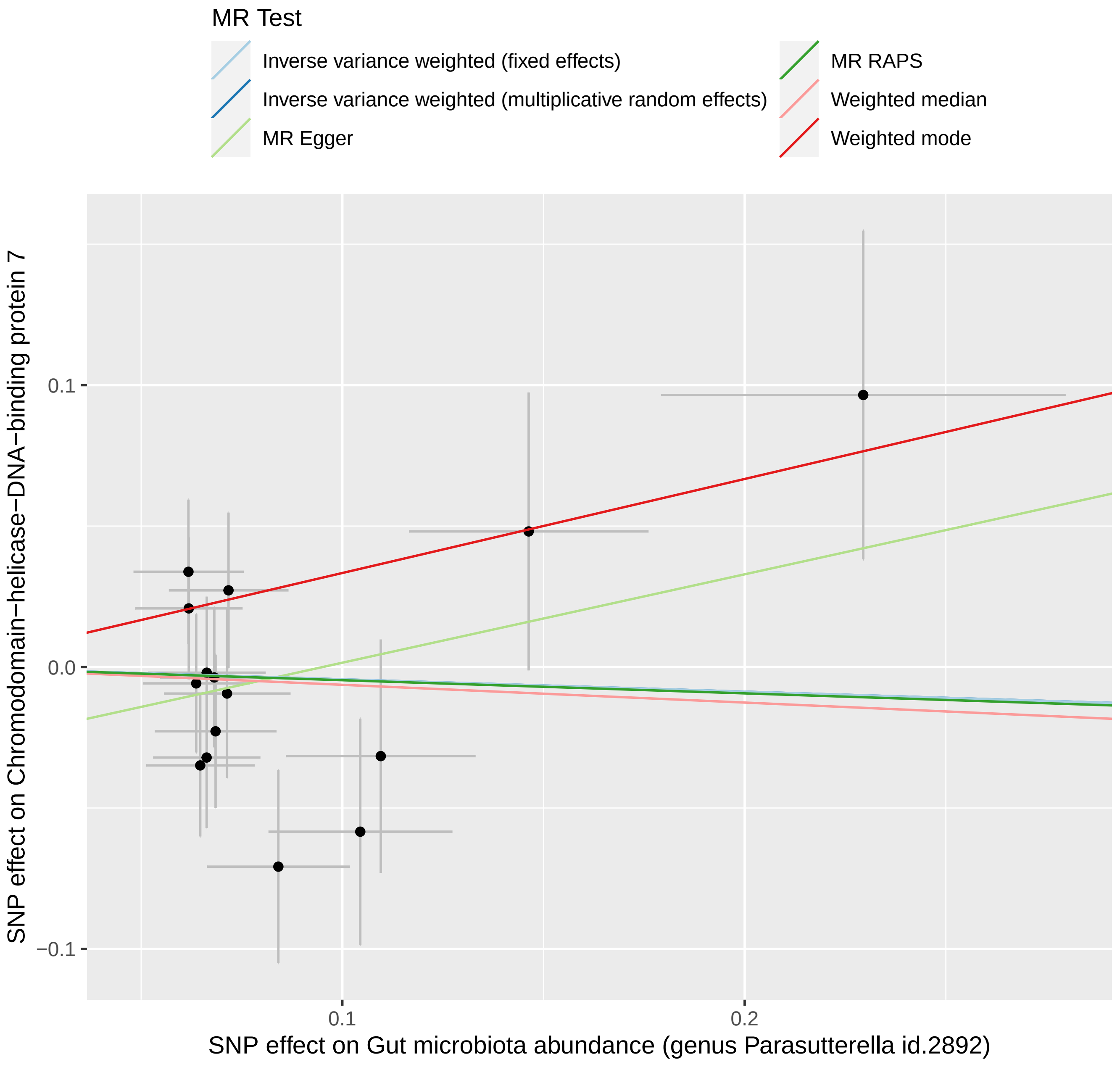

Batch 2−1073 : Gut microbiota abundance (genus Catenibacterium id.2153) on Serine/threonine−protein kinase Chk1
with a threshold of 1e−05

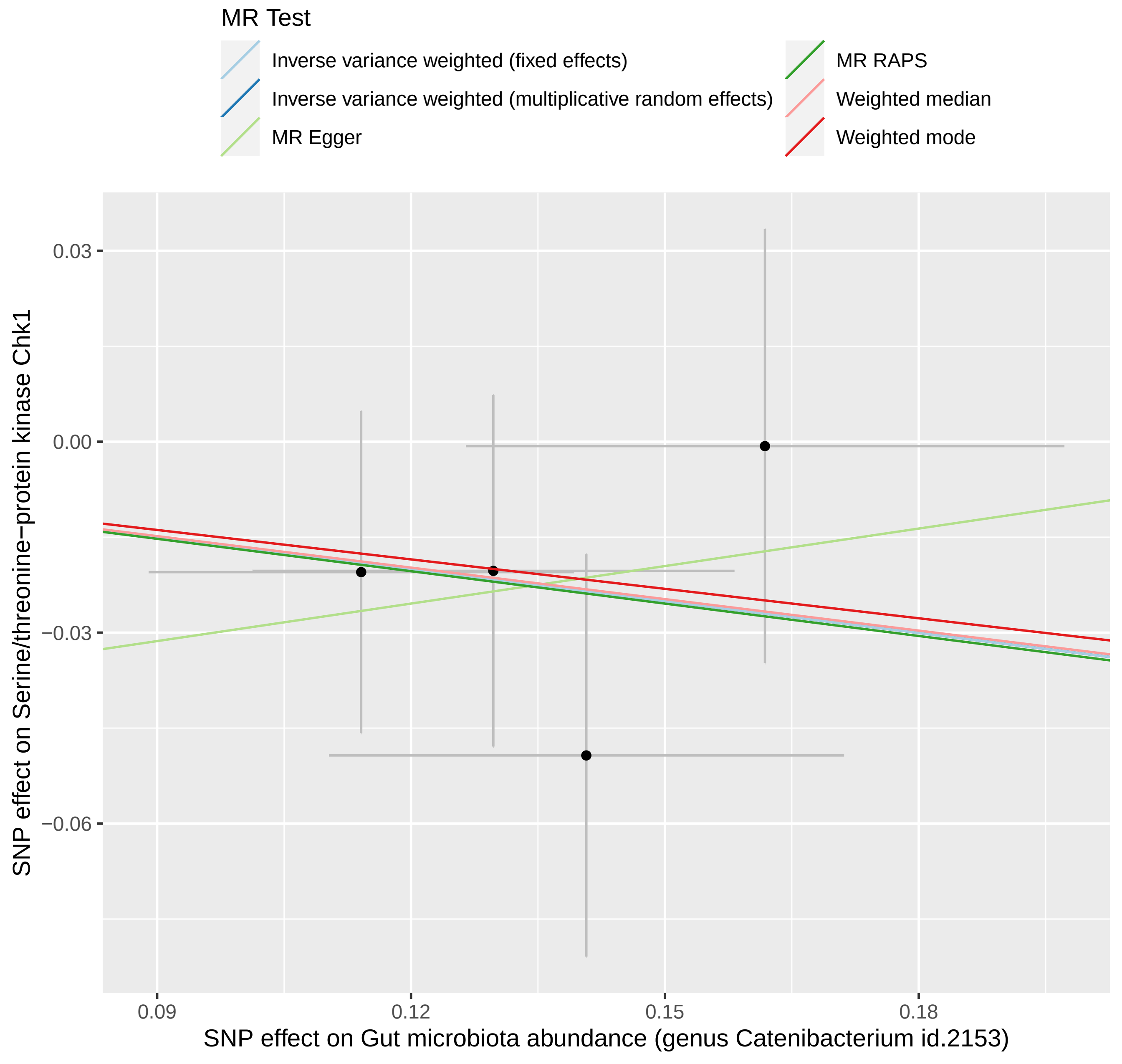

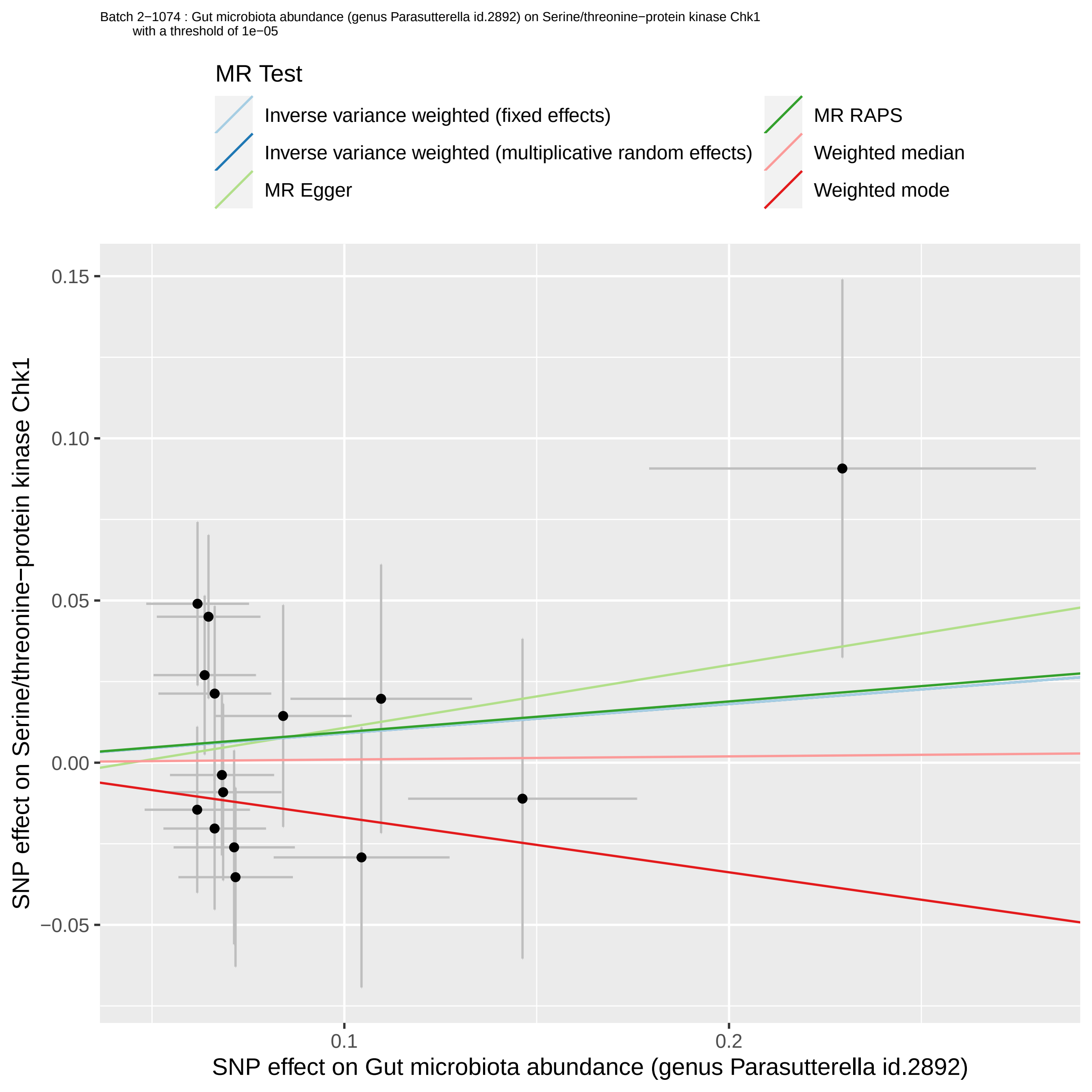
Batch 2−1074 : Gut microbiota abundance (genus Parasutterella id.2892) on Serine/threonine−protein kinase Chk1
with a threshold of 1e−05
MR Test
Inverse variance weighted (fixed effects)
Inverse variance weighted (multiplicative random effects)
MR Egger
MR RAPS
Weighted median
Weighted mode
0.15
0.10
0.05
0.00
−0.05
0.1
0.2
SNP effect on Serine/threonine−protein kinase Chk1
SNP effect on Gut microbiota abundance (genus Parasutterella id.2892)

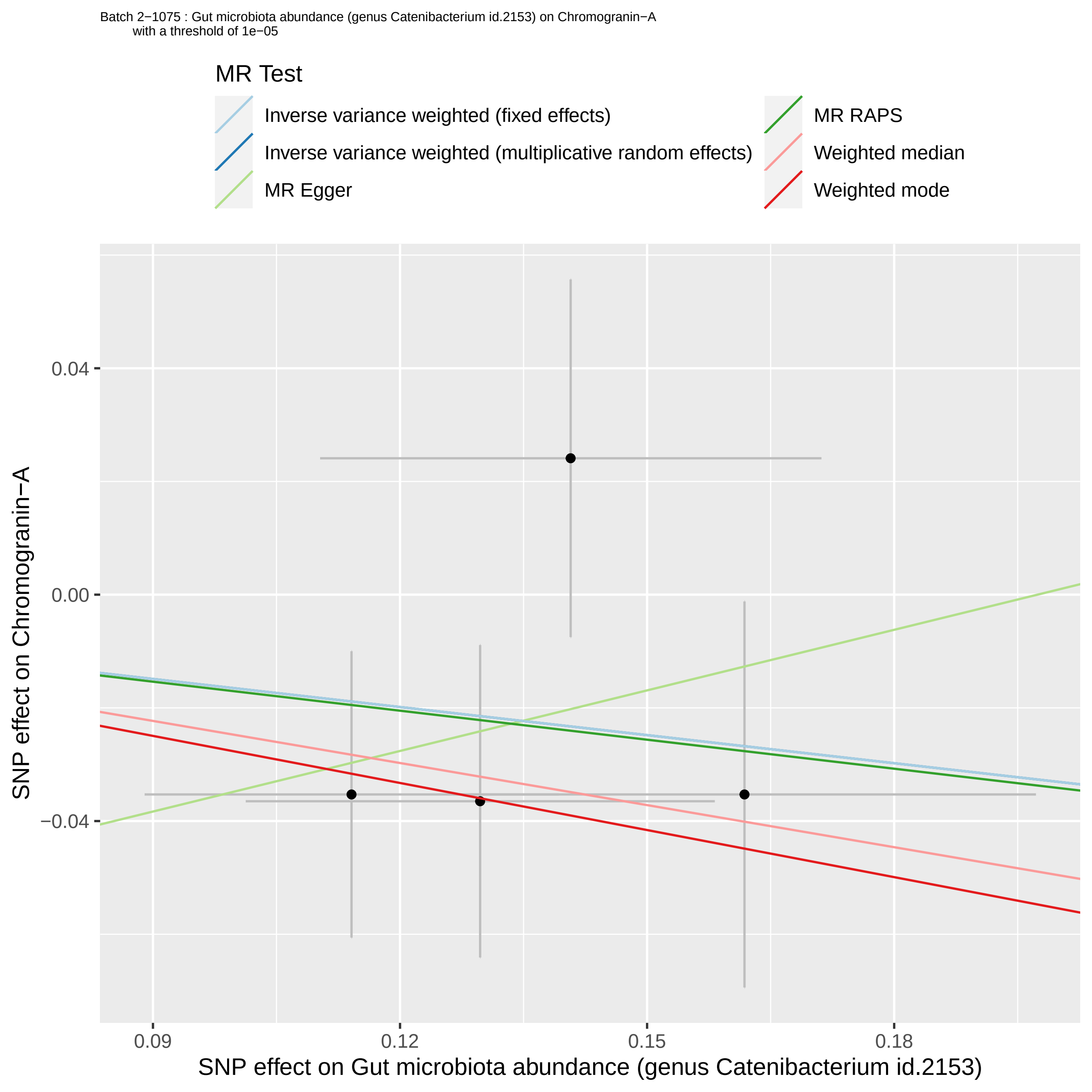

Batch 2−1075 : Gut microbiota abundance (genus Catenibacterium id.2153) on Chromogranin−A
with a threshold of 1e−05
MR Test
Inverse variance weighted (fixed effects)
Inverse variance weighted (multiplicative random effects)
MR Egger
MR RAPS
Weighted median
Weighted mode
0.04
0.00
−0.04
0.09
0.12
0.15
0.18
SNP effect on Chromogranin−A
SNP effect on Gut microbiota abundance (genus Catenibacterium id.2153)

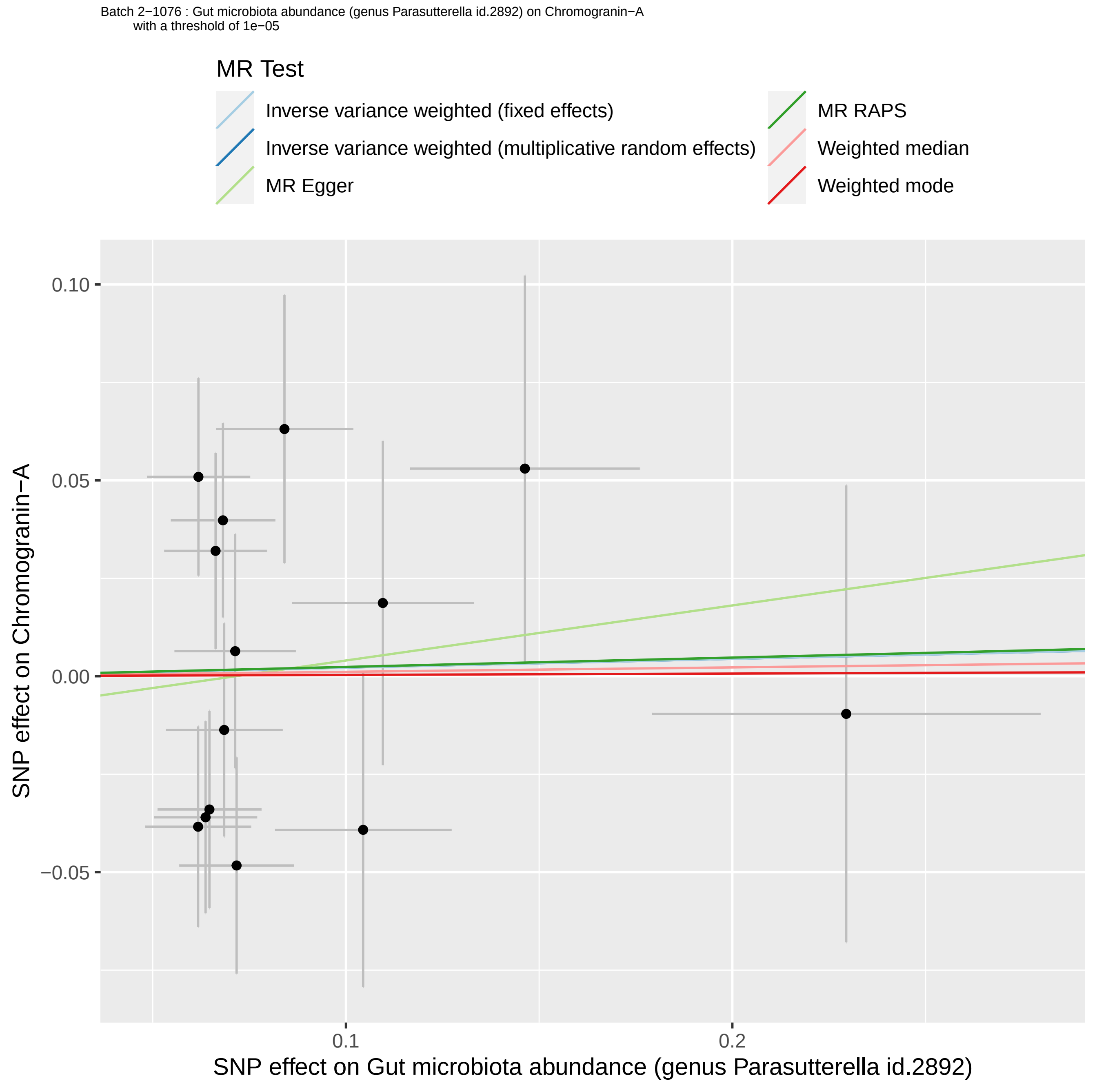

Batch 2−1076 : Gut microbiota abundance (genus Parasutterella id.2892) on Chromogranin−A
with a threshold of 1e−05
MR Test
Inverse variance weighted (fixed effects)
Inverse variance weighted (multiplicative random effects)
MR Egger
MR RAPS
Weighted median
Weighted mode
0.10
0.05
0.00
−0.05
0.1
0.2
SNP effect on Chromogranin−A
SNP effect on Gut microbiota abundance (genus Parasutterella id.2892)

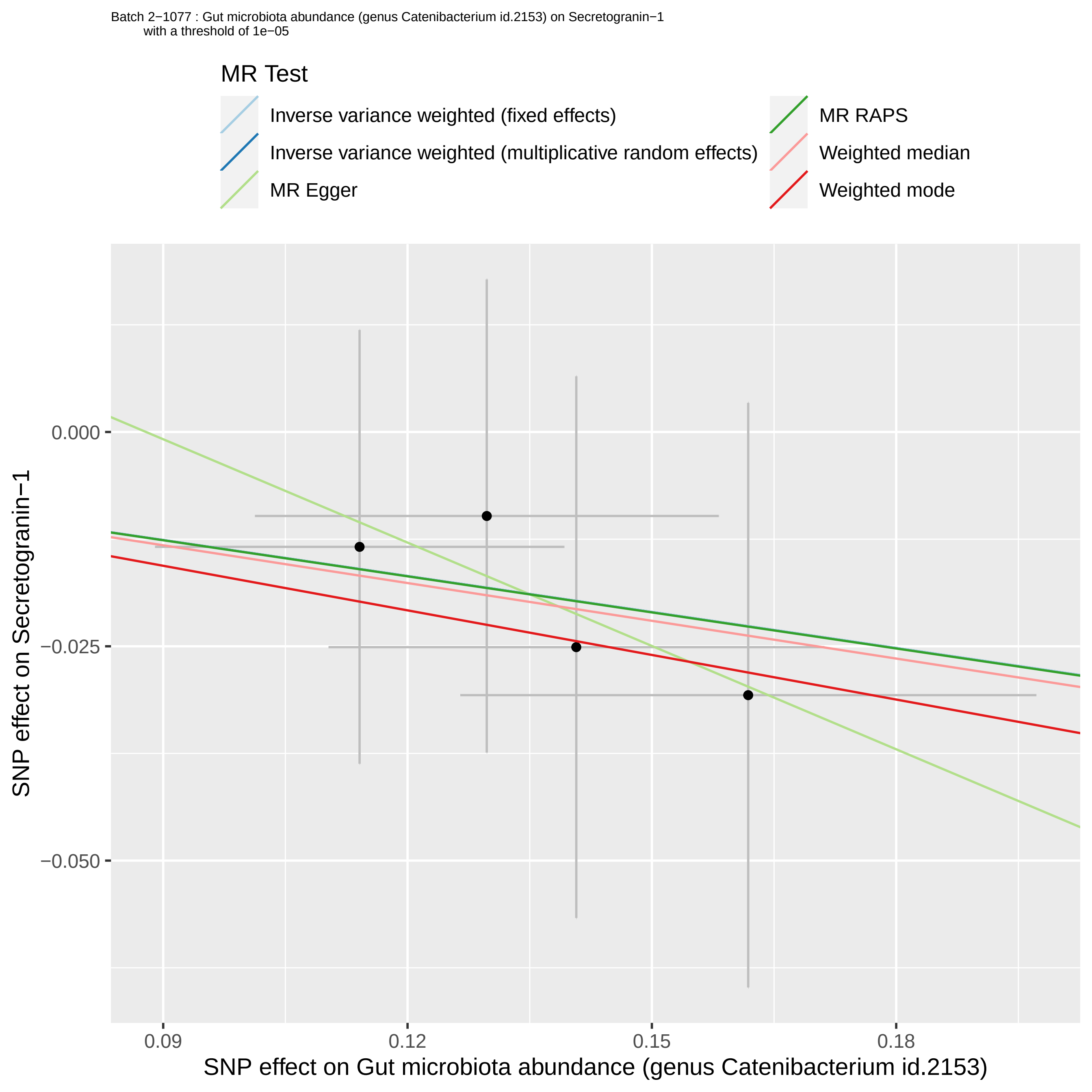

Batch 2−1077 : Gut microbiota abundance (genus Catenibacterium id.2153) on Secretogranin−1
with a threshold of 1e−05
MR Test
Inverse variance weighted (fixed effects)
Inverse variance weighted (multiplicative random effects)
MR Egger
MR RAPS
Weighted median
Weighted mode
0.000
−0.025
−0.050
0.09
0.12
0.15
0.18
SNP effect on Secretogranin−1
SNP effect on Gut microbiota abundance (genus Catenibacterium id.2153)

Batch 2−1078 : Gut microbiota abundance (genus Parasutterella id.2892) on Secretogranin−1
with a threshold of 1e−05

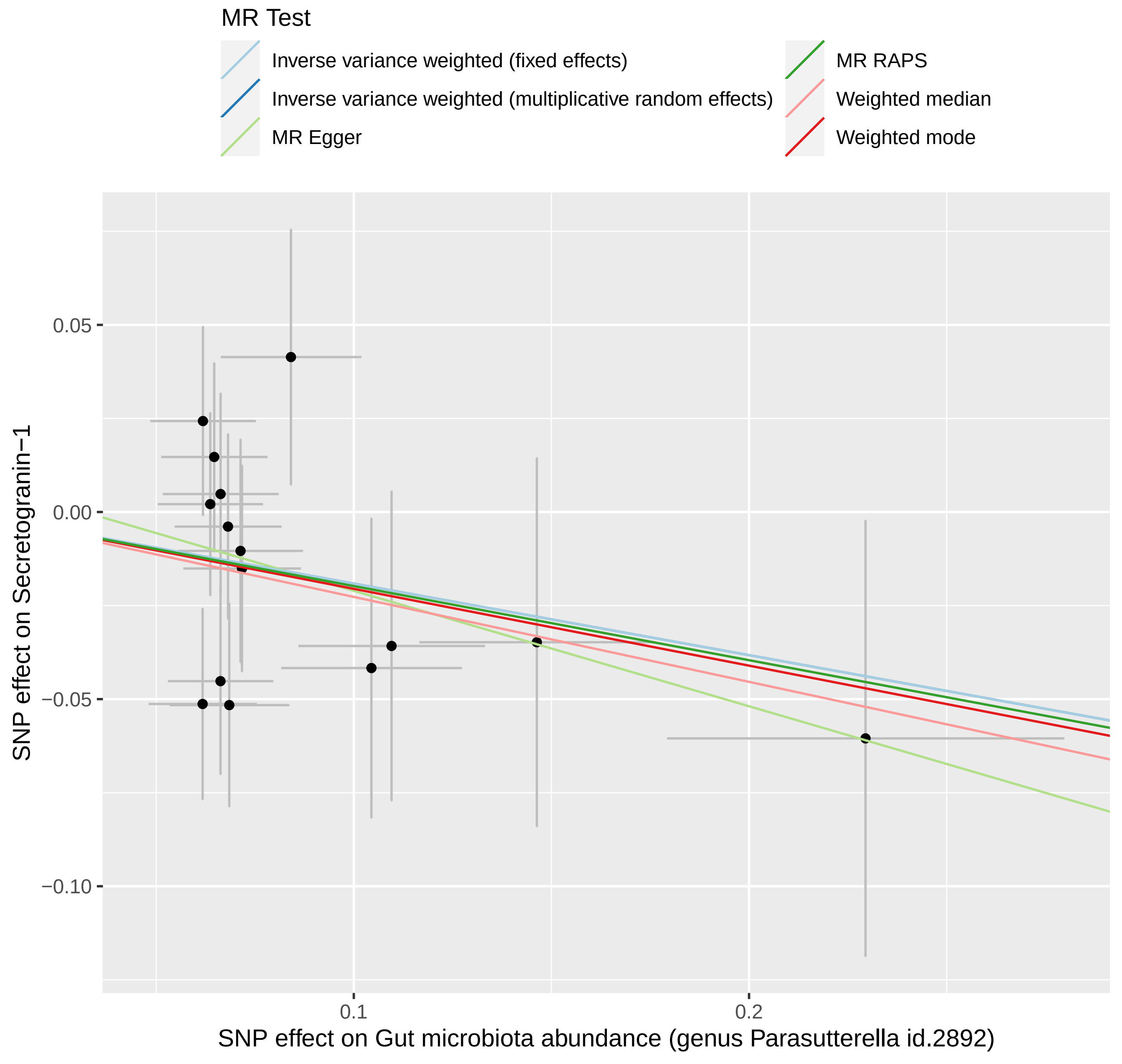

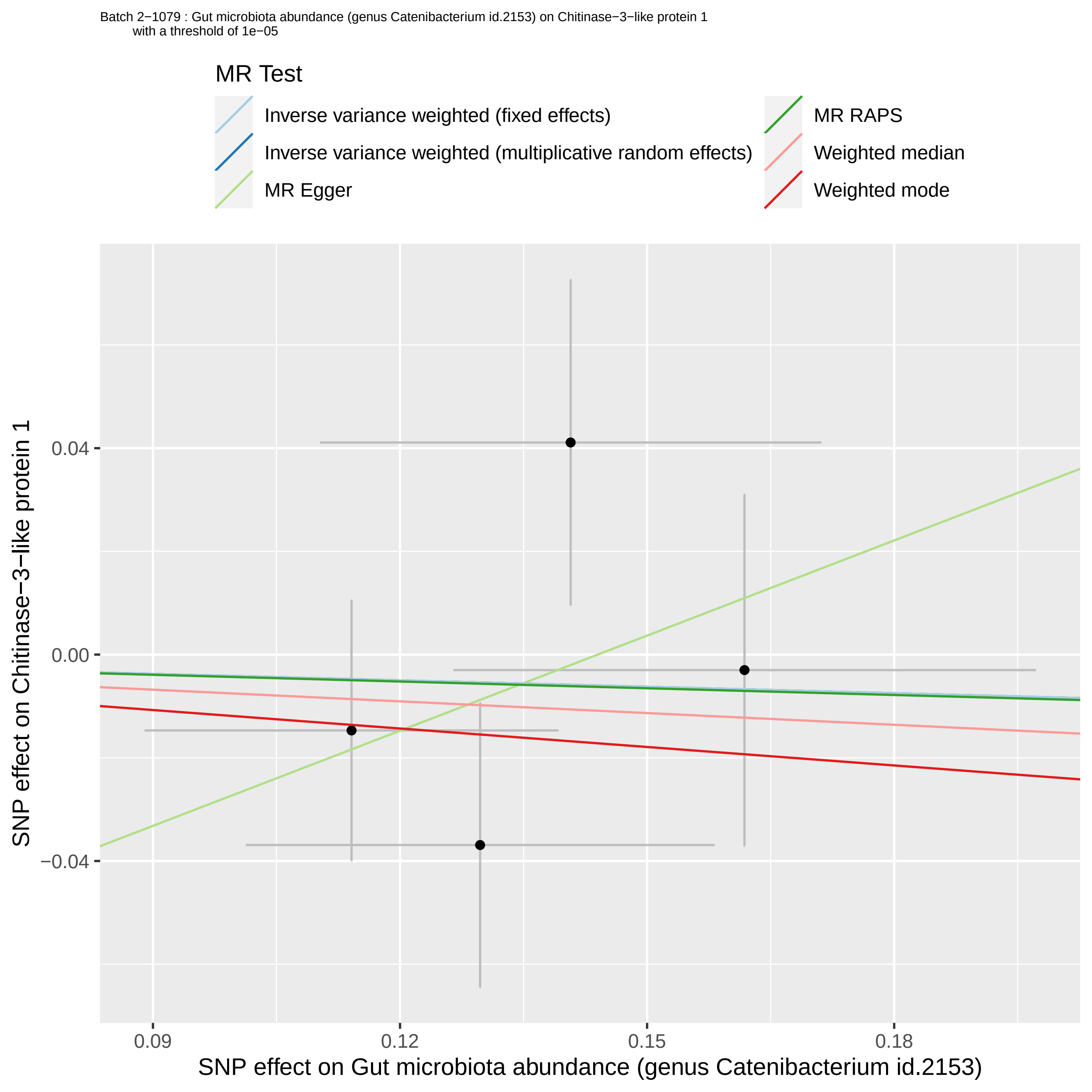

Batch 2−1079 : Gut microbiota abundance (genus Catenibacterium id.2153) on Chitinase−3−like protein 1
with a threshold of 1e−05
MR Test
Inverse variance weighted (fixed effects)
Inverse variance weighted (multiplicative random effects)
MR Egger
MR RAPS
Weighted median
Weighted mode
0.04
0.00
−0.04
0.09
0.12
0.15
0.18
SNP effect on Chitinase−3−like protein 1
SNP effect on Gut microbiota abundance (genus Catenibacterium id.2153)

Batch 2−1080 : Gut microbiota abundance (genus Parasutterella id.2892) on Chitinase−3−like protein 1
with a threshold of 1e−05

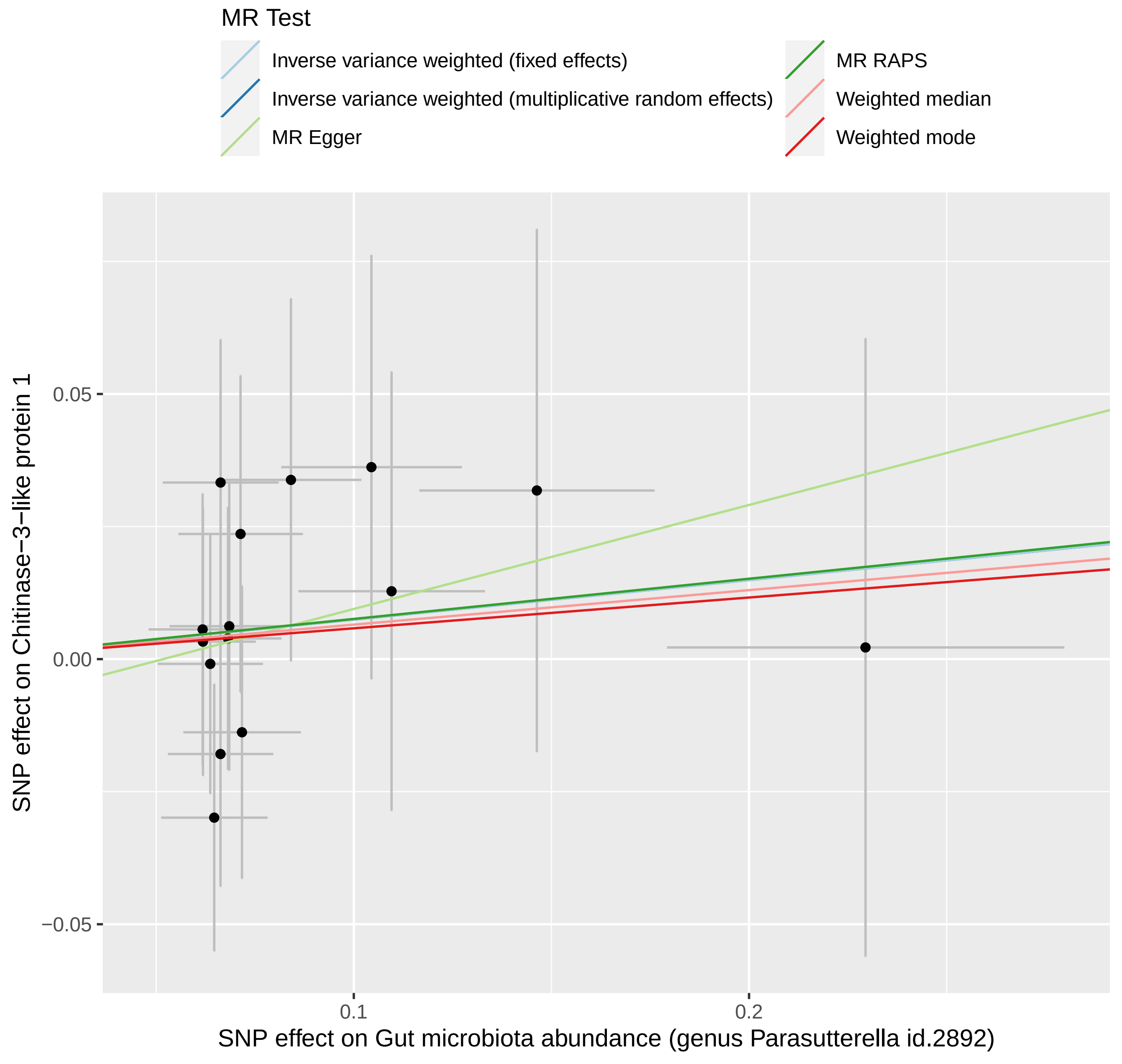

Batch 2−1081 : Gut microbiota abundance (genus Catenibacterium id.2153) on Chitinase−3−like protein 2
with a threshold of 1e−05

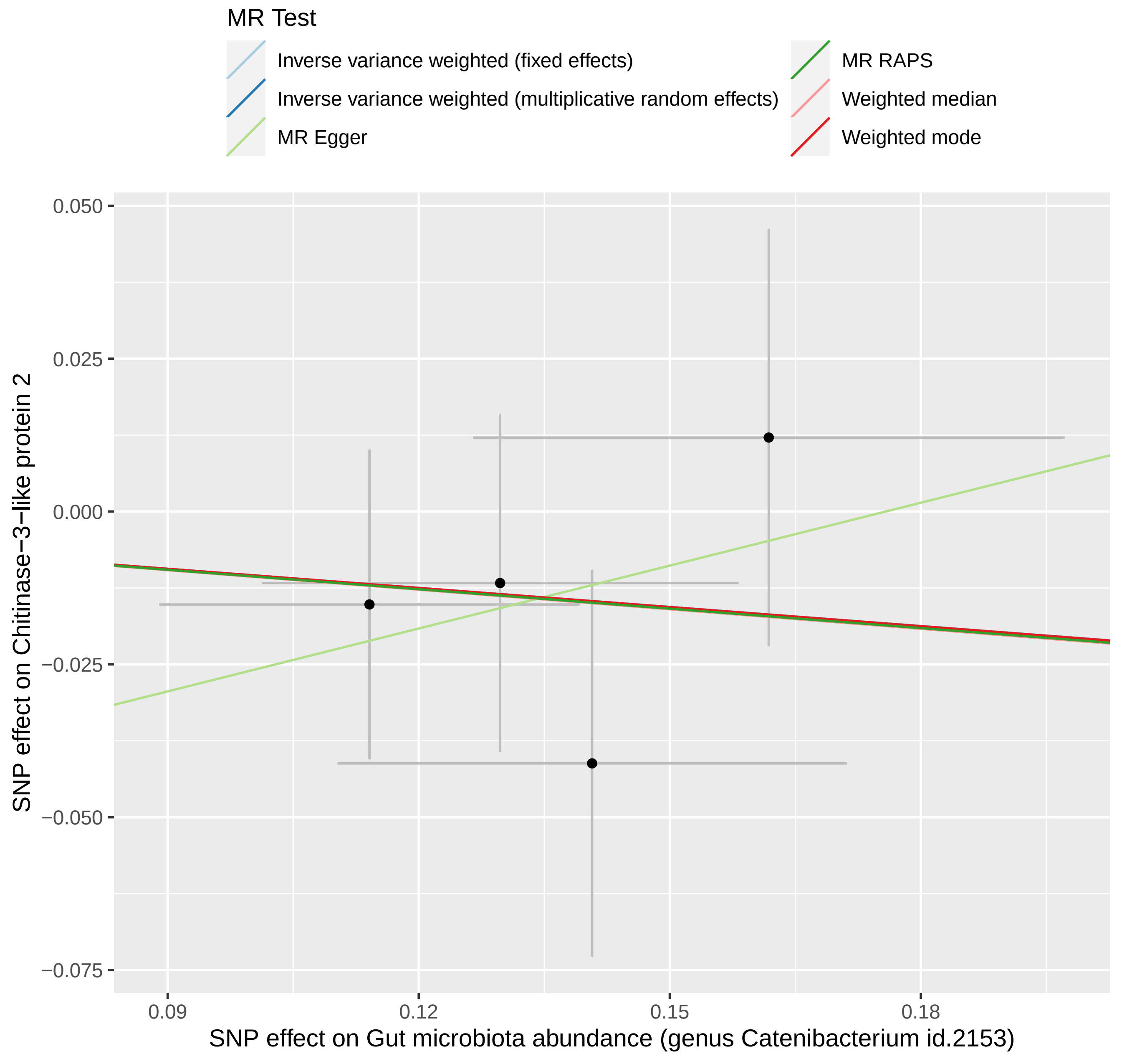

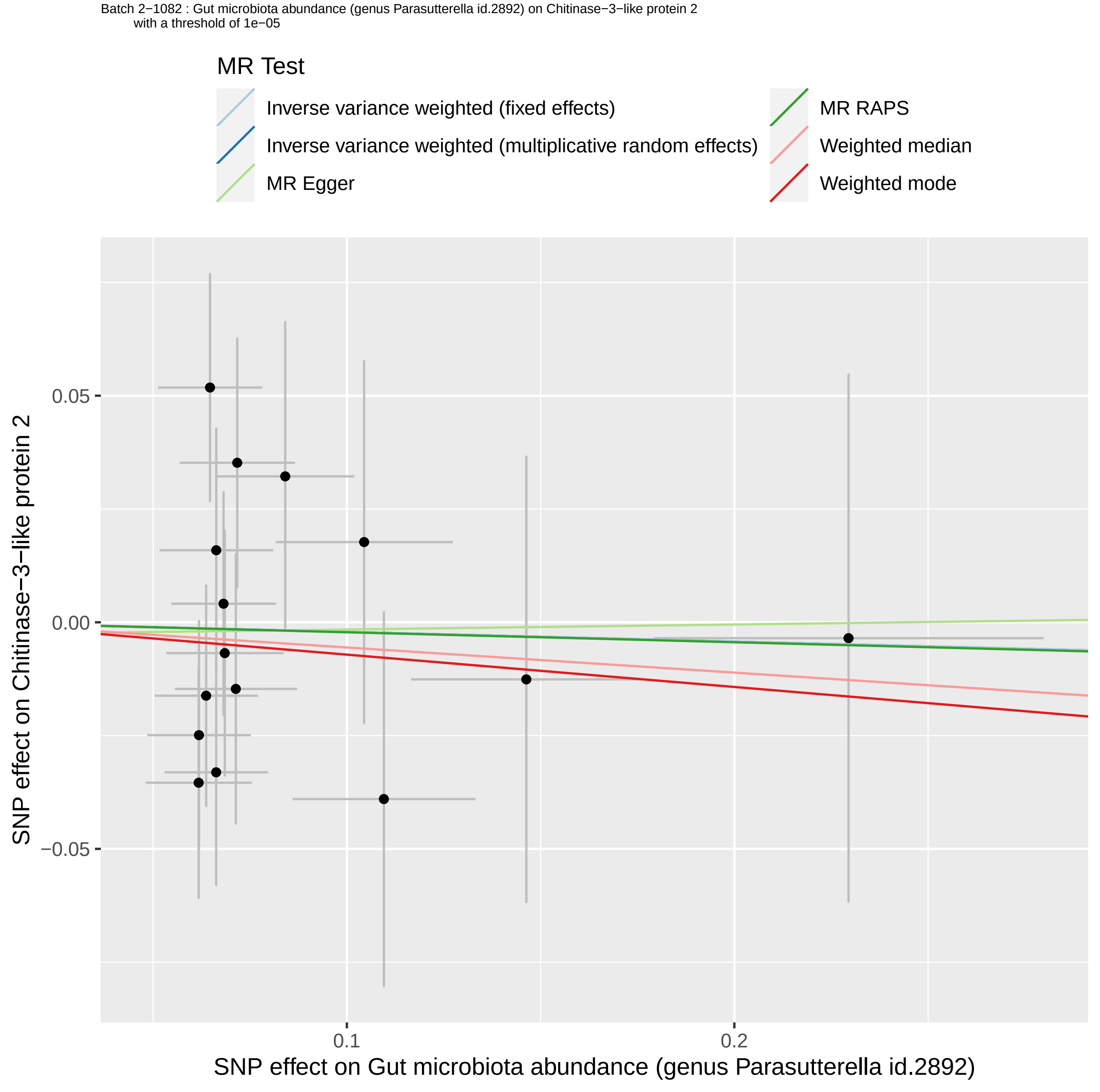
Batch 2−1082 : Gut microbiota abundance (genus Parasutterella id.2892) on Chitinase−3−like protein 2
with a threshold of 1e−05
MR Test
Inverse variance weighted (fixed effects)
Inverse variance weighted (multiplicative random effects)
MR Egger
MR RAPS
Weighted median
Weighted mode
0.05
0.00
−0.05
0.1
0.2
SNP effect on Chitinase−3−like protein 2
SNP effect on Gut microbiota abundance (genus Parasutterella id.2892)

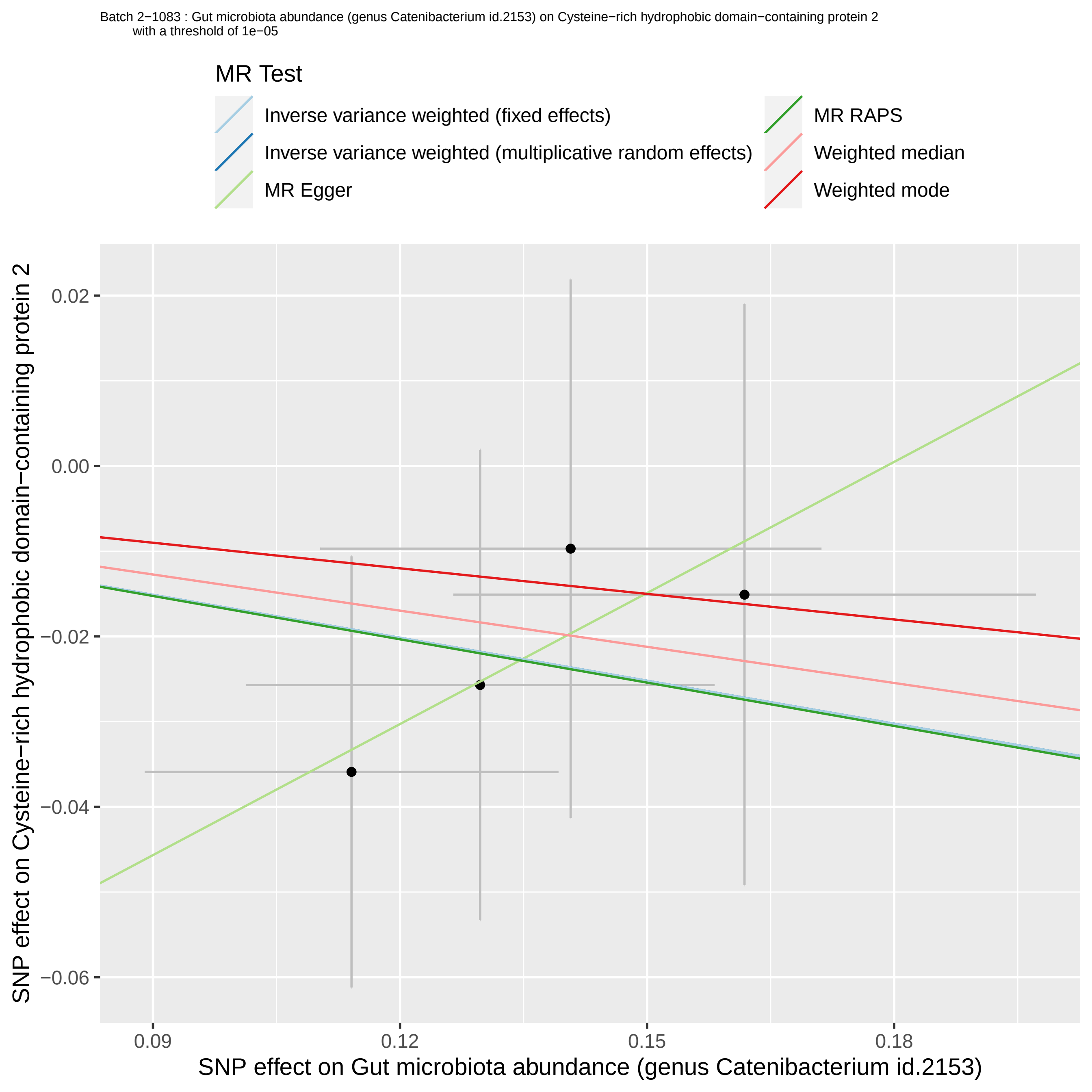
Batch 2−1083 : Gut microbiota abundance (genus Catenibacterium id.2153) on Cysteine−rich hydrophobic domain−containing protein 2
with a threshold of 1e−05
MR Test
Inverse variance weighted (fixed effects)
Inverse variance weighted (multiplicative random effects)
MR Egger
MR RAPS
Weighted median
Weighted mode
0.02
0.00
−0.02
−0.04
−0.06
0.09
0.12
0.15
0.18
SNP effect on Cysteine−rich hydrophobic domain−containing protein 2
SNP effect on Gut microbiota abundance (genus Catenibacterium id.2153)

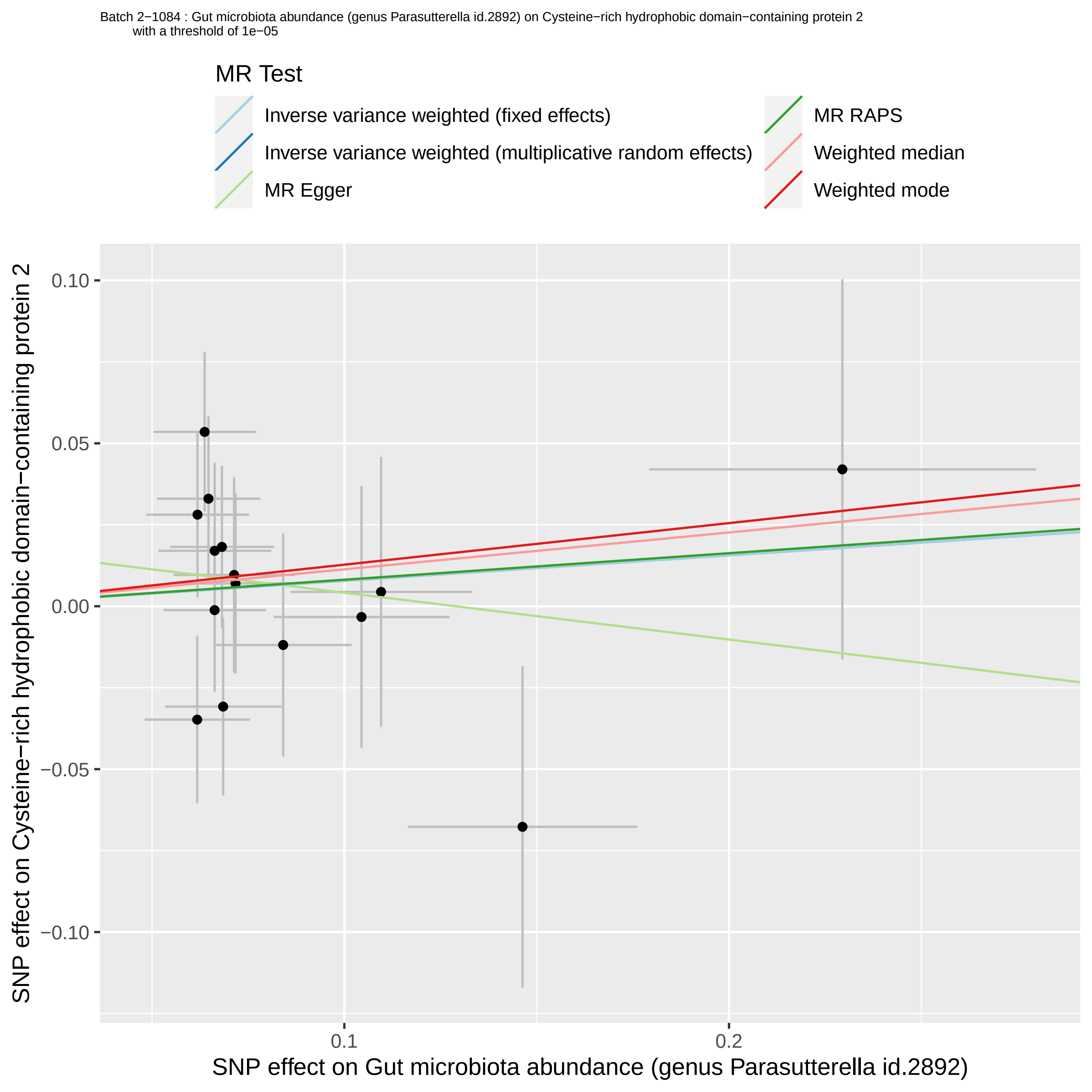
Batch 2−1084 : Gut microbiota abundance (genus Parasutterella id.2892) on Cysteine−rich hydrophobic domain−containing protein 2
with a threshold of 1e−05
MR Test
Inverse variance weighted (fixed effects)
Inverse variance weighted (multiplicative random effects)
MR Egger
MR RAPS
Weighted median
Weighted mode
0.10
0.05
0.00
−0.05
−0.10
0.1
0.2
SNP effect on Cysteine−rich hydrophobic domain−containing protein 2
SNP effect on Gut microbiota abundance (genus Parasutterella id.2892)

Batch 2−1085 : Gut microbiota abundance (genus Catenibacterium id.2153) on Chitotriosidase−1
with a threshold of 1e−05

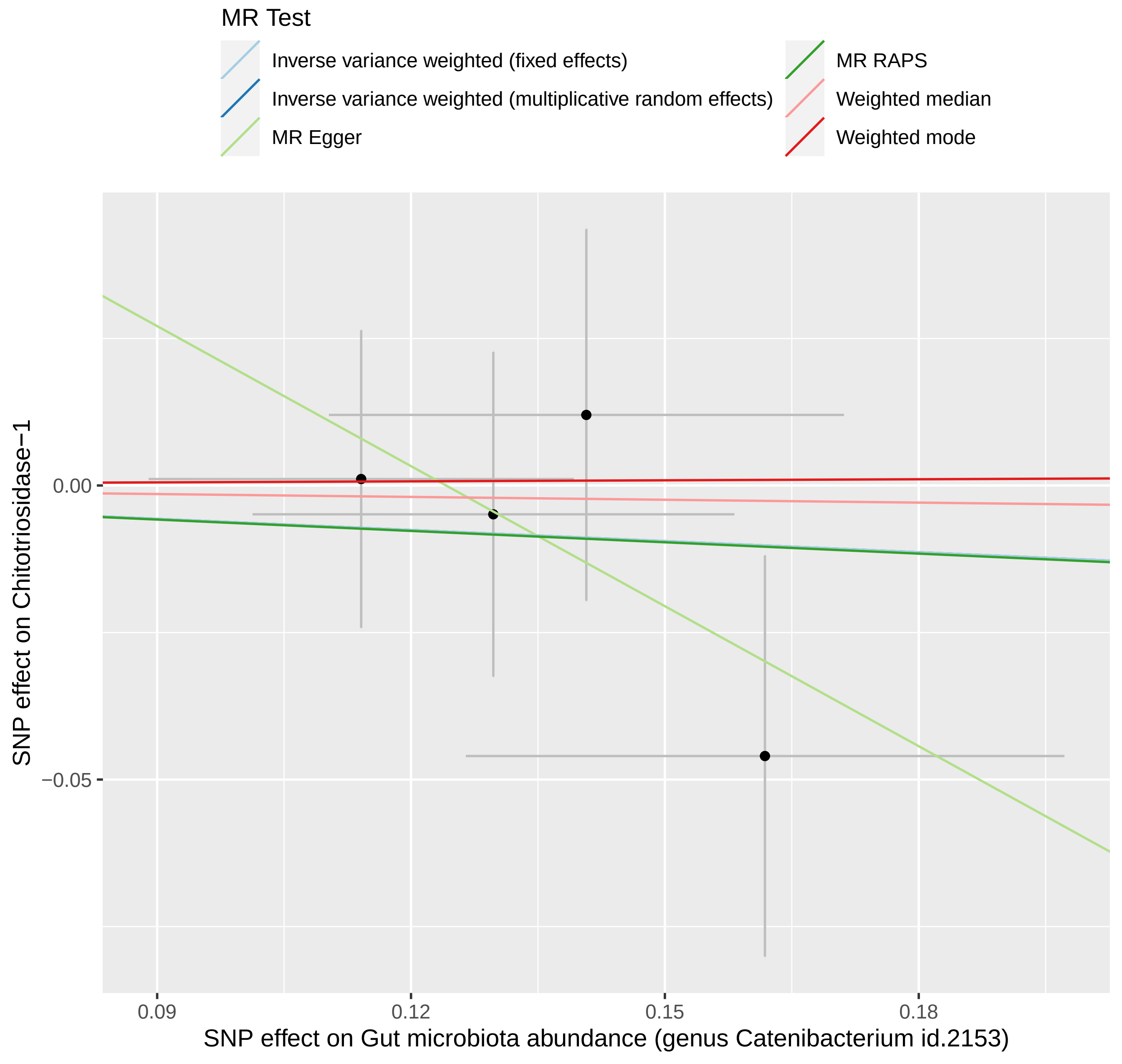

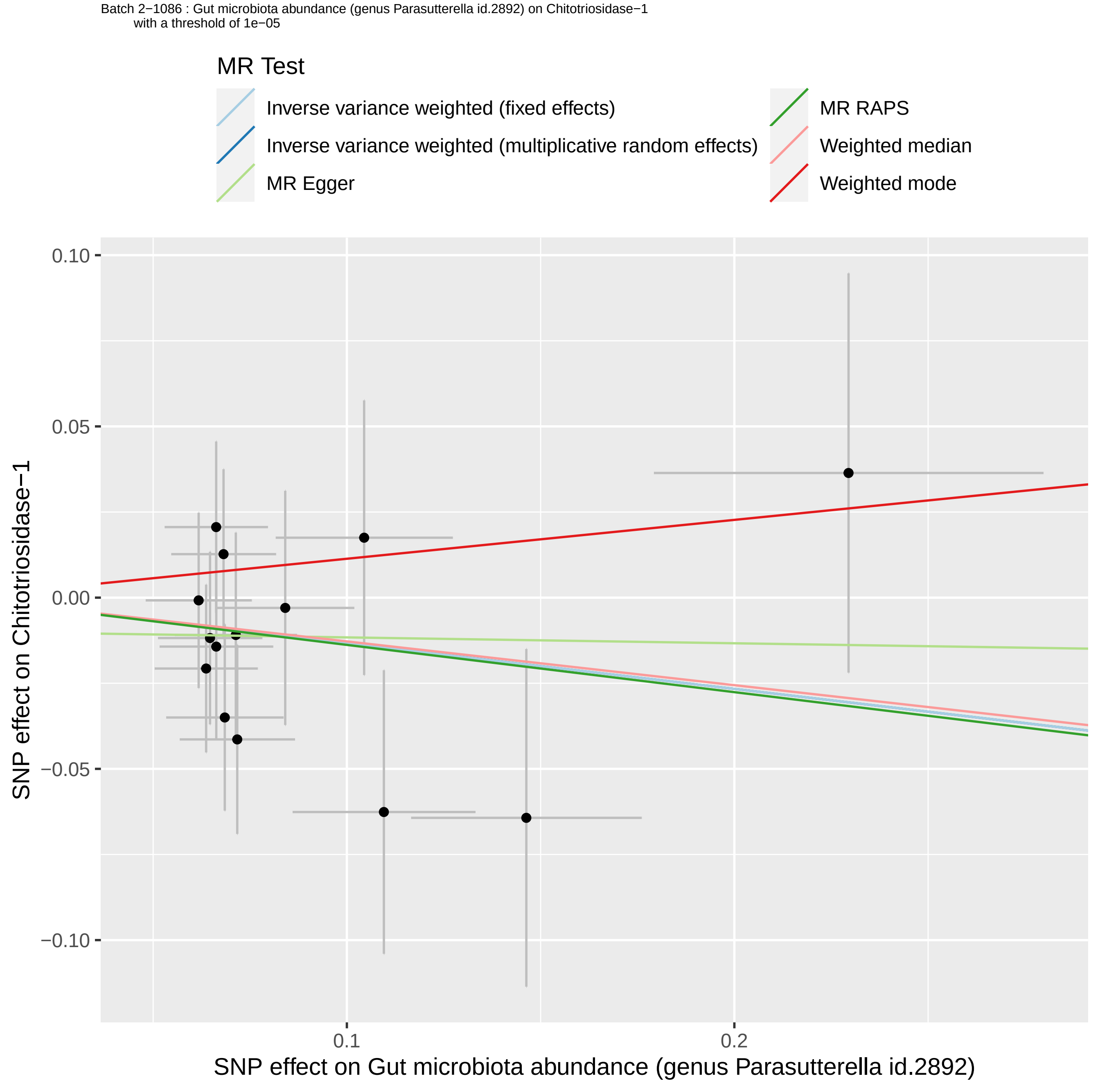

Batch 2−1086 : Gut microbiota abundance (genus Parasutterella id.2892) on Chitotriosidase−1
with a threshold of 1e−05
MR Test
Inverse variance weighted (fixed effects)
Inverse variance weighted (multiplicative random effects)
MR Egger
MR RAPS
Weighted median
Weighted mode
0.10
0.05
0.00
−0.05
−0.10
0.1
0.2
SNP effect on Chitotriosidase−1
SNP effect on Gut microbiota abundance (genus Parasutterella id.2892)

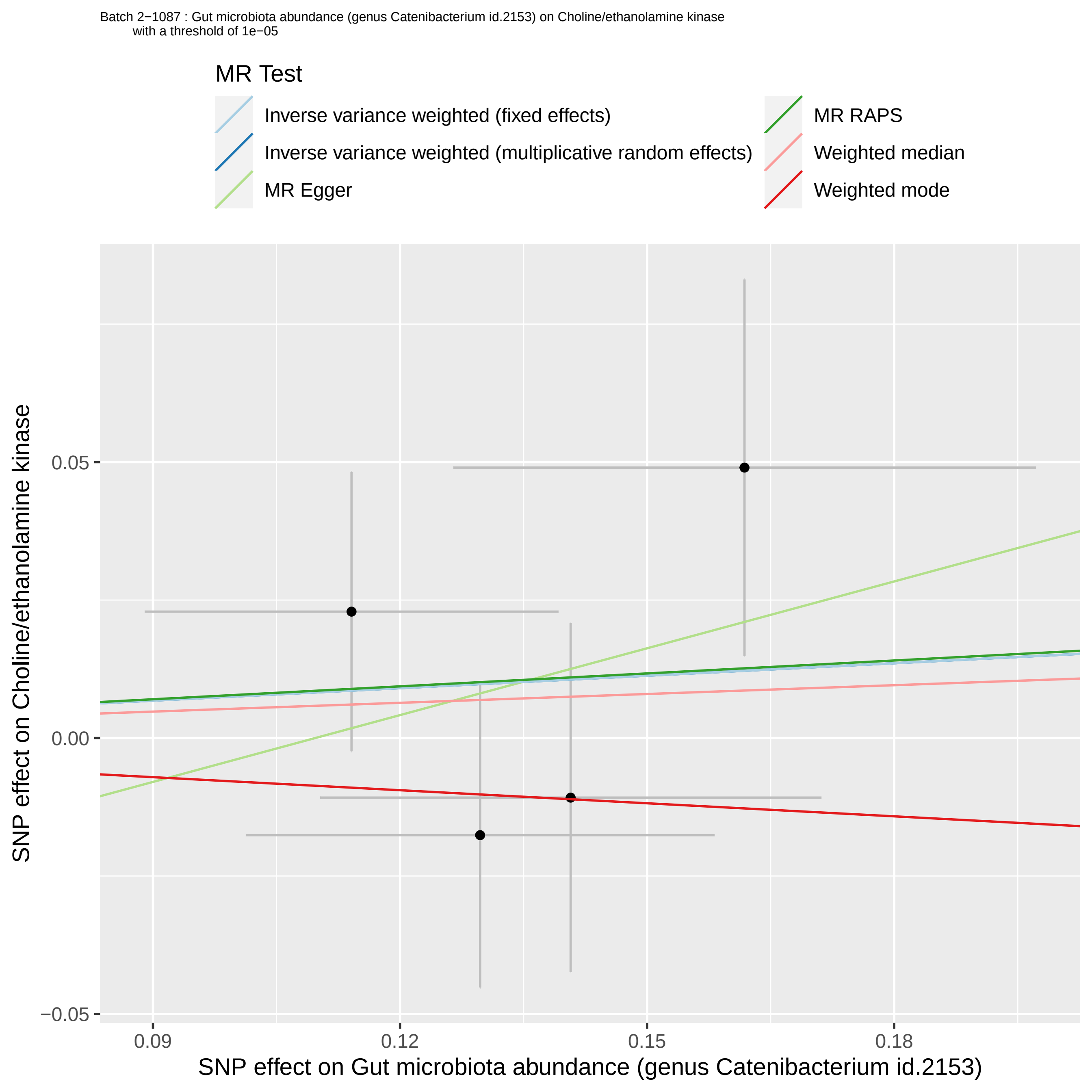
Batch 2−1087 : Gut microbiota abundance (genus Catenibacterium id.2153) on Choline/ethanolamine kinase
with a threshold of 1e−05
MR Test
Inverse variance weighted (fixed effects)
Inverse variance weighted (multiplicative random effects)
MR Egger
MR RAPS
Weighted median
Weighted mode
0.05
0.00
−0.05
0.09
0.12
0.15
0.18
SNP effect on Choline/ethanolamine kinase
SNP effect on Gut microbiota abundance (genus Catenibacterium id.2153)

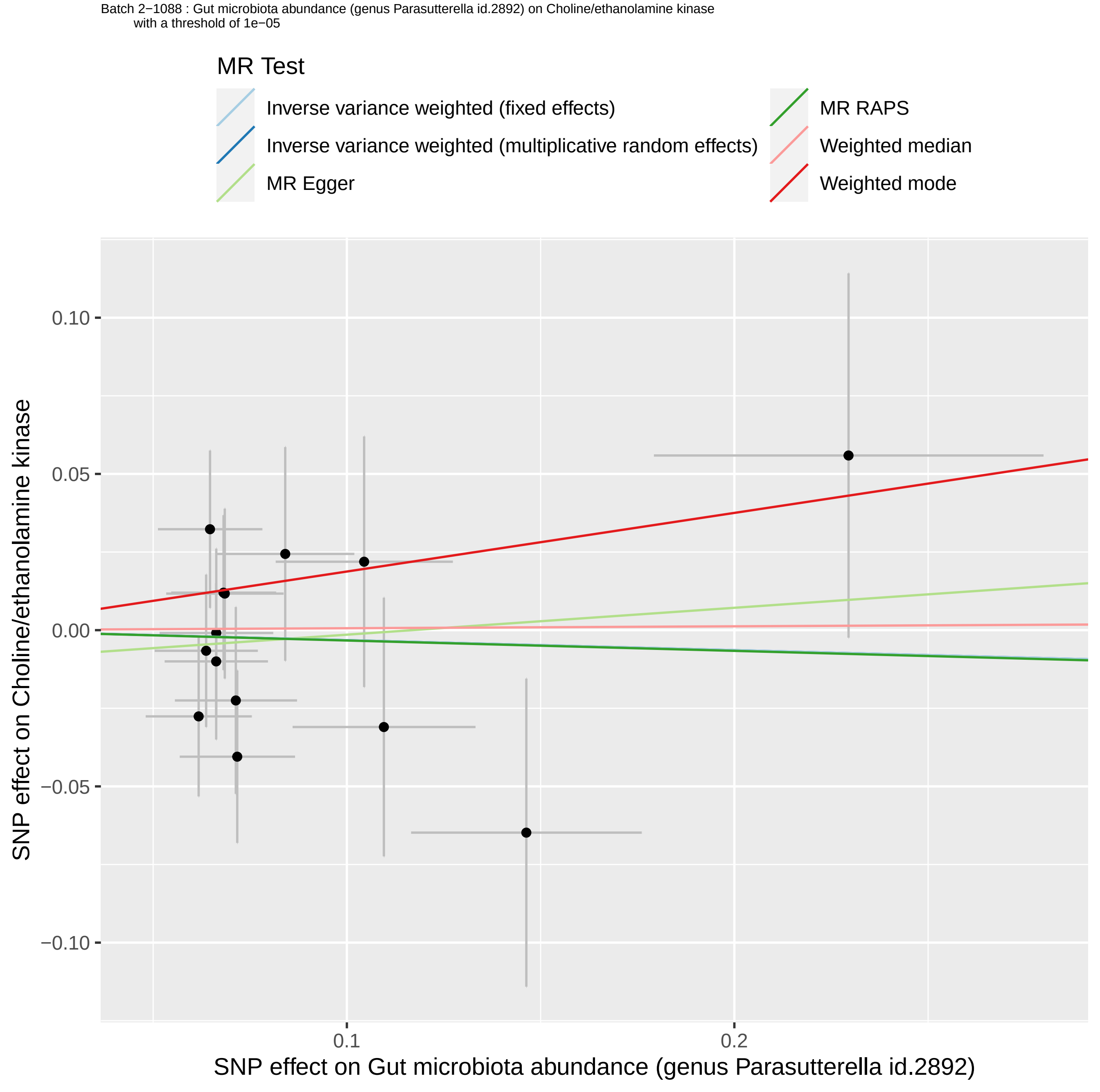

Batch 2−1088 : Gut microbiota abundance (genus Parasutterella id.2892) on Choline/ethanolamine kinase
with a threshold of 1e−05
MR Test
Inverse variance weighted (fixed effects)
Inverse variance weighted (multiplicative random effects)
MR Egger
MR RAPS
Weighted median
Weighted mode
SNP effect on Choline/ethanolamine kinase
0.10
0.05
0.00
−0.05
−0.10
0.1
0.2
SNP effect on Gut microbiota abundance (genus Parasutterella id.2892)

Batch 2−1089 : Gut microbiota abundance (genus Catenibacterium id.2153) on Neural cell adhesion molecule L1−like protein
with a threshold of 1e−05

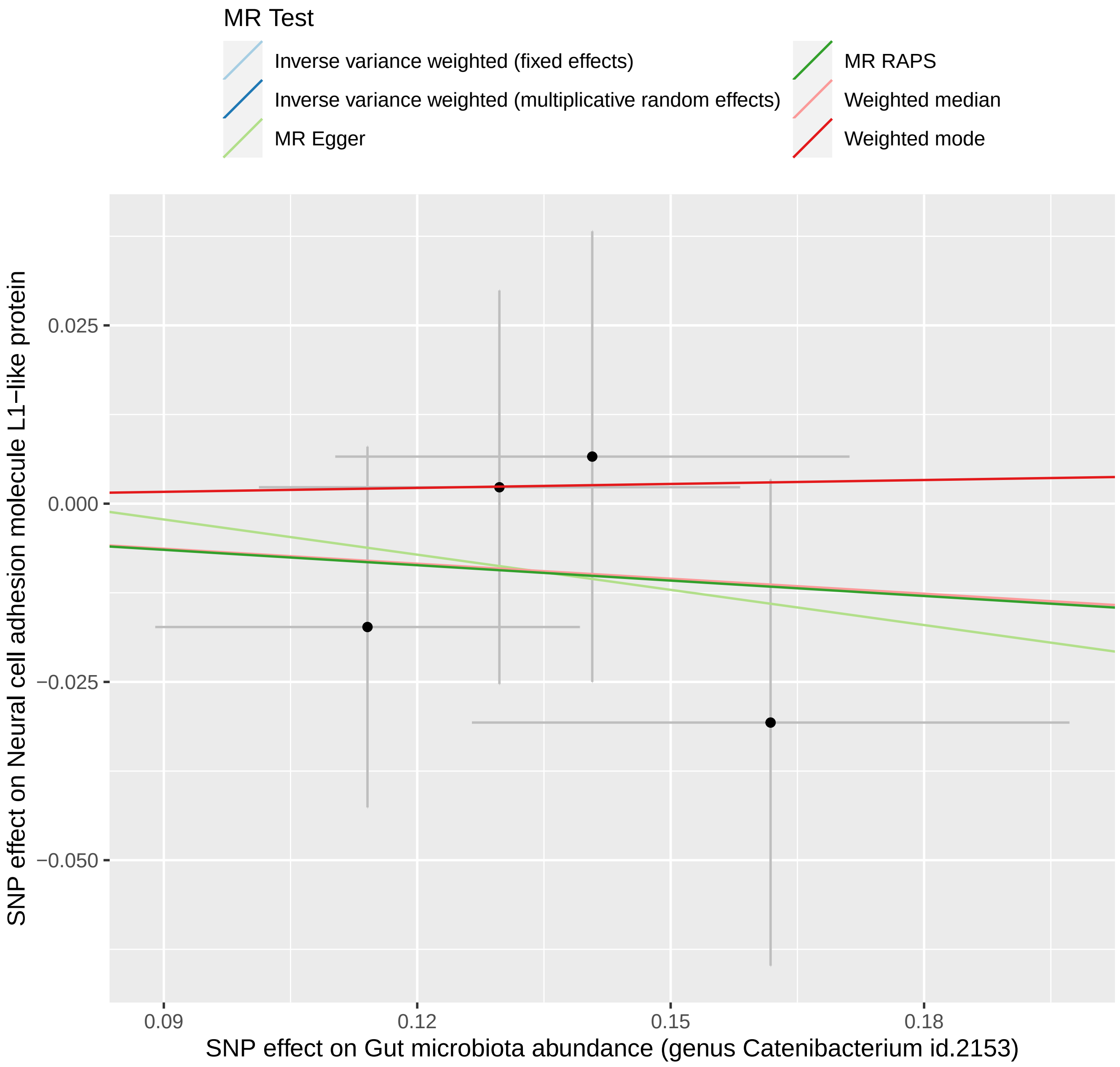

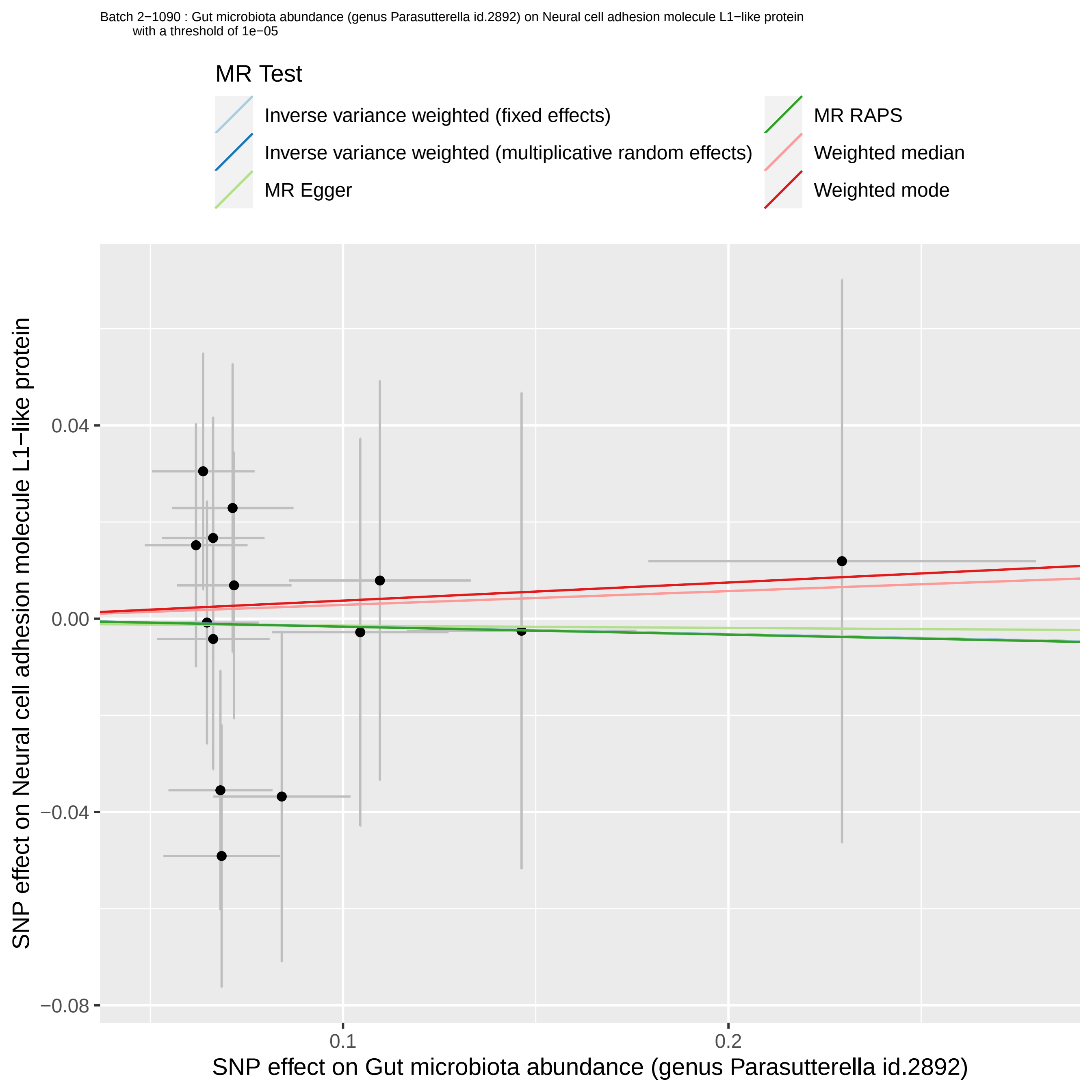
Batch 2−1090 : Gut microbiota abundance (genus Parasutterella id.2892) on Neural cell adhesion molecule L1−like protein
with a threshold of 1e−05
MR Test
Inverse variance weighted (fixed effects)
Inverse variance weighted (multiplicative random effects)
MR Egger
MR RAPS
Weighted median
Weighted mode
0.04
0.00
−0.04
−0.08
0.1
0.2
SNP effect on Neural cell adhesion molecule L1−like protein
SNP effect on Gut microbiota abundance (genus Parasutterella id.2892)

Batch 2−1091 : Gut microbiota abundance (genus Catenibacterium id.2153) on Calcineurin B homologous protein 1
with a threshold of 1e−05

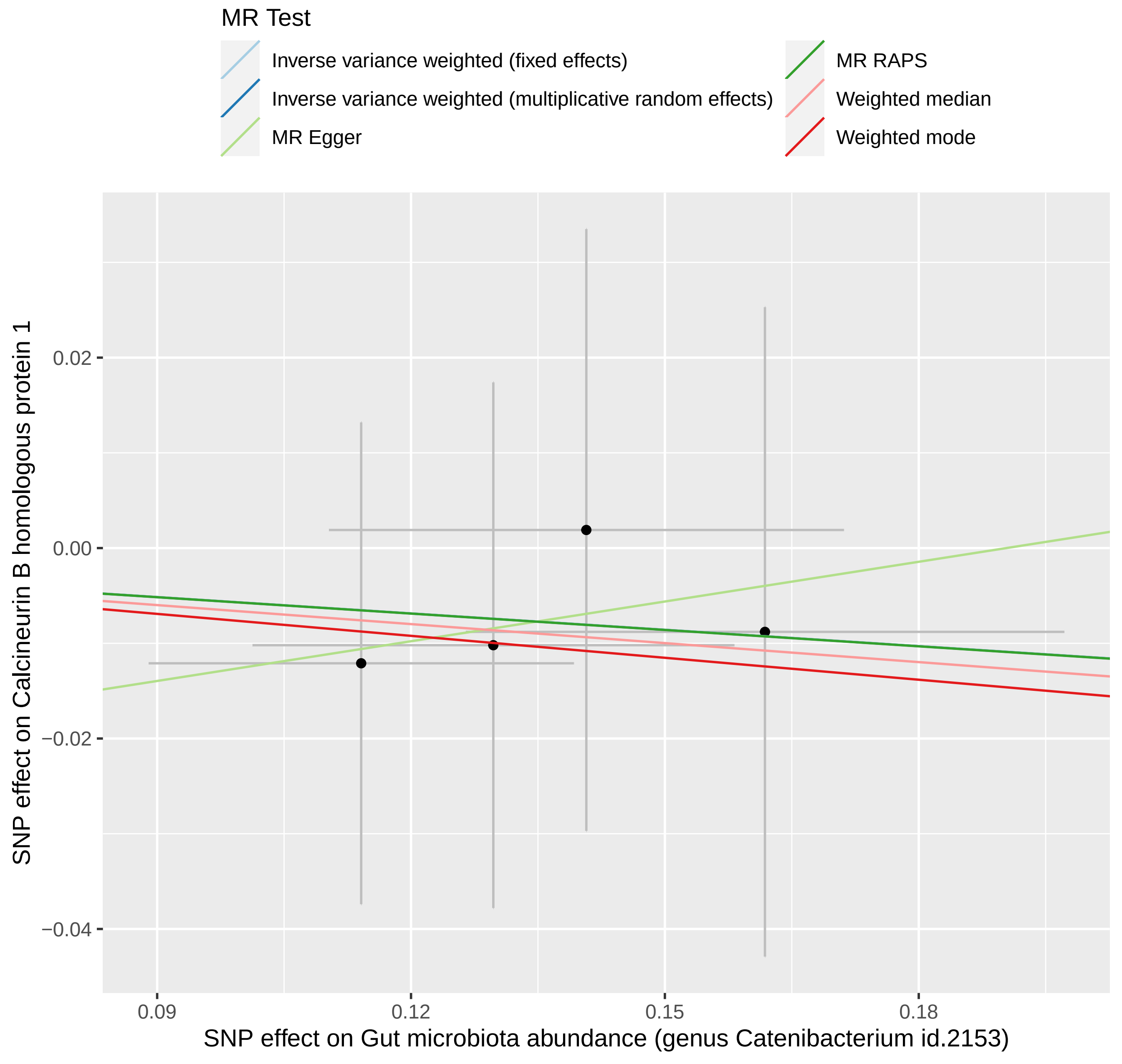

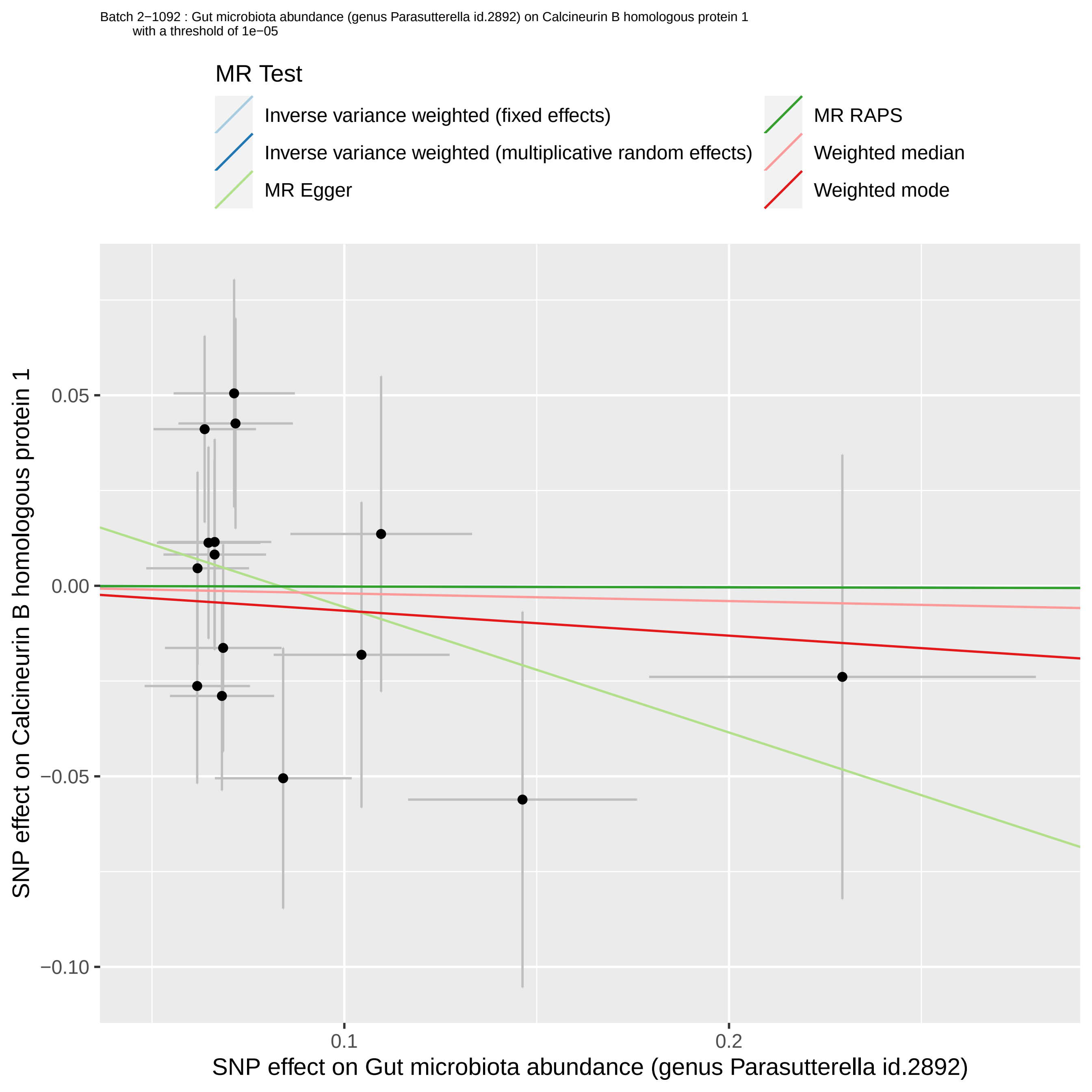
Batch 2−1092 : Gut microbiota abundance (genus Parasutterella id.2892) on Calcineurin B homologous protein 1
with a threshold of 1e−05
MR Test
Inverse variance weighted (fixed effects)
Inverse variance weighted (multiplicative random effects)
MR Egger
MR RAPS
Weighted median
Weighted mode
SNP effect on Calcineurin B homologous protein 1
0.05
0.00
−0.05
−0.10
0.1
0.2
SNP effect on Gut microbiota abundance (genus Parasutterella id.2892)

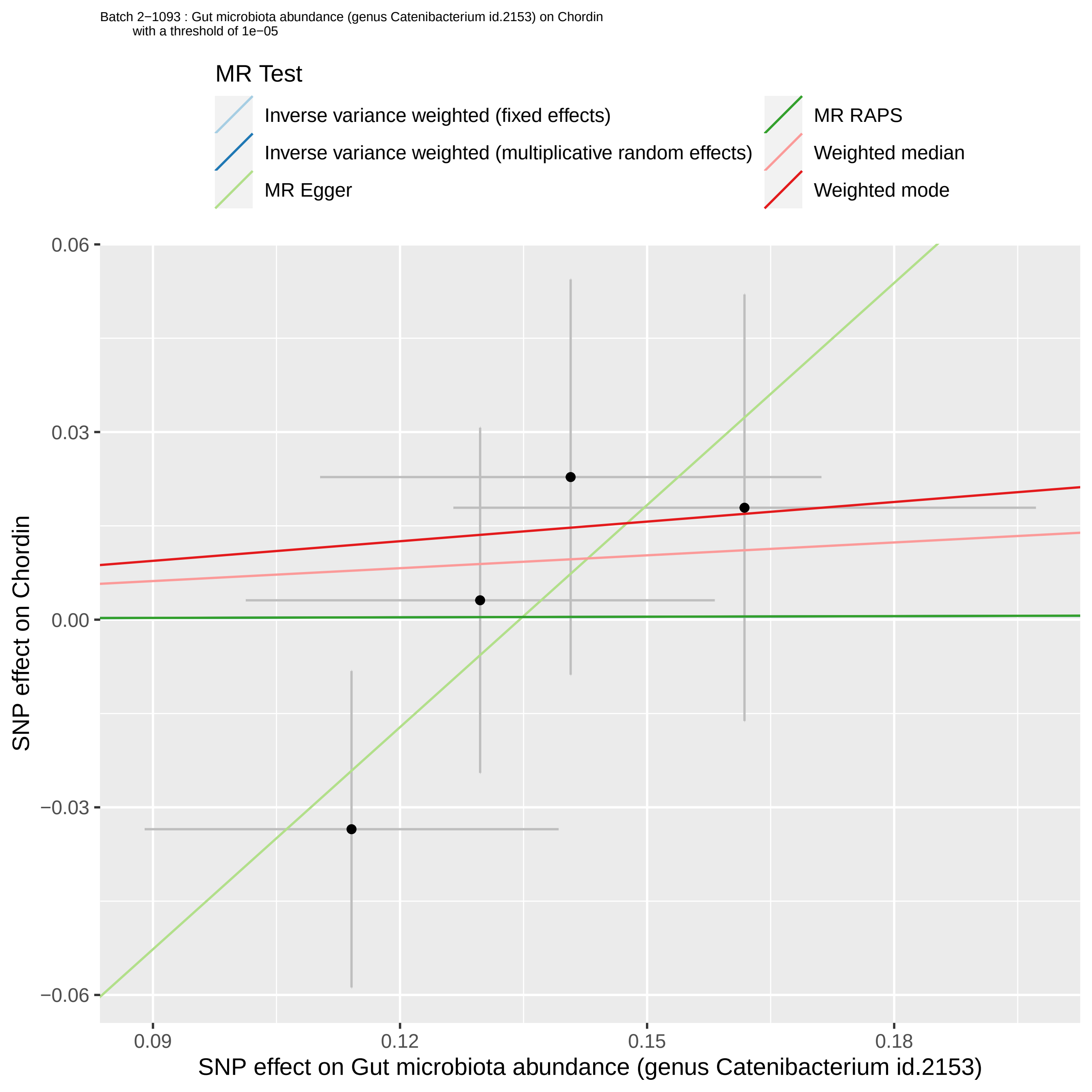

Batch 2−1093 : Gut microbiota abundance (genus Catenibacterium id.2153) on Chordin
with a threshold of 1e−05
MR Test
Inverse variance weighted (fixed effects)
Inverse variance weighted (multiplicative random effects)
MR Egger
MR RAPS
Weighted median
Weighted mode
0.06
0.03
0.00
−0.03
−0.06
0.09
0.12
0.15
0.18
SNP effect on Chordin
SNP effect on Gut microbiota abundance (genus Catenibacterium id.2153)

Batch 2−1094 : Gut microbiota abundance (genus Parasutterella id.2892) on Chordin
with a threshold of 1e−05

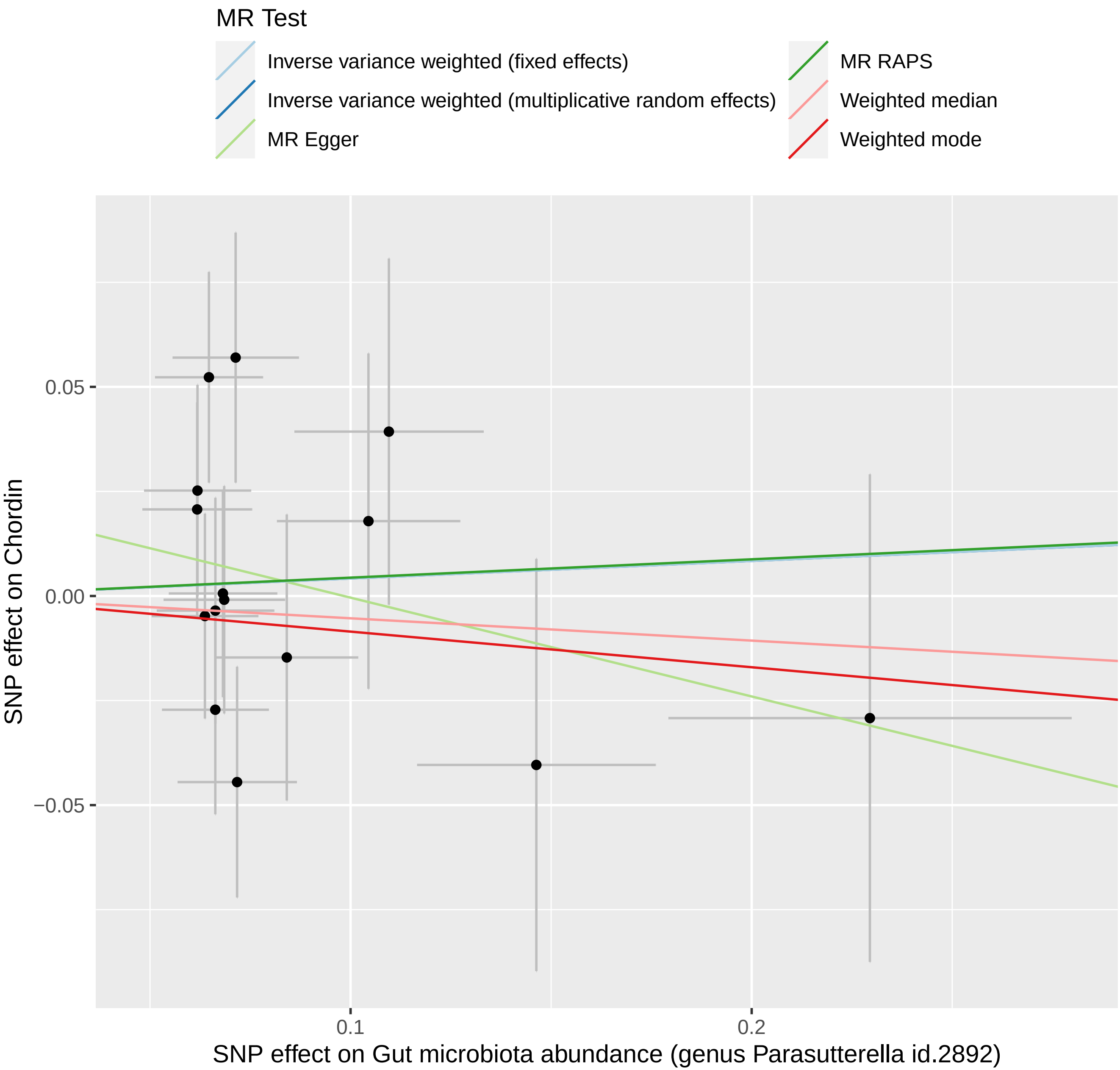

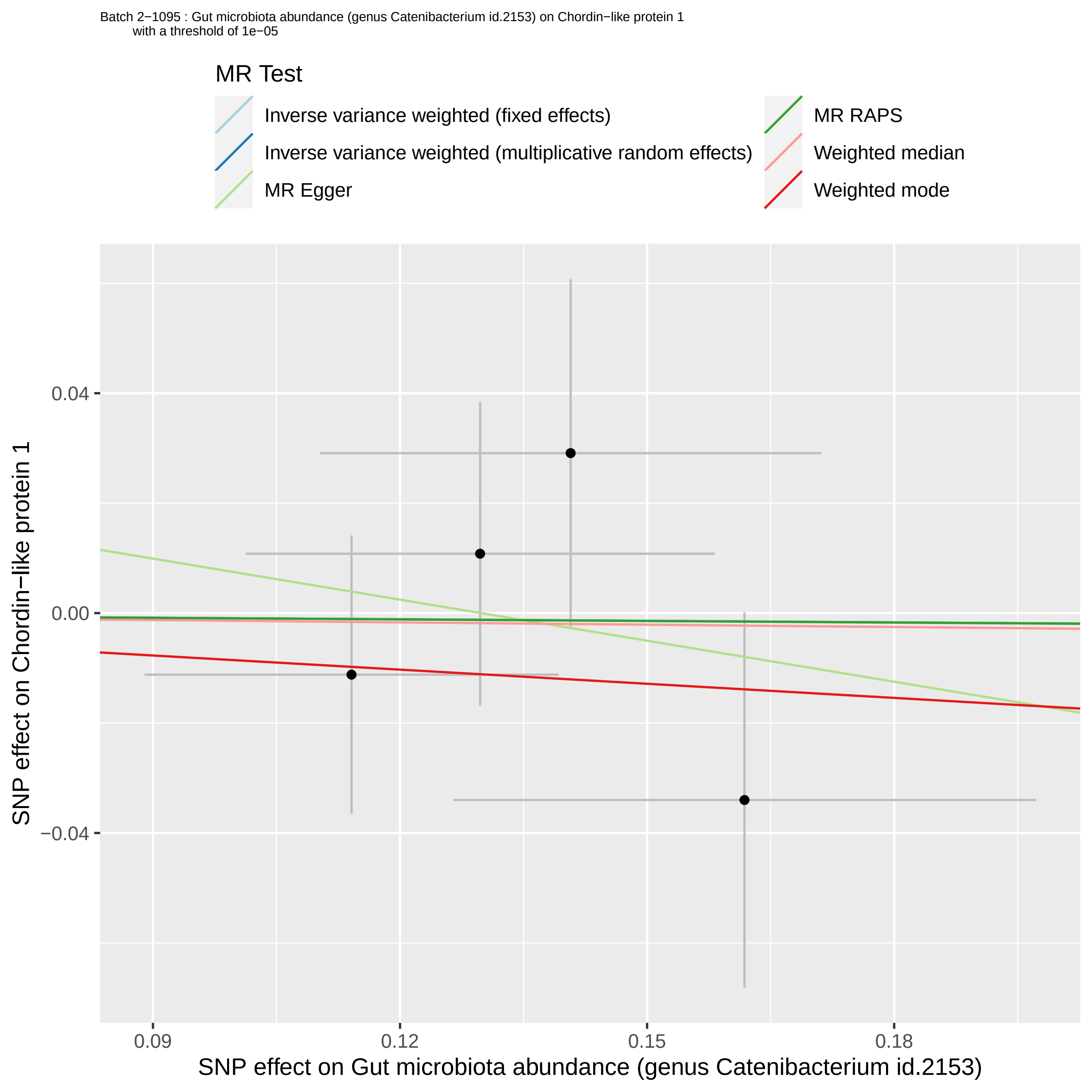
Batch 2−1095 : Gut microbiota abundance (genus Catenibacterium id.2153) on Chordin−like protein 1
with a threshold of 1e−05
MR Test
Inverse variance weighted (fixed effects)
Inverse variance weighted (multiplicative random effects)
MR Egger
MR RAPS
Weighted median
Weighted mode
0.04
0.00
−0.04
0.09
0.12
0.15
0.18
SNP effect on Chordin−like protein 1
SNP effect on Gut microbiota abundance (genus Catenibacterium id.2153)

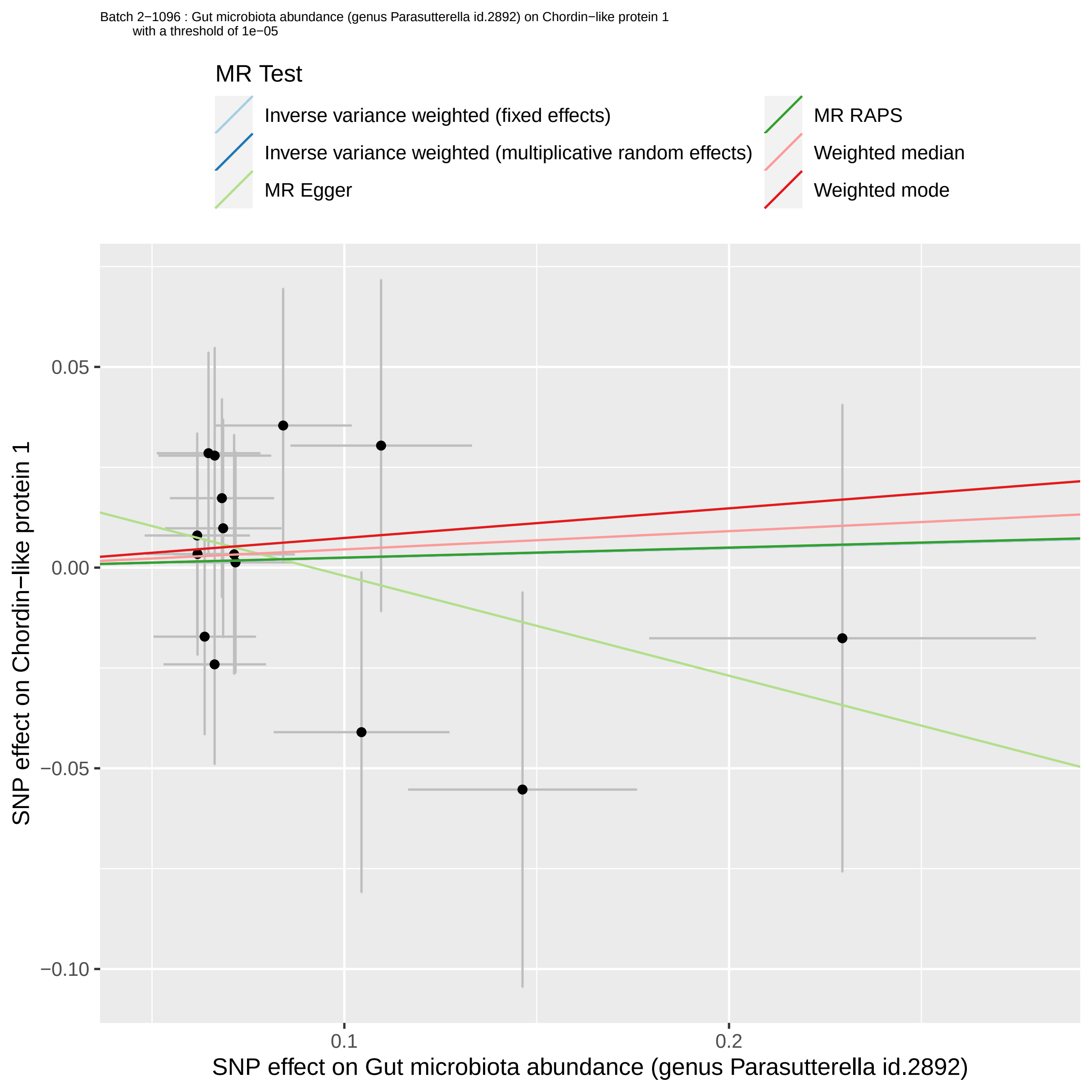
Batch 2−1096 : Gut microbiota abundance (genus Parasutterella id.2892) on Chordin−like protein 1
with a threshold of 1e−05
MR Test
Inverse variance weighted (fixed effects)
Inverse variance weighted (multiplicative random effects)
MR Egger
MR RAPS
Weighted median
Weighted mode
0.05
0.00
−0.05
−0.10
0.1
0.2
SNP effect on Chordin−like protein 1
SNP effect on Gut microbiota abundance (genus Parasutterella id.2892)

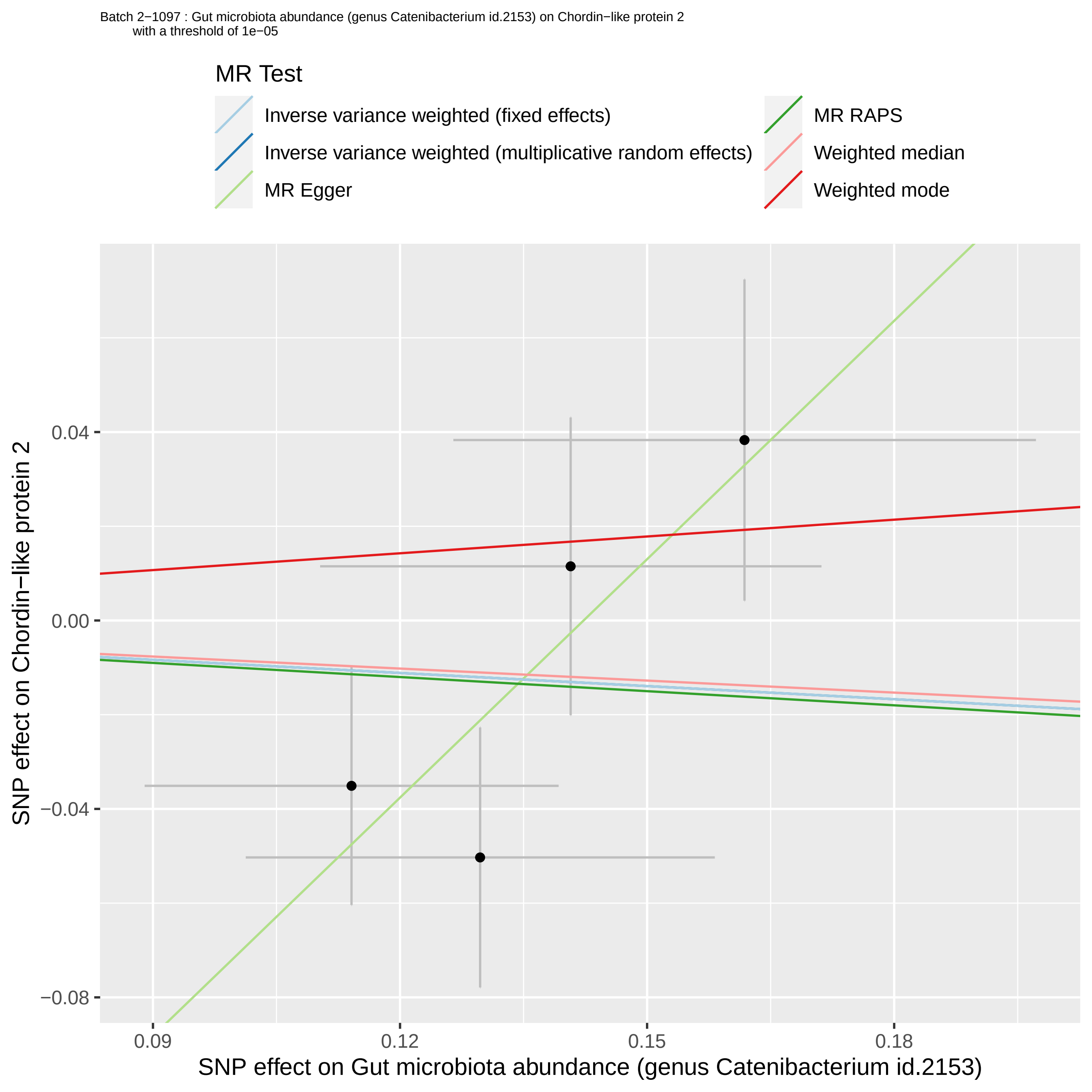

Batch 2−1097 : Gut microbiota abundance (genus Catenibacterium id.2153) on Chordin−like protein 2
with a threshold of 1e−05
MR Test
Inverse variance weighted (fixed effects)
Inverse variance weighted (multiplicative random effects)
MR Egger
MR RAPS
Weighted median
Weighted mode
SNP effect on Chordin−like protein 2
0.04
0.00
−0.04
−0.08
0.09
0.12
0.15
0.18
SNP effect on Gut microbiota abundance (genus Catenibacterium id.2153)

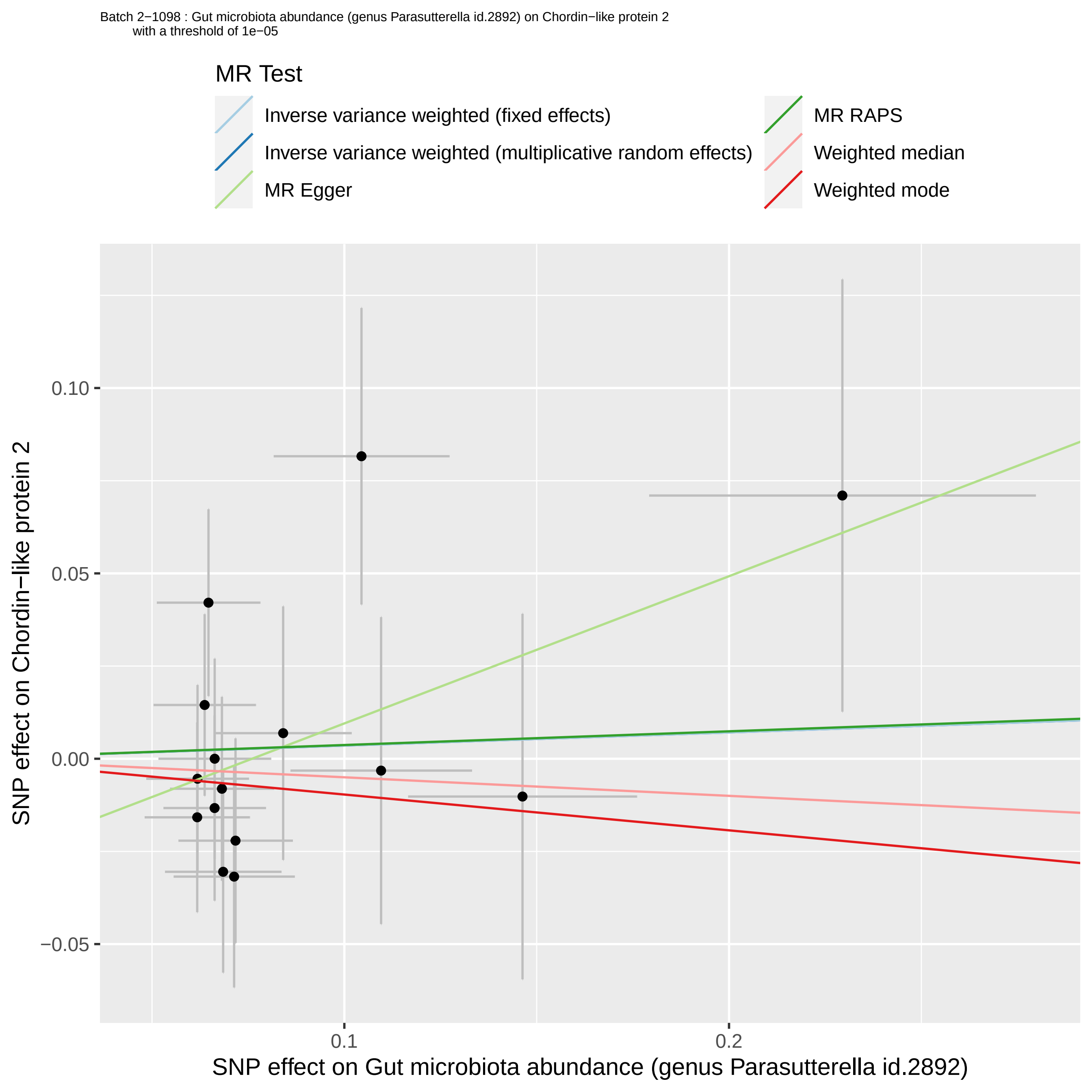

Batch 2−1098 : Gut microbiota abundance (genus Parasutterella id.2892) on Chordin−like protein 2
with a threshold of 1e−05
MR Test
Inverse variance weighted (fixed effects)
Inverse variance weighted (multiplicative random effects)
MR Egger
MR RAPS
Weighted median
Weighted mode
0.10
0.05
0.00
−0.05
SNP effect on Chordin−like protein 2
0.1
0.2
SNP effect on Gut microbiota abundance (genus Parasutterella id.2892)

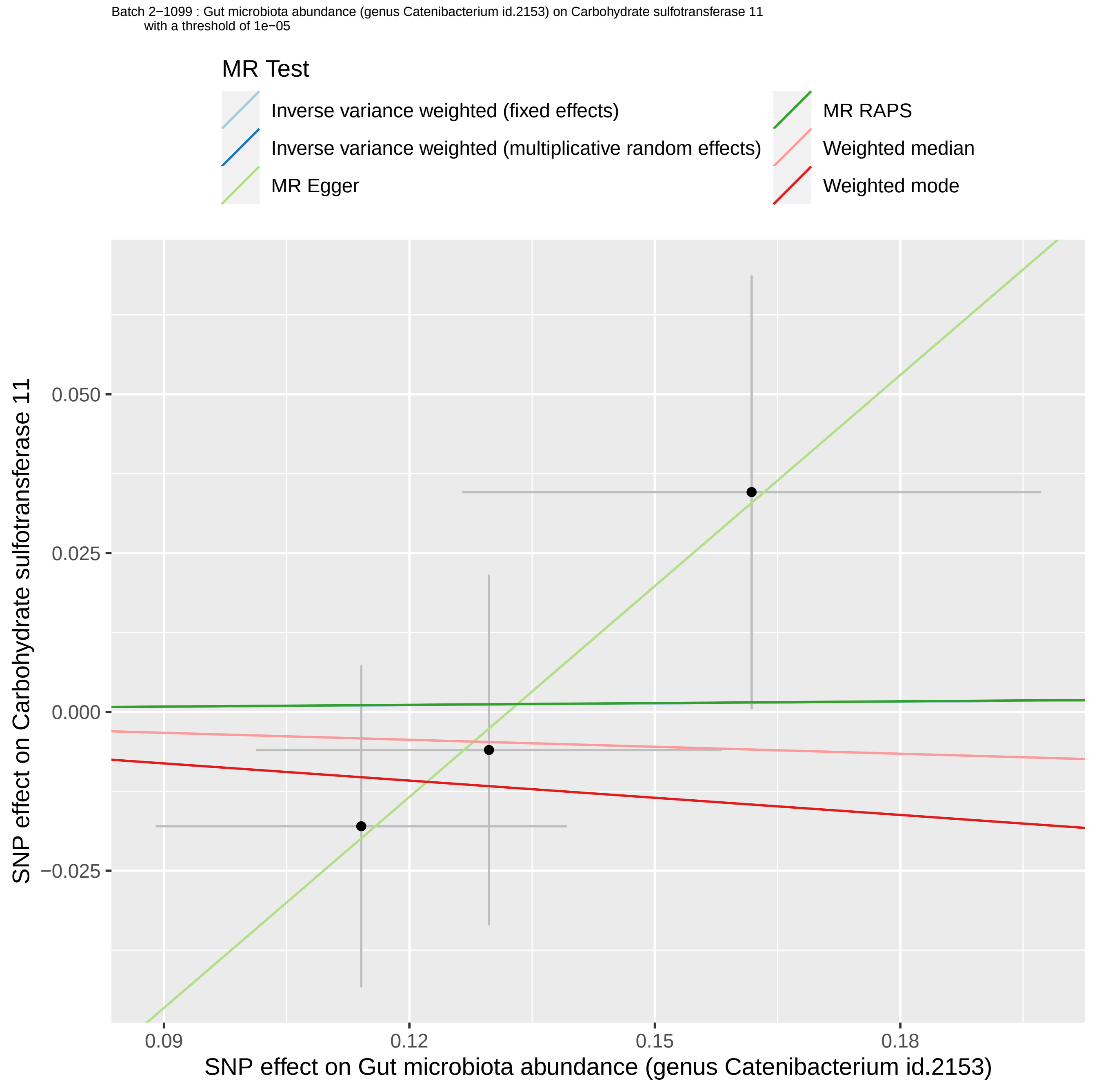
Batch 2−1099 : Gut microbiota abundance (genus Catenibacterium id.2153) on Carbohydrate sulfotransferase 11
with a threshold of 1e−05
MR Test
Inverse variance weighted (fixed effects)
Inverse variance weighted (multiplicative random effects)
MR Egger
MR RAPS
Weighted median
Weighted mode
0.050
0.025
0.000
−0.025
0.09
0.12
0.15
0.18
SNP effect on Carbohydrate sulfotransferase 11
SNP effect on Gut microbiota abundance (genus Catenibacterium id.2153)

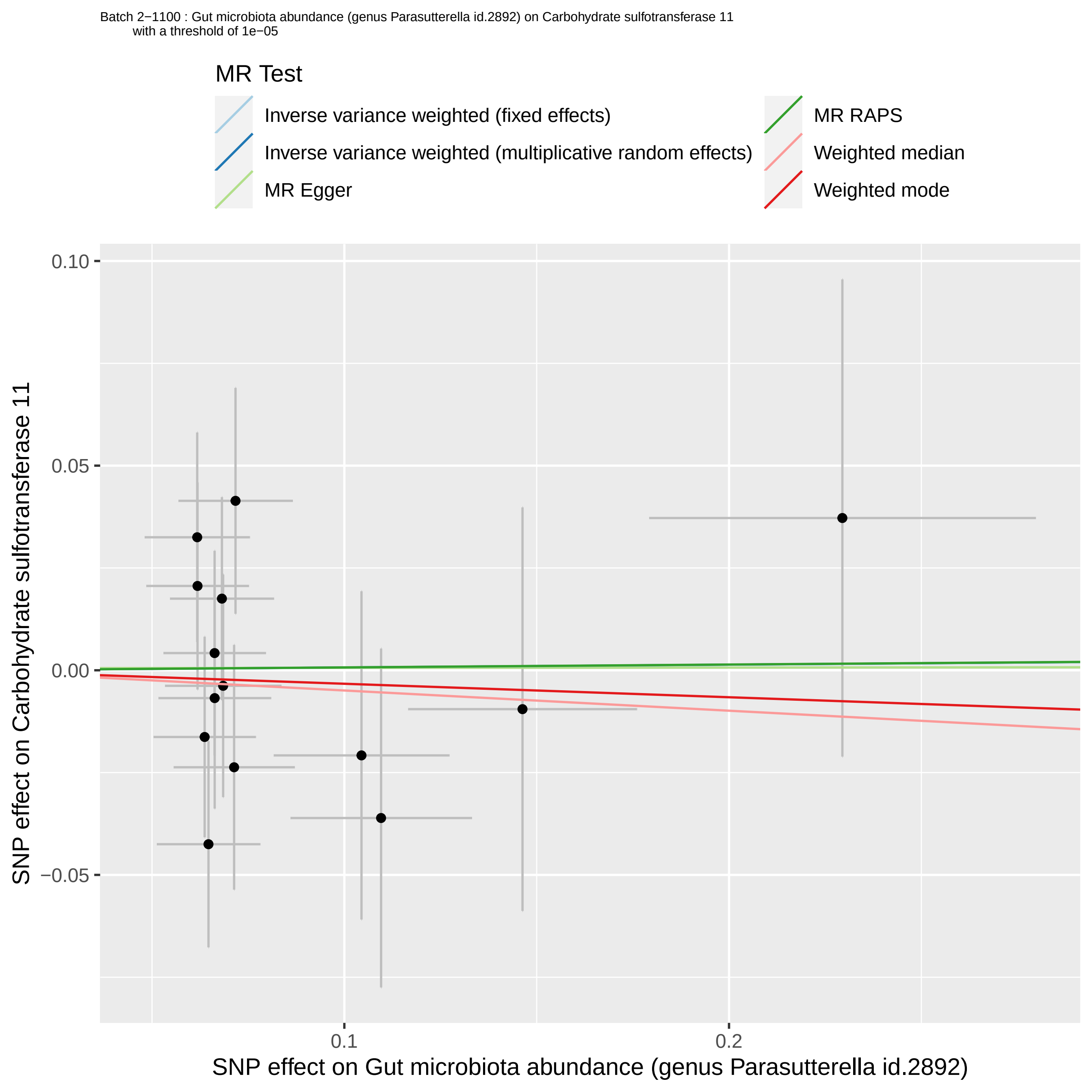

Batch 2−1100 : Gut microbiota abundance (genus Parasutterella id.2892) on Carbohydrate sulfotransferase 11
with a threshold of 1e−05
MR Test
Inverse variance weighted (fixed effects)
Inverse variance weighted (multiplicative random effects)
MR Egger
MR RAPS
Weighted median
Weighted mode
0.10
0.05
0.00
−0.05
0.1
0.2
SNP effect on Carbohydrate sulfotransferase 11
SNP effect on Gut microbiota abundance (genus Parasutterella id.2892)

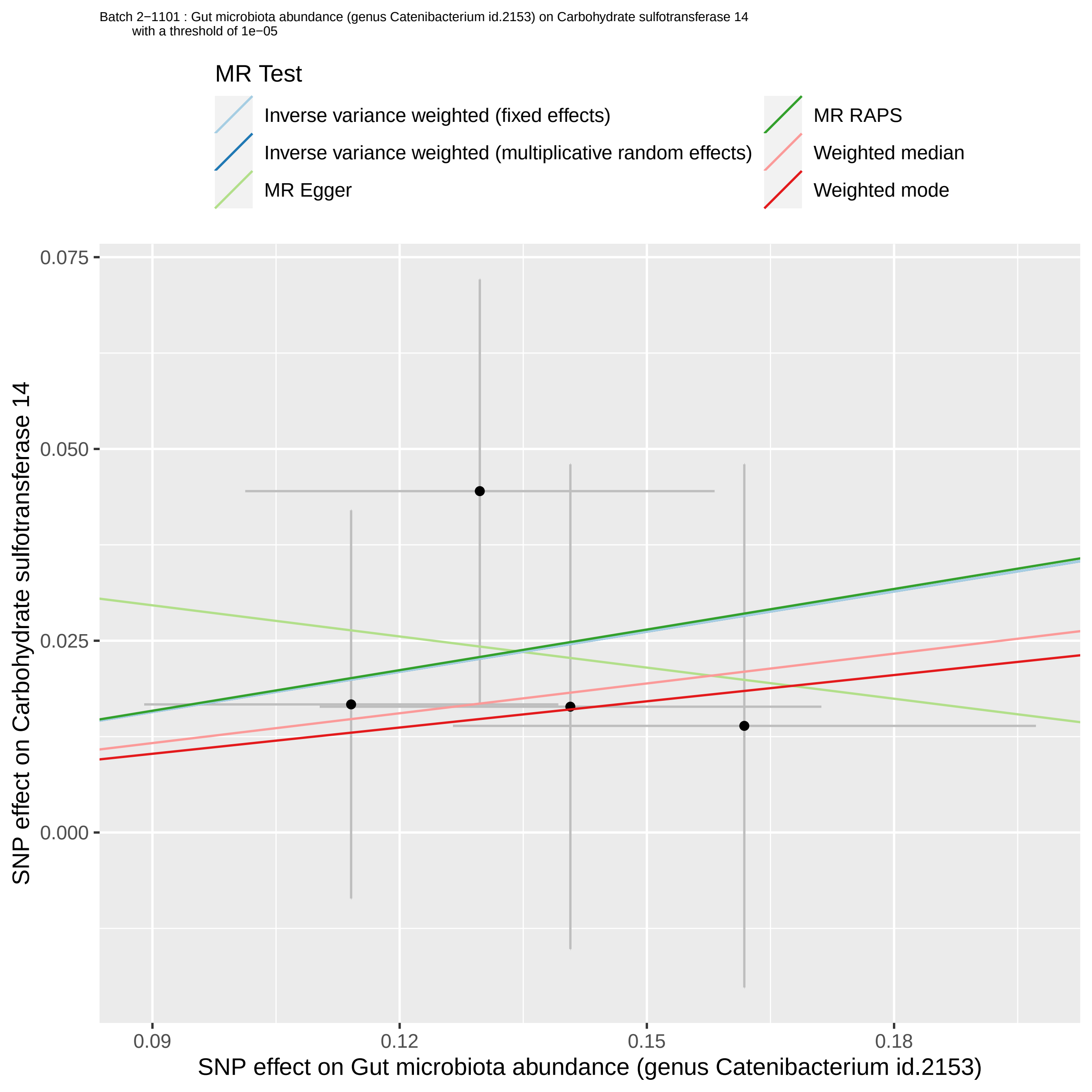

Batch 2−1101 : Gut microbiota abundance (genus Catenibacterium id.2153) on Carbohydrate sulfotransferase 14
with a threshold of 1e−05
MR Test
Inverse variance weighted (fixed effects)
Inverse variance weighted (multiplicative random effects)
MR Egger
MR RAPS
Weighted median
Weighted mode
0.075
0.050
0.025
0.000
0.09
0.12
0.15
0.18
SNP effect on Carbohydrate sulfotransferase 14
SNP effect on Gut microbiota abundance (genus Catenibacterium id.2153)

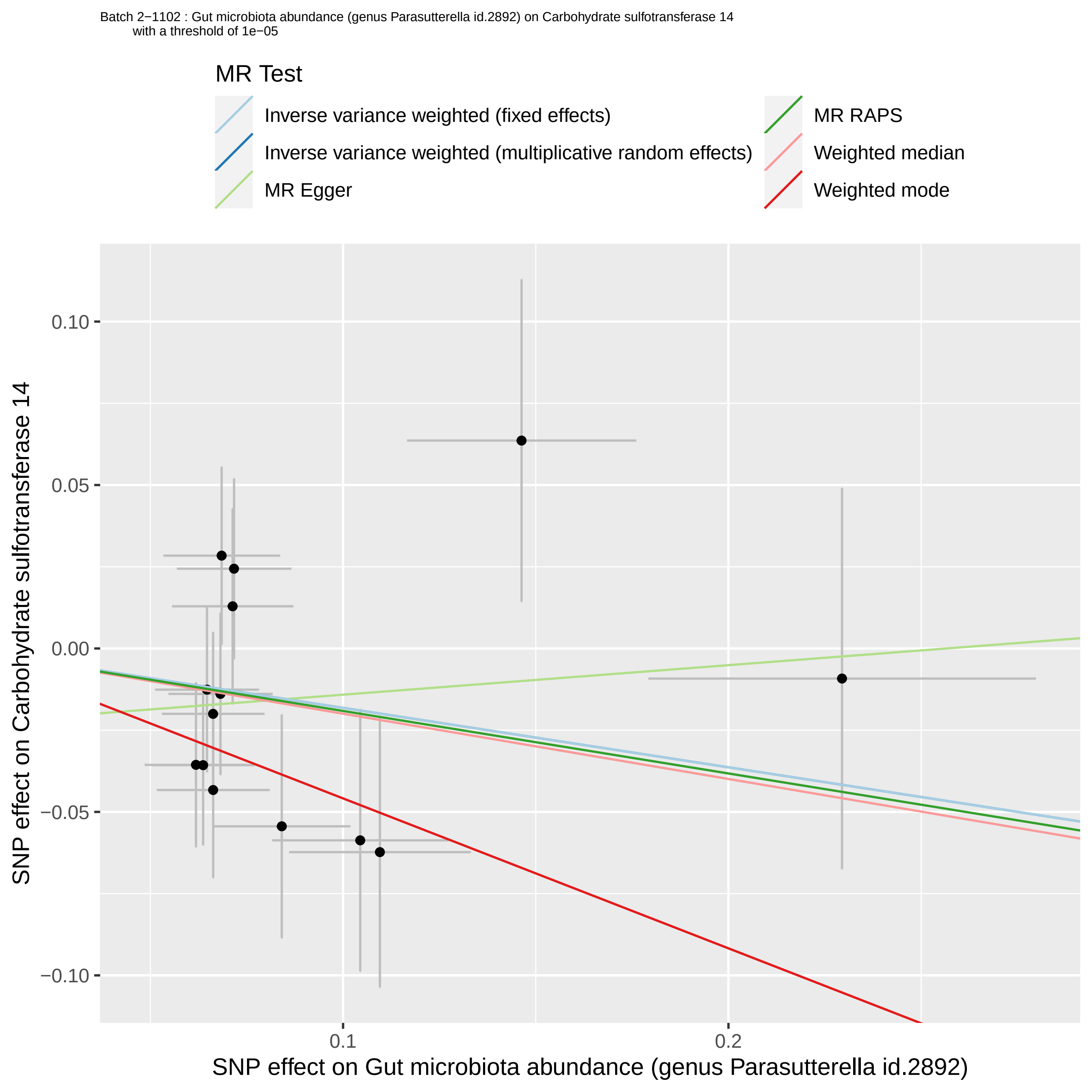
Batch 2−1102 : Gut microbiota abundance (genus Parasutterella id.2892) on Carbohydrate sulfotransferase 14
with a threshold of 1e−05
MR Test
Inverse variance weighted (fixed effects)
Inverse variance weighted (multiplicative random effects)
MR Egger
MR RAPS
Weighted median
Weighted mode
0.10
0.05
0.00
−0.05
−0.10
0.1
0.2
SNP effect on Carbohydrate sulfotransferase 14
SNP effect on Gut microbiota abundance (genus Parasutterella id.2892)

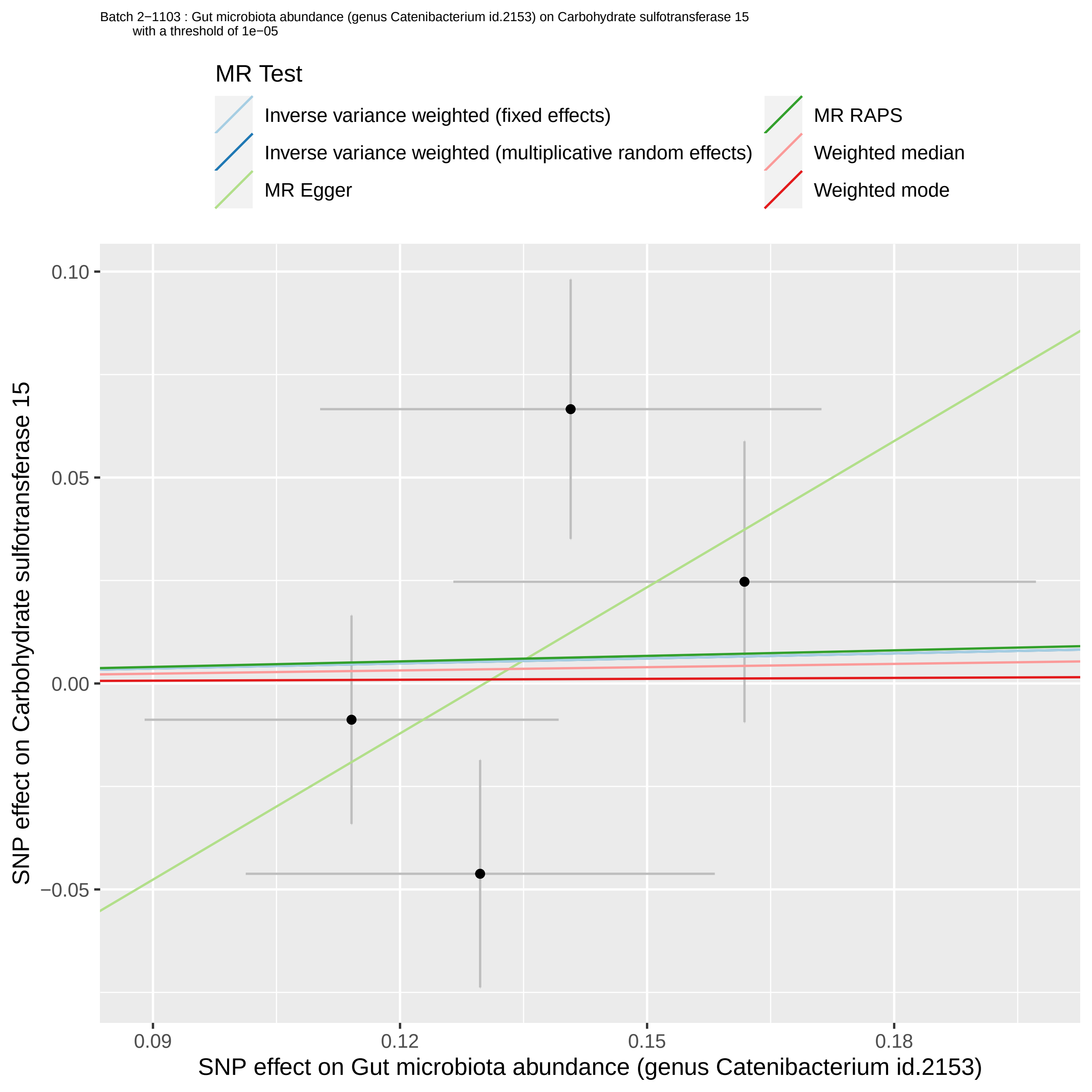
Batch 2−1103 : Gut microbiota abundance (genus Catenibacterium id.2153) on Carbohydrate sulfotransferase 15
with a threshold of 1e−05
MR Test
Inverse variance weighted (fixed effects)
Inverse variance weighted (multiplicative random effects)
MR Egger
MR RAPS
Weighted median
Weighted mode
0.10
0.05
0.00
−0.05
0.09
0.12
0.15
0.18
SNP effect on Carbohydrate sulfotransferase 15
SNP effect on Gut microbiota abundance (genus Catenibacterium id.2153)

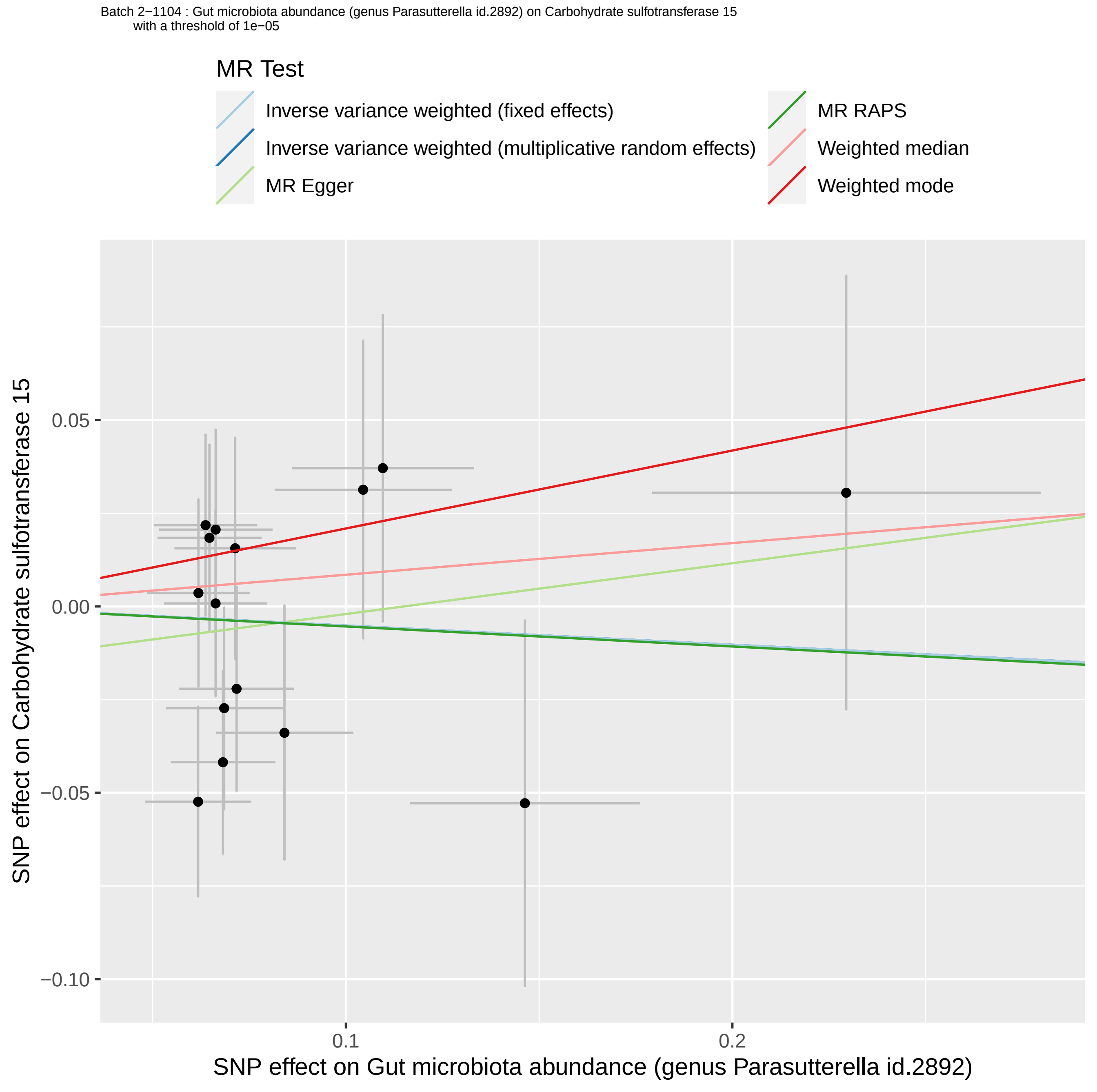
Batch 2−1104 : Gut microbiota abundance (genus Parasutterella id.2892) on Carbohydrate sulfotransferase 15
with a threshold of 1e−05
MR Test
Inverse variance weighted (fixed effects)
Inverse variance weighted (multiplicative random effects)
MR Egger
MR RAPS
Weighted median
Weighted mode
0.05
0.00
−0.05
−0.10
0.1
0.2
SNP effect on Carbohydrate sulfotransferase 15
SNP effect on Gut microbiota abundance (genus Parasutterella id.2892)

Batch 2−1105 : Gut microbiota abundance (genus Catenibacterium id.2153) on Carbohydrate sulfotransferase 15
with a threshold of 1e−05

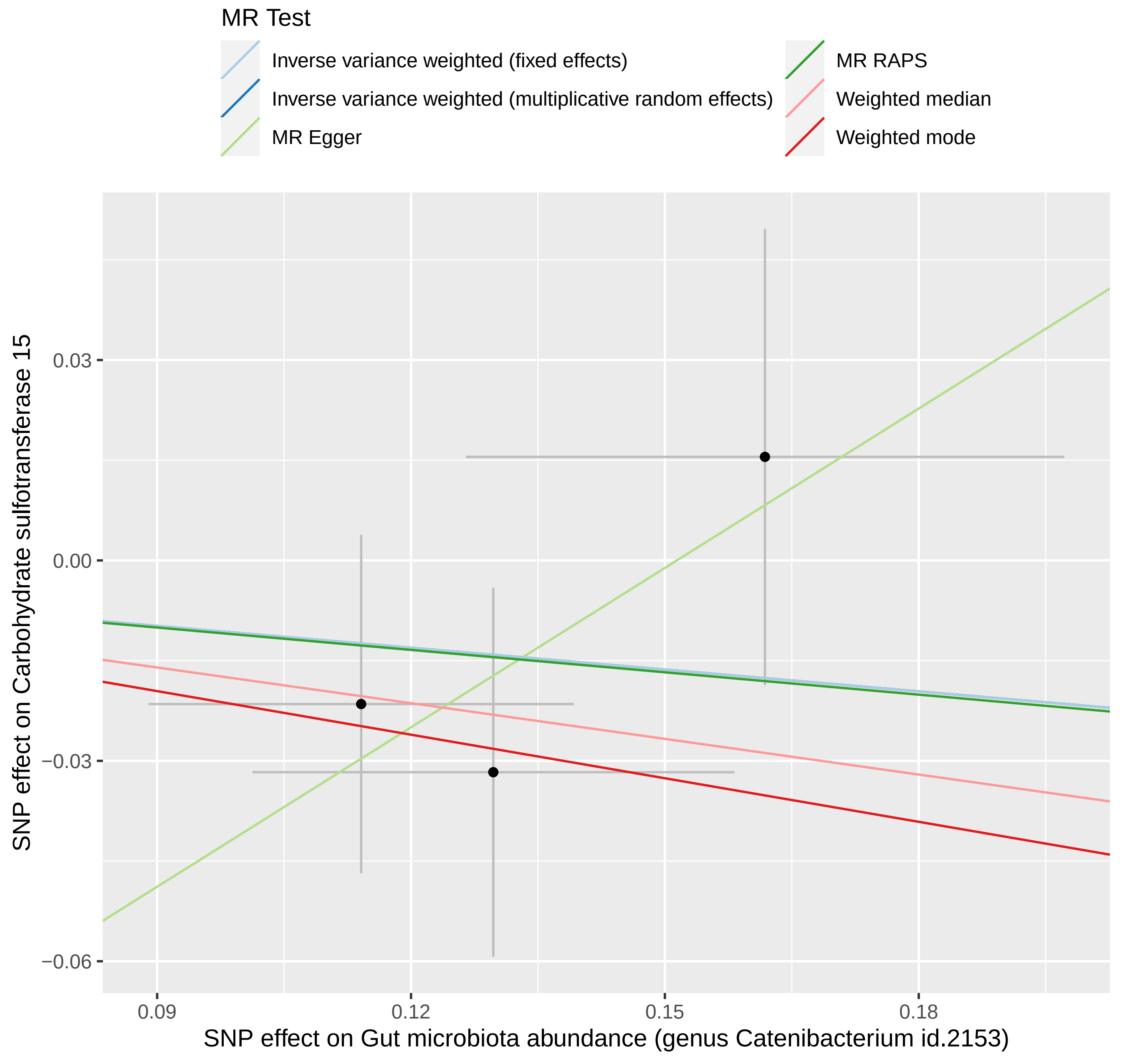

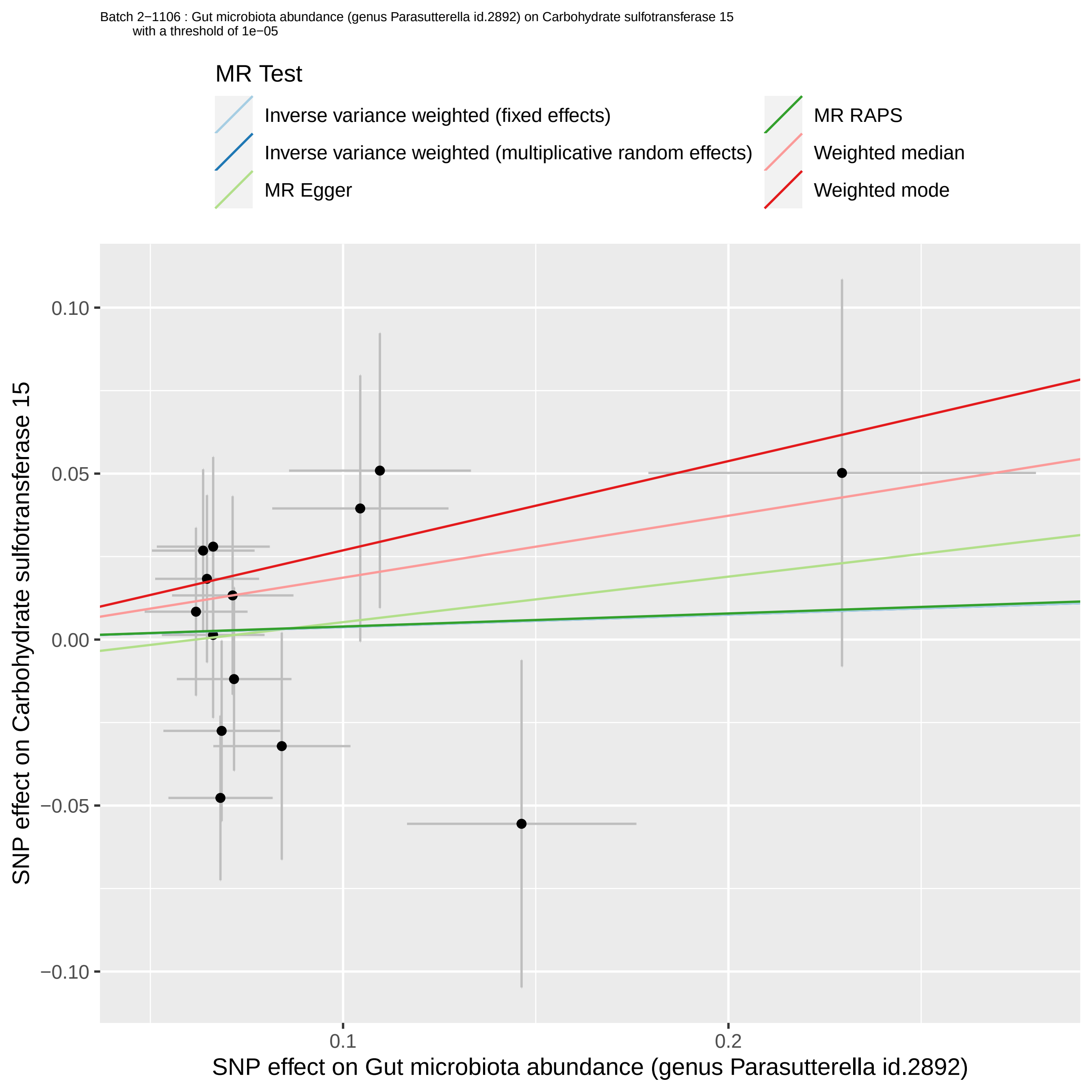
Batch 2−1106 : Gut microbiota abundance (genus Parasutterella id.2892) on Carbohydrate sulfotransferase 15
with a threshold of 1e−05
MR Test
Inverse variance weighted (fixed effects)
Inverse variance weighted (multiplicative random effects)
MR Egger
MR RAPS
Weighted median
Weighted mode
0.10
0.05
0.00
−0.05
−0.10
0.1
0.2
SNP effect on Carbohydrate sulfotransferase 15
SNP effect on Gut microbiota abundance (genus Parasutterella id.2892)

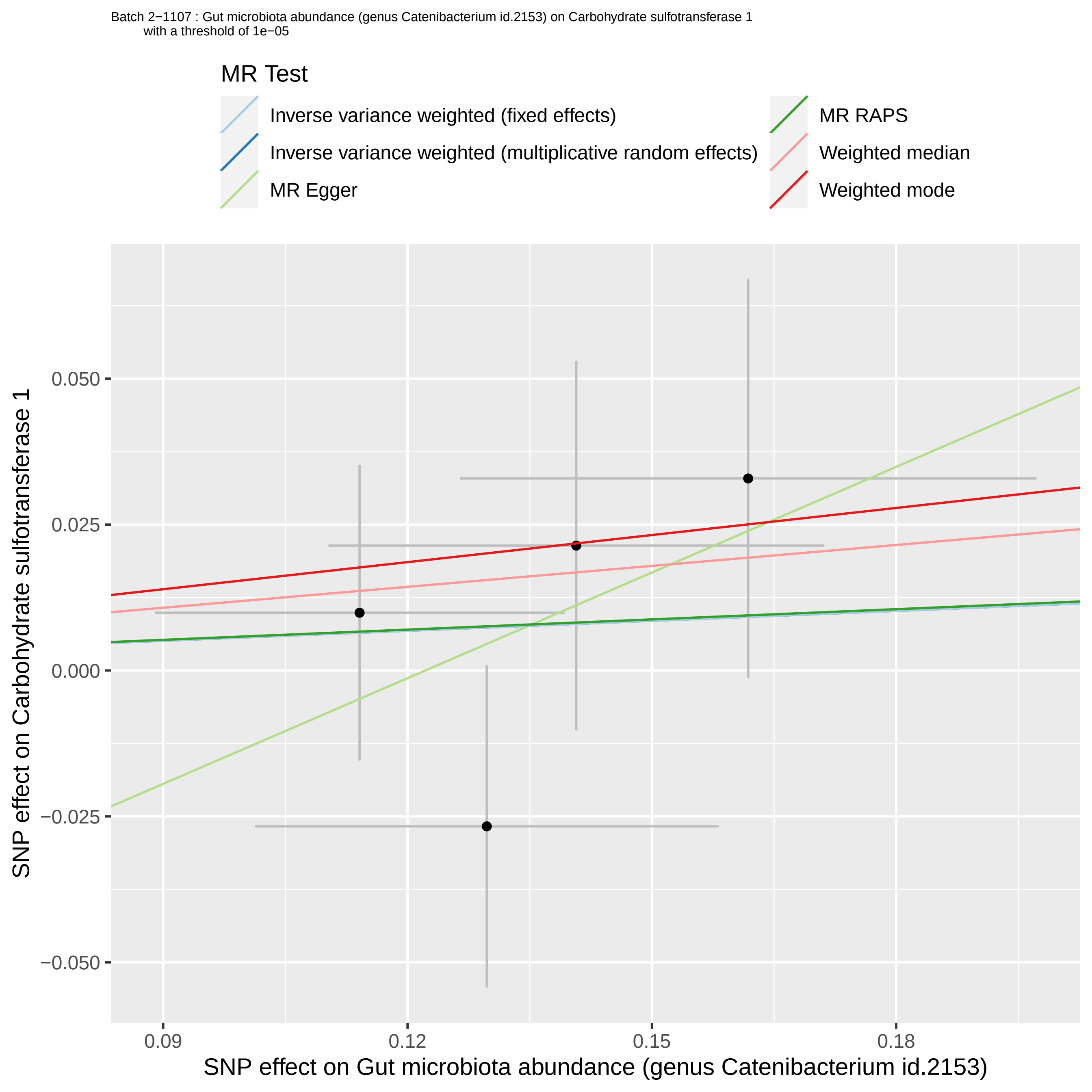
Batch 2−1107 : Gut microbiota abundance (genus Catenibacterium id.2153) on Carbohydrate sulfotransferase 1
with a threshold of 1e−05
MR Test
Inverse variance weighted (fixed effects)
Inverse variance weighted (multiplicative random effects)
MR Egger
MR RAPS
Weighted median
Weighted mode
SNP effect on Carbohydrate sulfotransferase 1
0.050
0.025
0.000
−0.025
−0.050
0.09
0.12
0.15
0.18
SNP effect on Gut microbiota abundance (genus Catenibacterium id.2153)

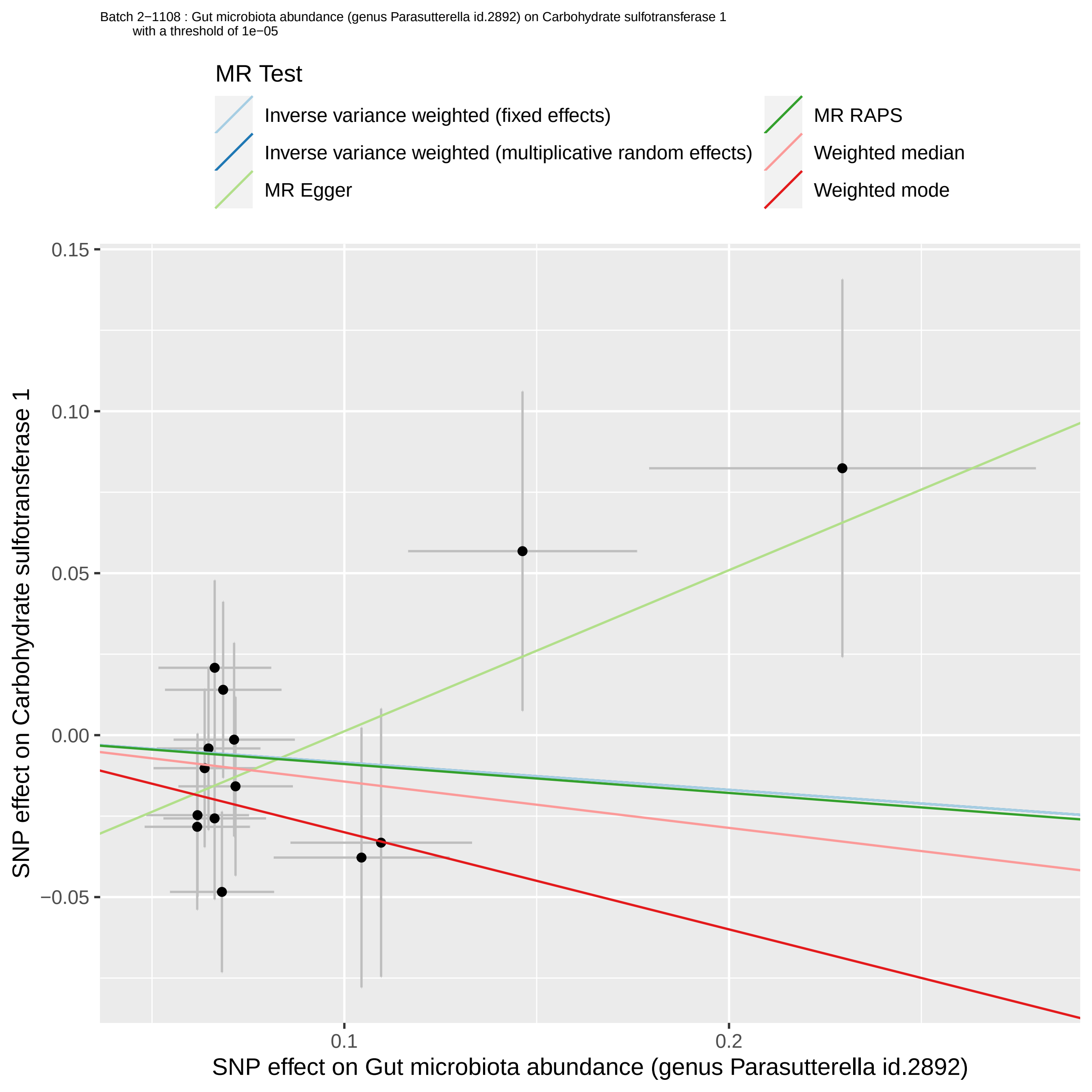
Batch 2−1108 : Gut microbiota abundance (genus Parasutterella id.2892) on Carbohydrate sulfotransferase 1
with a threshold of 1e−05
MR Test
Inverse variance weighted (fixed effects)
Inverse variance weighted (multiplicative random effects)
MR Egger
MR RAPS
Weighted median
Weighted mode
0.15
0.10
0.05
0.00
−0.05
0.1
0.2
SNP effect on Carbohydrate sulfotransferase 1
SNP effect on Gut microbiota abundance (genus Parasutterella id.2892)

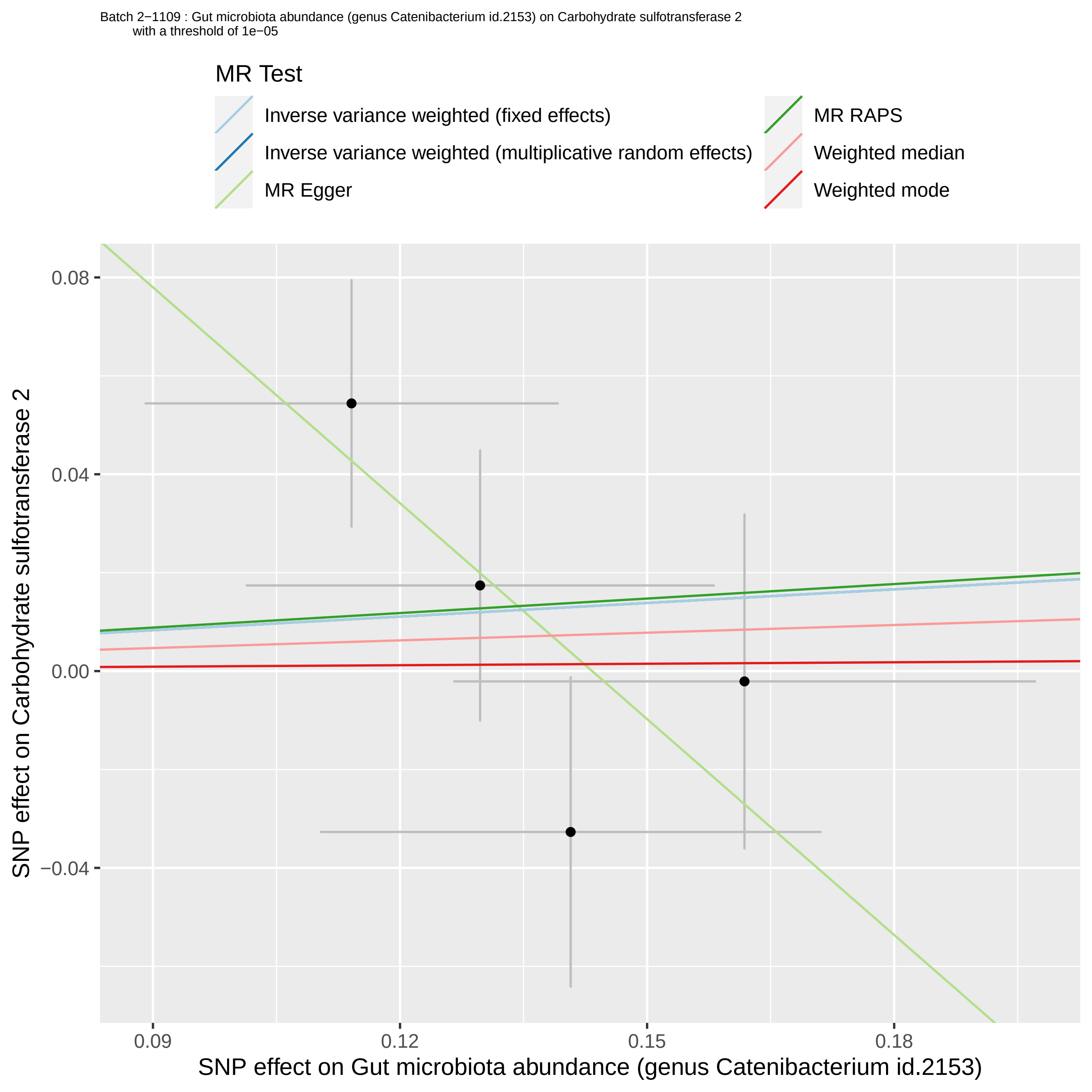
Batch 2−1109 : Gut microbiota abundance (genus Catenibacterium id.2153) on Carbohydrate sulfotransferase 2
with a threshold of 1e−05
MR Test
Inverse variance weighted (fixed effects)
Inverse variance weighted (multiplicative random effects)
MR Egger
MR RAPS
Weighted median
Weighted mode
0.08
0.04
0.00
−0.04
SNP effect on Carbohydrate sulfotransferase 2
0.09
0.12
0.15
0.18
SNP effect on Gut microbiota abundance (genus Catenibacterium id.2153)

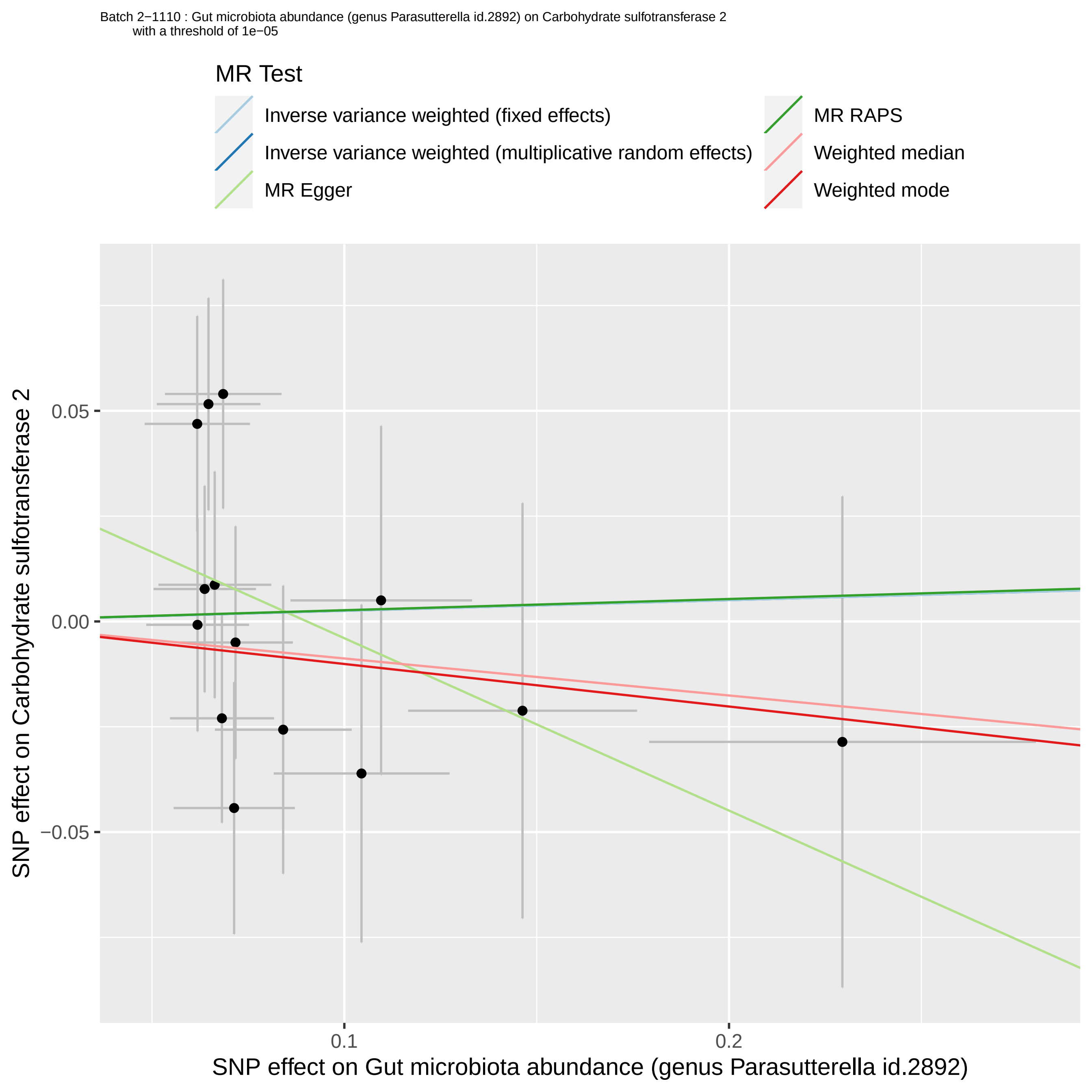
Batch 2−1110 : Gut microbiota abundance (genus Parasutterella id.2892) on Carbohydrate sulfotransferase 2
with a threshold of 1e−05
MR Test
Inverse variance weighted (fixed effects)
Inverse variance weighted (multiplicative random effects)
MR Egger
MR RAPS
Weighted median
Weighted mode
SNP effect on Carbohydrate sulfotransferase 2
0.05
0.00
−0.05
0.1
0.2
SNP effect on Gut microbiota abundance (genus Parasutterella id.2892)

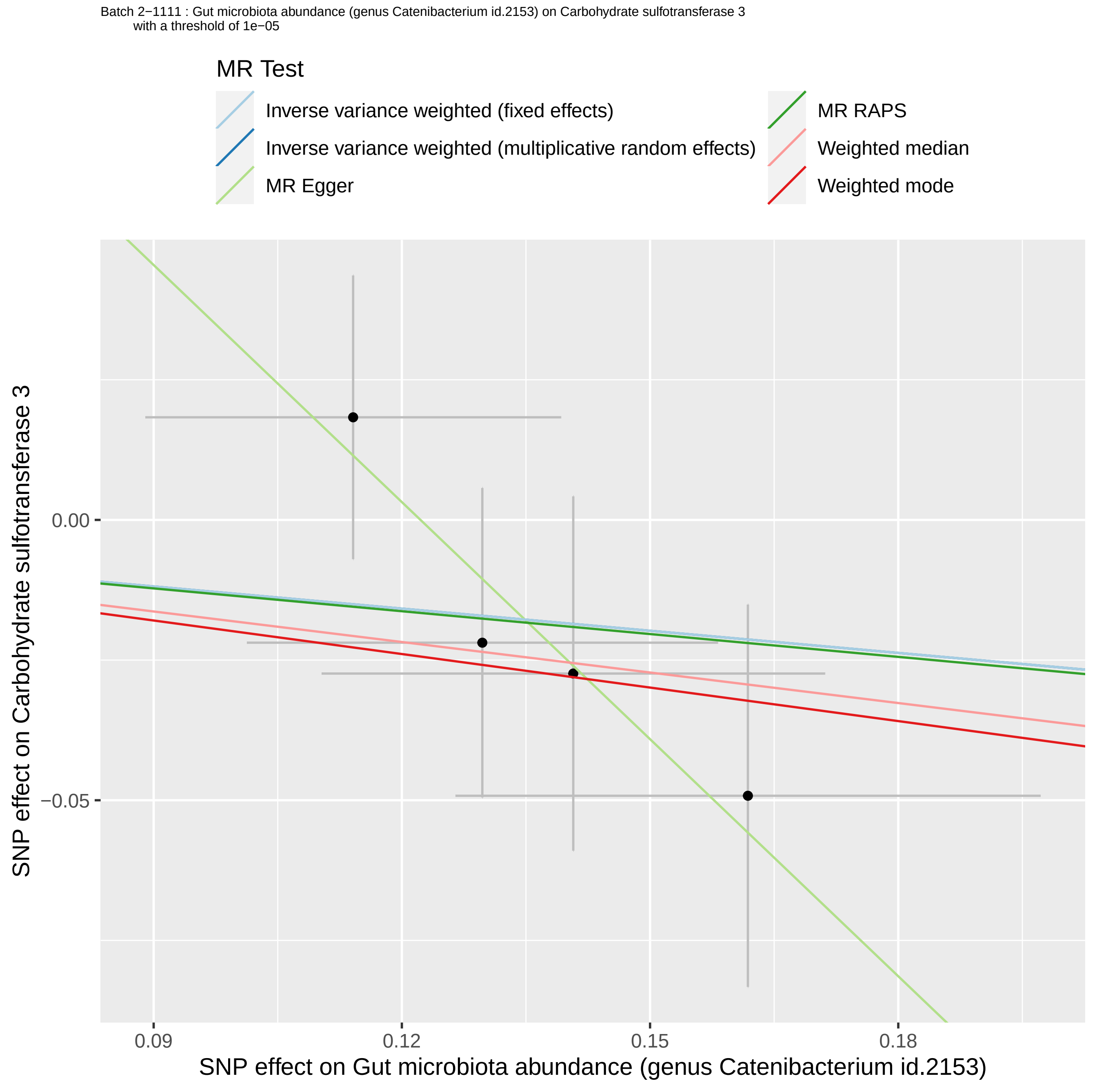
Batch 2−1111 : Gut microbiota abundance (genus Catenibacterium id.2153) on Carbohydrate sulfotransferase 3
with a threshold of 1e−05
MR Test
Inverse variance weighted (fixed effects)
Inverse variance weighted (multiplicative random effects)
MR Egger
MR RAPS
Weighted median
Weighted mode
SNP effect on Carbohydrate sulfotransferase 3
0.00
−0.05
0.09
0.12
0.15
0.18
SNP effect on Gut microbiota abundance (genus Catenibacterium id.2153)

Batch 2−1112 : Gut microbiota abundance (genus Parasutterella id.2892) on Carbohydrate sulfotransferase 3
with a threshold of 1e−05

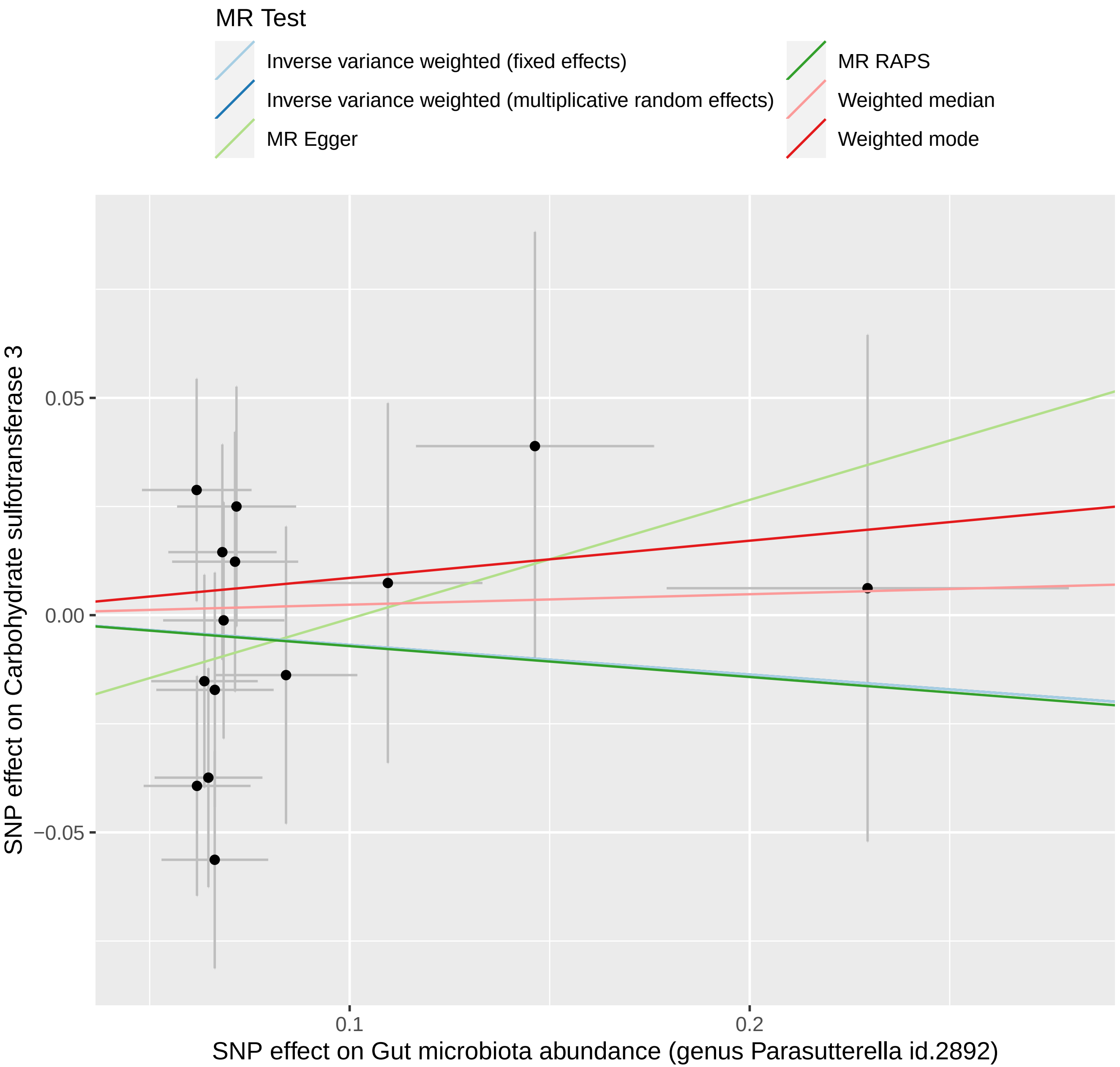

Batch 2−1113 : Gut microbiota abundance (genus Catenibacterium id.2153) on Carbohydrate sulfotransferase 5
with a threshold of 1e−05

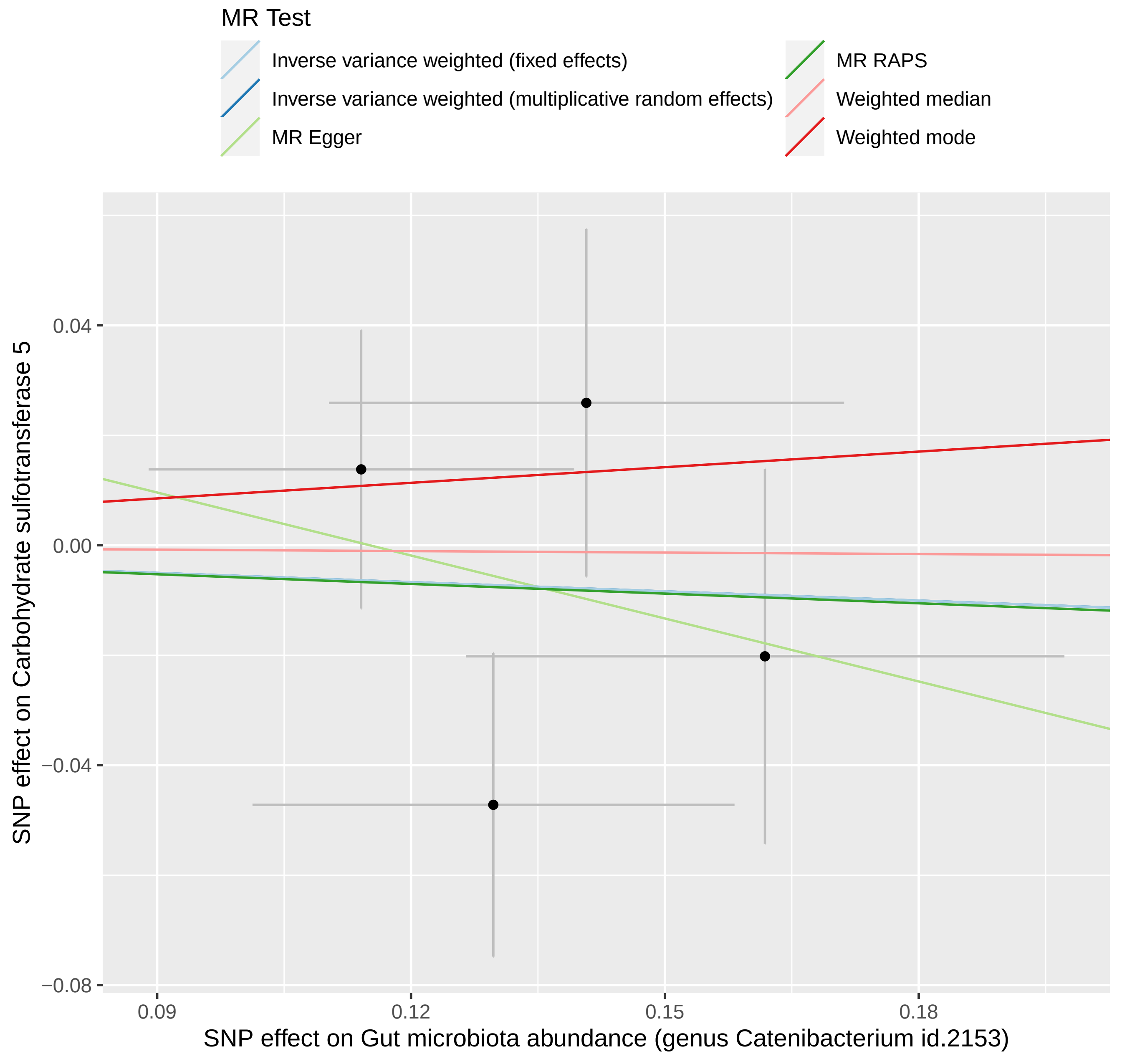

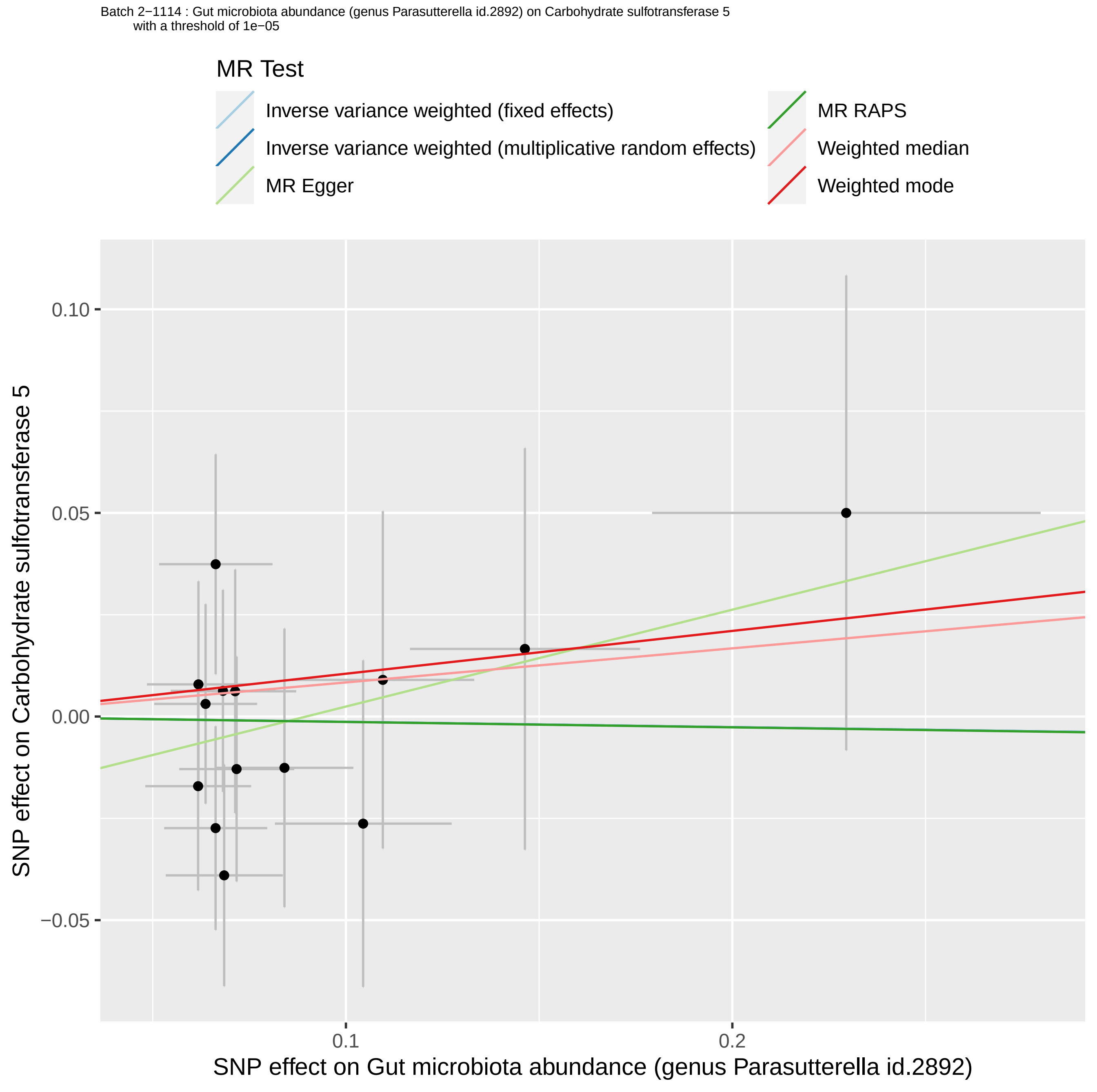

Batch 2−1114 : Gut microbiota abundance (genus Parasutterella id.2892) on Carbohydrate sulfotransferase 5
with a threshold of 1e−05
MR Test
Inverse variance weighted (fixed effects)
Inverse variance weighted (multiplicative random effects)
MR Egger
MR RAPS
Weighted median
Weighted mode
0.10
0.05
0.00
−0.05
0.1
0.2
SNP effect on Carbohydrate sulfotransferase 5
SNP effect on Gut microbiota abundance (genus Parasutterella id.2892)

Batch 2−1115 : Gut microbiota abundance (genus Catenibacterium id.2153) on Carbohydrate sulfotransferase 6
with a threshold of 1e−05

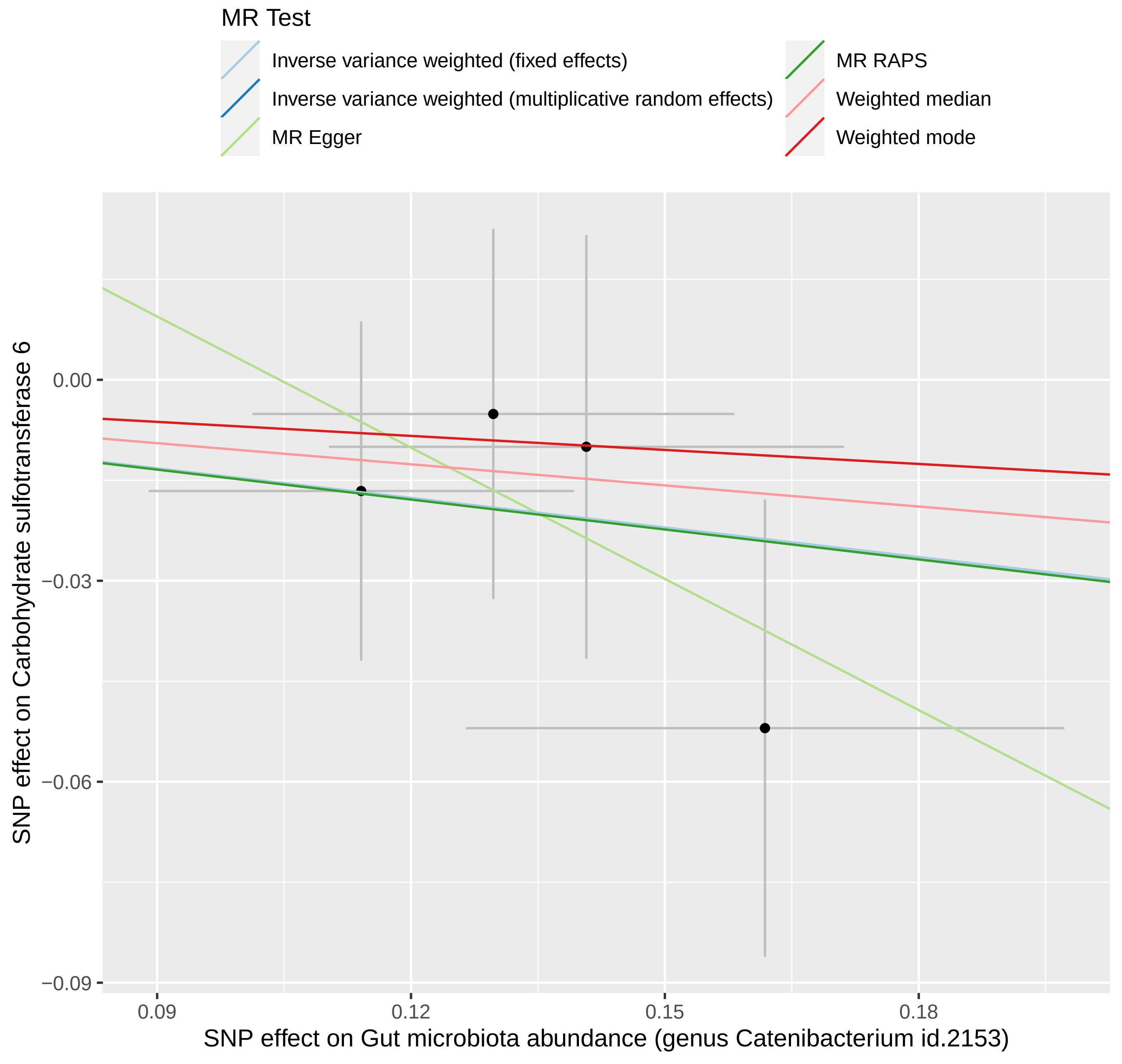

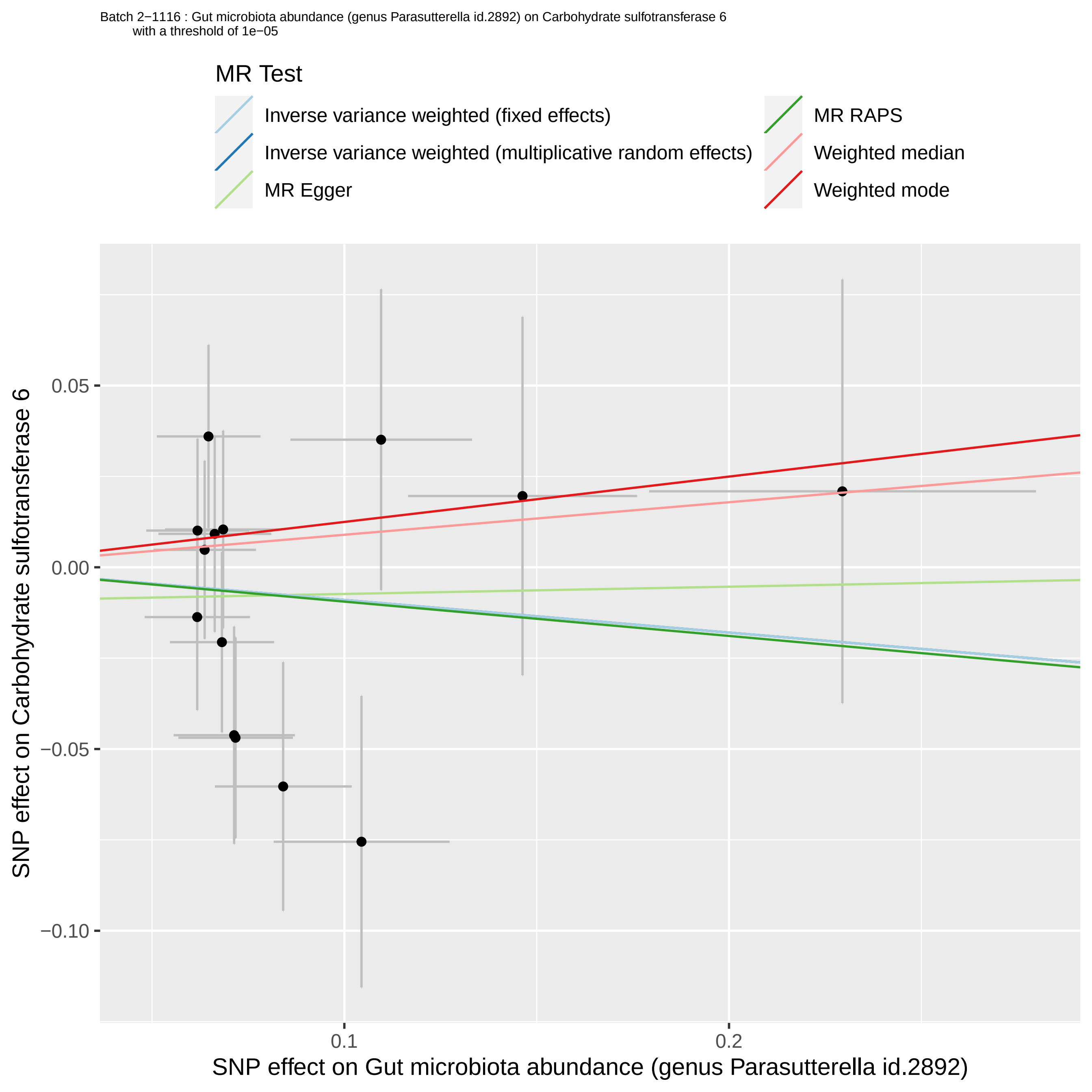
Batch 2−1116 : Gut microbiota abundance (genus Parasutterella id.2892) on Carbohydrate sulfotransferase 6
with a threshold of 1e−05
MR Test
Inverse variance weighted (fixed effects)
Inverse variance weighted (multiplicative random effects)
MR Egger
MR RAPS
Weighted median
Weighted mode
0.05
0.00
−0.05
−0.10
0.1
0.2
SNP effect on Carbohydrate sulfotransferase 6
SNP effect on Gut microbiota abundance (genus Parasutterella id.2892)

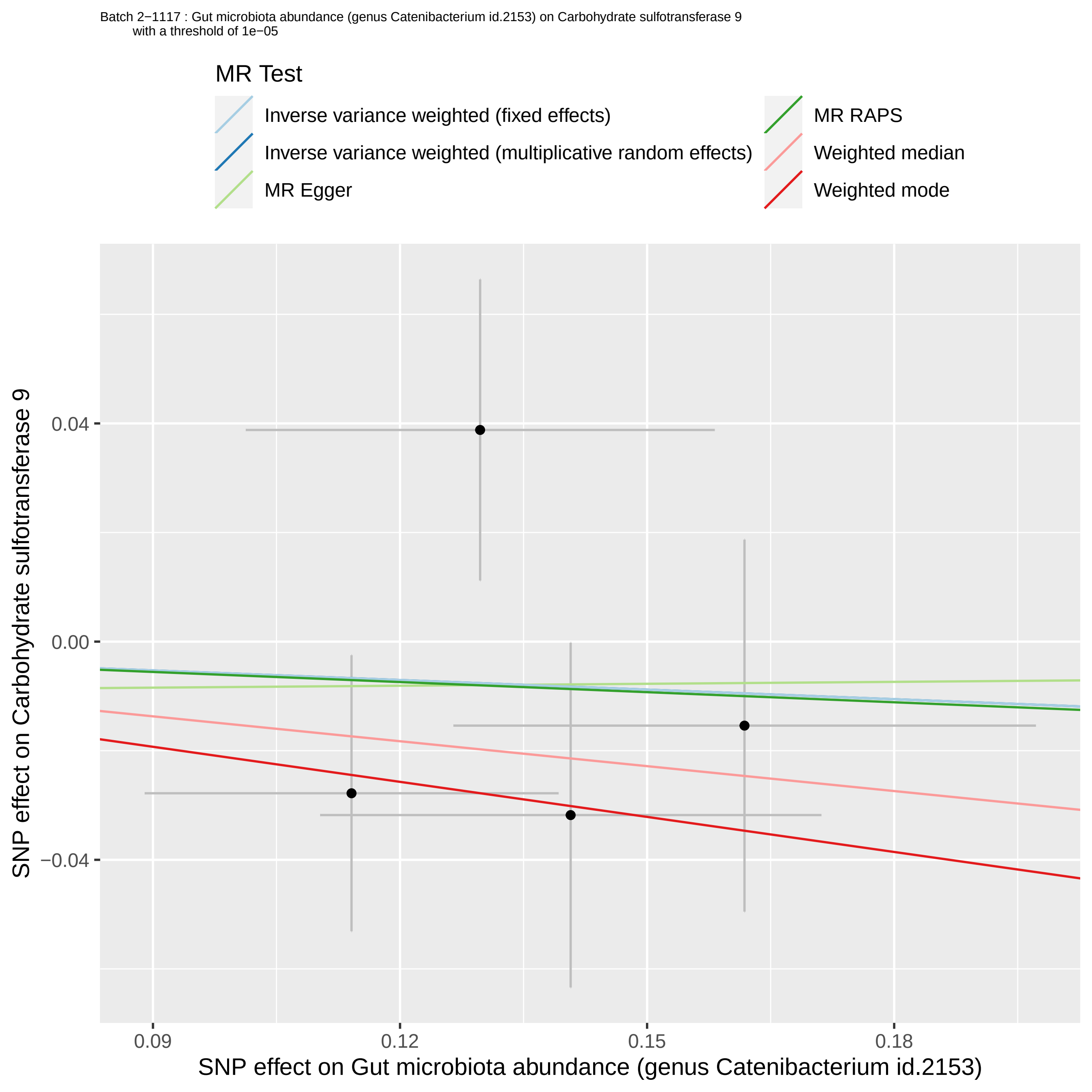

Batch 2−1117 : Gut microbiota abundance (genus Catenibacterium id.2153) on Carbohydrate sulfotransferase 9
with a threshold of 1e−05
MR Test
Inverse variance weighted (fixed effects)
Inverse variance weighted (multiplicative random effects)
MR Egger
MR RAPS
Weighted median
Weighted mode
0.04
0.00
−0.04
0.09
0.12
0.15
0.18
SNP effect on Carbohydrate sulfotransferase 9
SNP effect on Gut microbiota abundance (genus Catenibacterium id.2153)

Batch 2−1118 : Gut microbiota abundance (genus Parasutterella id.2892) on Carbohydrate sulfotransferase 9
with a threshold of 1e−05

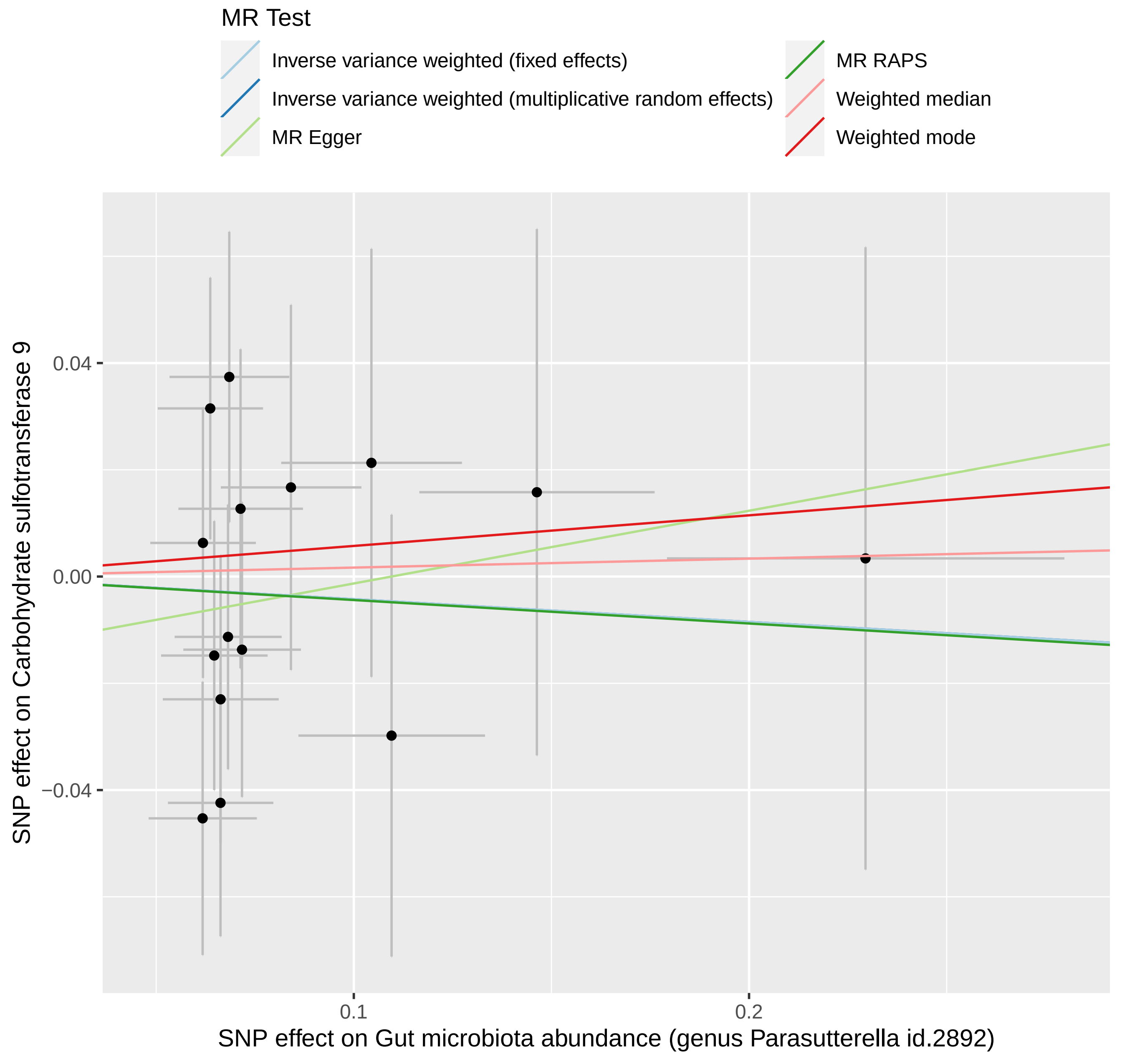

Batch 2−1119 : Gut microbiota abundance (genus Catenibacterium id.2153) on Cartilage intermediate layer protein 1
with a threshold of 1e−05

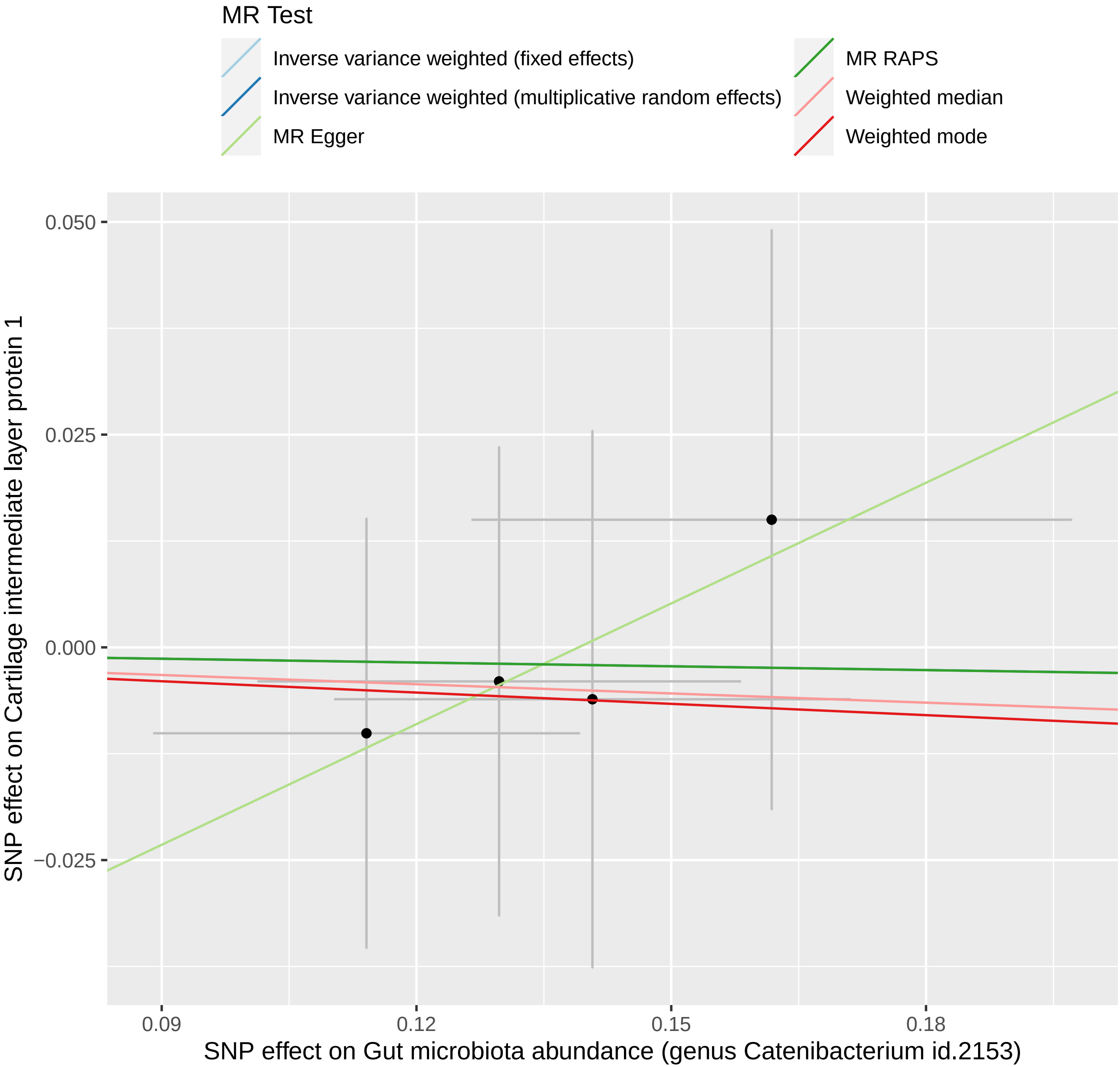

Batch 2−1120 : Gut microbiota abundance (genus Parasutterella id.2892) on Cartilage intermediate layer protein 1
with a threshold of 1e−05

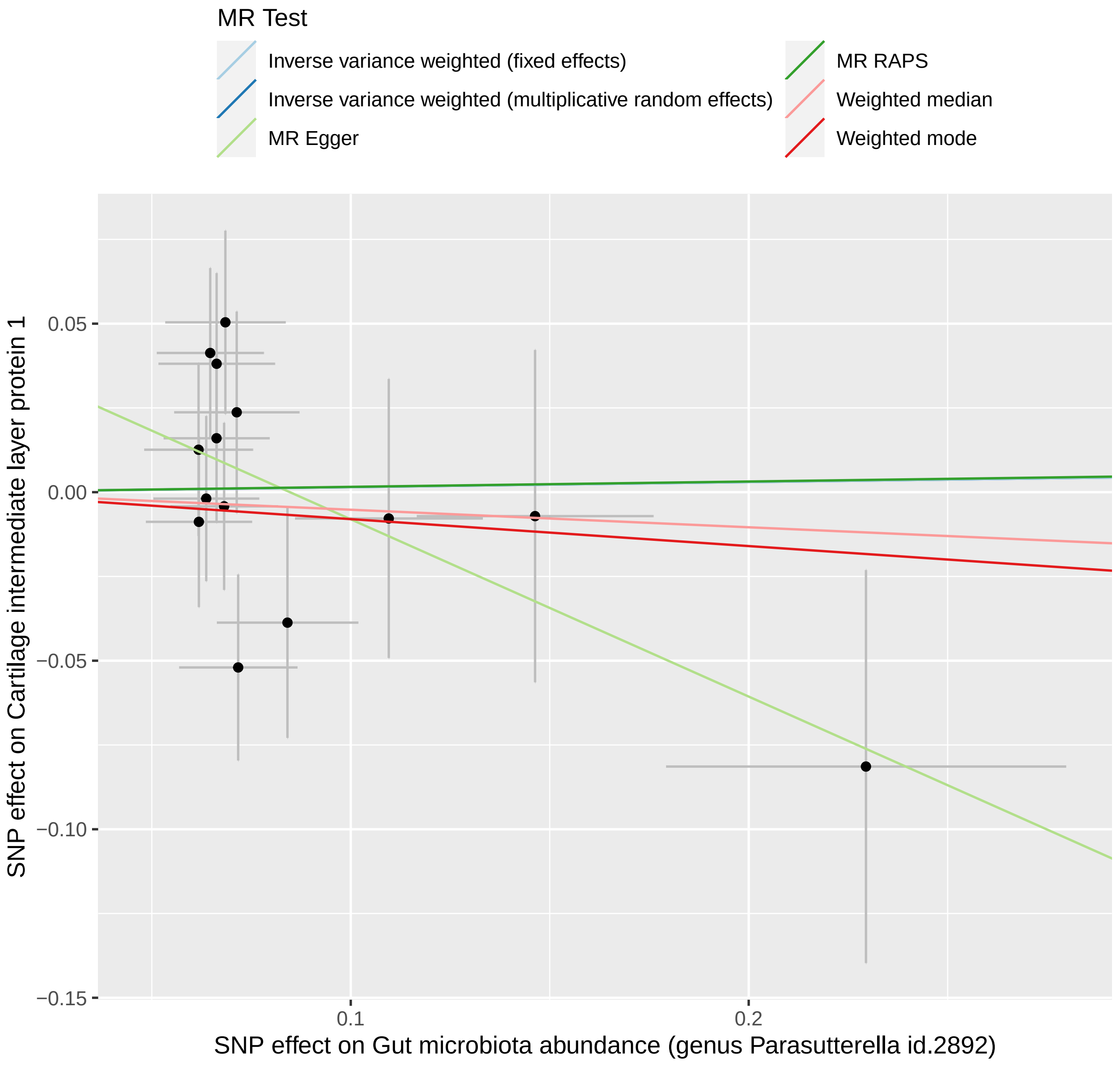

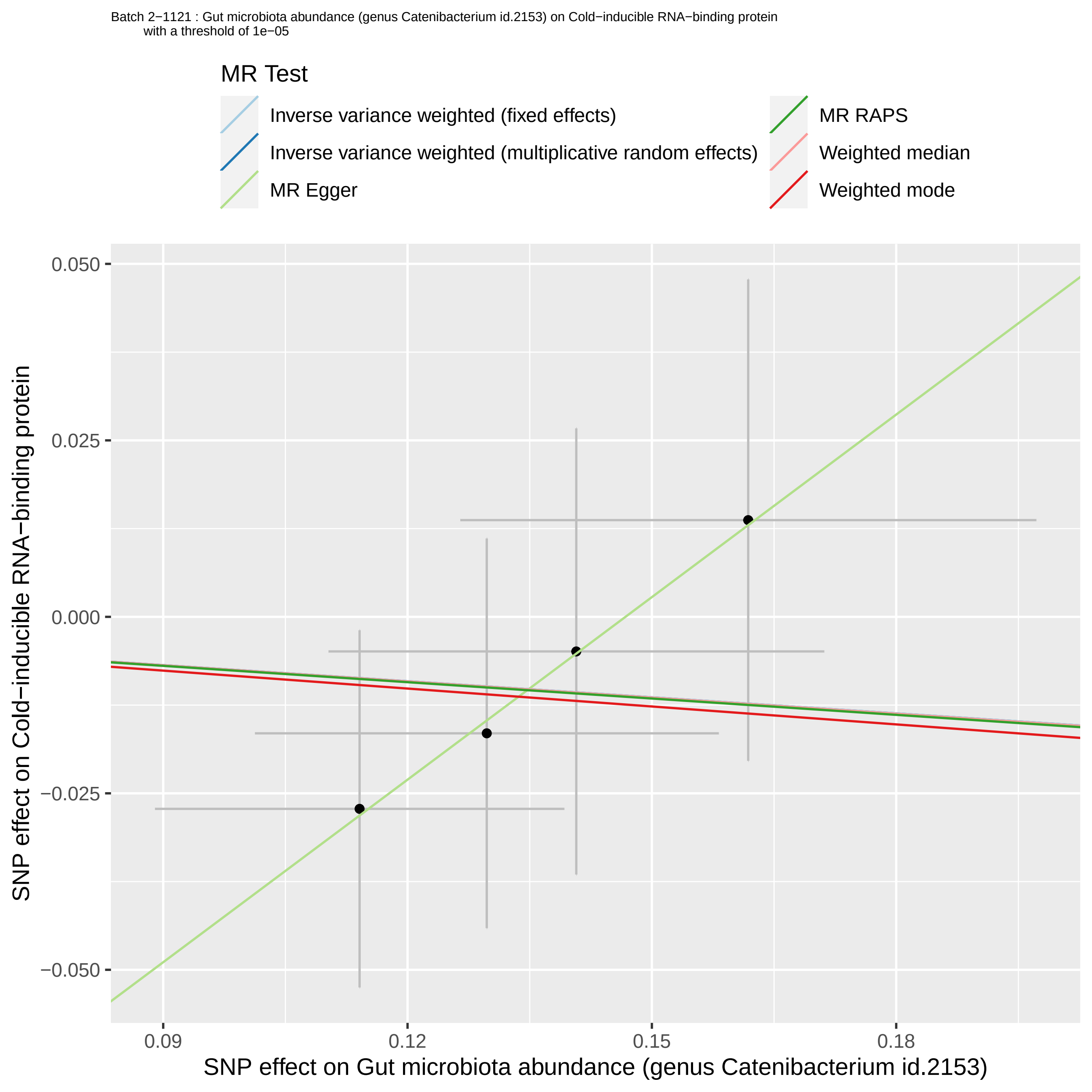
Batch 2−1121 : Gut microbiota abundance (genus Catenibacterium id.2153) on Cold−inducible RNA−binding protein
with a threshold of 1e−05
MR Test
Inverse variance weighted (fixed effects)
Inverse variance weighted (multiplicative random effects)
MR Egger
MR RAPS
Weighted median
Weighted mode
0.050
0.025
0.000
−0.025
−0.050
0.09
0.12
0.15
0.18
SNP effect on Cold−inducible RNA−binding protein
SNP effect on Gut microbiota abundance (genus Catenibacterium id.2153)

Batch 2−1122 : Gut microbiota abundance (genus Parasutterella id.2892) on Cold−inducible RNA−binding protein
with a threshold of 1e−05

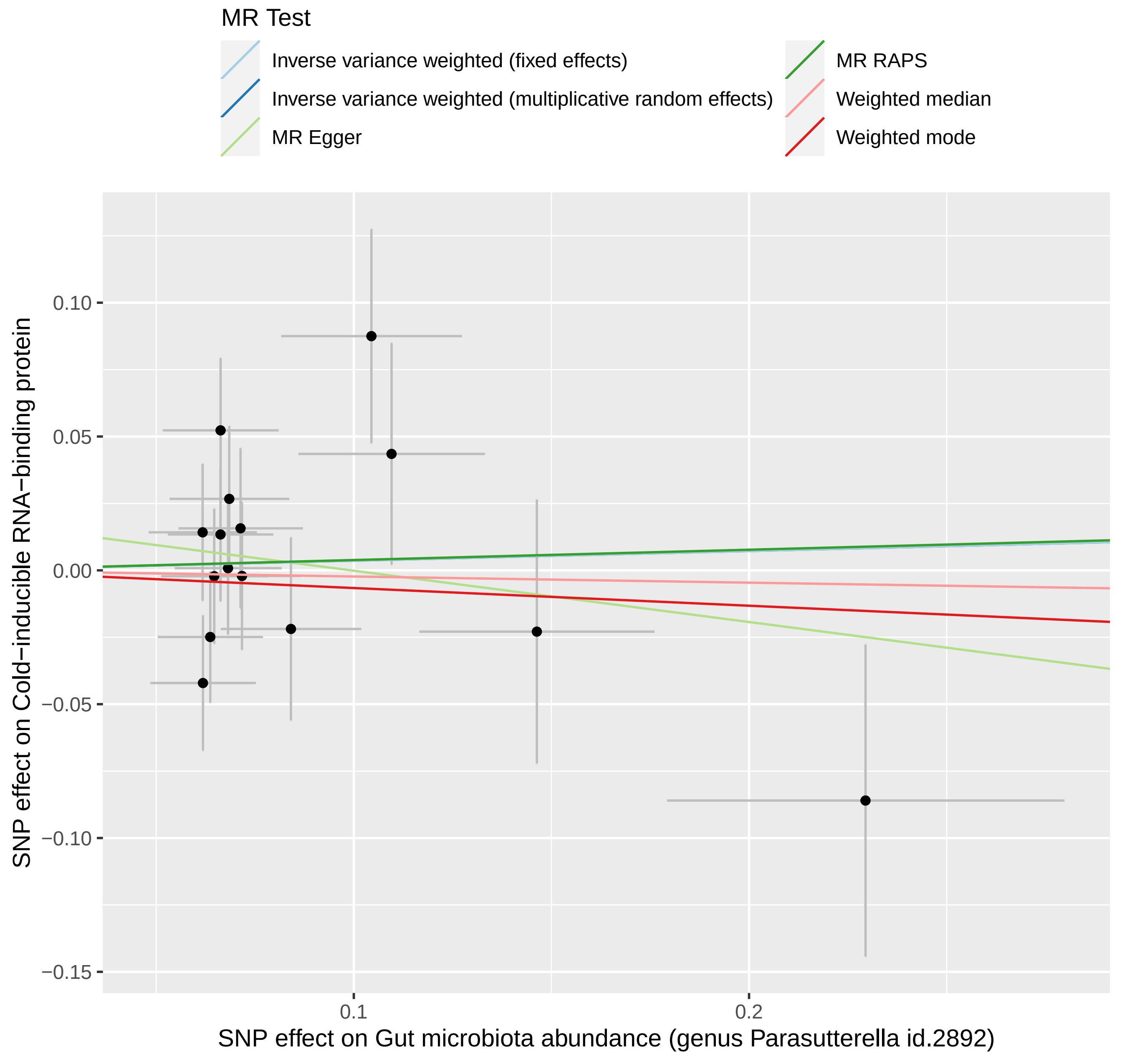

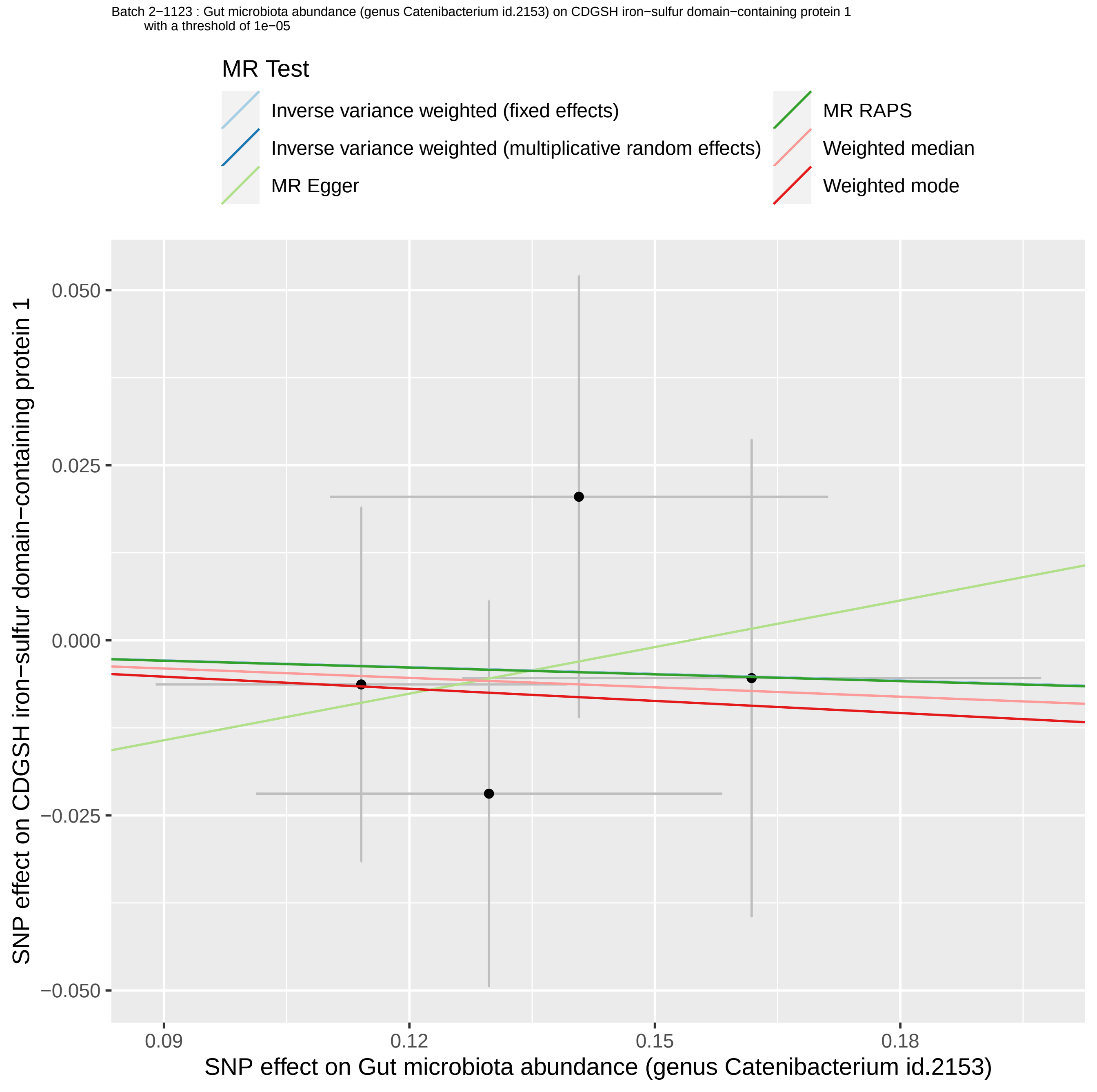

Batch 2−1123 : Gut microbiota abundance (genus Catenibacterium id.2153) on CDGSH iron−sulfur domain−containing protein 1
with a threshold of 1e−05
MR Test
Inverse variance weighted (fixed effects)
Inverse variance weighted (multiplicative random effects)
MR Egger
MR RAPS
Weighted median
Weighted mode
0.050
0.025
0.000
−0.025
−0.050
0.09
0.12
0.15
0.18
SNP effect on CDGSH iron−sulfur domain−containing protein 1
SNP effect on Gut microbiota abundance (genus Catenibacterium id.2153)

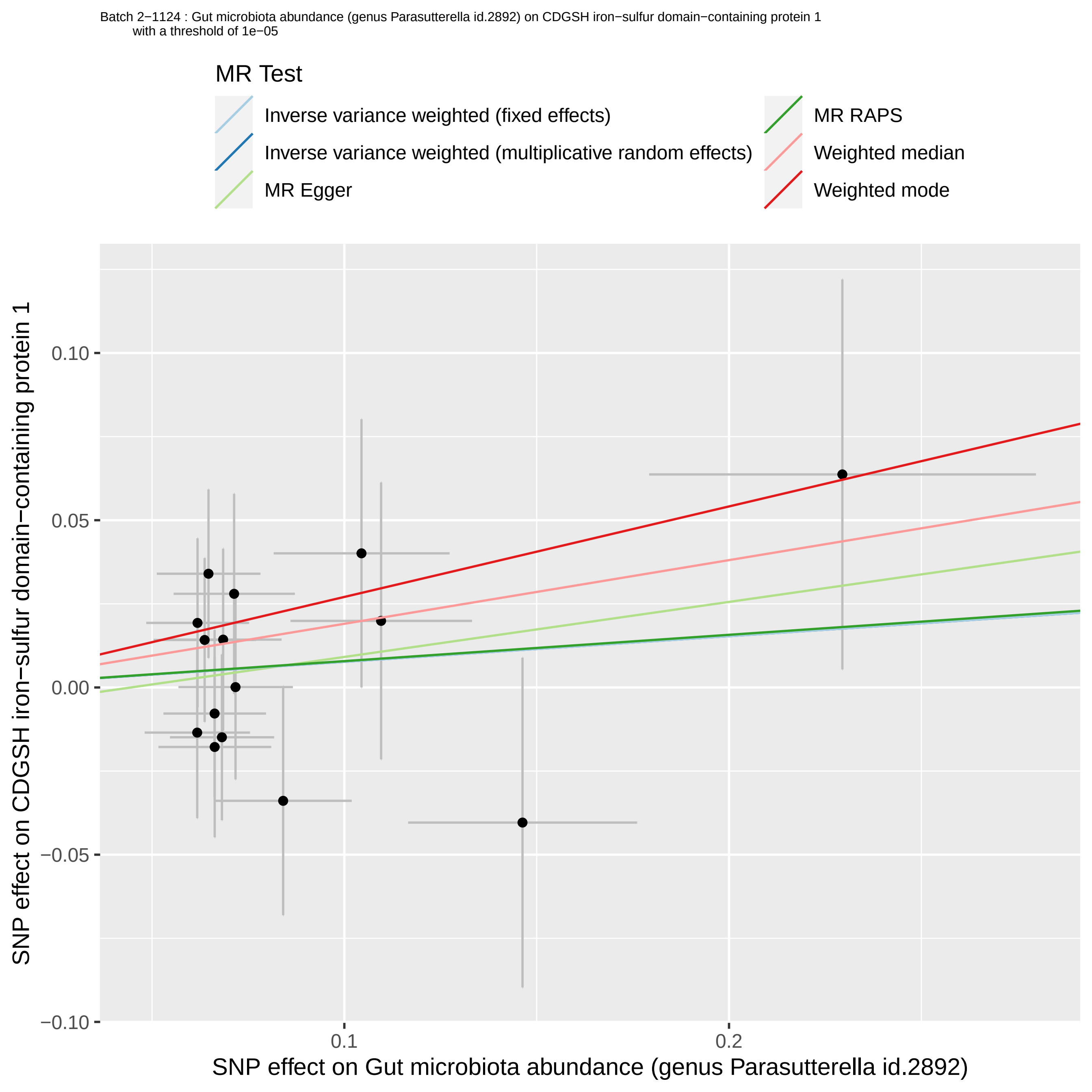
Batch 2−1124 : Gut microbiota abundance (genus Parasutterella id.2892) on CDGSH iron−sulfur domain−containing protein 1
with a threshold of 1e−05
MR Test
Inverse variance weighted (fixed effects)
Inverse variance weighted (multiplicative random effects)
MR Egger
MR RAPS
Weighted median
Weighted mode
0.10
0.05
0.00
−0.05
−0.10
0.1
0.2
SNP effect on CDGSH iron−sulfur domain−containing protein 1
SNP effect on Gut microbiota abundance (genus Parasutterella id.2892)

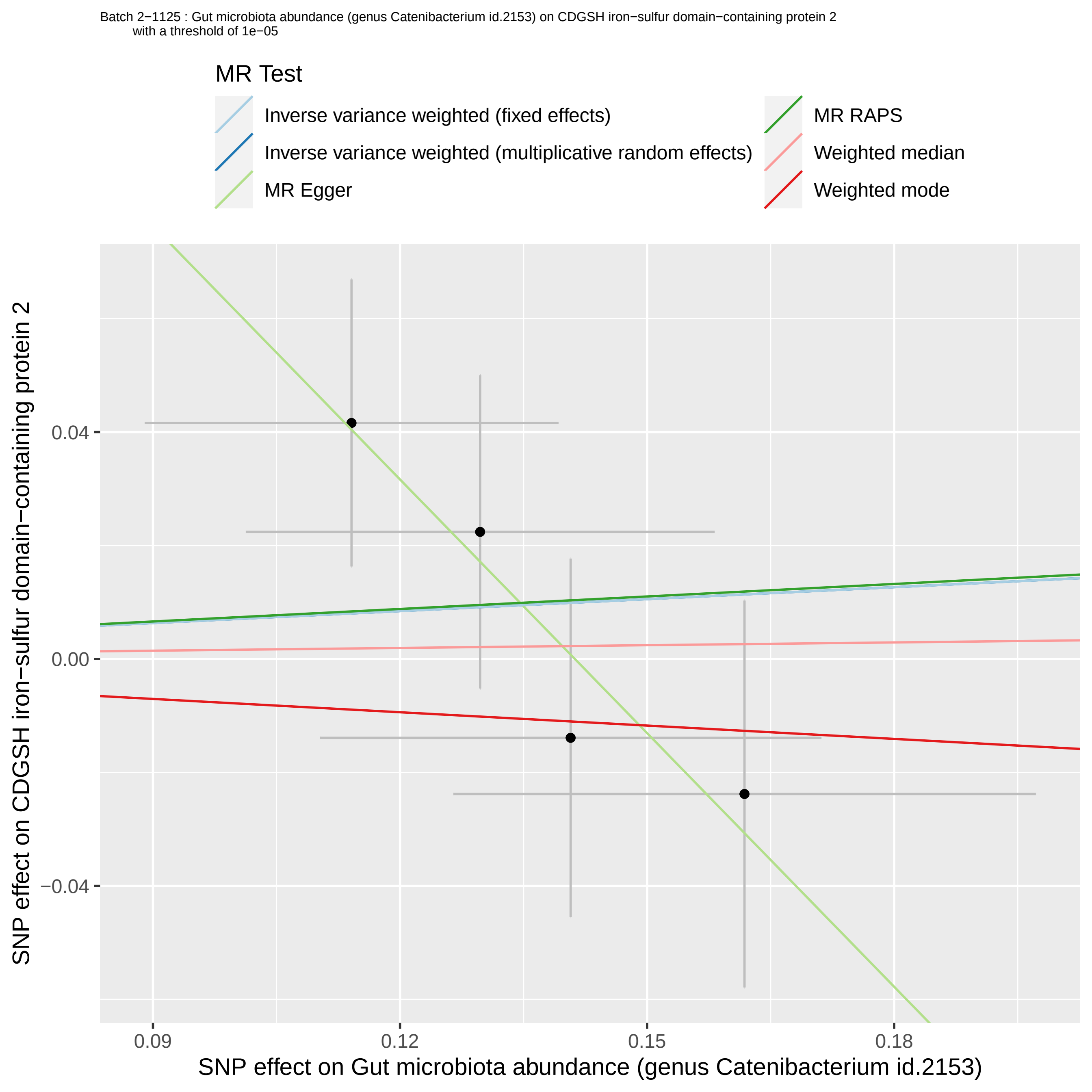

Batch 2−1125 : Gut microbiota abundance (genus Catenibacterium id.2153) on CDGSH iron−sulfur domain−containing protein 2
with a threshold of 1e−05
MR Test
Inverse variance weighted (fixed effects)
Inverse variance weighted (multiplicative random effects)
MR Egger
MR RAPS
Weighted median
Weighted mode
SNP effect on CDGSH iron−sulfur domain−containing protein 2
0.04
0.00
−0.04
0.09
0.12
0.15
0.18
SNP effect on Gut microbiota abundance (genus Catenibacterium id.2153)

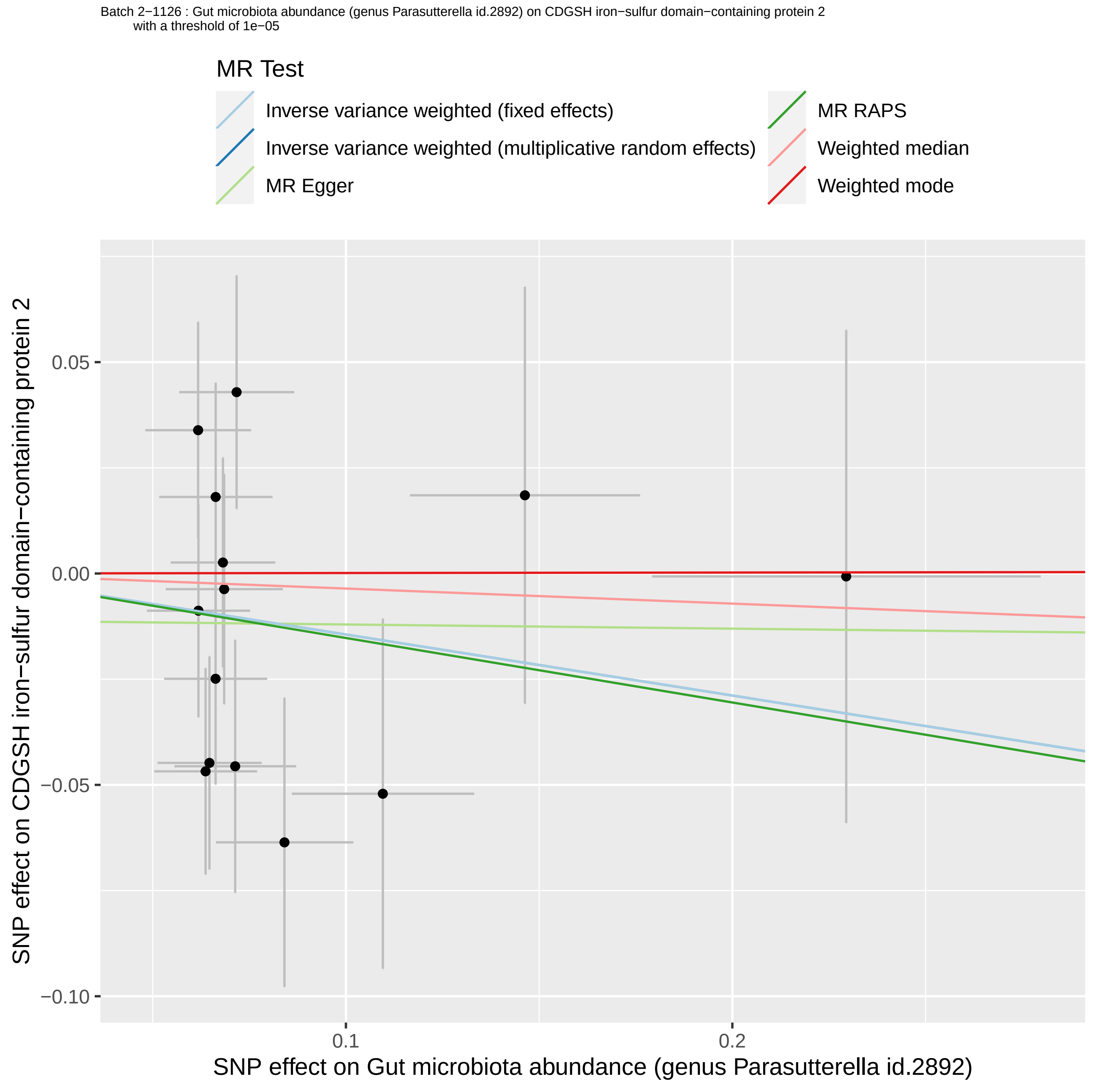

Batch 2−1126 : Gut microbiota abundance (genus Parasutterella id.2892) on CDGSH iron−sulfur domain−containing protein 2
with a threshold of 1e−05
MR Test
Inverse variance weighted (fixed effects)
Inverse variance weighted (multiplicative random effects)
MR Egger
MR RAPS
Weighted median
Weighted mode
0.05
0.00
−0.05
−0.10
0.1
0.2
SNP effect on CDGSH iron−sulfur domain−containing protein 2
SNP effect on Gut microbiota abundance (genus Parasutterella id.2892)

Batch 2−1127 : Gut microbiota abundance (genus Catenibacterium id.2153) on Cytoskeleton−associated protein 2
with a threshold of 1e−05

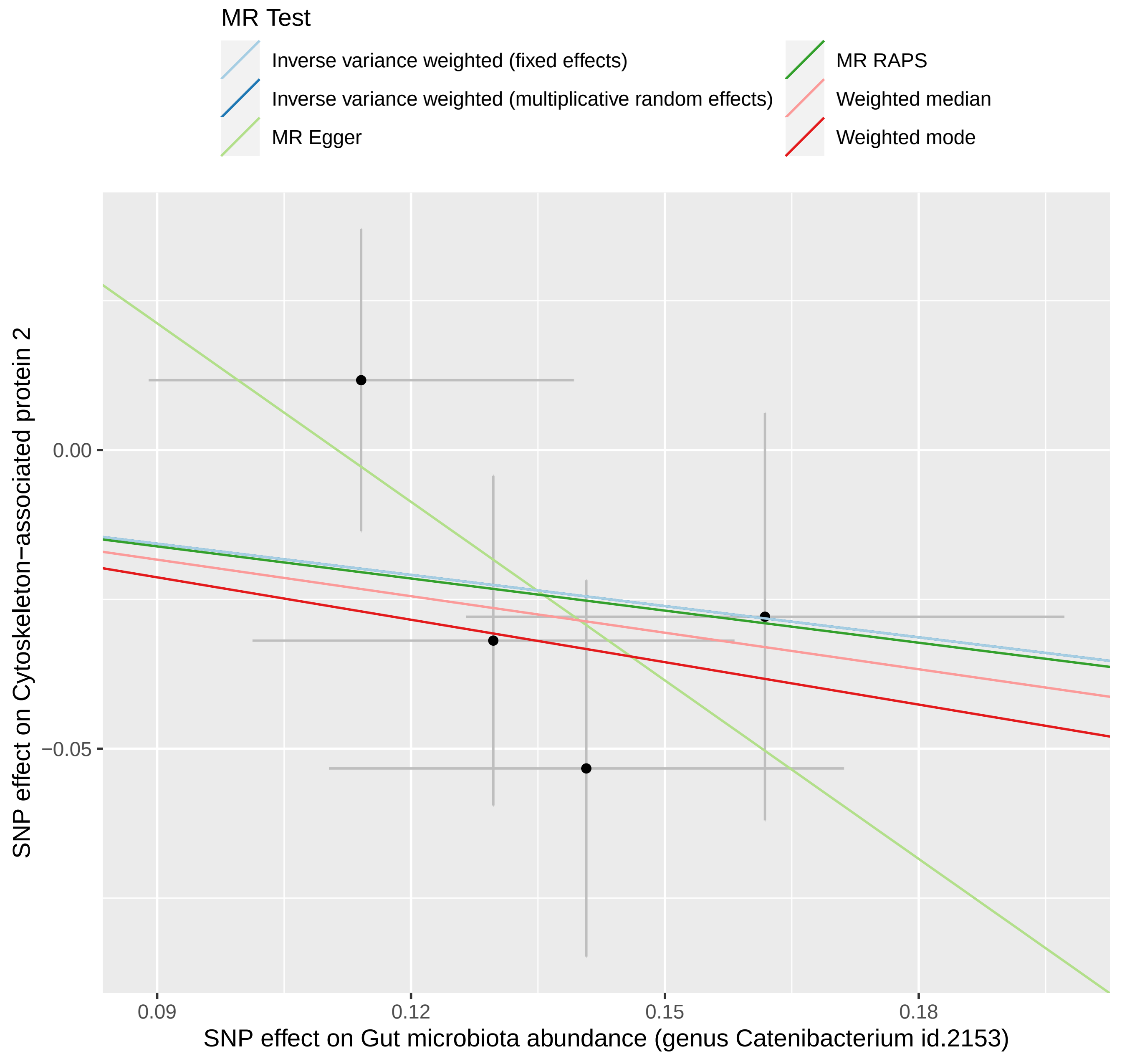

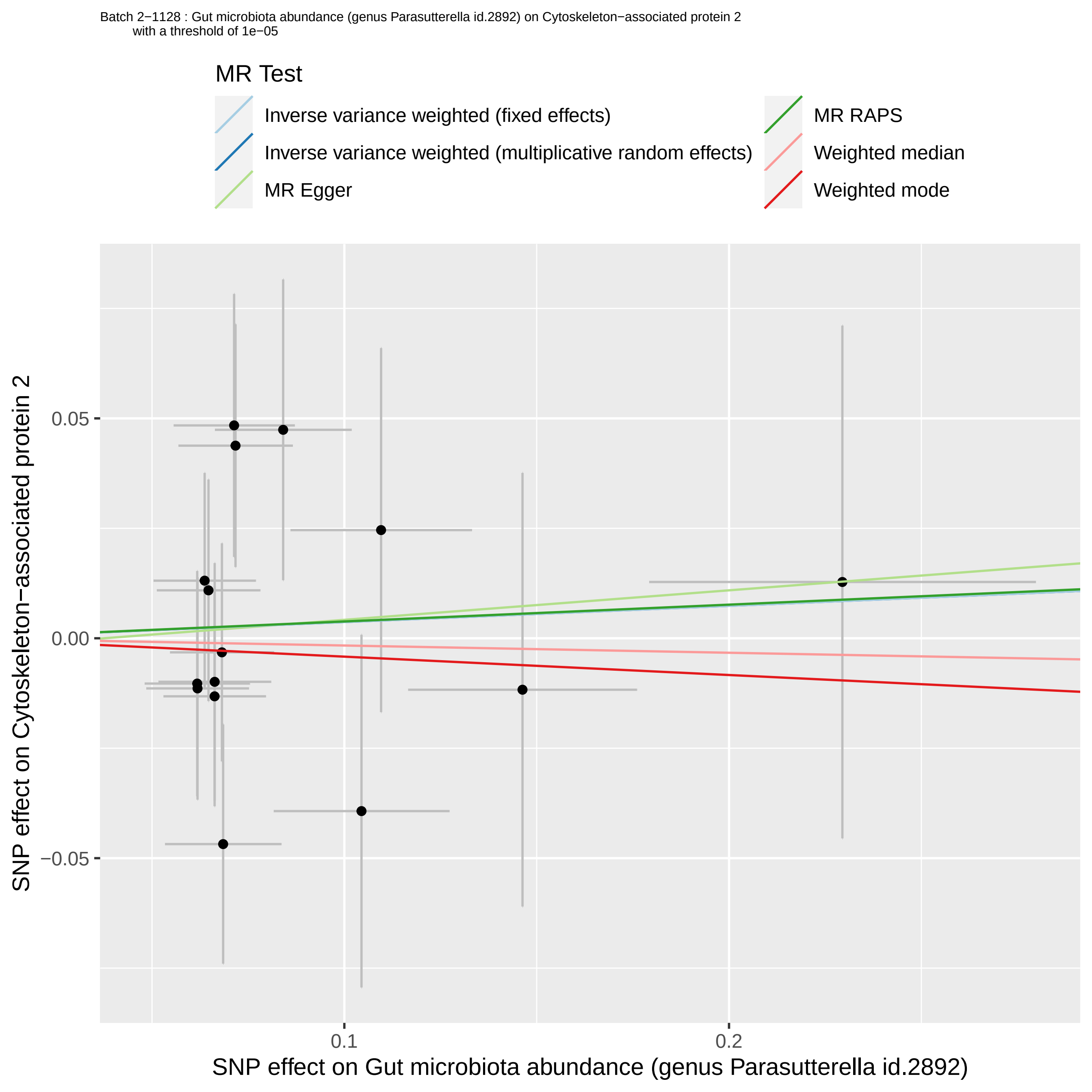

Batch 2−1128 : Gut microbiota abundance (genus Parasutterella id.2892) on Cytoskeleton−associated protein 2
with a threshold of 1e−05
MR Test
Inverse variance weighted (fixed effects)
Inverse variance weighted (multiplicative random effects)
MR Egger
MR RAPS
Weighted median
Weighted mode
SNP effect on Cytoskeleton−associated protein 2
0.05
0.00
−0.05
0.1
0.2
SNP effect on Gut microbiota abundance (genus Parasutterella id.2892)

Batch 2−1129 : Gut microbiota abundance (genus Catenibacterium id.2153) on Creatine kinase B−type
with a threshold of 1e−05

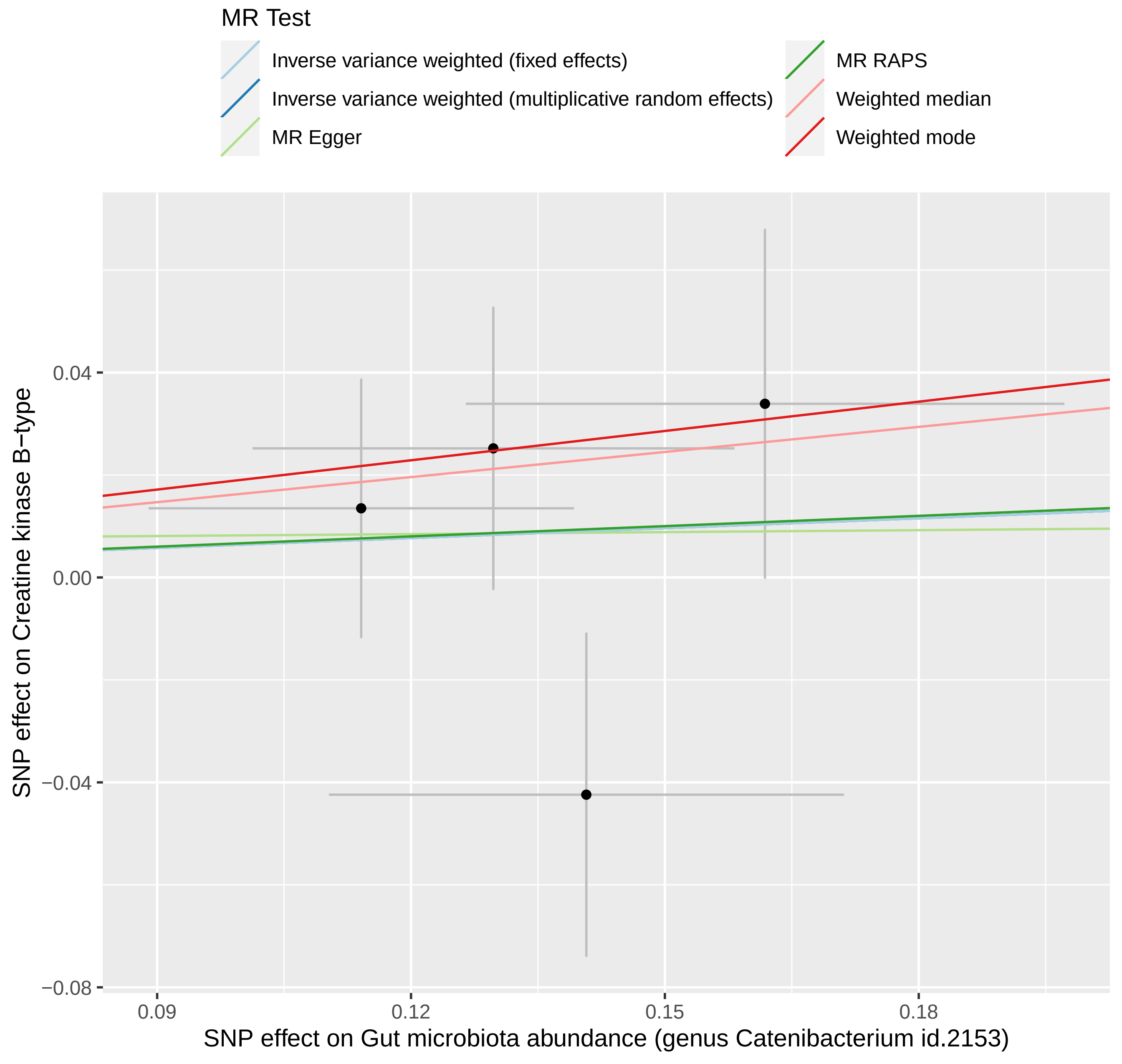

Batch 2−1130 : Gut microbiota abundance (genus Parasutterella id.2892) on Creatine kinase B−type
with a threshold of 1e−05

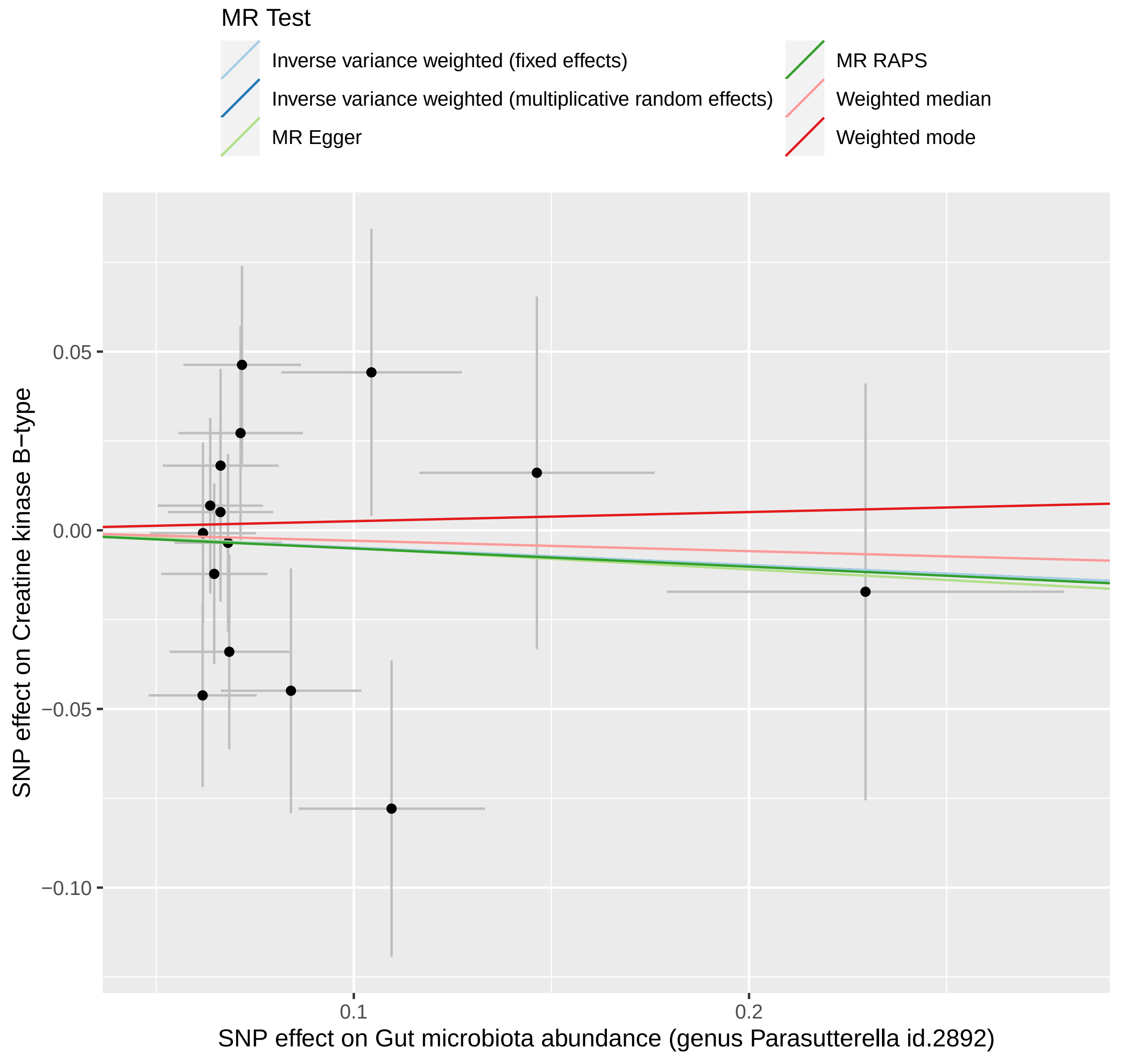

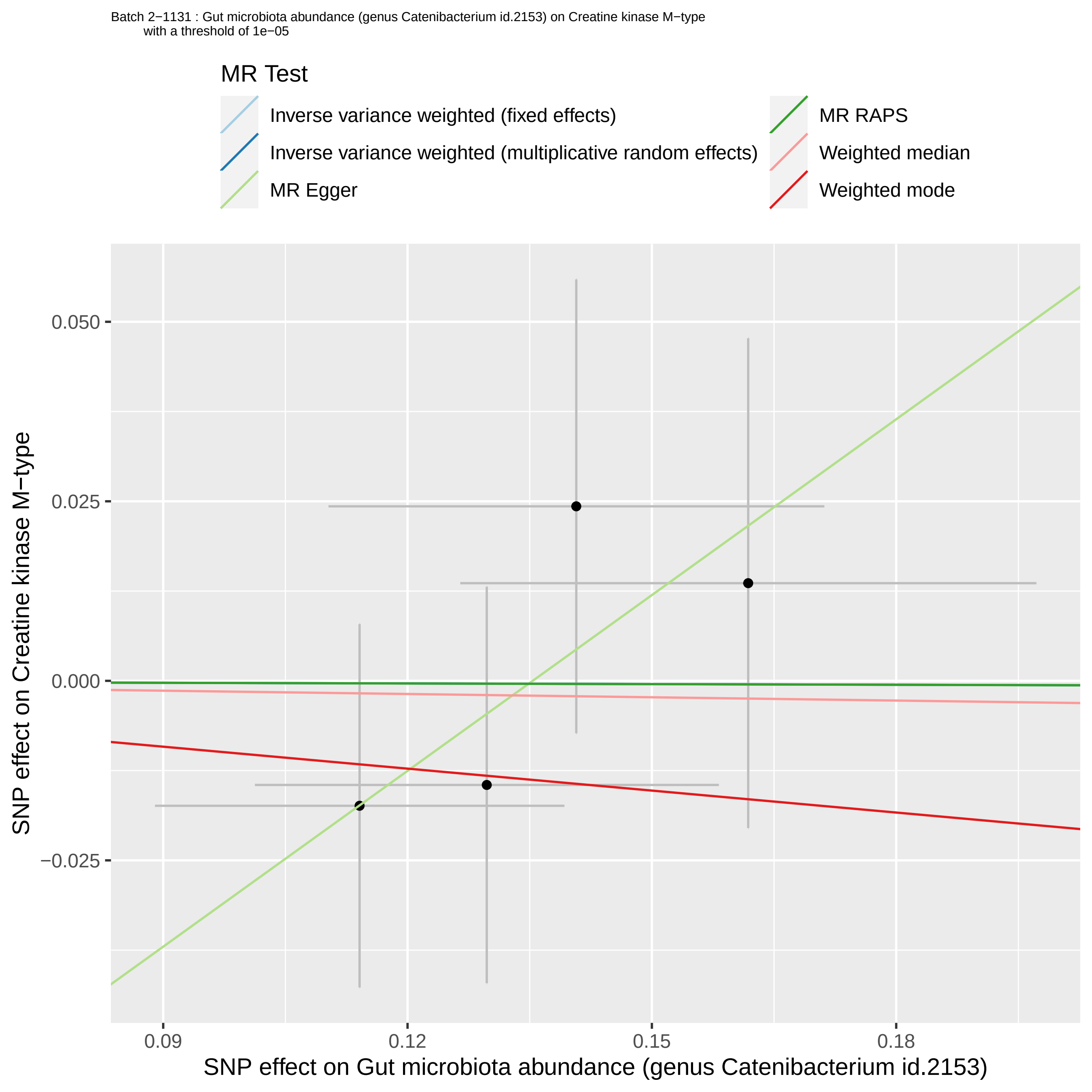

Batch 2−1131 : Gut microbiota abundance (genus Catenibacterium id.2153) on Creatine kinase M−type
with a threshold of 1e−05
MR Test
Inverse variance weighted (fixed effects)
Inverse variance weighted (multiplicative random effects)
MR Egger
MR RAPS
Weighted median
Weighted mode
0.050
0.025
0.000
−0.025
0.09
0.12
0.15
0.18
SNP effect on Creatine kinase M−type
SNP effect on Gut microbiota abundance (genus Catenibacterium id.2153)

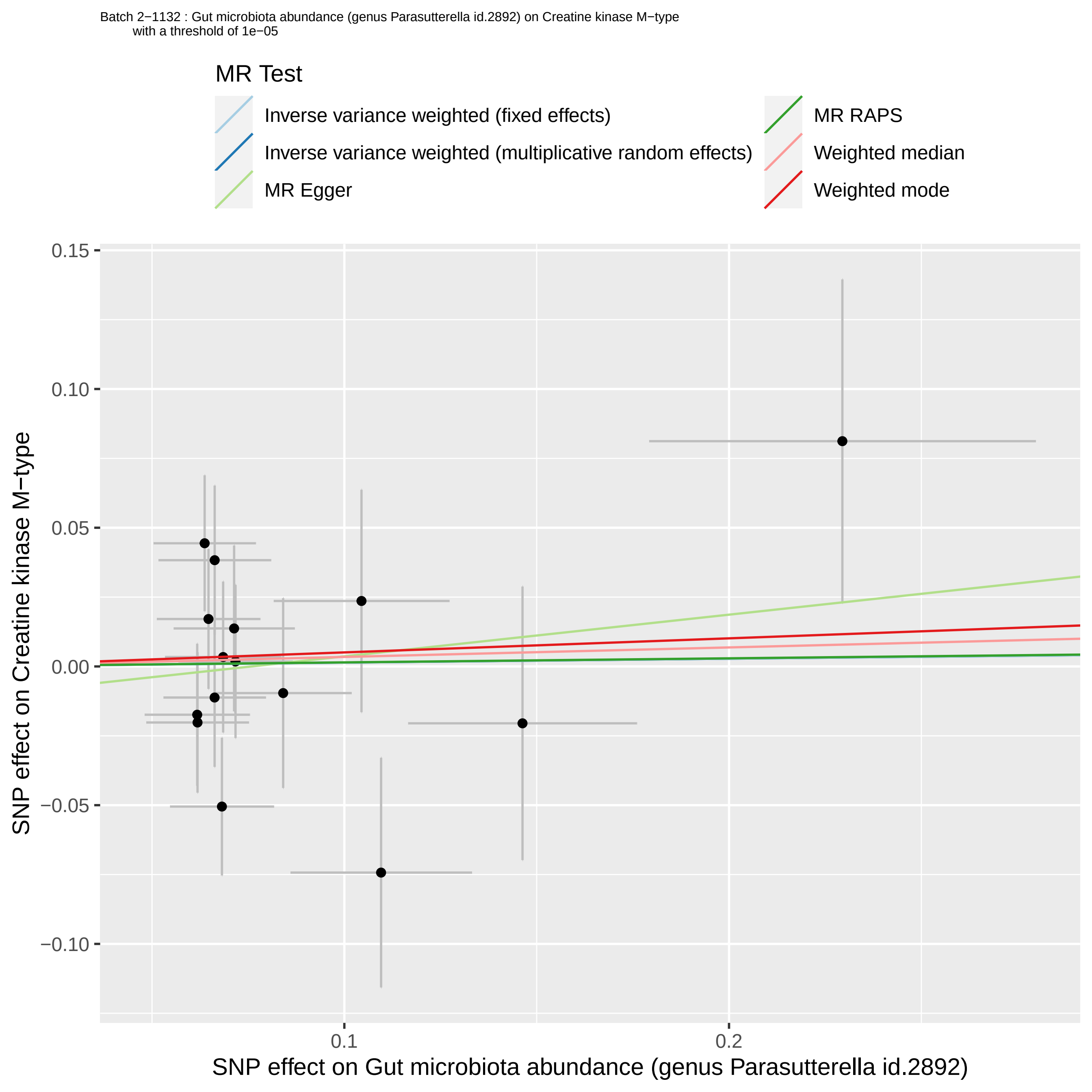
Batch 2−1132 : Gut microbiota abundance (genus Parasutterella id.2892) on Creatine kinase M−type
with a threshold of 1e−05
MR Test
Inverse variance weighted (fixed effects)
Inverse variance weighted (multiplicative random effects)
MR Egger
MR RAPS
Weighted median
Weighted mode
0.15
0.10
0.05
0.00
−0.05
−0.10
0.1
0.2
SNP effect on Creatine kinase M−type
SNP effect on Gut microbiota abundance (genus Parasutterella id.2892)

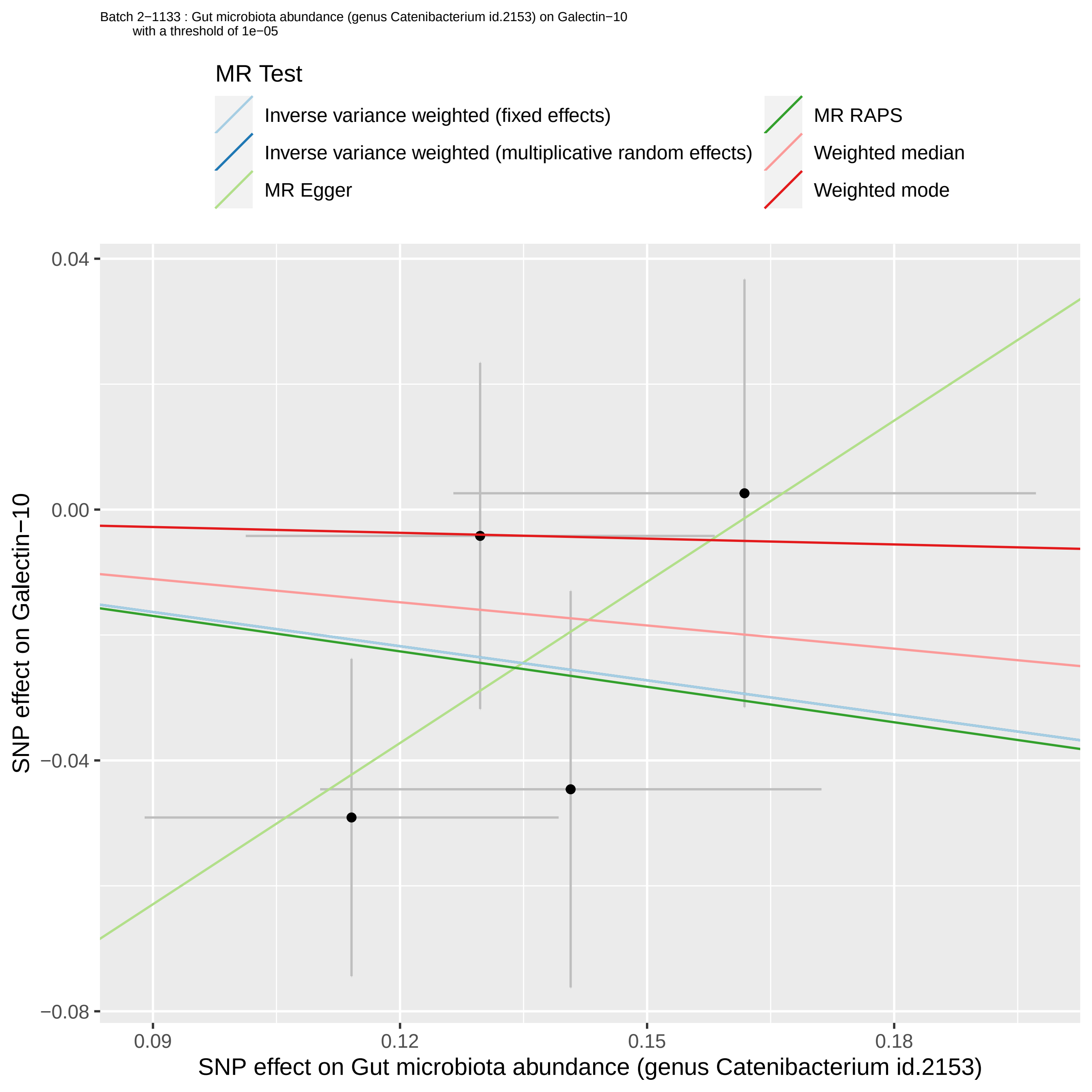

Batch 2−1133 : Gut microbiota abundance (genus Catenibacterium id.2153) on Galectin−10
with a threshold of 1e−05
MR Test
Inverse variance weighted (fixed effects)
Inverse variance weighted (multiplicative random effects)
MR Egger
MR RAPS
Weighted median
Weighted mode
0.04
0.00
−0.04
−0.08
0.09
0.12
0.15
0.18
SNP effect on Galectin−10
SNP effect on Gut microbiota abundance (genus Catenibacterium id.2153)

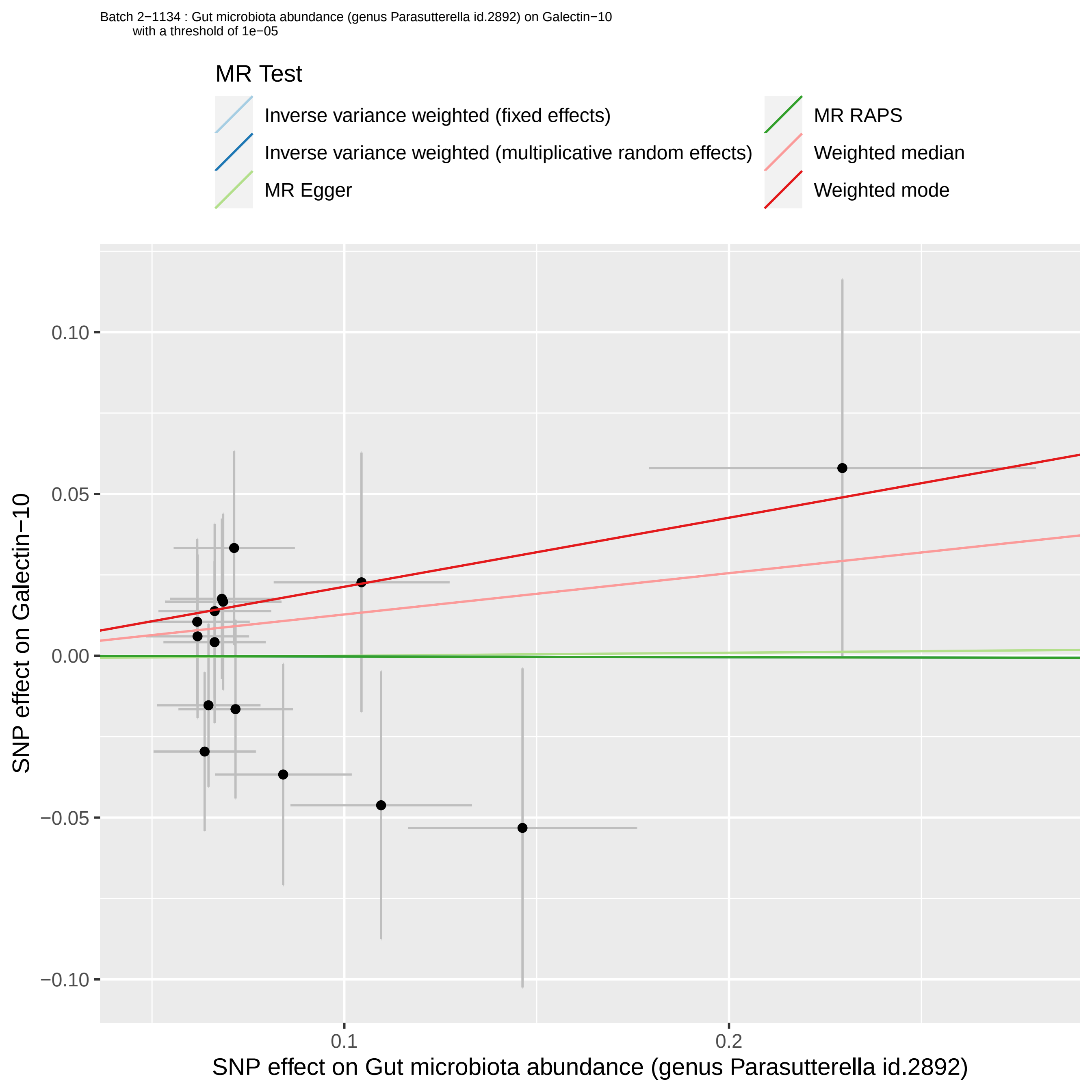
Batch 2−1134 : Gut microbiota abundance (genus Parasutterella id.2892) on Galectin−10
with a threshold of 1e−05
MR Test
Inverse variance weighted (fixed effects)
Inverse variance weighted (multiplicative random effects)
MR Egger
MR RAPS
Weighted median
Weighted mode
0.10
0.05
0.00
−0.05
−0.10
0.1
0.2
SNP effect on Galectin−10
SNP effect on Gut microbiota abundance (genus Parasutterella id.2892)

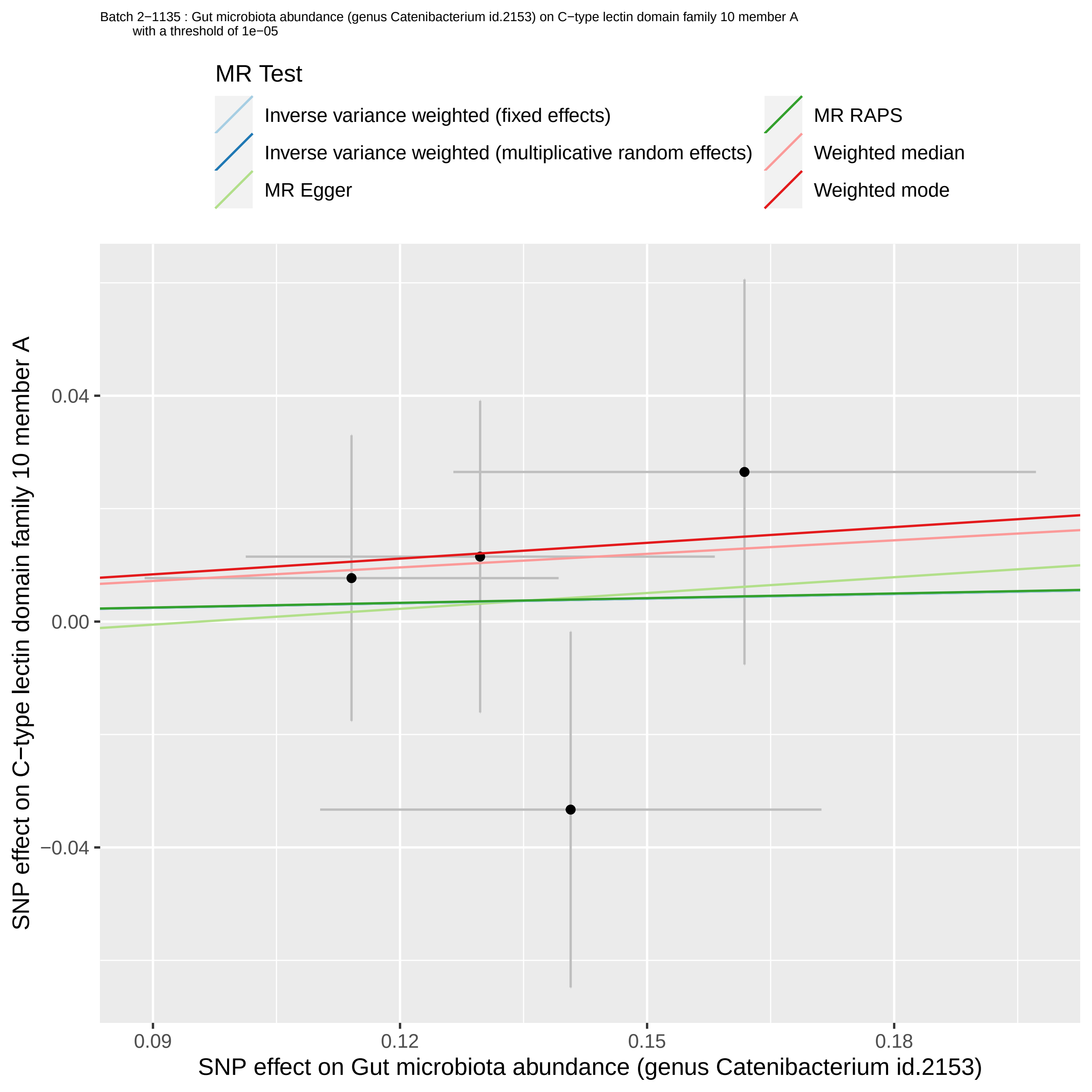

Batch 2−1135 : Gut microbiota abundance (genus Catenibacterium id.2153) on C−type lectin domain family 10 member A
with a threshold of 1e−05
MR Test
Inverse variance weighted (fixed effects)
Inverse variance weighted (multiplicative random effects)
MR Egger
MR RAPS
Weighted median
Weighted mode
SNP effect on C−type lectin domain family 10 member A
0.04
0.00
−0.04
0.09
0.12
0.15
0.18
SNP effect on Gut microbiota abundance (genus Catenibacterium id.2153)

Batch 2−1136 : Gut microbiota abundance (genus Parasutterella id.2892) on C−type lectin domain family 10 member A
with a threshold of 1e−05

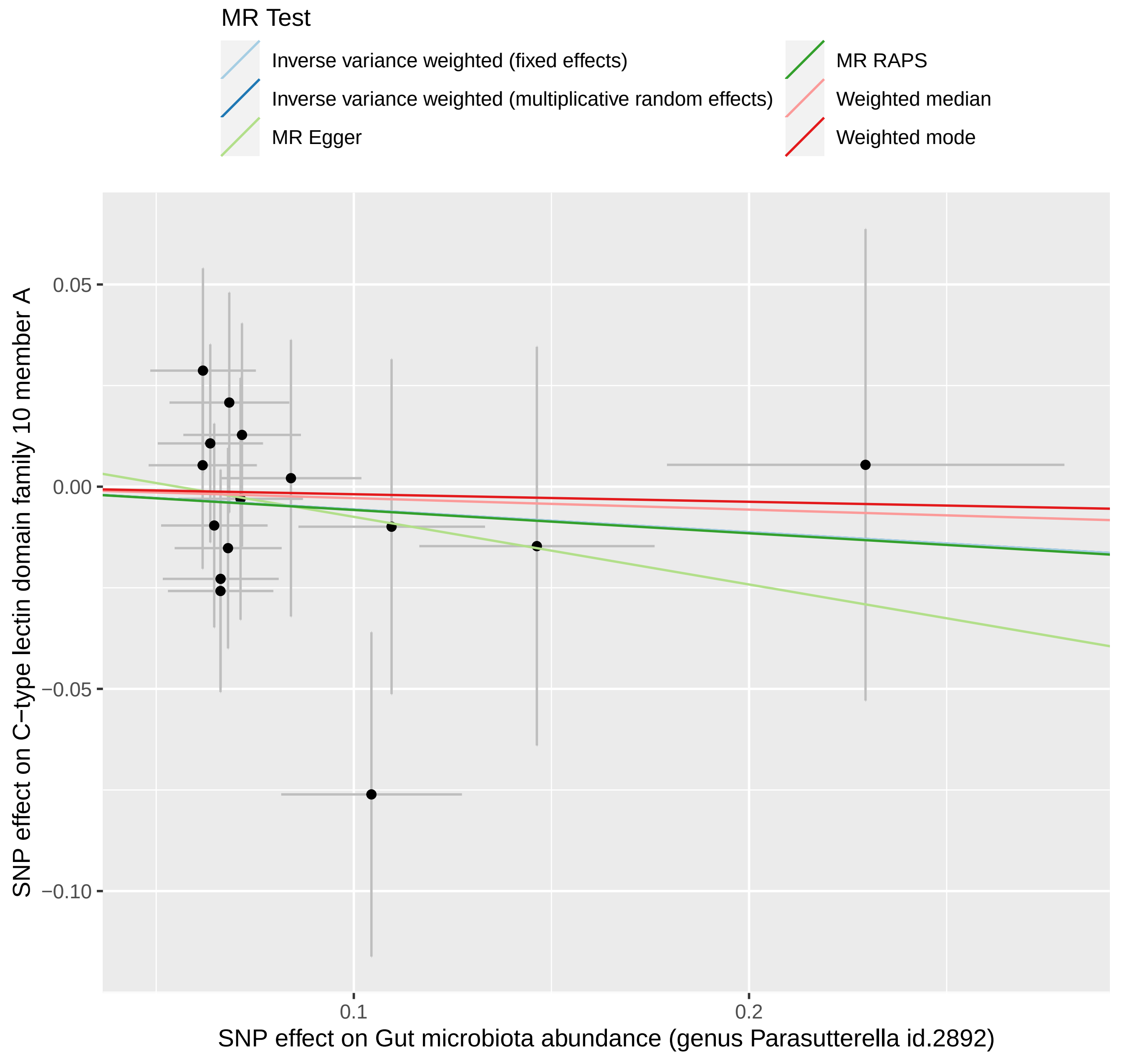

Batch 2−1137 : Gut microbiota abundance (genus Catenibacterium id.2153) on Stem Cell Growth Factor−beta
with a threshold of 1e−05

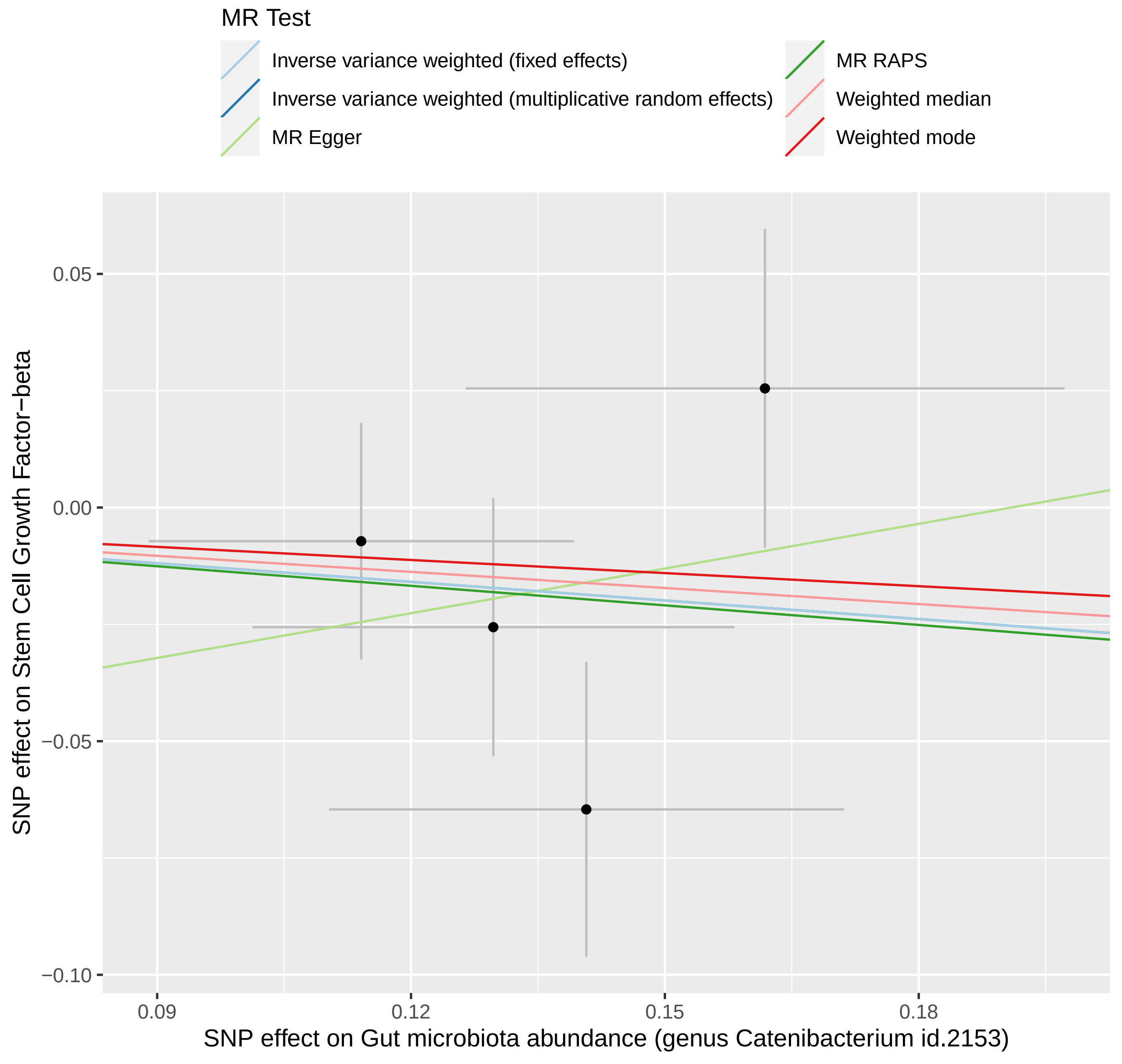

Batch 2−1138 : Gut microbiota abundance (genus Parasutterella id.2892) on Stem Cell Growth Factor−beta
with a threshold of 1e−05

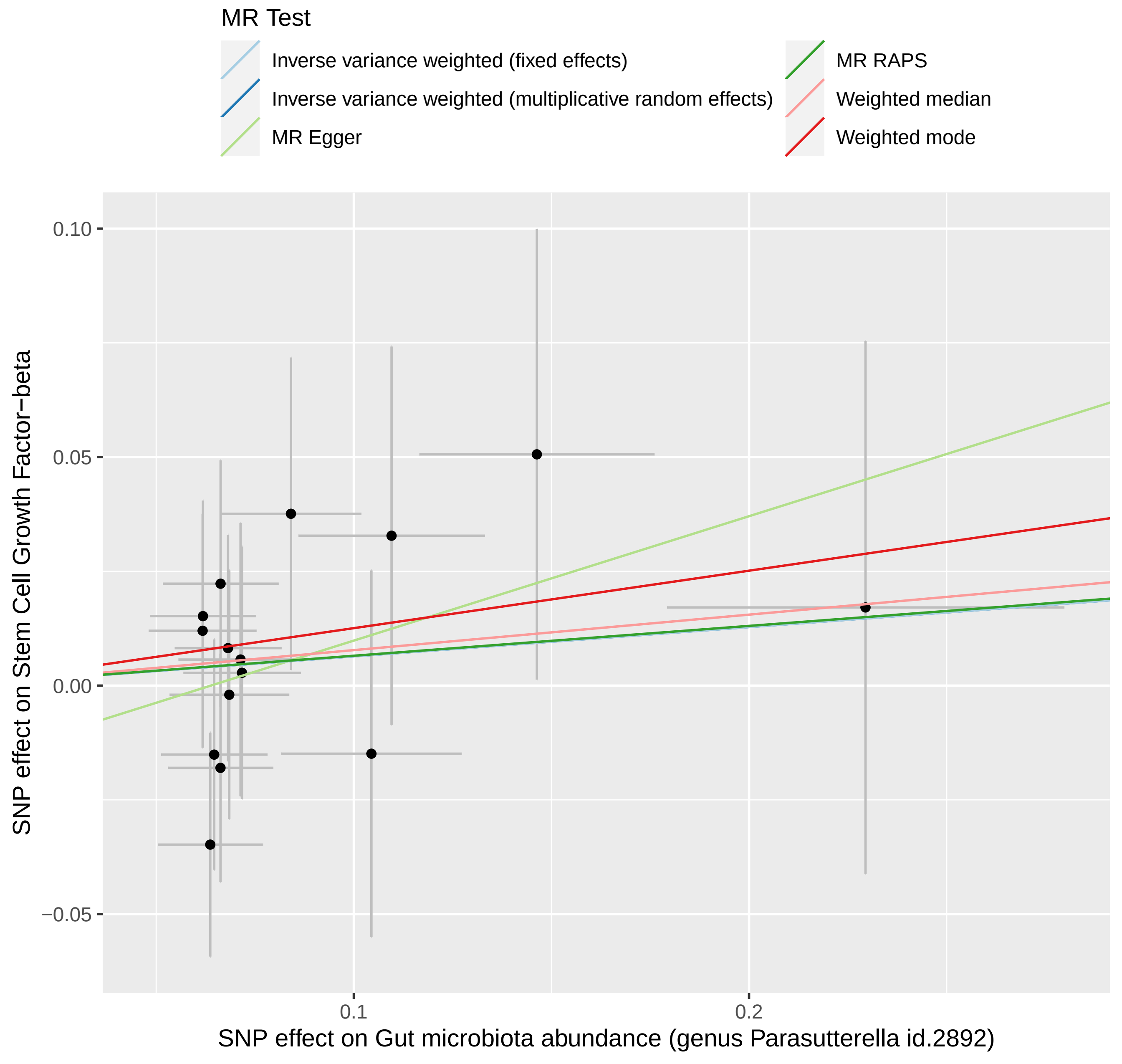

Batch 2−1139 : Gut microbiota abundance (genus Catenibacterium id.2153) on C−type lectin domain family 12 member A with a threshold of 1e−05

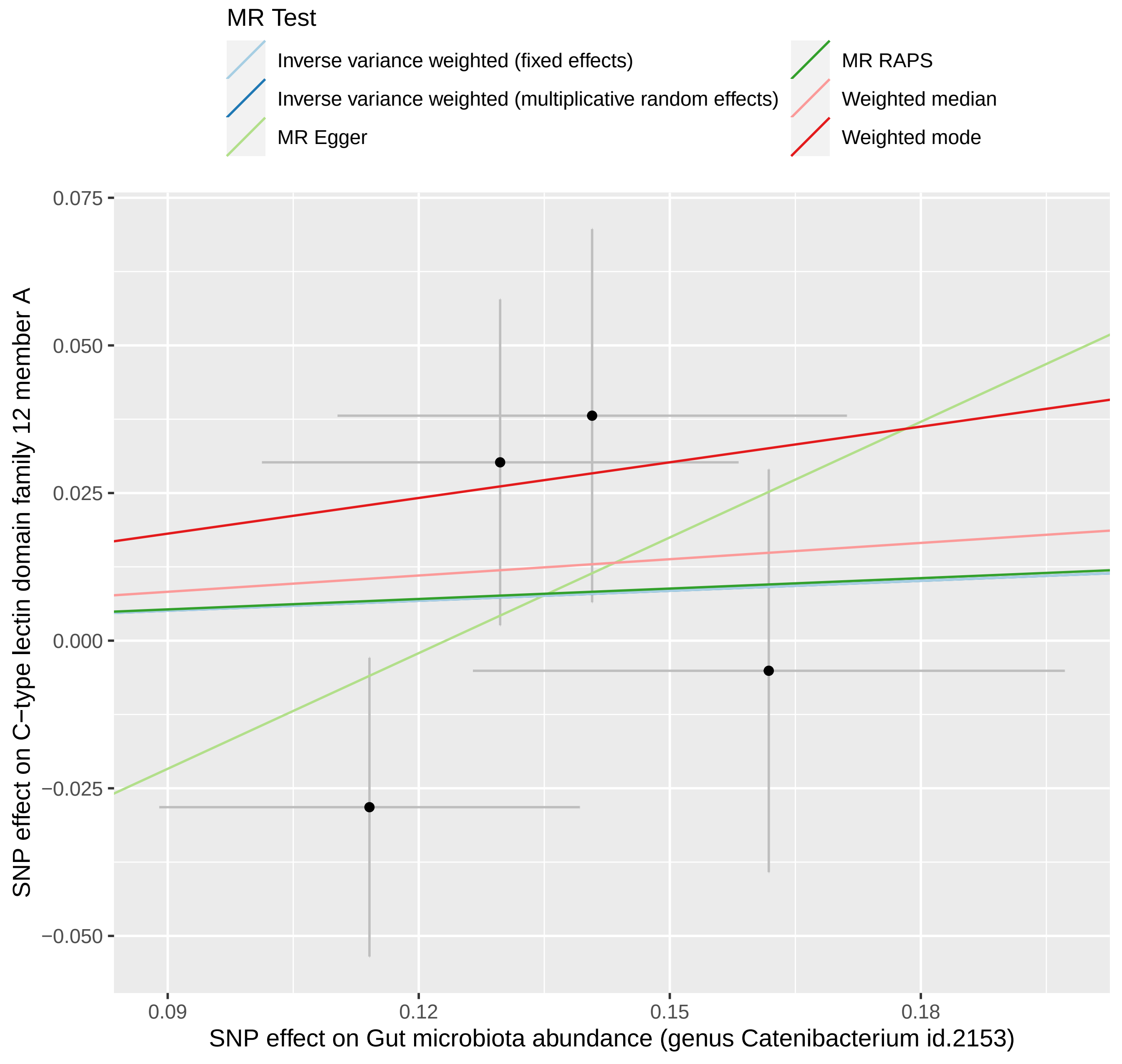

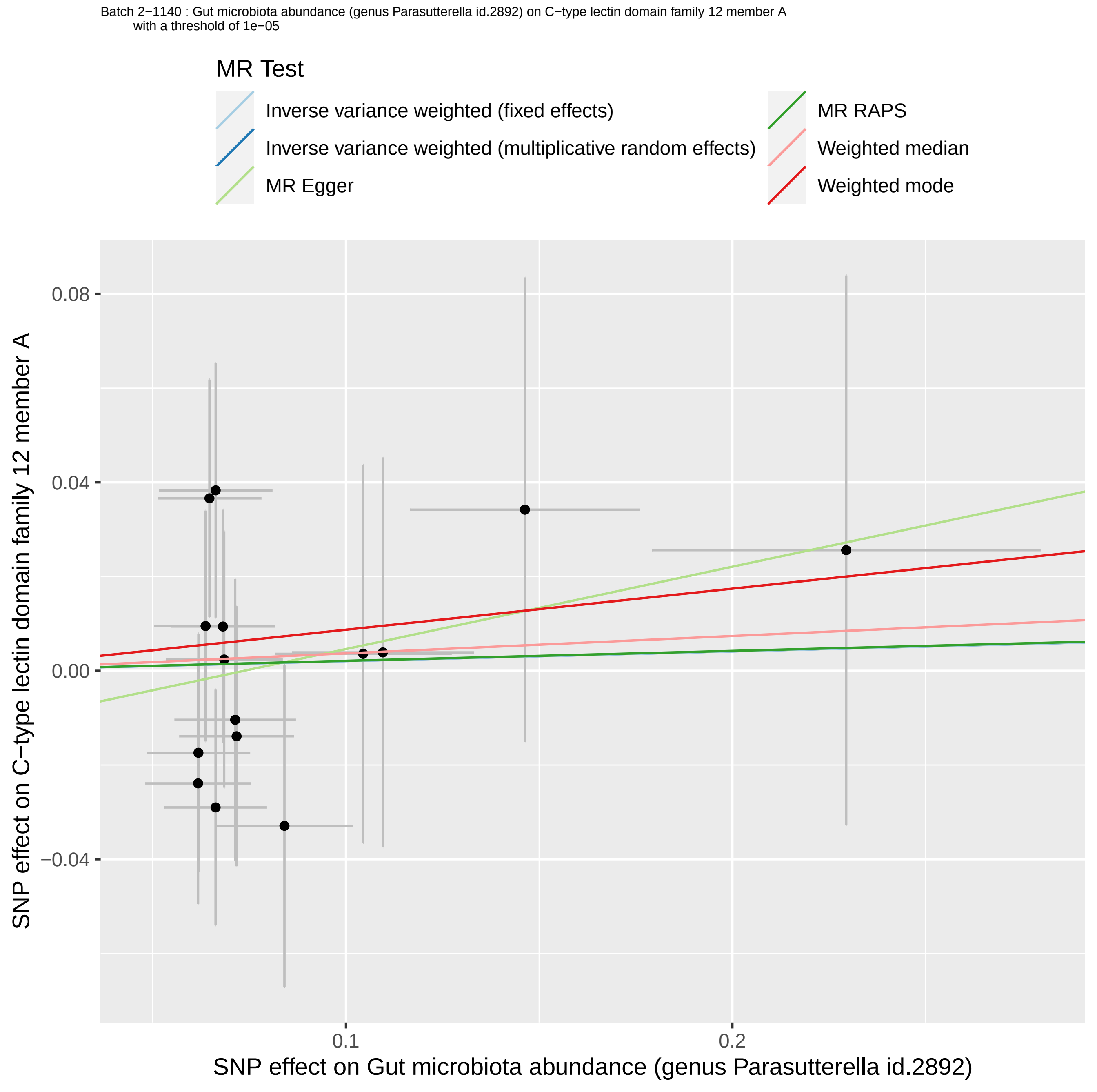

Batch 2−1140 : Gut microbiota abundance (genus Parasutterella id.2892) on C−type lectin domain family 12 member A
with a threshold of 1e−05
MR Test
Inverse variance weighted (fixed effects)
Inverse variance weighted (multiplicative random effects)
MR Egger
MR RAPS
Weighted median
Weighted mode
0.08
0.04
0.00
−0.04
0.1
0.2
SNP effect on C−type lectin domain family 12 member A
SNP effect on Gut microbiota abundance (genus Parasutterella id.2892)

Batch 2−1141 : Gut microbiota abundance (genus Catenibacterium id.2153) on C−type lectin domain family 14 member A with a threshold of 1e−05

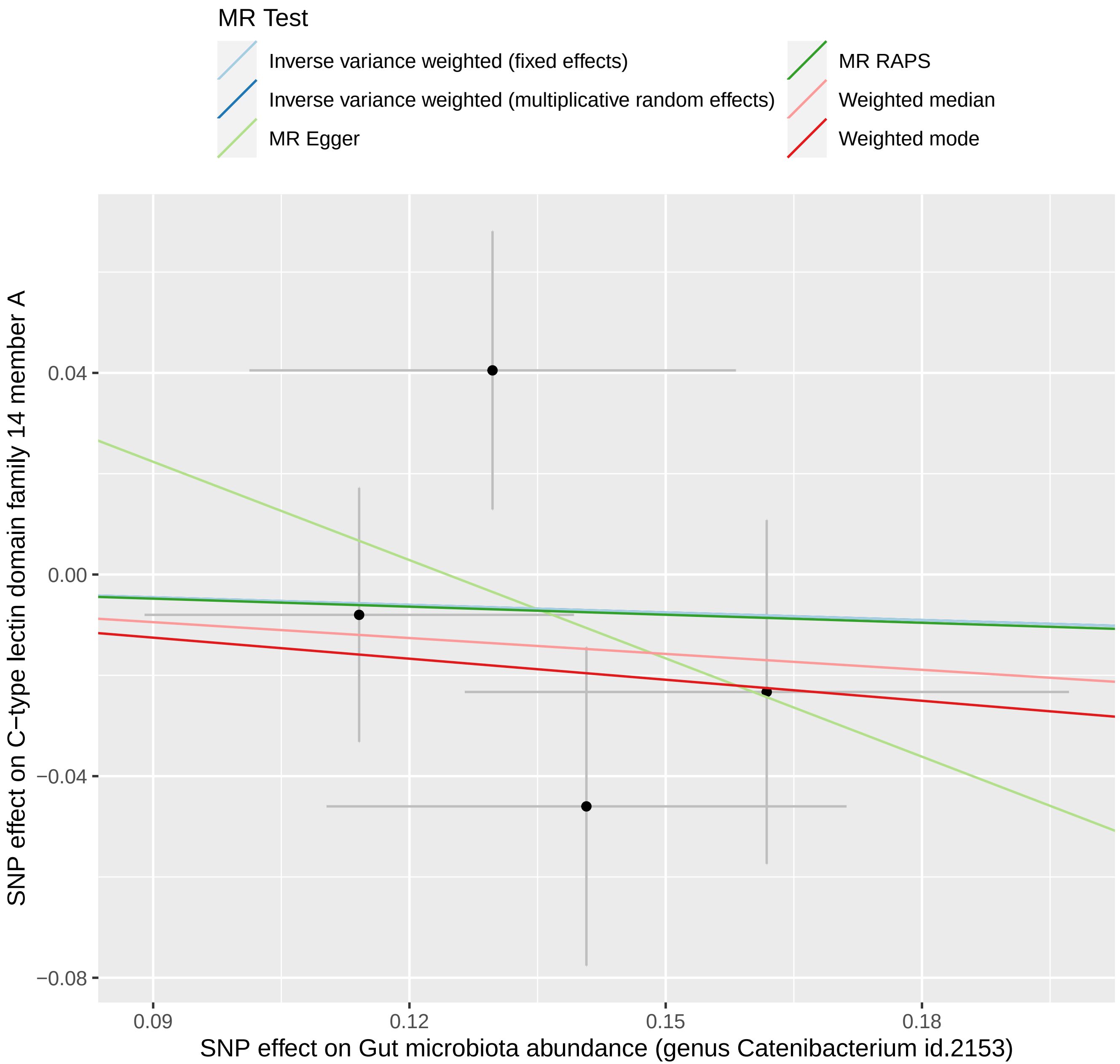

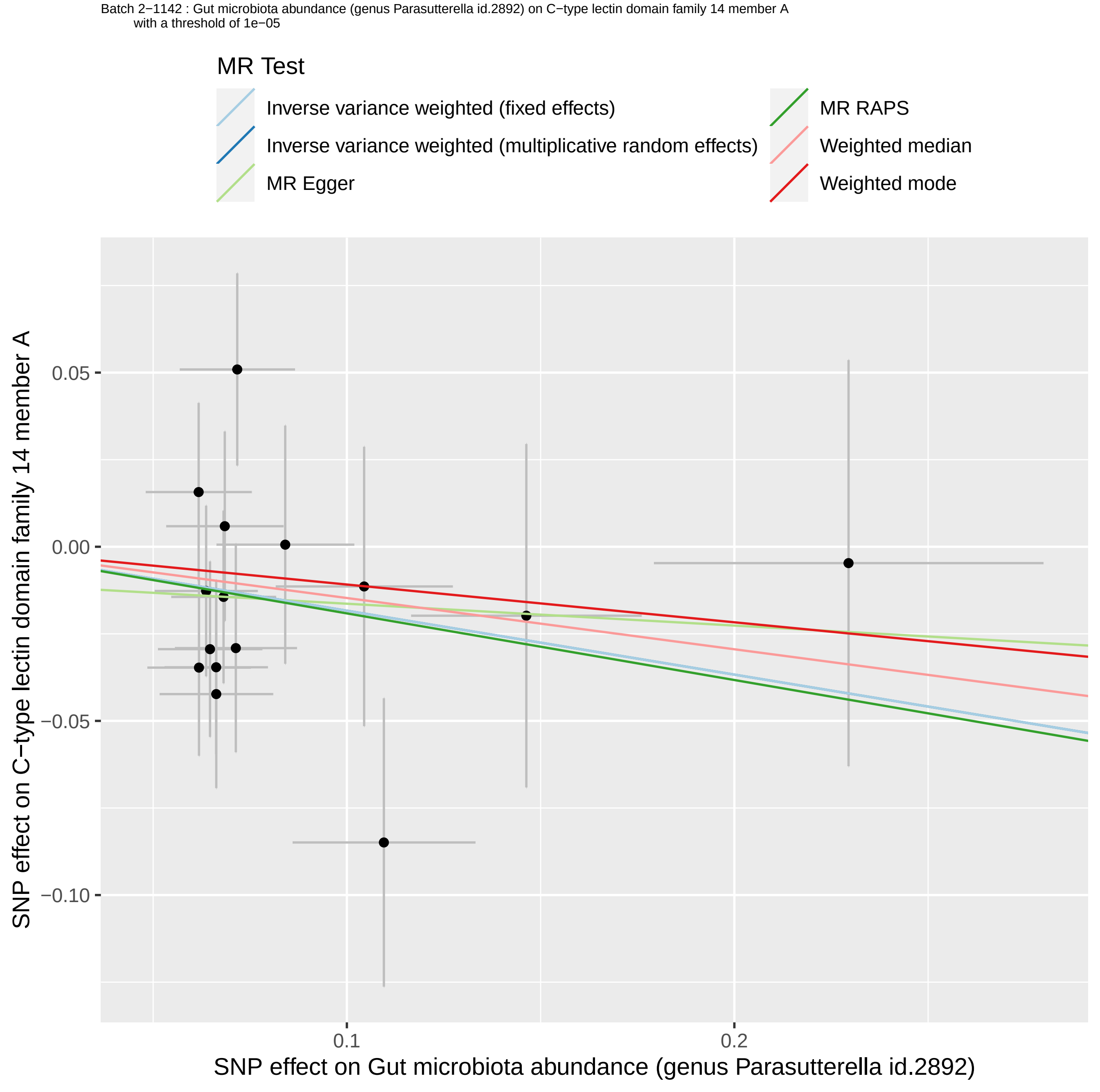
Batch 2−1142 : Gut microbiota abundance (genus Parasutterella id.2892) on C−type lectin domain family 14 member A
with a threshold of 1e−05
MR Test
Inverse variance weighted (fixed effects)
Inverse variance weighted (multiplicative random effects)
MR Egger
MR RAPS
Weighted median
Weighted mode
0.05
0.00
−0.05
−0.10
0.1
0.2
SNP effect on C−type lectin domain family 14 member A
SNP effect on Gut microbiota abundance (genus Parasutterella id.2892)

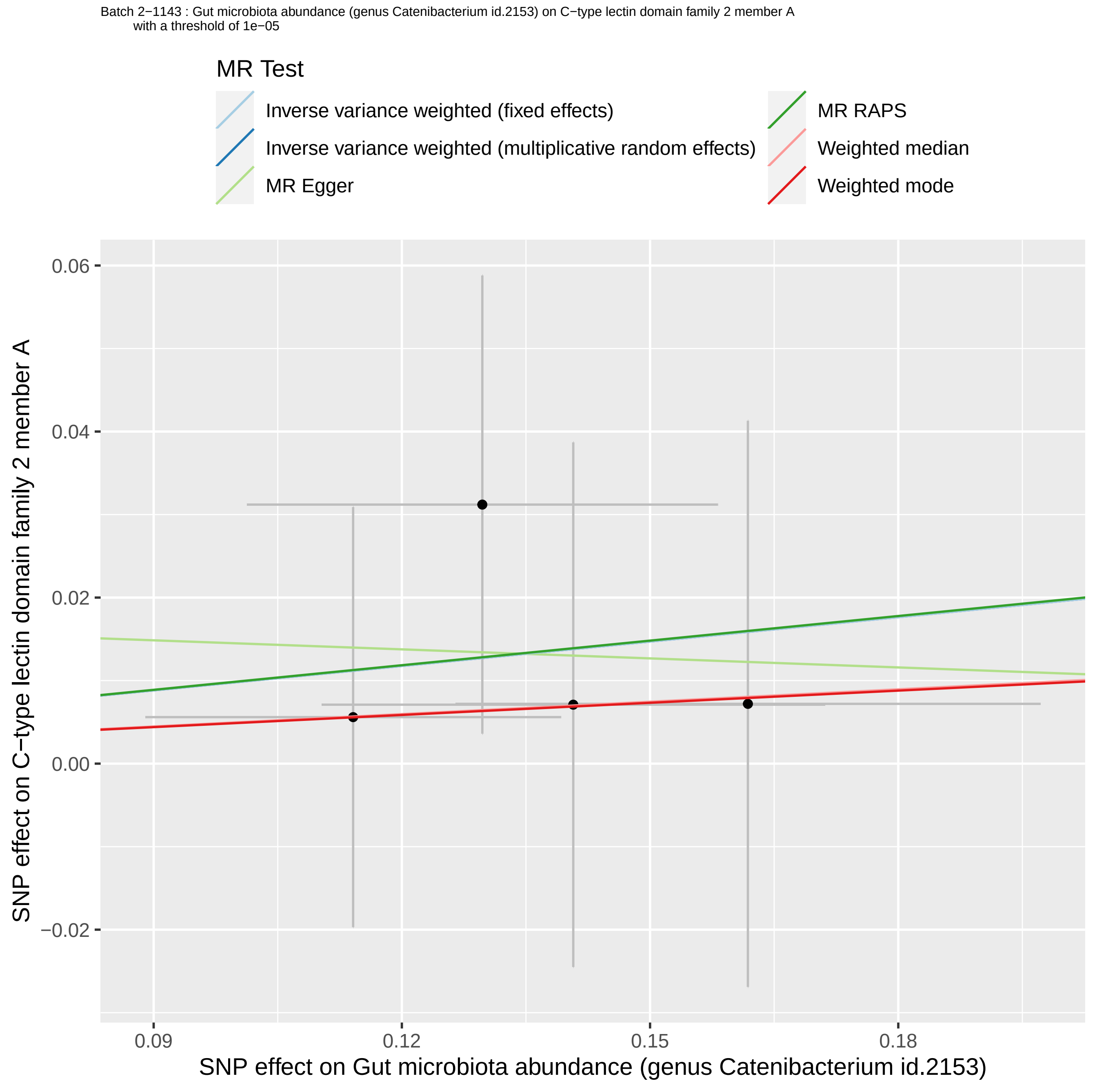
Batch 2−1143 : Gut microbiota abundance (genus Catenibacterium id.2153) on C−type lectin domain family 2 member A
with a threshold of 1e−05
MR Test
Inverse variance weighted (fixed effects)
Inverse variance weighted (multiplicative random effects)
MR Egger
MR RAPS
Weighted median
Weighted mode
SNP effect on C−type lectin domain family 2 member A
0.06
0.04
0.02
0.00
−0.02
0.09
0.12
0.15
0.18
SNP effect on Gut microbiota abundance (genus Catenibacterium id.2153)

Batch 2−1144 : Gut microbiota abundance (genus Parasutterella id.2892) on C−type lectin domain family 2 member A
with a threshold of 1e−05

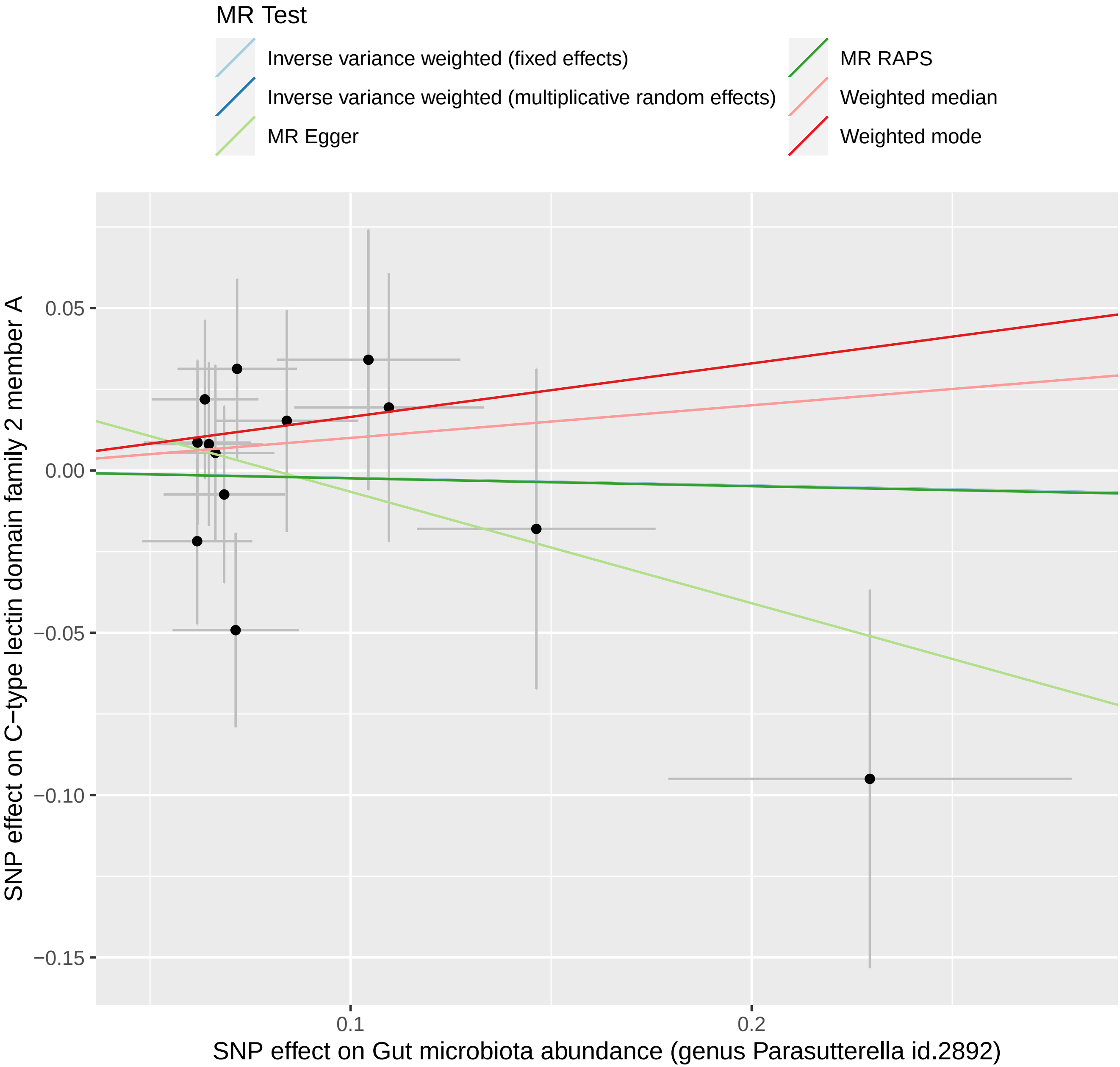

Batch 2−1145 : Gut microbiota abundance (genus Catenibacterium id.2153) on C−type lectin domain family 2 member B
with a threshold of 1e−05

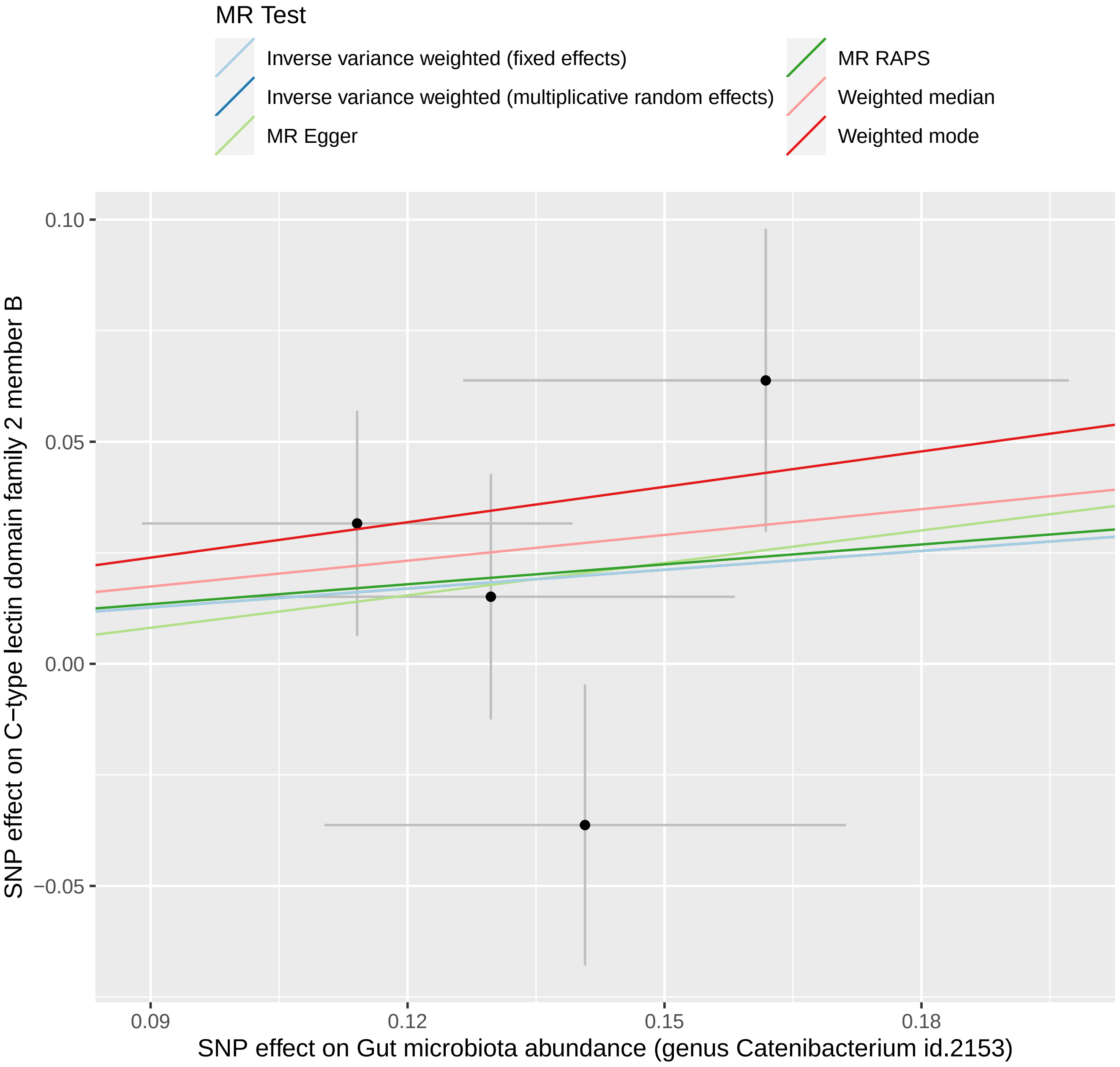

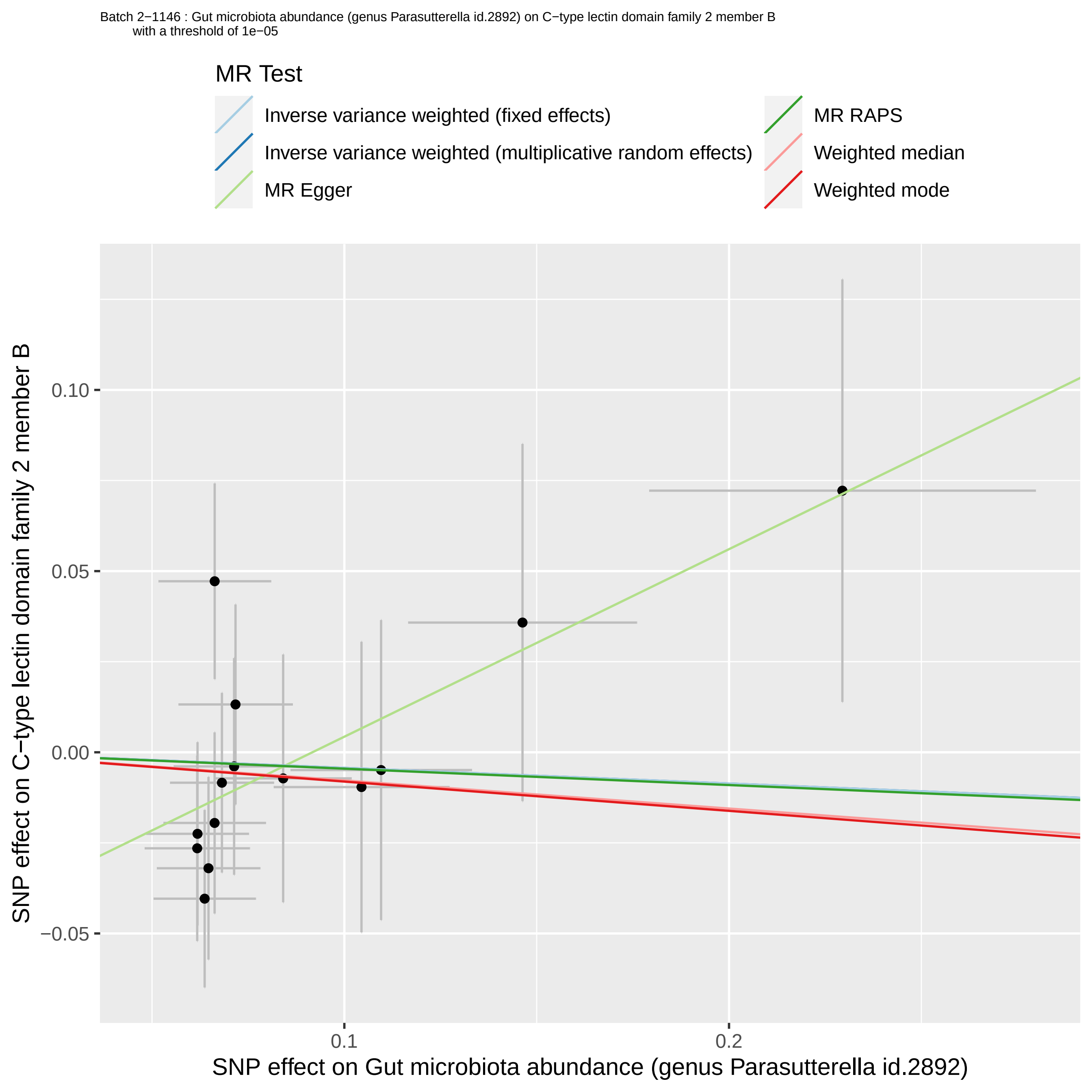
Batch 2−1146 : Gut microbiota abundance (genus Parasutterella id.2892) on C−type lectin domain family 2 member B
with a threshold of 1e−05
MR Test
Inverse variance weighted (fixed effects)
Inverse variance weighted (multiplicative random effects)
MR Egger
MR RAPS
Weighted median
Weighted mode
SNP effect on C−type lectin domain family 2 member B
0.10
0.05
0.00
−0.05
0.1
0.2
SNP effect on Gut microbiota abundance (genus Parasutterella id.2892)

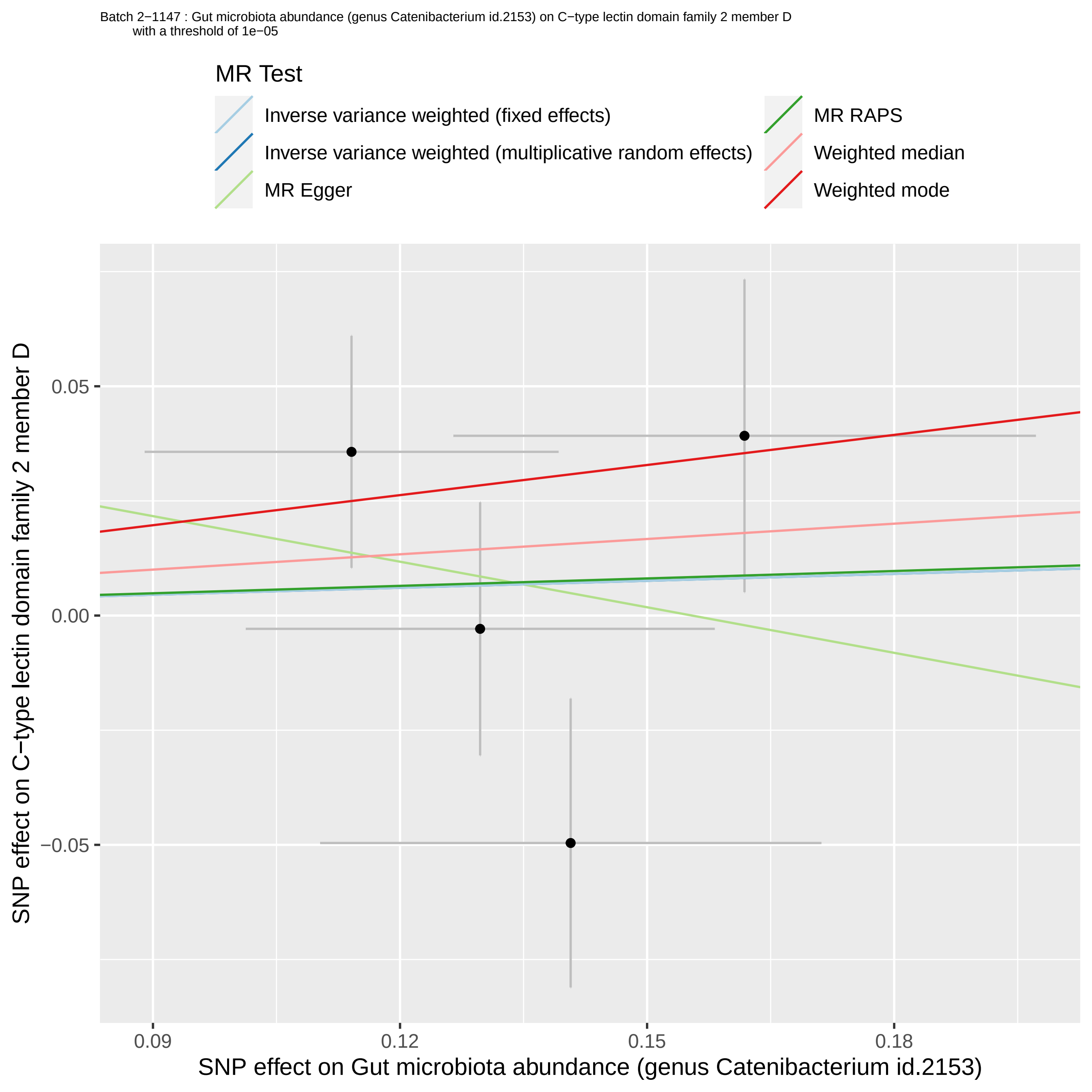

Batch 2−1147 : Gut microbiota abundance (genus Catenibacterium id.2153) on C−type lectin domain family 2 member D
with a threshold of 1e−05
MR Test
Inverse variance weighted (fixed effects)
Inverse variance weighted (multiplicative random effects)
MR Egger
MR RAPS
Weighted median
Weighted mode
0.05
0.00
−0.05
0.09
0.12
0.15
0.18
SNP effect on C−type lectin domain family 2 member D
SNP effect on Gut microbiota abundance (genus Catenibacterium id.2153)

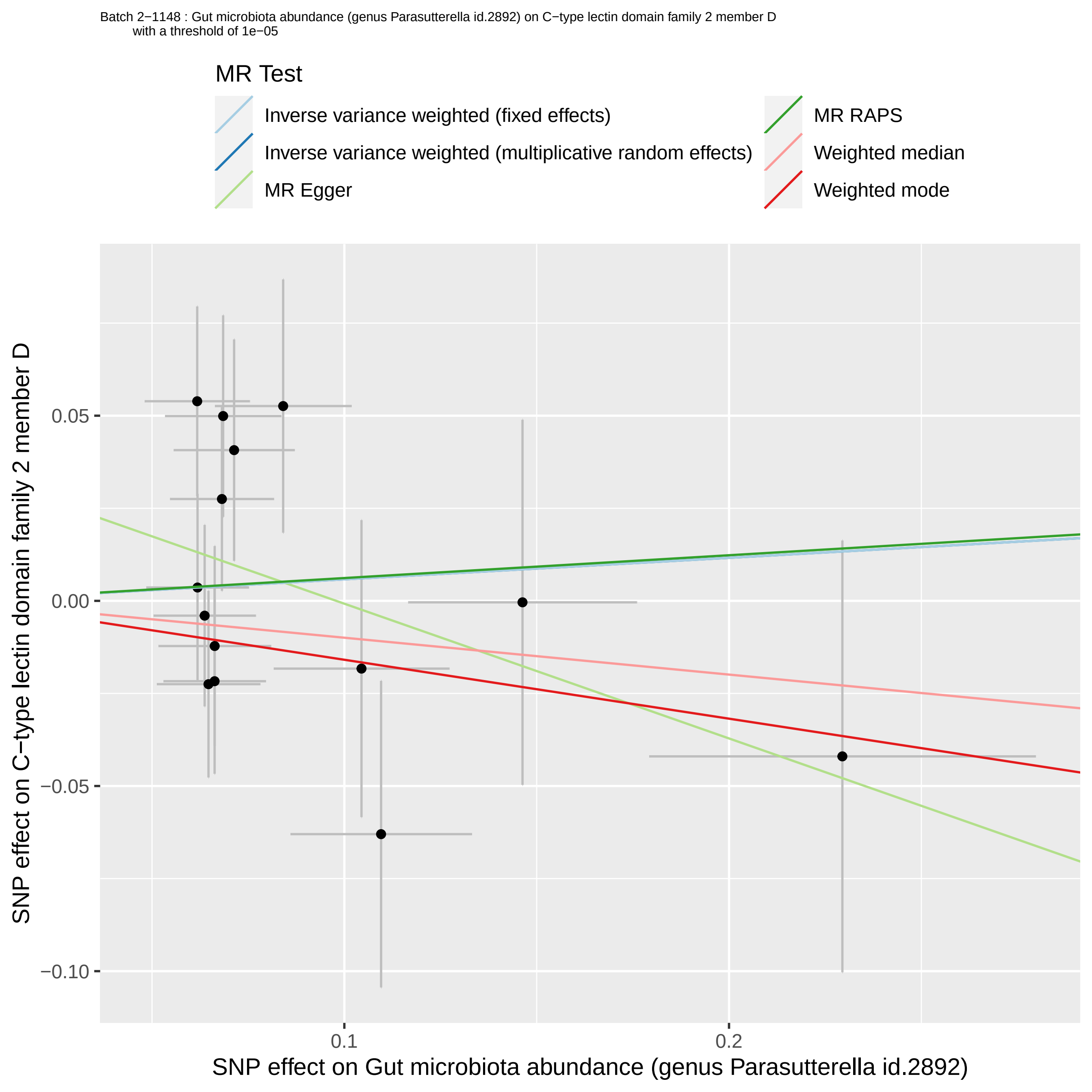
Batch 2−1148 : Gut microbiota abundance (genus Parasutterella id.2892) on C−type lectin domain family 2 member D
with a threshold of 1e−05
MR Test
Inverse variance weighted (fixed effects)
Inverse variance weighted (multiplicative random effects)
MR Egger
MR RAPS
Weighted median
Weighted mode
0.05
0.00
−0.05
−0.10
0.1
0.2
SNP effect on C−type lectin domain family 2 member D
SNP effect on Gut microbiota abundance (genus Parasutterella id.2892)

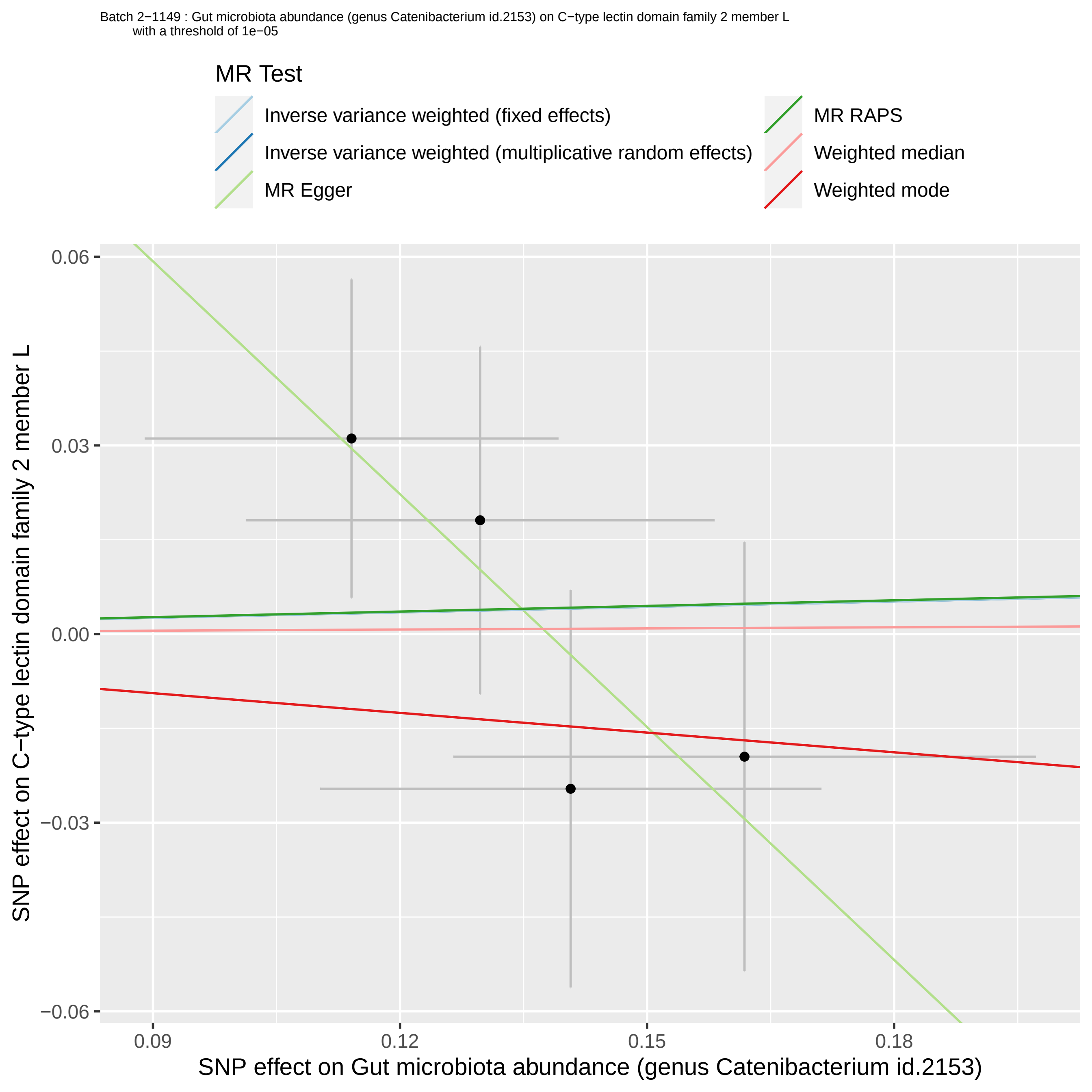
Batch 2−1149 : Gut microbiota abundance (genus Catenibacterium id.2153) on C−type lectin domain family 2 member L
with a threshold of 1e−05
MR Test
Inverse variance weighted (fixed effects)
Inverse variance weighted (multiplicative random effects)
MR Egger
MR RAPS
Weighted median
Weighted mode
0.06
0.03
0.00
−0.03
−0.06
0.09
0.12
0.15
0.18
SNP effect on C−type lectin domain family 2 member L
SNP effect on Gut microbiota abundance (genus Catenibacterium id.2153)

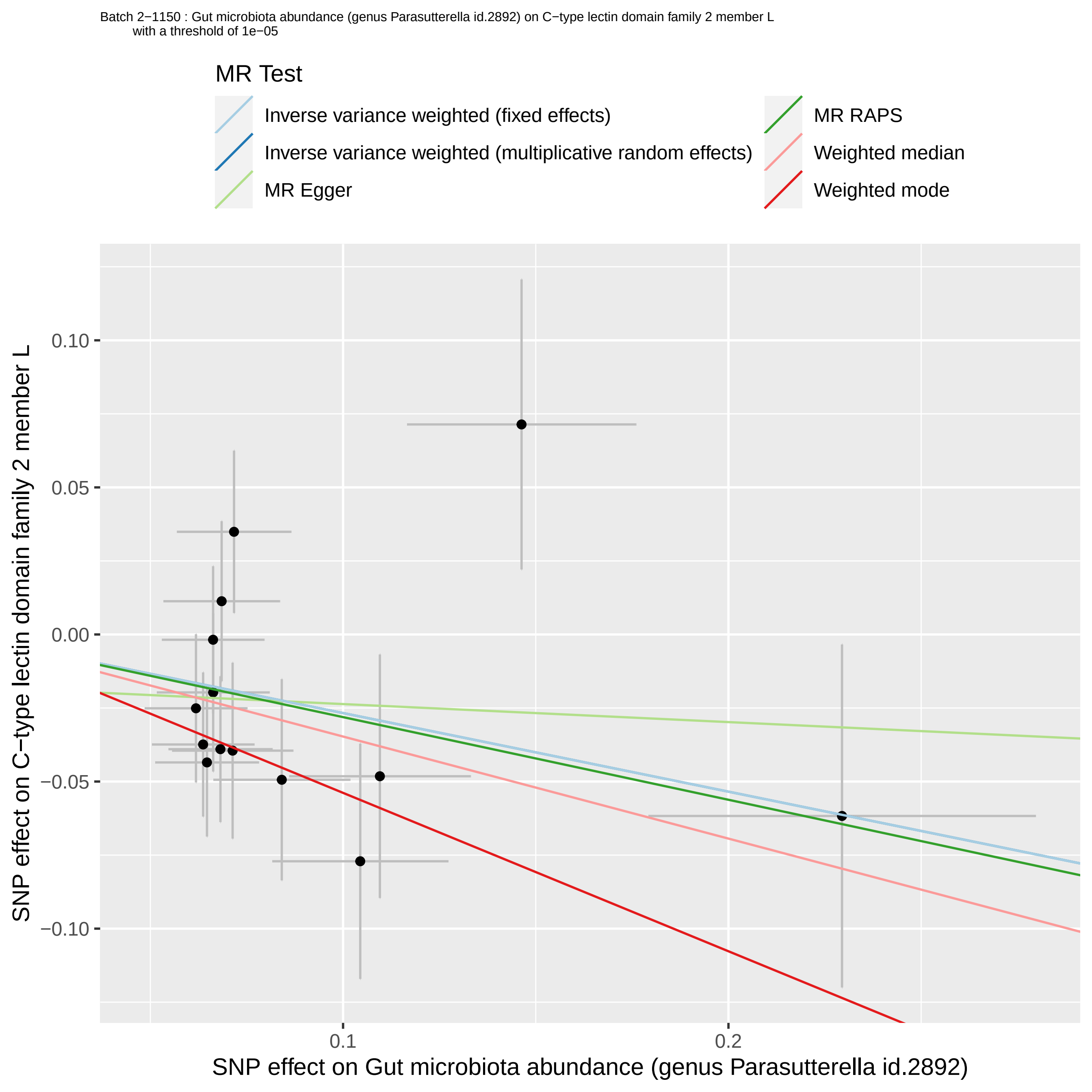

Batch 2−1150 : Gut microbiota abundance (genus Parasutterella id.2892) on C−type lectin domain family 2 member L
with a threshold of 1e−05
MR Test
Inverse variance weighted (fixed effects)
Inverse variance weighted (multiplicative random effects)
MR Egger
MR RAPS
Weighted median
Weighted mode
SNP effect on C−type lectin domain family 2 member L
0.10
0.05
0.00
−0.05
−0.10
0.1
0.2
SNP effect on Gut microbiota abundance (genus Parasutterella id.2892)

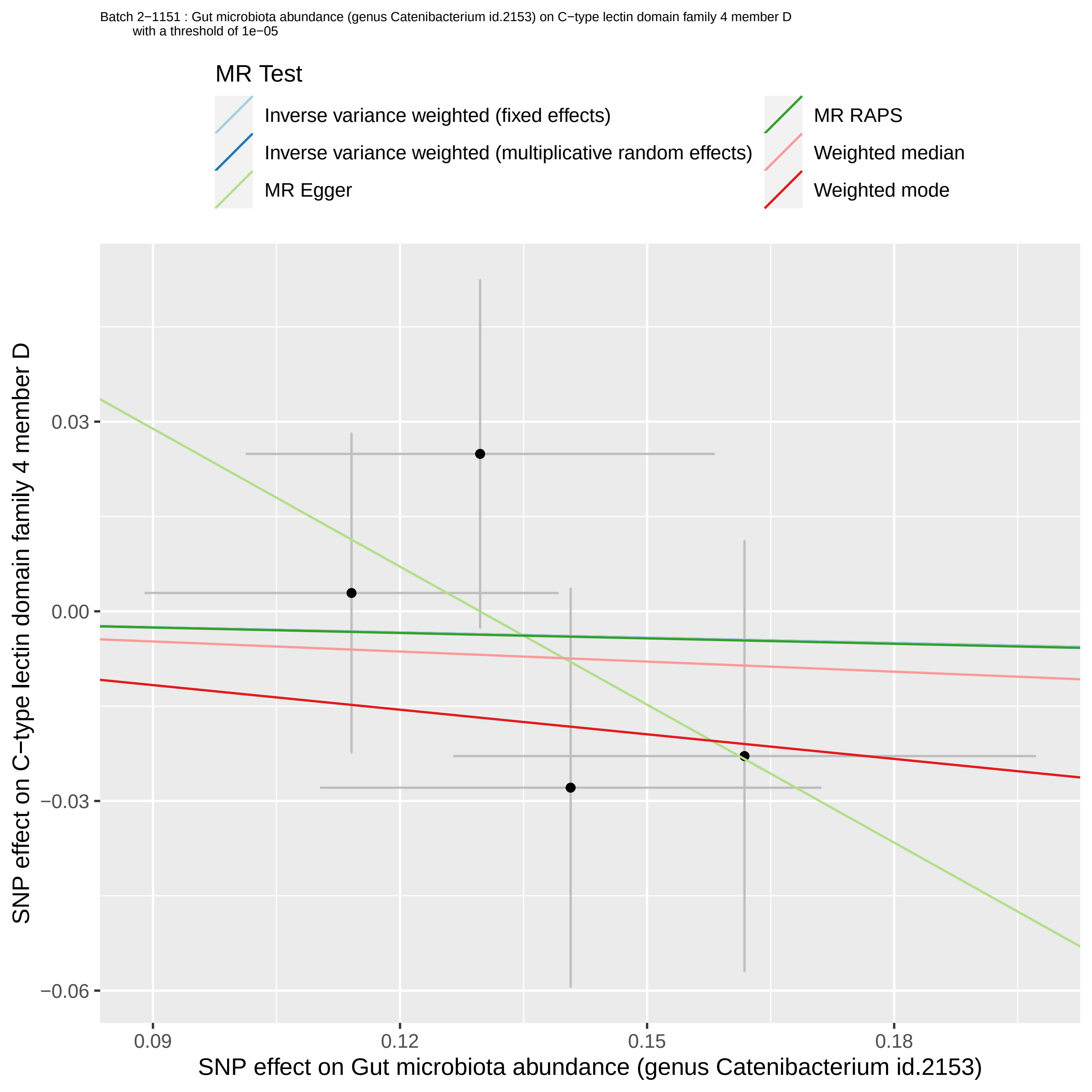

Batch 2−1151 : Gut microbiota abundance (genus Catenibacterium id.2153) on C−type lectin domain family 4 member D
with a threshold of 1e−05
MR Test
Inverse variance weighted (fixed effects)
Inverse variance weighted (multiplicative random effects)
MR Egger
MR RAPS
Weighted median
Weighted mode
SNP effect on C−type lectin domain family 4 member D
0.03
0.00
−0.03
−0.06
0.09
0.12
0.15
0.18
SNP effect on Gut microbiota abundance (genus Catenibacterium id.2153)

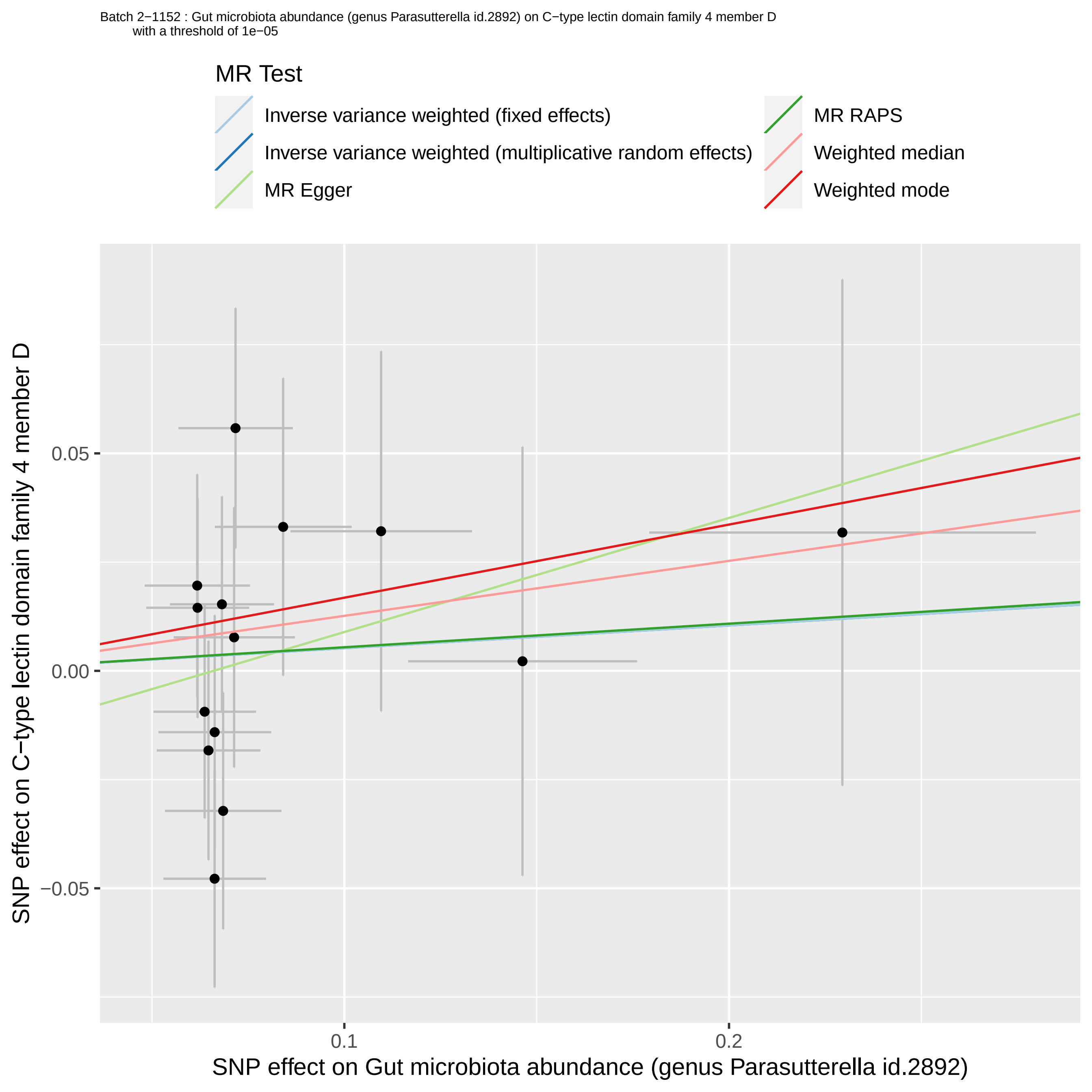

Batch 2−1152 : Gut microbiota abundance (genus Parasutterella id.2892) on C−type lectin domain family 4 member D
with a threshold of 1e−05
MR Test
Inverse variance weighted (fixed effects)
Inverse variance weighted (multiplicative random effects)
MR Egger
MR RAPS
Weighted median
Weighted mode
0.05
0.00
−0.05
0.1
0.2
SNP effect on C−type lectin domain family 4 member D
SNP effect on Gut microbiota abundance (genus Parasutterella id.2892)

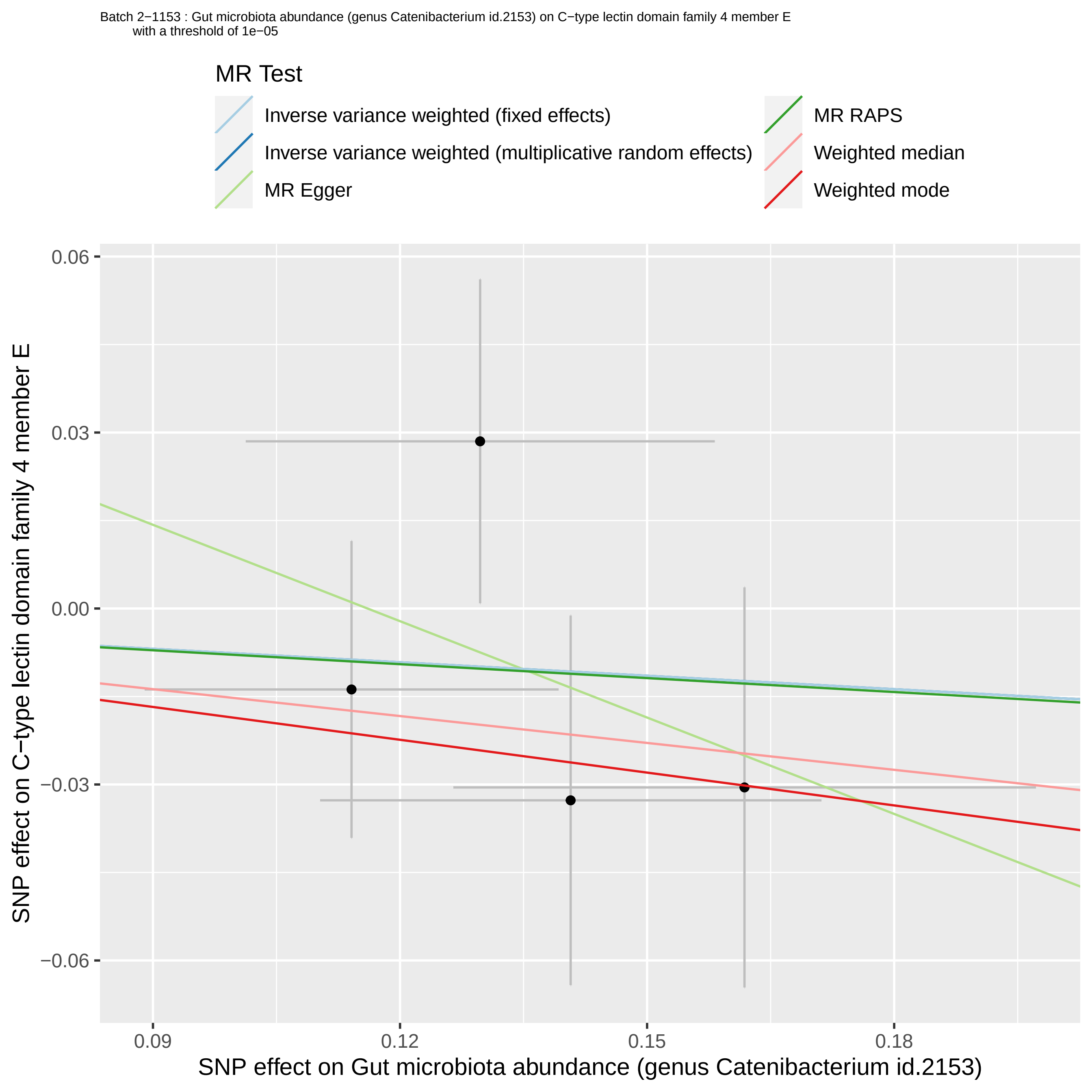

Batch 2−1153 : Gut microbiota abundance (genus Catenibacterium id.2153) on C−type lectin domain family 4 member E
with a threshold of 1e−05
MR Test
Inverse variance weighted (fixed effects)
Inverse variance weighted (multiplicative random effects)
MR Egger
MR RAPS
Weighted median
Weighted mode
0.06
0.03
0.00
−0.03
−0.06
0.09
0.12
0.15
0.18
SNP effect on C−type lectin domain family 4 member E
SNP effect on Gut microbiota abundance (genus Catenibacterium id.2153)

Batch 2−1154 : Gut microbiota abundance (genus Parasutterella id.2892) on C−type lectin domain family 4 member E
with a threshold of 1e−05

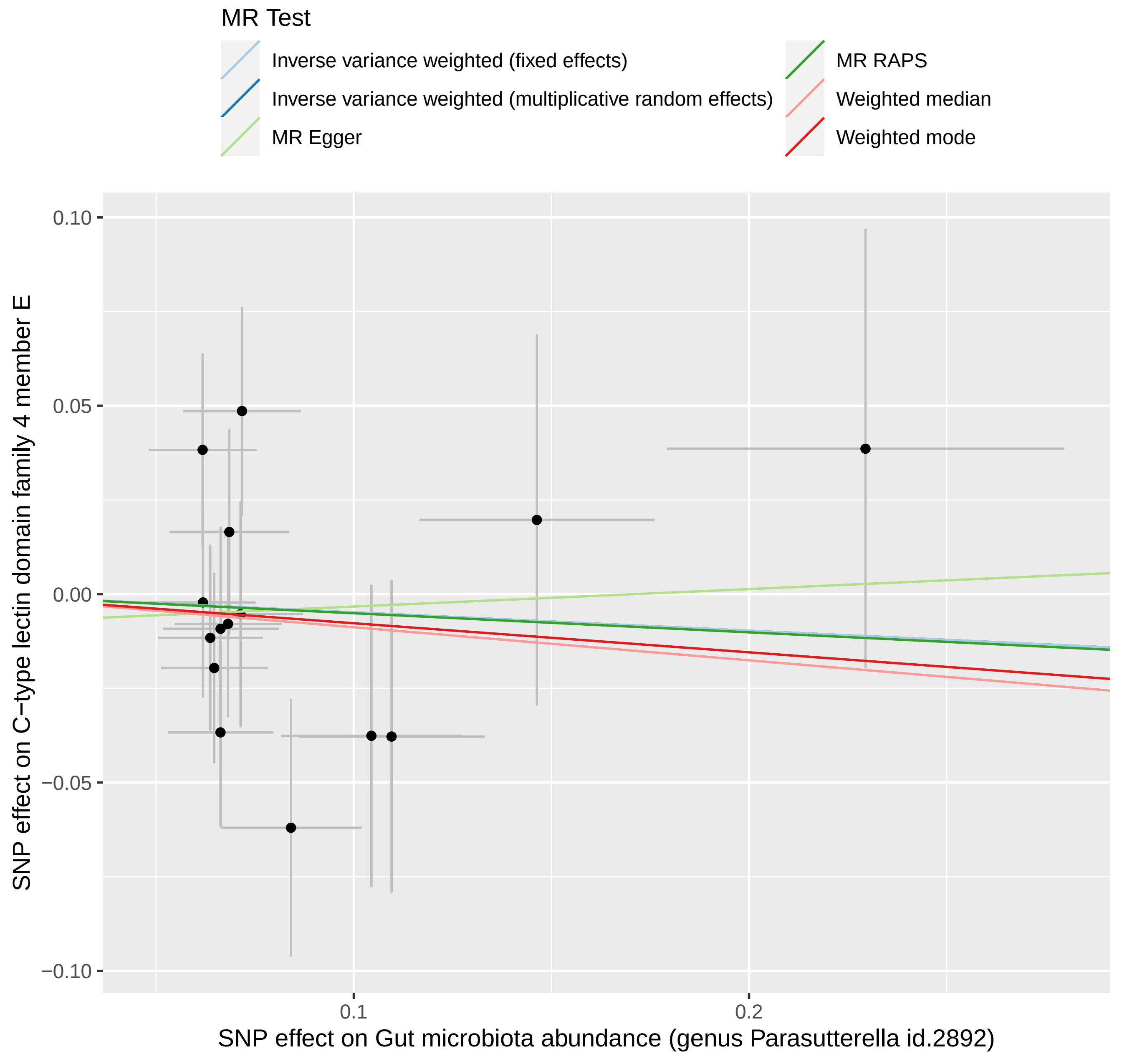

Batch 2−1155 : Gut microbiota abundance (genus Catenibacterium id.2153) on C−type lectin domain family 4 member M
with a threshold of 1e−05

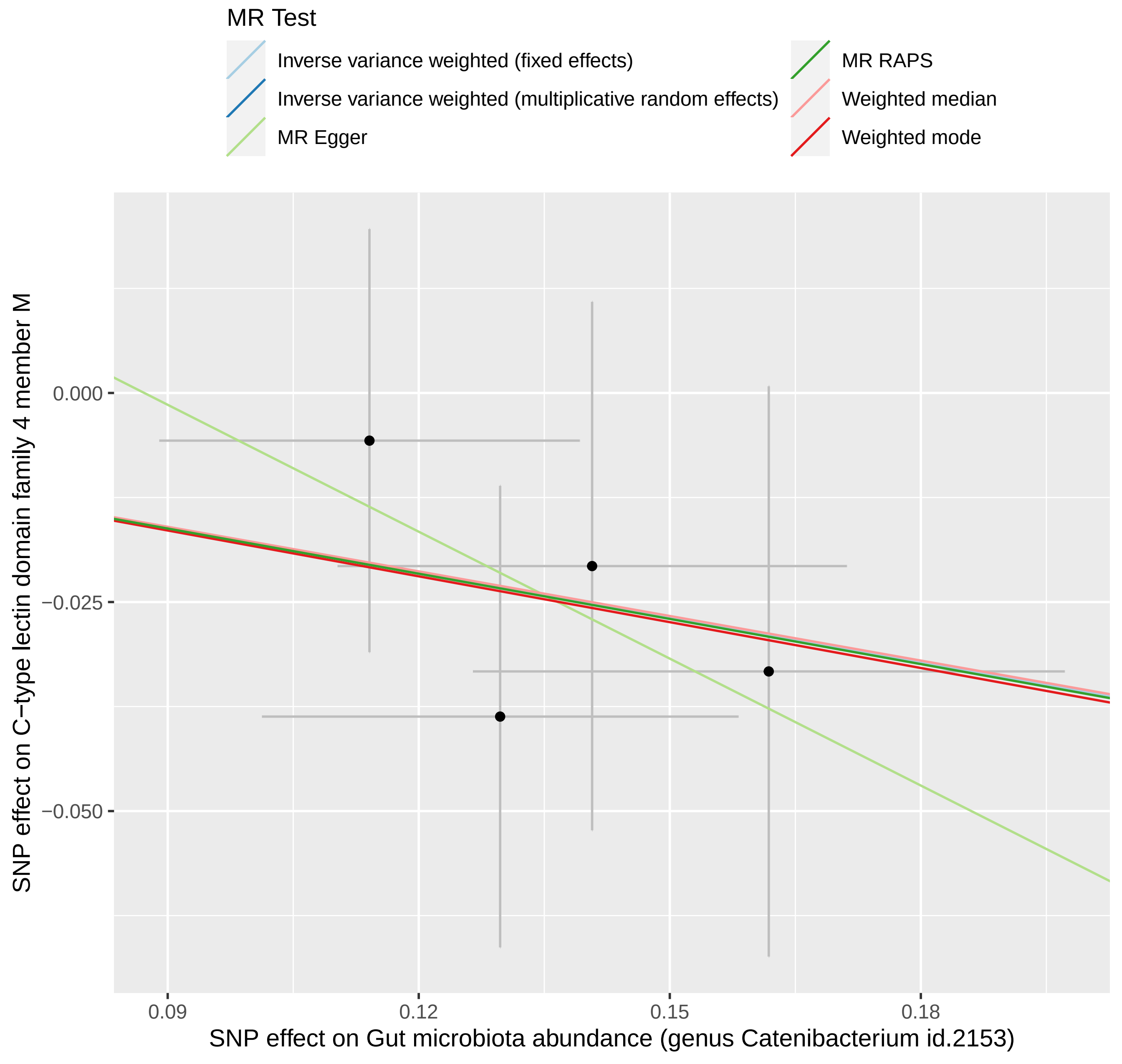

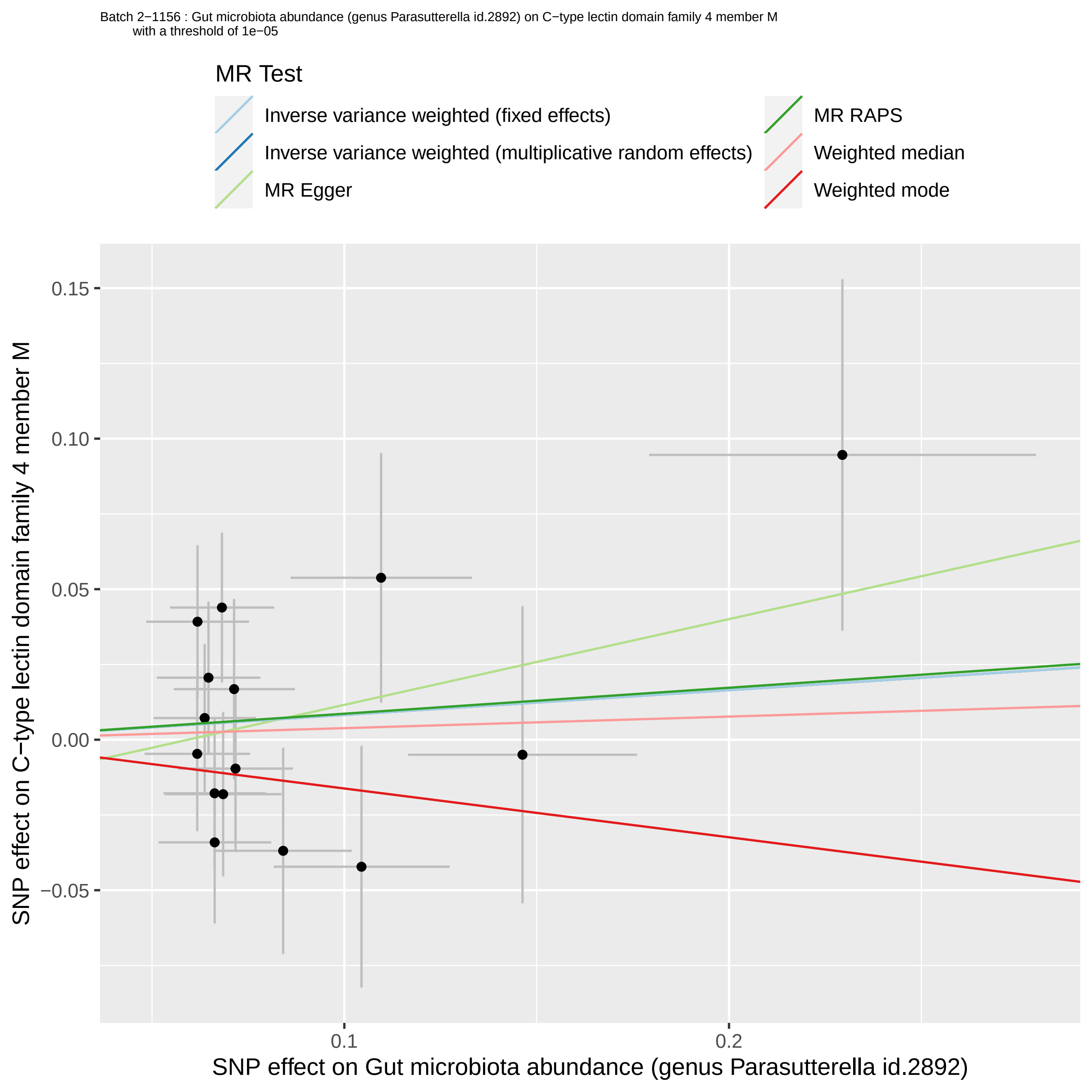

Batch 2−1156 : Gut microbiota abundance (genus Parasutterella id.2892) on C−type lectin domain family 4 member M
with a threshold of 1e−05
MR Test
Inverse variance weighted (fixed effects)
Inverse variance weighted (multiplicative random effects)
MR Egger
MR RAPS
Weighted median
Weighted mode
0.15
0.10
0.05
0.00
−0.05
0.1
0.2
SNP effect on C−type lectin domain family 4 member M
SNP effect on Gut microbiota abundance (genus Parasutterella id.2892)

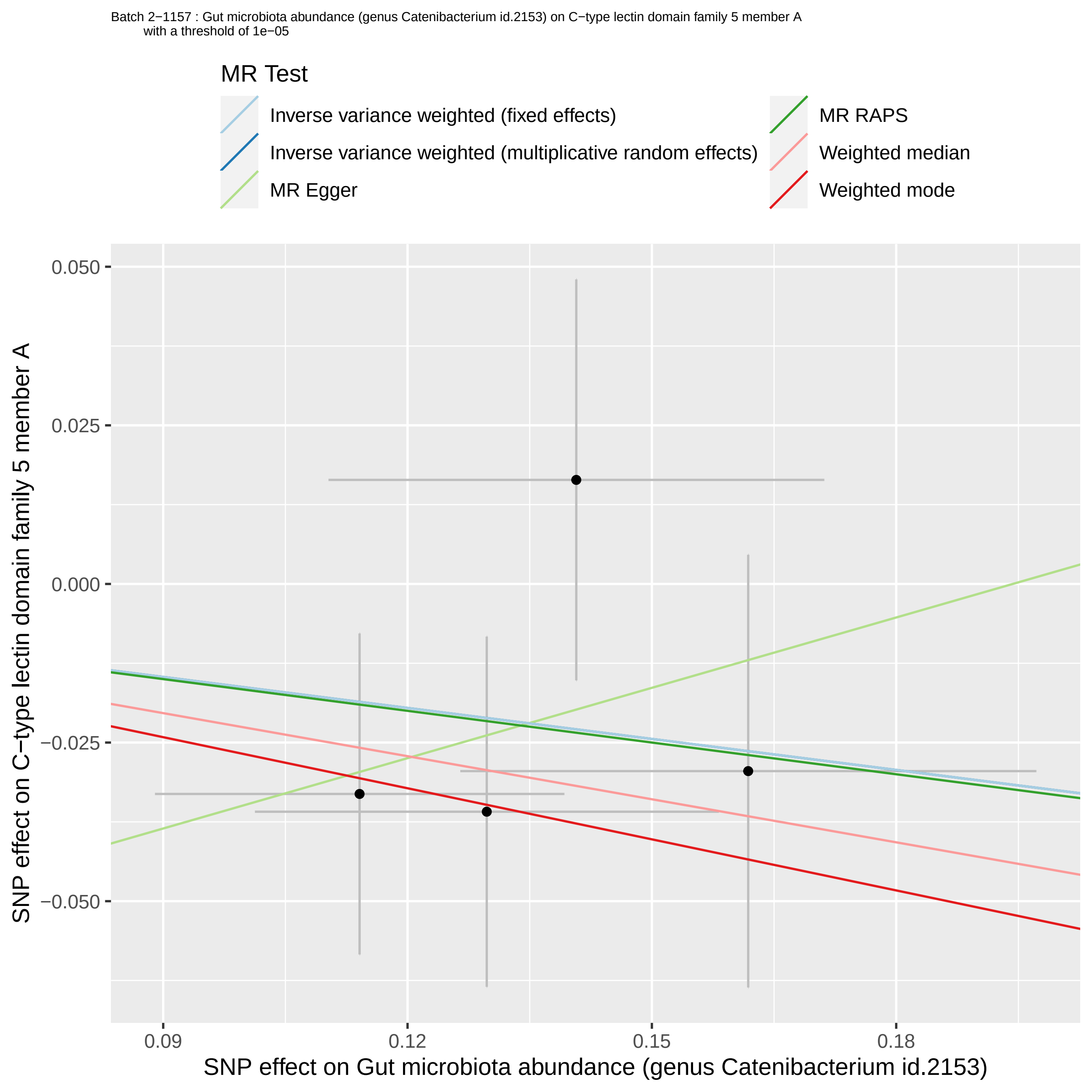
Batch 2−1157 : Gut microbiota abundance (genus Catenibacterium id.2153) on C−type lectin domain family 5 member A
with a threshold of 1e−05
MR Test
Inverse variance weighted (fixed effects)
Inverse variance weighted (multiplicative random effects)
MR Egger
MR RAPS
Weighted median
Weighted mode
0.050
0.025
0.000
−0.025
−0.050
0.09
0.12
0.15
0.18
SNP effect on C−type lectin domain family 5 member A
SNP effect on Gut microbiota abundance (genus Catenibacterium id.2153)

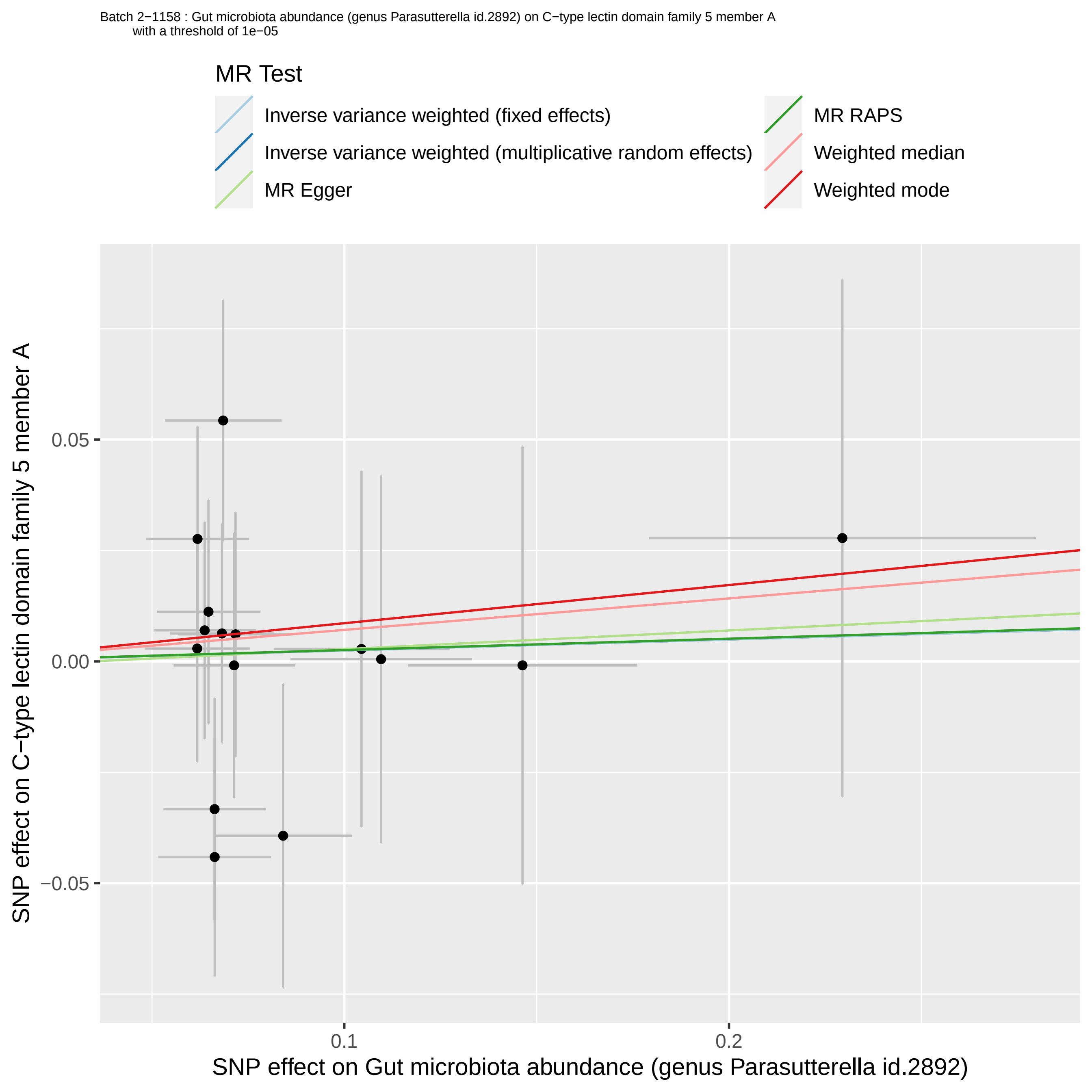
Batch 2−1158 : Gut microbiota abundance (genus Parasutterella id.2892) on C−type lectin domain family 5 member A
with a threshold of 1e−05
MR Test
Inverse variance weighted (fixed effects)
Inverse variance weighted (multiplicative random effects)
MR Egger
MR RAPS
Weighted median
Weighted mode
0.05
0.00
−0.05
0.1
0.2
SNP effect on C−type lectin domain family 5 member A
SNP effect on Gut microbiota abundance (genus Parasutterella id.2892)

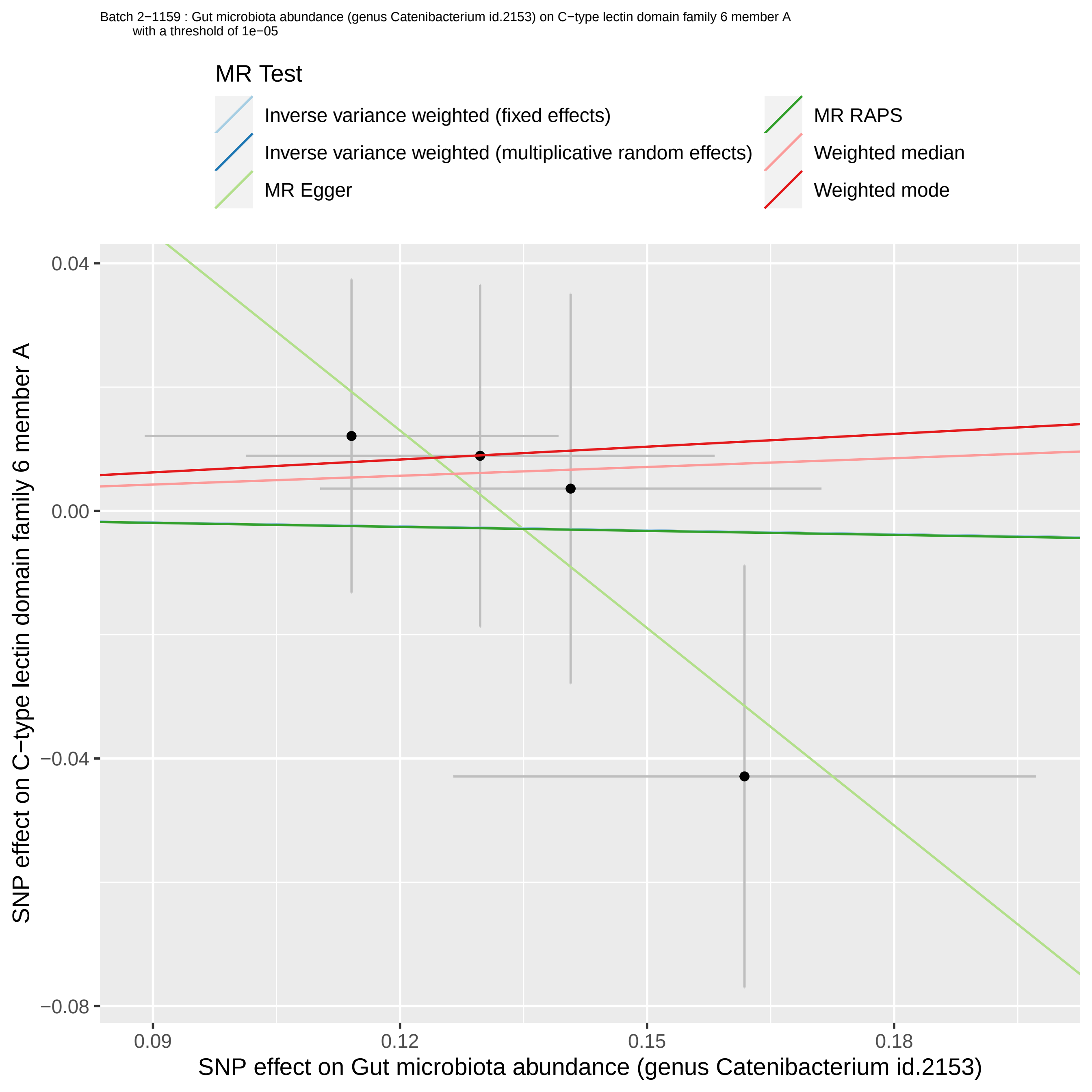

Batch 2−1159 : Gut microbiota abundance (genus Catenibacterium id.2153) on C−type lectin domain family 6 member A
with a threshold of 1e−05
MR Test
Inverse variance weighted (fixed effects)
Inverse variance weighted (multiplicative random effects)
MR Egger
MR RAPS
Weighted median
Weighted mode
0.04
0.00
−0.04
−0.08
0.09
0.12
0.15
0.18
SNP effect on C−type lectin domain family 6 member A
SNP effect on Gut microbiota abundance (genus Catenibacterium id.2153)

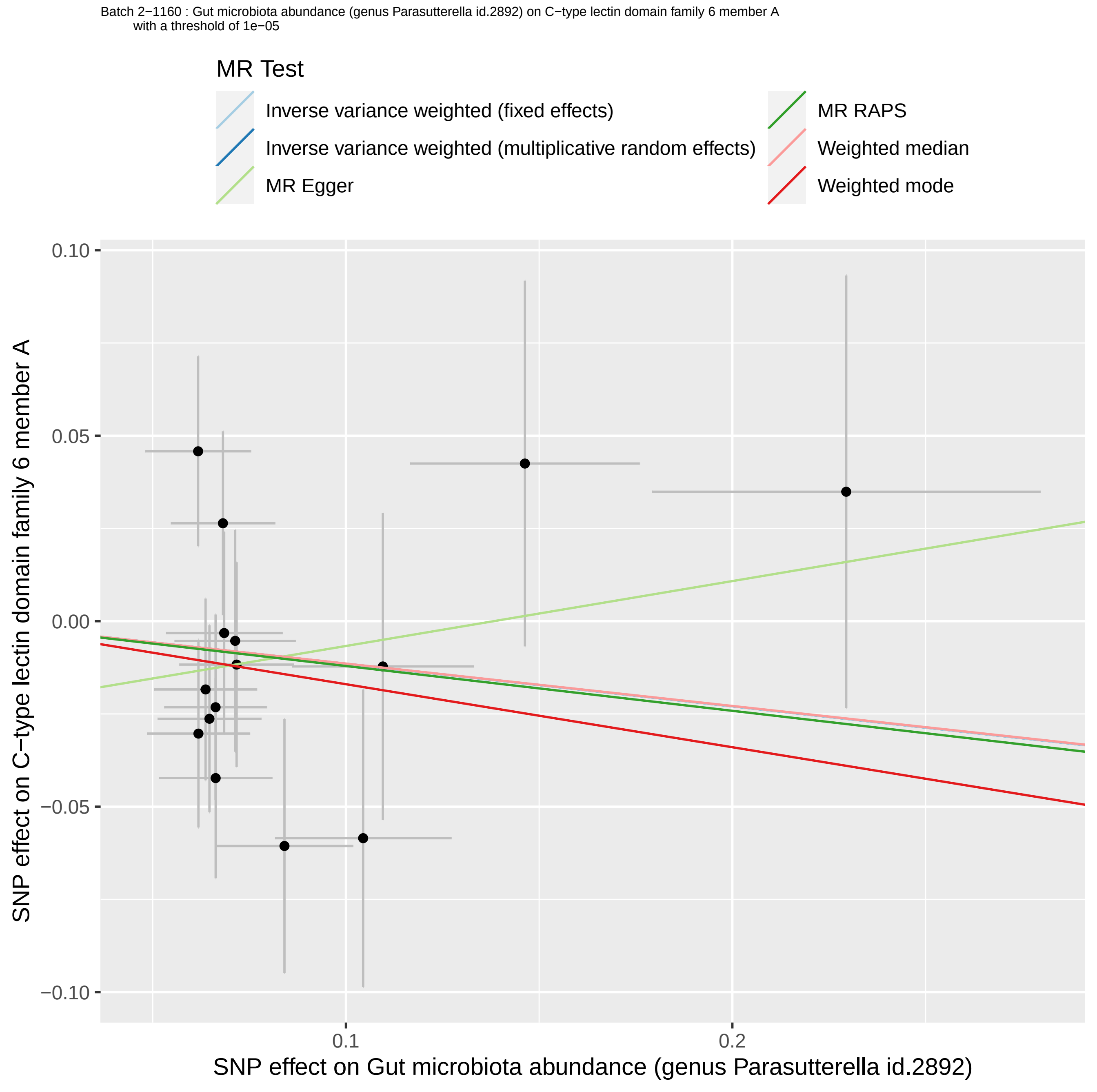
Batch 2−1160 : Gut microbiota abundance (genus Parasutterella id.2892) on C−type lectin domain family 6 member A
with a threshold of 1e−05
MR Test
Inverse variance weighted (fixed effects)
Inverse variance weighted (multiplicative random effects)
MR Egger
MR RAPS
Weighted median
Weighted mode
0.10
0.05
0.00
−0.05
−0.10
0.1
0.2
SNP effect on C−type lectin domain family 6 member A
SNP effect on Gut microbiota abundance (genus Parasutterella id.2892)

Batch 2−1161 : Gut microbiota abundance (genus Catenibacterium id.2153) on C−type lectin domain family 7 member A
with a threshold of 1e−05

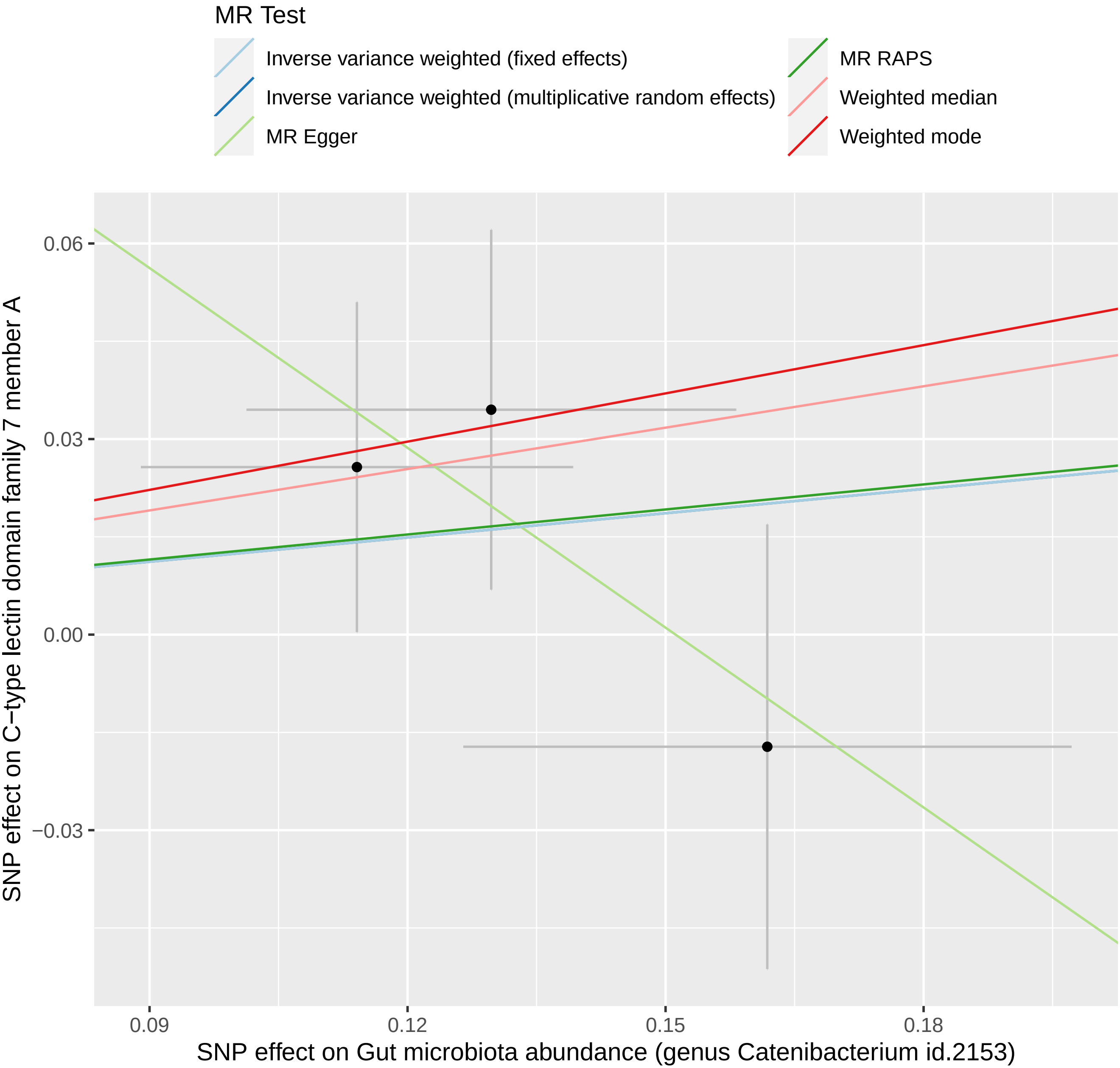

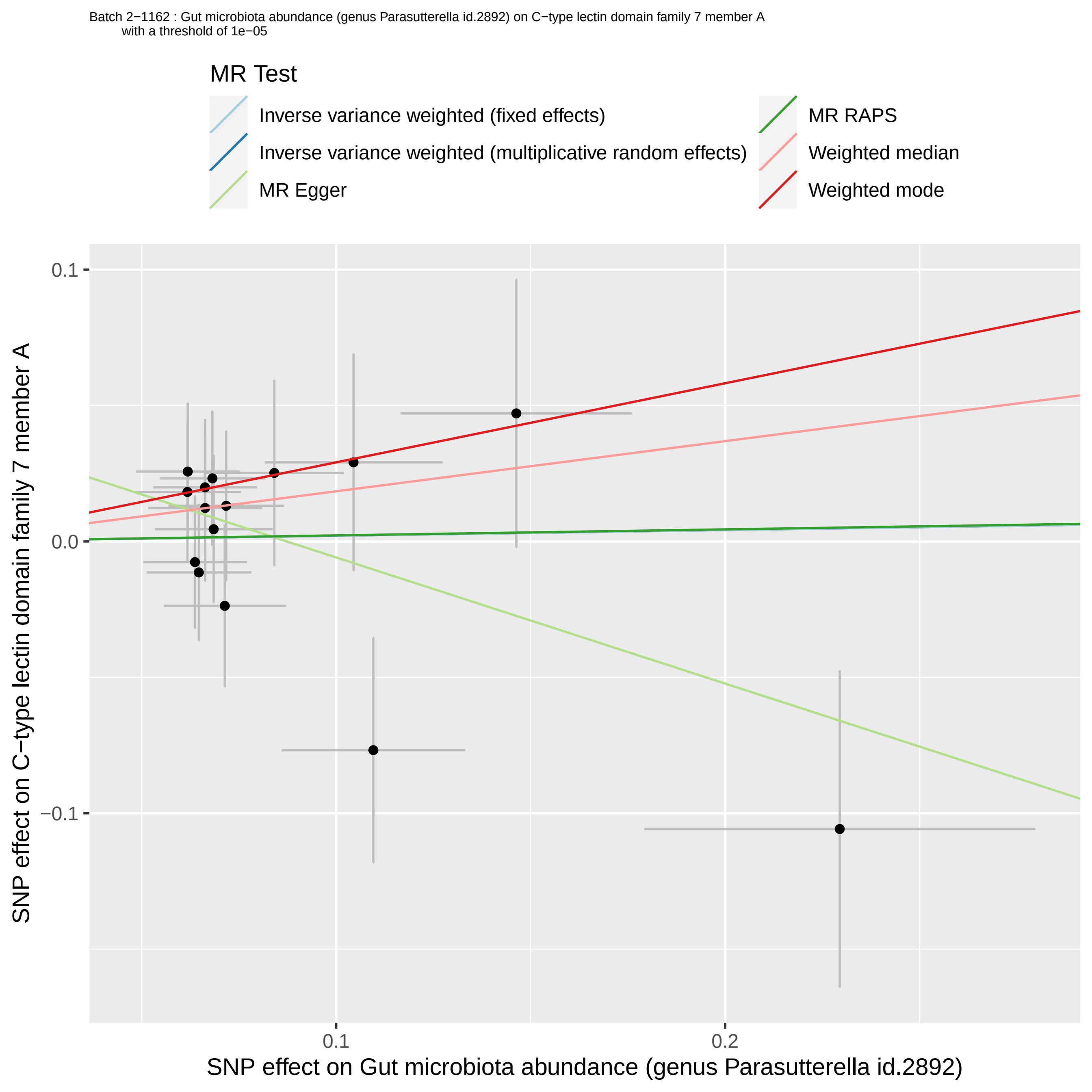
Batch 2−1162 : Gut microbiota abundance (genus Parasutterella id.2892) on C−type lectin domain family 7 member A
with a threshold of 1e−05
MR Test
Inverse variance weighted (fixed effects)
Inverse variance weighted (multiplicative random effects)
MR Egger
MR RAPS
Weighted median
Weighted mode
0.1
0.0
−0.1
0.1
0.2
SNP effect on C−type lectin domain family 7 member A
SNP effect on Gut microbiota abundance (genus Parasutterella id.2892)

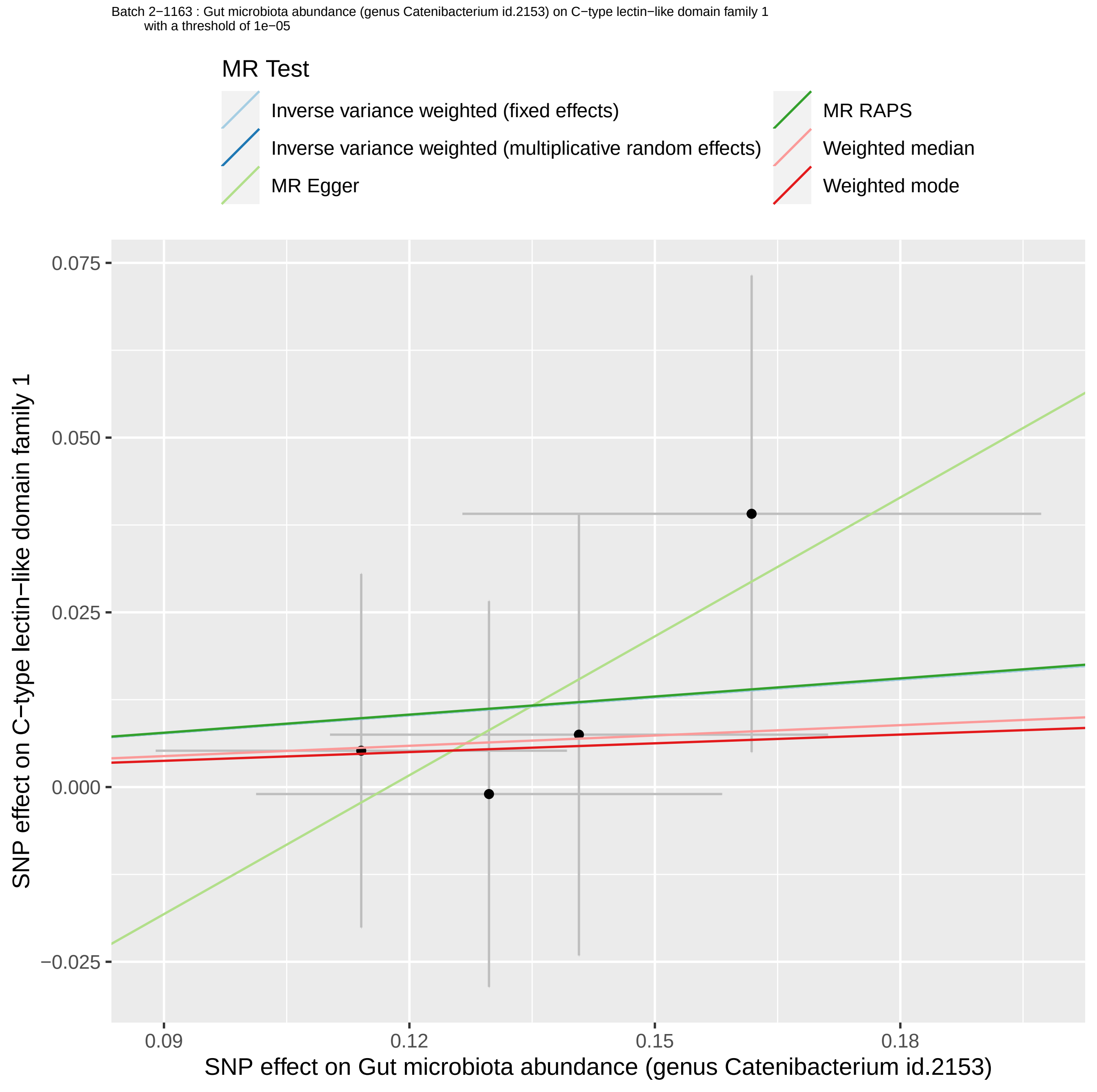

Batch 2−1163 : Gut microbiota abundance (genus Catenibacterium id.2153) on C−type lectin−like domain family 1
with a threshold of 1e−05
MR Test
Inverse variance weighted (fixed effects)
Inverse variance weighted (multiplicative random effects)
MR Egger
MR RAPS
Weighted median
Weighted mode
0.075
0.050
0.025
0.000
−0.025
0.09
0.12
0.15
0.18
SNP effect on C−type lectin−like domain family 1
SNP effect on Gut microbiota abundance (genus Catenibacterium id.2153)

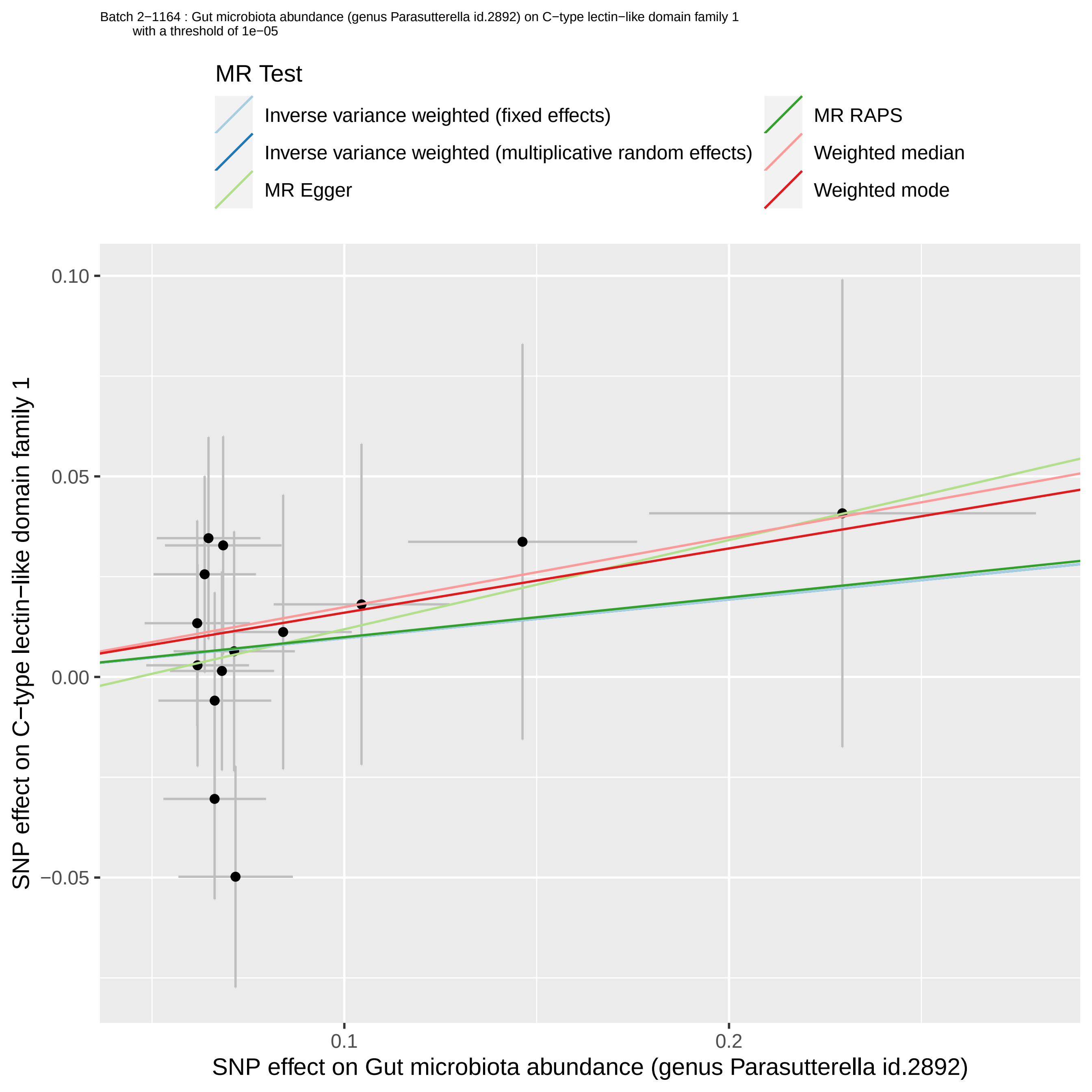
Batch 2−1164 : Gut microbiota abundance (genus Parasutterella id.2892) on C−type lectin−like domain family 1
with a threshold of 1e−05
MR Test
Inverse variance weighted (fixed effects)
Inverse variance weighted (multiplicative random effects)
MR Egger
MR RAPS
Weighted median
Weighted mode
0.10
0.05
0.00
−0.05
0.1
0.2
SNP effect on C−type lectin−like domain family 1
SNP effect on Gut microbiota abundance (genus Parasutterella id.2892)

Batch 2−1165 : Gut microbiota abundance (genus Catenibacterium id.2153) on Chloride intracellular channel protein 4 with a threshold of 1e−05

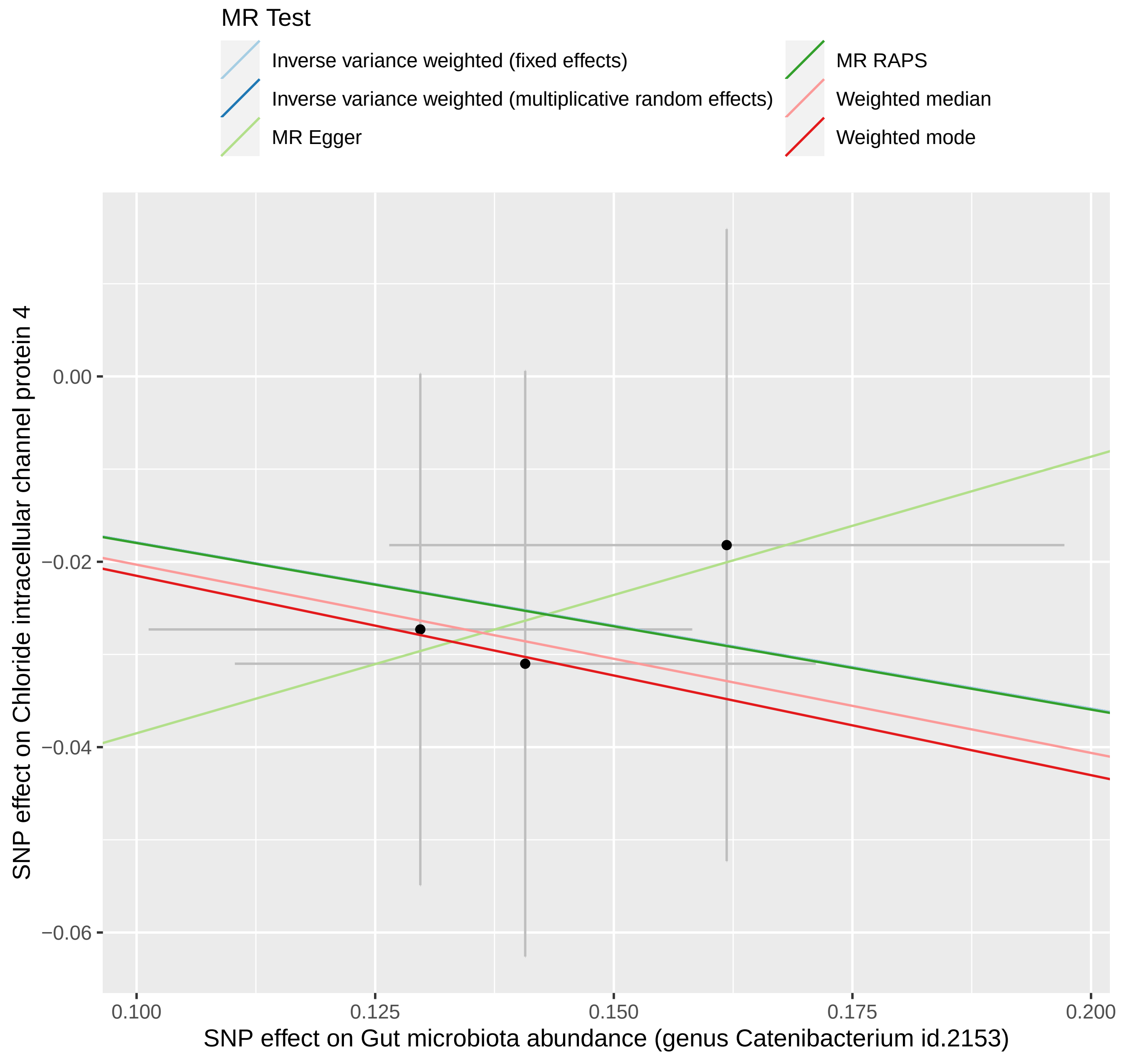

Batch 2−1166 : Gut microbiota abundance (genus Parasutterella id.2892) on Chloride intracellular channel protein 4 with a threshold of 1e−05

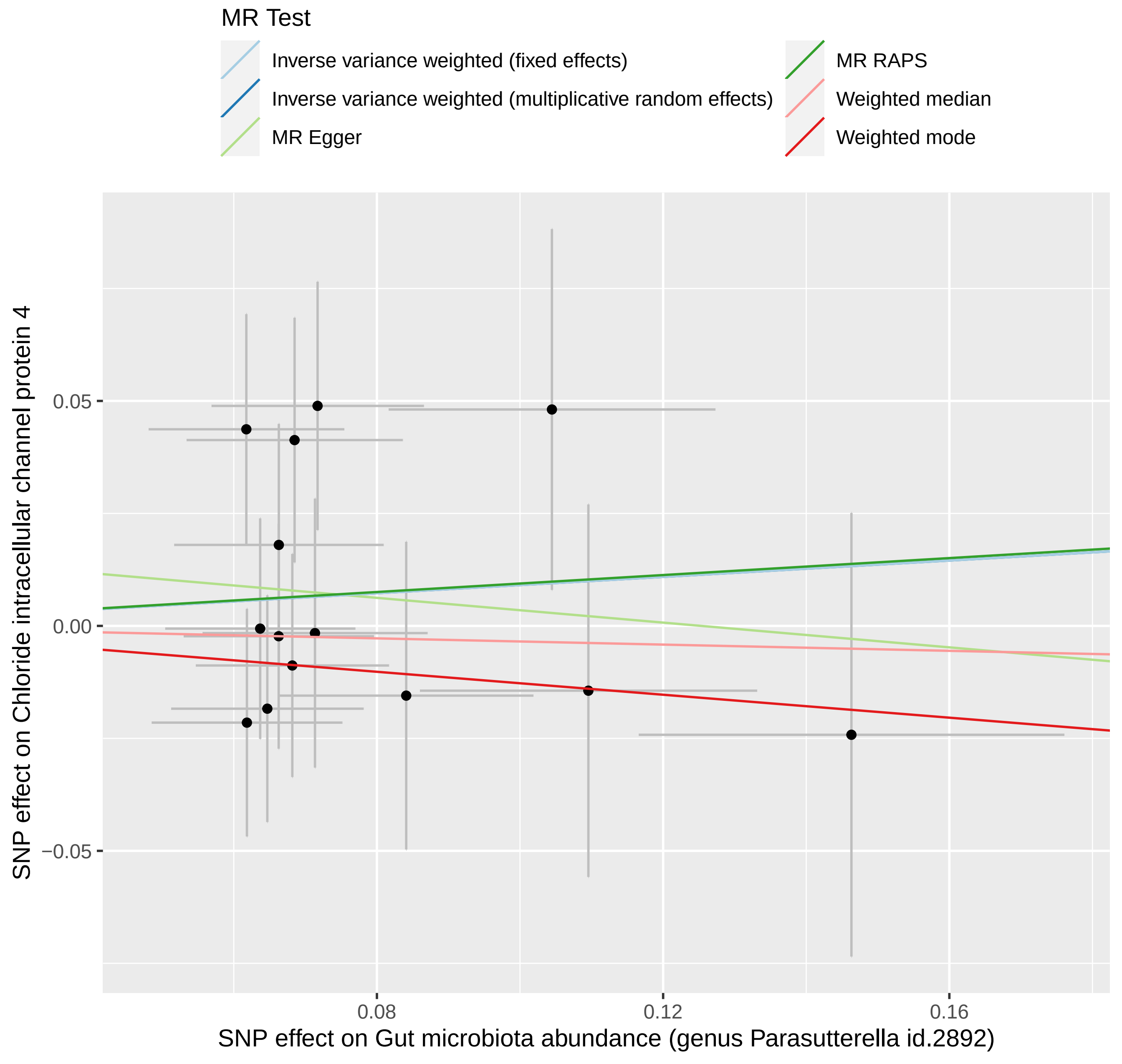

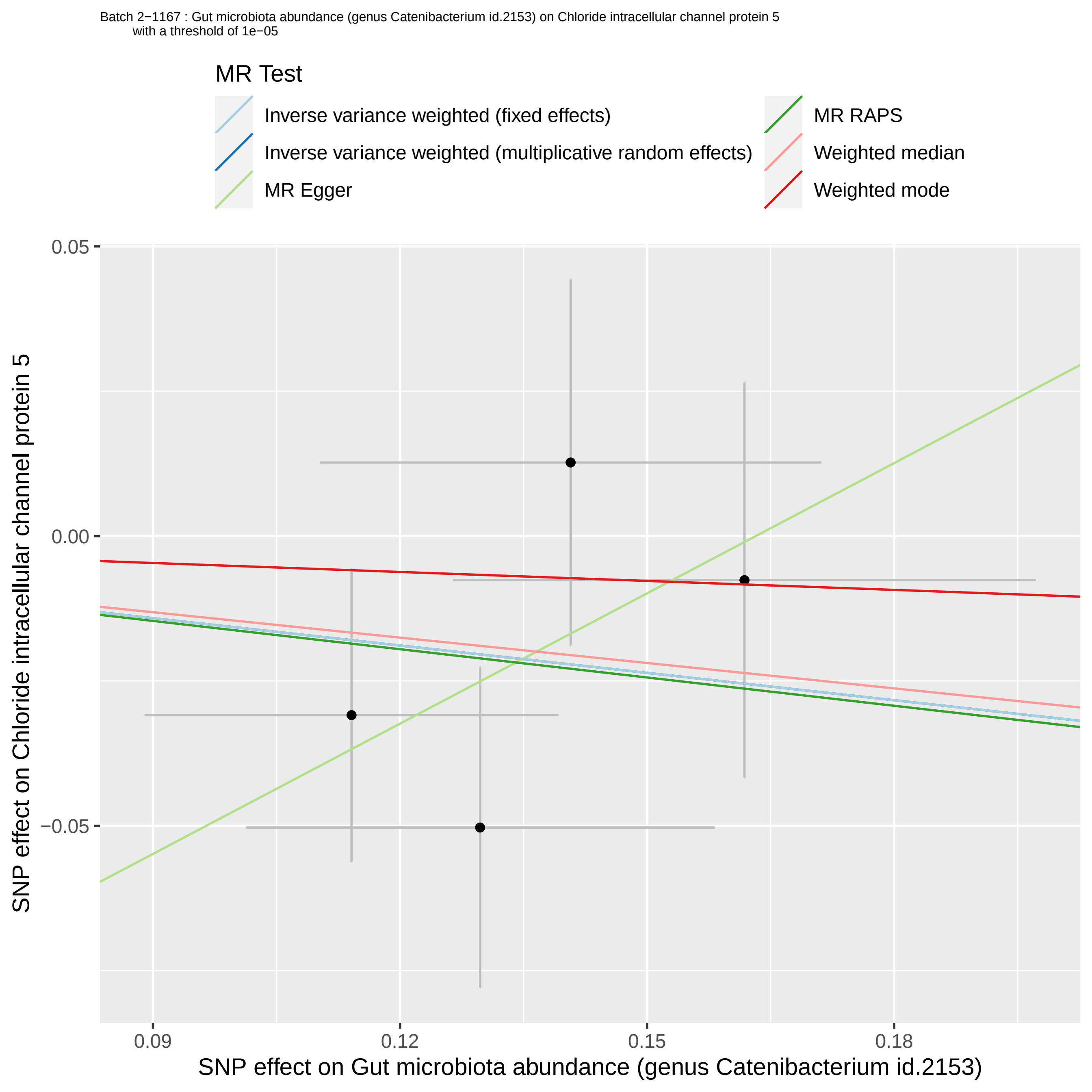

Batch 2−1167 : Gut microbiota abundance (genus Catenibacterium id.2153) on Chloride intracellular channel protein 5
with a threshold of 1e−05
MR Test
Inverse variance weighted (fixed effects)
Inverse variance weighted (multiplicative random effects)
MR Egger
MR RAPS
Weighted median
Weighted mode
0.05
0.00
−0.05
0.09
0.12
0.15
0.18
SNP effect on Chloride intracellular channel protein 5
SNP effect on Gut microbiota abundance (genus Catenibacterium id.2153)

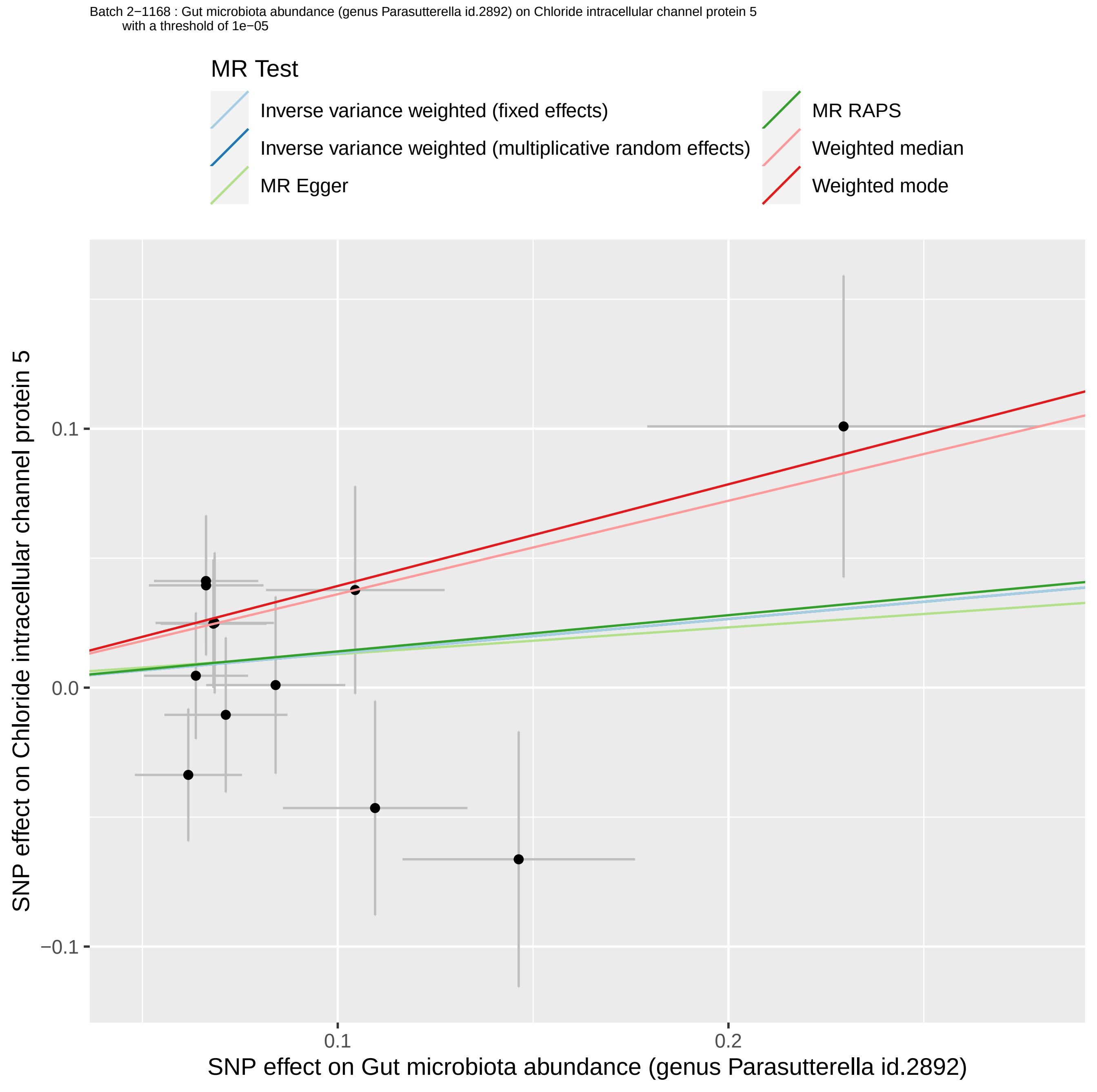
Batch 2−1168 : Gut microbiota abundance (genus Parasutterella id.2892) on Chloride intracellular channel protein 5
with a threshold of 1e−05
MR Test
Inverse variance weighted (fixed effects)
Inverse variance weighted (multiplicative random effects)
MR Egger
MR RAPS
Weighted median
Weighted mode
SNP effect on Chloride intracellular channel protein 5
0.1
0.0
−0.1
0.1
0.2
SNP effect on Gut microbiota abundance (genus Parasutterella id.2892)

Batch 2−1169 : Gut microbiota abundance (genus Catenibacterium id.2153) on Clathrin interactor 1
with a threshold of 1e−05

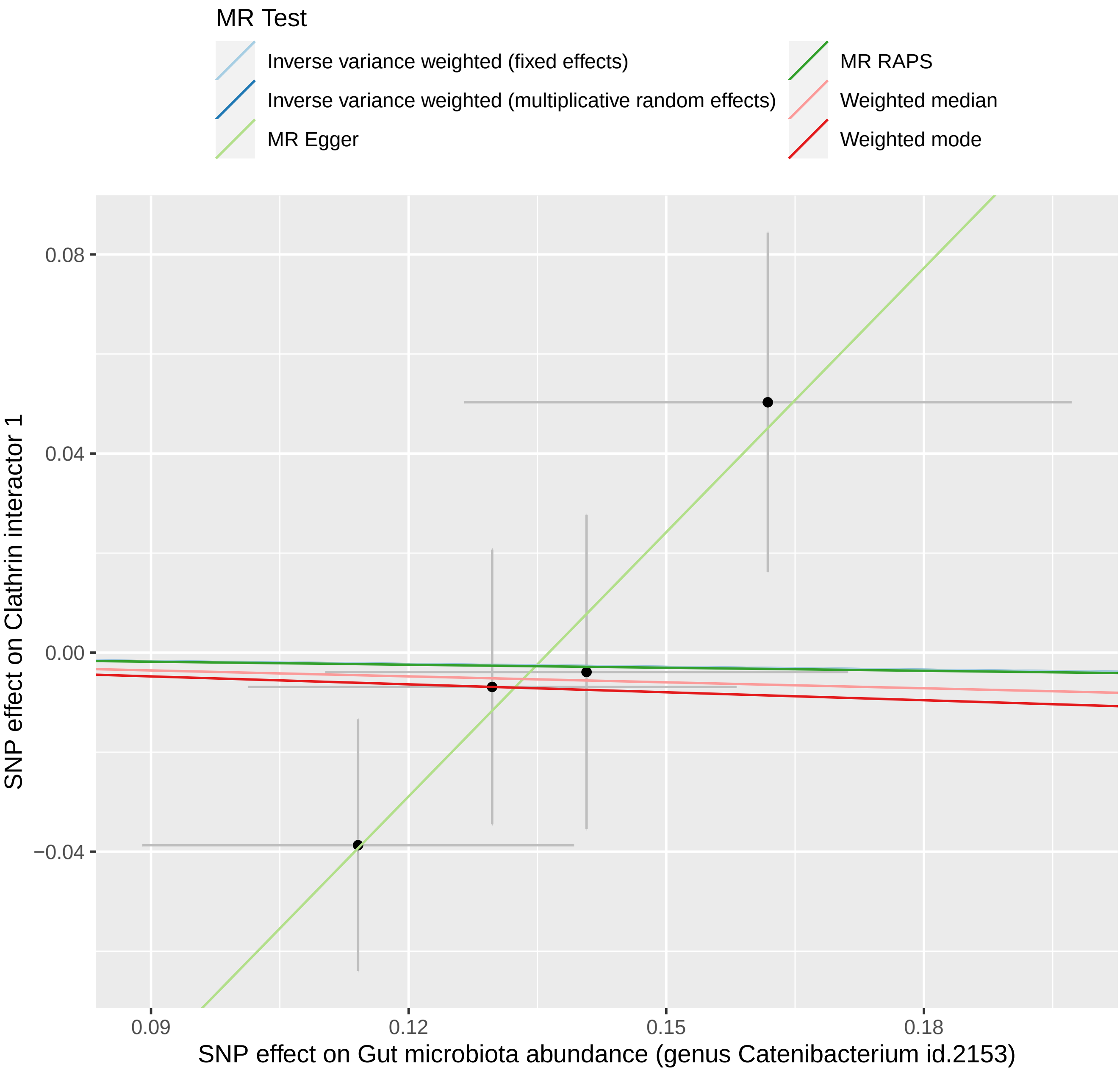

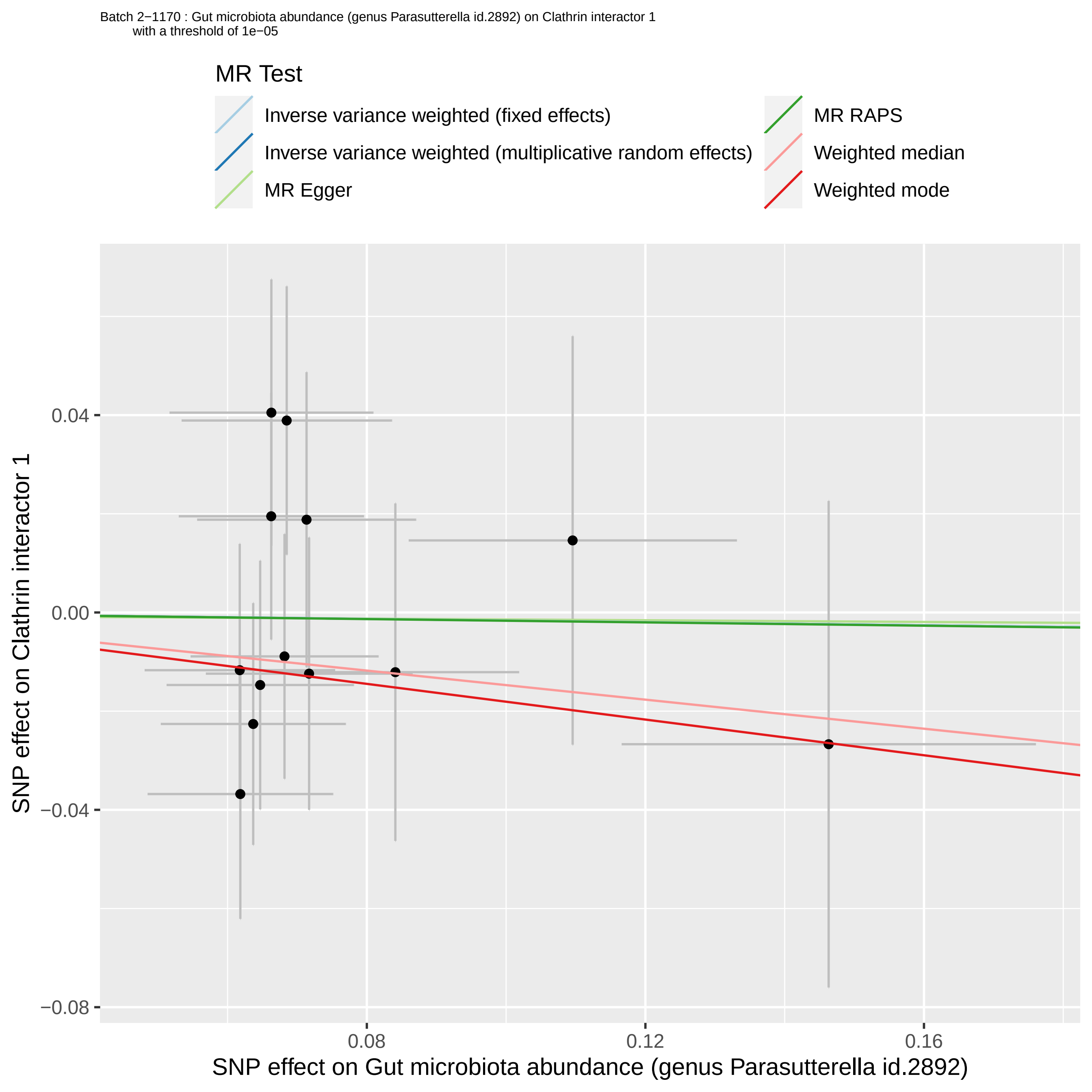
Batch 2−1170 : Gut microbiota abundance (genus Parasutterella id.2892) on Clathrin interactor 1
with a threshold of 1e−05
MR Test
Inverse variance weighted (fixed effects)
Inverse variance weighted (multiplicative random effects)
MR Egger
MR RAPS
Weighted median
Weighted mode
0.04
0.00
−0.04
−0.08
0.08
0.12
0.16
SNP effect on Clathrin interactor 1
SNP effect on Gut microbiota abundance (genus Parasutterella id.2892)

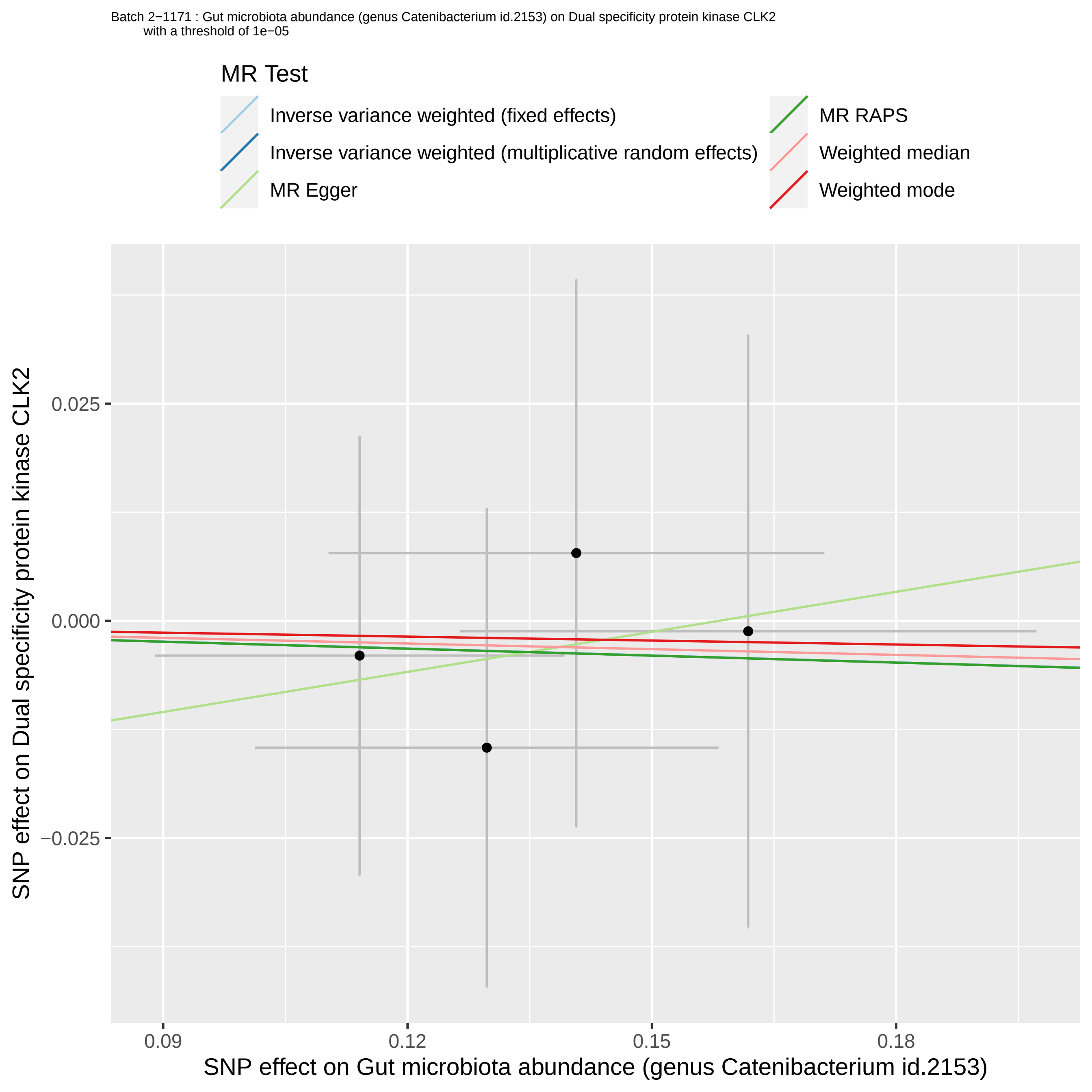
Batch 2−1171 : Gut microbiota abundance (genus Catenibacterium id.2153) on Dual specificity protein kinase CLK2
with a threshold of 1e−05
MR Test
Inverse variance weighted (fixed effects)
Inverse variance weighted (multiplicative random effects)
MR Egger
MR RAPS
Weighted median
Weighted mode
0.025
0.000
−0.025
0.09
0.12
0.15
0.18
SNP effect on Dual specificity protein kinase CLK2
SNP effect on Gut microbiota abundance (genus Catenibacterium id.2153)

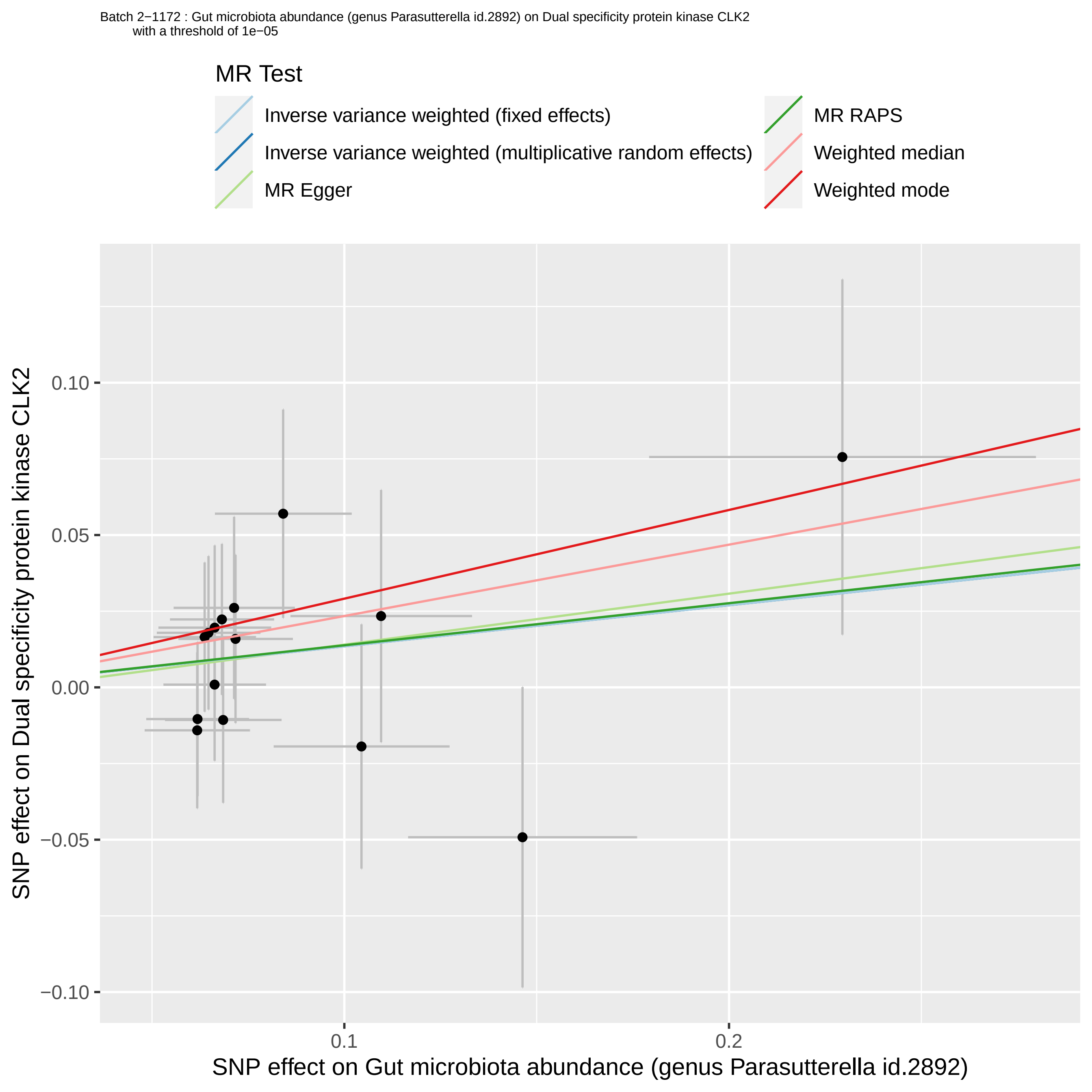

Batch 2−1172 : Gut microbiota abundance (genus Parasutterella id.2892) on Dual specificity protein kinase CLK2
with a threshold of 1e−05
MR Test
Inverse variance weighted (fixed effects)
Inverse variance weighted (multiplicative random effects)
MR Egger
MR RAPS
Weighted median
Weighted mode
0.10
0.05
0.00
−0.05
−0.10
0.1
0.2
SNP effect on Dual specificity protein kinase CLK2
SNP effect on Gut microbiota abundance (genus Parasutterella id.2892)

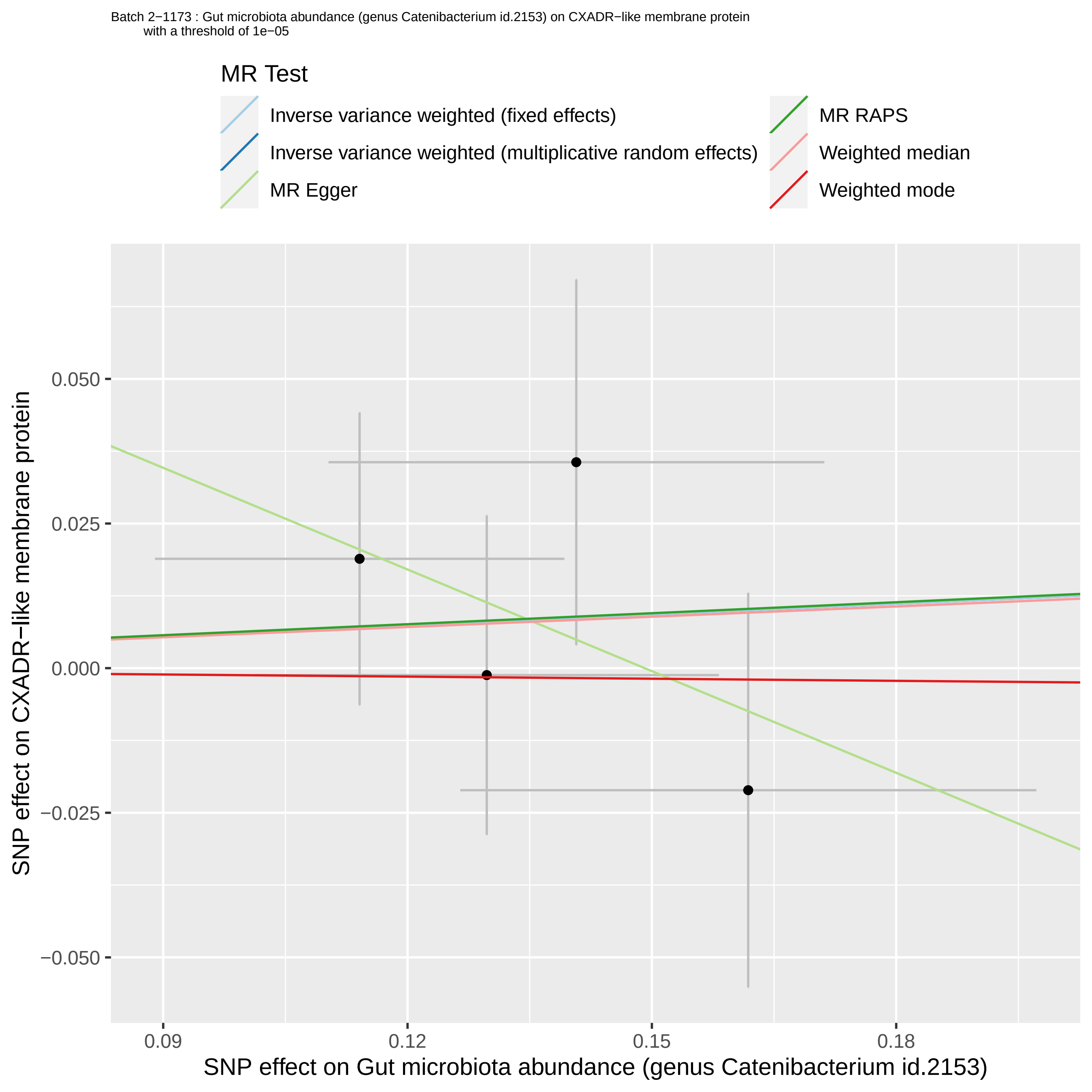

Batch 2−1173 : Gut microbiota abundance (genus Catenibacterium id.2153) on CXADR−like membrane protein
with a threshold of 1e−05
MR Test
Inverse variance weighted (fixed effects)
Inverse variance weighted (multiplicative random effects)
MR Egger
MR RAPS
Weighted median
Weighted mode
0.050
0.025
0.000
−0.025
−0.050
SNP effect on CXADR−like membrane protein
0.09
0.12
0.15
0.18
SNP effect on Gut microbiota abundance (genus Catenibacterium id.2153)

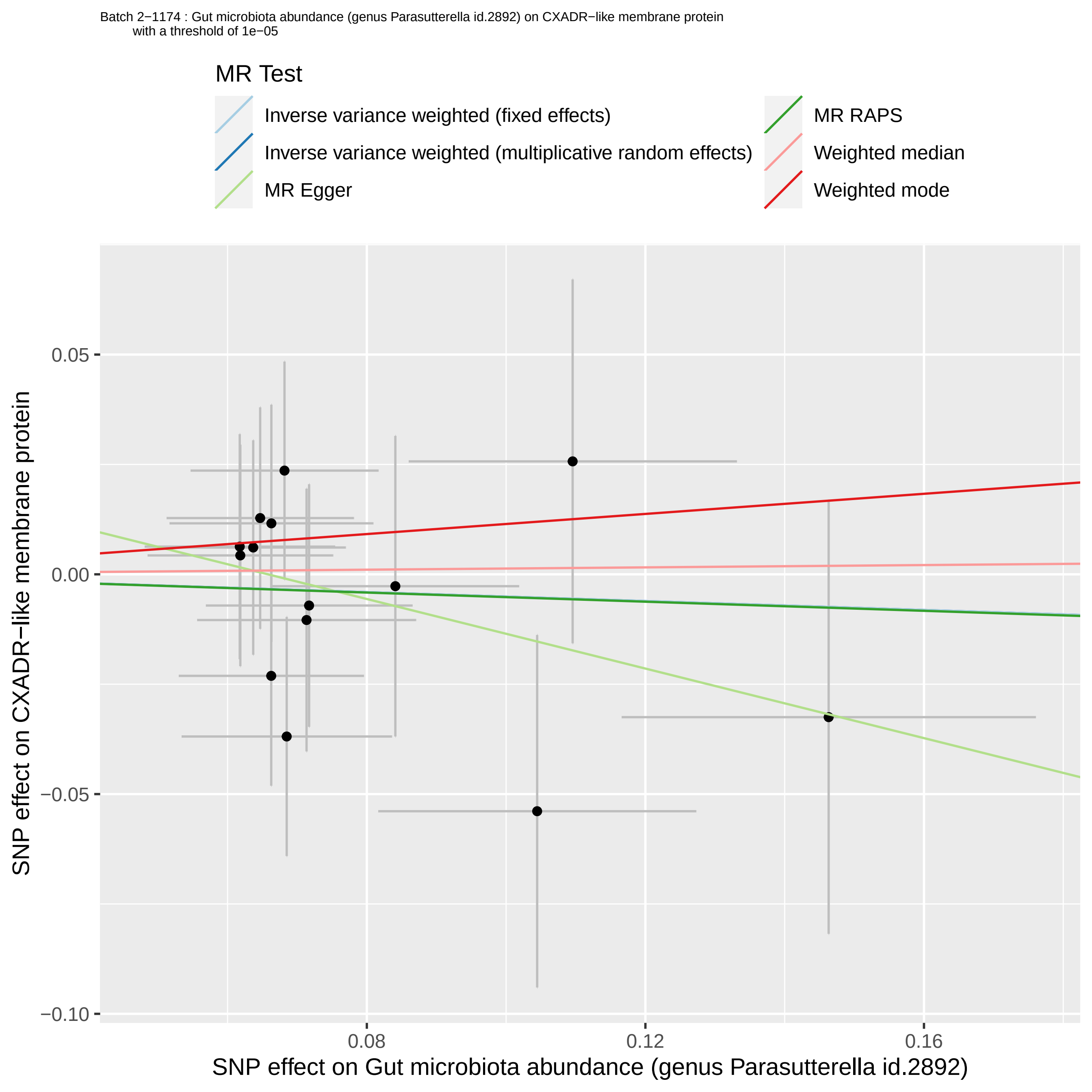
Batch 2−1174 : Gut microbiota abundance (genus Parasutterella id.2892) on CXADR−like membrane protein
with a threshold of 1e−05
MR Test
Inverse variance weighted (fixed effects)
Inverse variance weighted (multiplicative random effects)
MR Egger
MR RAPS
Weighted median
Weighted mode
0.05
0.00
−0.05
−0.10
0.08
0.12
0.16
SNP effect on CXADR−like membrane protein
SNP effect on Gut microbiota abundance (genus Parasutterella id.2892)

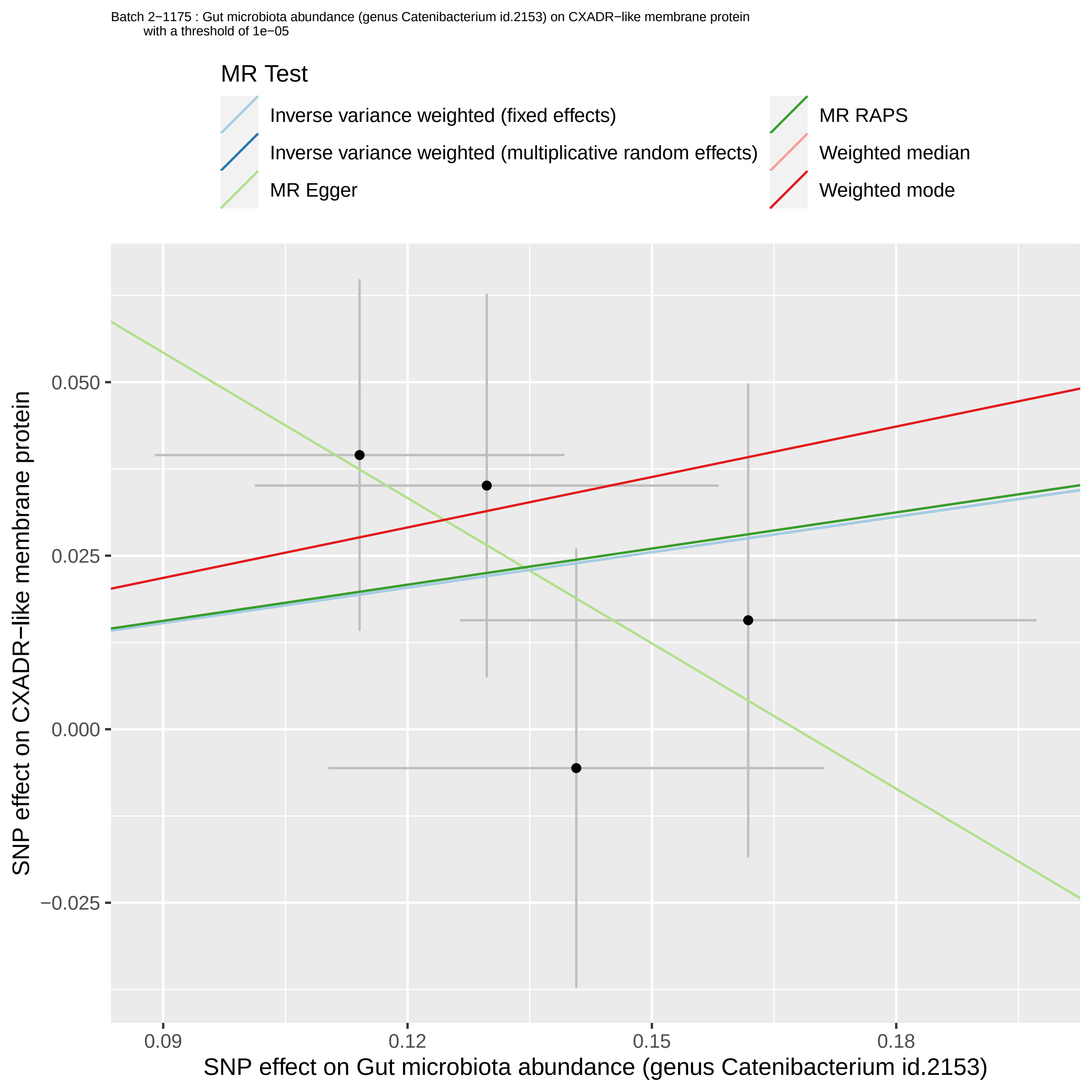

Batch 2−1175 : Gut microbiota abundance (genus Catenibacterium id.2153) on CXADR−like membrane protein with a threshold of 1e−05
MR Test
Inverse variance weighted (fixed effects)
Inverse variance weighted (multiplicative random effects)
MR Egger
MR RAPS
Weighted median
Weighted mode
SNP effect on CXADR−like membrane protein
0.050
0.025
0.000
−0.025
0.09
0.12
0.15
0.18
SNP effect on Gut microbiota abundance (genus Catenibacterium id.2153)

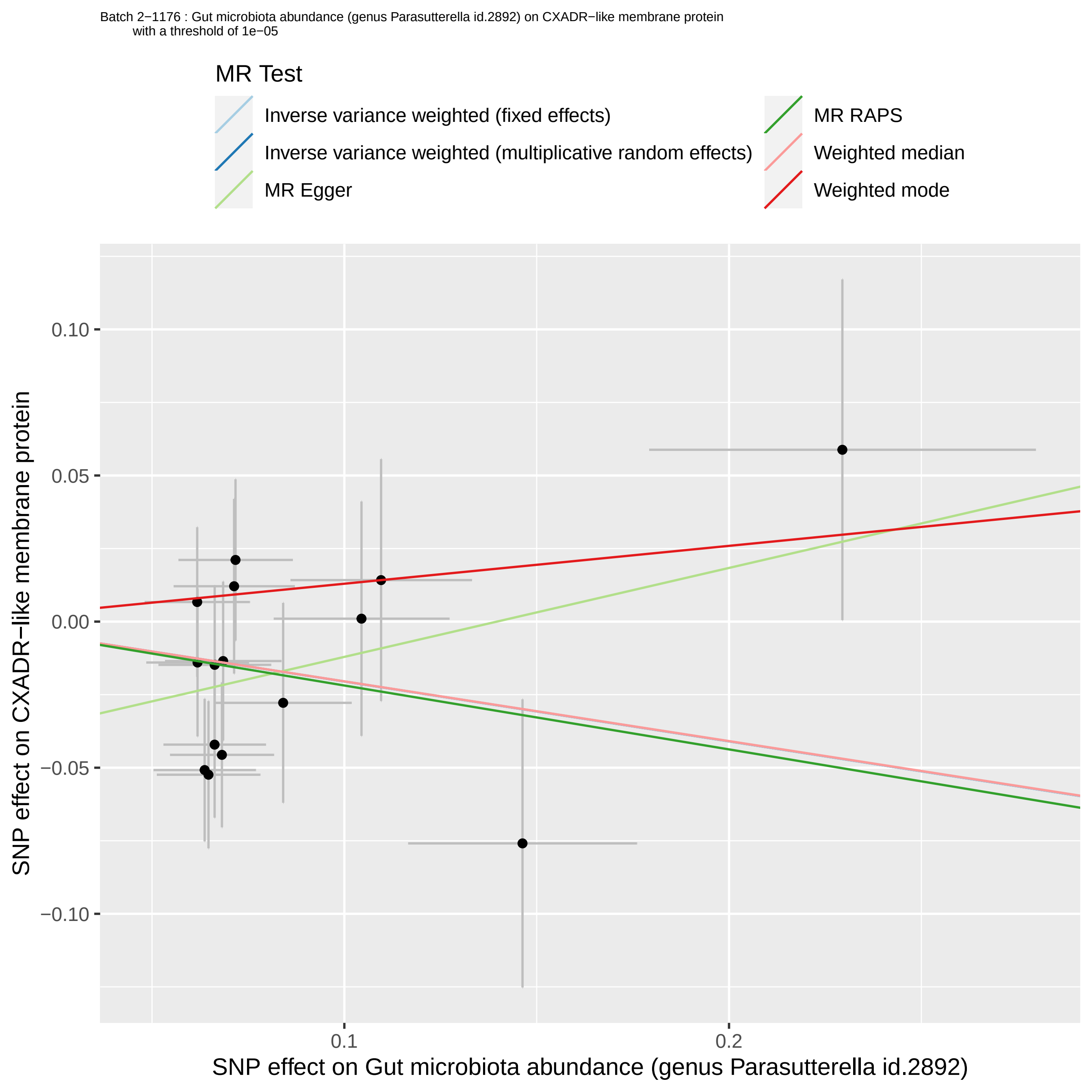
Batch 2−1176 : Gut microbiota abundance (genus Parasutterella id.2892) on CXADR−like membrane protein
with a threshold of 1e−05
MR Test
Inverse variance weighted (fixed effects)
Inverse variance weighted (multiplicative random effects)
MR Egger
MR RAPS
Weighted median
Weighted mode
SNP effect on CXADR−like membrane protein
0.10
0.05
0.00
−0.05
−0.10
0.1
0.2
SNP effect on Gut microbiota abundance (genus Parasutterella id.2892)

Batch 2−1177 : Gut microbiota abundance (genus Catenibacterium id.2153) on Ceroid−lipofuscinosis neuronal protein 5
with a threshold of 1e−05

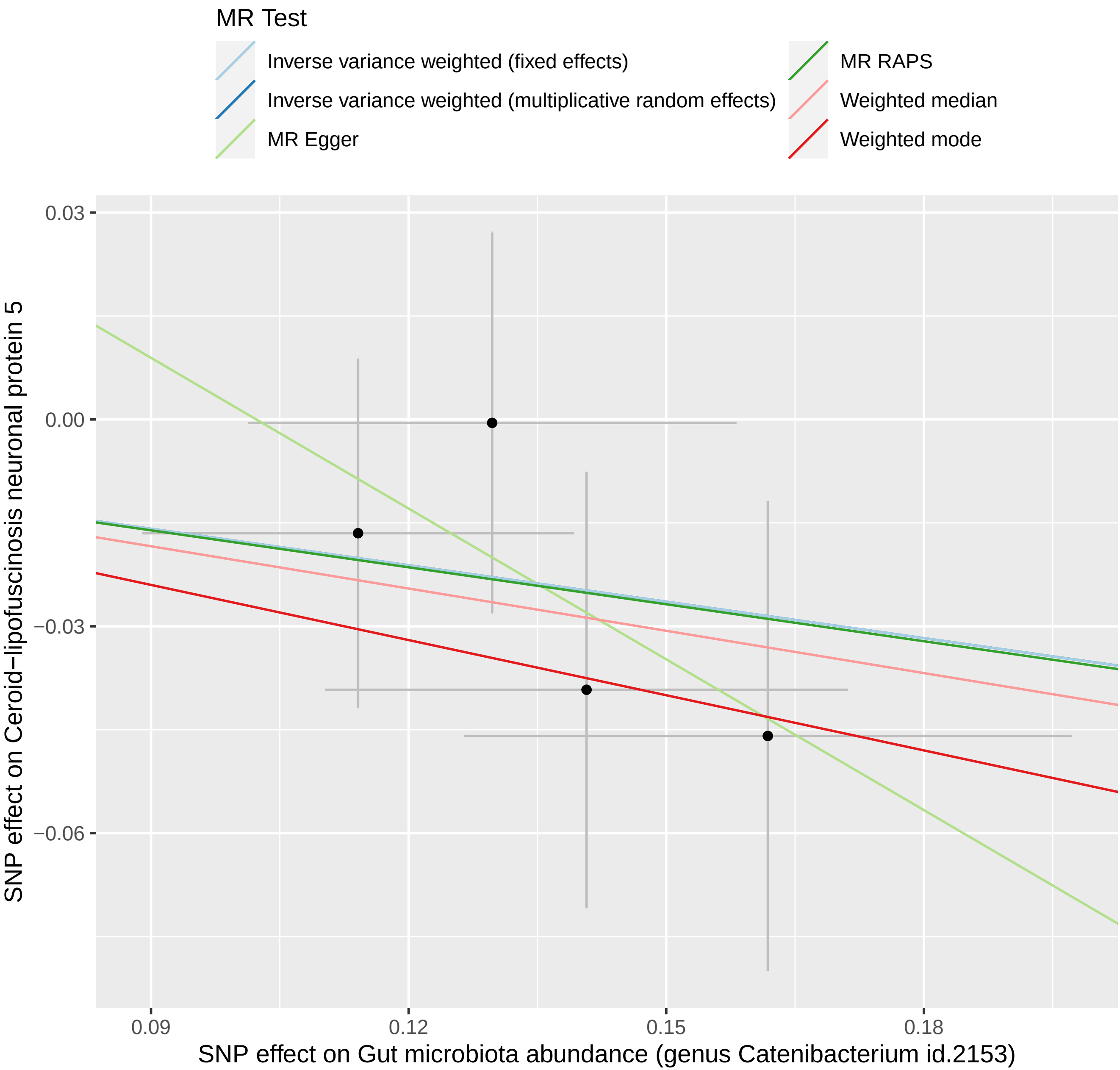

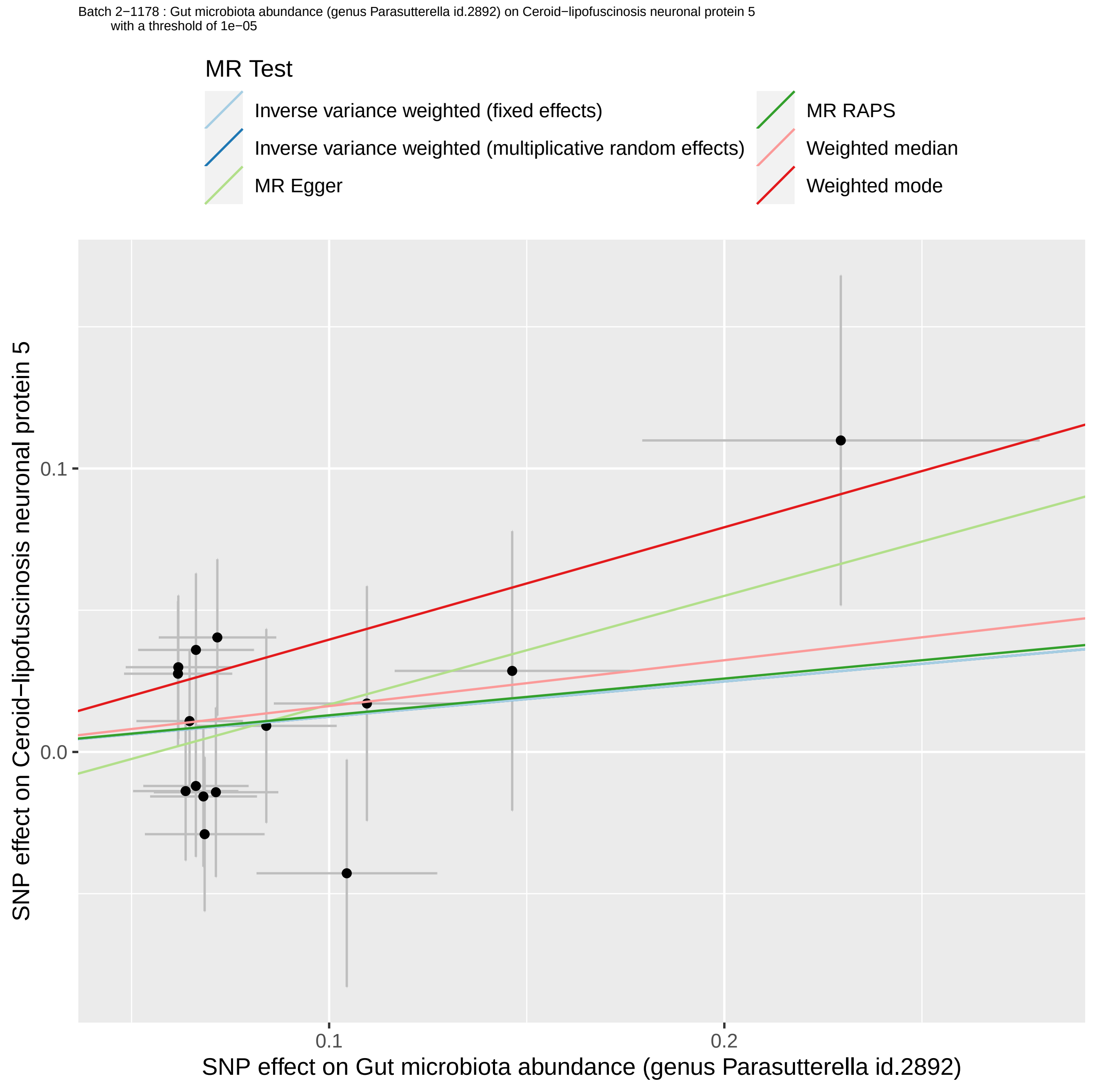
Batch 2−1178 : Gut microbiota abundance (genus Parasutterella id.2892) on Ceroid−lipofuscinosis neuronal protein 5
with a threshold of 1e−05
MR Test
Inverse variance weighted (fixed effects)
Inverse variance weighted (multiplicative random effects)
MR Egger
MR RAPS
Weighted median
Weighted mode
SNP effect on Ceroid−lipofuscinosis neuronal protein 5
0.1
0.0
0.1
0.2
SNP effect on Gut microbiota abundance (genus Parasutterella id.2892)

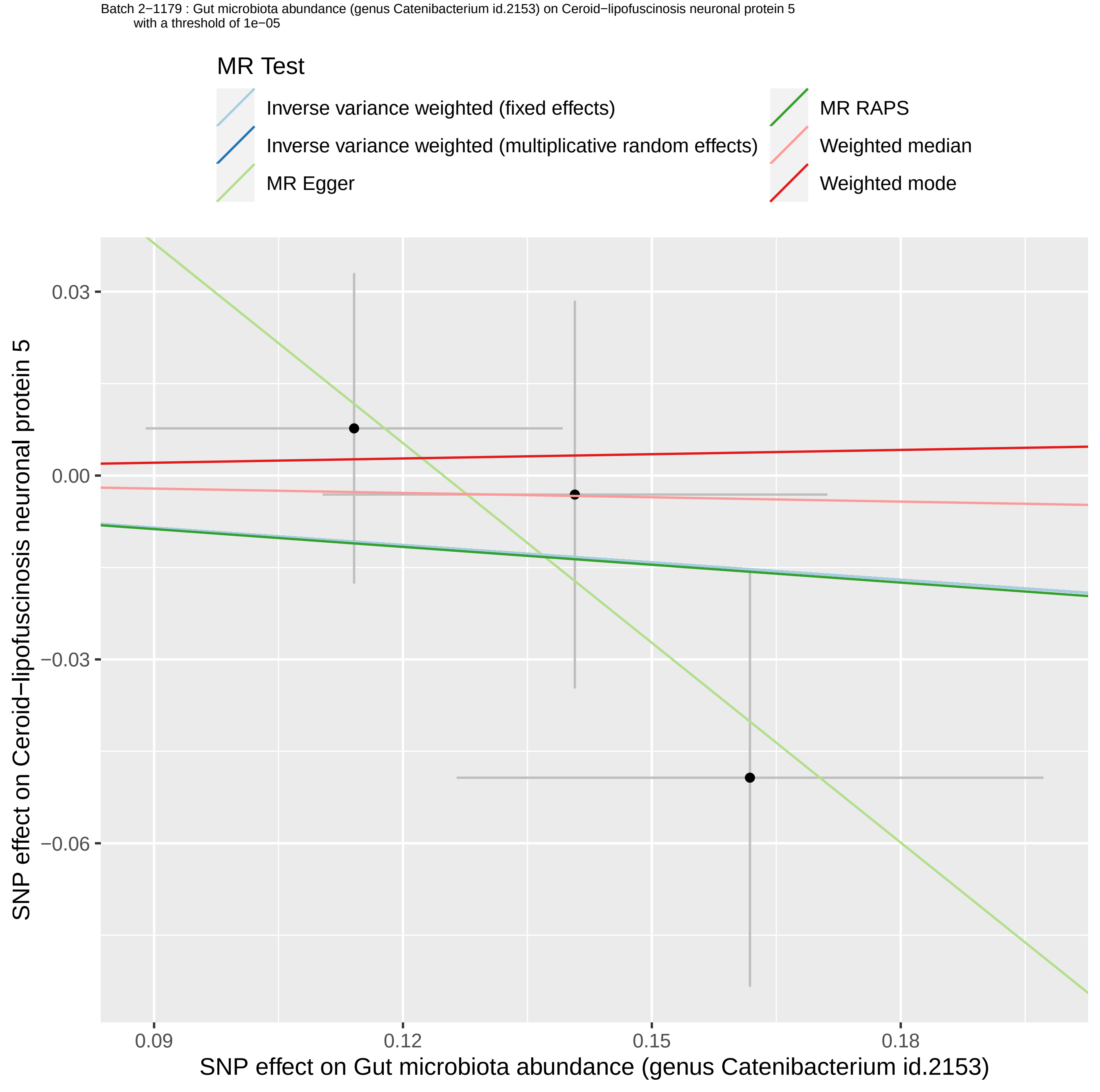

Batch 2−1179 : Gut microbiota abundance (genus Catenibacterium id.2153) on Ceroid−lipofuscinosis neuronal protein 5
with a threshold of 1e−05
MR Test
Inverse variance weighted (fixed effects)
Inverse variance weighted (multiplicative random effects)
MR Egger
MR RAPS
Weighted median
Weighted mode
0.03
0.00
−0.03
−0.06
SNP effect on Ceroid−lipofuscinosis neuronal protein 5
0.09
0.12
0.15
0.18
SNP effect on Gut microbiota abundance (genus Catenibacterium id.2153)

Batch 2−1180 : Gut microbiota abundance (genus Parasutterella id.2892) on Ceroid−lipofuscinosis neuronal protein 5
with a threshold of 1e−05

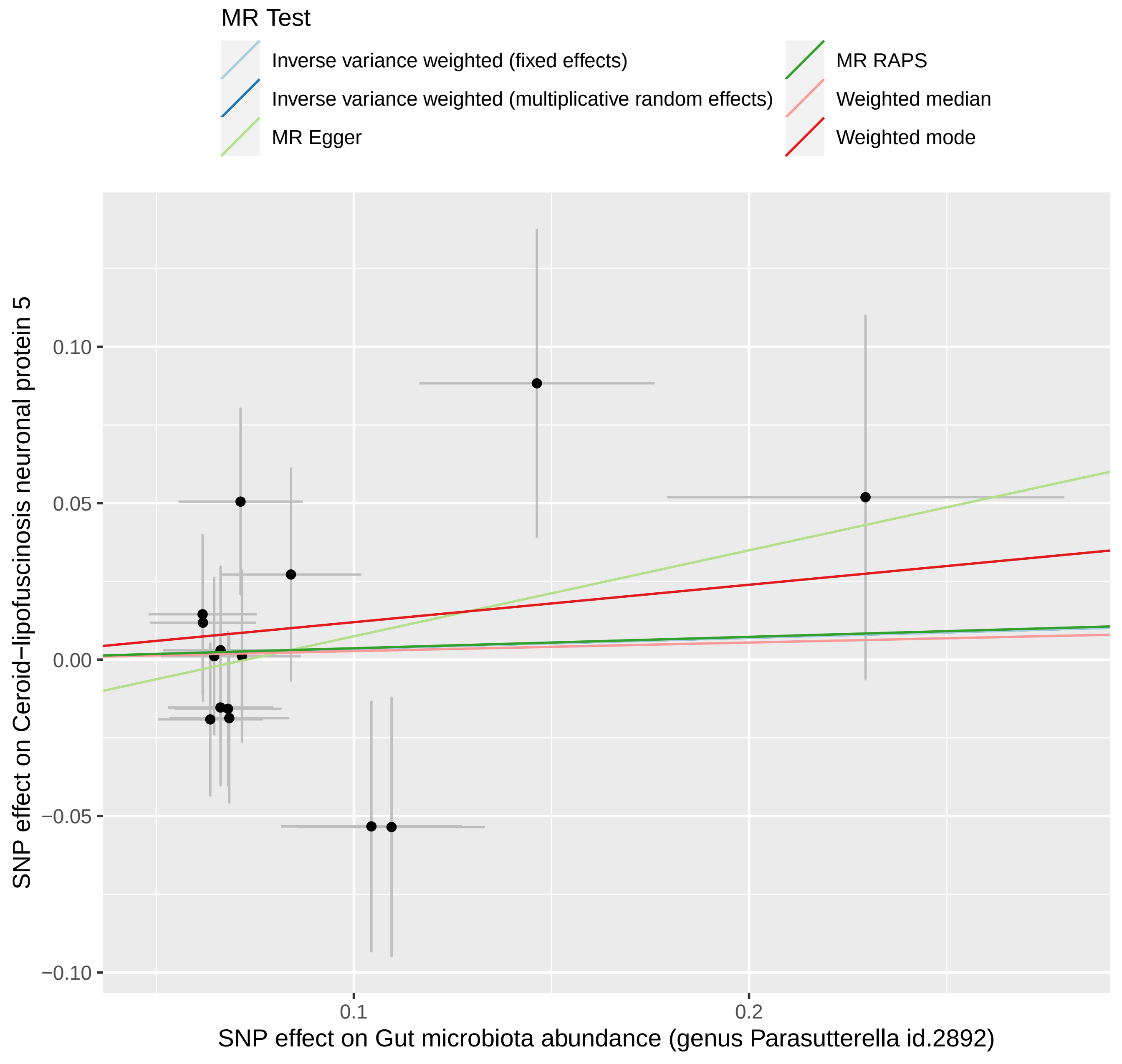

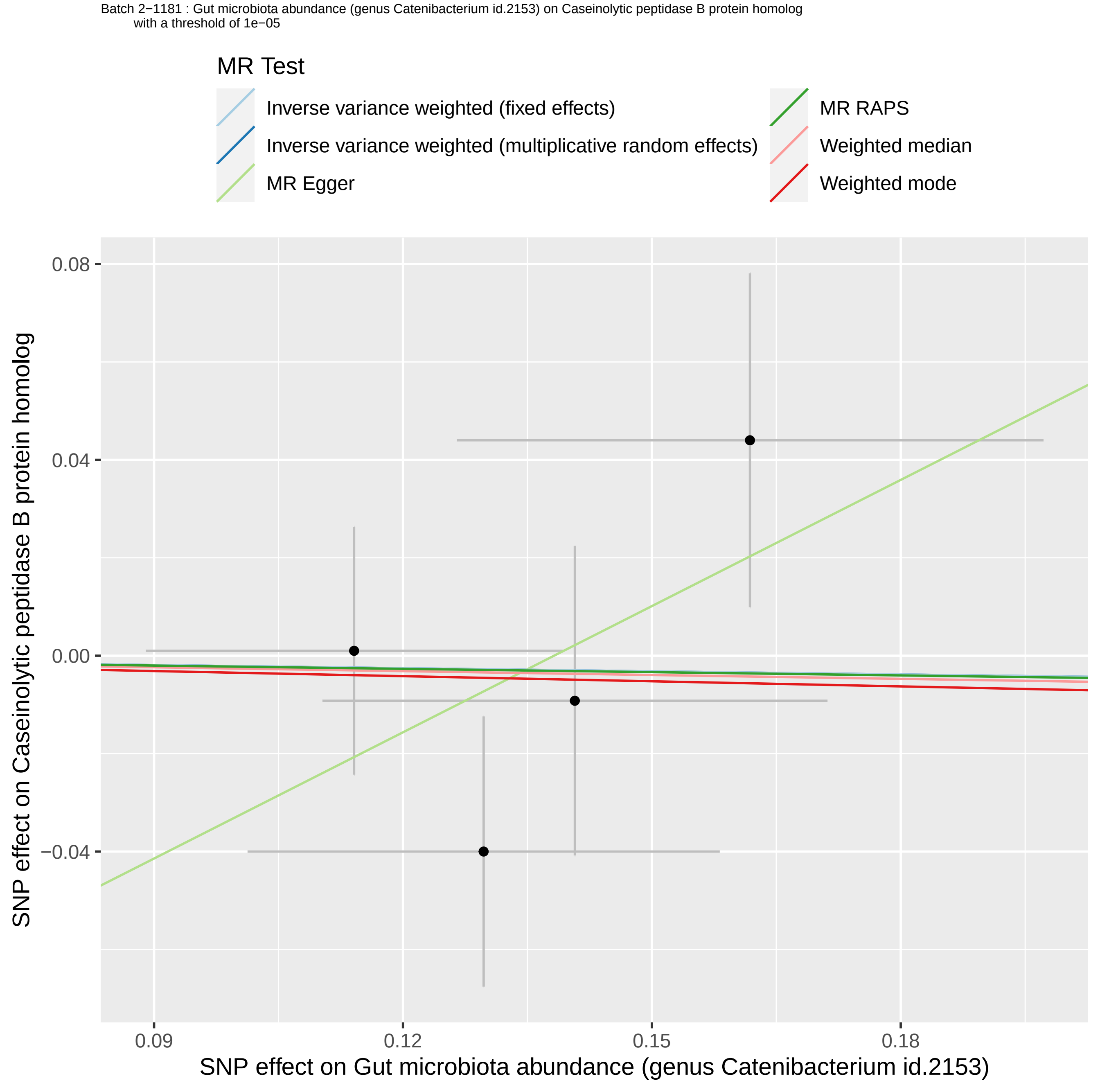

Batch 2−1181 : Gut microbiota abundance (genus Catenibacterium id.2153) on Caseinolytic peptidase B protein homolog
with a threshold of 1e−05
MR Test
Inverse variance weighted (fixed effects)
Inverse variance weighted (multiplicative random effects)
MR Egger
MR RAPS
Weighted median
Weighted mode
0.08
0.04
0.00
−0.04
0.09
0.12
0.15
0.18
SNP effect on Caseinolytic peptidase B protein homolog
SNP effect on Gut microbiota abundance (genus Catenibacterium id.2153)

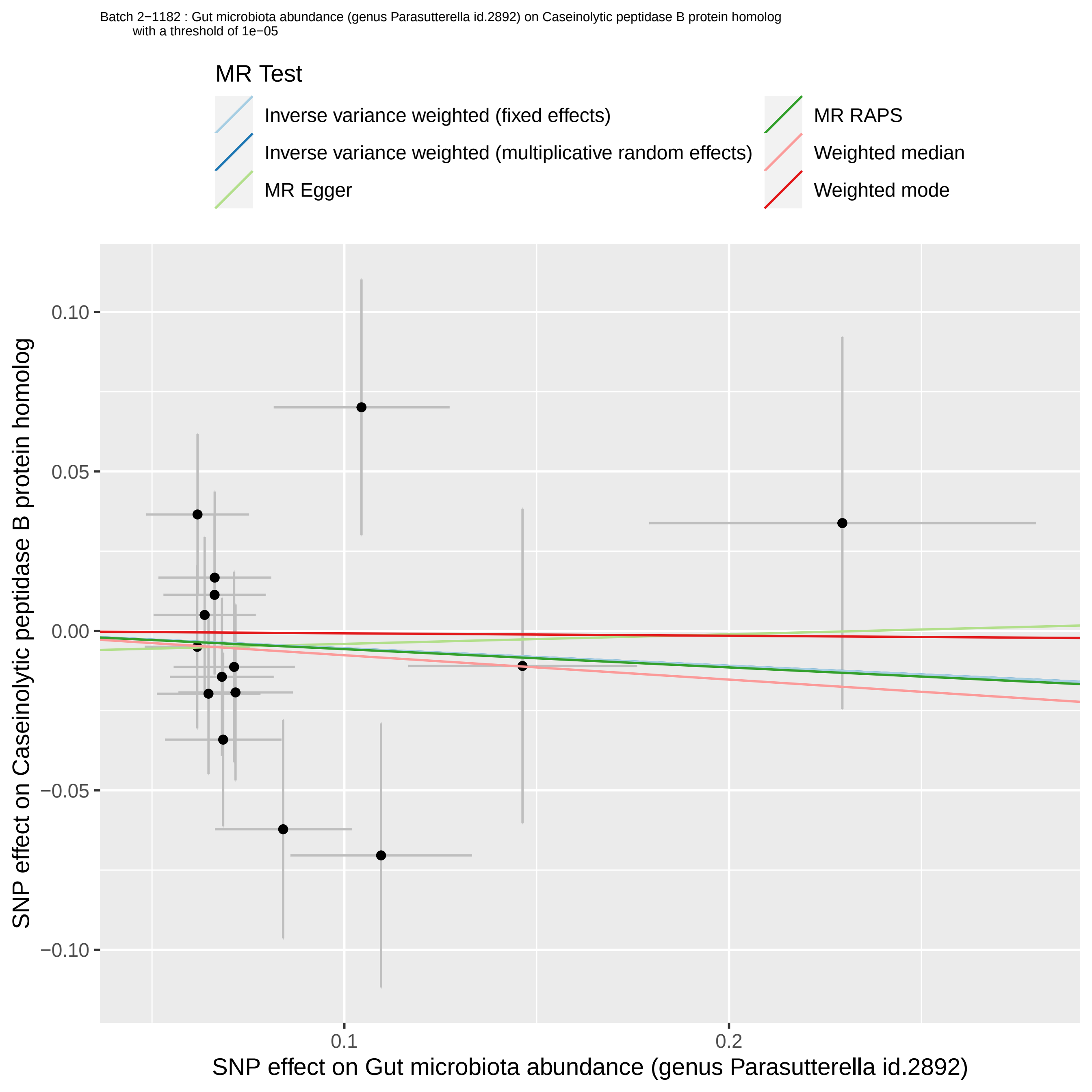
Batch 2−1182 : Gut microbiota abundance (genus Parasutterella id.2892) on Caseinolytic peptidase B protein homolog
with a threshold of 1e−05
MR Test
Inverse variance weighted (fixed effects)
Inverse variance weighted (multiplicative random effects)
MR Egger
MR RAPS
Weighted median
Weighted mode
0.10
0.05
0.00
−0.05
−0.10
0.1
0.2
SNP effect on Caseinolytic peptidase B protein homolog
SNP effect on Gut microbiota abundance (genus Parasutterella id.2892)

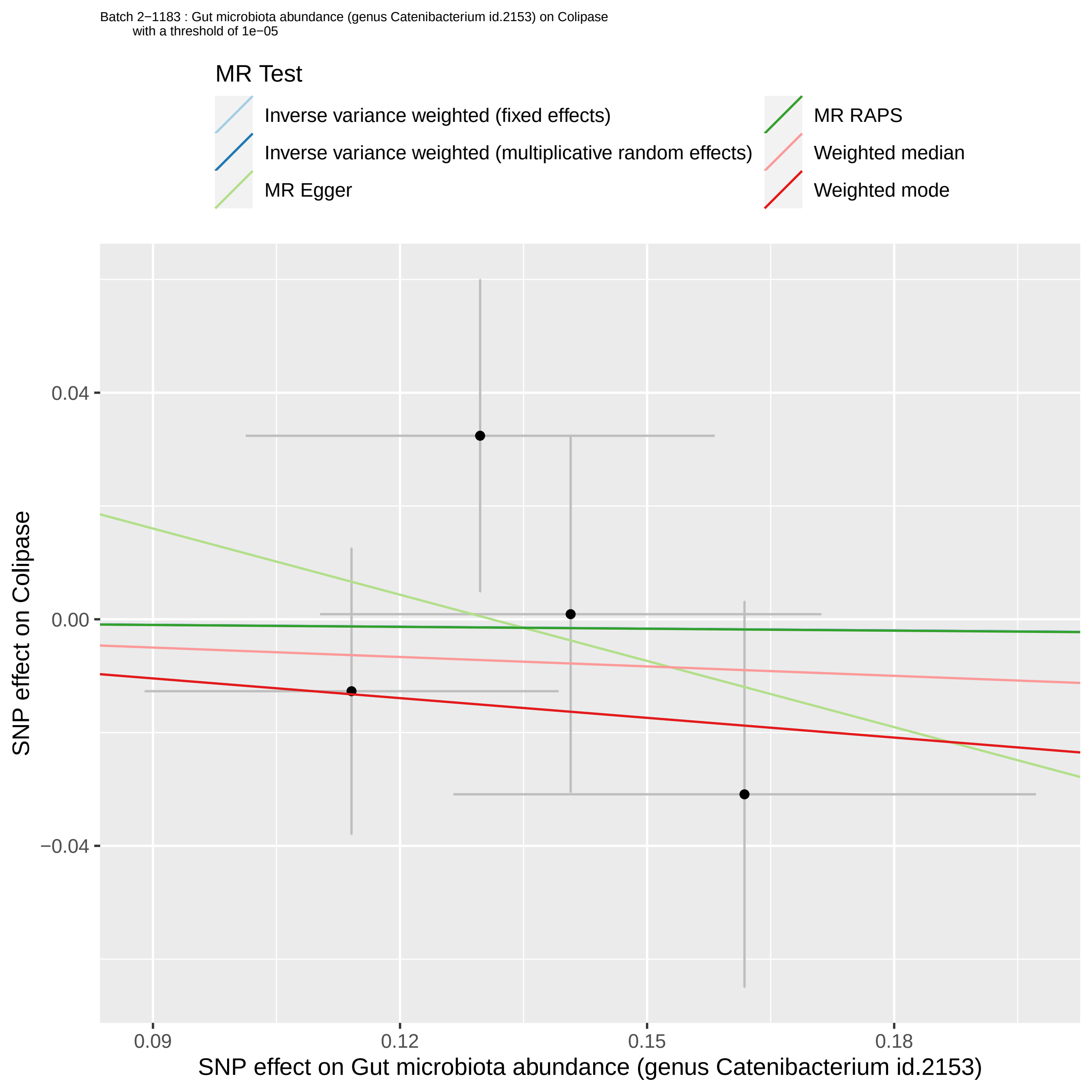
Batch 2−1183 : Gut microbiota abundance (genus Catenibacterium id.2153) on Colipase
with a threshold of 1e−05
MR Test
Inverse variance weighted (fixed effects)
Inverse variance weighted (multiplicative random effects)
MR Egger
MR RAPS
Weighted median
Weighted mode
0.04
0.00
−0.04
0.09
0.12
0.15
0.18
SNP effect on Colipase
SNP effect on Gut microbiota abundance (genus Catenibacterium id.2153)

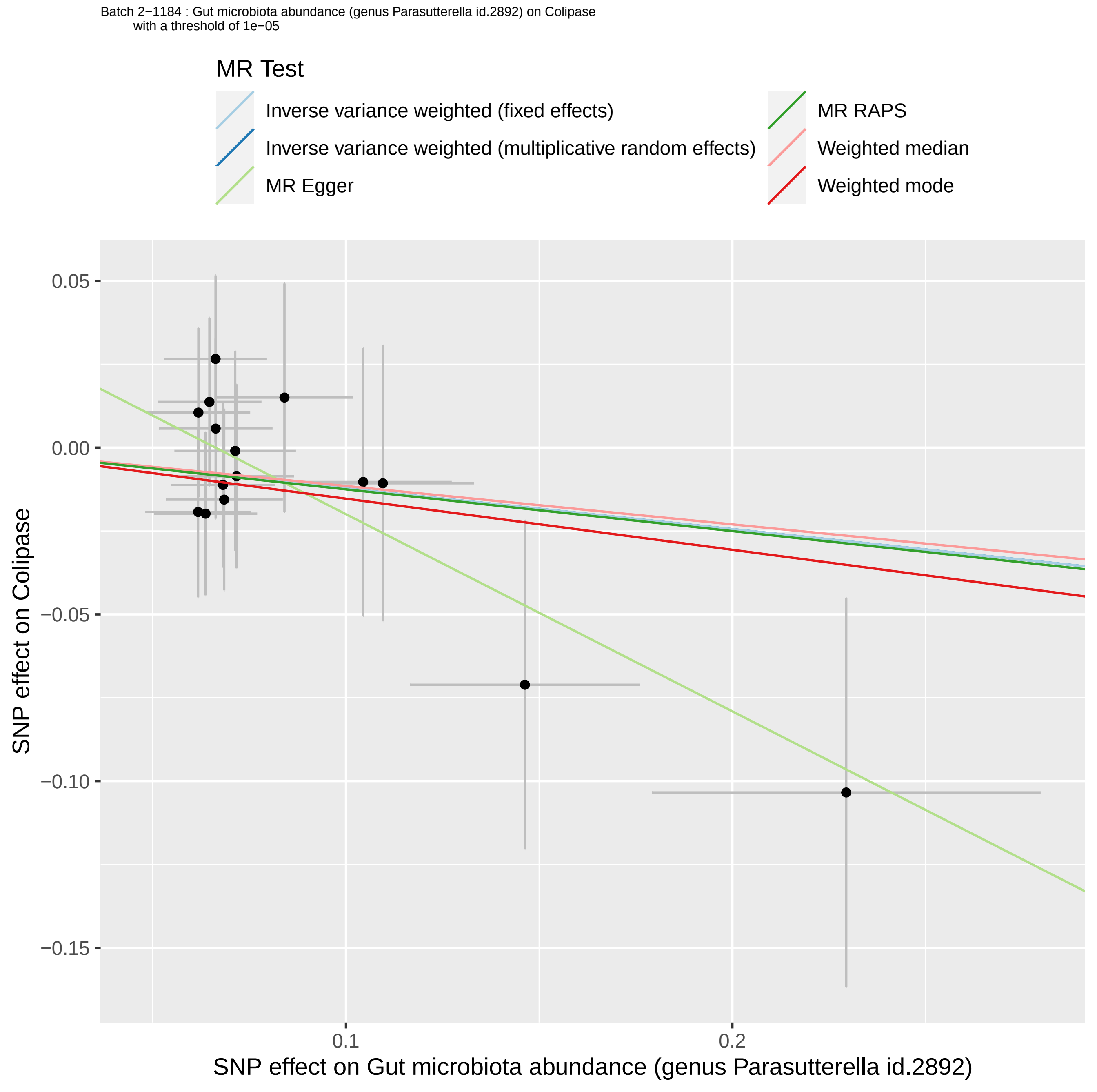

Batch 2−1184 : Gut microbiota abundance (genus Parasutterella id.2892) on Colipase
with a threshold of 1e−05
MR Test
Inverse variance weighted (fixed effects)
Inverse variance weighted (multiplicative random effects)
MR Egger
MR RAPS
Weighted median
Weighted mode
0.05
0.00
−0.05
−0.10
−0.15
SNP effect on Colipase
0.1
0.2
SNP effect on Gut microbiota abundance (genus Parasutterella id.2892)

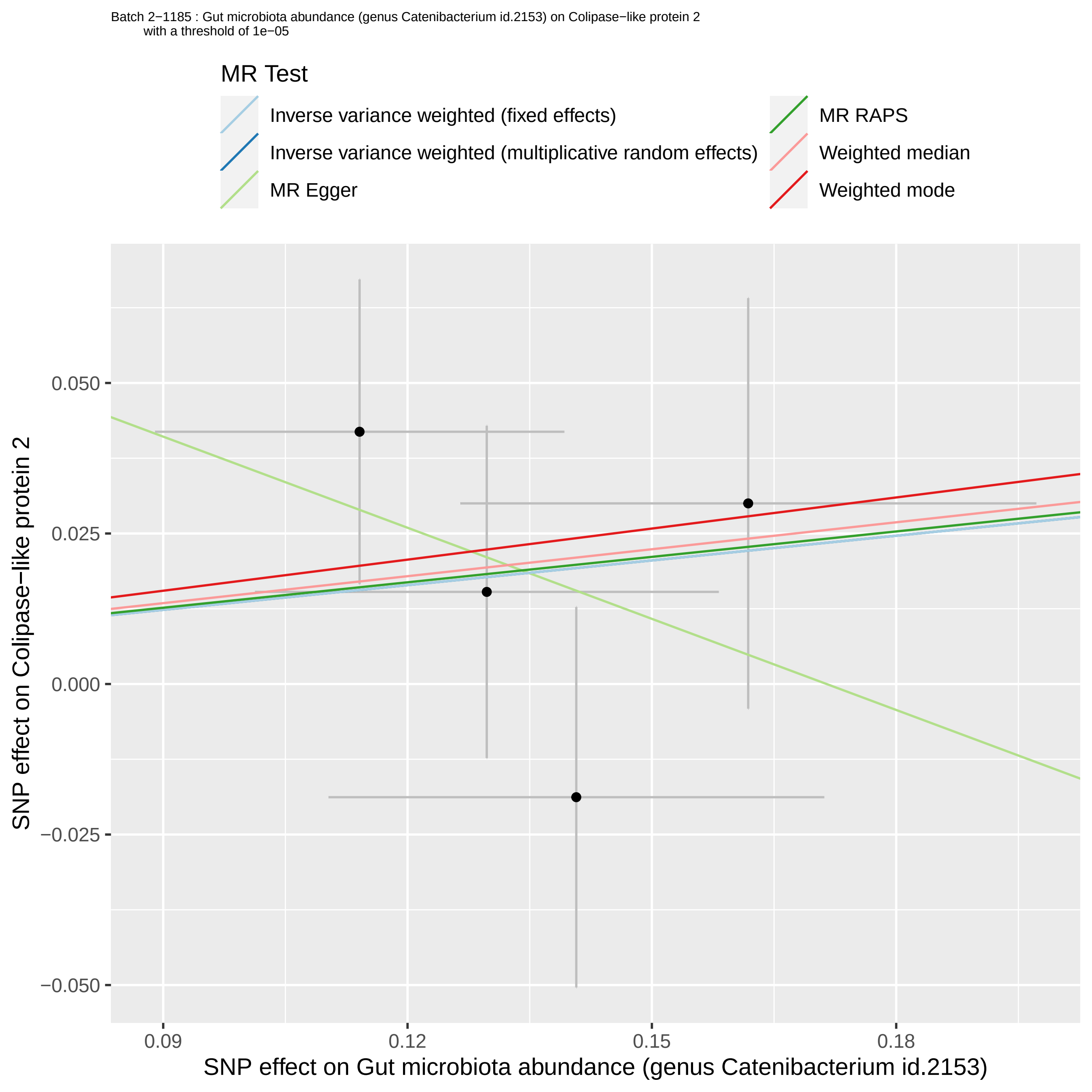
Batch 2−1185 : Gut microbiota abundance (genus Catenibacterium id.2153) on Colipase−like protein 2
with a threshold of 1e−05
MR Test
Inverse variance weighted (fixed effects)
Inverse variance weighted (multiplicative random effects)
MR Egger
MR RAPS
Weighted median
Weighted mode
0.050
0.025
0.000
−0.025
−0.050
SNP effect on Colipase−like protein 2
0.09
0.12
0.15
0.18
SNP effect on Gut microbiota abundance (genus Catenibacterium id.2153)

Batch 2−1186 : Gut microbiota abundance (genus Parasutterella id.2892) on Colipase−like protein 2
with a threshold of 1e−05

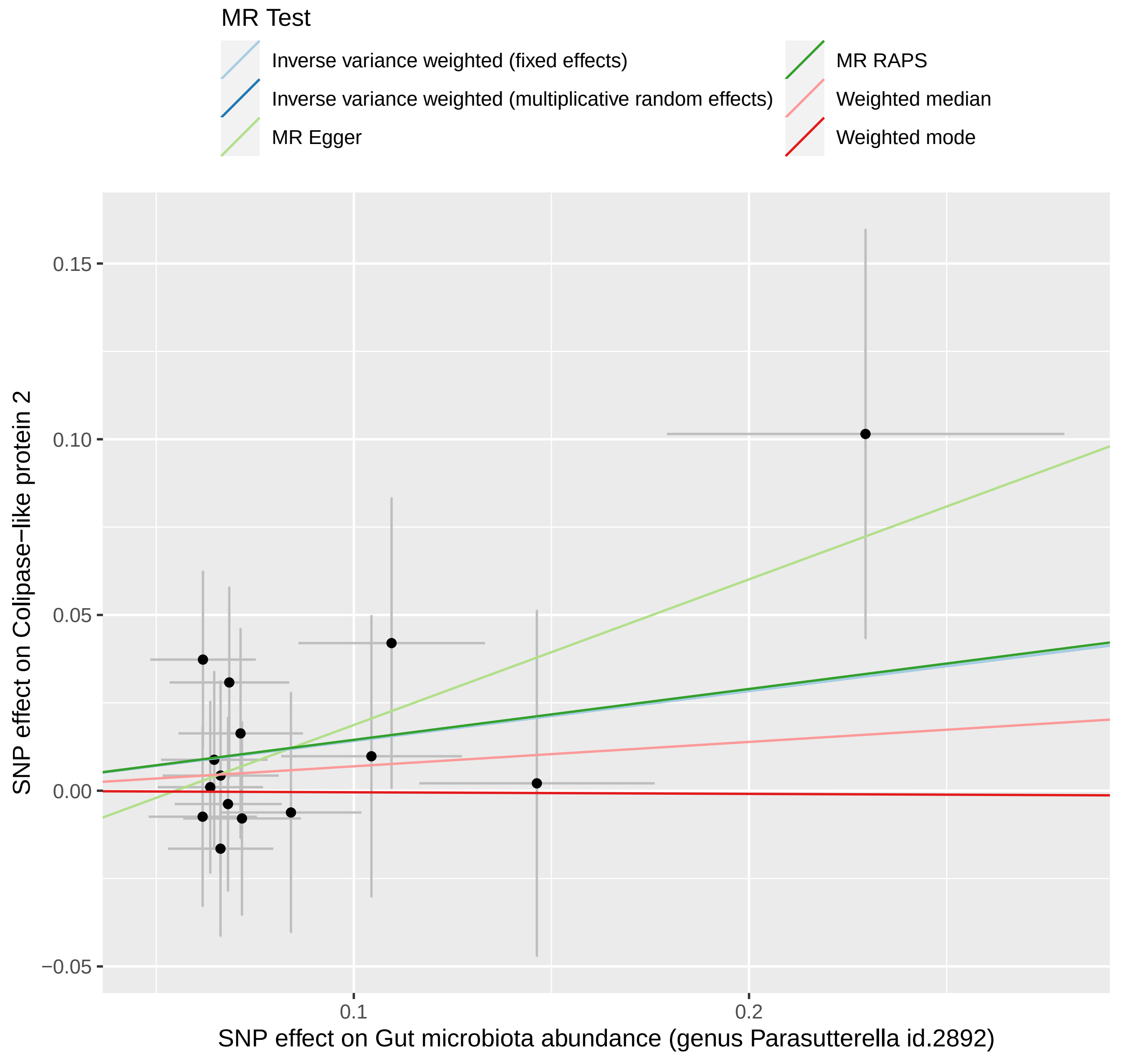

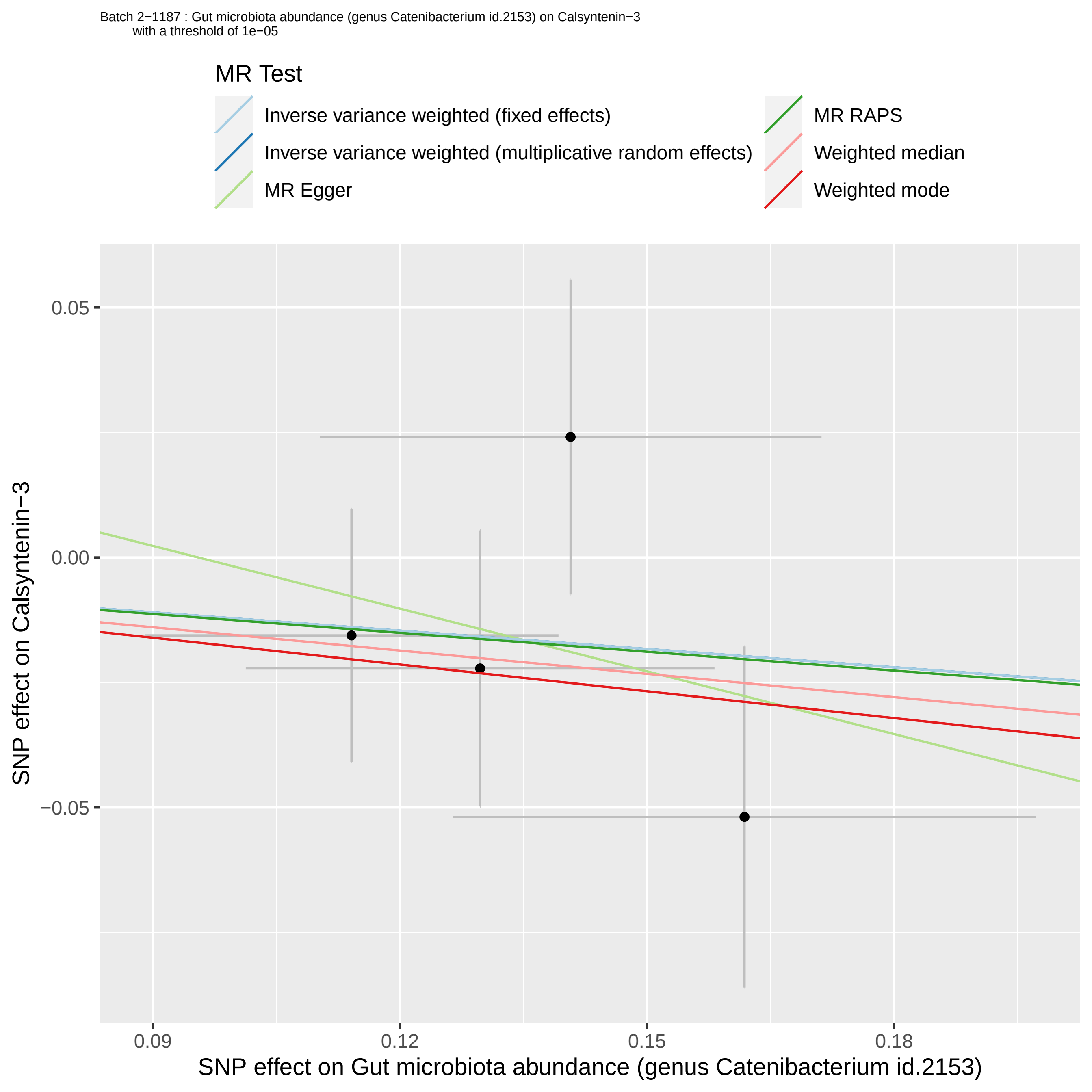
Batch 2−1187 : Gut microbiota abundance (genus Catenibacterium id.2153) on Calsyntenin−3
with a threshold of 1e−05
MR Test
Inverse variance weighted (fixed effects)
Inverse variance weighted (multiplicative random effects)
MR Egger
MR RAPS
Weighted median
Weighted mode
0.05
0.00
−0.05
SNP effect on Calsyntenin−3
0.09
0.12
0.15
0.18
SNP effect on Gut microbiota abundance (genus Catenibacterium id.2153)

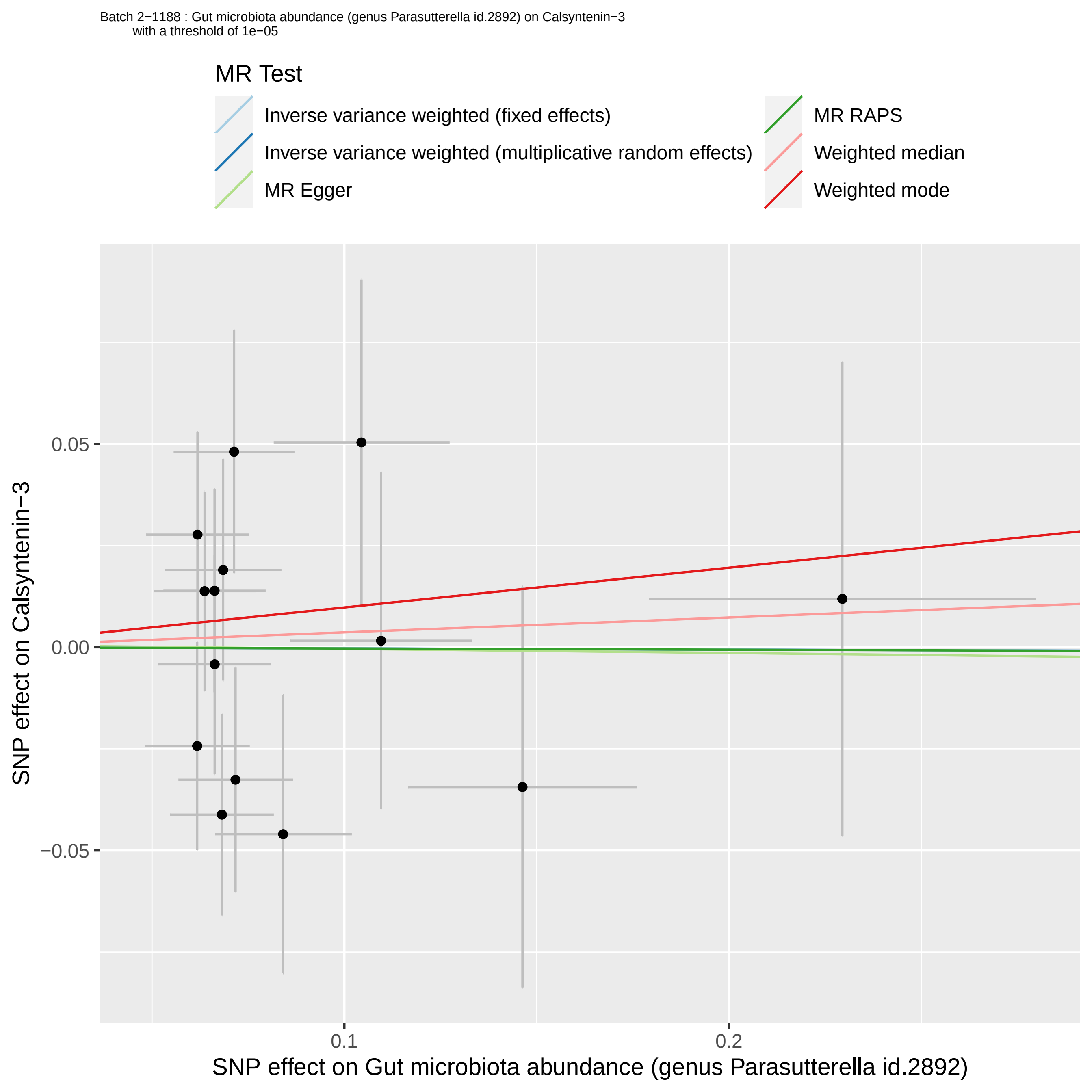
Batch 2−1188 : Gut microbiota abundance (genus Parasutterella id.2892) on Calsyntenin−3
with a threshold of 1e−05
MR Test
Inverse variance weighted (fixed effects)
Inverse variance weighted (multiplicative random effects)
MR Egger
MR RAPS
Weighted median
Weighted mode
0.05
0.00
−0.05
0.1
0.2
SNP effect on Calsyntenin−3
SNP effect on Gut microbiota abundance (genus Parasutterella id.2892)

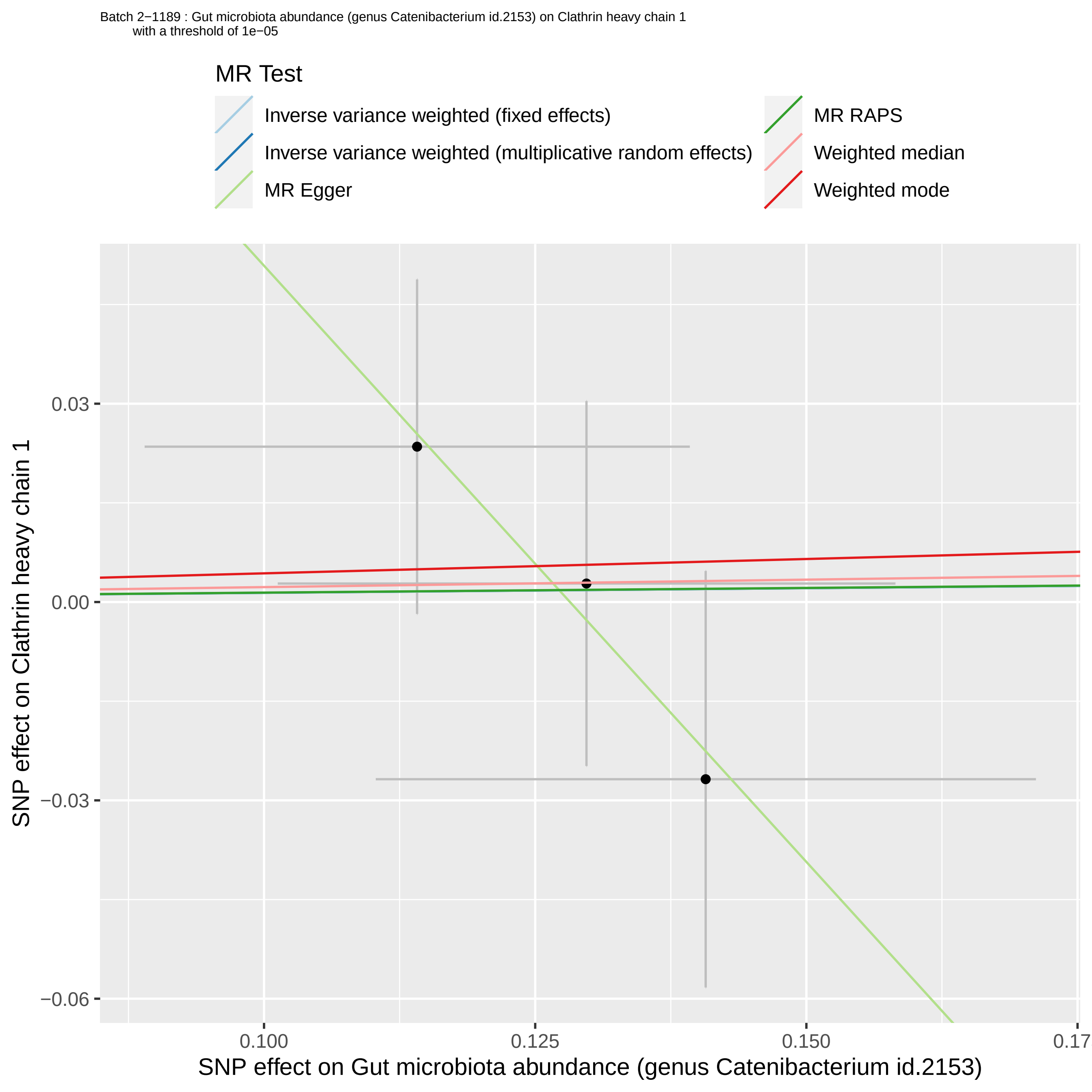
Batch 2−1189 : Gut microbiota abundance (genus Catenibacterium id.2153) on Clathrin heavy chain 1
with a threshold of 1e−05
MR Test
Inverse variance weighted (fixed effects)
Inverse variance weighted (multiplicative random effects)
MR Egger
MR RAPS
Weighted median
Weighted mode
0.03
0.00
−0.03
−0.06
0.100
0.125
0.150
0.17
SNP effect on Clathrin heavy chain 1
SNP effect on Gut microbiota abundance (genus Catenibacterium id.2153)

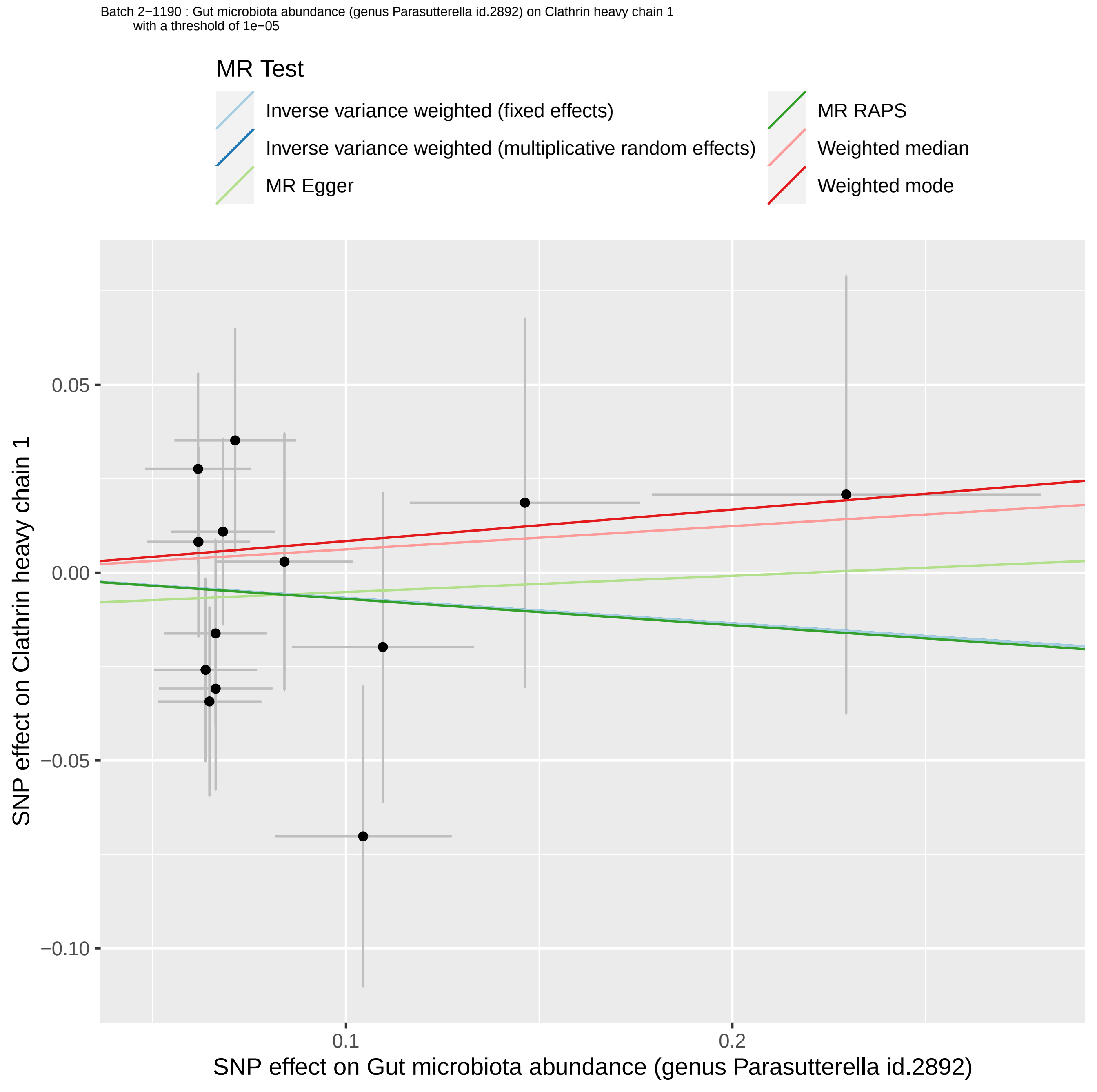
Batch 2−1190 : Gut microbiota abundance (genus Parasutterella id.2892) on Clathrin heavy chain 1
with a threshold of 1e−05
MR Test
Inverse variance weighted (fixed effects)
Inverse variance weighted (multiplicative random effects)
MR Egger
MR RAPS
Weighted median
Weighted mode
0.05
0.00
−0.05
−0.10
0.1
0.2
SNP effect on Clathrin heavy chain 1
SNP effect on Gut microbiota abundance (genus Parasutterella id.2892)

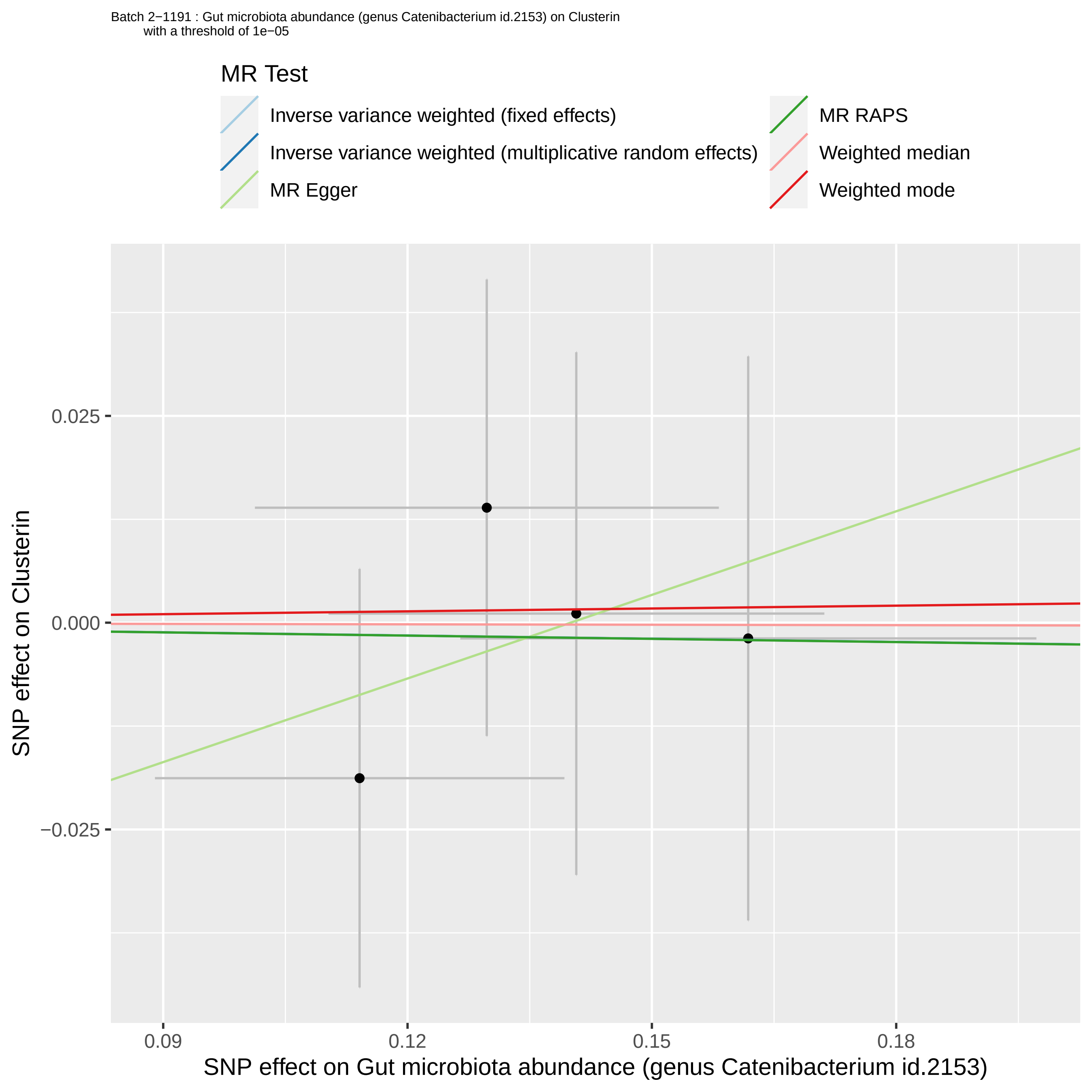
Batch 2−1191 : Gut microbiota abundance (genus Catenibacterium id.2153) on Clusterin
with a threshold of 1e−05
MR Test
Inverse variance weighted (fixed effects)
Inverse variance weighted (multiplicative random effects)
MR Egger
MR RAPS
Weighted median
Weighted mode
0.025
0.000
−0.025
SNP effect on Clusterin
0.09
0.12
0.15
0.18
SNP effect on Gut microbiota abundance (genus Catenibacterium id.2153)

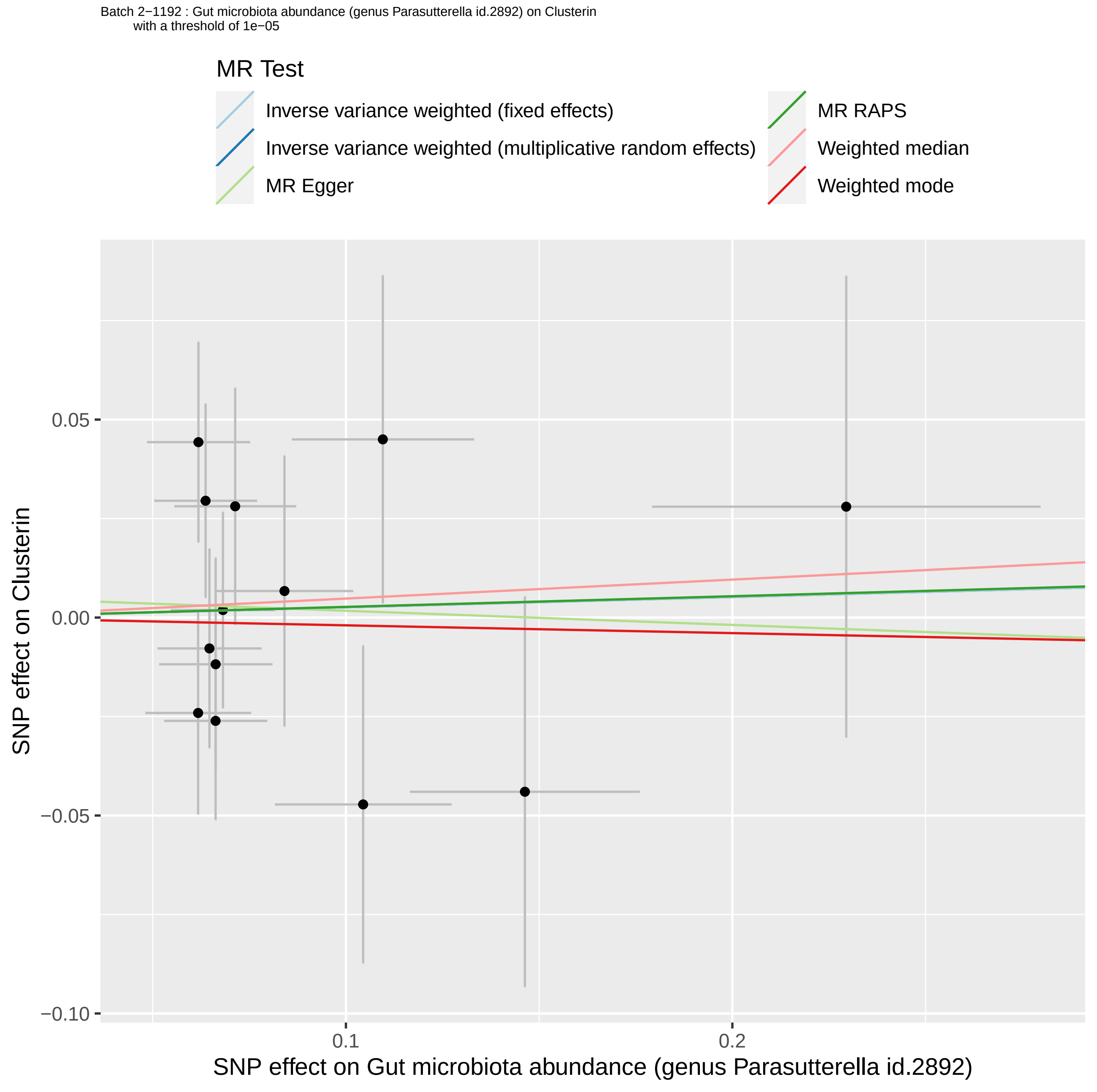
Batch 2−1192 : Gut microbiota abundance (genus Parasutterella id.2892) on Clusterin
with a threshold of 1e−05
MR Test
Inverse variance weighted (fixed effects)
Inverse variance weighted (multiplicative random effects)
MR Egger
MR RAPS
Weighted median
Weighted mode
SNP effect on Clusterin
0.05
0.00
−0.05
−0.10
0.1
0.2
SNP effect on Gut microbiota abundance (genus Parasutterella id.2892)

Batch 2−1193 : Gut microbiota abundance (genus Catenibacterium id.2153) on Chymase
with a threshold of 1e−05

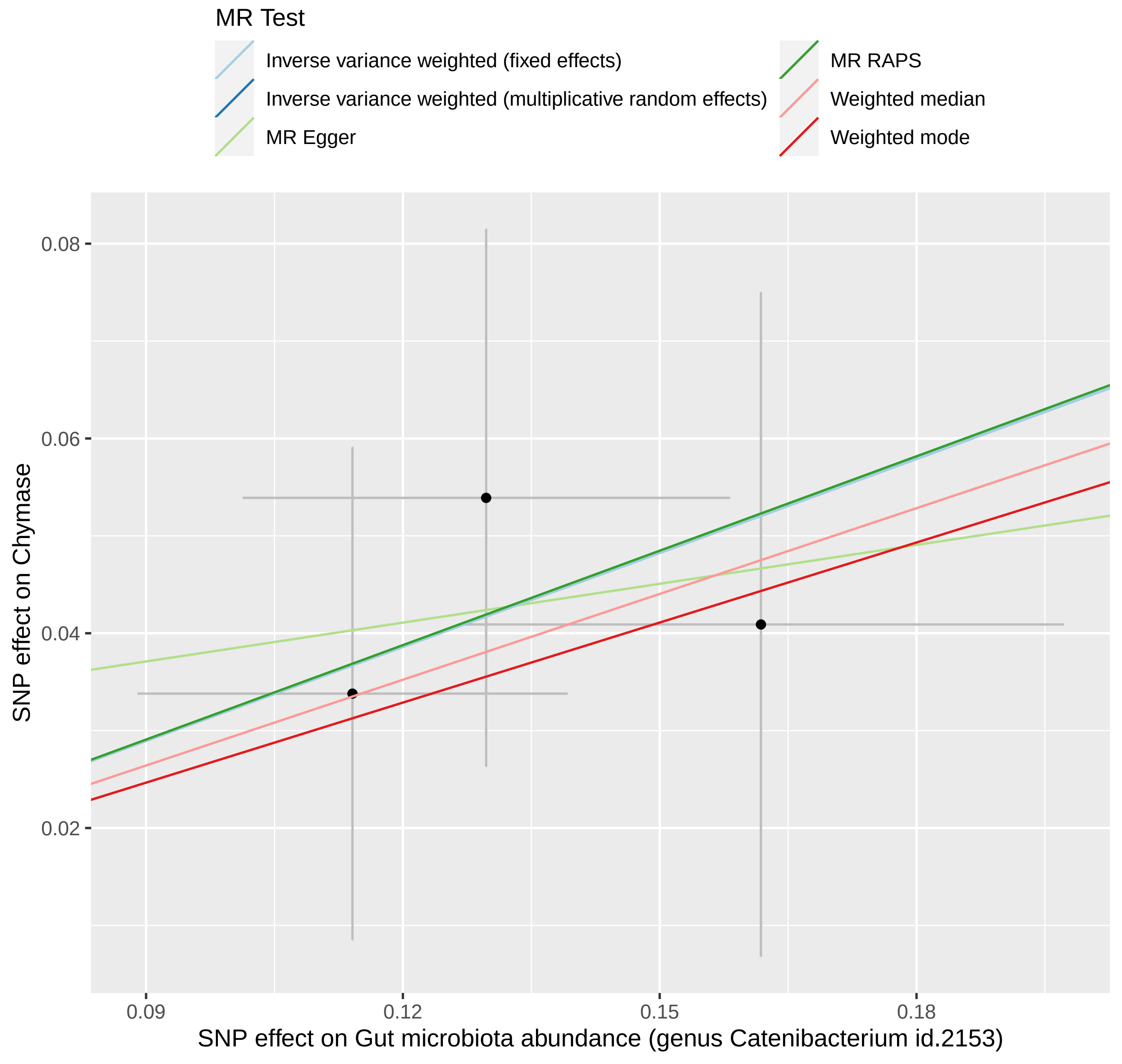

Batch 2−1194 : Gut microbiota abundance (genus Parasutterella id.2892) on Chymase
with a threshold of 1e−05

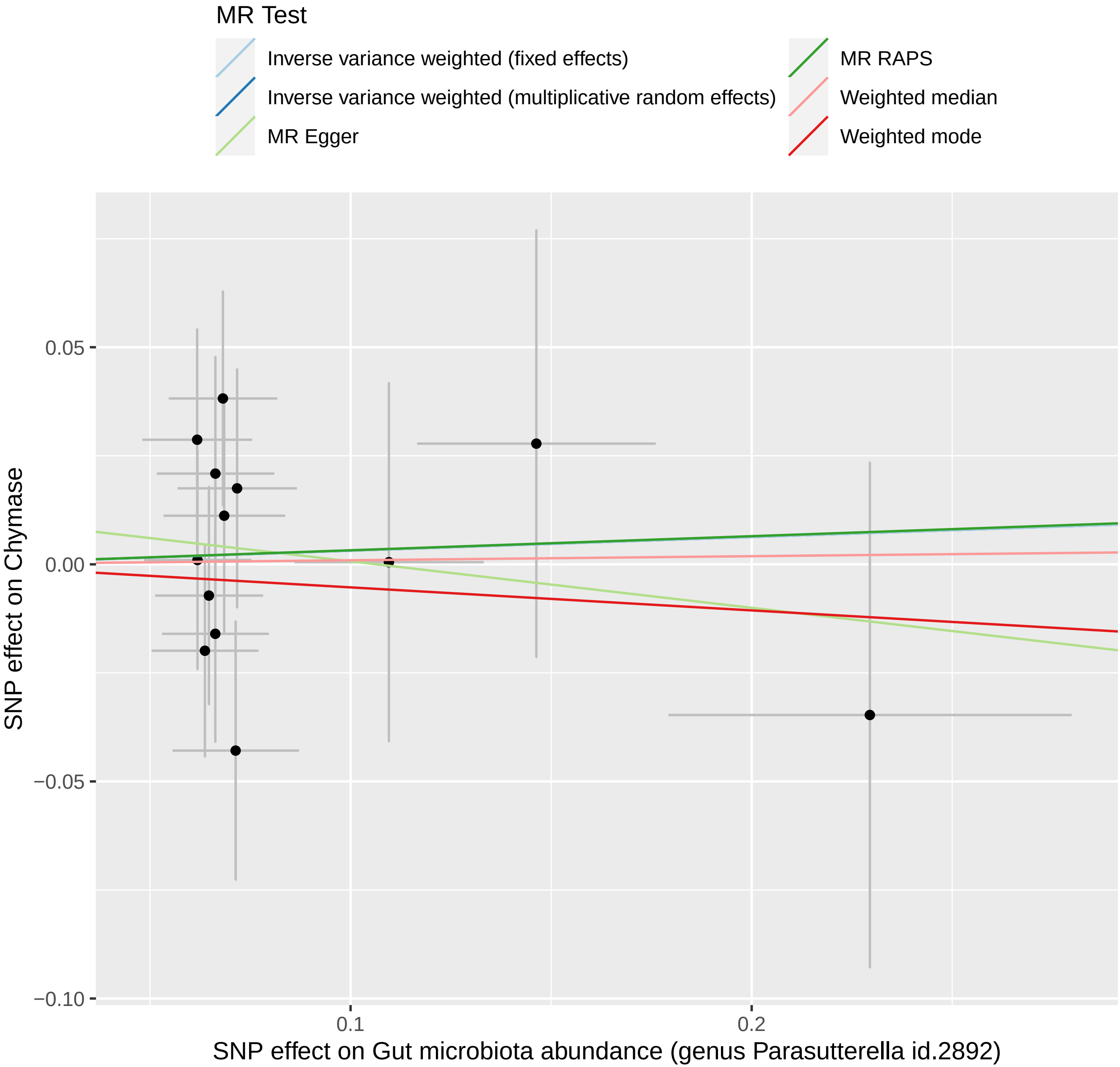

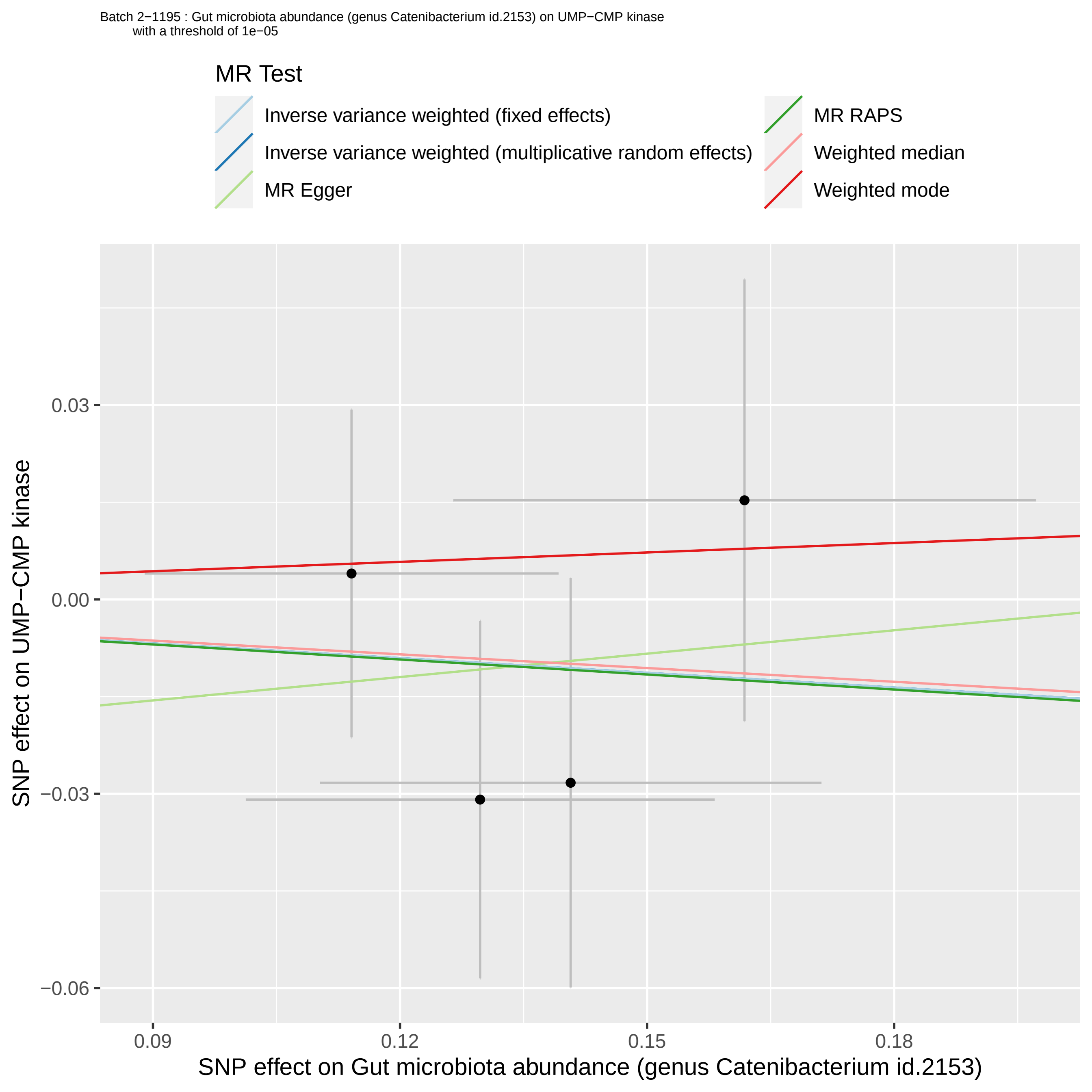
Batch 2−1195 : Gut microbiota abundance (genus Catenibacterium id.2153) on UMP−CMP kinase
with a threshold of 1e−05
MR Test
Inverse variance weighted (fixed effects)
Inverse variance weighted (multiplicative random effects)
MR Egger
MR RAPS
Weighted median
Weighted mode
SNP effect on UMP−CMP kinase
0.03
0.00
−0.03
−0.06
0.09
0.12
0.15
0.18
SNP effect on Gut microbiota abundance (genus Catenibacterium id.2153)

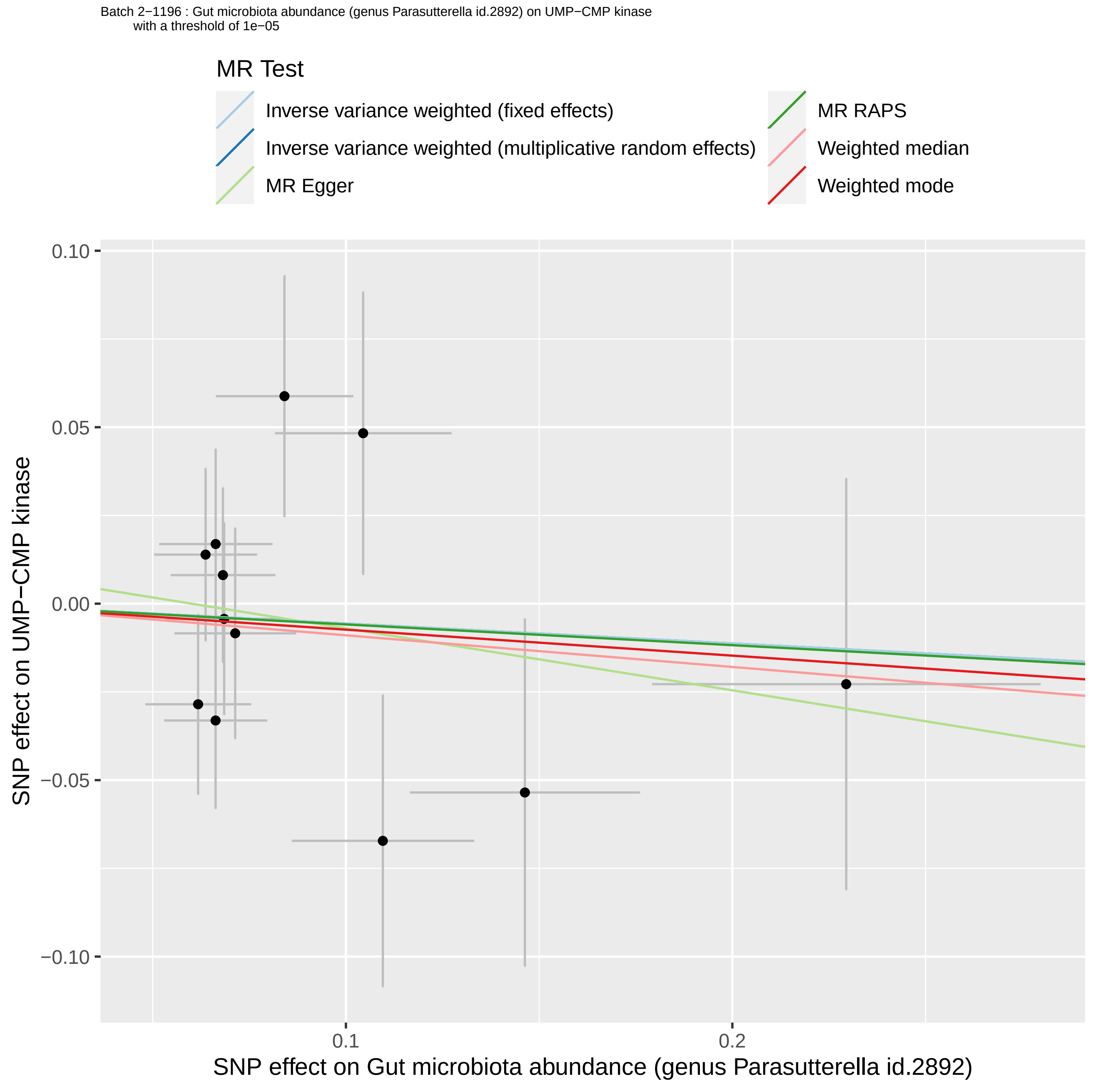
Batch 2−1196 : Gut microbiota abundance (genus Parasutterella id.2892) on UMP−CMP kinase
with a threshold of 1e−05
MR Test
Inverse variance weighted (fixed effects)
Inverse variance weighted (multiplicative random effects)
MR Egger
MR RAPS
Weighted median
Weighted mode
0.10
0.05
0.00
−0.05
−0.10
0.1
0.2
SNP effect on UMP−CMP kinase
SNP effect on Gut microbiota abundance (genus Parasutterella id.2892)

Batch 2−1197 : Gut microbiota abundance (genus Catenibacterium id.2153) on UMP−CMP kinase
with a threshold of 1e−05

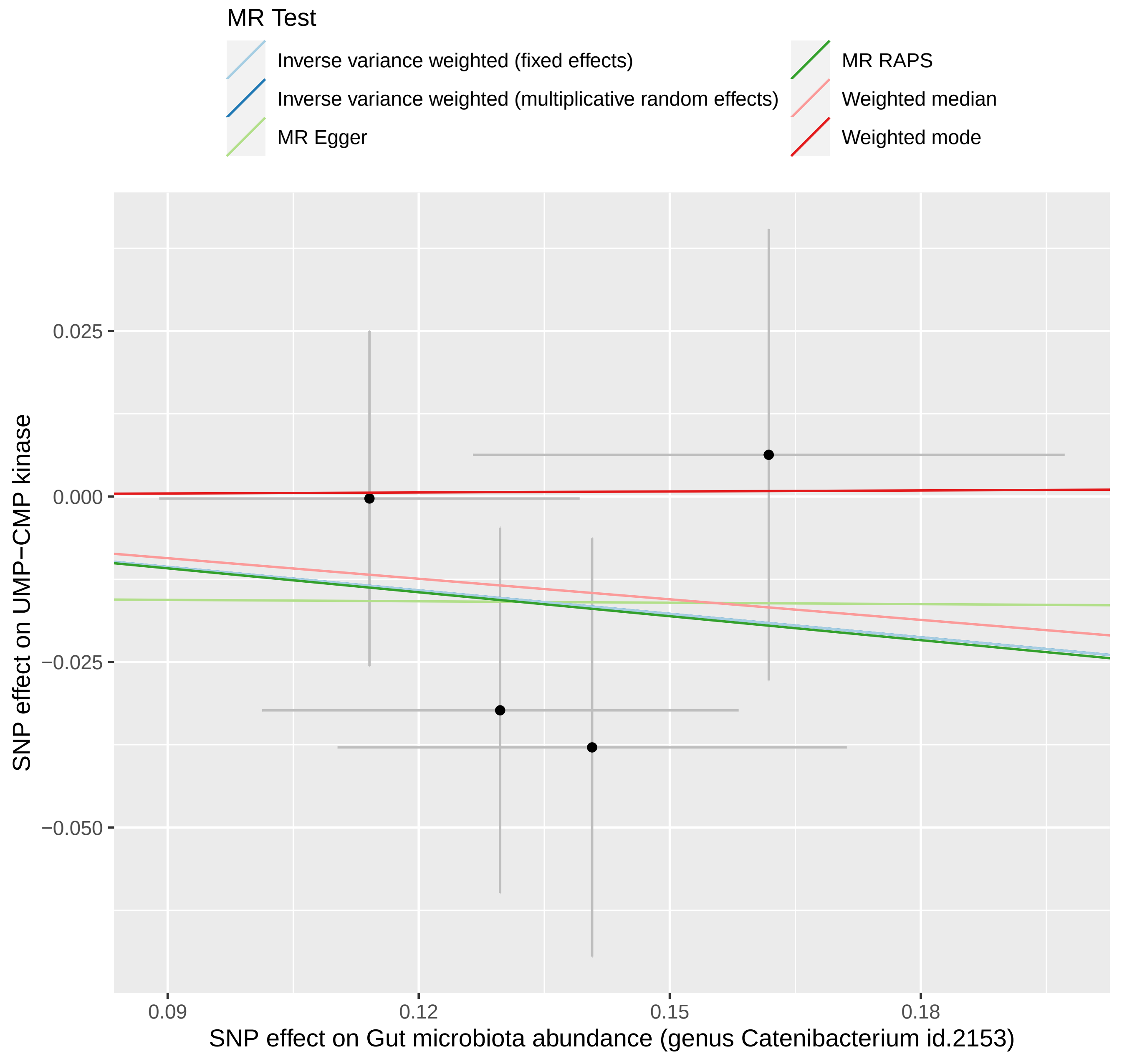

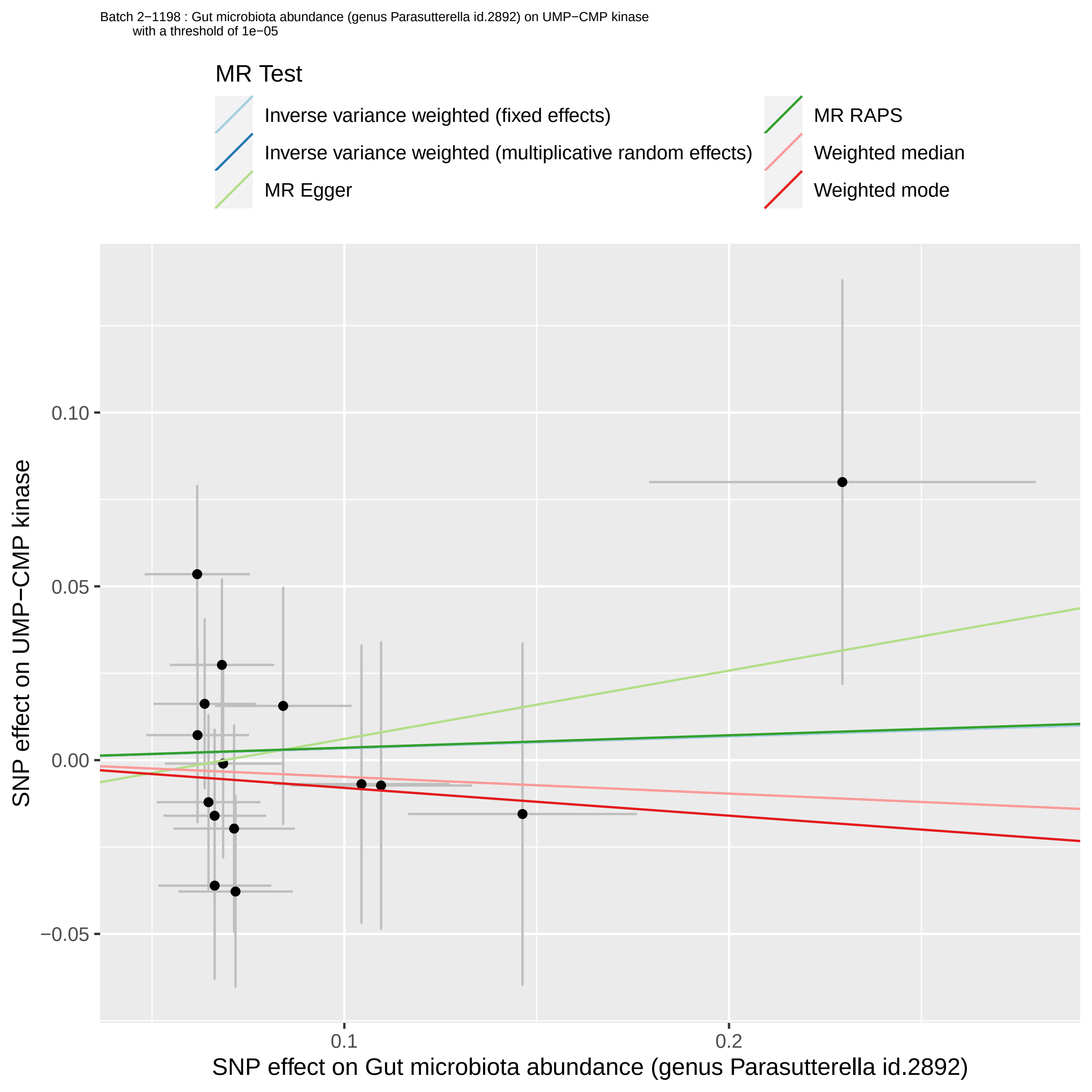
Batch 2−1198 : Gut microbiota abundance (genus Parasutterella id.2892) on UMP−CMP kinase
with a threshold of 1e−05
MR Test
Inverse variance weighted (fixed effects)
Inverse variance weighted (multiplicative random effects)
MR Egger
MR RAPS
Weighted median
Weighted mode
0.10
0.05
0.00
−0.05
0.1
0.2
SNP effect on UMP−CMP kinase
SNP effect on Gut microbiota abundance (genus Parasutterella id.2892)

Batch 2−1199 : Gut microbiota abundance (genus Catenibacterium id.2153) on Cytosolic non−specific dipeptidase
with a threshold of 1e−05

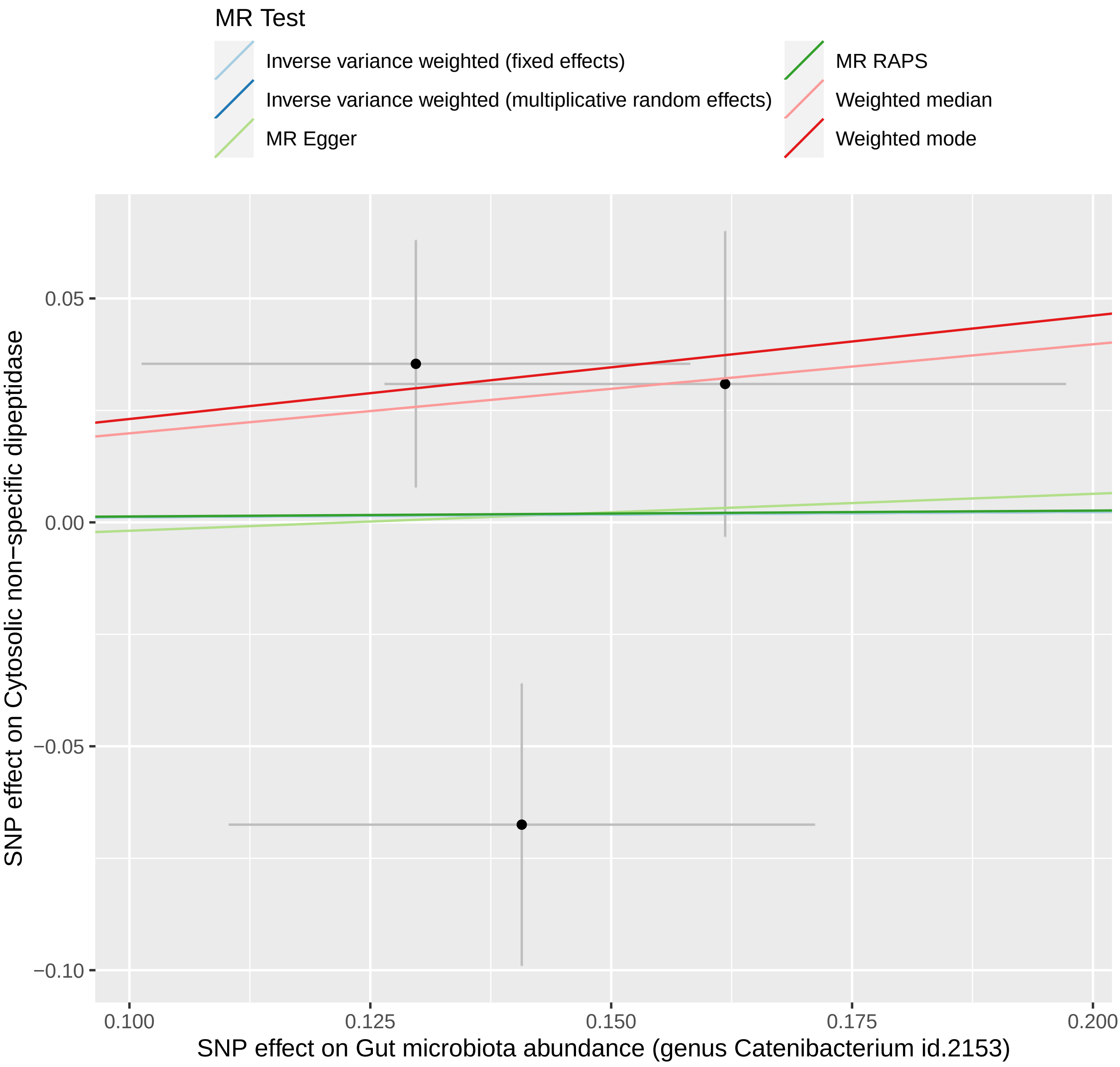

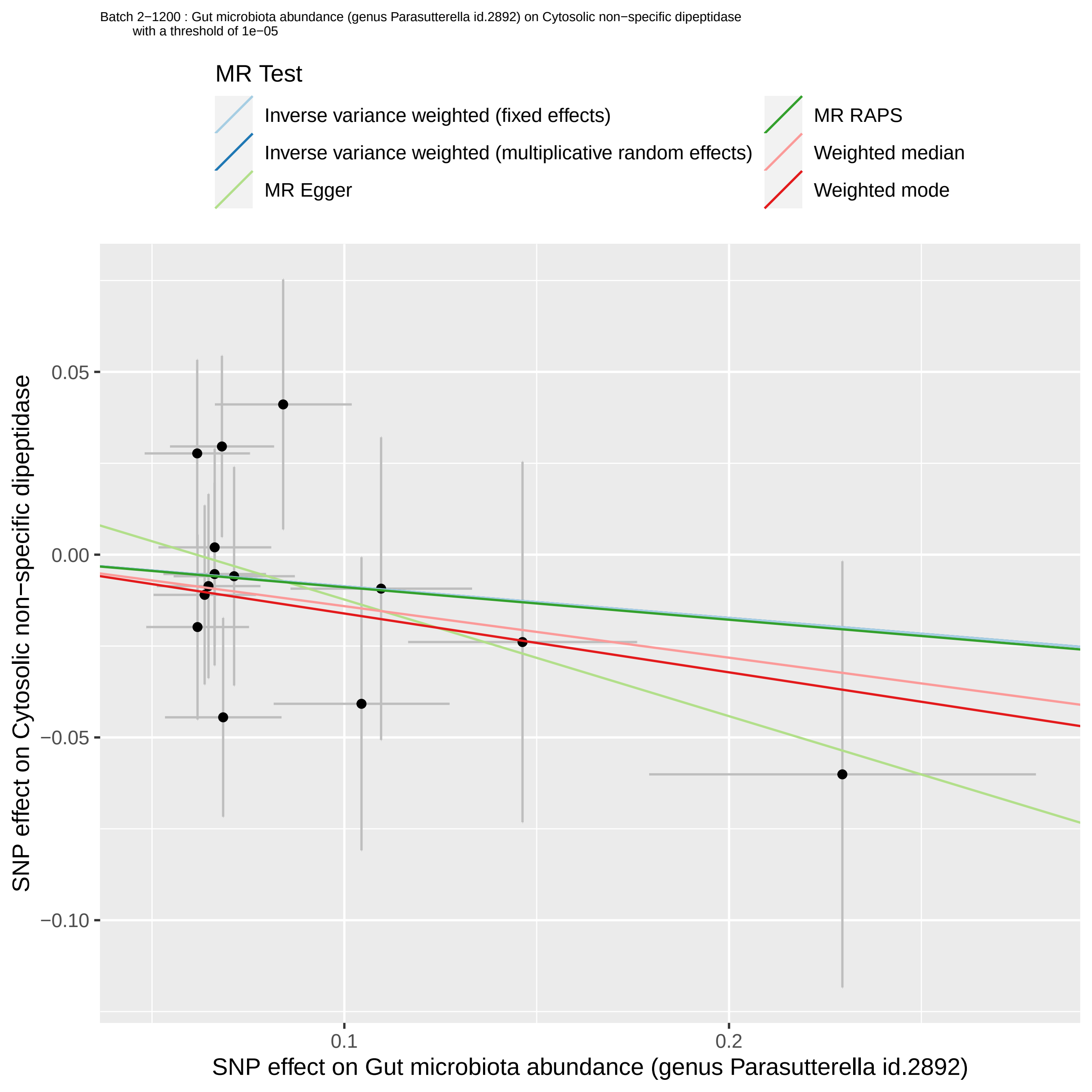
Batch 2−1200 : Gut microbiota abundance (genus Parasutterella id.2892) on Cytosolic non−specific dipeptidase
with a threshold of 1e−05
MR Test
Inverse variance weighted (fixed effects)
Inverse variance weighted (multiplicative random effects)
MR Egger
MR RAPS
Weighted median
Weighted mode
0.05
0.00
−0.05
−0.10
0.1
0.2
SNP effect on Cytosolic non−specific dipeptidase
SNP effect on Gut microbiota abundance (genus Parasutterella id.2892)

Batch 2−1201 : Gut microbiota abundance (genus Catenibacterium id.2153) on Nuclear envelope phosphatase−regulatory subunit 1 with a threshold of 1e−05

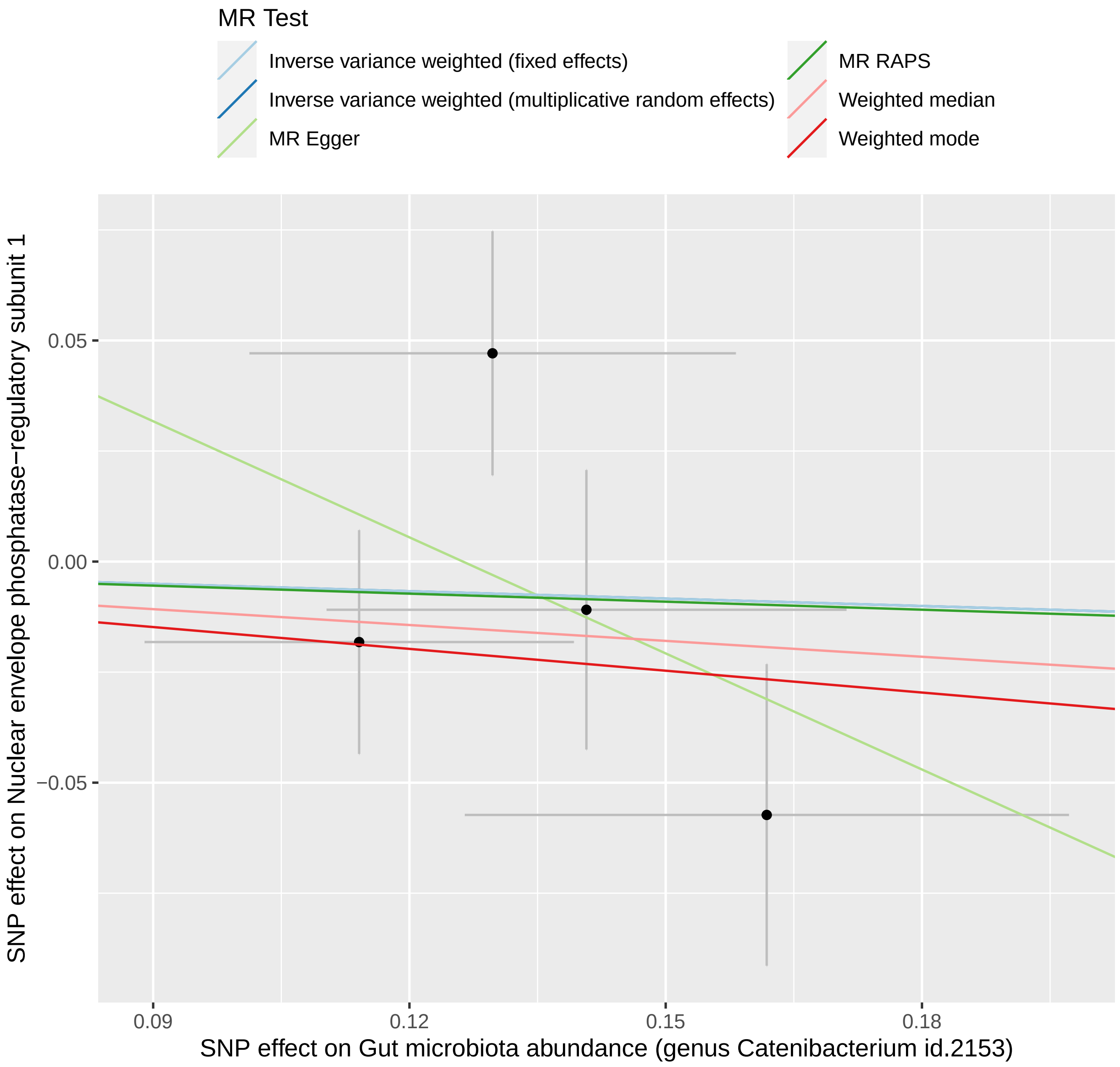

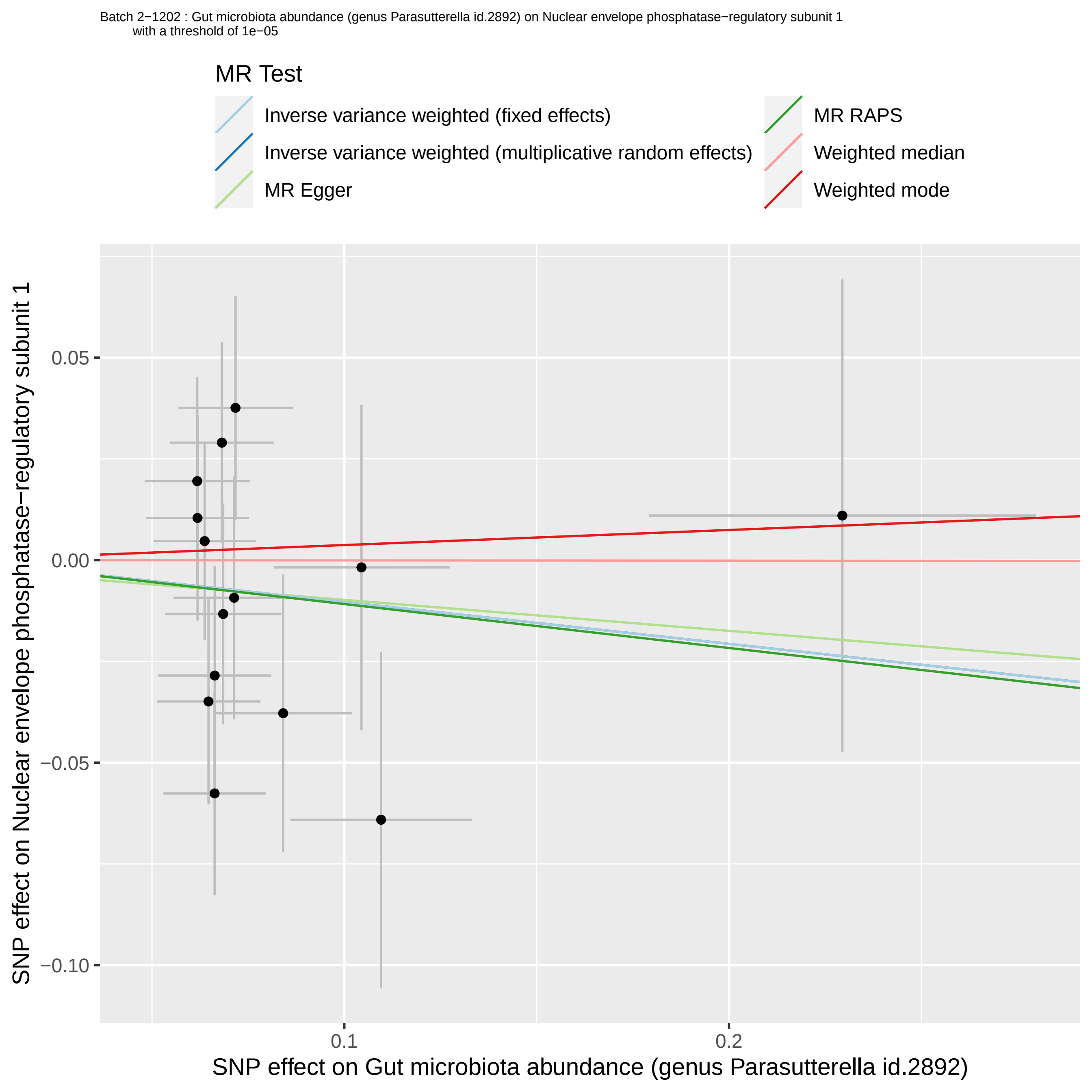
Batch 2−1202 : Gut microbiota abundance (genus Parasutterella id.2892) on Nuclear envelope phosphatase−regulatory subunit 1
with a threshold of 1e−05
MR Test
Inverse variance weighted (fixed effects)
Inverse variance weighted (multiplicative random effects)
MR Egger
MR RAPS
Weighted median
Weighted mode
SNP effect on Nuclear envelope phosphatase−regulatory subunit 1
0.05
0.00
−0.05
−0.10
0.1
0.2
SNP effect on Gut microbiota abundance (genus Parasutterella id.2892)

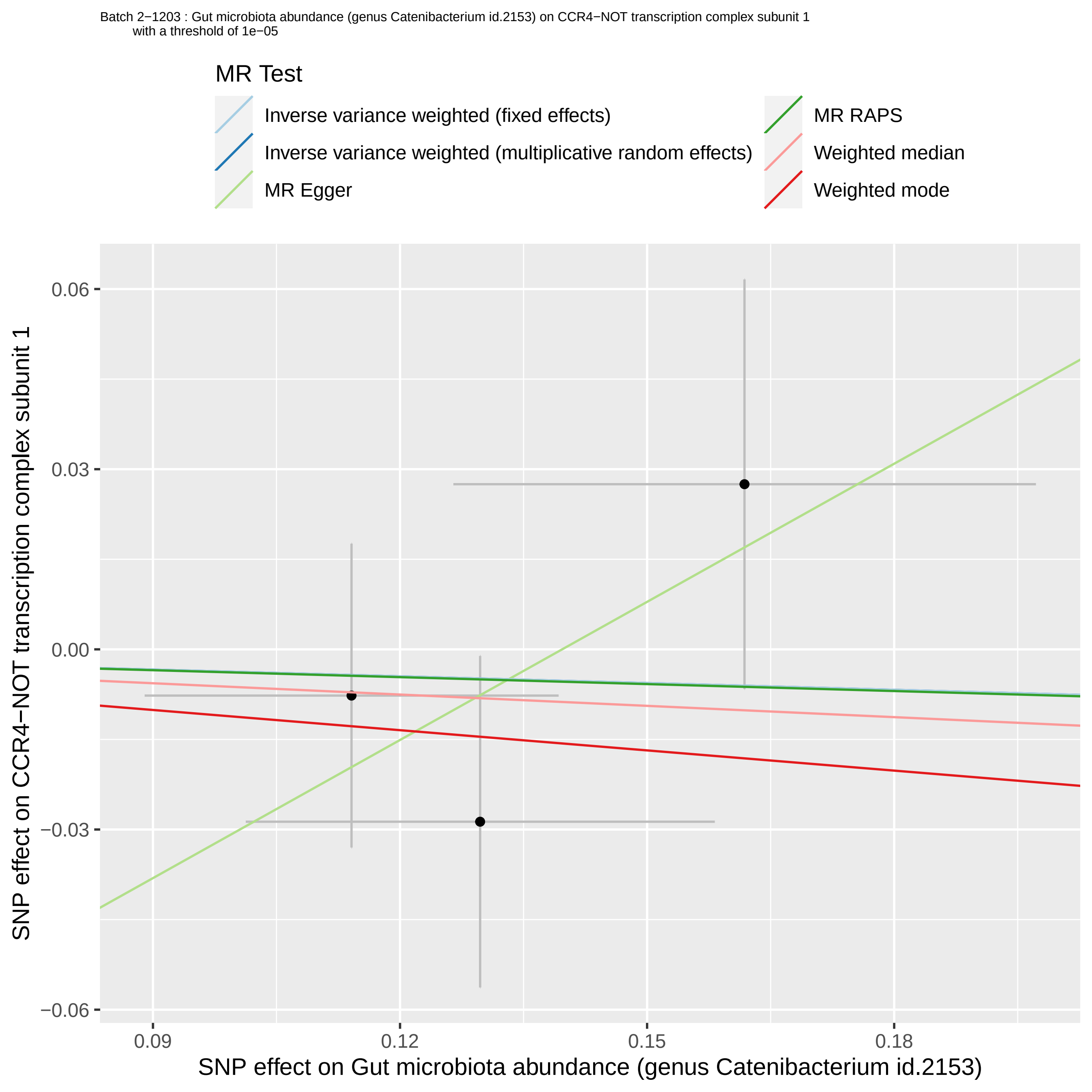

Batch 2−1203 : Gut microbiota abundance (genus Catenibacterium id.2153) on CCR4−NOT transcription complex subunit 1
with a threshold of 1e−05
MR Test
Inverse variance weighted (fixed effects)
Inverse variance weighted (multiplicative random effects)
MR Egger
MR RAPS
Weighted median
Weighted mode
0.06
0.03
0.00
−0.03
−0.06
0.09
0.12
0.15
0.18
SNP effect on CCR4−NOT transcription complex subunit 1
SNP effect on Gut microbiota abundance (genus Catenibacterium id.2153)

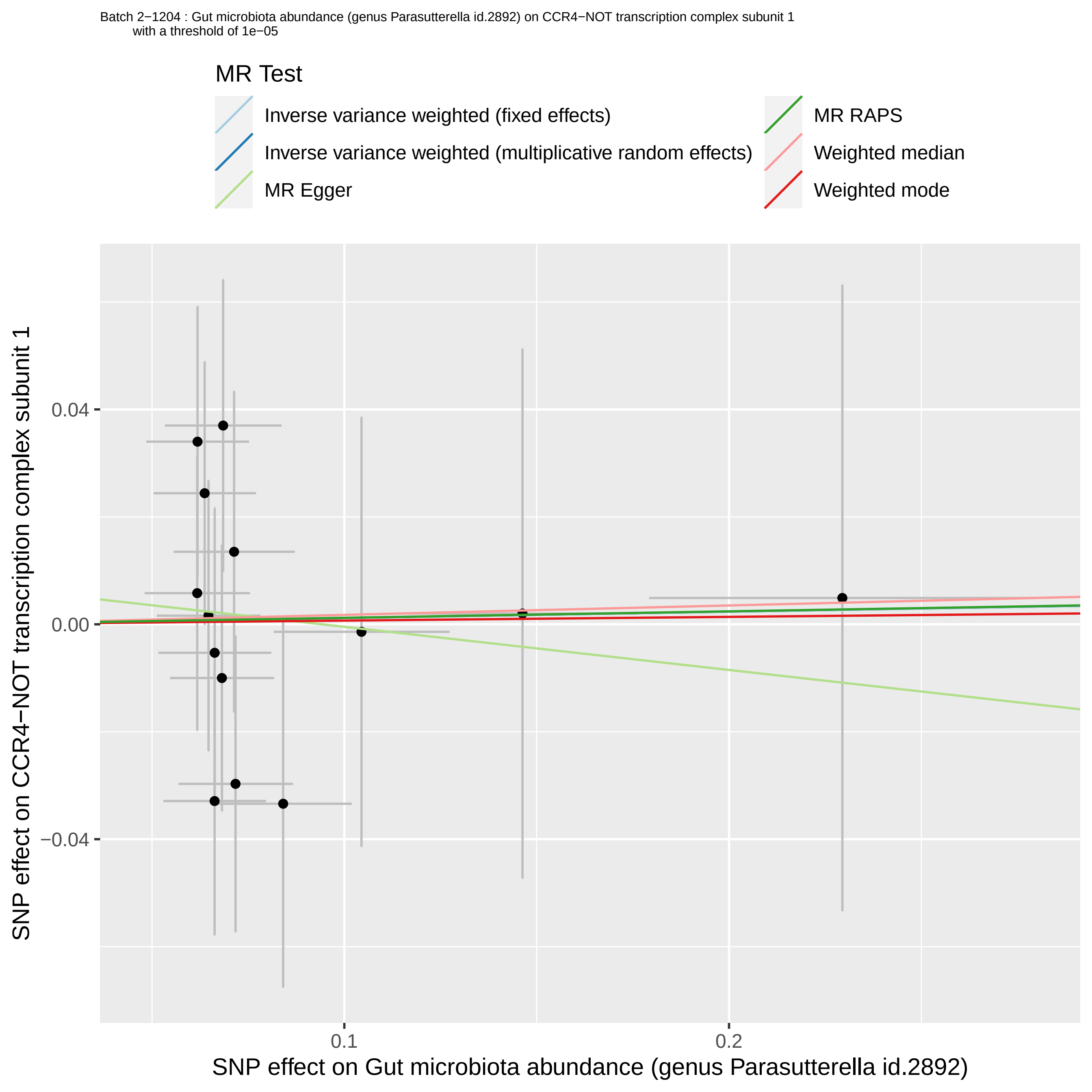
Batch 2−1204 : Gut microbiota abundance (genus Parasutterella id.2892) on CCR4−NOT transcription complex subunit 1
with a threshold of 1e−05
MR Test
Inverse variance weighted (fixed effects)
Inverse variance weighted (multiplicative random effects)
MR Egger
MR RAPS
Weighted median
Weighted mode
0.04
0.00
−0.04
0.1
0.2
SNP effect on CCR4−NOT transcription complex subunit 1
SNP effect on Gut microbiota abundance (genus Parasutterella id.2892)

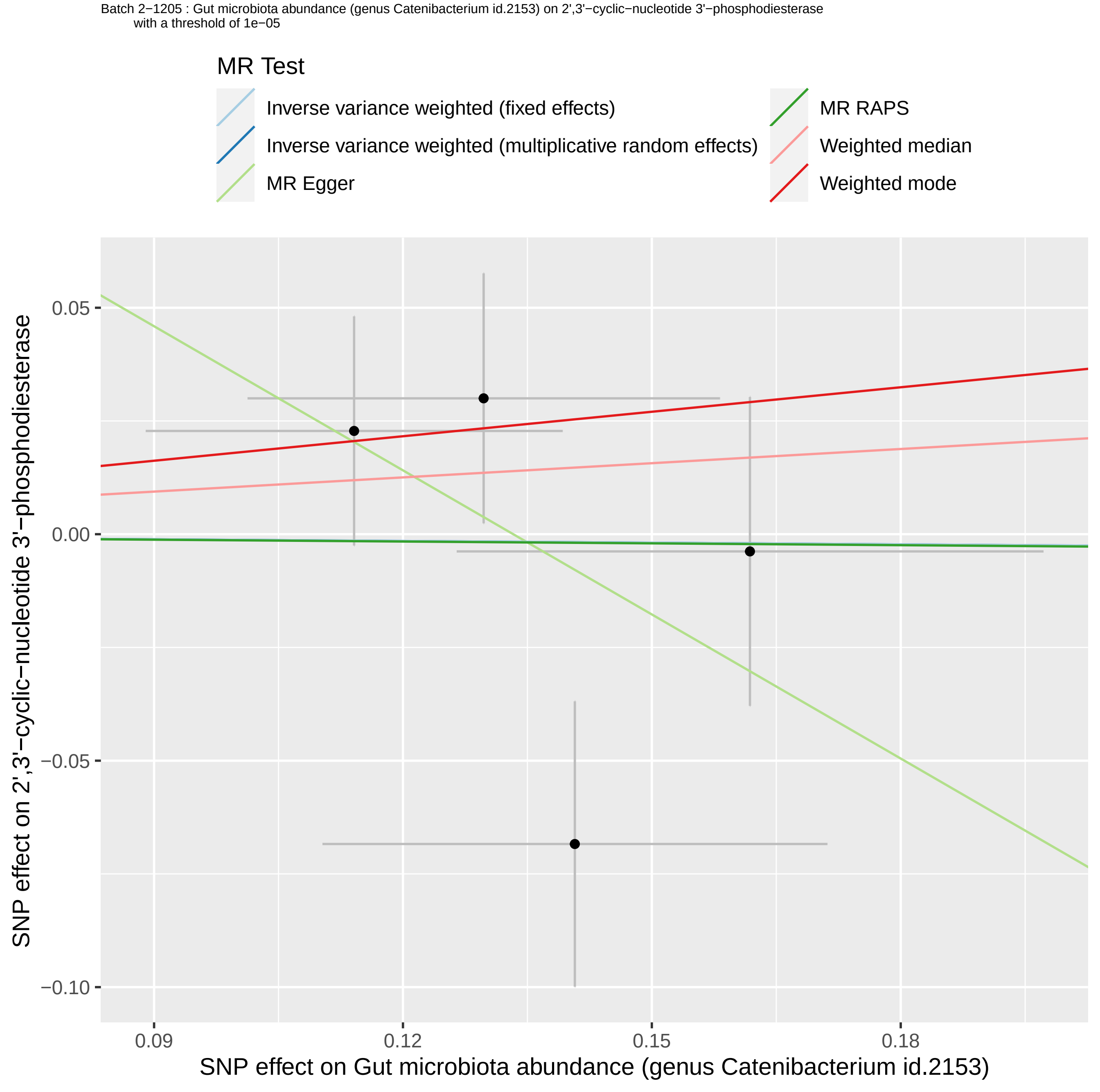

Batch 2−1205 : Gut microbiota abundance (genus Catenibacterium id.2153) on 2',3'−cyclic−nucleotide 3'−phosphodiesterase
with a threshold of 1e−05
MR Test
Inverse variance weighted (fixed effects)
Inverse variance weighted (multiplicative random effects)
MR Egger
MR RAPS
Weighted median
Weighted mode
0.05
0.00
−0.05
−0.10
0.09
0.12
0.15
0.18
SNP effect on 2',3'−cyclic−nucleotide 3'−phosphodiesterase
SNP effect on Gut microbiota abundance (genus Catenibacterium id.2153)

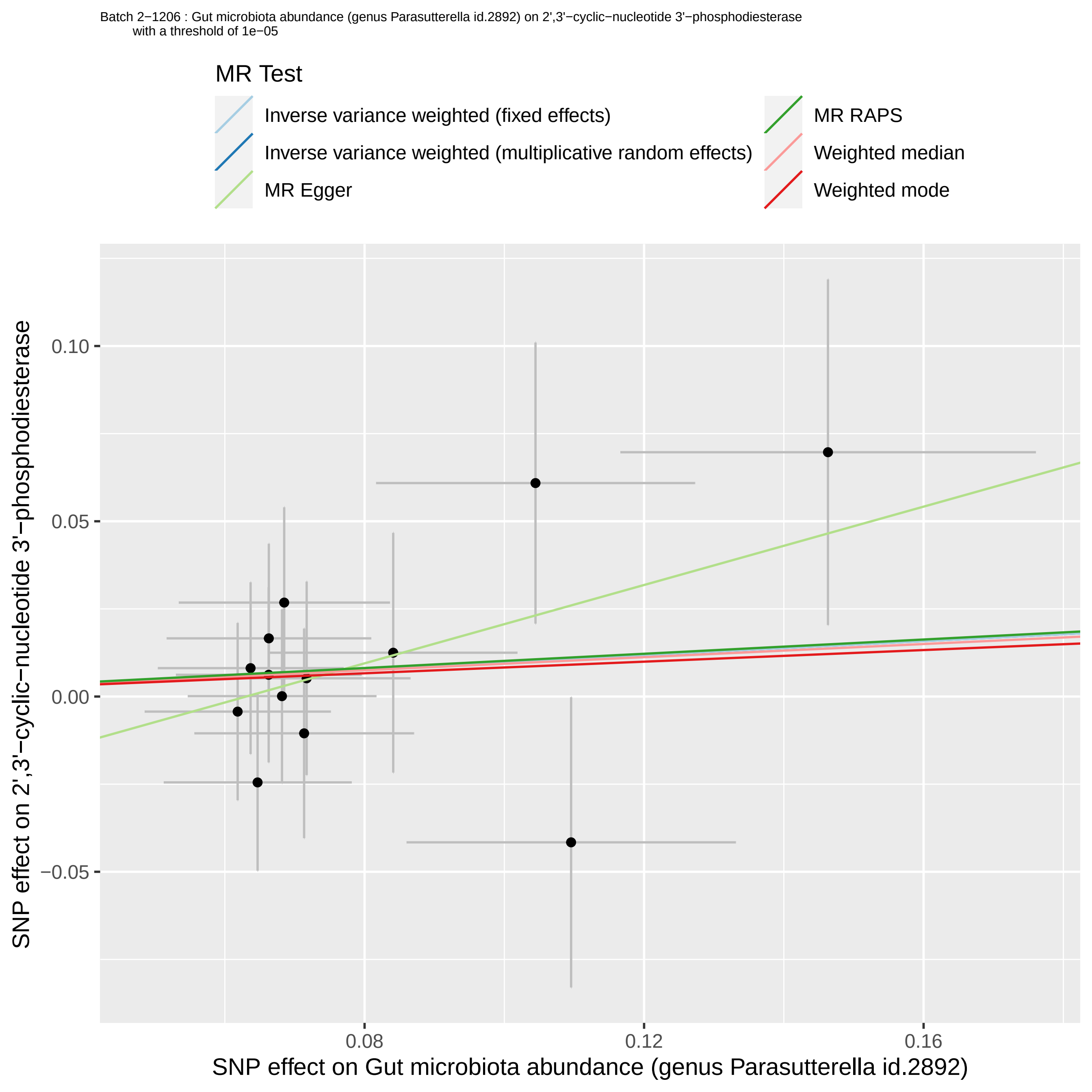

Batch 2−1206 : Gut microbiota abundance (genus Parasutterella id.2892) on 2',3'−cyclic−nucleotide 3'−phosphodiesterase with a threshold of 1e−05
MR Test
Inverse variance weighted (fixed effects)
Inverse variance weighted (multiplicative random effects)
MR Egger
MR RAPS
Weighted median
Weighted mode
0.10
0.05
0.00
−0.05
0.08
0.12
0.16
SNP effect on 2',3'−cyclic−nucleotide 3'−phosphodiesterase
SNP effect on Gut microbiota abundance (genus Parasutterella id.2892)

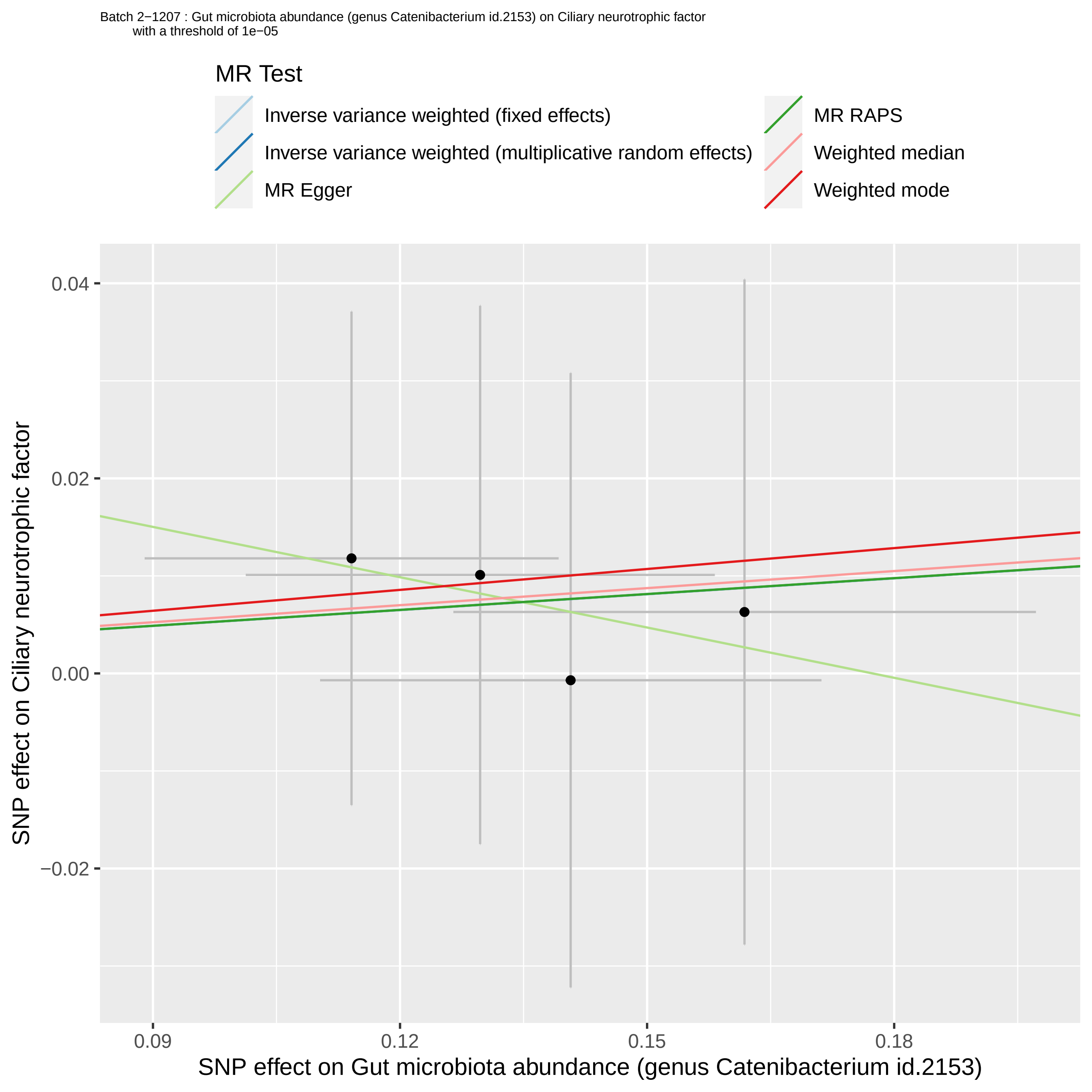
Batch 2−1207 : Gut microbiota abundance (genus Catenibacterium id.2153) on Ciliary neurotrophic factor
with a threshold of 1e−05
MR Test
Inverse variance weighted (fixed effects)
Inverse variance weighted (multiplicative random effects)
MR Egger
MR RAPS
Weighted median
Weighted mode
SNP effect on Ciliary neurotrophic factor
0.04
0.02
0.00
−0.02
0.09
0.12
0.15
0.18
SNP effect on Gut microbiota abundance (genus Catenibacterium id.2153)

Batch 2−1208 : Gut microbiota abundance (genus Parasutterella id.2892) on Ciliary neurotrophic factor
with a threshold of 1e−05

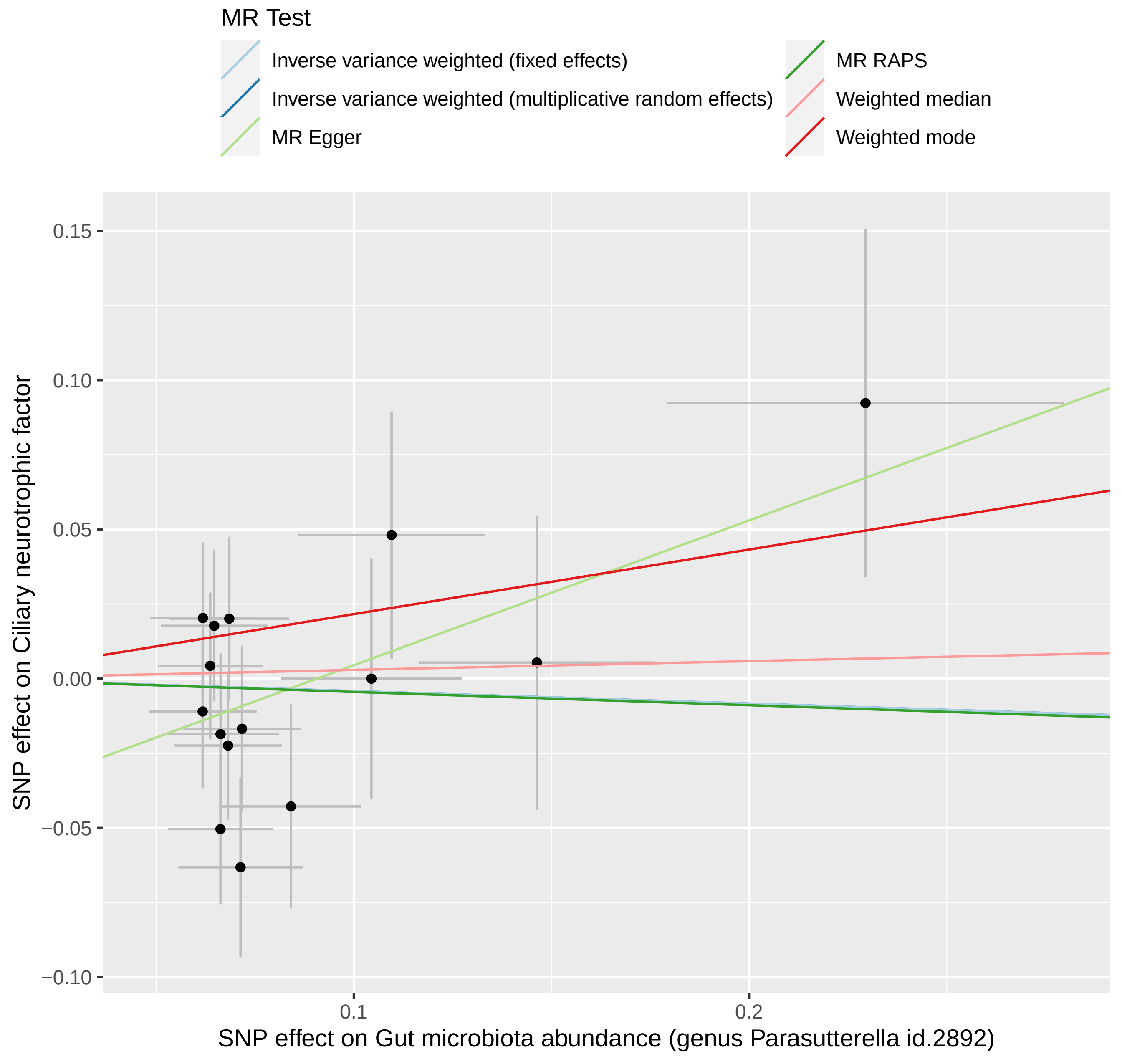

Batch 2−1209 : Gut microbiota abundance (genus Catenibacterium id.2153) on Ciliary neurotrophic factor receptor subunit alpha
with a threshold of 1e−05

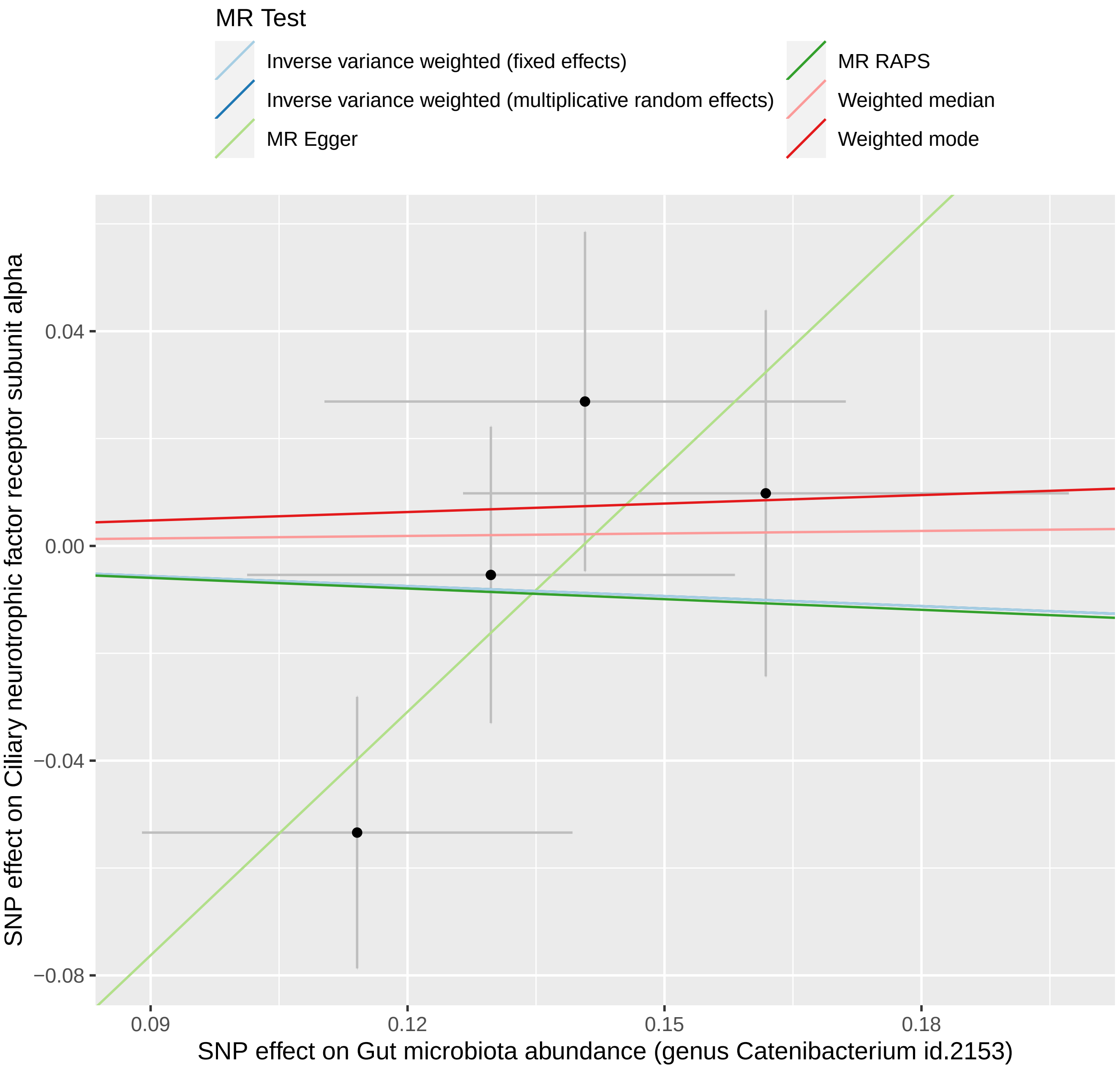

Batch 2−1210 : Gut microbiota abundance (genus Parasutterella id.2892) on Ciliary neurotrophic factor receptor subunit alpha
with a threshold of 1e−05

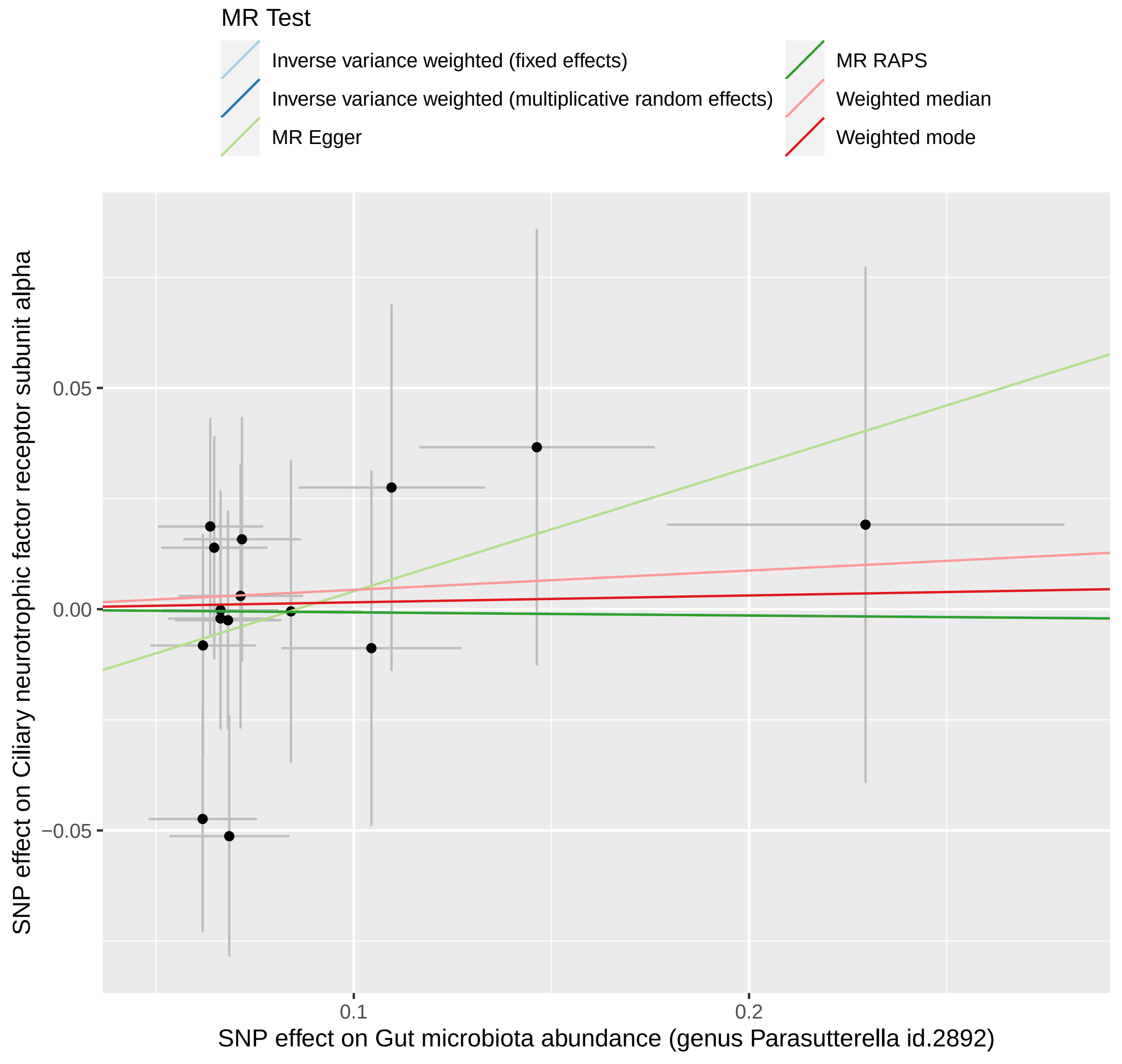

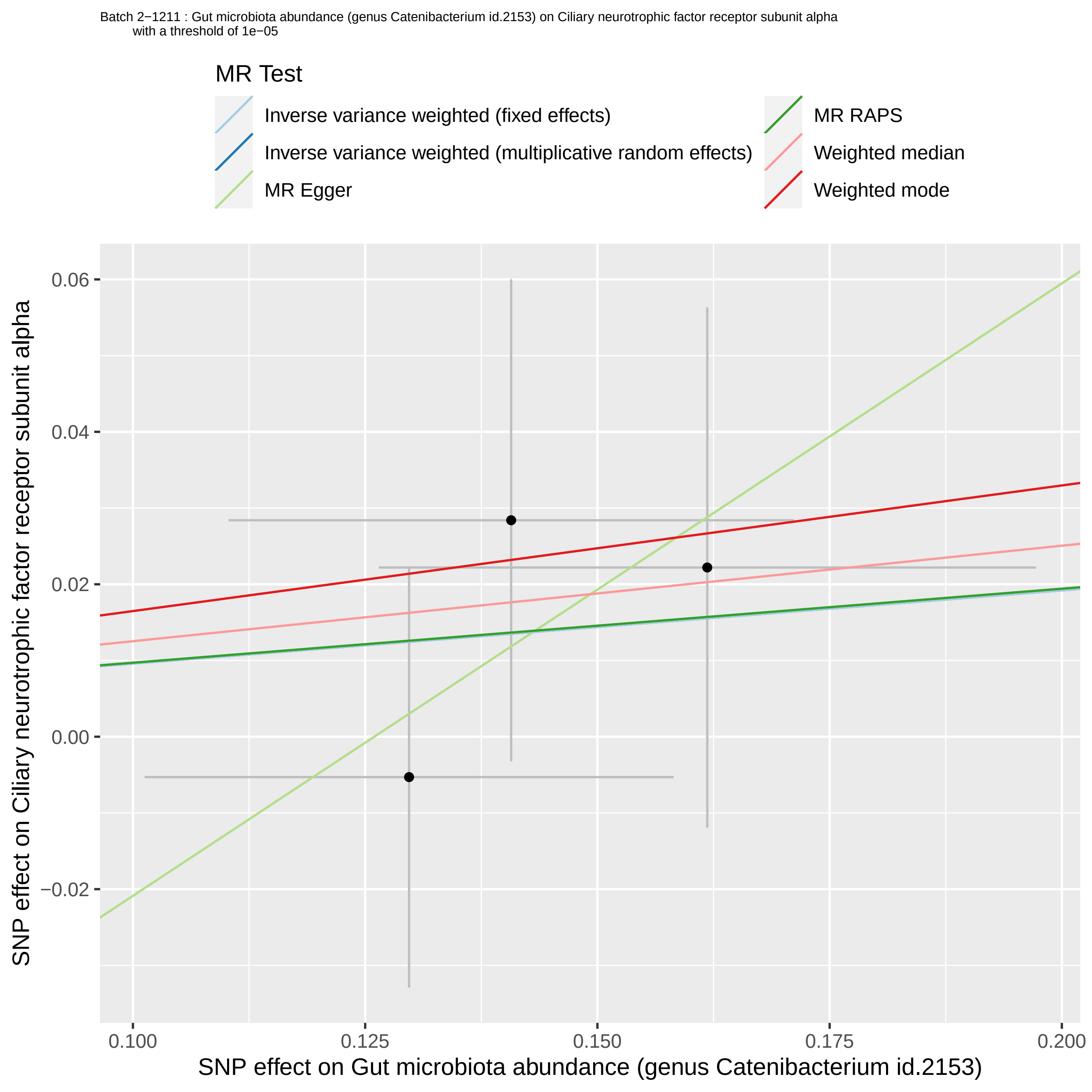
Batch 2−1211 : Gut microbiota abundance (genus Catenibacterium id.2153) on Ciliary neurotrophic factor receptor subunit alpha
with a threshold of 1e−05
MR Test
Inverse variance weighted (fixed effects)
Inverse variance weighted (multiplicative random effects)
MR Egger
MR RAPS
Weighted median
Weighted mode
0.06
0.04
0.02
0.00
−0.02
0.100
0.125
0.150
0.175
0.200
SNP effect on Ciliary neurotrophic factor receptor subunit alpha
SNP effect on Gut microbiota abundance (genus Catenibacterium id.2153)

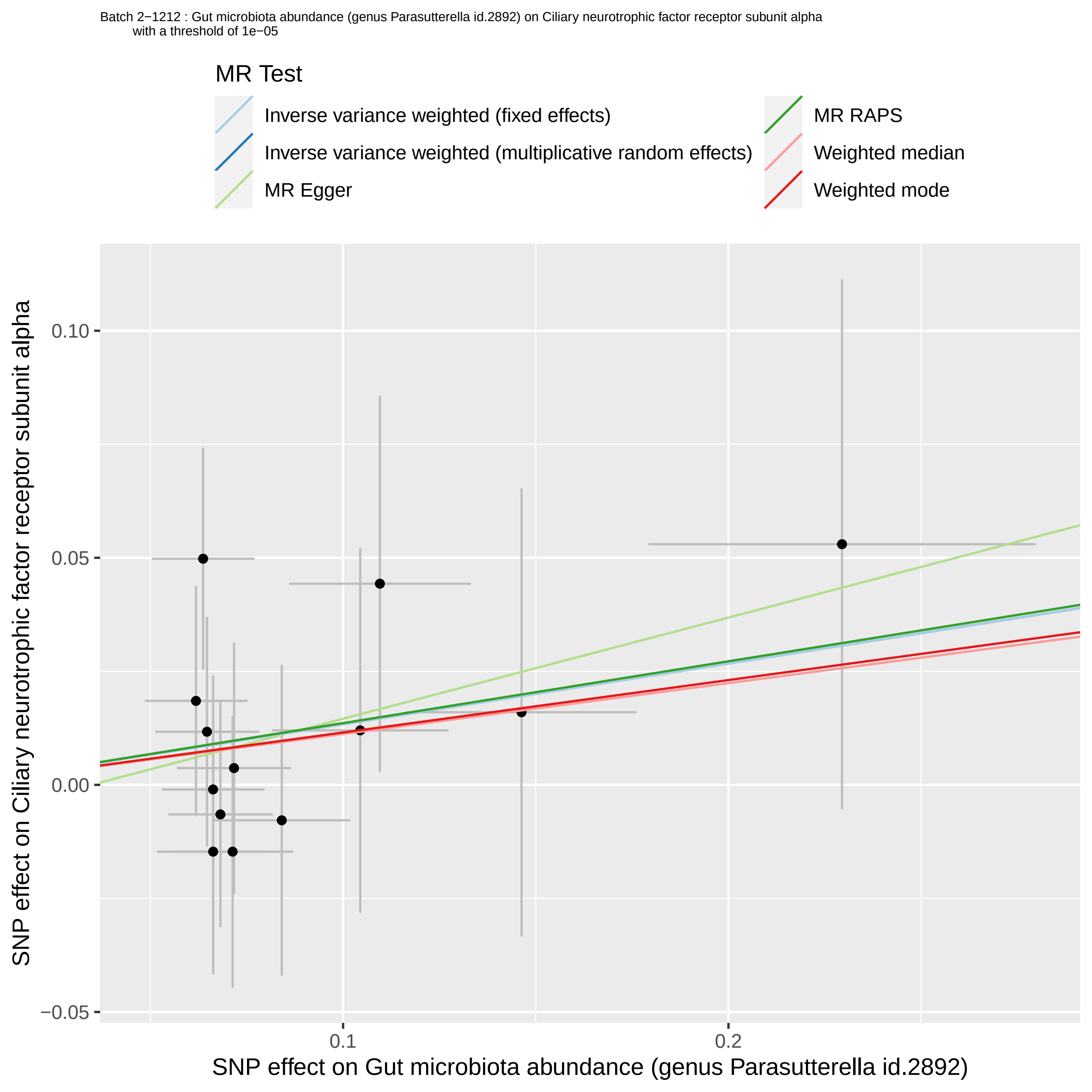

Batch 2−1212 : Gut microbiota abundance (genus Parasutterella id.2892) on Ciliary neurotrophic factor receptor subunit alpha
with a threshold of 1e−05
MR Test
Inverse variance weighted (fixed effects)
Inverse variance weighted (multiplicative random effects)
MR Egger
MR RAPS
Weighted median
Weighted mode
SNP effect on Ciliary neurotrophic factor receptor subunit alpha
0.10
0.05
0.00
−0.05
0.1
0.2
SNP effect on Gut microbiota abundance (genus Parasutterella id.2892)

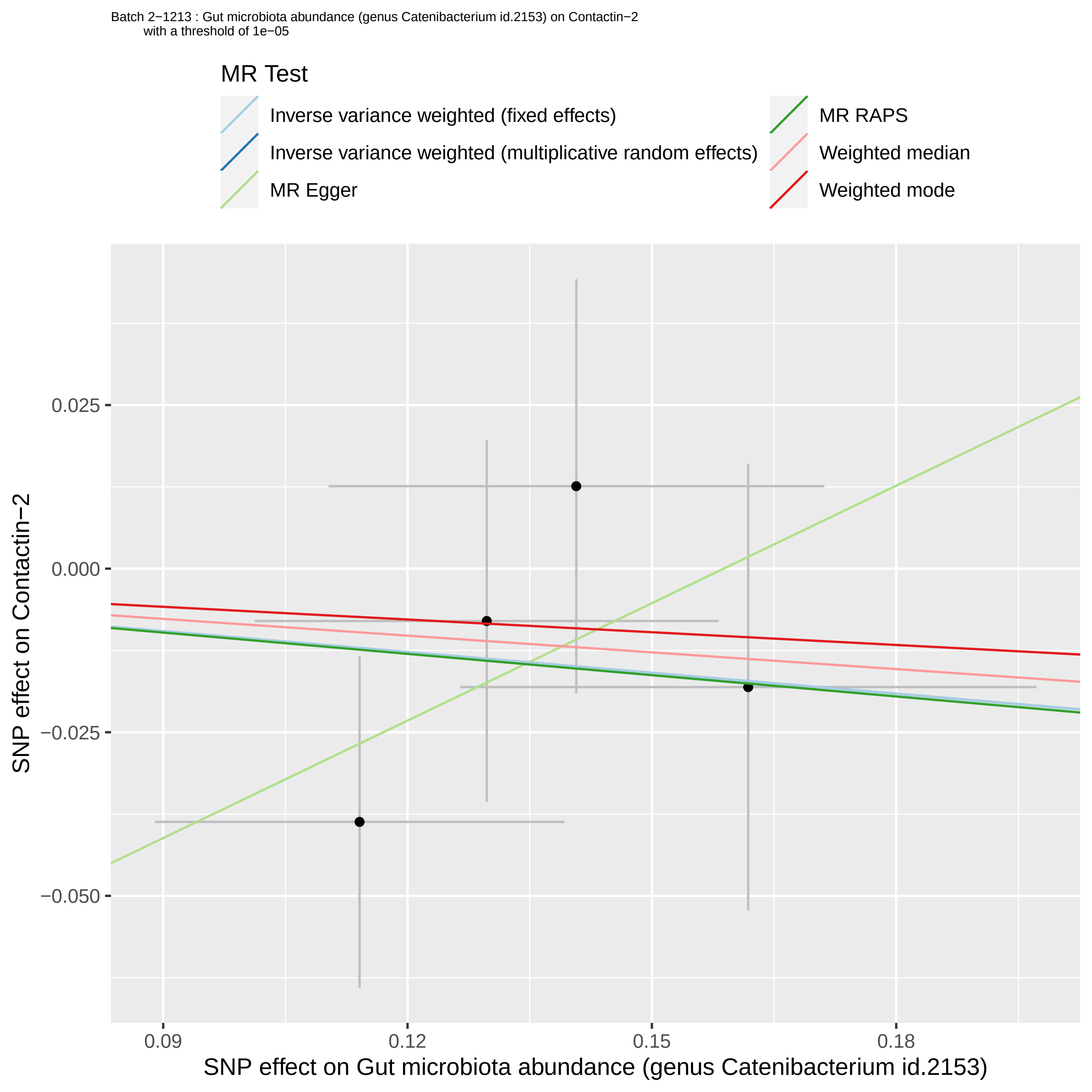
Batch 2−1213 : Gut microbiota abundance (genus Catenibacterium id.2153) on Contactin−2
with a threshold of 1e−05
MR Test
Inverse variance weighted (fixed effects)
Inverse variance weighted (multiplicative random effects)
MR Egger
MR RAPS
Weighted median
Weighted mode
0.025
0.000
−0.025
−0.050
0.09
0.12
0.15
0.18
SNP effect on Contactin−2
SNP effect on Gut microbiota abundance (genus Catenibacterium id.2153)

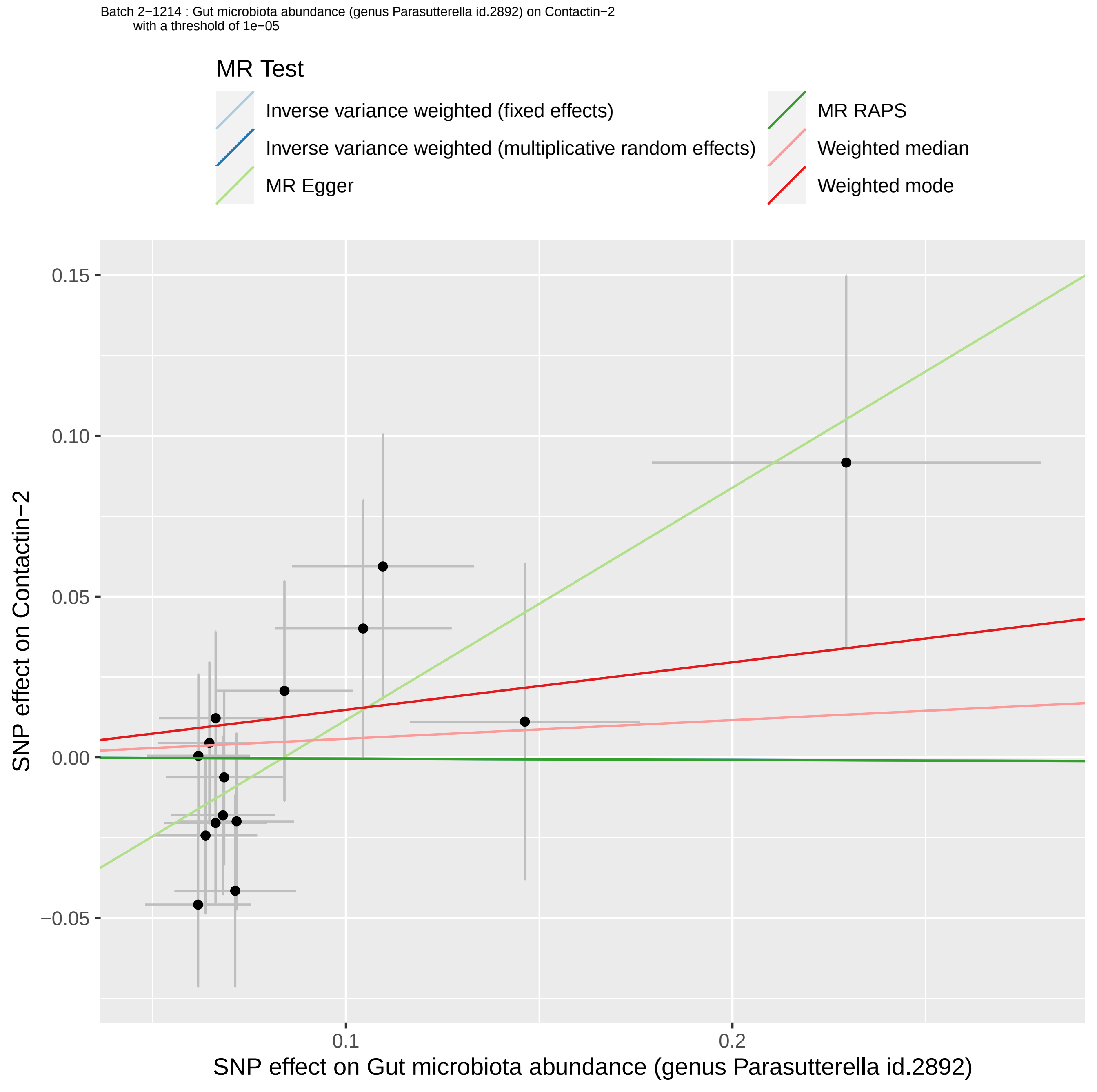
Batch 2−1214 : Gut microbiota abundance (genus Parasutterella id.2892) on Contactin−2
with a threshold of 1e−05
MR Test
Inverse variance weighted (fixed effects)
Inverse variance weighted (multiplicative random effects)
MR Egger
MR RAPS
Weighted median
Weighted mode
0.15
0.10
0.05
0.00
−0.05
0.1
0.2
SNP effect on Contactin−2
SNP effect on Gut microbiota abundance (genus Parasutterella id.2892)

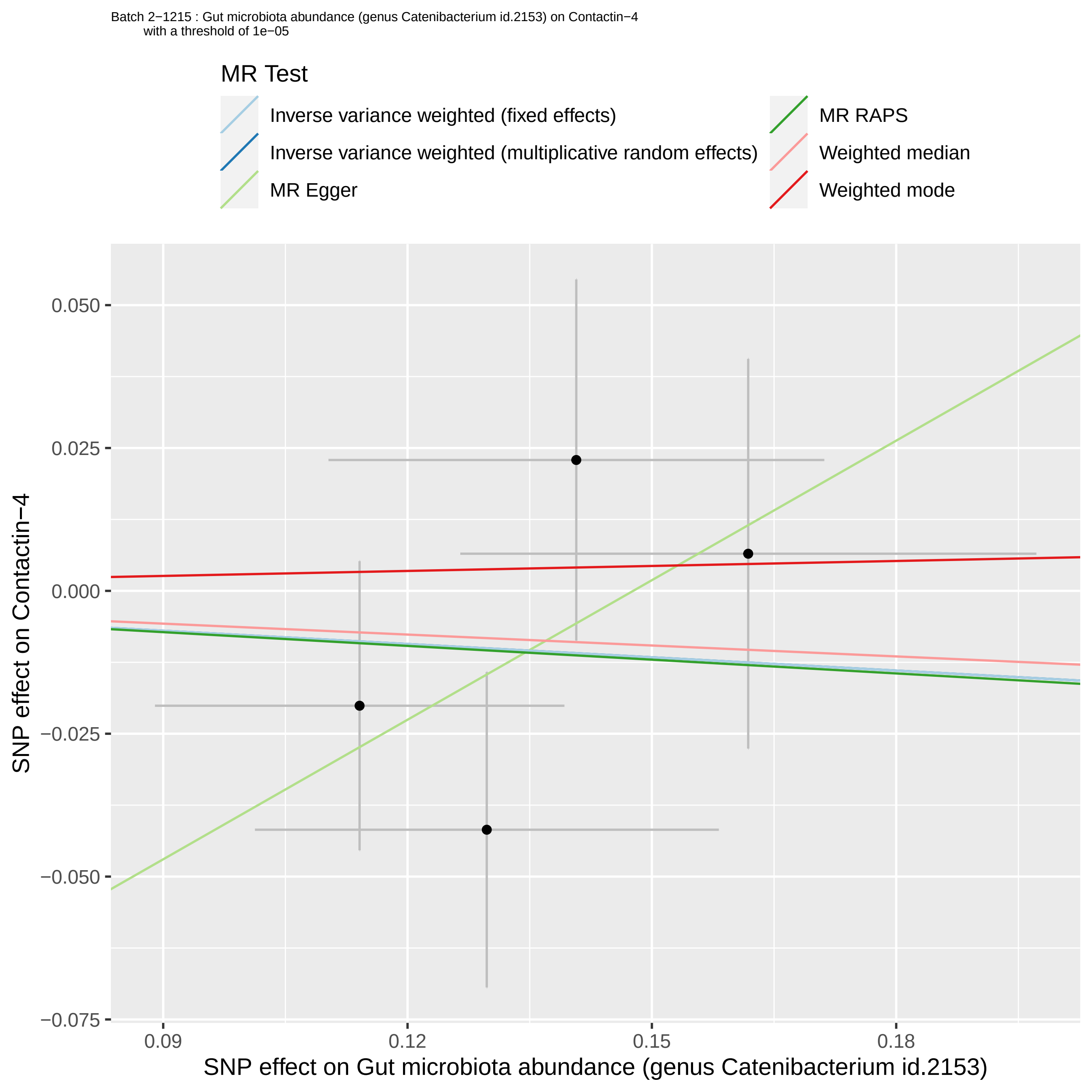

Batch 2−1215 : Gut microbiota abundance (genus Catenibacterium id.2153) on Contactin−4
with a threshold of 1e−05
MR Test
Inverse variance weighted (fixed effects)
Inverse variance weighted (multiplicative random effects)
MR Egger
MR RAPS
Weighted median
Weighted mode
0.050
0.025
0.000
−0.025
−0.050
−0.075
0.09
0.12
0.15
0.18
SNP effect on Contactin−4
SNP effect on Gut microbiota abundance (genus Catenibacterium id.2153)

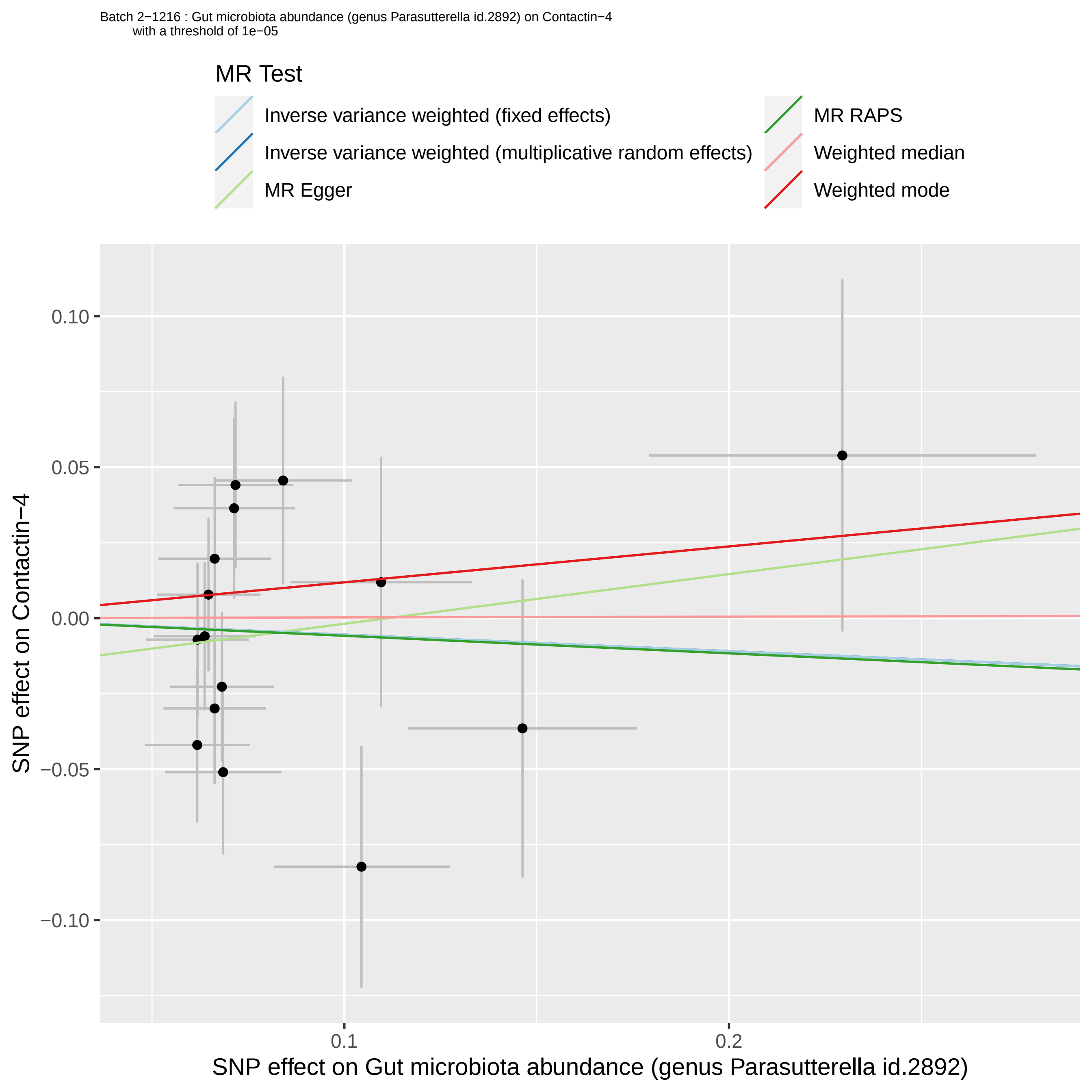

Batch 2−1216 : Gut microbiota abundance (genus Parasutterella id.2892) on Contactin−4
with a threshold of 1e−05
MR Test
Inverse variance weighted (fixed effects)
Inverse variance weighted (multiplicative random effects)
MR Egger
MR RAPS
Weighted median
Weighted mode
0.10
0.05
0.00
−0.05
−0.10
SNP effect on Contactin−4
0.1
0.2
SNP effect on Gut microbiota abundance (genus Parasutterella id.2892)

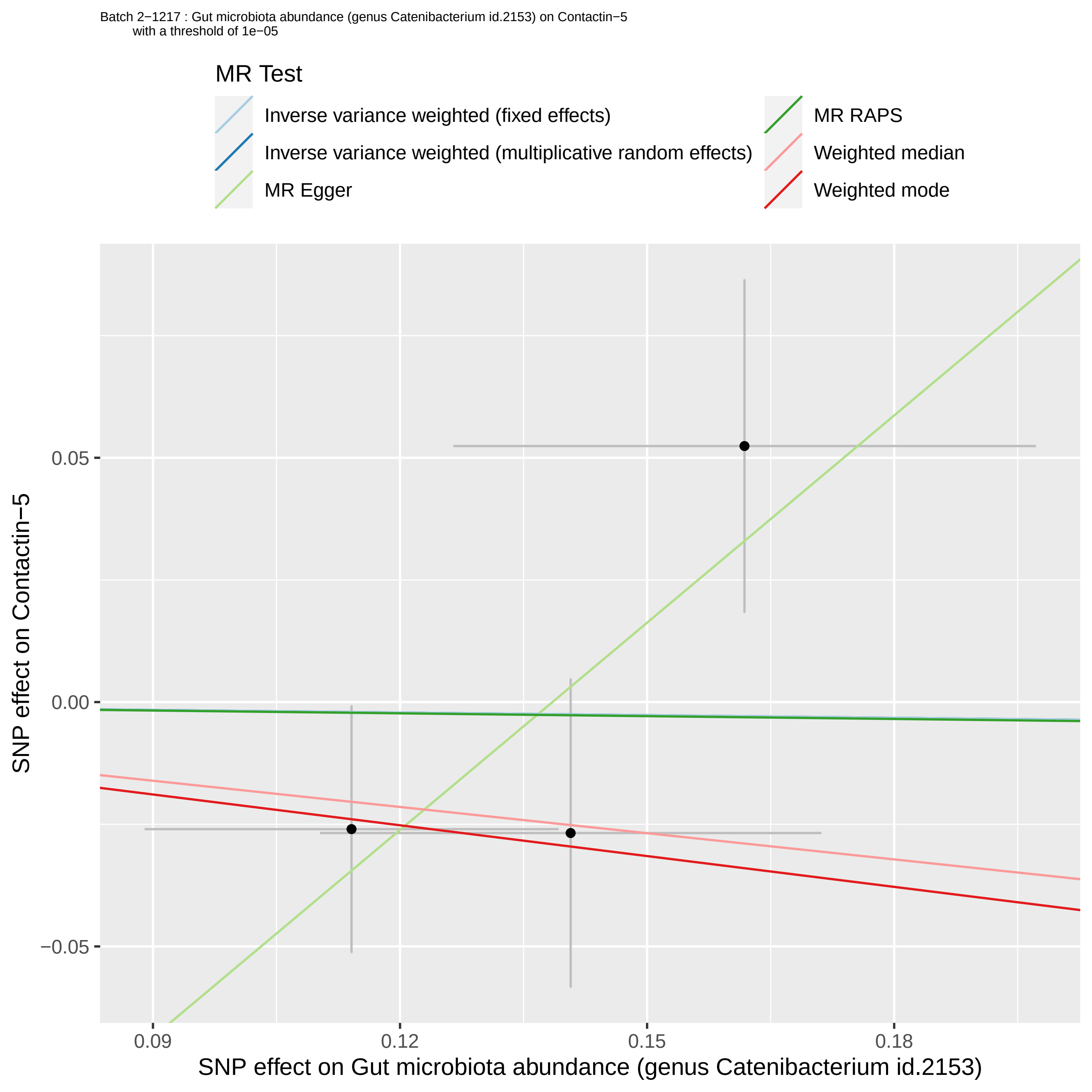
Batch 2−1217 : Gut microbiota abundance (genus Catenibacterium id.2153) on Contactin−5
with a threshold of 1e−05
MR Test
Inverse variance weighted (fixed effects)
Inverse variance weighted (multiplicative random effects)
MR Egger
MR RAPS
Weighted median
Weighted mode
0.05
0.00
−0.05
0.09
0.12
0.15
0.18
SNP effect on Contactin−5
SNP effect on Gut microbiota abundance (genus Catenibacterium id.2153)

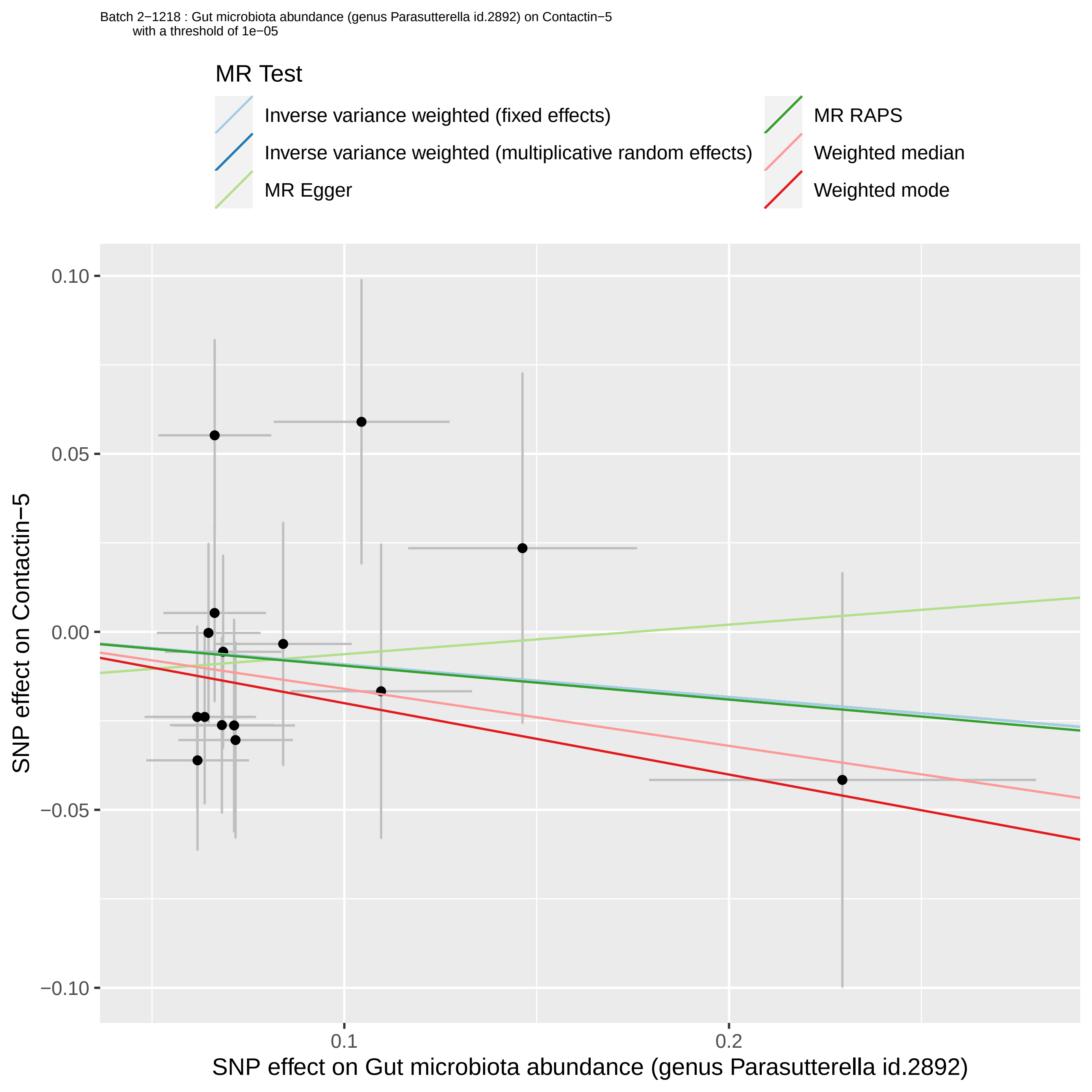
Batch 2−1218 : Gut microbiota abundance (genus Parasutterella id.2892) on Contactin−5
with a threshold of 1e−05
MR Test
Inverse variance weighted (fixed effects)
Inverse variance weighted (multiplicative random effects)
MR Egger
MR RAPS
Weighted median
Weighted mode
0.10
0.05
0.00
−0.05
−0.10
0.1
0.2
SNP effect on Contactin−5
SNP effect on Gut microbiota abundance (genus Parasutterella id.2892)

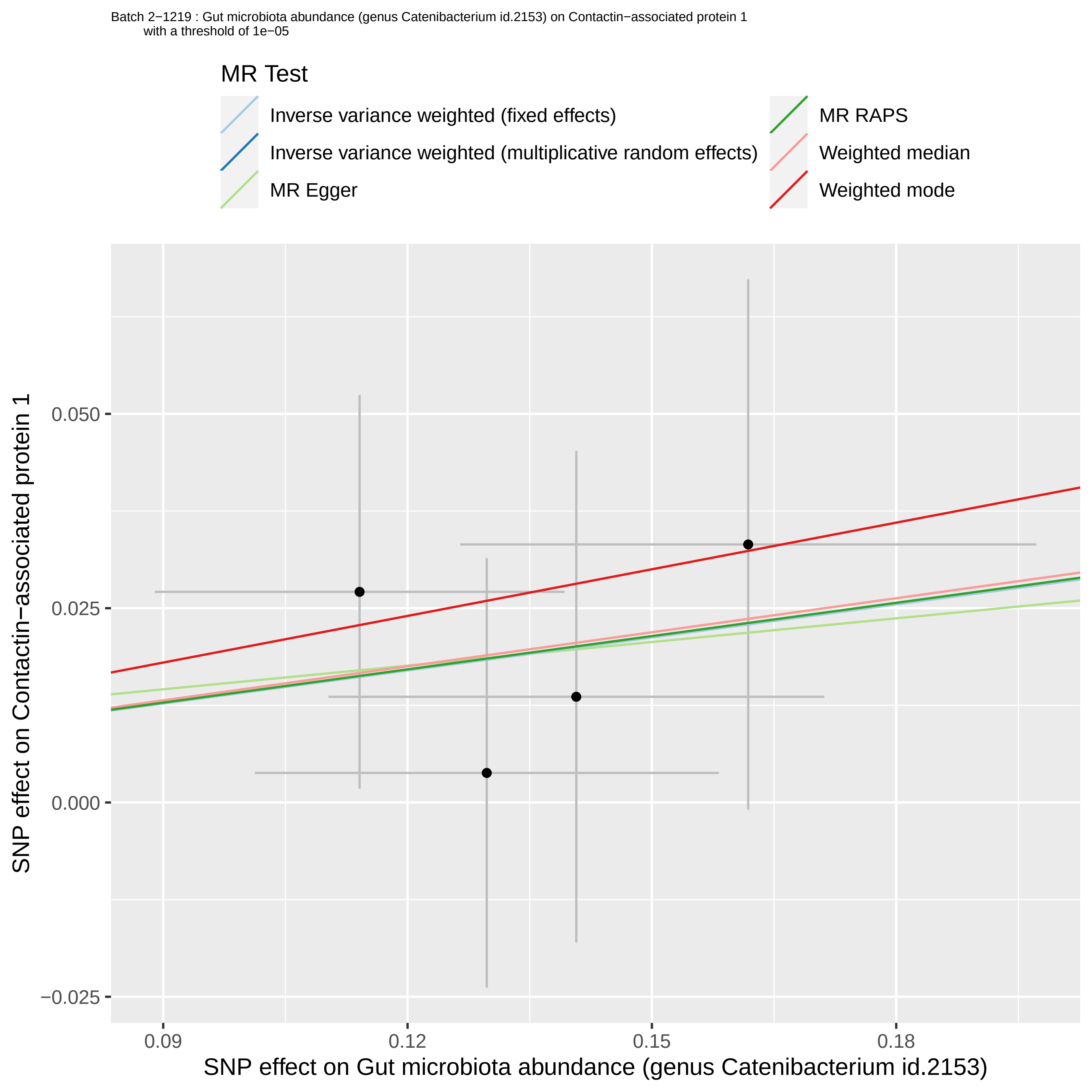

Batch 2−1219 : Gut microbiota abundance (genus Catenibacterium id.2153) on Contactin−associated protein 1
with a threshold of 1e−05
MR Test
Inverse variance weighted (fixed effects)
Inverse variance weighted (multiplicative random effects)
MR Egger
MR RAPS
Weighted median
Weighted mode
0.050
0.025
0.000
−0.025
0.09
0.12
0.15
0.18
SNP effect on Contactin−associated protein 1
SNP effect on Gut microbiota abundance (genus Catenibacterium id.2153)

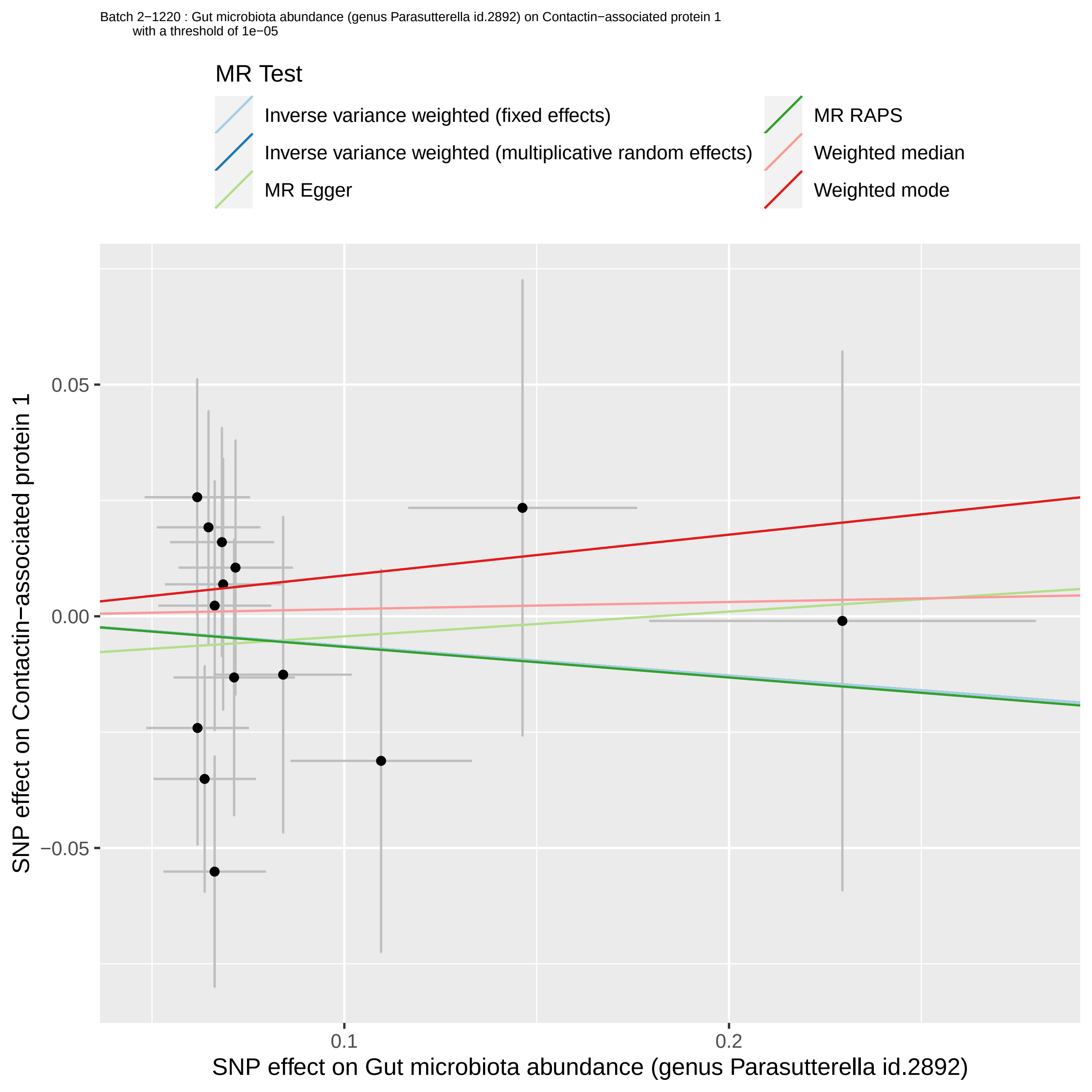
Batch 2−1220 : Gut microbiota abundance (genus Parasutterella id.2892) on Contactin−associated protein 1
with a threshold of 1e−05
MR Test
Inverse variance weighted (fixed effects)
Inverse variance weighted (multiplicative random effects)
MR Egger
MR RAPS
Weighted median
Weighted mode
0.05
0.00
−0.05
0.1
0.2
SNP effect on Contactin−associated protein 1
SNP effect on Gut microbiota abundance (genus Parasutterella id.2892)

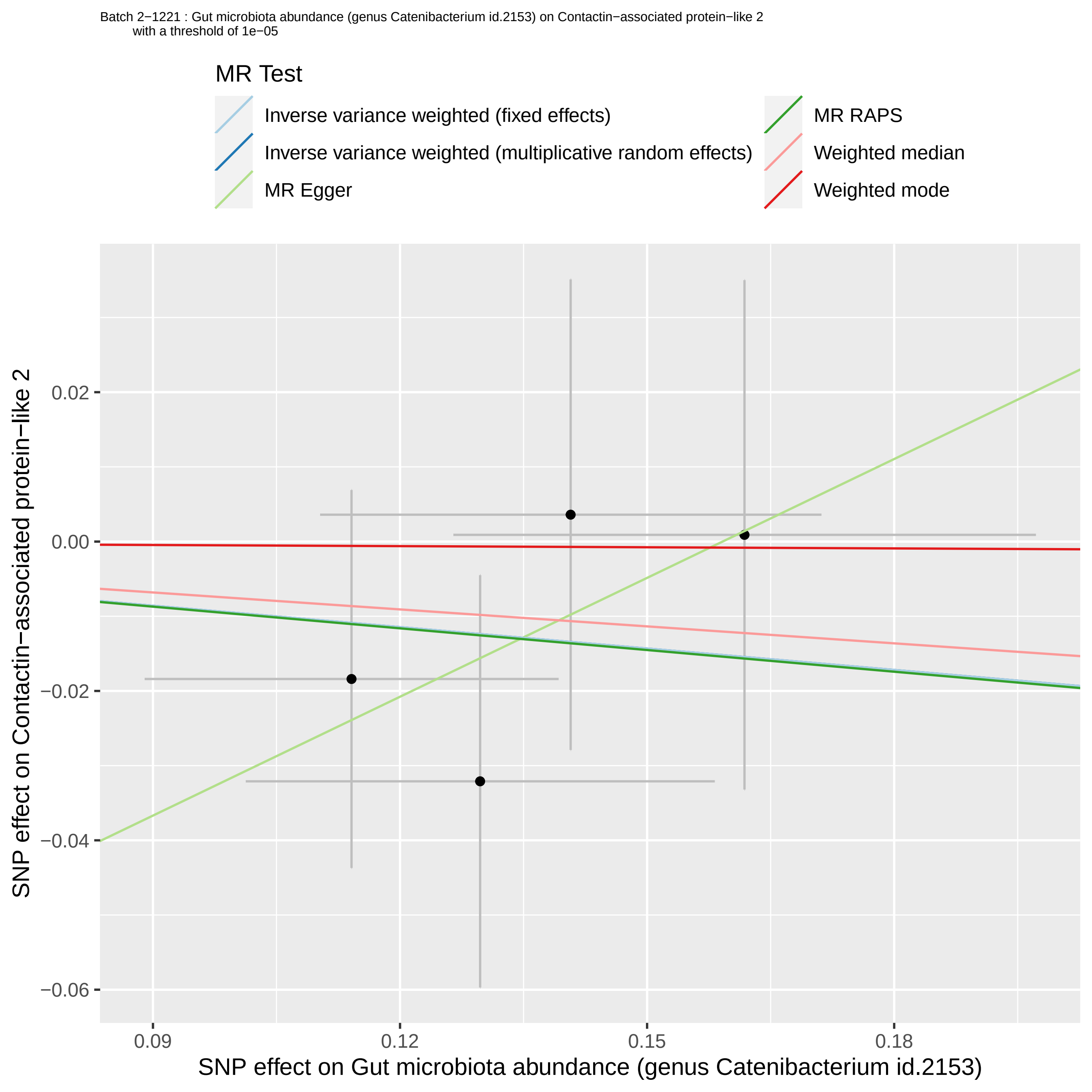

Batch 2−1221 : Gut microbiota abundance (genus Catenibacterium id.2153) on Contactin−associated protein−like 2
with a threshold of 1e−05
MR Test
Inverse variance weighted (fixed effects)
Inverse variance weighted (multiplicative random effects)
MR Egger
MR RAPS
Weighted median
Weighted mode
0.02
0.00
−0.02
−0.04
−0.06
SNP effect on Contactin−associated protein−like 2
0.09
0.12
0.15
0.18
SNP effect on Gut microbiota abundance (genus Catenibacterium id.2153)

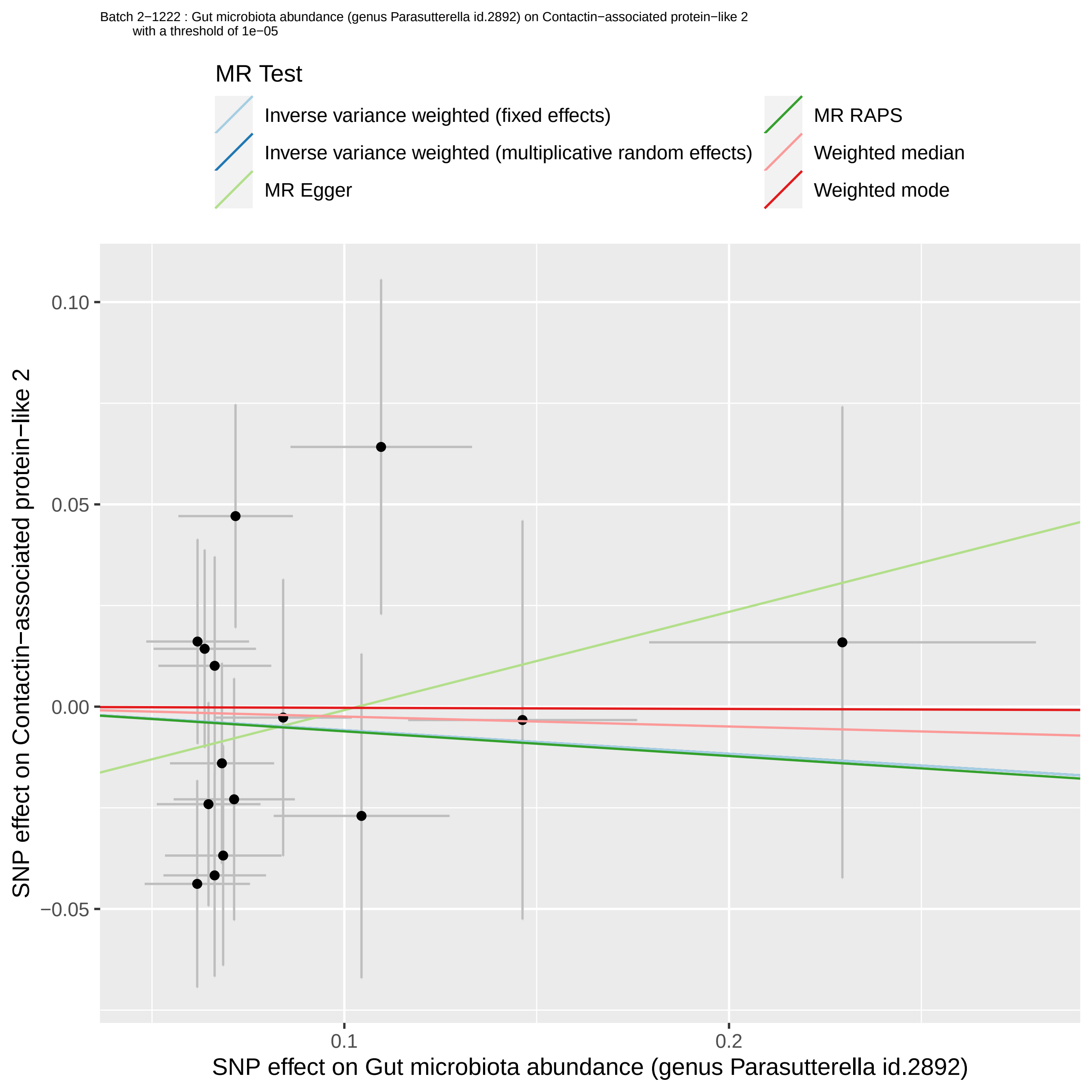
Batch 2−1222 : Gut microbiota abundance (genus Parasutterella id.2892) on Contactin−associated protein−like 2
with a threshold of 1e−05
MR Test
Inverse variance weighted (fixed effects)
Inverse variance weighted (multiplicative random effects)
MR Egger
MR RAPS
Weighted median
Weighted mode
0.10
0.05
0.00
−0.05
0.1
0.2
SNP effect on Contactin−associated protein−like 2
SNP effect on Gut microbiota abundance (genus Parasutterella id.2892)

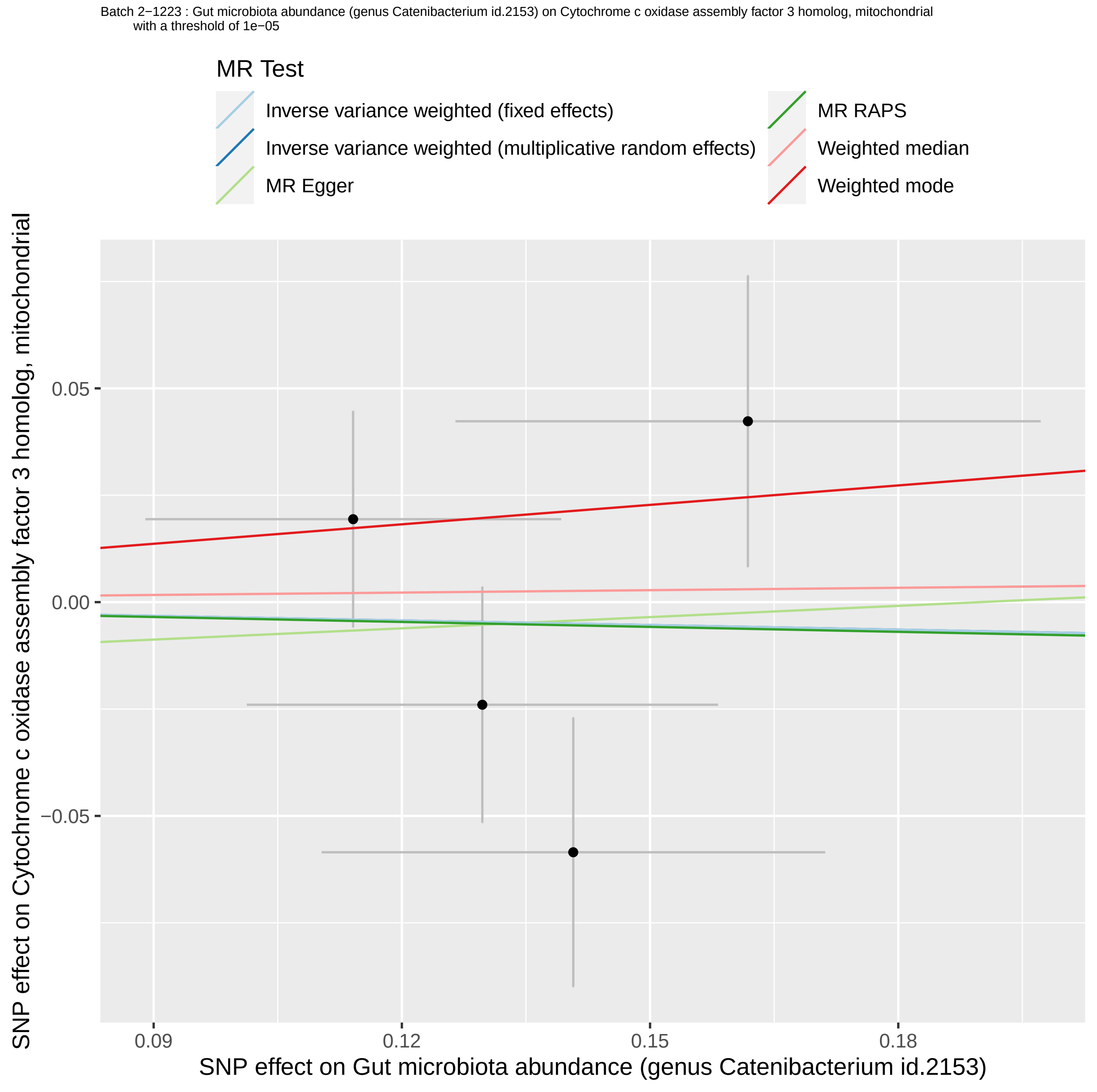

Batch 2−1223 : Gut microbiota abundance (genus Catenibacterium id.2153) on Cytochrome c oxidase assembly factor 3 homolog, mitochondrial
with a threshold of 1e−05
MR Test
Inverse variance weighted (fixed effects)
Inverse variance weighted (multiplicative random effects)
MR Egger
MR RAPS
Weighted median
Weighted mode
0.05
0.00
−0.05
0.09
0.12
0.15
0.18
SNP effect on Cytochrome c oxidase assembly factor 3 homolog, mitochondrial
SNP effect on Gut microbiota abundance (genus Catenibacterium id.2153)

Batch 2−1224 : Gut microbiota abundance (genus Parasutterella id.2892) on Cytochrome c oxidase assembly factor 3 homolog, mitochondrial with a threshold of 1e−05

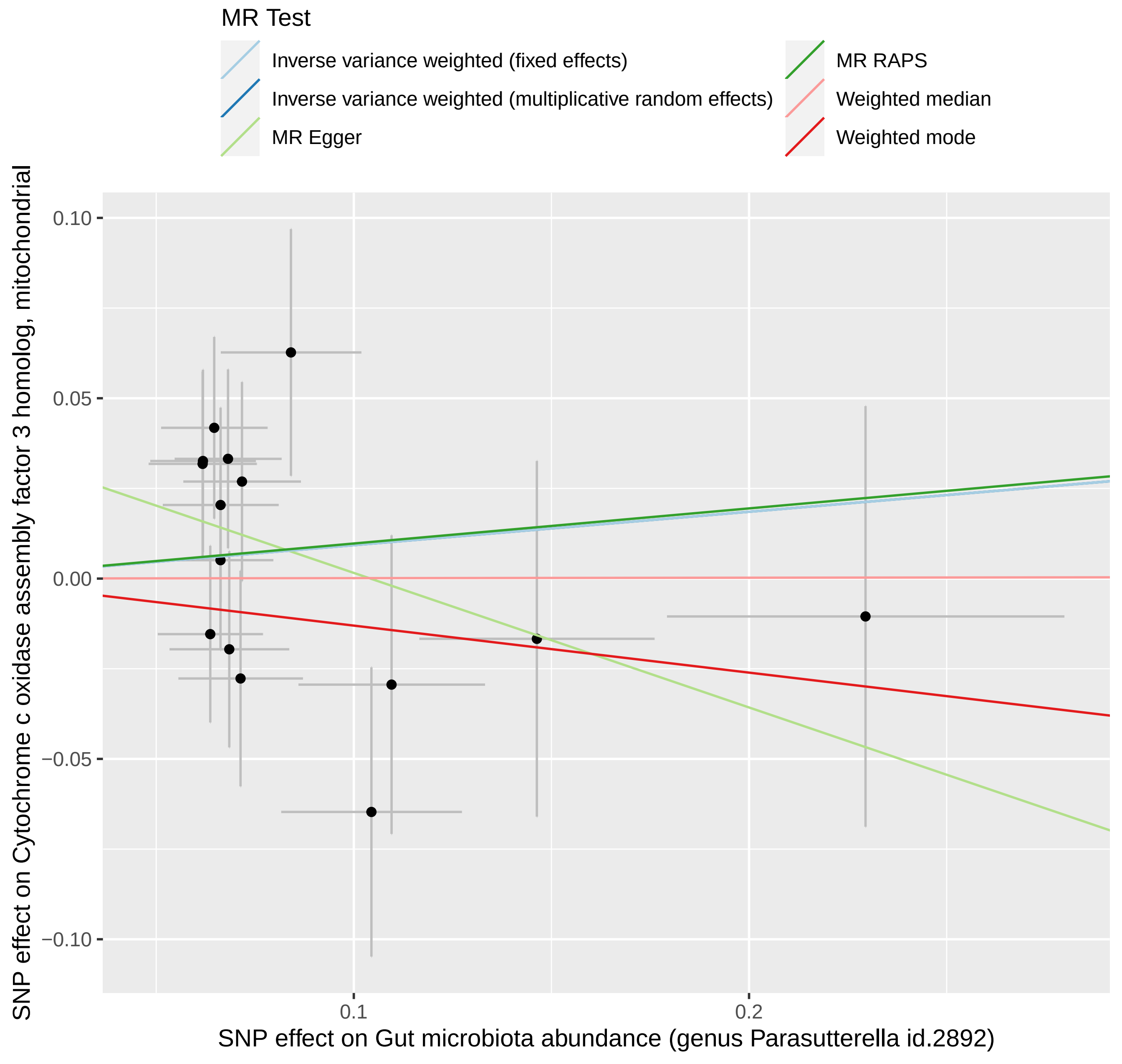

Batch 2−1225 : Gut microbiota abundance (genus Catenibacterium id.2153) on Cochlin
with a threshold of 1e−05

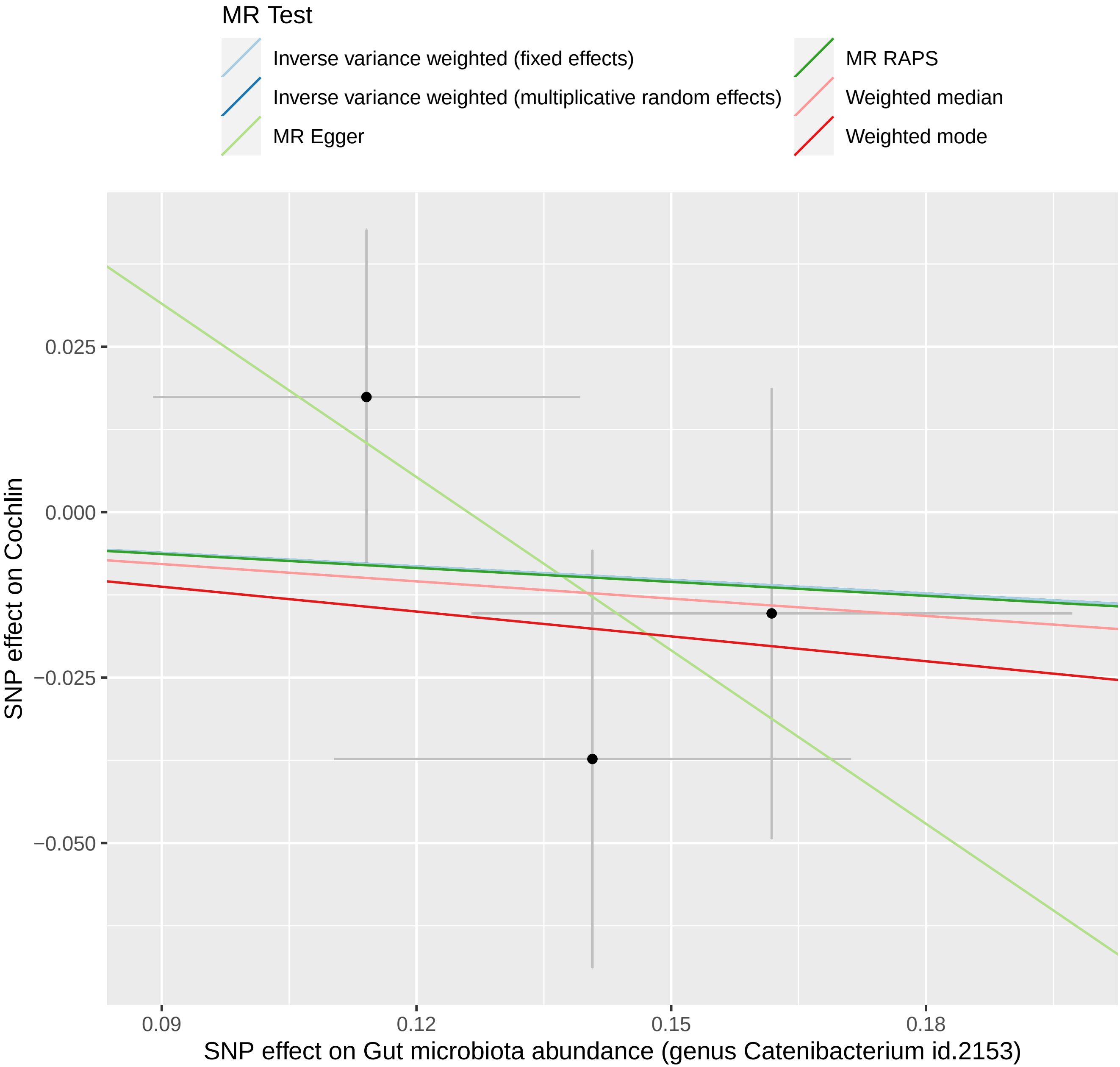

Batch 2−1226 : Gut microbiota abundance (genus Parasutterella id.2892) on Cochlin
with a threshold of 1e−05

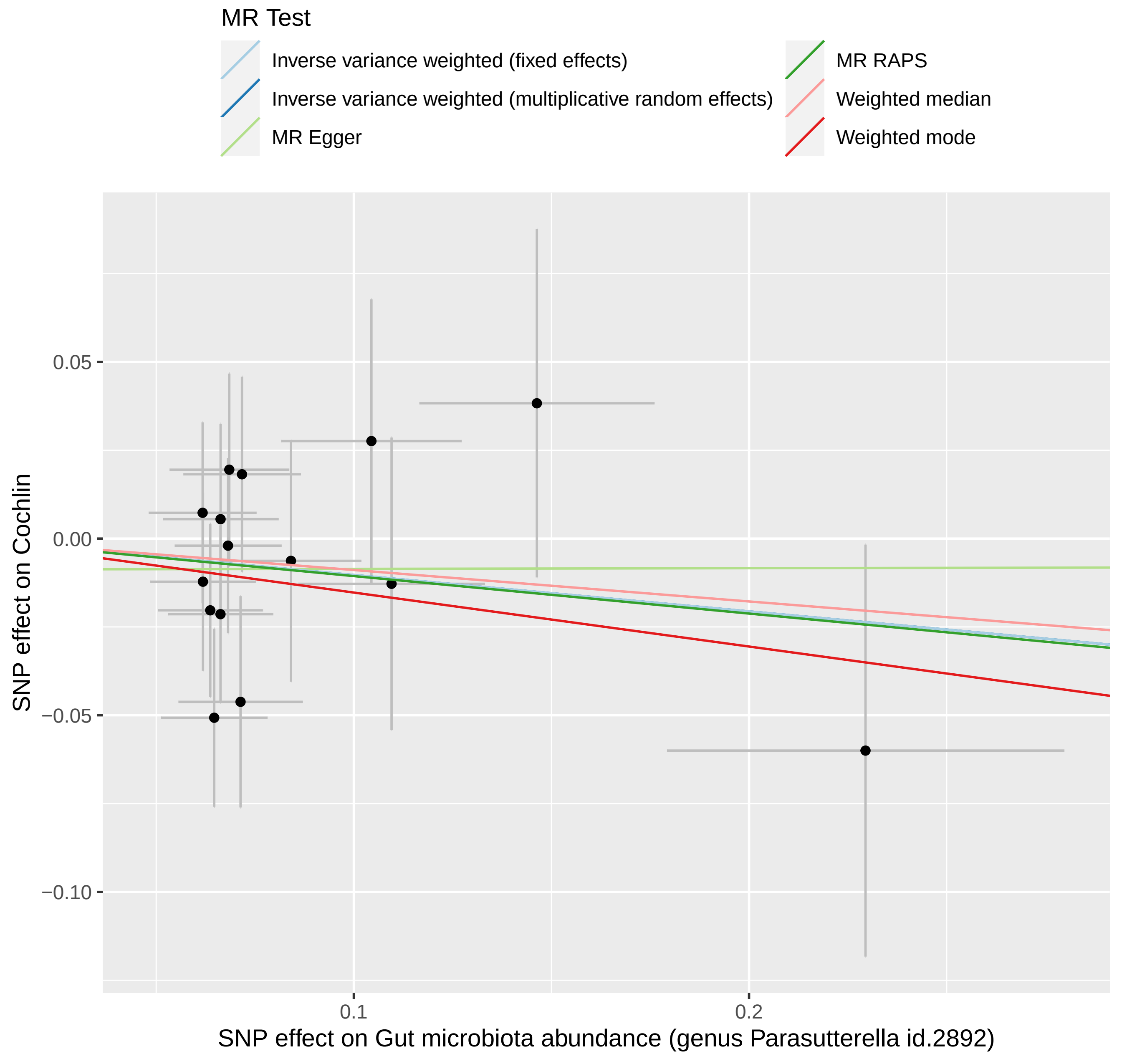

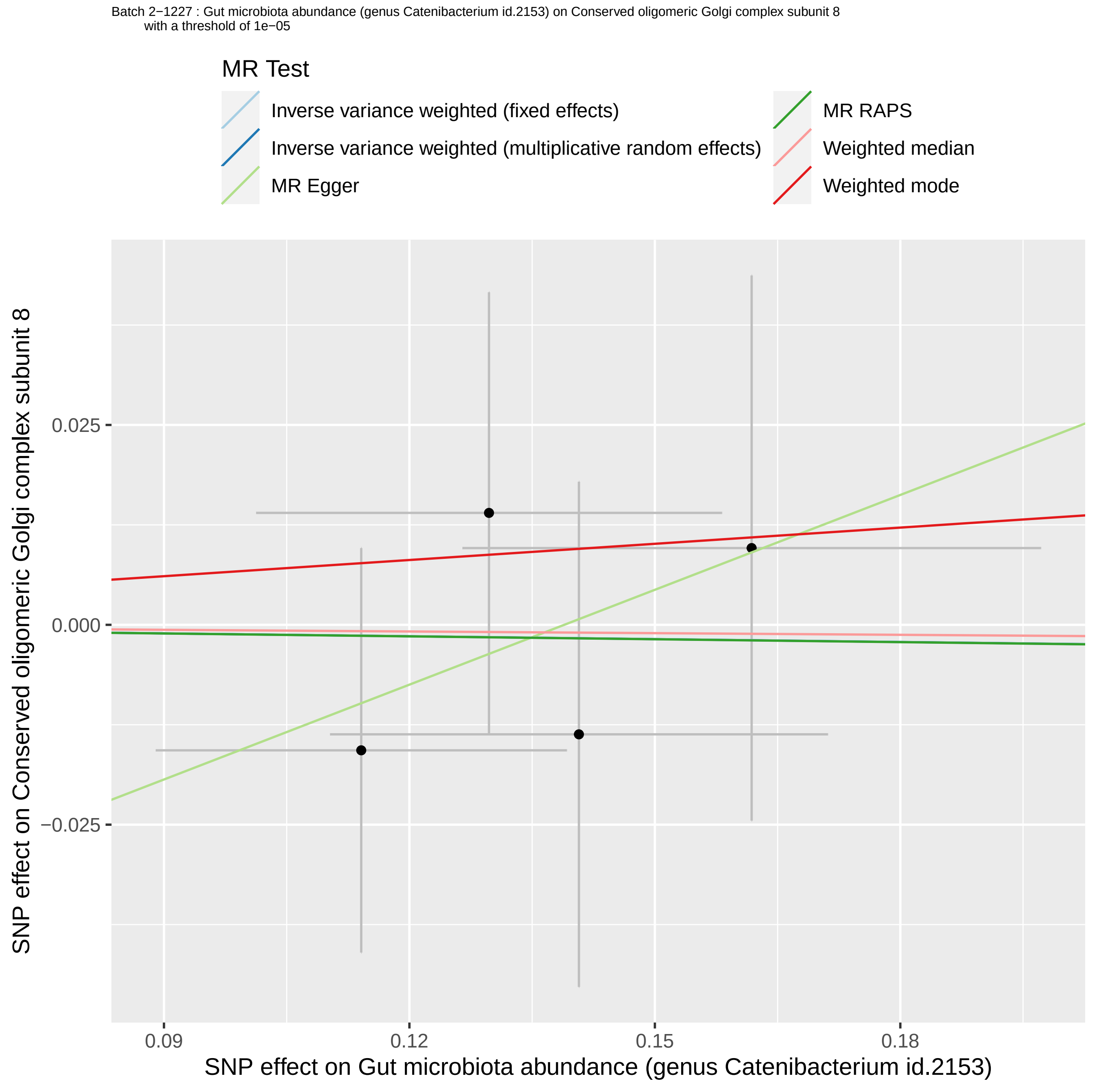
Batch 2−1227 : Gut microbiota abundance (genus Catenibacterium id.2153) on Conserved oligomeric Golgi complex subunit 8
with a threshold of 1e−05
MR Test
Inverse variance weighted (fixed effects)
Inverse variance weighted (multiplicative random effects)
MR Egger
MR RAPS
Weighted median
Weighted mode
0.025
0.000
−0.025
0.09
0.12
0.15
0.18
SNP effect on Gut microbiota abundance (genus Catenibacterium id.2153)
SNP effect on Conserved oligomeric Golgi complex subunit 8

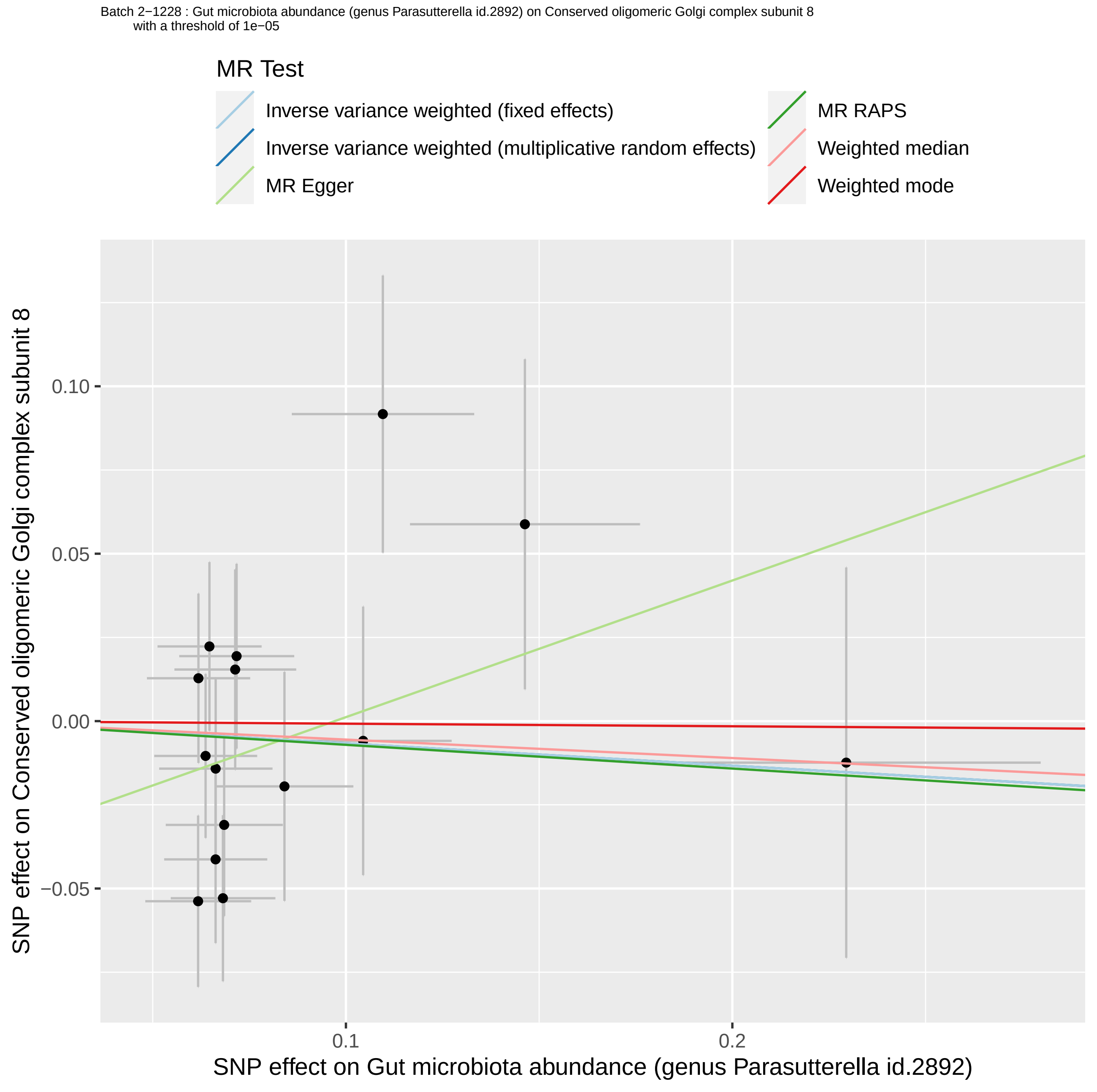

Batch 2−1228 : Gut microbiota abundance (genus Parasutterella id.2892) on Conserved oligomeric Golgi complex subunit 8
with a threshold of 1e−05
MR Test
Inverse variance weighted (fixed effects)
Inverse variance weighted (multiplicative random effects)
MR Egger
MR RAPS
Weighted median
Weighted mode
0.10
0.05
0.00
−0.05
0.1
0.2
SNP effect on Conserved oligomeric Golgi complex subunit 8
SNP effect on Gut microbiota abundance (genus Parasutterella id.2892)

Batch 2−1229 : Gut microbiota abundance (genus Catenibacterium id.2153) on Collagen alpha−2(XI) chain
with a threshold of 1e−05

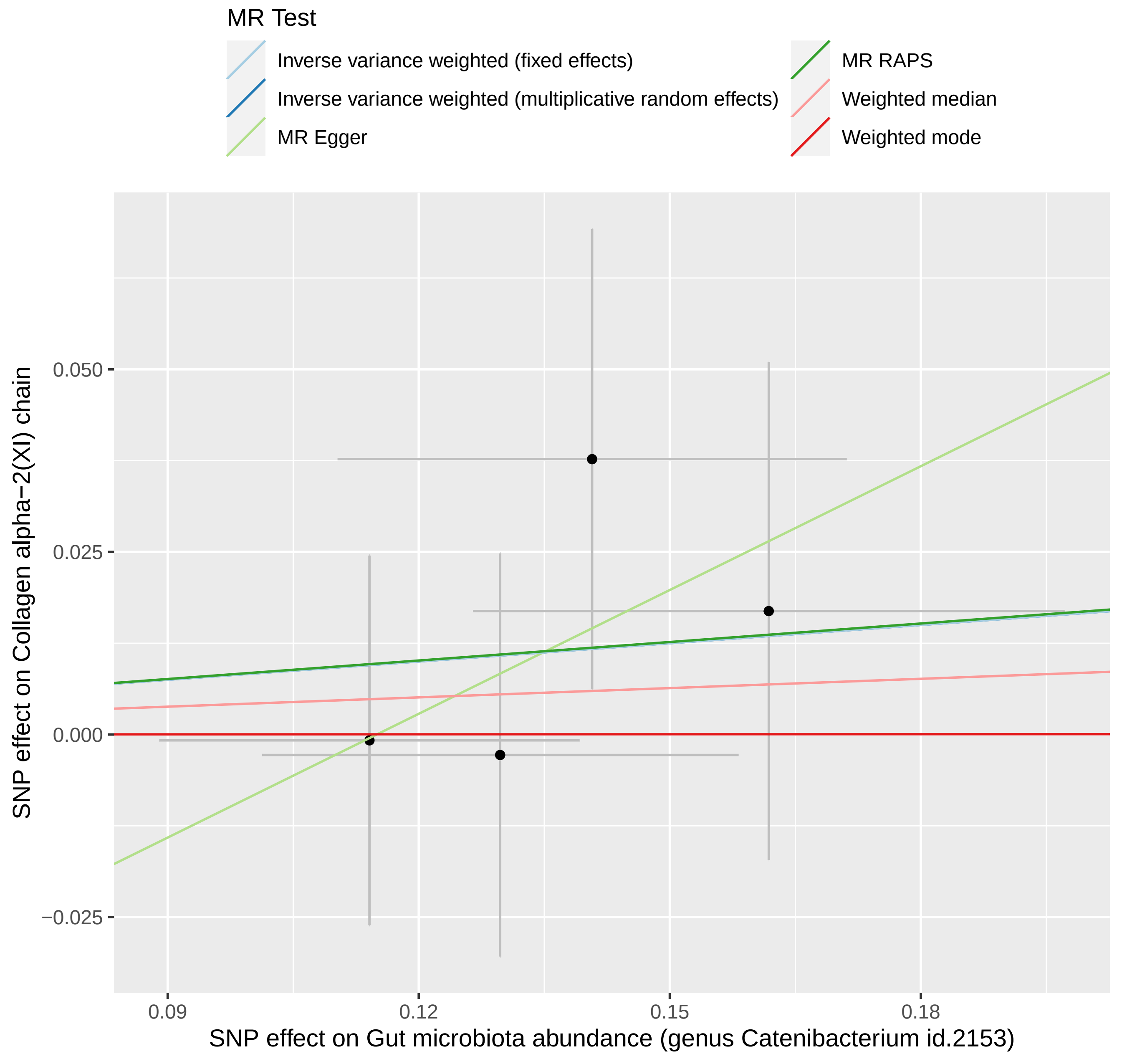

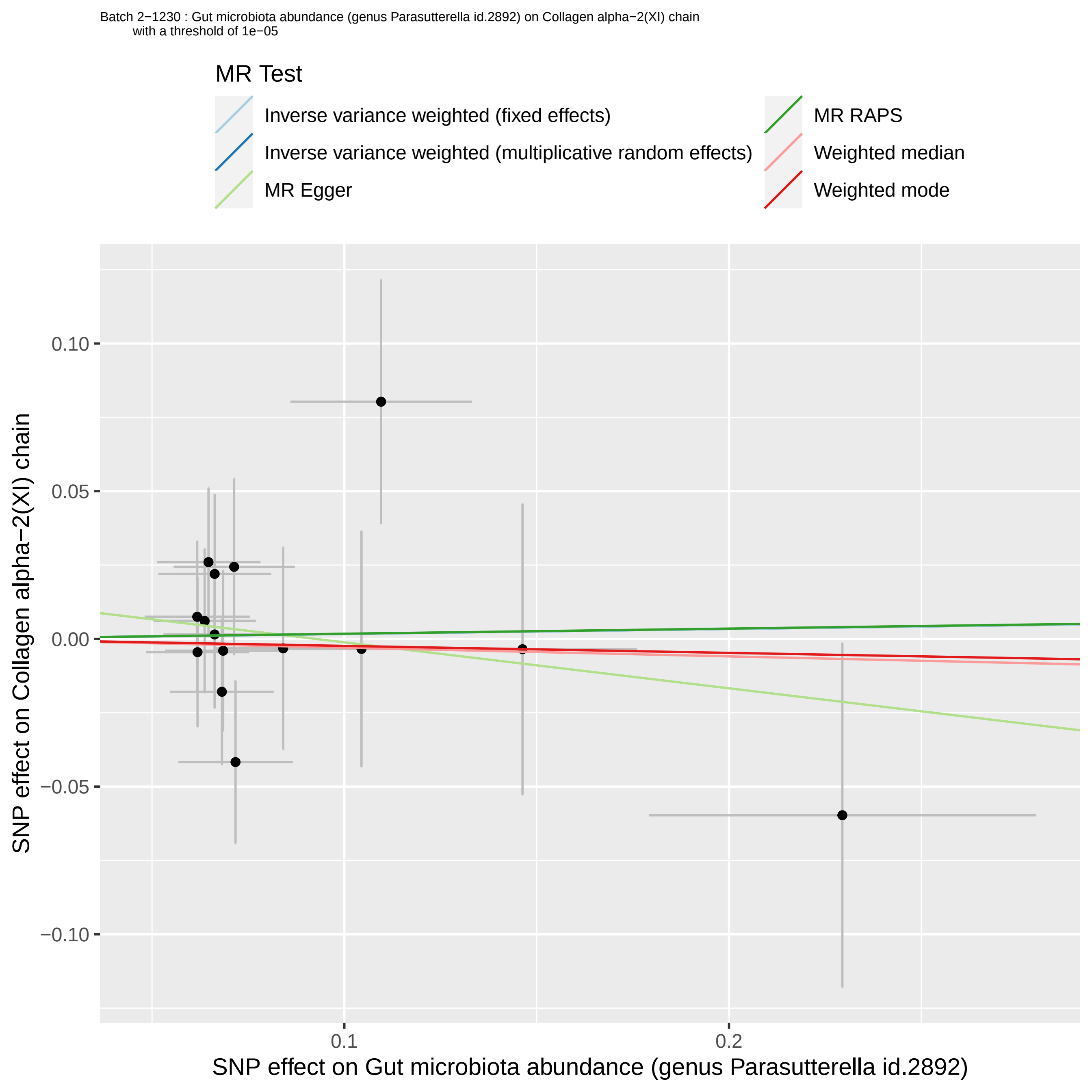

Batch 2−1230 : Gut microbiota abundance (genus Parasutterella id.2892) on Collagen alpha−2(XI) chain
with a threshold of 1e−05
MR Test
Inverse variance weighted (fixed effects)
Inverse variance weighted (multiplicative random effects)
MR Egger
MR RAPS
Weighted median
Weighted mode
0.10
0.05
0.00
−0.05
−0.10
0.1
0.2
SNP effect on Collagen alpha−2(XI) chain
SNP effect on Gut microbiota abundance (genus Parasutterella id.2892)

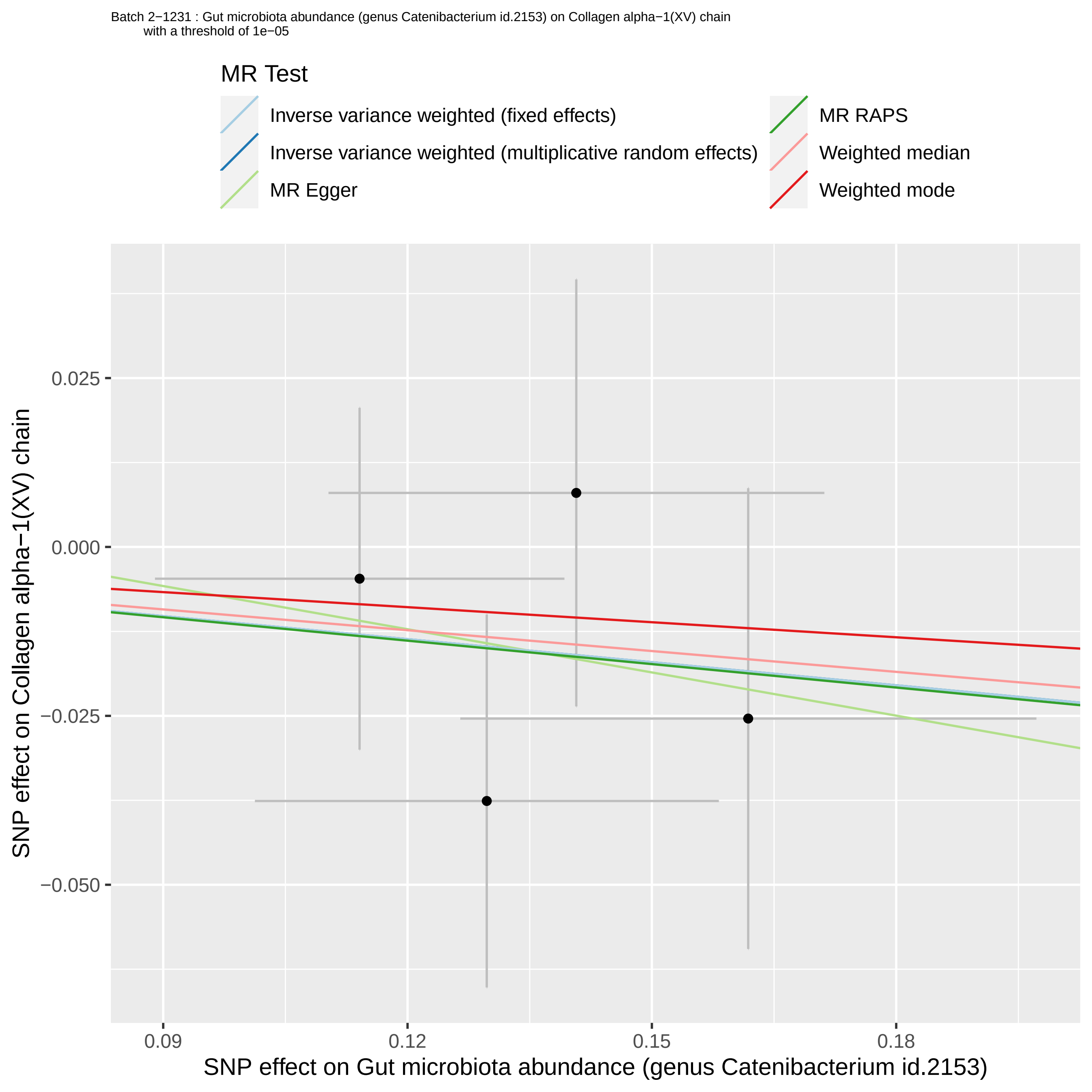

Batch 2−1231 : Gut microbiota abundance (genus Catenibacterium id.2153) on Collagen alpha−1(XV) chain
with a threshold of 1e−05
MR Test
Inverse variance weighted (fixed effects)
Inverse variance weighted (multiplicative random effects)
MR Egger
MR RAPS
Weighted median
Weighted mode
0.025
0.000
−0.025
−0.050
0.09
0.12
0.15
0.18
SNP effect on Collagen alpha−1(XV) chain
SNP effect on Gut microbiota abundance (genus Catenibacterium id.2153)

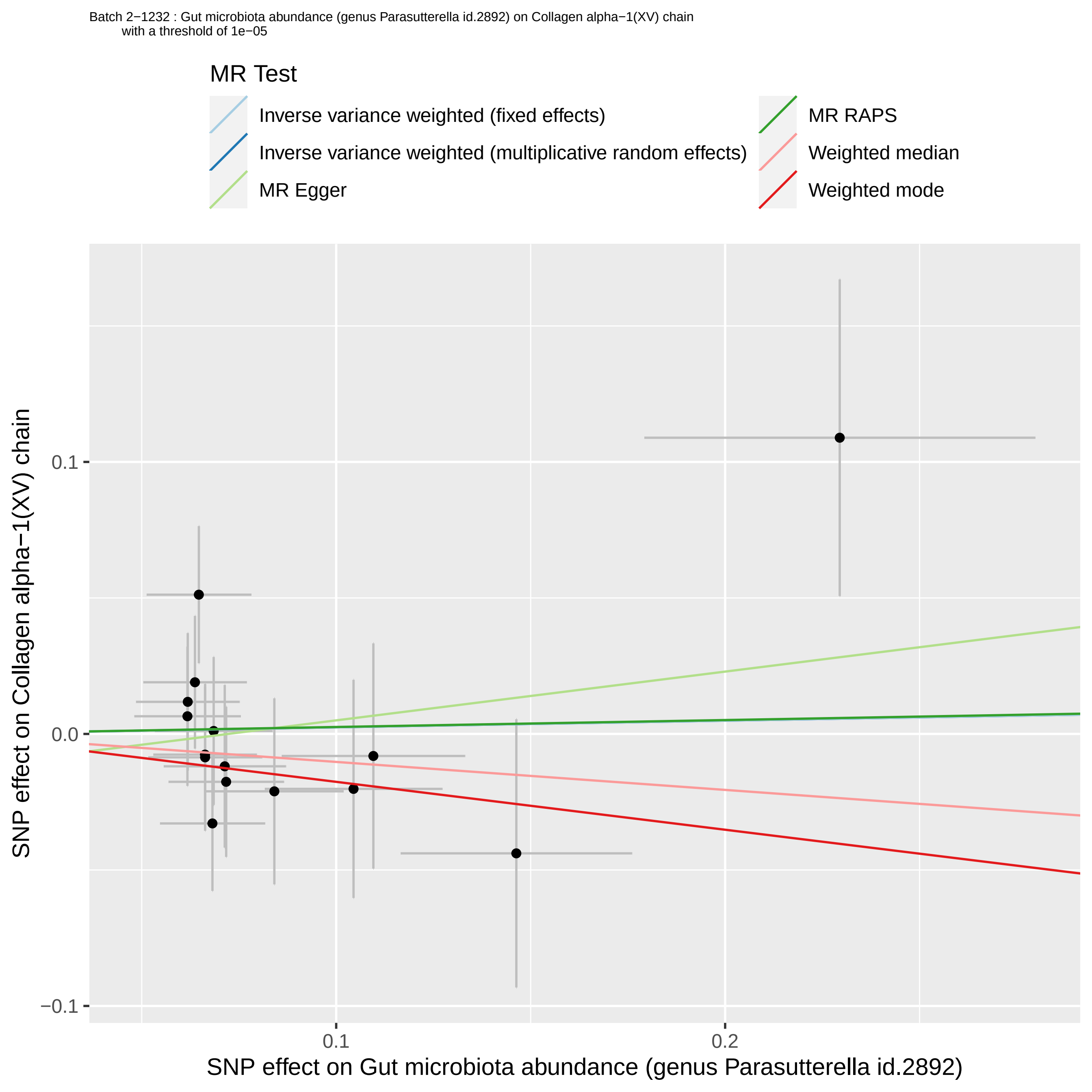

Batch 2−1232 : Gut microbiota abundance (genus Parasutterella id.2892) on Collagen alpha−1(XV) chain
with a threshold of 1e−05
MR Test
Inverse variance weighted (fixed effects)
Inverse variance weighted (multiplicative random effects)
MR Egger
MR RAPS
Weighted median
Weighted mode
0.1
0.0
−0.1
0.1
0.2
SNP effect on Collagen alpha−1(XV) chain
SNP effect on Gut microbiota abundance (genus Parasutterella id.2892)

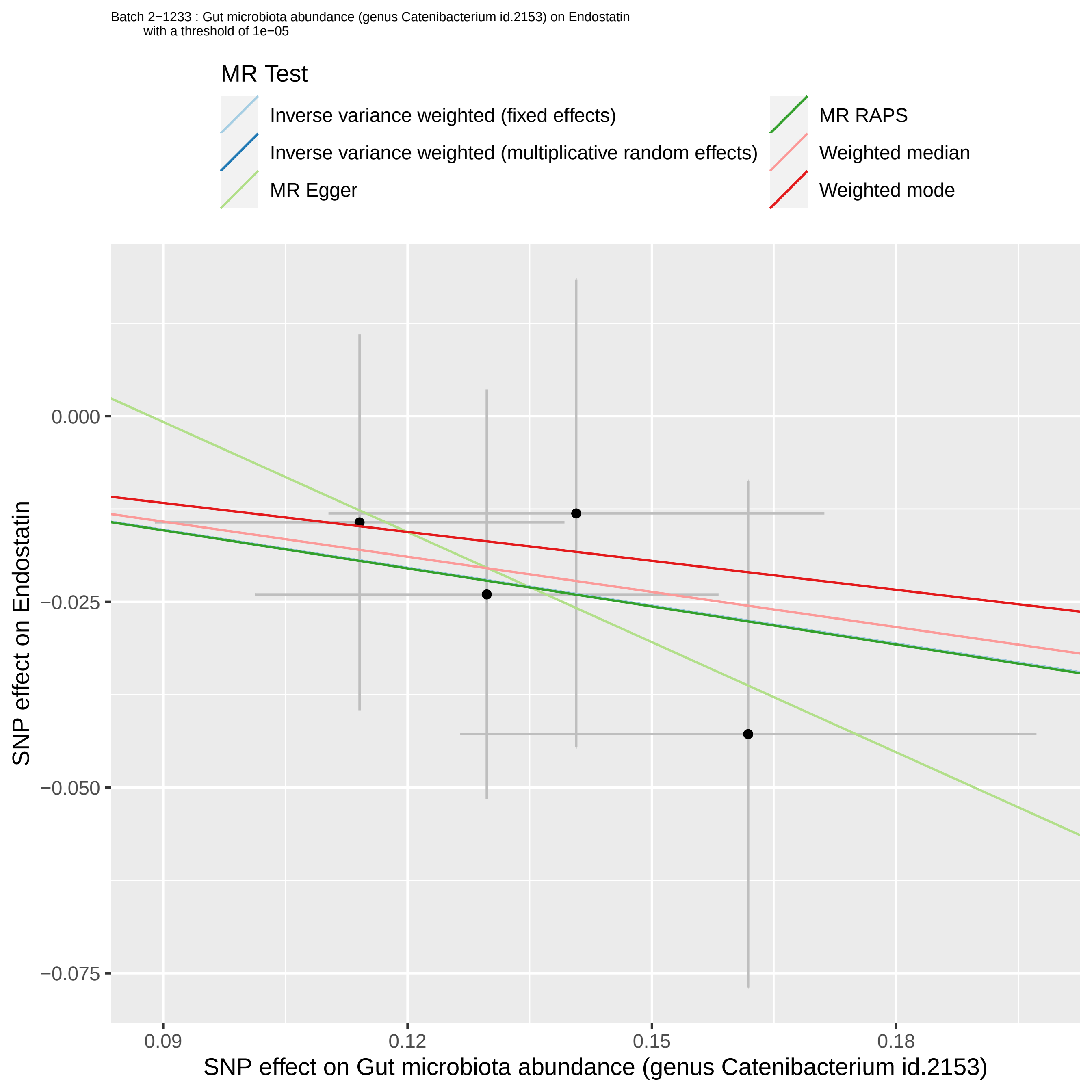

Batch 2−1233 : Gut microbiota abundance (genus Catenibacterium id.2153) on Endostatin
with a threshold of 1e−05
MR Test
Inverse variance weighted (fixed effects)
Inverse variance weighted (multiplicative random effects)
MR Egger
MR RAPS
Weighted median
Weighted mode
0.000
−0.025
−0.050
−0.075
0.09
0.12
0.15
0.18
SNP effect on Endostatin
SNP effect on Gut microbiota abundance (genus Catenibacterium id.2153)

Batch 2−1234 : Gut microbiota abundance (genus Parasutterella id.2892) on Endostatin
with a threshold of 1e−05

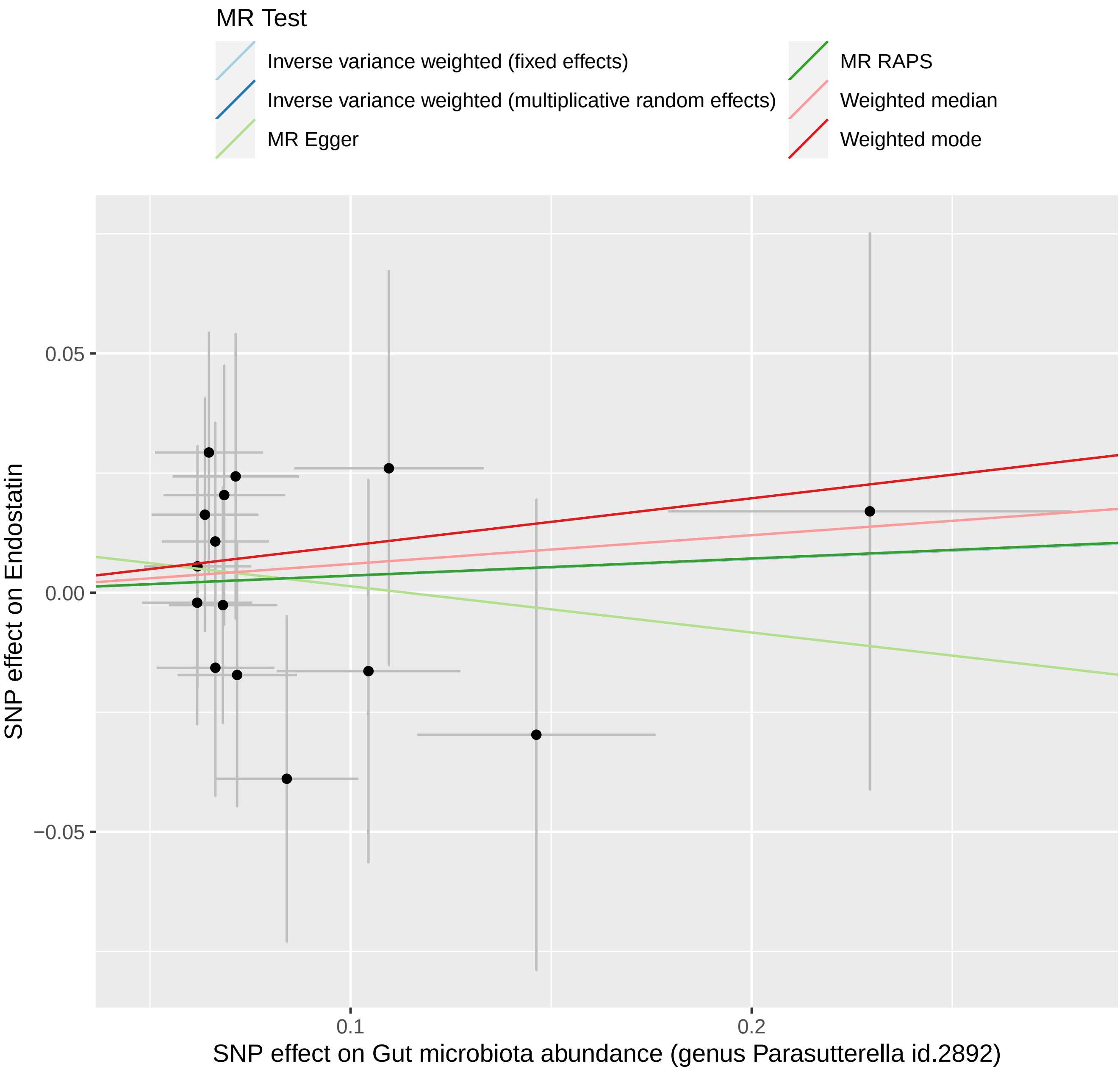

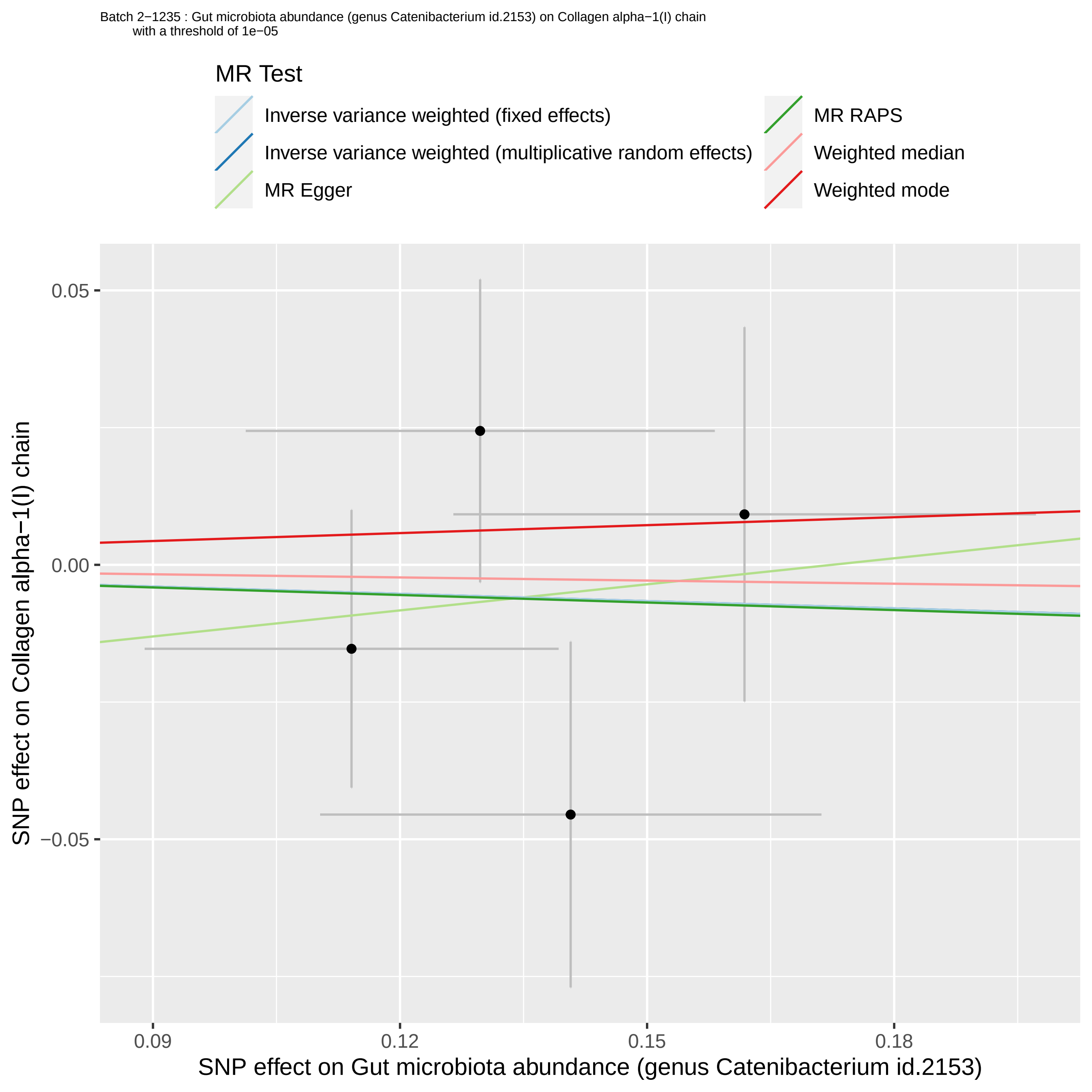
Batch 2−1235 : Gut microbiota abundance (genus Catenibacterium id.2153) on Collagen alpha−1(I) chain
with a threshold of 1e−05
MR Test
Inverse variance weighted (fixed effects)
Inverse variance weighted (multiplicative random effects)
MR Egger
MR RAPS
Weighted median
Weighted mode
0.05
0.00
−0.05
0.09
0.12
0.15
0.18
SNP effect on Collagen alpha−1(I) chain
SNP effect on Gut microbiota abundance (genus Catenibacterium id.2153)

Batch 2−1236 : Gut microbiota abundance (genus Parasutterella id.2892) on Collagen alpha−1(I) chain
with a threshold of 1e−05

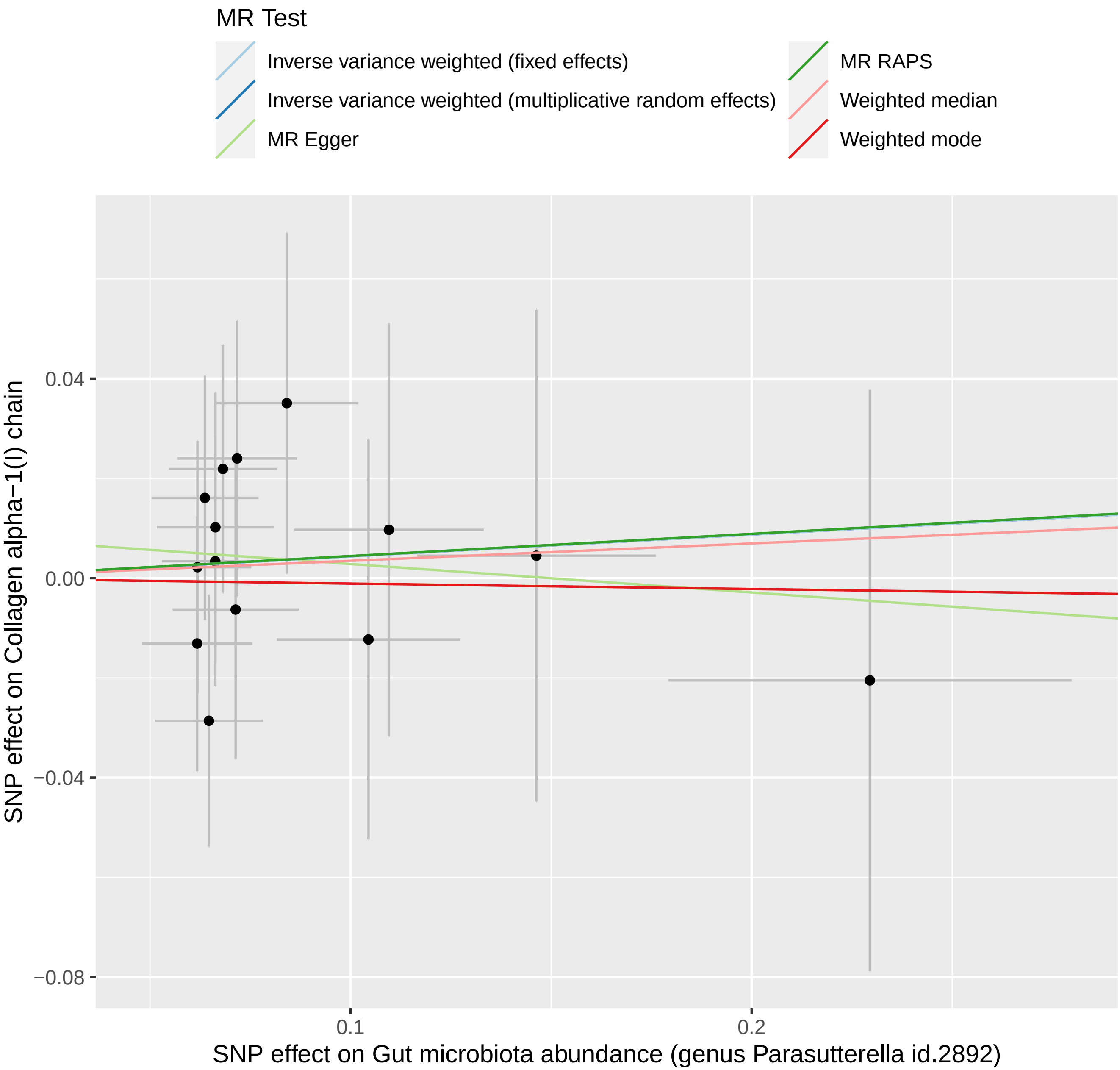

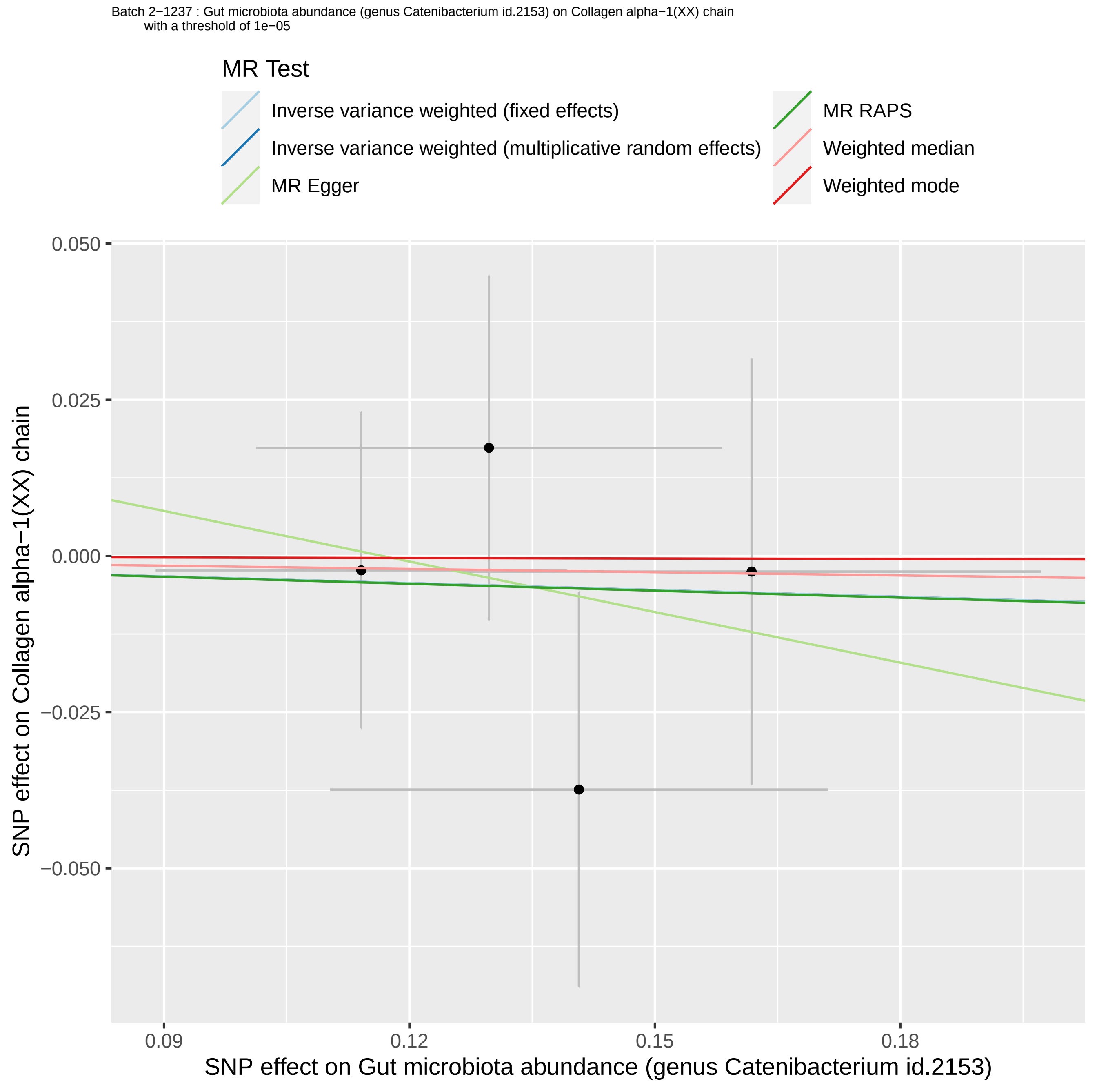

Batch 2−1237 : Gut microbiota abundance (genus Catenibacterium id.2153) on Collagen alpha−1(XX) chain
with a threshold of 1e−05
MR Test
Inverse variance weighted (fixed effects)
Inverse variance weighted (multiplicative random effects)
MR Egger
MR RAPS
Weighted median
Weighted mode
0.050
0.025
0.000
−0.025
−0.050
0.09
0.12
0.15
0.18
SNP effect on Collagen alpha−1(XX) chain
SNP effect on Gut microbiota abundance (genus Catenibacterium id.2153)

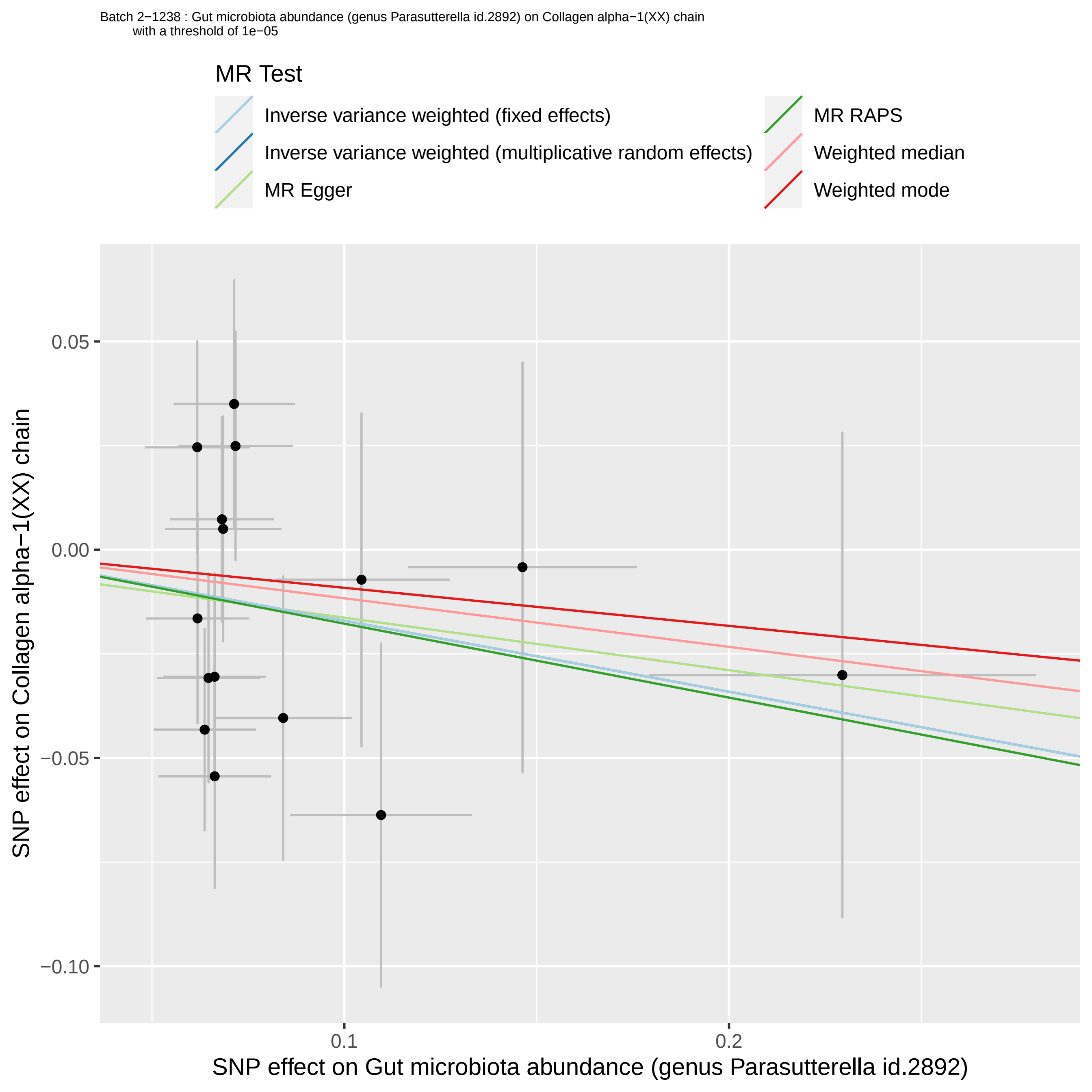

Batch 2−1238 : Gut microbiota abundance (genus Parasutterella id.2892) on Collagen alpha−1(XX) chain
with a threshold of 1e−05
MR Test
Inverse variance weighted (fixed effects)
Inverse variance weighted (multiplicative random effects)
MR Egger
MR RAPS
Weighted median
Weighted mode
0.05
0.00
−0.05
−0.10
0.1
0.2
SNP effect on Collagen alpha−1(XX) chain
SNP effect on Gut microbiota abundance (genus Parasutterella id.2892)

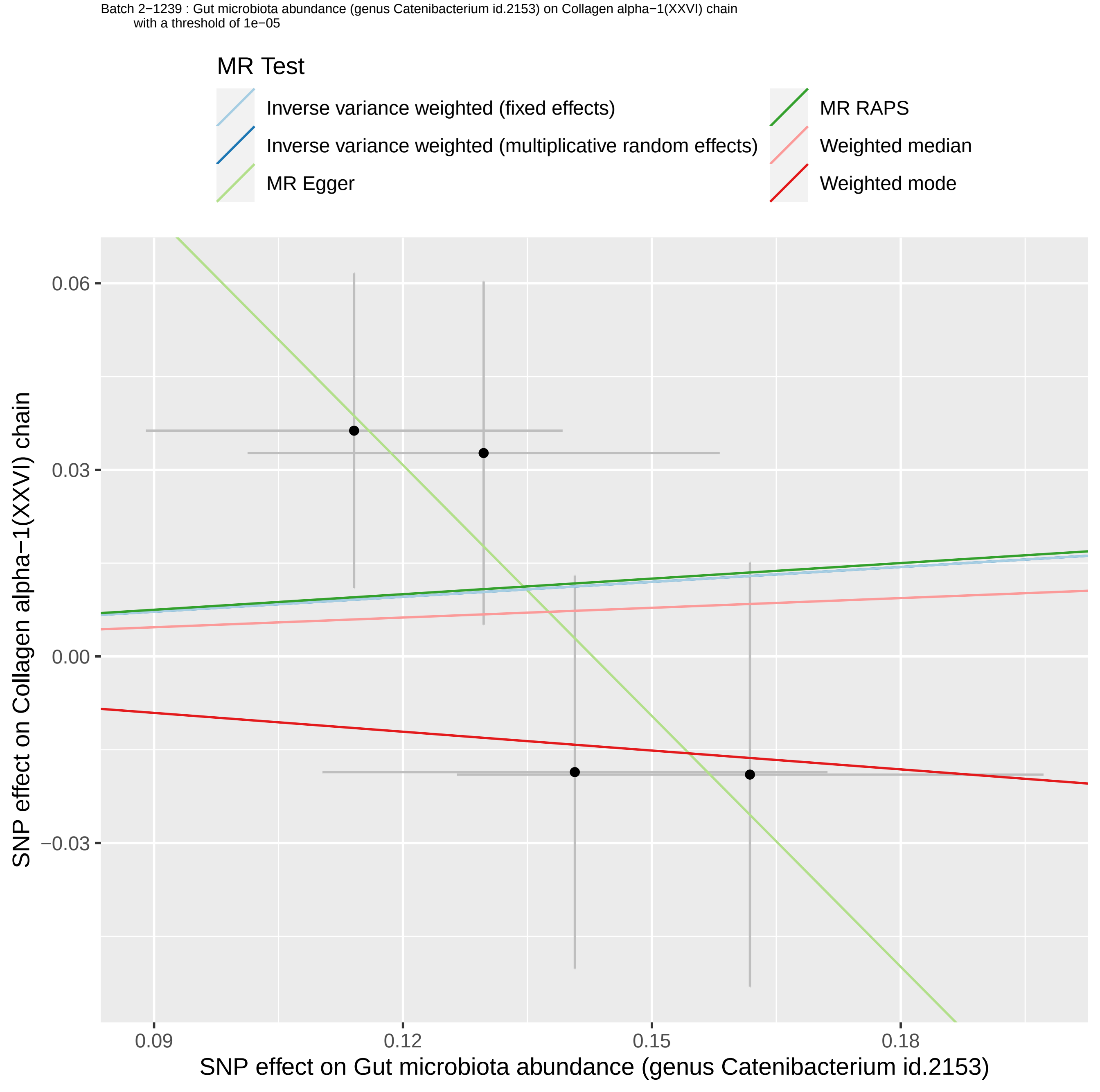
Batch 2−1239 : Gut microbiota abundance (genus Catenibacterium id.2153) on Collagen alpha−1(XXVI) chain
with a threshold of 1e−05
MR Test
Inverse variance weighted (fixed effects)
Inverse variance weighted (multiplicative random effects)
MR Egger
MR RAPS
Weighted median
Weighted mode
0.06
0.03
0.00
−0.03
0.09
0.12
0.15
0.18
SNP effect on Collagen alpha−1(XXVI) chain
SNP effect on Gut microbiota abundance (genus Catenibacterium id.2153)

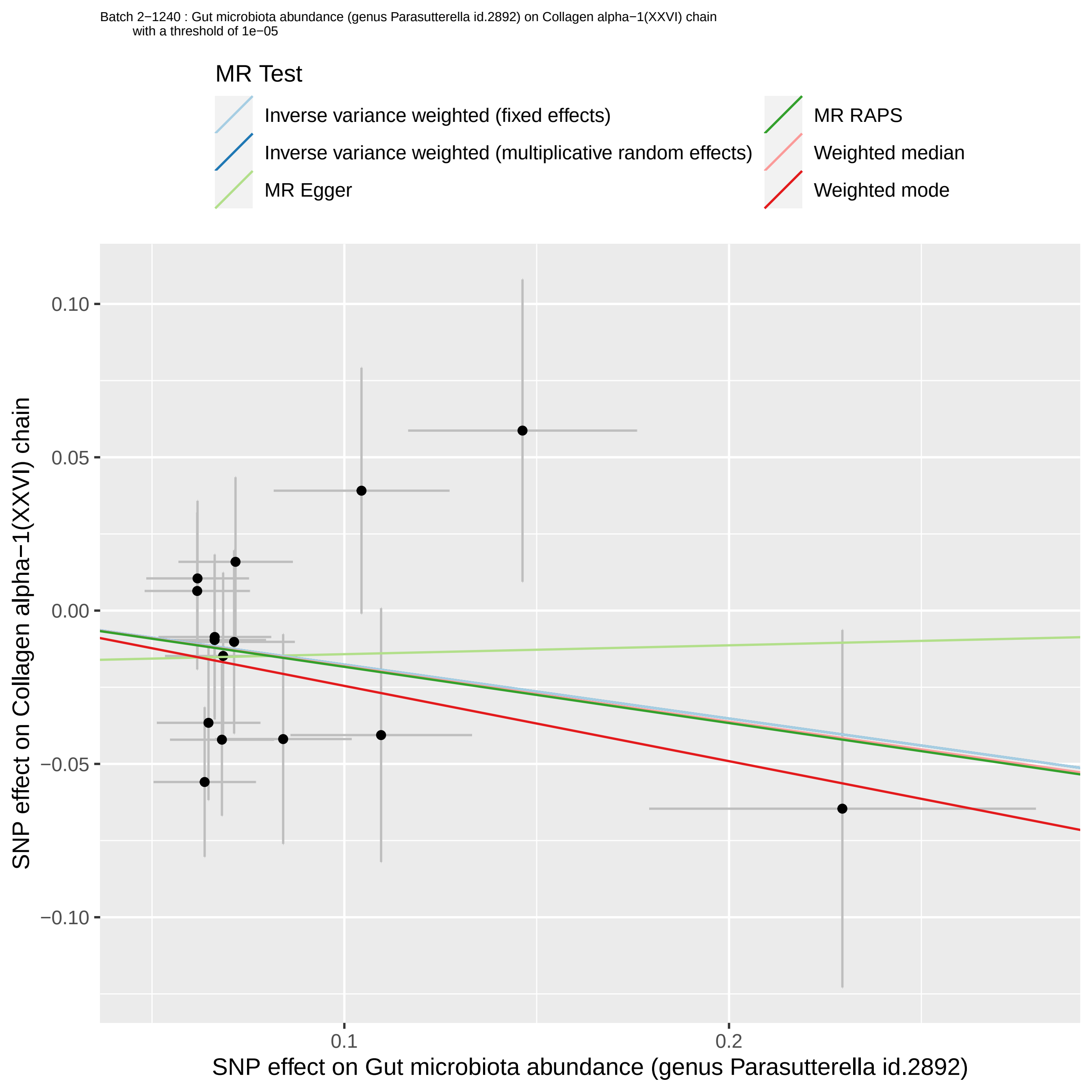

Batch 2−1240 : Gut microbiota abundance (genus Parasutterella id.2892) on Collagen alpha−1(XXVI) chain
with a threshold of 1e−05
MR Test
Inverse variance weighted (fixed effects)
Inverse variance weighted (multiplicative random effects)
MR Egger
MR RAPS
Weighted median
Weighted mode
0.10
0.05
0.00
−0.05
−0.10
0.1
0.2
SNP effect on Collagen alpha−1(XXVI) chain
SNP effect on Gut microbiota abundance (genus Parasutterella id.2892)

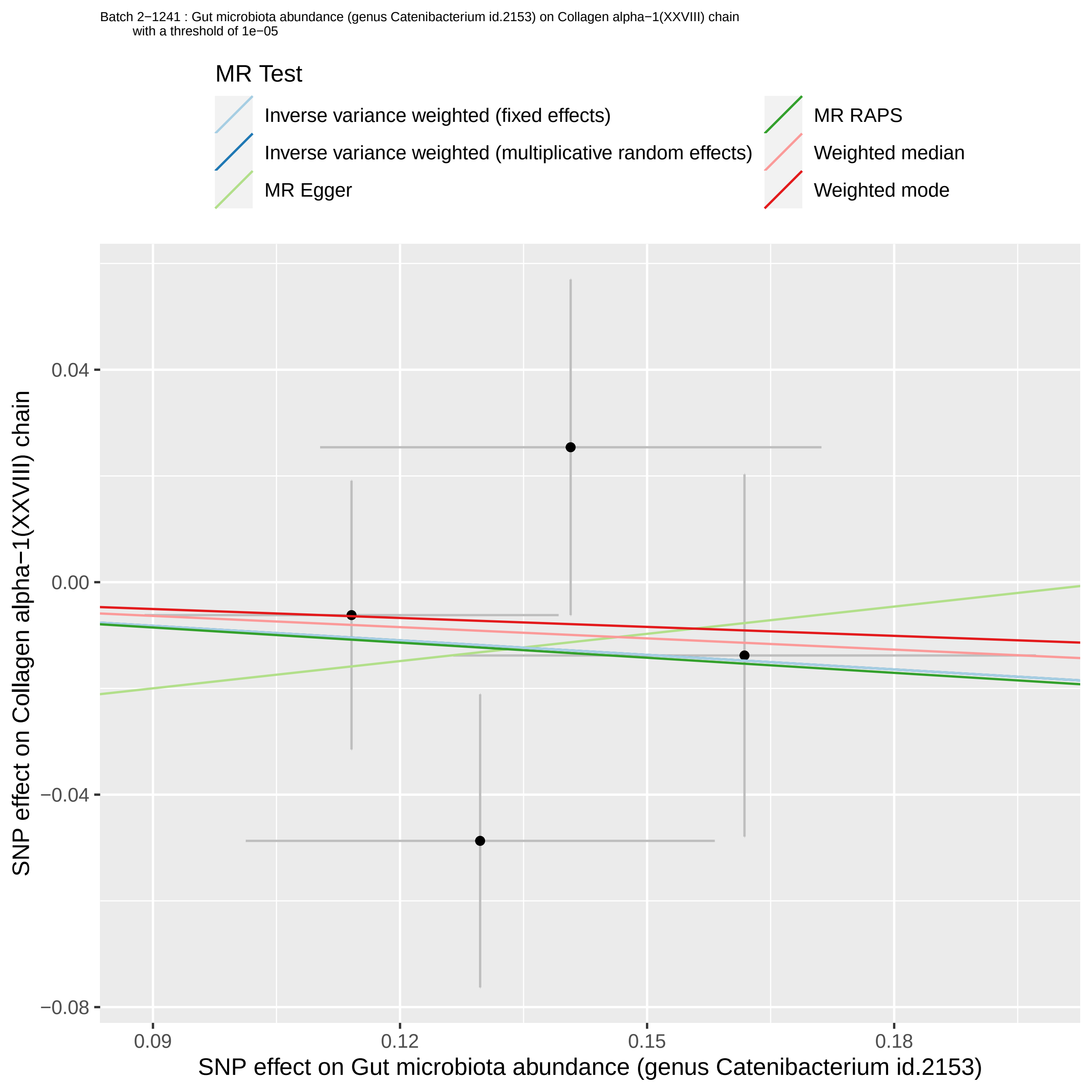

Batch 2−1241 : Gut microbiota abundance (genus Catenibacterium id.2153) on Collagen alpha−1(XXVIII) chain
with a threshold of 1e−05
MR Test
Inverse variance weighted (fixed effects)
Inverse variance weighted (multiplicative random effects)
MR Egger
MR RAPS
Weighted median
Weighted mode
0.04
0.00
−0.04
−0.08
0.09
0.12
0.15
0.18
SNP effect on Collagen alpha−1(XXVIII) chain
SNP effect on Gut microbiota abundance (genus Catenibacterium id.2153)

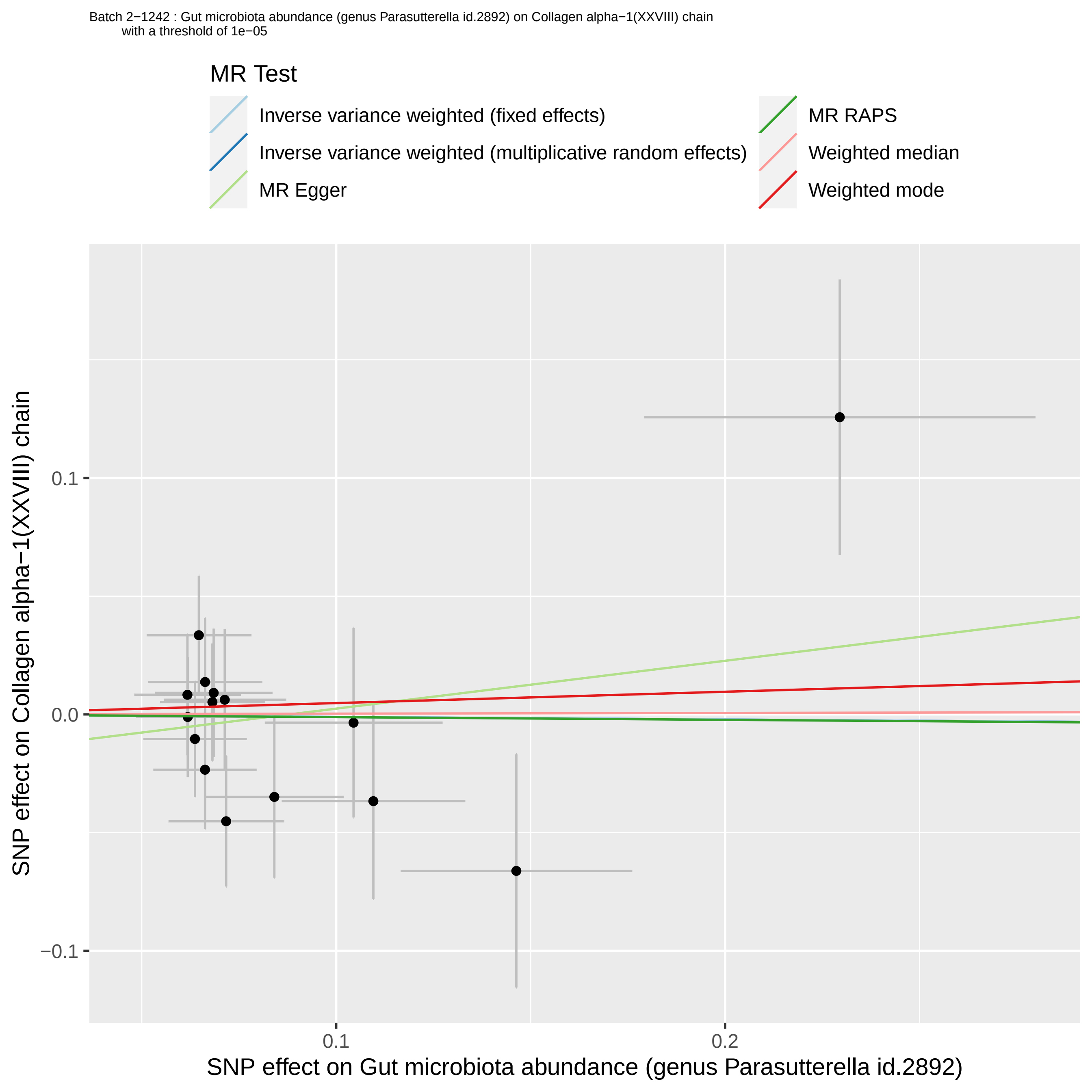
Batch 2−1242 : Gut microbiota abundance (genus Parasutterella id.2892) on Collagen alpha−1(XXVIII) chain
with a threshold of 1e−05
MR Test
Inverse variance weighted (fixed effects)
Inverse variance weighted (multiplicative random effects)
MR Egger
MR RAPS
Weighted median
Weighted mode
0.1
0.0
−0.1
0.1
0.2
SNP effect on Collagen alpha−1(XXVIII) chain
SNP effect on Gut microbiota abundance (genus Parasutterella id.2892)

Batch 2−1243 : Gut microbiota abundance (genus Catenibacterium id.2153) on Collagen alpha−1(VI) chain
with a threshold of 1e−05

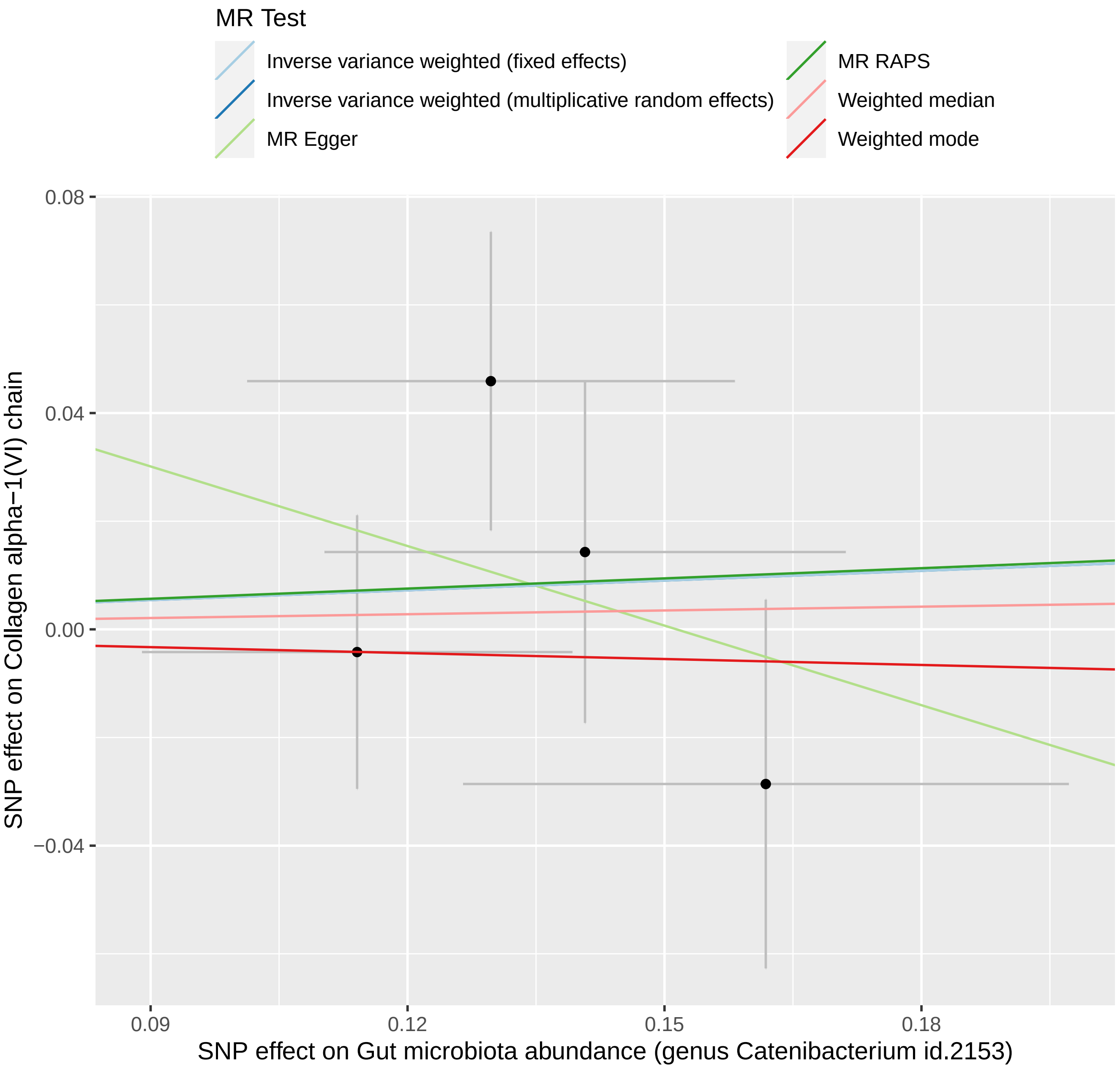

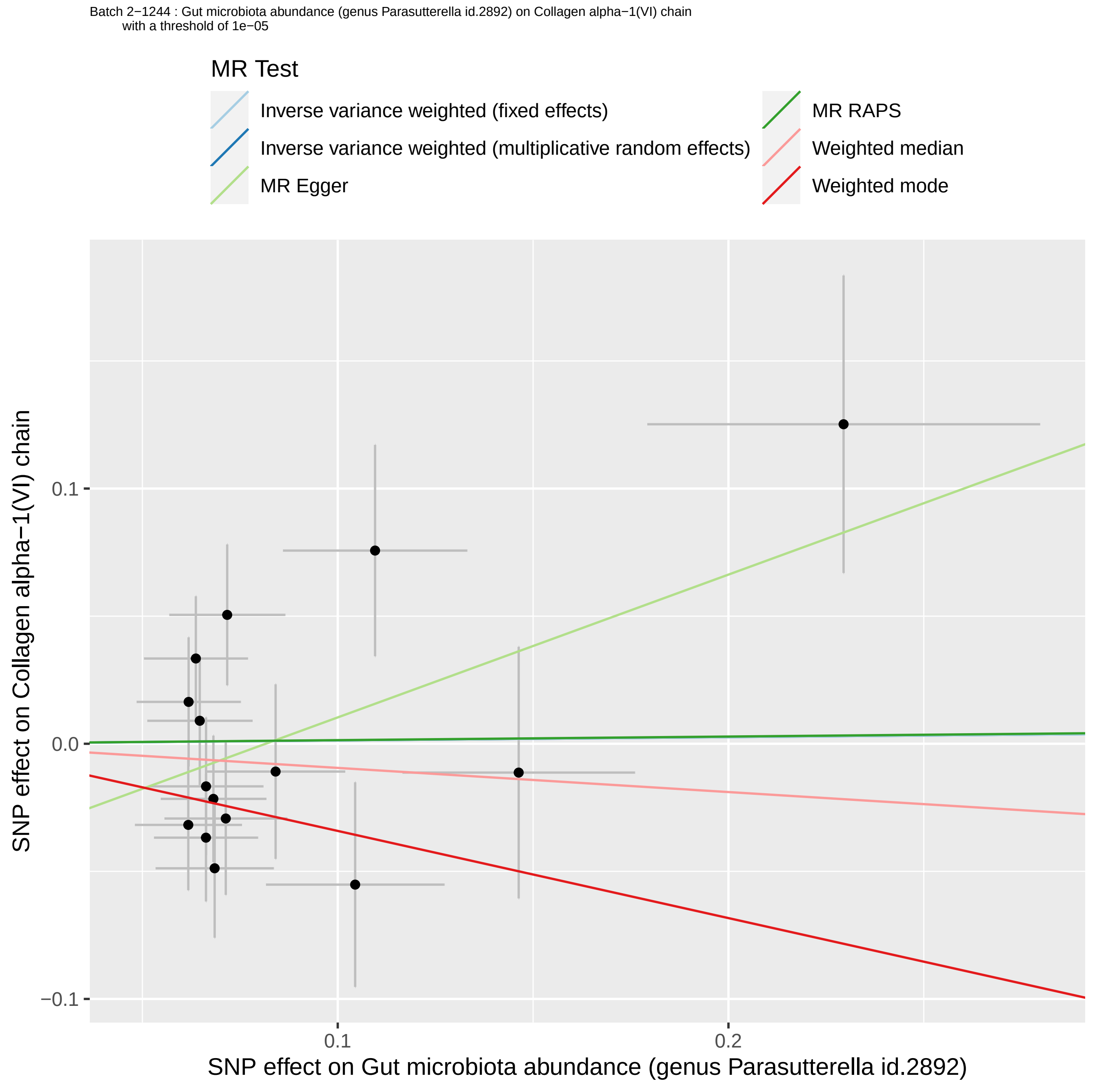

Batch 2−1244 : Gut microbiota abundance (genus Parasutterella id.2892) on Collagen alpha−1(VI) chain
with a threshold of 1e−05
MR Test
Inverse variance weighted (fixed effects)
Inverse variance weighted (multiplicative random effects)
MR Egger
MR RAPS
Weighted median
Weighted mode
0.1
0.0
−0.1
0.1
0.2
SNP effect on Collagen alpha−1(VI) chain
SNP effect on Gut microbiota abundance (genus Parasutterella id.2892)

Batch 2−1245 : Gut microbiota abundance (genus Catenibacterium id.2153) on Collagen alpha−3(VI) chain
with a threshold of 1e−05

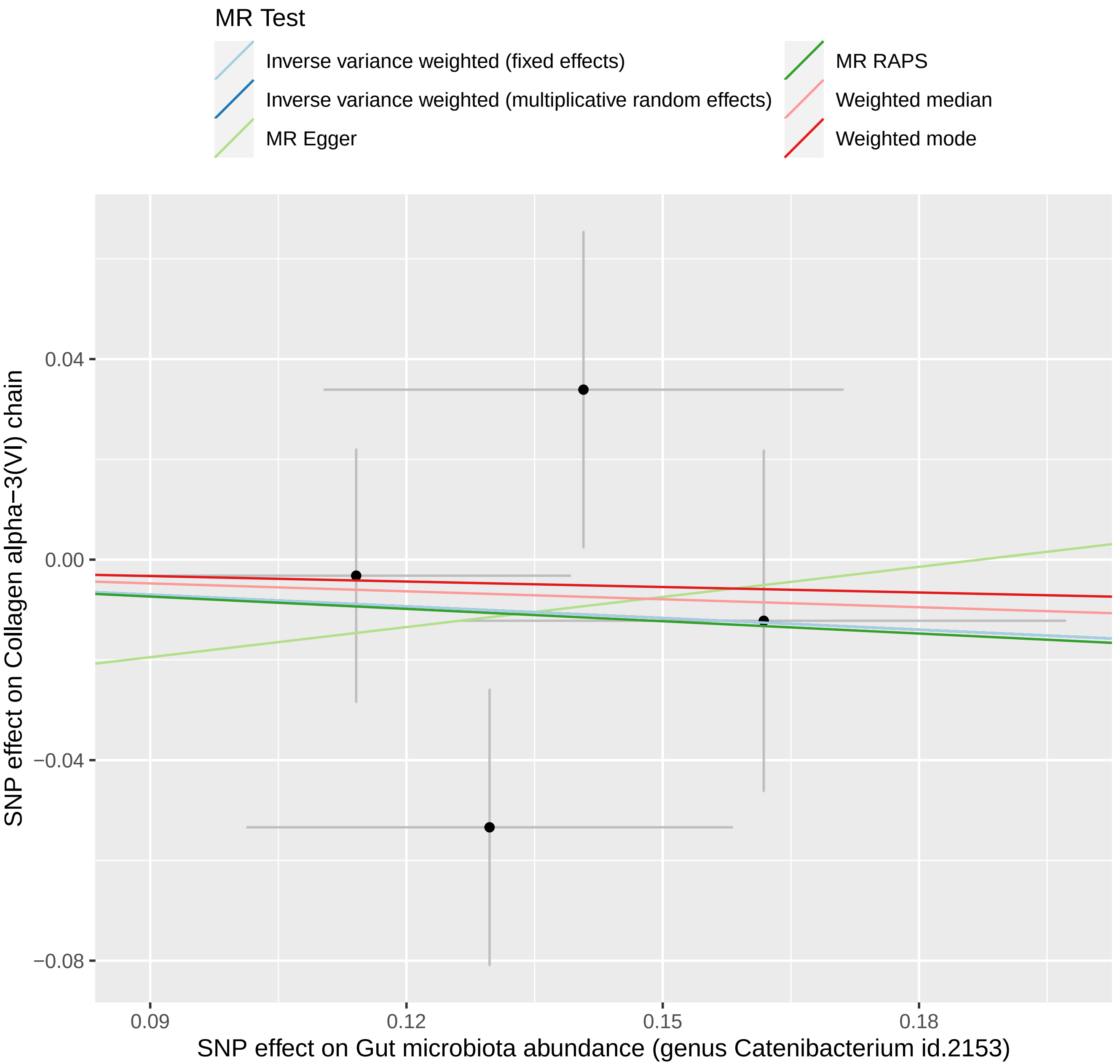

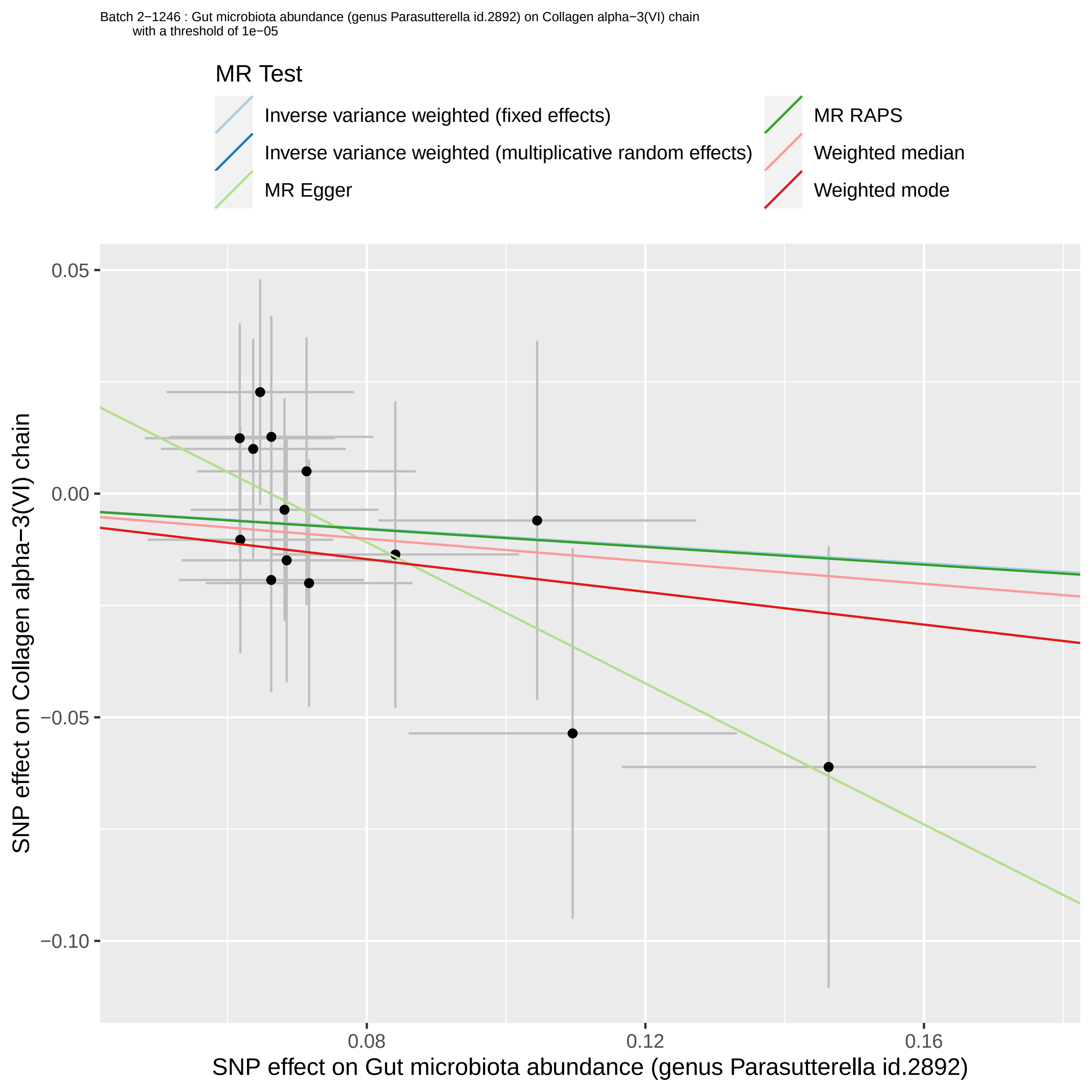

Batch 2−1246 : Gut microbiota abundance (genus Parasutterella id.2892) on Collagen alpha−3(VI) chain
with a threshold of 1e−05
MR Test
Inverse variance weighted (fixed effects)
Inverse variance weighted (multiplicative random effects)
MR Egger
MR RAPS
Weighted median
Weighted mode
0.05
0.00
−0.05
−0.10
0.08
0.12
0.16
SNP effect on Collagen alpha−3(VI) chain
SNP effect on Gut microbiota abundance (genus Parasutterella id.2892)

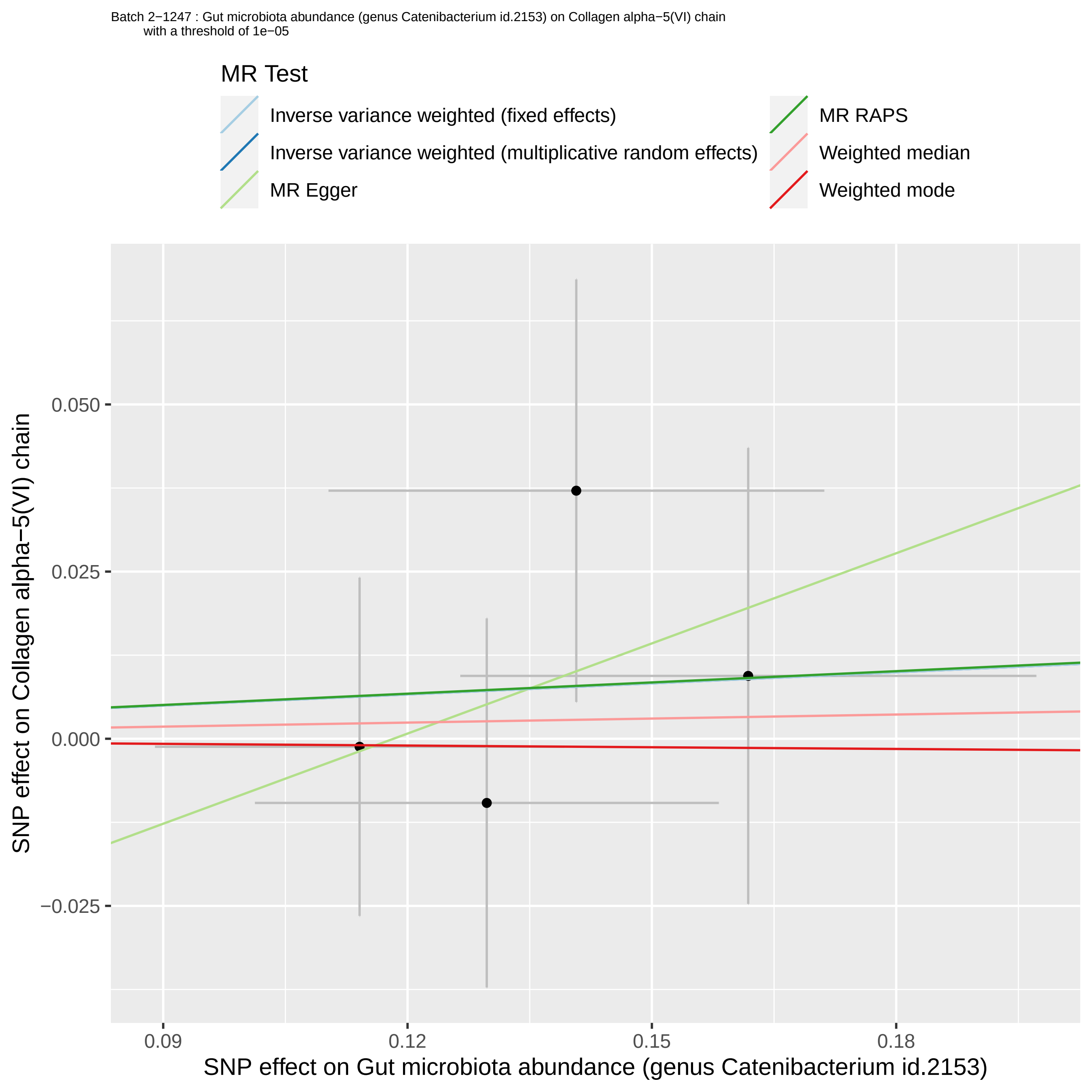
Batch 2−1247 : Gut microbiota abundance (genus Catenibacterium id.2153) on Collagen alpha−5(VI) chain
with a threshold of 1e−05
MR Test
Inverse variance weighted (fixed effects)
Inverse variance weighted (multiplicative random effects)
MR Egger
MR RAPS
Weighted median
Weighted mode
0.050
0.025
0.000
−0.025
0.09
0.12
0.15
0.18
SNP effect on Collagen alpha−5(VI) chain
SNP effect on Gut microbiota abundance (genus Catenibacterium id.2153)

Batch 2−1248 : Gut microbiota abundance (genus Parasutterella id.2892) on Collagen alpha−5(VI) chain
with a threshold of 1e−05

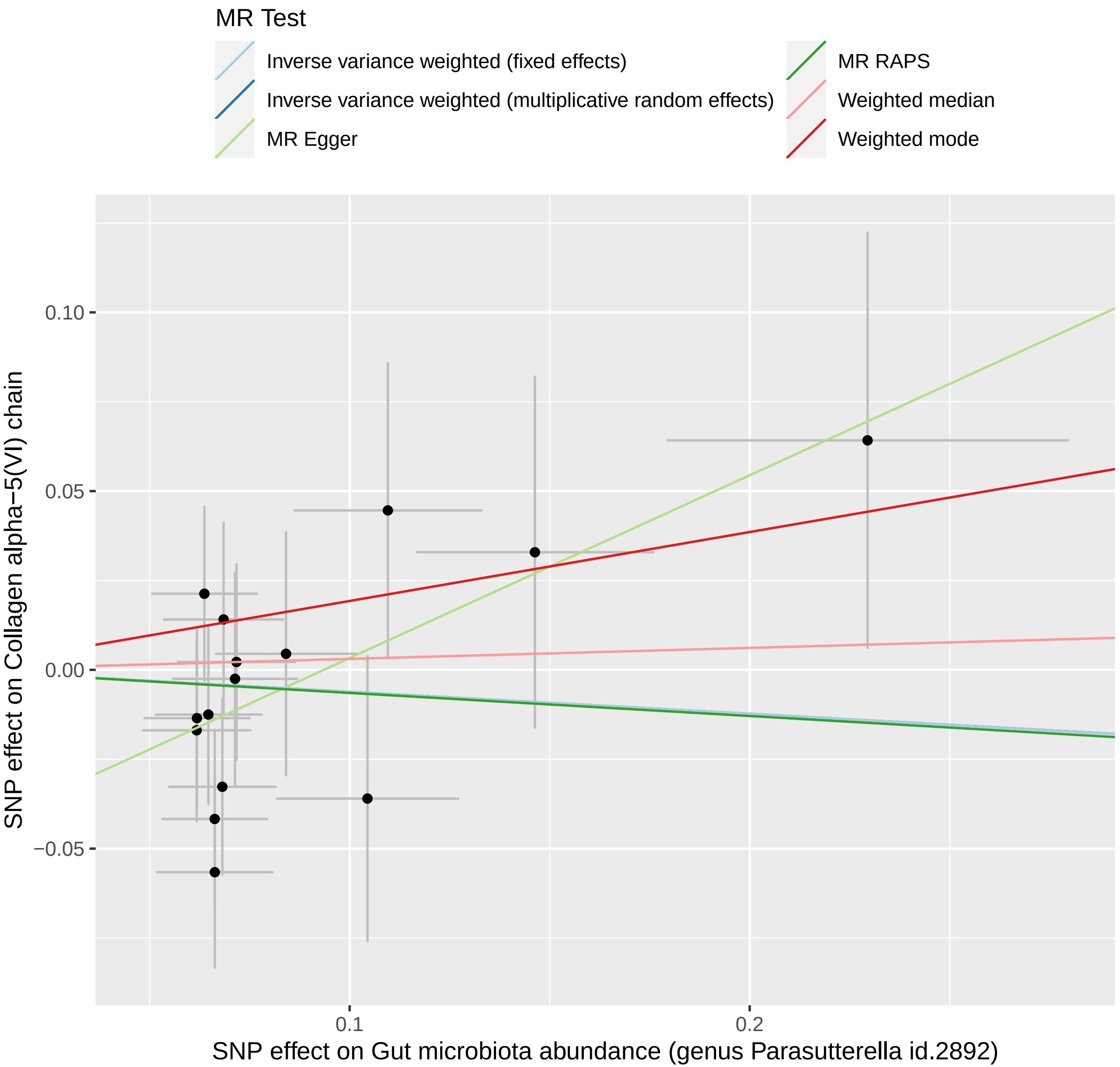

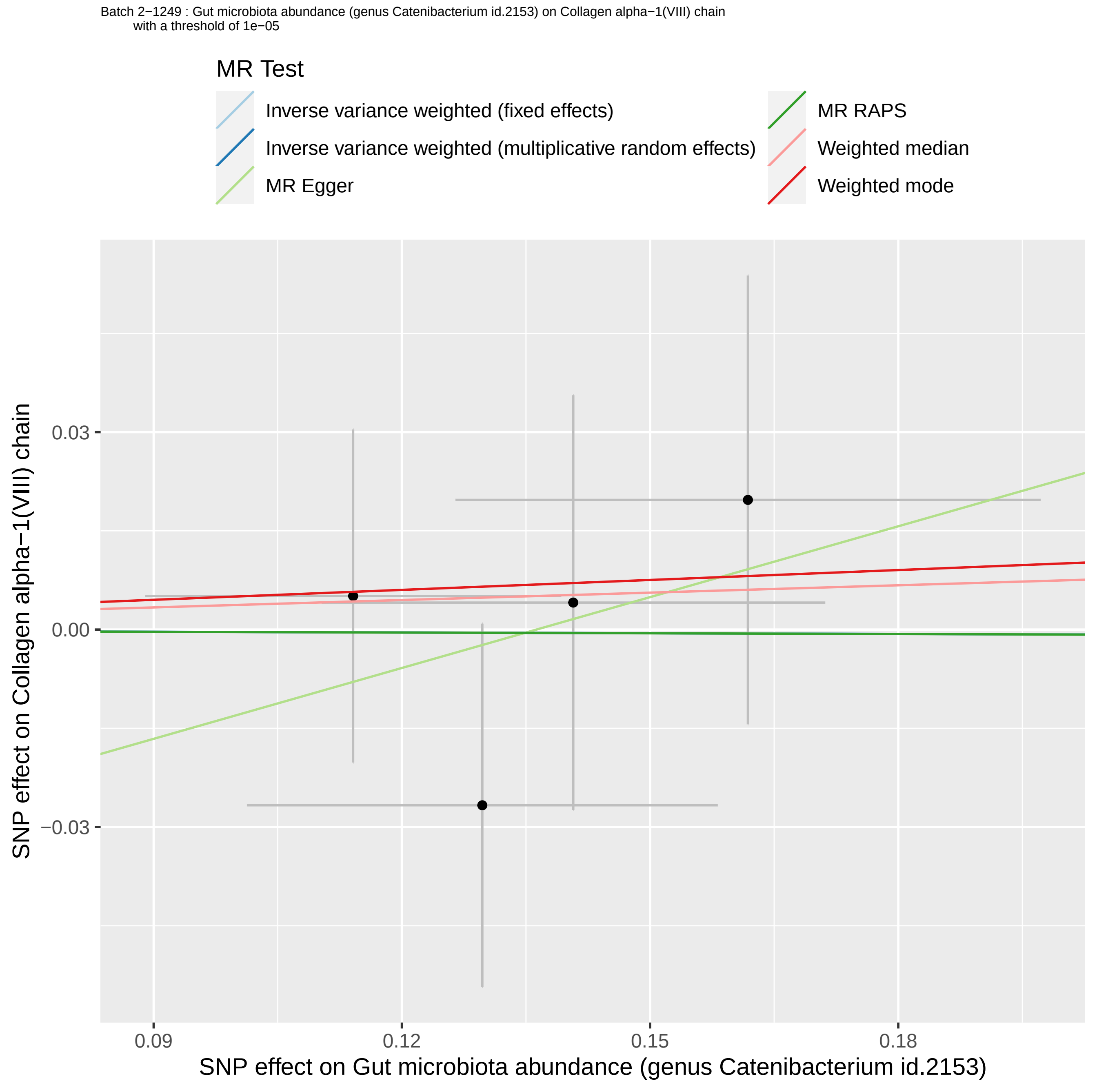
Batch 2−1249 : Gut microbiota abundance (genus Catenibacterium id.2153) on Collagen alpha−1(VIII) chain
with a threshold of 1e−05
MR Test
Inverse variance weighted (fixed effects)
Inverse variance weighted (multiplicative random effects)
MR Egger
MR RAPS
Weighted median
Weighted mode
0.03
0.00
−0.03
0.09
0.12
0.15
0.18
SNP effect on Collagen alpha−1(VIII) chain
SNP effect on Gut microbiota abundance (genus Catenibacterium id.2153)

Batch 2−1250 : Gut microbiota abundance (genus Parasutterella id.2892) on Collagen alpha−1(VIII) chain
with a threshold of 1e−05

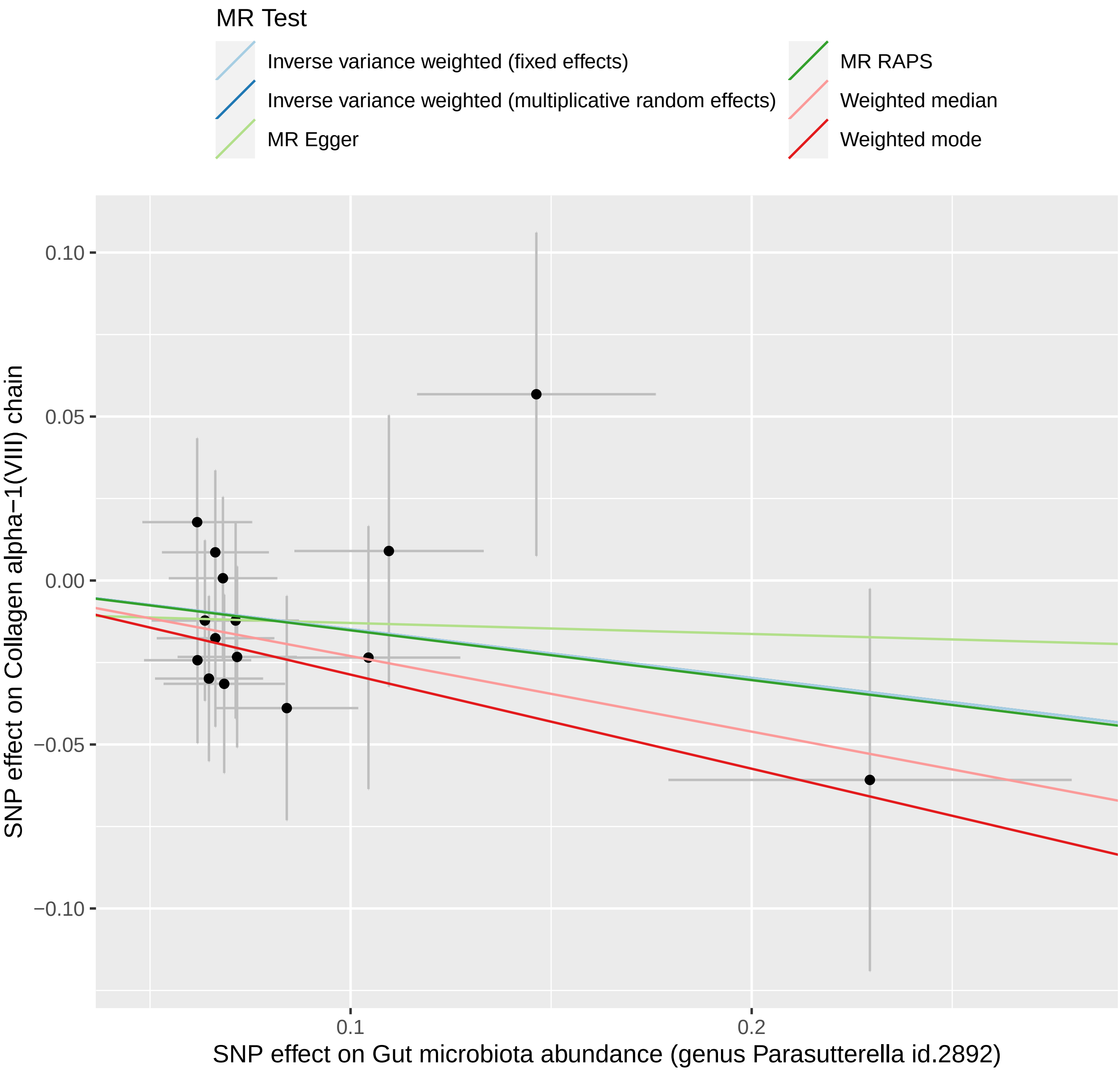

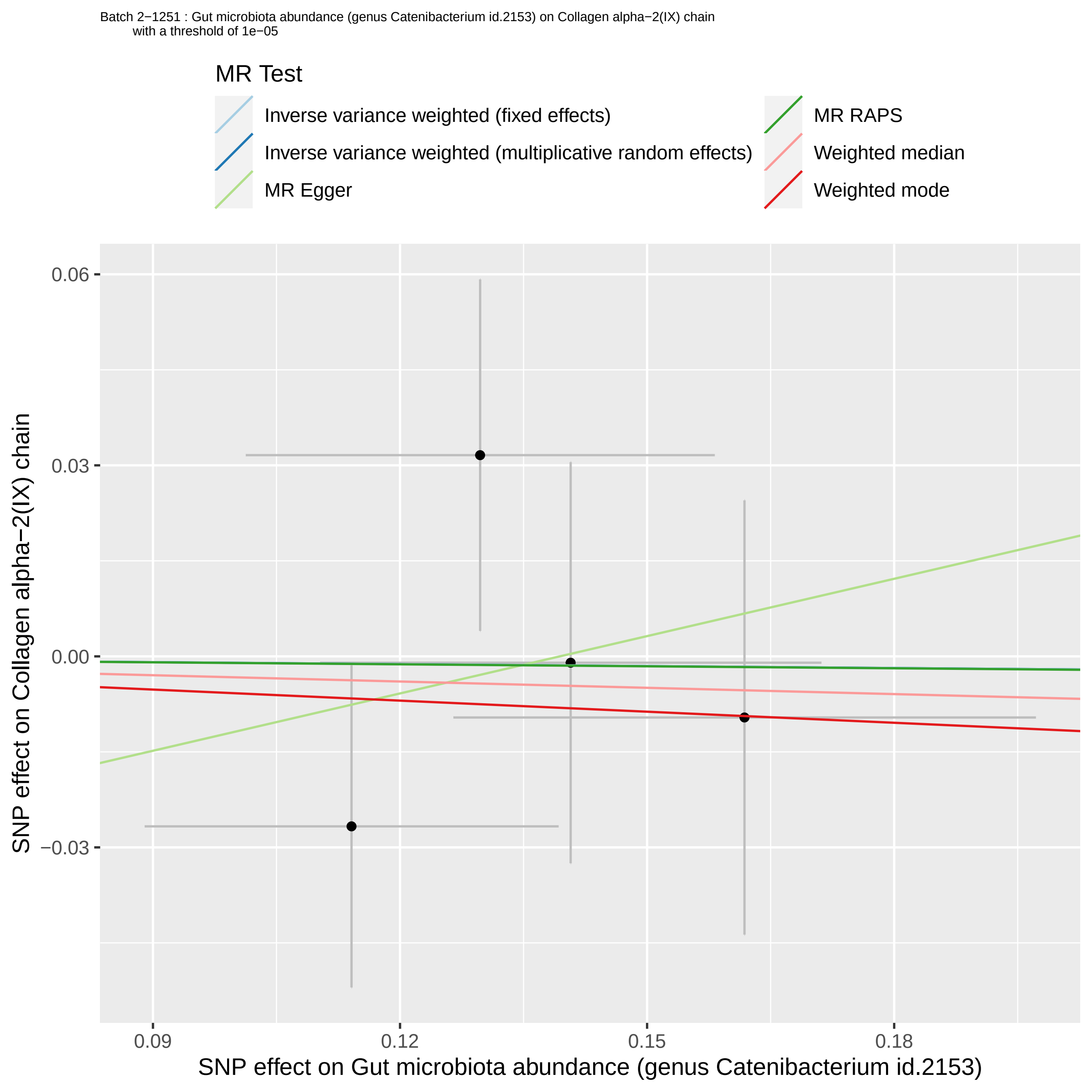
Batch 2−1251 : Gut microbiota abundance (genus Catenibacterium id.2153) on Collagen alpha−2(IX) chain
with a threshold of 1e−05
MR Test
Inverse variance weighted (fixed effects)
Inverse variance weighted (multiplicative random effects)
MR Egger
MR RAPS
Weighted median
Weighted mode
0.06
0.03
0.00
−0.03
0.09
0.12
0.15
0.18
SNP effect on Collagen alpha−2(IX) chain
SNP effect on Gut microbiota abundance (genus Catenibacterium id.2153)

Batch 2−1252 : Gut microbiota abundance (genus Parasutterella id.2892) on Collagen alpha−2(IX) chain with a threshold of 1e−05

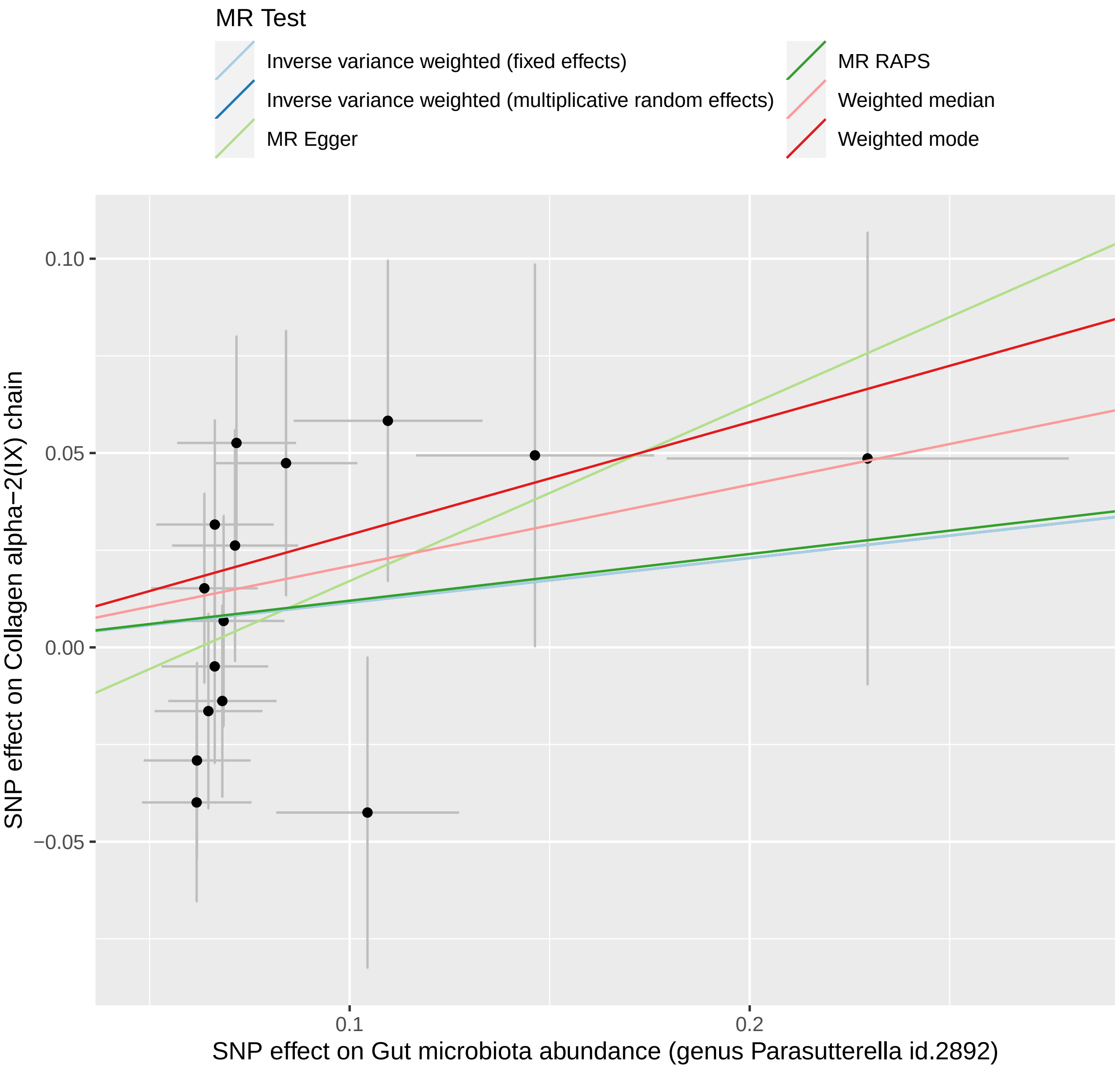

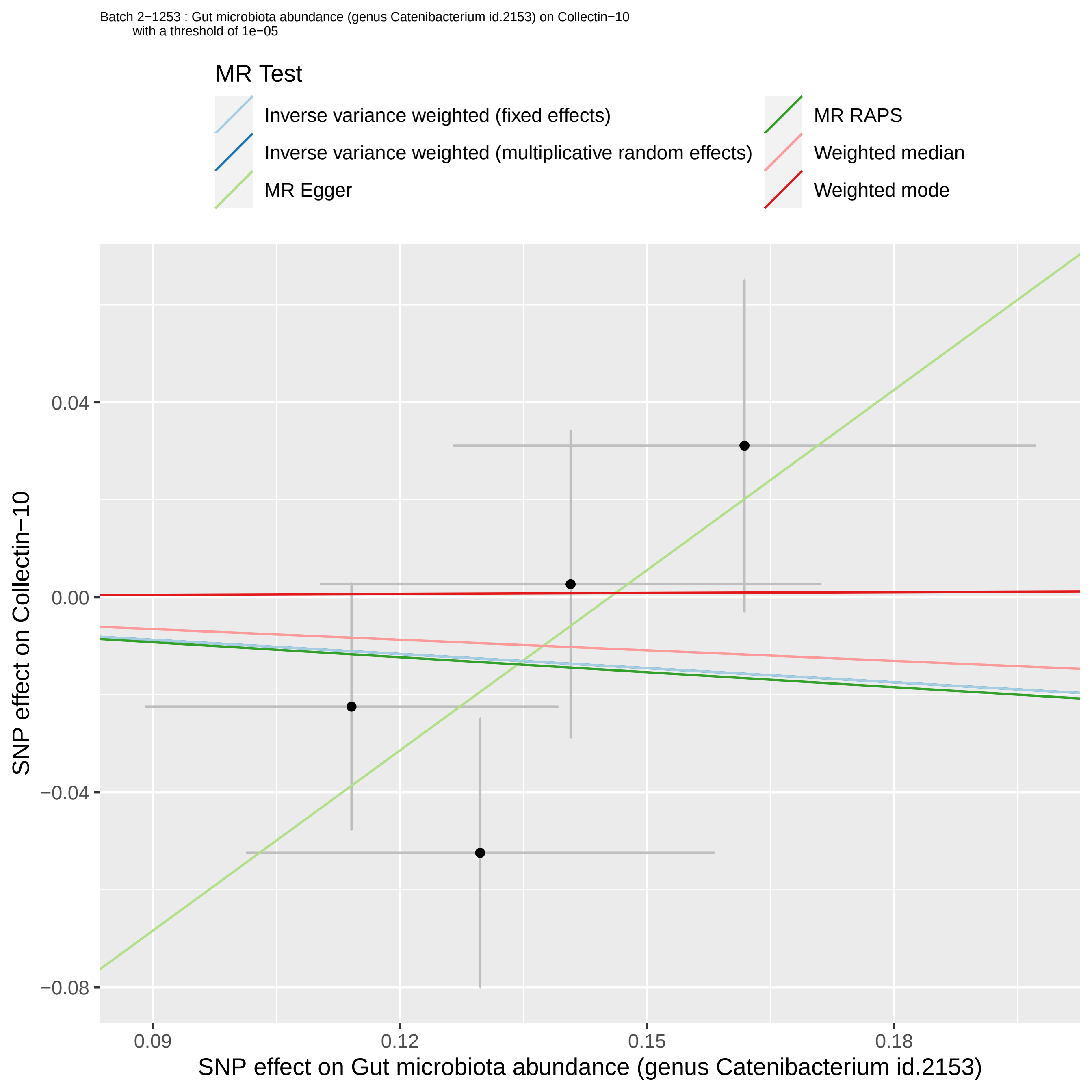
Batch 2−1253 : Gut microbiota abundance (genus Catenibacterium id.2153) on Collectin−10
with a threshold of 1e−05
MR Test
Inverse variance weighted (fixed effects)
Inverse variance weighted (multiplicative random effects)
MR Egger
MR RAPS
Weighted median
Weighted mode
0.04
0.00
−0.04
−0.08
0.09
0.12
0.15
0.18
SNP effect on Collectin−10
SNP effect on Gut microbiota abundance (genus Catenibacterium id.2153)

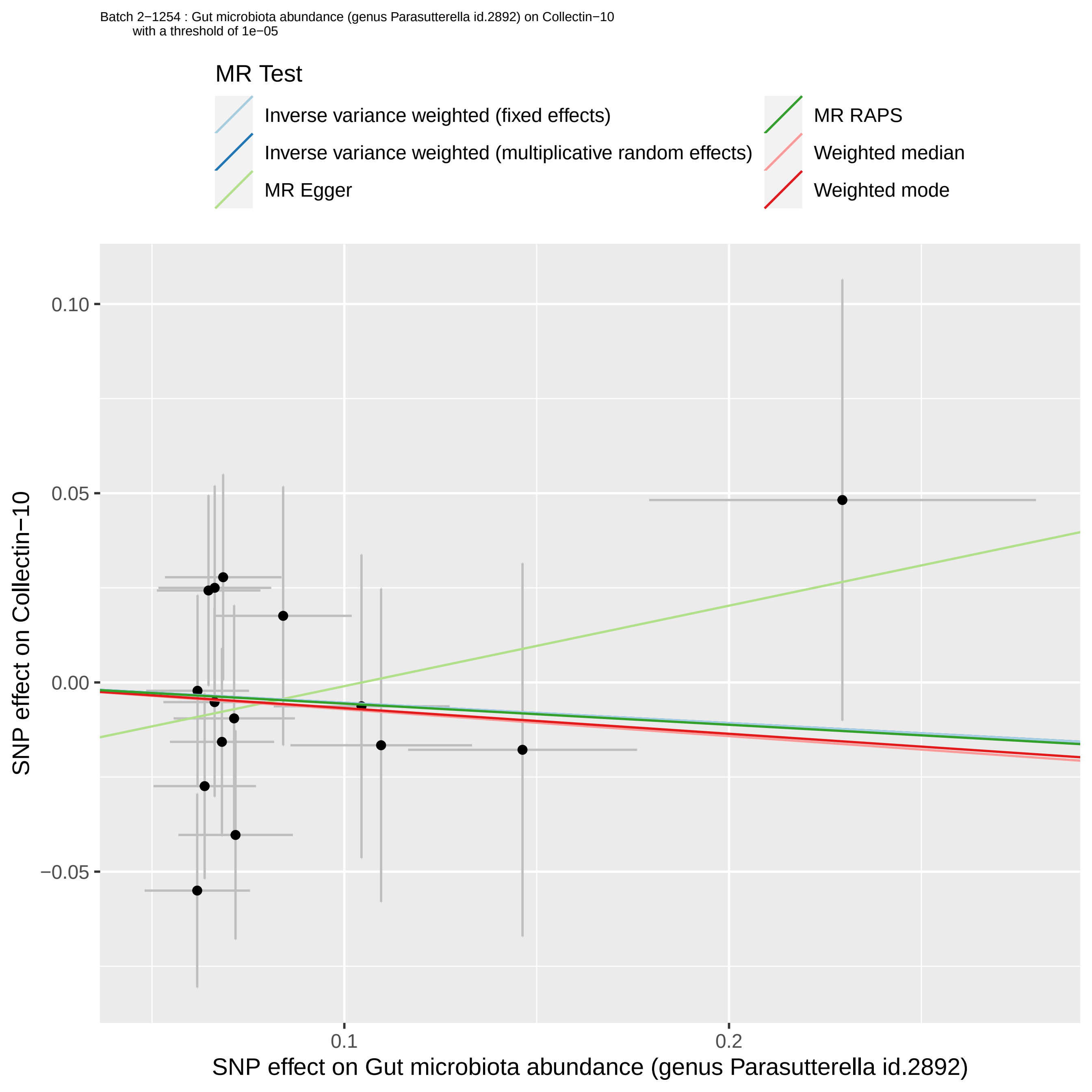

Batch 2−1254 : Gut microbiota abundance (genus Parasutterella id.2892) on Collectin−10
with a threshold of 1e−05
MR Test
Inverse variance weighted (fixed effects)
Inverse variance weighted (multiplicative random effects)
MR Egger
MR RAPS
Weighted median
Weighted mode
0.10
0.05
0.00
−0.05
0.1
0.2
SNP effect on Collectin−10
SNP effect on Gut microbiota abundance (genus Parasutterella id.2892)

Batch 2−1255 : Gut microbiota abundance (genus Catenibacterium id.2153) on Collectin−11
with a threshold of 1e−05

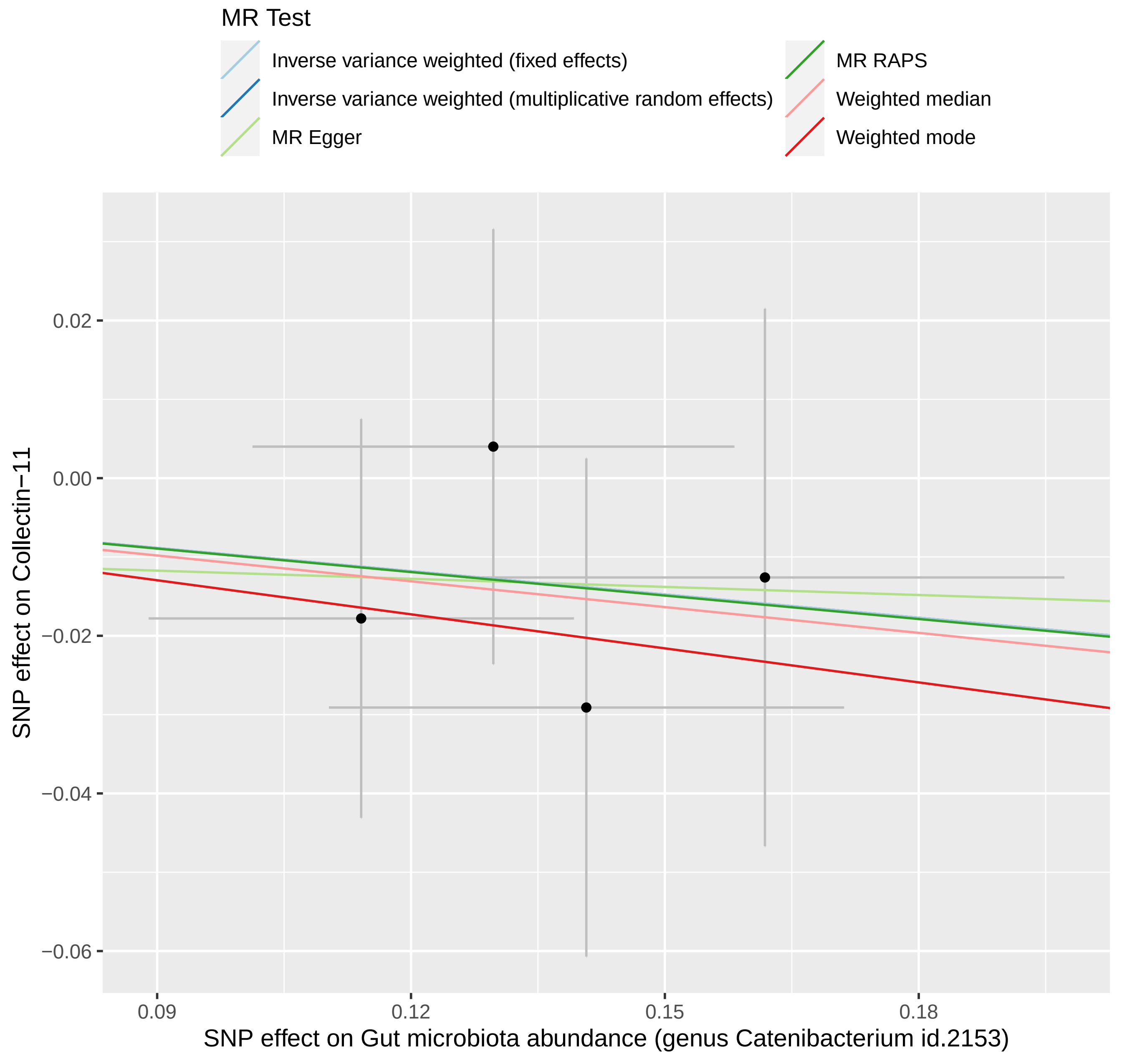

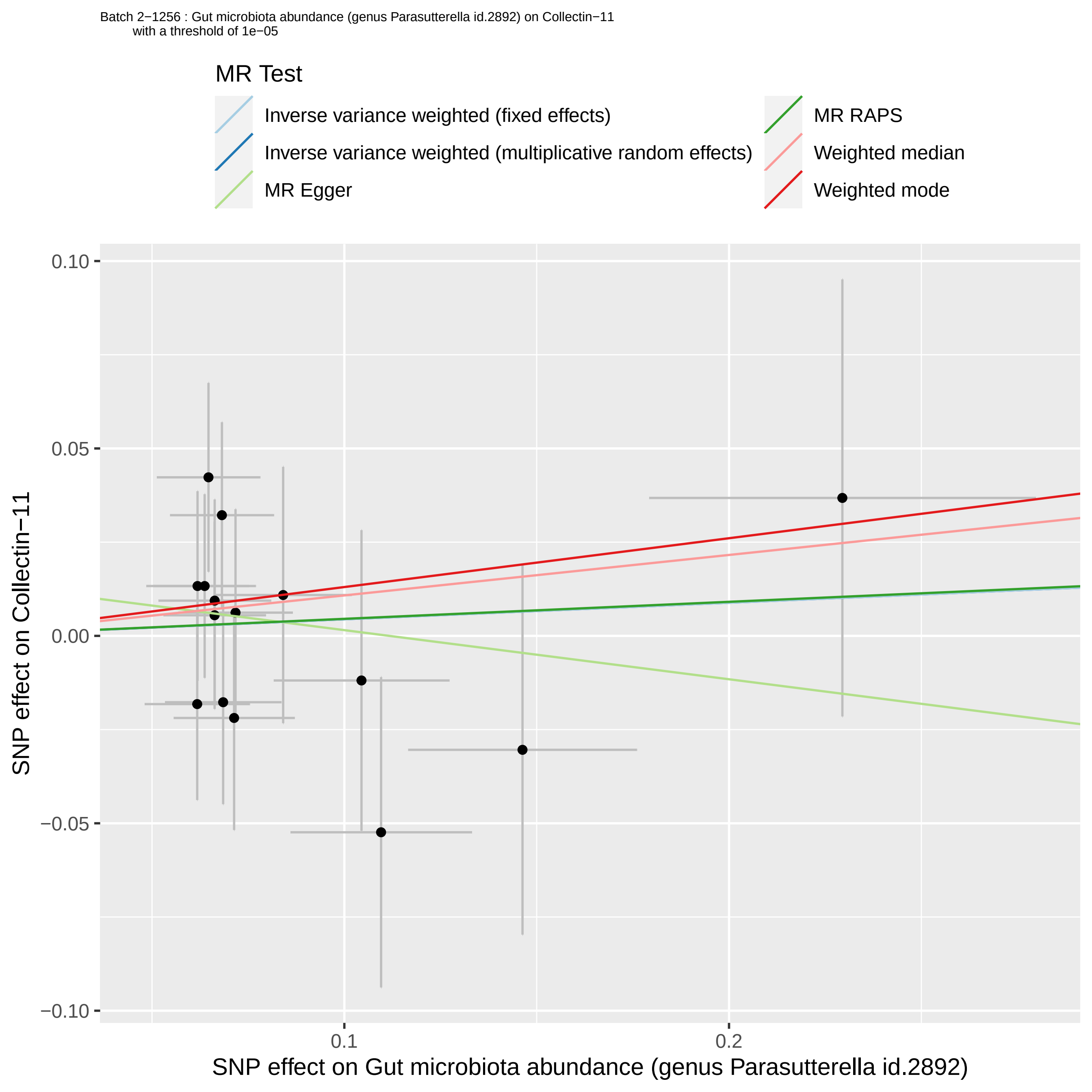
Batch 2−1256 : Gut microbiota abundance (genus Parasutterella id.2892) on Collectin−11
with a threshold of 1e−05
MR Test
Inverse variance weighted (fixed effects)
Inverse variance weighted (multiplicative random effects)
MR Egger
MR RAPS
Weighted median
Weighted mode
0.10
0.05
0.00
−0.05
−0.10
SNP effect on Collectin−11
0.1
0.2
SNP effect on Gut microbiota abundance (genus Parasutterella id.2892)

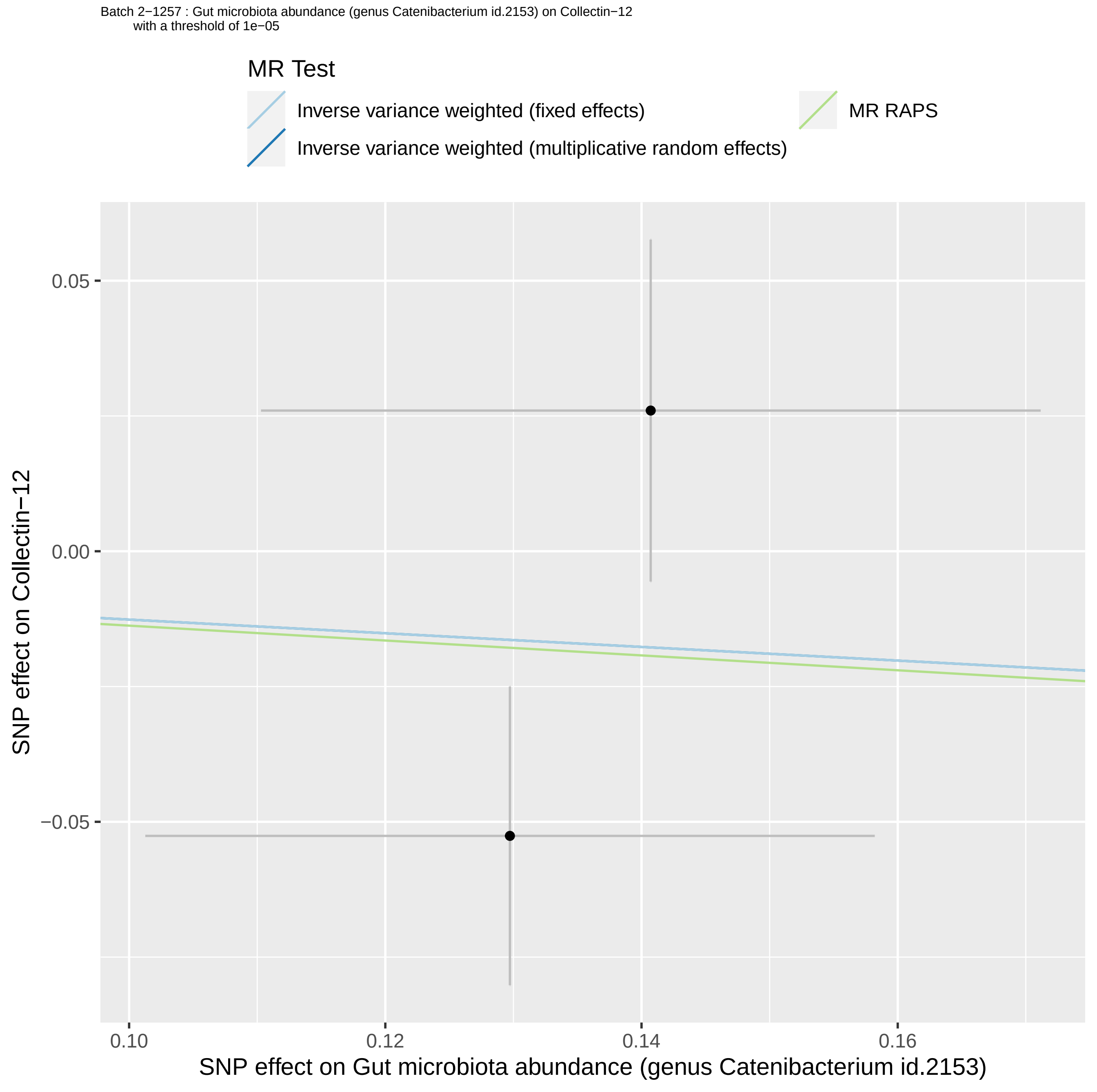
Batch 2−1257 : Gut microbiota abundance (genus Catenibacterium id.2153) on Collectin−12
with a threshold of 1e−05
MR Test
Inverse variance weighted (fixed effects)
Inverse variance weighted (multiplicative random effects)
MR RAPS
0.05
0.00
−0.05
0.10
0.12
0.14
0.16
SNP effect on Collectin−12
SNP effect on Gut microbiota abundance (genus Catenibacterium id.2153)

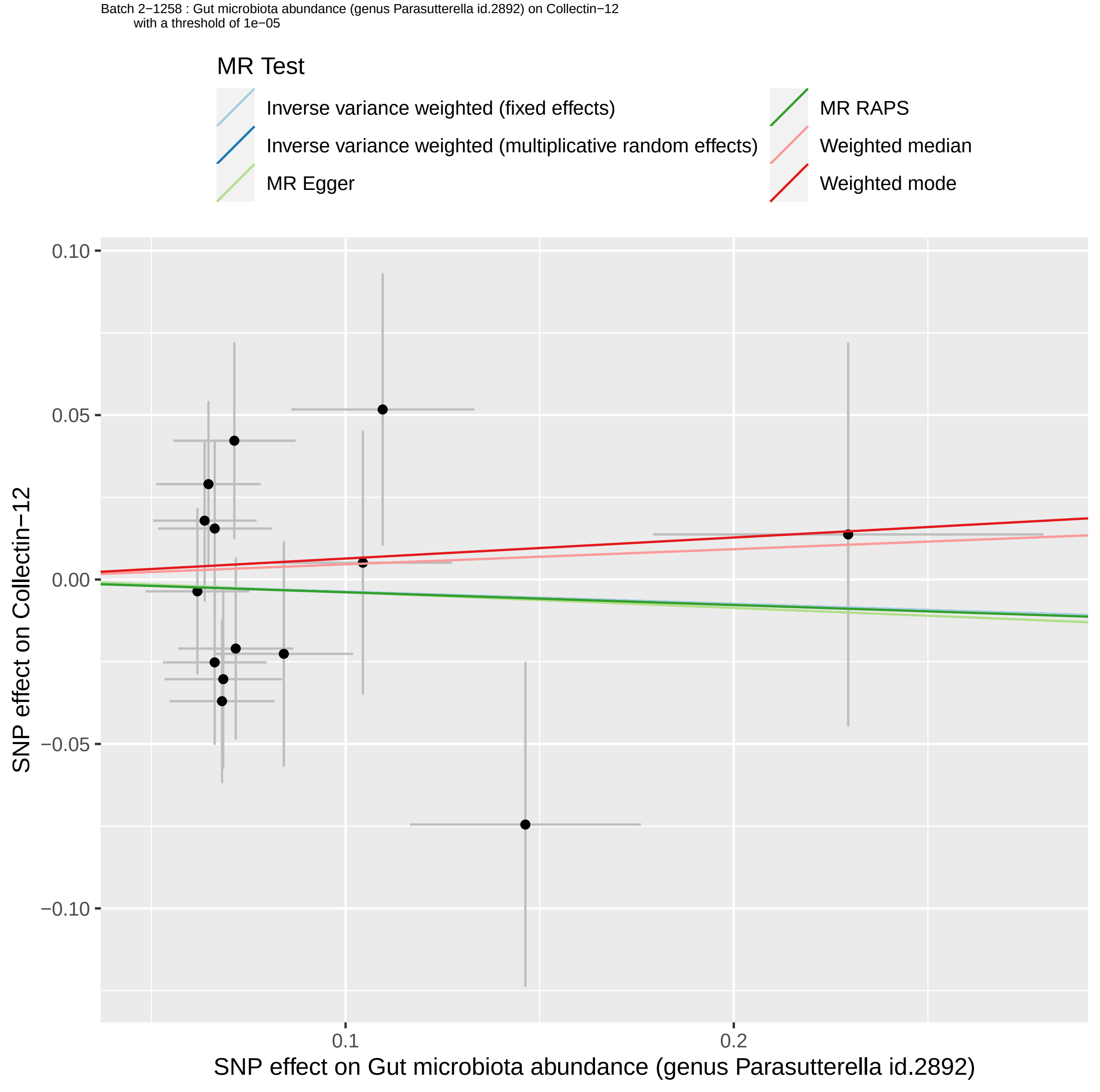

Batch 2−1258 : Gut microbiota abundance (genus Parasutterella id.2892) on Collectin−12
with a threshold of 1e−05
MR Test
Inverse variance weighted (fixed effects)
Inverse variance weighted (multiplicative random effects)
MR Egger
MR RAPS
Weighted median
Weighted mode
0.10
0.05
0.00
−0.05
−0.10
0.1
0.2
SNP effect on Collectin−12
SNP effect on Gut microbiota abundance (genus Parasutterella id.2892)

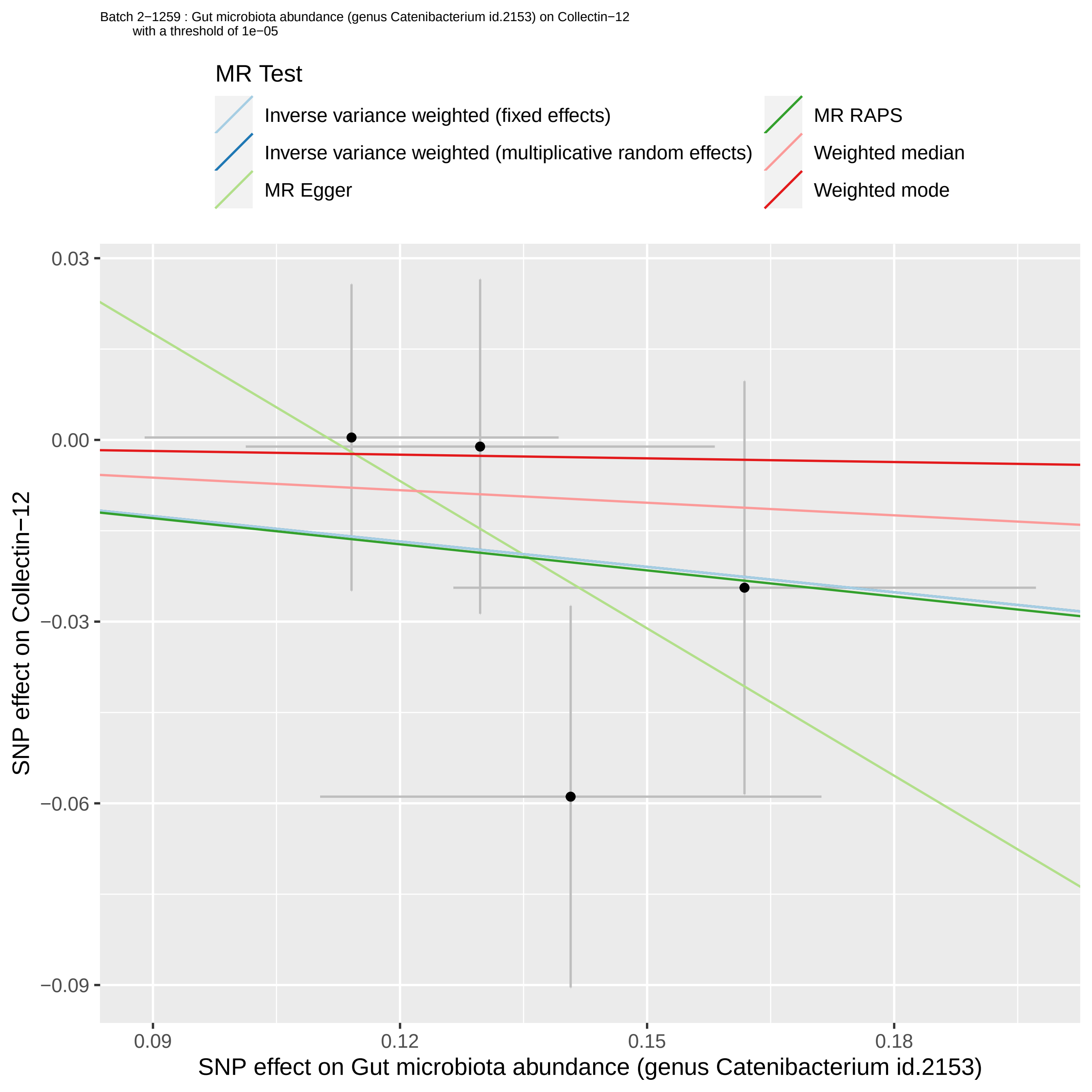

Batch 2−1259 : Gut microbiota abundance (genus Catenibacterium id.2153) on Collectin−12
with a threshold of 1e−05
MR Test
Inverse variance weighted (fixed effects)
Inverse variance weighted (multiplicative random effects)
MR Egger
MR RAPS
Weighted median
Weighted mode
0.03
0.00
−0.03
−0.06
−0.09
0.09
0.12
0.15
0.18
SNP effect on Collectin−12
SNP effect on Gut microbiota abundance (genus Catenibacterium id.2153)

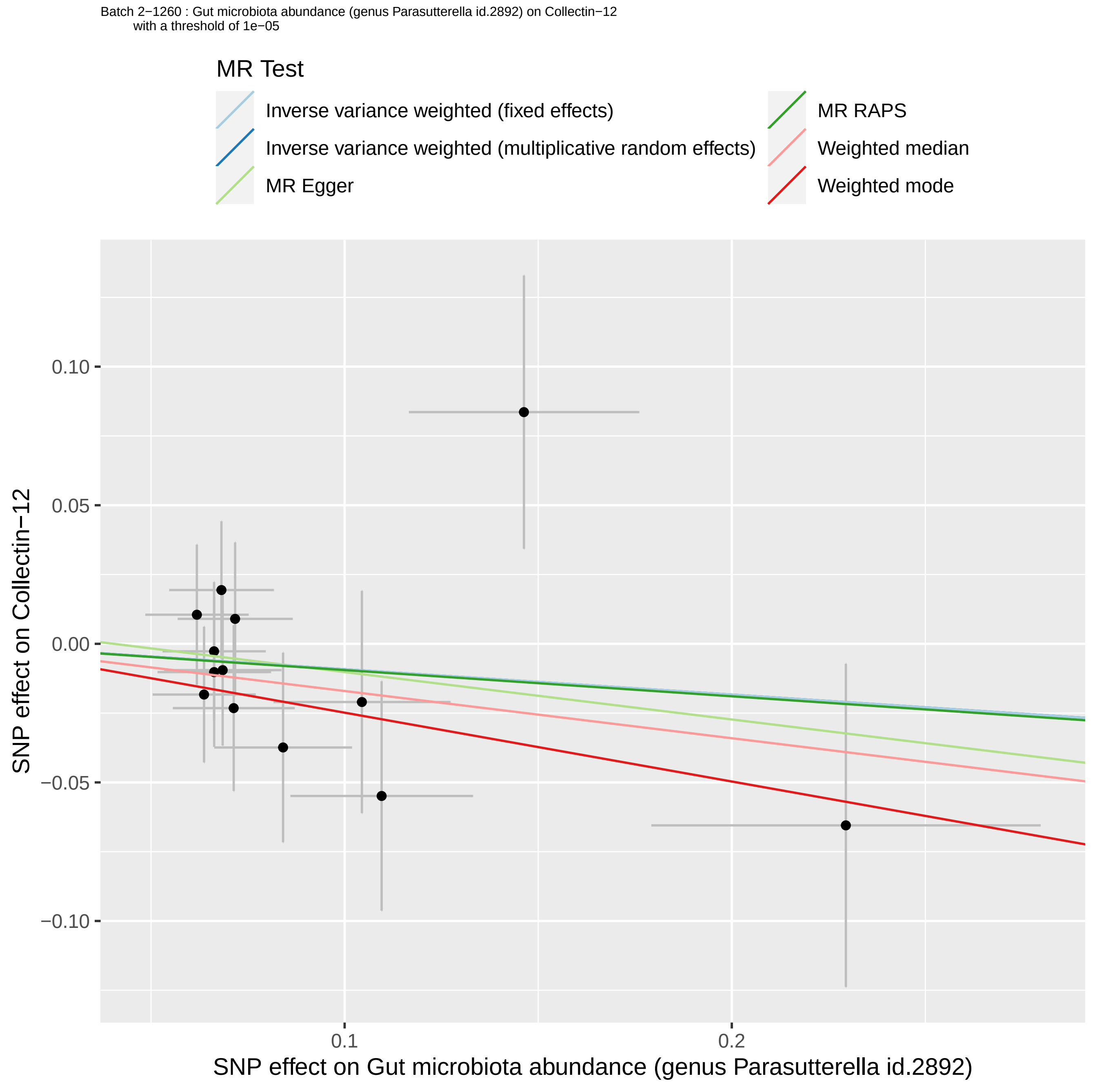

Batch 2−1260 : Gut microbiota abundance (genus Parasutterella id.2892) on Collectin−12
with a threshold of 1e−05
MR Test
Inverse variance weighted (fixed effects)
Inverse variance weighted (multiplicative random effects)
MR Egger
MR RAPS
Weighted median
Weighted mode
0.10
0.05
0.00
−0.05
−0.10
SNP effect on Collectin−12
0.1
0.2
SNP effect on Gut microbiota abundance (genus Parasutterella id.2892)

Batch 2−1261 : Gut microbiota abundance (genus Catenibacterium id.2153) on Procollagen galactosyltransferase 1
with a threshold of 1e−05

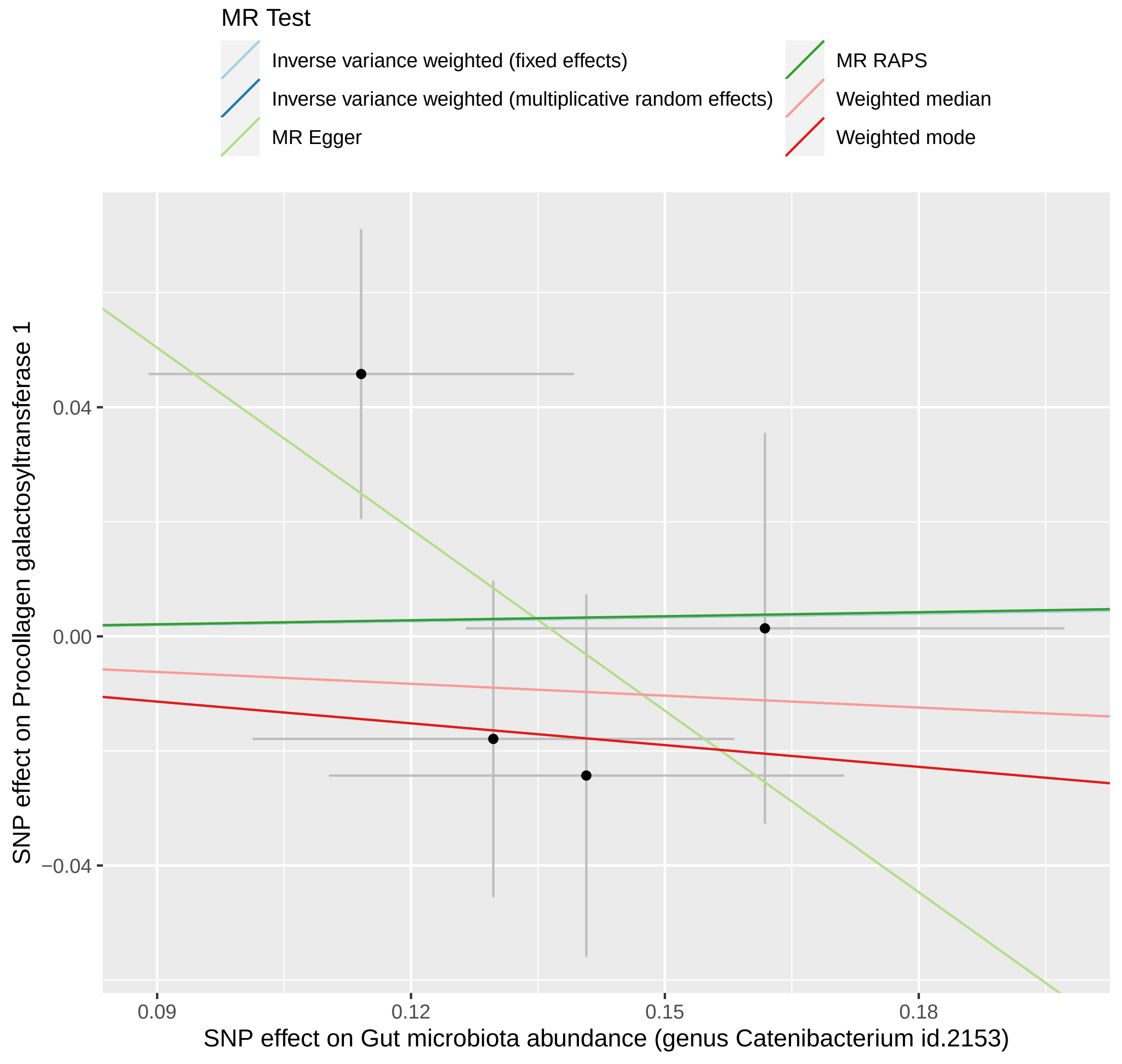

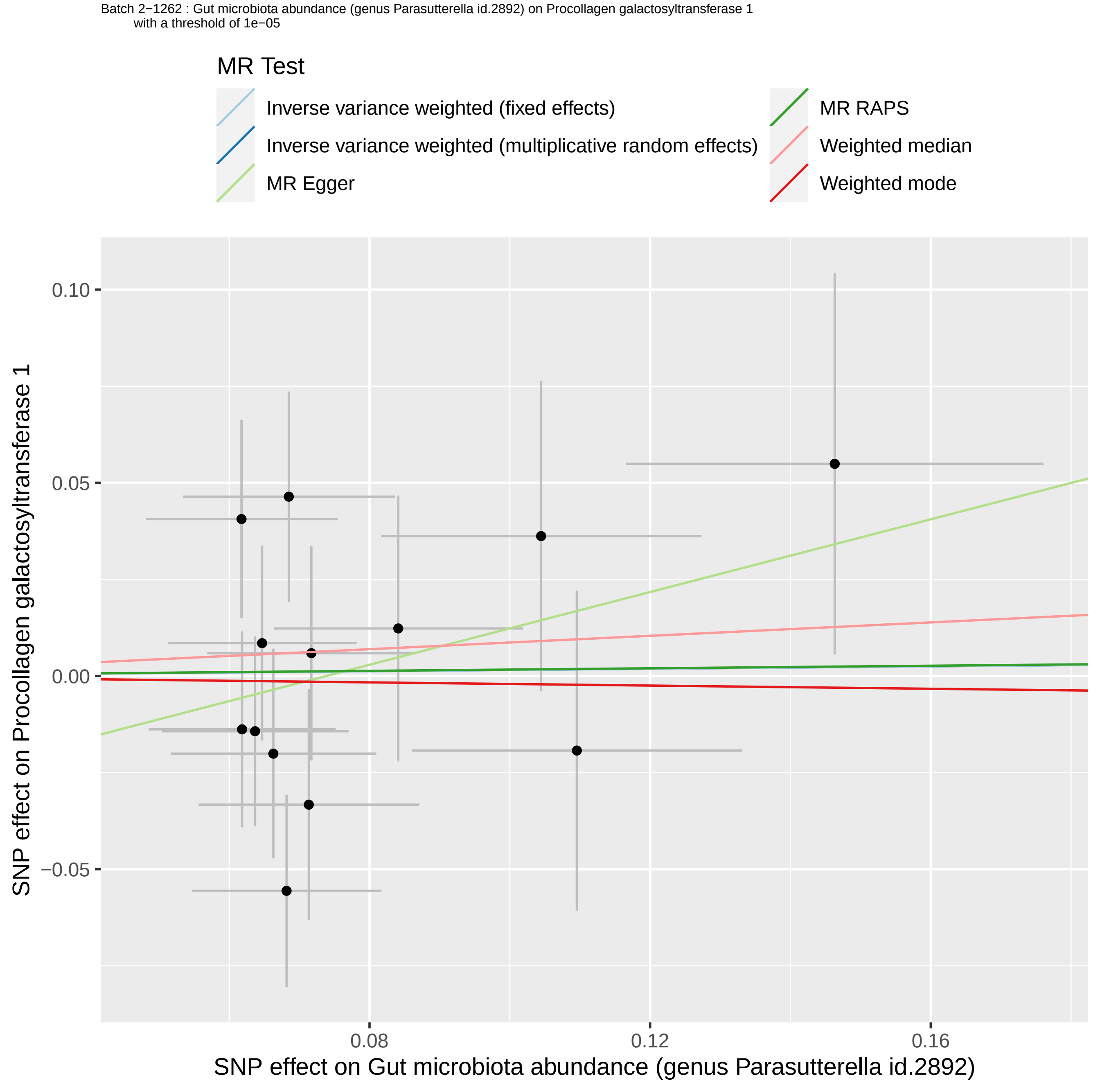
Batch 2−1262 : Gut microbiota abundance (genus Parasutterella id.2892) on Procollagen galactosyltransferase 1
with a threshold of 1e−05
MR Test
Inverse variance weighted (fixed effects)
Inverse variance weighted (multiplicative random effects)
MR Egger
MR RAPS
Weighted median
Weighted mode
0.10
0.05
0.00
−0.05
0.08
0.12
0.16
SNP effect on Procollagen galactosyltransferase 1
SNP effect on Gut microbiota abundance (genus Parasutterella id.2892)

Batch 2−1263 : Gut microbiota abundance (genus Catenibacterium id.2153) on Procollagen galactosyltransferase 2
with a threshold of 1e−05

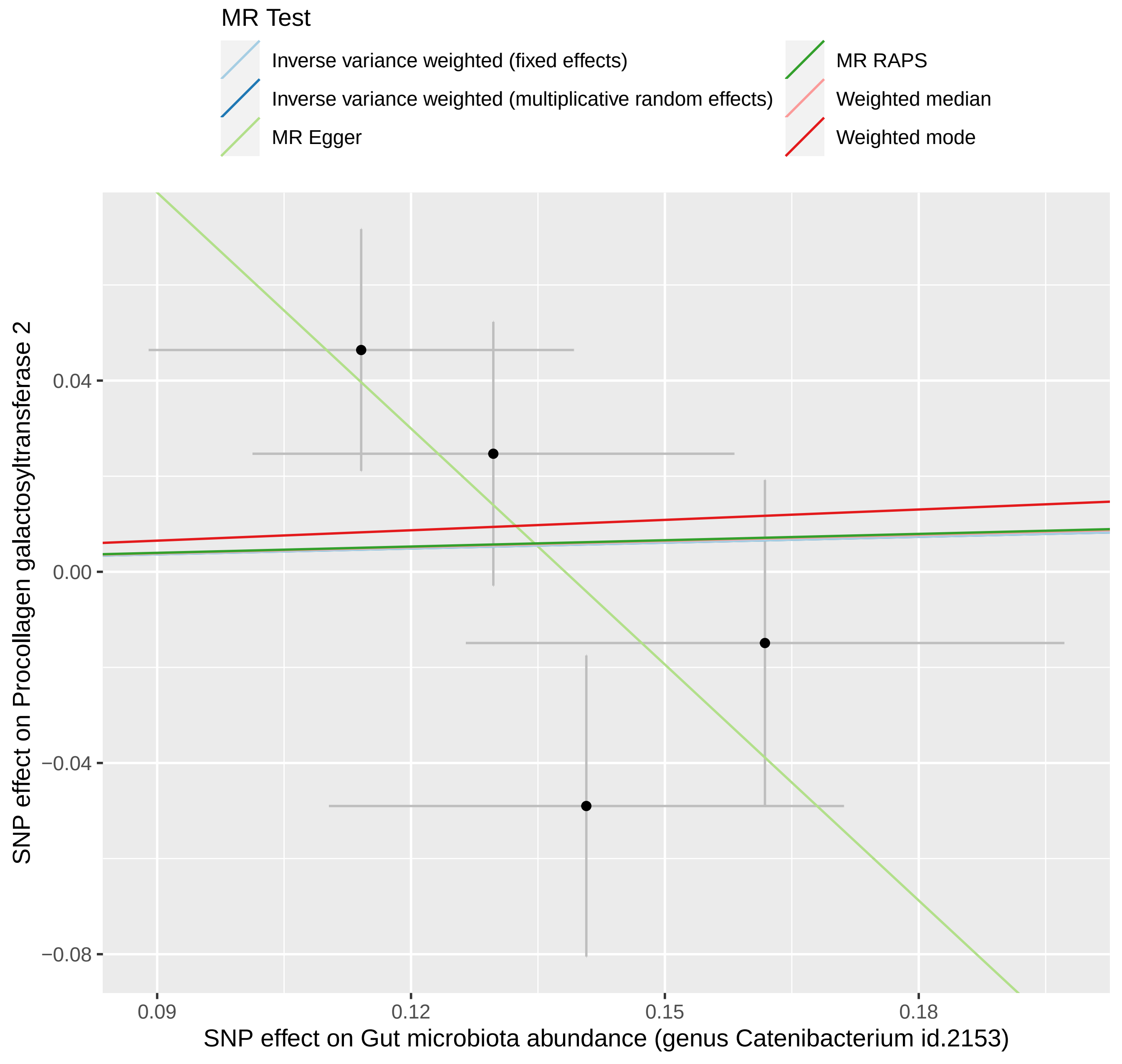

Batch 2−1264 : Gut microbiota abundance (genus Parasutterella id.2892) on Procollagen galactosyltransferase 2 with a threshold of 1e−05

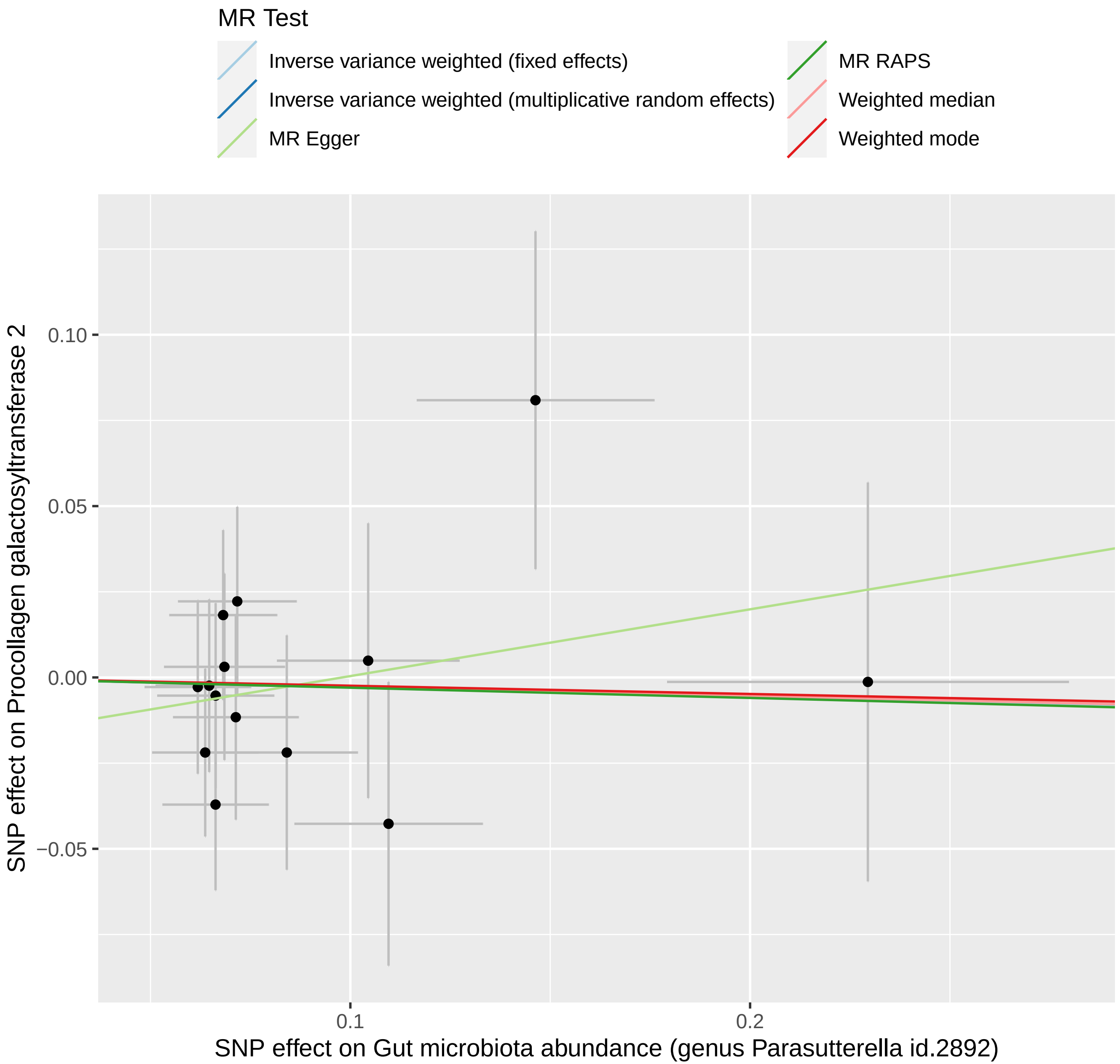

Batch 2−1265 : Gut microbiota abundance (genus Catenibacterium id.2153) on COMM domain−containing protein 1
with a threshold of 1e−05

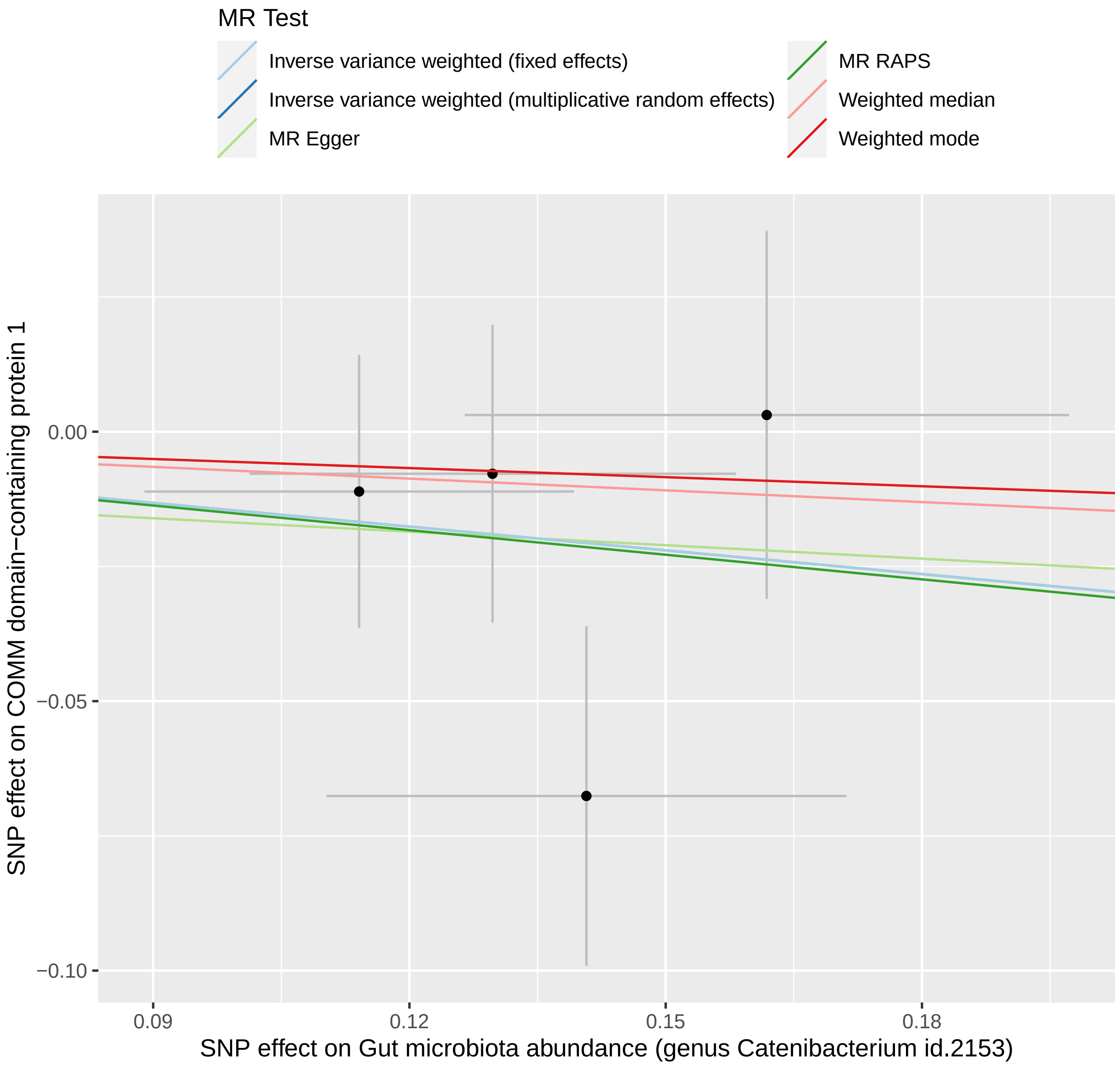

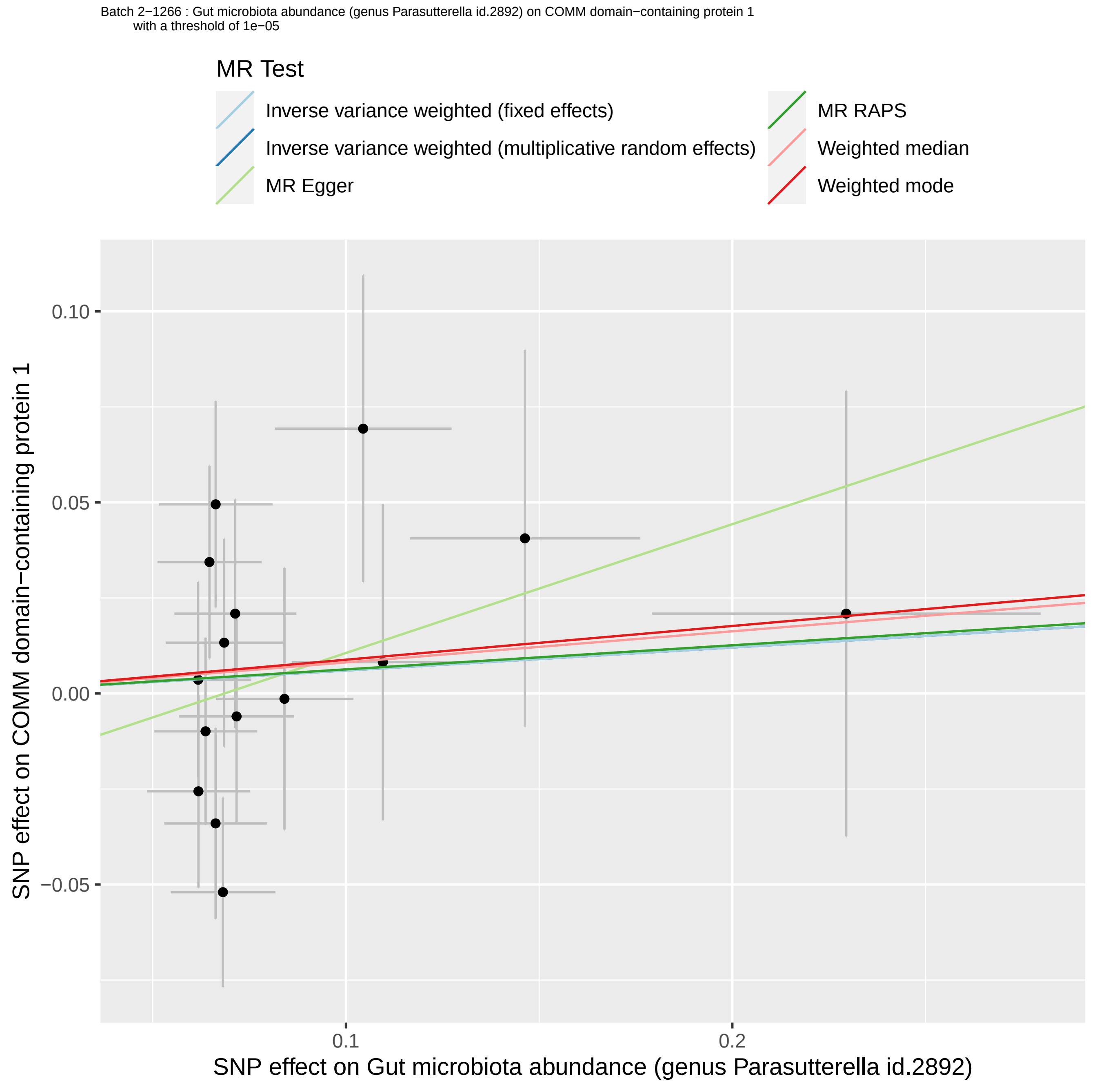

Batch 2−1266 : Gut microbiota abundance (genus Parasutterella id.2892) on COMM domain−containing protein 1
with a threshold of 1e−05
MR Test
Inverse variance weighted (fixed effects)
Inverse variance weighted (multiplicative random effects)
MR Egger
MR RAPS
Weighted median
Weighted mode
SNP effect on COMM domain−containing protein 1
0.10
0.05
0.00
−0.05
0.1
0.2
SNP effect on Gut microbiota abundance (genus Parasutterella id.2892)

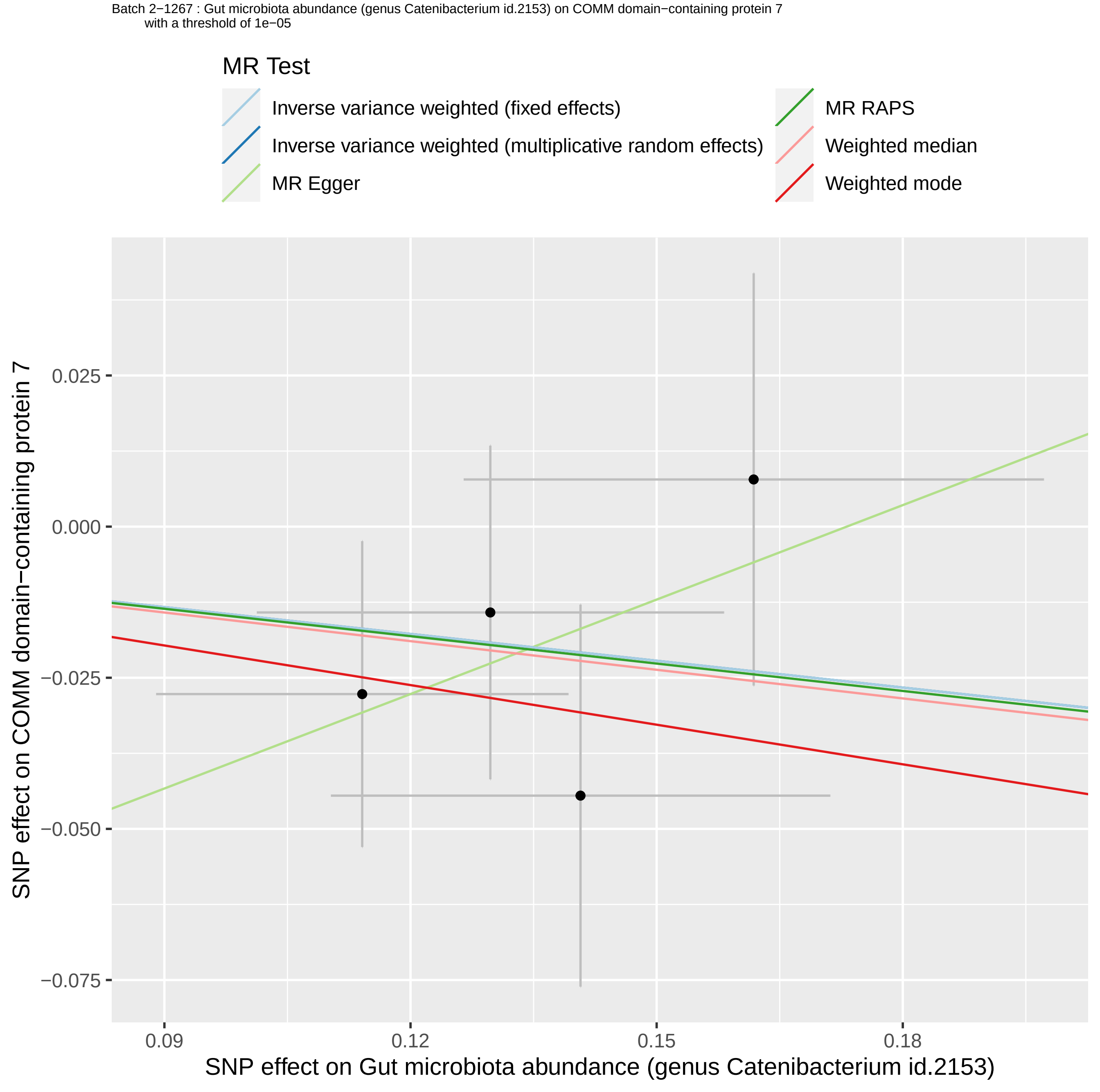
Batch 2−1267 : Gut microbiota abundance (genus Catenibacterium id.2153) on COMM domain−containing protein 7
with a threshold of 1e−05
MR Test
Inverse variance weighted (fixed effects)
Inverse variance weighted (multiplicative random effects)
MR Egger
MR RAPS
Weighted median
Weighted mode
SNP effect on COMM domain−containing protein 7
0.025
0.000
−0.025
−0.050
−0.075
0.09
0.12
0.15
0.18
SNP effect on Gut microbiota abundance (genus Catenibacterium id.2153)

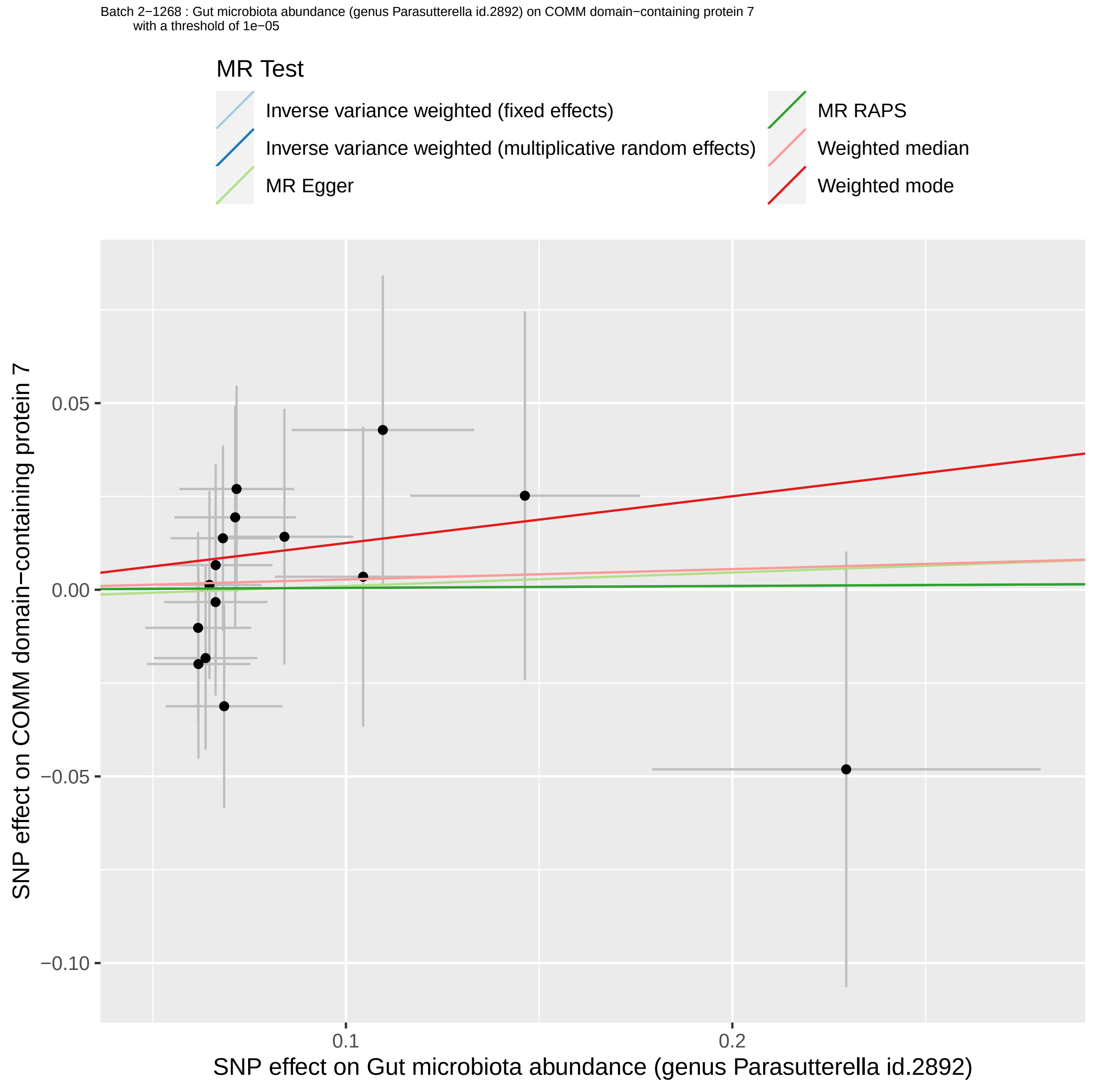
Batch 2−1268 : Gut microbiota abundance (genus Parasutterella id.2892) on COMM domain−containing protein 7
with a threshold of 1e−05
MR Test
Inverse variance weighted (fixed effects)
Inverse variance weighted (multiplicative random effects)
MR Egger
MR RAPS
Weighted median
Weighted mode
SNP effect on COMM domain−containing protein 7
0.05
0.00
−0.05
−0.10
0.1
0.2
SNP effect on Gut microbiota abundance (genus Parasutterella id.2892)

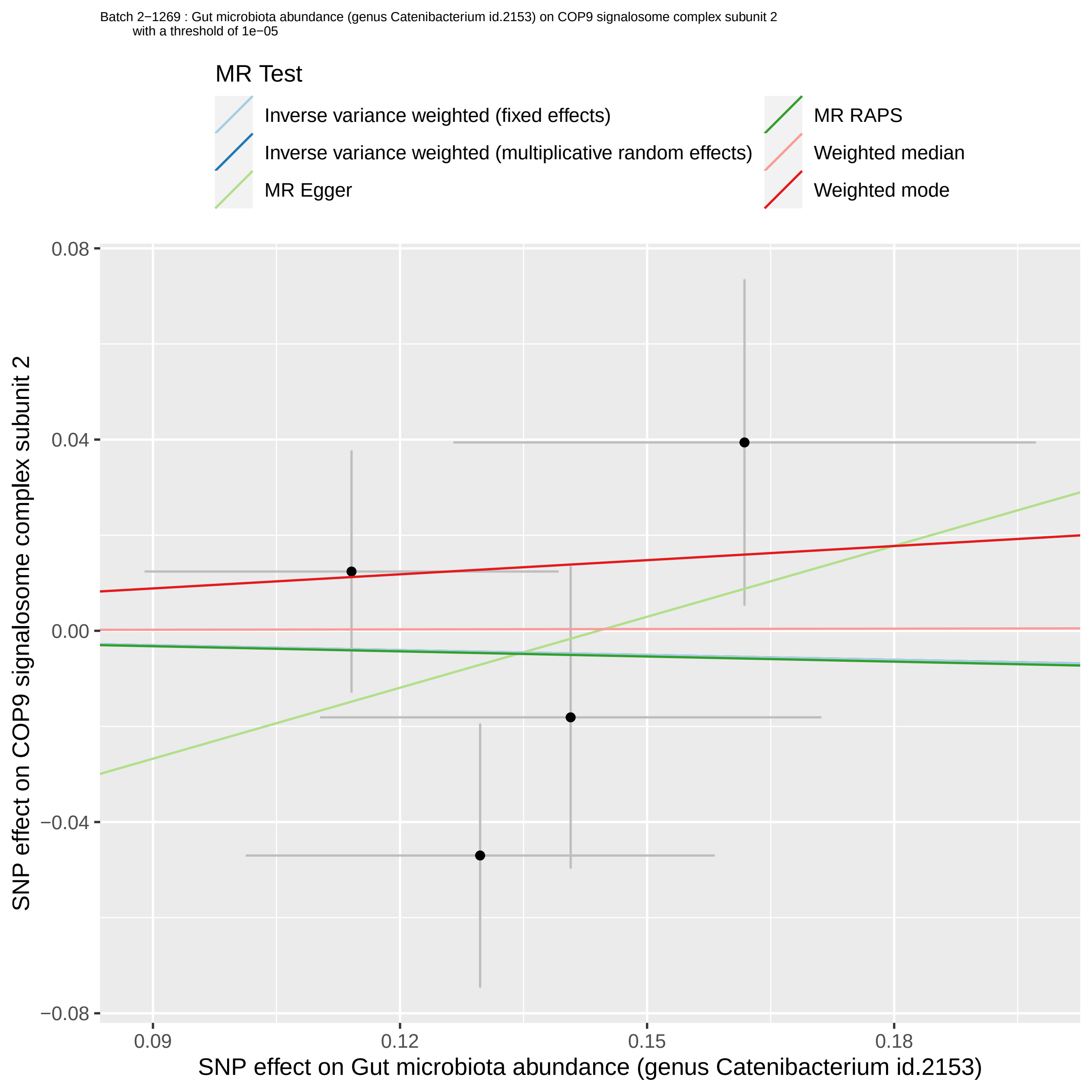
Batch 2−1269 : Gut microbiota abundance (genus Catenibacterium id.2153) on COP9 signalosome complex subunit 2
with a threshold of 1e−05
MR Test
Inverse variance weighted (fixed effects)
Inverse variance weighted (multiplicative random effects)
MR Egger
MR RAPS
Weighted median
Weighted mode
0.08
0.04
0.00
−0.04
−0.08
0.09
0.12
0.15
0.18
SNP effect on COP9 signalosome complex subunit 2
SNP effect on Gut microbiota abundance (genus Catenibacterium id.2153)

Batch 2−1270 : Gut microbiota abundance (genus Parasutterella id.2892) on COP9 signalosome complex subunit 2
with a threshold of 1e−05

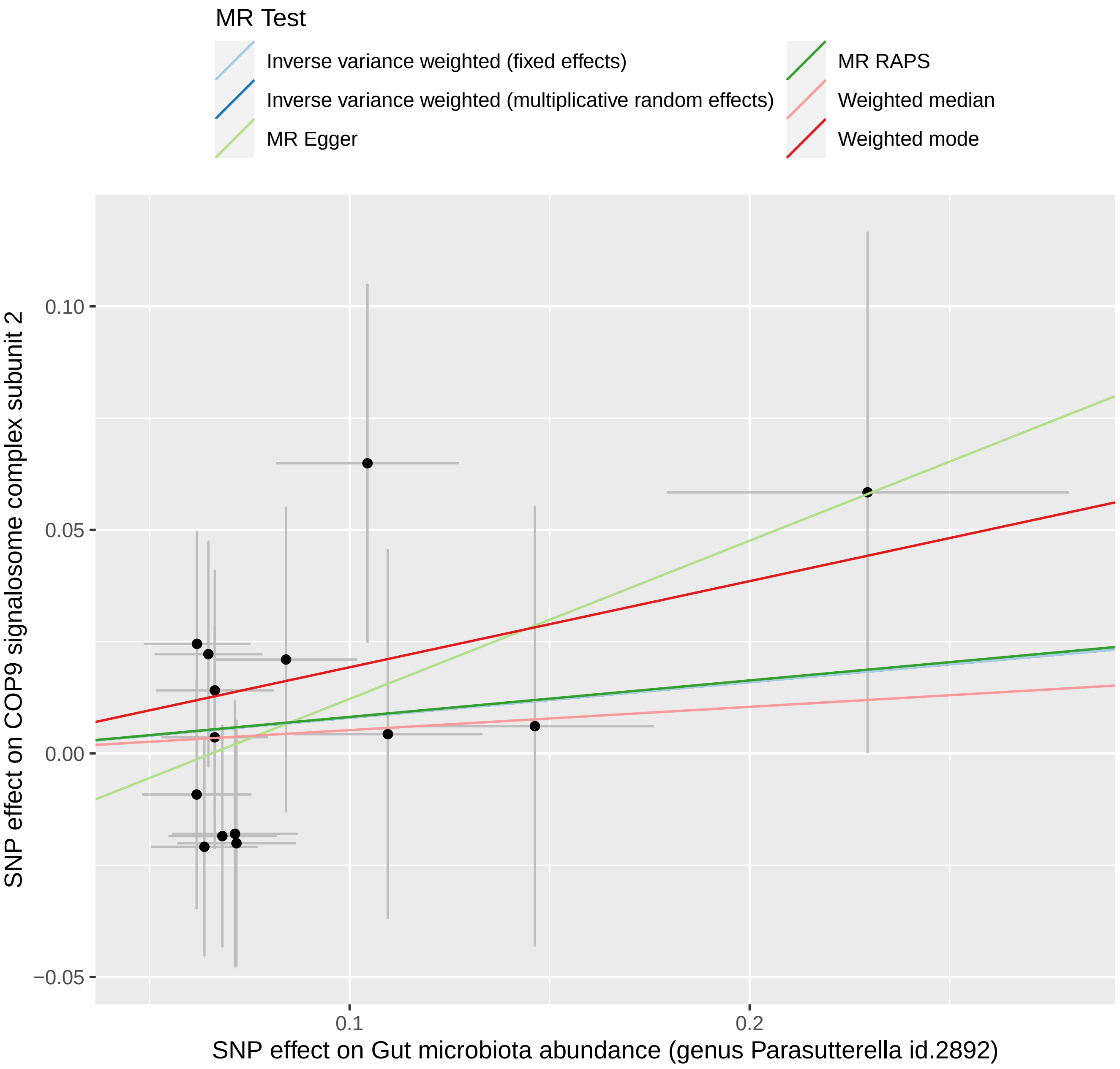

Batch 2−1271 : Gut microbiota abundance (genus Catenibacterium id.2153) on Coactosin−like protein
with a threshold of 1e−05

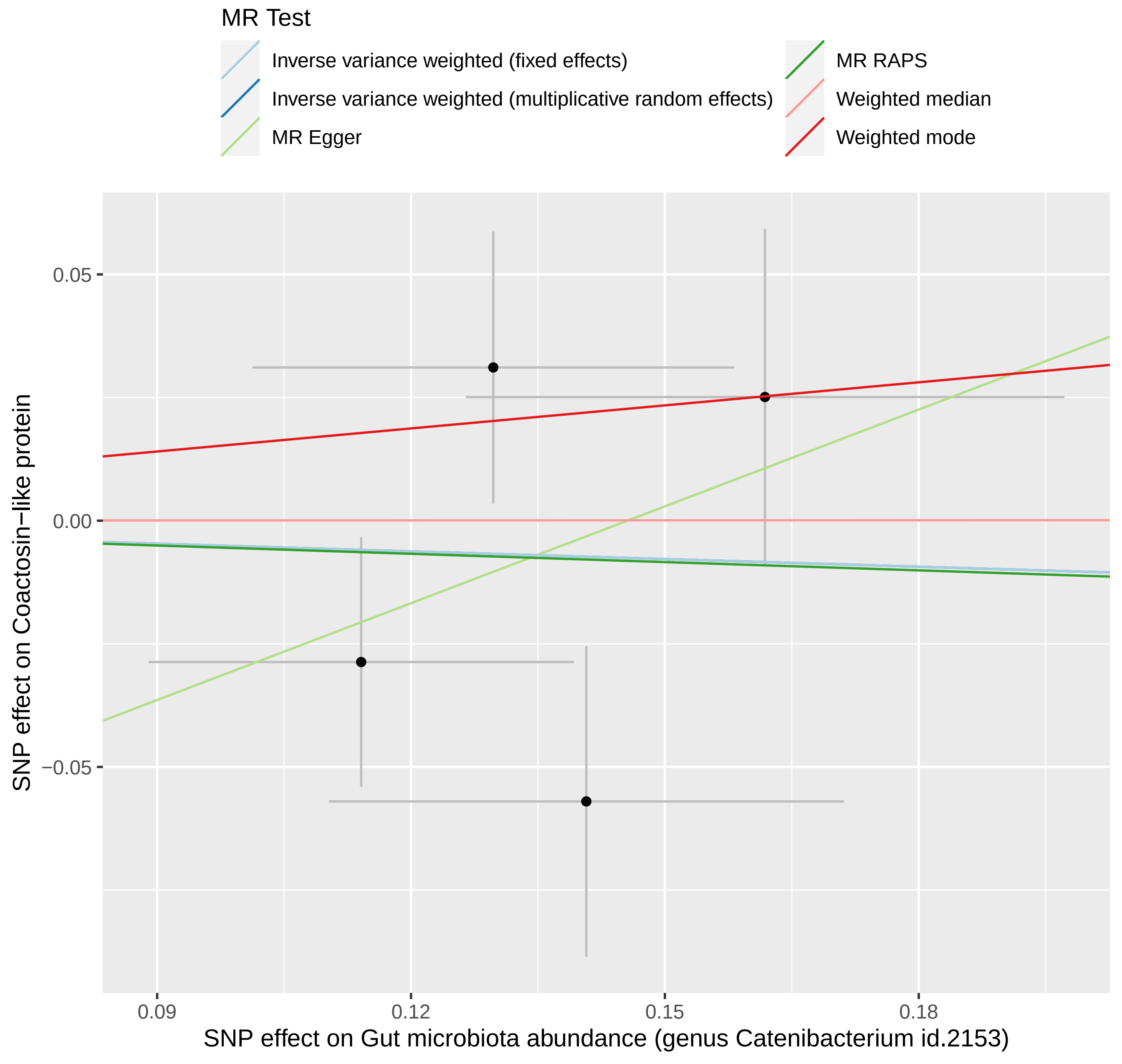

Batch 2−1272 : Gut microbiota abundance (genus Parasutterella id.2892) on Coactosin−like protein
with a threshold of 1e−05

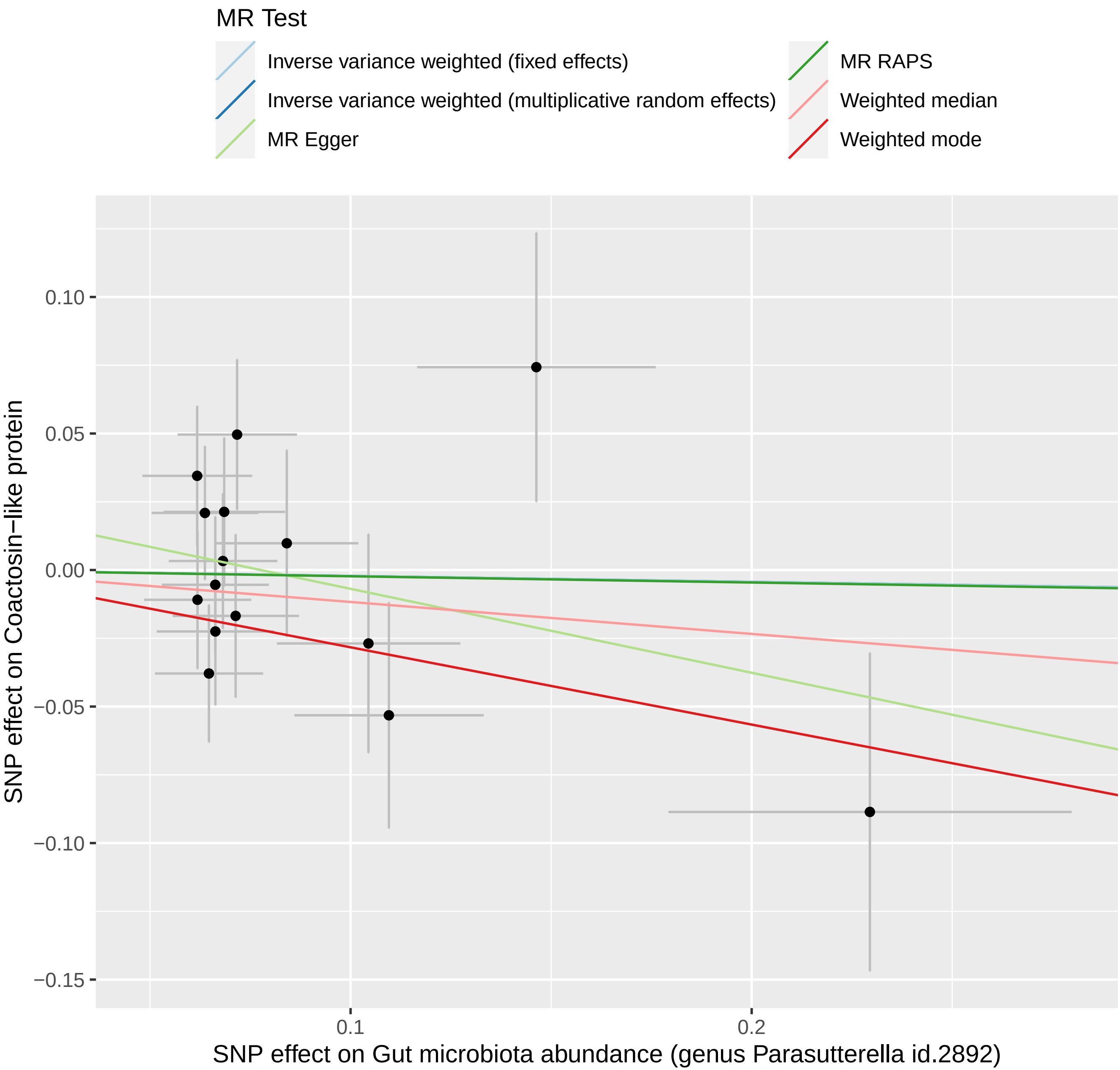

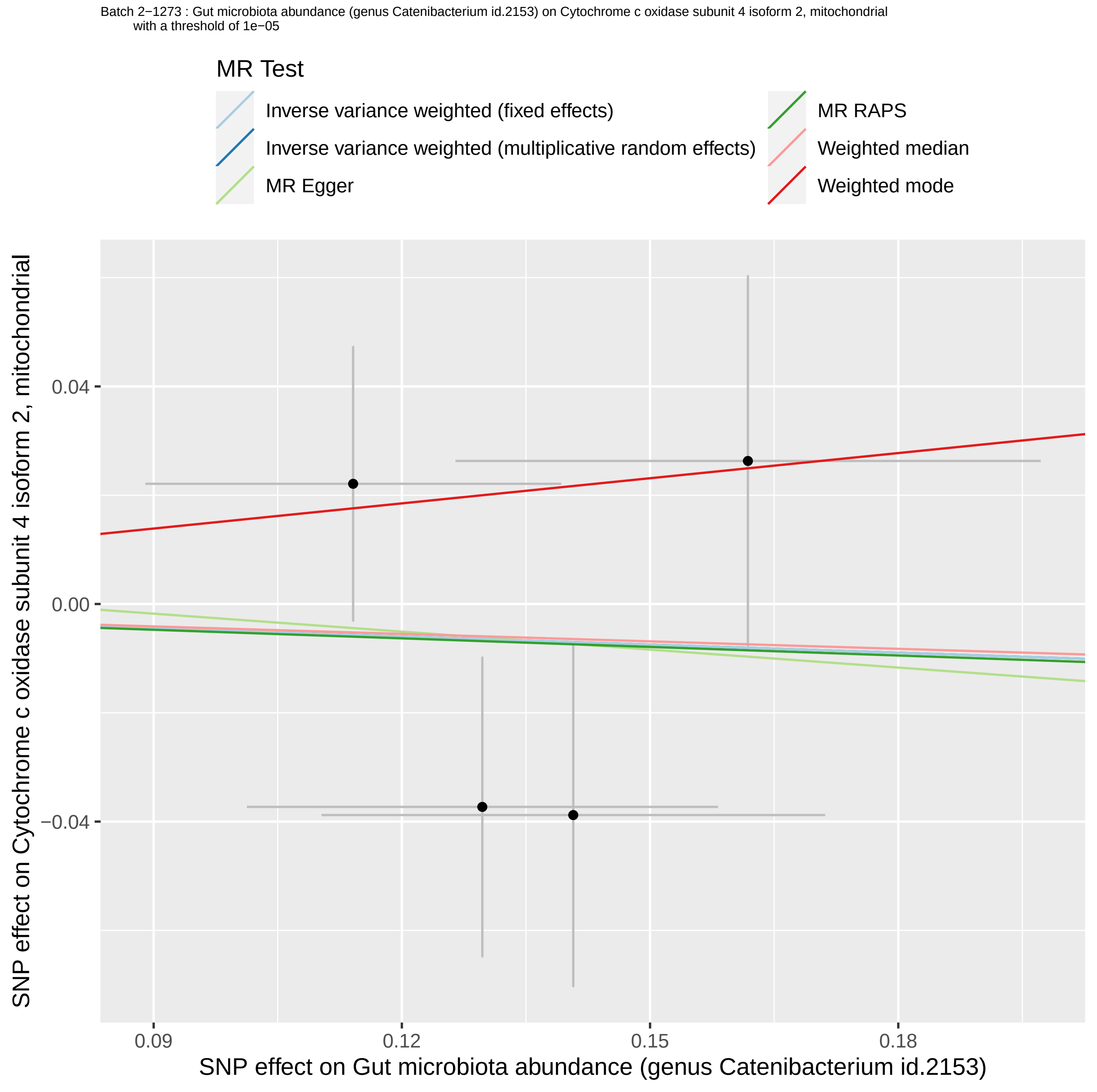

Batch 2−1273 : Gut microbiota abundance (genus Catenibacterium id.2153) on Cytochrome c oxidase subunit 4 isoform 2, mitochondrial
with a threshold of 1e−05
MR Test
Inverse variance weighted (fixed effects)
Inverse variance weighted (multiplicative random effects)
MR Egger
MR RAPS
Weighted median
Weighted mode
0.04
0.00
−0.04
0.09
0.12
0.15
0.18
SNP effect on Cytochrome c oxidase subunit 4 isoform 2, mitochondrial
SNP effect on Gut microbiota abundance (genus Catenibacterium id.2153)

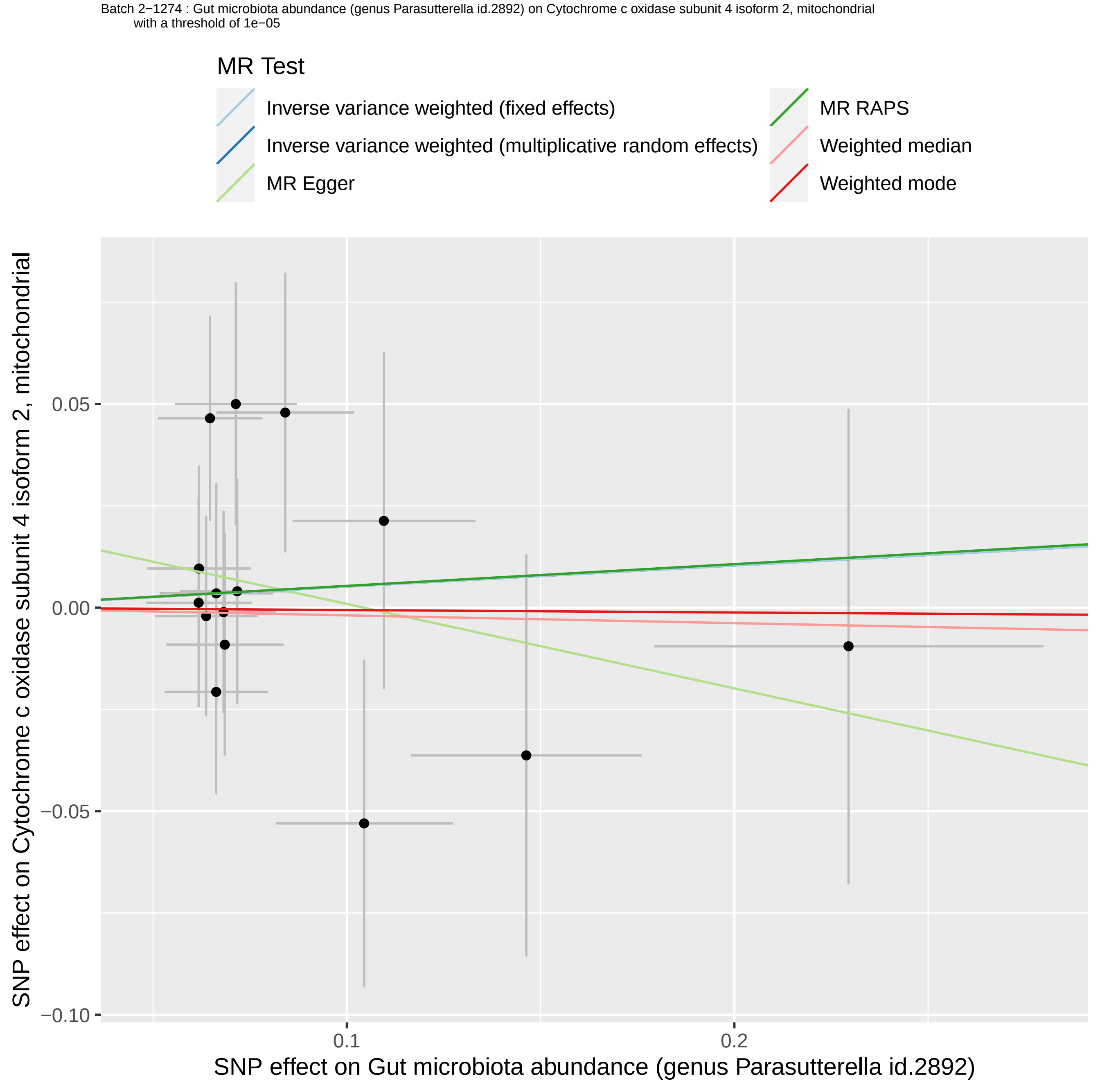
Batch 2−1274 : Gut microbiota abundance (genus Parasutterella id.2892) on Cytochrome c oxidase subunit 4 isoform 2, mitochondrial
with a threshold of 1e−05
MR Test
Inverse variance weighted (fixed effects)
Inverse variance weighted (multiplicative random effects)
MR Egger
MR RAPS
Weighted median
Weighted mode
SNP effect on Cytochrome c oxidase subunit 4 isoform 2, mitochondrial
0.05
0.00
−0.05
−0.10
0.1
0.2
SNP effect on Gut microbiota abundance (genus Parasutterella id.2892)

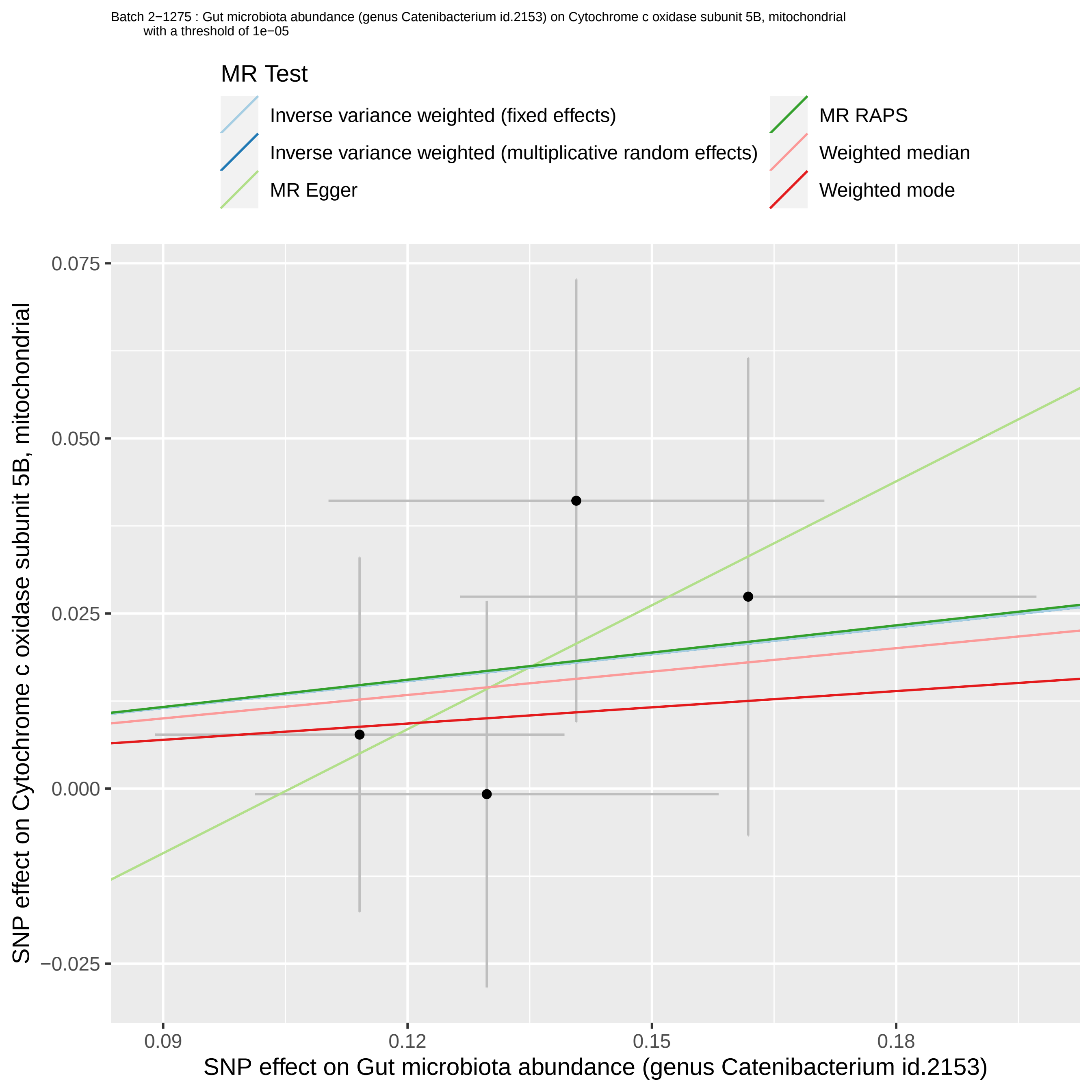
Batch 2−1275 : Gut microbiota abundance (genus Catenibacterium id.2153) on Cytochrome c oxidase subunit 5B, mitochondrial
with a threshold of 1e−05
MR Test
Inverse variance weighted (fixed effects)
Inverse variance weighted (multiplicative random effects)
MR Egger
MR RAPS
Weighted median
Weighted mode
0.075
0.050
0.025
0.000
−0.025
0.09
0.12
0.15
0.18
SNP effect on Cytochrome c oxidase subunit 5B, mitochondrial
SNP effect on Gut microbiota abundance (genus Catenibacterium id.2153)

Batch 2−1276 : Gut microbiota abundance (genus Parasutterella id.2892) on Cytochrome c oxidase subunit 5B, mitochondrial
with a threshold of 1e−05

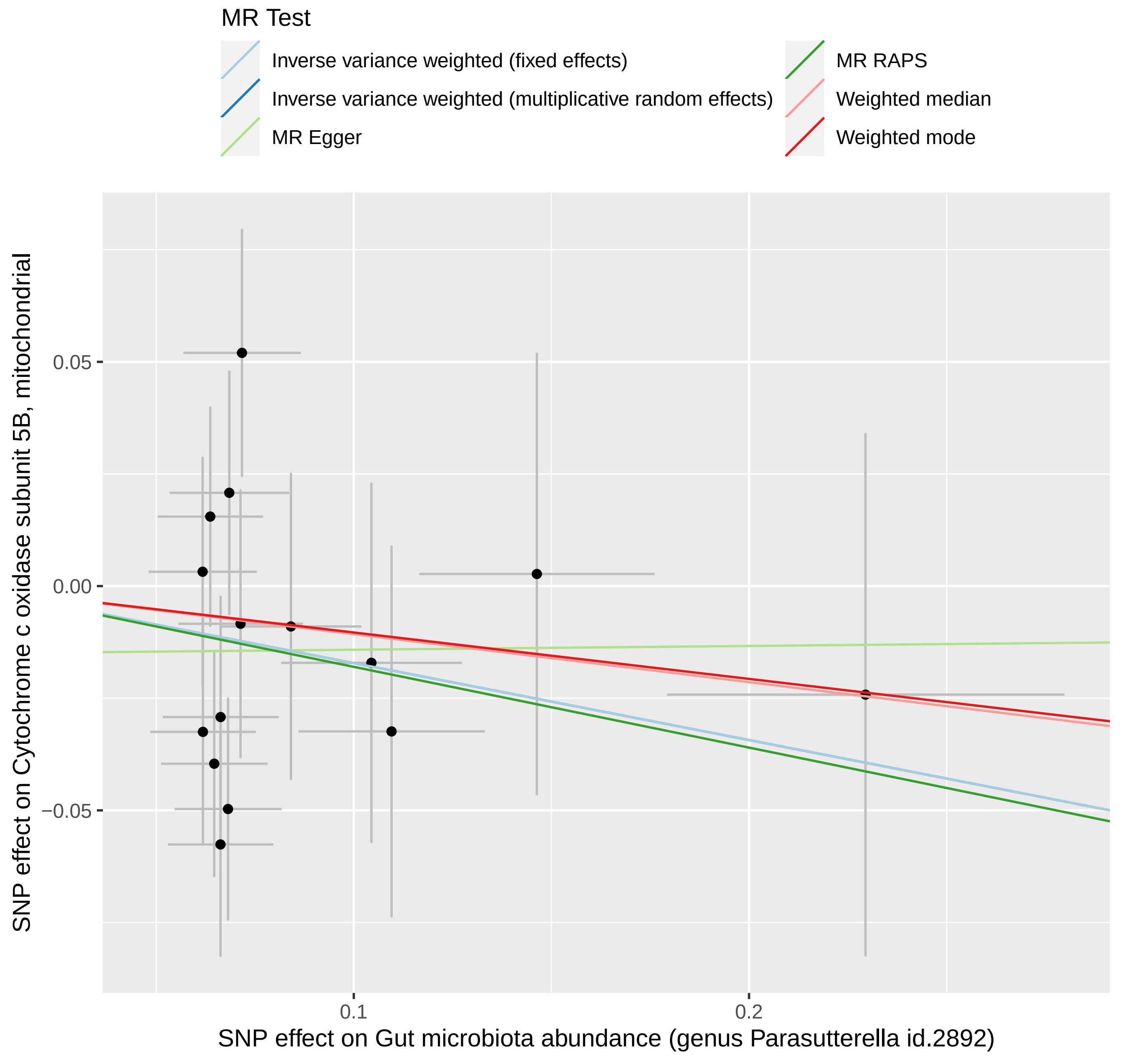

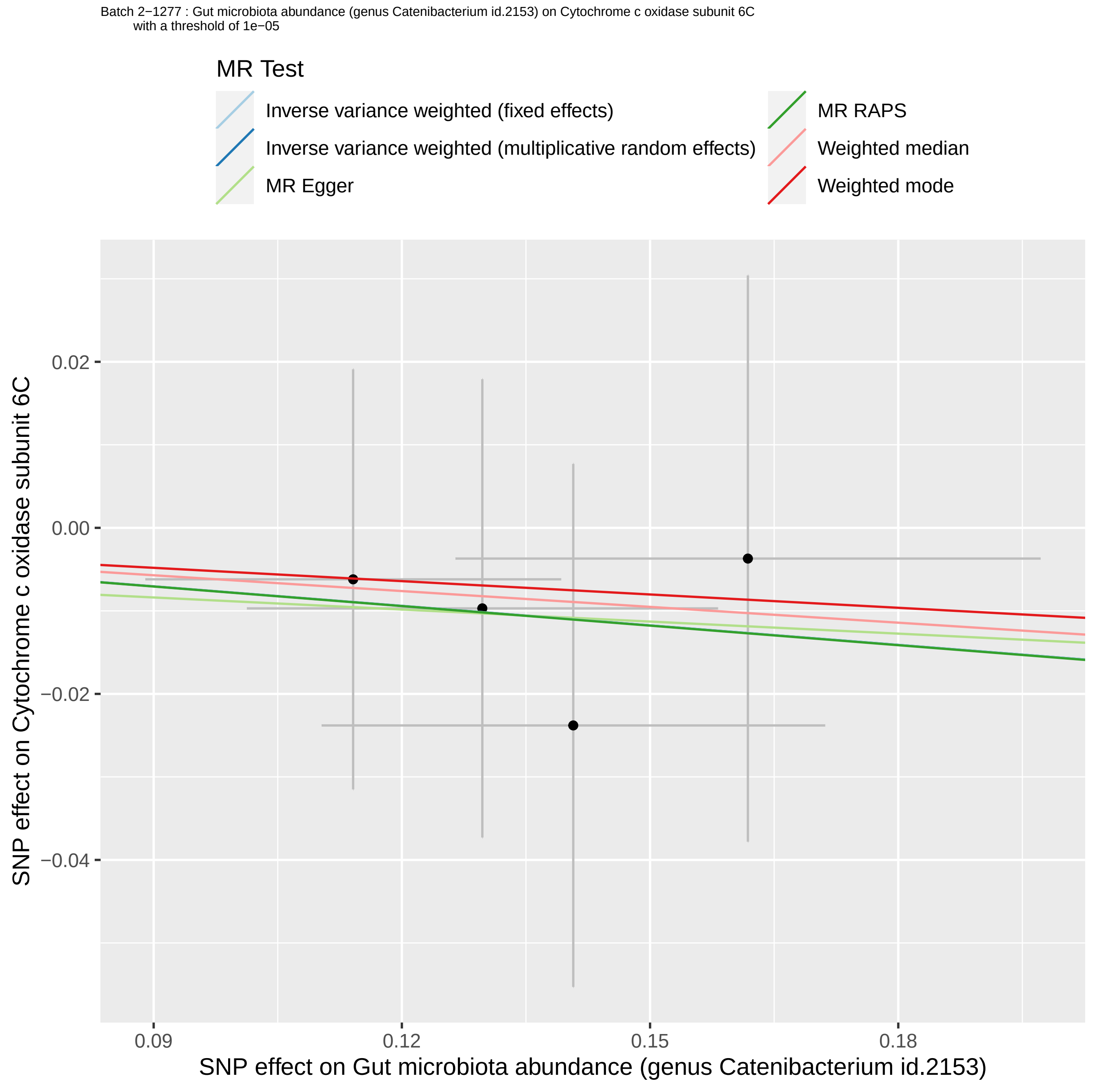
Batch 2−1277 : Gut microbiota abundance (genus Catenibacterium id.2153) on Cytochrome c oxidase subunit 6C
with a threshold of 1e−05
MR Test
Inverse variance weighted (fixed effects)
Inverse variance weighted (multiplicative random effects)
MR Egger
MR RAPS
Weighted median
Weighted mode
0.02
0.00
−0.02
−0.04
0.09
0.12
0.15
0.18
SNP effect on Cytochrome c oxidase subunit 6C
SNP effect on Gut microbiota abundance (genus Catenibacterium id.2153)

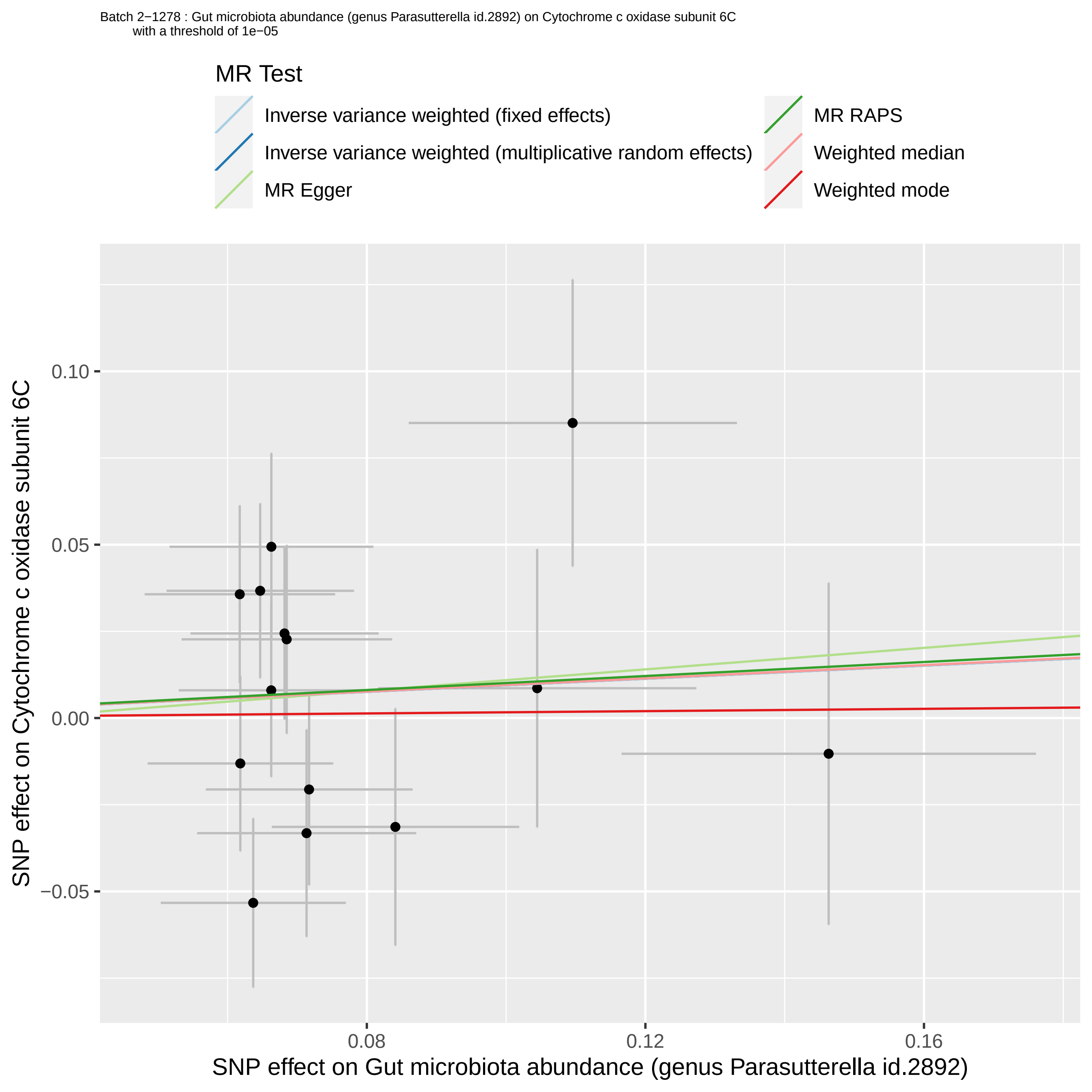

Batch 2−1278 : Gut microbiota abundance (genus Parasutterella id.2892) on Cytochrome c oxidase subunit 6C
with a threshold of 1e−05
MR Test
Inverse variance weighted (fixed effects)
Inverse variance weighted (multiplicative random effects)
MR Egger
MR RAPS
Weighted median
Weighted mode
0.10
0.05
0.00
−0.05
0.08
0.12
0.16
SNP effect on Cytochrome c oxidase subunit 6C
SNP effect on Gut microbiota abundance (genus Parasutterella id.2892)

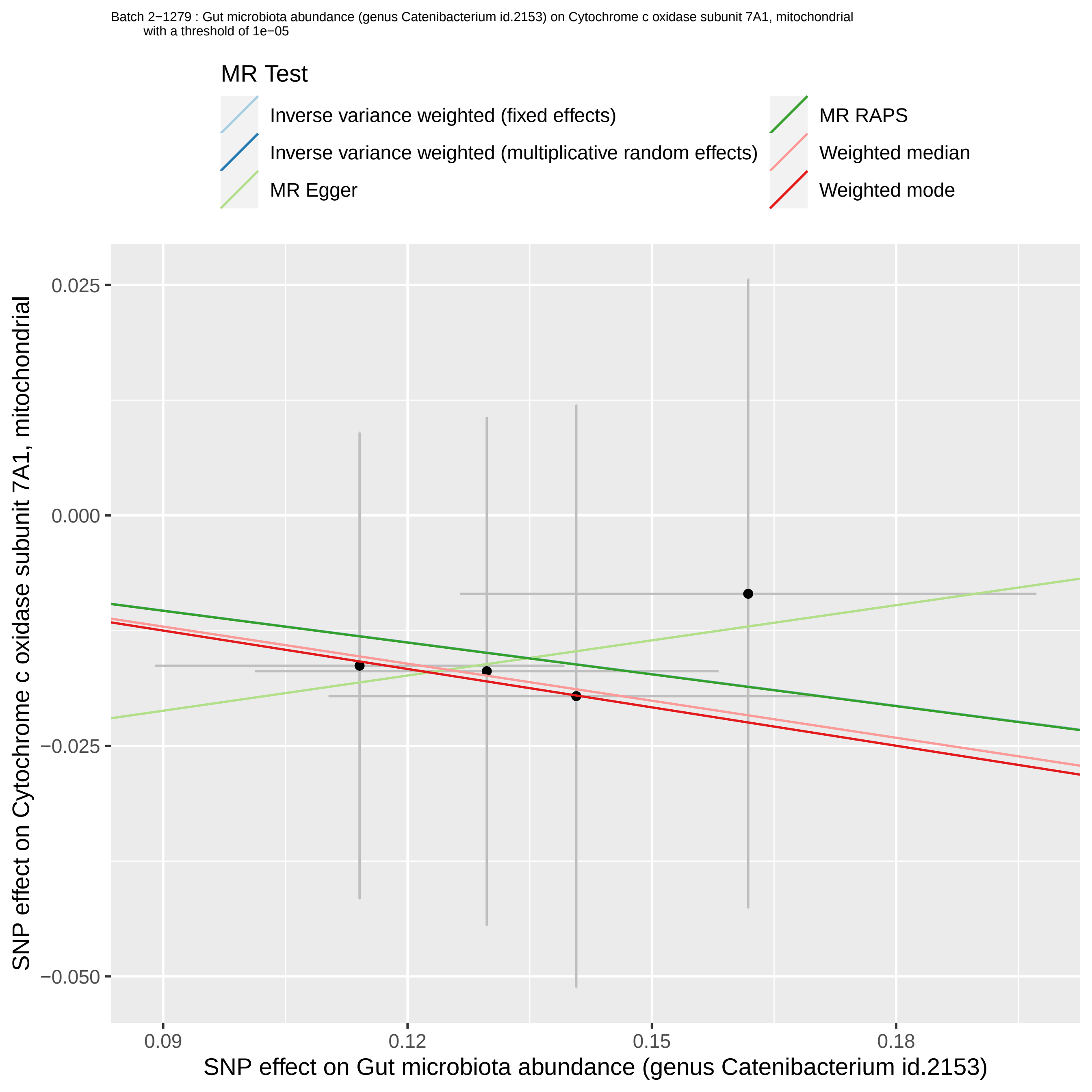

Batch 2−1279 : Gut microbiota abundance (genus Catenibacterium id.2153) on Cytochrome c oxidase subunit 7A1, mitochondrial with a threshold of 1e−05
MR Test
Inverse variance weighted (fixed effects)
Inverse variance weighted (multiplicative random effects)
MR Egger
MR RAPS
Weighted median
Weighted mode
0.025
0.000
−0.025
−0.050
0.09
0.12
0.15
0.18
SNP effect on Cytochrome c oxidase subunit 7A1, mitochondrial
SNP effect on Gut microbiota abundance (genus Catenibacterium id.2153)

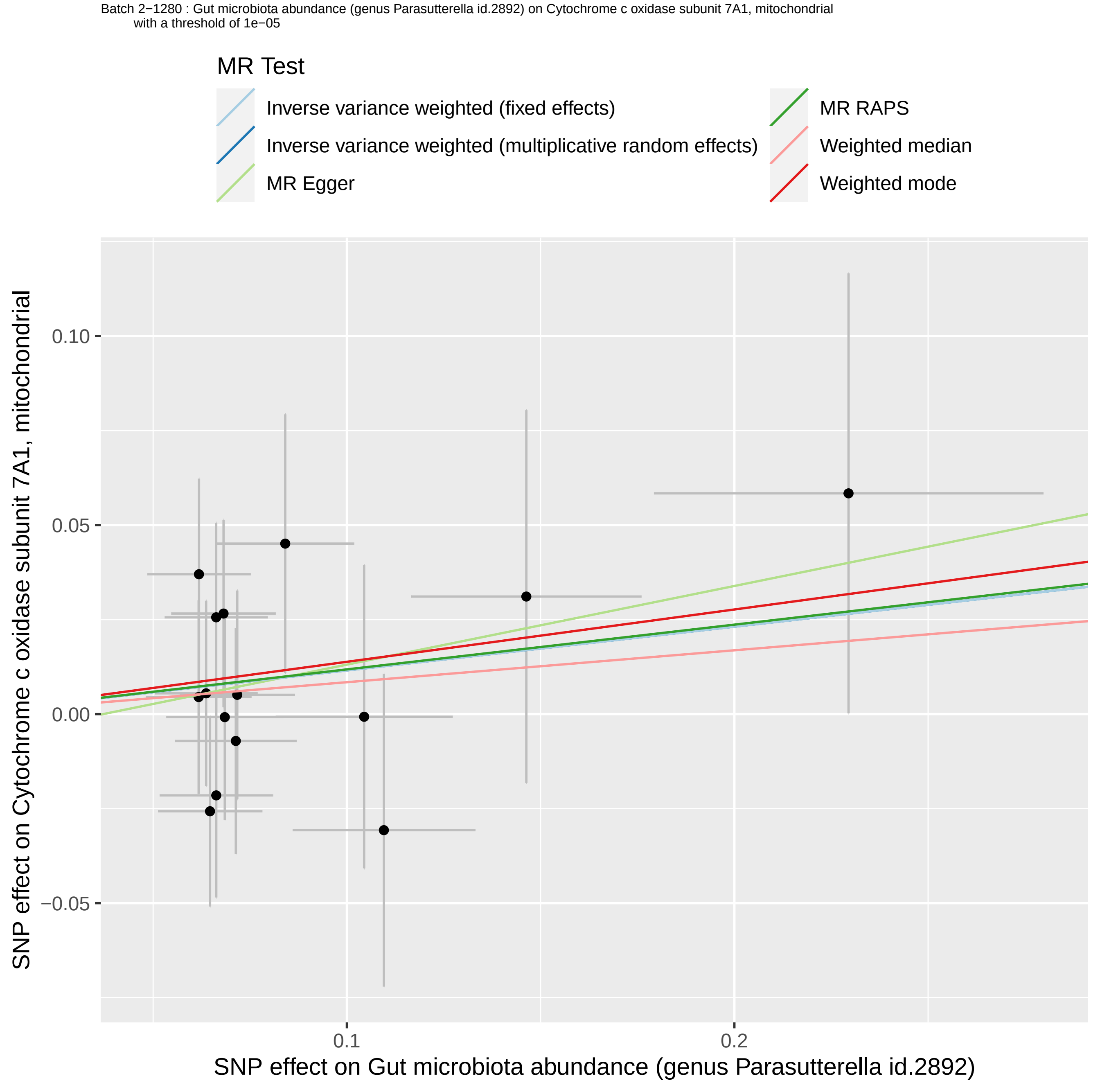

Batch 2−1280 : Gut microbiota abundance (genus Parasutterella id.2892) on Cytochrome c oxidase subunit 7A1, mitochondrial
with a threshold of 1e−05
MR Test
Inverse variance weighted (fixed effects)
Inverse variance weighted (multiplicative random effects)
MR Egger
MR RAPS
Weighted median
Weighted mode
0.10
0.05
0.00
−0.05
0.1
0.2
SNP effect on Cytochrome c oxidase subunit 7A1, mitochondrial
SNP effect on Gut microbiota abundance (genus Parasutterella id.2892)

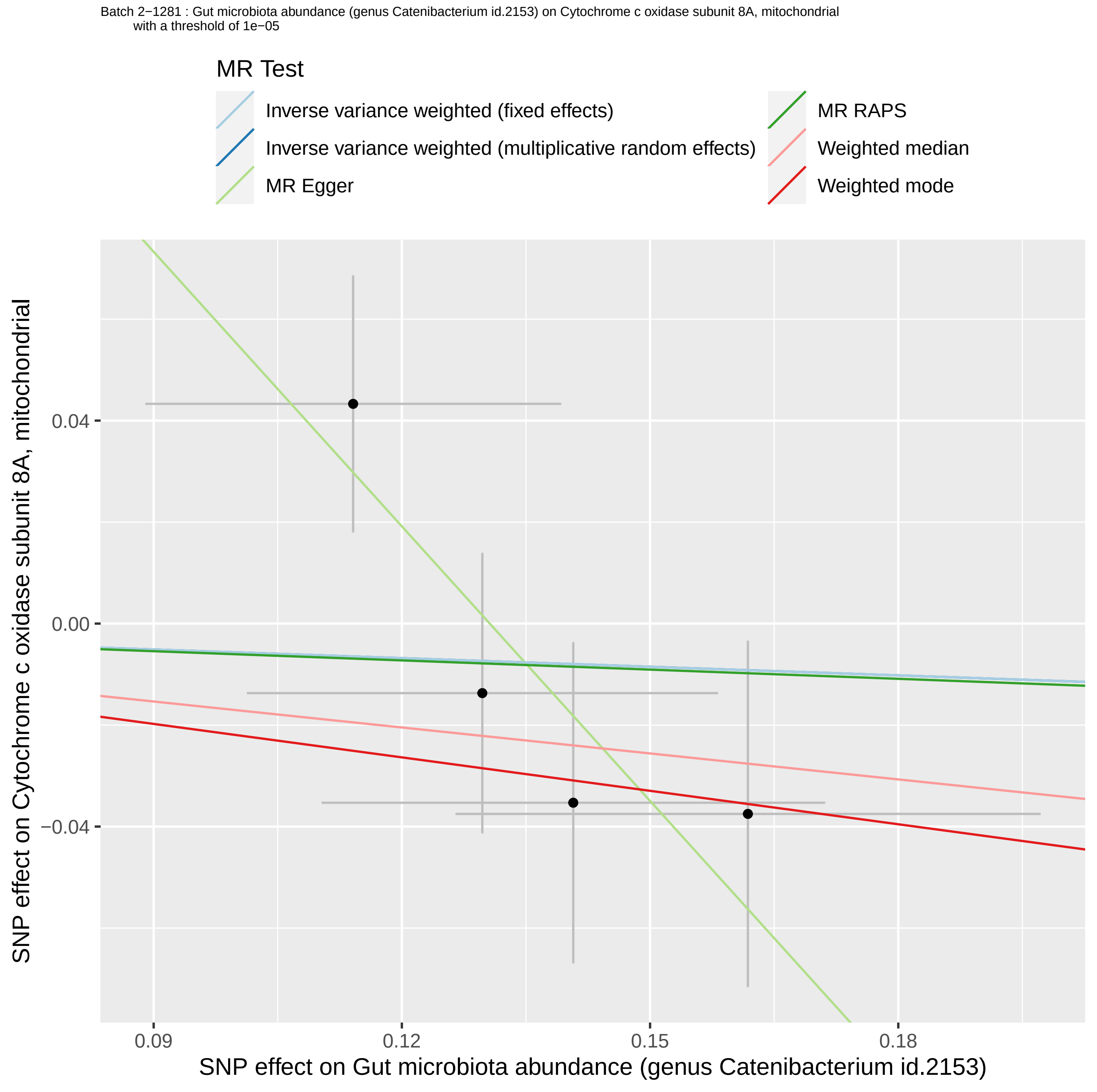
Batch 2−1281 : Gut microbiota abundance (genus Catenibacterium id.2153) on Cytochrome c oxidase subunit 8A, mitochondrial
with a threshold of 1e−05
MR Test
Inverse variance weighted (fixed effects)
Inverse variance weighted (multiplicative random effects)
MR Egger
MR RAPS
Weighted median
Weighted mode
0.04
0.00
−0.04
0.09
0.12
0.15
0.18
SNP effect on Cytochrome c oxidase subunit 8A, mitochondrial
SNP effect on Gut microbiota abundance (genus Catenibacterium id.2153)

Batch 2−1282 : Gut microbiota abundance (genus Parasutterella id.2892) on Cytochrome c oxidase subunit 8A, mitochondrial
with a threshold of 1e−05

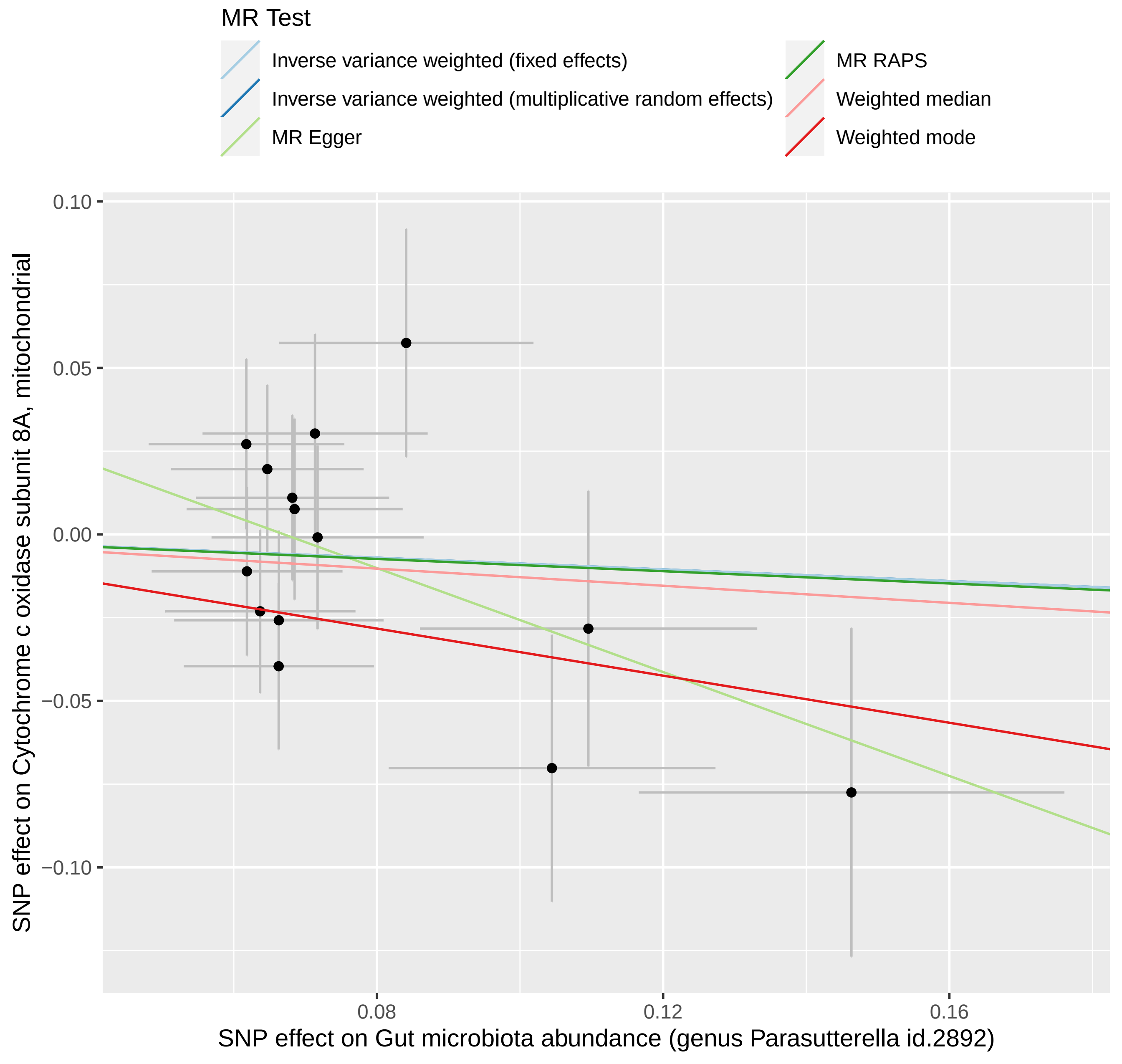

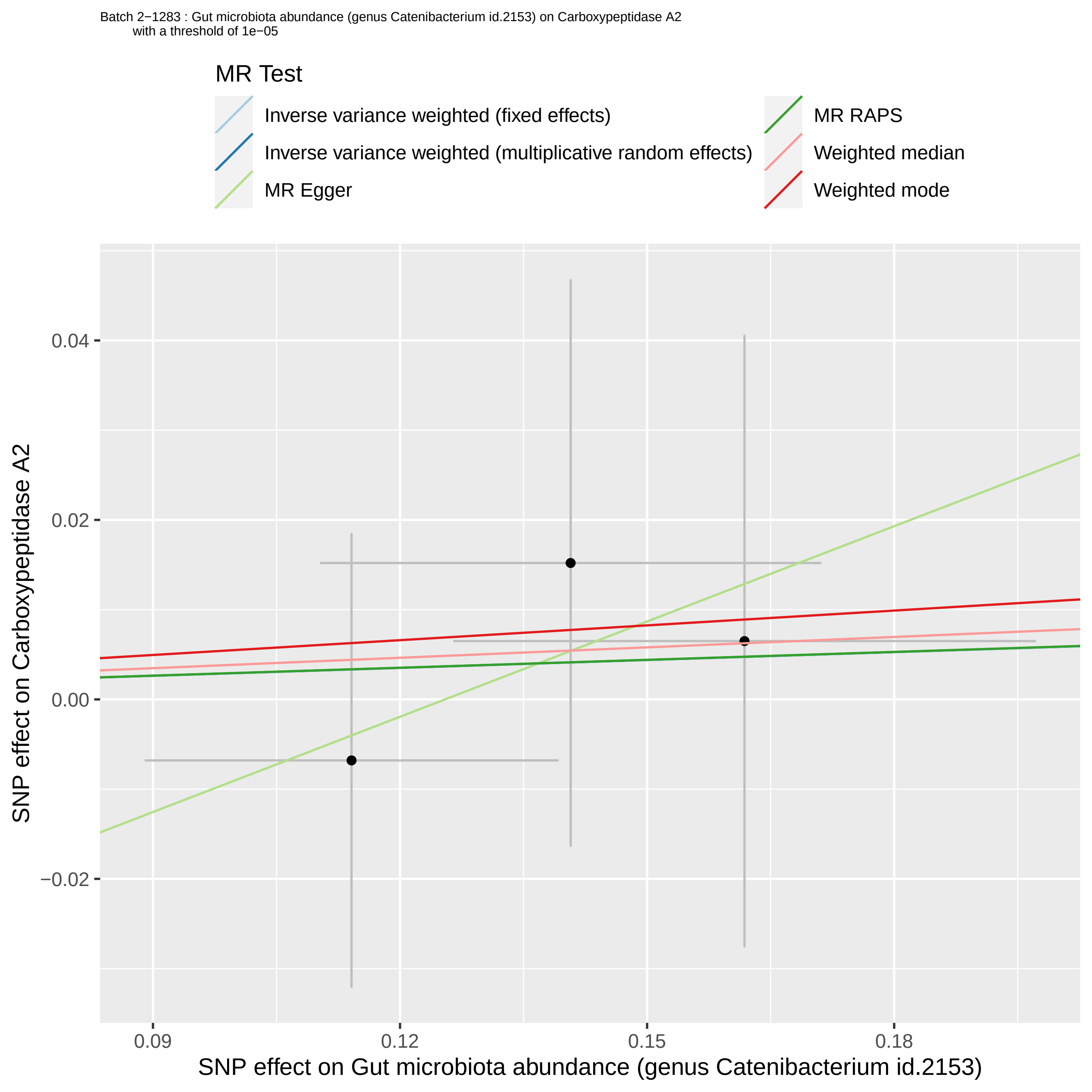

Batch 2−1283 : Gut microbiota abundance (genus Catenibacterium id.2153) on Carboxypeptidase A2
with a threshold of 1e−05
MR Test
Inverse variance weighted (fixed effects)
Inverse variance weighted (multiplicative random effects)
MR Egger
MR RAPS
Weighted median
Weighted mode
0.04
0.02
0.00
−0.02
SNP effect on Carboxypeptidase A2
0.09
0.12
0.15
0.18
SNP effect on Gut microbiota abundance (genus Catenibacterium id.2153)

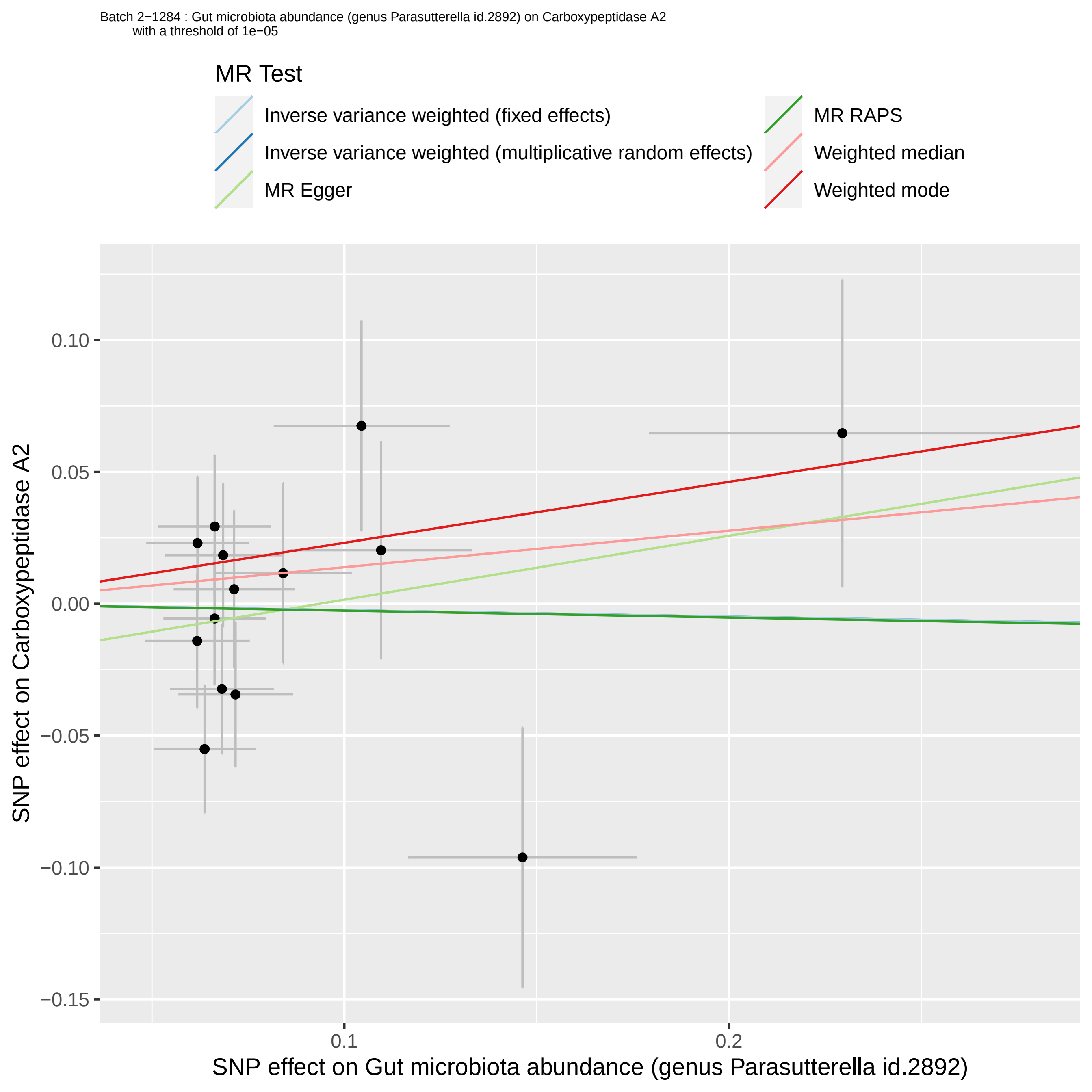

Batch 2−1284 : Gut microbiota abundance (genus Parasutterella id.2892) on Carboxypeptidase A2
with a threshold of 1e−05
MR Test
Inverse variance weighted (fixed effects)
Inverse variance weighted (multiplicative random effects)
MR Egger
MR RAPS
Weighted median
Weighted mode
0.10
0.05
0.00
−0.05
−0.10
−0.15
0.1
0.2
SNP effect on Carboxypeptidase A2
SNP effect on Gut microbiota abundance (genus Parasutterella id.2892)

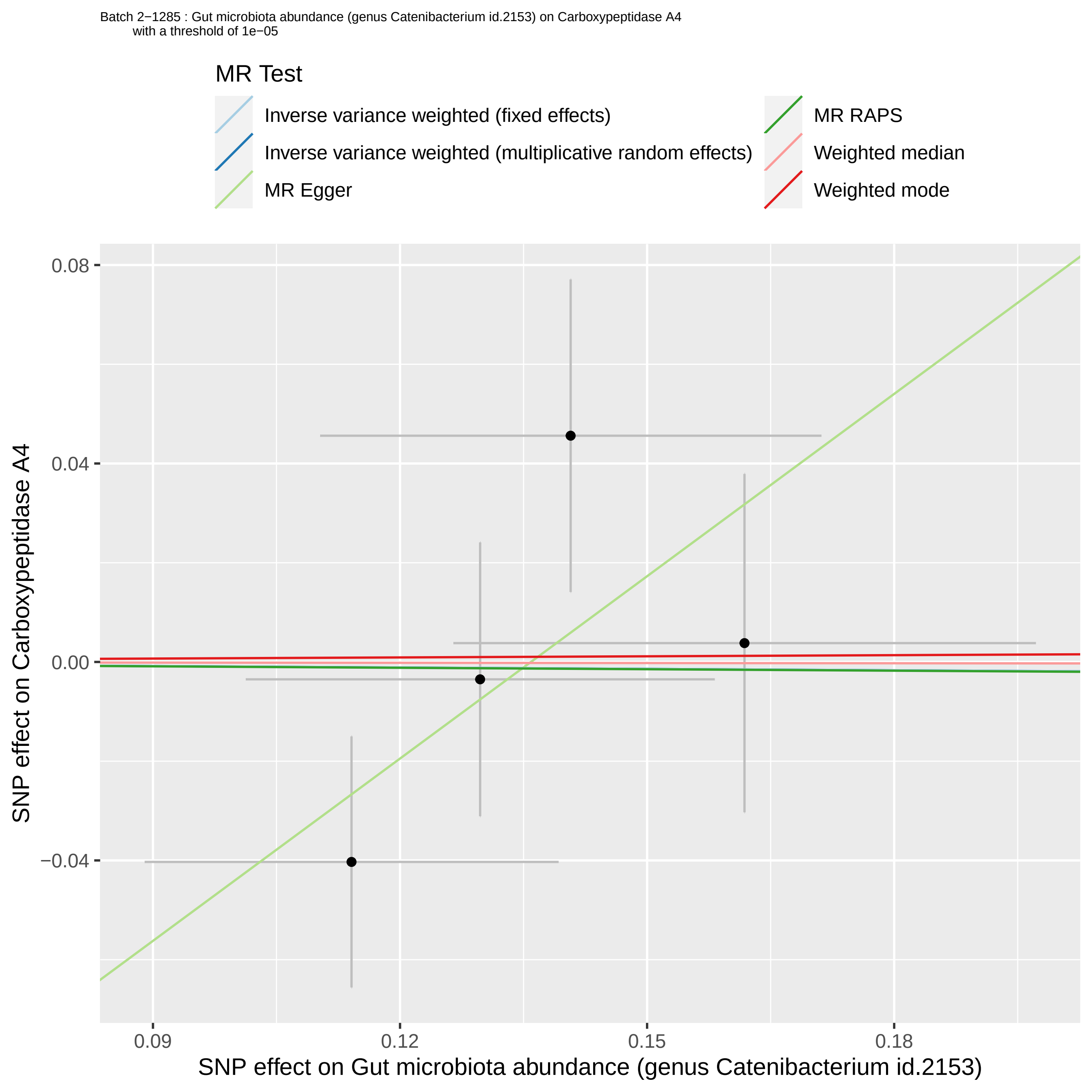

Batch 2−1285 : Gut microbiota abundance (genus Catenibacterium id.2153) on Carboxypeptidase A4
with a threshold of 1e−05
MR Test
Inverse variance weighted (fixed effects)
Inverse variance weighted (multiplicative random effects)
MR Egger
MR RAPS
Weighted median
Weighted mode
0.08
0.04
0.00
−0.04
0.09
0.12
0.15
0.18
SNP effect on Carboxypeptidase A4
SNP effect on Gut microbiota abundance (genus Catenibacterium id.2153)

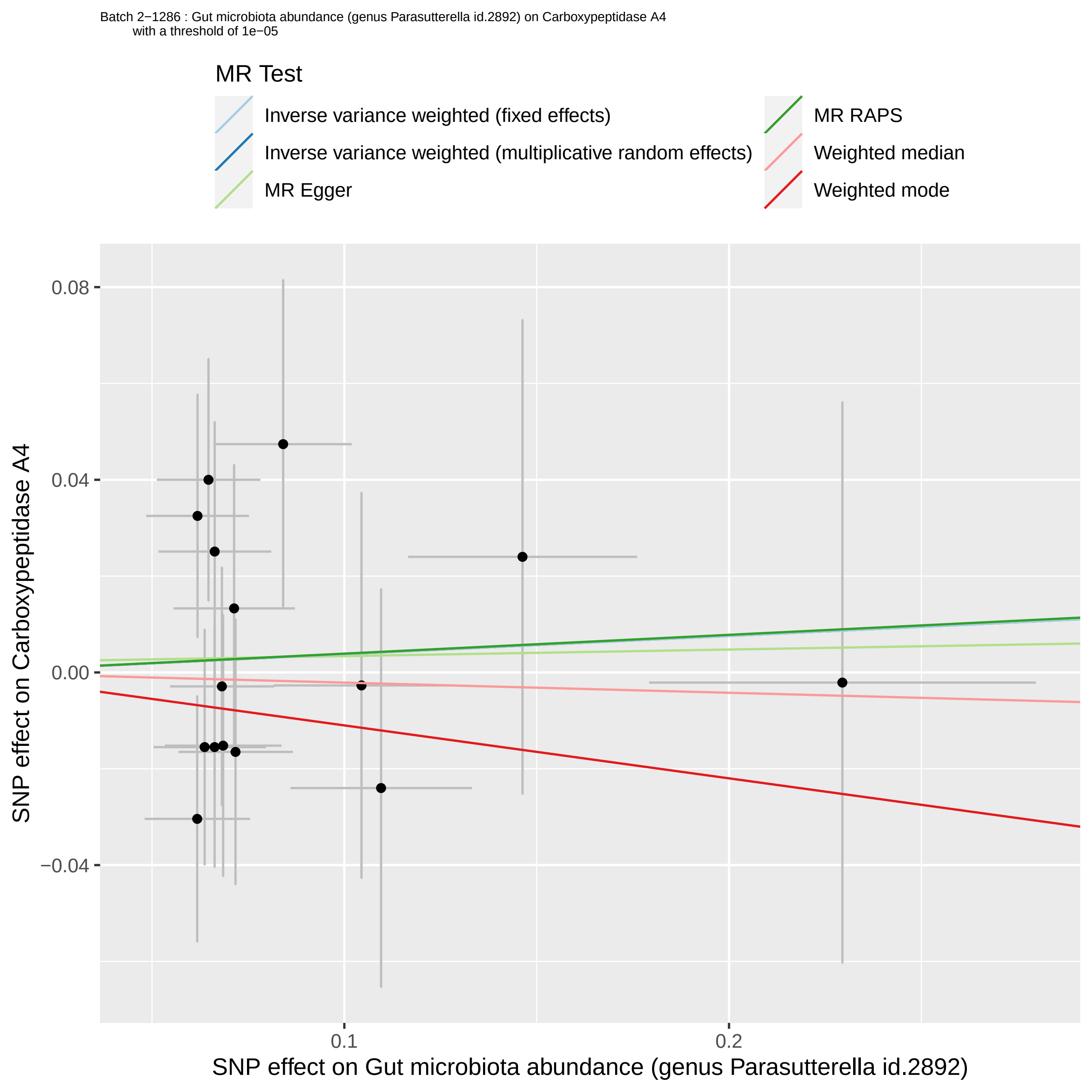
Batch 2−1286 : Gut microbiota abundance (genus Parasutterella id.2892) on Carboxypeptidase A4
with a threshold of 1e−05
MR Test
Inverse variance weighted (fixed effects)
Inverse variance weighted (multiplicative random effects)
MR Egger
MR RAPS
Weighted median
Weighted mode
0.08
0.04
0.00
−0.04
0.1
0.2
SNP effect on Carboxypeptidase A4
SNP effect on Gut microbiota abundance (genus Parasutterella id.2892)

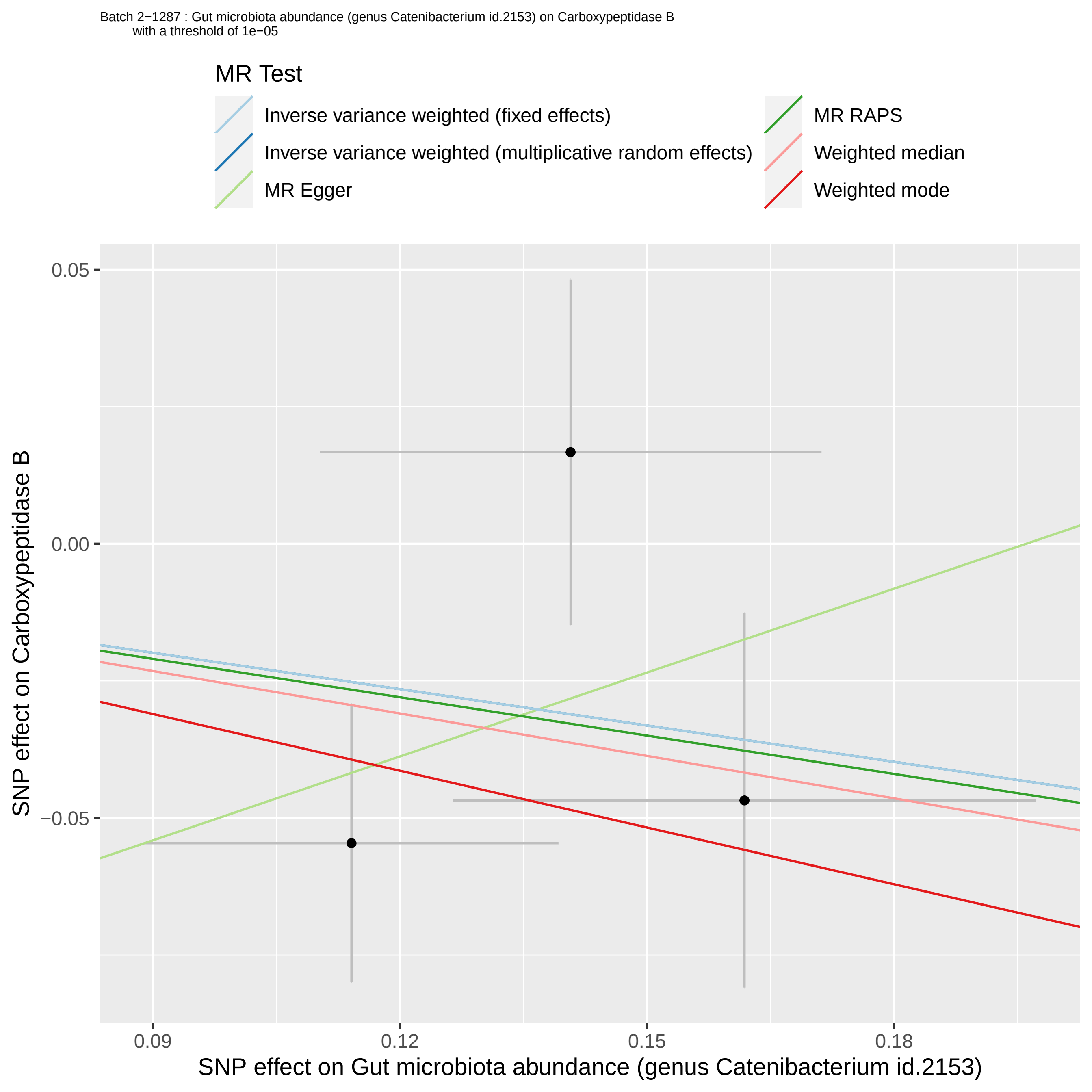

Batch 2−1287 : Gut microbiota abundance (genus Catenibacterium id.2153) on Carboxypeptidase B
with a threshold of 1e−05
MR Test
Inverse variance weighted (fixed effects)
Inverse variance weighted (multiplicative random effects)
MR Egger
MR RAPS
Weighted median
Weighted mode
0.05
0.00
−0.05
0.09
0.12
0.15
0.18
SNP effect on Carboxypeptidase B
SNP effect on Gut microbiota abundance (genus Catenibacterium id.2153)

Batch 2−1288 : Gut microbiota abundance (genus Parasutterella id.2892) on Carboxypeptidase B
with a threshold of 1e−05

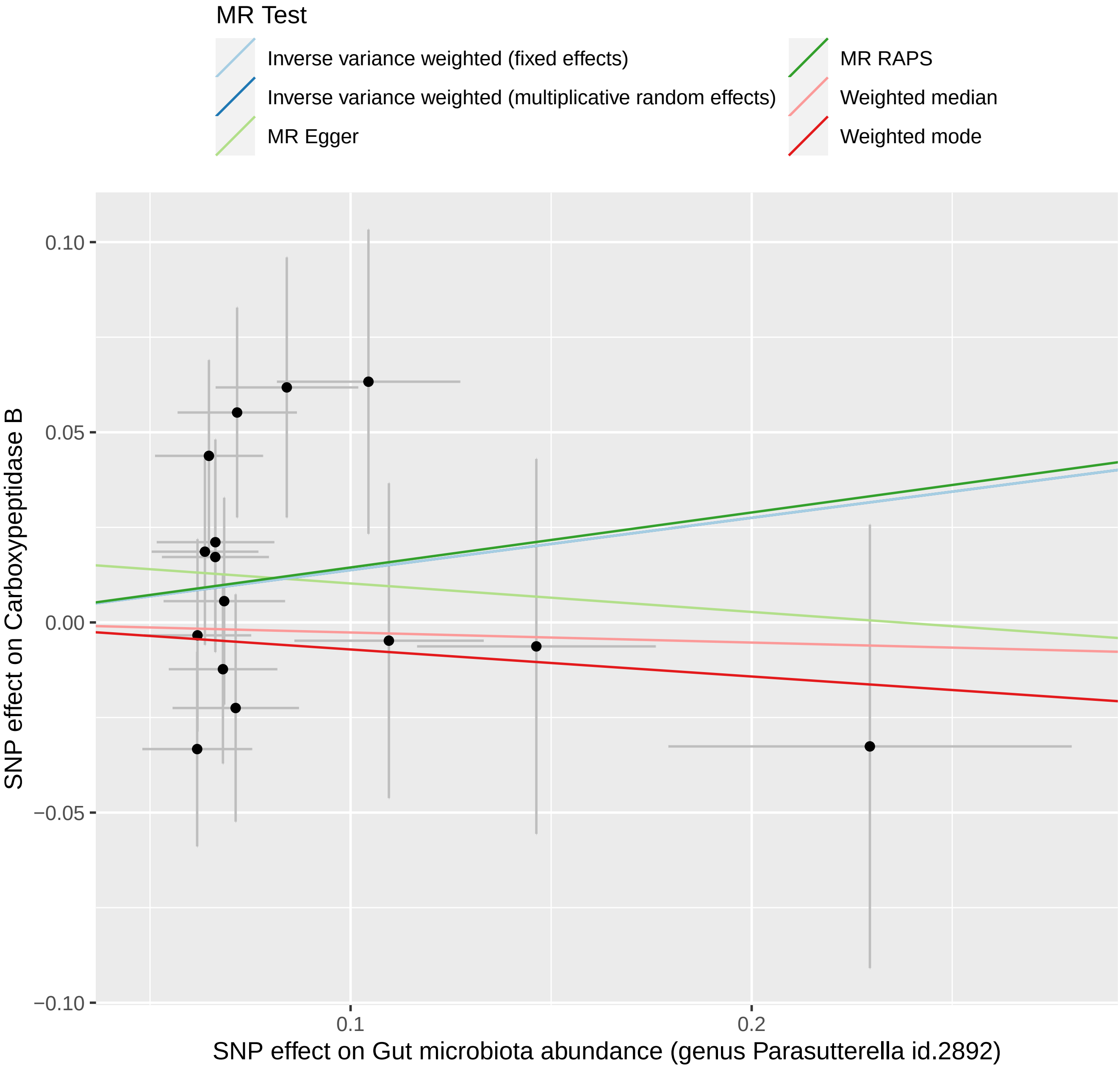

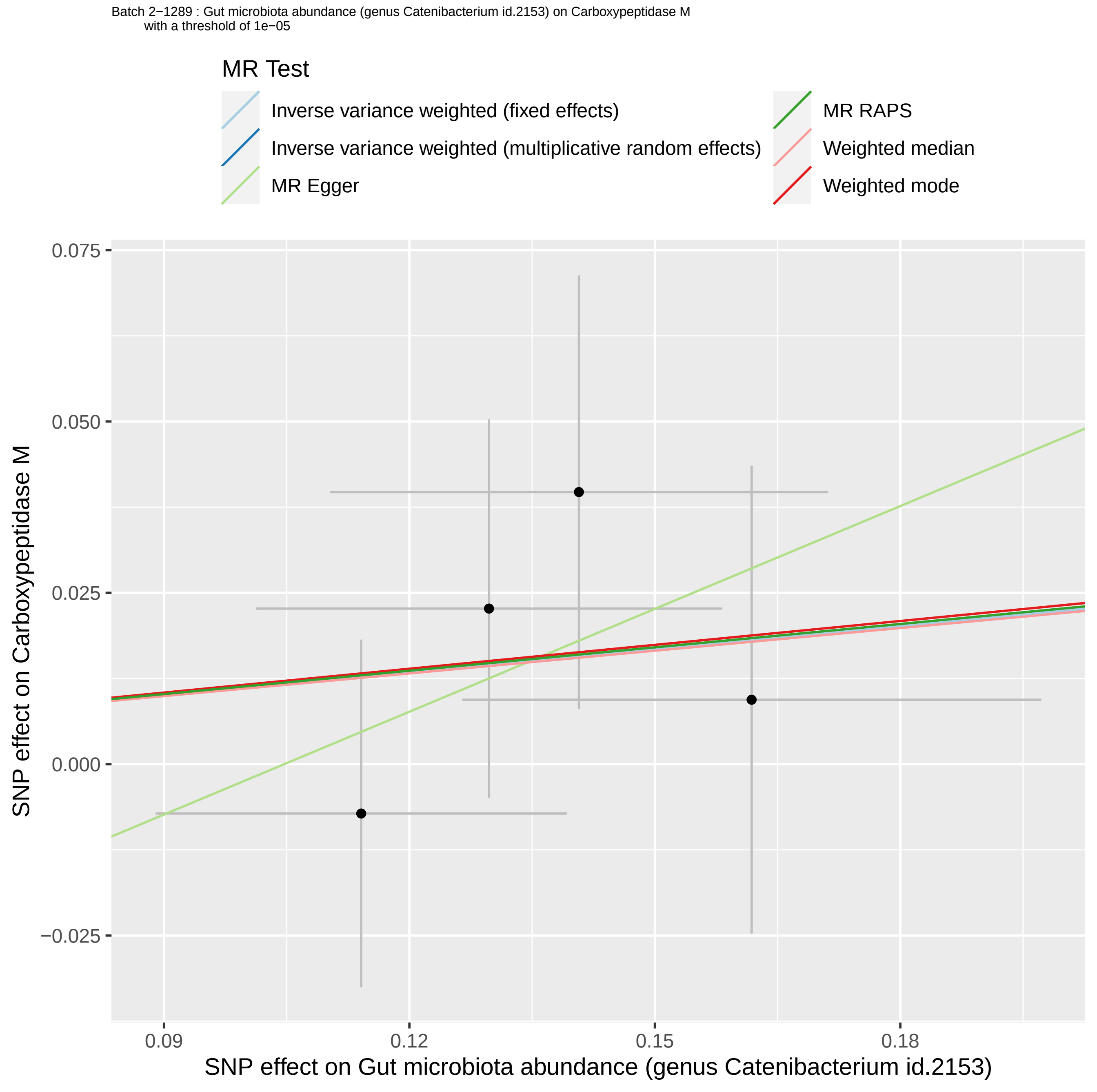
Batch 2−1289 : Gut microbiota abundance (genus Catenibacterium id.2153) on Carboxypeptidase M
with a threshold of 1e−05
MR Test
Inverse variance weighted (fixed effects)
Inverse variance weighted (multiplicative random effects)
MR Egger
MR RAPS
Weighted median
Weighted mode
0.075
0.050
0.025
0.000
−0.025
0.09
0.12
0.15
0.18
SNP effect on Carboxypeptidase M
SNP effect on Gut microbiota abundance (genus Catenibacterium id.2153)

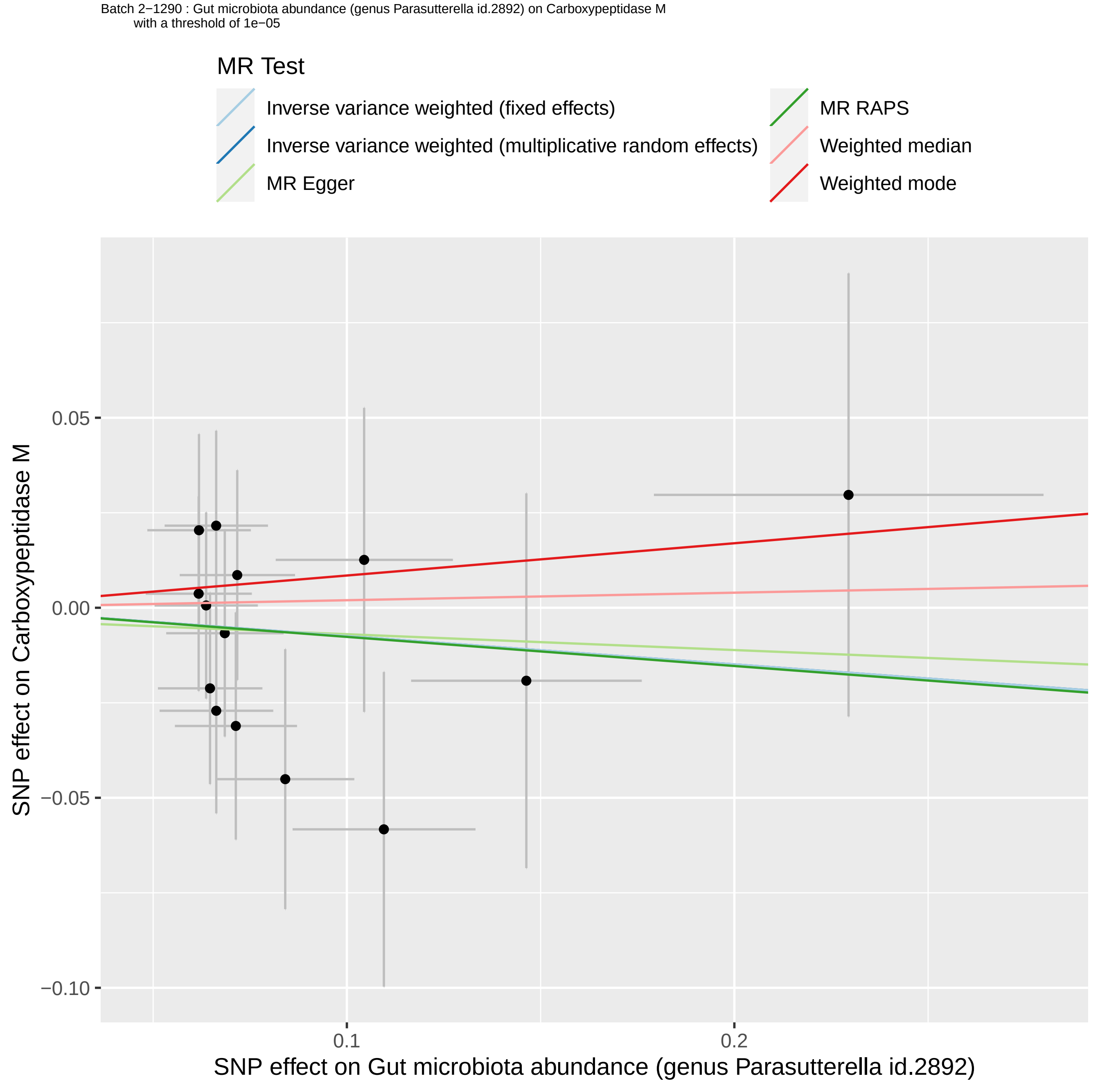
Batch 2−1290 : Gut microbiota abundance (genus Parasutterella id.2892) on Carboxypeptidase M
with a threshold of 1e−05
MR Test
Inverse variance weighted (fixed effects)
Inverse variance weighted (multiplicative random effects)
MR Egger
MR RAPS
Weighted median
Weighted mode
SNP effect on Carboxypeptidase M
0.05
0.00
−0.05
−0.10
0.1
0.2
SNP effect on Gut microbiota abundance (genus Parasutterella id.2892)

Batch 2−1291 : Gut microbiota abundance (genus Catenibacterium id.2153) on Copine−1
with a threshold of 1e−05

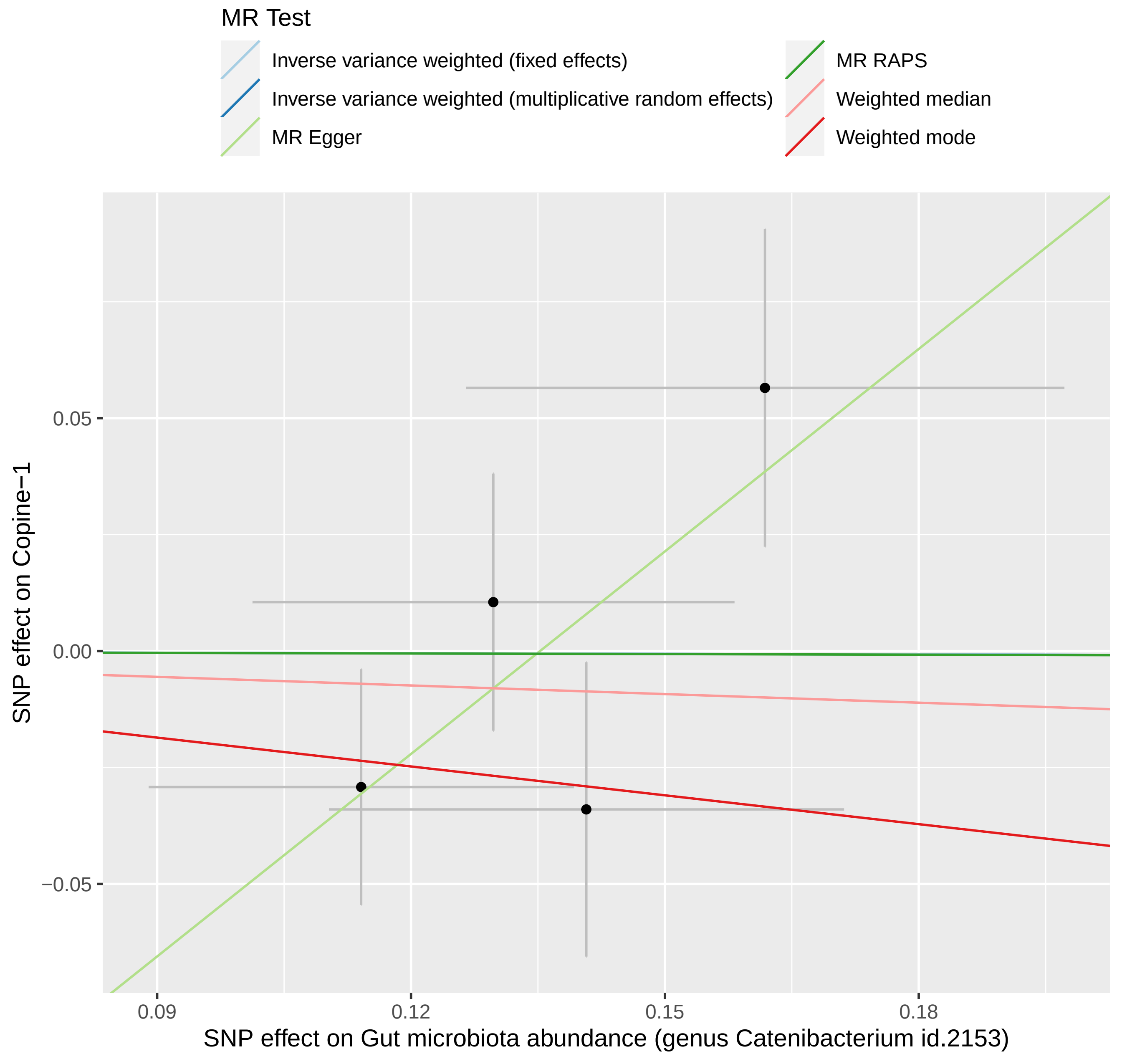

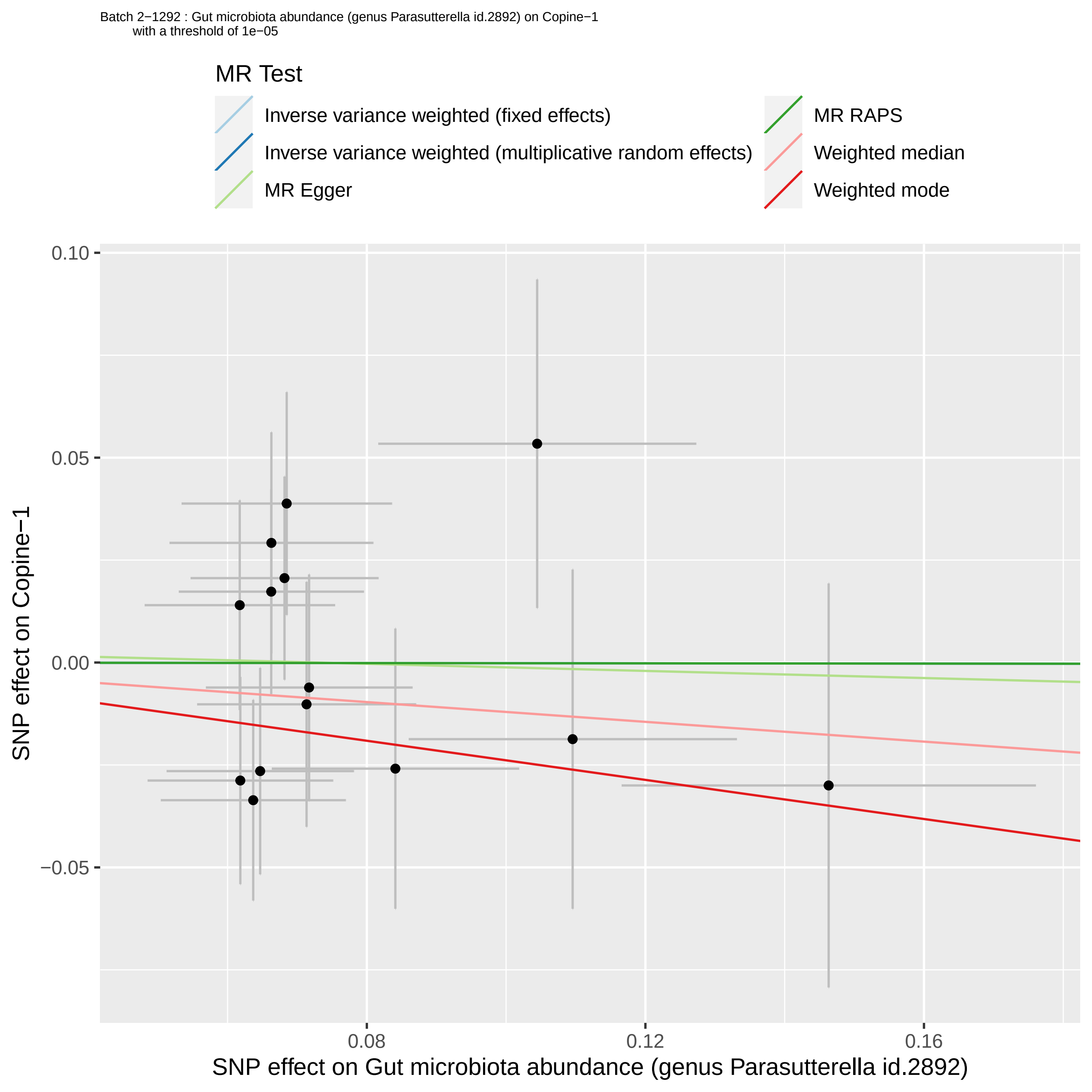
Batch 2−1292 : Gut microbiota abundance (genus Parasutterella id.2892) on Copine−1
with a threshold of 1e−05
MR Test
Inverse variance weighted (fixed effects)
Inverse variance weighted (multiplicative random effects)
MR Egger
MR RAPS
Weighted median
Weighted mode
0.10
0.05
0.00
−0.05
0.08
0.12
0.16
SNP effect on Copine−1
SNP effect on Gut microbiota abundance (genus Parasutterella id.2892)

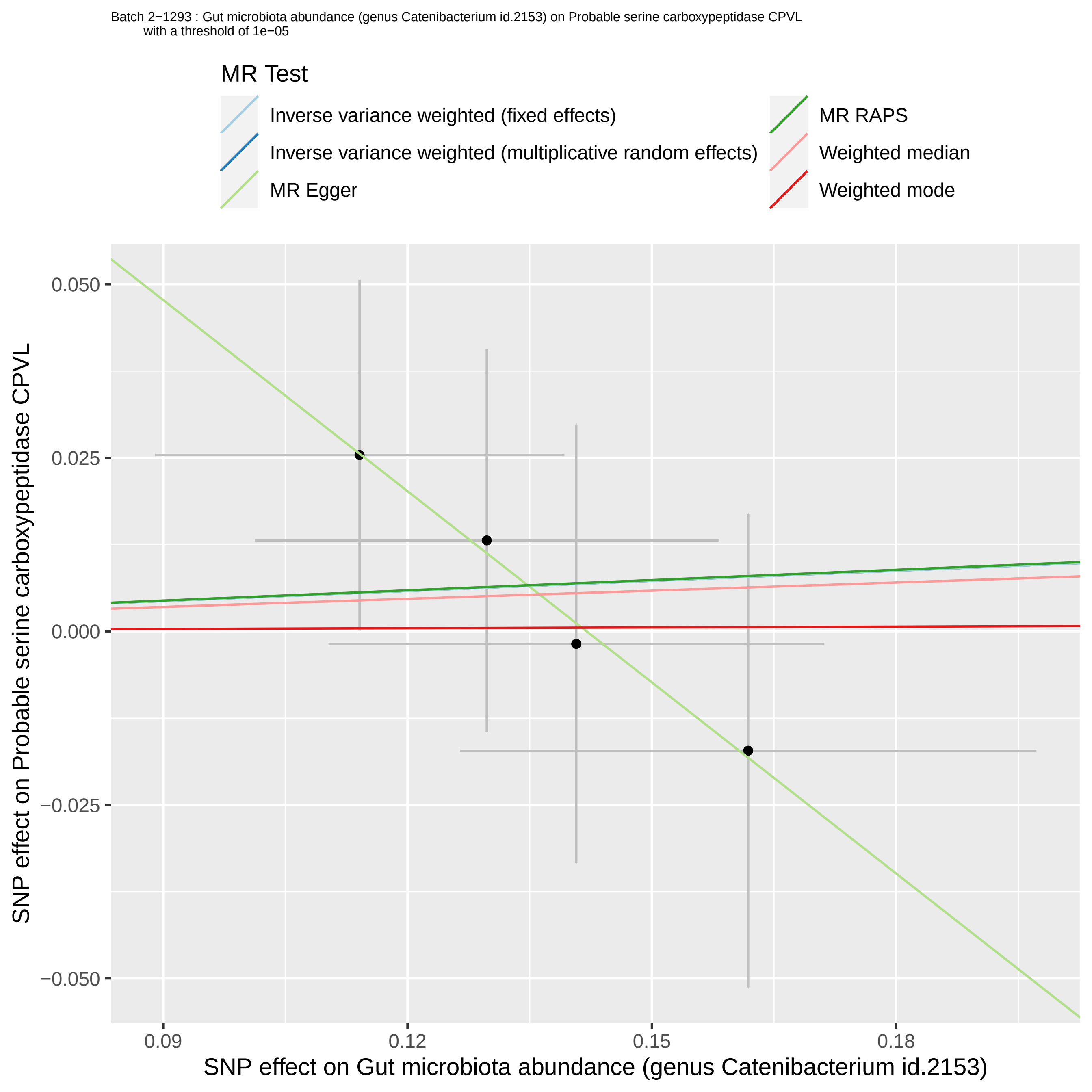
Batch 2−1293 : Gut microbiota abundance (genus Catenibacterium id.2153) on Probable serine carboxypeptidase CPVL
with a threshold of 1e−05
MR Test
Inverse variance weighted (fixed effects)
Inverse variance weighted (multiplicative random effects)
MR Egger
MR RAPS
Weighted median
Weighted mode
0.050
0.025
0.000
−0.025
−0.050
0.09
0.12
0.15
0.18
SNP effect on Probable serine carboxypeptidase CPVL
SNP effect on Gut microbiota abundance (genus Catenibacterium id.2153)

Batch 2−1294 : Gut microbiota abundance (genus Parasutterella id.2892) on Probable serine carboxypeptidase CPVL
with a threshold of 1e−05

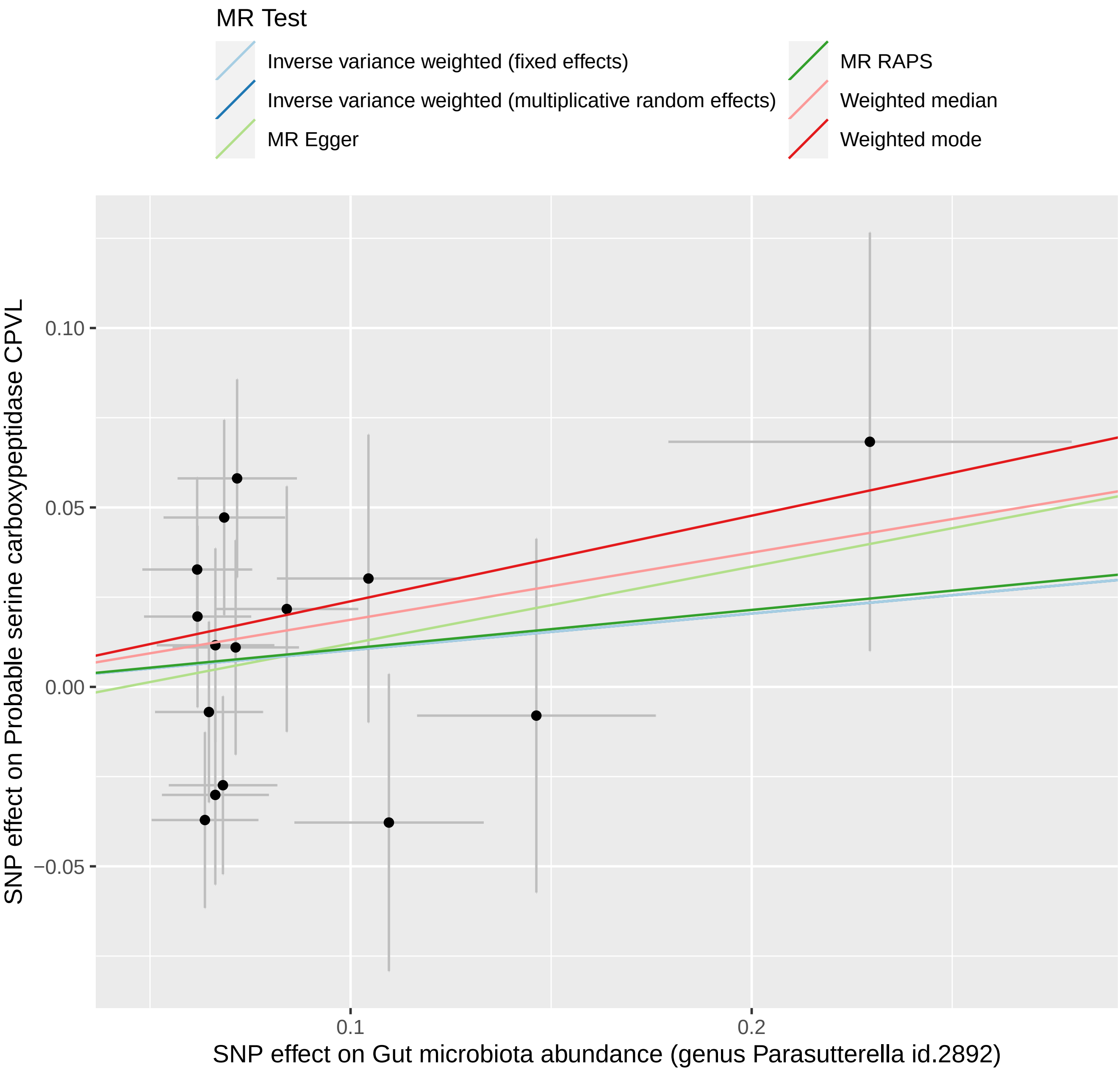

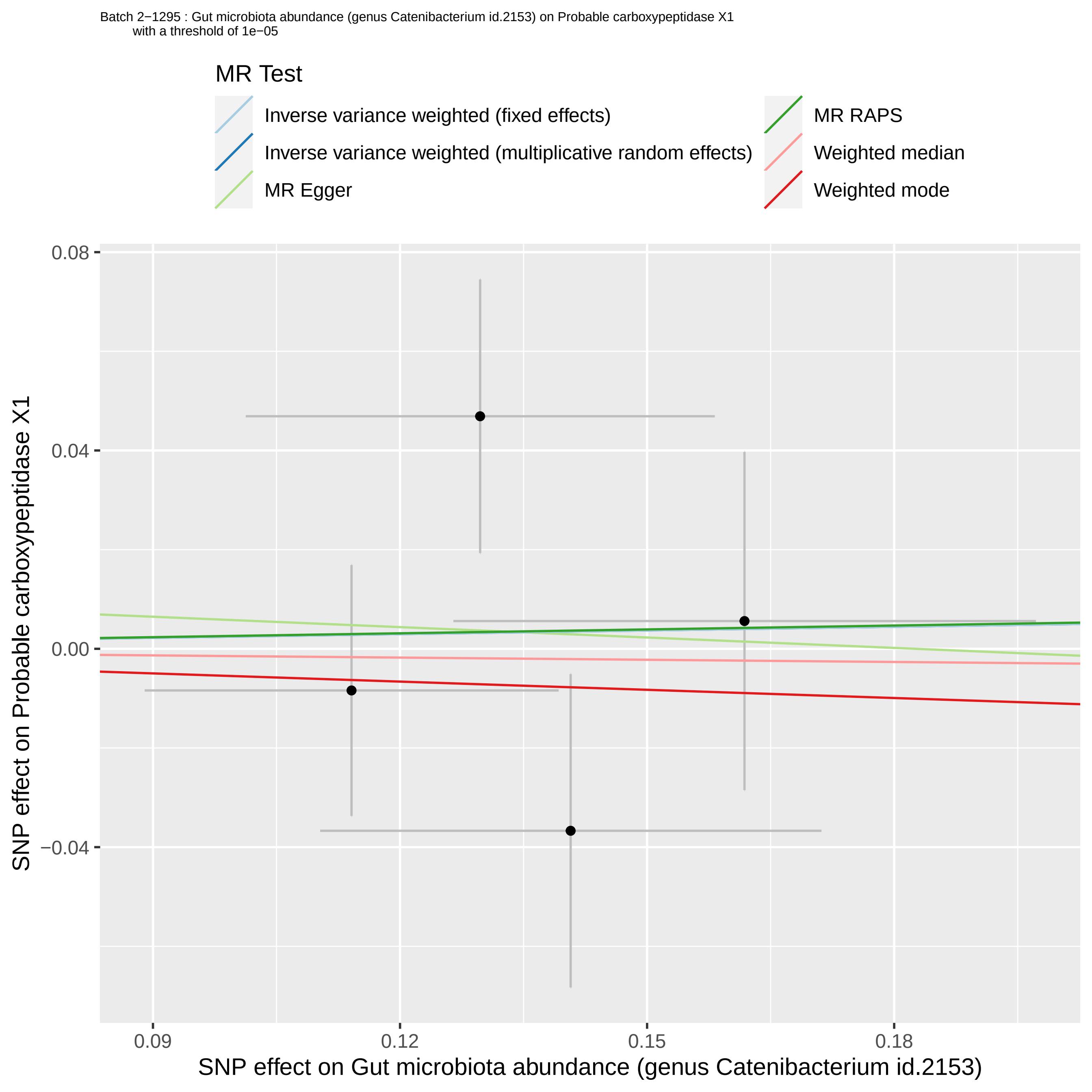

Batch 2−1295 : Gut microbiota abundance (genus Catenibacterium id.2153) on Probable carboxypeptidase X1
with a threshold of 1e−05
MR Test
Inverse variance weighted (fixed effects)
Inverse variance weighted (multiplicative random effects)
MR Egger
MR RAPS
Weighted median
Weighted mode
0.08
0.04
0.00
−0.04
0.09
0.12
0.15
0.18
SNP effect on Probable carboxypeptidase X1
SNP effect on Gut microbiota abundance (genus Catenibacterium id.2153)

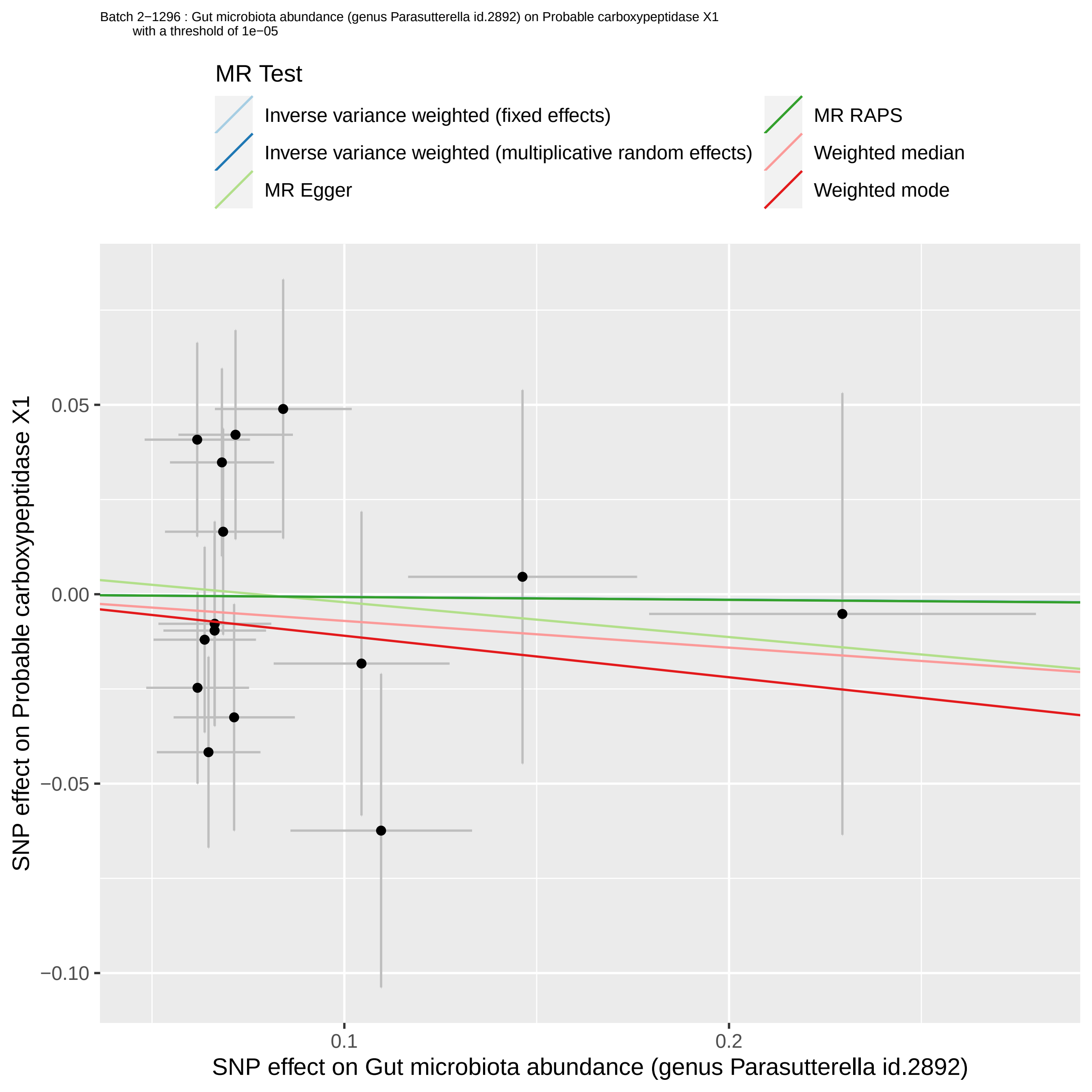
Batch 2−1296 : Gut microbiota abundance (genus Parasutterella id.2892) on Probable carboxypeptidase X1
with a threshold of 1e−05
MR Test
Inverse variance weighted (fixed effects)
Inverse variance weighted (multiplicative random effects)
MR Egger
MR RAPS
Weighted median
Weighted mode
SNP effect on Probable carboxypeptidase X1
0.05
0.00
−0.05
−0.10
0.1
0.2
SNP effect on Gut microbiota abundance (genus Parasutterella id.2892)

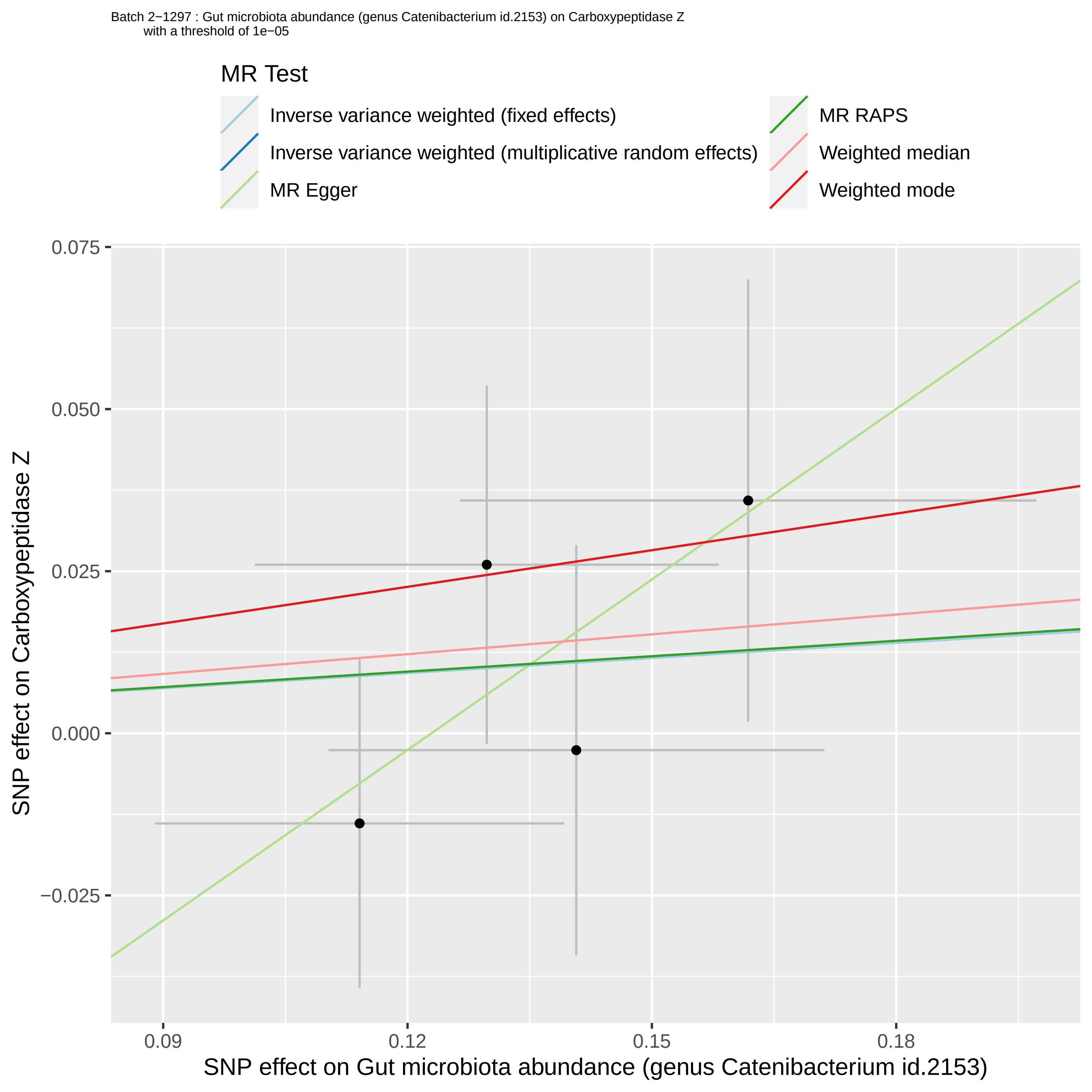
Batch 2−1297 : Gut microbiota abundance (genus Catenibacterium id.2153) on Carboxypeptidase Z
with a threshold of 1e−05
MR Test
Inverse variance weighted (fixed effects)
Inverse variance weighted (multiplicative random effects)
MR Egger
MR RAPS
Weighted median
Weighted mode
0.075
0.050
0.025
0.000
−0.025
0.09
0.12
0.15
0.18
SNP effect on Carboxypeptidase Z
SNP effect on Gut microbiota abundance (genus Catenibacterium id.2153)

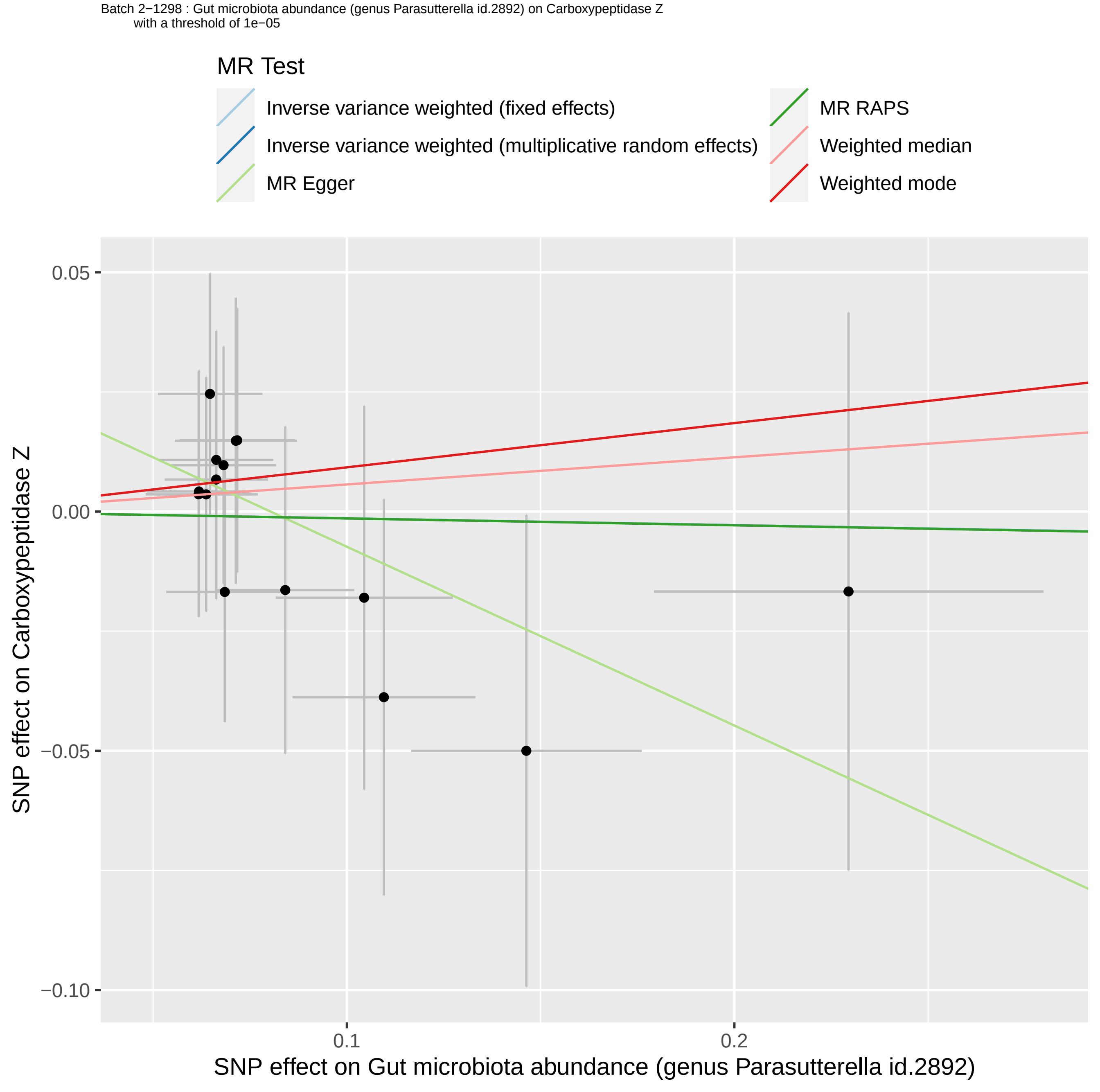

Batch 2−1298 : Gut microbiota abundance (genus Parasutterella id.2892) on Carboxypeptidase Z
with a threshold of 1e−05
MR Test
Inverse variance weighted (fixed effects)
Inverse variance weighted (multiplicative random effects)
MR Egger
MR RAPS
Weighted median
Weighted mode
0.05
0.00
−0.05
−0.10
0.1
0.2
SNP effect on Carboxypeptidase Z
SNP effect on Gut microbiota abundance (genus Parasutterella id.2892)

Batch 2−1299 : Gut microbiota abundance (genus Catenibacterium id.2153) on Cellular retinoic acid−binding protein 1 with a threshold of 1e−05

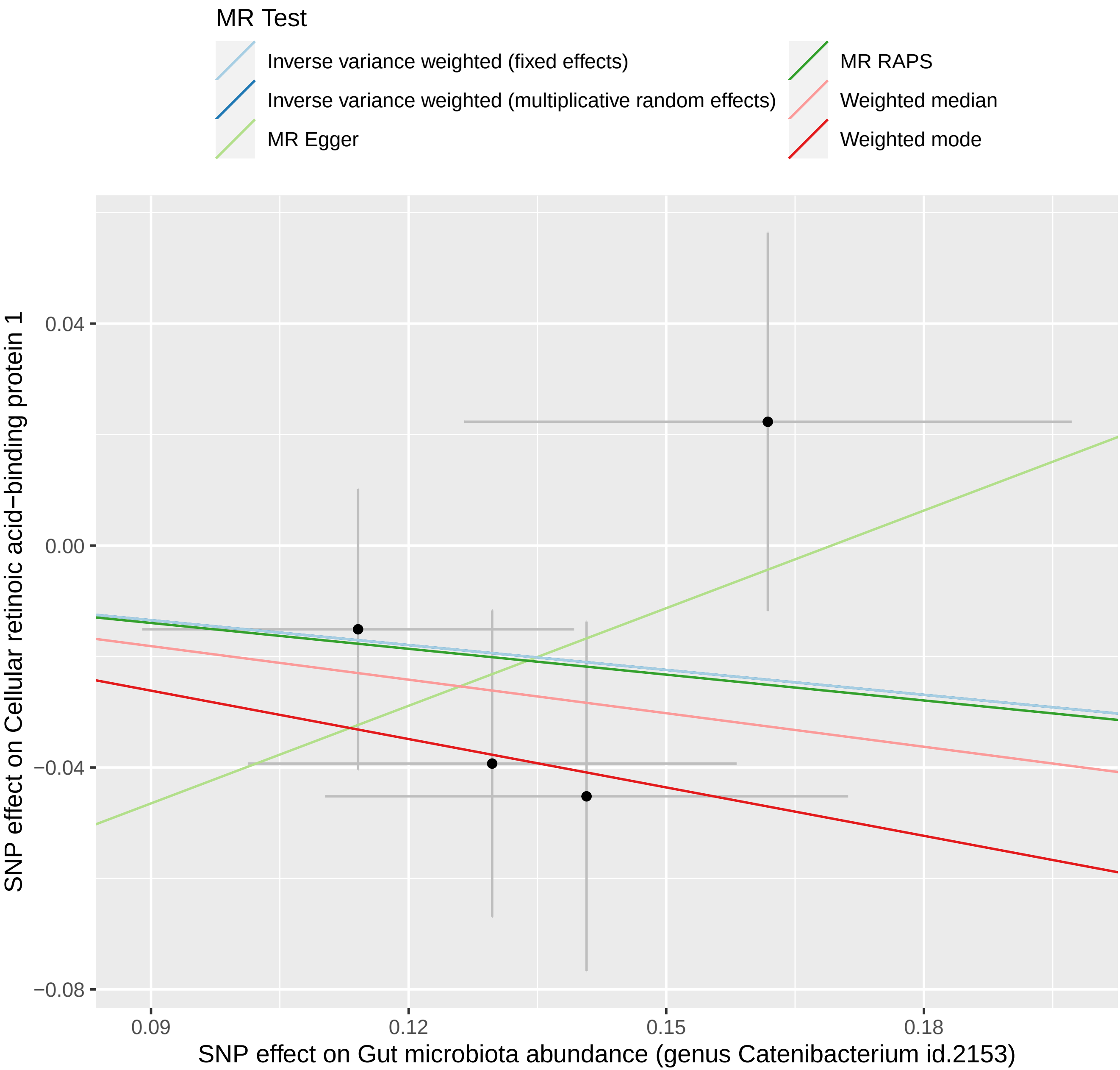

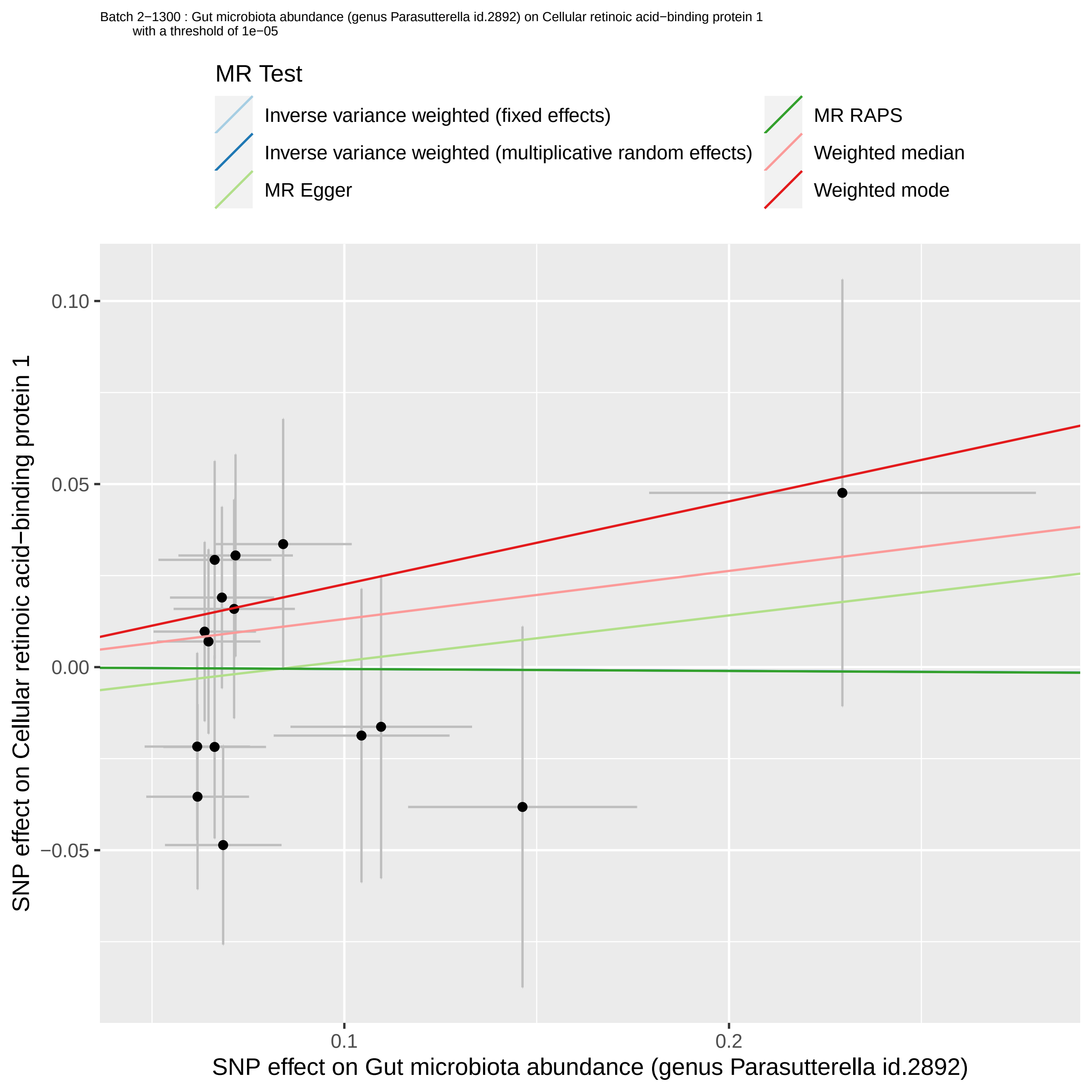
Batch 2−1300 : Gut microbiota abundance (genus Parasutterella id.2892) on Cellular retinoic acid−binding protein 1
with a threshold of 1e−05
MR Test
Inverse variance weighted (fixed effects)
Inverse variance weighted (multiplicative random effects)
MR Egger
MR RAPS
Weighted median
Weighted mode
0.10
0.05
0.00
−0.05
0.1
0.2
SNP effect on Cellular retinoic acid−binding protein 1
SNP effect on Gut microbiota abundance (genus Parasutterella id.2892)

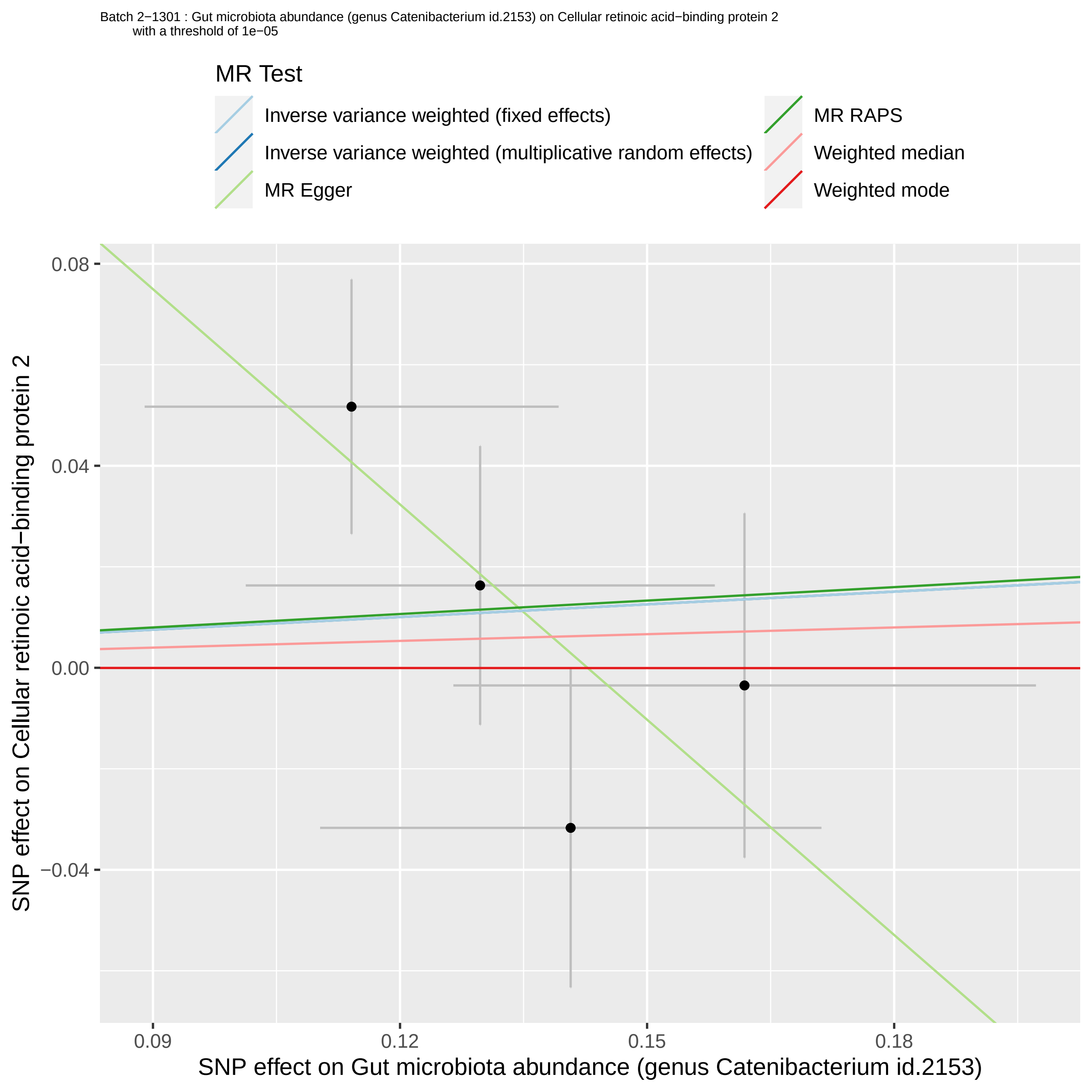

Batch 2−1301 : Gut microbiota abundance (genus Catenibacterium id.2153) on Cellular retinoic acid−binding protein 2
with a threshold of 1e−05
MR Test
Inverse variance weighted (fixed effects)
Inverse variance weighted (multiplicative random effects)
MR Egger
MR RAPS
Weighted median
Weighted mode
0.08
0.04
0.00
−0.04
0.09
0.12
0.15
0.18
SNP effect on Cellular retinoic acid−binding protein 2
SNP effect on Gut microbiota abundance (genus Catenibacterium id.2153)

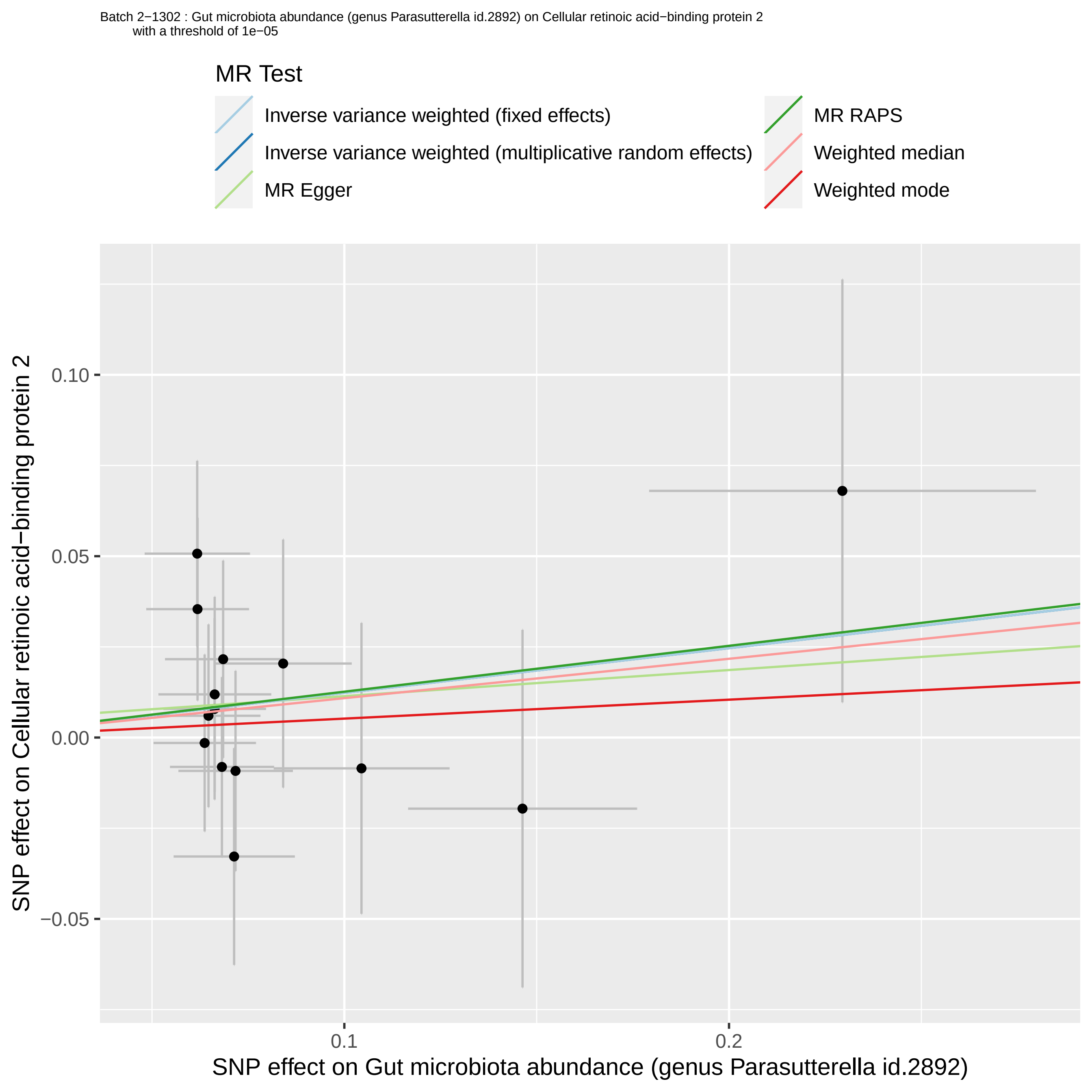
Batch 2−1302 : Gut microbiota abundance (genus Parasutterella id.2892) on Cellular retinoic acid−binding protein 2
with a threshold of 1e−05
MR Test
Inverse variance weighted (fixed effects)
Inverse variance weighted (multiplicative random effects)
MR Egger
MR RAPS
Weighted median
Weighted mode
SNP effect on Cellular retinoic acid−binding protein 2
0.10
0.05
0.00
−0.05
0.1
0.2
SNP effect on Gut microbiota abundance (genus Parasutterella id.2892)

Batch 2−1303 : Gut microbiota abundance (genus Catenibacterium id.2153) on Protein crumbs homolog 1
with a threshold of 1e−05

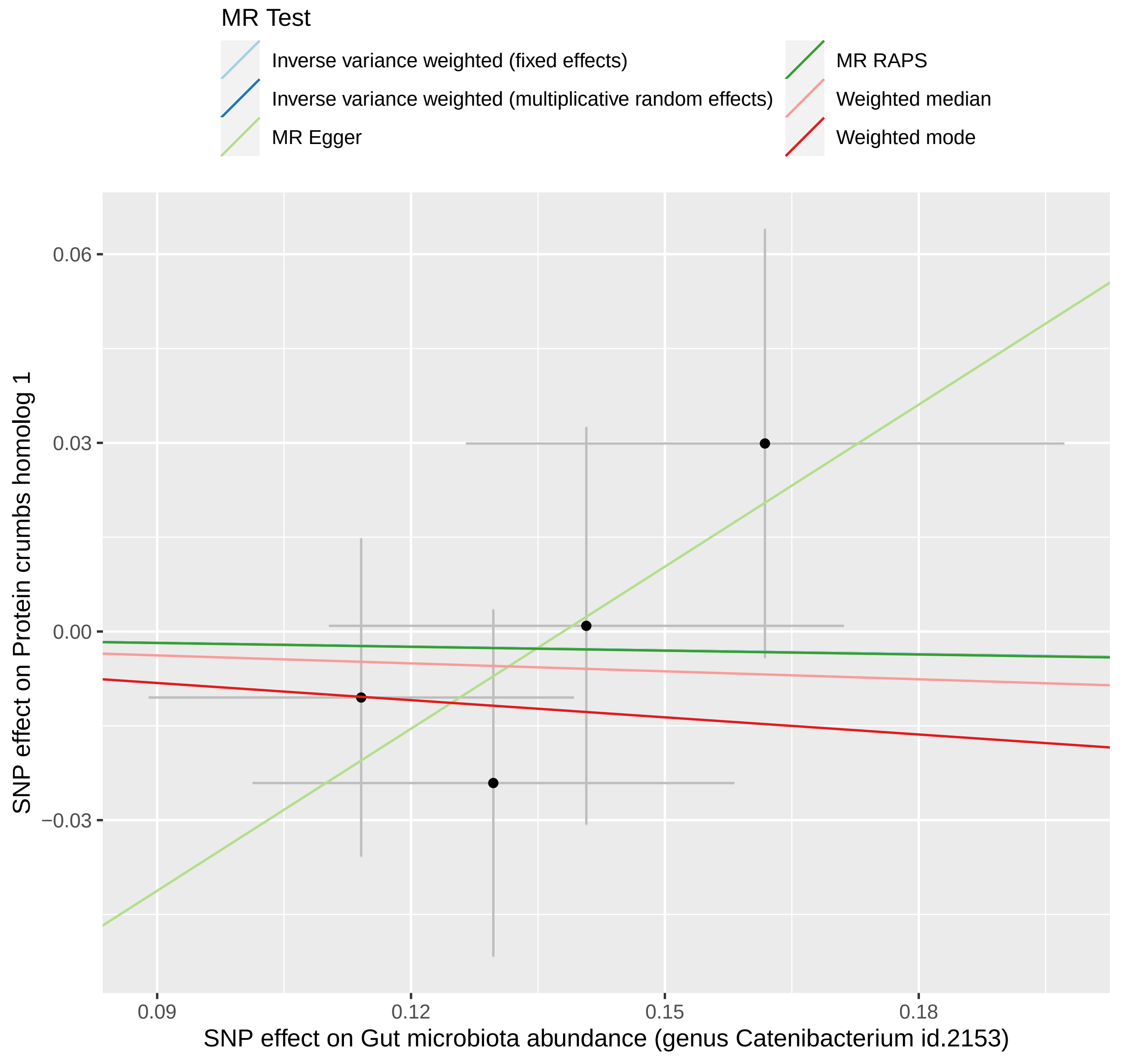

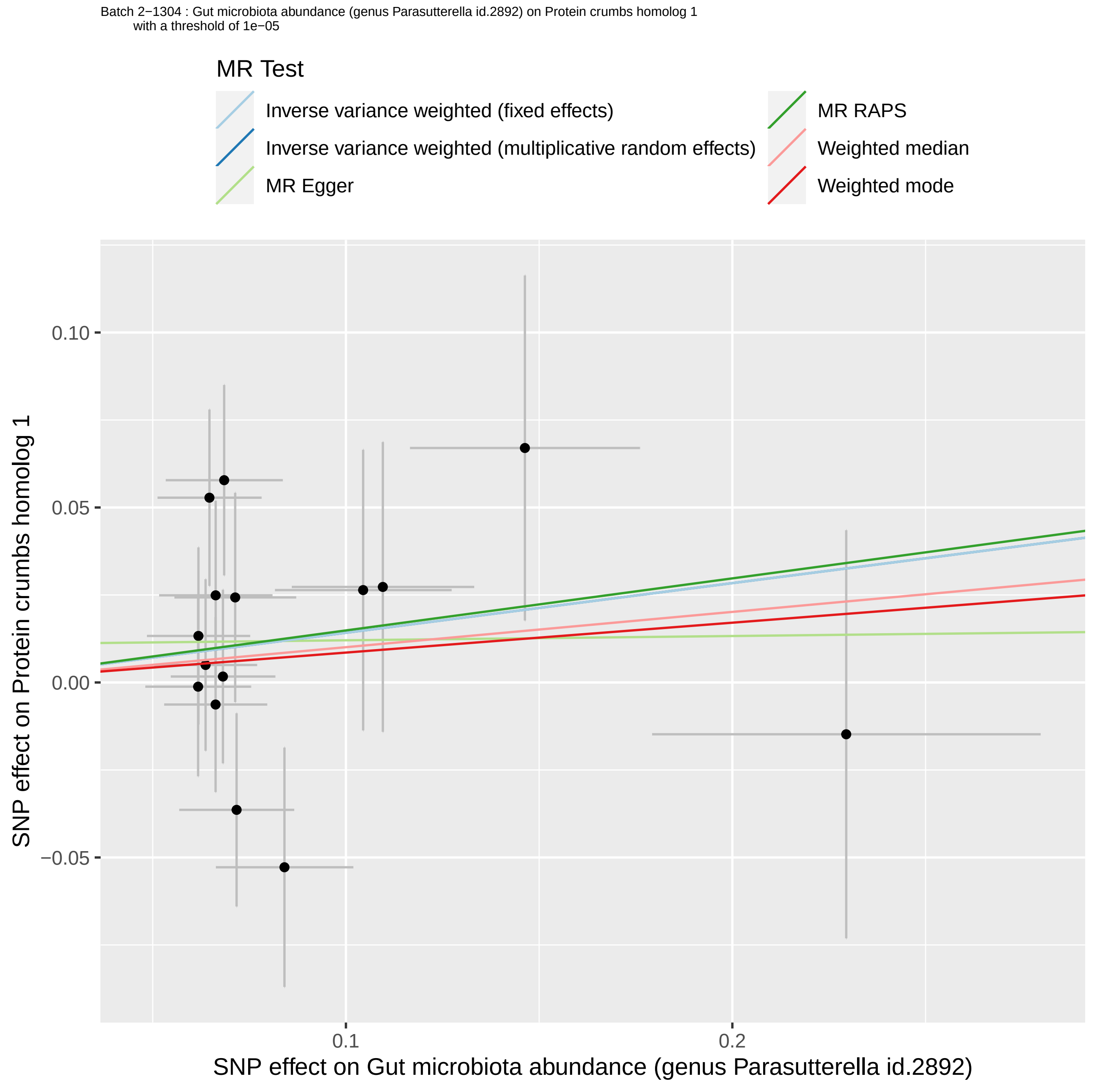
Batch 2−1304 : Gut microbiota abundance (genus Parasutterella id.2892) on Protein crumbs homolog 1
with a threshold of 1e−05
MR Test
Inverse variance weighted (fixed effects)
Inverse variance weighted (multiplicative random effects)
MR Egger
MR RAPS
Weighted median
Weighted mode
0.10
0.05
0.00
−0.05
0.1
0.2
SNP effect on Protein crumbs homolog 1
SNP effect on Gut microbiota abundance (genus Parasutterella id.2892)

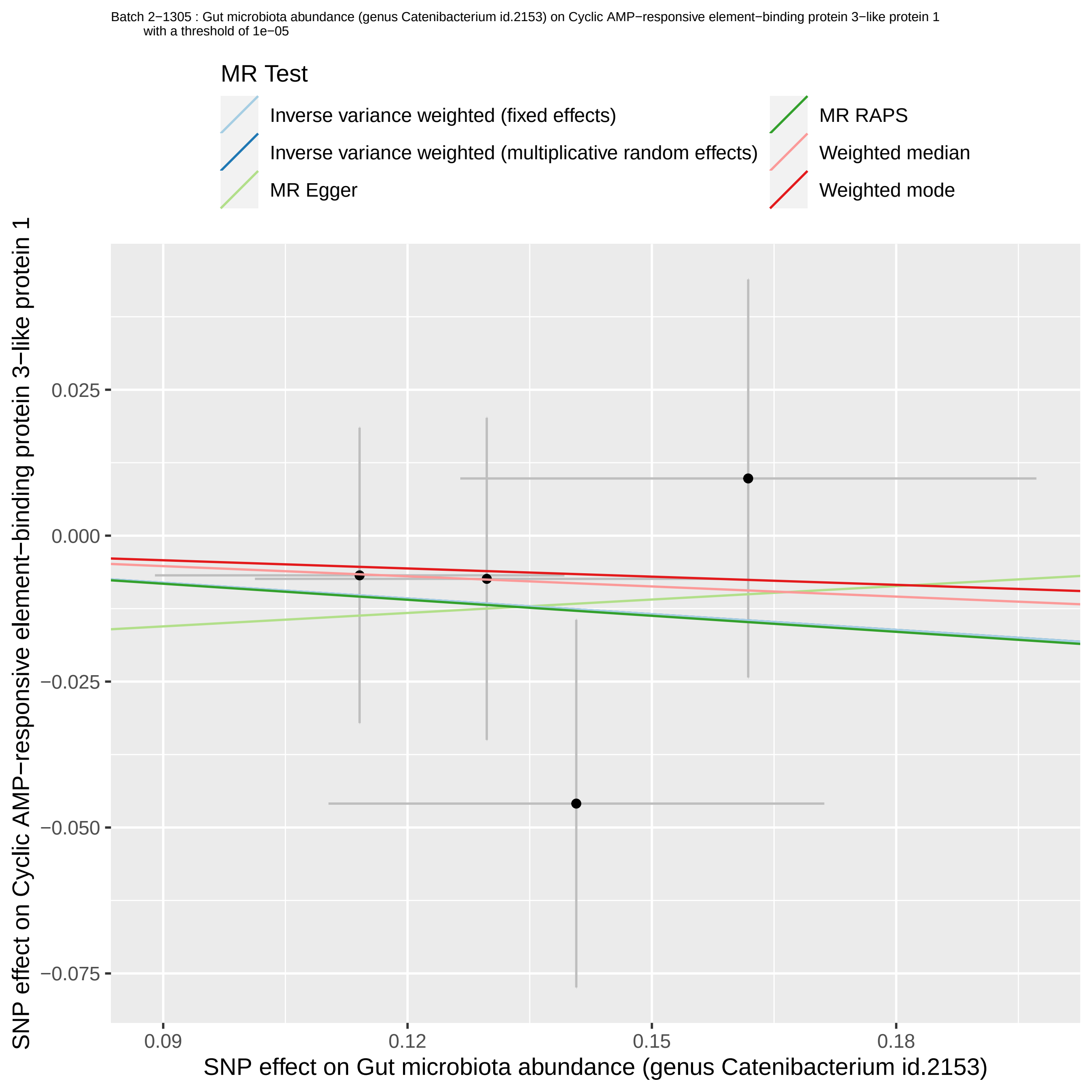
Batch 2−1305 : Gut microbiota abundance (genus Catenibacterium id.2153) on Cyclic AMP−responsive element−binding protein 3−like protein 1
with a threshold of 1e−05
MR Test
Inverse variance weighted (fixed effects)
Inverse variance weighted (multiplicative random effects)
MR Egger
MR RAPS
Weighted median
Weighted mode
0.025
0.000
−0.025
−0.050
−0.075
0.09
0.12
0.15
0.18
SNP effect on Gut microbiota abundance (genus Catenibacterium id.2153)
SNP effect on Cyclic AMP−responsive element−binding protein 3−like protein 1

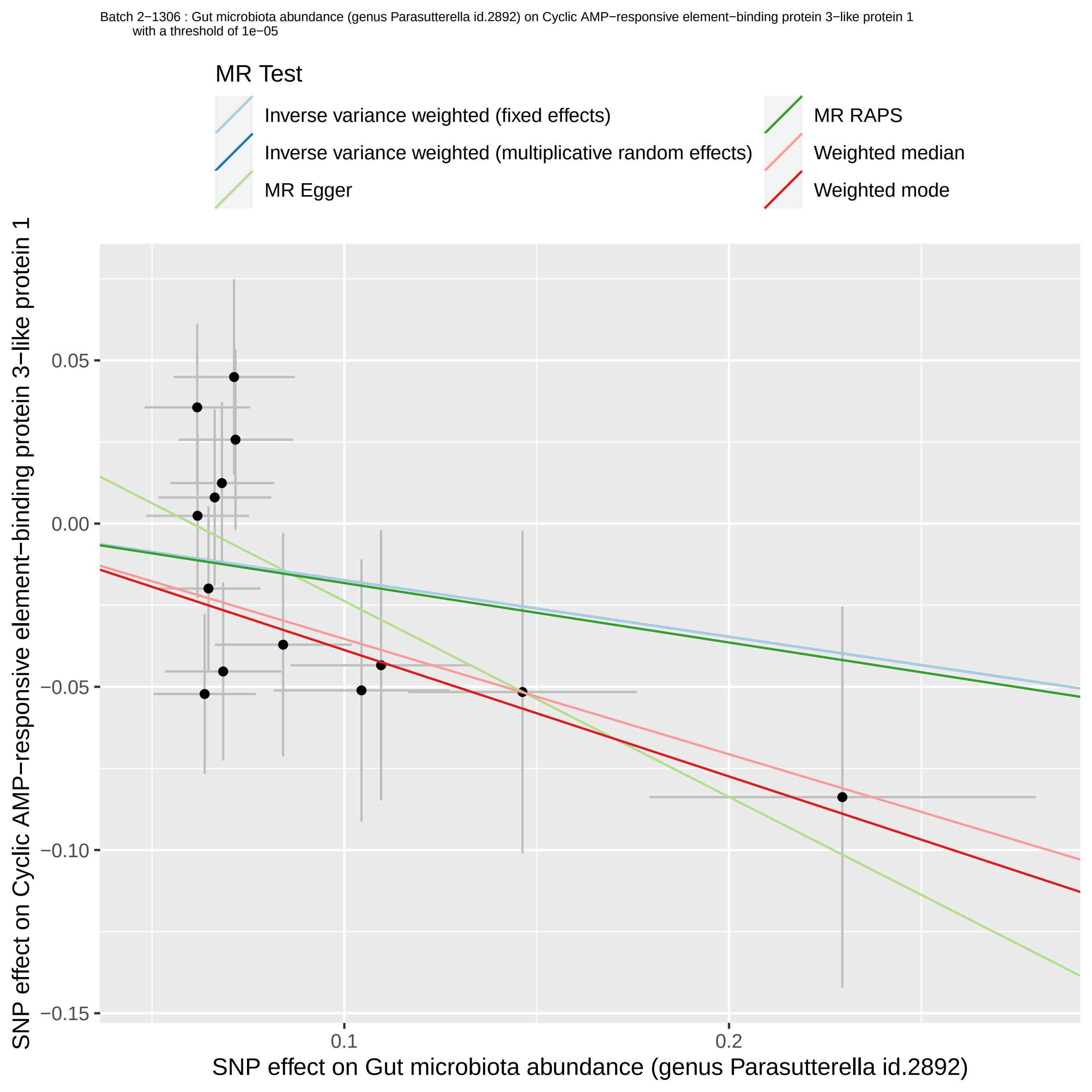
Batch 2−1306 : Gut microbiota abundance (genus Parasutterella id.2892) on Cyclic AMP−responsive element−binding protein 3−like protein 1
with a threshold of 1e−05
MR Test
Inverse variance weighted (fixed effects)
Inverse variance weighted (multiplicative random effects)
MR Egger
MR RAPS
Weighted median
Weighted mode
0.05
0.00
−0.05
−0.10
−0.15
0.1
0.2
SNP effect on Cyclic AMP−responsive element−binding protein 3−like protein 1
SNP effect on Gut microbiota abundance (genus Parasutterella id.2892)

Batch 2−1307 : Gut microbiota abundance (genus Catenibacterium id.2153) on Cyclic AMP−responsive element−binding protein 3−like protein 4 with a threshold of 1e−05

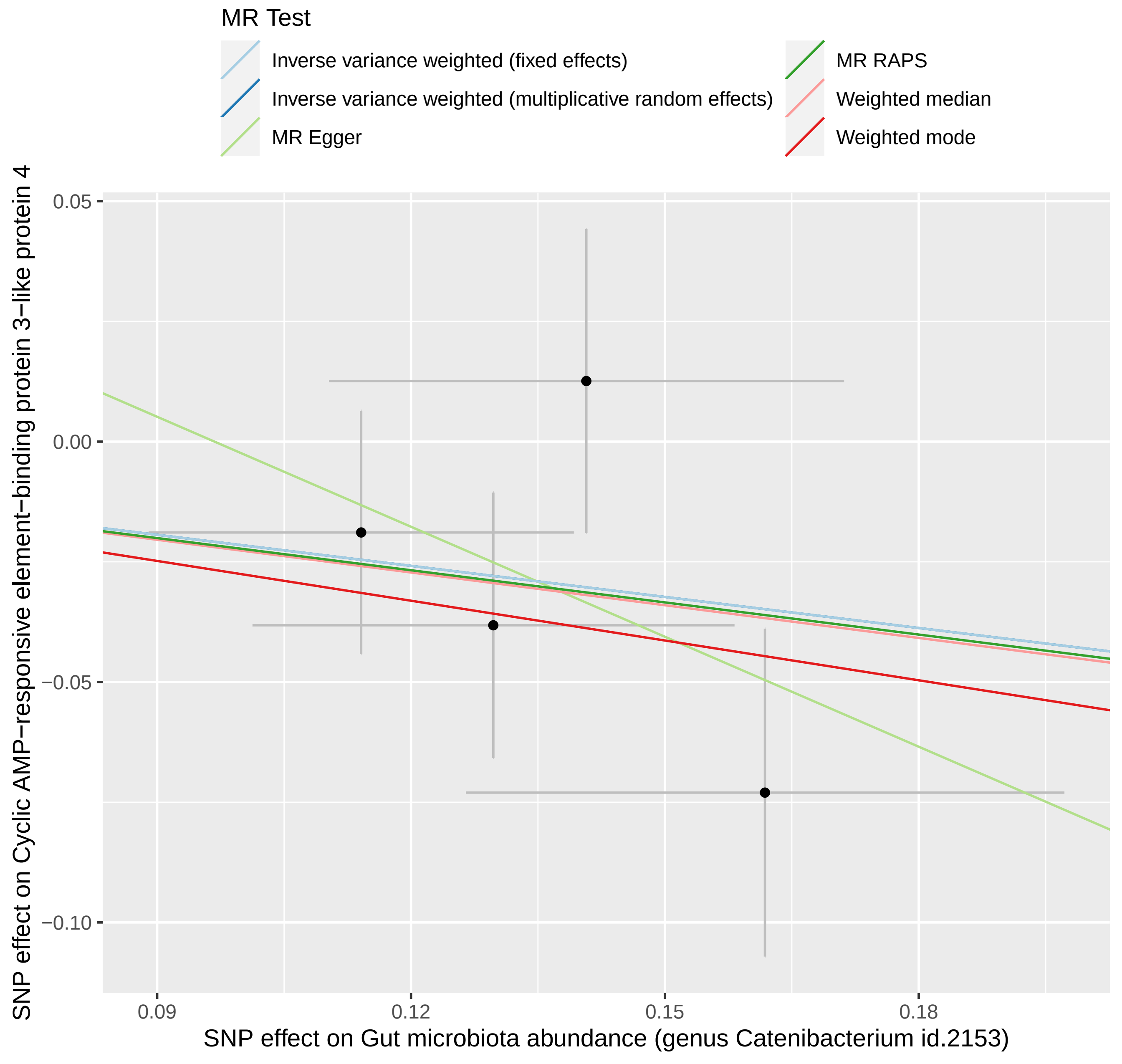

Batch 2−1308 : Gut microbiota abundance (genus Parasutterella id.2892) on Cyclic AMP−responsive element−binding protein 3−like protein 4 with a threshold of 1e−05

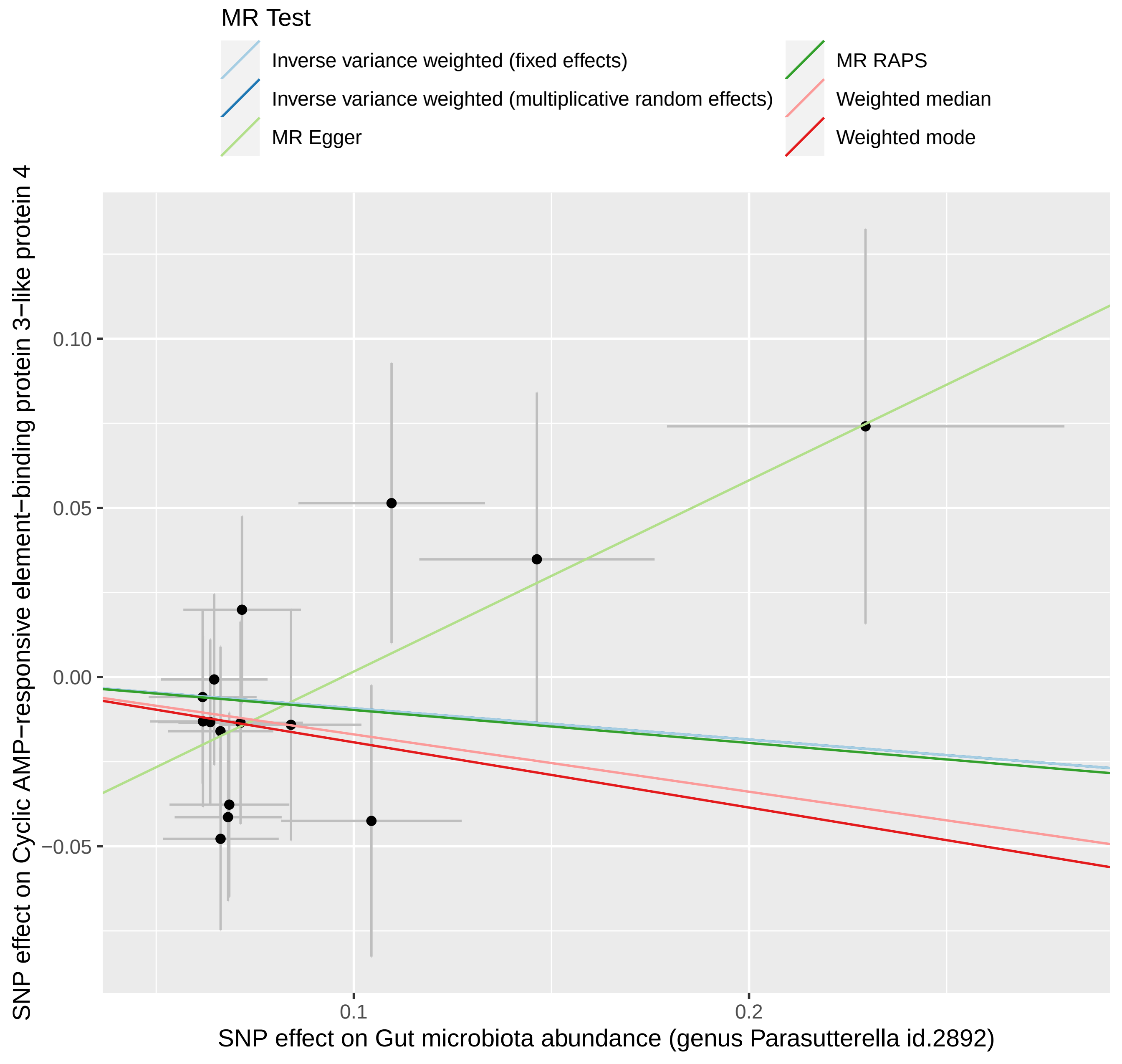

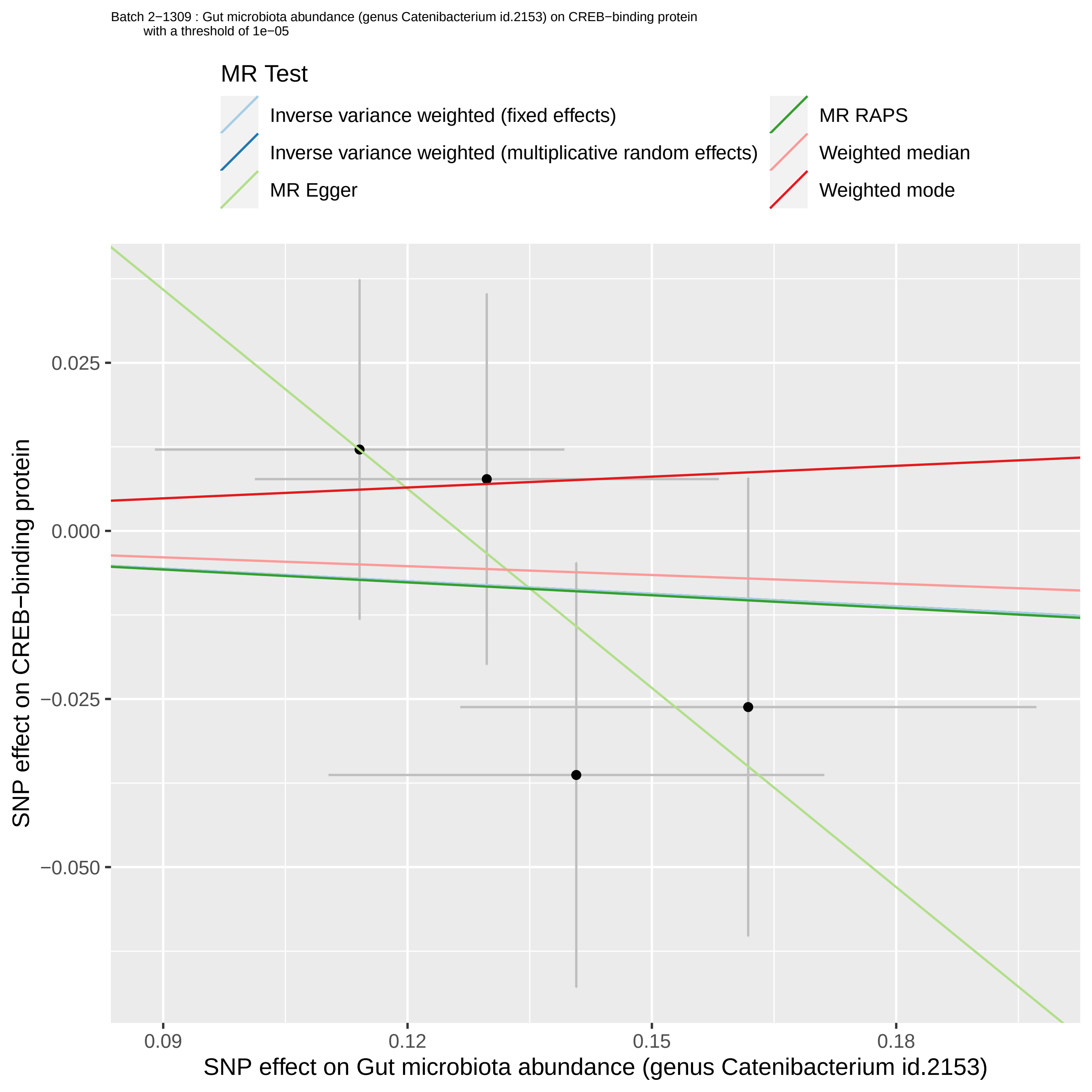
Batch 2−1309 : Gut microbiota abundance (genus Catenibacterium id.2153) on CREB−binding protein
with a threshold of 1e−05
MR Test
Inverse variance weighted (fixed effects)
Inverse variance weighted (multiplicative random effects)
MR Egger
MR RAPS
Weighted median
Weighted mode
0.025
0.000
−0.025
−0.050
0.09
0.12
0.15
0.18
SNP effect on CREB−binding protein
SNP effect on Gut microbiota abundance (genus Catenibacterium id.2153)

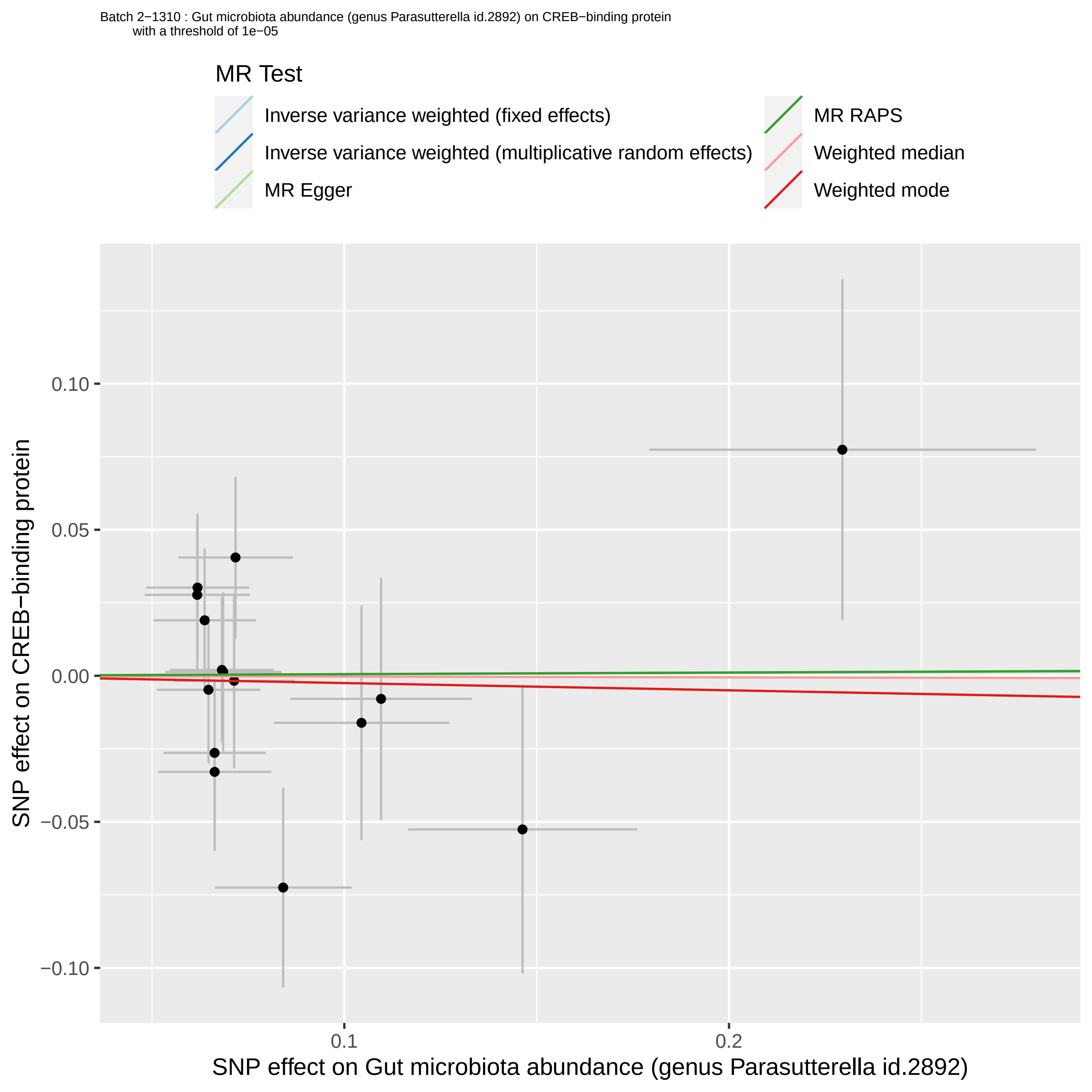

Batch 2−1310 : Gut microbiota abundance (genus Parasutterella id.2892) on CREB−binding protein
with a threshold of 1e−05
MR Test
Inverse variance weighted (fixed effects)
Inverse variance weighted (multiplicative random effects)
MR Egger
MR RAPS
Weighted median
Weighted mode
0.10
0.05
0.00
−0.05
−0.10
0.1
0.2
SNP effect on CREB−binding protein
SNP effect on Gut microbiota abundance (genus Parasutterella id.2892)

Batch 2−1311 : Gut microbiota abundance (genus Catenibacterium id.2153) on Protein CREG1
with a threshold of 1e−05

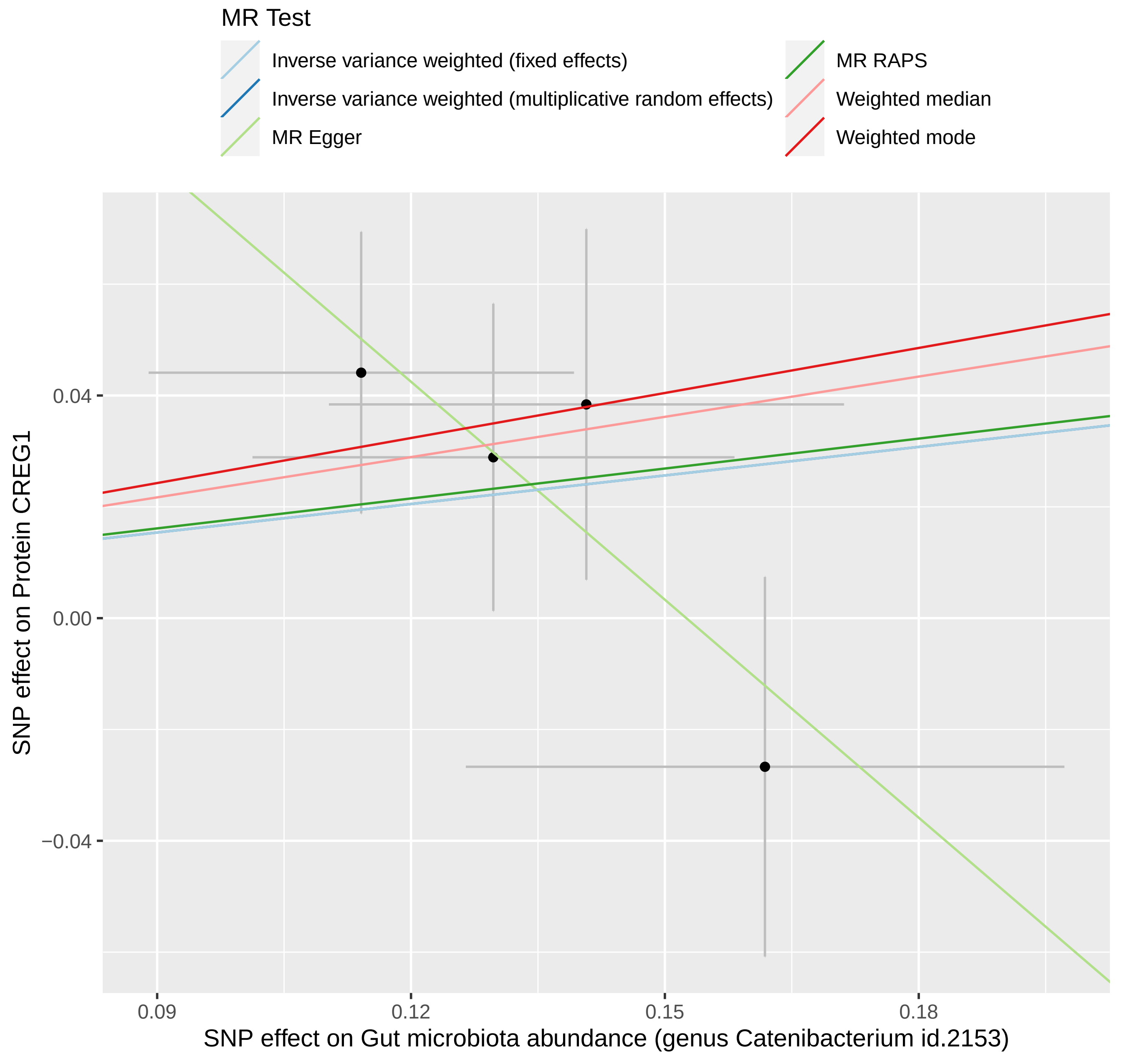

Batch 2−1312 : Gut microbiota abundance (genus Parasutterella id.2892) on Protein CREG1
with a threshold of 1e−05

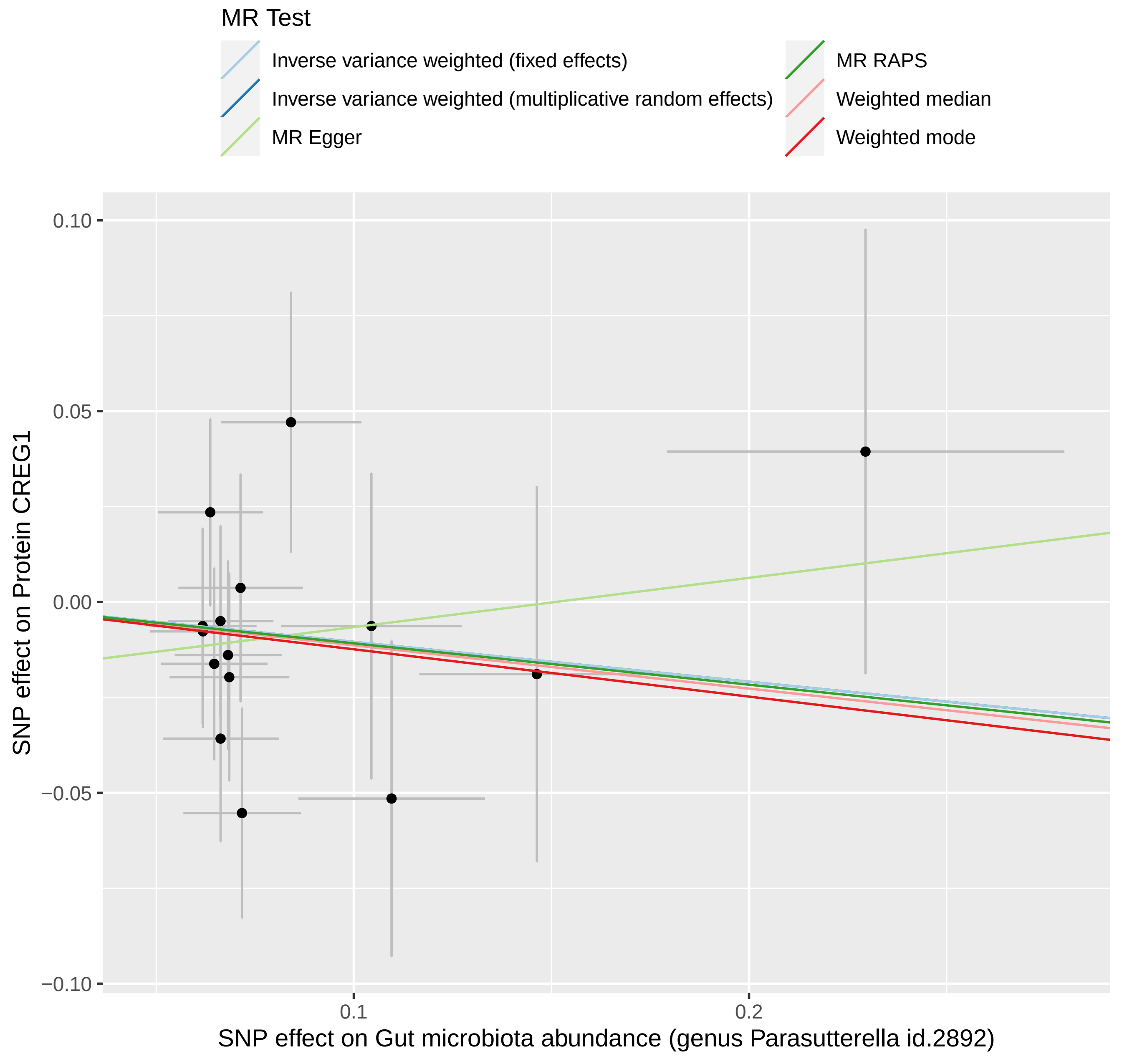

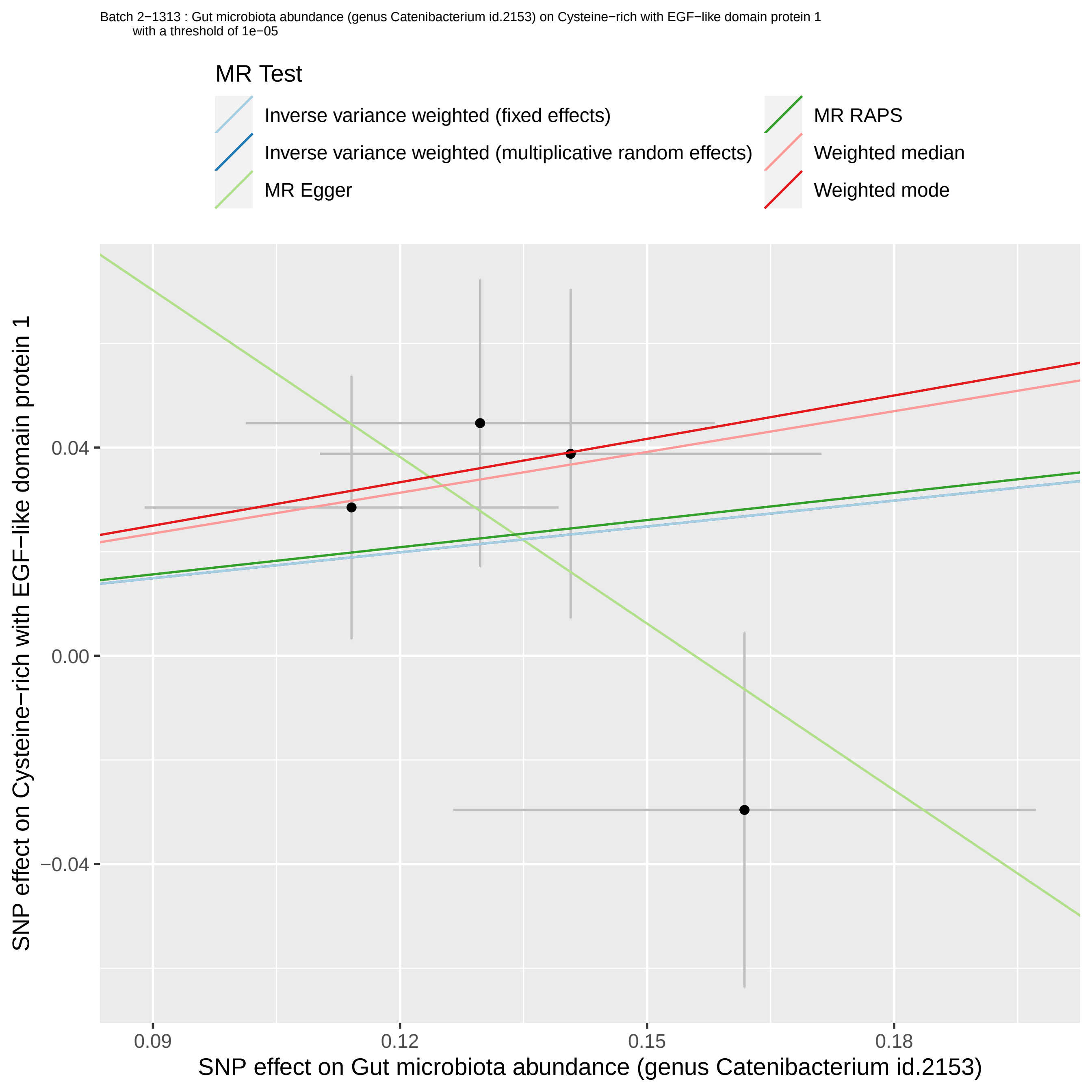
Batch 2−1313 : Gut microbiota abundance (genus Catenibacterium id.2153) on Cysteine−rich with EGF−like domain protein 1
with a threshold of 1e−05
MR Test
Inverse variance weighted (fixed effects)
Inverse variance weighted (multiplicative random effects)
MR Egger
MR RAPS
Weighted median
Weighted mode
0.04
0.00
−0.04
0.09
0.12
0.15
0.18
SNP effect on Cysteine−rich with EGF−like domain protein 1
SNP effect on Gut microbiota abundance (genus Catenibacterium id.2153)

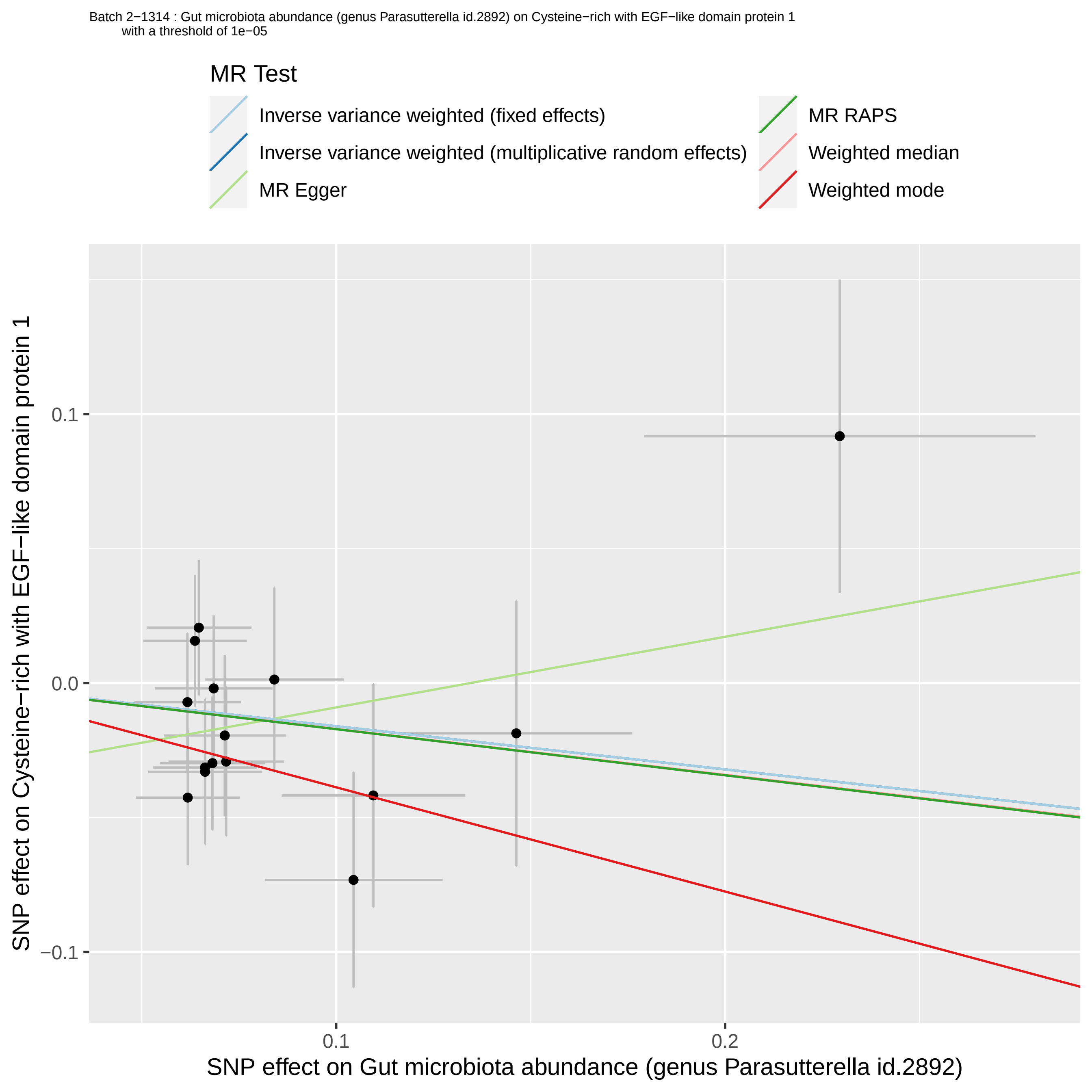

Batch 2−1314 : Gut microbiota abundance (genus Parasutterella id.2892) on Cysteine−rich with EGF−like domain protein 1
with a threshold of 1e−05
MR Test
Inverse variance weighted (fixed effects)
Inverse variance weighted (multiplicative random effects)
MR Egger
MR RAPS
Weighted median
Weighted mode
SNP effect on Cysteine−rich with EGF−like domain protein 1
0.1
0.0
−0.1
0.1
0.2
SNP effect on Gut microbiota abundance (genus Parasutterella id.2892)

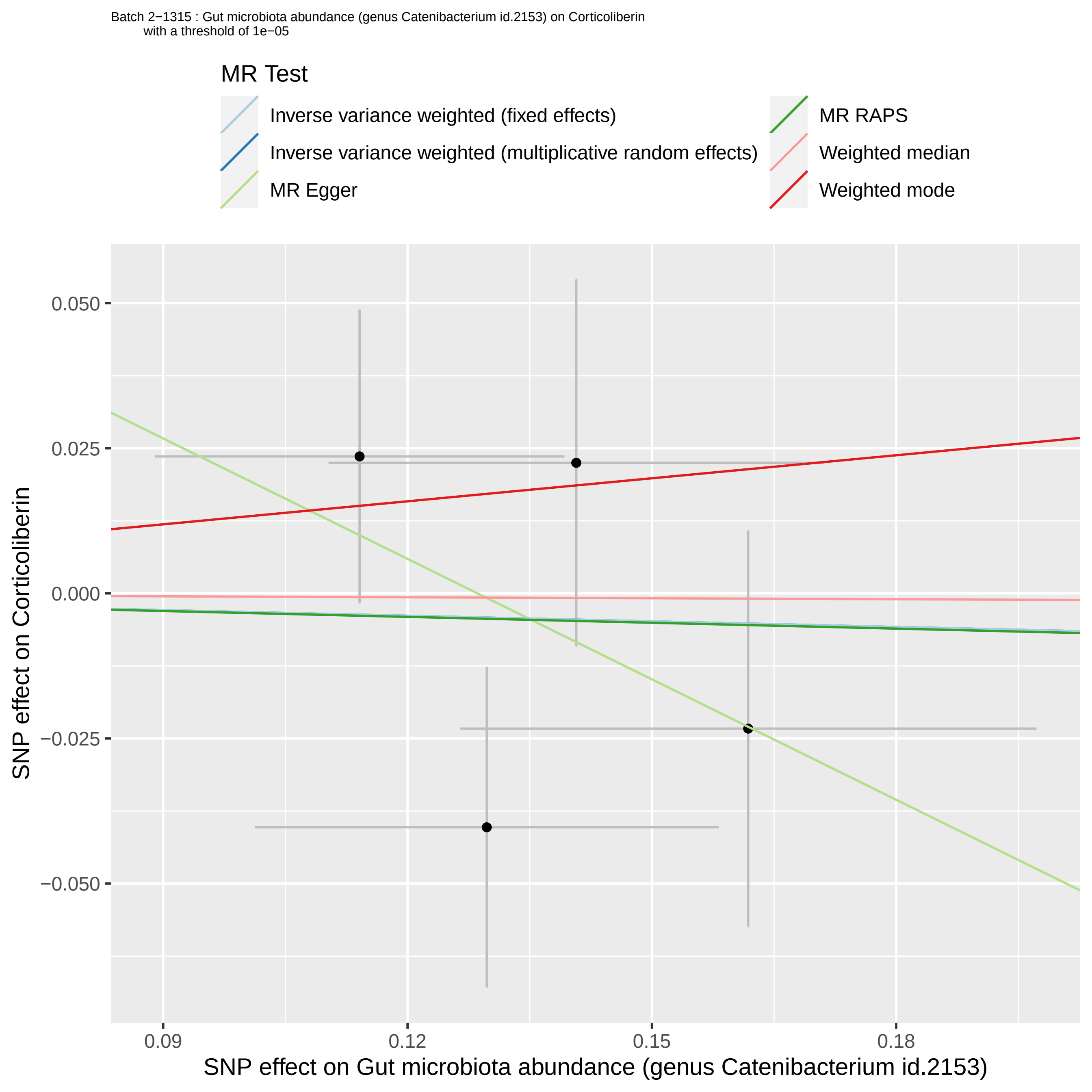

Batch 2−1315 : Gut microbiota abundance (genus Catenibacterium id.2153) on Corticoliberin
with a threshold of 1e−05
MR Test
Inverse variance weighted (fixed effects)
Inverse variance weighted (multiplicative random effects)
MR Egger
MR RAPS
Weighted median
Weighted mode
0.050
0.025
0.000
−0.025
−0.050
0.09
0.12
0.15
0.18
SNP effect on Corticoliberin
SNP effect on Gut microbiota abundance (genus Catenibacterium id.2153)

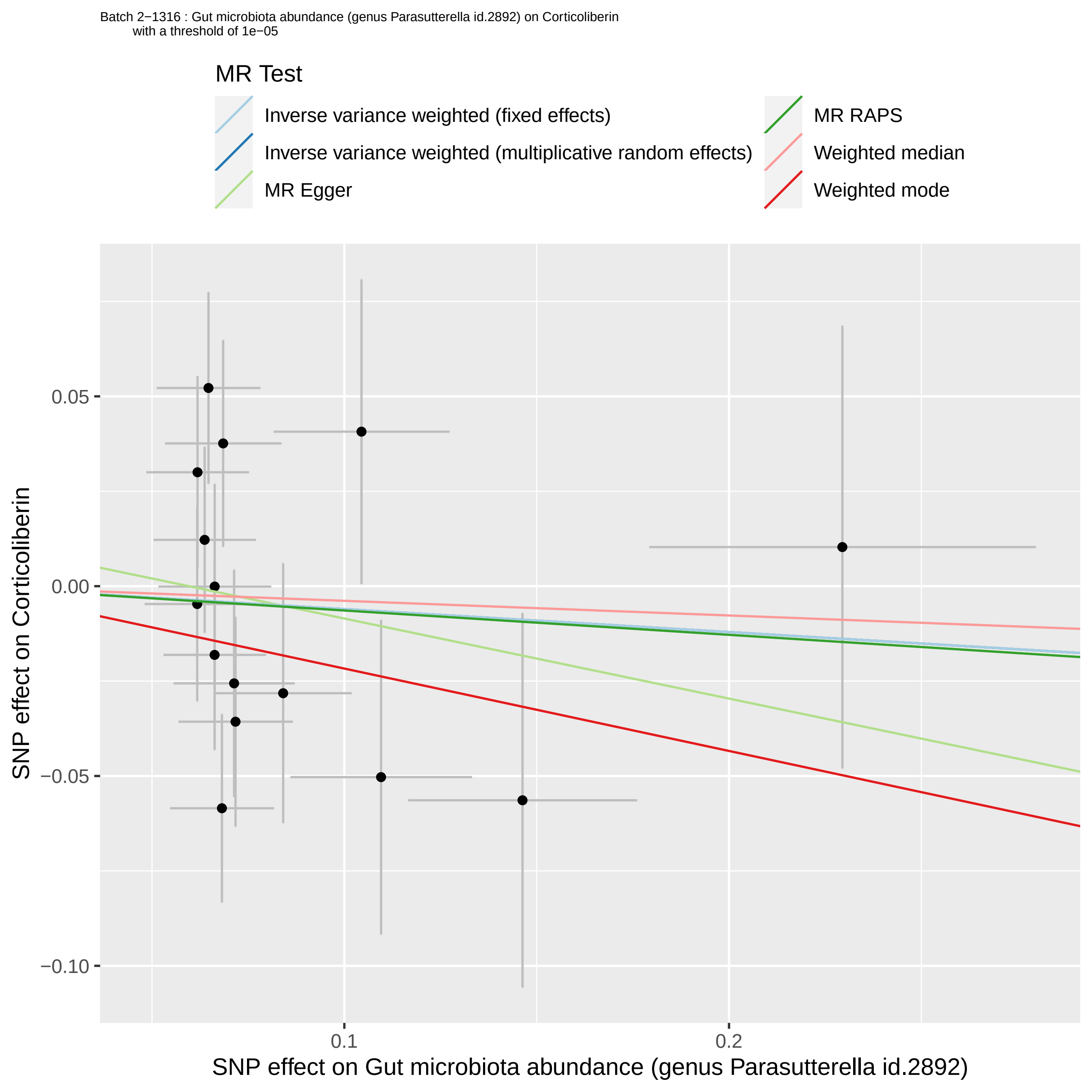
Batch 2−1316 : Gut microbiota abundance (genus Parasutterella id.2892) on Corticoliberin
with a threshold of 1e−05
MR Test
Inverse variance weighted (fixed effects)
Inverse variance weighted (multiplicative random effects)
MR Egger
MR RAPS
Weighted median
Weighted mode
0.05
0.00
−0.05
−0.10
0.1
0.2
SNP effect on Corticoliberin
SNP effect on Gut microbiota abundance (genus Parasutterella id.2892)

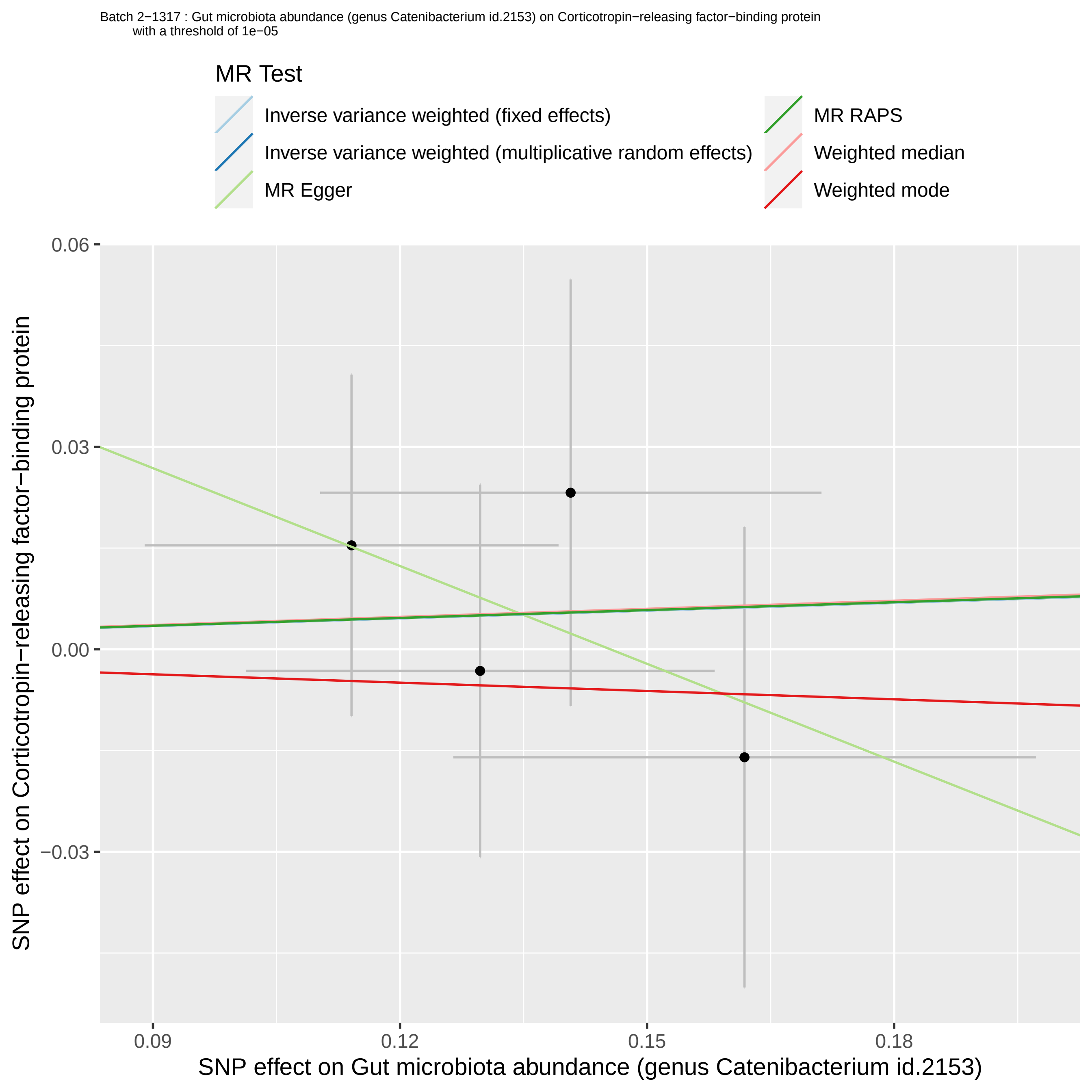

Batch 2−1317 : Gut microbiota abundance (genus Catenibacterium id.2153) on Corticotropin−releasing factor−binding protein
with a threshold of 1e−05
MR Test
Inverse variance weighted (fixed effects)
Inverse variance weighted (multiplicative random effects)
MR Egger
MR RAPS
Weighted median
Weighted mode
0.06
0.03
0.00
−0.03
0.09
0.12
0.15
0.18
SNP effect on Corticotropin−releasing factor−binding protein
SNP effect on Gut microbiota abundance (genus Catenibacterium id.2153)

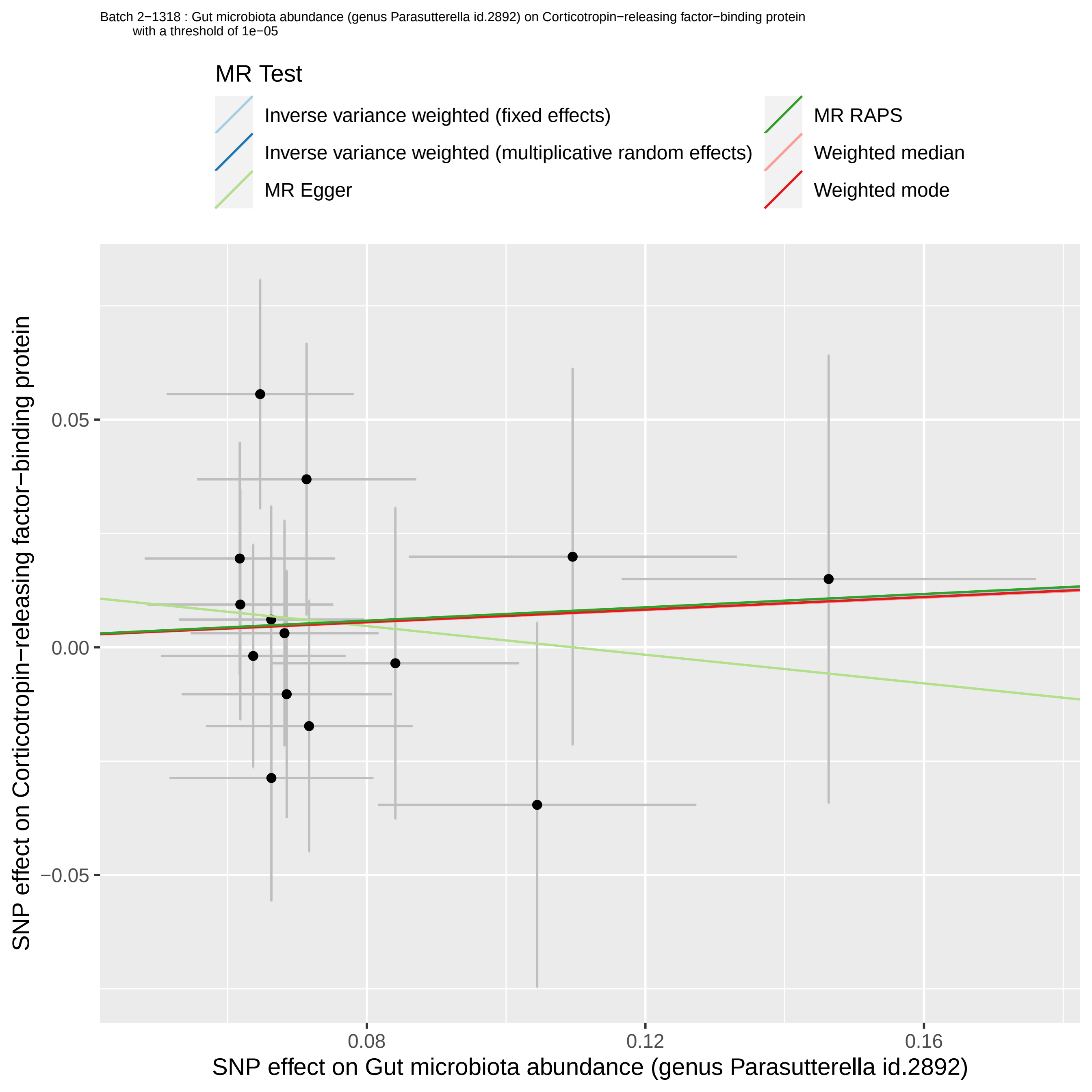

Batch 2−1318 : Gut microbiota abundance (genus Parasutterella id.2892) on Corticotropin−releasing factor−binding protein
with a threshold of 1e−05
MR Test
Inverse variance weighted (fixed effects)
Inverse variance weighted (multiplicative random effects)
MR Egger
MR RAPS
Weighted median
Weighted mode
0.05
0.00
−0.05
0.08
0.12
0.16
SNP effect on Corticotropin−releasing factor−binding protein
SNP effect on Gut microbiota abundance (genus Parasutterella id.2892)

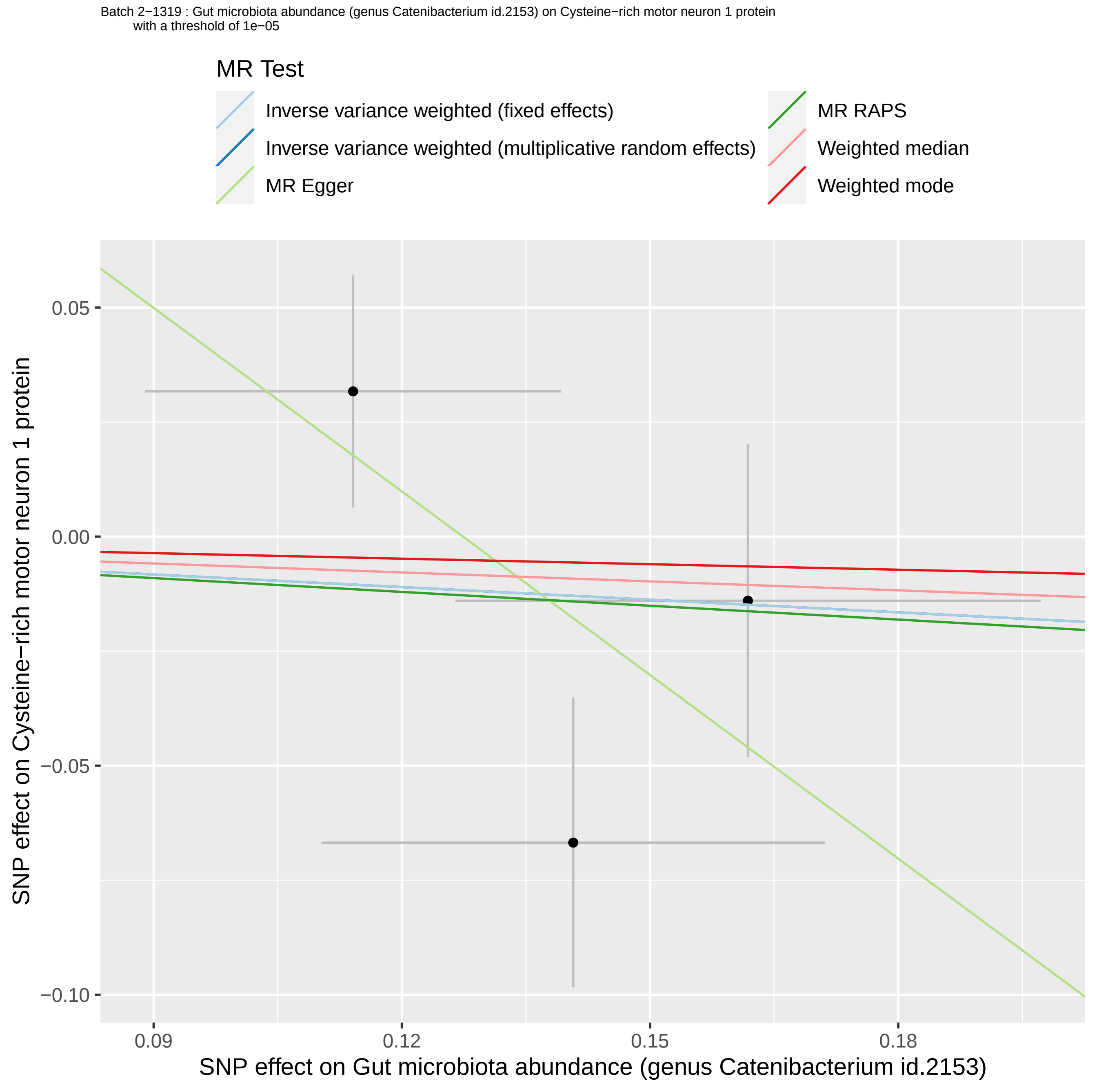
Batch 2−1319 : Gut microbiota abundance (genus Catenibacterium id.2153) on Cysteine−rich motor neuron 1 protein
with a threshold of 1e−05
MR Test
Inverse variance weighted (fixed effects)
Inverse variance weighted (multiplicative random effects)
MR Egger
MR RAPS
Weighted median
Weighted mode
0.05
0.00
−0.05
−0.10
0.09
0.12
0.15
0.18
SNP effect on Cysteine−rich motor neuron 1 protein
SNP effect on Gut microbiota abundance (genus Catenibacterium id.2153)

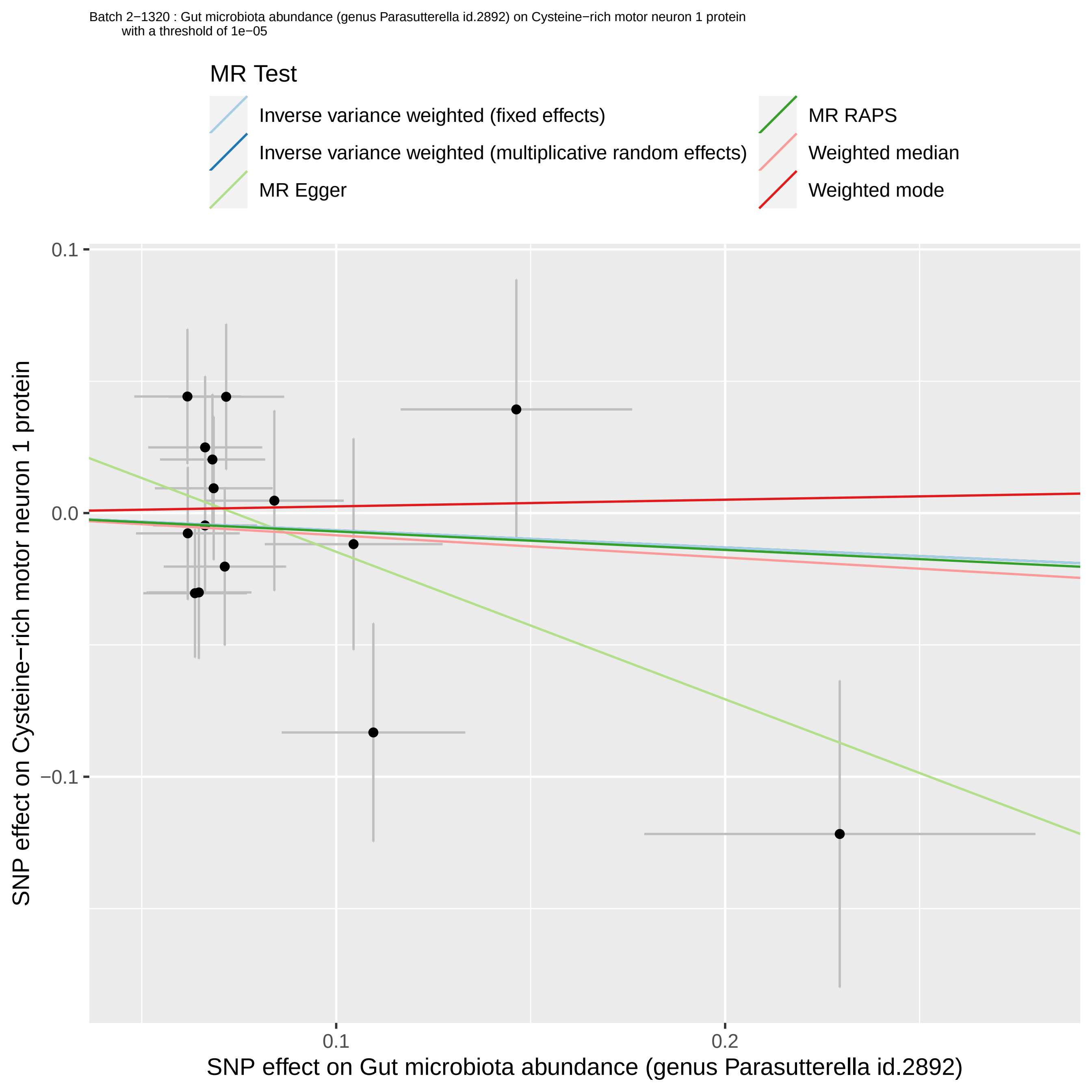

Batch 2−1320 : Gut microbiota abundance (genus Parasutterella id.2892) on Cysteine−rich motor neuron 1 protein
with a threshold of 1e−05
MR Test
Inverse variance weighted (fixed effects)
Inverse variance weighted (multiplicative random effects)
MR Egger
MR RAPS
Weighted median
Weighted mode
0.1
0.0
−0.1
0.1
0.2
SNP effect on Cysteine−rich motor neuron 1 protein
SNP effect on Gut microbiota abundance (genus Parasutterella id.2892)

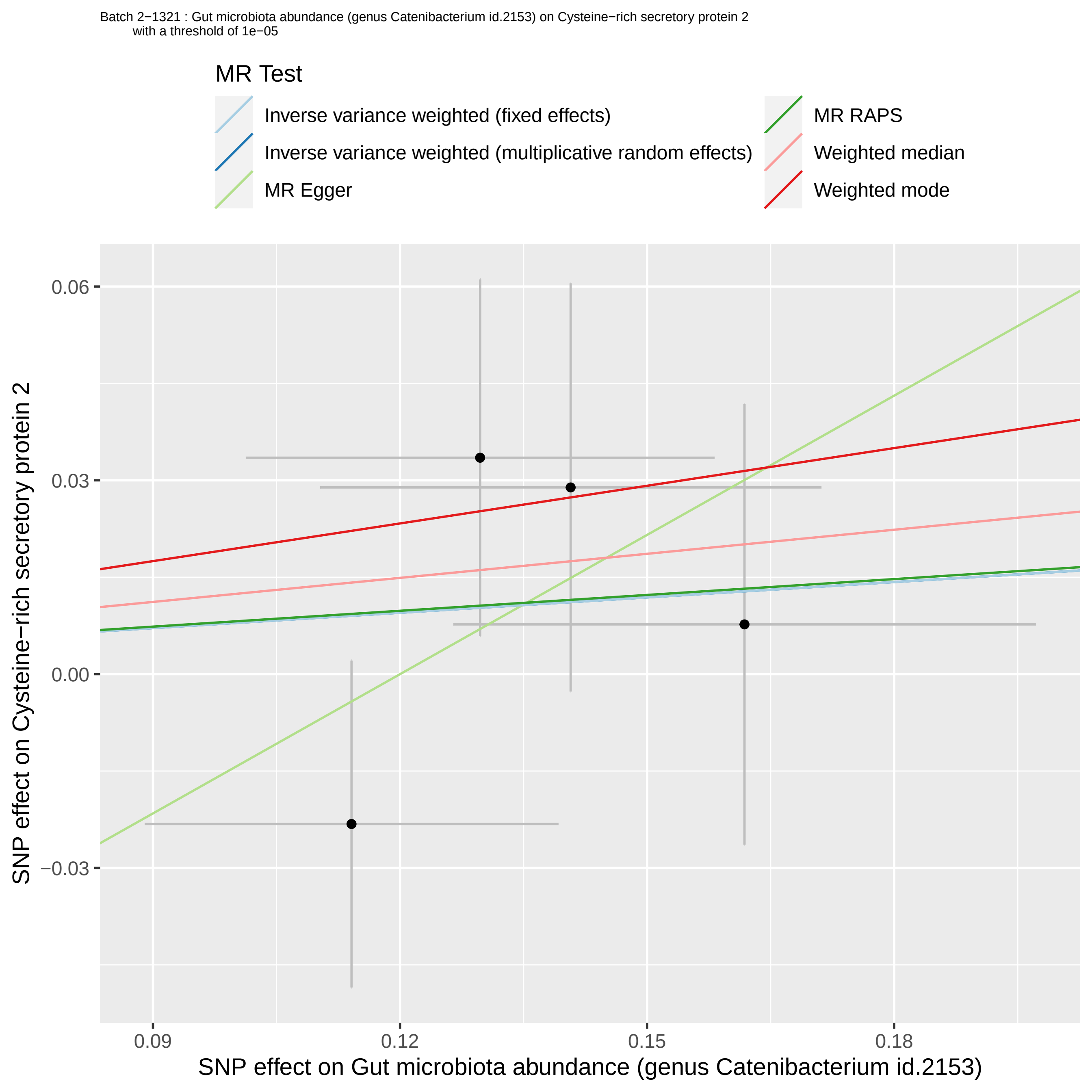

Batch 2−1321 : Gut microbiota abundance (genus Catenibacterium id.2153) on Cysteine−rich secretory protein 2
with a threshold of 1e−05
MR Test
Inverse variance weighted (fixed effects)
Inverse variance weighted (multiplicative random effects)
MR Egger
MR RAPS
Weighted median
Weighted mode
0.06
0.03
0.00
−0.03
0.09
0.12
0.15
0.18
SNP effect on Cysteine−rich secretory protein 2
SNP effect on Gut microbiota abundance (genus Catenibacterium id.2153)

Batch 2−1322 : Gut microbiota abundance (genus Parasutterella id.2892) on Cysteine−rich secretory protein 2
with a threshold of 1e−05

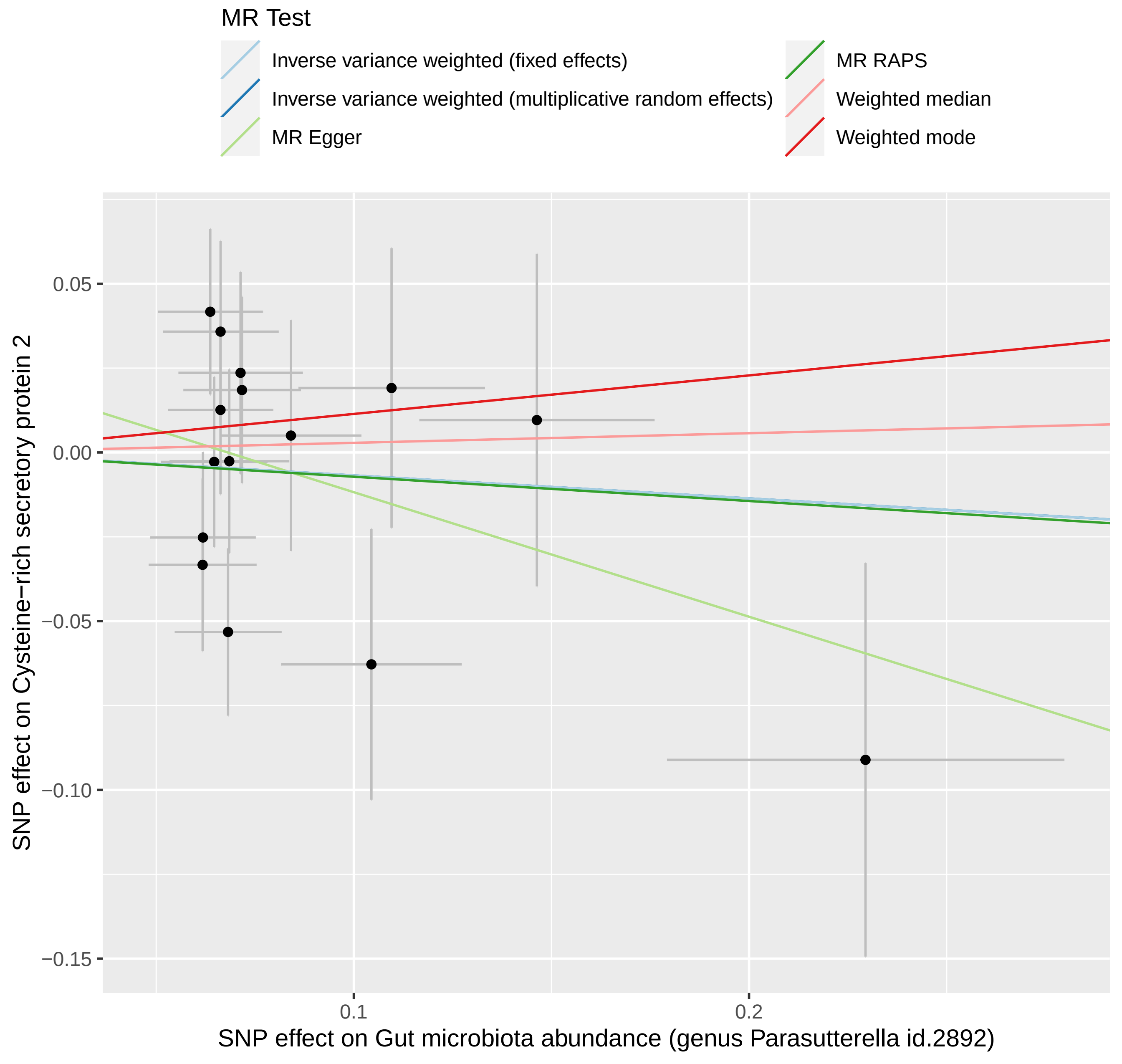

Batch 2−1323 : Gut microbiota abundance (genus Catenibacterium id.2153) on Cysteine−rich secretory protein 3
with a threshold of 1e−05

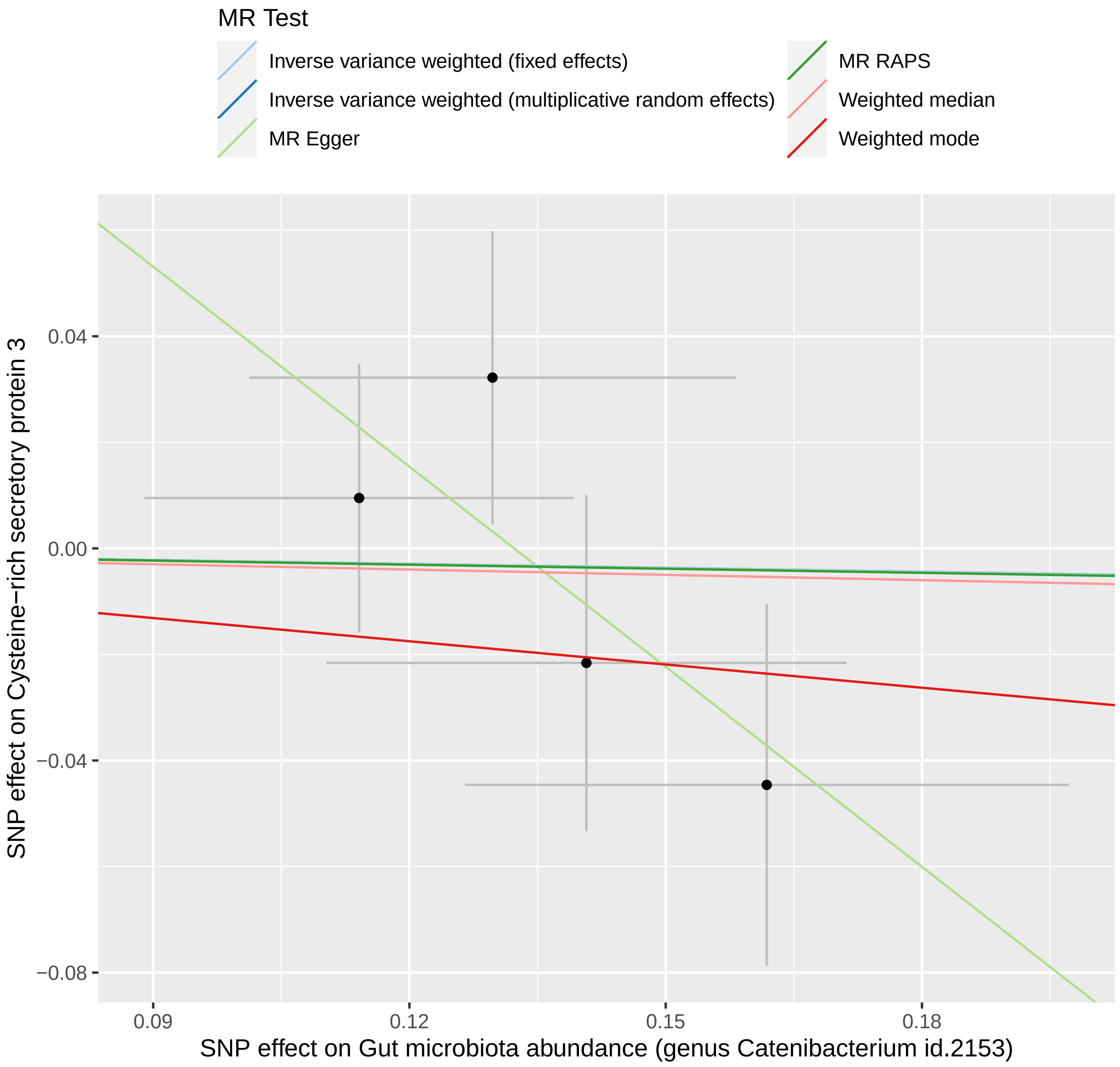

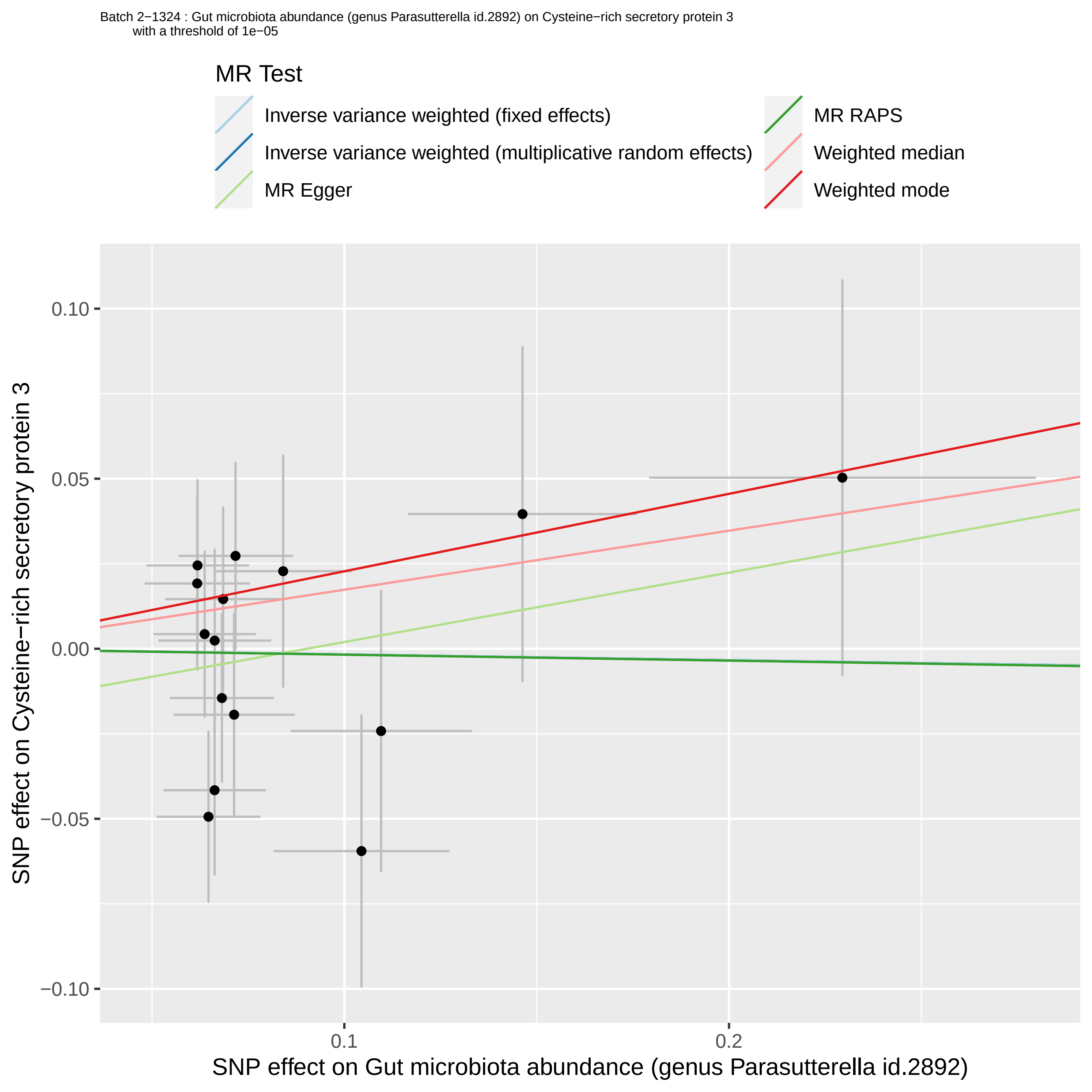
Batch 2−1324 : Gut microbiota abundance (genus Parasutterella id.2892) on Cysteine−rich secretory protein 3
with a threshold of 1e−05
MR Test
Inverse variance weighted (fixed effects)
Inverse variance weighted (multiplicative random effects)
MR Egger
MR RAPS
Weighted median
Weighted mode
SNP effect on Cysteine−rich secretory protein 3
0.10
0.05
0.00
−0.05
−0.10
0.1
0.2
SNP effect on Gut microbiota abundance (genus Parasutterella id.2892)

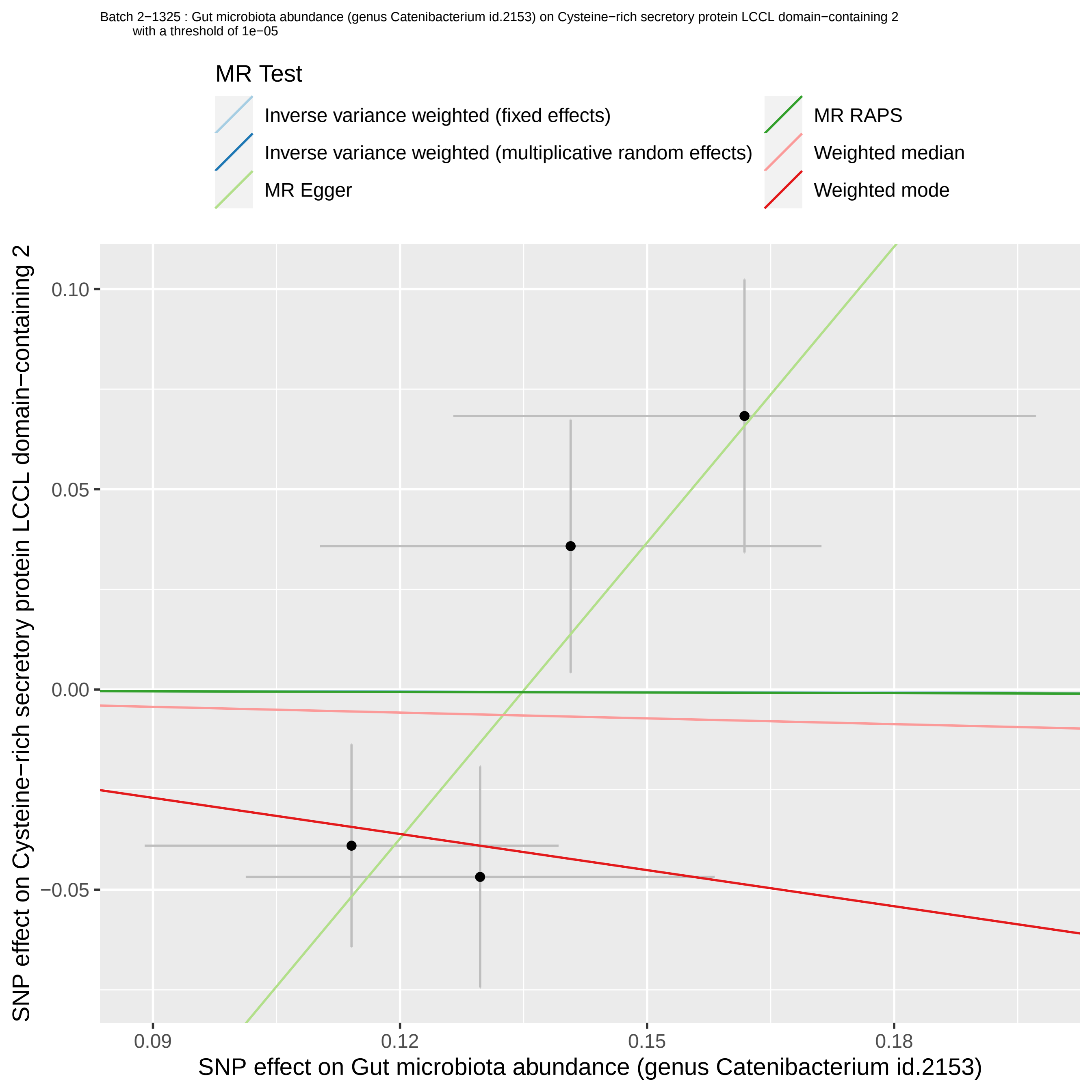

Batch 2−1325 : Gut microbiota abundance (genus Catenibacterium id.2153) on Cysteine−rich secretory protein LCCL domain−containing 2
with a threshold of 1e−05
MR Test
Inverse variance weighted (fixed effects)
Inverse variance weighted (multiplicative random effects)
MR Egger
MR RAPS
Weighted median
Weighted mode
0.10
0.05
0.00
−0.05
0.09
0.12
0.15
0.18
SNP effect on Cysteine−rich secretory protein LCCL domain−containing 2
SNP effect on Gut microbiota abundance (genus Catenibacterium id.2153)

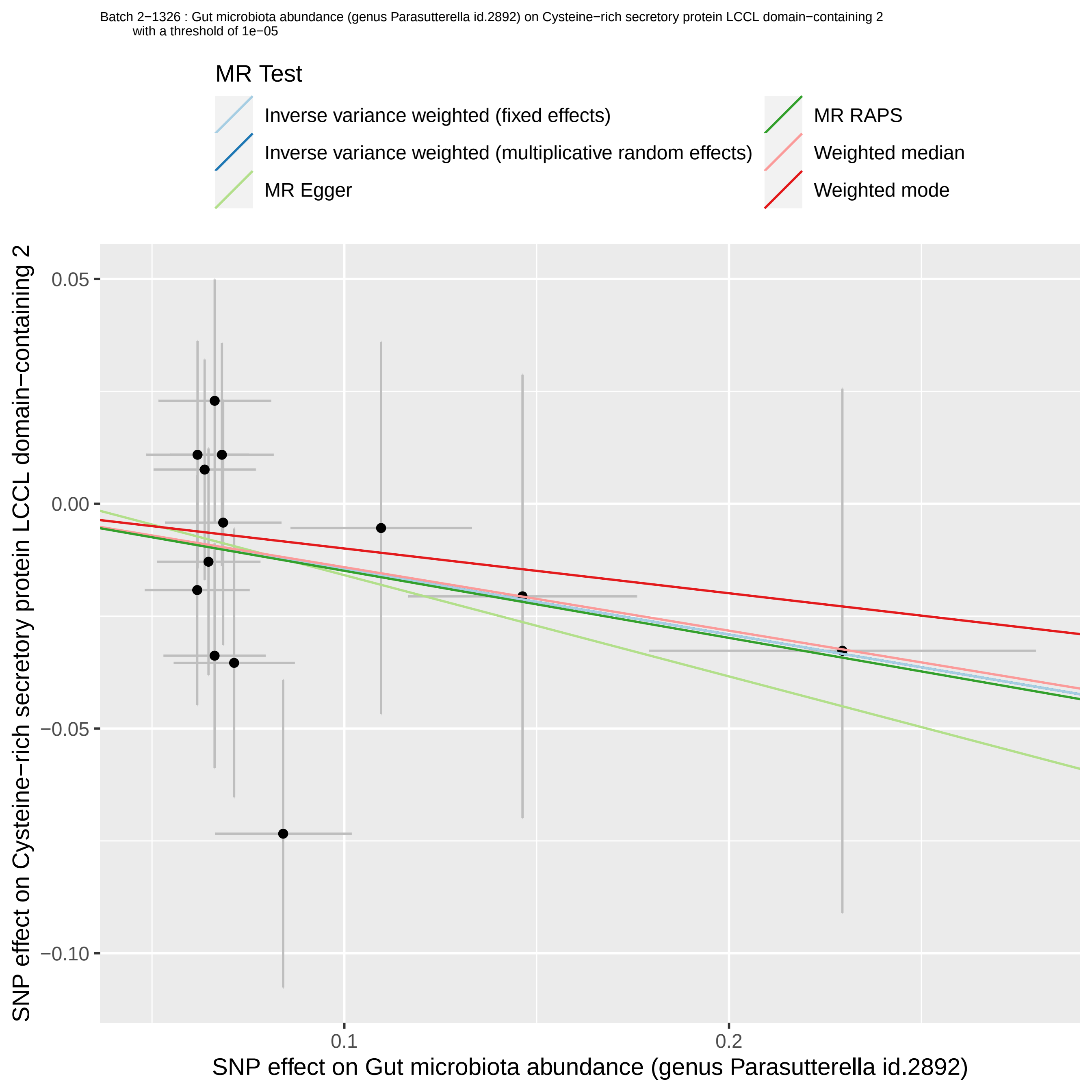

Batch 2−1326 : Gut microbiota abundance (genus Parasutterella id.2892) on Cysteine−rich secretory protein LCCL domain−containing 2
with a threshold of 1e−05
MR Test
Inverse variance weighted (fixed effects)
Inverse variance weighted (multiplicative random effects)
MR Egger
MR RAPS
Weighted median
Weighted mode
0.05
0.00
−0.05
−0.10
0.1
0.2
SNP effect on Cysteine−rich secretory protein LCCL domain−containing 2
SNP effect on Gut microbiota abundance (genus Parasutterella id.2892)

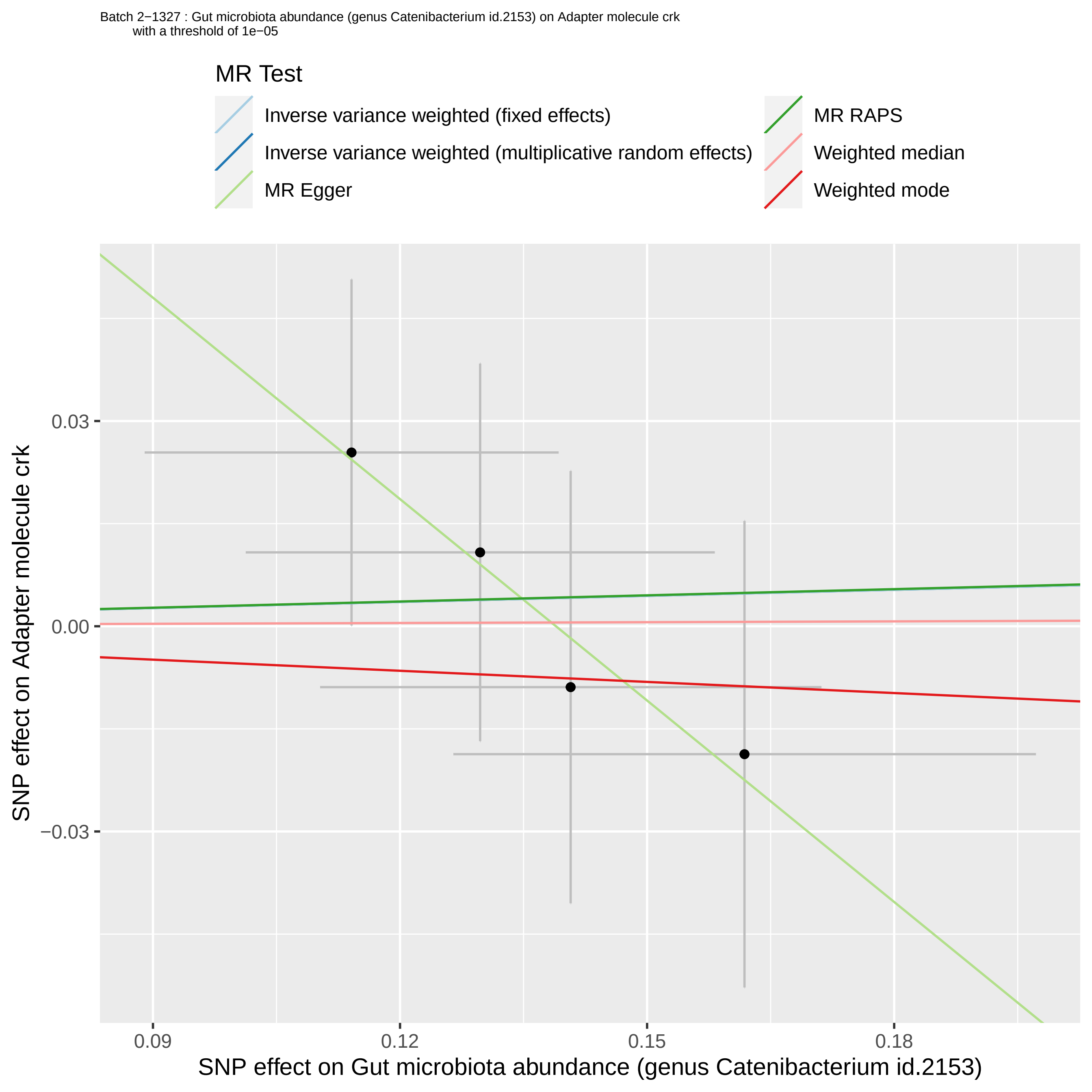
Batch 2−1327 : Gut microbiota abundance (genus Catenibacterium id.2153) on Adapter molecule crk
with a threshold of 1e−05
MR Test
Inverse variance weighted (fixed effects)
Inverse variance weighted (multiplicative random effects)
MR Egger
MR RAPS
Weighted median
Weighted mode
0.03
0.00
−0.03
0.09
0.12
0.15
0.18
SNP effect on Adapter molecule crk
SNP effect on Gut microbiota abundance (genus Catenibacterium id.2153)

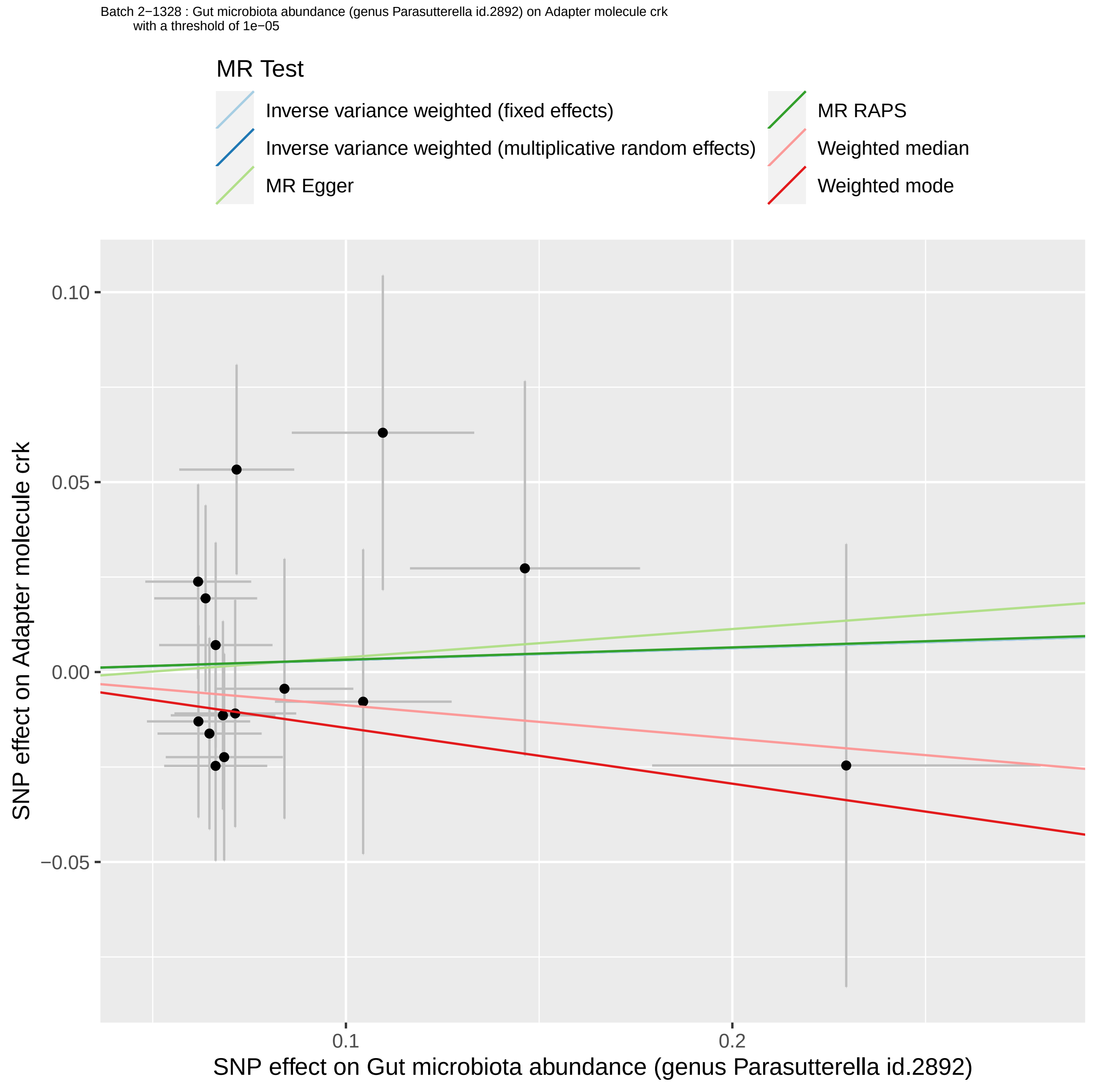
Batch 2−1328 : Gut microbiota abundance (genus Parasutterella id.2892) on Adapter molecule crk
with a threshold of 1e−05
MR Test
Inverse variance weighted (fixed effects)
Inverse variance weighted (multiplicative random effects)
MR Egger
MR RAPS
Weighted median
Weighted mode
SNP effect on Adapter molecule crk
0.10
0.05
0.00
−0.05
0.1
0.2
SNP effect on Gut microbiota abundance (genus Parasutterella id.2892)

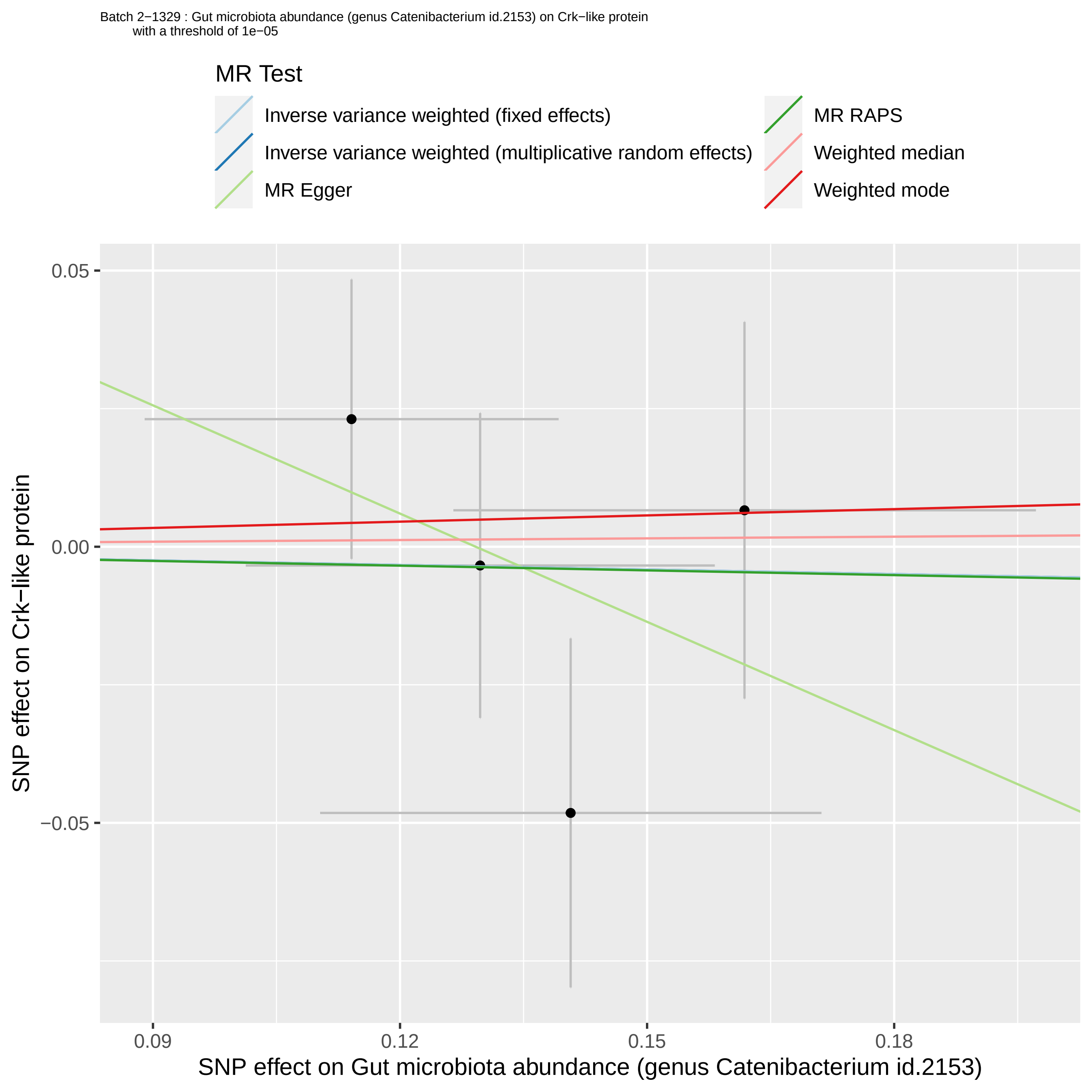
Batch 2−1329 : Gut microbiota abundance (genus Catenibacterium id.2153) on Crk−like protein
with a threshold of 1e−05
MR Test
Inverse variance weighted (fixed effects)
Inverse variance weighted (multiplicative random effects)
MR Egger
MR RAPS
Weighted median
Weighted mode
0.05
0.00
−0.05
0.09
0.12
0.15
0.18
SNP effect on Crk−like protein
SNP effect on Gut microbiota abundance (genus Catenibacterium id.2153)

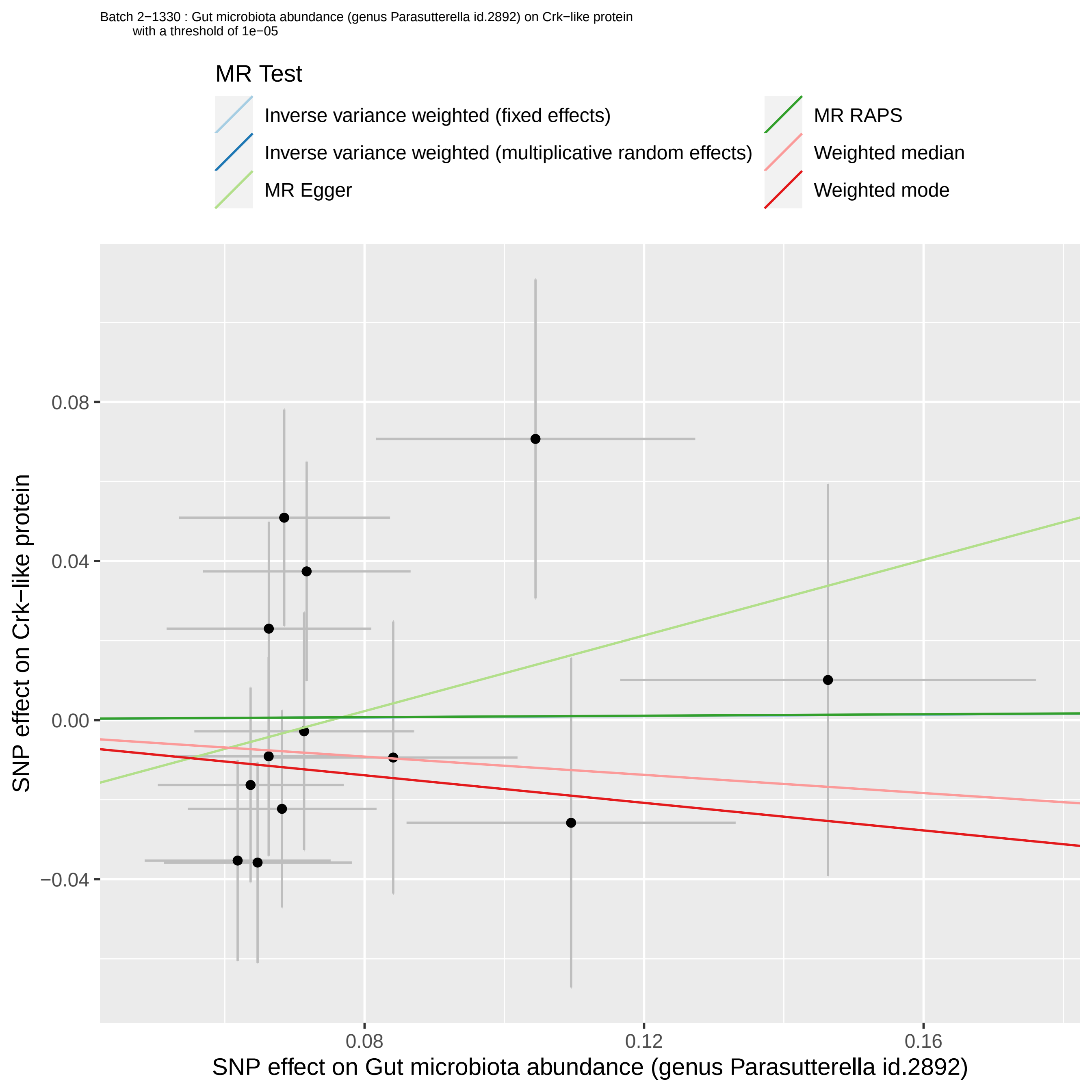

Batch 2−1330 : Gut microbiota abundance (genus Parasutterella id.2892) on Crk−like protein
with a threshold of 1e−05
MR Test
Inverse variance weighted (fixed effects)
Inverse variance weighted (multiplicative random effects)
MR Egger
MR RAPS
Weighted median
Weighted mode
0.08
0.04
0.00
−0.04
0.08
0.12
0.16
SNP effect on Crk−like protein
SNP effect on Gut microbiota abundance (genus Parasutterella id.2892)

Batch 2−1331 : Gut microbiota abundance (genus Catenibacterium id.2153) on Cytokine receptor−like factor 1:Cardiotrophin−like cytokine factor 1 Complex with a threshold of 1e−05

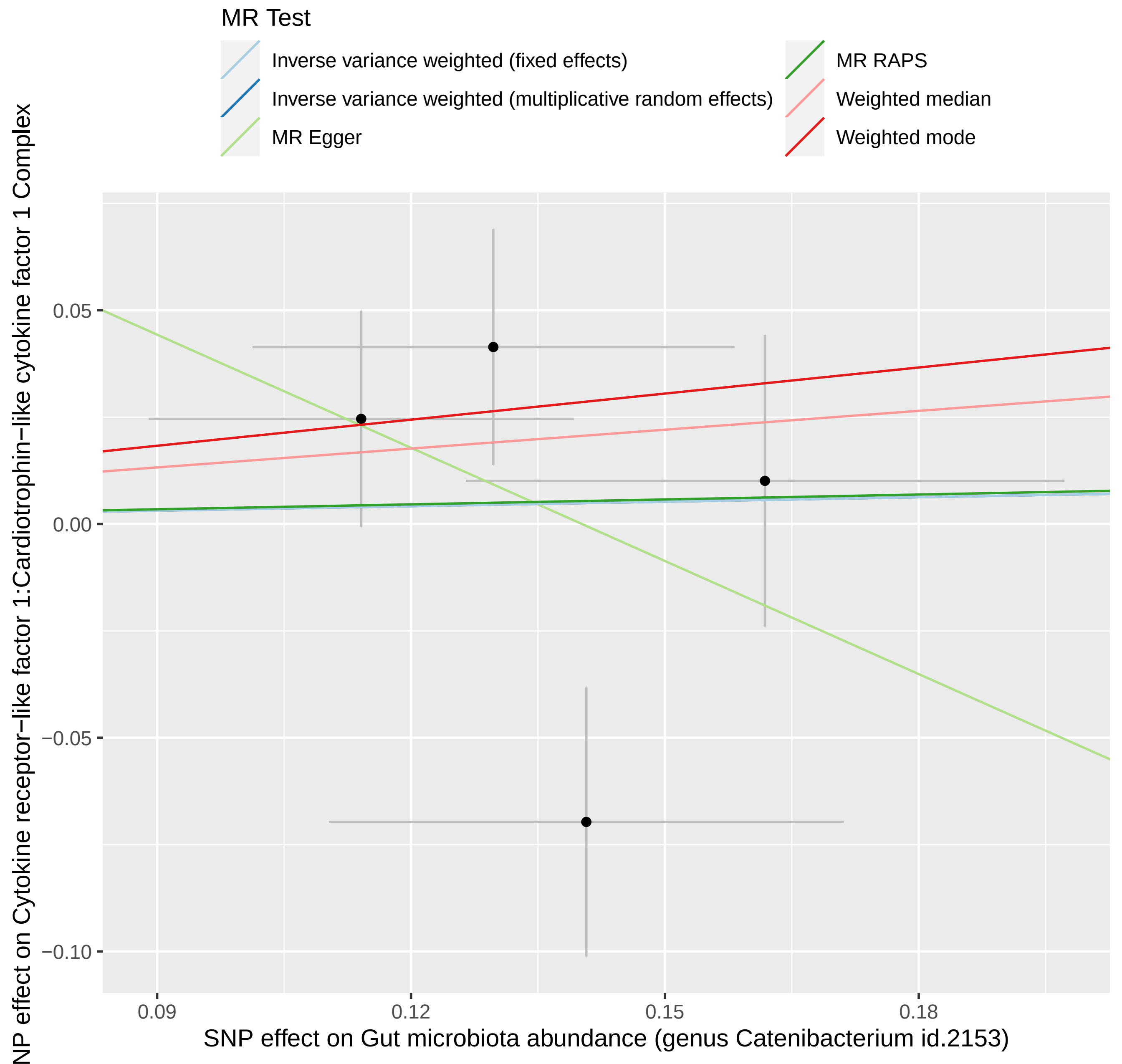

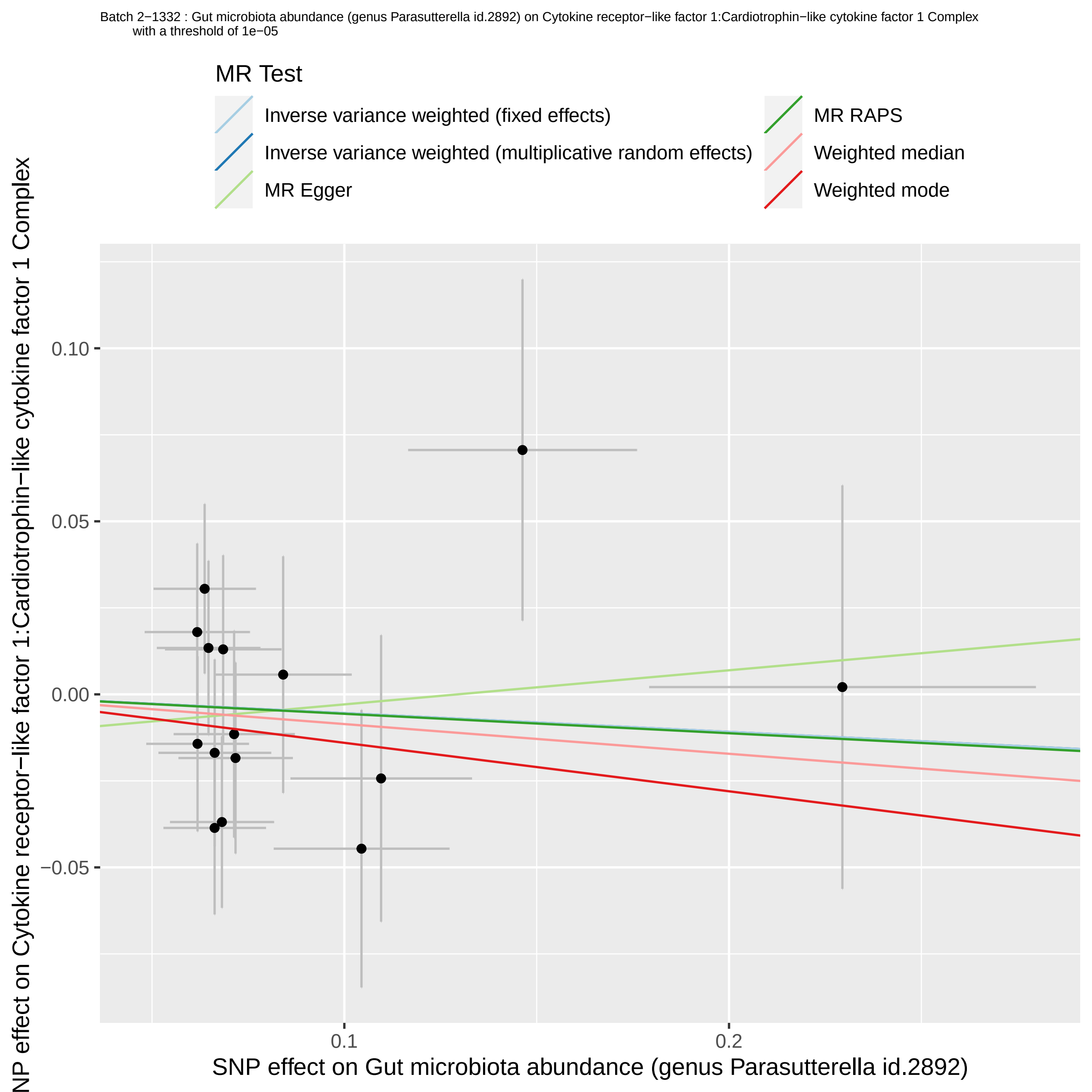

Batch 2−1332 : Gut microbiota abundance (genus Parasutterella id.2892) on Cytokine receptor−like factor 1:Cardiotrophin−like cytokine factor 1 Complex with a threshold of 1e−05
MR Test
Inverse variance weighted (fixed effects)
Inverse variance weighted (multiplicative random effects)
MR Egger
MR RAPS
Weighted median
Weighted mode
0.10
0.05
0.00
−0.05
0.1
0.2
SNP effect on Gut microbiota abundance (genus Parasutterella id.2892)
SNP effect on Cytokine receptor−like factor 1:Cardiotrophin−like cytokine factor 1 Complex

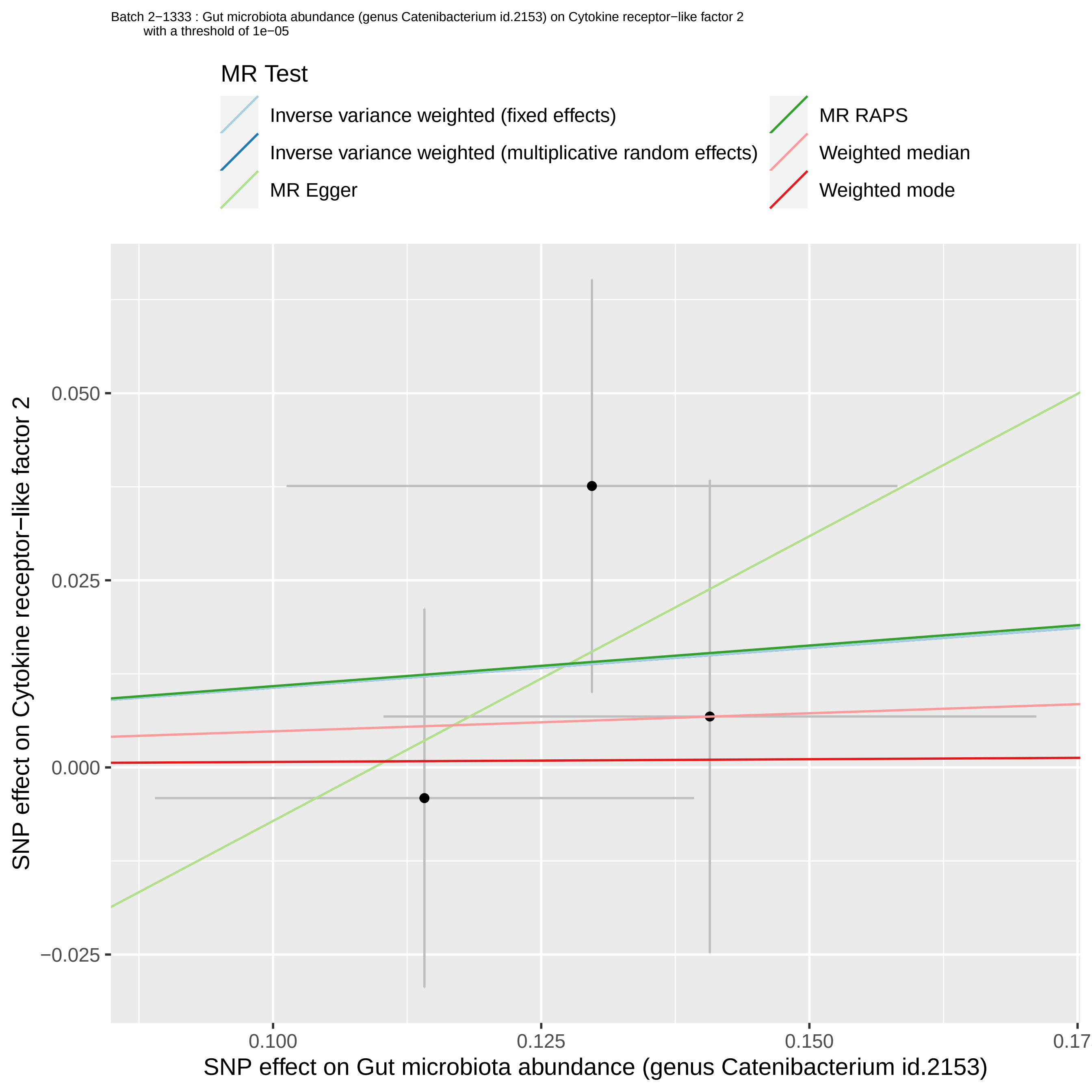

Batch 2−1333 : Gut microbiota abundance (genus Catenibacterium id.2153) on Cytokine receptor−like factor 2
with a threshold of 1e−05
MR Test
Inverse variance weighted (fixed effects)
Inverse variance weighted (multiplicative random effects)
MR Egger
MR RAPS
Weighted median
Weighted mode
0.050
0.025
0.000
−0.025
0.100
0.125
0.150
0.17
SNP effect on Cytokine receptor−like factor 2
SNP effect on Gut microbiota abundance (genus Catenibacterium id.2153)

Batch 2−1334 : Gut microbiota abundance (genus Parasutterella id.2892) on Cytokine receptor−like factor 2
with a threshold of 1e−05

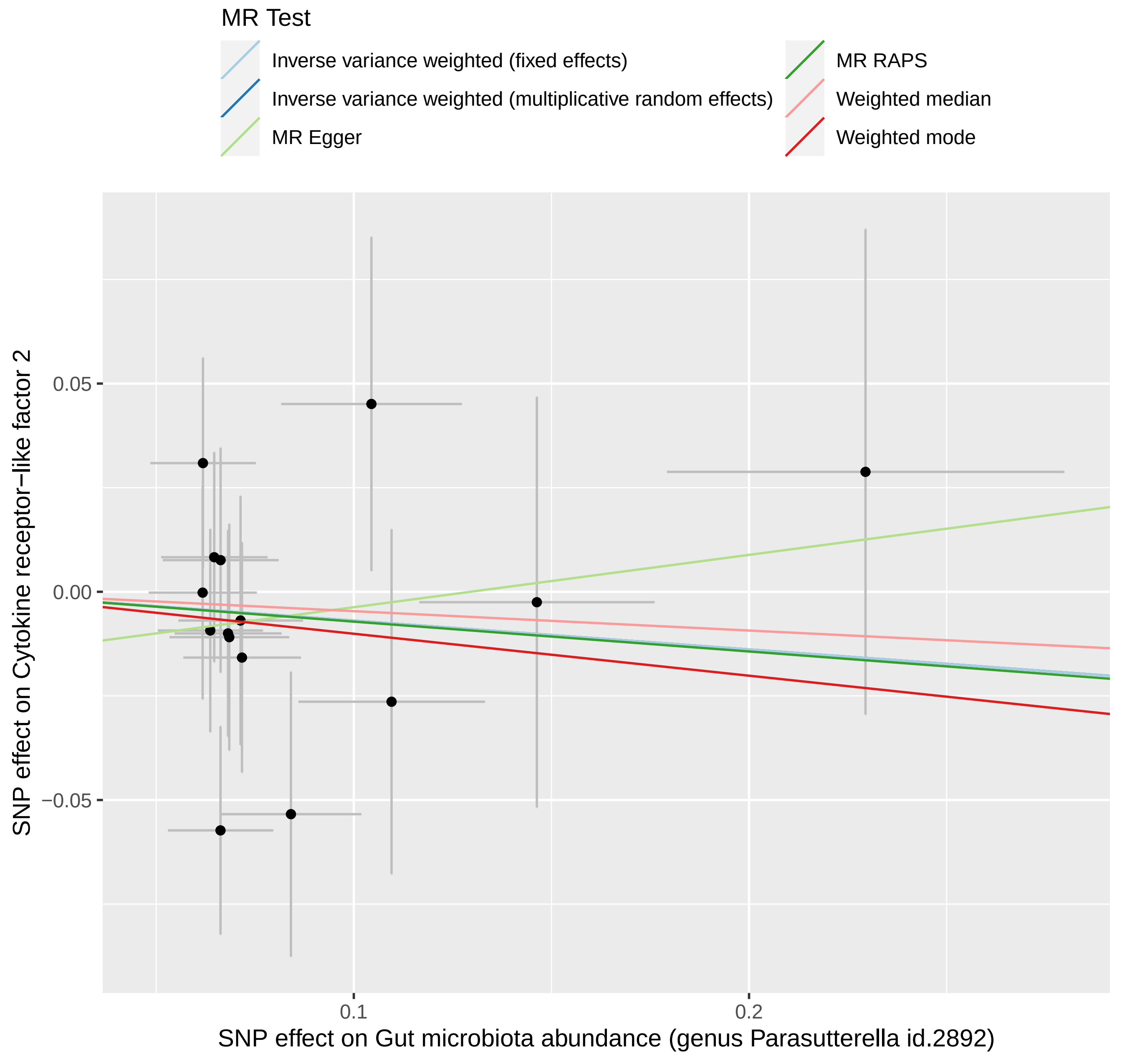

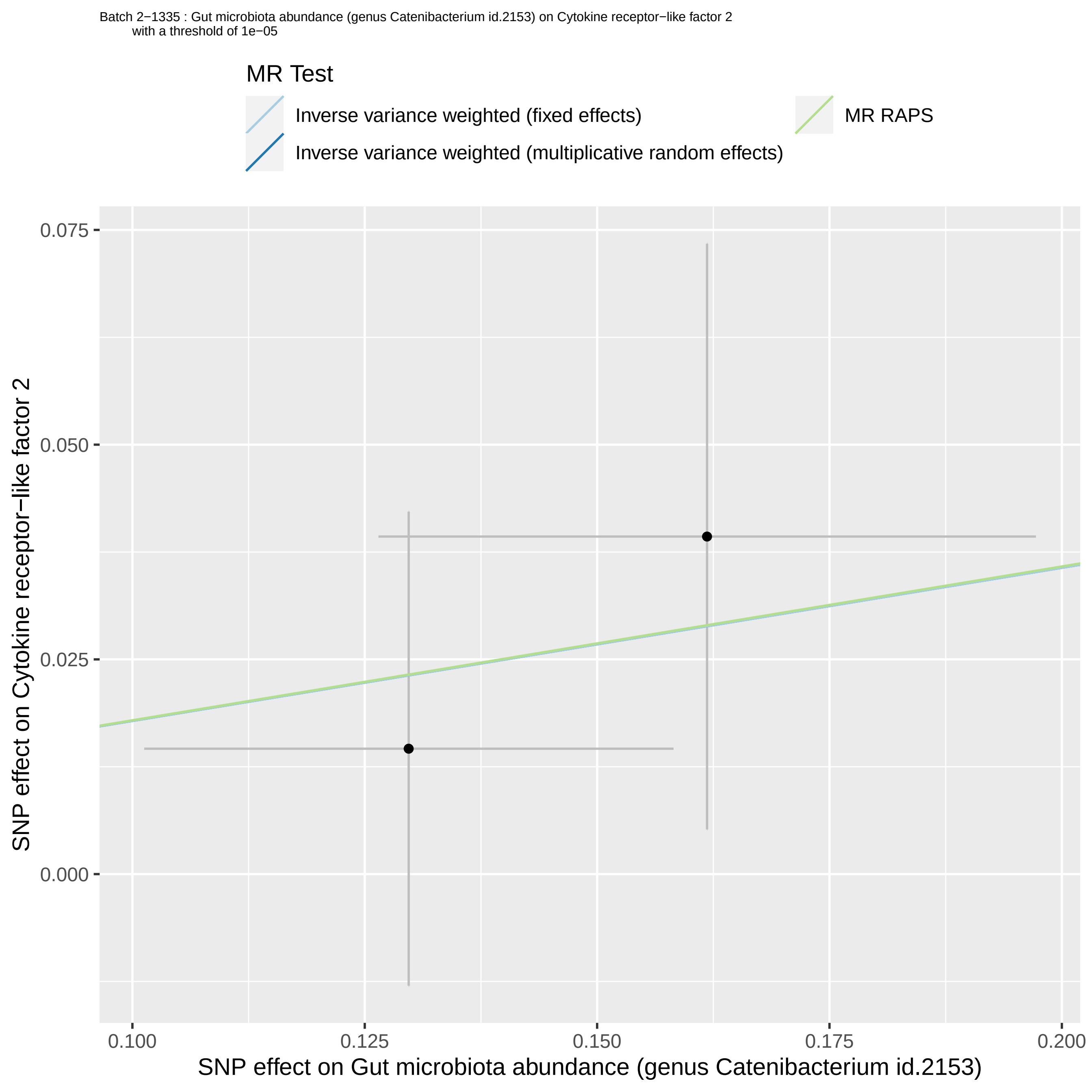
Batch 2−1335 : Gut microbiota abundance (genus Catenibacterium id.2153) on Cytokine receptor−like factor 2
with a threshold of 1e−05
MR Test
Inverse variance weighted (fixed effects)
Inverse variance weighted (multiplicative random effects)
MR RAPS
0.075
0.050
0.025
0.000
0.100
0.125
0.150
0.175
0.200
SNP effect on Cytokine receptor−like factor 2
SNP effect on Gut microbiota abundance (genus Catenibacterium id.2153)

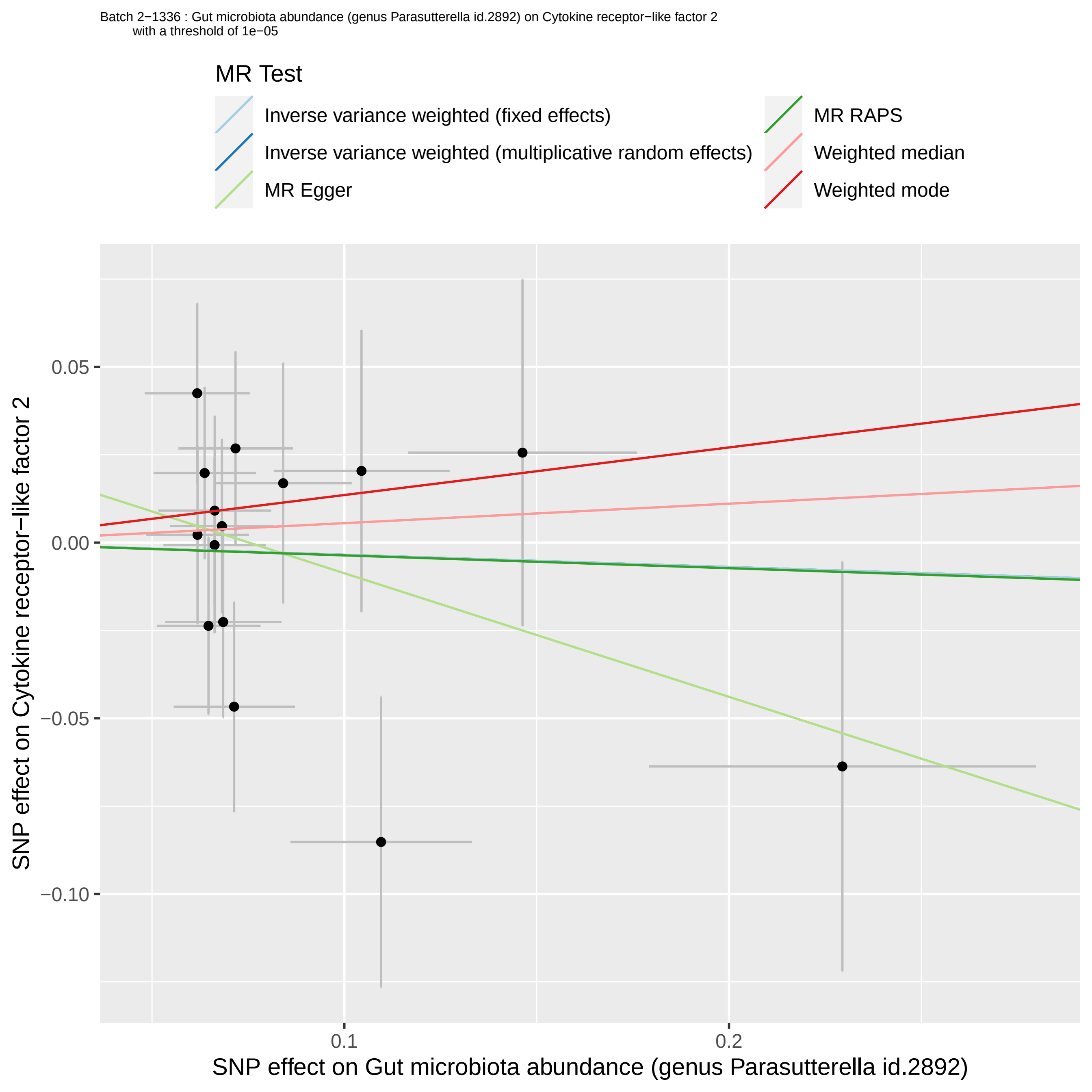
Batch 2−1336 : Gut microbiota abundance (genus Parasutterella id.2892) on Cytokine receptor−like factor 2
with a threshold of 1e−05
MR Test
Inverse variance weighted (fixed effects)
Inverse variance weighted (multiplicative random effects)
MR Egger
MR RAPS
Weighted median
Weighted mode
0.05
0.00
−0.05
−0.10
0.1
0.2
SNP effect on Cytokine receptor−like factor 2
SNP effect on Gut microbiota abundance (genus Parasutterella id.2892)

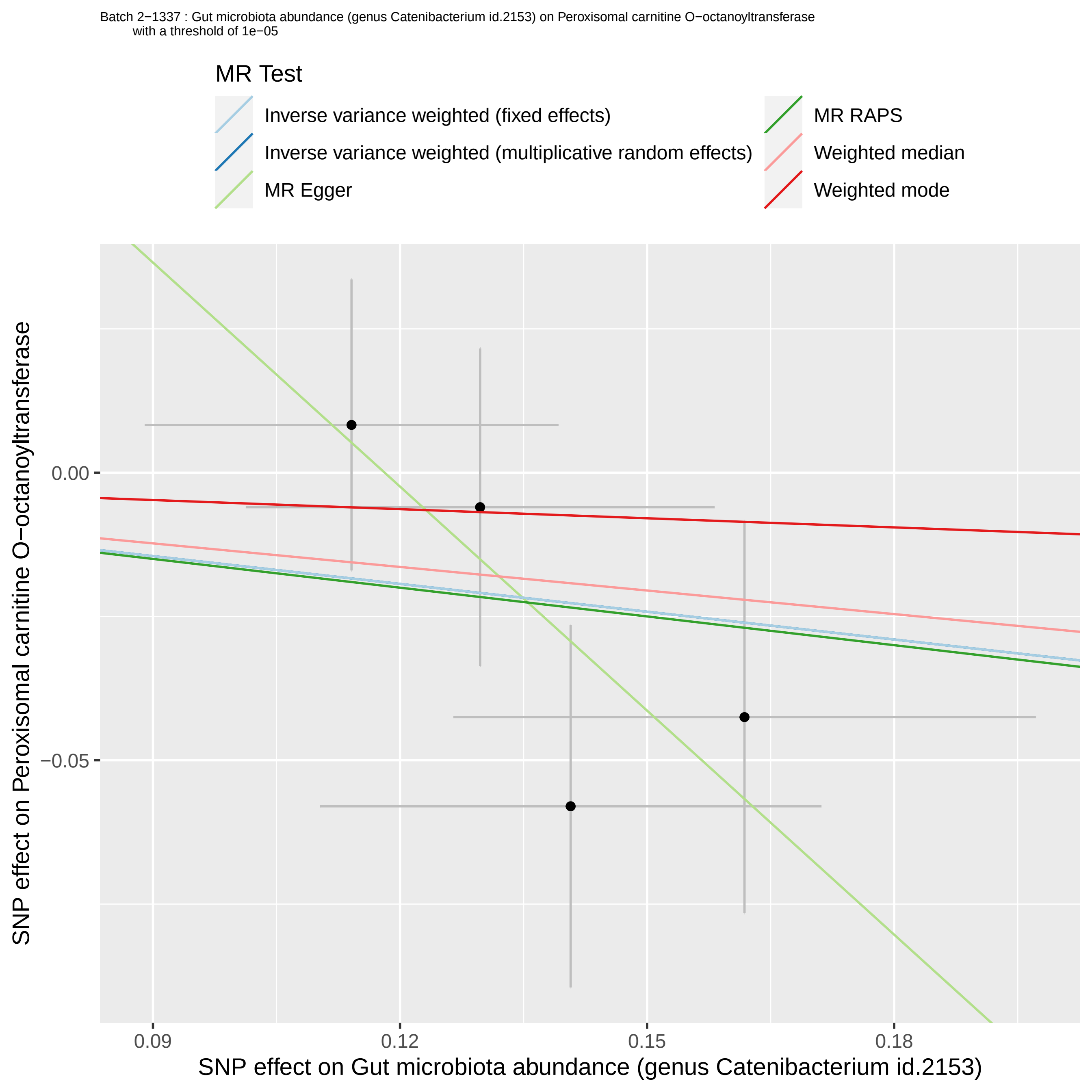
Batch 2−1337 : Gut microbiota abundance (genus Catenibacterium id.2153) on Peroxisomal carnitine O−octanoyltransferase
with a threshold of 1e−05
MR Test
Inverse variance weighted (fixed effects)
Inverse variance weighted (multiplicative random effects)
MR Egger
MR RAPS
Weighted median
Weighted mode
SNP effect on Peroxisomal carnitine O−octanoyltransferase
0.00
−0.05
0.09
0.12
0.15
0.18
SNP effect on Gut microbiota abundance (genus Catenibacterium id.2153)

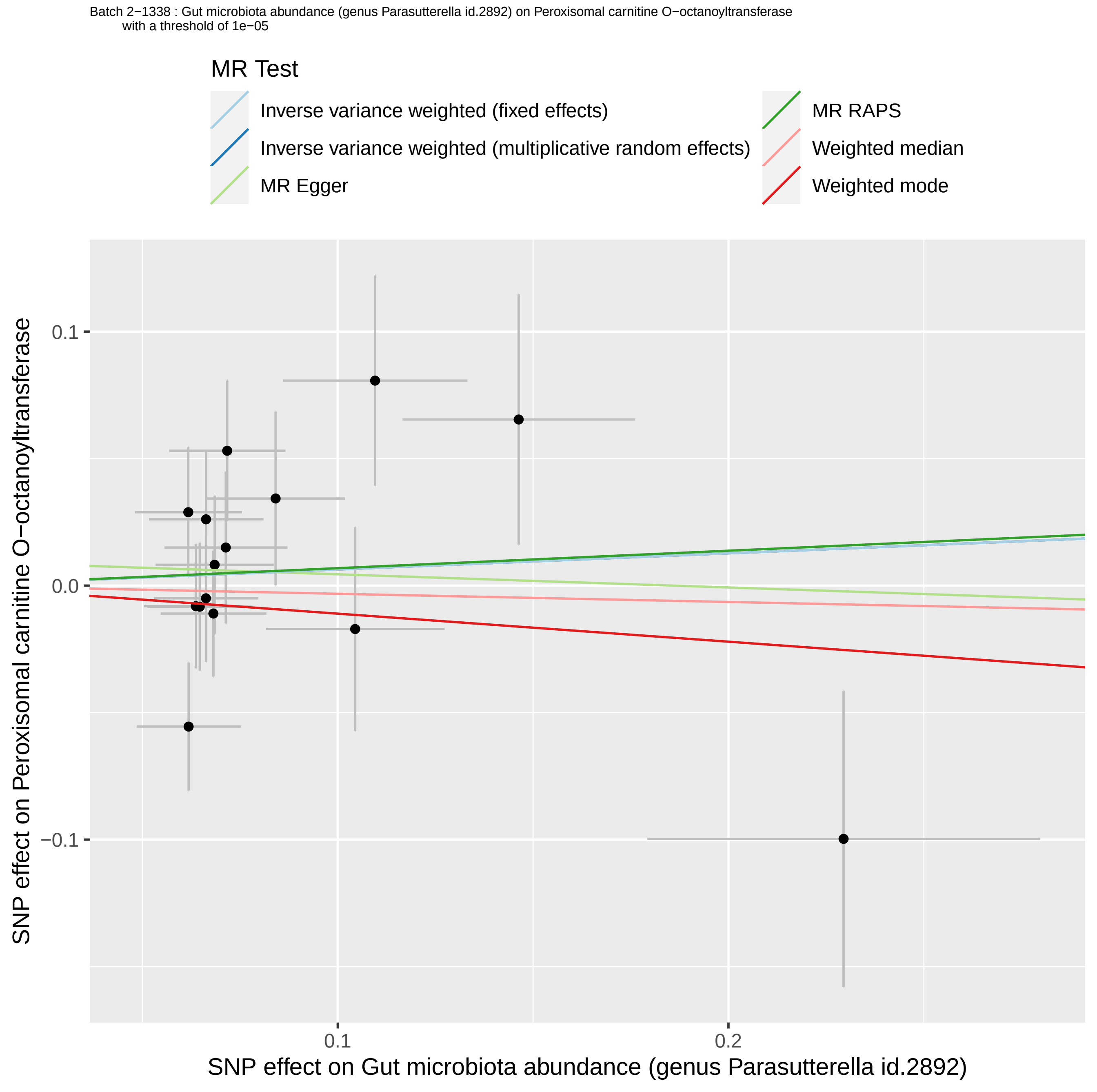

Batch 2−1338 : Gut microbiota abundance (genus Parasutterella id.2892) on Peroxisomal carnitine O−octanoyltransferase
with a threshold of 1e−05
MR Test
Inverse variance weighted (fixed effects)
Inverse variance weighted (multiplicative random effects)
MR Egger
MR RAPS
Weighted median
Weighted mode
0.1
0.0
−0.1
0.1
0.2
SNP effect on Peroxisomal carnitine O−octanoyltransferase
SNP effect on Gut microbiota abundance (genus Parasutterella id.2892)

Batch 2−1339 : Gut microbiota abundance (genus Catenibacterium id.2153) on C−reactive protein
with a threshold of 1e−05

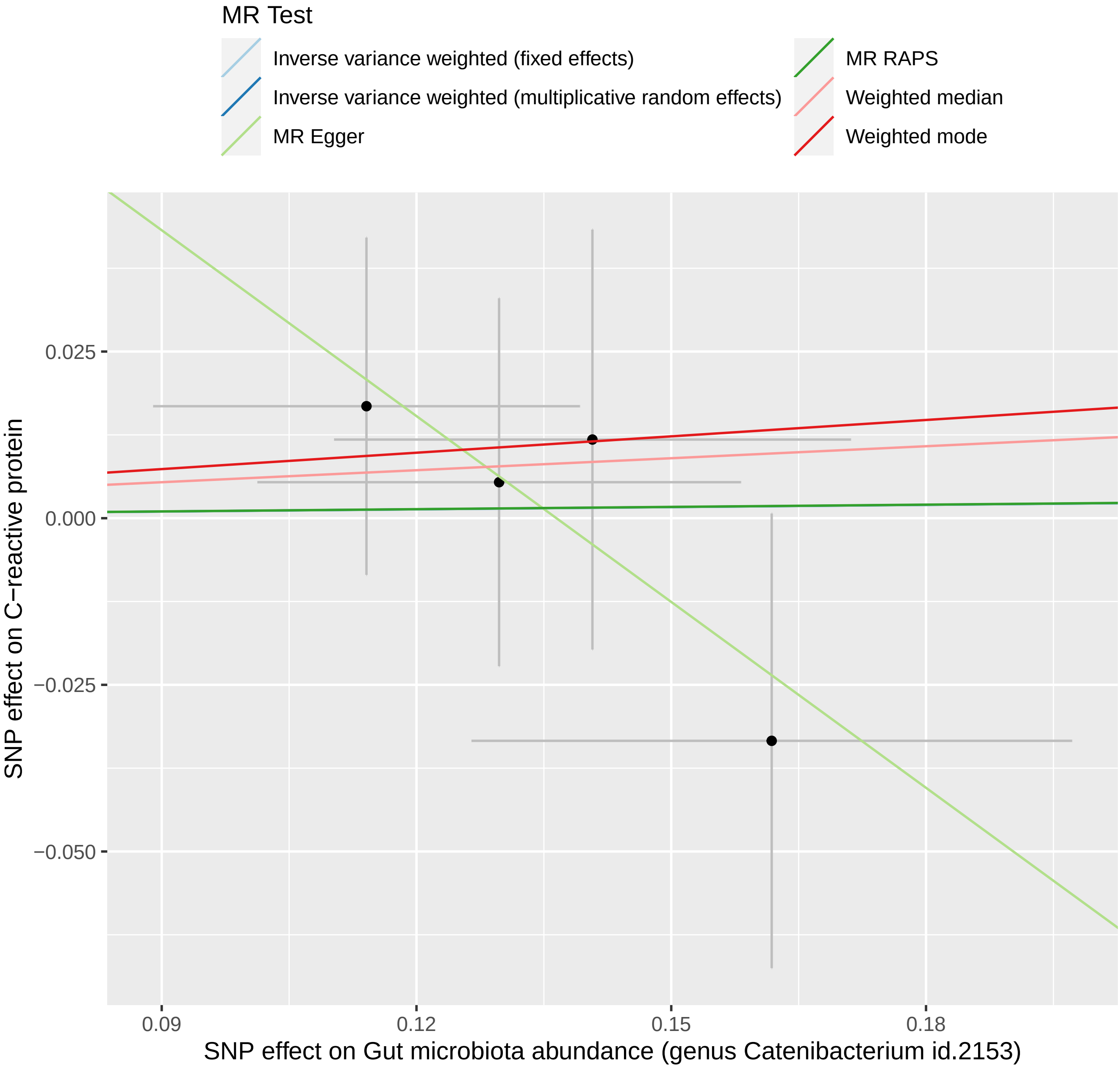

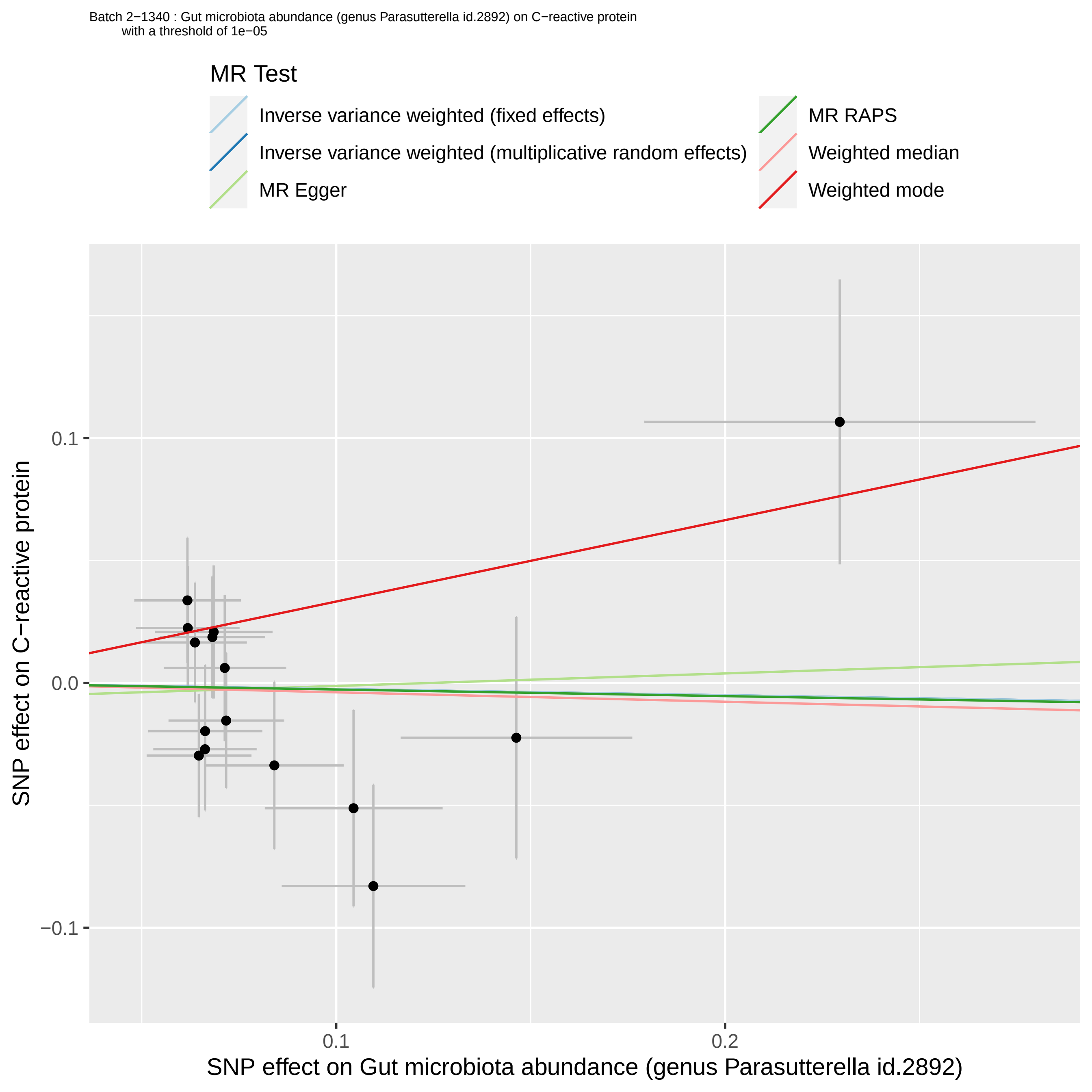

Batch 2−1340 : Gut microbiota abundance (genus Parasutterella id.2892) on C−reactive protein
with a threshold of 1e−05
MR Test
Inverse variance weighted (fixed effects)
Inverse variance weighted (multiplicative random effects)
MR Egger
MR RAPS
Weighted median
Weighted mode
0.1
0.0
−0.1
0.1
0.2
SNP effect on C−reactive protein
SNP effect on Gut microbiota abundance (genus Parasutterella id.2892)

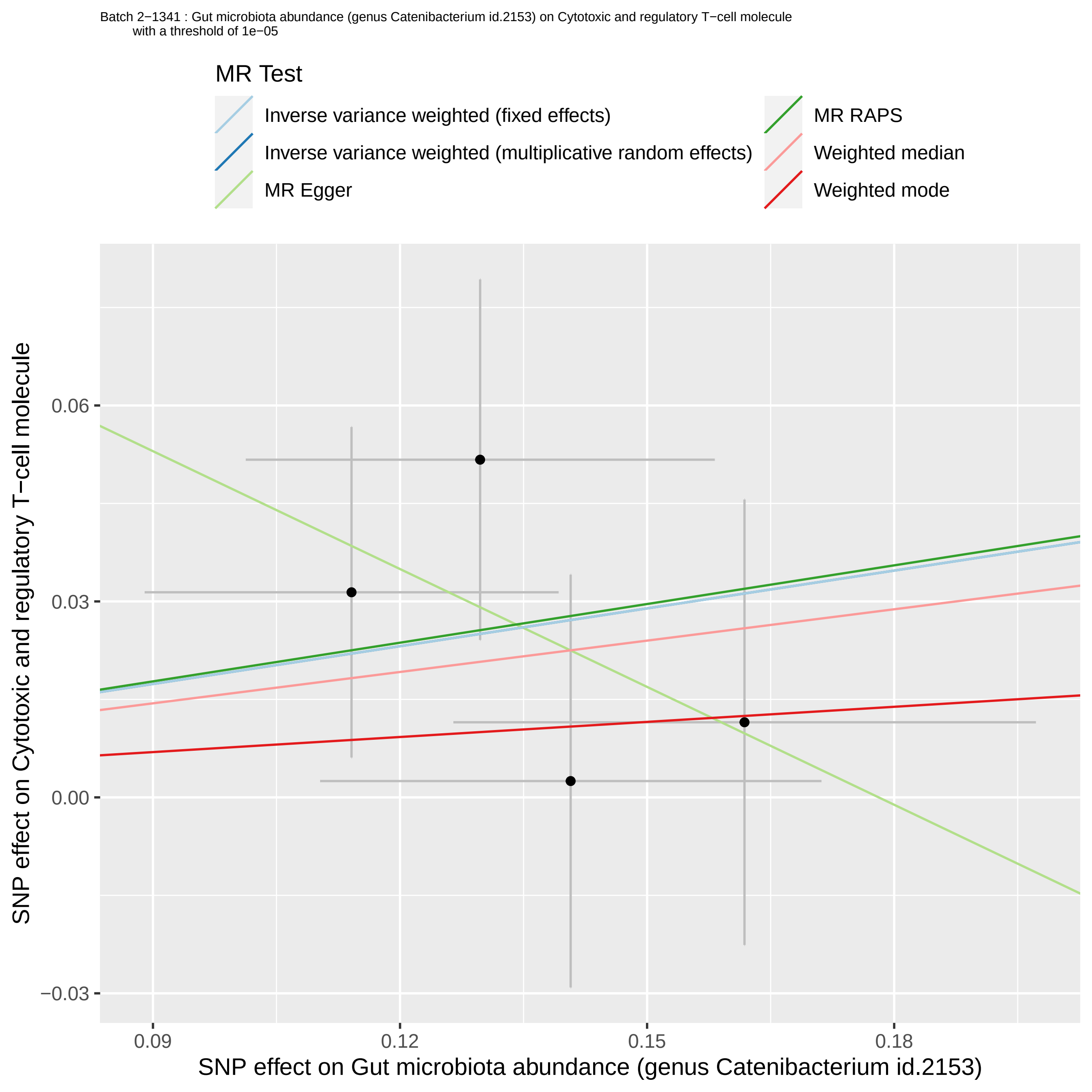
Batch 2−1341 : Gut microbiota abundance (genus Catenibacterium id.2153) on Cytotoxic and regulatory T−cell molecule
with a threshold of 1e−05
MR Test
Inverse variance weighted (fixed effects)
Inverse variance weighted (multiplicative random effects)
MR Egger
MR RAPS
Weighted median
Weighted mode
0.06
0.03
0.00
−0.03
0.09
0.12
0.15
0.18
SNP effect on Cytotoxic and regulatory T−cell molecule
SNP effect on Gut microbiota abundance (genus Catenibacterium id.2153)

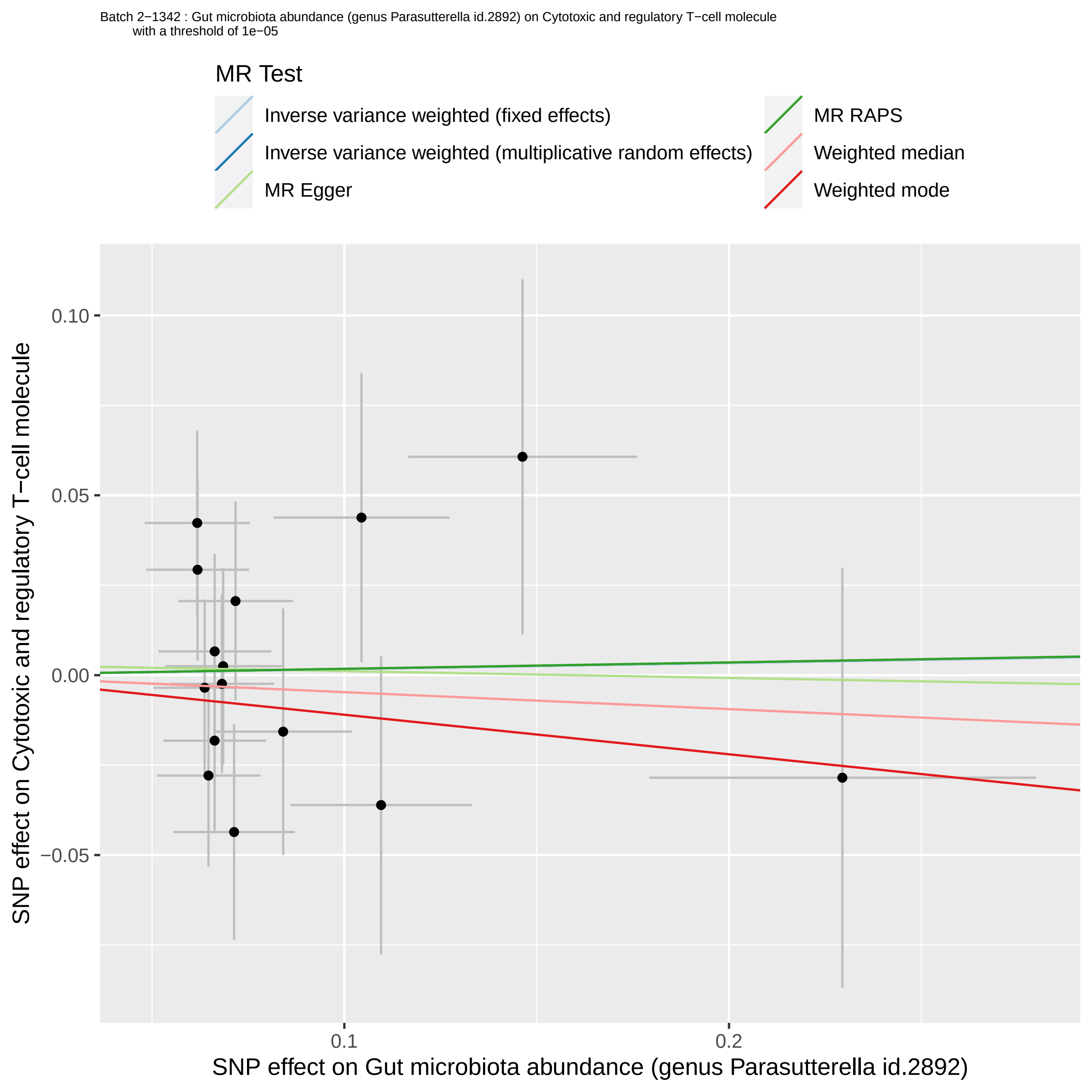

Batch 2−1342 : Gut microbiota abundance (genus Parasutterella id.2892) on Cytotoxic and regulatory T−cell molecule
with a threshold of 1e−05
MR Test
Inverse variance weighted (fixed effects)
Inverse variance weighted (multiplicative random effects)
MR Egger
MR RAPS
Weighted median
Weighted mode
0.10
0.05
0.00
−0.05
0.1
0.2
SNP effect on Cytotoxic and regulatory T−cell molecule
SNP effect on Gut microbiota abundance (genus Parasutterella id.2892)

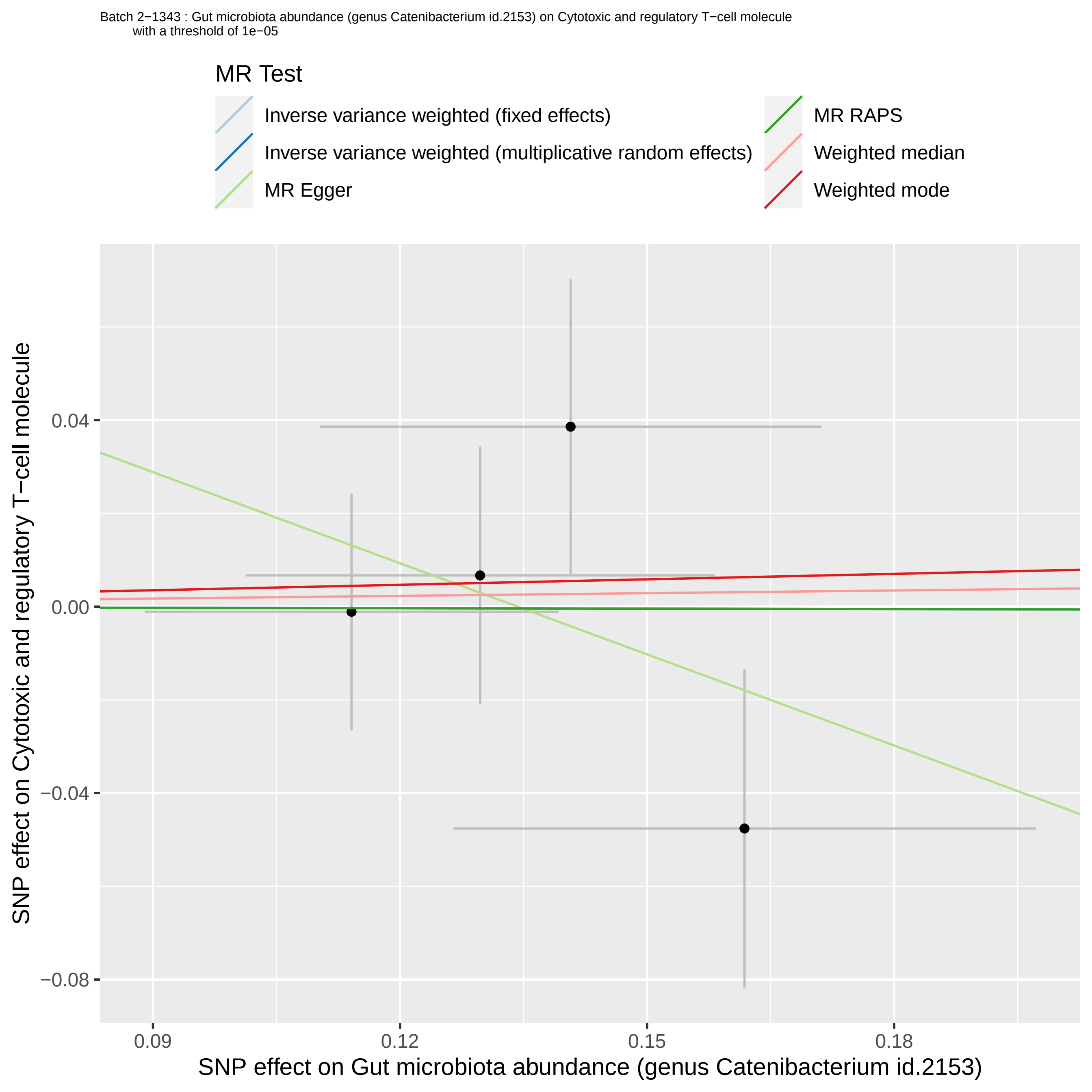
Batch 2−1343 : Gut microbiota abundance (genus Catenibacterium id.2153) on Cytotoxic and regulatory T−cell molecule
with a threshold of 1e−05
MR Test
Inverse variance weighted (fixed effects)
Inverse variance weighted (multiplicative random effects)
MR Egger
MR RAPS
Weighted median
Weighted mode
SNP effect on Cytotoxic and regulatory T−cell molecule
0.04
0.00
−0.04
−0.08
0.09
0.12
0.15
0.18
SNP effect on Gut microbiota abundance (genus Catenibacterium id.2153)

Batch 2−1344 : Gut microbiota abundance (genus Parasutterella id.2892) on Cytotoxic and regulatory T−cell molecule
with a threshold of 1e−05

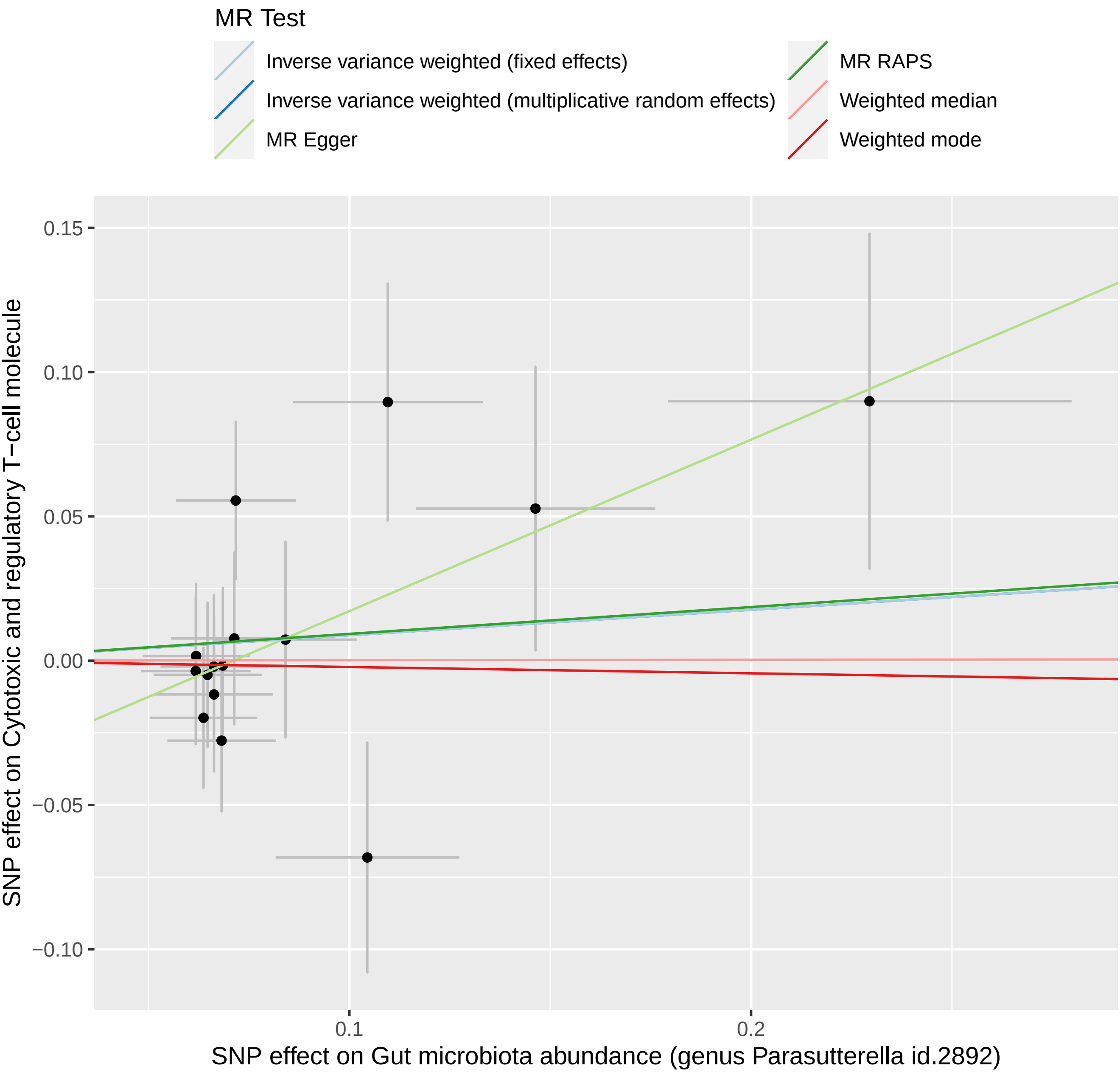

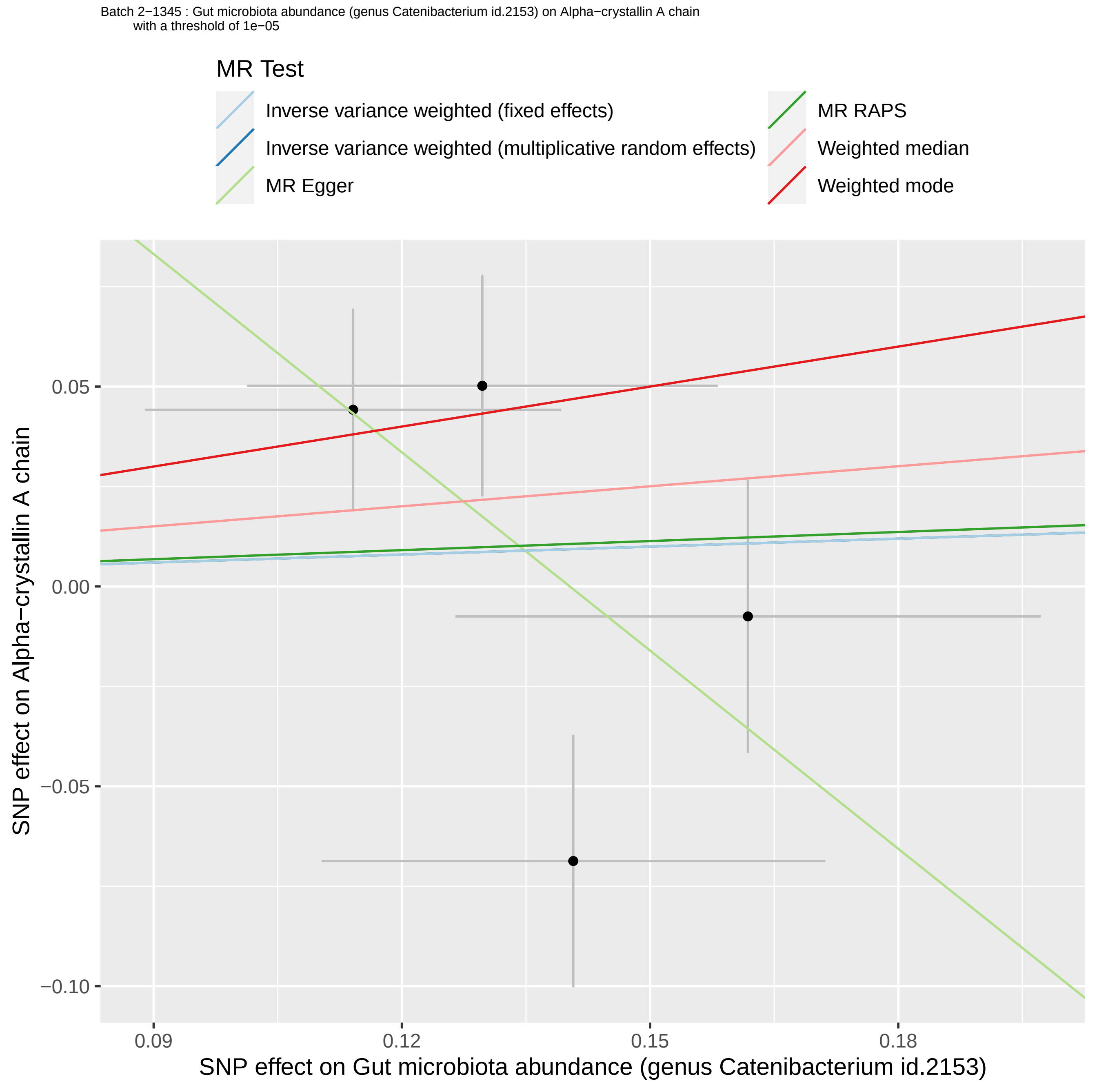

Batch 2−1345 : Gut microbiota abundance (genus Catenibacterium id.2153) on Alpha−crystallin A chain
with a threshold of 1e−05
MR Test
Inverse variance weighted (fixed effects)
Inverse variance weighted (multiplicative random effects)
MR Egger
MR RAPS
Weighted median
Weighted mode
0.05
0.00
−0.05
−0.10
0.09
0.12
0.15
0.18
SNP effect on Alpha−crystallin A chain
SNP effect on Gut microbiota abundance (genus Catenibacterium id.2153)

Batch 2−1346 : Gut microbiota abundance (genus Parasutterella id.2892) on Alpha−crystallin A chain
with a threshold of 1e−05

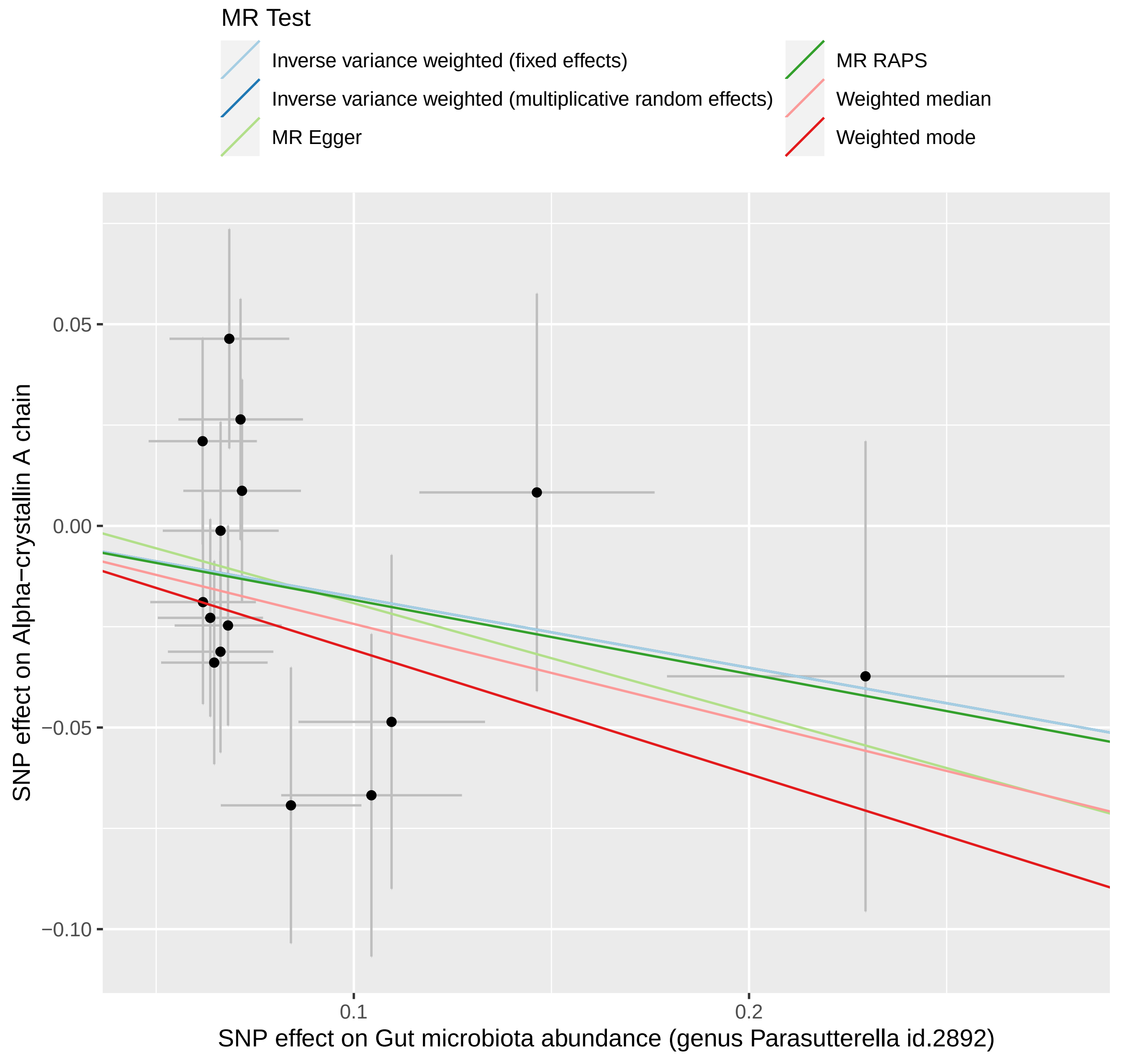

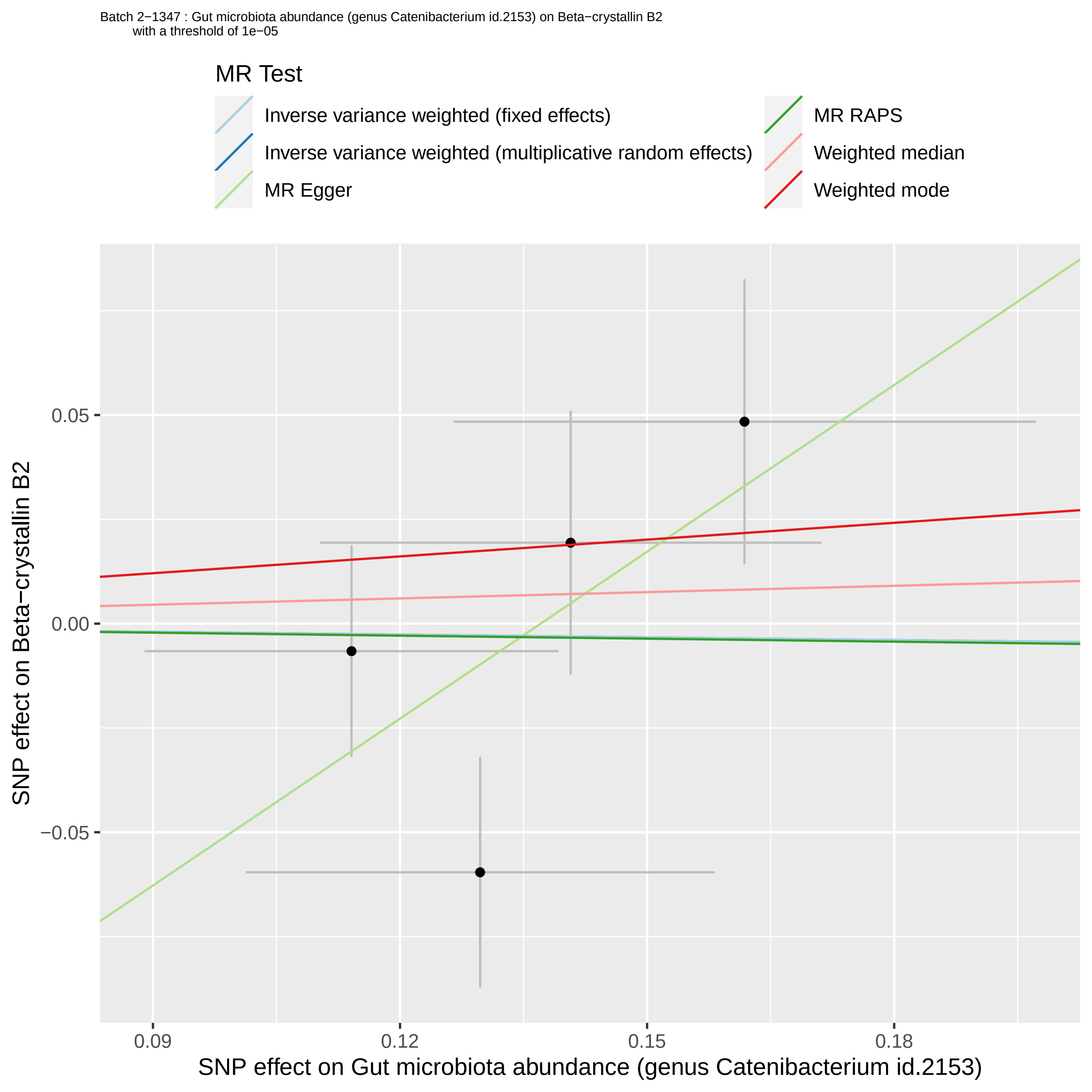
Batch 2−1347 : Gut microbiota abundance (genus Catenibacterium id.2153) on Beta−crystallin B2
with a threshold of 1e−05
MR Test
Inverse variance weighted (fixed effects)
Inverse variance weighted (multiplicative random effects)
MR Egger
MR RAPS
Weighted median
Weighted mode
0.05
0.00
−0.05
0.09
0.12
0.15
0.18
SNP effect on Beta−crystallin B2
SNP effect on Gut microbiota abundance (genus Catenibacterium id.2153)

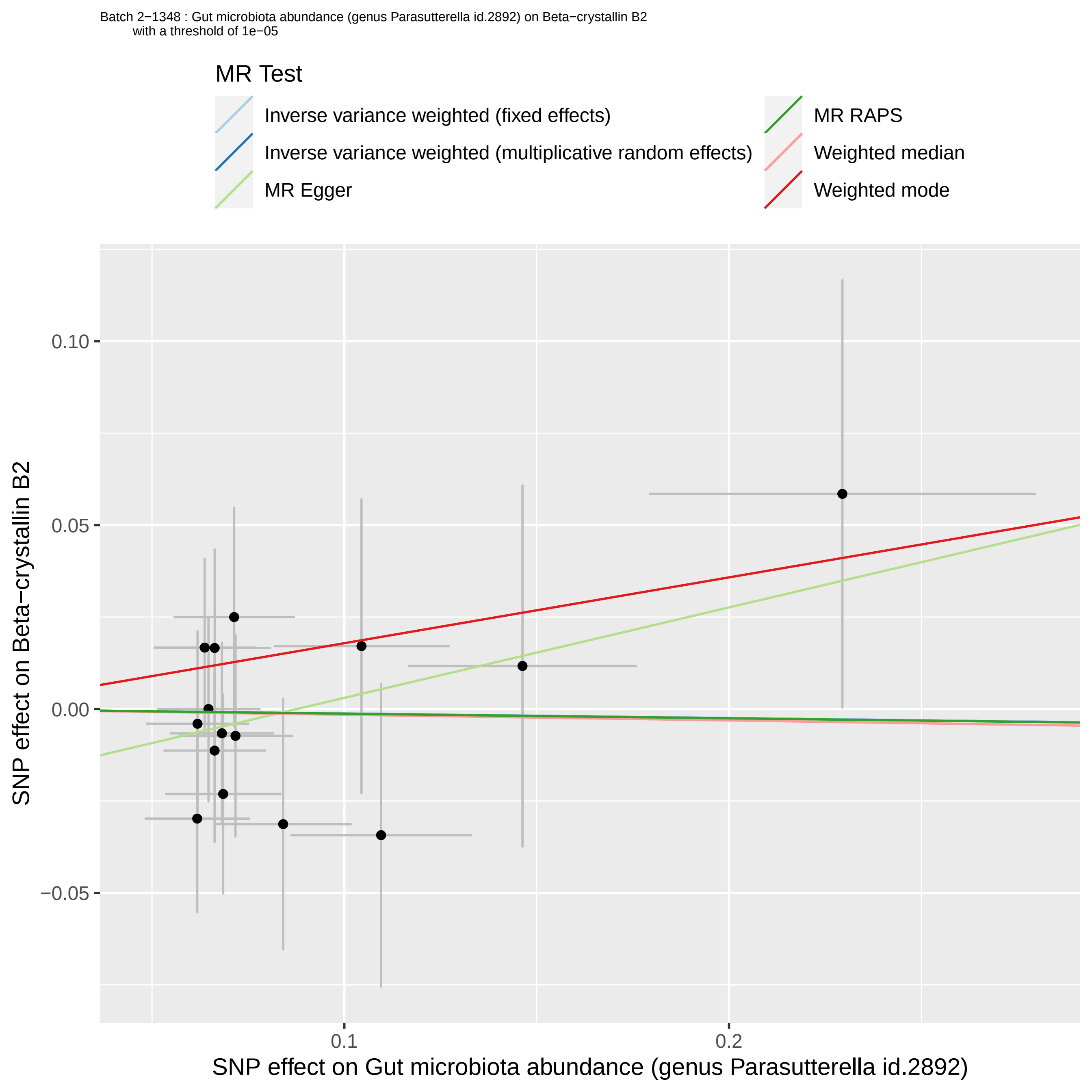
Batch 2−1348 : Gut microbiota abundance (genus Parasutterella id.2892) on Beta−crystallin B2
with a threshold of 1e−05
MR Test
Inverse variance weighted (fixed effects)
Inverse variance weighted (multiplicative random effects)
MR Egger
MR RAPS
Weighted median
Weighted mode
SNP effect on Beta−crystallin B2
0.10
0.05
0.00
−0.05
0.1
0.2
SNP effect on Gut microbiota abundance (genus Parasutterella id.2892)

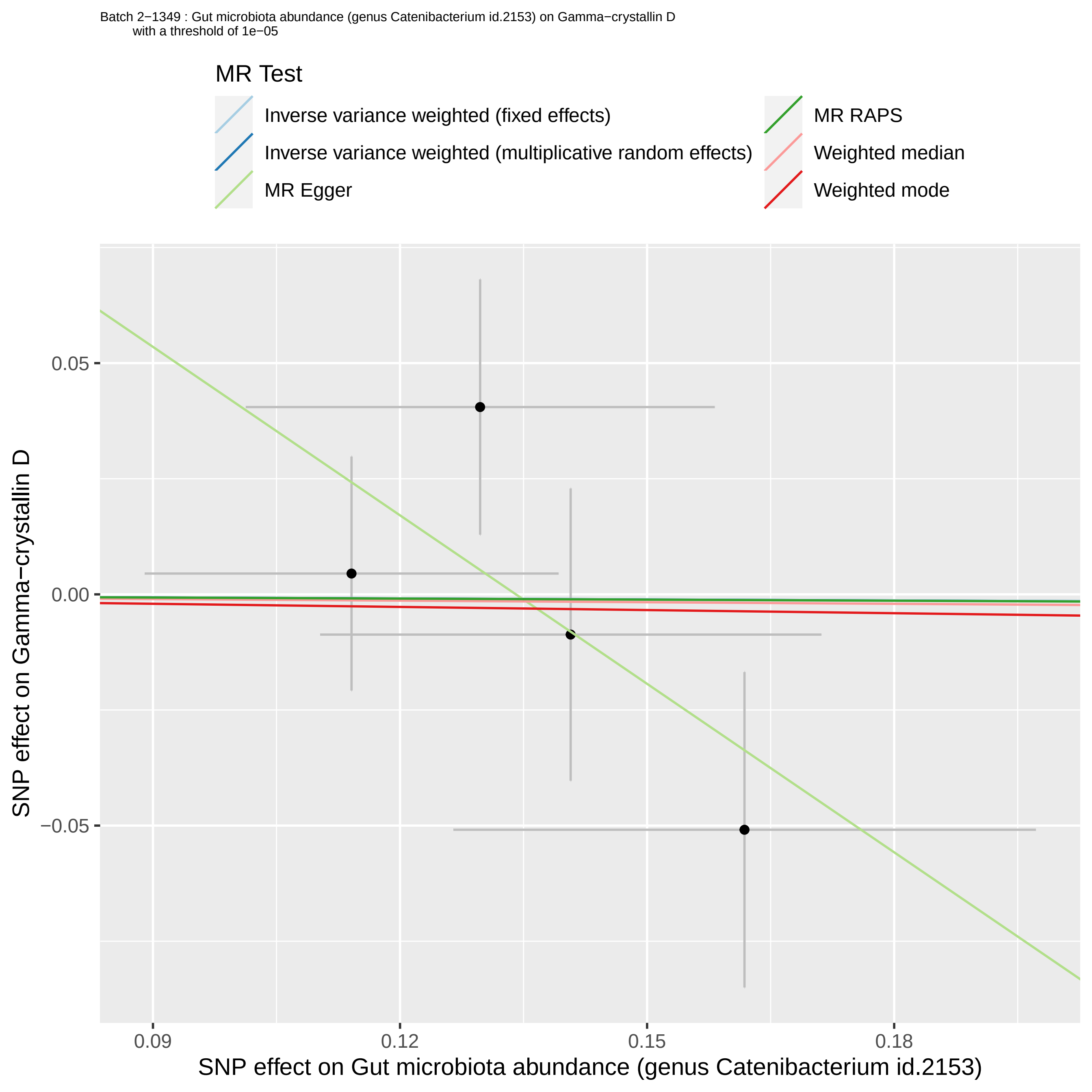
Batch 2−1349 : Gut microbiota abundance (genus Catenibacterium id.2153) on Gamma−crystallin D
with a threshold of 1e−05
MR Test
Inverse variance weighted (fixed effects)
Inverse variance weighted (multiplicative random effects)
MR Egger
MR RAPS
Weighted median
Weighted mode
0.05
0.00
−0.05
0.09
0.12
0.15
0.18
SNP effect on Gamma−crystallin D
SNP effect on Gut microbiota abundance (genus Catenibacterium id.2153)

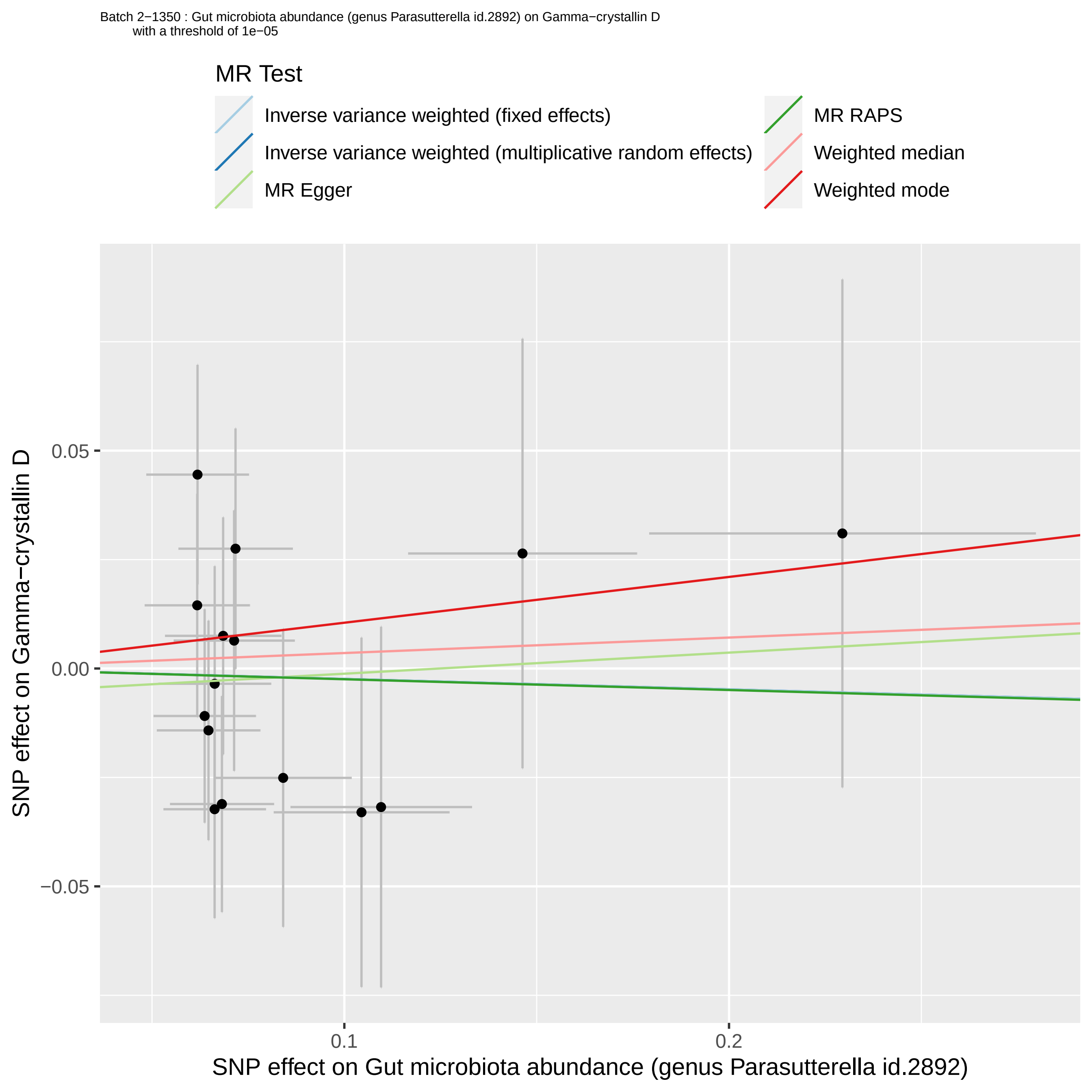
Batch 2−1350 : Gut microbiota abundance (genus Parasutterella id.2892) on Gamma−crystallin D
with a threshold of 1e−05
MR Test
Inverse variance weighted (fixed effects)
Inverse variance weighted (multiplicative random effects)
MR Egger
MR RAPS
Weighted median
Weighted mode
0.05
0.00
−0.05
0.1
0.2
SNP effect on Gamma−crystallin D
SNP effect on Gut microbiota abundance (genus Parasutterella id.2892)

Batch 2−1351 : Gut microbiota abundance (genus Catenibacterium id.2153) on Quinone oxidoreductase−like protein 1
with a threshold of 1e−05

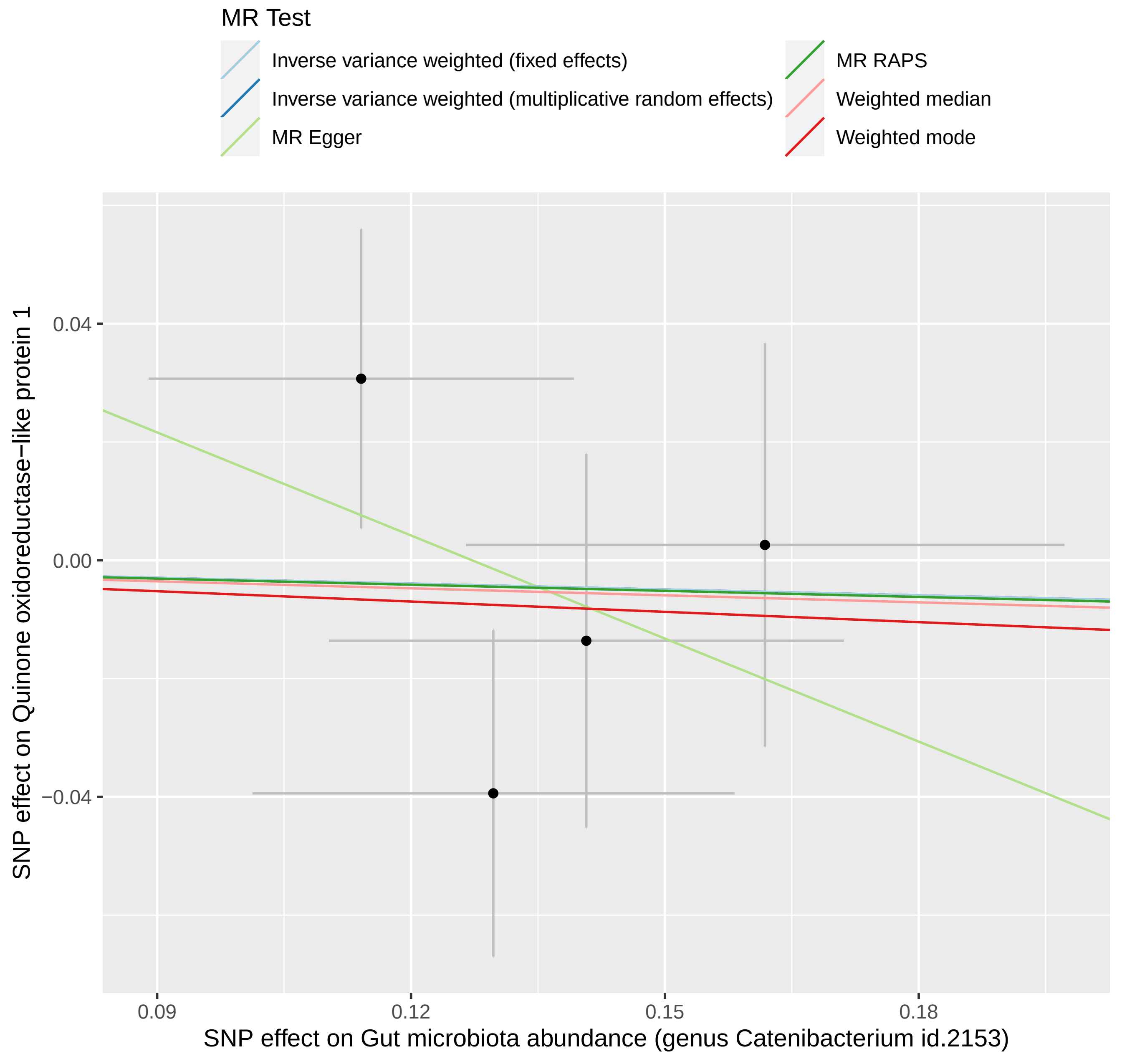

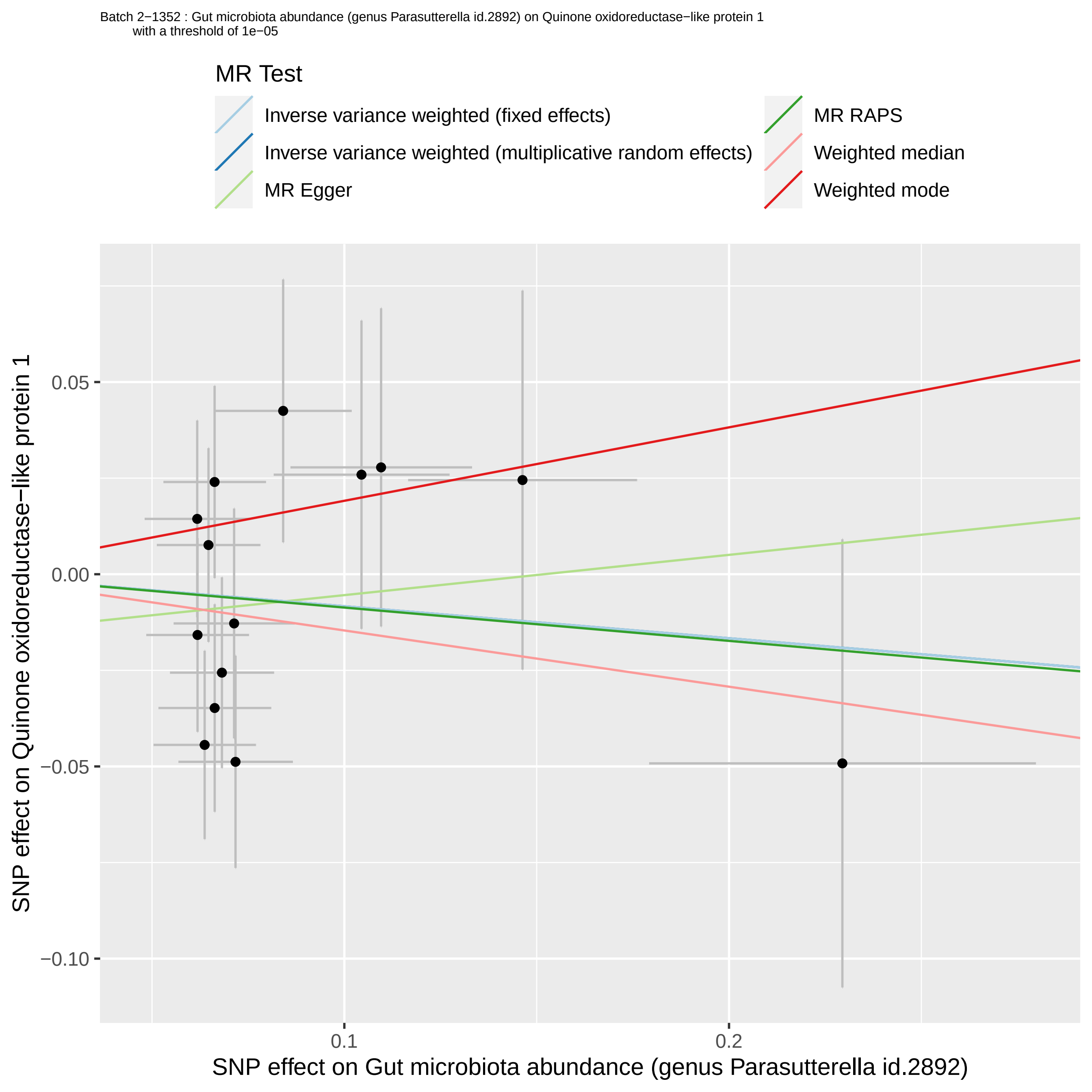
Batch 2−1352 : Gut microbiota abundance (genus Parasutterella id.2892) on Quinone oxidoreductase−like protein 1
with a threshold of 1e−05
MR Test
Inverse variance weighted (fixed effects)
Inverse variance weighted (multiplicative random effects)
MR Egger
MR RAPS
Weighted median
Weighted mode
0.05
0.00
−0.05
−0.10
0.1
0.2
SNP effect on Quinone oxidoreductase−like protein 1
SNP effect on Gut microbiota abundance (genus Parasutterella id.2892)

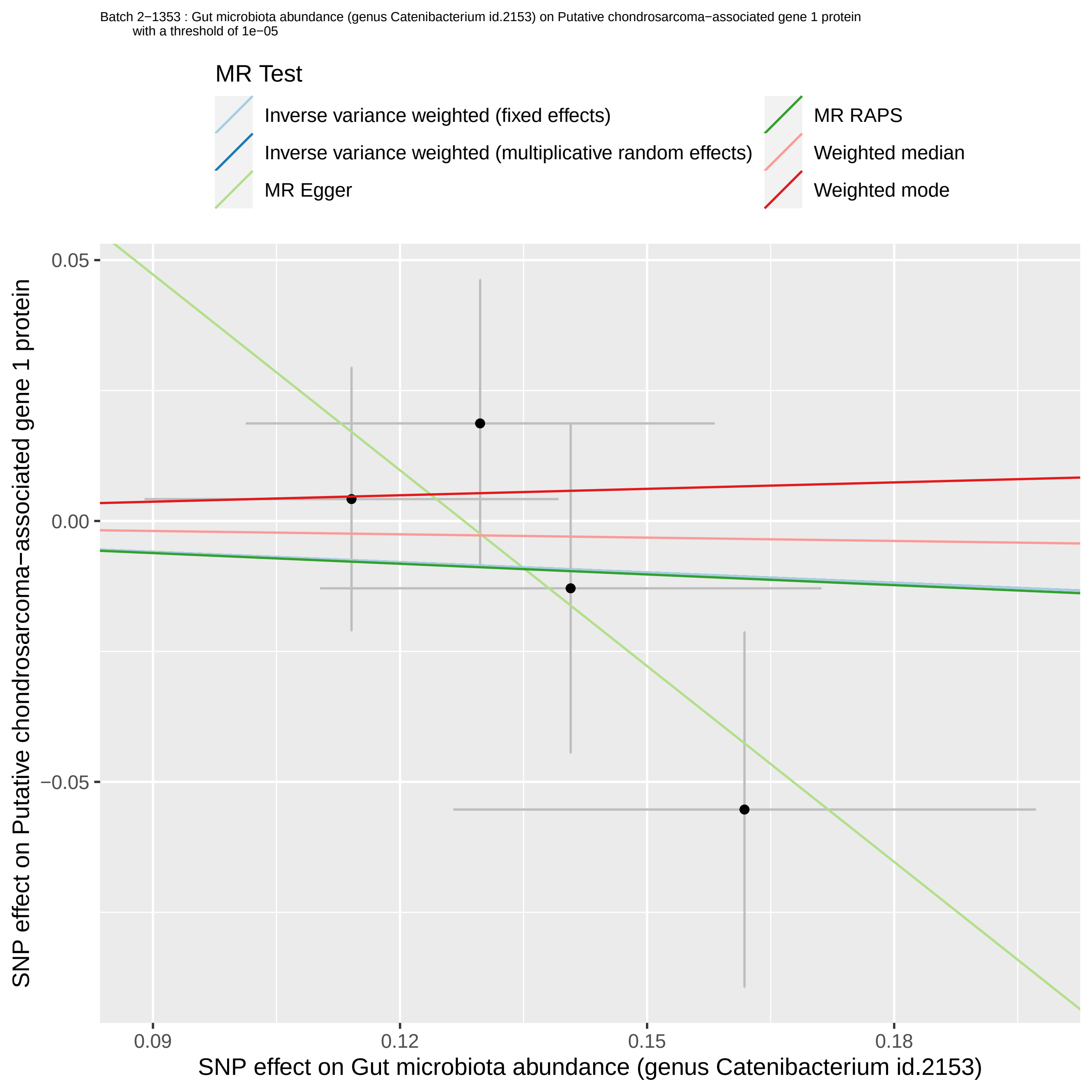

Batch 2−1353 : Gut microbiota abundance (genus Catenibacterium id.2153) on Putative chondrosarcoma−associated gene 1 protein
with a threshold of 1e−05
MR Test
Inverse variance weighted (fixed effects)
Inverse variance weighted (multiplicative random effects)
MR Egger
MR RAPS
Weighted median
Weighted mode
0.05
0.00
−0.05
0.09
0.12
0.15
0.18
SNP effect on Putative chondrosarcoma−associated gene 1 protein
SNP effect on Gut microbiota abundance (genus Catenibacterium id.2153)

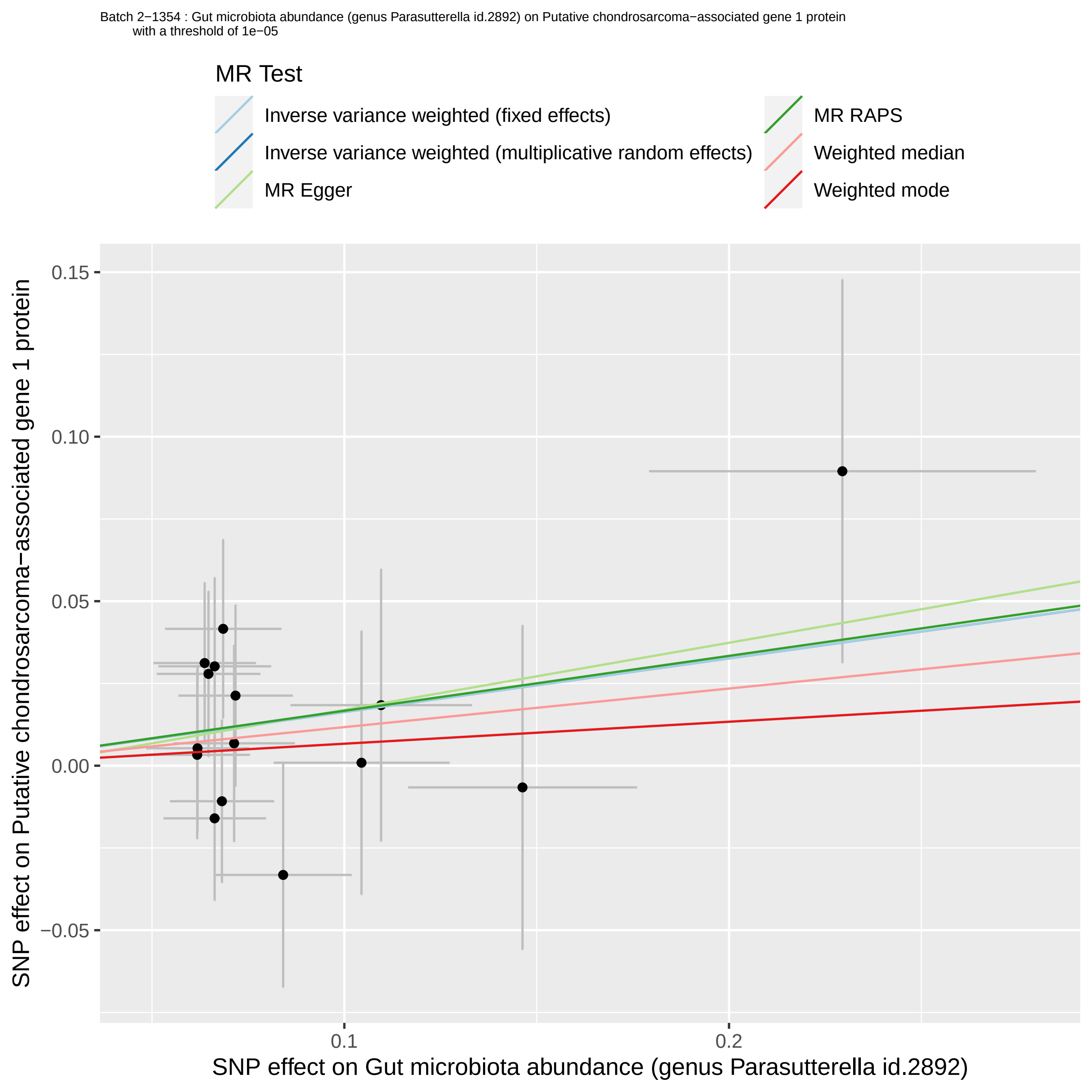
Batch 2−1354 : Gut microbiota abundance (genus Parasutterella id.2892) on Putative chondrosarcoma−associated gene 1 protein
with a threshold of 1e−05
MR Test
Inverse variance weighted (fixed effects)
Inverse variance weighted (multiplicative random effects)
MR Egger
MR RAPS
Weighted median
Weighted mode
0.15
0.10
0.05
0.00
−0.05
0.1
0.2
SNP effect on Putative chondrosarcoma−associated gene 1 protein
SNP effect on Gut microbiota abundance (genus Parasutterella id.2892)

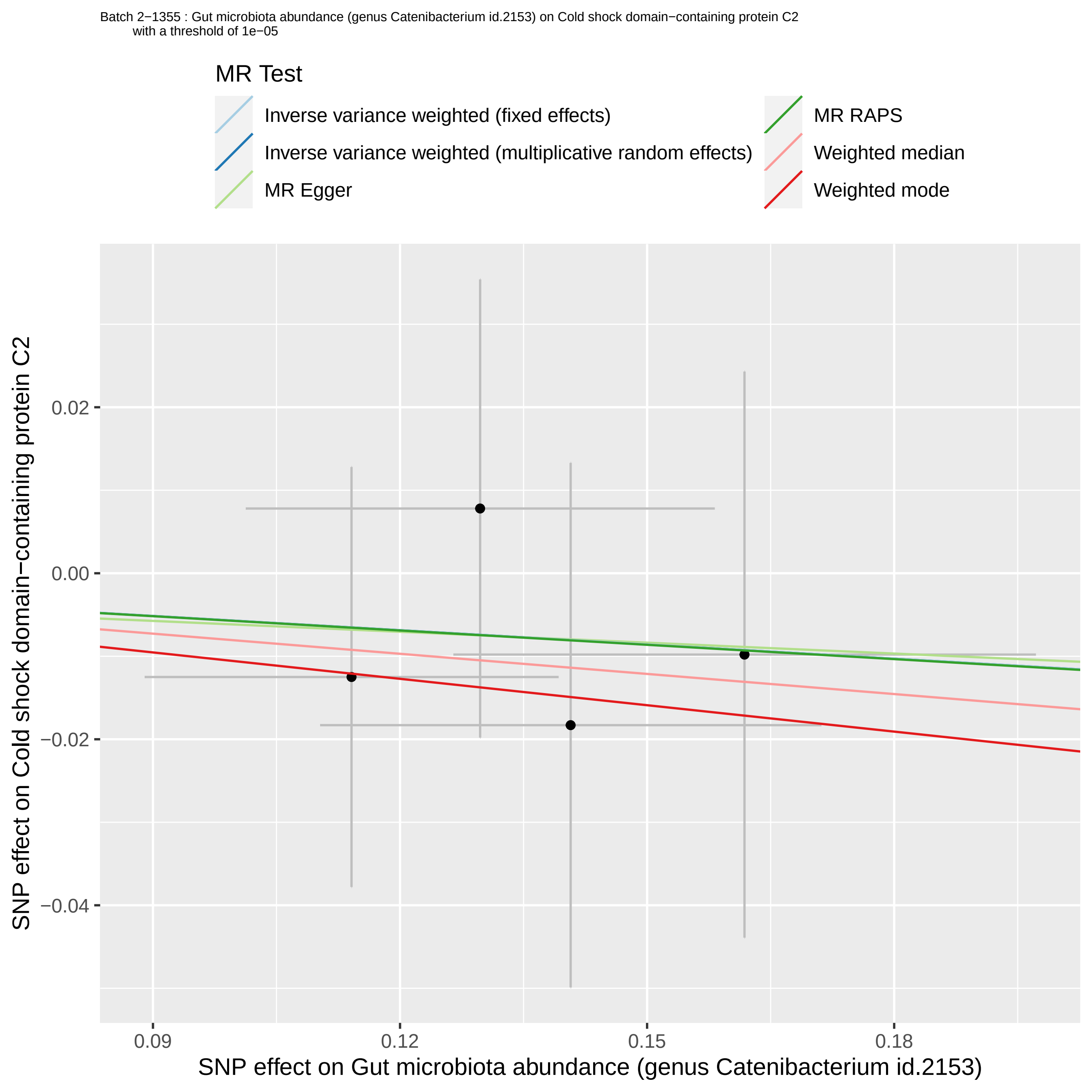

Batch 2−1355 : Gut microbiota abundance (genus Catenibacterium id.2153) on Cold shock domain−containing protein C2
with a threshold of 1e−05
MR Test
Inverse variance weighted (fixed effects)
Inverse variance weighted (multiplicative random effects)
MR Egger
MR RAPS
Weighted median
Weighted mode
SNP effect on Cold shock domain−containing protein C2
0.02
0.00
−0.02
−0.04
0.09
0.12
0.15
0.18
SNP effect on Gut microbiota abundance (genus Catenibacterium id.2153)

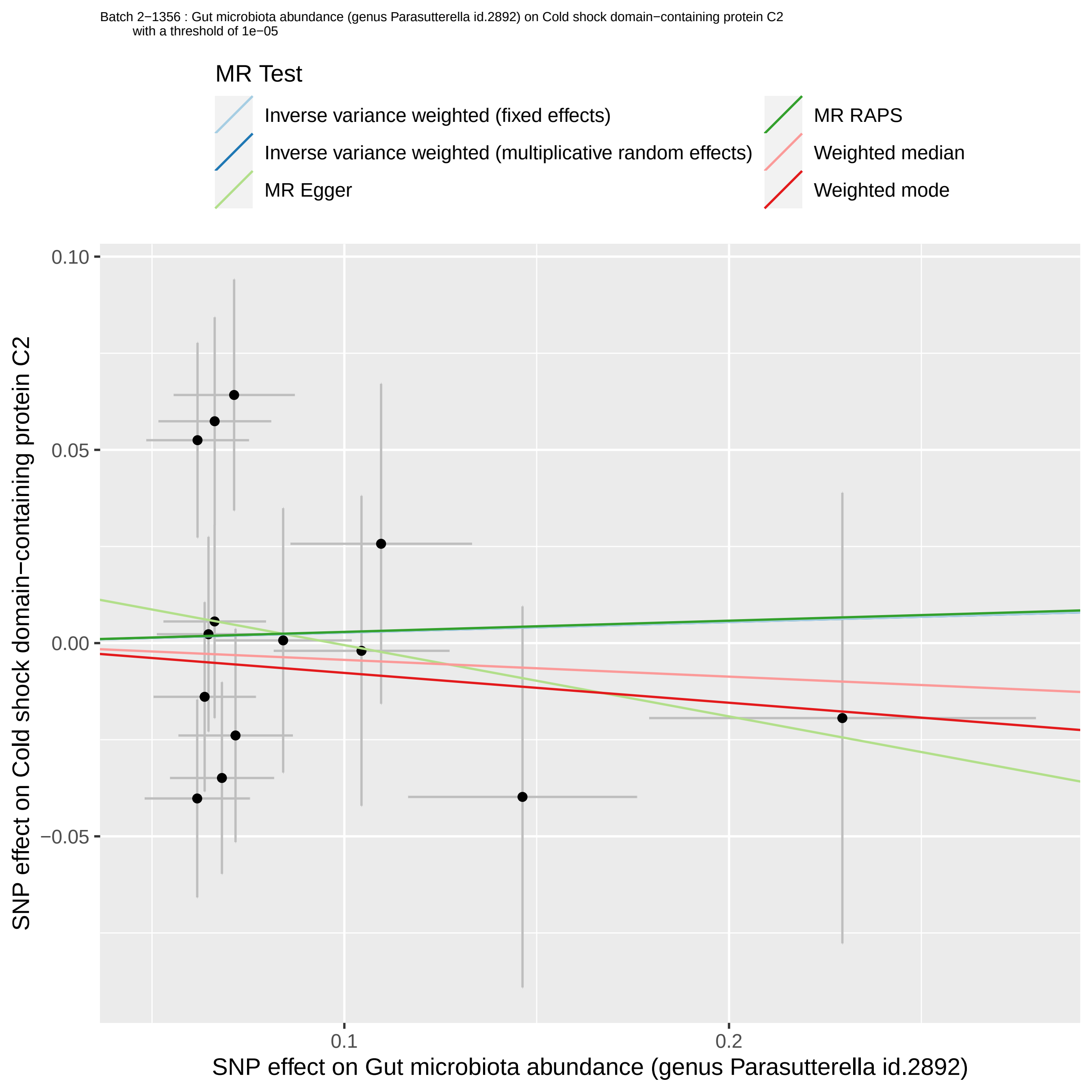
Batch 2−1356 : Gut microbiota abundance (genus Parasutterella id.2892) on Cold shock domain−containing protein C2
with a threshold of 1e−05
MR Test
Inverse variance weighted (fixed effects)
Inverse variance weighted (multiplicative random effects)
MR Egger
MR RAPS
Weighted median
Weighted mode
0.10
0.05
0.00
−0.05
0.1
0.2
SNP effect on Cold shock domain−containing protein C2
SNP effect on Gut microbiota abundance (genus Parasutterella id.2892)

Batch 2−1357 : Gut microbiota abundance (genus Catenibacterium id.2153) on Cold shock domain−containing protein E1
with a threshold of 1e−05

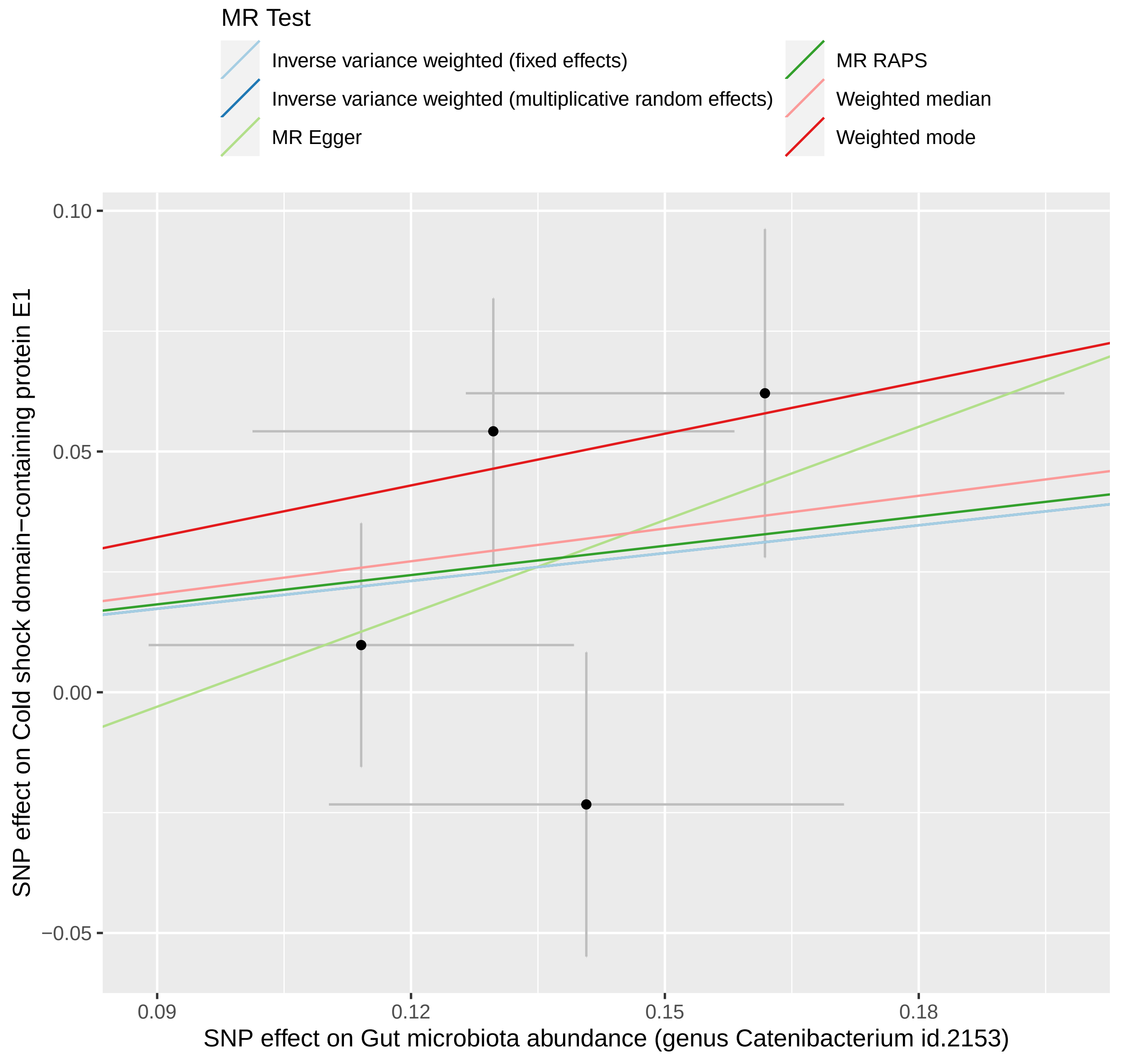

Batch 2−1358 : Gut microbiota abundance (genus Parasutterella id.2892) on Cold shock domain−containing protein E1
with a threshold of 1e−05

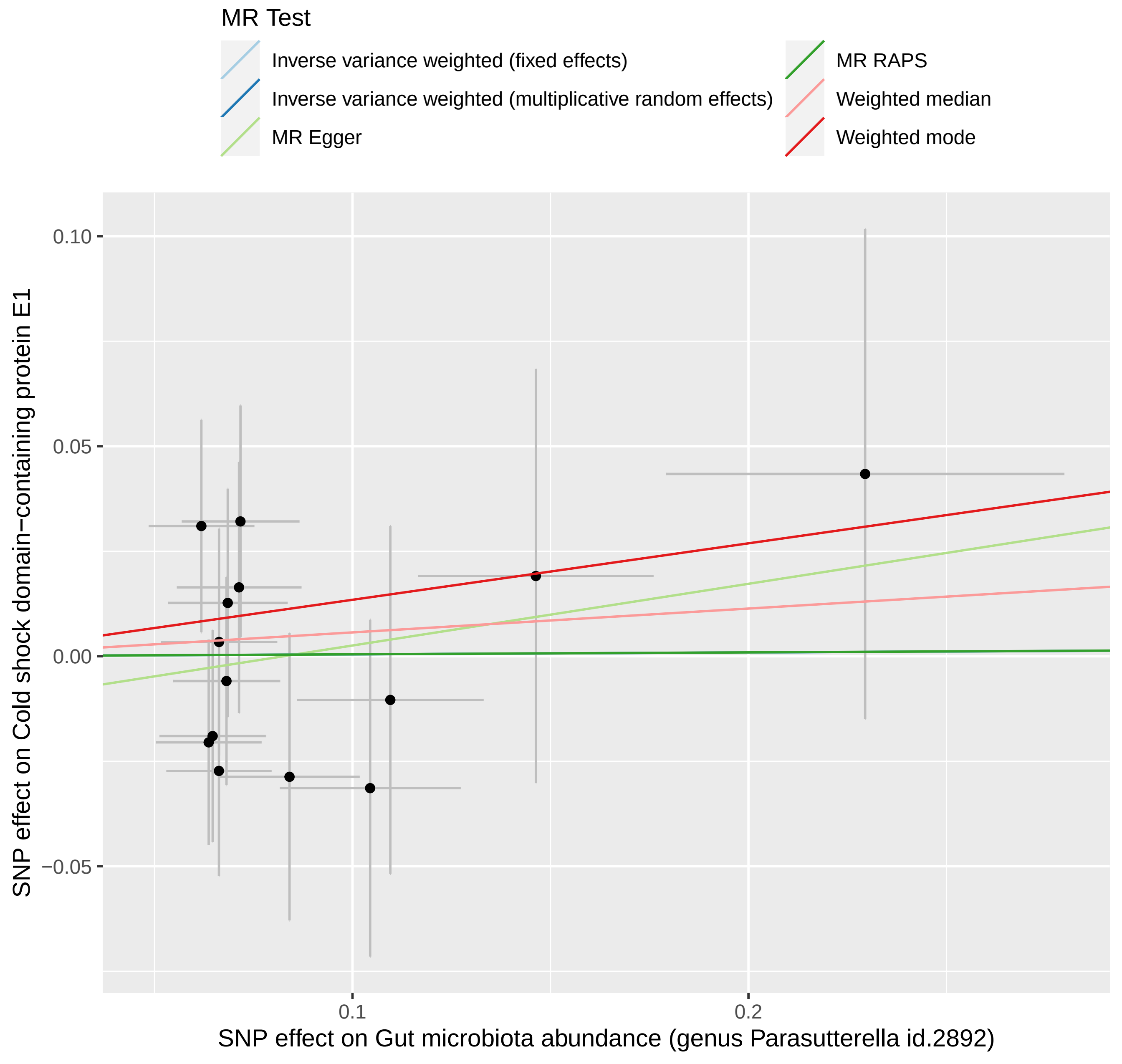

Batch 2−1359 : Gut microbiota abundance (genus Catenibacterium id.2153) on Macrophage colony−stimulating factor 1
with a threshold of 1e−05

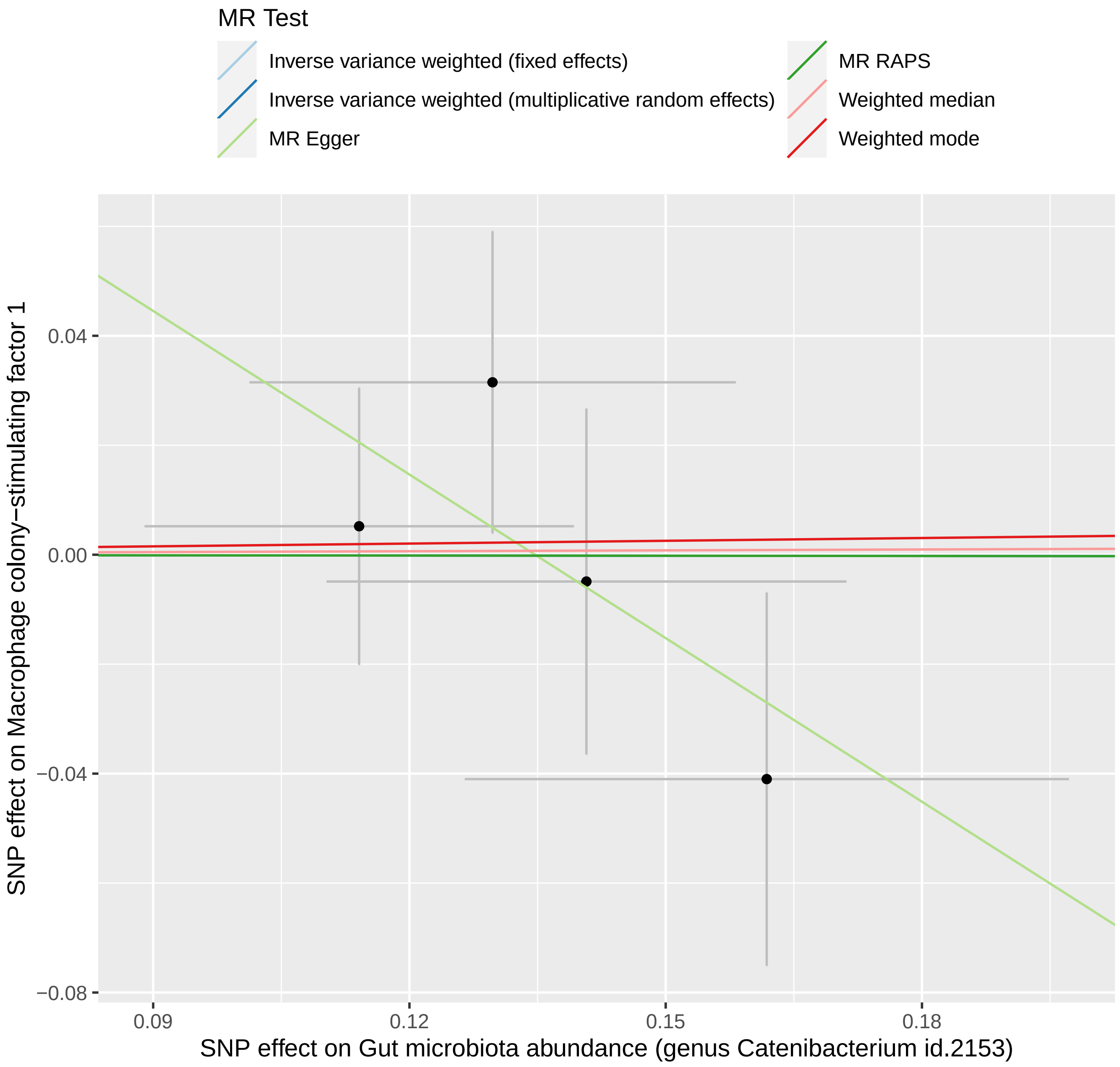

Batch 2−1360 : Gut microbiota abundance (genus Parasutterella id.2892) on Macrophage colony−stimulating factor 1 with a threshold of 1e−05

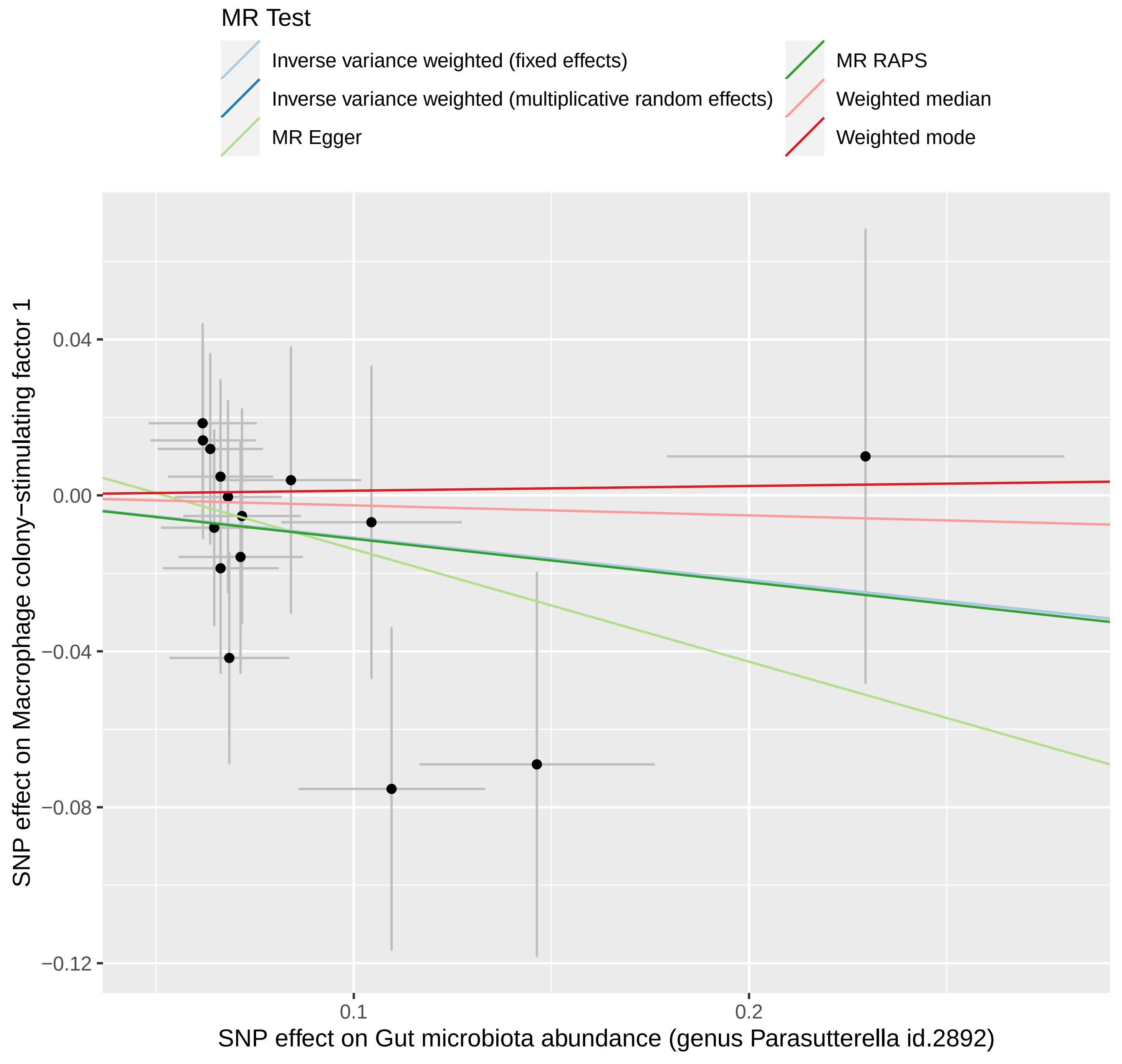

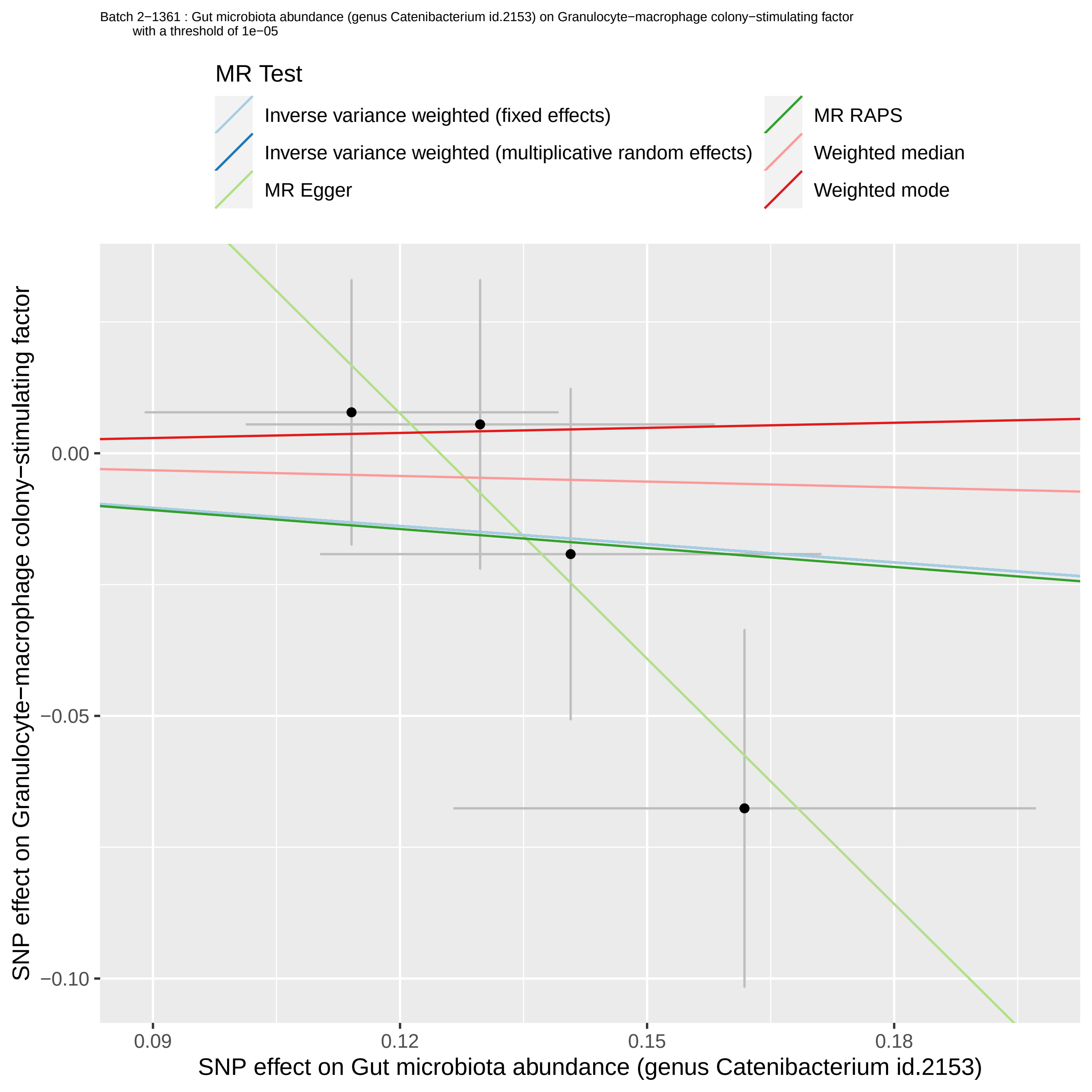
Batch 2−1361 : Gut microbiota abundance (genus Catenibacterium id.2153) on Granulocyte−macrophage colony−stimulating factor
with a threshold of 1e−05
MR Test
Inverse variance weighted (fixed effects)
Inverse variance weighted (multiplicative random effects)
MR Egger
MR RAPS
Weighted median
Weighted mode
SNP effect on Granulocyte−macrophage colony−stimulating factor
0.00
−0.05
−0.10
0.09
0.12
0.15
0.18
SNP effect on Gut microbiota abundance (genus Catenibacterium id.2153)

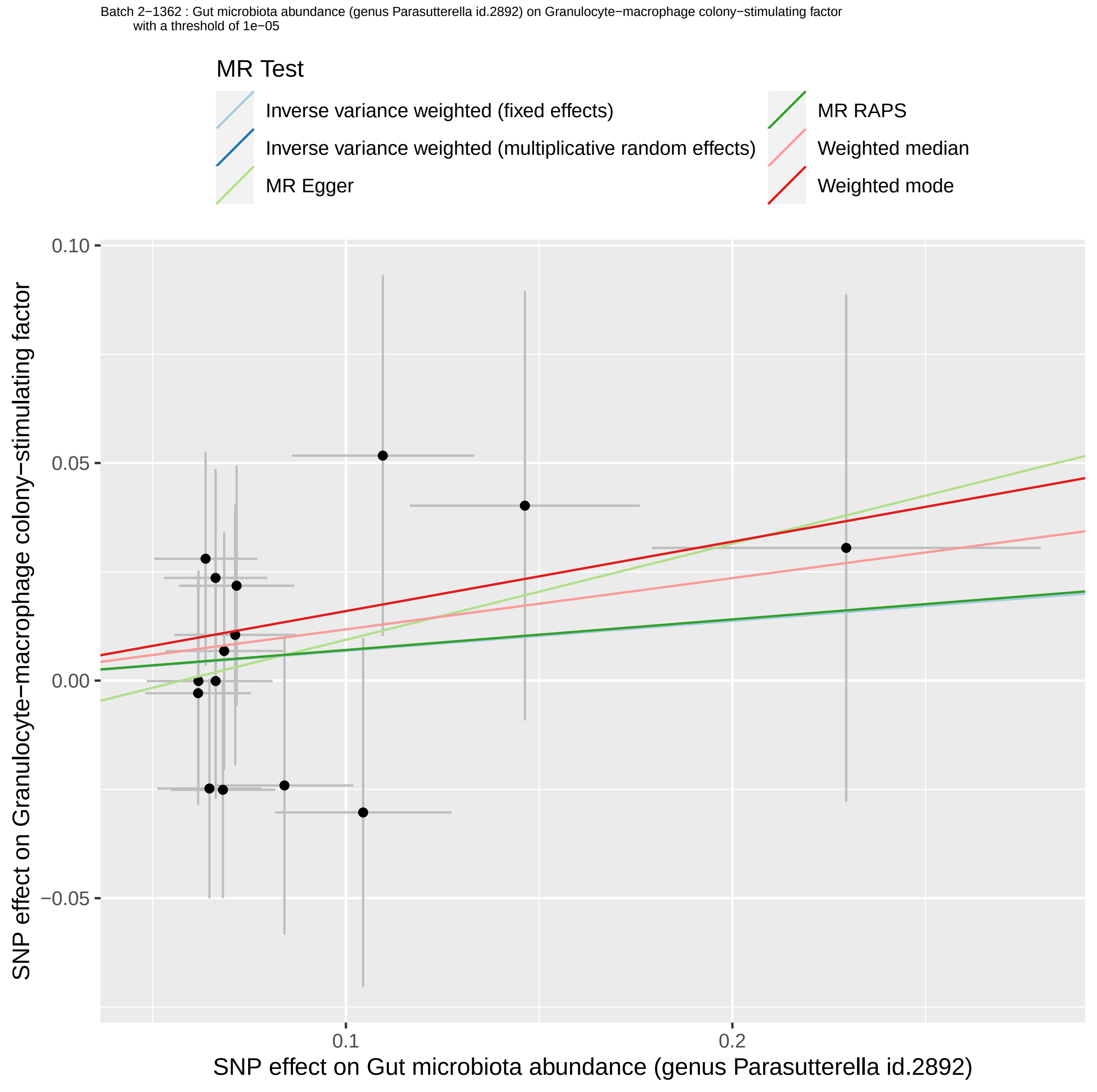

Batch 2−1362 : Gut microbiota abundance (genus Parasutterella id.2892) on Granulocyte−macrophage colony−stimulating factor
with a threshold of 1e−05
MR Test
Inverse variance weighted (fixed effects)
Inverse variance weighted (multiplicative random effects)
MR Egger
MR RAPS
Weighted median
Weighted mode
0.10
0.05
0.00
−0.05
0.1
0.2
SNP effect on Granulocyte−macrophage colony−stimulating factor
SNP effect on Gut microbiota abundance (genus Parasutterella id.2892)

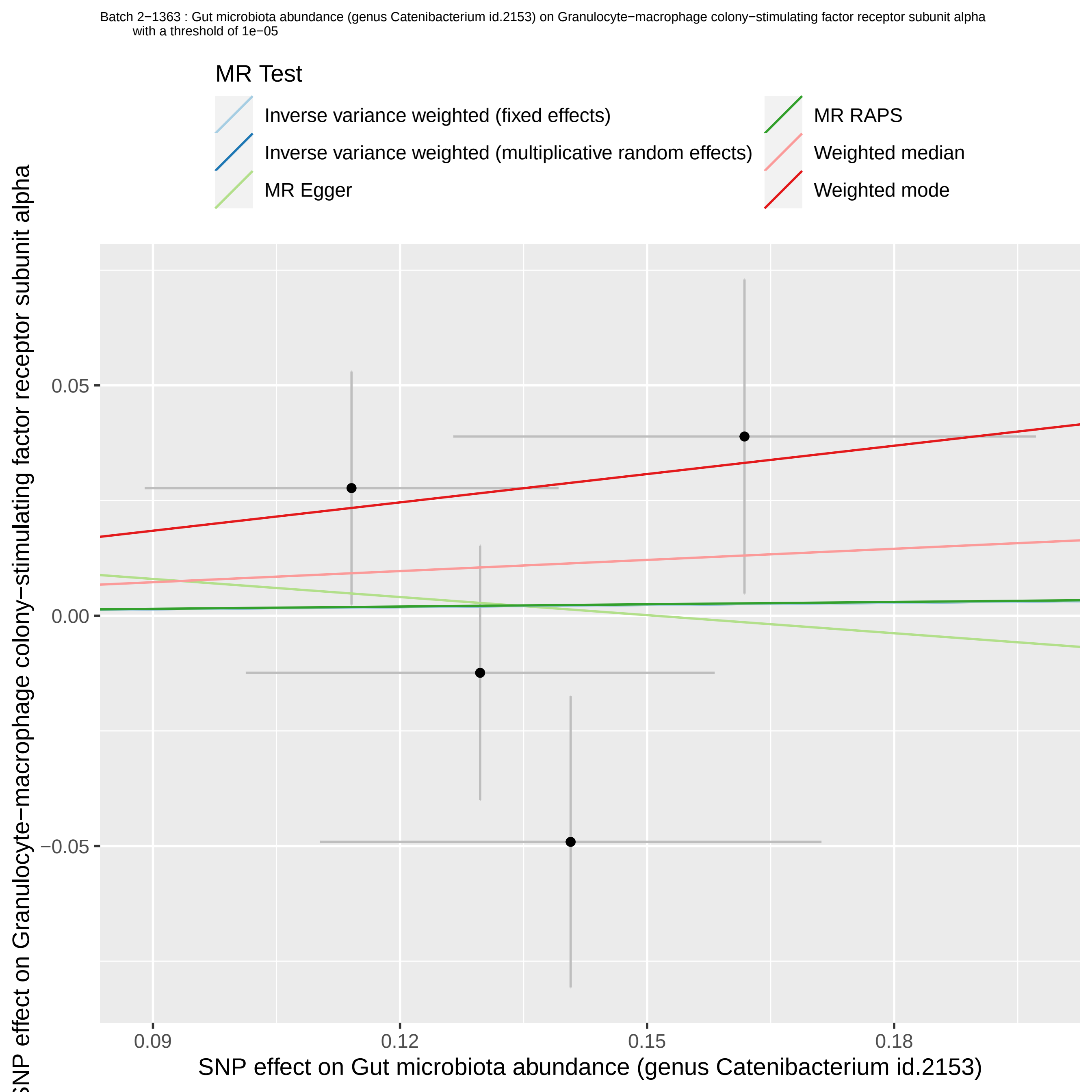
Batch 2−1363 : Gut microbiota abundance (genus Catenibacterium id.2153) on Granulocyte−macrophage colony−stimulating factor receptor subunit alpha with a threshold of 1e−05
MR Test
Inverse variance weighted (fixed effects)
Inverse variance weighted (multiplicative random effects)
MR Egger
MR RAPS
Weighted median
Weighted mode
0.05
0.00
−0.05
0.09
0.12
0.15
0.18
SNP effect on Gut microbiota abundance (genus Catenibacterium id.2153)
SNP effect on Granulocyte−macrophage colony−stimulating factor receptor subunit alpha

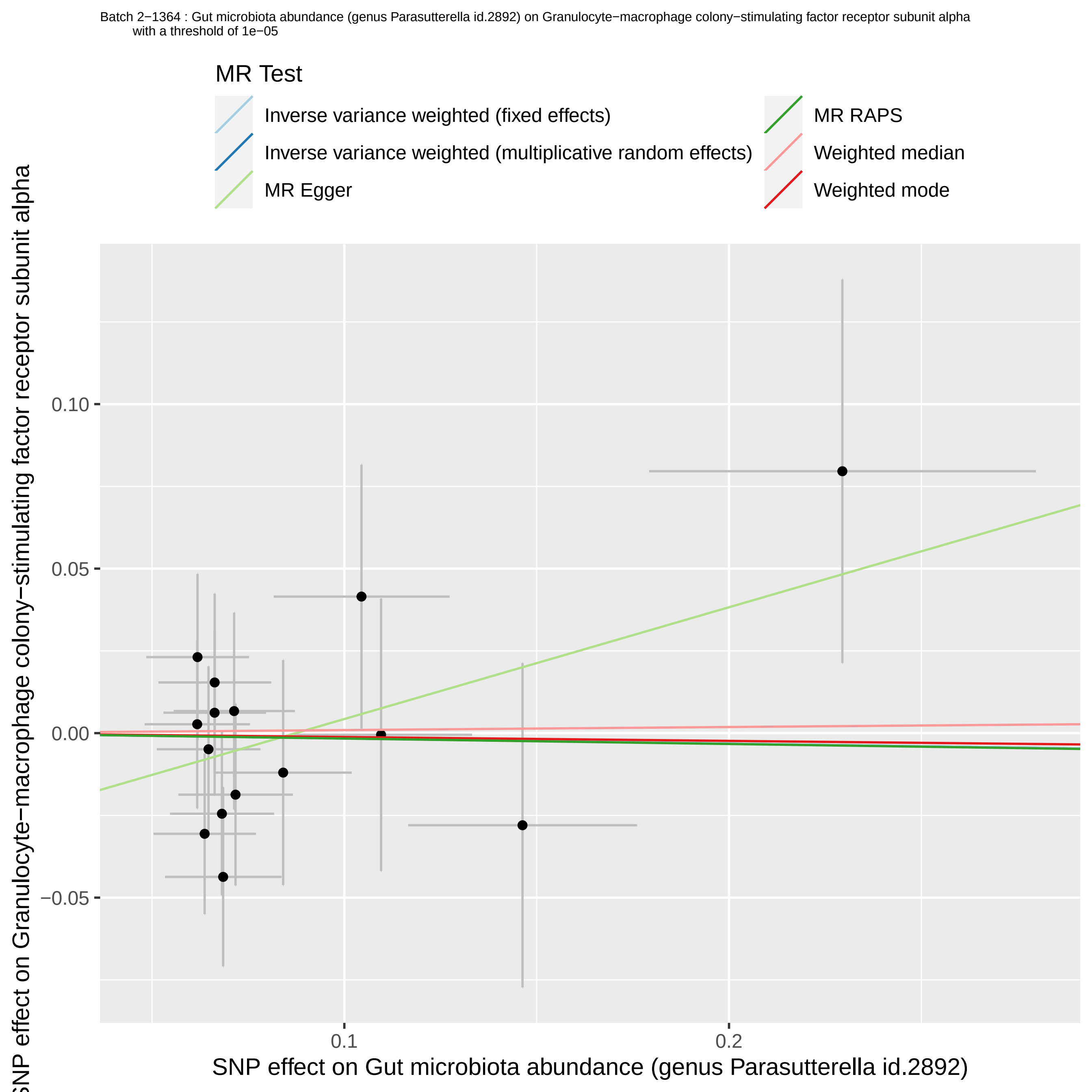
Batch 2−1364 : Gut microbiota abundance (genus Parasutterella id.2892) on Granulocyte−macrophage colony−stimulating factor receptor subunit alpha
with a threshold of 1e−05
MR Test
Inverse variance weighted (fixed effects)
Inverse variance weighted (multiplicative random effects)
MR Egger
MR RAPS
Weighted median
Weighted mode
0.10
0.05
0.00
−0.05
0.1
0.2
SNP effect on Gut microbiota abundance (genus Parasutterella id.2892)
SNP effect on Granulocyte−macrophage colony−stimulating factor receptor subunit alpha

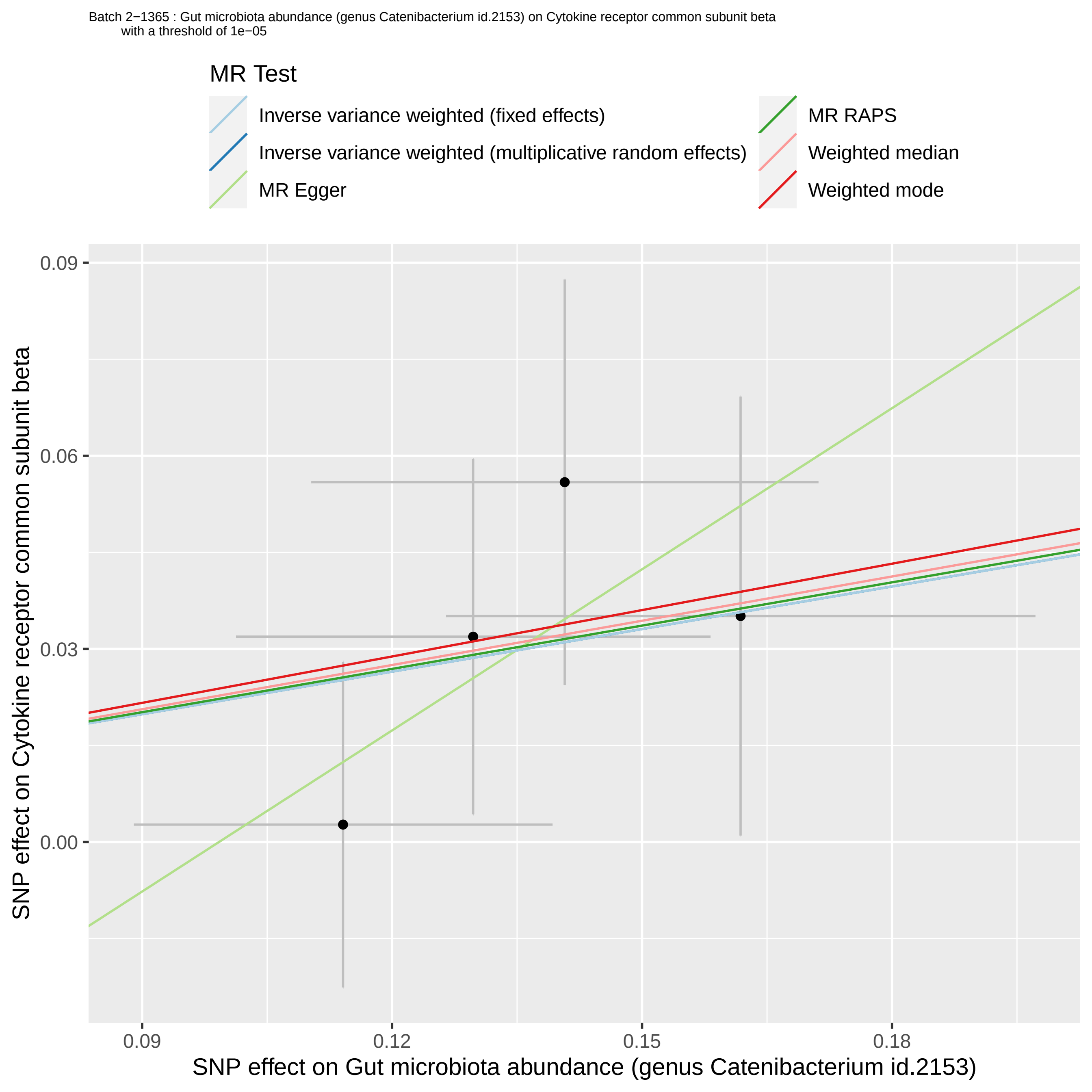
Batch 2−1365 : Gut microbiota abundance (genus Catenibacterium id.2153) on Cytokine receptor common subunit beta
with a threshold of 1e−05
MR Test
Inverse variance weighted (fixed effects)
Inverse variance weighted (multiplicative random effects)
MR Egger
MR RAPS
Weighted median
Weighted mode
0.09
0.06
0.03
0.00
0.09
0.12
0.15
0.18
SNP effect on Cytokine receptor common subunit beta
SNP effect on Gut microbiota abundance (genus Catenibacterium id.2153)

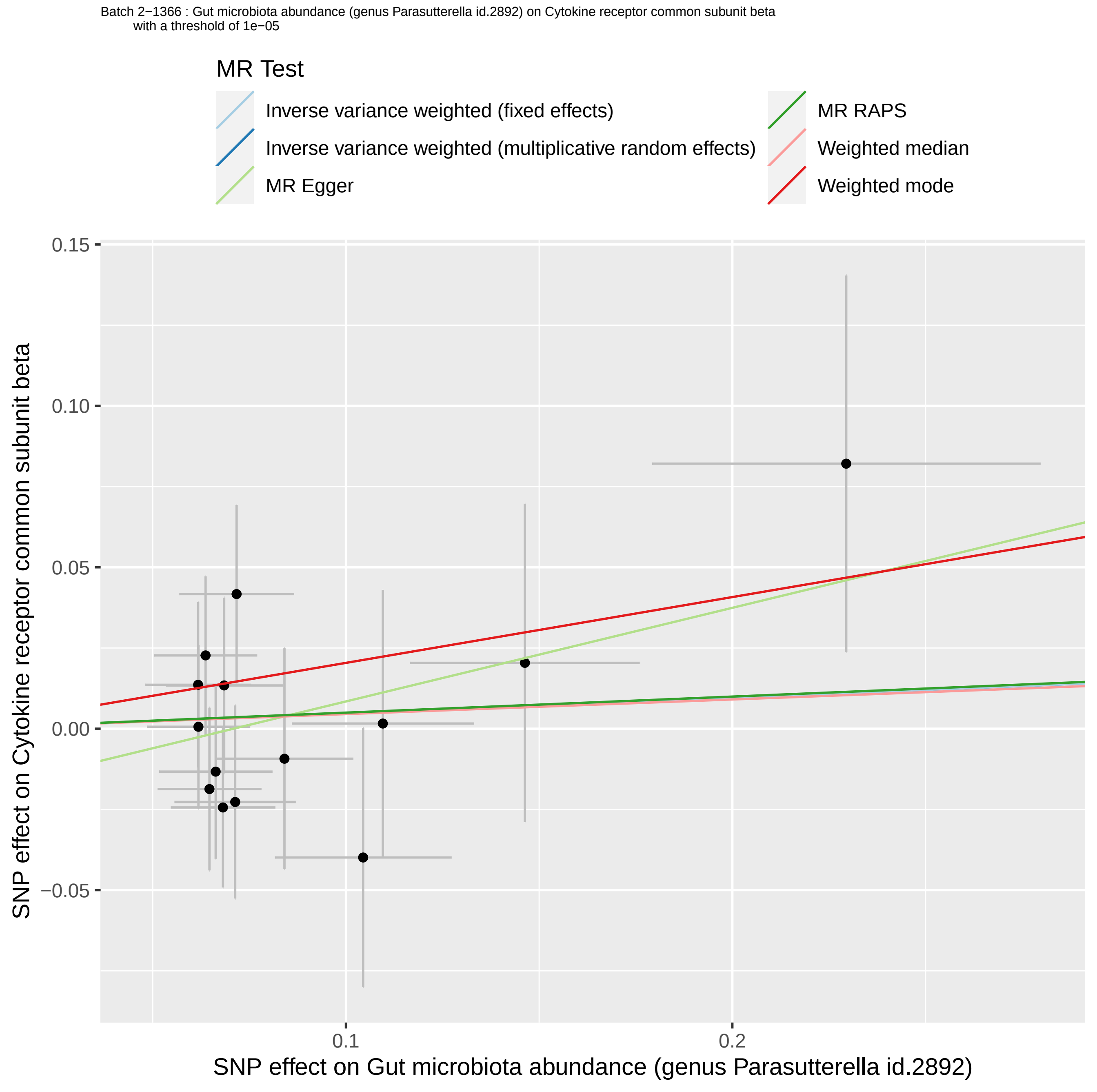
Batch 2−1366 : Gut microbiota abundance (genus Parasutterella id.2892) on Cytokine receptor common subunit beta
with a threshold of 1e−05
MR Test
Inverse variance weighted (fixed effects)
Inverse variance weighted (multiplicative random effects)
MR Egger
MR RAPS
Weighted median
Weighted mode
0.15
0.10
0.05
0.00
−0.05
0.1
0.2
SNP effect on Cytokine receptor common subunit beta
SNP effect on Gut microbiota abundance (genus Parasutterella id.2892)

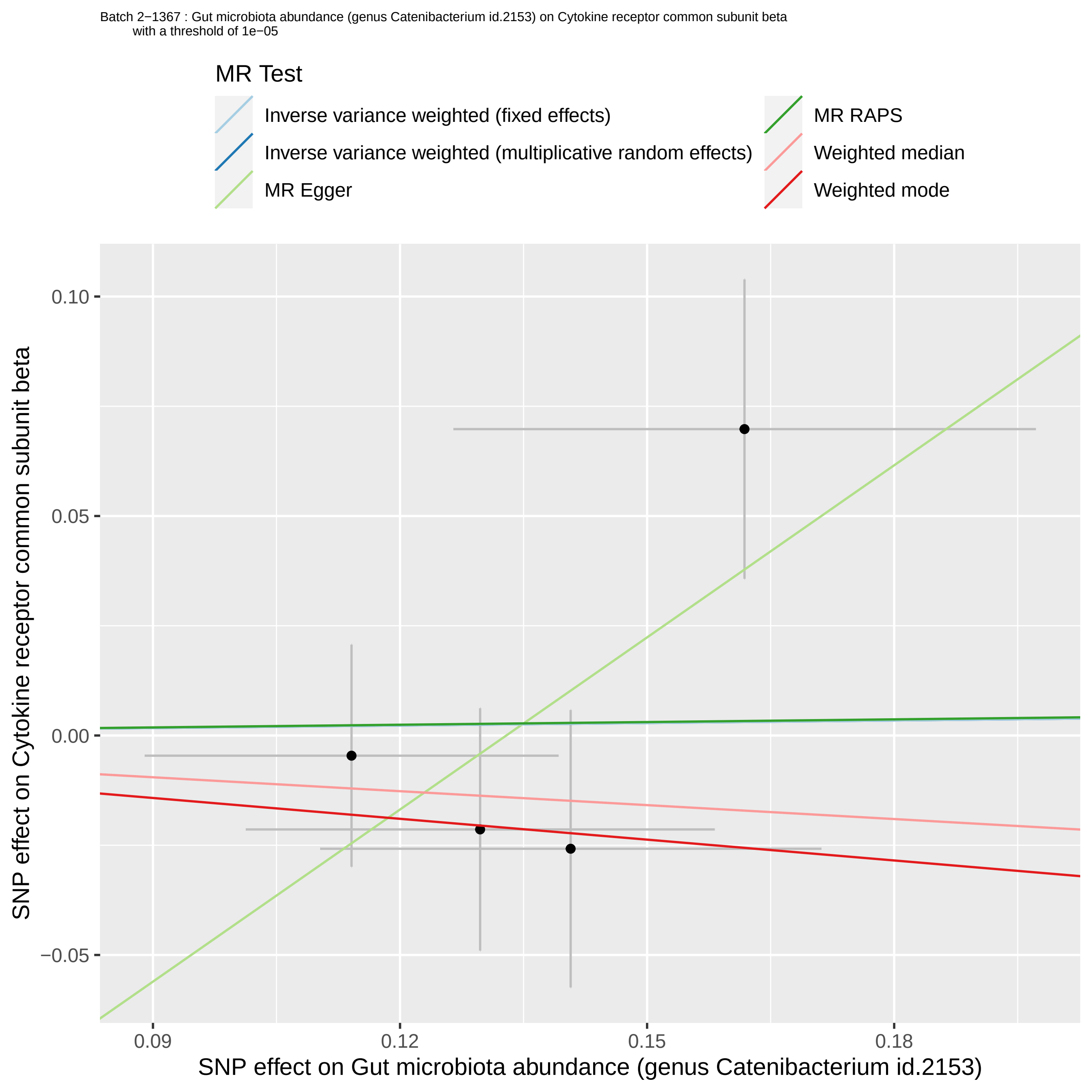

Batch 2−1367 : Gut microbiota abundance (genus Catenibacterium id.2153) on Cytokine receptor common subunit beta
with a threshold of 1e−05
MR Test
Inverse variance weighted (fixed effects)
Inverse variance weighted (multiplicative random effects)
MR Egger
MR RAPS
Weighted median
Weighted mode
0.10
0.05
0.00
−0.05
0.09
0.12
0.15
0.18
SNP effect on Cytokine receptor common subunit beta
SNP effect on Gut microbiota abundance (genus Catenibacterium id.2153)

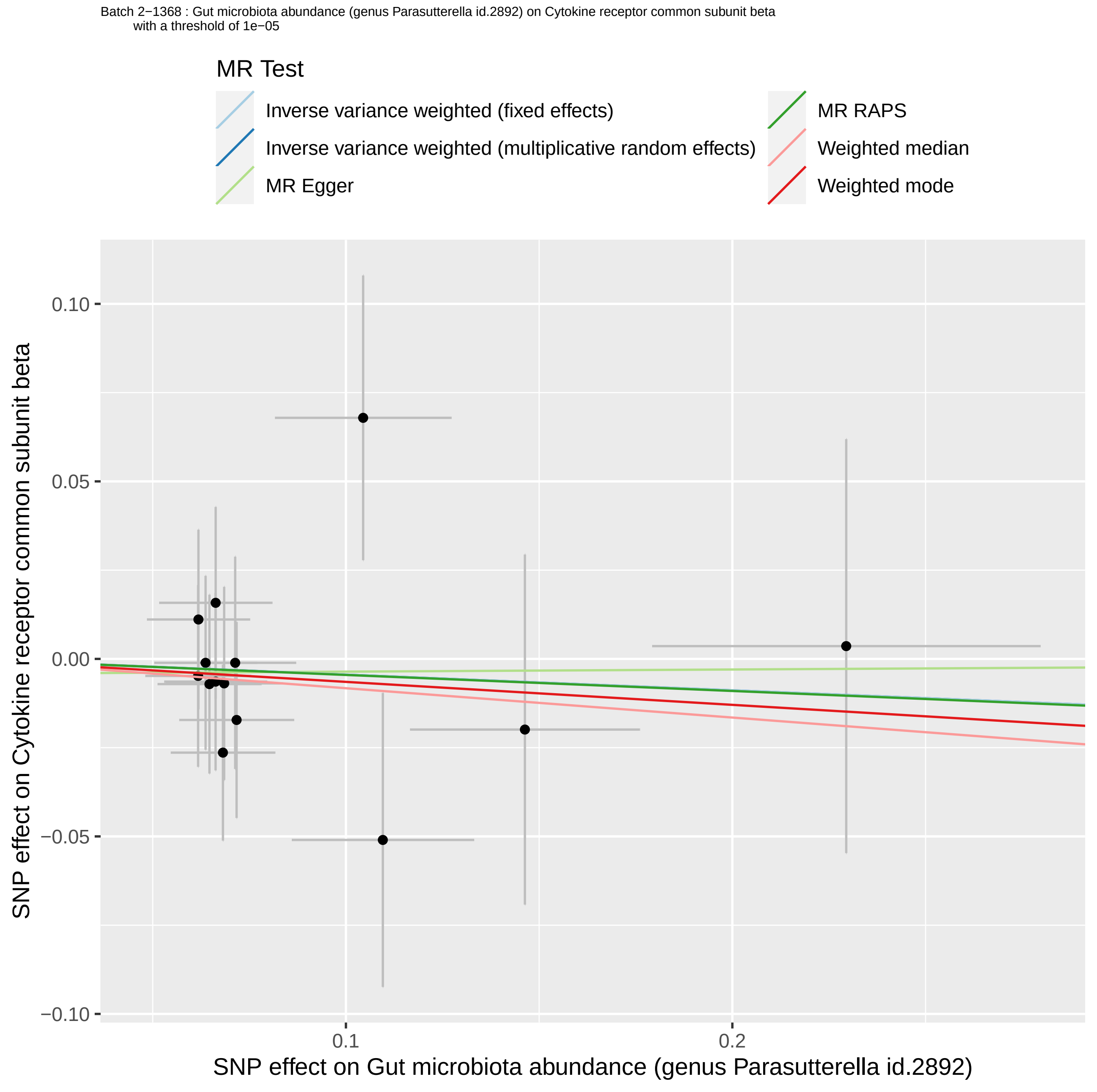
Batch 2−1368 : Gut microbiota abundance (genus Parasutterella id.2892) on Cytokine receptor common subunit beta
with a threshold of 1e−05
MR Test
Inverse variance weighted (fixed effects)
Inverse variance weighted (multiplicative random effects)
MR Egger
MR RAPS
Weighted median
Weighted mode
0.10
0.05
0.00
−0.05
−0.10
0.1
0.2
SNP effect on Cytokine receptor common subunit beta
SNP effect on Gut microbiota abundance (genus Parasutterella id.2892)

Batch 2−1369 : Gut microbiota abundance (genus Catenibacterium id.2153) on Granulocyte colony−stimulating factor
with a threshold of 1e−05

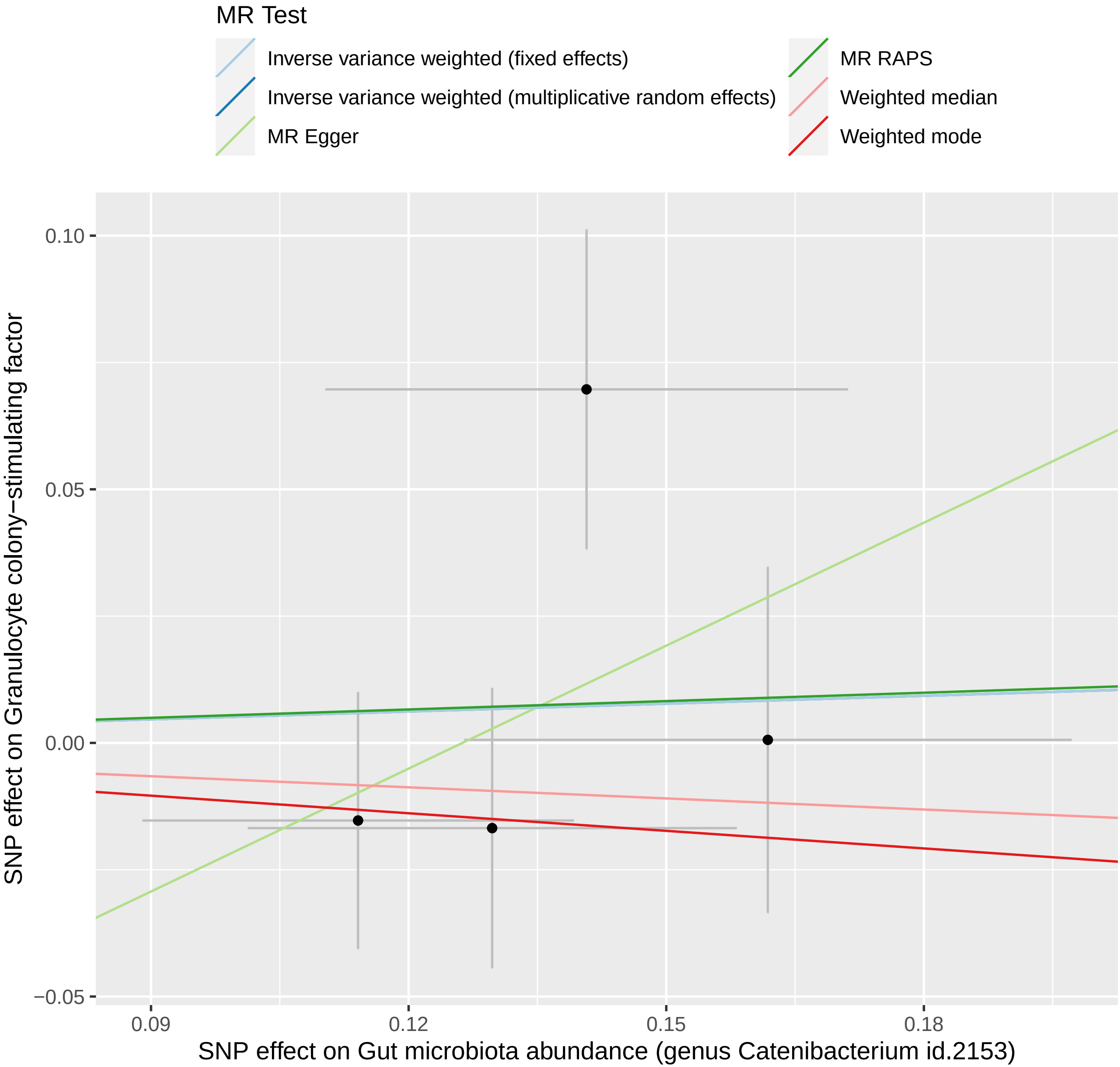

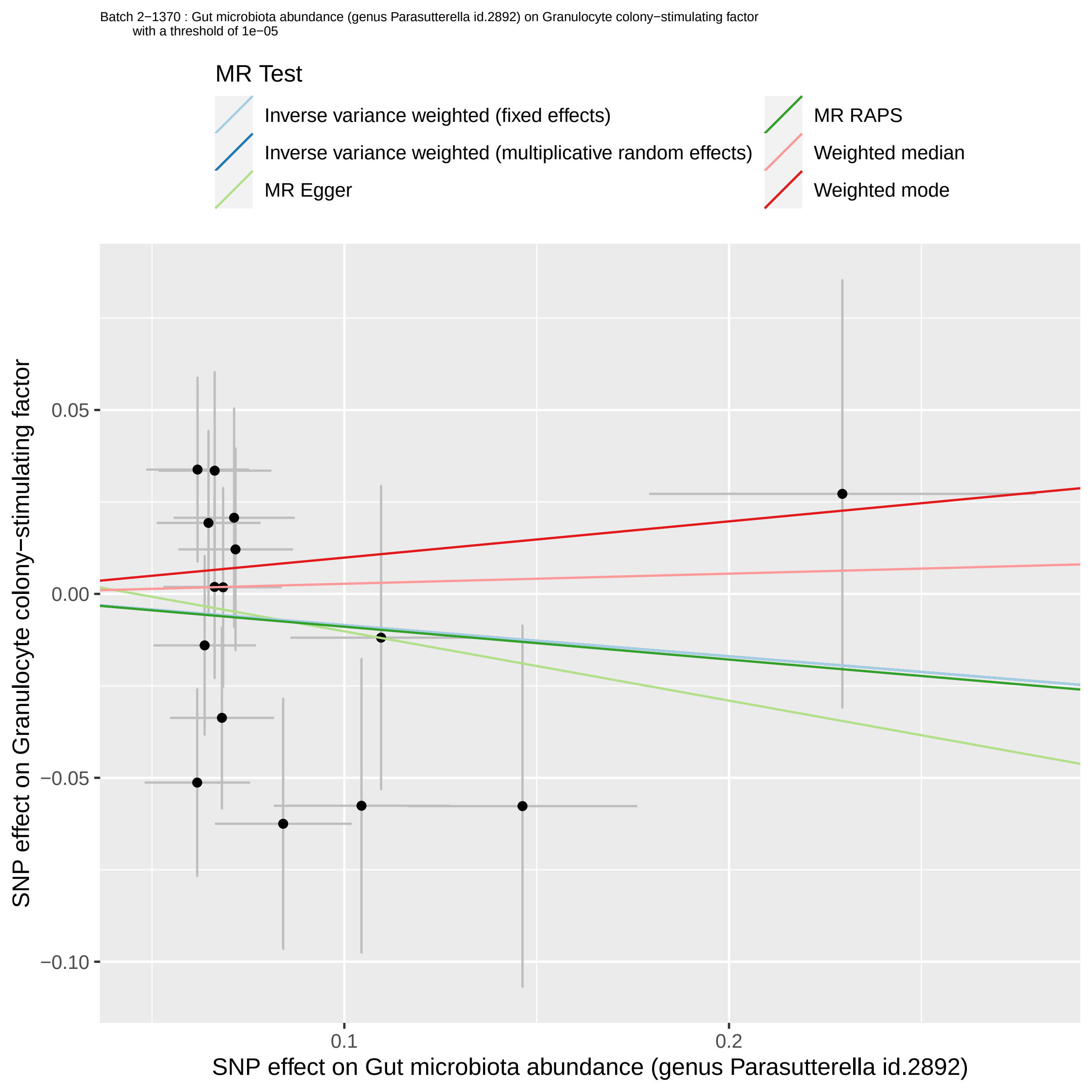
Batch 2−1370 : Gut microbiota abundance (genus Parasutterella id.2892) on Granulocyte colony−stimulating factor
with a threshold of 1e−05
MR Test
Inverse variance weighted (fixed effects)
Inverse variance weighted (multiplicative random effects)
MR Egger
MR RAPS
Weighted median
Weighted mode
0.05
0.00
−0.05
−0.10
0.1
0.2
SNP effect on Granulocyte colony−stimulating factor
SNP effect on Gut microbiota abundance (genus Parasutterella id.2892)

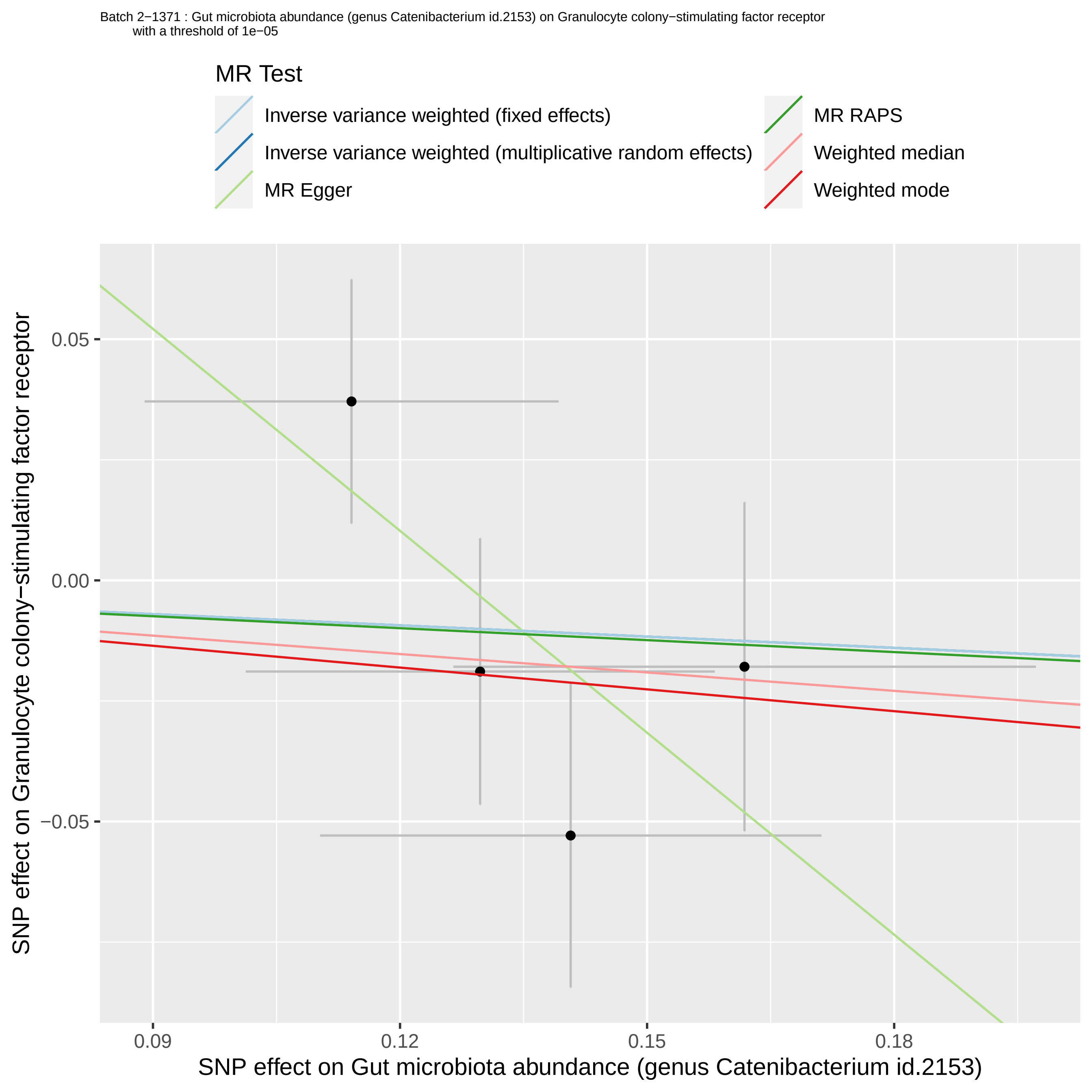
Batch 2−1371 : Gut microbiota abundance (genus Catenibacterium id.2153) on Granulocyte colony−stimulating factor receptor
with a threshold of 1e−05
MR Test
Inverse variance weighted (fixed effects)
Inverse variance weighted (multiplicative random effects)
MR Egger
MR RAPS
Weighted median
Weighted mode
0.05
0.00
−0.05
0.09
0.12
0.15
0.18
SNP effect on Granulocyte colony−stimulating factor receptor
SNP effect on Gut microbiota abundance (genus Catenibacterium id.2153)

Batch 2−1372 : Gut microbiota abundance (genus Parasutterella id.2892) on Granulocyte colony−stimulating factor receptor
with a threshold of 1e−05

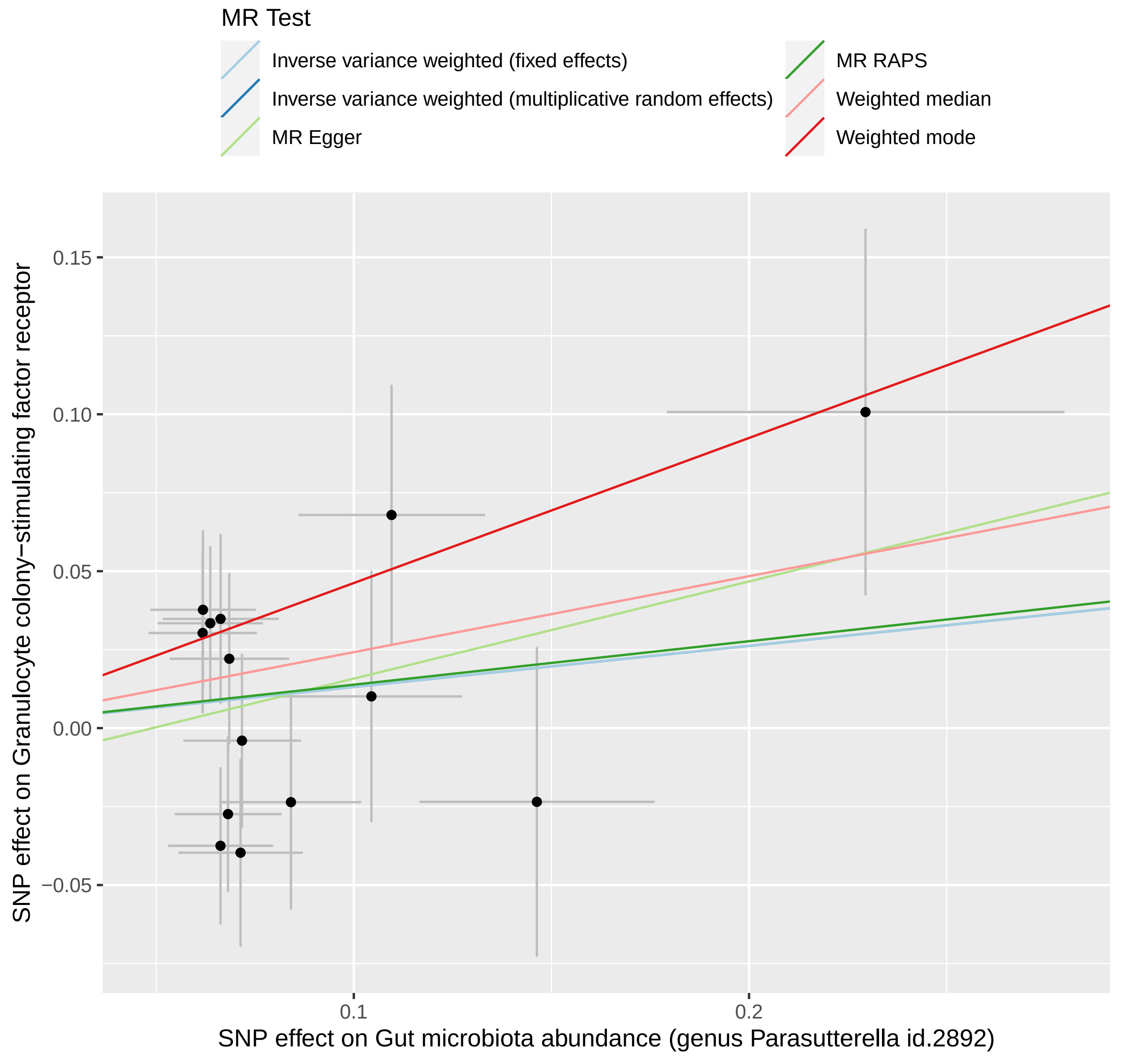

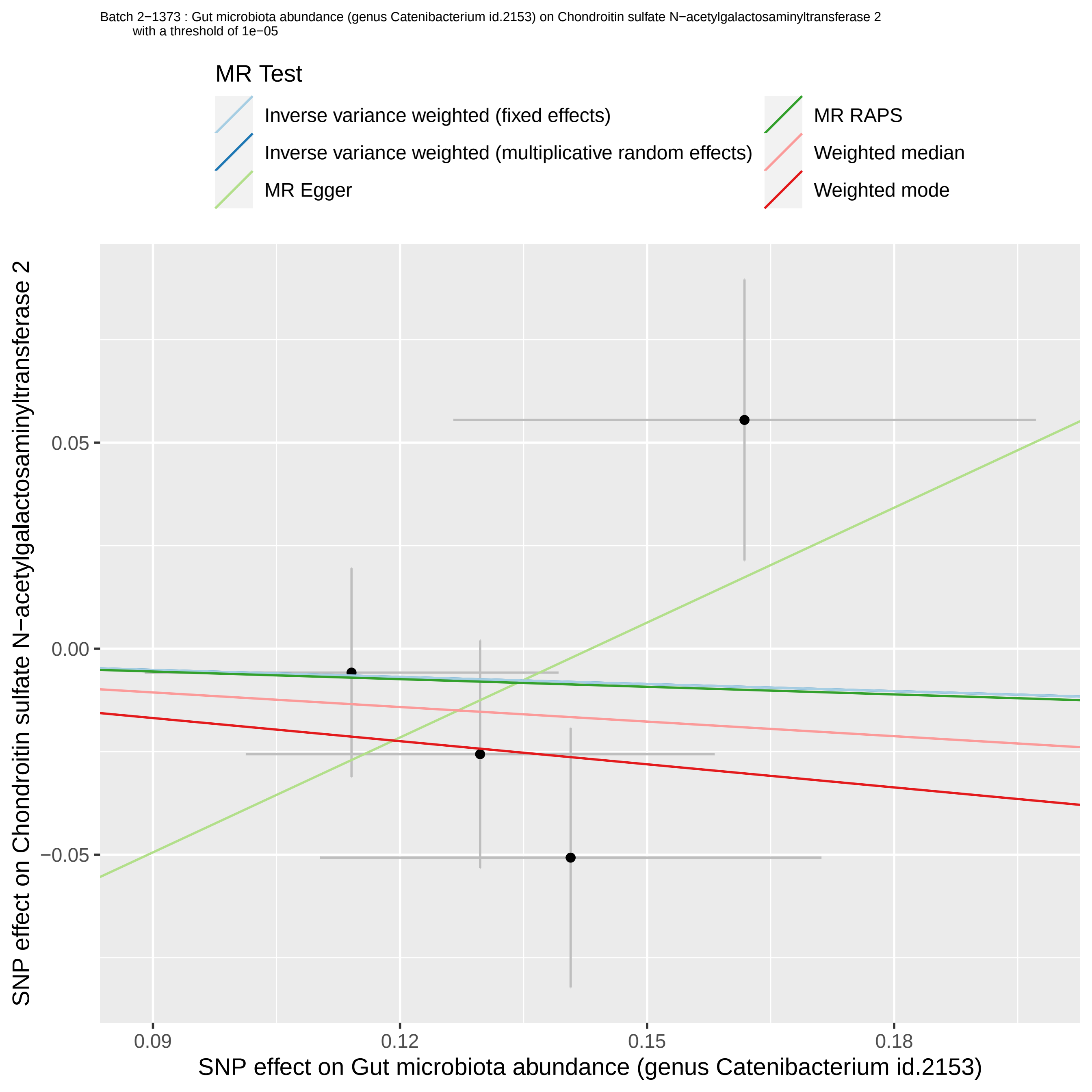
Batch 2−1373 : Gut microbiota abundance (genus Catenibacterium id.2153) on Chondroitin sulfate N−acetylgalactosaminyltransferase 2
with a threshold of 1e−05
MR Test
Inverse variance weighted (fixed effects)
Inverse variance weighted (multiplicative random effects)
MR Egger
MR RAPS
Weighted median
Weighted mode
SNP effect on Chondroitin sulfate N−acetylgalactosaminyltransferase 2
0.05
0.00
−0.05
0.09
0.12
0.15
0.18
SNP effect on Gut microbiota abundance (genus Catenibacterium id.2153)

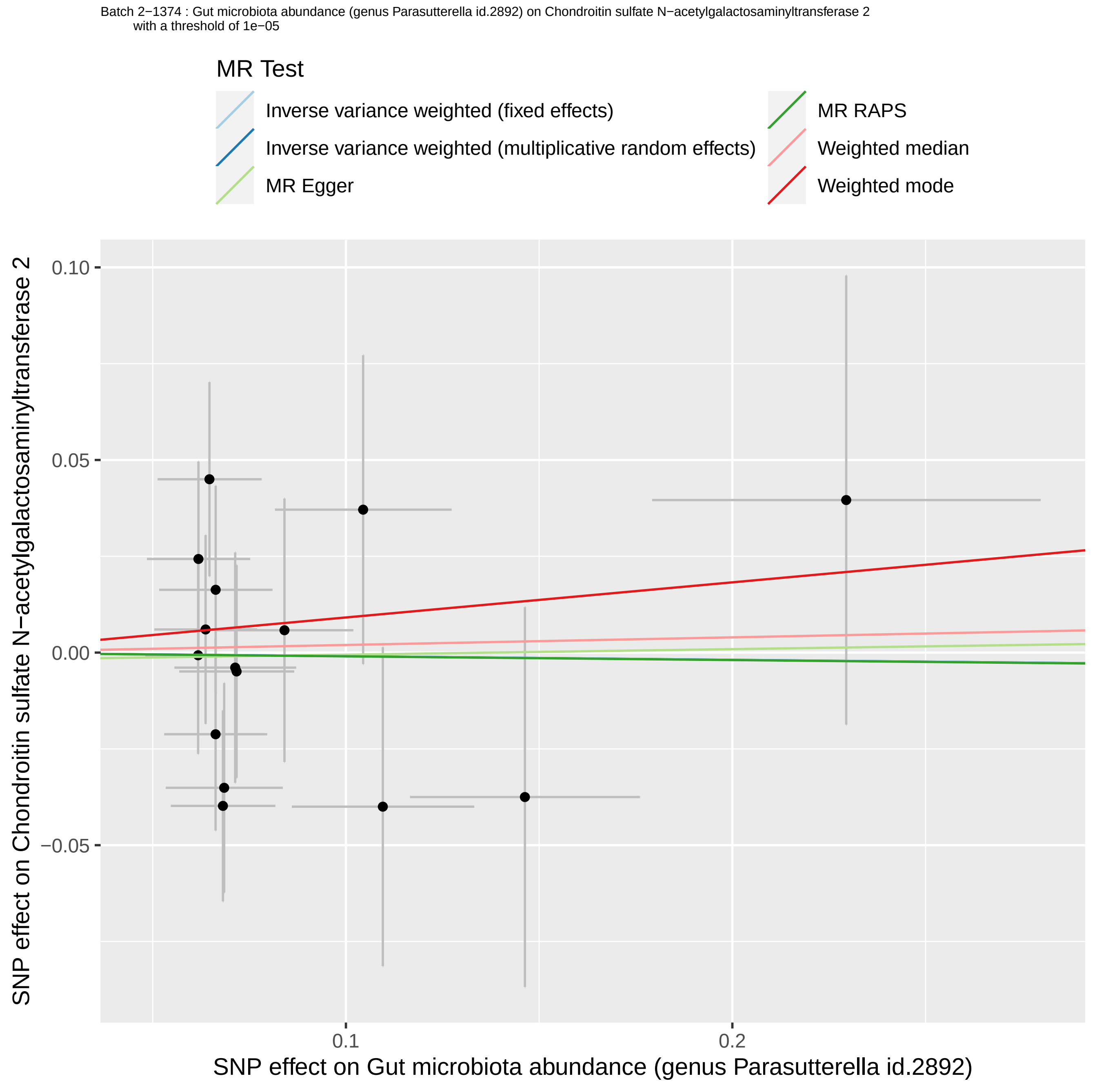
Batch 2−1374 : Gut microbiota abundance (genus Parasutterella id.2892) on Chondroitin sulfate N−acetylgalactosaminyltransferase 2
with a threshold of 1e−05
MR Test
Inverse variance weighted (fixed effects)
Inverse variance weighted (multiplicative random effects)
MR Egger
MR RAPS
Weighted median
Weighted mode
0.10
0.05
0.00
−0.05
0.1
0.2
SNP effect on Chondroitin sulfate N−acetylgalactosaminyltransferase 2
SNP effect on Gut microbiota abundance (genus Parasutterella id.2892)

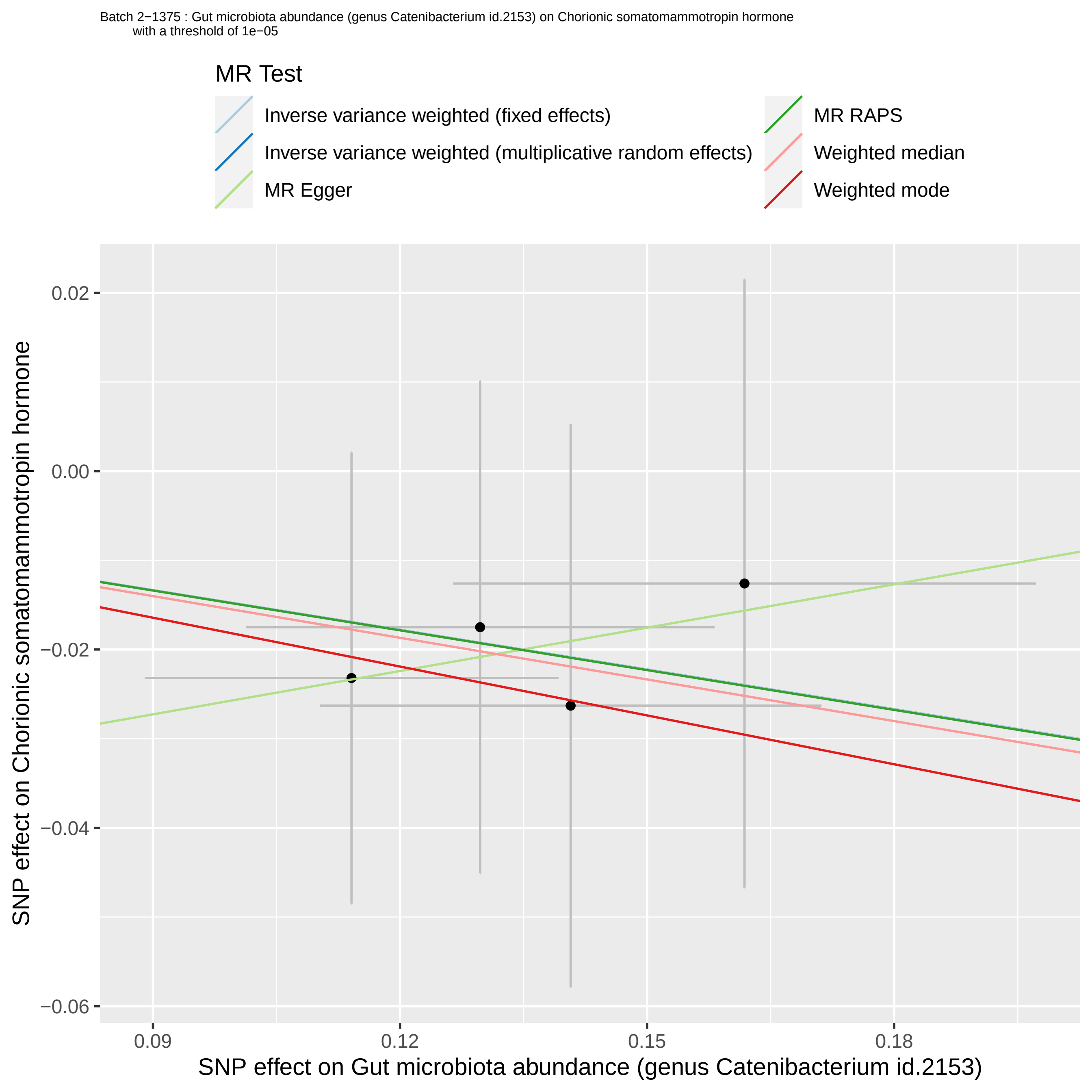

Batch 2−1375 : Gut microbiota abundance (genus Catenibacterium id.2153) on Chorionic somatomammotropin hormone
with a threshold of 1e−05
MR Test
Inverse variance weighted (fixed effects)
Inverse variance weighted (multiplicative random effects)
MR Egger
MR RAPS
Weighted median
Weighted mode
0.02
0.00
−0.02
−0.04
−0.06
0.09
0.12
0.15
0.18
SNP effect on Chorionic somatomammotropin hormone
SNP effect on Gut microbiota abundance (genus Catenibacterium id.2153)

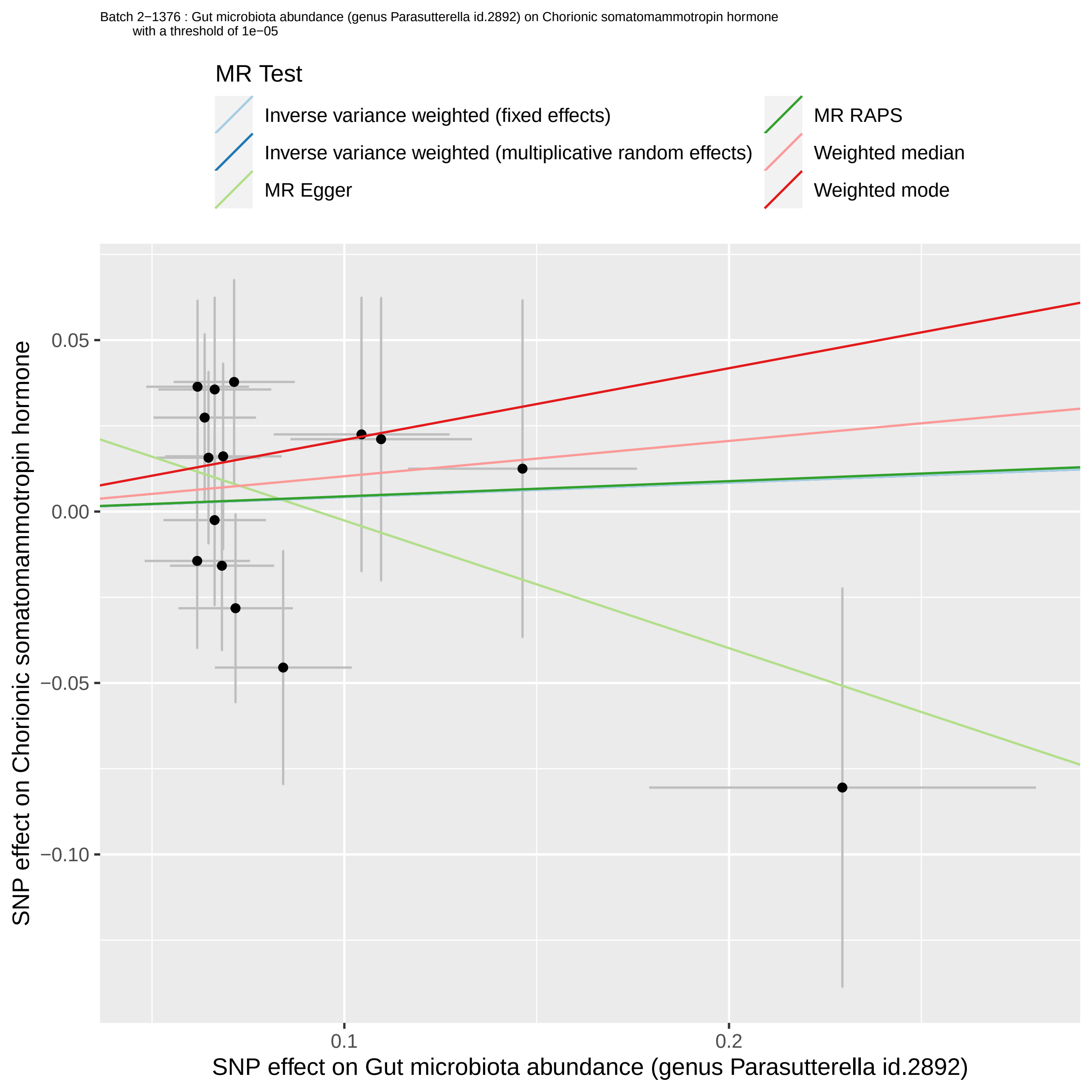
Batch 2−1376 : Gut microbiota abundance (genus Parasutterella id.2892) on Chorionic somatomammotropin hormone
with a threshold of 1e−05
MR Test
Inverse variance weighted (fixed effects)
Inverse variance weighted (multiplicative random effects)
MR Egger
MR RAPS
Weighted median
Weighted mode
0.05
0.00
−0.05
−0.10
0.1
0.2
SNP effect on Chorionic somatomammotropin hormone
SNP effect on Gut microbiota abundance (genus Parasutterella id.2892)

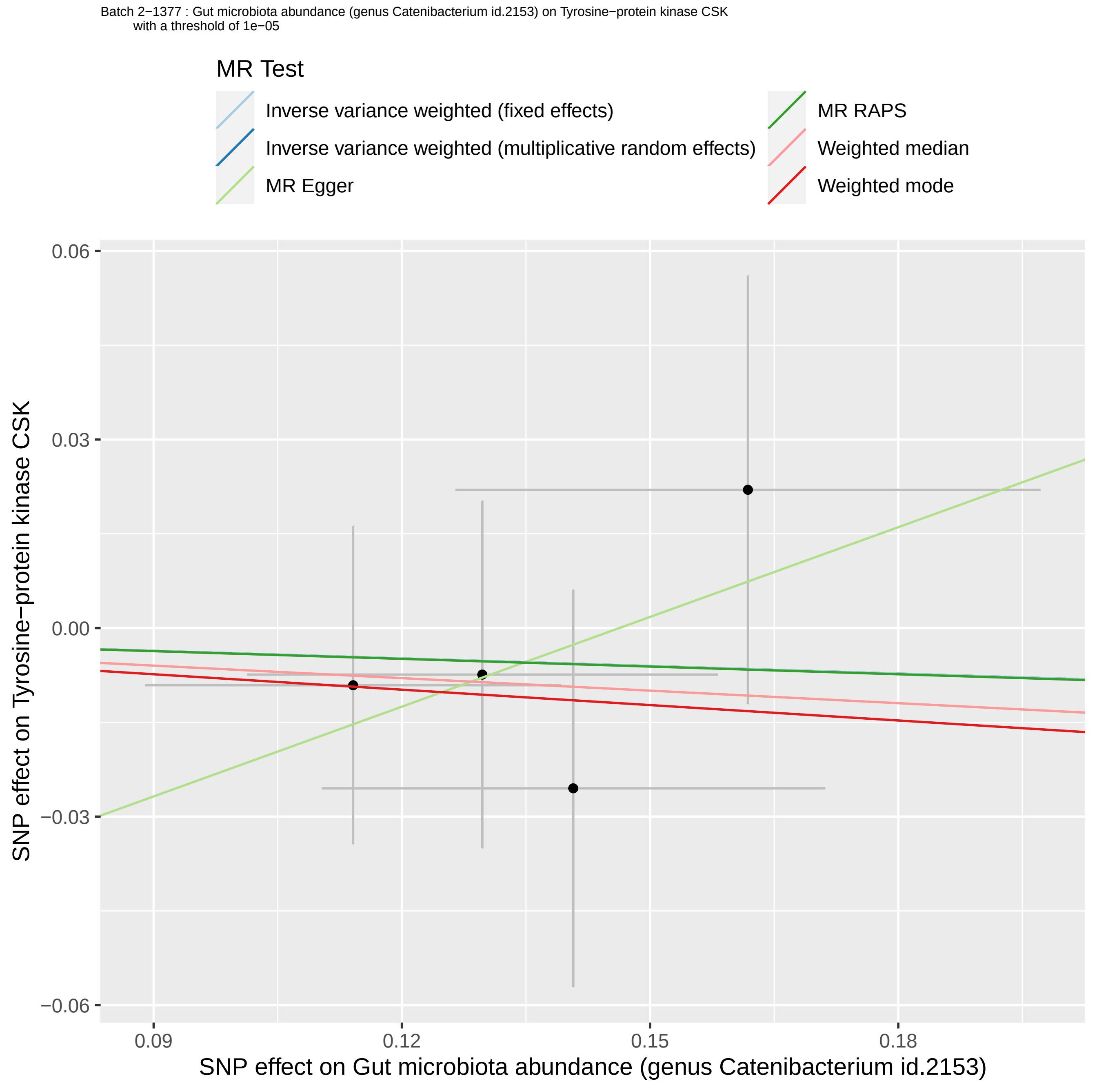
Batch 2−1377 : Gut microbiota abundance (genus Catenibacterium id.2153) on Tyrosine−protein kinase CSK
with a threshold of 1e−05
MR Test
Inverse variance weighted (fixed effects)
Inverse variance weighted (multiplicative random effects)
MR Egger
MR RAPS
Weighted median
Weighted mode
0.06
0.03
0.00
−0.03
−0.06
0.09
0.12
0.15
0.18
SNP effect on Tyrosine−protein kinase CSK
SNP effect on Gut microbiota abundance (genus Catenibacterium id.2153)

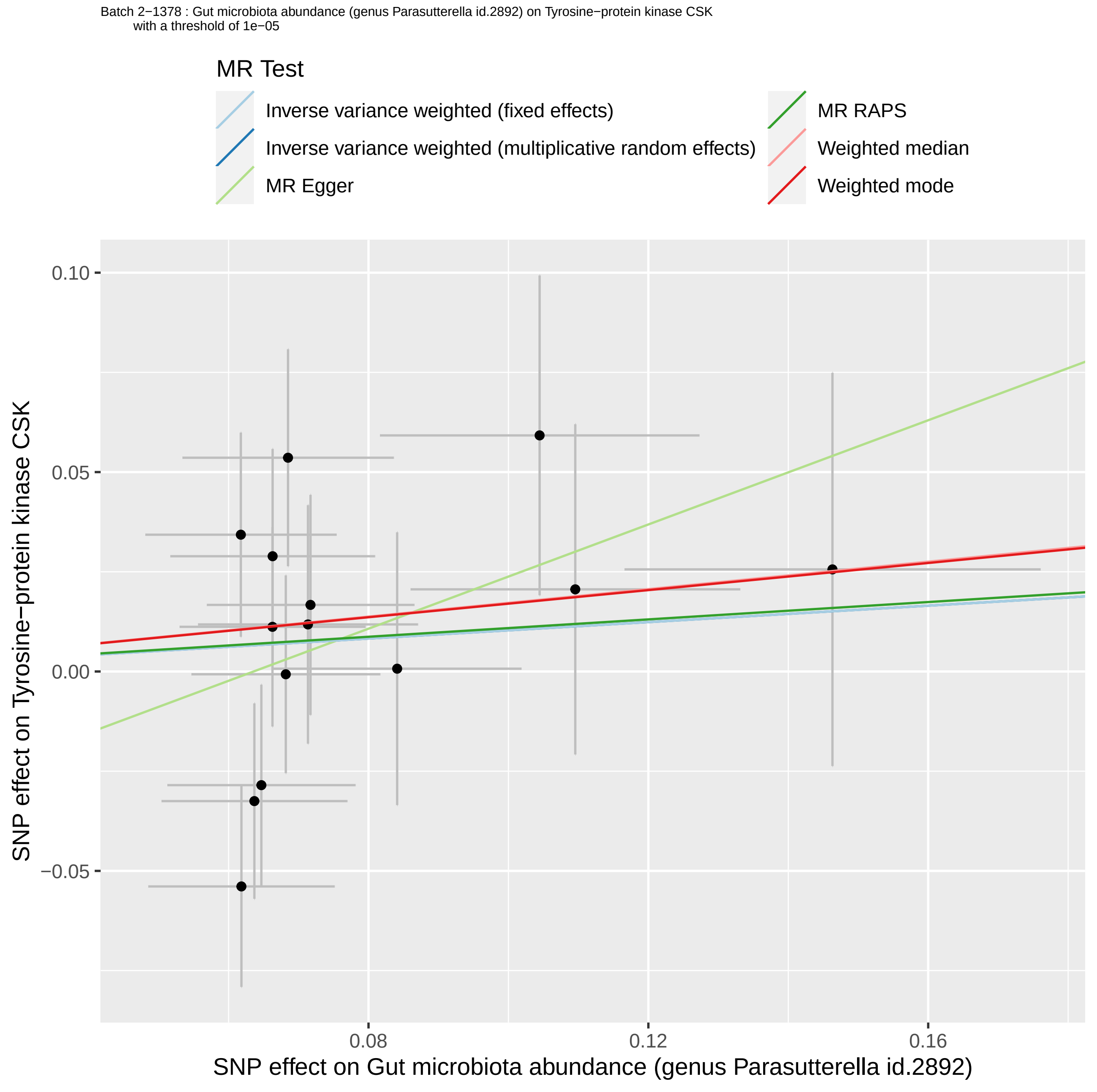
Batch 2−1378 : Gut microbiota abundance (genus Parasutterella id.2892) on Tyrosine−protein kinase CSK
with a threshold of 1e−05
MR Test
Inverse variance weighted (fixed effects)
Inverse variance weighted (multiplicative random effects)
MR Egger
MR RAPS
Weighted median
Weighted mode
0.10
0.05
0.00
−0.05
0.08
0.12
0.16
SNP effect on Tyrosine−protein kinase CSK
SNP effect on Gut microbiota abundance (genus Parasutterella id.2892)

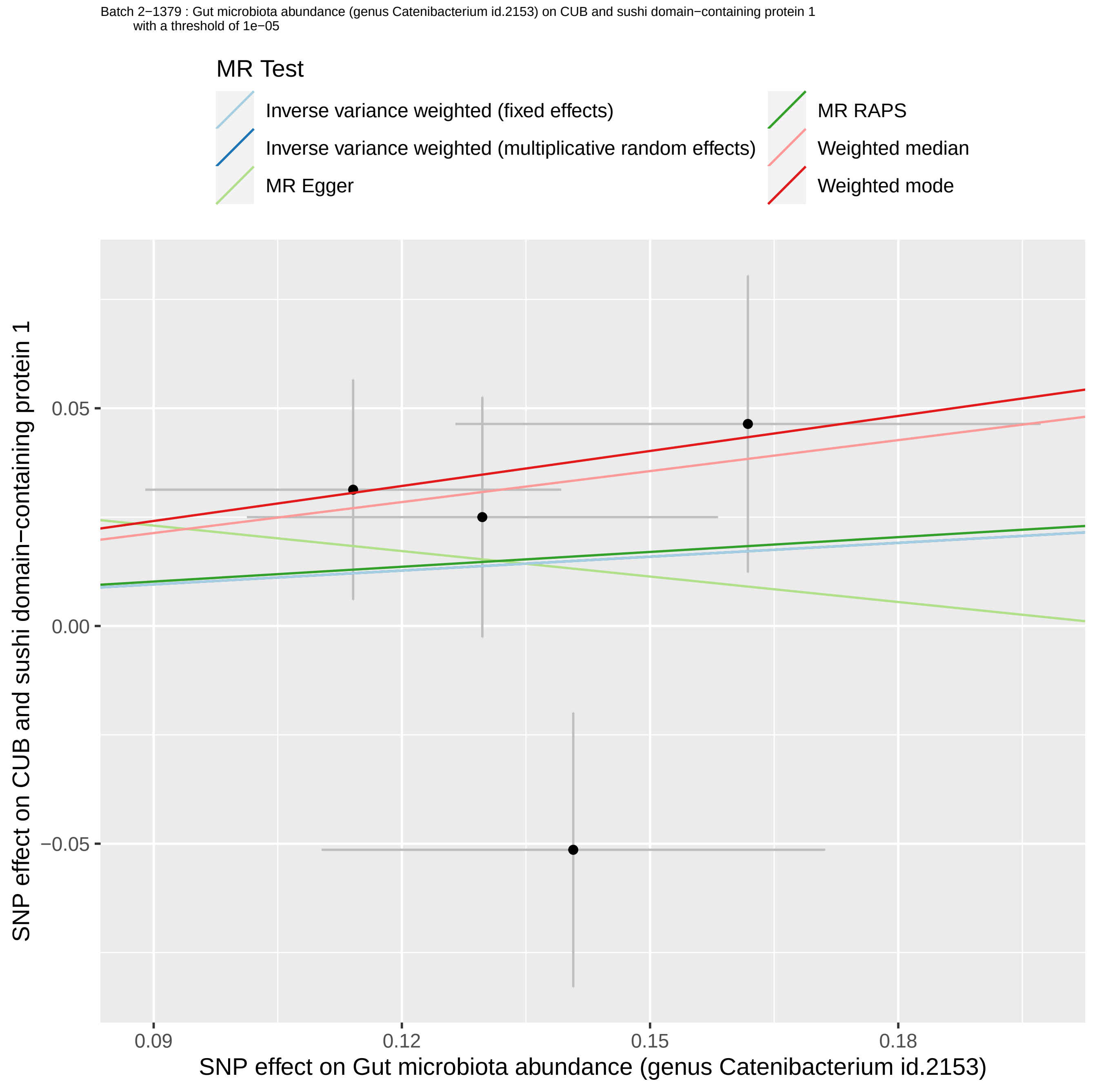
Batch 2−1379 : Gut microbiota abundance (genus Catenibacterium id.2153) on CUB and sushi domain−containing protein 1
with a threshold of 1e−05
MR Test
Inverse variance weighted (fixed effects)
Inverse variance weighted (multiplicative random effects)
MR Egger
MR RAPS
Weighted median
Weighted mode
0.05
0.00
−0.05
0.09
0.12
0.15
0.18
SNP effect on CUB and sushi domain−containing protein 1
SNP effect on Gut microbiota abundance (genus Catenibacterium id.2153)

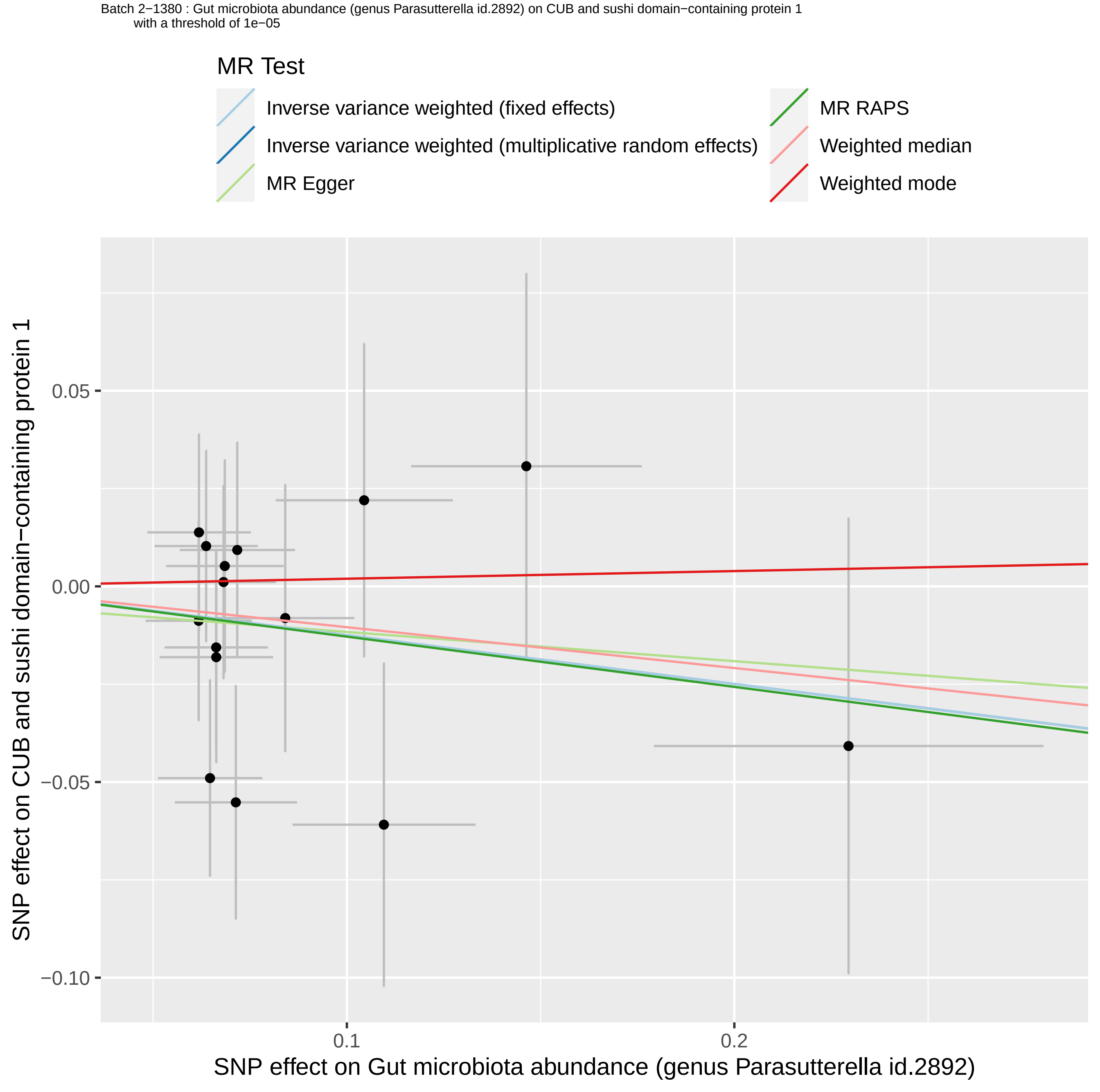

Batch 2−1380 : Gut microbiota abundance (genus Parasutterella id.2892) on CUB and sushi domain−containing protein 1
with a threshold of 1e−05
MR Test
Inverse variance weighted (fixed effects)
Inverse variance weighted (multiplicative random effects)
MR Egger
MR RAPS
Weighted median
Weighted mode
0.05
0.00
−0.05
−0.10
0.1
0.2
SNP effect on CUB and sushi domain−containing protein 1
SNP effect on Gut microbiota abundance (genus Parasutterella id.2892)

Batch 2−1381 : Gut microbiota abundance (genus Catenibacterium id.2153) on CUB and sushi domain−containing protein 2
with a threshold of 1e−05

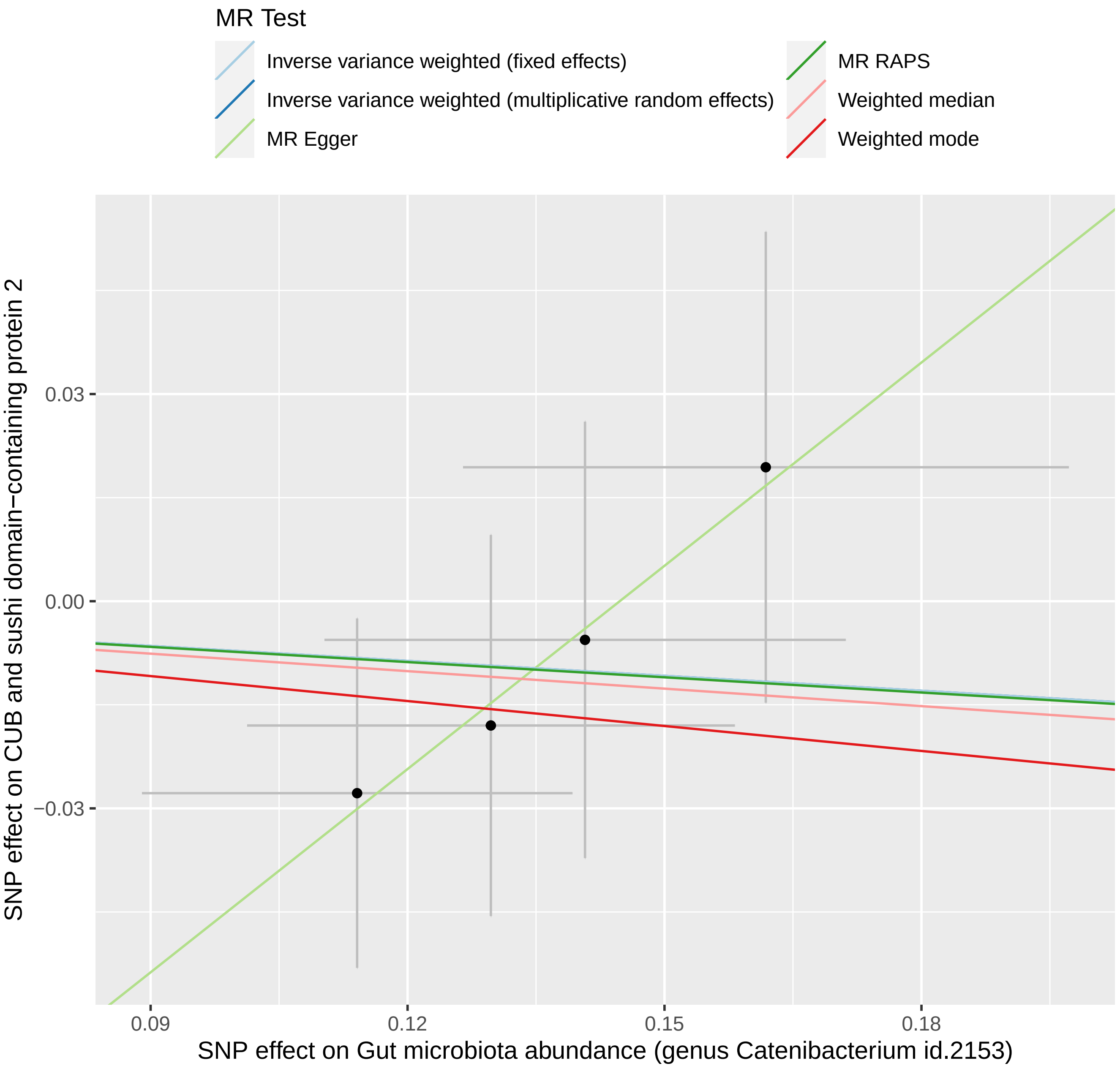

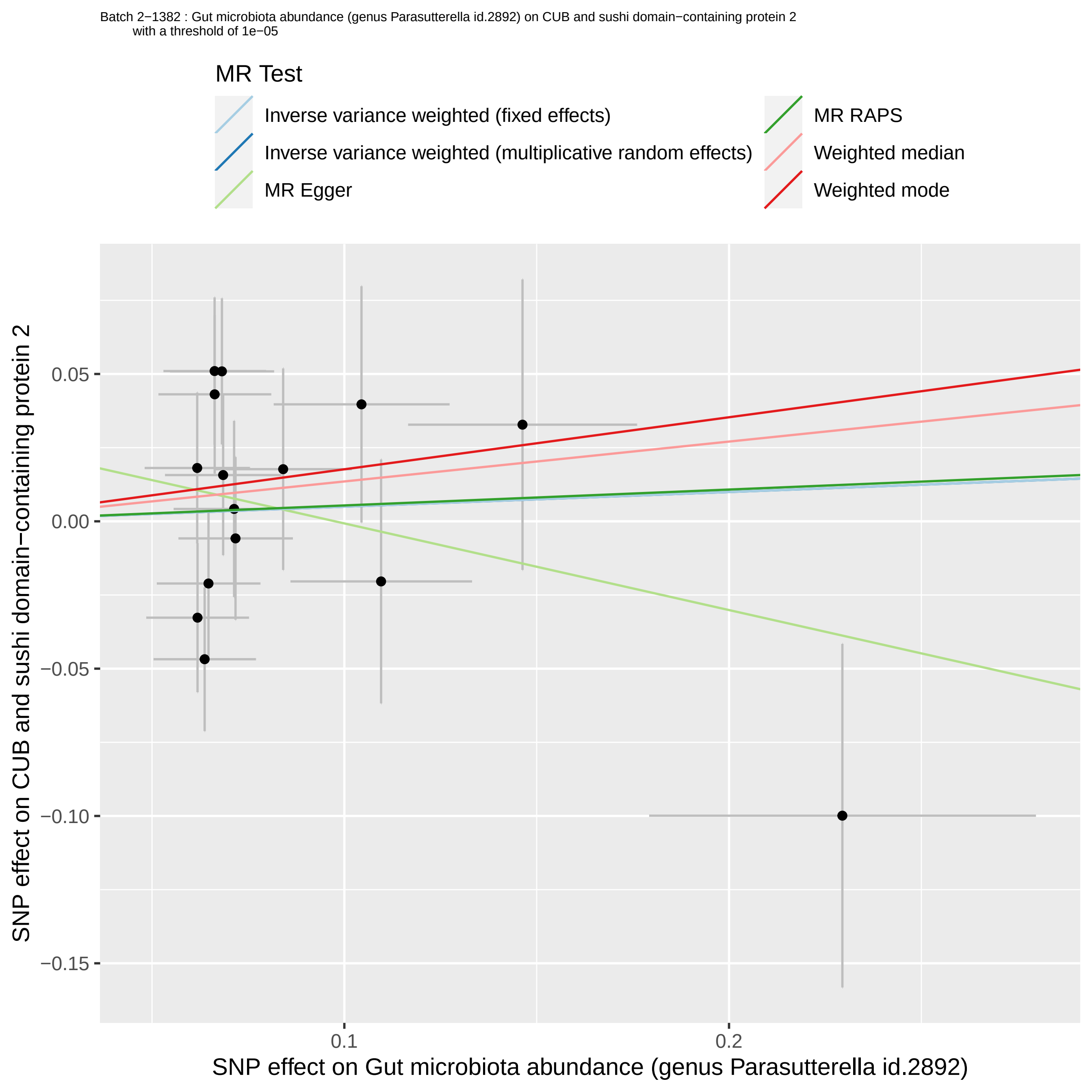
Batch 2−1382 : Gut microbiota abundance (genus Parasutterella id.2892) on CUB and sushi domain−containing protein 2
with a threshold of 1e−05
MR Test
Inverse variance weighted (fixed effects)
Inverse variance weighted (multiplicative random effects)
MR Egger
MR RAPS
Weighted median
Weighted mode
SNP effect on CUB and sushi domain−containing protein 2
0.05
0.00
−0.05
−0.10
−0.15
0.1
0.2
SNP effect on Gut microbiota abundance (genus Parasutterella id.2892)

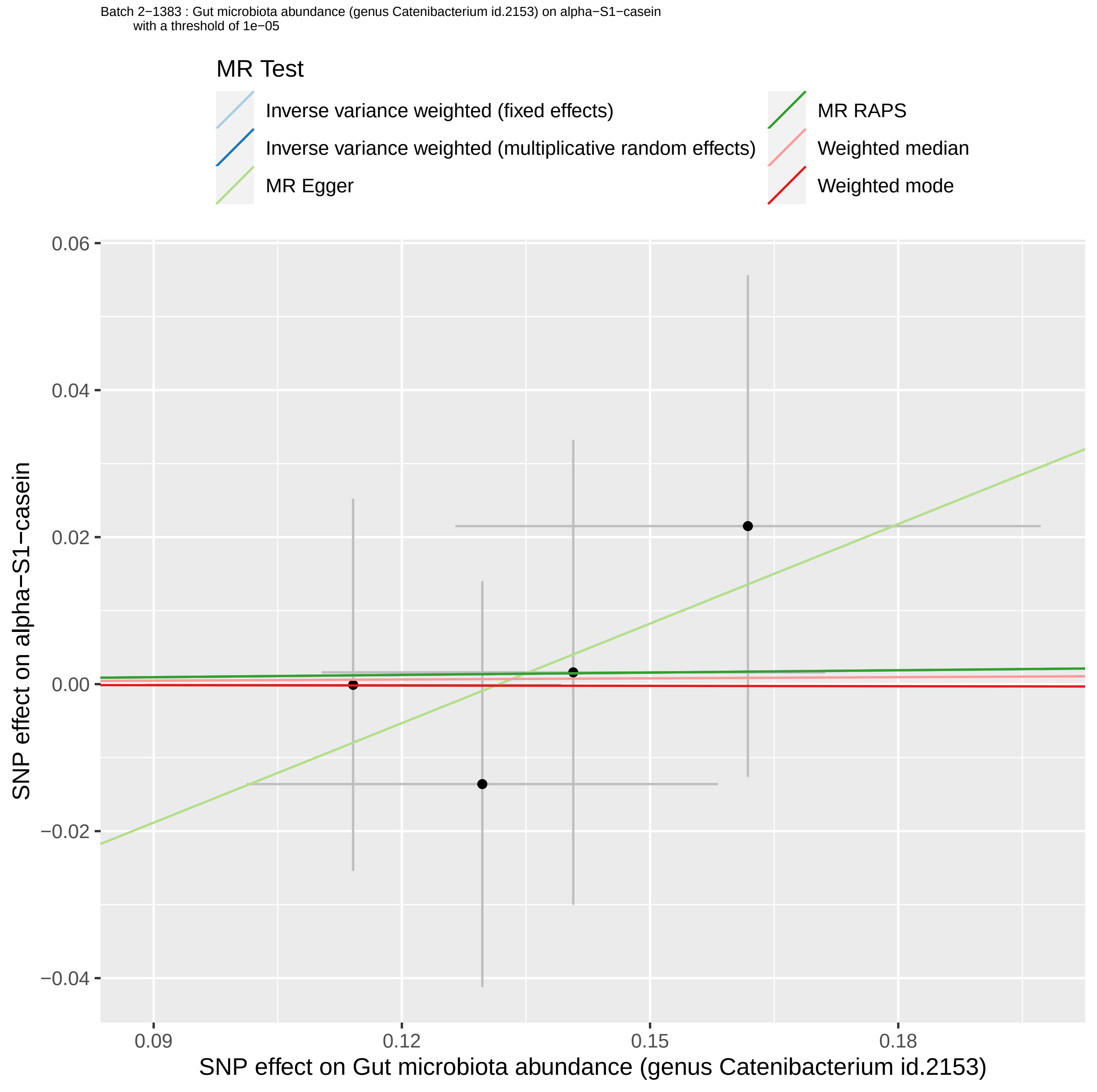

Batch 2−1383 : Gut microbiota abundance (genus Catenibacterium id.2153) on alpha−S1−casein
with a threshold of 1e−05
MR Test
Inverse variance weighted (fixed effects)
Inverse variance weighted (multiplicative random effects)
MR Egger
MR RAPS
Weighted median
Weighted mode
0.06
0.04
0.02
0.00
−0.02
−0.04
0.09
0.12
0.15
0.18
SNP effect on alpha−S1−casein
SNP effect on Gut microbiota abundance (genus Catenibacterium id.2153)

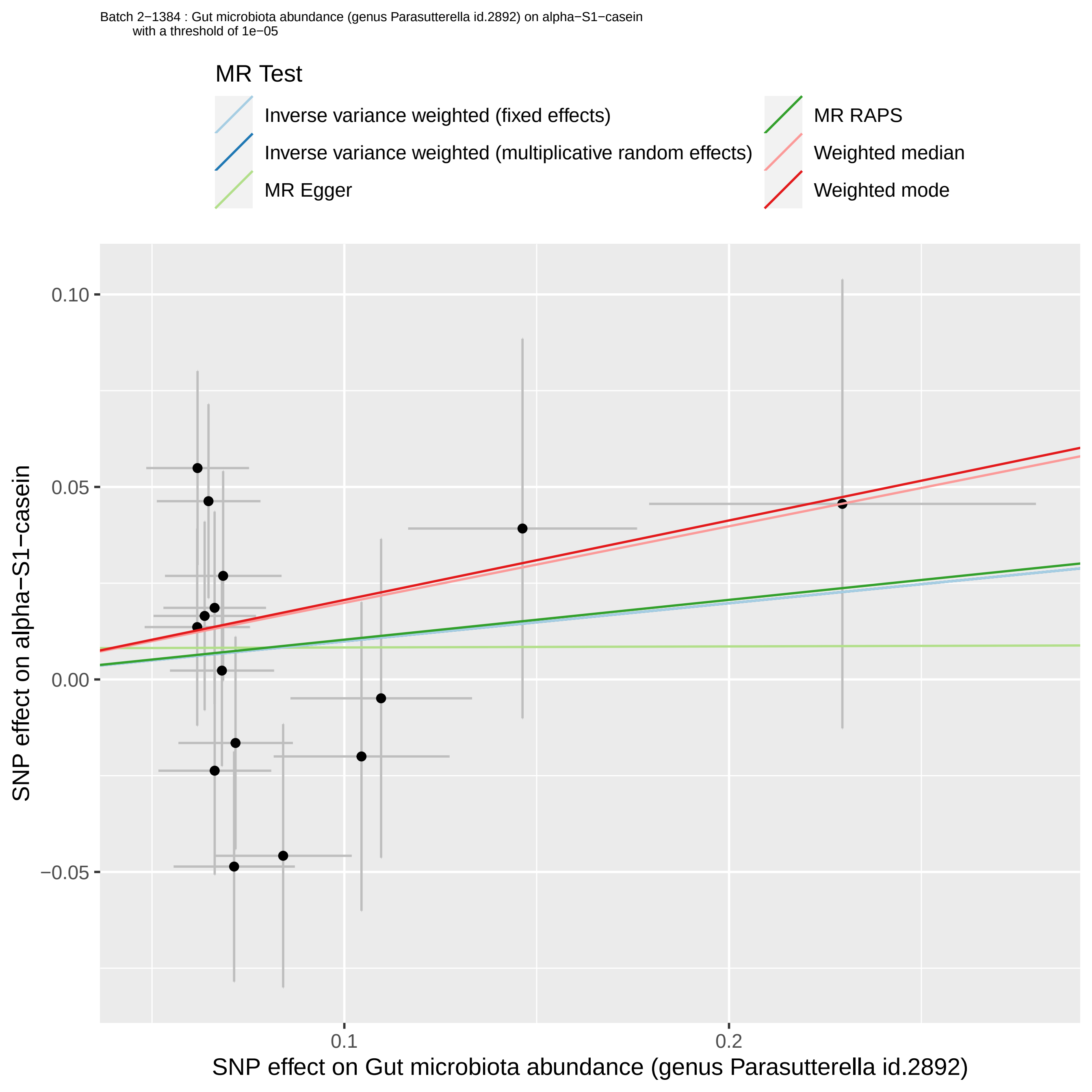
Batch 2−1384 : Gut microbiota abundance (genus Parasutterella id.2892) on alpha−S1−casein
with a threshold of 1e−05
MR Test
Inverse variance weighted (fixed effects)
Inverse variance weighted (multiplicative random effects)
MR Egger
MR RAPS
Weighted median
Weighted mode
0.10
0.05
0.00
−0.05
0.1
0.2
SNP effect on alpha−S1−casein
SNP effect on Gut microbiota abundance (genus Parasutterella id.2892)

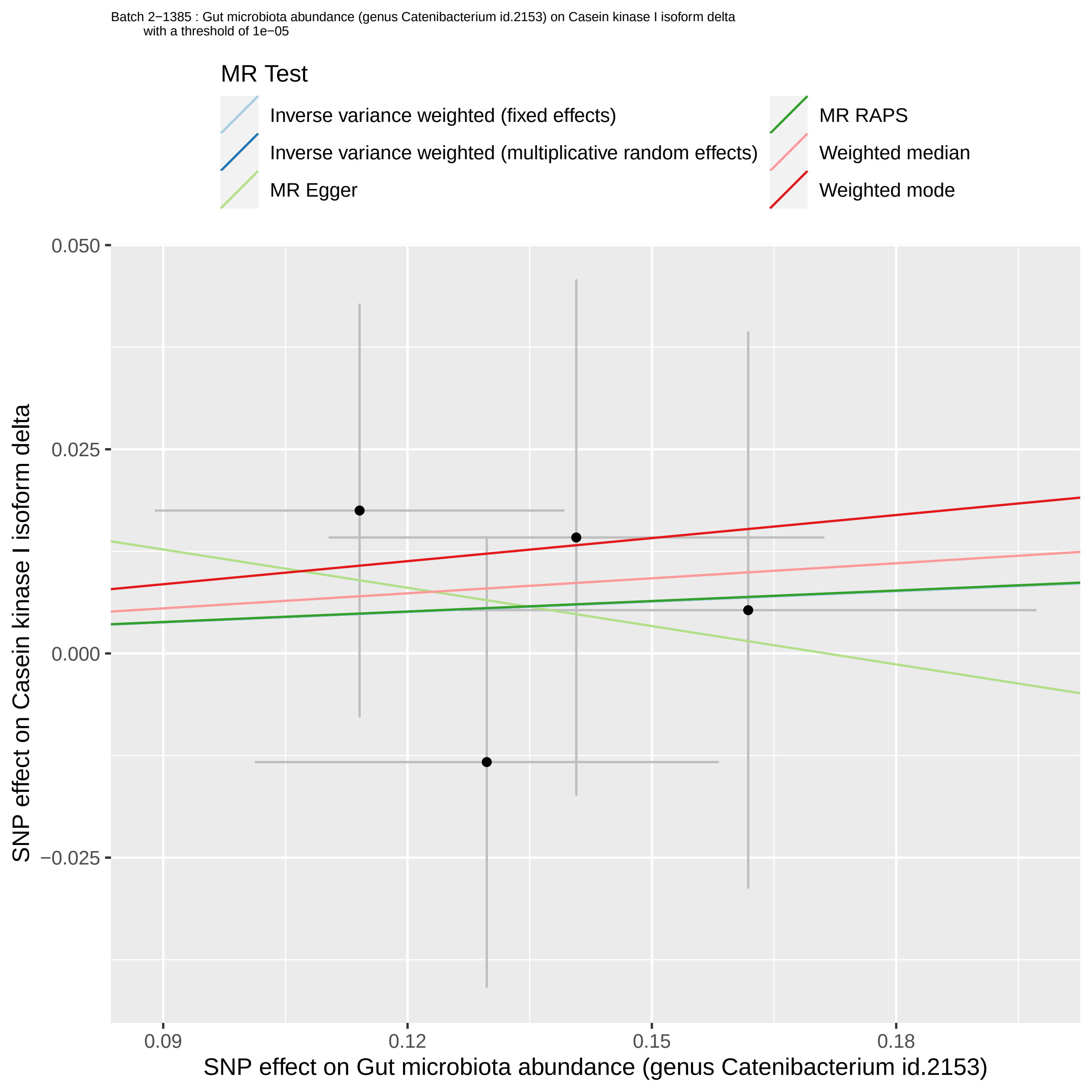

Batch 2−1385 : Gut microbiota abundance (genus Catenibacterium id.2153) on Casein kinase I isoform delta
with a threshold of 1e−05
MR Test
Inverse variance weighted (fixed effects)
Inverse variance weighted (multiplicative random effects)
MR Egger
MR RAPS
Weighted median
Weighted mode
0.050
0.025
0.000
−0.025
0.09
0.12
0.15
0.18
SNP effect on Casein kinase I isoform delta
SNP effect on Gut microbiota abundance (genus Catenibacterium id.2153)

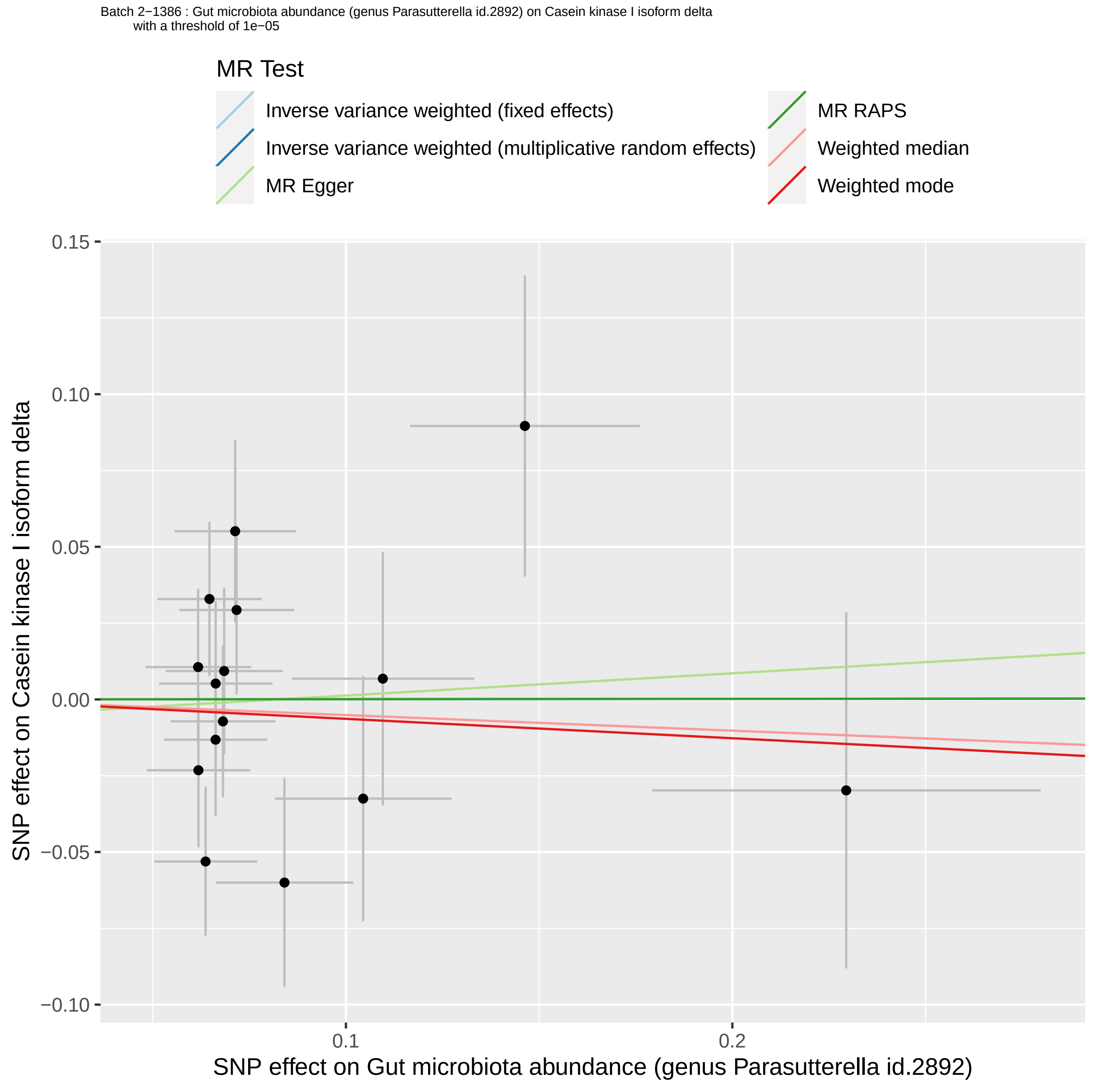
Batch 2−1386 : Gut microbiota abundance (genus Parasutterella id.2892) on Casein kinase I isoform delta
with a threshold of 1e−05
MR Test
Inverse variance weighted (fixed effects)
Inverse variance weighted (multiplicative random effects)
MR Egger
MR RAPS
Weighted median
Weighted mode
0.15
0.10
0.05
0.00
−0.05
−0.10
0.1
0.2
SNP effect on Casein kinase I isoform delta
SNP effect on Gut microbiota abundance (genus Parasutterella id.2892)

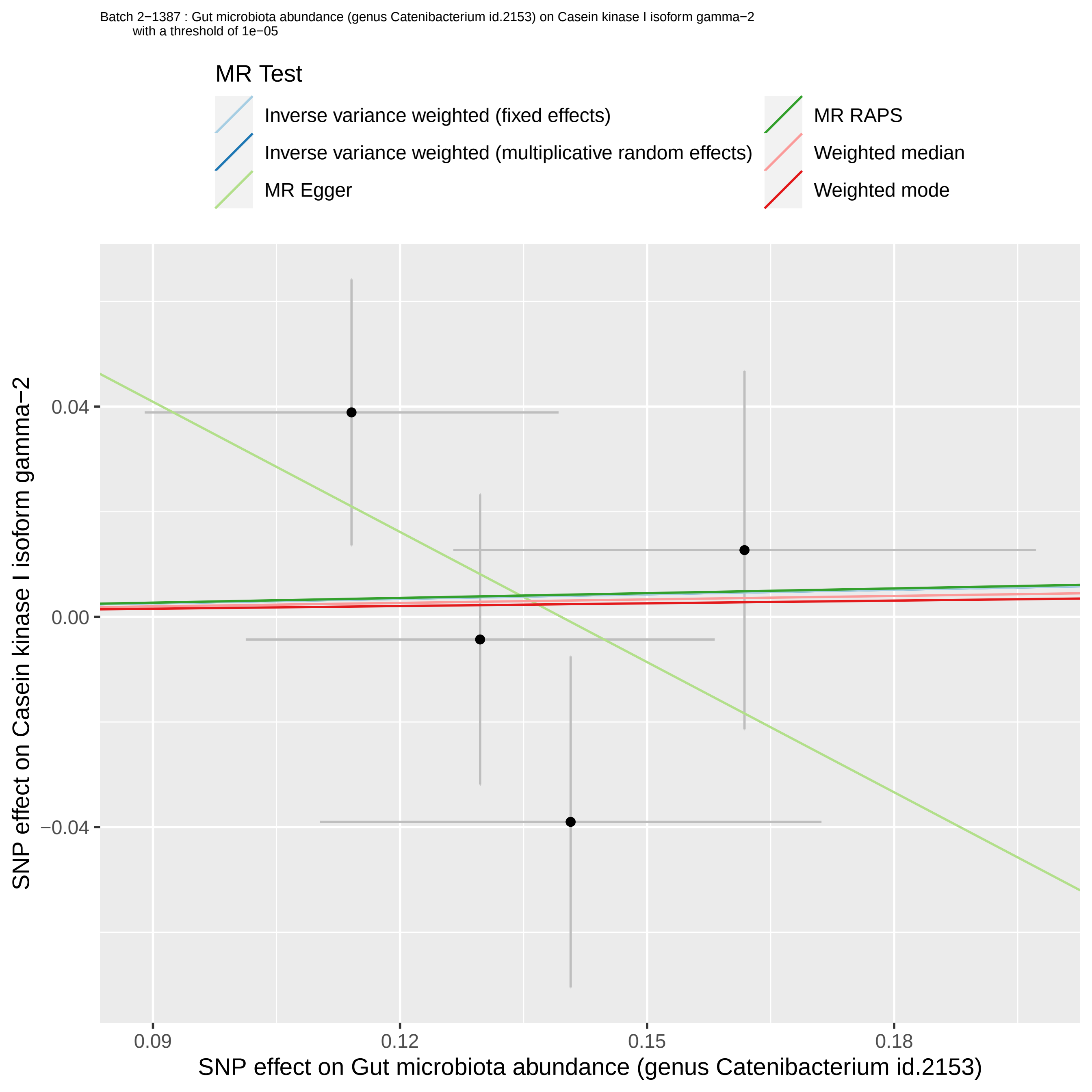

Batch 2−1387 : Gut microbiota abundance (genus Catenibacterium id.2153) on Casein kinase I isoform gamma−2
with a threshold of 1e−05
MR Test
Inverse variance weighted (fixed effects)
Inverse variance weighted (multiplicative random effects)
MR Egger
MR RAPS
Weighted median
Weighted mode
0.04
0.00
−0.04
0.09
0.12
0.15
0.18
SNP effect on Casein kinase I isoform gamma−2
SNP effect on Gut microbiota abundance (genus Catenibacterium id.2153)

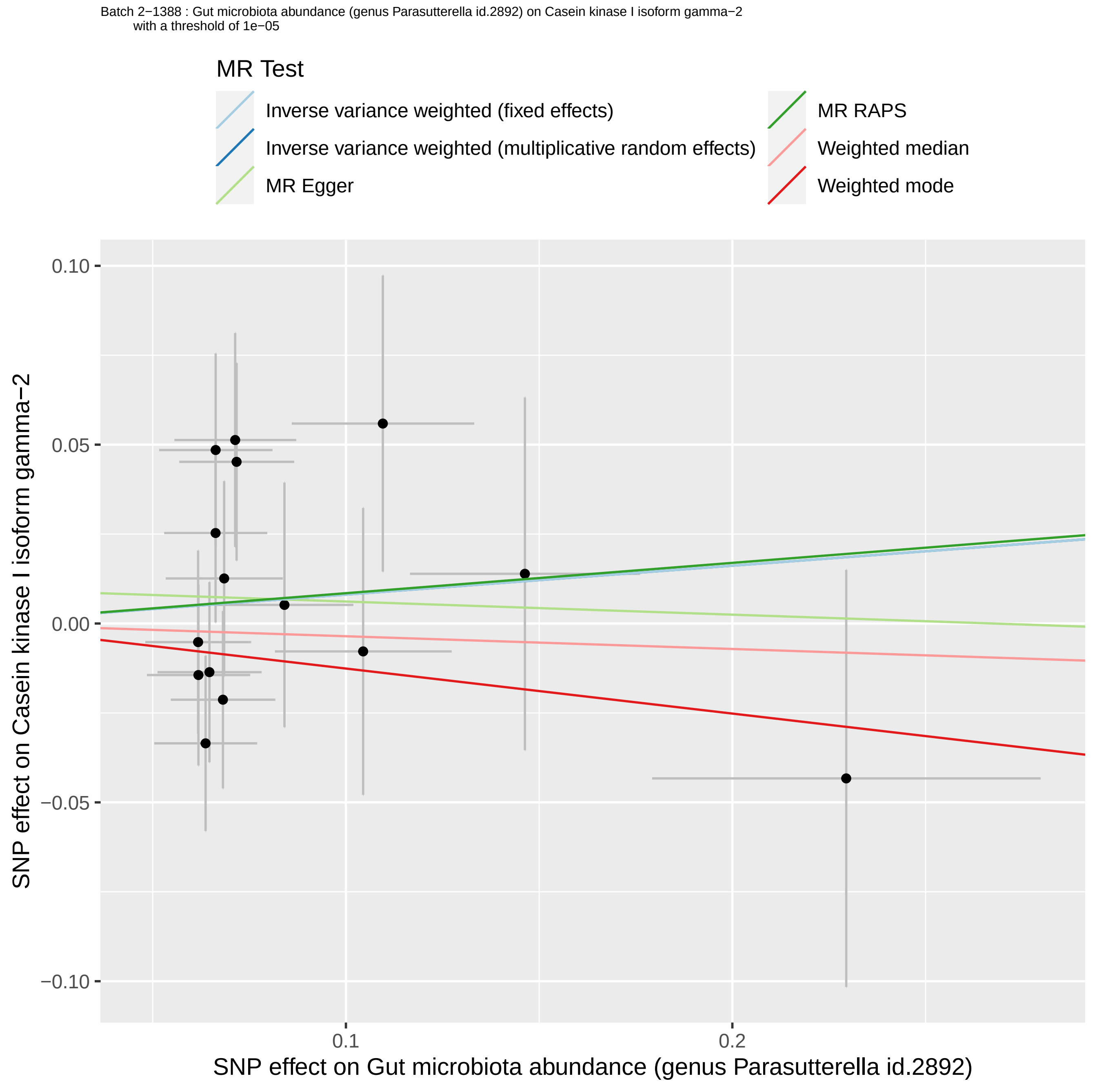
Batch 2−1388 : Gut microbiota abundance (genus Parasutterella id.2892) on Casein kinase I isoform gamma−2
with a threshold of 1e−05
MR Test
Inverse variance weighted (fixed effects)
Inverse variance weighted (multiplicative random effects)
MR Egger
MR RAPS
Weighted median
Weighted mode
0.10
0.05
0.00
−0.05
−0.10
0.1
0.2
SNP effect on Casein kinase I isoform gamma−2
SNP effect on Gut microbiota abundance (genus Parasutterella id.2892)

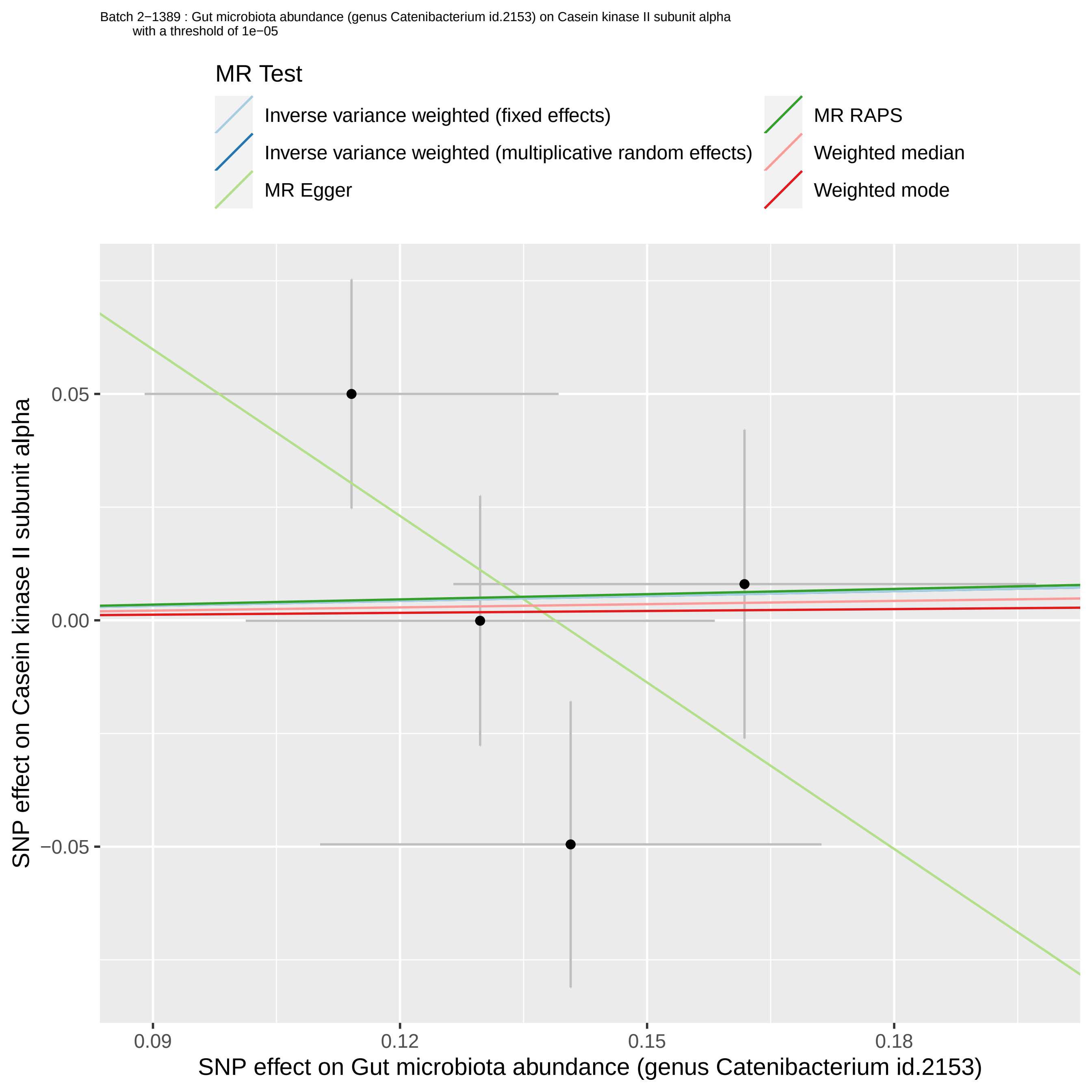

Batch 2−1389 : Gut microbiota abundance (genus Catenibacterium id.2153) on Casein kinase II subunit alpha
with a threshold of 1e−05
MR Test
Inverse variance weighted (fixed effects)
Inverse variance weighted (multiplicative random effects)
MR Egger
MR RAPS
Weighted median
Weighted mode
0.05
0.00
−0.05
0.09
0.12
0.15
0.18
SNP effect on Casein kinase II subunit alpha
SNP effect on Gut microbiota abundance (genus Catenibacterium id.2153)

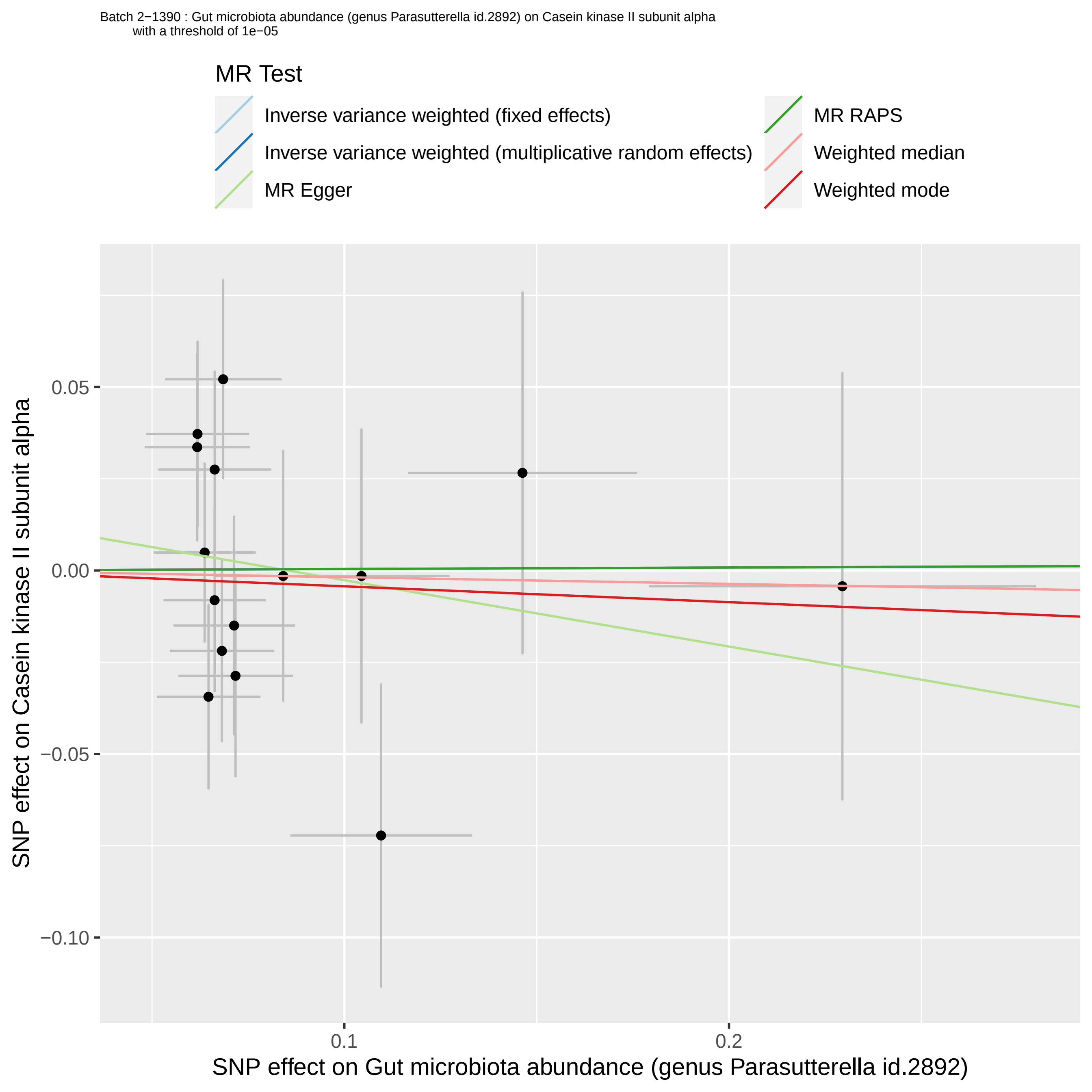
Batch 2−1390 : Gut microbiota abundance (genus Parasutterella id.2892) on Casein kinase II subunit alpha
with a threshold of 1e−05
MR Test
Inverse variance weighted (fixed effects)
Inverse variance weighted (multiplicative random effects)
MR Egger
MR RAPS
Weighted median
Weighted mode
0.05
0.00
−0.05
−0.10
0.1
0.2
SNP effect on Casein kinase II subunit alpha
SNP effect on Gut microbiota abundance (genus Parasutterella id.2892)

Batch 2−1391 : Gut microbiota abundance (genus Catenibacterium id.2153) on Casein kinase II subunit alpha
with a threshold of 1e−05

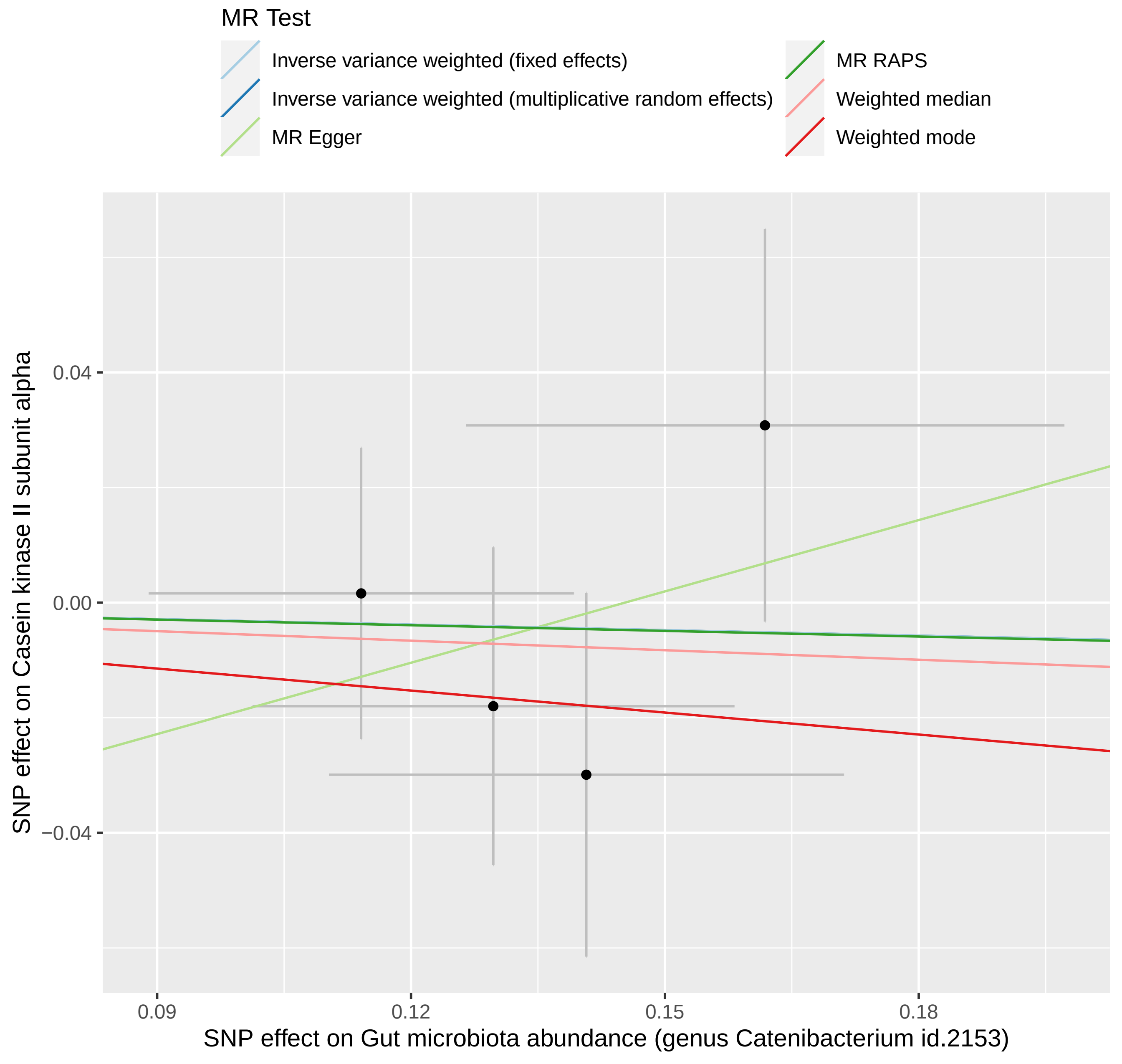

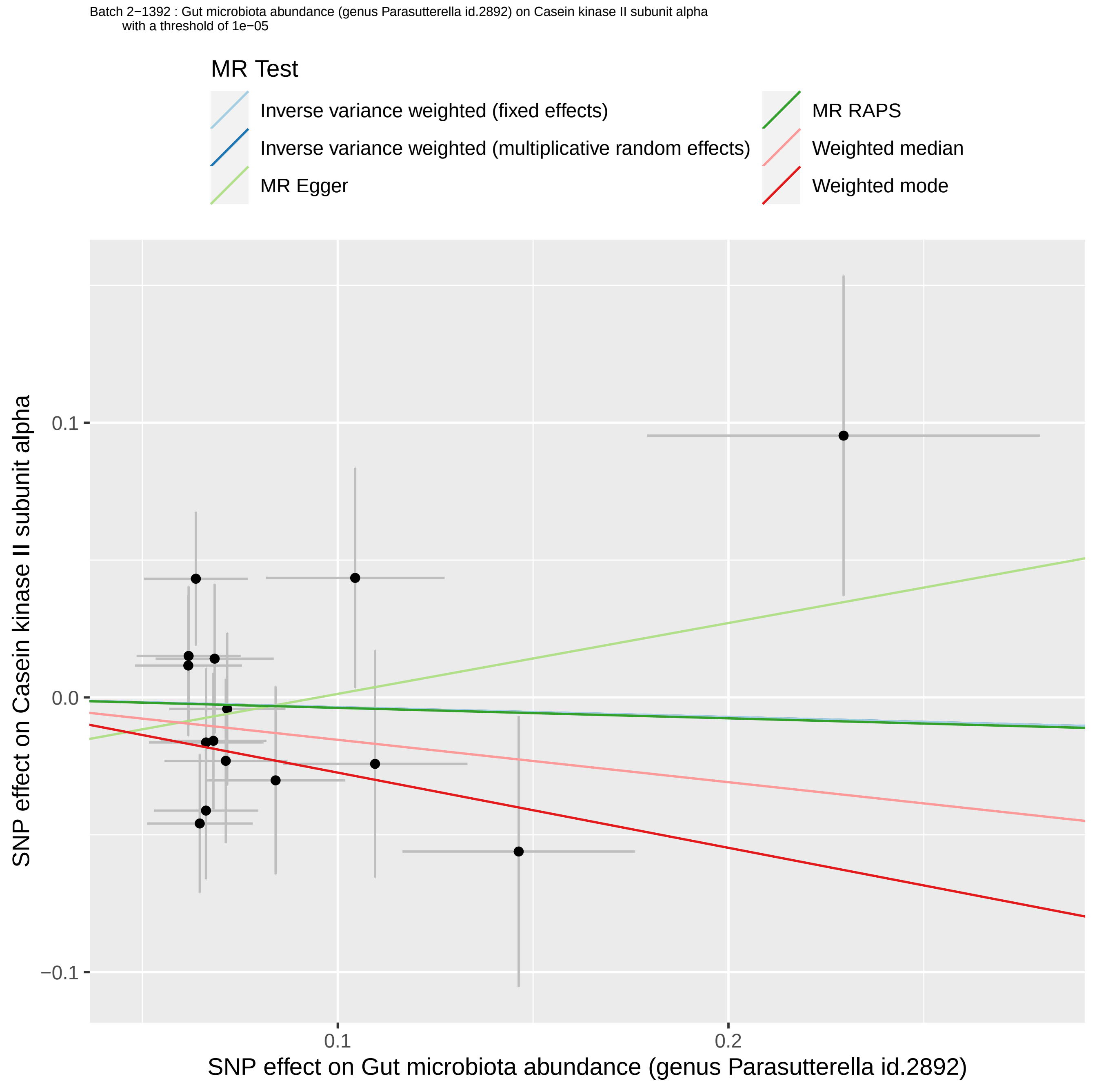

Batch 2−1392 : Gut microbiota abundance (genus Parasutterella id.2892) on Casein kinase II subunit alpha
with a threshold of 1e−05
MR Test
Inverse variance weighted (fixed effects)
Inverse variance weighted (multiplicative random effects)
MR Egger
MR RAPS
Weighted median
Weighted mode
0.1
0.0
−0.1
0.1
0.2
SNP effect on Casein kinase II subunit alpha
SNP effect on Gut microbiota abundance (genus Parasutterella id.2892)

Batch 2−1393 : Gut microbiota abundance (genus Catenibacterium id.2153) on Casein kinase II 2−alpha:2−beta heterotetramer with a threshold of 1e−05

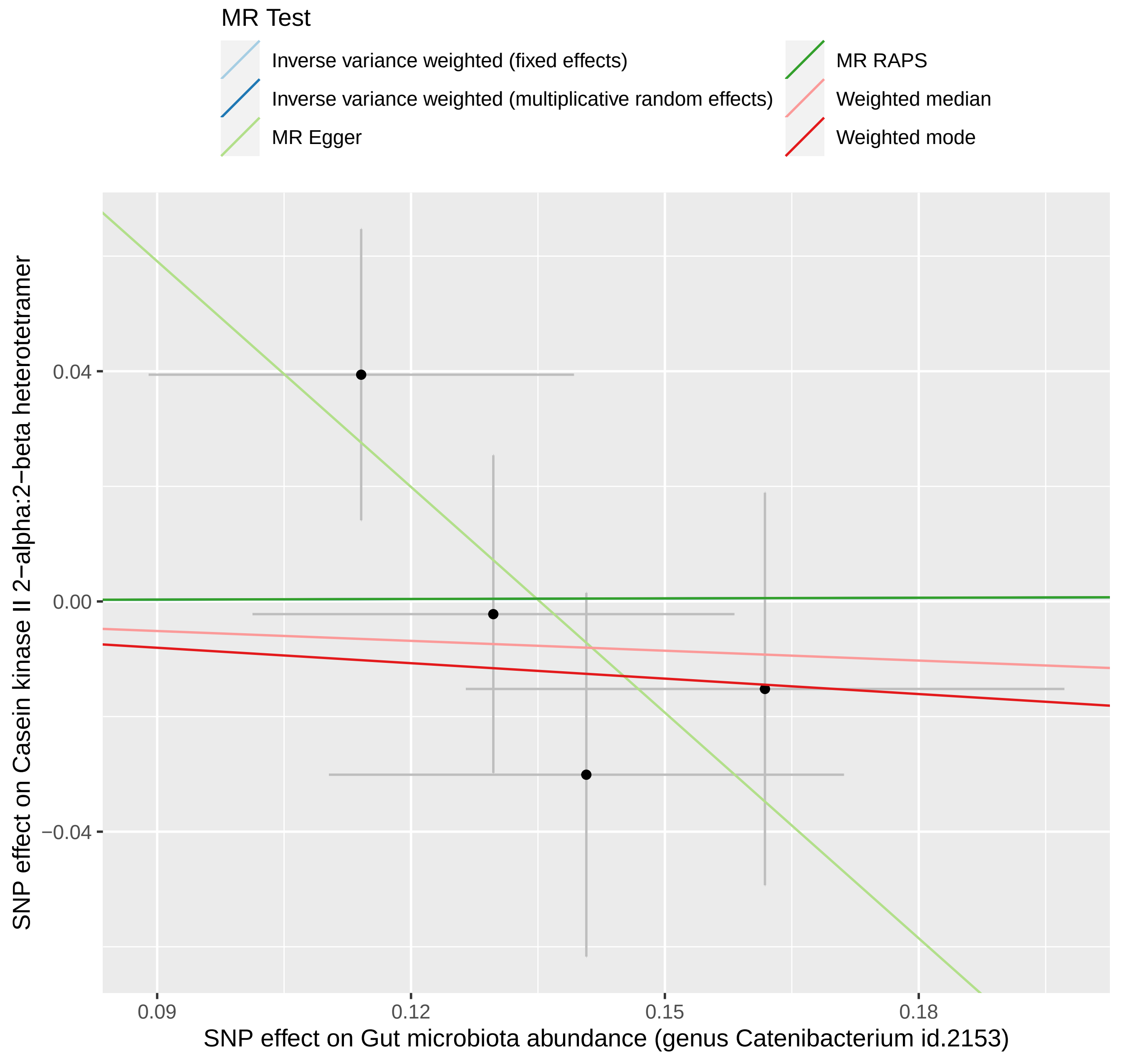

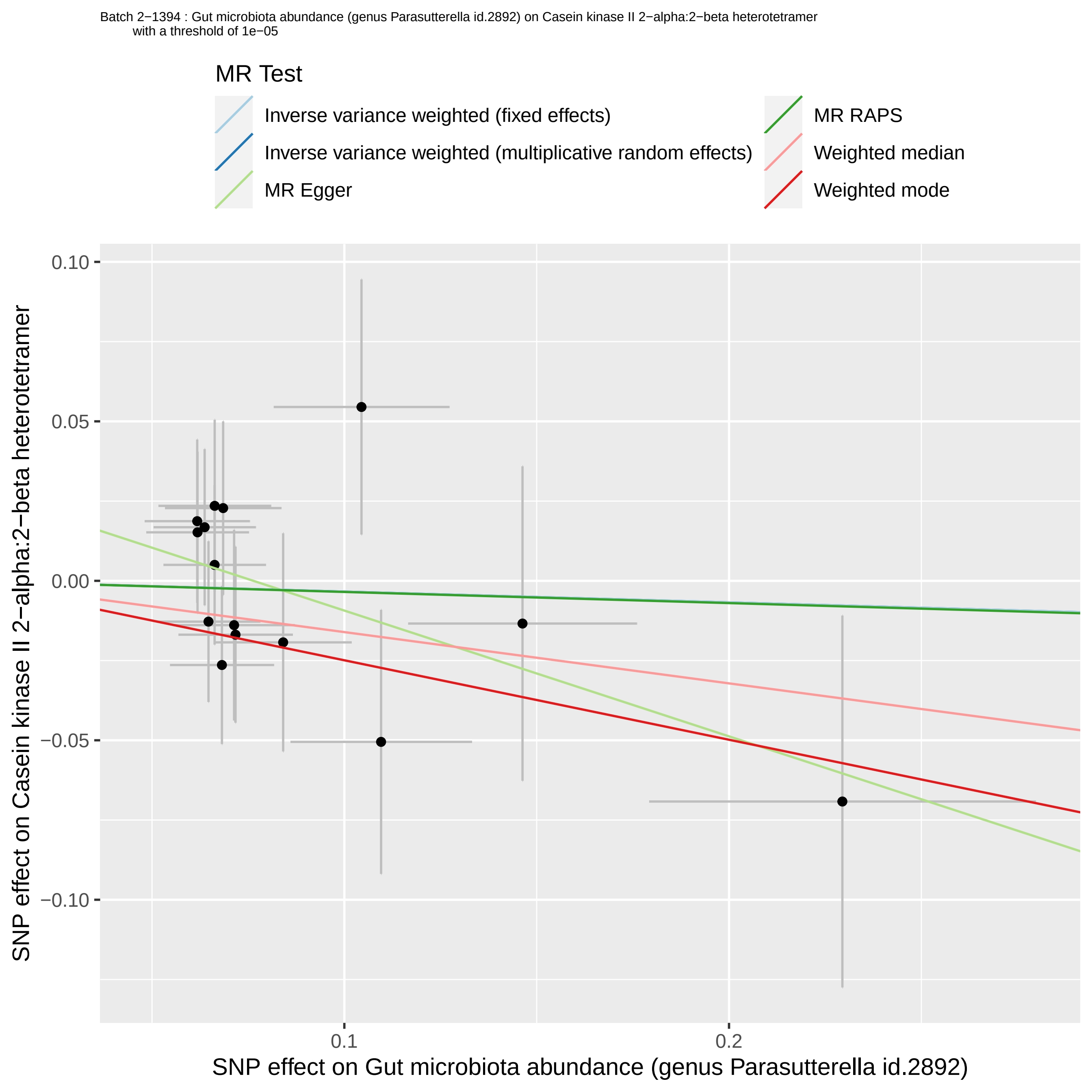

Batch 2−1394 : Gut microbiota abundance (genus Parasutterella id.2892) on Casein kinase II 2−alpha:2−beta heterotetramer
with a threshold of 1e−05
MR Test
Inverse variance weighted (fixed effects)
Inverse variance weighted (multiplicative random effects)
MR Egger
MR RAPS
Weighted median
Weighted mode
0.10
0.05
0.00
−0.05
−0.10
0.1
0.2
SNP effect on Casein kinase II 2−alpha:2−beta heterotetramer
SNP effect on Gut microbiota abundance (genus Parasutterella id.2892)

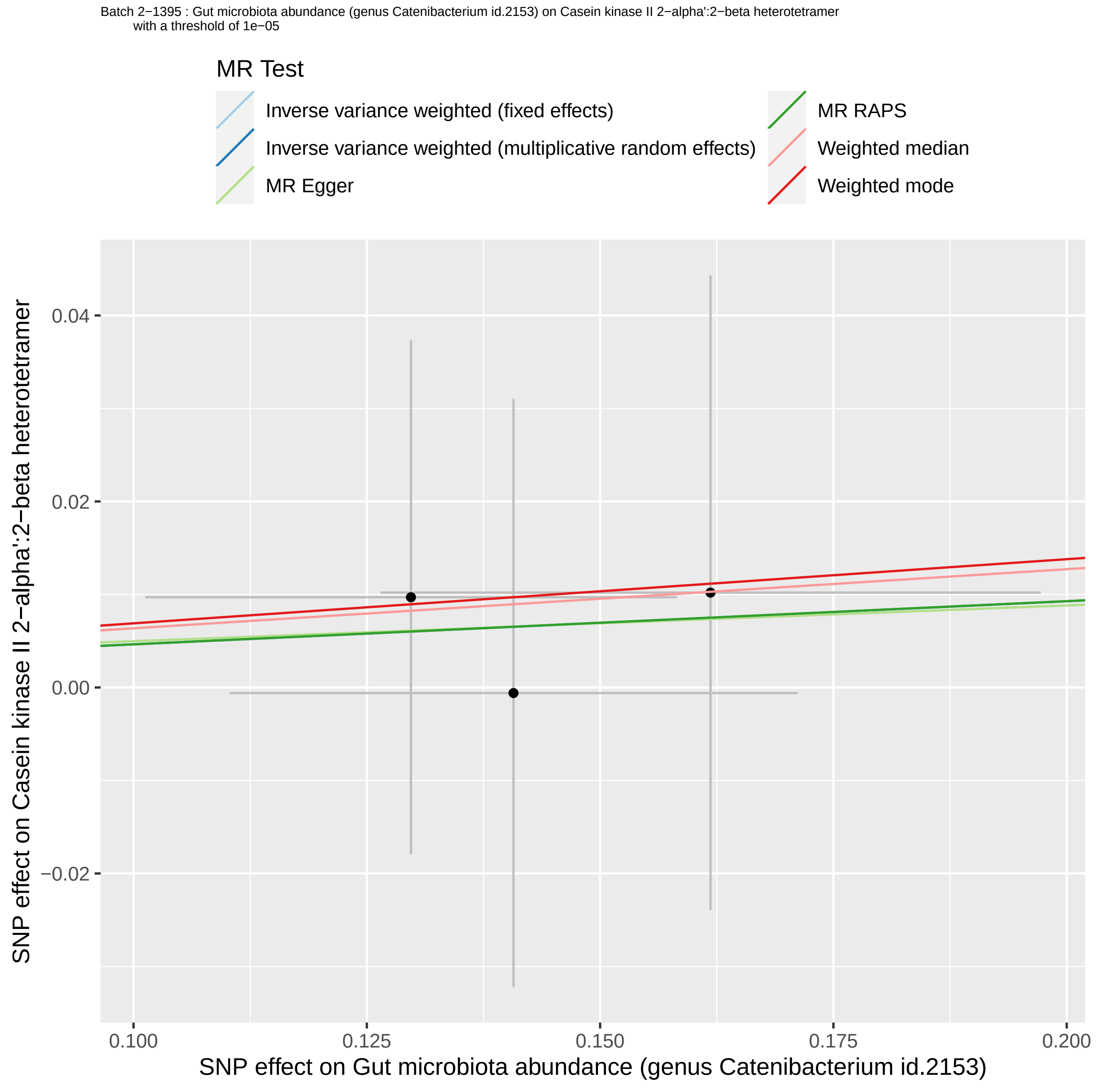

Batch 2−1395 : Gut microbiota abundance (genus Catenibacterium id.2153) on Casein kinase II 2−alpha':2−beta heterotetramer
with a threshold of 1e−05
MR Test
Inverse variance weighted (fixed effects)
Inverse variance weighted (multiplicative random effects)
MR Egger
MR RAPS
Weighted median
Weighted mode
0.04
0.02
0.00
−0.02
0.100
0.125
0.150
0.175
0.200
SNP effect on Casein kinase II 2−alpha':2−beta heterotetramer
SNP effect on Gut microbiota abundance (genus Catenibacterium id.2153)

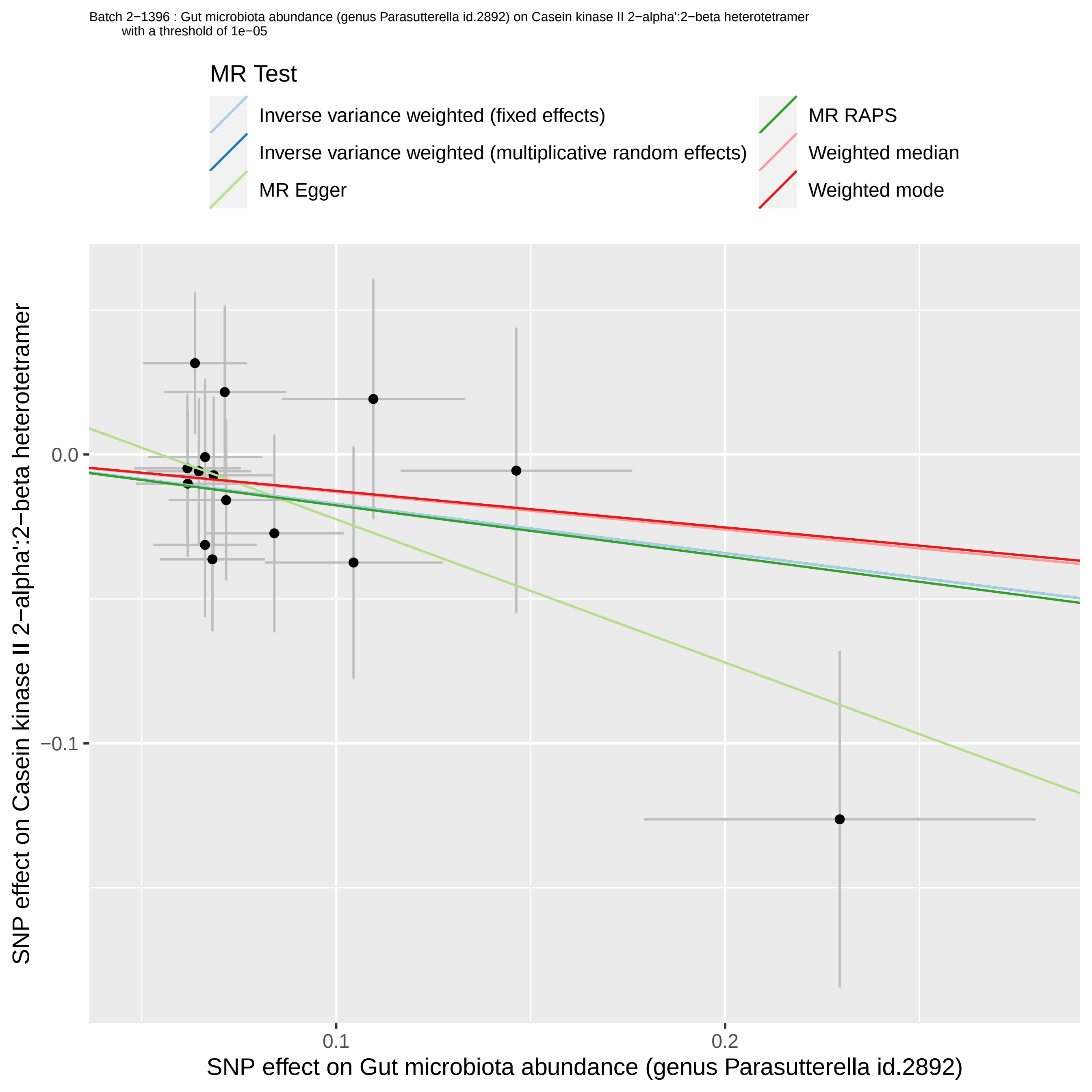
Batch 2−1396 : Gut microbiota abundance (genus Parasutterella id.2892) on Casein kinase II 2−alpha':2−beta heterotetramer
with a threshold of 1e−05
MR Test
Inverse variance weighted (fixed effects)
Inverse variance weighted (multiplicative random effects)
MR Egger
MR RAPS
Weighted median
Weighted mode
0.0
−0.1
0.1
0.2
SNP effect on Gut microbiota abundance (genus Parasutterella id.2892)
SNP effect on Casein kinase II 2−alpha':2−beta heterotetramer

Batch 2−1397 : Gut microbiota abundance (genus Catenibacterium id.2153) on Chondroitin sulfate proteoglycan 4
with a threshold of 1e−05

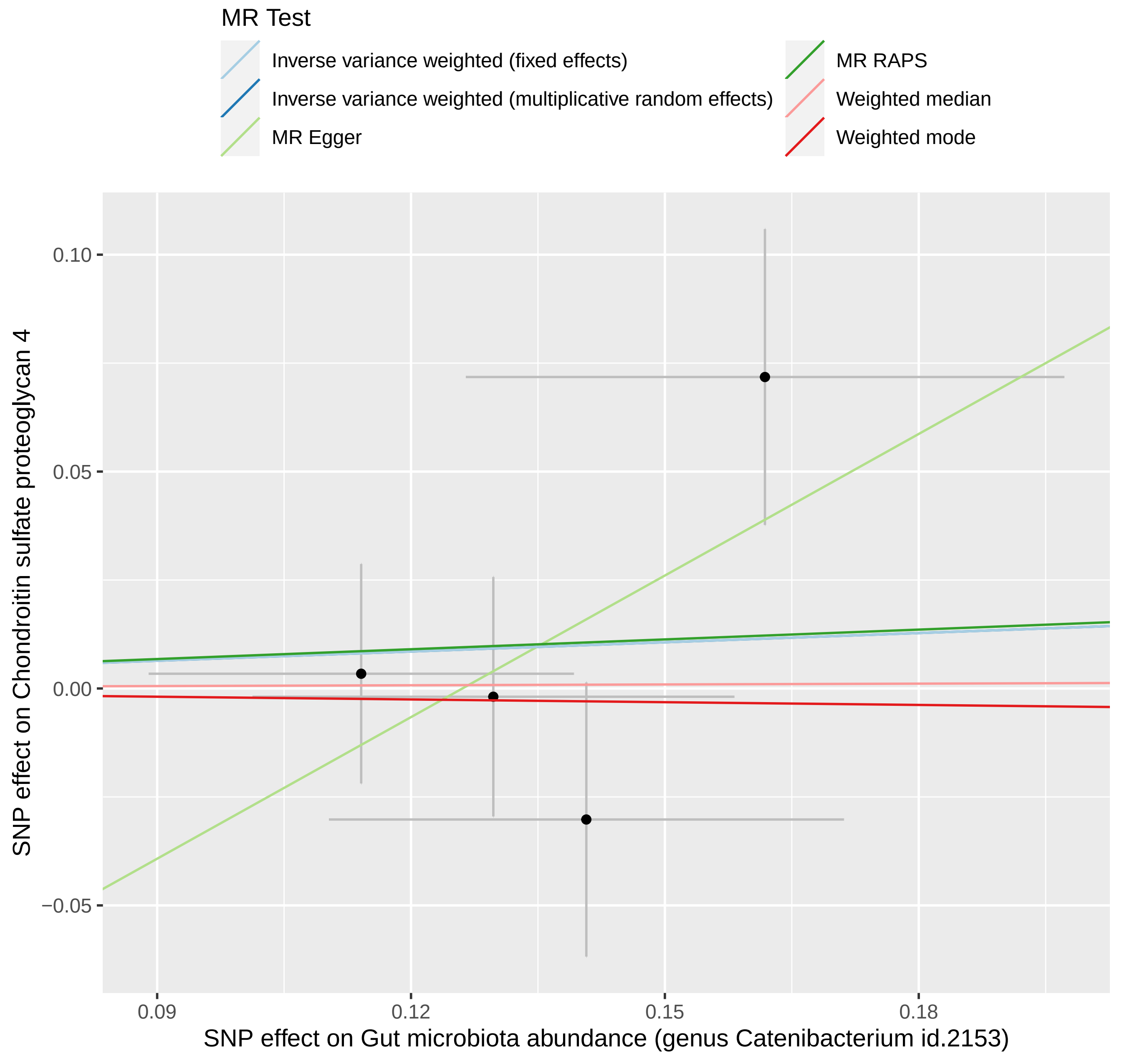

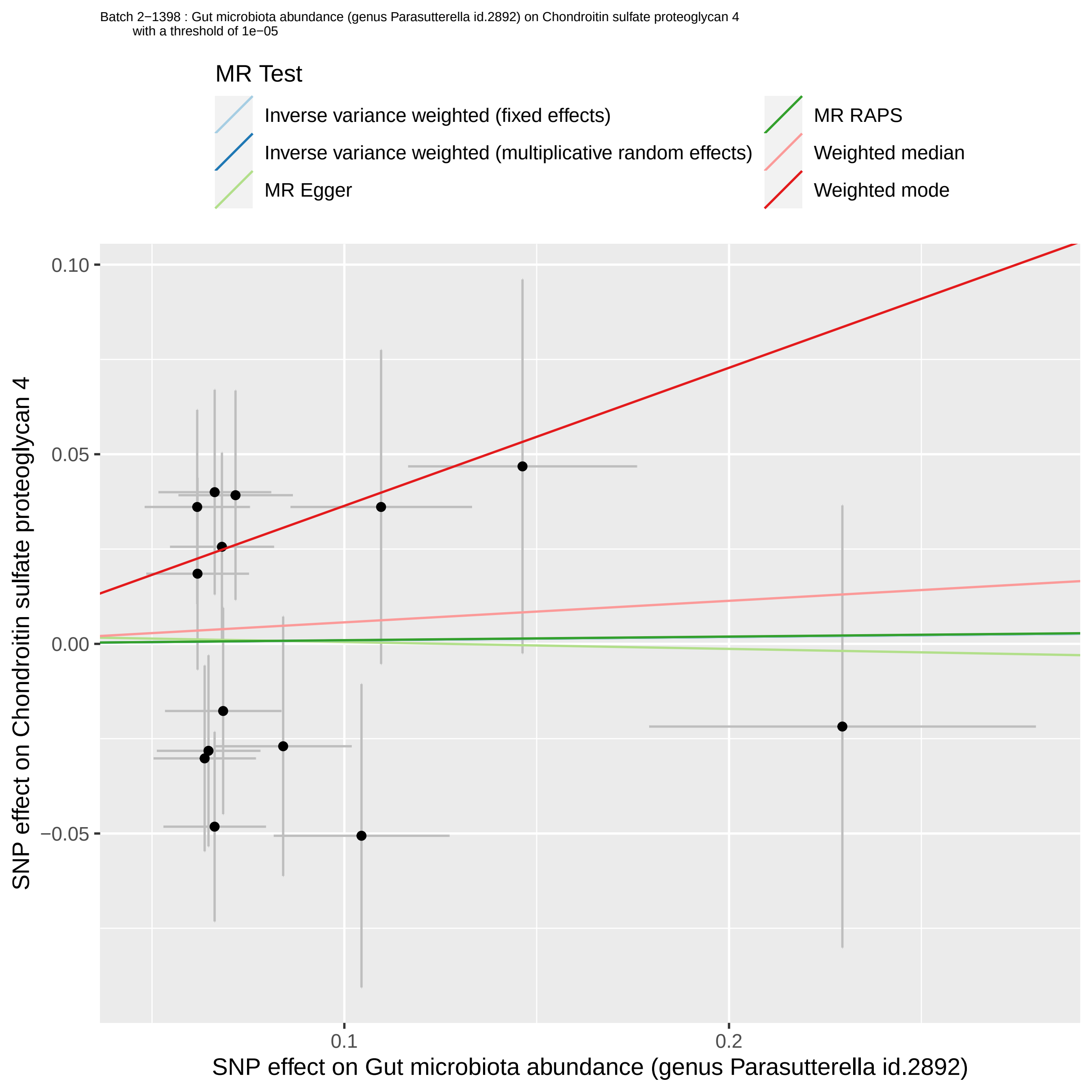

Batch 2−1398 : Gut microbiota abundance (genus Parasutterella id.2892) on Chondroitin sulfate proteoglycan 4
with a threshold of 1e−05
MR Test
Inverse variance weighted (fixed effects)
Inverse variance weighted (multiplicative random effects)
MR Egger
MR RAPS
Weighted median
Weighted mode
0.10
0.05
0.00
−0.05
0.1
0.2
SNP effect on Chondroitin sulfate proteoglycan 4
SNP effect on Gut microbiota abundance (genus Parasutterella id.2892)

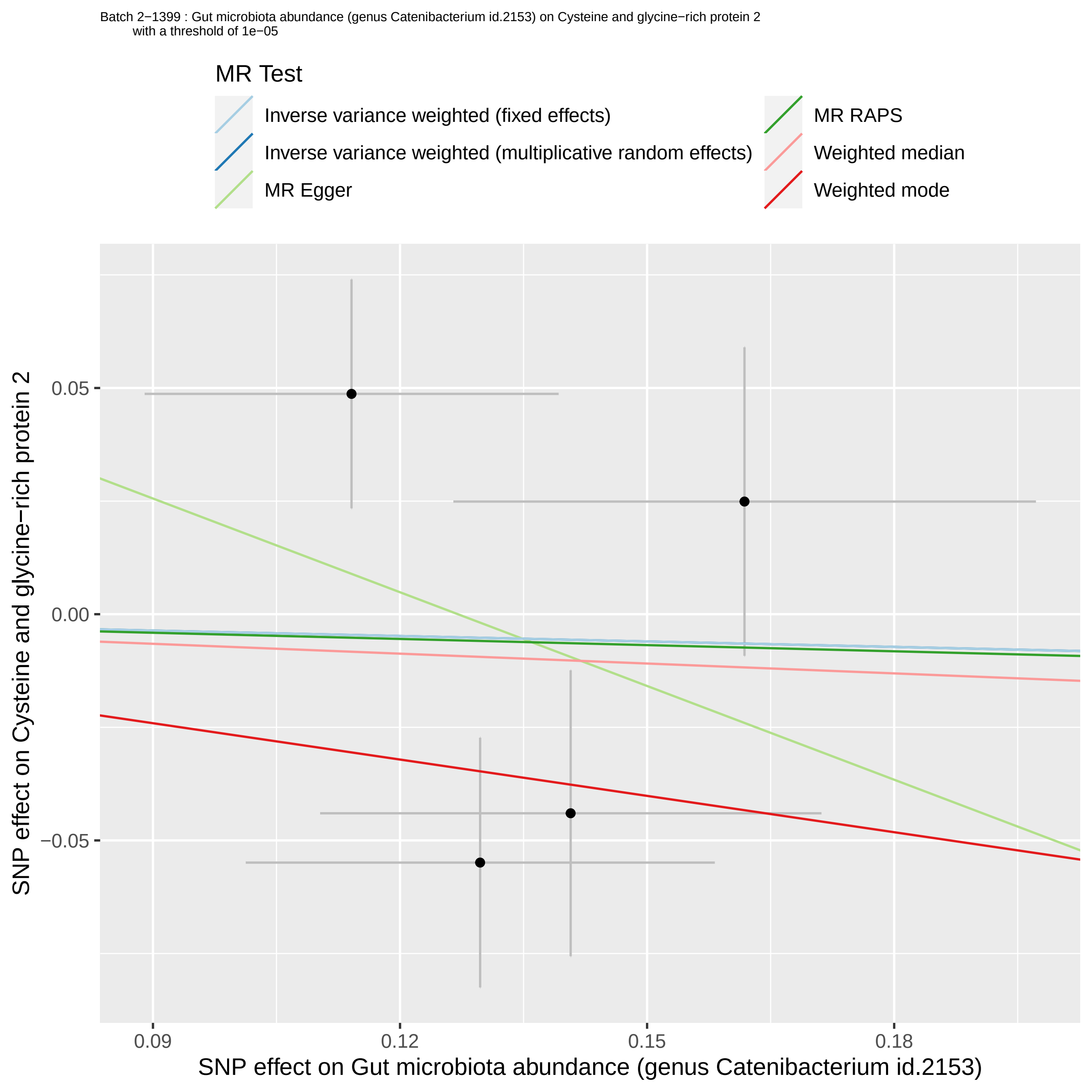
Batch 2−1399 : Gut microbiota abundance (genus Catenibacterium id.2153) on Cysteine and glycine−rich protein 2
with a threshold of 1e−05
MR Test
Inverse variance weighted (fixed effects)
Inverse variance weighted (multiplicative random effects)
MR Egger
MR RAPS
Weighted median
Weighted mode
0.05
0.00
−0.05
0.09
0.12
0.15
0.18
SNP effect on Cysteine and glycine−rich protein 2
SNP effect on Gut microbiota abundance (genus Catenibacterium id.2153)

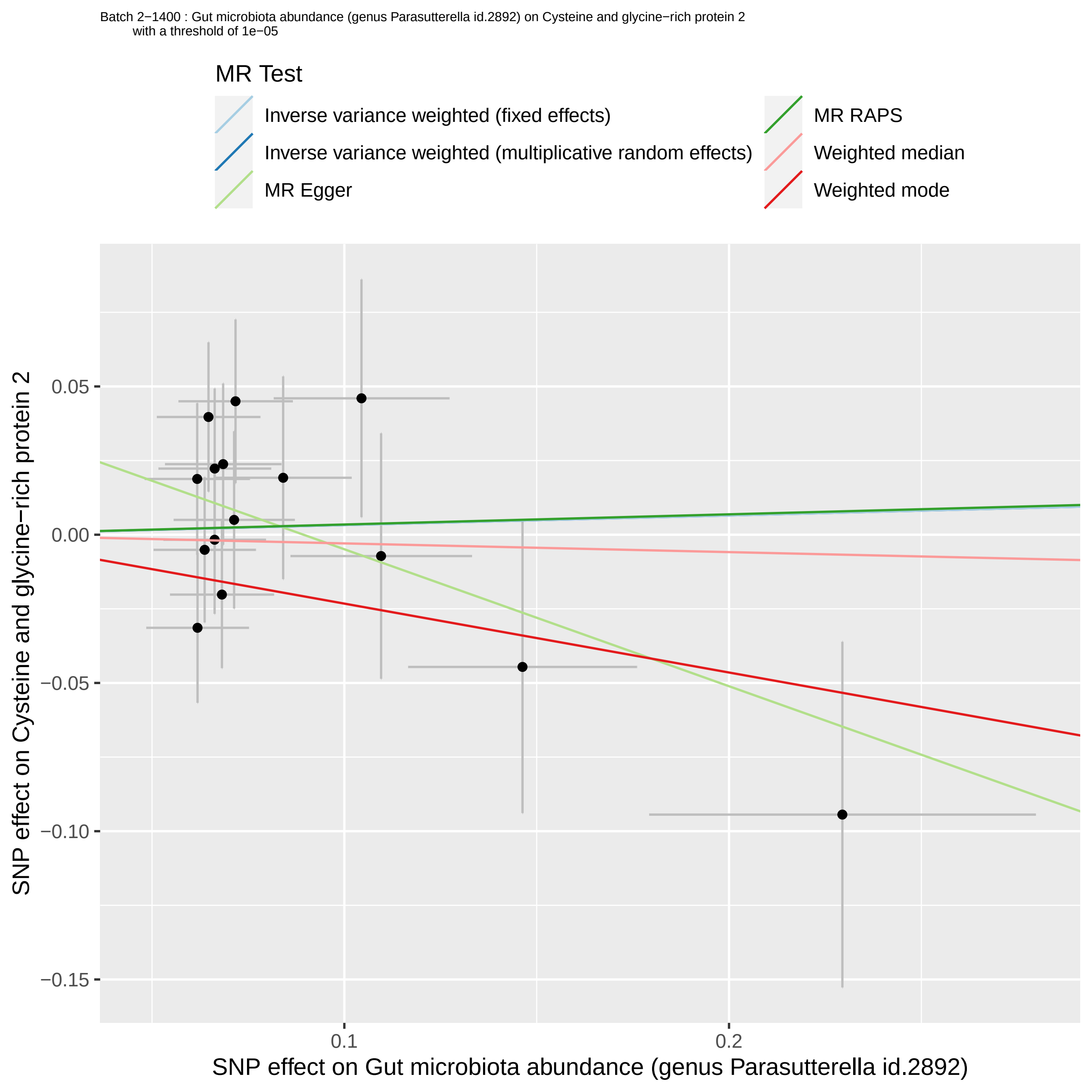
Batch 2−1400 : Gut microbiota abundance (genus Parasutterella id.2892) on Cysteine and glycine−rich protein 2
with a threshold of 1e−05
MR Test
Inverse variance weighted (fixed effects)
Inverse variance weighted (multiplicative random effects)
MR Egger
MR RAPS
Weighted median
Weighted mode
SNP effect on Cysteine and glycine−rich protein 2
0.05
0.00
−0.05
−0.10
−0.15
0.1
0.2
SNP effect on Gut microbiota abundance (genus Parasutterella id.2892)

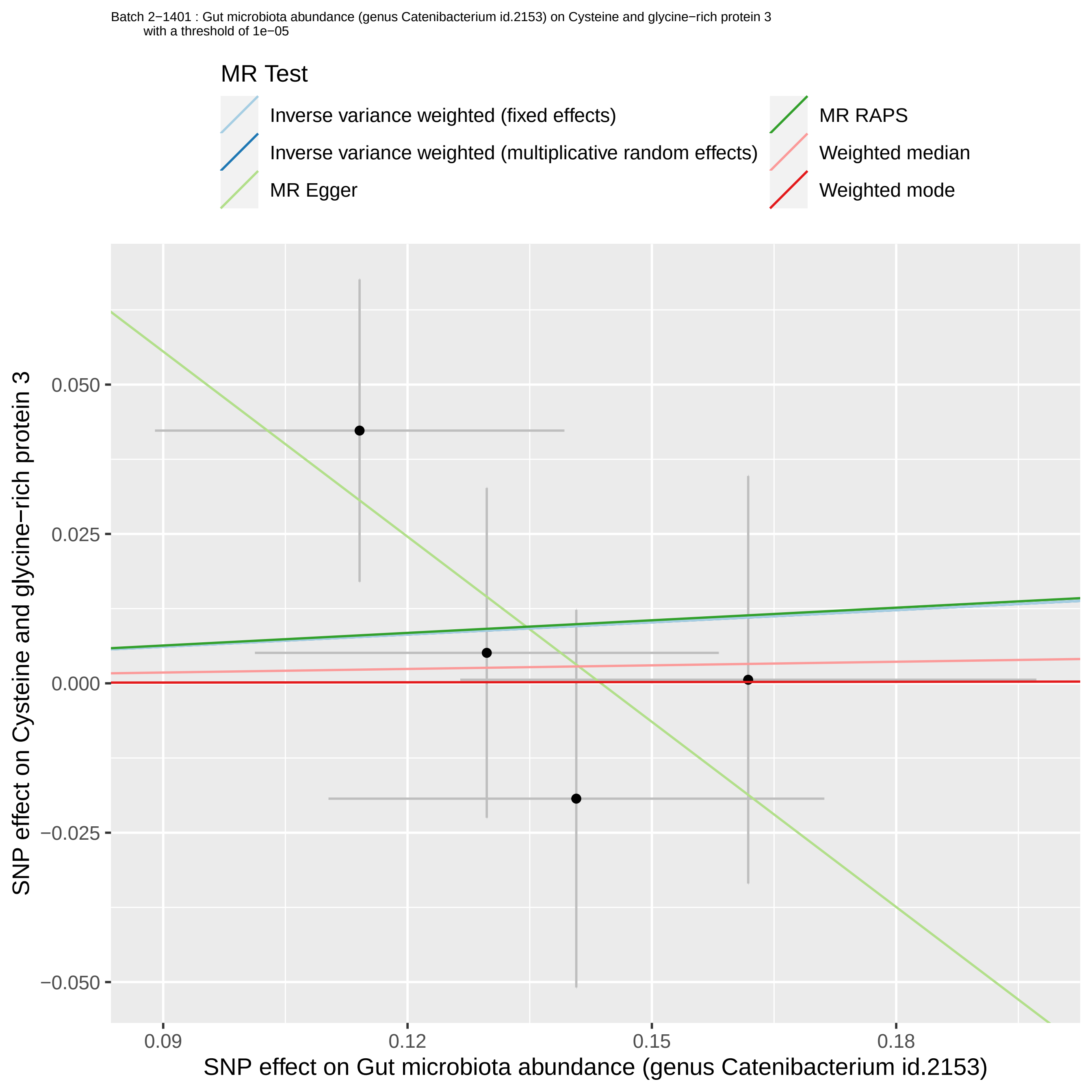
Batch 2−1401 : Gut microbiota abundance (genus Catenibacterium id.2153) on Cysteine and glycine−rich protein 3
with a threshold of 1e−05
MR Test
Inverse variance weighted (fixed effects)
Inverse variance weighted (multiplicative random effects)
MR Egger
MR RAPS
Weighted median
Weighted mode
0.050
0.025
0.000
−0.025
−0.050
0.09
0.12
0.15
0.18
SNP effect on Cysteine and glycine−rich protein 3
SNP effect on Gut microbiota abundance (genus Catenibacterium id.2153)

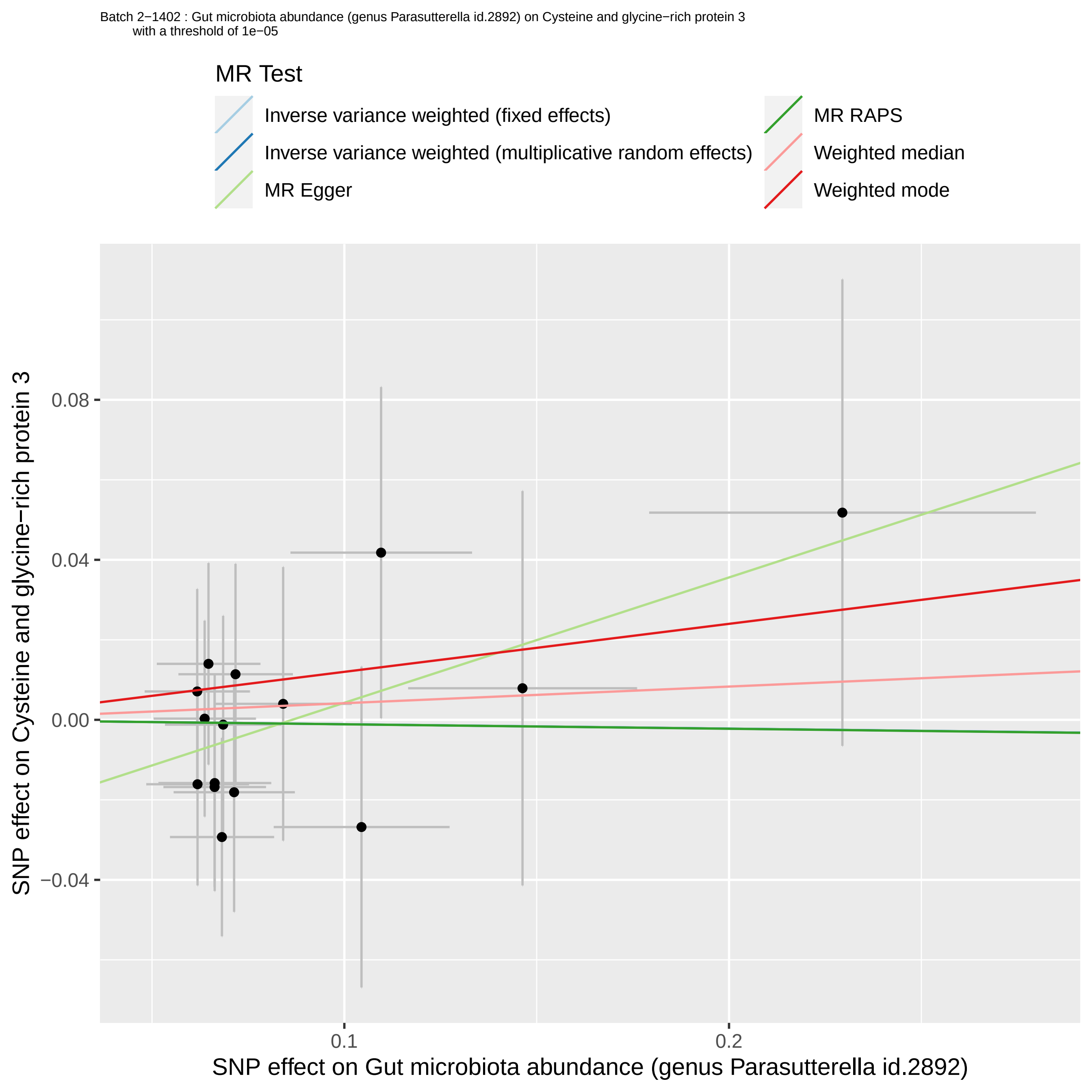

Batch 2−1402 : Gut microbiota abundance (genus Parasutterella id.2892) on Cysteine and glycine−rich protein 3
with a threshold of 1e−05
MR Test
Inverse variance weighted (fixed effects)
Inverse variance weighted (multiplicative random effects)
MR Egger
MR RAPS
Weighted median
Weighted mode
0.08
0.04
0.00
−0.04
0.1
0.2
SNP effect on Cysteine and glycine−rich protein 3
SNP effect on Gut microbiota abundance (genus Parasutterella id.2892)

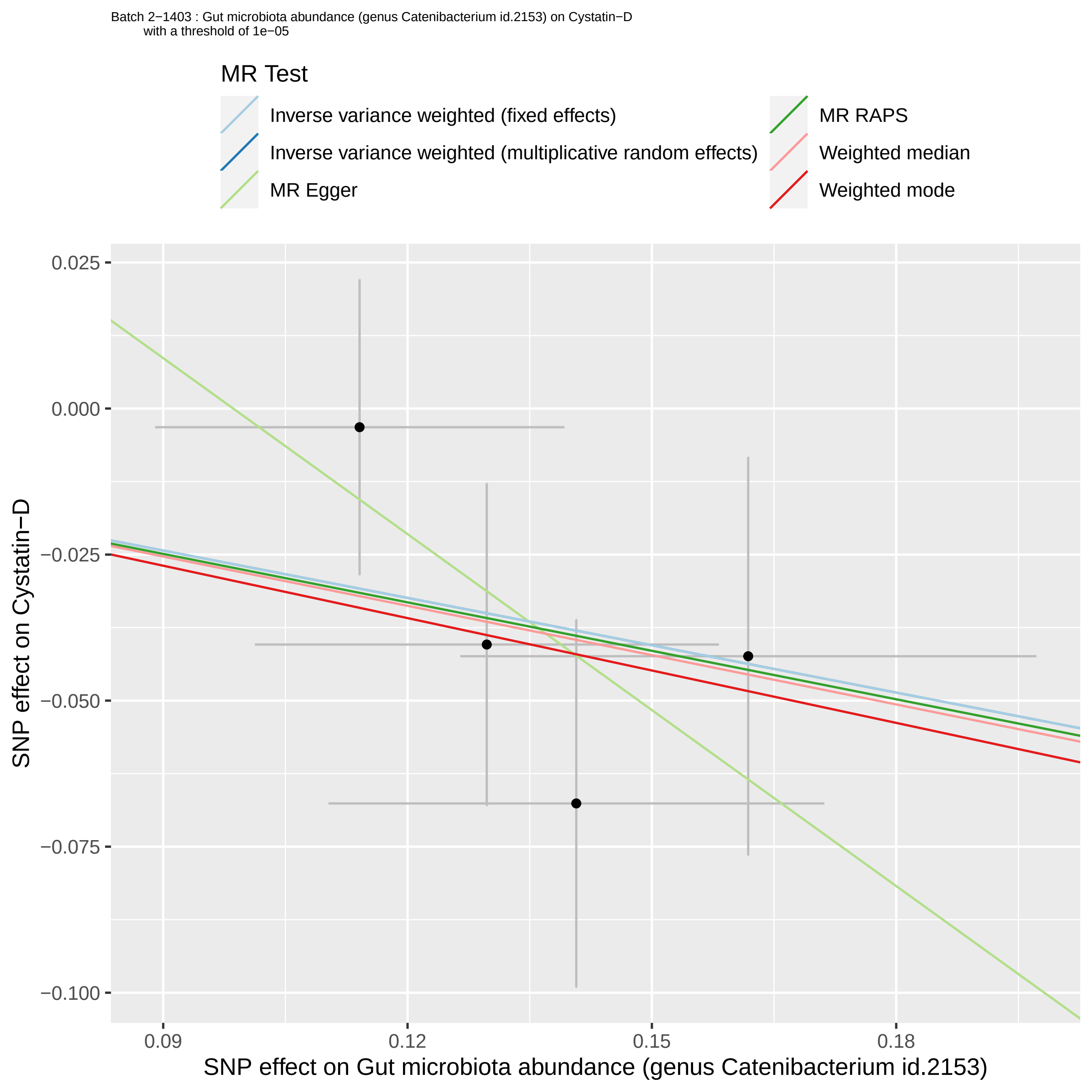

Batch 2−1403 : Gut microbiota abundance (genus Catenibacterium id.2153) on Cystatin−D
with a threshold of 1e−05
MR Test
Inverse variance weighted (fixed effects)
Inverse variance weighted (multiplicative random effects)
MR Egger
MR RAPS
Weighted median
Weighted mode
0.025
0.000
−0.025
−0.050
−0.075
−0.100
0.09
0.12
0.15
0.18
SNP effect on Cystatin−D
SNP effect on Gut microbiota abundance (genus Catenibacterium id.2153)

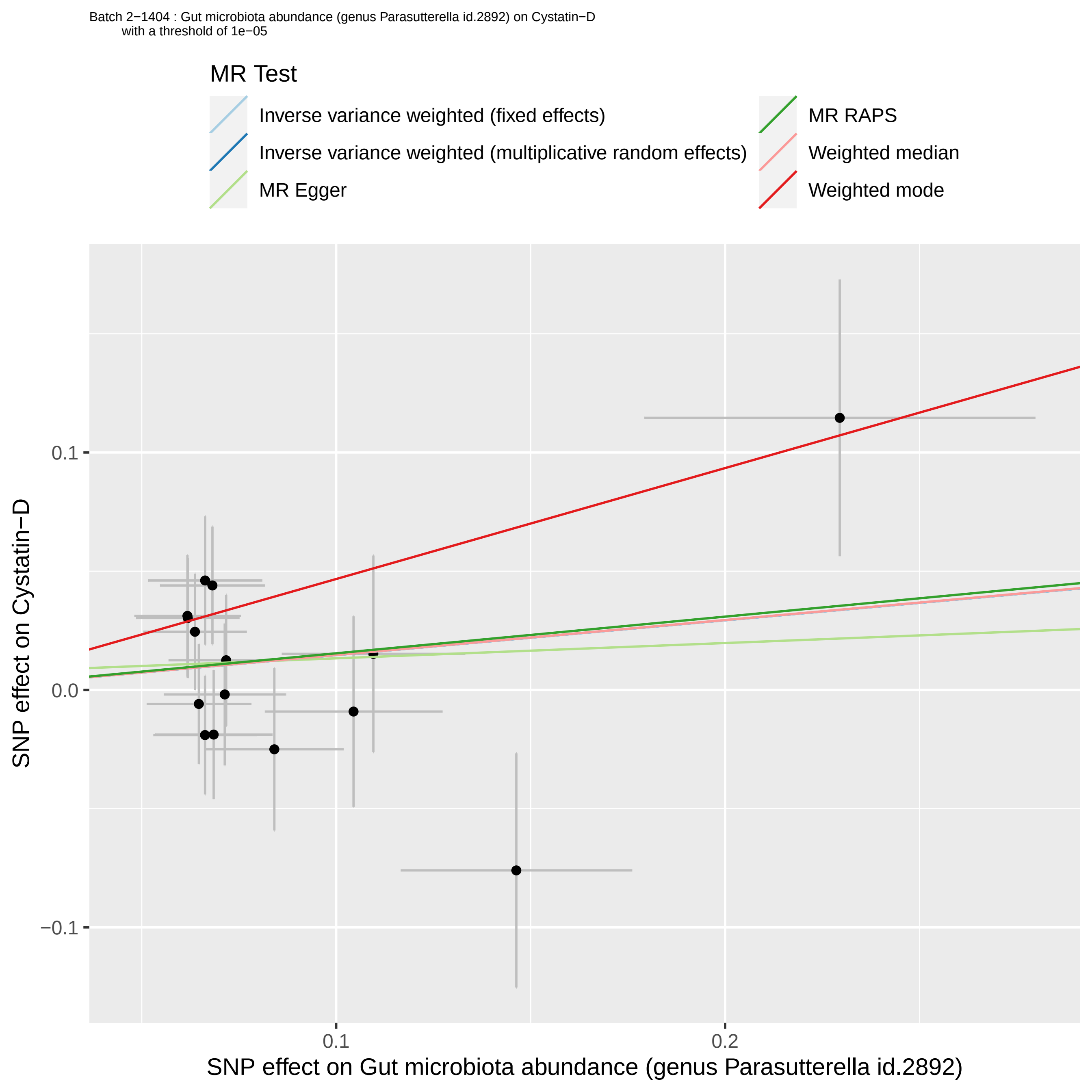
Batch 2−1404 : Gut microbiota abundance (genus Parasutterella id.2892) on Cystatin−D
with a threshold of 1e−05
MR Test
Inverse variance weighted (fixed effects)
Inverse variance weighted (multiplicative random effects)
MR Egger
MR RAPS
Weighted median
Weighted mode
0.1
0.0
−0.1
SNP effect on Cystatin−D
0.1
0.2
SNP effect on Gut microbiota abundance (genus Parasutterella id.2892)

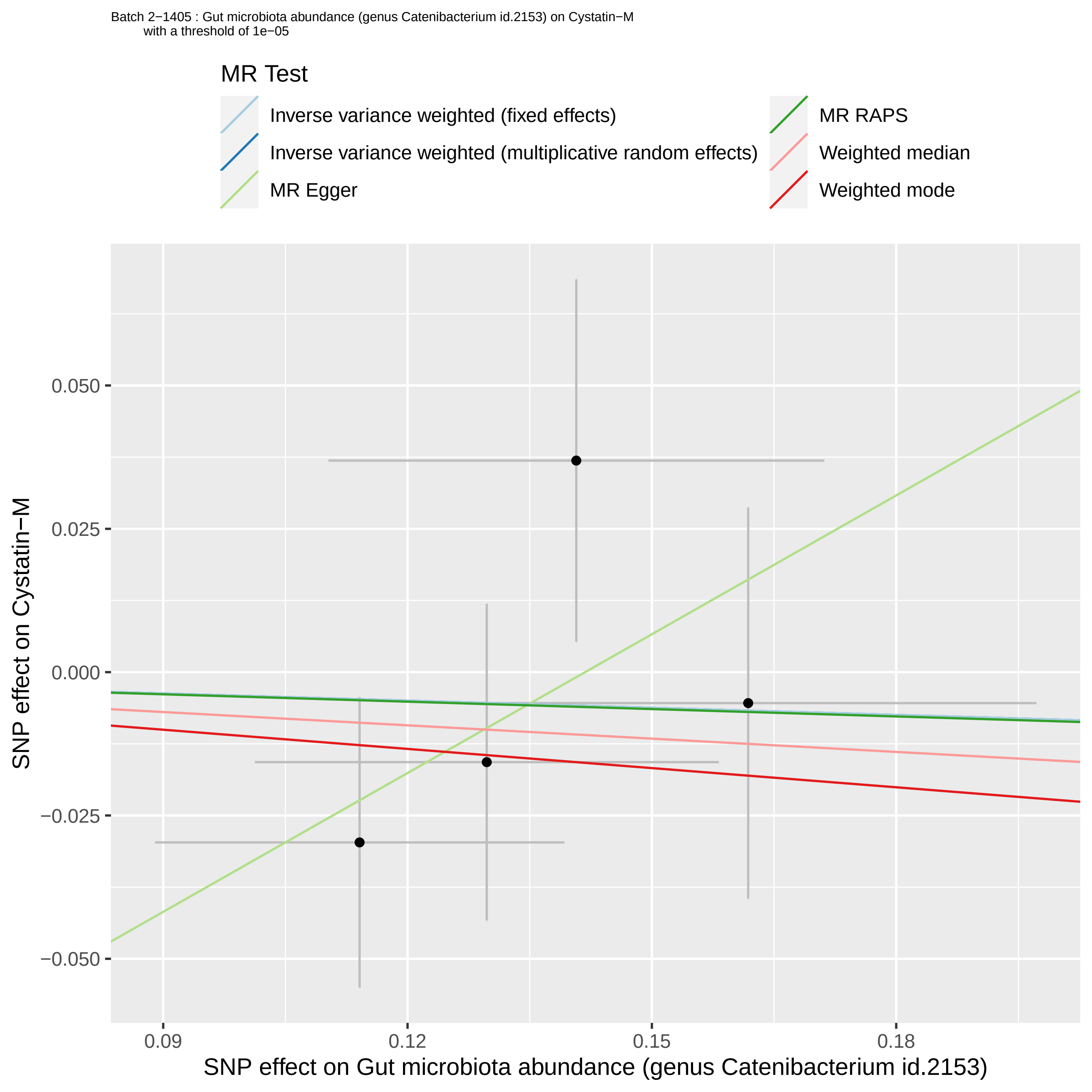

Batch 2−1405 : Gut microbiota abundance (genus Catenibacterium id.2153) on Cystatin−M
with a threshold of 1e−05
MR Test
Inverse variance weighted (fixed effects)
Inverse variance weighted (multiplicative random effects)
MR Egger
MR RAPS
Weighted median
Weighted mode
0.050
0.025
0.000
−0.025
−0.050
SNP effect on Cystatin−M
0.09
0.12
0.15
0.18
SNP effect on Gut microbiota abundance (genus Catenibacterium id.2153)

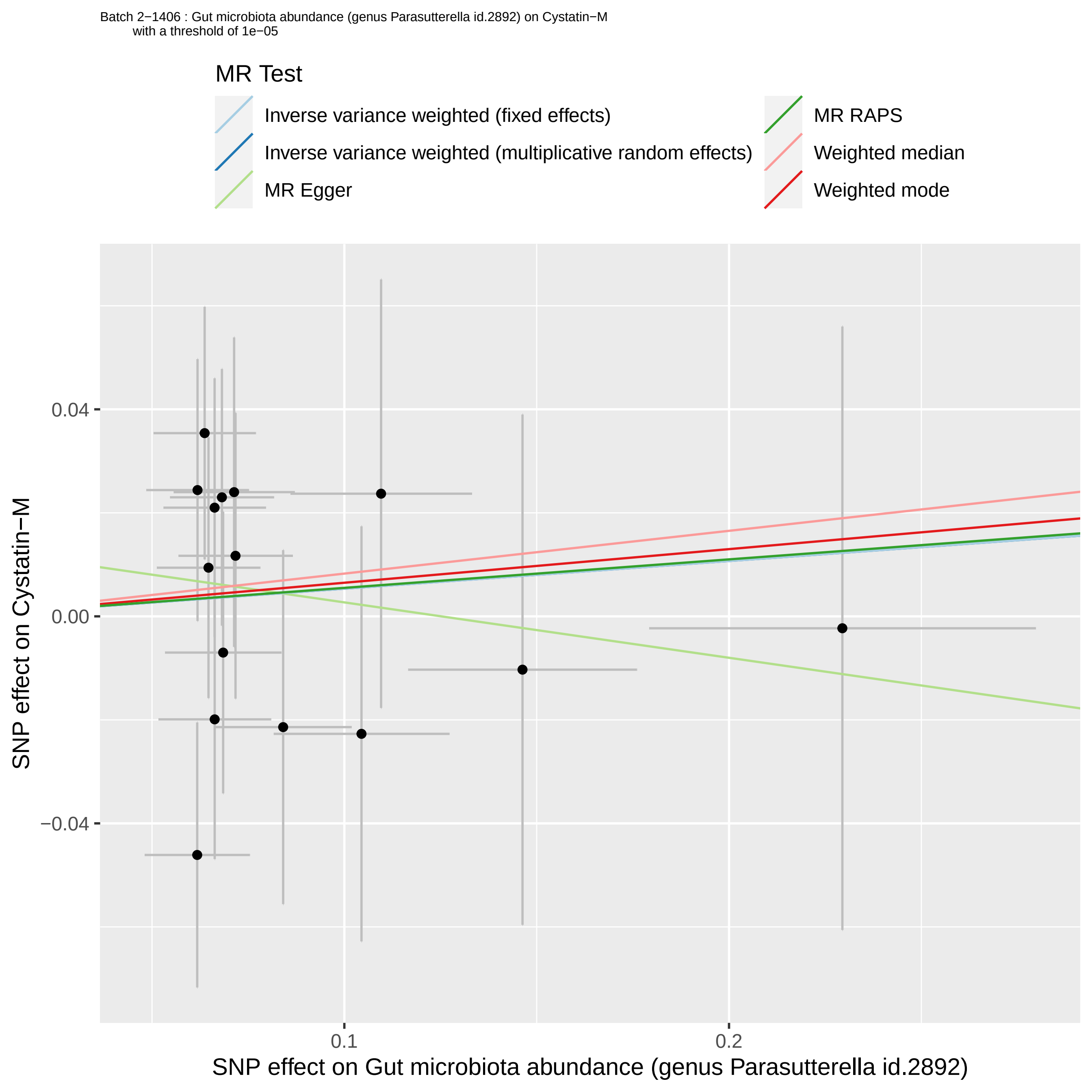

Batch 2−1406 : Gut microbiota abundance (genus Parasutterella id.2892) on Cystatin−M
with a threshold of 1e−05
MR Test
Inverse variance weighted (fixed effects)
Inverse variance weighted (multiplicative random effects)
MR Egger
MR RAPS
Weighted median
Weighted mode
0.04
0.00
−0.04
0.1
0.2
SNP effect on Cystatin−M
SNP effect on Gut microbiota abundance (genus Parasutterella id.2892)

Batch 2−1407 : Gut microbiota abundance (genus Catenibacterium id.2153) on Cystatin−M
with a threshold of 1e−05

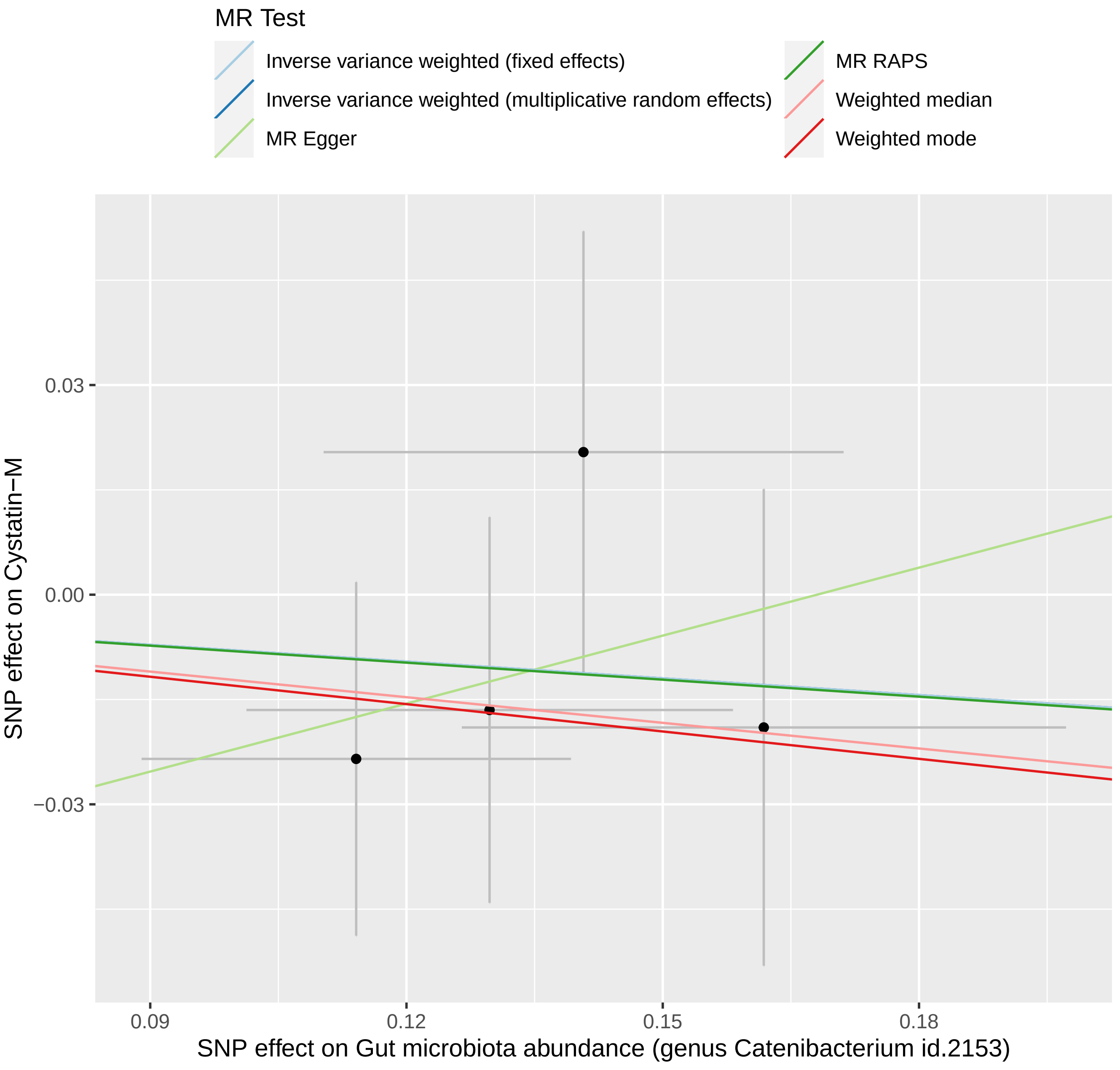

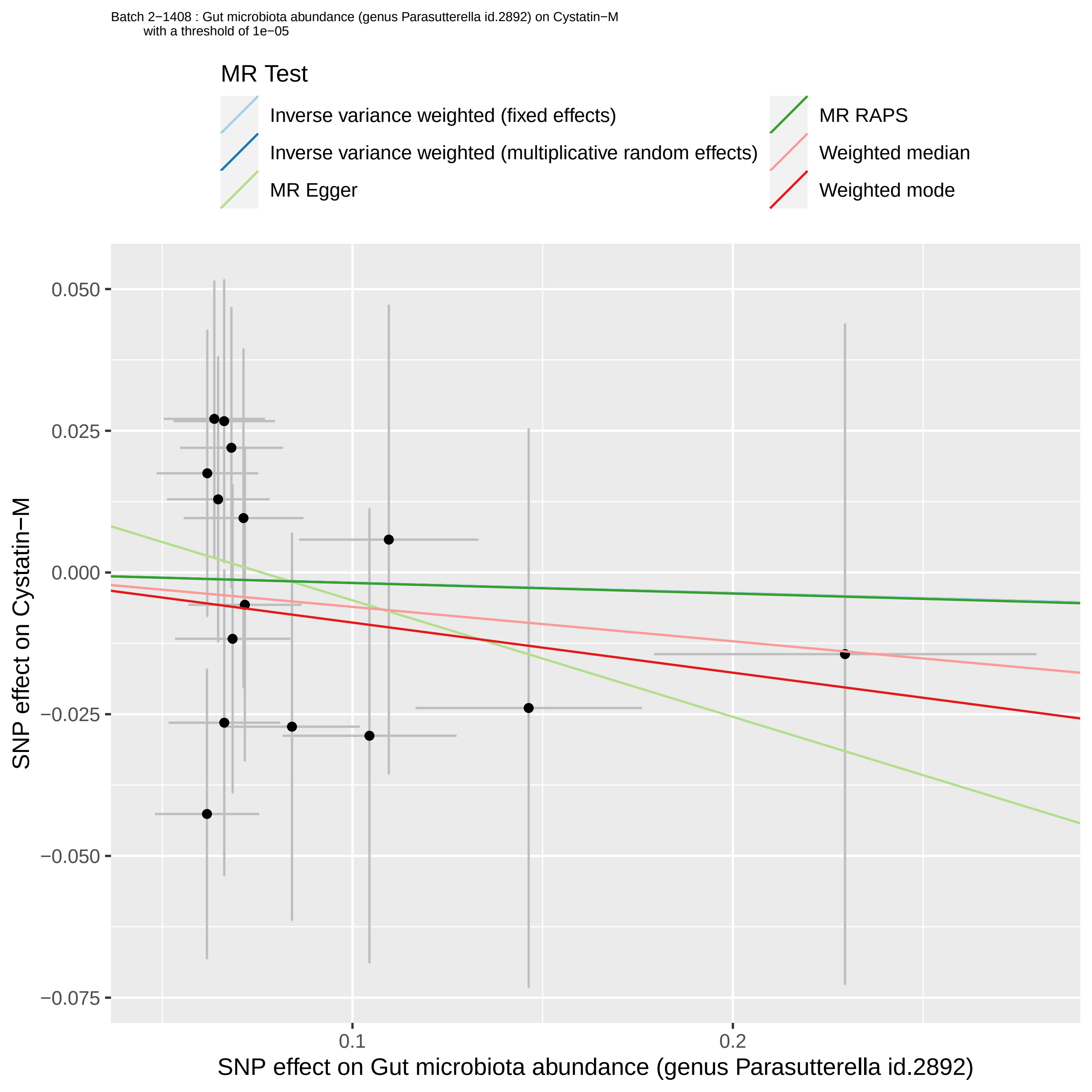
Batch 2−1408 : Gut microbiota abundance (genus Parasutterella id.2892) on Cystatin−M
with a threshold of 1e−05
MR Test
Inverse variance weighted (fixed effects)
Inverse variance weighted (multiplicative random effects)
MR Egger
MR RAPS
Weighted median
Weighted mode
0.050
0.025
0.000
−0.025
−0.050
−0.075
0.1
0.2
SNP effect on Cystatin−M
SNP effect on Gut microbiota abundance (genus Parasutterella id.2892)

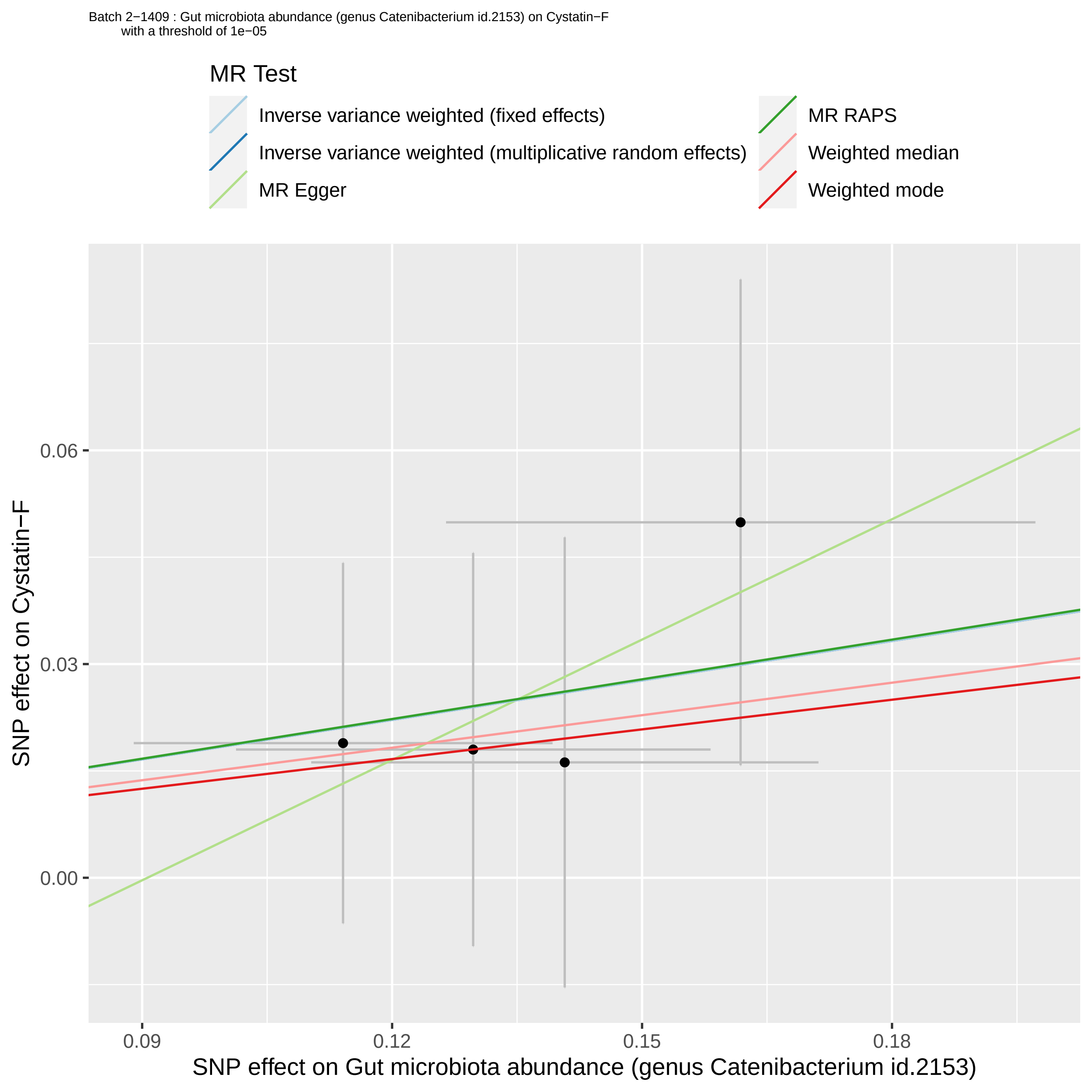

Batch 2−1409 : Gut microbiota abundance (genus Catenibacterium id.2153) on Cystatin−F
with a threshold of 1e−05
MR Test
Inverse variance weighted (fixed effects)
Inverse variance weighted (multiplicative random effects)
MR Egger
MR RAPS
Weighted median
Weighted mode
0.06
0.03
0.00
0.09
0.12
0.15
0.18
SNP effect on Cystatin−F
SNP effect on Gut microbiota abundance (genus Catenibacterium id.2153)

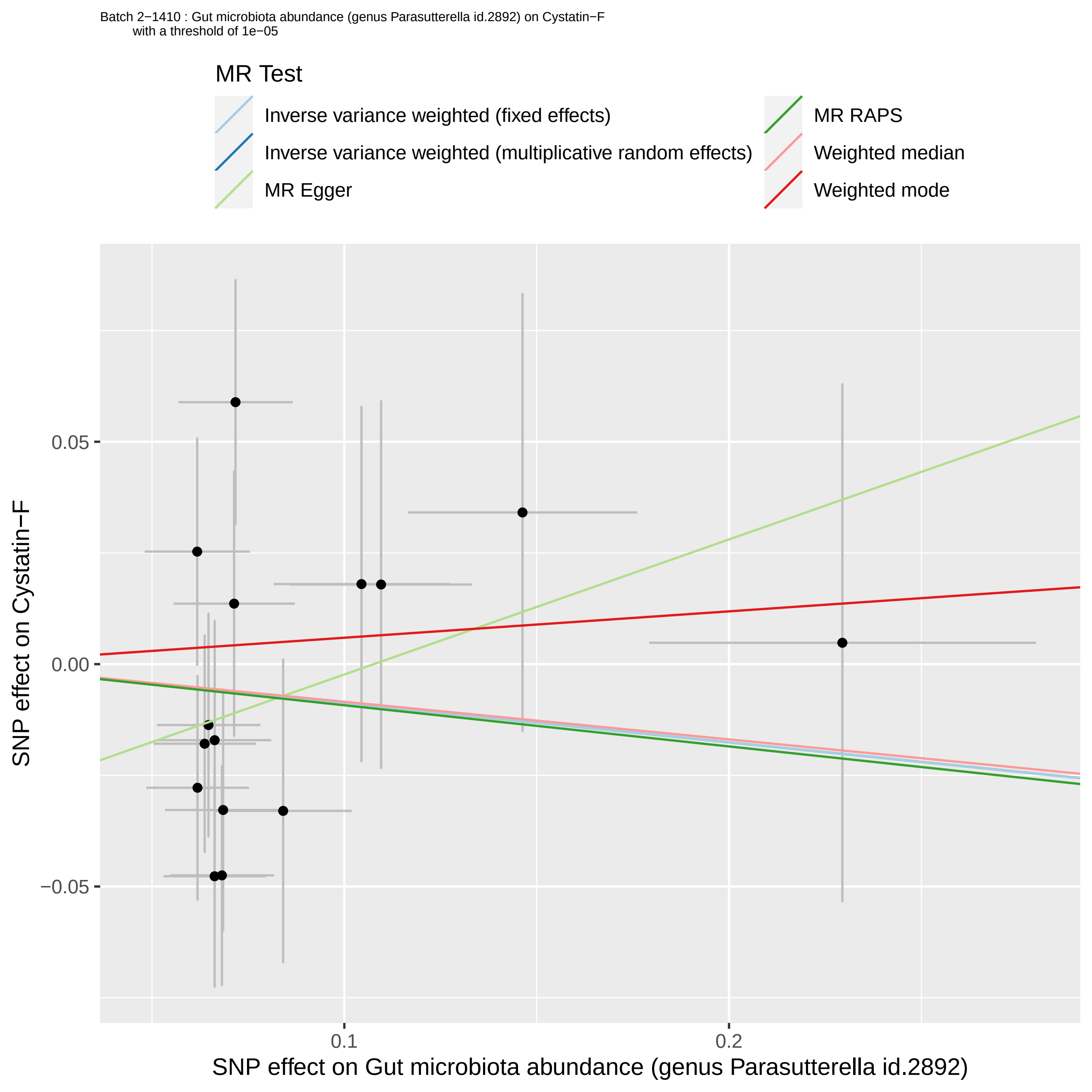
Batch 2−1410 : Gut microbiota abundance (genus Parasutterella id.2892) on Cystatin−F
with a threshold of 1e−05
MR Test
Inverse variance weighted (fixed effects)
Inverse variance weighted (multiplicative random effects)
MR Egger
MR RAPS
Weighted median
Weighted mode
0.05
0.00
−0.05
0.1
0.2
SNP effect on Cystatin−F
SNP effect on Gut microbiota abundance (genus Parasutterella id.2892)

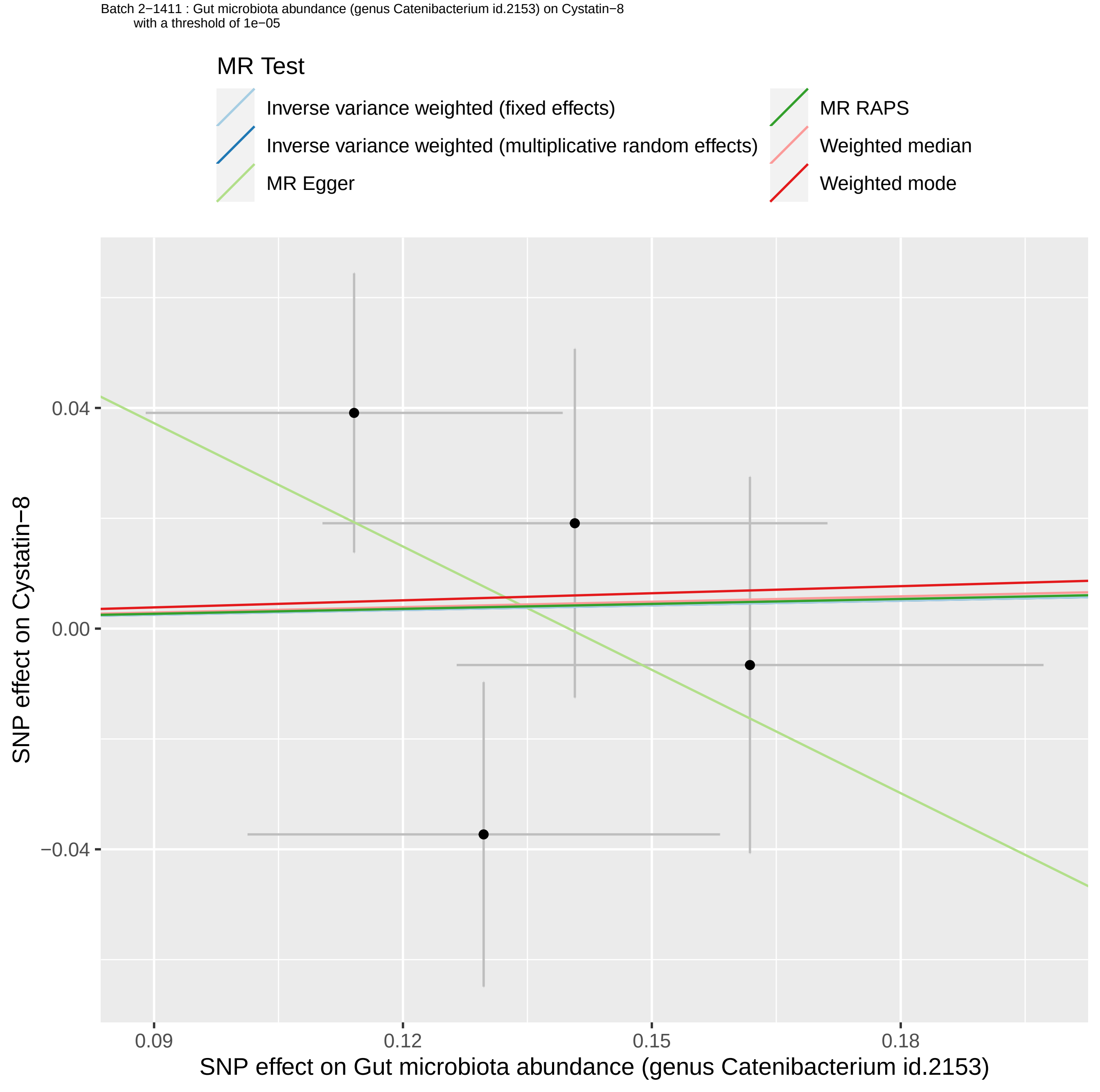
Batch 2−1411 : Gut microbiota abundance (genus Catenibacterium id.2153) on Cystatin−8
with a threshold of 1e−05
MR Test
Inverse variance weighted (fixed effects)
Inverse variance weighted (multiplicative random effects)
MR Egger
MR RAPS
Weighted median
Weighted mode
0.04
0.00
−0.04
0.09
0.12
0.15
0.18
SNP effect on Cystatin−8
SNP effect on Gut microbiota abundance (genus Catenibacterium id.2153)

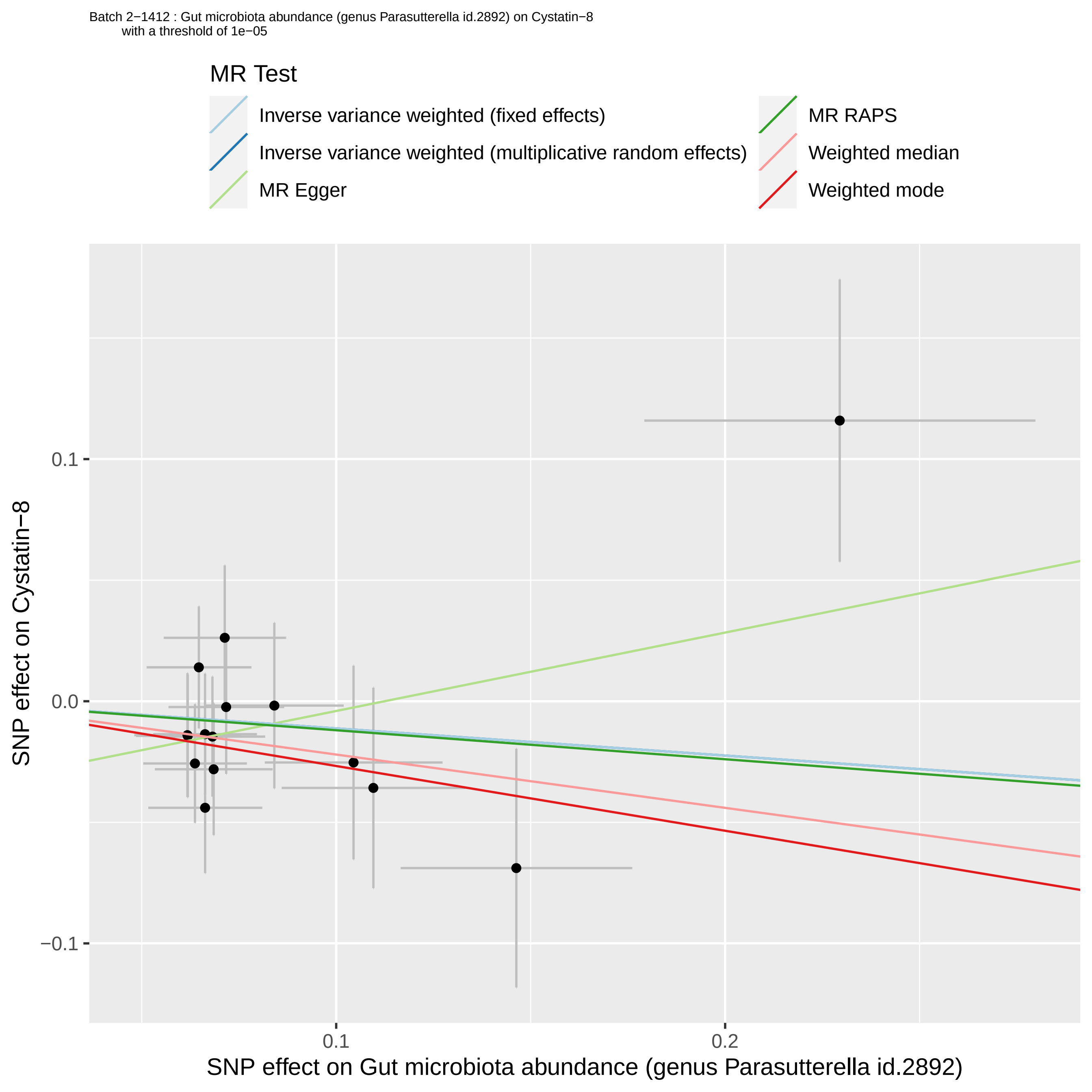
Batch 2−1412 : Gut microbiota abundance (genus Parasutterella id.2892) on Cystatin−8
with a threshold of 1e−05
MR Test
Inverse variance weighted (fixed effects)
Inverse variance weighted (multiplicative random effects)
MR Egger
MR RAPS
Weighted median
Weighted mode
0.1
0.0
−0.1
0.1
0.2
SNP effect on Cystatin−8
SNP effect on Gut microbiota abundance (genus Parasutterella id.2892)

Batch 2−1413 : Gut microbiota abundance (genus Catenibacterium id.2153) on Cancer/testis antigen 55
with a threshold of 1e−05

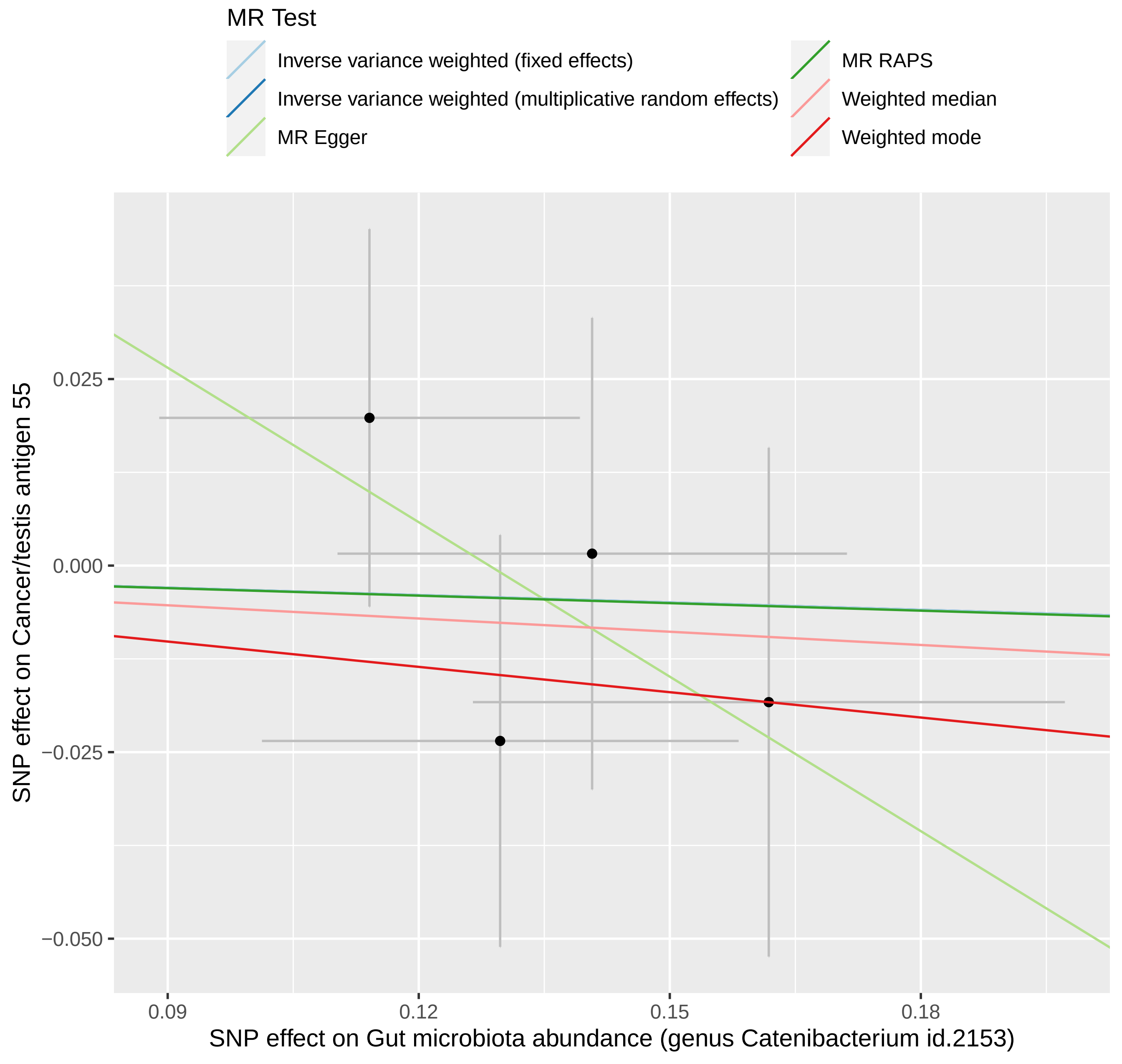

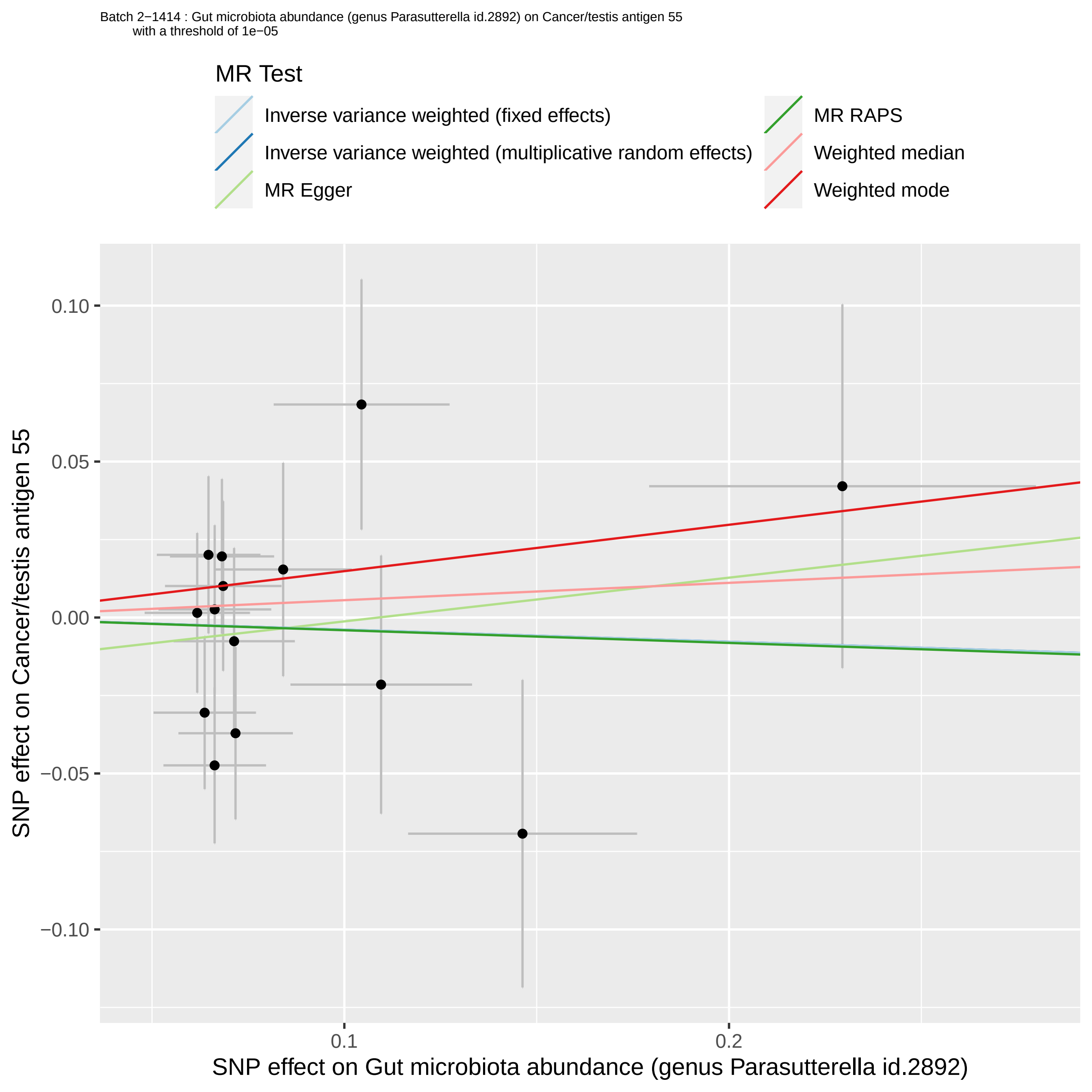
Batch 2−1414 : Gut microbiota abundance (genus Parasutterella id.2892) on Cancer/testis antigen 55
with a threshold of 1e−05
MR Test
Inverse variance weighted (fixed effects)
Inverse variance weighted (multiplicative random effects)
MR Egger
MR RAPS
Weighted median
Weighted mode
0.10
0.05
0.00
−0.05
−0.10
0.1
0.2
SNP effect on Cancer/testis antigen 55
SNP effect on Gut microbiota abundance (genus Parasutterella id.2892)

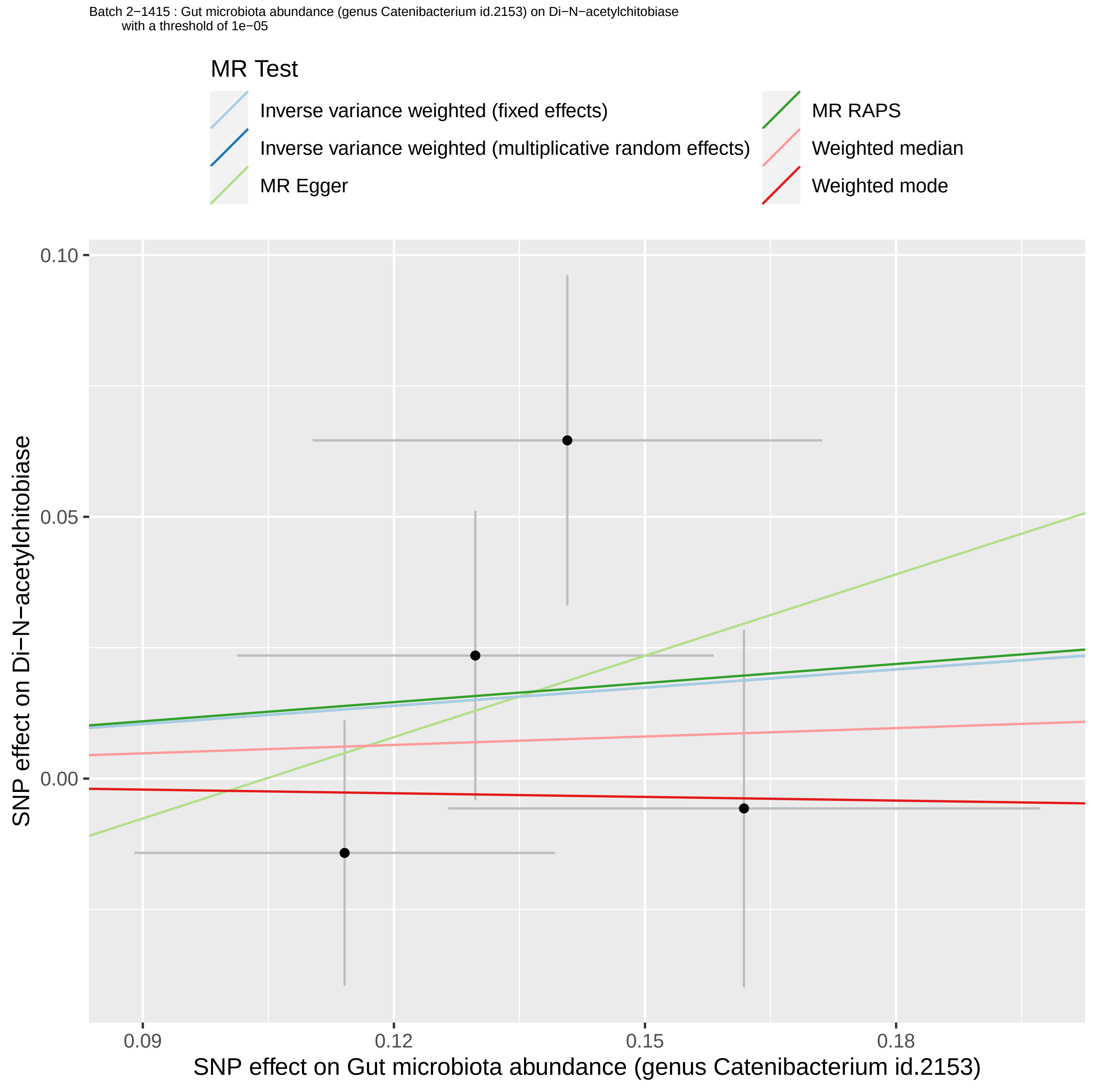
Batch 2−1415 : Gut microbiota abundance (genus Catenibacterium id.2153) on Di−N−acetylchitobiase
with a threshold of 1e−05
MR Test
Inverse variance weighted (fixed effects)
Inverse variance weighted (multiplicative random effects)
MR Egger
MR RAPS
Weighted median
Weighted mode
0.10
0.05
0.00
SNP effect on Di−N−acetylchitobiase
0.09
0.12
0.15
0.18
SNP effect on Gut microbiota abundance (genus Catenibacterium id.2153)

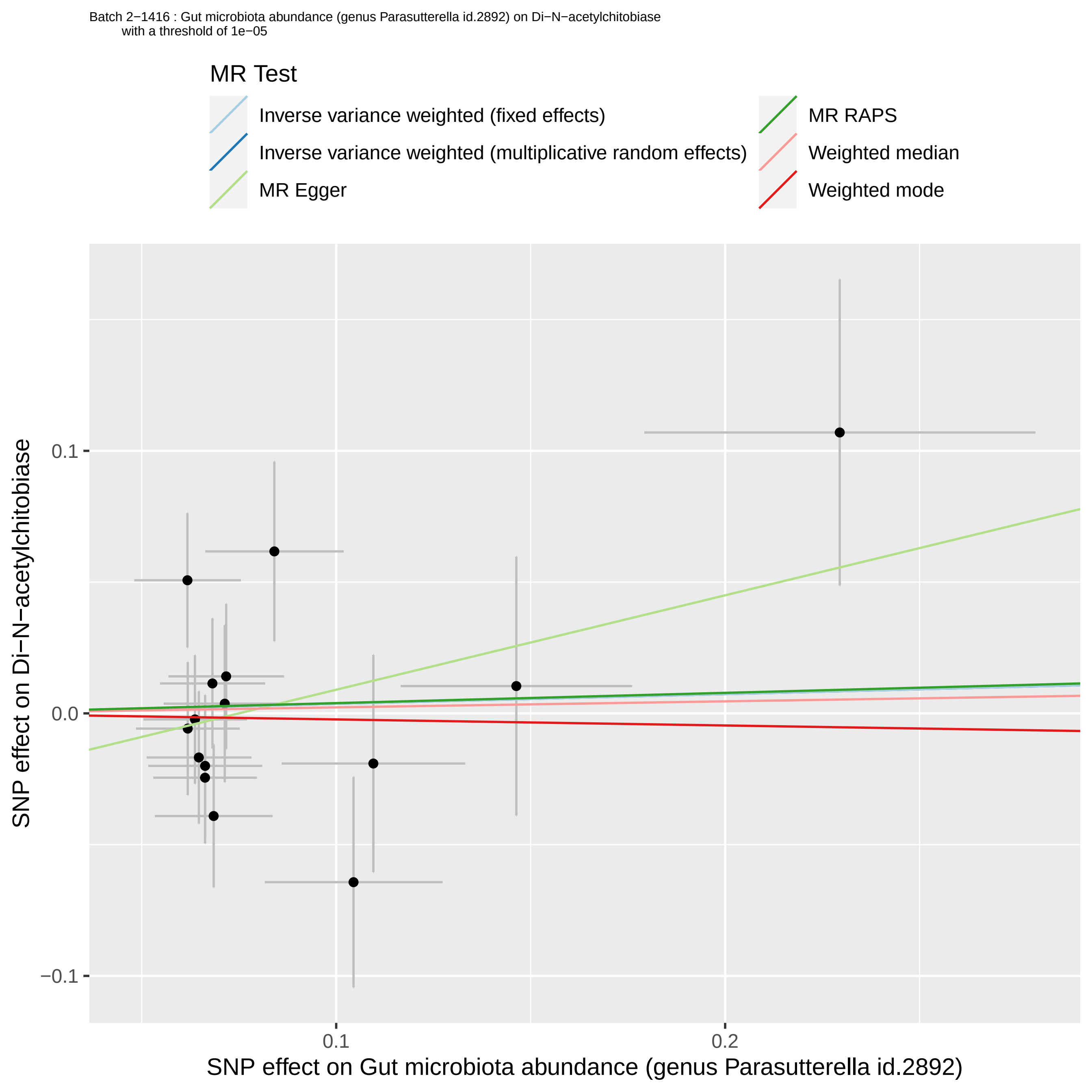

Batch 2−1416 : Gut microbiota abundance (genus Parasutterella id.2892) on Di−N−acetylchitobiase
with a threshold of 1e−05
MR Test
Inverse variance weighted (fixed effects)
Inverse variance weighted (multiplicative random effects)
MR Egger
MR RAPS
Weighted median
Weighted mode
0.1
0.0
−0.1
0.1
0.2
SNP effect on Di−N−acetylchitobiase
SNP effect on Gut microbiota abundance (genus Parasutterella id.2892)

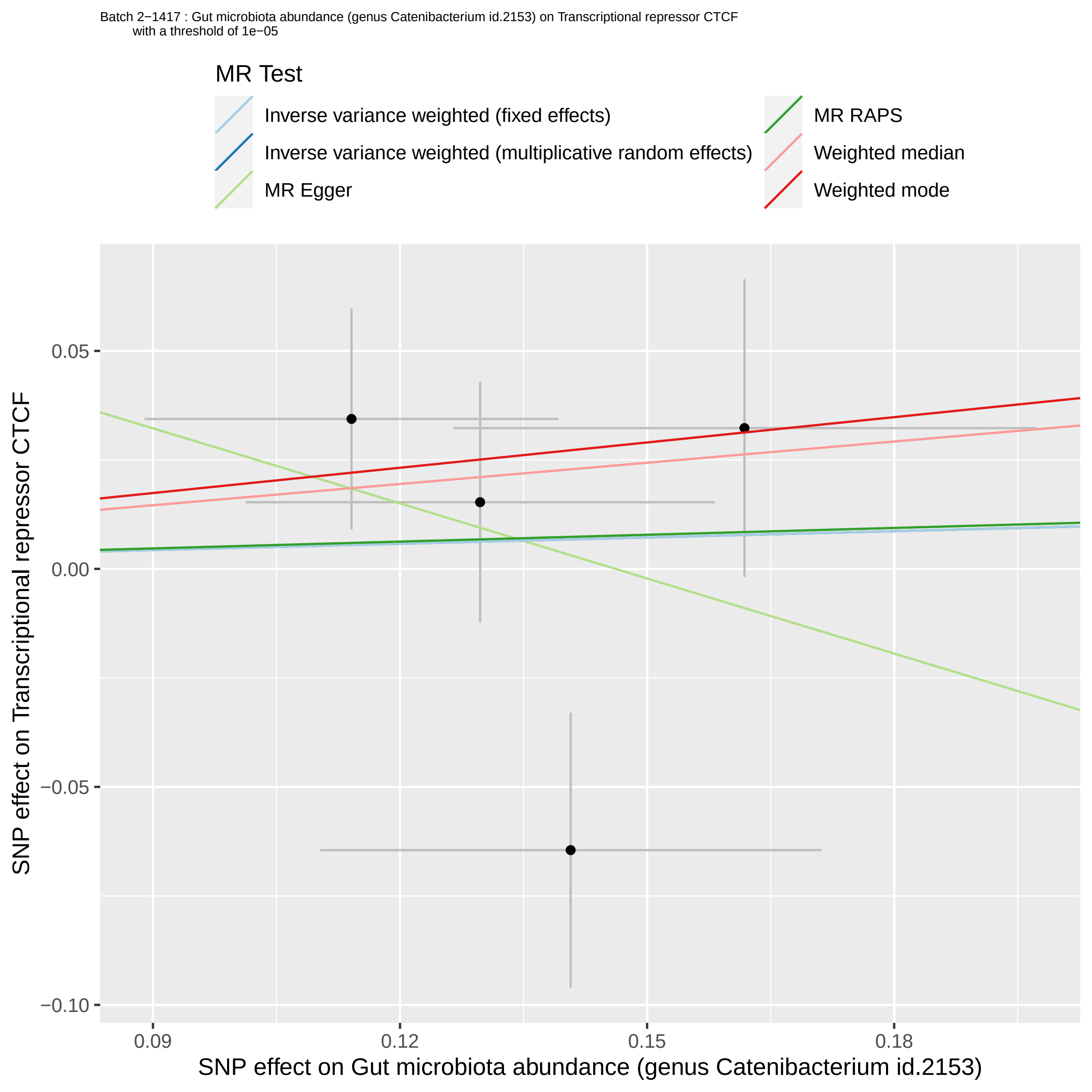

Batch 2−1417 : Gut microbiota abundance (genus Catenibacterium id.2153) on Transcriptional repressor CTCF
with a threshold of 1e−05
MR Test
Inverse variance weighted (fixed effects)
Inverse variance weighted (multiplicative random effects)
MR Egger
MR RAPS
Weighted median
Weighted mode
0.05
0.00
−0.05
−0.10
0.09
0.12
0.15
0.18
SNP effect on Transcriptional repressor CTCF
SNP effect on Gut microbiota abundance (genus Catenibacterium id.2153)

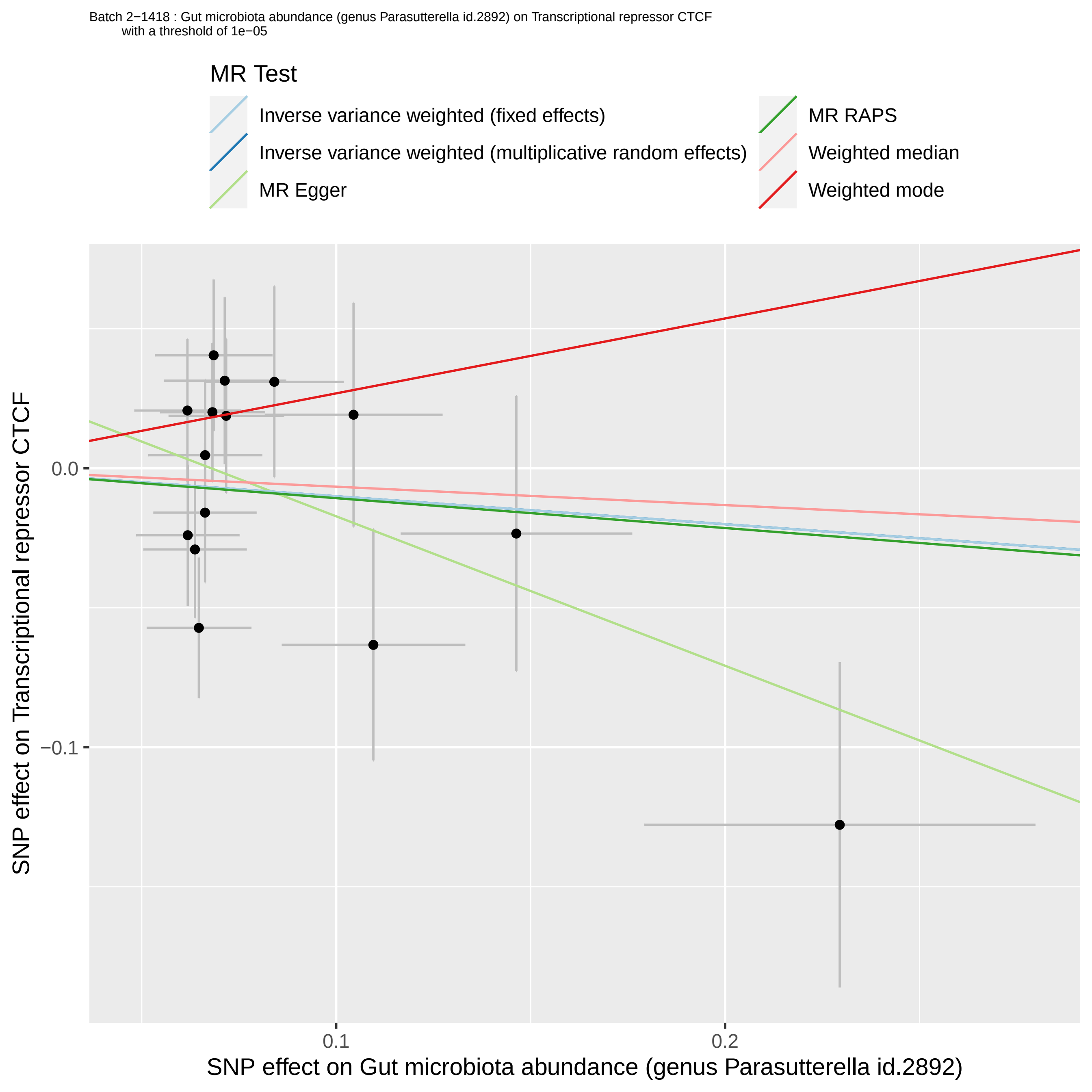

Batch 2−1418 : Gut microbiota abundance (genus Parasutterella id.2892) on Transcriptional repressor CTCF
with a threshold of 1e−05
MR Test
Inverse variance weighted (fixed effects)
Inverse variance weighted (multiplicative random effects)
MR Egger
MR RAPS
Weighted median
Weighted mode
SNP effect on Transcriptional repressor CTCF
0.0
−0.1
0.1
0.2
SNP effect on Gut microbiota abundance (genus Parasutterella id.2892)

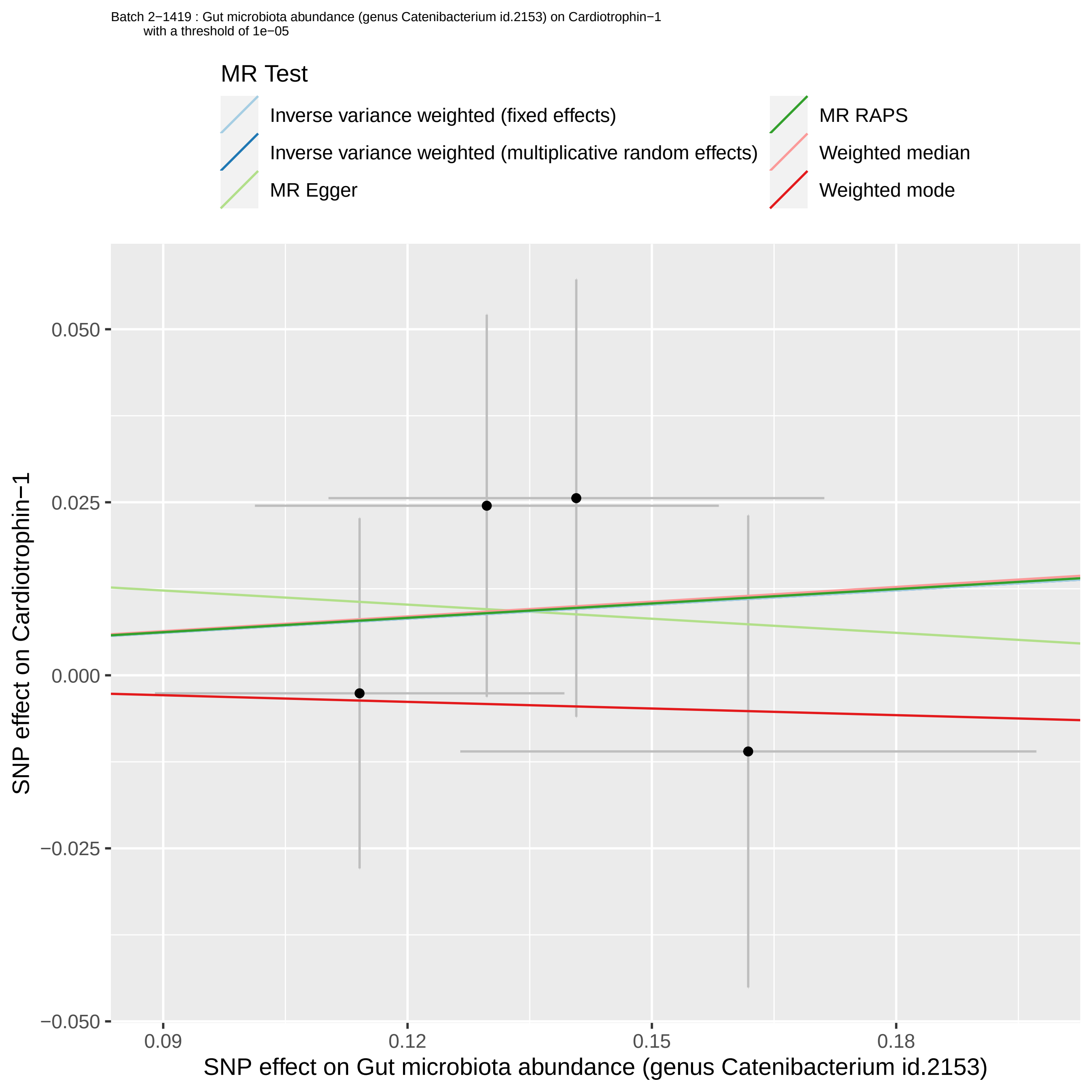

Batch 2−1419 : Gut microbiota abundance (genus Catenibacterium id.2153) on Cardiotrophin−1
with a threshold of 1e−05
MR Test
Inverse variance weighted (fixed effects)
Inverse variance weighted (multiplicative random effects)
MR Egger
MR RAPS
Weighted median
Weighted mode
SNP effect on Cardiotrophin−1
0.050
0.025
0.000
−0.025
−0.050
0.09
0.12
0.15
0.18
SNP effect on Gut microbiota abundance (genus Catenibacterium id.2153)

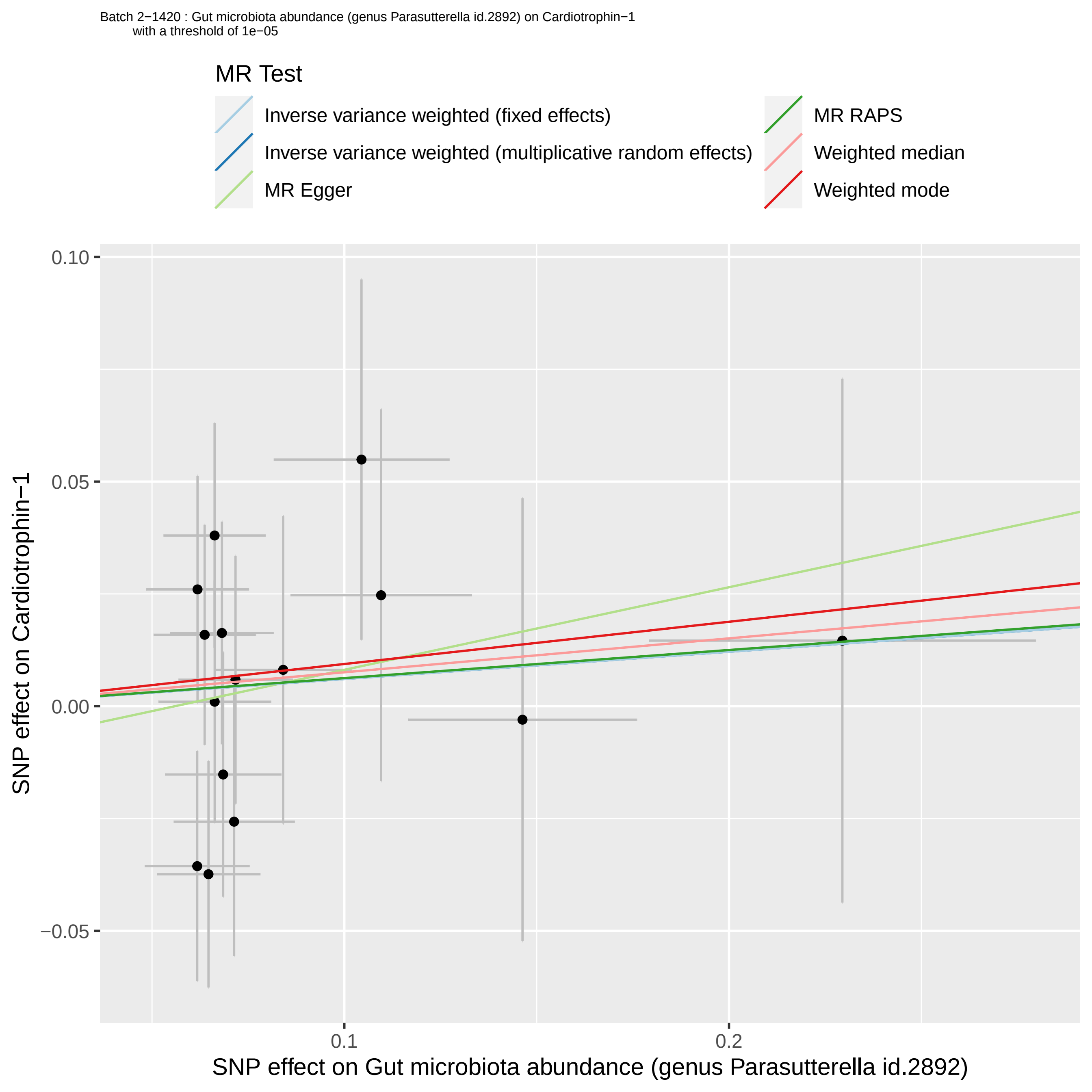

Batch 2−1420 : Gut microbiota abundance (genus Parasutterella id.2892) on Cardiotrophin−1
with a threshold of 1e−05
MR Test
Inverse variance weighted (fixed effects)
Inverse variance weighted (multiplicative random effects)
MR Egger
MR RAPS
Weighted median
Weighted mode
0.10
0.05
0.00
−0.05
0.1
0.2
SNP effect on Cardiotrophin−1
SNP effect on Gut microbiota abundance (genus Parasutterella id.2892)

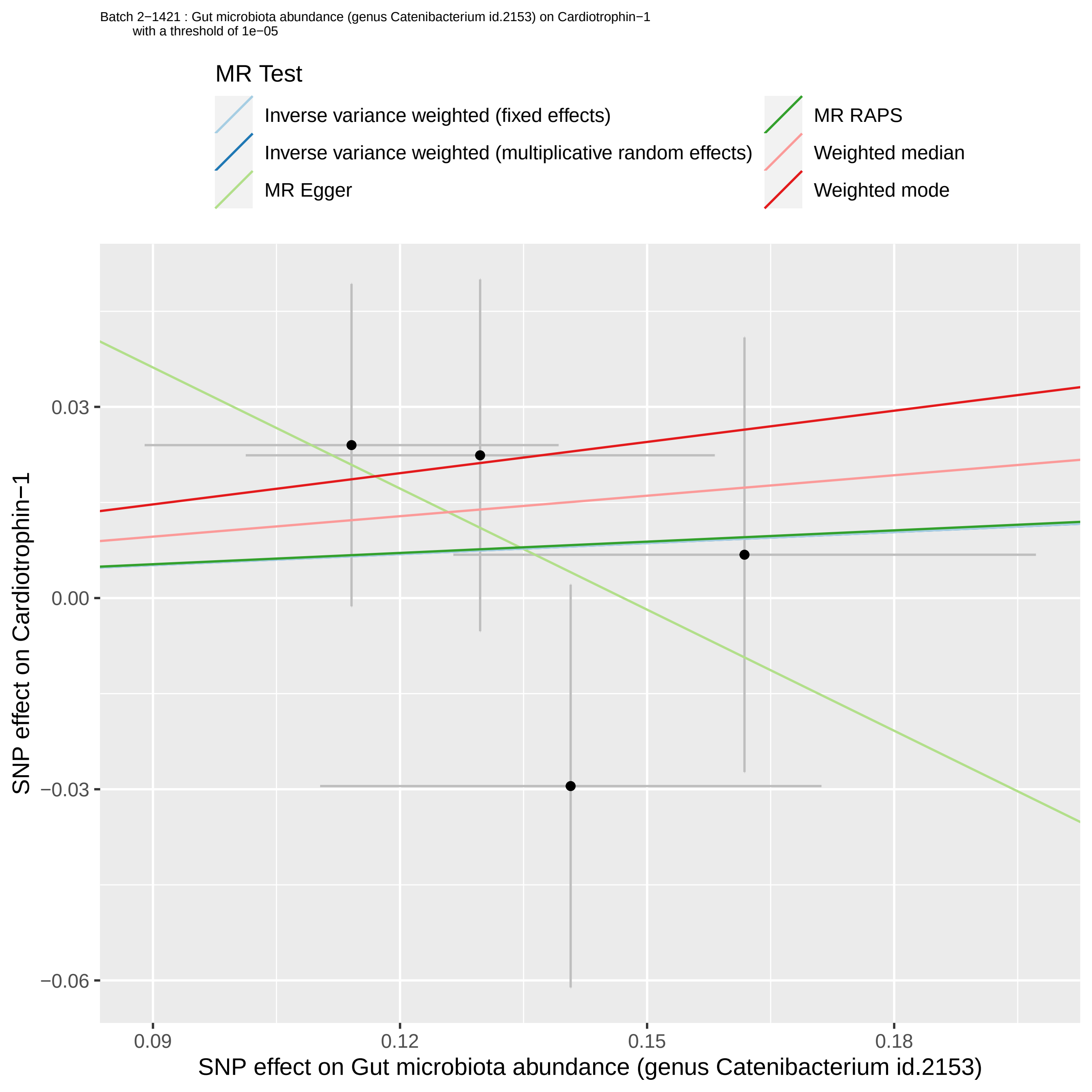

Batch 2−1421 : Gut microbiota abundance (genus Catenibacterium id.2153) on Cardiotrophin−1
with a threshold of 1e−05
MR Test
Inverse variance weighted (fixed effects)
Inverse variance weighted (multiplicative random effects)
MR Egger
MR RAPS
Weighted median
Weighted mode
SNP effect on Cardiotrophin−1
0.03
0.00
−0.03
−0.06
0.09
0.12
0.15
0.18
SNP effect on Gut microbiota abundance (genus Catenibacterium id.2153)

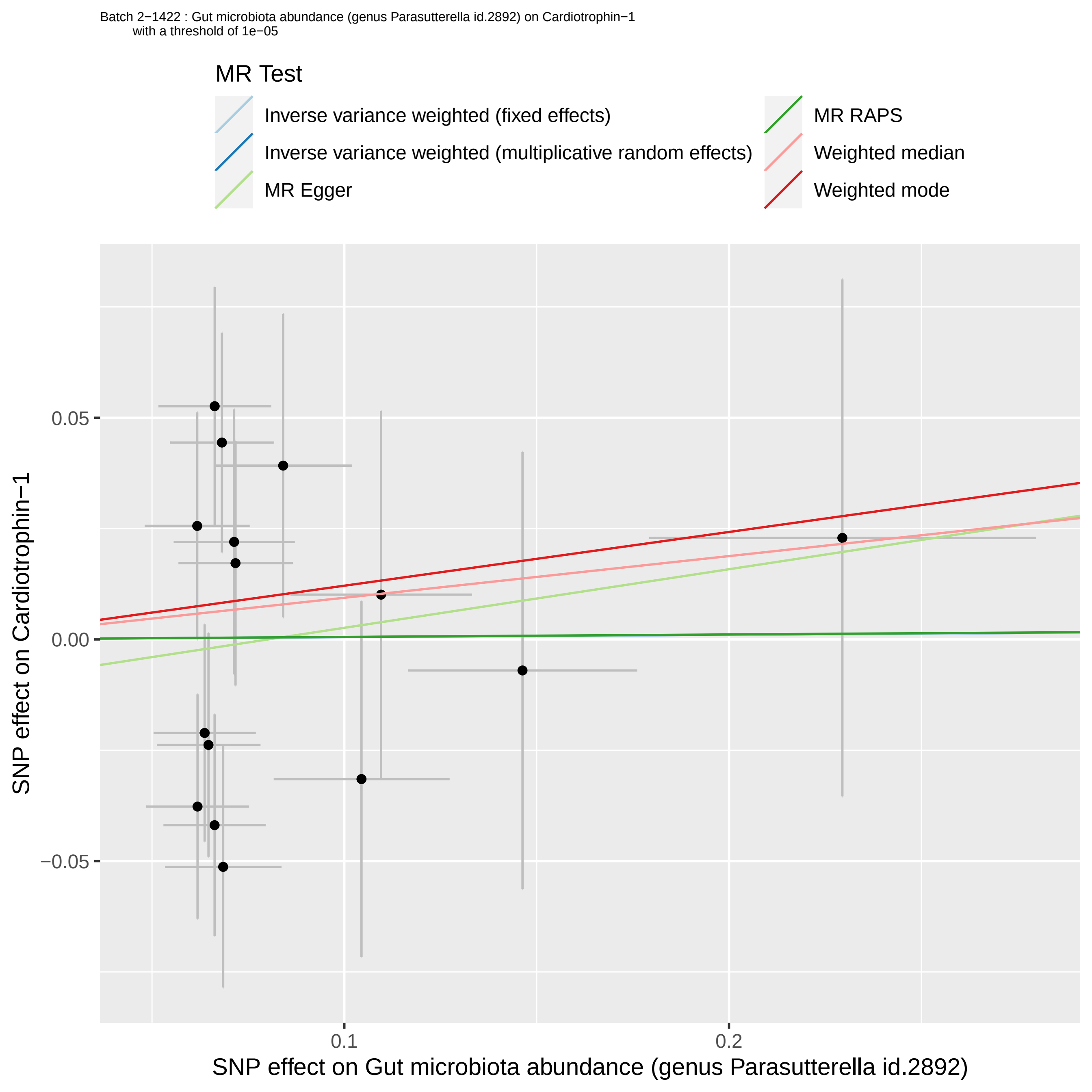

Batch 2−1422 : Gut microbiota abundance (genus Parasutterella id.2892) on Cardiotrophin−1
with a threshold of 1e−05
MR Test
Inverse variance weighted (fixed effects)
Inverse variance weighted (multiplicative random effects)
MR Egger
MR RAPS
Weighted median
Weighted mode
0.05
0.00
−0.05
0.1
0.2
SNP effect on Cardiotrophin−1
SNP effect on Gut microbiota abundance (genus Parasutterella id.2892)

Batch 2−1423 : Gut microbiota abundance (genus Catenibacterium id.2153) on Connective tissue growth factor
with a threshold of 1e−05

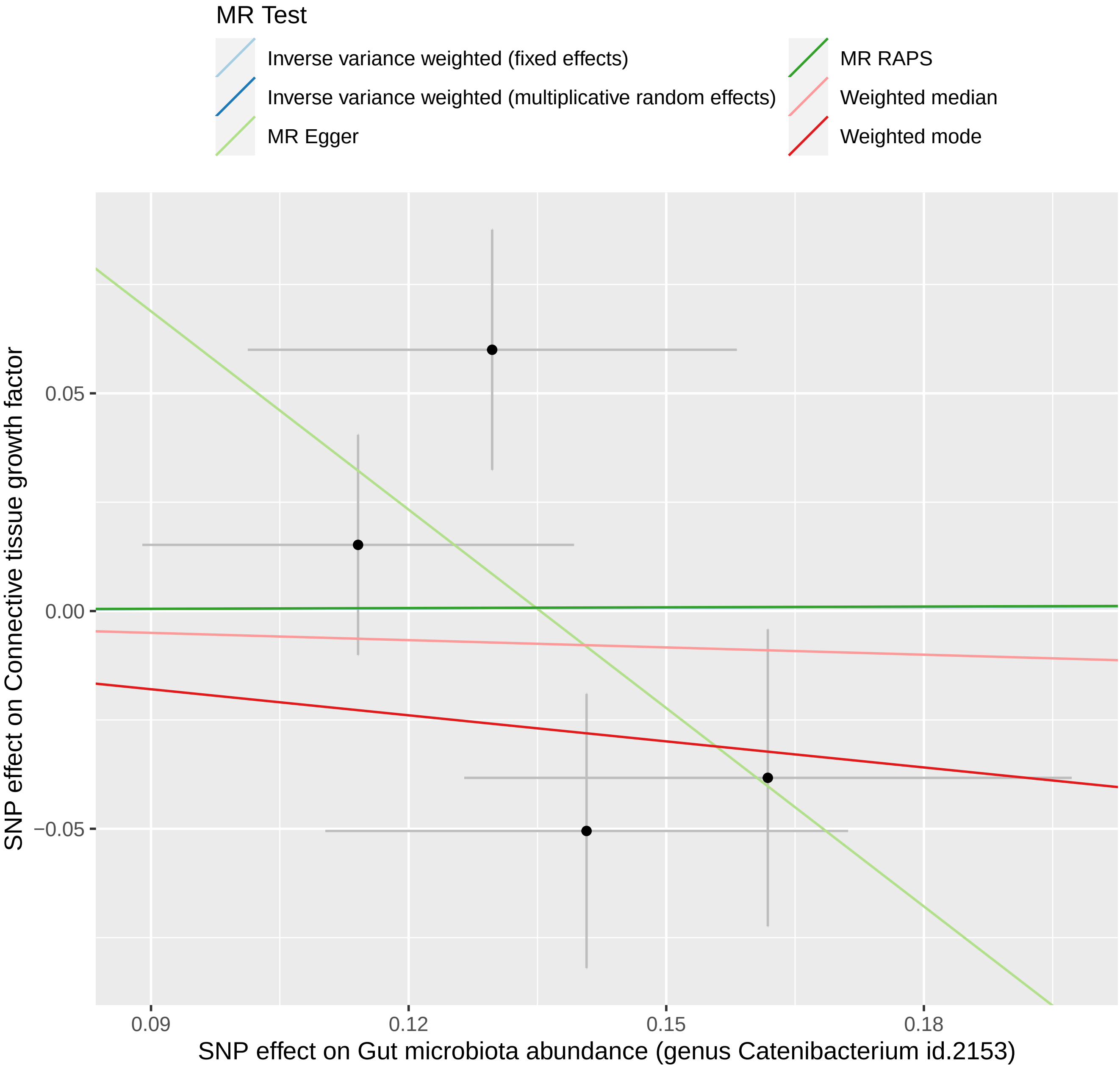

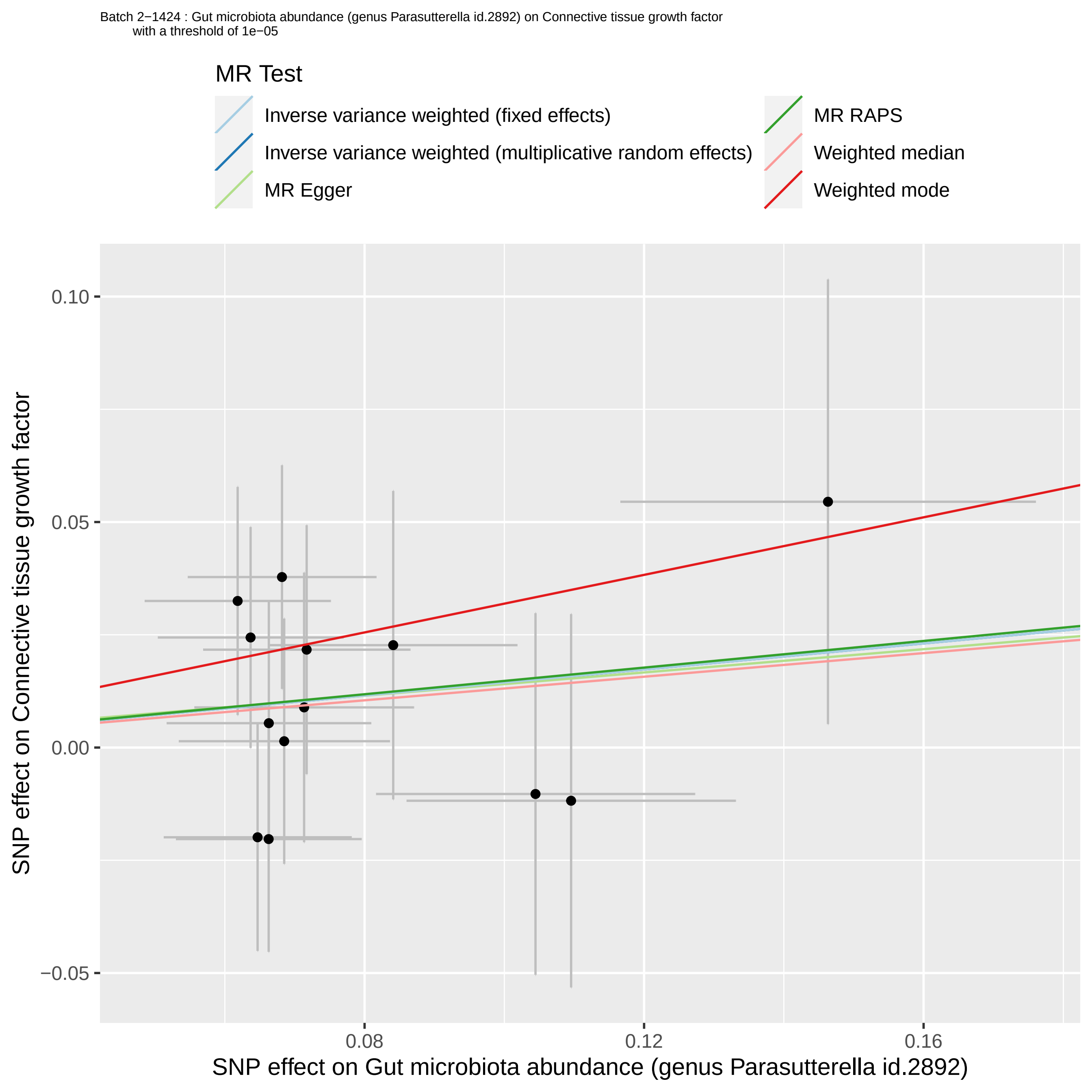
Batch 2−1424 : Gut microbiota abundance (genus Parasutterella id.2892) on Connective tissue growth factor
with a threshold of 1e−05
MR Test
Inverse variance weighted (fixed effects)
Inverse variance weighted (multiplicative random effects)
MR Egger
MR RAPS
Weighted median
Weighted mode
0.10
0.05
0.00
−0.05
0.08
0.12
0.16
SNP effect on Connective tissue growth factor
SNP effect on Gut microbiota abundance (genus Parasutterella id.2892)

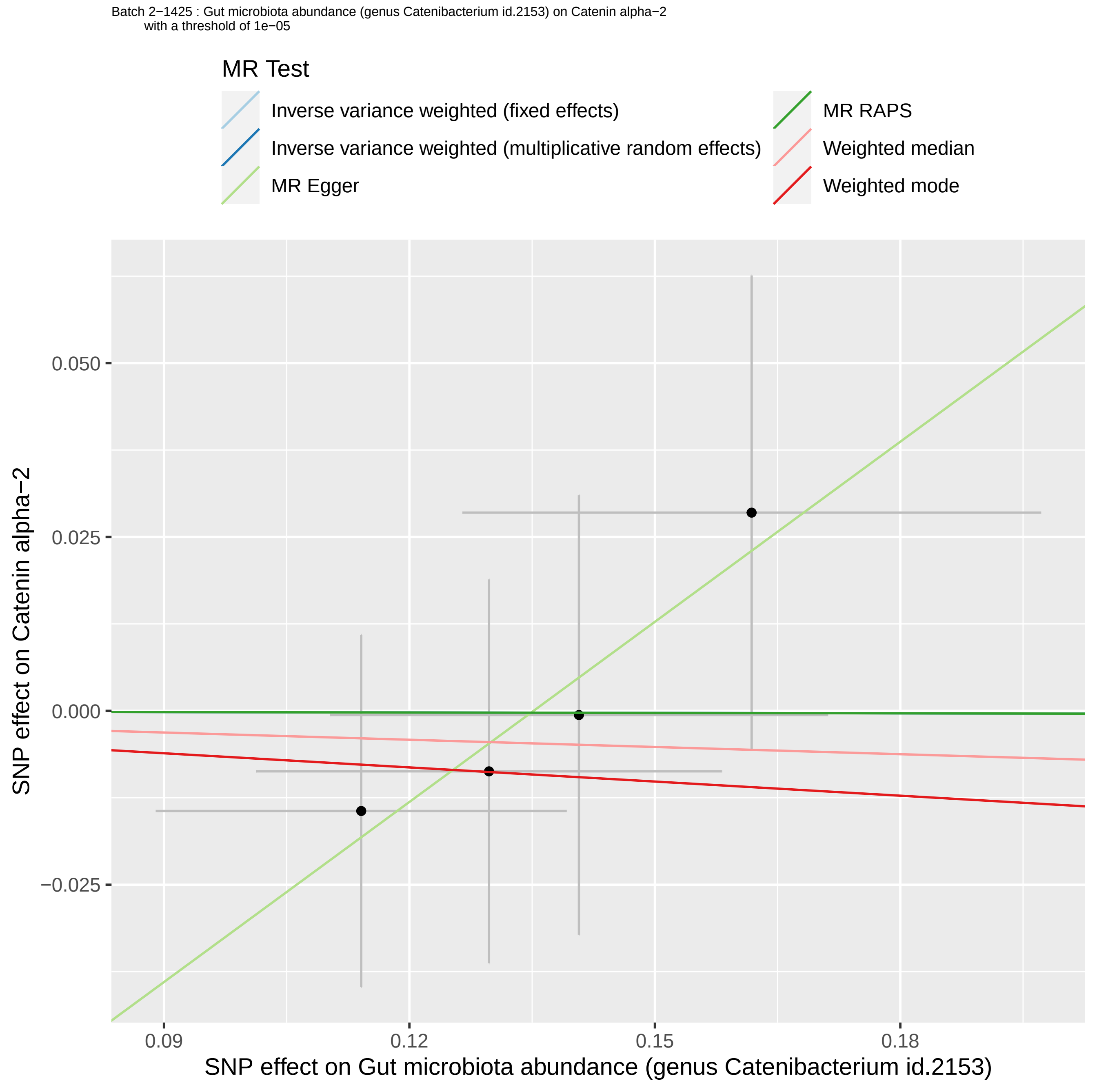
Batch 2−1425 : Gut microbiota abundance (genus Catenibacterium id.2153) on Catenin alpha−2
with a threshold of 1e−05
MR Test
Inverse variance weighted (fixed effects)
Inverse variance weighted (multiplicative random effects)
MR Egger
MR RAPS
Weighted median
Weighted mode
0.050
0.025
0.000
−0.025
0.09
0.12
0.15
0.18
SNP effect on Catenin alpha−2
SNP effect on Gut microbiota abundance (genus Catenibacterium id.2153)

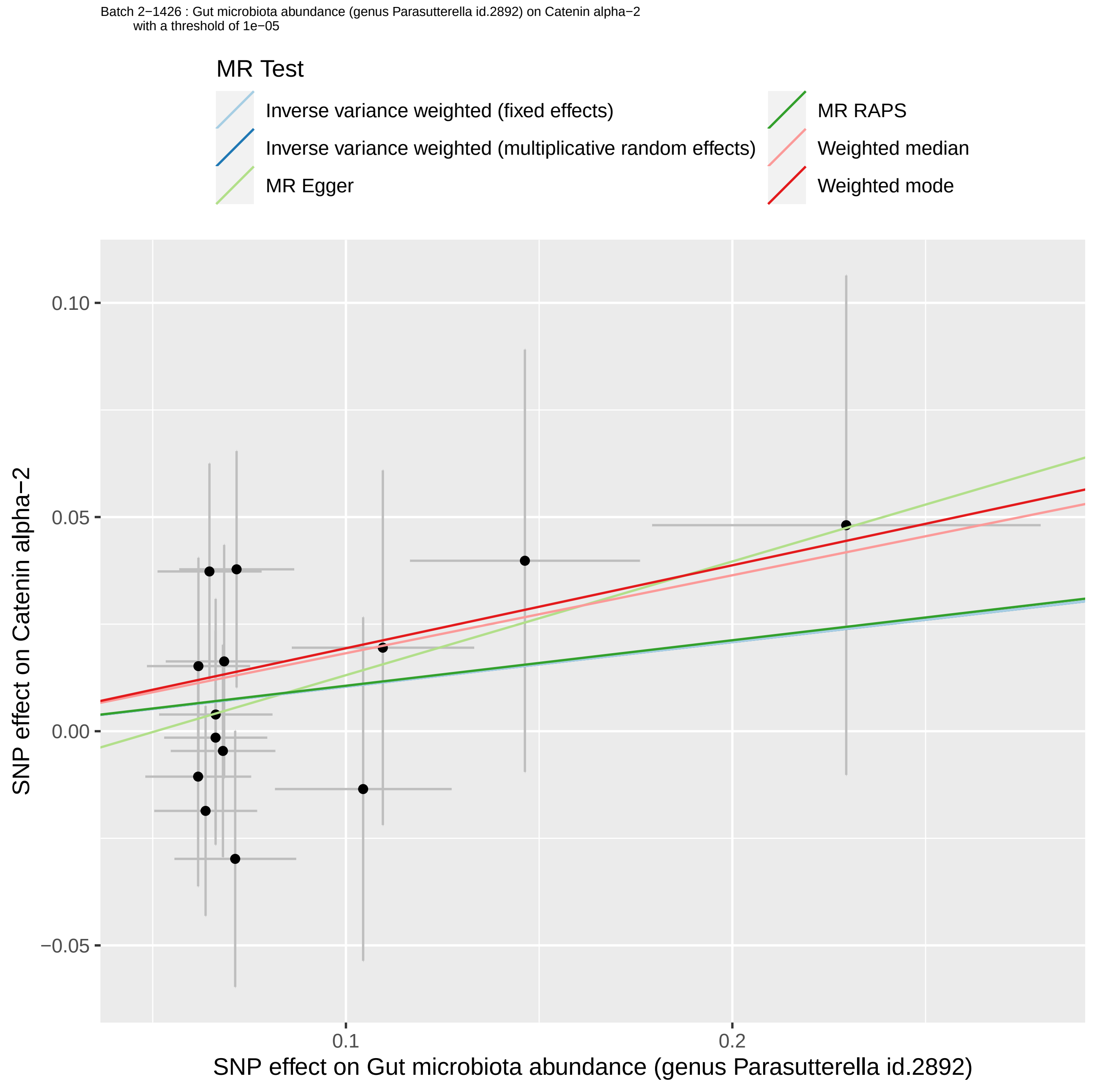

Batch 2−1426 : Gut microbiota abundance (genus Parasutterella id.2892) on Catenin alpha−2
with a threshold of 1e−05
MR Test
Inverse variance weighted (fixed effects)
Inverse variance weighted (multiplicative random effects)
MR Egger
MR RAPS
Weighted median
Weighted mode
0.10
0.05
0.00
−0.05
0.1
0.2
SNP effect on Catenin alpha−2
SNP effect on Gut microbiota abundance (genus Parasutterella id.2892)

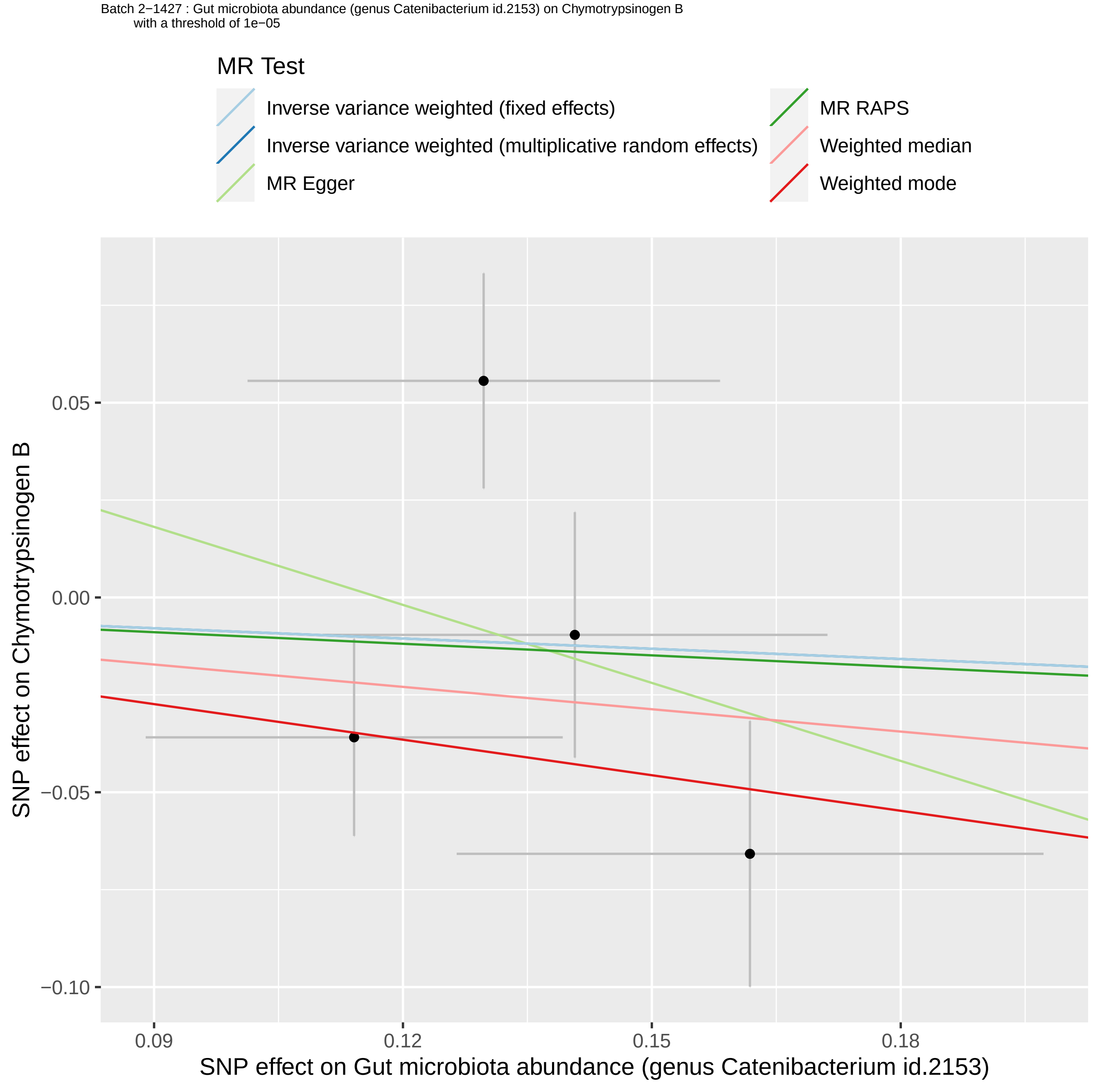

Batch 2−1427 : Gut microbiota abundance (genus Catenibacterium id.2153) on Chymotrypsinogen B
with a threshold of 1e−05
MR Test
Inverse variance weighted (fixed effects)
Inverse variance weighted (multiplicative random effects)
MR Egger
MR RAPS
Weighted median
Weighted mode
0.05
0.00
−0.05
−0.10
SNP effect on Chymotrypsinogen B
0.09
0.12
0.15
0.18
SNP effect on Gut microbiota abundance (genus Catenibacterium id.2153)

Batch 2−1428 : Gut microbiota abundance (genus Parasutterella id.2892) on Chymotrypsinogen B
with a threshold of 1e−05

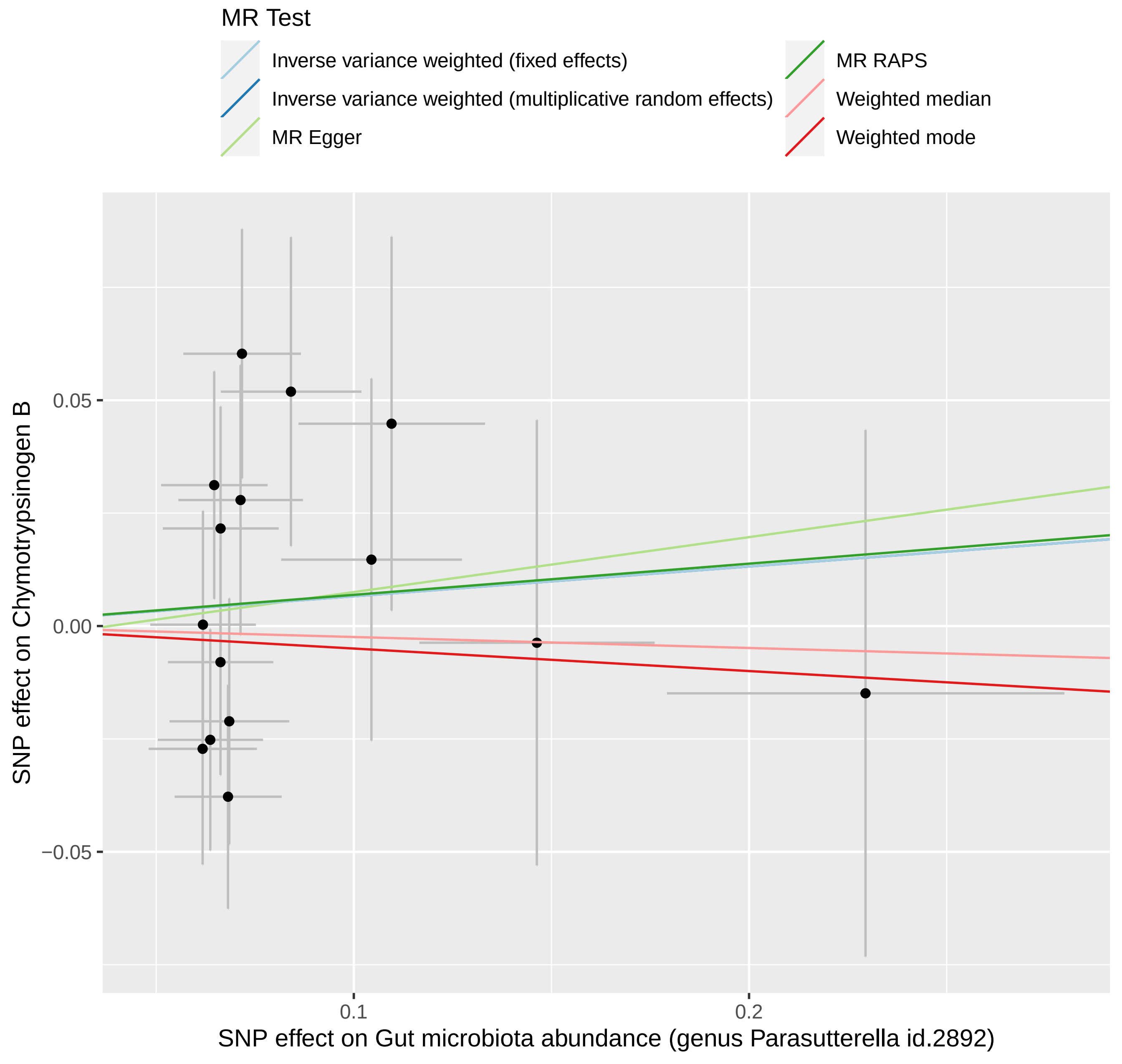

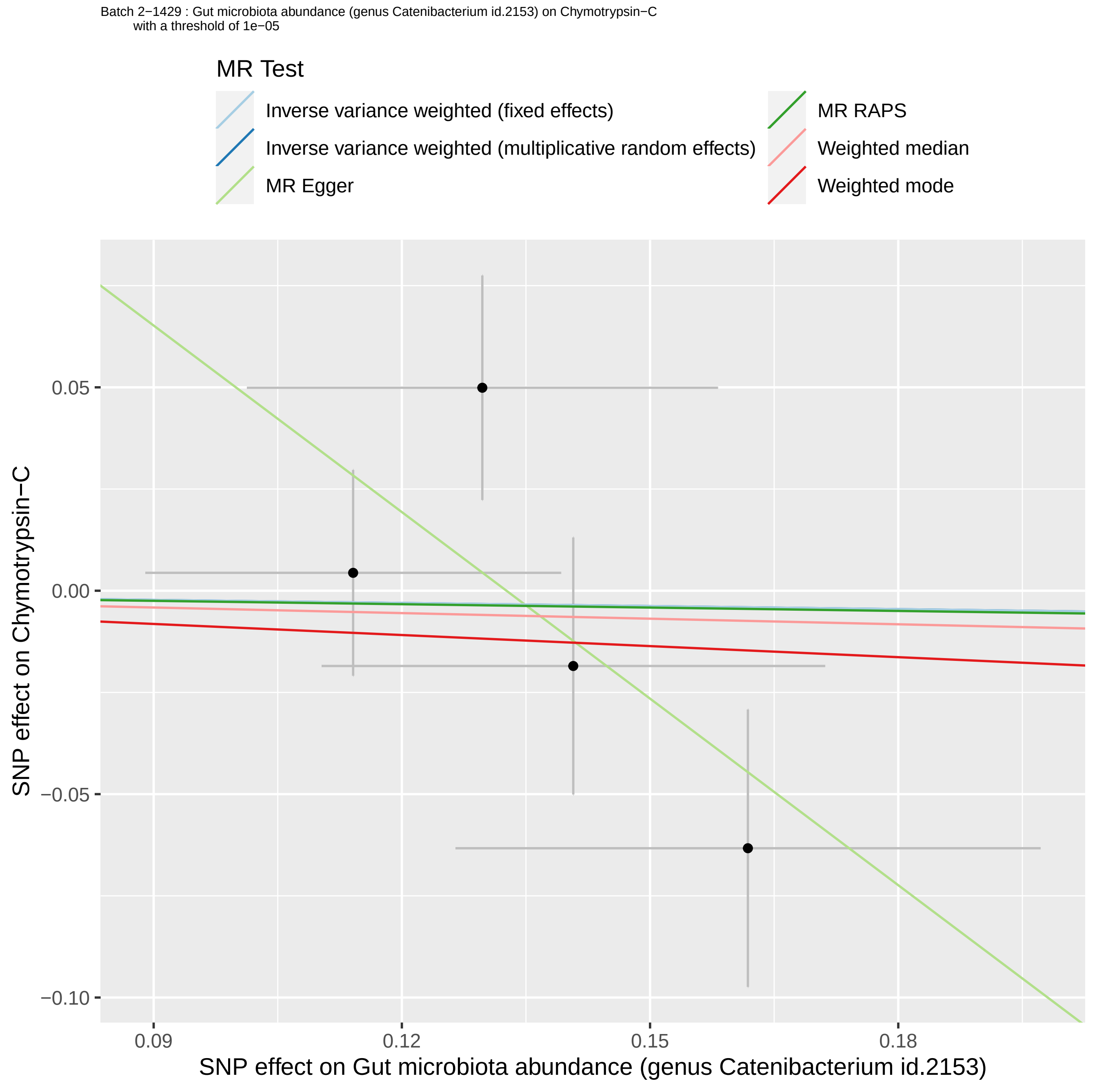
Batch 2−1429 : Gut microbiota abundance (genus Catenibacterium id.2153) on Chymotrypsin−C
with a threshold of 1e−05
MR Test
Inverse variance weighted (fixed effects)
Inverse variance weighted (multiplicative random effects)
MR Egger
MR RAPS
Weighted median
Weighted mode
0.05
0.00
−0.05
−0.10
0.09
0.12
0.15
0.18
SNP effect on Chymotrypsin−C
SNP effect on Gut microbiota abundance (genus Catenibacterium id.2153)

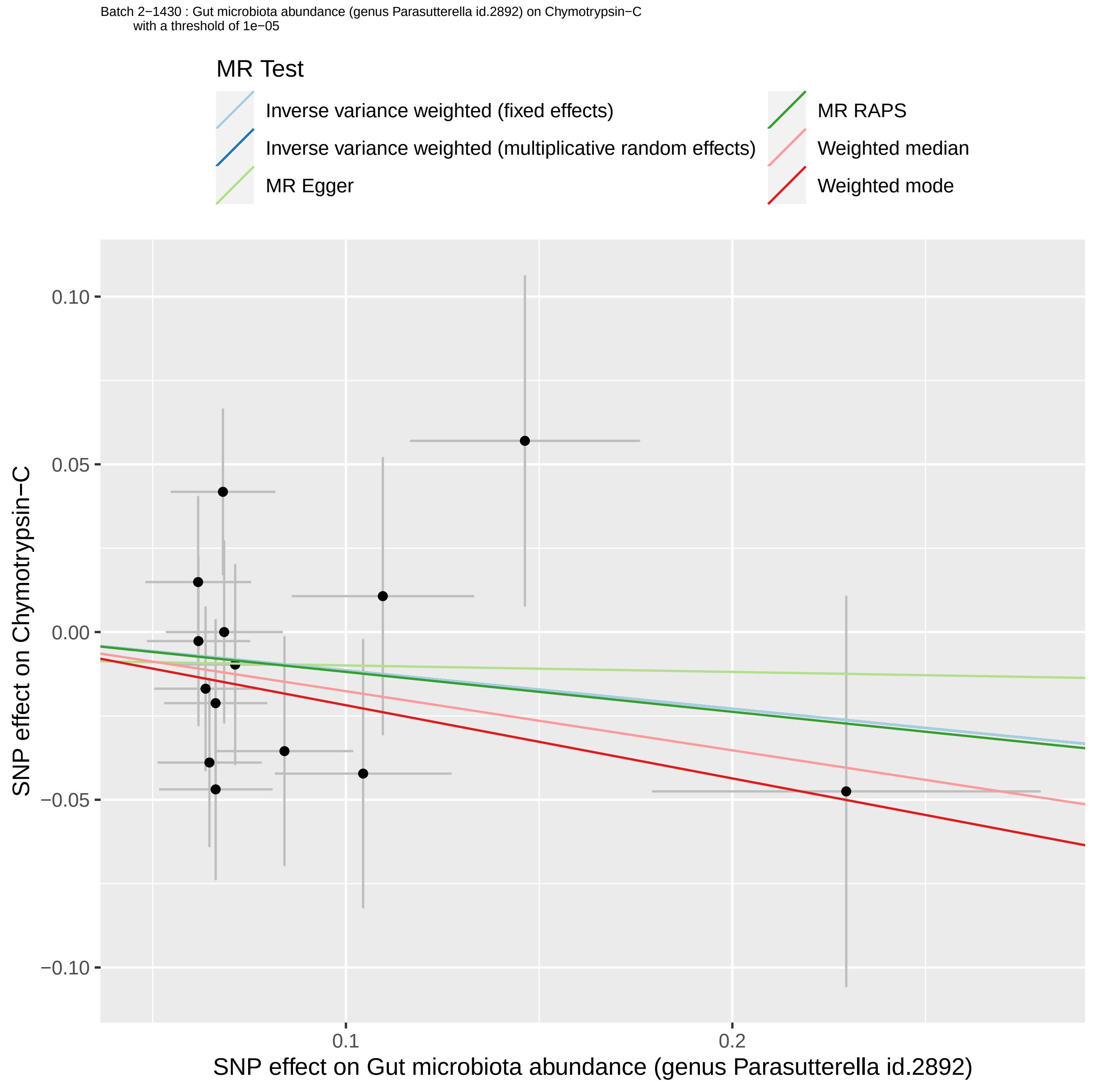
Batch 2−1430 : Gut microbiota abundance (genus Parasutterella id.2892) on Chymotrypsin−C
with a threshold of 1e−05
MR Test
Inverse variance weighted (fixed effects)
Inverse variance weighted (multiplicative random effects)
MR Egger
MR RAPS
Weighted median
Weighted mode
0.10
0.05
0.00
−0.05
−0.10
0.1
0.2
SNP effect on Chymotrypsin−C
SNP effect on Gut microbiota abundance (genus Parasutterella id.2892)

Batch 2−1431 : Gut microbiota abundance (genus Catenibacterium id.2153) on Chymotrypsin−like protease CTRL−1 with a threshold of 1e−05

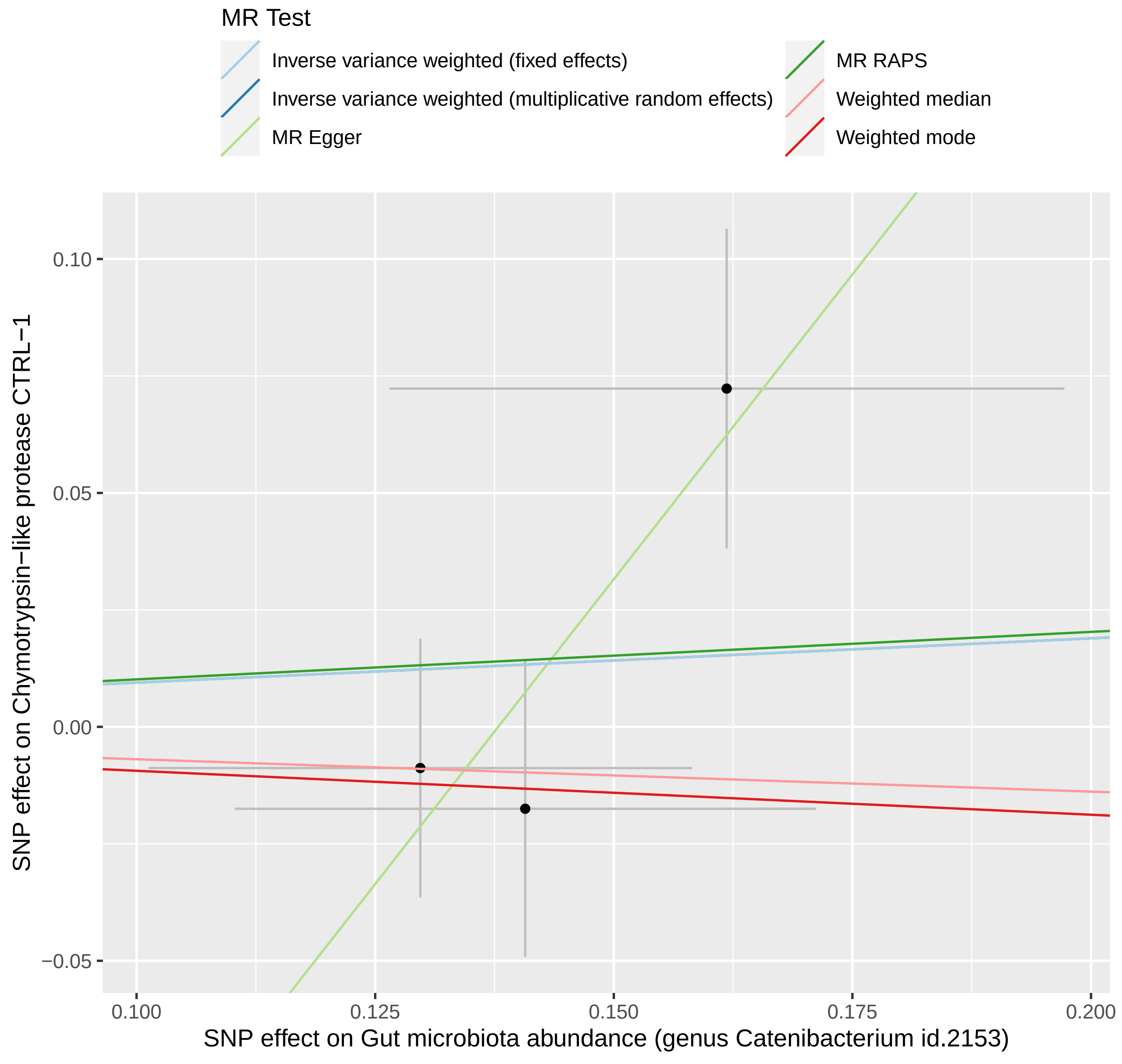

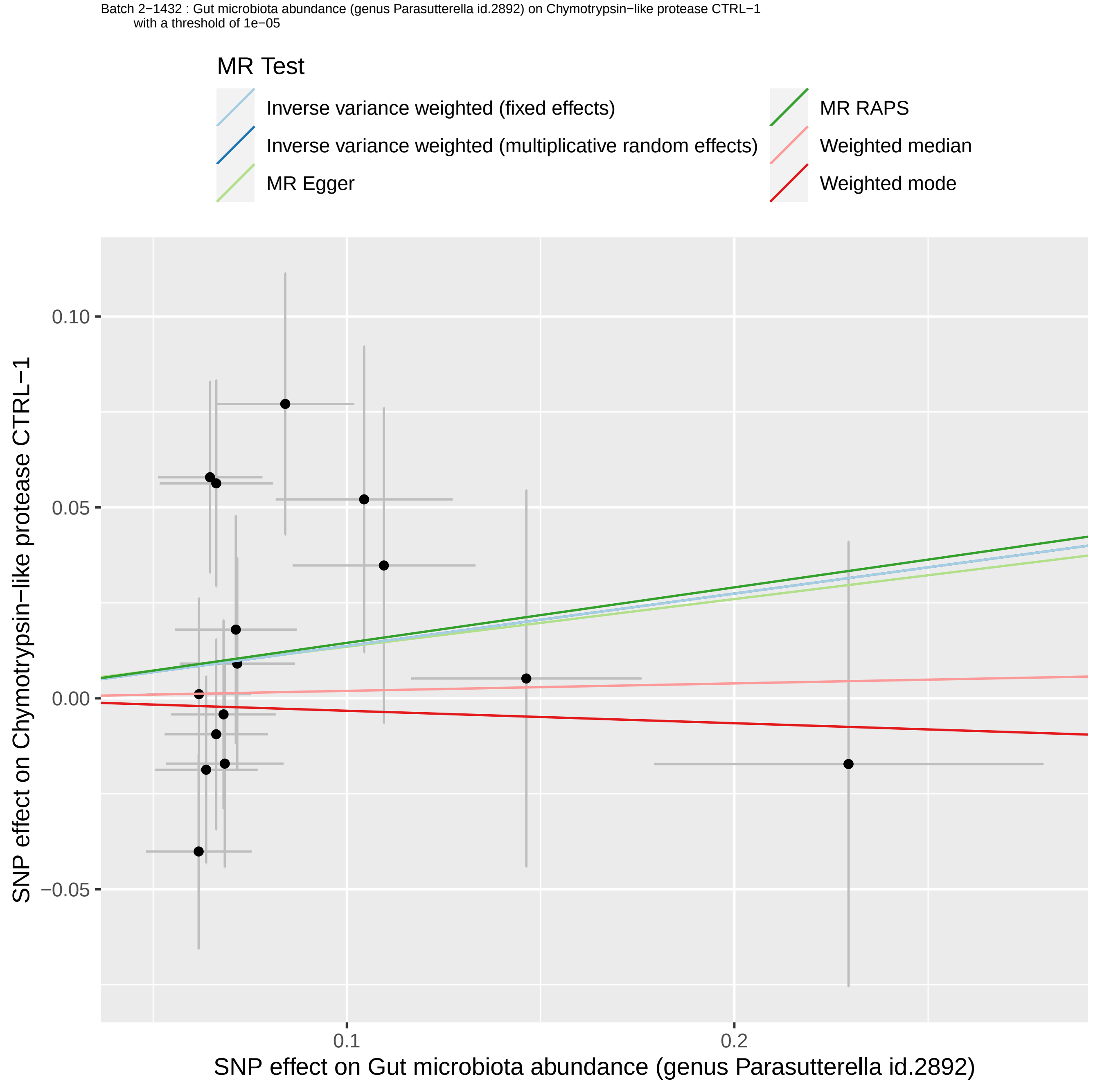

Batch 2−1432 : Gut microbiota abundance (genus Parasutterella id.2892) on Chymotrypsin−like protease CTRL−1
with a threshold of 1e−05
MR Test
Inverse variance weighted (fixed effects)
Inverse variance weighted (multiplicative random effects)
MR Egger
MR RAPS
Weighted median
Weighted mode
0.10
0.05
0.00
−0.05
0.1
0.2
SNP effect on Chymotrypsin−like protease CTRL−1
SNP effect on Gut microbiota abundance (genus Parasutterella id.2892)

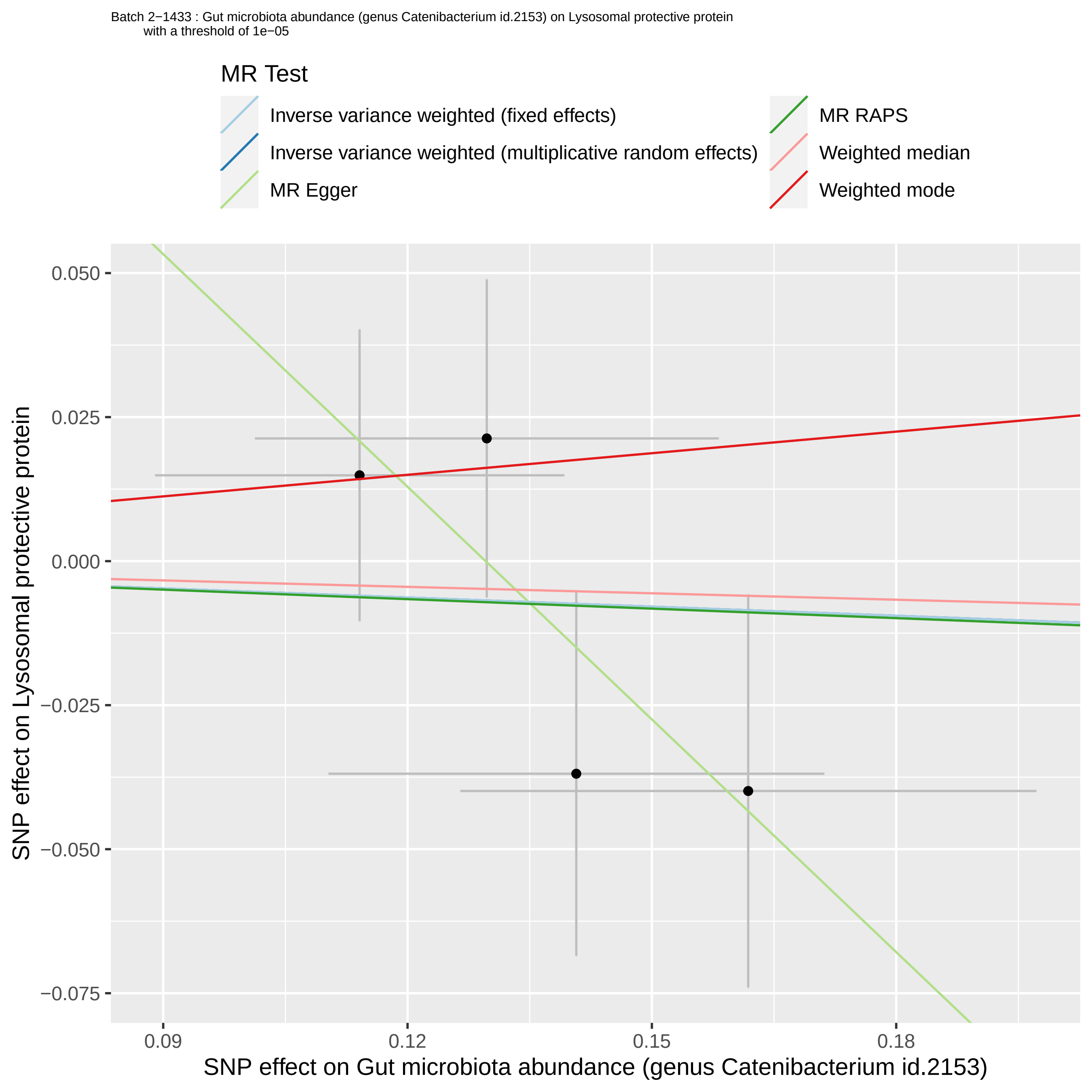

Batch 2−1433 : Gut microbiota abundance (genus Catenibacterium id.2153) on Lysosomal protective protein
with a threshold of 1e−05
MR Test
Inverse variance weighted (fixed effects)
Inverse variance weighted (multiplicative random effects)
MR Egger
MR RAPS
Weighted median
Weighted mode
0.050
0.025
0.000
−0.025
−0.050
−0.075
0.09
0.12
0.15
0.18
SNP effect on Lysosomal protective protein
SNP effect on Gut microbiota abundance (genus Catenibacterium id.2153)

Batch 2−1434 : Gut microbiota abundance (genus Parasutterella id.2892) on Lysosomal protective protein
with a threshold of 1e−05

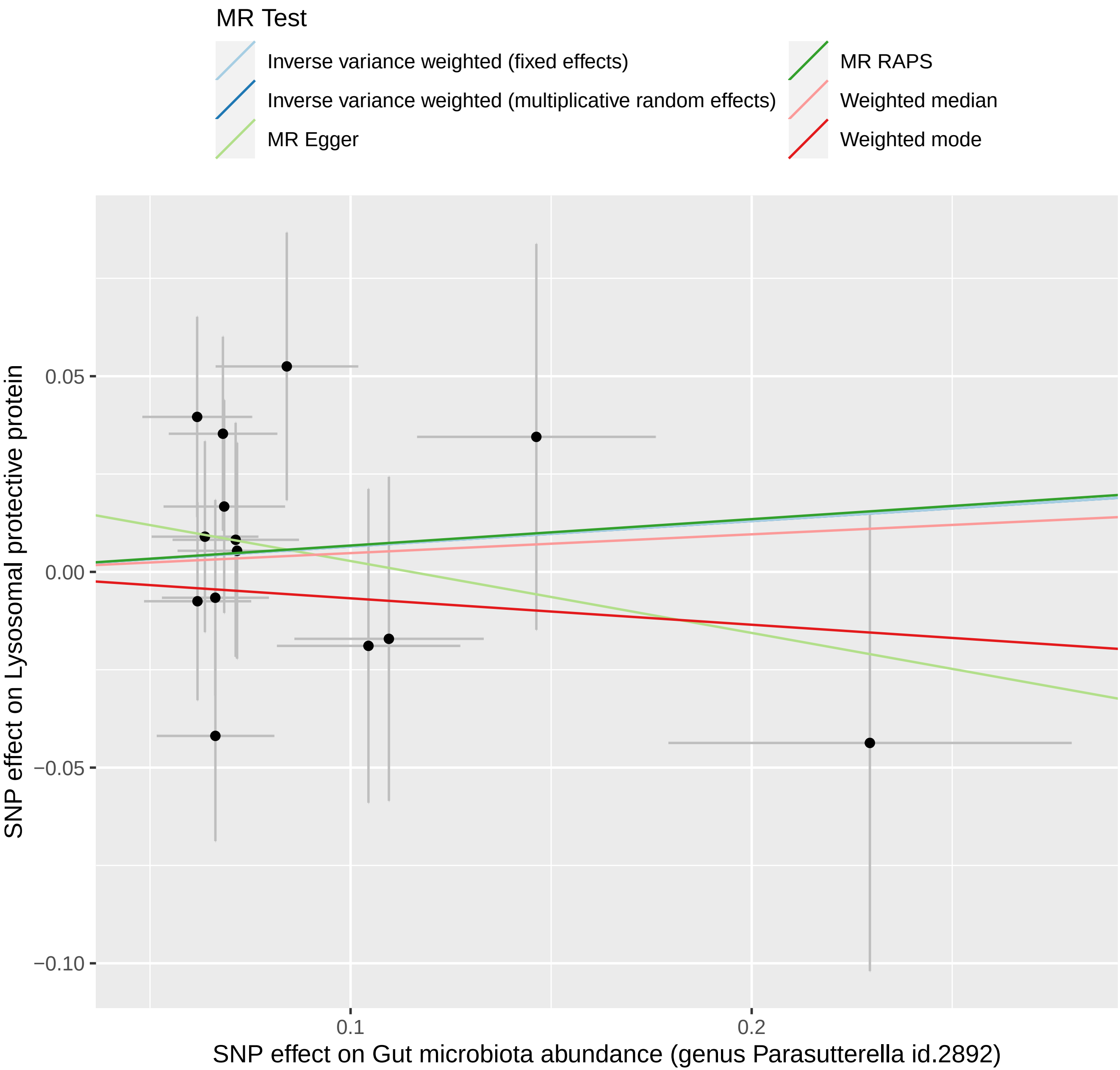

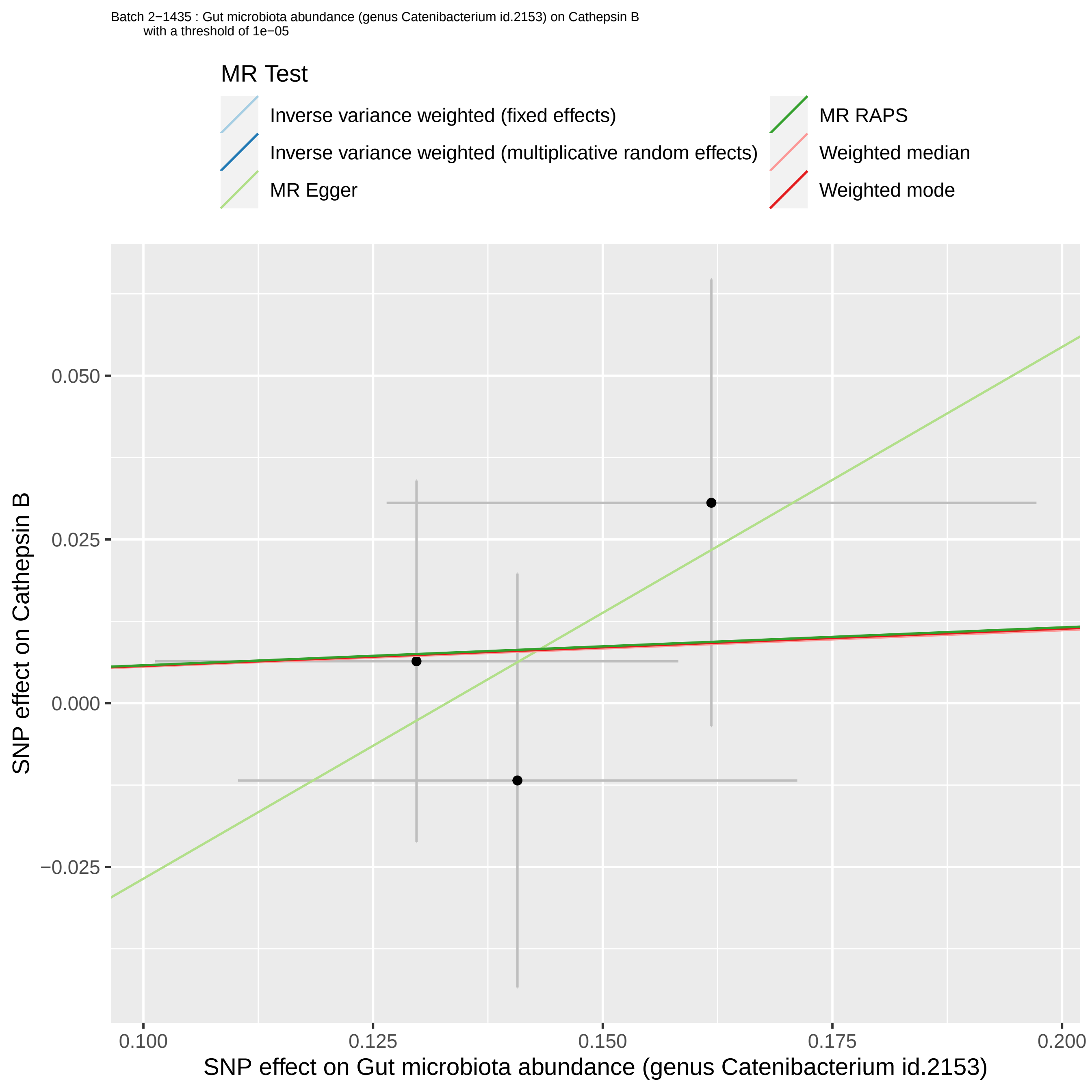
Batch 2−1435 : Gut microbiota abundance (genus Catenibacterium id.2153) on Cathepsin B
with a threshold of 1e−05
MR Test
Inverse variance weighted (fixed effects)
Inverse variance weighted (multiplicative random effects)
MR Egger
MR RAPS
Weighted median
Weighted mode
SNP effect on Cathepsin B
0.050
0.025
0.000
−0.025
0.100
0.125
0.150
0.175
0.200
SNP effect on Gut microbiota abundance (genus Catenibacterium id.2153)

Batch 2−1436 : Gut microbiota abundance (genus Parasutterella id.2892) on Cathepsin B
with a threshold of 1e−05

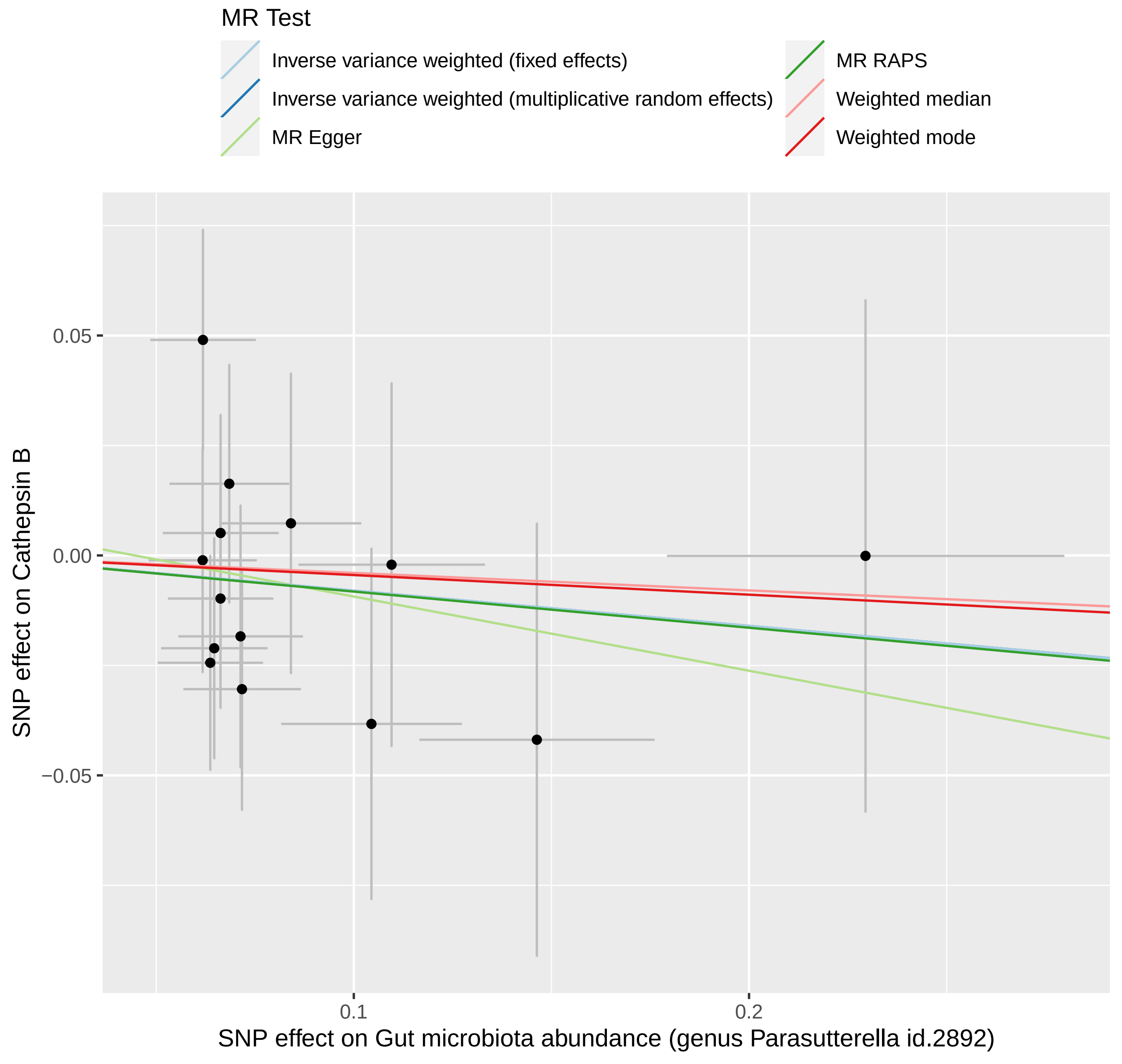

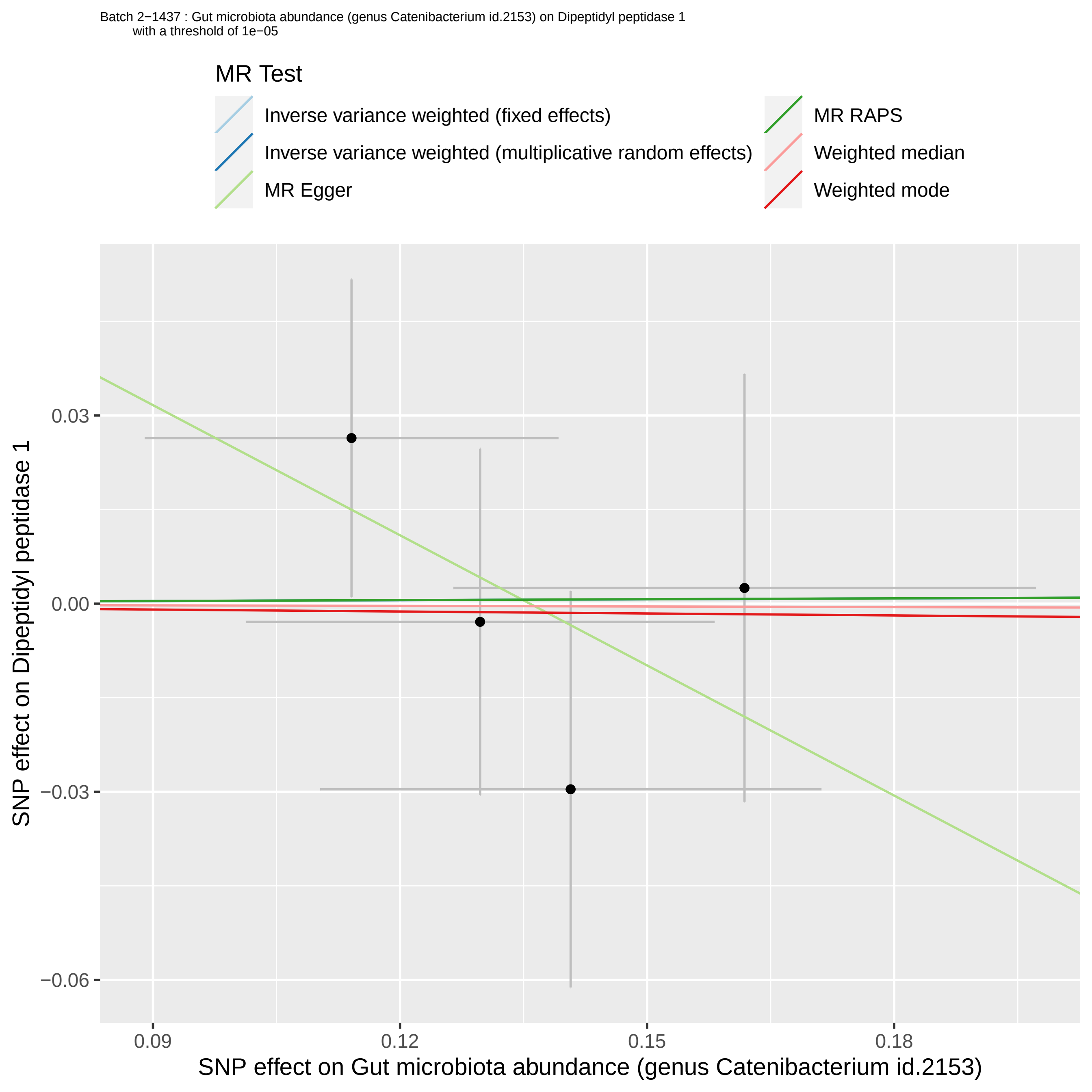
Batch 2−1437 : Gut microbiota abundance (genus Catenibacterium id.2153) on Dipeptidyl peptidase 1
with a threshold of 1e−05
MR Test
Inverse variance weighted (fixed effects)
Inverse variance weighted (multiplicative random effects)
MR Egger
MR RAPS
Weighted median
Weighted mode
0.03
0.00
−0.03
−0.06
0.09
0.12
0.15
0.18
SNP effect on Dipeptidyl peptidase 1
SNP effect on Gut microbiota abundance (genus Catenibacterium id.2153)

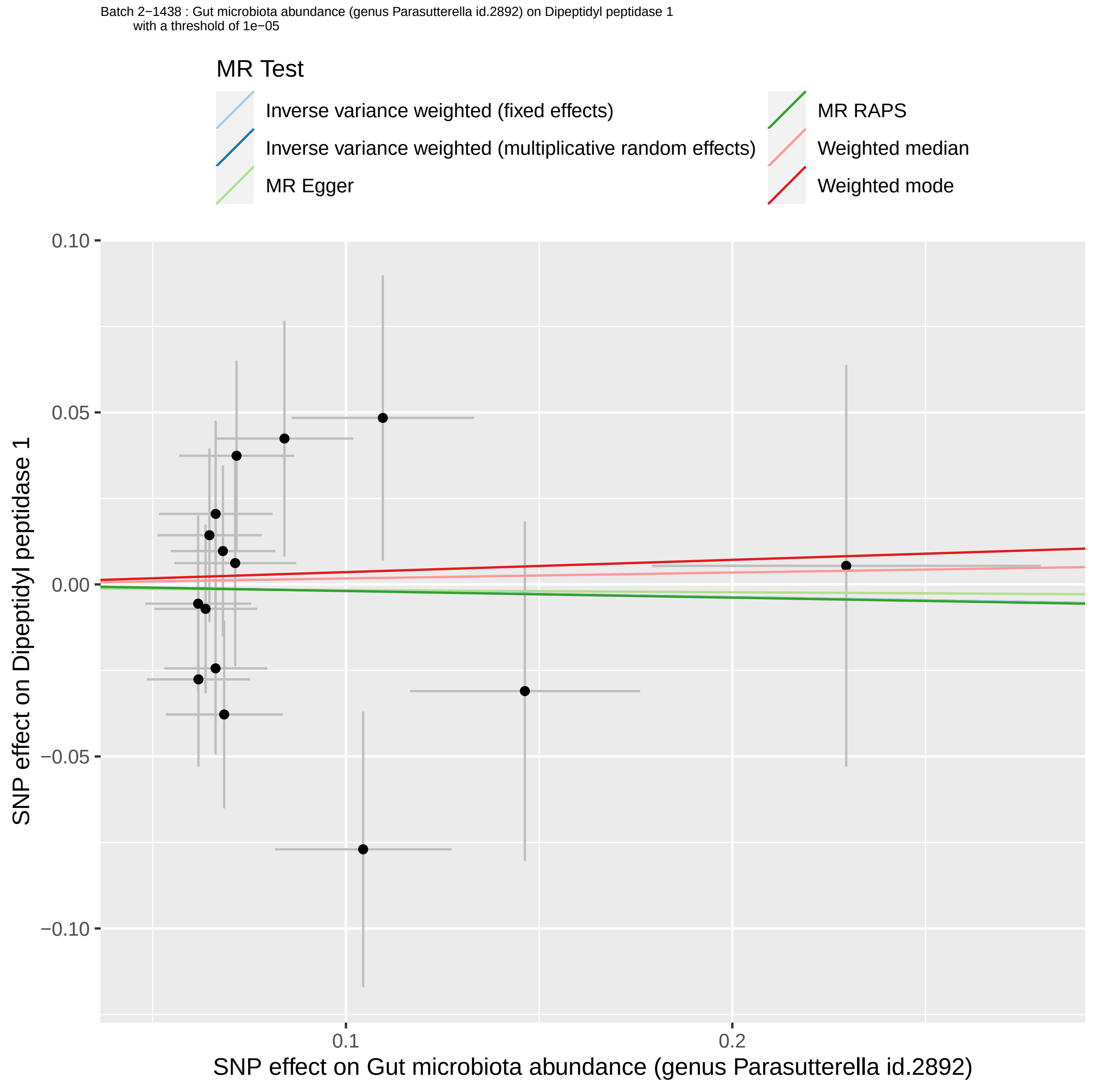
Batch 2−1438 : Gut microbiota abundance (genus Parasutterella id.2892) on Dipeptidyl peptidase 1
with a threshold of 1e−05
MR Test
Inverse variance weighted (fixed effects)
Inverse variance weighted (multiplicative random effects)
MR Egger
MR RAPS
Weighted median
Weighted mode
0.10
0.05
0.00
−0.05
−0.10
0.1
0.2
SNP effect on Dipeptidyl peptidase 1
SNP effect on Gut microbiota abundance (genus Parasutterella id.2892)

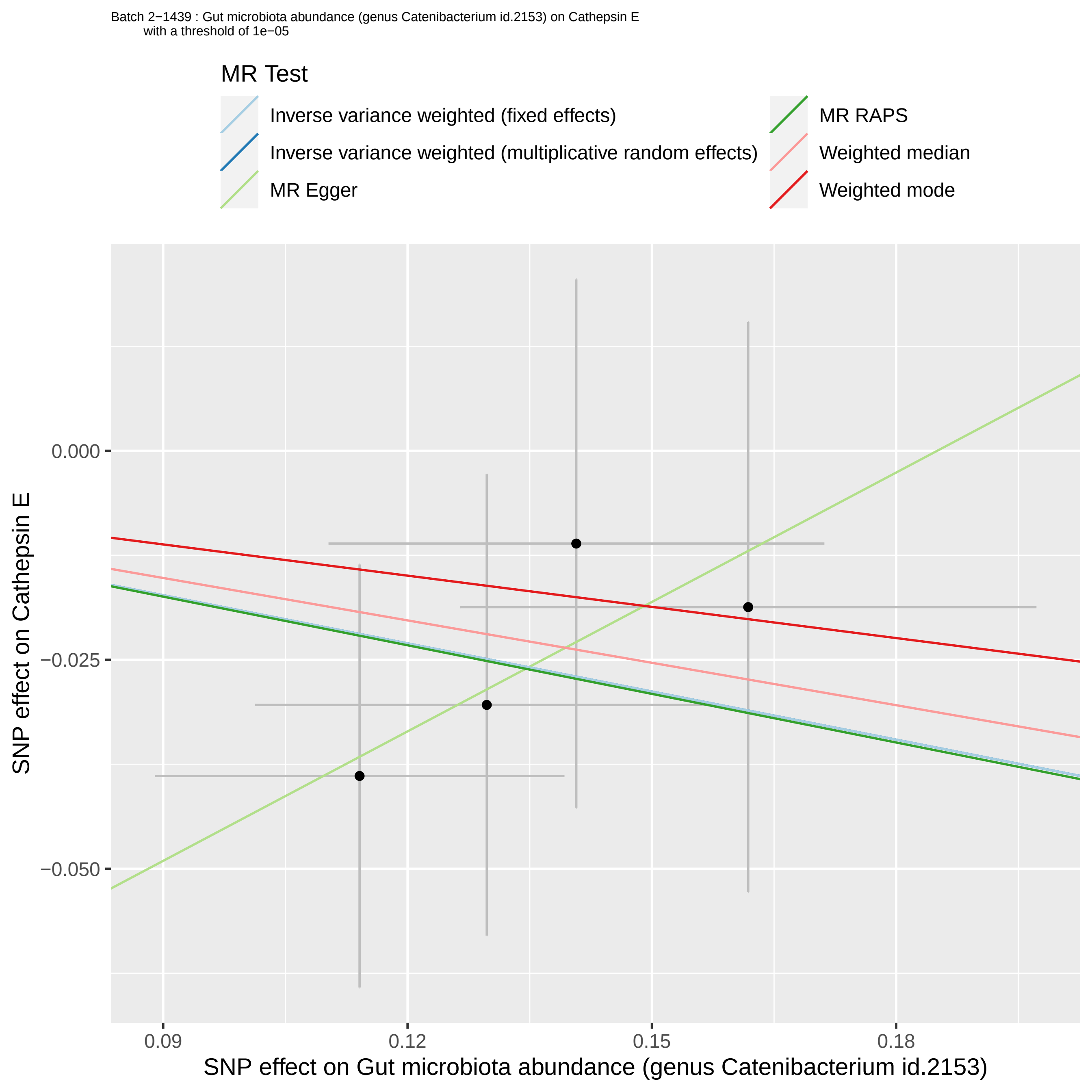
Batch 2−1439 : Gut microbiota abundance (genus Catenibacterium id.2153) on Cathepsin E
with a threshold of 1e−05
MR Test
Inverse variance weighted (fixed effects)
Inverse variance weighted (multiplicative random effects)
MR Egger
MR RAPS
Weighted median
Weighted mode
0.000
−0.025
−0.050
SNP effect on Cathepsin E
0.09
0.12
0.15
0.18
SNP effect on Gut microbiota abundance (genus Catenibacterium id.2153)

Batch 2−1440 : Gut microbiota abundance (genus Parasutterella id.2892) on Cathepsin E
with a threshold of 1e−05

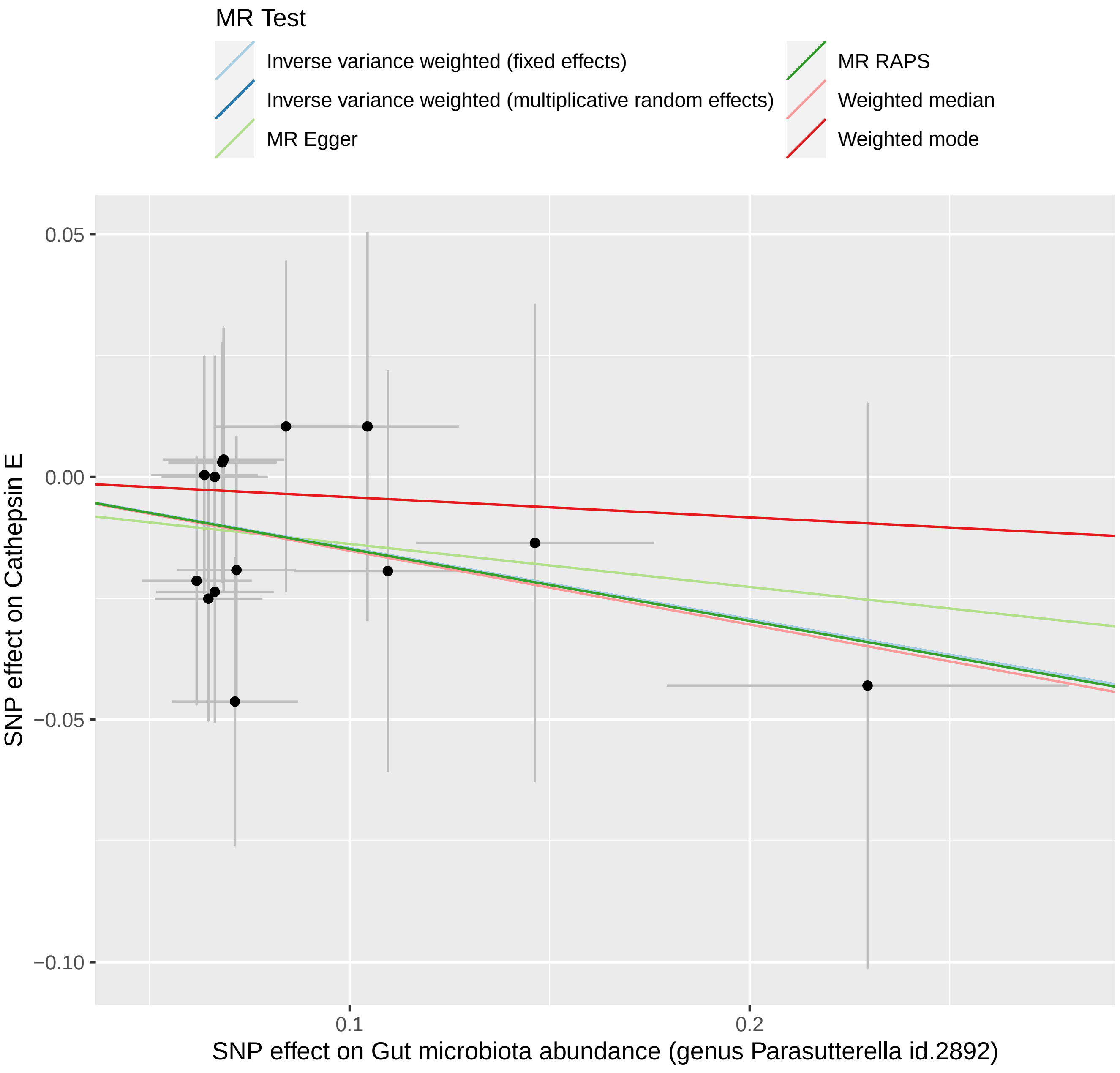

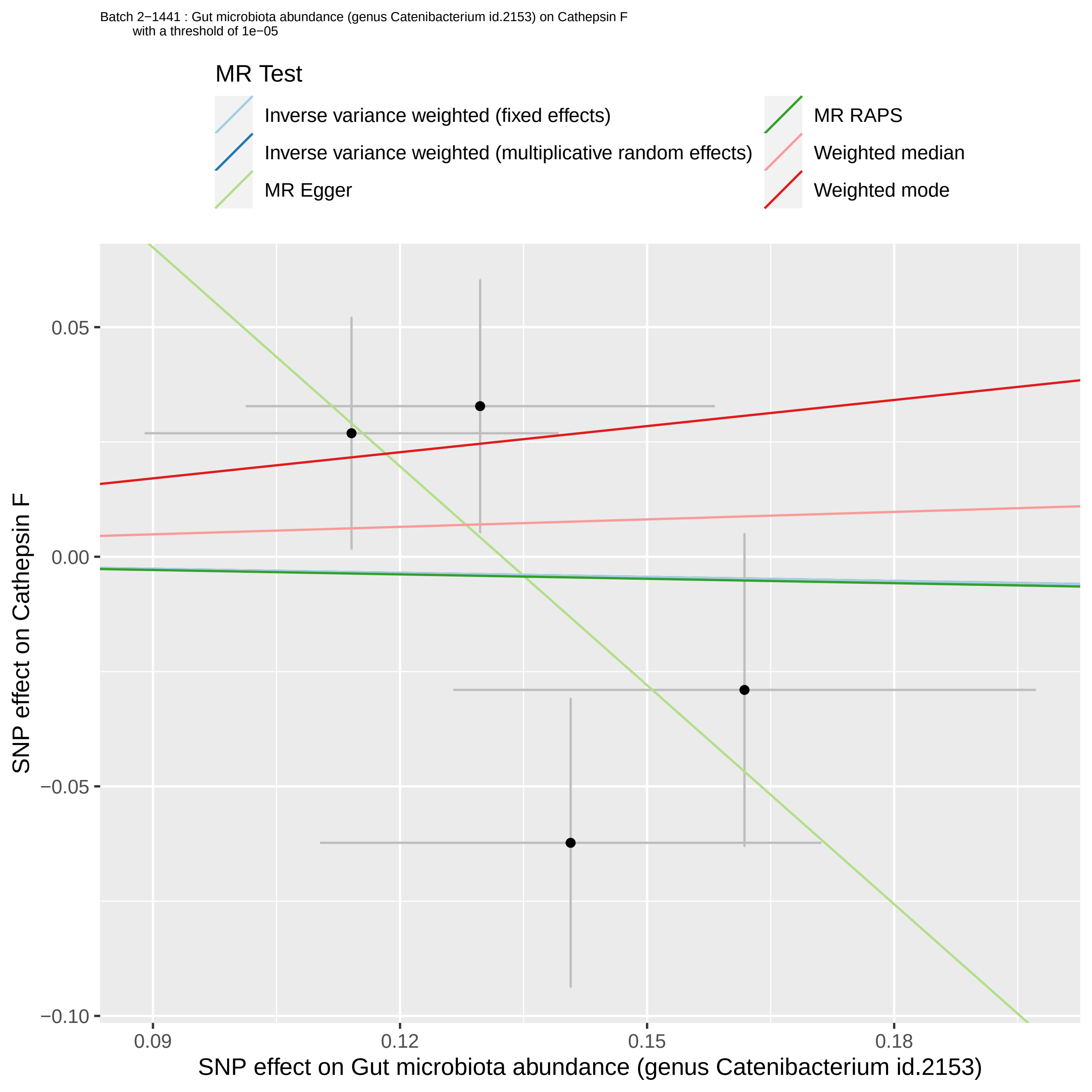
Batch 2−1441 : Gut microbiota abundance (genus Catenibacterium id.2153) on Cathepsin F
with a threshold of 1e−05
MR Test
Inverse variance weighted (fixed effects)
Inverse variance weighted (multiplicative random effects)
MR Egger
MR RAPS
Weighted median
Weighted mode
0.05
0.00
−0.05
−0.10
0.09
0.12
0.15
0.18
SNP effect on Cathepsin F
SNP effect on Gut microbiota abundance (genus Catenibacterium id.2153)

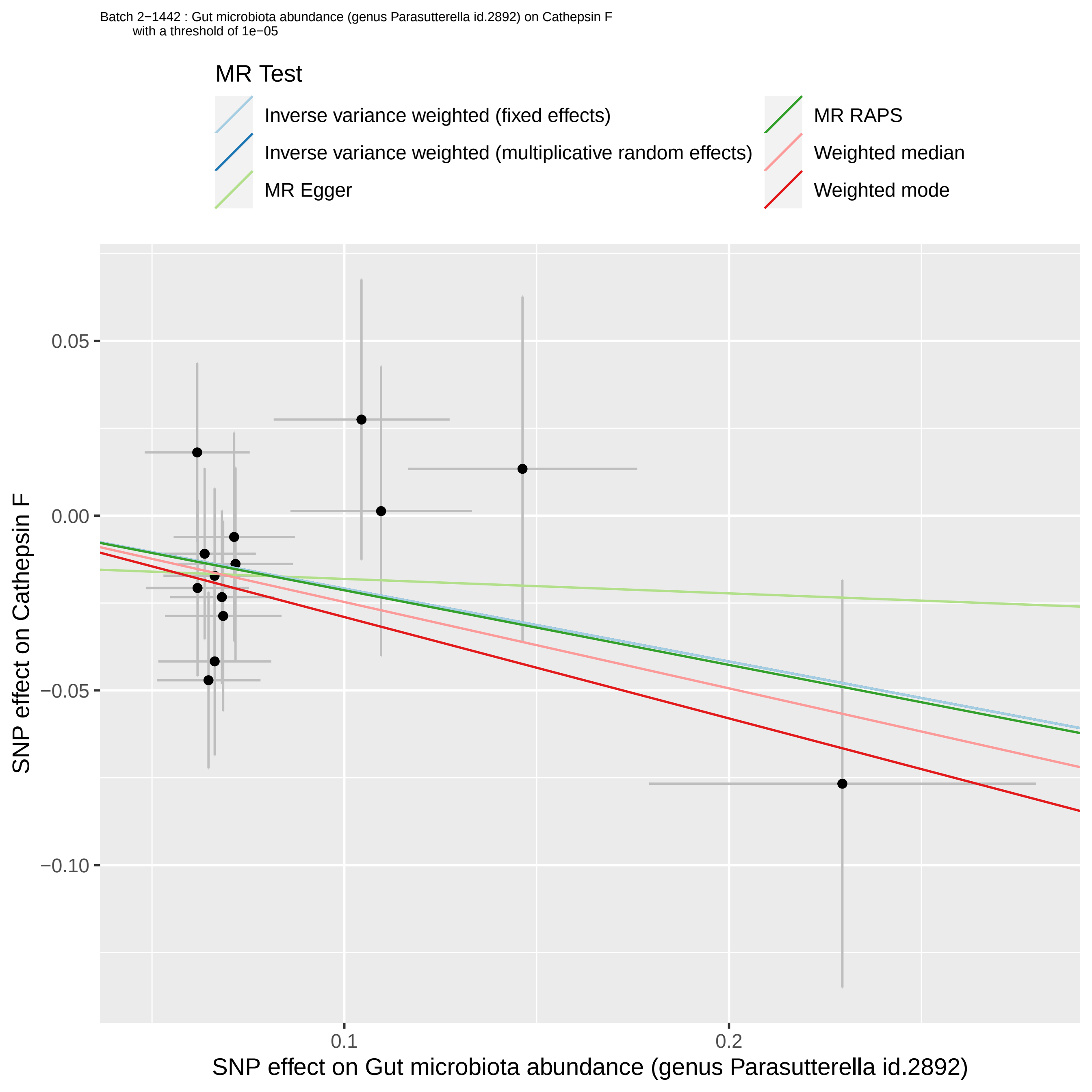
Batch 2−1442 : Gut microbiota abundance (genus Parasutterella id.2892) on Cathepsin F
with a threshold of 1e−05
MR Test
Inverse variance weighted (fixed effects)
Inverse variance weighted (multiplicative random effects)
MR Egger
MR RAPS
Weighted median
Weighted mode
0.05
0.00
−0.05
−0.10
0.1
0.2
SNP effect on Cathepsin F
SNP effect on Gut microbiota abundance (genus Parasutterella id.2892)

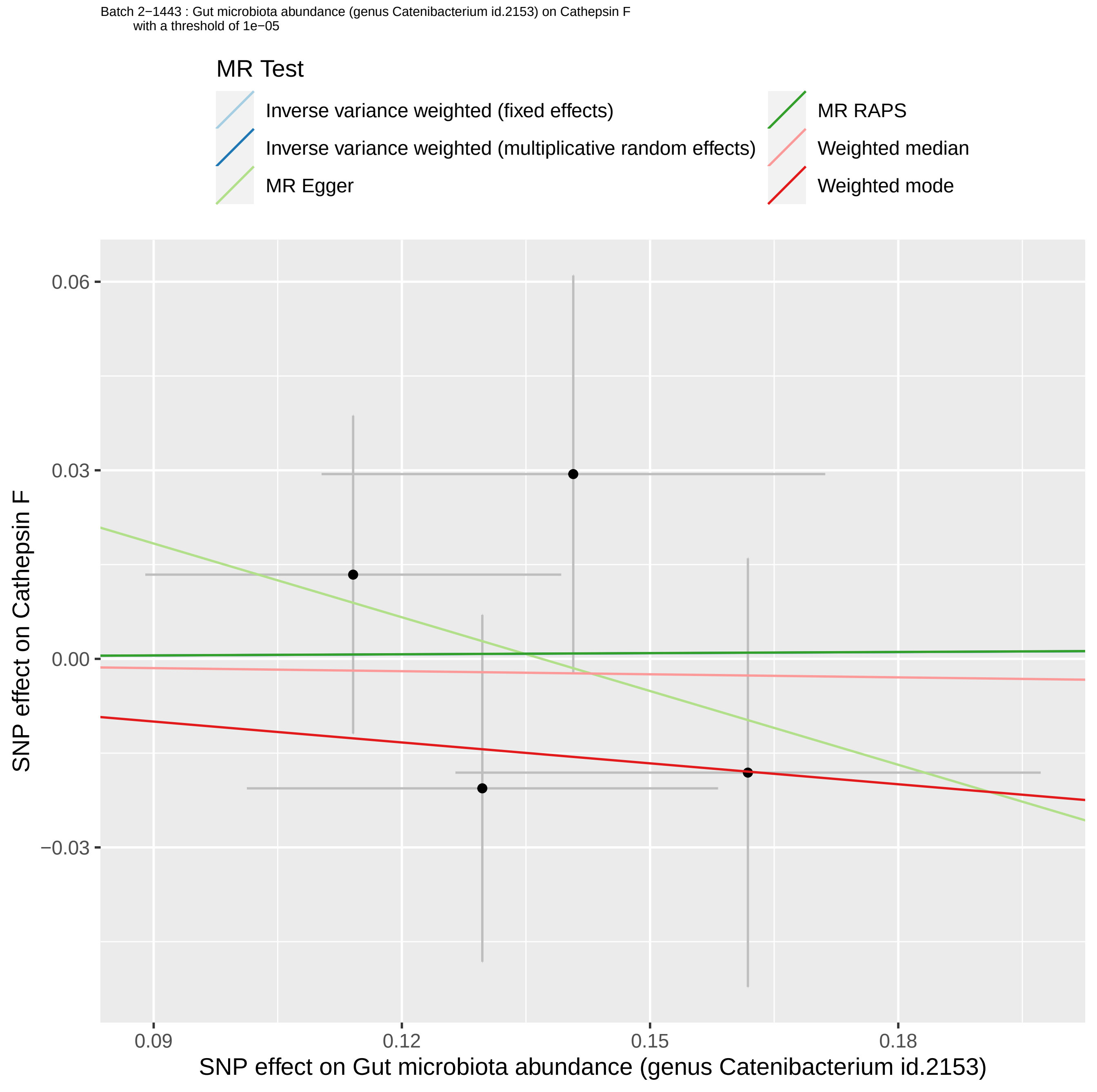
Batch 2−1443 : Gut microbiota abundance (genus Catenibacterium id.2153) on Cathepsin F
with a threshold of 1e−05
MR Test
Inverse variance weighted (fixed effects)
Inverse variance weighted (multiplicative random effects)
MR Egger
MR RAPS
Weighted median
Weighted mode
0.06
0.03
0.00
−0.03
SNP effect on Cathepsin F
0.09
0.12
0.15
0.18
SNP effect on Gut microbiota abundance (genus Catenibacterium id.2153)

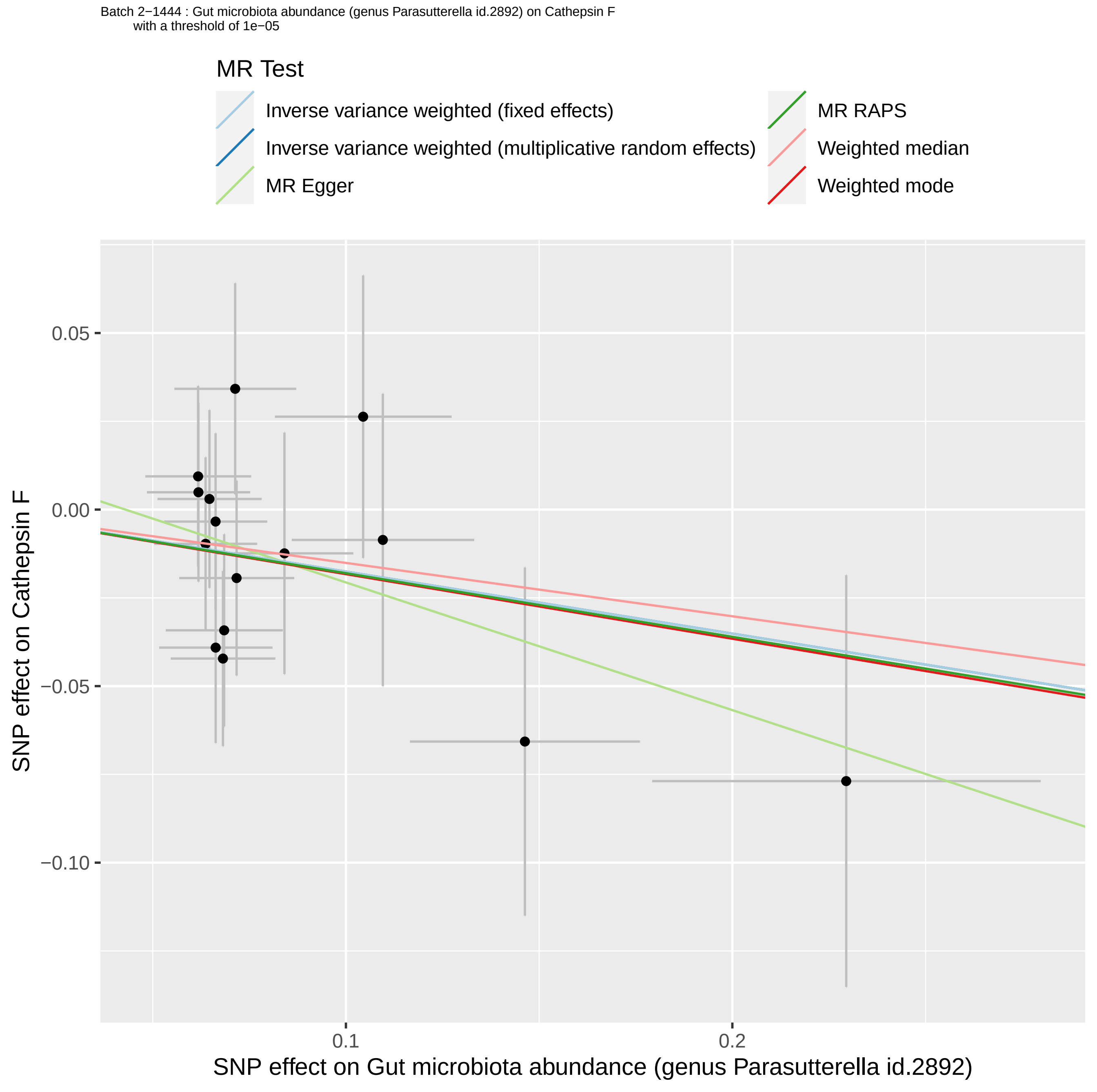

Batch 2−1444 : Gut microbiota abundance (genus Parasutterella id.2892) on Cathepsin F
with a threshold of 1e−05
MR Test
Inverse variance weighted (fixed effects)
Inverse variance weighted (multiplicative random effects)
MR Egger
MR RAPS
Weighted median
Weighted mode
0.05
0.00
−0.05
−0.10
0.1
0.2
SNP effect on Cathepsin F
SNP effect on Gut microbiota abundance (genus Parasutterella id.2892)

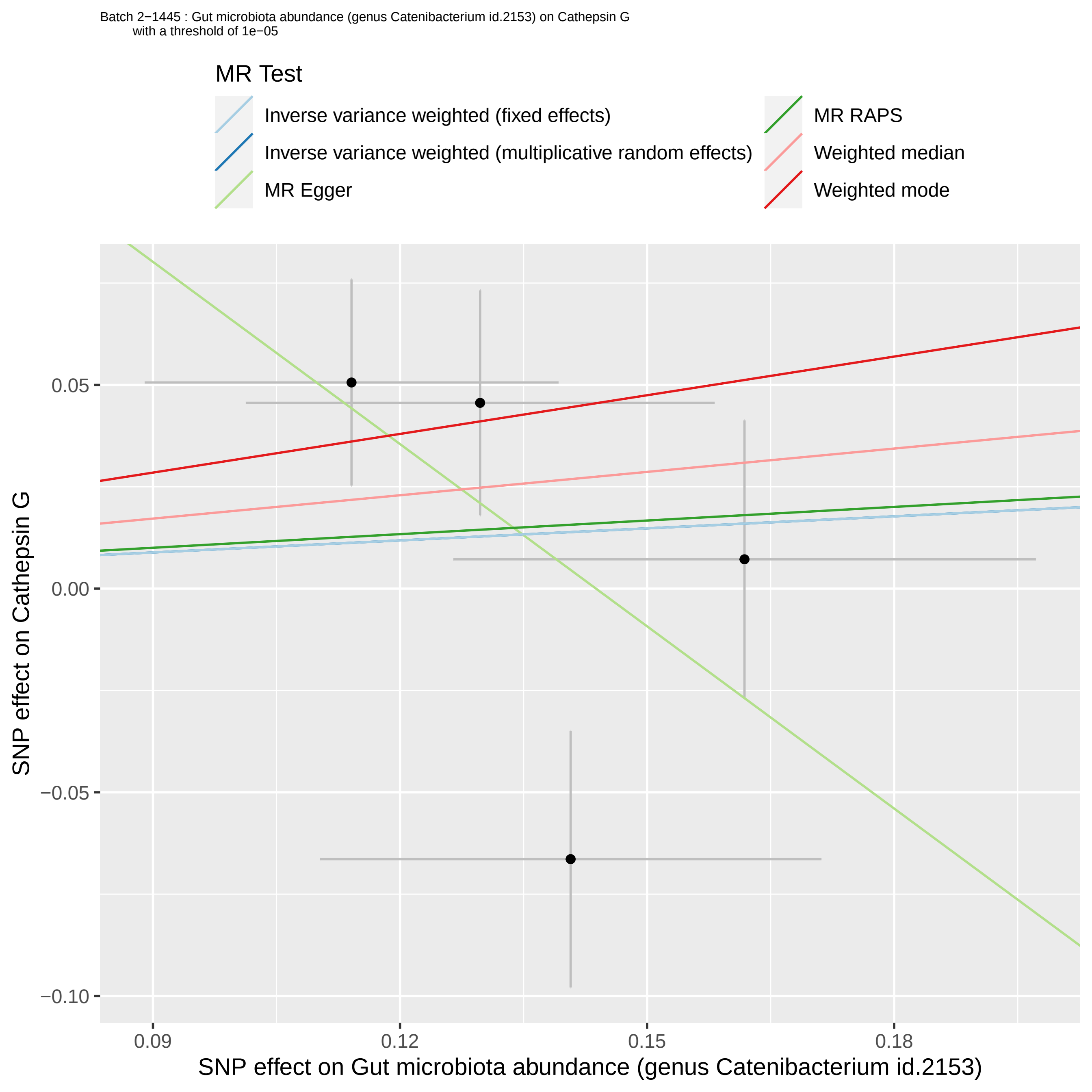
Batch 2−1445 : Gut microbiota abundance (genus Catenibacterium id.2153) on Cathepsin G
with a threshold of 1e−05
MR Test
Inverse variance weighted (fixed effects)
Inverse variance weighted (multiplicative random effects)
MR Egger
MR RAPS
Weighted median
Weighted mode
0.05
0.00
−0.05
−0.10
SNP effect on Cathepsin G
0.09
0.12
0.15
0.18
SNP effect on Gut microbiota abundance (genus Catenibacterium id.2153)

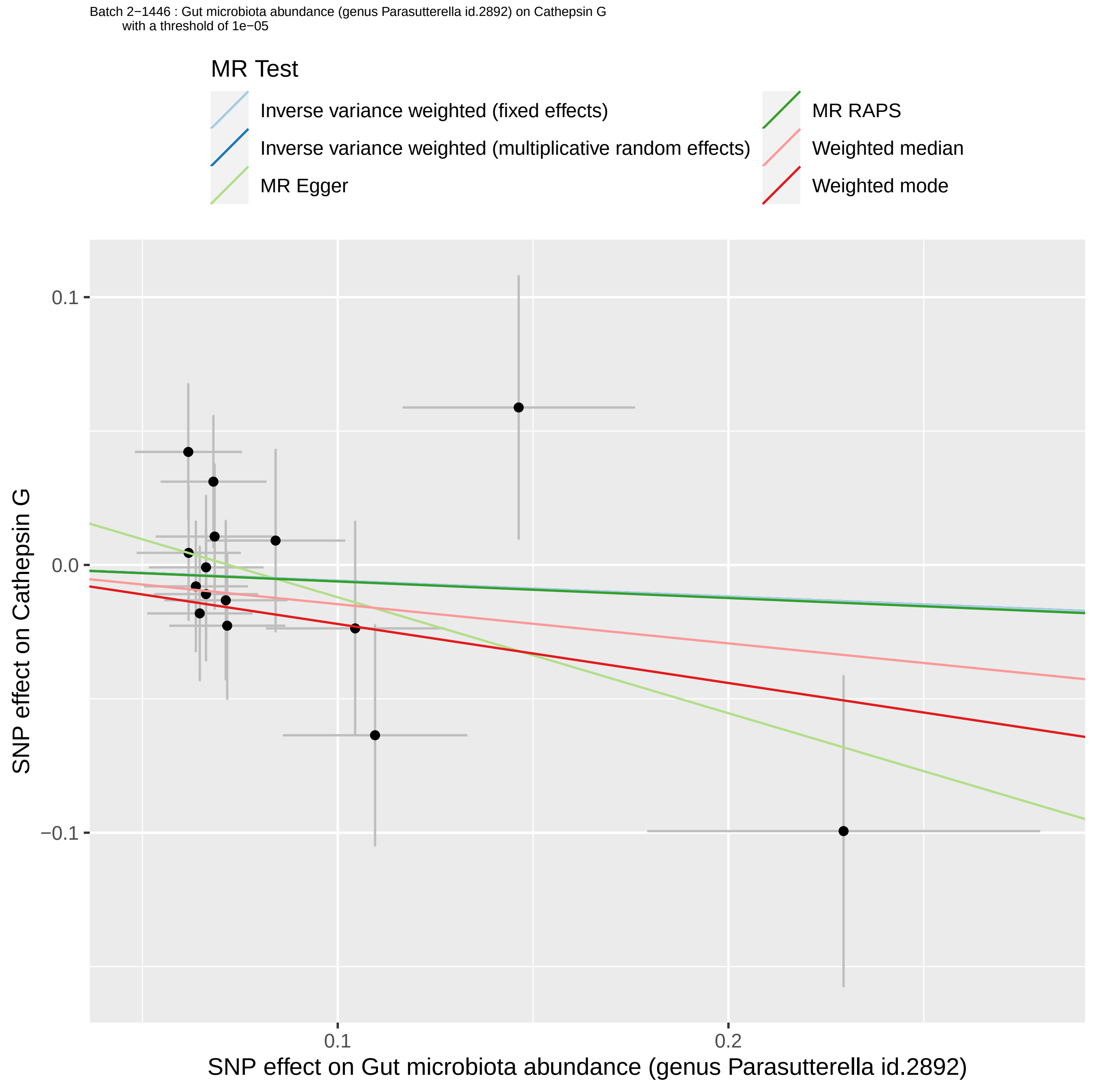

Batch 2−1446 : Gut microbiota abundance (genus Parasutterella id.2892) on Cathepsin G
with a threshold of 1e−05
MR Test
Inverse variance weighted (fixed effects)
Inverse variance weighted (multiplicative random effects)
MR Egger
MR RAPS
Weighted median
Weighted mode
0.1
0.0
−0.1
SNP effect on Cathepsin G
0.1
0.2
SNP effect on Gut microbiota abundance (genus Parasutterella id.2892)

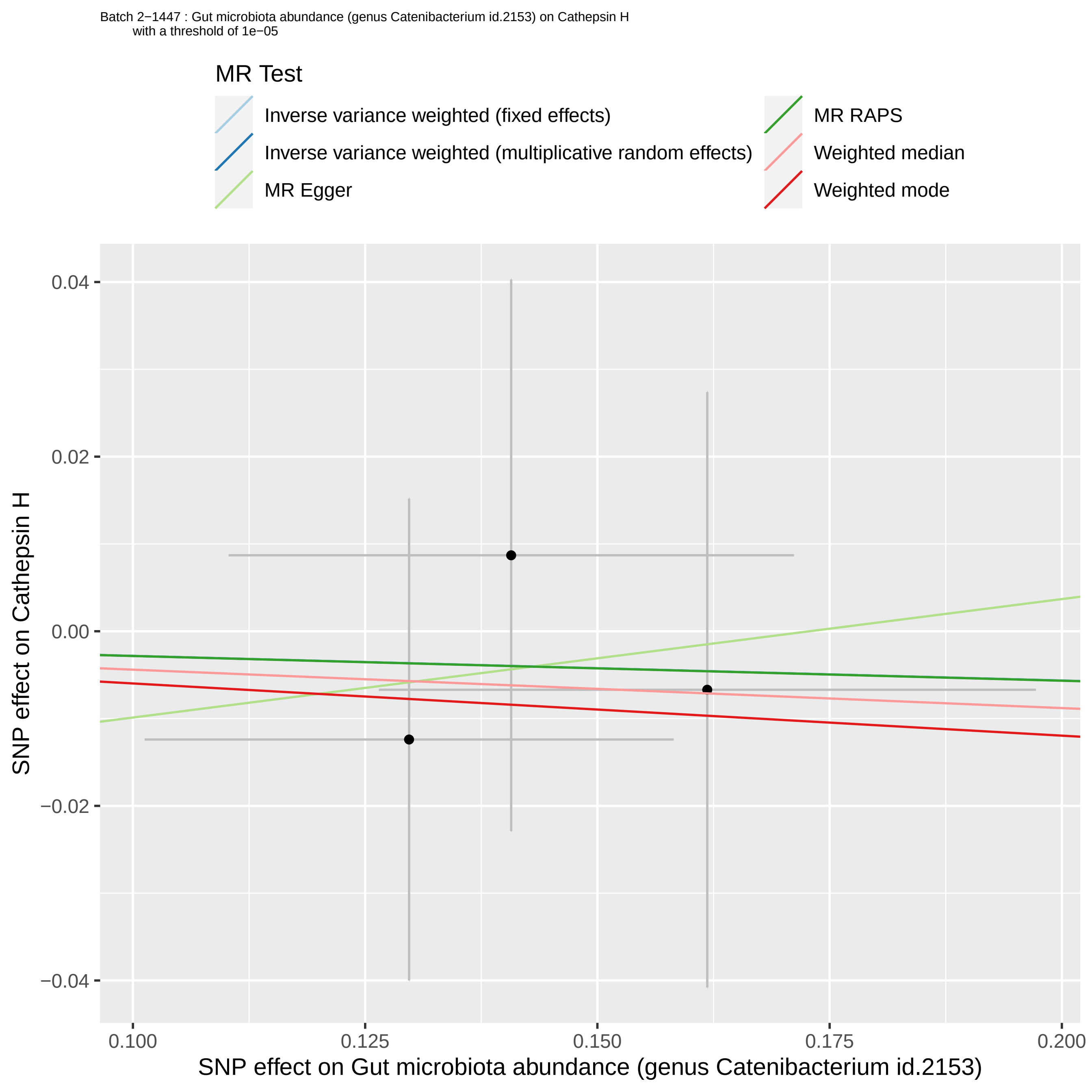
Batch 2−1447 : Gut microbiota abundance (genus Catenibacterium id.2153) on Cathepsin H
with a threshold of 1e−05
MR Test
Inverse variance weighted (fixed effects)
Inverse variance weighted (multiplicative random effects)
MR Egger
MR RAPS
Weighted median
Weighted mode
0.04
0.02
0.00
−0.02
−0.04
SNP effect on Cathepsin H
0.100
0.125
0.150
0.175
0.200
SNP effect on Gut microbiota abundance (genus Catenibacterium id.2153)

Batch 2−1448 : Gut microbiota abundance (genus Parasutterella id.2892) on Cathepsin H
with a threshold of 1e−05

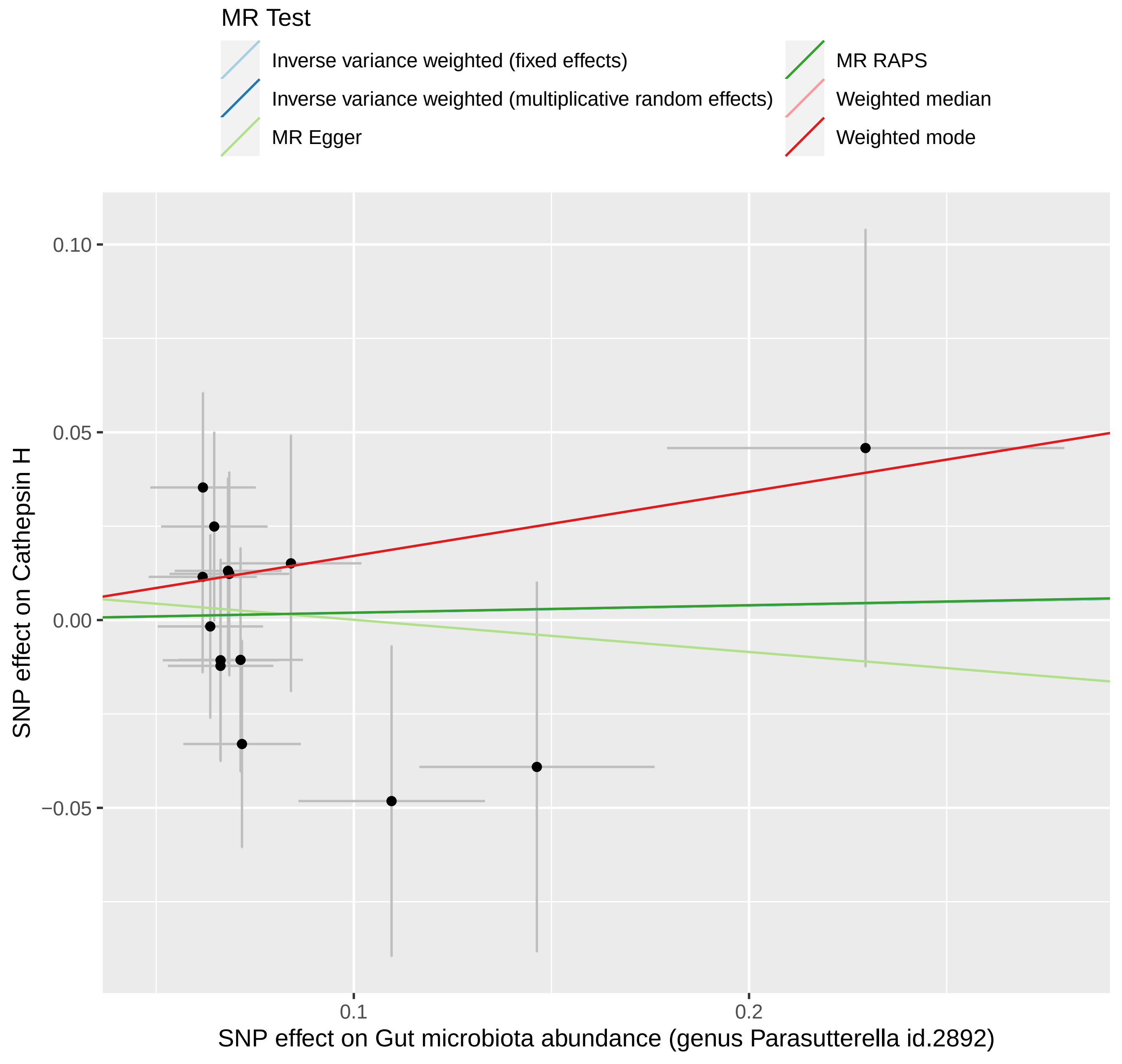

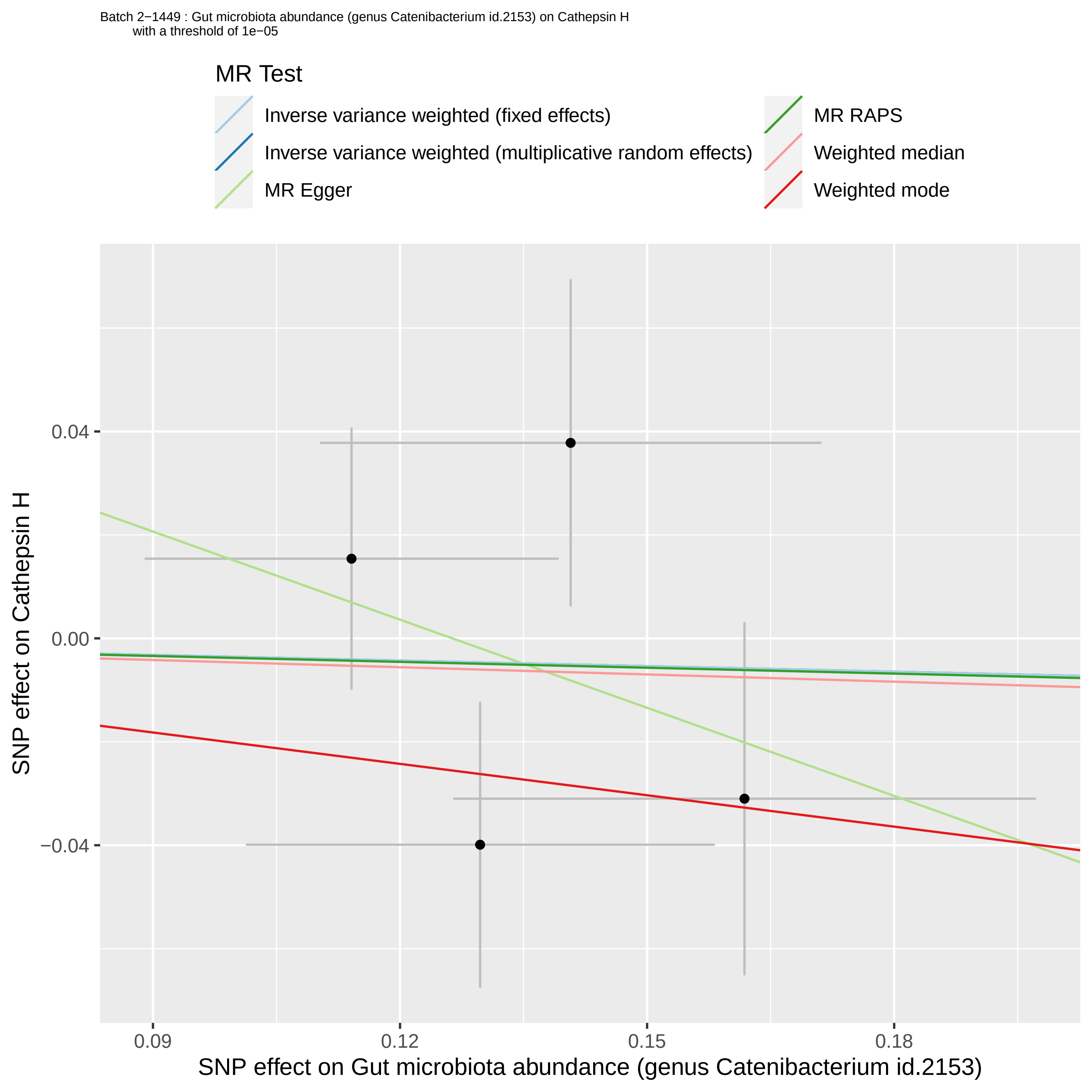

Batch 2−1449 : Gut microbiota abundance (genus Catenibacterium id.2153) on Cathepsin H
with a threshold of 1e−05
MR Test
Inverse variance weighted (fixed effects)
Inverse variance weighted (multiplicative random effects)
MR Egger
MR RAPS
Weighted median
Weighted mode
SNP effect on Cathepsin H
0.04
0.00
−0.04
0.09
0.12
0.15
0.18
SNP effect on Gut microbiota abundance (genus Catenibacterium id.2153)

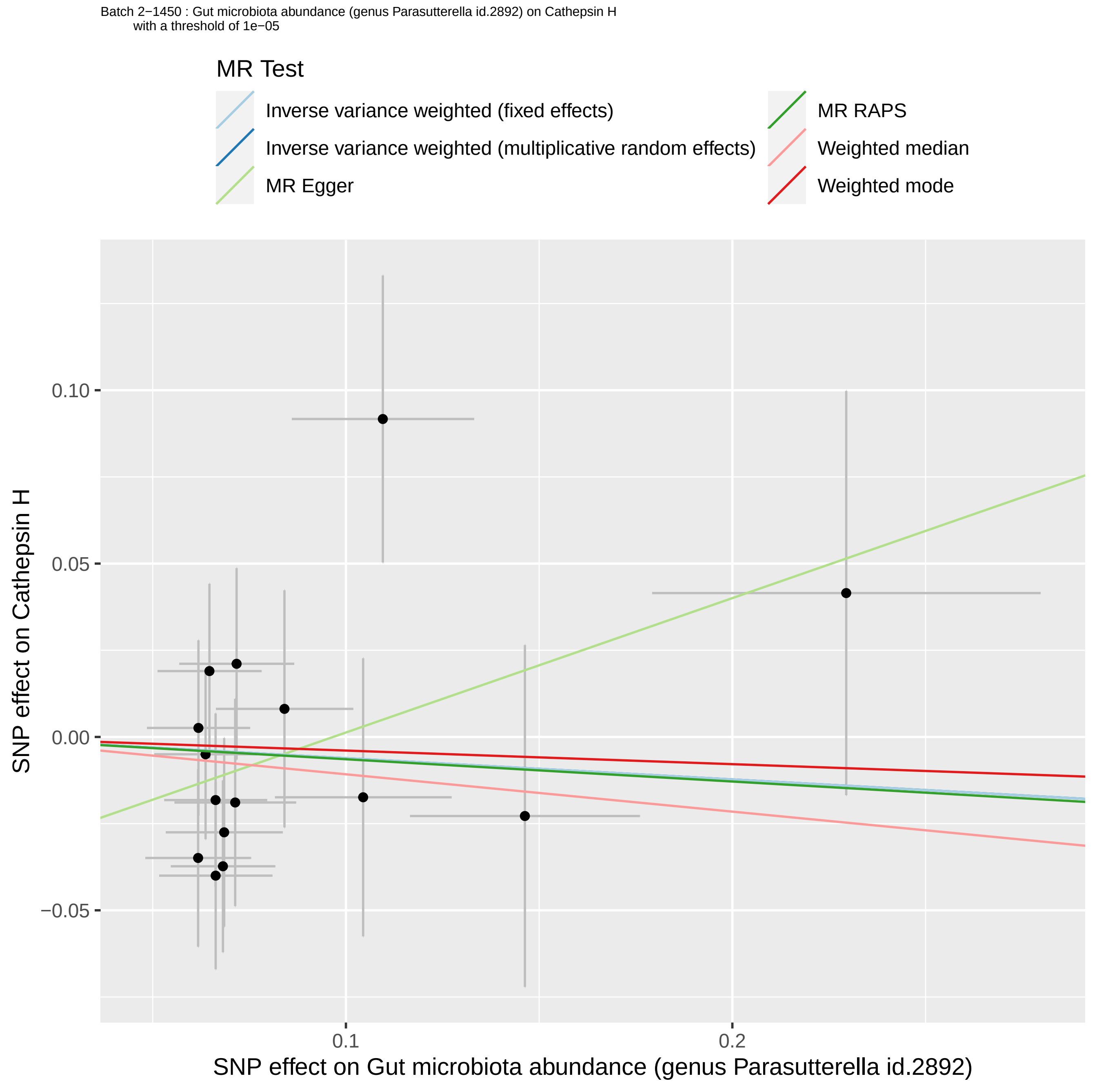
Batch 2−1450 : Gut microbiota abundance (genus Parasutterella id.2892) on Cathepsin H
with a threshold of 1e−05
MR Test
Inverse variance weighted (fixed effects)
Inverse variance weighted (multiplicative random effects)
MR Egger
MR RAPS
Weighted median
Weighted mode
0.10
0.05
0.00
−0.05
SNP effect on Cathepsin H
0.1
0.2
SNP effect on Gut microbiota abundance (genus Parasutterella id.2892)

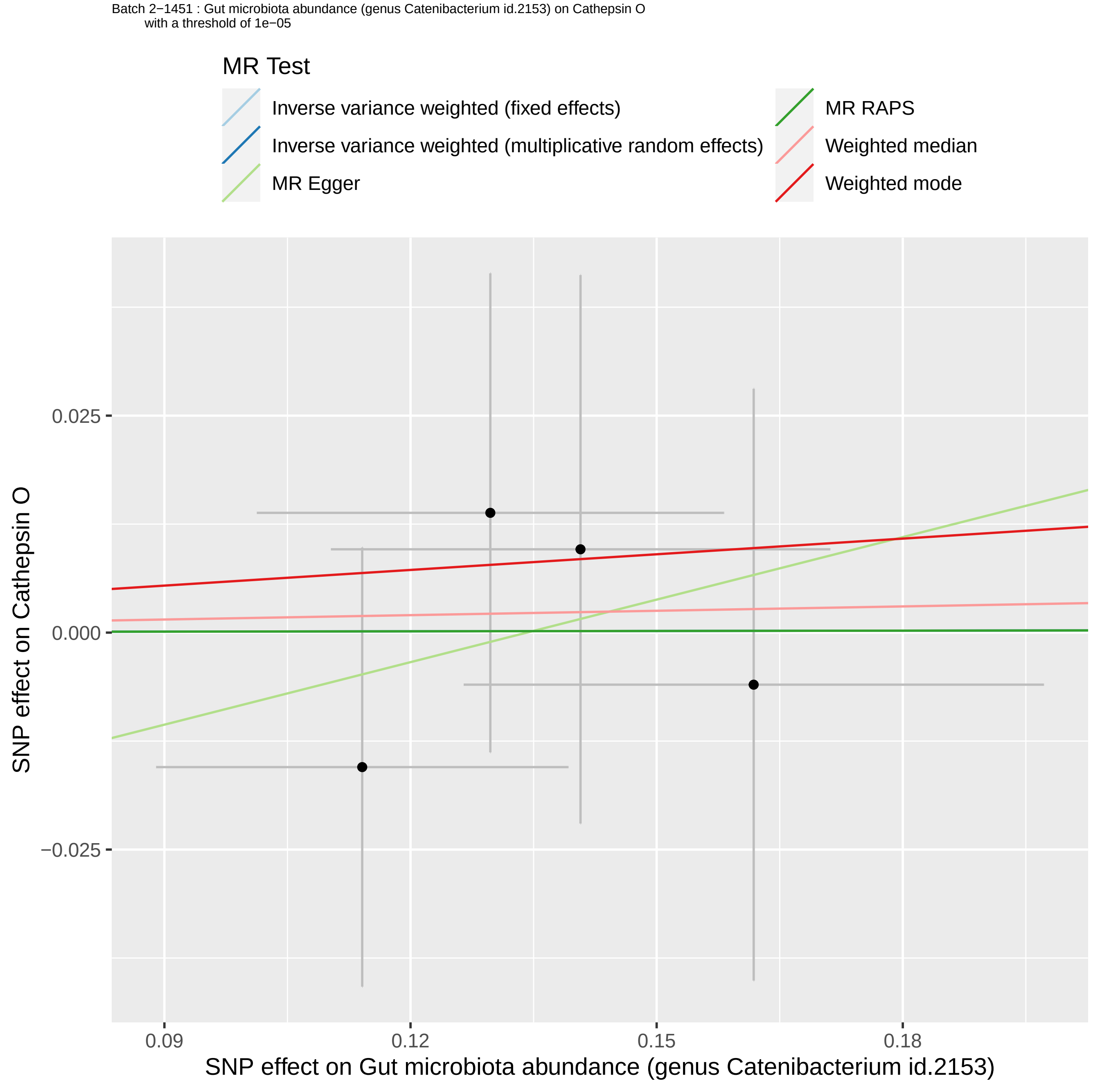
Batch 2−1451 : Gut microbiota abundance (genus Catenibacterium id.2153) on Cathepsin O
with a threshold of 1e−05
MR Test
Inverse variance weighted (fixed effects)
Inverse variance weighted (multiplicative random effects)
MR Egger
MR RAPS
Weighted median
Weighted mode
0.025
0.000
−0.025
0.09
0.12
0.15
0.18
SNP effect on Cathepsin O
SNP effect on Gut microbiota abundance (genus Catenibacterium id.2153)

Batch 2−1452 : Gut microbiota abundance (genus Parasutterella id.2892) on Cathepsin O
with a threshold of 1e−05

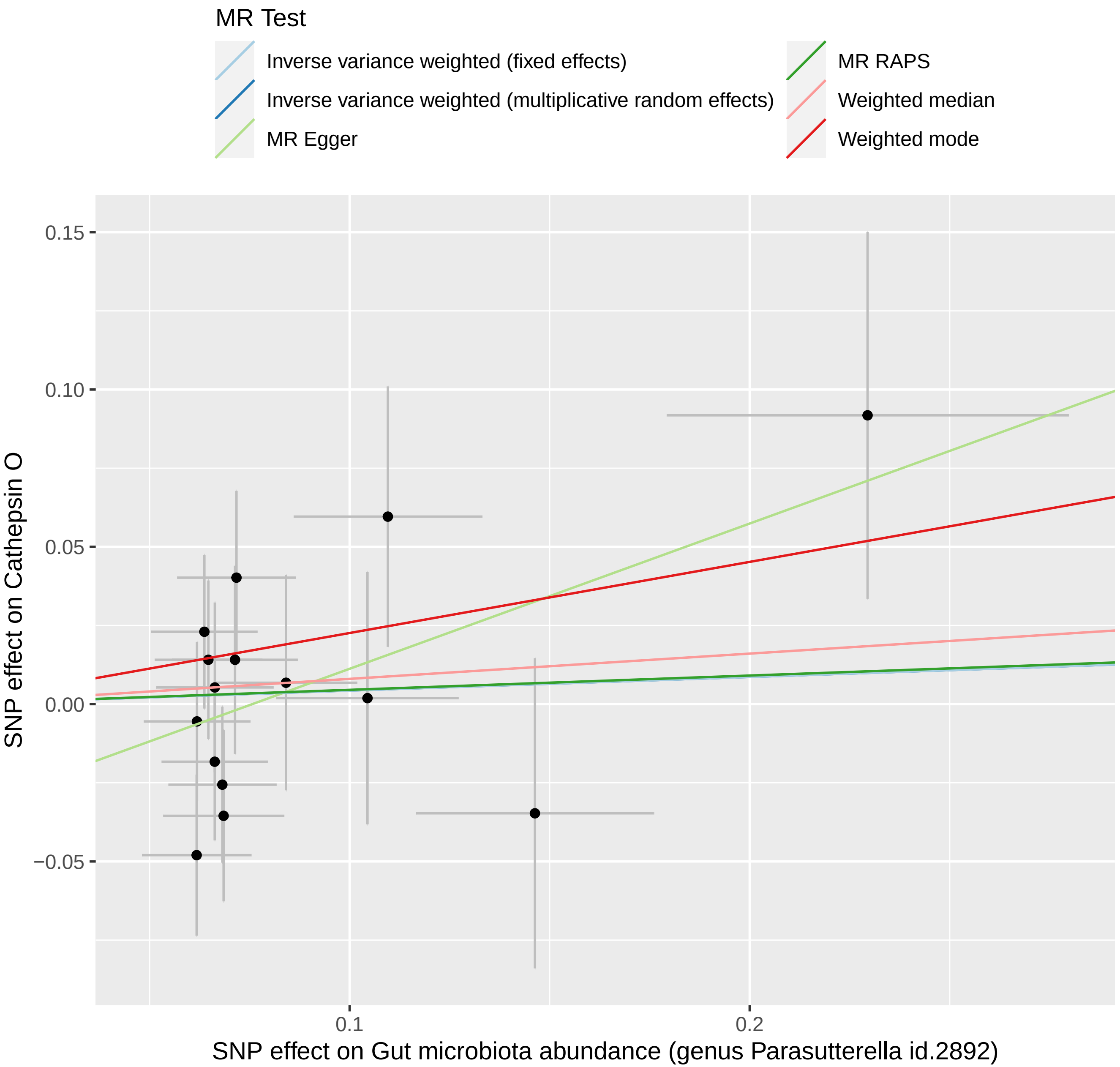

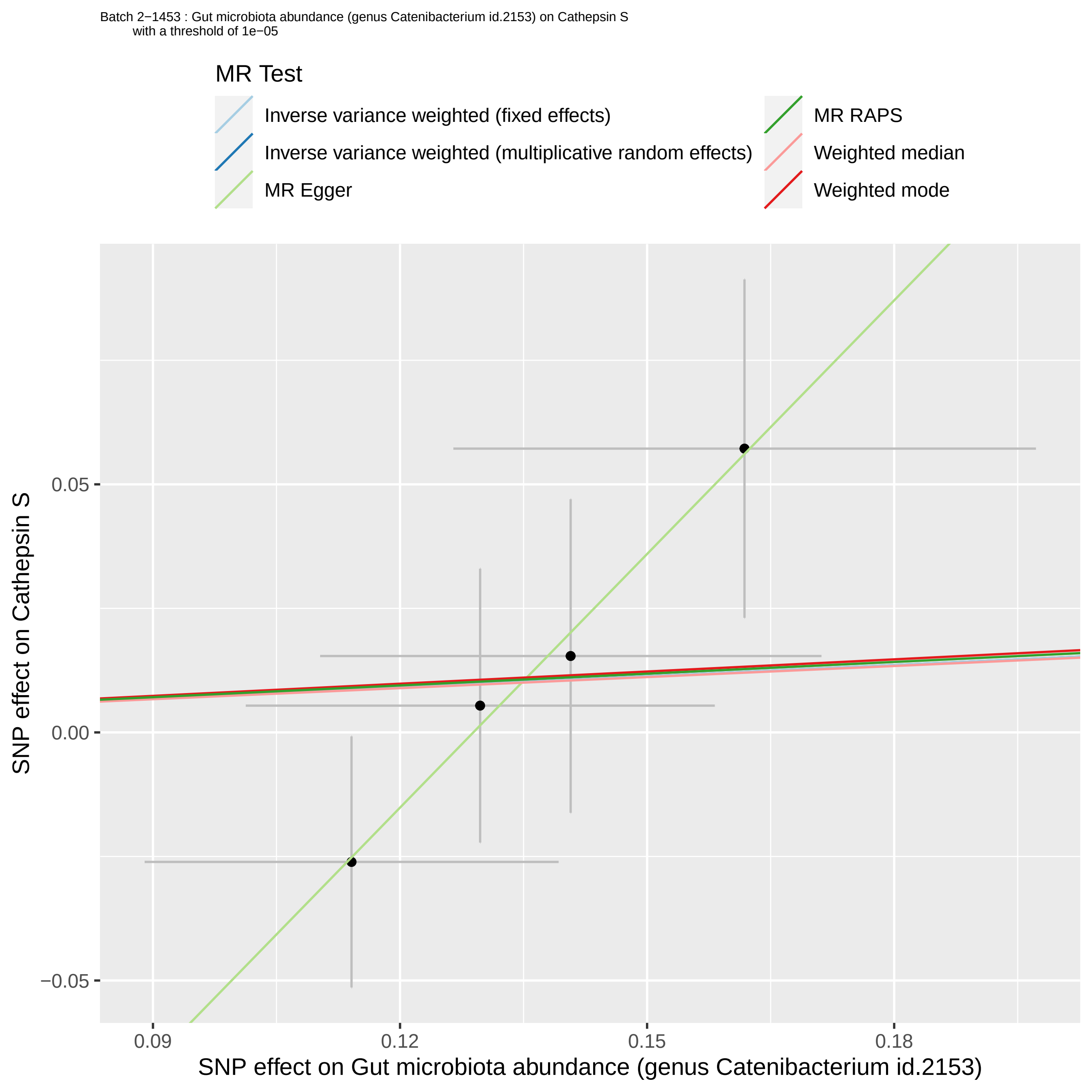
Batch 2−1453 : Gut microbiota abundance (genus Catenibacterium id.2153) on Cathepsin S
with a threshold of 1e−05
MR Test
Inverse variance weighted (fixed effects)
Inverse variance weighted (multiplicative random effects)
MR Egger
MR RAPS
Weighted median
Weighted mode
0.05
0.00
−0.05
SNP effect on Cathepsin S
0.09
0.12
0.15
0.18
SNP effect on Gut microbiota abundance (genus Catenibacterium id.2153)

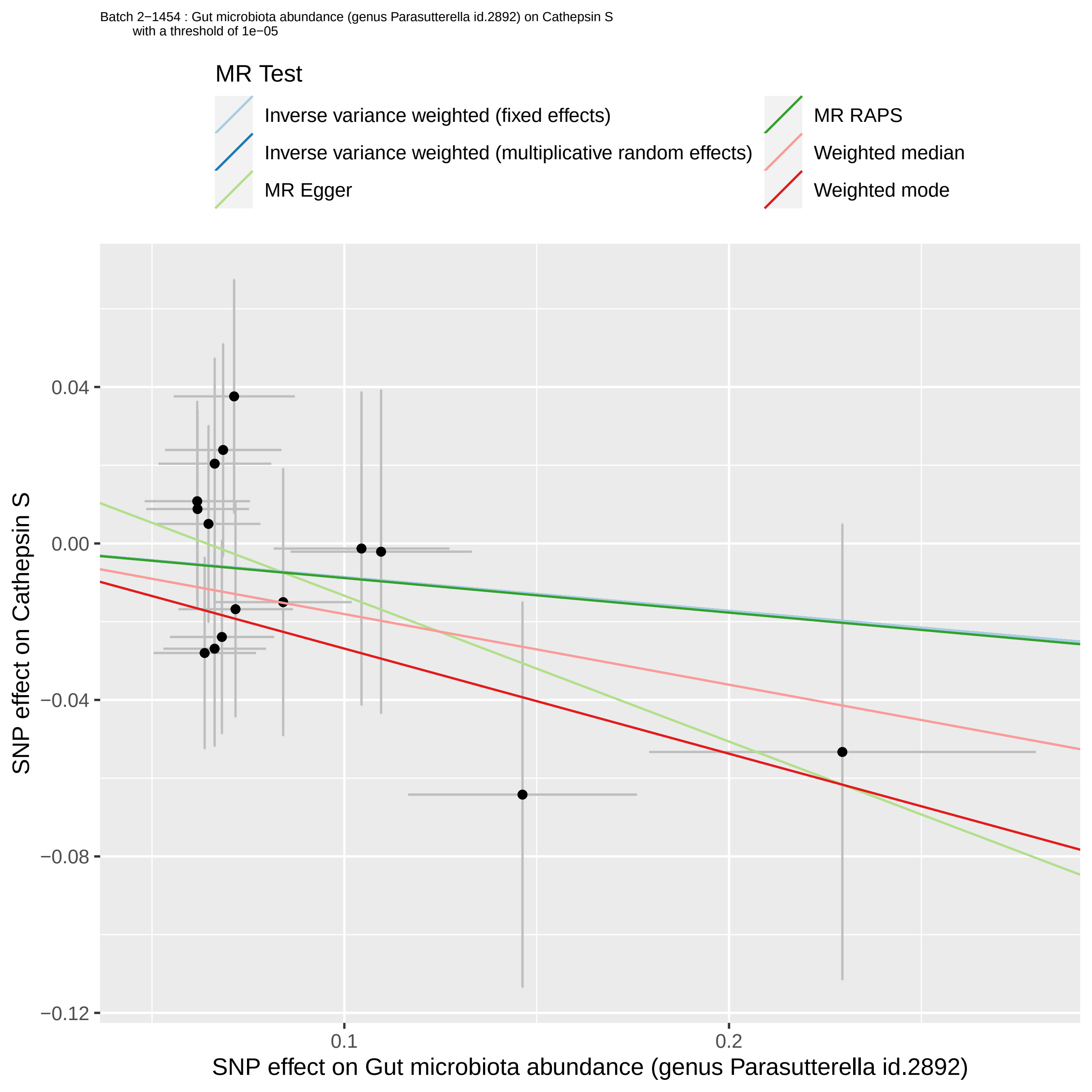

Batch 2−1454 : Gut microbiota abundance (genus Parasutterella id.2892) on Cathepsin S
with a threshold of 1e−05
MR Test
Inverse variance weighted (fixed effects)
Inverse variance weighted (multiplicative random effects)
MR Egger
MR RAPS
Weighted median
Weighted mode
0.04
0.00
−0.04
−0.08
−0.12
SNP effect on Cathepsin S
0.1
0.2
SNP effect on Gut microbiota abundance (genus Parasutterella id.2892)

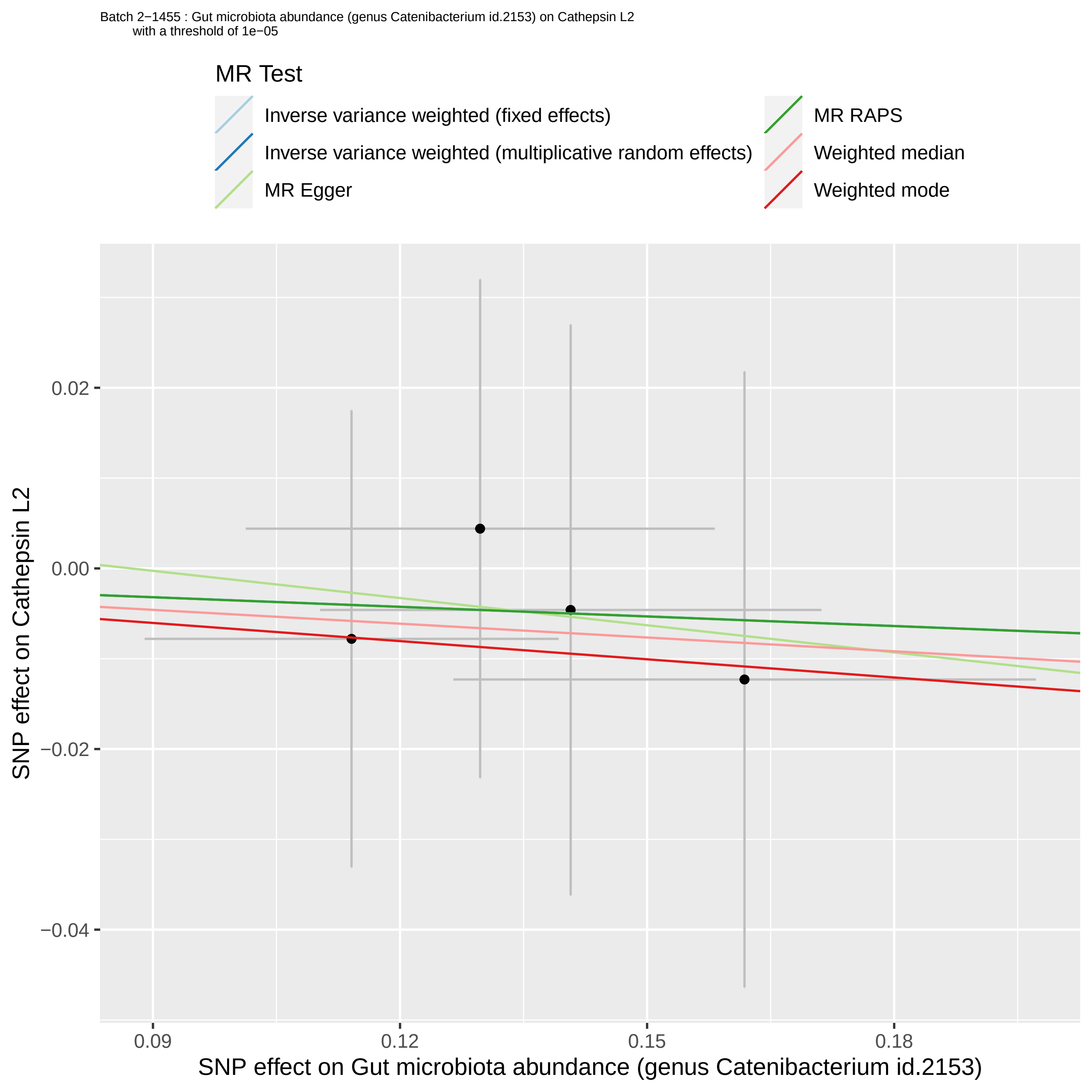
Batch 2−1455 : Gut microbiota abundance (genus Catenibacterium id.2153) on Cathepsin L2
with a threshold of 1e−05
MR Test
Inverse variance weighted (fixed effects)
Inverse variance weighted (multiplicative random effects)
MR Egger
MR RAPS
Weighted median
Weighted mode
SNP effect on Cathepsin L2
0.02
0.00
−0.02
−0.04
0.09
0.12
0.15
0.18
SNP effect on Gut microbiota abundance (genus Catenibacterium id.2153)

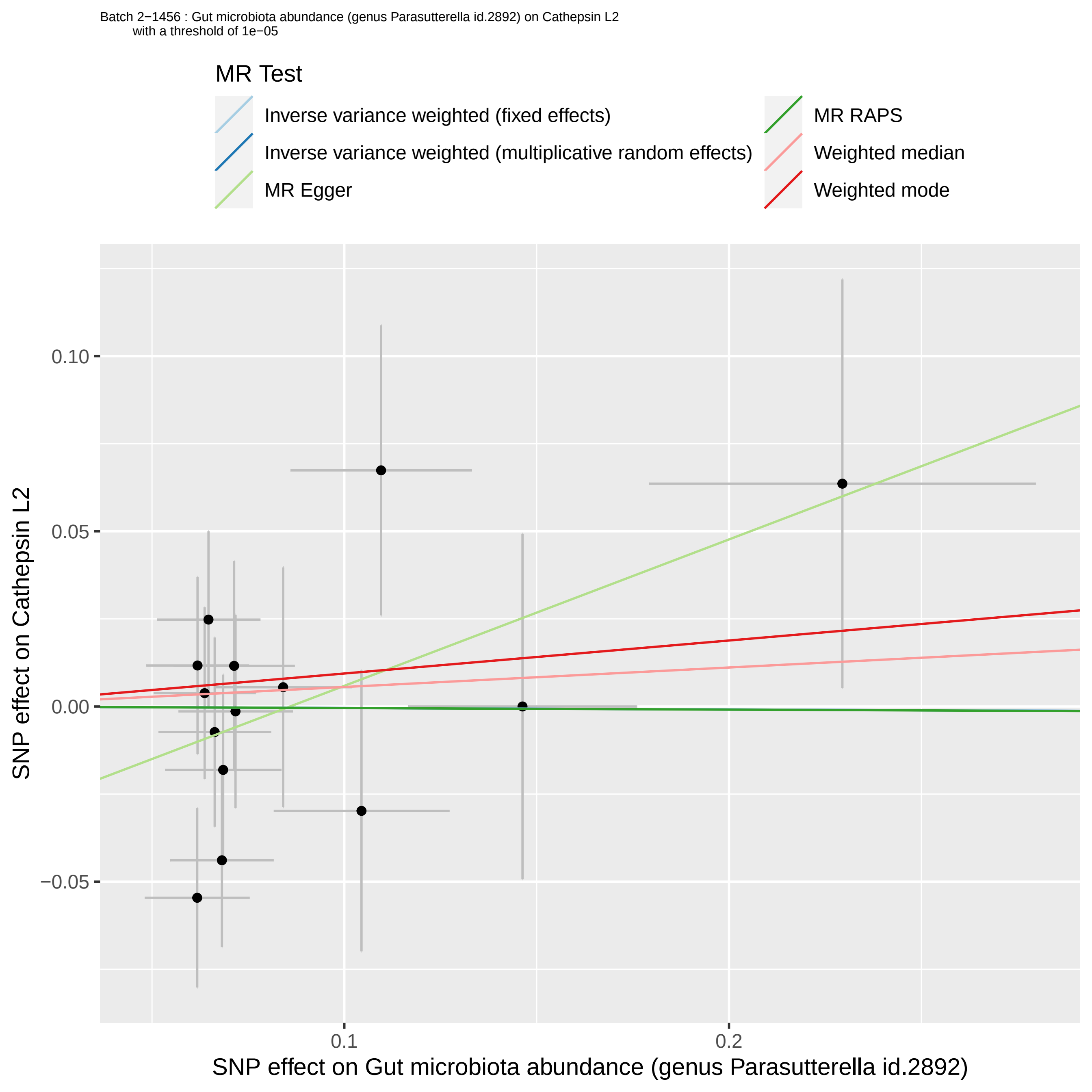
Batch 2−1456 : Gut microbiota abundance (genus Parasutterella id.2892) on Cathepsin L2
with a threshold of 1e−05
MR Test
Inverse variance weighted (fixed effects)
Inverse variance weighted (multiplicative random effects)
MR Egger
MR RAPS
Weighted median
Weighted mode
0.10
0.05
0.00
−0.05
SNP effect on Cathepsin L2
0.1
0.2
SNP effect on Gut microbiota abundance (genus Parasutterella id.2892)

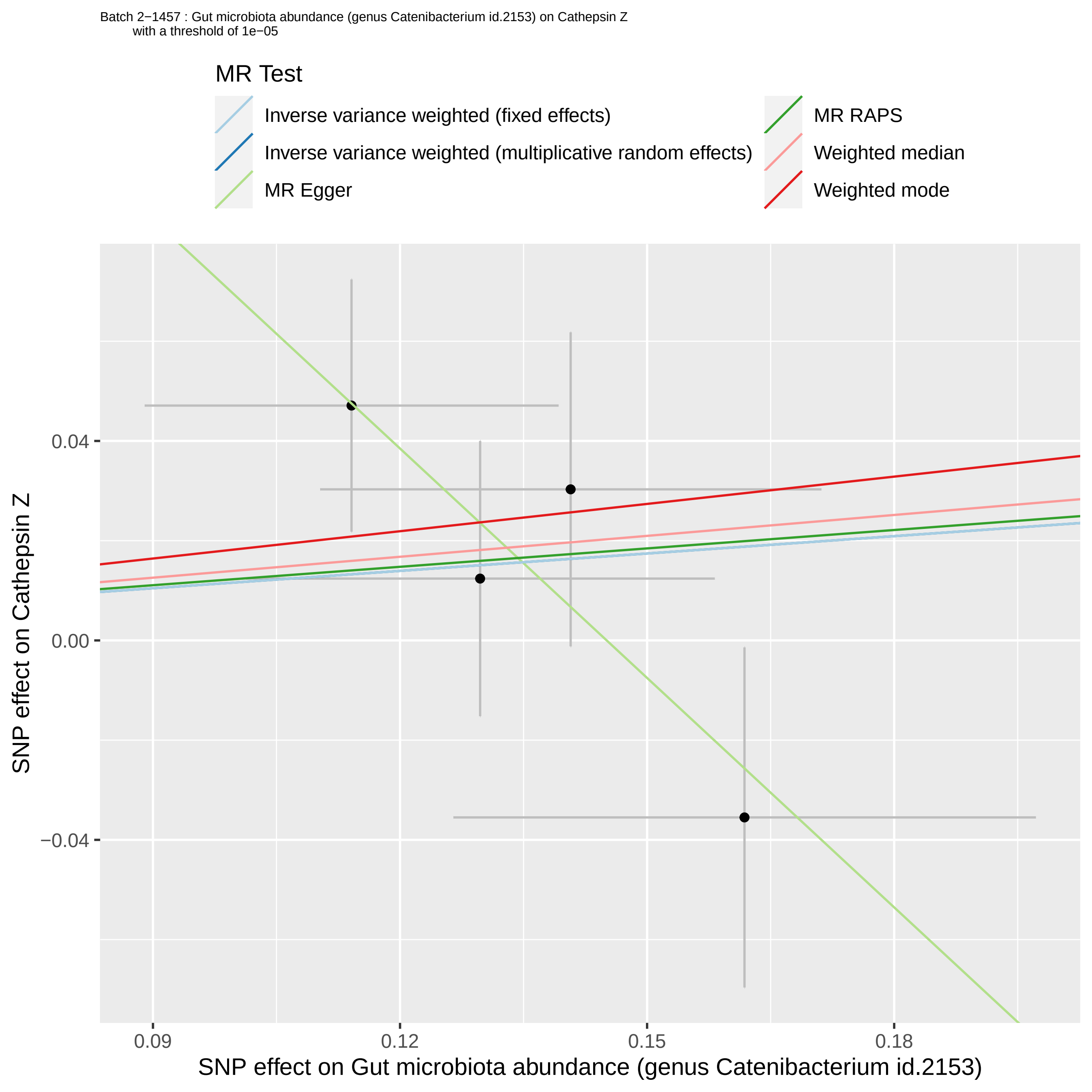
Batch 2−1457 : Gut microbiota abundance (genus Catenibacterium id.2153) on Cathepsin Z
with a threshold of 1e−05
MR Test
Inverse variance weighted (fixed effects)
Inverse variance weighted (multiplicative random effects)
MR Egger
MR RAPS
Weighted median
Weighted mode
0.04
0.00
−0.04
0.09
0.12
0.15
0.18
SNP effect on Cathepsin Z
SNP effect on Gut microbiota abundance (genus Catenibacterium id.2153)

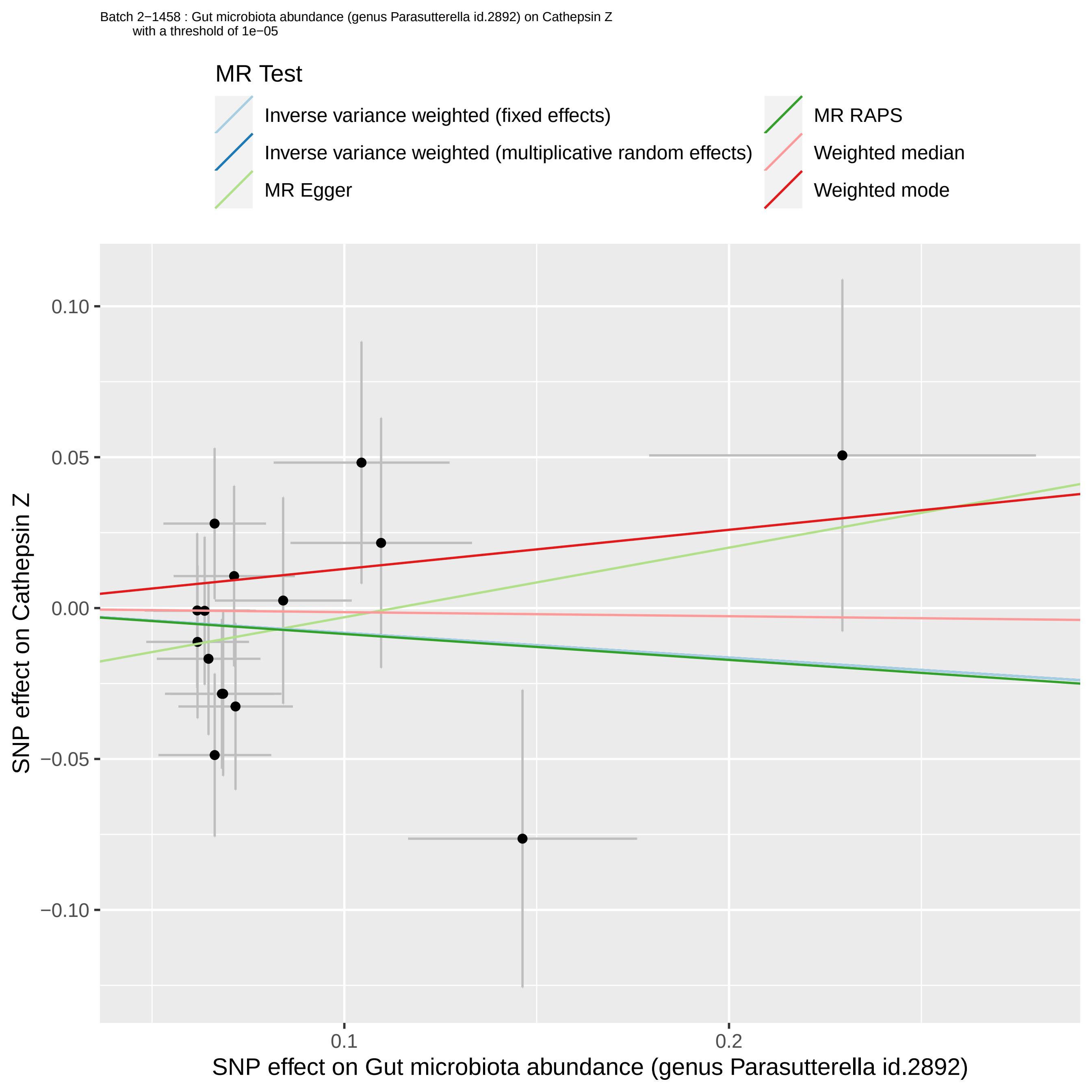
Batch 2−1458 : Gut microbiota abundance (genus Parasutterella id.2892) on Cathepsin Z
with a threshold of 1e−05
MR Test
Inverse variance weighted (fixed effects)
Inverse variance weighted (multiplicative random effects)
MR Egger
MR RAPS
Weighted median
Weighted mode
0.10
0.05
0.00
−0.05
−0.10
0.1
0.2
SNP effect on Cathepsin Z
SNP effect on Gut microbiota abundance (genus Parasutterella id.2892)

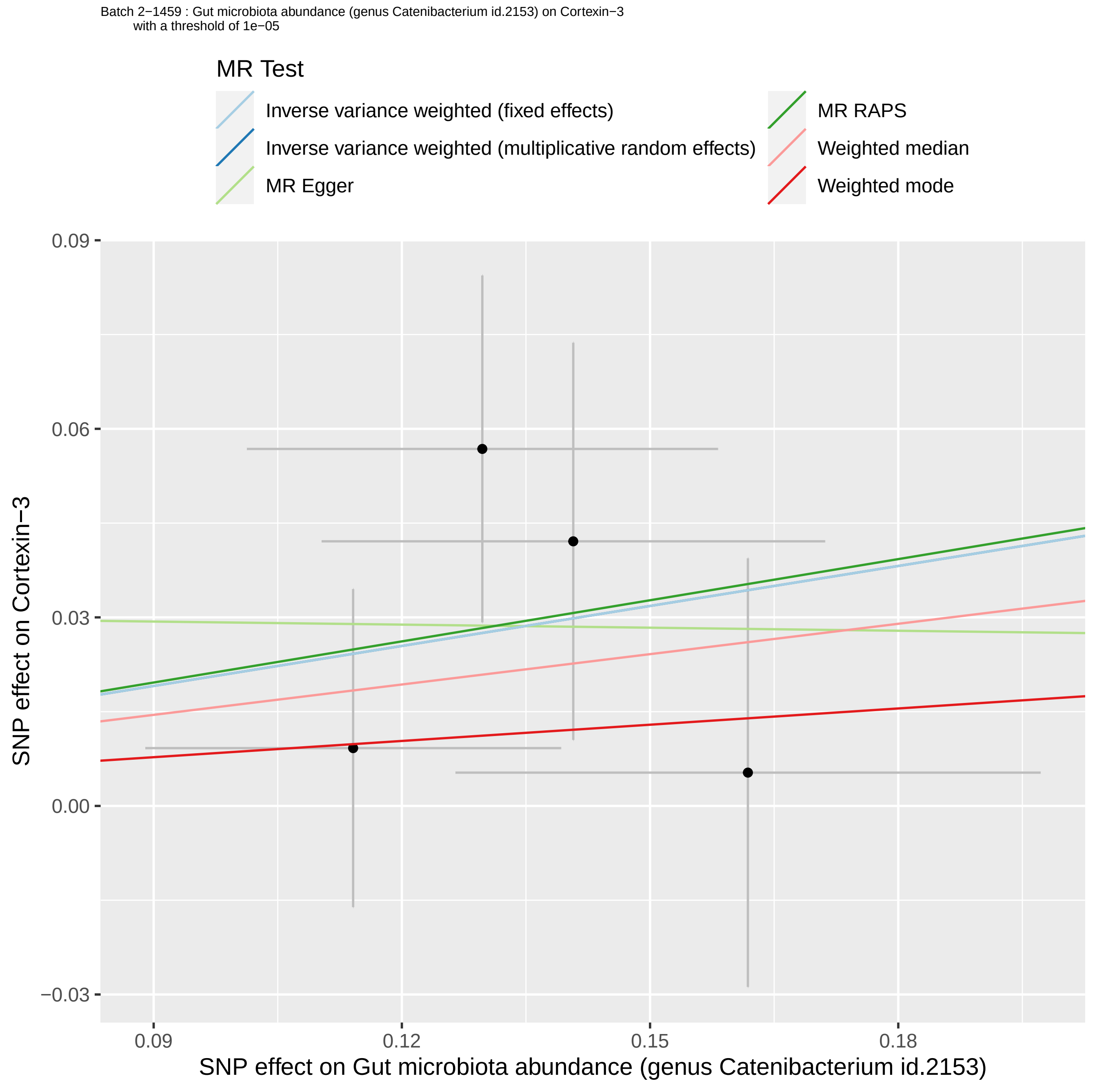
Batch 2−1459 : Gut microbiota abundance (genus Catenibacterium id.2153) on Cortexin−3
with a threshold of 1e−05
MR Test
Inverse variance weighted (fixed effects)
Inverse variance weighted (multiplicative random effects)
MR Egger
MR RAPS
Weighted median
Weighted mode
0.09
0.06
0.03
0.00
−0.03
0.09
0.12
0.15
0.18
SNP effect on Cortexin−3
SNP effect on Gut microbiota abundance (genus Catenibacterium id.2153)

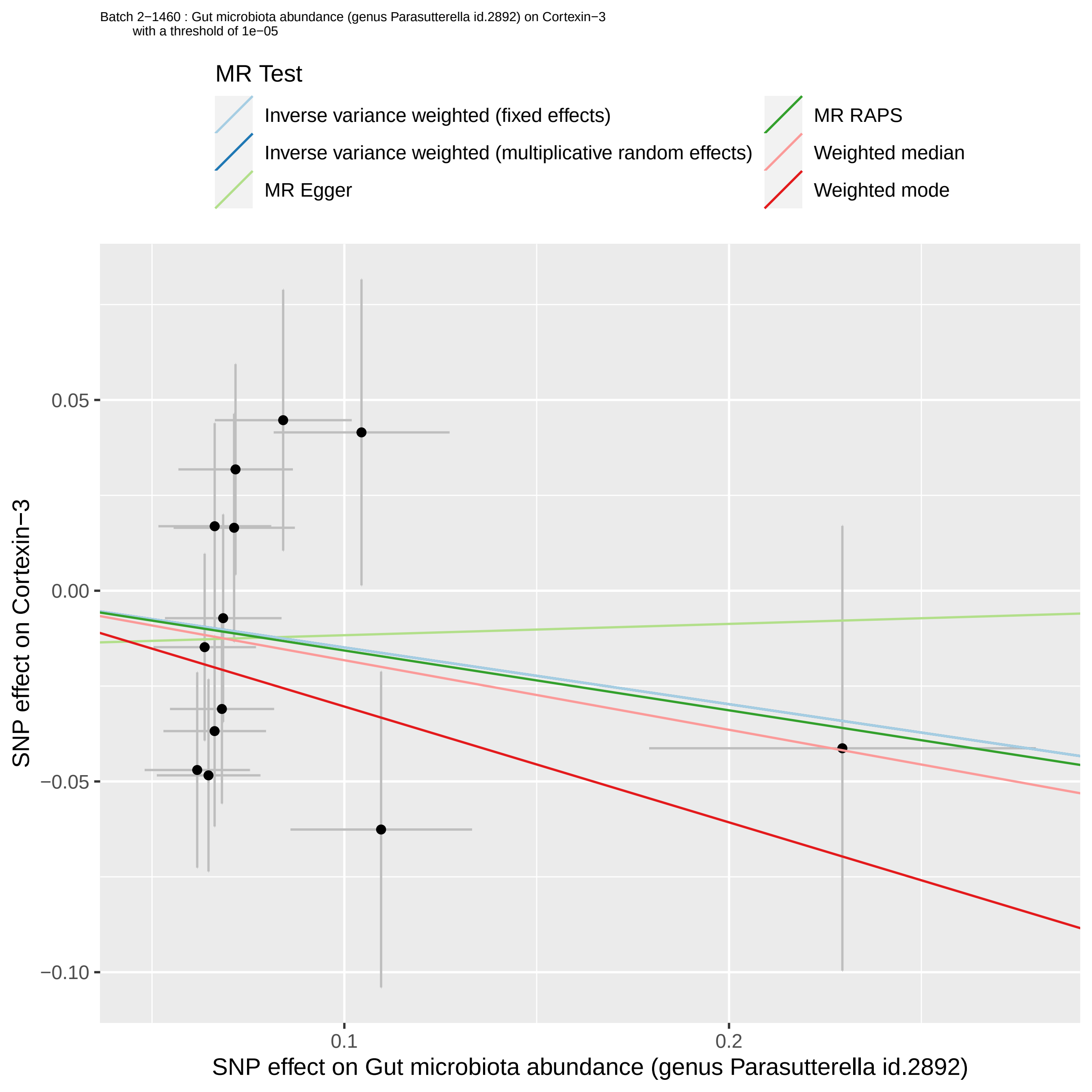

Batch 2−1460 : Gut microbiota abundance (genus Parasutterella id.2892) on Cortexin−3
with a threshold of 1e−05
MR Test
Inverse variance weighted (fixed effects)
Inverse variance weighted (multiplicative random effects)
MR Egger
MR RAPS
Weighted median
Weighted mode
0.05
0.00
−0.05
−0.10
0.1
0.2
SNP effect on Cortexin−3
SNP effect on Gut microbiota abundance (genus Parasutterella id.2892)

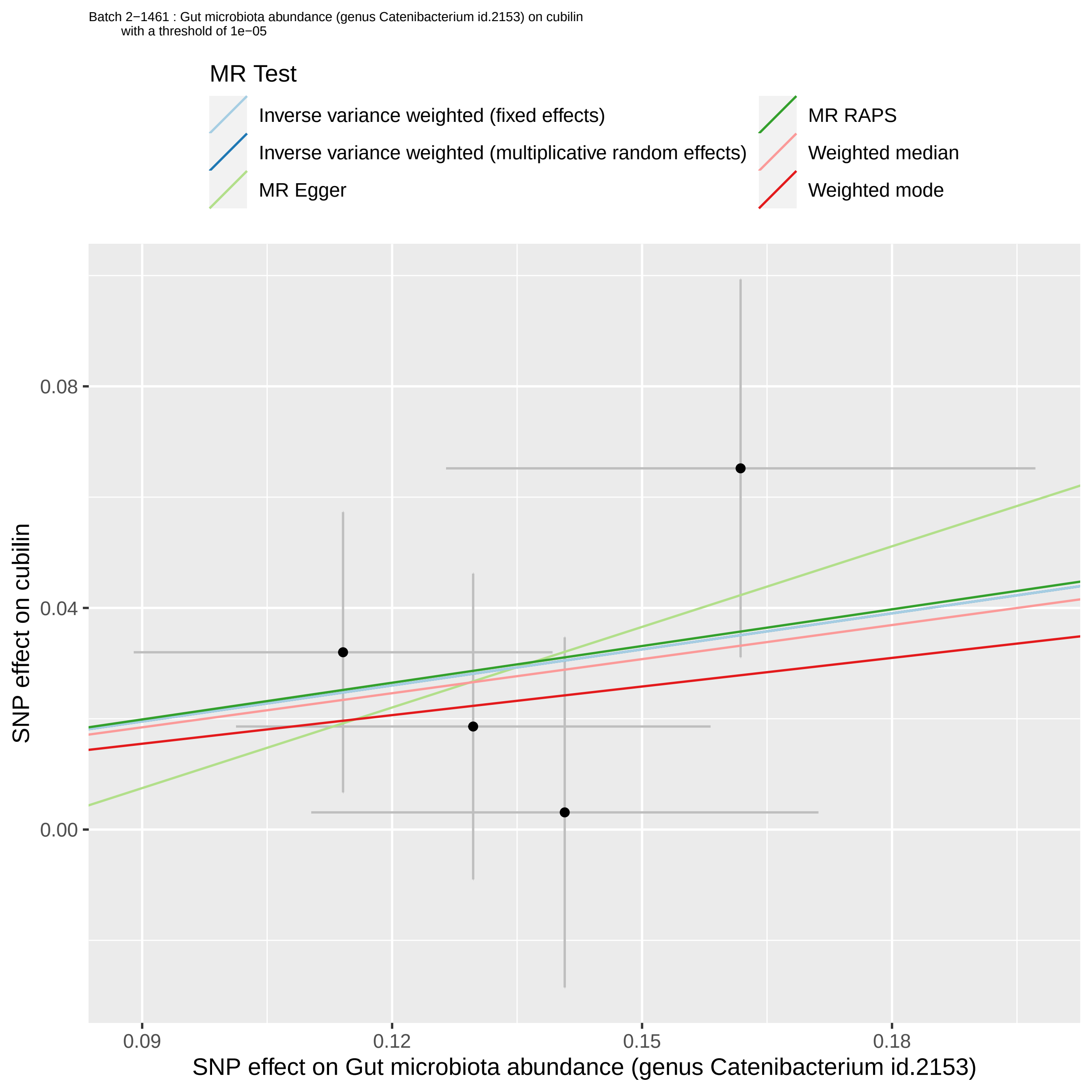
Batch 2−1461 : Gut microbiota abundance (genus Catenibacterium id.2153) on cubilin
with a threshold of 1e−05
MR Test
Inverse variance weighted (fixed effects)
Inverse variance weighted (multiplicative random effects)
MR Egger
MR RAPS
Weighted median
Weighted mode
0.08
0.04
0.00
SNP effect on cubilin
0.09
0.12
0.15
0.18
SNP effect on Gut microbiota abundance (genus Catenibacterium id.2153)

Batch 2−1462 : Gut microbiota abundance (genus Parasutterella id.2892) on cubilin
with a threshold of 1e−05

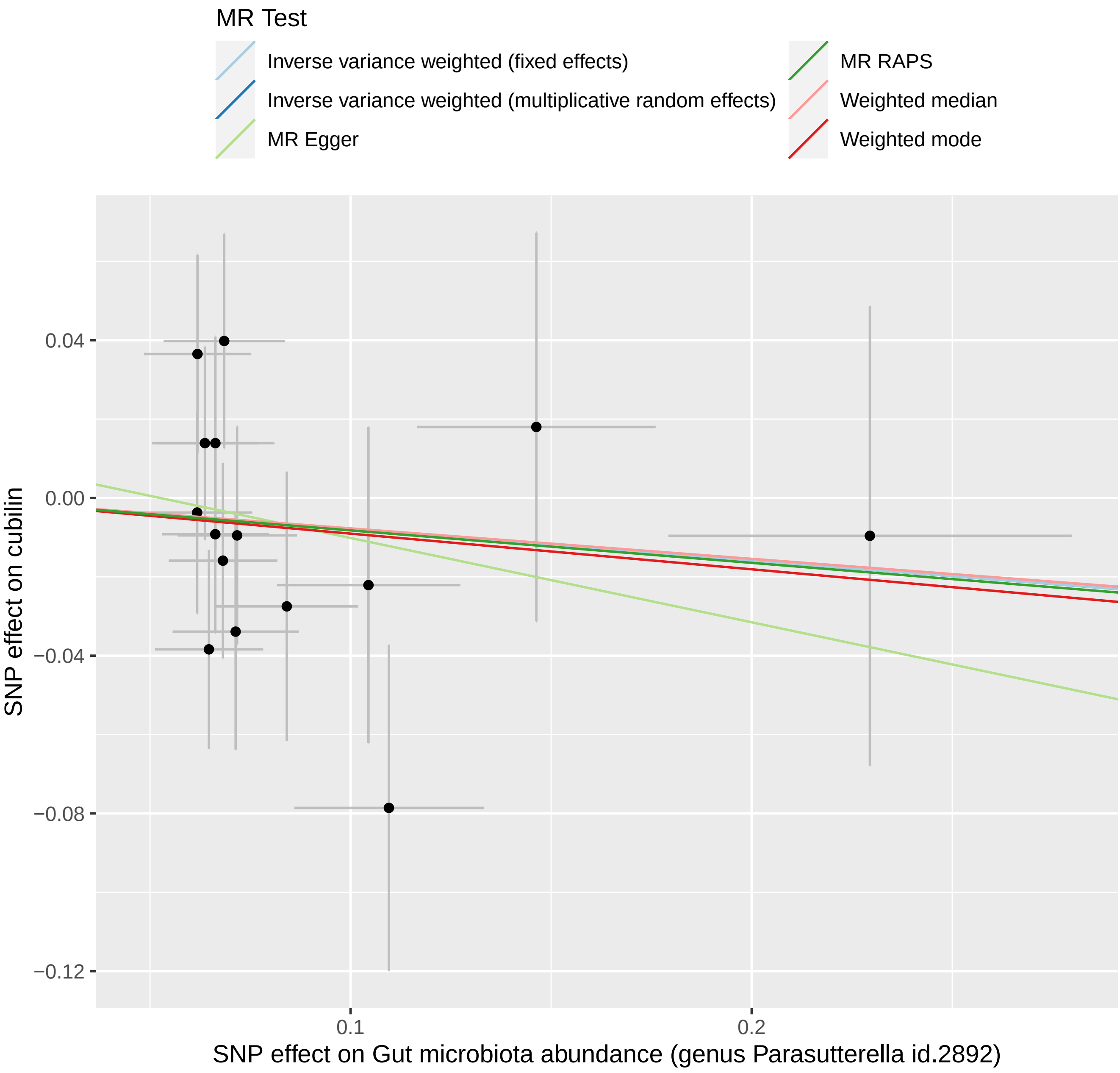

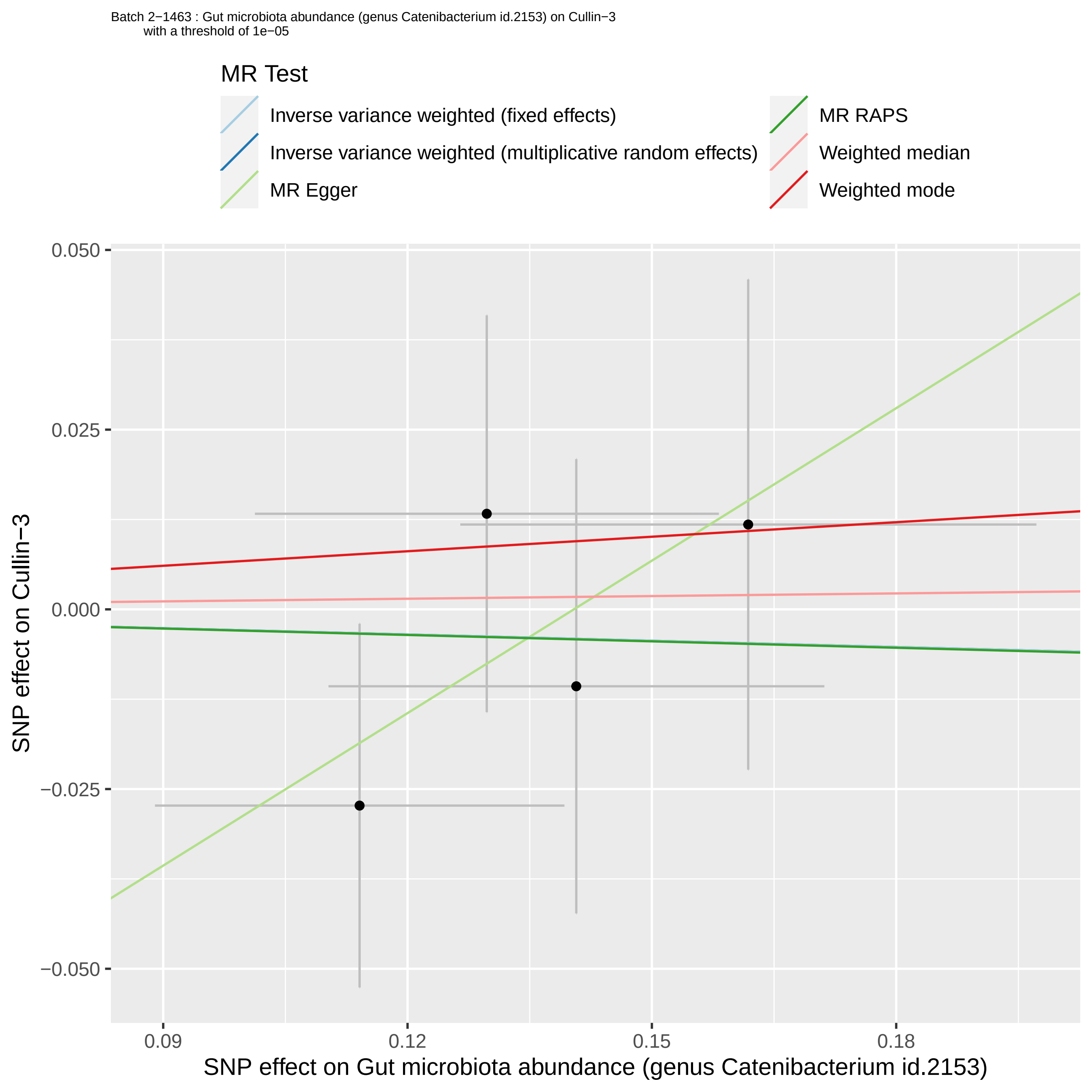
Batch 2−1463 : Gut microbiota abundance (genus Catenibacterium id.2153) on Cullin−3
with a threshold of 1e−05
MR Test
Inverse variance weighted (fixed effects)
Inverse variance weighted (multiplicative random effects)
MR Egger
MR RAPS
Weighted median
Weighted mode
0.050
0.025
0.000
−0.025
−0.050
0.09
0.12
0.15
0.18
SNP effect on Cullin−3
SNP effect on Gut microbiota abundance (genus Catenibacterium id.2153)

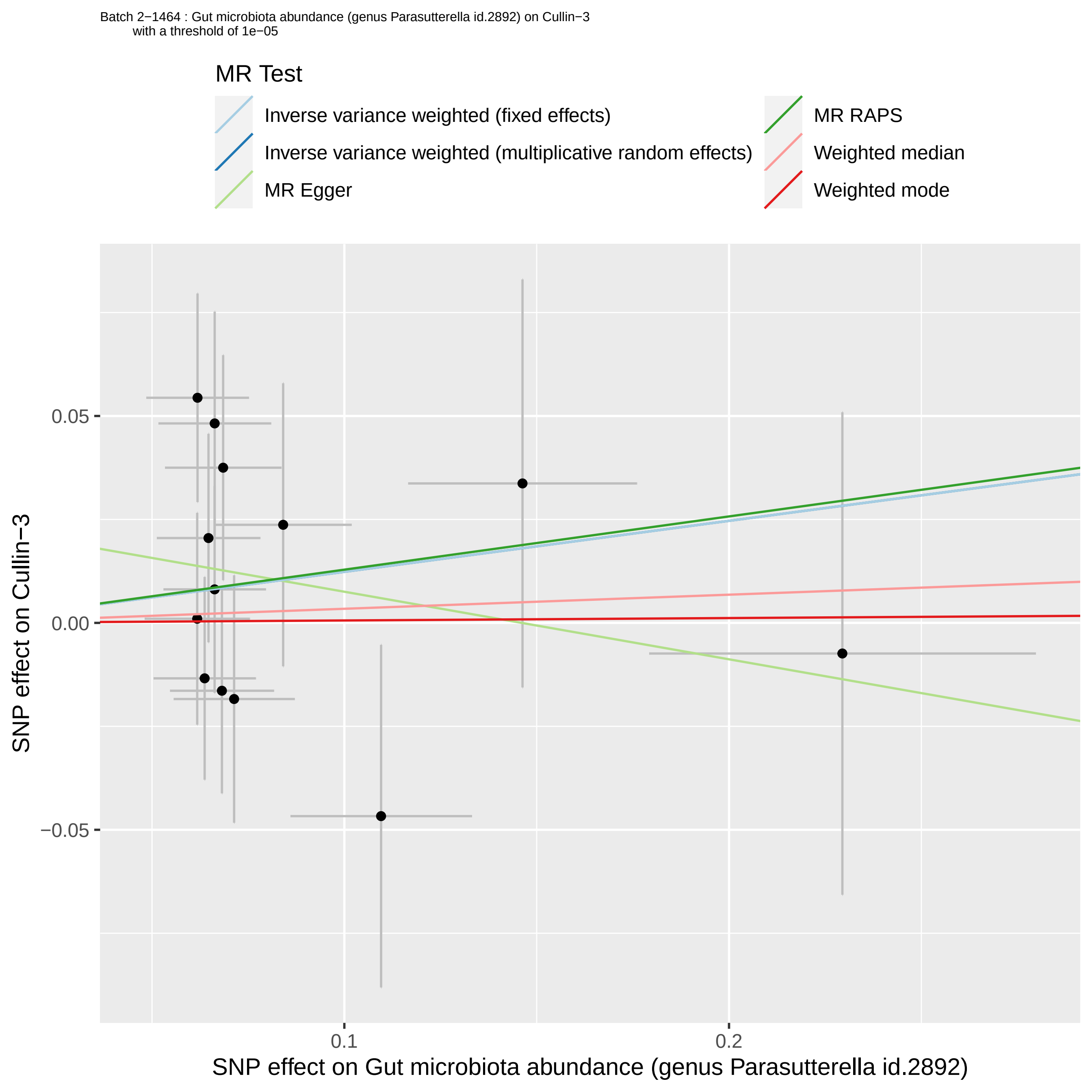
Batch 2−1464 : Gut microbiota abundance (genus Parasutterella id.2892) on Cullin−3
with a threshold of 1e−05
MR Test
Inverse variance weighted (fixed effects)
Inverse variance weighted (multiplicative random effects)
MR Egger
MR RAPS
Weighted median
Weighted mode
0.05
0.00
−0.05
SNP effect on Cullin−3
0.1
0.2
SNP effect on Gut microbiota abundance (genus Parasutterella id.2892)

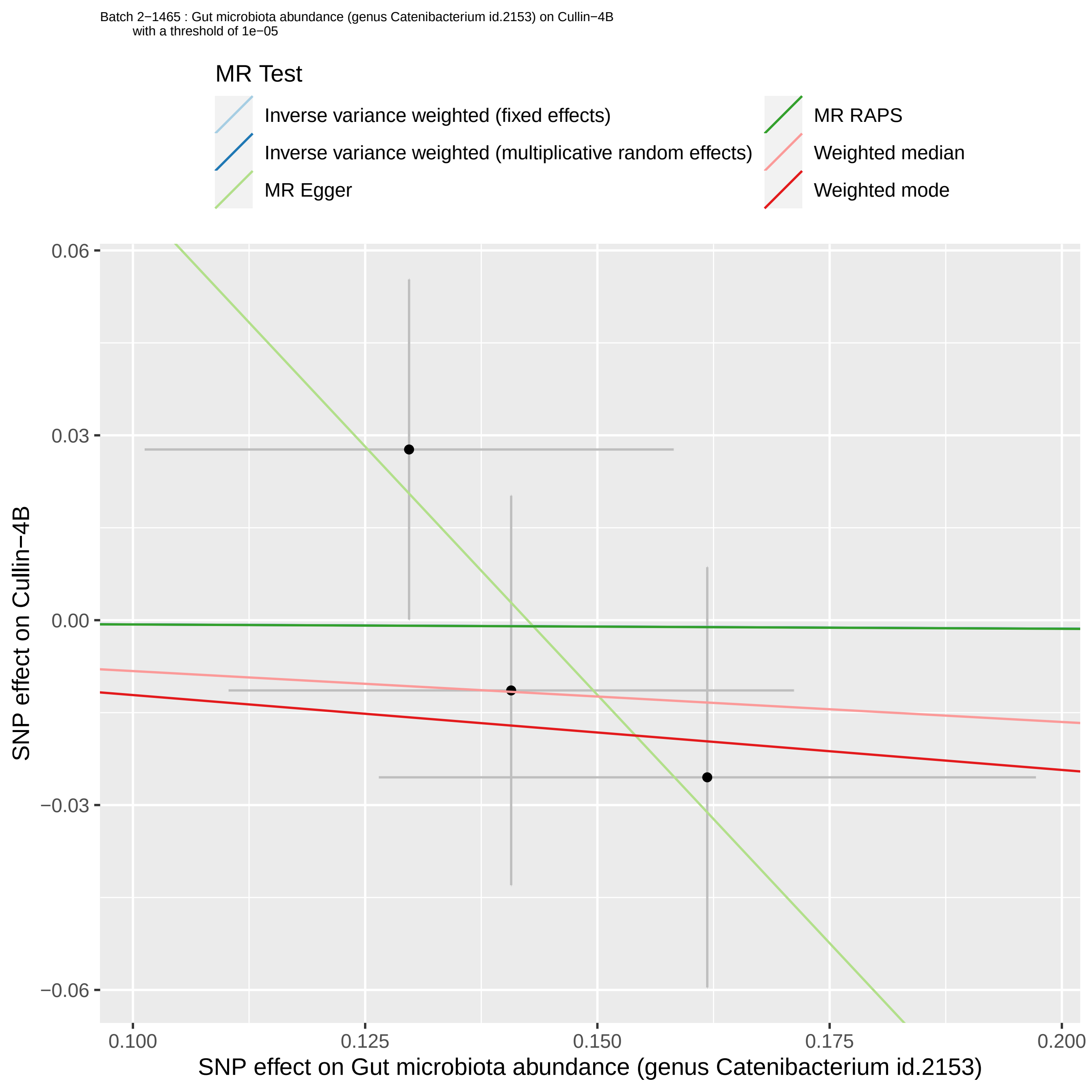
Batch 2−1465 : Gut microbiota abundance (genus Catenibacterium id.2153) on Cullin−4B
with a threshold of 1e−05
MR Test
Inverse variance weighted (fixed effects)
Inverse variance weighted (multiplicative random effects)
MR Egger
MR RAPS
Weighted median
Weighted mode
0.06
0.03
0.00
−0.03
−0.06
0.100
0.125
0.150
0.175
0.200
SNP effect on Cullin−4B
SNP effect on Gut microbiota abundance (genus Catenibacterium id.2153)

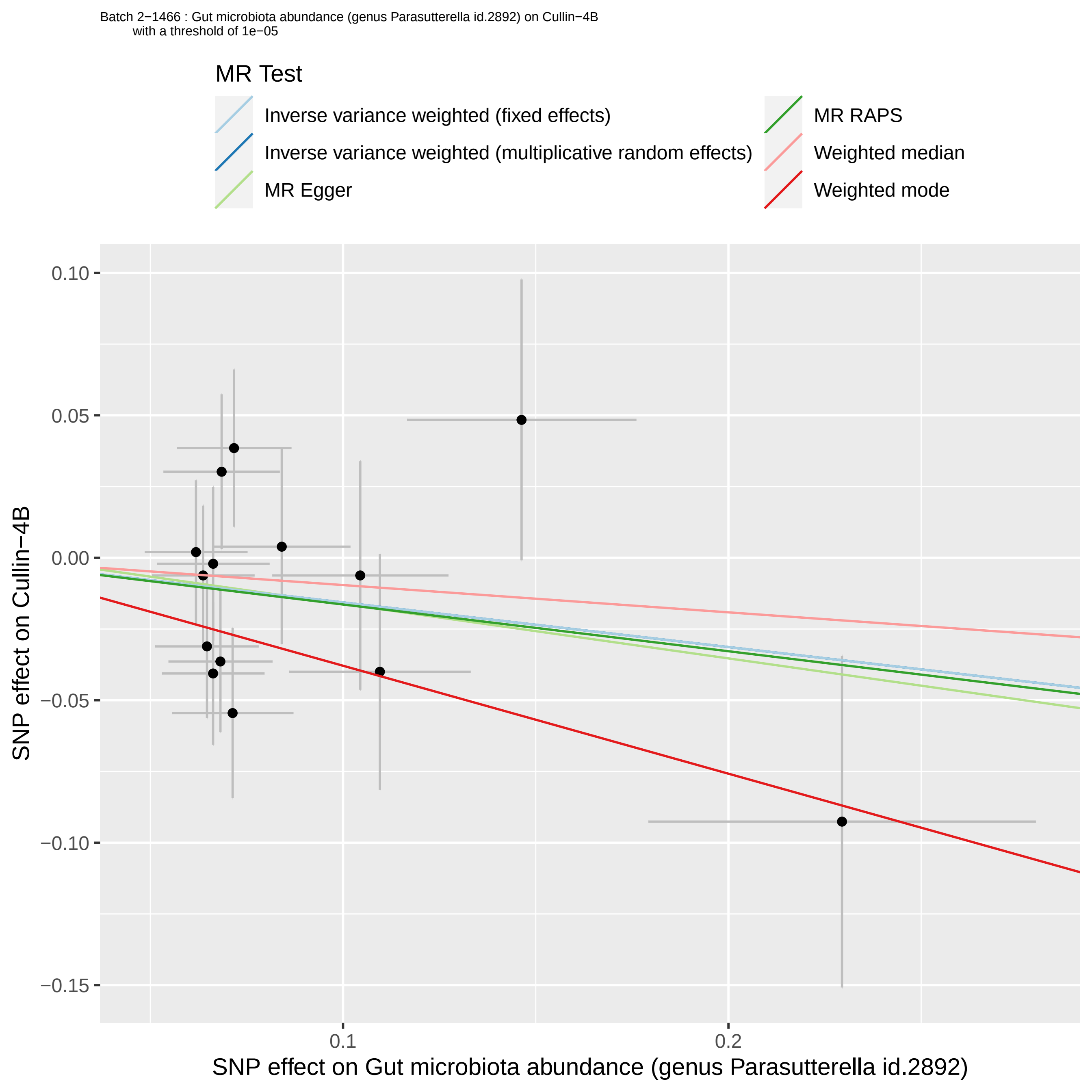
Batch 2−1466 : Gut microbiota abundance (genus Parasutterella id.2892) on Cullin−4B
with a threshold of 1e−05
MR Test
Inverse variance weighted (fixed effects)
Inverse variance weighted (multiplicative random effects)
MR Egger
MR RAPS
Weighted median
Weighted mode
0.10
0.05
0.00
−0.05
−0.10
−0.15
0.1
0.2
SNP effect on Cullin−4B
SNP effect on Gut microbiota abundance (genus Parasutterella id.2892)

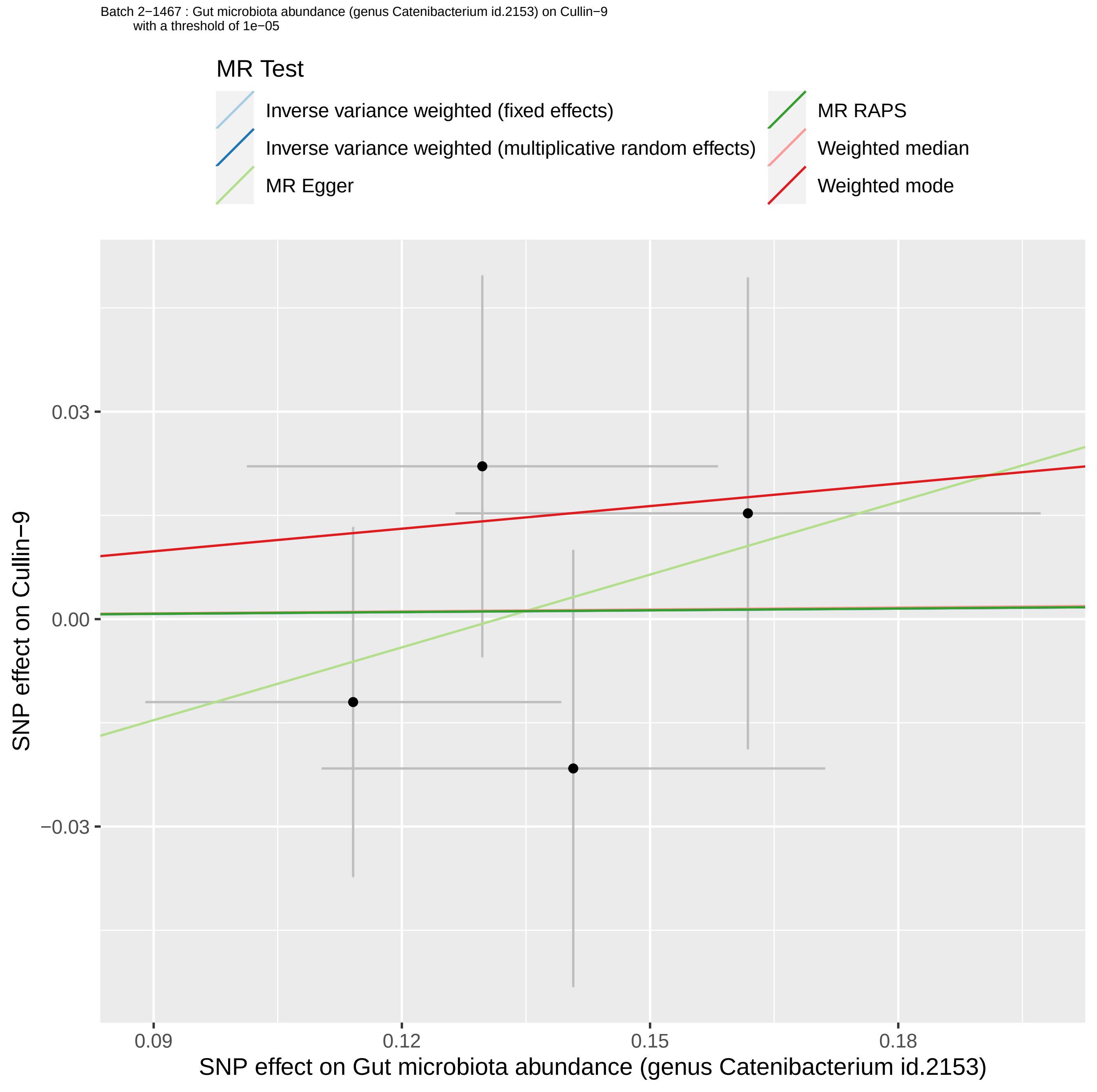

Batch 2−1467 : Gut microbiota abundance (genus Catenibacterium id.2153) on Cullin−9
with a threshold of 1e−05
MR Test
Inverse variance weighted (fixed effects)
Inverse variance weighted (multiplicative random effects)
MR Egger
MR RAPS
Weighted median
Weighted mode
0.03
0.00
−0.03
0.09
0.12
0.15
0.18
SNP effect on Cullin−9
SNP effect on Gut microbiota abundance (genus Catenibacterium id.2153)

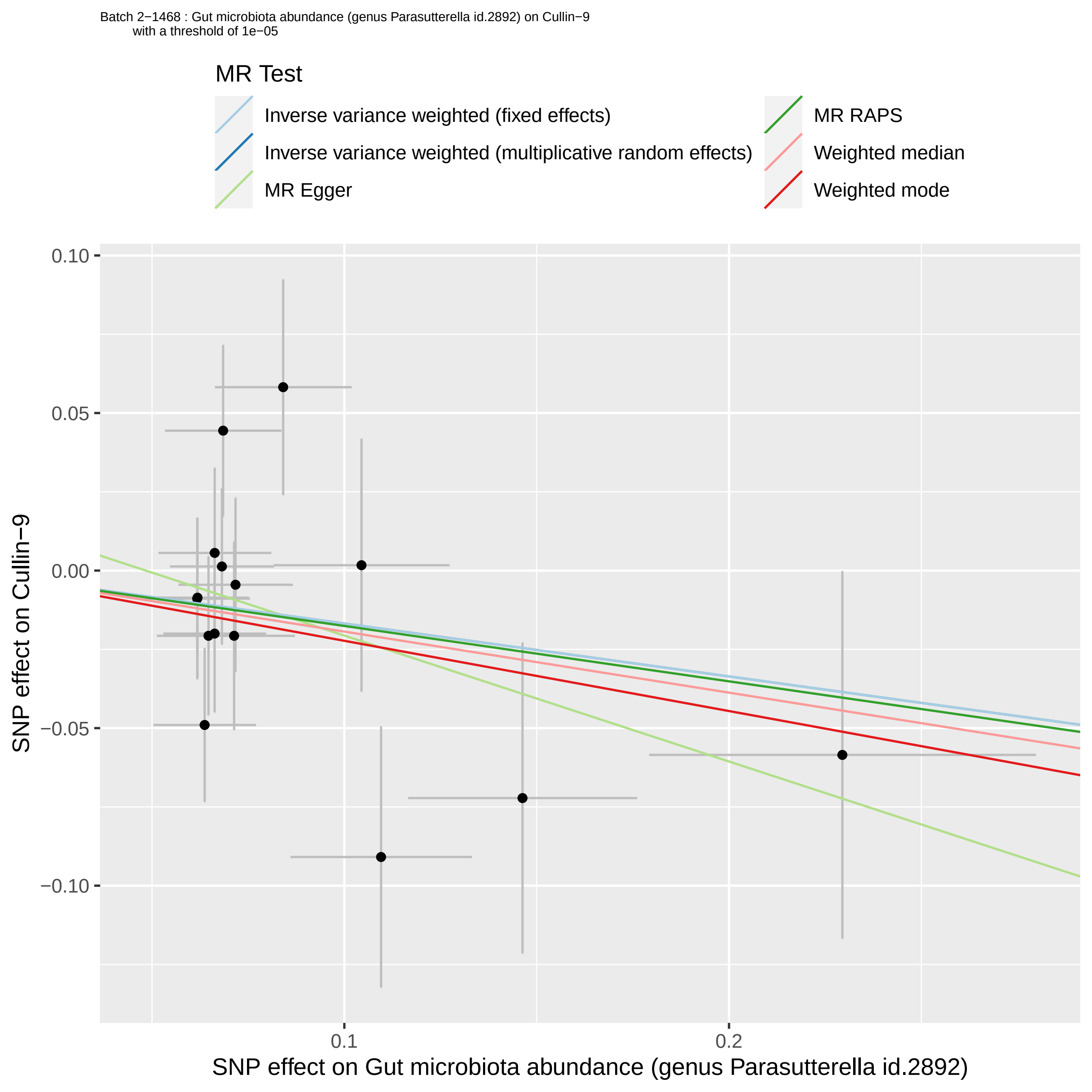
Batch 2−1468 : Gut microbiota abundance (genus Parasutterella id.2892) on Cullin−9
with a threshold of 1e−05
MR Test
Inverse variance weighted (fixed effects)
Inverse variance weighted (multiplicative random effects)
MR Egger
MR RAPS
Weighted median
Weighted mode
0.10
0.05
0.00
−0.05
−0.10
SNP effect on Cullin−9
0.1
0.2
SNP effect on Gut microbiota abundance (genus Parasutterella id.2892)

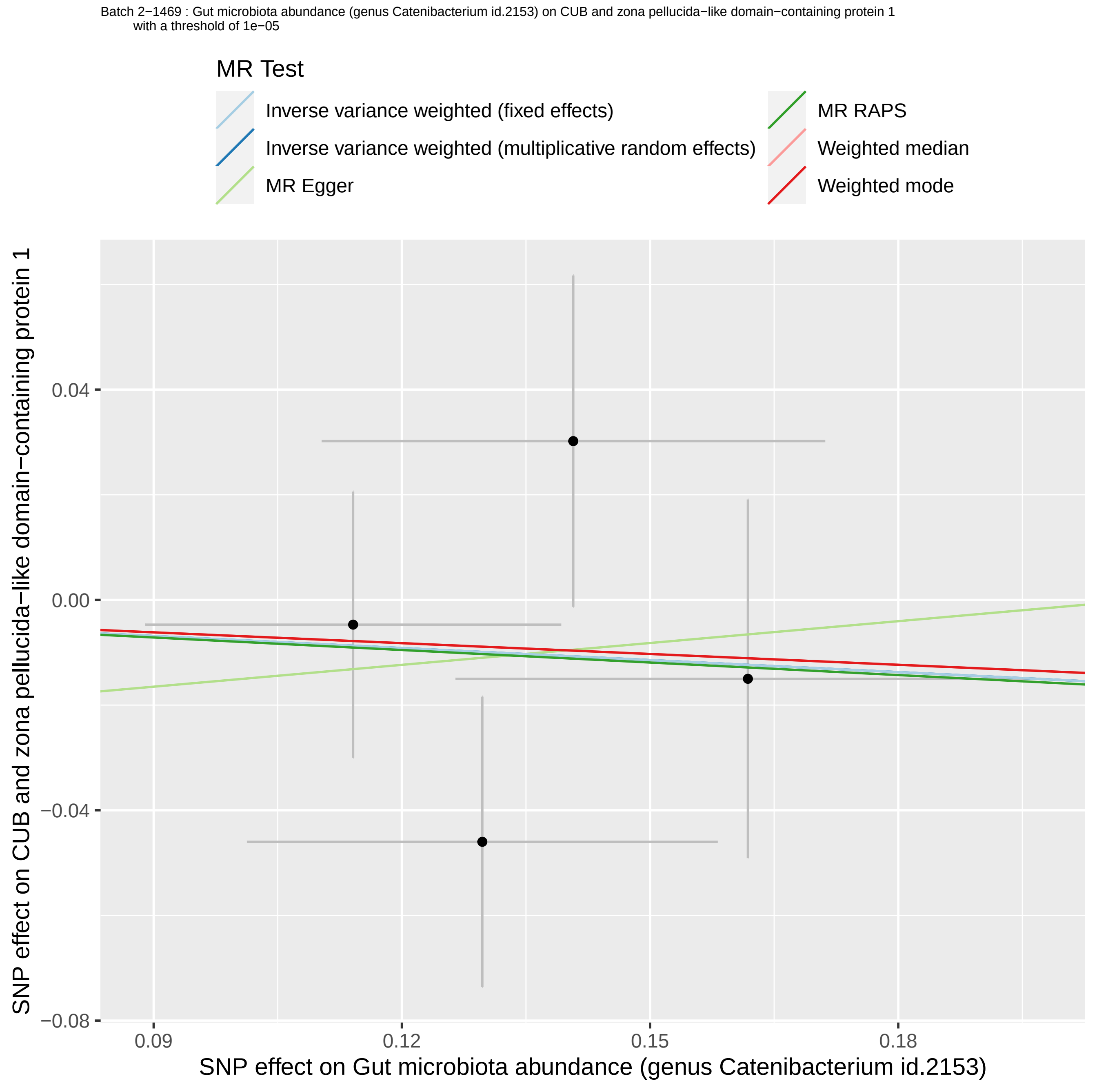

Batch 2−1469 : Gut microbiota abundance (genus Catenibacterium id.2153) on CUB and zona pellucida−like domain−containing protein 1 with a threshold of 1e−05
MR Test
Inverse variance weighted (fixed effects)
Inverse variance weighted (multiplicative random effects)
MR Egger
MR RAPS
Weighted median
Weighted mode
SNP effect on CUB and zona pellucida−like domain−containing protein 1
0.04
0.00
−0.04
−0.08
0.09
0.12
0.15
0.18
SNP effect on Gut microbiota abundance (genus Catenibacterium id.2153)

Batch 2−1470 : Gut microbiota abundance (genus Parasutterella id.2892) on CUB and zona pellucida−like domain−containing protein 1 with a threshold of 1e−05

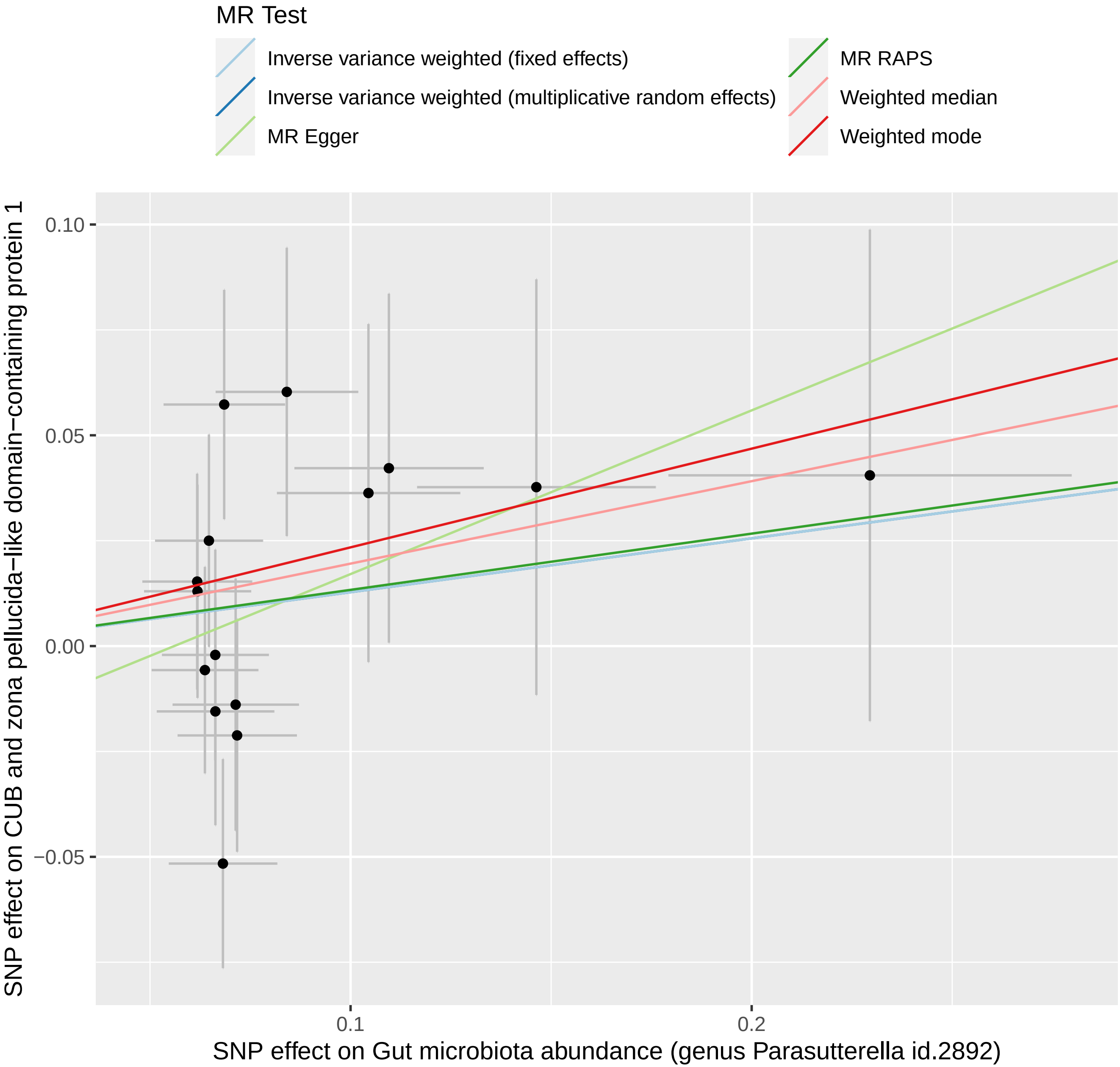

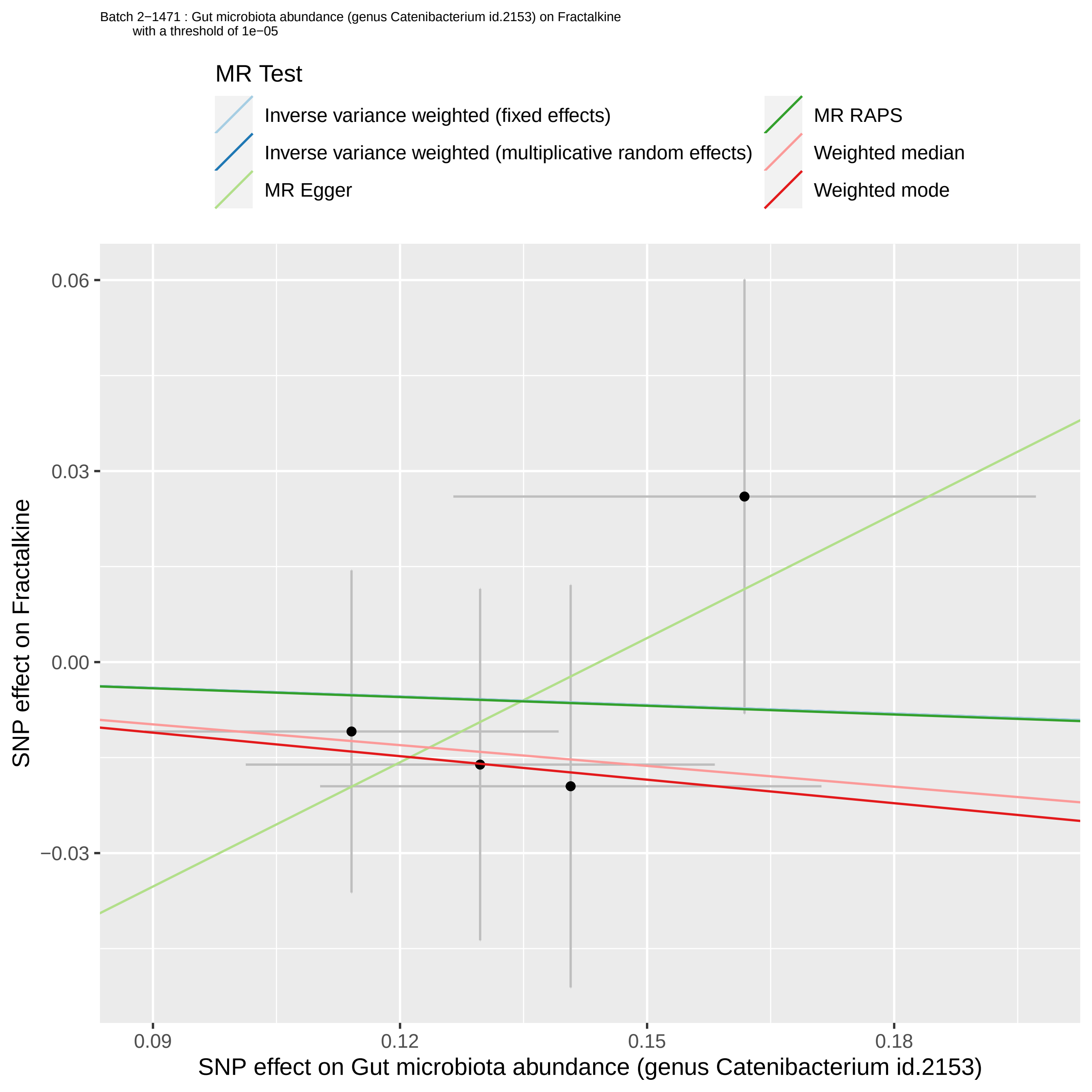
Batch 2−1471 : Gut microbiota abundance (genus Catenibacterium id.2153) on Fractalkine
with a threshold of 1e−05
MR Test
Inverse variance weighted (fixed effects)
Inverse variance weighted (multiplicative random effects)
MR Egger
MR RAPS
Weighted median
Weighted mode
0.06
0.03
0.00
−0.03
0.09
0.12
0.15
0.18
SNP effect on Fractalkine
SNP effect on Gut microbiota abundance (genus Catenibacterium id.2153)

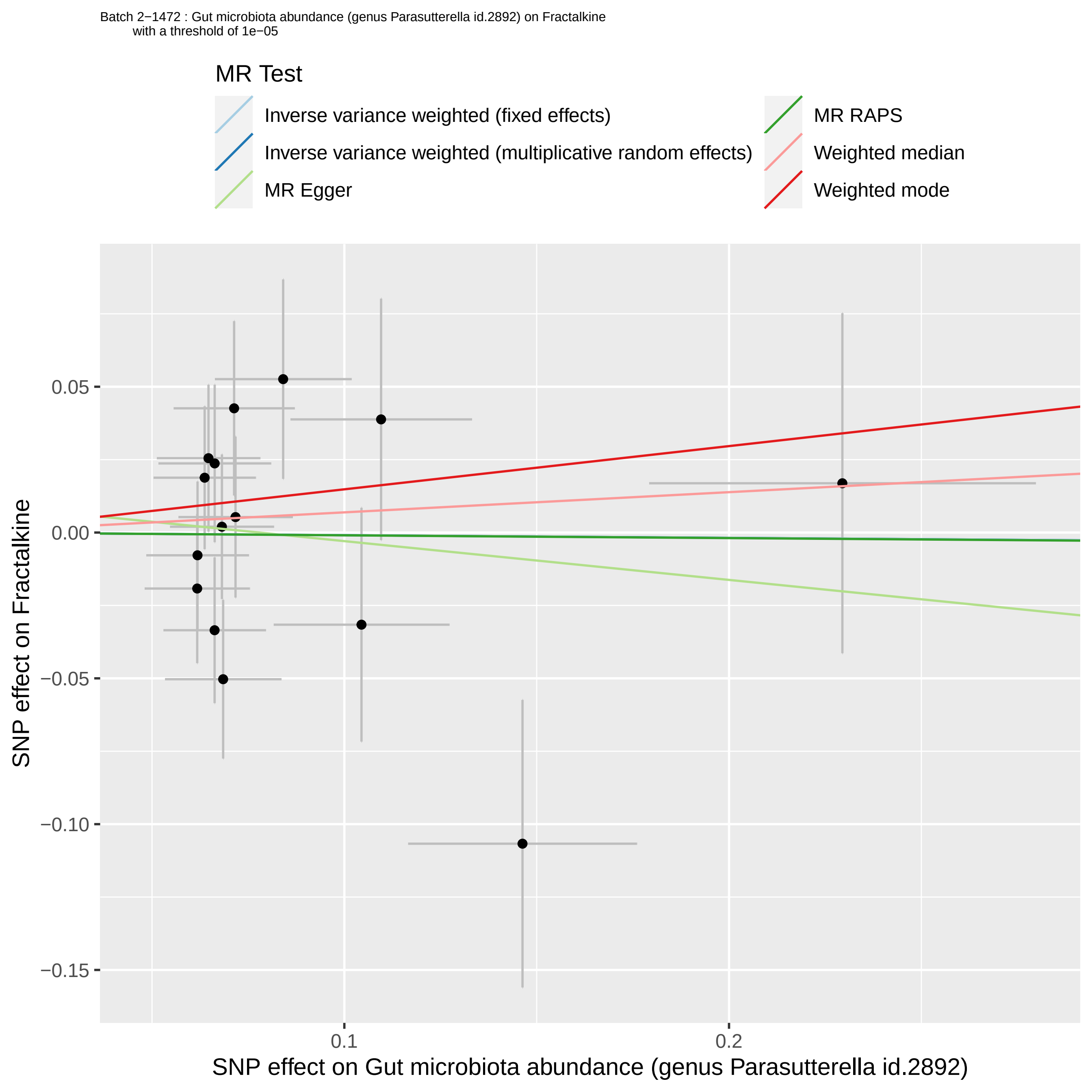
Batch 2−1472 : Gut microbiota abundance (genus Parasutterella id.2892) on Fractalkine
with a threshold of 1e−05
MR Test
Inverse variance weighted (fixed effects)
Inverse variance weighted (multiplicative random effects)
MR Egger
MR RAPS
Weighted median
Weighted mode
SNP effect on Fractalkine
0.05
0.00
−0.05
−0.10
−0.15
0.1
0.2
SNP effect on Gut microbiota abundance (genus Parasutterella id.2892)

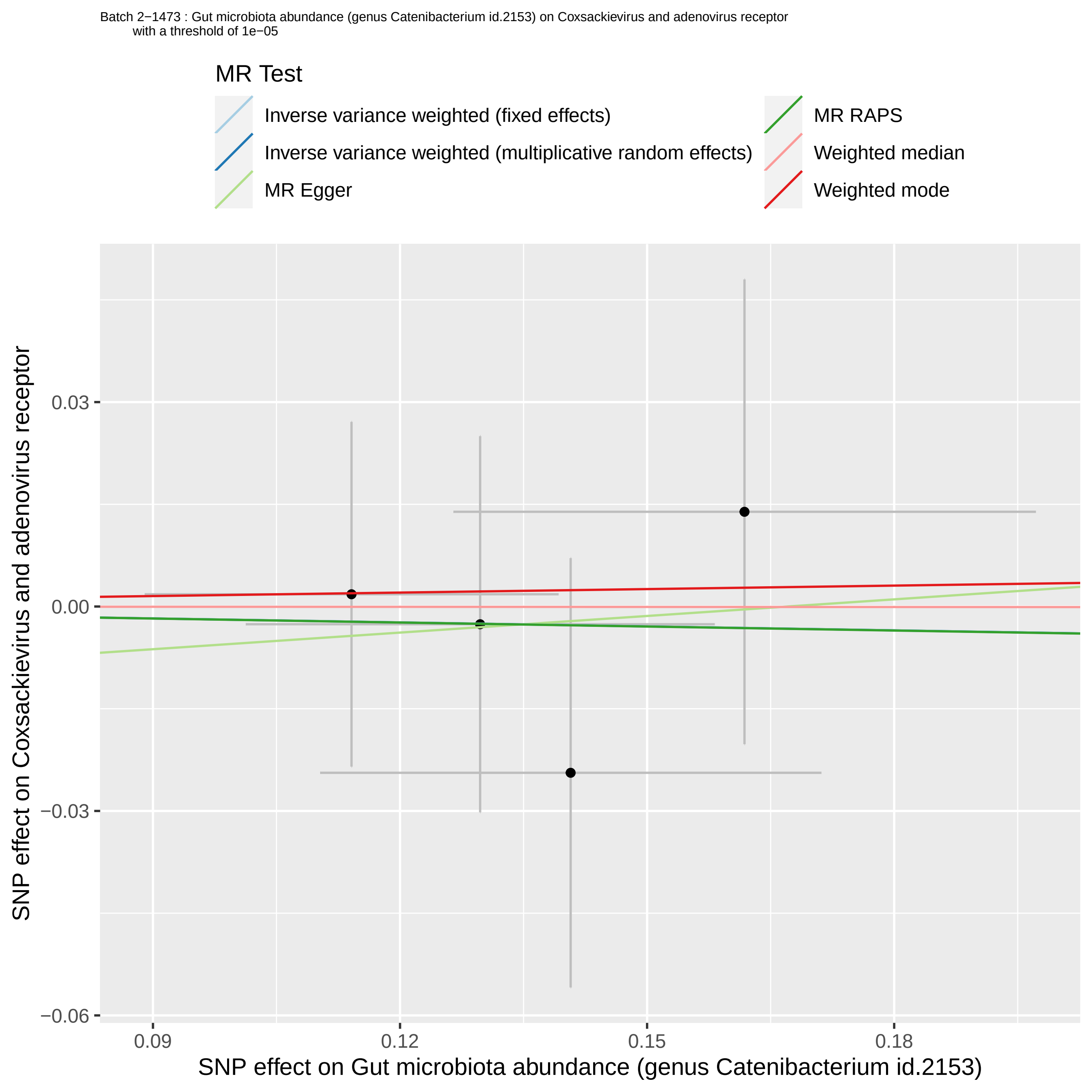

Batch 2−1473 : Gut microbiota abundance (genus Catenibacterium id.2153) on Coxsackievirus and adenovirus receptor
with a threshold of 1e−05
MR Test
Inverse variance weighted (fixed effects)
Inverse variance weighted (multiplicative random effects)
MR Egger
MR RAPS
Weighted median
Weighted mode
SNP effect on Coxsackievirus and adenovirus receptor
0.03
0.00
−0.03
−0.06
0.09
0.12
0.15
0.18
SNP effect on Gut microbiota abundance (genus Catenibacterium id.2153)

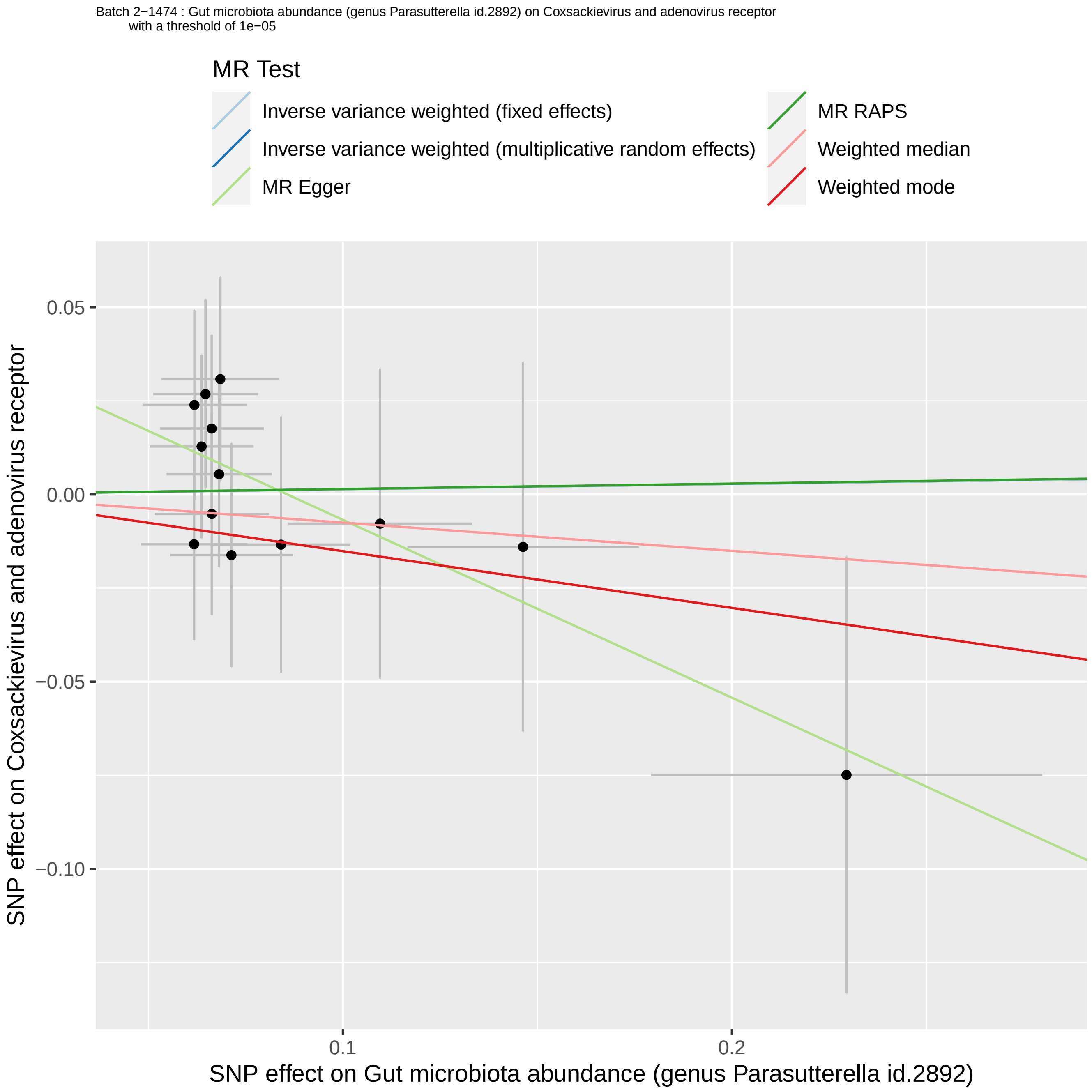
Batch 2−1474 : Gut microbiota abundance (genus Parasutterella id.2892) on Coxsackievirus and adenovirus receptor
with a threshold of 1e−05
MR Test
Inverse variance weighted (fixed effects)
Inverse variance weighted (multiplicative random effects)
MR Egger
MR RAPS
Weighted median
Weighted mode
0.05
0.00
−0.05
−0.10
0.1
0.2
SNP effect on Coxsackievirus and adenovirus receptor
SNP effect on Gut microbiota abundance (genus Parasutterella id.2892)

Batch 2−1475 : Gut microbiota abundance (genus Catenibacterium id.2153) on C−X−C motif chemokine 10
with a threshold of 1e−05

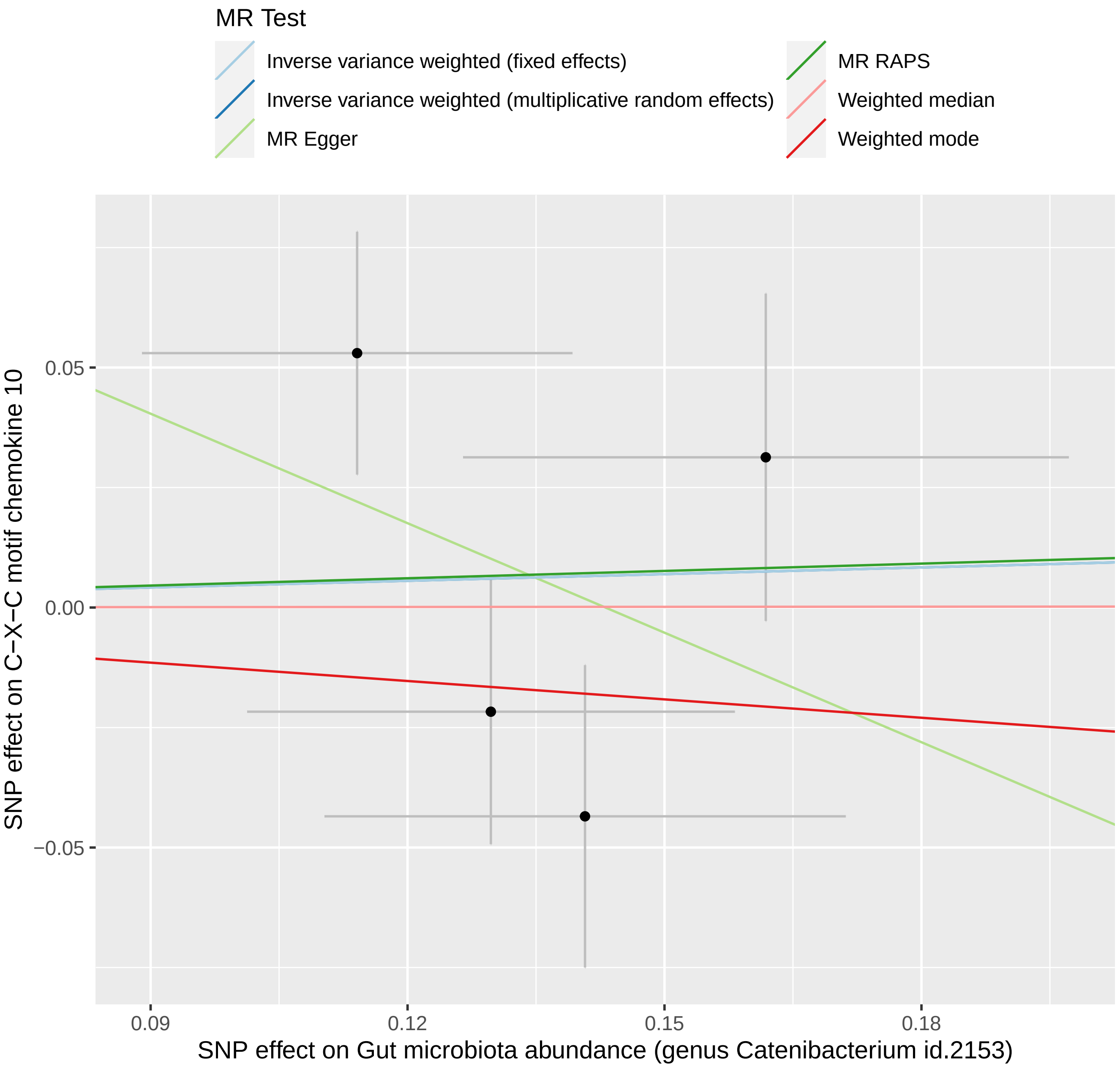

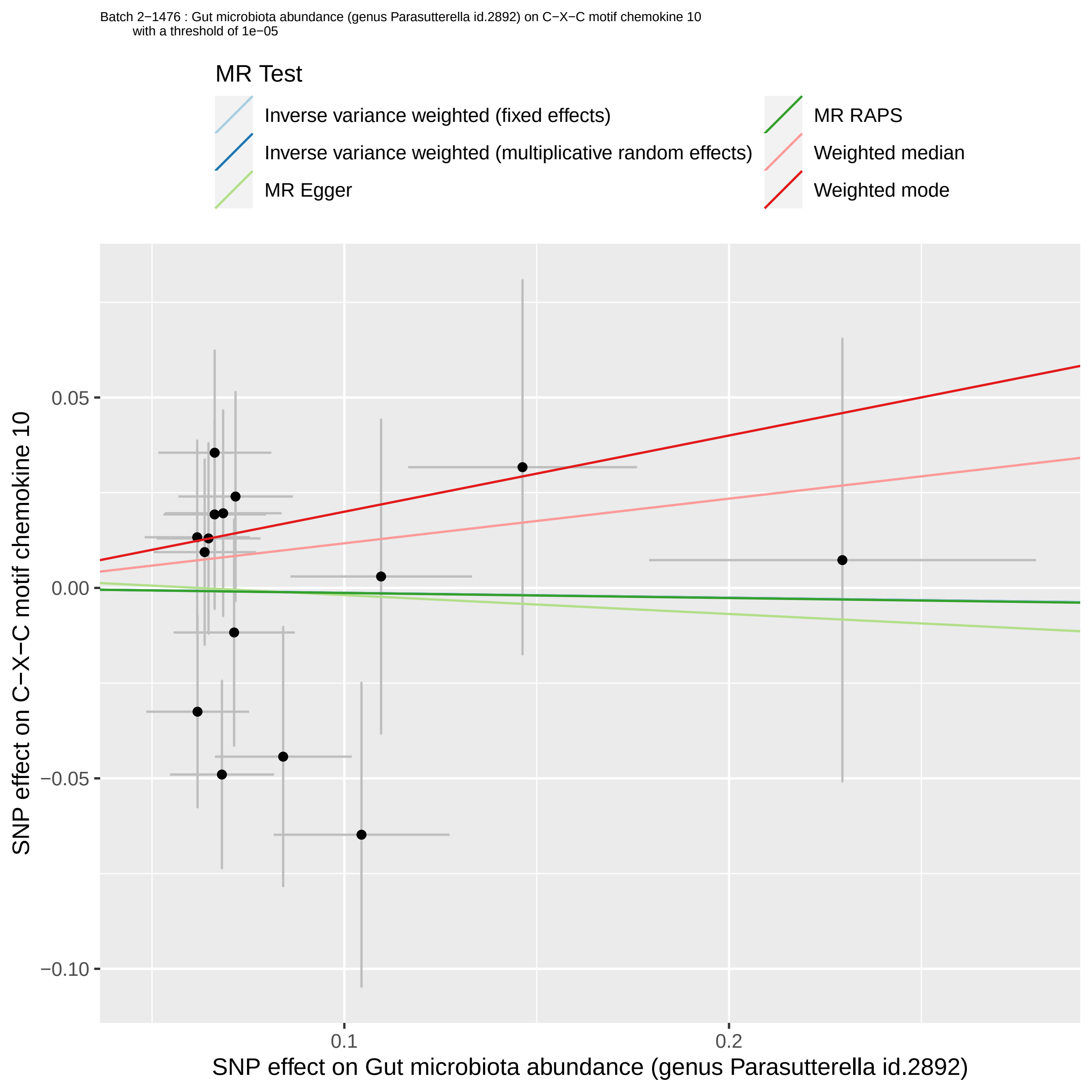

Batch 2−1476 : Gut microbiota abundance (genus Parasutterella id.2892) on C−X−C motif chemokine 10
with a threshold of 1e−05
MR Test
Inverse variance weighted (fixed effects)
Inverse variance weighted (multiplicative random effects)
MR Egger
MR RAPS
Weighted median
Weighted mode
SNP effect on C−X−C motif chemokine 10
0.05
0.00
−0.05
−0.10
0.1
0.2
SNP effect on Gut microbiota abundance (genus Parasutterella id.2892)

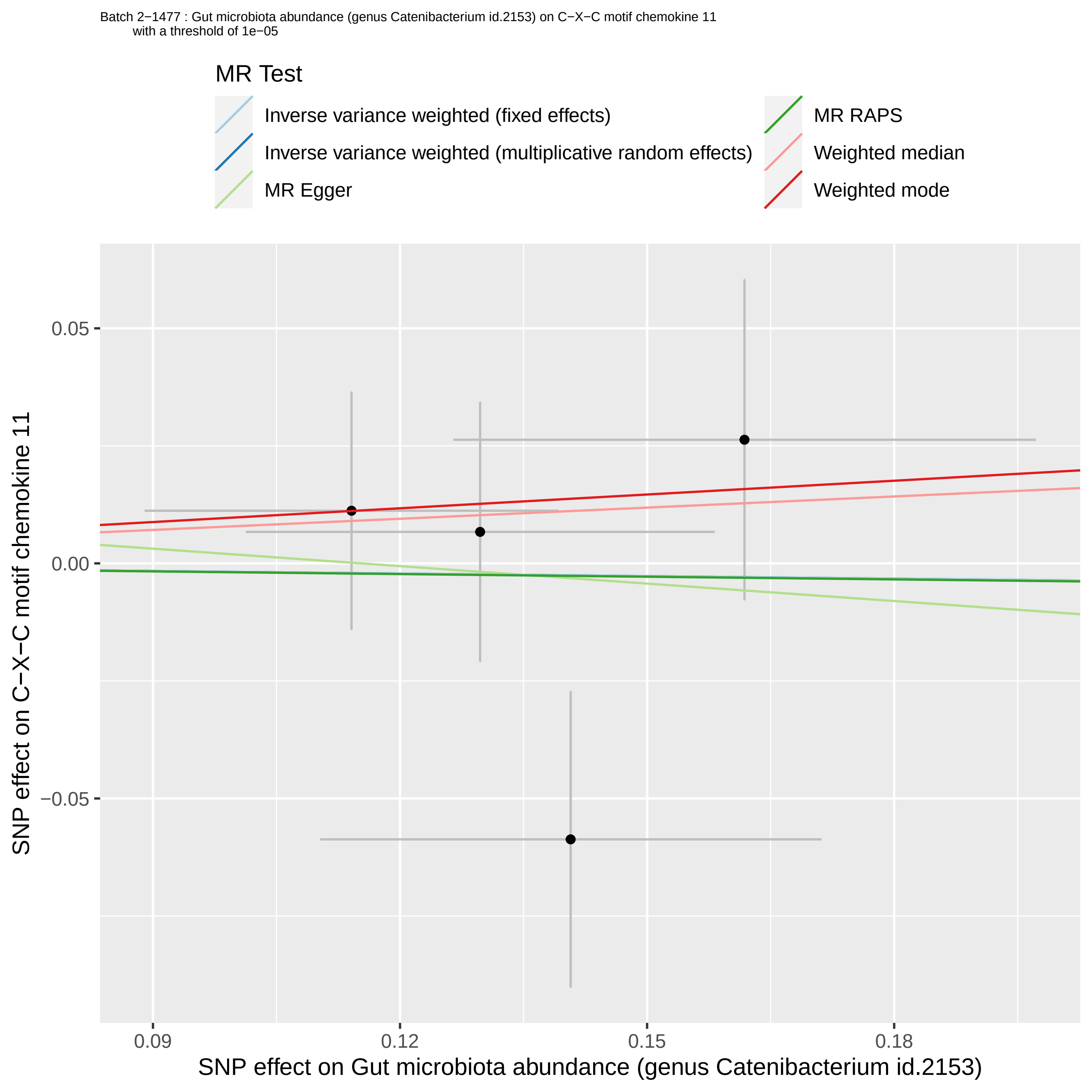

Batch 2−1477 : Gut microbiota abundance (genus Catenibacterium id.2153) on C−X−C motif chemokine 11
with a threshold of 1e−05
MR Test
Inverse variance weighted (fixed effects)
Inverse variance weighted (multiplicative random effects)
MR Egger
MR RAPS
Weighted median
Weighted mode
0.05
0.00
−0.05
0.09
0.12
0.15
0.18
SNP effect on C−X−C motif chemokine 11
SNP effect on Gut microbiota abundance (genus Catenibacterium id.2153)

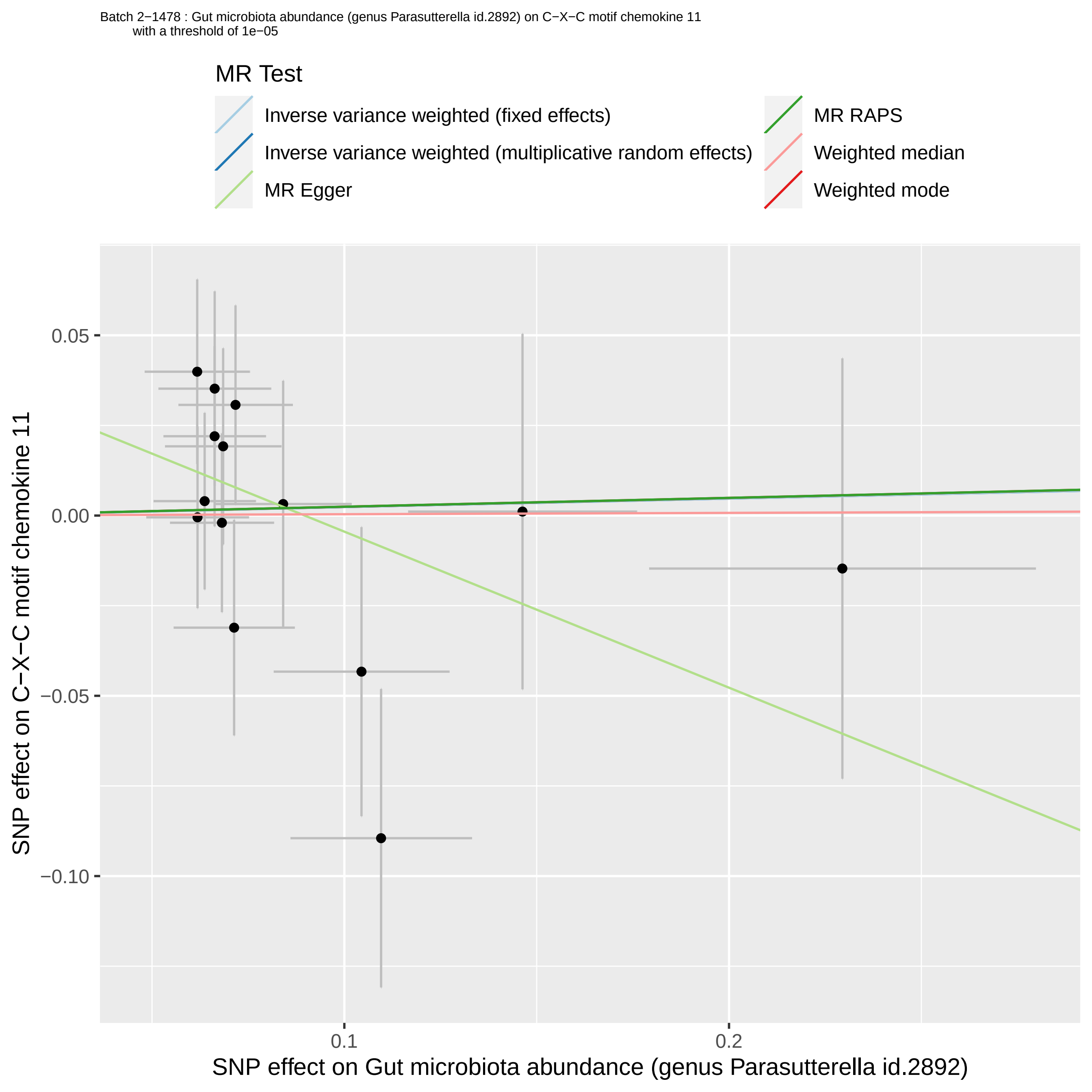
Batch 2−1478 : Gut microbiota abundance (genus Parasutterella id.2892) on C−X−C motif chemokine 11
with a threshold of 1e−05
MR Test
Inverse variance weighted (fixed effects)
Inverse variance weighted (multiplicative random effects)
MR Egger
MR RAPS
Weighted median
Weighted mode
0.05
0.00
−0.05
−0.10
0.1
0.2
SNP effect on C−X−C motif chemokine 11
SNP effect on Gut microbiota abundance (genus Parasutterella id.2892)

Batch 2−1479 : Gut microbiota abundance (genus Catenibacterium id.2153) on Stromal cell−derived factor 1
with a threshold of 1e−05

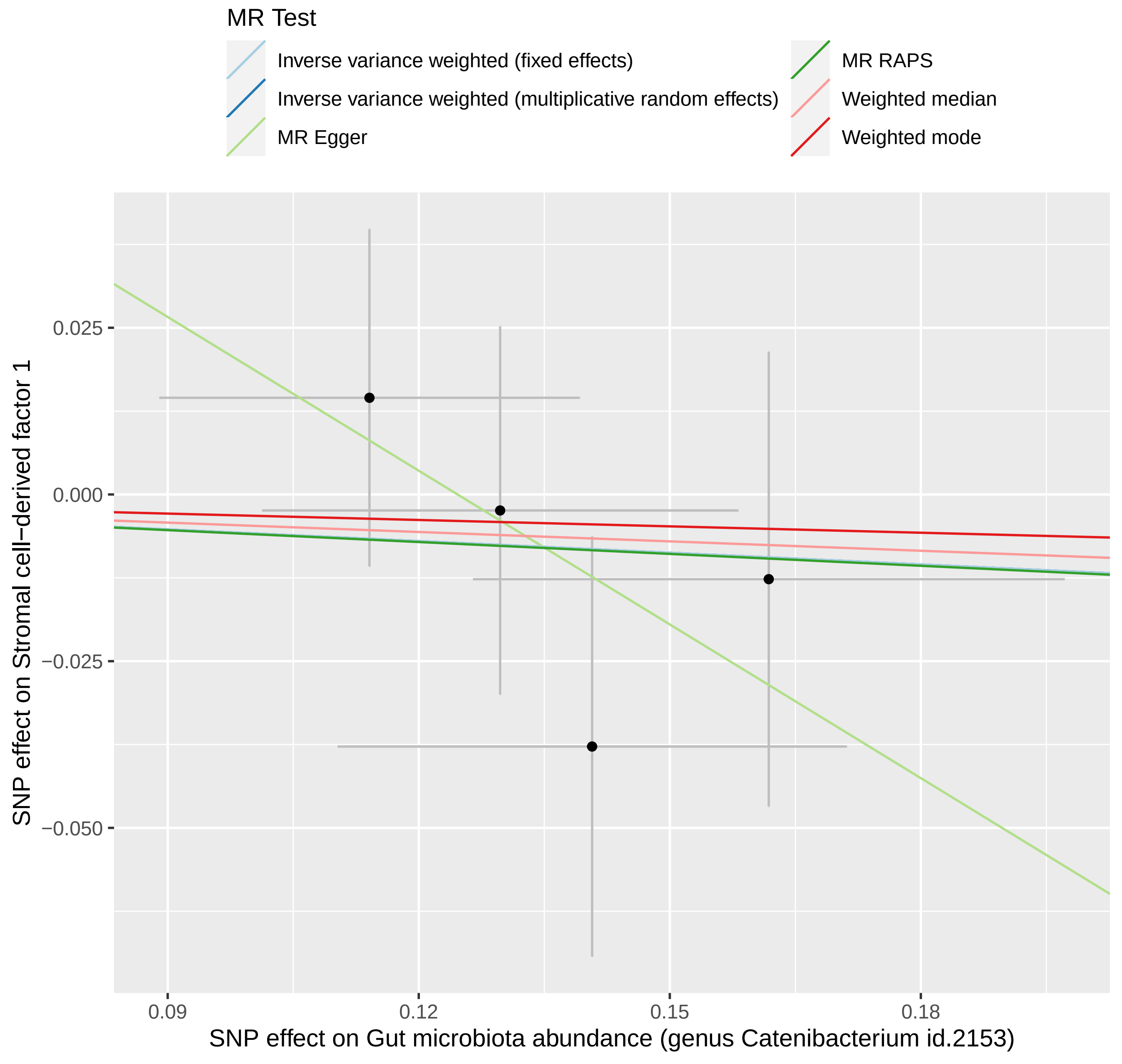

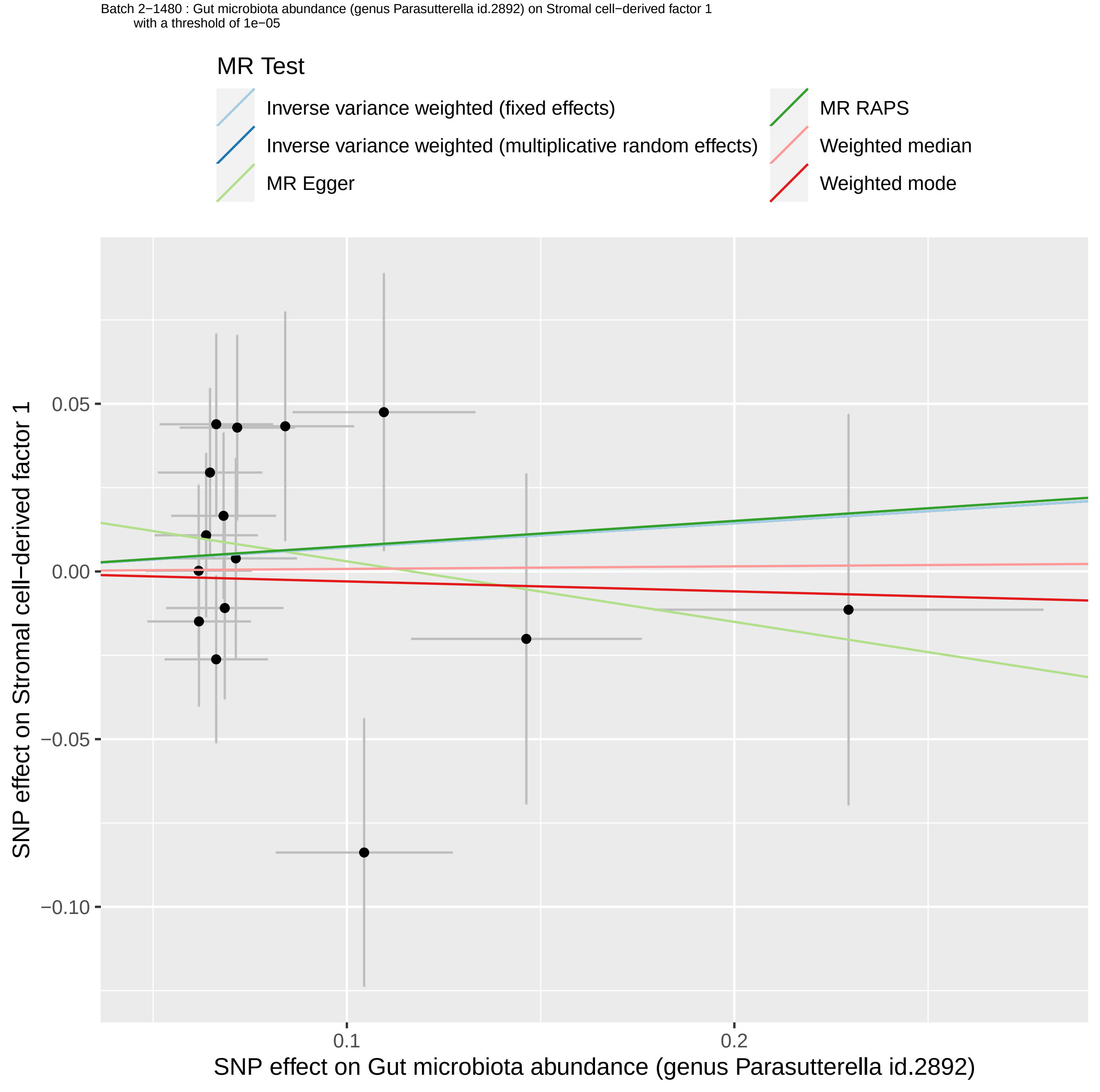

Batch 2−1480 : Gut microbiota abundance (genus Parasutterella id.2892) on Stromal cell−derived factor 1
with a threshold of 1e−05
MR Test
Inverse variance weighted (fixed effects)
Inverse variance weighted (multiplicative random effects)
MR Egger
MR RAPS
Weighted median
Weighted mode
0.05
0.00
−0.05
−0.10
0.1
0.2
SNP effect on Stromal cell−derived factor 1
SNP effect on Gut microbiota abundance (genus Parasutterella id.2892)

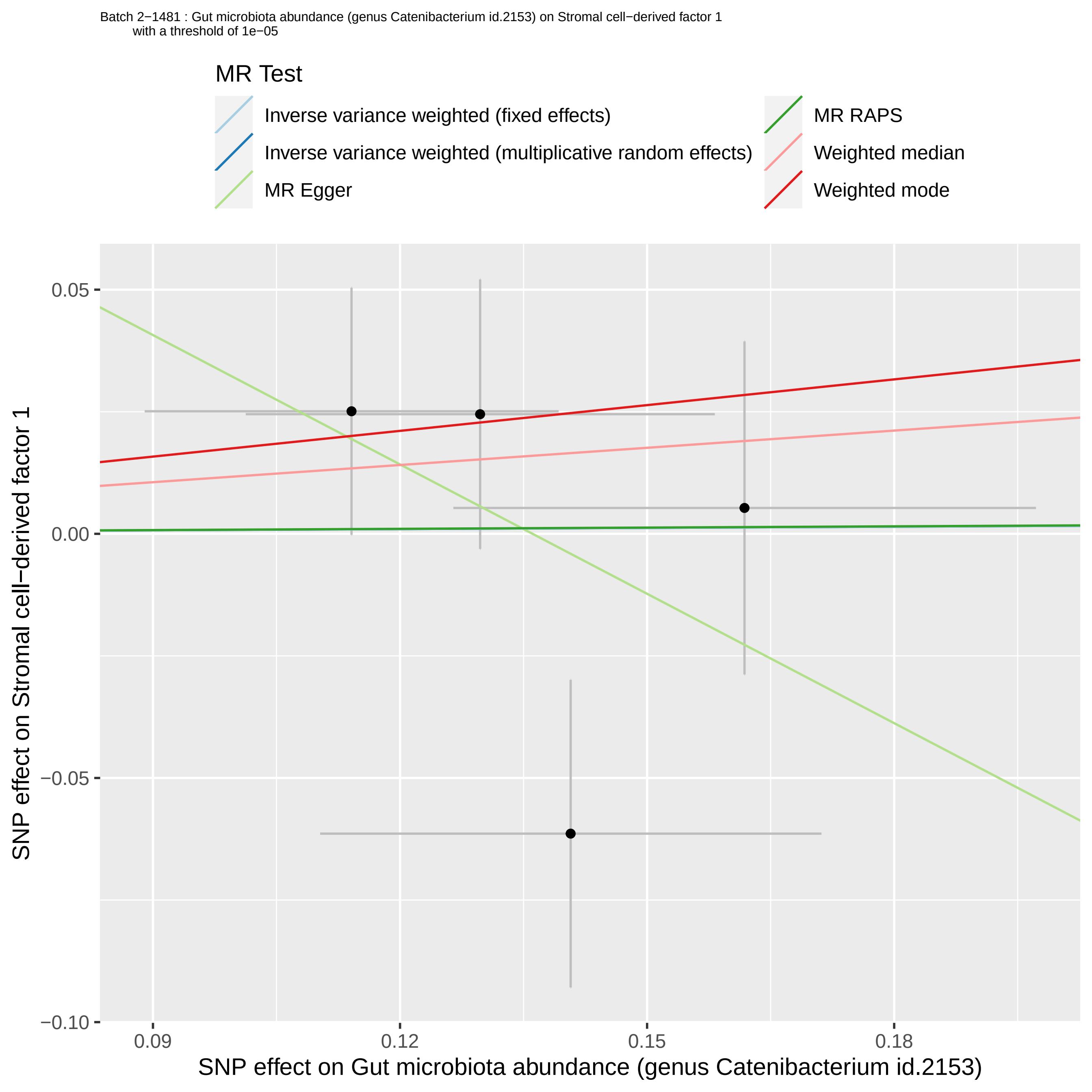

Batch 2−1481 : Gut microbiota abundance (genus Catenibacterium id.2153) on Stromal cell−derived factor 1
with a threshold of 1e−05
MR Test
Inverse variance weighted (fixed effects)
Inverse variance weighted (multiplicative random effects)
MR Egger
MR RAPS
Weighted median
Weighted mode
0.05
0.00
−0.05
−0.10
0.09
0.12
0.15
0.18
SNP effect on Stromal cell−derived factor 1
SNP effect on Gut microbiota abundance (genus Catenibacterium id.2153)

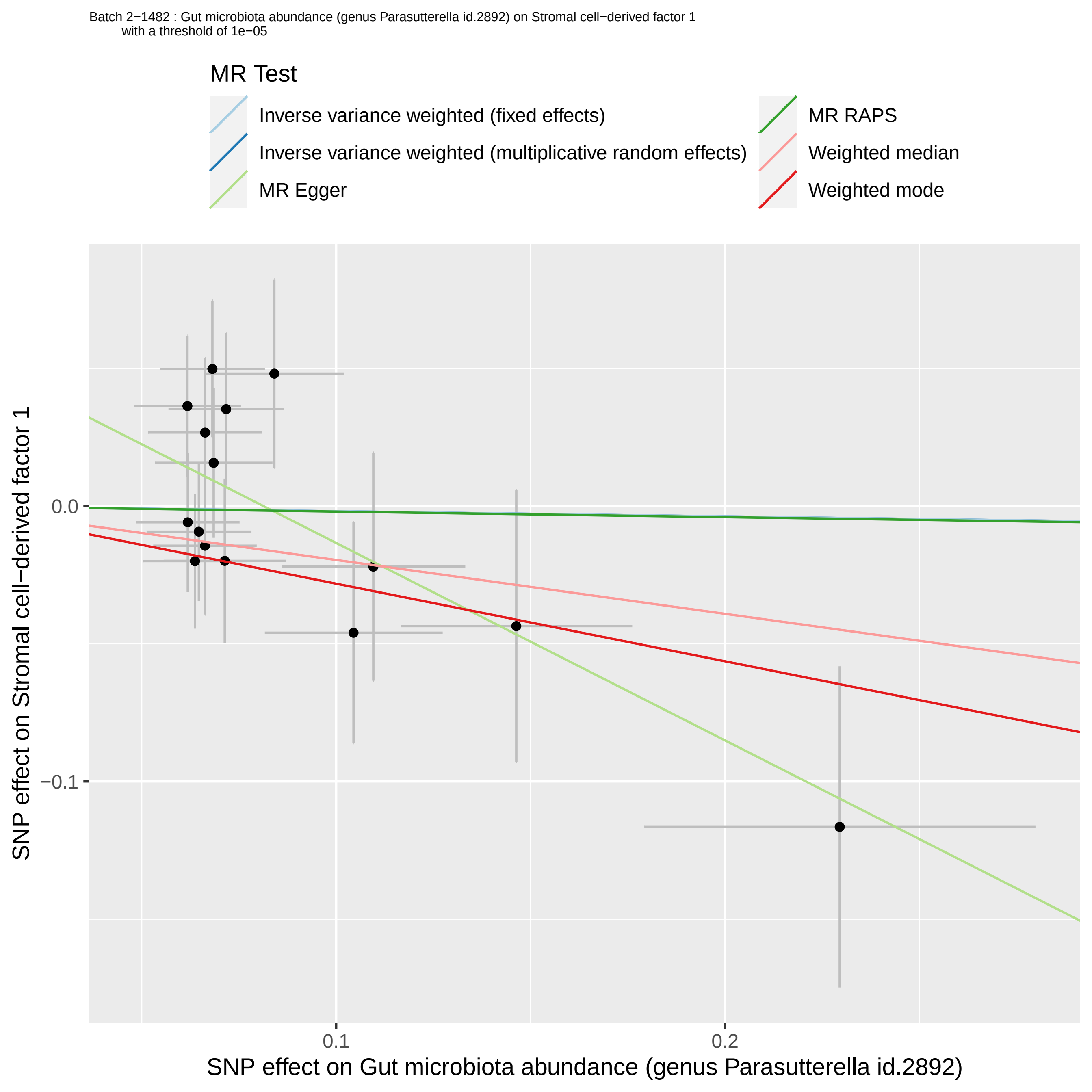

Batch 2−1482 : Gut microbiota abundance (genus Parasutterella id.2892) on Stromal cell−derived factor 1
with a threshold of 1e−05
MR Test
Inverse variance weighted (fixed effects)
Inverse variance weighted (multiplicative random effects)
MR Egger
MR RAPS
Weighted median
Weighted mode
0.0
−0.1
0.1
0.2
SNP effect on Stromal cell−derived factor 1
SNP effect on Gut microbiota abundance (genus Parasutterella id.2892)

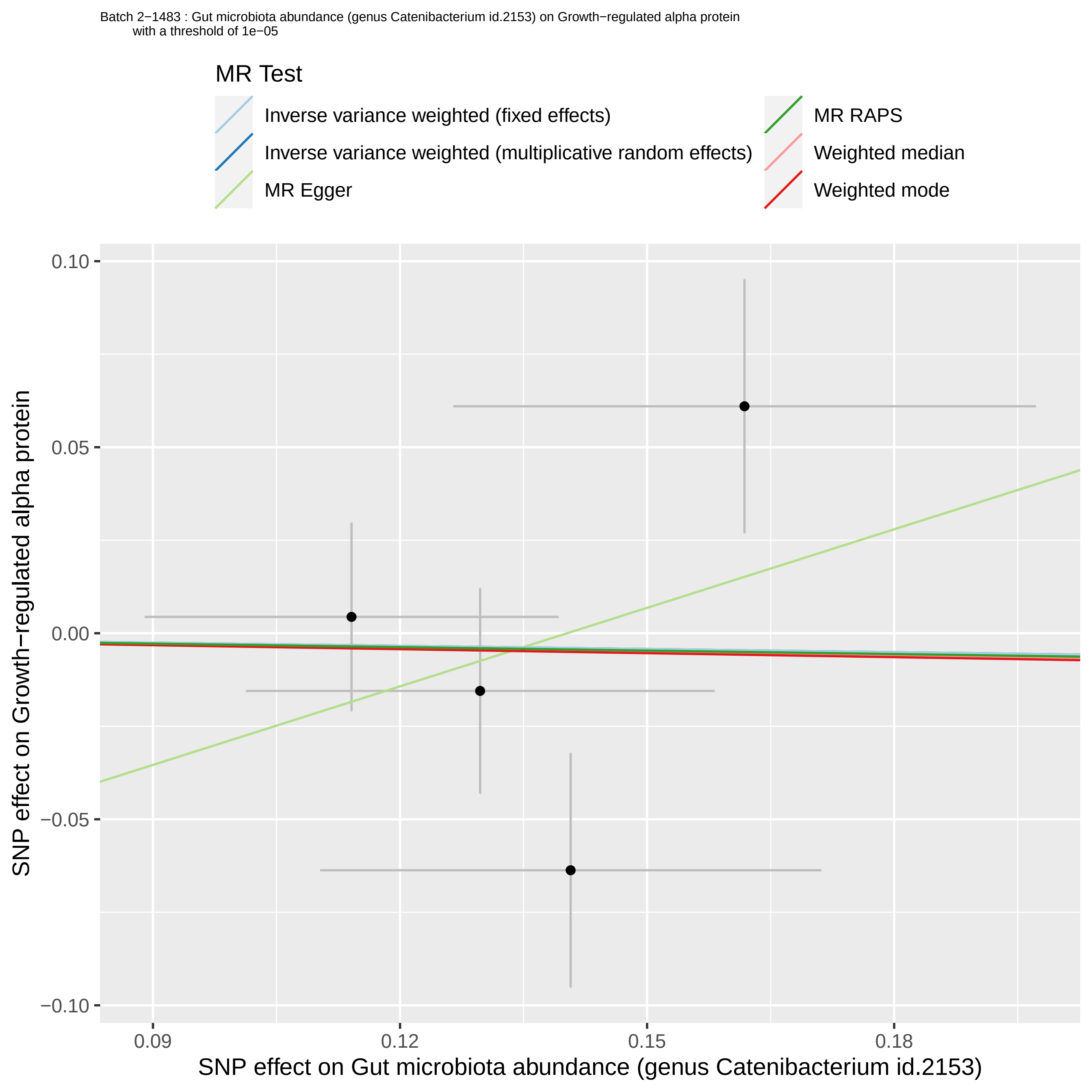
Batch 2−1483 : Gut microbiota abundance (genus Catenibacterium id.2153) on Growth−regulated alpha protein
with a threshold of 1e−05
MR Test
Inverse variance weighted (fixed effects)
Inverse variance weighted (multiplicative random effects)
MR Egger
MR RAPS
Weighted median
Weighted mode
0.10
0.05
0.00
−0.05
−0.10
0.09
0.12
0.15
0.18
SNP effect on Growth−regulated alpha protein
SNP effect on Gut microbiota abundance (genus Catenibacterium id.2153)

Batch 2−1484 : Gut microbiota abundance (genus Parasutterella id.2892) on Growth−regulated alpha protein
with a threshold of 1e−05

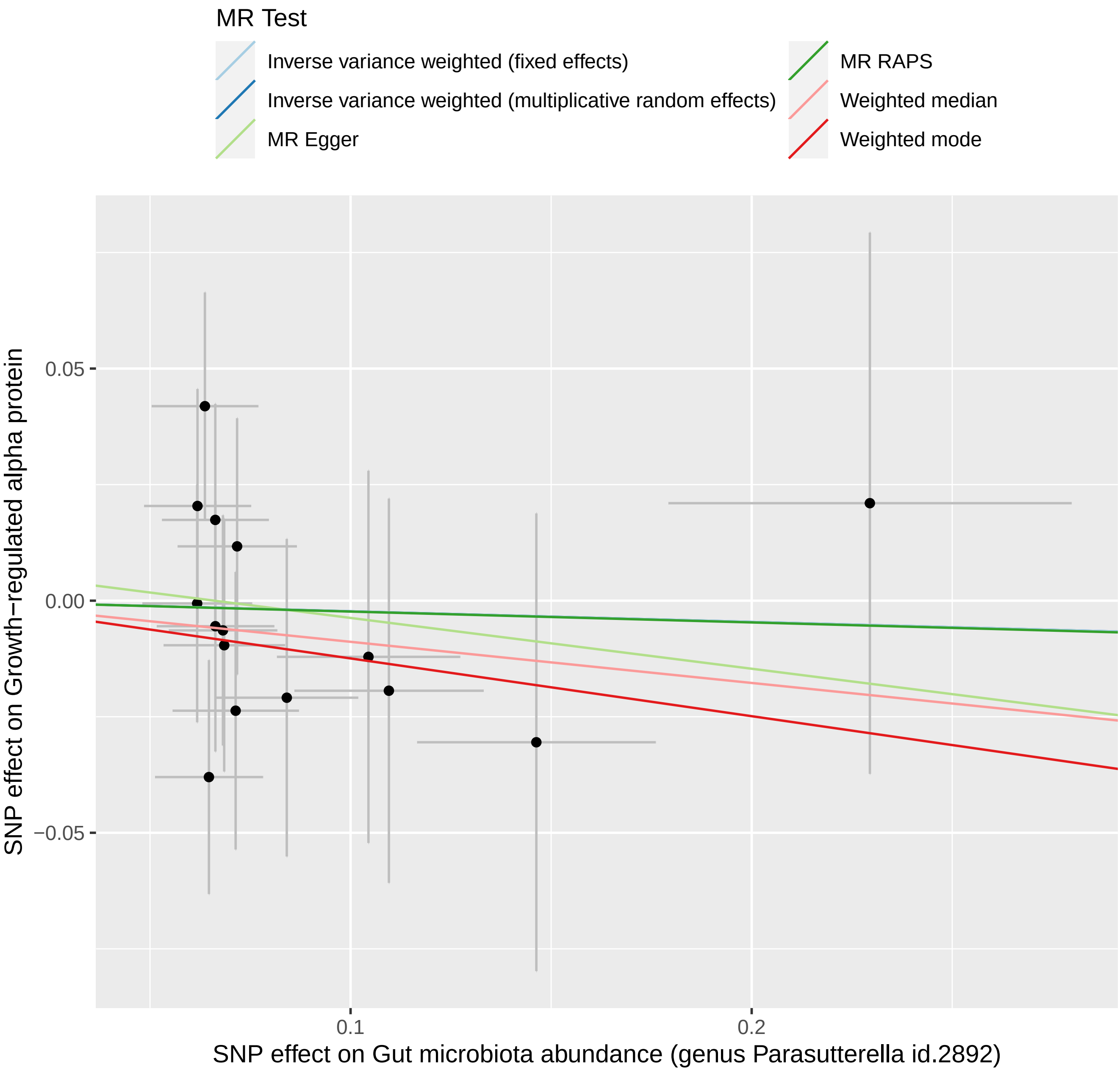

Batch 2−1485 : Gut microbiota abundance (genus Catenibacterium id.2153) on C−X−C motif chemokine 13
with a threshold of 1e−05

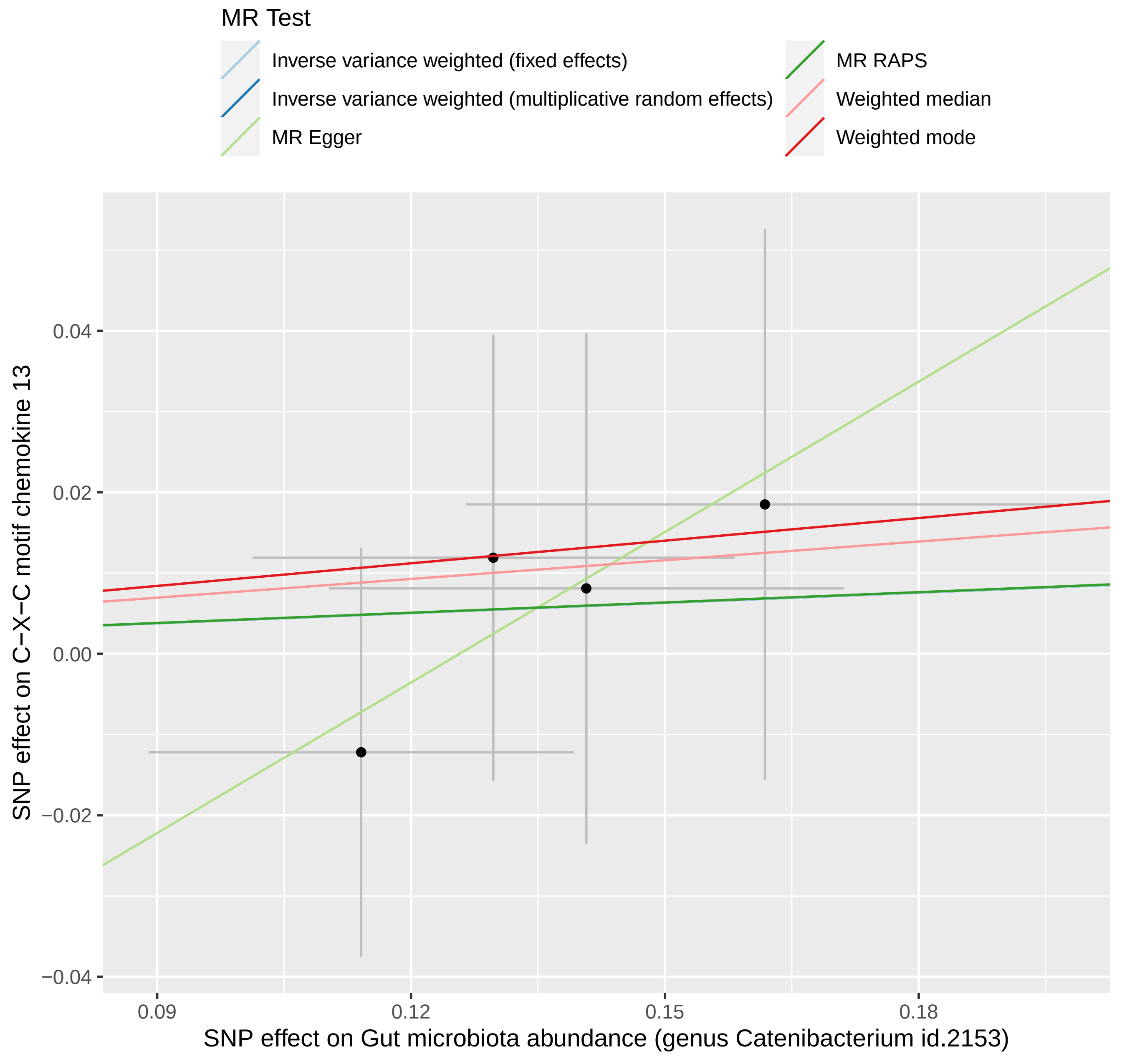

Batch 2−1486 : Gut microbiota abundance (genus Parasutterella id.2892) on C−X−C motif chemokine 13
with a threshold of 1e−05

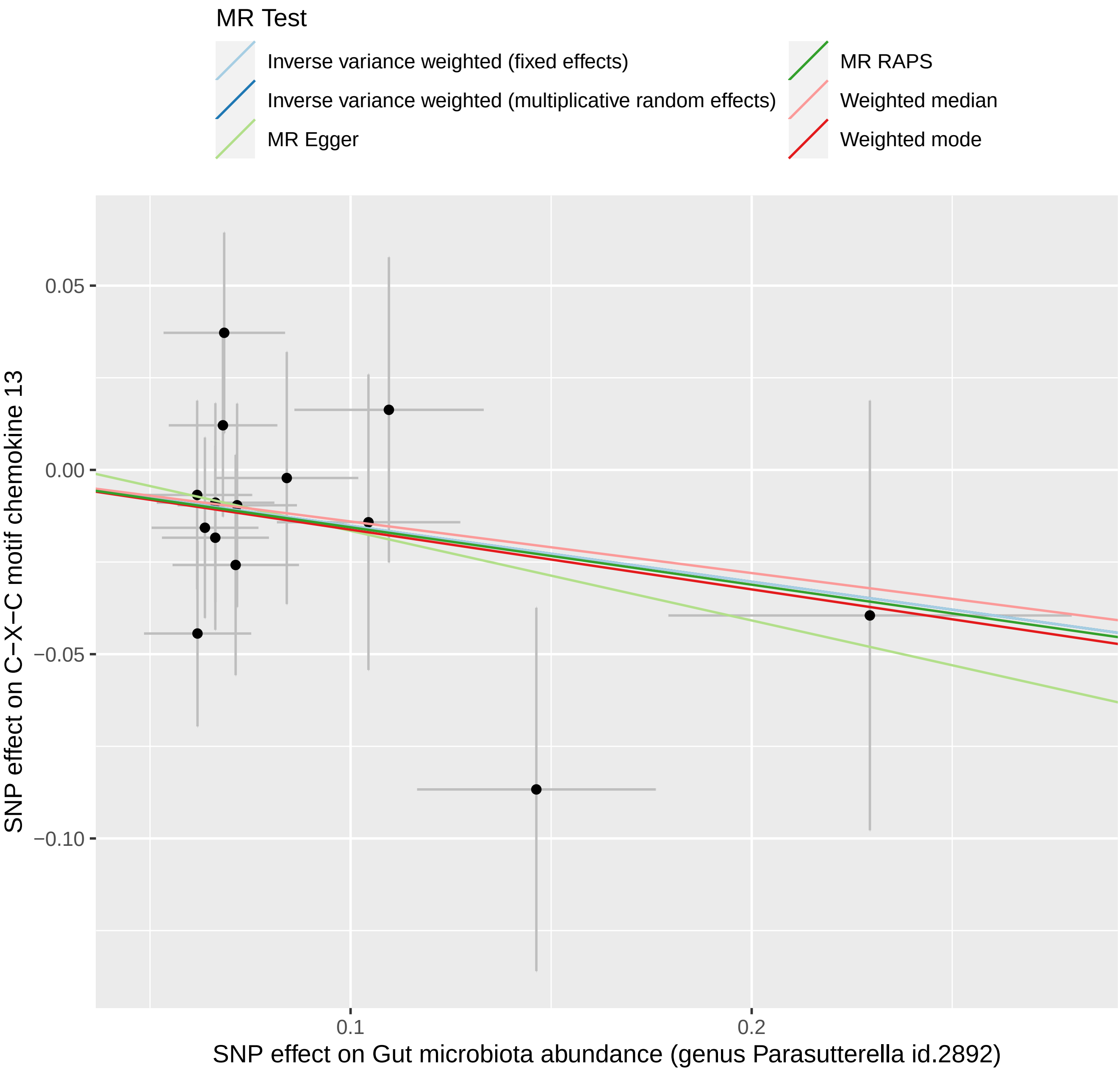

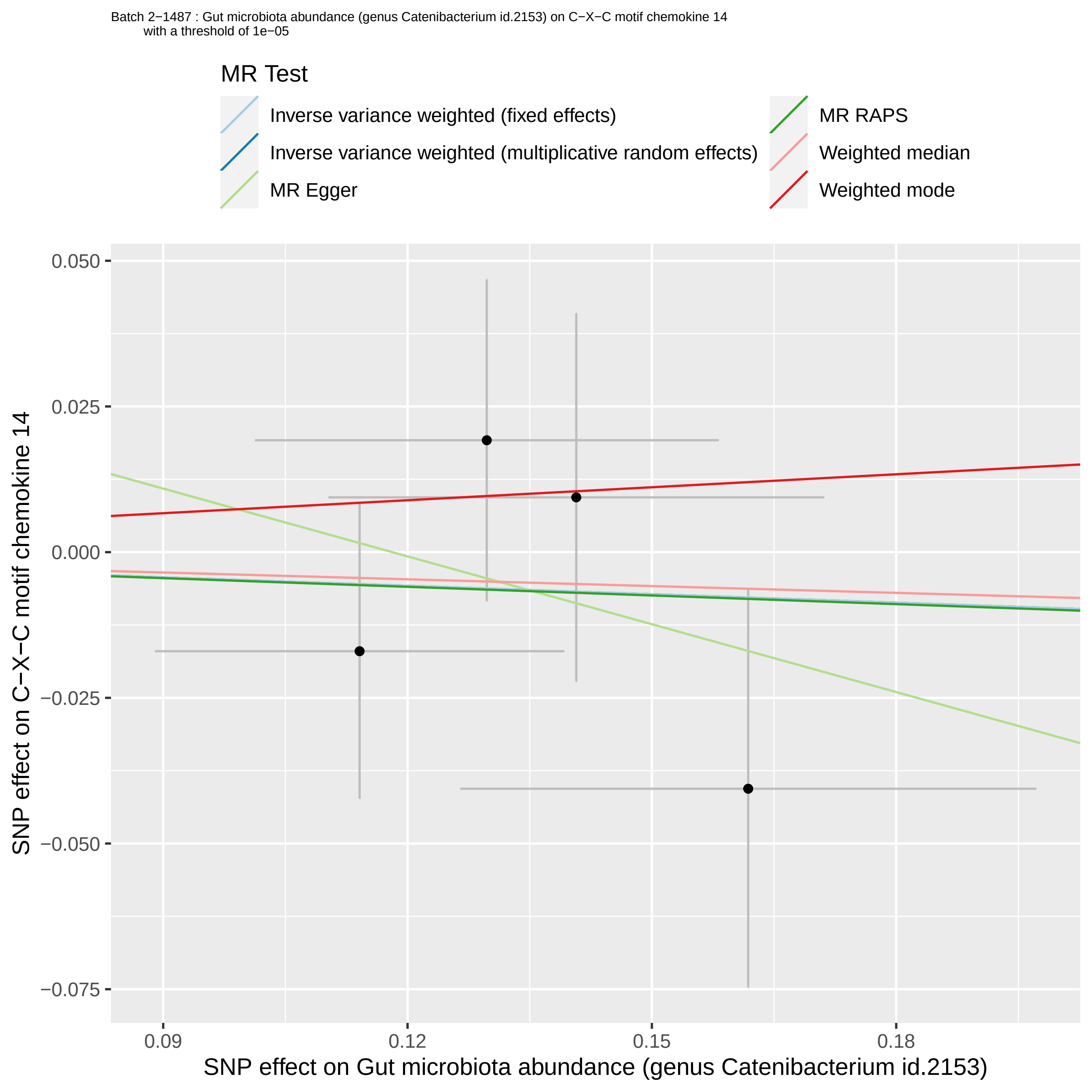

Batch 2−1487 : Gut microbiota abundance (genus Catenibacterium id.2153) on C−X−C motif chemokine 14
with a threshold of 1e−05
MR Test
Inverse variance weighted (fixed effects)
Inverse variance weighted (multiplicative random effects)
MR Egger
MR RAPS
Weighted median
Weighted mode
0.050
0.025
0.000
−0.025
−0.050
−0.075
0.09
0.12
0.15
0.18
SNP effect on C−X−C motif chemokine 14
SNP effect on Gut microbiota abundance (genus Catenibacterium id.2153)

Batch 2−1488 : Gut microbiota abundance (genus Parasutterella id.2892) on C−X−C motif chemokine 14
with a threshold of 1e−05

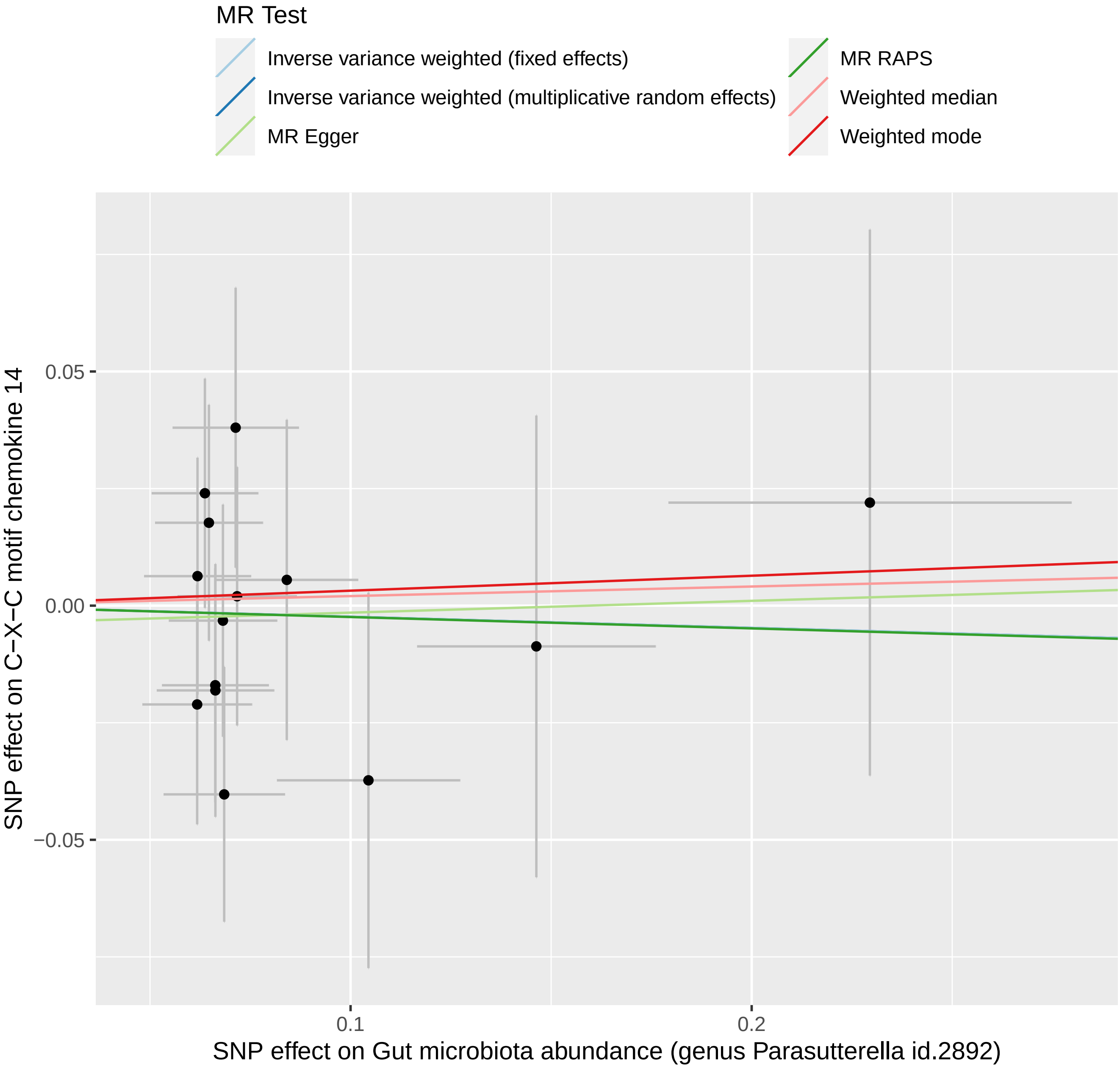

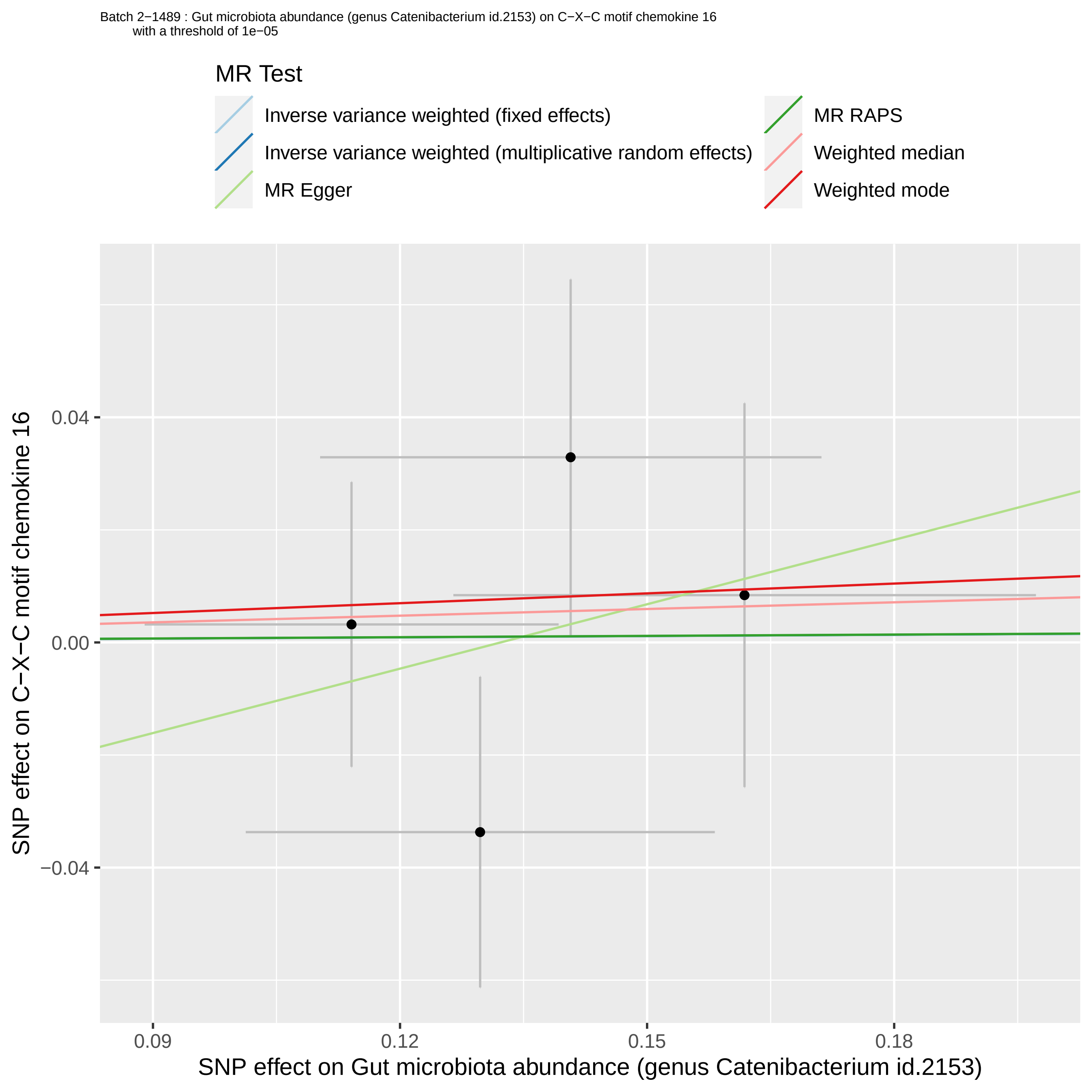
Batch 2−1489 : Gut microbiota abundance (genus Catenibacterium id.2153) on C−X−C motif chemokine 16
with a threshold of 1e−05
MR Test
Inverse variance weighted (fixed effects)
Inverse variance weighted (multiplicative random effects)
MR Egger
MR RAPS
Weighted median
Weighted mode
SNP effect on C−X−C motif chemokine 16
0.04
0.00
−0.04
0.09
0.12
0.15
0.18
SNP effect on Gut microbiota abundance (genus Catenibacterium id.2153)

Batch 2−1490 : Gut microbiota abundance (genus Parasutterella id.2892) on C−X−C motif chemokine 16
with a threshold of 1e−05

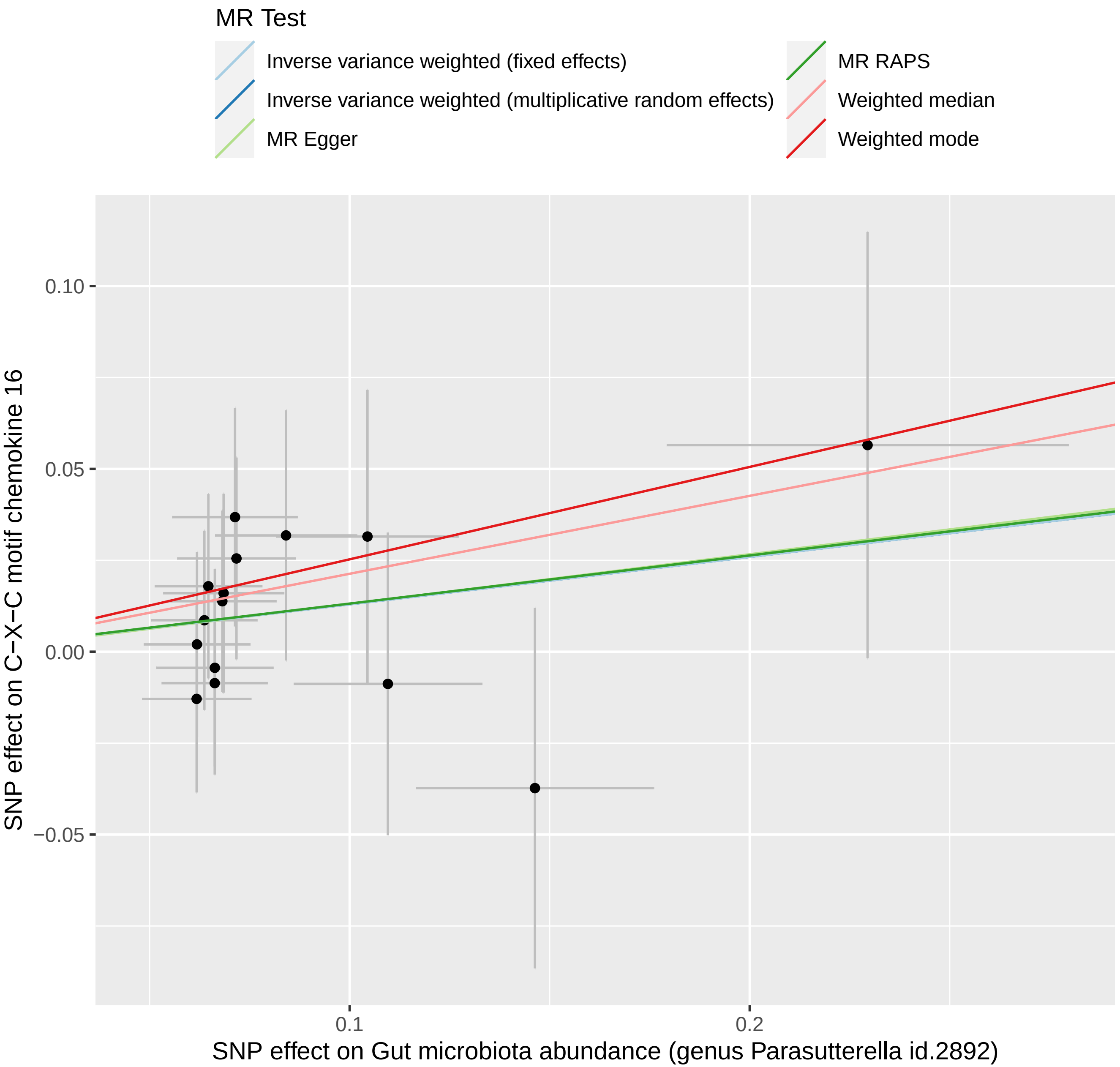

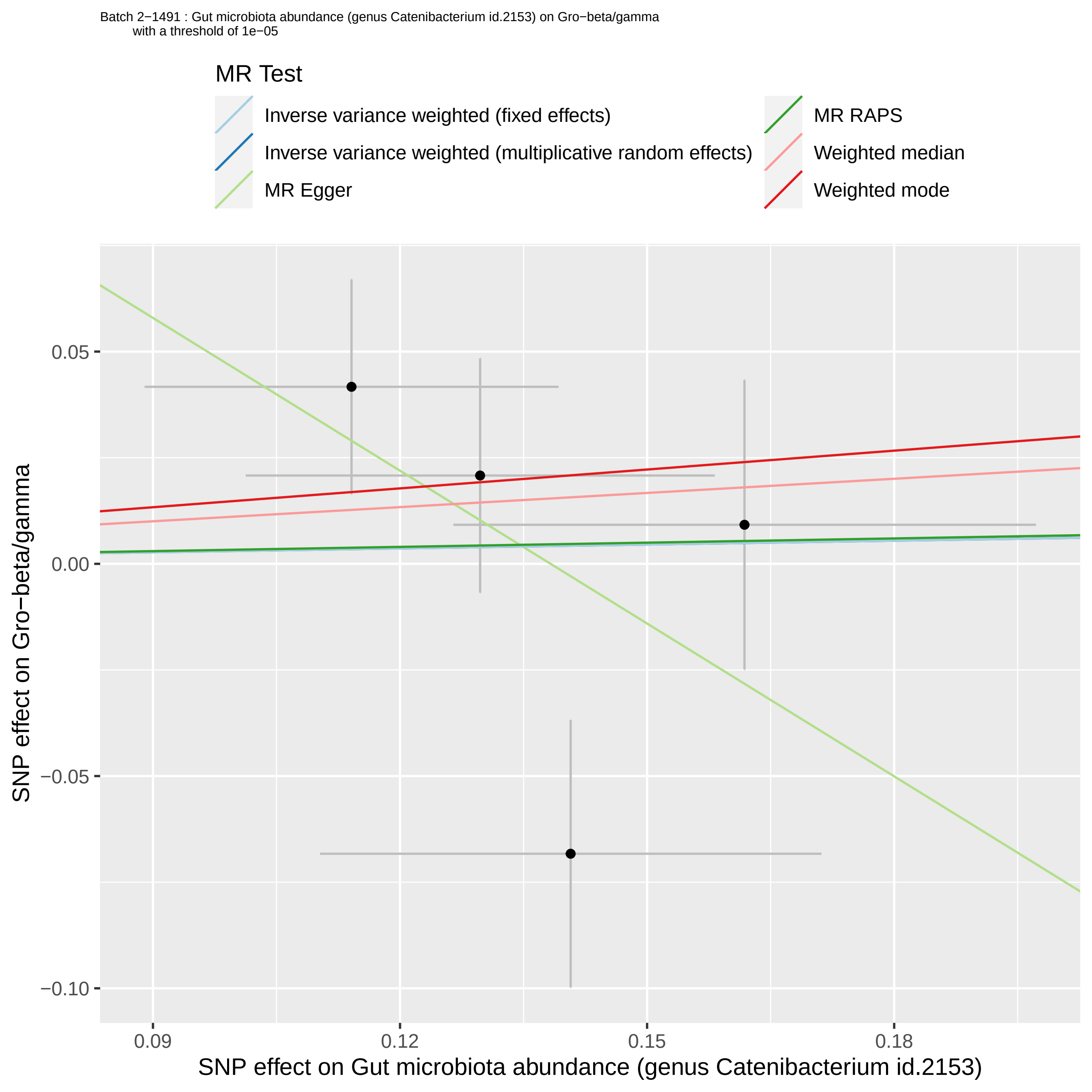

Batch 2−1491 : Gut microbiota abundance (genus Catenibacterium id.2153) on Gro−beta/gamma
with a threshold of 1e−05
MR Test
Inverse variance weighted (fixed effects)
Inverse variance weighted (multiplicative random effects)
MR Egger
MR RAPS
Weighted median
Weighted mode
0.05
0.00
−0.05
−0.10
0.09
0.12
0.15
0.18
SNP effect on Gro−beta/gamma
SNP effect on Gut microbiota abundance (genus Catenibacterium id.2153)

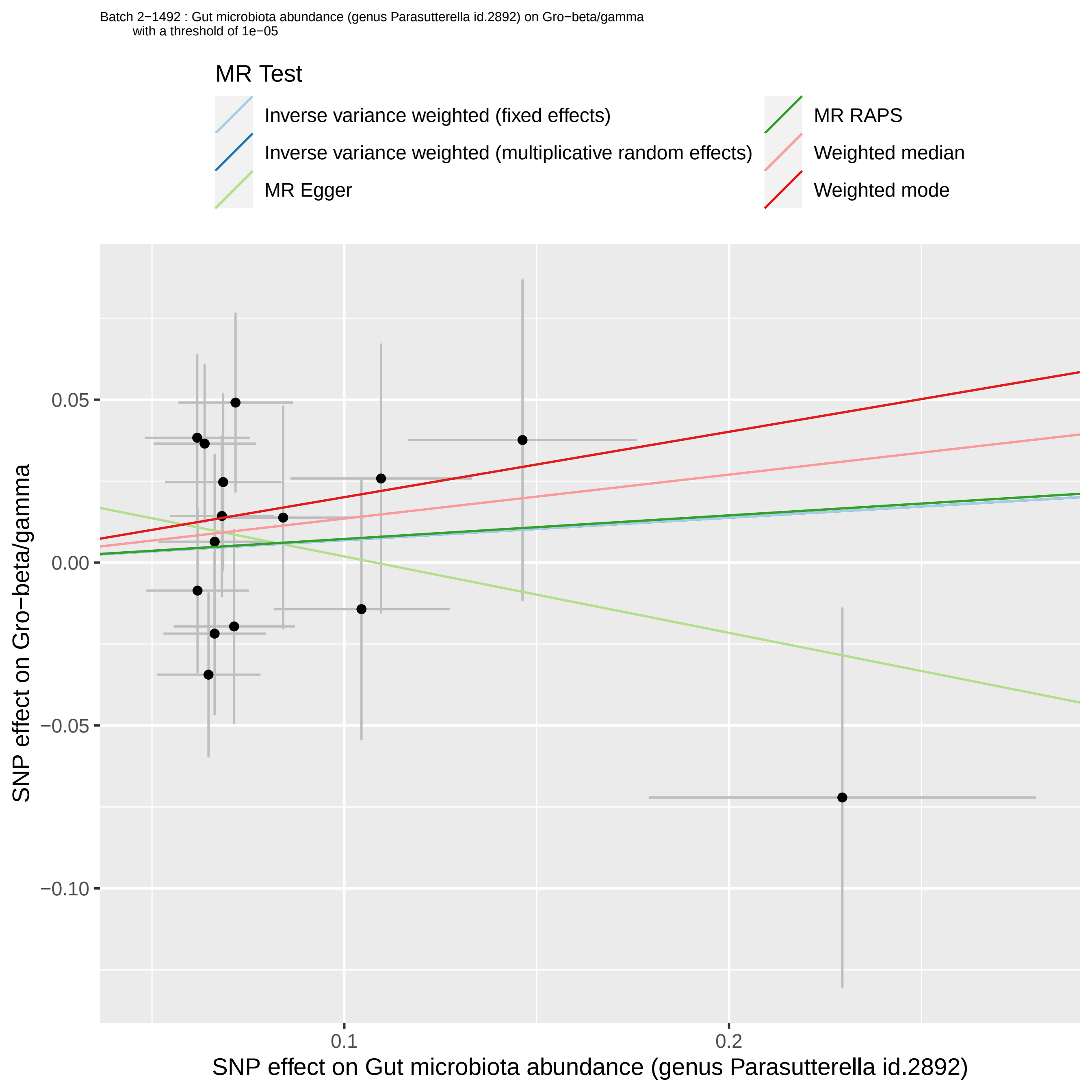
Batch 2−1492 : Gut microbiota abundance (genus Parasutterella id.2892) on Gro−beta/gamma
with a threshold of 1e−05
MR Test
Inverse variance weighted (fixed effects)
Inverse variance weighted (multiplicative random effects)
MR Egger
MR RAPS
Weighted median
Weighted mode
SNP effect on Gro−beta/gamma
0.05
0.00
−0.05
−0.10
0.1
0.2
SNP effect on Gut microbiota abundance (genus Parasutterella id.2892)

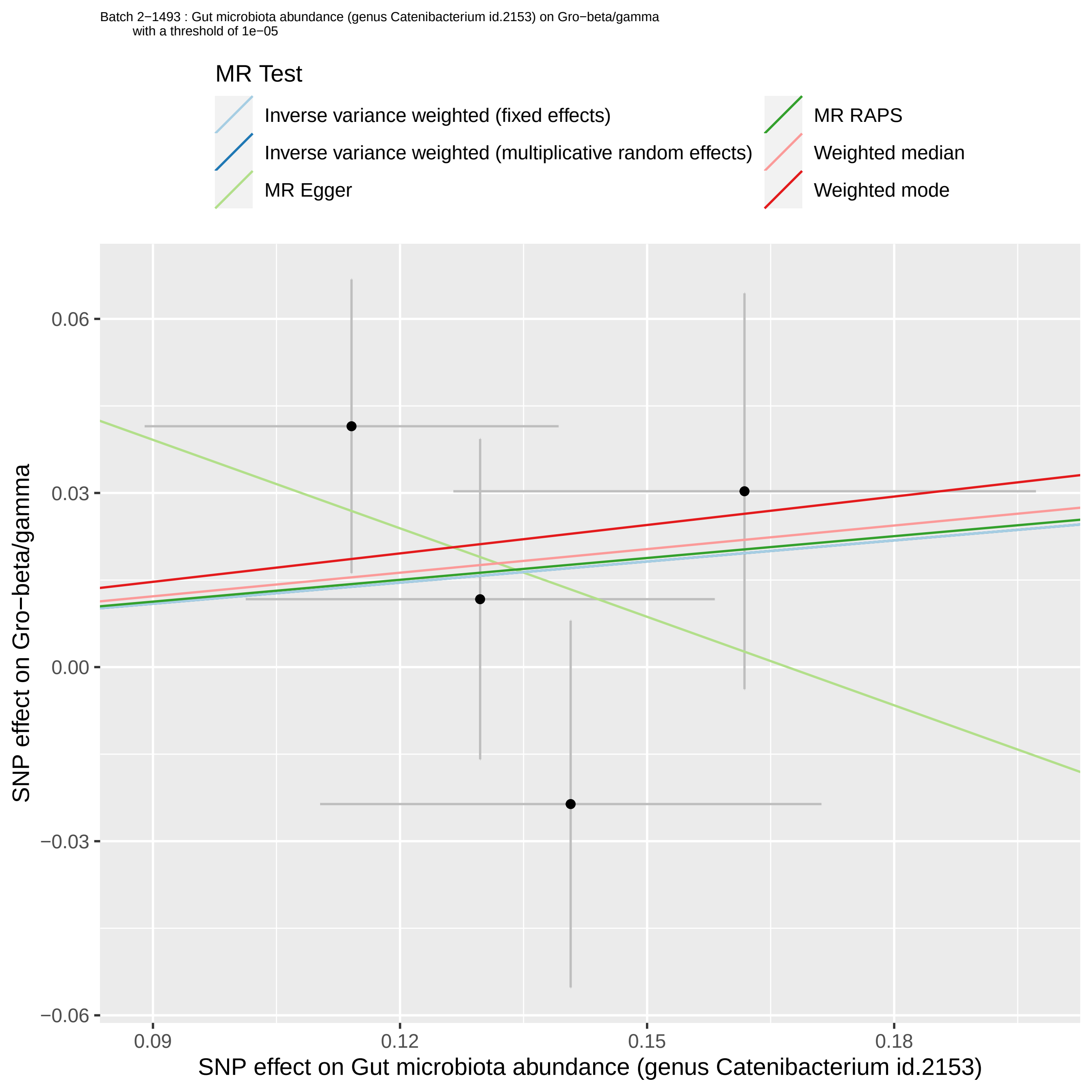
Batch 2−1493 : Gut microbiota abundance (genus Catenibacterium id.2153) on Gro−beta/gamma
with a threshold of 1e−05
MR Test
Inverse variance weighted (fixed effects)
Inverse variance weighted (multiplicative random effects)
MR Egger
MR RAPS
Weighted median
Weighted mode
0.06
0.03
0.00
−0.03
−0.06
0.09
0.12
0.15
0.18
SNP effect on Gro−beta/gamma
SNP effect on Gut microbiota abundance (genus Catenibacterium id.2153)

Batch 2−1494 : Gut microbiota abundance (genus Parasutterella id.2892) on Gro−beta/gamma
with a threshold of 1e−05

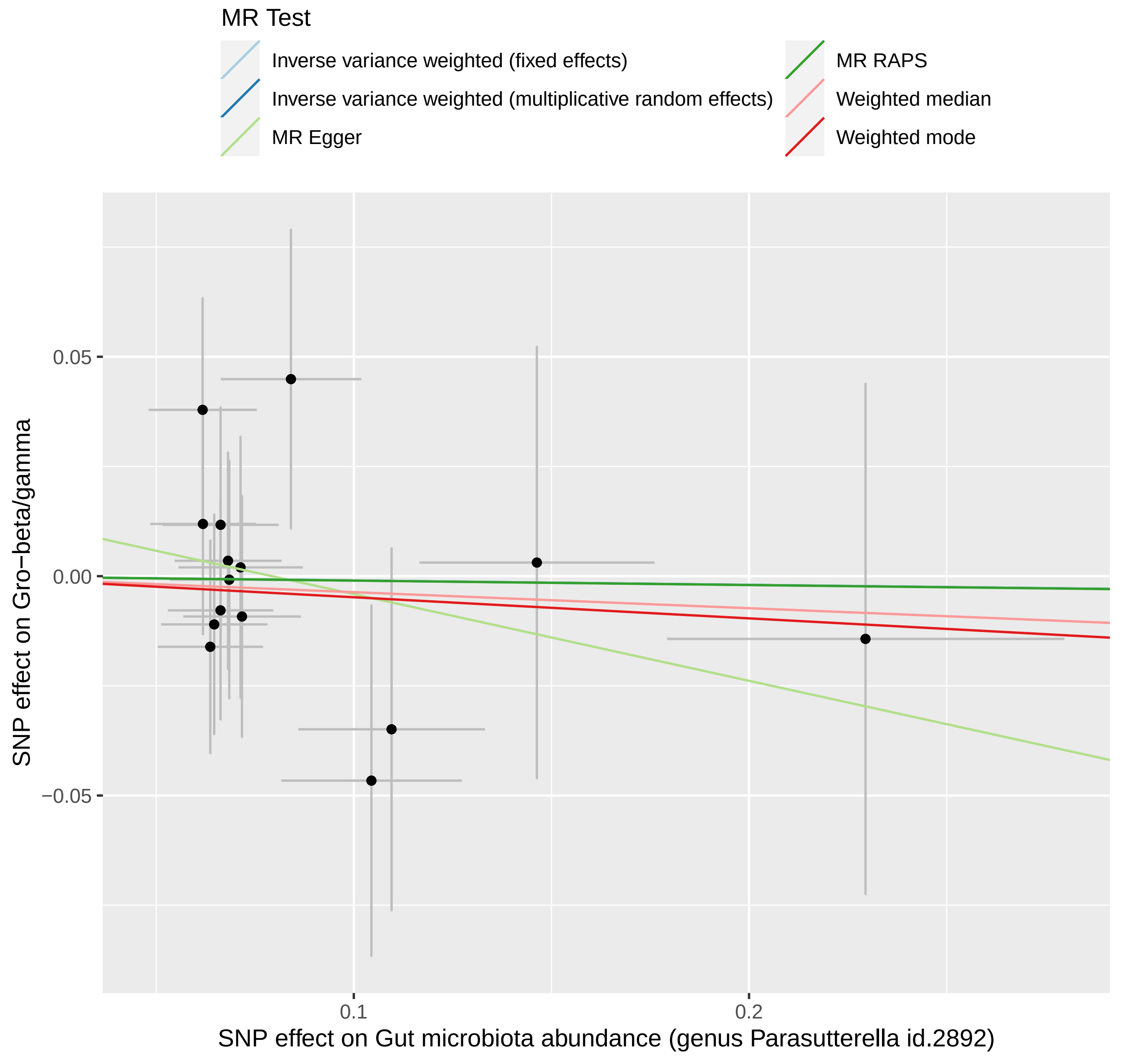

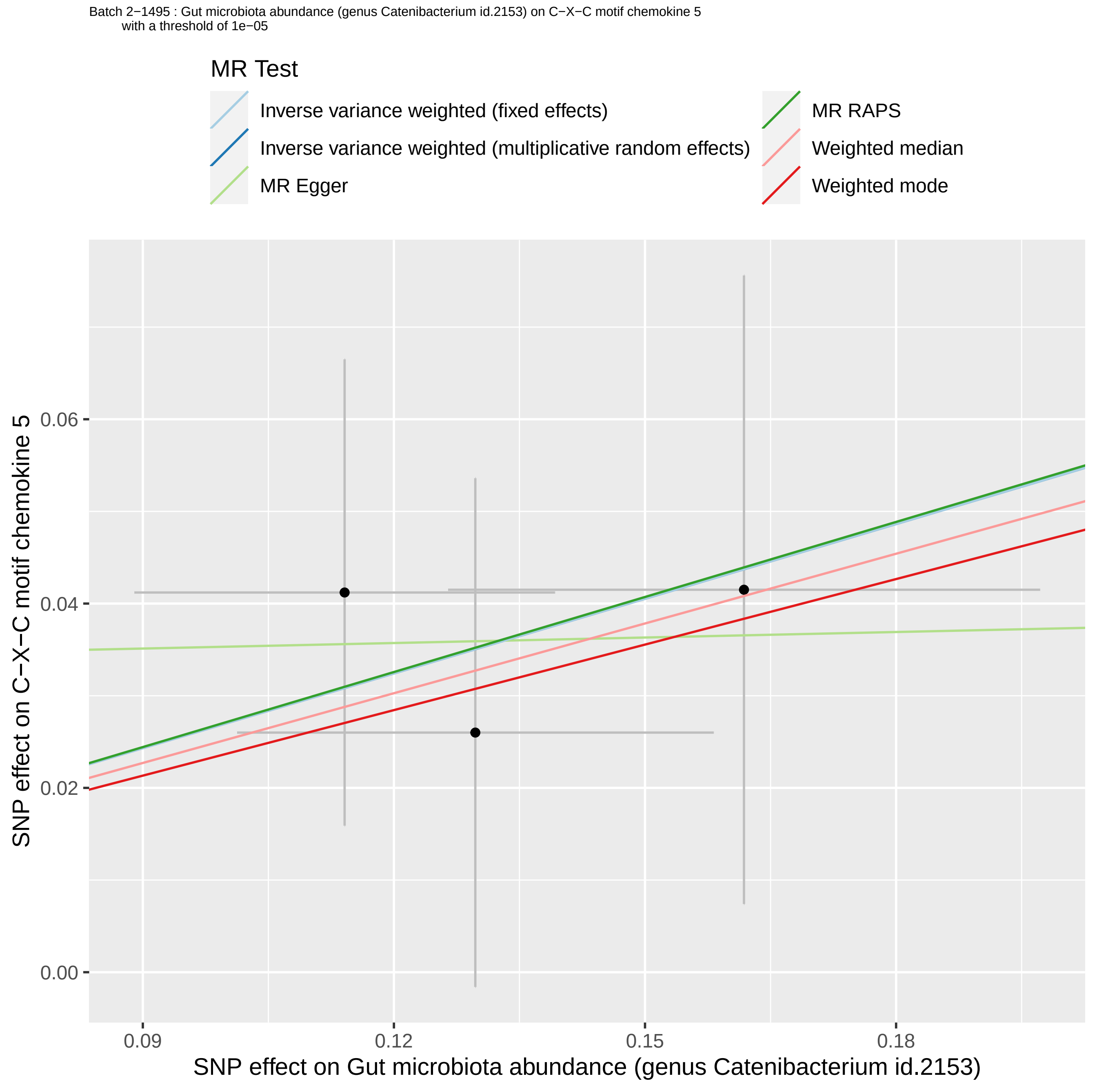
Batch 2−1495 : Gut microbiota abundance (genus Catenibacterium id.2153) on C−X−C motif chemokine 5
with a threshold of 1e−05
MR Test
Inverse variance weighted (fixed effects)
Inverse variance weighted (multiplicative random effects)
MR Egger
MR RAPS
Weighted median
Weighted mode
0.06
0.04
0.02
0.00
0.09
0.12
0.15
0.18
SNP effect on C−X−C motif chemokine 5
SNP effect on Gut microbiota abundance (genus Catenibacterium id.2153)

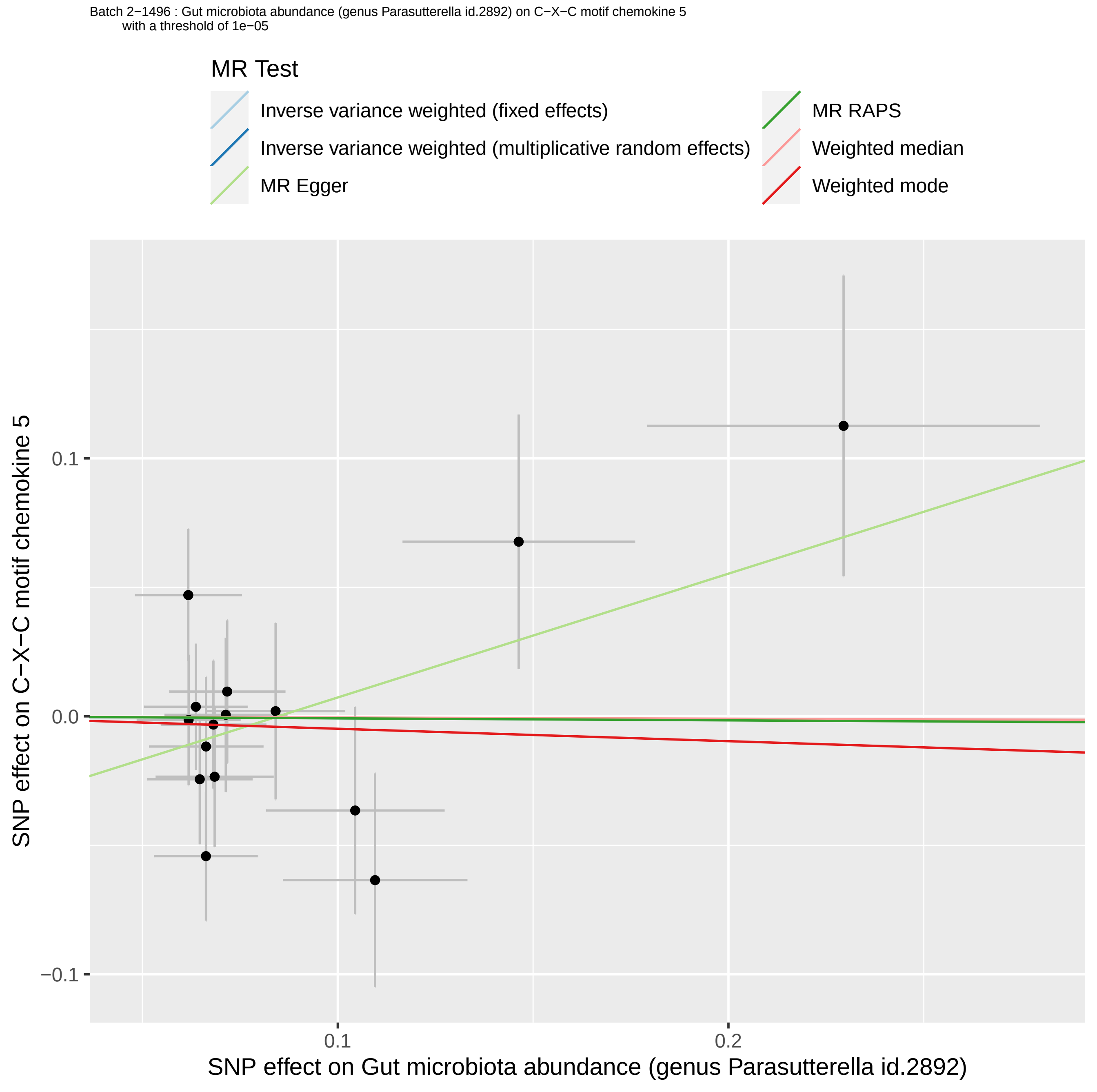
Batch 2−1496 : Gut microbiota abundance (genus Parasutterella id.2892) on C−X−C motif chemokine 5
with a threshold of 1e−05
MR Test
Inverse variance weighted (fixed effects)
Inverse variance weighted (multiplicative random effects)
MR Egger
MR RAPS
Weighted median
Weighted mode
SNP effect on C−X−C motif chemokine 5
0.1
0.0
−0.1
0.1
0.2
SNP effect on Gut microbiota abundance (genus Parasutterella id.2892)

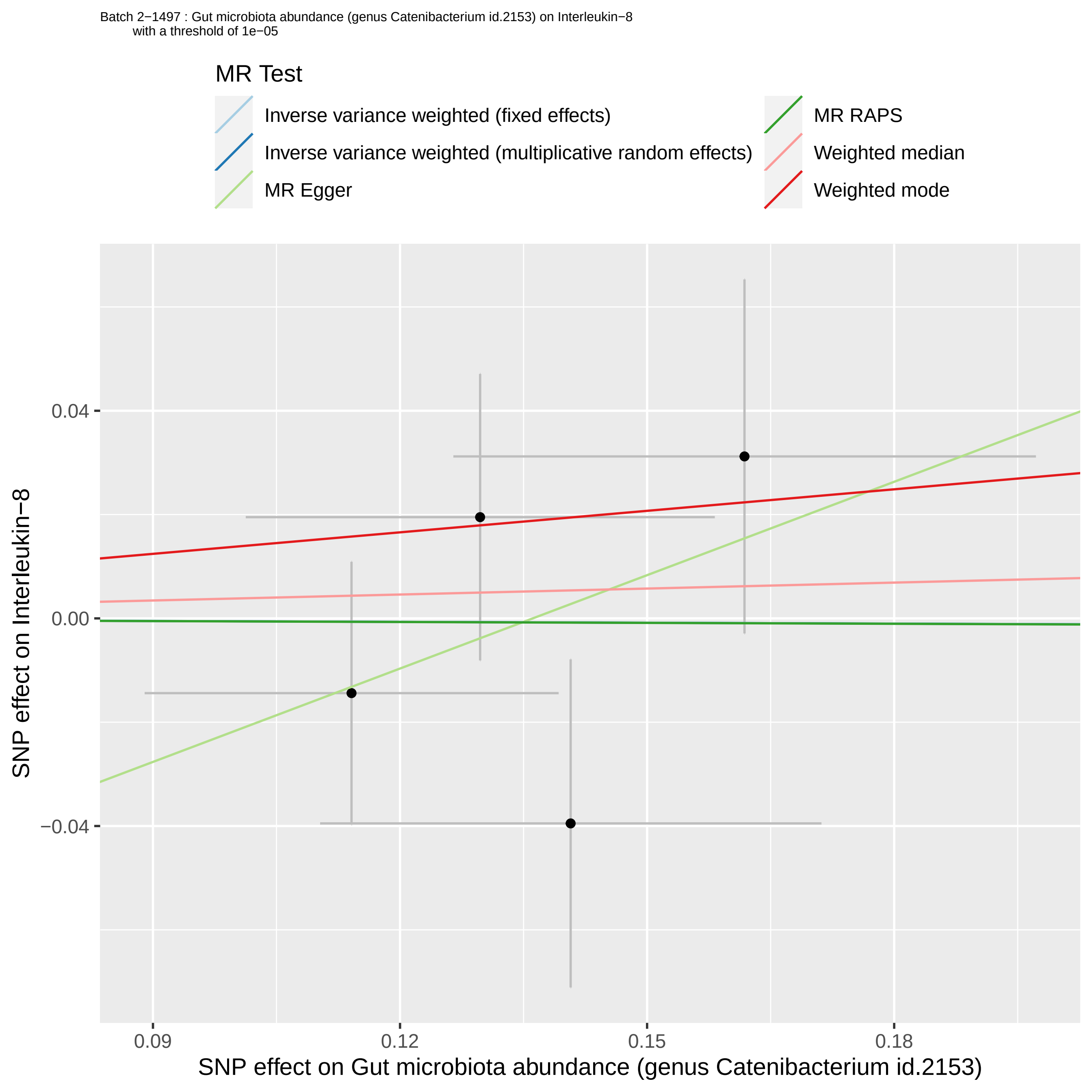
Batch 2−1497 : Gut microbiota abundance (genus Catenibacterium id.2153) on Interleukin−8
with a threshold of 1e−05
MR Test
Inverse variance weighted (fixed effects)
Inverse variance weighted (multiplicative random effects)
MR Egger
MR RAPS
Weighted median
Weighted mode
0.04
0.00
−0.04
SNP effect on Interleukin−8
0.09
0.12
0.15
0.18
SNP effect on Gut microbiota abundance (genus Catenibacterium id.2153)

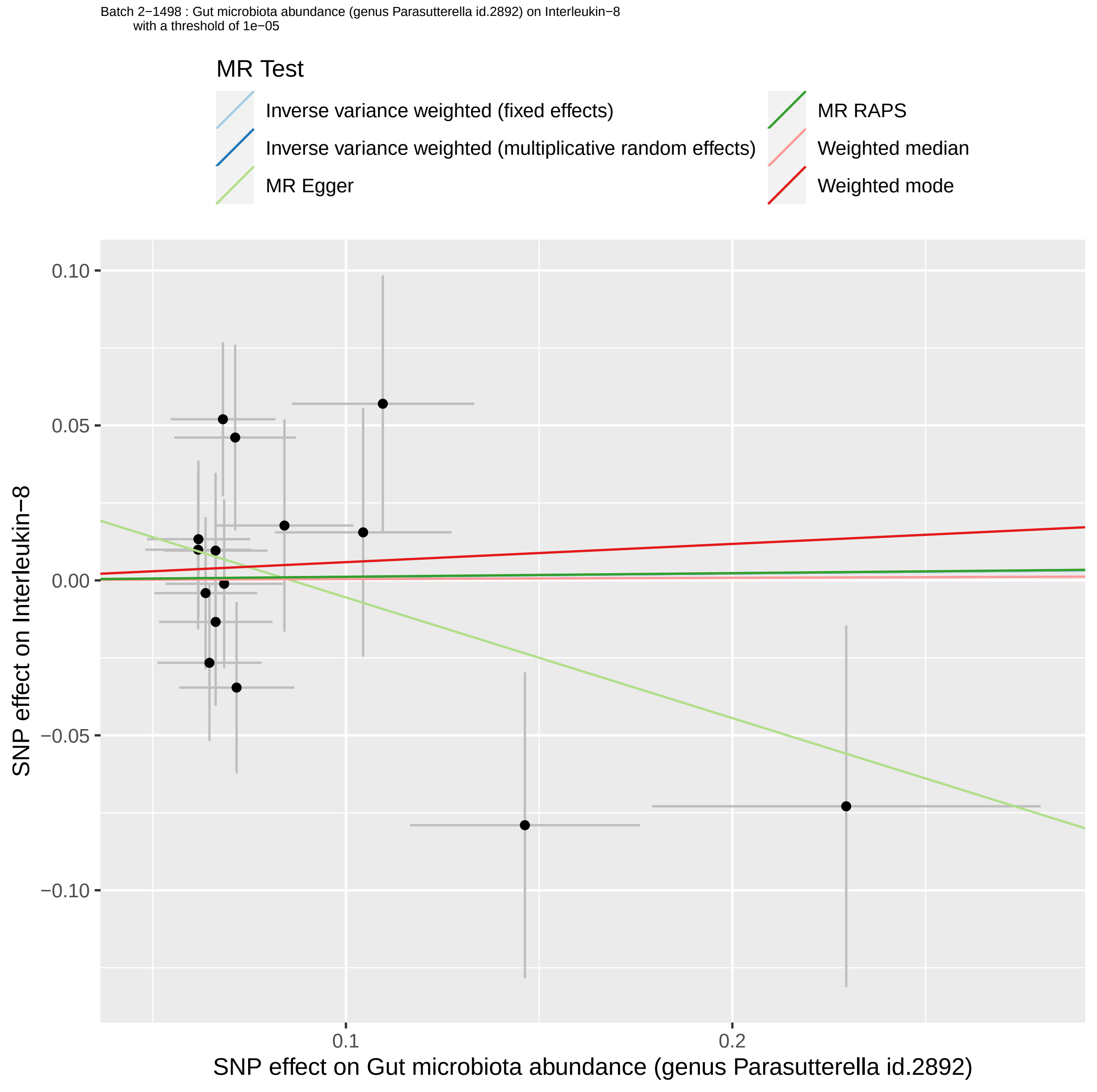
Batch 2−1498 : Gut microbiota abundance (genus Parasutterella id.2892) on Interleukin−8
with a threshold of 1e−05
MR Test
Inverse variance weighted (fixed effects)
Inverse variance weighted (multiplicative random effects)
MR Egger
MR RAPS
Weighted median
Weighted mode
0.10
0.05
0.00
−0.05
−0.10
0.1
0.2
SNP effect on Interleukin−8
SNP effect on Gut microbiota abundance (genus Parasutterella id.2892)

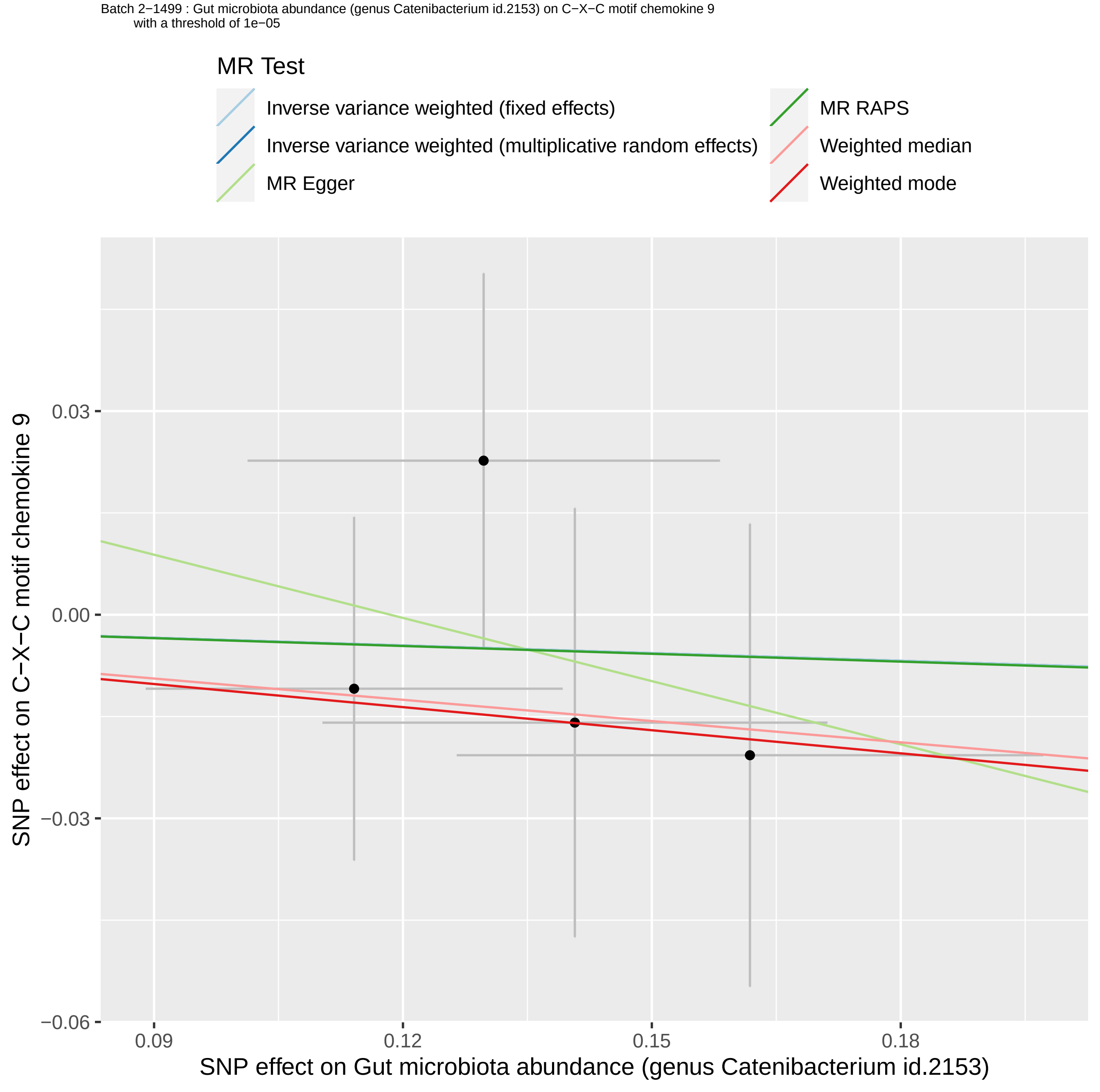
Batch 2−1499 : Gut microbiota abundance (genus Catenibacterium id.2153) on C−X−C motif chemokine 9
with a threshold of 1e−05
MR Test
Inverse variance weighted (fixed effects)
Inverse variance weighted (multiplicative random effects)
MR Egger
MR RAPS
Weighted median
Weighted mode
0.03
0.00
−0.03
−0.06
0.09
0.12
0.15
0.18
SNP effect on C−X−C motif chemokine 9
SNP effect on Gut microbiota abundance (genus Catenibacterium id.2153)

Batch 2−1500 : Gut microbiota abundance (genus Parasutterella id.2892) on C−X−C motif chemokine 9
with a threshold of 1e−05

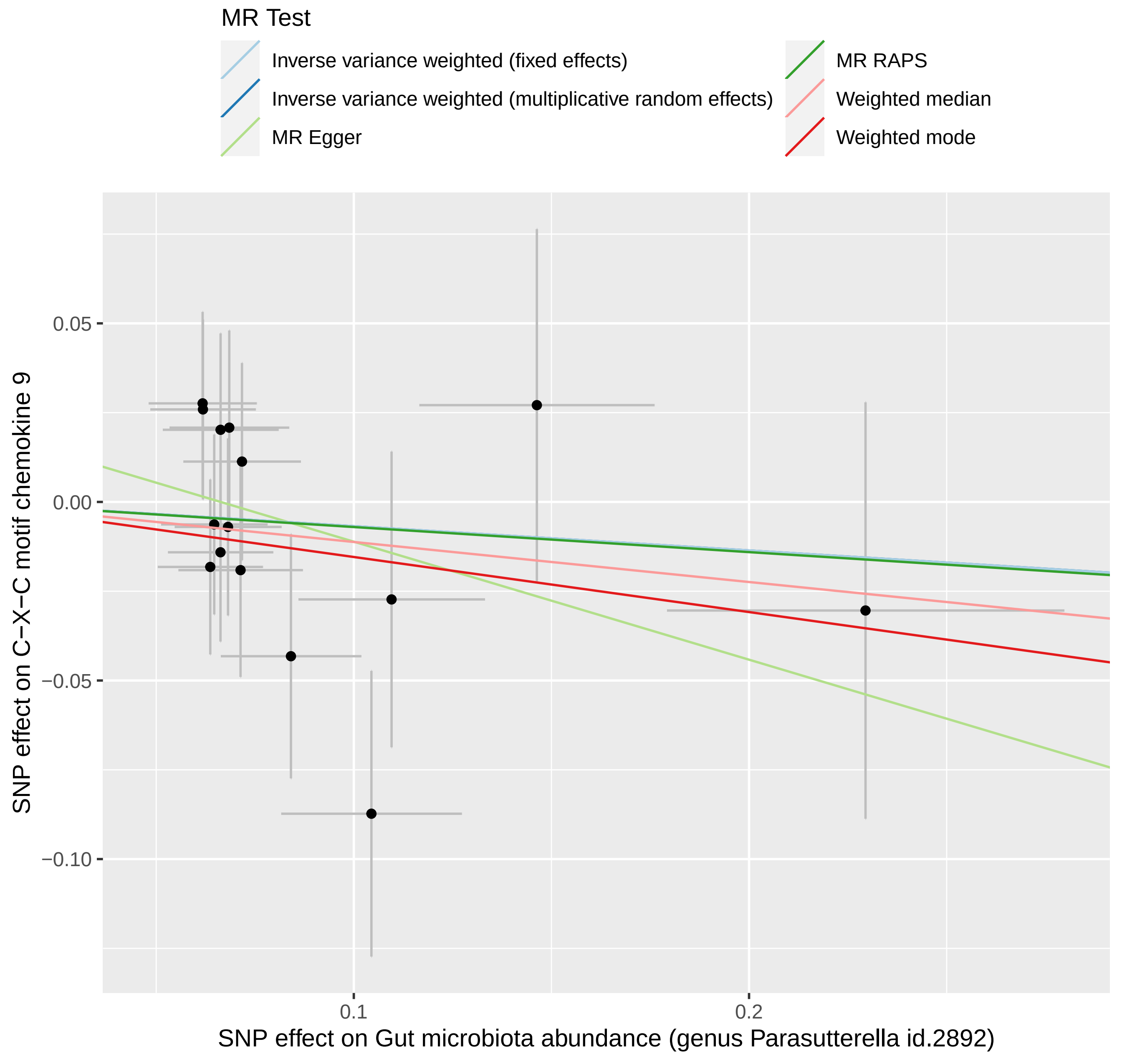

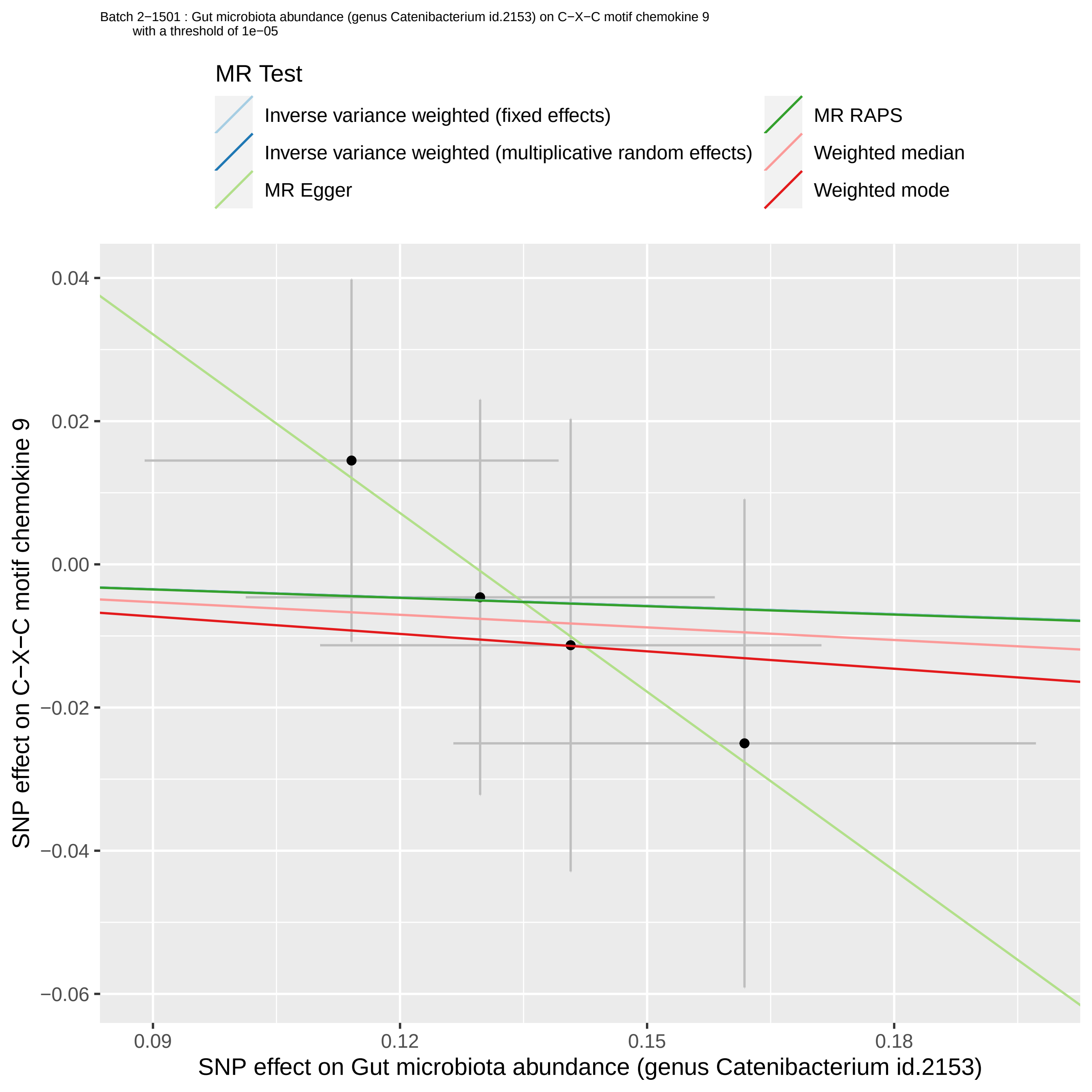
Batch 2−1501 : Gut microbiota abundance (genus Catenibacterium id.2153) on C−X−C motif chemokine 9
with a threshold of 1e−05
MR Test
Inverse variance weighted (fixed effects)
Inverse variance weighted (multiplicative random effects)
MR Egger
MR RAPS
Weighted median
Weighted mode
0.04
0.02
0.00
−0.02
−0.04
−0.06
0.09
0.12
0.15
0.18
SNP effect on C−X−C motif chemokine 9
SNP effect on Gut microbiota abundance (genus Catenibacterium id.2153)

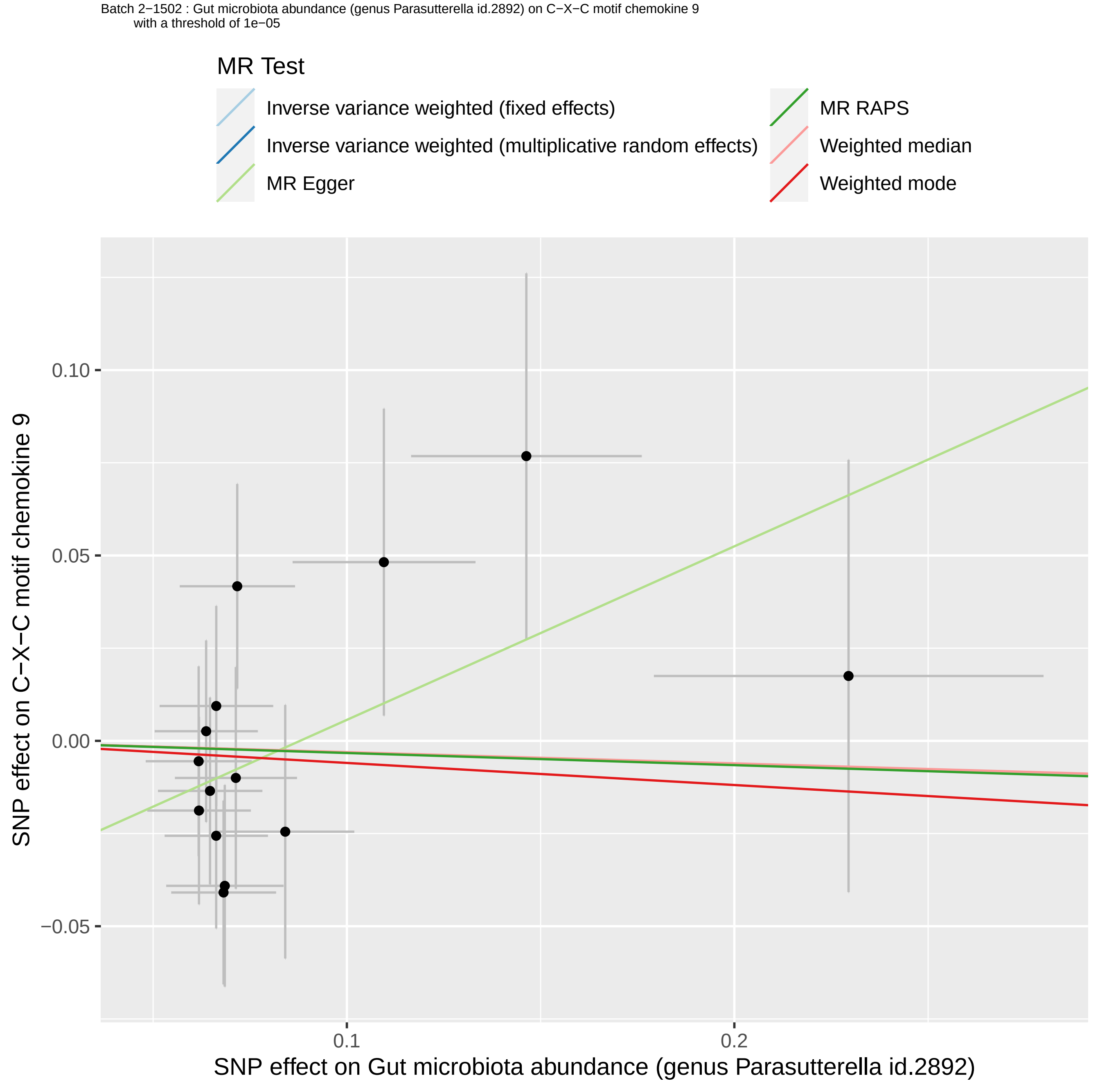
Batch 2−1502 : Gut microbiota abundance (genus Parasutterella id.2892) on C−X−C motif chemokine 9
with a threshold of 1e−05
MR Test
Inverse variance weighted (fixed effects)
Inverse variance weighted (multiplicative random effects)
MR Egger
MR RAPS
Weighted median
Weighted mode
0.10
0.05
0.00
−0.05
0.1
0.2
SNP effect on C−X−C motif chemokine 9
SNP effect on Gut microbiota abundance (genus Parasutterella id.2892)

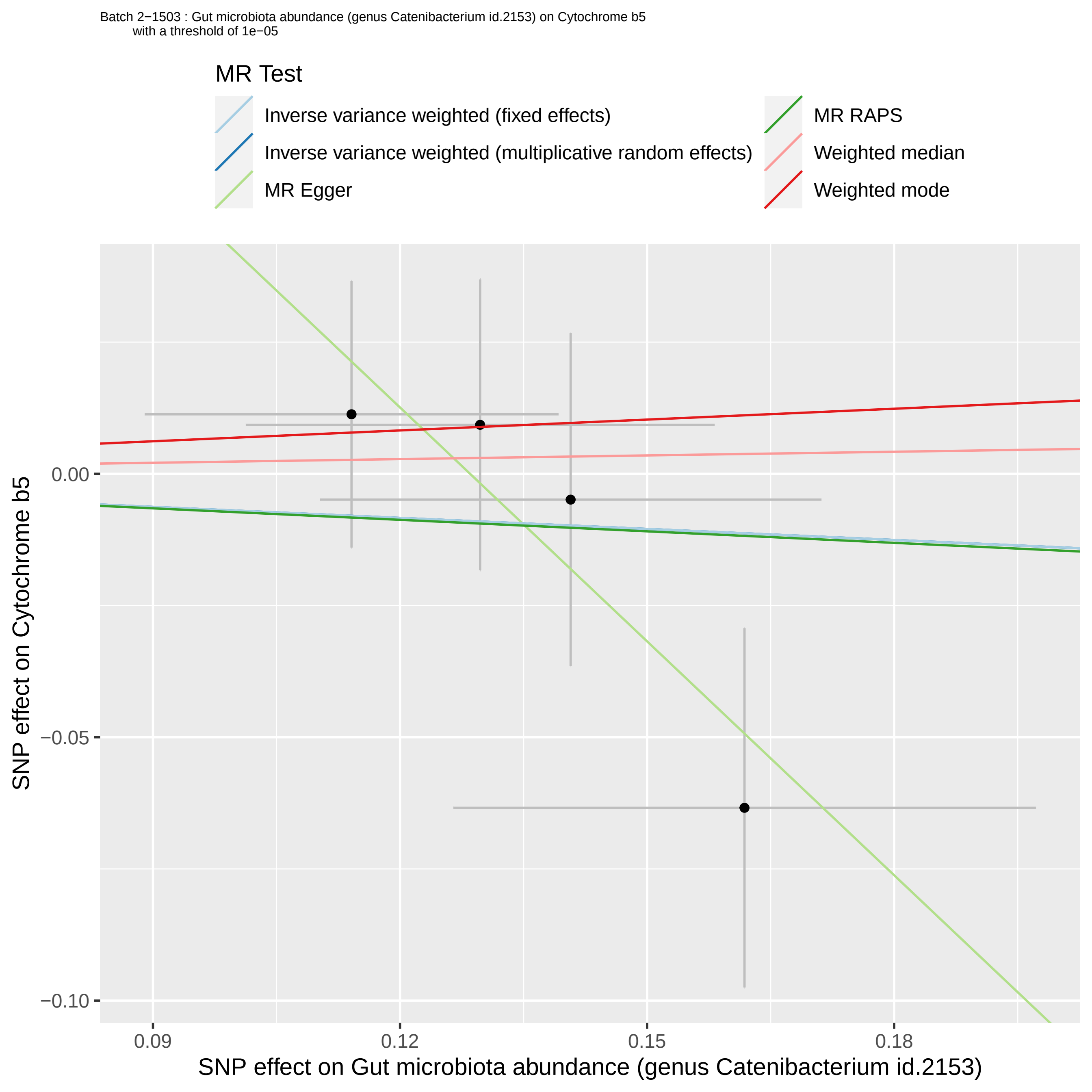

Batch 2−1503 : Gut microbiota abundance (genus Catenibacterium id.2153) on Cytochrome b5
with a threshold of 1e−05
MR Test
Inverse variance weighted (fixed effects)
Inverse variance weighted (multiplicative random effects)
MR Egger
MR RAPS
Weighted median
Weighted mode
0.00
−0.05
−0.10
0.09
0.12
0.15
0.18
SNP effect on Cytochrome b5
SNP effect on Gut microbiota abundance (genus Catenibacterium id.2153)

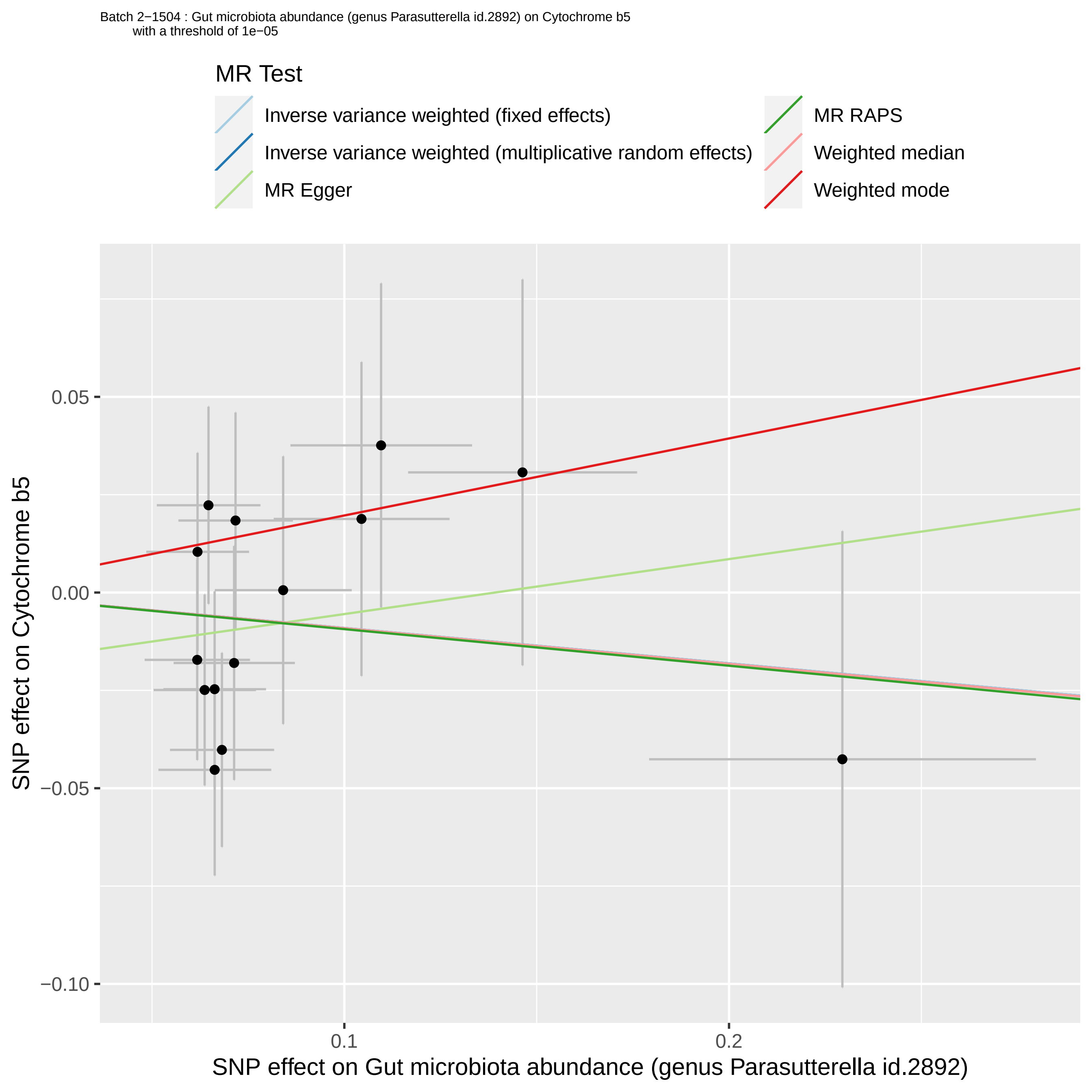
Batch 2−1504 : Gut microbiota abundance (genus Parasutterella id.2892) on Cytochrome b5
with a threshold of 1e−05
MR Test
Inverse variance weighted (fixed effects)
Inverse variance weighted (multiplicative random effects)
MR Egger
MR RAPS
Weighted median
Weighted mode
0.05
0.00
−0.05
−0.10
0.1
0.2
SNP effect on Cytochrome b5
SNP effect on Gut microbiota abundance (genus Parasutterella id.2892)

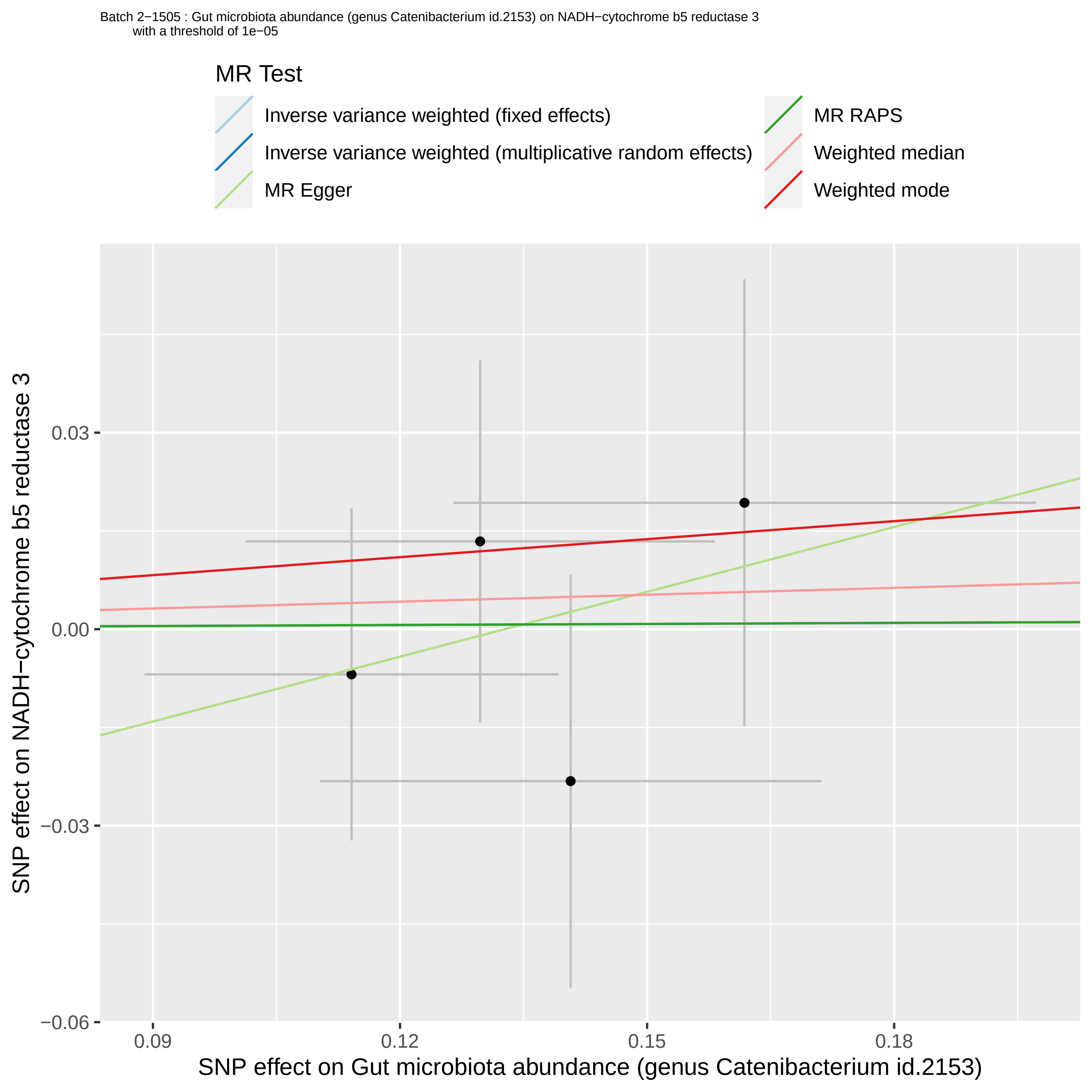
Batch 2−1505 : Gut microbiota abundance (genus Catenibacterium id.2153) on NADH−cytochrome b5 reductase 3
with a threshold of 1e−05
MR Test
Inverse variance weighted (fixed effects)
Inverse variance weighted (multiplicative random effects)
MR Egger
MR RAPS
Weighted median
Weighted mode
SNP effect on NADH−cytochrome b5 reductase 3
0.03
0.00
−0.03
−0.06
0.09
0.12
0.15
0.18
SNP effect on Gut microbiota abundance (genus Catenibacterium id.2153)

Batch 2−1506 : Gut microbiota abundance (genus Parasutterella id.2892) on NADH−cytochrome b5 reductase 3
with a threshold of 1e−05

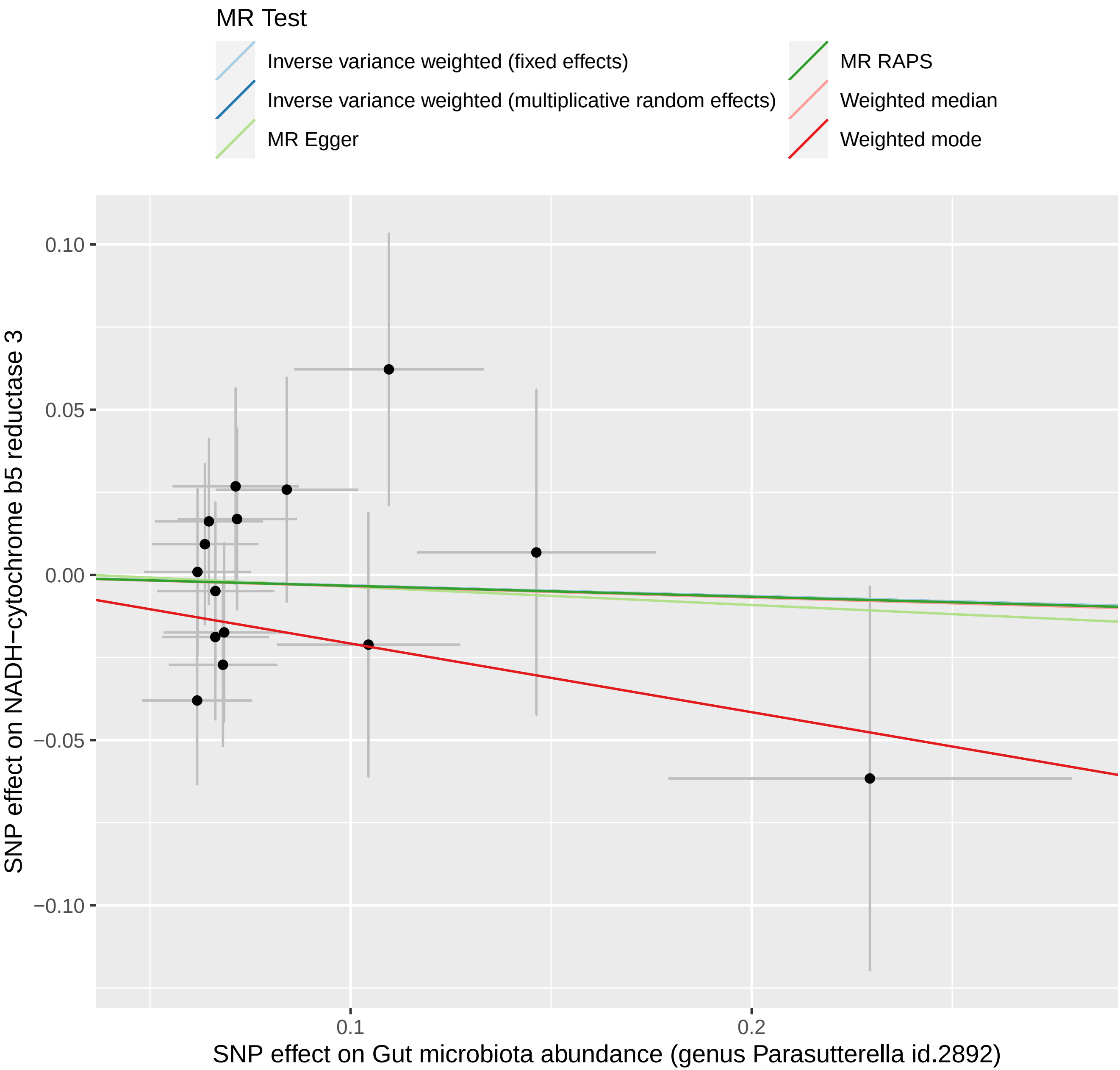

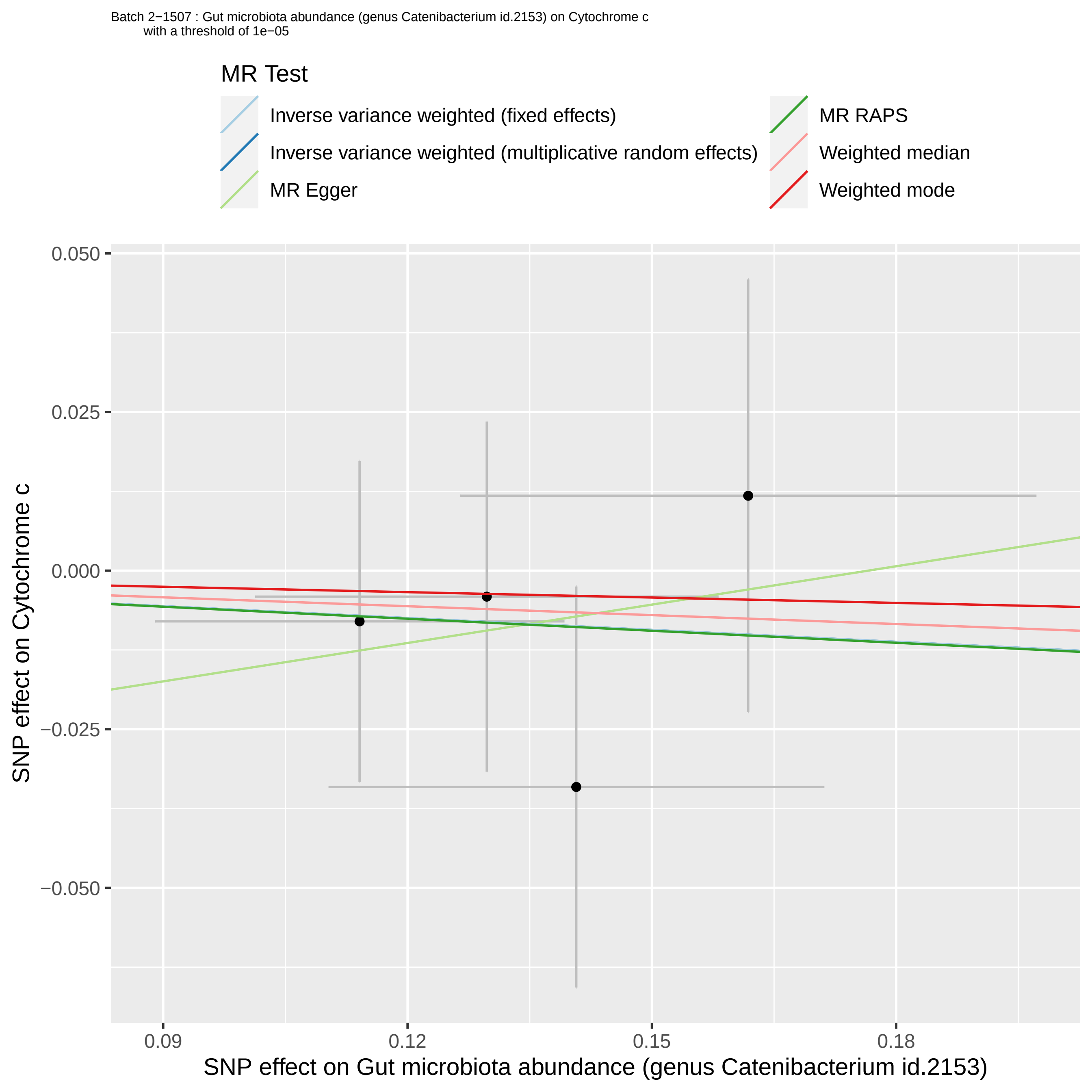
Batch 2−1507 : Gut microbiota abundance (genus Catenibacterium id.2153) on Cytochrome c
with a threshold of 1e−05
MR Test
Inverse variance weighted (fixed effects)
Inverse variance weighted (multiplicative random effects)
MR Egger
MR RAPS
Weighted median
Weighted mode
0.050
0.025
0.000
−0.025
−0.050
0.09
0.12
0.15
0.18
SNP effect on Cytochrome c
SNP effect on Gut microbiota abundance (genus Catenibacterium id.2153)

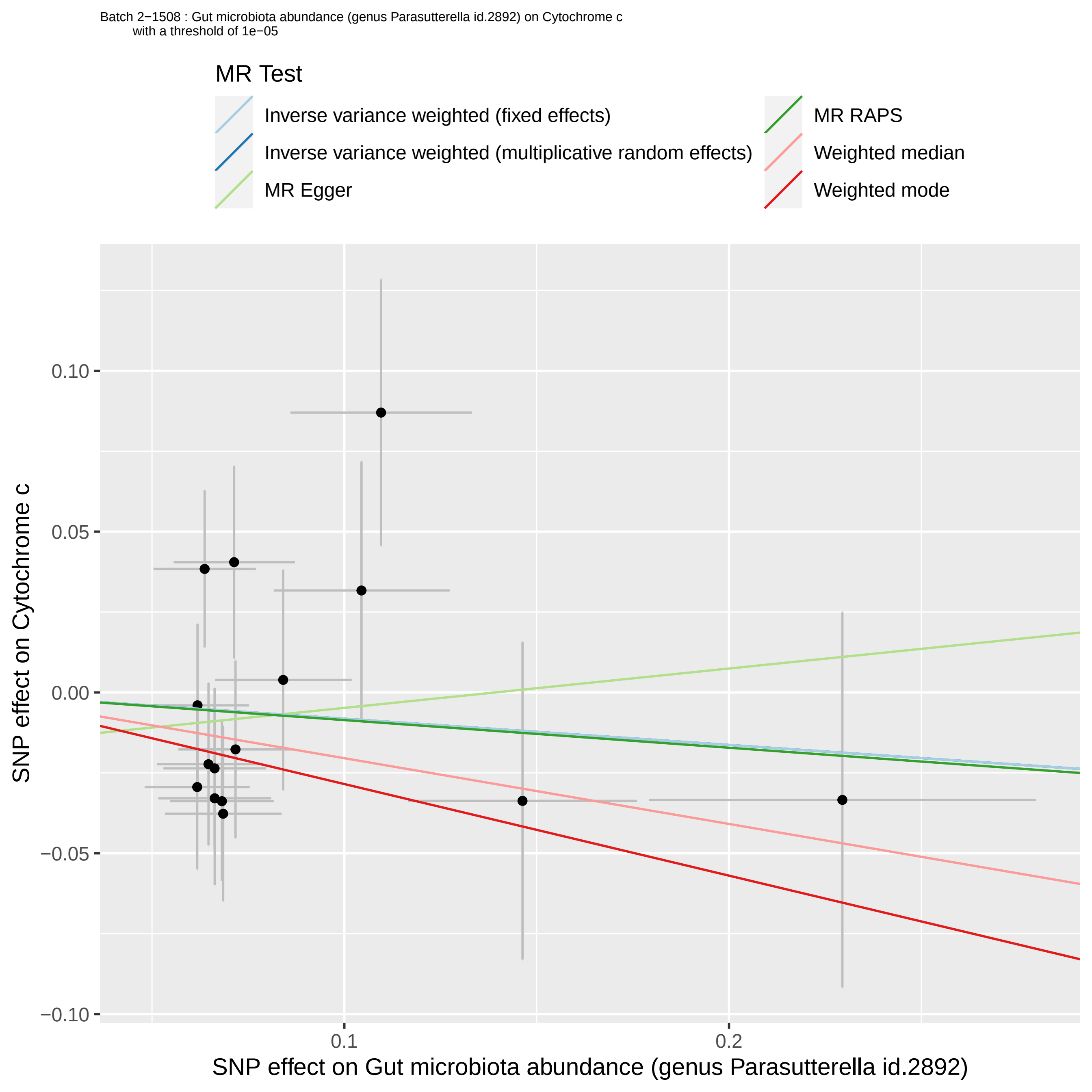

Batch 2−1508 : Gut microbiota abundance (genus Parasutterella id.2892) on Cytochrome c
with a threshold of 1e−05
MR Test
Inverse variance weighted (fixed effects)
Inverse variance weighted (multiplicative random effects)
MR Egger
MR RAPS
Weighted median
Weighted mode
0.10
0.05
0.00
−0.05
−0.10
0.1
0.2
SNP effect on Cytochrome c
SNP effect on Gut microbiota abundance (genus Parasutterella id.2892)

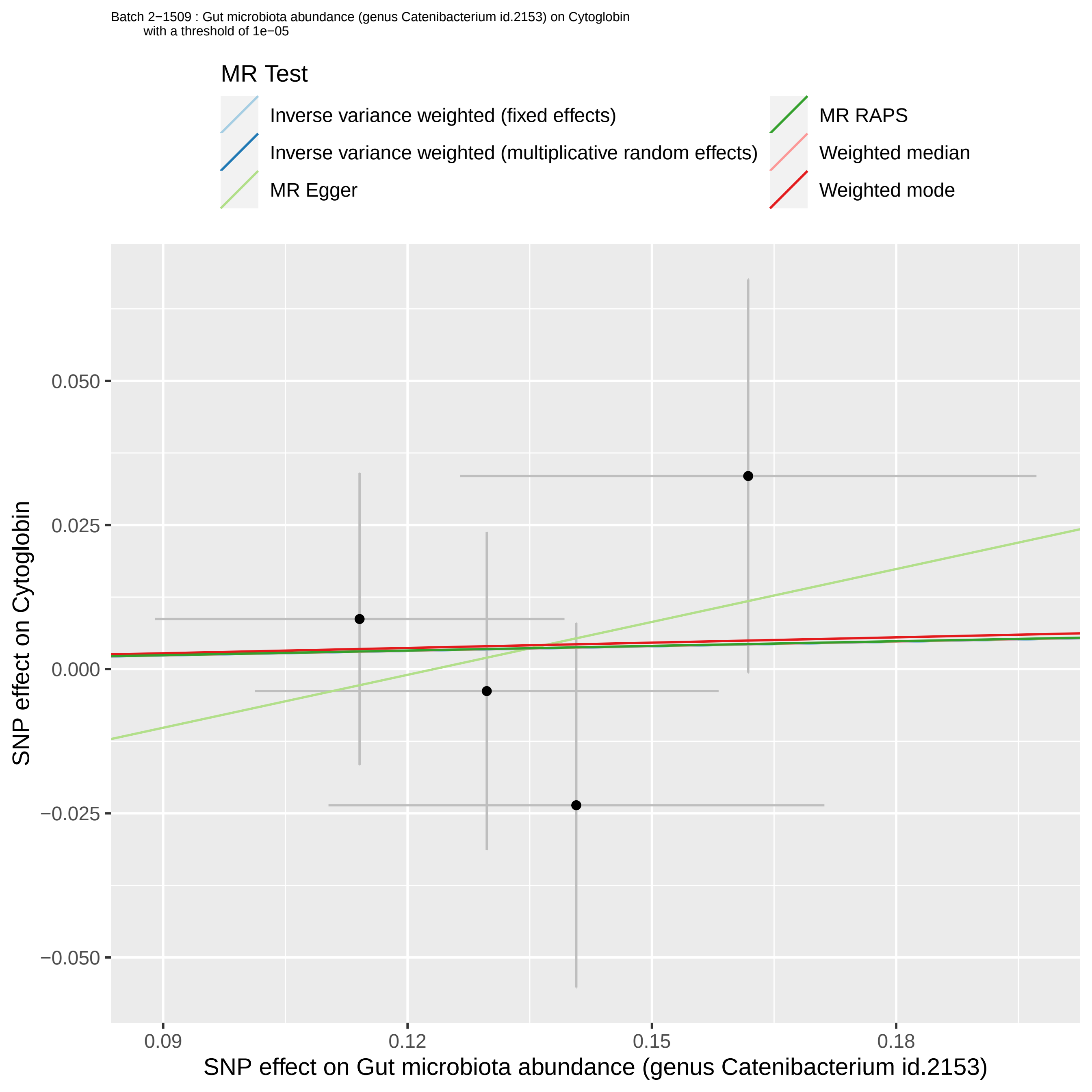
Batch 2−1509 : Gut microbiota abundance (genus Catenibacterium id.2153) on Cytoglobin
with a threshold of 1e−05
MR Test
Inverse variance weighted (fixed effects)
Inverse variance weighted (multiplicative random effects)
MR Egger
MR RAPS
Weighted median
Weighted mode
0.050
0.025
0.000
−0.025
−0.050
0.09
0.12
0.15
0.18
SNP effect on Cytoglobin
SNP effect on Gut microbiota abundance (genus Catenibacterium id.2153)

Batch 2−1510 : Gut microbiota abundance (genus Parasutterella id.2892) on Cytoglobin
with a threshold of 1e−05

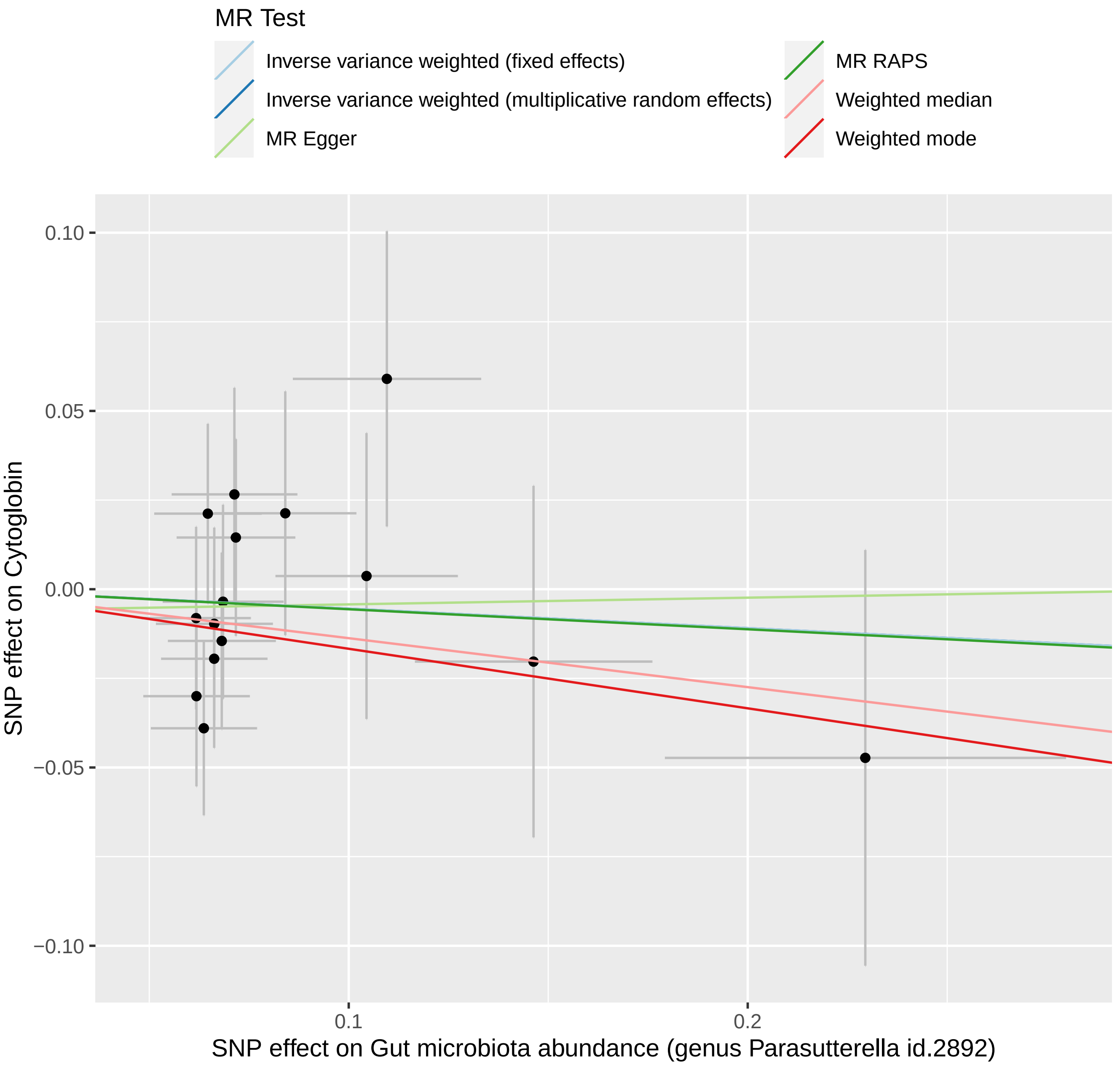

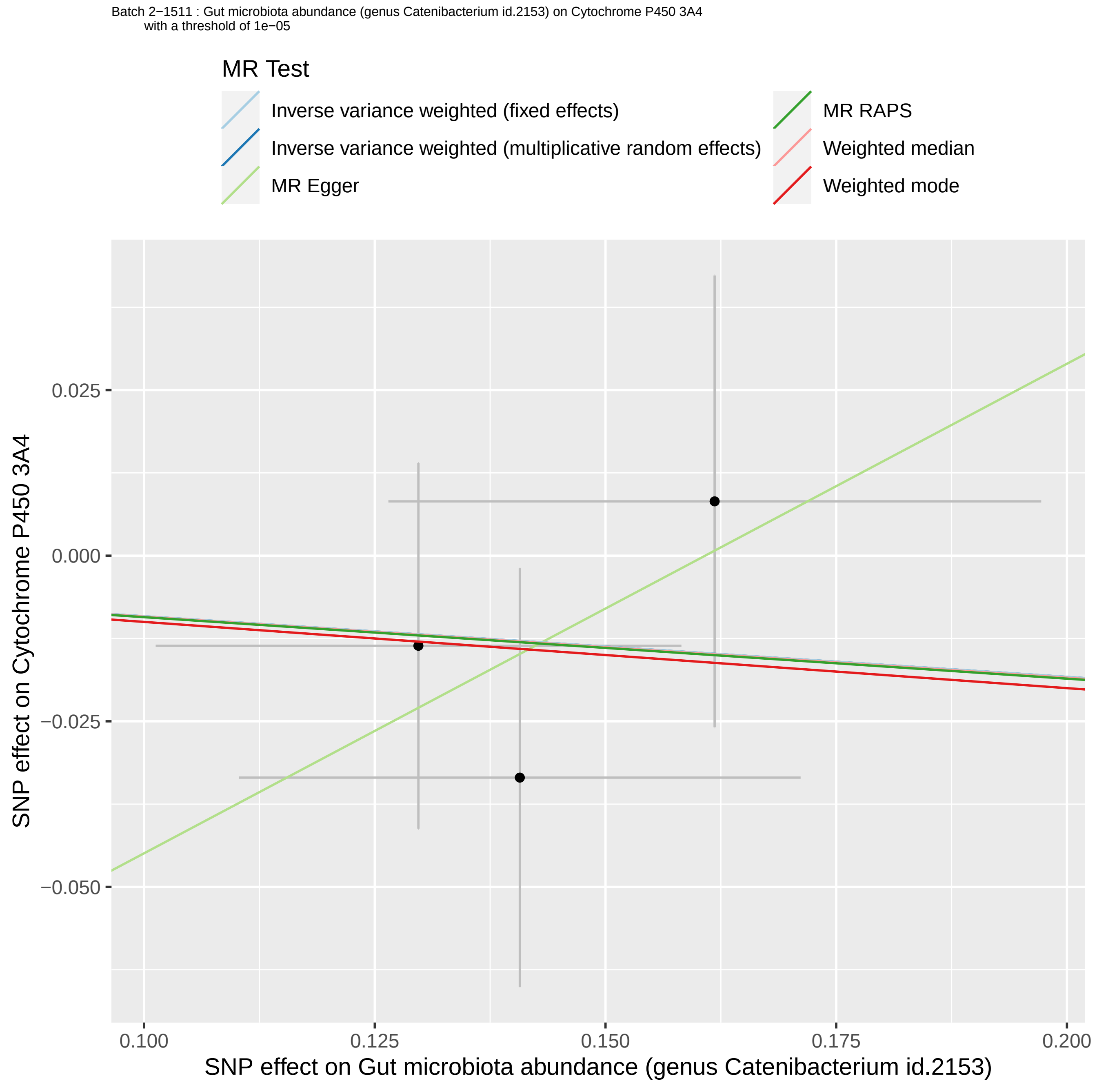

Batch 2−1511 : Gut microbiota abundance (genus Catenibacterium id.2153) on Cytochrome P450 3A4
with a threshold of 1e−05
MR Test
Inverse variance weighted (fixed effects)
Inverse variance weighted (multiplicative random effects)
MR Egger
MR RAPS
Weighted median
Weighted mode
0.025
0.000
−0.025
−0.050
0.100
0.125
0.150
0.175
0.200
SNP effect on Cytochrome P450 3A4
SNP effect on Gut microbiota abundance (genus Catenibacterium id.2153)

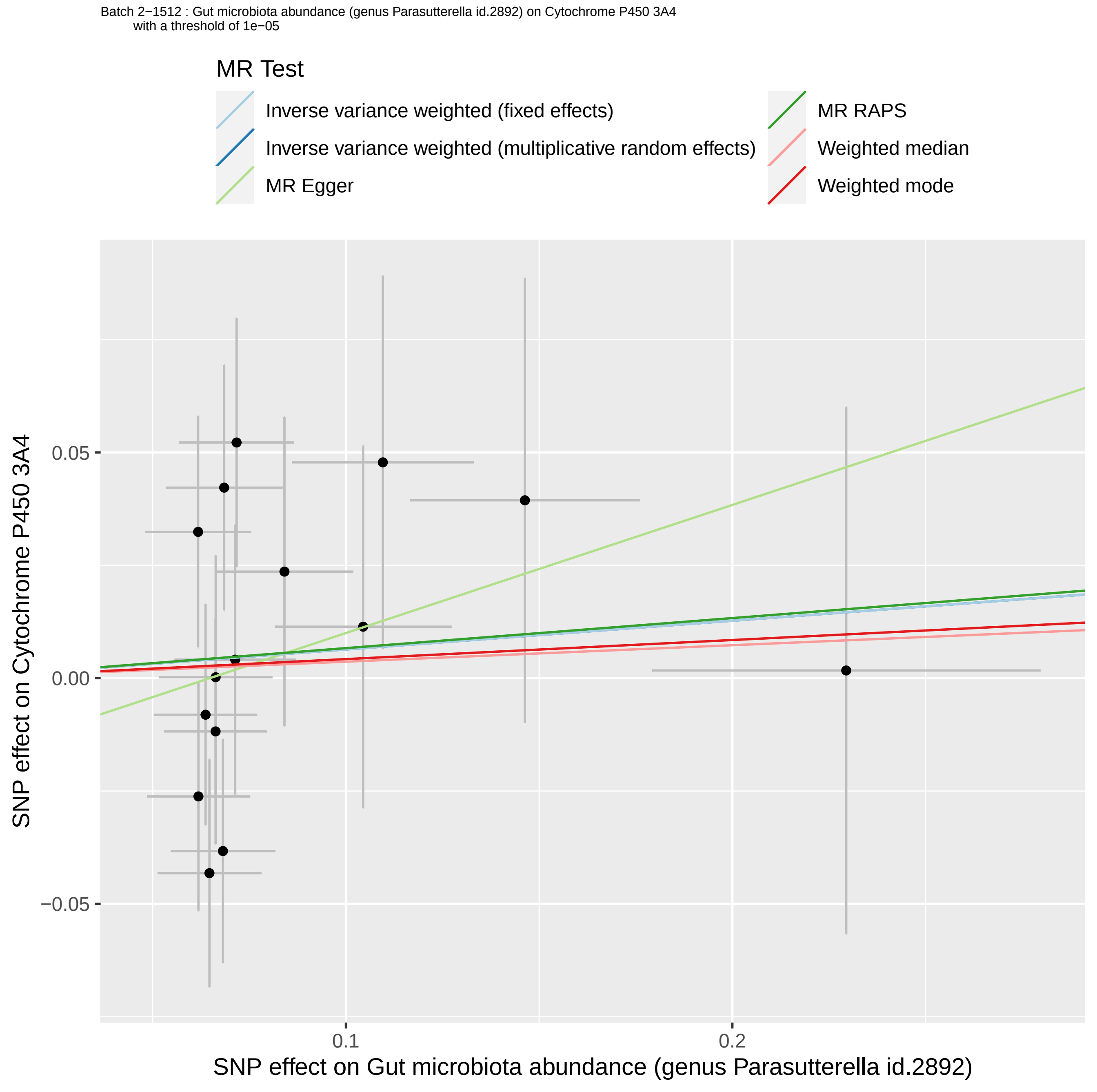

Batch 2−1512 : Gut microbiota abundance (genus Parasutterella id.2892) on Cytochrome P450 3A4
with a threshold of 1e−05
MR Test
Inverse variance weighted (fixed effects)
Inverse variance weighted (multiplicative random effects)
MR Egger
MR RAPS
Weighted median
Weighted mode
0.05
0.00
−0.05
0.1
0.2
SNP effect on Cytochrome P450 3A4
SNP effect on Gut microbiota abundance (genus Parasutterella id.2892)

Batch 2−1513 : Gut microbiota abundance (genus Catenibacterium id.2153) on Cytochrome P450 3A4
with a threshold of 1e−05

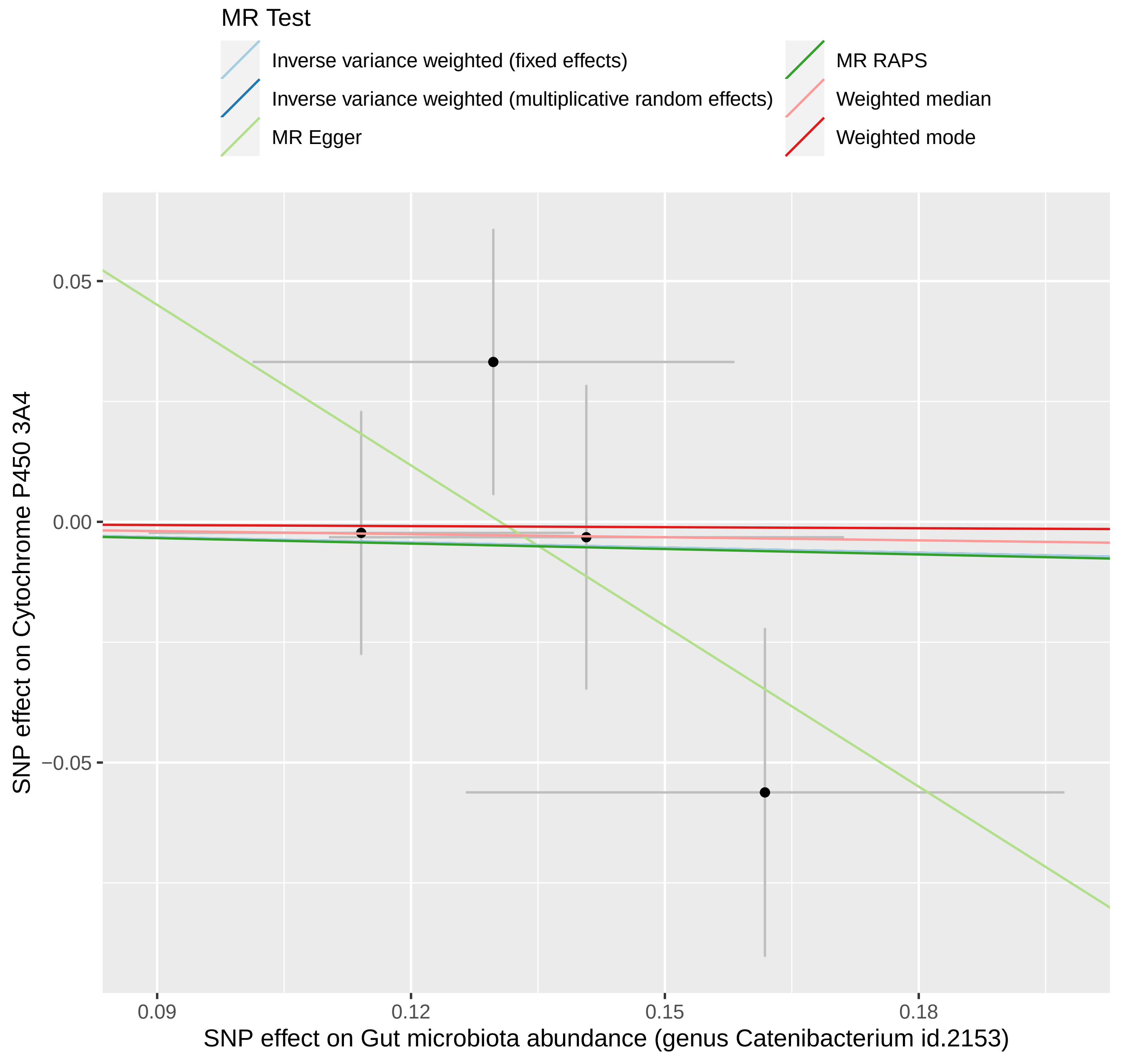

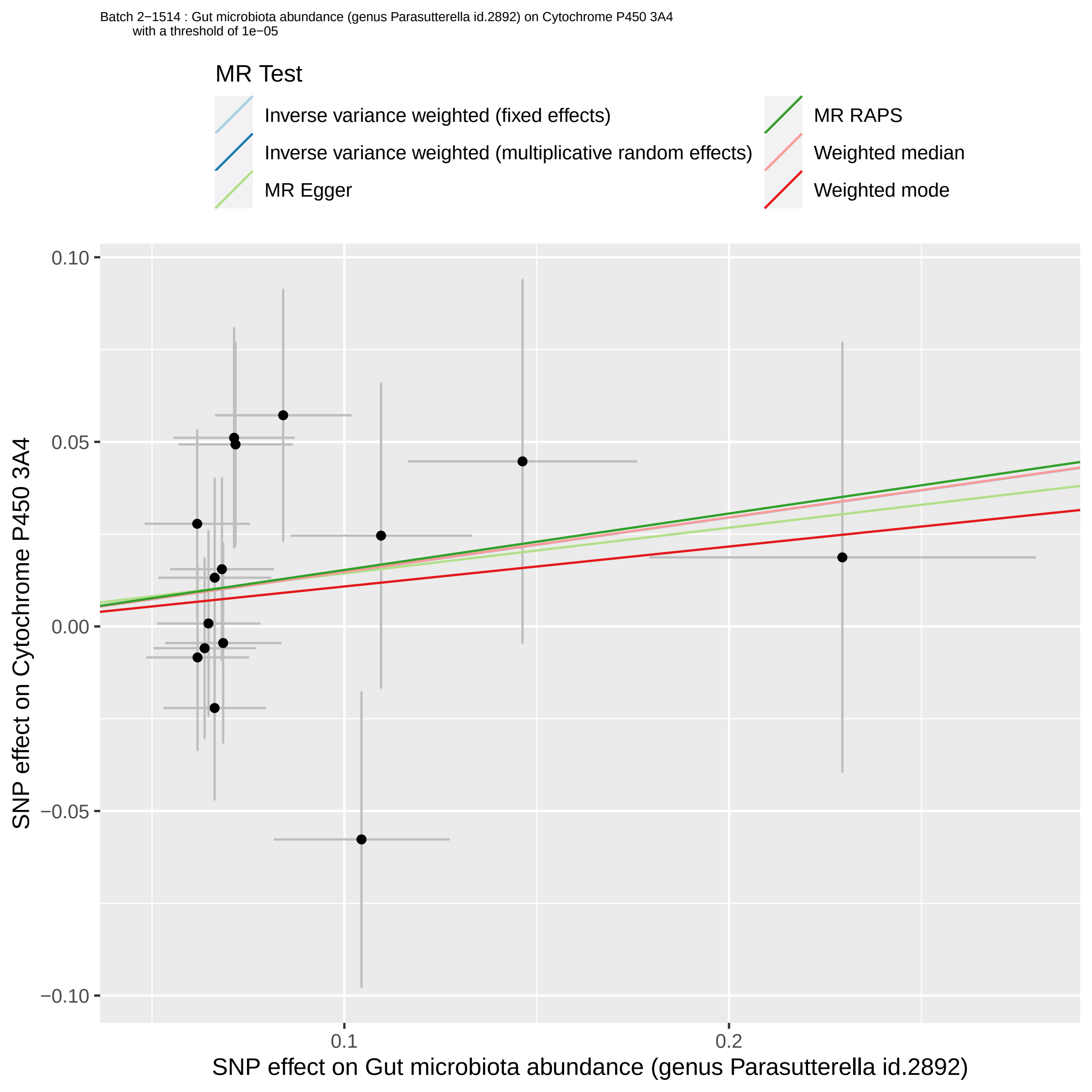
Batch 2−1514 : Gut microbiota abundance (genus Parasutterella id.2892) on Cytochrome P450 3A4
with a threshold of 1e−05
MR Test
Inverse variance weighted (fixed effects)
Inverse variance weighted (multiplicative random effects)
MR Egger
MR RAPS
Weighted median
Weighted mode
0.10
0.05
0.00
−0.05
−0.10
0.1
0.2
SNP effect on Cytochrome P450 3A4
SNP effect on Gut microbiota abundance (genus Parasutterella id.2892)

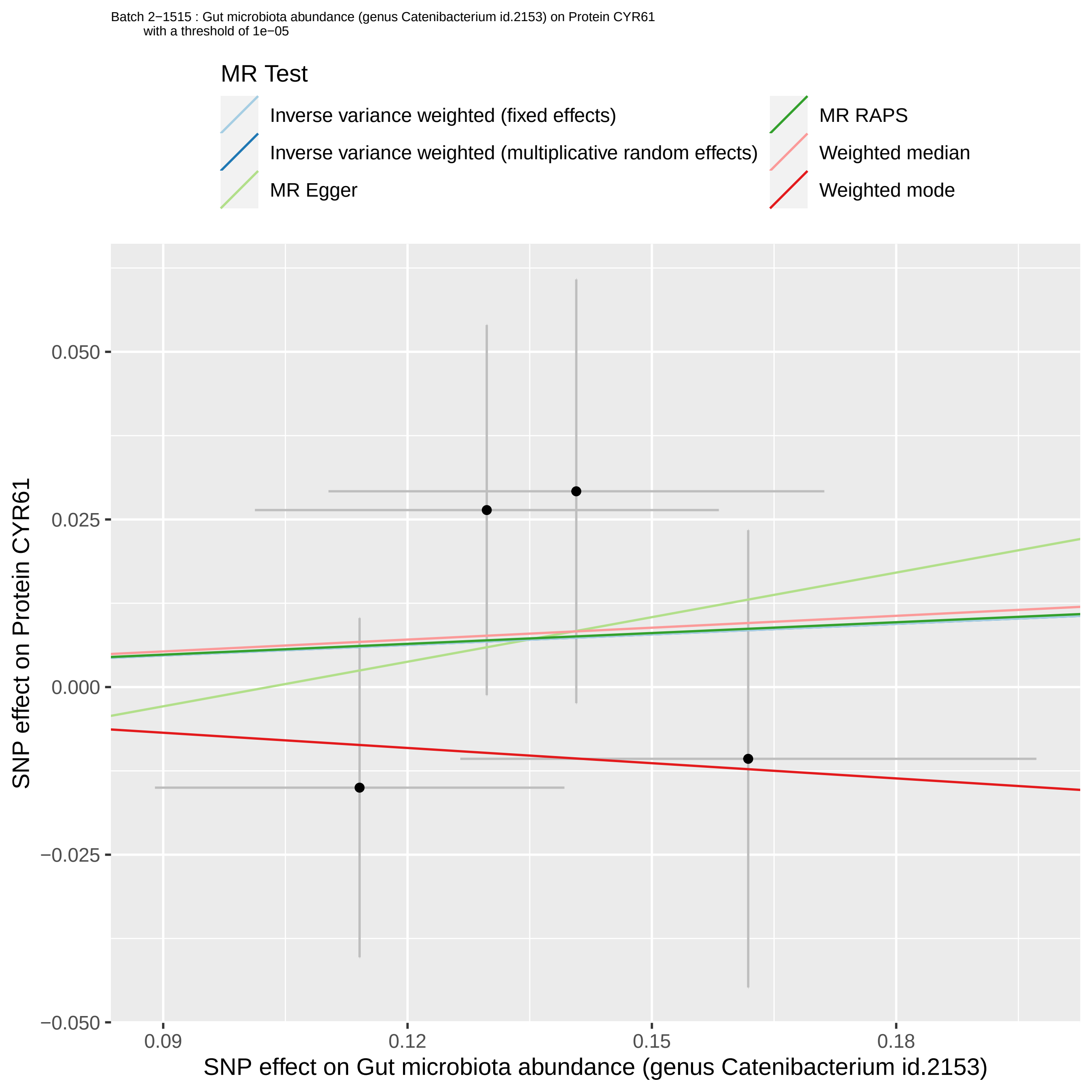
Batch 2−1515 : Gut microbiota abundance (genus Catenibacterium id.2153) on Protein CYR61
with a threshold of 1e−05
MR Test
Inverse variance weighted (fixed effects)
Inverse variance weighted (multiplicative random effects)
MR Egger
MR RAPS
Weighted median
Weighted mode
0.050
0.025
0.000
−0.025
−0.050
0.09
0.12
0.15
0.18
SNP effect on Protein CYR61
SNP effect on Gut microbiota abundance (genus Catenibacterium id.2153)

Batch 2−1516 : Gut microbiota abundance (genus Parasutterella id.2892) on Protein CYR61
with a threshold of 1e−05

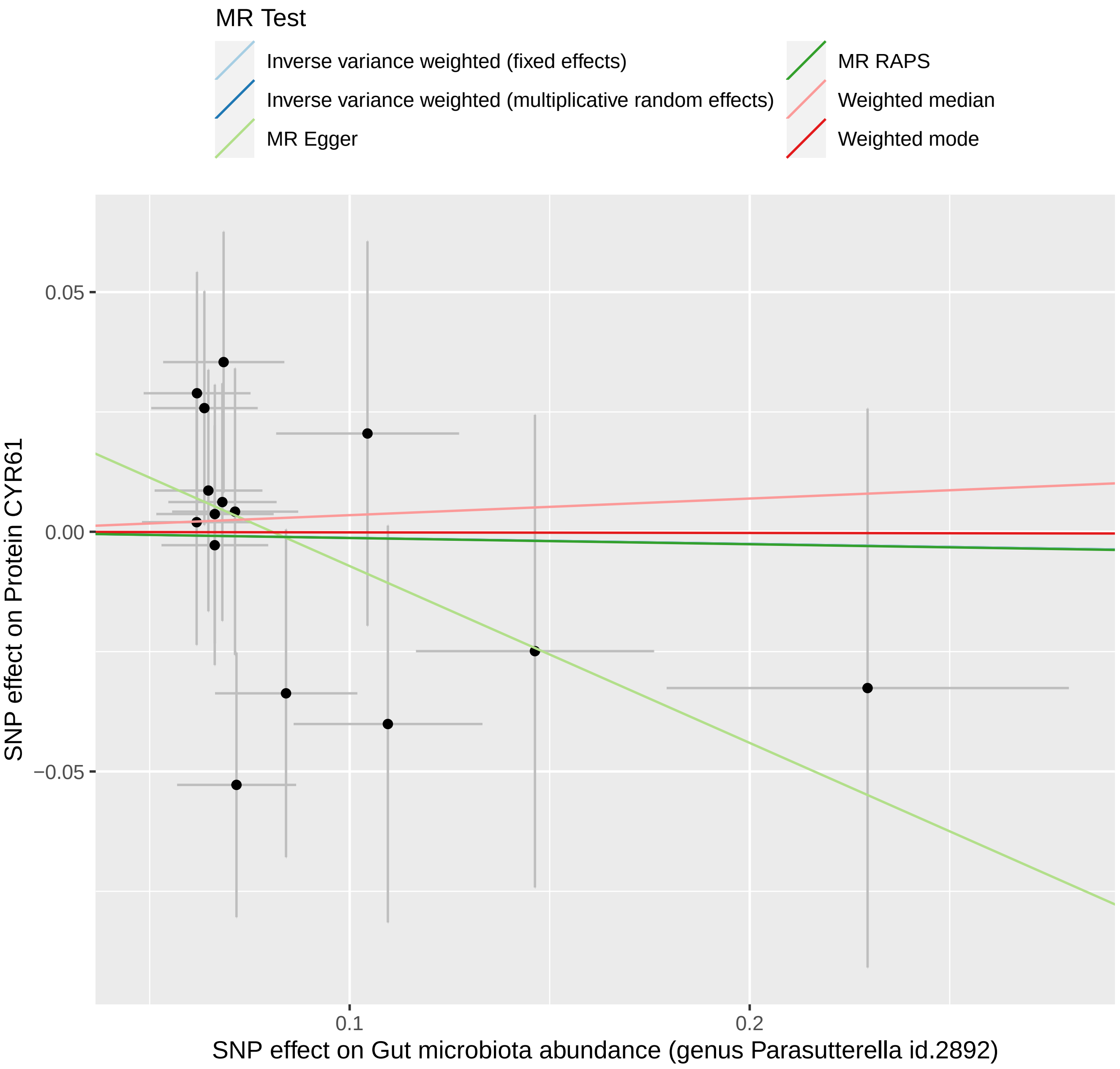

Batch 2−1517 : Gut microbiota abundance (genus Catenibacterium id.2153) on Cytohesin−2
with a threshold of 1e−05

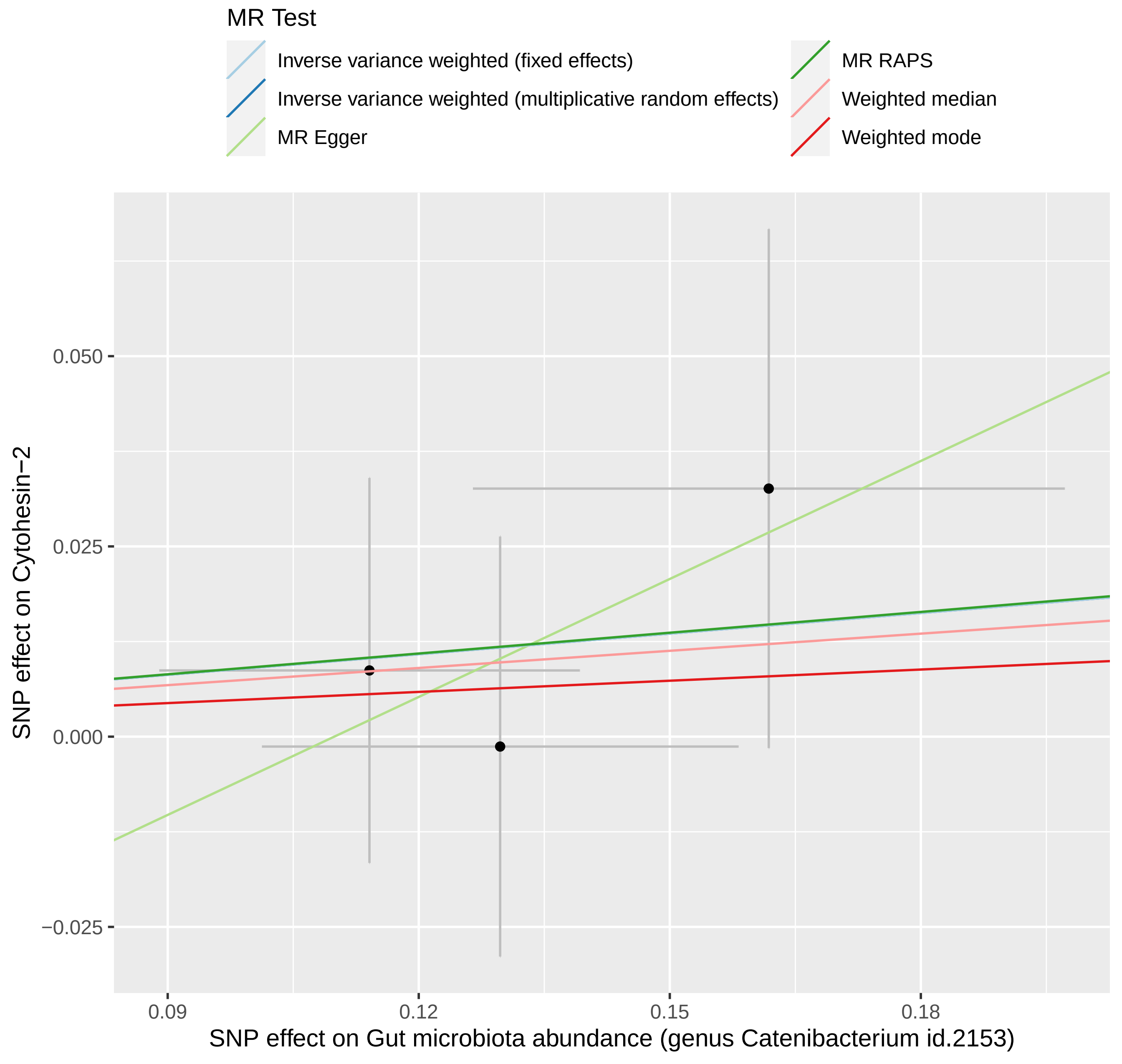

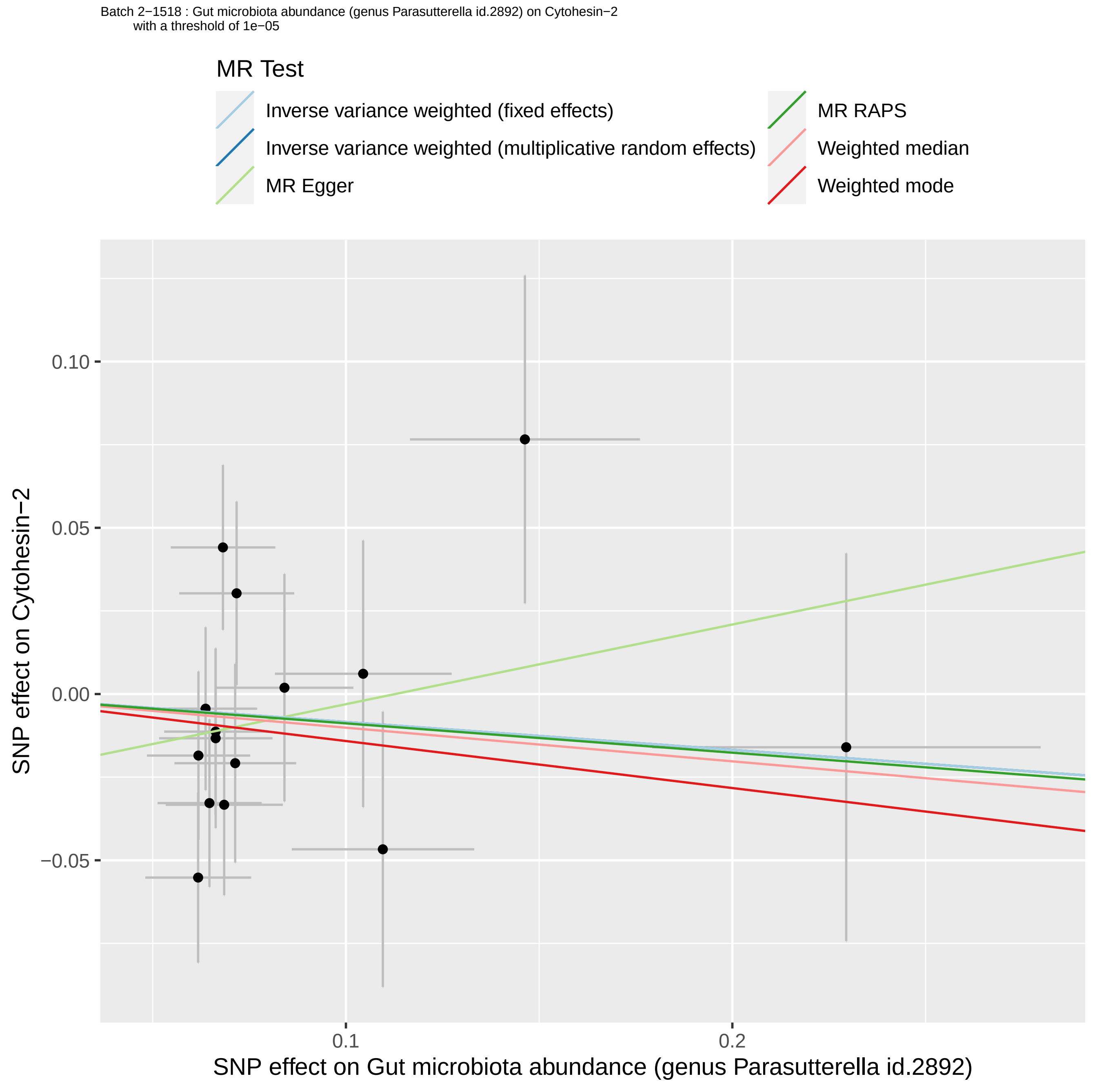

Batch 2−1518 : Gut microbiota abundance (genus Parasutterella id.2892) on Cytohesin−2
with a threshold of 1e−05
MR Test
Inverse variance weighted (fixed effects)
Inverse variance weighted (multiplicative random effects)
MR Egger
MR RAPS
Weighted median
Weighted mode
0.10
0.05
0.00
−0.05
0.1
0.2
SNP effect on Cytohesin−2
SNP effect on Gut microbiota abundance (genus Parasutterella id.2892)

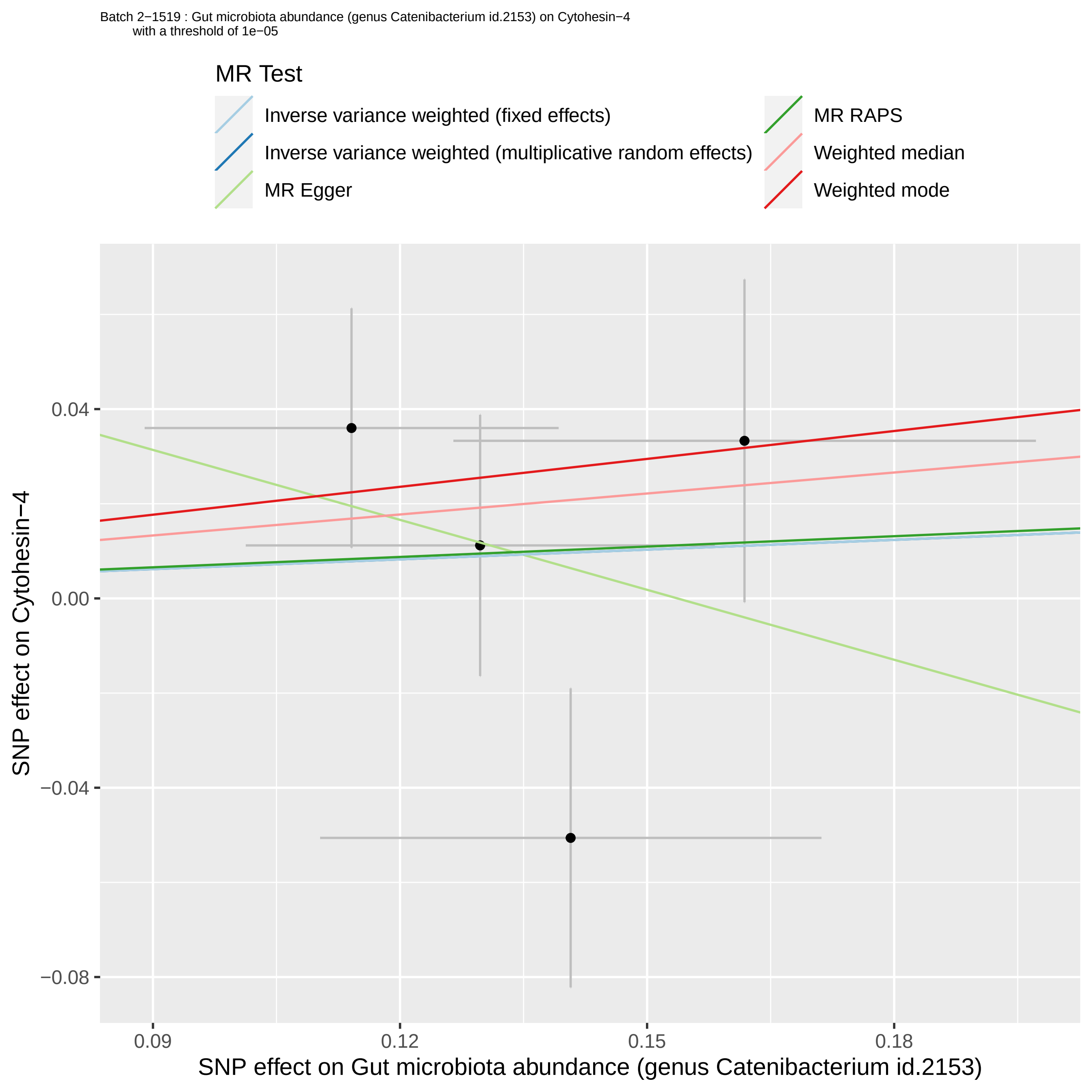
Batch 2−1519 : Gut microbiota abundance (genus Catenibacterium id.2153) on Cytohesin−4
with a threshold of 1e−05
MR Test
Inverse variance weighted (fixed effects)
Inverse variance weighted (multiplicative random effects)
MR Egger
MR RAPS
Weighted median
Weighted mode
SNP effect on Cytohesin−4
0.04
0.00
−0.04
−0.08
0.09
0.12
0.15
0.18
SNP effect on Gut microbiota abundance (genus Catenibacterium id.2153)

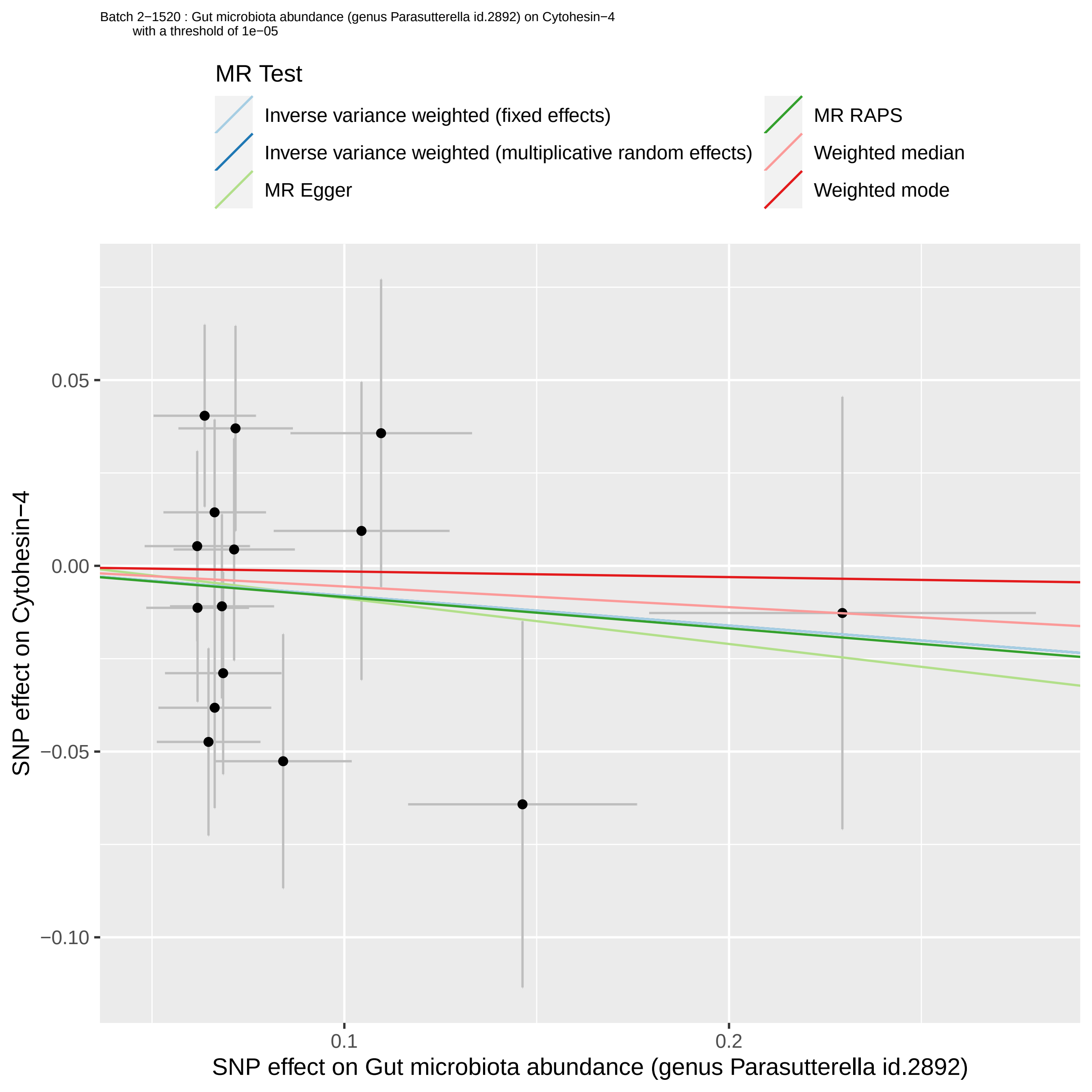
Batch 2−1520 : Gut microbiota abundance (genus Parasutterella id.2892) on Cytohesin−4
with a threshold of 1e−05
MR Test
Inverse variance weighted (fixed effects)
Inverse variance weighted (multiplicative random effects)
MR Egger
MR RAPS
Weighted median
Weighted mode
0.05
0.00
−0.05
−0.10
0.1
0.2
SNP effect on Cytohesin−4
SNP effect on Gut microbiota abundance (genus Parasutterella id.2892)

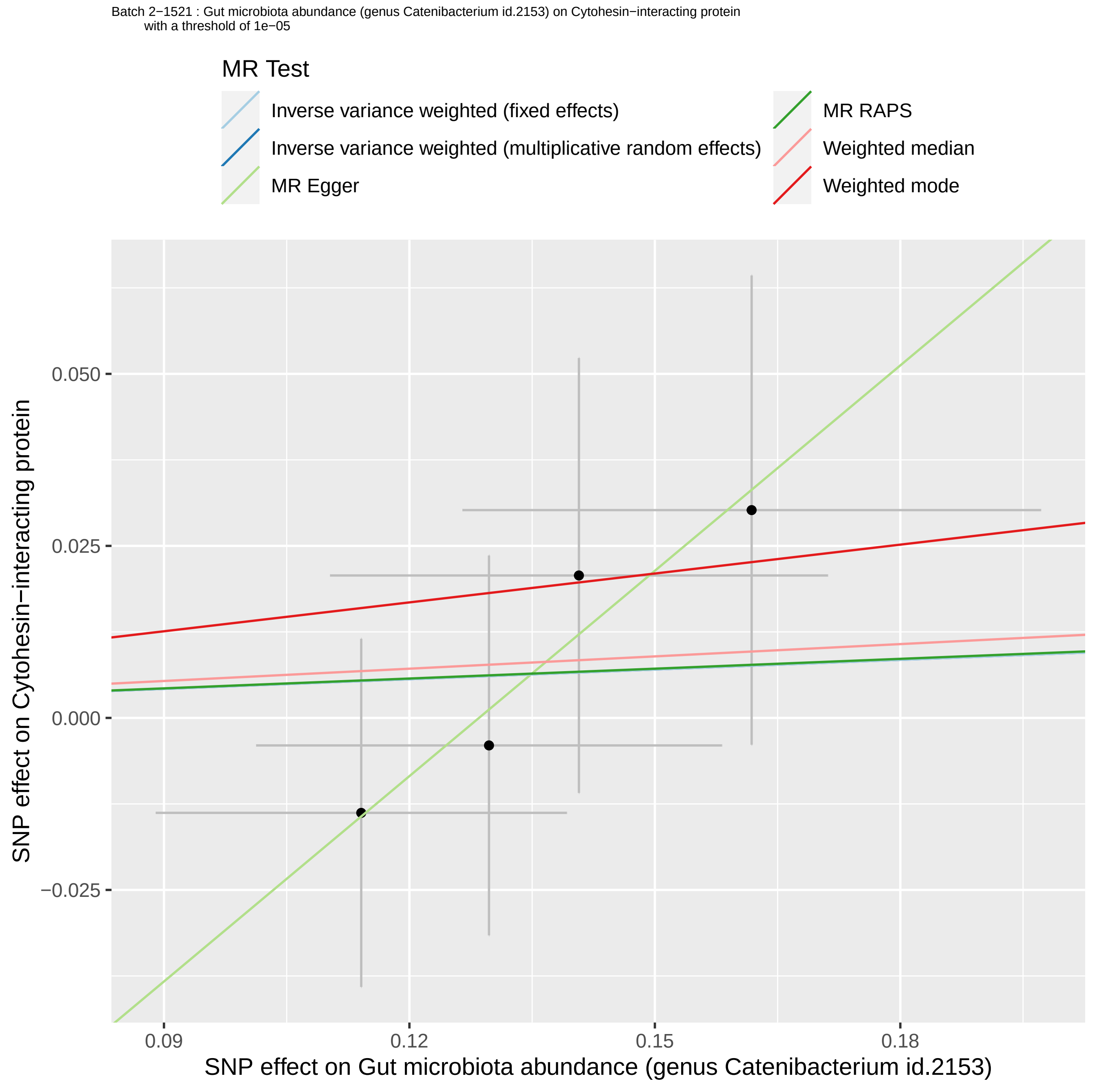

Batch 2−1521 : Gut microbiota abundance (genus Catenibacterium id.2153) on Cytohesin−interacting protein
with a threshold of 1e−05
MR Test
Inverse variance weighted (fixed effects)
Inverse variance weighted (multiplicative random effects)
MR Egger
MR RAPS
Weighted median
Weighted mode
0.050
0.025
0.000
−0.025
0.09
0.12
0.15
0.18
SNP effect on Cytohesin−interacting protein
SNP effect on Gut microbiota abundance (genus Catenibacterium id.2153)

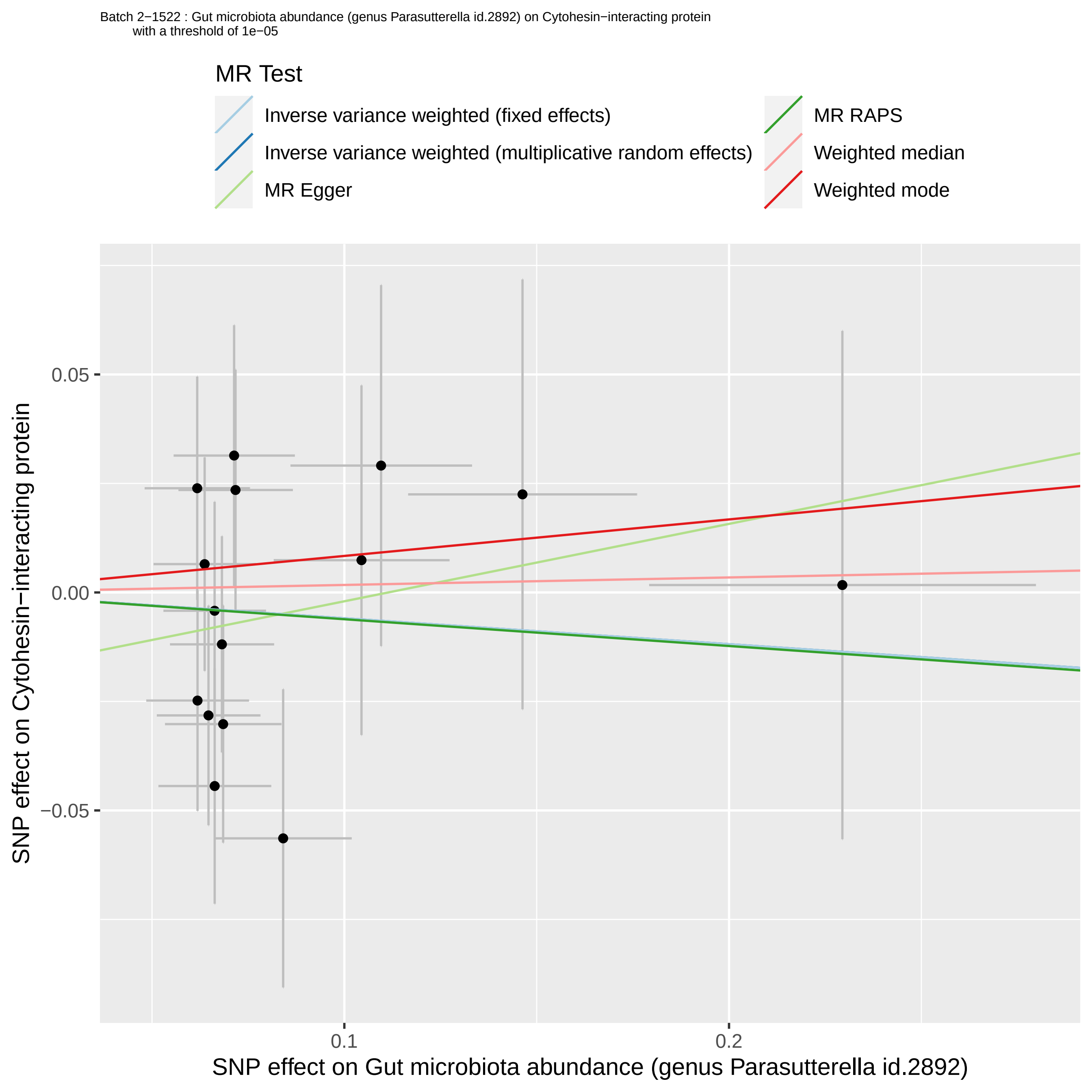
Batch 2−1522 : Gut microbiota abundance (genus Parasutterella id.2892) on Cytohesin−interacting protein
with a threshold of 1e−05
MR Test
Inverse variance weighted (fixed effects)
Inverse variance weighted (multiplicative random effects)
MR Egger
MR RAPS
Weighted median
Weighted mode
0.05
0.00
−0.05
0.1
0.2
SNP effect on Cytohesin−interacting protein
SNP effect on Gut microbiota abundance (genus Parasutterella id.2892)

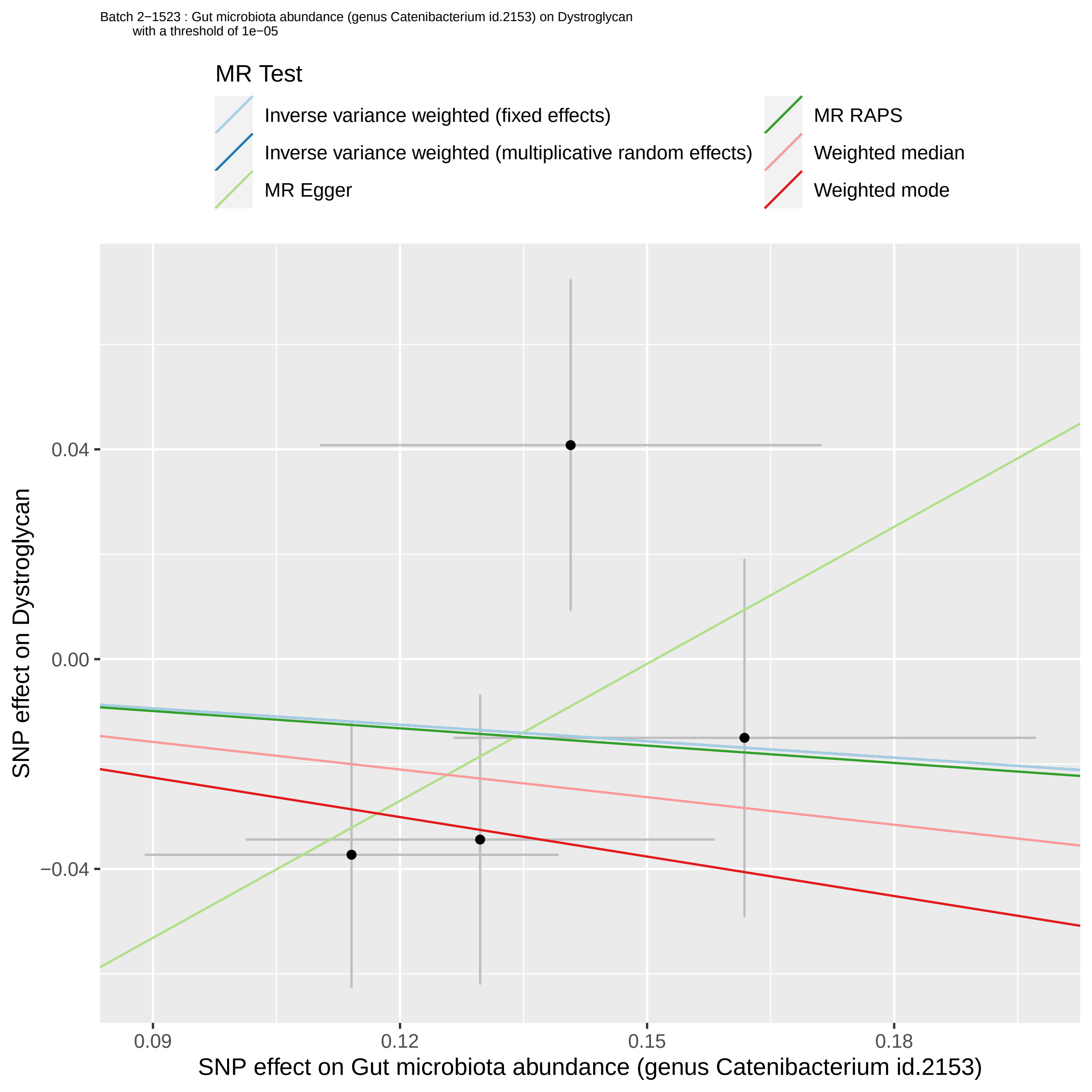

Batch 2−1523 : Gut microbiota abundance (genus Catenibacterium id.2153) on Dystroglycan
with a threshold of 1e−05
MR Test
Inverse variance weighted (fixed effects)
Inverse variance weighted (multiplicative random effects)
MR Egger
MR RAPS
Weighted median
Weighted mode
0.04
0.00
−0.04
0.09
0.12
0.15
0.18
SNP effect on Dystroglycan
SNP effect on Gut microbiota abundance (genus Catenibacterium id.2153)

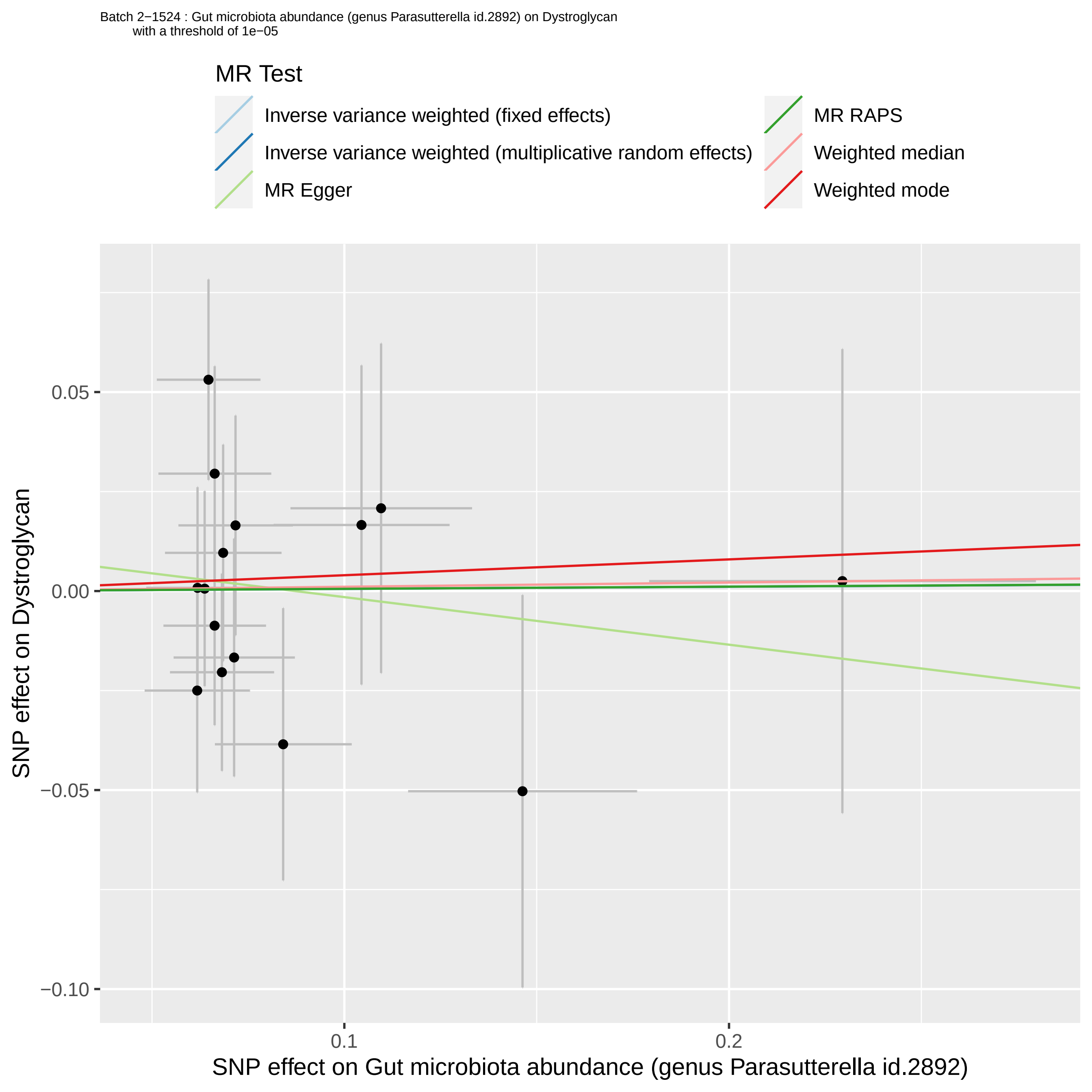
Batch 2−1524 : Gut microbiota abundance (genus Parasutterella id.2892) on Dystroglycan
with a threshold of 1e−05
MR Test
Inverse variance weighted (fixed effects)
Inverse variance weighted (multiplicative random effects)
MR Egger
MR RAPS
Weighted median
Weighted mode
0.05
0.00
−0.05
−0.10
0.1
0.2
SNP effect on Dystroglycan
SNP effect on Gut microbiota abundance (genus Parasutterella id.2892)

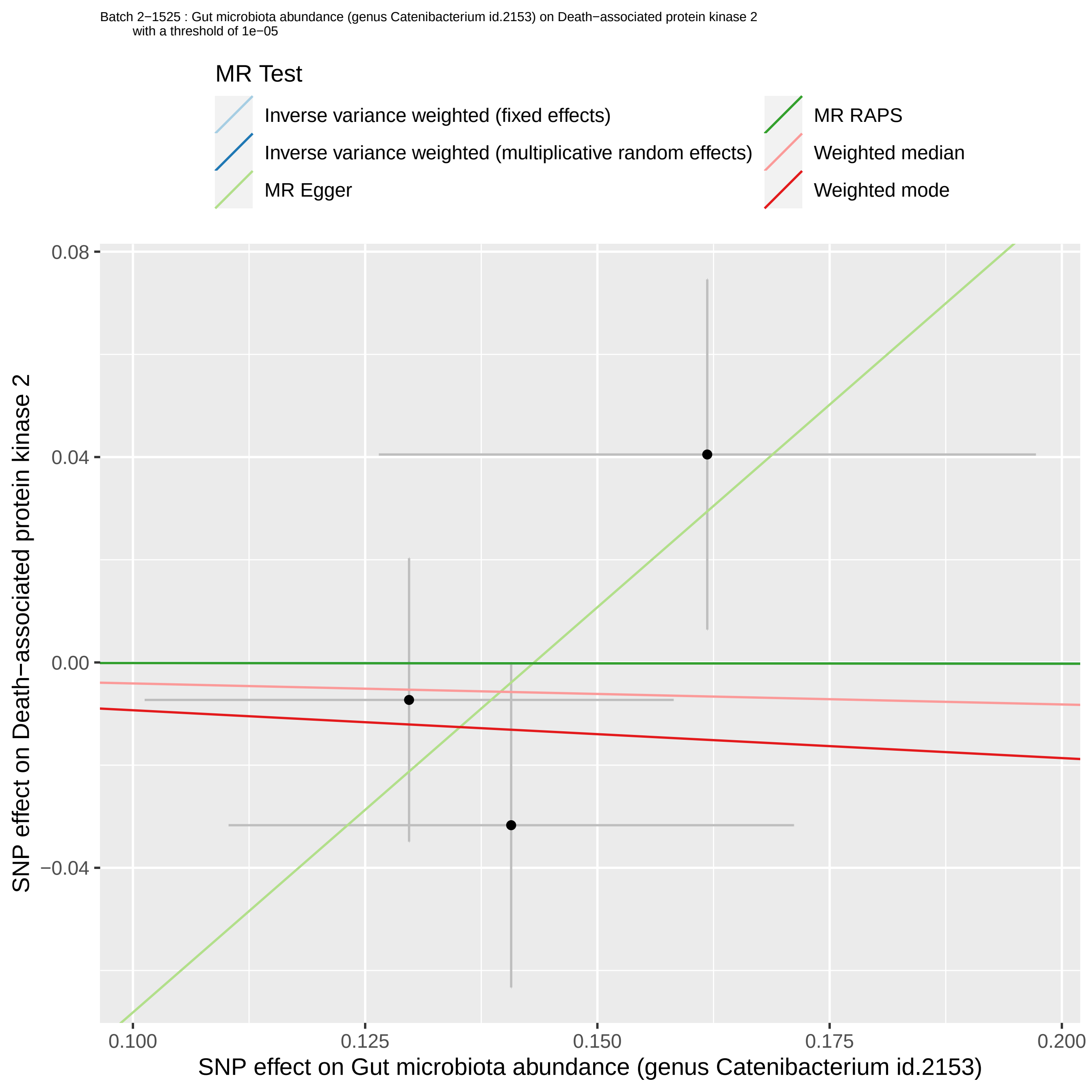
Batch 2−1525 : Gut microbiota abundance (genus Catenibacterium id.2153) on Death−associated protein kinase 2
with a threshold of 1e−05
MR Test
Inverse variance weighted (fixed effects)
Inverse variance weighted (multiplicative random effects)
MR Egger
MR RAPS
Weighted median
Weighted mode
0.08
0.04
0.00
−0.04
0.100
0.125
0.150
0.175
0.200
SNP effect on Death−associated protein kinase 2
SNP effect on Gut microbiota abundance (genus Catenibacterium id.2153)

Batch 2−1526 : Gut microbiota abundance (genus Parasutterella id.2892) on Death−associated protein kinase 2
with a threshold of 1e−05

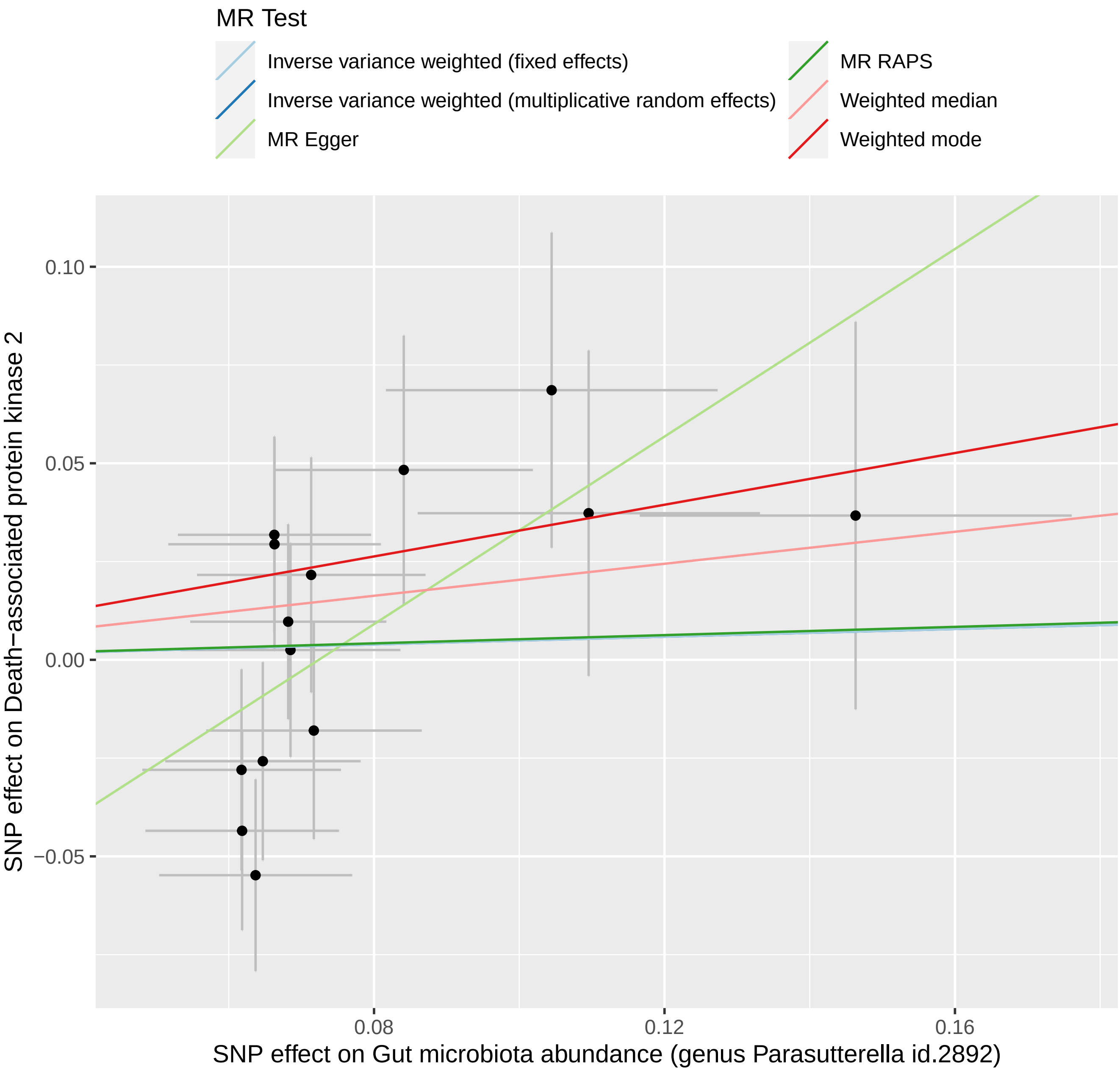

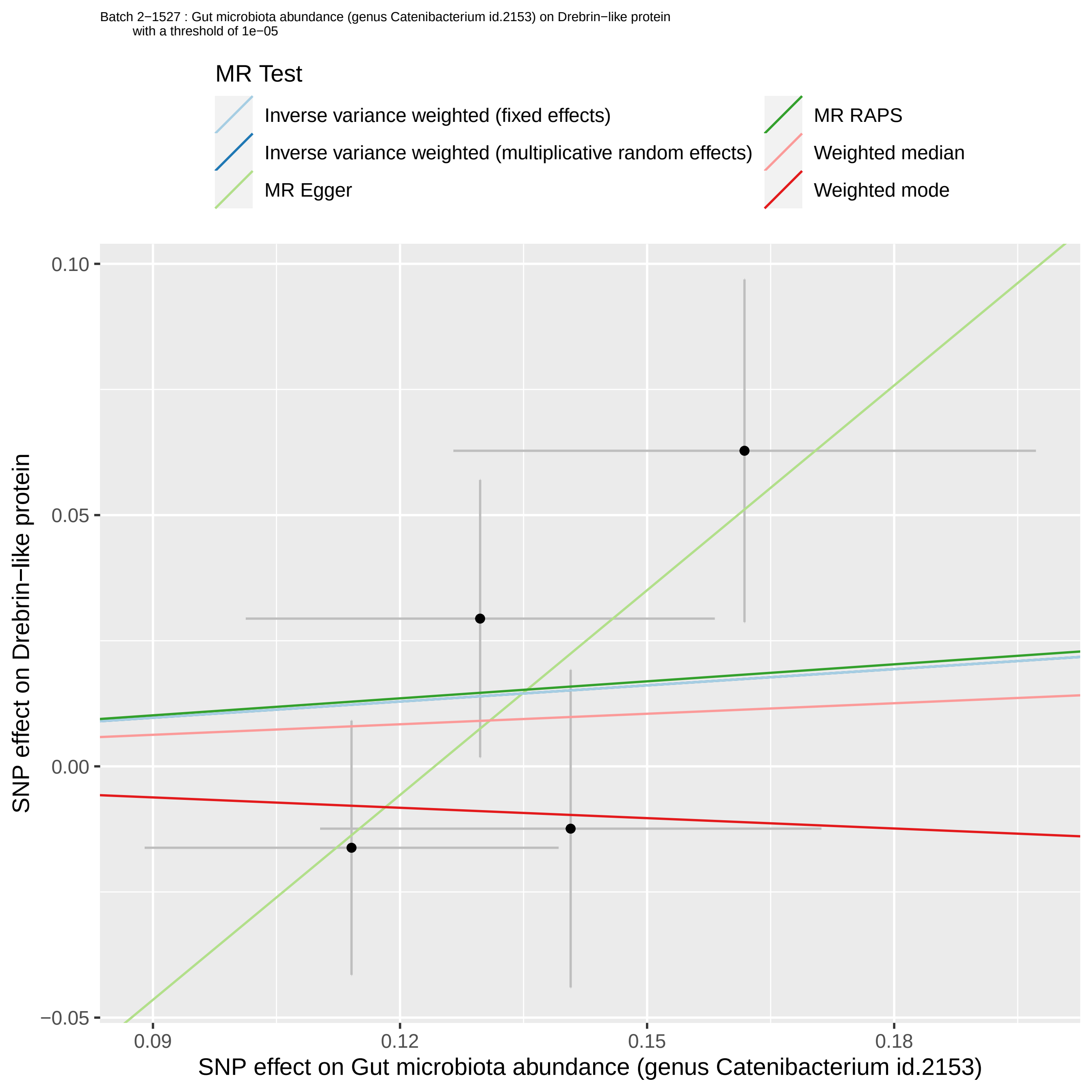
Batch 2−1527 : Gut microbiota abundance (genus Catenibacterium id.2153) on Drebrin−like protein
with a threshold of 1e−05
MR Test
Inverse variance weighted (fixed effects)
Inverse variance weighted (multiplicative random effects)
MR Egger
MR RAPS
Weighted median
Weighted mode
SNP effect on Drebrin−like protein
0.10
0.05
0.00
−0.05
0.09
0.12
0.15
0.18
SNP effect on Gut microbiota abundance (genus Catenibacterium id.2153)

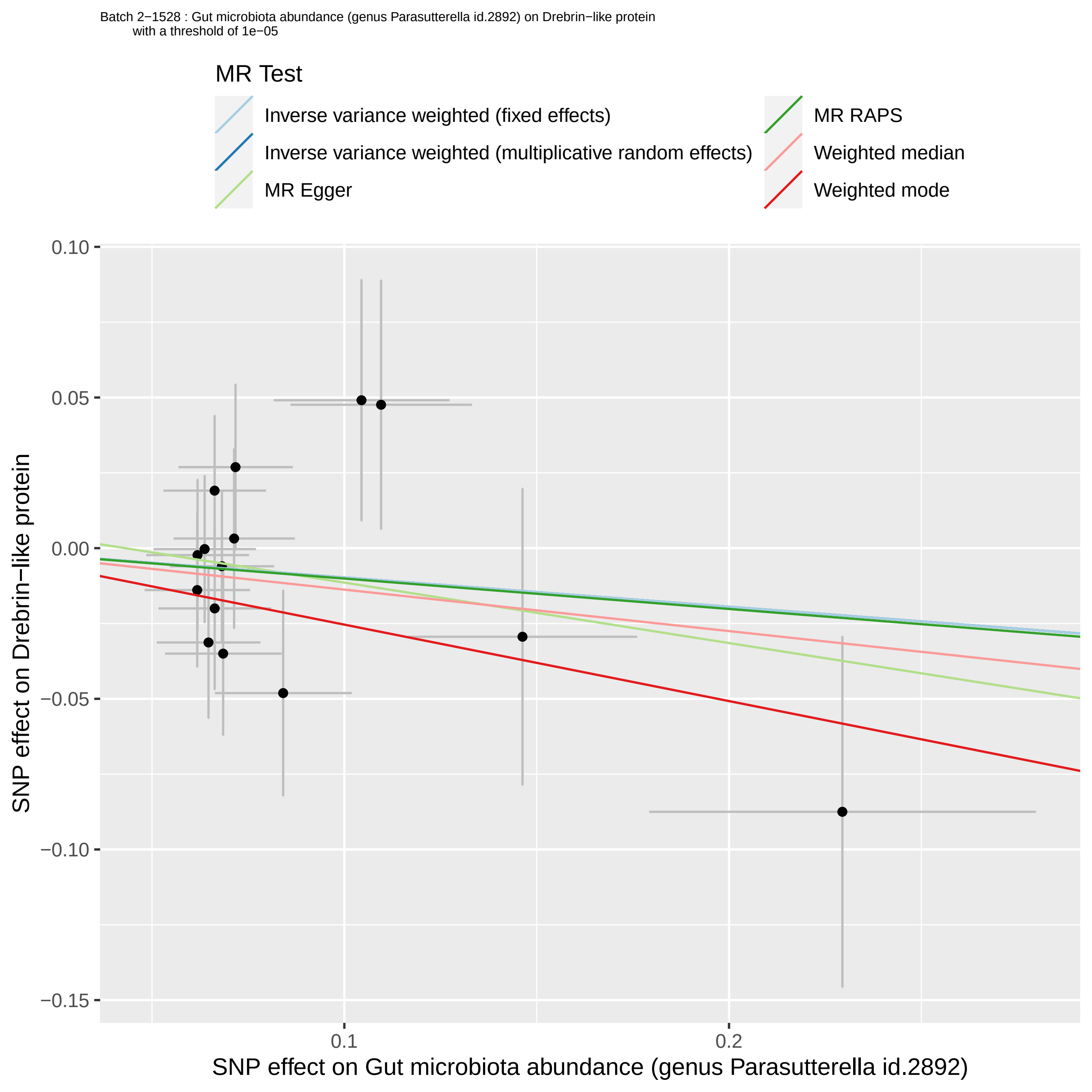
Batch 2−1528 : Gut microbiota abundance (genus Parasutterella id.2892) on Drebrin−like protein
with a threshold of 1e−05
MR Test
Inverse variance weighted (fixed effects)
Inverse variance weighted (multiplicative random effects)
MR Egger
MR RAPS
Weighted median
Weighted mode
0.10
0.05
0.00
−0.05
−0.10
−0.15
SNP effect on Drebrin−like protein
0.1
0.2
SNP effect on Gut microbiota abundance (genus Parasutterella id.2892)

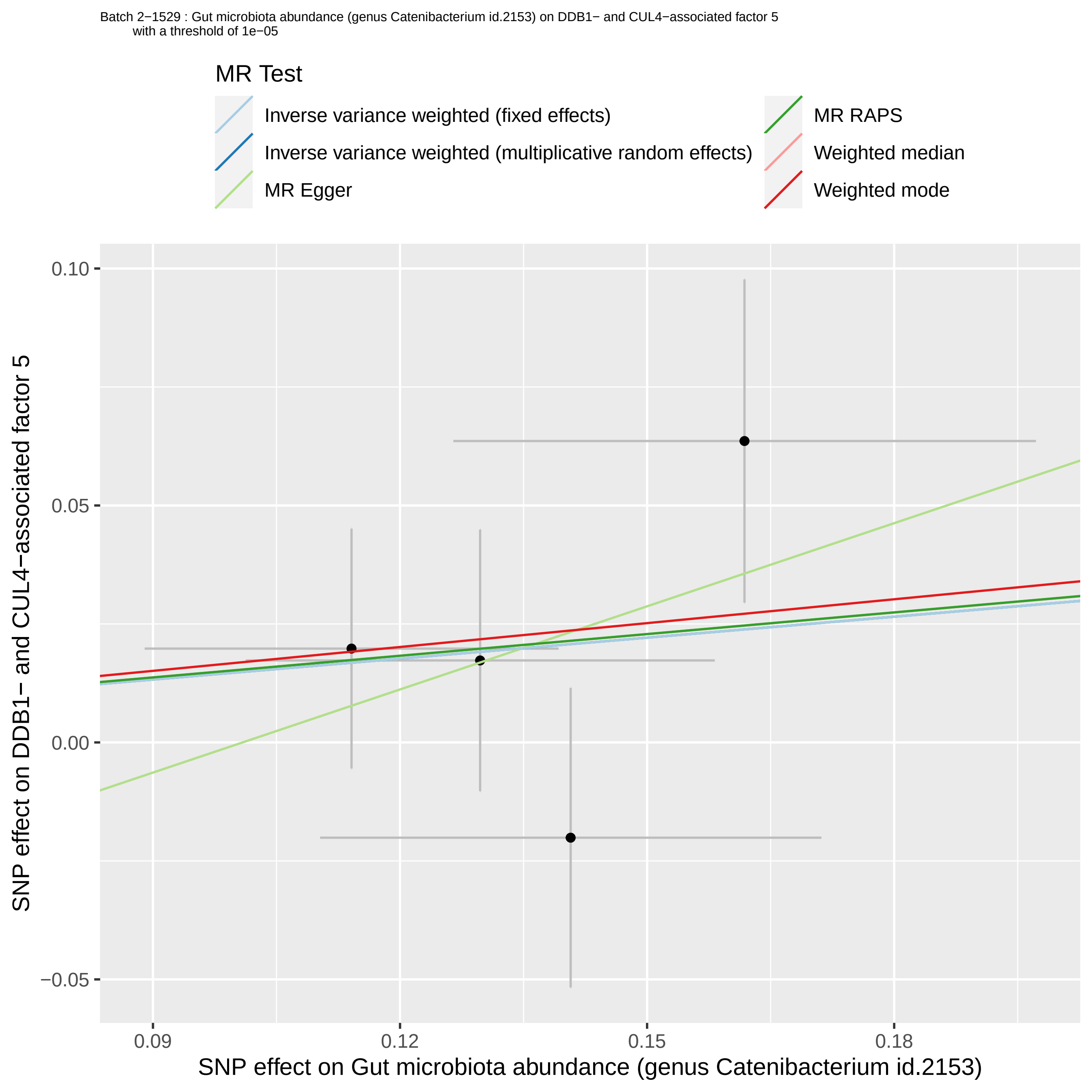
Batch 2−1529 : Gut microbiota abundance (genus Catenibacterium id.2153) on DDB1− and CUL4−associated factor 5
with a threshold of 1e−05
MR Test
Inverse variance weighted (fixed effects)
Inverse variance weighted (multiplicative random effects)
MR Egger
MR RAPS
Weighted median
Weighted mode
0.10
0.05
0.00
−0.05
0.09
0.12
0.15
0.18
SNP effect on DDB1− and CUL4−associated factor 5
SNP effect on Gut microbiota abundance (genus Catenibacterium id.2153)

Batch 2−1530 : Gut microbiota abundance (genus Parasutterella id.2892) on DDB1− and CUL4−associated factor 5
with a threshold of 1e−05

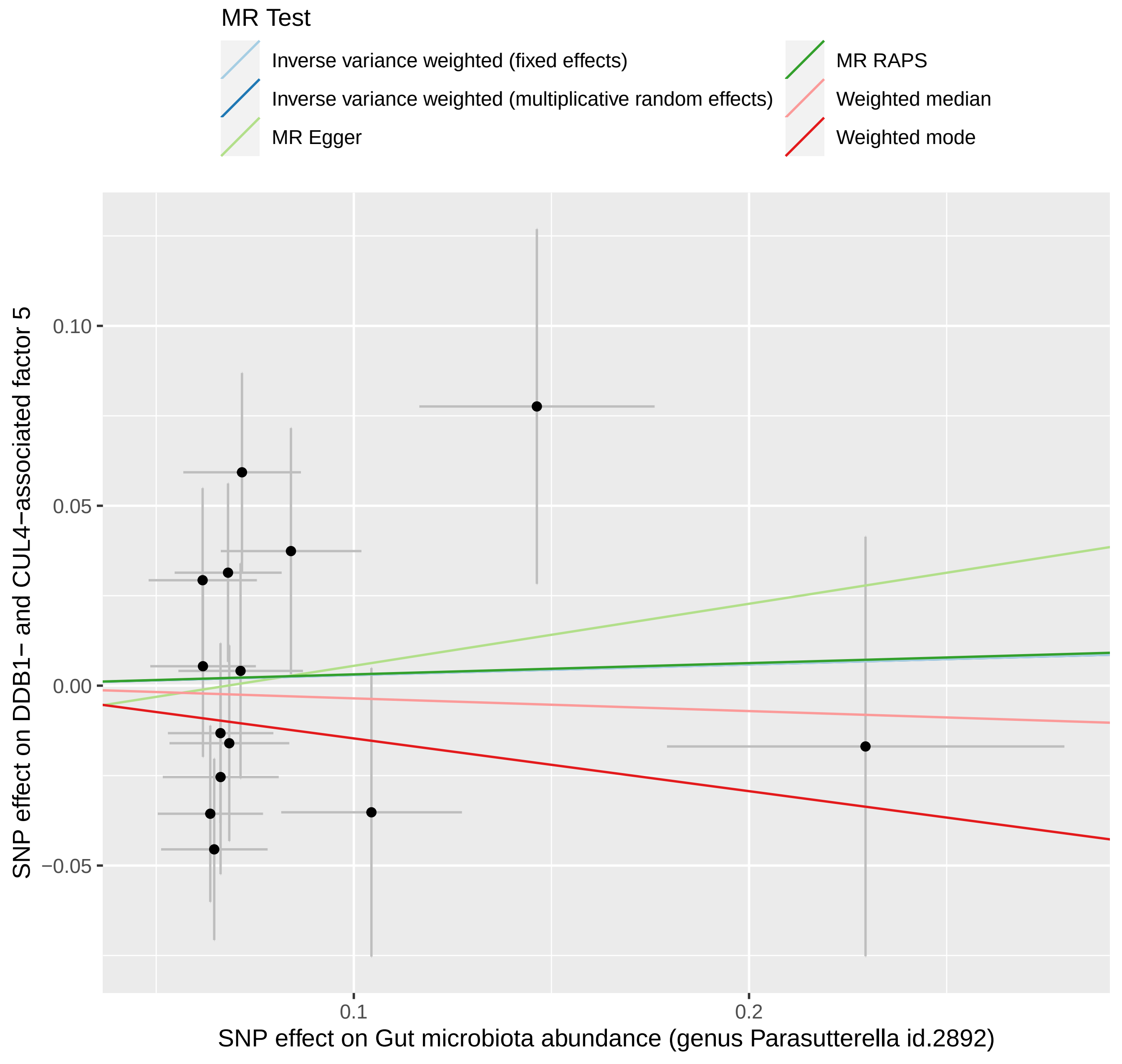

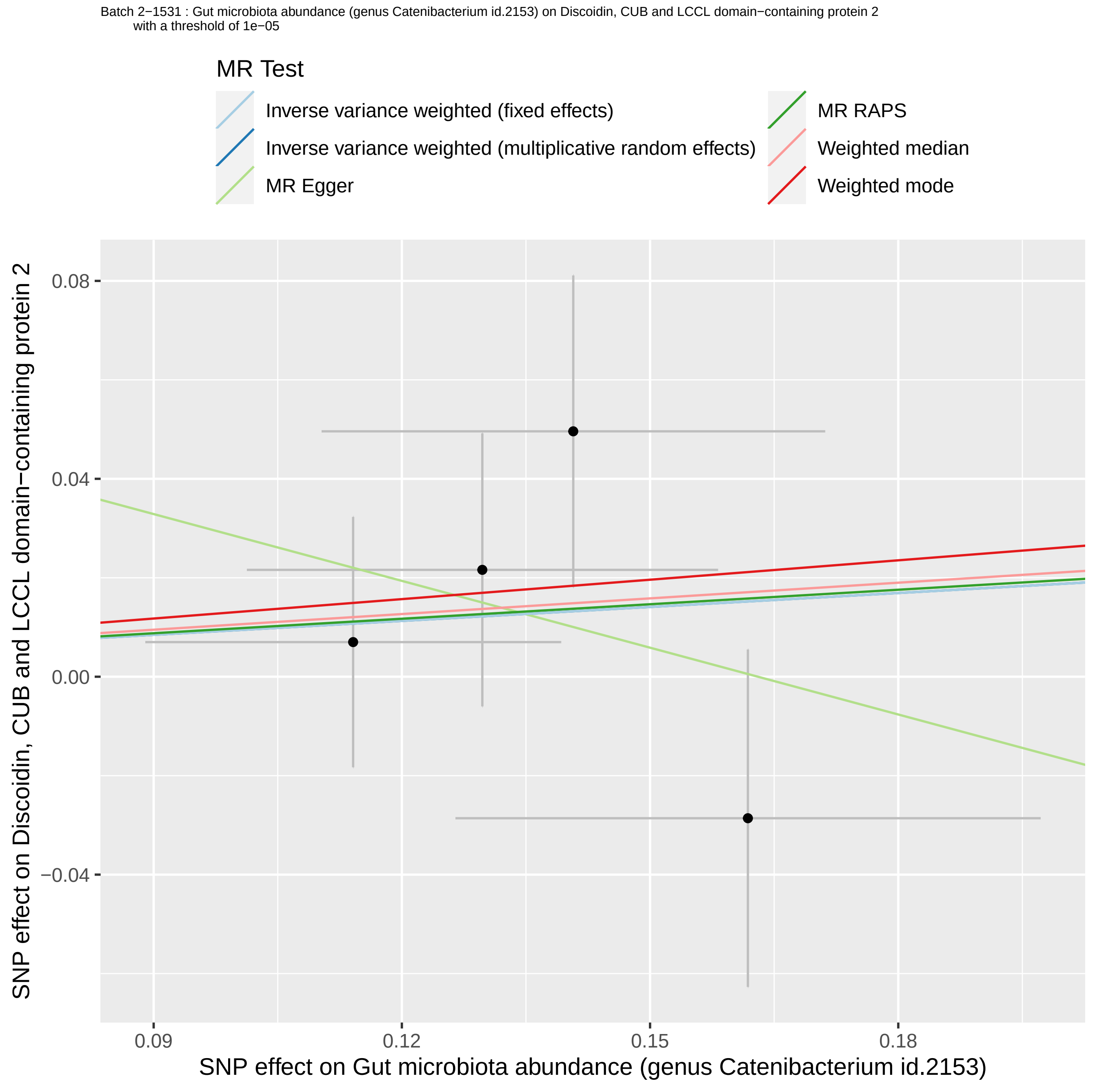
Batch 2−1531 : Gut microbiota abundance (genus Catenibacterium id.2153) on Discoidin, CUB and LCCL domain−containing protein 2
with a threshold of 1e−05
MR Test
Inverse variance weighted (fixed effects)
Inverse variance weighted (multiplicative random effects)
MR Egger
MR RAPS
Weighted median
Weighted mode
0.08
0.04
0.00
−0.04
0.09
0.12
0.15
0.18
SNP effect on Discoidin, CUB and LCCL domain−containing protein 2
SNP effect on Gut microbiota abundance (genus Catenibacterium id.2153)

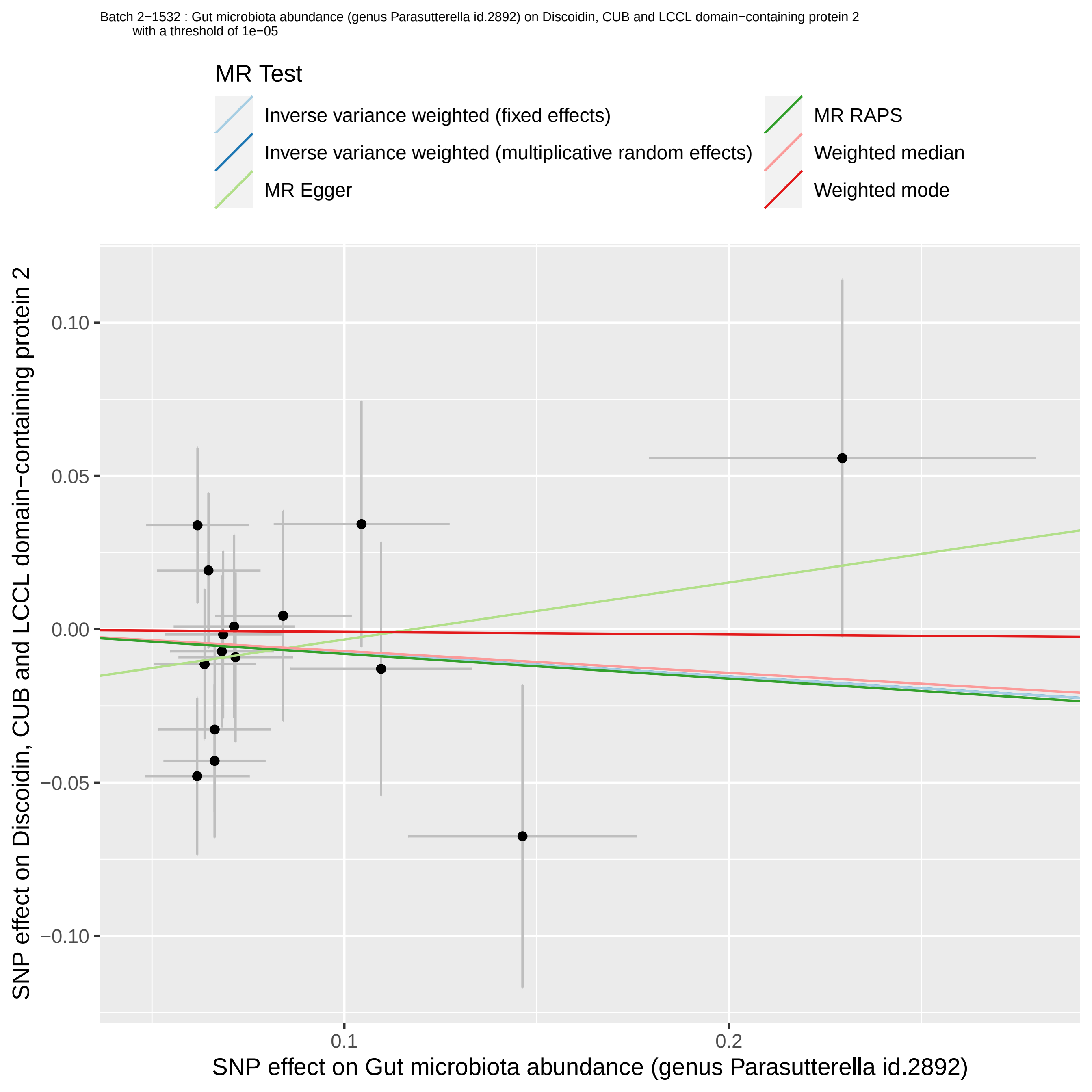
Batch 2−1532 : Gut microbiota abundance (genus Parasutterella id.2892) on Discoidin, CUB and LCCL domain−containing protein 2
with a threshold of 1e−05
MR Test
Inverse variance weighted (fixed effects)
Inverse variance weighted (multiplicative random effects)
MR Egger
MR RAPS
Weighted median
Weighted mode
0.10
0.05
0.00
−0.05
−0.10
0.1
0.2
SNP effect on Discoidin, CUB and LCCL domain−containing protein 2
SNP effect on Gut microbiota abundance (genus Parasutterella id.2892)

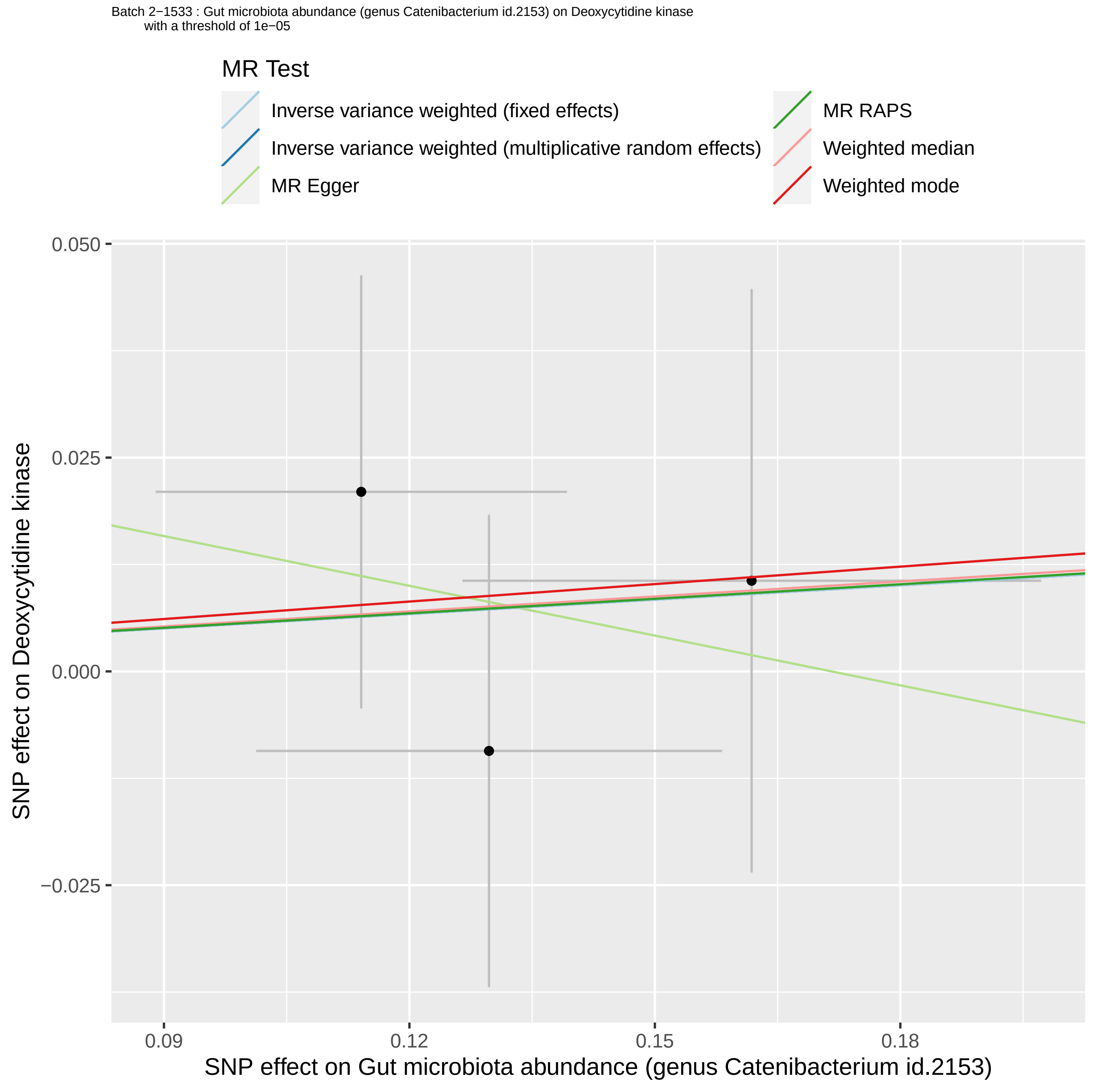

Batch 2−1533 : Gut microbiota abundance (genus Catenibacterium id.2153) on Deoxycytidine kinase
with a threshold of 1e−05
MR Test
Inverse variance weighted (fixed effects)
Inverse variance weighted (multiplicative random effects)
MR Egger
MR RAPS
Weighted median
Weighted mode
0.050
0.025
0.000
−0.025
SNP effect on Deoxycytidine kinase
0.09
0.12
0.15
0.18
SNP effect on Gut microbiota abundance (genus Catenibacterium id.2153)

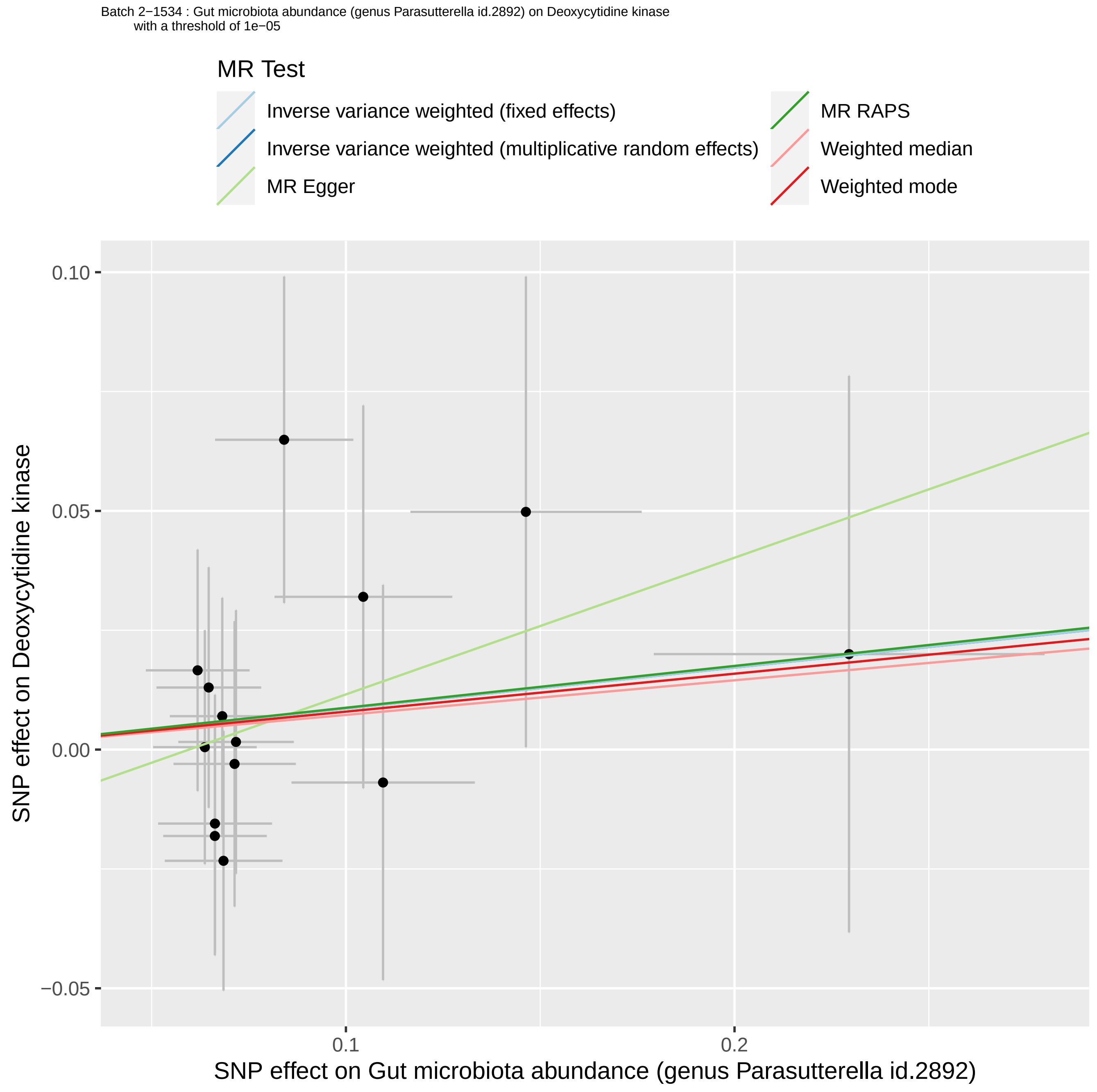
Batch 2−1534 : Gut microbiota abundance (genus Parasutterella id.2892) on Deoxycytidine kinase
with a threshold of 1e−05
MR Test
Inverse variance weighted (fixed effects)
Inverse variance weighted (multiplicative random effects)
MR Egger
MR RAPS
Weighted median
Weighted mode
0.10
0.05
0.00
−0.05
0.1
0.2
SNP effect on Deoxycytidine kinase
SNP effect on Gut microbiota abundance (genus Parasutterella id.2892)

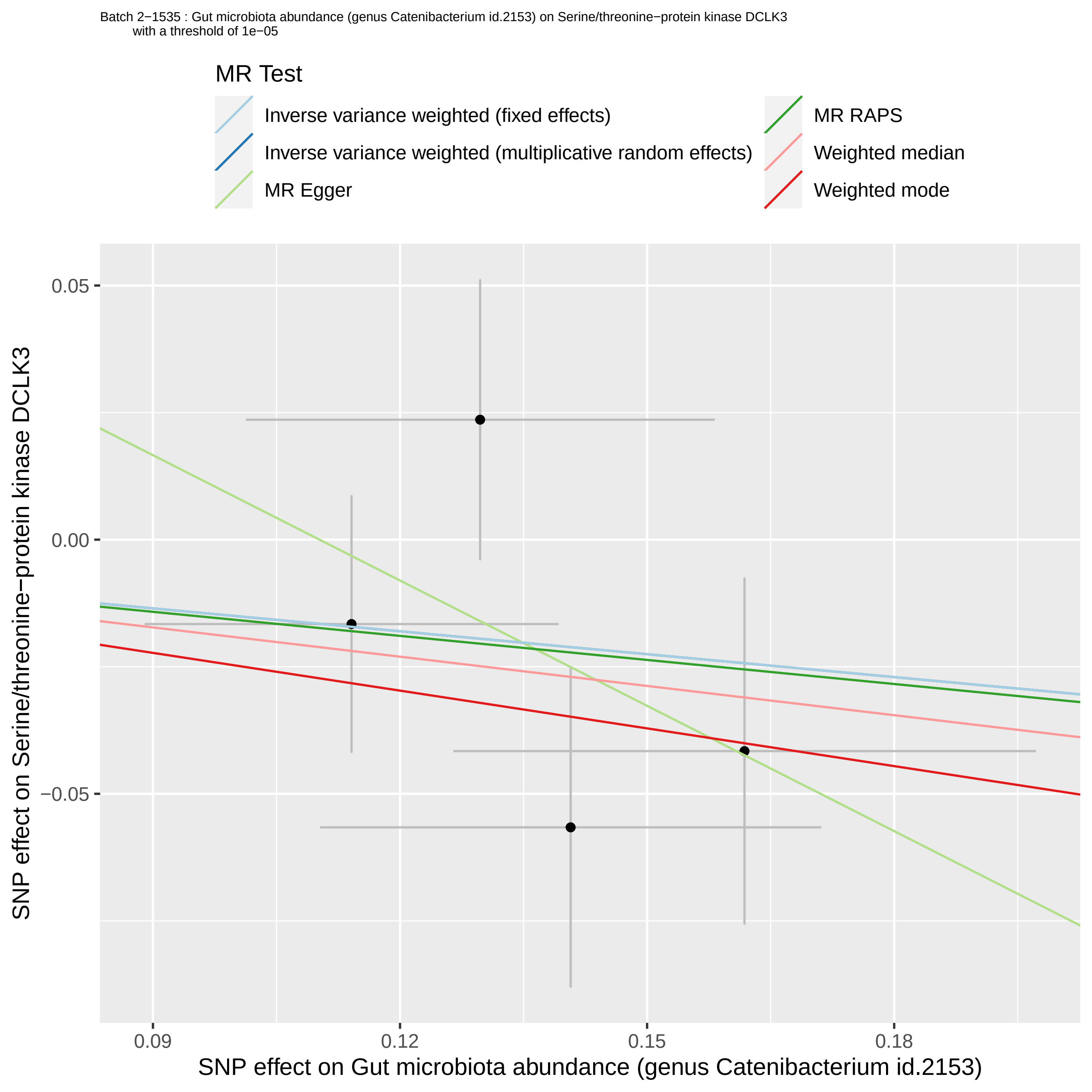

Batch 2−1535 : Gut microbiota abundance (genus Catenibacterium id.2153) on Serine/threonine−protein kinase DCLK3
with a threshold of 1e−05
MR Test
Inverse variance weighted (fixed effects)
Inverse variance weighted (multiplicative random effects)
MR Egger
MR RAPS
Weighted median
Weighted mode
0.05
0.00
−0.05
0.09
0.12
0.15
0.18
SNP effect on Serine/threonine−protein kinase DCLK3
SNP effect on Gut microbiota abundance (genus Catenibacterium id.2153)

Batch 2−1536 : Gut microbiota abundance (genus Parasutterella id.2892) on Serine/threonine−protein kinase DCLK3
with a threshold of 1e−05

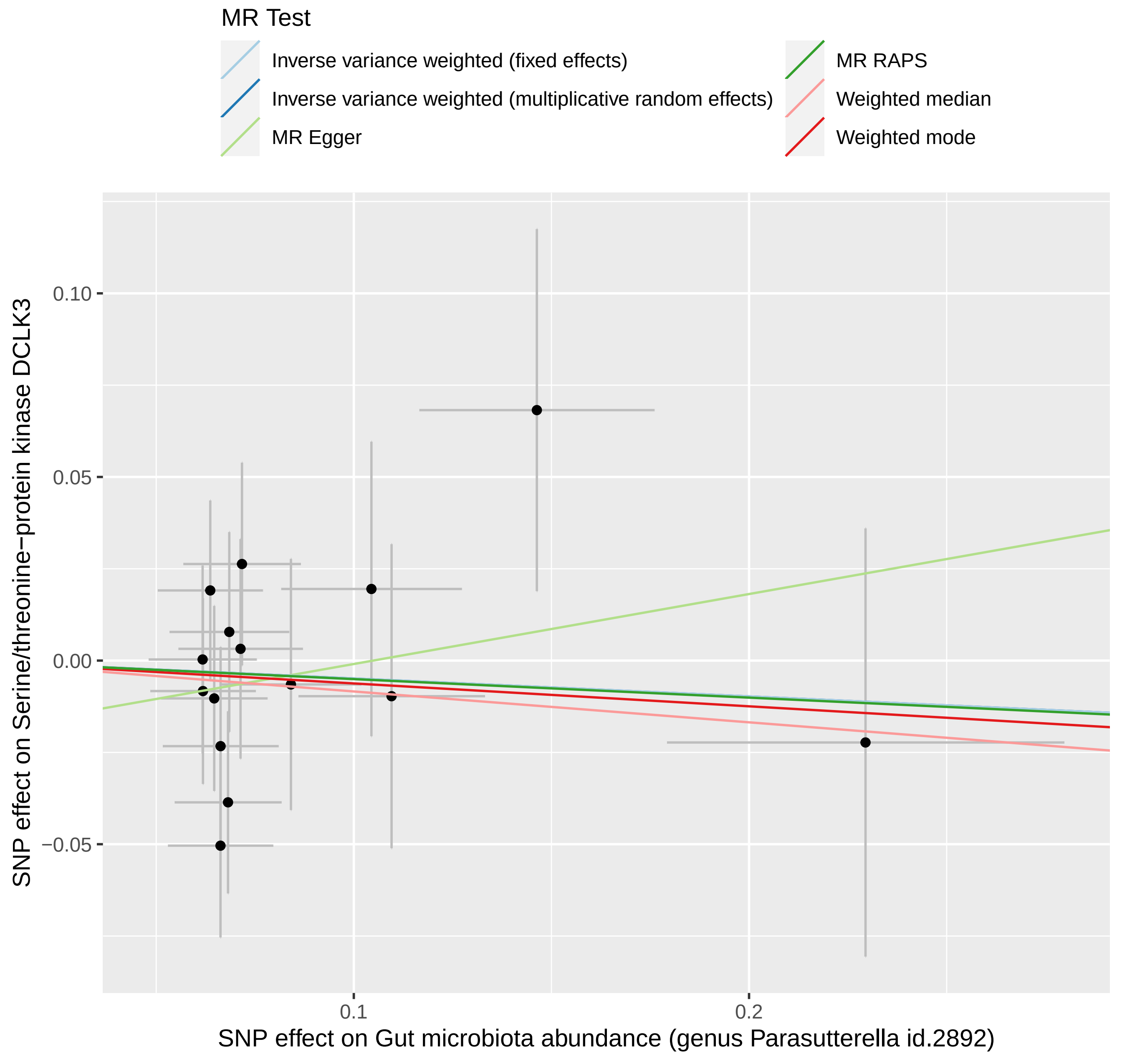

Batch 2−1537 : Gut microbiota abundance (genus Catenibacterium id.2153) on Decorin
with a threshold of 1e−05

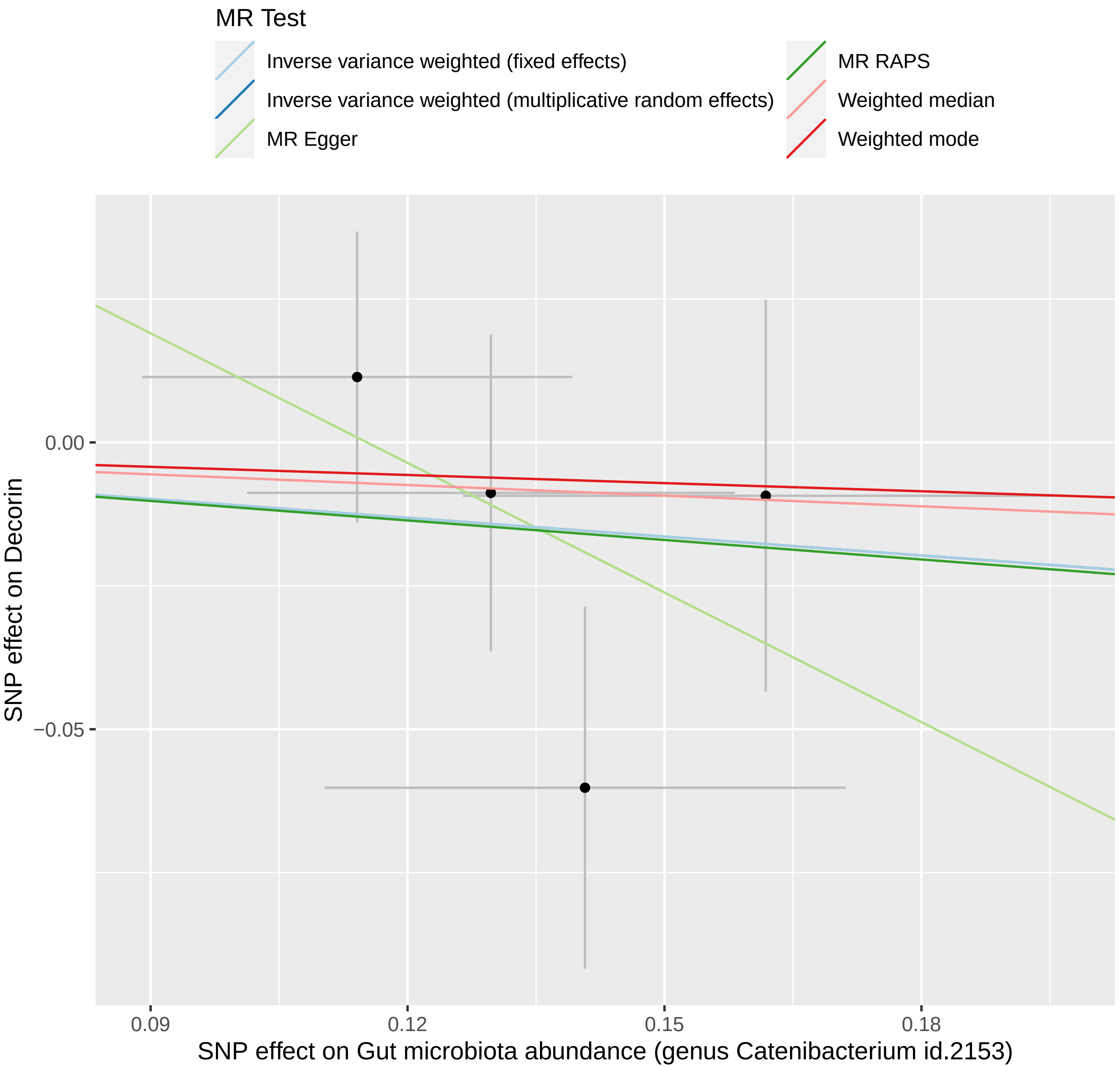

Batch 2−1538 : Gut microbiota abundance (genus Parasutterella id.2892) on Decorin
with a threshold of 1e−05

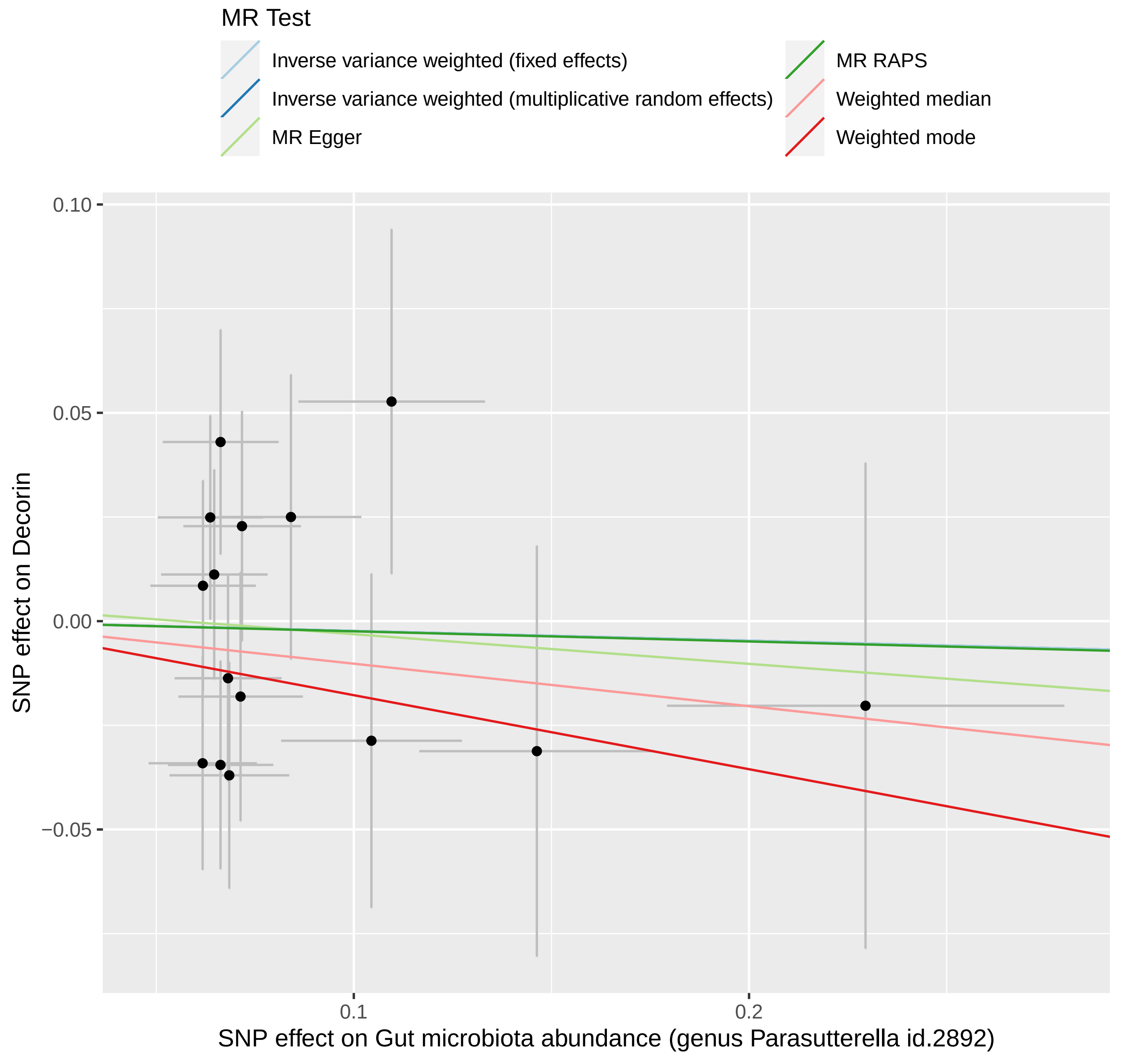

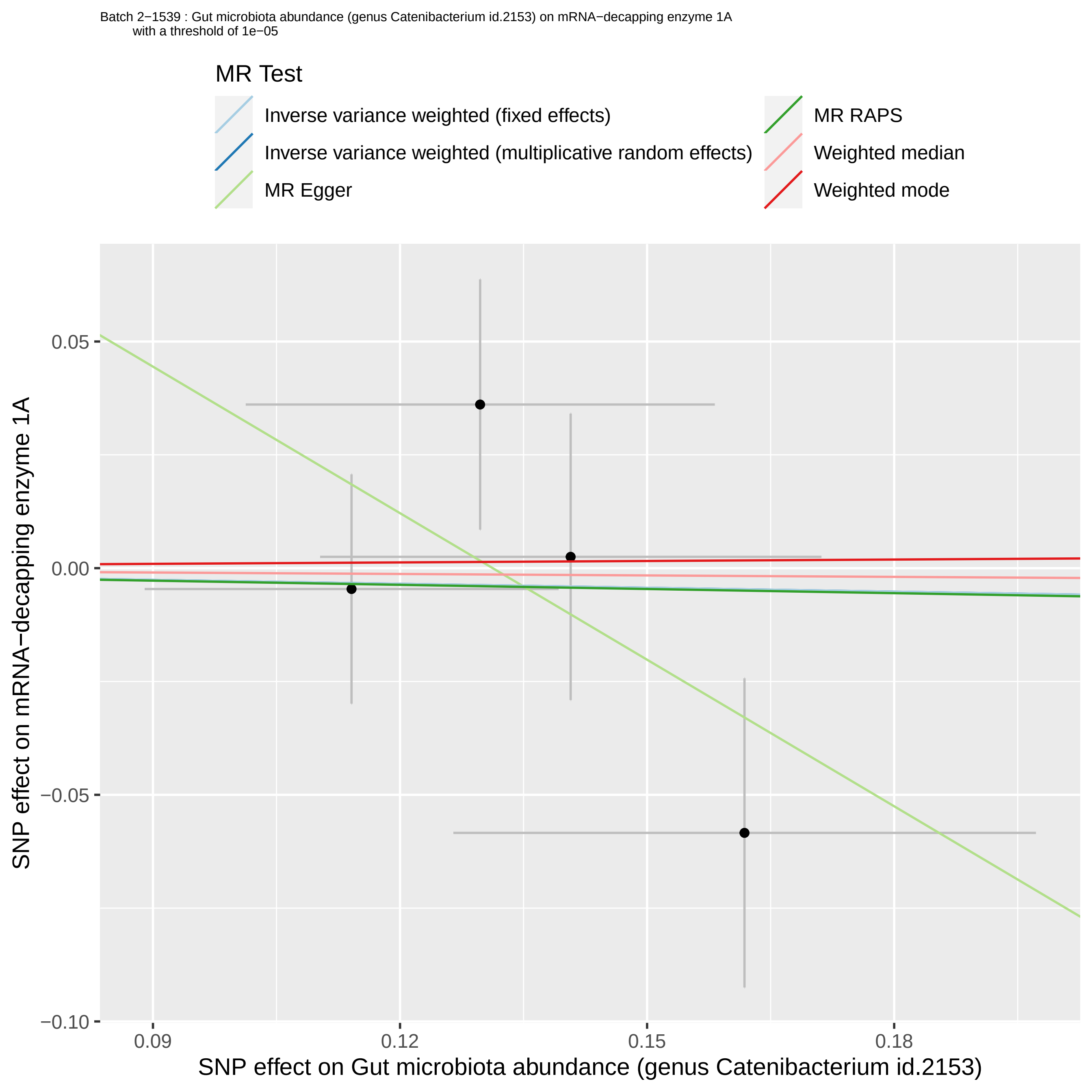
Batch 2−1539 : Gut microbiota abundance (genus Catenibacterium id.2153) on mRNA−decapping enzyme 1A
with a threshold of 1e−05
MR Test
Inverse variance weighted (fixed effects)
Inverse variance weighted (multiplicative random effects)
MR Egger
MR RAPS
Weighted median
Weighted mode
0.05
0.00
−0.05
−0.10
0.09
0.12
0.15
0.18
SNP effect on mRNA−decapping enzyme 1A
SNP effect on Gut microbiota abundance (genus Catenibacterium id.2153)

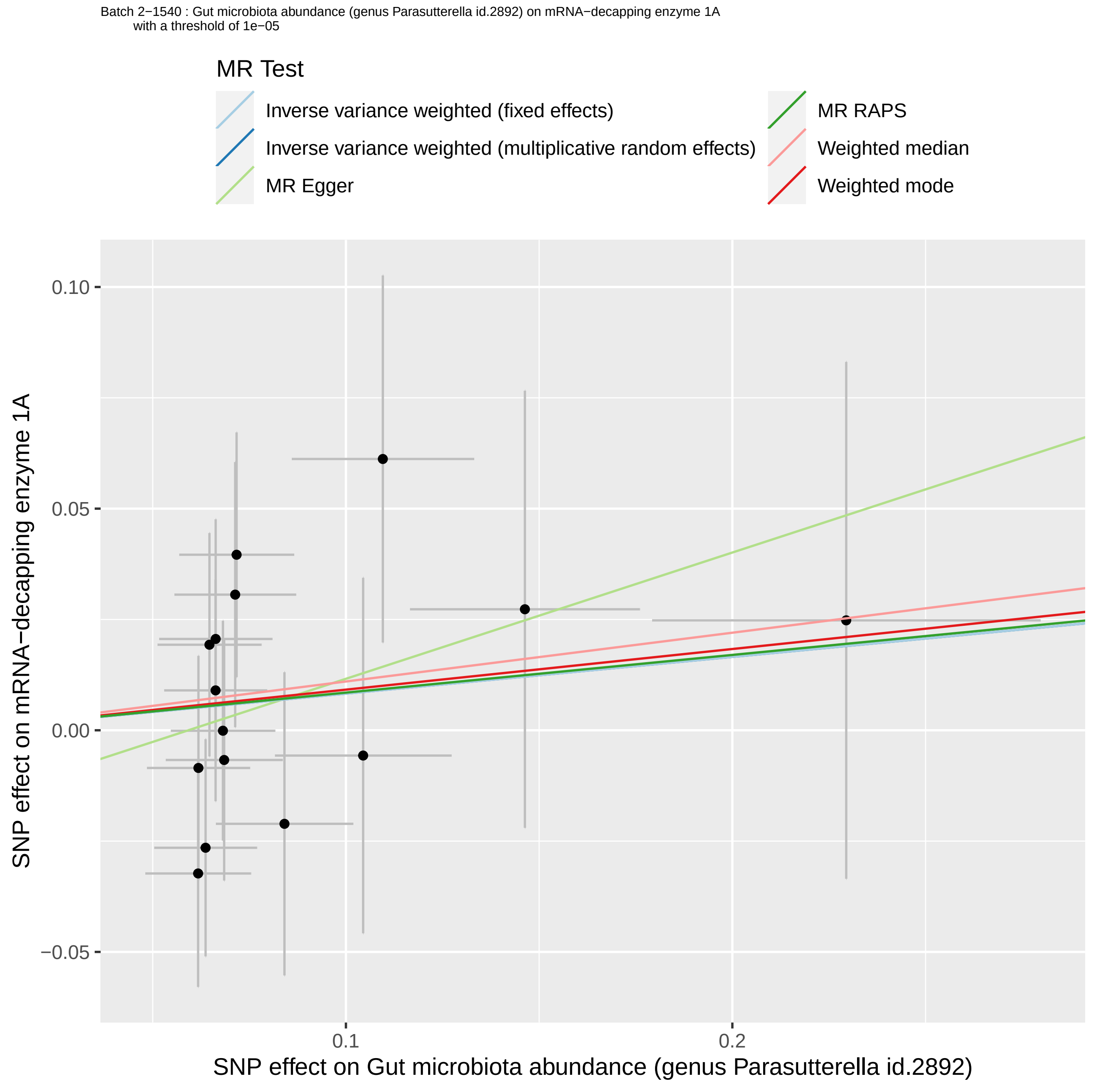
Batch 2−1540 : Gut microbiota abundance (genus Parasutterella id.2892) on mRNA−decapping enzyme 1A
with a threshold of 1e−05
MR Test
Inverse variance weighted (fixed effects)
Inverse variance weighted (multiplicative random effects)
MR Egger
MR RAPS
Weighted median
Weighted mode
0.10
0.05
0.00
−0.05
0.1
0.2
SNP effect on mRNA−decapping enzyme 1A
SNP effect on Gut microbiota abundance (genus Parasutterella id.2892)

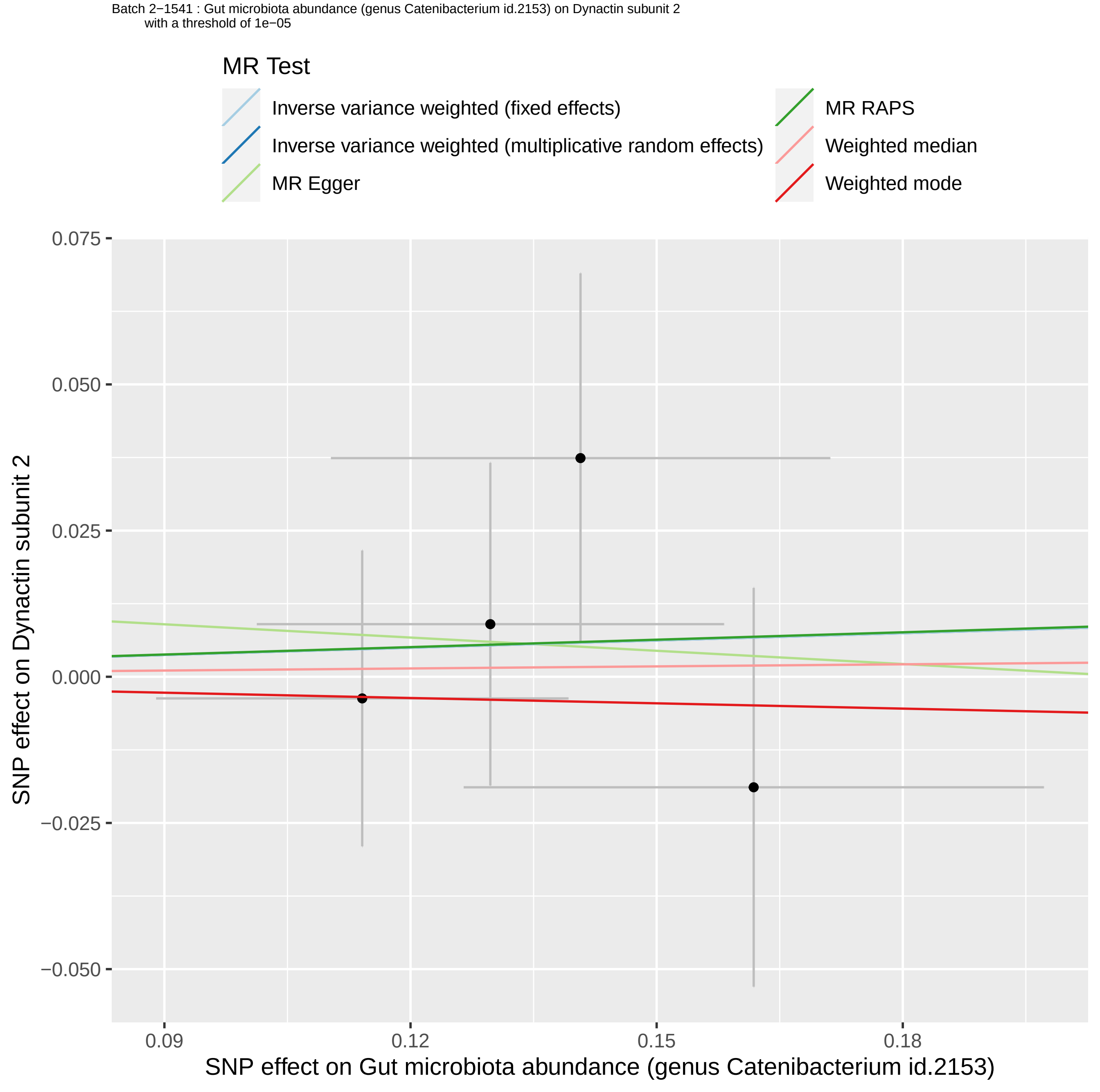
Batch 2−1541 : Gut microbiota abundance (genus Catenibacterium id.2153) on Dynactin subunit 2
with a threshold of 1e−05
MR Test
Inverse variance weighted (fixed effects)
Inverse variance weighted (multiplicative random effects)
MR Egger
MR RAPS
Weighted median
Weighted mode
0.075
0.050
0.025
0.000
−0.025
−0.050
SNP effect on Dynactin subunit 2
0.09
0.12
0.15
0.18
SNP effect on Gut microbiota abundance (genus Catenibacterium id.2153)

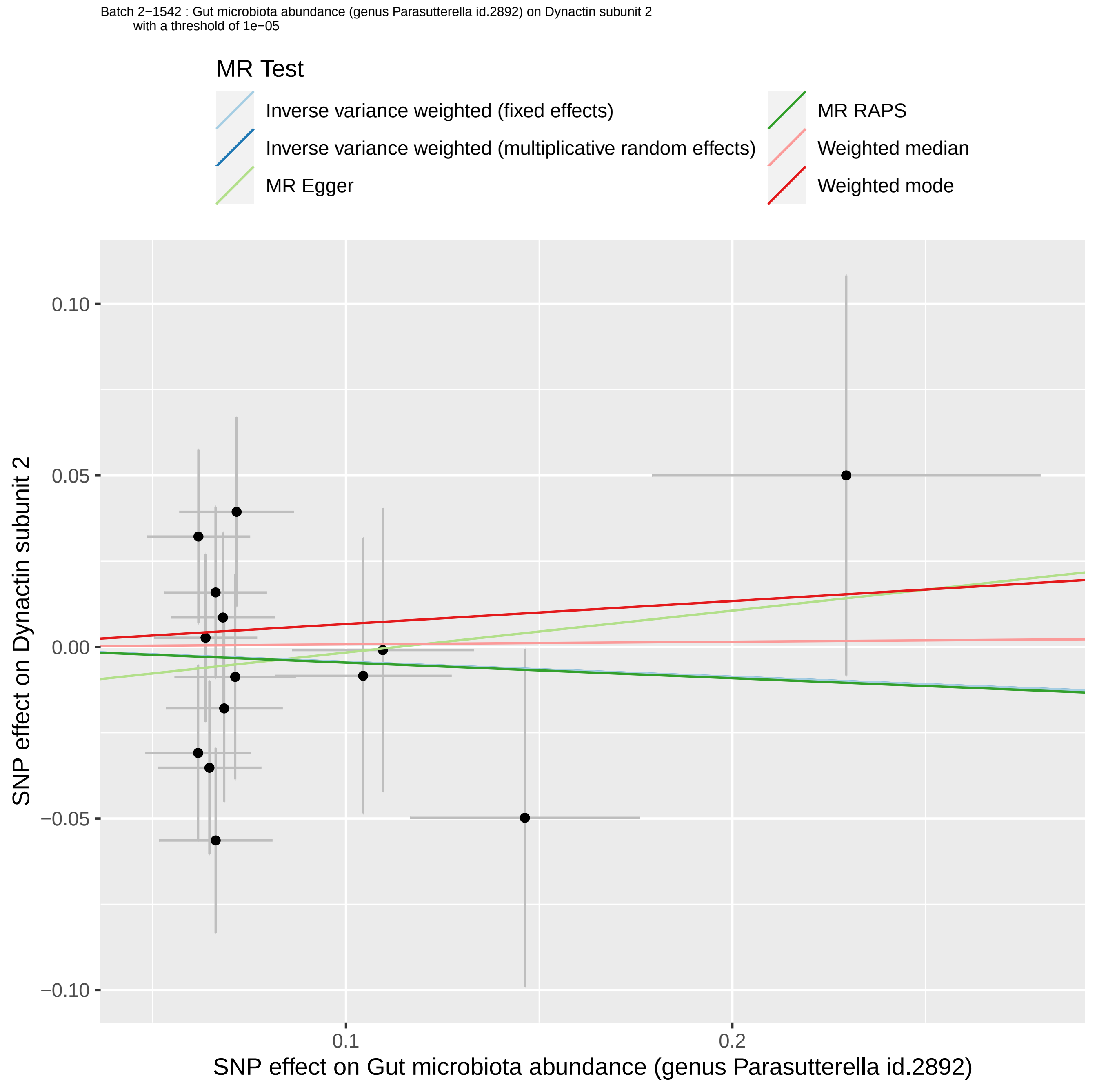

Batch 2−1542 : Gut microbiota abundance (genus Parasutterella id.2892) on Dynactin subunit 2
with a threshold of 1e−05
MR Test
Inverse variance weighted (fixed effects)
Inverse variance weighted (multiplicative random effects)
MR Egger
MR RAPS
Weighted median
Weighted mode
0.10
0.05
0.00
−0.05
−0.10
0.1
0.2
SNP effect on Dynactin subunit 2
SNP effect on Gut microbiota abundance (genus Parasutterella id.2892)

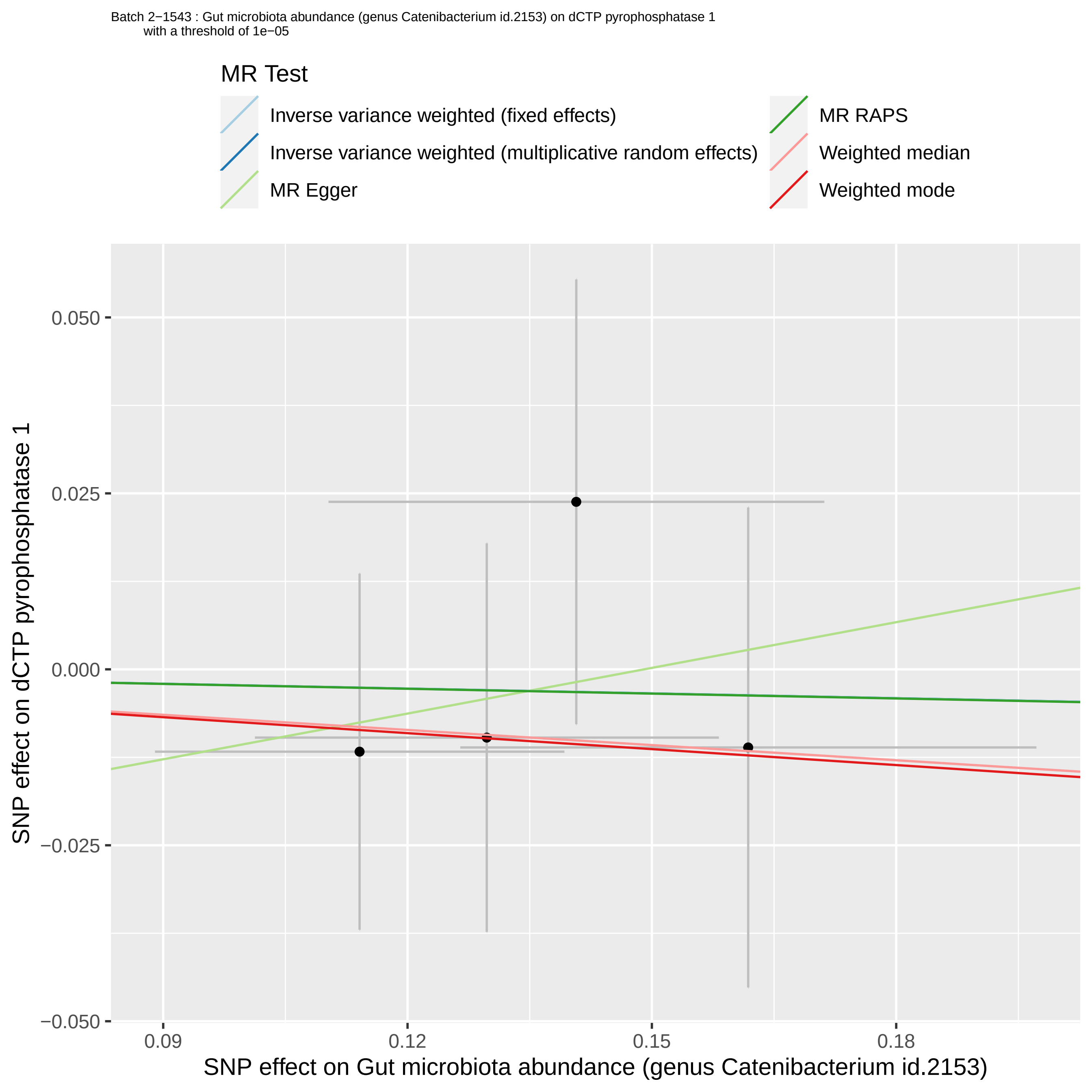
Batch 2−1543 : Gut microbiota abundance (genus Catenibacterium id.2153) on dCTP pyrophosphatase 1
with a threshold of 1e−05
MR Test
Inverse variance weighted (fixed effects)
Inverse variance weighted (multiplicative random effects)
MR Egger
MR RAPS
Weighted median
Weighted mode
SNP effect on dCTP pyrophosphatase 1
0.050
0.025
0.000
−0.025
−0.050
0.09
0.12
0.15
0.18
SNP effect on Gut microbiota abundance (genus Catenibacterium id.2153)

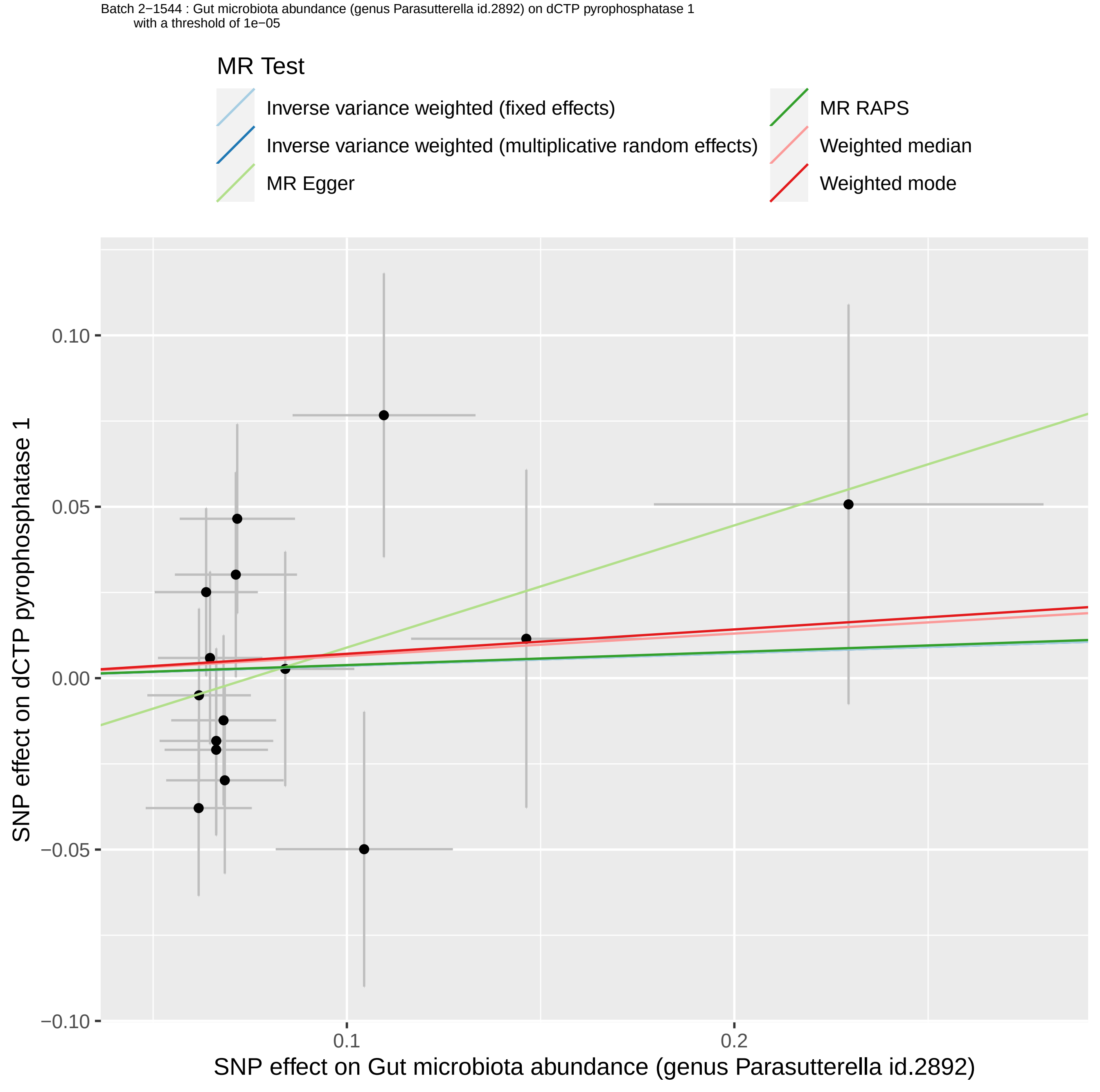

Batch 2−1544 : Gut microbiota abundance (genus Parasutterella id.2892) on dCTP pyrophosphatase 1
with a threshold of 1e−05
MR Test
Inverse variance weighted (fixed effects)
Inverse variance weighted (multiplicative random effects)
MR Egger
MR RAPS
Weighted median
Weighted mode
0.10
0.05
0.00
−0.05
−0.10
0.1
0.2
SNP effect on dCTP pyrophosphatase 1
SNP effect on Gut microbiota abundance (genus Parasutterella id.2892)

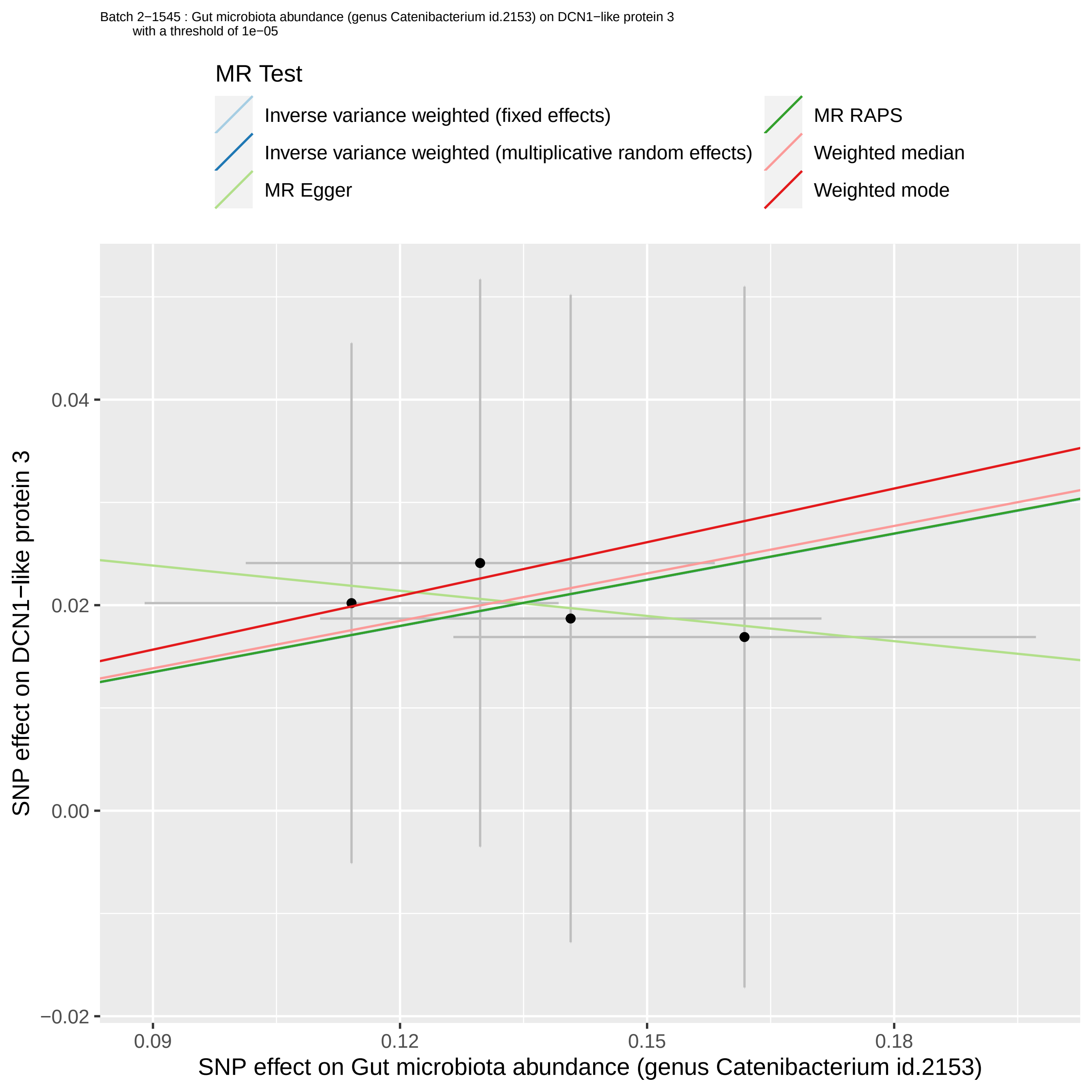
Batch 2−1545 : Gut microbiota abundance (genus Catenibacterium id.2153) on DCN1−like protein 3
with a threshold of 1e−05
MR Test
Inverse variance weighted (fixed effects)
Inverse variance weighted (multiplicative random effects)
MR Egger
MR RAPS
Weighted median
Weighted mode
SNP effect on DCN1−like protein 3
0.04
0.02
0.00
−0.02
0.09
0.12
0.15
0.18
SNP effect on Gut microbiota abundance (genus Catenibacterium id.2153)

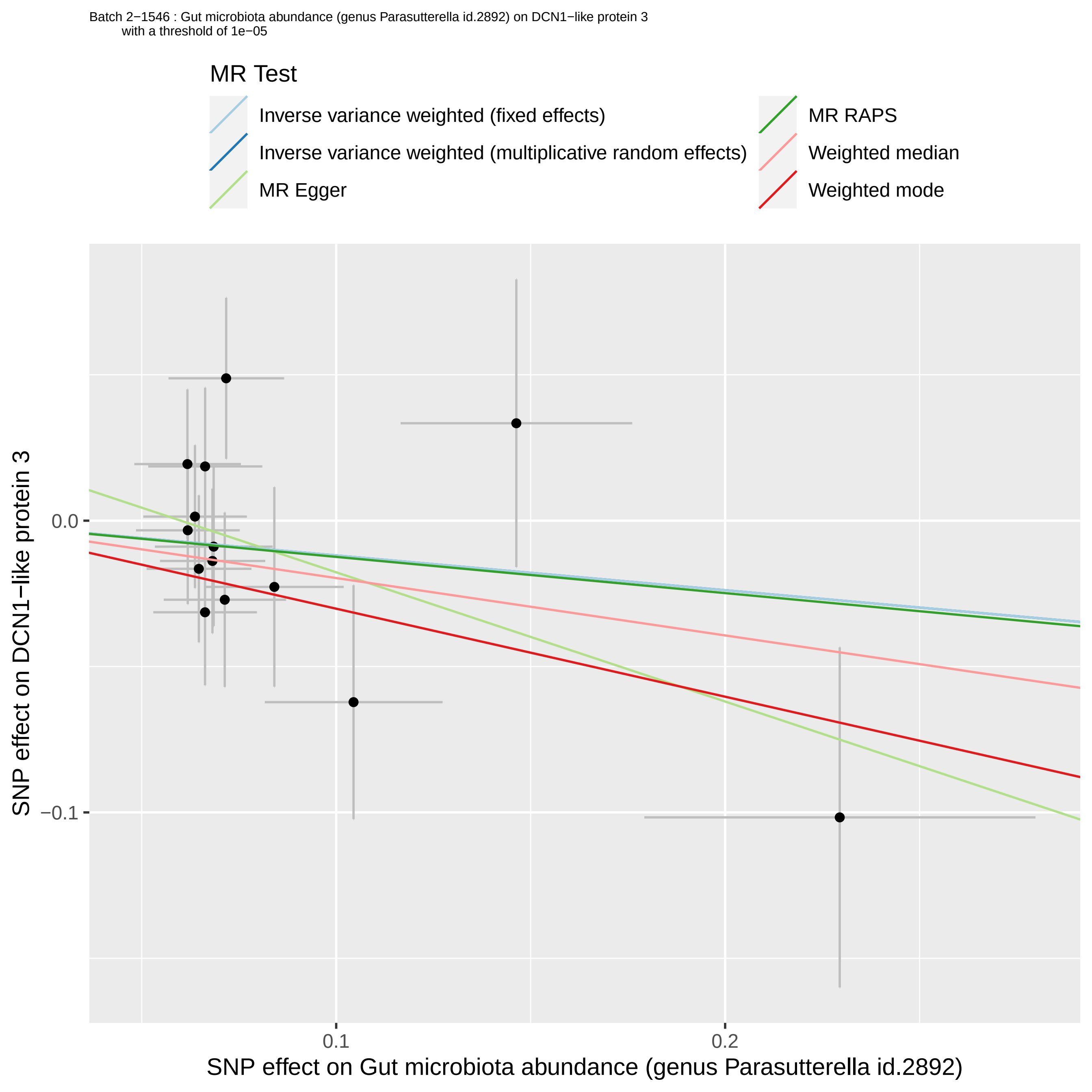
Batch 2−1546 : Gut microbiota abundance (genus Parasutterella id.2892) on DCN1−like protein 3
with a threshold of 1e−05
MR Test
Inverse variance weighted (fixed effects)
Inverse variance weighted (multiplicative random effects)
MR Egger
MR RAPS
Weighted median
Weighted mode
0.0
−0.1
0.1
0.2
SNP effect on DCN1−like protein 3
SNP effect on Gut microbiota abundance (genus Parasutterella id.2892)

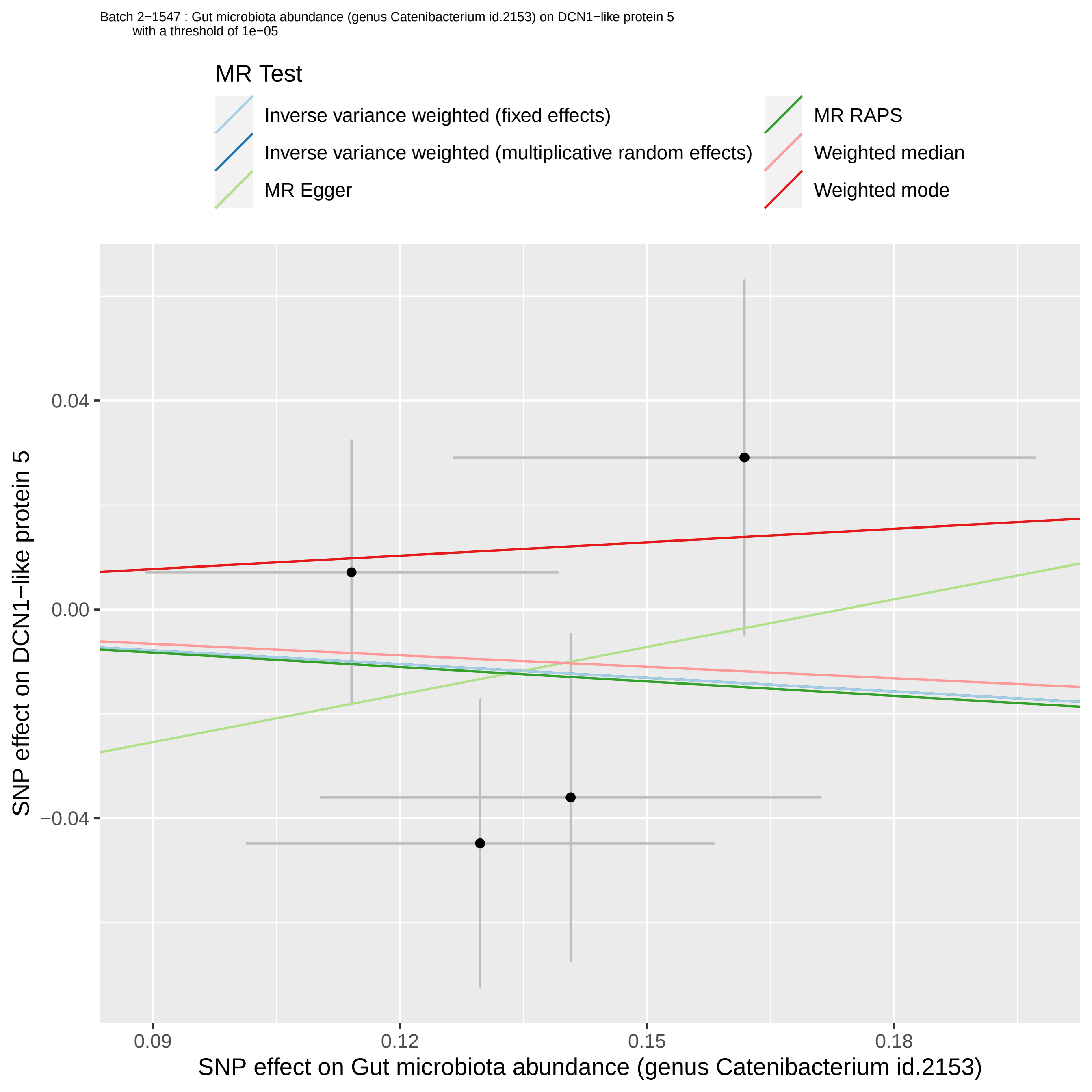

Batch 2−1547 : Gut microbiota abundance (genus Catenibacterium id.2153) on DCN1−like protein 5
with a threshold of 1e−05
MR Test
Inverse variance weighted (fixed effects)
Inverse variance weighted (multiplicative random effects)
MR Egger
MR RAPS
Weighted median
Weighted mode
0.04
0.00
−0.04
0.09
0.12
0.15
0.18
SNP effect on DCN1−like protein 5
SNP effect on Gut microbiota abundance (genus Catenibacterium id.2153)

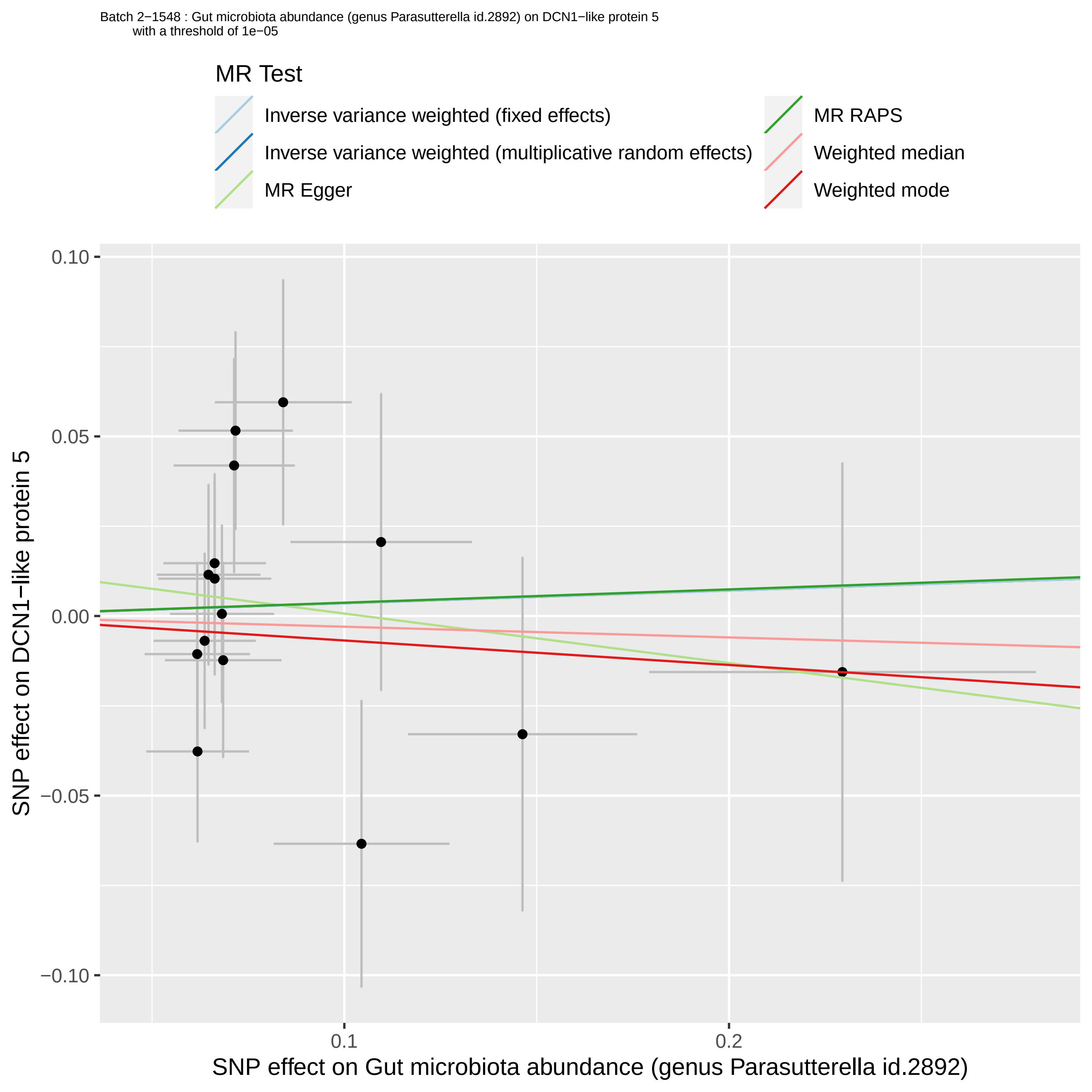
Batch 2−1548 : Gut microbiota abundance (genus Parasutterella id.2892) on DCN1−like protein 5
with a threshold of 1e−05
MR Test
Inverse variance weighted (fixed effects)
Inverse variance weighted (multiplicative random effects)
MR Egger
MR RAPS
Weighted median
Weighted mode
0.10
0.05
0.00
−0.05
−0.10
0.1
0.2
SNP effect on DCN1−like protein 5
SNP effect on Gut microbiota abundance (genus Parasutterella id.2892)

Batch 2−1549 : Gut microbiota abundance (genus Catenibacterium id.2153) on Aromatic−L−amino−acid decarboxylase
with a threshold of 1e−05

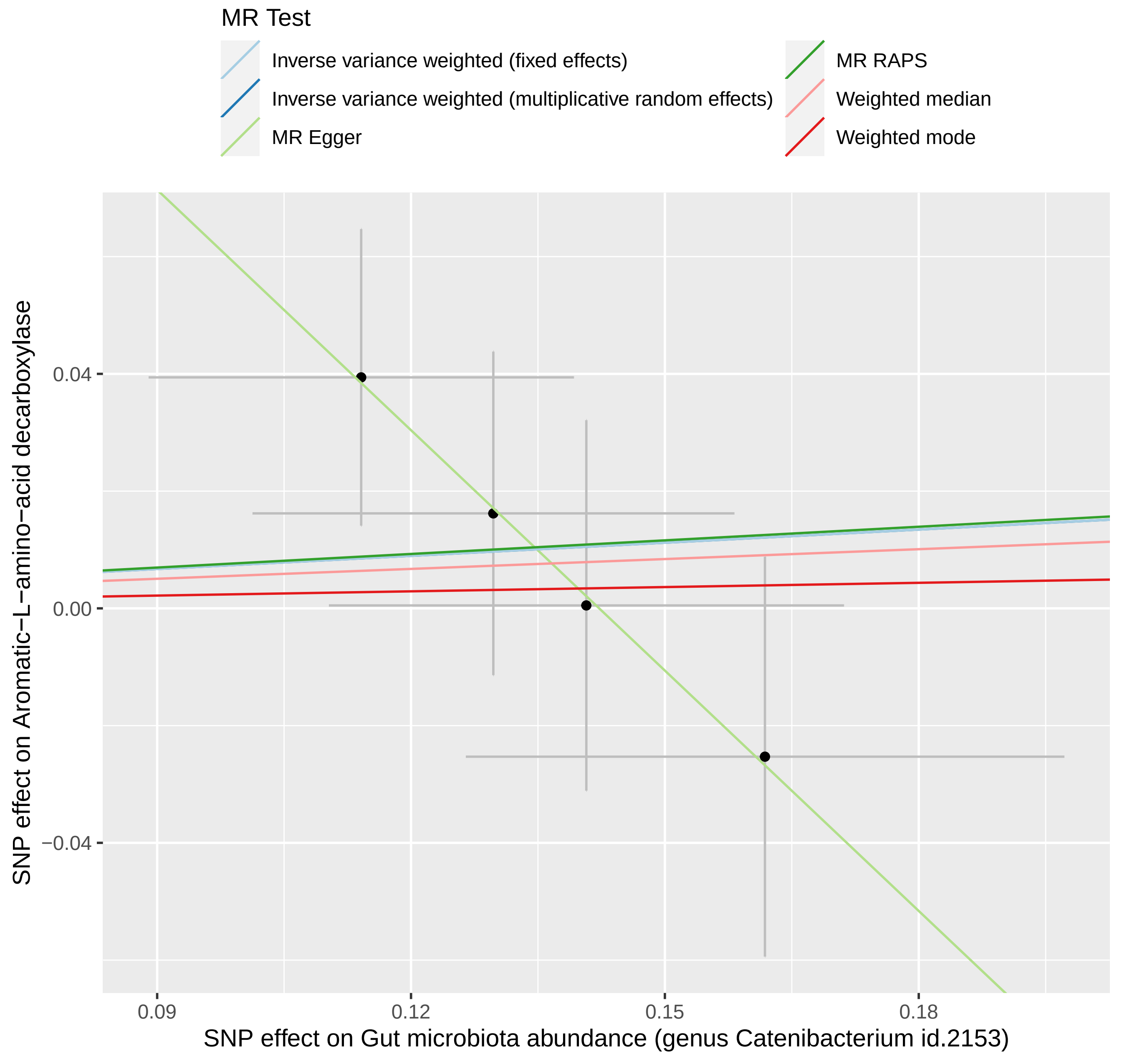

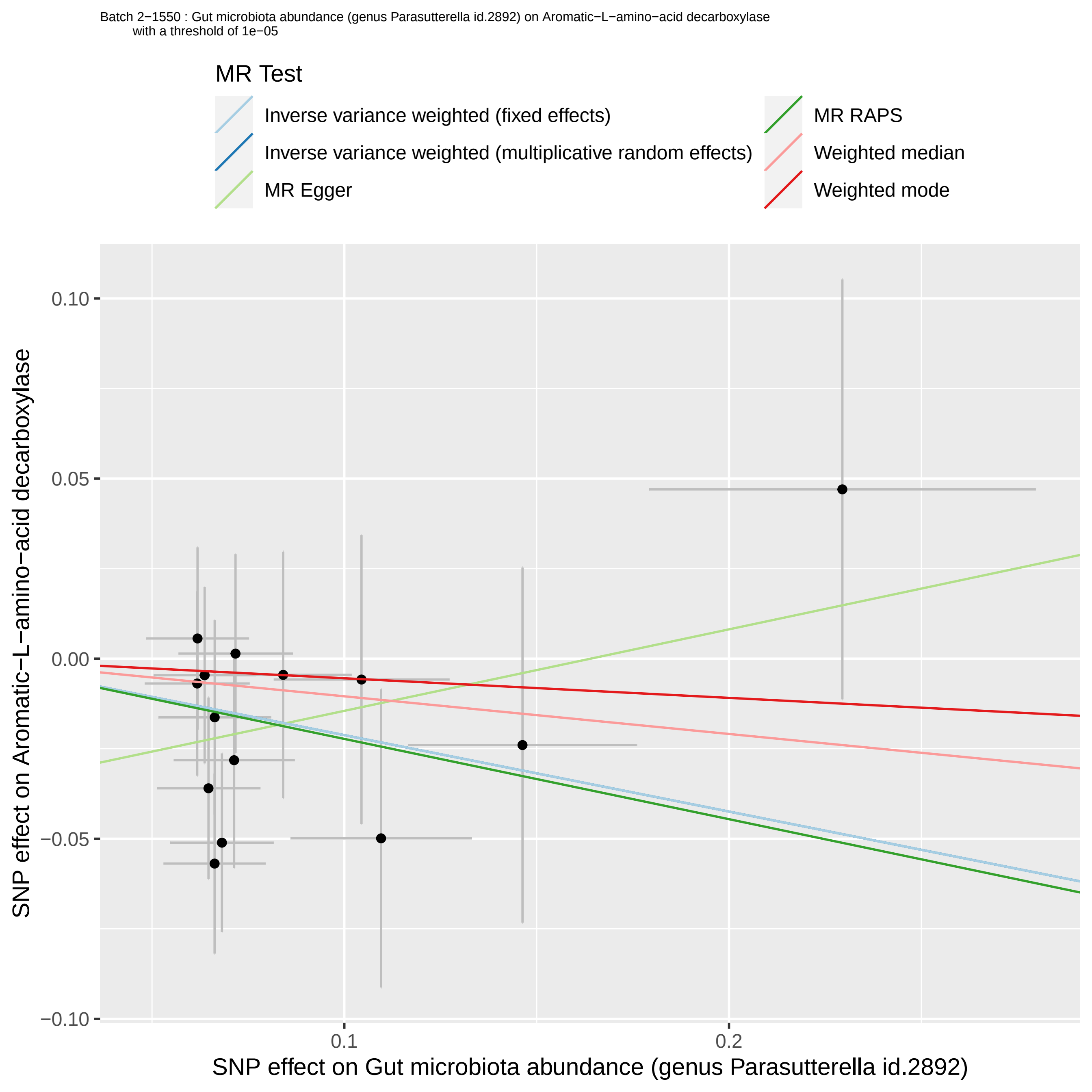
Batch 2−1550 : Gut microbiota abundance (genus Parasutterella id.2892) on Aromatic−L−amino−acid decarboxylase
with a threshold of 1e−05
MR Test
Inverse variance weighted (fixed effects)
Inverse variance weighted (multiplicative random effects)
MR Egger
MR RAPS
Weighted median
Weighted mode
0.10
0.05
0.00
−0.05
−0.10
0.1
0.2
SNP effect on Aromatic−L−amino−acid decarboxylase
SNP effect on Gut microbiota abundance (genus Parasutterella id.2892)

Batch 2−1551 : Gut microbiota abundance (genus Catenibacterium id.2153) on Discoidin domain−containing receptor 2
with a threshold of 1e−05

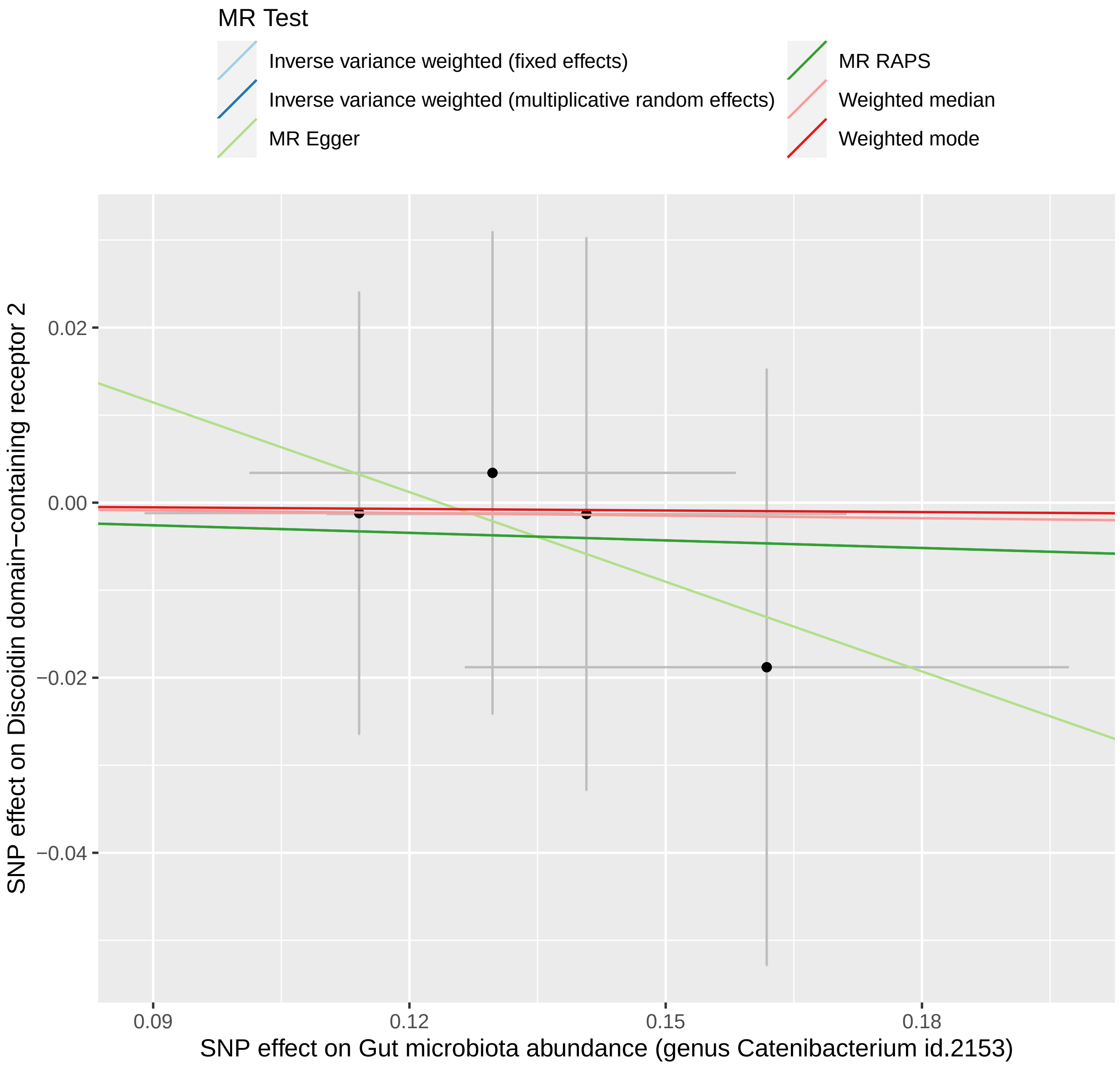

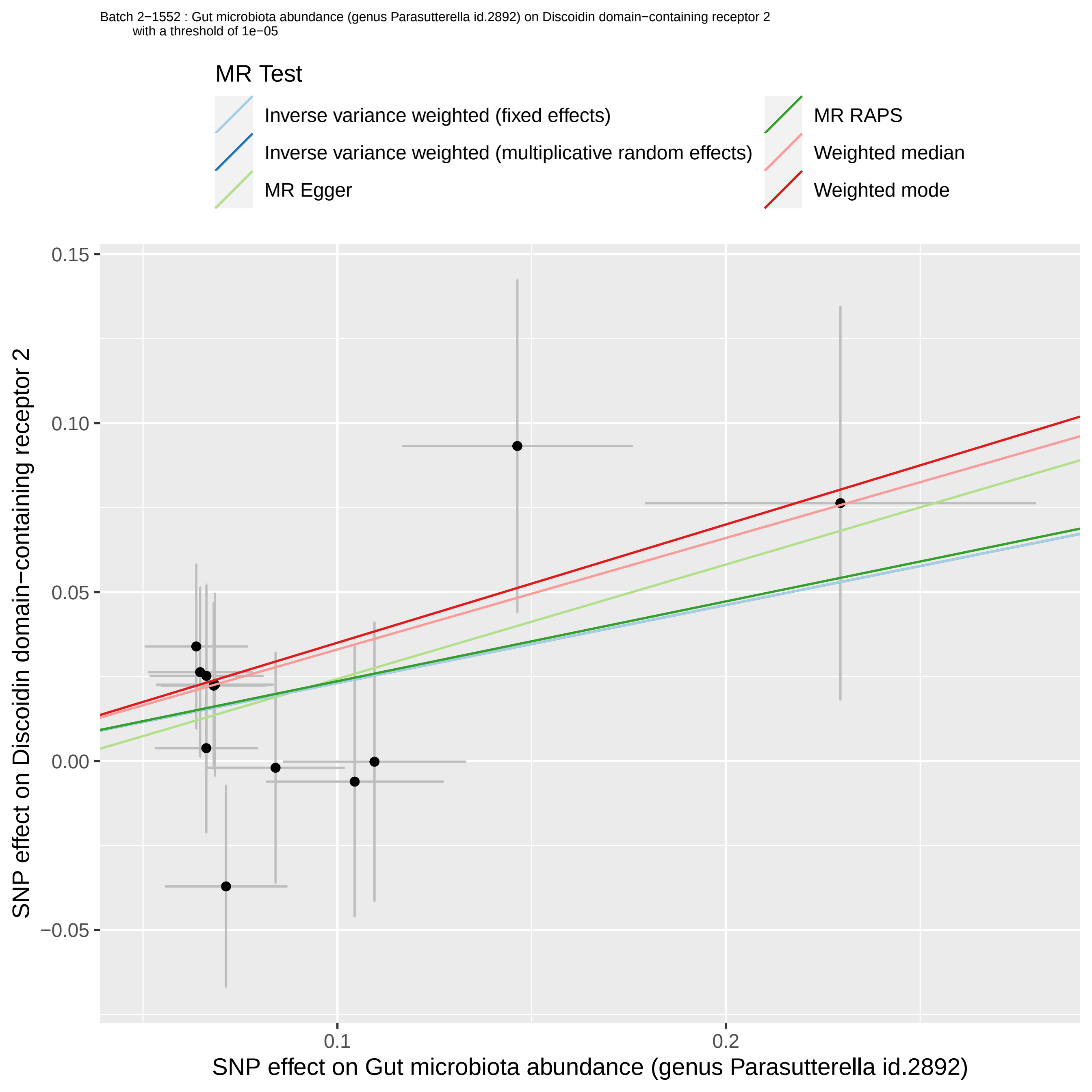
Batch 2−1552 : Gut microbiota abundance (genus Parasutterella id.2892) on Discoidin domain−containing receptor 2
with a threshold of 1e−05
MR Test
Inverse variance weighted (fixed effects)
Inverse variance weighted (multiplicative random effects)
MR Egger
MR RAPS
Weighted median
Weighted mode
0.15
0.10
0.05
0.00
−0.05
0.1
0.2
SNP effect on Discoidin domain−containing receptor 2
SNP effect on Gut microbiota abundance (genus Parasutterella id.2892)

Batch 2−1553 : Gut microbiota abundance (genus Catenibacterium id.2153) on ATP−dependent RNA helicase DDX25
with a threshold of 1e−05

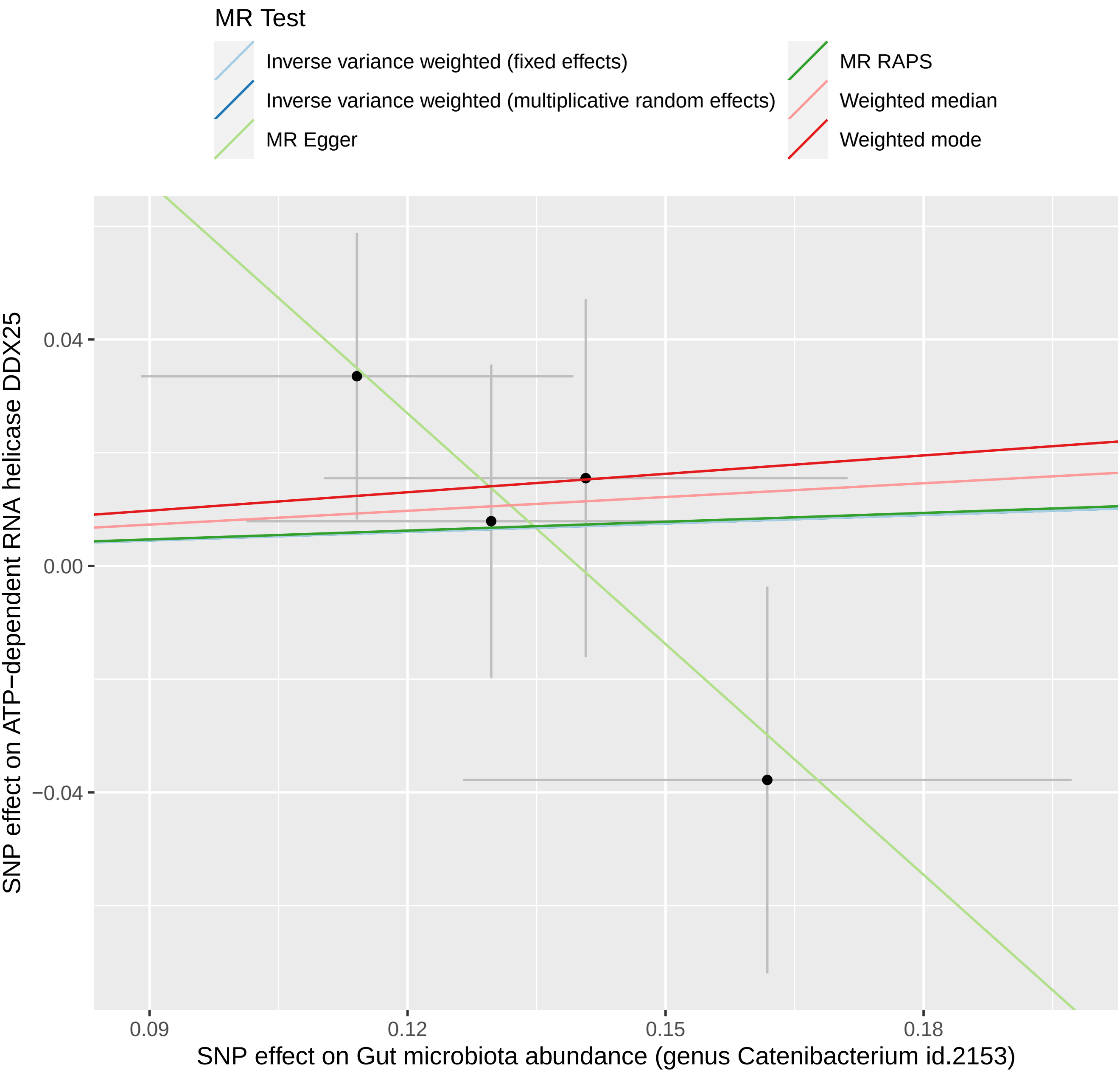

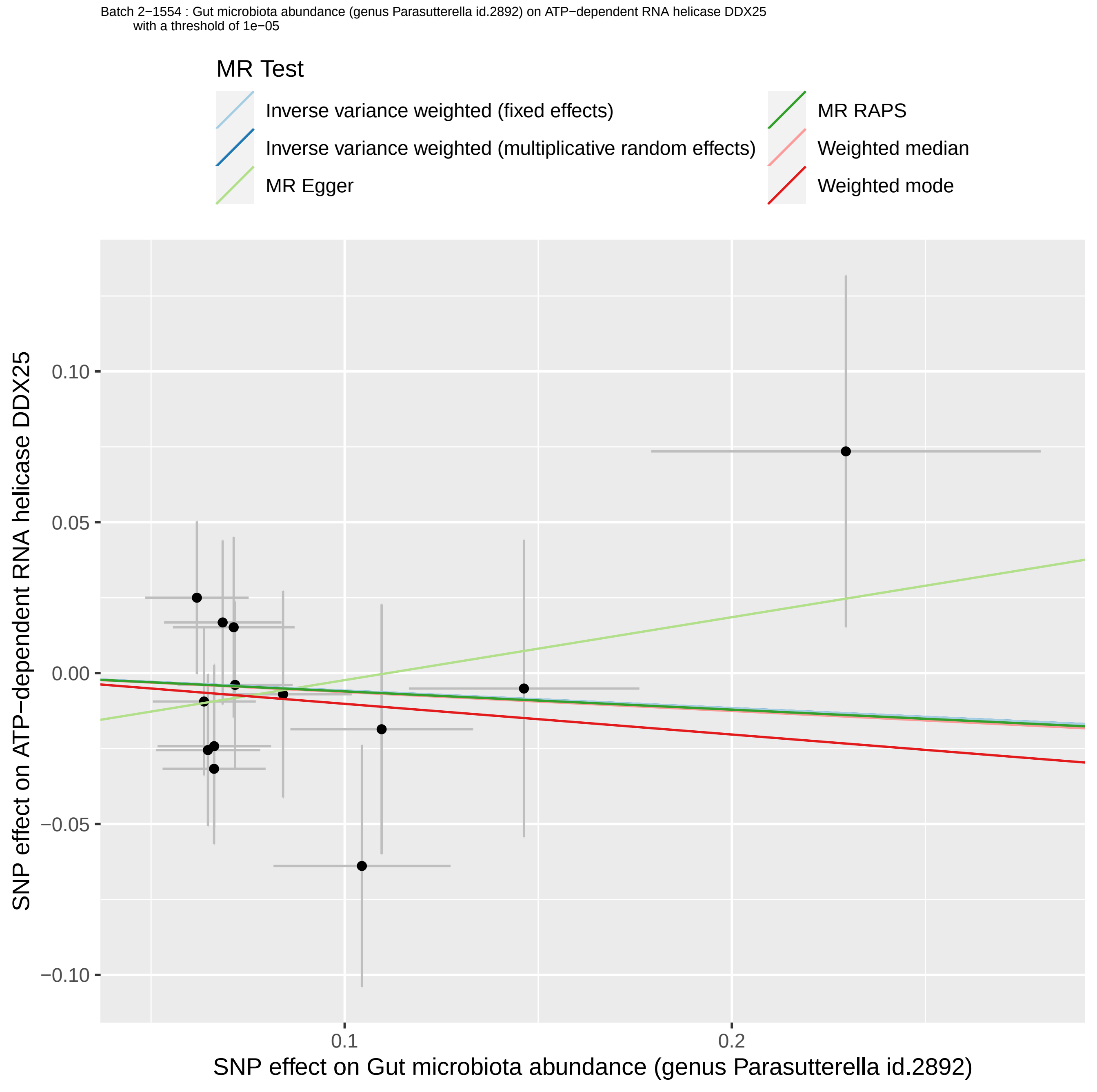
Batch 2−1554 : Gut microbiota abundance (genus Parasutterella id.2892) on ATP−dependent RNA helicase DDX25
with a threshold of 1e−05
MR Test
Inverse variance weighted (fixed effects)
Inverse variance weighted (multiplicative random effects)
MR Egger
MR RAPS
Weighted median
Weighted mode
0.10
0.05
0.00
−0.05
−0.10
0.1
0.2
SNP effect on ATP−dependent RNA helicase DDX25
SNP effect on Gut microbiota abundance (genus Parasutterella id.2892)

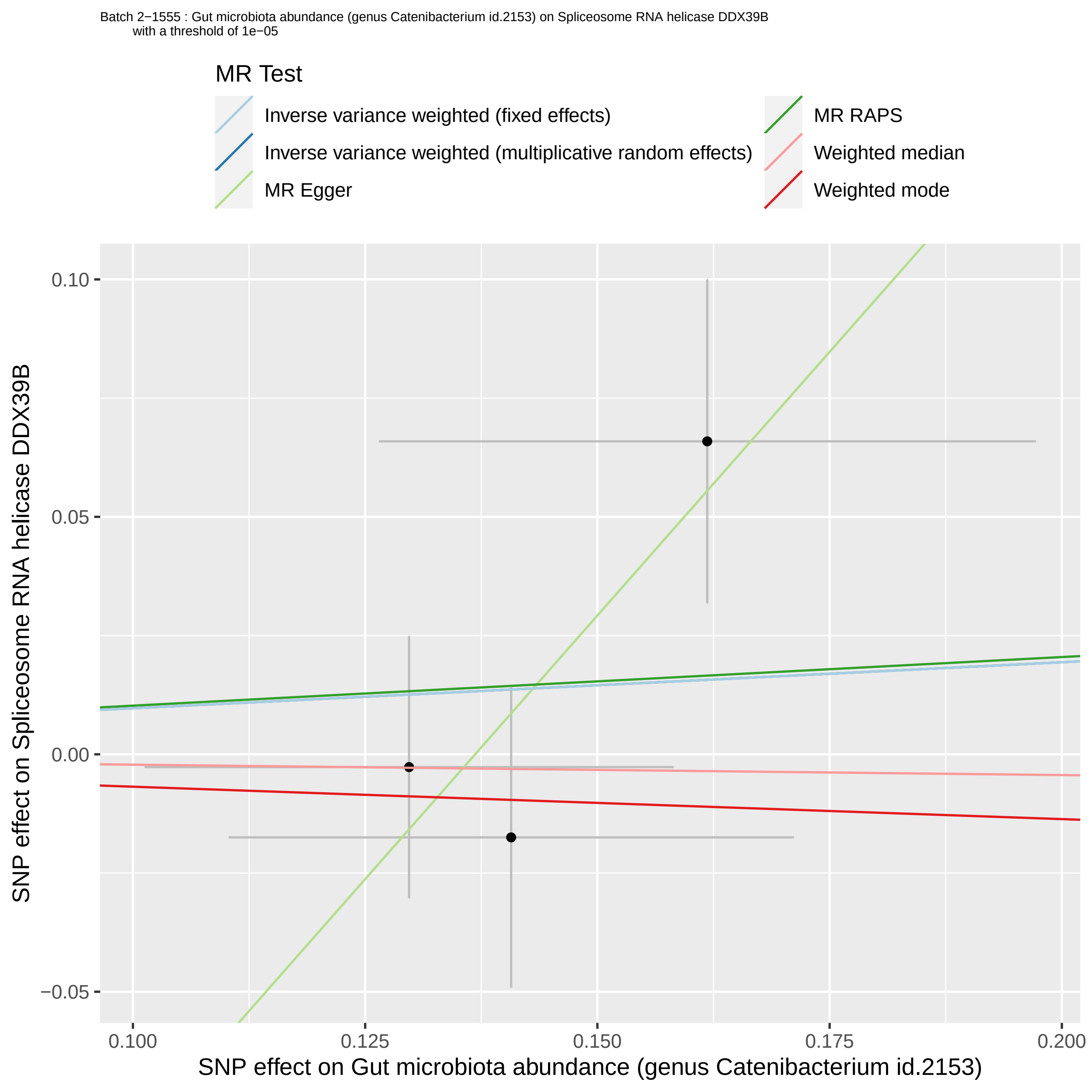
Batch 2−1555 : Gut microbiota abundance (genus Catenibacterium id.2153) on Spliceosome RNA helicase DDX39B
with a threshold of 1e−05
MR Test
Inverse variance weighted (fixed effects)
Inverse variance weighted (multiplicative random effects)
MR Egger
MR RAPS
Weighted median
Weighted mode
0.10
0.05
0.00
−0.05
0.100
0.125
0.150
0.175
0.200
SNP effect on Spliceosome RNA helicase DDX39B
SNP effect on Gut microbiota abundance (genus Catenibacterium id.2153)

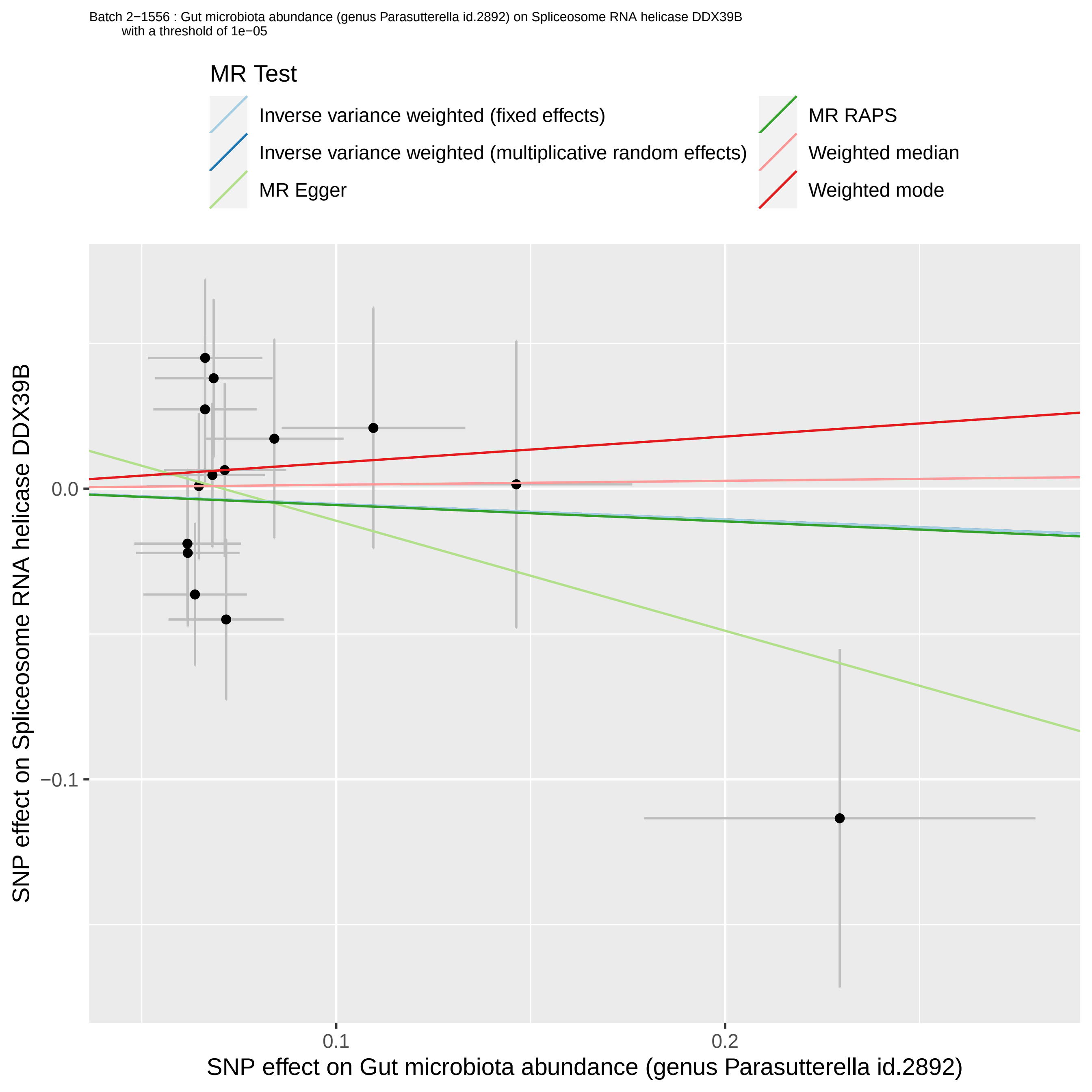

Batch 2−1556 : Gut microbiota abundance (genus Parasutterella id.2892) on Spliceosome RNA helicase DDX39B
with a threshold of 1e−05
MR Test
Inverse variance weighted (fixed effects)
Inverse variance weighted (multiplicative random effects)
MR Egger
MR RAPS
Weighted median
Weighted mode
0.0
−0.1
0.1
0.2
SNP effect on Spliceosome RNA helicase DDX39B
SNP effect on Gut microbiota abundance (genus Parasutterella id.2892)

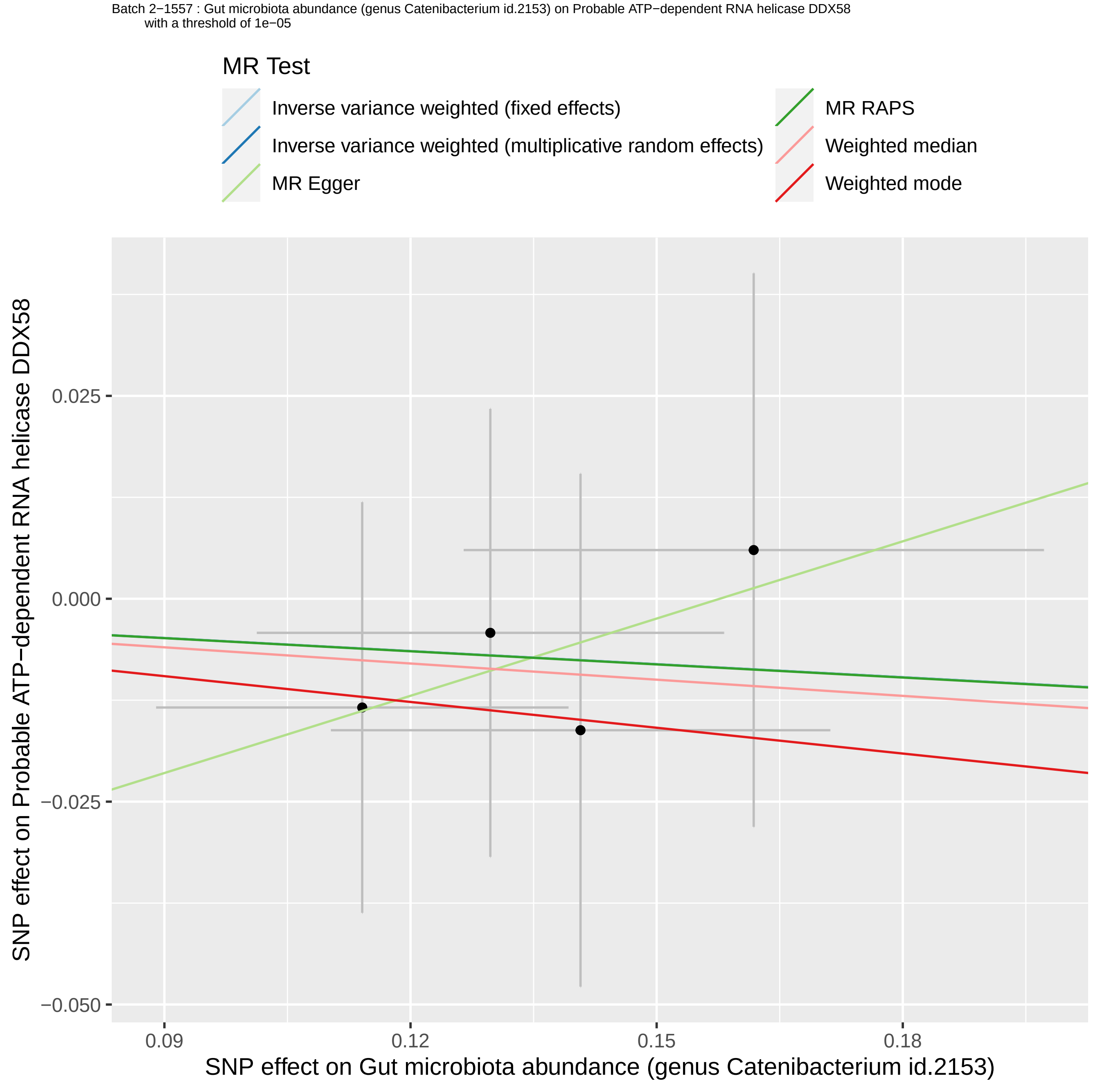

Batch 2−1557 : Gut microbiota abundance (genus Catenibacterium id.2153) on Probable ATP−dependent RNA helicase DDX58
with a threshold of 1e−05
MR Test
Inverse variance weighted (fixed effects)
Inverse variance weighted (multiplicative random effects)
MR Egger
MR RAPS
Weighted median
Weighted mode
SNP effect on Probable ATP−dependent RNA helicase DDX58
0.025
0.000
−0.025
−0.050
0.09
0.12
0.15
0.18
SNP effect on Gut microbiota abundance (genus Catenibacterium id.2153)

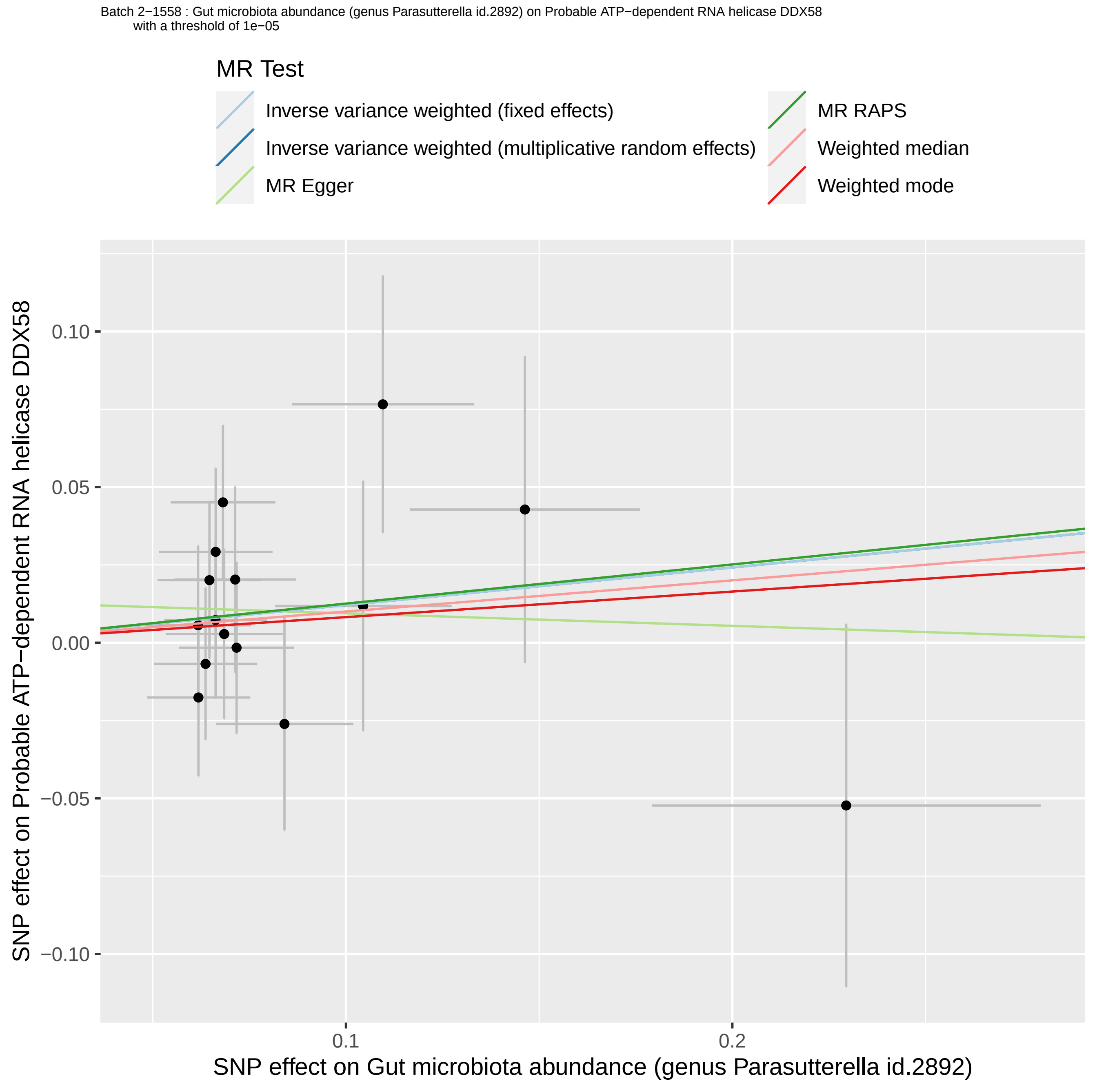
Batch 2−1558 : Gut microbiota abundance (genus Parasutterella id.2892) on Probable ATP−dependent RNA helicase DDX58
with a threshold of 1e−05
MR Test
Inverse variance weighted (fixed effects)
Inverse variance weighted (multiplicative random effects)
MR Egger
MR RAPS
Weighted median
Weighted mode
0.10
0.05
0.00
−0.05
−0.10
0.1
0.2
SNP effect on Probable ATP−dependent RNA helicase DDX58
SNP effect on Gut microbiota abundance (genus Parasutterella id.2892)

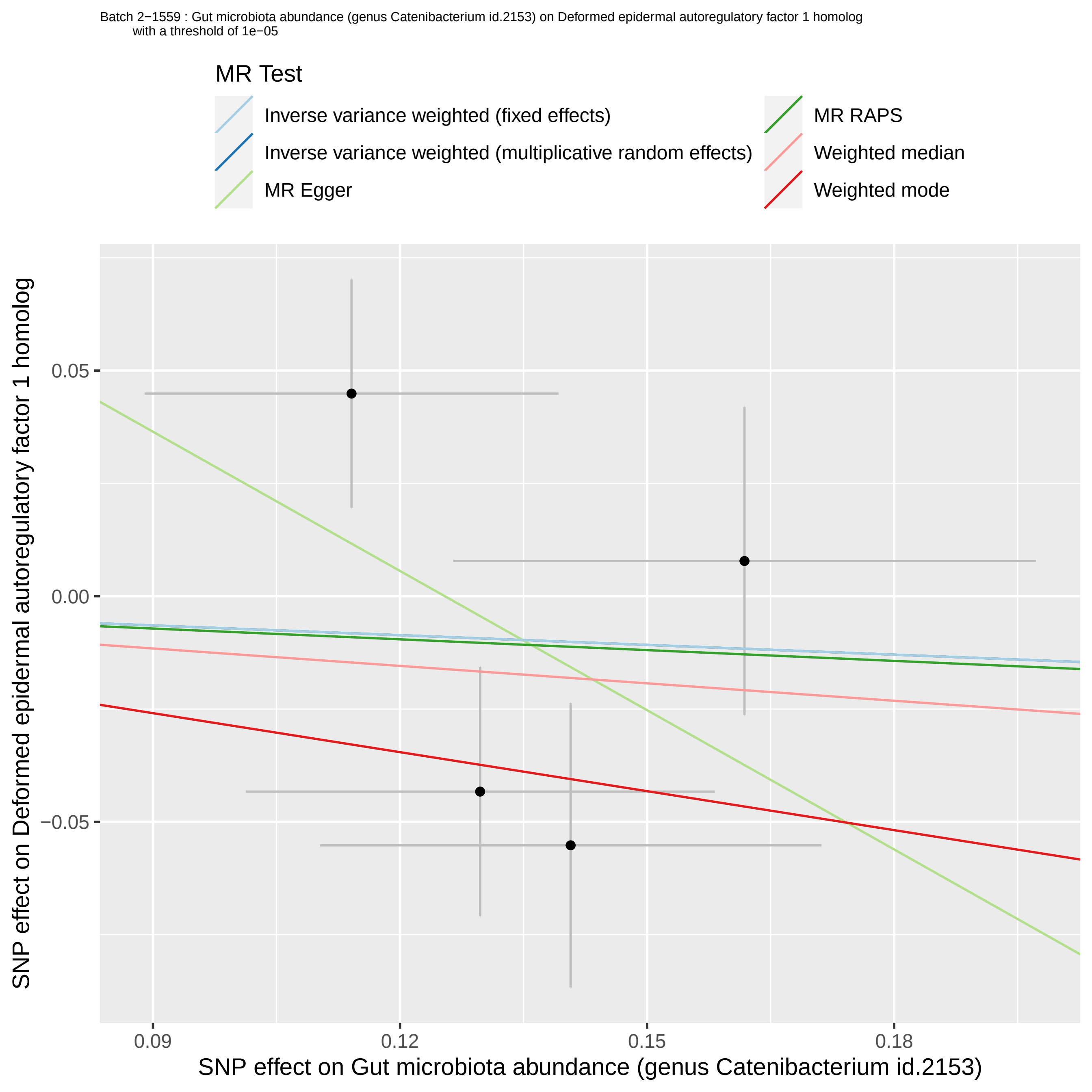
Batch 2−1559 : Gut microbiota abundance (genus Catenibacterium id.2153) on Deformed epidermal autoregulatory factor 1 homolog
with a threshold of 1e−05
MR Test
Inverse variance weighted (fixed effects)
Inverse variance weighted (multiplicative random effects)
MR Egger
MR RAPS
Weighted median
Weighted mode
0.05
0.00
−0.05
0.09
0.12
0.15
0.18
SNP effect on Deformed epidermal autoregulatory factor 1 homolog
SNP effect on Gut microbiota abundance (genus Catenibacterium id.2153)

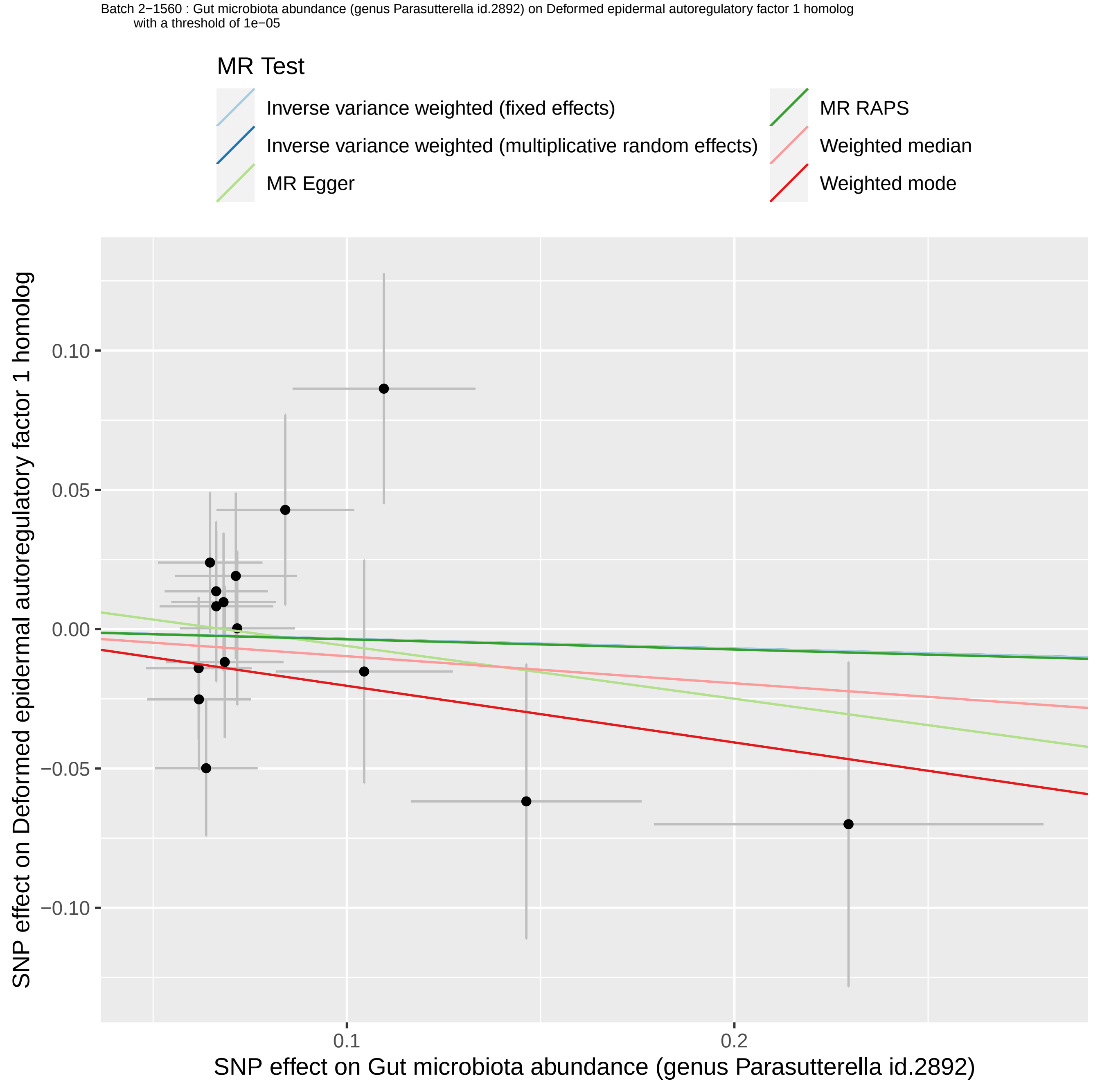

Batch 2−1560 : Gut microbiota abundance (genus Parasutterella id.2892) on Deformed epidermal autoregulatory factor 1 homolog with a threshold of 1e−05
MR Test
Inverse variance weighted (fixed effects)
Inverse variance weighted (multiplicative random effects)
MR Egger
MR RAPS
Weighted median
Weighted mode
SNP effect on Deformed epidermal autoregulatory factor 1 homolog
0.10
0.05
0.00
−0.05
−0.10
0.1
0.2
SNP effect on Gut microbiota abundance (genus Parasutterella id.2892)

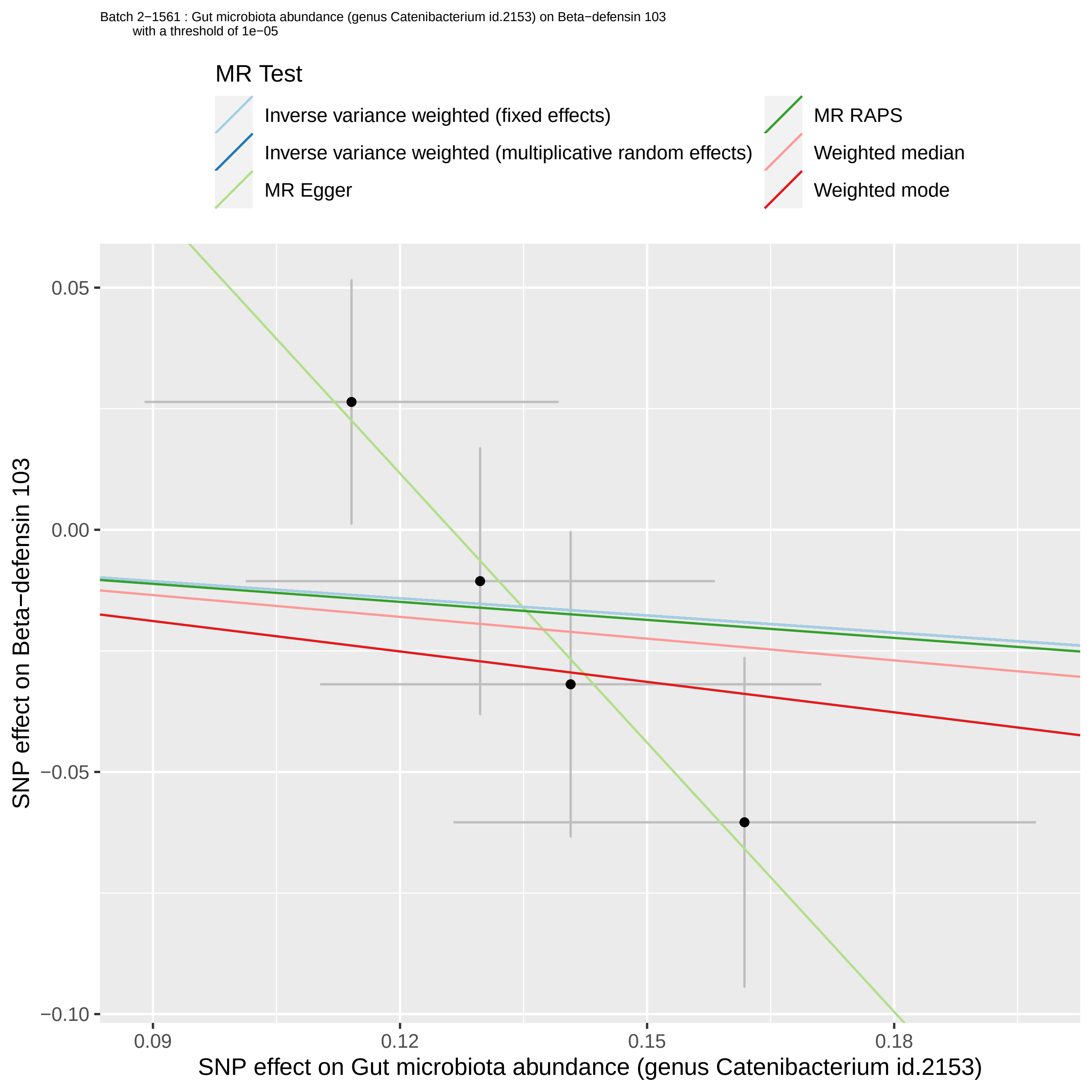
Batch 2−1561 : Gut microbiota abundance (genus Catenibacterium id.2153) on Beta−defensin 103
with a threshold of 1e−05
MR Test
Inverse variance weighted (fixed effects)
Inverse variance weighted (multiplicative random effects)
MR Egger
MR RAPS
Weighted median
Weighted mode
0.05
0.00
−0.05
−0.10
0.09
0.12
0.15
0.18
SNP effect on Beta−defensin 103
SNP effect on Gut microbiota abundance (genus Catenibacterium id.2153)

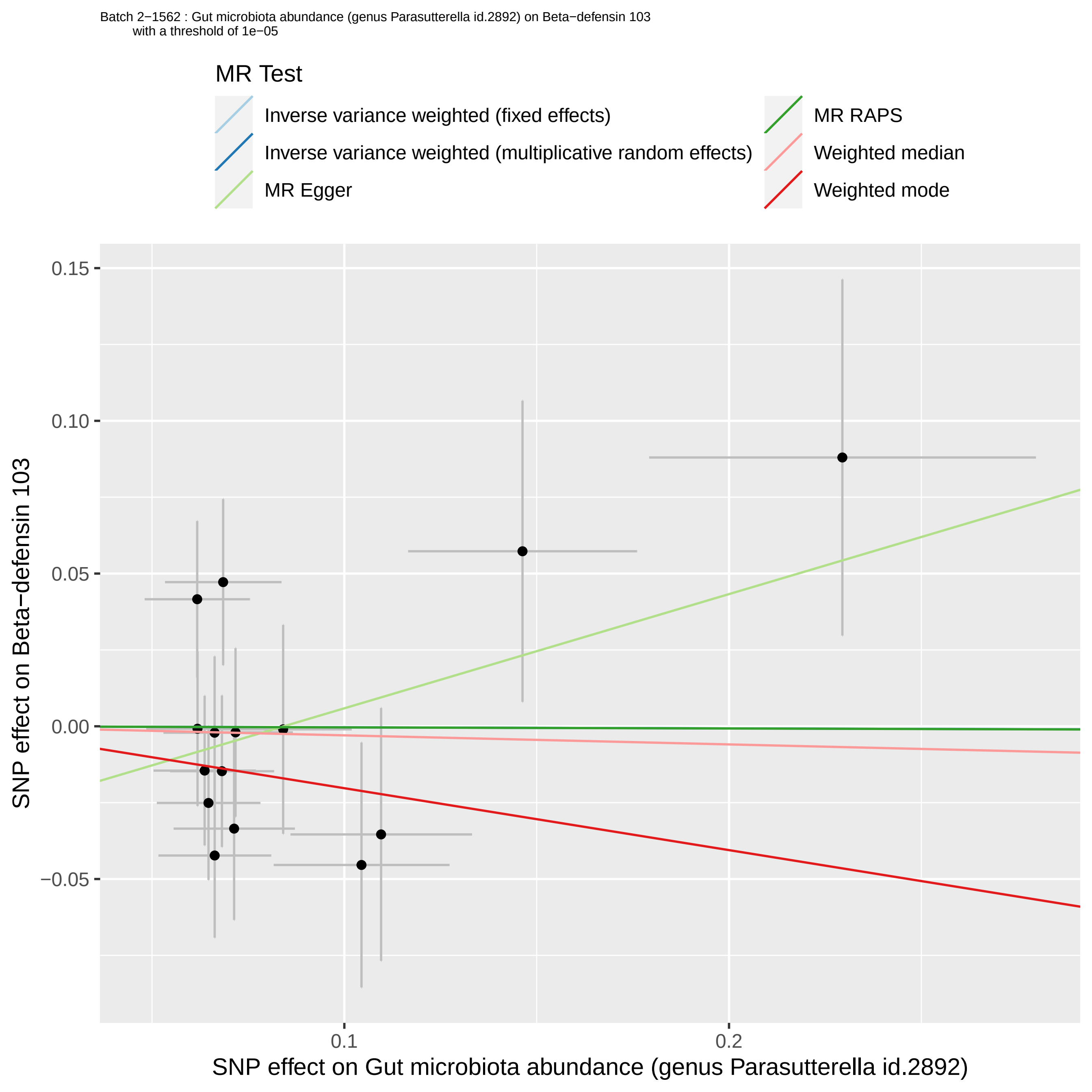
Batch 2−1562 : Gut microbiota abundance (genus Parasutterella id.2892) on Beta−defensin 103
with a threshold of 1e−05
MR Test
Inverse variance weighted (fixed effects)
Inverse variance weighted (multiplicative random effects)
MR Egger
MR RAPS
Weighted median
Weighted mode
0.15
0.10
0.05
0.00
−0.05
0.1
0.2
SNP effect on Beta−defensin 103
SNP effect on Gut microbiota abundance (genus Parasutterella id.2892)

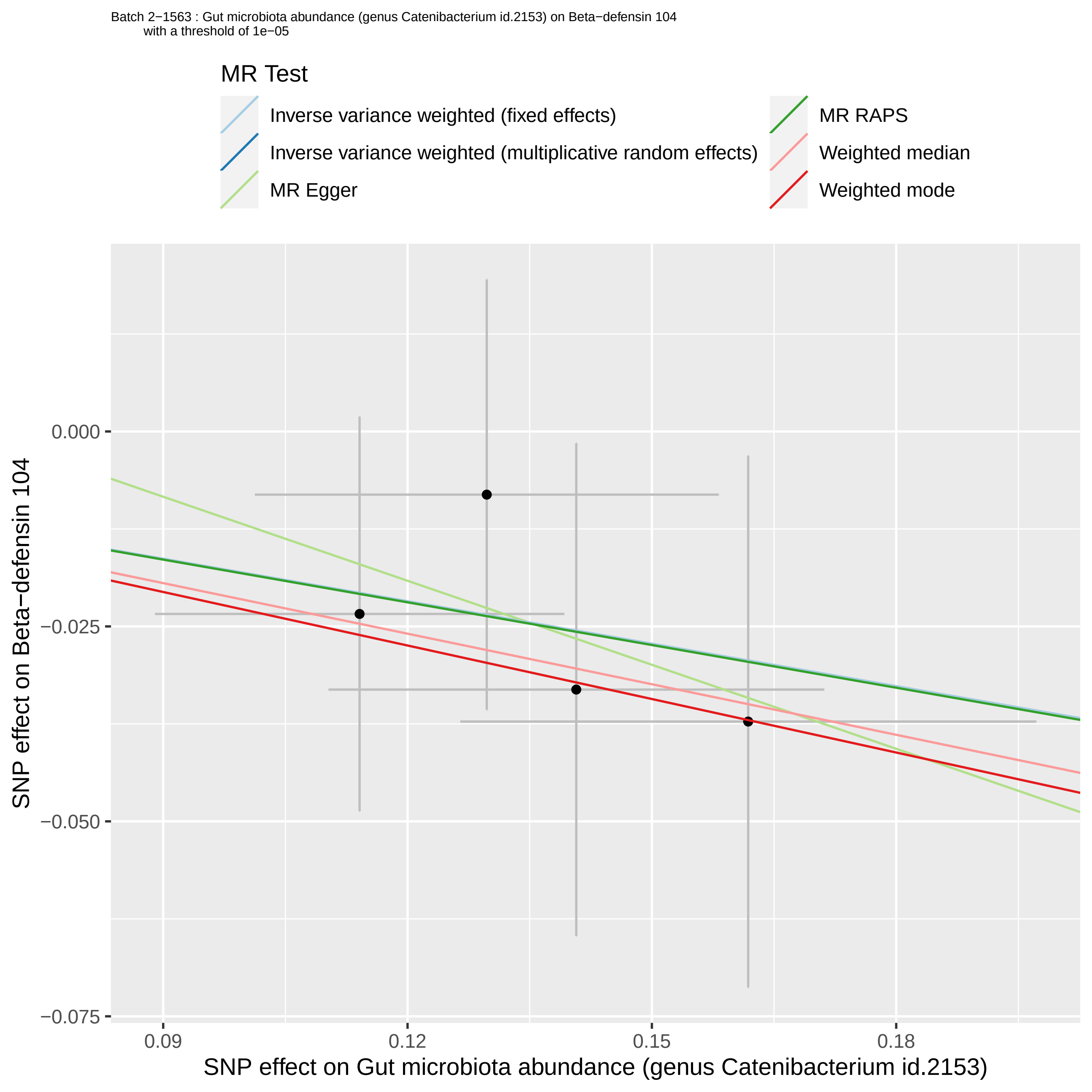
Batch 2−1563 : Gut microbiota abundance (genus Catenibacterium id.2153) on Beta−defensin 104
with a threshold of 1e−05
MR Test
Inverse variance weighted (fixed effects)
Inverse variance weighted (multiplicative random effects)
MR Egger
MR RAPS
Weighted median
Weighted mode
0.000
−0.025
−0.050
−0.075
0.09
0.12
0.15
0.18
SNP effect on Beta−defensin 104
SNP effect on Gut microbiota abundance (genus Catenibacterium id.2153)

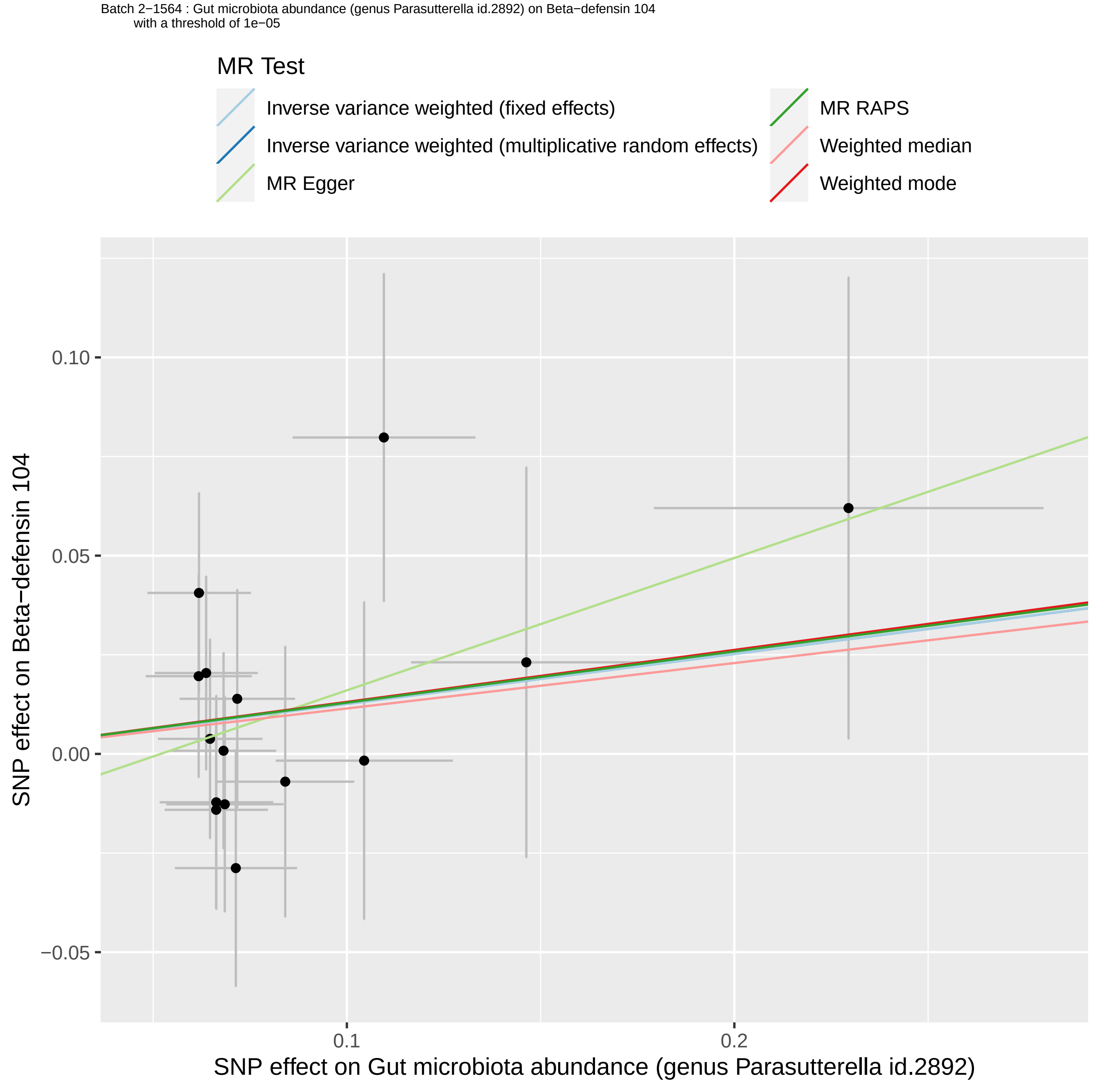

Batch 2−1564 : Gut microbiota abundance (genus Parasutterella id.2892) on Beta−defensin 104
with a threshold of 1e−05
MR Test
Inverse variance weighted (fixed effects)
Inverse variance weighted (multiplicative random effects)
MR Egger
MR RAPS
Weighted median
Weighted mode
SNP effect on Beta−defensin 104
0.10
0.05
0.00
−0.05
0.1
0.2
SNP effect on Gut microbiota abundance (genus Parasutterella id.2892)

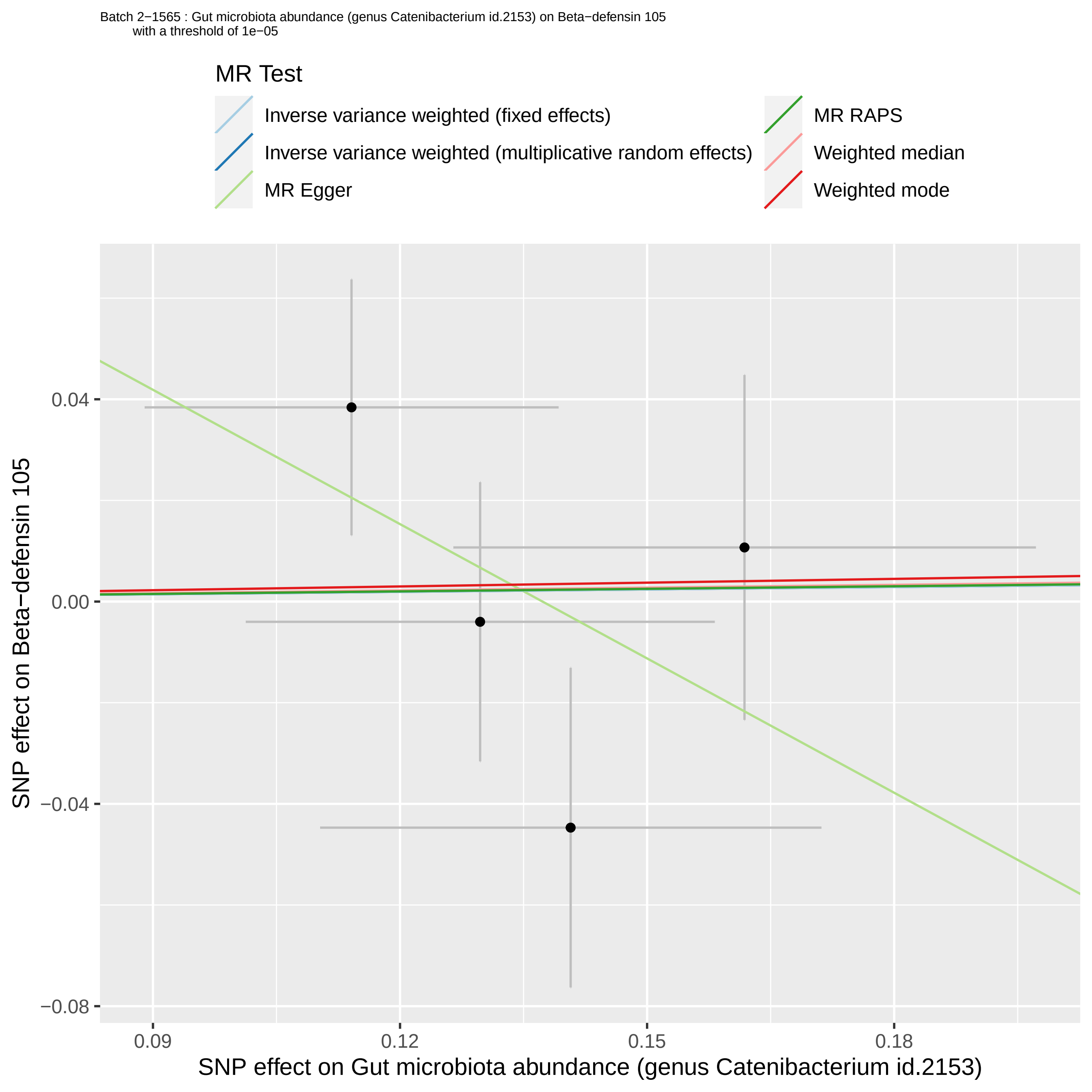
Batch 2−1565 : Gut microbiota abundance (genus Catenibacterium id.2153) on Beta−defensin 105
with a threshold of 1e−05
MR Test
Inverse variance weighted (fixed effects)
Inverse variance weighted (multiplicative random effects)
MR Egger
MR RAPS
Weighted median
Weighted mode
0.04
0.00
−0.04
−0.08
0.09
0.12
0.15
0.18
SNP effect on Beta−defensin 105
SNP effect on Gut microbiota abundance (genus Catenibacterium id.2153)

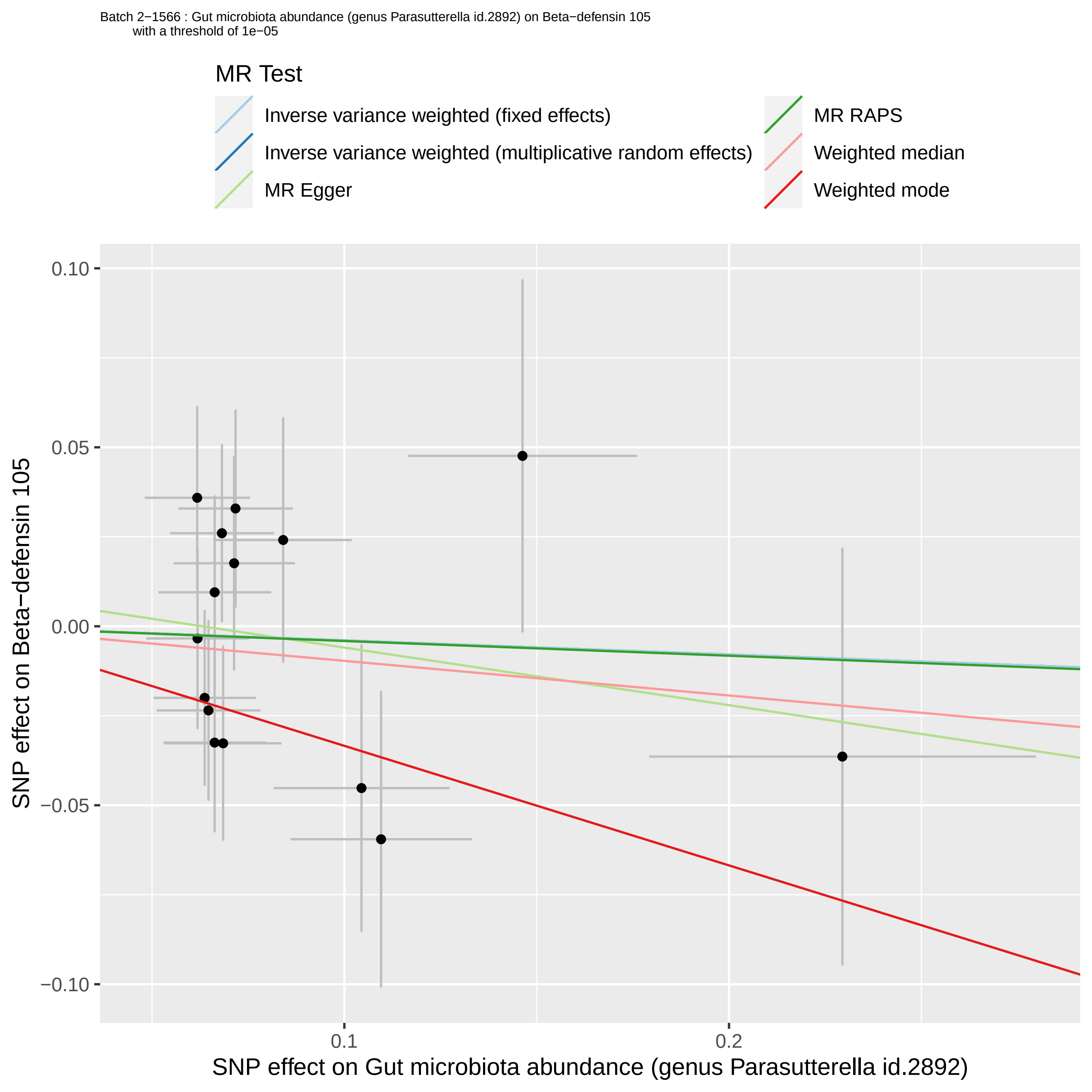
Batch 2−1566 : Gut microbiota abundance (genus Parasutterella id.2892) on Beta−defensin 105
with a threshold of 1e−05
MR Test
Inverse variance weighted (fixed effects)
Inverse variance weighted (multiplicative random effects)
MR Egger
MR RAPS
Weighted median
Weighted mode
0.10
0.05
0.00
−0.05
−0.10
SNP effect on Beta−defensin 105
0.1
0.2
SNP effect on Gut microbiota abundance (genus Parasutterella id.2892)

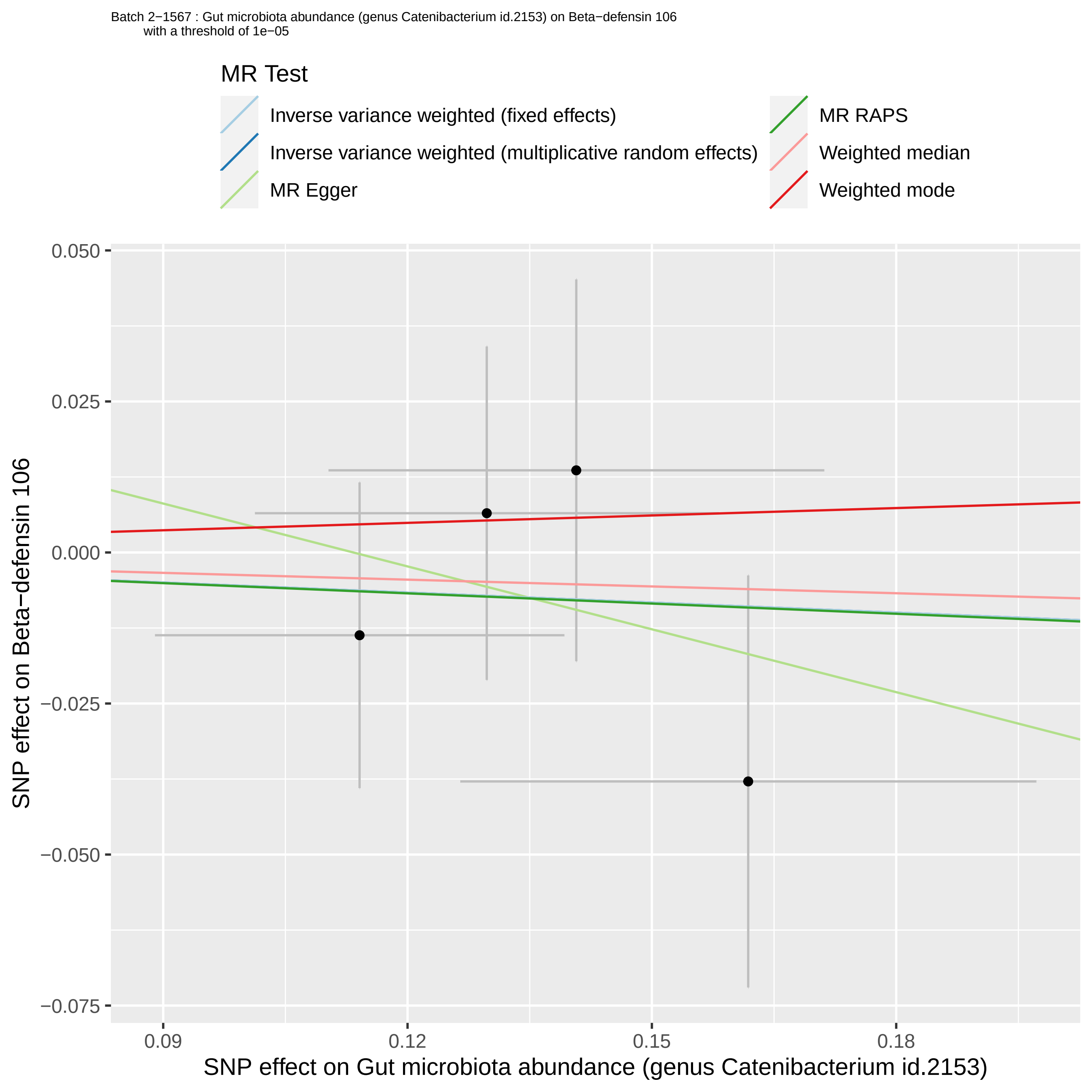
Batch 2−1567 : Gut microbiota abundance (genus Catenibacterium id.2153) on Beta−defensin 106
with a threshold of 1e−05
MR Test
Inverse variance weighted (fixed effects)
Inverse variance weighted (multiplicative random effects)
MR Egger
MR RAPS
Weighted median
Weighted mode
0.050
0.025
0.000
−0.025
−0.050
−0.075
0.09
0.12
0.15
0.18
SNP effect on Beta−defensin 106
SNP effect on Gut microbiota abundance (genus Catenibacterium id.2153)

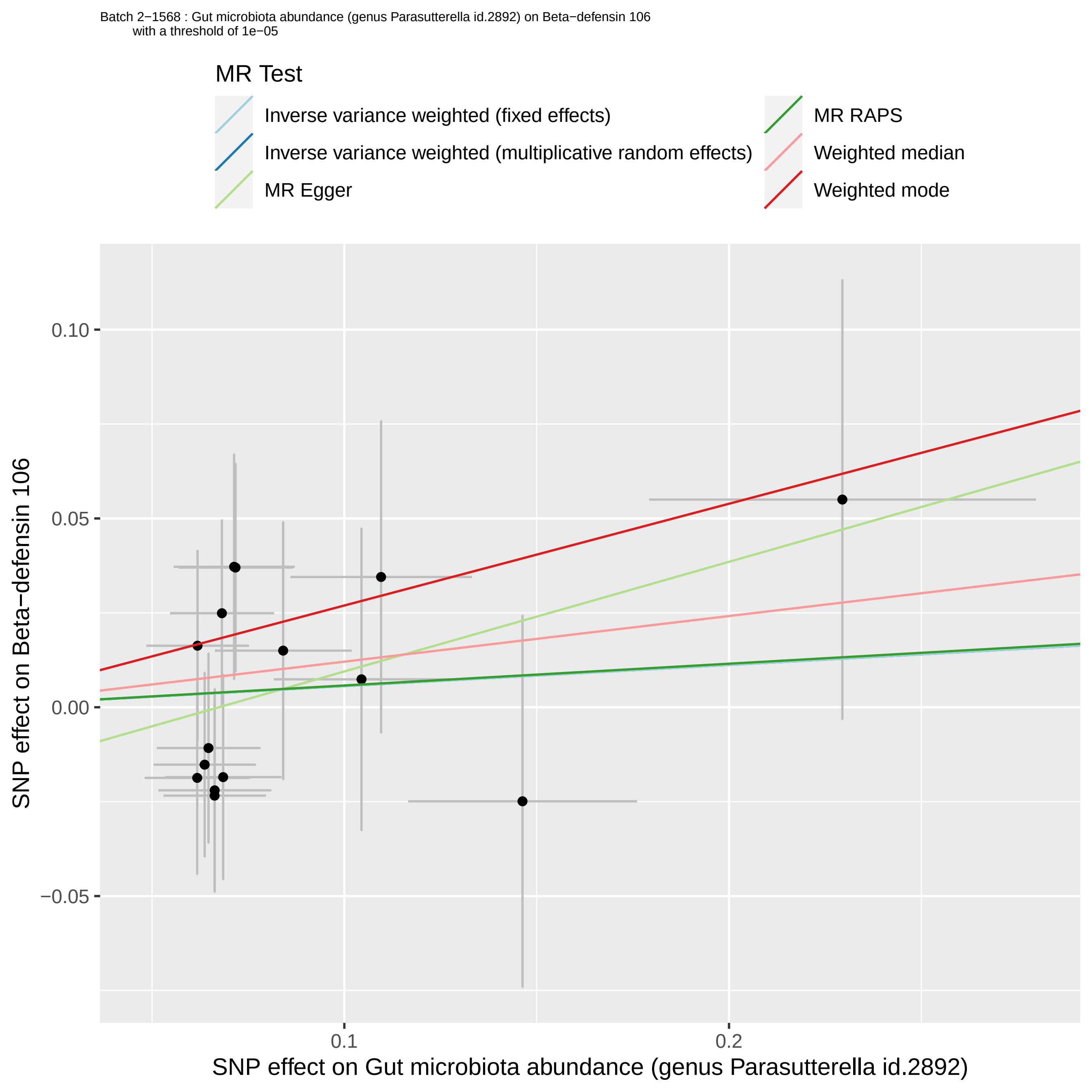
Batch 2−1568 : Gut microbiota abundance (genus Parasutterella id.2892) on Beta−defensin 106
with a threshold of 1e−05
MR Test
Inverse variance weighted (fixed effects)
Inverse variance weighted (multiplicative random effects)
MR Egger
MR RAPS
Weighted median
Weighted mode
0.10
0.05
0.00
−0.05
0.1
0.2
SNP effect on Beta−defensin 106
SNP effect on Gut microbiota abundance (genus Parasutterella id.2892)

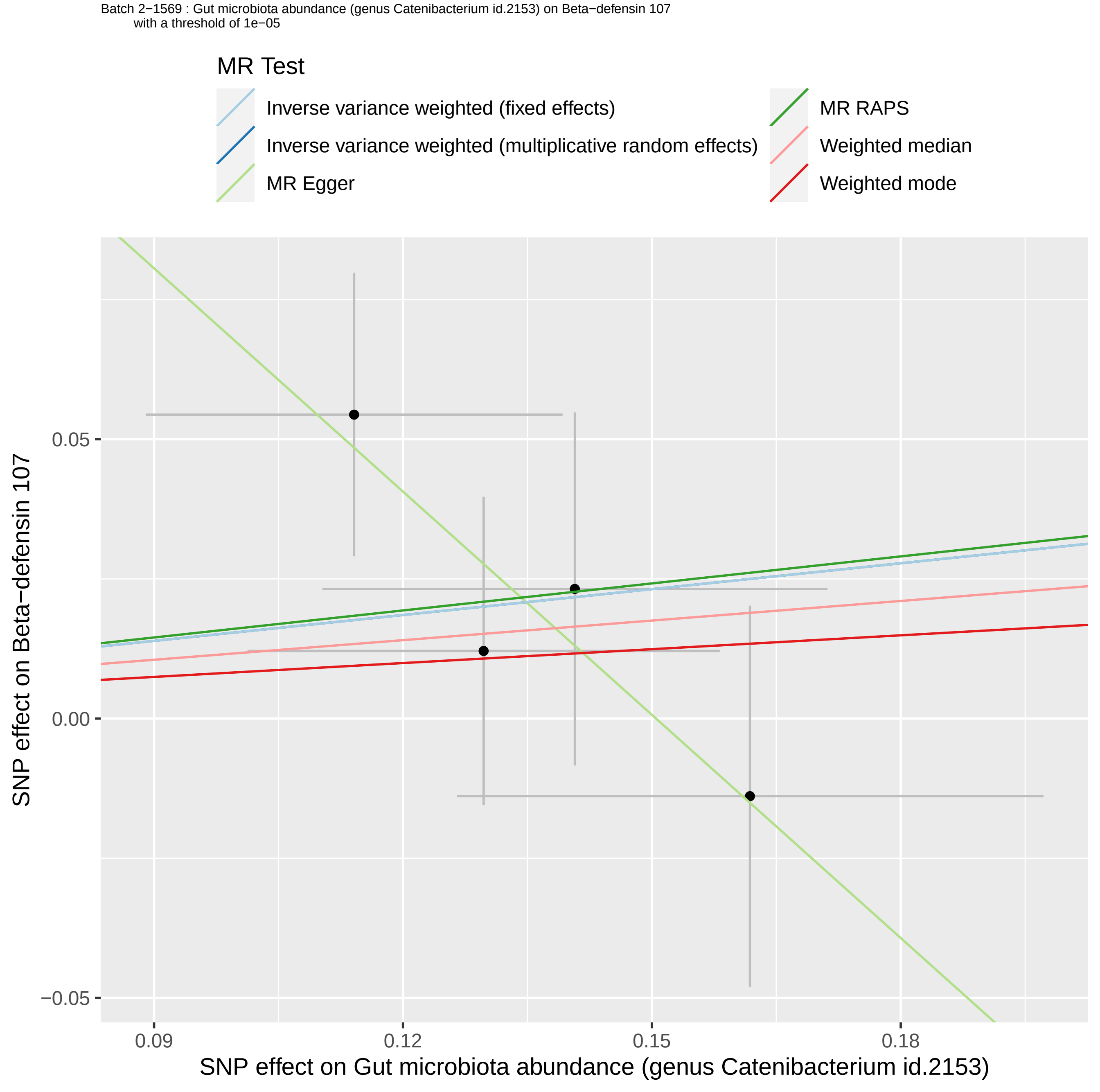

Batch 2−1569 : Gut microbiota abundance (genus Catenibacterium id.2153) on Beta−defensin 107
with a threshold of 1e−05
MR Test
Inverse variance weighted (fixed effects)
Inverse variance weighted (multiplicative random effects)
MR Egger
MR RAPS
Weighted median
Weighted mode
0.05
0.00
−0.05
0.09
0.12
0.15
0.18
SNP effect on Beta−defensin 107
SNP effect on Gut microbiota abundance (genus Catenibacterium id.2153)

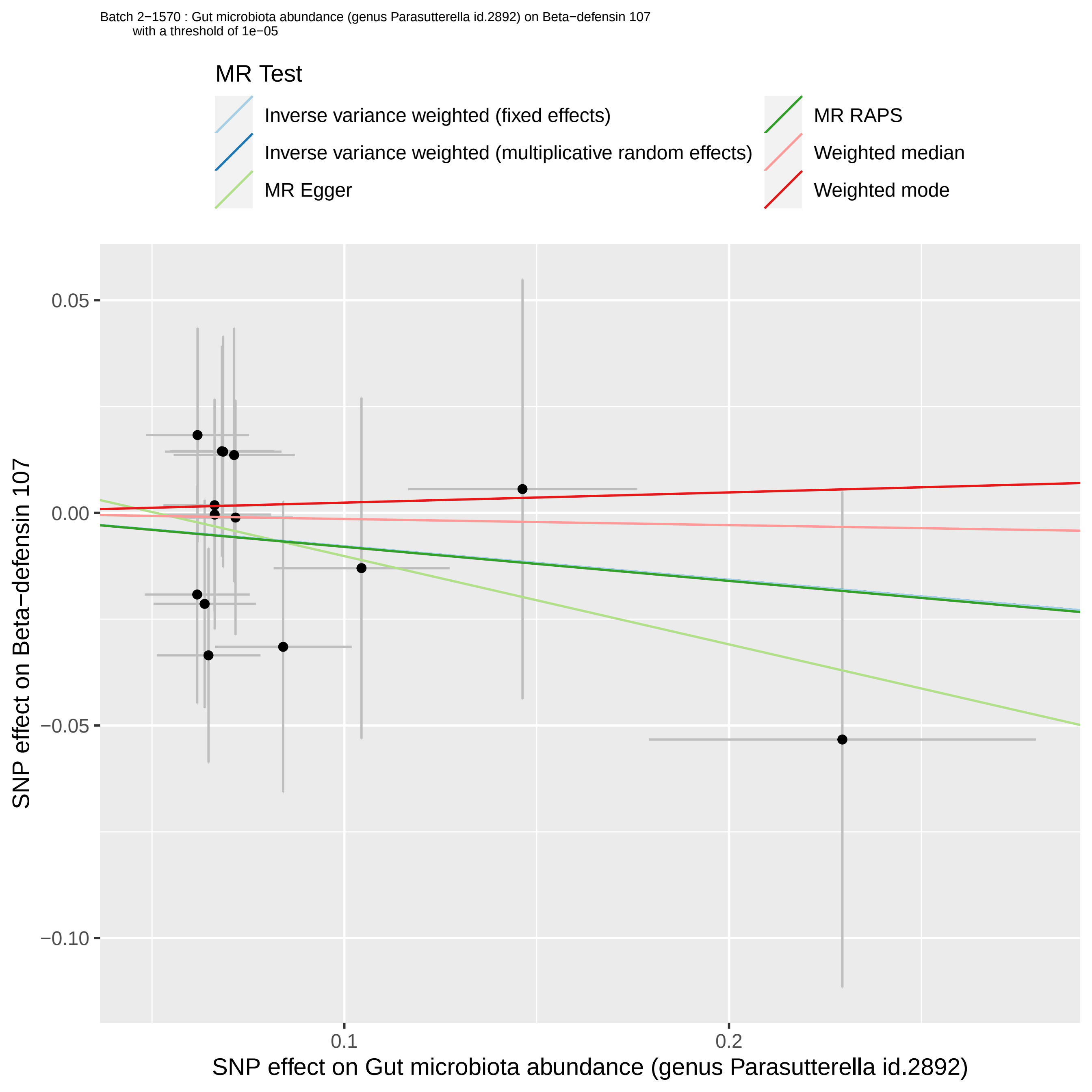
Batch 2−1570 : Gut microbiota abundance (genus Parasutterella id.2892) on Beta−defensin 107
with a threshold of 1e−05
MR Test
Inverse variance weighted (fixed effects)
Inverse variance weighted (multiplicative random effects)
MR Egger
MR RAPS
Weighted median
Weighted mode
0.05
0.00
−0.05
−0.10
0.1
0.2
SNP effect on Beta−defensin 107
SNP effect on Gut microbiota abundance (genus Parasutterella id.2892)

Batch 2−1571 : Gut microbiota abundance (genus Catenibacterium id.2153) on Beta−defensin 108B
with a threshold of 1e−05

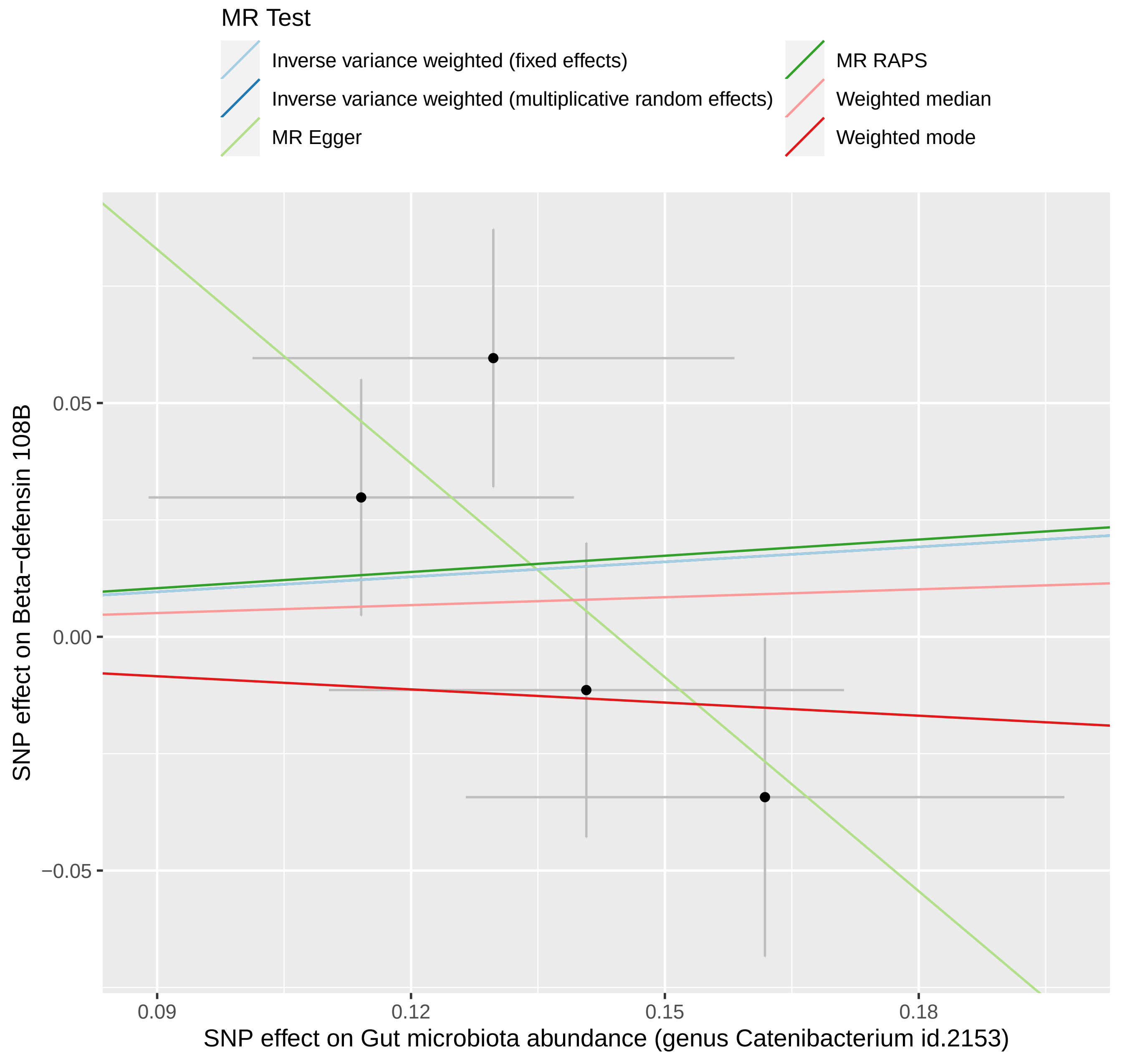

Batch 2−1572 : Gut microbiota abundance (genus Parasutterella id.2892) on Beta−defensin 108B
with a threshold of 1e−05

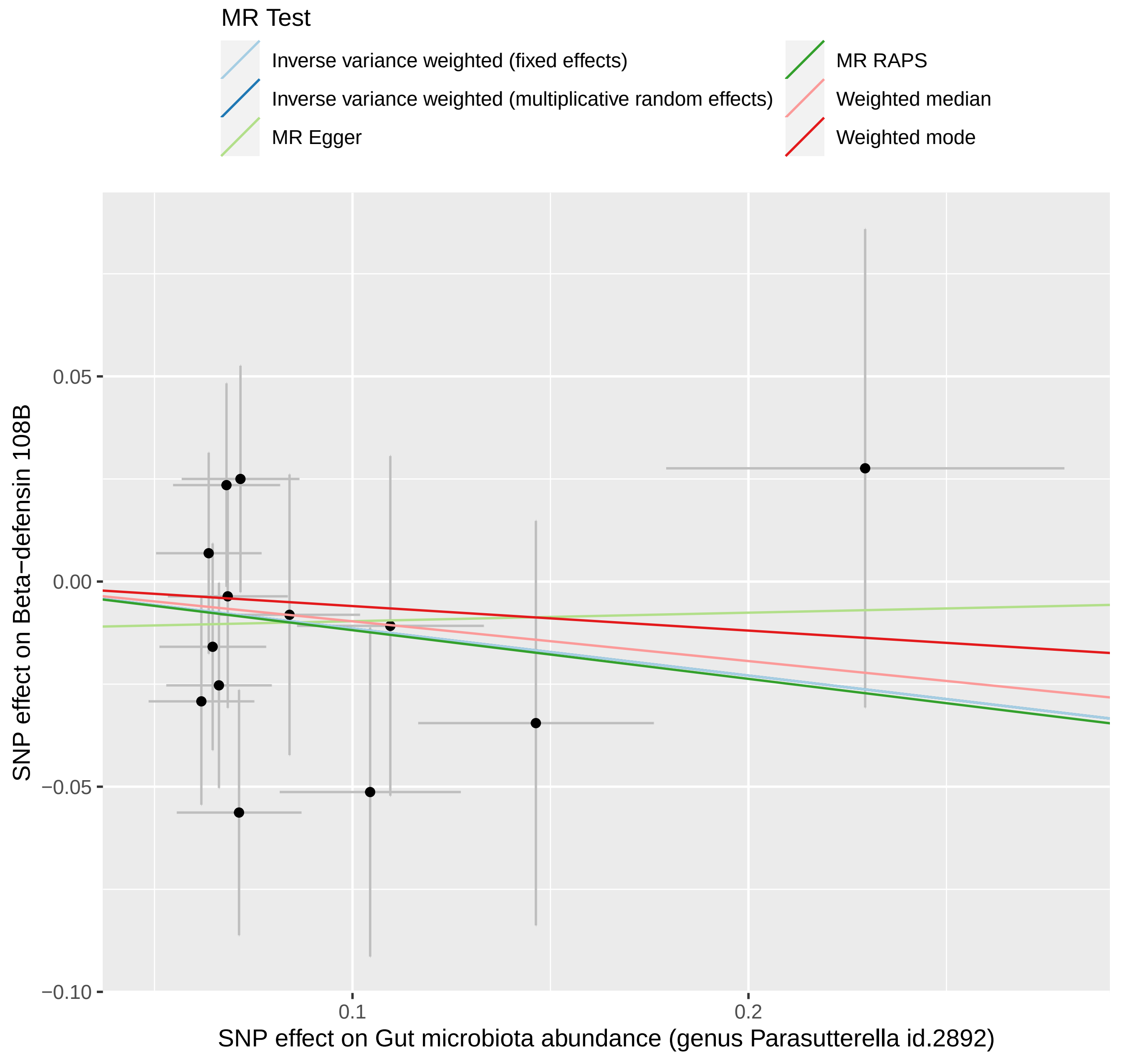

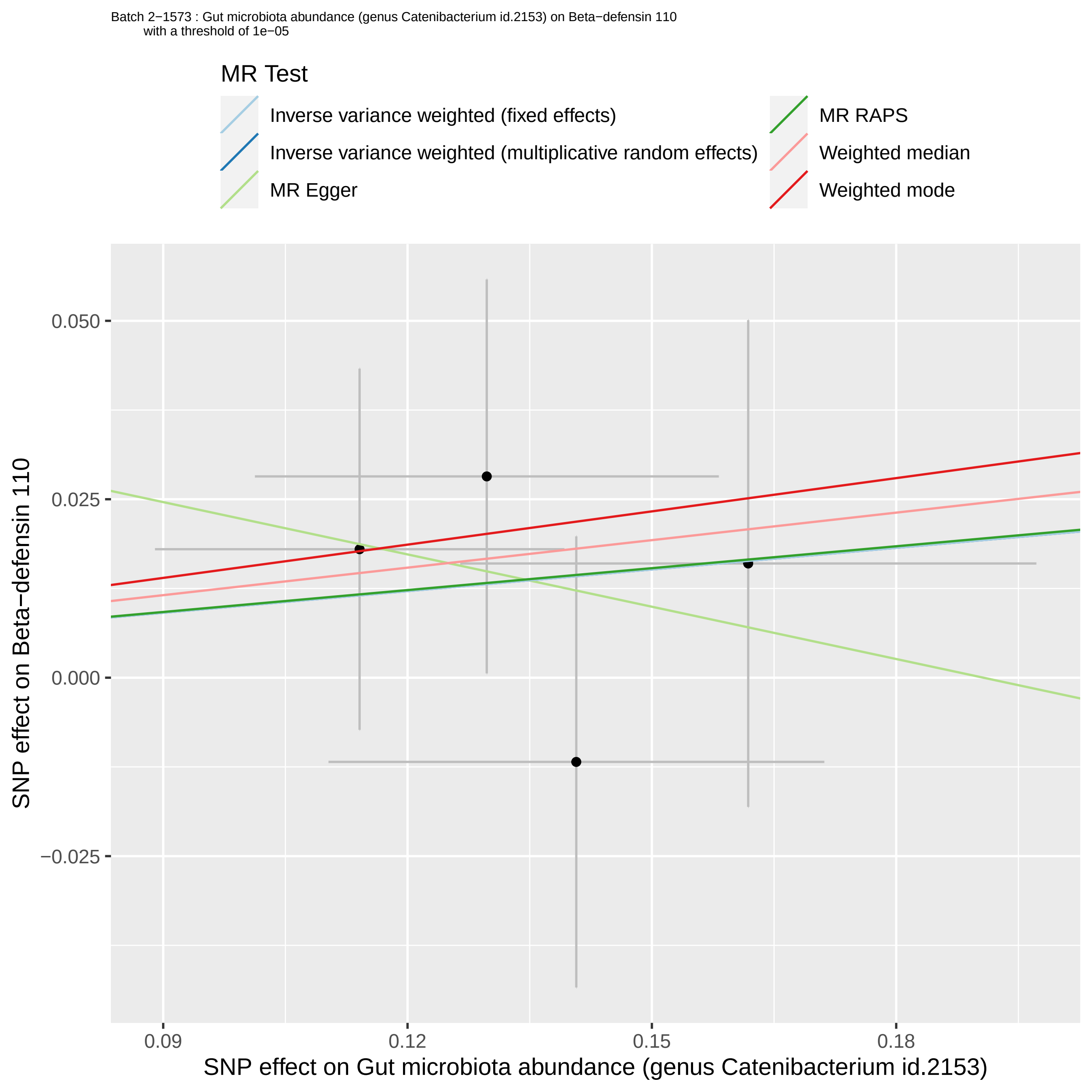
Batch 2−1573 : Gut microbiota abundance (genus Catenibacterium id.2153) on Beta−defensin 110
with a threshold of 1e−05
MR Test
Inverse variance weighted (fixed effects)
Inverse variance weighted (multiplicative random effects)
MR Egger
MR RAPS
Weighted median
Weighted mode
SNP effect on Beta−defensin 110
0.050
0.025
0.000
−0.025
0.09
0.12
0.15
0.18
SNP effect on Gut microbiota abundance (genus Catenibacterium id.2153)

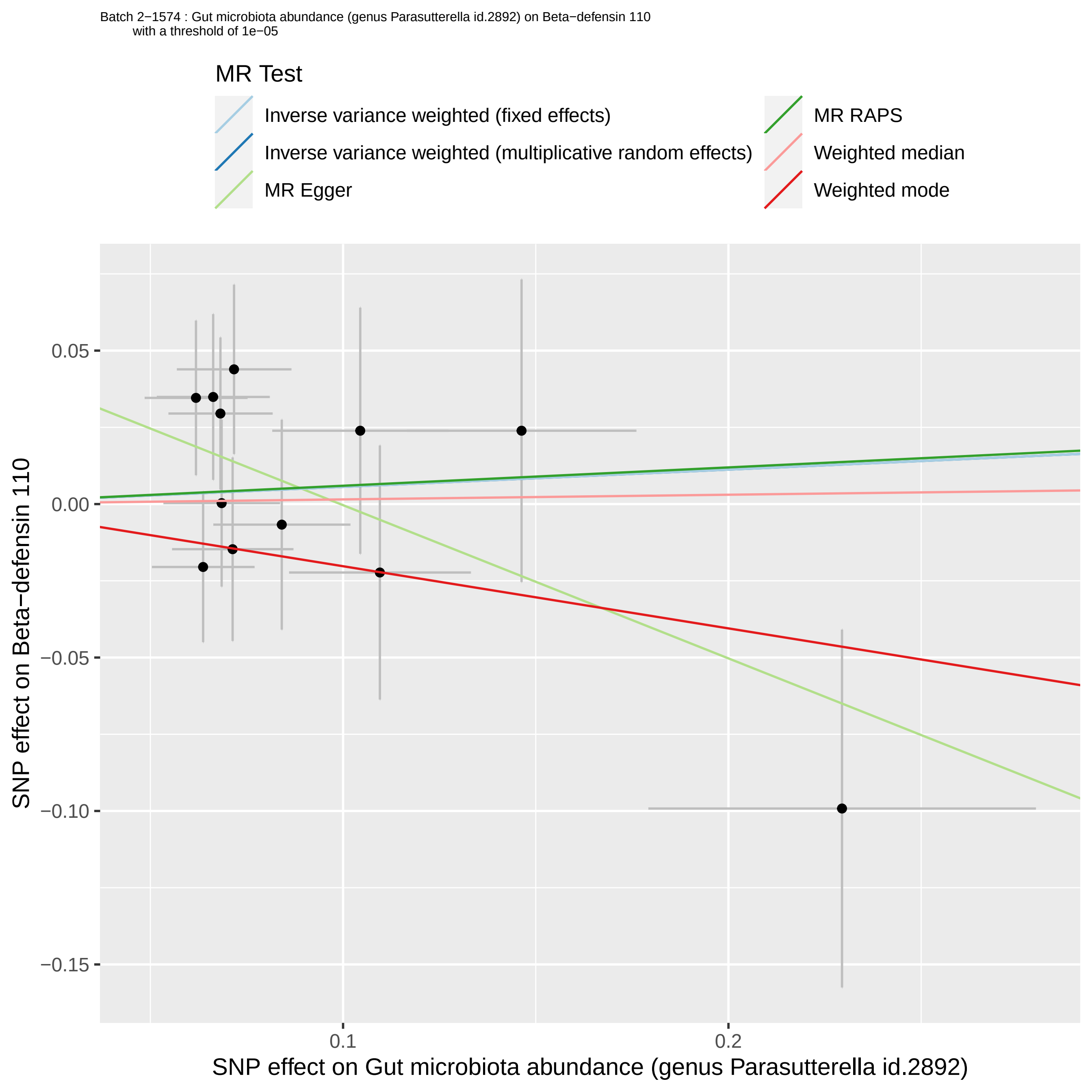
Batch 2−1574 : Gut microbiota abundance (genus Parasutterella id.2892) on Beta−defensin 110
with a threshold of 1e−05
MR Test
Inverse variance weighted (fixed effects)
Inverse variance weighted (multiplicative random effects)
MR Egger
MR RAPS
Weighted median
Weighted mode
SNP effect on Beta−defensin 110
0.05
0.00
−0.05
−0.10
−0.15
0.1
0.2
SNP effect on Gut microbiota abundance (genus Parasutterella id.2892)

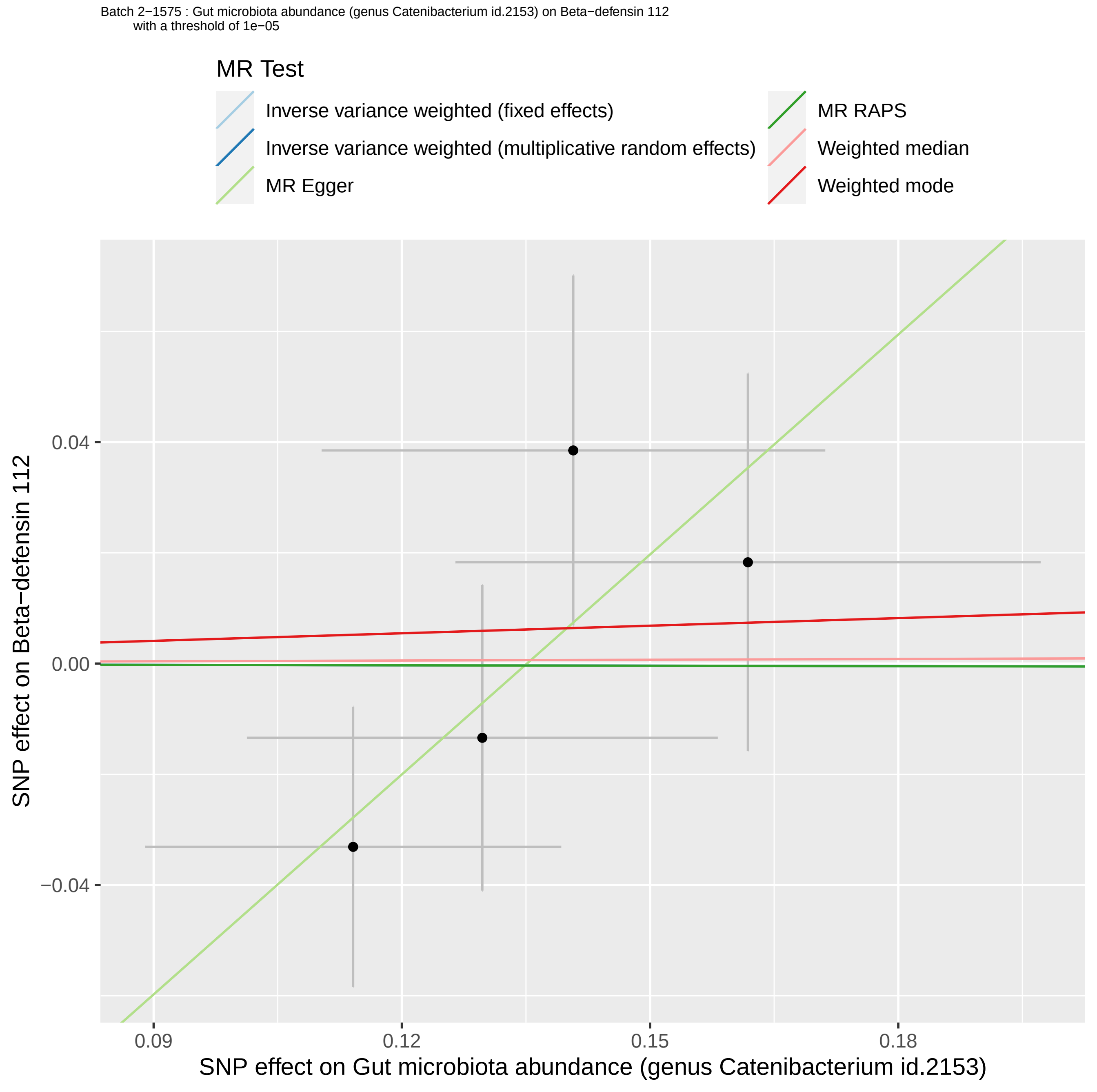
Batch 2−1575 : Gut microbiota abundance (genus Catenibacterium id.2153) on Beta−defensin 112
with a threshold of 1e−05
MR Test
Inverse variance weighted (fixed effects)
Inverse variance weighted (multiplicative random effects)
MR Egger
MR RAPS
Weighted median
Weighted mode
SNP effect on Beta−defensin 112
0.04
0.00
−0.04
0.09
0.12
0.15
0.18
SNP effect on Gut microbiota abundance (genus Catenibacterium id.2153)

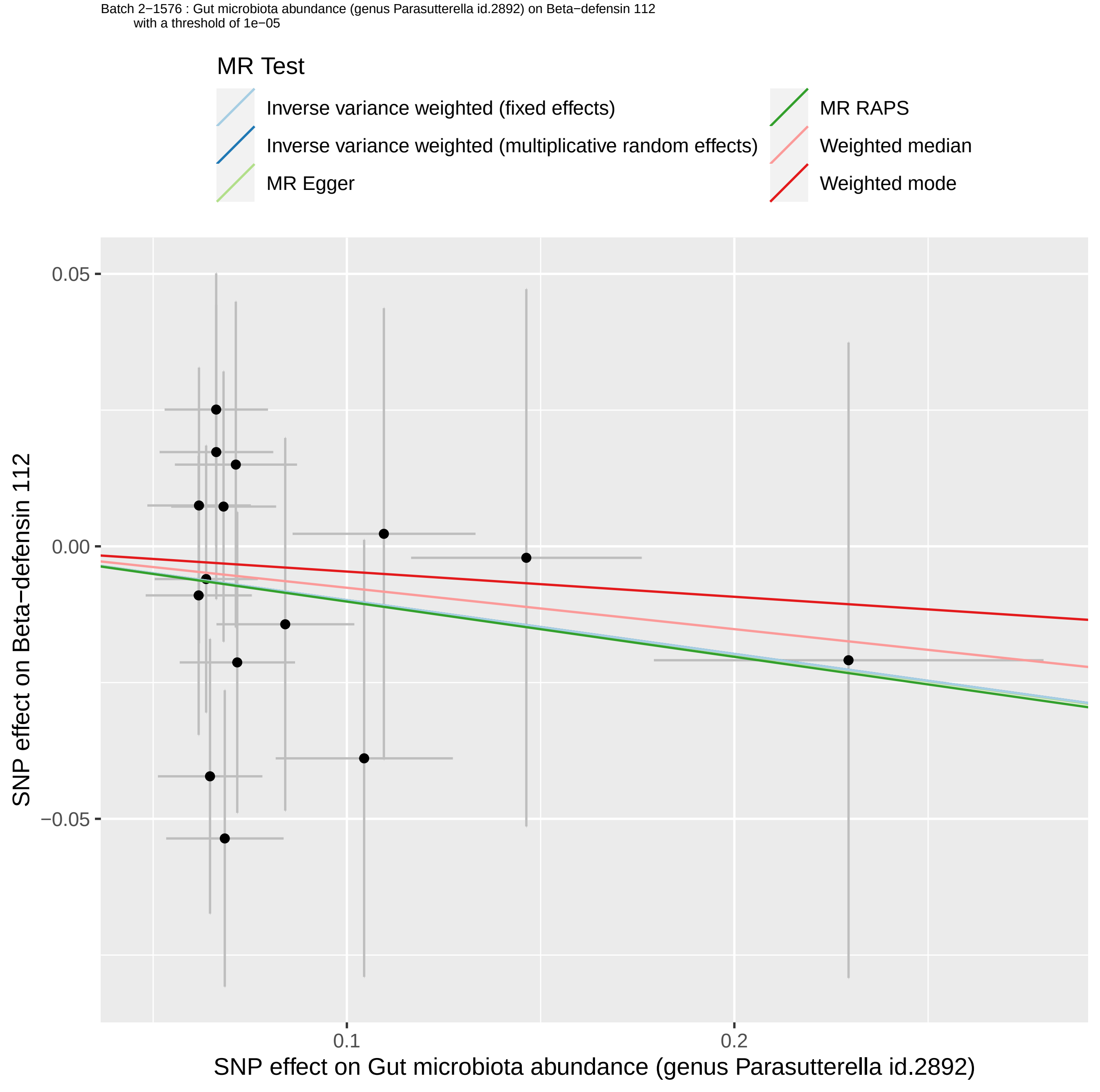

Batch 2−1576 : Gut microbiota abundance (genus Parasutterella id.2892) on Beta−defensin 112
with a threshold of 1e−05
MR Test
Inverse variance weighted (fixed effects)
Inverse variance weighted (multiplicative random effects)
MR Egger
MR RAPS
Weighted median
Weighted mode
0.05
0.00
−0.05
0.1
0.2
SNP effect on Beta−defensin 112
SNP effect on Gut microbiota abundance (genus Parasutterella id.2892)

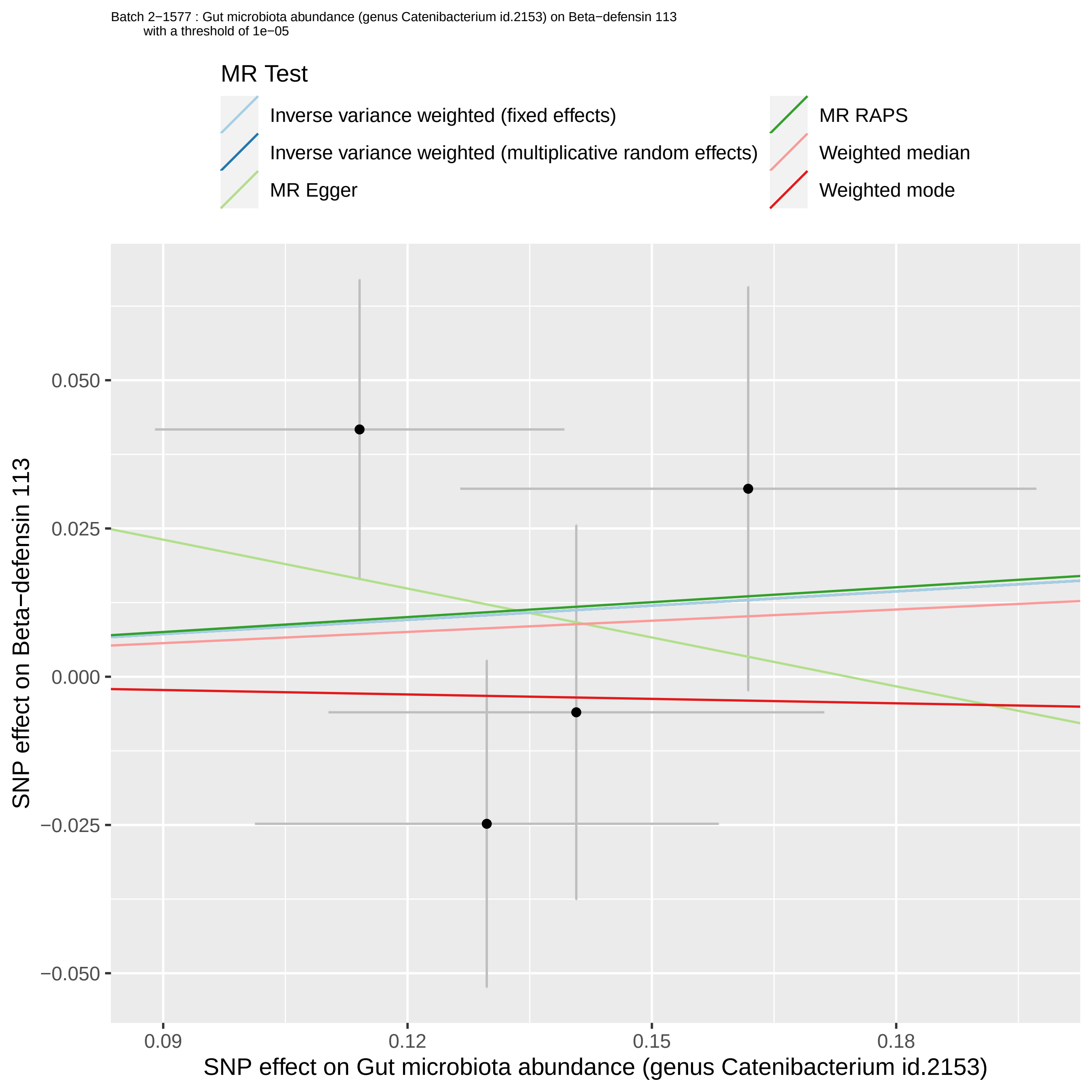
Batch 2−1577 : Gut microbiota abundance (genus Catenibacterium id.2153) on Beta−defensin 113
with a threshold of 1e−05
MR Test
Inverse variance weighted (fixed effects)
Inverse variance weighted (multiplicative random effects)
MR Egger
MR RAPS
Weighted median
Weighted mode
SNP effect on Beta−defensin 113
0.050
0.025
0.000
−0.025
−0.050
0.09
0.12
0.15
0.18
SNP effect on Gut microbiota abundance (genus Catenibacterium id.2153)

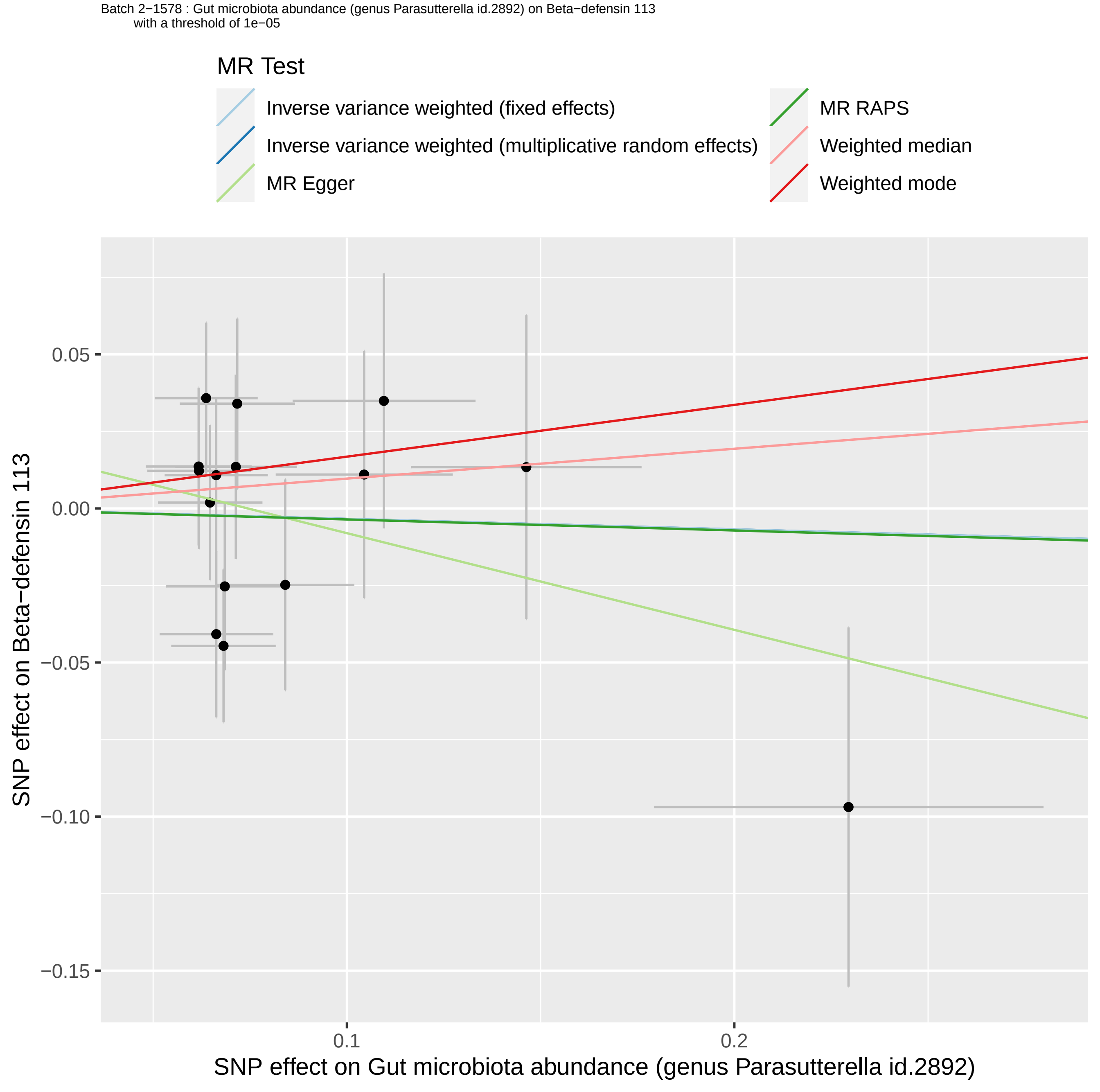
Batch 2−1578 : Gut microbiota abundance (genus Parasutterella id.2892) on Beta−defensin 113
with a threshold of 1e−05
MR Test
Inverse variance weighted (fixed effects)
Inverse variance weighted (multiplicative random effects)
MR Egger
MR RAPS
Weighted median
Weighted mode
SNP effect on Beta−defensin 113
0.05
0.00
−0.05
−0.10
−0.15
0.1
0.2
SNP effect on Gut microbiota abundance (genus Parasutterella id.2892)

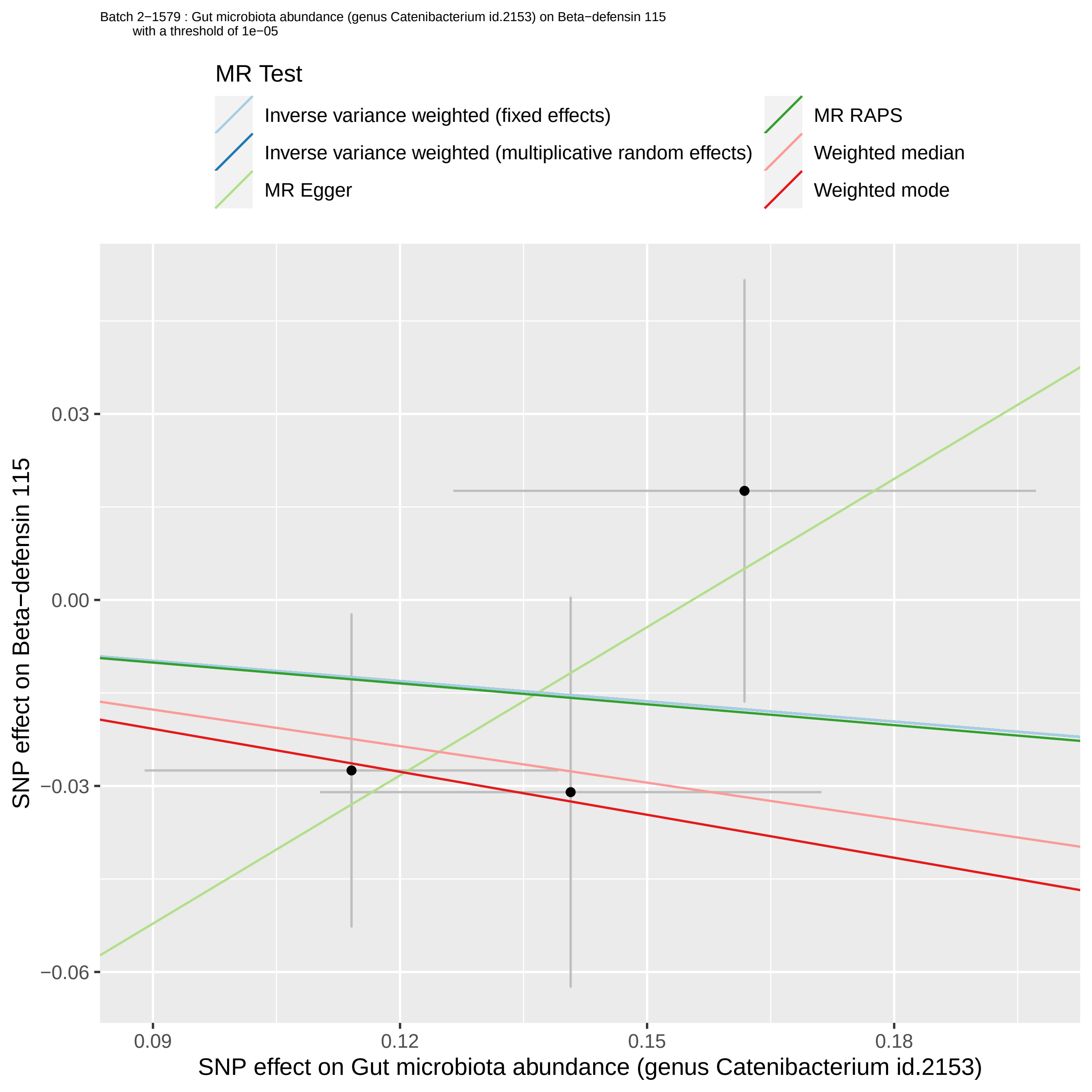
Batch 2−1579 : Gut microbiota abundance (genus Catenibacterium id.2153) on Beta−defensin 115
with a threshold of 1e−05
MR Test
Inverse variance weighted (fixed effects)
Inverse variance weighted (multiplicative random effects)
MR Egger
MR RAPS
Weighted median
Weighted mode
0.03
0.00
−0.03
−0.06
0.09
0.12
0.15
0.18
SNP effect on Beta−defensin 115
SNP effect on Gut microbiota abundance (genus Catenibacterium id.2153)

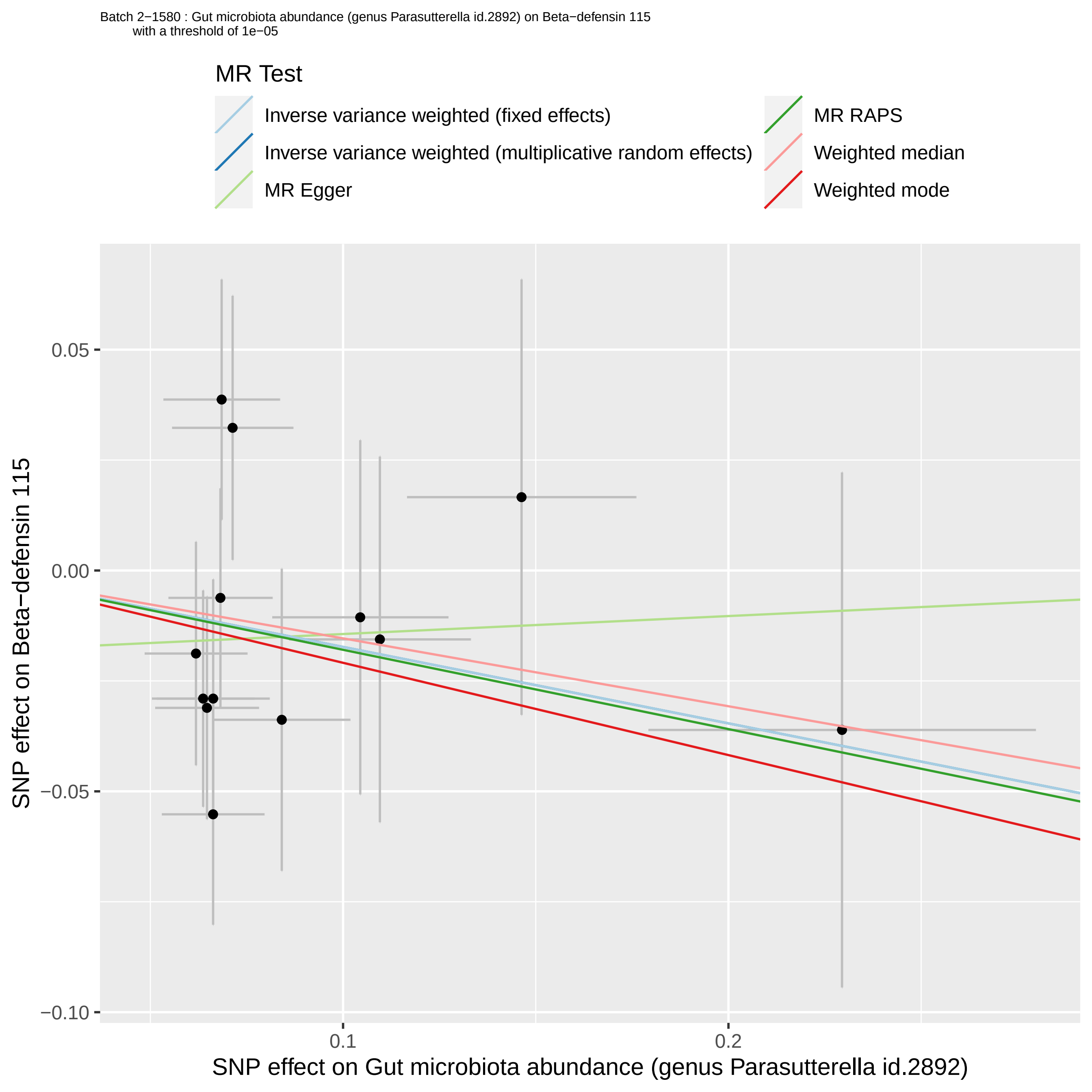
Batch 2−1580 : Gut microbiota abundance (genus Parasutterella id.2892) on Beta−defensin 115
with a threshold of 1e−05
MR Test
Inverse variance weighted (fixed effects)
Inverse variance weighted (multiplicative random effects)
MR Egger
MR RAPS
Weighted median
Weighted mode
SNP effect on Beta−defensin 115
0.05
0.00
−0.05
−0.10
0.1
0.2
SNP effect on Gut microbiota abundance (genus Parasutterella id.2892)

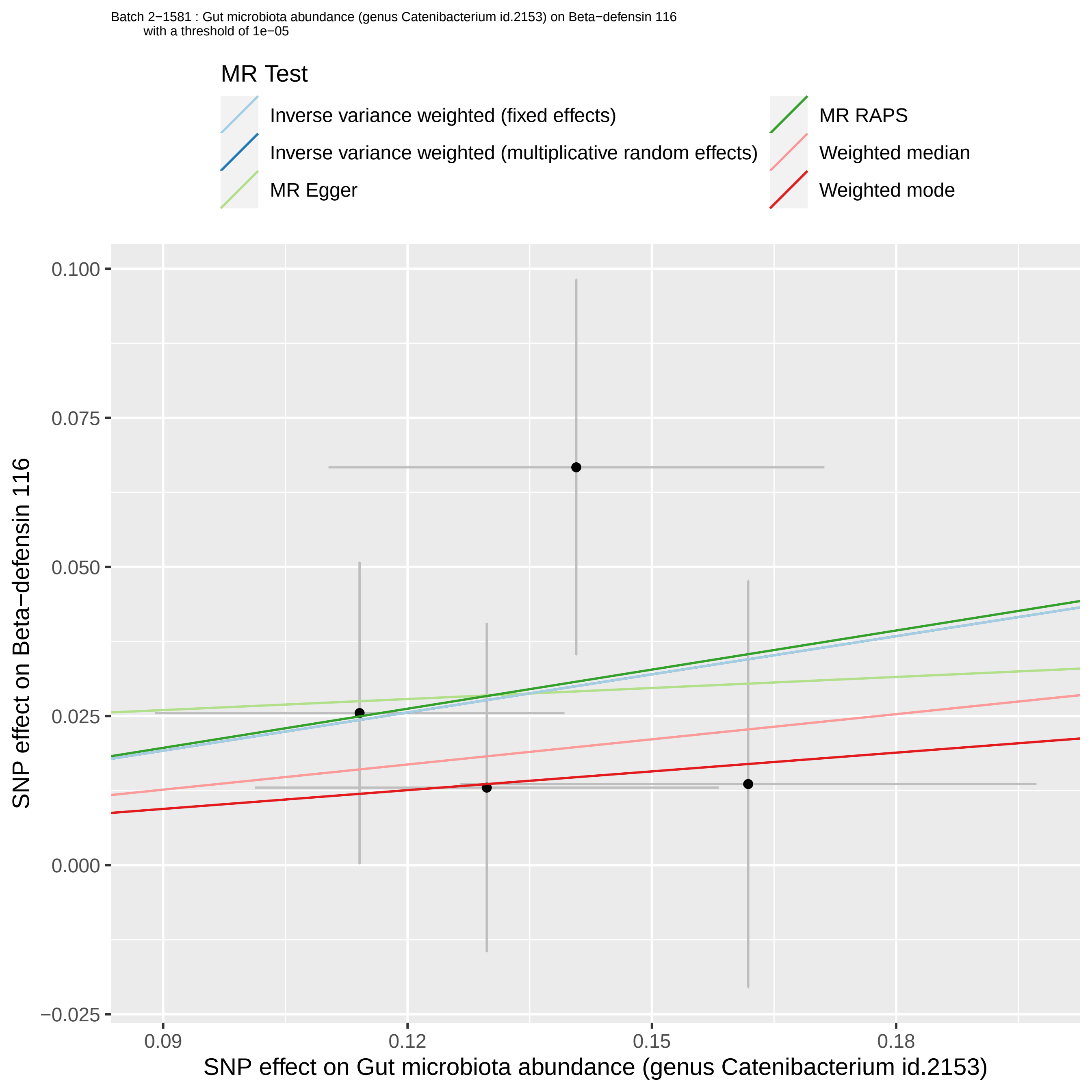

Batch 2−1581 : Gut microbiota abundance (genus Catenibacterium id.2153) on Beta−defensin 116
with a threshold of 1e−05
MR Test
Inverse variance weighted (fixed effects)
Inverse variance weighted (multiplicative random effects)
MR Egger
MR RAPS
Weighted median
Weighted mode
0.100
0.075
0.050
0.025
0.000
−0.025
0.09
0.12
0.15
0.18
SNP effect on Beta−defensin 116
SNP effect on Gut microbiota abundance (genus Catenibacterium id.2153)

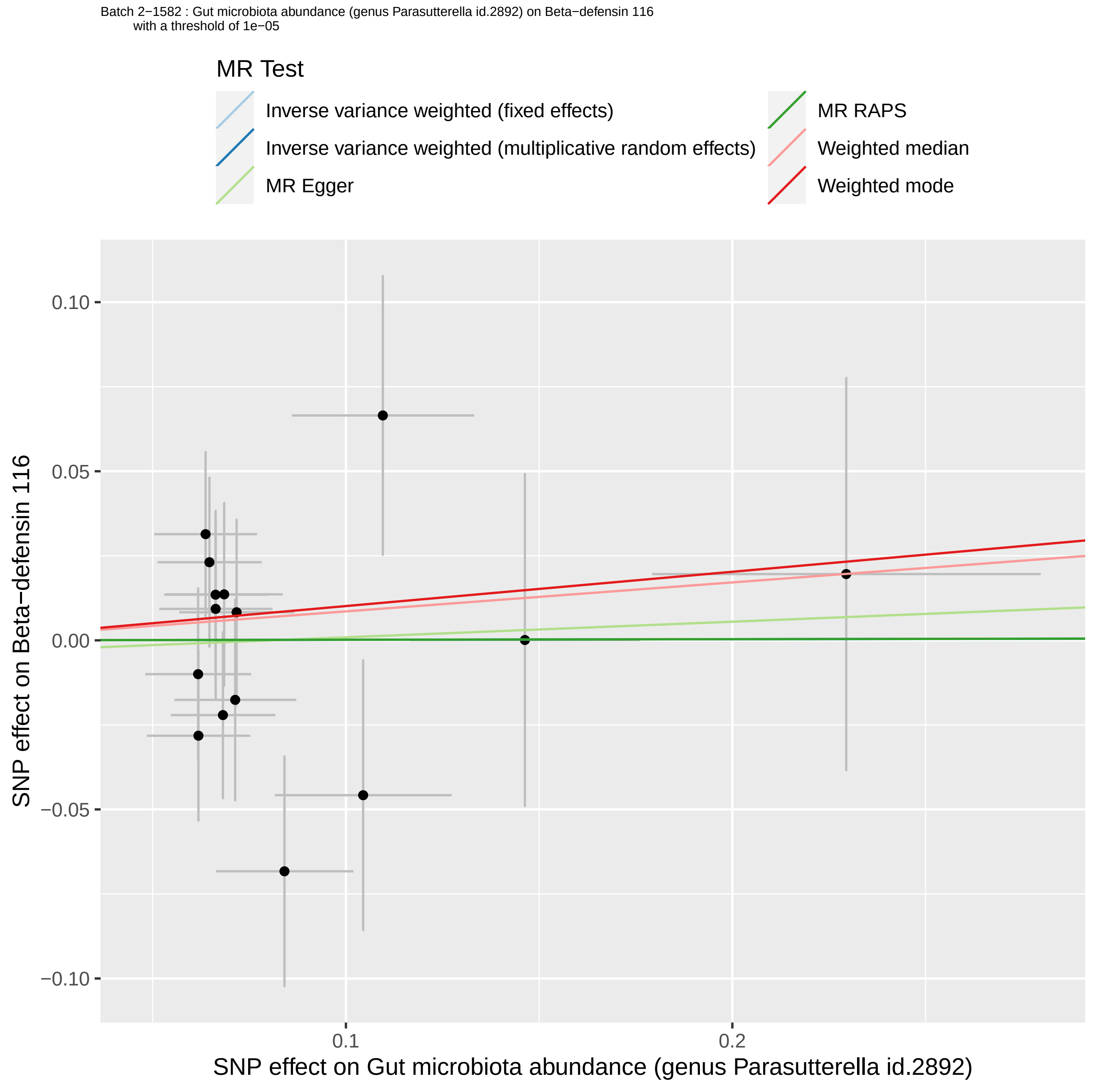
Batch 2−1582 : Gut microbiota abundance (genus Parasutterella id.2892) on Beta−defensin 116
with a threshold of 1e−05
MR Test
Inverse variance weighted (fixed effects)
Inverse variance weighted (multiplicative random effects)
MR Egger
MR RAPS
Weighted median
Weighted mode
SNP effect on Beta−defensin 116
0.10
0.05
0.00
−0.05
−0.10
0.1
0.2
SNP effect on Gut microbiota abundance (genus Parasutterella id.2892)

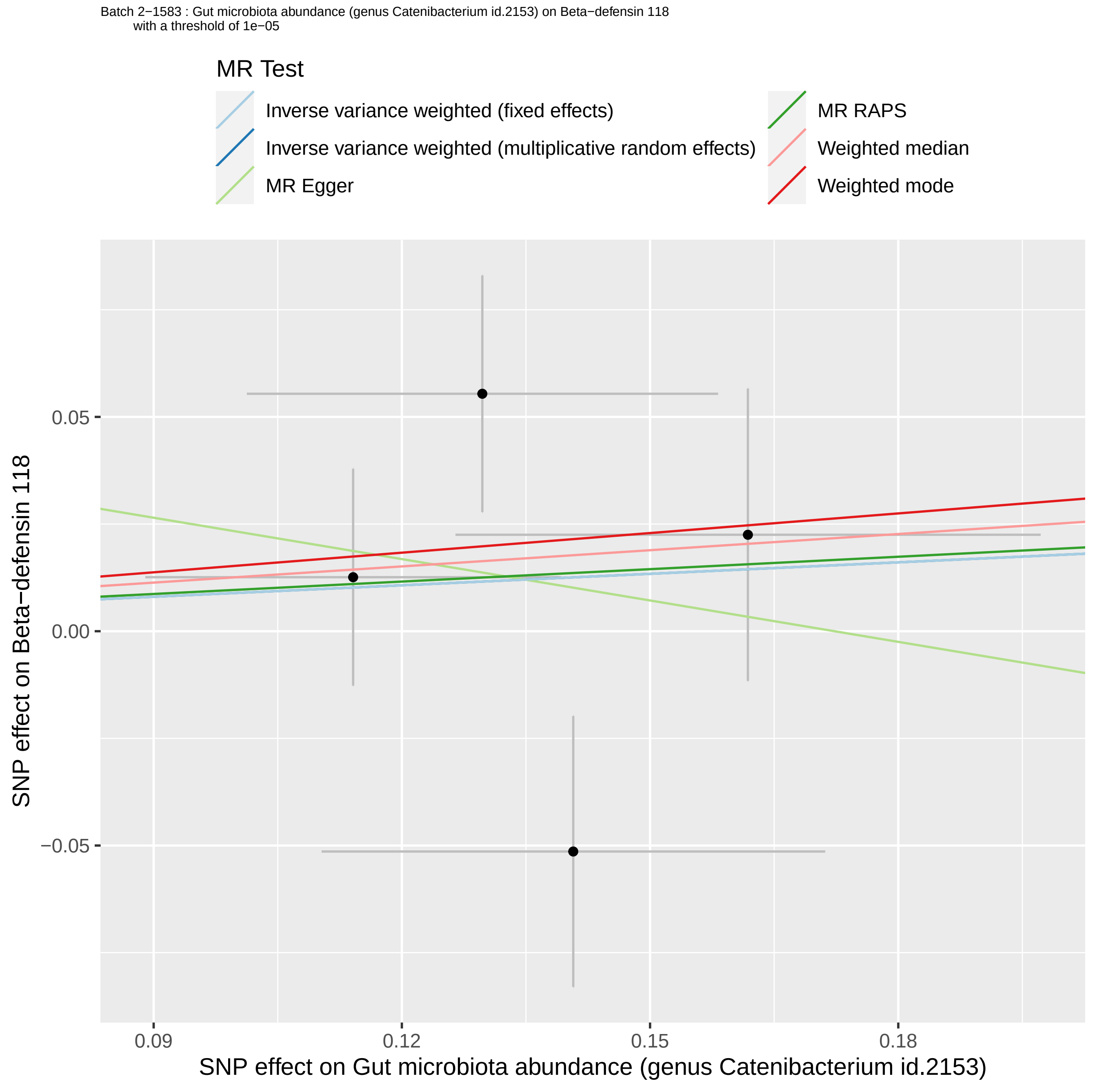
Batch 2−1583 : Gut microbiota abundance (genus Catenibacterium id.2153) on Beta−defensin 118
with a threshold of 1e−05
MR Test
Inverse variance weighted (fixed effects)
Inverse variance weighted (multiplicative random effects)
MR Egger
MR RAPS
Weighted median
Weighted mode
0.05
0.00
−0.05
0.09
0.12
0.15
0.18
SNP effect on Beta−defensin 118
SNP effect on Gut microbiota abundance (genus Catenibacterium id.2153)

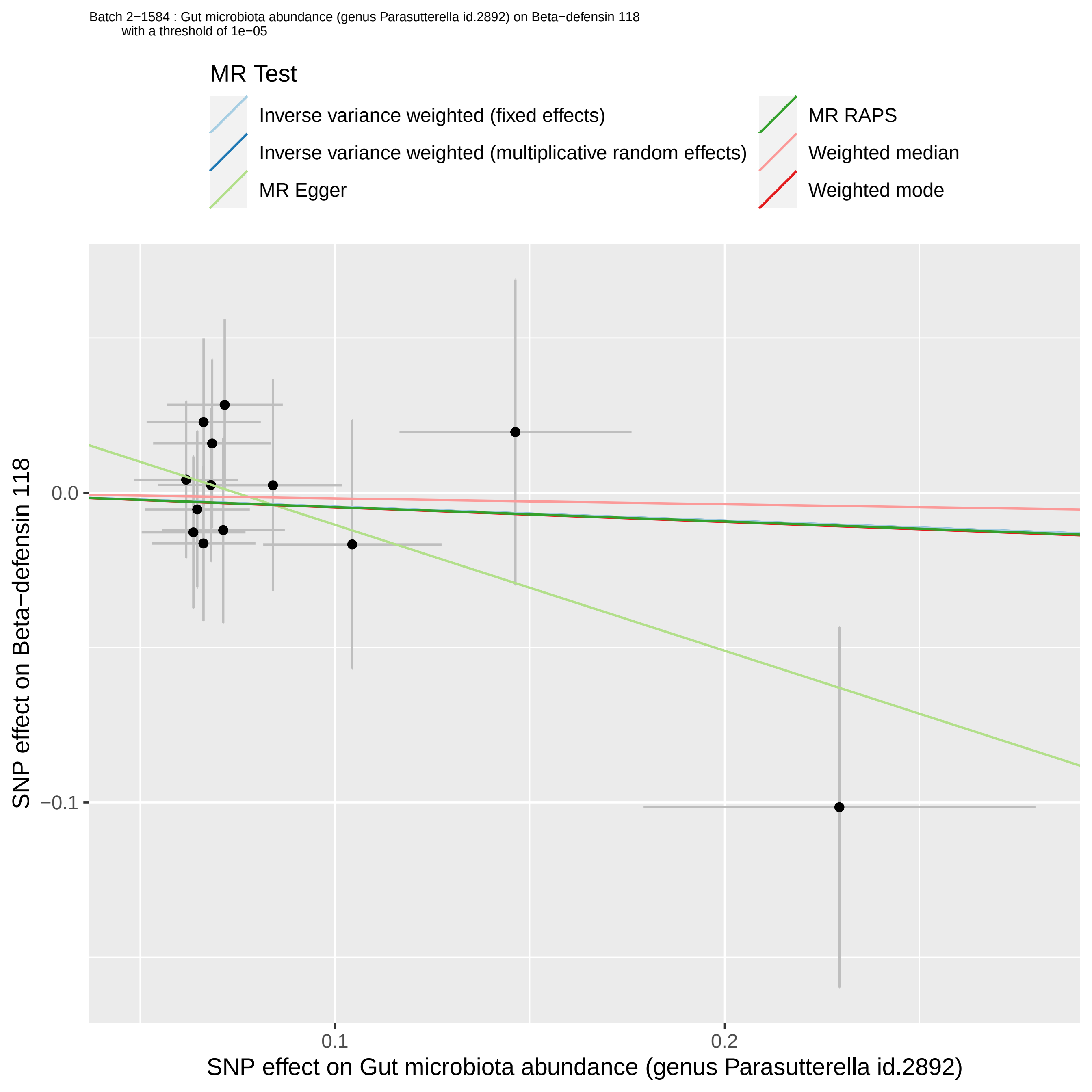

Batch 2−1584 : Gut microbiota abundance (genus Parasutterella id.2892) on Beta−defensin 118
with a threshold of 1e−05
MR Test
Inverse variance weighted (fixed effects)
Inverse variance weighted (multiplicative random effects)
MR Egger
MR RAPS
Weighted median
Weighted mode
SNP effect on Beta−defensin 118
0.0
−0.1
0.1
0.2
SNP effect on Gut microbiota abundance (genus Parasutterella id.2892)

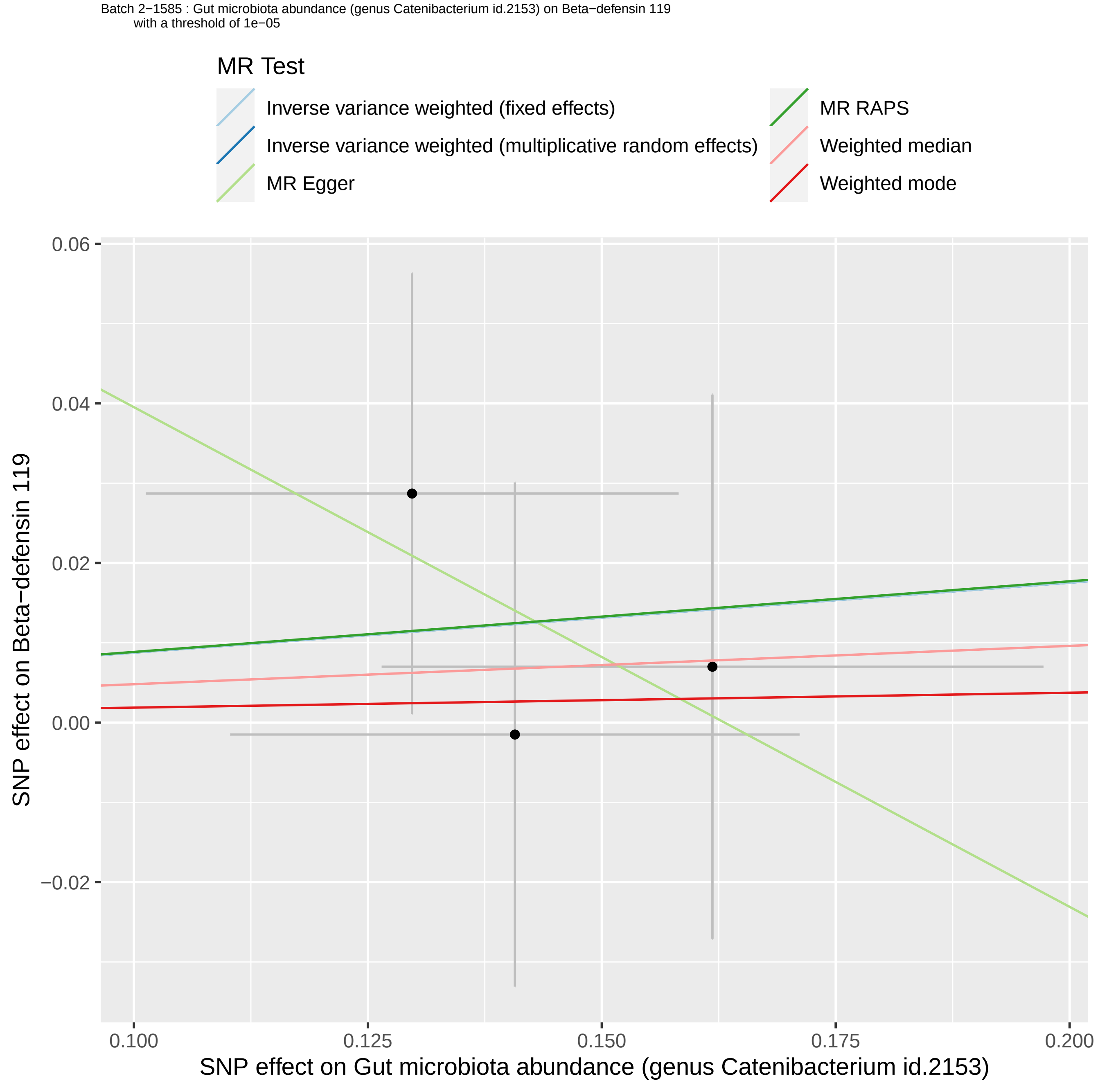
Batch 2−1585 : Gut microbiota abundance (genus Catenibacterium id.2153) on Beta−defensin 119
with a threshold of 1e−05
MR Test
Inverse variance weighted (fixed effects)
Inverse variance weighted (multiplicative random effects)
MR Egger
MR RAPS
Weighted median
Weighted mode
0.06
0.04
0.02
0.00
−0.02
0.100
0.125
0.150
0.175
0.200
SNP effect on Beta−defensin 119
SNP effect on Gut microbiota abundance (genus Catenibacterium id.2153)

Batch 2−1586 : Gut microbiota abundance (genus Parasutterella id.2892) on Beta−defensin 119
with a threshold of 1e−05

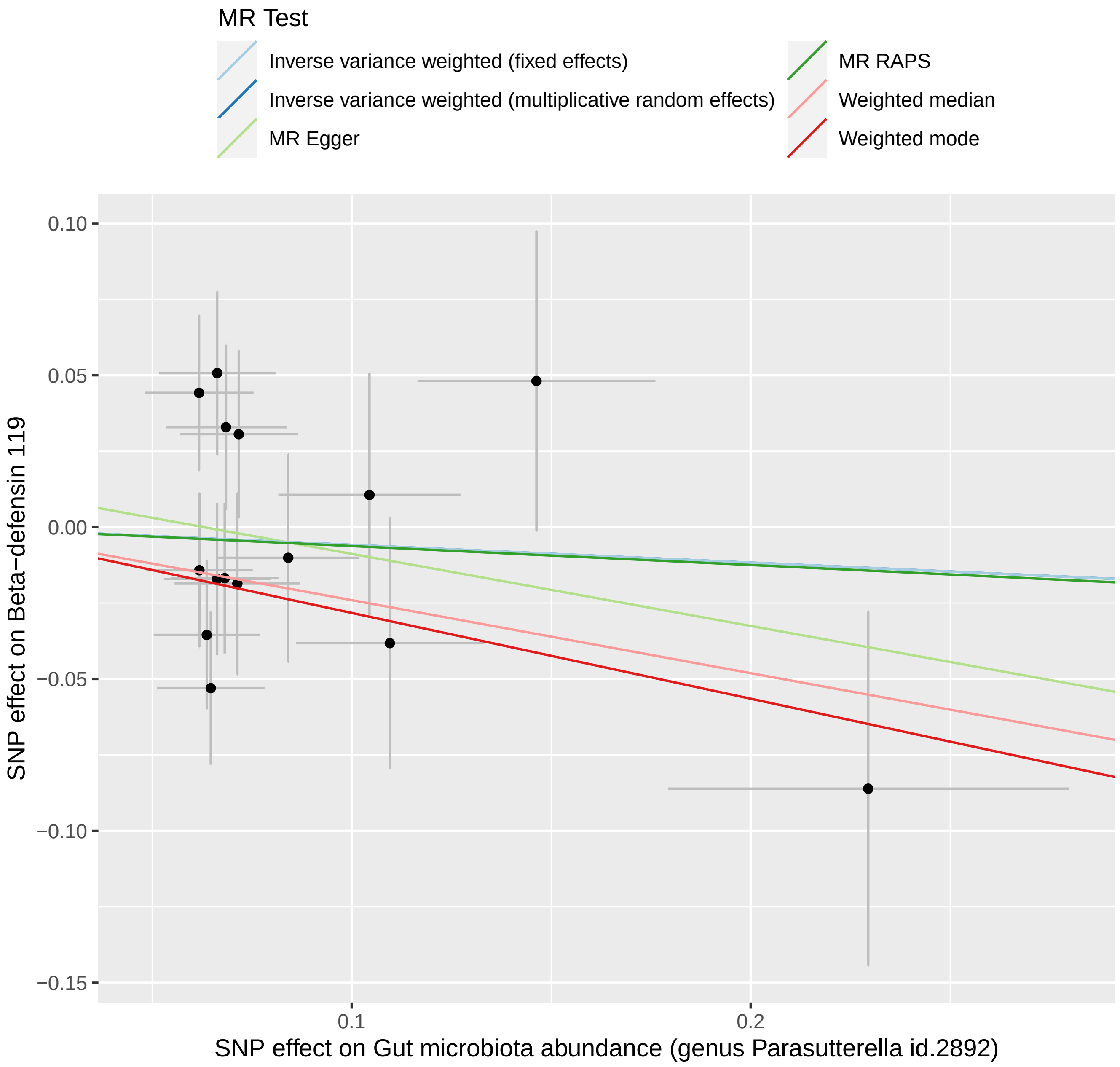

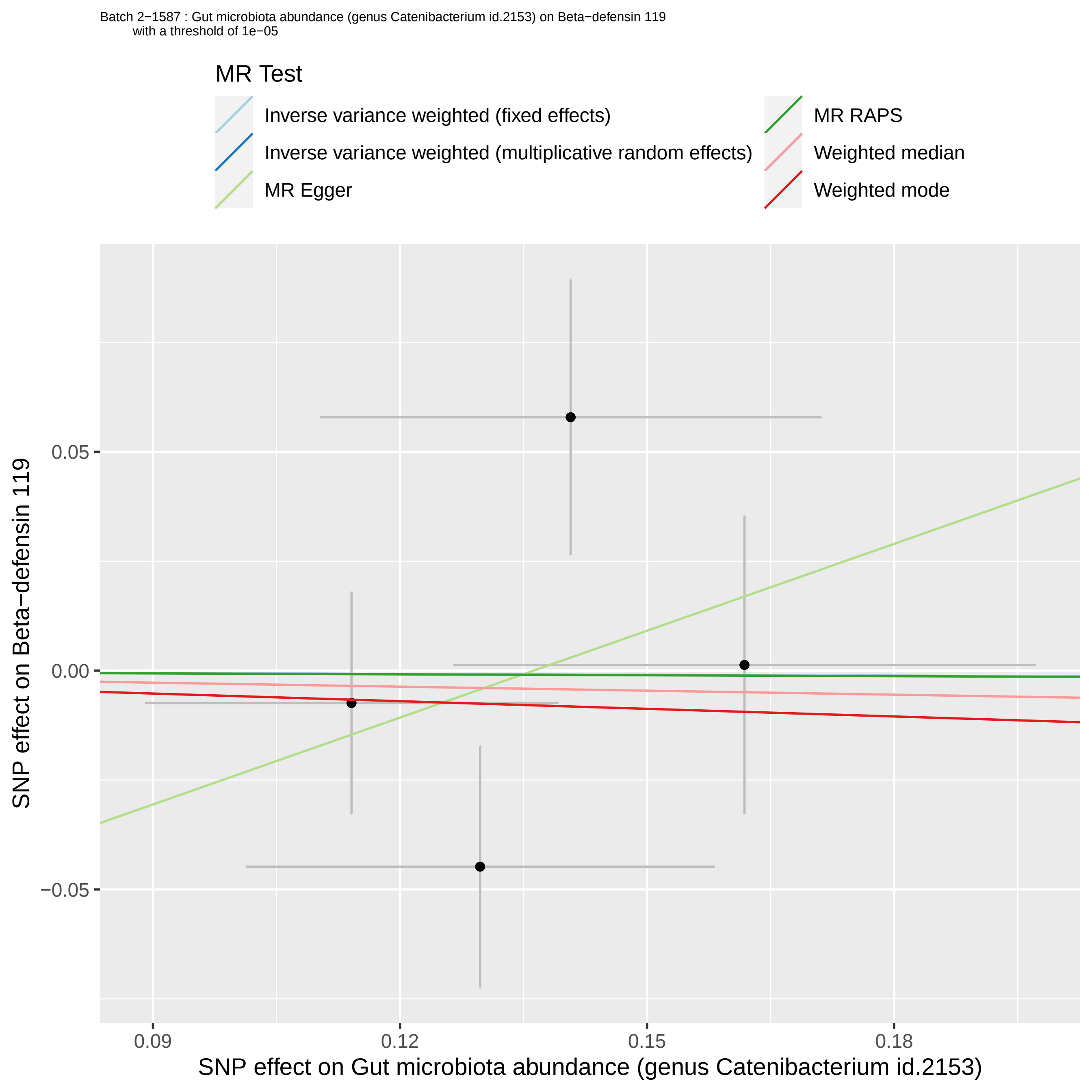
Batch 2−1587 : Gut microbiota abundance (genus Catenibacterium id.2153) on Beta−defensin 119
with a threshold of 1e−05
MR Test
Inverse variance weighted (fixed effects)
Inverse variance weighted (multiplicative random effects)
MR Egger
MR RAPS
Weighted median
Weighted mode
0.05
0.00
−0.05
0.09
0.12
0.15
0.18
SNP effect on Beta−defensin 119
SNP effect on Gut microbiota abundance (genus Catenibacterium id.2153)

Batch 2−1588 : Gut microbiota abundance (genus Parasutterella id.2892) on Beta−defensin 119
with a threshold of 1e−05

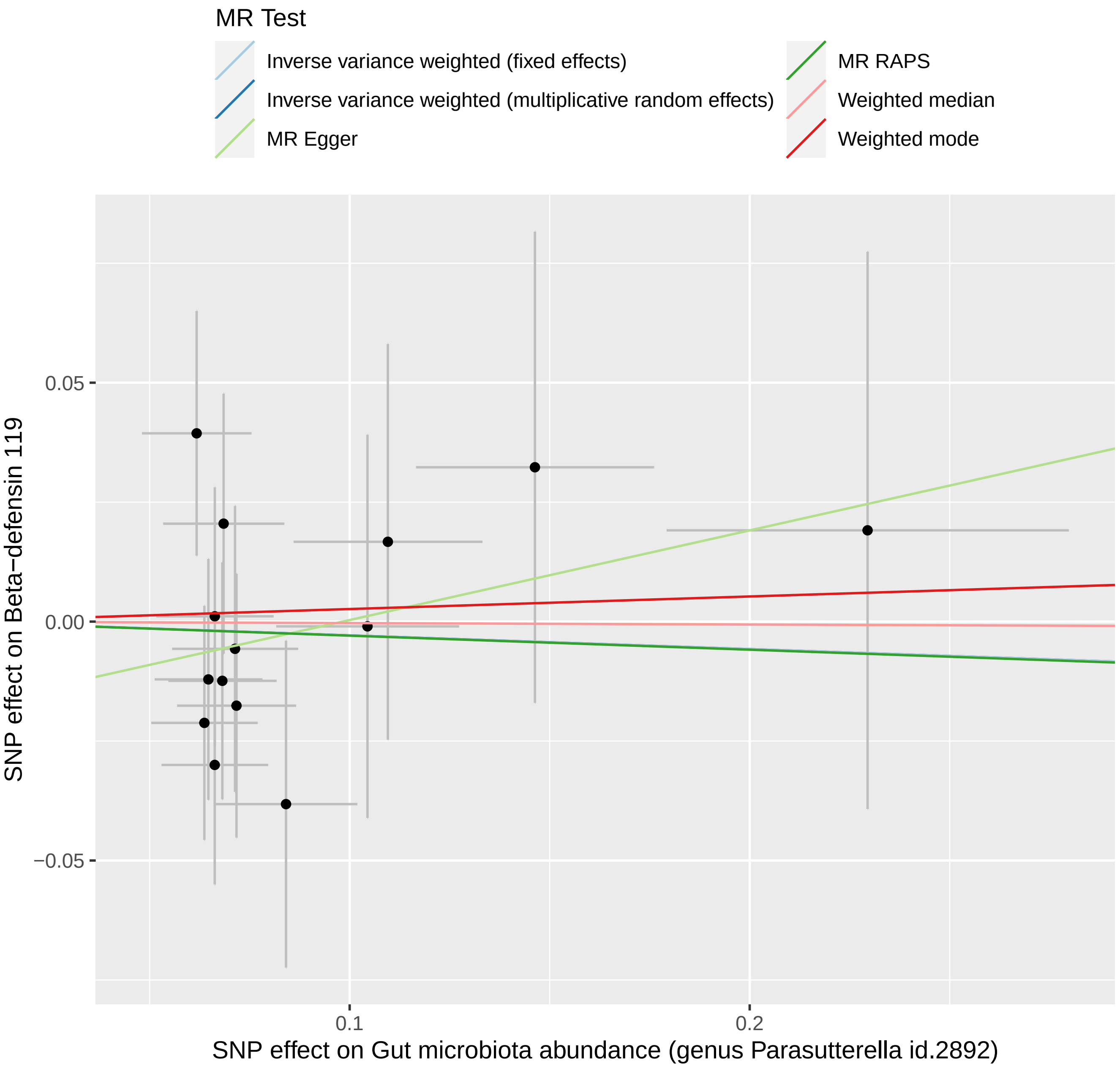

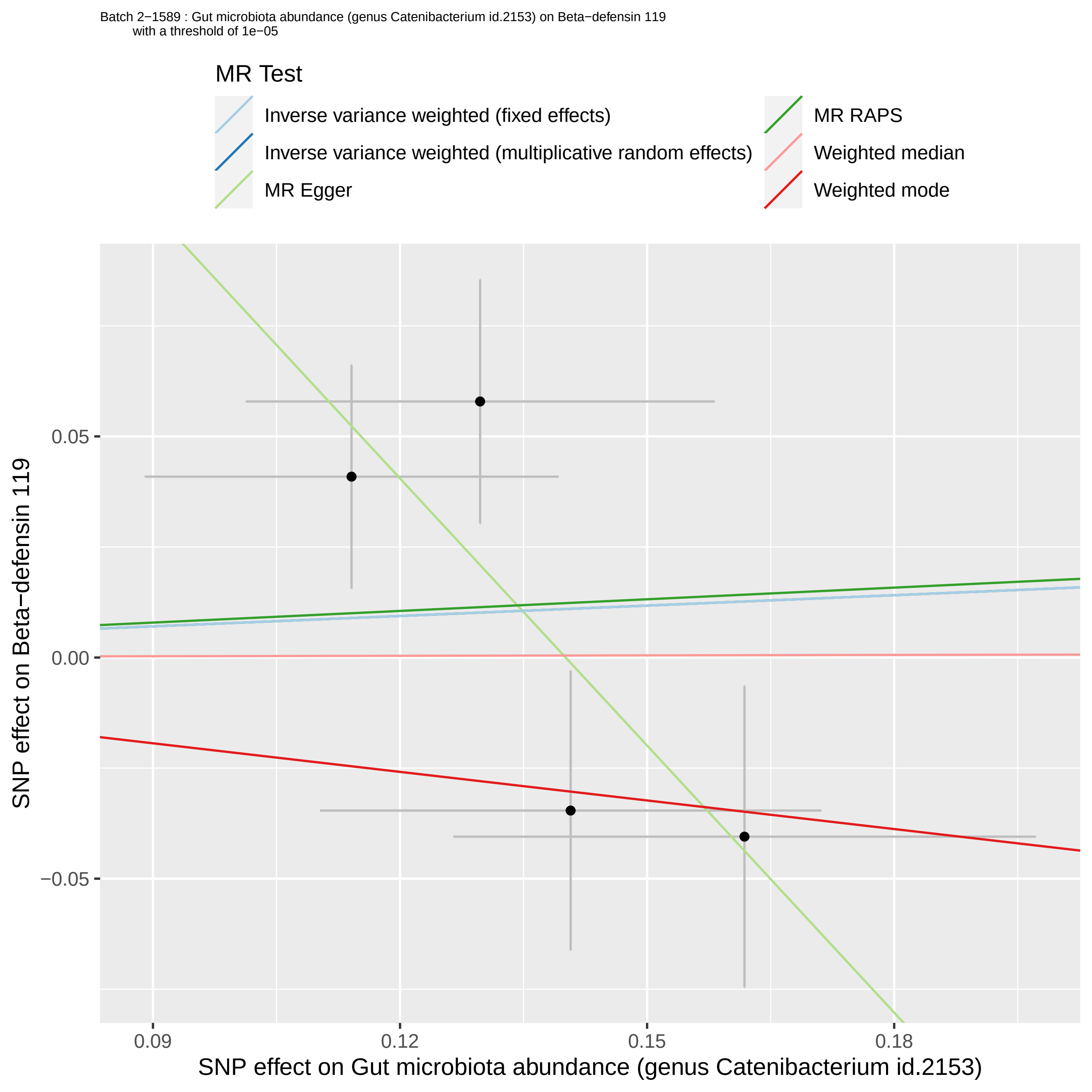
Batch 2−1589 : Gut microbiota abundance (genus Catenibacterium id.2153) on Beta−defensin 119
with a threshold of 1e−05
MR Test
Inverse variance weighted (fixed effects)
Inverse variance weighted (multiplicative random effects)
MR Egger
MR RAPS
Weighted median
Weighted mode
0.05
0.00
−0.05
0.09
0.12
0.15
0.18
SNP effect on Beta−defensin 119
SNP effect on Gut microbiota abundance (genus Catenibacterium id.2153)

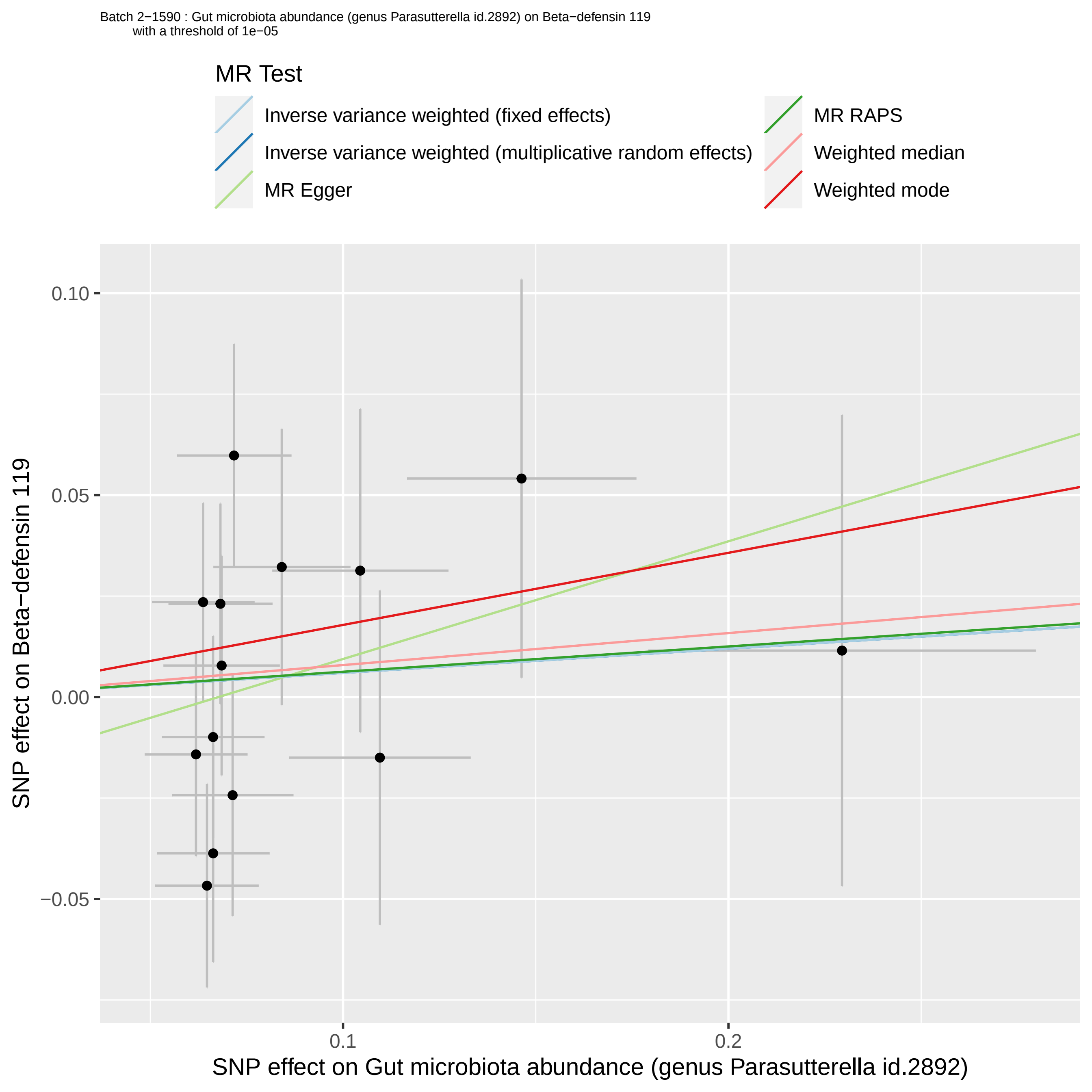
Batch 2−1590 : Gut microbiota abundance (genus Parasutterella id.2892) on Beta−defensin 119
with a threshold of 1e−05
MR Test
Inverse variance weighted (fixed effects)
Inverse variance weighted (multiplicative random effects)
MR Egger
MR RAPS
Weighted median
Weighted mode
0.10
0.05
0.00
−0.05
0.1
0.2
SNP effect on Beta−defensin 119
SNP effect on Gut microbiota abundance (genus Parasutterella id.2892)

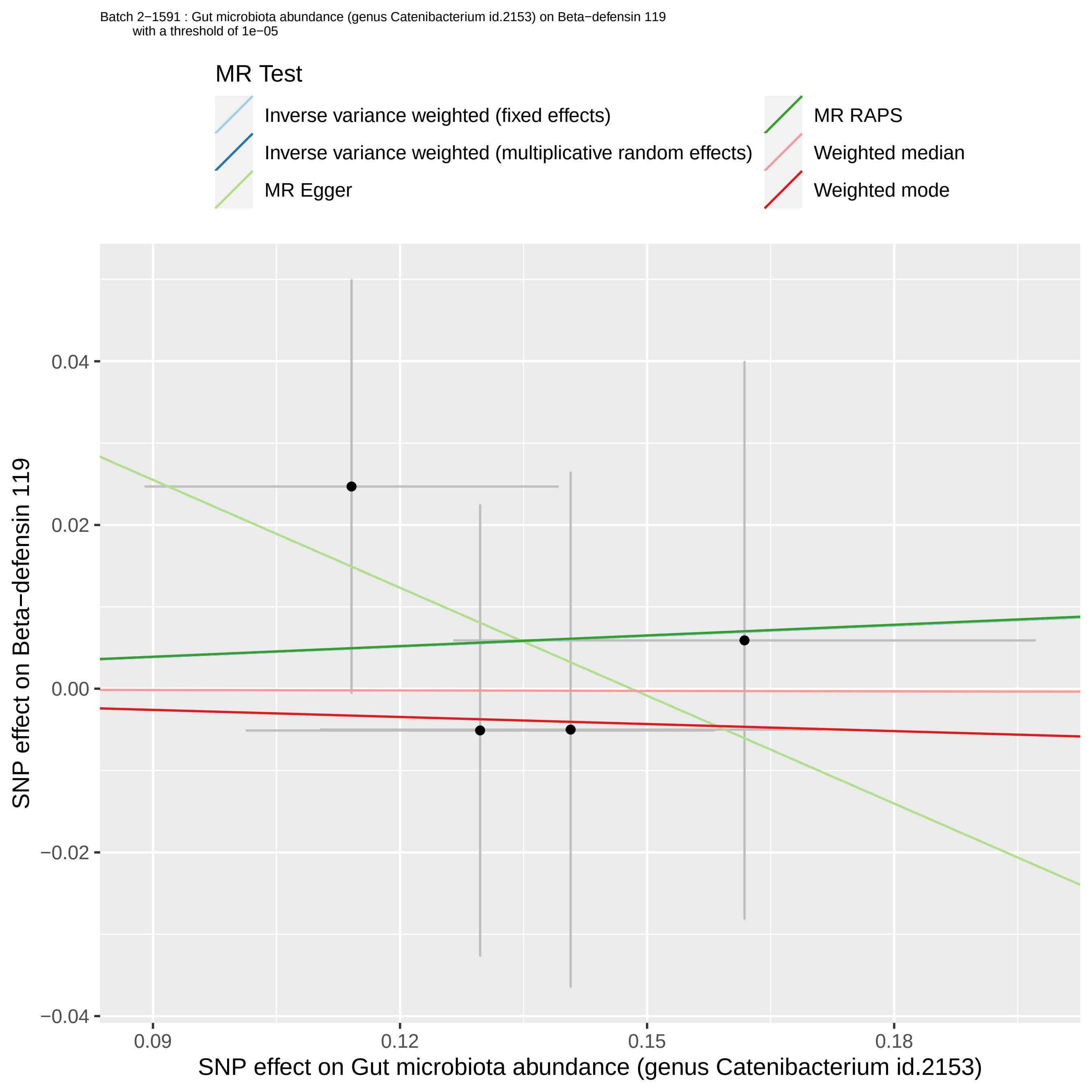

Batch 2−1591 : Gut microbiota abundance (genus Catenibacterium id.2153) on Beta−defensin 119
with a threshold of 1e−05
MR Test
Inverse variance weighted (fixed effects)
Inverse variance weighted (multiplicative random effects)
MR Egger
MR RAPS
Weighted median
Weighted mode
0.04
0.02
0.00
−0.02
−0.04
0.09
0.12
0.15
0.18
SNP effect on Beta−defensin 119
SNP effect on Gut microbiota abundance (genus Catenibacterium id.2153)

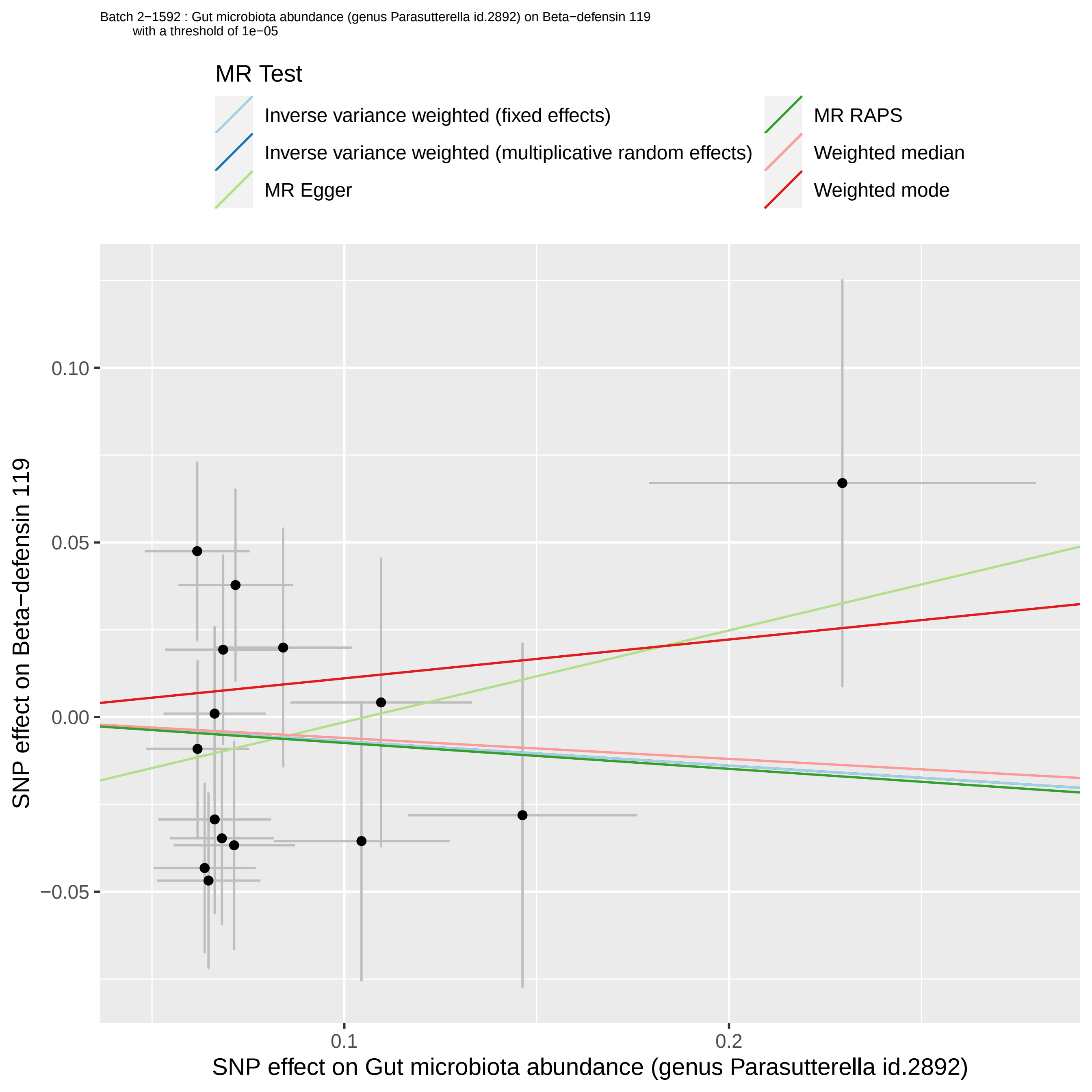

Batch 2−1592 : Gut microbiota abundance (genus Parasutterella id.2892) on Beta−defensin 119
with a threshold of 1e−05
MR Test
Inverse variance weighted (fixed effects)
Inverse variance weighted (multiplicative random effects)
MR Egger
MR RAPS
Weighted median
Weighted mode
0.10
0.05
0.00
−0.05
0.1
0.2
SNP effect on Beta−defensin 119
SNP effect on Gut microbiota abundance (genus Parasutterella id.2892)

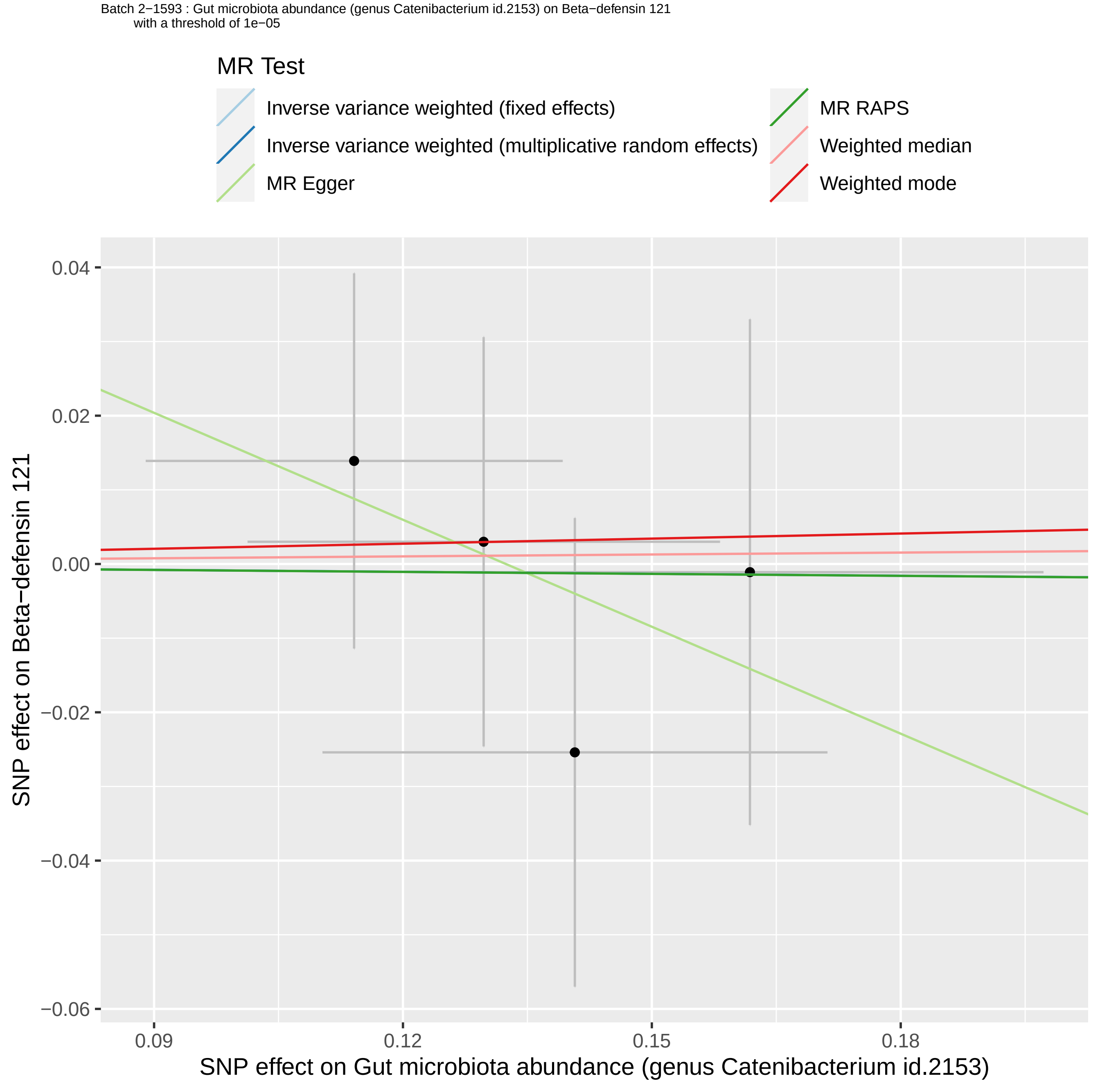
Batch 2−1593 : Gut microbiota abundance (genus Catenibacterium id.2153) on Beta−defensin 121
with a threshold of 1e−05
MR Test
Inverse variance weighted (fixed effects)
Inverse variance weighted (multiplicative random effects)
MR Egger
MR RAPS
Weighted median
Weighted mode
0.04
0.02
0.00
−0.02
−0.04
−0.06
0.09
0.12
0.15
0.18
SNP effect on Beta−defensin 121
SNP effect on Gut microbiota abundance (genus Catenibacterium id.2153)

Batch 2−1594 : Gut microbiota abundance (genus Parasutterella id.2892) on Beta−defensin 121
with a threshold of 1e−05

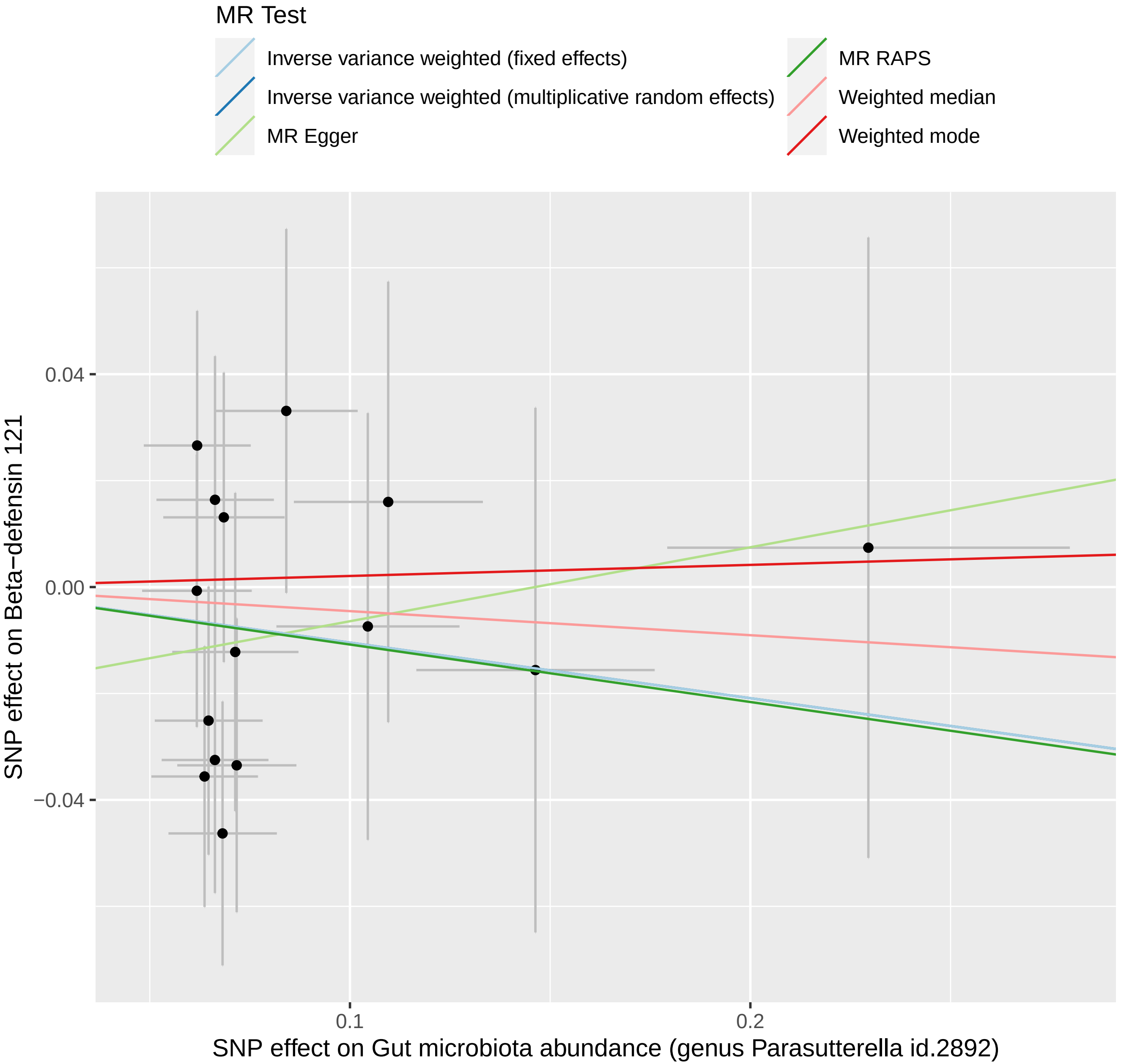

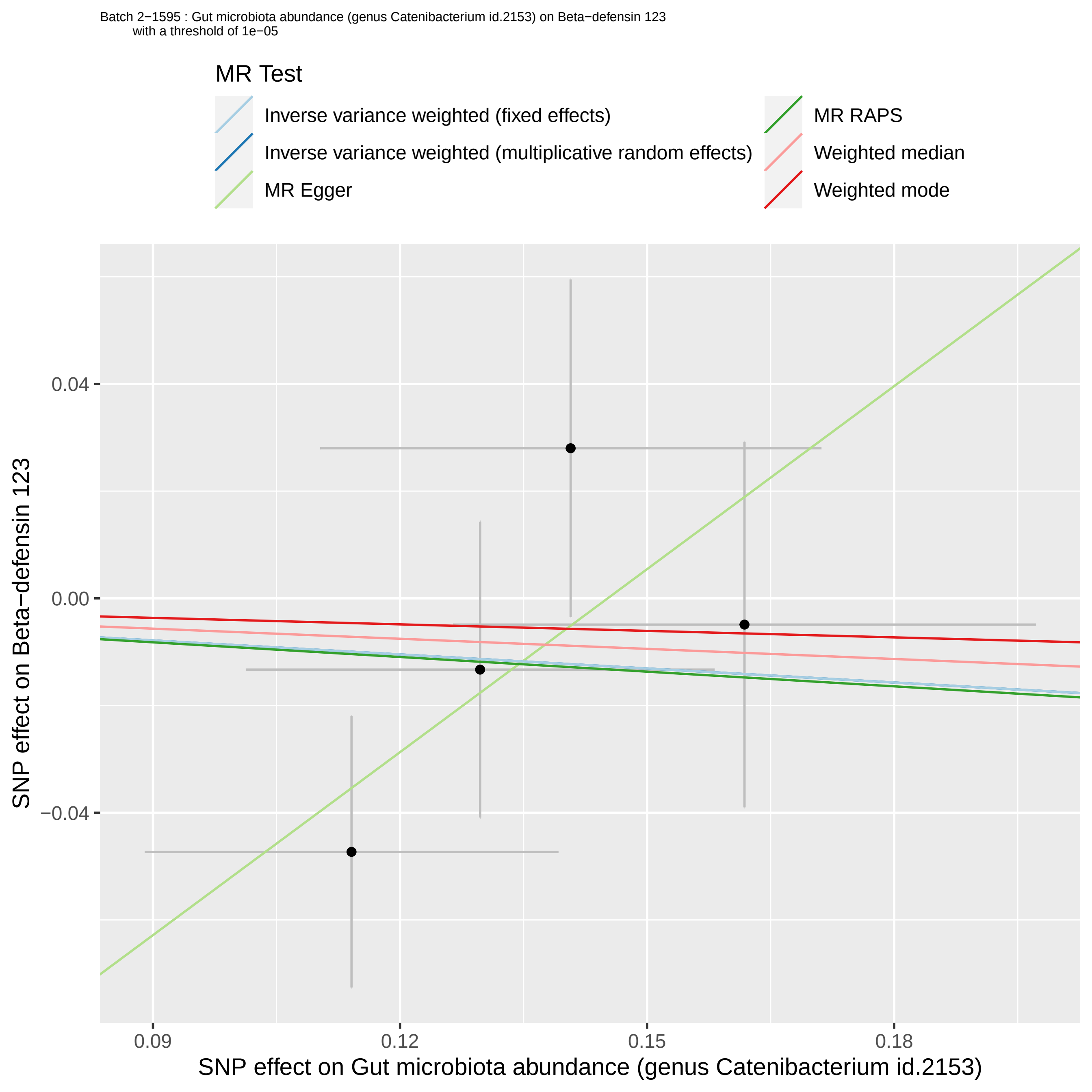

Batch 2−1595 : Gut microbiota abundance (genus Catenibacterium id.2153) on Beta−defensin 123
with a threshold of 1e−05
MR Test
Inverse variance weighted (fixed effects)
Inverse variance weighted (multiplicative random effects)
MR Egger
MR RAPS
Weighted median
Weighted mode
0.04
0.00
−0.04
0.09
0.12
0.15
0.18
SNP effect on Beta−defensin 123
SNP effect on Gut microbiota abundance (genus Catenibacterium id.2153)

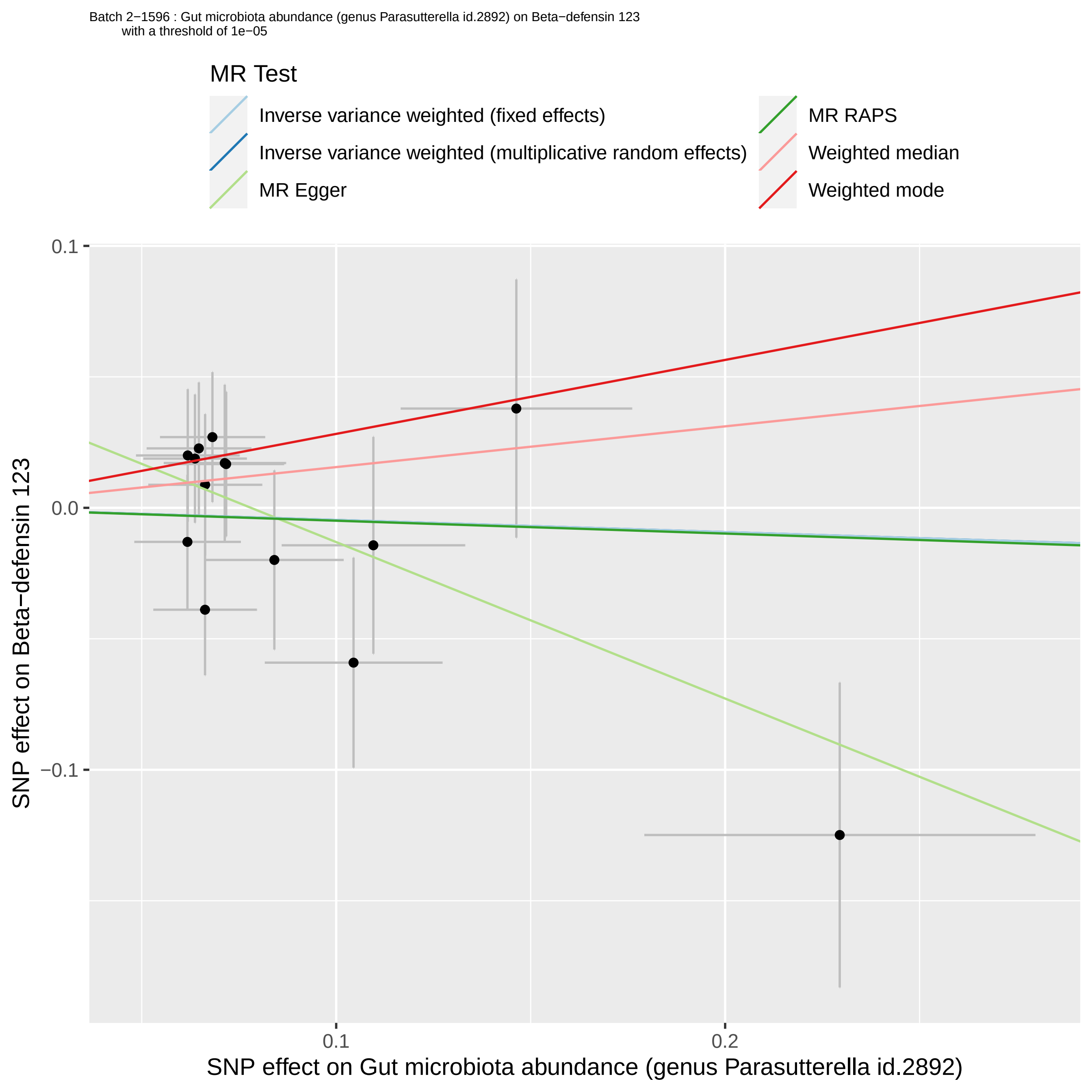

Batch 2−1596 : Gut microbiota abundance (genus Parasutterella id.2892) on Beta−defensin 123
with a threshold of 1e−05
MR Test
Inverse variance weighted (fixed effects)
Inverse variance weighted (multiplicative random effects)
MR Egger
MR RAPS
Weighted median
Weighted mode
0.1
0.0
−0.1
0.1
0.2
SNP effect on Beta−defensin 123
SNP effect on Gut microbiota abundance (genus Parasutterella id.2892)

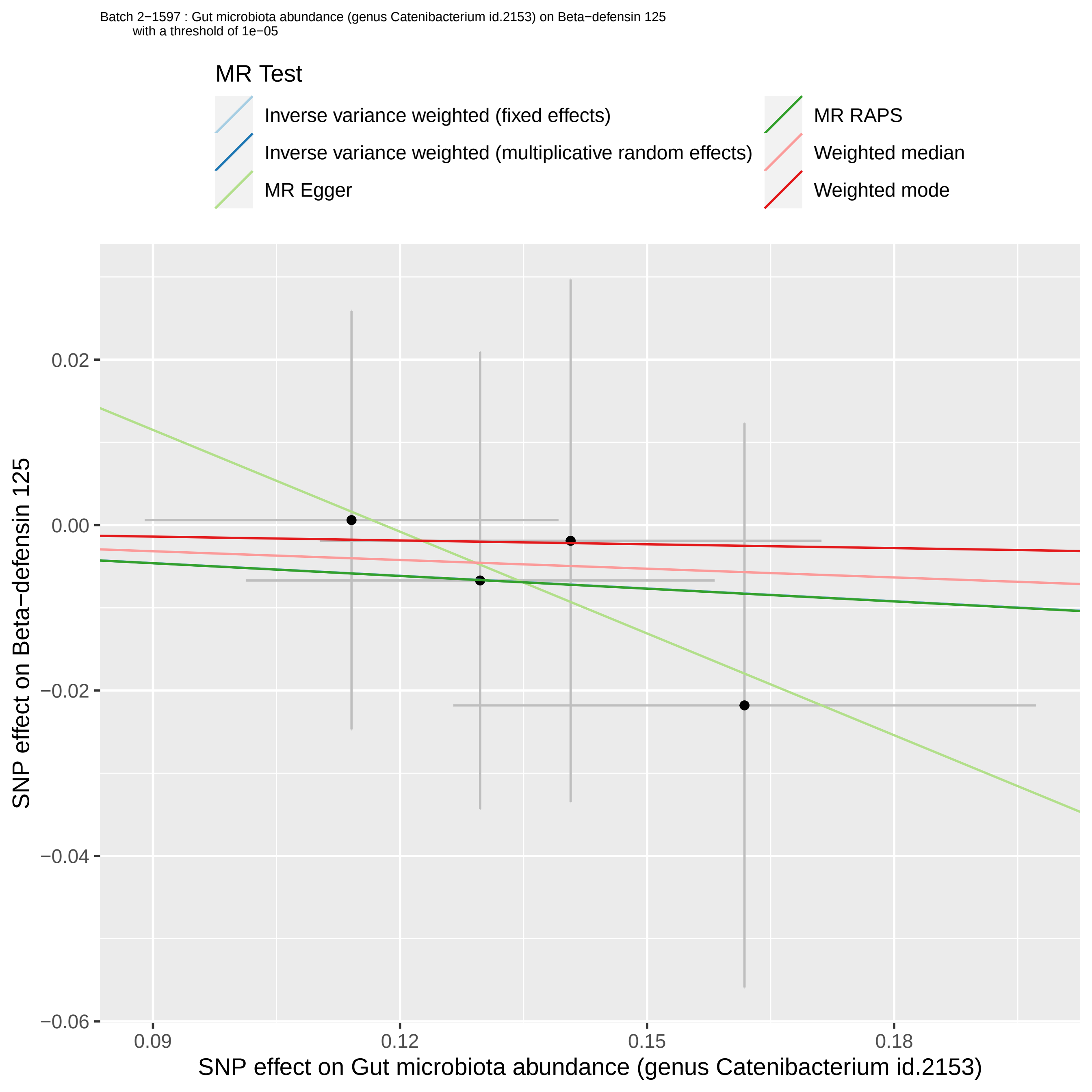

Batch 2−1597 : Gut microbiota abundance (genus Catenibacterium id.2153) on Beta−defensin 125
with a threshold of 1e−05
MR Test
Inverse variance weighted (fixed effects)
Inverse variance weighted (multiplicative random effects)
MR Egger
MR RAPS
Weighted median
Weighted mode
SNP effect on Beta−defensin 125
0.02
0.00
−0.02
−0.04
−0.06
0.09
0.12
0.15
0.18
SNP effect on Gut microbiota abundance (genus Catenibacterium id.2153)

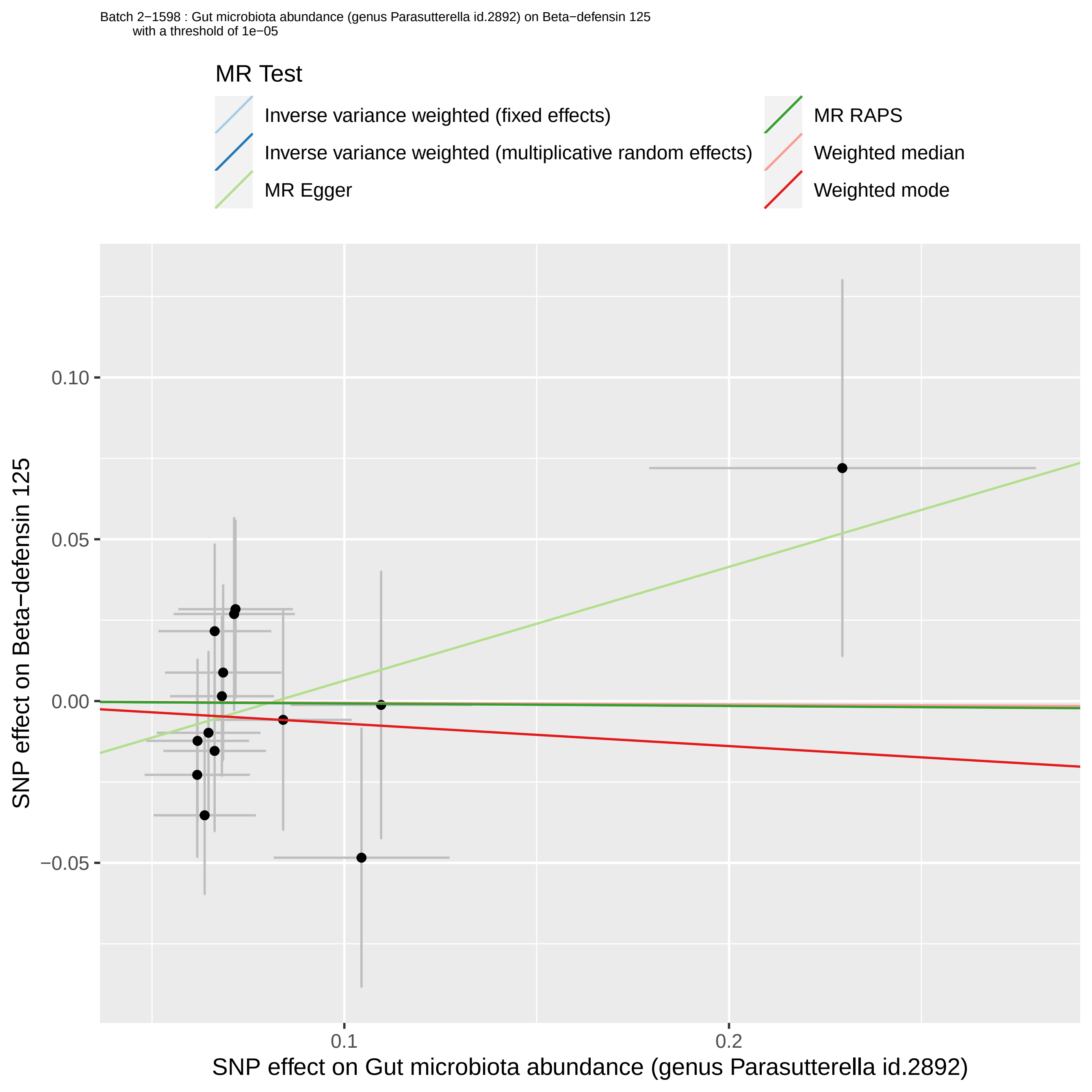
Batch 2−1598 : Gut microbiota abundance (genus Parasutterella id.2892) on Beta−defensin 125
with a threshold of 1e−05
MR Test
Inverse variance weighted (fixed effects)
Inverse variance weighted (multiplicative random effects)
MR Egger
MR RAPS
Weighted median
Weighted mode
0.10
0.05
0.00
−0.05
0.1
0.2
SNP effect on Beta−defensin 125
SNP effect on Gut microbiota abundance (genus Parasutterella id.2892)

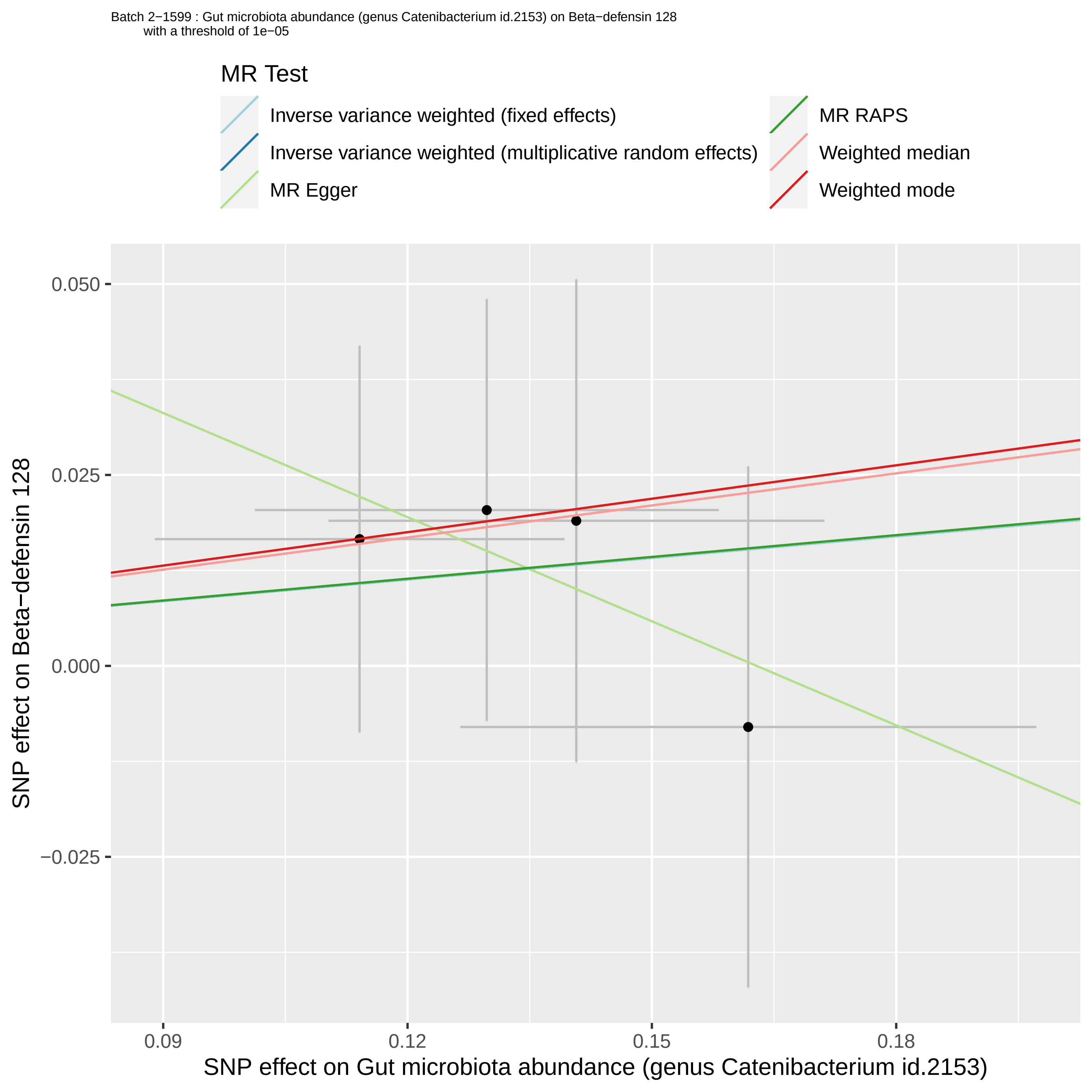

Batch 2−1599 : Gut microbiota abundance (genus Catenibacterium id.2153) on Beta−defensin 128
with a threshold of 1e−05
MR Test
Inverse variance weighted (fixed effects)
Inverse variance weighted (multiplicative random effects)
MR Egger
MR RAPS
Weighted median
Weighted mode
SNP effect on Beta−defensin 128
0.050
0.025
0.000
−0.025
0.09
0.12
0.15
0.18
SNP effect on Gut microbiota abundance (genus Catenibacterium id.2153)

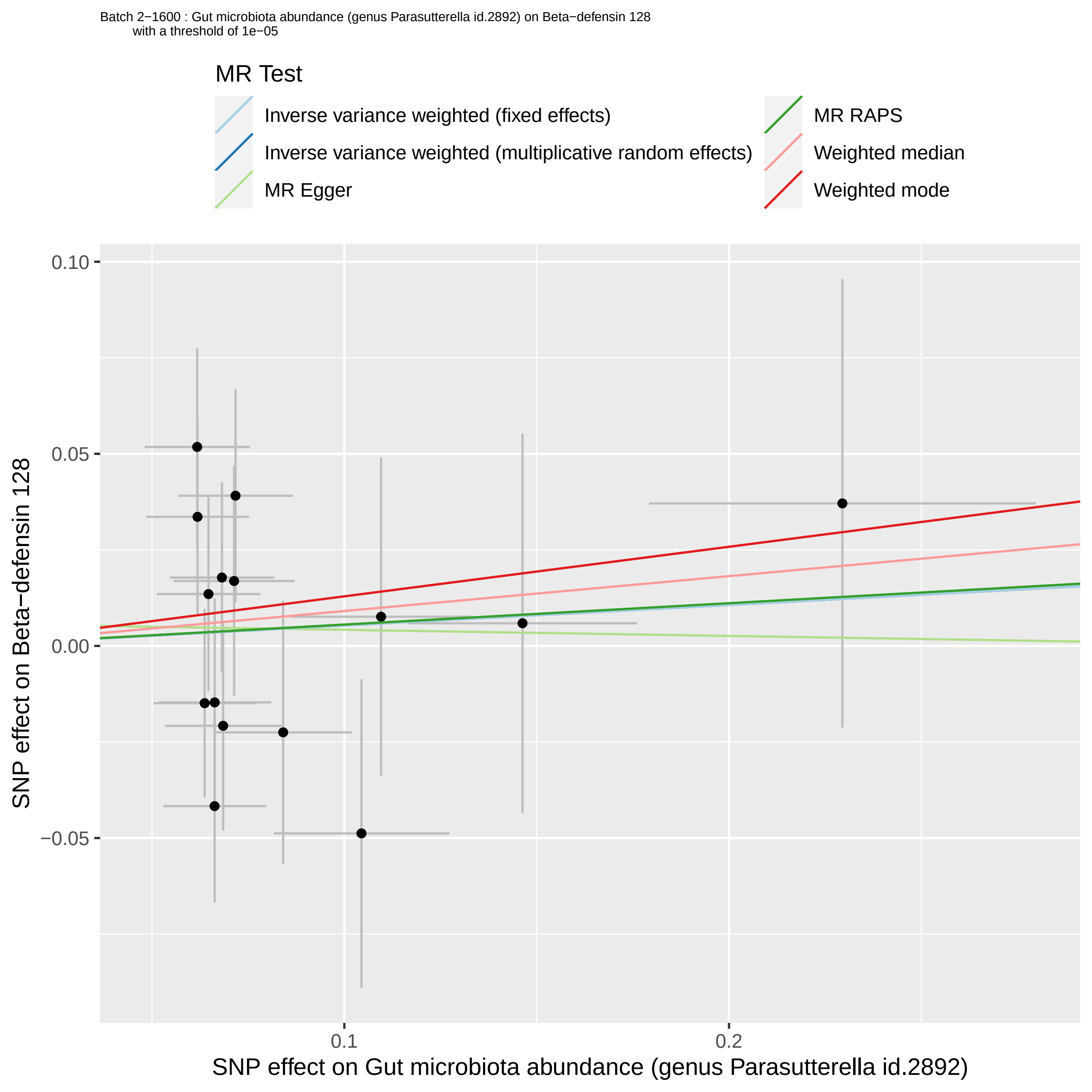

Batch 2−1600 : Gut microbiota abundance (genus Parasutterella id.2892) on Beta−defensin 128
with a threshold of 1e−05
MR Test
Inverse variance weighted (fixed effects)
Inverse variance weighted (multiplicative random effects)
MR Egger
MR RAPS
Weighted median
Weighted mode
0.10
0.05
0.00
−0.05
0.1
0.2
SNP effect on Beta−defensin 128
SNP effect on Gut microbiota abundance (genus Parasutterella id.2892)

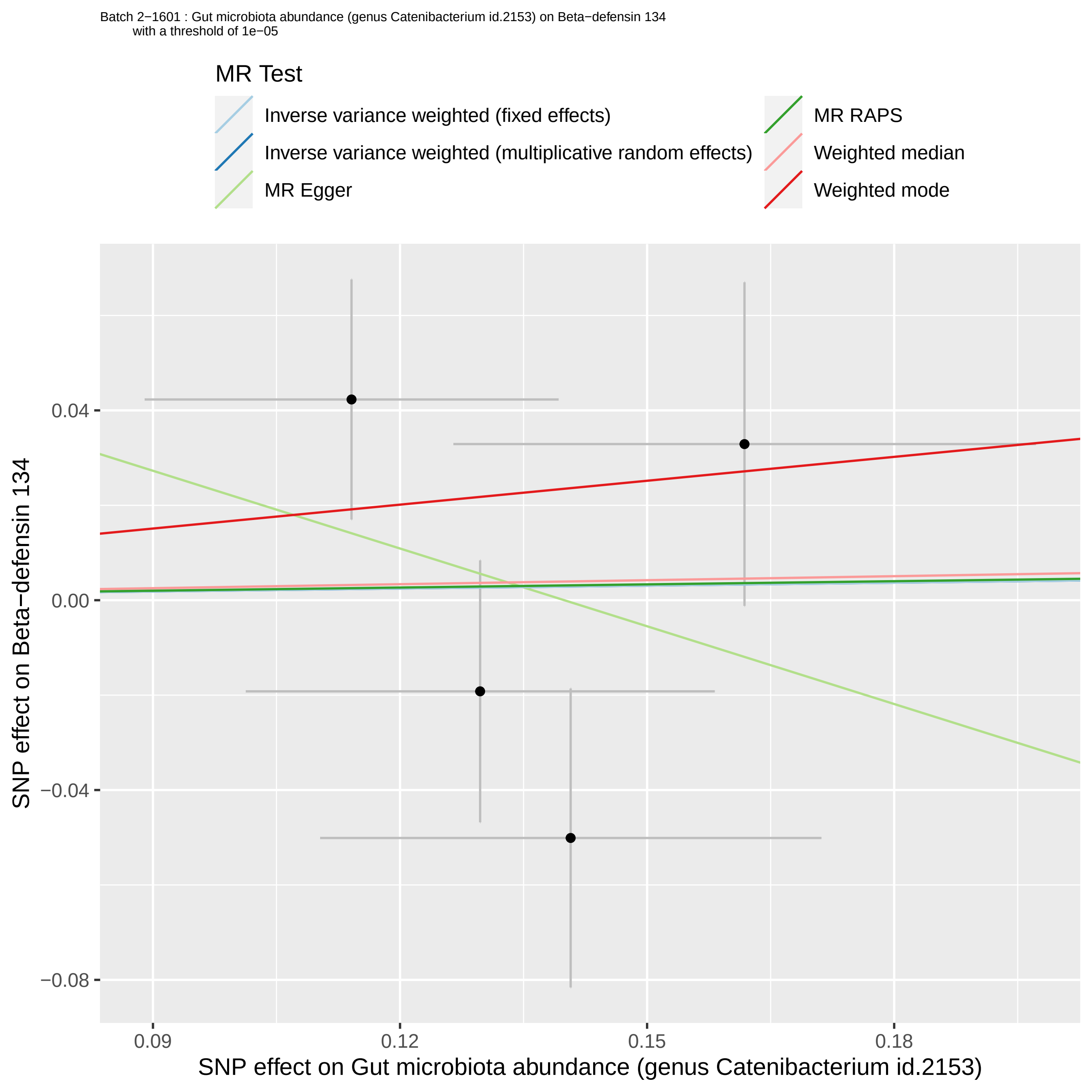

Batch 2−1601 : Gut microbiota abundance (genus Catenibacterium id.2153) on Beta−defensin 134
with a threshold of 1e−05
MR Test
Inverse variance weighted (fixed effects)
Inverse variance weighted (multiplicative random effects)
MR Egger
MR RAPS
Weighted median
Weighted mode
SNP effect on Beta−defensin 134
0.04
0.00
−0.04
−0.08
0.09
0.12
0.15
0.18
SNP effect on Gut microbiota abundance (genus Catenibacterium id.2153)

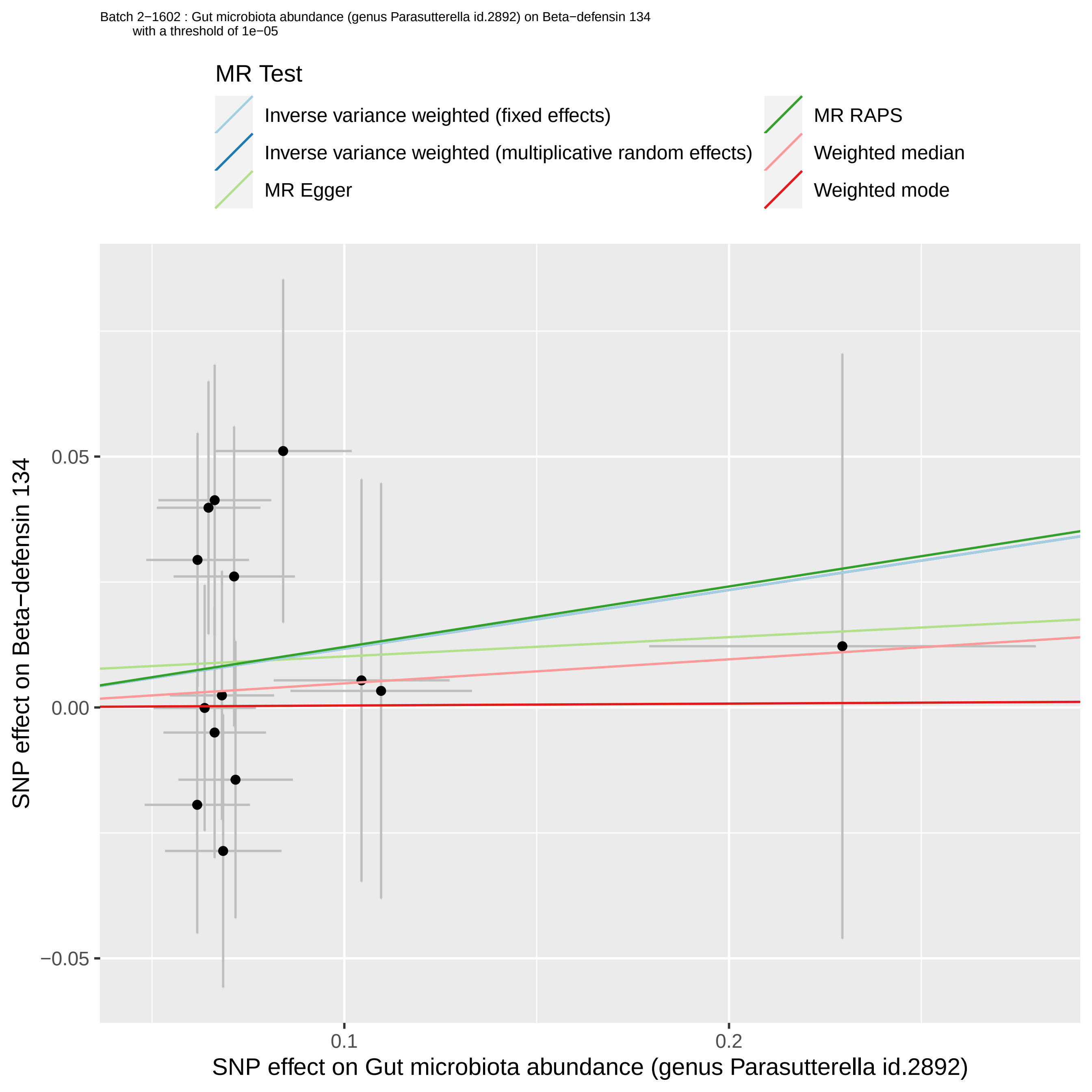

Batch 2−1602 : Gut microbiota abundance (genus Parasutterella id.2892) on Beta−defensin 134
with a threshold of 1e−05
MR Test
Inverse variance weighted (fixed effects)
Inverse variance weighted (multiplicative random effects)
MR Egger
MR RAPS
Weighted median
Weighted mode
0.05
0.00
−0.05
0.1
0.2
SNP effect on Beta−defensin 134
SNP effect on Gut microbiota abundance (genus Parasutterella id.2892)

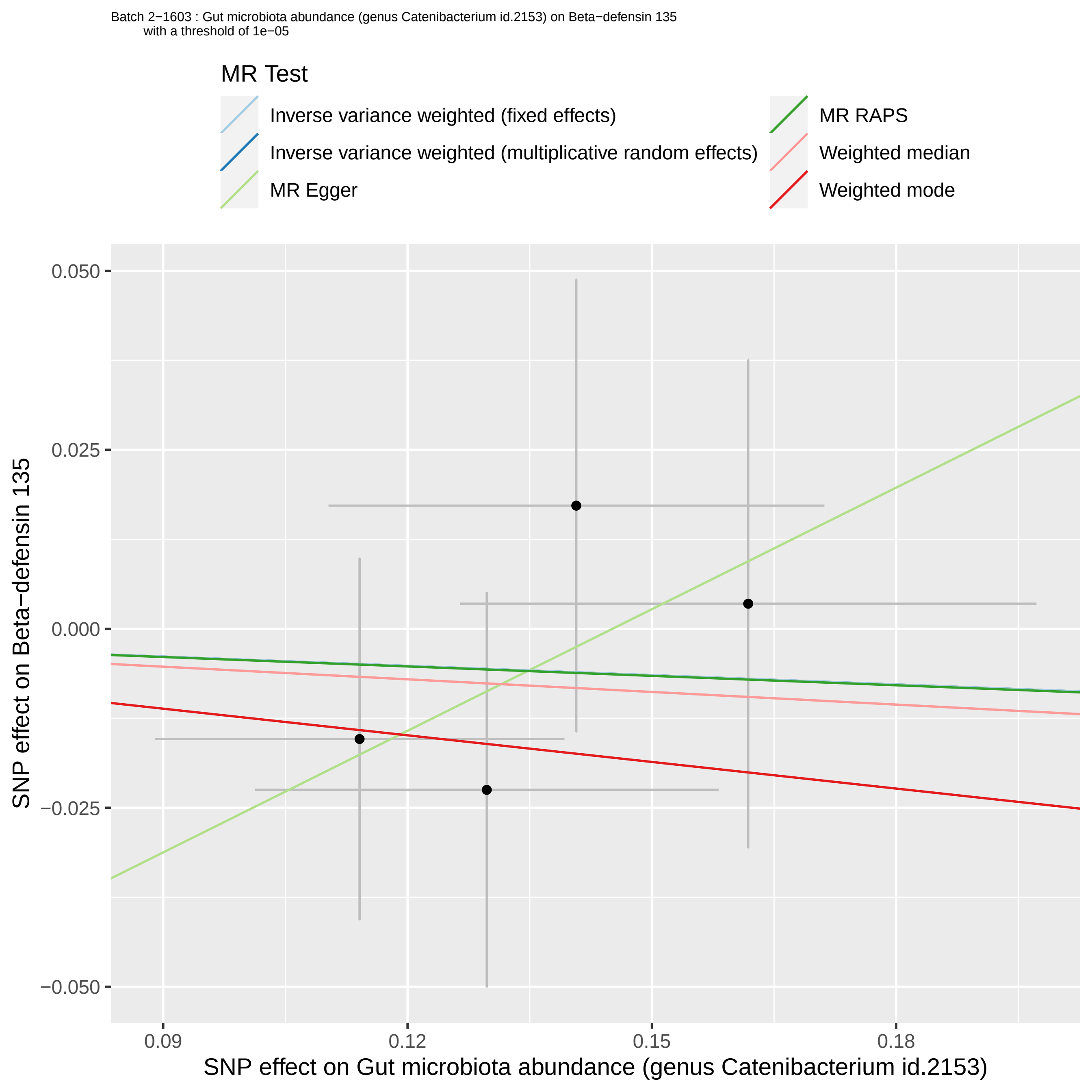
Batch 2−1603 : Gut microbiota abundance (genus Catenibacterium id.2153) on Beta−defensin 135
with a threshold of 1e−05
MR Test
Inverse variance weighted (fixed effects)
Inverse variance weighted (multiplicative random effects)
MR Egger
MR RAPS
Weighted median
Weighted mode
0.050
0.025
0.000
−0.025
−0.050
0.09
0.12
0.15
0.18
SNP effect on Beta−defensin 135
SNP effect on Gut microbiota abundance (genus Catenibacterium id.2153)

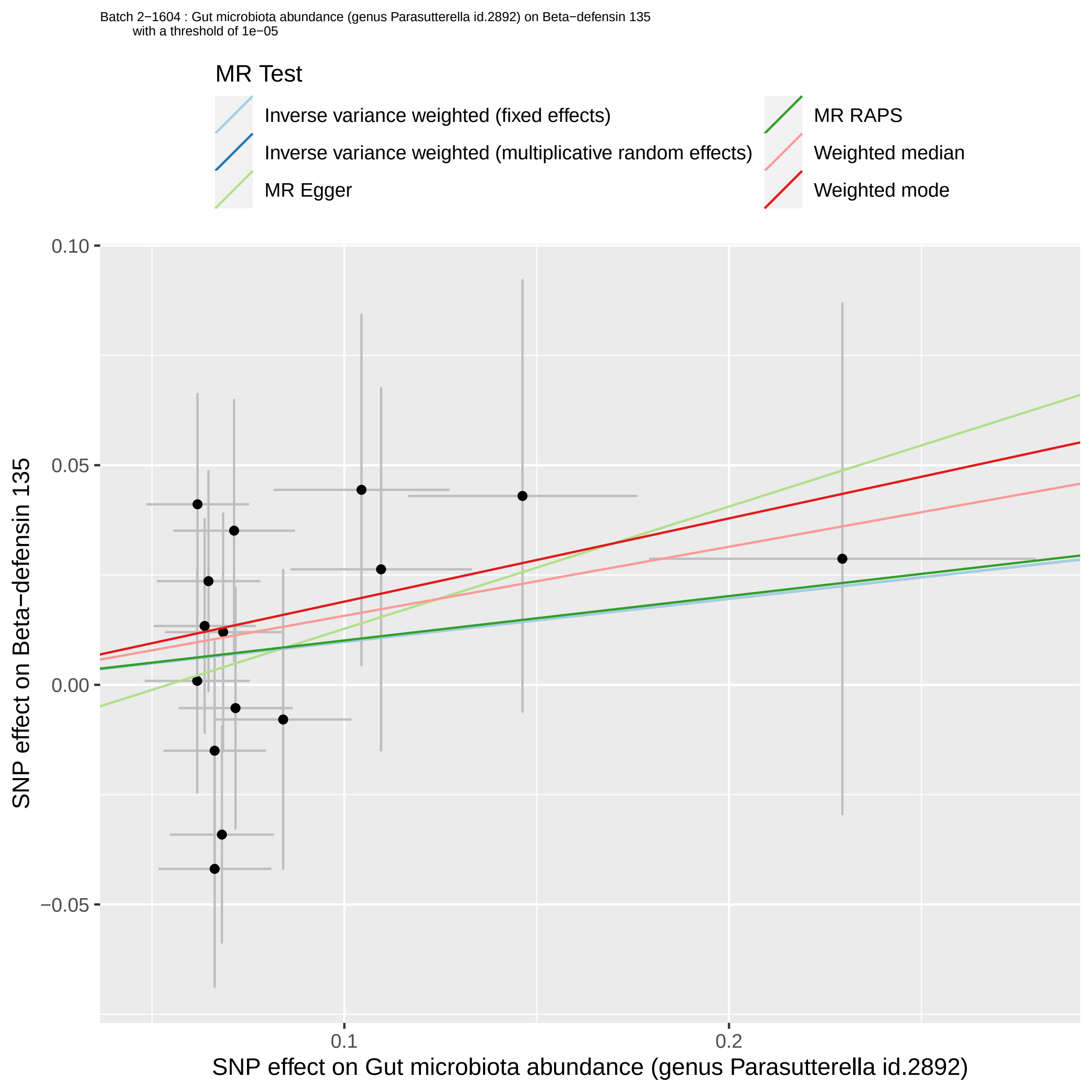

Batch 2−1604 : Gut microbiota abundance (genus Parasutterella id.2892) on Beta−defensin 135
with a threshold of 1e−05
MR Test
Inverse variance weighted (fixed effects)
Inverse variance weighted (multiplicative random effects)
MR Egger
MR RAPS
Weighted median
Weighted mode
0.10
0.05
0.00
−0.05
0.1
0.2
SNP effect on Beta−defensin 135
SNP effect on Gut microbiota abundance (genus Parasutterella id.2892)

Batch 2−1605 : Gut microbiota abundance (genus Catenibacterium id.2153) on Beta−defensin 136
with a threshold of 1e−05

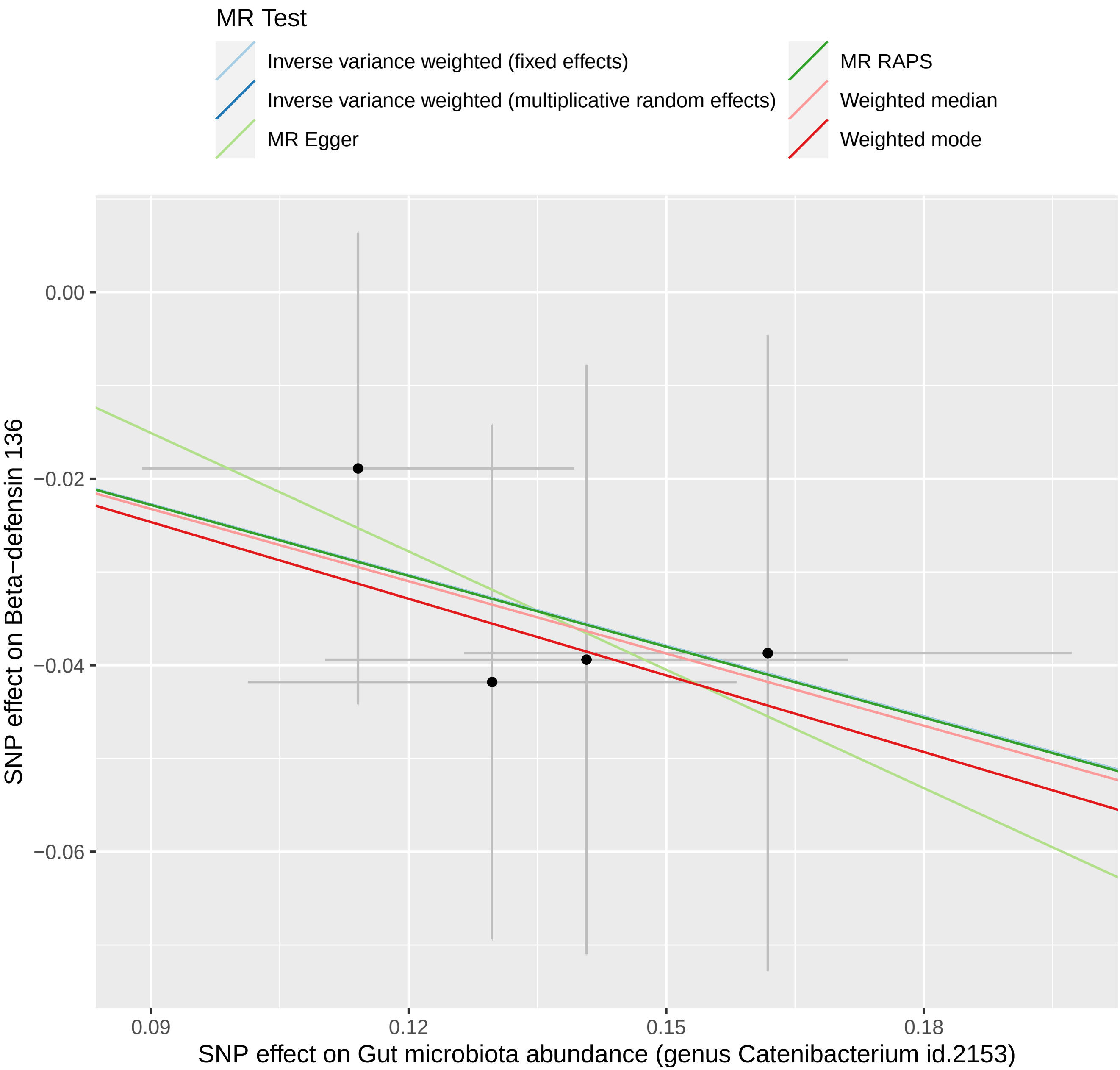

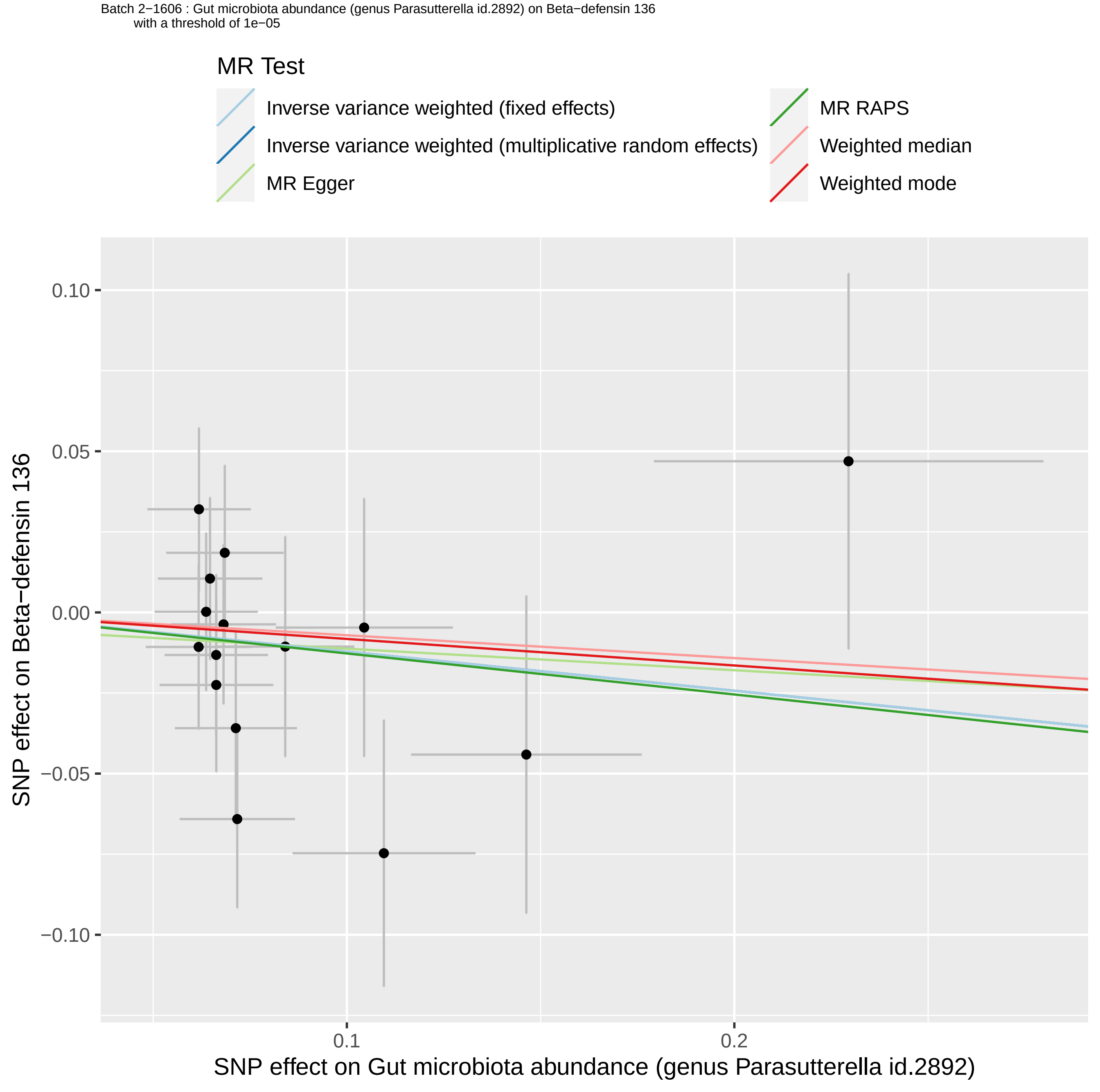
Batch 2−1606 : Gut microbiota abundance (genus Parasutterella id.2892) on Beta−defensin 136
with a threshold of 1e−05
MR Test
Inverse variance weighted (fixed effects)
Inverse variance weighted (multiplicative random effects)
MR Egger
MR RAPS
Weighted median
Weighted mode
0.10
0.05
0.00
−0.05
−0.10
0.1
0.2
SNP effect on Beta−defensin 136
SNP effect on Gut microbiota abundance (genus Parasutterella id.2892)

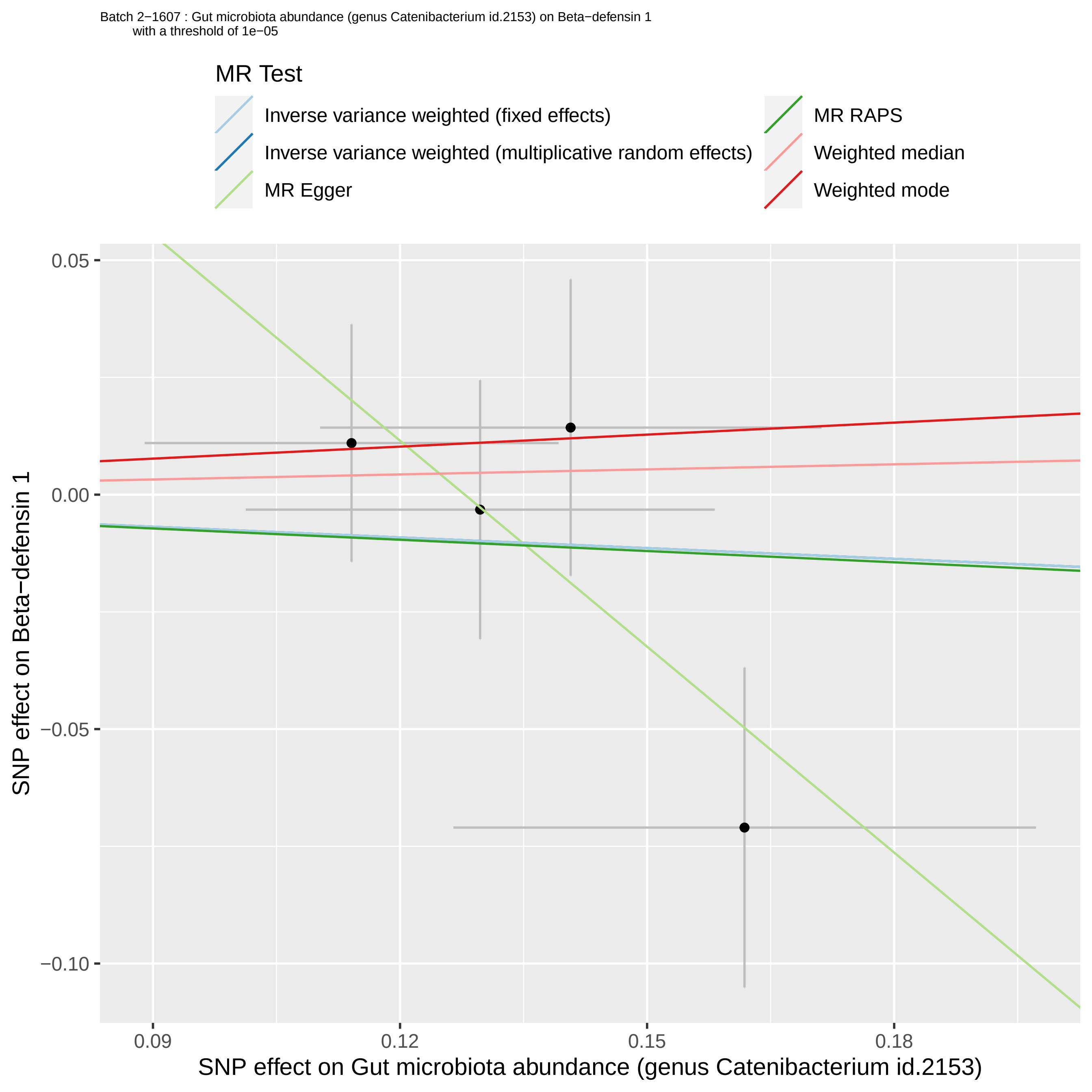
Batch 2−1607 : Gut microbiota abundance (genus Catenibacterium id.2153) on Beta−defensin 1
with a threshold of 1e−05
MR Test
Inverse variance weighted (fixed effects)
Inverse variance weighted (multiplicative random effects)
MR Egger
MR RAPS
Weighted median
Weighted mode
0.05
0.00
−0.05
−0.10
0.09
0.12
0.15
0.18
SNP effect on Beta−defensin 1
SNP effect on Gut microbiota abundance (genus Catenibacterium id.2153)

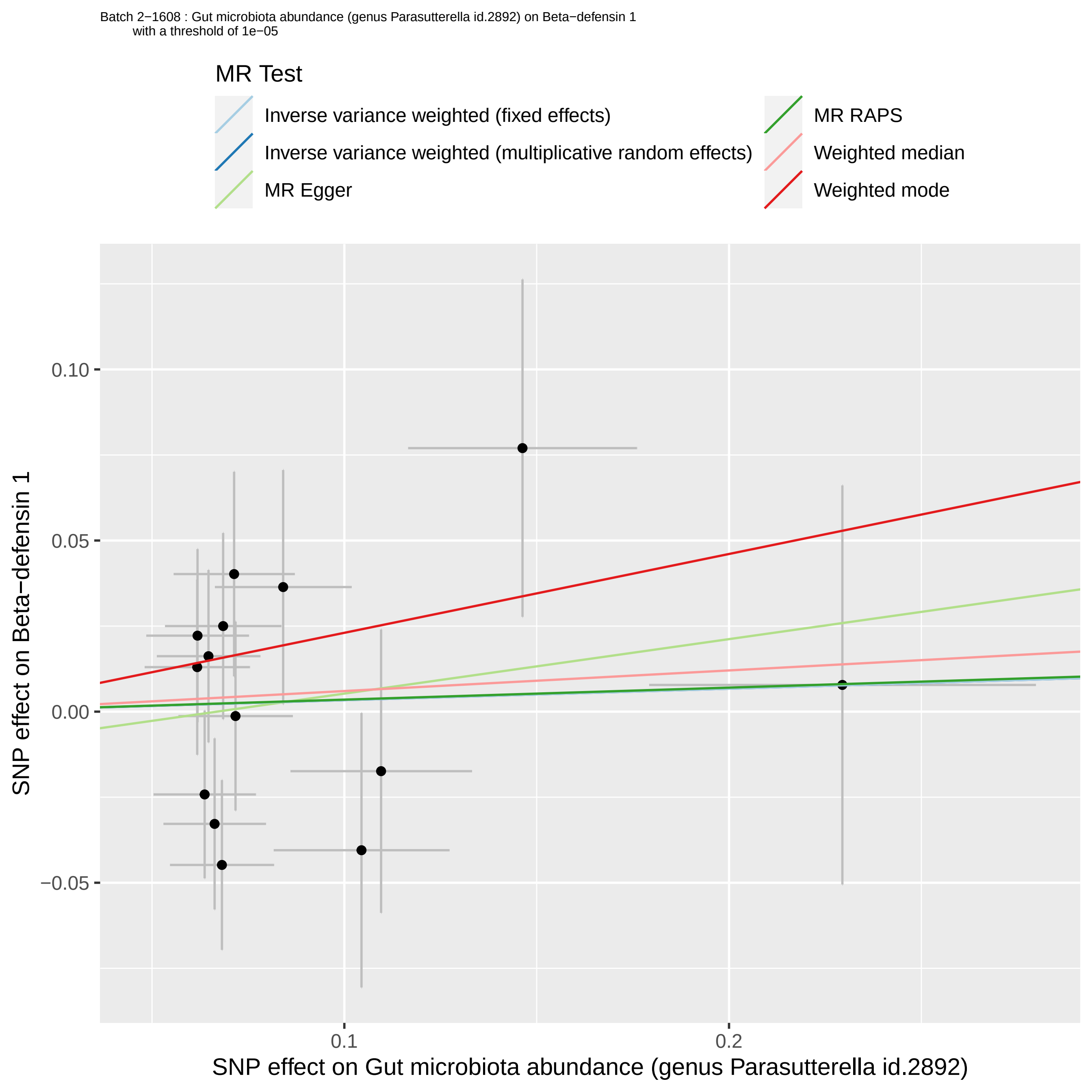
Batch 2−1608 : Gut microbiota abundance (genus Parasutterella id.2892) on Beta−defensin 1
with a threshold of 1e−05
MR Test
Inverse variance weighted (fixed effects)
Inverse variance weighted (multiplicative random effects)
MR Egger
MR RAPS
Weighted median
Weighted mode
SNP effect on Beta−defensin 1
0.10
0.05
0.00
−0.05
0.1
0.2
SNP effect on Gut microbiota abundance (genus Parasutterella id.2892)

Batch 2−1609 : Gut microbiota abundance (genus Catenibacterium id.2153) on Protein DEPP
with a threshold of 1e−05

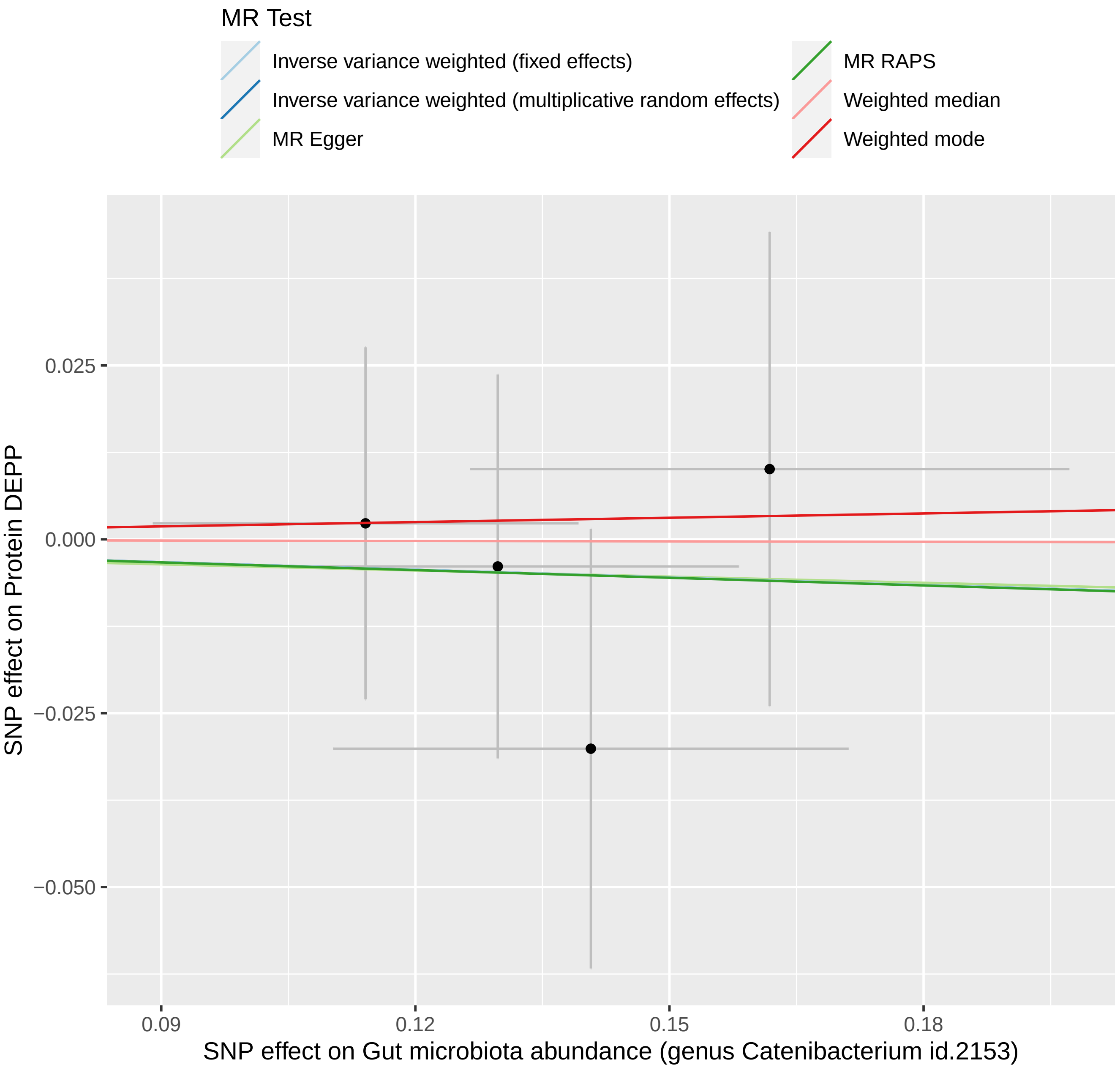

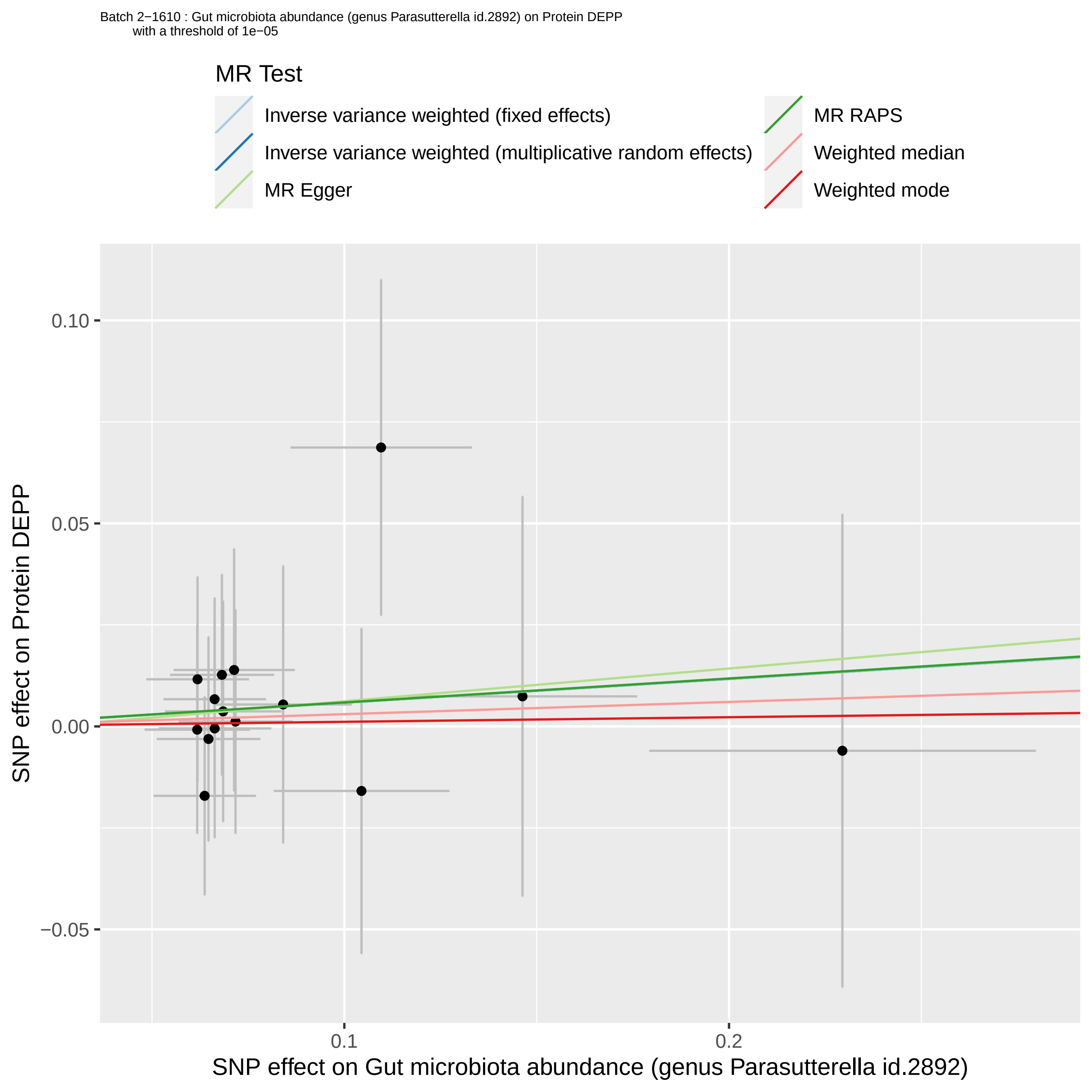

Batch 2−1610 : Gut microbiota abundance (genus Parasutterella id.2892) on Protein DEPP
with a threshold of 1e−05
MR Test
Inverse variance weighted (fixed effects)
Inverse variance weighted (multiplicative random effects)
MR Egger
MR RAPS
Weighted median
Weighted mode
0.10
0.05
0.00
−0.05
0.1
0.2
SNP effect on Protein DEPP
SNP effect on Gut microbiota abundance (genus Parasutterella id.2892)

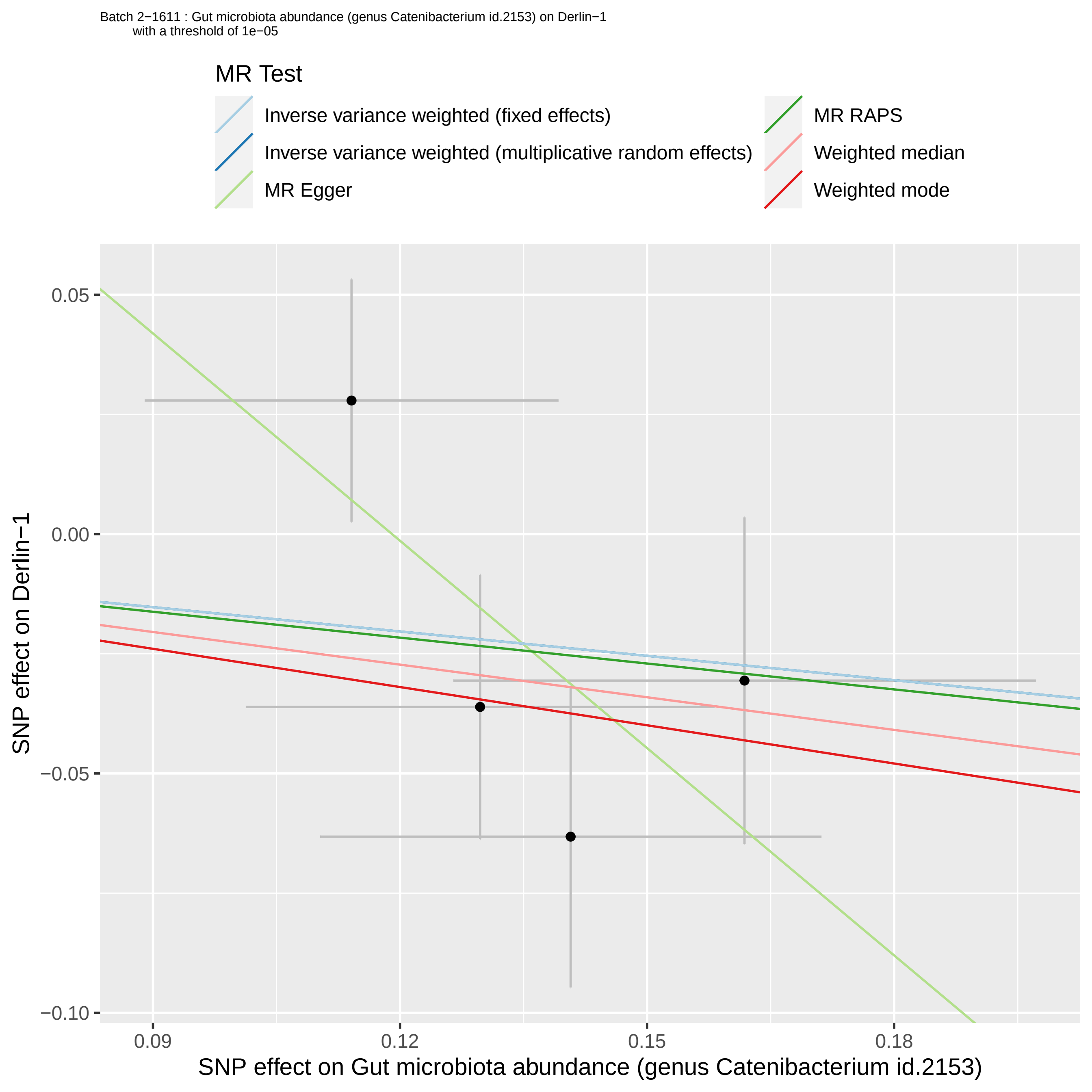
Batch 2−1611 : Gut microbiota abundance (genus Catenibacterium id.2153) on Derlin−1
with a threshold of 1e−05
MR Test
Inverse variance weighted (fixed effects)
Inverse variance weighted (multiplicative random effects)
MR Egger
MR RAPS
Weighted median
Weighted mode
0.05
0.00
−0.05
−0.10
0.09
0.12
0.15
0.18
SNP effect on Derlin−1
SNP effect on Gut microbiota abundance (genus Catenibacterium id.2153)

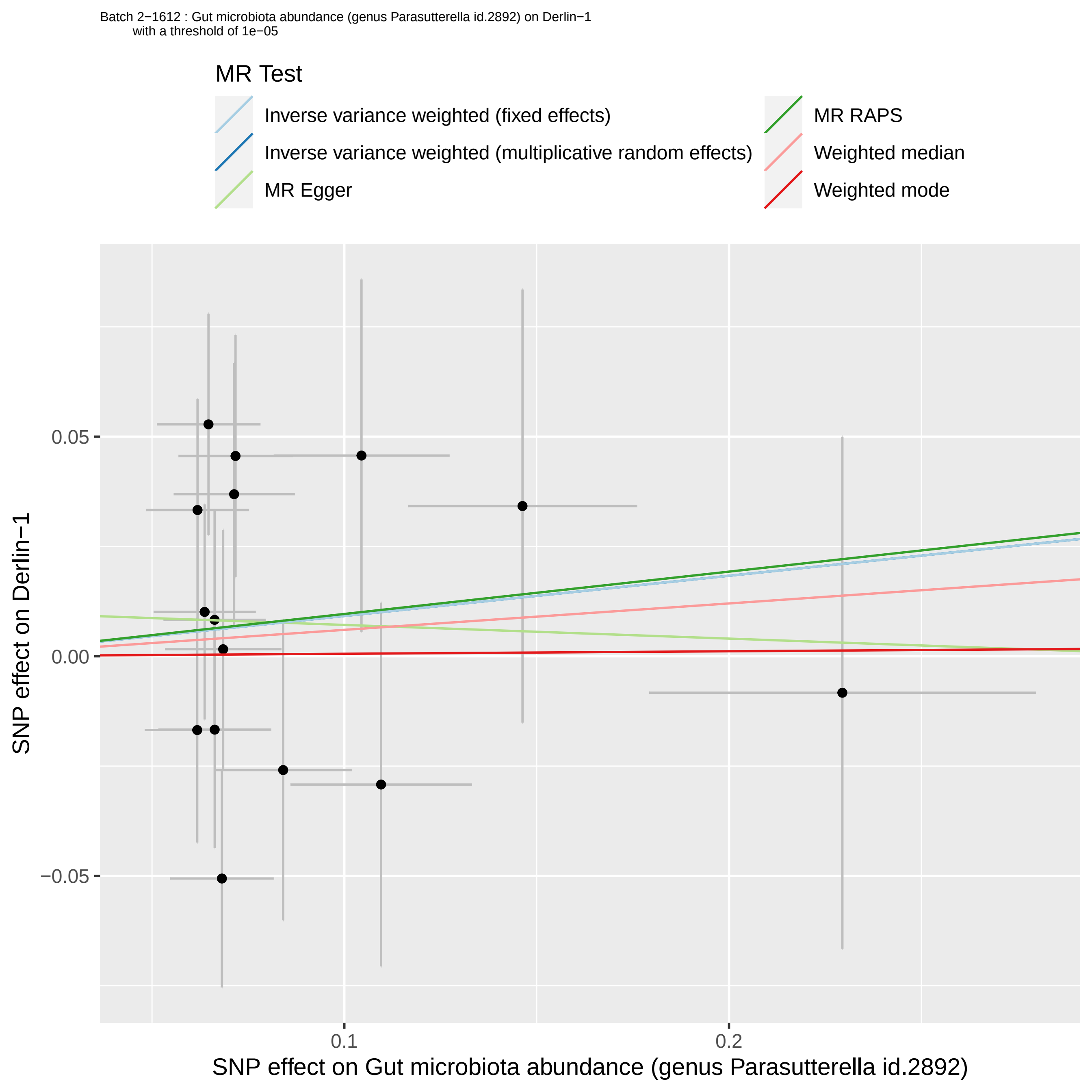
Batch 2−1612 : Gut microbiota abundance (genus Parasutterella id.2892) on Derlin−1
with a threshold of 1e−05
MR Test
Inverse variance weighted (fixed effects)
Inverse variance weighted (multiplicative random effects)
MR Egger
MR RAPS
Weighted median
Weighted mode
0.05
0.00
−0.05
0.1
0.2
SNP effect on Derlin−1
SNP effect on Gut microbiota abundance (genus Parasutterella id.2892)

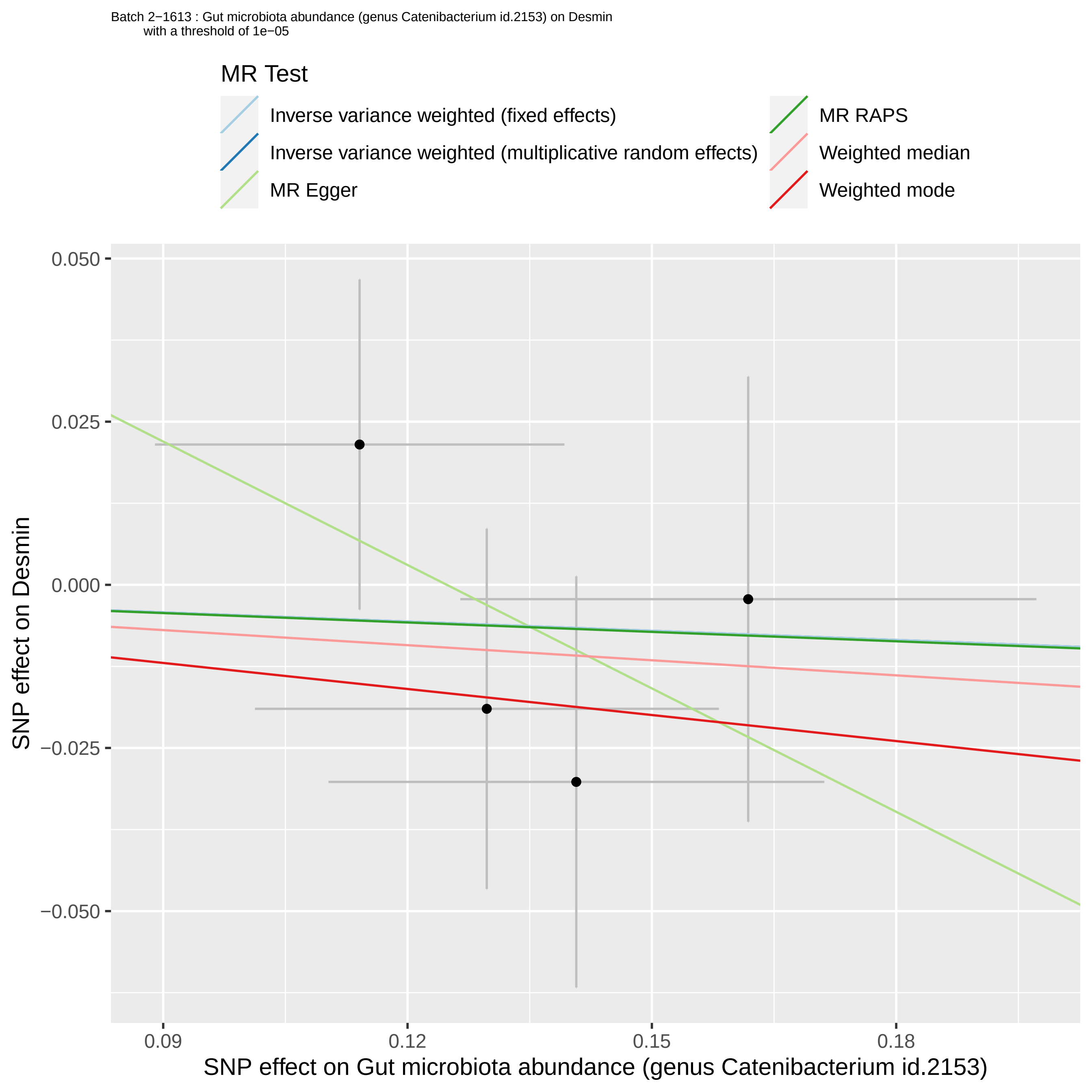

Batch 2−1613 : Gut microbiota abundance (genus Catenibacterium id.2153) on Desmin
with a threshold of 1e−05
MR Test
Inverse variance weighted (fixed effects)
Inverse variance weighted (multiplicative random effects)
MR Egger
MR RAPS
Weighted median
Weighted mode
0.050
0.025
0.000
−0.025
−0.050
0.09
0.12
0.15
0.18
SNP effect on Desmin
SNP effect on Gut microbiota abundance (genus Catenibacterium id.2153)

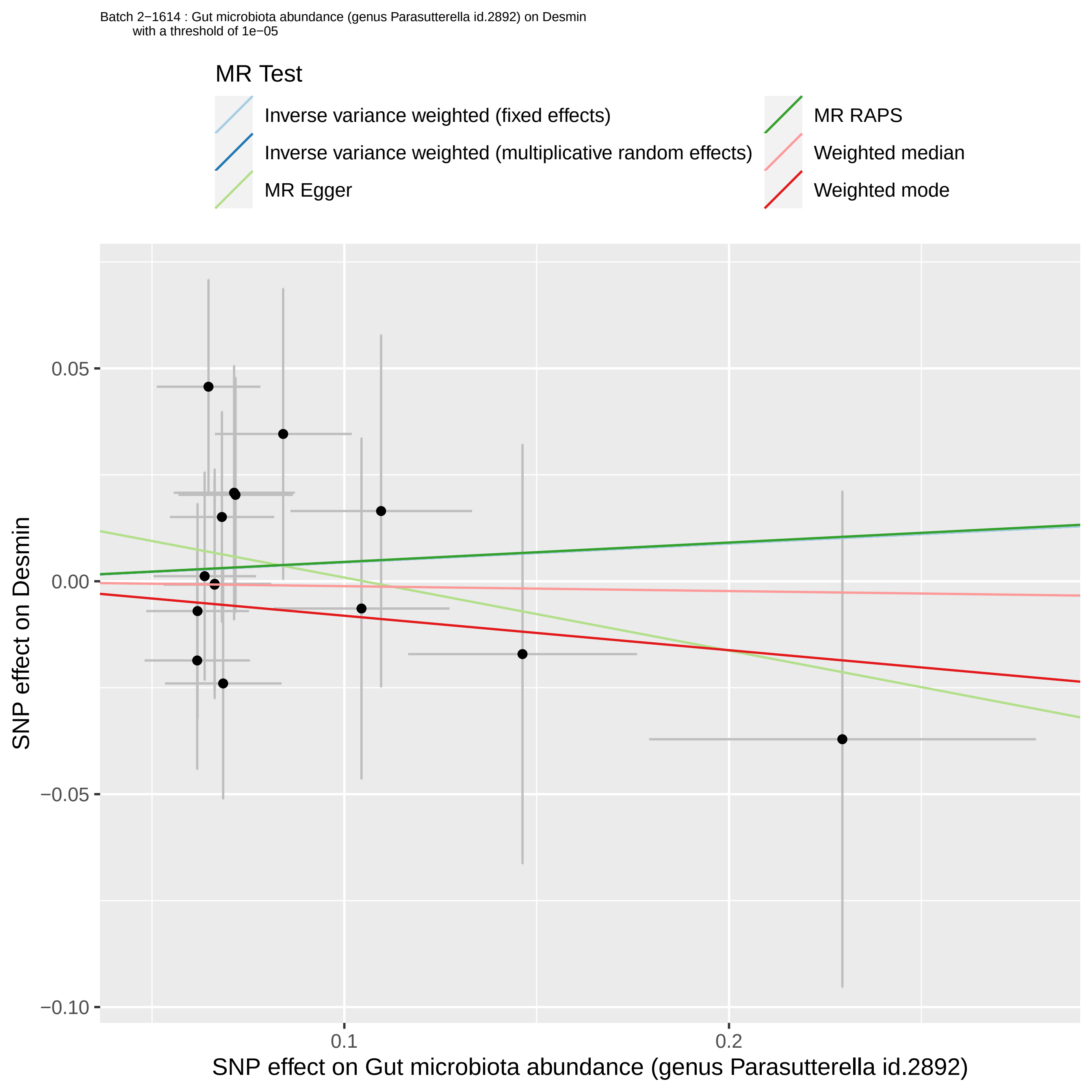

Batch 2−1614 : Gut microbiota abundance (genus Parasutterella id.2892) on Desmin
with a threshold of 1e−05
MR Test
Inverse variance weighted (fixed effects)
Inverse variance weighted (multiplicative random effects)
MR Egger
MR RAPS
Weighted median
Weighted mode
0.05
0.00
−0.05
−0.10
0.1
0.2
SNP effect on Desmin
SNP effect on Gut microbiota abundance (genus Parasutterella id.2892)

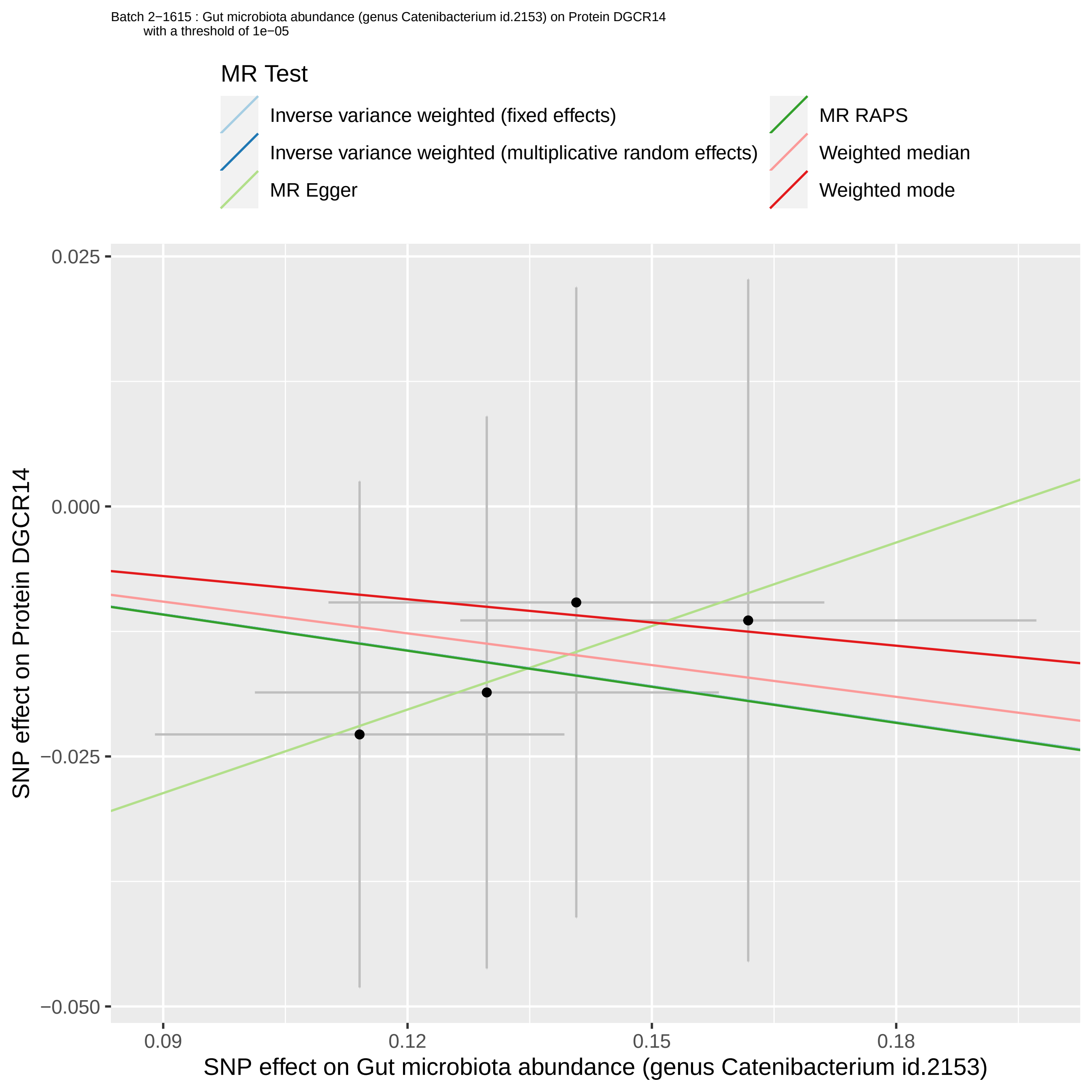
Batch 2−1615 : Gut microbiota abundance (genus Catenibacterium id.2153) on Protein DGCR14
with a threshold of 1e−05
MR Test
Inverse variance weighted (fixed effects)
Inverse variance weighted (multiplicative random effects)
MR Egger
MR RAPS
Weighted median
Weighted mode
0.025
0.000
−0.025
−0.050
0.09
0.12
0.15
0.18
SNP effect on Protein DGCR14
SNP effect on Gut microbiota abundance (genus Catenibacterium id.2153)

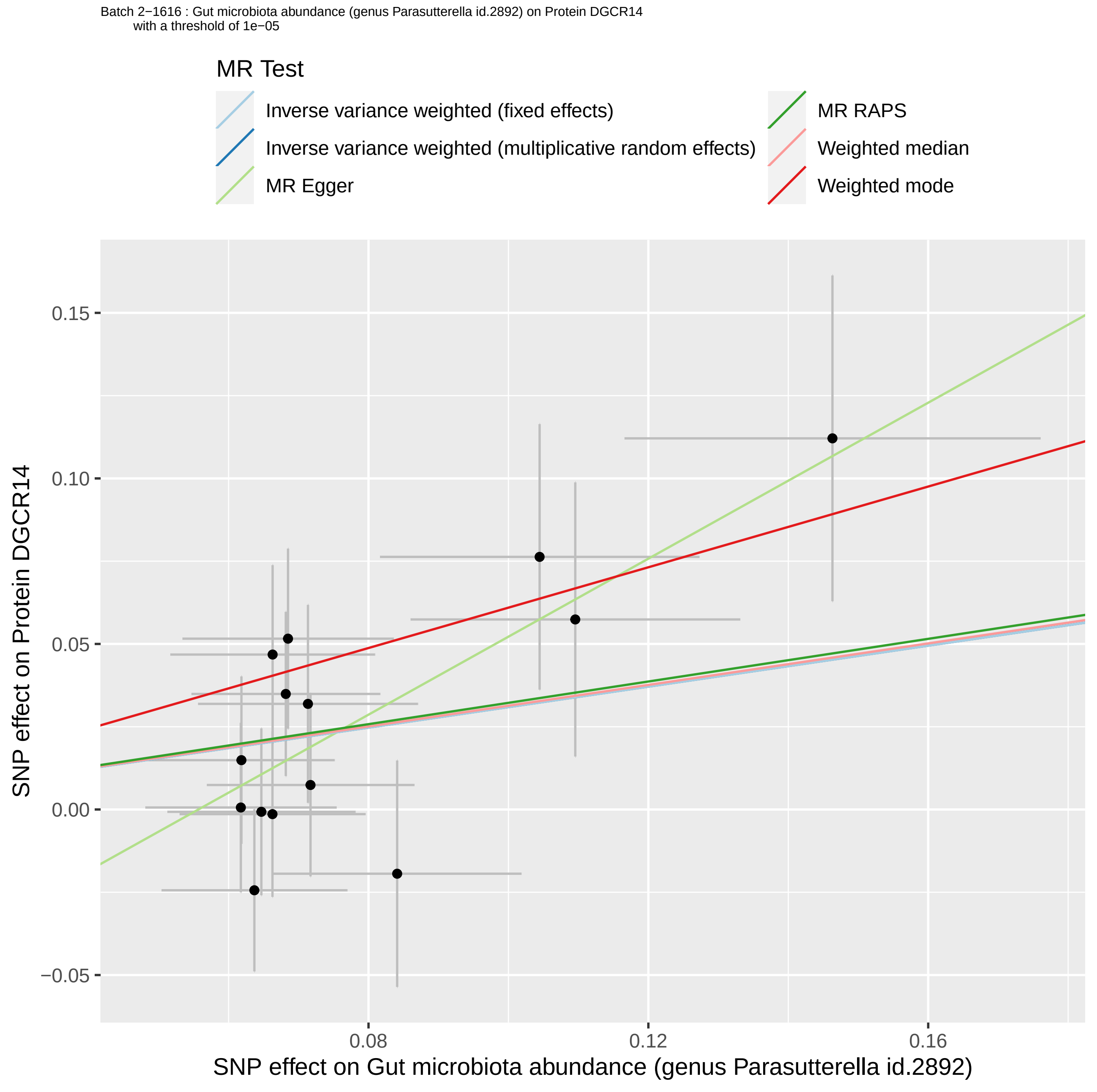

Batch 2−1616 : Gut microbiota abundance (genus Parasutterella id.2892) on Protein DGCR14
with a threshold of 1e−05
MR Test
Inverse variance weighted (fixed effects)
Inverse variance weighted (multiplicative random effects)
MR Egger
MR RAPS
Weighted median
Weighted mode
0.15
0.10
0.05
0.00
−0.05
0.08
0.12
0.16
SNP effect on Protein DGCR14
SNP effect on Gut microbiota abundance (genus Parasutterella id.2892)

Batch 2−1617 : Gut microbiota abundance (genus Catenibacterium id.2153) on Integral membrane protein DGCR2/IDD
with a threshold of 1e−05

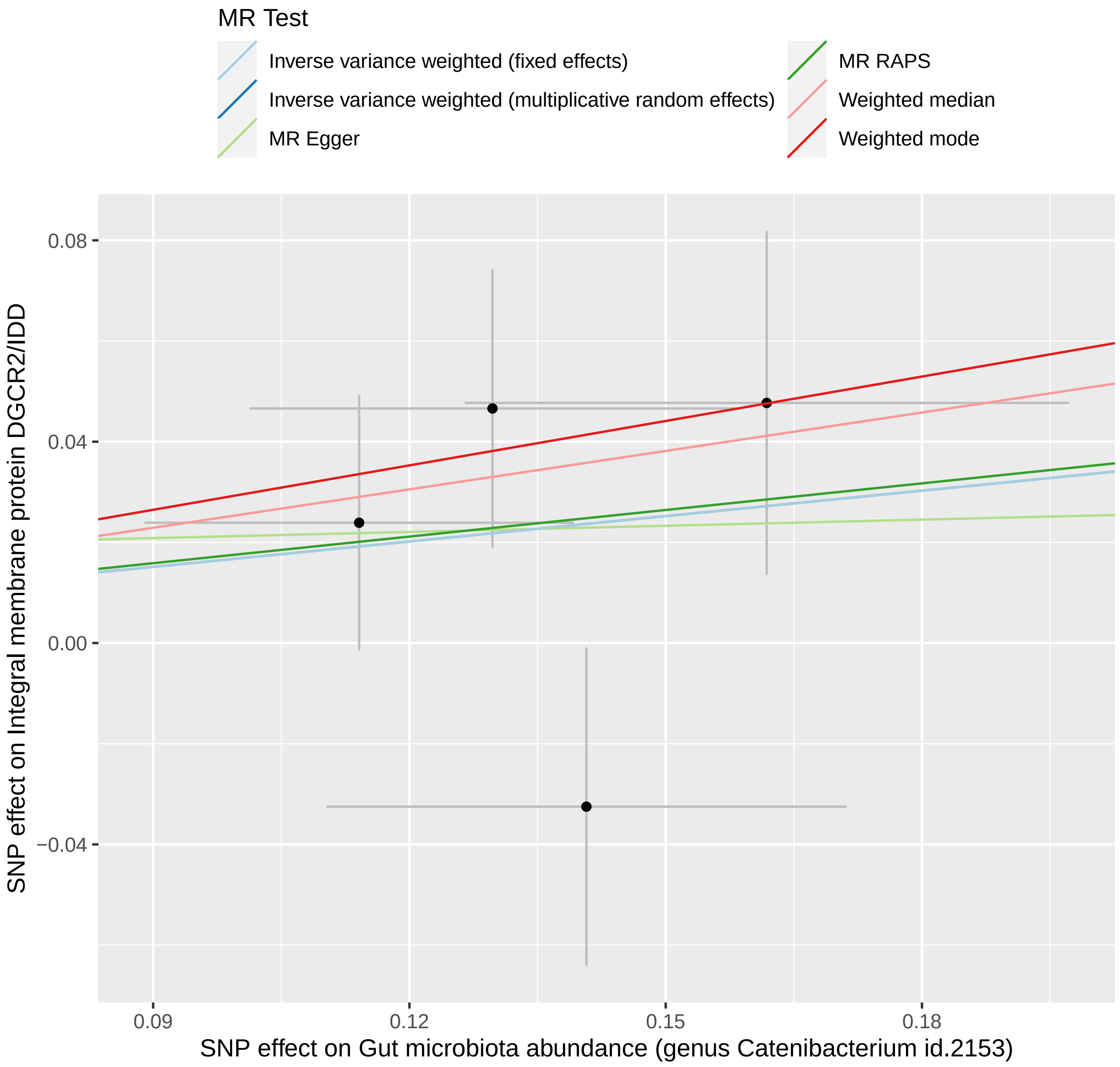

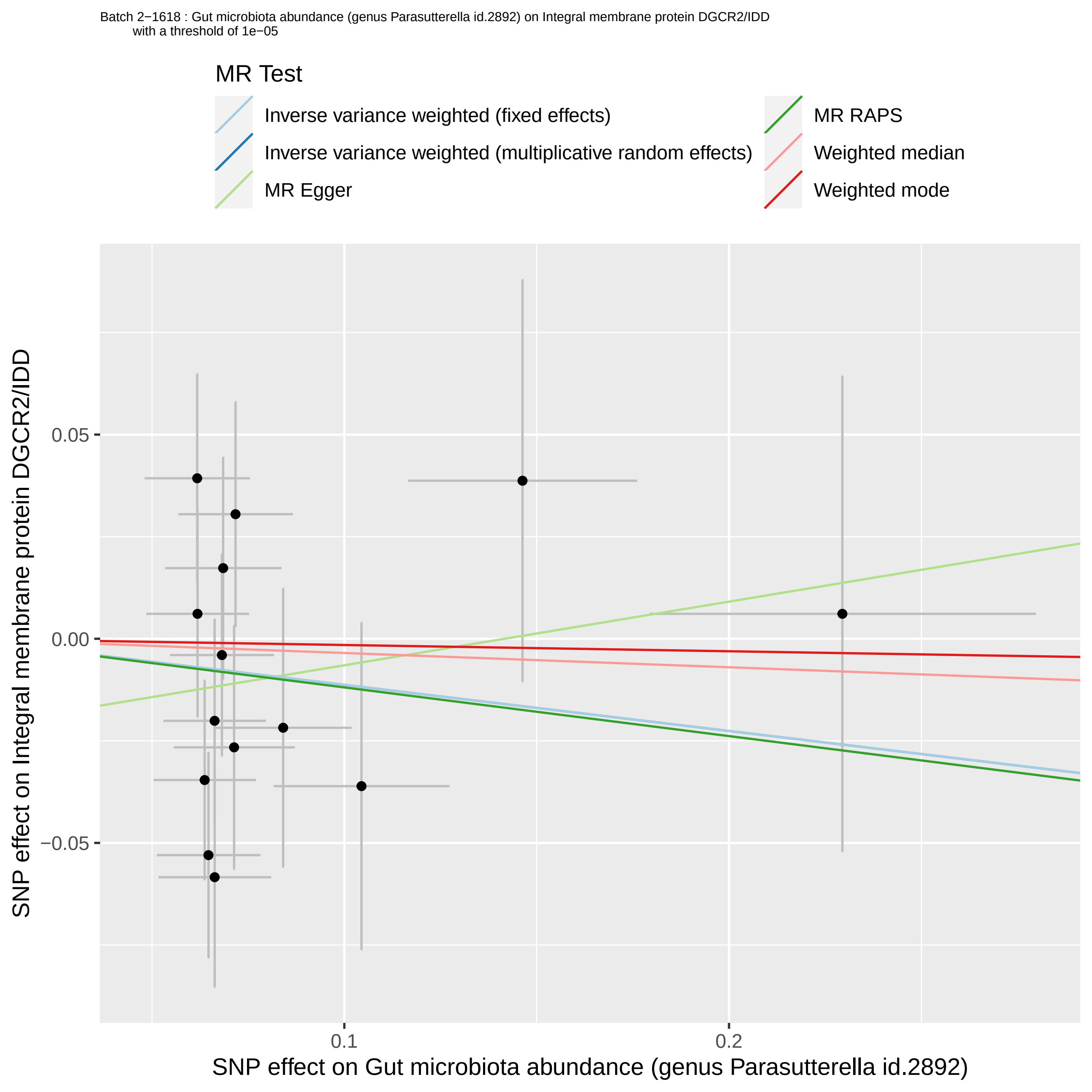
Batch 2−1618 : Gut microbiota abundance (genus Parasutterella id.2892) on Integral membrane protein DGCR2/IDD
with a threshold of 1e−05
MR Test
Inverse variance weighted (fixed effects)
Inverse variance weighted (multiplicative random effects)
MR Egger
MR RAPS
Weighted median
Weighted mode
0.05
0.00
−0.05
0.1
0.2
SNP effect on Integral membrane protein DGCR2/IDD
SNP effect on Gut microbiota abundance (genus Parasutterella id.2892)

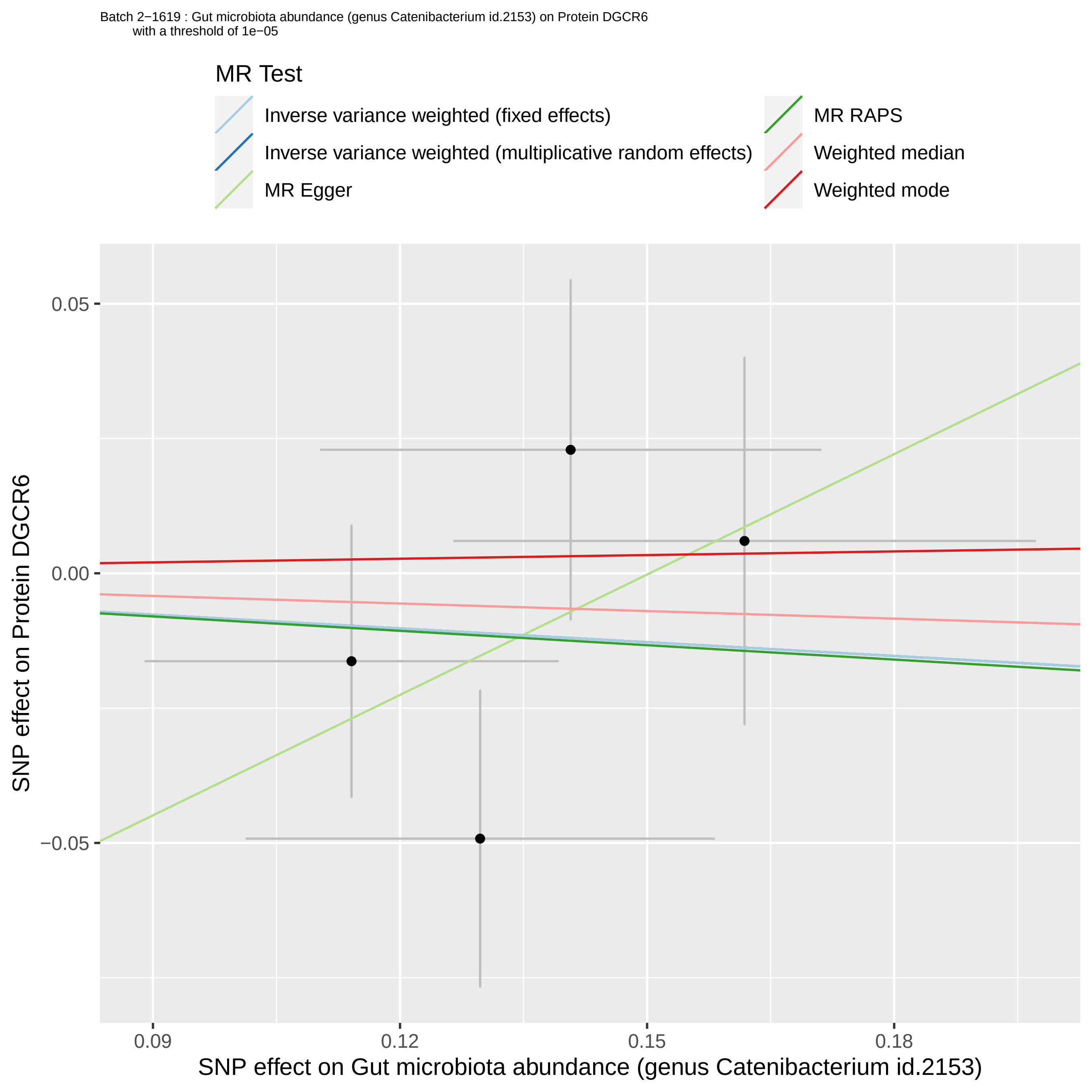
Batch 2−1619 : Gut microbiota abundance (genus Catenibacterium id.2153) on Protein DGCR6
with a threshold of 1e−05
MR Test
Inverse variance weighted (fixed effects)
Inverse variance weighted (multiplicative random effects)
MR Egger
MR RAPS
Weighted median
Weighted mode
0.05
0.00
−0.05
0.09
0.12
0.15
0.18
SNP effect on Protein DGCR6
SNP effect on Gut microbiota abundance (genus Catenibacterium id.2153)

Batch 2−1620 : Gut microbiota abundance (genus Parasutterella id.2892) on Protein DGCR6
with a threshold of 1e−05

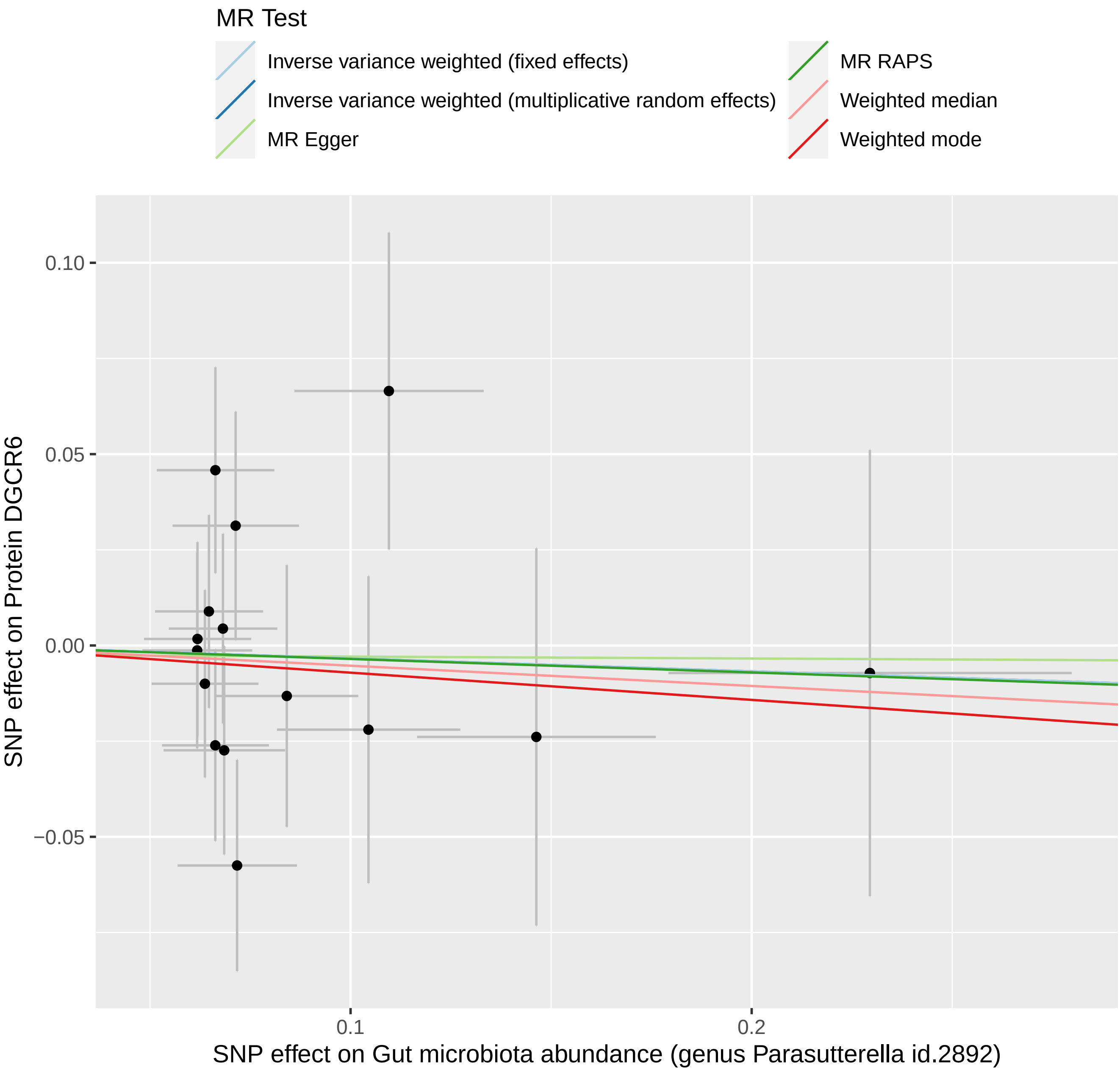

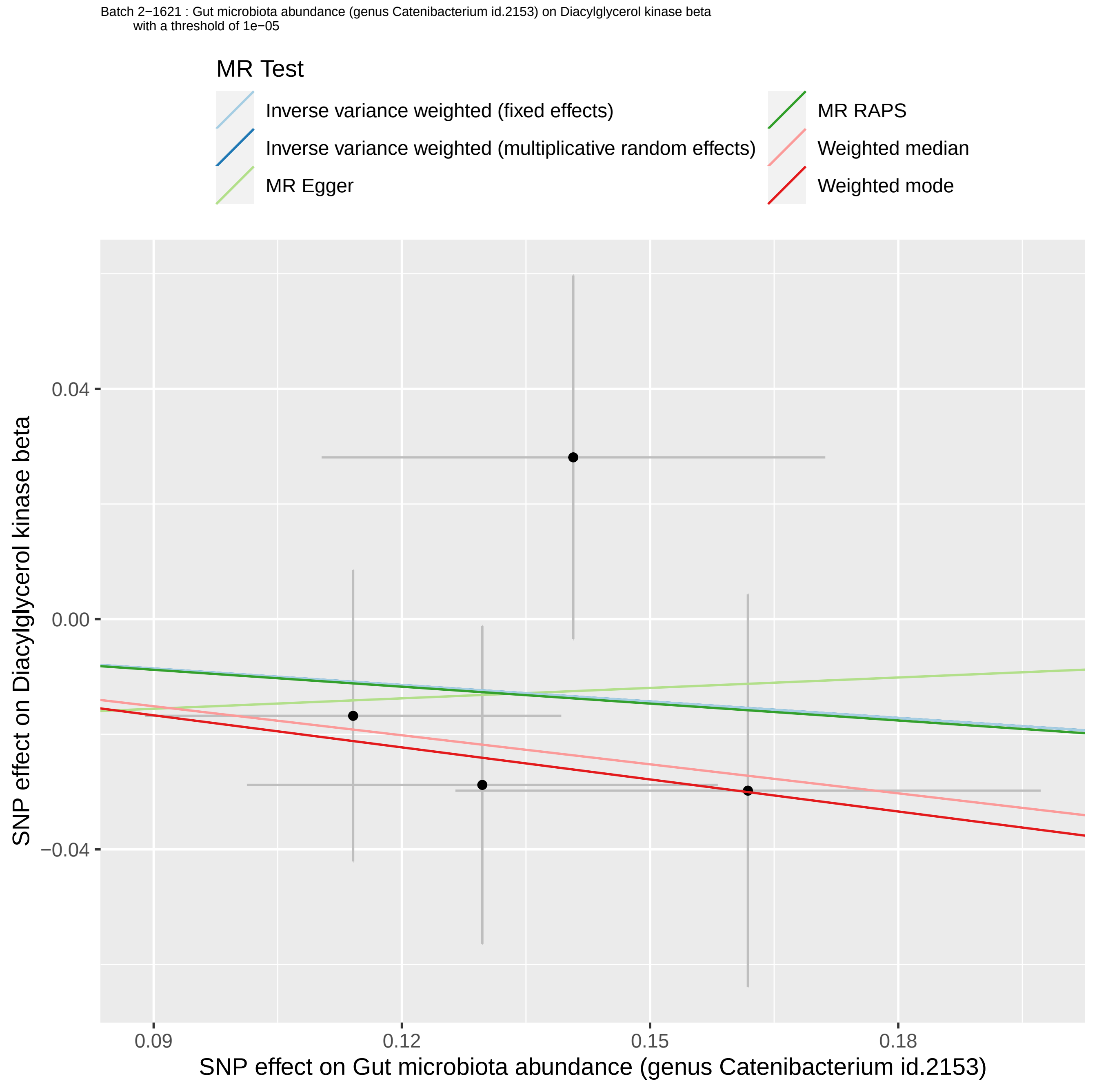

Batch 2−1621 : Gut microbiota abundance (genus Catenibacterium id.2153) on Diacylglycerol kinase beta
with a threshold of 1e−05
MR Test
Inverse variance weighted (fixed effects)
Inverse variance weighted (multiplicative random effects)
MR Egger
MR RAPS
Weighted median
Weighted mode
0.04
0.00
−0.04
0.09
0.12
0.15
0.18
SNP effect on Diacylglycerol kinase beta
SNP effect on Gut microbiota abundance (genus Catenibacterium id.2153)

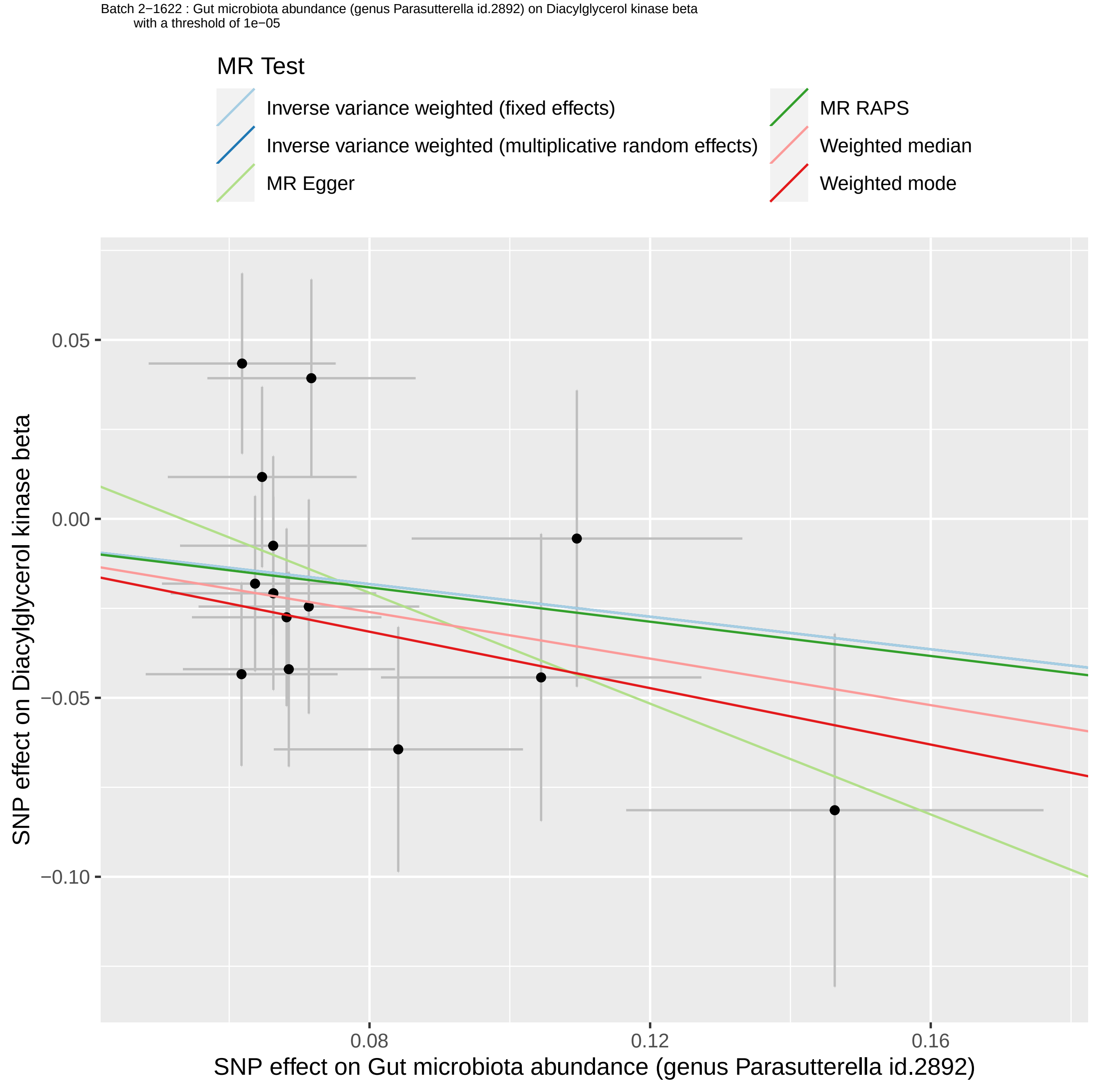
Batch 2−1622 : Gut microbiota abundance (genus Parasutterella id.2892) on Diacylglycerol kinase beta
with a threshold of 1e−05
MR Test
Inverse variance weighted (fixed effects)
Inverse variance weighted (multiplicative random effects)
MR Egger
MR RAPS
Weighted median
Weighted mode
0.05
0.00
−0.05
−0.10
0.08
0.12
0.16
SNP effect on Diacylglycerol kinase beta
SNP effect on Gut microbiota abundance (genus Parasutterella id.2892)

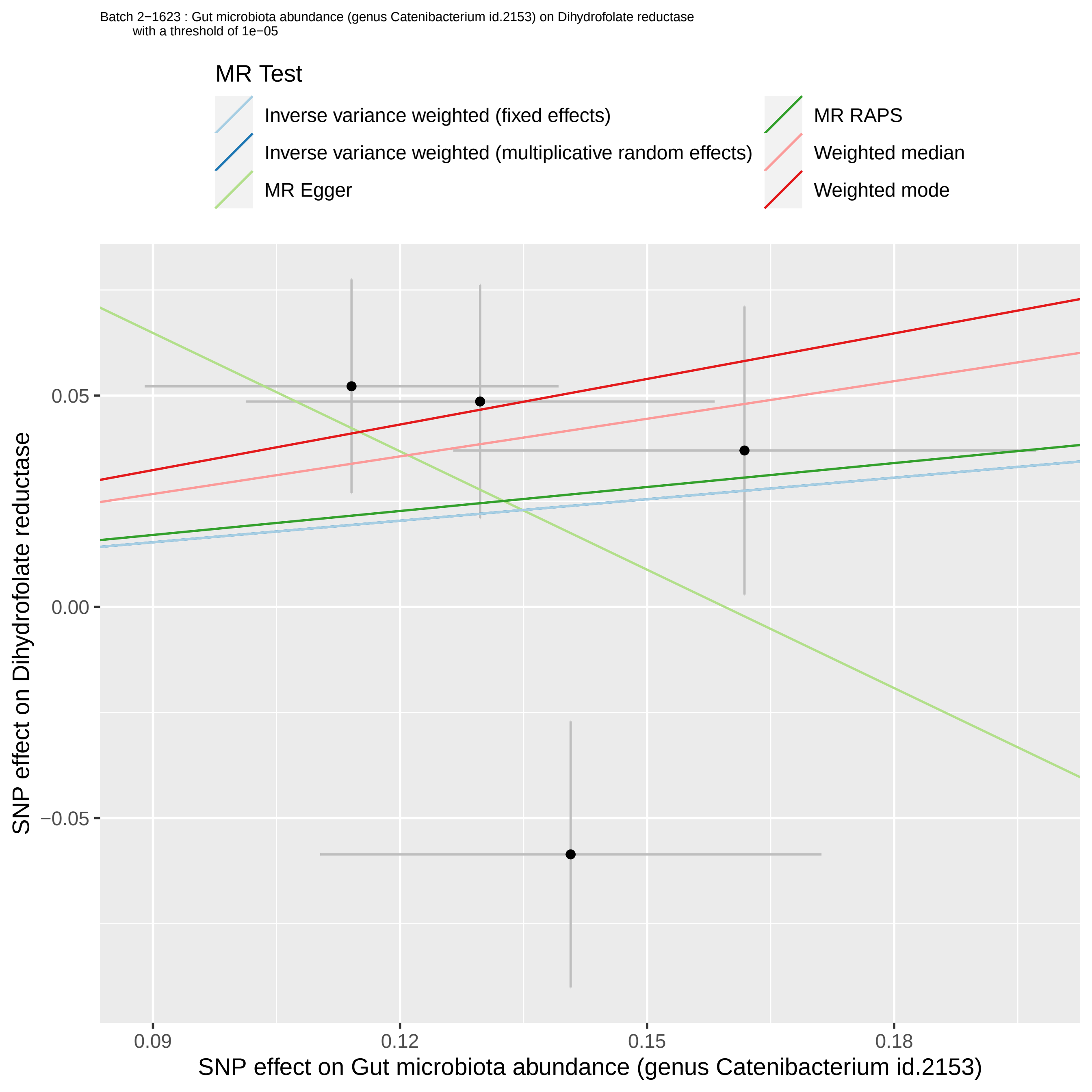

Batch 2−1623 : Gut microbiota abundance (genus Catenibacterium id.2153) on Dihydrofolate reductase
with a threshold of 1e−05
MR Test
Inverse variance weighted (fixed effects)
Inverse variance weighted (multiplicative random effects)
MR Egger
MR RAPS
Weighted median
Weighted mode
0.05
0.00
−0.05
0.09
0.12
0.15
0.18
SNP effect on Dihydrofolate reductase
SNP effect on Gut microbiota abundance (genus Catenibacterium id.2153)

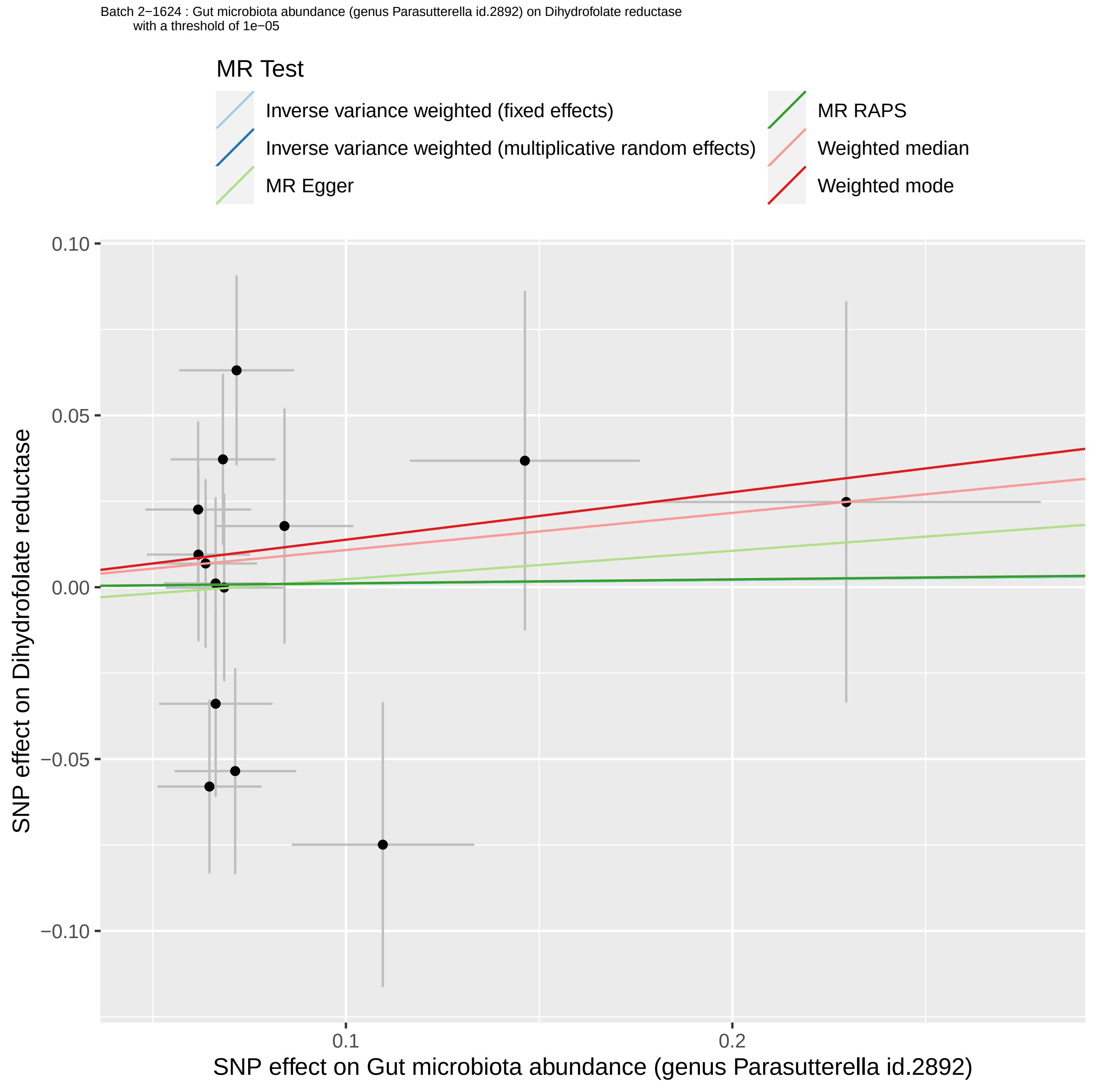
Batch 2−1624 : Gut microbiota abundance (genus Parasutterella id.2892) on Dihydrofolate reductase
with a threshold of 1e−05
MR Test
Inverse variance weighted (fixed effects)
Inverse variance weighted (multiplicative random effects)
MR Egger
MR RAPS
Weighted median
Weighted mode
0.10
0.05
0.00
−0.05
−0.10
0.1
0.2
SNP effect on Dihydrofolate reductase
SNP effect on Gut microbiota abundance (genus Parasutterella id.2892)

Batch 2−1625 : Gut microbiota abundance (genus Catenibacterium id.2153) on Desert hedgehog protein N−product
with a threshold of 1e−05

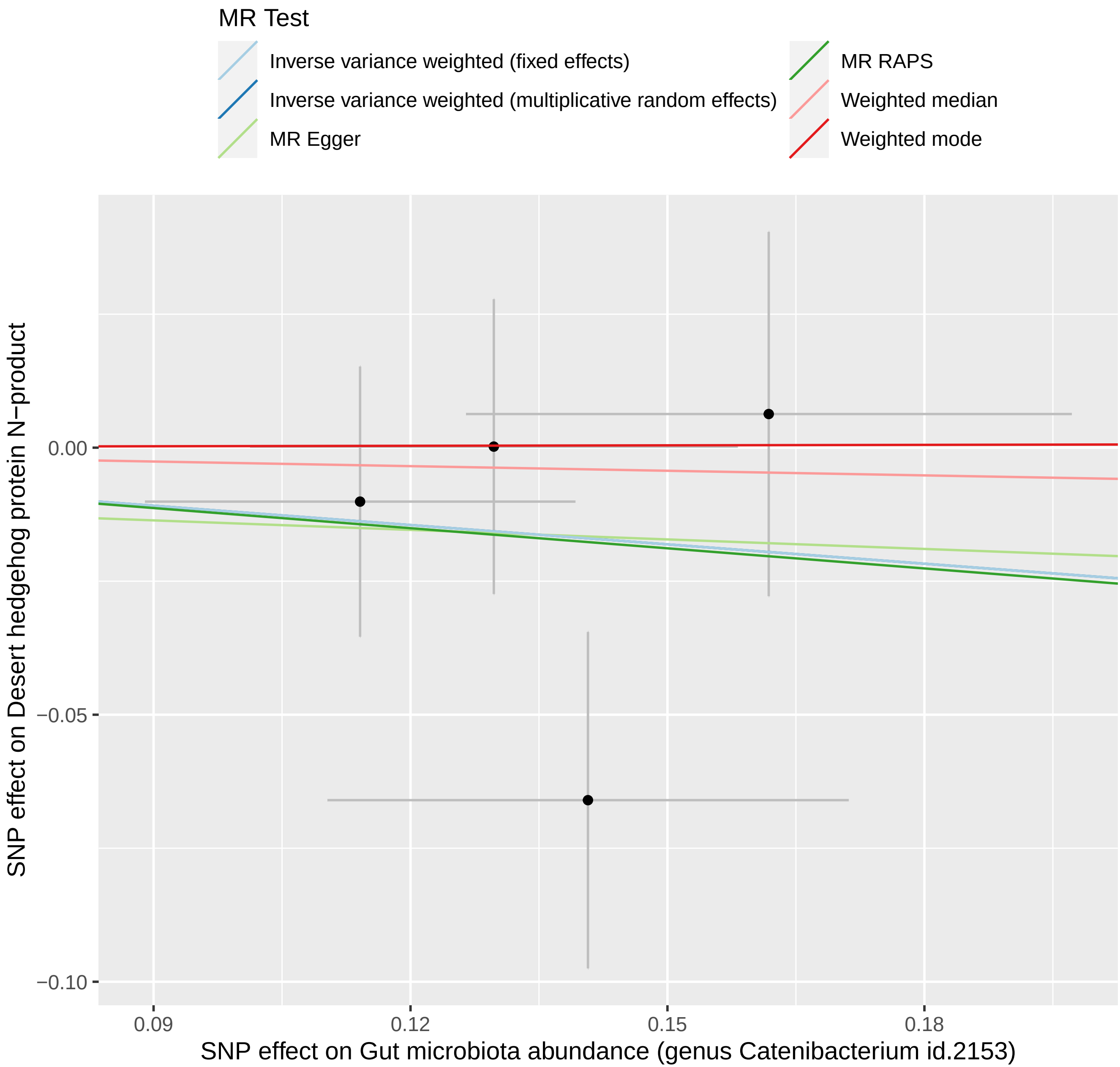

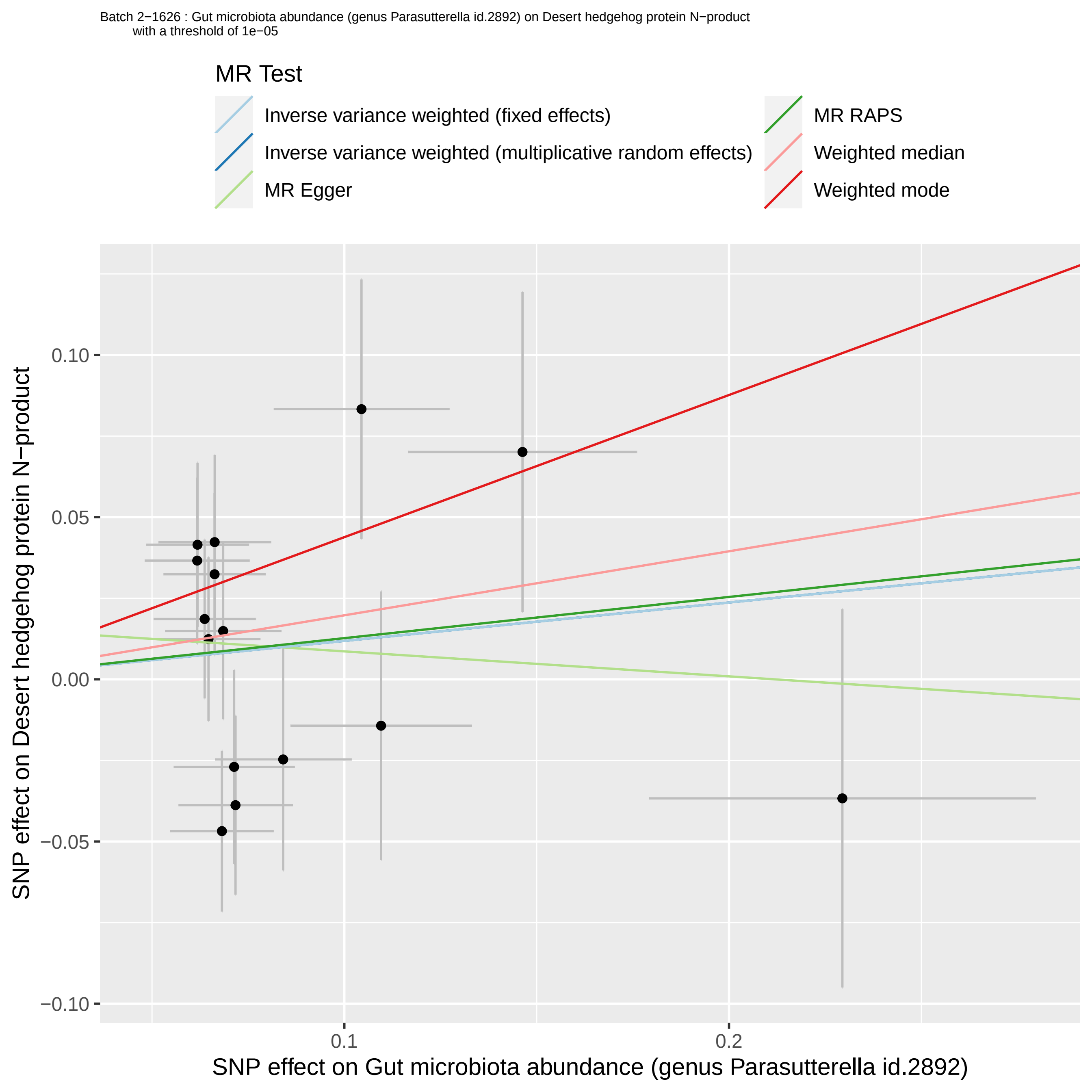
Batch 2−1626 : Gut microbiota abundance (genus Parasutterella id.2892) on Desert hedgehog protein N−product
with a threshold of 1e−05
MR Test
Inverse variance weighted (fixed effects)
Inverse variance weighted (multiplicative random effects)
MR Egger
MR RAPS
Weighted median
Weighted mode
0.10
0.05
0.00
−0.05
−0.10
0.1
0.2
SNP effect on Desert hedgehog protein N−product
SNP effect on Gut microbiota abundance (genus Parasutterella id.2892)

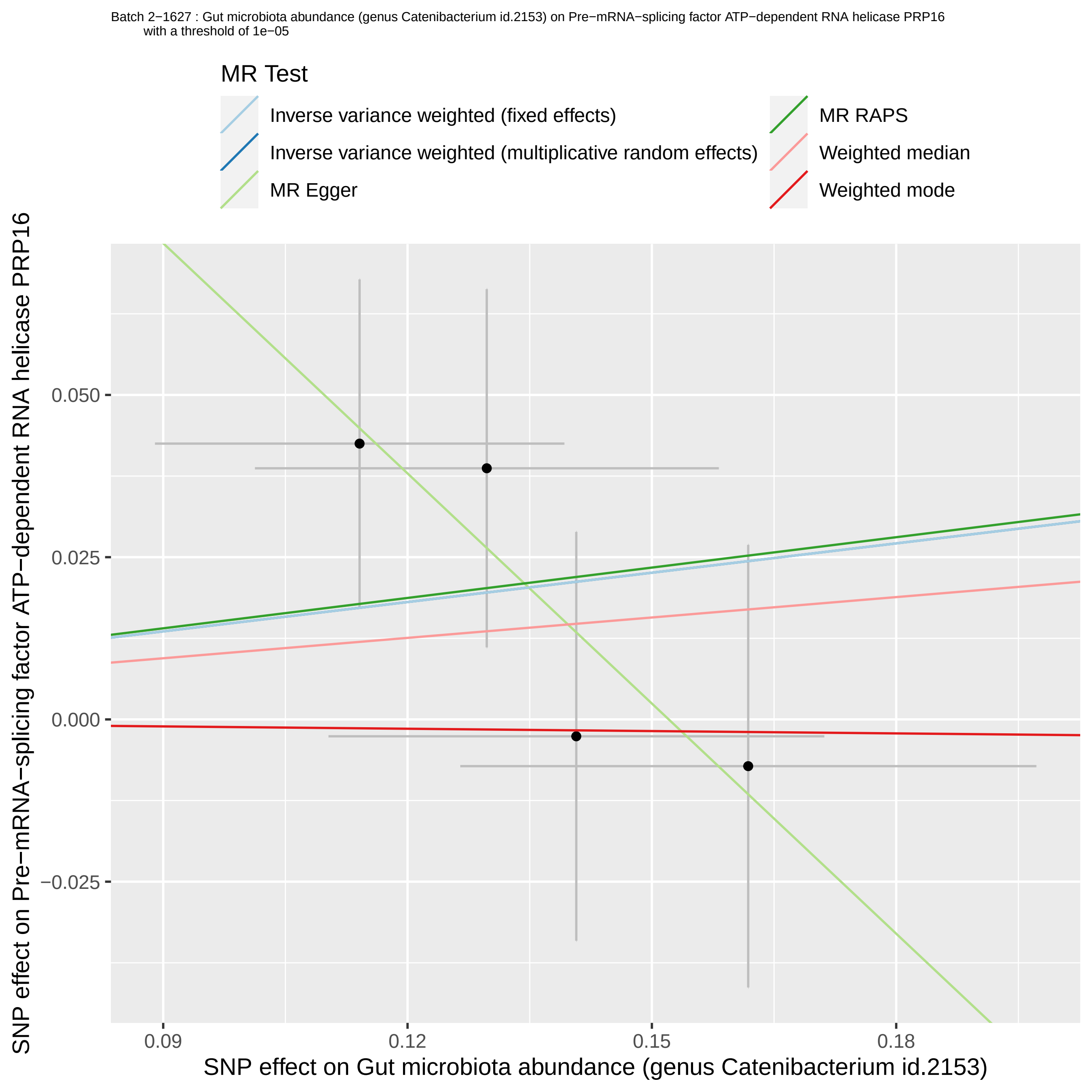

Batch 2−1627 : Gut microbiota abundance (genus Catenibacterium id.2153) on Pre−mRNA−splicing factor ATP−dependent RNA helicase PRP16 with a threshold of 1e−05
MR Test
Inverse variance weighted (fixed effects)
Inverse variance weighted (multiplicative random effects)
MR Egger
MR RAPS
Weighted median
Weighted mode
0.050
0.025
0.000
−0.025
0.09
0.12
0.15
0.18
SNP effect on Pre−mRNA−splicing factor ATP−dependent RNA helicase PRP16
SNP effect on Gut microbiota abundance (genus Catenibacterium id.2153)

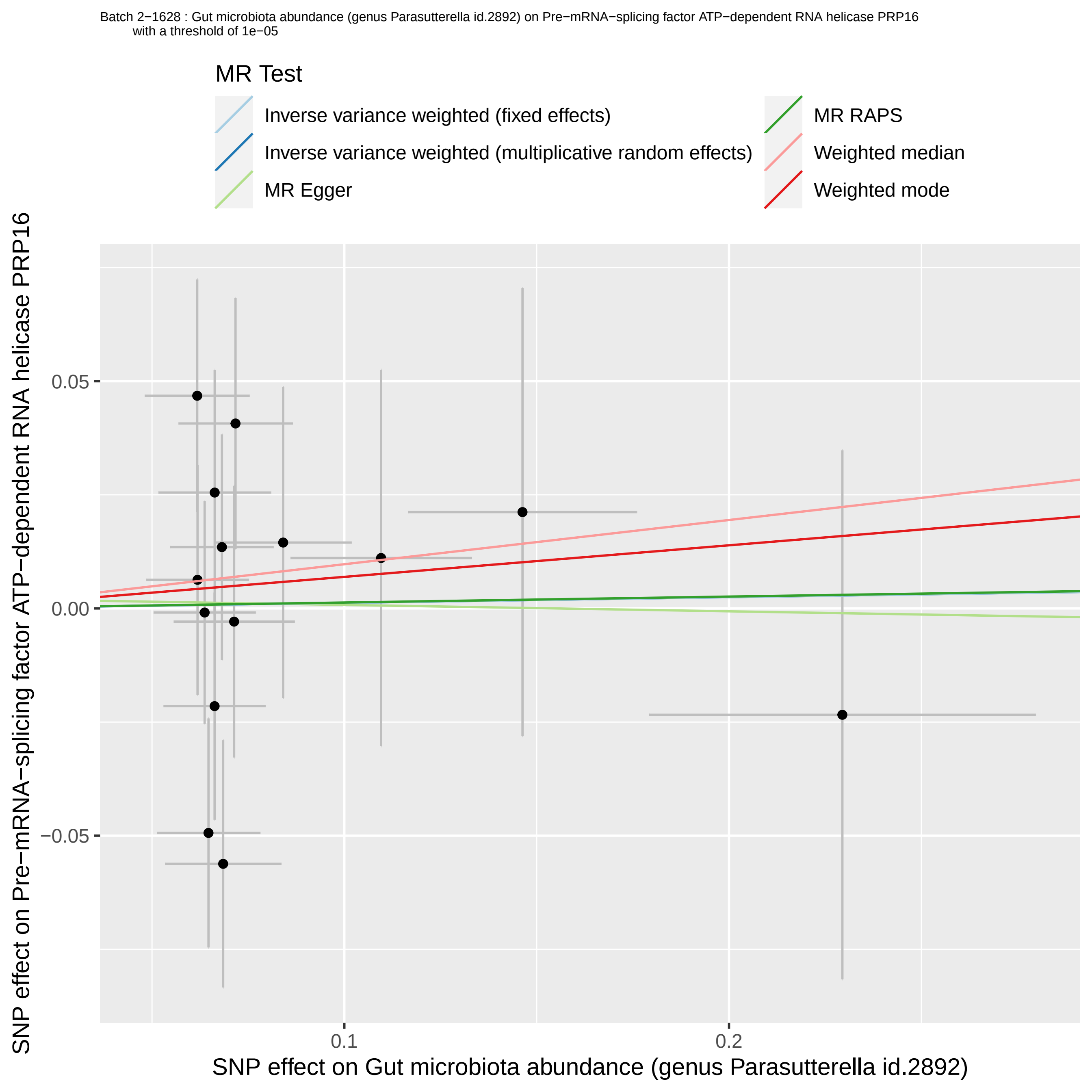

Batch 2−1628 : Gut microbiota abundance (genus Parasutterella id.2892) on Pre−mRNA−splicing factor ATP−dependent RNA helicase PRP16
with a threshold of 1e−05
MR Test
Inverse variance weighted (fixed effects)
Inverse variance weighted (multiplicative random effects)
MR Egger
MR RAPS
Weighted median
Weighted mode
0.05
0.00
−0.05
0.1
0.2
SNP effect on Pre−mRNA−splicing factor ATP−dependent RNA helicase PRP16
SNP effect on Gut microbiota abundance (genus Parasutterella id.2892)

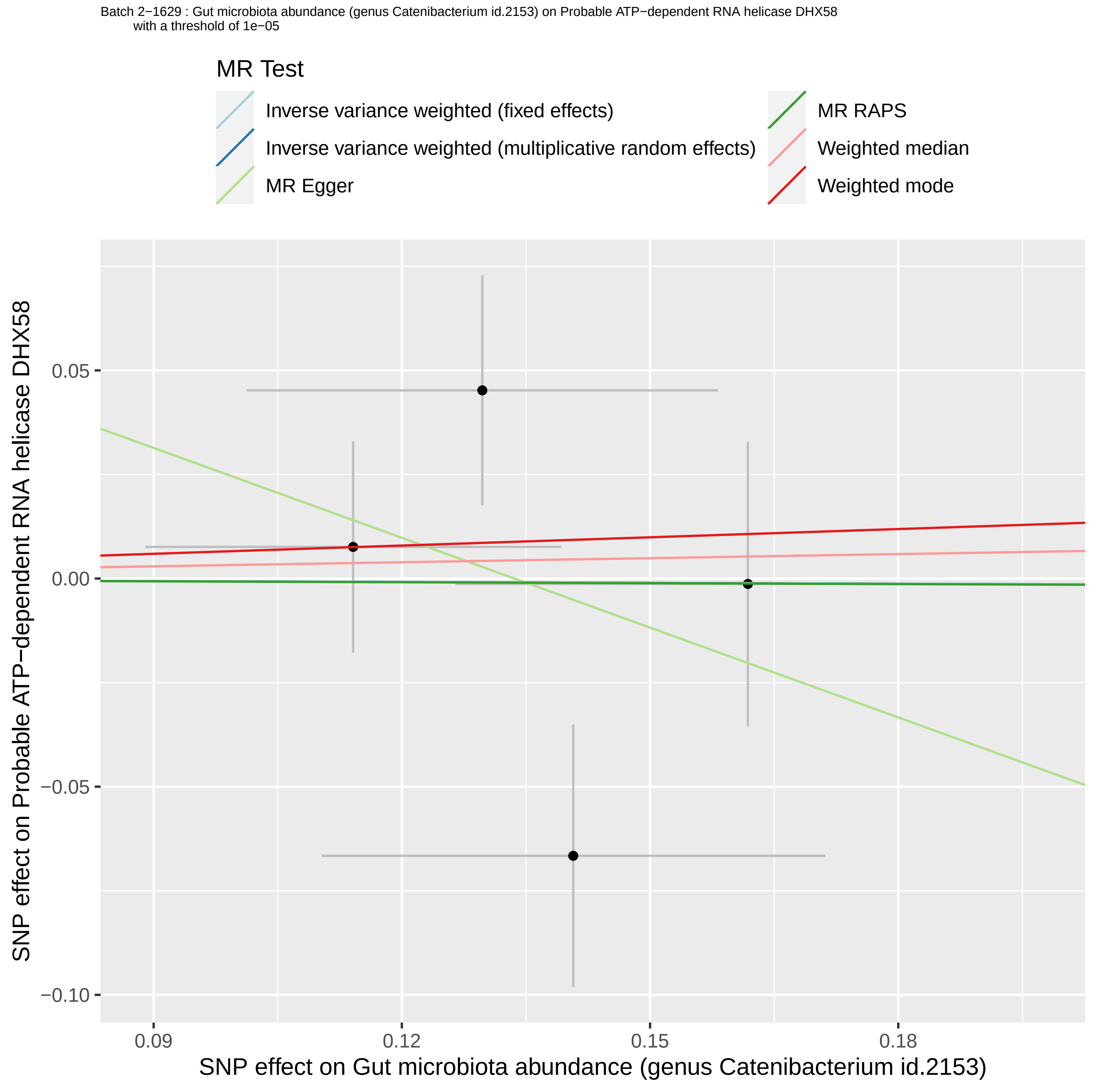

Batch 2−1629 : Gut microbiota abundance (genus Catenibacterium id.2153) on Probable ATP−dependent RNA helicase DHX58
with a threshold of 1e−05
MR Test
Inverse variance weighted (fixed effects)
Inverse variance weighted (multiplicative random effects)
MR Egger
MR RAPS
Weighted median
Weighted mode
0.05
0.00
−0.05
−0.10
0.09
0.12
0.15
0.18
SNP effect on Probable ATP−dependent RNA helicase DHX58
SNP effect on Gut microbiota abundance (genus Catenibacterium id.2153)

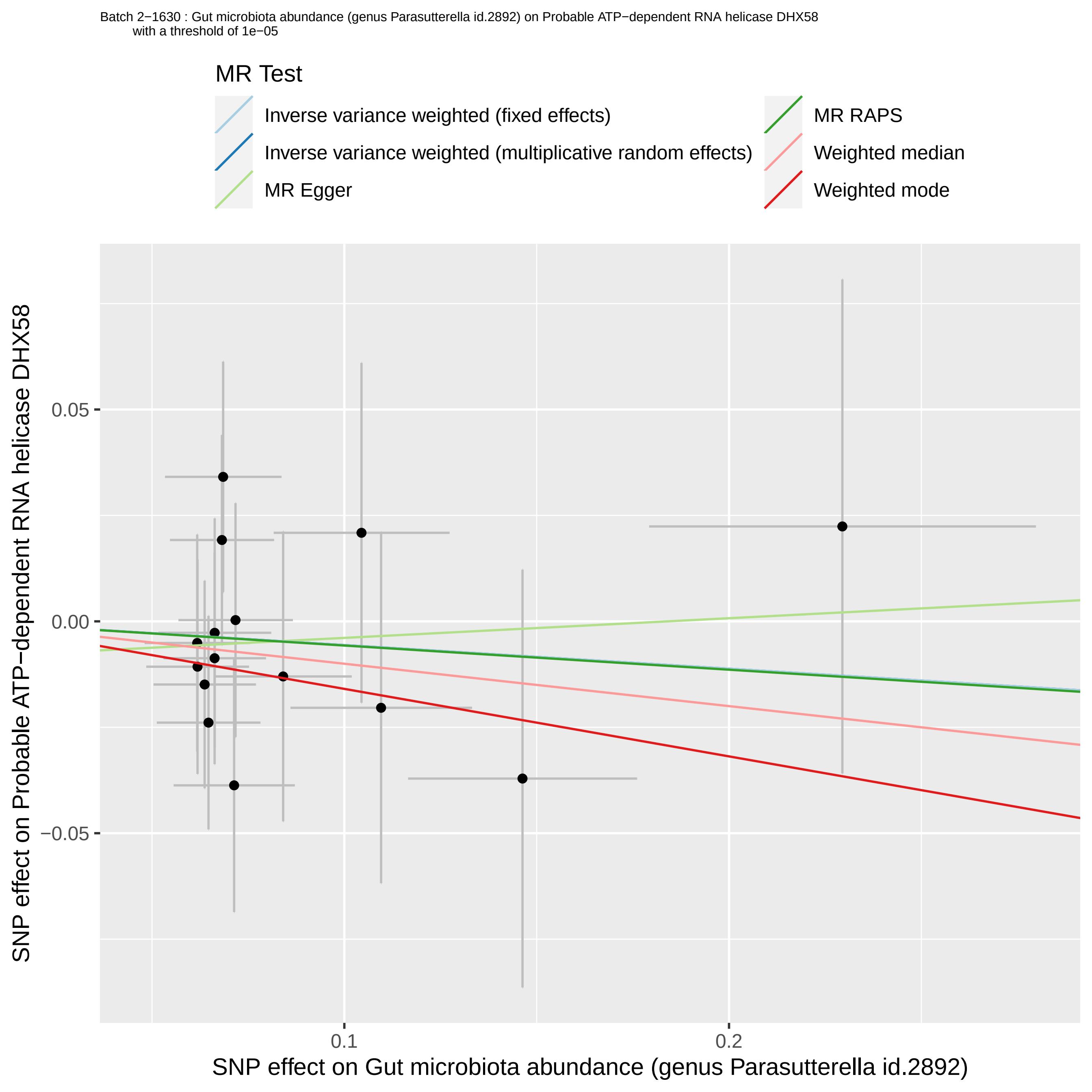

Batch 2−1630 : Gut microbiota abundance (genus Parasutterella id.2892) on Probable ATP−dependent RNA helicase DHX58
with a threshold of 1e−05
MR Test
Inverse variance weighted (fixed effects)
Inverse variance weighted (multiplicative random effects)
MR Egger
MR RAPS
Weighted median
Weighted mode
0.05
0.00
−0.05
0.1
0.2
SNP effect on Probable ATP−dependent RNA helicase DHX58
SNP effect on Gut microbiota abundance (genus Parasutterella id.2892)

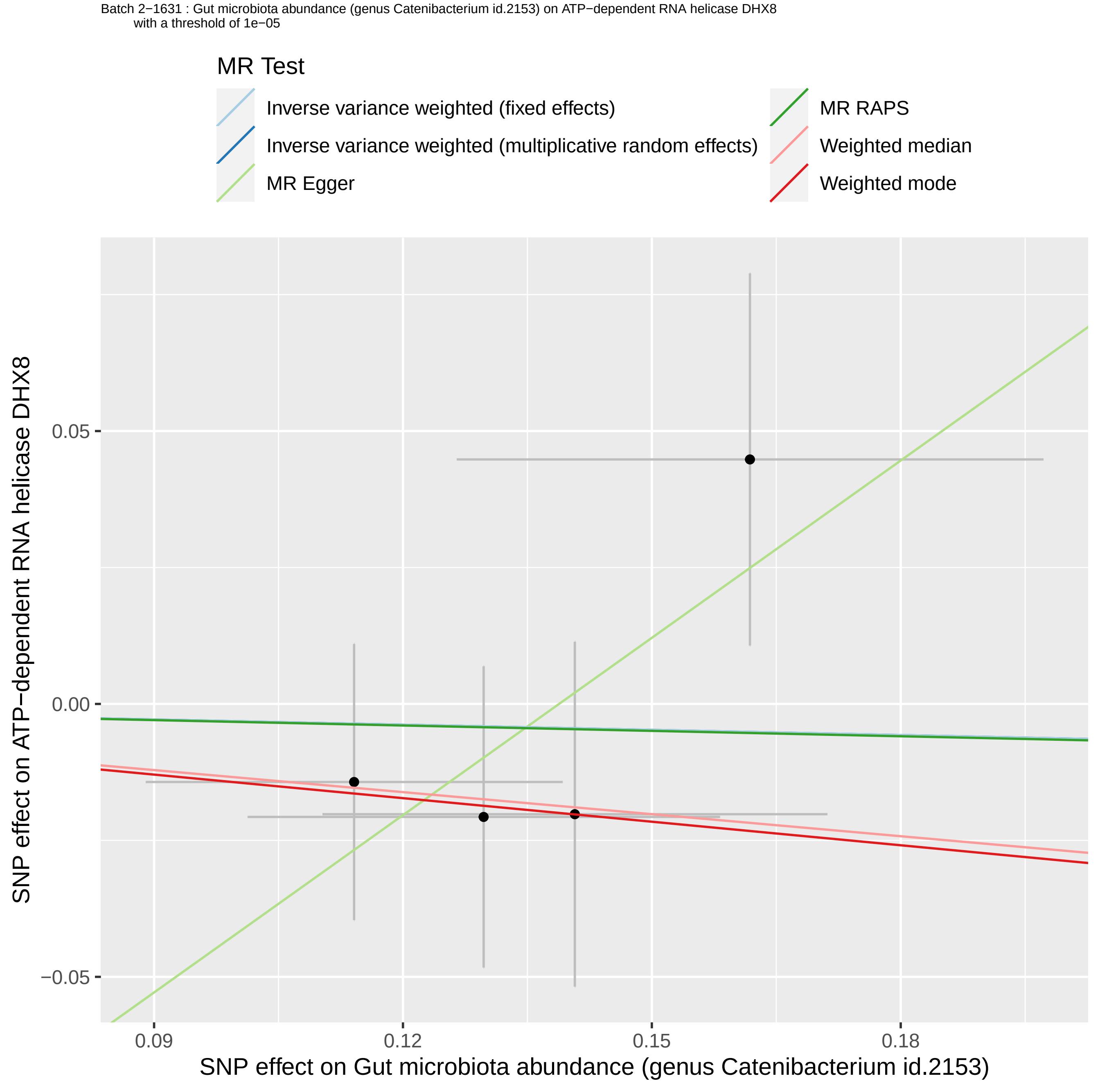
Batch 2−1631 : Gut microbiota abundance (genus Catenibacterium id.2153) on ATP−dependent RNA helicase DHX8
with a threshold of 1e−05
MR Test
Inverse variance weighted (fixed effects)
Inverse variance weighted (multiplicative random effects)
MR Egger
MR RAPS
Weighted median
Weighted mode
0.05
0.00
−0.05
0.09
0.12
0.15
0.18
SNP effect on ATP−dependent RNA helicase DHX8
SNP effect on Gut microbiota abundance (genus Catenibacterium id.2153)

Batch 2−1632 : Gut microbiota abundance (genus Parasutterella id.2892) on ATP−dependent RNA helicase DHX8
with a threshold of 1e−05

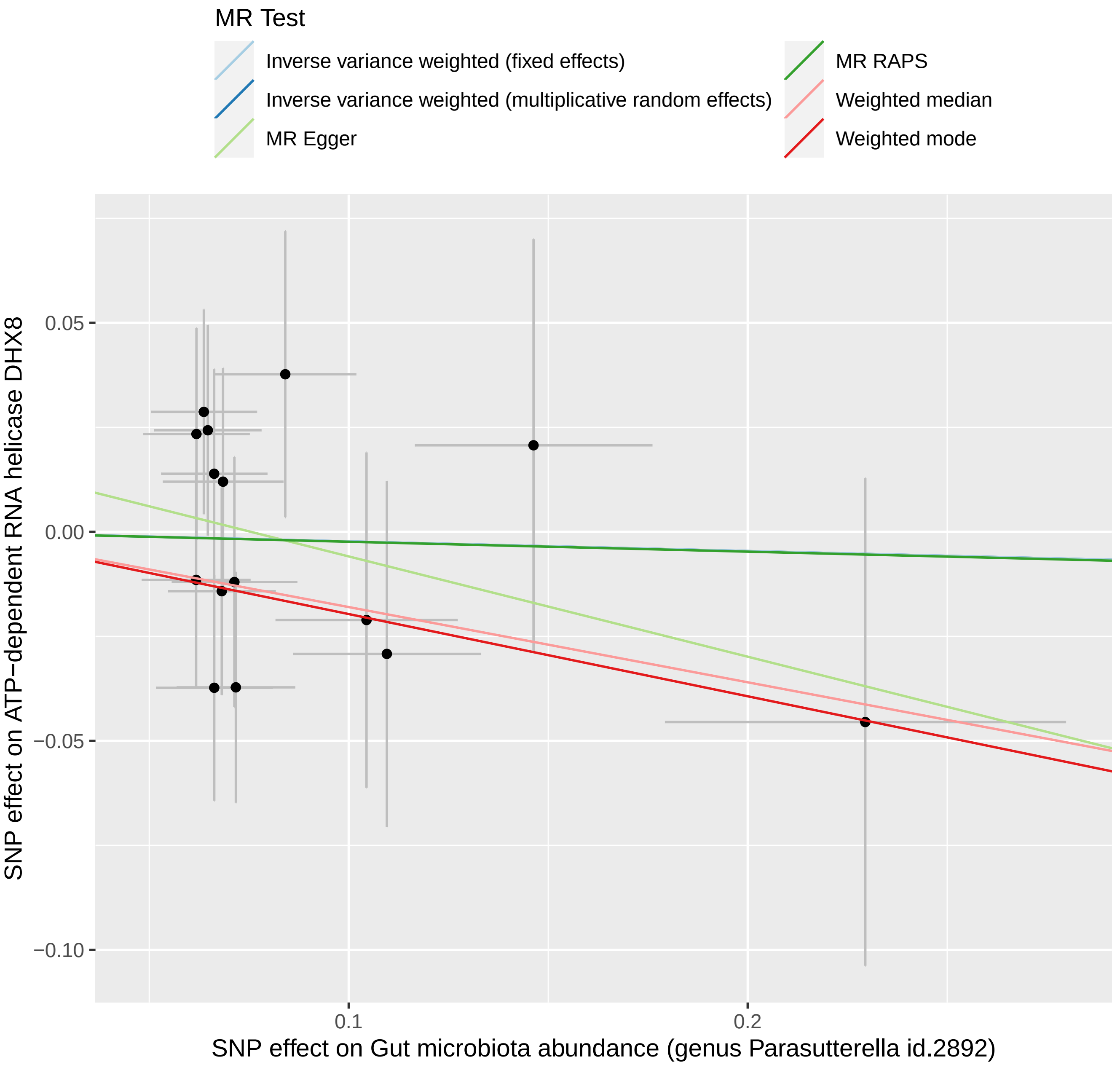

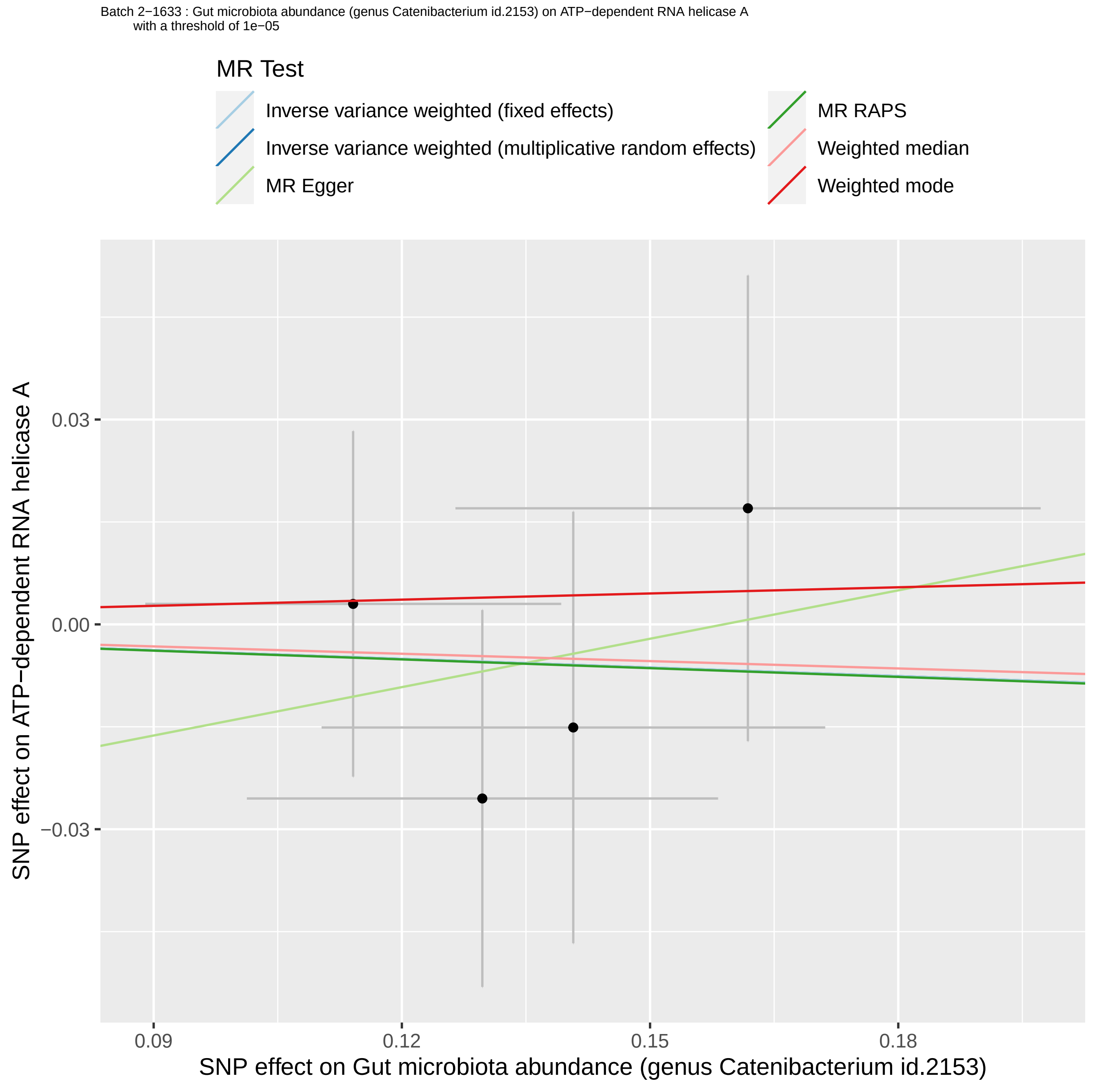

Batch 2−1633 : Gut microbiota abundance (genus Catenibacterium id.2153) on ATP−dependent RNA helicase A
with a threshold of 1e−05
MR Test
Inverse variance weighted (fixed effects)
Inverse variance weighted (multiplicative random effects)
MR Egger
MR RAPS
Weighted median
Weighted mode
0.03
0.00
−0.03
0.09
0.12
0.15
0.18
SNP effect on ATP−dependent RNA helicase A
SNP effect on Gut microbiota abundance (genus Catenibacterium id.2153)

Batch 2−1634 : Gut microbiota abundance (genus Parasutterella id.2892) on ATP−dependent RNA helicase A
with a threshold of 1e−05

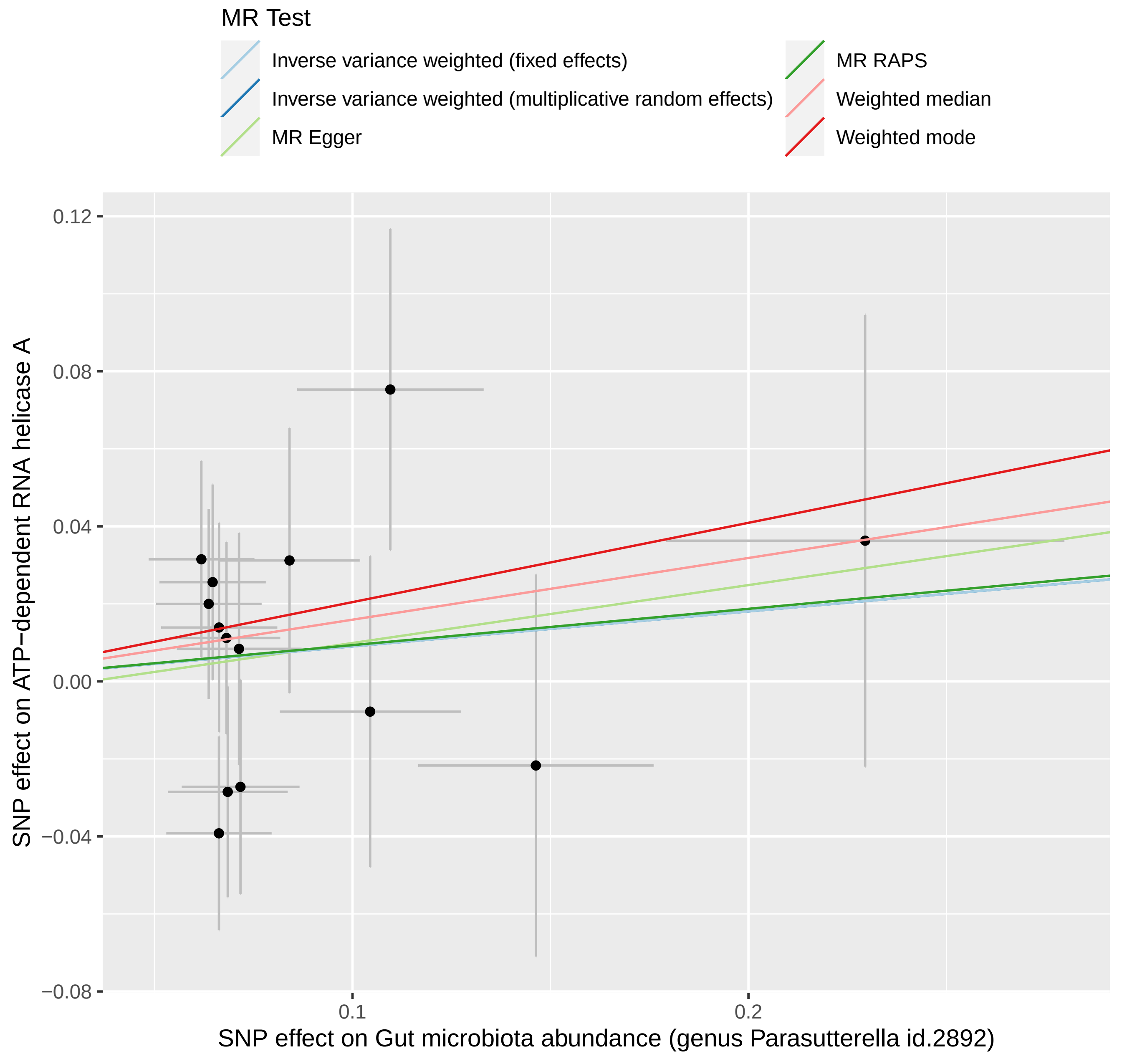

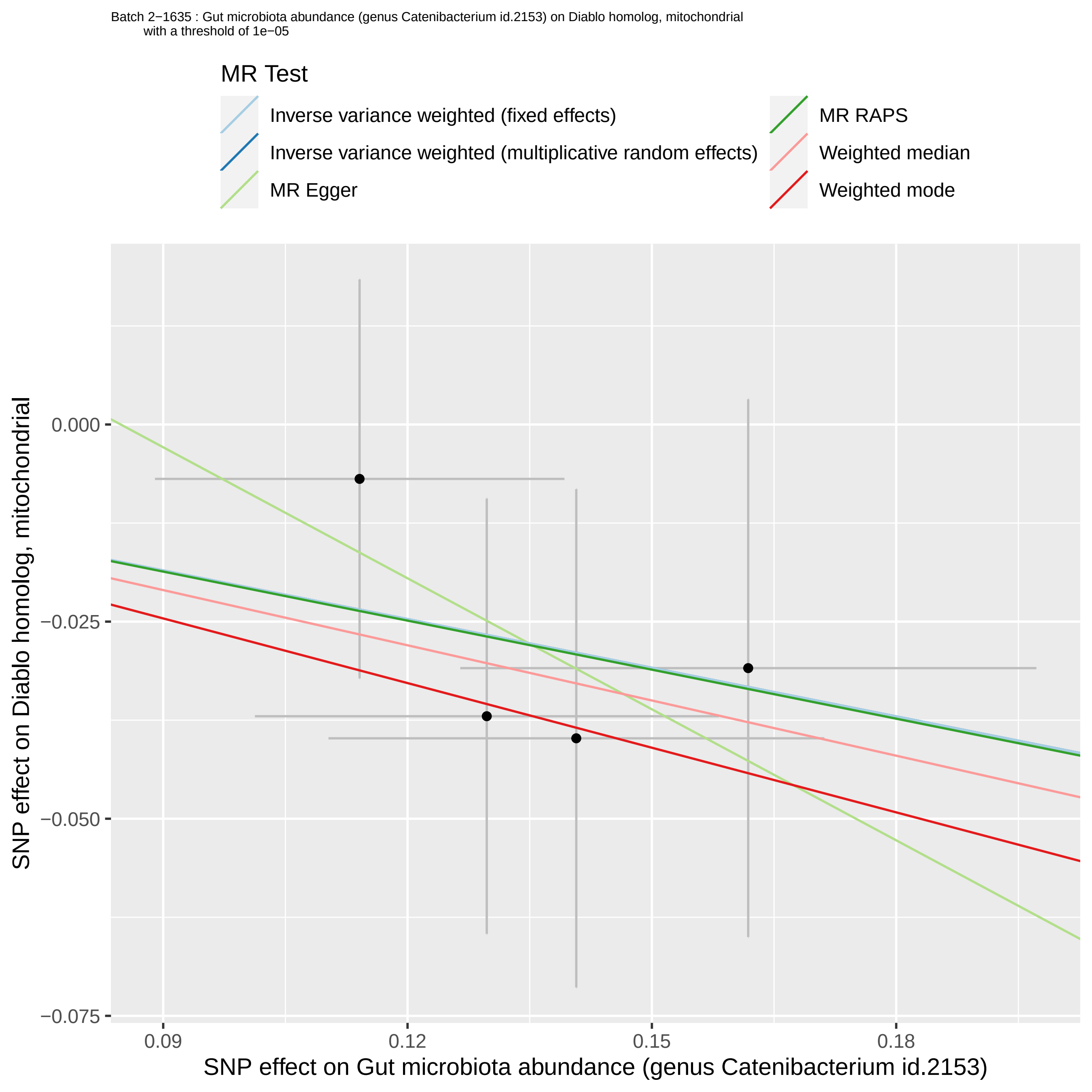

Batch 2−1635 : Gut microbiota abundance (genus Catenibacterium id.2153) on Diablo homolog, mitochondrial
with a threshold of 1e−05
MR Test
Inverse variance weighted (fixed effects)
Inverse variance weighted (multiplicative random effects)
MR Egger
MR RAPS
Weighted median
Weighted mode
SNP effect on Diablo homolog, mitochondrial
0.000
−0.025
−0.050
−0.075
0.09
0.12
0.15
0.18
SNP effect on Gut microbiota abundance (genus Catenibacterium id.2153)

Batch 2−1636 : Gut microbiota abundance (genus Parasutterella id.2892) on Diablo homolog, mitochondrial
with a threshold of 1e−05

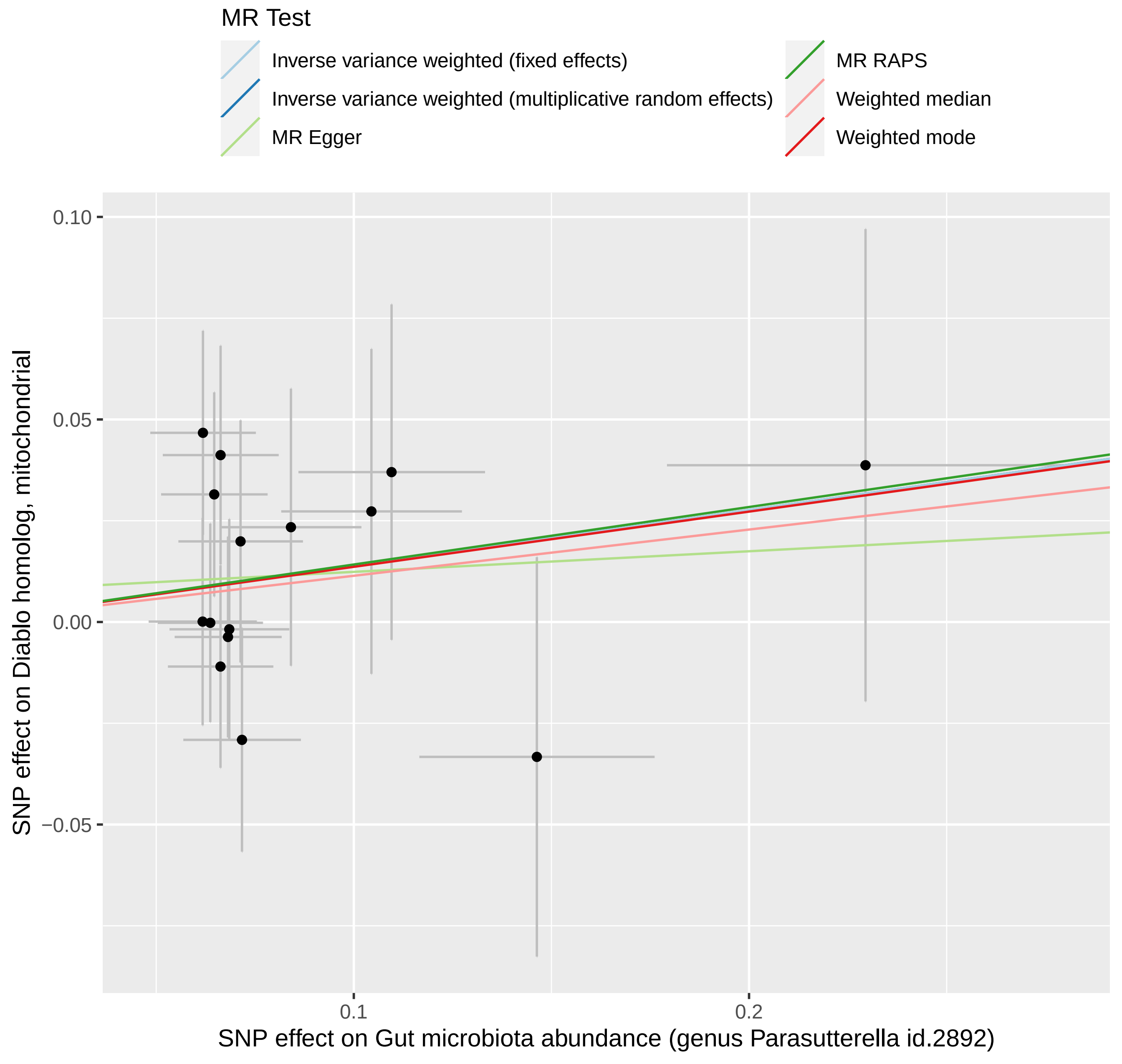

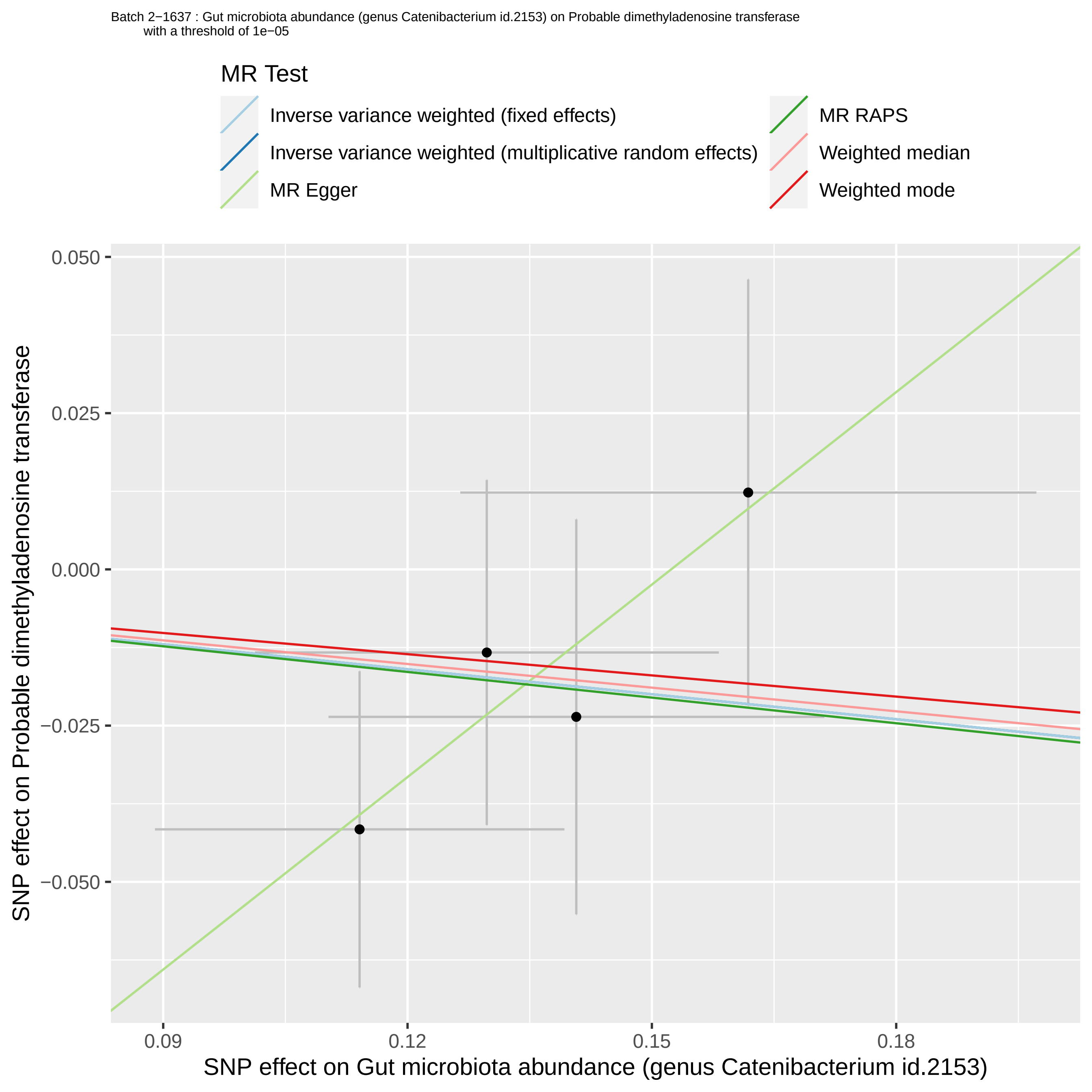
Batch 2−1637 : Gut microbiota abundance (genus Catenibacterium id.2153) on Probable dimethyladenosine transferase
with a threshold of 1e−05
MR Test
Inverse variance weighted (fixed effects)
Inverse variance weighted (multiplicative random effects)
MR Egger
MR RAPS
Weighted median
Weighted mode
0.050
0.025
0.000
−0.025
−0.050
0.09
0.12
0.15
0.18
SNP effect on Probable dimethyladenosine transferase
SNP effect on Gut microbiota abundance (genus Catenibacterium id.2153)

Batch 2−1638 : Gut microbiota abundance (genus Parasutterella id.2892) on Probable dimethyladenosine transferase
with a threshold of 1e−05

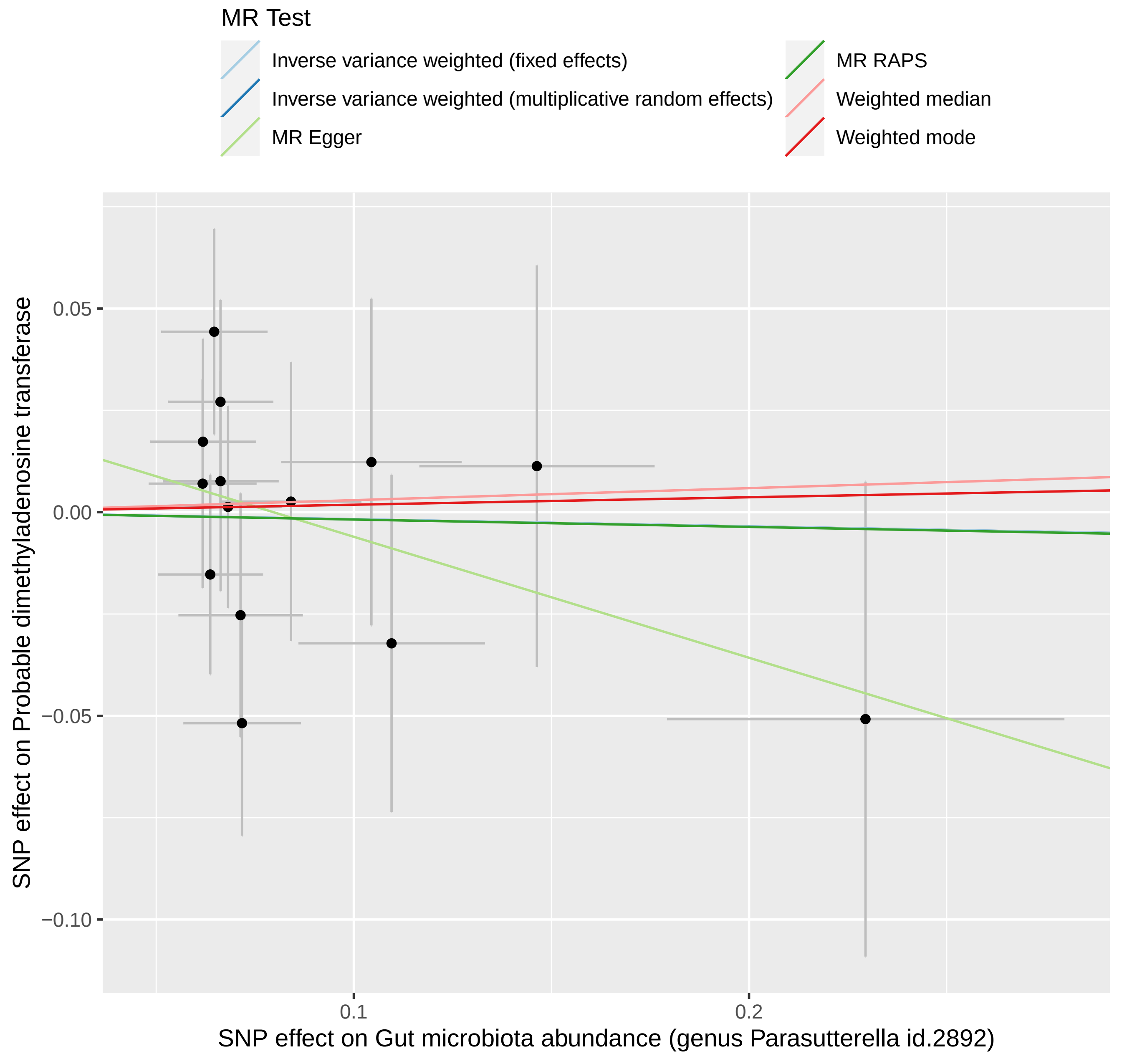

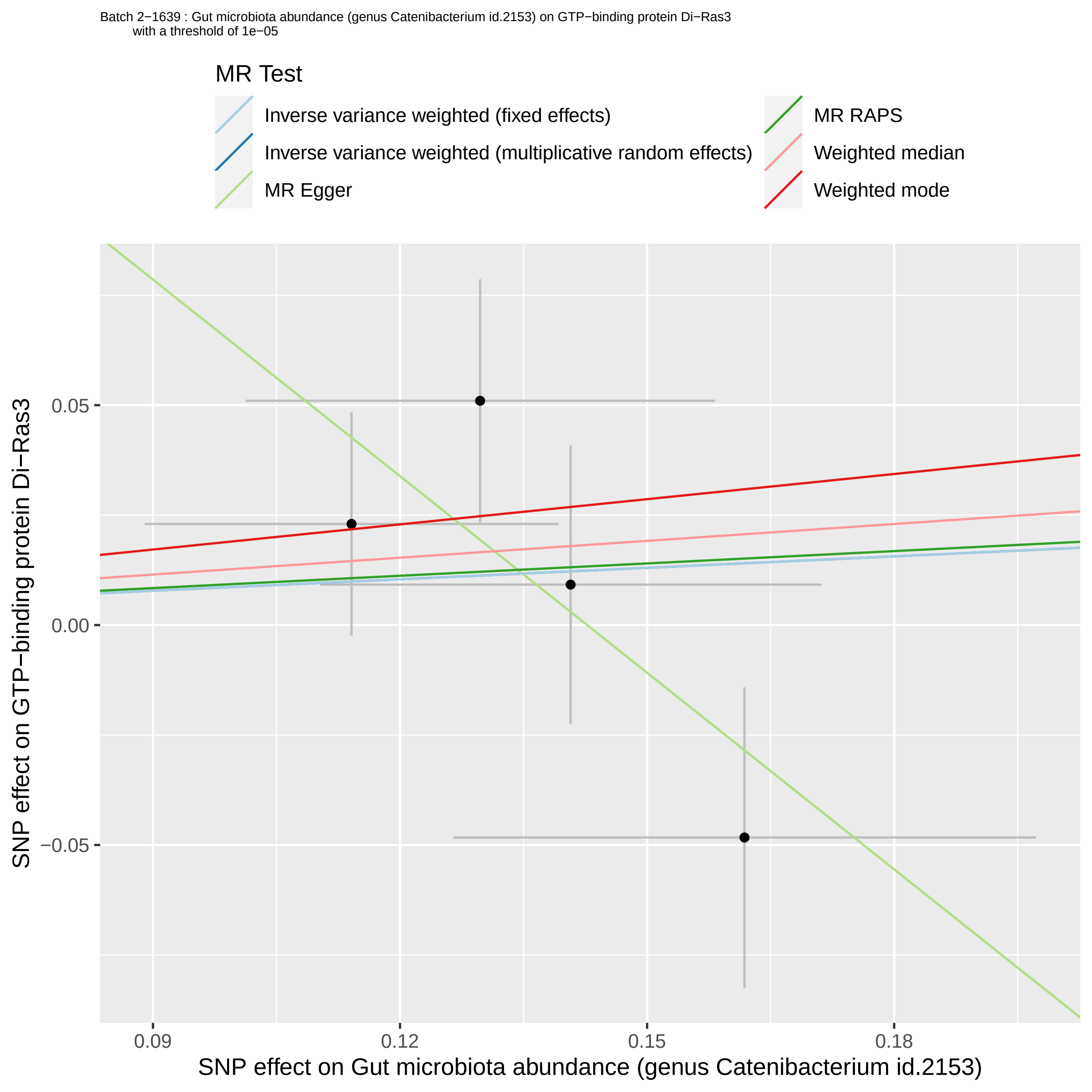
Batch 2−1639 : Gut microbiota abundance (genus Catenibacterium id.2153) on GTP−binding protein Di−Ras3
with a threshold of 1e−05
MR Test
Inverse variance weighted (fixed effects)
Inverse variance weighted (multiplicative random effects)
MR Egger
MR RAPS
Weighted median
Weighted mode
0.05
0.00
−0.05
0.09
0.12
0.15
0.18
SNP effect on GTP−binding protein Di−Ras3
SNP effect on Gut microbiota abundance (genus Catenibacterium id.2153)

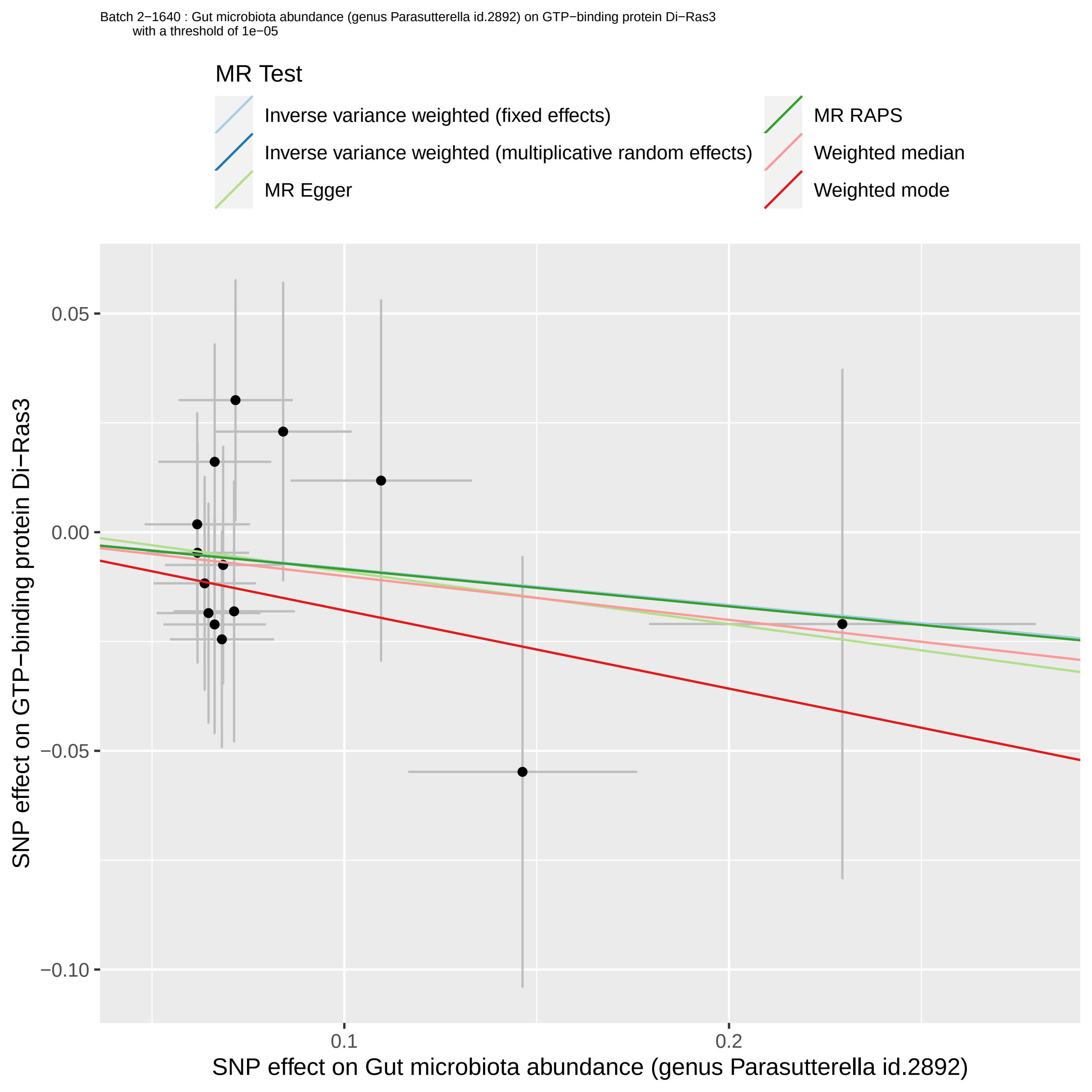
Batch 2−1640 : Gut microbiota abundance (genus Parasutterella id.2892) on GTP−binding protein Di−Ras3
with a threshold of 1e−05
MR Test
Inverse variance weighted (fixed effects)
Inverse variance weighted (multiplicative random effects)
MR Egger
MR RAPS
Weighted median
Weighted mode
0.05
0.00
−0.05
−0.10
0.1
0.2
SNP effect on GTP−binding protein Di−Ras3
SNP effect on Gut microbiota abundance (genus Parasutterella id.2892)

Batch 2−1641 : Gut microbiota abundance (genus Catenibacterium id.2153) on Dickkopf−related protein 1
with a threshold of 1e−05

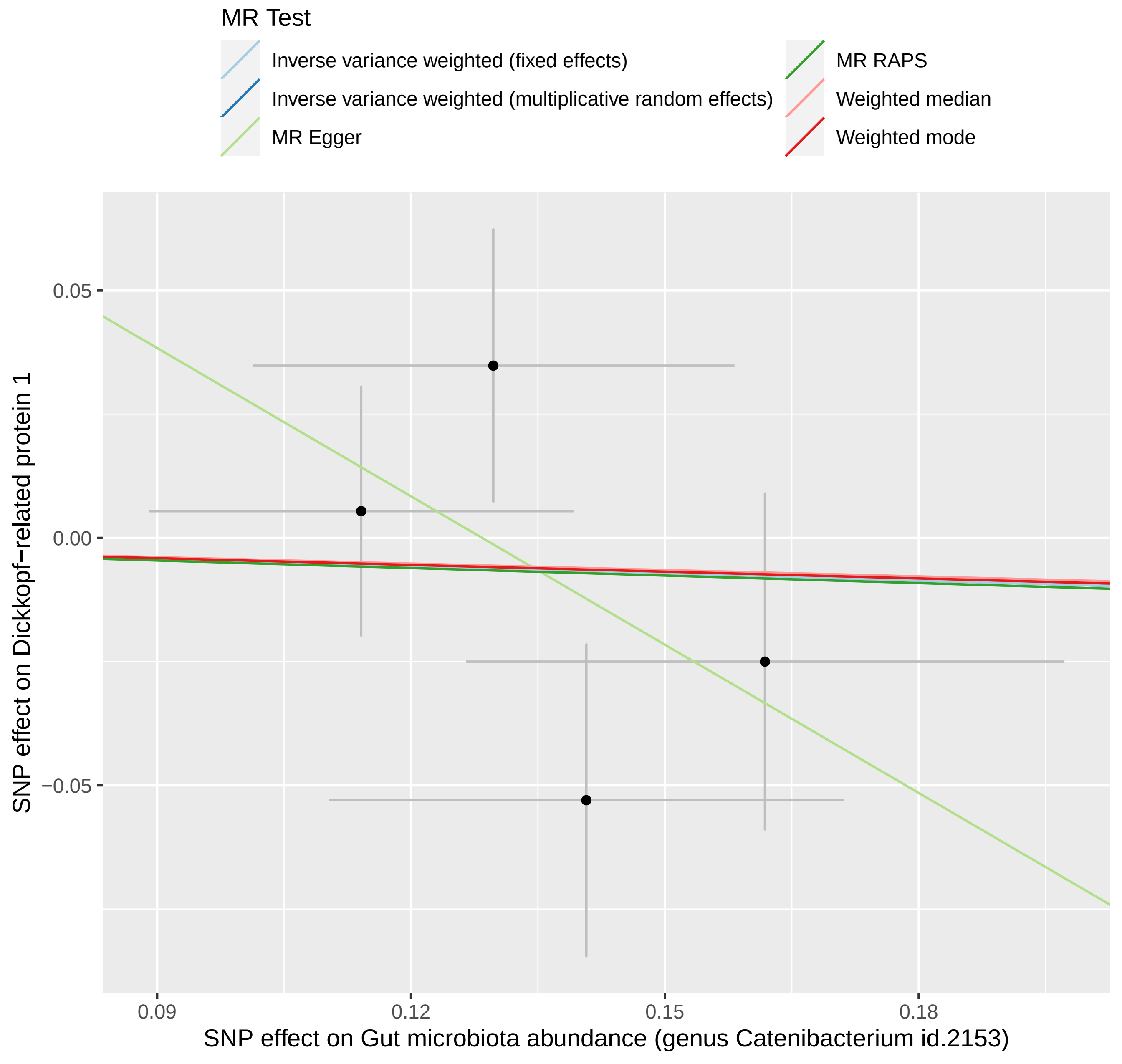

Batch 2−1642 : Gut microbiota abundance (genus Parasutterella id.2892) on Dickkopf−related protein 1
with a threshold of 1e−05

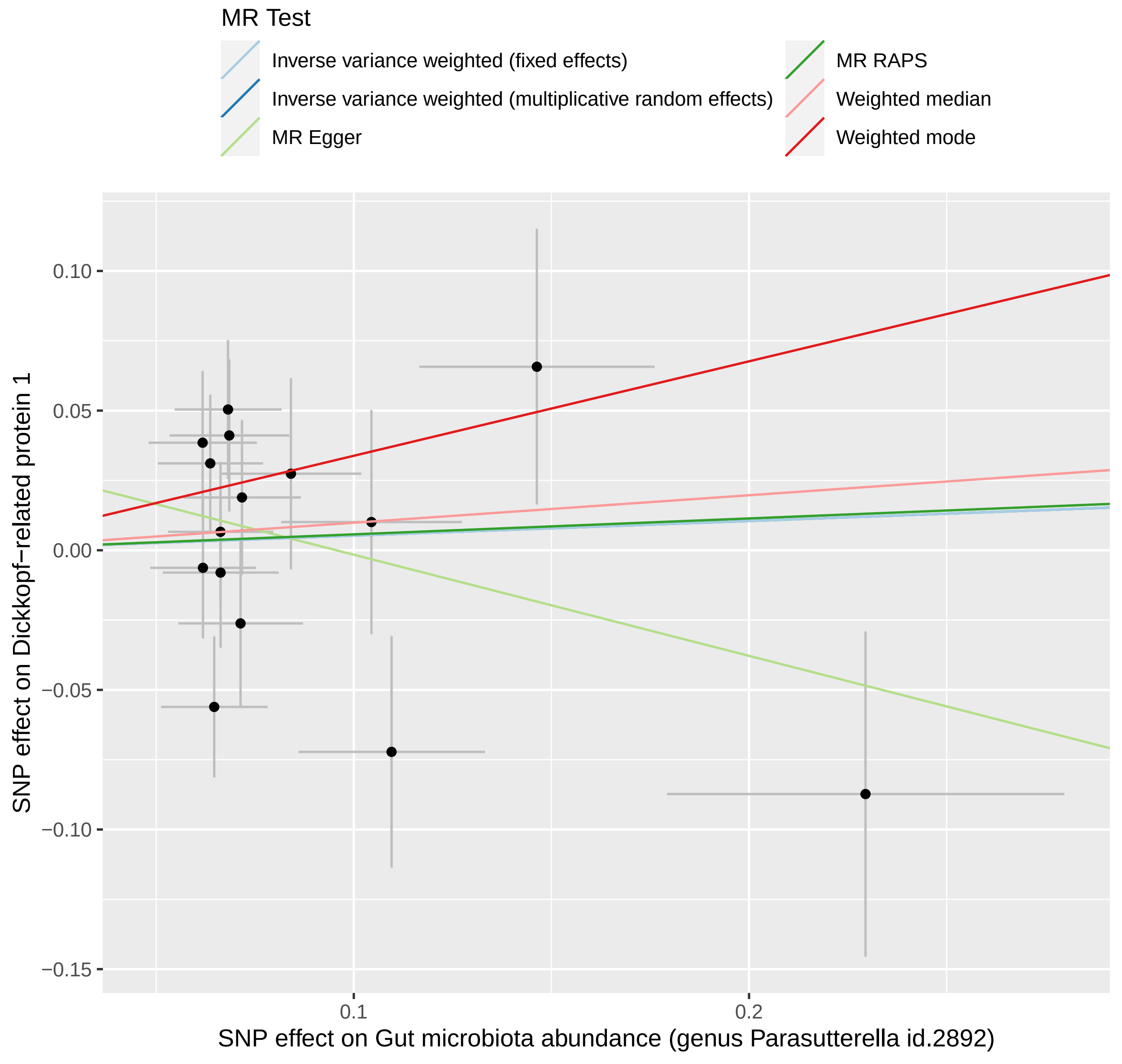

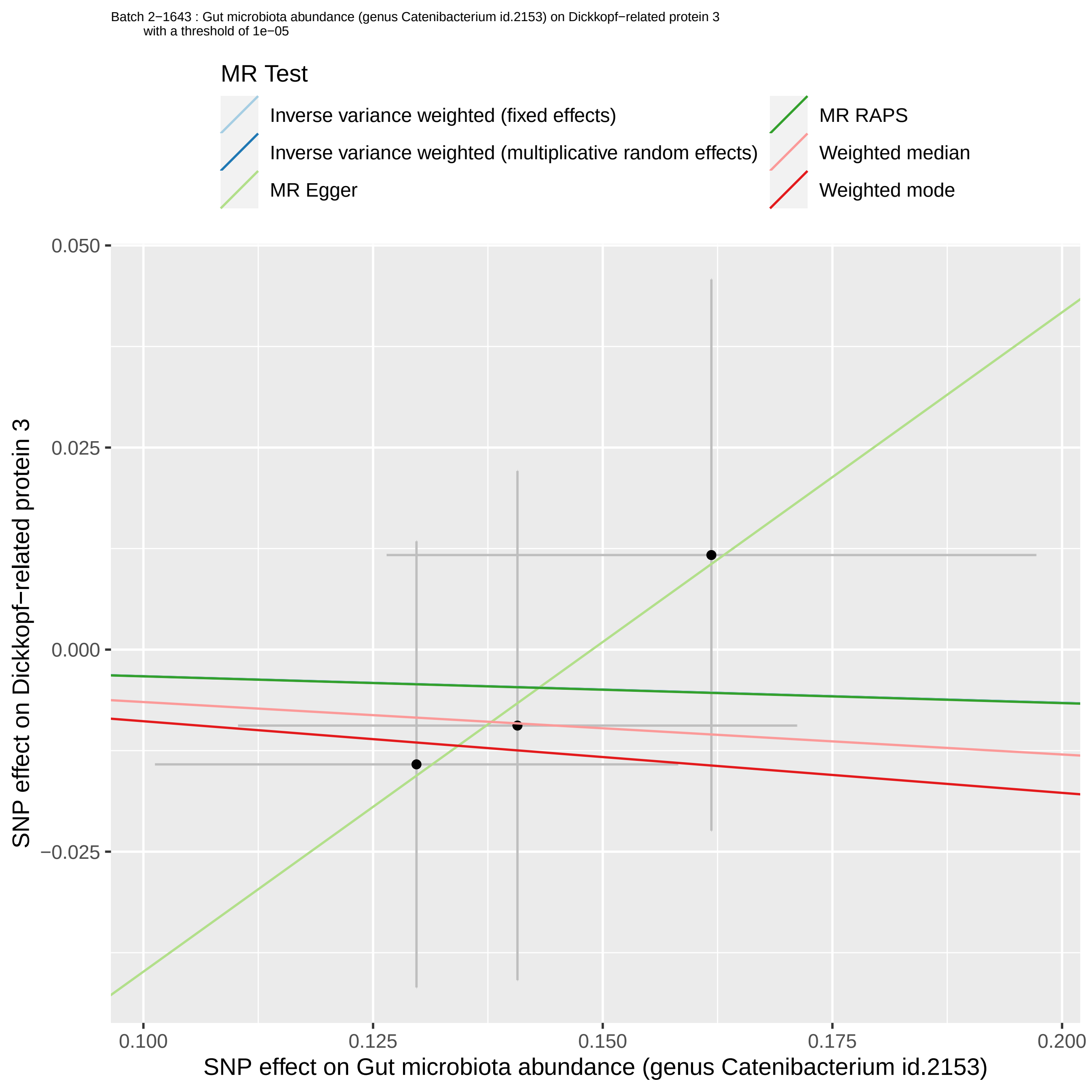
Batch 2−1643 : Gut microbiota abundance (genus Catenibacterium id.2153) on Dickkopf−related protein 3
with a threshold of 1e−05
MR Test
Inverse variance weighted (fixed effects)
Inverse variance weighted (multiplicative random effects)
MR Egger
MR RAPS
Weighted median
Weighted mode
0.050
0.025
0.000
−0.025
0.100
0.125
0.150
0.175
0.200
SNP effect on Dickkopf−related protein 3
SNP effect on Gut microbiota abundance (genus Catenibacterium id.2153)

Batch 2−1644 : Gut microbiota abundance (genus Parasutterella id.2892) on Dickkopf−related protein 3
with a threshold of 1e−05

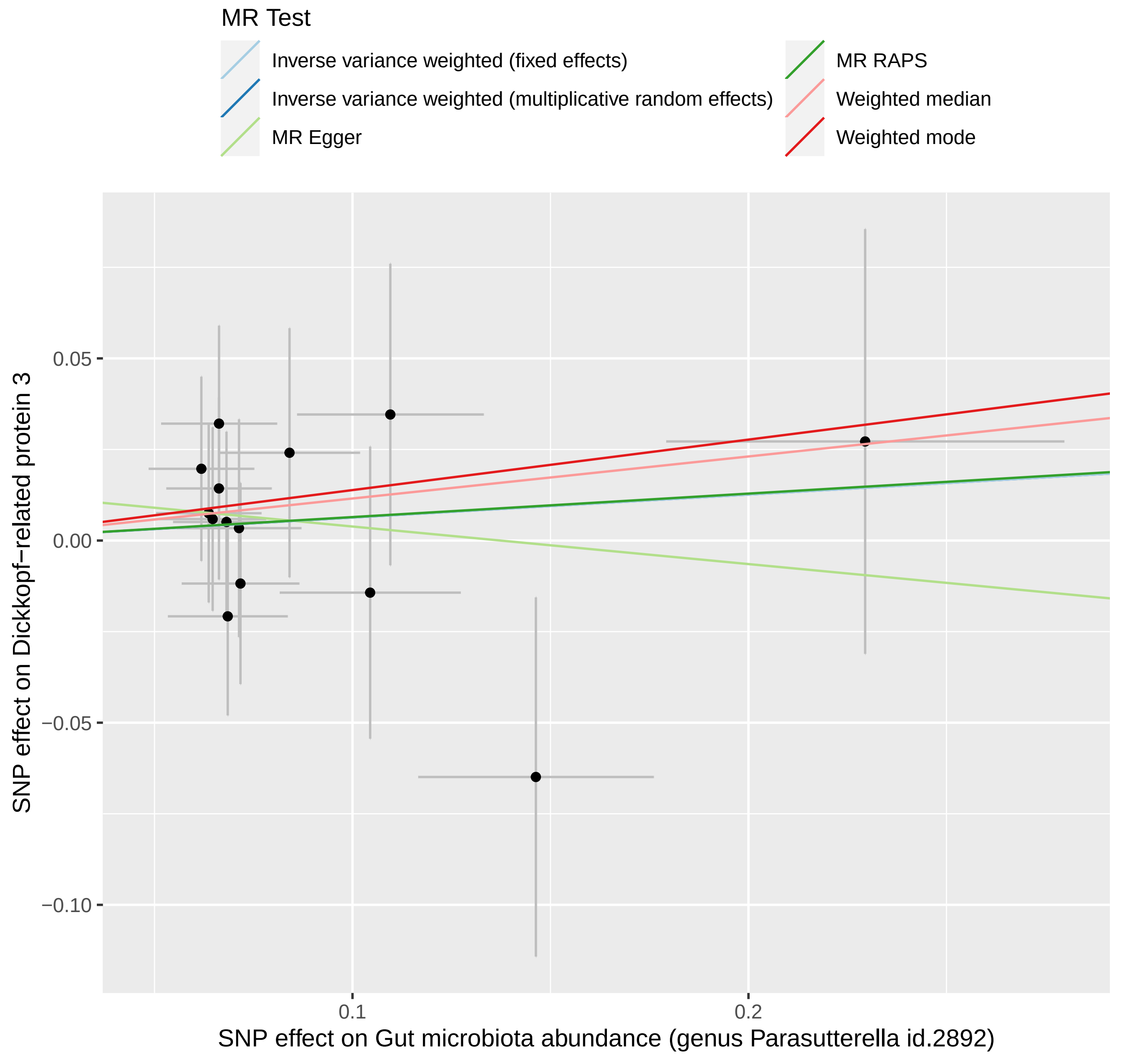

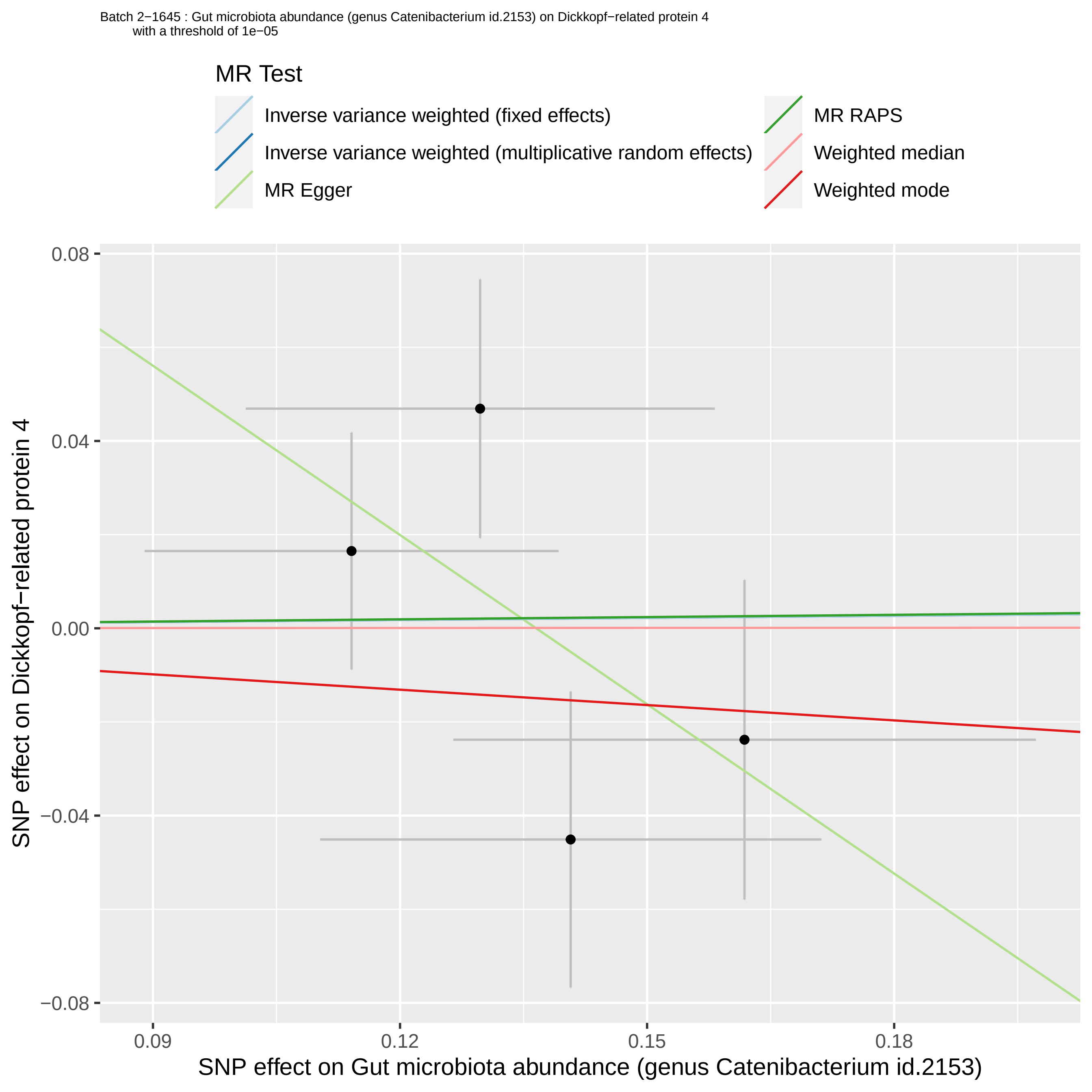
Batch 2−1645 : Gut microbiota abundance (genus Catenibacterium id.2153) on Dickkopf−related protein 4
with a threshold of 1e−05
MR Test
Inverse variance weighted (fixed effects)
Inverse variance weighted (multiplicative random effects)
MR Egger
MR RAPS
Weighted median
Weighted mode
0.08
0.04
0.00
−0.04
−0.08
0.09
0.12
0.15
0.18
SNP effect on Dickkopf−related protein 4
SNP effect on Gut microbiota abundance (genus Catenibacterium id.2153)

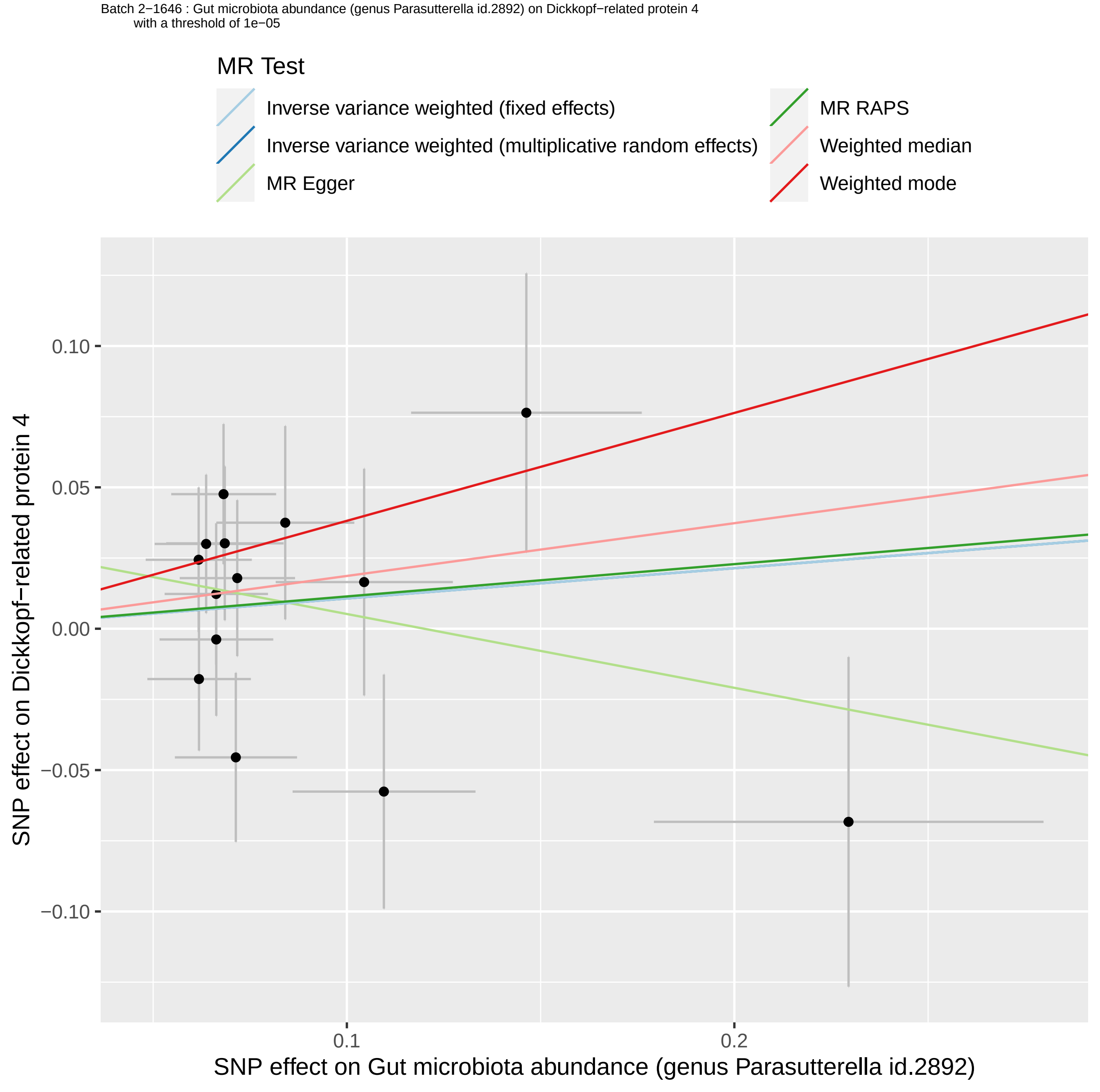
Batch 2−1646 : Gut microbiota abundance (genus Parasutterella id.2892) on Dickkopf−related protein 4
with a threshold of 1e−05
MR Test
Inverse variance weighted (fixed effects)
Inverse variance weighted (multiplicative random effects)
MR Egger
MR RAPS
Weighted median
Weighted mode
0.10
0.05
0.00
−0.05
−0.10
SNP effect on Dickkopf−related protein 4
0.1
0.2
SNP effect on Gut microbiota abundance (genus Parasutterella id.2892)

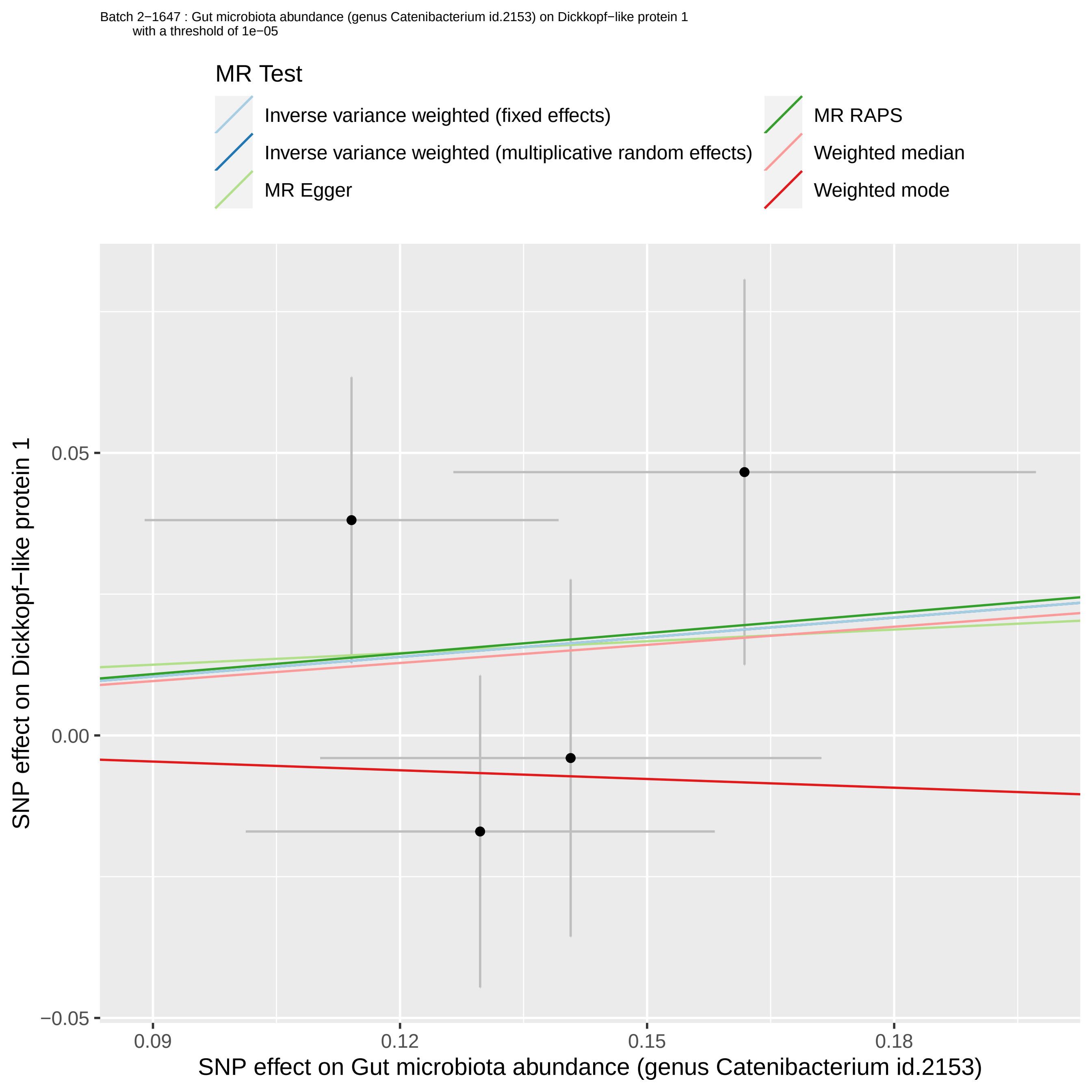
Batch 2−1647 : Gut microbiota abundance (genus Catenibacterium id.2153) on Dickkopf−like protein 1
with a threshold of 1e−05
MR Test
Inverse variance weighted (fixed effects)
Inverse variance weighted (multiplicative random effects)
MR Egger
MR RAPS
Weighted median
Weighted mode
0.05
0.00
−0.05
0.09
0.12
0.15
0.18
SNP effect on Dickkopf−like protein 1
SNP effect on Gut microbiota abundance (genus Catenibacterium id.2153)

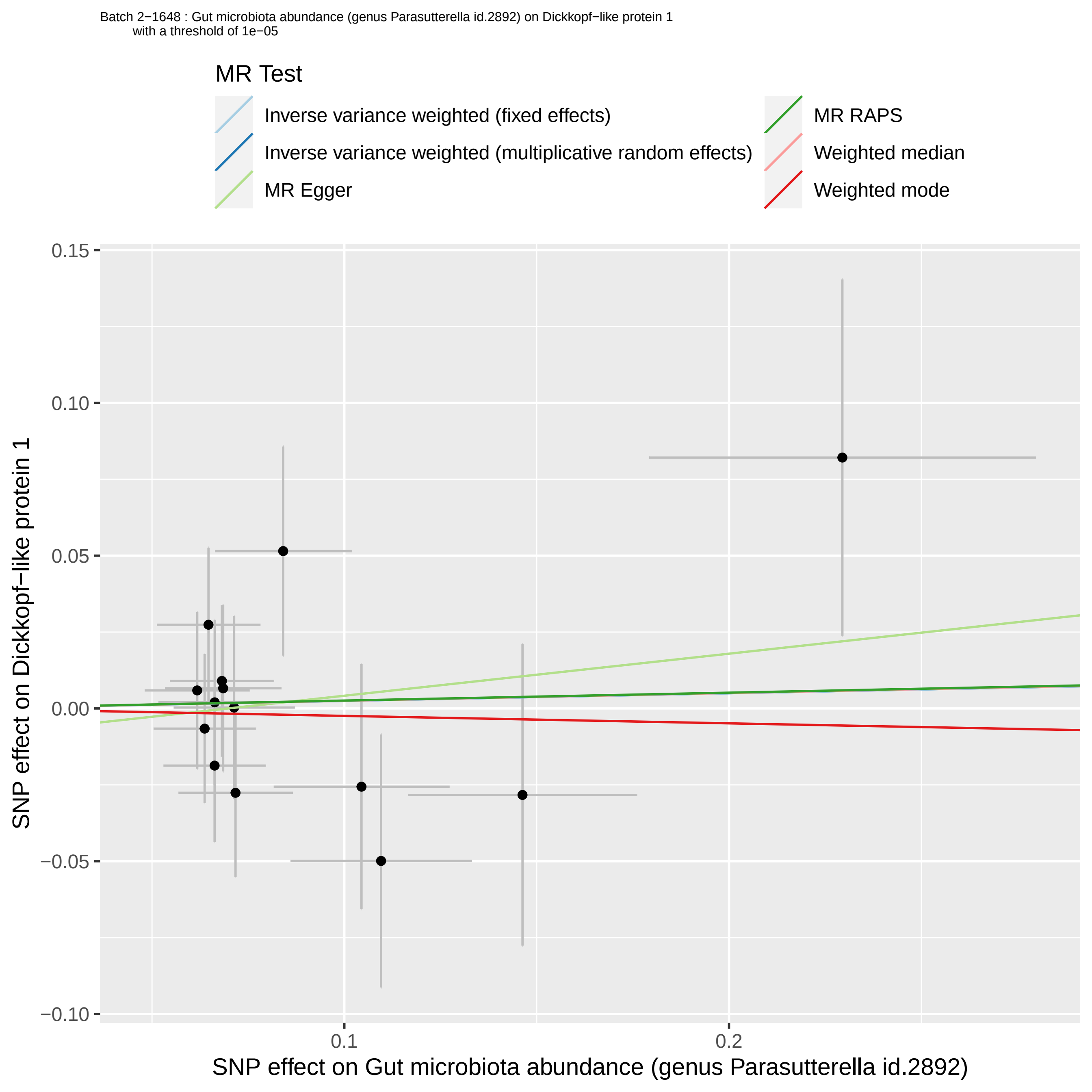
Batch 2−1648 : Gut microbiota abundance (genus Parasutterella id.2892) on Dickkopf−like protein 1
with a threshold of 1e−05
MR Test
Inverse variance weighted (fixed effects)
Inverse variance weighted (multiplicative random effects)
MR Egger
MR RAPS
Weighted median
Weighted mode
0.15
0.10
0.05
0.00
−0.05
−0.10
0.1
0.2
SNP effect on Dickkopf−like protein 1
SNP effect on Gut microbiota abundance (genus Parasutterella id.2892)

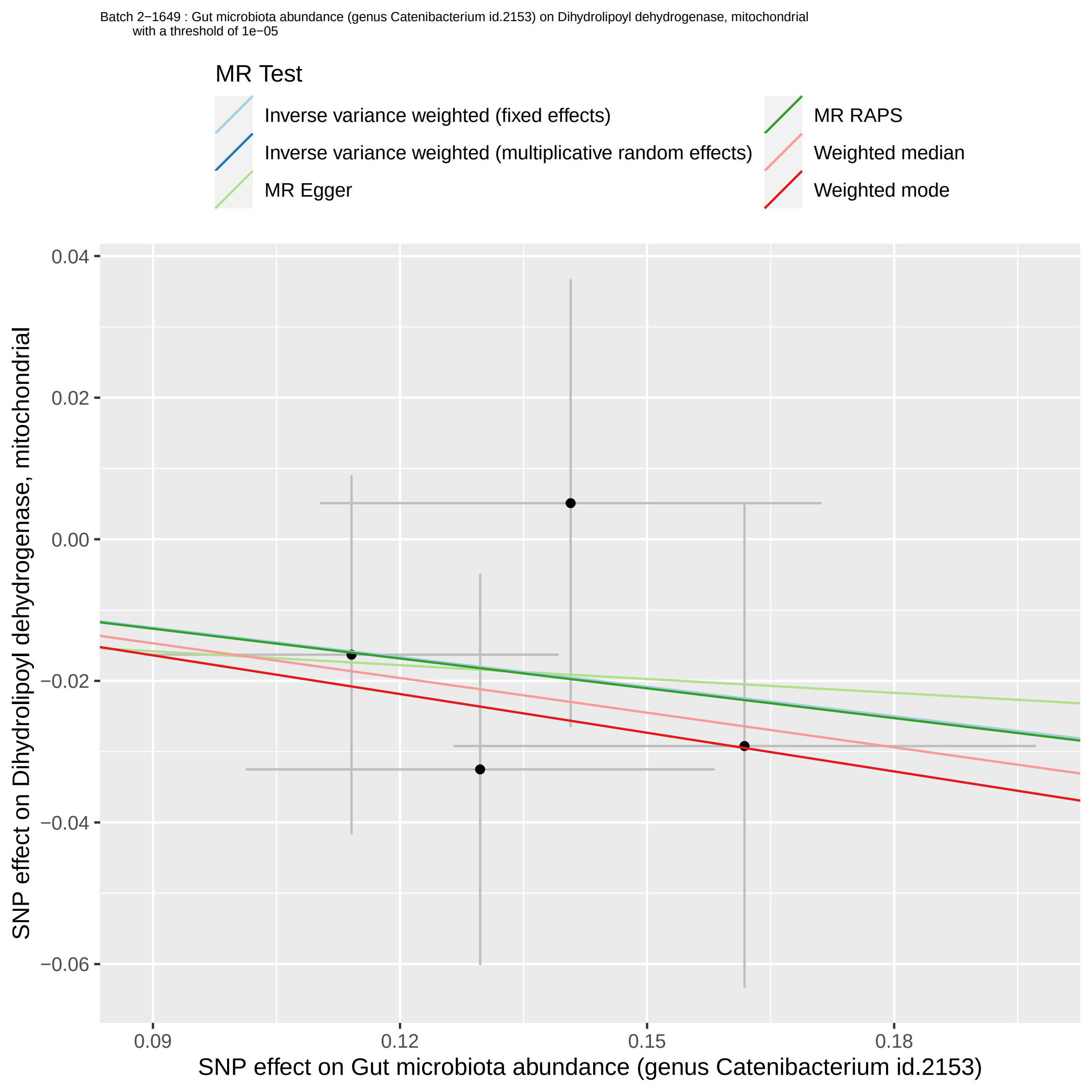
Batch 2−1649 : Gut microbiota abundance (genus Catenibacterium id.2153) on Dihydrolipoyl dehydrogenase, mitochondrial
with a threshold of 1e−05
MR Test
Inverse variance weighted (fixed effects)
Inverse variance weighted (multiplicative random effects)
MR Egger
MR RAPS
Weighted median
Weighted mode
0.04
0.02
0.00
−0.02
−0.04
−0.06
0.09
0.12
0.15
0.18
SNP effect on Dihydrolipoyl dehydrogenase, mitochondrial
SNP effect on Gut microbiota abundance (genus Catenibacterium id.2153)

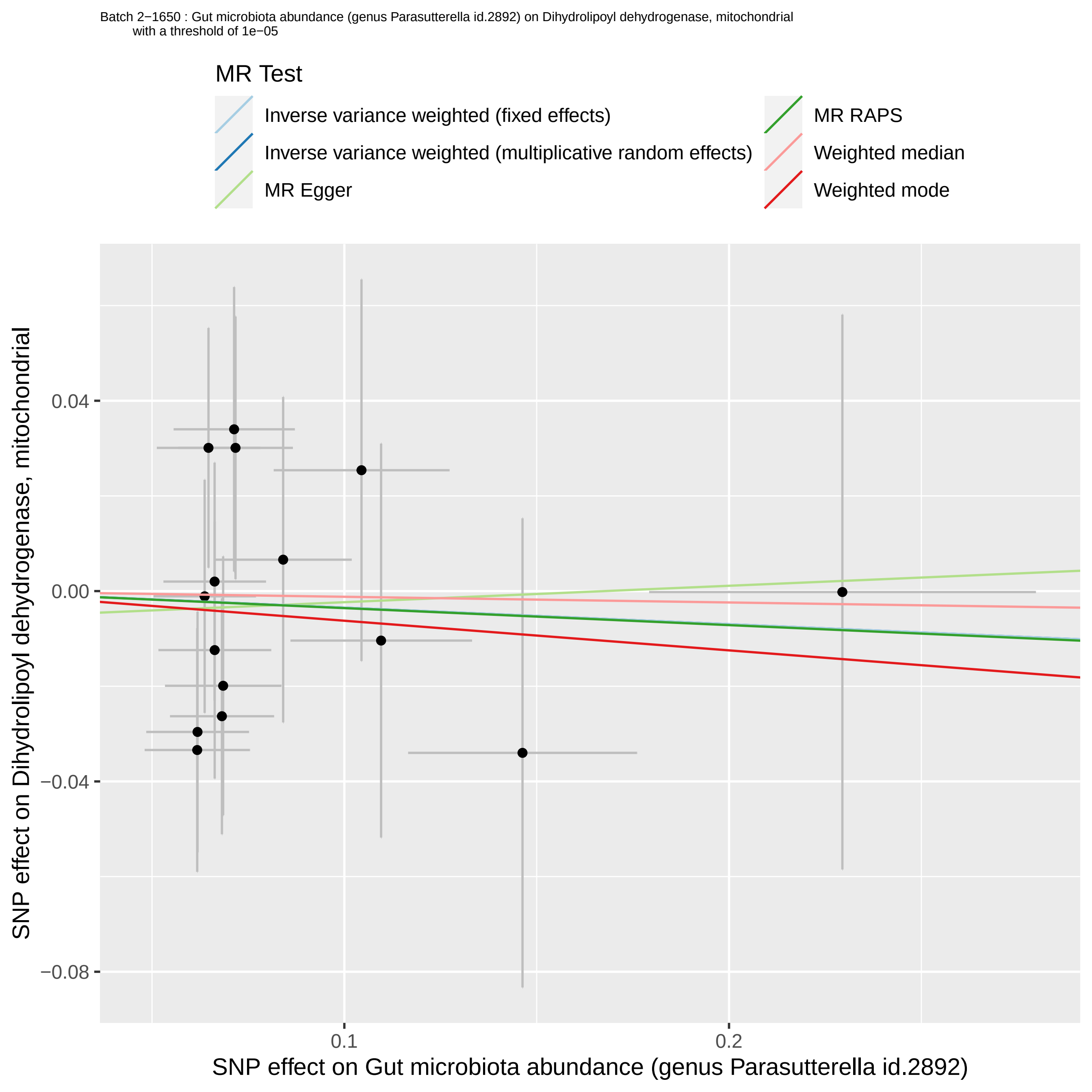

Batch 2−1650 : Gut microbiota abundance (genus Parasutterella id.2892) on Dihydrolipoyl dehydrogenase, mitochondrial
with a threshold of 1e−05
MR Test
Inverse variance weighted (fixed effects)
Inverse variance weighted (multiplicative random effects)
MR Egger
MR RAPS
Weighted median
Weighted mode
0.04
0.00
−0.04
−0.08
0.1
0.2
SNP effect on Dihydrolipoyl dehydrogenase, mitochondrial
SNP effect on Gut microbiota abundance (genus Parasutterella id.2892)

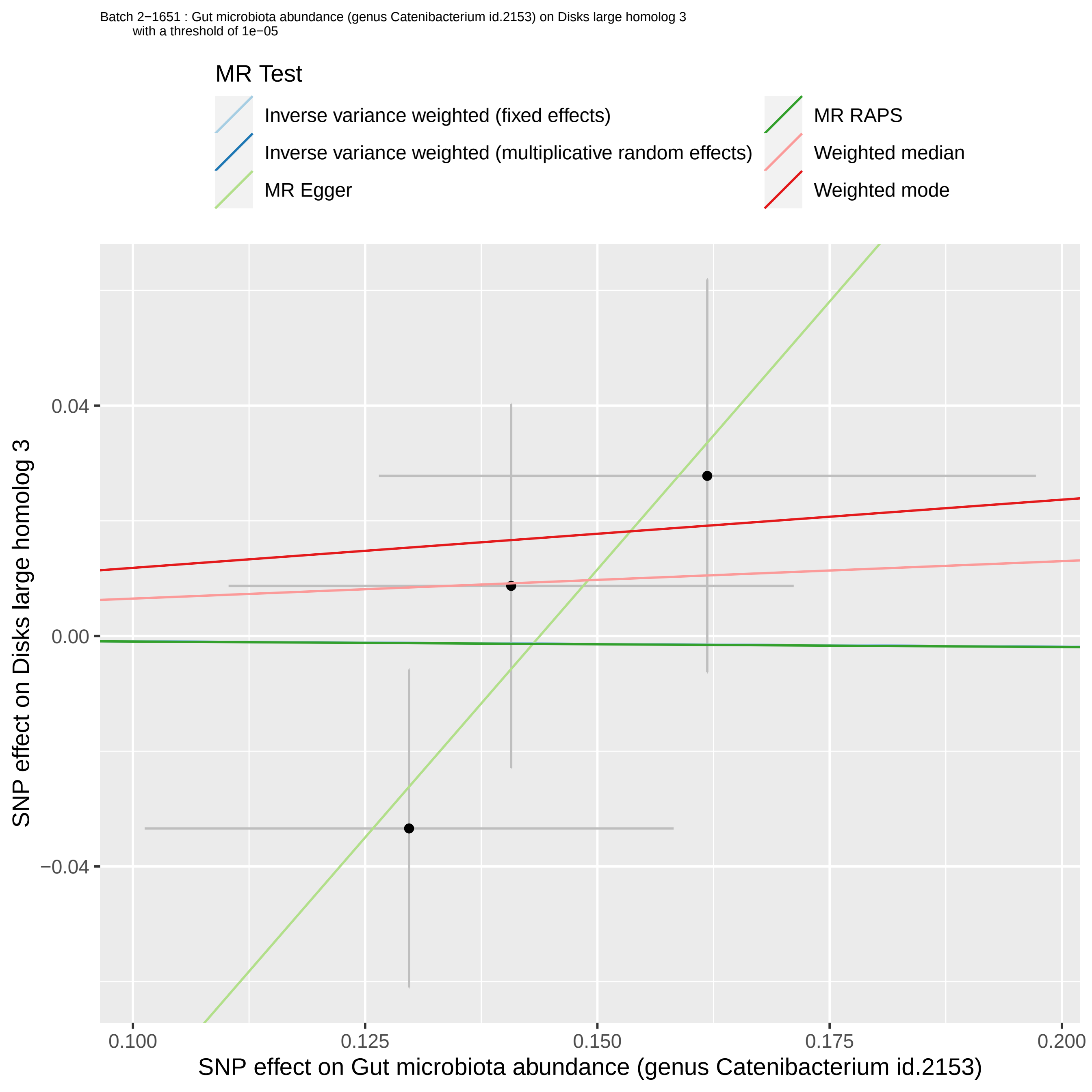
Batch 2−1651 : Gut microbiota abundance (genus Catenibacterium id.2153) on Disks large homolog 3
with a threshold of 1e−05
MR Test
Inverse variance weighted (fixed effects)
Inverse variance weighted (multiplicative random effects)
MR Egger
MR RAPS
Weighted median
Weighted mode
SNP effect on Disks large homolog 3
0.04
0.00
−0.04
0.100
0.125
0.150
0.175
0.200
SNP effect on Gut microbiota abundance (genus Catenibacterium id.2153)

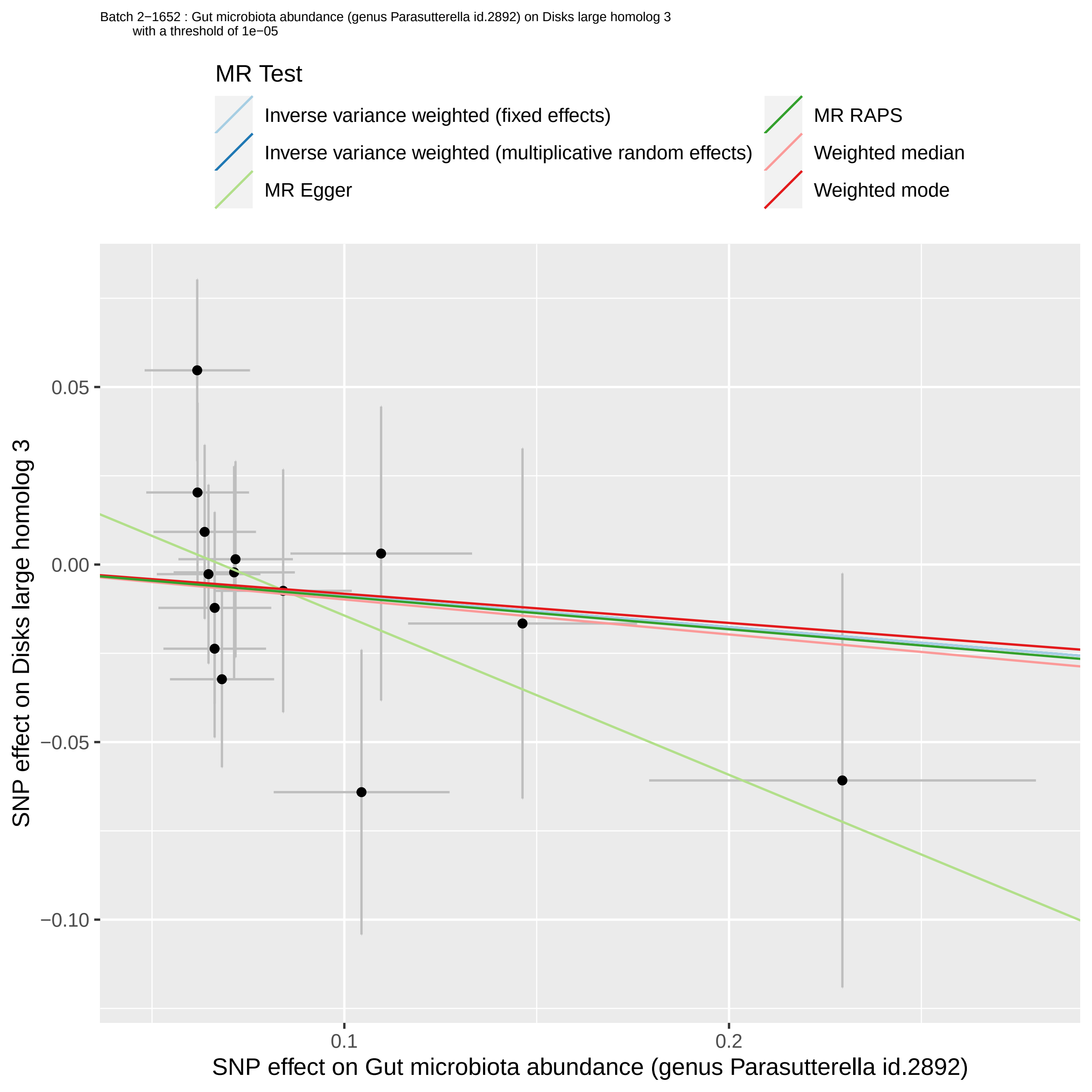
Batch 2−1652 : Gut microbiota abundance (genus Parasutterella id.2892) on Disks large homolog 3
with a threshold of 1e−05
MR Test
Inverse variance weighted (fixed effects)
Inverse variance weighted (multiplicative random effects)
MR Egger
MR RAPS
Weighted median
Weighted mode
0.05
0.00
−0.05
−0.10
0.1
0.2
SNP effect on Disks large homolog 3
SNP effect on Gut microbiota abundance (genus Parasutterella id.2892)

Batch 2−1653 : Gut microbiota abundance (genus Catenibacterium id.2153) on Protein delta homolog 1
with a threshold of 1e−05

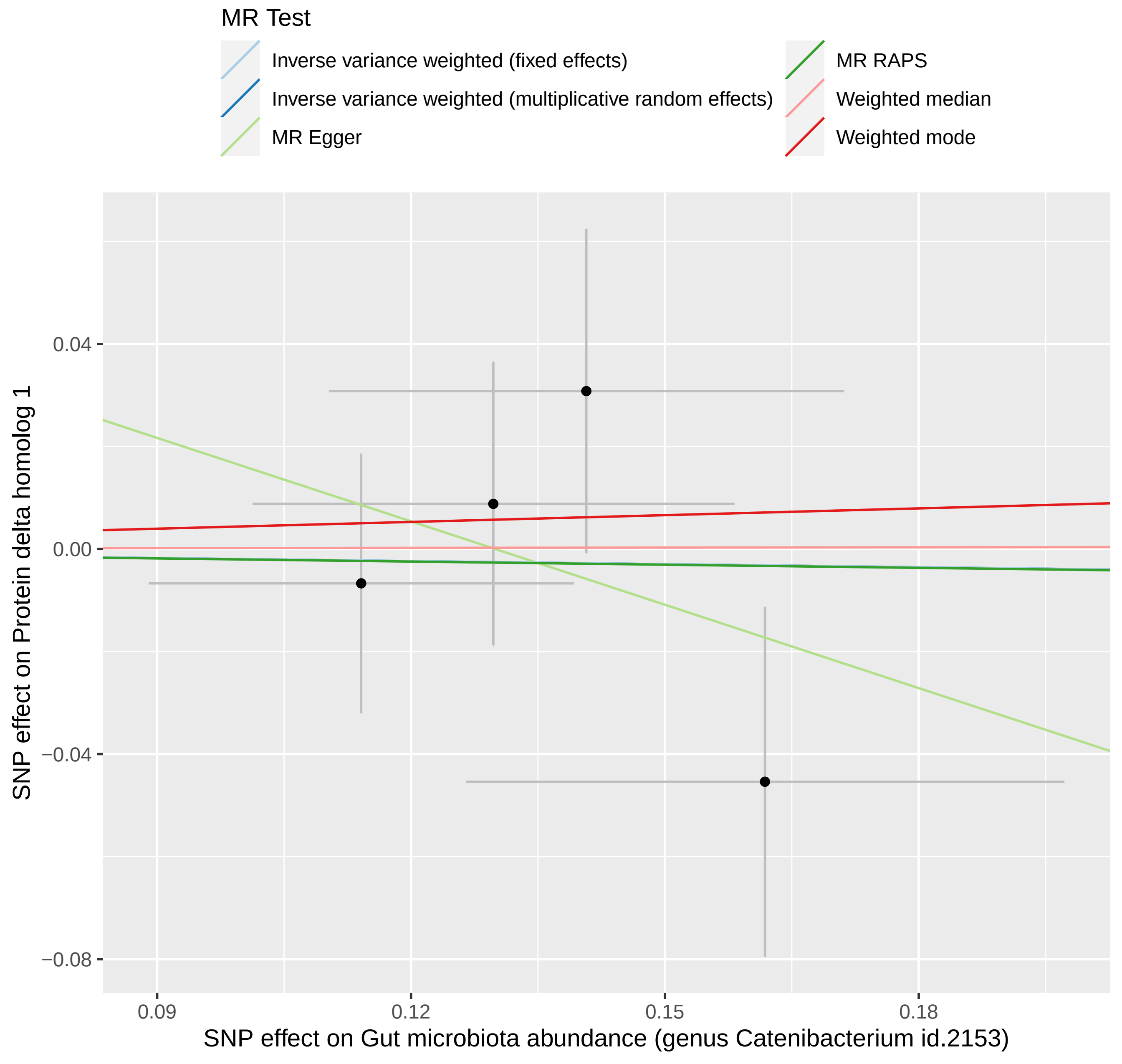

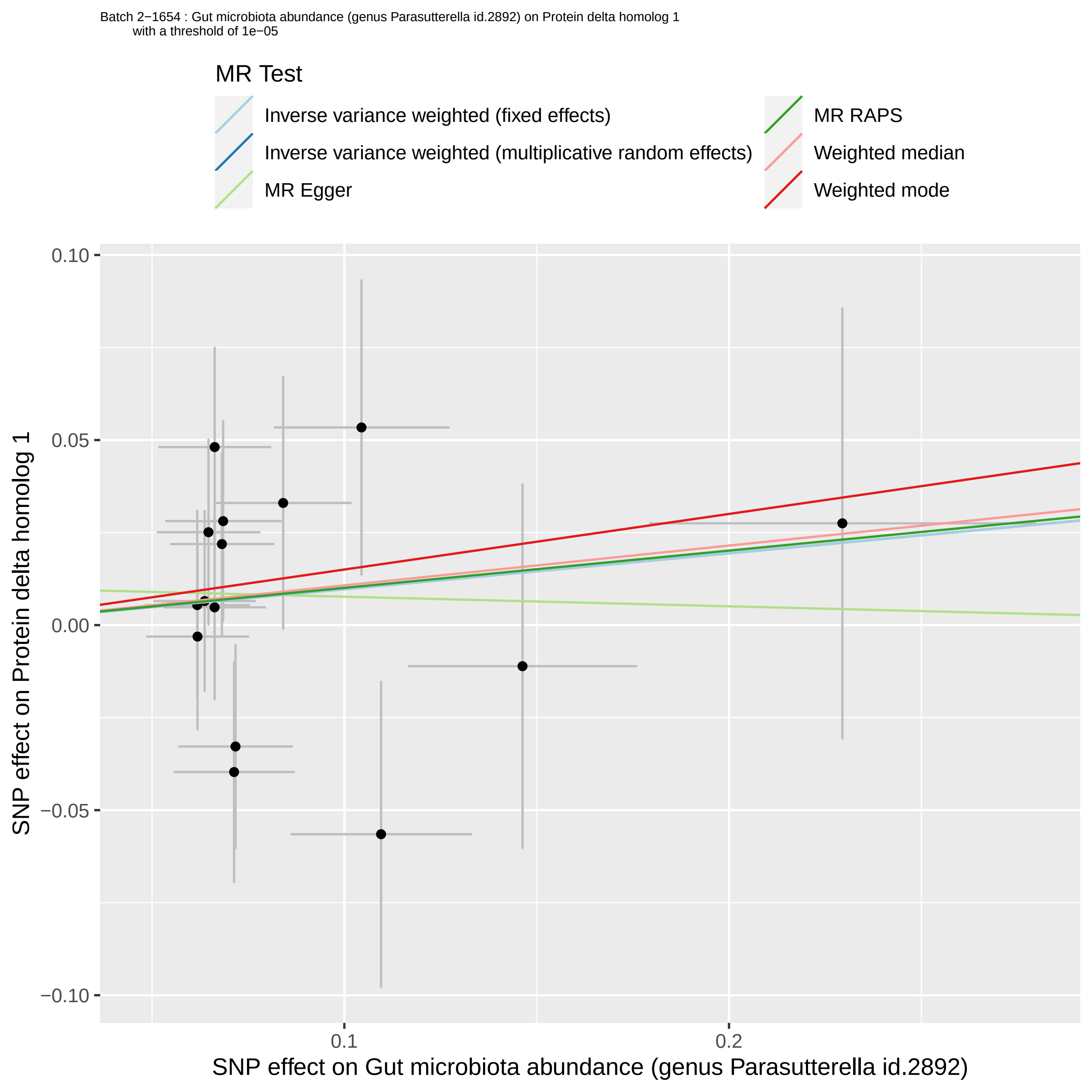
Batch 2−1654 : Gut microbiota abundance (genus Parasutterella id.2892) on Protein delta homolog 1
with a threshold of 1e−05
MR Test
Inverse variance weighted (fixed effects)
Inverse variance weighted (multiplicative random effects)
MR Egger
MR RAPS
Weighted median
Weighted mode
0.10
0.05
0.00
−0.05
−0.10
0.1
0.2
SNP effect on Protein delta homolog 1
SNP effect on Gut microbiota abundance (genus Parasutterella id.2892)

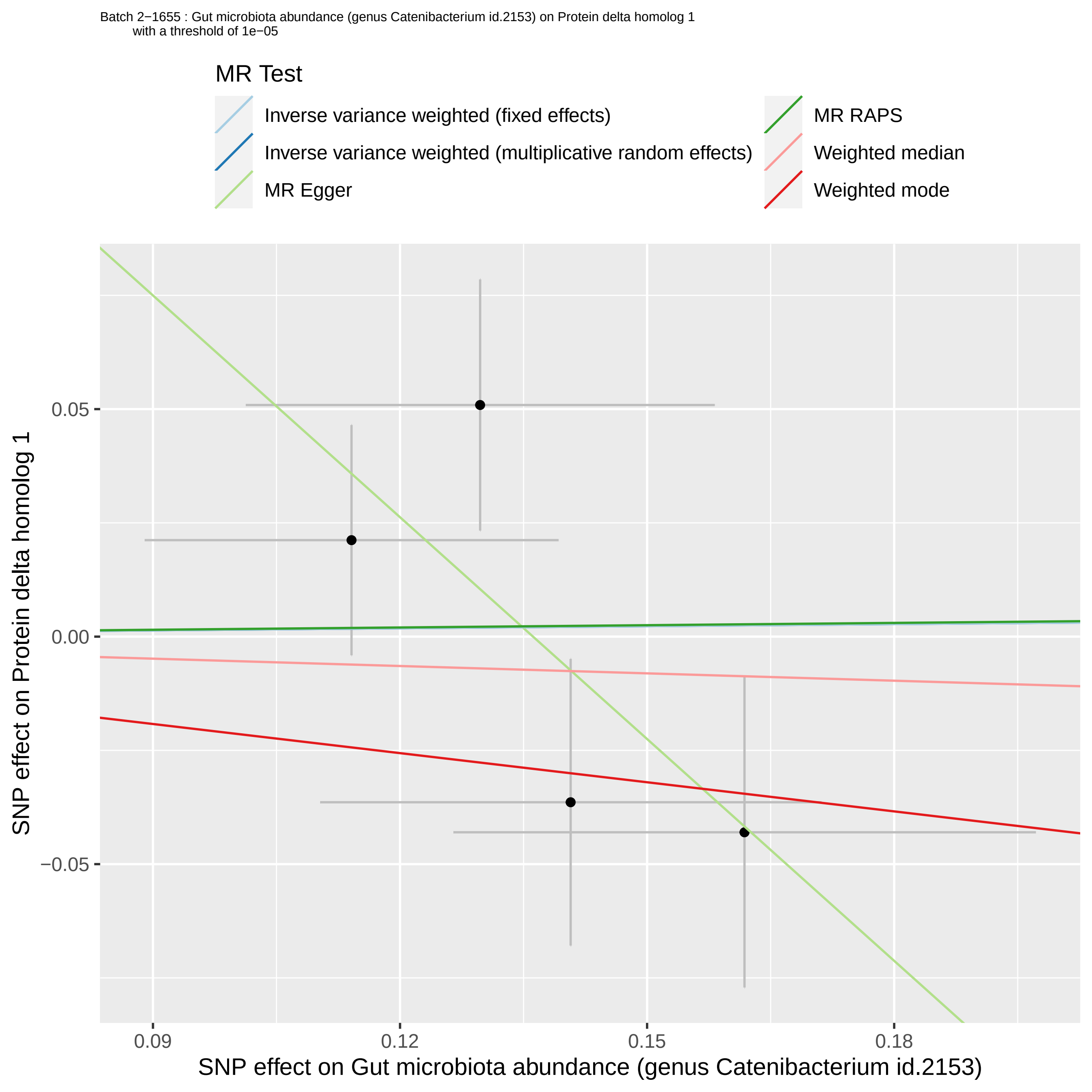
Batch 2−1655 : Gut microbiota abundance (genus Catenibacterium id.2153) on Protein delta homolog 1
with a threshold of 1e−05
MR Test
Inverse variance weighted (fixed effects)
Inverse variance weighted (multiplicative random effects)
MR Egger
MR RAPS
Weighted median
Weighted mode
0.05
0.00
−0.05
0.09
0.12
0.15
0.18
SNP effect on Protein delta homolog 1
SNP effect on Gut microbiota abundance (genus Catenibacterium id.2153)

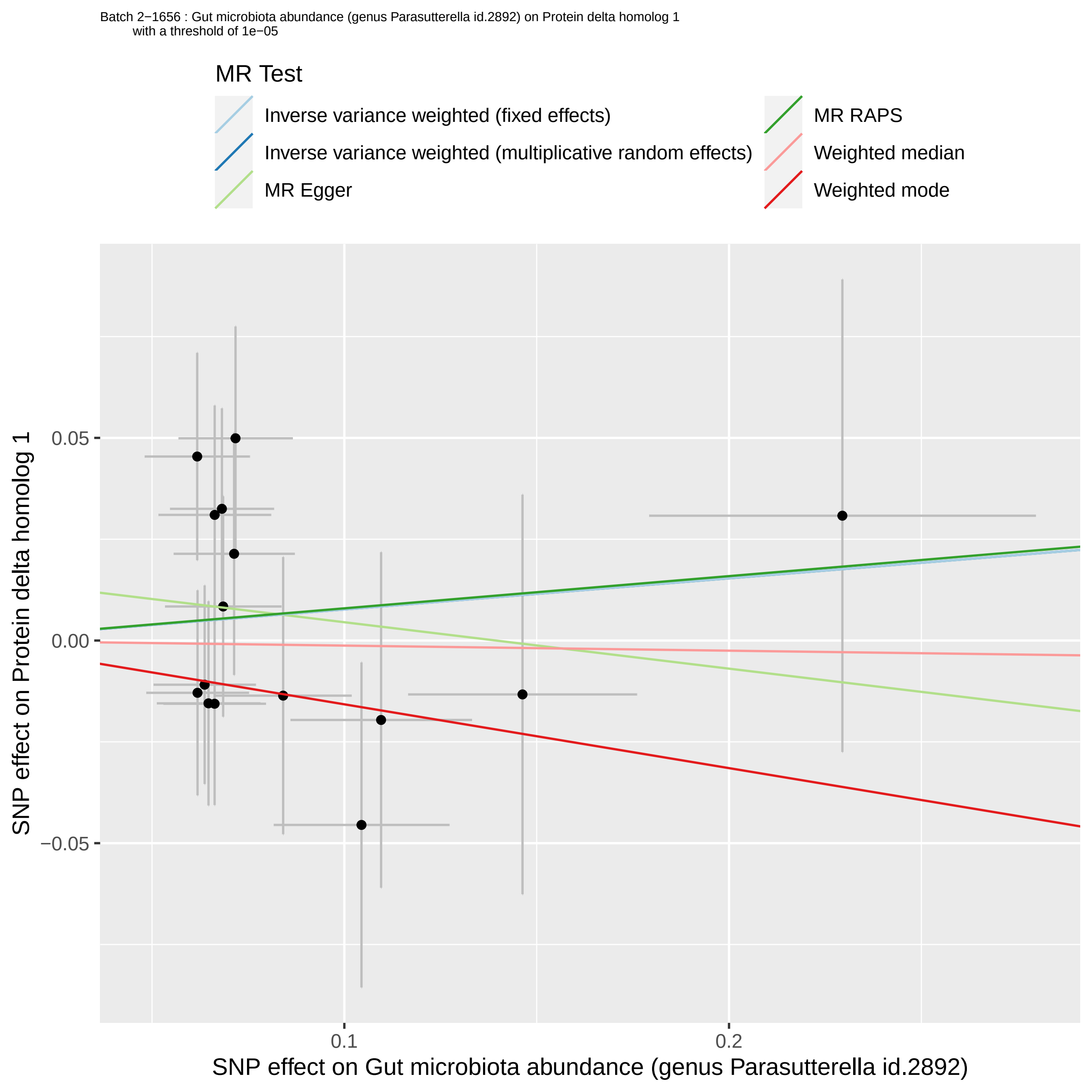
Batch 2−1656 : Gut microbiota abundance (genus Parasutterella id.2892) on Protein delta homolog 1
with a threshold of 1e−05
MR Test
Inverse variance weighted (fixed effects)
Inverse variance weighted (multiplicative random effects)
MR Egger
MR RAPS
Weighted median
Weighted mode
0.05
0.00
−0.05
0.1
0.2
SNP effect on Protein delta homolog 1
SNP effect on Gut microbiota abundance (genus Parasutterella id.2892)

Batch 2−1657 : Gut microbiota abundance (genus Catenibacterium id.2153) on Protein delta homolog 2
with a threshold of 1e−05

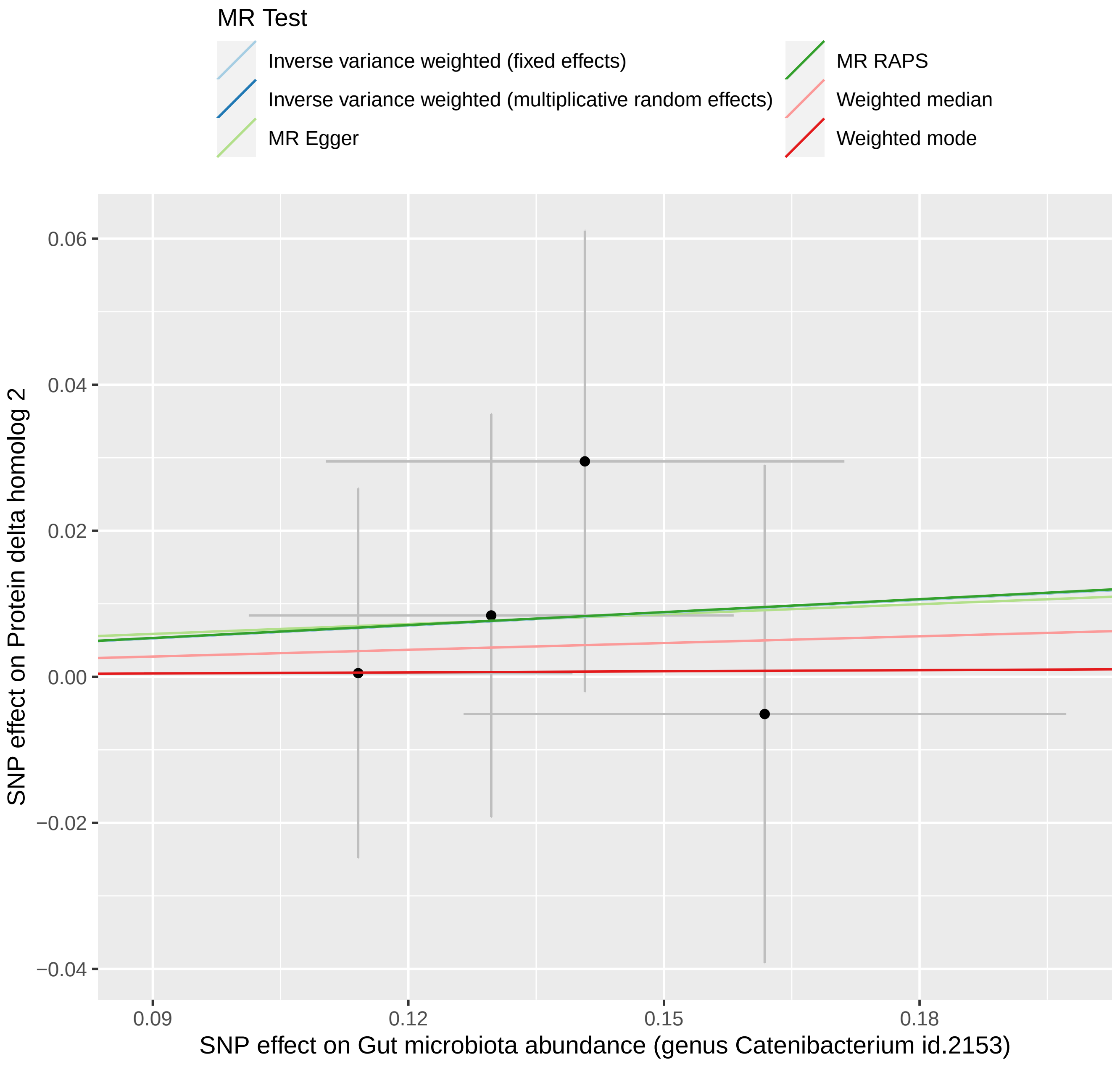

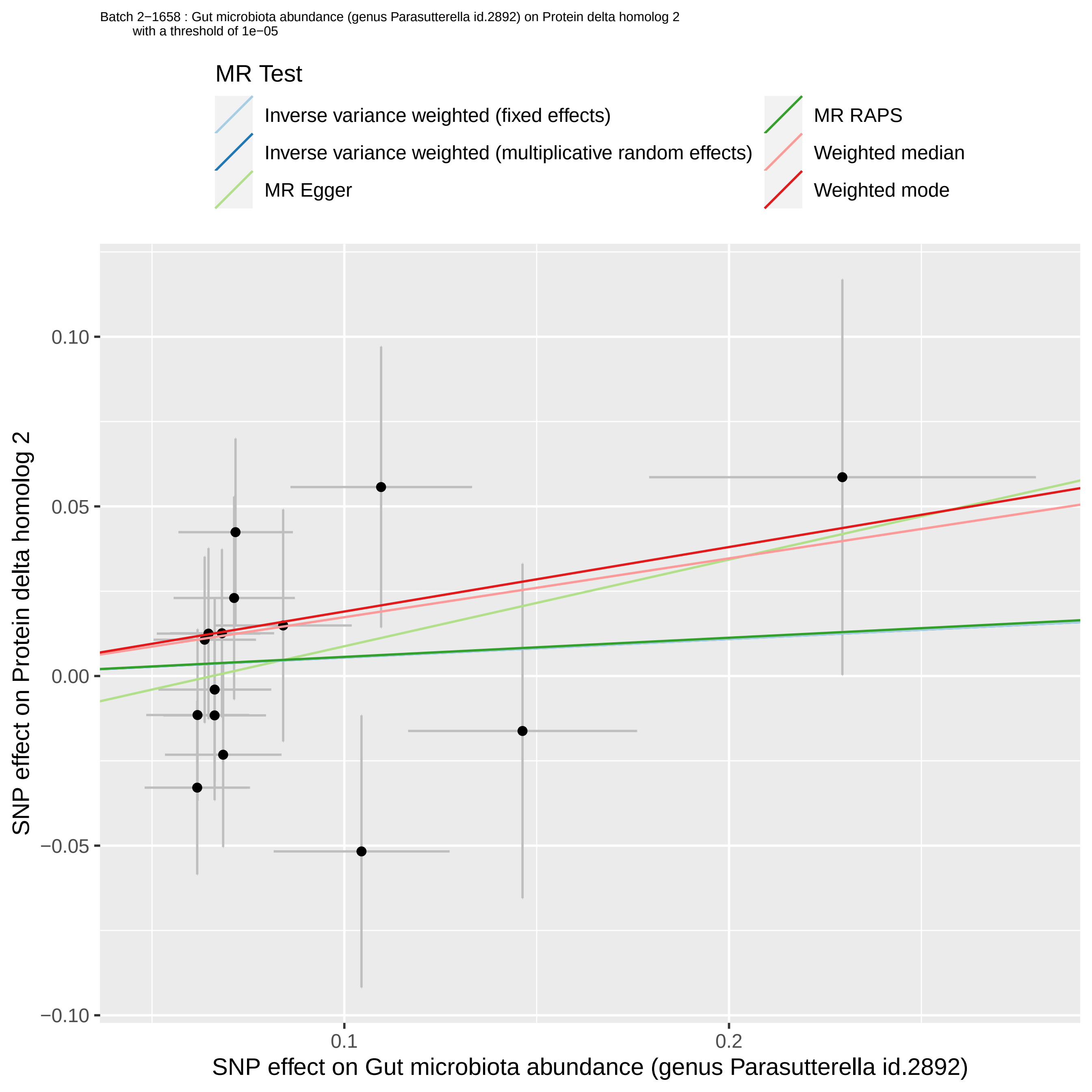
Batch 2−1658 : Gut microbiota abundance (genus Parasutterella id.2892) on Protein delta homolog 2
with a threshold of 1e−05
MR Test
Inverse variance weighted (fixed effects)
Inverse variance weighted (multiplicative random effects)
MR Egger
MR RAPS
Weighted median
Weighted mode
0.10
0.05
0.00
−0.05
−0.10
0.1
0.2
SNP effect on Protein delta homolog 2
SNP effect on Gut microbiota abundance (genus Parasutterella id.2892)

Batch 2−1659 : Gut microbiota abundance (genus Catenibacterium id.2153) on Delta−like protein 1
with a threshold of 1e−05

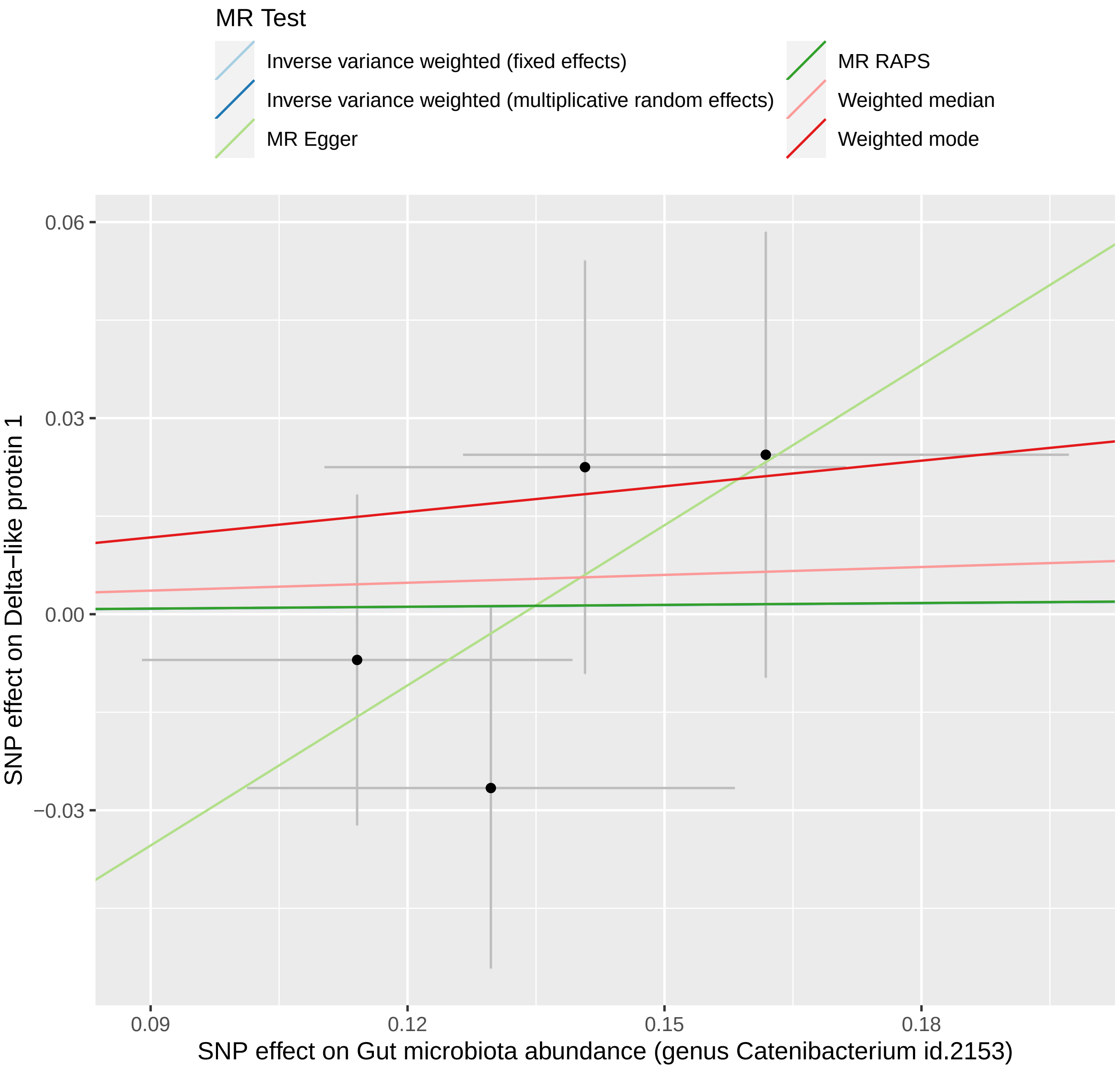

Batch 2−1660 : Gut microbiota abundance (genus Parasutterella id.2892) on Delta−like protein 1
with a threshold of 1e−05

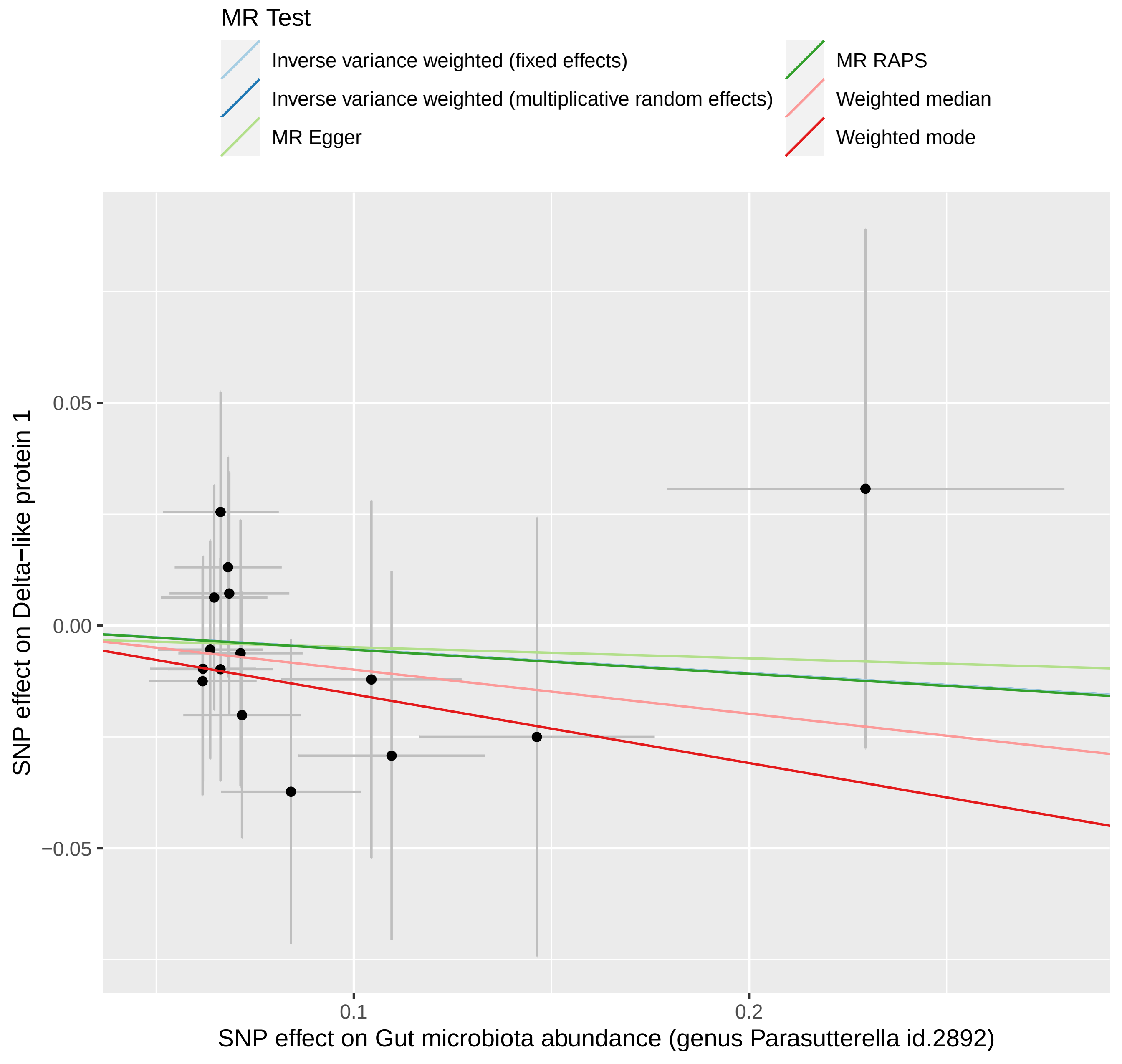

Batch 2−1661 : Gut microbiota abundance (genus Catenibacterium id.2153) on Delta−like protein 1
with a threshold of 1e−05

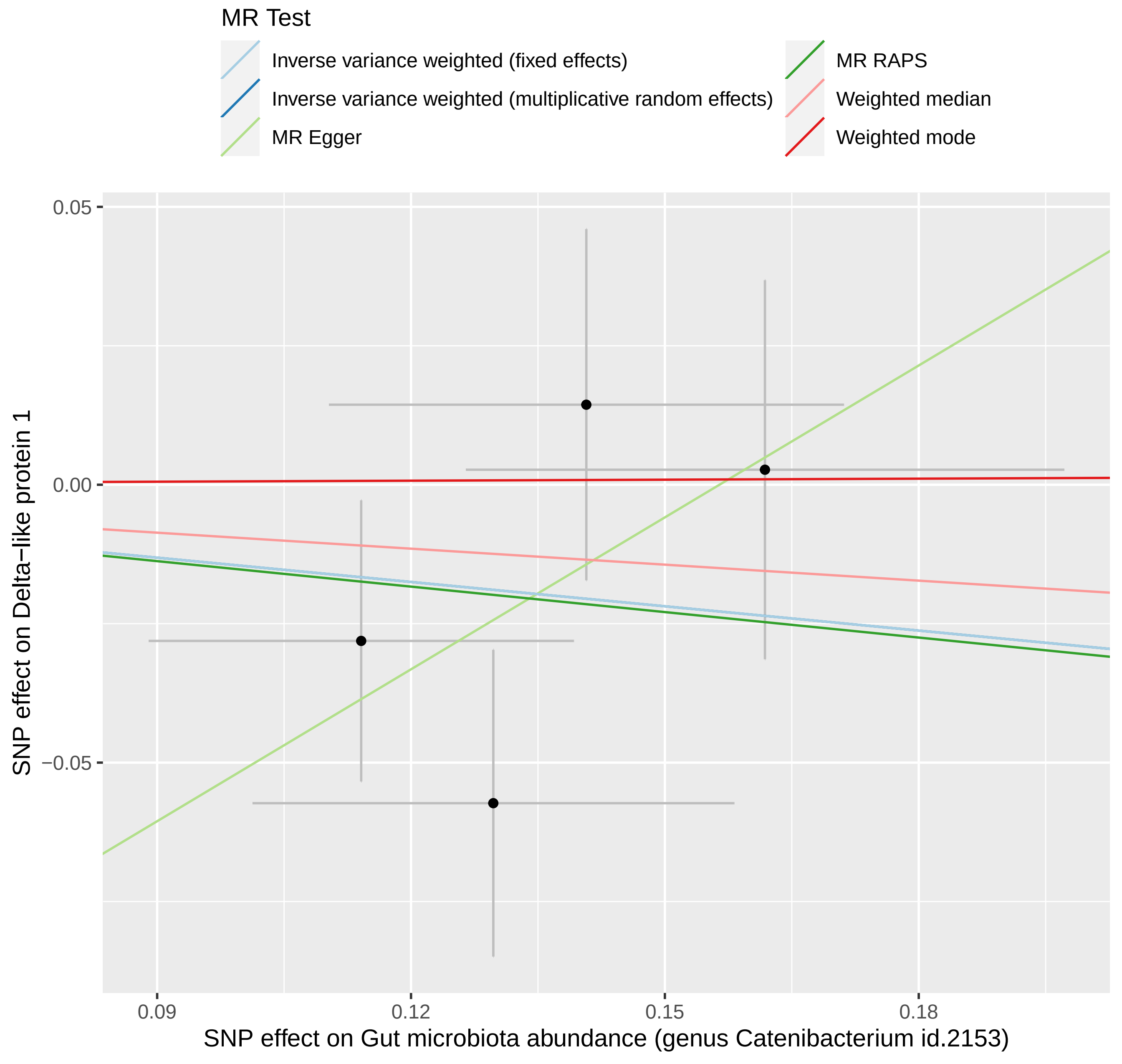

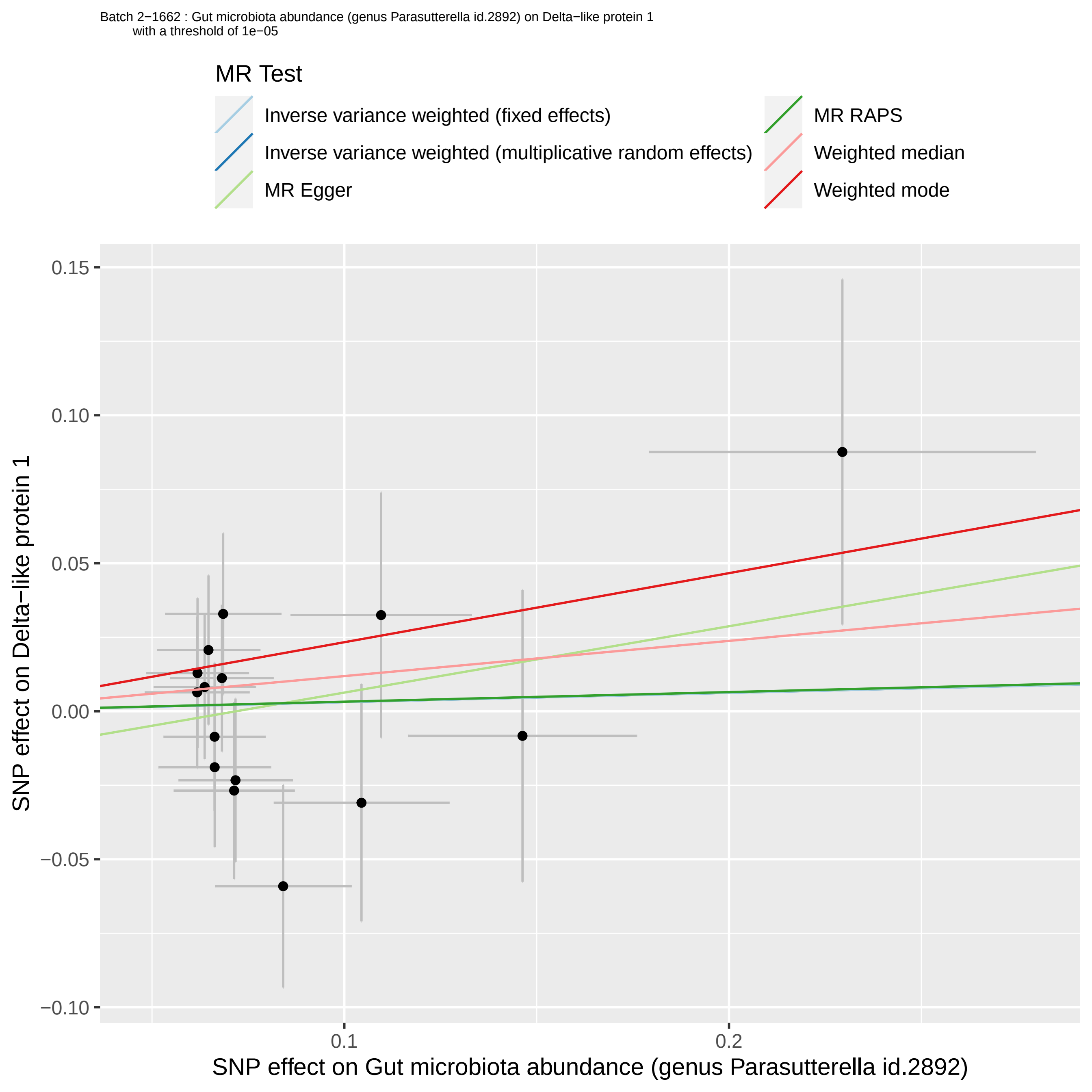
Batch 2−1662 : Gut microbiota abundance (genus Parasutterella id.2892) on Delta−like protein 1
with a threshold of 1e−05
MR Test
Inverse variance weighted (fixed effects)
Inverse variance weighted (multiplicative random effects)
MR Egger
MR RAPS
Weighted median
Weighted mode
0.15
0.10
0.05
0.00
−0.05
−0.10
0.1
0.2
SNP effect on Delta−like protein 1
SNP effect on Gut microbiota abundance (genus Parasutterella id.2892)

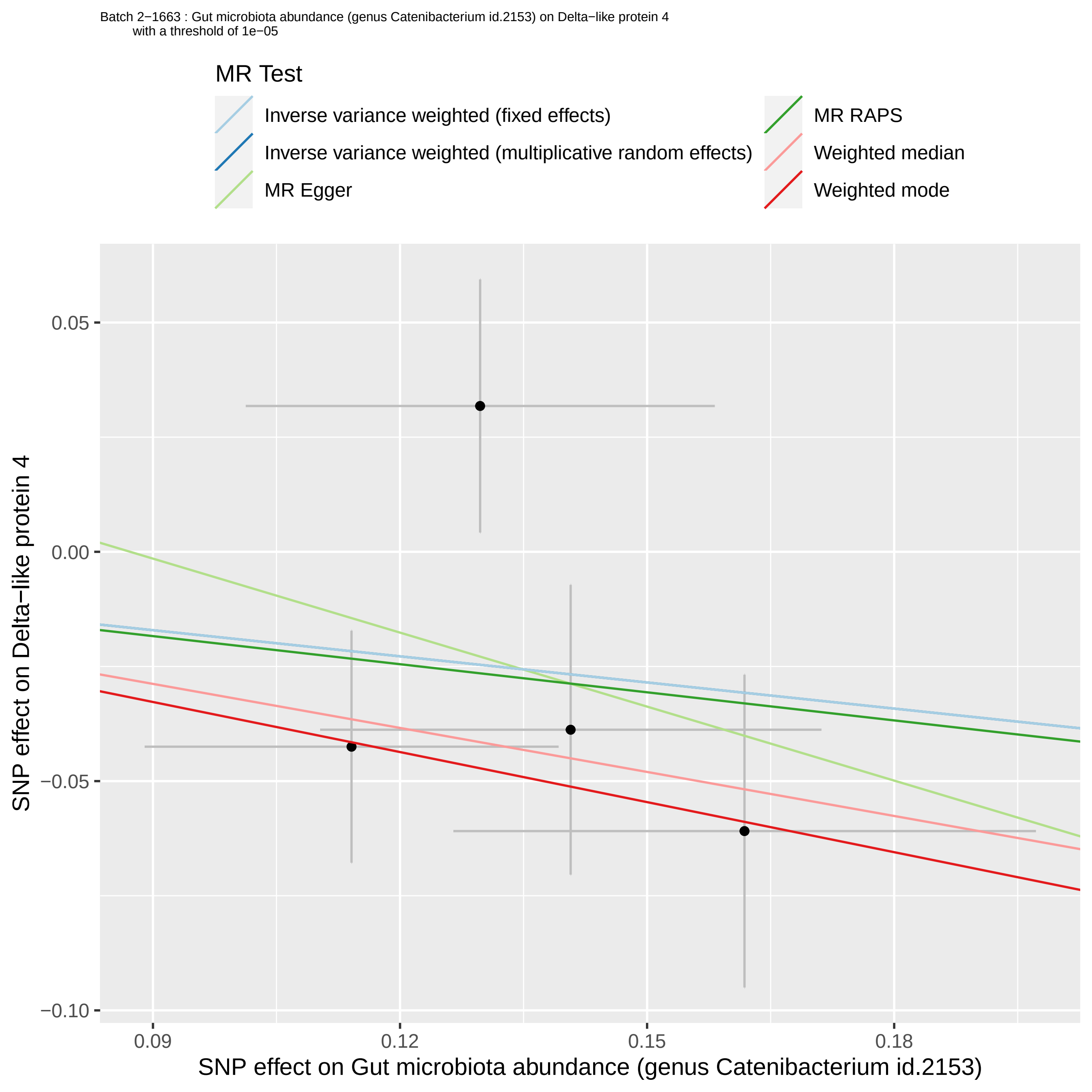
Batch 2−1663 : Gut microbiota abundance (genus Catenibacterium id.2153) on Delta−like protein 4
with a threshold of 1e−05
MR Test
Inverse variance weighted (fixed effects)
Inverse variance weighted (multiplicative random effects)
MR Egger
MR RAPS
Weighted median
Weighted mode
0.05
0.00
−0.05
−0.10
0.09
0.12
0.15
0.18
SNP effect on Delta−like protein 4
SNP effect on Gut microbiota abundance (genus Catenibacterium id.2153)

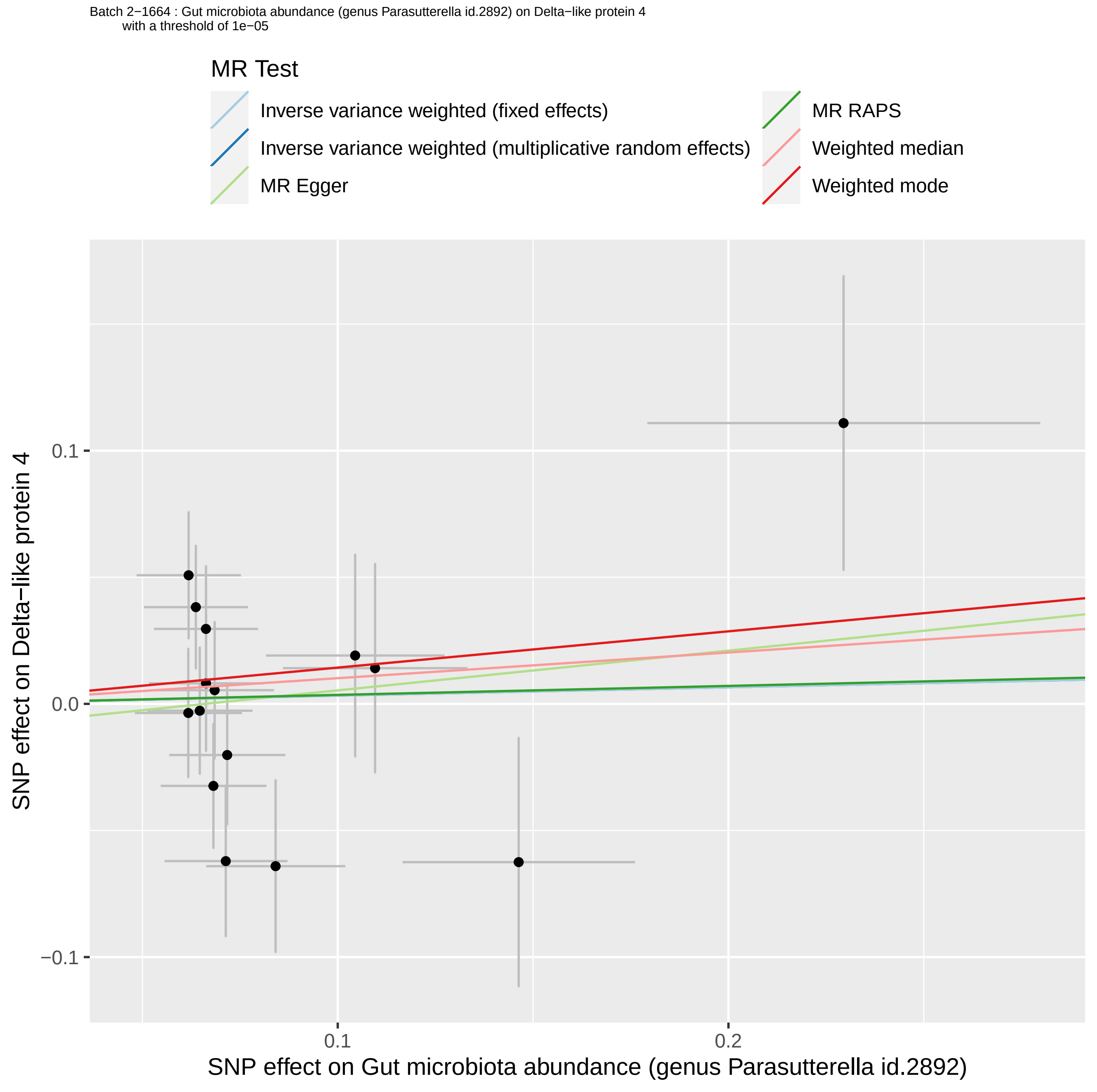

Batch 2−1664 : Gut microbiota abundance (genus Parasutterella id.2892) on Delta−like protein 4
with a threshold of 1e−05
MR Test
Inverse variance weighted (fixed effects)
Inverse variance weighted (multiplicative random effects)
MR Egger
MR RAPS
Weighted median
Weighted mode
SNP effect on Delta−like protein 4
0.1
0.0
−0.1
0.1
0.2
SNP effect on Gut microbiota abundance (genus Parasutterella id.2892)

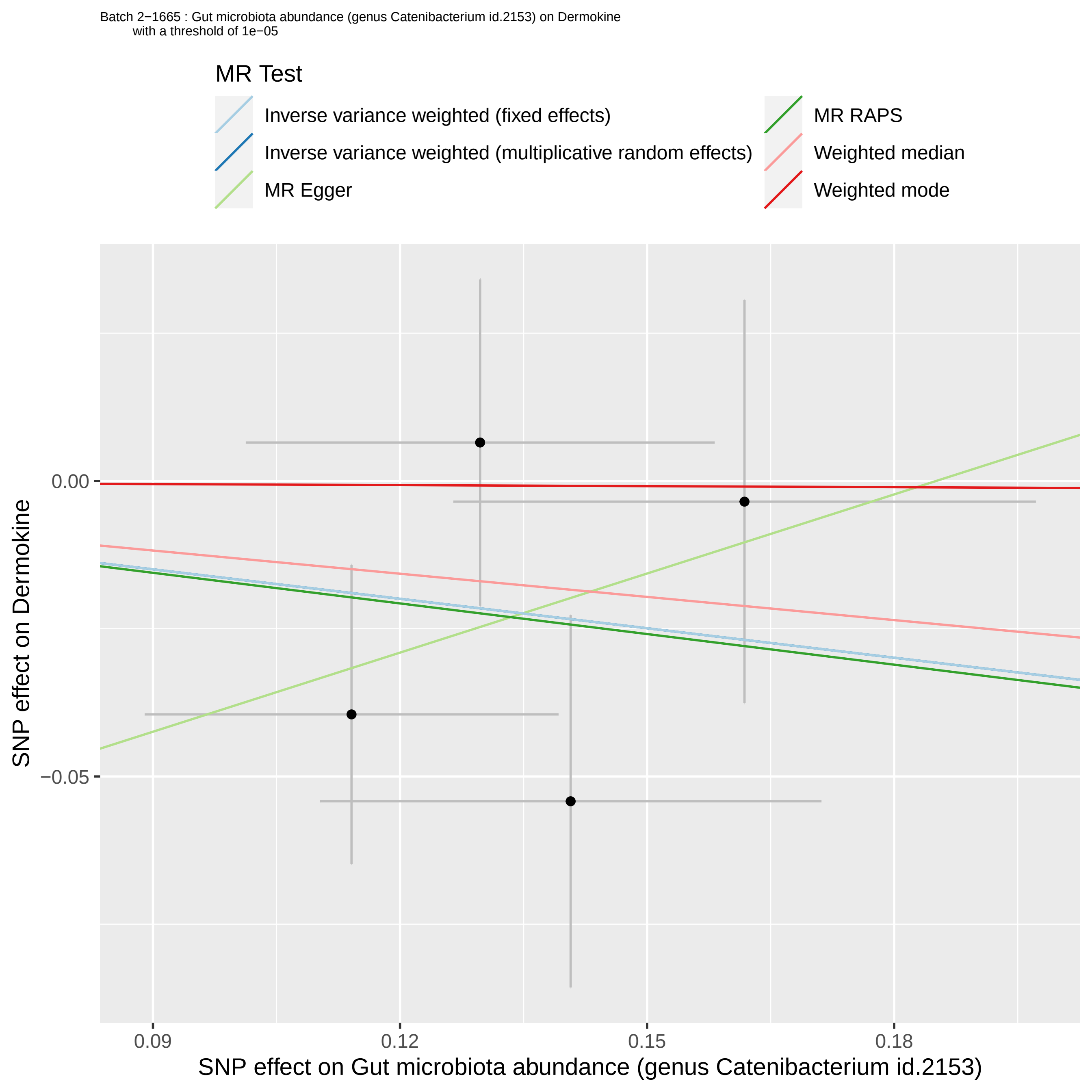
Batch 2−1665 : Gut microbiota abundance (genus Catenibacterium id.2153) on Dermokine
with a threshold of 1e−05
MR Test
Inverse variance weighted (fixed effects)
Inverse variance weighted (multiplicative random effects)
MR Egger
MR RAPS
Weighted median
Weighted mode
0.00
−0.05
0.09
0.12
0.15
0.18
SNP effect on Dermokine
SNP effect on Gut microbiota abundance (genus Catenibacterium id.2153)

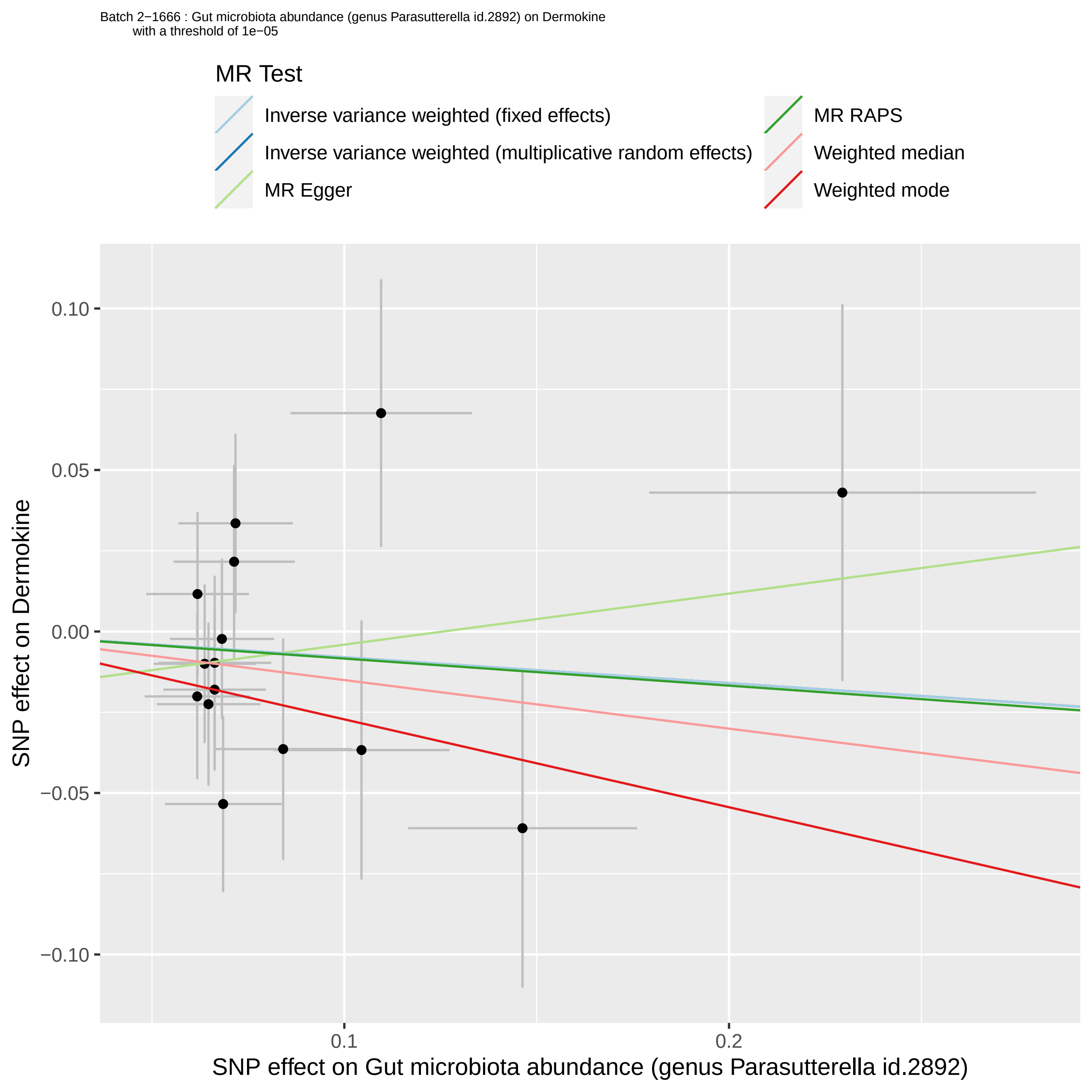

Batch 2−1666 : Gut microbiota abundance (genus Parasutterella id.2892) on Dermokine
with a threshold of 1e−05
MR Test
Inverse variance weighted (fixed effects)
Inverse variance weighted (multiplicative random effects)
MR Egger
MR RAPS
Weighted median
Weighted mode
0.10
0.05
0.00
−0.05
−0.10
SNP effect on Dermokine
0.1
0.2
SNP effect on Gut microbiota abundance (genus Parasutterella id.2892)

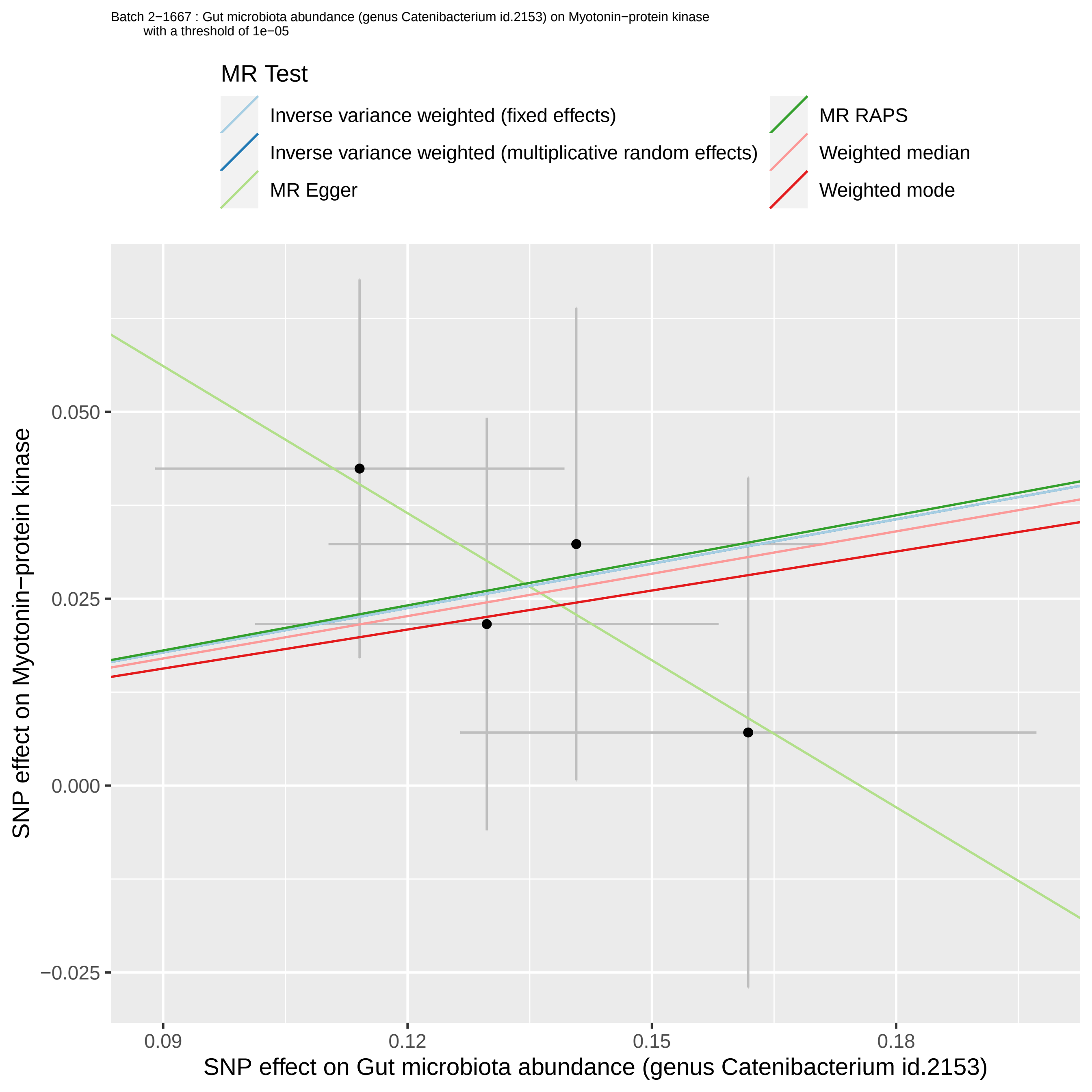
Batch 2−1667 : Gut microbiota abundance (genus Catenibacterium id.2153) on Myotonin−protein kinase
with a threshold of 1e−05
MR Test
Inverse variance weighted (fixed effects)
Inverse variance weighted (multiplicative random effects)
MR Egger
MR RAPS
Weighted median
Weighted mode
SNP effect on Myotonin−protein kinase
0.050
0.025
0.000
−0.025
0.09
0.12
0.15
0.18
SNP effect on Gut microbiota abundance (genus Catenibacterium id.2153)

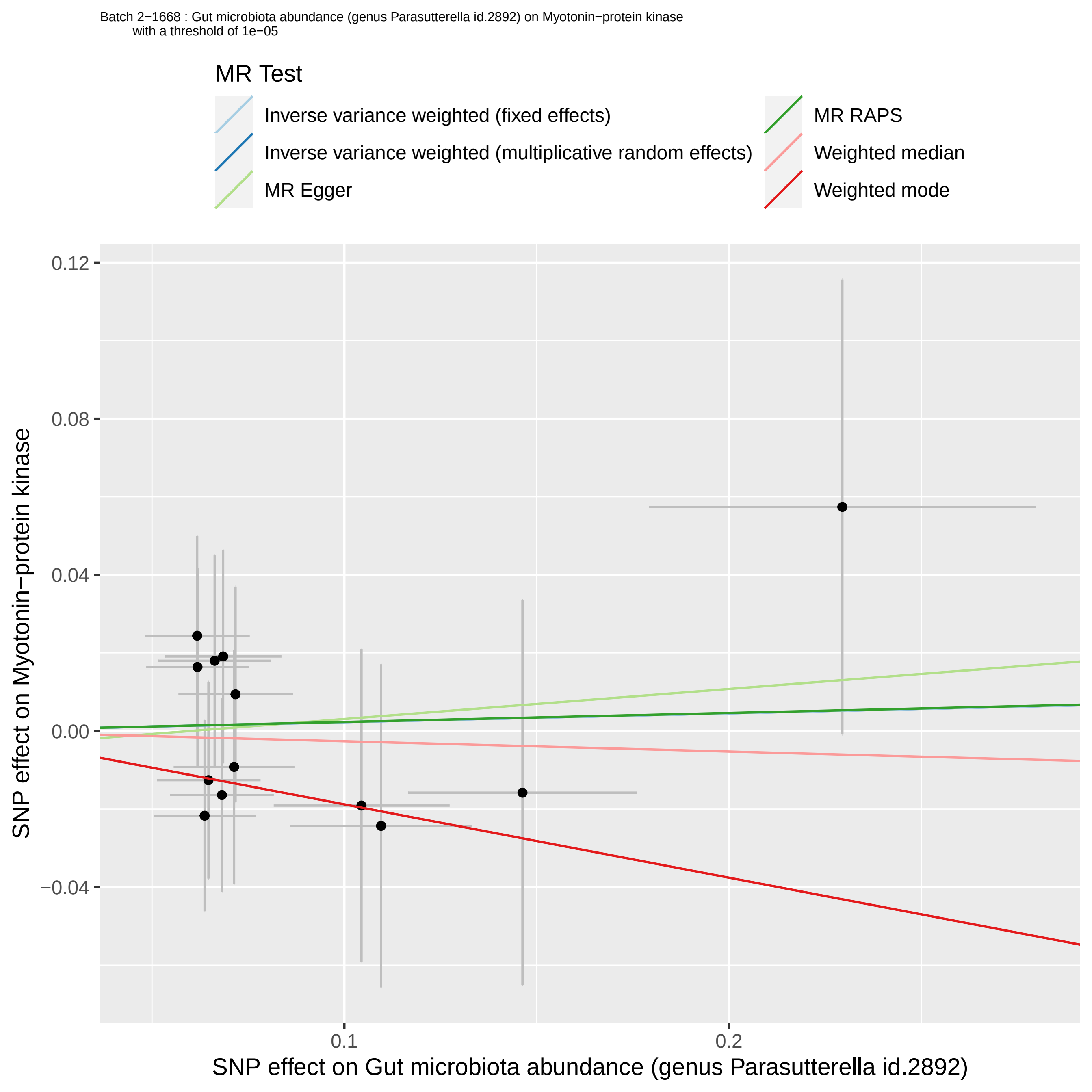

Batch 2−1668 : Gut microbiota abundance (genus Parasutterella id.2892) on Myotonin−protein kinase
with a threshold of 1e−05
MR Test
Inverse variance weighted (fixed effects)
Inverse variance weighted (multiplicative random effects)
MR Egger
MR RAPS
Weighted median
Weighted mode
0.12
0.08
0.04
0.00
−0.04
0.1
0.2
SNP effect on Myotonin−protein kinase
SNP effect on Gut microbiota abundance (genus Parasutterella id.2892)

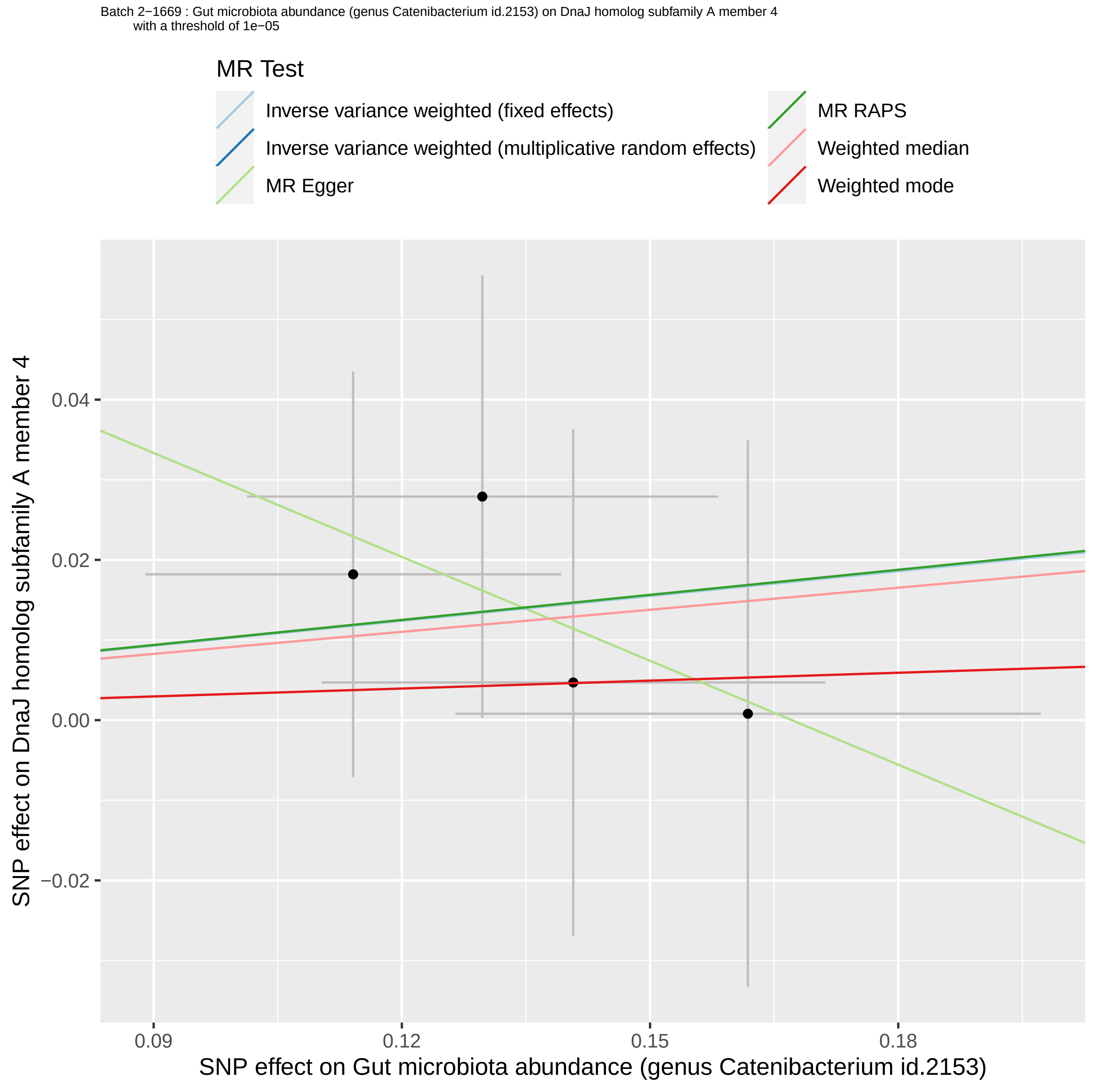

Batch 2−1669 : Gut microbiota abundance (genus Catenibacterium id.2153) on DnaJ homolog subfamily A member 4
with a threshold of 1e−05
MR Test
Inverse variance weighted (fixed effects)
Inverse variance weighted (multiplicative random effects)
MR Egger
MR RAPS
Weighted median
Weighted mode
0.04
0.02
0.00
−0.02
0.09
0.12
0.15
0.18
SNP effect on DnaJ homolog subfamily A member 4
SNP effect on Gut microbiota abundance (genus Catenibacterium id.2153)

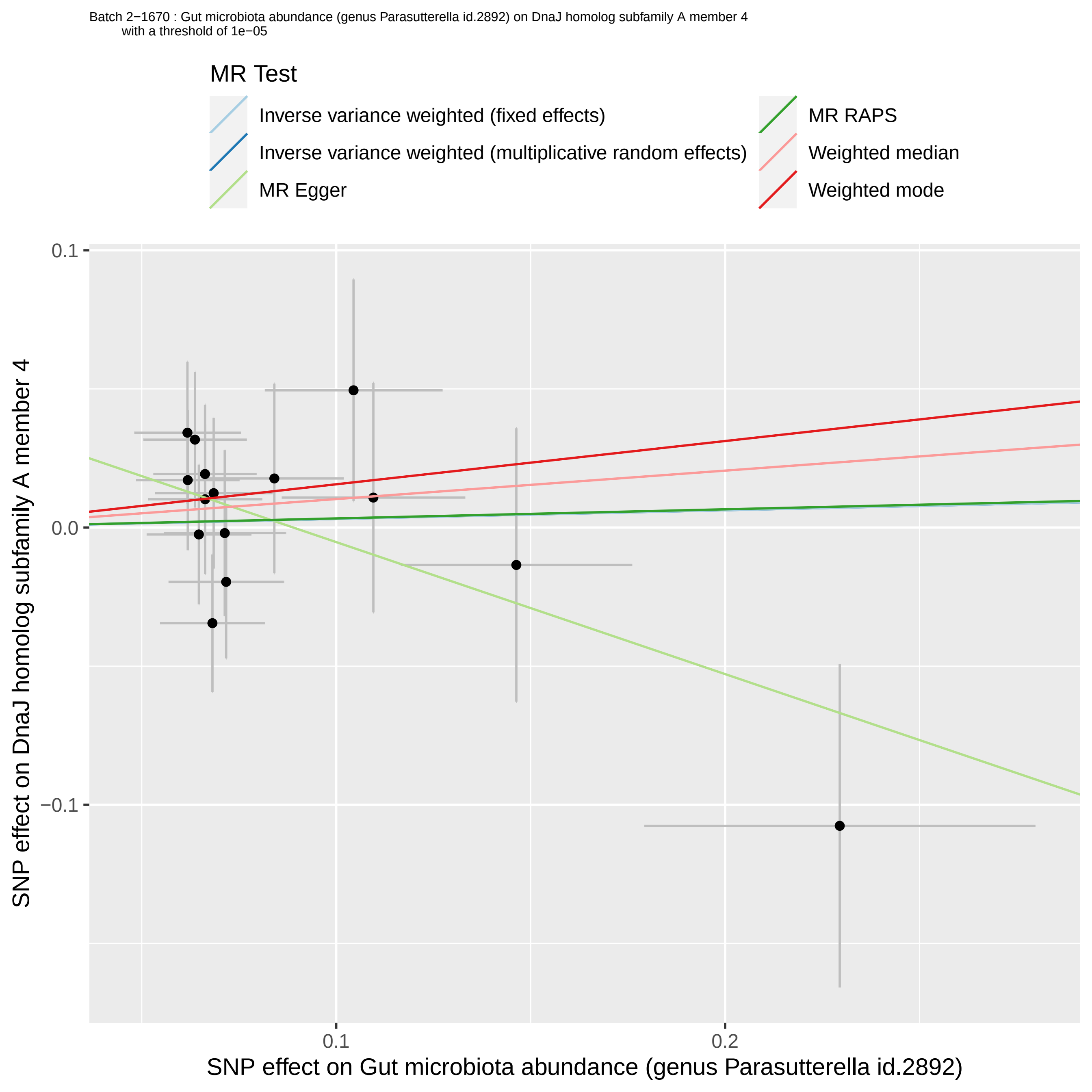
Batch 2−1670 : Gut microbiota abundance (genus Parasutterella id.2892) on DnaJ homolog subfamily A member 4
with a threshold of 1e−05
MR Test
Inverse variance weighted (fixed effects)
Inverse variance weighted (multiplicative random effects)
MR Egger
MR RAPS
Weighted median
Weighted mode
0.1
0.0
−0.1
0.1
0.2
SNP effect on DnaJ homolog subfamily A member 4
SNP effect on Gut microbiota abundance (genus Parasutterella id.2892)

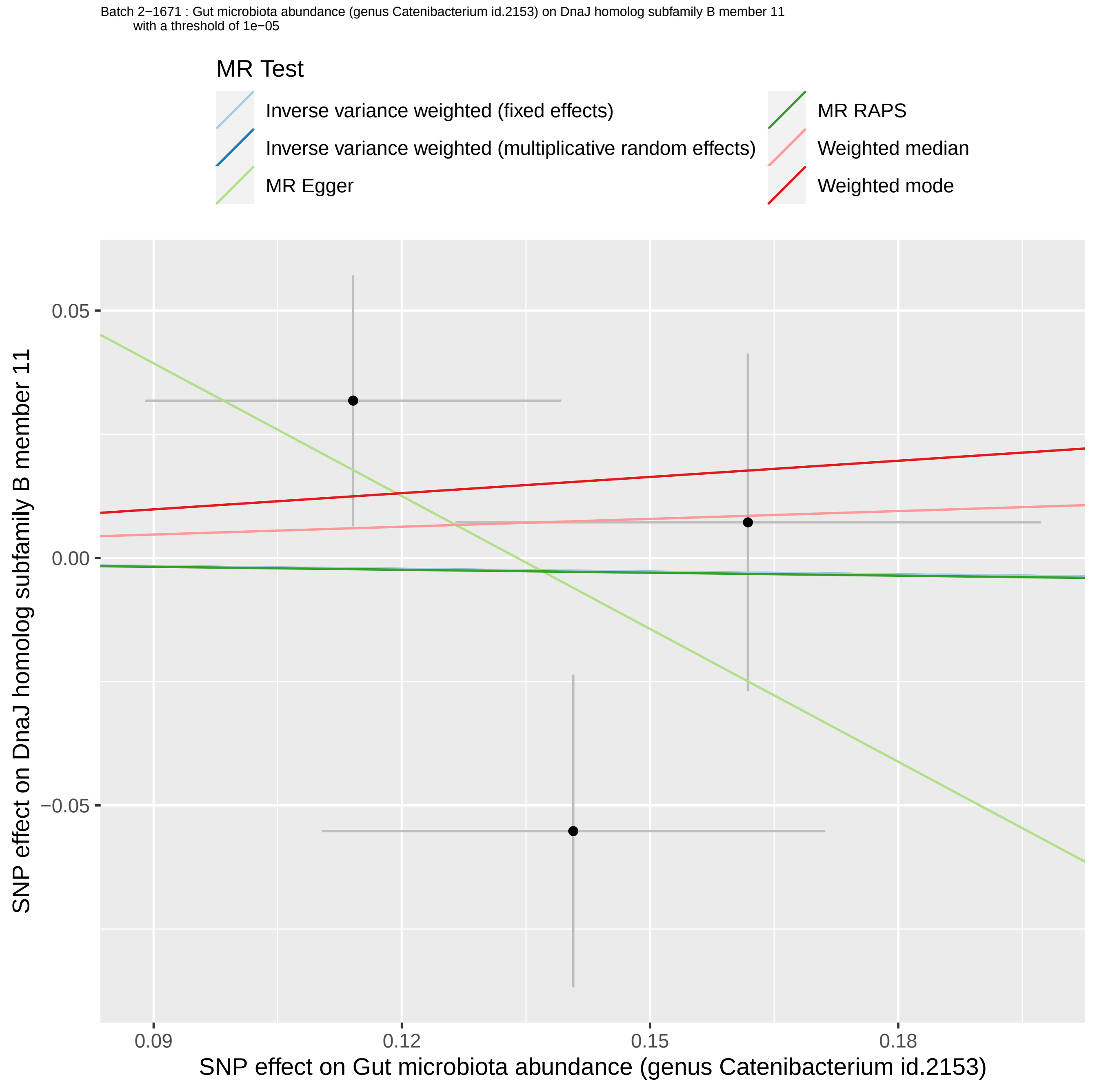

Batch 2−1671 : Gut microbiota abundance (genus Catenibacterium id.2153) on DnaJ homolog subfamily B member 11
with a threshold of 1e−05
MR Test
Inverse variance weighted (fixed effects)
Inverse variance weighted (multiplicative random effects)
MR Egger
MR RAPS
Weighted median
Weighted mode
SNP effect on DnaJ homolog subfamily B member 11
0.05
0.00
−0.05
0.09
0.12
0.15
0.18
SNP effect on Gut microbiota abundance (genus Catenibacterium id.2153)

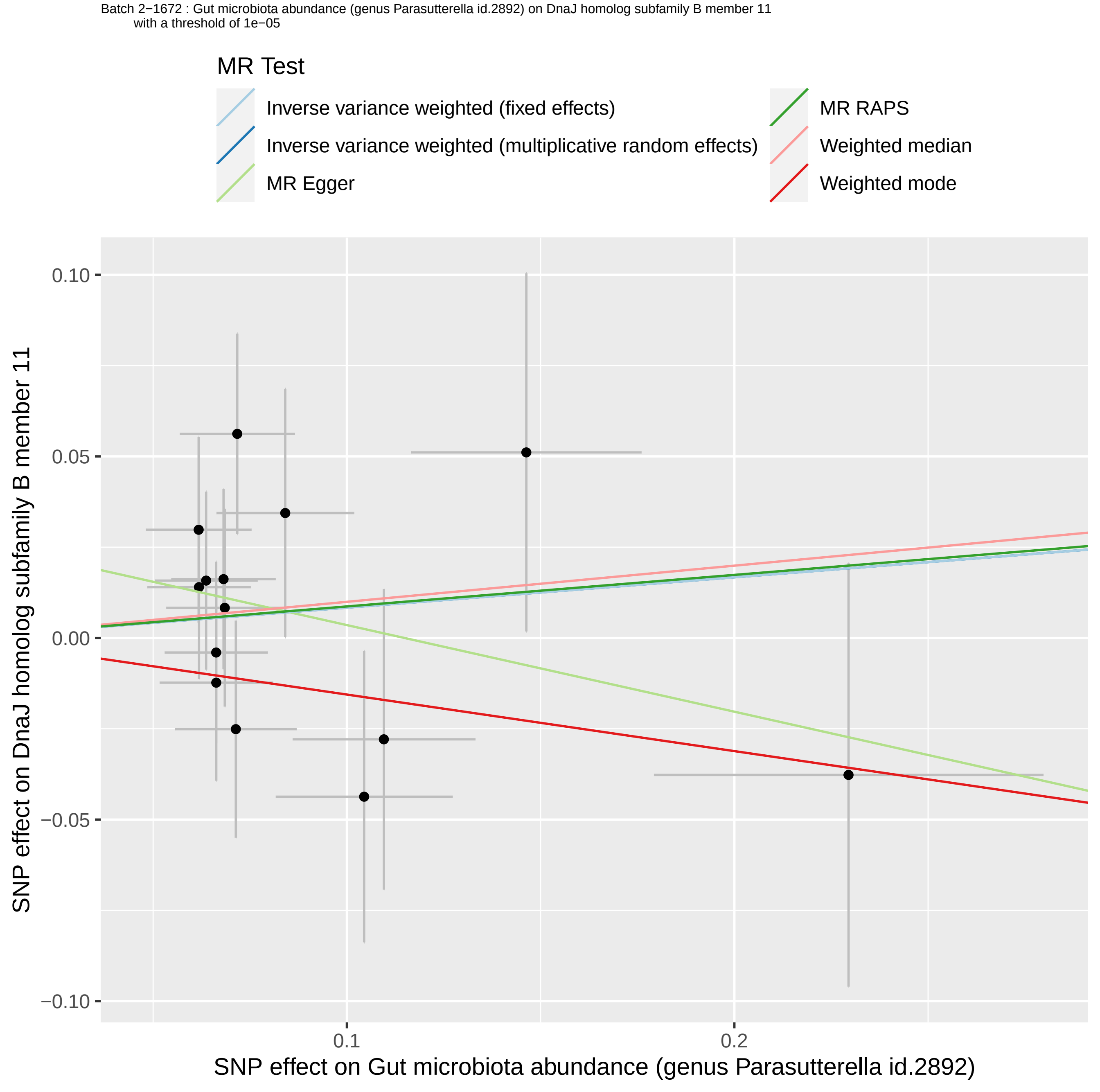

Batch 2−1672 : Gut microbiota abundance (genus Parasutterella id.2892) on DnaJ homolog subfamily B member 11
with a threshold of 1e−05
MR Test
Inverse variance weighted (fixed effects)
Inverse variance weighted (multiplicative random effects)
MR Egger
MR RAPS
Weighted median
Weighted mode
0.10
0.05
0.00
−0.05
−0.10
0.1
0.2
SNP effect on DnaJ homolog subfamily B member 11
SNP effect on Gut microbiota abundance (genus Parasutterella id.2892)

Batch 2−1673 : Gut microbiota abundance (genus Catenibacterium id.2153) on DnaJ homolog subfamily B member 12
with a threshold of 1e−05

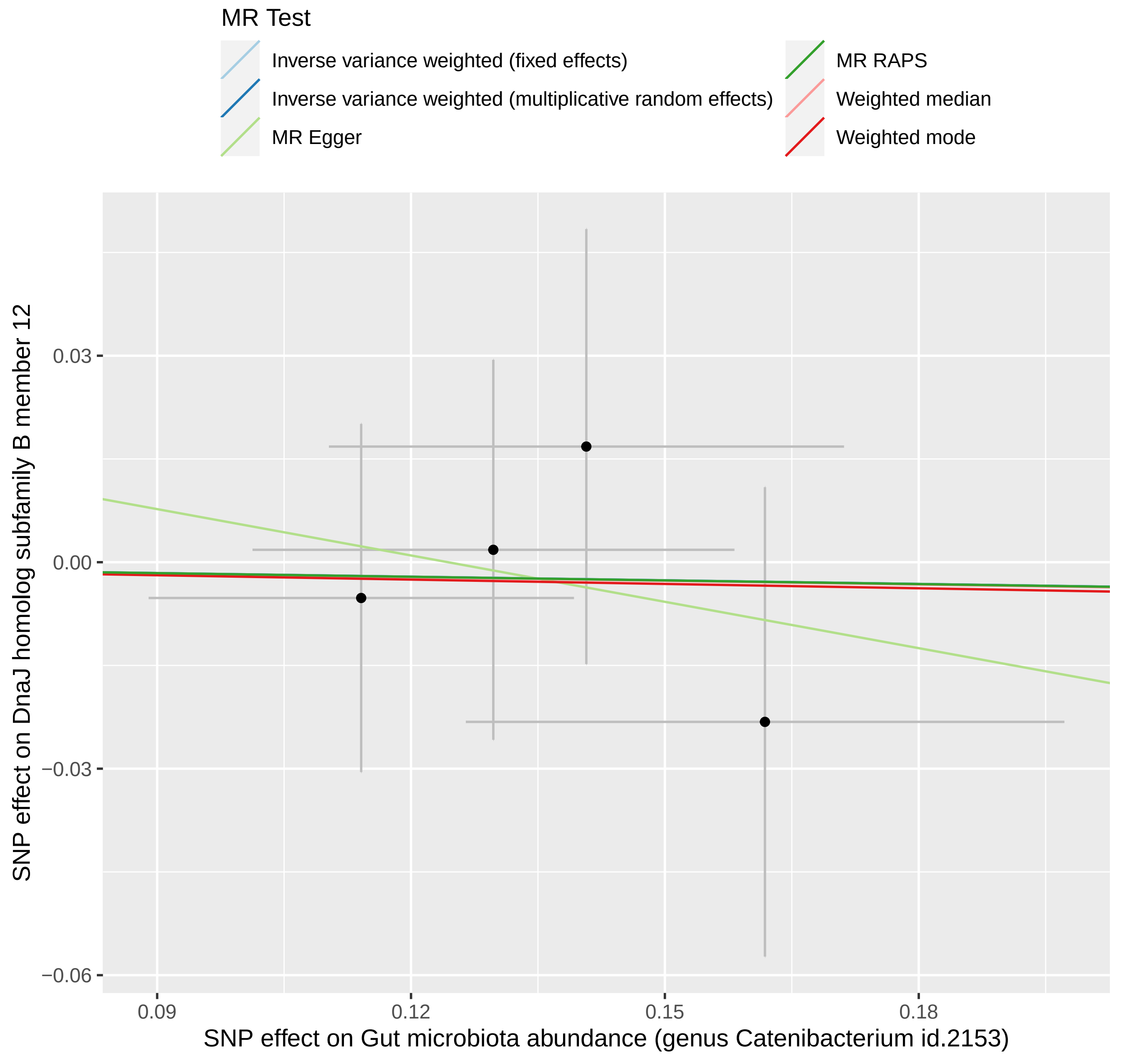

Batch 2−1674 : Gut microbiota abundance (genus Parasutterella id.2892) on DnaJ homolog subfamily B member 12
with a threshold of 1e−05

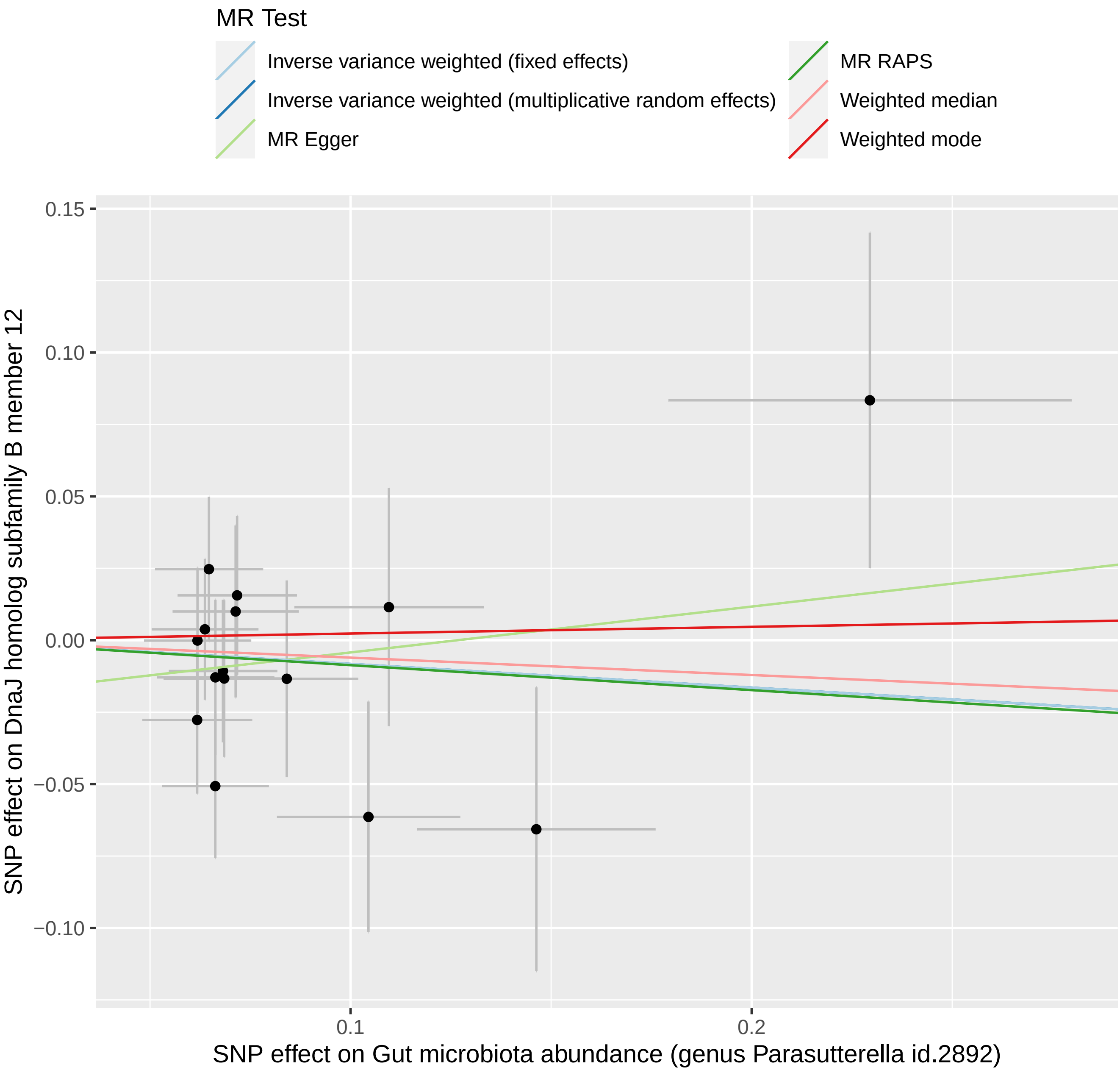

Batch 2−1675 : Gut microbiota abundance (genus Catenibacterium id.2153) on DnaJ homolog subfamily B member 1
with a threshold of 1e−05

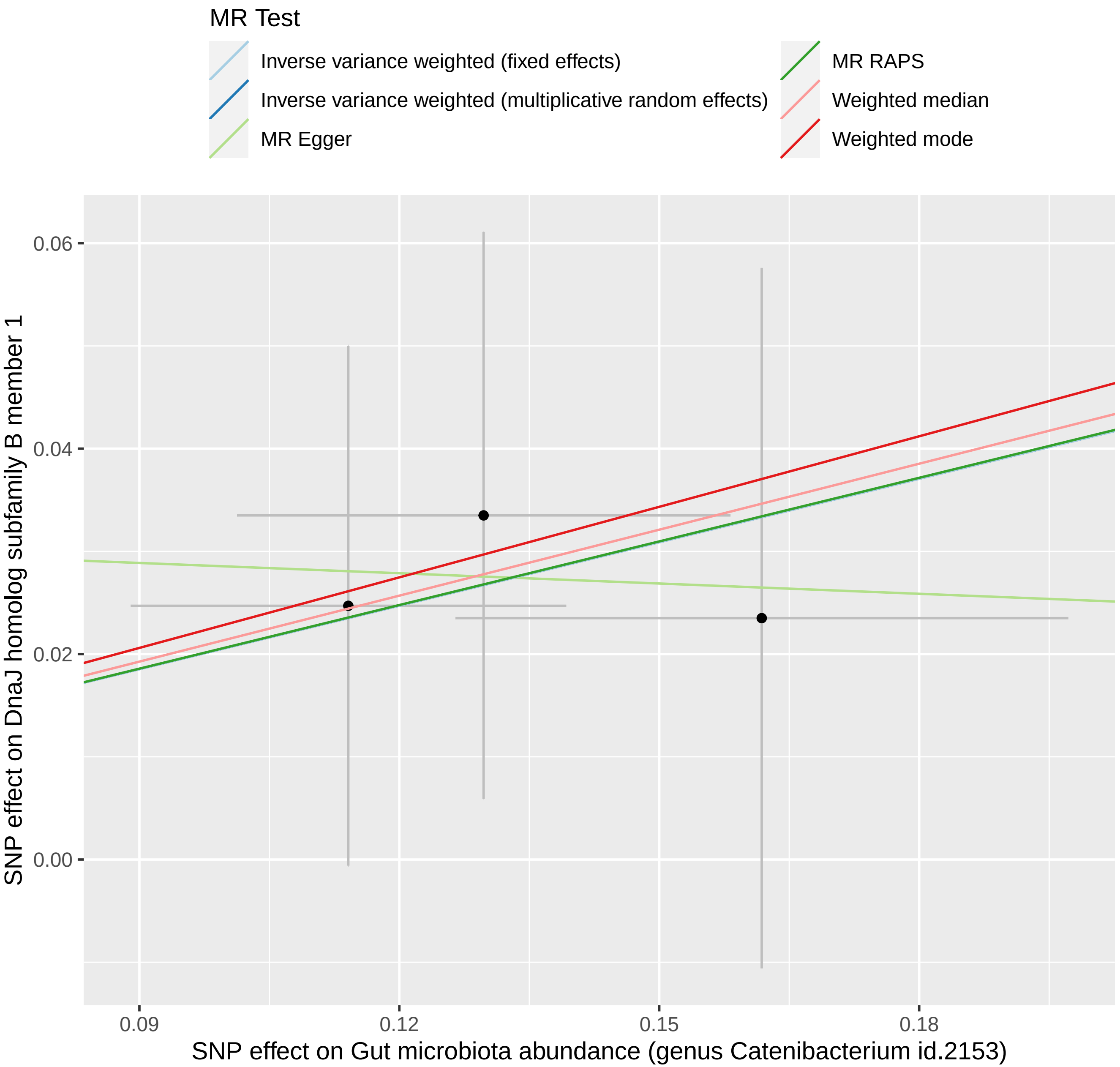

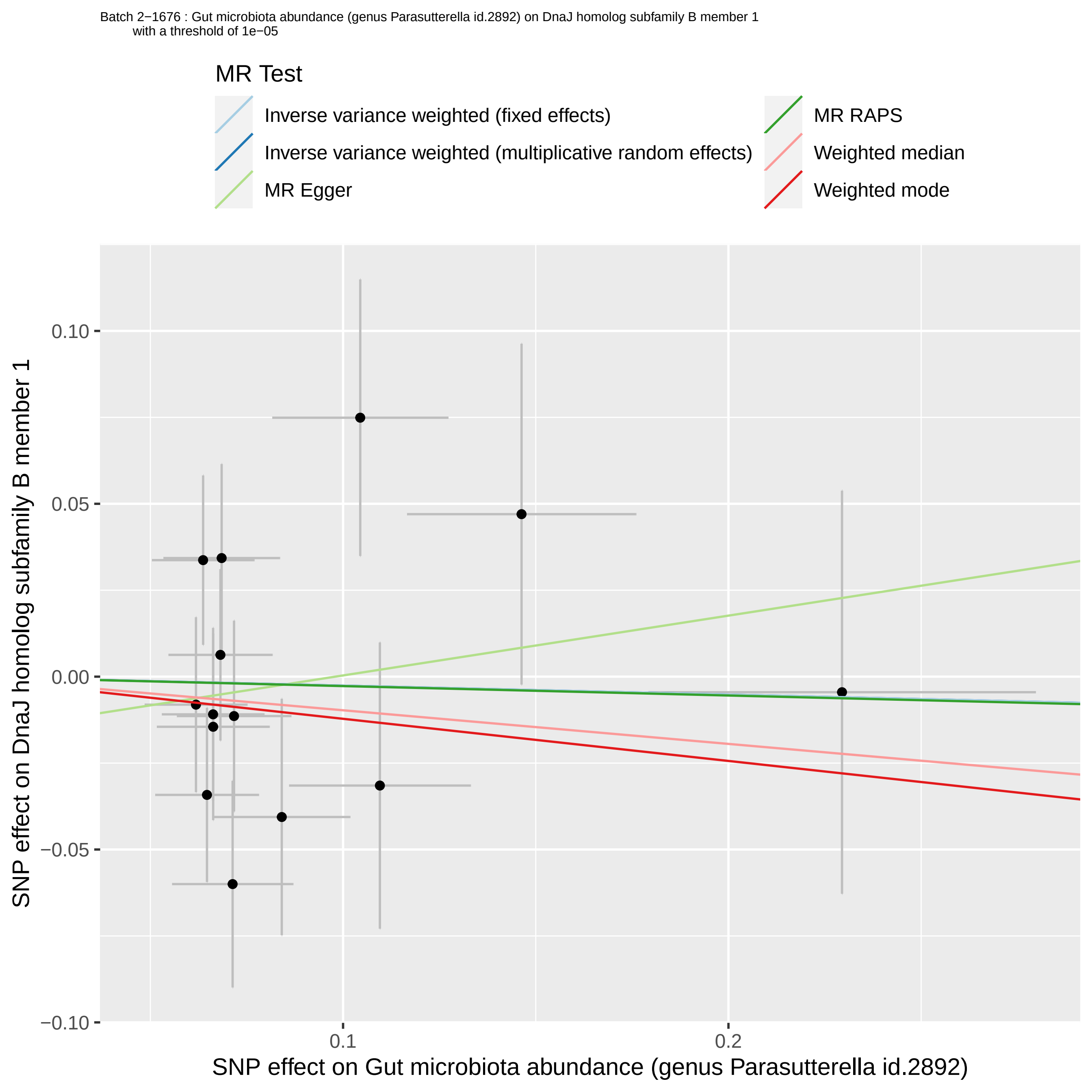

Batch 2−1676 : Gut microbiota abundance (genus Parasutterella id.2892) on DnaJ homolog subfamily B member 1
with a threshold of 1e−05
MR Test
Inverse variance weighted (fixed effects)
Inverse variance weighted (multiplicative random effects)
MR Egger
MR RAPS
Weighted median
Weighted mode
0.10
0.05
0.00
−0.05
−0.10
0.1
0.2
SNP effect on DnaJ homolog subfamily B member 1
SNP effect on Gut microbiota abundance (genus Parasutterella id.2892)

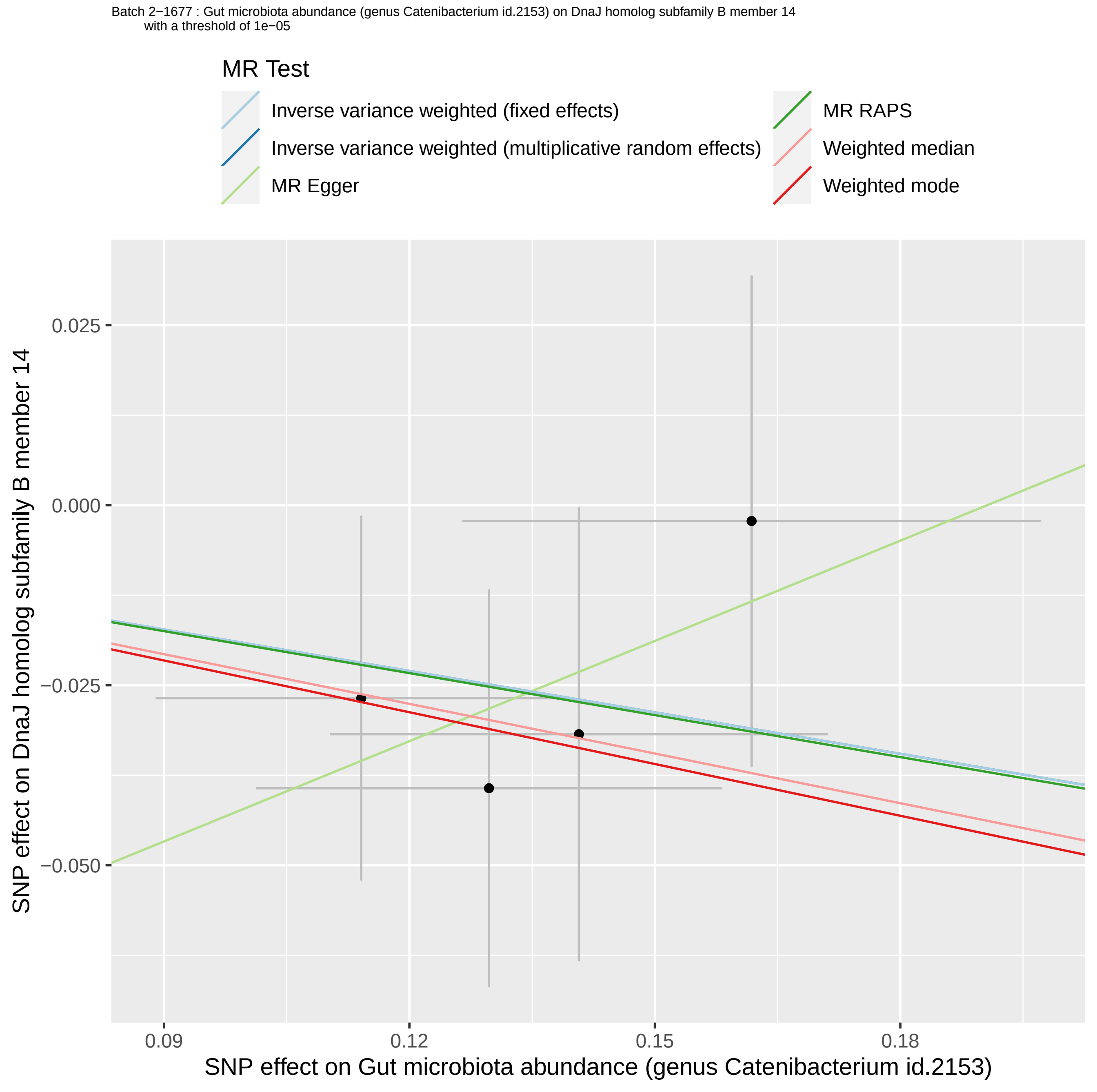
Batch 2−1677 : Gut microbiota abundance (genus Catenibacterium id.2153) on DnaJ homolog subfamily B member 14
with a threshold of 1e−05
MR Test
Inverse variance weighted (fixed effects)
Inverse variance weighted (multiplicative random effects)
MR Egger
MR RAPS
Weighted median
Weighted mode
0.025
0.000
−0.025
−0.050
0.09
0.12
0.15
0.18
SNP effect on DnaJ homolog subfamily B member 14
SNP effect on Gut microbiota abundance (genus Catenibacterium id.2153)

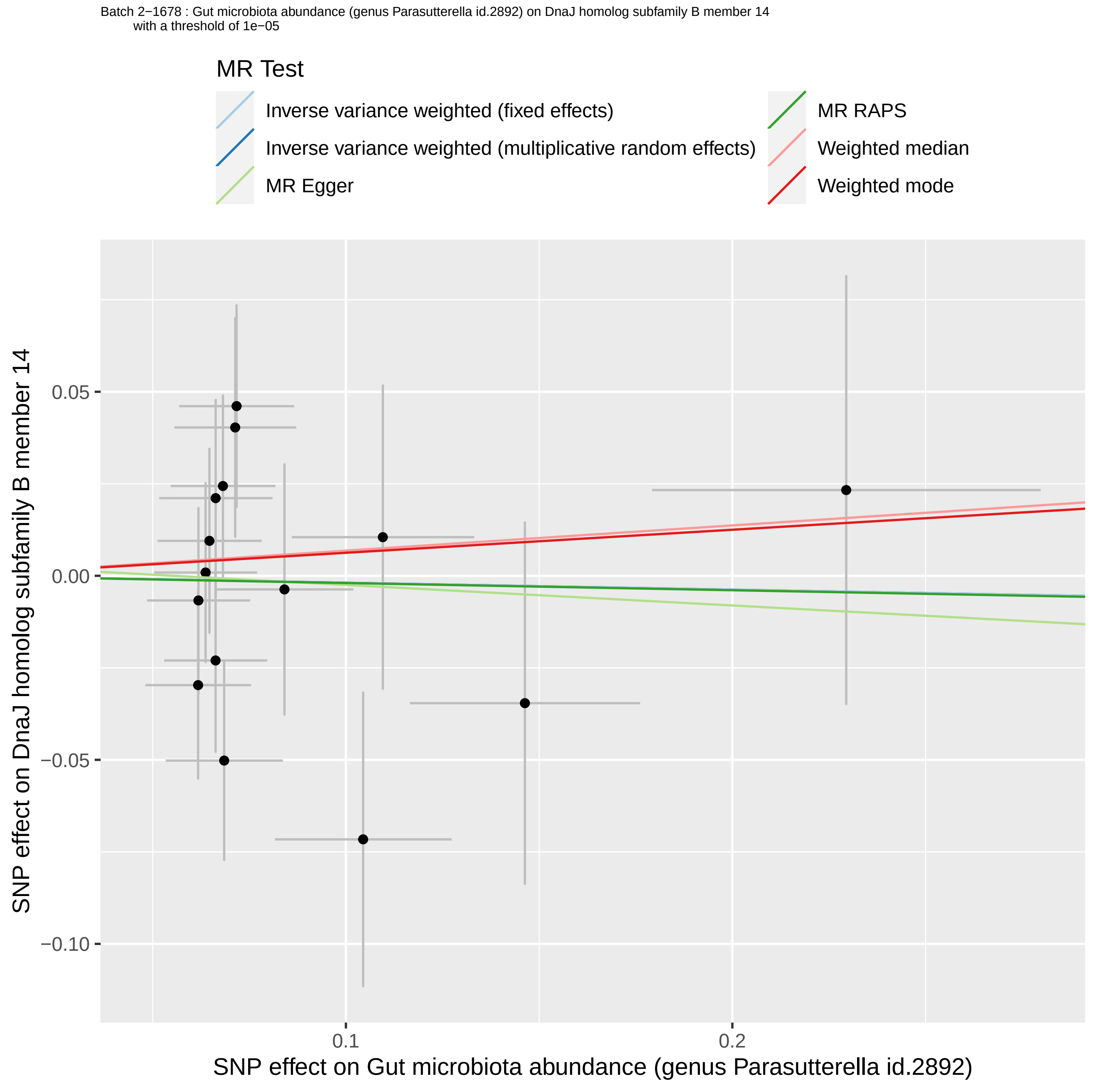

Batch 2−1678 : Gut microbiota abundance (genus Parasutterella id.2892) on DnaJ homolog subfamily B member 14
with a threshold of 1e−05
MR Test
Inverse variance weighted (fixed effects)
Inverse variance weighted (multiplicative random effects)
MR Egger
MR RAPS
Weighted median
Weighted mode
SNP effect on DnaJ homolog subfamily B member 14
0.05
0.00
−0.05
−0.10
0.1
0.2
SNP effect on Gut microbiota abundance (genus Parasutterella id.2892)

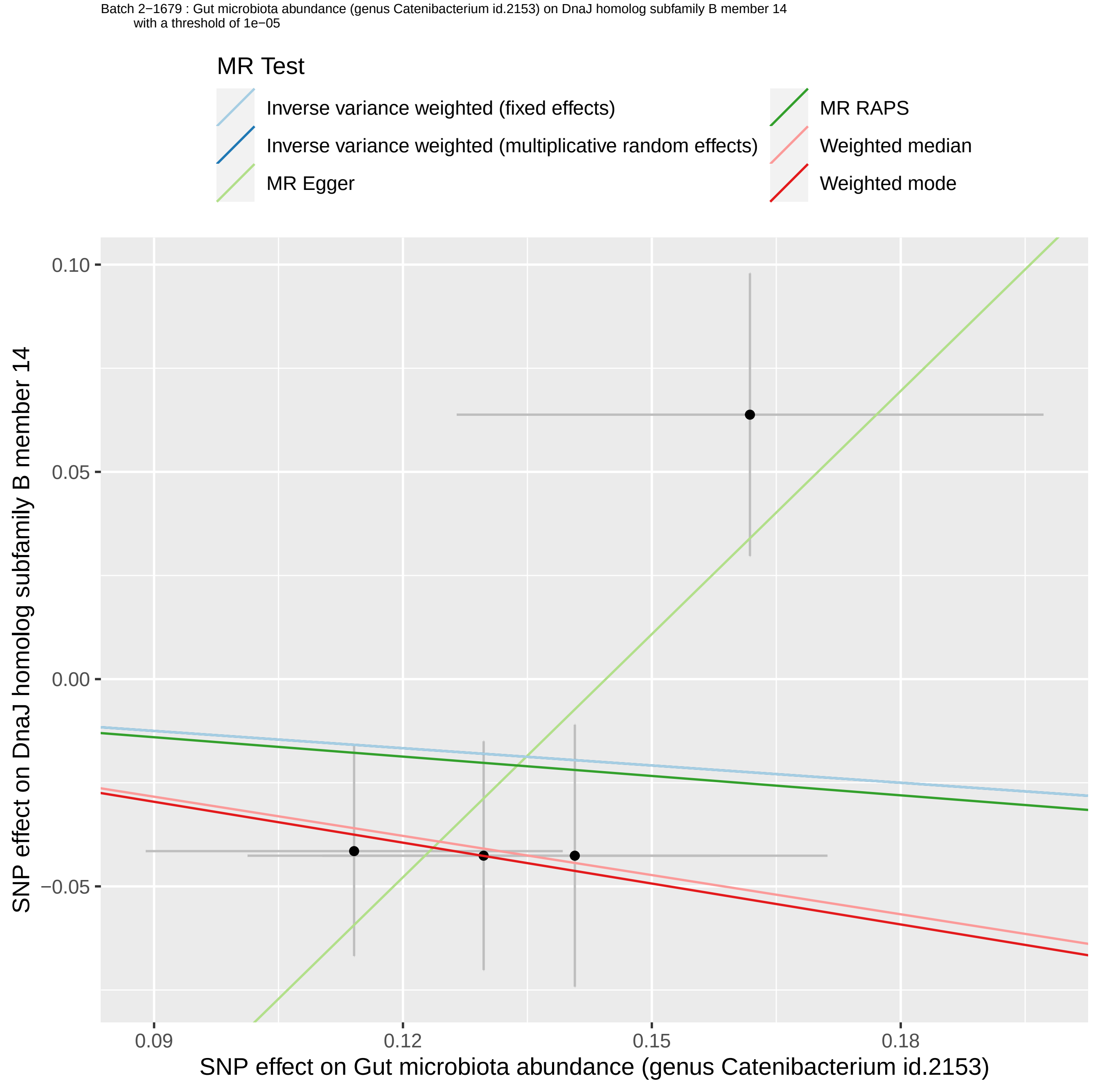
Batch 2−1679 : Gut microbiota abundance (genus Catenibacterium id.2153) on DnaJ homolog subfamily B member 14
with a threshold of 1e−05
MR Test
Inverse variance weighted (fixed effects)
Inverse variance weighted (multiplicative random effects)
MR Egger
MR RAPS
Weighted median
Weighted mode
0.10
0.05
0.00
−0.05
0.09
0.12
0.15
0.18
SNP effect on DnaJ homolog subfamily B member 14
SNP effect on Gut microbiota abundance (genus Catenibacterium id.2153)

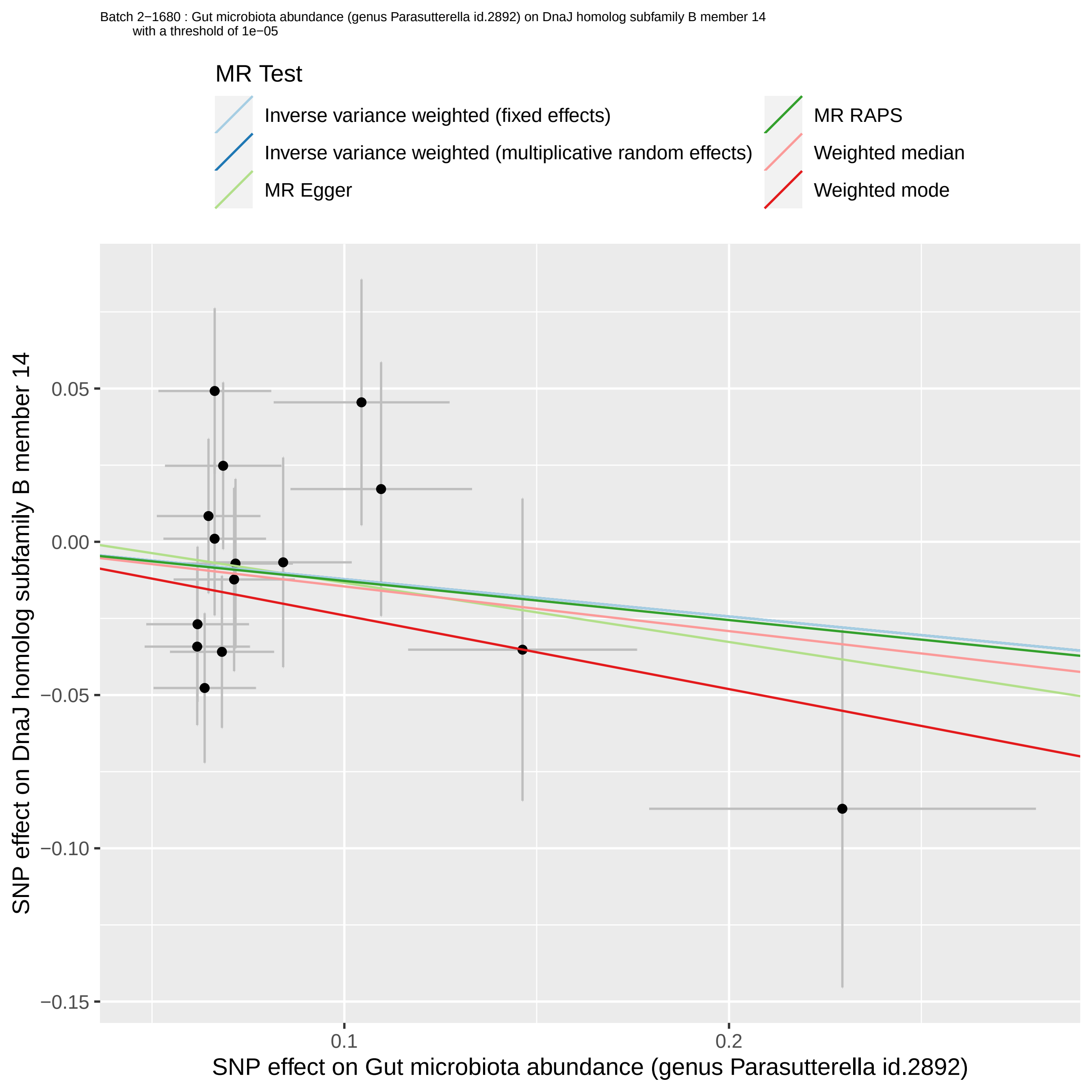
Batch 2−1680 : Gut microbiota abundance (genus Parasutterella id.2892) on DnaJ homolog subfamily B member 14
with a threshold of 1e−05
MR Test
Inverse variance weighted (fixed effects)
Inverse variance weighted (multiplicative random effects)
MR Egger
MR RAPS
Weighted median
Weighted mode
0.05
0.00
−0.05
−0.10
−0.15
0.1
0.2
SNP effect on DnaJ homolog subfamily B member 14
SNP effect on Gut microbiota abundance (genus Parasutterella id.2892)

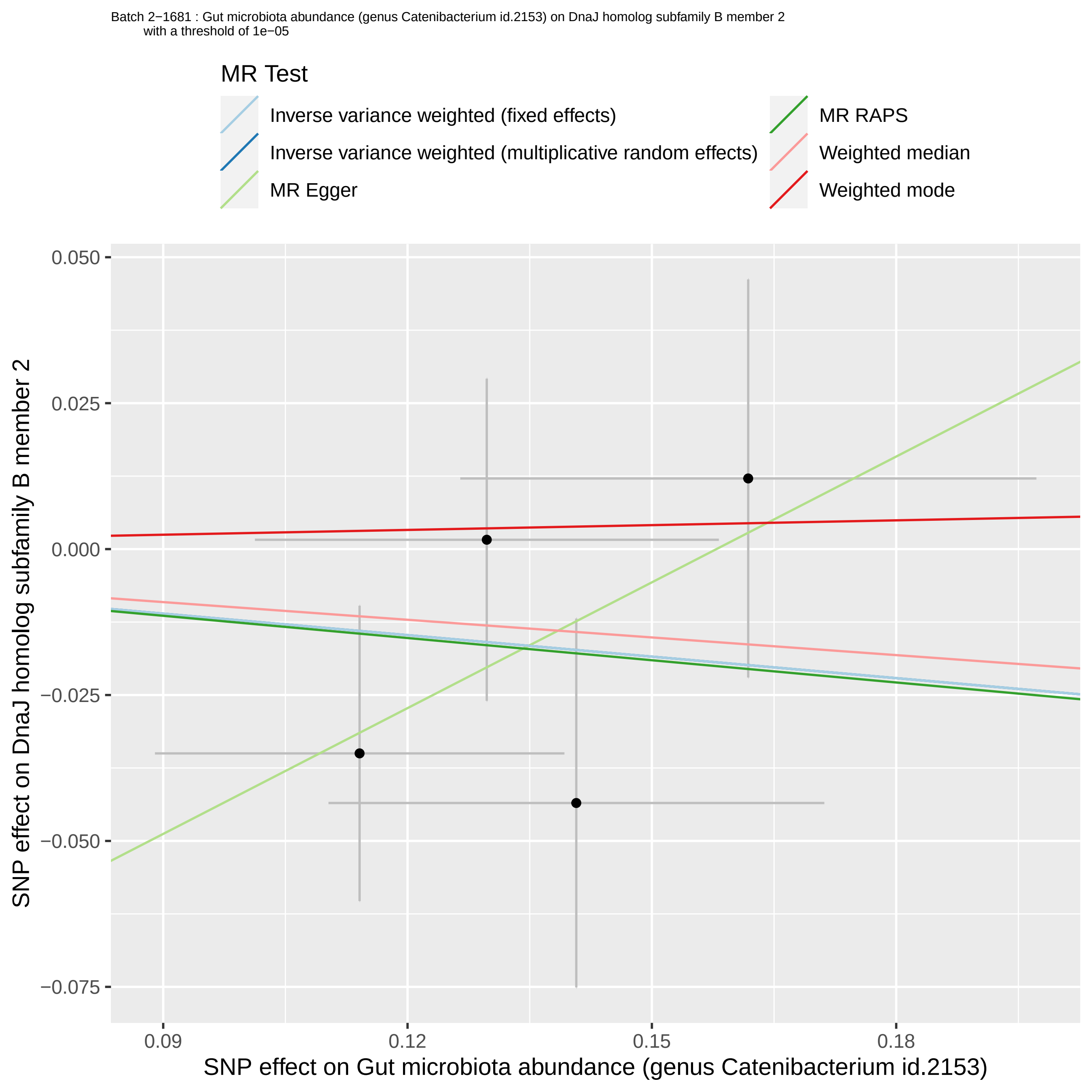
Batch 2−1681 : Gut microbiota abundance (genus Catenibacterium id.2153) on DnaJ homolog subfamily B member 2
with a threshold of 1e−05
MR Test
Inverse variance weighted (fixed effects)
Inverse variance weighted (multiplicative random effects)
MR Egger
MR RAPS
Weighted median
Weighted mode
0.050
0.025
0.000
−0.025
−0.050
−0.075
0.09
0.12
0.15
0.18
SNP effect on DnaJ homolog subfamily B member 2
SNP effect on Gut microbiota abundance (genus Catenibacterium id.2153)

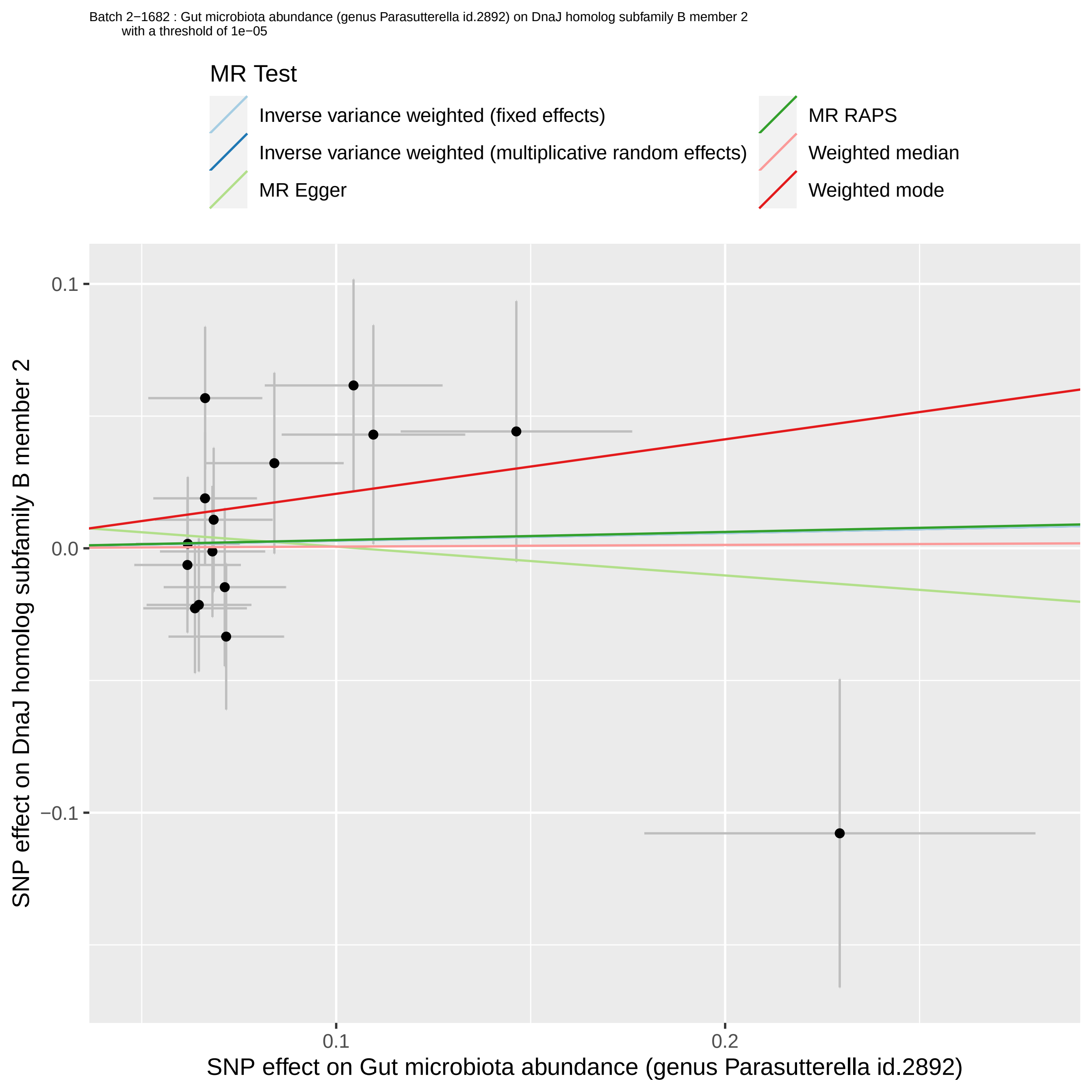
Batch 2−1682 : Gut microbiota abundance (genus Parasutterella id.2892) on DnaJ homolog subfamily B member 2
with a threshold of 1e−05
MR Test
Inverse variance weighted (fixed effects)
Inverse variance weighted (multiplicative random effects)
MR Egger
MR RAPS
Weighted median
Weighted mode
0.1
0.0
−0.1
0.1
0.2
SNP effect on DnaJ homolog subfamily B member 2
SNP effect on Gut microbiota abundance (genus Parasutterella id.2892)

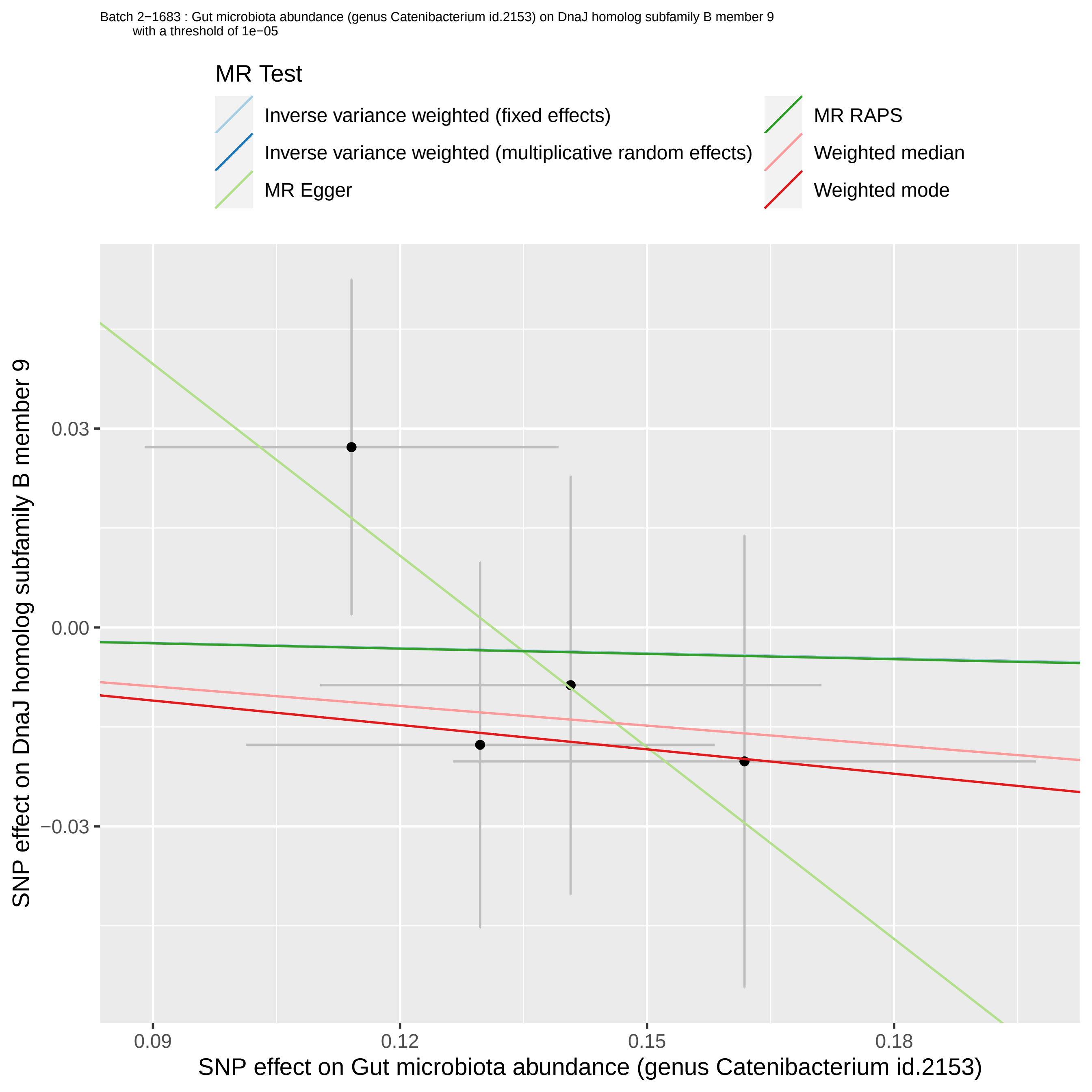

Batch 2−1683 : Gut microbiota abundance (genus Catenibacterium id.2153) on DnaJ homolog subfamily B member 9
with a threshold of 1e−05
MR Test
Inverse variance weighted (fixed effects)
Inverse variance weighted (multiplicative random effects)
MR Egger
MR RAPS
Weighted median
Weighted mode
SNP effect on DnaJ homolog subfamily B member 9
0.03
0.00
−0.03
0.09
0.12
0.15
0.18
SNP effect on Gut microbiota abundance (genus Catenibacterium id.2153)

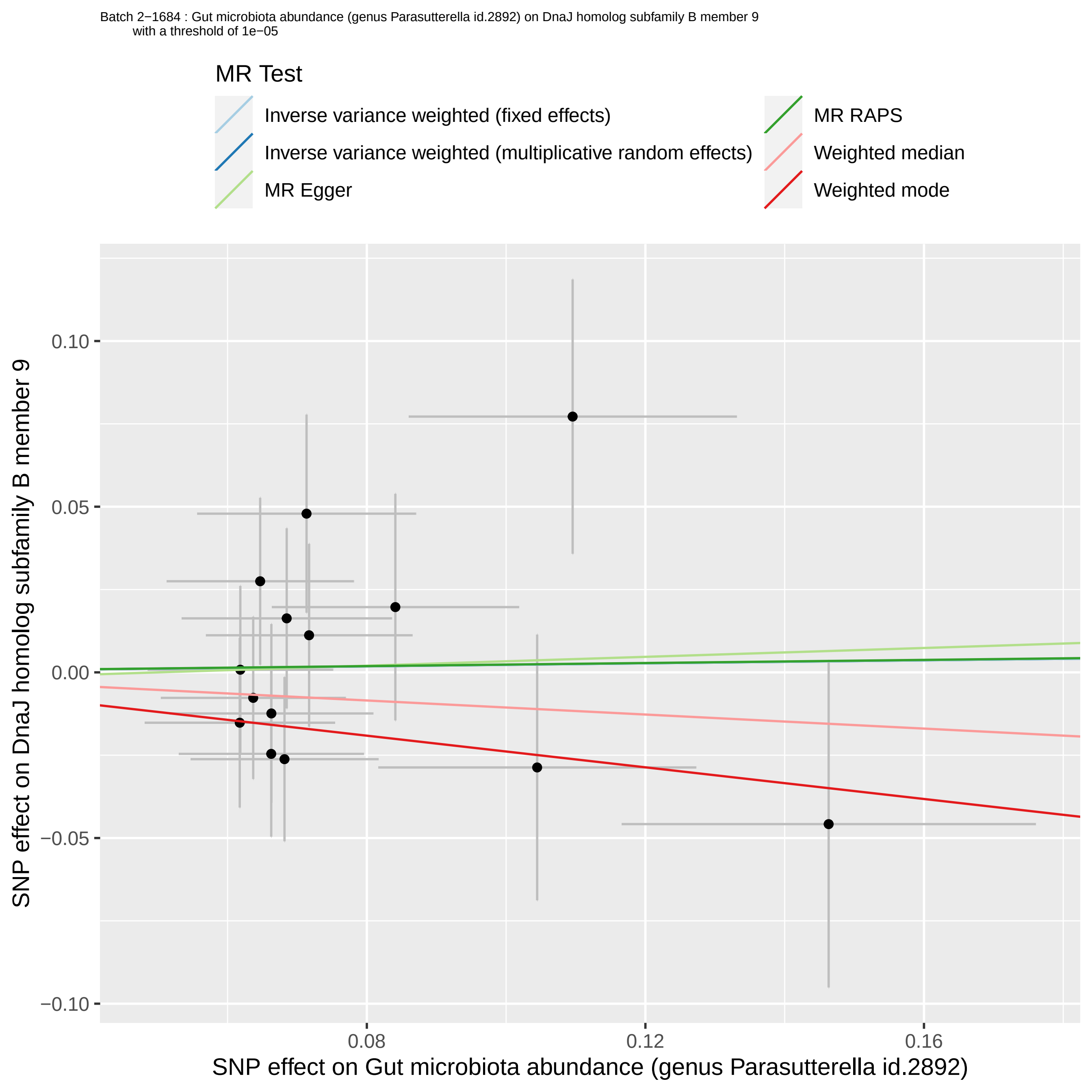

Batch 2−1684 : Gut microbiota abundance (genus Parasutterella id.2892) on DnaJ homolog subfamily B member 9
with a threshold of 1e−05
MR Test
Inverse variance weighted (fixed effects)
Inverse variance weighted (multiplicative random effects)
MR Egger
MR RAPS
Weighted median
Weighted mode
0.10
0.05
0.00
−0.05
−0.10
0.08
0.12
0.16
SNP effect on DnaJ homolog subfamily B member 9
SNP effect on Gut microbiota abundance (genus Parasutterella id.2892)

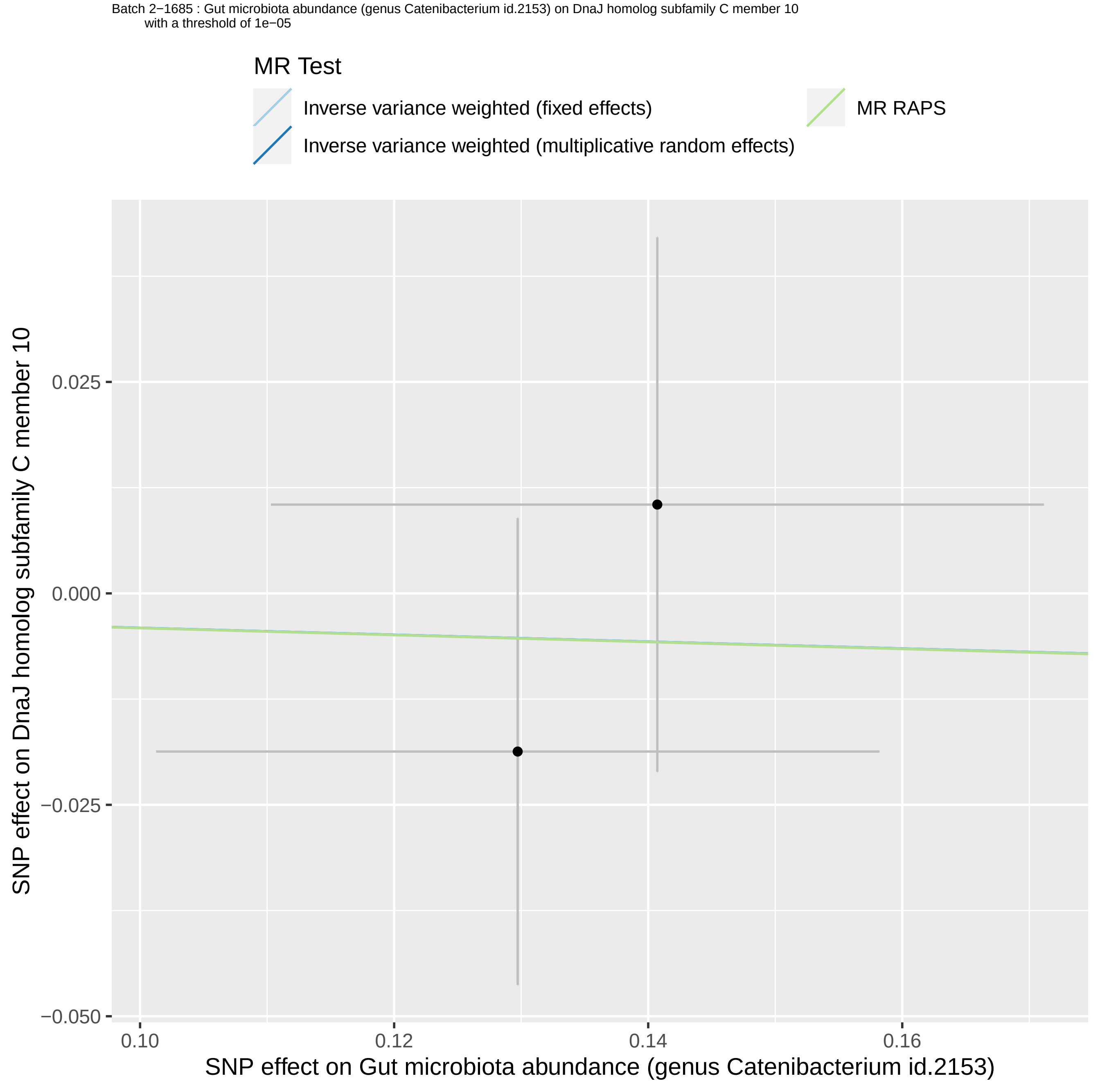
Batch 2−1685 : Gut microbiota abundance (genus Catenibacterium id.2153) on DnaJ homolog subfamily C member 10
with a threshold of 1e−05
MR Test
Inverse variance weighted (fixed effects)
Inverse variance weighted (multiplicative random effects)
MR RAPS
SNP effect on DnaJ homolog subfamily C member 10
0.025
0.000
−0.025
−0.050
0.10
0.12
0.14
0.16
SNP effect on Gut microbiota abundance (genus Catenibacterium id.2153)

Batch 2−1686 : Gut microbiota abundance (genus Parasutterella id.2892) on DnaJ homolog subfamily C member 10
with a threshold of 1e−05

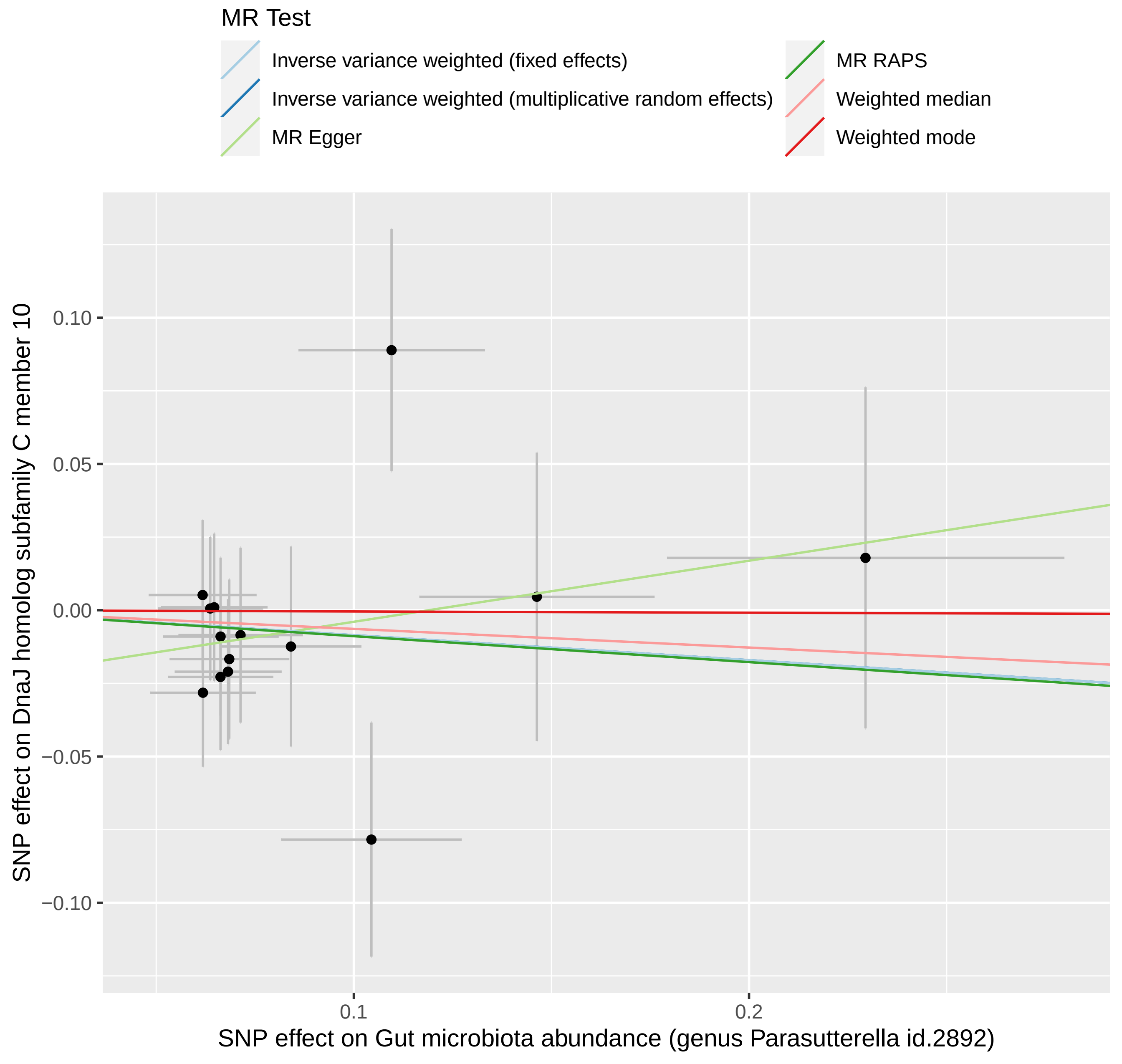

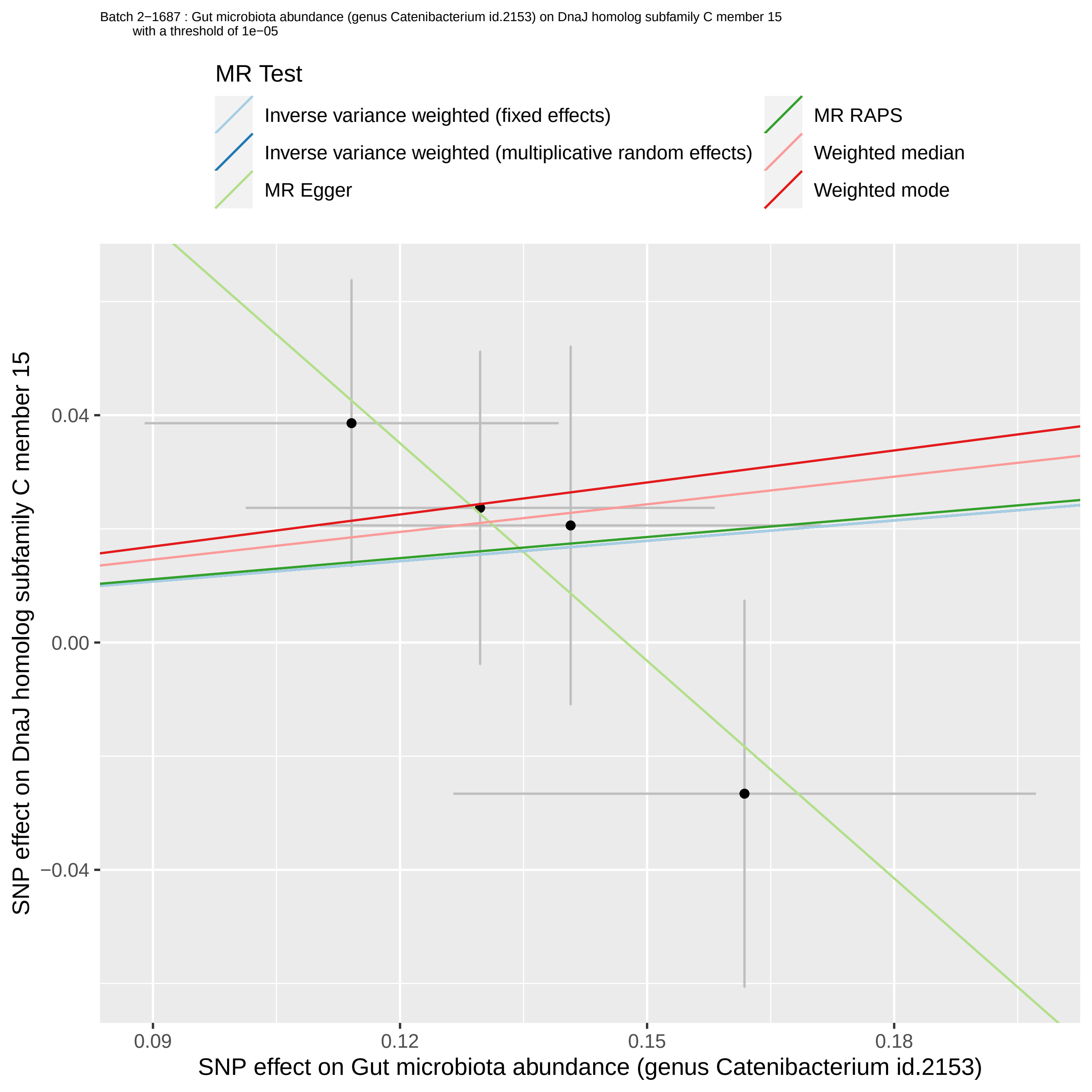
Batch 2−1687 : Gut microbiota abundance (genus Catenibacterium id.2153) on DnaJ homolog subfamily C member 15
with a threshold of 1e−05
MR Test
Inverse variance weighted (fixed effects)
Inverse variance weighted (multiplicative random effects)
MR Egger
MR RAPS
Weighted median
Weighted mode
0.04
0.00
−0.04
0.09
0.12
0.15
0.18
SNP effect on DnaJ homolog subfamily C member 15
SNP effect on Gut microbiota abundance (genus Catenibacterium id.2153)

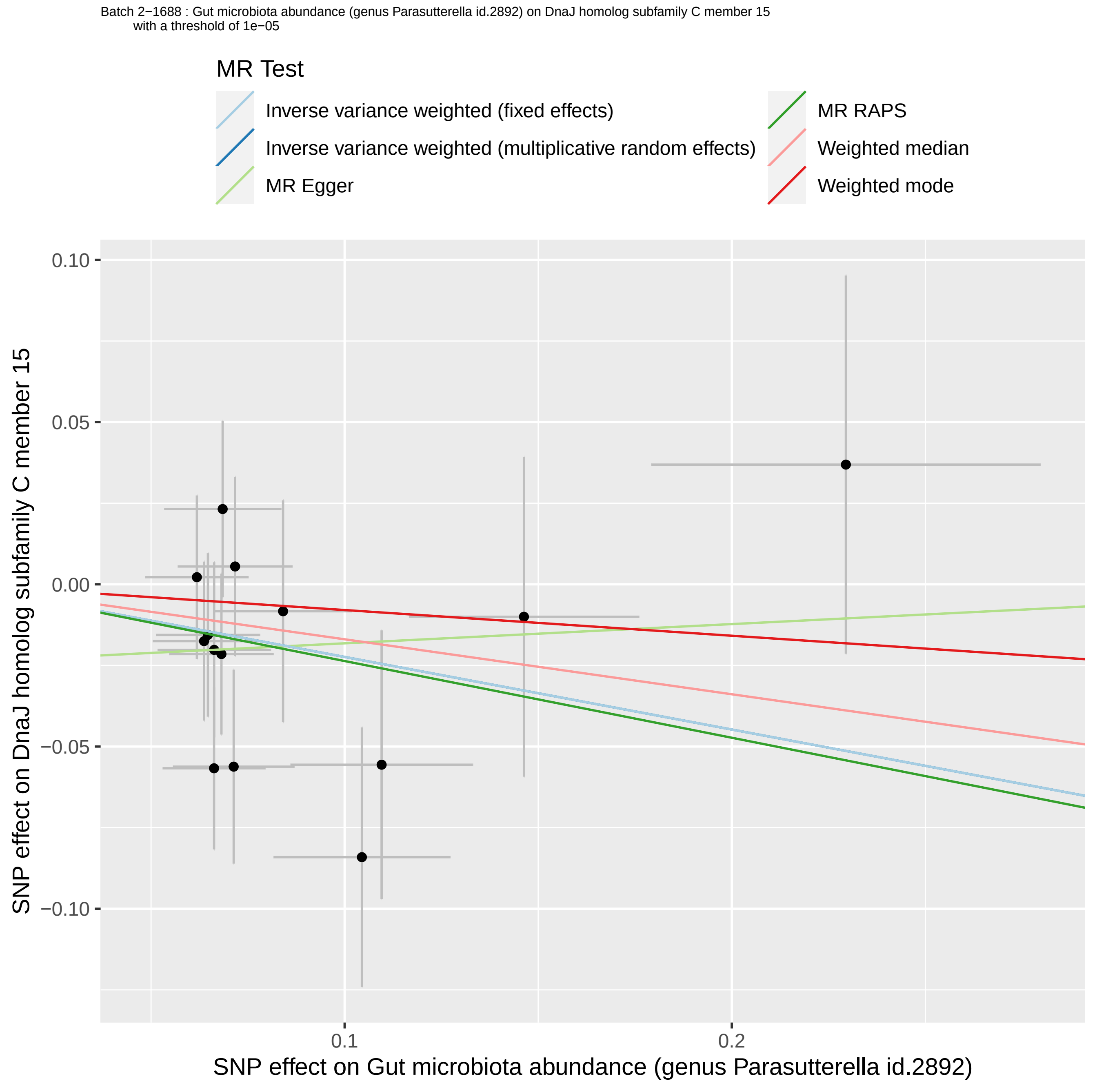

Batch 2−1688 : Gut microbiota abundance (genus Parasutterella id.2892) on DnaJ homolog subfamily C member 15
with a threshold of 1e−05
MR Test
Inverse variance weighted (fixed effects)
Inverse variance weighted (multiplicative random effects)
MR Egger
MR RAPS
Weighted median
Weighted mode
0.10
0.05
0.00
−0.05
−0.10
0.1
0.2
SNP effect on DnaJ homolog subfamily C member 15
SNP effect on Gut microbiota abundance (genus Parasutterella id.2892)

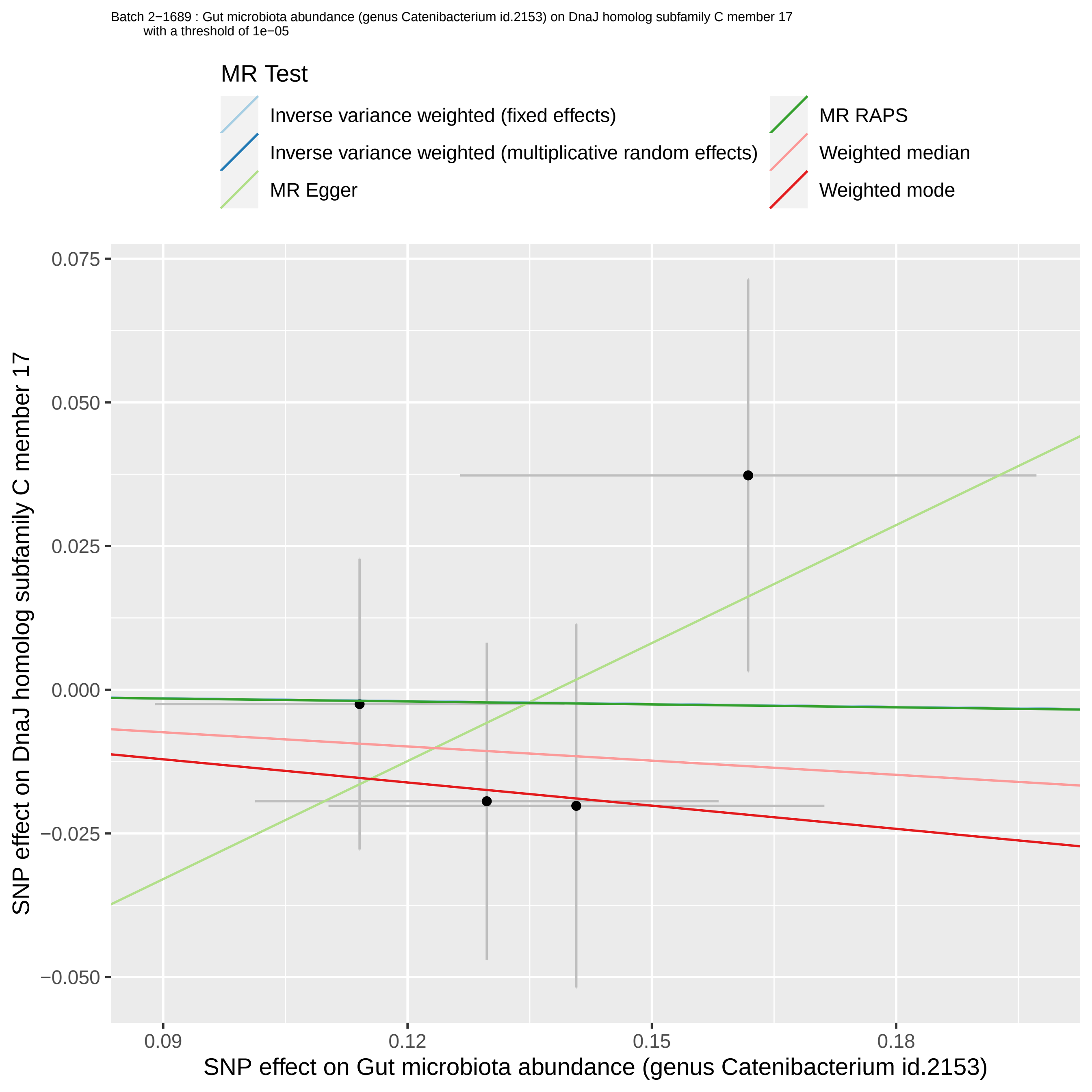

Batch 2−1689 : Gut microbiota abundance (genus Catenibacterium id.2153) on DnaJ homolog subfamily C member 17
with a threshold of 1e−05
MR Test
Inverse variance weighted (fixed effects)
Inverse variance weighted (multiplicative random effects)
MR Egger
MR RAPS
Weighted median
Weighted mode
0.075
0.050
0.025
0.000
−0.025
−0.050
SNP effect on DnaJ homolog subfamily C member 17
0.09
0.12
0.15
0.18
SNP effect on Gut microbiota abundance (genus Catenibacterium id.2153)

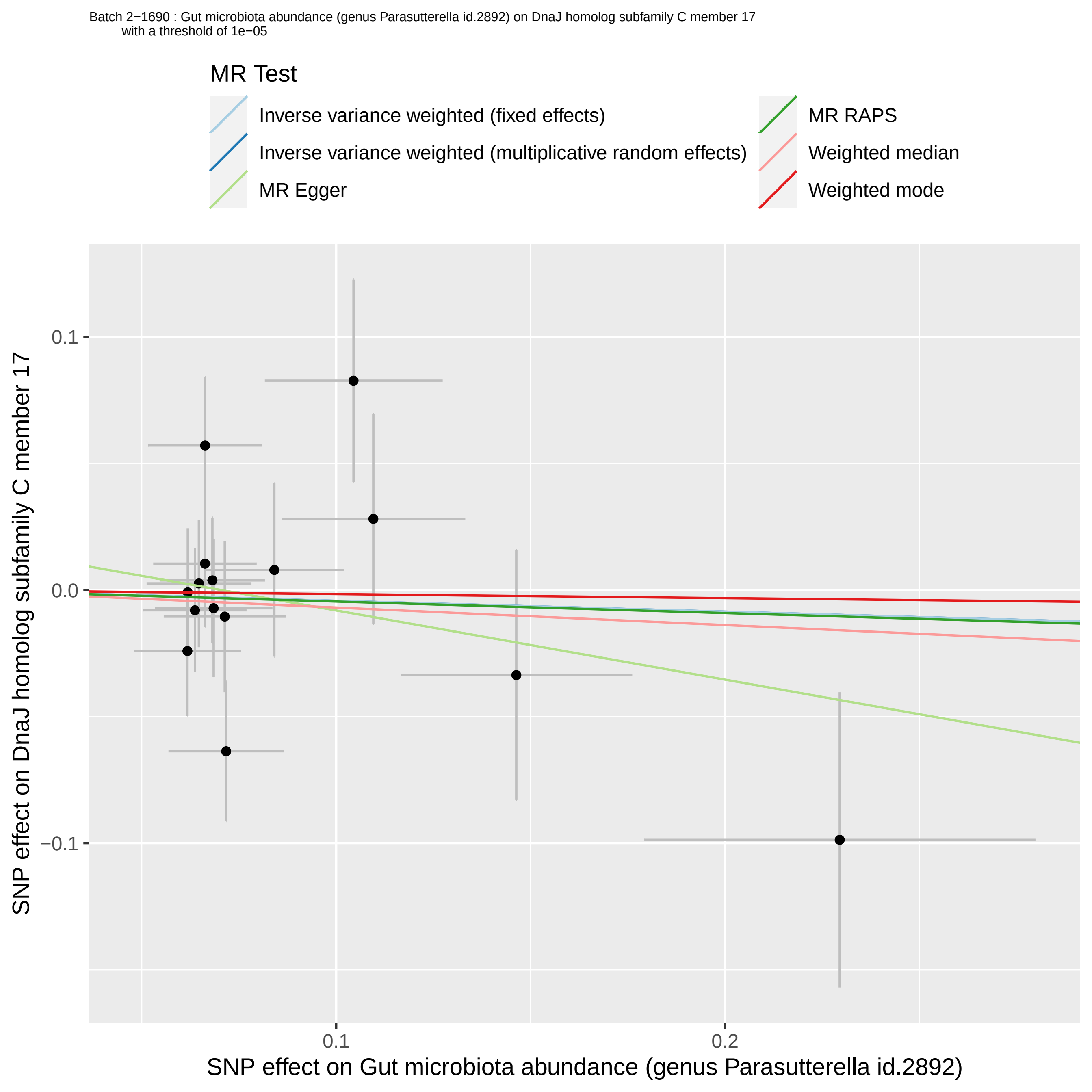

Batch 2−1690 : Gut microbiota abundance (genus Parasutterella id.2892) on DnaJ homolog subfamily C member 17
with a threshold of 1e−05
MR Test
Inverse variance weighted (fixed effects)
Inverse variance weighted (multiplicative random effects)
MR Egger
MR RAPS
Weighted median
Weighted mode
0.1
0.0
−0.1
0.1
0.2
SNP effect on DnaJ homolog subfamily C member 17
SNP effect on Gut microbiota abundance (genus Parasutterella id.2892)

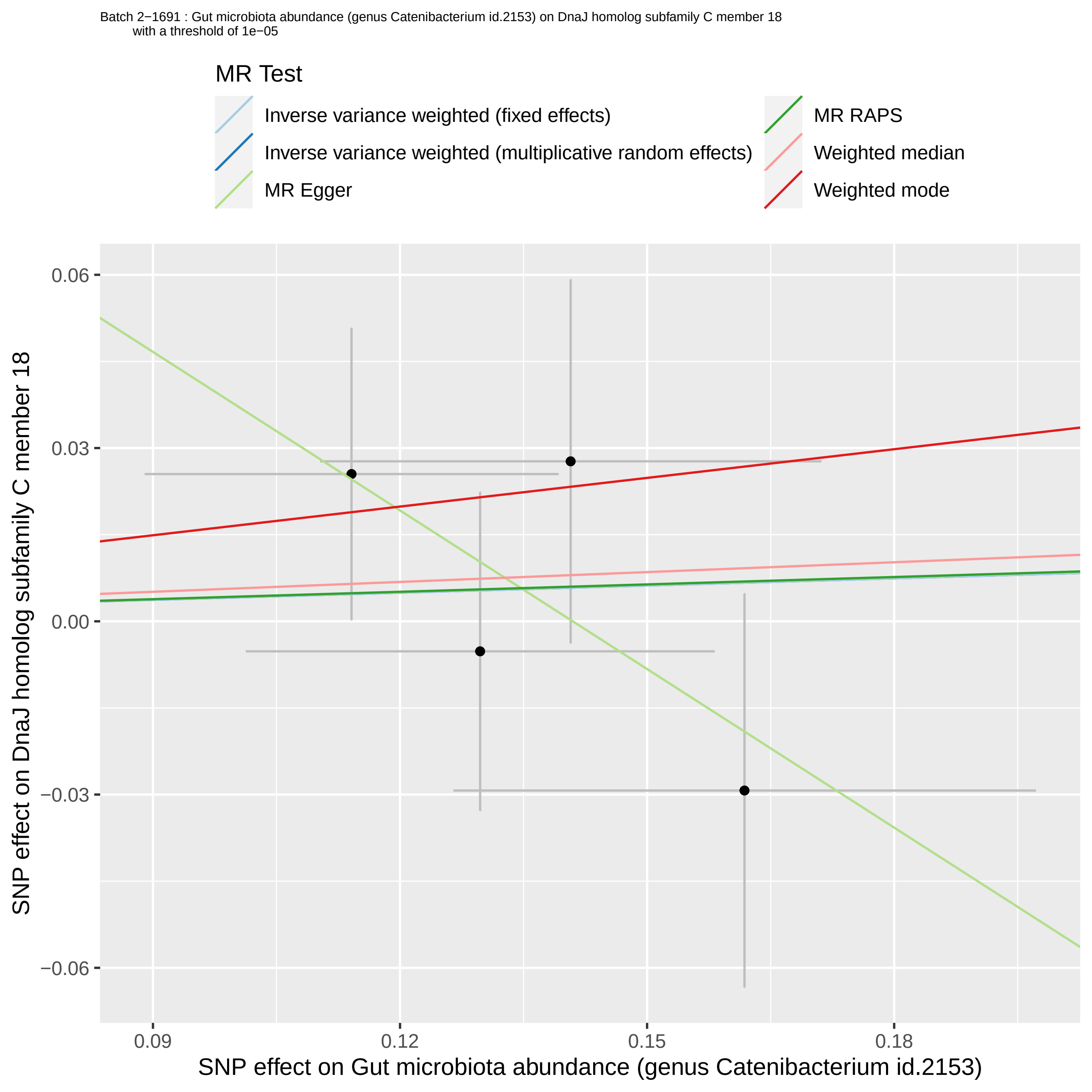
Batch 2−1691 : Gut microbiota abundance (genus Catenibacterium id.2153) on DnaJ homolog subfamily C member 18
with a threshold of 1e−05
MR Test
Inverse variance weighted (fixed effects)
Inverse variance weighted (multiplicative random effects)
MR Egger
MR RAPS
Weighted median
Weighted mode
0.06
0.03
0.00
−0.03
−0.06
0.09
0.12
0.15
0.18
SNP effect on DnaJ homolog subfamily C member 18
SNP effect on Gut microbiota abundance (genus Catenibacterium id.2153)

Batch 2−1692 : Gut microbiota abundance (genus Parasutterella id.2892) on DnaJ homolog subfamily C member 18
with a threshold of 1e−05

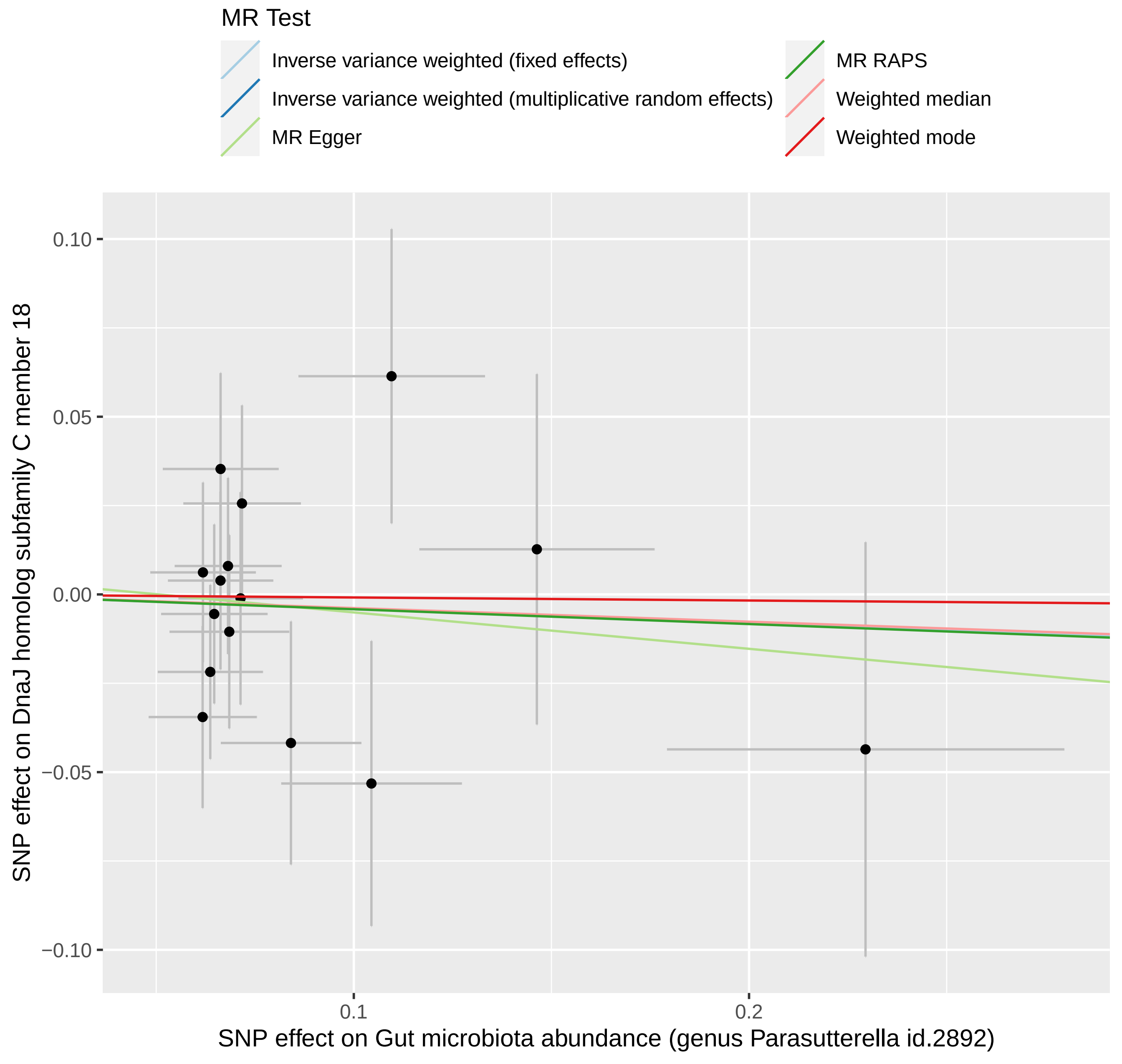

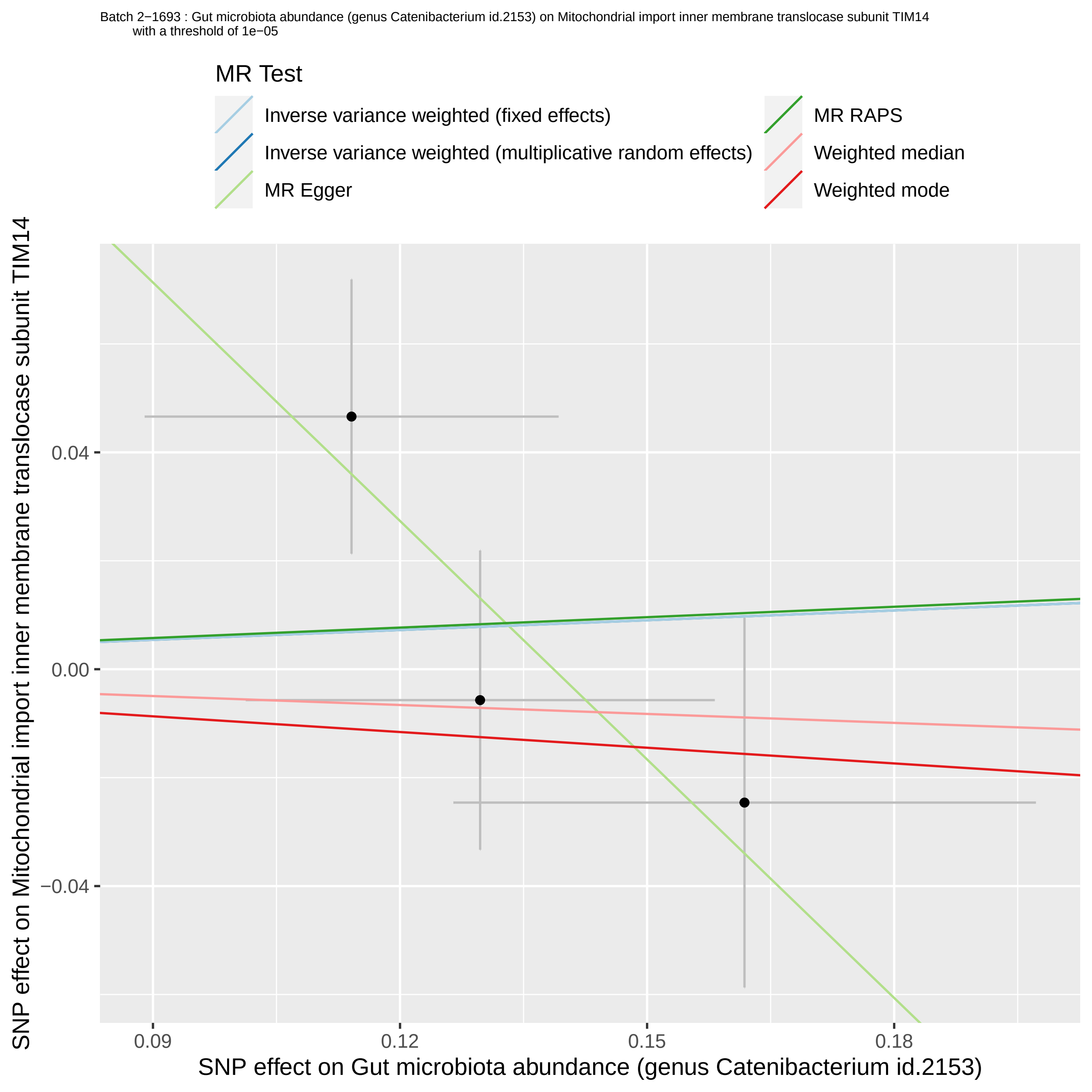
Batch 2−1693 : Gut microbiota abundance (genus Catenibacterium id.2153) on Mitochondrial import inner membrane translocase subunit TIM14
with a threshold of 1e−05
MR Test
Inverse variance weighted (fixed effects)
Inverse variance weighted (multiplicative random effects)
MR Egger
MR RAPS
Weighted median
Weighted mode
0.04
0.00
−0.04
0.09
0.12
0.15
0.18
SNP effect on Gut microbiota abundance (genus Catenibacterium id.2153)
SNP effect on Mitochondrial import inner membrane translocase subunit TIM14

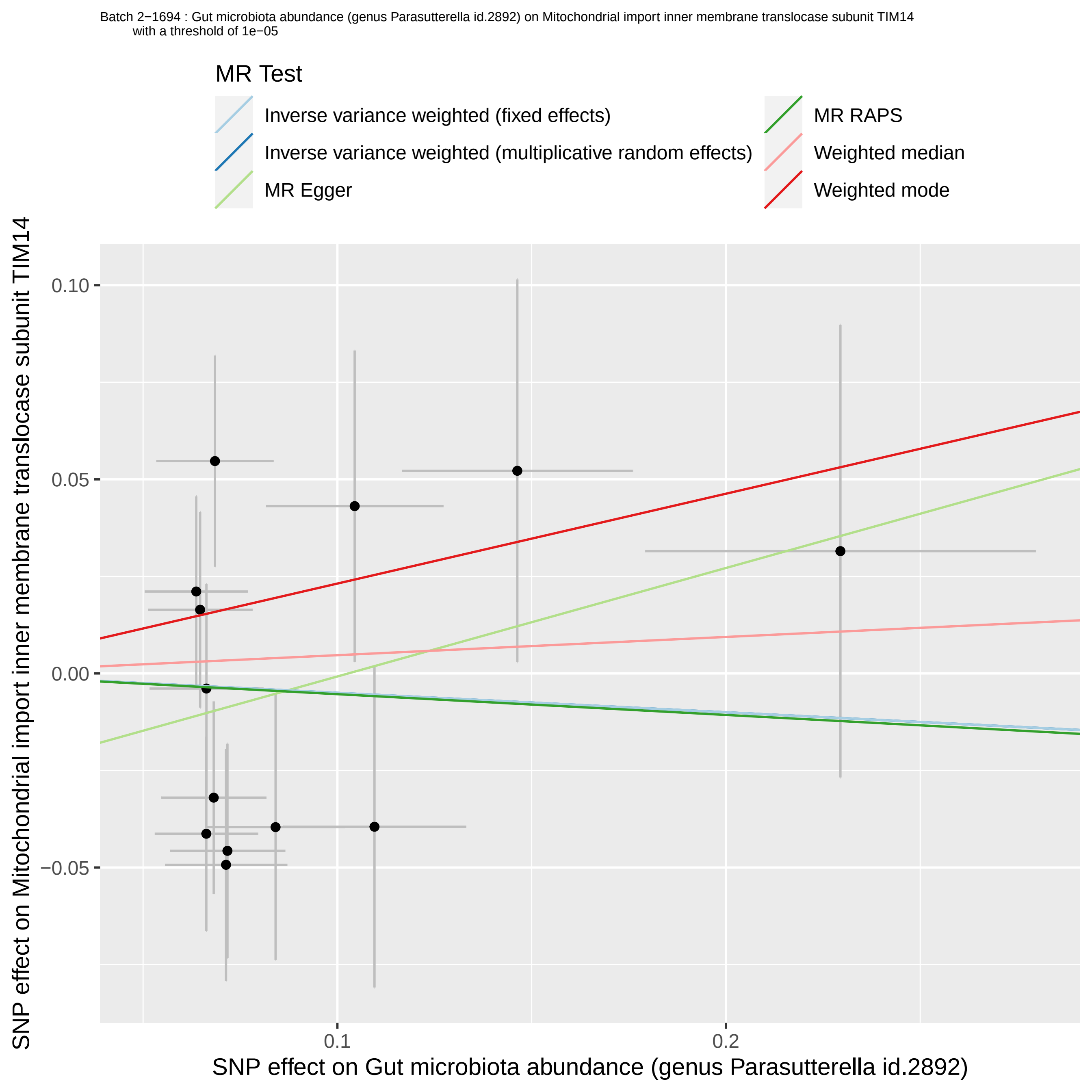
Batch 2−1694 : Gut microbiota abundance (genus Parasutterella id.2892) on Mitochondrial import inner membrane translocase subunit TIM14
with a threshold of 1e−05
MR Test
Inverse variance weighted (fixed effects)
Inverse variance weighted (multiplicative random effects)
MR Egger
MR RAPS
Weighted median
Weighted mode
0.10
0.05
0.00
−0.05
0.1
0.2
SNP effect on Gut microbiota abundance (genus Parasutterella id.2892)
SNP effect on Mitochondrial import inner membrane translocase subunit TIM14

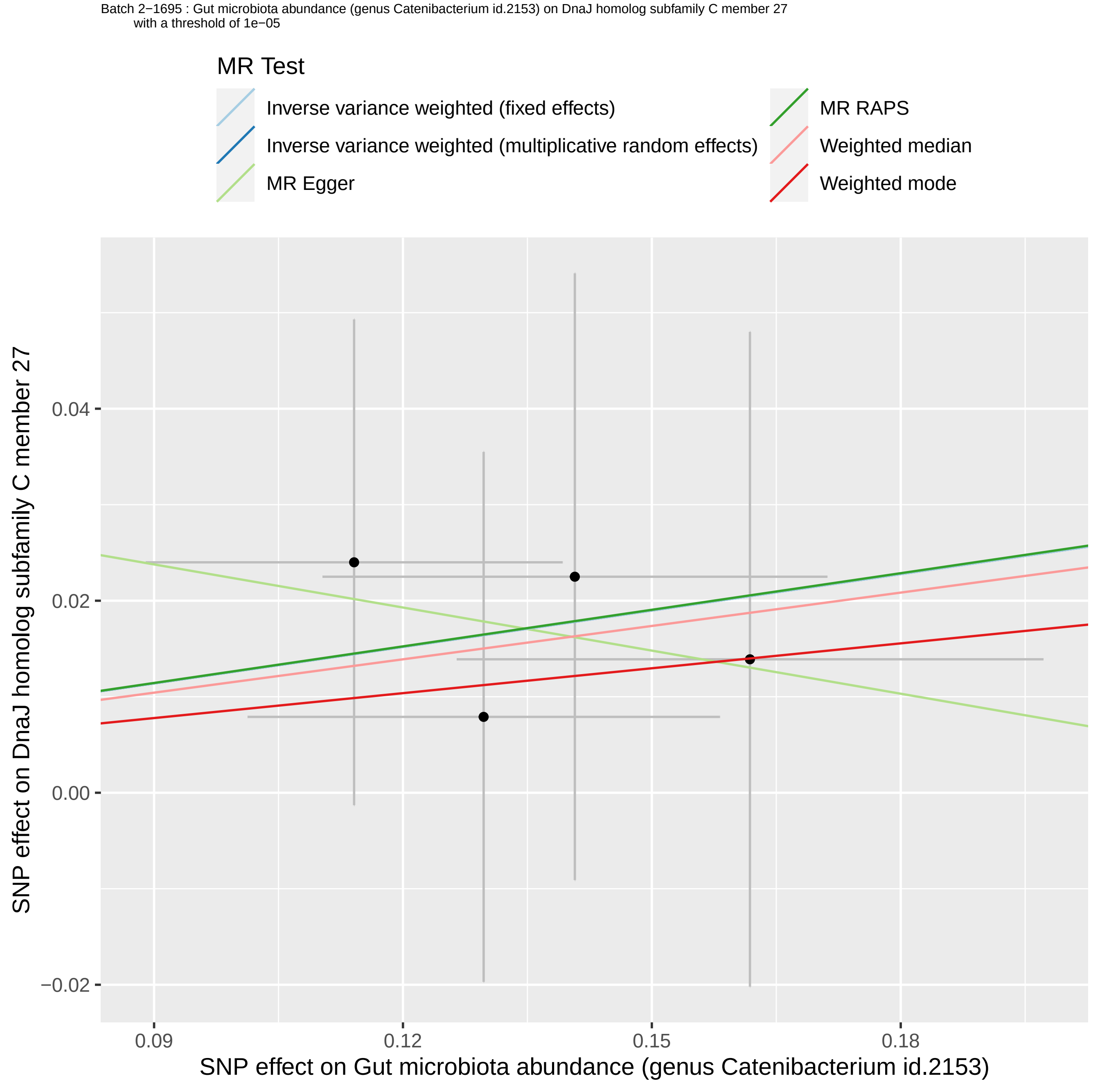

Batch 2−1695 : Gut microbiota abundance (genus Catenibacterium id.2153) on DnaJ homolog subfamily C member 27
with a threshold of 1e−05
MR Test
Inverse variance weighted (fixed effects)
Inverse variance weighted (multiplicative random effects)
MR Egger
MR RAPS
Weighted median
Weighted mode
SNP effect on DnaJ homolog subfamily C member 27
0.04
0.02
0.00
−0.02
0.09
0.12
0.15
0.18
SNP effect on Gut microbiota abundance (genus Catenibacterium id.2153)

Batch 2−1696 : Gut microbiota abundance (genus Parasutterella id.2892) on DnaJ homolog subfamily C member 27
with a threshold of 1e−05

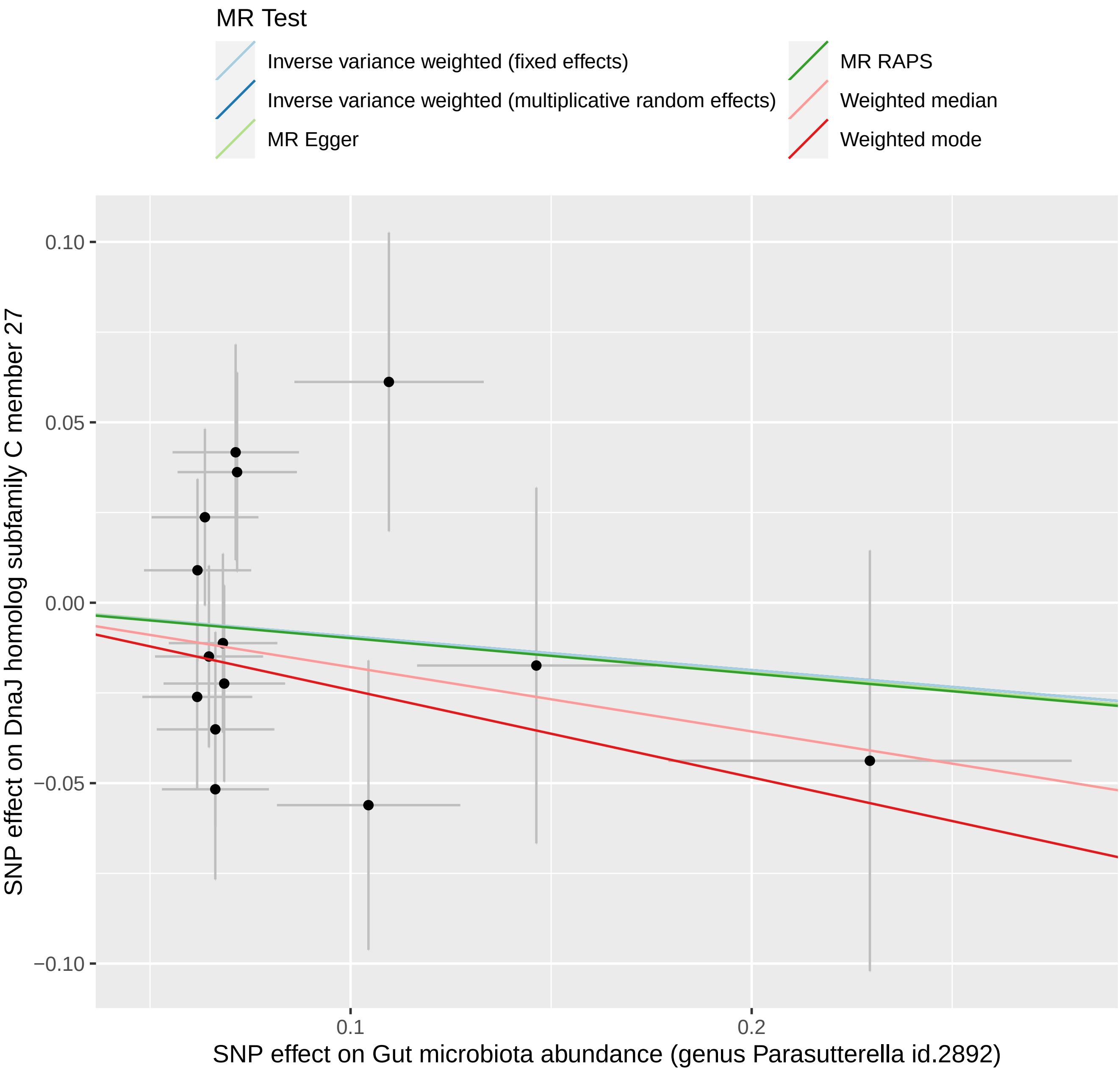

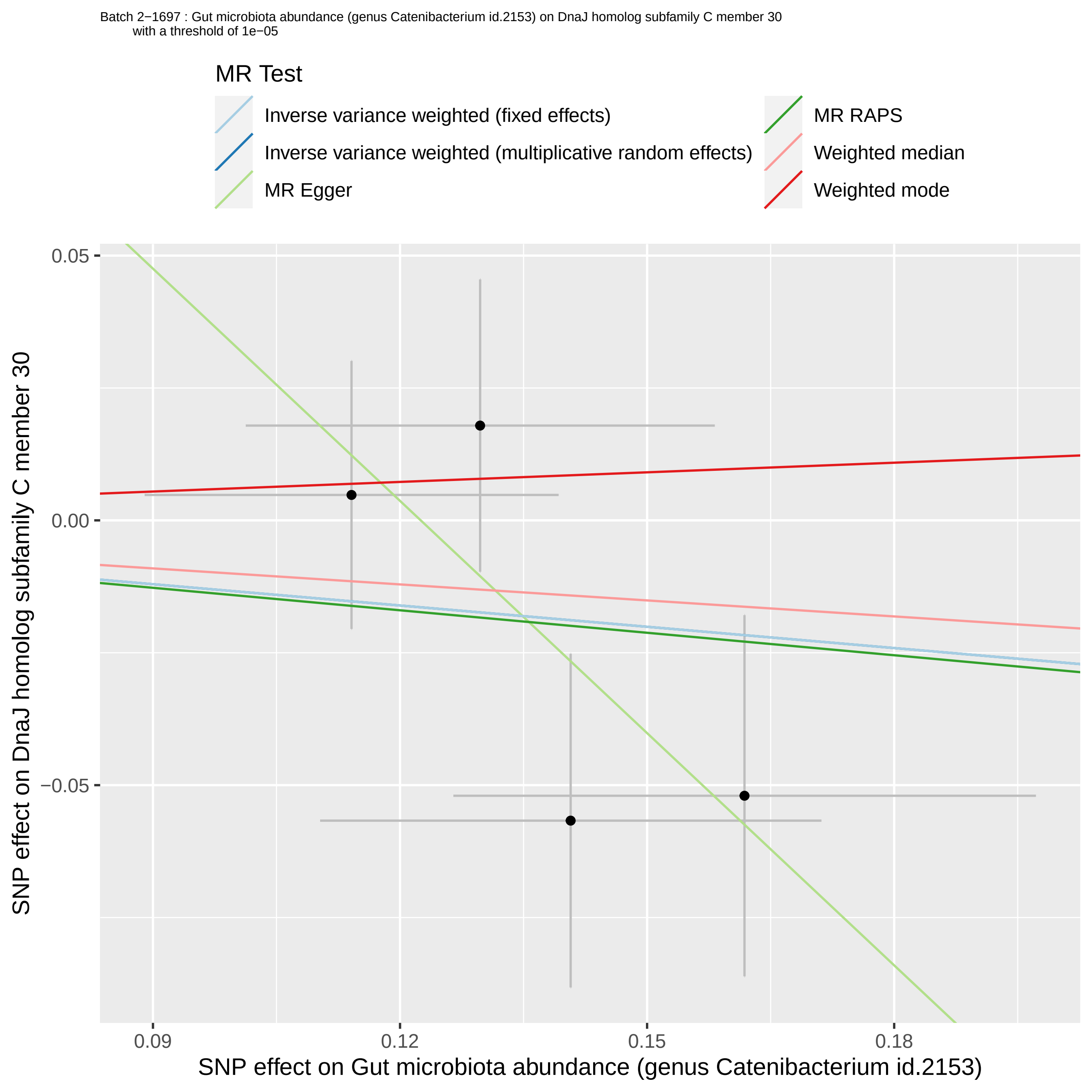

Batch 2−1697 : Gut microbiota abundance (genus Catenibacterium id.2153) on DnaJ homolog subfamily C member 30
with a threshold of 1e−05
MR Test
Inverse variance weighted (fixed effects)
Inverse variance weighted (multiplicative random effects)
MR Egger
MR RAPS
Weighted median
Weighted mode
0.05
0.00
−0.05
0.09
0.12
0.15
0.18
SNP effect on DnaJ homolog subfamily C member 30
SNP effect on Gut microbiota abundance (genus Catenibacterium id.2153)

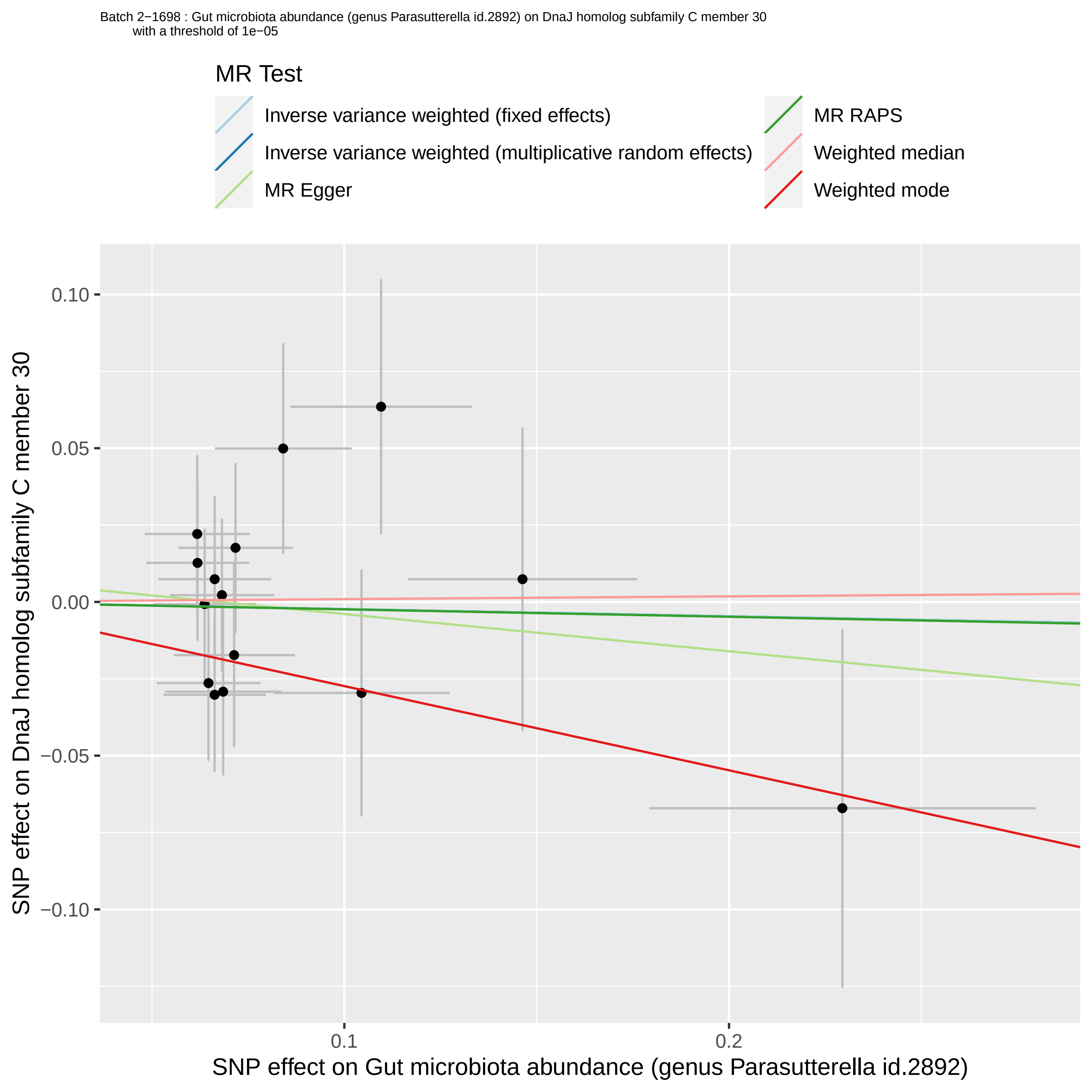

Batch 2−1698 : Gut microbiota abundance (genus Parasutterella id.2892) on DnaJ homolog subfamily C member 30
with a threshold of 1e−05
MR Test
Inverse variance weighted (fixed effects)
Inverse variance weighted (multiplicative random effects)
MR Egger
MR RAPS
Weighted median
Weighted mode
0.10
0.05
0.00
−0.05
−0.10
0.1
0.2
SNP effect on DnaJ homolog subfamily C member 30
SNP effect on Gut microbiota abundance (genus Parasutterella id.2892)

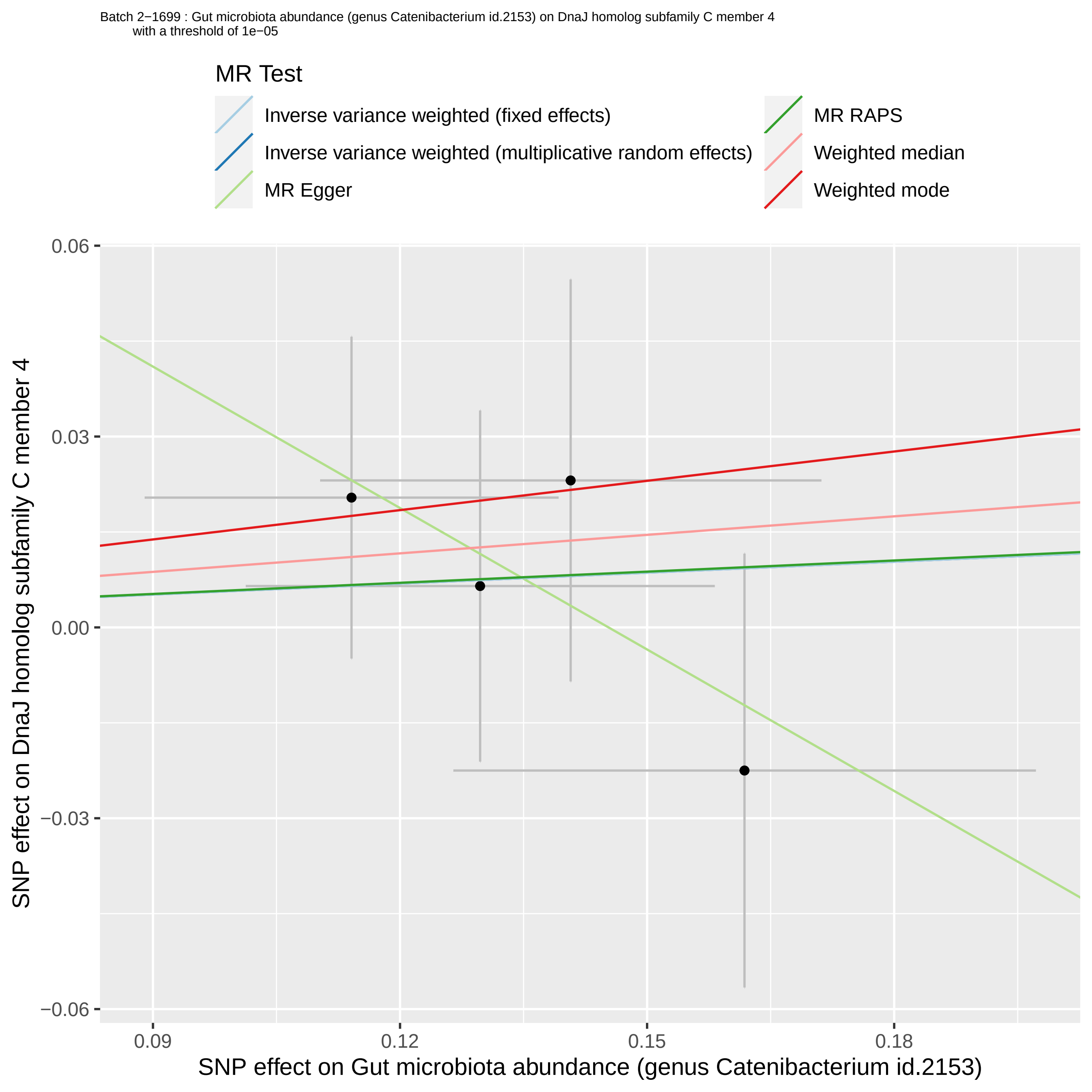

Batch 2−1699 : Gut microbiota abundance (genus Catenibacterium id.2153) on DnaJ homolog subfamily C member 4
with a threshold of 1e−05
MR Test
Inverse variance weighted (fixed effects)
Inverse variance weighted (multiplicative random effects)
MR Egger
MR RAPS
Weighted median
Weighted mode
0.06
0.03
0.00
−0.03
−0.06
0.09
0.12
0.15
0.18
SNP effect on DnaJ homolog subfamily C member 4
SNP effect on Gut microbiota abundance (genus Catenibacterium id.2153)

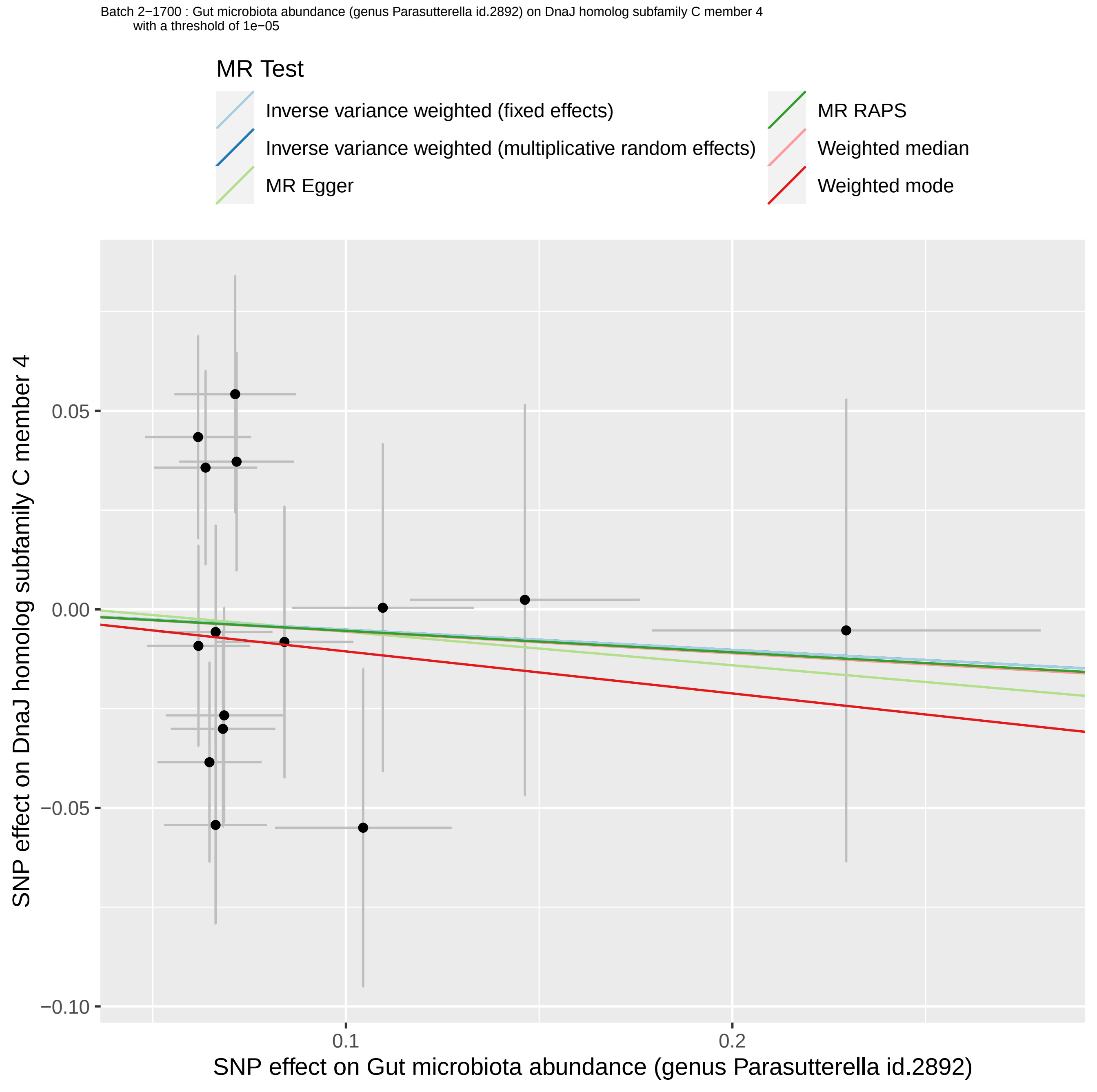

Batch 2−1700 : Gut microbiota abundance (genus Parasutterella id.2892) on DnaJ homolog subfamily C member 4
with a threshold of 1e−05
MR Test
Inverse variance weighted (fixed effects)
Inverse variance weighted (multiplicative random effects)
MR Egger
MR RAPS
Weighted median
Weighted mode
0.05
0.00
−0.05
−0.10
0.1
0.2
SNP effect on DnaJ homolog subfamily C member 4
SNP effect on Gut microbiota abundance (genus Parasutterella id.2892)

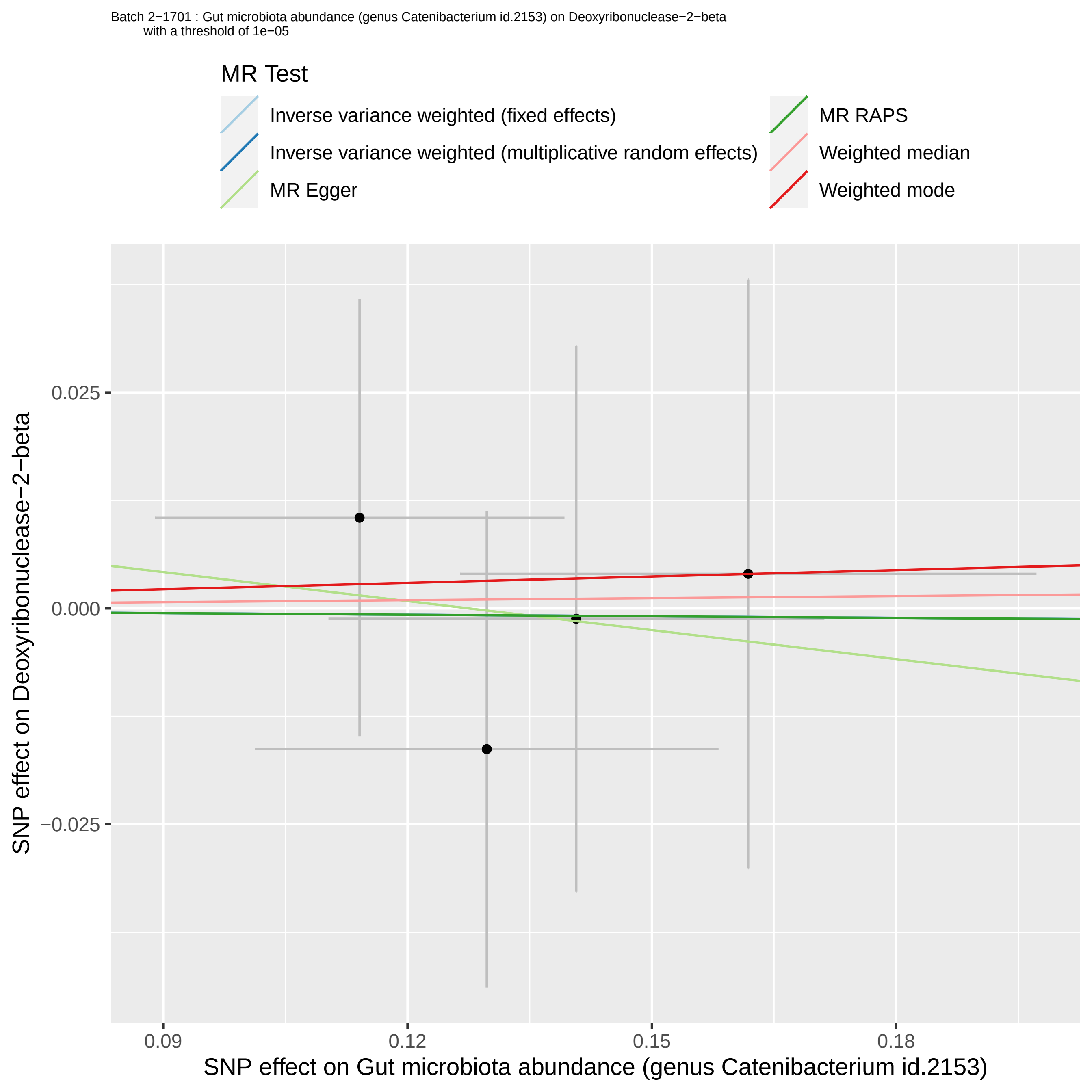
Batch 2−1701 : Gut microbiota abundance (genus Catenibacterium id.2153) on Deoxyribonuclease−2−beta
with a threshold of 1e−05
MR Test
Inverse variance weighted (fixed effects)
Inverse variance weighted (multiplicative random effects)
MR Egger
MR RAPS
Weighted median
Weighted mode
0.025
0.000
−0.025
SNP effect on Deoxyribonuclease−2−beta
0.09
0.12
0.15
0.18
SNP effect on Gut microbiota abundance (genus Catenibacterium id.2153)

Batch 2−1702 : Gut microbiota abundance (genus Parasutterella id.2892) on Deoxyribonuclease−2−beta
with a threshold of 1e−05

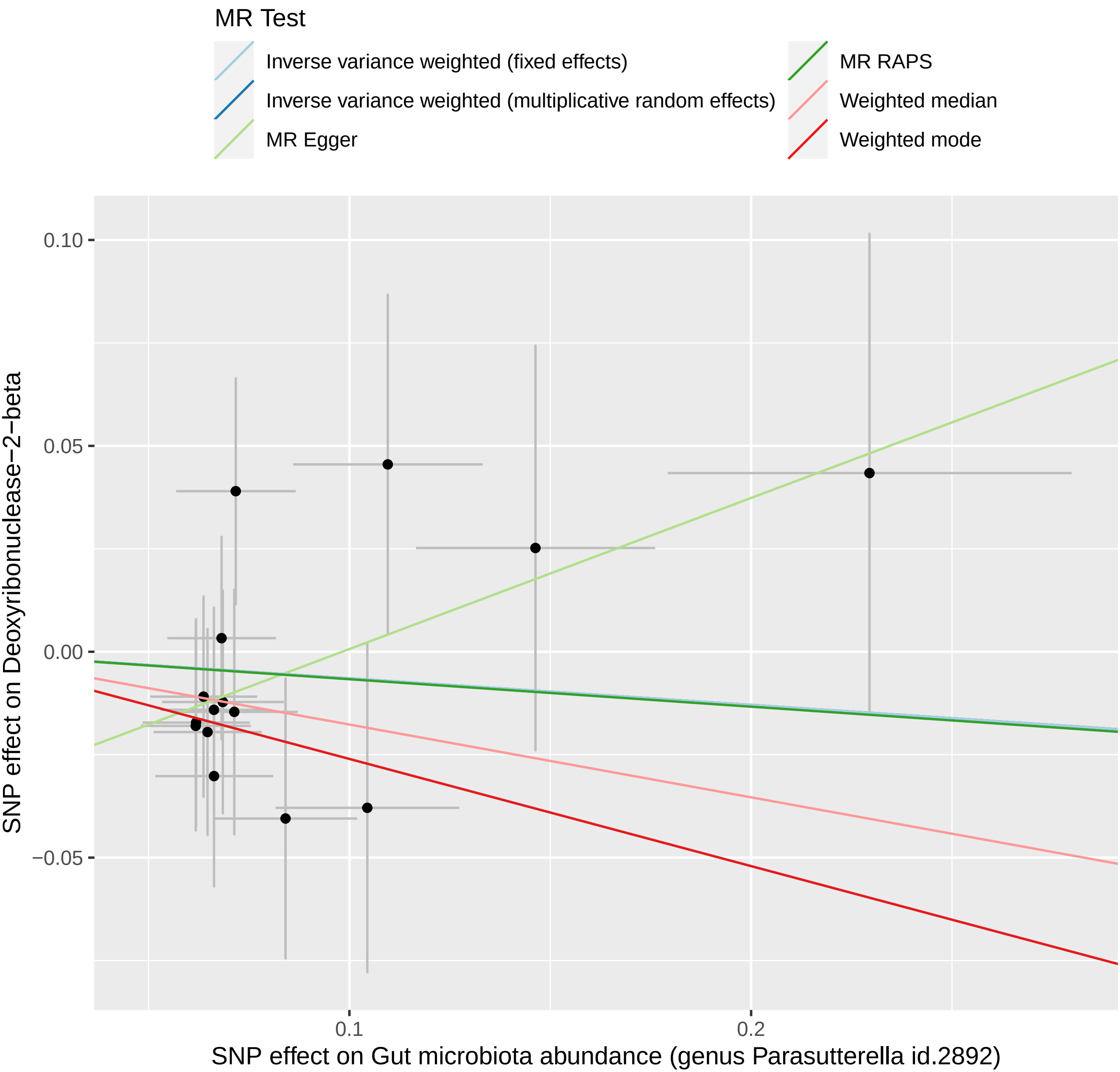

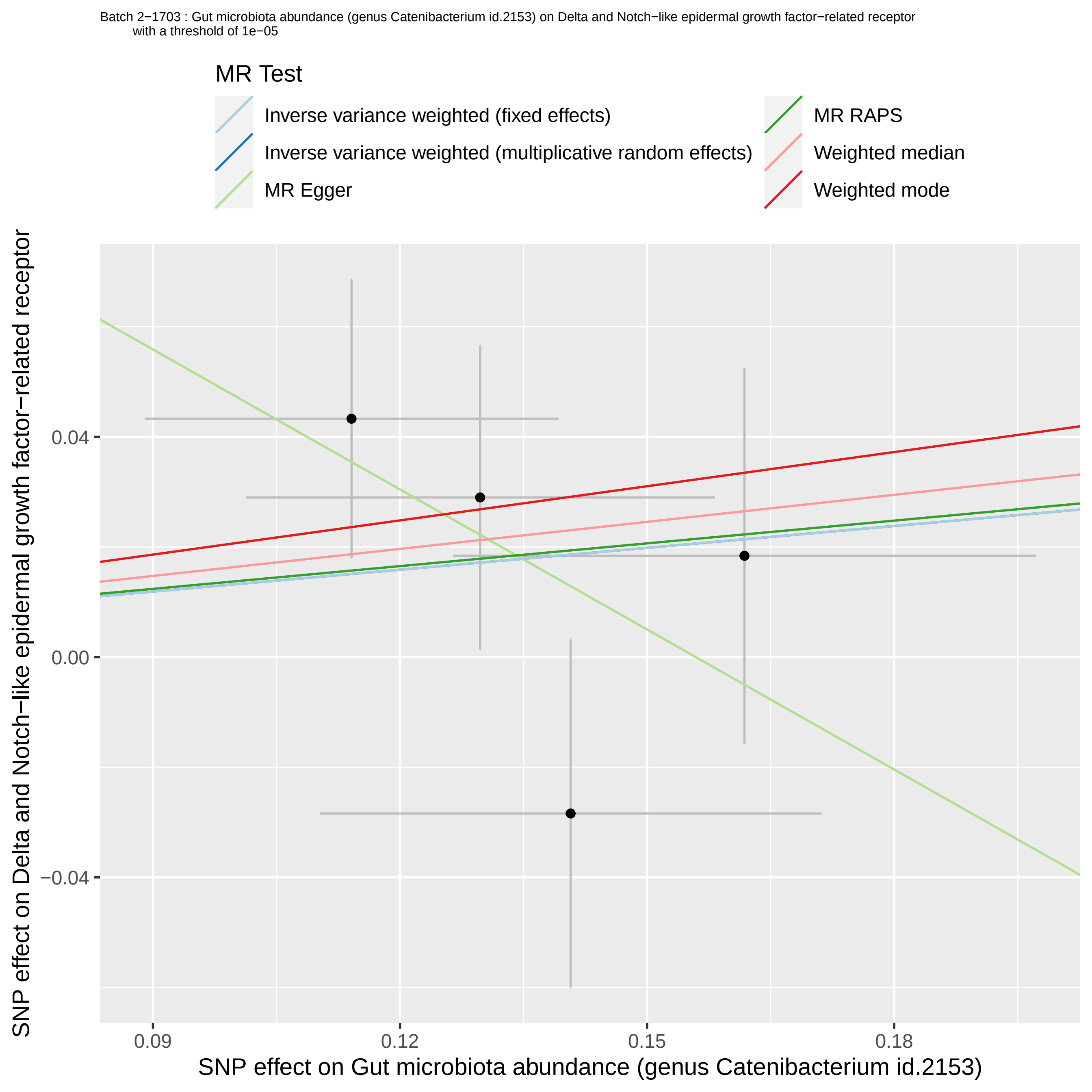
Batch 2−1703 : Gut microbiota abundance (genus Catenibacterium id.2153) on Delta and Notch−like epidermal growth factor−related receptor with a threshold of 1e−05
MR Test
Inverse variance weighted (fixed effects)
Inverse variance weighted (multiplicative random effects)
MR Egger
MR RAPS
Weighted median
Weighted mode
SNP effect on Delta and Notch−like epidermal growth factor−related receptor
0.04
0.00
−0.04
0.09
0.12
0.15
0.18
SNP effect on Gut microbiota abundance (genus Catenibacterium id.2153)

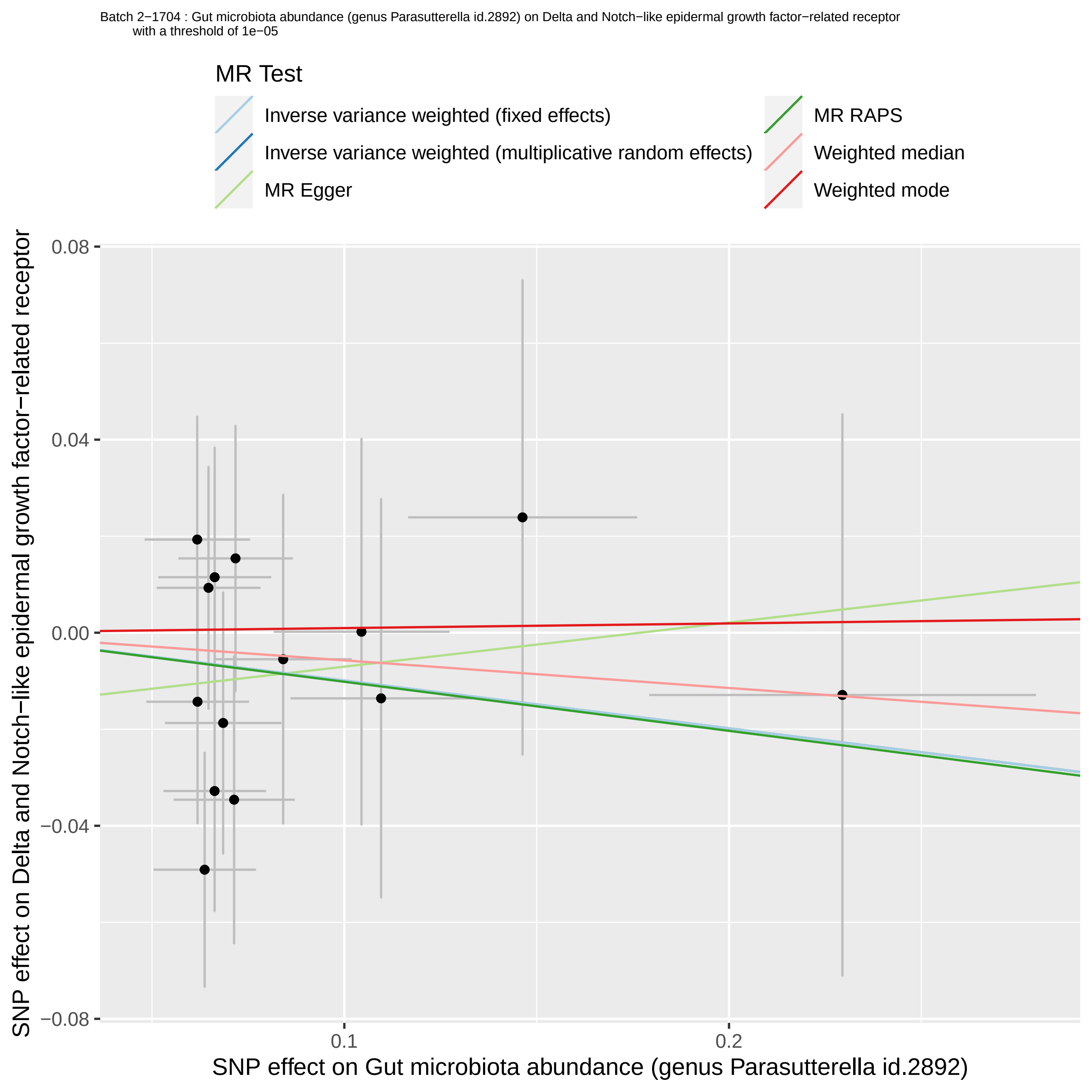
Batch 2−1704 : Gut microbiota abundance (genus Parasutterella id.2892) on Delta and Notch−like epidermal growth factor−related receptor
with a threshold of 1e−05
MR Test
Inverse variance weighted (fixed effects)
Inverse variance weighted (multiplicative random effects)
MR Egger
MR RAPS
Weighted median
Weighted mode
0.08
0.04
0.00
−0.04
−0.08
0.1
0.2
SNP effect on Delta and Notch−like epidermal growth factor−related receptor
SNP effect on Gut microbiota abundance (genus Parasutterella id.2892)

Batch 2−1705 : Gut microbiota abundance (genus Catenibacterium id.2153) on Delta and Notch−like epidermal growth factor−related receptor with a threshold of 1e−05

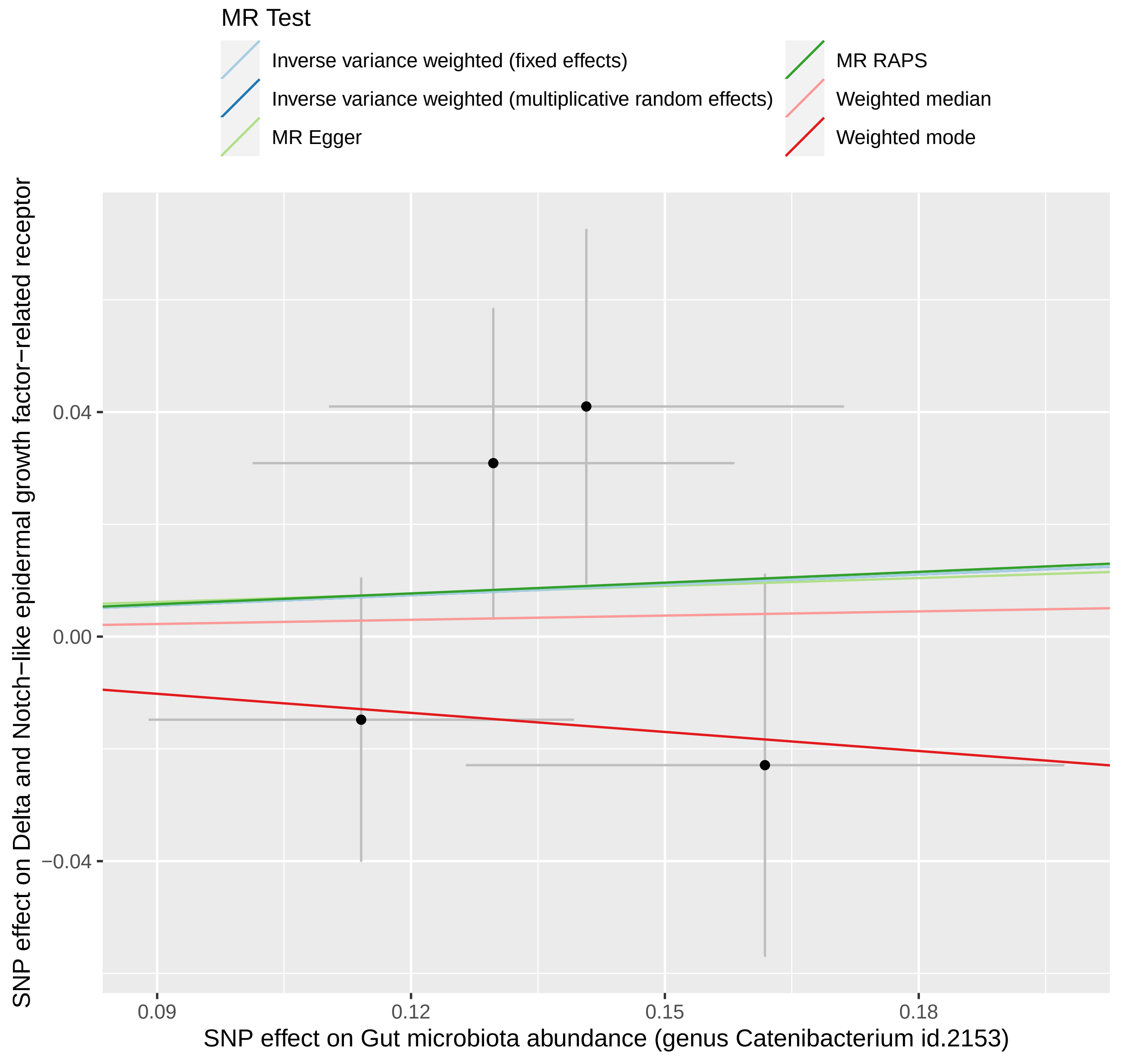

Batch 2−1706 : Gut microbiota abundance (genus Parasutterella id.2892) on Delta and Notch−like epidermal growth factor−related receptor
with a threshold of 1e−05

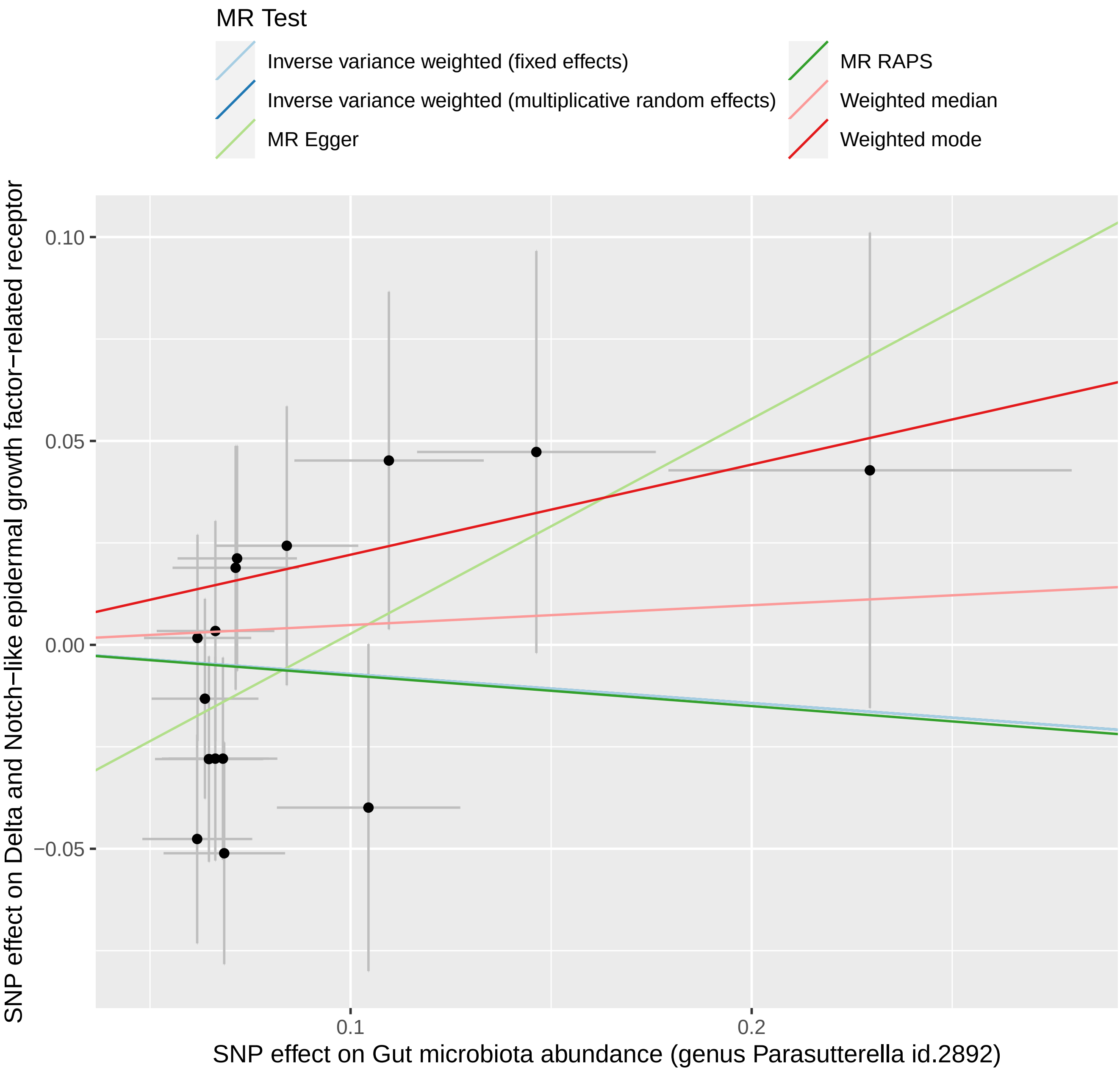

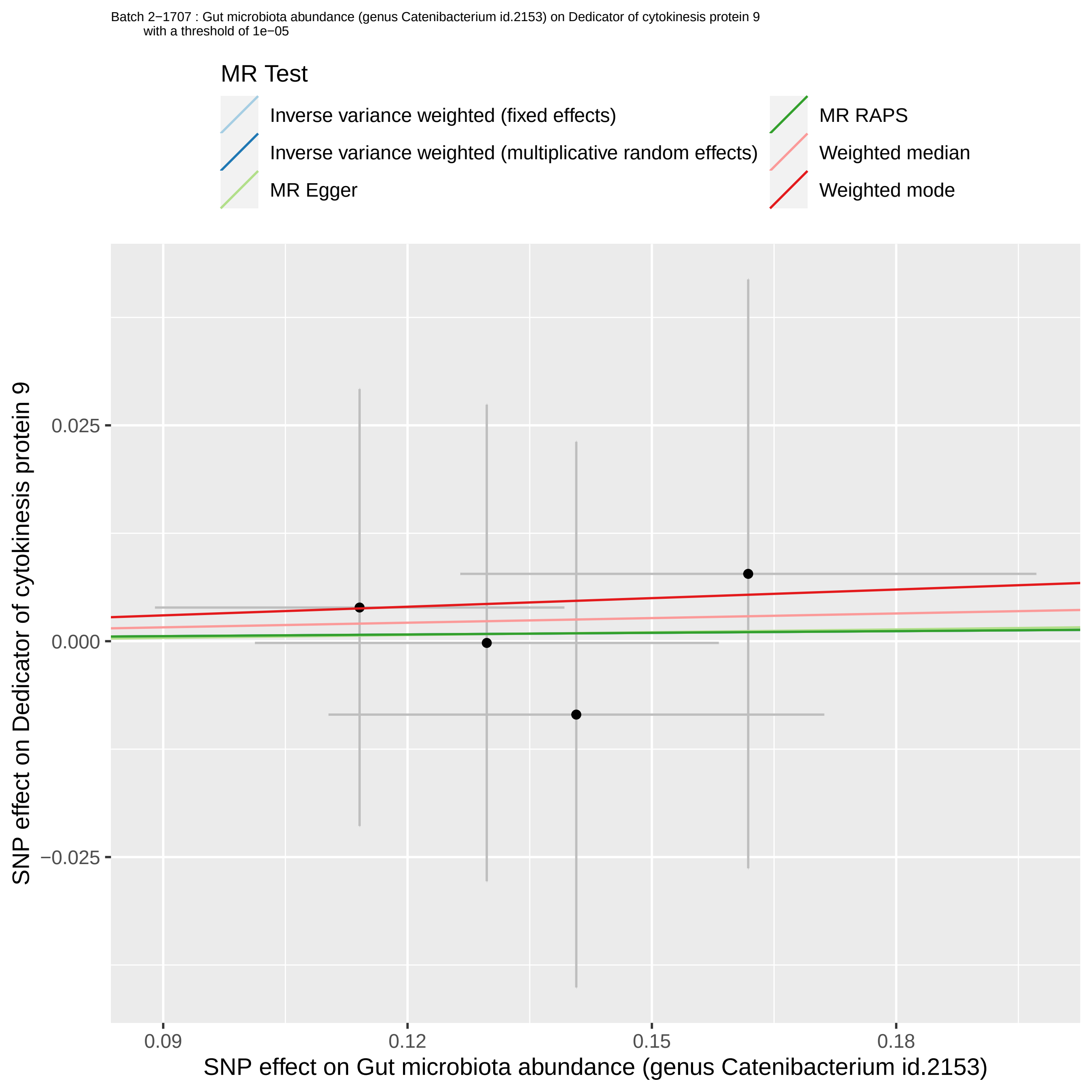

Batch 2−1707 : Gut microbiota abundance (genus Catenibacterium id.2153) on Dedicator of cytokinesis protein 9
with a threshold of 1e−05
MR Test
Inverse variance weighted (fixed effects)
Inverse variance weighted (multiplicative random effects)
MR Egger
MR RAPS
Weighted median
Weighted mode
SNP effect on Dedicator of cytokinesis protein 9
0.025
0.000
−0.025
0.09
0.12
0.15
0.18
SNP effect on Gut microbiota abundance (genus Catenibacterium id.2153)

Batch 2−1708 : Gut microbiota abundance (genus Parasutterella id.2892) on Dedicator of cytokinesis protein 9
with a threshold of 1e−05

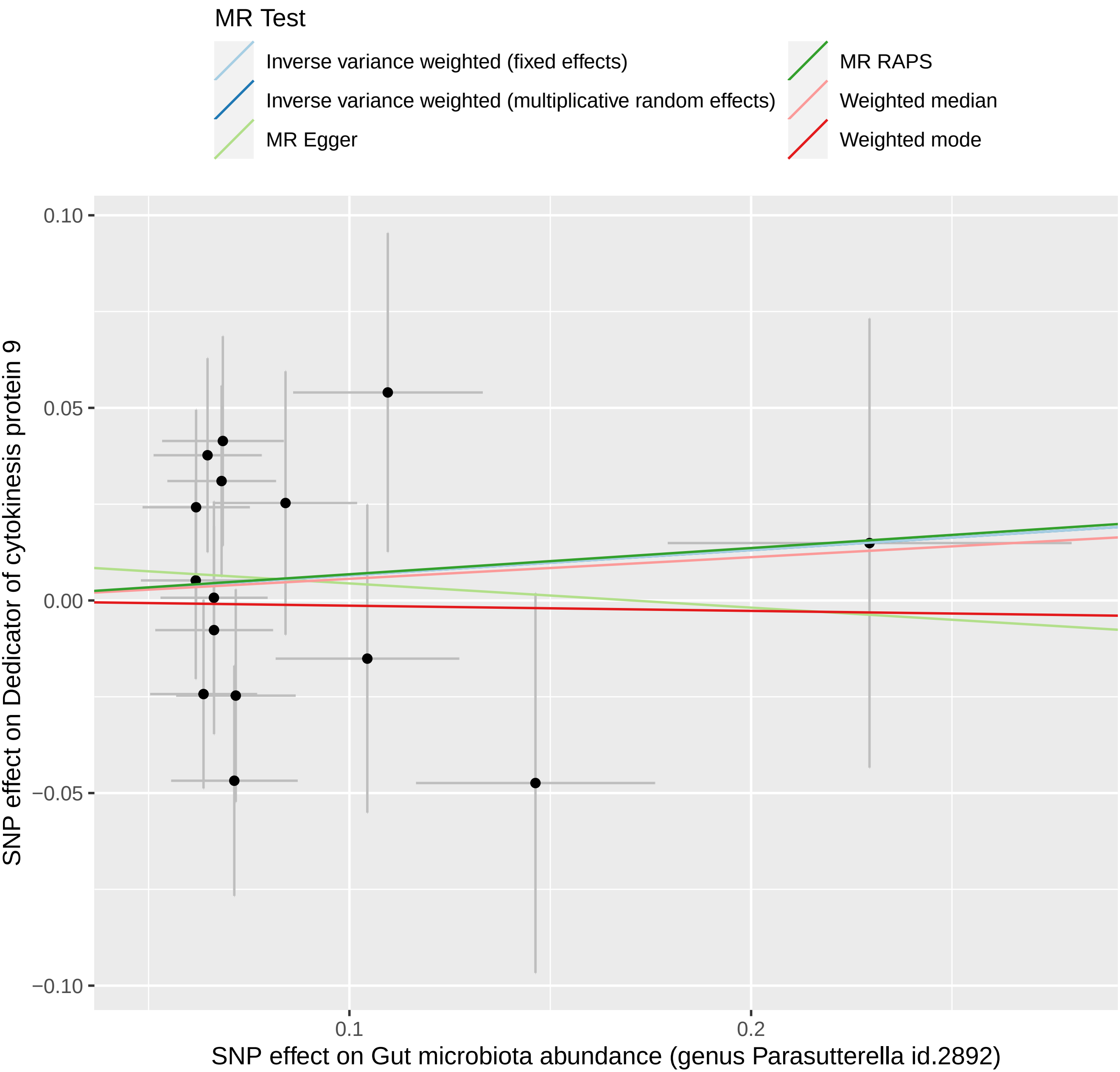

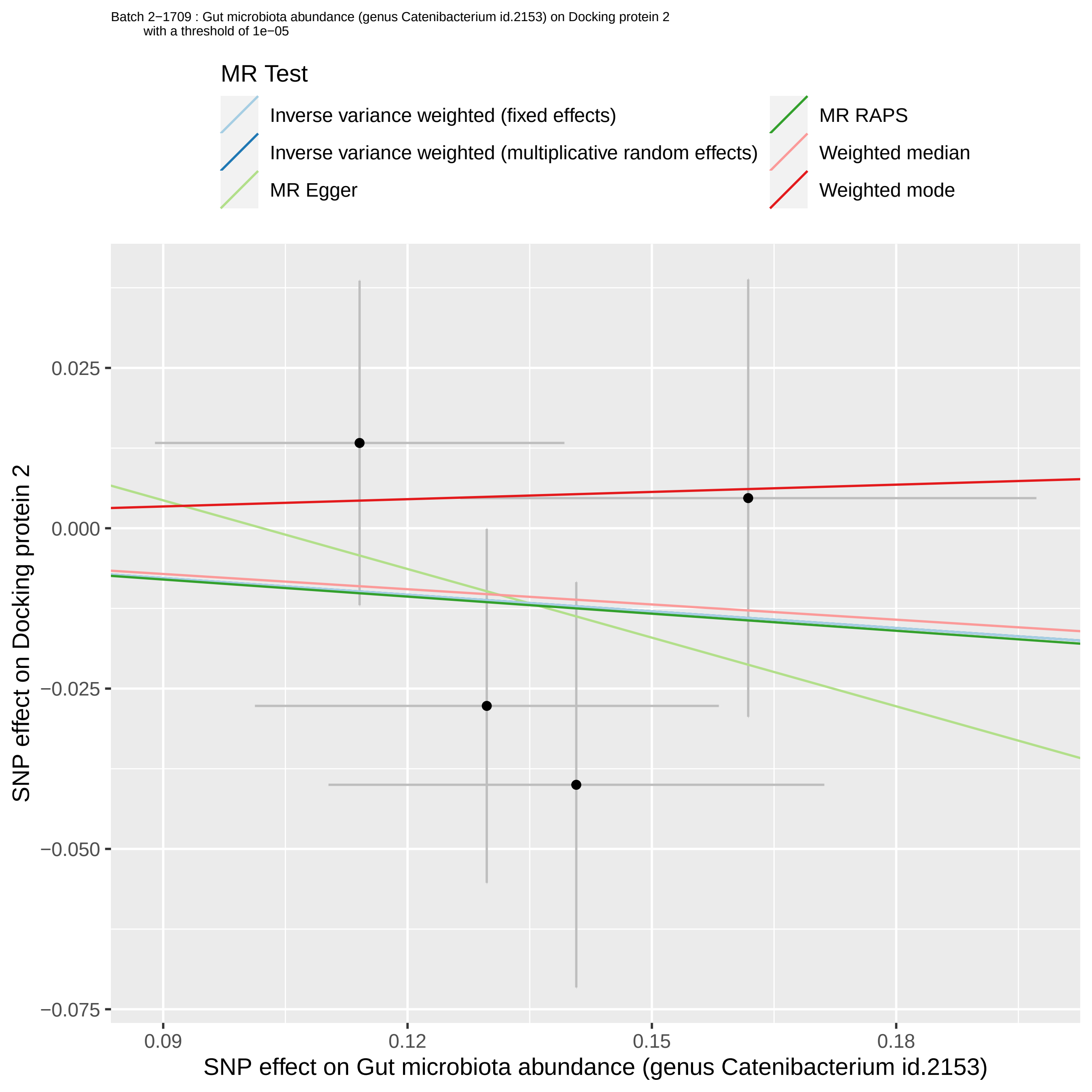

Batch 2−1709 : Gut microbiota abundance (genus Catenibacterium id.2153) on Docking protein 2
with a threshold of 1e−05
MR Test
Inverse variance weighted (fixed effects)
Inverse variance weighted (multiplicative random effects)
MR Egger
MR RAPS
Weighted median
Weighted mode
0.025
0.000
−0.025
−0.050
−0.075
SNP effect on Docking protein 2
0.09
0.12
0.15
0.18
SNP effect on Gut microbiota abundance (genus Catenibacterium id.2153)

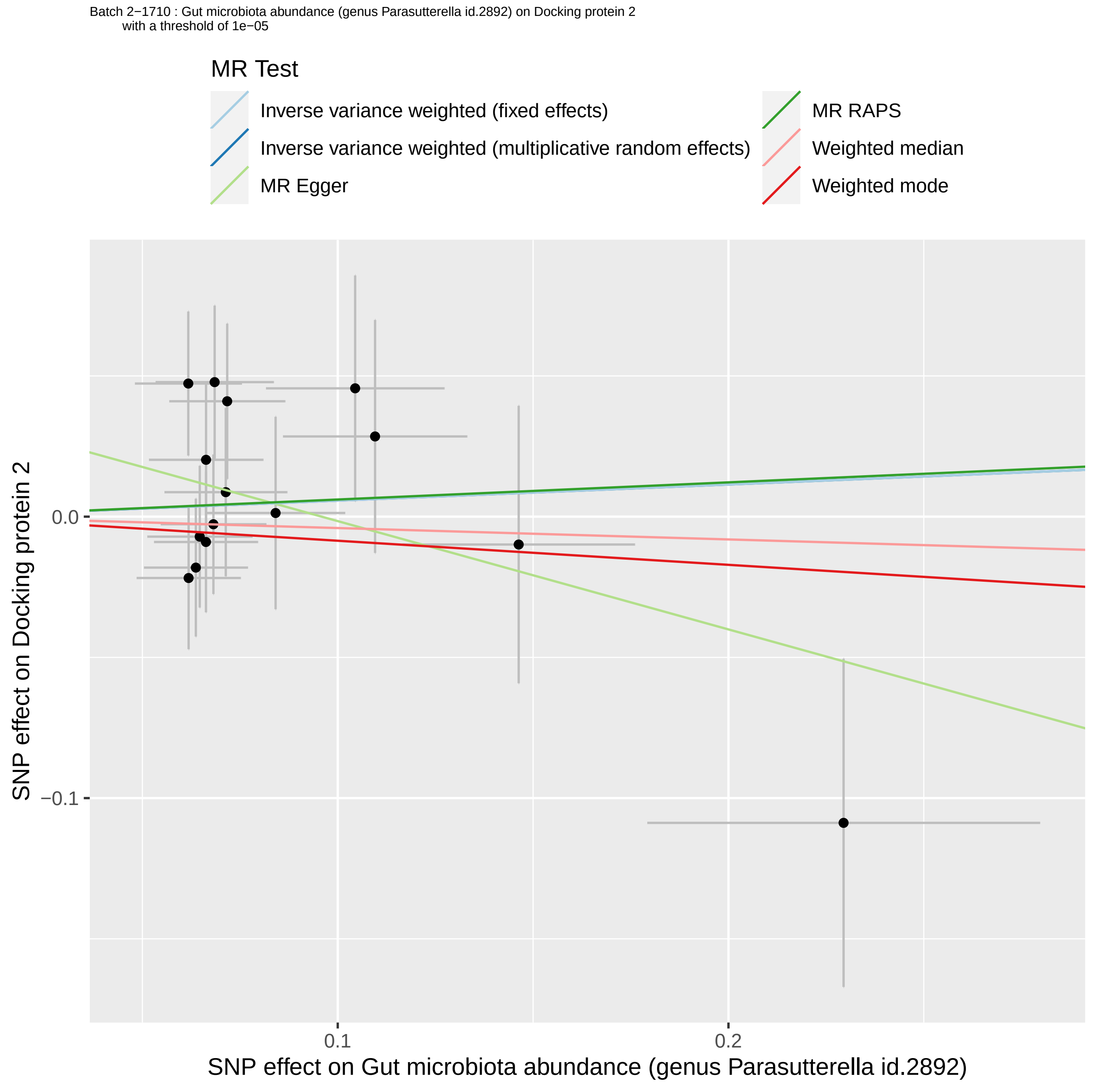

Batch 2−1710 : Gut microbiota abundance (genus Parasutterella id.2892) on Docking protein 2
with a threshold of 1e−05
MR Test
Inverse variance weighted (fixed effects)
Inverse variance weighted (multiplicative random effects)
MR Egger
MR RAPS
Weighted median
Weighted mode
SNP effect on Docking protein 2
0.0
−0.1
0.1
0.2
SNP effect on Gut microbiota abundance (genus Parasutterella id.2892)

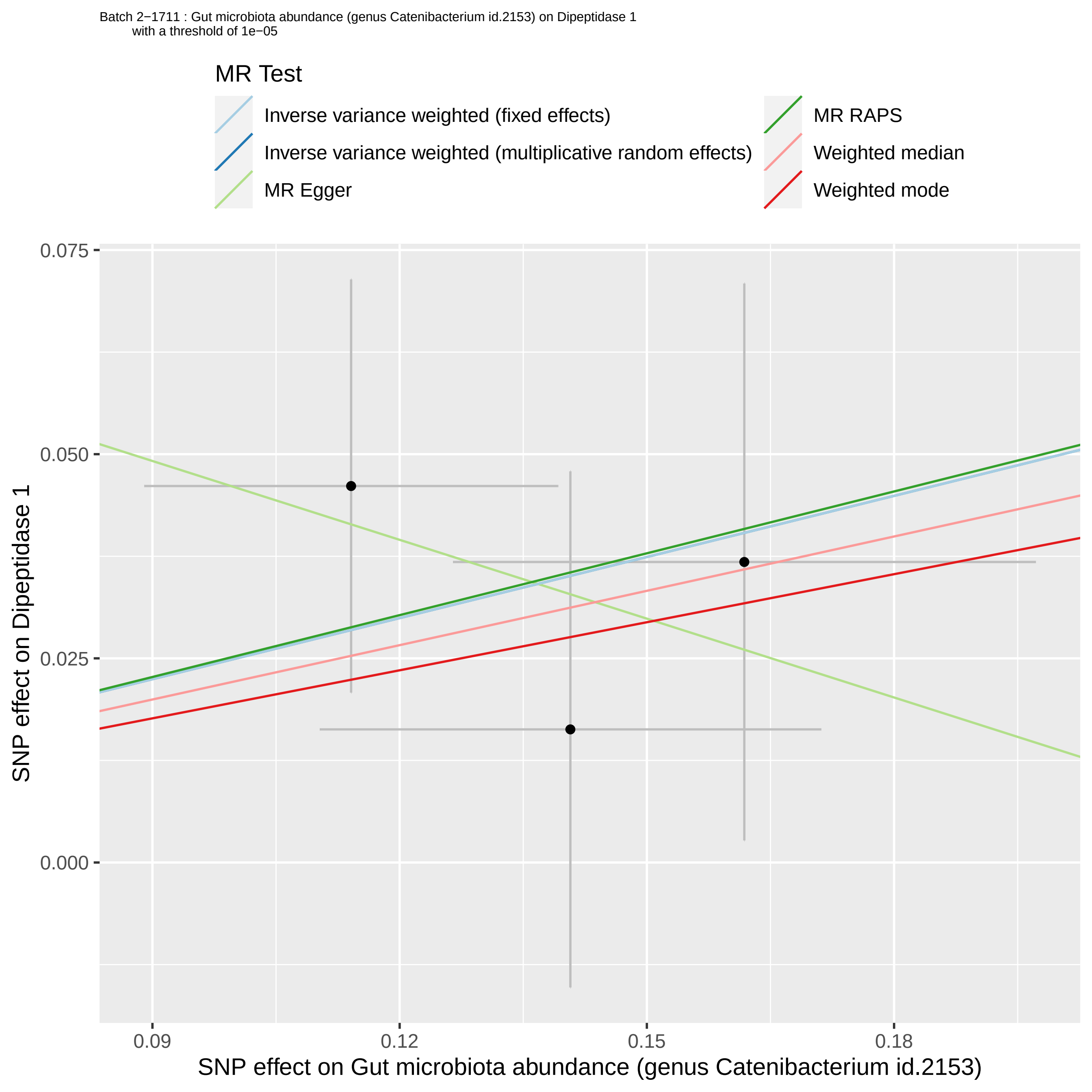

Batch 2−1711 : Gut microbiota abundance (genus Catenibacterium id.2153) on Dipeptidase 1
with a threshold of 1e−05
MR Test
Inverse variance weighted (fixed effects)
Inverse variance weighted (multiplicative random effects)
MR Egger
MR RAPS
Weighted median
Weighted mode
0.075
0.050
0.025
0.000
0.09
0.12
0.15
0.18
SNP effect on Dipeptidase 1
SNP effect on Gut microbiota abundance (genus Catenibacterium id.2153)

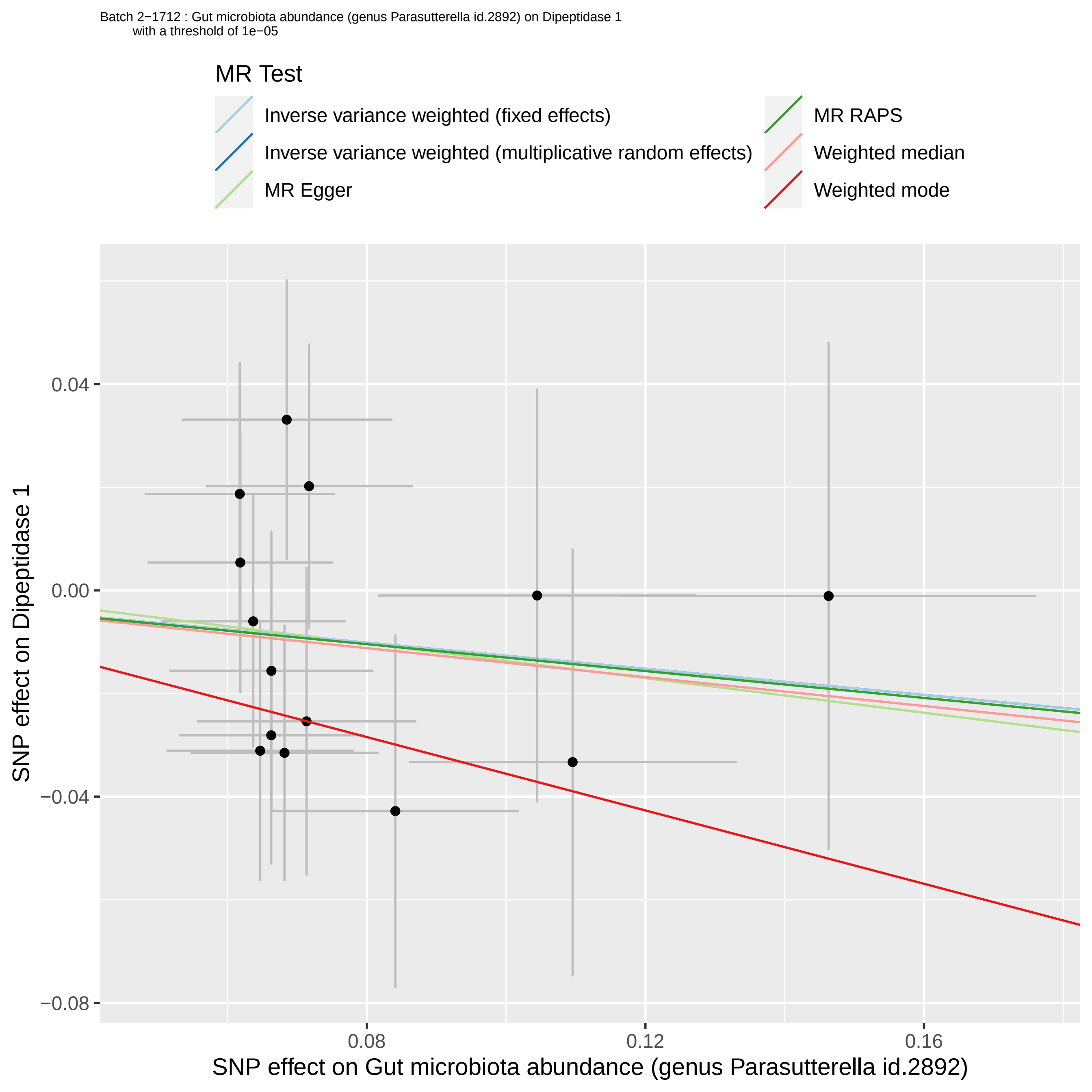
Batch 2−1712 : Gut microbiota abundance (genus Parasutterella id.2892) on Dipeptidase 1
with a threshold of 1e−05
MR Test
Inverse variance weighted (fixed effects)
Inverse variance weighted (multiplicative random effects)
MR Egger
MR RAPS
Weighted median
Weighted mode
SNP effect on Dipeptidase 1
0.04
0.00
−0.04
−0.08
0.08
0.12
0.16
SNP effect on Gut microbiota abundance (genus Parasutterella id.2892)

Batch 2−1713 : Gut microbiota abundance (genus Catenibacterium id.2153) on Dipeptidase 2
with a threshold of 1e−05

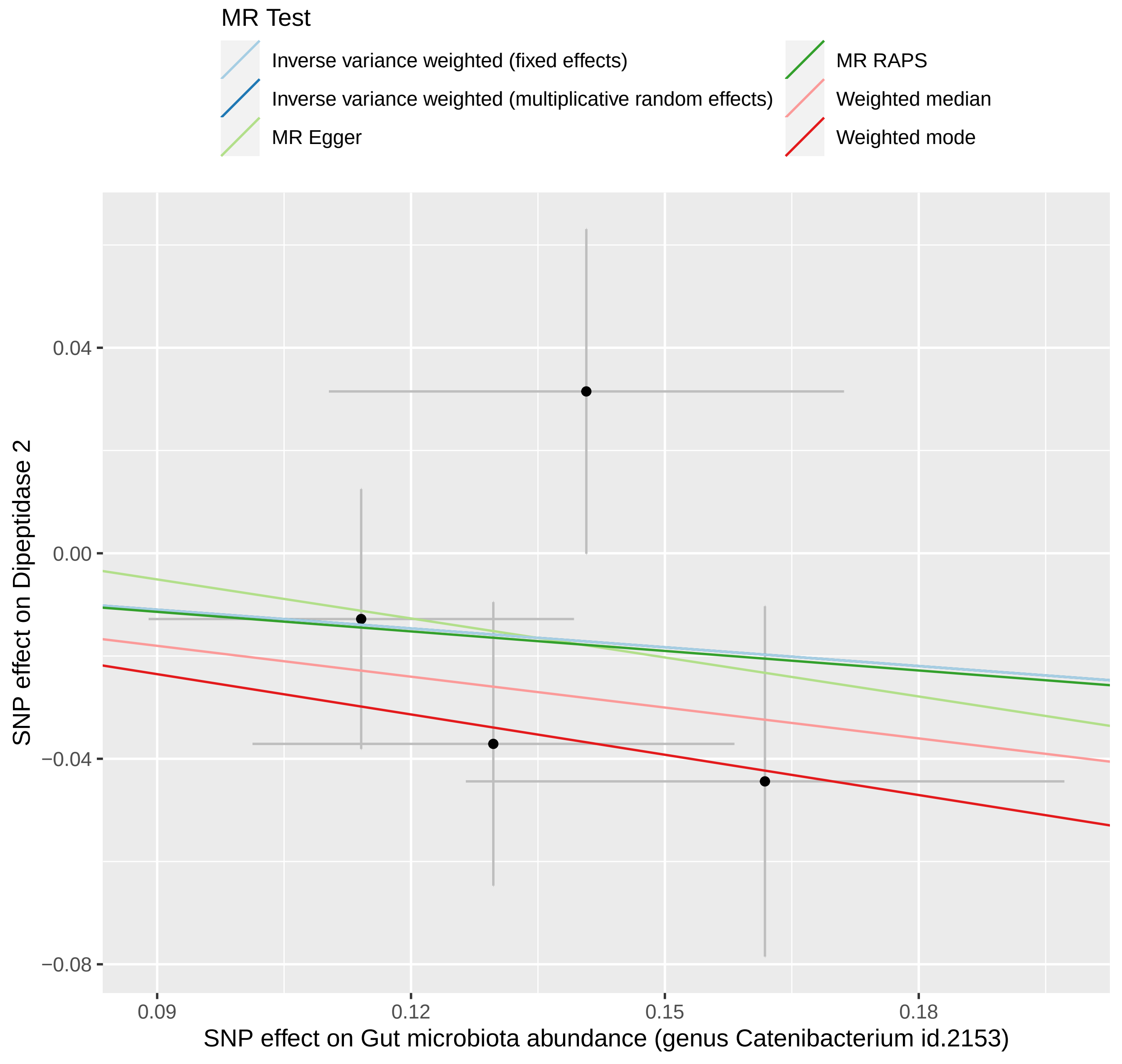

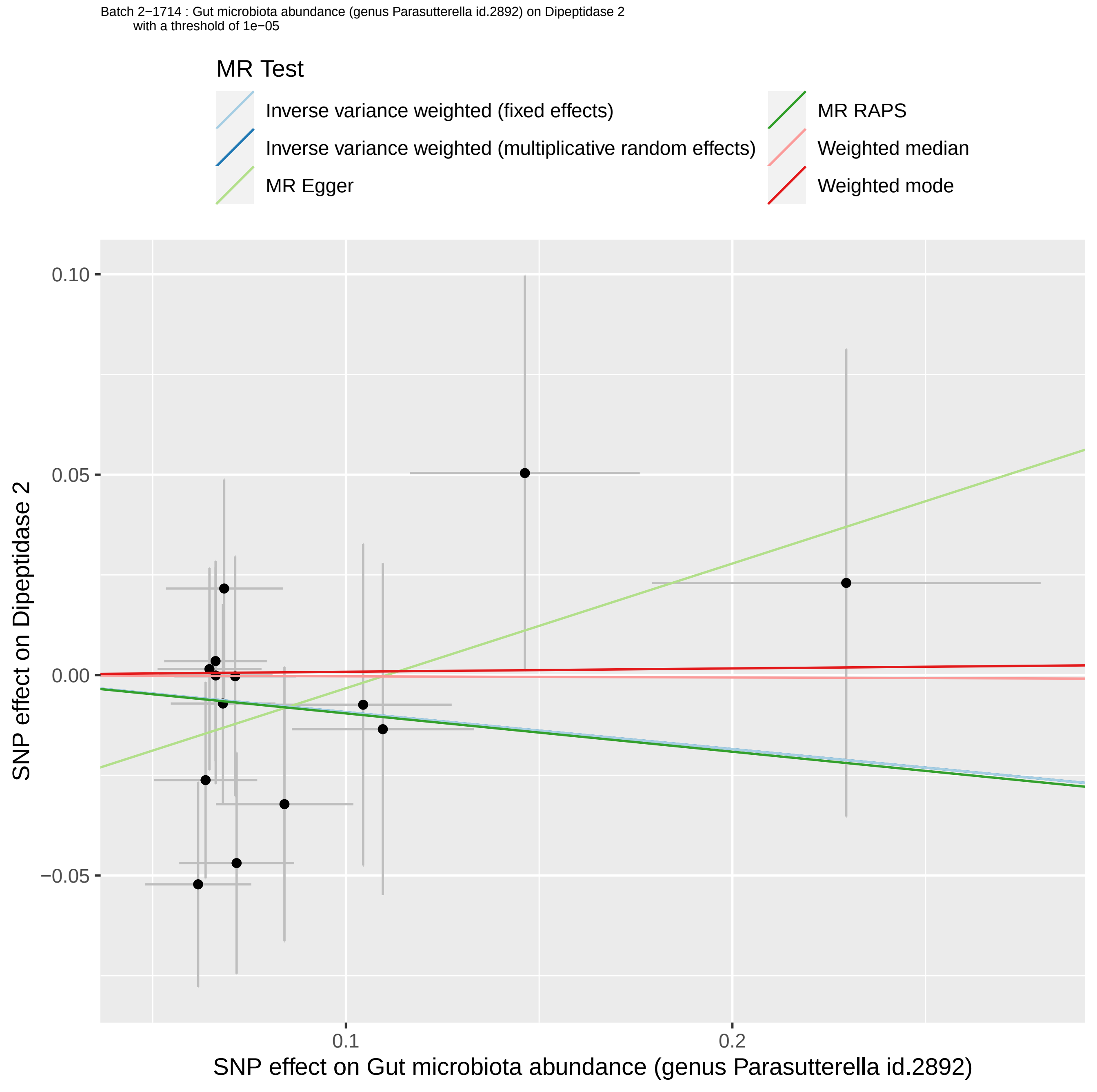

Batch 2−1714 : Gut microbiota abundance (genus Parasutterella id.2892) on Dipeptidase 2
with a threshold of 1e−05
MR Test
Inverse variance weighted (fixed effects)
Inverse variance weighted (multiplicative random effects)
MR Egger
MR RAPS
Weighted median
Weighted mode
0.10
0.05
0.00
−0.05
0.1
0.2
SNP effect on Dipeptidase 2
SNP effect on Gut microbiota abundance (genus Parasutterella id.2892)

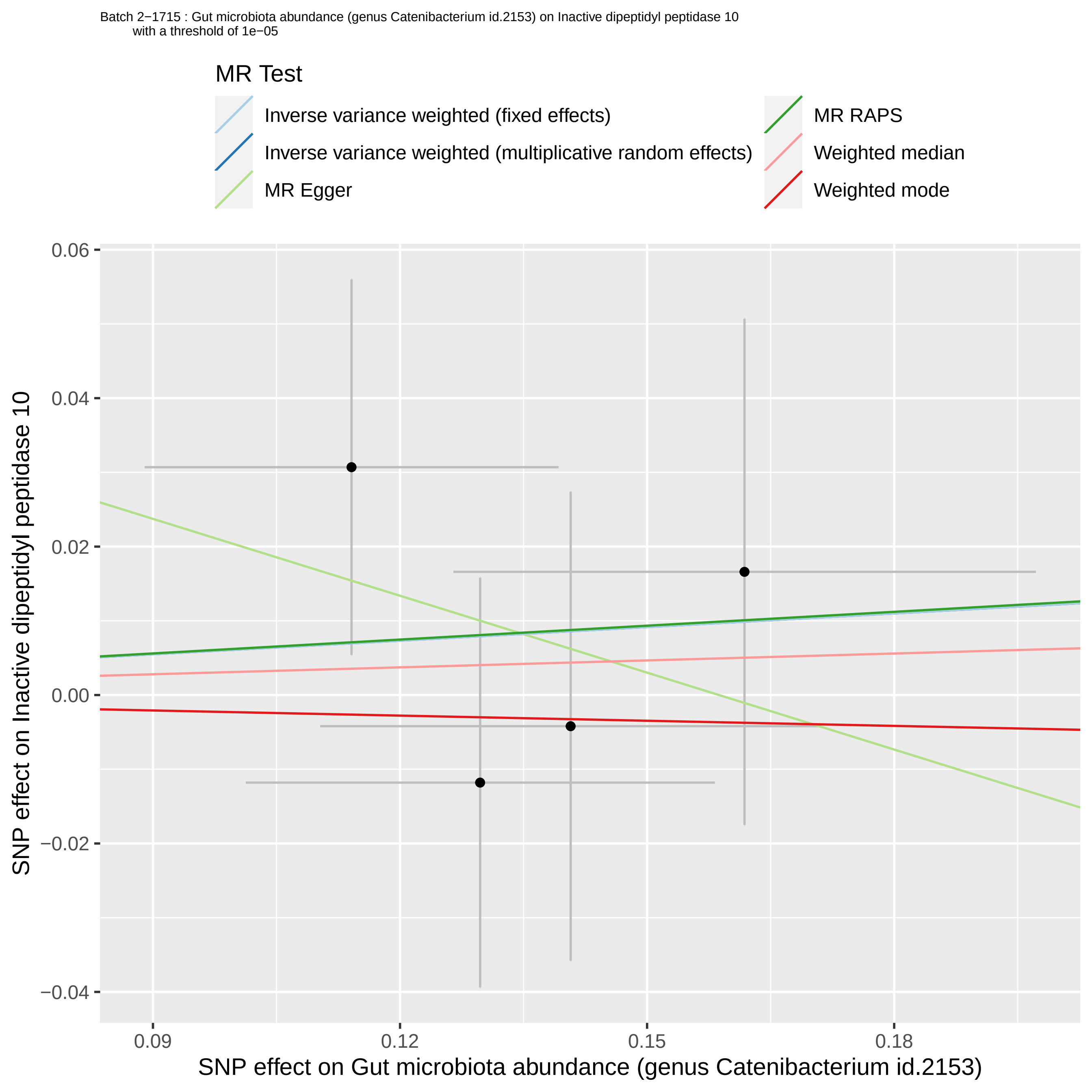
Batch 2−1715 : Gut microbiota abundance (genus Catenibacterium id.2153) on Inactive dipeptidyl peptidase 10
with a threshold of 1e−05
MR Test
Inverse variance weighted (fixed effects)
Inverse variance weighted (multiplicative random effects)
MR Egger
MR RAPS
Weighted median
Weighted mode
0.06
0.04
0.02
0.00
−0.02
−0.04
0.09
0.12
0.15
0.18
SNP effect on Inactive dipeptidyl peptidase 10
SNP effect on Gut microbiota abundance (genus Catenibacterium id.2153)

Batch 2−1716 : Gut microbiota abundance (genus Parasutterella id.2892) on Inactive dipeptidyl peptidase 10
with a threshold of 1e−05

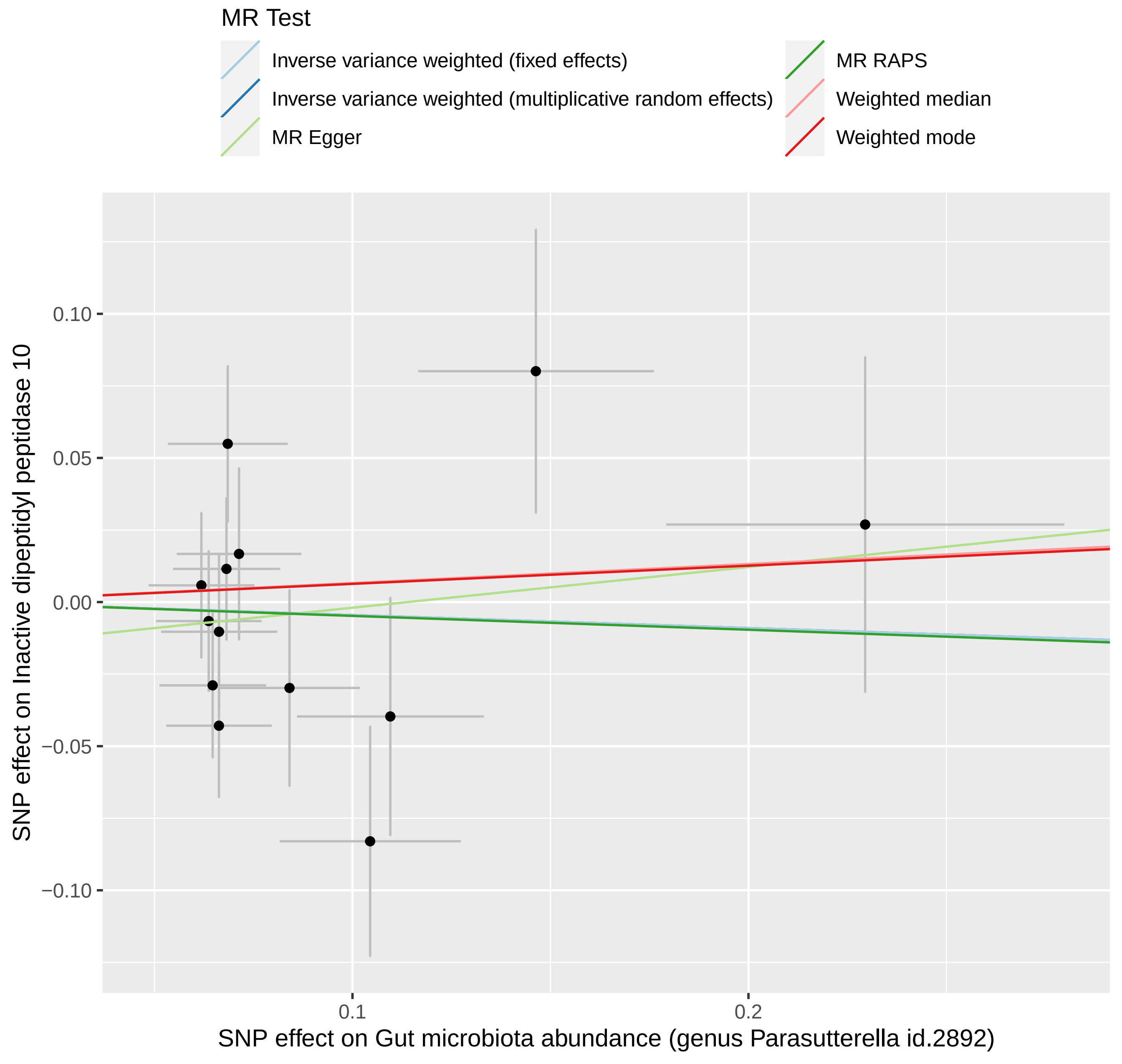

Batch 2−1717 : Gut microbiota abundance (genus Catenibacterium id.2153) on Dipeptidyl peptidase 2
with a threshold of 1e−05

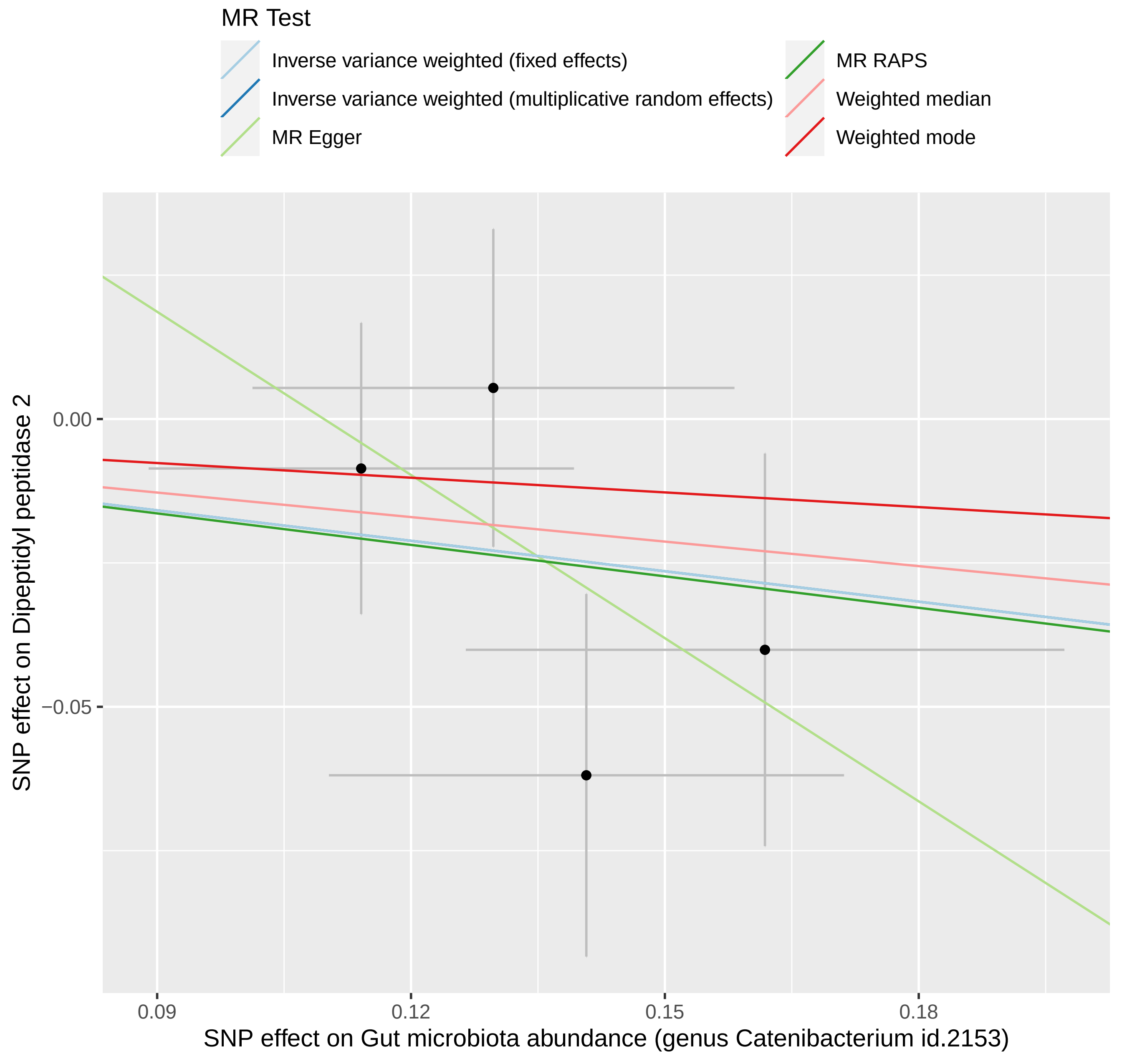

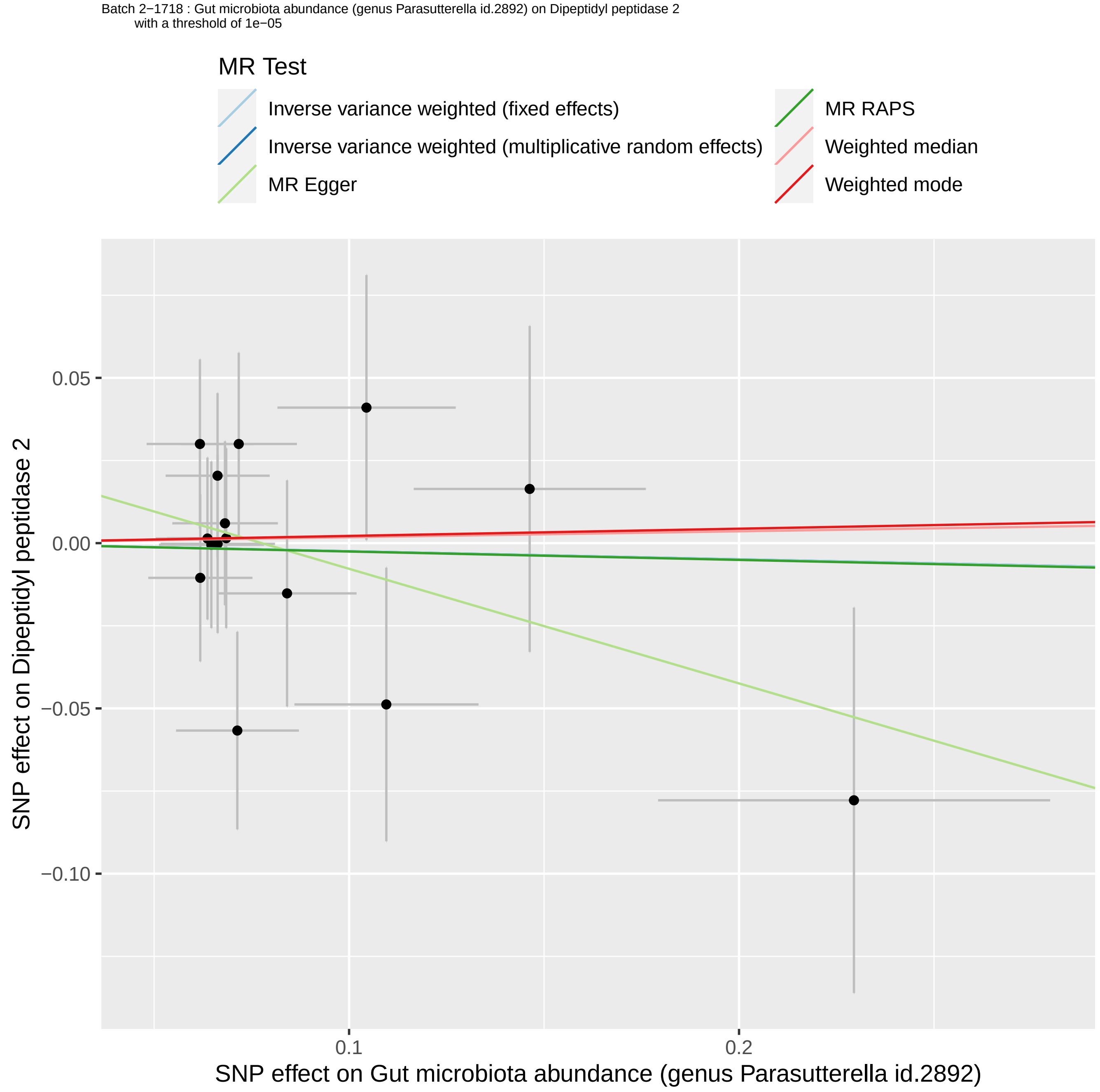
Batch 2−1718 : Gut microbiota abundance (genus Parasutterella id.2892) on Dipeptidyl peptidase 2
with a threshold of 1e−05
MR Test
Inverse variance weighted (fixed effects)
Inverse variance weighted (multiplicative random effects)
MR Egger
MR RAPS
Weighted median
Weighted mode
0.05
0.00
−0.05
−0.10
0.1
0.2
SNP effect on Dipeptidyl peptidase 2
SNP effect on Gut microbiota abundance (genus Parasutterella id.2892)

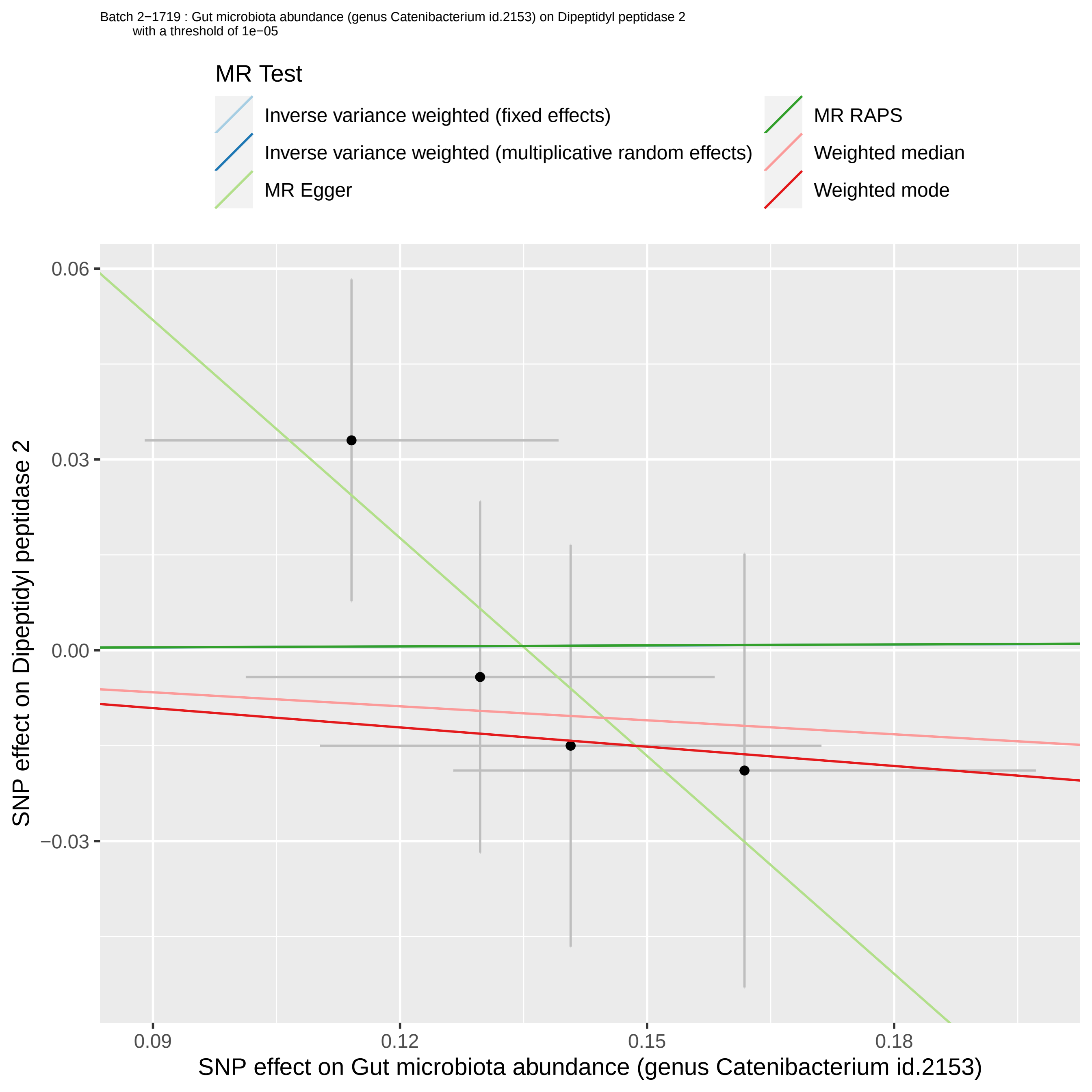
Batch 2−1719 : Gut microbiota abundance (genus Catenibacterium id.2153) on Dipeptidyl peptidase 2
with a threshold of 1e−05
MR Test
Inverse variance weighted (fixed effects)
Inverse variance weighted (multiplicative random effects)
MR Egger
MR RAPS
Weighted median
Weighted mode
0.06
0.03
0.00
−0.03
0.09
0.12
0.15
0.18
SNP effect on Dipeptidyl peptidase 2
SNP effect on Gut microbiota abundance (genus Catenibacterium id.2153)

Batch 2−1720 : Gut microbiota abundance (genus Parasutterella id.2892) on Dipeptidyl peptidase 2
with a threshold of 1e−05

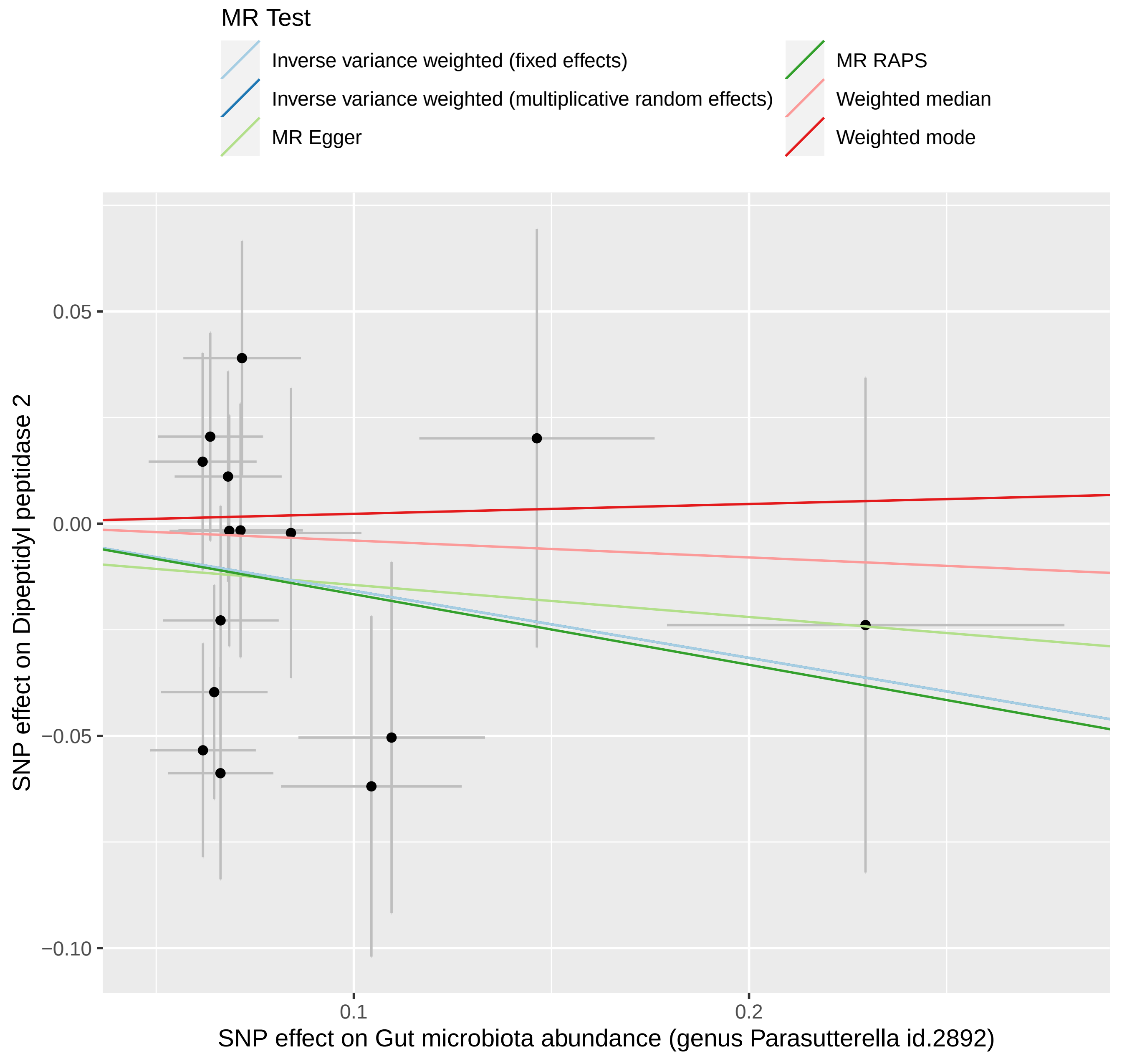

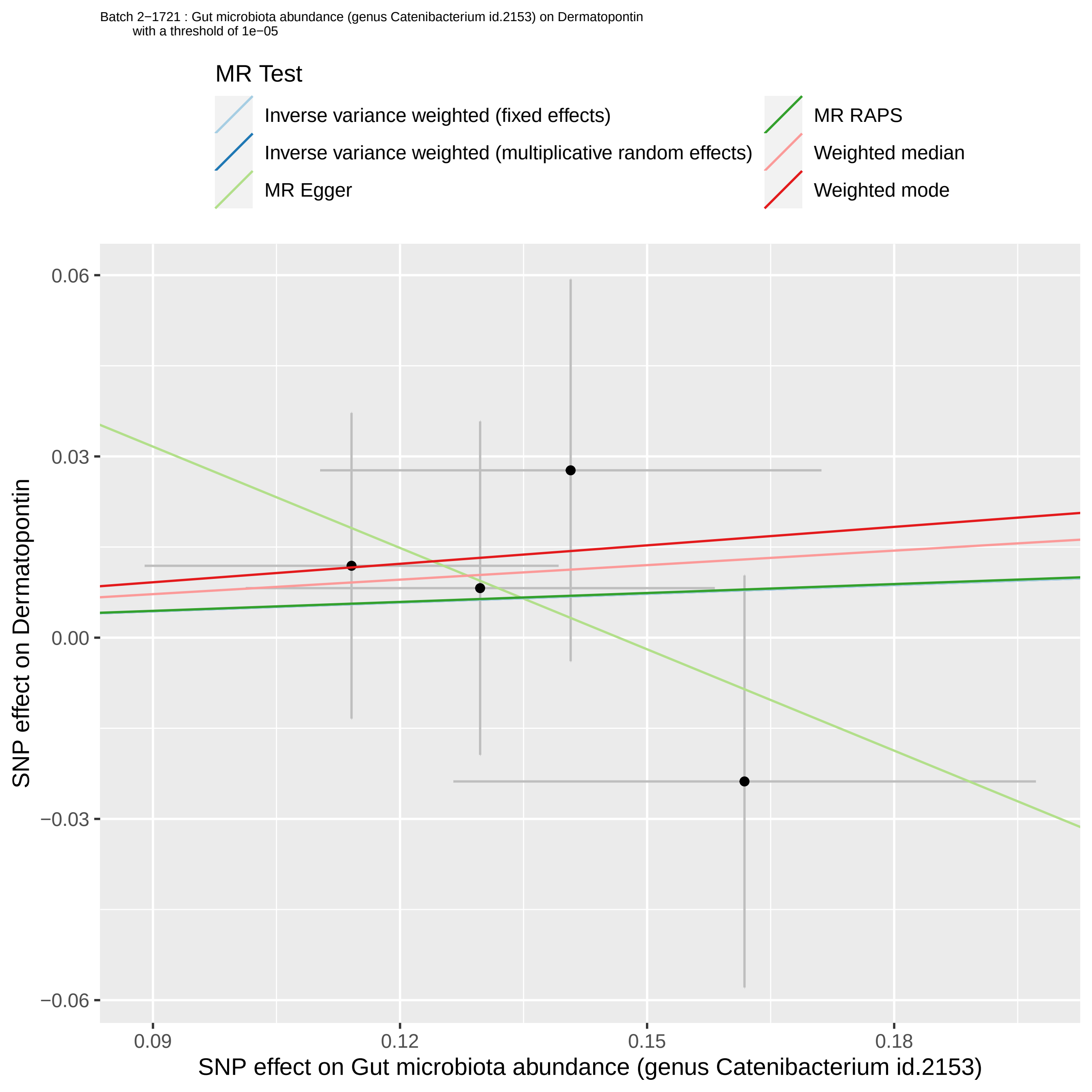
Batch 2−1721 : Gut microbiota abundance (genus Catenibacterium id.2153) on Dermatopontin
with a threshold of 1e−05
MR Test
Inverse variance weighted (fixed effects)
Inverse variance weighted (multiplicative random effects)
MR Egger
MR RAPS
Weighted median
Weighted mode
0.06
0.03
0.00
−0.03
−0.06
SNP effect on Dermatopontin
0.09
0.12
0.15
0.18
SNP effect on Gut microbiota abundance (genus Catenibacterium id.2153)

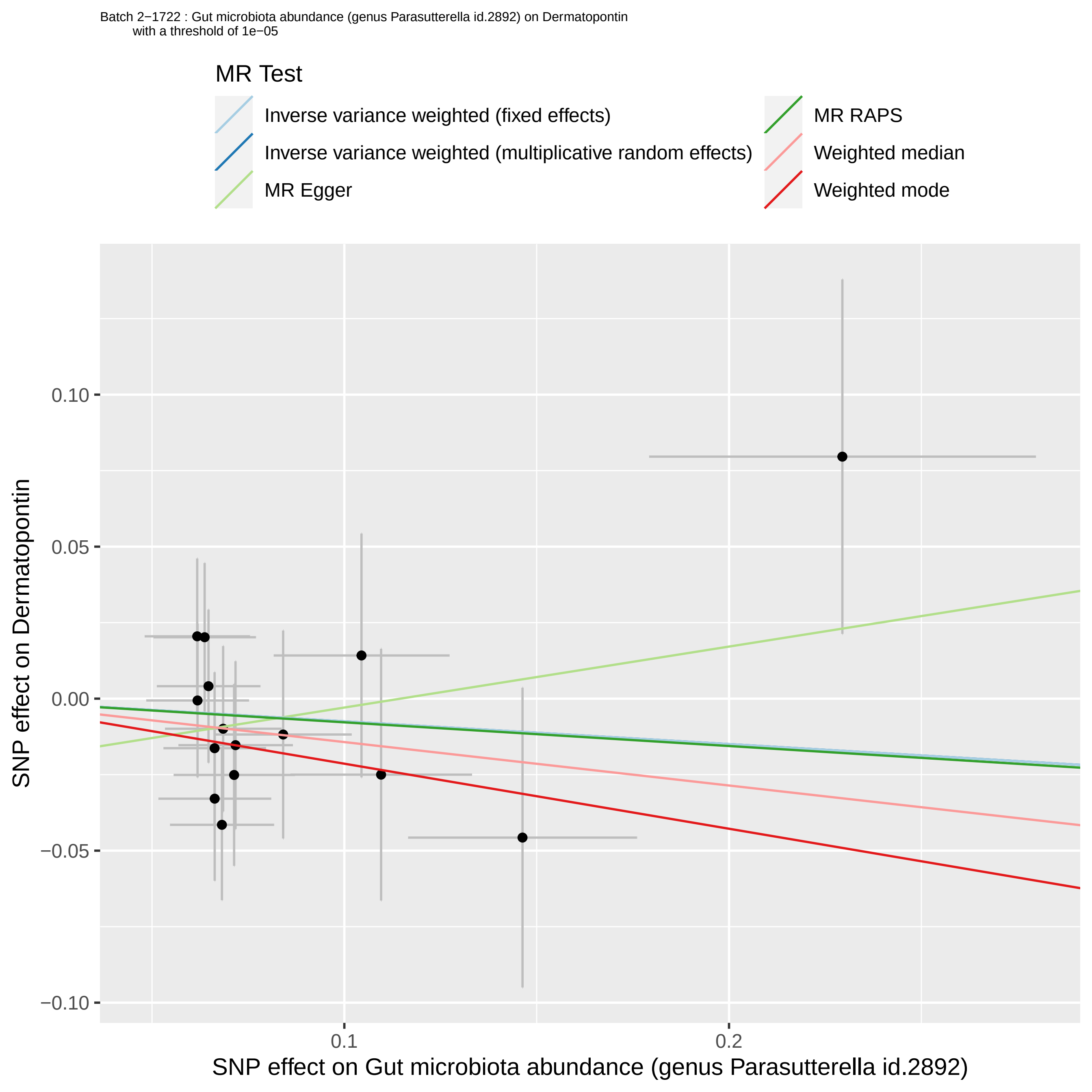
Batch 2−1722 : Gut microbiota abundance (genus Parasutterella id.2892) on Dermatopontin
with a threshold of 1e−05
MR Test
Inverse variance weighted (fixed effects)
Inverse variance weighted (multiplicative random effects)
MR Egger
MR RAPS
Weighted median
Weighted mode
0.10
0.05
0.00
−0.05
−0.10
0.1
0.2
SNP effect on Dermatopontin
SNP effect on Gut microbiota abundance (genus Parasutterella id.2892)

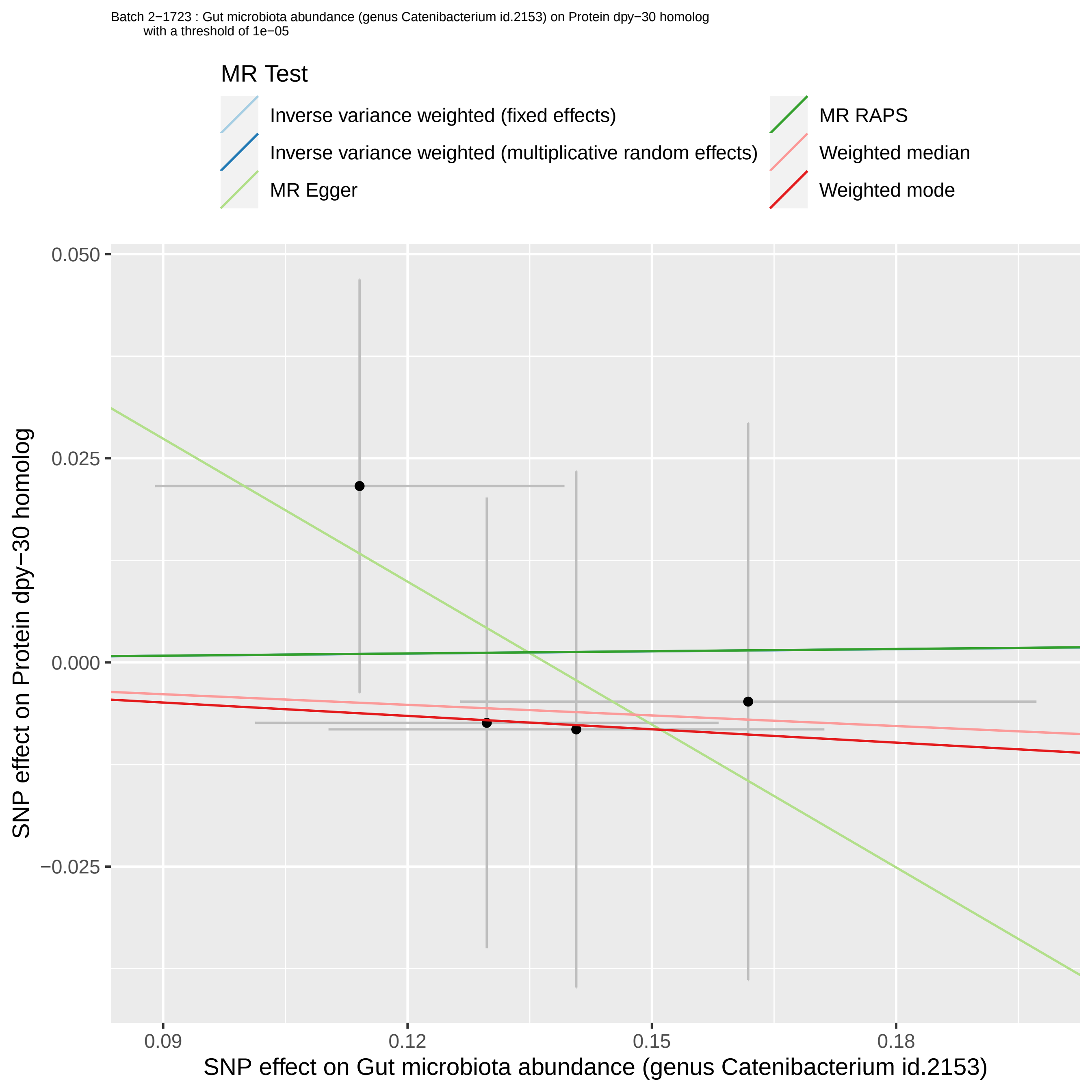

Batch 2−1723 : Gut microbiota abundance (genus Catenibacterium id.2153) on Protein dpy−30 homolog
with a threshold of 1e−05
MR Test
Inverse variance weighted (fixed effects)
Inverse variance weighted (multiplicative random effects)
MR Egger
MR RAPS
Weighted median
Weighted mode
0.050
0.025
0.000
−0.025
0.09
0.12
0.15
0.18
SNP effect on Protein dpy−30 homolog
SNP effect on Gut microbiota abundance (genus Catenibacterium id.2153)

Batch 2−1724 : Gut microbiota abundance (genus Parasutterella id.2892) on Protein dpy−30 homolog
with a threshold of 1e−05

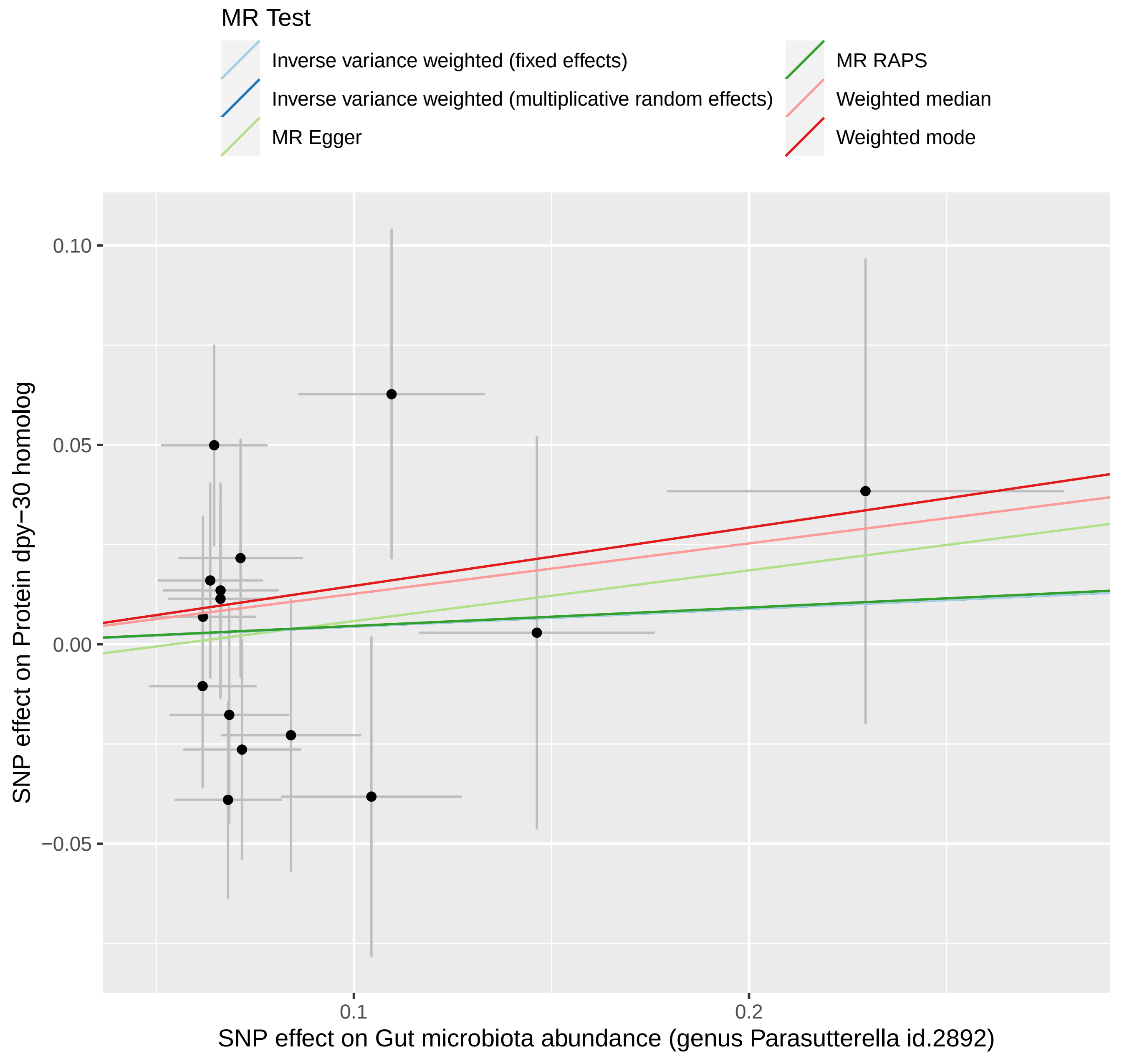

Batch 2−1725 : Gut microbiota abundance (genus Catenibacterium id.2153) on Dihydropyrimidinase−related protein 3
with a threshold of 1e−05

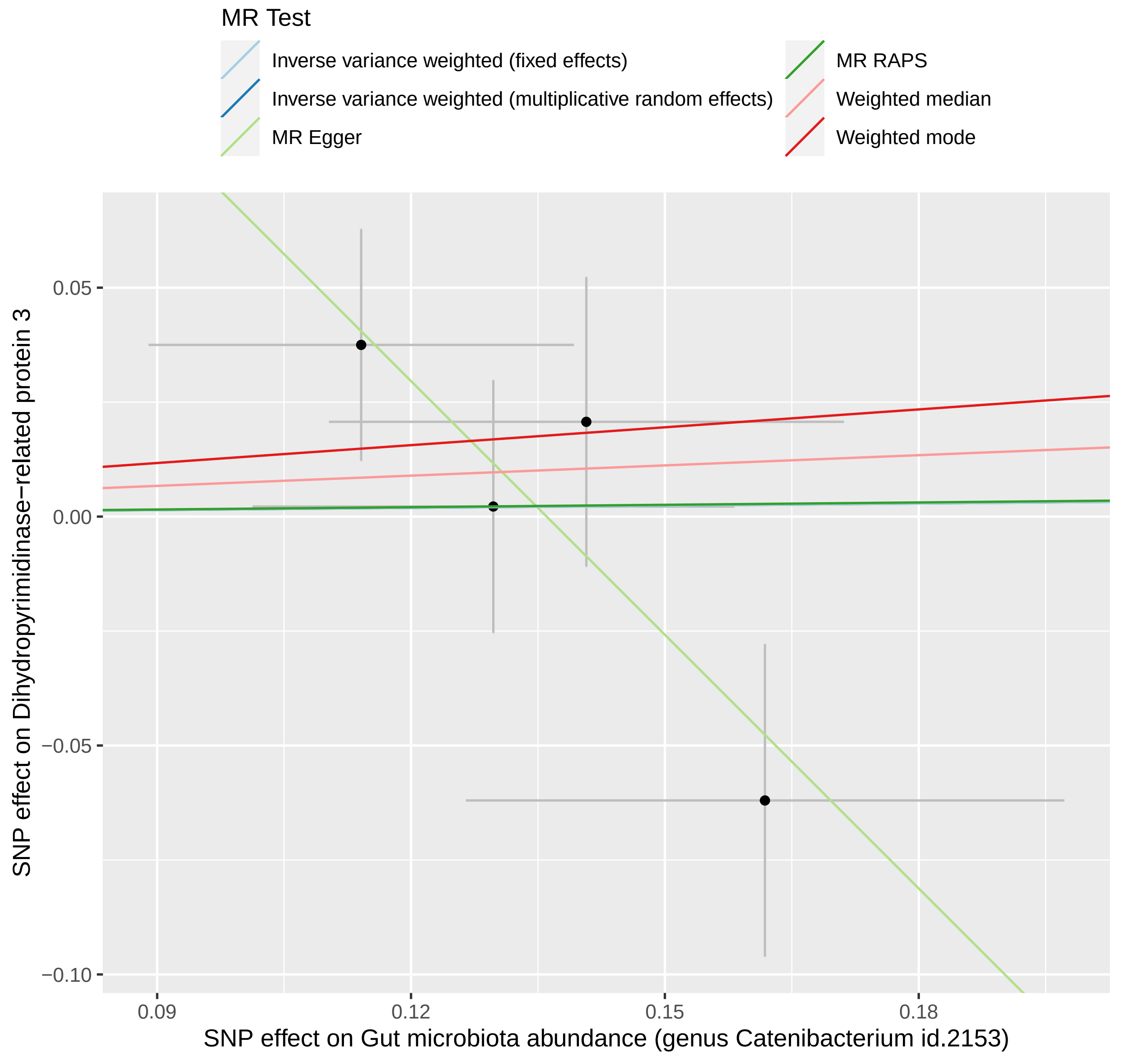

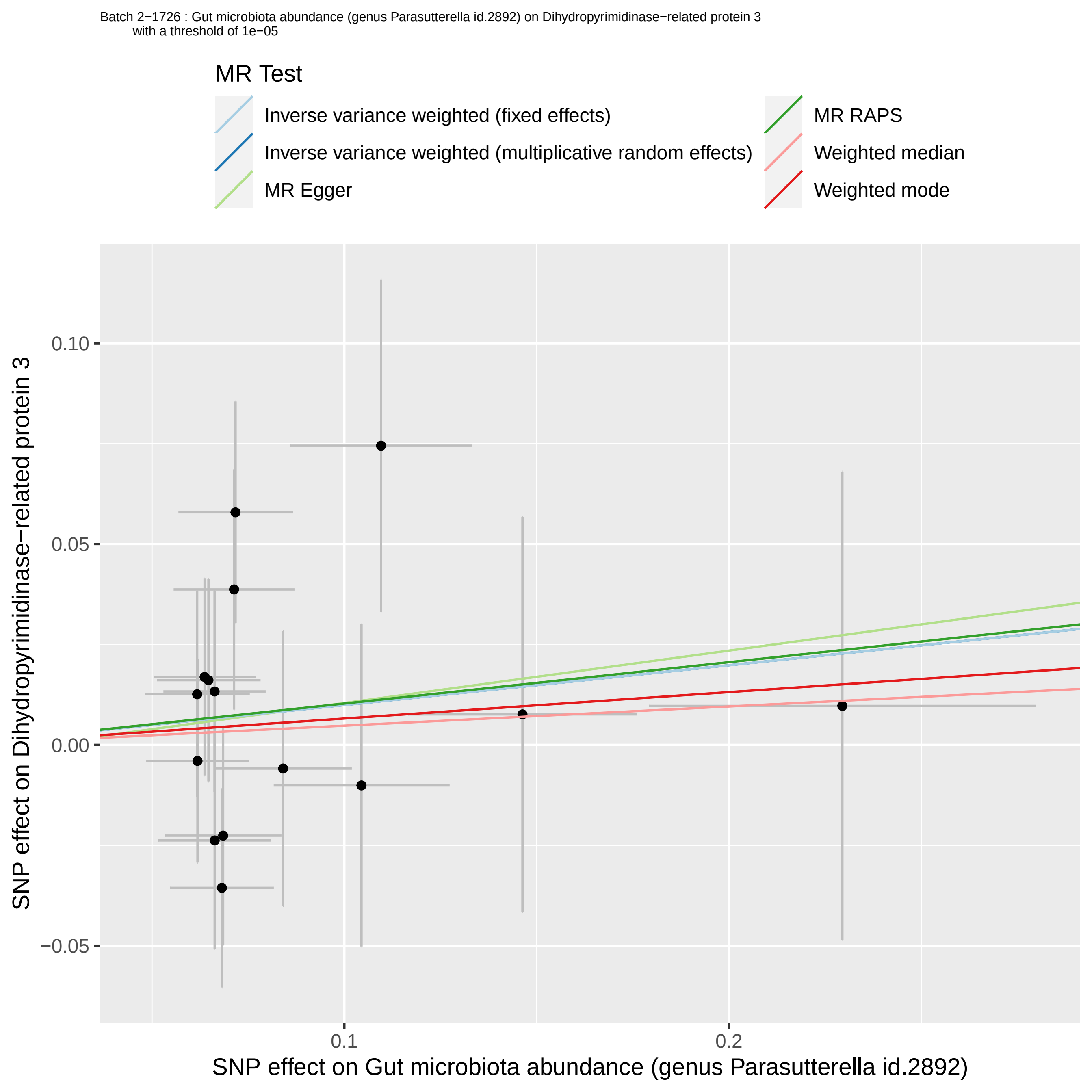

Batch 2−1726 : Gut microbiota abundance (genus Parasutterella id.2892) on Dihydropyrimidinase−related protein 3
with a threshold of 1e−05
MR Test
Inverse variance weighted (fixed effects)
Inverse variance weighted (multiplicative random effects)
MR Egger
MR RAPS
Weighted median
Weighted mode
0.10
0.05
0.00
−0.05
0.1
0.2
SNP effect on Dihydropyrimidinase−related protein 3
SNP effect on Gut microbiota abundance (genus Parasutterella id.2892)

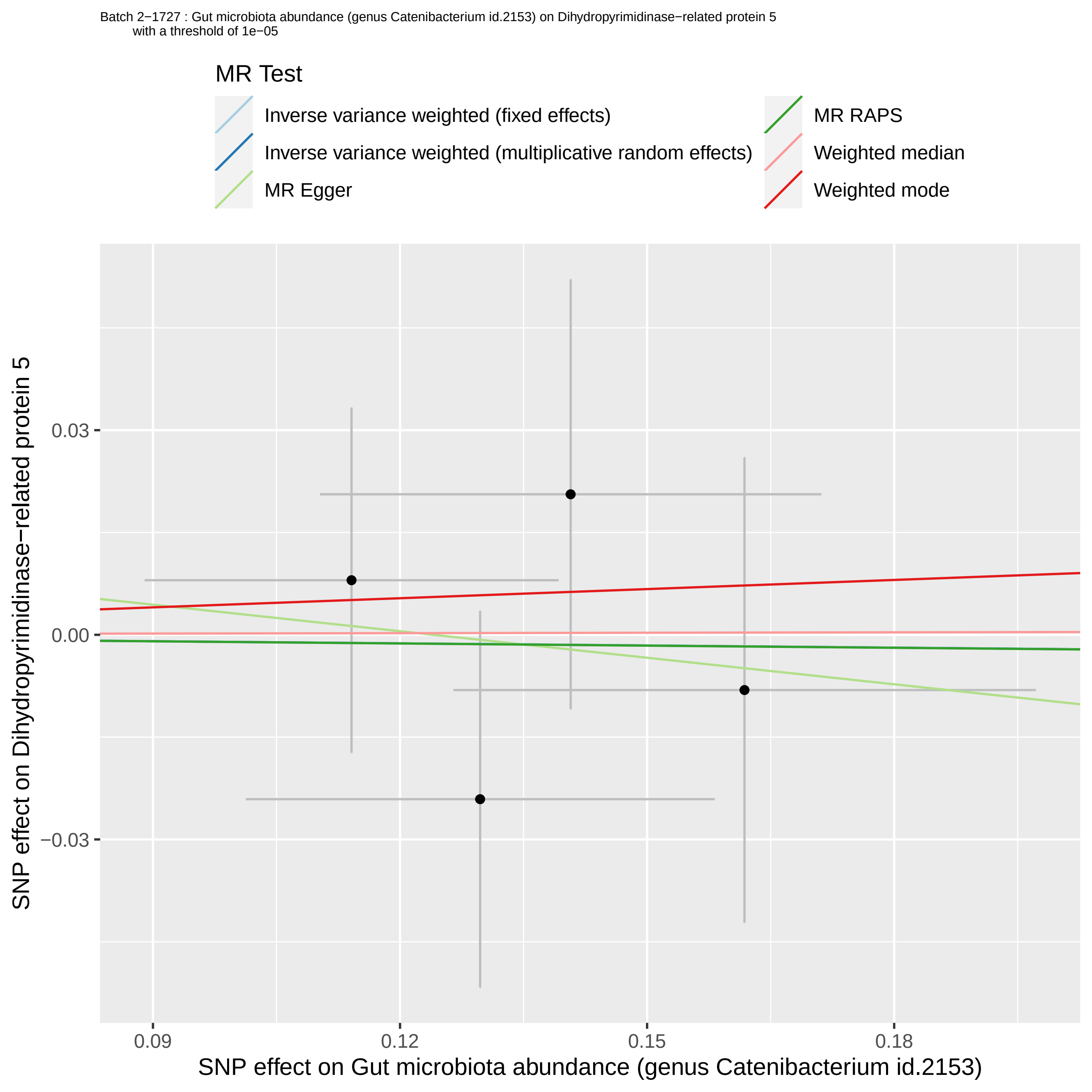
Batch 2−1727 : Gut microbiota abundance (genus Catenibacterium id.2153) on Dihydropyrimidinase−related protein 5
with a threshold of 1e−05
MR Test
Inverse variance weighted (fixed effects)
Inverse variance weighted (multiplicative random effects)
MR Egger
MR RAPS
Weighted median
Weighted mode
0.03
0.00
−0.03
0.09
0.12
0.15
0.18
SNP effect on Dihydropyrimidinase−related protein 5
SNP effect on Gut microbiota abundance (genus Catenibacterium id.2153)

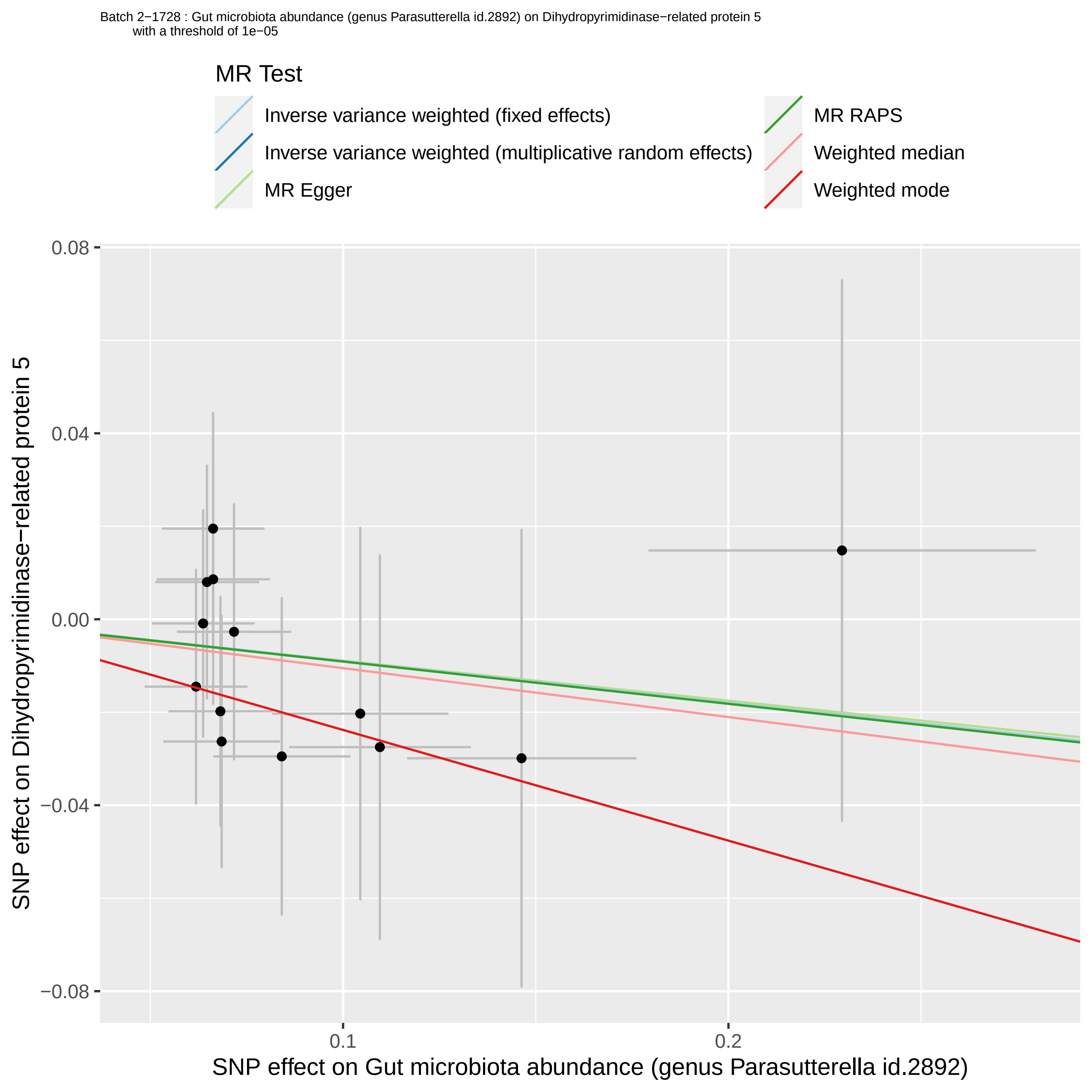

Batch 2−1728 : Gut microbiota abundance (genus Parasutterella id.2892) on Dihydropyrimidinase−related protein 5
with a threshold of 1e−05
MR Test
Inverse variance weighted (fixed effects)
Inverse variance weighted (multiplicative random effects)
MR Egger
MR RAPS
Weighted median
Weighted mode
0.08
0.04
0.00
−0.04
−0.08
0.1
0.2
SNP effect on Dihydropyrimidinase−related protein 5
SNP effect on Gut microbiota abundance (genus Parasutterella id.2892)

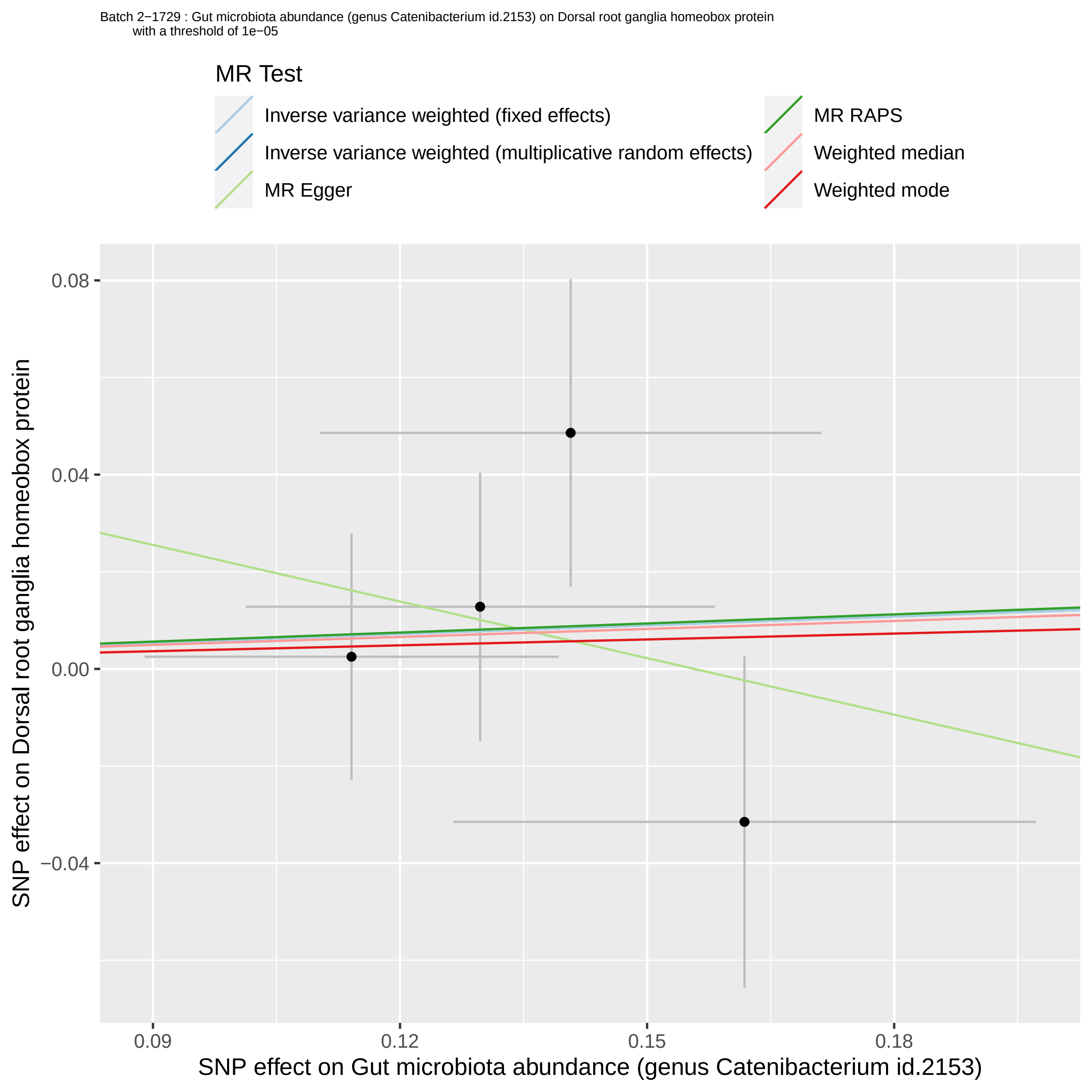

Batch 2−1729 : Gut microbiota abundance (genus Catenibacterium id.2153) on Dorsal root ganglia homeobox protein
with a threshold of 1e−05
MR Test
Inverse variance weighted (fixed effects)
Inverse variance weighted (multiplicative random effects)
MR Egger
MR RAPS
Weighted median
Weighted mode
0.08
0.04
0.00
−0.04
0.09
0.12
0.15
0.18
SNP effect on Dorsal root ganglia homeobox protein
SNP effect on Gut microbiota abundance (genus Catenibacterium id.2153)

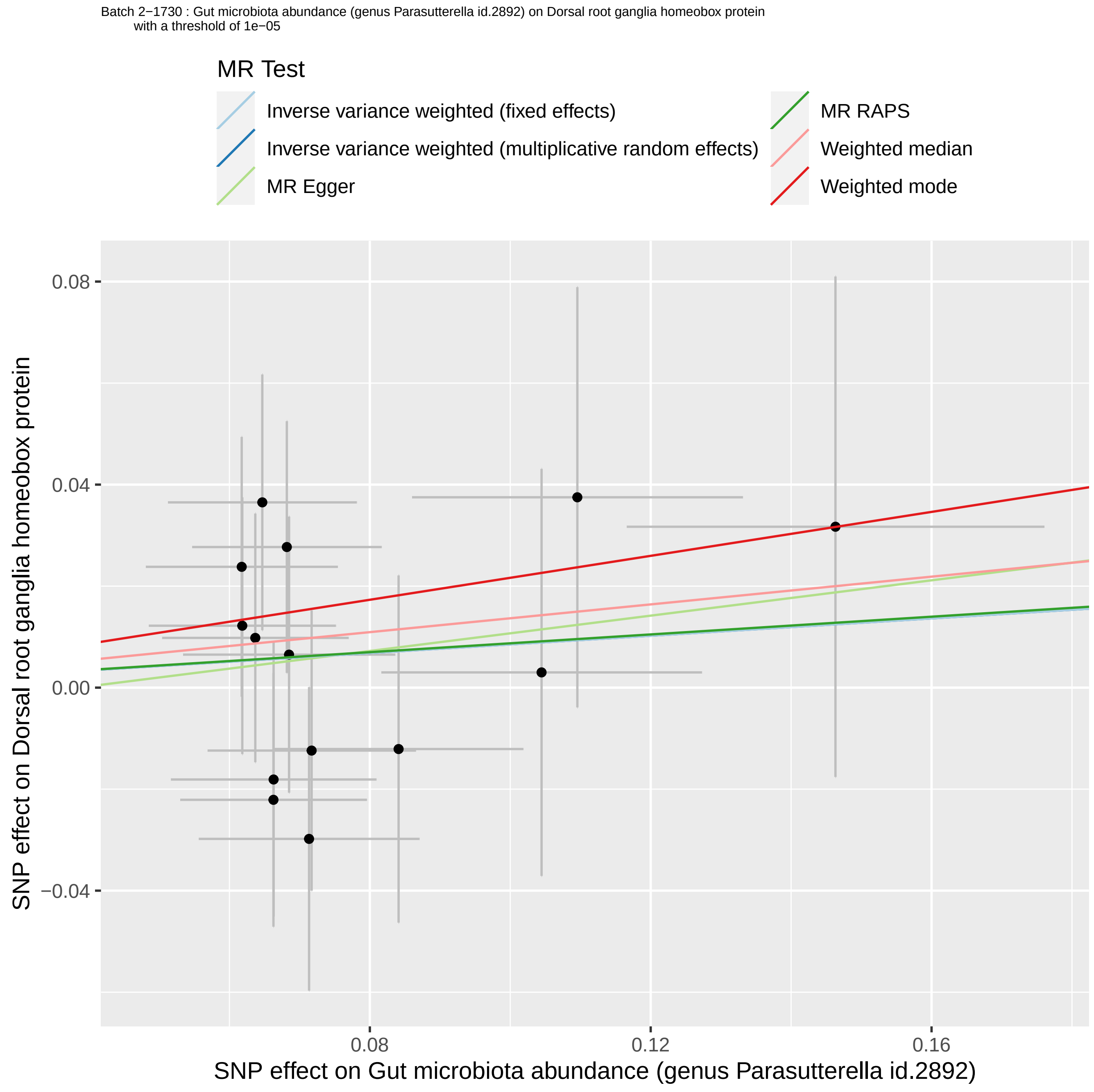

Batch 2−1730 : Gut microbiota abundance (genus Parasutterella id.2892) on Dorsal root ganglia homeobox protein
with a threshold of 1e−05
MR Test
Inverse variance weighted (fixed effects)
Inverse variance weighted (multiplicative random effects)
MR Egger
MR RAPS
Weighted median
Weighted mode
0.08
0.04
0.00
−0.04
0.08
0.12
0.16
SNP effect on Dorsal root ganglia homeobox protein
SNP effect on Gut microbiota abundance (genus Parasutterella id.2892)

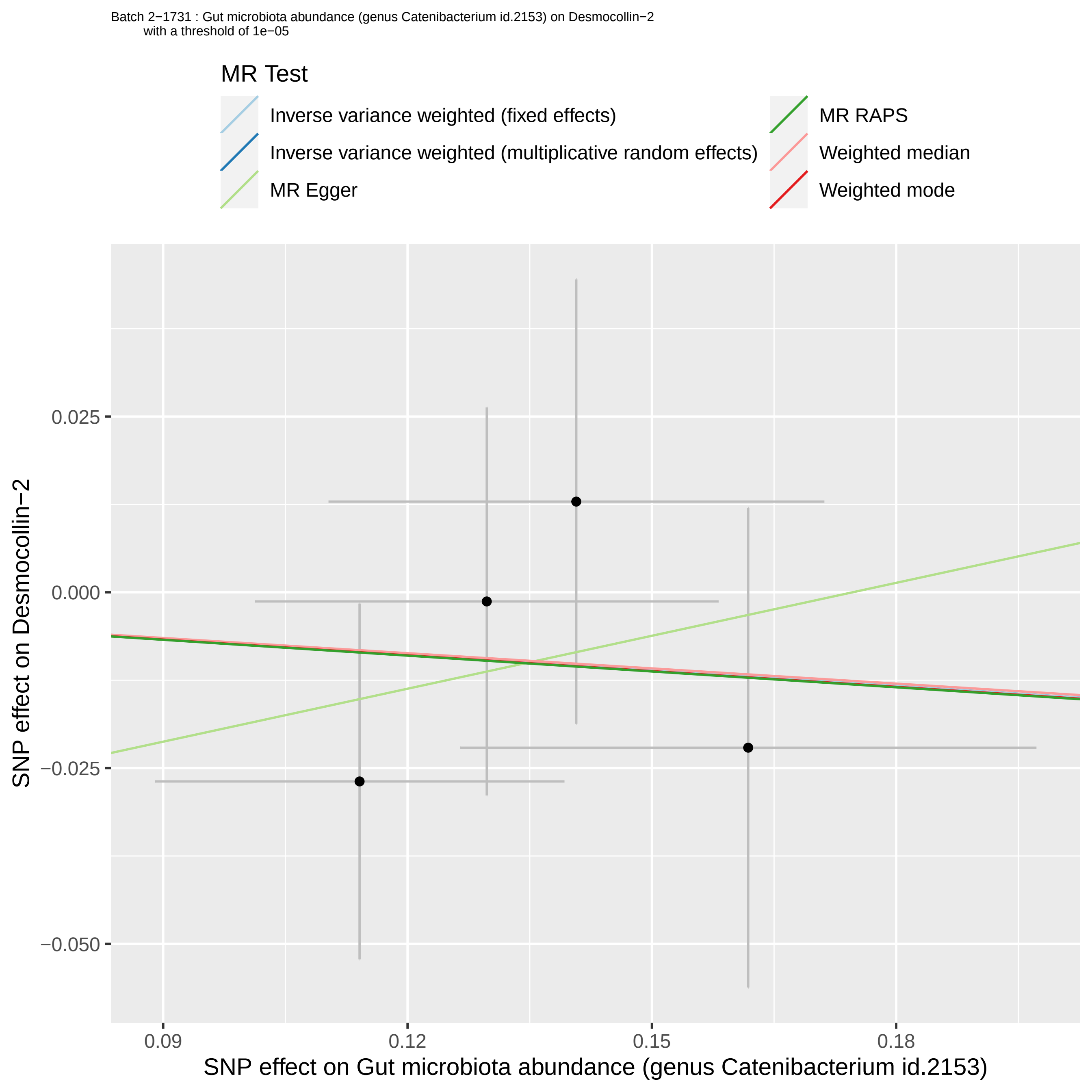
Batch 2−1731 : Gut microbiota abundance (genus Catenibacterium id.2153) on Desmocollin−2
with a threshold of 1e−05
MR Test
Inverse variance weighted (fixed effects)
Inverse variance weighted (multiplicative random effects)
MR Egger
MR RAPS
Weighted median
Weighted mode
0.025
0.000
−0.025
−0.050
0.09
0.12
0.15
0.18
SNP effect on Desmocollin−2
SNP effect on Gut microbiota abundance (genus Catenibacterium id.2153)

Batch 2−1732 : Gut microbiota abundance (genus Parasutterella id.2892) on Desmocollin−2
with a threshold of 1e−05

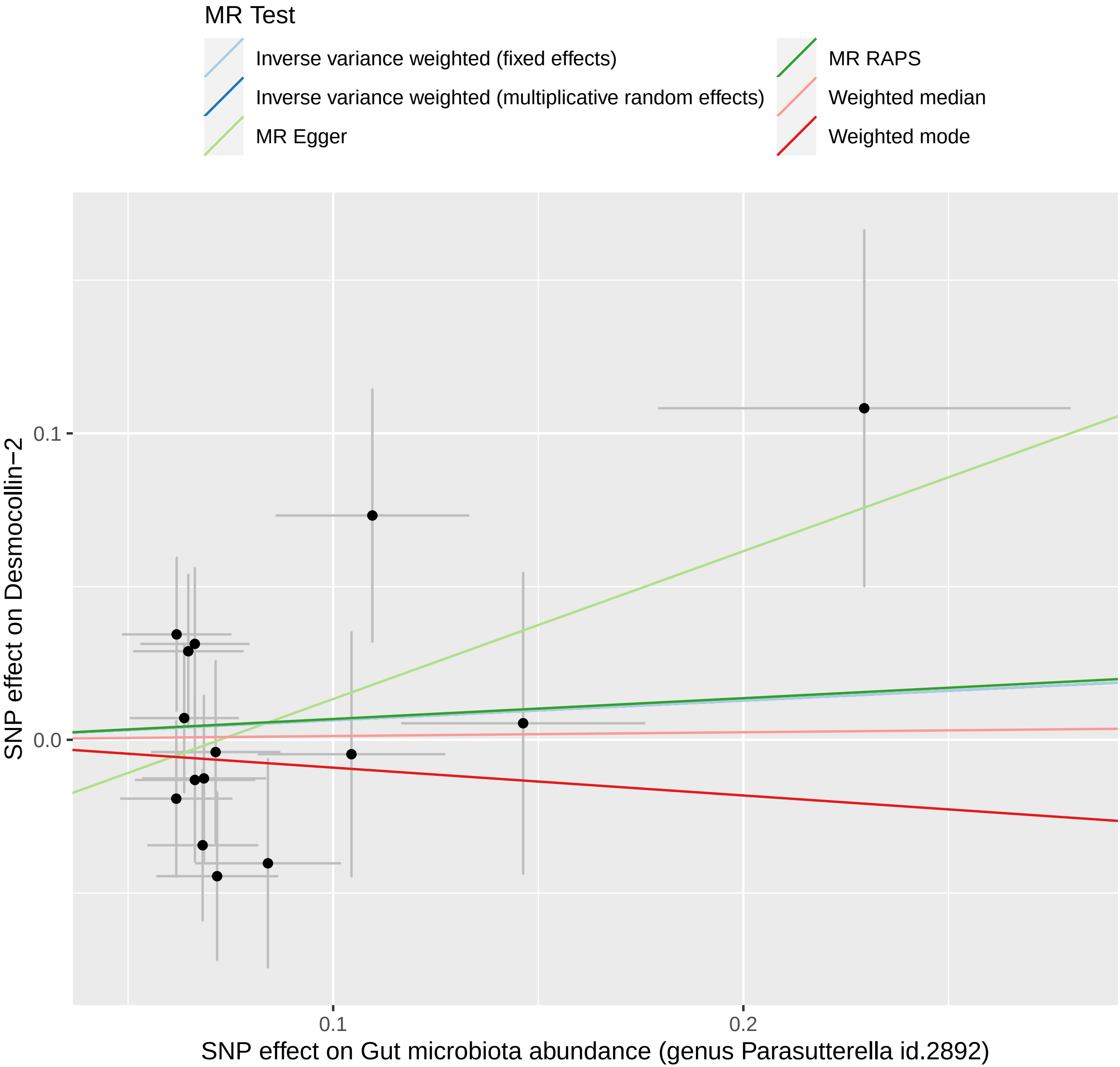

Batch 2−1733 : Gut microbiota abundance (genus Catenibacterium id.2153) on Desmocollin−3
with a threshold of 1e−05

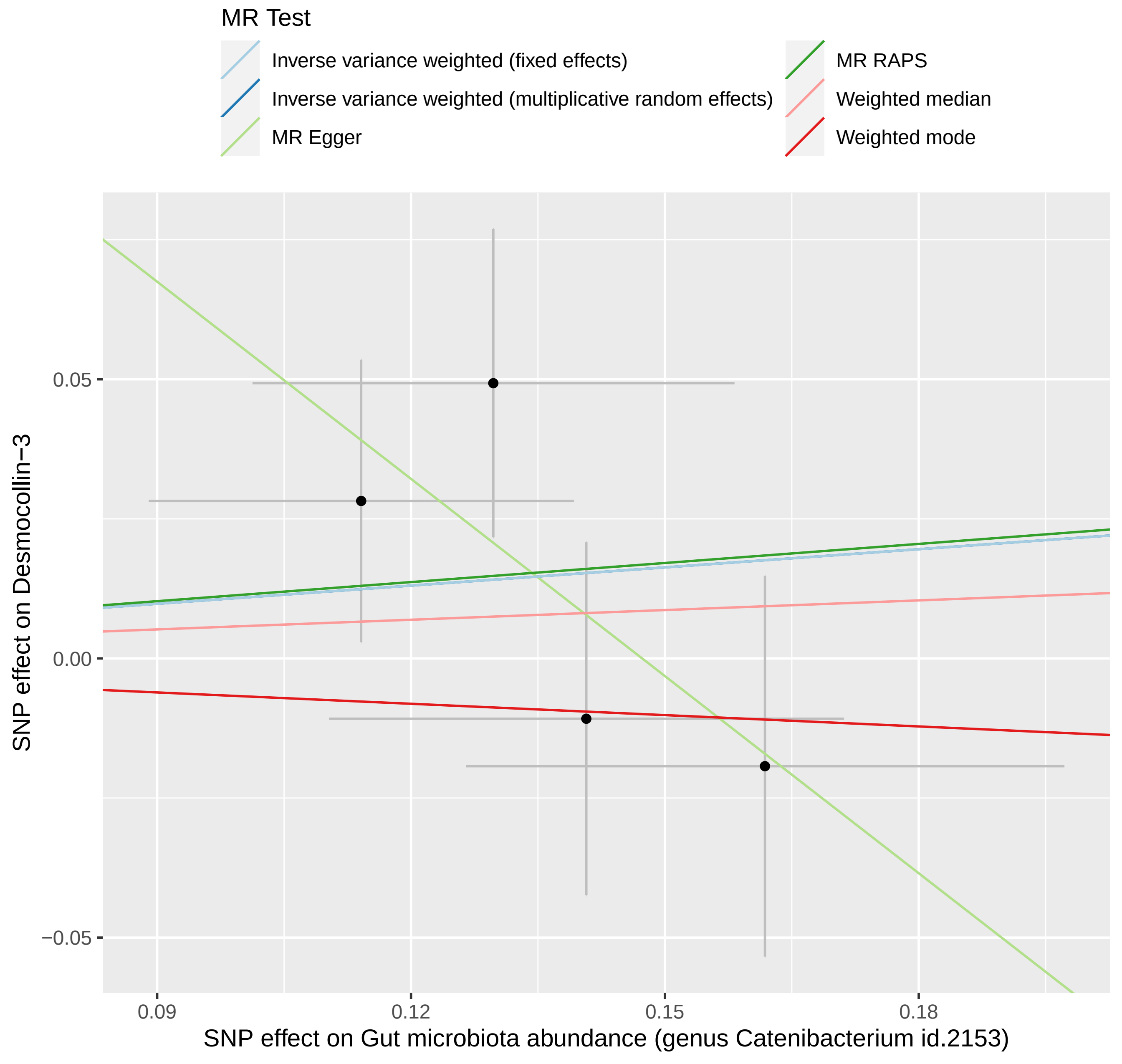

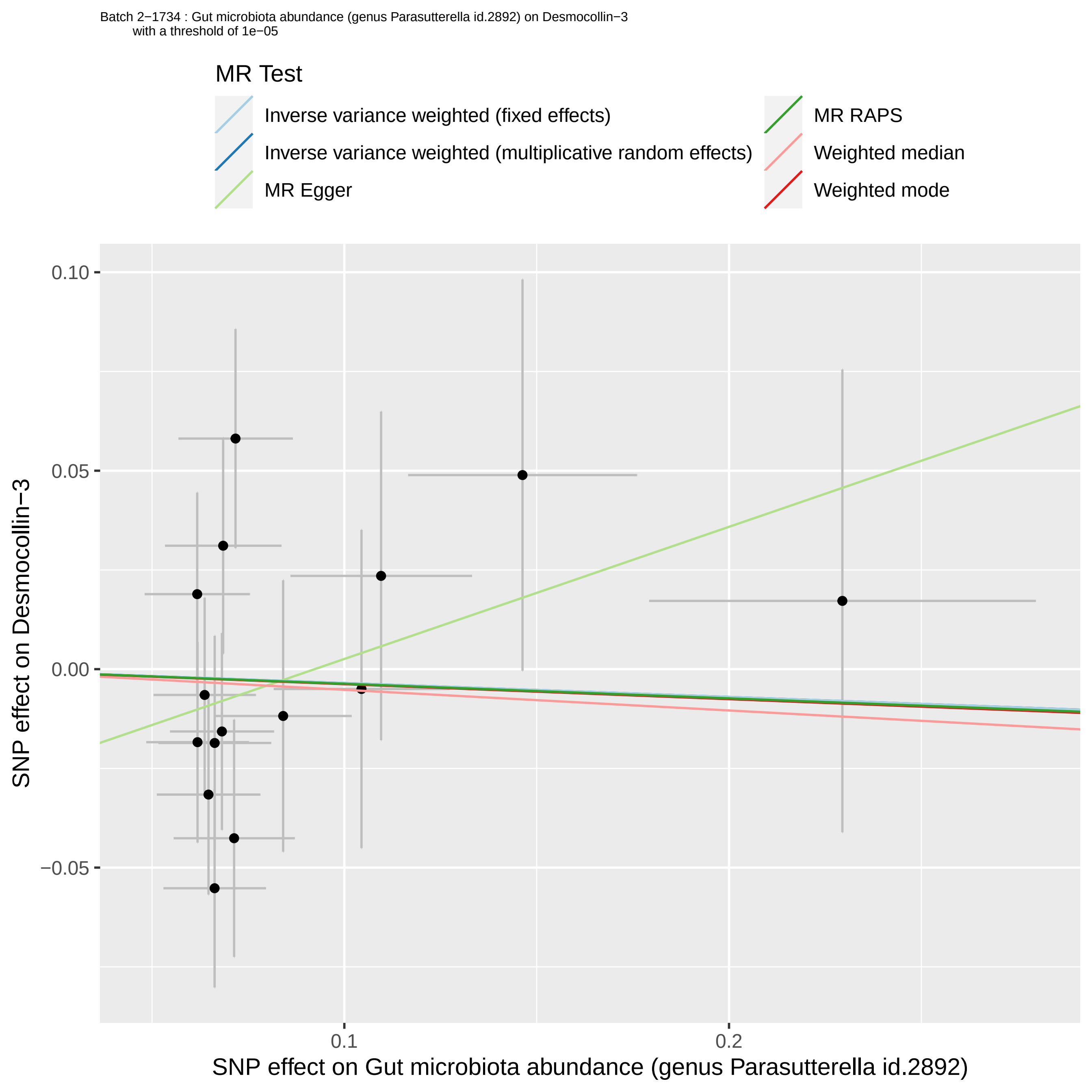
Batch 2−1734 : Gut microbiota abundance (genus Parasutterella id.2892) on Desmocollin−3
with a threshold of 1e−05
MR Test
Inverse variance weighted (fixed effects)
Inverse variance weighted (multiplicative random effects)
MR Egger
MR RAPS
Weighted median
Weighted mode
0.10
0.05
0.00
−0.05
0.1
0.2
SNP effect on Desmocollin−3
SNP effect on Gut microbiota abundance (genus Parasutterella id.2892)

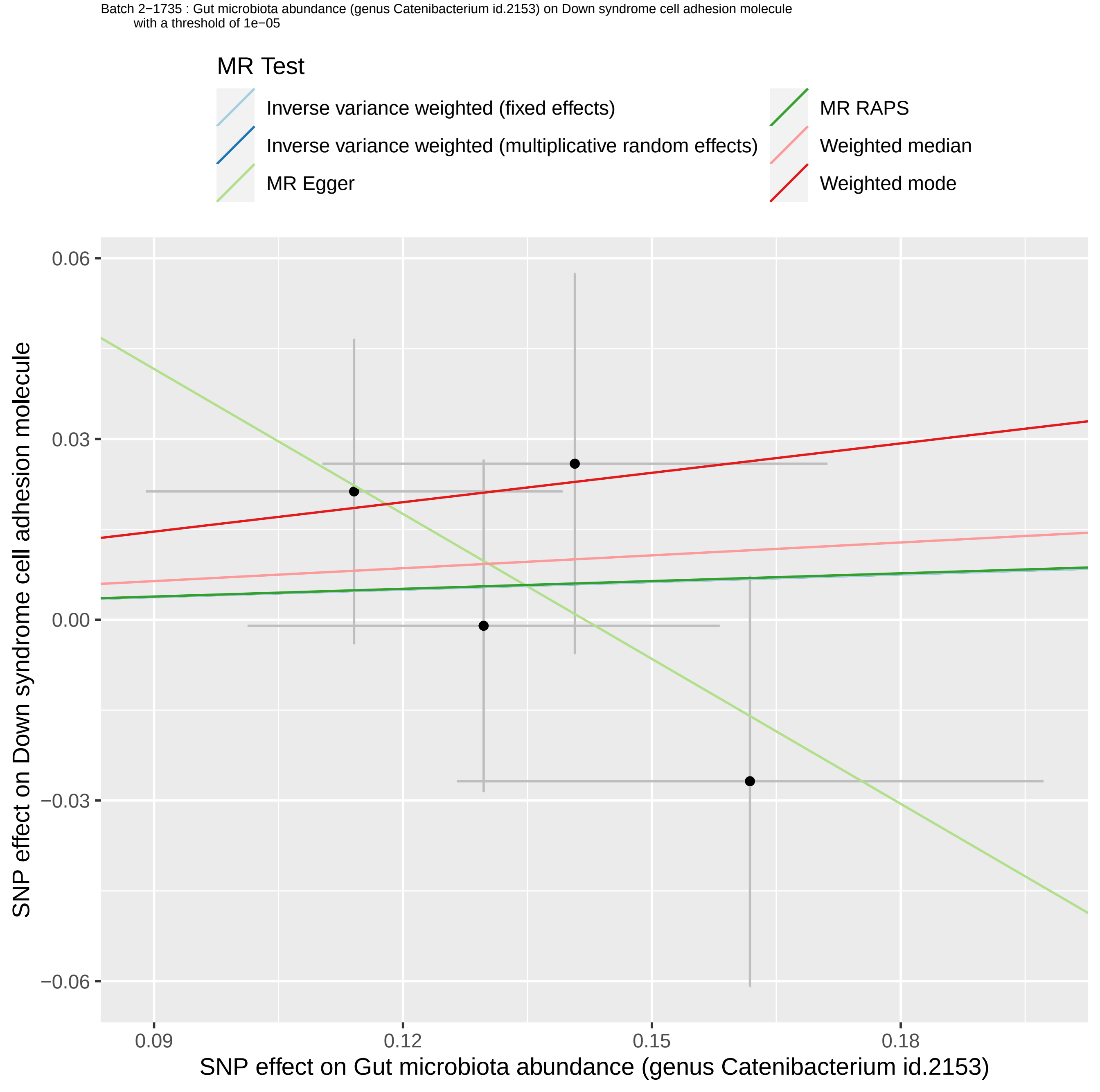

Batch 2−1735 : Gut microbiota abundance (genus Catenibacterium id.2153) on Down syndrome cell adhesion molecule
with a threshold of 1e−05
MR Test
Inverse variance weighted (fixed effects)
Inverse variance weighted (multiplicative random effects)
MR Egger
MR RAPS
Weighted median
Weighted mode
0.06
0.03
0.00
−0.03
−0.06
0.09
0.12
0.15
0.18
SNP effect on Down syndrome cell adhesion molecule
SNP effect on Gut microbiota abundance (genus Catenibacterium id.2153)

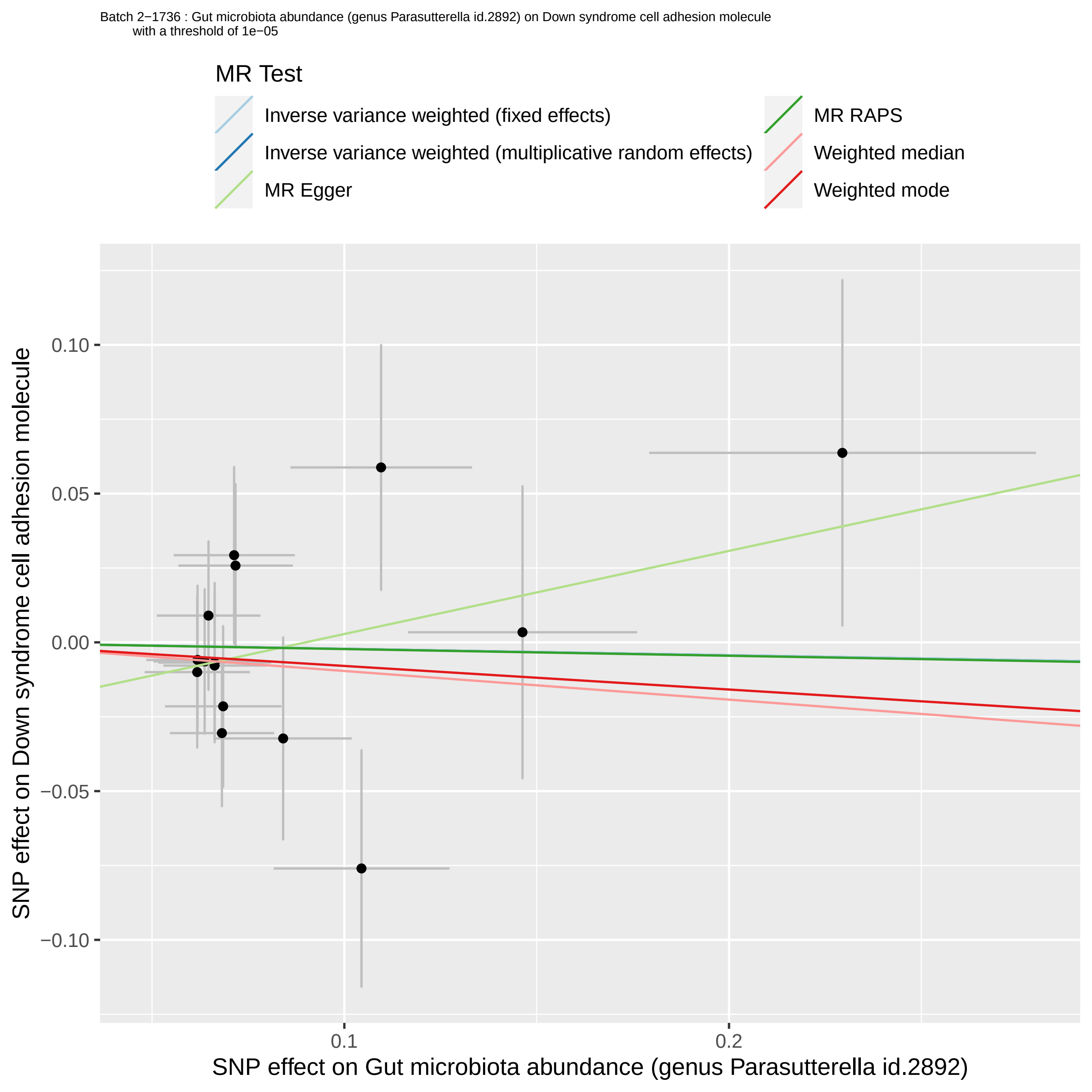

Batch 2−1736 : Gut microbiota abundance (genus Parasutterella id.2892) on Down syndrome cell adhesion molecule
with a threshold of 1e−05
MR Test
Inverse variance weighted (fixed effects)
Inverse variance weighted (multiplicative random effects)
MR Egger
MR RAPS
Weighted median
Weighted mode
0.10
0.05
0.00
−0.05
−0.10
0.1
0.2
SNP effect on Down syndrome cell adhesion molecule
SNP effect on Gut microbiota abundance (genus Parasutterella id.2892)

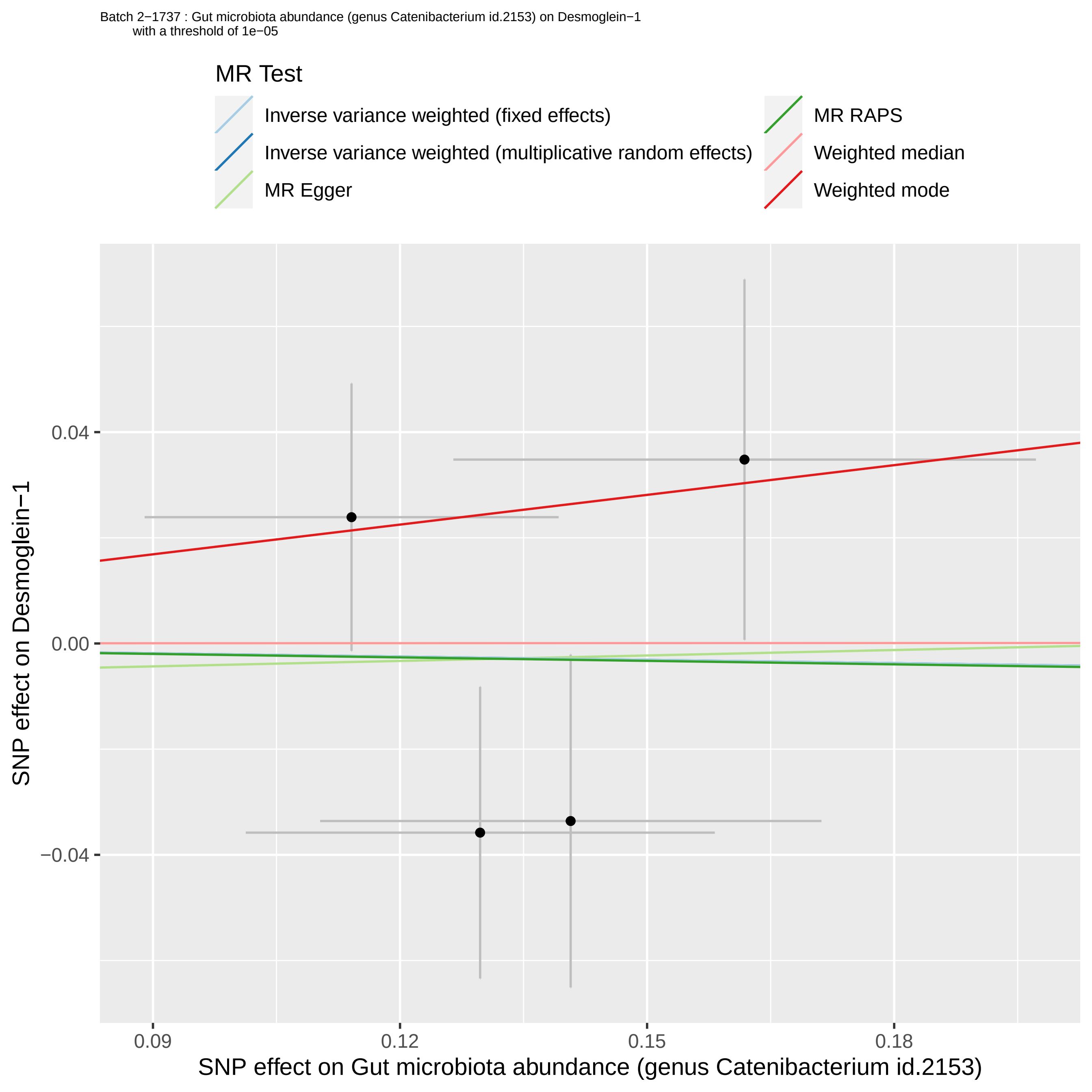
Batch 2−1737 : Gut microbiota abundance (genus Catenibacterium id.2153) on Desmoglein−1
with a threshold of 1e−05
MR Test
Inverse variance weighted (fixed effects)
Inverse variance weighted (multiplicative random effects)
MR Egger
MR RAPS
Weighted median
Weighted mode
0.04
0.00
−0.04
0.09
0.12
0.15
0.18
SNP effect on Desmoglein−1
SNP effect on Gut microbiota abundance (genus Catenibacterium id.2153)

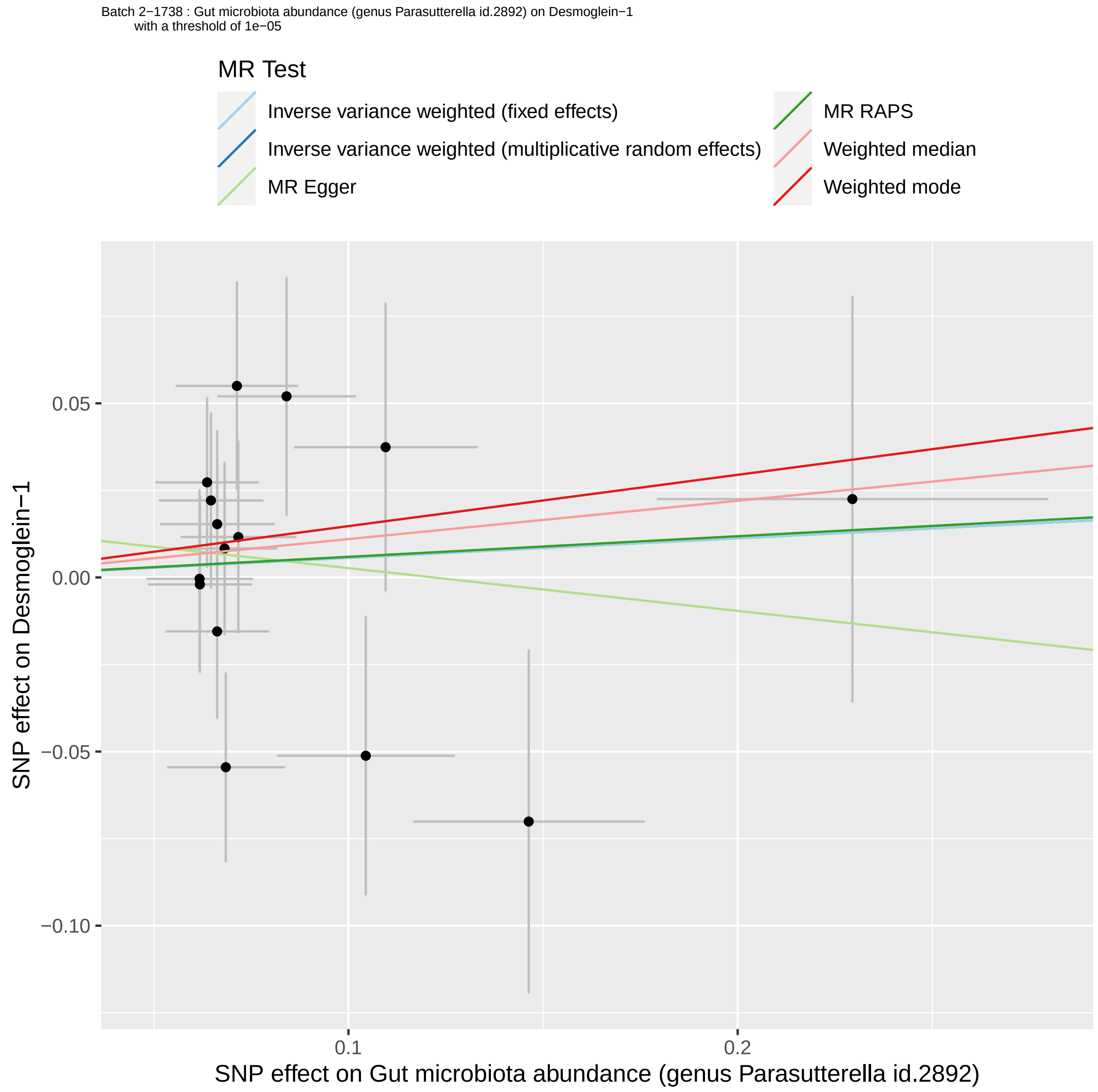

Batch 2−1738 : Gut microbiota abundance (genus Parasutterella id.2892) on Desmoglein−1
with a threshold of 1e−05
MR Test
Inverse variance weighted (fixed effects)
Inverse variance weighted (multiplicative random effects)
MR Egger
MR RAPS
Weighted median
Weighted mode
0.05
0.00
−0.05
−0.10
0.1
0.2
SNP effect on Desmoglein−1
SNP effect on Gut microbiota abundance (genus Parasutterella id.2892)

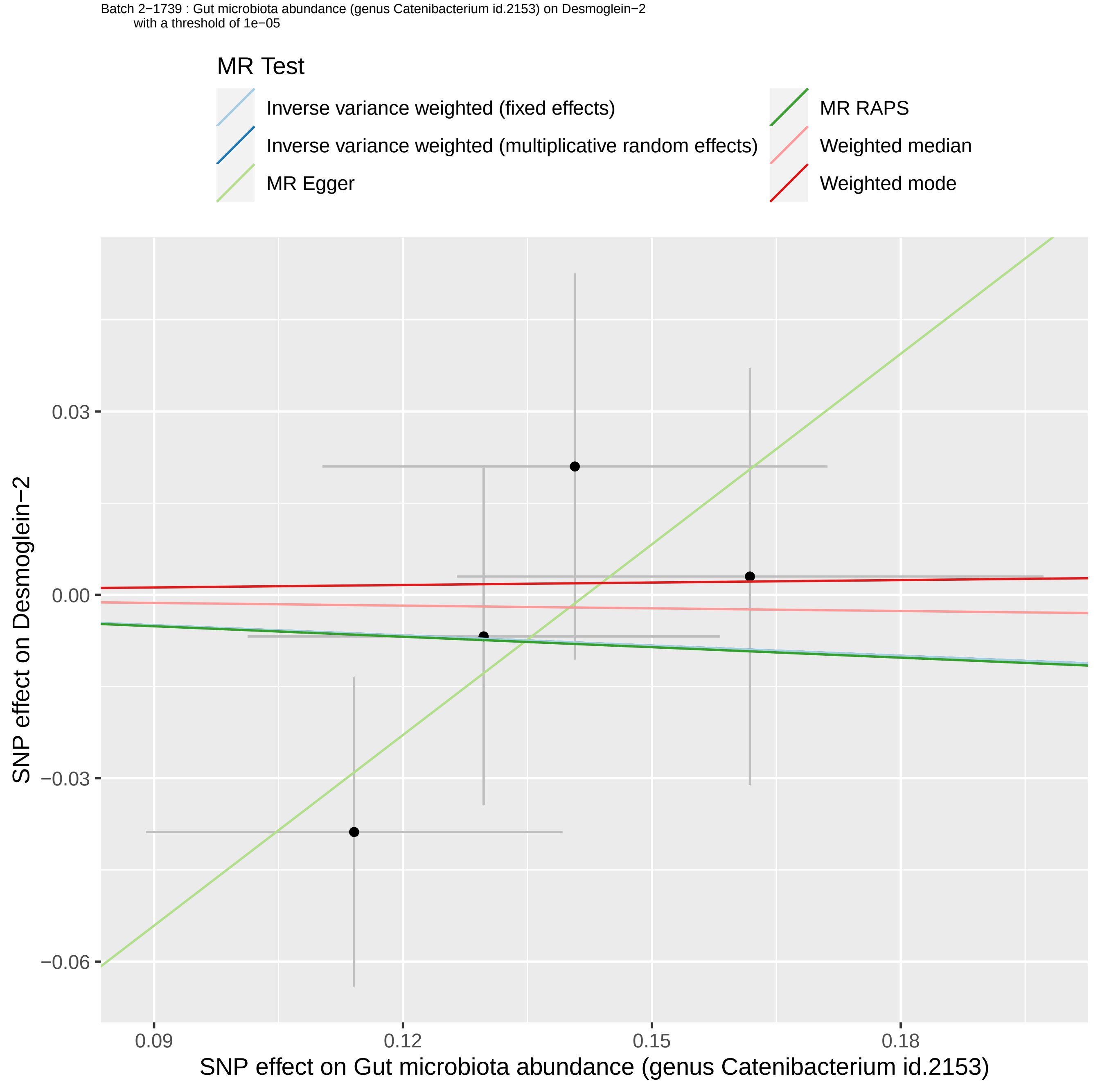
Batch 2−1739 : Gut microbiota abundance (genus Catenibacterium id.2153) on Desmoglein−2
with a threshold of 1e−05
MR Test
Inverse variance weighted (fixed effects)
Inverse variance weighted (multiplicative random effects)
MR Egger
MR RAPS
Weighted median
Weighted mode
0.03
0.00
−0.03
−0.06
SNP effect on Desmoglein−2
0.09
0.12
0.15
0.18
SNP effect on Gut microbiota abundance (genus Catenibacterium id.2153)

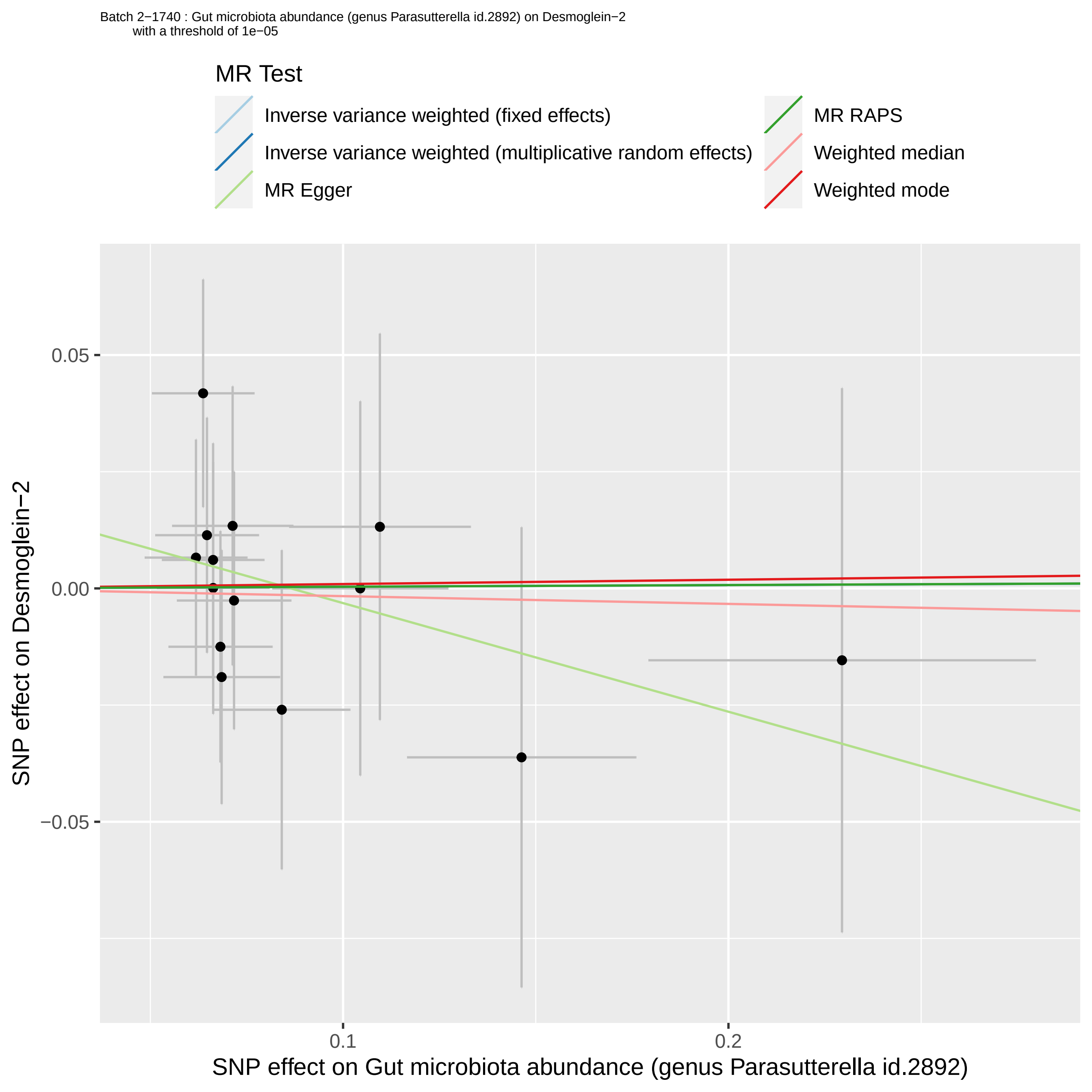
Batch 2−1740 : Gut microbiota abundance (genus Parasutterella id.2892) on Desmoglein−2
with a threshold of 1e−05
MR Test
Inverse variance weighted (fixed effects)
Inverse variance weighted (multiplicative random effects)
MR Egger
MR RAPS
Weighted median
Weighted mode
0.05
0.00
−0.05
0.1
0.2
SNP effect on Desmoglein−2
SNP effect on Gut microbiota abundance (genus Parasutterella id.2892)

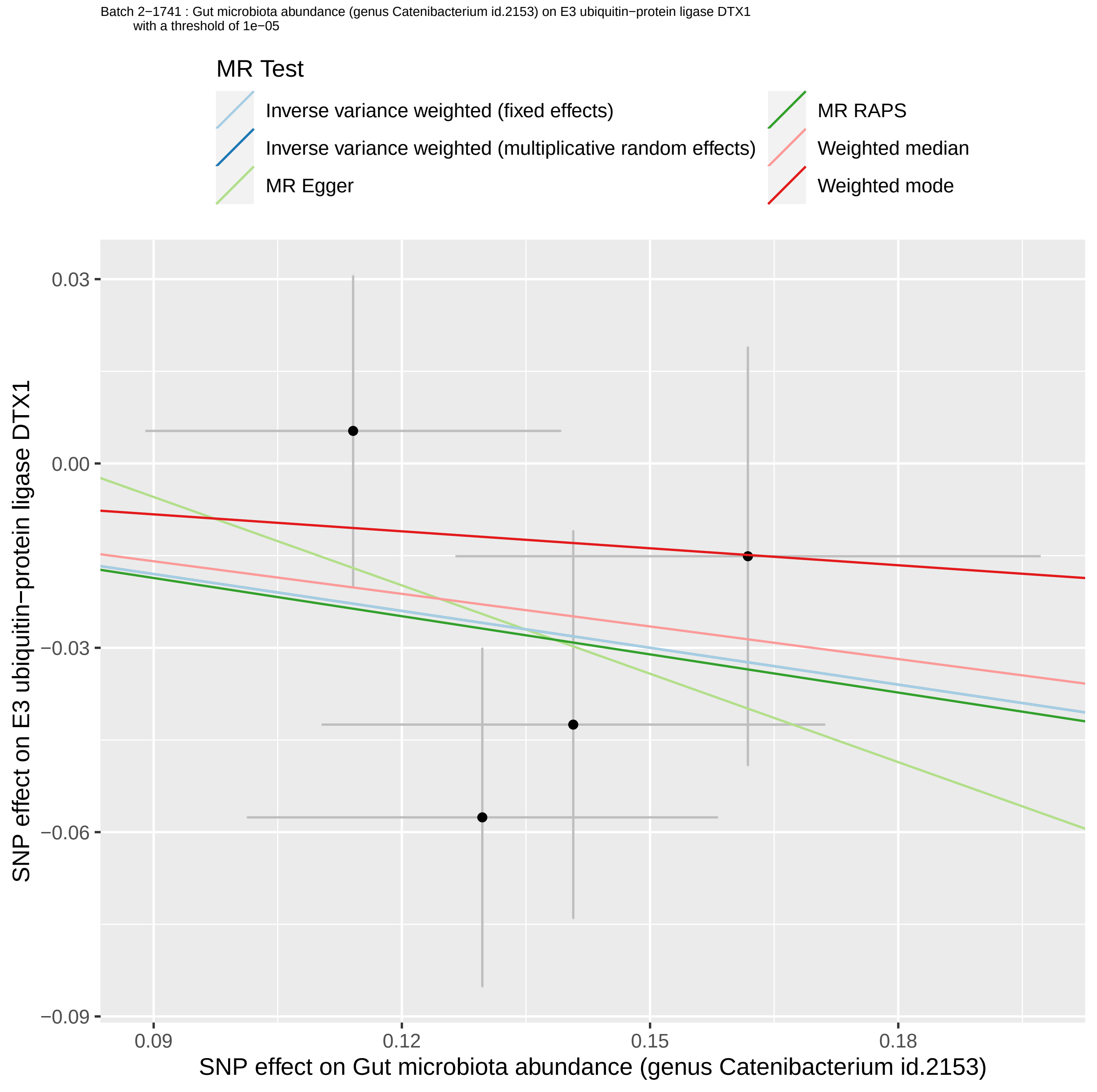
Batch 2−1741 : Gut microbiota abundance (genus Catenibacterium id.2153) on E3 ubiquitin−protein ligase DTX1
with a threshold of 1e−05
MR Test
Inverse variance weighted (fixed effects)
Inverse variance weighted (multiplicative random effects)
MR Egger
MR RAPS
Weighted median
Weighted mode
0.03
0.00
−0.03
−0.06
−0.09
0.09
0.12
0.15
0.18
SNP effect on E3 ubiquitin−protein ligase DTX1
SNP effect on Gut microbiota abundance (genus Catenibacterium id.2153)

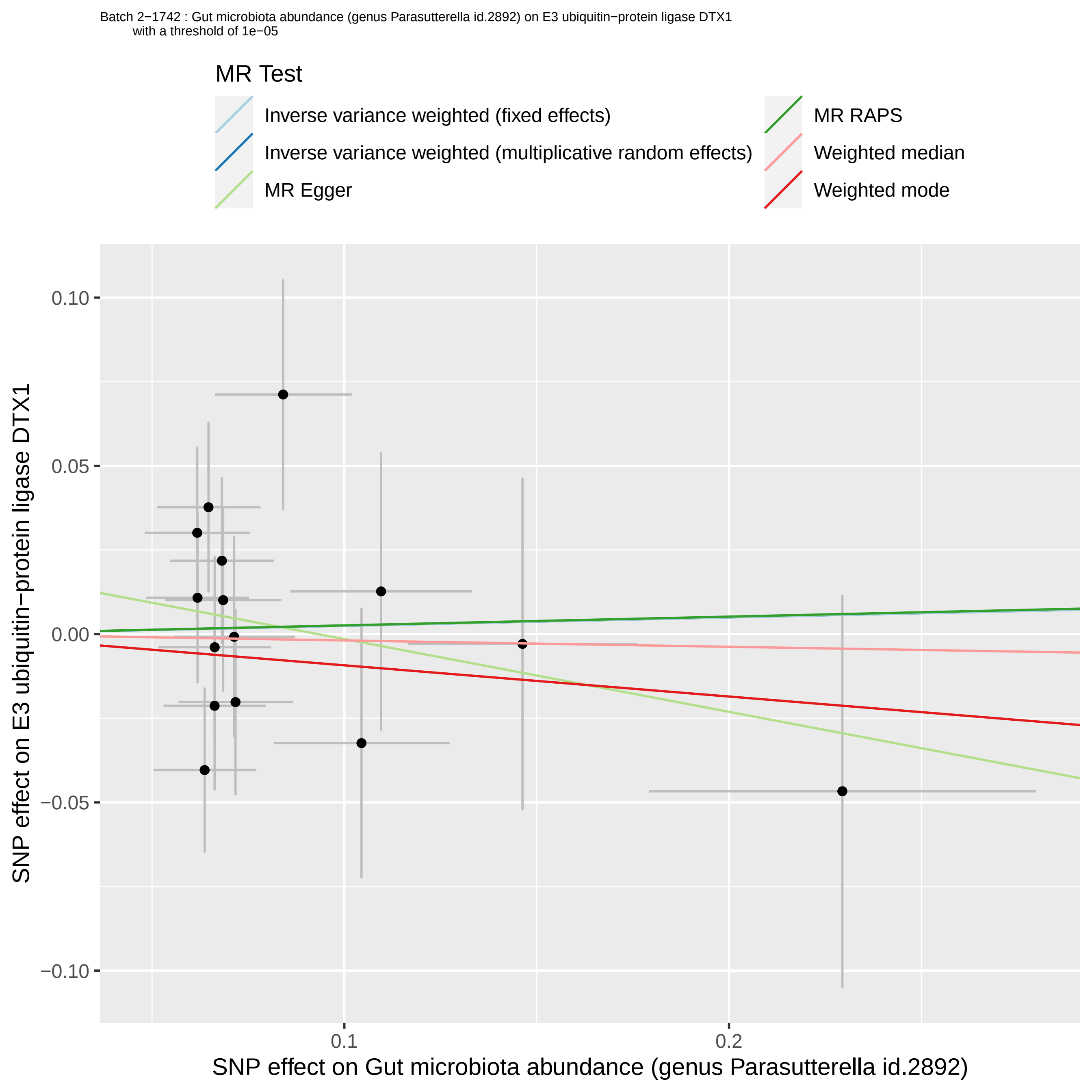

Batch 2−1742 : Gut microbiota abundance (genus Parasutterella id.2892) on E3 ubiquitin−protein ligase DTX1
with a threshold of 1e−05
MR Test
Inverse variance weighted (fixed effects)
Inverse variance weighted (multiplicative random effects)
MR Egger
MR RAPS
Weighted median
Weighted mode
0.10
0.05
0.00
−0.05
−0.10
0.1
0.2
SNP effect on E3 ubiquitin−protein ligase DTX1
SNP effect on Gut microbiota abundance (genus Parasutterella id.2892)

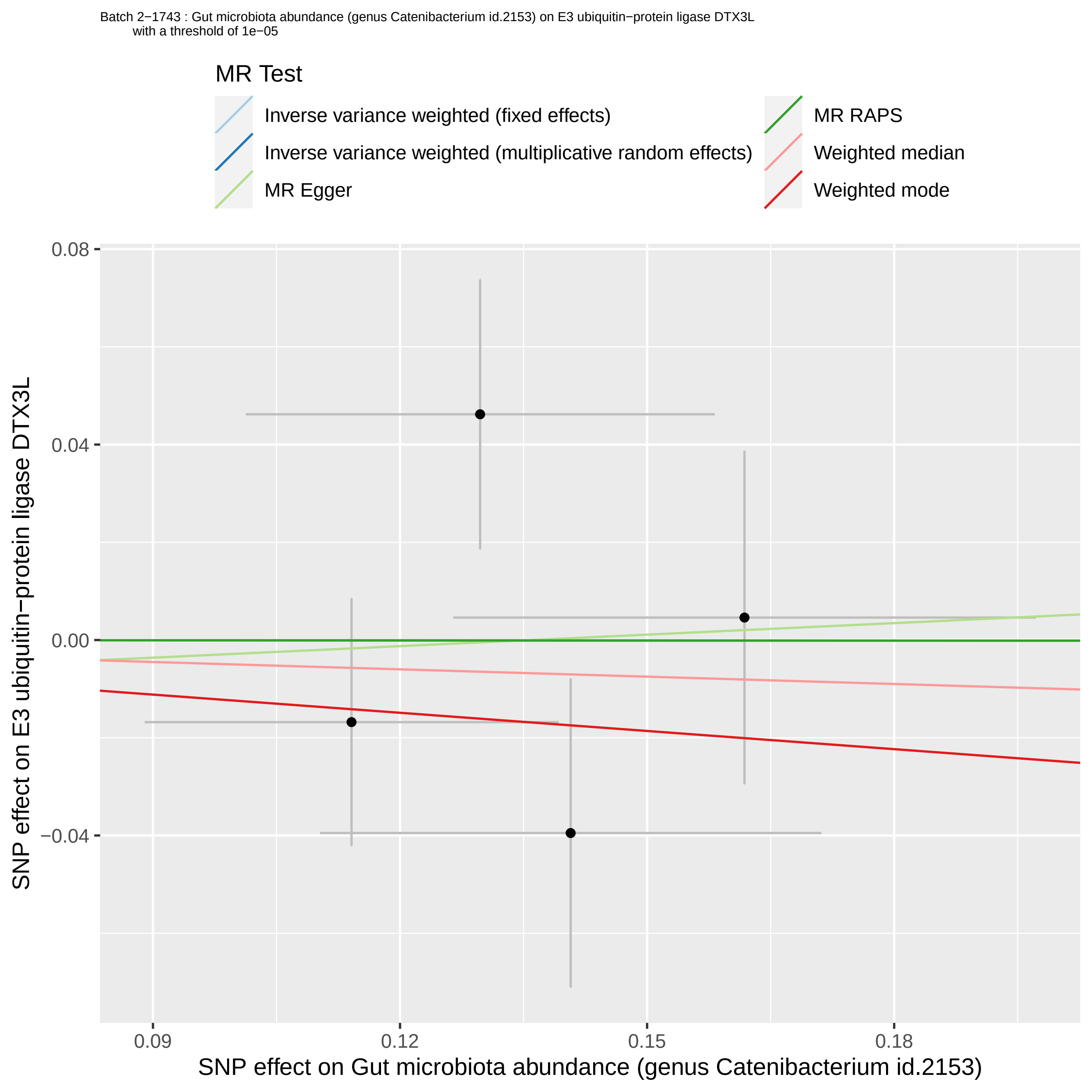
Batch 2−1743 : Gut microbiota abundance (genus Catenibacterium id.2153) on E3 ubiquitin−protein ligase DTX3L
with a threshold of 1e−05
MR Test
Inverse variance weighted (fixed effects)
Inverse variance weighted (multiplicative random effects)
MR Egger
MR RAPS
Weighted median
Weighted mode
0.08
0.04
0.00
−0.04
0.09
0.12
0.15
0.18
SNP effect on E3 ubiquitin−protein ligase DTX3L
SNP effect on Gut microbiota abundance (genus Catenibacterium id.2153)

Batch 2−1744 : Gut microbiota abundance (genus Parasutterella id.2892) on E3 ubiquitin−protein ligase DTX3L
with a threshold of 1e−05

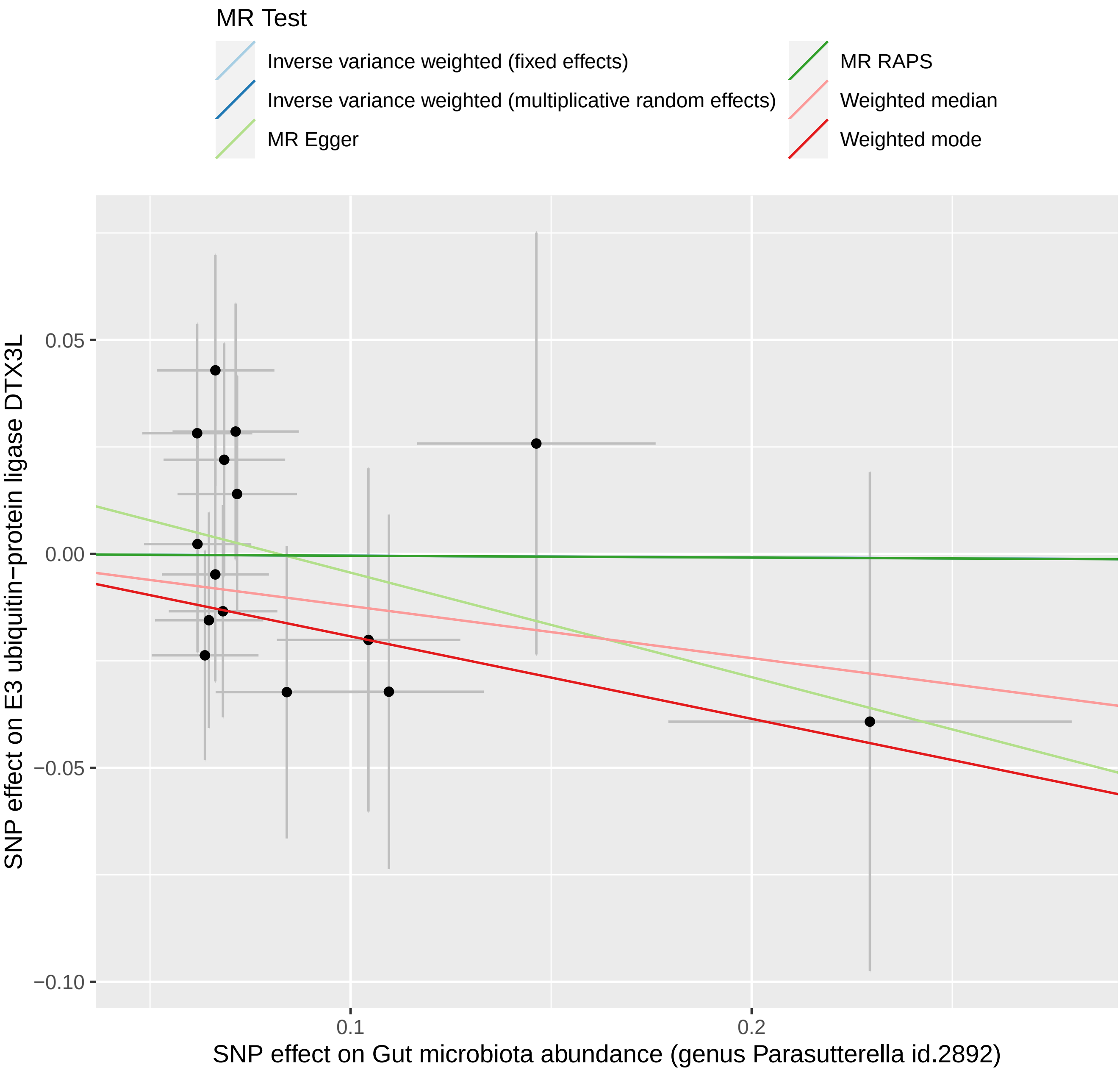

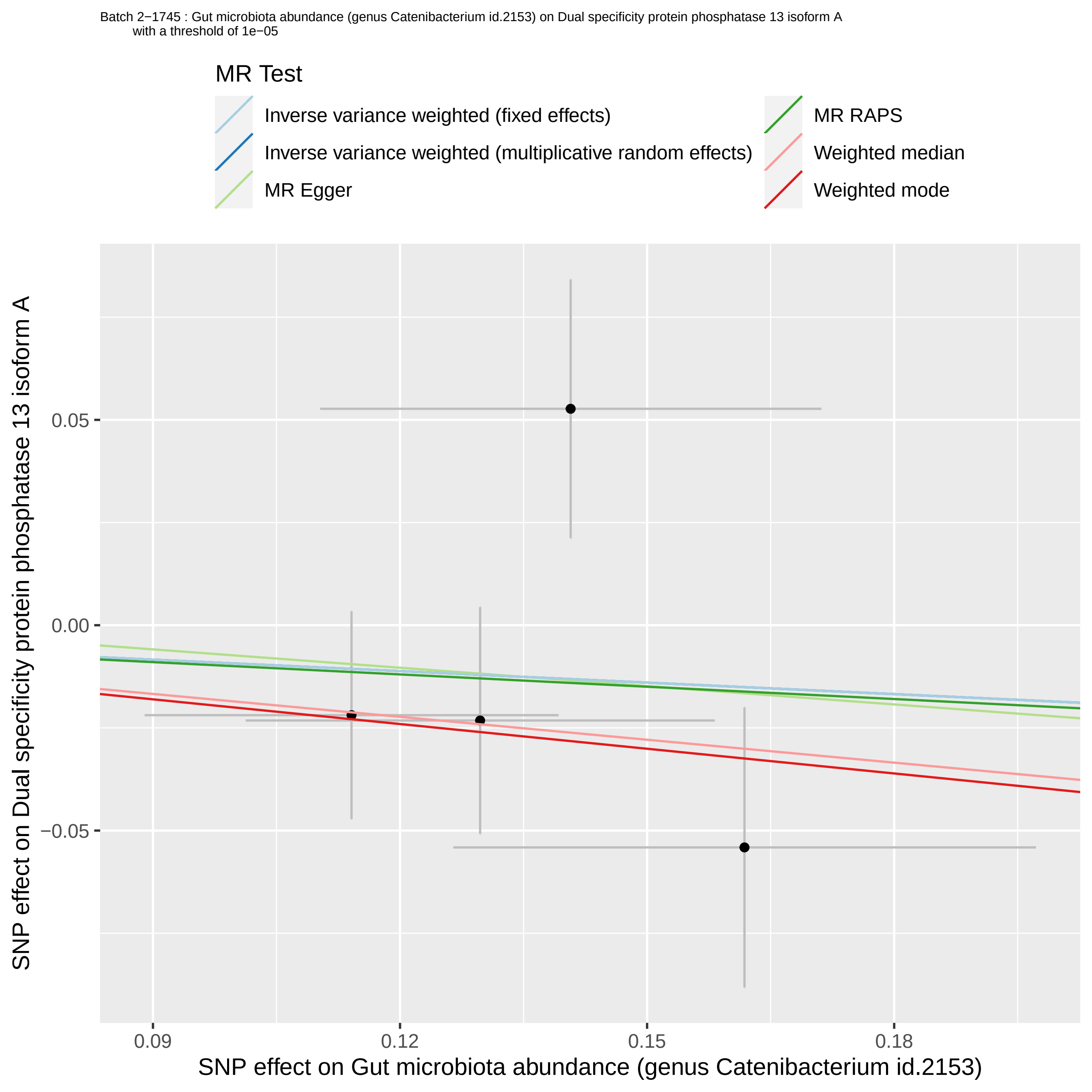
Batch 2−1745 : Gut microbiota abundance (genus Catenibacterium id.2153) on Dual specificity protein phosphatase 13 isoform A
with a threshold of 1e−05
MR Test
Inverse variance weighted (fixed effects)
Inverse variance weighted (multiplicative random effects)
MR Egger
MR RAPS
Weighted median
Weighted mode
0.05
0.00
−0.05
0.09
0.12
0.15
0.18
SNP effect on Dual specificity protein phosphatase 13 isoform A
SNP effect on Gut microbiota abundance (genus Catenibacterium id.2153)

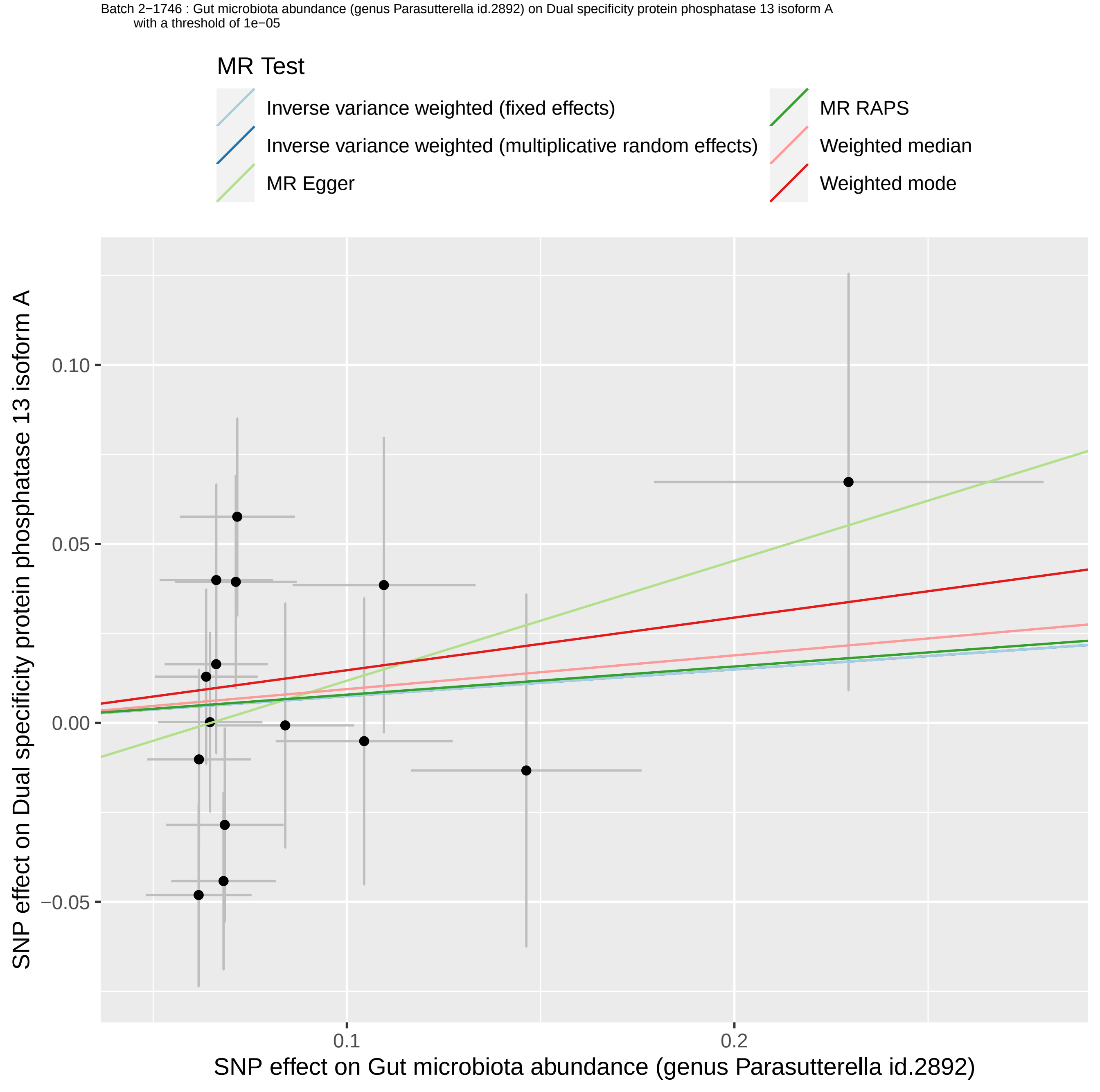

Batch 2−1746 : Gut microbiota abundance (genus Parasutterella id.2892) on Dual specificity protein phosphatase 13 isoform A
with a threshold of 1e−05
MR Test
Inverse variance weighted (fixed effects)
Inverse variance weighted (multiplicative random effects)
MR Egger
MR RAPS
Weighted median
Weighted mode
SNP effect on Dual specificity protein phosphatase 13 isoform A
0.10
0.05
0.00
−0.05
0.1
0.2
SNP effect on Gut microbiota abundance (genus Parasutterella id.2892)

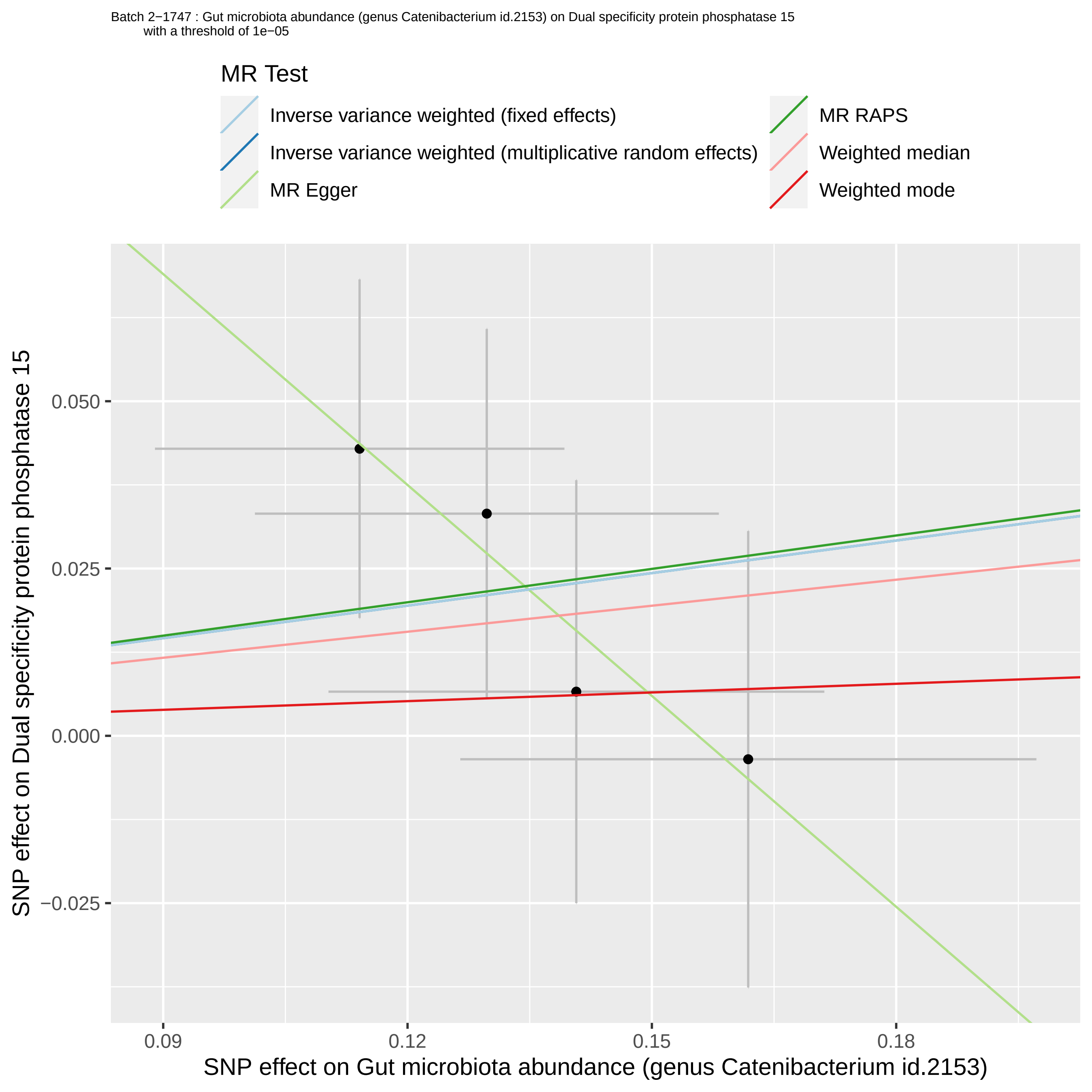
Batch 2−1747 : Gut microbiota abundance (genus Catenibacterium id.2153) on Dual specificity protein phosphatase 15
with a threshold of 1e−05
MR Test
Inverse variance weighted (fixed effects)
Inverse variance weighted (multiplicative random effects)
MR Egger
MR RAPS
Weighted median
Weighted mode
0.050
0.025
0.000
−0.025
0.09
0.12
0.15
0.18
SNP effect on Dual specificity protein phosphatase 15
SNP effect on Gut microbiota abundance (genus Catenibacterium id.2153)

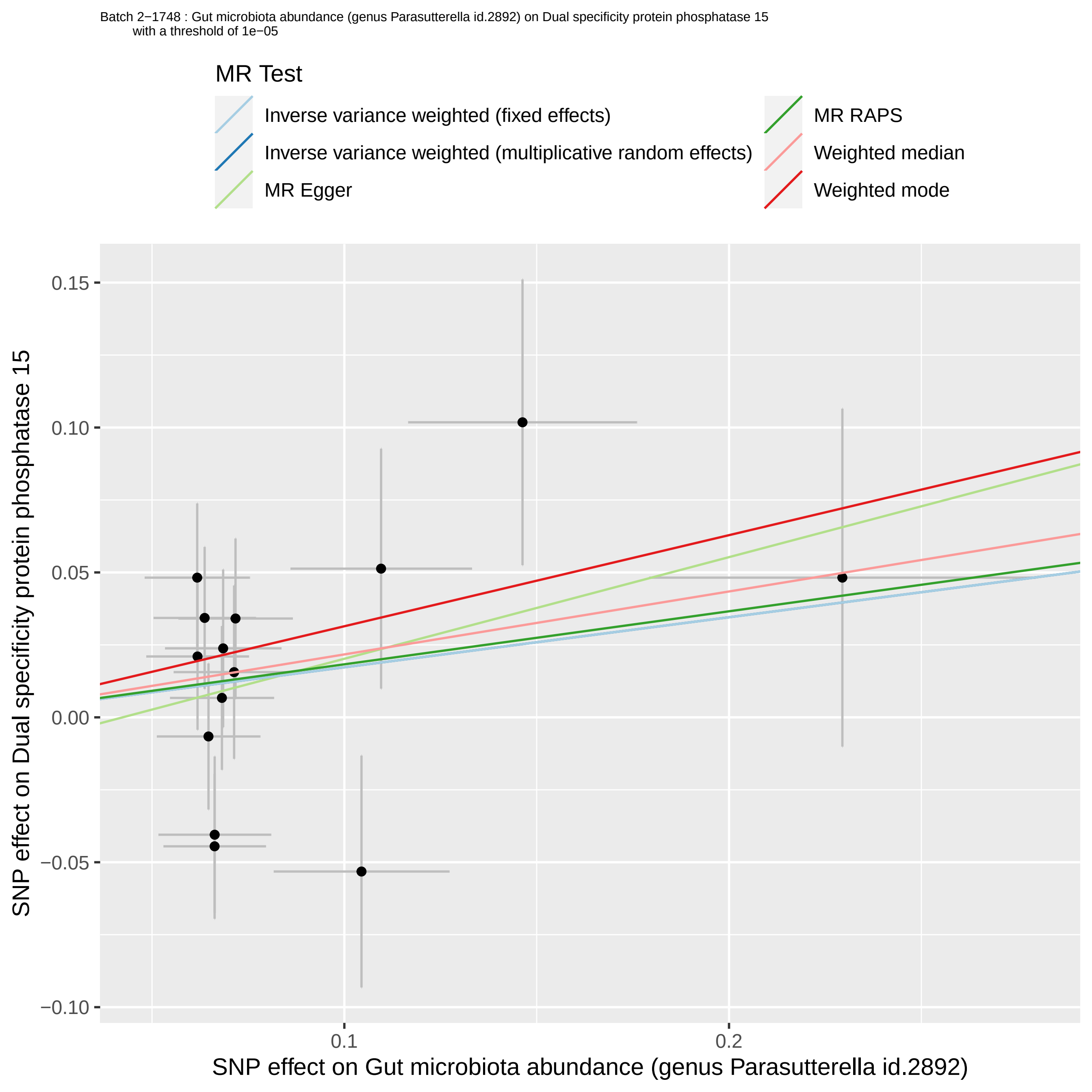
Batch 2−1748 : Gut microbiota abundance (genus Parasutterella id.2892) on Dual specificity protein phosphatase 15
with a threshold of 1e−05
MR Test
Inverse variance weighted (fixed effects)
Inverse variance weighted (multiplicative random effects)
MR Egger
MR RAPS
Weighted median
Weighted mode
SNP effect on Dual specificity protein phosphatase 15
0.15
0.10
0.05
0.00
−0.05
−0.10
0.1
0.2
SNP effect on Gut microbiota abundance (genus Parasutterella id.2892)

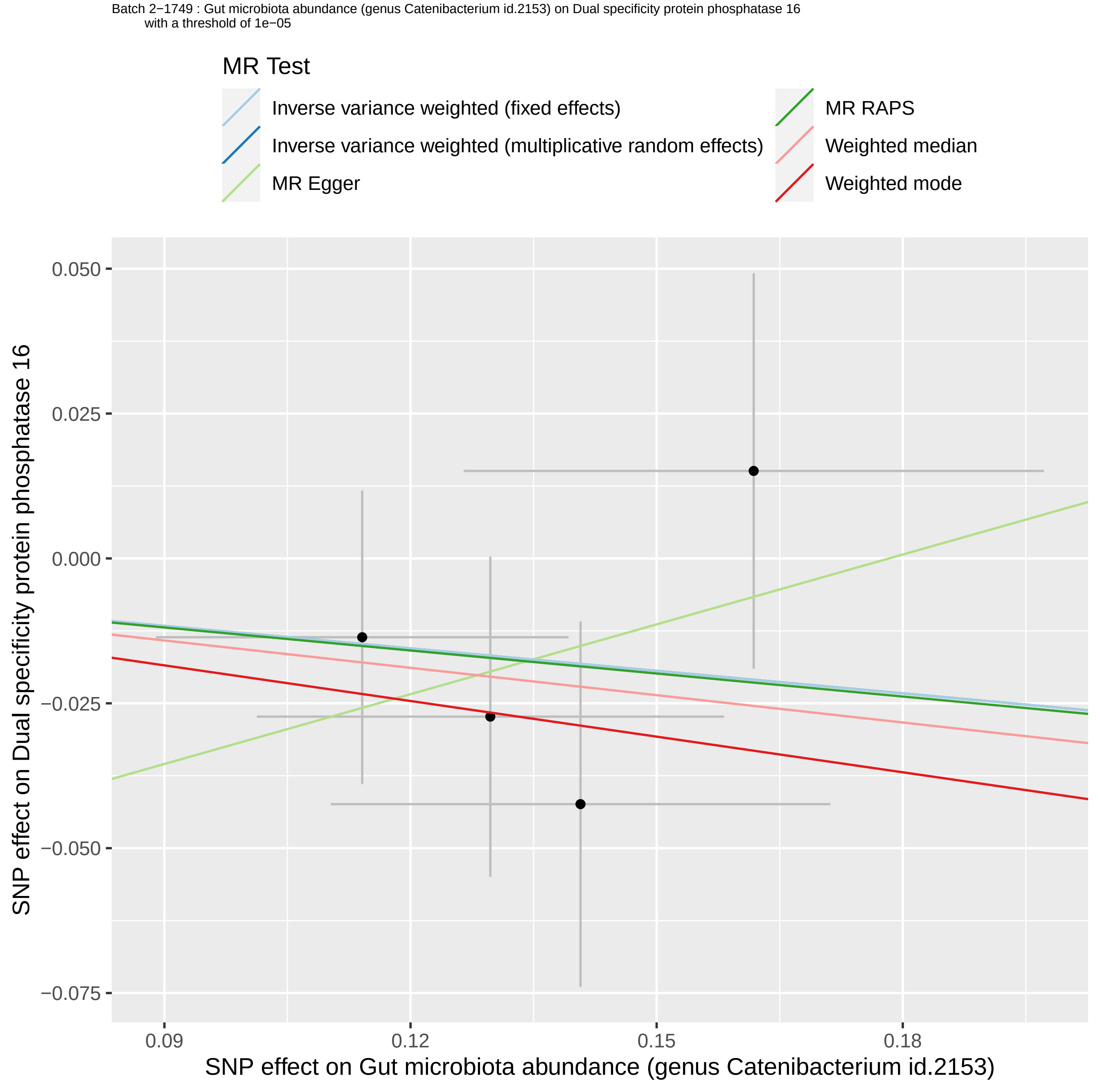
Batch 2−1749 : Gut microbiota abundance (genus Catenibacterium id.2153) on Dual specificity protein phosphatase 16
with a threshold of 1e−05
MR Test
Inverse variance weighted (fixed effects)
Inverse variance weighted (multiplicative random effects)
MR Egger
MR RAPS
Weighted median
Weighted mode
0.050
0.025
0.000
−0.025
−0.050
−0.075
0.09
0.12
0.15
0.18
SNP effect on Dual specificity protein phosphatase 16
SNP effect on Gut microbiota abundance (genus Catenibacterium id.2153)

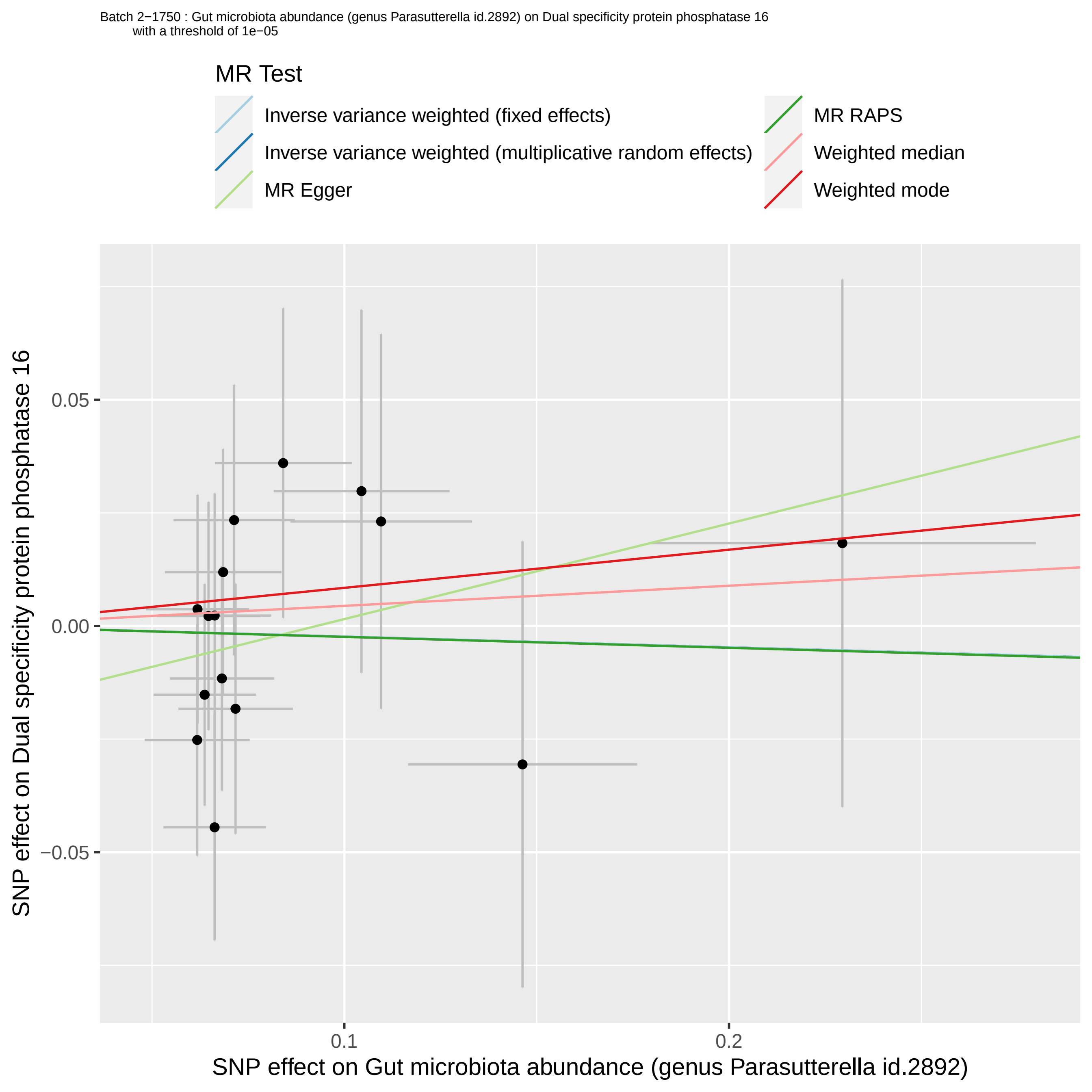
Batch 2−1750 : Gut microbiota abundance (genus Parasutterella id.2892) on Dual specificity protein phosphatase 16
with a threshold of 1e−05
MR Test
Inverse variance weighted (fixed effects)
Inverse variance weighted (multiplicative random effects)
MR Egger
MR RAPS
Weighted median
Weighted mode
0.05
0.00
−0.05
0.1
0.2
SNP effect on Dual specificity protein phosphatase 16
SNP effect on Gut microbiota abundance (genus Parasutterella id.2892)

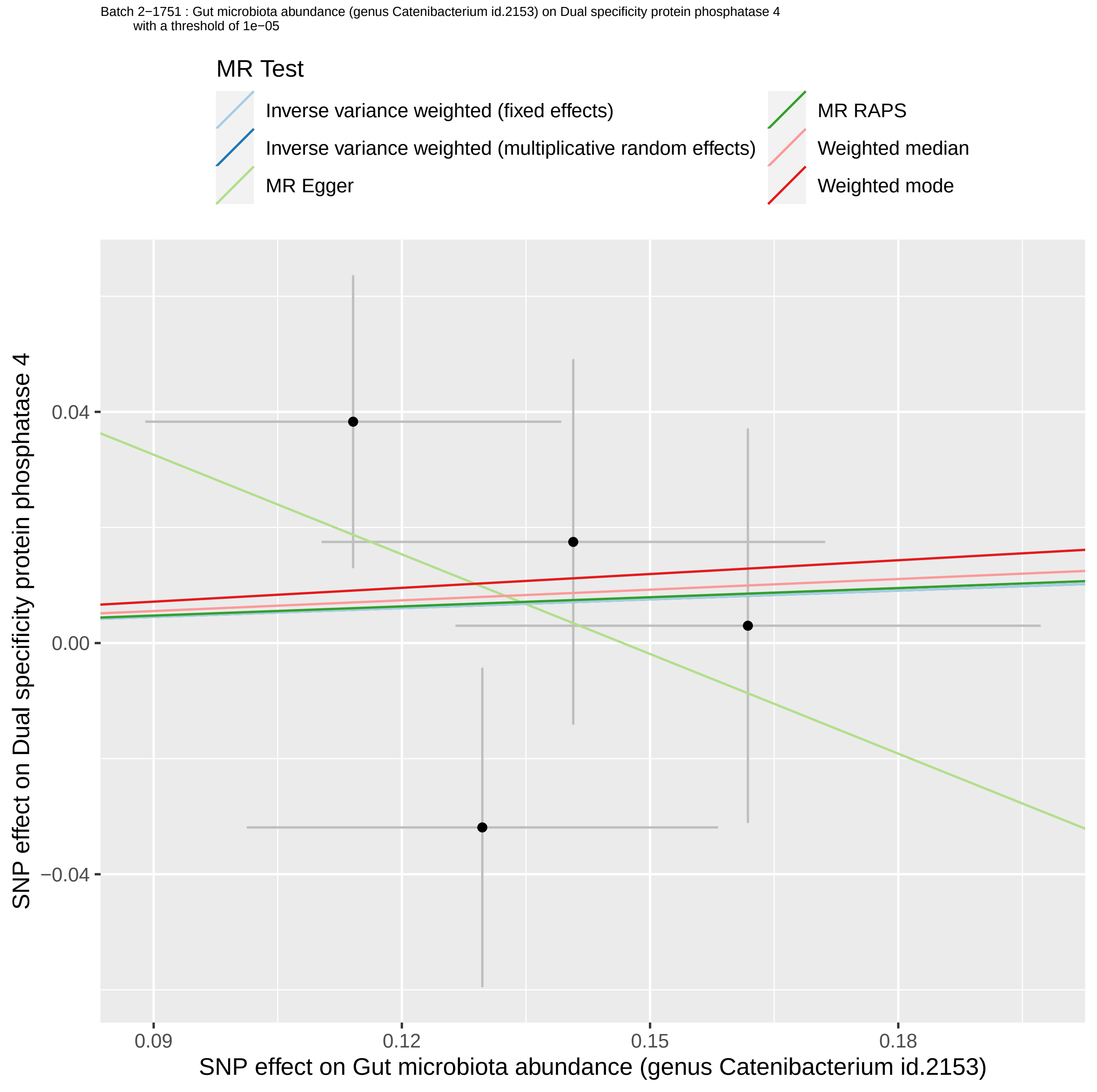

Batch 2−1751 : Gut microbiota abundance (genus Catenibacterium id.2153) on Dual specificity protein phosphatase 4
with a threshold of 1e−05
MR Test
Inverse variance weighted (fixed effects)
Inverse variance weighted (multiplicative random effects)
MR Egger
MR RAPS
Weighted median
Weighted mode
0.04
0.00
−0.04
0.09
0.12
0.15
0.18
SNP effect on Dual specificity protein phosphatase 4
SNP effect on Gut microbiota abundance (genus Catenibacterium id.2153)

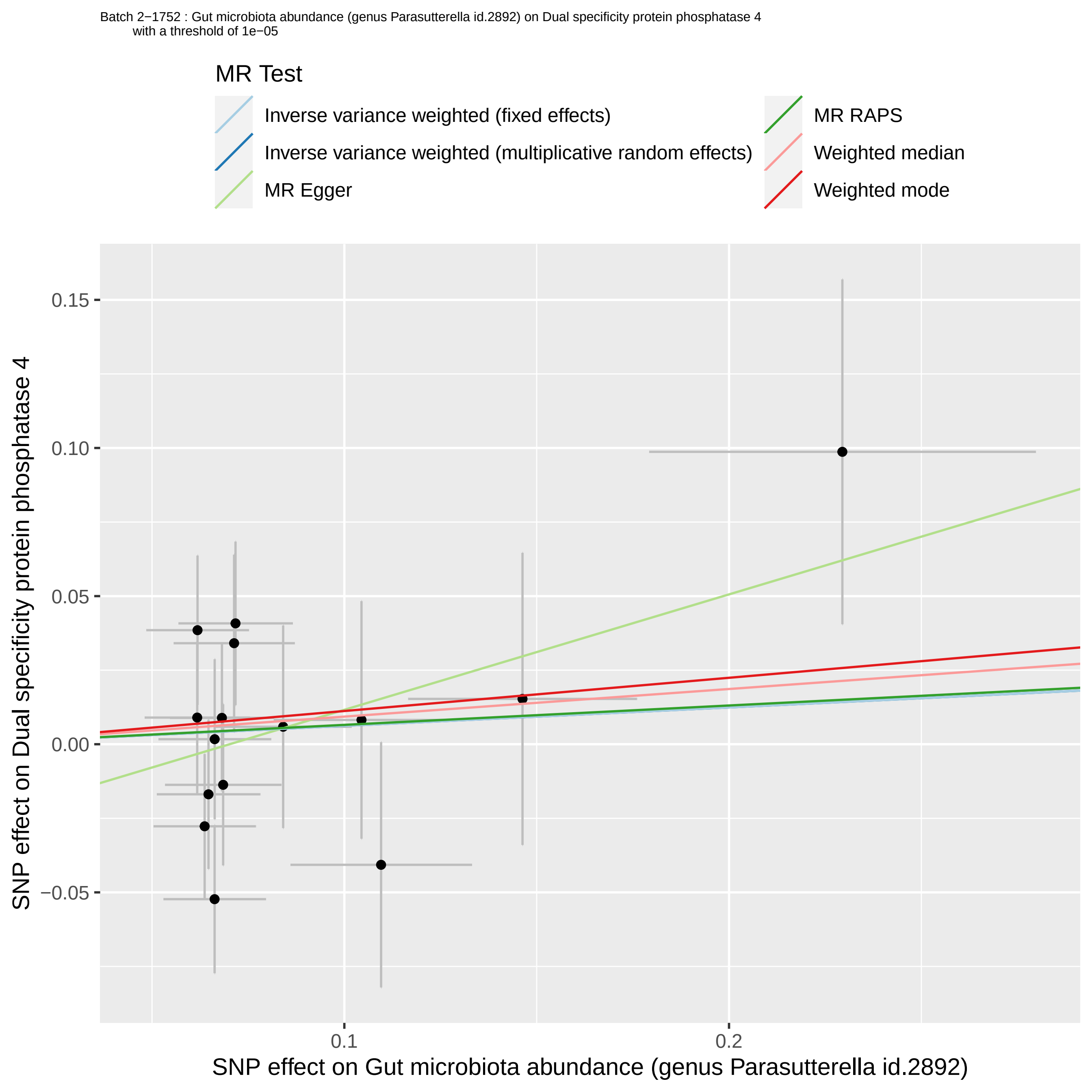
Batch 2−1752 : Gut microbiota abundance (genus Parasutterella id.2892) on Dual specificity protein phosphatase 4
with a threshold of 1e−05
MR Test
Inverse variance weighted (fixed effects)
Inverse variance weighted (multiplicative random effects)
MR Egger
MR RAPS
Weighted median
Weighted mode
0.15
0.10
0.05
0.00
−0.05
0.1
0.2
SNP effect on Dual specificity protein phosphatase 4
SNP effect on Gut microbiota abundance (genus Parasutterella id.2892)

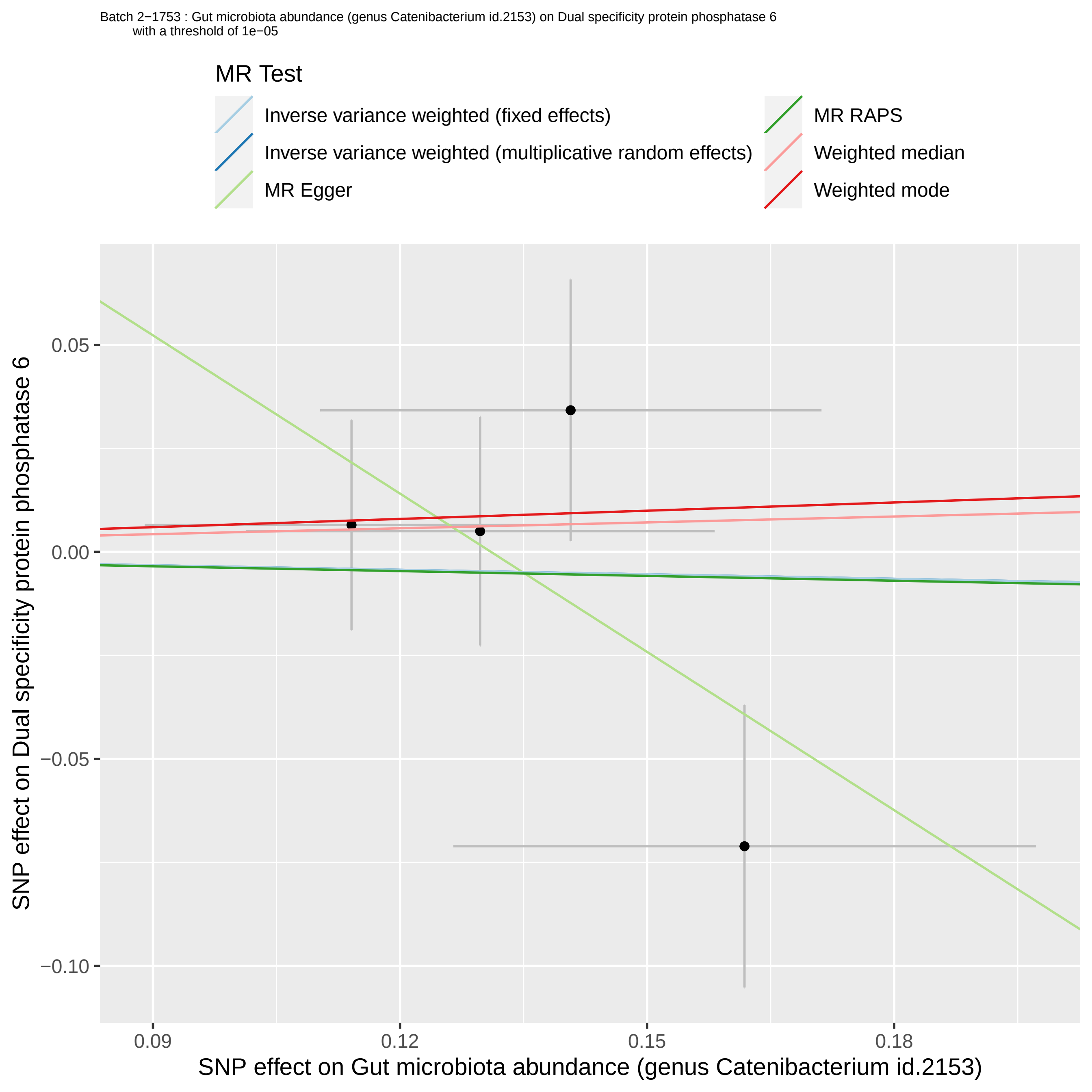

Batch 2−1753 : Gut microbiota abundance (genus Catenibacterium id.2153) on Dual specificity protein phosphatase 6
with a threshold of 1e−05
MR Test
Inverse variance weighted (fixed effects)
Inverse variance weighted (multiplicative random effects)
MR Egger
MR RAPS
Weighted median
Weighted mode
0.05
0.00
−0.05
−0.10
0.09
0.12
0.15
0.18
SNP effect on Dual specificity protein phosphatase 6
SNP effect on Gut microbiota abundance (genus Catenibacterium id.2153)

Batch 2−1754 : Gut microbiota abundance (genus Parasutterella id.2892) on Dual specificity protein phosphatase 6
with a threshold of 1e−05

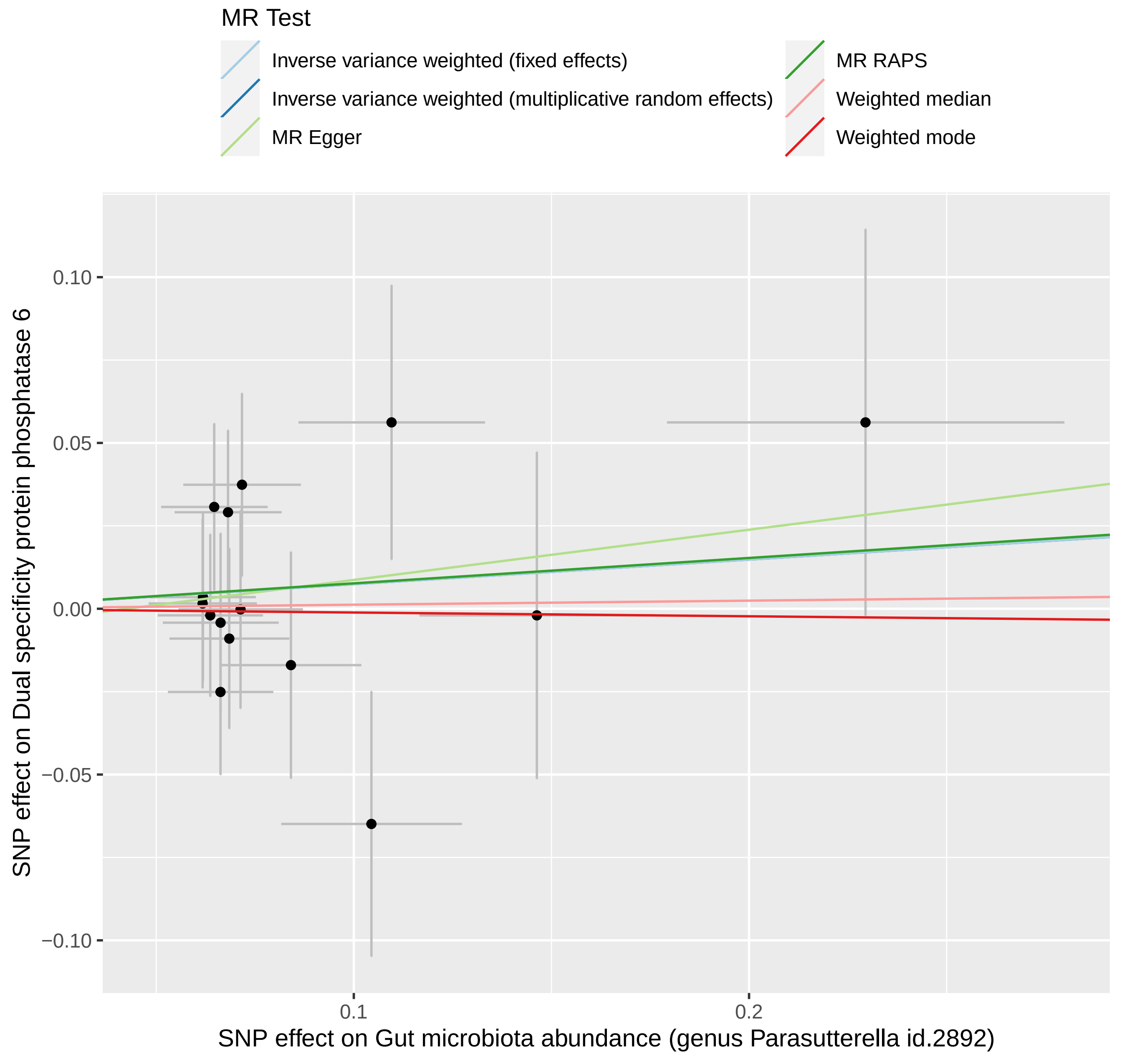

Batch 2−1755 : Gut microbiota abundance (genus Catenibacterium id.2153) on Segment polarity protein dishevelled homolog DVL−2 with a threshold of 1e−05

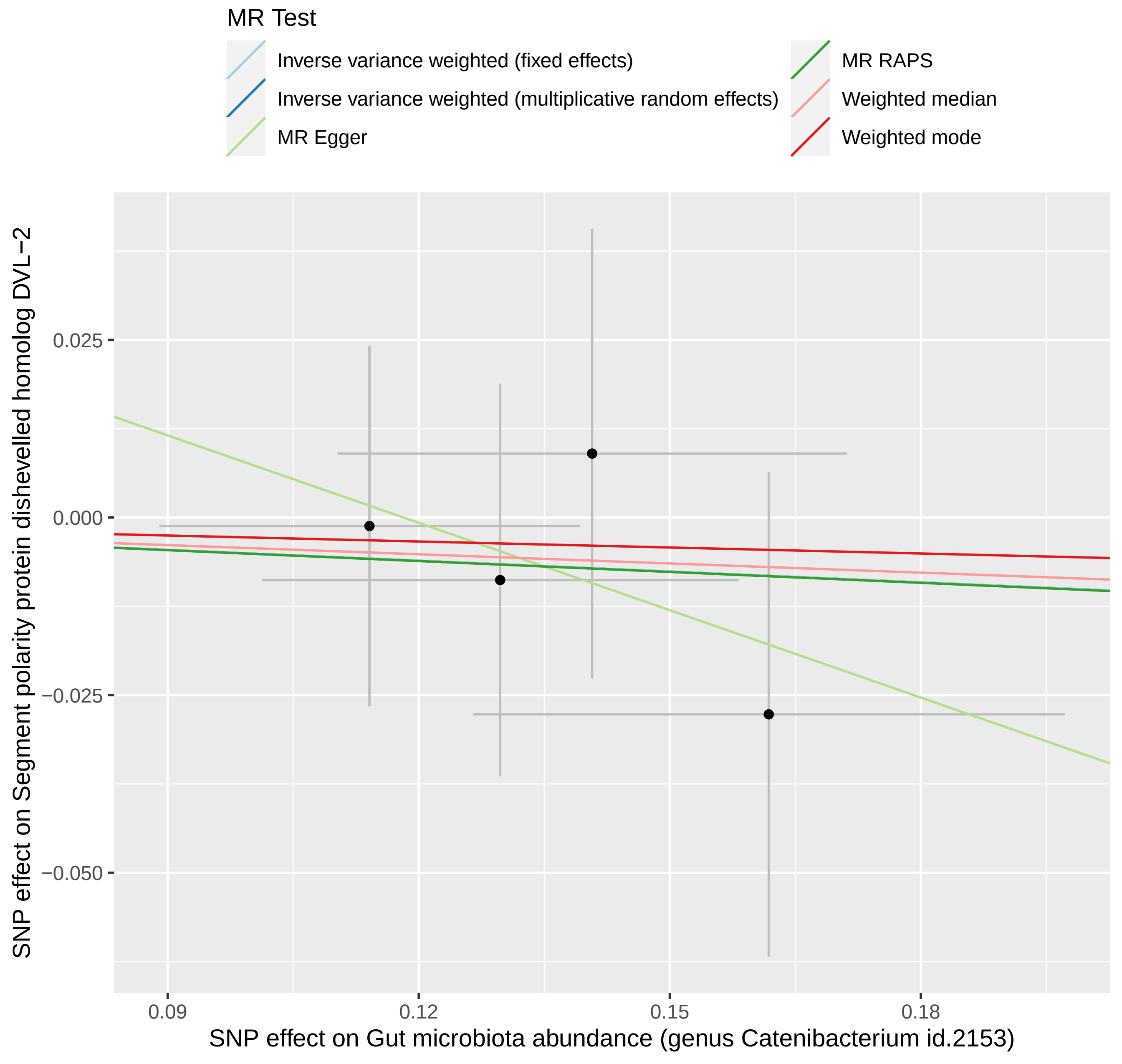

Batch 2−1756 : Gut microbiota abundance (genus Parasutterella id.2892) on Segment polarity protein dishevelled homolog DVL−2
with a threshold of 1e−05

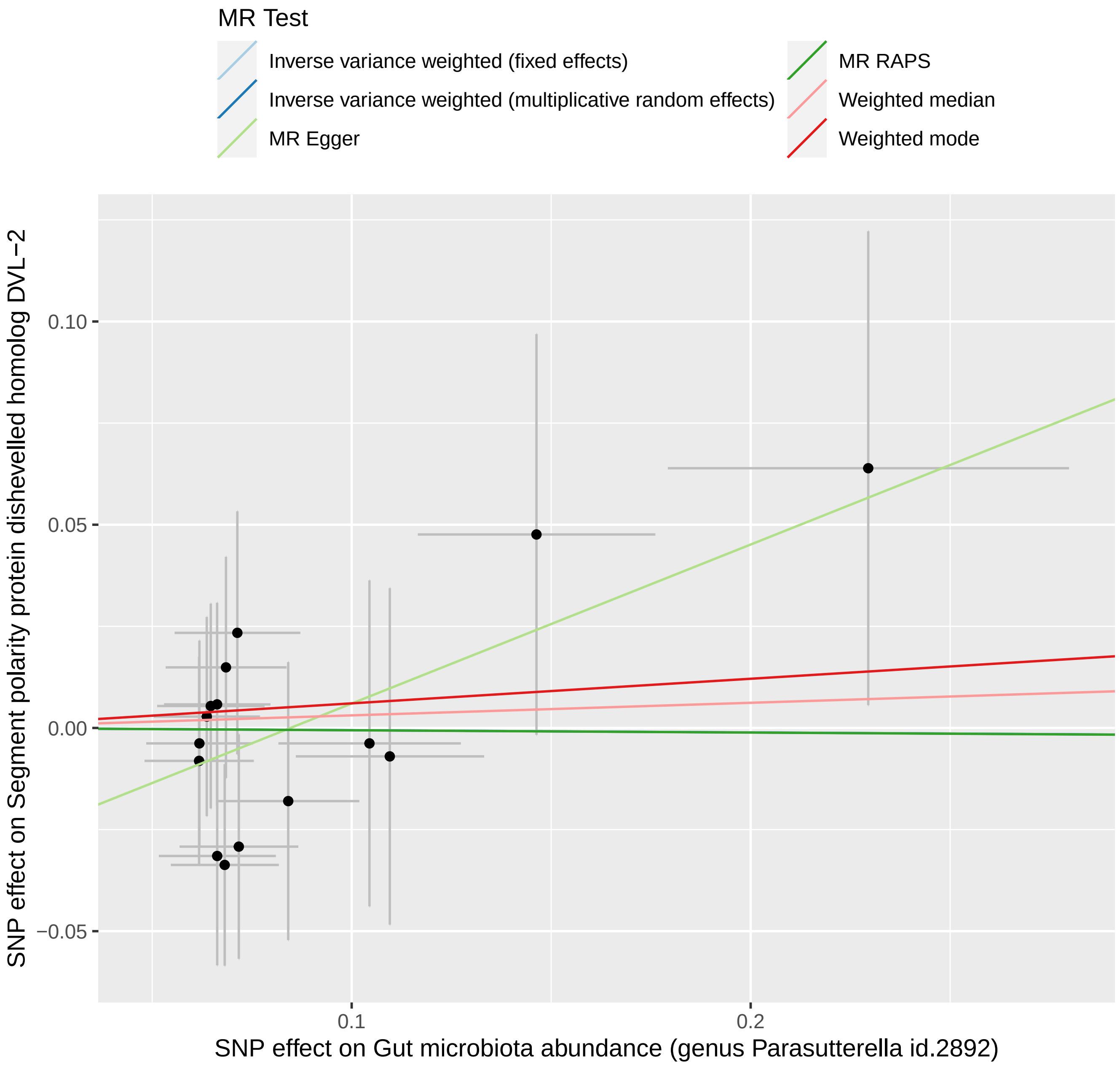

Batch 2−1757 : Gut microbiota abundance (genus Catenibacterium id.2153) on Dynactin−associated protein
with a threshold of 1e−05

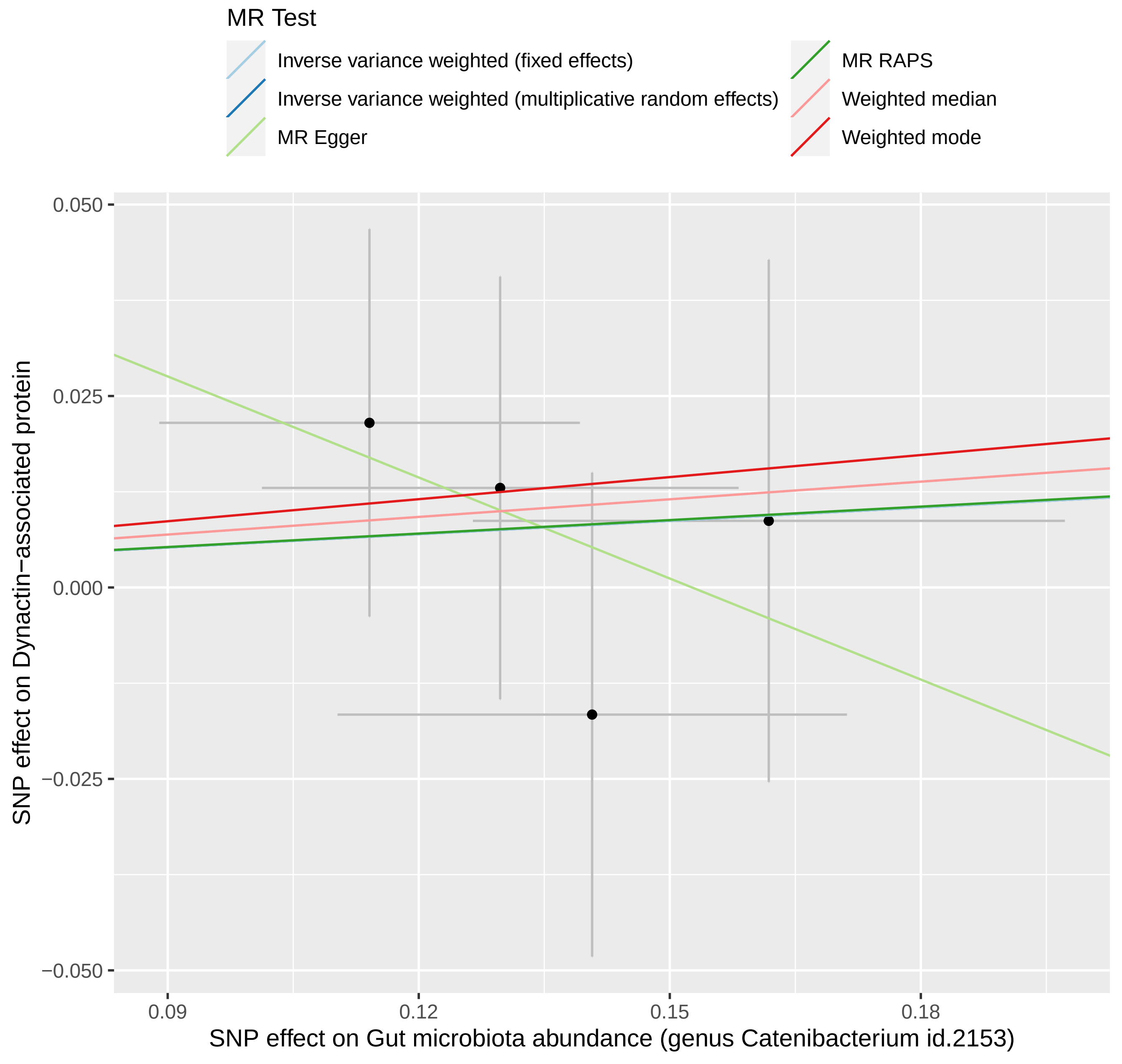

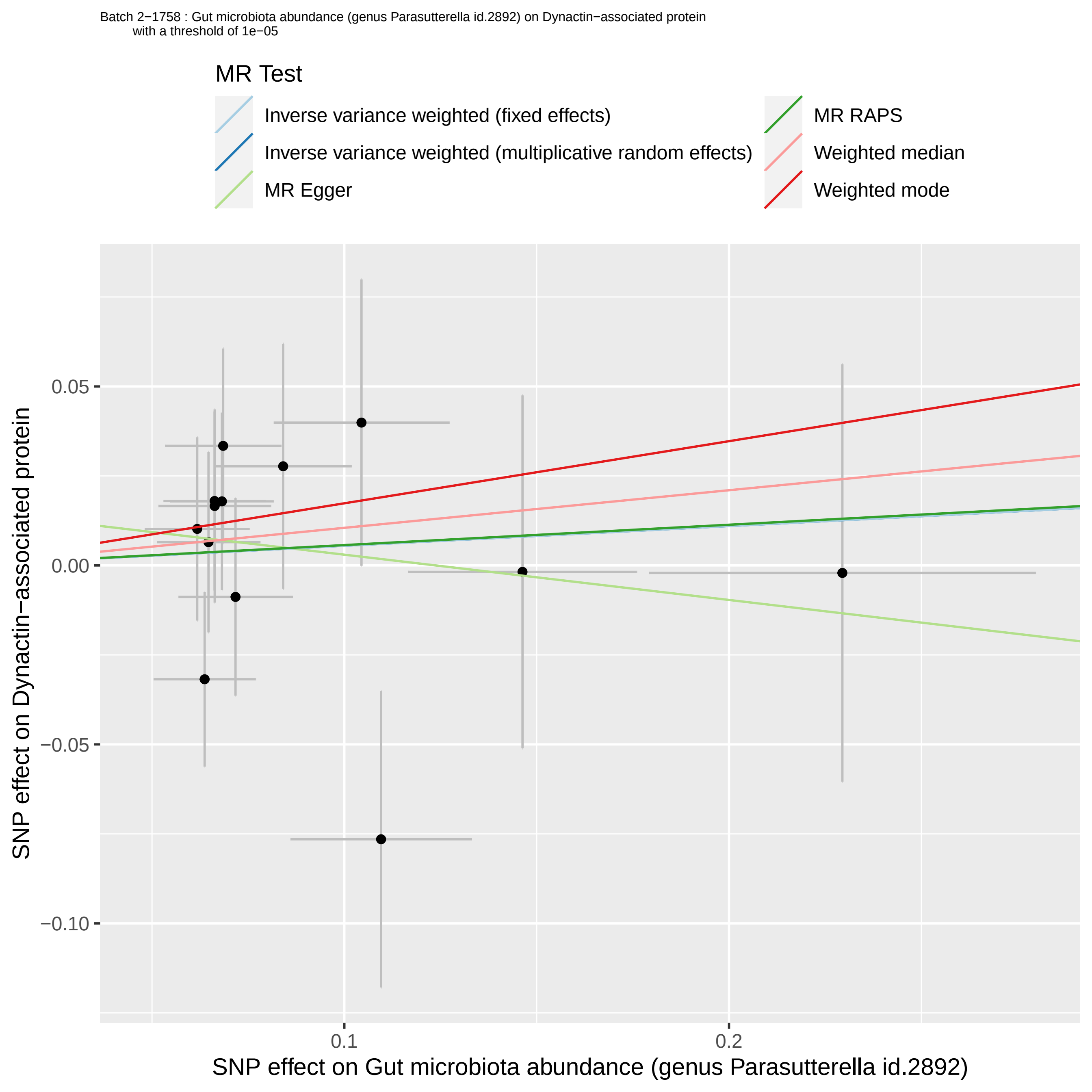

Batch 2−1758 : Gut microbiota abundance (genus Parasutterella id.2892) on Dynactin−associated protein
with a threshold of 1e−05
MR Test
Inverse variance weighted (fixed effects)
Inverse variance weighted (multiplicative random effects)
MR Egger
MR RAPS
Weighted median
Weighted mode
0.05
0.00
−0.05
−0.10
0.1
0.2
SNP effect on Dynactin−associated protein
SNP effect on Gut microbiota abundance (genus Parasutterella id.2892)

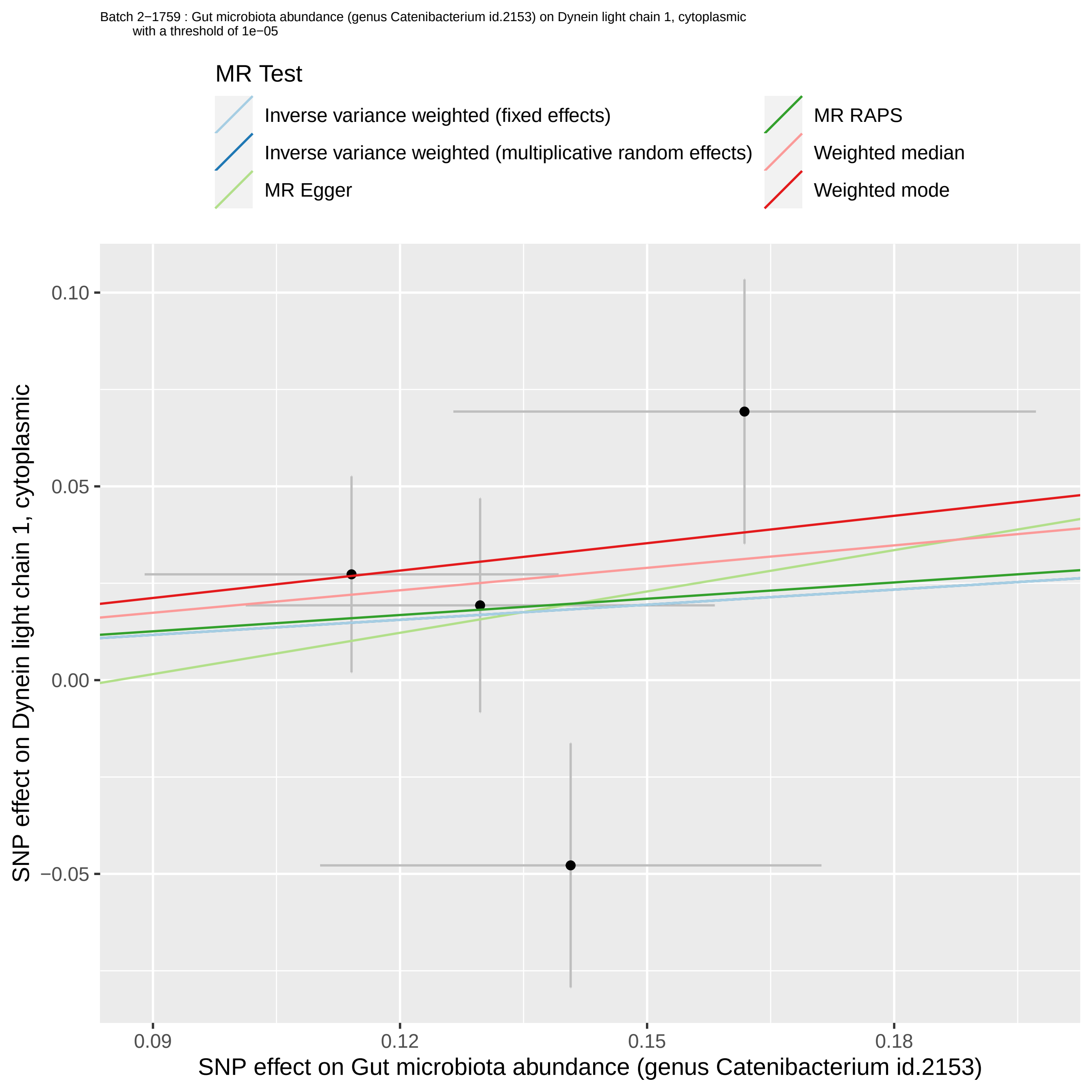
Batch 2−1759 : Gut microbiota abundance (genus Catenibacterium id.2153) on Dynein light chain 1, cytoplasmic
with a threshold of 1e−05
MR Test
Inverse variance weighted (fixed effects)
Inverse variance weighted (multiplicative random effects)
MR Egger
MR RAPS
Weighted median
Weighted mode
0.10
0.05
0.00
−0.05
0.09
0.12
0.15
0.18
SNP effect on Dynein light chain 1, cytoplasmic
SNP effect on Gut microbiota abundance (genus Catenibacterium id.2153)

Batch 2−1760 : Gut microbiota abundance (genus Parasutterella id.2892) on Dynein light chain 1, cytoplasmic
with a threshold of 1e−05

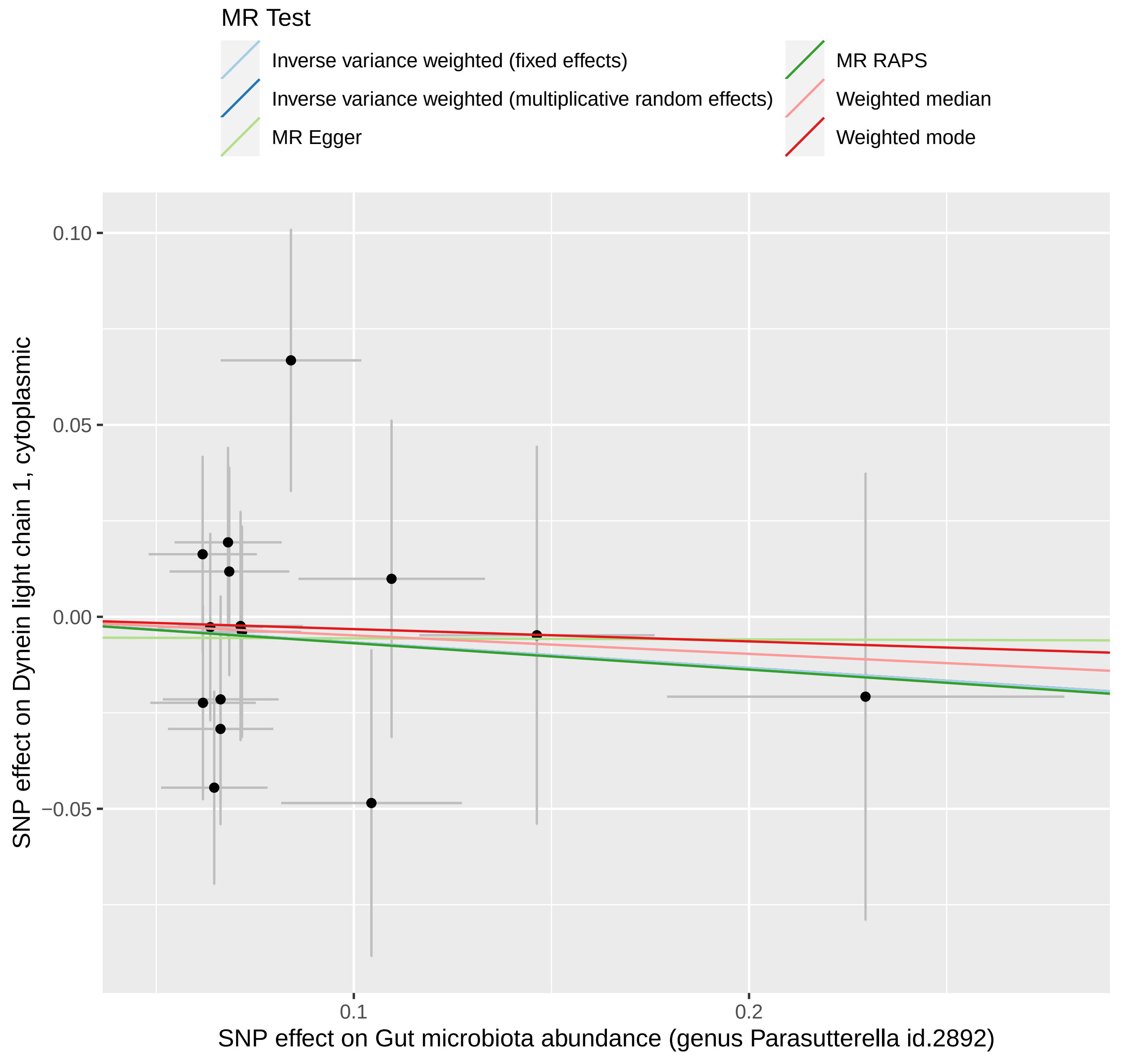

Batch 2−1761 : Gut microbiota abundance (genus Catenibacterium id.2153) on Dynein light chain Tctex−type 3
with a threshold of 1e−05

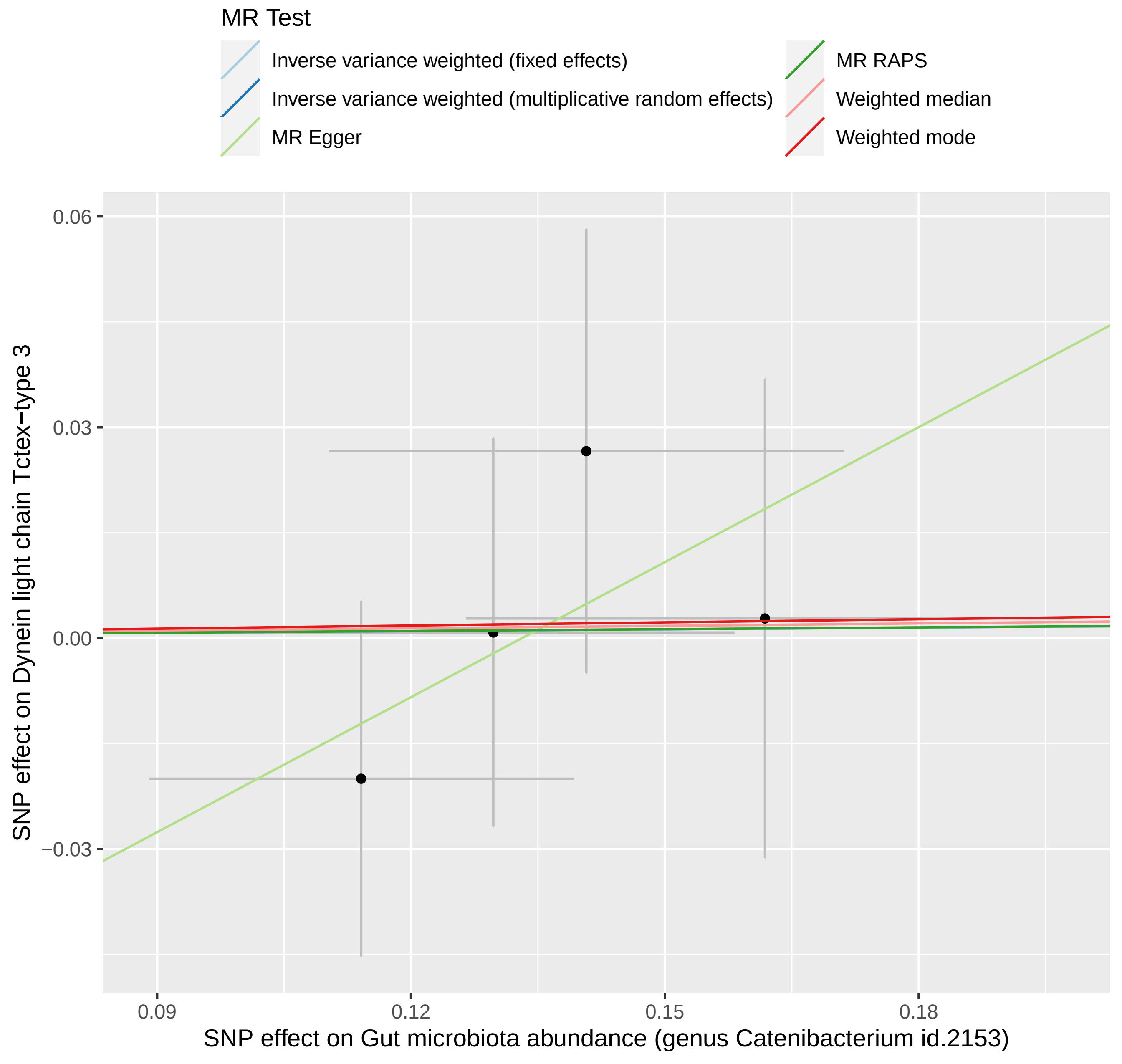

Batch 2−1762 : Gut microbiota abundance (genus Parasutterella id.2892) on Dynein light chain Tctex−type 3
with a threshold of 1e−05

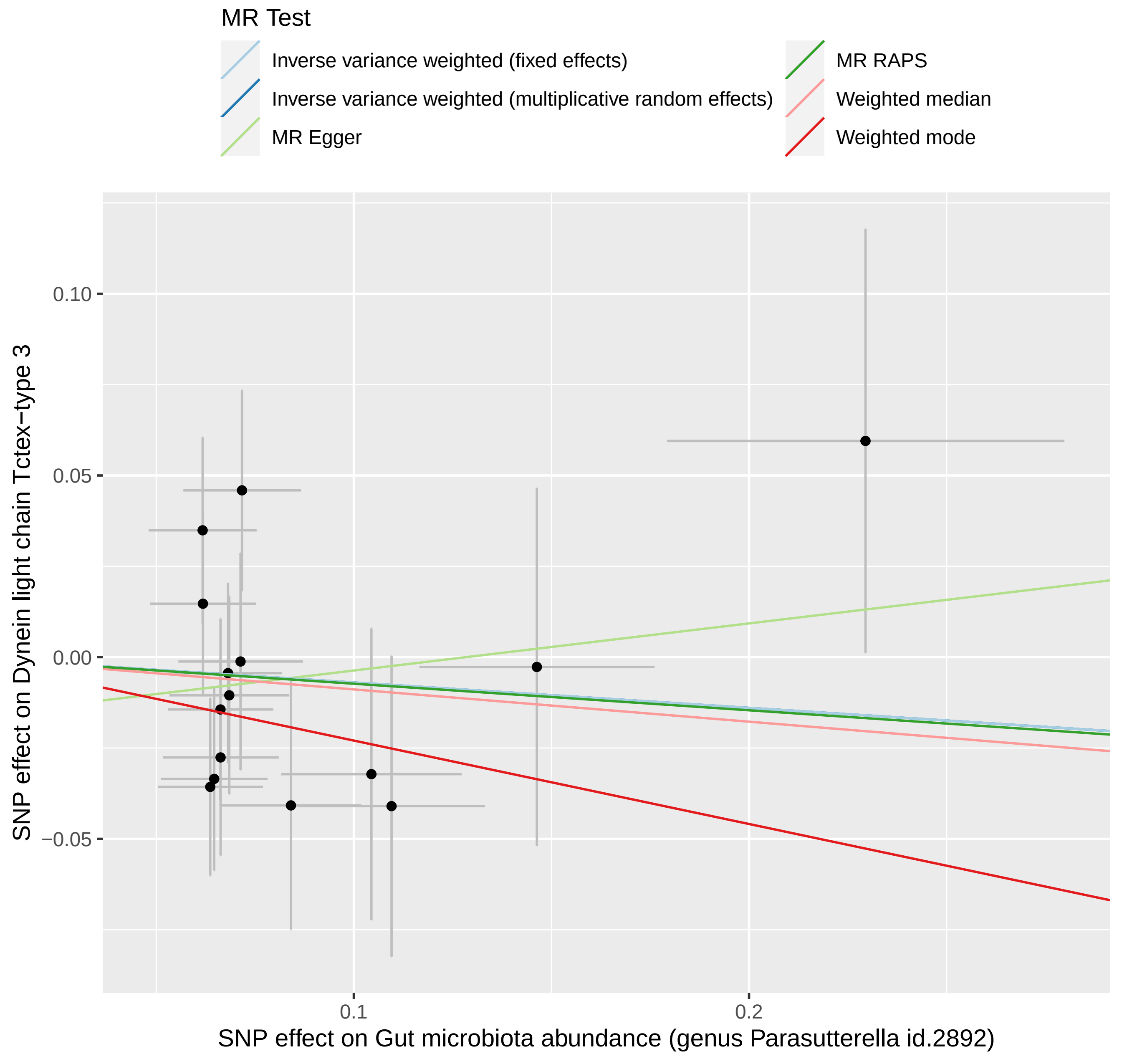

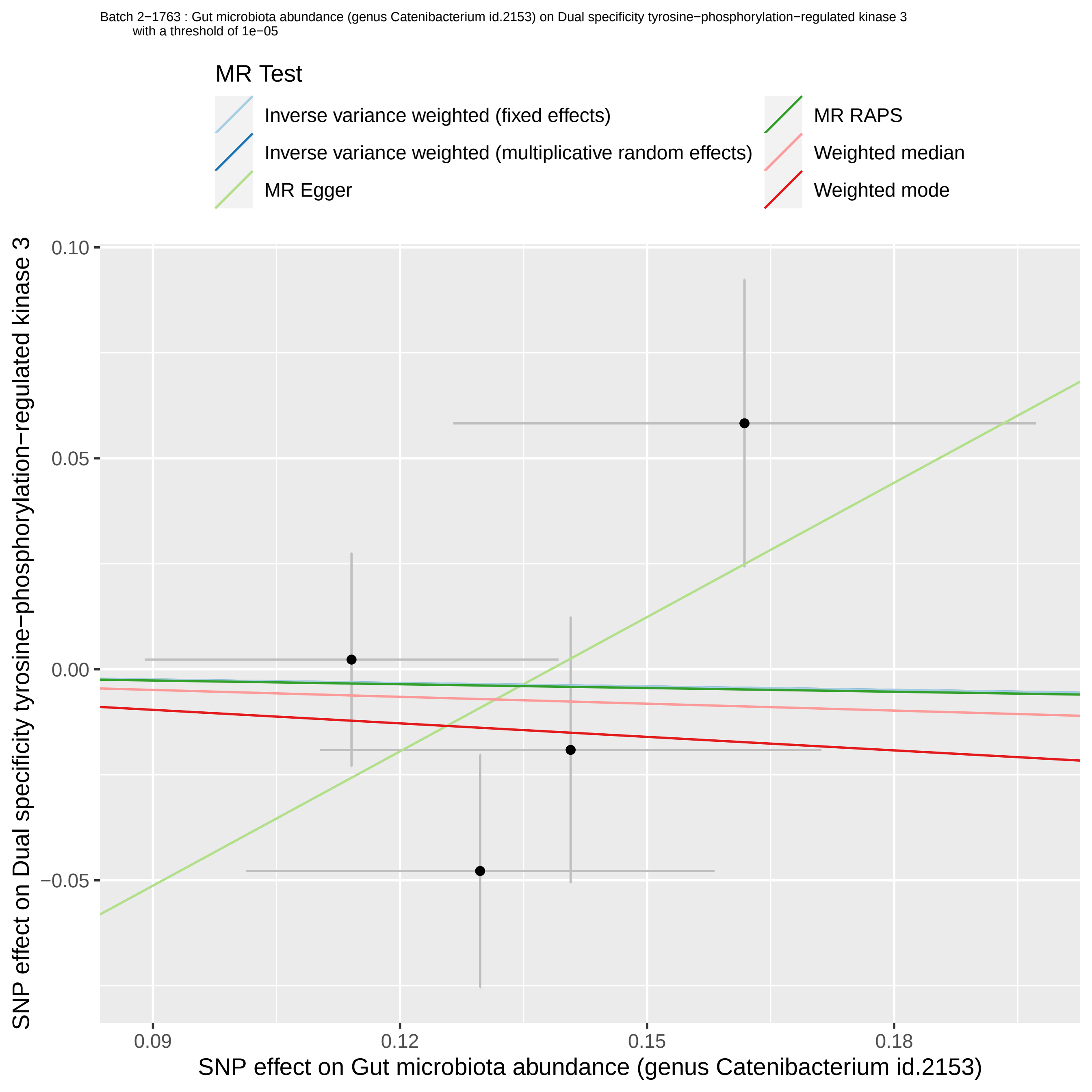
Batch 2−1763 : Gut microbiota abundance (genus Catenibacterium id.2153) on Dual specificity tyrosine−phosphorylation−regulated kinase 3
with a threshold of 1e−05
MR Test
Inverse variance weighted (fixed effects)
Inverse variance weighted (multiplicative random effects)
MR Egger
MR RAPS
Weighted median
Weighted mode
0.10
0.05
0.00
−0.05
0.09
0.12
0.15
0.18
SNP effect on Dual specificity tyrosine−phosphorylation−regulated kinase 3
SNP effect on Gut microbiota abundance (genus Catenibacterium id.2153)

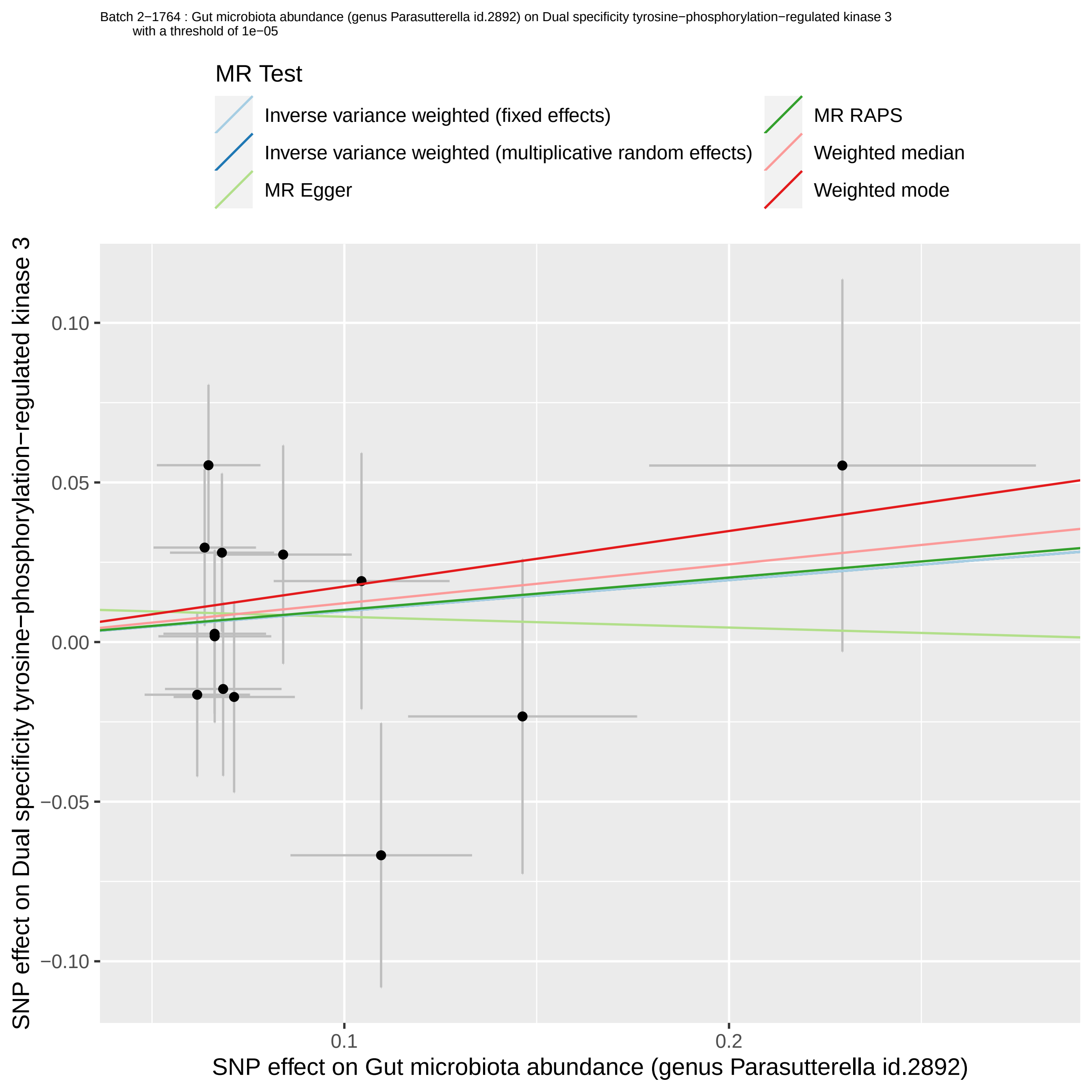
Batch 2−1764 : Gut microbiota abundance (genus Parasutterella id.2892) on Dual specificity tyrosine−phosphorylation−regulated kinase 3
with a threshold of 1e−05
MR Test
Inverse variance weighted (fixed effects)
Inverse variance weighted (multiplicative random effects)
MR Egger
MR RAPS
Weighted median
Weighted mode
0.10
0.05
0.00
−0.05
−0.10
0.1
0.2
SNP effect on Dual specificity tyrosine−phosphorylation−regulated kinase 3
SNP effect on Gut microbiota abundance (genus Parasutterella id.2892)

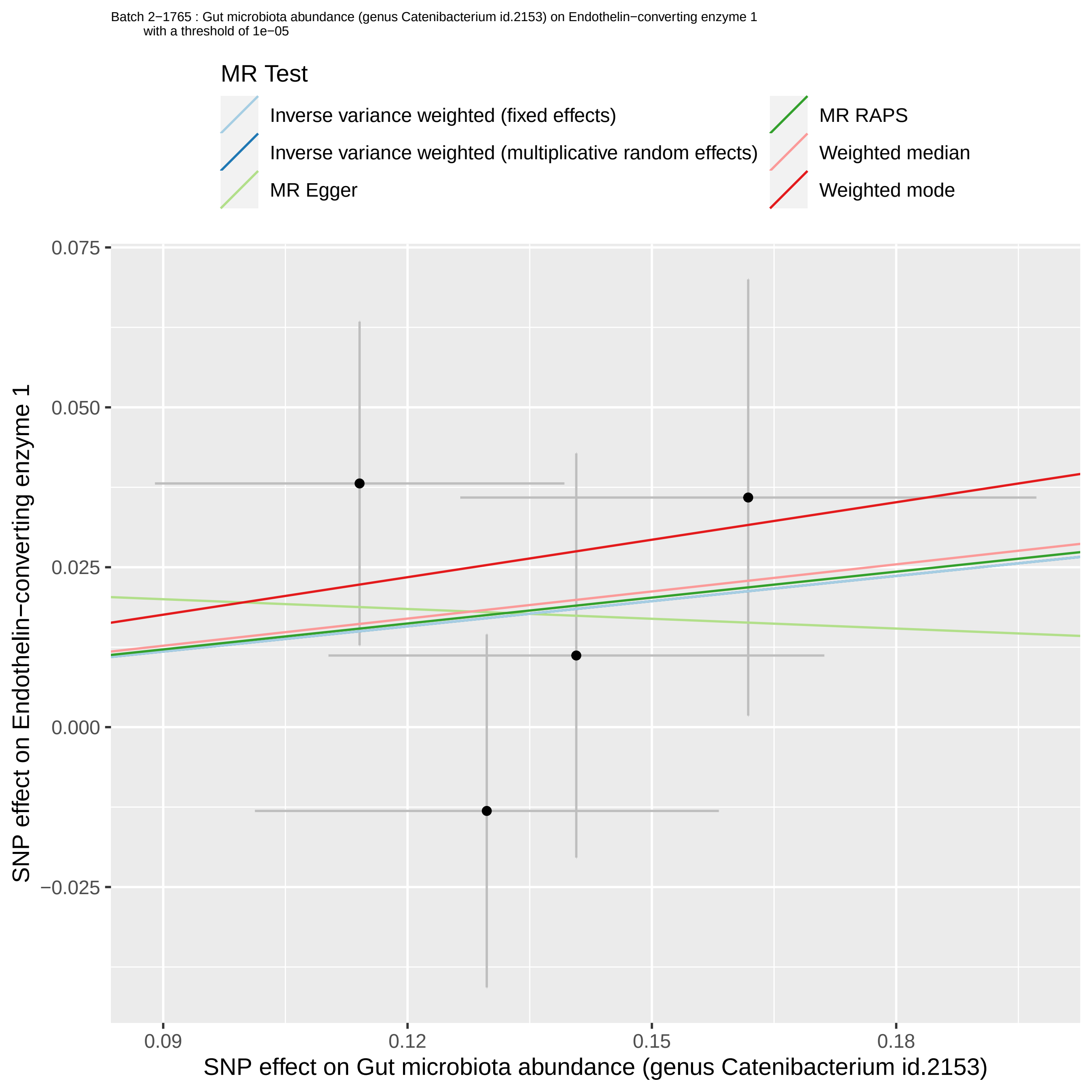

Batch 2−1765 : Gut microbiota abundance (genus Catenibacterium id.2153) on Endothelin−converting enzyme 1
with a threshold of 1e−05
MR Test
Inverse variance weighted (fixed effects)
Inverse variance weighted (multiplicative random effects)
MR Egger
MR RAPS
Weighted median
Weighted mode
0.075
0.050
0.025
0.000
−0.025
0.09
0.12
0.15
0.18
SNP effect on Endothelin−converting enzyme 1
SNP effect on Gut microbiota abundance (genus Catenibacterium id.2153)

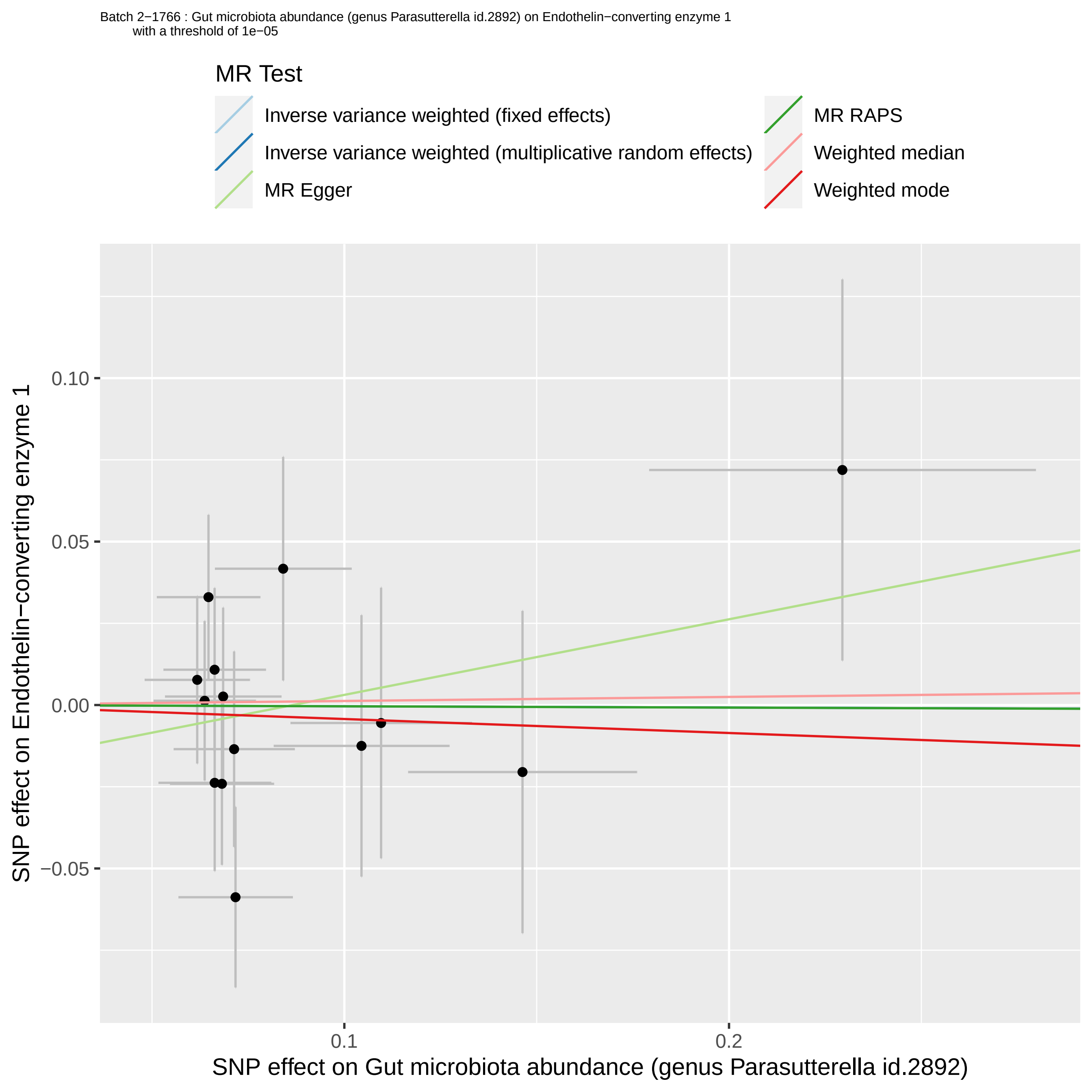
Batch 2−1766 : Gut microbiota abundance (genus Parasutterella id.2892) on Endothelin−converting enzyme 1
with a threshold of 1e−05
MR Test
Inverse variance weighted (fixed effects)
Inverse variance weighted (multiplicative random effects)
MR Egger
MR RAPS
Weighted median
Weighted mode
0.10
0.05
0.00
−0.05
0.1
0.2
SNP effect on Endothelin−converting enzyme 1
SNP effect on Gut microbiota abundance (genus Parasutterella id.2892)

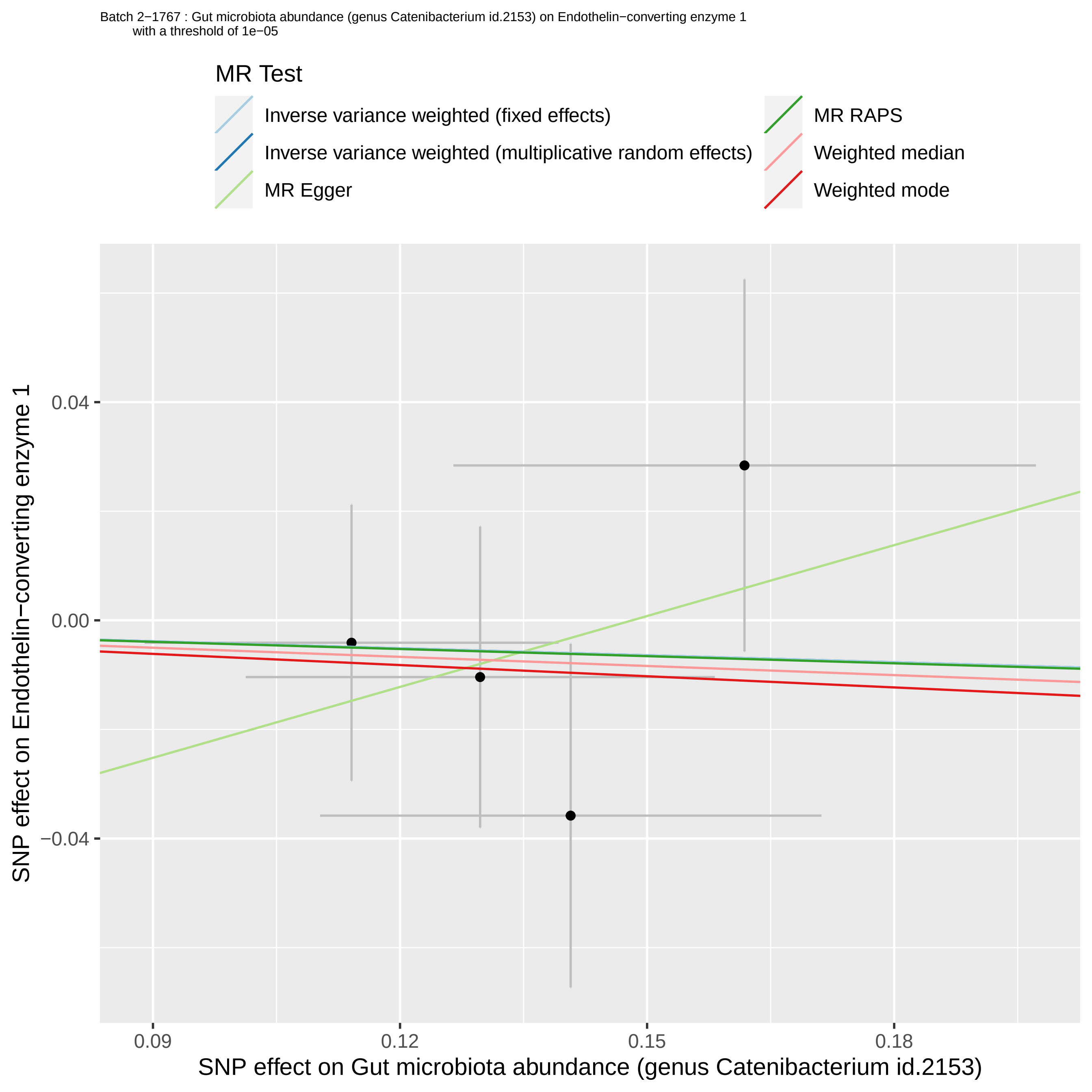
Batch 2−1767 : Gut microbiota abundance (genus Catenibacterium id.2153) on Endothelin−converting enzyme 1
with a threshold of 1e−05
MR Test
Inverse variance weighted (fixed effects)
Inverse variance weighted (multiplicative random effects)
MR Egger
MR RAPS
Weighted median
Weighted mode
0.04
0.00
−0.04
0.09
0.12
0.15
0.18
SNP effect on Endothelin−converting enzyme 1
SNP effect on Gut microbiota abundance (genus Catenibacterium id.2153)

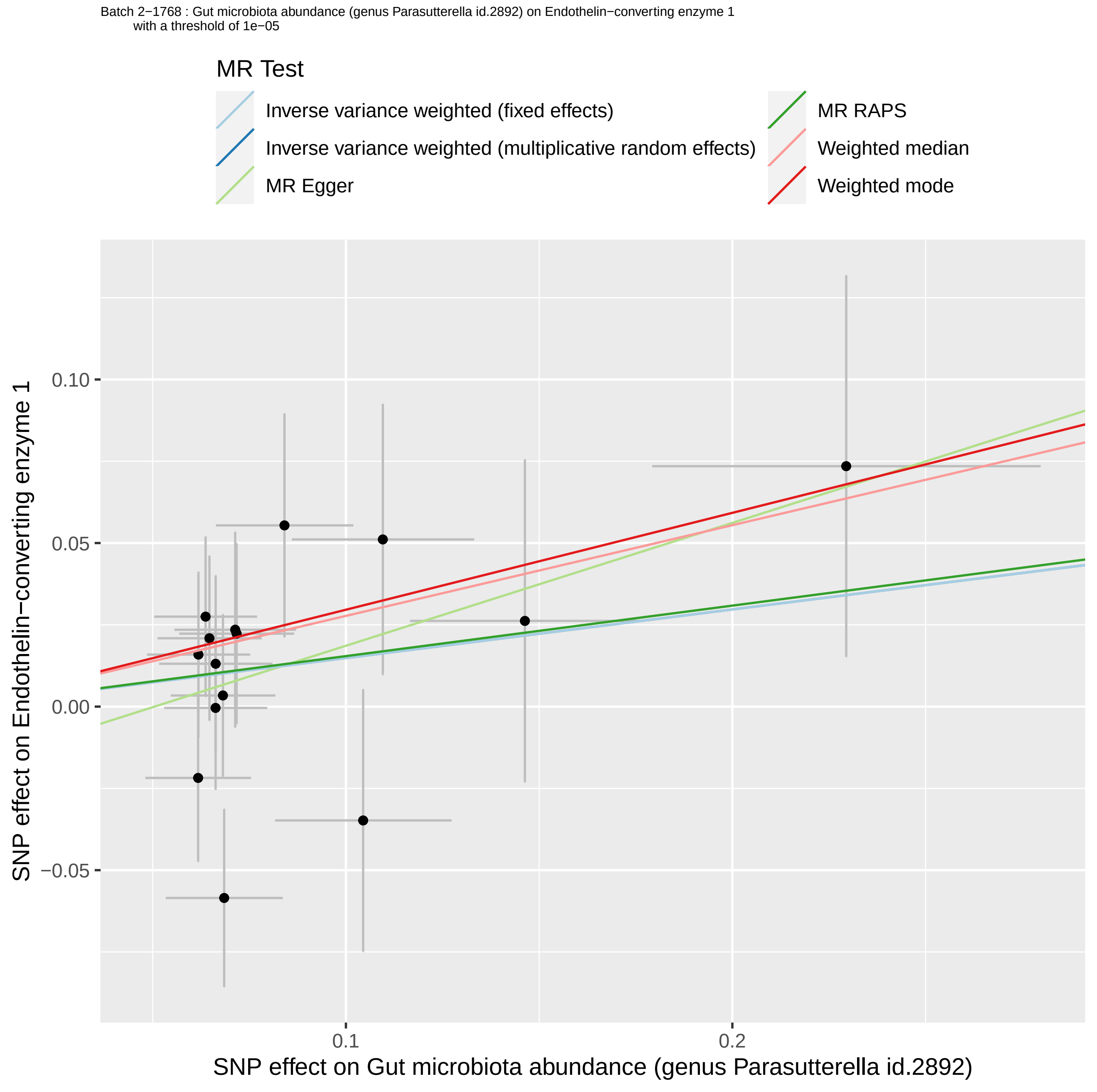
Batch 2−1768 : Gut microbiota abundance (genus Parasutterella id.2892) on Endothelin−converting enzyme 1
with a threshold of 1e−05
MR Test
Inverse variance weighted (fixed effects)
Inverse variance weighted (multiplicative random effects)
MR Egger
MR RAPS
Weighted median
Weighted mode
0.10
0.05
0.00
−0.05
0.1
0.2
SNP effect on Endothelin−converting enzyme 1
SNP effect on Gut microbiota abundance (genus Parasutterella id.2892)

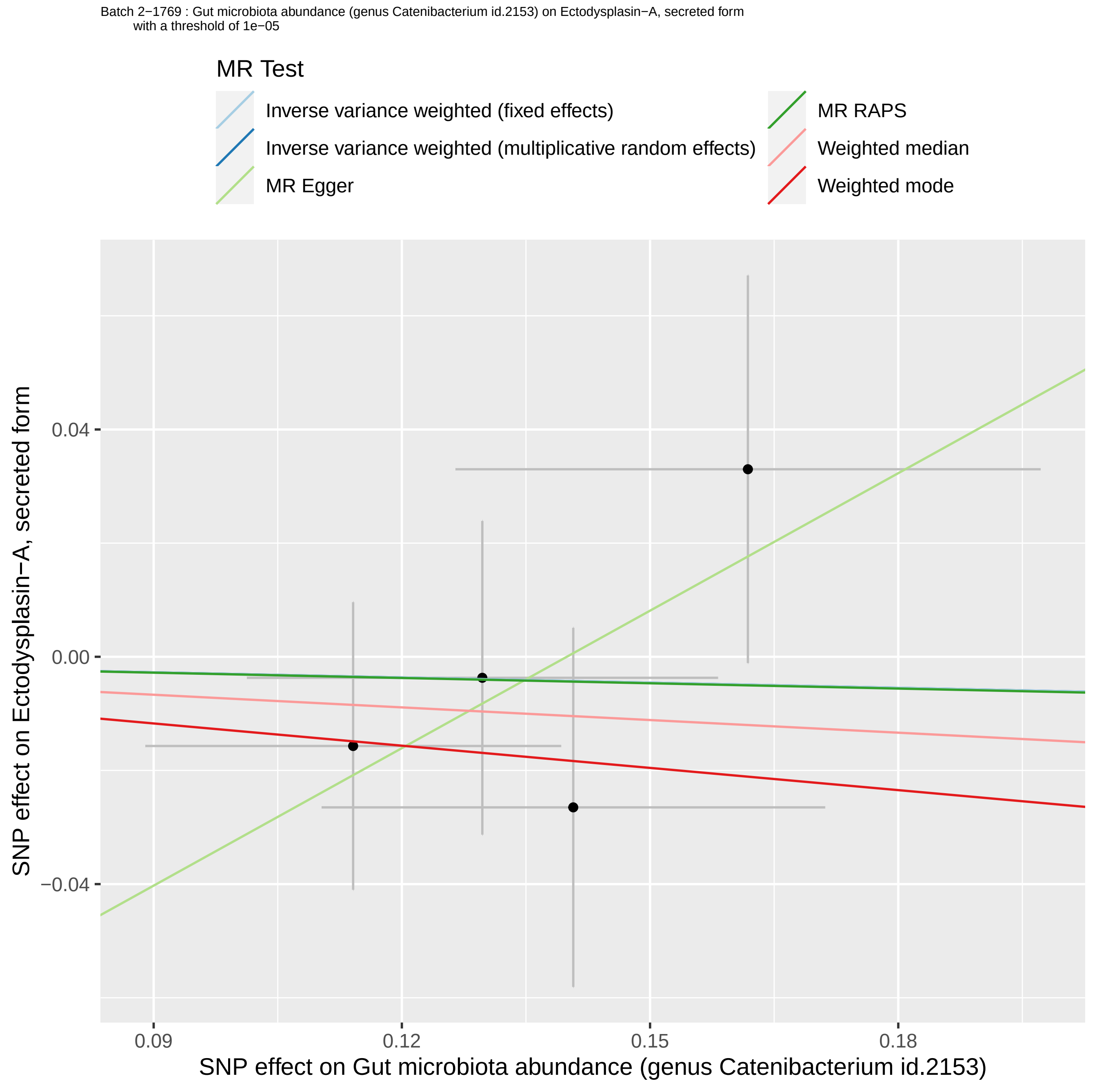
Batch 2−1769 : Gut microbiota abundance (genus Catenibacterium id.2153) on Ectodysplasin−A, secreted form
with a threshold of 1e−05
MR Test
Inverse variance weighted (fixed effects)
Inverse variance weighted (multiplicative random effects)
MR Egger
MR RAPS
Weighted median
Weighted mode
0.04
0.00
−0.04
0.09
0.12
0.15
0.18
SNP effect on Ectodysplasin−A, secreted form
SNP effect on Gut microbiota abundance (genus Catenibacterium id.2153)

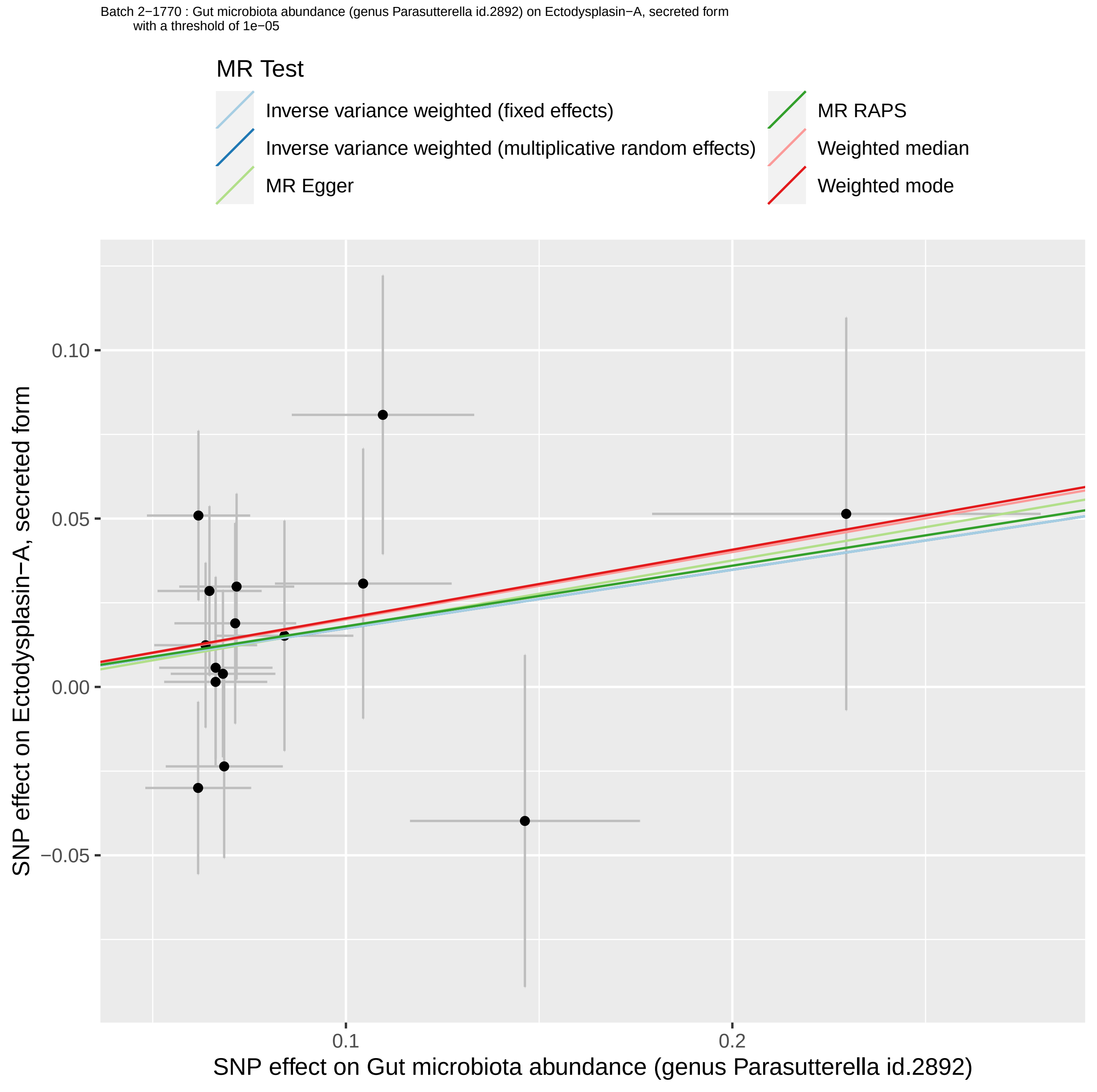
Batch 2−1770 : Gut microbiota abundance (genus Parasutterella id.2892) on Ectodysplasin−A, secreted form
with a threshold of 1e−05
MR Test
Inverse variance weighted (fixed effects)
Inverse variance weighted (multiplicative random effects)
MR Egger
MR RAPS
Weighted median
Weighted mode
0.10
0.05
0.00
−0.05
0.1
0.2
SNP effect on Ectodysplasin−A, secreted form
SNP effect on Gut microbiota abundance (genus Parasutterella id.2892)

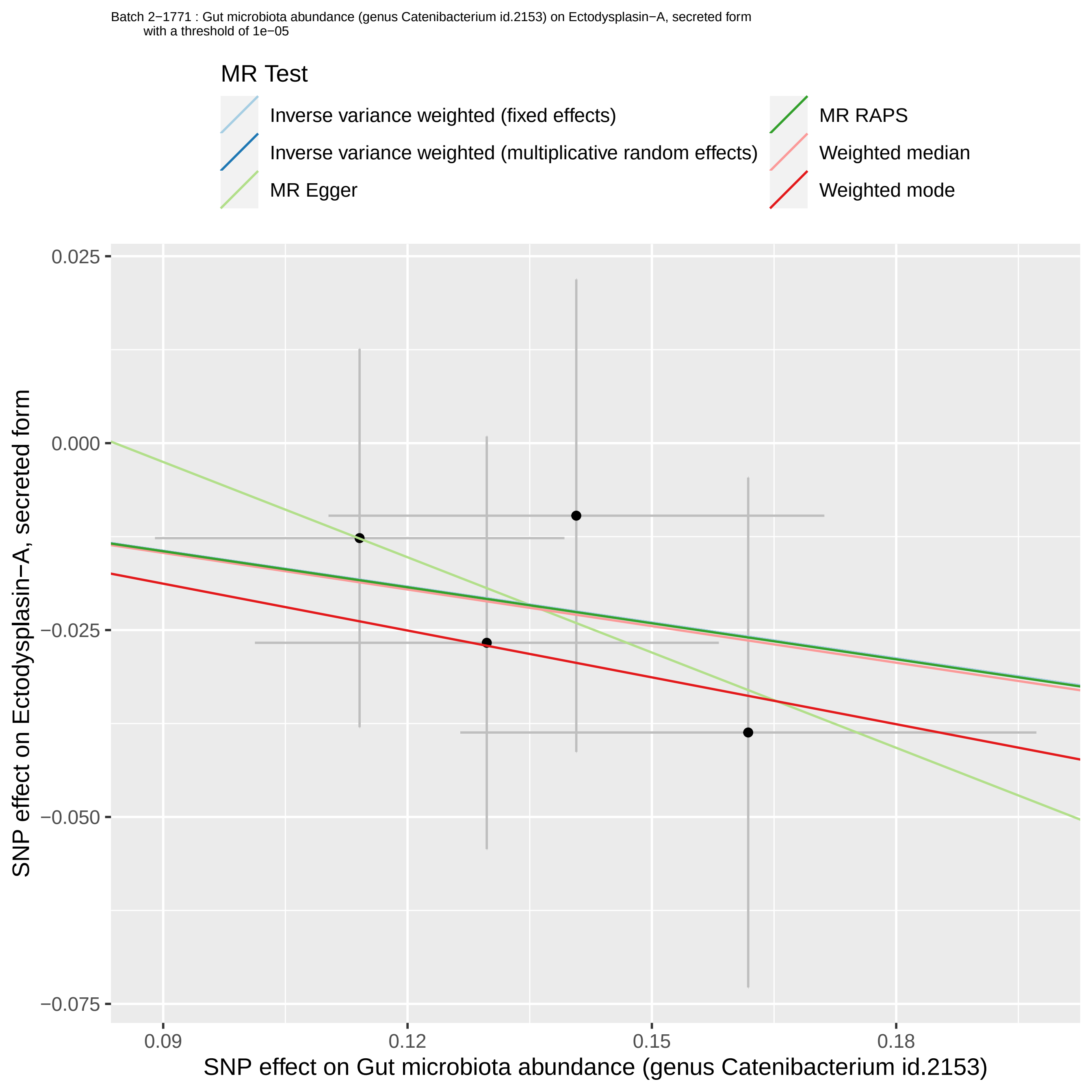
Batch 2−1771 : Gut microbiota abundance (genus Catenibacterium id.2153) on Ectodysplasin−A, secreted form
with a threshold of 1e−05
MR Test
Inverse variance weighted (fixed effects)
Inverse variance weighted (multiplicative random effects)
MR Egger
MR RAPS
Weighted median
Weighted mode
0.025
0.000
−0.025
−0.050
−0.075
0.09
0.12
0.15
0.18
SNP effect on Gut microbiota abundance (genus Catenibacterium id.2153)
SNP effect on Ectodysplasin−A, secreted form

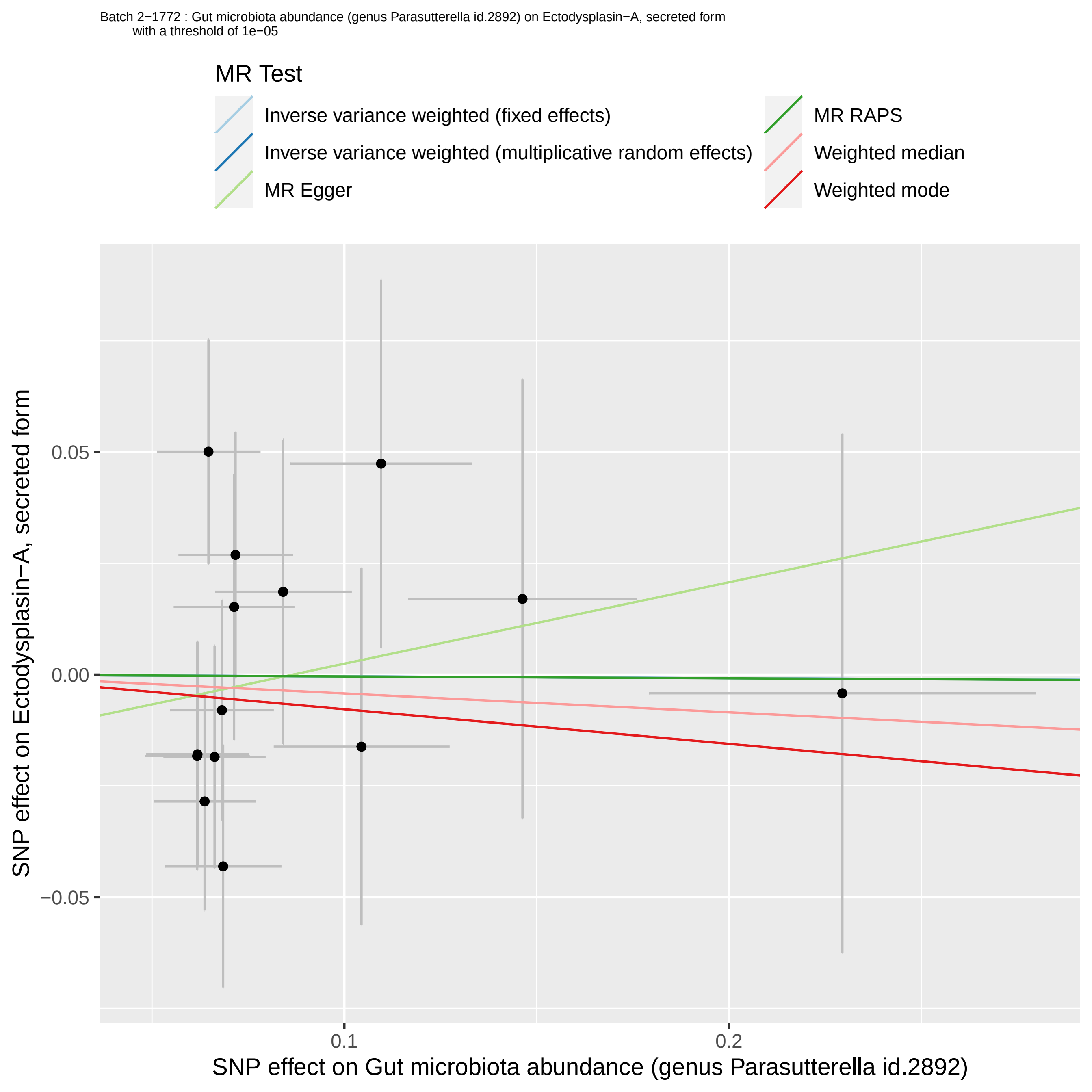

Batch 2−1772 : Gut microbiota abundance (genus Parasutterella id.2892) on Ectodysplasin−A, secreted form
with a threshold of 1e−05
MR Test
Inverse variance weighted (fixed effects)
Inverse variance weighted (multiplicative random effects)
MR Egger
MR RAPS
Weighted median
Weighted mode
SNP effect on Ectodysplasin−A, secreted form
0.05
0.00
−0.05
0.1
0.2
SNP effect on Gut microbiota abundance (genus Parasutterella id.2892)

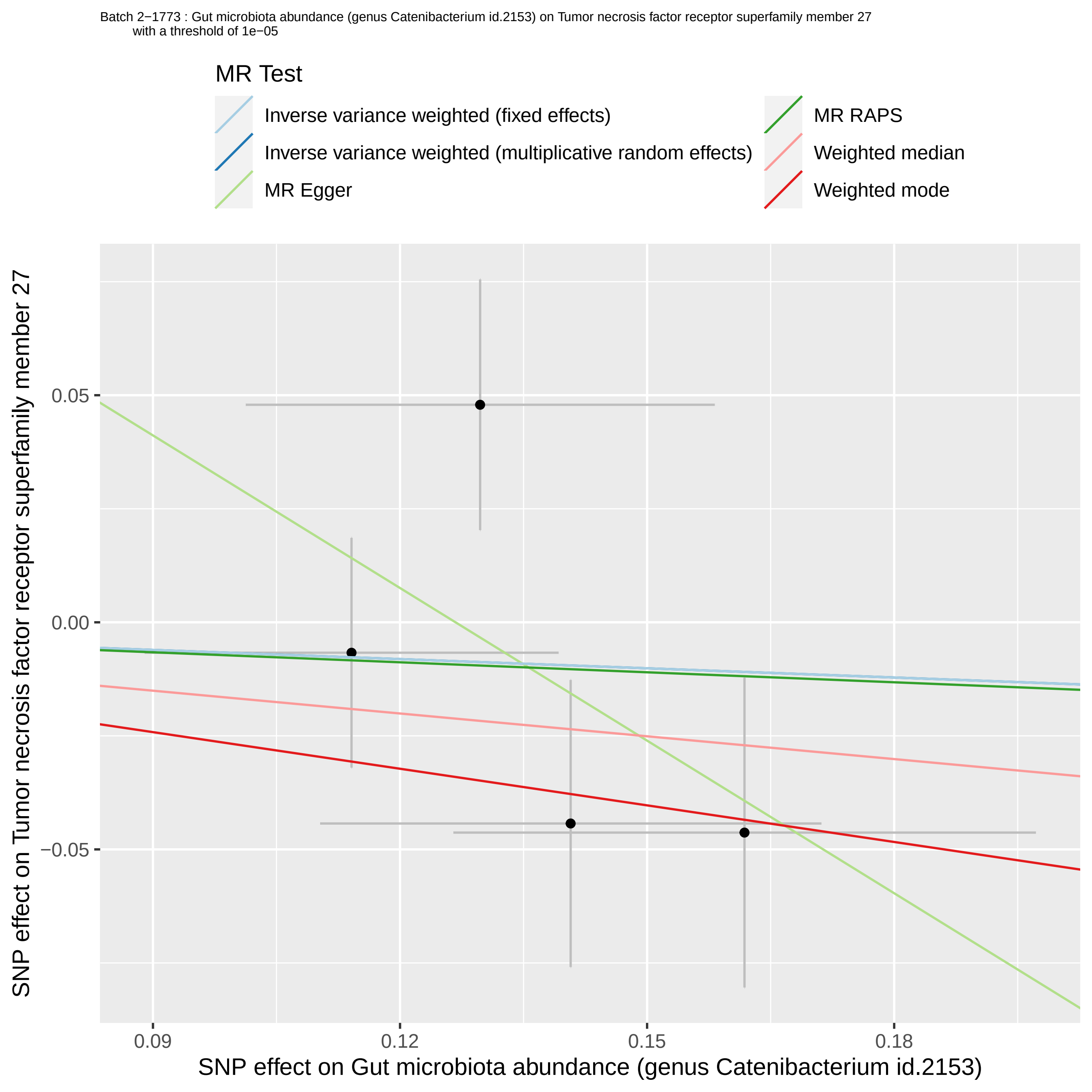
Batch 2−1773 : Gut microbiota abundance (genus Catenibacterium id.2153) on Tumor necrosis factor receptor superfamily member 27
with a threshold of 1e−05
MR Test
Inverse variance weighted (fixed effects)
Inverse variance weighted (multiplicative random effects)
MR Egger
MR RAPS
Weighted median
Weighted mode
0.05
0.00
−0.05
0.09
0.12
0.15
0.18
SNP effect on Tumor necrosis factor receptor superfamily member 27
SNP effect on Gut microbiota abundance (genus Catenibacterium id.2153)

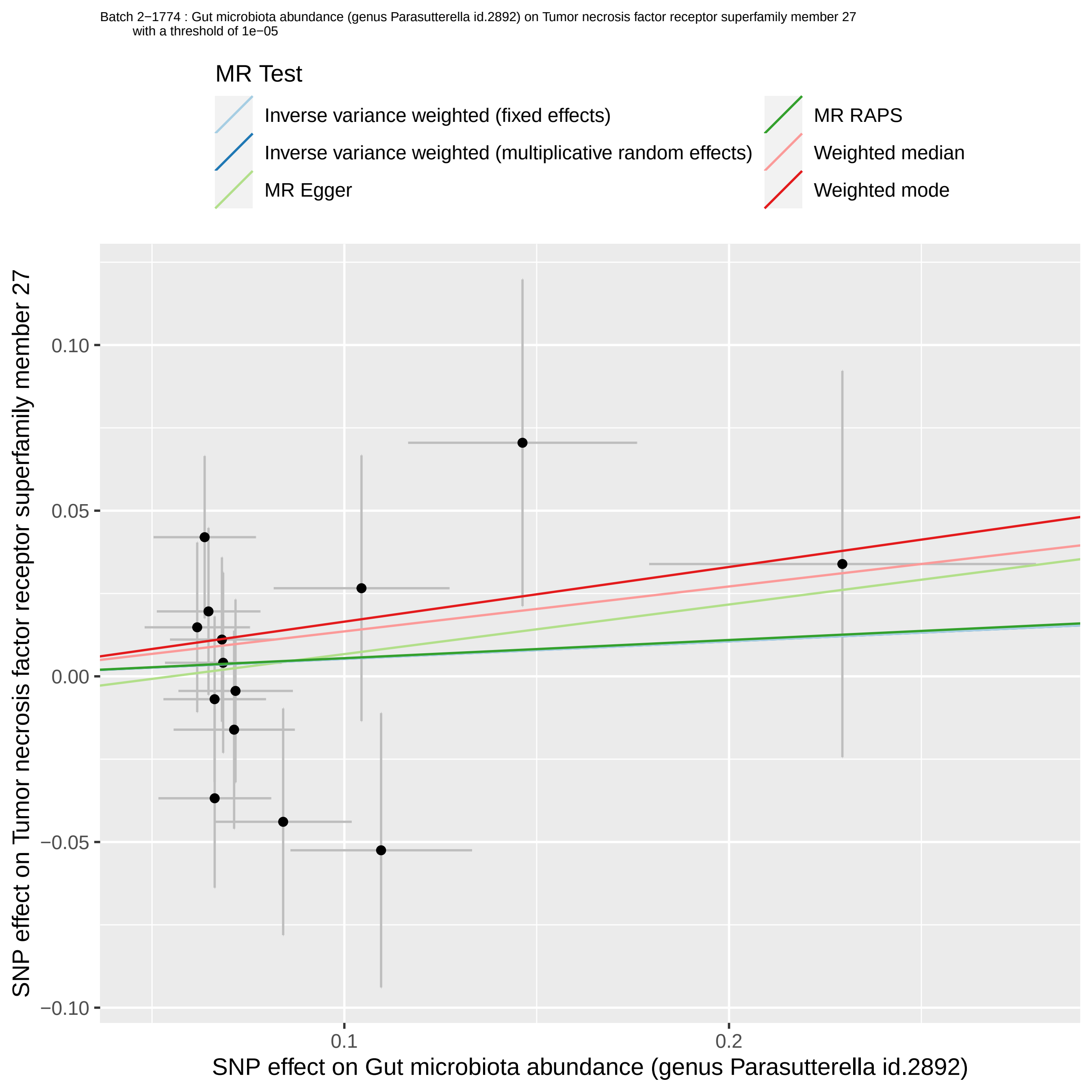
Batch 2−1774 : Gut microbiota abundance (genus Parasutterella id.2892) on Tumor necrosis factor receptor superfamily member 27
with a threshold of 1e−05
MR Test
Inverse variance weighted (fixed effects)
Inverse variance weighted (multiplicative random effects)
MR Egger
MR RAPS
Weighted median
Weighted mode
0.10
0.05
0.00
−0.05
−0.10
0.1
0.2
SNP effect on Tumor necrosis factor receptor superfamily member 27
SNP effect on Gut microbiota abundance (genus Parasutterella id.2892)

Batch 2−1775 : Gut microbiota abundance (genus Catenibacterium id.2153) on Tumor necrosis factor receptor superfamily member EDAR with a threshold of 1e−05

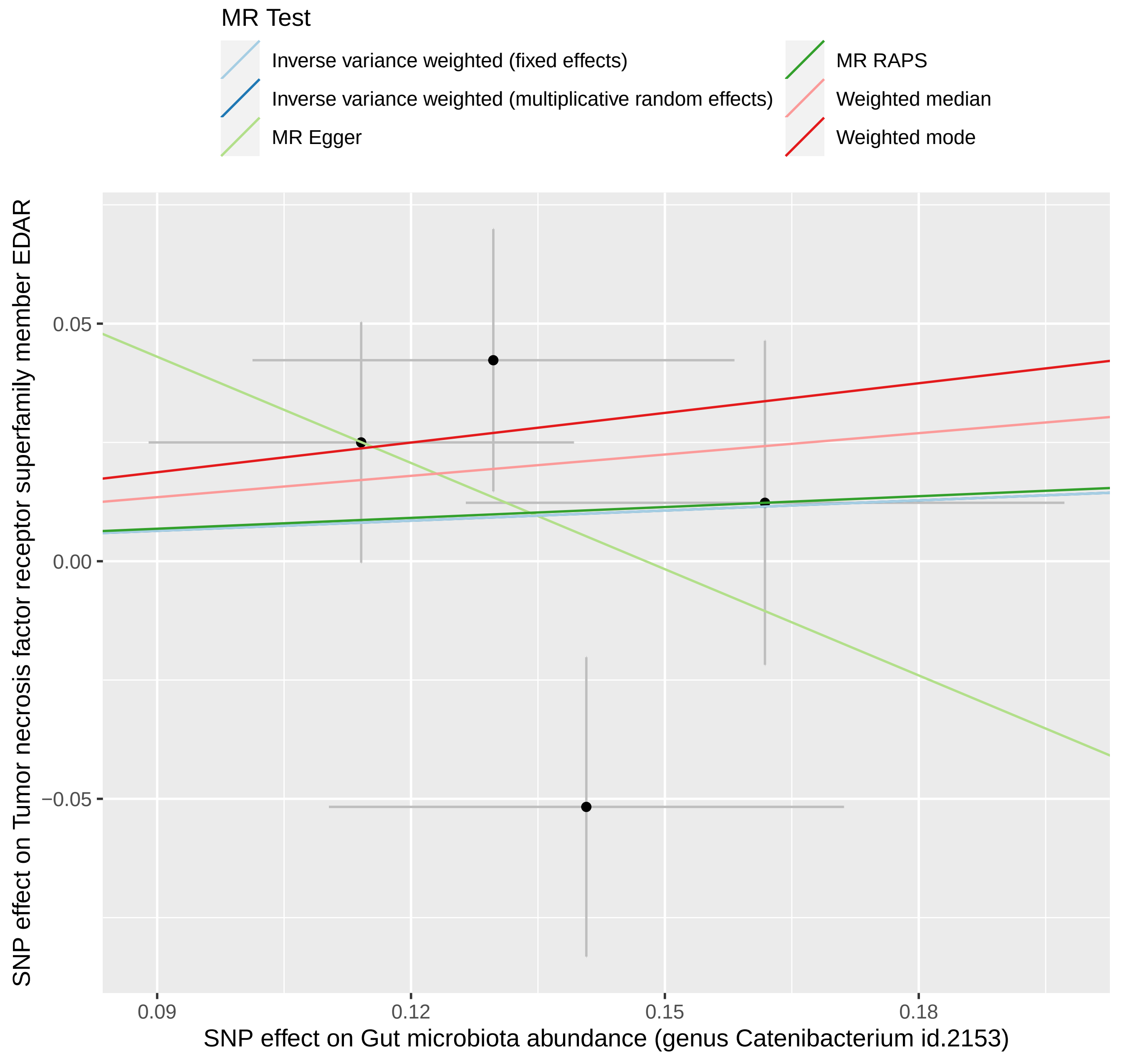

Batch 2−1776 : Gut microbiota abundance (genus Parasutterella id.2892) on Tumor necrosis factor receptor superfamily member EDAR
with a threshold of 1e−05

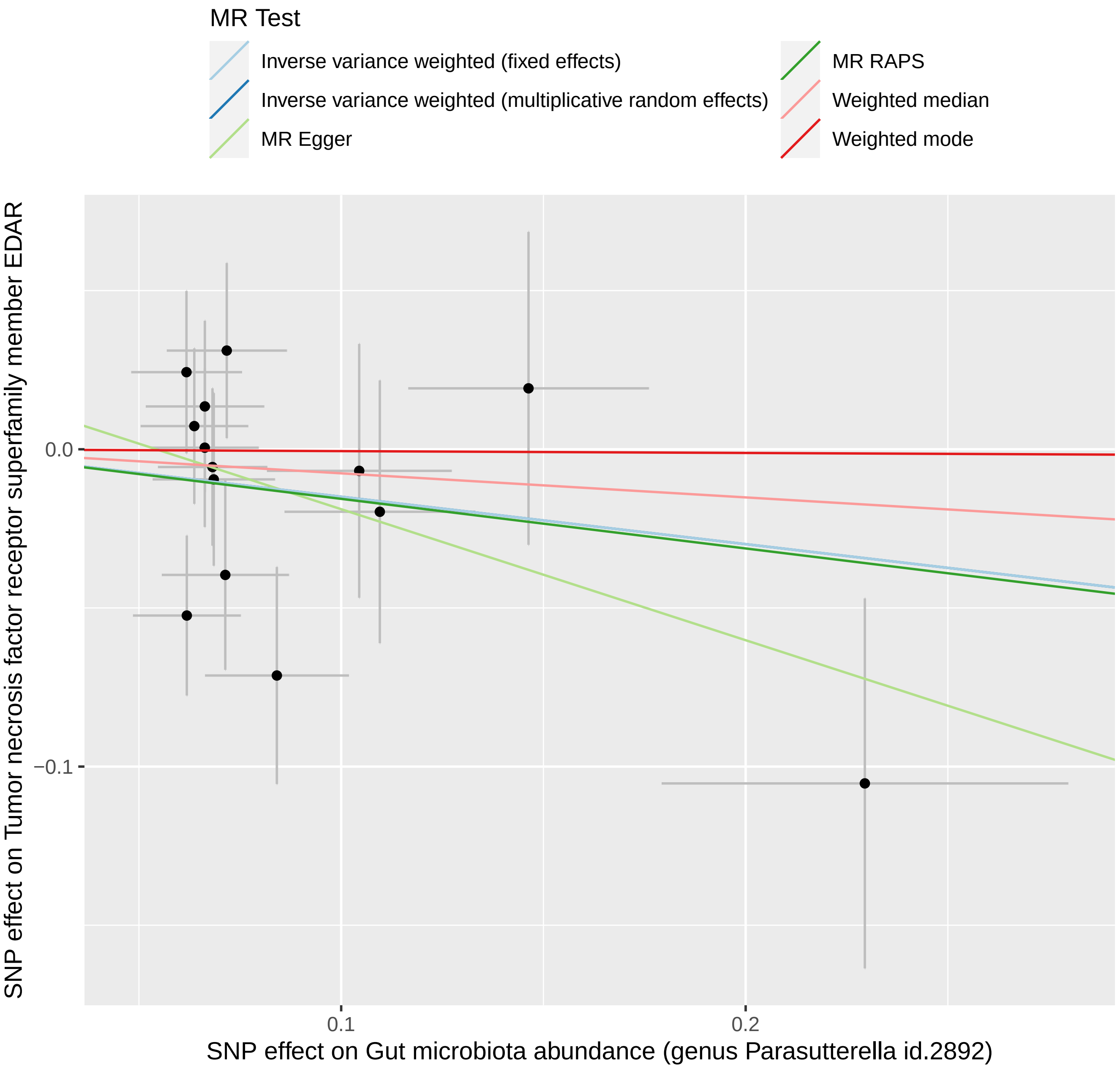

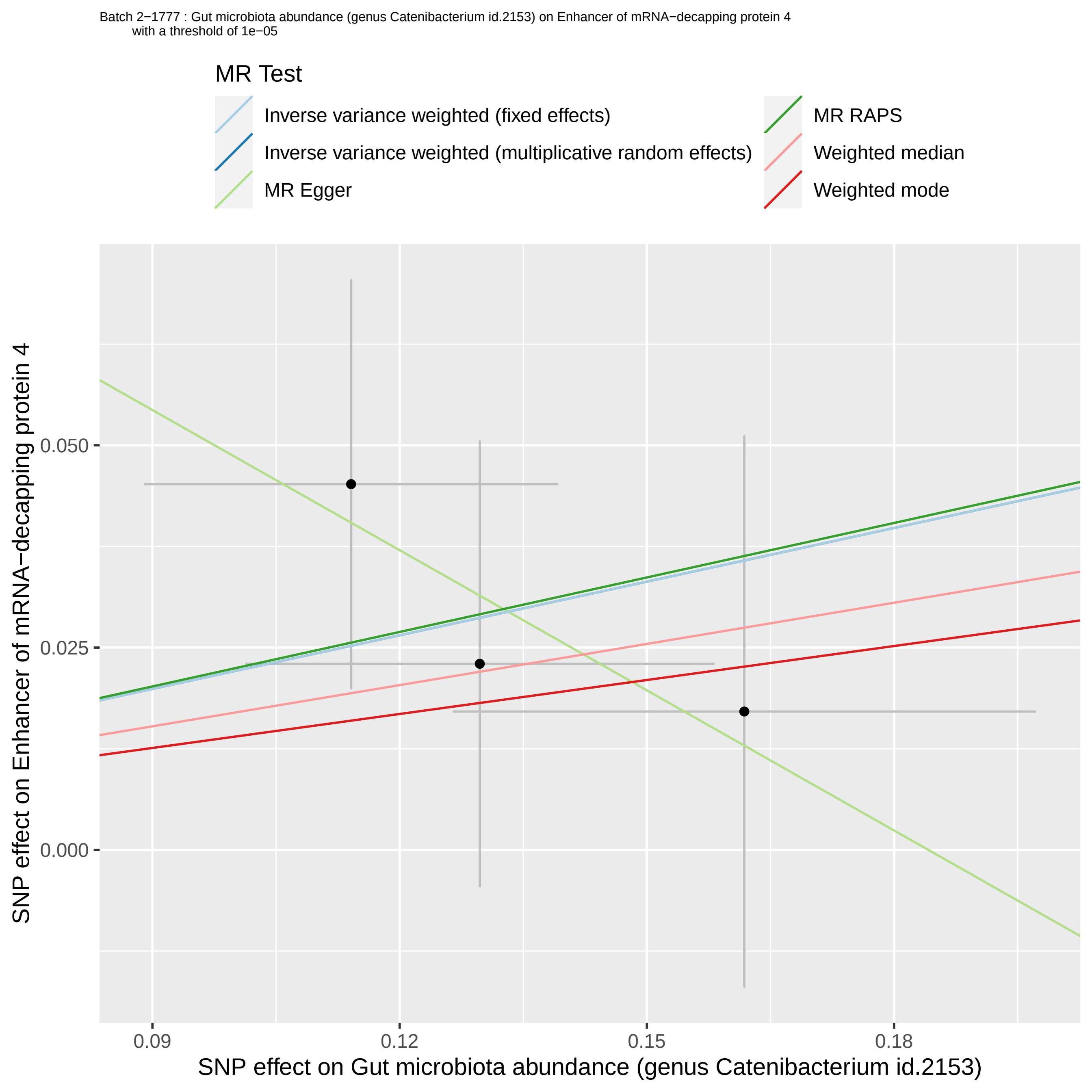

Batch 2−1777 : Gut microbiota abundance (genus Catenibacterium id.2153) on Enhancer of mRNA−decapping protein 4
with a threshold of 1e−05
MR Test
Inverse variance weighted (fixed effects)
Inverse variance weighted (multiplicative random effects)
MR Egger
MR RAPS
Weighted median
Weighted mode
0.050
0.025
0.000
0.09
0.12
0.15
0.18
SNP effect on Enhancer of mRNA−decapping protein 4
SNP effect on Gut microbiota abundance (genus Catenibacterium id.2153)

Batch 2−1778 : Gut microbiota abundance (genus Parasutterella id.2892) on Enhancer of mRNA−decapping protein 4
with a threshold of 1e−05

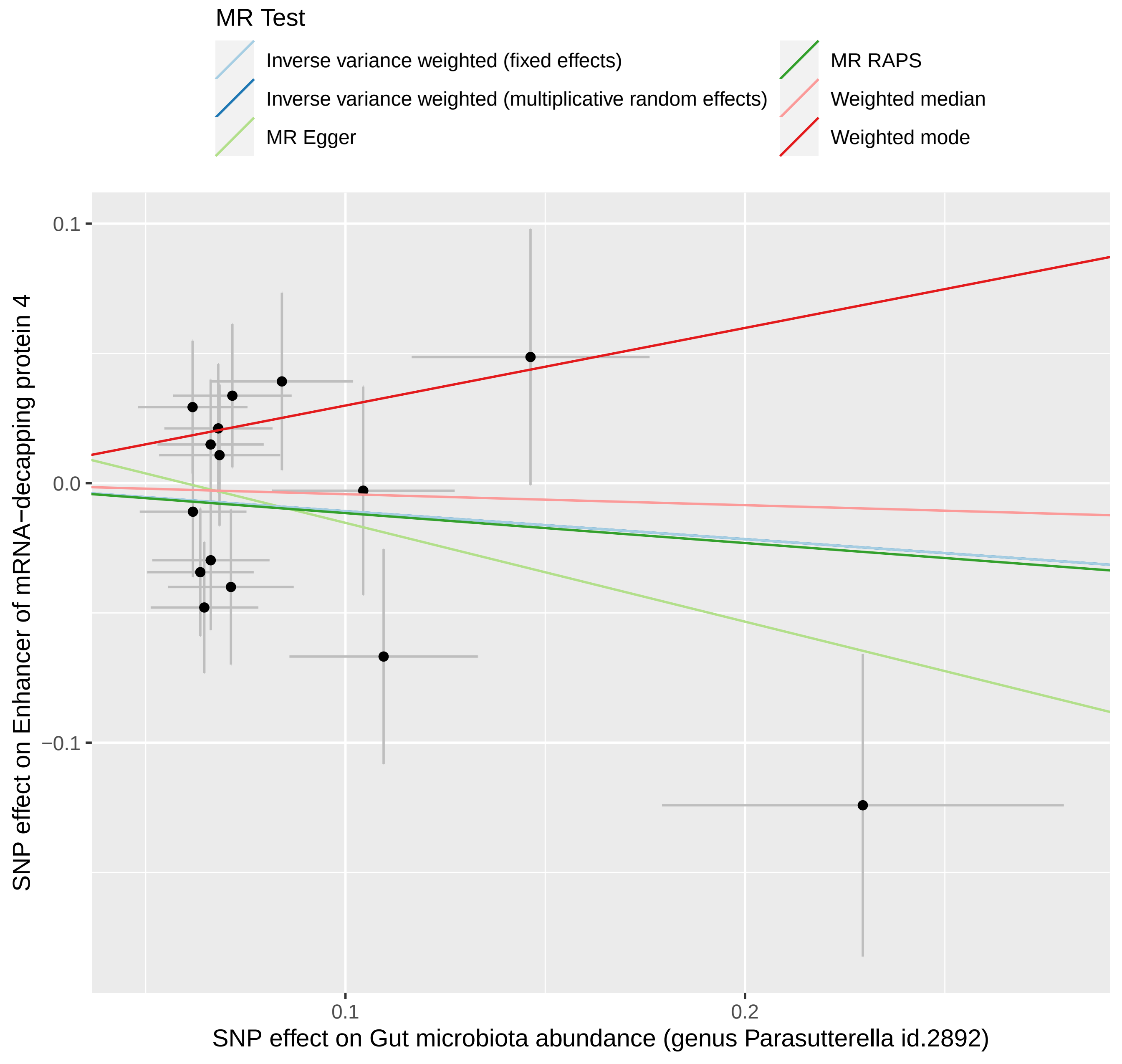

Batch 2−1779 : Gut microbiota abundance (genus Catenibacterium id.2153) on Epididymal secretory protein E3−beta
with a threshold of 1e−05

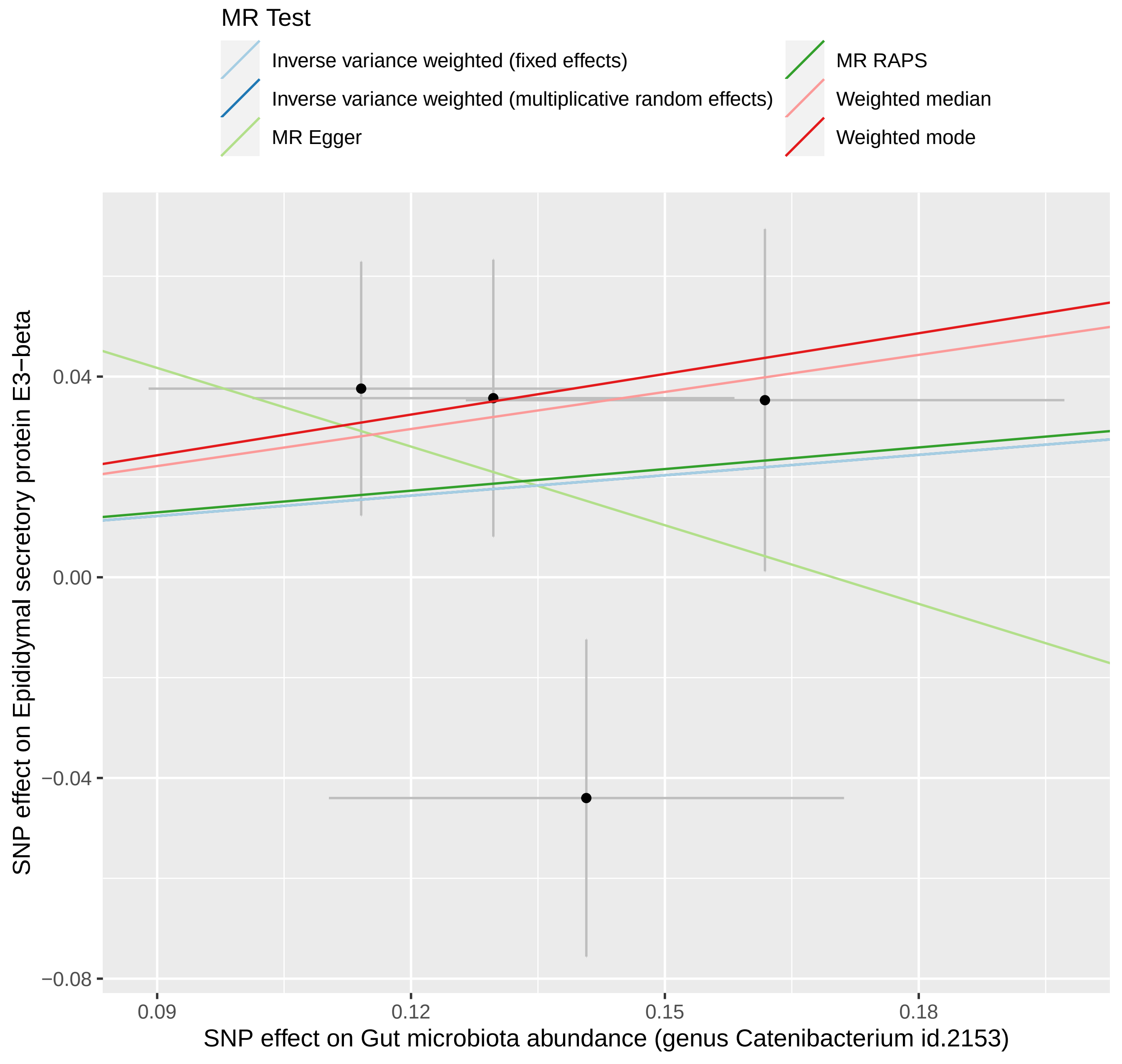

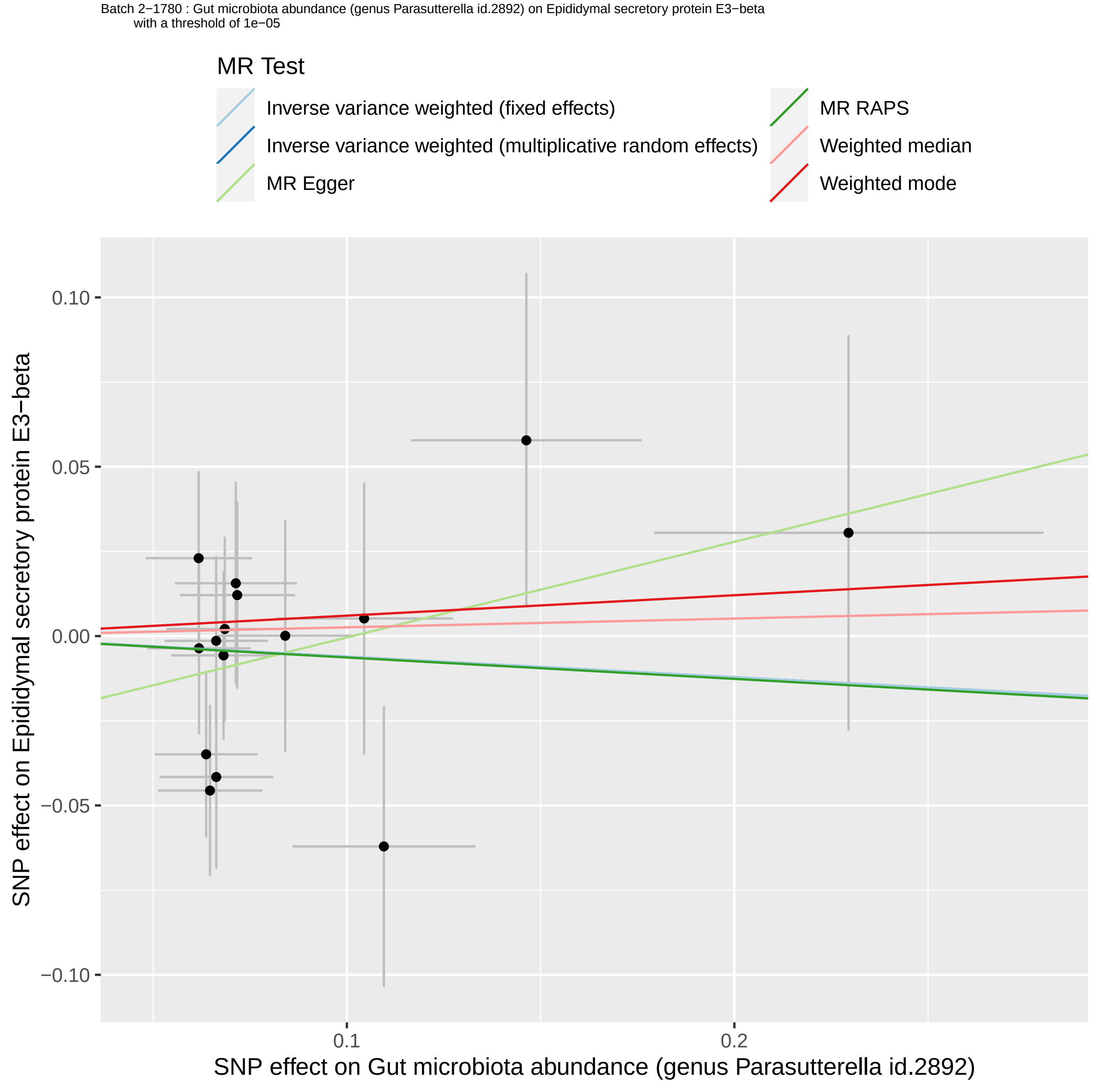

Batch 2−1780 : Gut microbiota abundance (genus Parasutterella id.2892) on Epididymal secretory protein E3−beta
with a threshold of 1e−05
MR Test
Inverse variance weighted (fixed effects)
Inverse variance weighted (multiplicative random effects)
MR Egger
MR RAPS
Weighted median
Weighted mode
0.10
0.05
0.00
−0.05
−0.10
0.1
0.2
SNP effect on Epididymal secretory protein E3−beta
SNP effect on Gut microbiota abundance (genus Parasutterella id.2892)

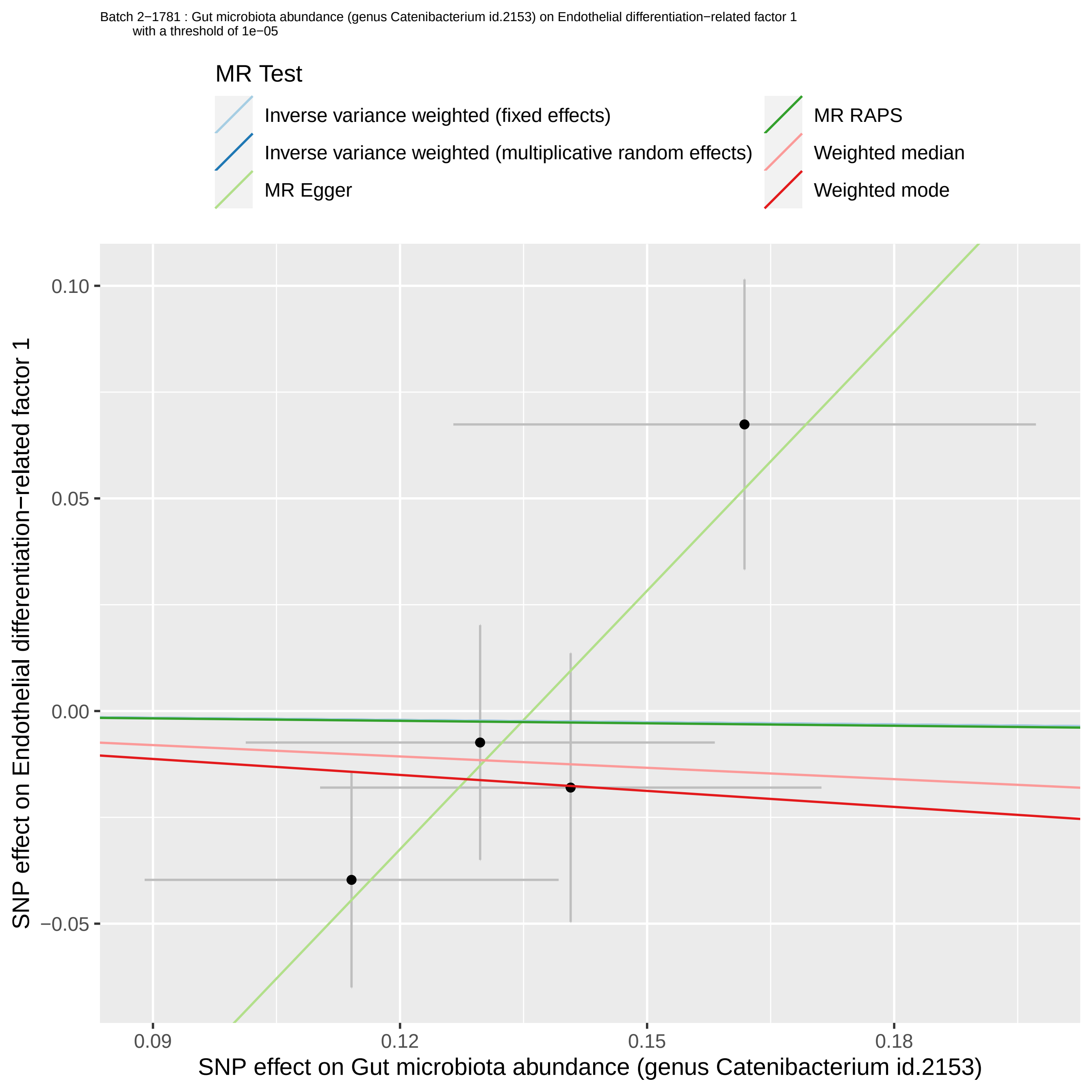
Batch 2−1781 : Gut microbiota abundance (genus Catenibacterium id.2153) on Endothelial differentiation−related factor 1
with a threshold of 1e−05
MR Test
Inverse variance weighted (fixed effects)
Inverse variance weighted (multiplicative random effects)
MR Egger
MR RAPS
Weighted median
Weighted mode
0.10
0.05
0.00
−0.05
0.09
0.12
0.15
0.18
SNP effect on Endothelial differentiation−related factor 1
SNP effect on Gut microbiota abundance (genus Catenibacterium id.2153)

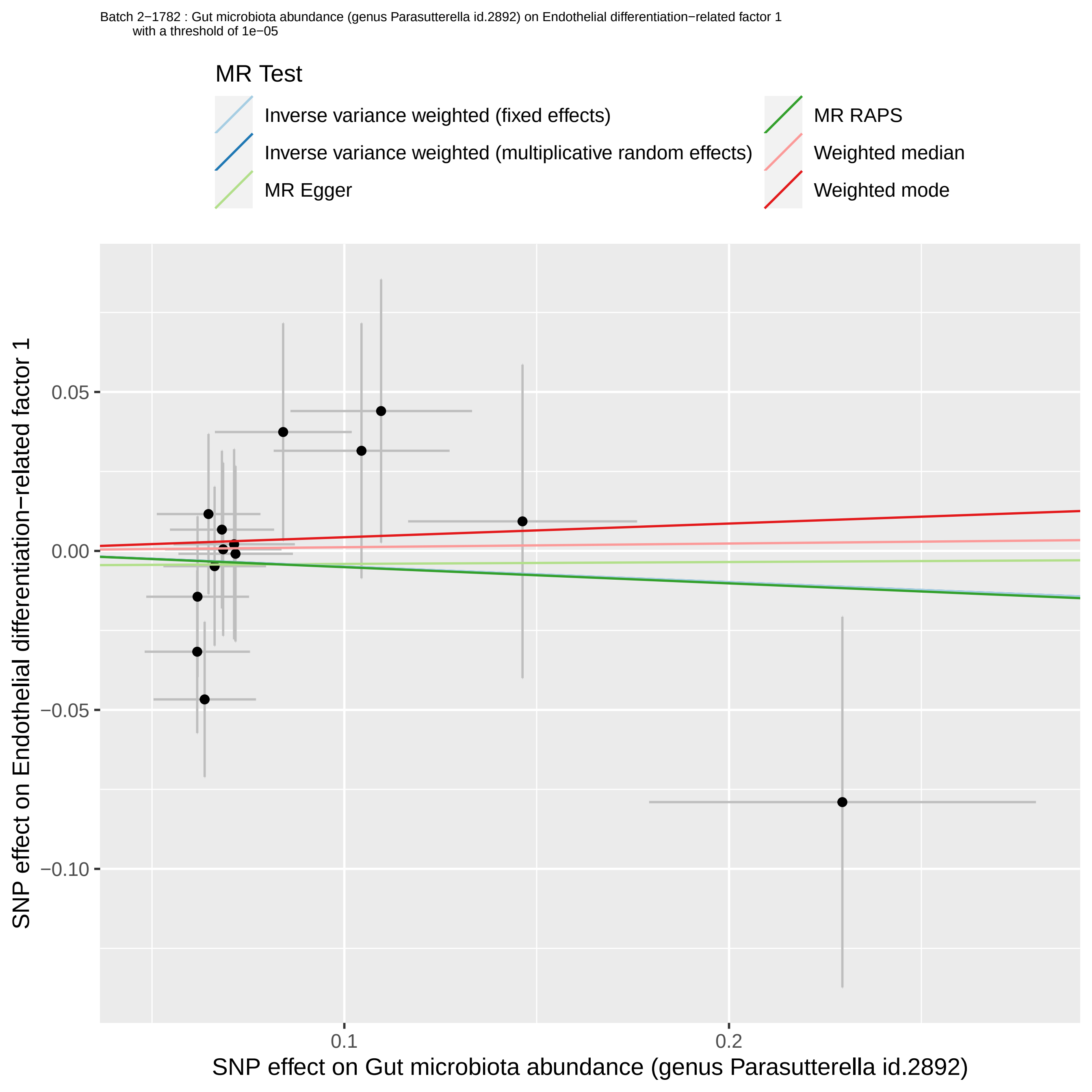

Batch 2−1782 : Gut microbiota abundance (genus Parasutterella id.2892) on Endothelial differentiation−related factor 1
with a threshold of 1e−05
MR Test
Inverse variance weighted (fixed effects)
Inverse variance weighted (multiplicative random effects)
MR Egger
MR RAPS
Weighted median
Weighted mode
0.05
0.00
−0.05
−0.10
0.1
0.2
SNP effect on Endothelial differentiation−related factor 1
SNP effect on Gut microbiota abundance (genus Parasutterella id.2892)

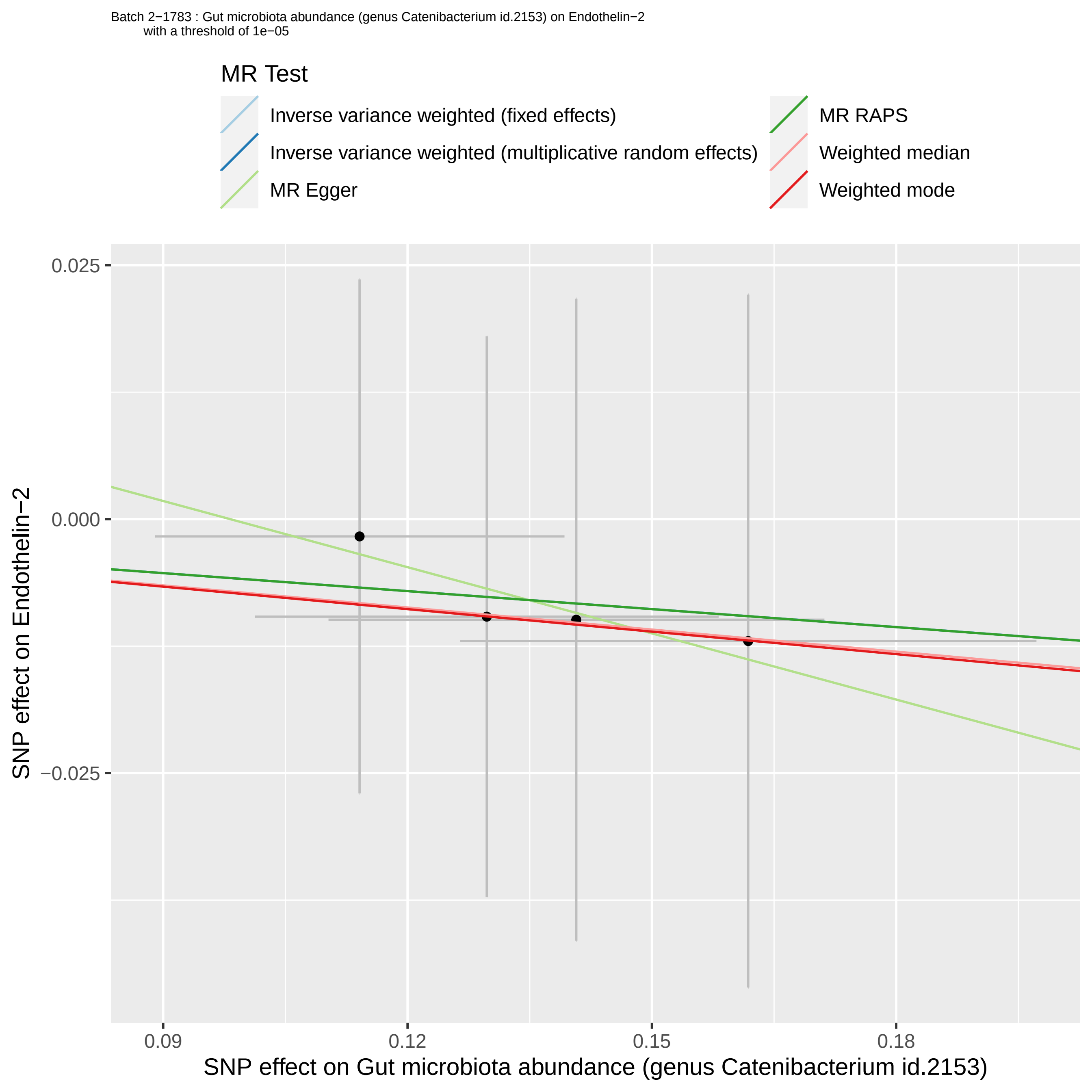

Batch 2−1783 : Gut microbiota abundance (genus Catenibacterium id.2153) on Endothelin−2
with a threshold of 1e−05
MR Test
Inverse variance weighted (fixed effects)
Inverse variance weighted (multiplicative random effects)
MR Egger
MR RAPS
Weighted median
Weighted mode
0.025
0.000
−0.025
0.09
0.12
0.15
0.18
SNP effect on Endothelin−2
SNP effect on Gut microbiota abundance (genus Catenibacterium id.2153)

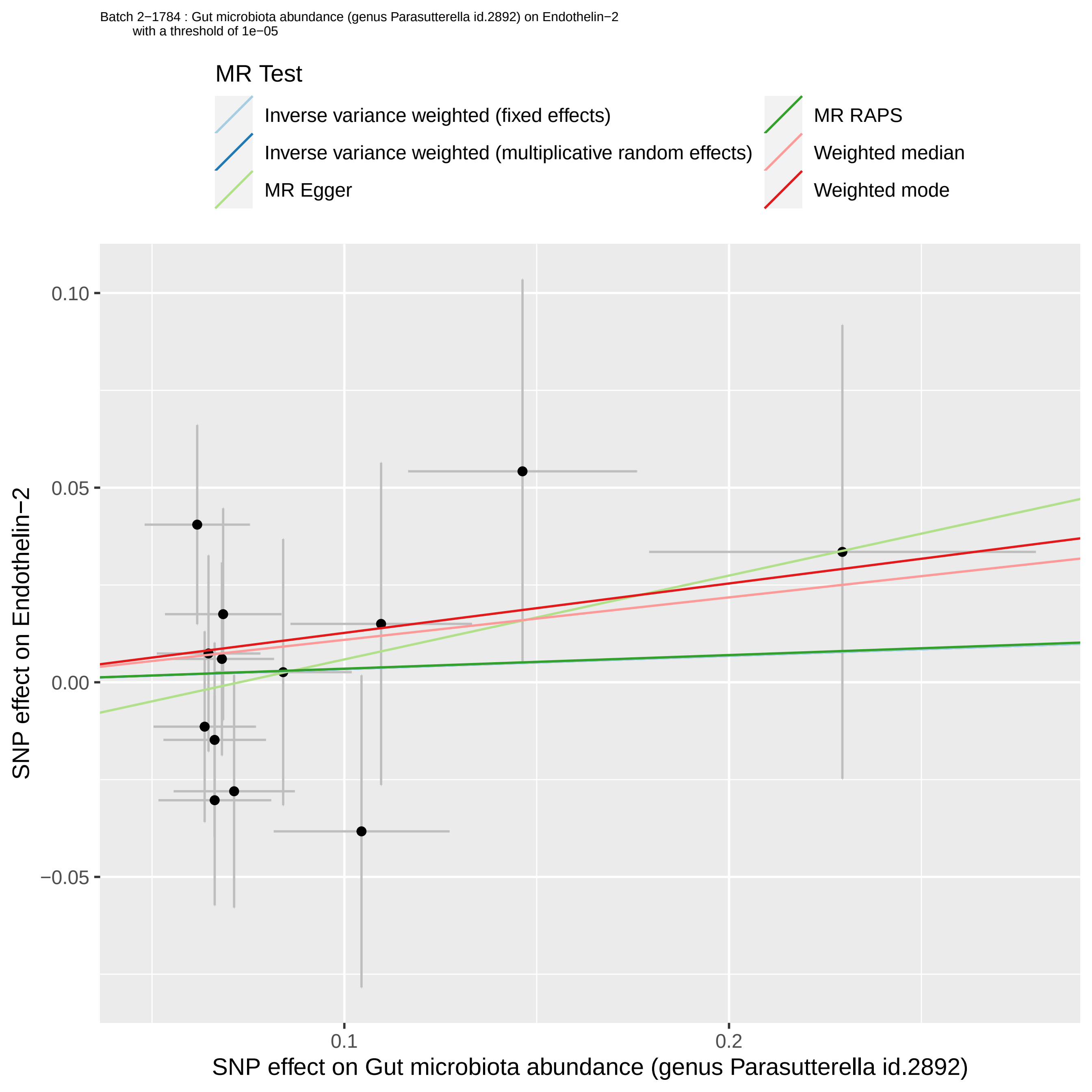
Batch 2−1784 : Gut microbiota abundance (genus Parasutterella id.2892) on Endothelin−2
with a threshold of 1e−05
MR Test
Inverse variance weighted (fixed effects)
Inverse variance weighted (multiplicative random effects)
MR Egger
MR RAPS
Weighted median
Weighted mode
0.10
0.05
0.00
−0.05
0.1
0.2
SNP effect on Endothelin−2
SNP effect on Gut microbiota abundance (genus Parasutterella id.2892)

Batch 2−1785 : Gut microbiota abundance (genus Catenibacterium id.2153) on Early endosome antigen 1
with a threshold of 1e−05

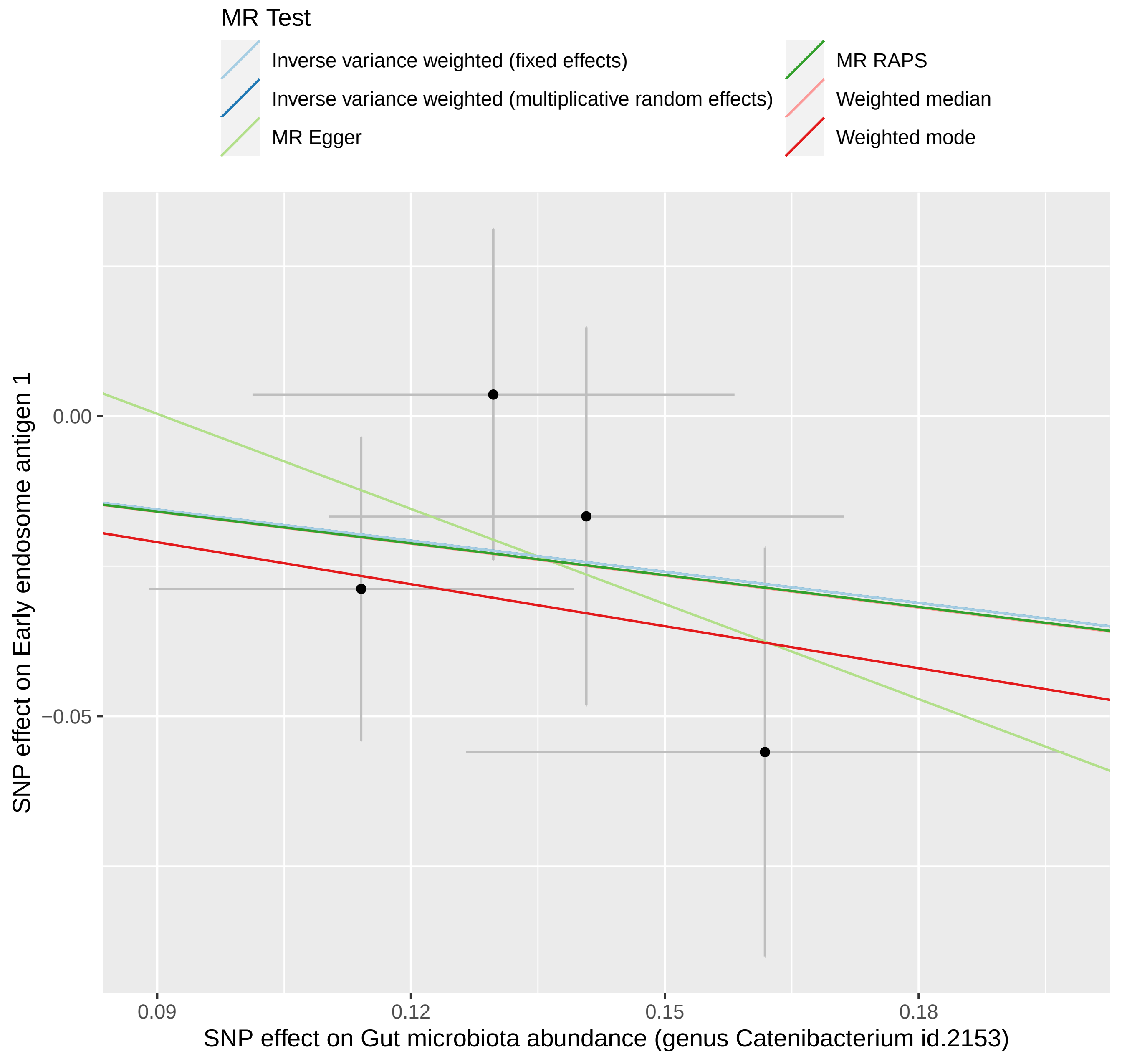

Batch 2−1786 : Gut microbiota abundance (genus Parasutterella id.2892) on Early endosome antigen 1
with a threshold of 1e−05

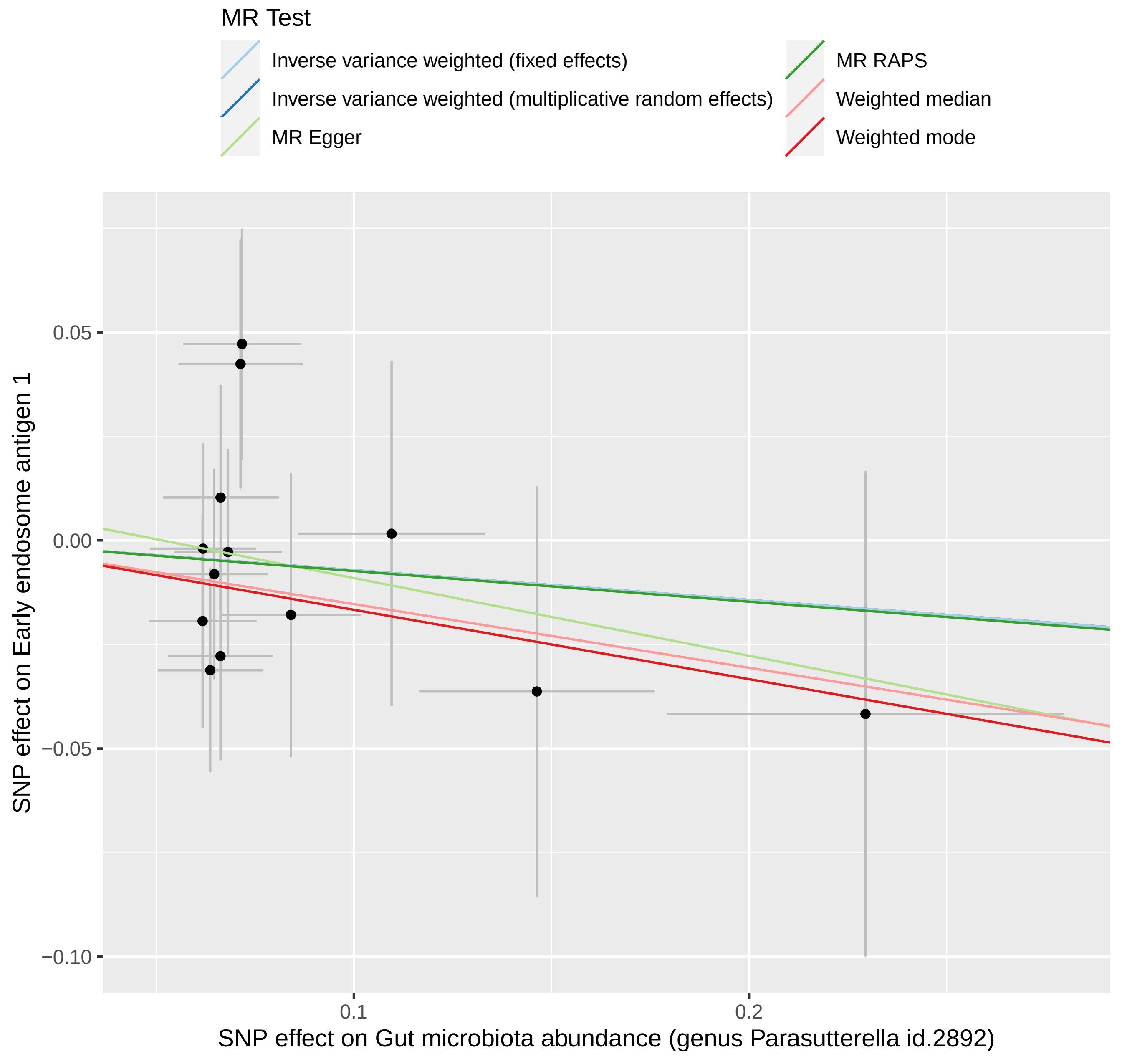

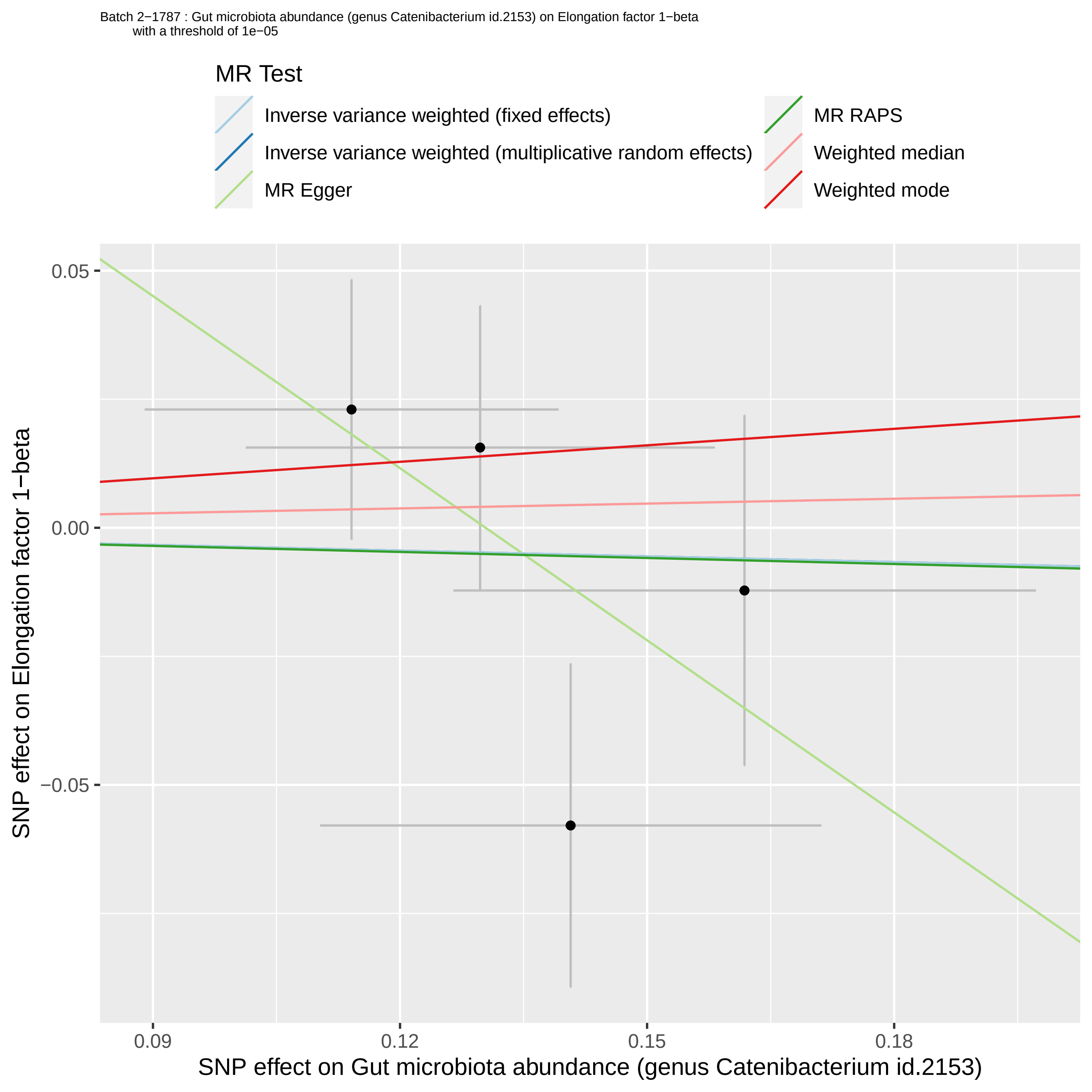

Batch 2−1787 : Gut microbiota abundance (genus Catenibacterium id.2153) on Elongation factor 1−beta
with a threshold of 1e−05
MR Test
Inverse variance weighted (fixed effects)
Inverse variance weighted (multiplicative random effects)
MR Egger
MR RAPS
Weighted median
Weighted mode
0.05
0.00
−0.05
0.09
0.12
0.15
0.18
SNP effect on Elongation factor 1−beta
SNP effect on Gut microbiota abundance (genus Catenibacterium id.2153)

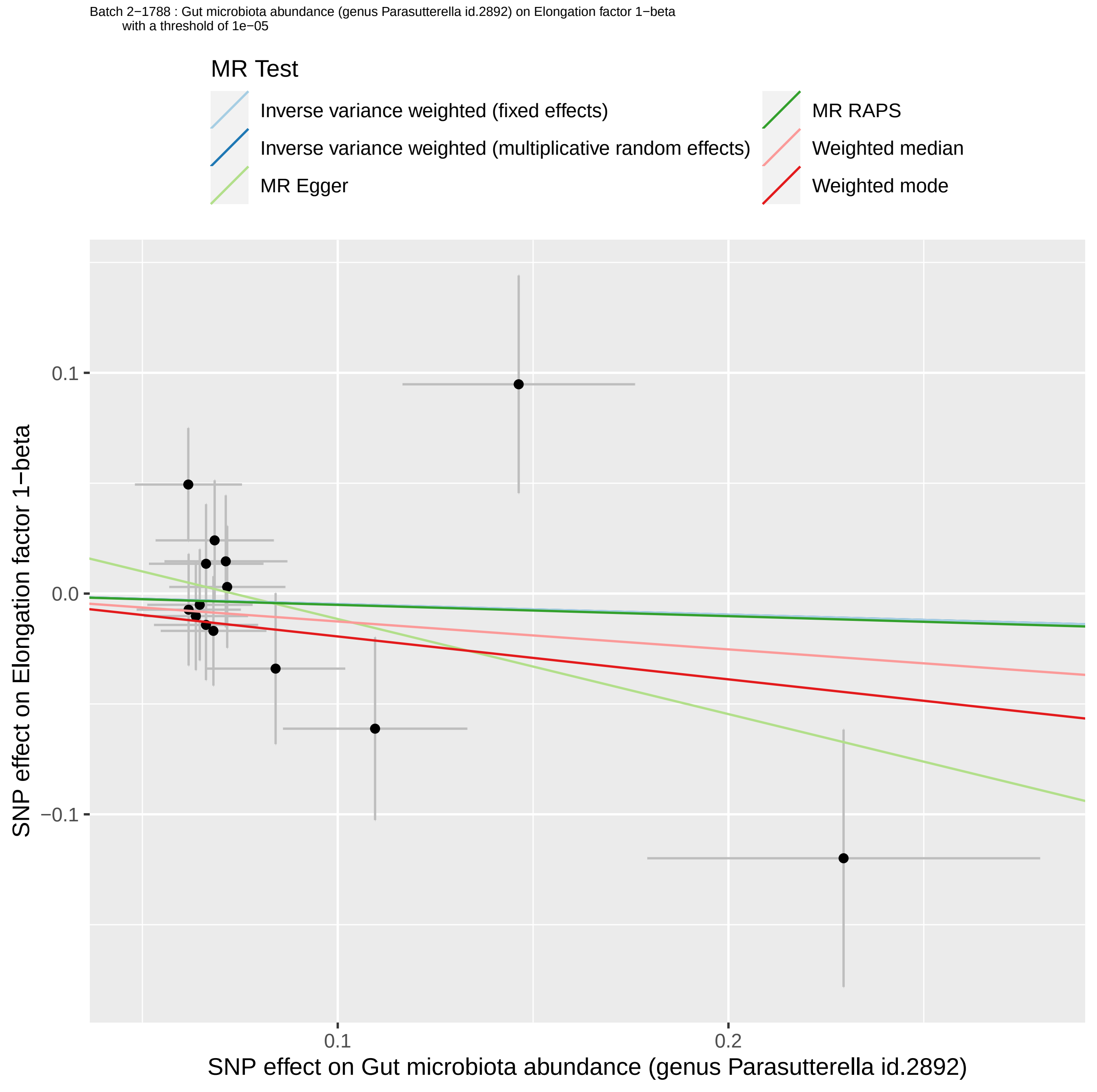
Batch 2−1788 : Gut microbiota abundance (genus Parasutterella id.2892) on Elongation factor 1−beta
with a threshold of 1e−05
MR Test
Inverse variance weighted (fixed effects)
Inverse variance weighted (multiplicative random effects)
MR Egger
MR RAPS
Weighted median
Weighted mode
0.1
0.0
−0.1
0.1
0.2
SNP effect on Elongation factor 1−beta
SNP effect on Gut microbiota abundance (genus Parasutterella id.2892)

Batch 2−1789 : Gut microbiota abundance (genus Catenibacterium id.2153) on EF−hand calcium−binding domain−containing protein 14 with a threshold of 1e−05

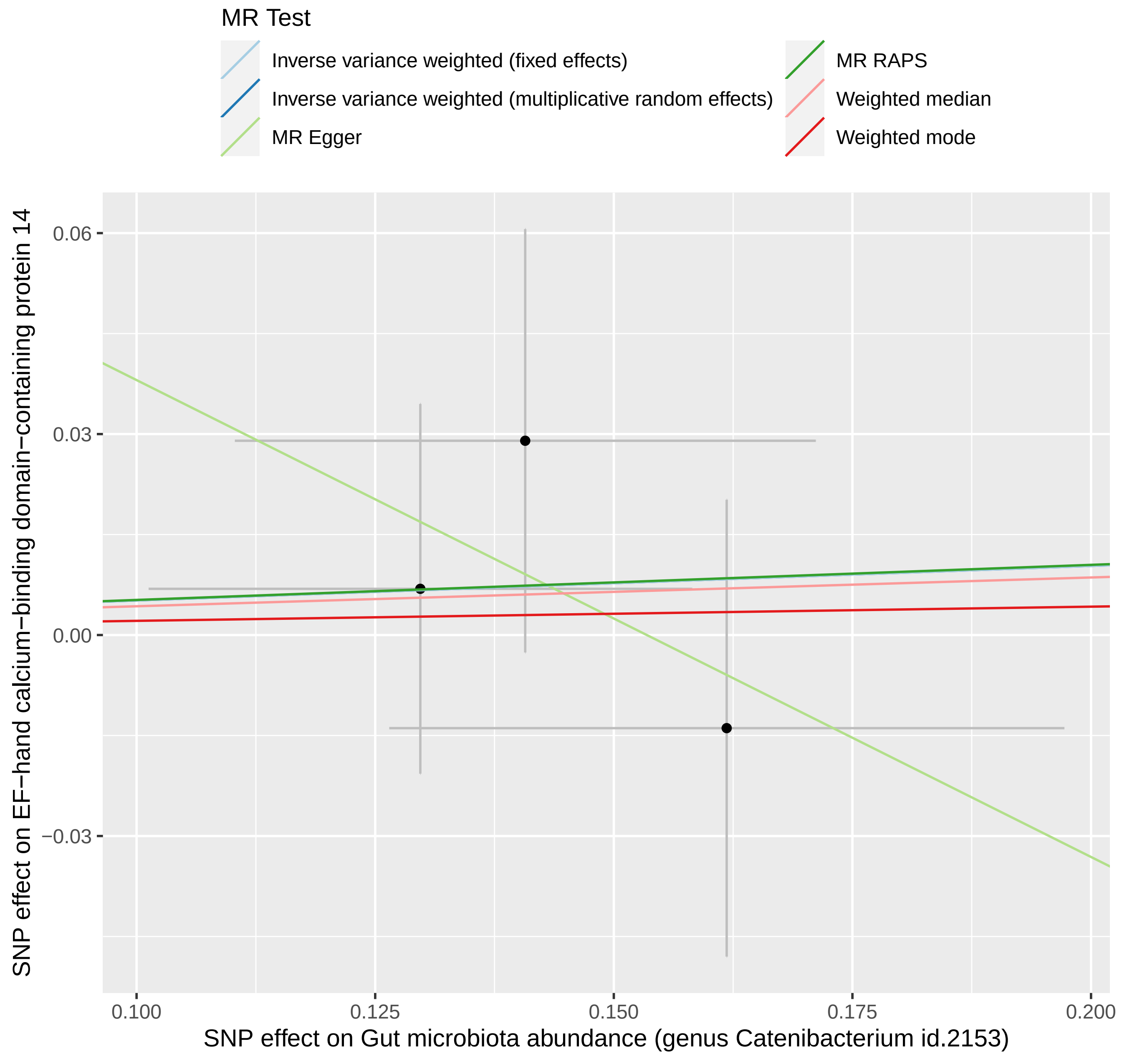

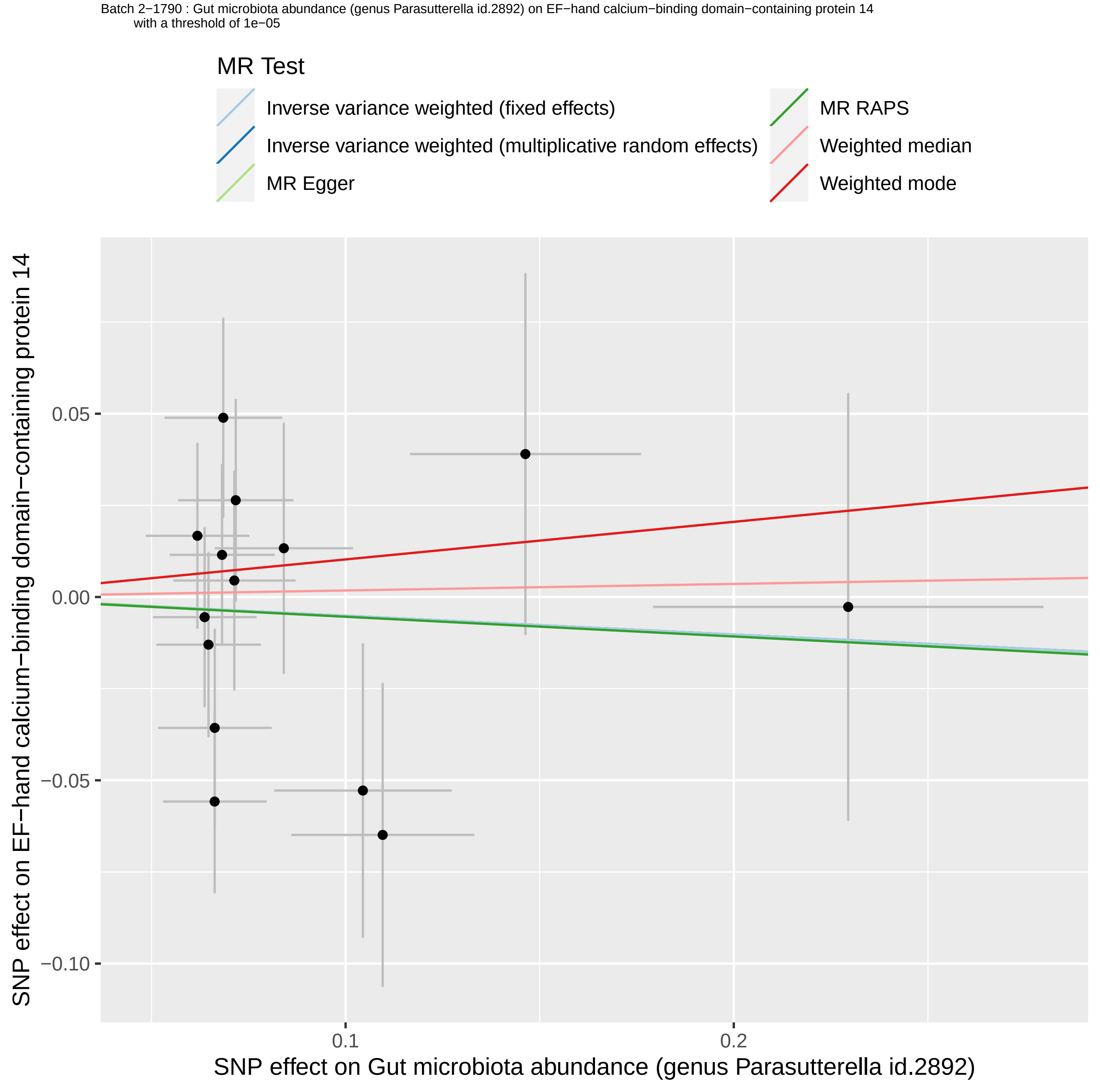
Batch 2−1790 : Gut microbiota abundance (genus Parasutterella id.2892) on EF−hand calcium−binding domain−containing protein 14
with a threshold of 1e−05
MR Test
Inverse variance weighted (fixed effects)
Inverse variance weighted (multiplicative random effects)
MR Egger
MR RAPS
Weighted median
Weighted mode
0.05
0.00
−0.05
−0.10
0.1
0.2
SNP effect on EF−hand calcium−binding domain−containing protein 14
SNP effect on Gut microbiota abundance (genus Parasutterella id.2892)

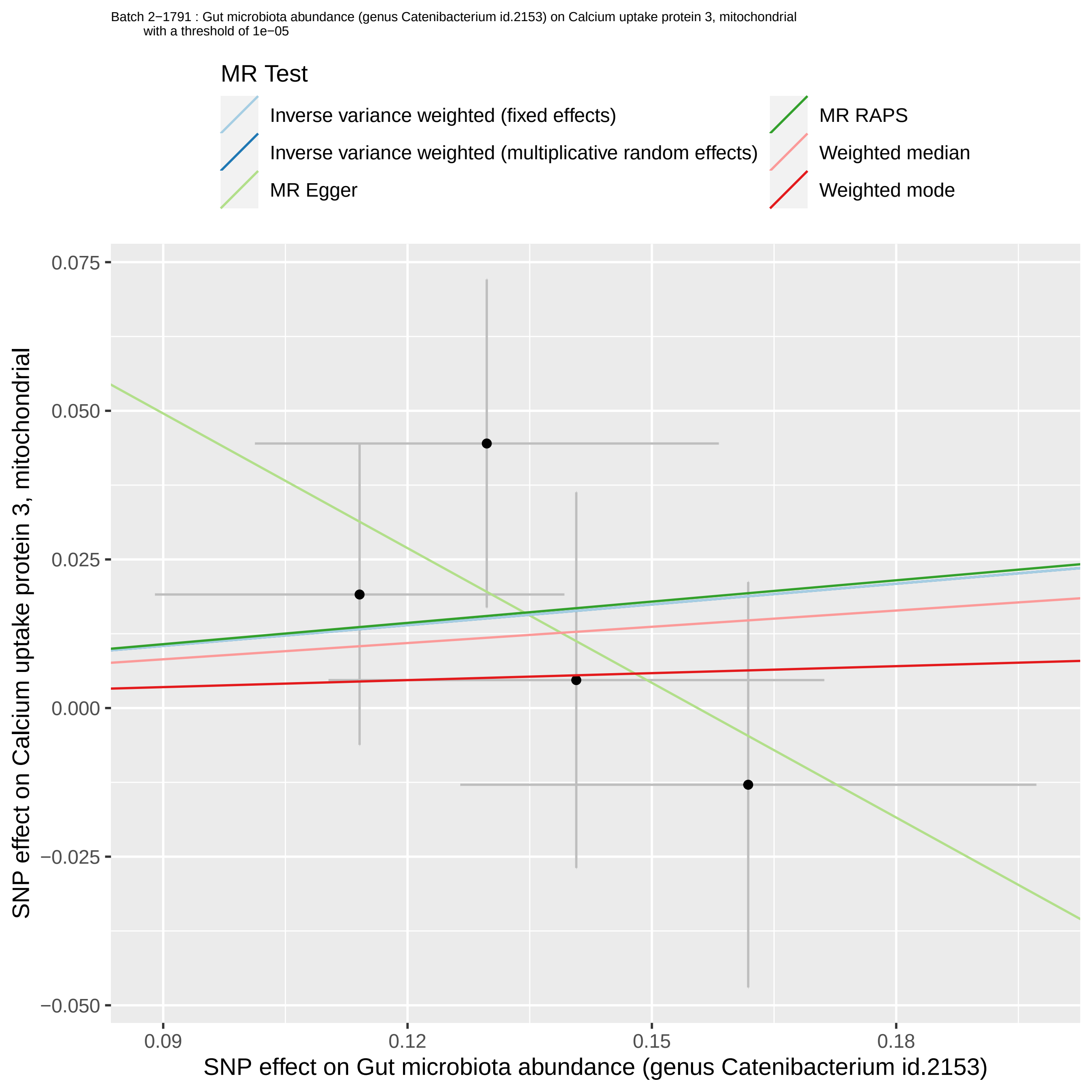
Batch 2−1791 : Gut microbiota abundance (genus Catenibacterium id.2153) on Calcium uptake protein 3, mitochondrial
with a threshold of 1e−05
MR Test
Inverse variance weighted (fixed effects)
Inverse variance weighted (multiplicative random effects)
MR Egger
MR RAPS
Weighted median
Weighted mode
0.075
0.050
0.025
0.000
−0.025
−0.050
0.09
0.12
0.15
0.18
SNP effect on Calcium uptake protein 3, mitochondrial
SNP effect on Gut microbiota abundance (genus Catenibacterium id.2153)

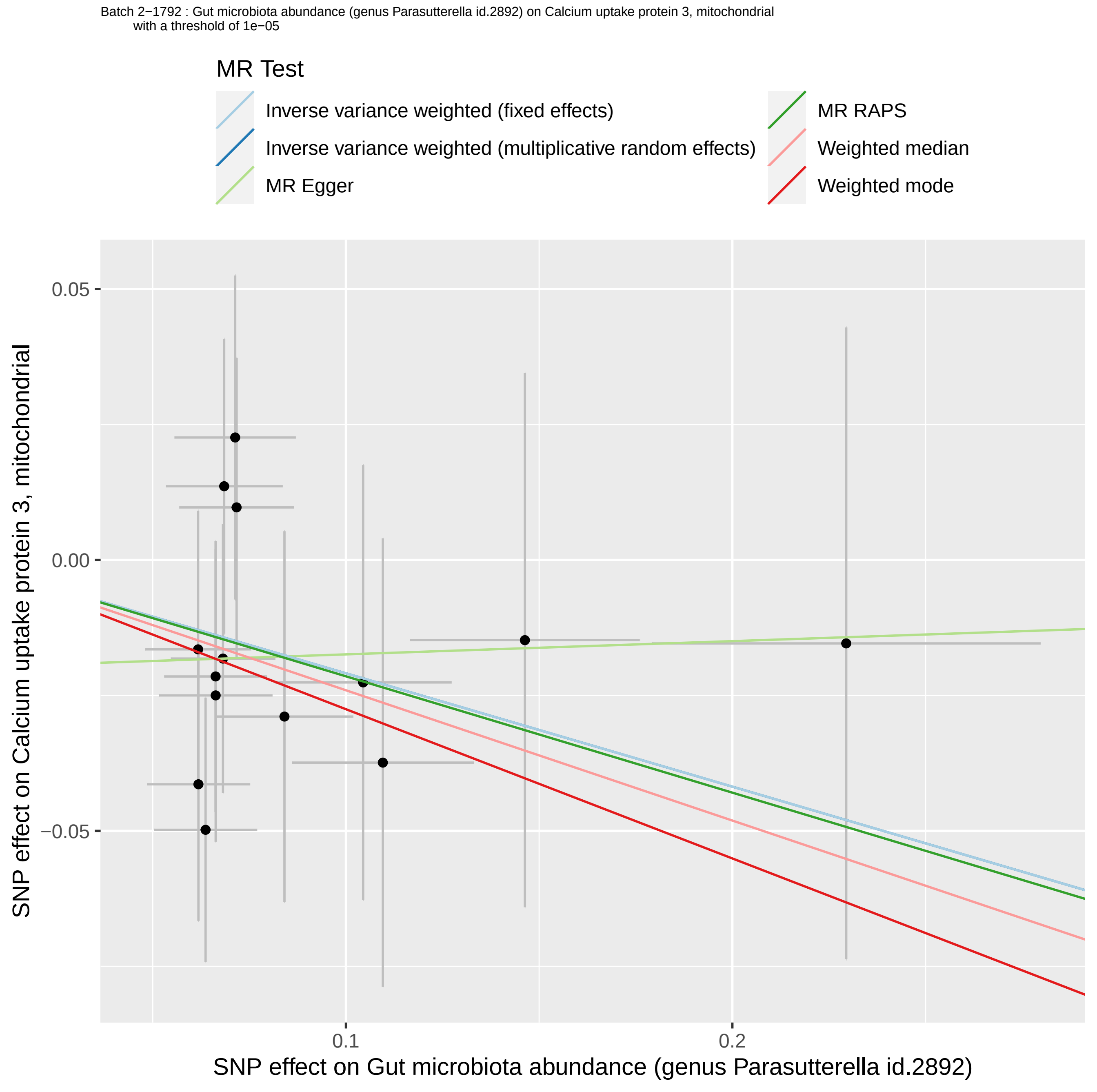

Batch 2−1792 : Gut microbiota abundance (genus Parasutterella id.2892) on Calcium uptake protein 3, mitochondrial
with a threshold of 1e−05
MR Test
Inverse variance weighted (fixed effects)
Inverse variance weighted (multiplicative random effects)
MR Egger
MR RAPS
Weighted median
Weighted mode
0.05
0.00
−0.05
0.1
0.2
SNP effect on Calcium uptake protein 3, mitochondrial
SNP effect on Gut microbiota abundance (genus Parasutterella id.2892)

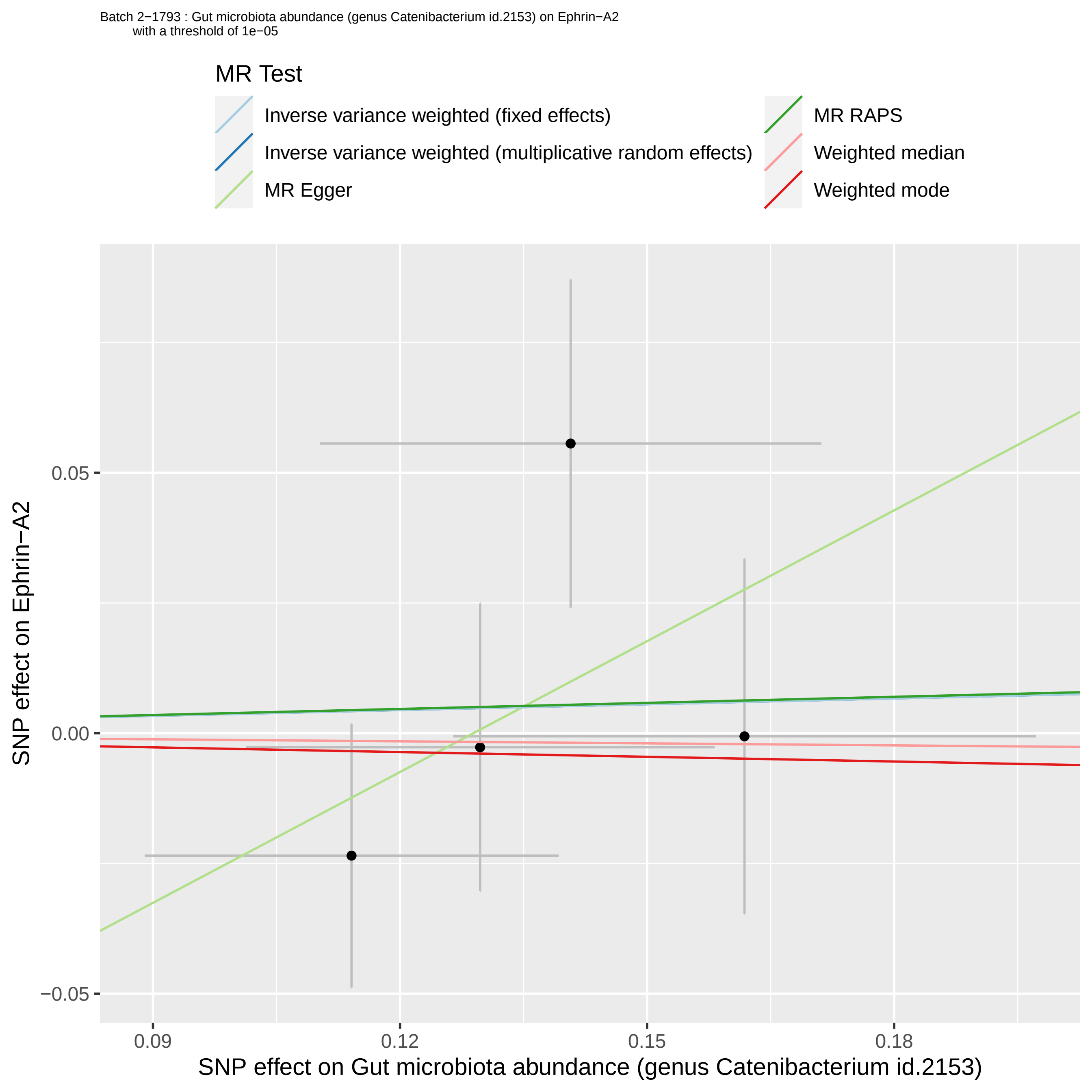
Batch 2−1793 : Gut microbiota abundance (genus Catenibacterium id.2153) on Ephrin−A2
with a threshold of 1e−05
MR Test
Inverse variance weighted (fixed effects)
Inverse variance weighted (multiplicative random effects)
MR Egger
MR RAPS
Weighted median
Weighted mode
SNP effect on Ephrin−A2
0.05
0.00
−0.05
0.09
0.12
0.15
0.18
SNP effect on Gut microbiota abundance (genus Catenibacterium id.2153)

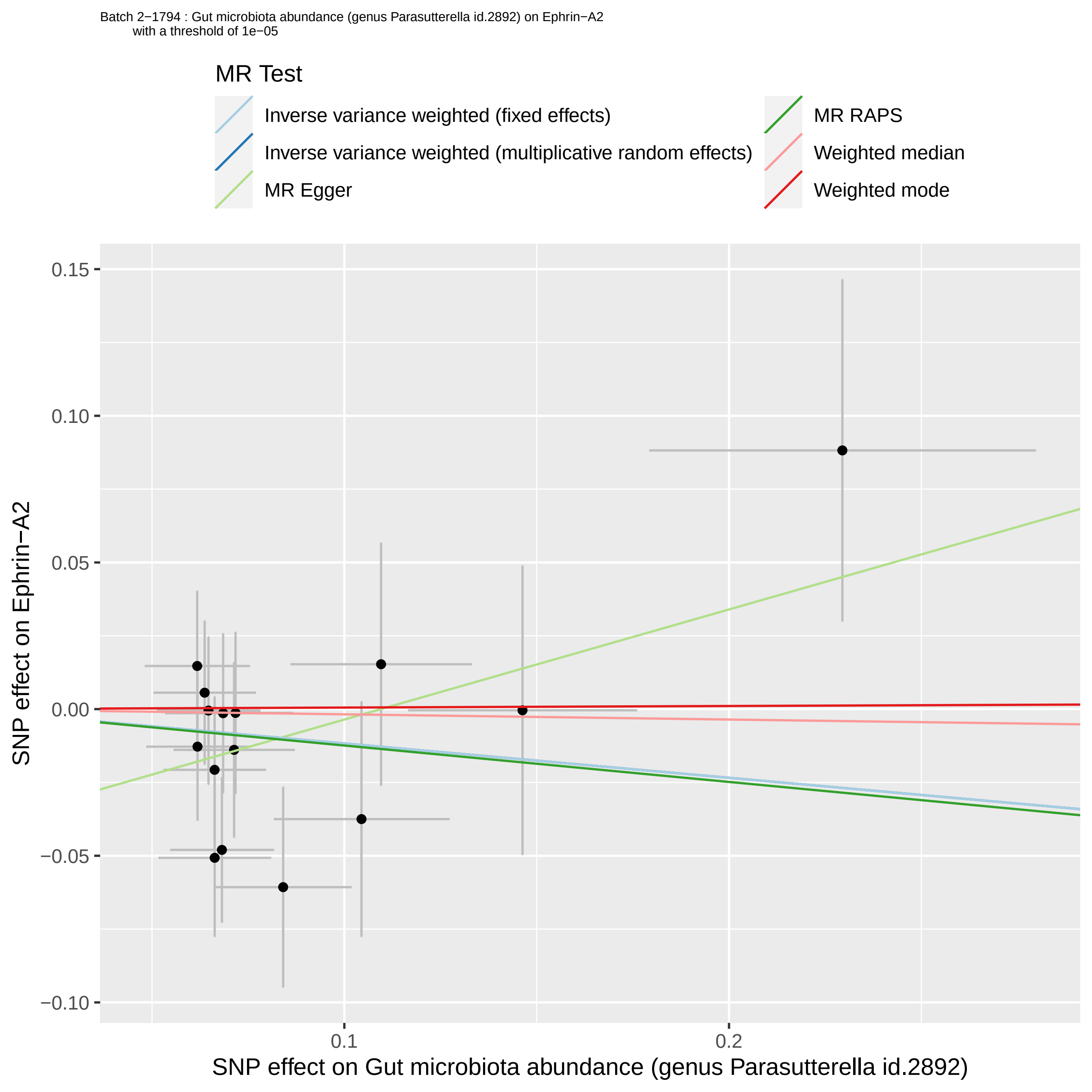

Batch 2−1794 : Gut microbiota abundance (genus Parasutterella id.2892) on Ephrin−A2
with a threshold of 1e−05
MR Test
Inverse variance weighted (fixed effects)
Inverse variance weighted (multiplicative random effects)
MR Egger
MR RAPS
Weighted median
Weighted mode
0.15
0.10
0.05
0.00
−0.05
−0.10
SNP effect on Ephrin−A2
0.1
0.2
SNP effect on Gut microbiota abundance (genus Parasutterella id.2892)

Batch 2−1795 : Gut microbiota abundance (genus Catenibacterium id.2153) on Ephrin−A3
with a threshold of 1e−05

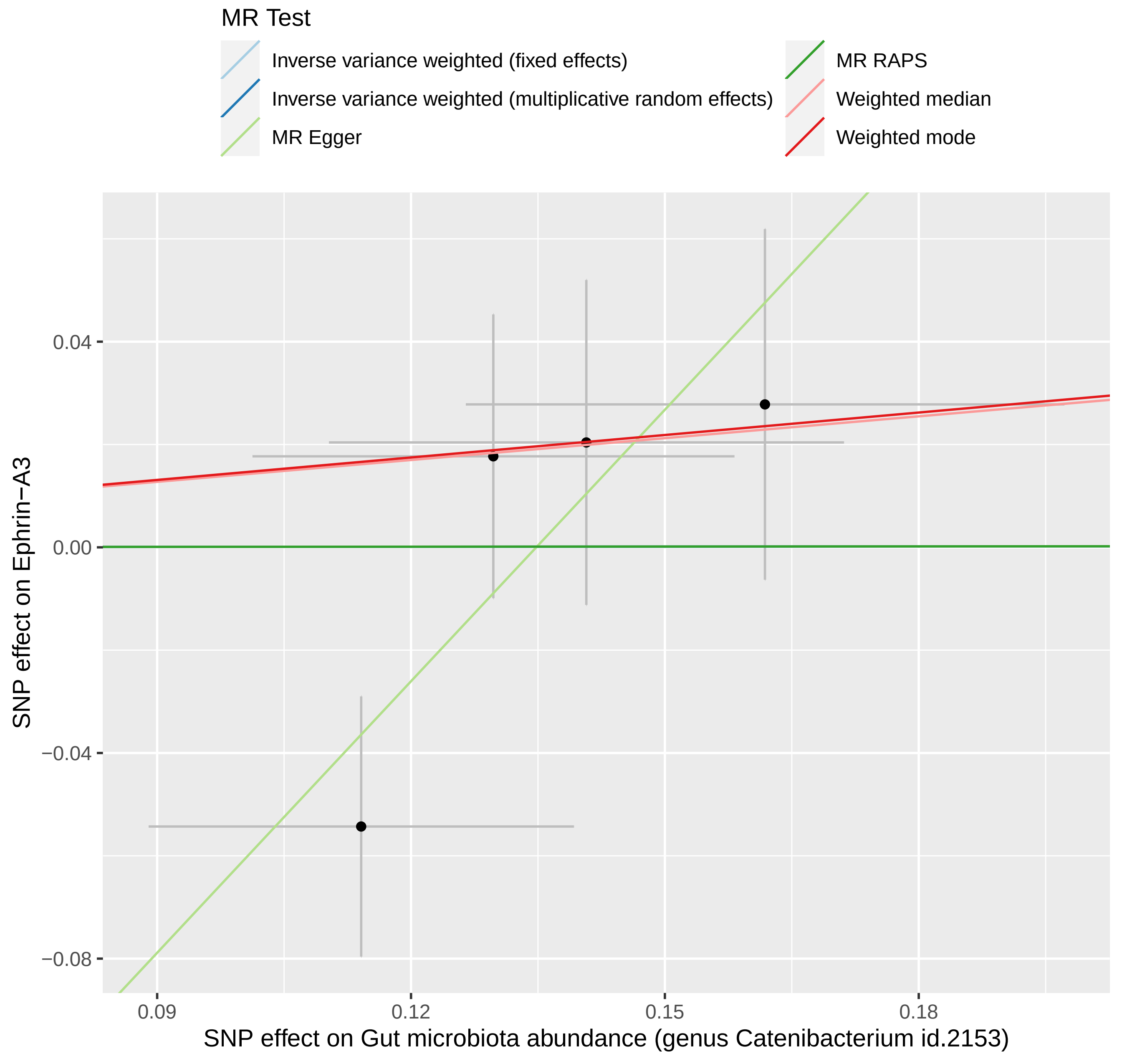

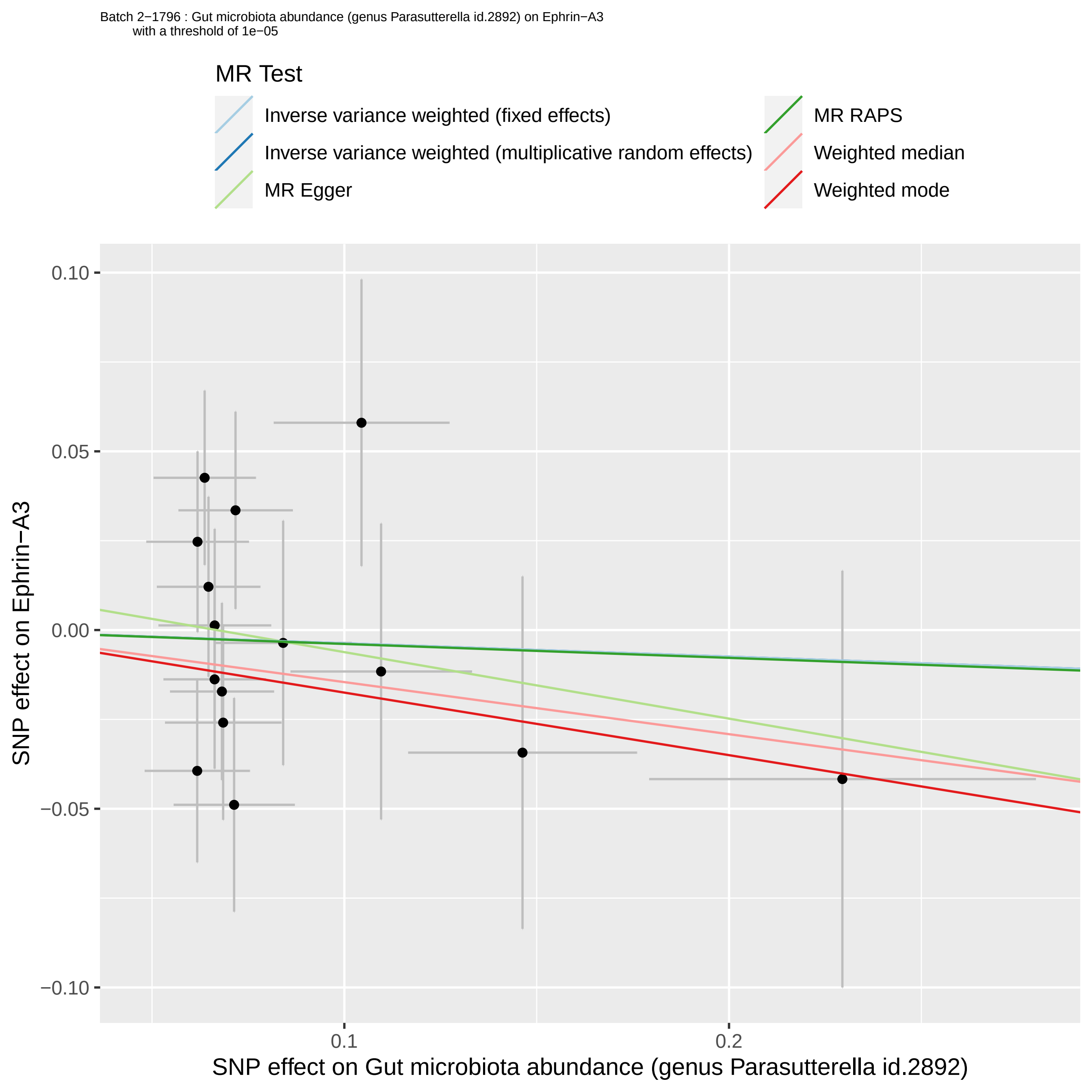

Batch 2−1796 : Gut microbiota abundance (genus Parasutterella id.2892) on Ephrin−A3
with a threshold of 1e−05
MR Test
Inverse variance weighted (fixed effects)
Inverse variance weighted (multiplicative random effects)
MR Egger
MR RAPS
Weighted median
Weighted mode
0.10
0.05
0.00
−0.05
−0.10
0.1
0.2
SNP effect on Ephrin−A3
SNP effect on Gut microbiota abundance (genus Parasutterella id.2892)

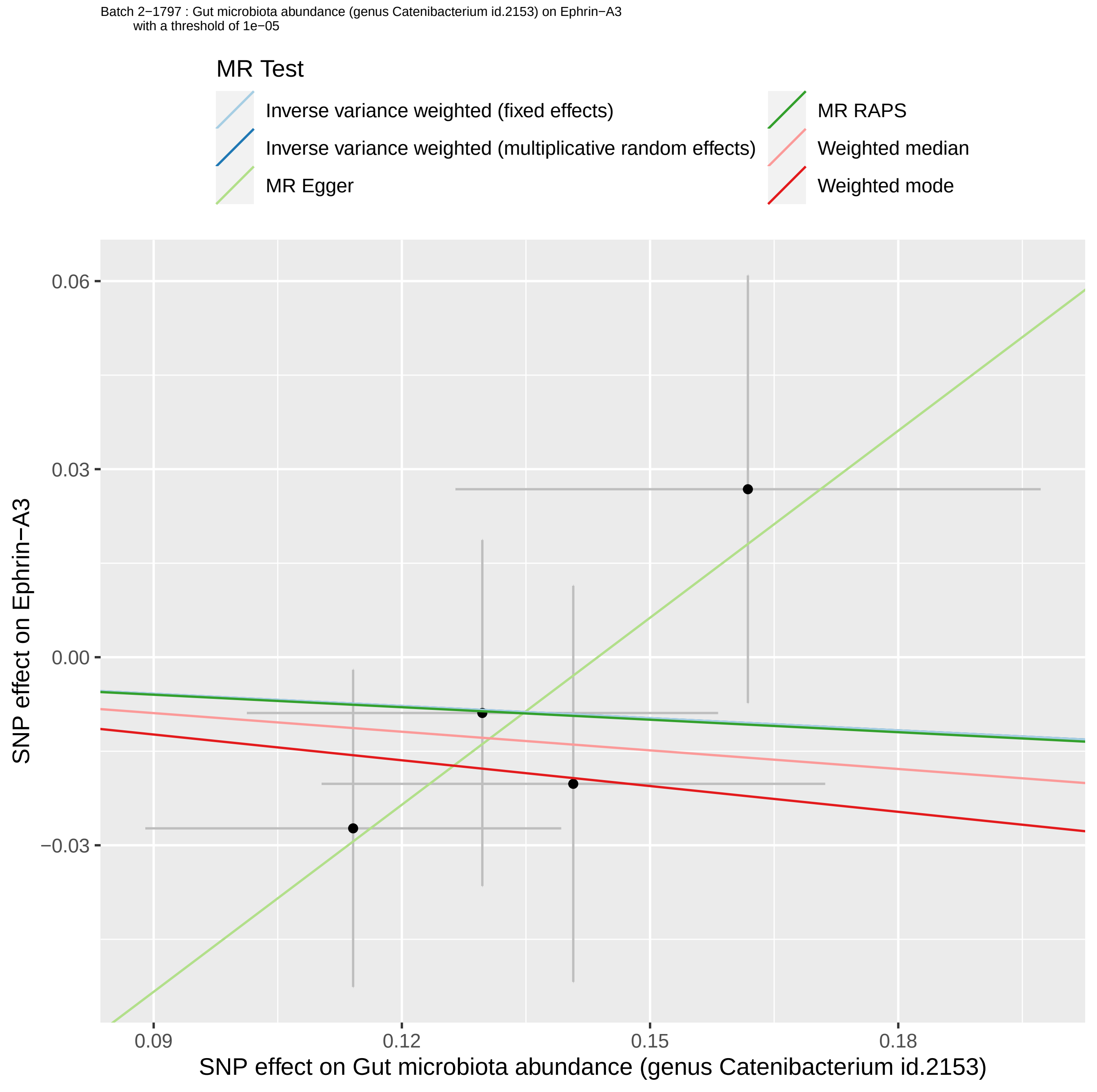
Batch 2−1797 : Gut microbiota abundance (genus Catenibacterium id.2153) on Ephrin−A3
with a threshold of 1e−05
MR Test
Inverse variance weighted (fixed effects)
Inverse variance weighted (multiplicative random effects)
MR Egger
MR RAPS
Weighted median
Weighted mode
0.06
0.03
0.00
−0.03
0.09
0.12
0.15
0.18
SNP effect on Ephrin−A3
SNP effect on Gut microbiota abundance (genus Catenibacterium id.2153)

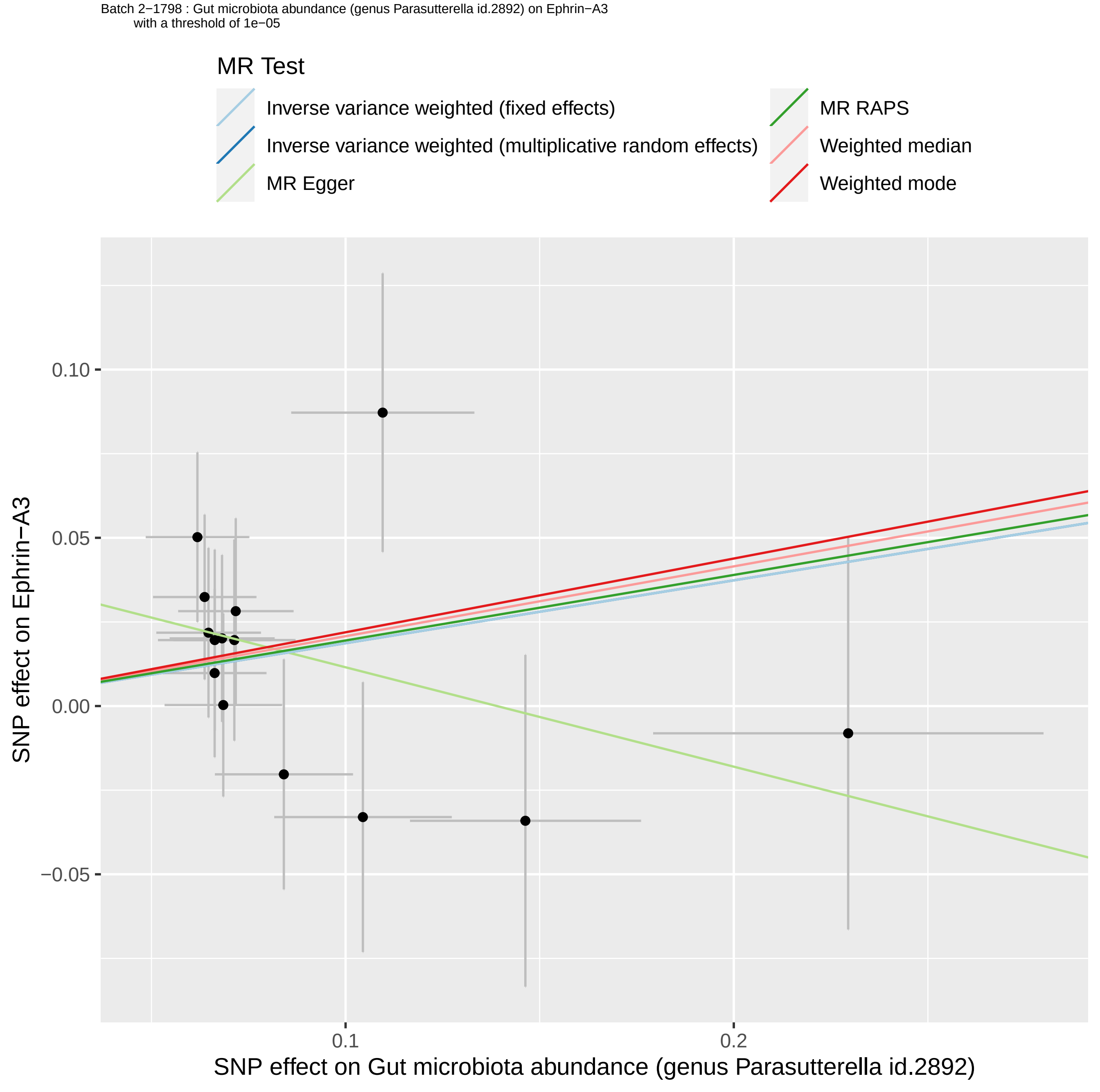

Batch 2−1798 : Gut microbiota abundance (genus Parasutterella id.2892) on Ephrin−A3
with a threshold of 1e−05
MR Test
Inverse variance weighted (fixed effects)
Inverse variance weighted (multiplicative random effects)
MR Egger
MR RAPS
Weighted median
Weighted mode
0.10
0.05
0.00
−0.05
0.1
0.2
SNP effect on Ephrin−A3
SNP effect on Gut microbiota abundance (genus Parasutterella id.2892)

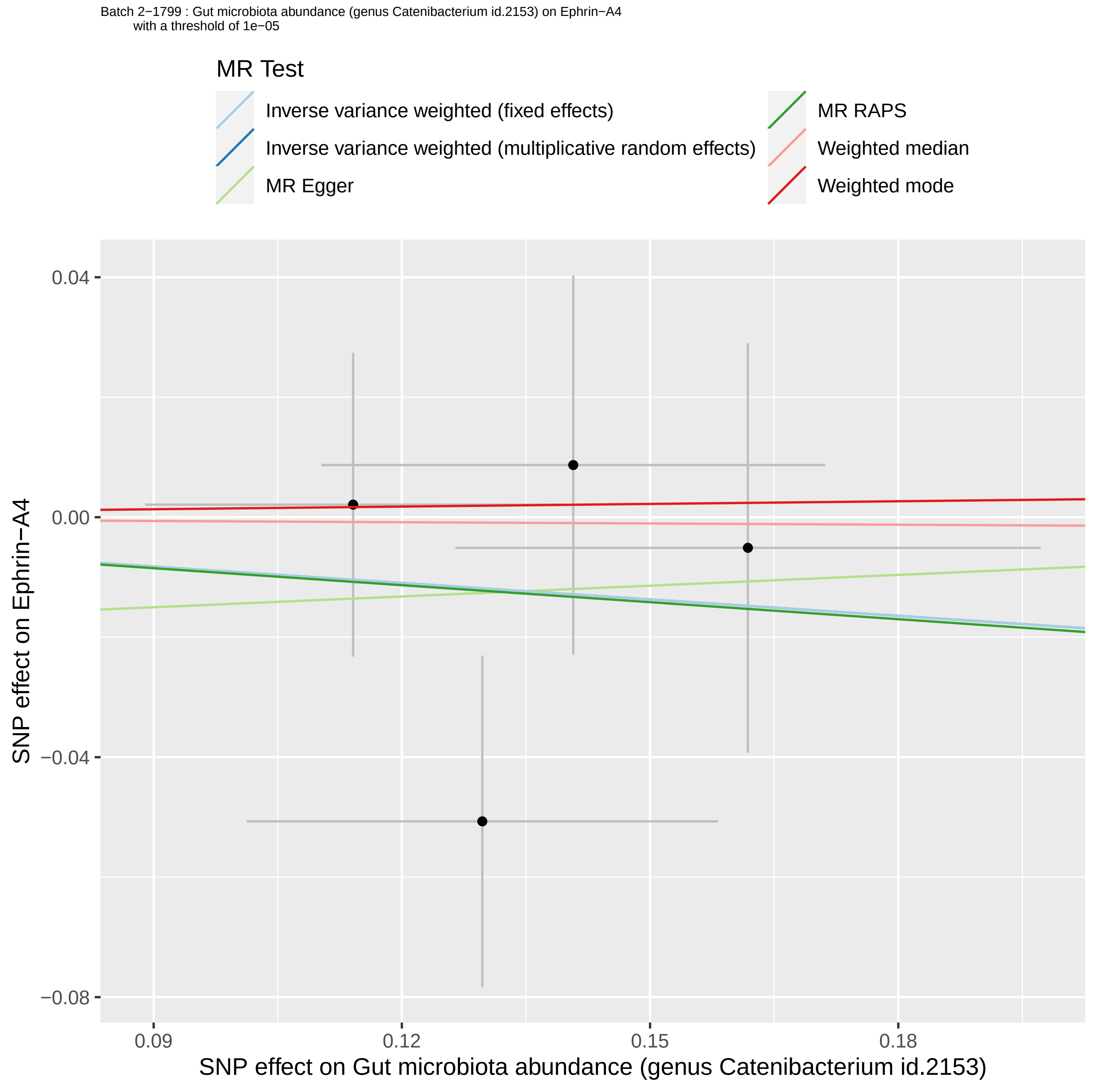
Batch 2−1799 : Gut microbiota abundance (genus Catenibacterium id.2153) on Ephrin−A4
with a threshold of 1e−05
MR Test
Inverse variance weighted (fixed effects)
Inverse variance weighted (multiplicative random effects)
MR Egger
MR RAPS
Weighted median
Weighted mode
0.04
0.00
−0.04
−0.08
0.09
0.12
0.15
0.18
SNP effect on Ephrin−A4
SNP effect on Gut microbiota abundance (genus Catenibacterium id.2153)

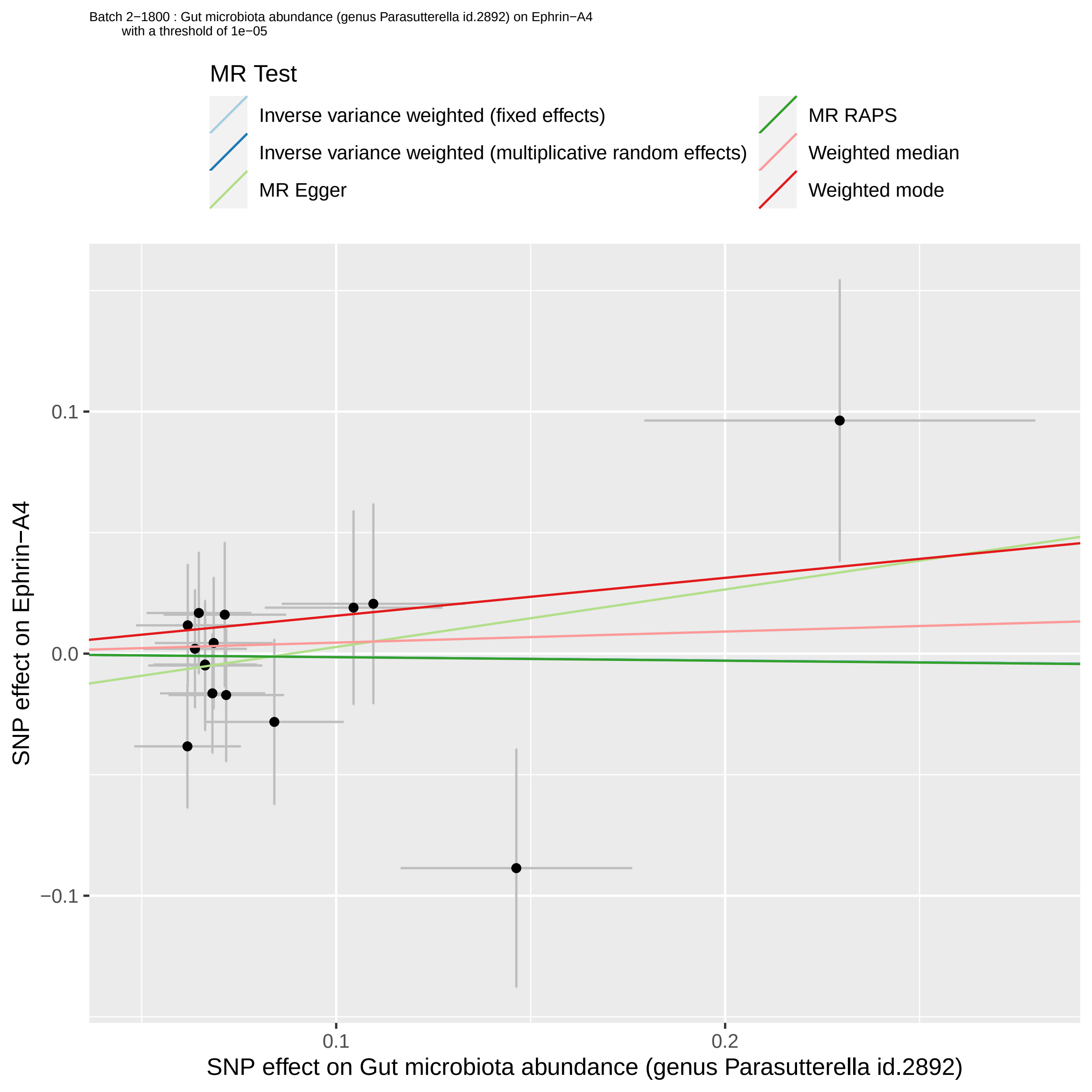
Batch 2−1800 : Gut microbiota abundance (genus Parasutterella id.2892) on Ephrin−A4
with a threshold of 1e−05
MR Test
Inverse variance weighted (fixed effects)
Inverse variance weighted (multiplicative random effects)
MR Egger
MR RAPS
Weighted median
Weighted mode
0.1
0.0
−0.1
0.1
0.2
SNP effect on Ephrin−A4
SNP effect on Gut microbiota abundance (genus Parasutterella id.2892)

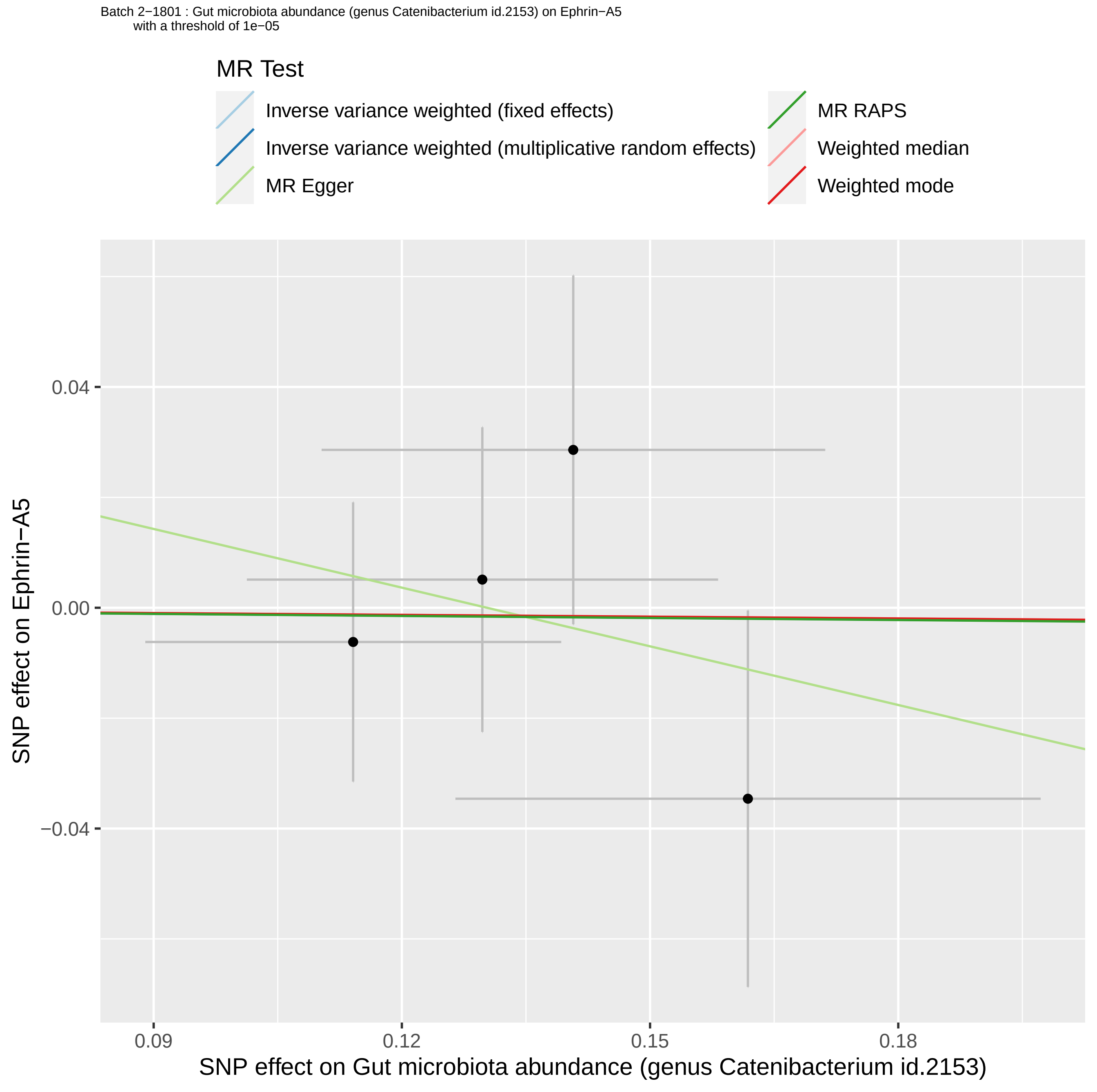
Batch 2−1801 : Gut microbiota abundance (genus Catenibacterium id.2153) on Ephrin−A5
with a threshold of 1e−05
MR Test
Inverse variance weighted (fixed effects)
Inverse variance weighted (multiplicative random effects)
MR Egger
MR RAPS
Weighted median
Weighted mode
0.04
0.00
−0.04
SNP effect on Ephrin−A5
0.09
0.12
0.15
0.18
SNP effect on Gut microbiota abundance (genus Catenibacterium id.2153)

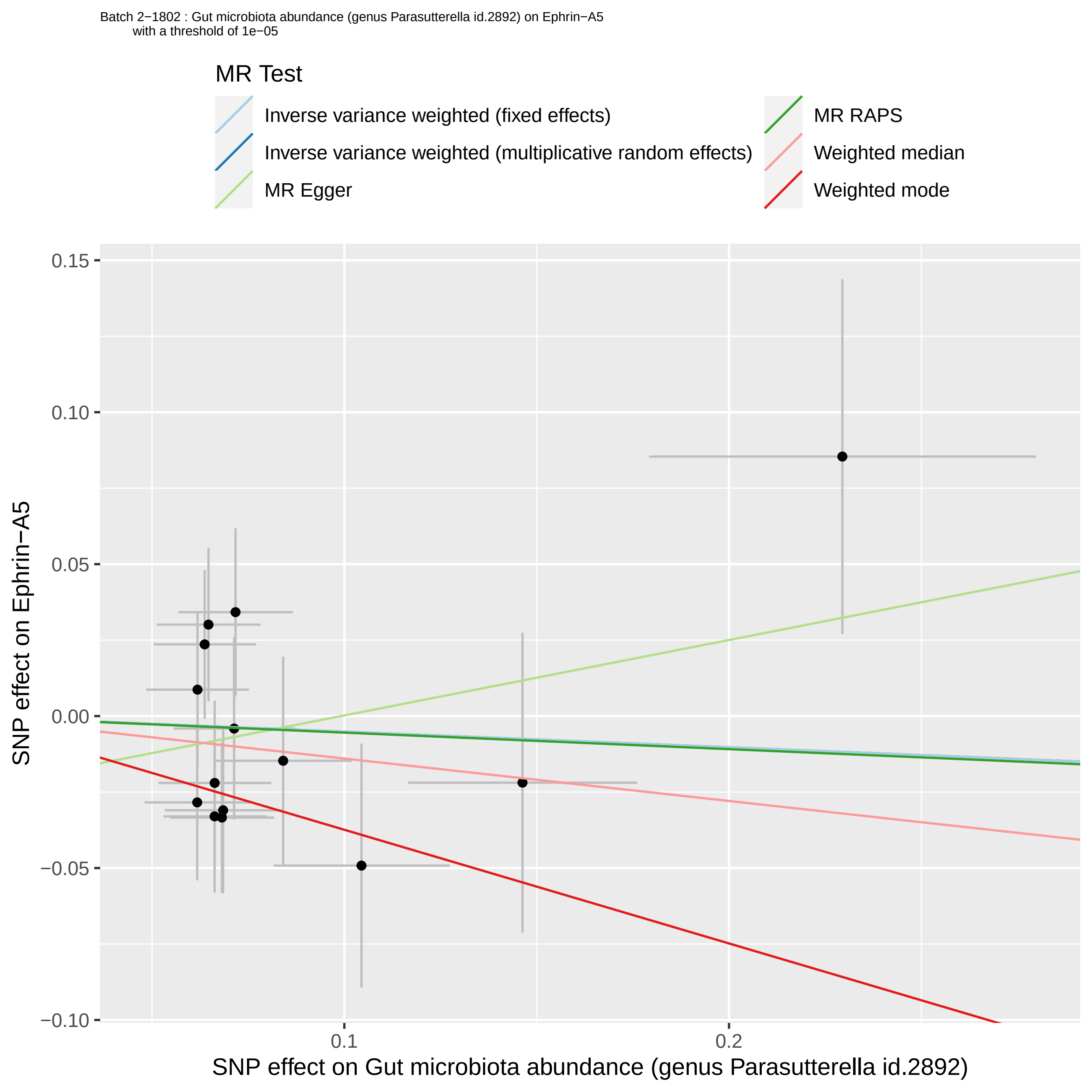
Batch 2−1802 : Gut microbiota abundance (genus Parasutterella id.2892) on Ephrin−A5
with a threshold of 1e−05
MR Test
Inverse variance weighted (fixed effects)
Inverse variance weighted (multiplicative random effects)
MR Egger
MR RAPS
Weighted median
Weighted mode
0.15
0.10
0.05
0.00
−0.05
−0.10
0.1
0.2
SNP effect on Ephrin−A5
SNP effect on Gut microbiota abundance (genus Parasutterella id.2892)

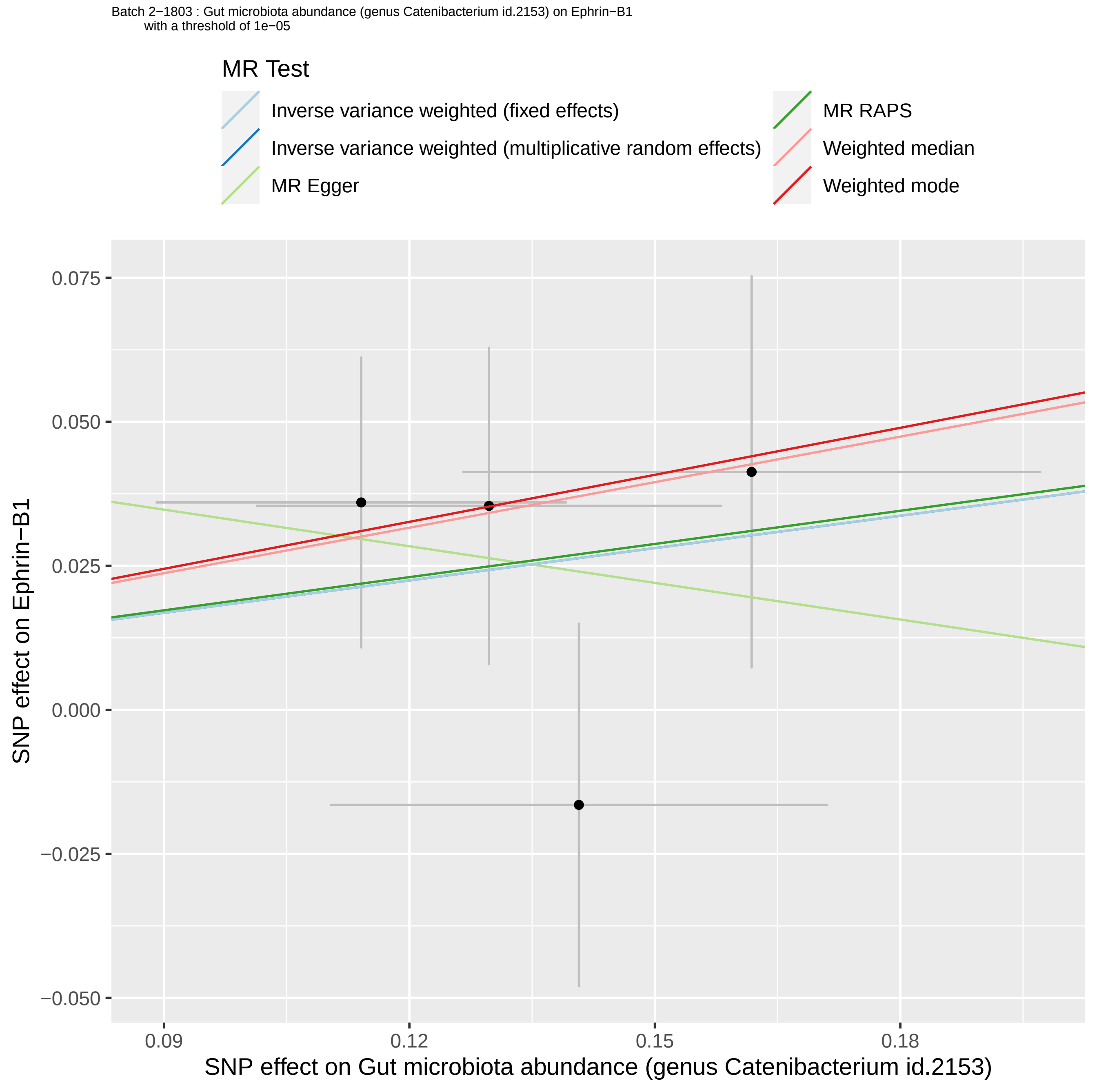

Batch 2−1803 : Gut microbiota abundance (genus Catenibacterium id.2153) on Ephrin−B1
with a threshold of 1e−05
MR Test
Inverse variance weighted (fixed effects)
Inverse variance weighted (multiplicative random effects)
MR Egger
MR RAPS
Weighted median
Weighted mode
0.075
0.050
0.025
0.000
−0.025
−0.050
0.09
0.12
0.15
0.18
SNP effect on Ephrin−B1
SNP effect on Gut microbiota abundance (genus Catenibacterium id.2153)

Batch 2−1804 : Gut microbiota abundance (genus Parasutterella id.2892) on Ephrin−B1
with a threshold of 1e−05

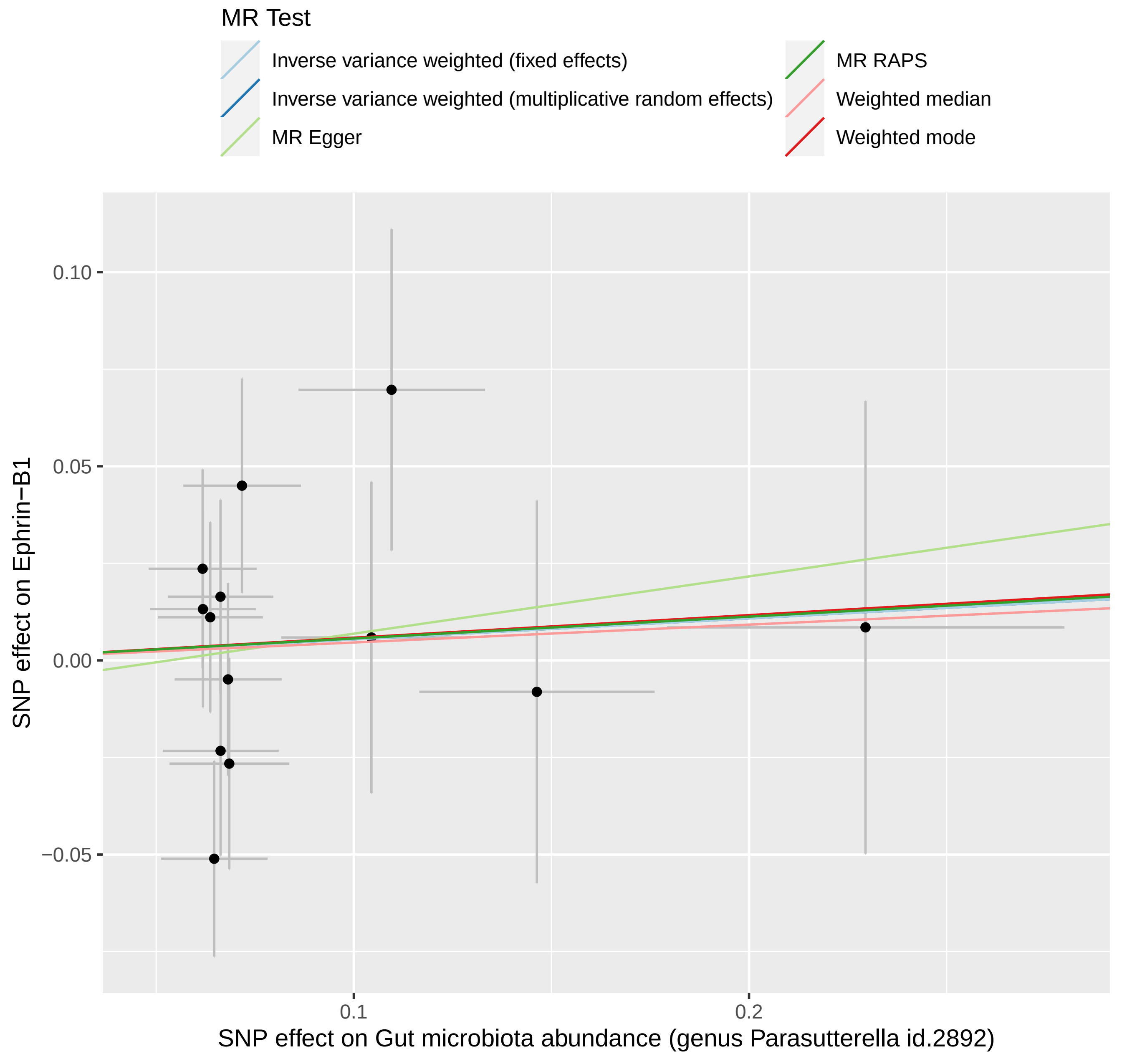

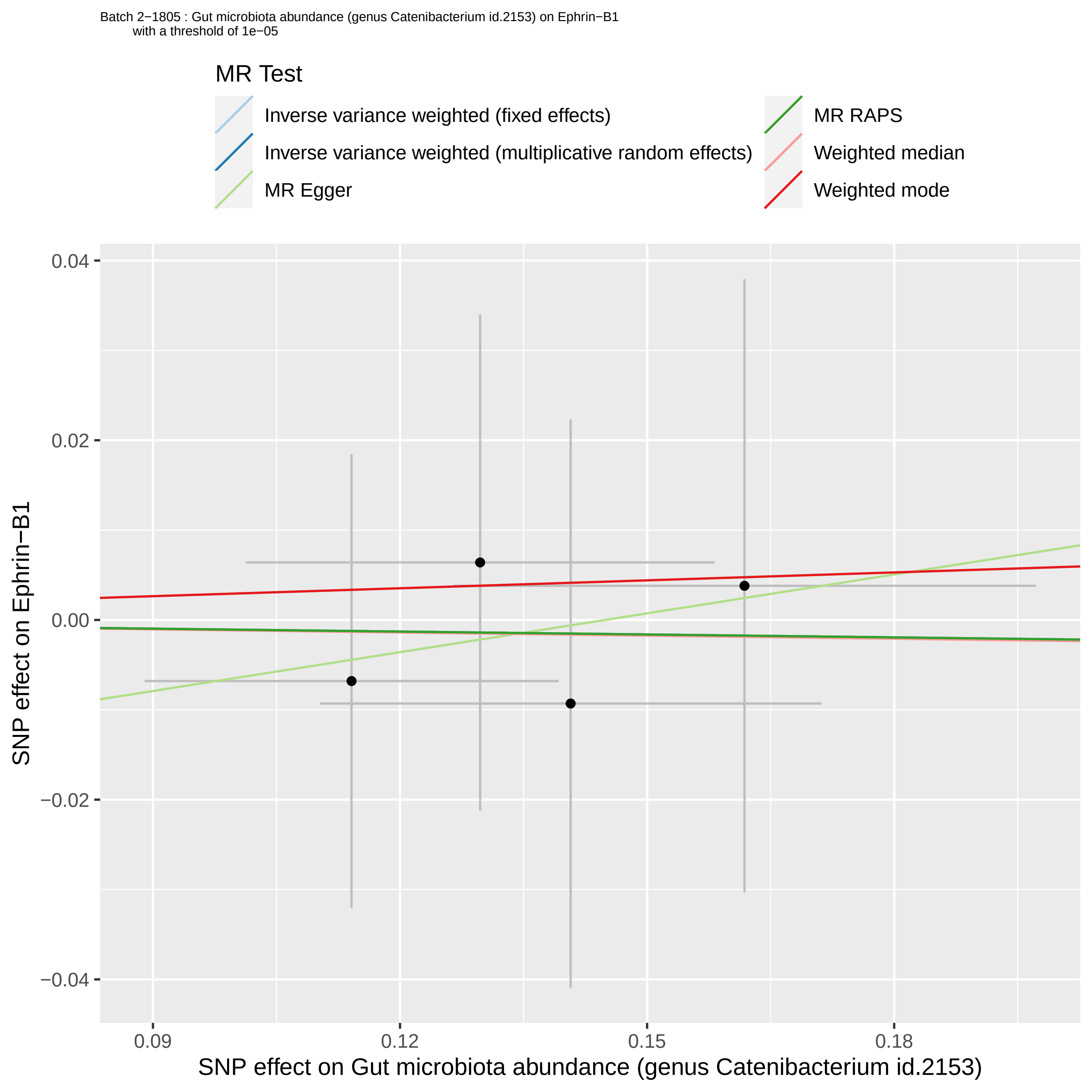

Batch 2−1805 : Gut microbiota abundance (genus Catenibacterium id.2153) on Ephrin−B1
with a threshold of 1e−05
MR Test
Inverse variance weighted (fixed effects)
Inverse variance weighted (multiplicative random effects)
MR Egger
MR RAPS
Weighted median
Weighted mode
0.04
0.02
0.00
−0.02
−0.04
SNP effect on Ephrin−B1
0.09
0.12
0.15
0.18
SNP effect on Gut microbiota abundance (genus Catenibacterium id.2153)

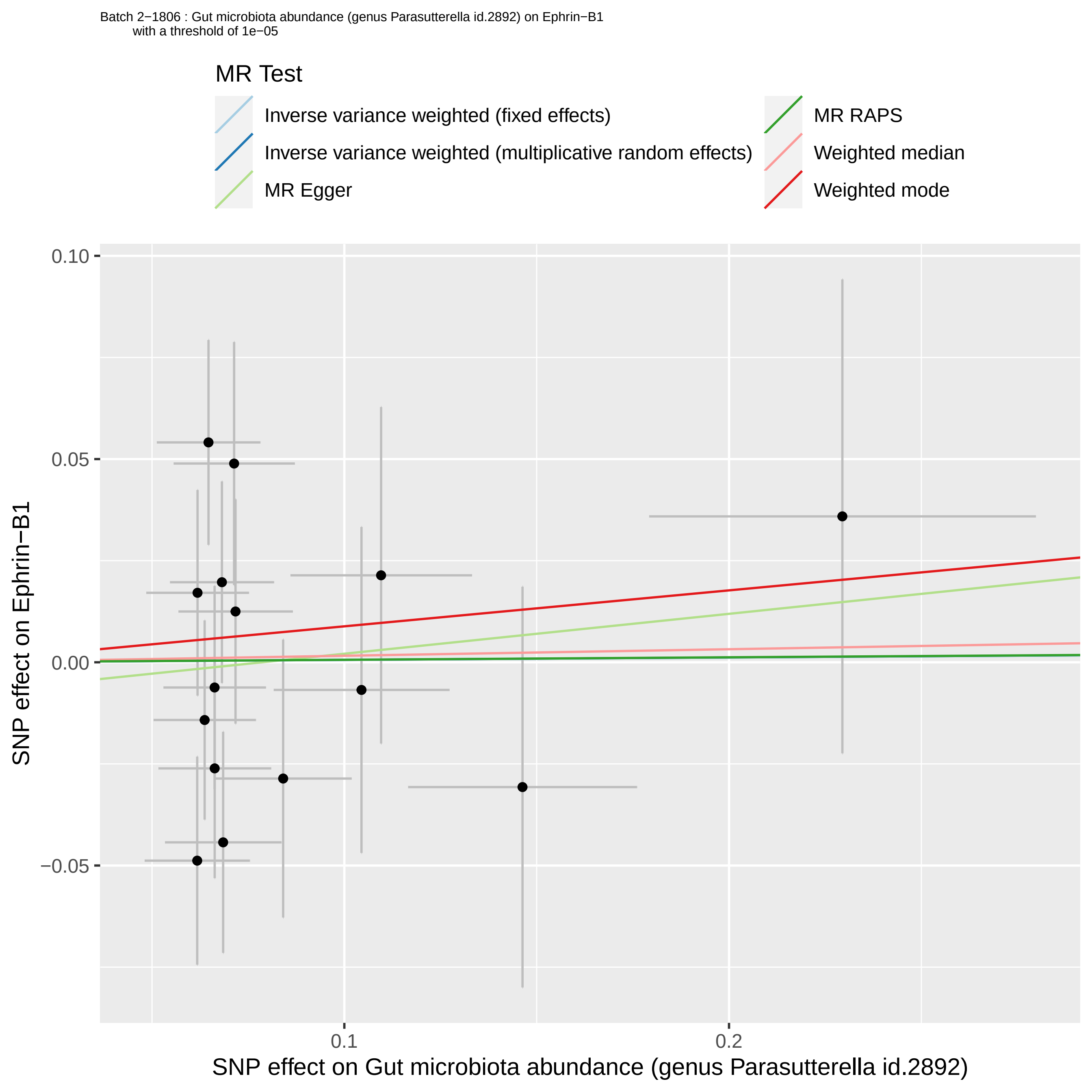
Batch 2−1806 : Gut microbiota abundance (genus Parasutterella id.2892) on Ephrin−B1
with a threshold of 1e−05
MR Test
Inverse variance weighted (fixed effects)
Inverse variance weighted (multiplicative random effects)
MR Egger
MR RAPS
Weighted median
Weighted mode
0.10
0.05
0.00
−0.05
0.1
0.2
SNP effect on Ephrin−B1
SNP effect on Gut microbiota abundance (genus Parasutterella id.2892)

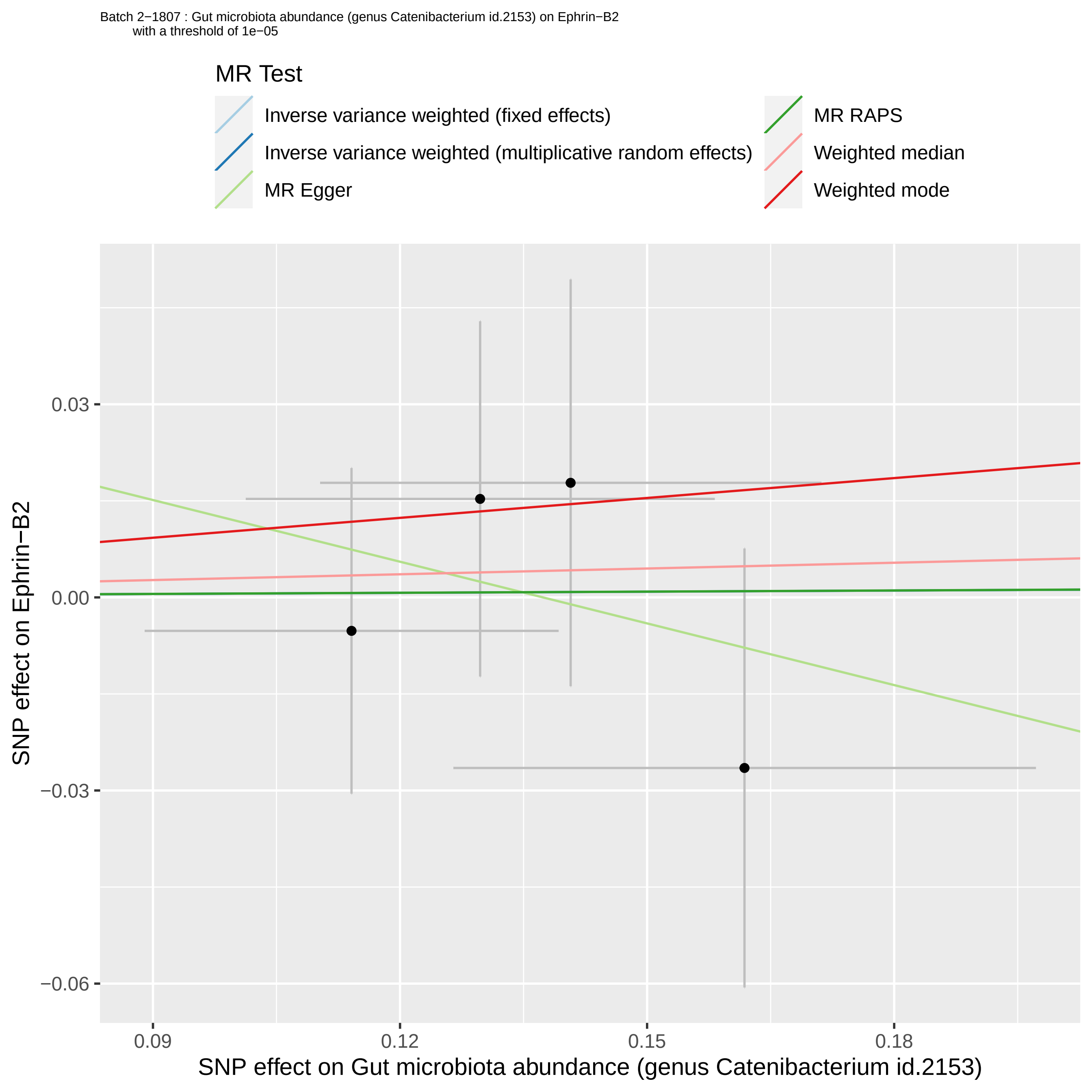
Batch 2−1807 : Gut microbiota abundance (genus Catenibacterium id.2153) on Ephrin−B2
with a threshold of 1e−05
MR Test
Inverse variance weighted (fixed effects)
Inverse variance weighted (multiplicative random effects)
MR Egger
MR RAPS
Weighted median
Weighted mode
SNP effect on Ephrin−B2
0.03
0.00
−0.03
−0.06
0.09
0.12
0.15
0.18
SNP effect on Gut microbiota abundance (genus Catenibacterium id.2153)

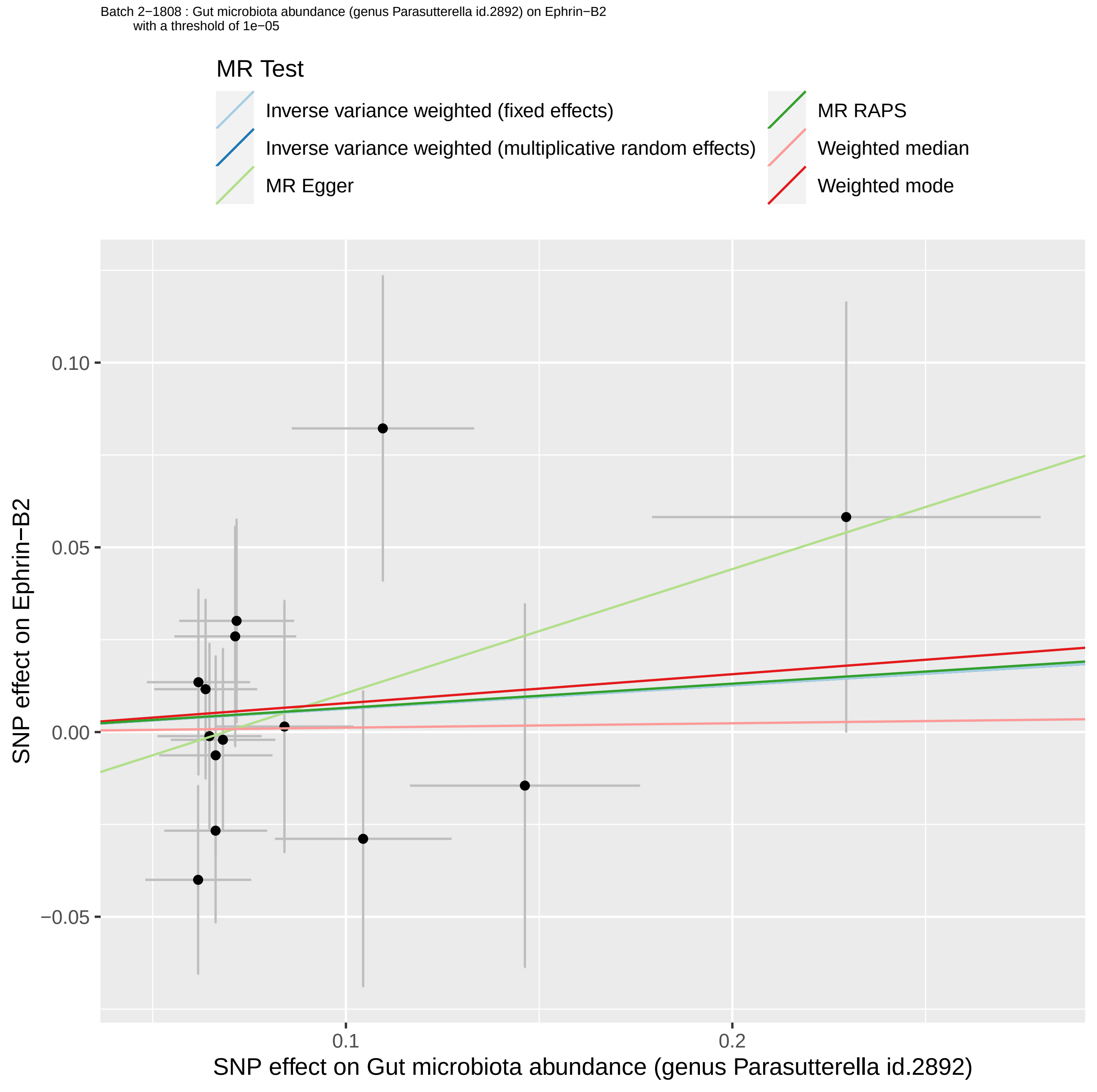
Batch 2−1808 : Gut microbiota abundance (genus Parasutterella id.2892) on Ephrin−B2
with a threshold of 1e−05
MR Test
Inverse variance weighted (fixed effects)
Inverse variance weighted (multiplicative random effects)
MR Egger
MR RAPS
Weighted median
Weighted mode
0.10
0.05
0.00
−0.05
0.1
0.2
SNP effect on Ephrin−B2
SNP effect on Gut microbiota abundance (genus Parasutterella id.2892)

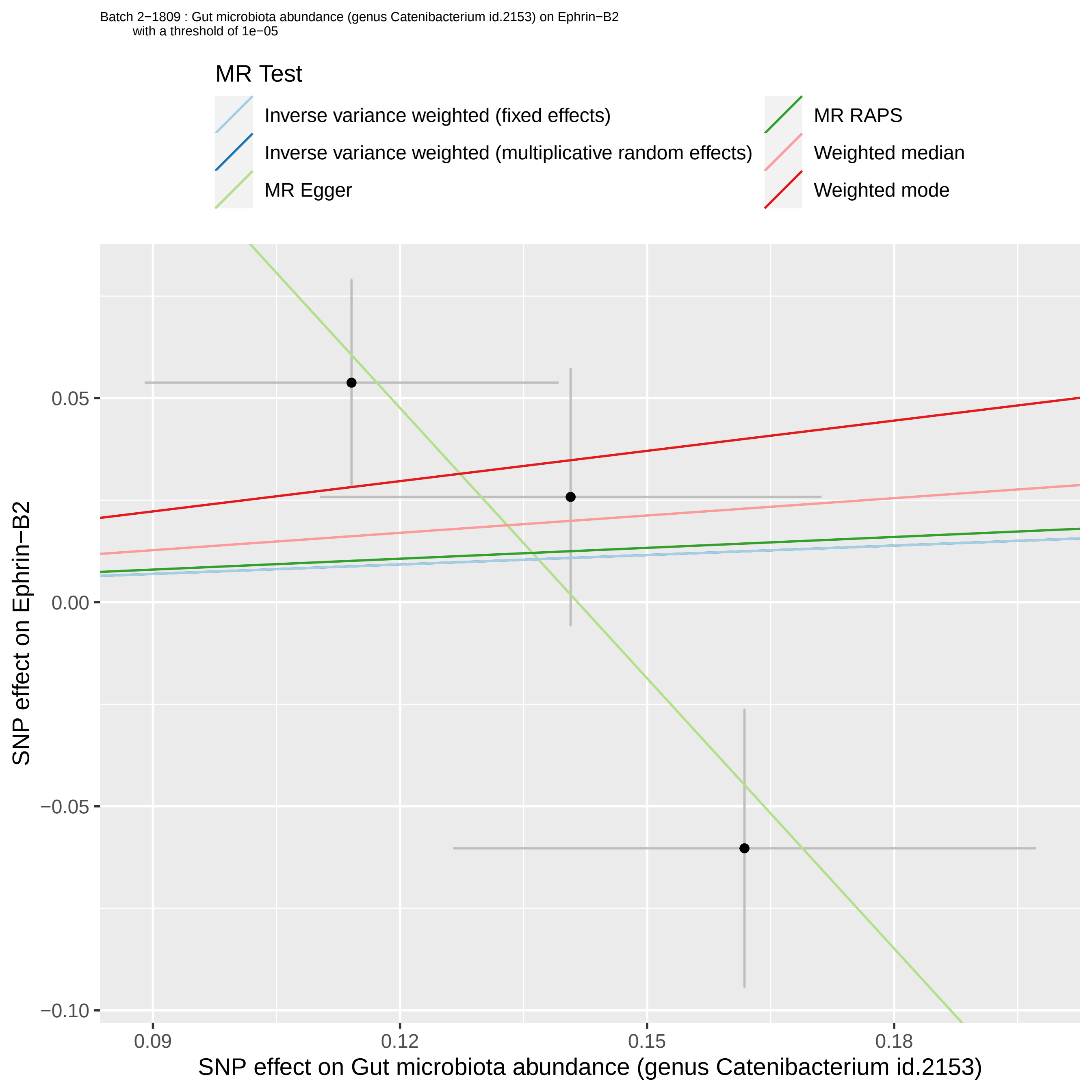
Batch 2−1809 : Gut microbiota abundance (genus Catenibacterium id.2153) on Ephrin−B2
with a threshold of 1e−05
MR Test
Inverse variance weighted (fixed effects)
Inverse variance weighted (multiplicative random effects)
MR Egger
MR RAPS
Weighted median
Weighted mode
SNP effect on Ephrin−B2
0.05
0.00
−0.05
−0.10
0.09
0.12
0.15
0.18
SNP effect on Gut microbiota abundance (genus Catenibacterium id.2153)

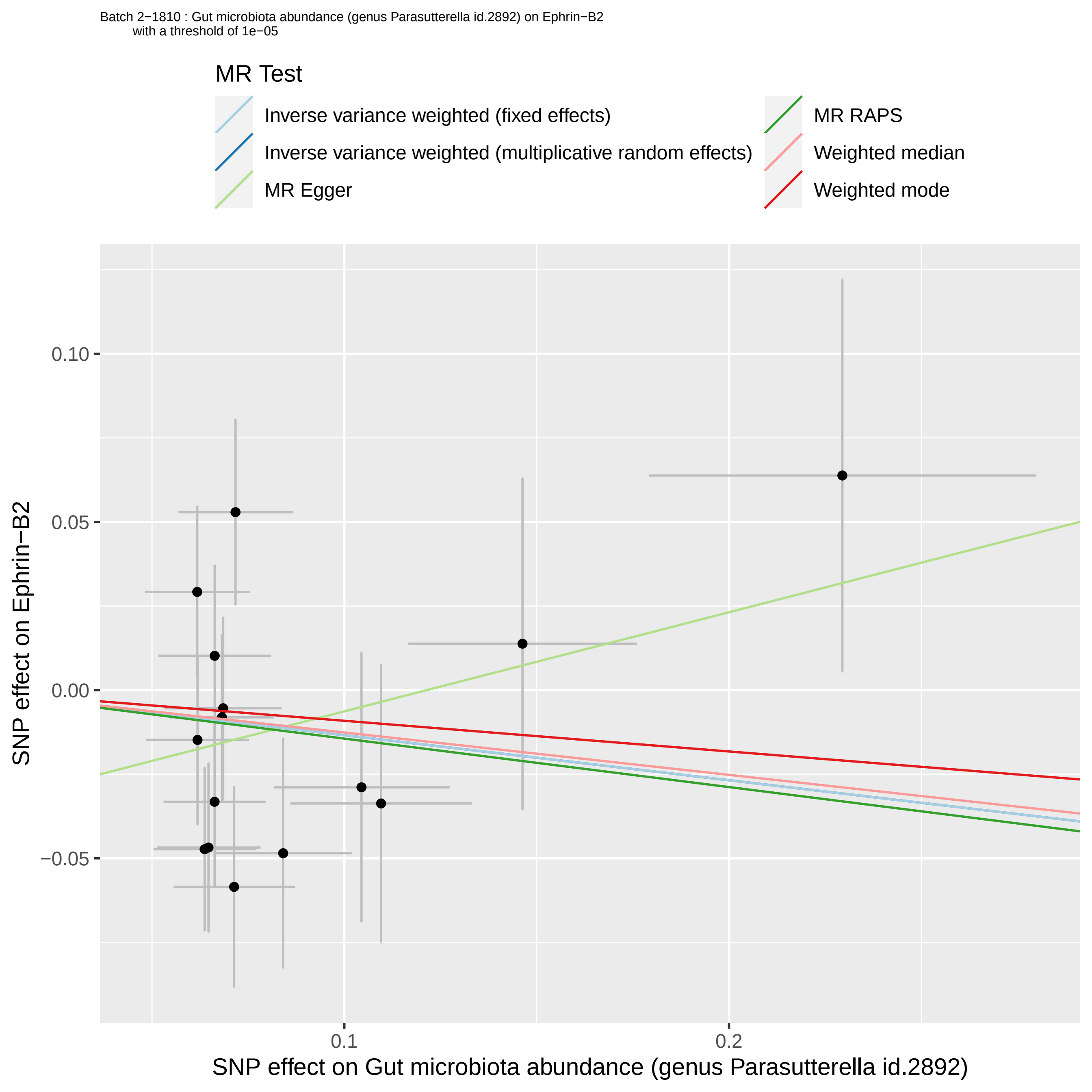
Batch 2−1810 : Gut microbiota abundance (genus Parasutterella id.2892) on Ephrin−B2
with a threshold of 1e−05
MR Test
Inverse variance weighted (fixed effects)
Inverse variance weighted (multiplicative random effects)
MR Egger
MR RAPS
Weighted median
Weighted mode
0.10
0.05
0.00
−0.05
0.1
0.2
SNP effect on Ephrin−B2
SNP effect on Gut microbiota abundance (genus Parasutterella id.2892)

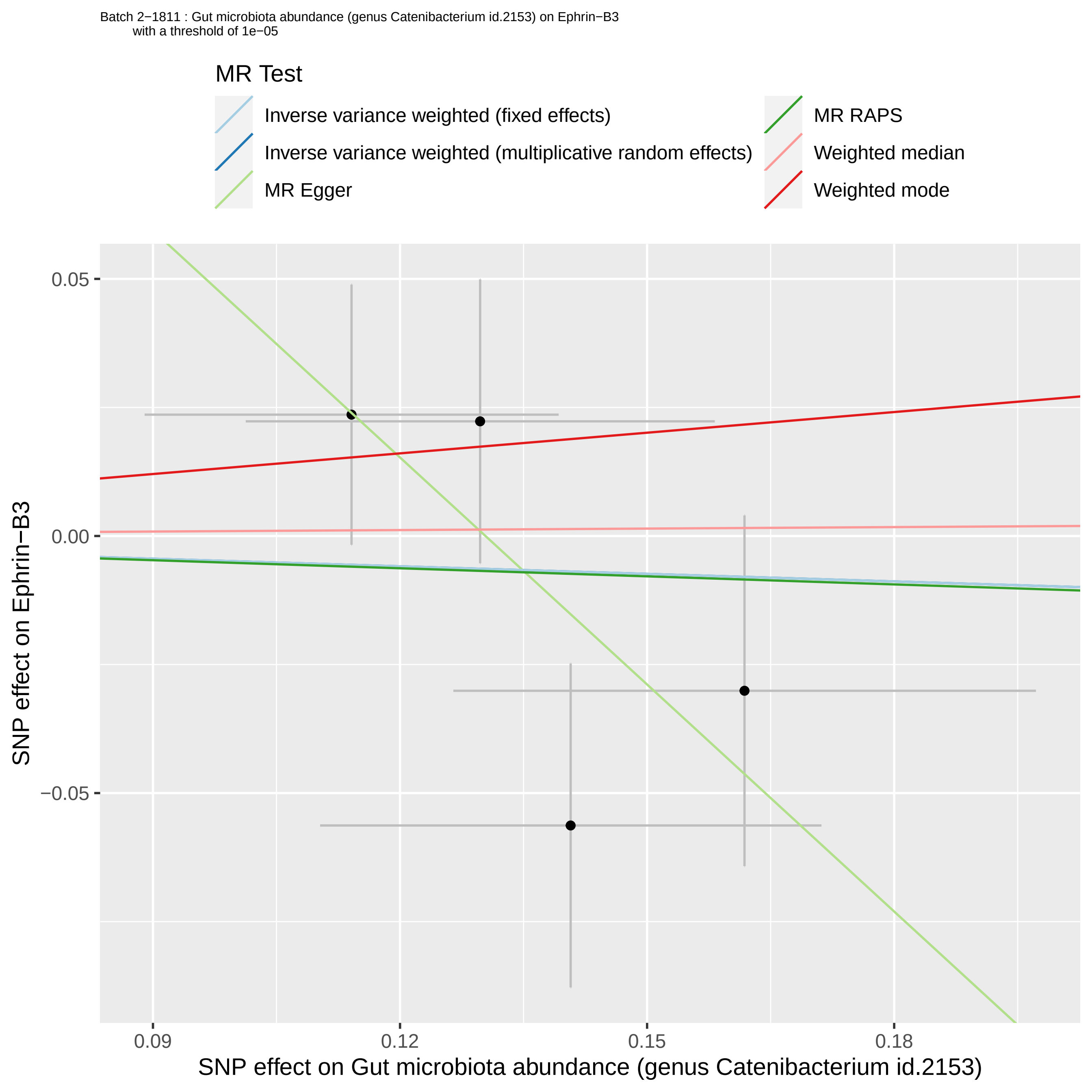

Batch 2−1811 : Gut microbiota abundance (genus Catenibacterium id.2153) on Ephrin−B3
with a threshold of 1e−05
MR Test
Inverse variance weighted (fixed effects)
Inverse variance weighted (multiplicative random effects)
MR Egger
MR RAPS
Weighted median
Weighted mode
0.05
0.00
−0.05
0.09
0.12
0.15
0.18
SNP effect on Ephrin−B3
SNP effect on Gut microbiota abundance (genus Catenibacterium id.2153)

Batch 2−1812 : Gut microbiota abundance (genus Parasutterella id.2892) on Ephrin−B3
with a threshold of 1e−05

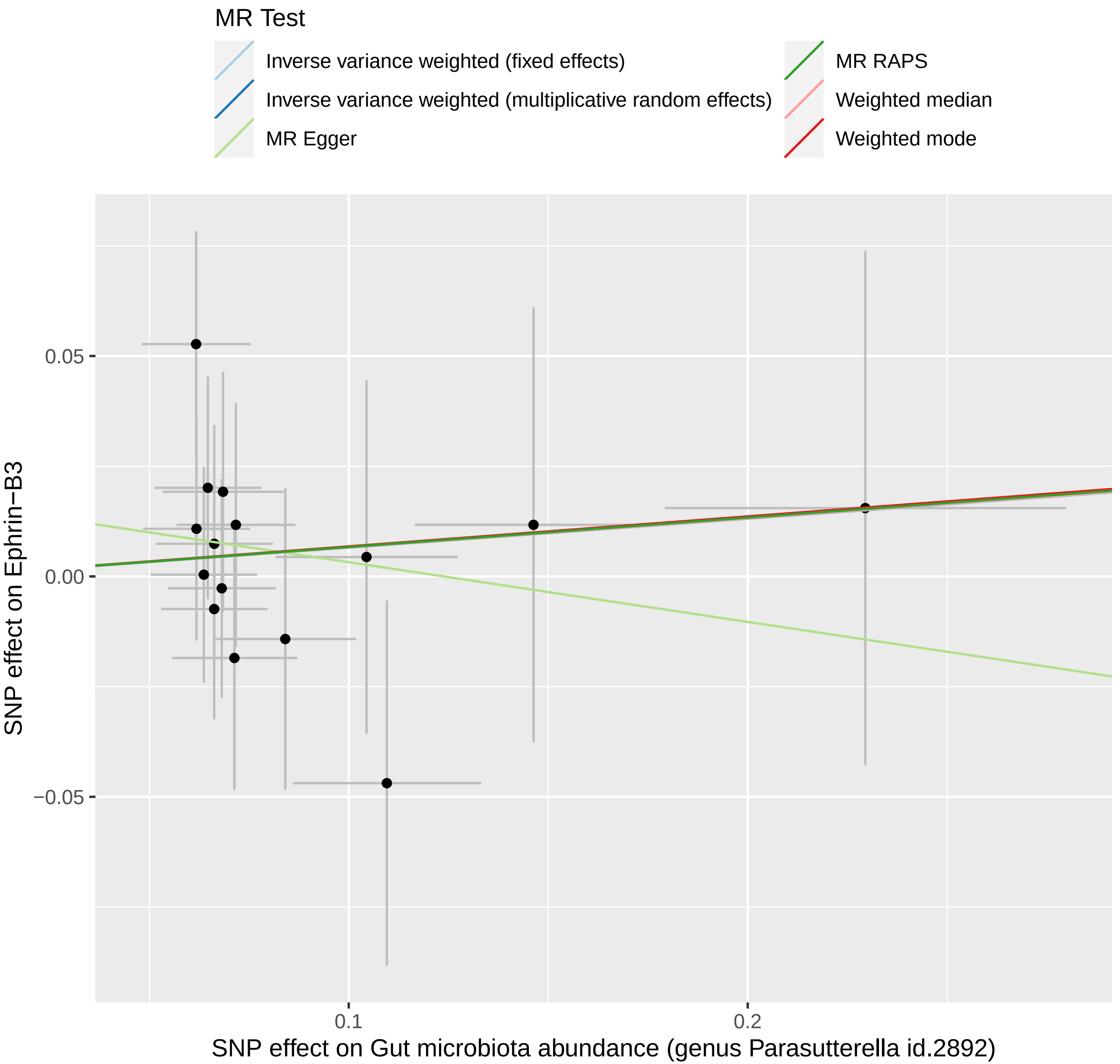

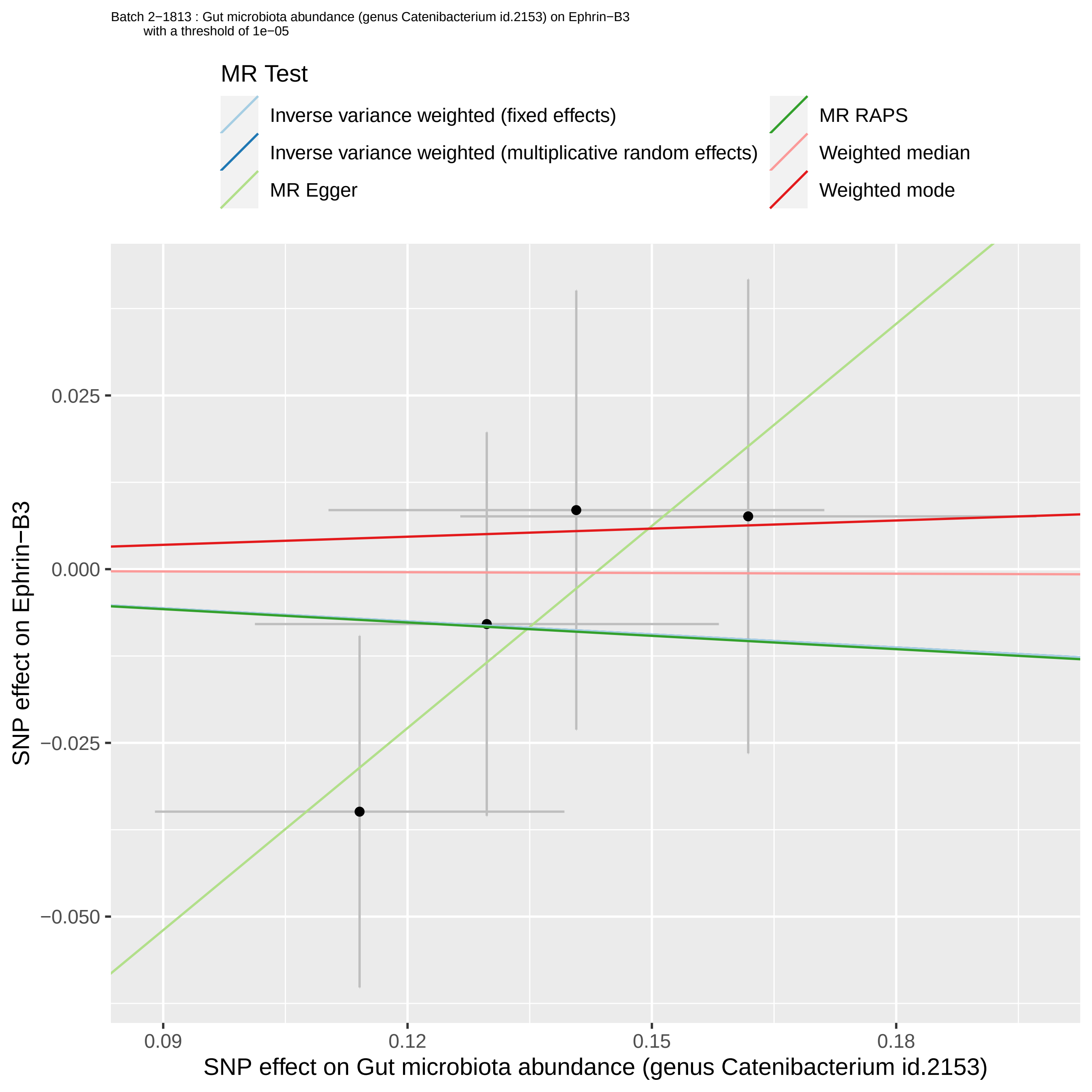
Batch 2−1813 : Gut microbiota abundance (genus Catenibacterium id.2153) on Ephrin−B3
with a threshold of 1e−05
MR Test
Inverse variance weighted (fixed effects)
Inverse variance weighted (multiplicative random effects)
MR Egger
MR RAPS
Weighted median
Weighted mode
0.025
0.000
−0.025
−0.050
0.09
0.12
0.15
0.18
SNP effect on Ephrin−B3
SNP effect on Gut microbiota abundance (genus Catenibacterium id.2153)

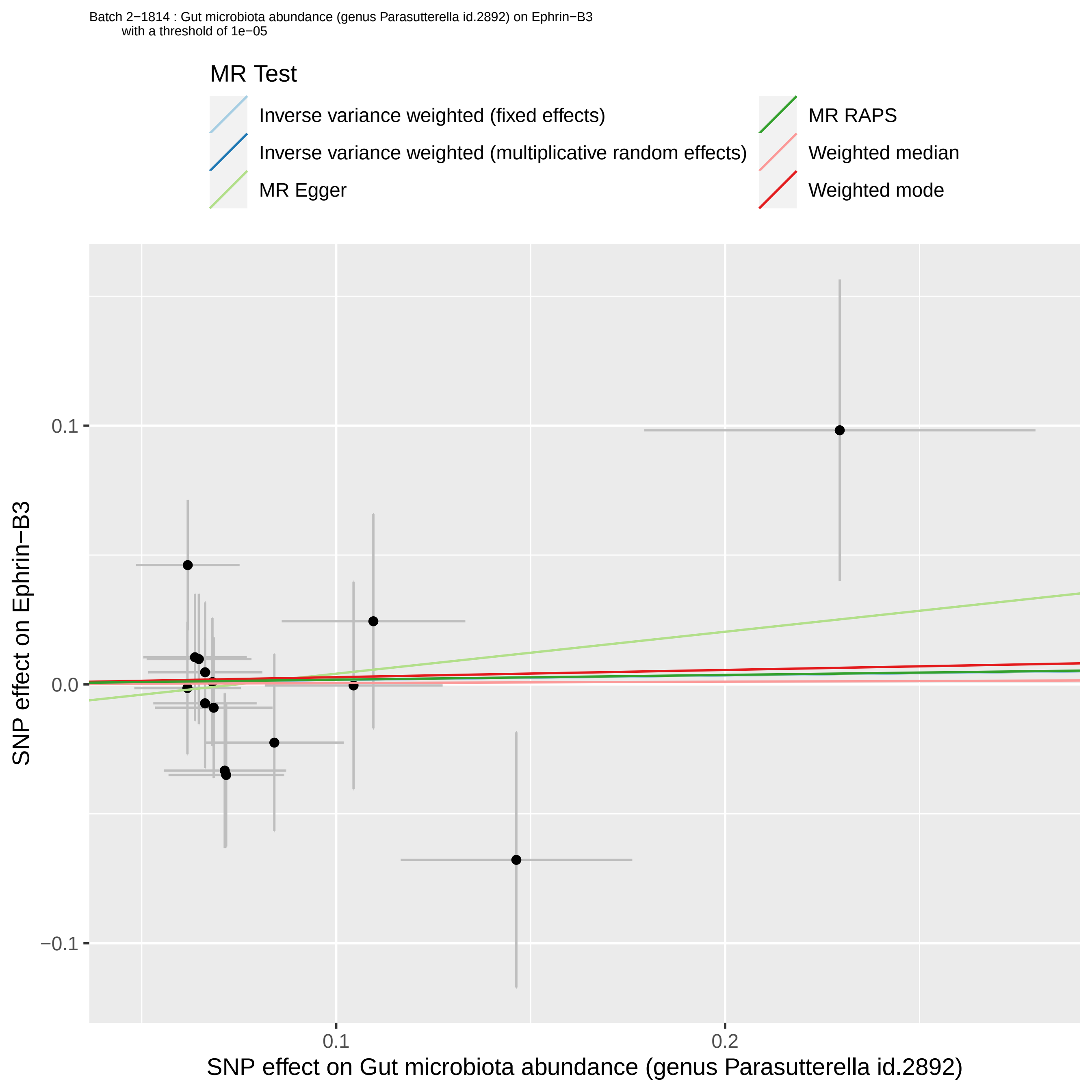

Batch 2−1814 : Gut microbiota abundance (genus Parasutterella id.2892) on Ephrin−B3
with a threshold of 1e−05
MR Test
Inverse variance weighted (fixed effects)
Inverse variance weighted (multiplicative random effects)
MR Egger
MR RAPS
Weighted median
Weighted mode
0.1
0.0
−0.1
0.1
0.2
SNP effect on Ephrin−B3
SNP effect on Gut microbiota abundance (genus Parasutterella id.2892)

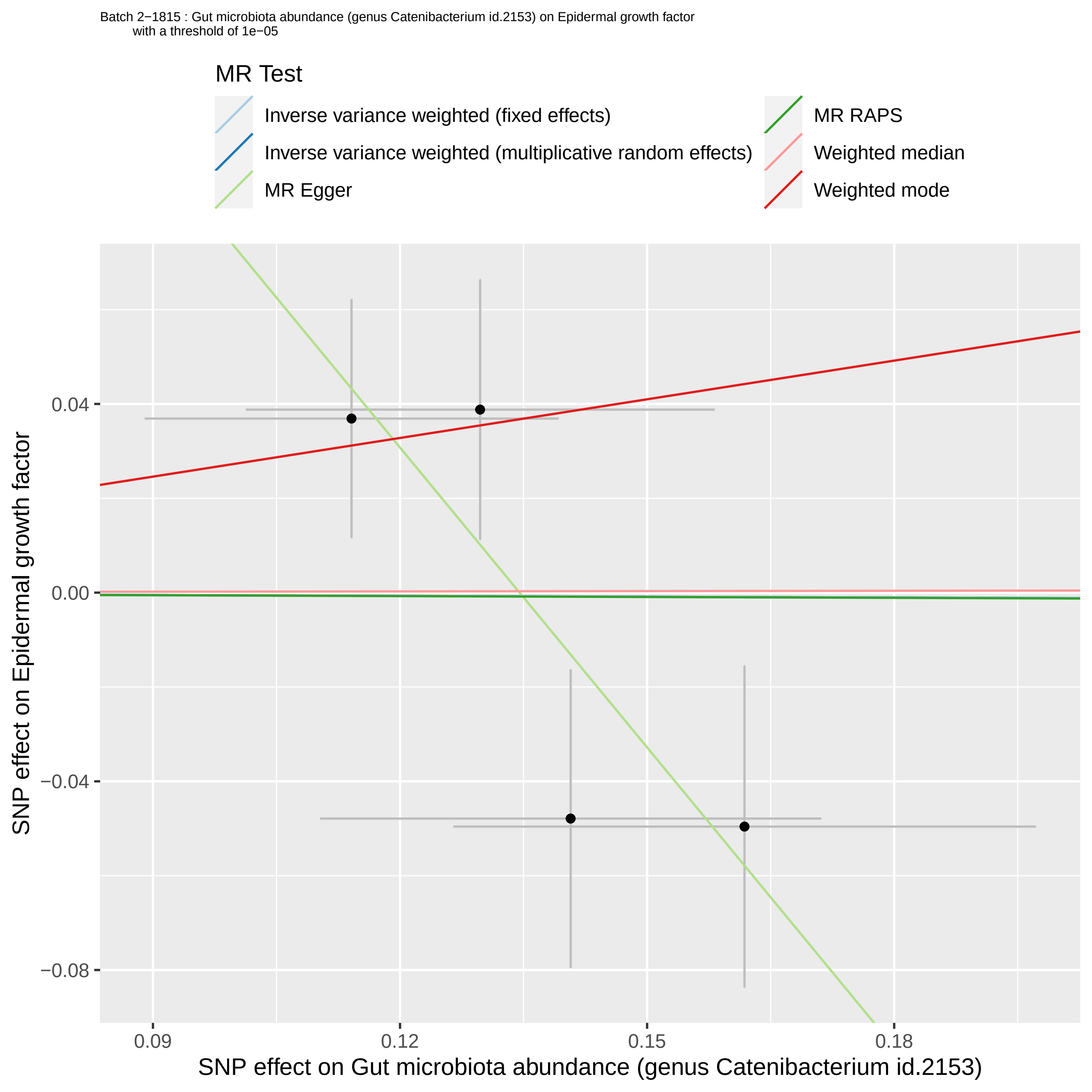
Batch 2−1815 : Gut microbiota abundance (genus Catenibacterium id.2153) on Epidermal growth factor
with a threshold of 1e−05
MR Test
Inverse variance weighted (fixed effects)
Inverse variance weighted (multiplicative random effects)
MR Egger
MR RAPS
Weighted median
Weighted mode
0.04
0.00
−0.04
−0.08
SNP effect on Epidermal growth factor
0.09
0.12
0.15
0.18
SNP effect on Gut microbiota abundance (genus Catenibacterium id.2153)

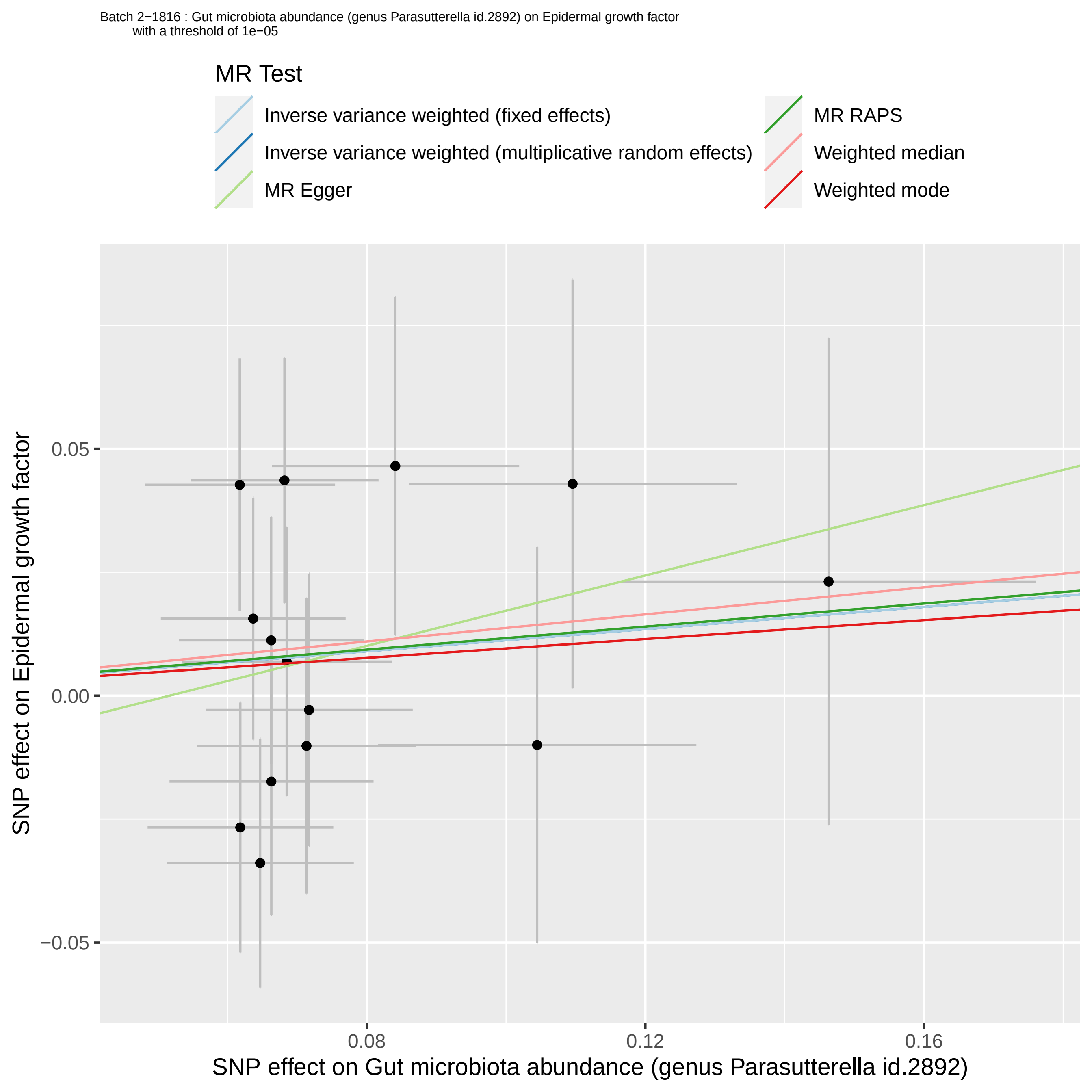
Batch 2−1816 : Gut microbiota abundance (genus Parasutterella id.2892) on Epidermal growth factor
with a threshold of 1e−05
MR Test
Inverse variance weighted (fixed effects)
Inverse variance weighted (multiplicative random effects)
MR Egger
MR RAPS
Weighted median
Weighted mode
SNP effect on Epidermal growth factor
0.05
0.00
−0.05
0.08
0.12
0.16
SNP effect on Gut microbiota abundance (genus Parasutterella id.2892)

Batch 2−1817 : Gut microbiota abundance (genus Catenibacterium id.2153) on Epidermal growth factor receptor
with a threshold of 1e−05

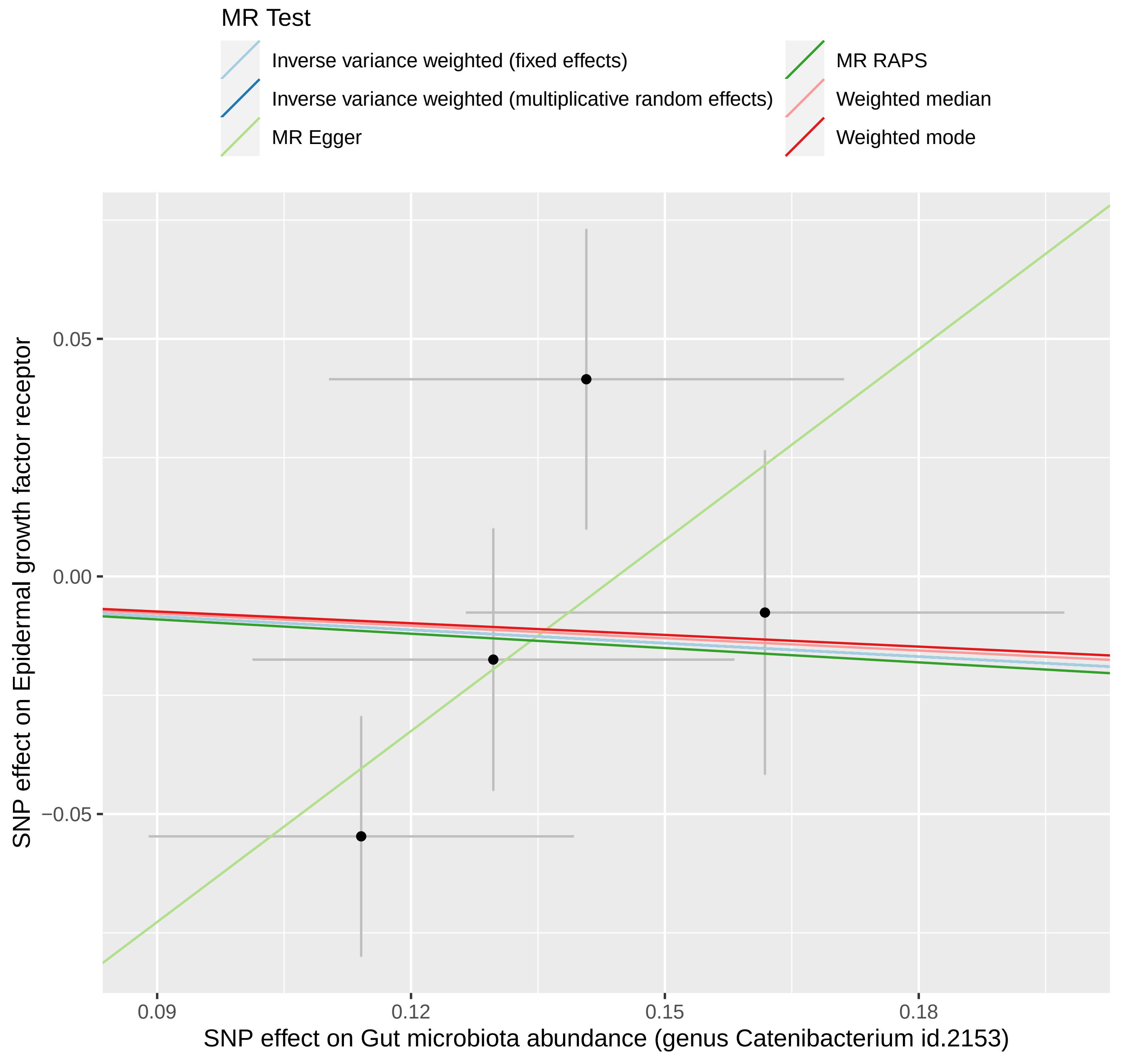

Batch 2−1818 : Gut microbiota abundance (genus Parasutterella id.2892) on Epidermal growth factor receptor
with a threshold of 1e−05

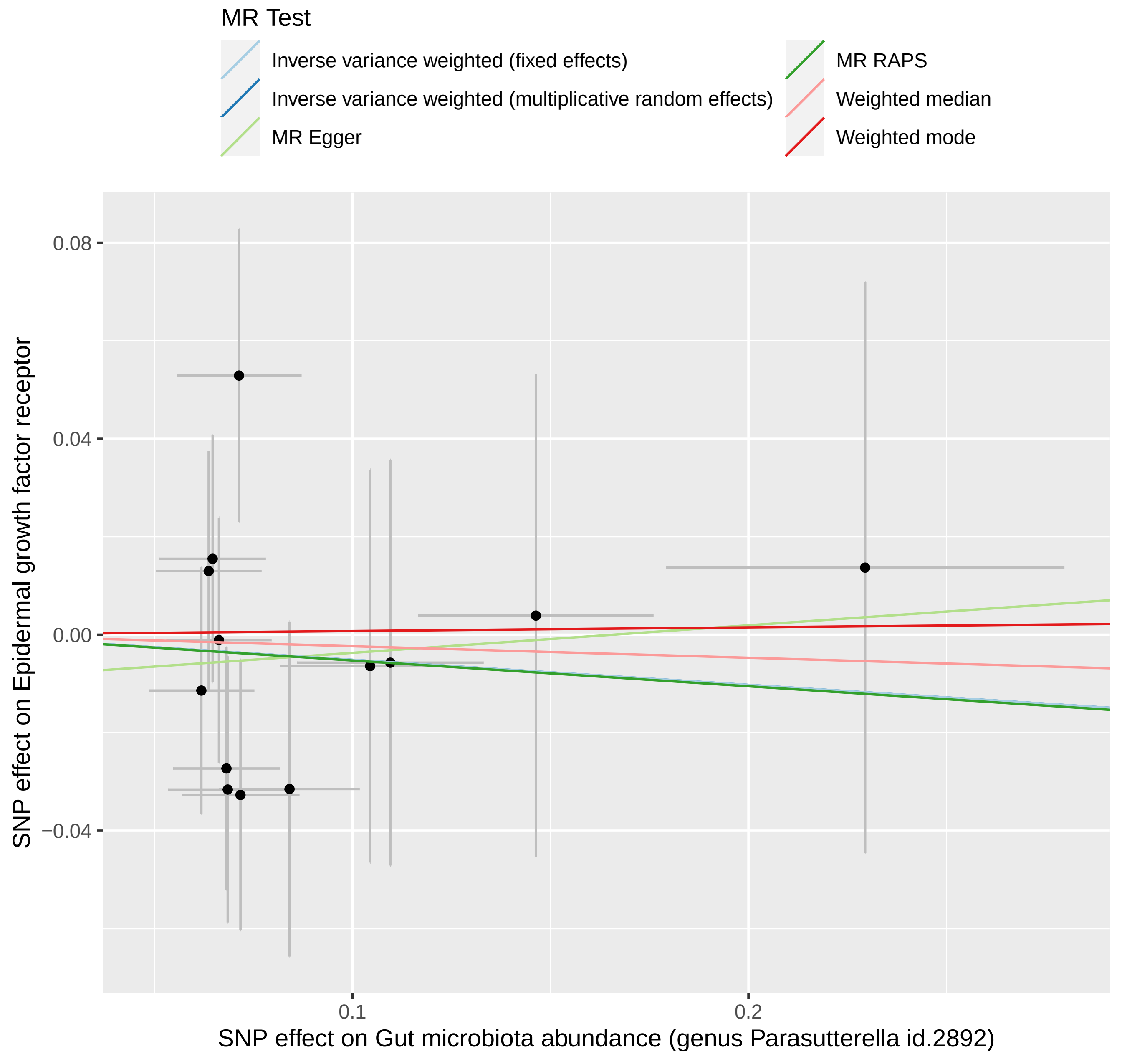

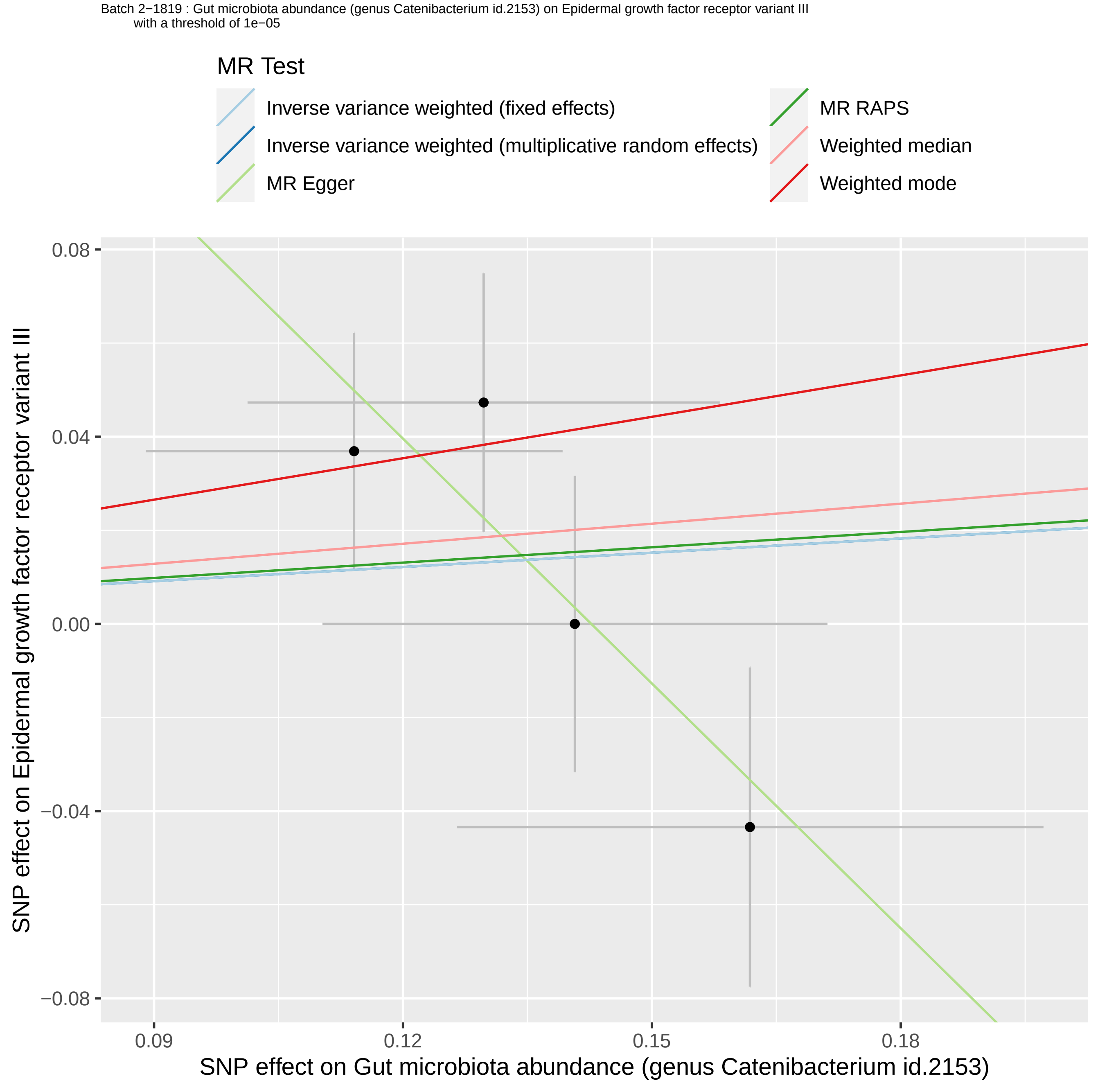

Batch 2−1819 : Gut microbiota abundance (genus Catenibacterium id.2153) on Epidermal growth factor receptor variant III
with a threshold of 1e−05
MR Test
Inverse variance weighted (fixed effects)
Inverse variance weighted (multiplicative random effects)
MR Egger
MR RAPS
Weighted median
Weighted mode
0.08
0.04
0.00
−0.04
−0.08
0.09
0.12
0.15
0.18
SNP effect on Epidermal growth factor receptor variant III
SNP effect on Gut microbiota abundance (genus Catenibacterium id.2153)

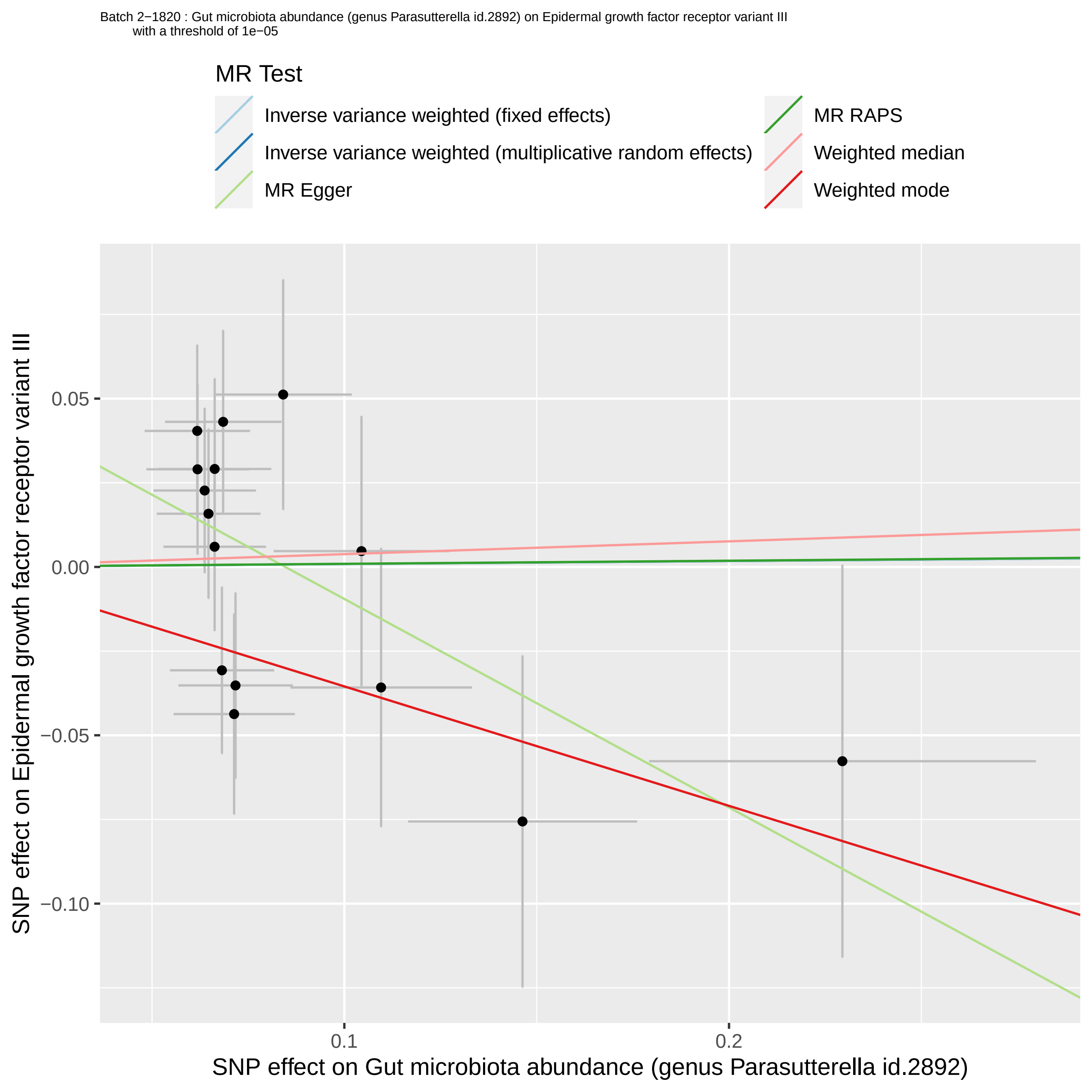
Batch 2−1820 : Gut microbiota abundance (genus Parasutterella id.2892) on Epidermal growth factor receptor variant III
with a threshold of 1e−05
MR Test
Inverse variance weighted (fixed effects)
Inverse variance weighted (multiplicative random effects)
MR Egger
MR RAPS
Weighted median
Weighted mode
0.05
0.00
−0.05
−0.10
0.1
0.2
SNP effect on Epidermal growth factor receptor variant III
SNP effect on Gut microbiota abundance (genus Parasutterella id.2892)

Batch 2−1821 : Gut microbiota abundance (genus Catenibacterium id.2153) on EH domain−binding protein 1
with a threshold of 1e−05

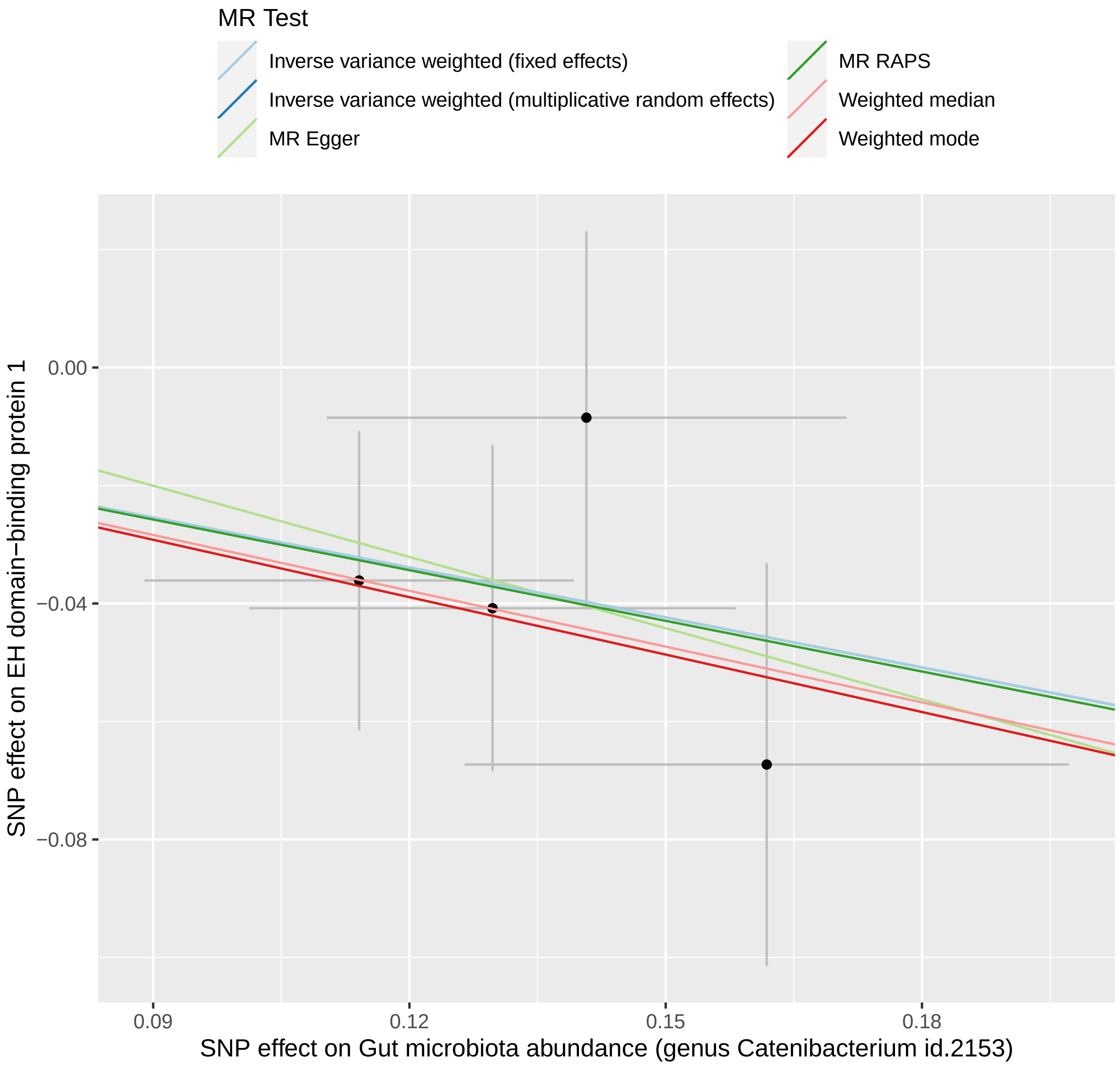

Batch 2−1822 : Gut microbiota abundance (genus Parasutterella id.2892) on EH domain−binding protein 1
with a threshold of 1e−05

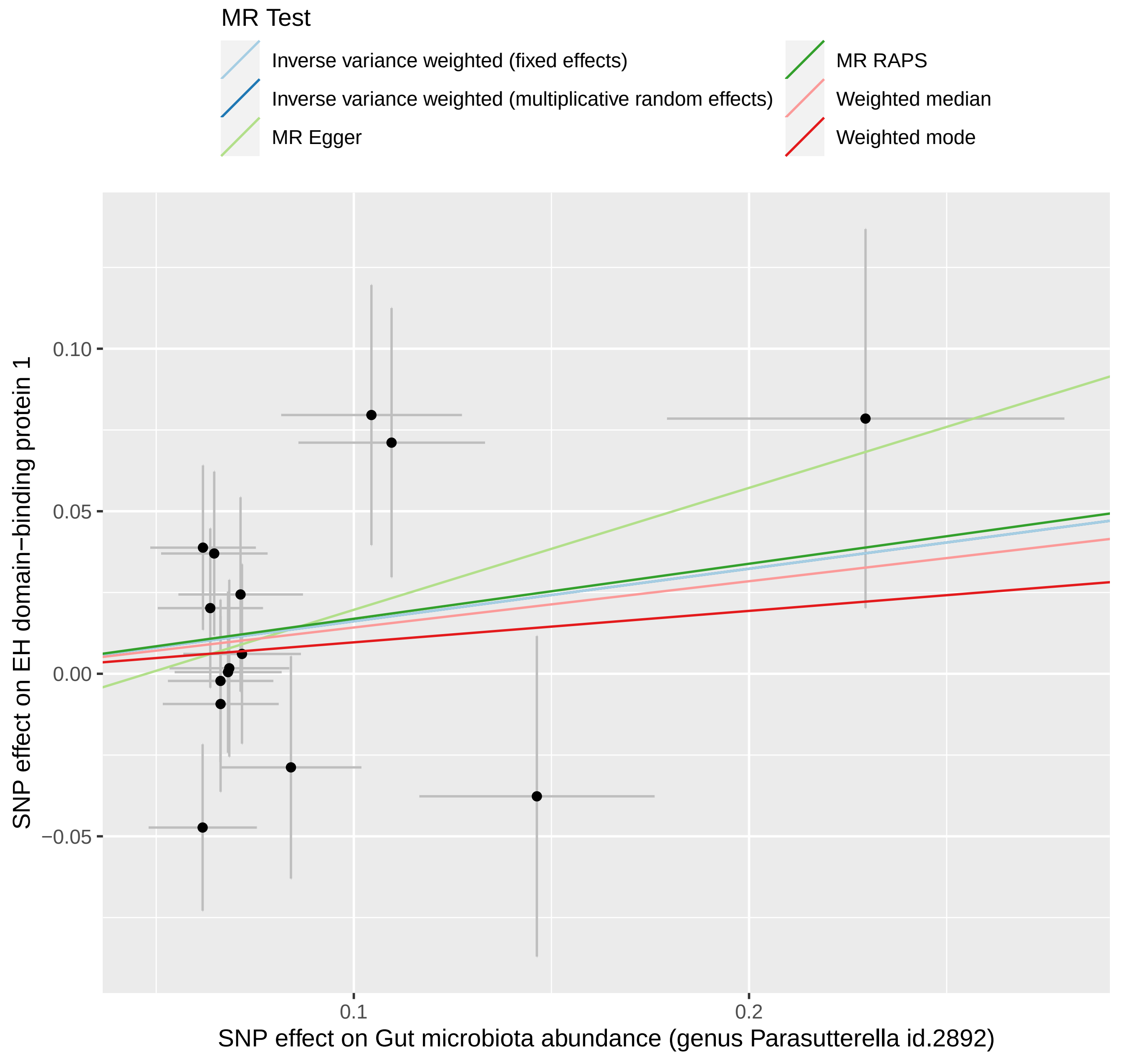

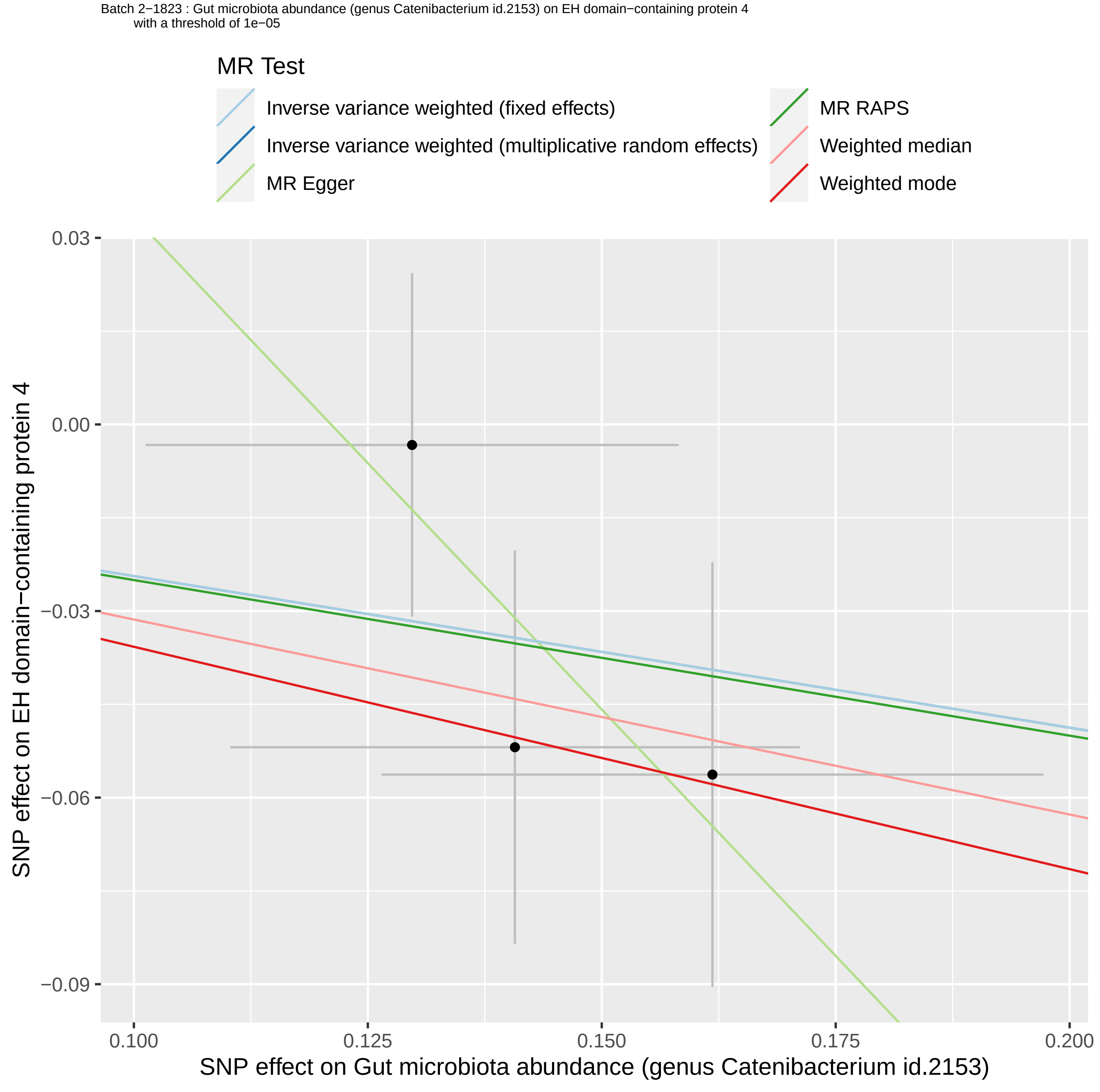

Batch 2−1823 : Gut microbiota abundance (genus Catenibacterium id.2153) on EH domain−containing protein 4
with a threshold of 1e−05
MR Test
Inverse variance weighted (fixed effects)
Inverse variance weighted (multiplicative random effects)
MR Egger
MR RAPS
Weighted median
Weighted mode
0.03
0.00
−0.03
−0.06
−0.09
0.100
0.125
0.150
0.175
0.200
SNP effect on EH domain−containing protein 4
SNP effect on Gut microbiota abundance (genus Catenibacterium id.2153)

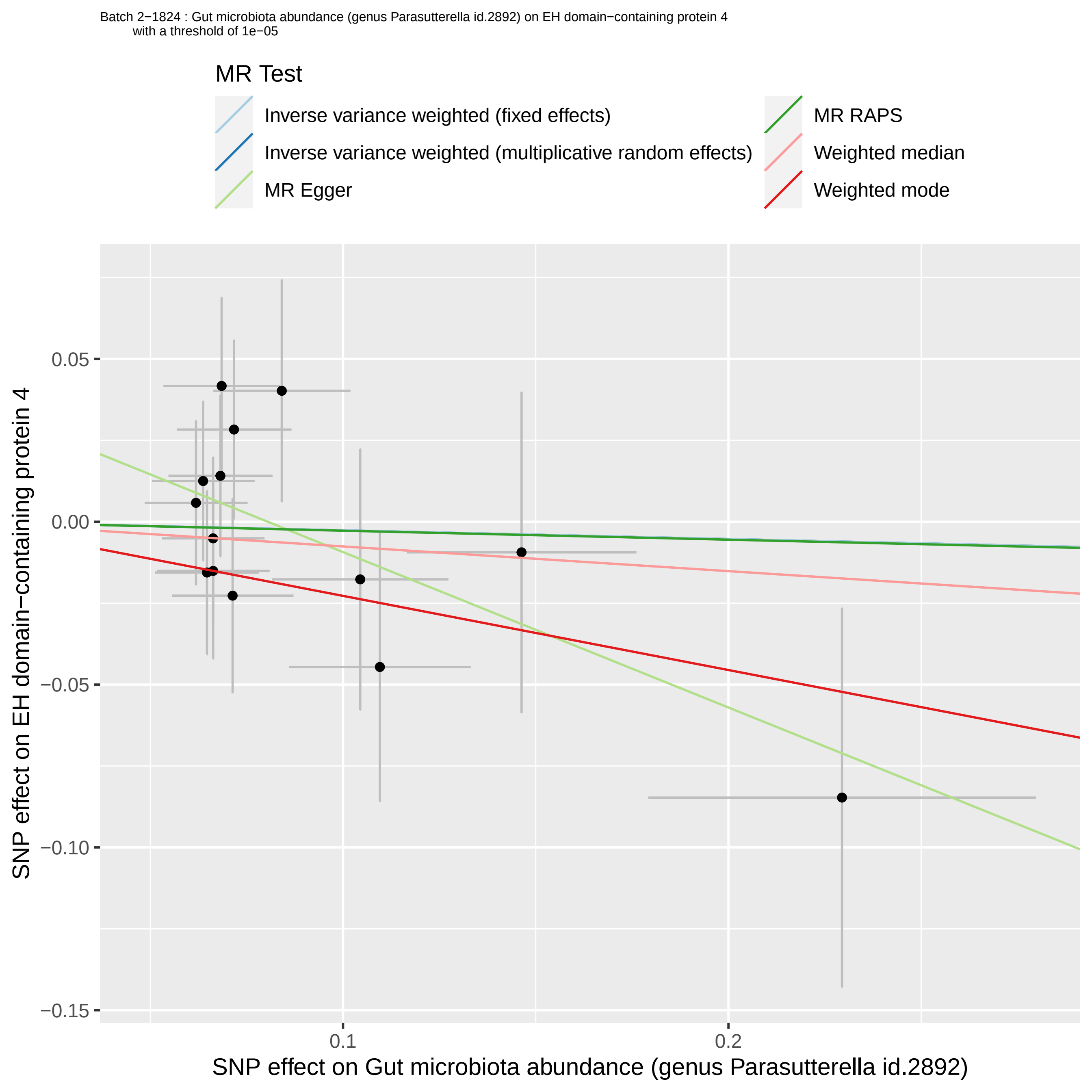
Batch 2−1824 : Gut microbiota abundance (genus Parasutterella id.2892) on EH domain−containing protein 4
with a threshold of 1e−05
MR Test
Inverse variance weighted (fixed effects)
Inverse variance weighted (multiplicative random effects)
MR Egger
MR RAPS
Weighted median
Weighted mode
0.05
0.00
−0.05
−0.10
−0.15
0.1
0.2
SNP effect on EH domain−containing protein 4
SNP effect on Gut microbiota abundance (genus Parasutterella id.2892)

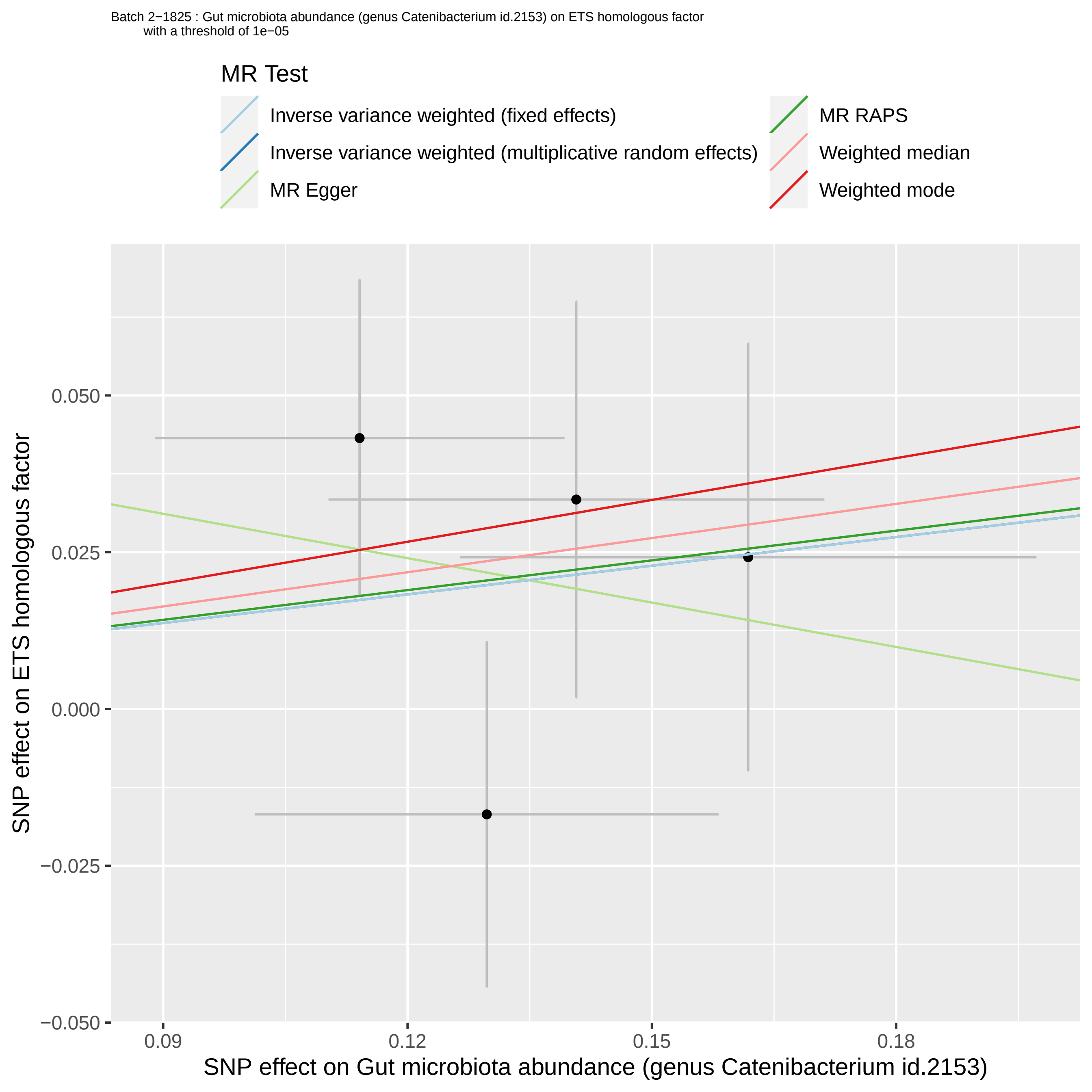

Batch 2−1825 : Gut microbiota abundance (genus Catenibacterium id.2153) on ETS homologous factor
with a threshold of 1e−05
MR Test
Inverse variance weighted (fixed effects)
Inverse variance weighted (multiplicative random effects)
MR Egger
MR RAPS
Weighted median
Weighted mode
0.050
0.025
0.000
−0.025
−0.050
0.09
0.12
0.15
0.18
SNP effect on ETS homologous factor
SNP effect on Gut microbiota abundance (genus Catenibacterium id.2153)

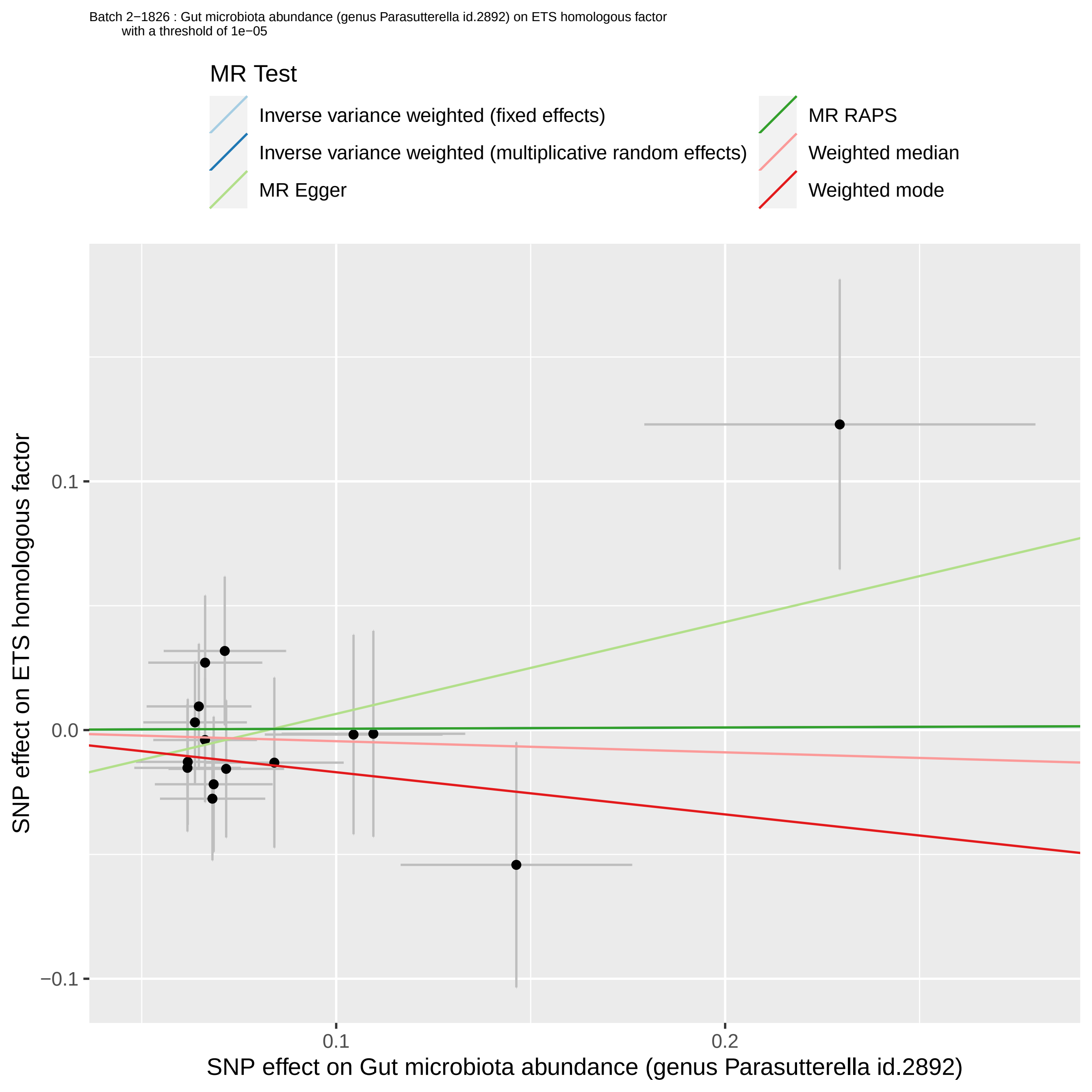

Batch 2−1826 : Gut microbiota abundance (genus Parasutterella id.2892) on ETS homologous factor
with a threshold of 1e−05
MR Test
Inverse variance weighted (fixed effects)
Inverse variance weighted (multiplicative random effects)
MR Egger
MR RAPS
Weighted median
Weighted mode
SNP effect on ETS homologous factor
0.1
0.0
−0.1
0.1
0.2
SNP effect on Gut microbiota abundance (genus Parasutterella id.2892)

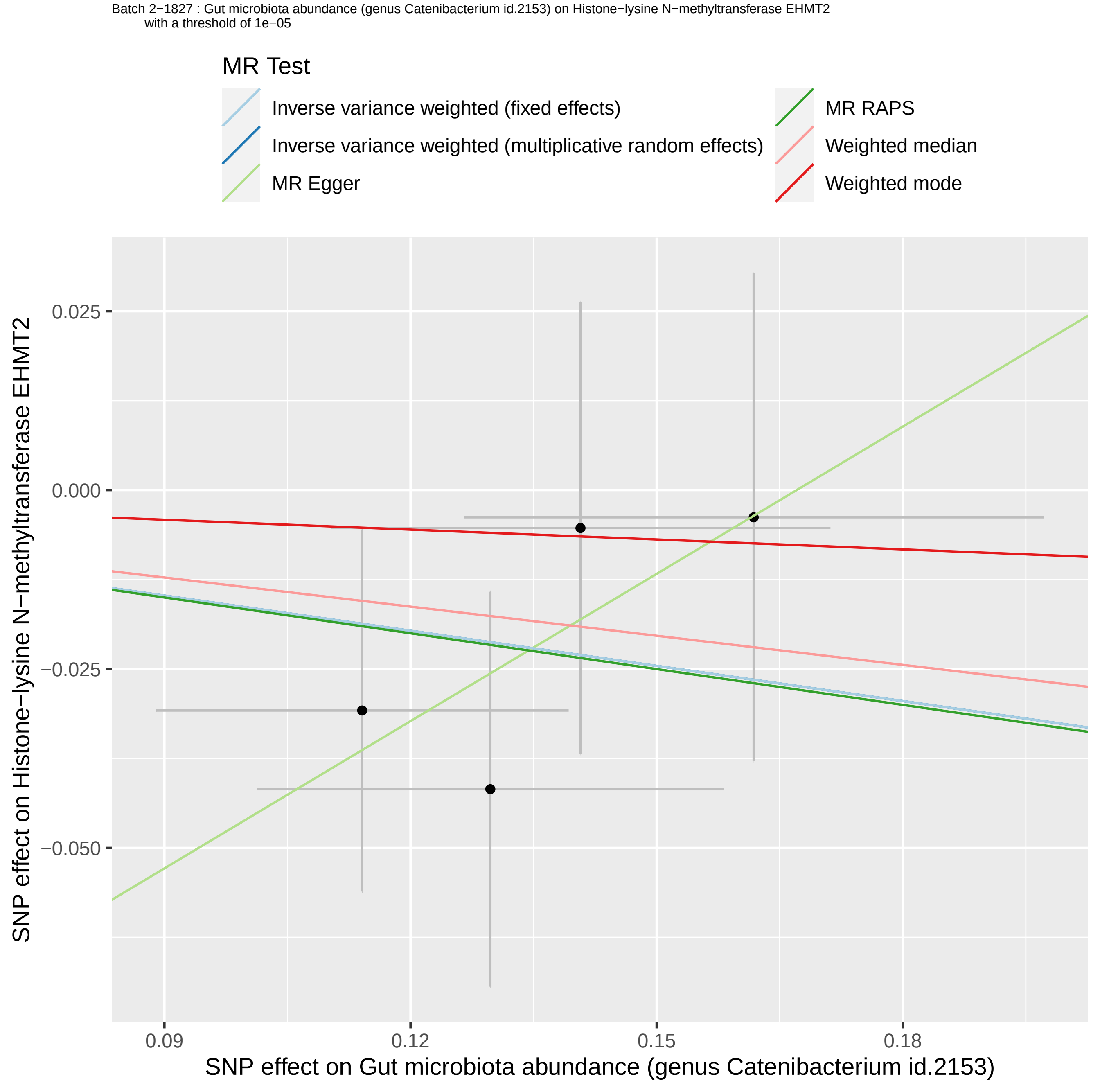

Batch 2−1827 : Gut microbiota abundance (genus Catenibacterium id.2153) on Histone−lysine N−methyltransferase EHMT2
with a threshold of 1e−05
MR Test
Inverse variance weighted (fixed effects)
Inverse variance weighted (multiplicative random effects)
MR Egger
MR RAPS
Weighted median
Weighted mode
0.025
0.000
−0.025
−0.050
0.09
0.12
0.15
0.18
SNP effect on Histone−lysine N−methyltransferase EHMT2
SNP effect on Gut microbiota abundance (genus Catenibacterium id.2153)

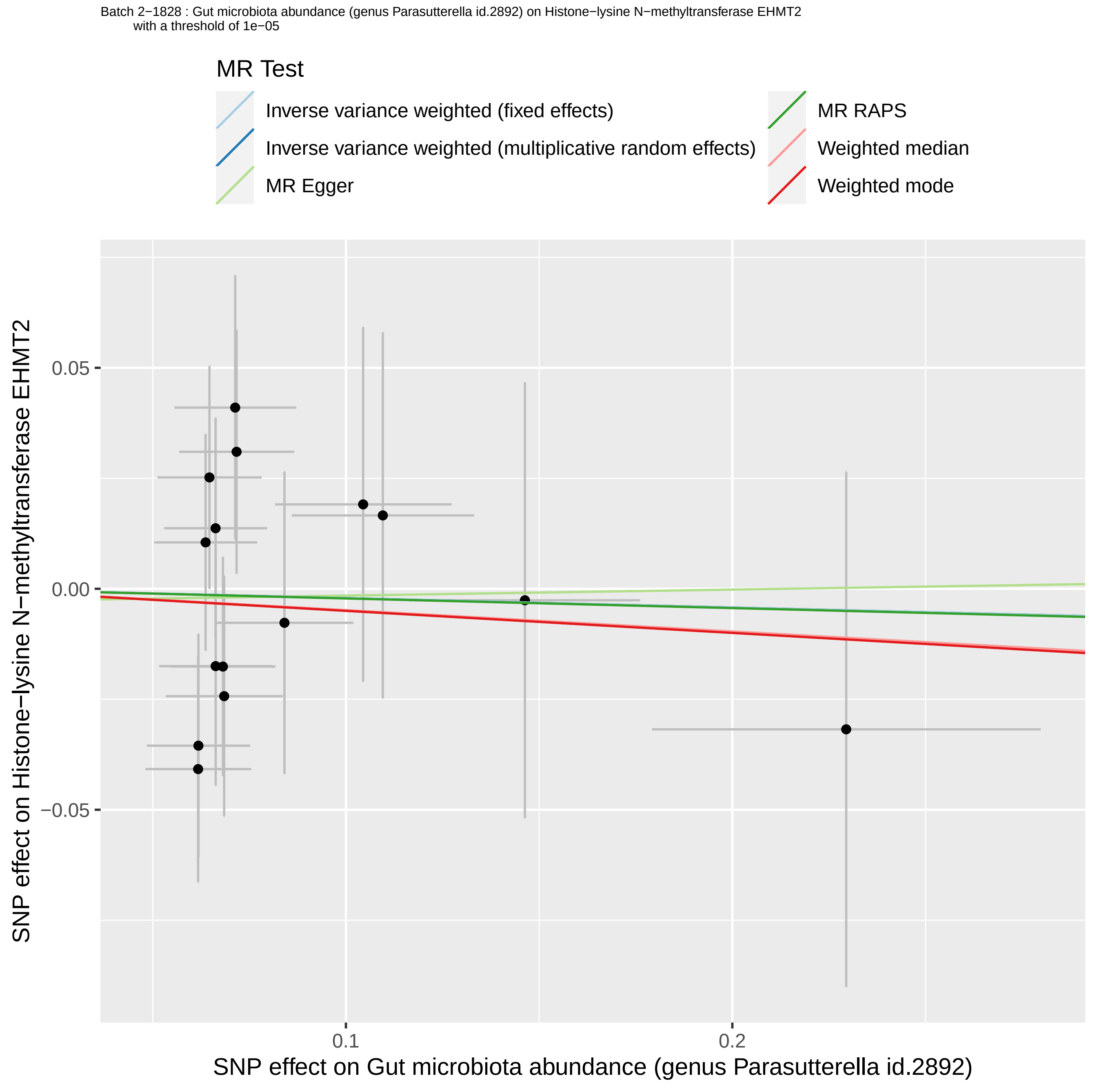

Batch 2−1828 : Gut microbiota abundance (genus Parasutterella id.2892) on Histone−lysine N−methyltransferase EHMT2
with a threshold of 1e−05
MR Test
Inverse variance weighted (fixed effects)
Inverse variance weighted (multiplicative random effects)
MR Egger
MR RAPS
Weighted median
Weighted mode
0.05
0.00
−0.05
0.1
0.2
SNP effect on Histone−lysine N−methyltransferase EHMT2
SNP effect on Gut microbiota abundance (genus Parasutterella id.2892)

Batch 2−1829 : Gut microbiota abundance (genus Catenibacterium id.2153) on EP300−interacting inhibitor of differentiation 3
with a threshold of 1e−05

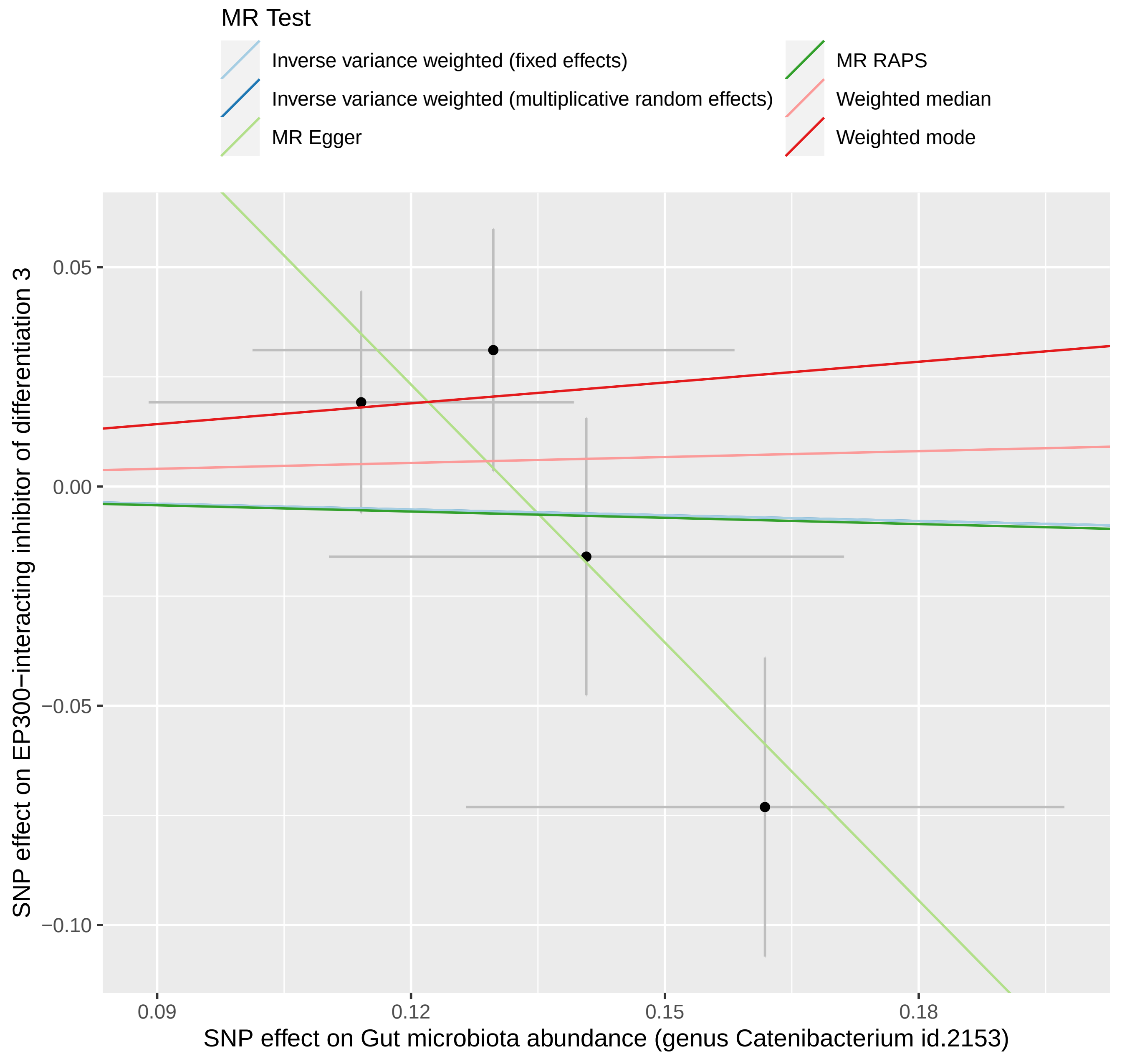

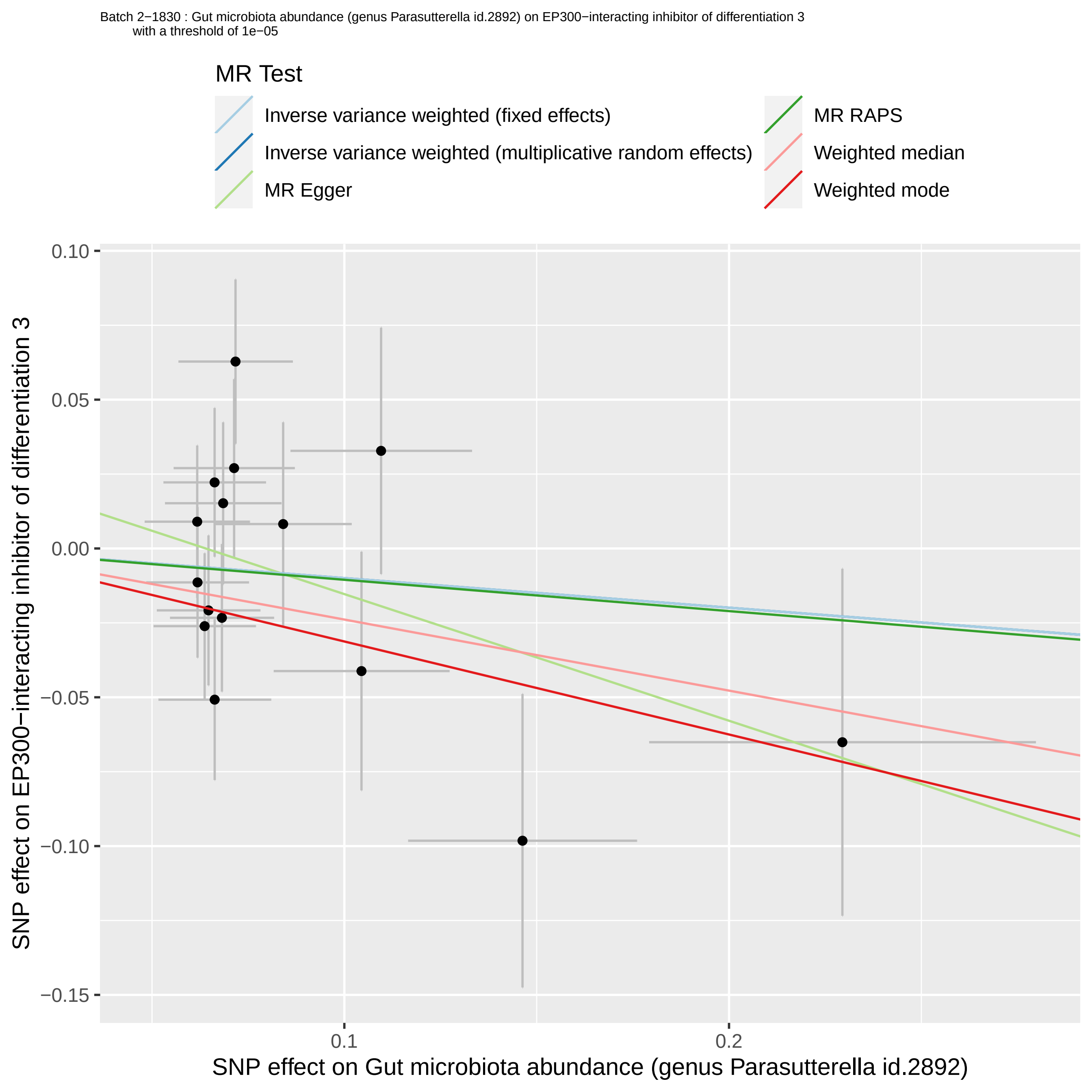
Batch 2−1830 : Gut microbiota abundance (genus Parasutterella id.2892) on EP300−interacting inhibitor of differentiation 3
with a threshold of 1e−05
MR Test
Inverse variance weighted (fixed effects)
Inverse variance weighted (multiplicative random effects)
MR Egger
MR RAPS
Weighted median
Weighted mode
0.10
0.05
0.00
−0.05
−0.10
−0.15
0.1
0.2
SNP effect on EP300−interacting inhibitor of differentiation 3
SNP effect on Gut microbiota abundance (genus Parasutterella id.2892)

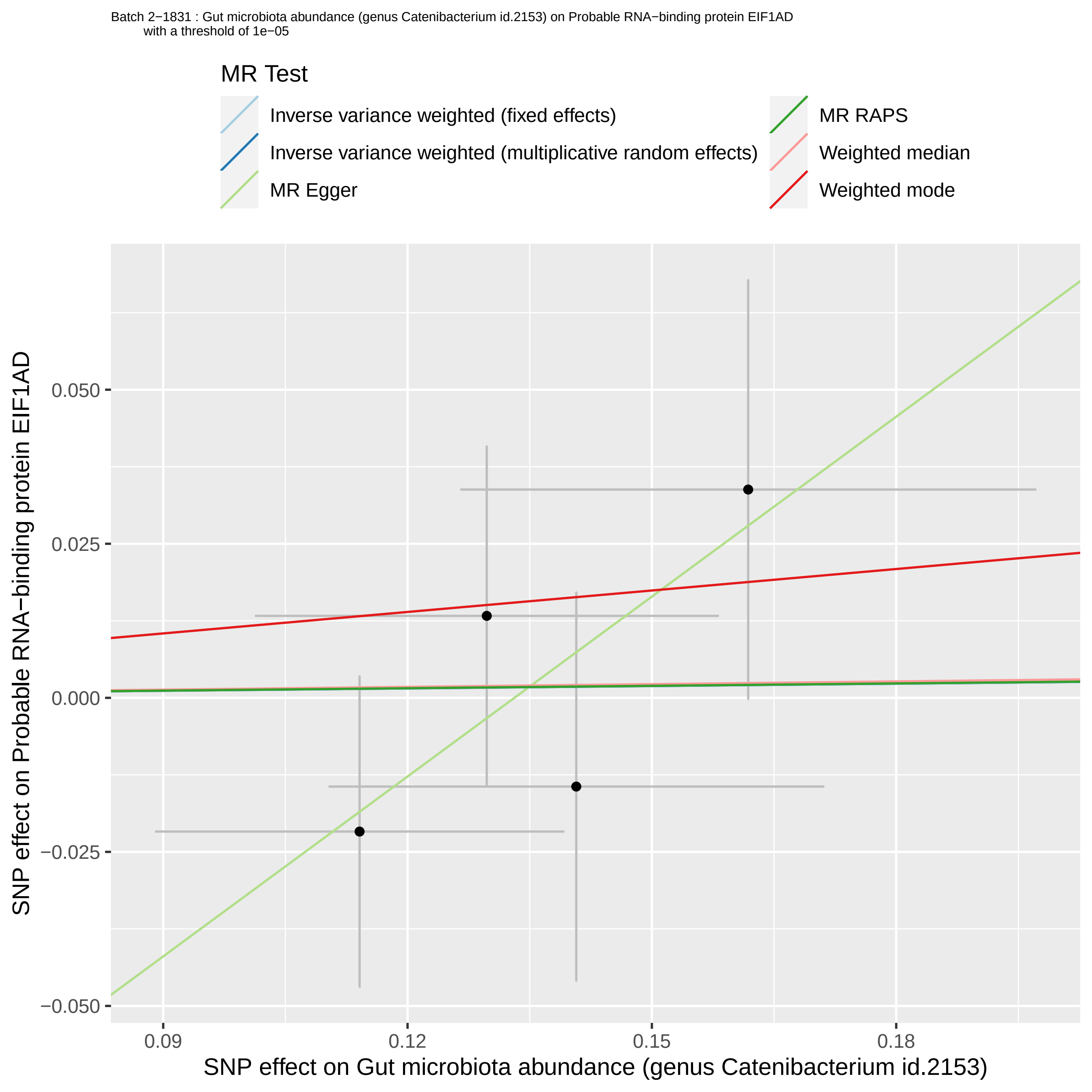

Batch 2−1831 : Gut microbiota abundance (genus Catenibacterium id.2153) on Probable RNA−binding protein EIF1AD
with a threshold of 1e−05
MR Test
Inverse variance weighted (fixed effects)
Inverse variance weighted (multiplicative random effects)
MR Egger
MR RAPS
Weighted median
Weighted mode
0.050
0.025
0.000
−0.025
−0.050
0.09
0.12
0.15
0.18
SNP effect on Probable RNA−binding protein EIF1AD
SNP effect on Gut microbiota abundance (genus Catenibacterium id.2153)

Batch 2−1832 : Gut microbiota abundance (genus Parasutterella id.2892) on Probable RNA−binding protein EIF1AD
with a threshold of 1e−05

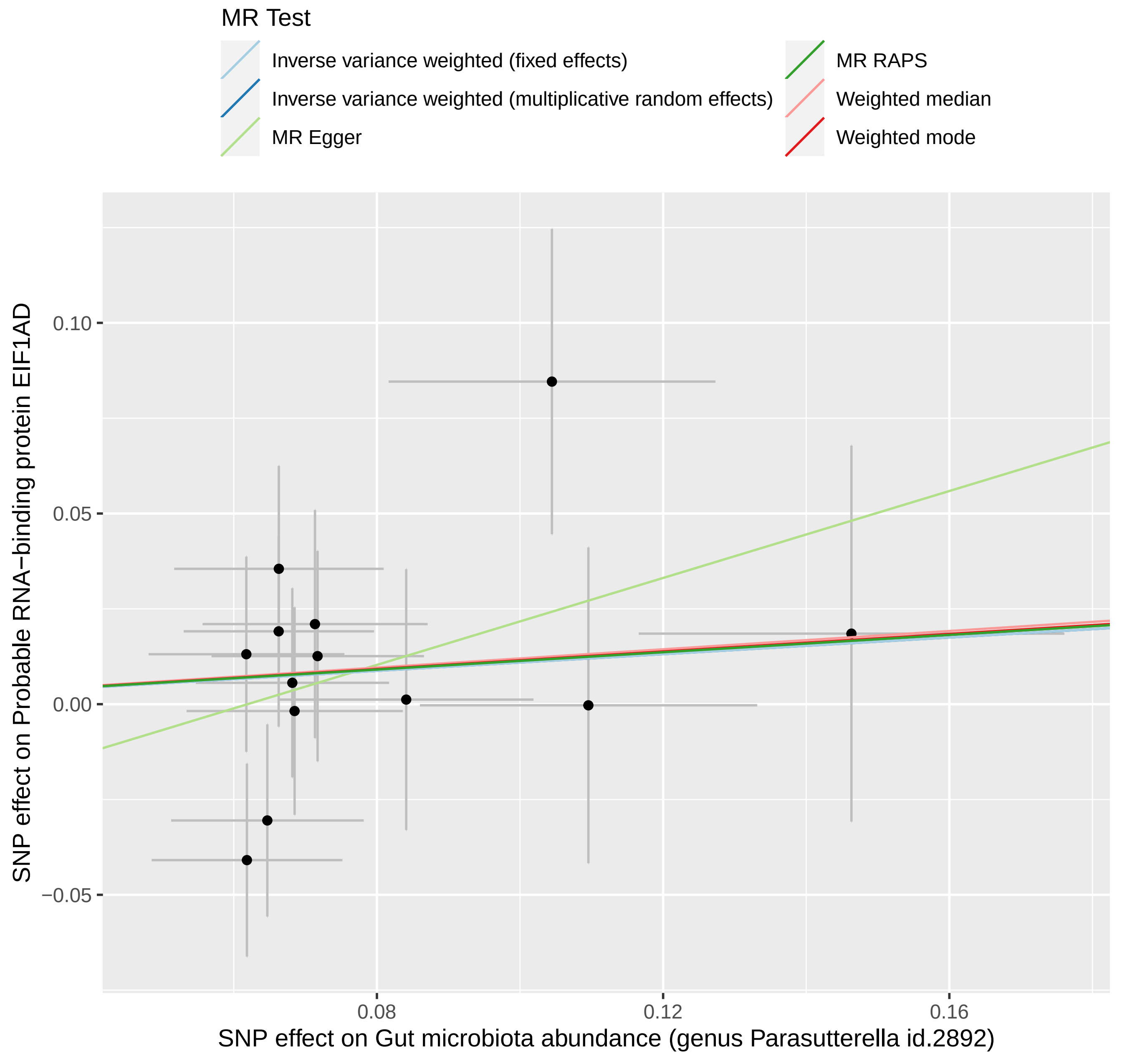

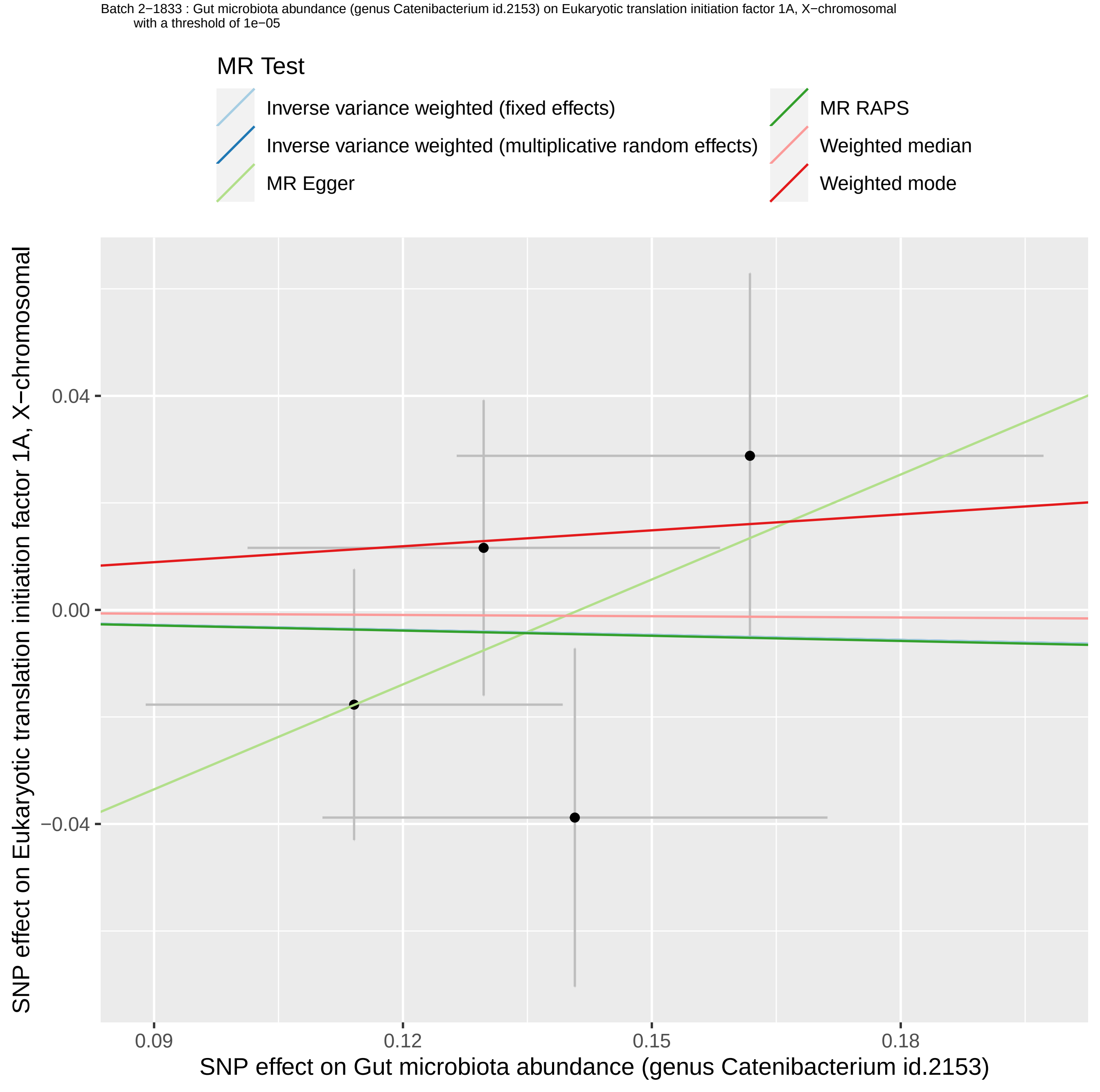
Batch 2−1833 : Gut microbiota abundance (genus Catenibacterium id.2153) on Eukaryotic translation initiation factor 1A, X−chromosomal
with a threshold of 1e−05
MR Test
Inverse variance weighted (fixed effects)
Inverse variance weighted (multiplicative random effects)
MR Egger
MR RAPS
Weighted median
Weighted mode
0.04
0.00
−0.04
0.09
0.12
0.15
0.18
SNP effect on Eukaryotic translation initiation factor 1A, X−chromosomal
SNP effect on Gut microbiota abundance (genus Catenibacterium id.2153)

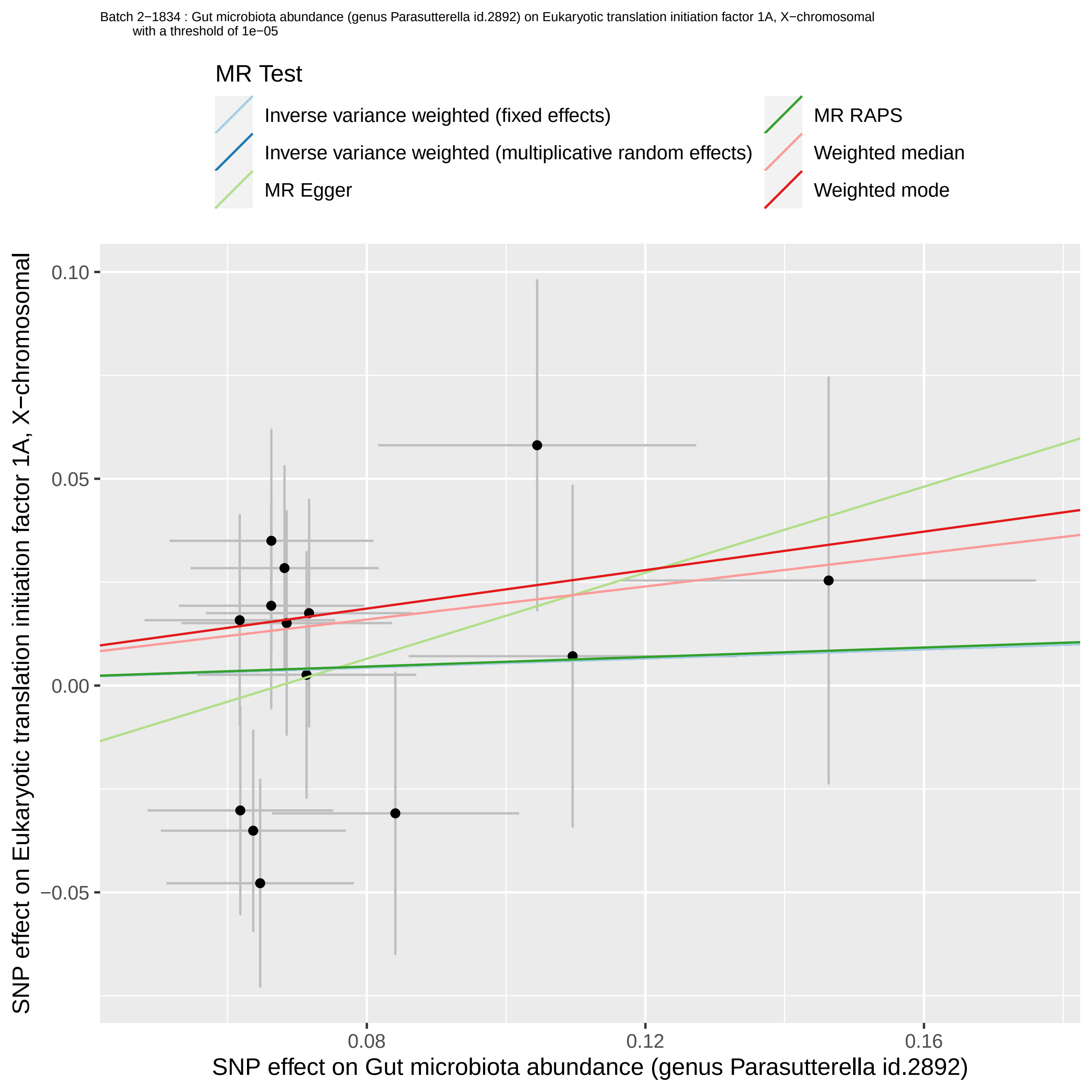

Batch 2−1834 : Gut microbiota abundance (genus Parasutterella id.2892) on Eukaryotic translation initiation factor 1A, X−chromosomal
with a threshold of 1e−05
MR Test
Inverse variance weighted (fixed effects)
Inverse variance weighted (multiplicative random effects)
MR Egger
MR RAPS
Weighted median
Weighted mode
0.10
0.05
0.00
−0.05
0.08
0.12
0.16
SNP effect on Eukaryotic translation initiation factor 1A, X−chromosomal
SNP effect on Gut microbiota abundance (genus Parasutterella id.2892)

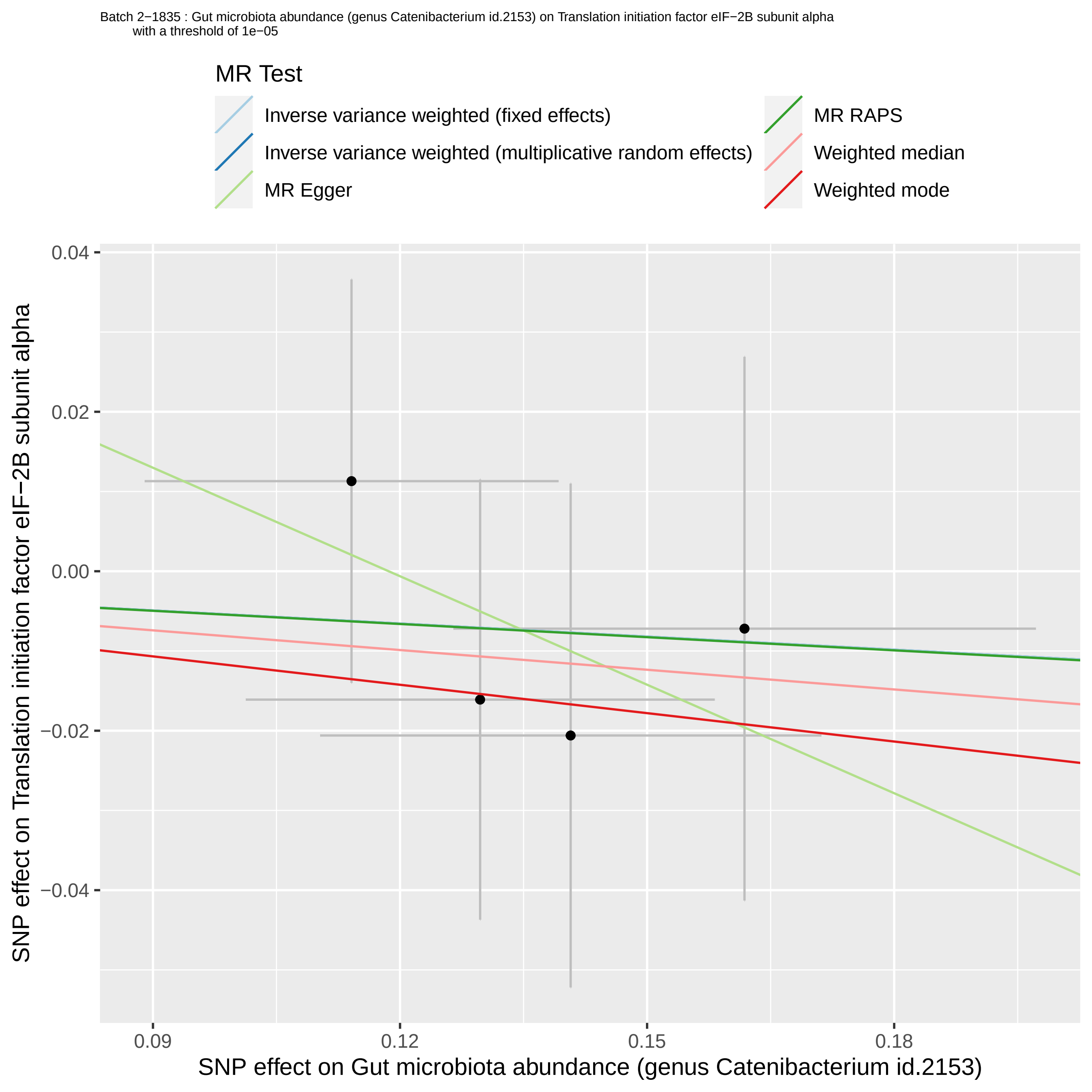
Batch 2−1835 : Gut microbiota abundance (genus Catenibacterium id.2153) on Translation initiation factor eIF−2B subunit alpha
with a threshold of 1e−05
MR Test
Inverse variance weighted (fixed effects)
Inverse variance weighted (multiplicative random effects)
MR Egger
MR RAPS
Weighted median
Weighted mode
0.04
0.02
0.00
−0.02
−0.04
0.09
0.12
0.15
0.18
SNP effect on Translation initiation factor eIF−2B subunit alpha
SNP effect on Gut microbiota abundance (genus Catenibacterium id.2153)

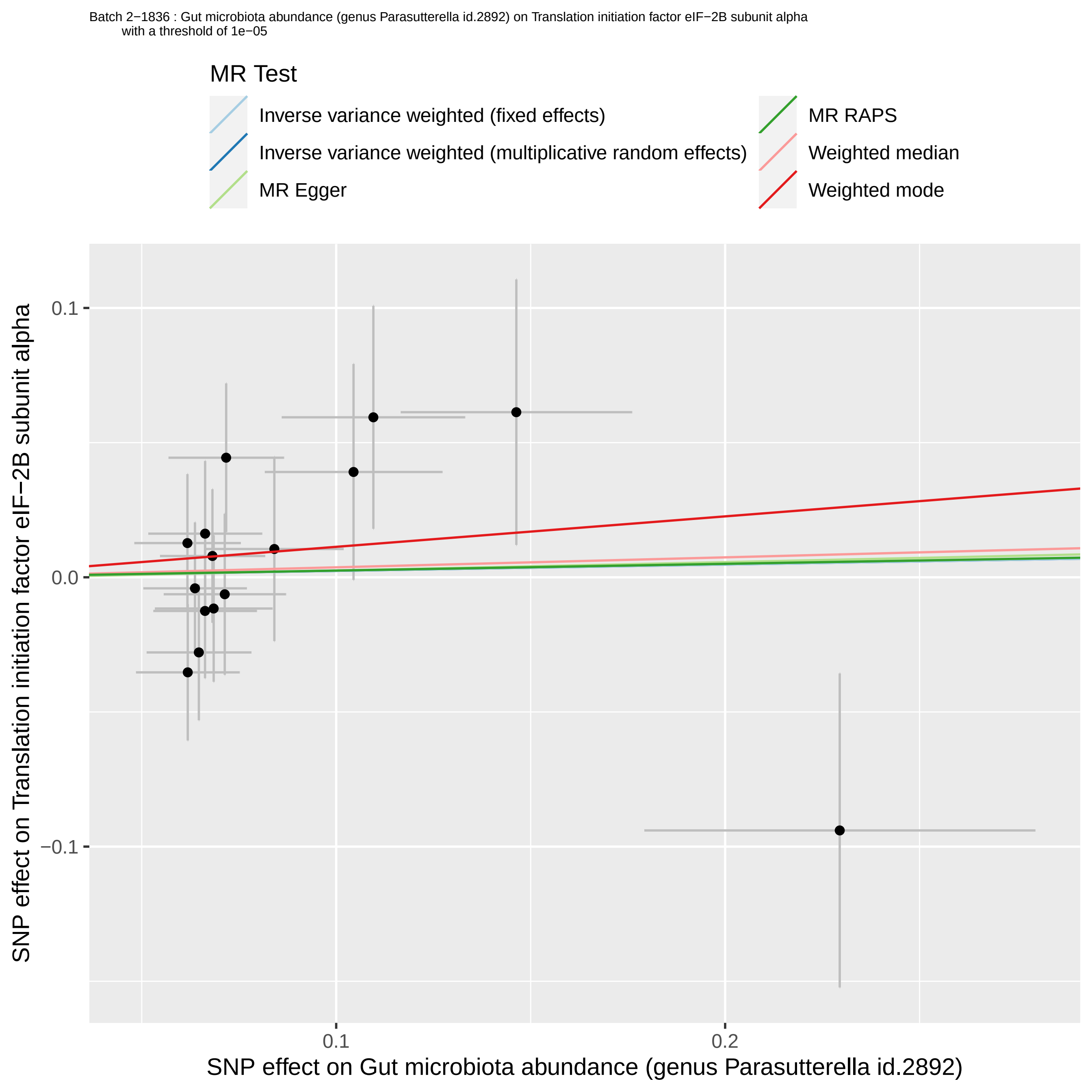

Batch 2−1836 : Gut microbiota abundance (genus Parasutterella id.2892) on Translation initiation factor eIF−2B subunit alpha
with a threshold of 1e−05
MR Test
Inverse variance weighted (fixed effects)
Inverse variance weighted (multiplicative random effects)
MR Egger
MR RAPS
Weighted median
Weighted mode
0.1
0.0
−0.1
0.1
0.2
SNP effect on Translation initiation factor eIF−2B subunit alpha
SNP effect on Gut microbiota abundance (genus Parasutterella id.2892)

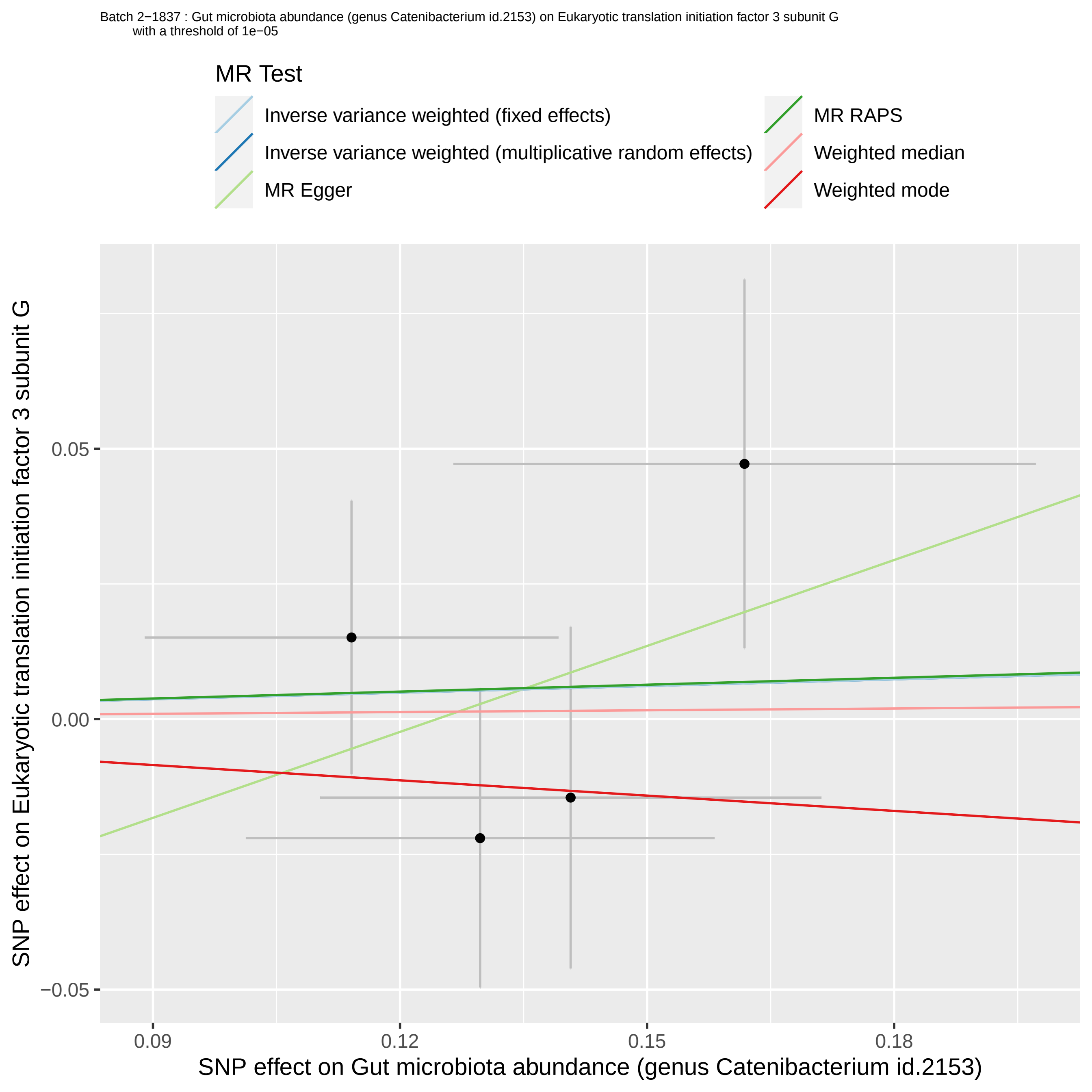
Batch 2−1837 : Gut microbiota abundance (genus Catenibacterium id.2153) on Eukaryotic translation initiation factor 3 subunit G
with a threshold of 1e−05
MR Test
Inverse variance weighted (fixed effects)
Inverse variance weighted (multiplicative random effects)
MR Egger
MR RAPS
Weighted median
Weighted mode
0.05
0.00
−0.05
0.09
0.12
0.15
0.18
SNP effect on Eukaryotic translation initiation factor 3 subunit G
SNP effect on Gut microbiota abundance (genus Catenibacterium id.2153)

Batch 2−1838 : Gut microbiota abundance (genus Parasutterella id.2892) on Eukaryotic translation initiation factor 3 subunit G
with a threshold of 1e−05

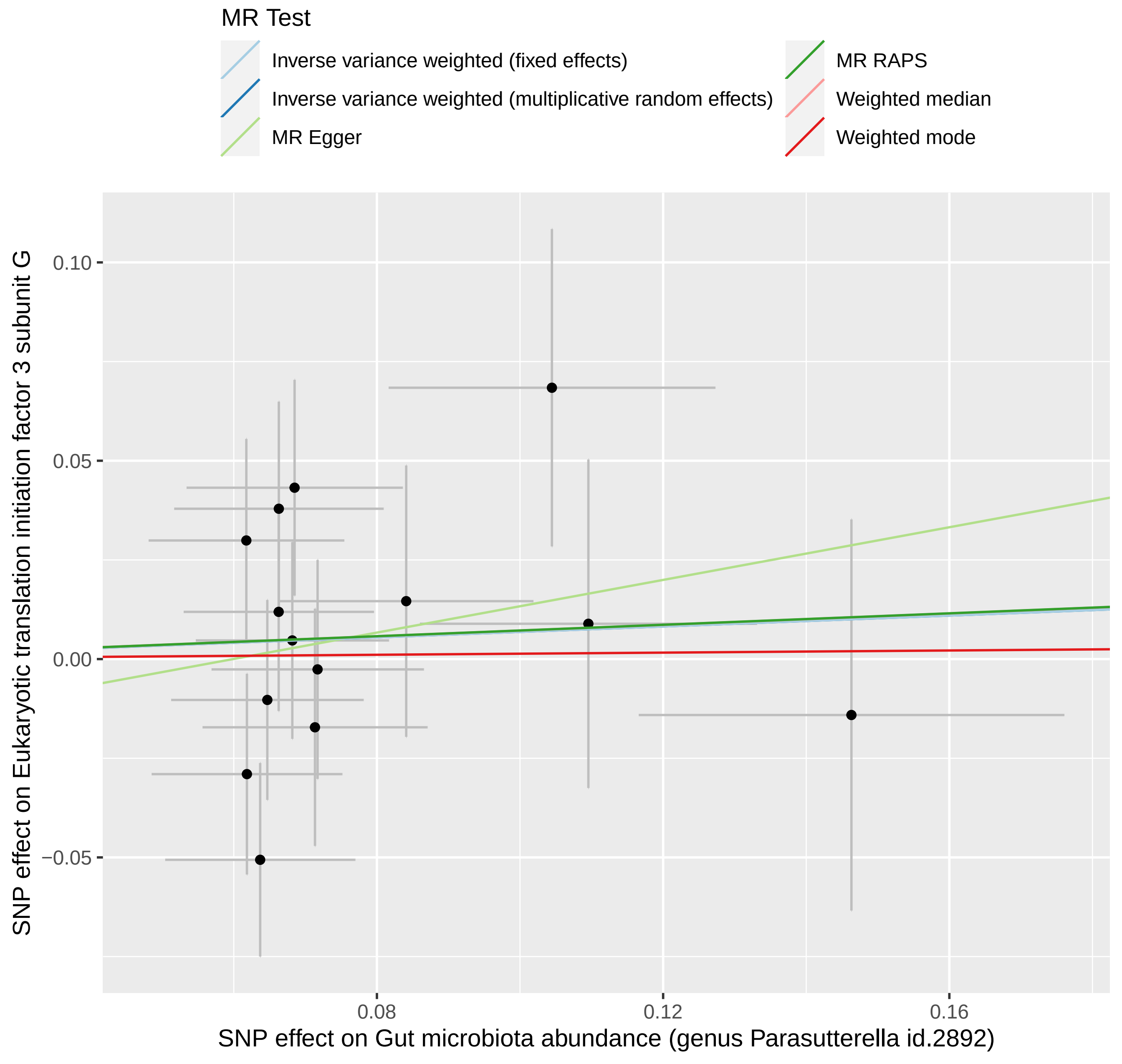

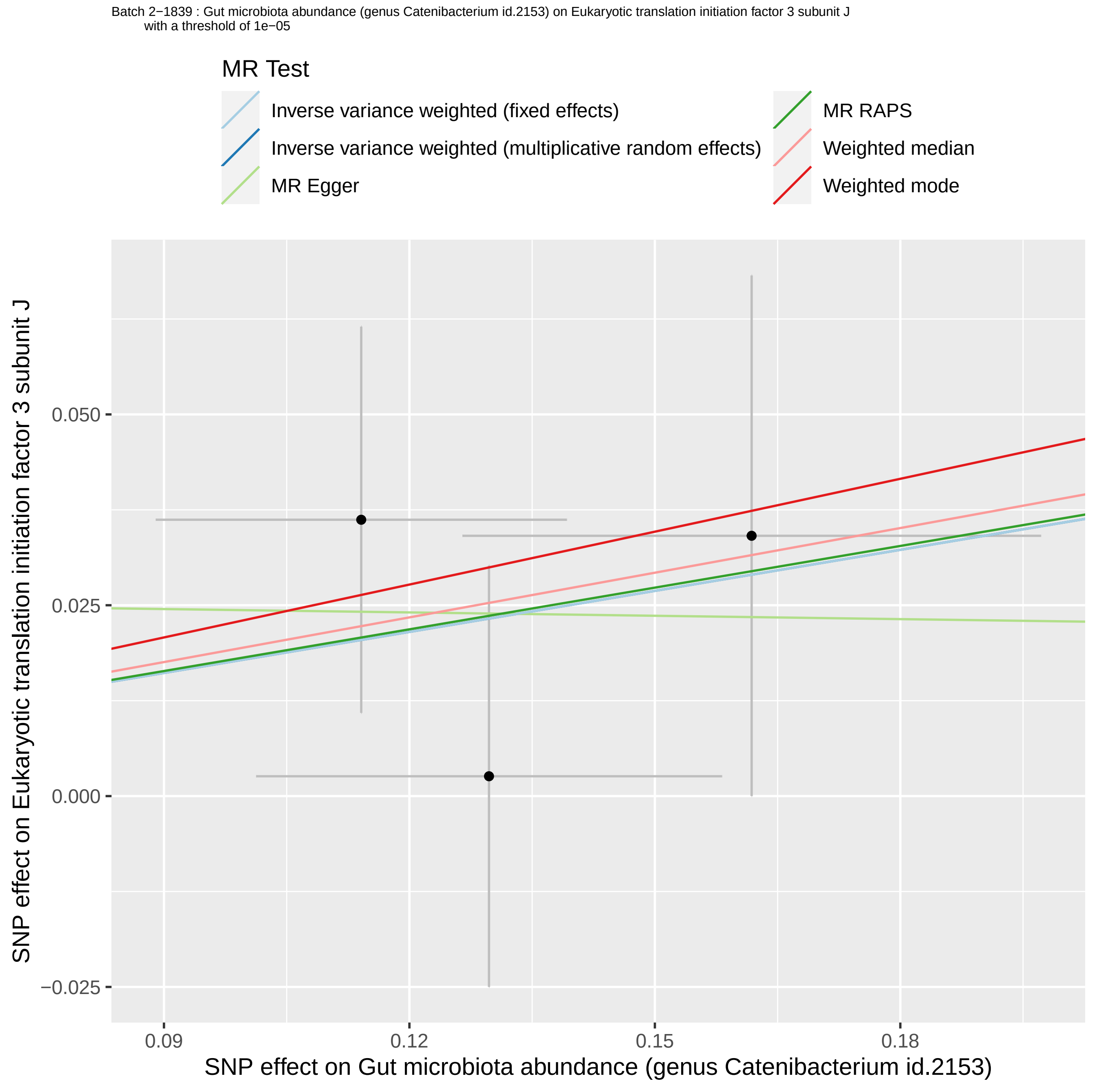

Batch 2−1839 : Gut microbiota abundance (genus Catenibacterium id.2153) on Eukaryotic translation initiation factor 3 subunit J
with a threshold of 1e−05
MR Test
Inverse variance weighted (fixed effects)
Inverse variance weighted (multiplicative random effects)
MR Egger
MR RAPS
Weighted median
Weighted mode
0.050
0.025
0.000
−0.025
0.09
0.12
0.15
0.18
SNP effect on Eukaryotic translation initiation factor 3 subunit J
SNP effect on Gut microbiota abundance (genus Catenibacterium id.2153)

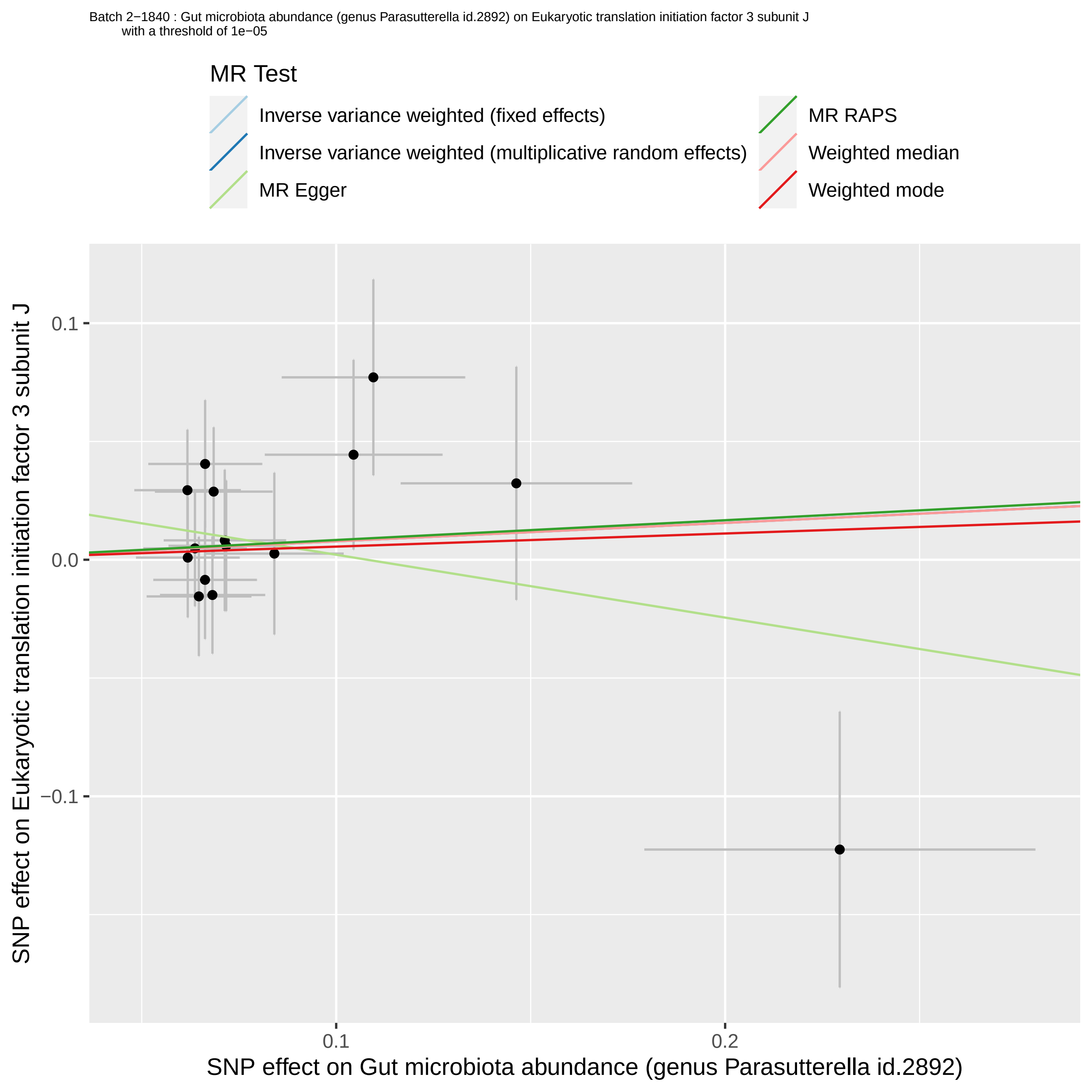
Batch 2−1840 : Gut microbiota abundance (genus Parasutterella id.2892) on Eukaryotic translation initiation factor 3 subunit J
with a threshold of 1e−05
MR Test
Inverse variance weighted (fixed effects)
Inverse variance weighted (multiplicative random effects)
MR Egger
MR RAPS
Weighted median
Weighted mode
0.1
0.0
−0.1
0.1
0.2
SNP effect on Eukaryotic translation initiation factor 3 subunit J
SNP effect on Gut microbiota abundance (genus Parasutterella id.2892)

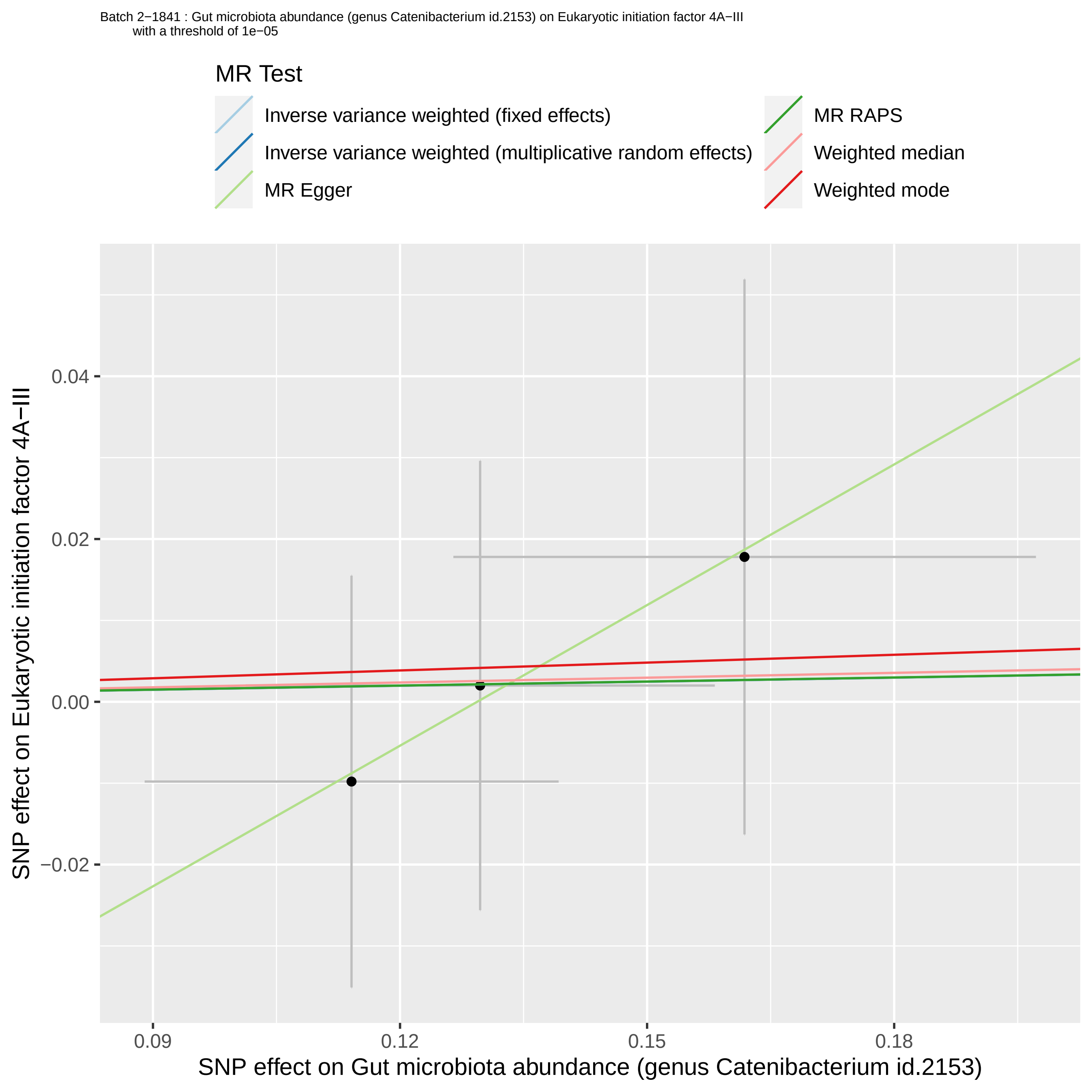
Batch 2−1841 : Gut microbiota abundance (genus Catenibacterium id.2153) on Eukaryotic initiation factor 4A−III
with a threshold of 1e−05
MR Test
Inverse variance weighted (fixed effects)
Inverse variance weighted (multiplicative random effects)
MR Egger
MR RAPS
Weighted median
Weighted mode
0.04
0.02
0.00
−0.02
0.09
0.12
0.15
0.18
SNP effect on Eukaryotic initiation factor 4A−III
SNP effect on Gut microbiota abundance (genus Catenibacterium id.2153)

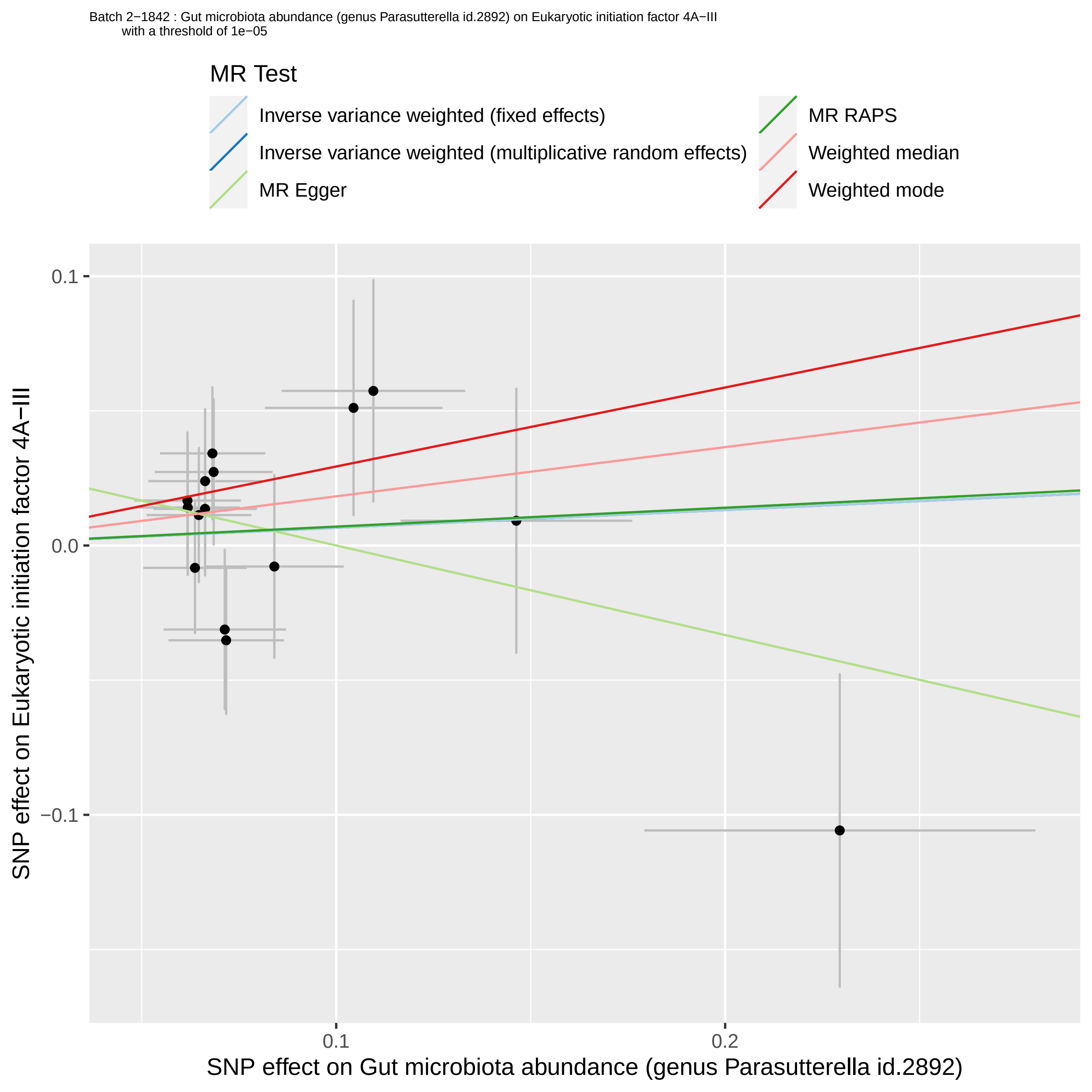

Batch 2−1842 : Gut microbiota abundance (genus Parasutterella id.2892) on Eukaryotic initiation factor 4A−III
with a threshold of 1e−05
MR Test
Inverse variance weighted (fixed effects)
Inverse variance weighted (multiplicative random effects)
MR Egger
MR RAPS
Weighted median
Weighted mode
0.1
0.0
−0.1
0.1
0.2
SNP effect on Eukaryotic initiation factor 4A−III
SNP effect on Gut microbiota abundance (genus Parasutterella id.2892)

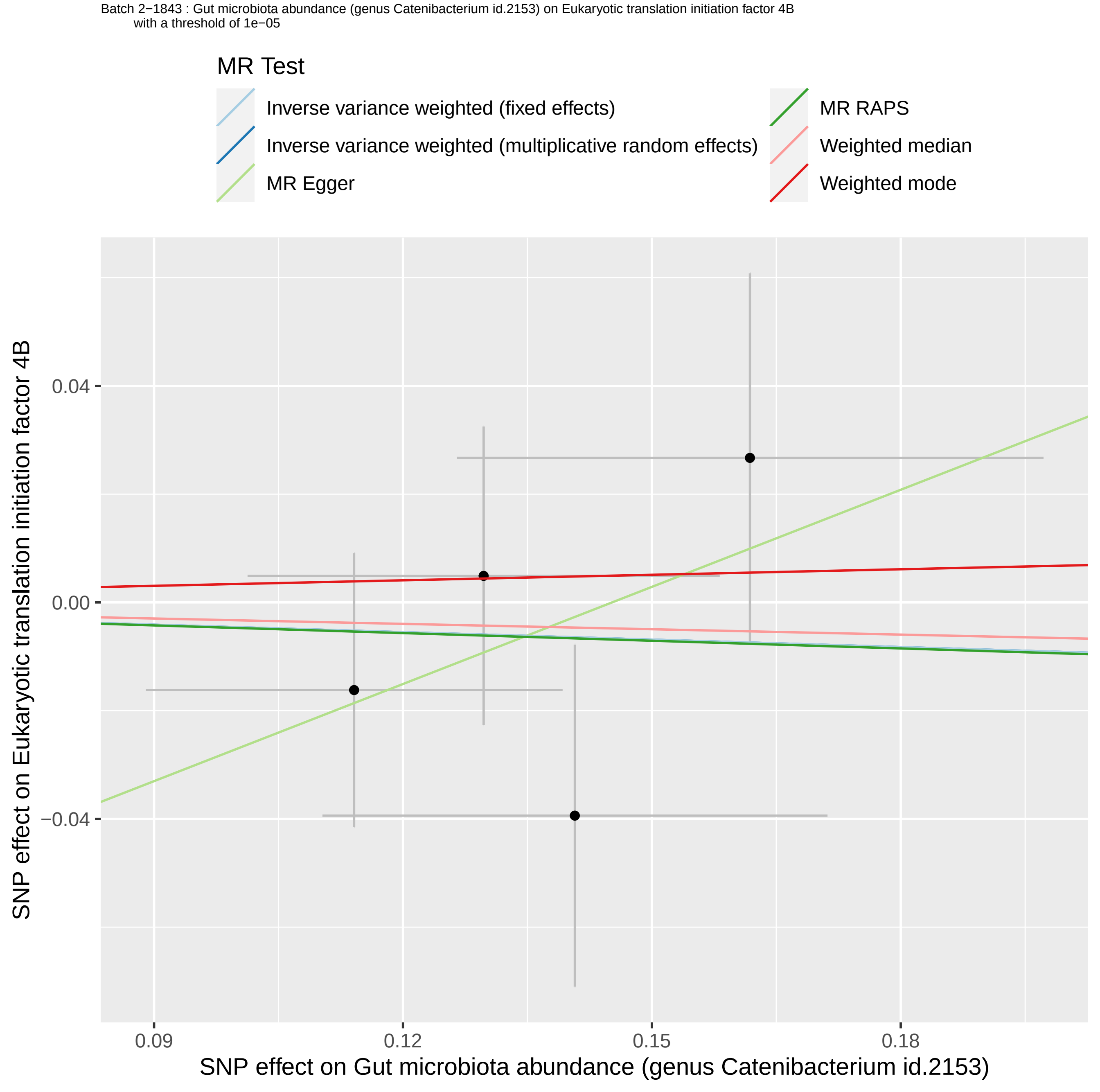

Batch 2−1843 : Gut microbiota abundance (genus Catenibacterium id.2153) on Eukaryotic translation initiation factor 4B
with a threshold of 1e−05
MR Test
Inverse variance weighted (fixed effects)
Inverse variance weighted (multiplicative random effects)
MR Egger
MR RAPS
Weighted median
Weighted mode
0.04
0.00
−0.04
0.09
0.12
0.15
0.18
SNP effect on Eukaryotic translation initiation factor 4B
SNP effect on Gut microbiota abundance (genus Catenibacterium id.2153)

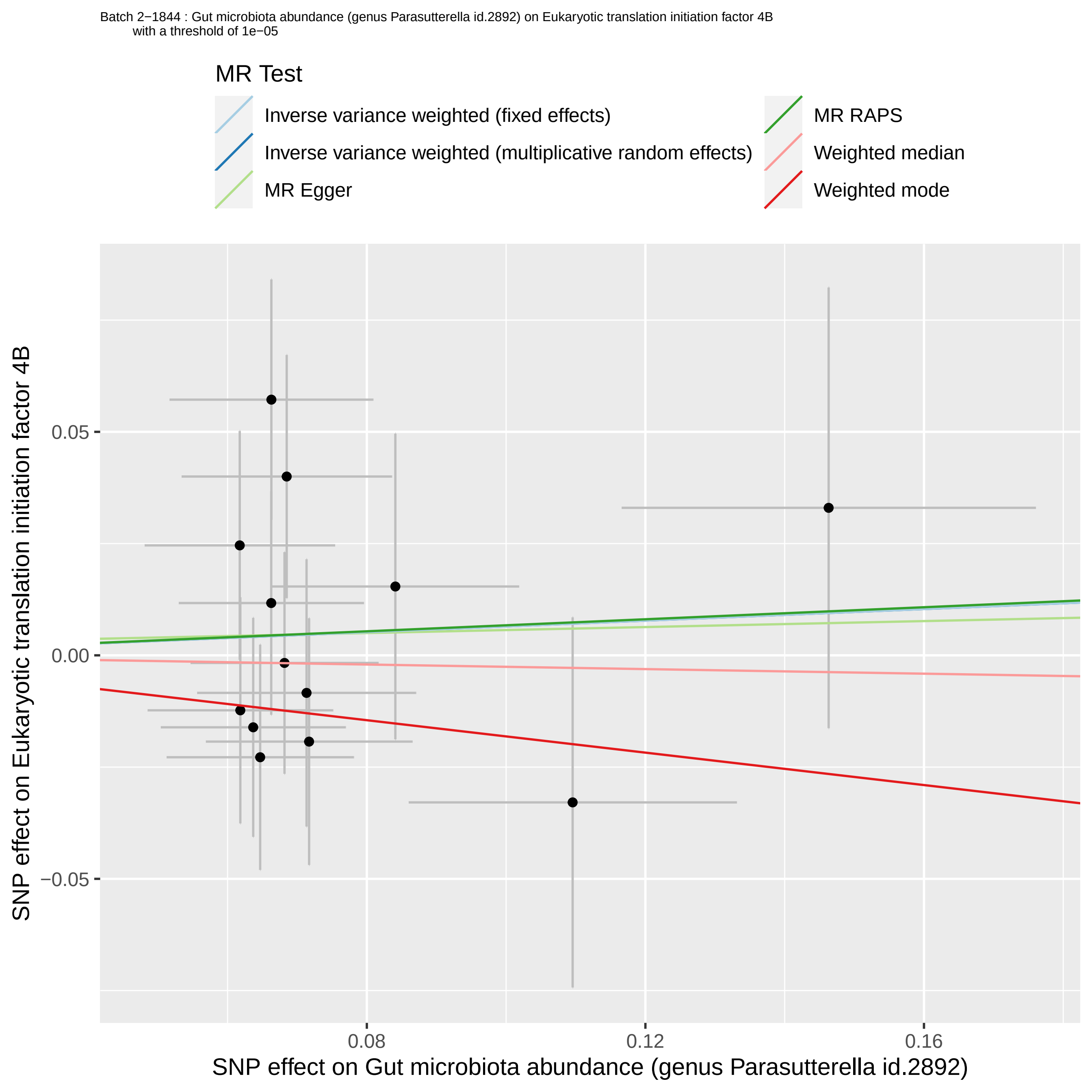
Batch 2−1844 : Gut microbiota abundance (genus Parasutterella id.2892) on Eukaryotic translation initiation factor 4B
with a threshold of 1e−05
MR Test
Inverse variance weighted (fixed effects)
Inverse variance weighted (multiplicative random effects)
MR Egger
MR RAPS
Weighted median
Weighted mode
0.05
0.00
−0.05
0.08
0.12
0.16
SNP effect on Eukaryotic translation initiation factor 4B
SNP effect on Gut microbiota abundance (genus Parasutterella id.2892)

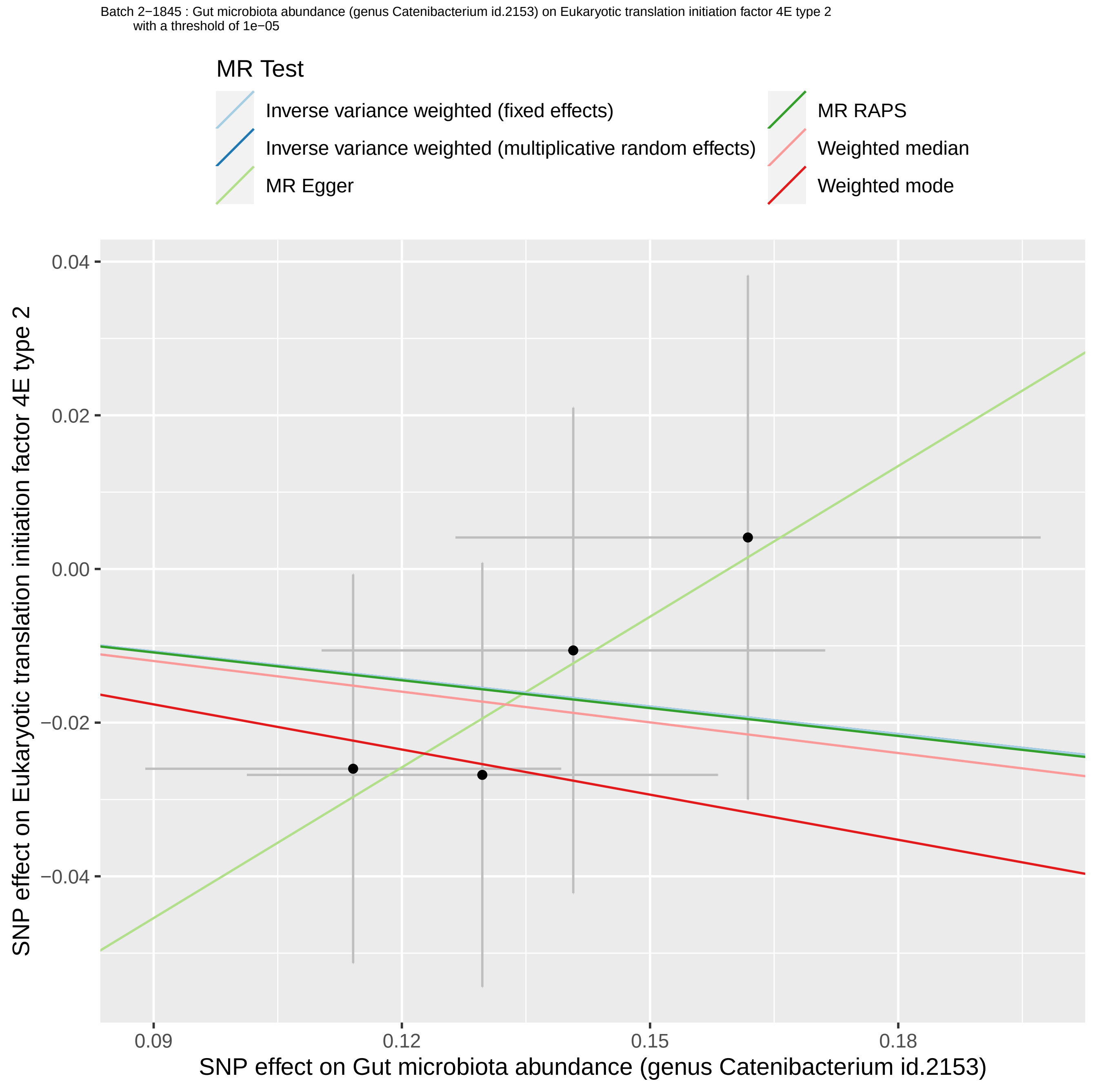

Batch 2−1845 : Gut microbiota abundance (genus Catenibacterium id.2153) on Eukaryotic translation initiation factor 4E type 2
with a threshold of 1e−05
MR Test
Inverse variance weighted (fixed effects)
Inverse variance weighted (multiplicative random effects)
MR Egger
MR RAPS
Weighted median
Weighted mode
0.04
0.02
0.00
−0.02
−0.04
0.09
0.12
0.15
0.18
SNP effect on Eukaryotic translation initiation factor 4E type 2
SNP effect on Gut microbiota abundance (genus Catenibacterium id.2153)

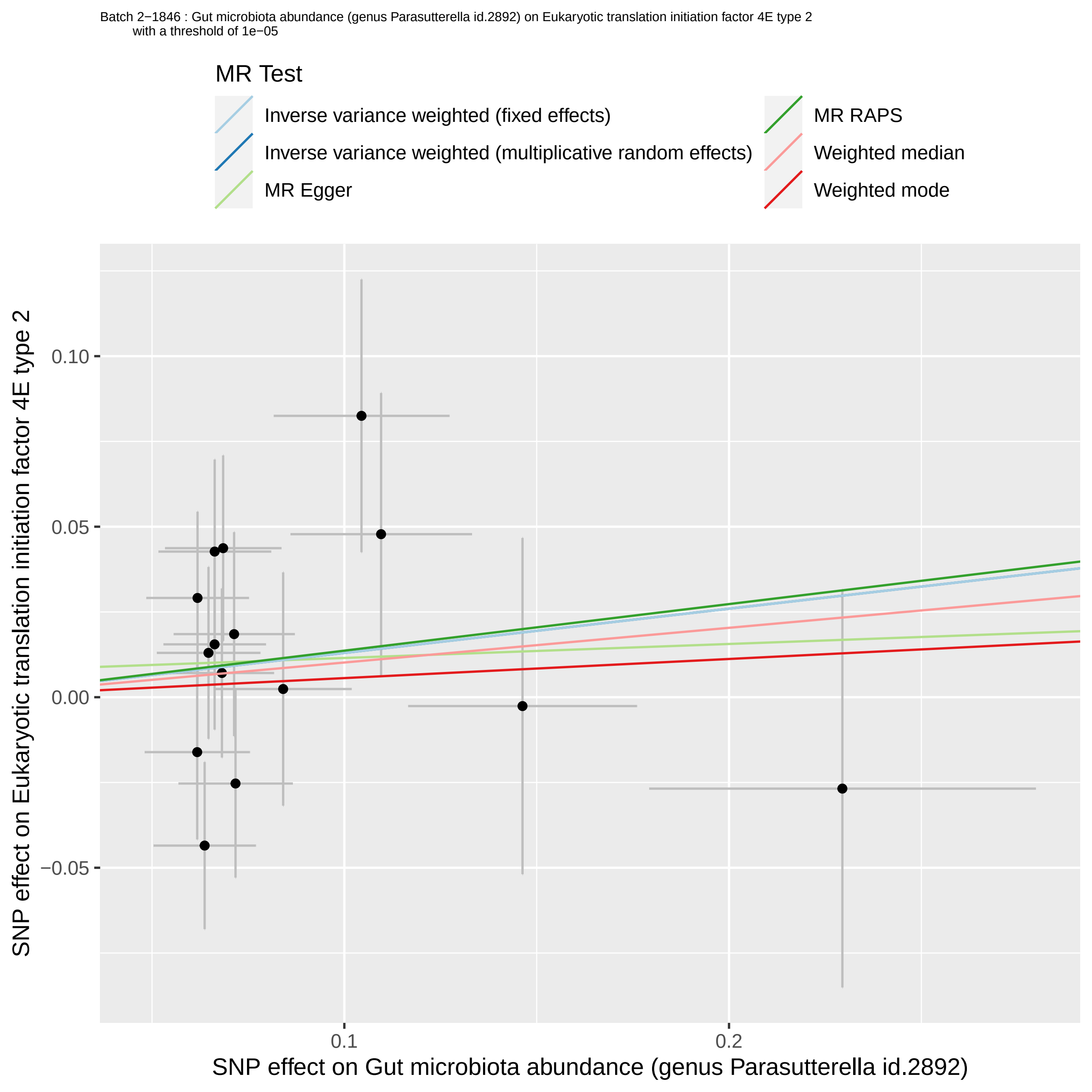
Batch 2−1846 : Gut microbiota abundance (genus Parasutterella id.2892) on Eukaryotic translation initiation factor 4E type 2
with a threshold of 1e−05
MR Test
Inverse variance weighted (fixed effects)
Inverse variance weighted (multiplicative random effects)
MR Egger
MR RAPS
Weighted median
Weighted mode
0.10
0.05
0.00
−0.05
0.1
0.2
SNP effect on Eukaryotic translation initiation factor 4E type 2
SNP effect on Gut microbiota abundance (genus Parasutterella id.2892)

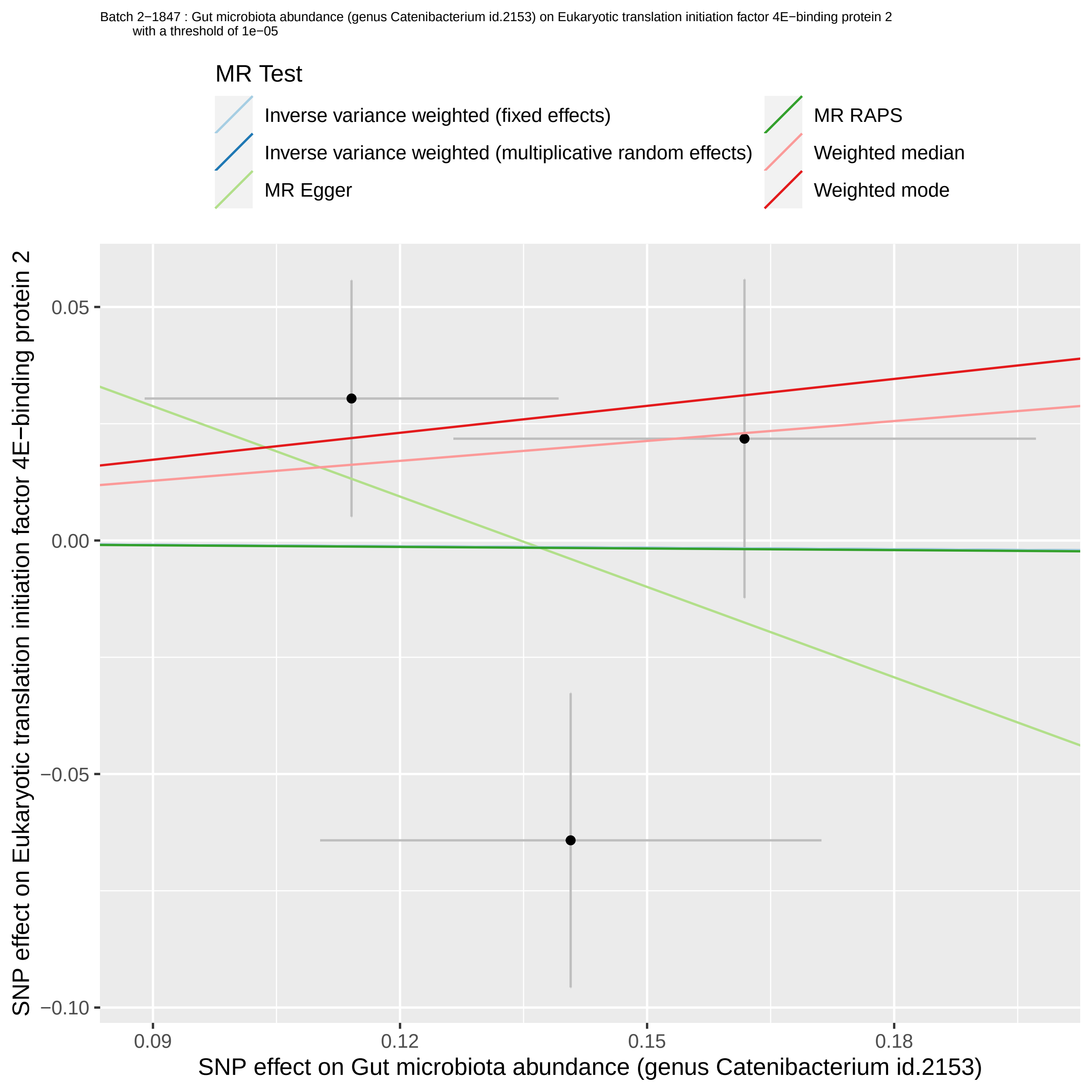
Batch 2−1847 : Gut microbiota abundance (genus Catenibacterium id.2153) on Eukaryotic translation initiation factor 4E−binding protein 2
with a threshold of 1e−05
MR Test
Inverse variance weighted (fixed effects)
Inverse variance weighted (multiplicative random effects)
MR Egger
MR RAPS
Weighted median
Weighted mode
SNP effect on Eukaryotic translation initiation factor 4E−binding protein 2
0.05
0.00
−0.05
−0.10
0.09
0.12
0.15
0.18
SNP effect on Gut microbiota abundance (genus Catenibacterium id.2153)

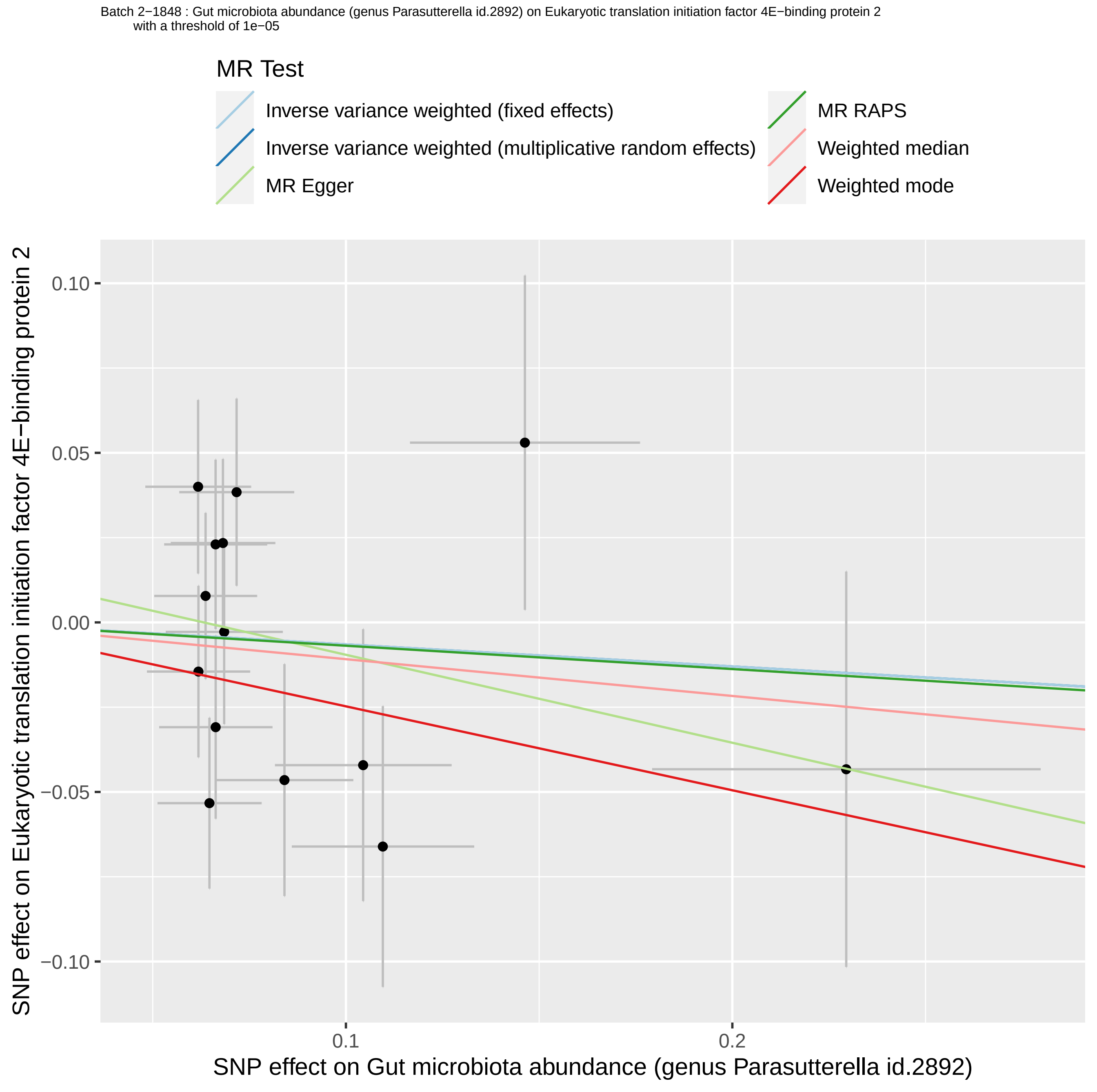
Batch 2−1848 : Gut microbiota abundance (genus Parasutterella id.2892) on Eukaryotic translation initiation factor 4E−binding protein 2
with a threshold of 1e−05
MR Test
Inverse variance weighted (fixed effects)
Inverse variance weighted (multiplicative random effects)
MR Egger
MR RAPS
Weighted median
Weighted mode
SNP effect on Eukaryotic translation initiation factor 4E−binding protein 2
0.10
0.05
0.00
−0.05
−0.10
0.1
0.2
SNP effect on Gut microbiota abundance (genus Parasutterella id.2892)

Batch 2−1849 : Gut microbiota abundance (genus Catenibacterium id.2153) on Eukaryotic translation initiation factor 4 gamma 3 with a threshold of 1e−05

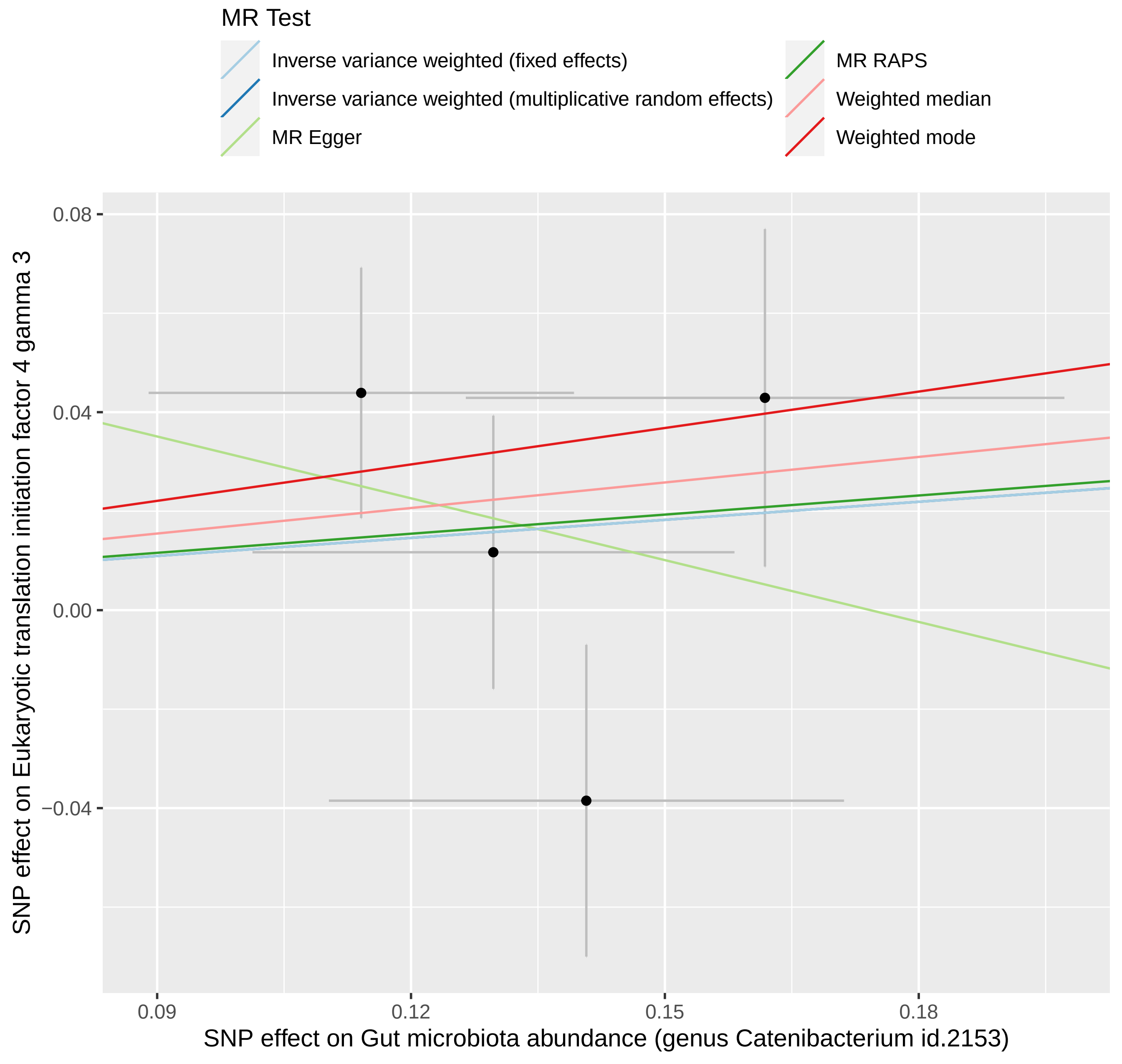

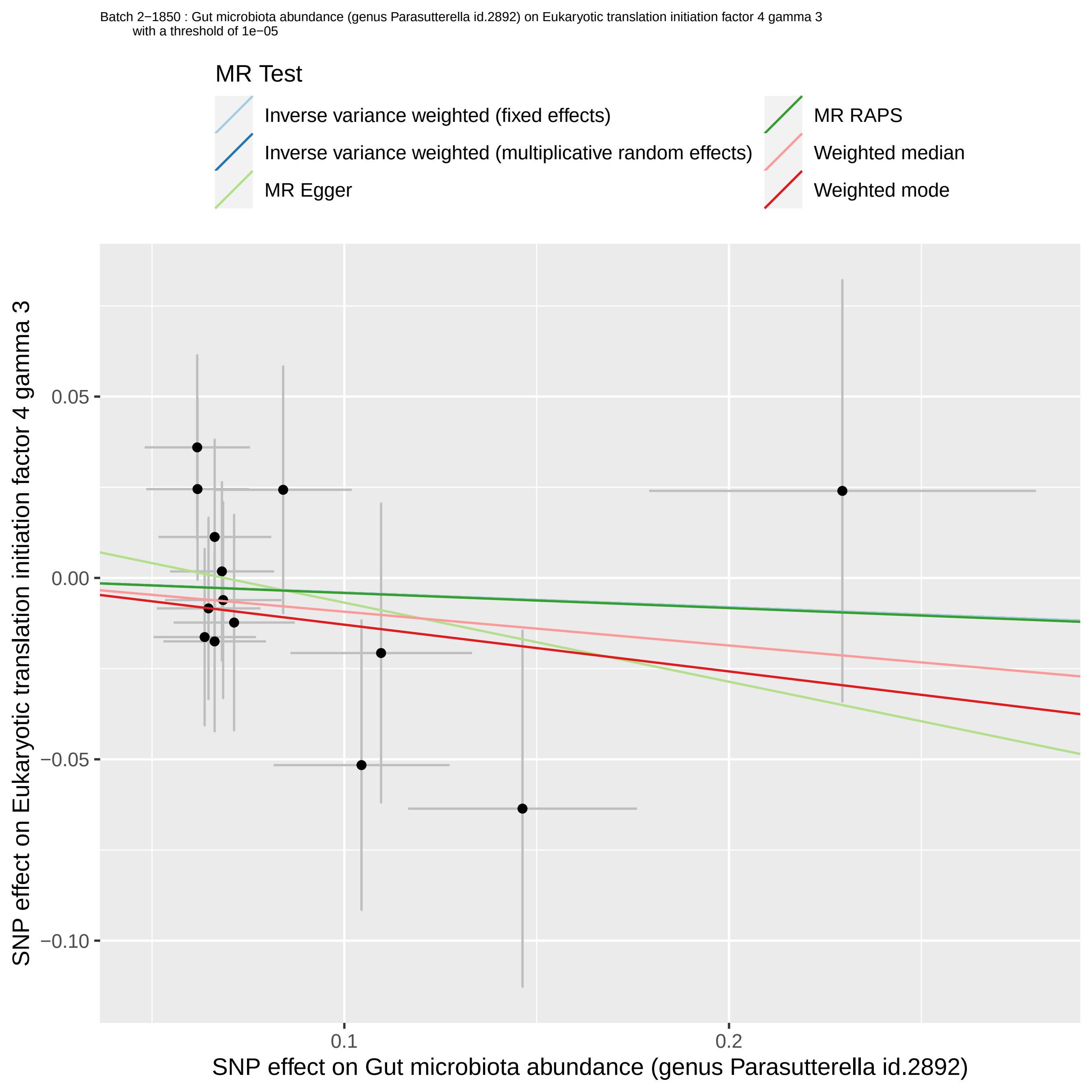

Batch 2−1850 : Gut microbiota abundance (genus Parasutterella id.2892) on Eukaryotic translation initiation factor 4 gamma 3
with a threshold of 1e−05
MR Test
Inverse variance weighted (fixed effects)
Inverse variance weighted (multiplicative random effects)
MR Egger
MR RAPS
Weighted median
Weighted mode
0.05
0.00
−0.05
−0.10
0.1
0.2
SNP effect on Eukaryotic translation initiation factor 4 gamma 3
SNP effect on Gut microbiota abundance (genus Parasutterella id.2892)

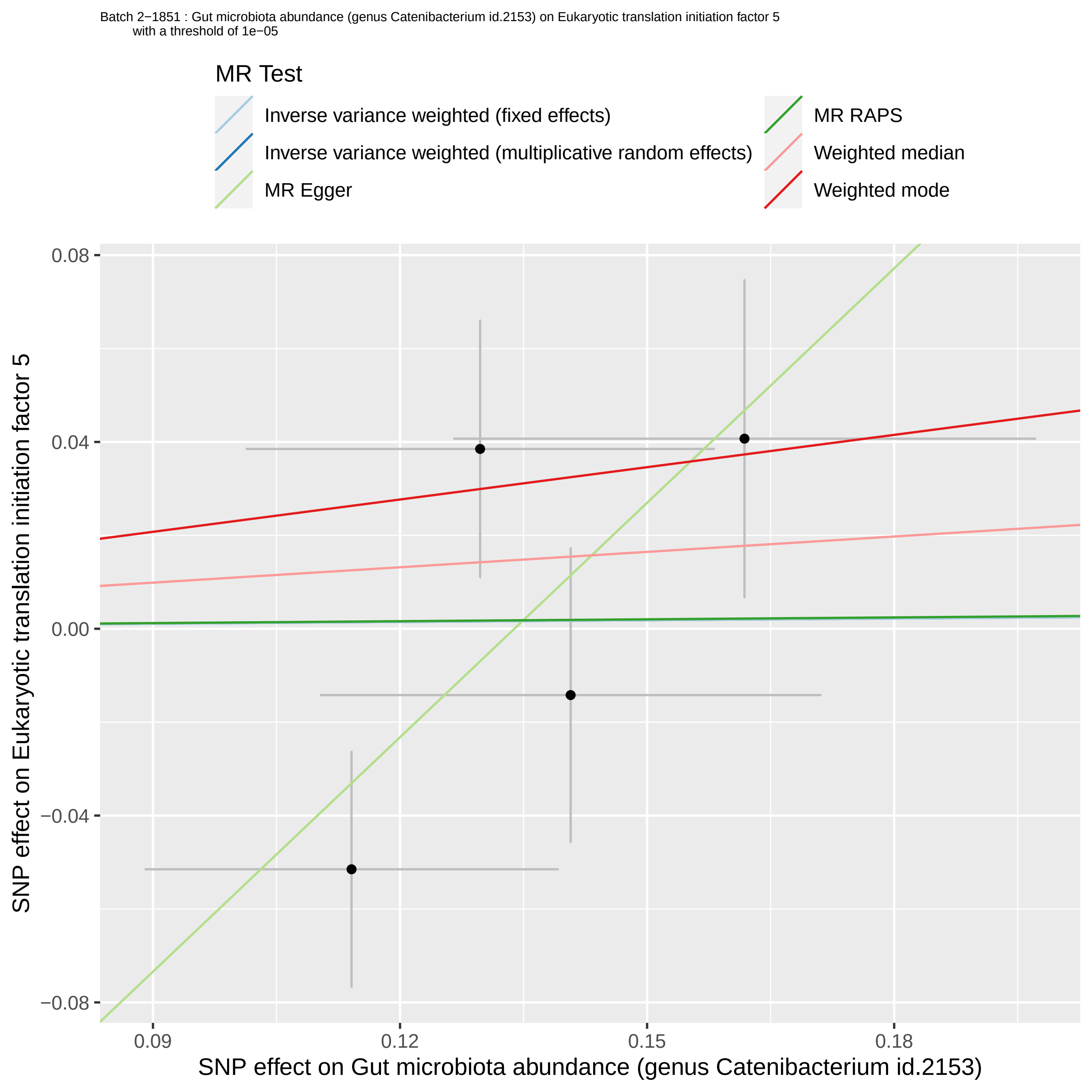

Batch 2−1851 : Gut microbiota abundance (genus Catenibacterium id.2153) on Eukaryotic translation initiation factor 5
with a threshold of 1e−05
MR Test
Inverse variance weighted (fixed effects)
Inverse variance weighted (multiplicative random effects)
MR Egger
MR RAPS
Weighted median
Weighted mode
0.08
0.04
0.00
−0.04
−0.08
0.09
0.12
0.15
0.18
SNP effect on Eukaryotic translation initiation factor 5
SNP effect on Gut microbiota abundance (genus Catenibacterium id.2153)

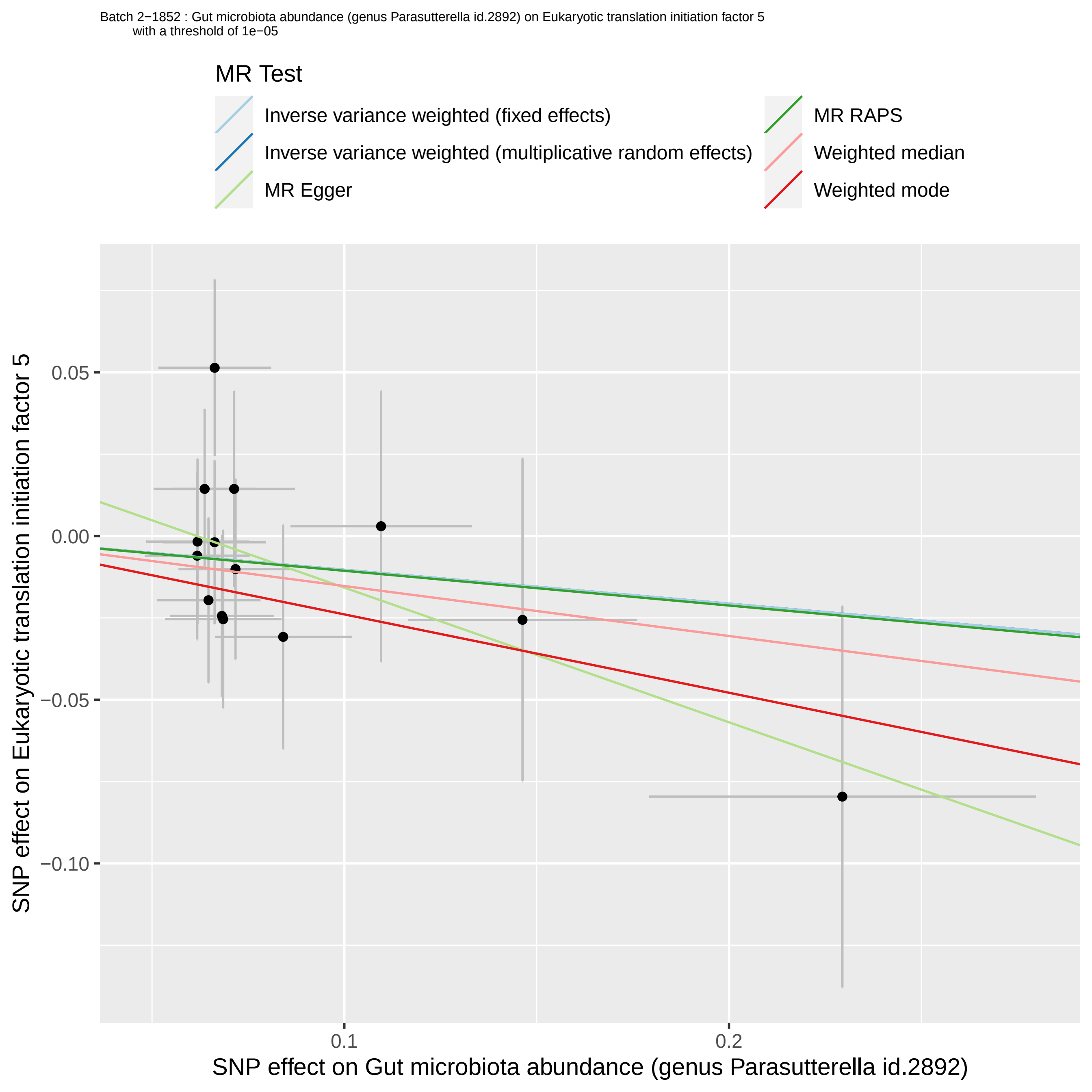

Batch 2−1852 : Gut microbiota abundance (genus Parasutterella id.2892) on Eukaryotic translation initiation factor 5
with a threshold of 1e−05
MR Test
Inverse variance weighted (fixed effects)
Inverse variance weighted (multiplicative random effects)
MR Egger
MR RAPS
Weighted median
Weighted mode
0.05
0.00
−0.05
−0.10
0.1
0.2
SNP effect on Eukaryotic translation initiation factor 5
SNP effect on Gut microbiota abundance (genus Parasutterella id.2892)

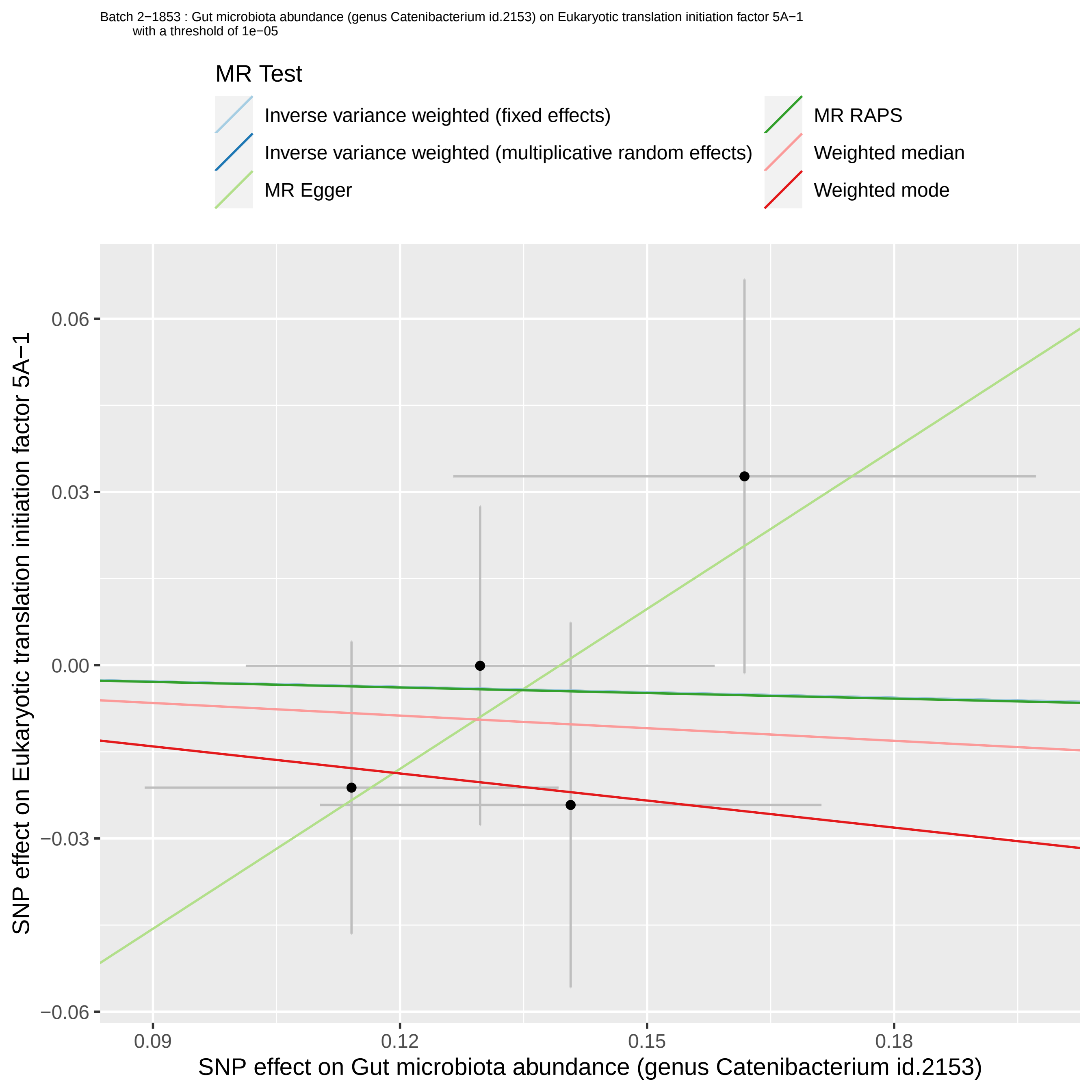
Batch 2−1853 : Gut microbiota abundance (genus Catenibacterium id.2153) on Eukaryotic translation initiation factor 5A−1
with a threshold of 1e−05
MR Test
Inverse variance weighted (fixed effects)
Inverse variance weighted (multiplicative random effects)
MR Egger
MR RAPS
Weighted median
Weighted mode
0.06
0.03
0.00
−0.03
−0.06
0.09
0.12
0.15
0.18
SNP effect on Eukaryotic translation initiation factor 5A−1
SNP effect on Gut microbiota abundance (genus Catenibacterium id.2153)

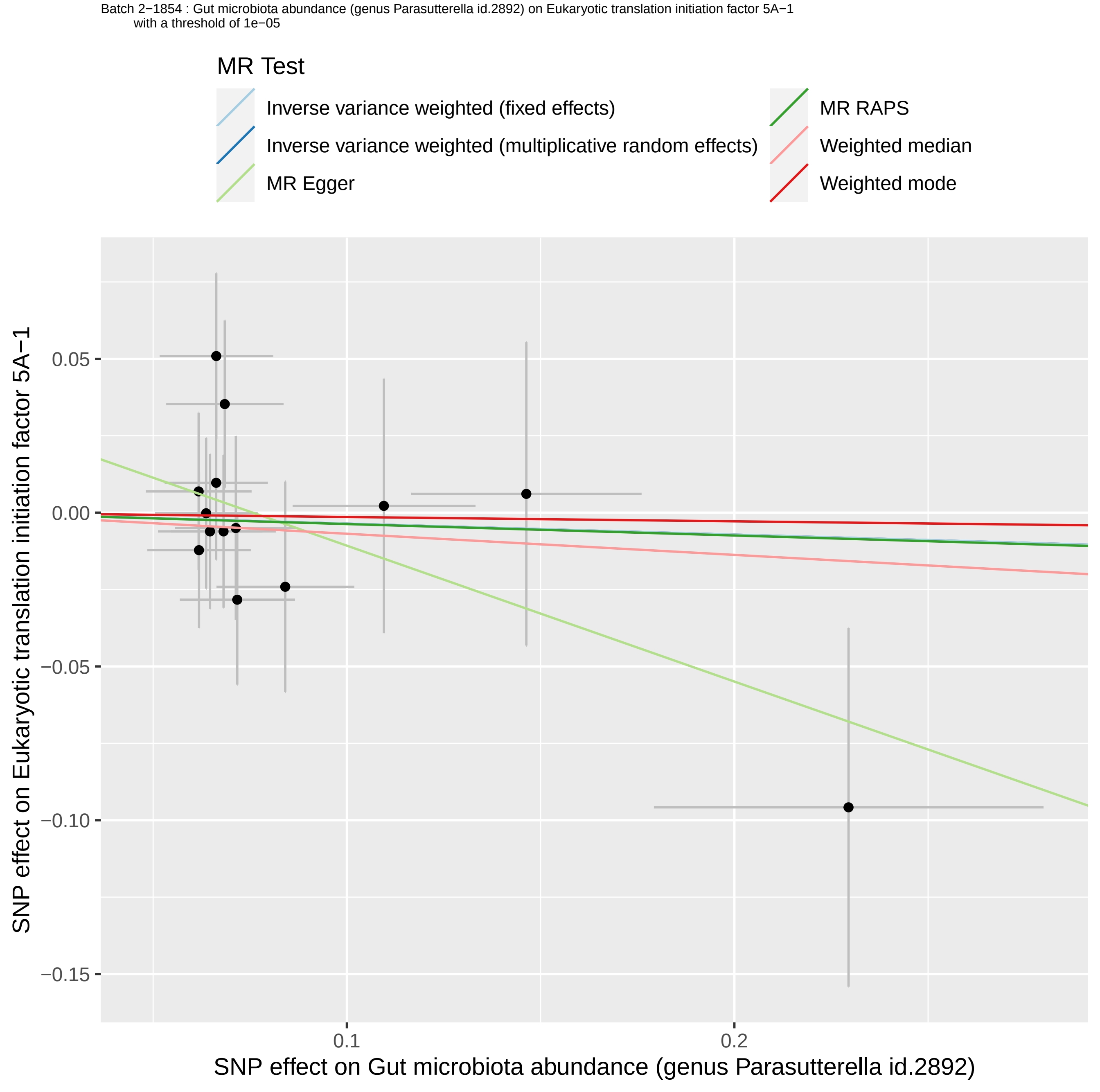

Batch 2−1854 : Gut microbiota abundance (genus Parasutterella id.2892) on Eukaryotic translation initiation factor 5A−1
with a threshold of 1e−05
MR Test
Inverse variance weighted (fixed effects)
Inverse variance weighted (multiplicative random effects)
MR Egger
MR RAPS
Weighted median
Weighted mode
SNP effect on Eukaryotic translation initiation factor 5A−1
0.05
0.00
−0.05
−0.10
−0.15
0.1
0.2
SNP effect on Gut microbiota abundance (genus Parasutterella id.2892)

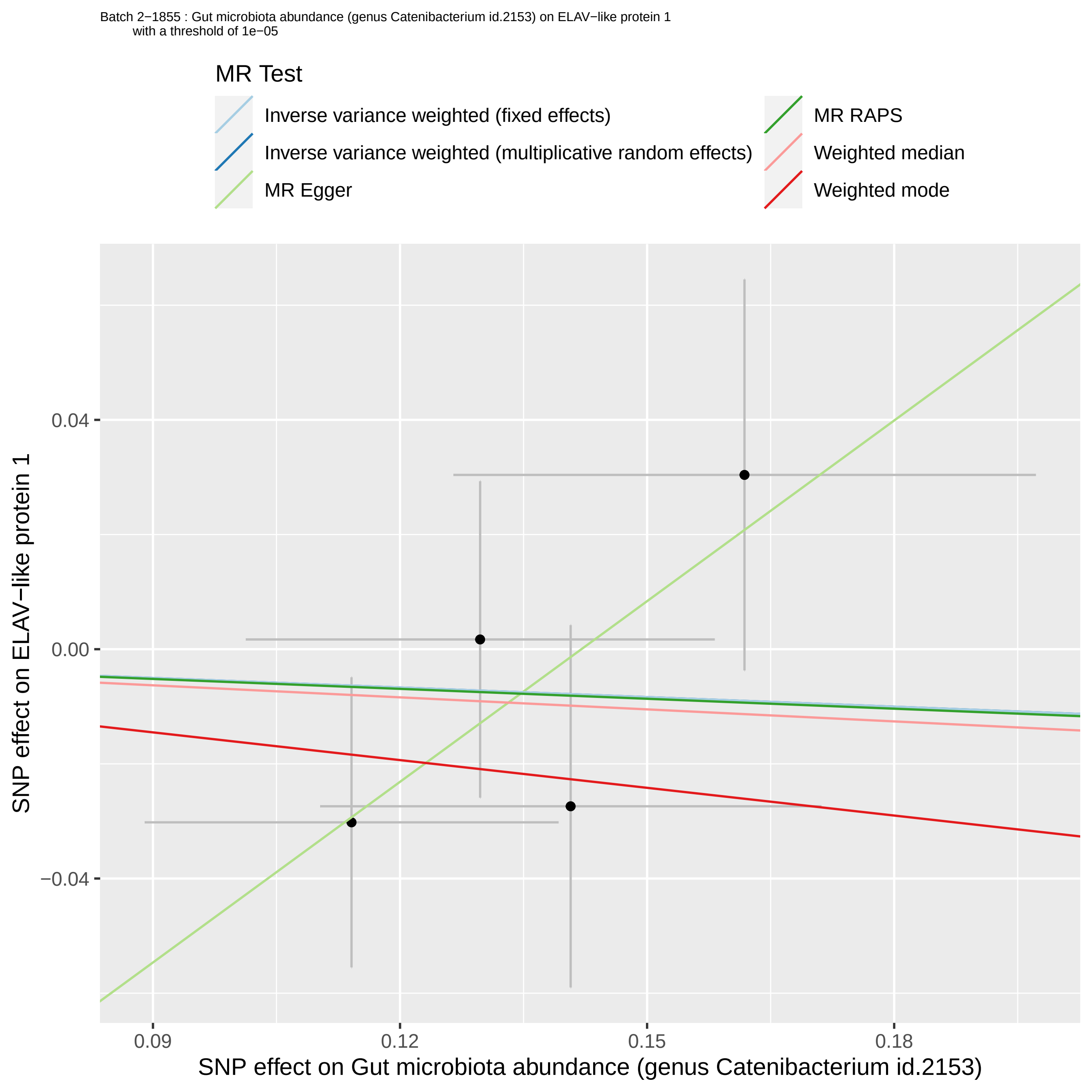
Batch 2−1855 : Gut microbiota abundance (genus Catenibacterium id.2153) on ELAV−like protein 1
with a threshold of 1e−05
MR Test
Inverse variance weighted (fixed effects)
Inverse variance weighted (multiplicative random effects)
MR Egger
MR RAPS
Weighted median
Weighted mode
0.04
0.00
−0.04
0.09
0.12
0.15
0.18
SNP effect on ELAV−like protein 1
SNP effect on Gut microbiota abundance (genus Catenibacterium id.2153)

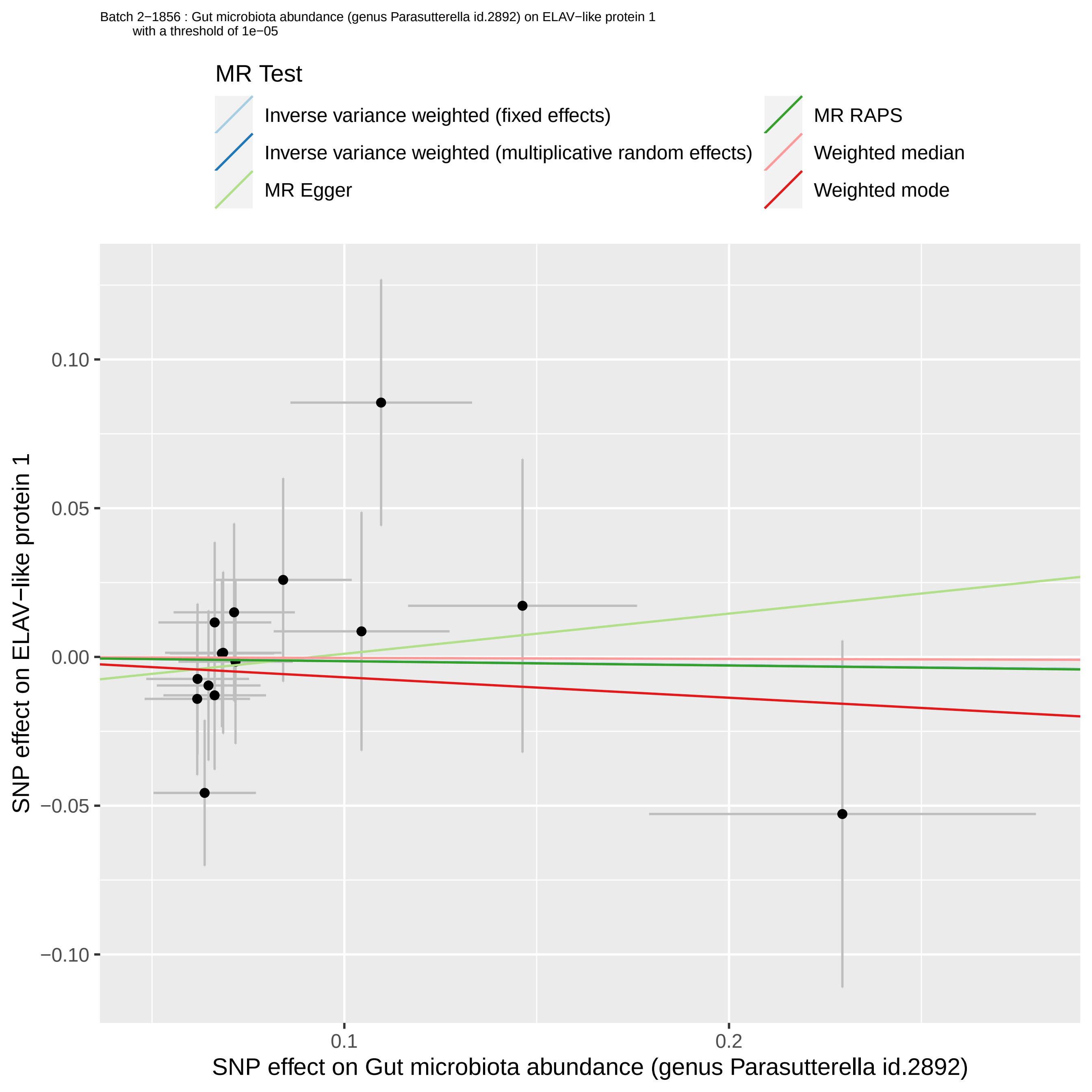

Batch 2−1856 : Gut microbiota abundance (genus Parasutterella id.2892) on ELAV−like protein 1
with a threshold of 1e−05
MR Test
Inverse variance weighted (fixed effects)
Inverse variance weighted (multiplicative random effects)
MR Egger
MR RAPS
Weighted median
Weighted mode
0.10
0.05
0.00
−0.05
−0.10
0.1
0.2
SNP effect on ELAV−like protein 1
SNP effect on Gut microbiota abundance (genus Parasutterella id.2892)

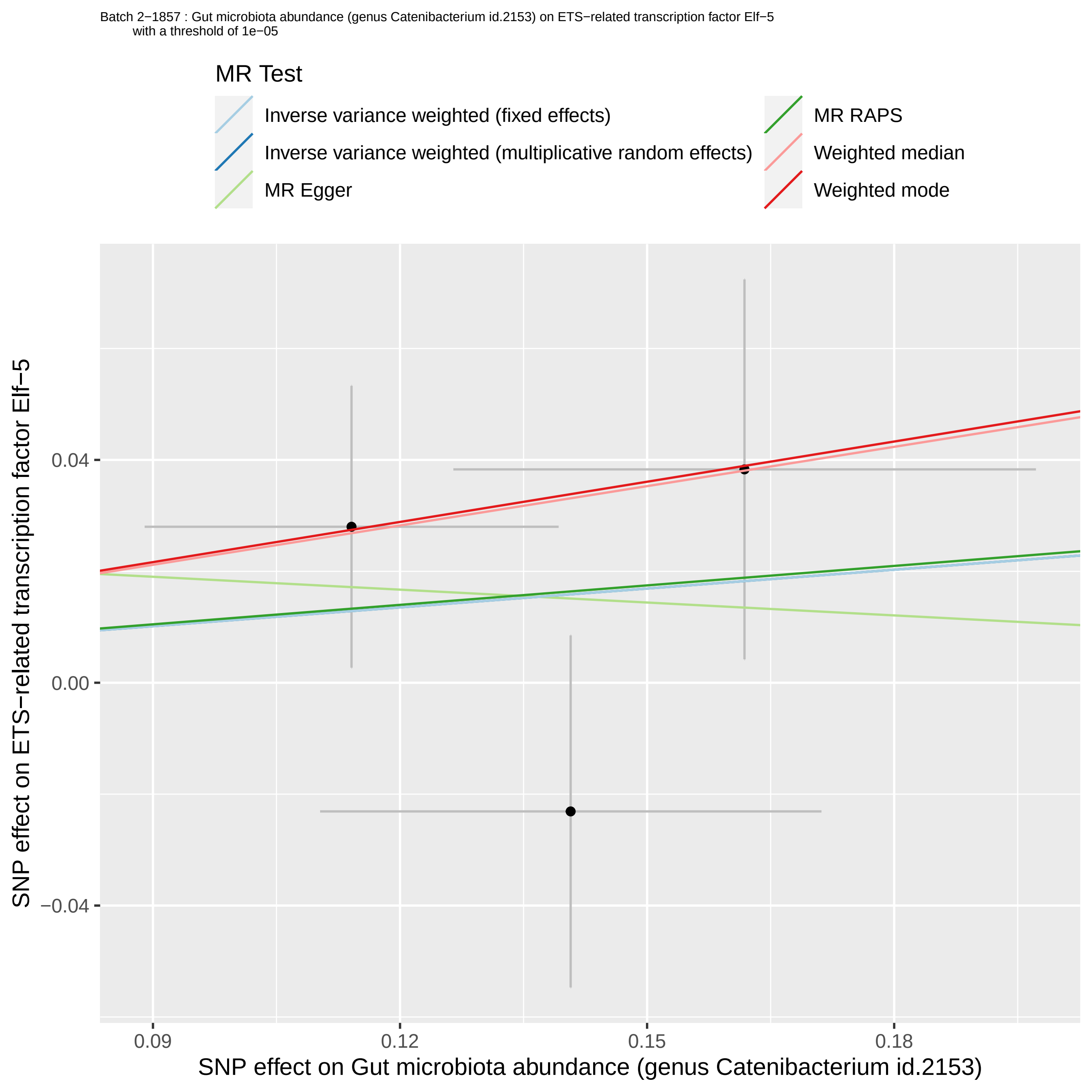

Batch 2−1857 : Gut microbiota abundance (genus Catenibacterium id.2153) on ETS−related transcription factor Elf−5
with a threshold of 1e−05
MR Test
Inverse variance weighted (fixed effects)
Inverse variance weighted (multiplicative random effects)
MR Egger
MR RAPS
Weighted median
Weighted mode
SNP effect on ETS−related transcription factor Elf−5
0.04
0.00
−0.04
0.09
0.12
0.15
0.18
SNP effect on Gut microbiota abundance (genus Catenibacterium id.2153)

Batch 2−1858 : Gut microbiota abundance (genus Parasutterella id.2892) on ETS−related transcription factor Elf−5
with a threshold of 1e−05

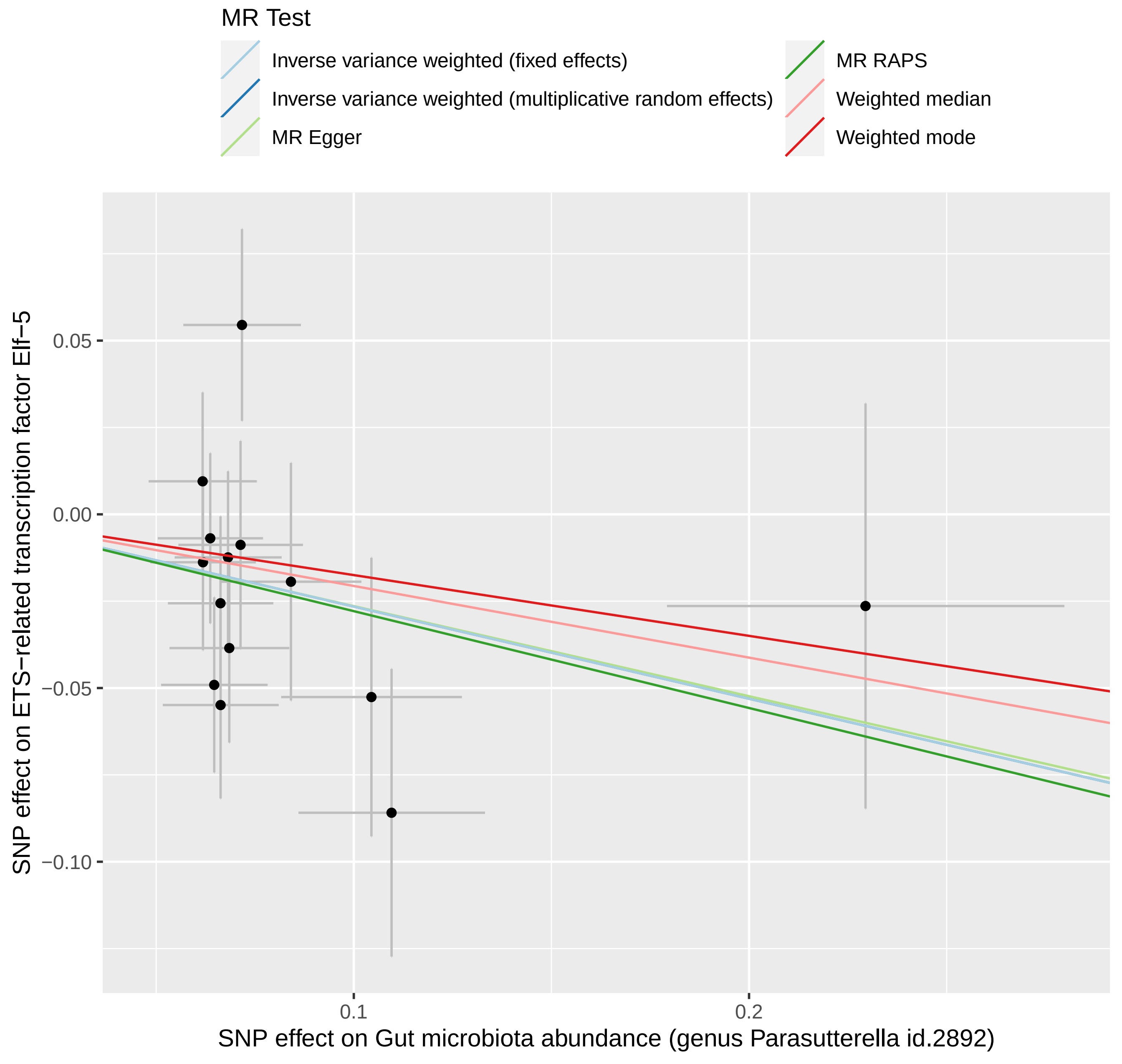

Batch 2−1859 : Gut microbiota abundance (genus Catenibacterium id.2153) on ETS domain−containing protein Elk−1
with a threshold of 1e−05

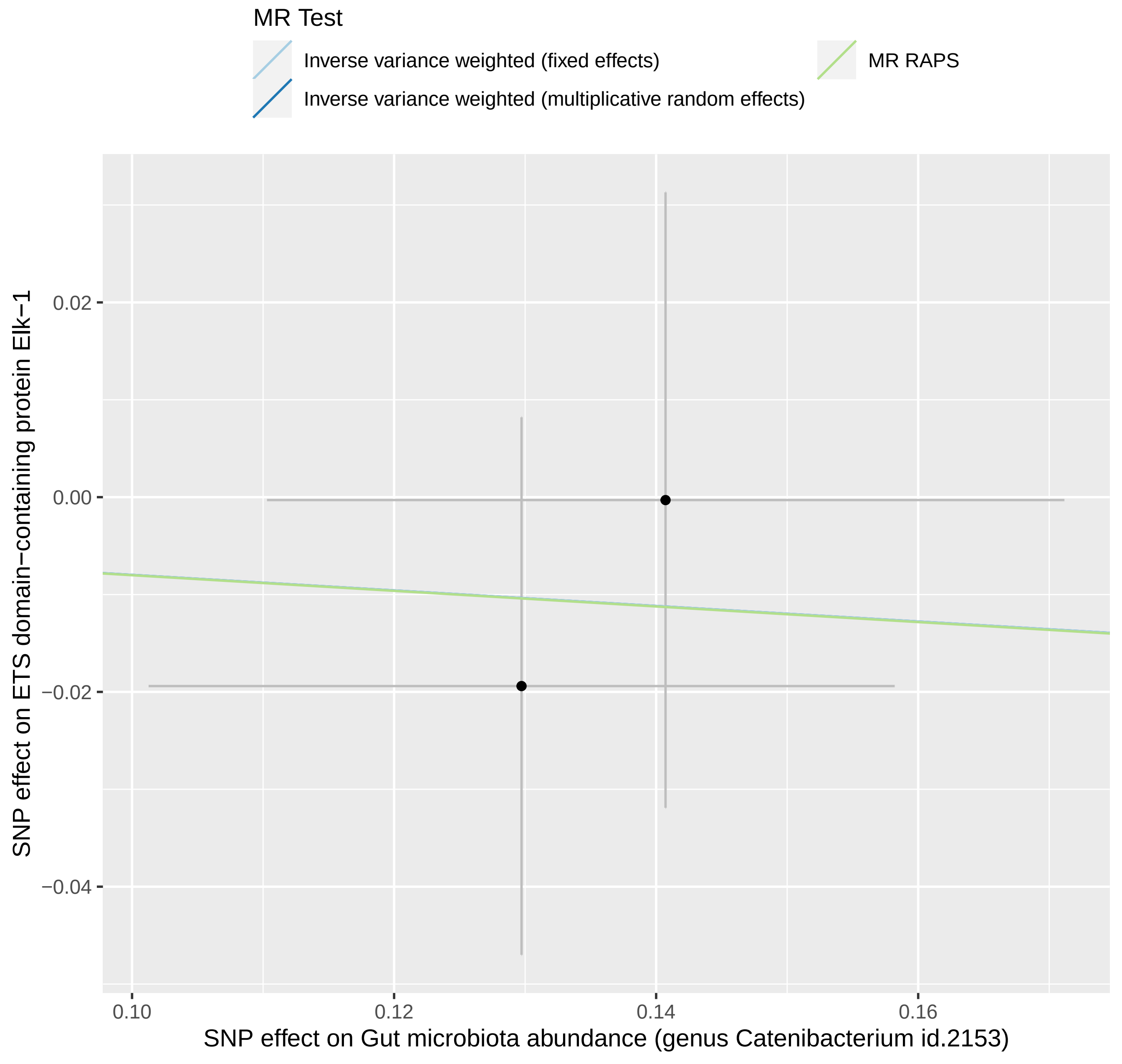

Batch 2−1860 : Gut microbiota abundance (genus Parasutterella id.2892) on ETS domain−containing protein Elk−1 with a threshold of 1e−05

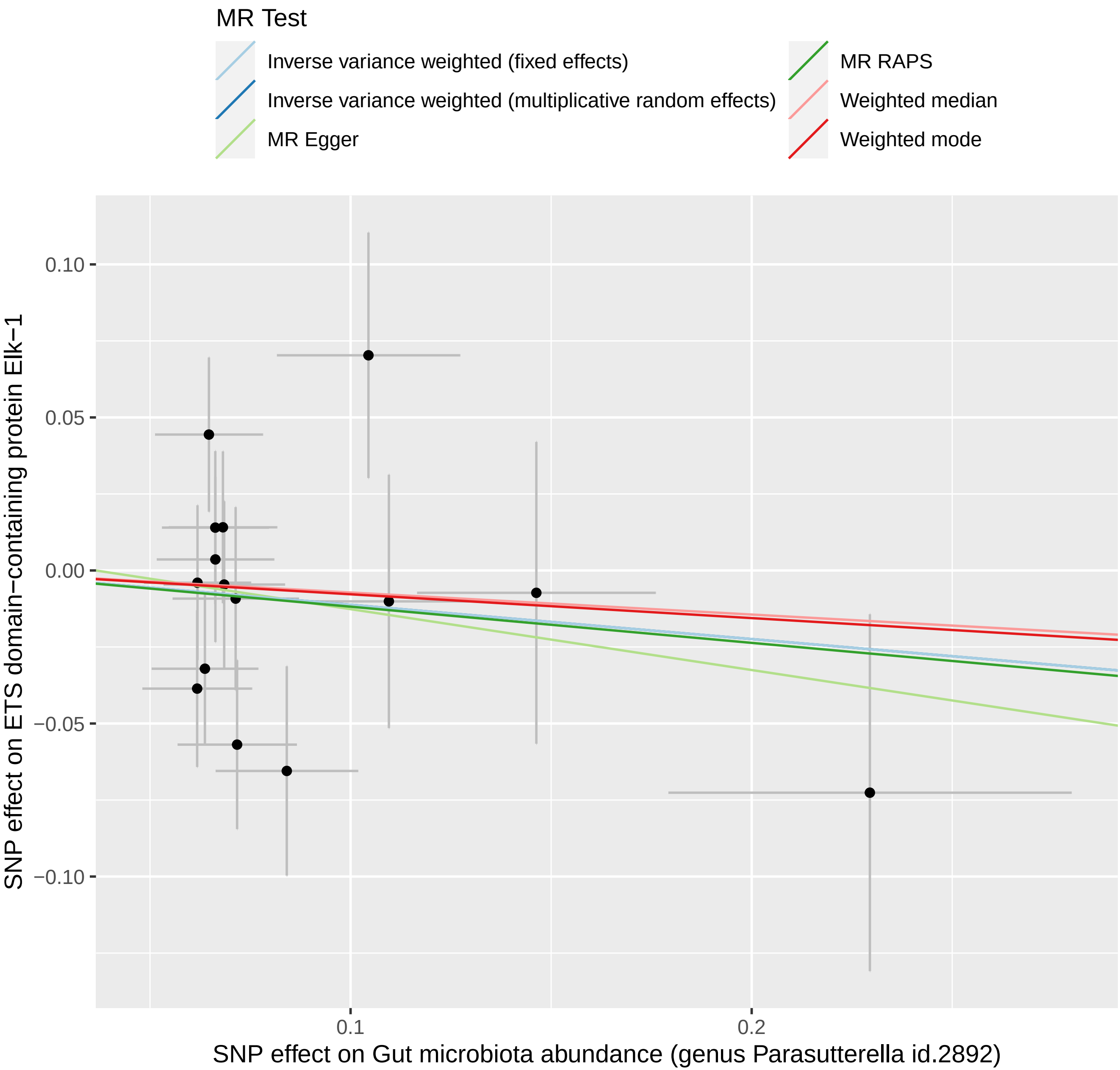

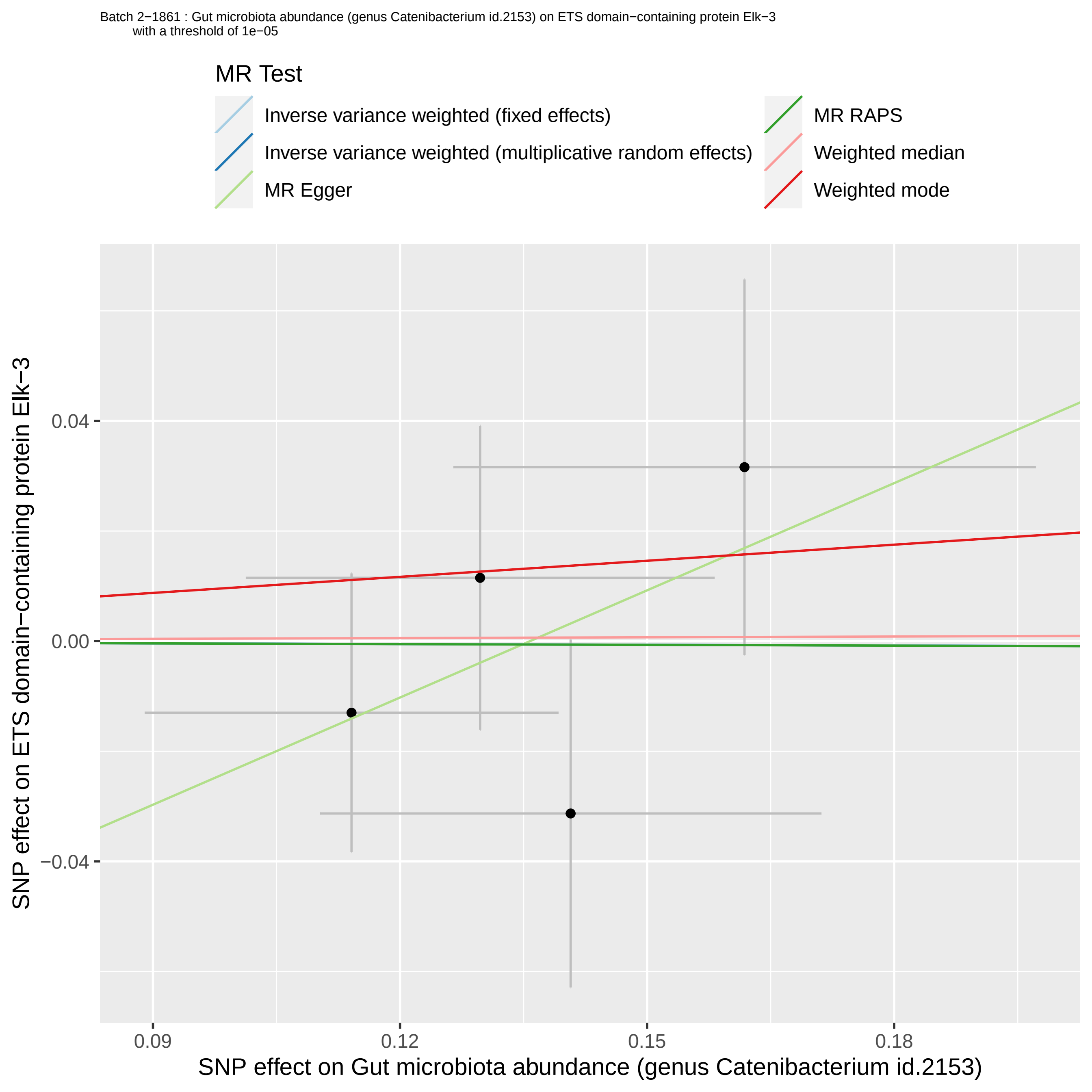
Batch 2−1861 : Gut microbiota abundance (genus Catenibacterium id.2153) on ETS domain−containing protein Elk−3
with a threshold of 1e−05
MR Test
Inverse variance weighted (fixed effects)
Inverse variance weighted (multiplicative random effects)
MR Egger
MR RAPS
Weighted median
Weighted mode
0.04
0.00
−0.04
0.09
0.12
0.15
0.18
SNP effect on Gut microbiota abundance (genus Catenibacterium id.2153)
SNP effect on ETS domain−containing protein Elk−3

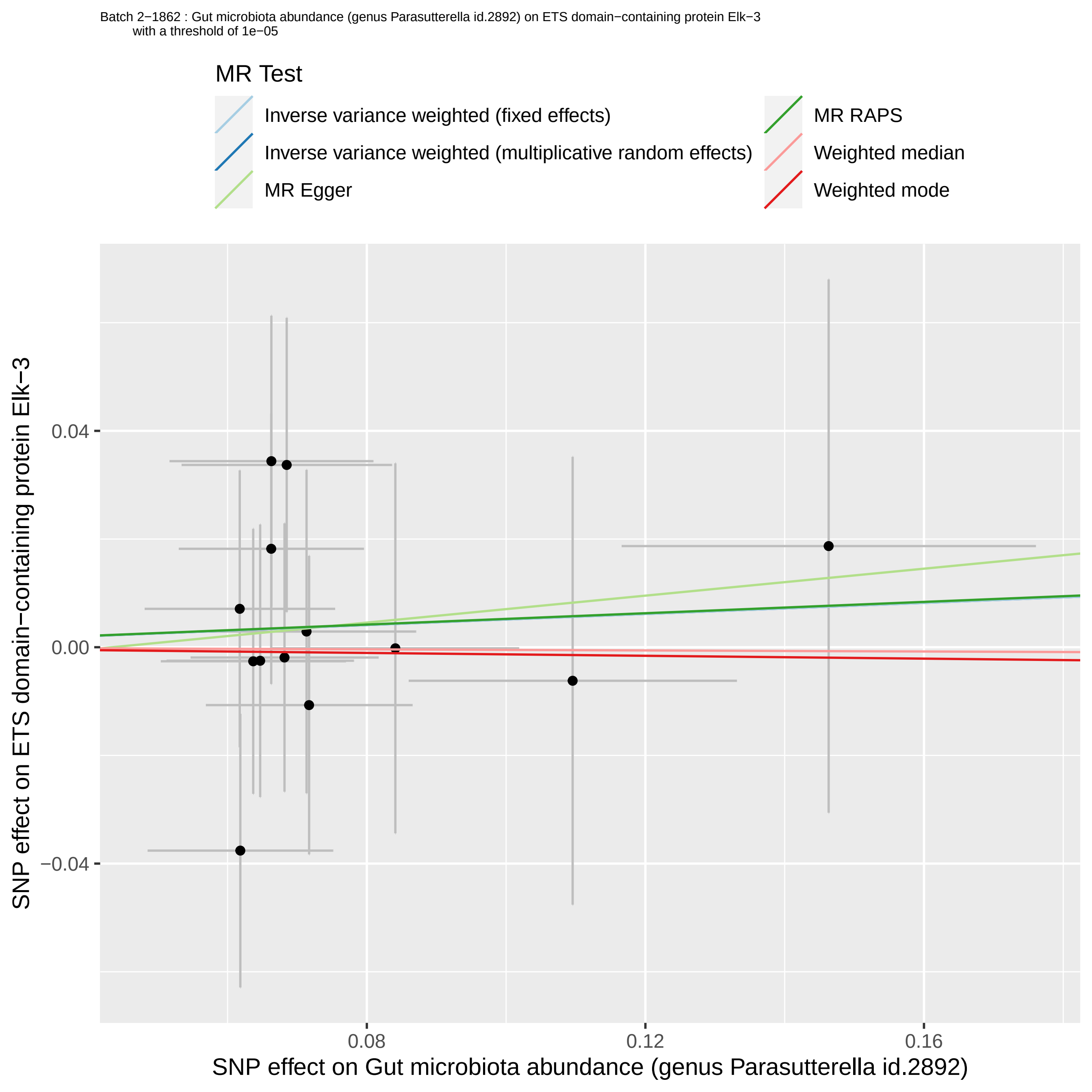

Batch 2−1862 : Gut microbiota abundance (genus Parasutterella id.2892) on ETS domain−containing protein Elk−3
with a threshold of 1e−05
MR Test
Inverse variance weighted (fixed effects)
Inverse variance weighted (multiplicative random effects)
MR Egger
MR RAPS
Weighted median
Weighted mode
SNP effect on ETS domain−containing protein Elk−3
0.04
0.00
−0.04
0.08
0.12
0.16
SNP effect on Gut microbiota abundance (genus Parasutterella id.2892)

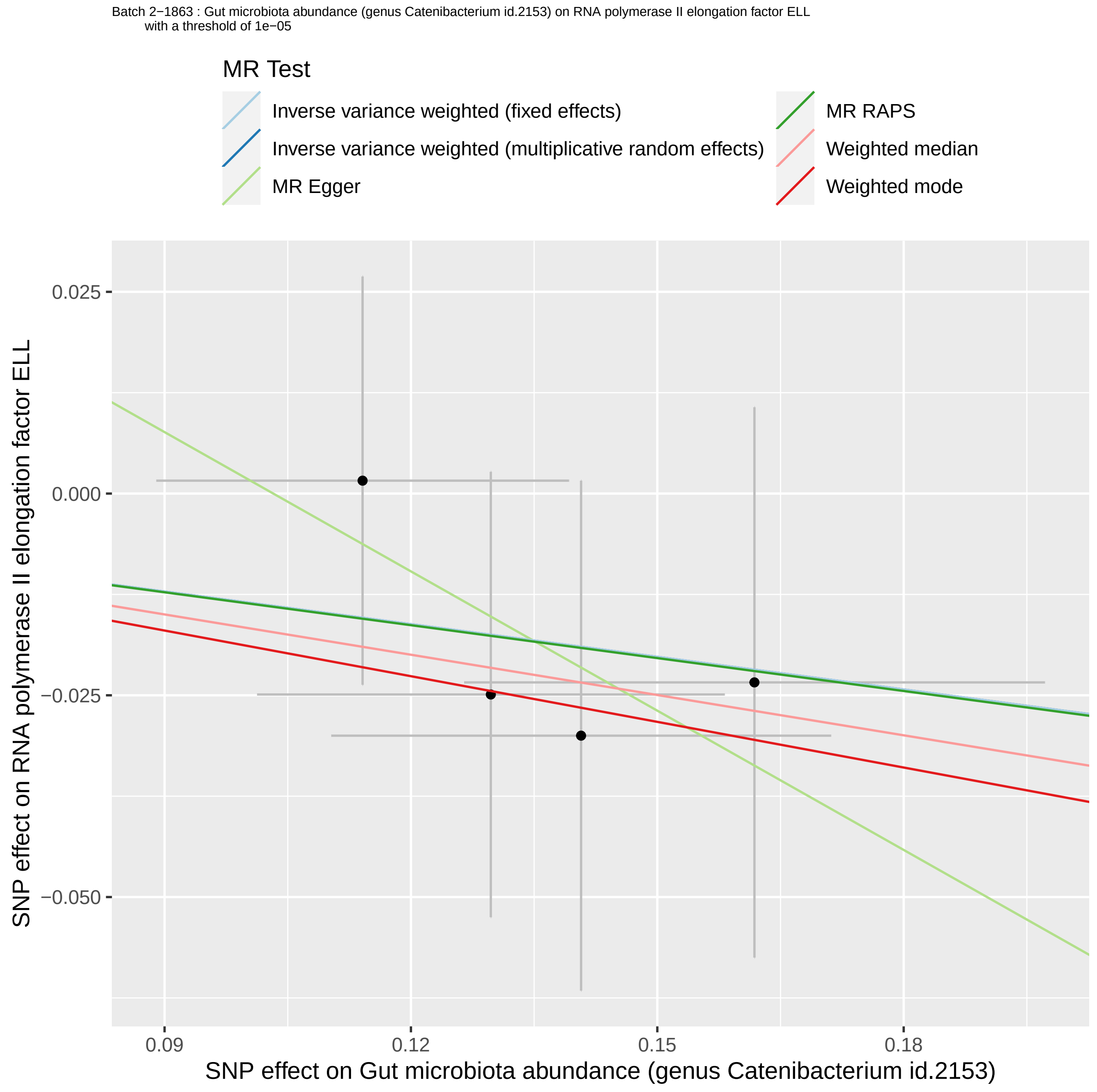
Batch 2−1863 : Gut microbiota abundance (genus Catenibacterium id.2153) on RNA polymerase II elongation factor ELL
with a threshold of 1e−05
MR Test
Inverse variance weighted (fixed effects)
Inverse variance weighted (multiplicative random effects)
MR Egger
MR RAPS
Weighted median
Weighted mode
0.025
0.000
−0.025
−0.050
0.09
0.12
0.15
0.18
SNP effect on RNA polymerase II elongation factor ELL
SNP effect on Gut microbiota abundance (genus Catenibacterium id.2153)

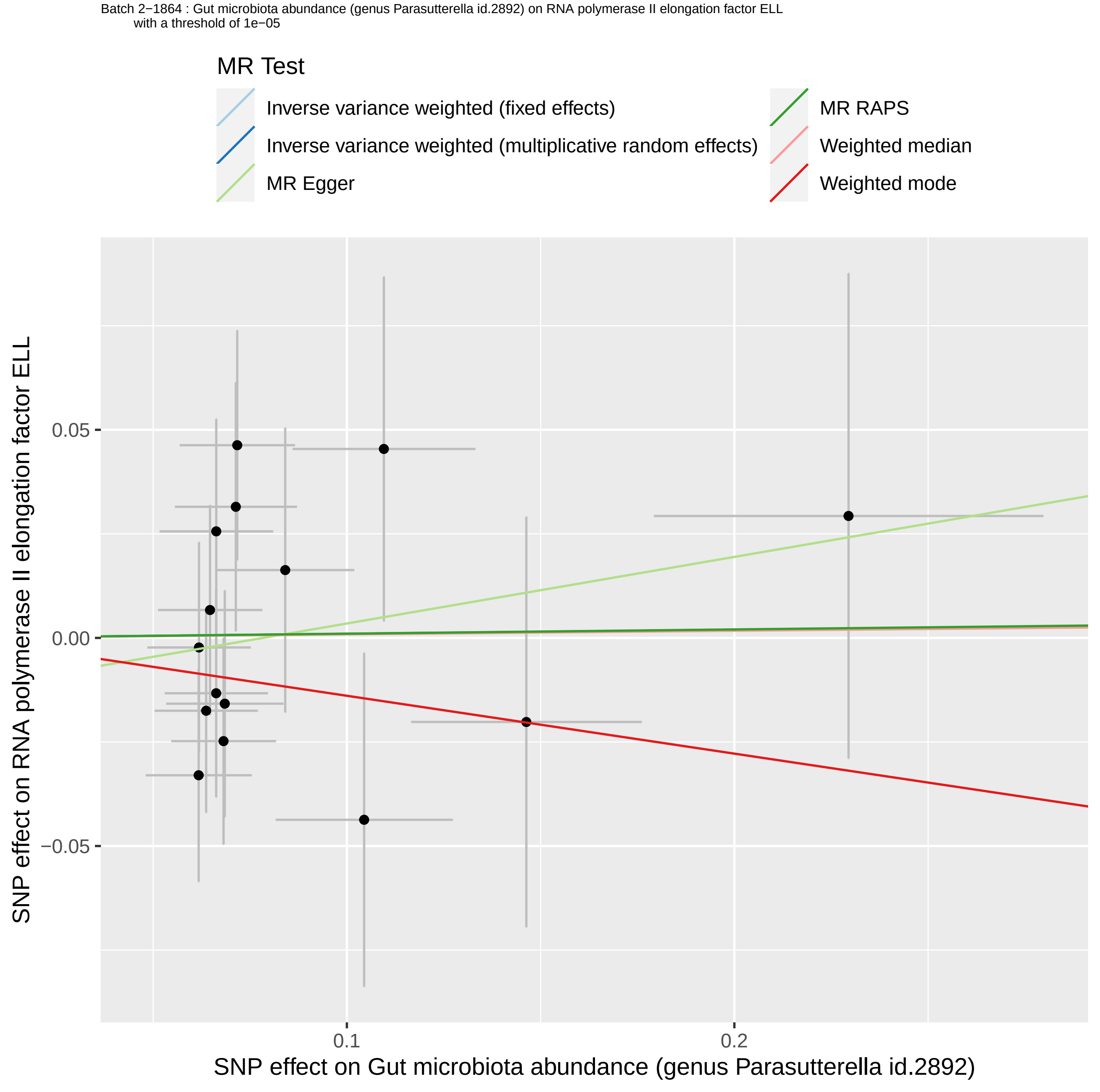

Batch 2−1864 : Gut microbiota abundance (genus Parasutterella id.2892) on RNA polymerase II elongation factor ELL
with a threshold of 1e−05
MR Test
Inverse variance weighted (fixed effects)
Inverse variance weighted (multiplicative random effects)
MR Egger
MR RAPS
Weighted median
Weighted mode
SNP effect on RNA polymerase II elongation factor ELL
0.05
0.00
−0.05
0.1
0.2
SNP effect on Gut microbiota abundance (genus Parasutterella id.2892)

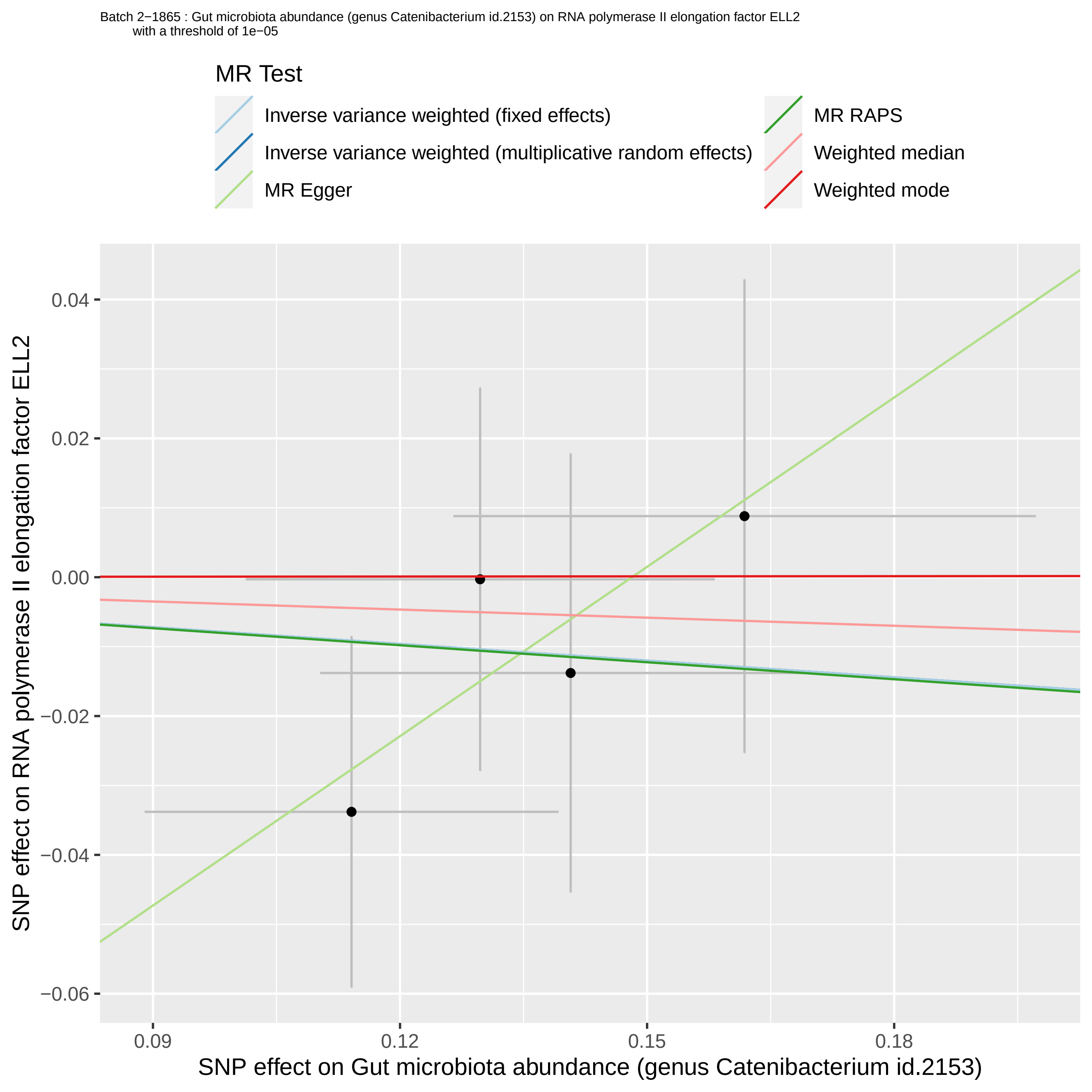
Batch 2−1865 : Gut microbiota abundance (genus Catenibacterium id.2153) on RNA polymerase II elongation factor ELL2
with a threshold of 1e−05
MR Test
Inverse variance weighted (fixed effects)
Inverse variance weighted (multiplicative random effects)
MR Egger
MR RAPS
Weighted median
Weighted mode
0.04
0.02
0.00
−0.02
−0.04
−0.06
0.09
0.12
0.15
0.18
SNP effect on RNA polymerase II elongation factor ELL2
SNP effect on Gut microbiota abundance (genus Catenibacterium id.2153)

Batch 2−1866 : Gut microbiota abundance (genus Parasutterella id.2892) on RNA polymerase II elongation factor ELL2
with a threshold of 1e−05

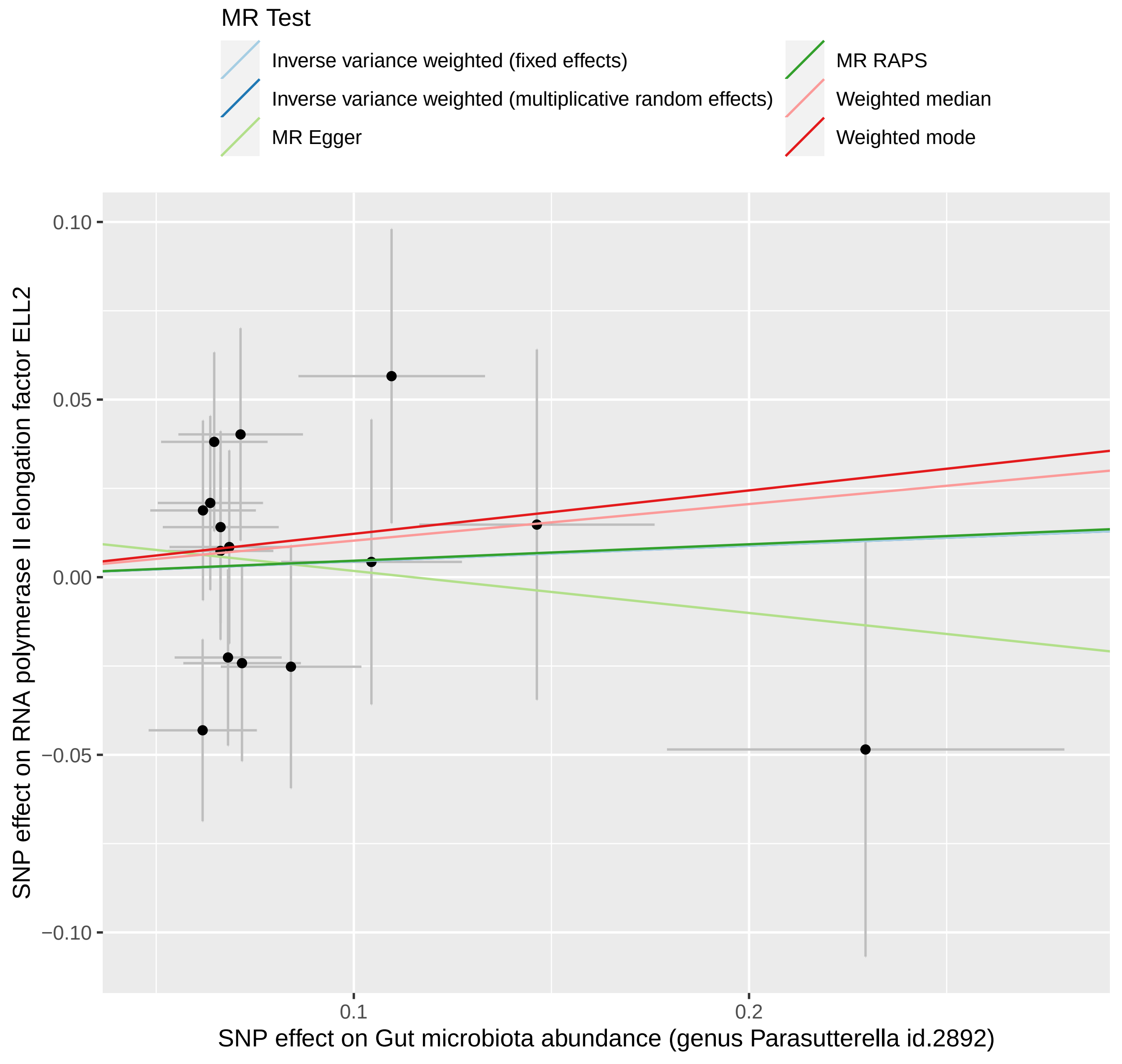

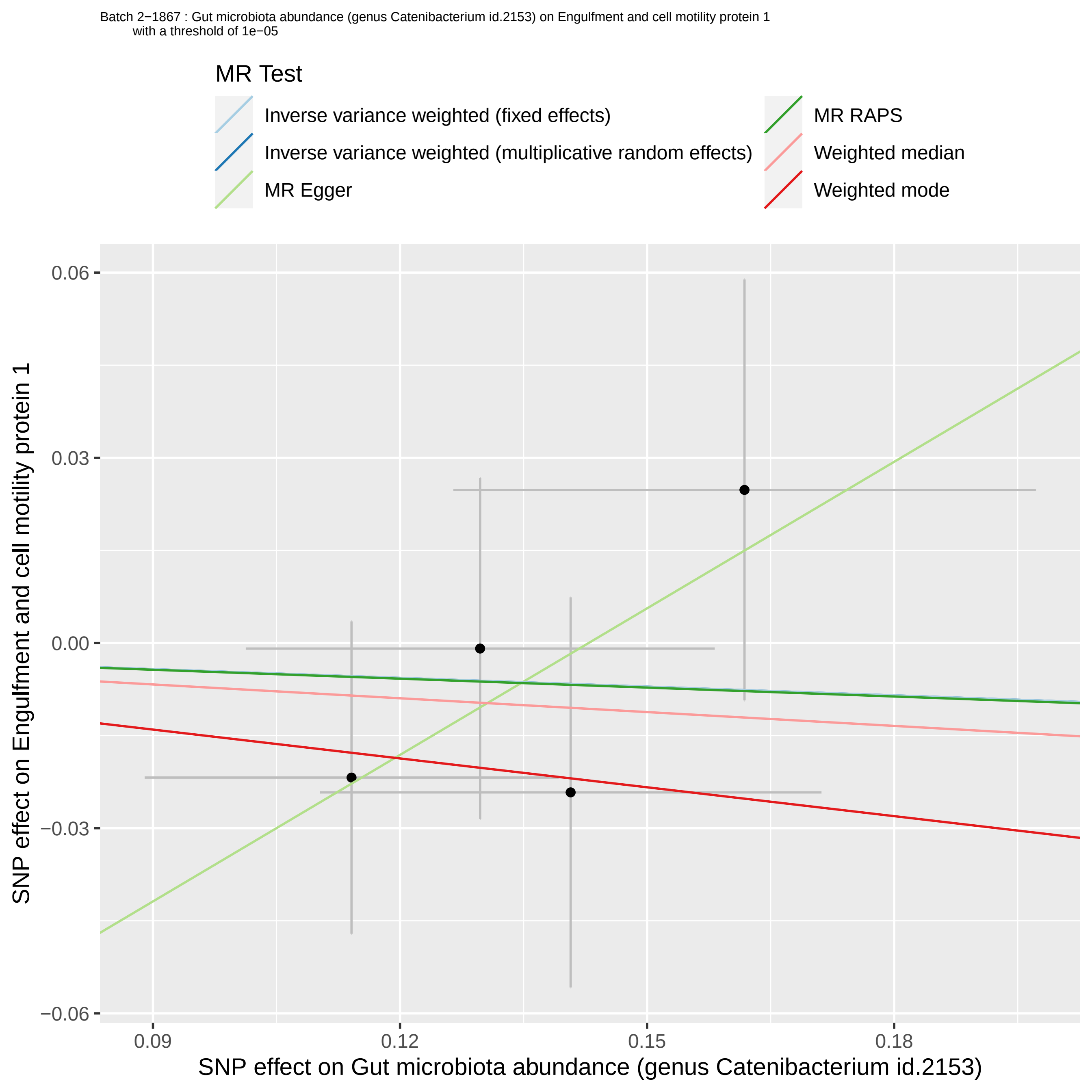

Batch 2−1867 : Gut microbiota abundance (genus Catenibacterium id.2153) on Engulfment and cell motility protein 1
with a threshold of 1e−05
MR Test
Inverse variance weighted (fixed effects)
Inverse variance weighted (multiplicative random effects)
MR Egger
MR RAPS
Weighted median
Weighted mode
0.06
0.03
0.00
−0.03
−0.06
0.09
0.12
0.15
0.18
SNP effect on Engulfment and cell motility protein 1
SNP effect on Gut microbiota abundance (genus Catenibacterium id.2153)

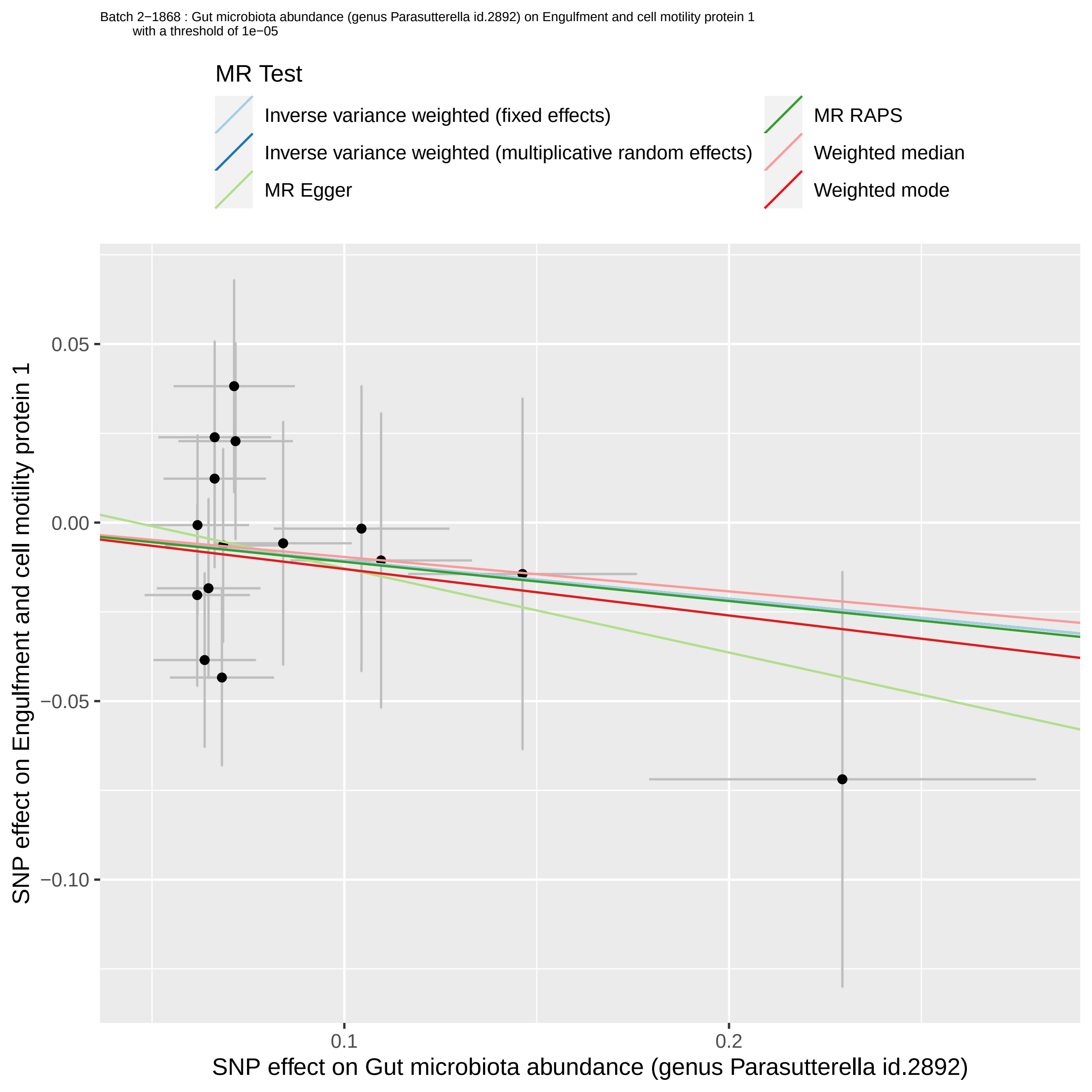
Batch 2−1868 : Gut microbiota abundance (genus Parasutterella id.2892) on Engulfment and cell motility protein 1
with a threshold of 1e−05
MR Test
Inverse variance weighted (fixed effects)
Inverse variance weighted (multiplicative random effects)
MR Egger
MR RAPS
Weighted median
Weighted mode
0.05
0.00
−0.05
−0.10
0.1
0.2
SNP effect on Engulfment and cell motility protein 1
SNP effect on Gut microbiota abundance (genus Parasutterella id.2892)

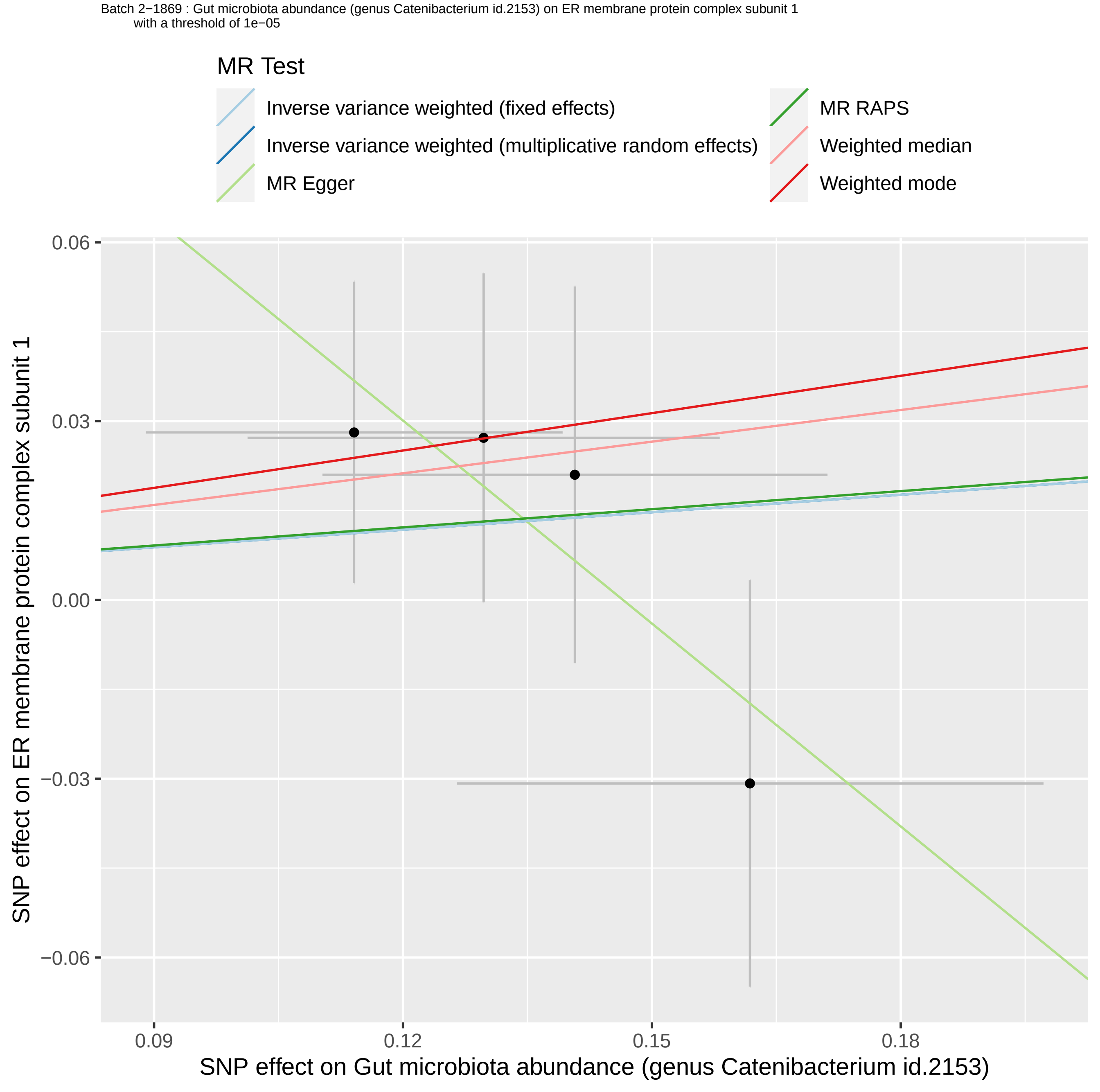

Batch 2−1869 : Gut microbiota abundance (genus Catenibacterium id.2153) on ER membrane protein complex subunit 1
with a threshold of 1e−05
MR Test
Inverse variance weighted (fixed effects)
Inverse variance weighted (multiplicative random effects)
MR Egger
MR RAPS
Weighted median
Weighted mode
0.06
0.03
0.00
−0.03
−0.06
0.09
0.12
0.15
0.18
SNP effect on ER membrane protein complex subunit 1
SNP effect on Gut microbiota abundance (genus Catenibacterium id.2153)

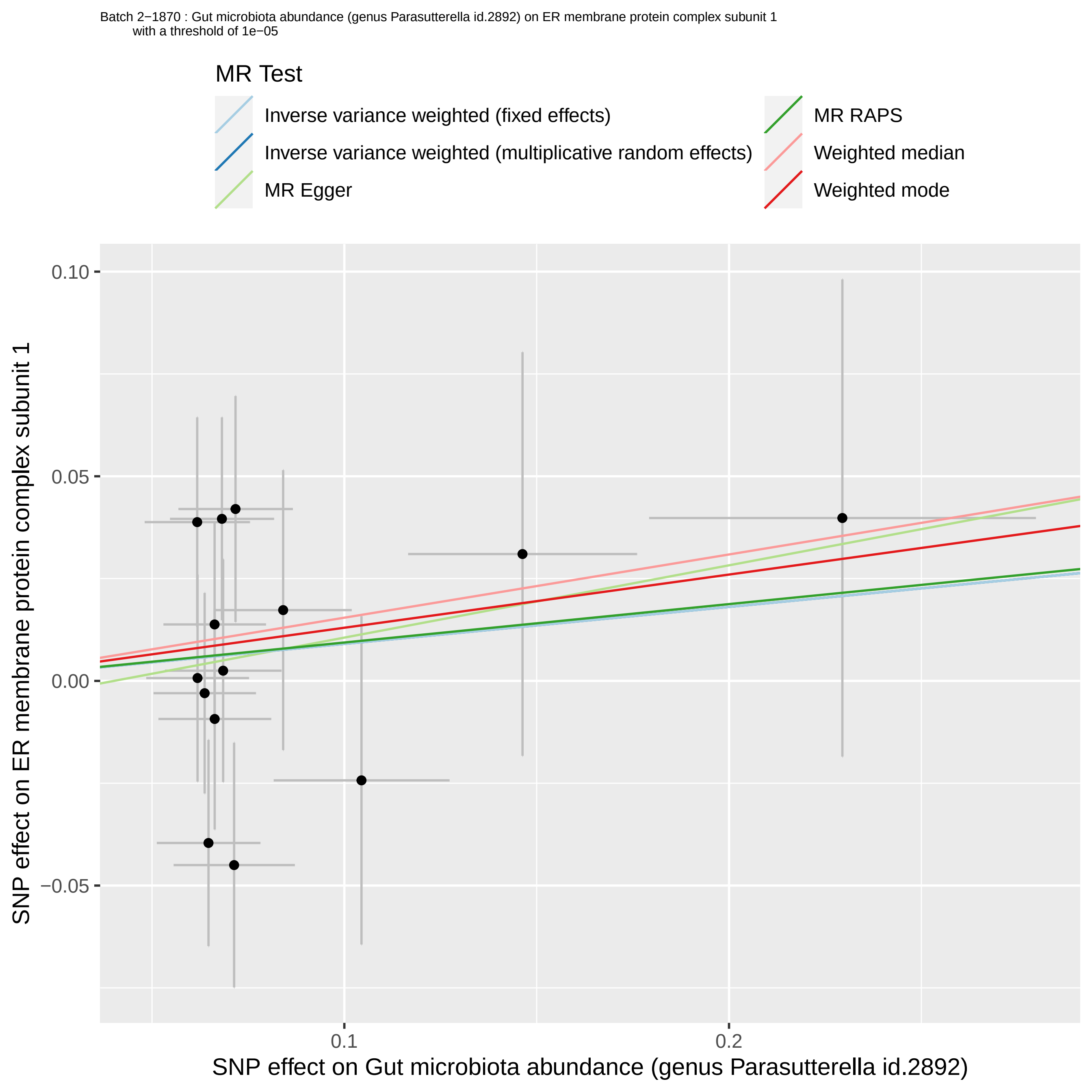

Batch 2−1870 : Gut microbiota abundance (genus Parasutterella id.2892) on ER membrane protein complex subunit 1
with a threshold of 1e−05
MR Test
Inverse variance weighted (fixed effects)
Inverse variance weighted (multiplicative random effects)
MR Egger
MR RAPS
Weighted median
Weighted mode
0.10
0.05
0.00
−0.05
0.1
0.2
SNP effect on ER membrane protein complex subunit 1
SNP effect on Gut microbiota abundance (genus Parasutterella id.2892)

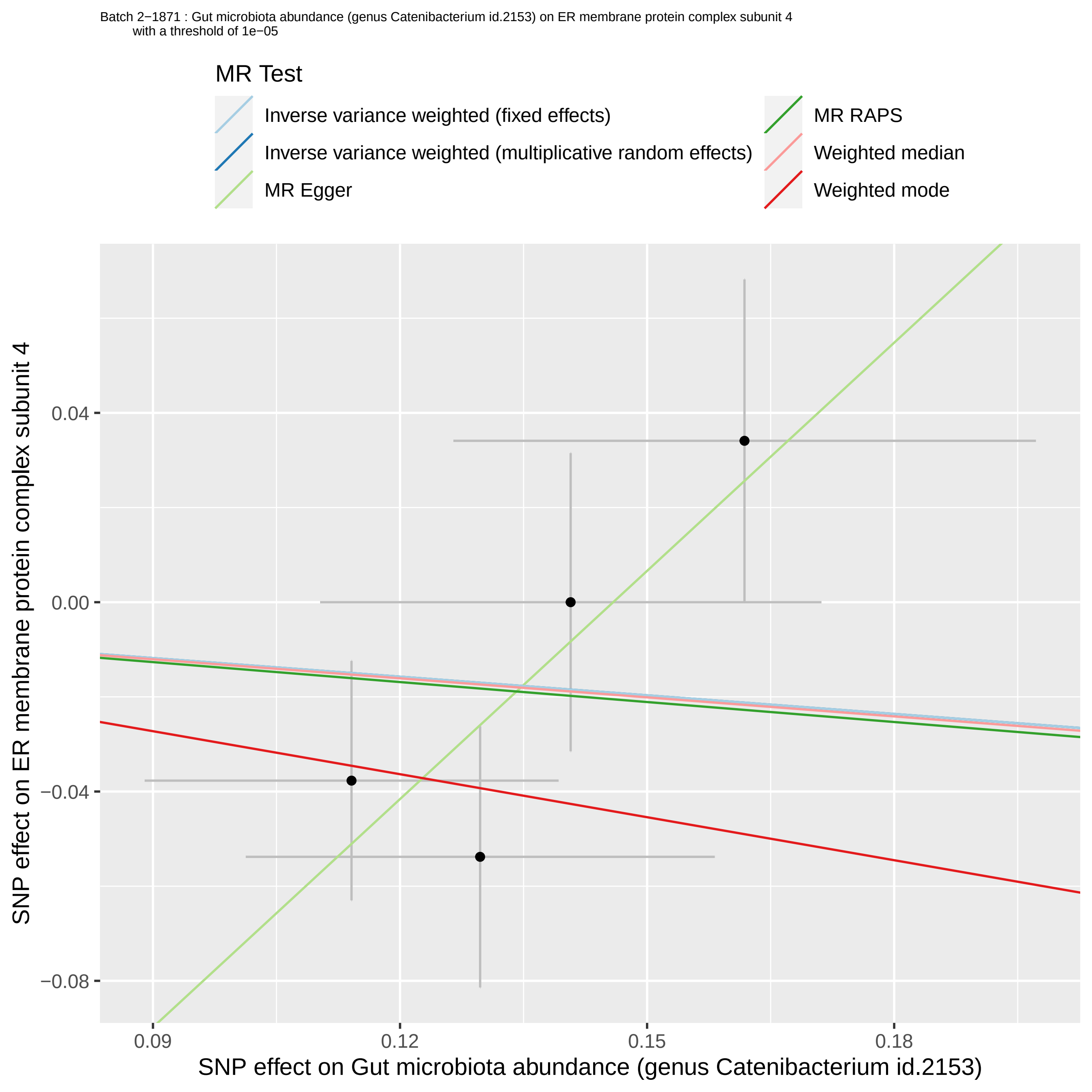

Batch 2−1871 : Gut microbiota abundance (genus Catenibacterium id.2153) on ER membrane protein complex subunit 4
with a threshold of 1e−05
MR Test
Inverse variance weighted (fixed effects)
Inverse variance weighted (multiplicative random effects)
MR Egger
MR RAPS
Weighted median
Weighted mode
SNP effect on ER membrane protein complex subunit 4
0.04
0.00
−0.04
−0.08
0.09
0.12
0.15
0.18
SNP effect on Gut microbiota abundance (genus Catenibacterium id.2153)

Batch 2−1872 : Gut microbiota abundance (genus Parasutterella id.2892) on ER membrane protein complex subunit 4
with a threshold of 1e−05

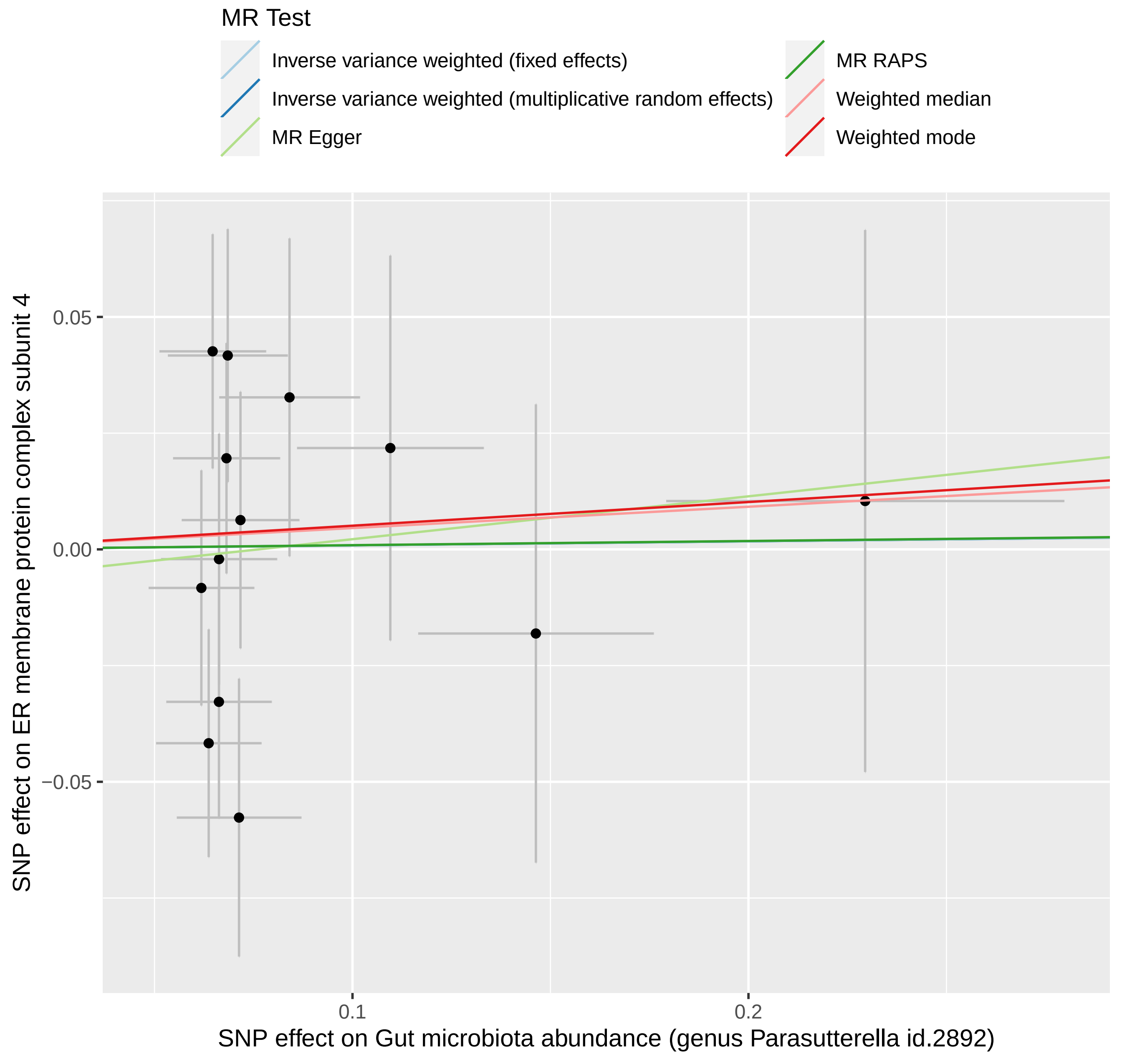

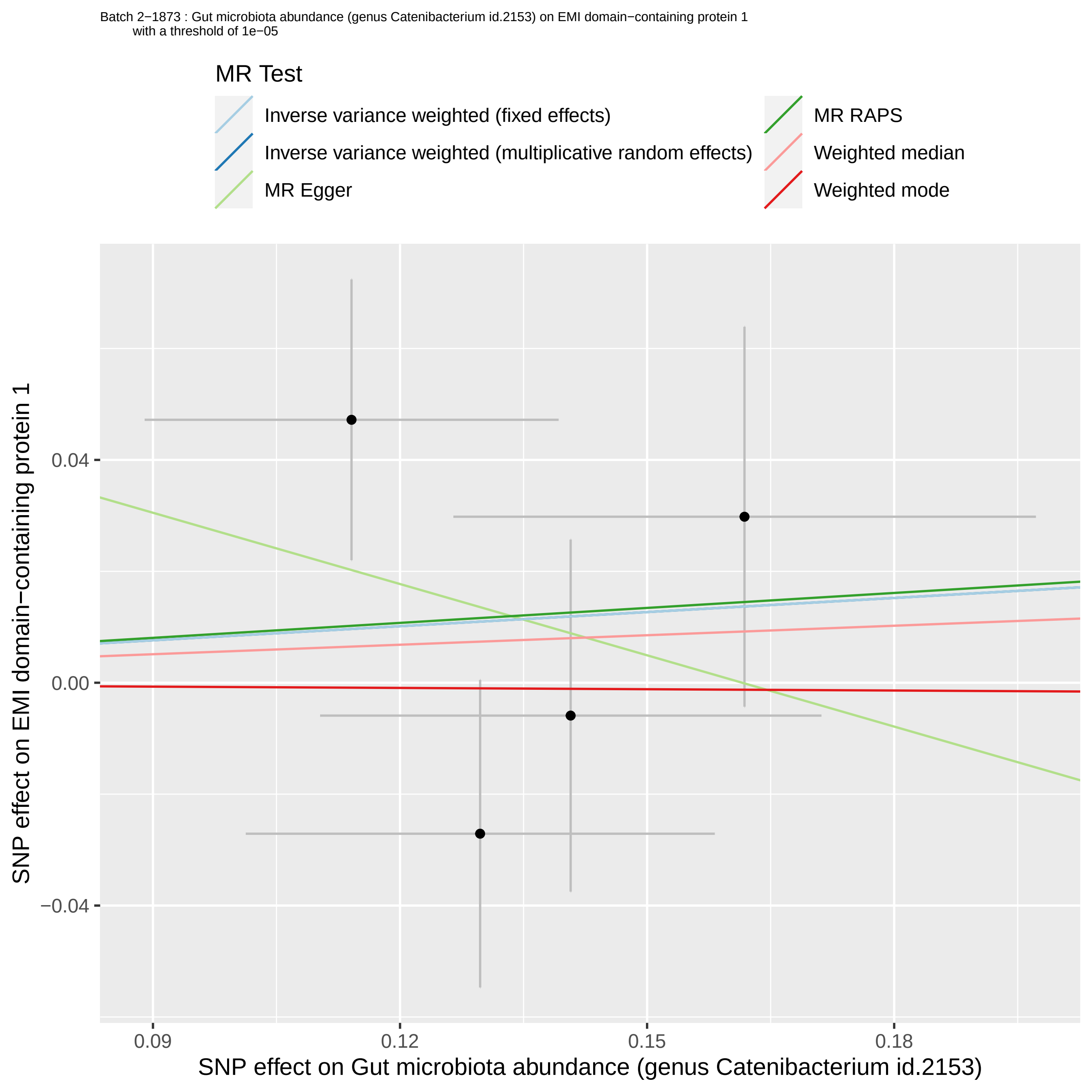

Batch 2−1873 : Gut microbiota abundance (genus Catenibacterium id.2153) on EMI domain−containing protein 1
with a threshold of 1e−05
MR Test
Inverse variance weighted (fixed effects)
Inverse variance weighted (multiplicative random effects)
MR Egger
MR RAPS
Weighted median
Weighted mode
0.04
0.00
−0.04
0.09
0.12
0.15
0.18
SNP effect on EMI domain−containing protein 1
SNP effect on Gut microbiota abundance (genus Catenibacterium id.2153)

Batch 2−1874 : Gut microbiota abundance (genus Parasutterella id.2892) on EMI domain−containing protein 1
with a threshold of 1e−05

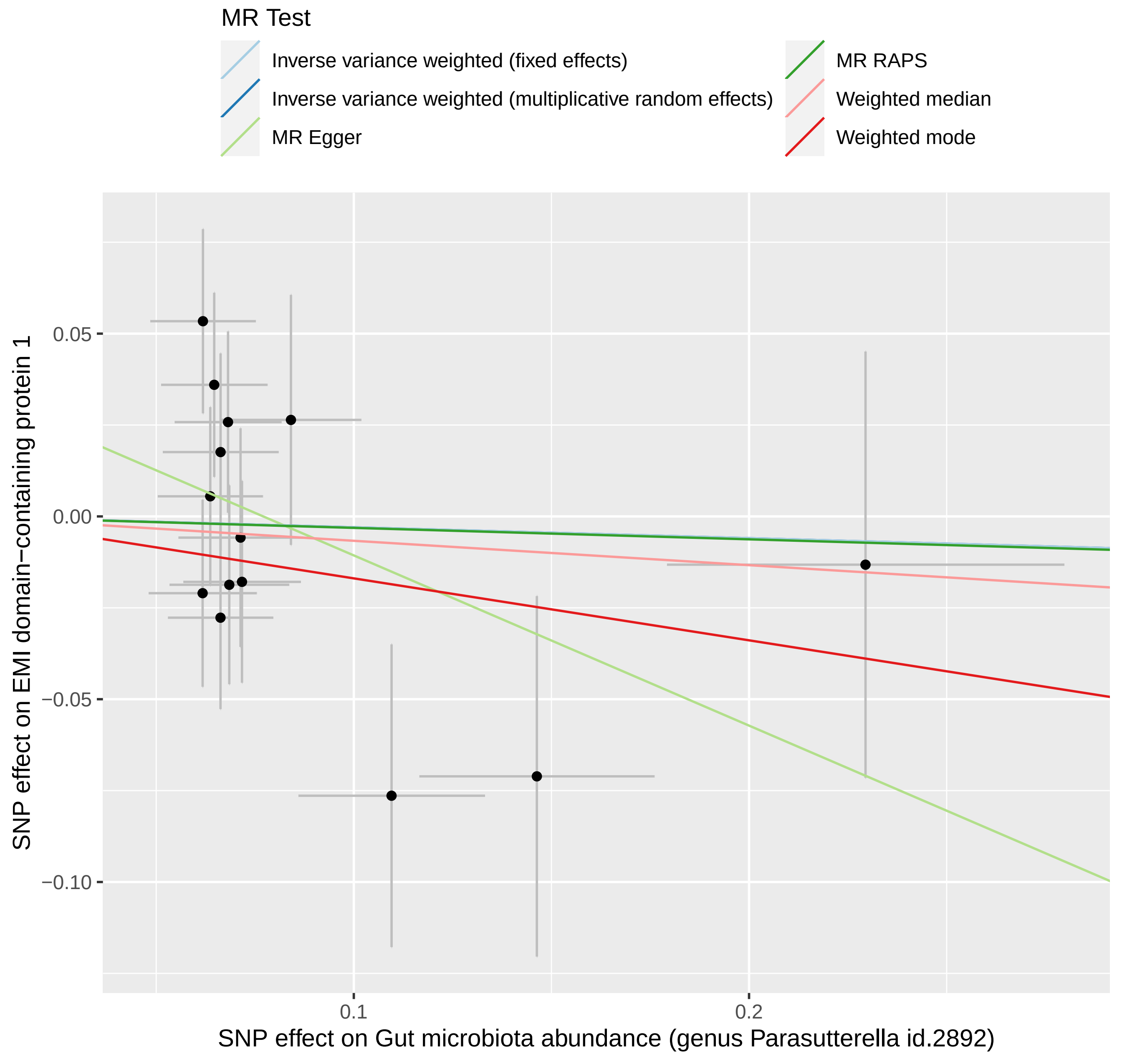

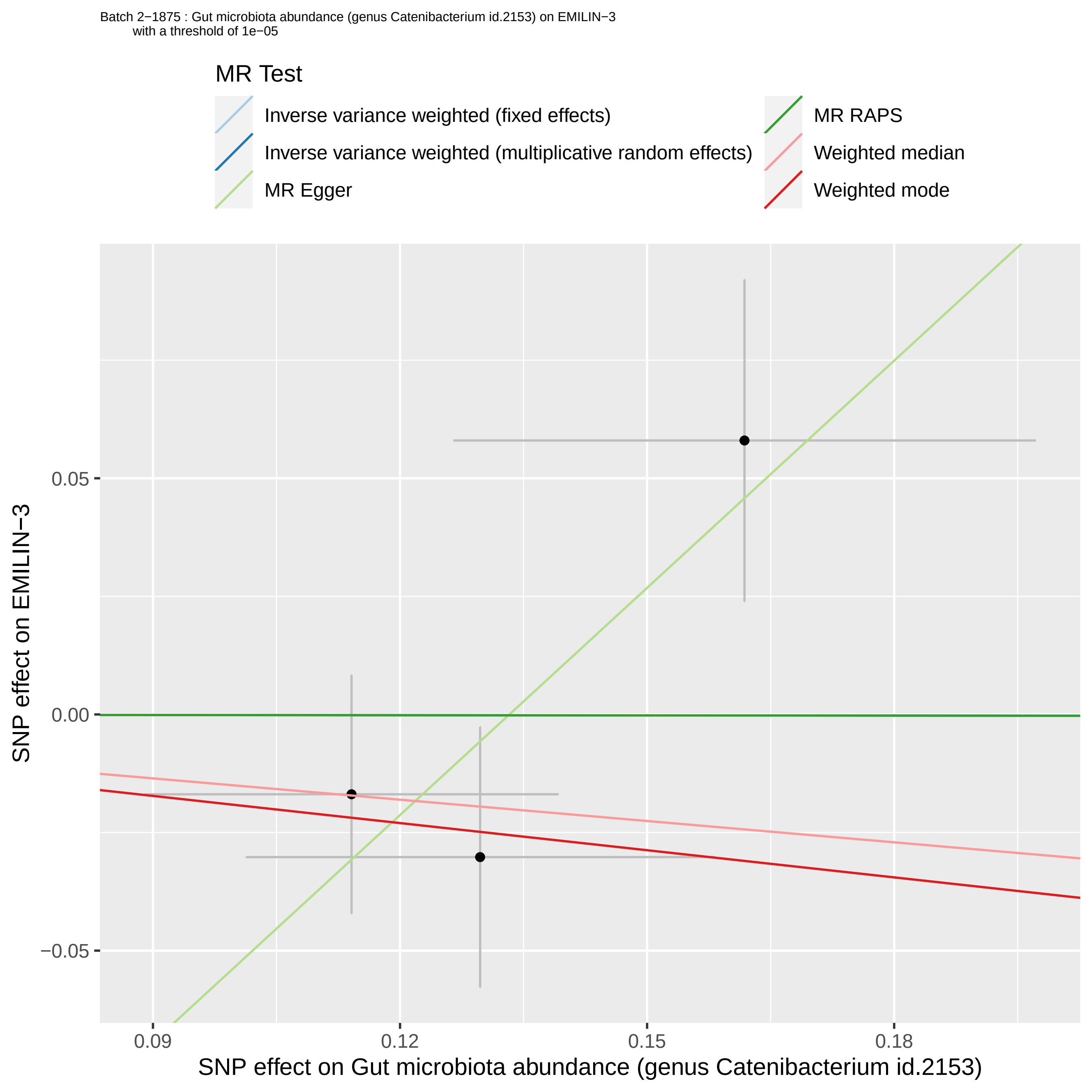
Batch 2−1875 : Gut microbiota abundance (genus Catenibacterium id.2153) on EMILIN−3
with a threshold of 1e−05
MR Test
Inverse variance weighted (fixed effects)
Inverse variance weighted (multiplicative random effects)
MR Egger
MR RAPS
Weighted median
Weighted mode
SNP effect on EMILIN−3
0.05
0.00
−0.05
0.09
0.12
0.15
0.18
SNP effect on Gut microbiota abundance (genus Catenibacterium id.2153)

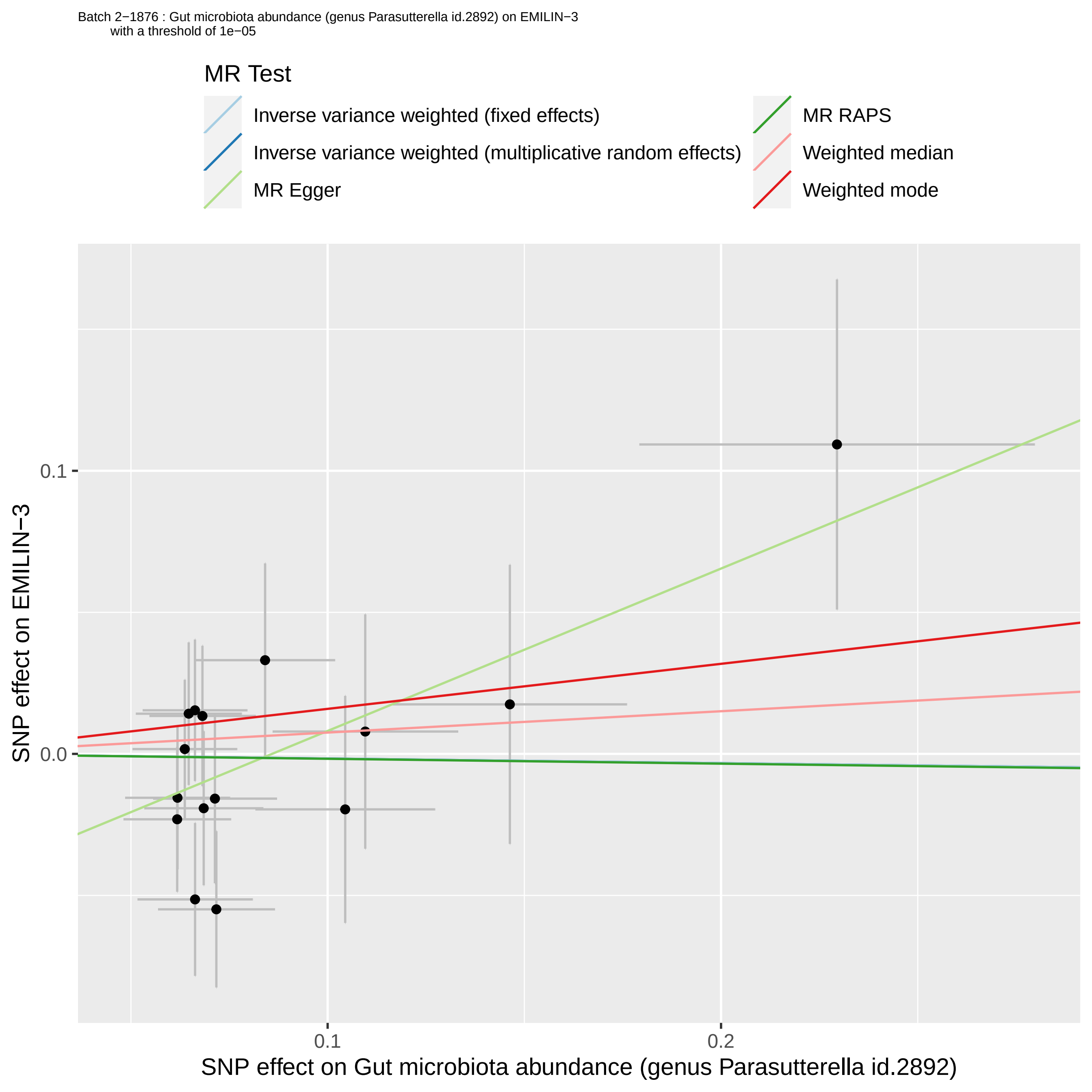

Batch 2−1876 : Gut microbiota abundance (genus Parasutterella id.2892) on EMILIN−3
with a threshold of 1e−05
MR Test
Inverse variance weighted (fixed effects)
Inverse variance weighted (multiplicative random effects)
MR Egger
MR RAPS
Weighted median
Weighted mode
0.1
0.0
0.1
0.2
SNP effect on EMILIN−3
SNP effect on Gut microbiota abundance (genus Parasutterella id.2892)

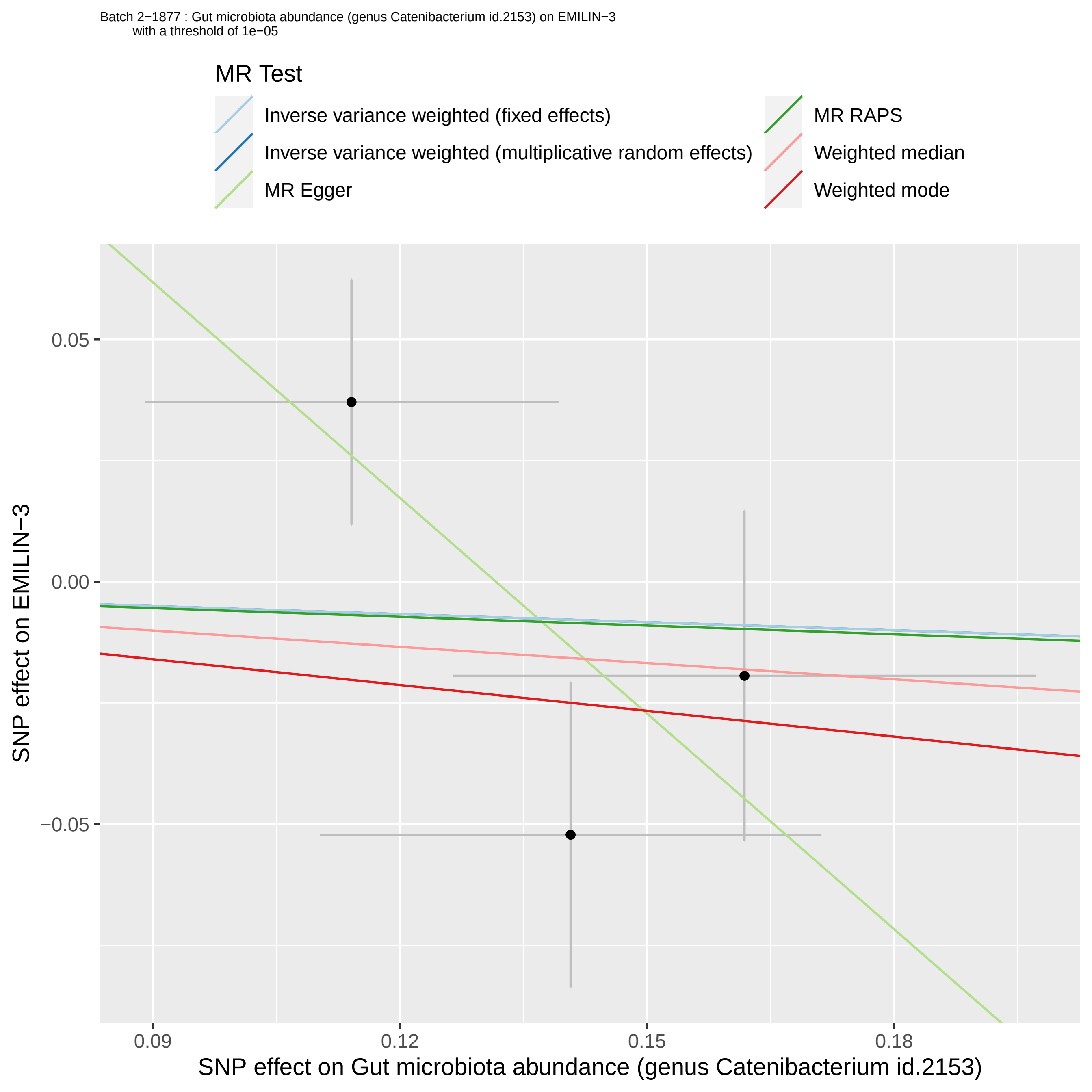
Batch 2−1877 : Gut microbiota abundance (genus Catenibacterium id.2153) on EMILIN−3
with a threshold of 1e−05
MR Test
Inverse variance weighted (fixed effects)
Inverse variance weighted (multiplicative random effects)
MR Egger
MR RAPS
Weighted median
Weighted mode
0.05
0.00
−0.05
0.09
0.12
0.15
0.18
SNP effect on EMILIN−3
SNP effect on Gut microbiota abundance (genus Catenibacterium id.2153)

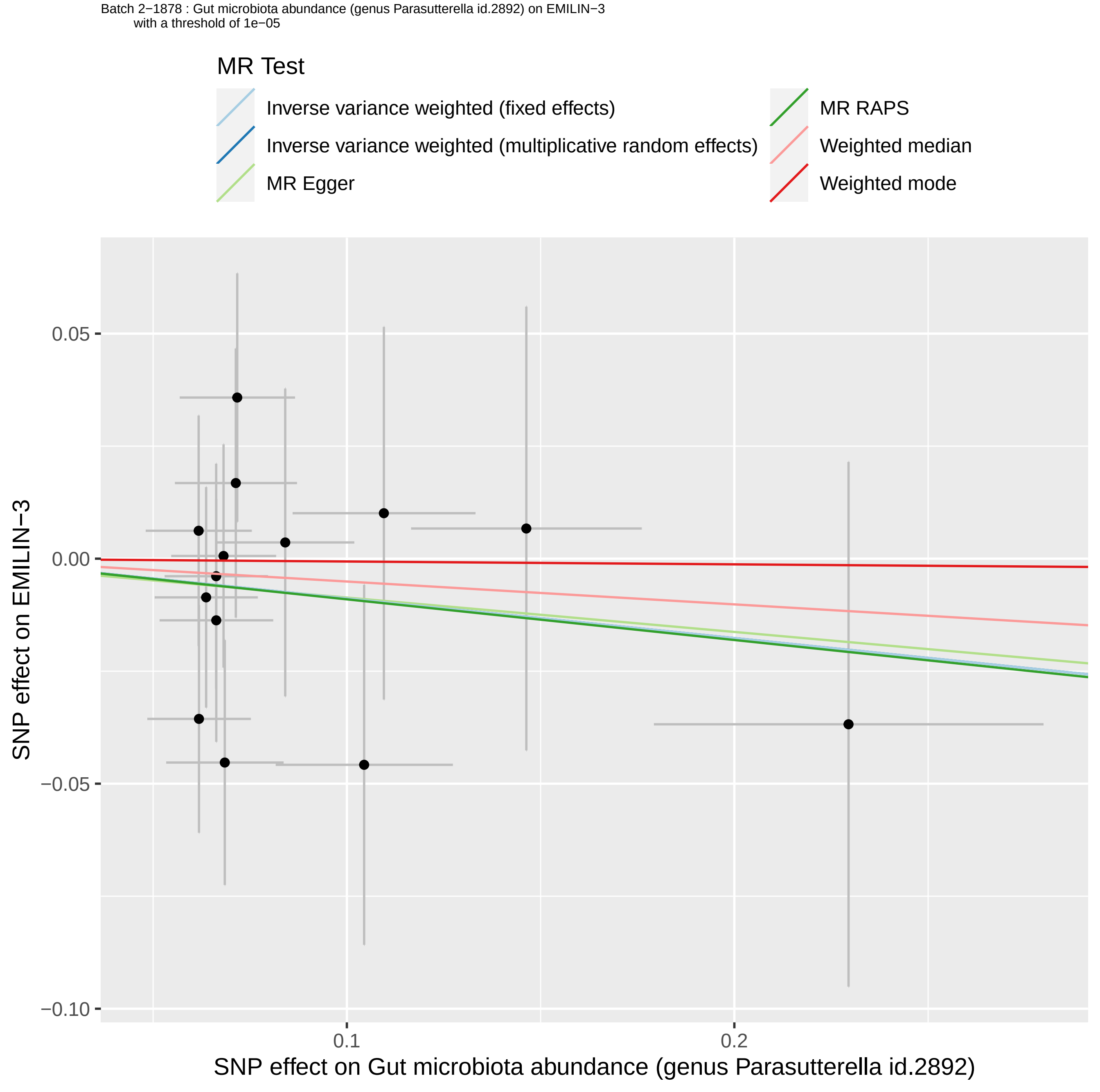

Batch 2−1878 : Gut microbiota abundance (genus Parasutterella id.2892) on EMILIN−3
with a threshold of 1e−05
MR Test
Inverse variance weighted (fixed effects)
Inverse variance weighted (multiplicative random effects)
MR Egger
MR RAPS
Weighted median
Weighted mode
0.05
0.00
−0.05
−0.10
0.1
0.2
SNP effect on EMILIN−3
SNP effect on Gut microbiota abundance (genus Parasutterella id.2892)

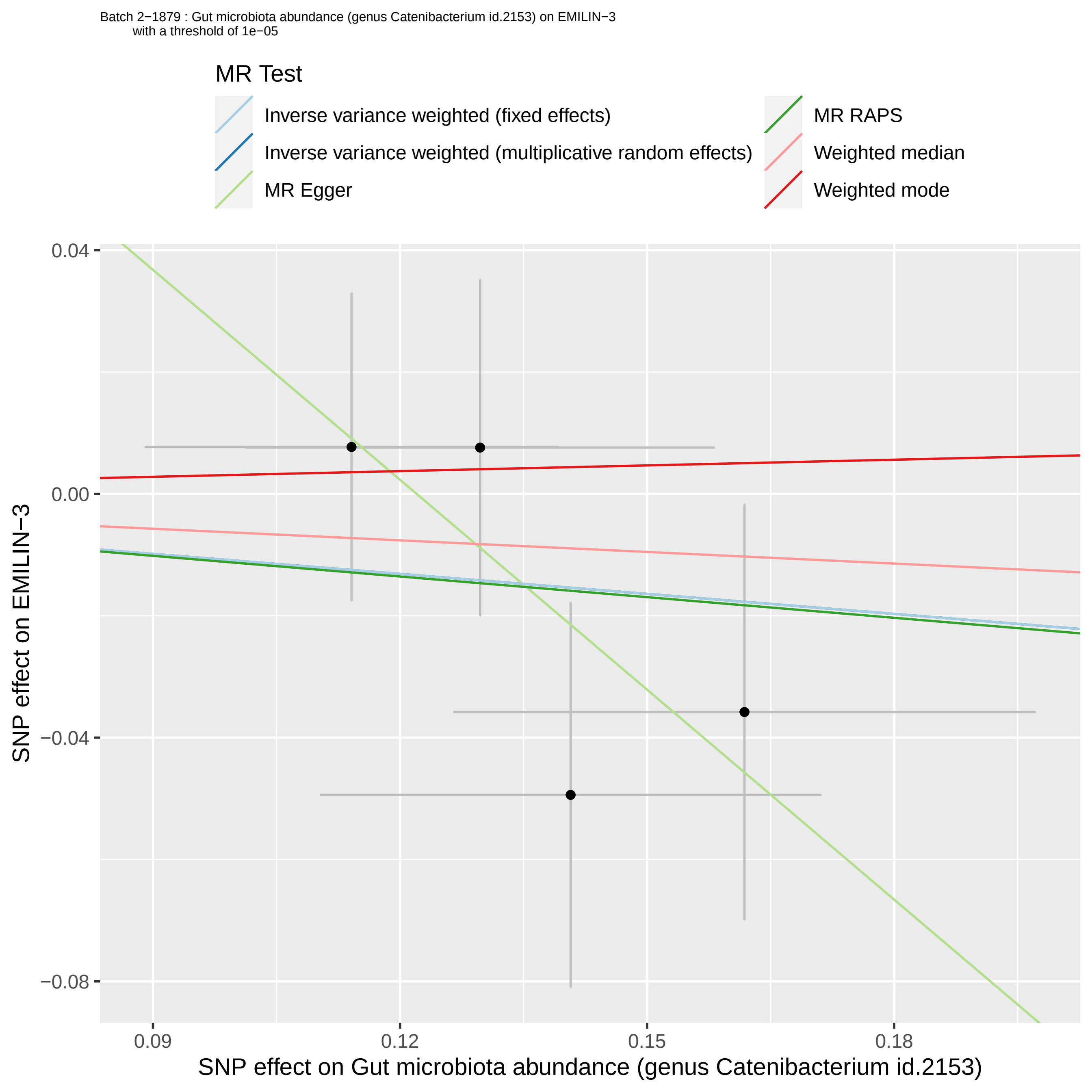

Batch 2−1879 : Gut microbiota abundance (genus Catenibacterium id.2153) on EMILIN−3
with a threshold of 1e−05
MR Test
Inverse variance weighted (fixed effects)
Inverse variance weighted (multiplicative random effects)
MR Egger
MR RAPS
Weighted median
Weighted mode
0.04
0.00
−0.04
−0.08
SNP effect on EMILIN−3
0.09
0.12
0.15
0.18
SNP effect on Gut microbiota abundance (genus Catenibacterium id.2153)

Batch 2−1880 : Gut microbiota abundance (genus Parasutterella id.2892) on EMILIN−3
with a threshold of 1e−05

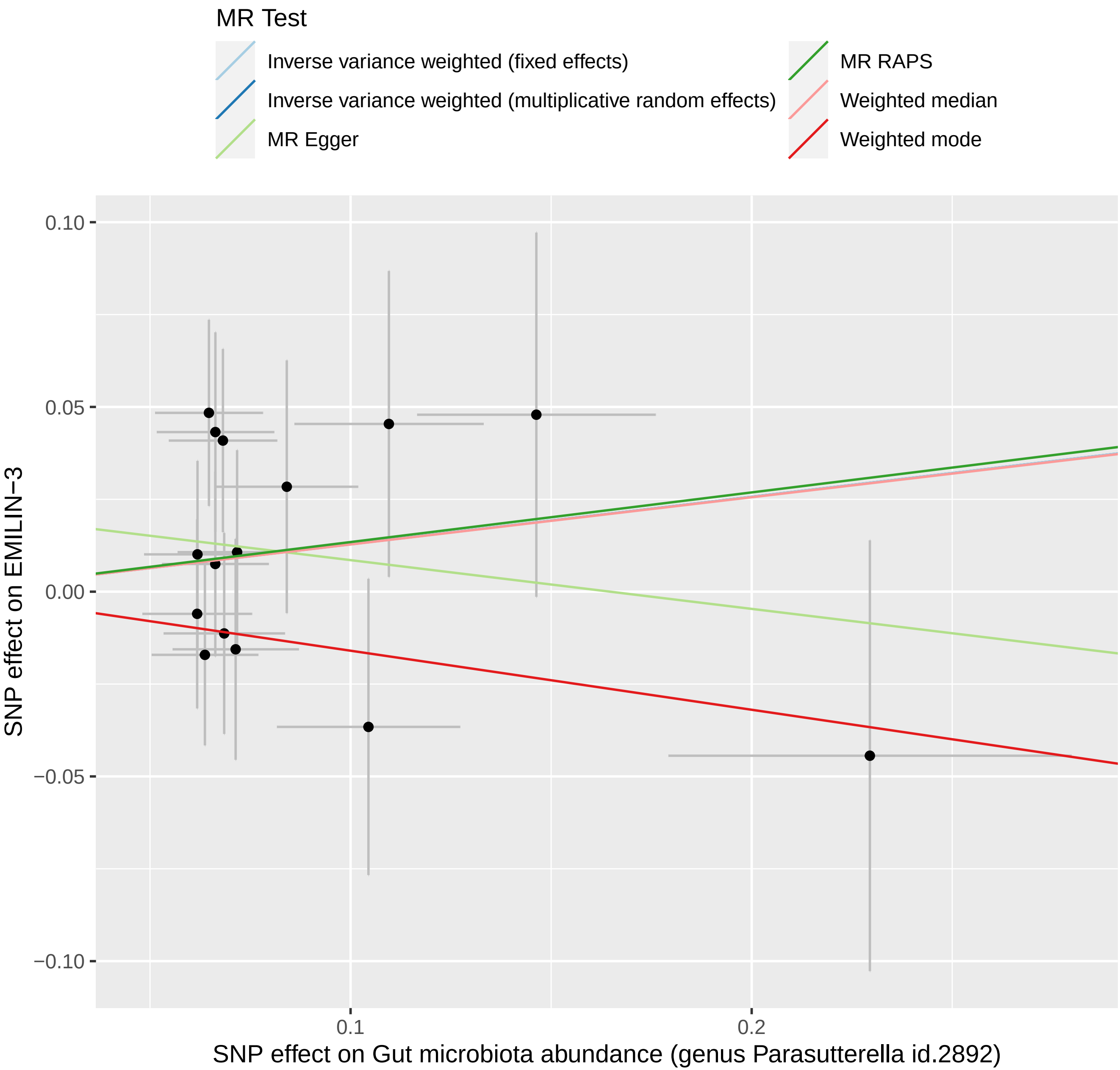

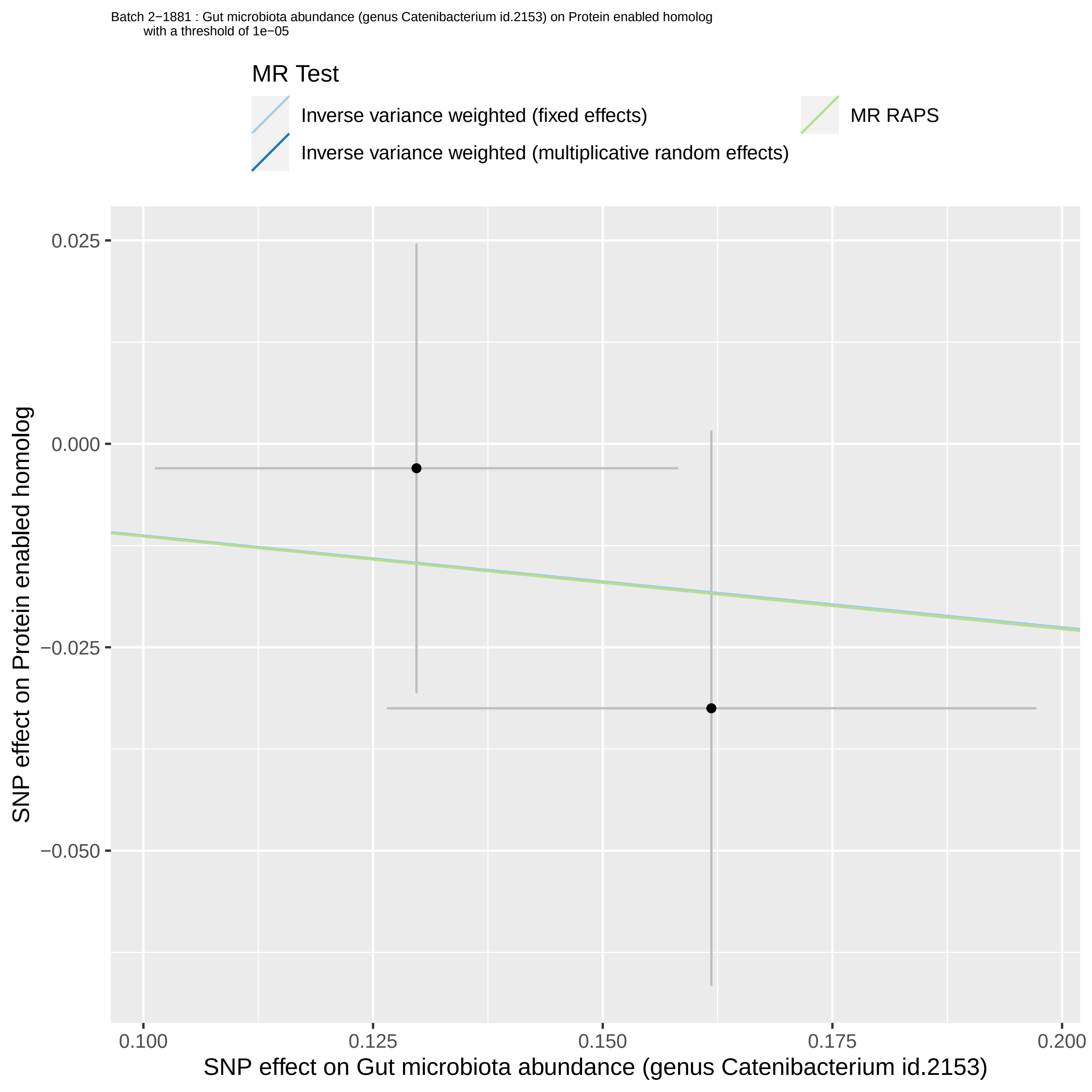
Batch 2−1881 : Gut microbiota abundance (genus Catenibacterium id.2153) on Protein enabled homolog
with a threshold of 1e−05
MR Test
Inverse variance weighted (fixed effects)
Inverse variance weighted (multiplicative random effects)
MR RAPS
0.025
0.000
−0.025
−0.050
SNP effect on Protein enabled homolog
0.100
0.125
0.150
0.175
0.200
SNP effect on Gut microbiota abundance (genus Catenibacterium id.2153)

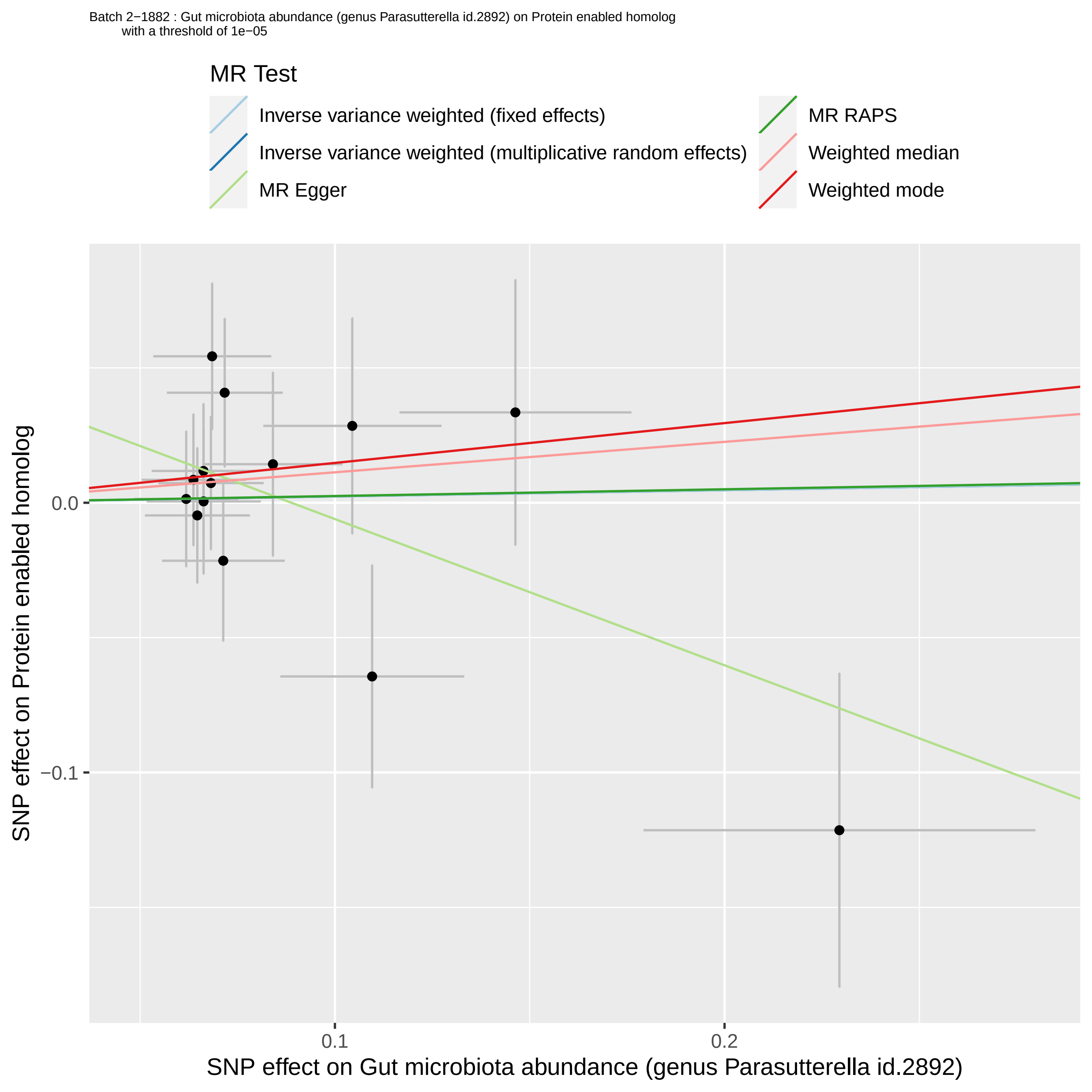
Batch 2−1882 : Gut microbiota abundance (genus Parasutterella id.2892) on Protein enabled homolog
with a threshold of 1e−05
MR Test
Inverse variance weighted (fixed effects)
Inverse variance weighted (multiplicative random effects)
MR Egger
MR RAPS
Weighted median
Weighted mode
0.0
−0.1
0.1
0.2
SNP effect on Protein enabled homolog
SNP effect on Gut microbiota abundance (genus Parasutterella id.2892)

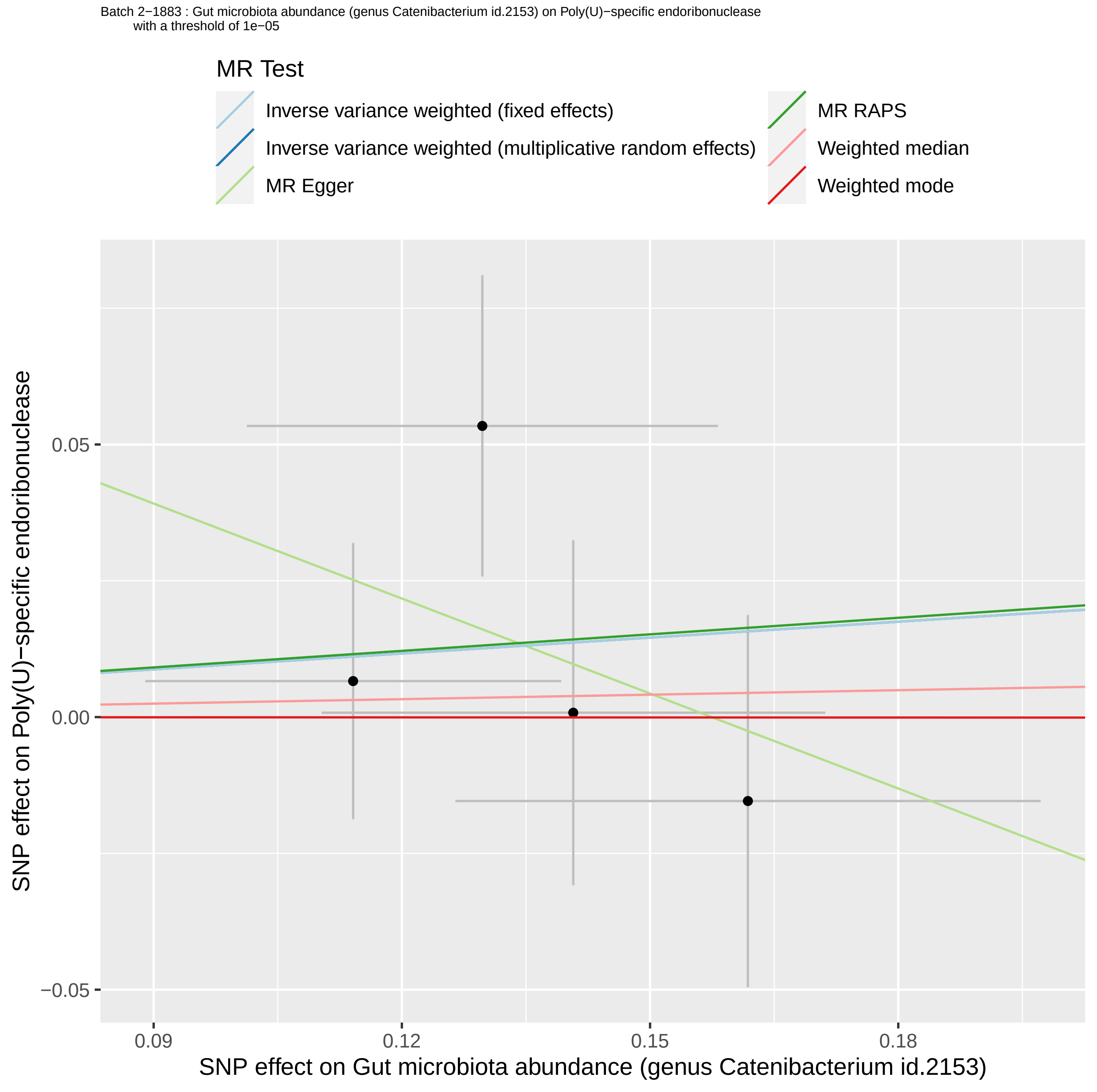

Batch 2−1883 : Gut microbiota abundance (genus Catenibacterium id.2153) on Poly(U)−specific endoribonuclease
with a threshold of 1e−05
MR Test
Inverse variance weighted (fixed effects)
Inverse variance weighted (multiplicative random effects)
MR Egger
MR RAPS
Weighted median
Weighted mode
0.05
0.00
−0.05
0.09
0.12
0.15
0.18
SNP effect on Poly(U)−specific endoribonuclease
SNP effect on Gut microbiota abundance (genus Catenibacterium id.2153)

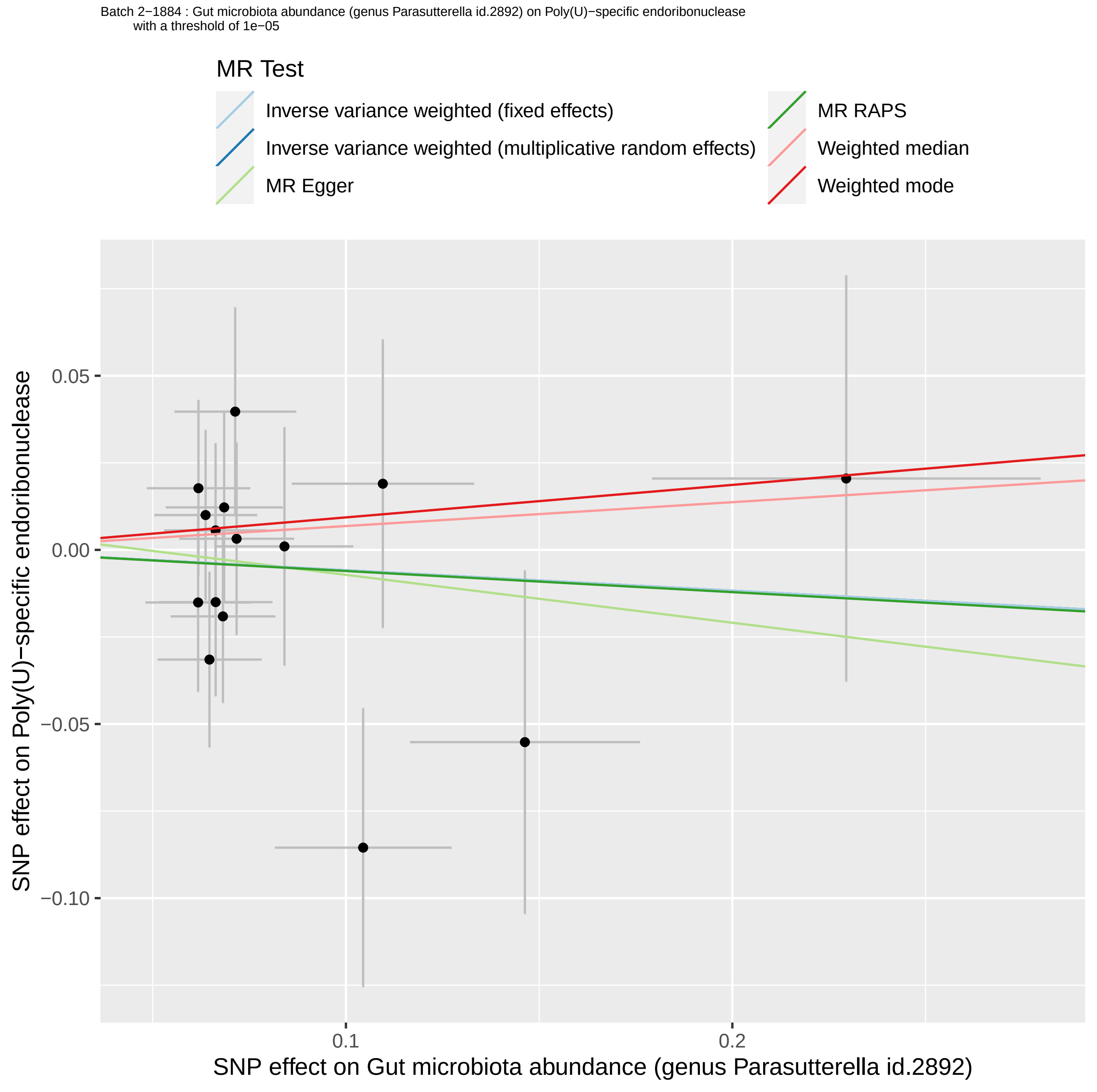

Batch 2−1884 : Gut microbiota abundance (genus Parasutterella id.2892) on Poly(U)−specific endoribonuclease
with a threshold of 1e−05
MR Test
Inverse variance weighted (fixed effects)
Inverse variance weighted (multiplicative random effects)
MR Egger
MR RAPS
Weighted median
Weighted mode
0.05
0.00
−0.05
−0.10
0.1
0.2
SNP effect on Poly(U)−specific endoribonuclease
SNP effect on Gut microbiota abundance (genus Parasutterella id.2892)

Batch 2−1885 : Gut microbiota abundance (genus Catenibacterium id.2153) on Endoglin
with a threshold of 1e−05

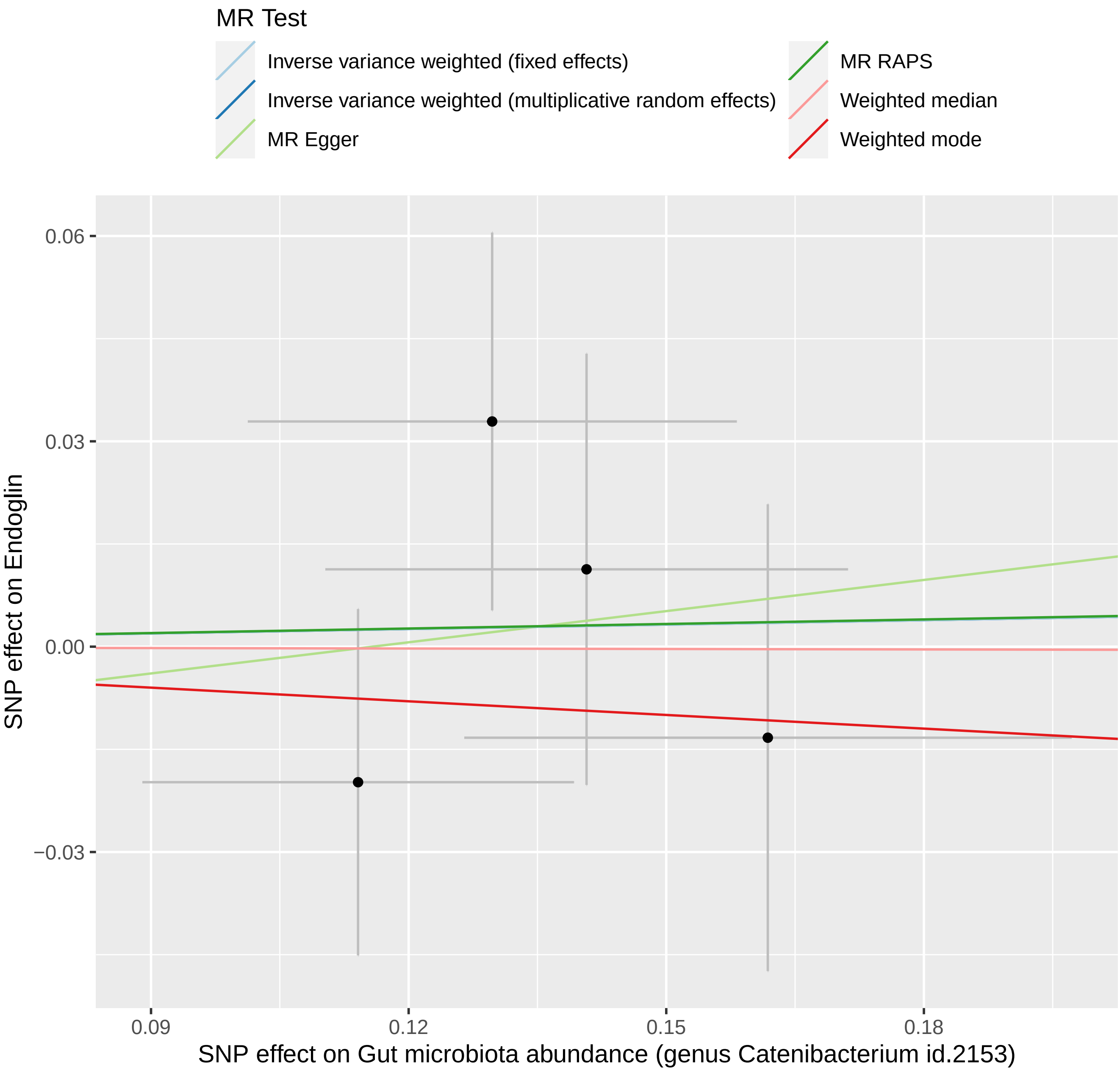

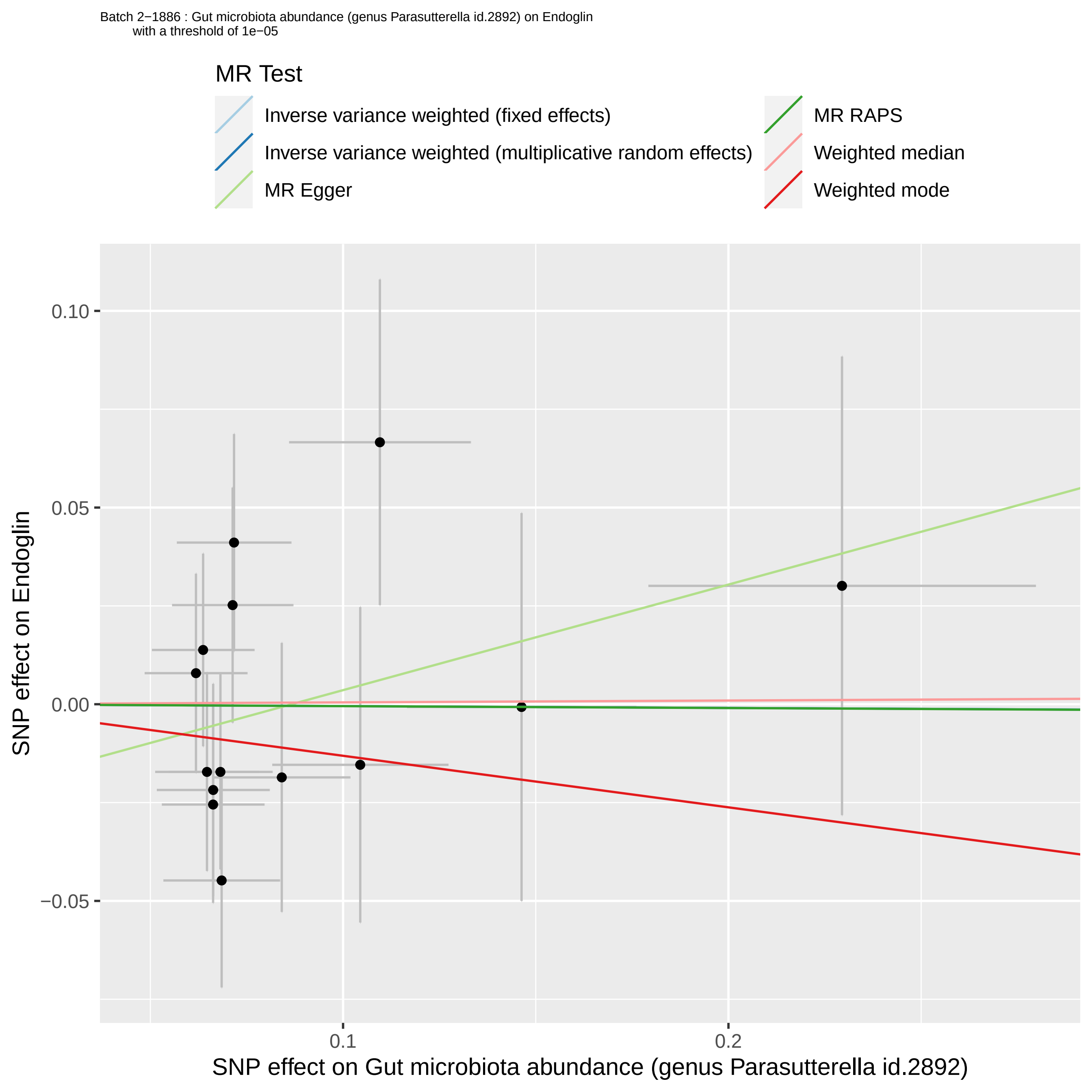

Batch 2−1886 : Gut microbiota abundance (genus Parasutterella id.2892) on Endoglin
with a threshold of 1e−05
MR Test
Inverse variance weighted (fixed effects)
Inverse variance weighted (multiplicative random effects)
MR Egger
MR RAPS
Weighted median
Weighted mode
0.10
0.05
0.00
−0.05
SNP effect on Endoglin
0.1
0.2
SNP effect on Gut microbiota abundance (genus Parasutterella id.2892)

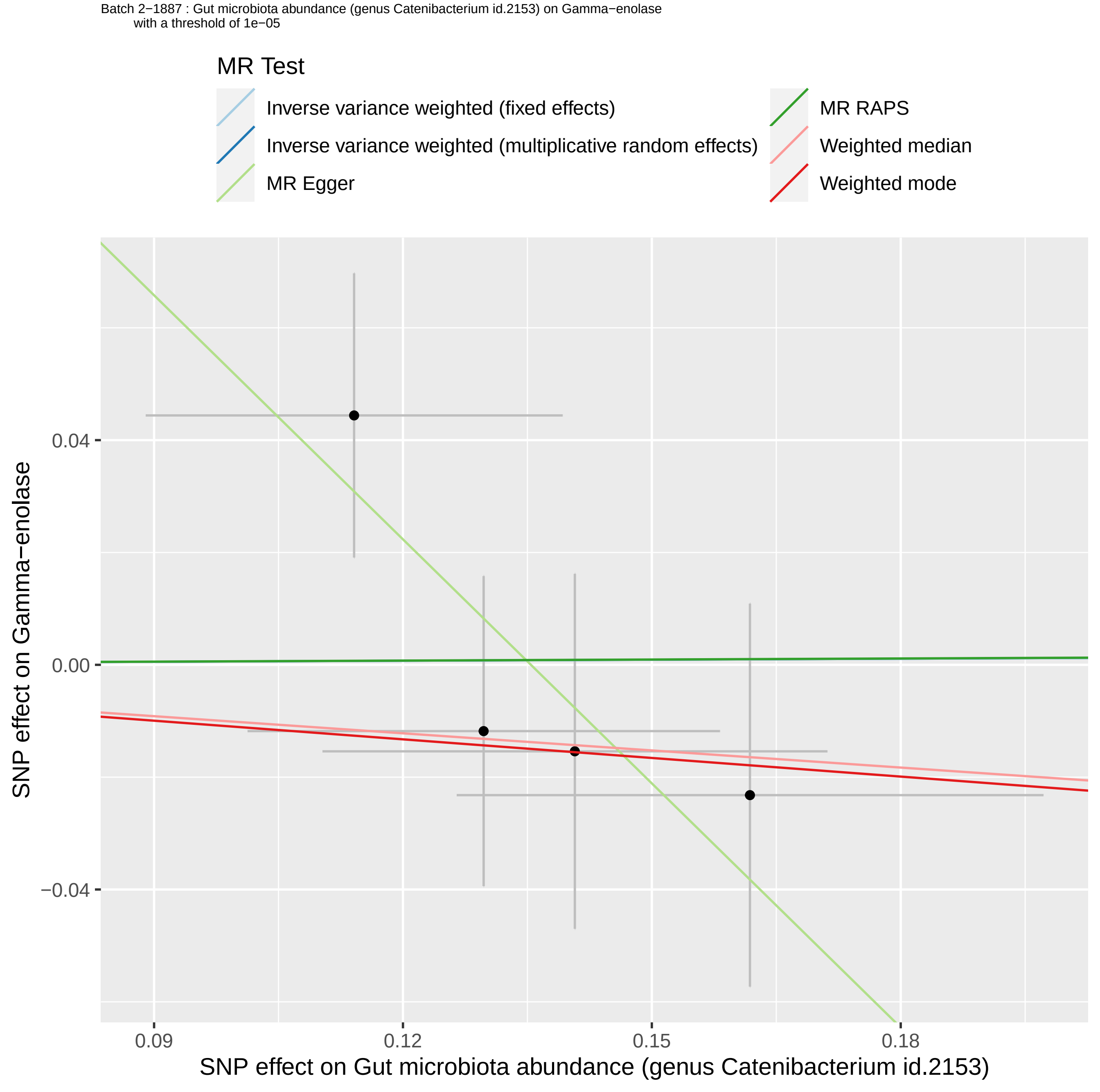

Batch 2−1887 : Gut microbiota abundance (genus Catenibacterium id.2153) on Gamma−enolase
with a threshold of 1e−05
MR Test
Inverse variance weighted (fixed effects)
Inverse variance weighted (multiplicative random effects)
MR Egger
MR RAPS
Weighted median
Weighted mode
0.04
0.00
−0.04
0.09
0.12
0.15
0.18
SNP effect on Gamma−enolase
SNP effect on Gut microbiota abundance (genus Catenibacterium id.2153)

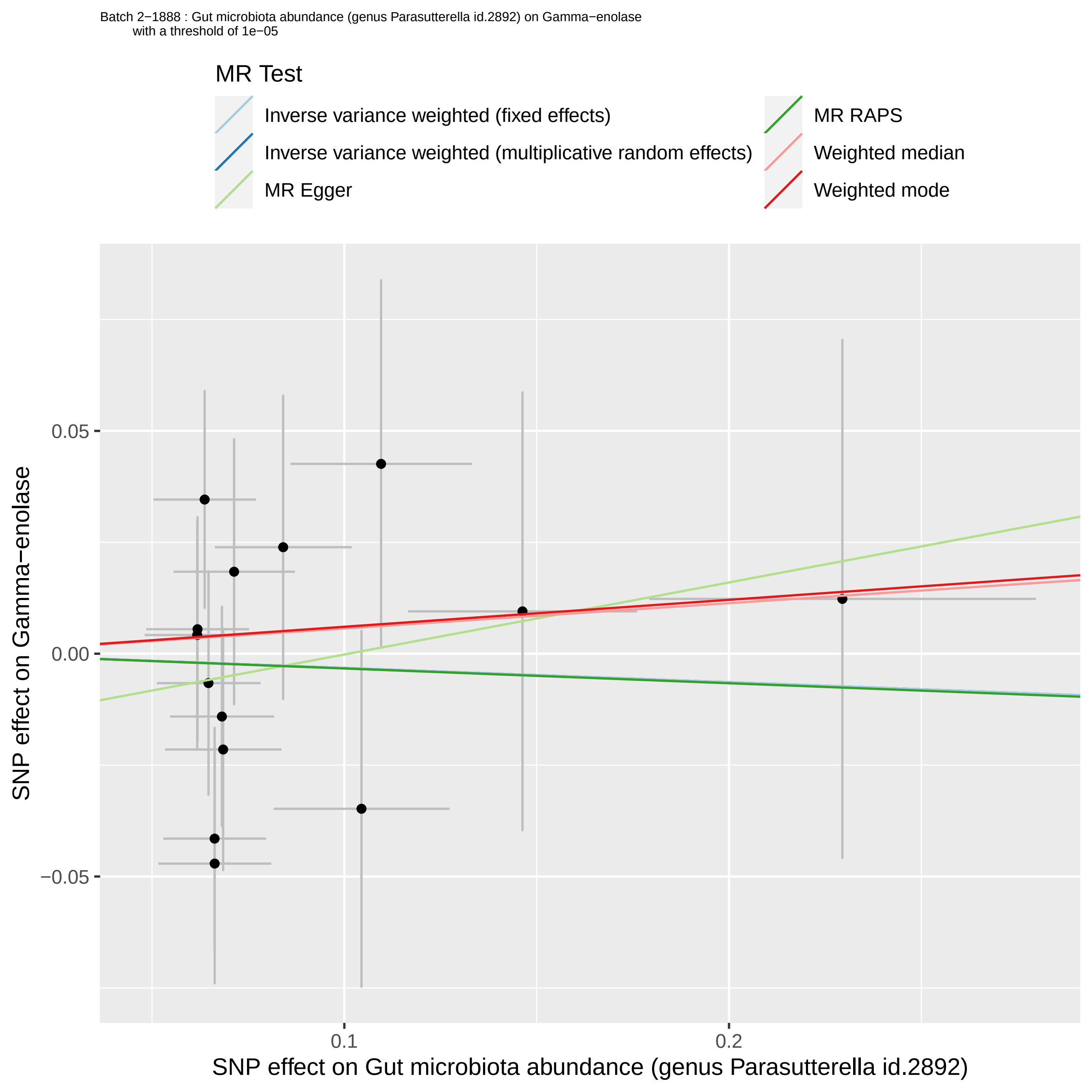
Batch 2−1888 : Gut microbiota abundance (genus Parasutterella id.2892) on Gamma−enolase
with a threshold of 1e−05
MR Test
Inverse variance weighted (fixed effects)
Inverse variance weighted (multiplicative random effects)
MR Egger
MR RAPS
Weighted median
Weighted mode
0.05
0.00
−0.05
0.1
0.2
SNP effect on Gamma−enolase
SNP effect on Gut microbiota abundance (genus Parasutterella id.2892)

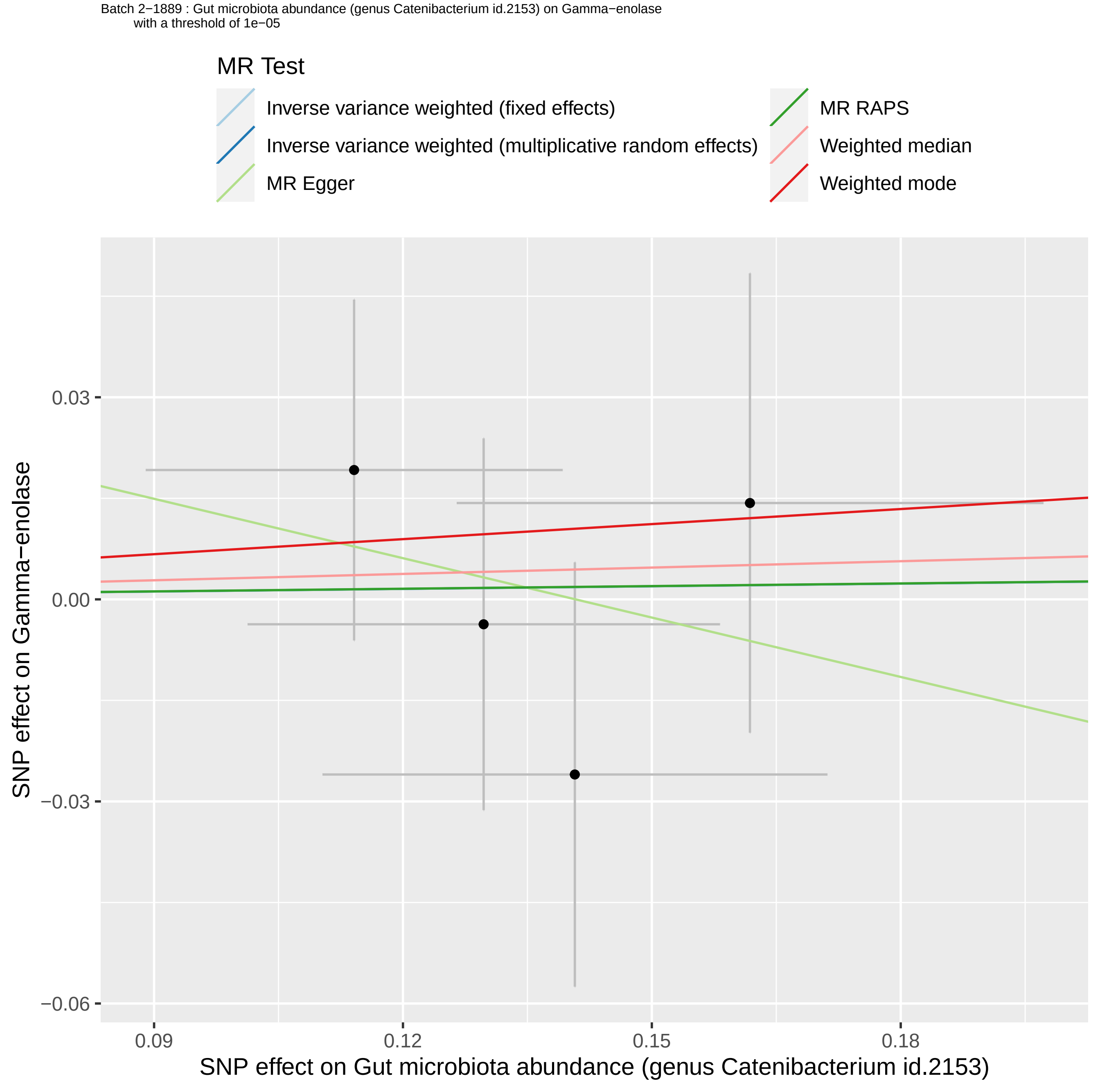

Batch 2−1889 : Gut microbiota abundance (genus Catenibacterium id.2153) on Gamma−enolase
with a threshold of 1e−05
MR Test
Inverse variance weighted (fixed effects)
Inverse variance weighted (multiplicative random effects)
MR Egger
MR RAPS
Weighted median
Weighted mode
0.03
0.00
−0.03
−0.06
0.09
0.12
0.15
0.18
SNP effect on Gamma−enolase
SNP effect on Gut microbiota abundance (genus Catenibacterium id.2153)

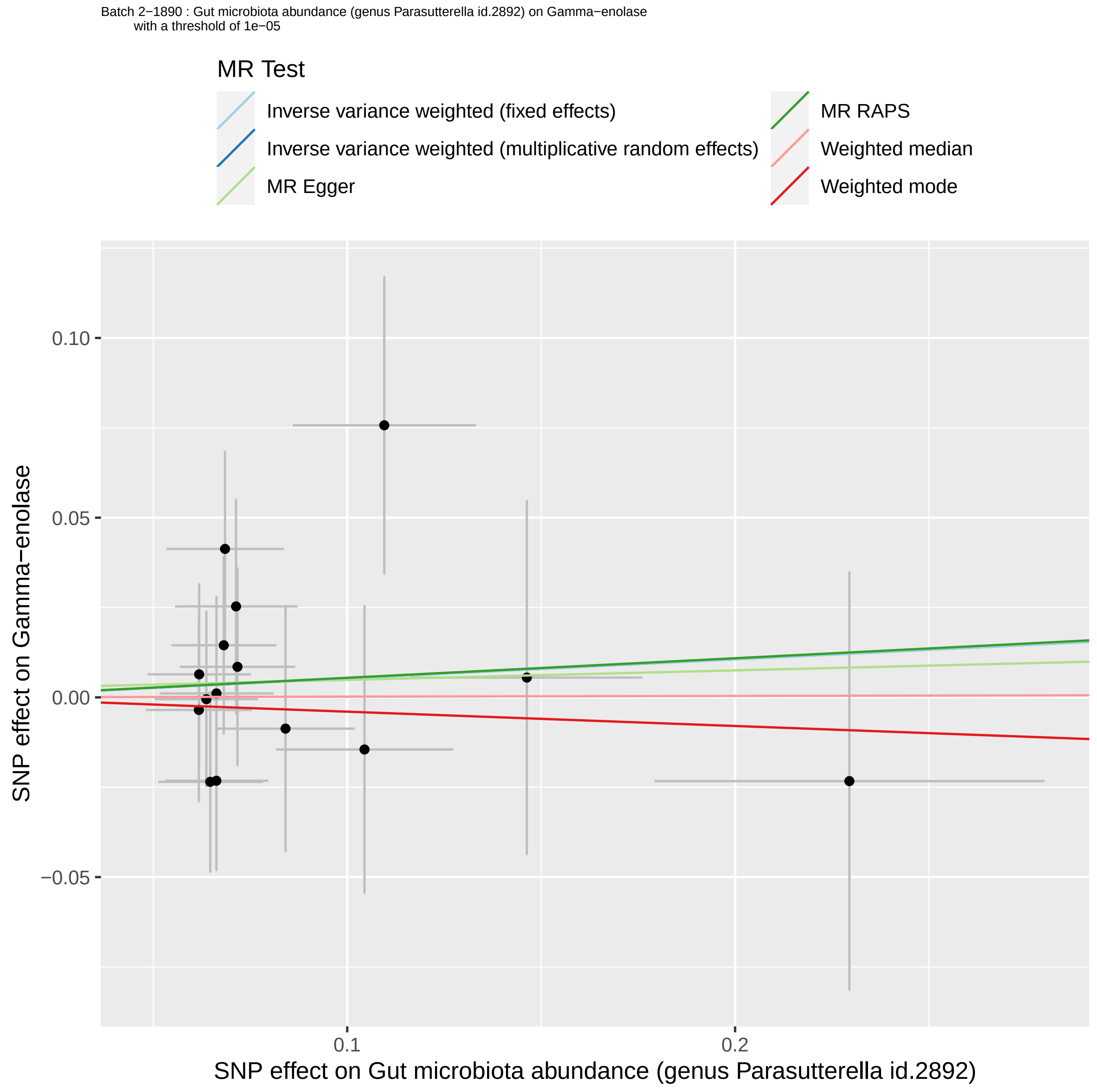
Batch 2−1890 : Gut microbiota abundance (genus Parasutterella id.2892) on Gamma−enolase
with a threshold of 1e−05
MR Test
Inverse variance weighted (fixed effects)
Inverse variance weighted (multiplicative random effects)
MR Egger
MR RAPS
Weighted median
Weighted mode
0.10
0.05
0.00
−0.05
0.1
0.2
SNP effect on Gamma−enolase
SNP effect on Gut microbiota abundance (genus Parasutterella id.2892)

Batch 2−1891 : Gut microbiota abundance (genus Catenibacterium id.2153) on Ecto−NOX disulfide−thiol exchanger 2
with a threshold of 1e−05

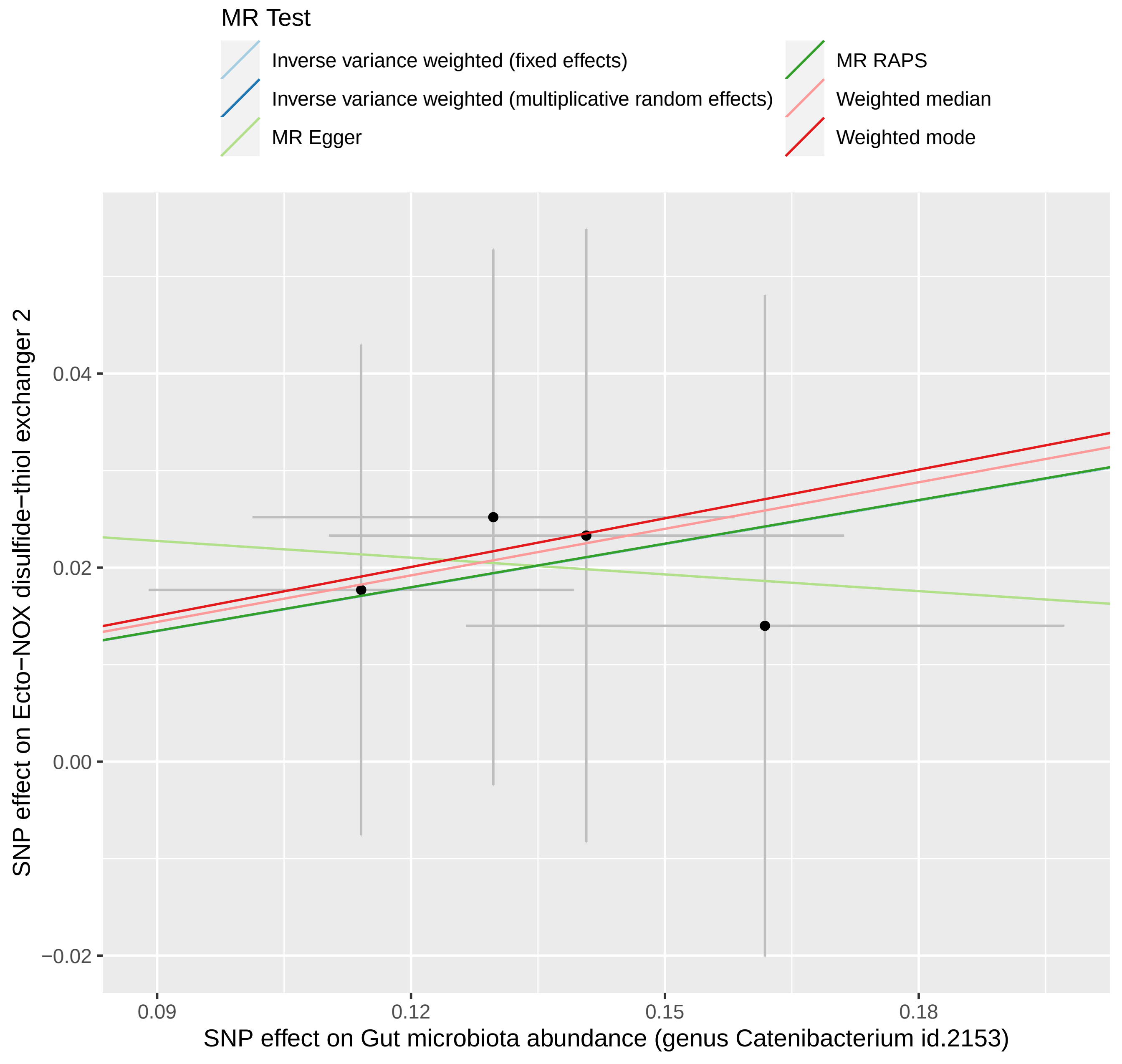

Batch 2−1892 : Gut microbiota abundance (genus Parasutterella id.2892) on Ecto−NOX disulfide−thiol exchanger 2
with a threshold of 1e−05

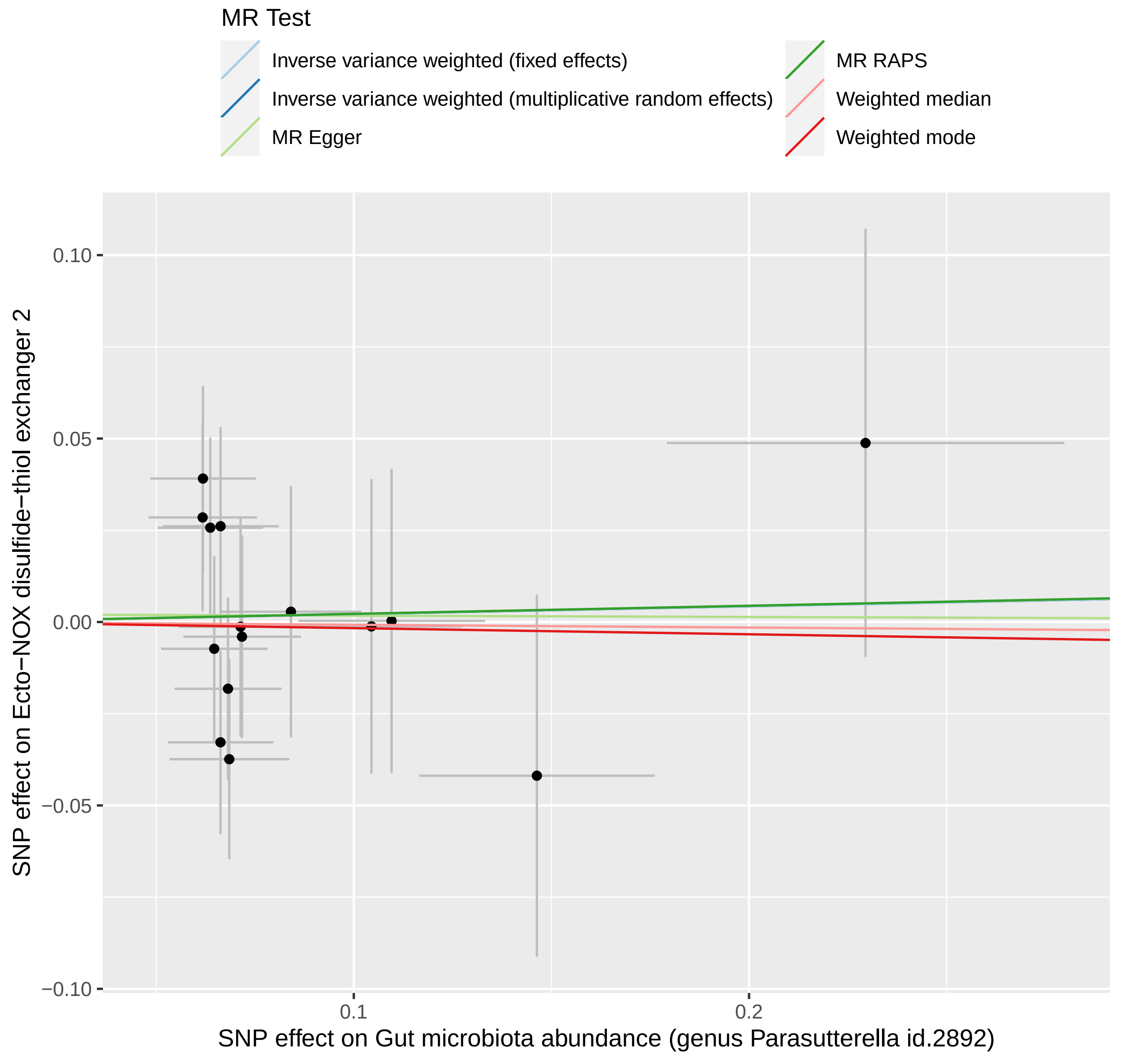

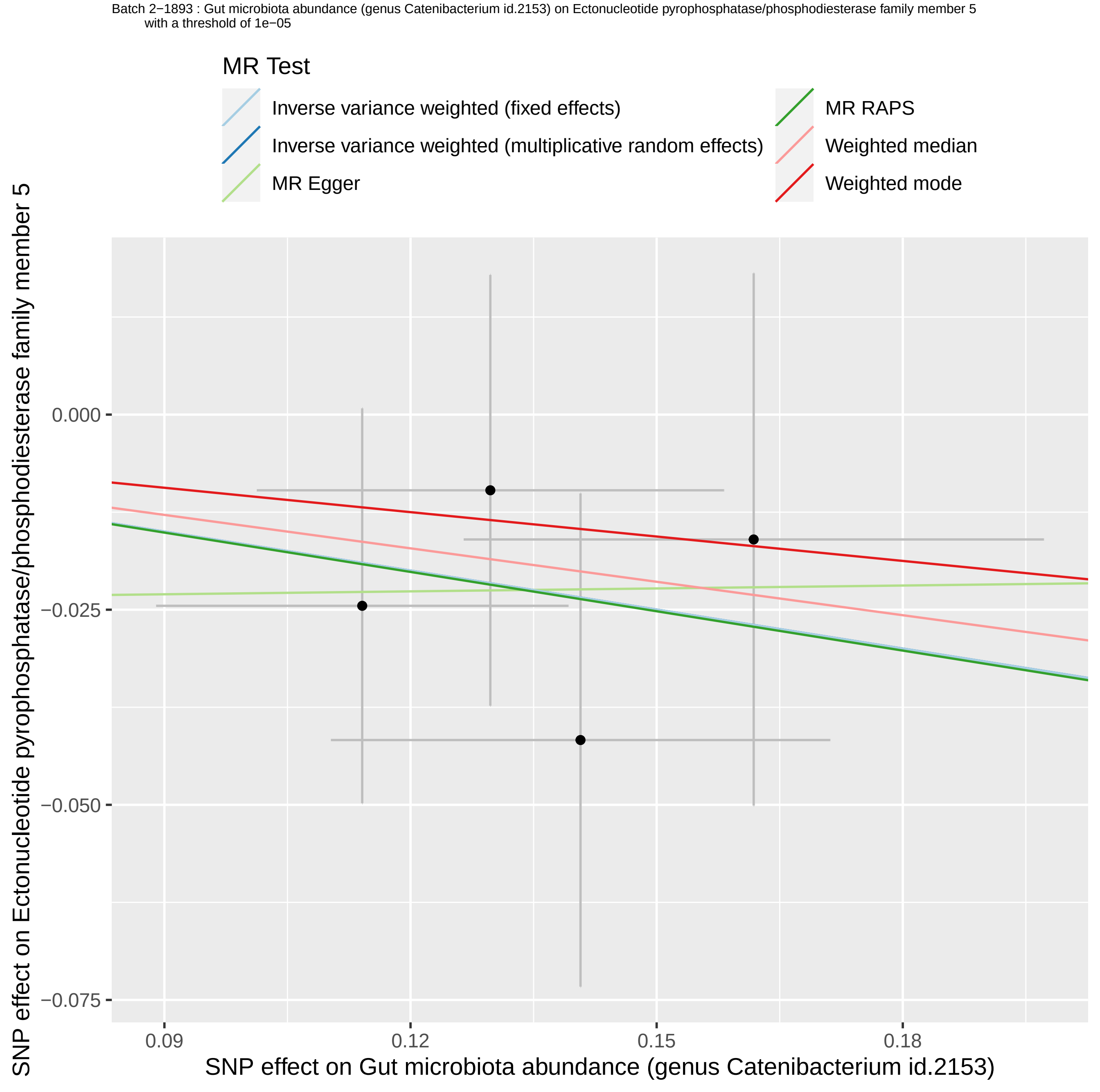
Batch 2−1893 : Gut microbiota abundance (genus Catenibacterium id.2153) on Ectonucleotide pyrophosphatase/phosphodiesterase family member 5 with a threshold of 1e−05
MR Test
Inverse variance weighted (fixed effects)
Inverse variance weighted (multiplicative random effects)
MR Egger
MR RAPS
Weighted median
Weighted mode
SNP effect on Ectonucleotide pyrophosphatase/phosphodiesterase family member 5
0.000
−0.025
−0.050
−0.075
0.09
0.12
0.15
0.18
SNP effect on Gut microbiota abundance (genus Catenibacterium id.2153)

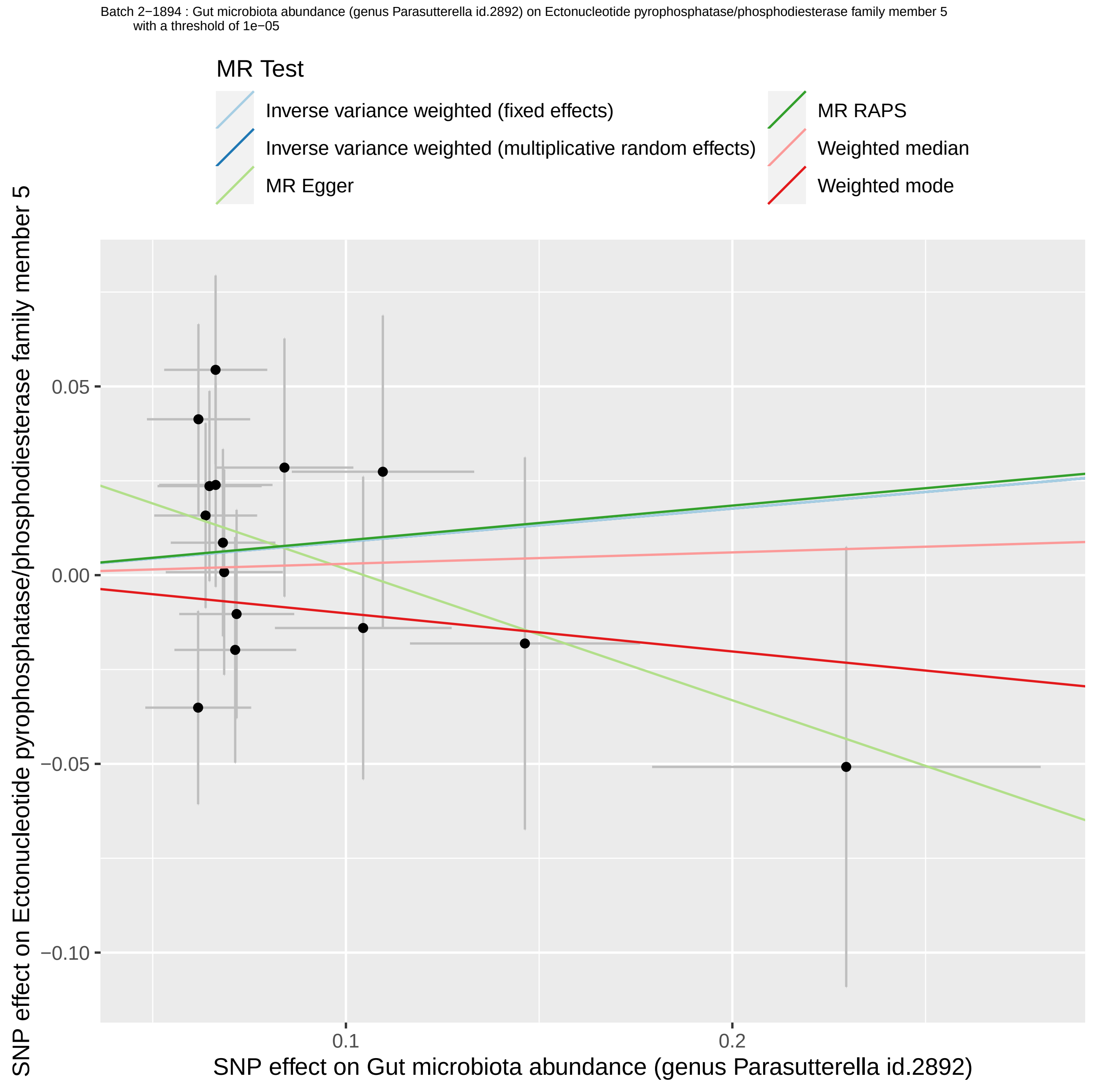

Batch 2−1894 : Gut microbiota abundance (genus Parasutterella id.2892) on Ectonucleotide pyrophosphatase/phosphodiesterase family member 5 with a threshold of 1e−05
MR Test
Inverse variance weighted (fixed effects)
Inverse variance weighted (multiplicative random effects)
MR Egger
MR RAPS
Weighted median
Weighted mode
0.05
0.00
−0.05
−0.10
0.1
0.2
SNP effect on Ectonucleotide pyrophosphatase/phosphodiesterase family member 5
SNP effect on Gut microbiota abundance (genus Parasutterella id.2892)

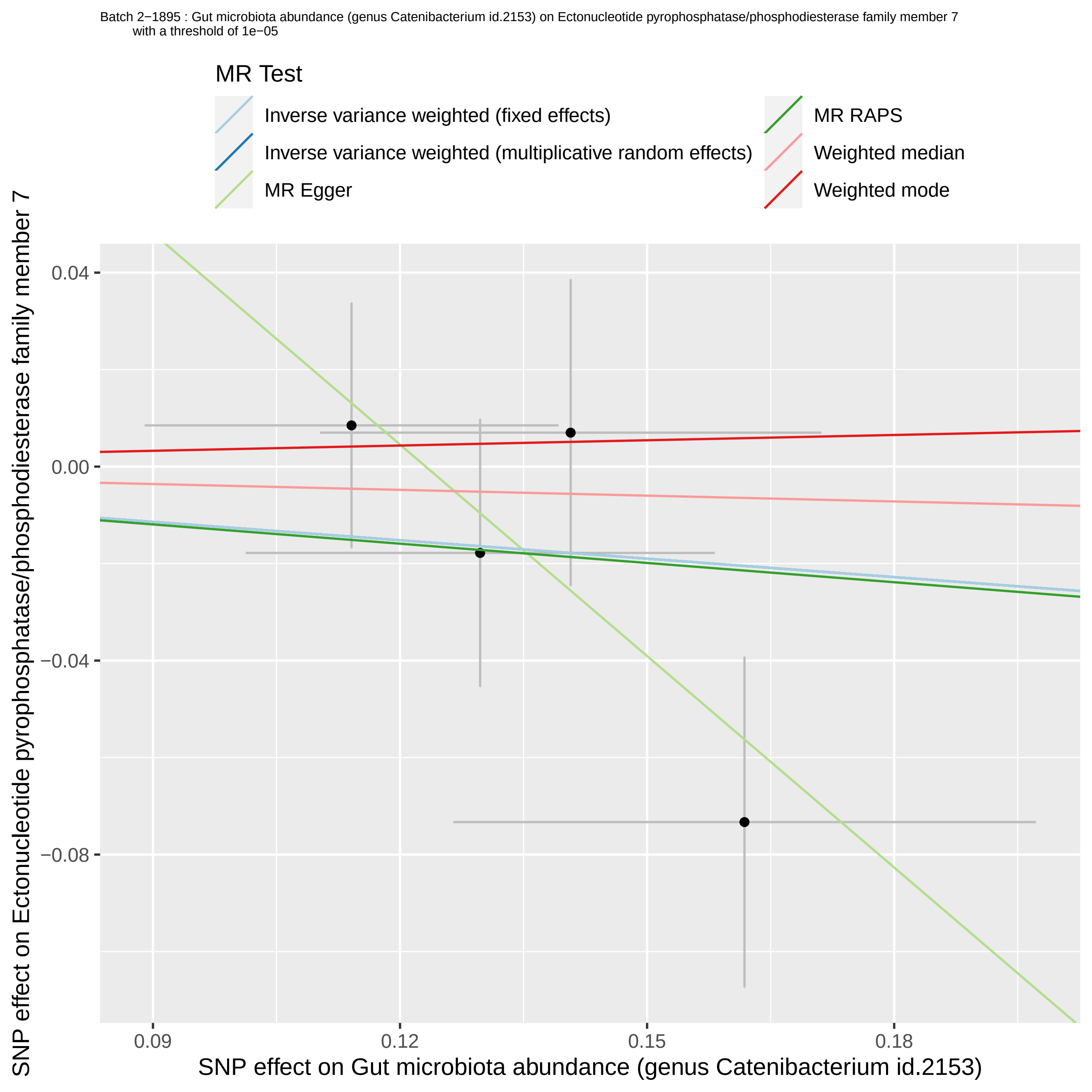

Batch 2−1895 : Gut microbiota abundance (genus Catenibacterium id.2153) on Ectonucleotide pyrophosphatase/phosphodiesterase family member 7 with a threshold of 1e−05
MR Test
Inverse variance weighted (fixed effects)
Inverse variance weighted (multiplicative random effects)
MR Egger
MR RAPS
Weighted median
Weighted mode
0.04
0.00
−0.04
−0.08
0.09
0.12
0.15
0.18
SNP effect on Gut microbiota abundance (genus Catenibacterium id.2153)
SNP effect on Ectonucleotide pyrophosphatase/phosphodiesterase family member 7

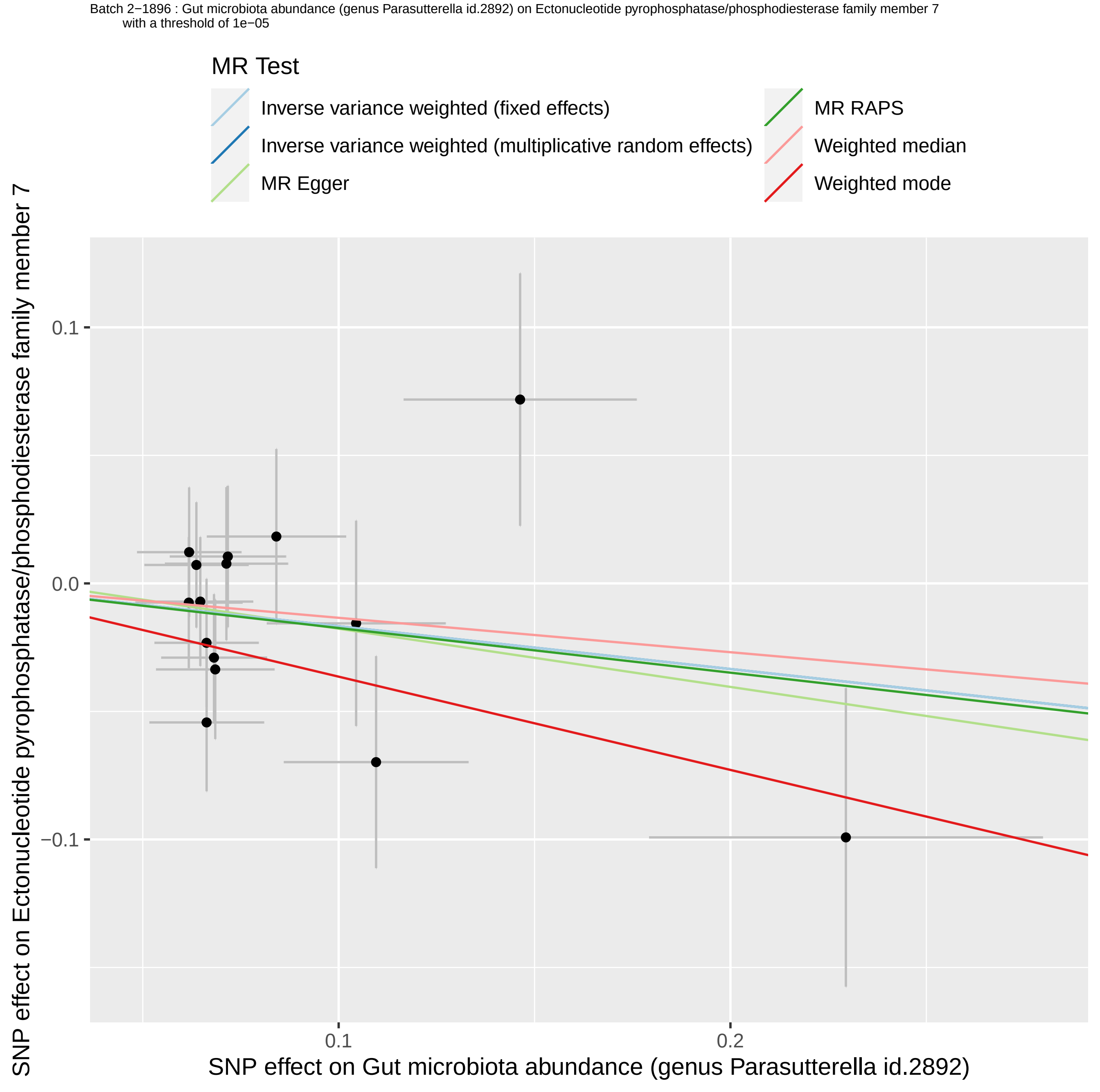

Batch 2−1896 : Gut microbiota abundance (genus Parasutterella id.2892) on Ectonucleotide pyrophosphatase/phosphodiesterase family member 7
with a threshold of 1e−05
MR Test
Inverse variance weighted (fixed effects)
Inverse variance weighted (multiplicative random effects)
MR Egger
MR RAPS
Weighted median
Weighted mode
0.1
0.0
−0.1
0.1
0.2
SNP effect on Ectonucleotide pyrophosphatase/phosphodiesterase family member 7
SNP effect on Gut microbiota abundance (genus Parasutterella id.2892)

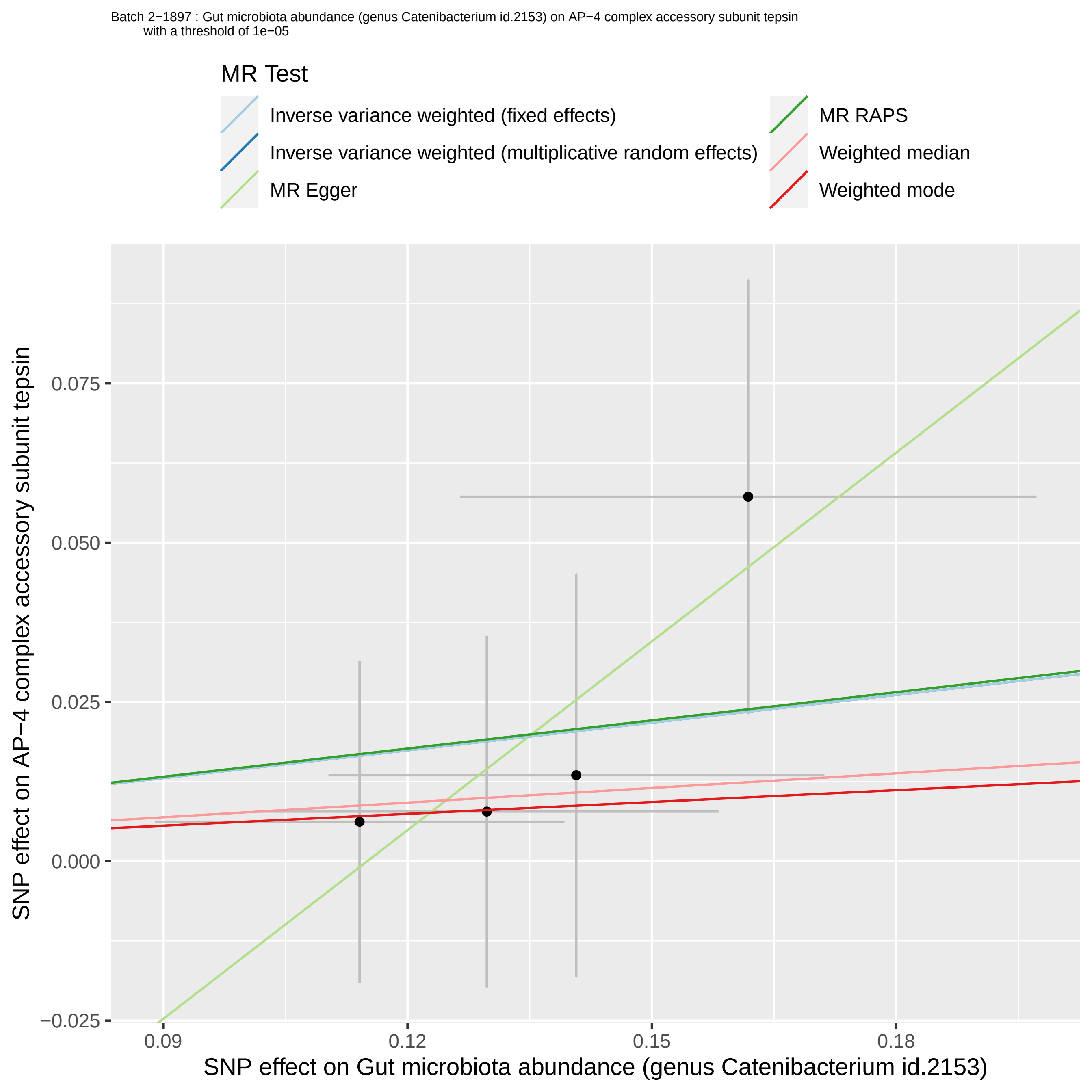
Batch 2−1897 : Gut microbiota abundance (genus Catenibacterium id.2153) on AP−4 complex accessory subunit tepsin
with a threshold of 1e−05
MR Test
Inverse variance weighted (fixed effects)
Inverse variance weighted (multiplicative random effects)
MR Egger
MR RAPS
Weighted median
Weighted mode
SNP effect on AP−4 complex accessory subunit tepsin
0.075
0.050
0.025
0.000
−0.025
0.09
0.12
0.15
0.18
SNP effect on Gut microbiota abundance (genus Catenibacterium id.2153)

Batch 2−1898 : Gut microbiota abundance (genus Parasutterella id.2892) on AP−4 complex accessory subunit tepsin
with a threshold of 1e−05

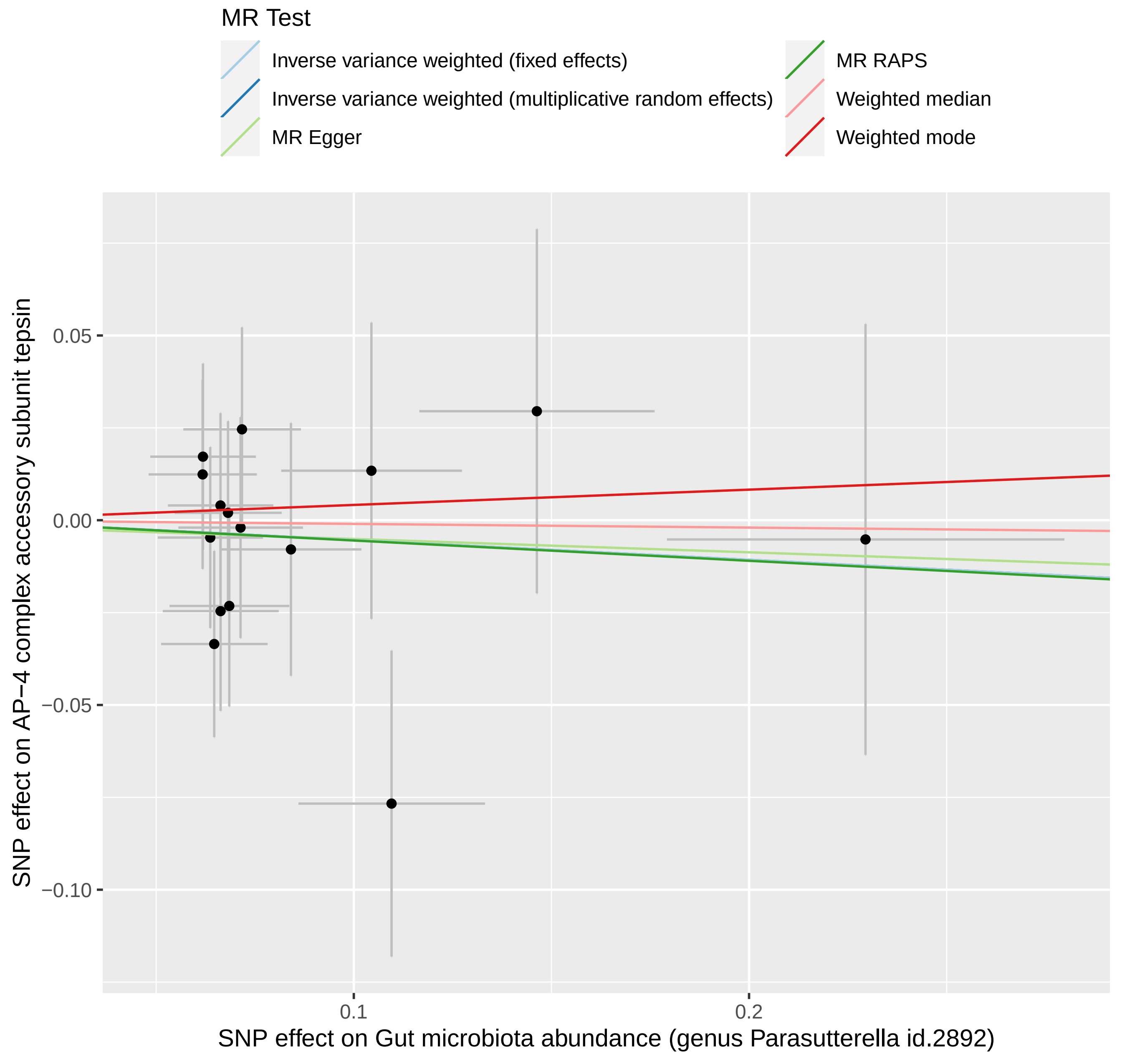

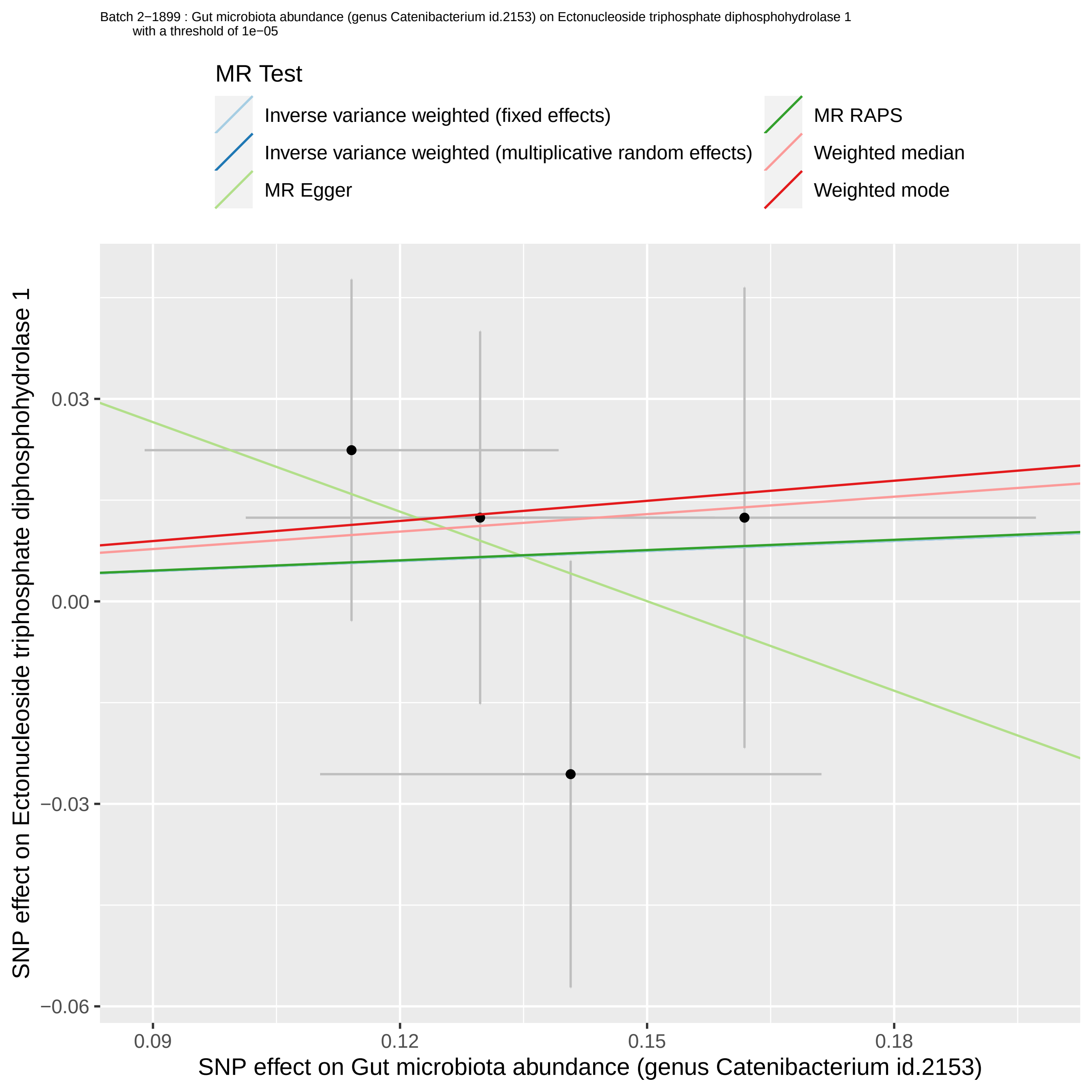
Batch 2−1899 : Gut microbiota abundance (genus Catenibacterium id.2153) on Ectonucleoside triphosphate diphosphohydrolase 1
with a threshold of 1e−05
MR Test
Inverse variance weighted (fixed effects)
Inverse variance weighted (multiplicative random effects)
MR Egger
MR RAPS
Weighted median
Weighted mode
0.03
0.00
−0.03
−0.06
0.09
0.12
0.15
0.18
SNP effect on Ectonucleoside triphosphate diphosphohydrolase 1
SNP effect on Gut microbiota abundance (genus Catenibacterium id.2153)

Batch 2−1900 : Gut microbiota abundance (genus Parasutterella id.2892) on Ectonucleoside triphosphate diphosphohydrolase 1
with a threshold of 1e−05

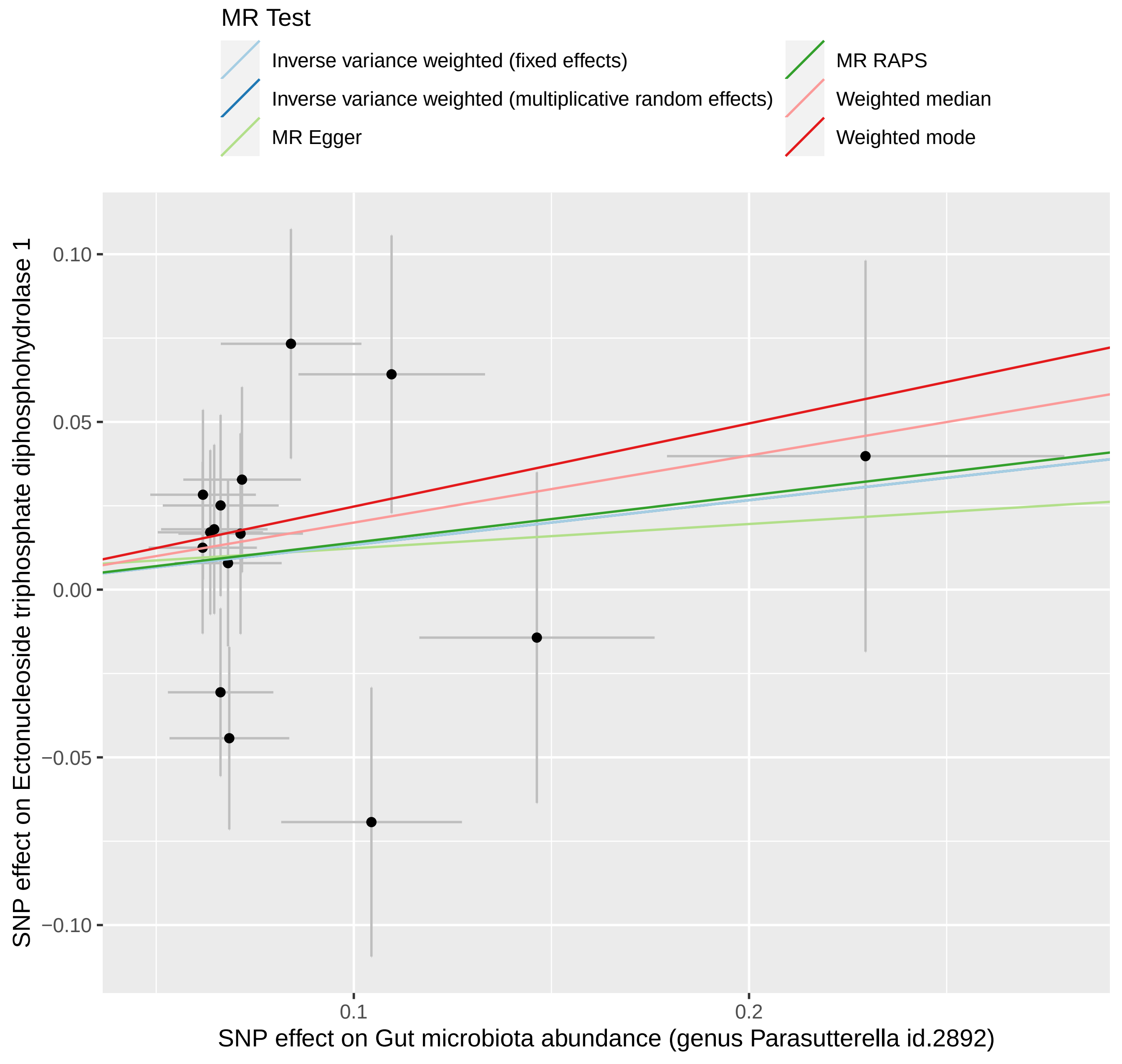

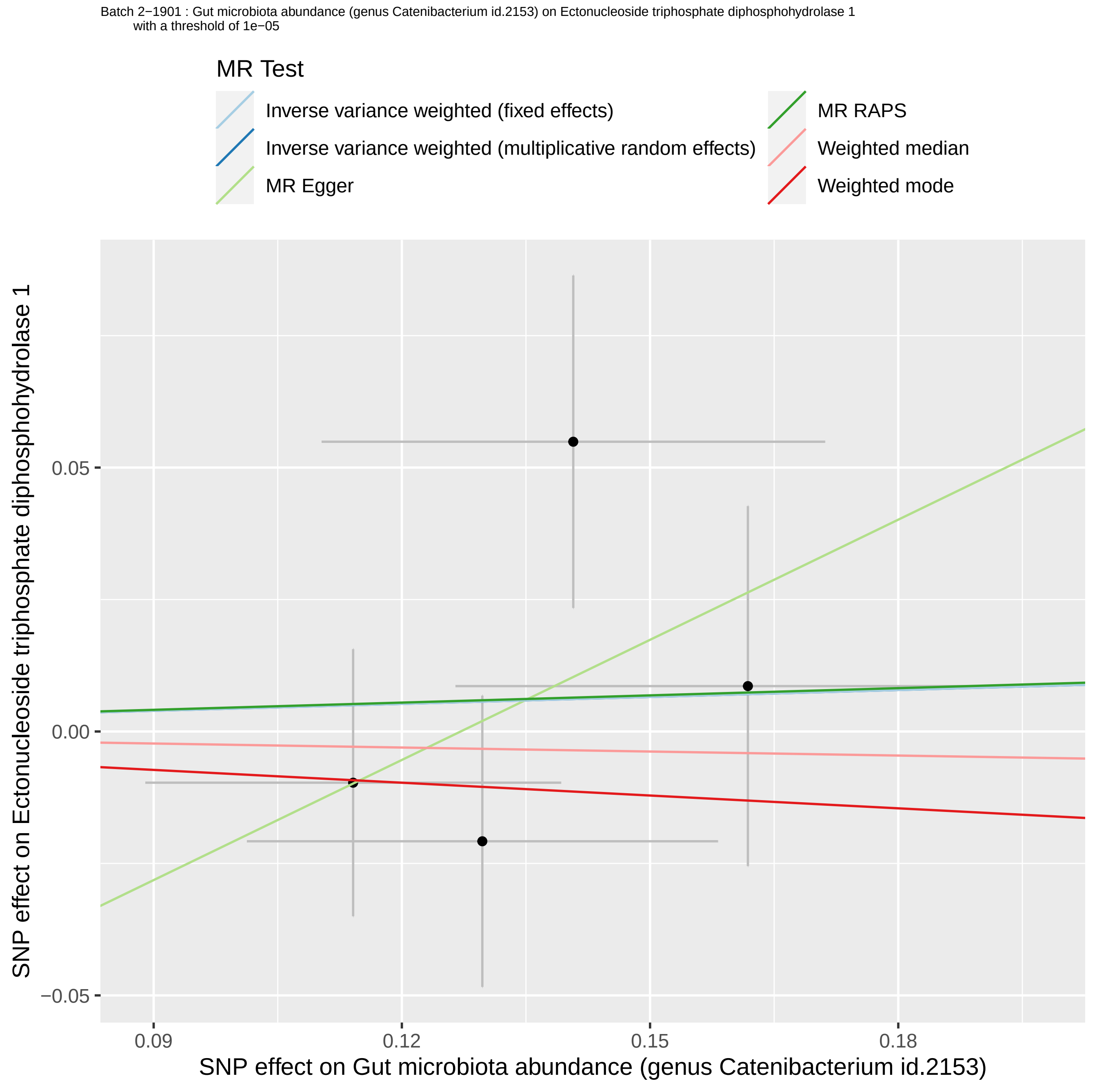

Batch 2−1901 : Gut microbiota abundance (genus Catenibacterium id.2153) on Ectonucleoside triphosphate diphosphohydrolase 1
with a threshold of 1e−05
MR Test
Inverse variance weighted (fixed effects)
Inverse variance weighted (multiplicative random effects)
MR Egger
MR RAPS
Weighted median
Weighted mode
0.05
0.00
−0.05
0.09
0.12
0.15
0.18
SNP effect on Ectonucleoside triphosphate diphosphohydrolase 1
SNP effect on Gut microbiota abundance (genus Catenibacterium id.2153)

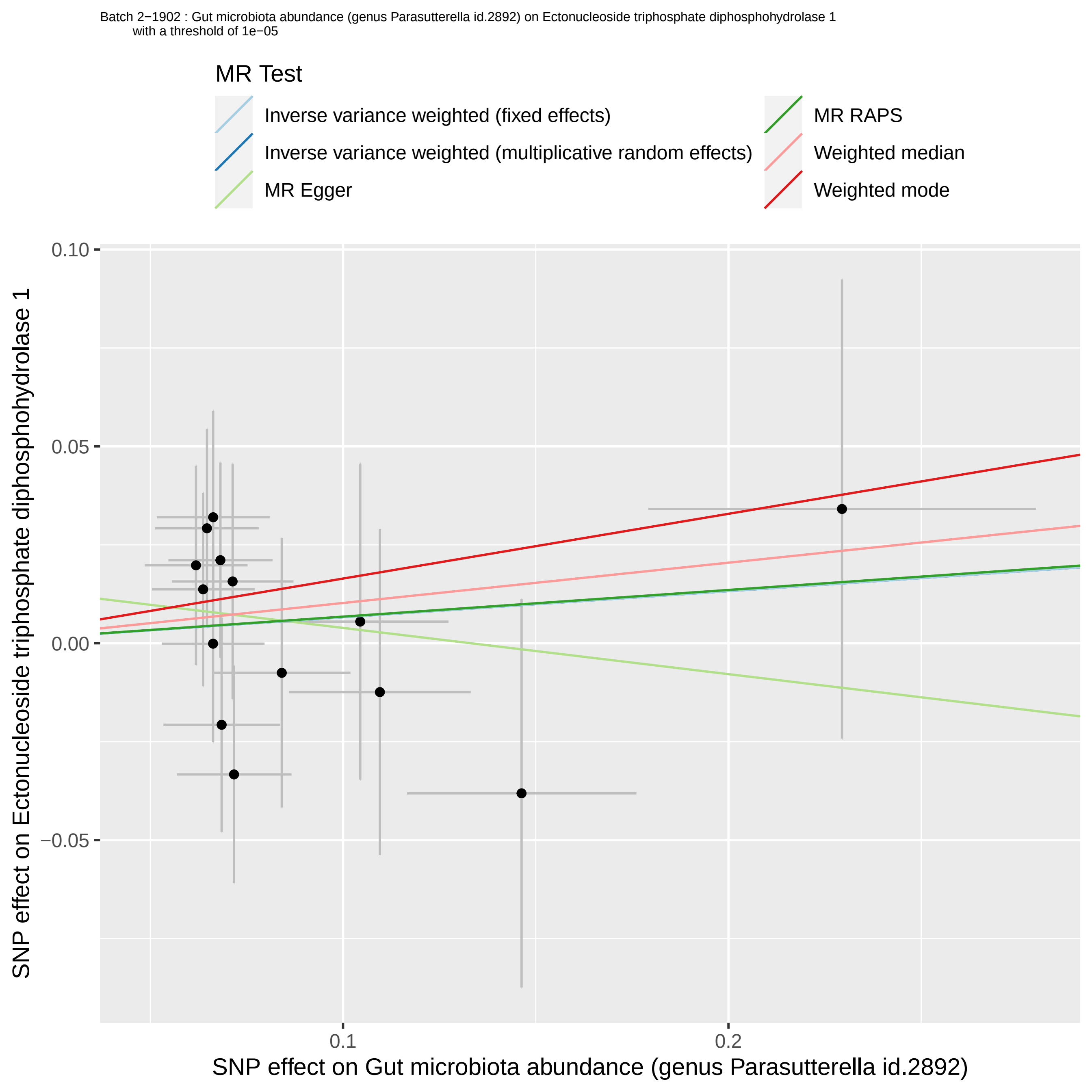
Batch 2−1902 : Gut microbiota abundance (genus Parasutterella id.2892) on Ectonucleoside triphosphate diphosphohydrolase 1
with a threshold of 1e−05
MR Test
Inverse variance weighted (fixed effects)
Inverse variance weighted (multiplicative random effects)
MR Egger
MR RAPS
Weighted median
Weighted mode
0.10
0.05
0.00
−0.05
0.1
0.2
SNP effect on Ectonucleoside triphosphate diphosphohydrolase 1
SNP effect on Gut microbiota abundance (genus Parasutterella id.2892)

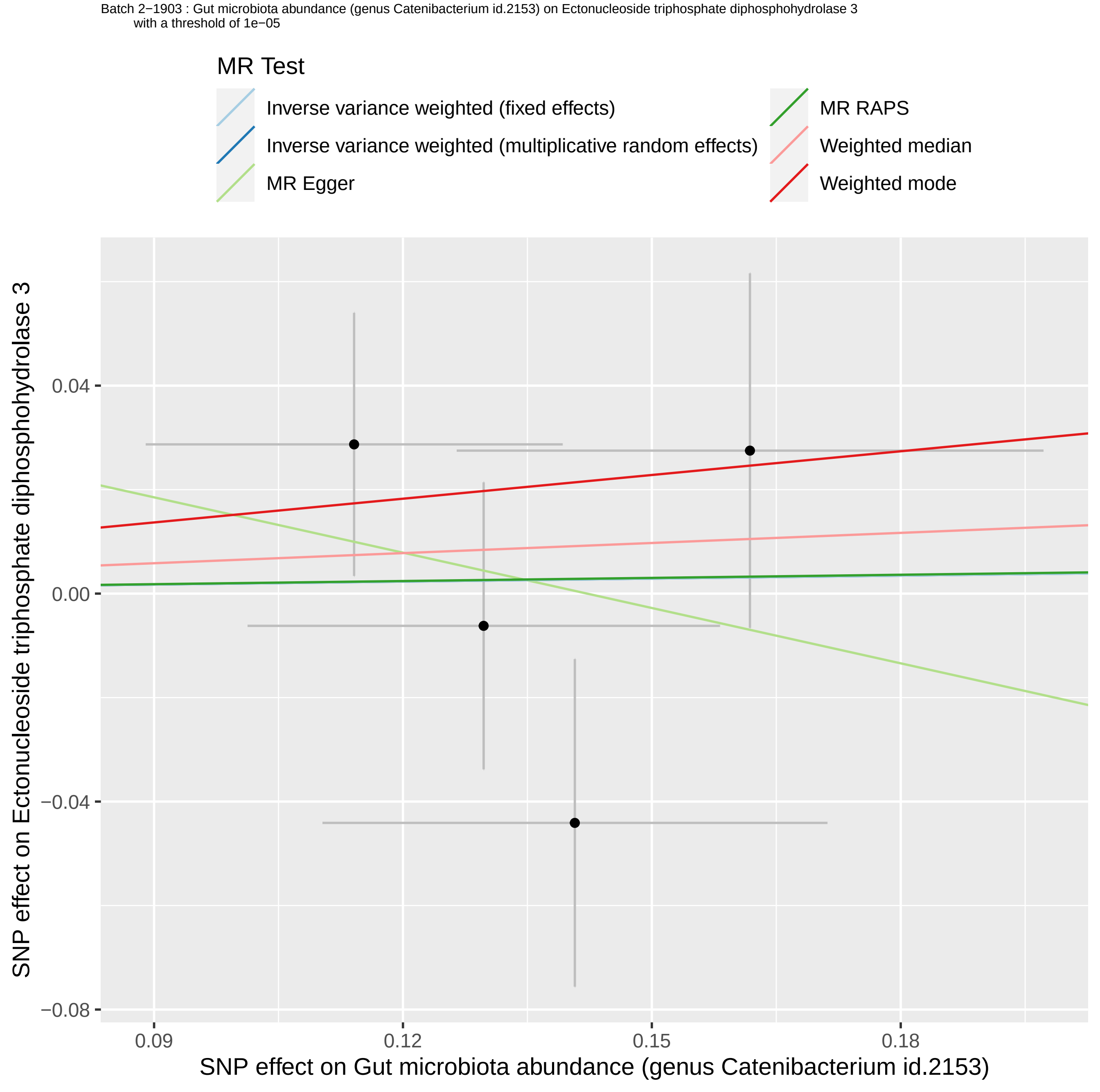
Batch 2−1903 : Gut microbiota abundance (genus Catenibacterium id.2153) on Ectonucleoside triphosphate diphosphohydrolase 3
with a threshold of 1e−05
MR Test
Inverse variance weighted (fixed effects)
Inverse variance weighted (multiplicative random effects)
MR Egger
MR RAPS
Weighted median
Weighted mode
0.04
0.00
−0.04
−0.08
0.09
0.12
0.15
0.18
SNP effect on Ectonucleoside triphosphate diphosphohydrolase 3
SNP effect on Gut microbiota abundance (genus Catenibacterium id.2153)

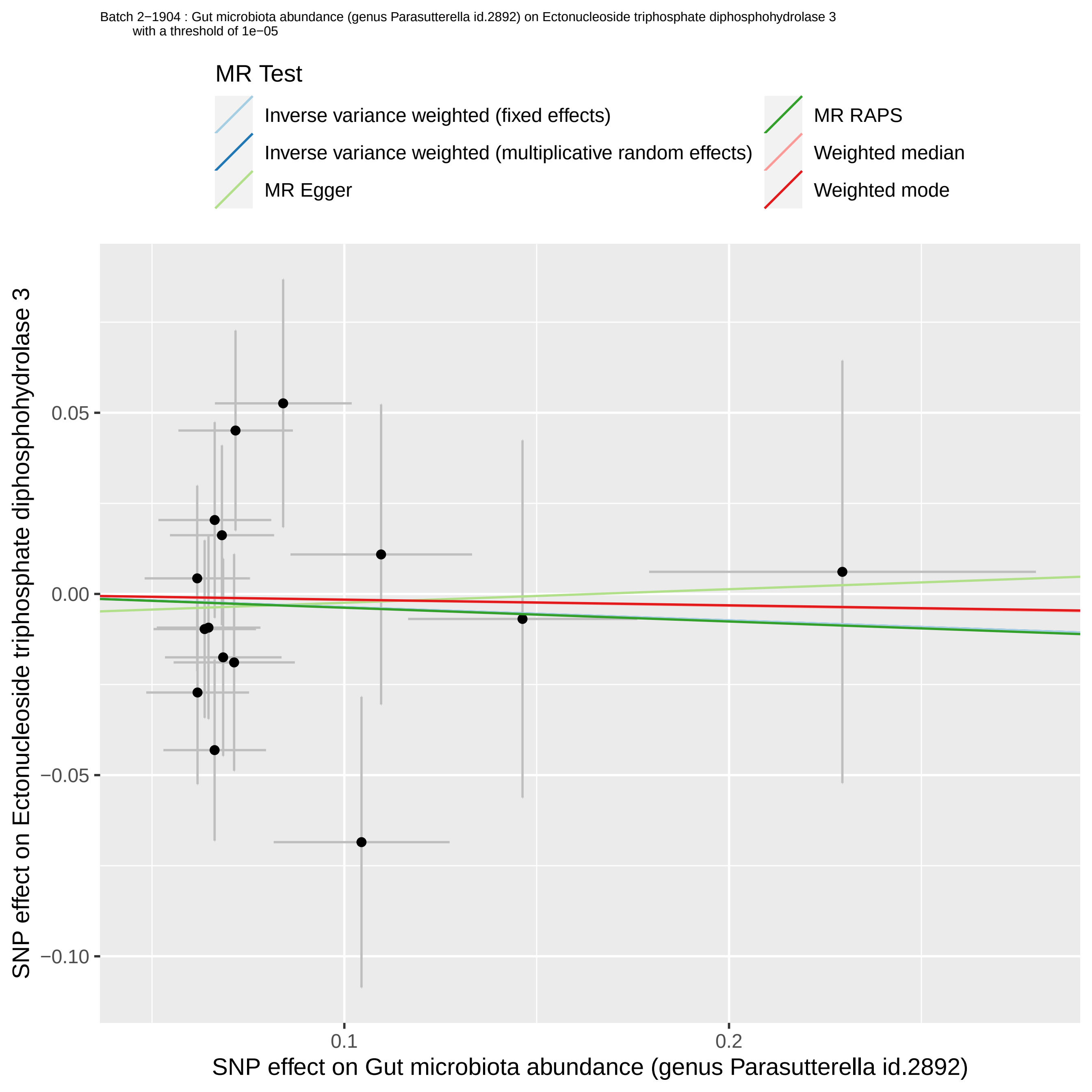
Batch 2−1904 : Gut microbiota abundance (genus Parasutterella id.2892) on Ectonucleoside triphosphate diphosphohydrolase 3
with a threshold of 1e−05
MR Test
Inverse variance weighted (fixed effects)
Inverse variance weighted (multiplicative random effects)
MR Egger
MR RAPS
Weighted median
Weighted mode
SNP effect on Ectonucleoside triphosphate diphosphohydrolase 3
0.05
0.00
−0.05
−0.10
0.1
0.2
SNP effect on Gut microbiota abundance (genus Parasutterella id.2892)

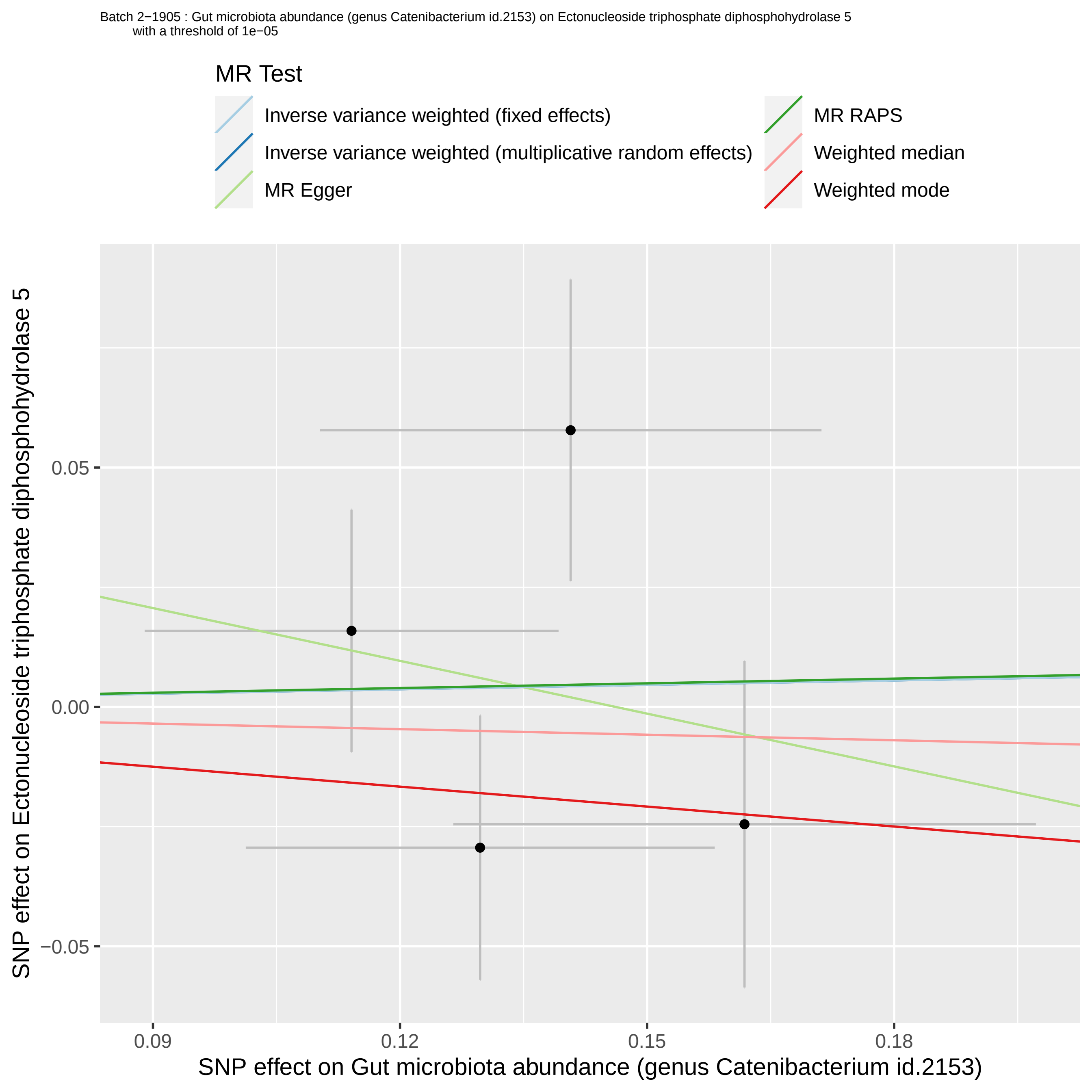
Batch 2−1905 : Gut microbiota abundance (genus Catenibacterium id.2153) on Ectonucleoside triphosphate diphosphohydrolase 5
with a threshold of 1e−05
MR Test
Inverse variance weighted (fixed effects)
Inverse variance weighted (multiplicative random effects)
MR Egger
MR RAPS
Weighted median
Weighted mode
0.05
0.00
−0.05
0.09
0.12
0.15
0.18
SNP effect on Ectonucleoside triphosphate diphosphohydrolase 5
SNP effect on Gut microbiota abundance (genus Catenibacterium id.2153)

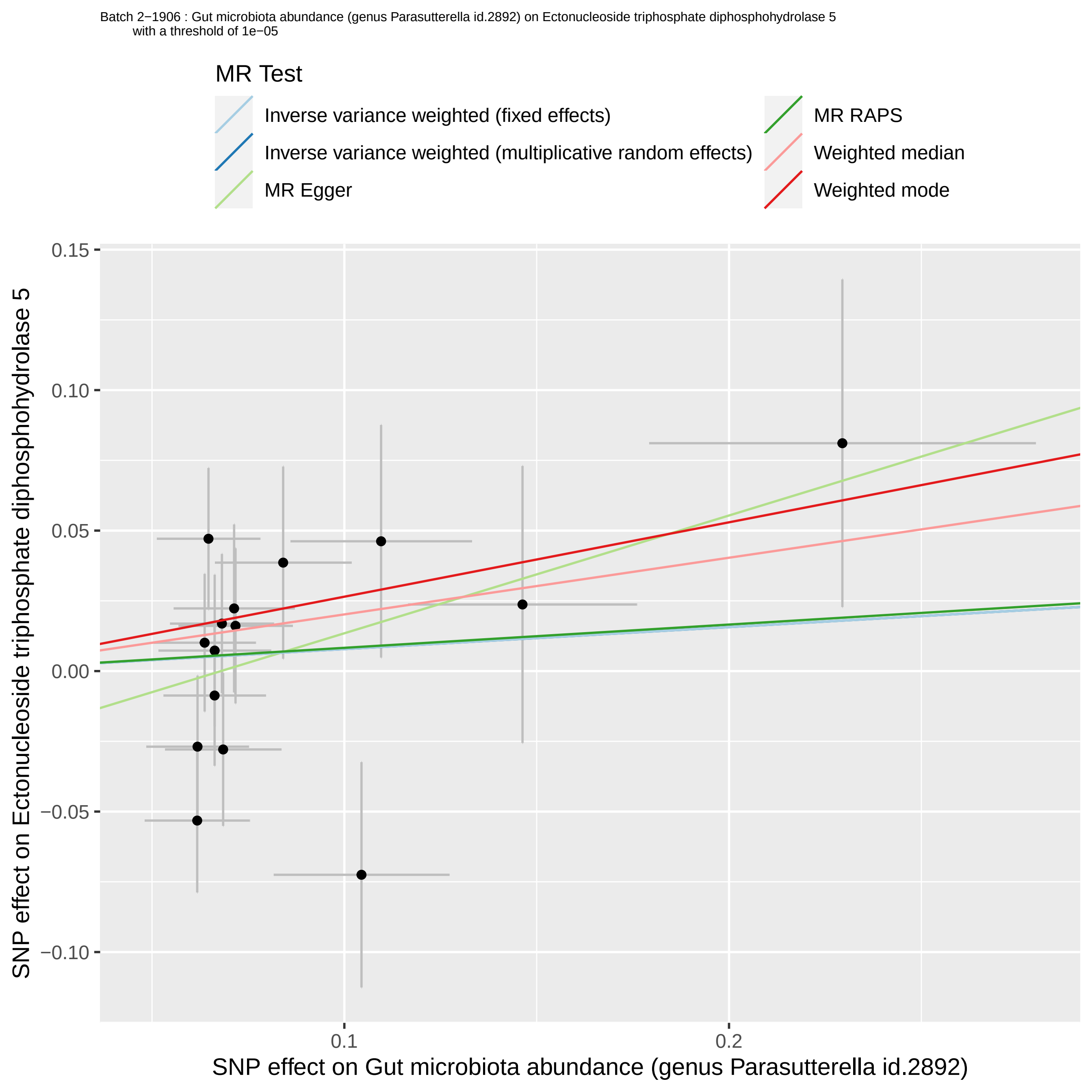

Batch 2−1906 : Gut microbiota abundance (genus Parasutterella id.2892) on Ectonucleoside triphosphate diphosphohydrolase 5
with a threshold of 1e−05
MR Test
Inverse variance weighted (fixed effects)
Inverse variance weighted (multiplicative random effects)
MR Egger
MR RAPS
Weighted median
Weighted mode
0.15
0.10
0.05
0.00
−0.05
−0.10
0.1
0.2
SNP effect on Ectonucleoside triphosphate diphosphohydrolase 5
SNP effect on Gut microbiota abundance (genus Parasutterella id.2892)

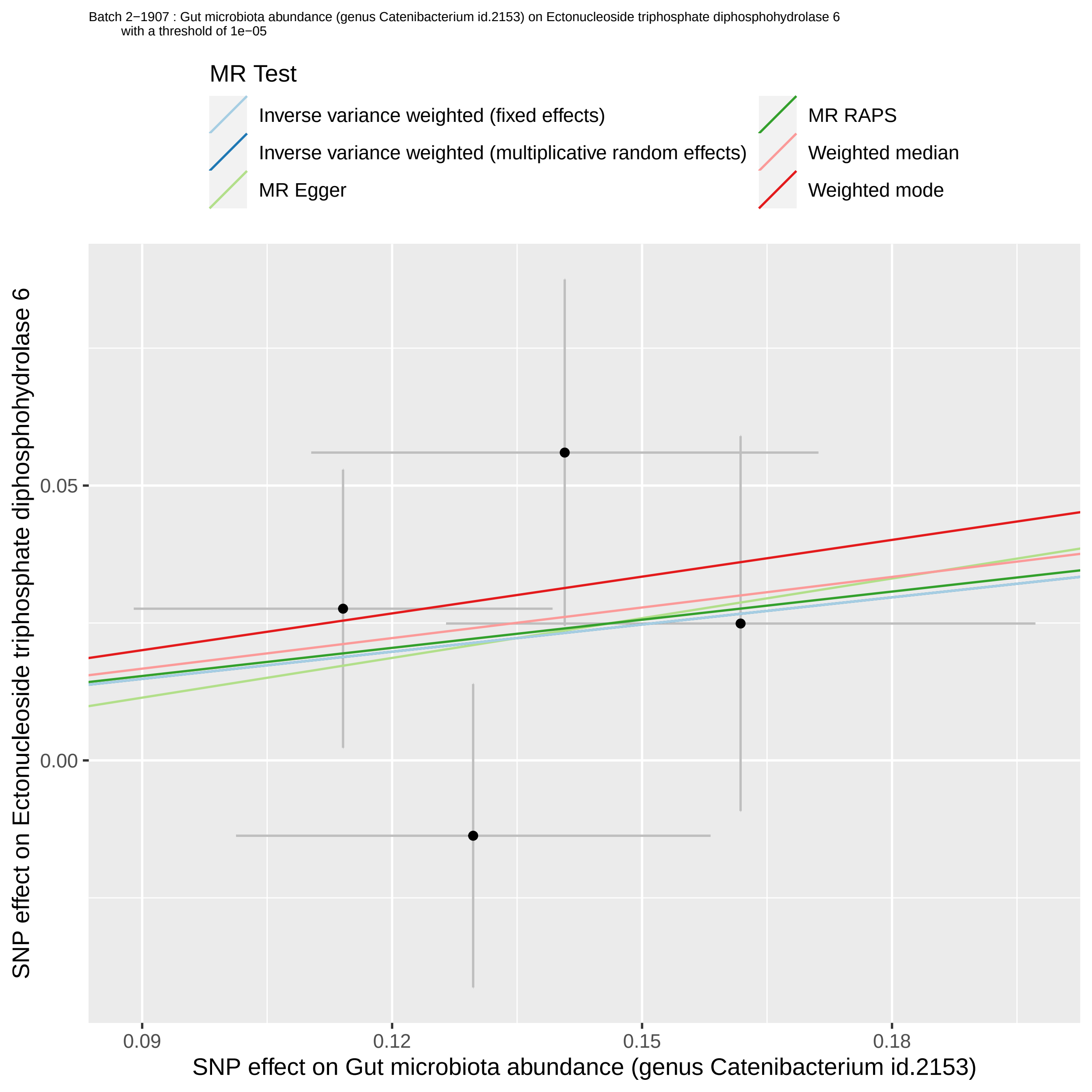

Batch 2−1907 : Gut microbiota abundance (genus Catenibacterium id.2153) on Ectonucleoside triphosphate diphosphohydrolase 6
with a threshold of 1e−05
MR Test
Inverse variance weighted (fixed effects)
Inverse variance weighted (multiplicative random effects)
MR Egger
MR RAPS
Weighted median
Weighted mode
SNP effect on Ectonucleoside triphosphate diphosphohydrolase 6
0.05
0.00
0.09
0.12
0.15
0.18
SNP effect on Gut microbiota abundance (genus Catenibacterium id.2153)

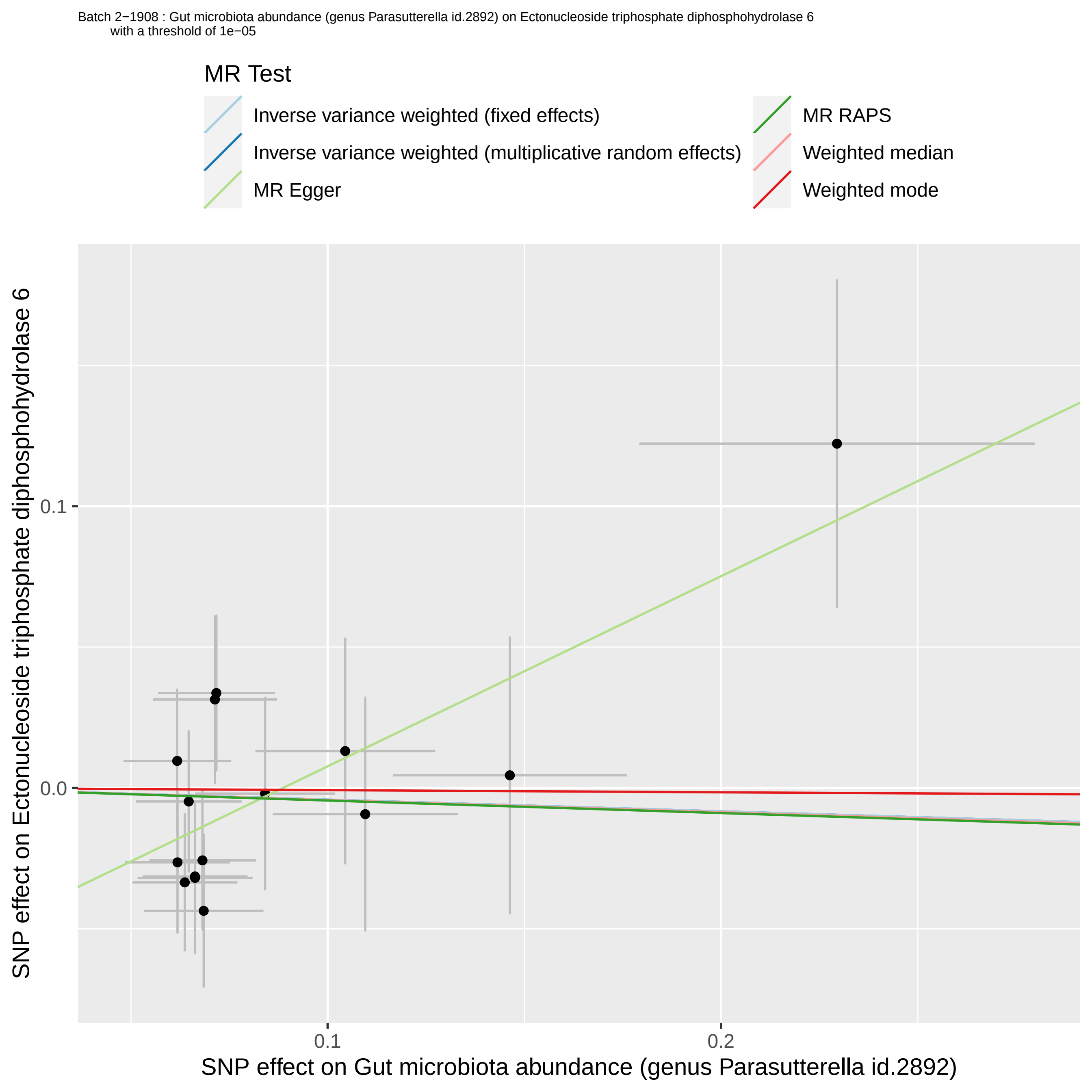
Batch 2−1908 : Gut microbiota abundance (genus Parasutterella id.2892) on Ectonucleoside triphosphate diphosphohydrolase 6
with a threshold of 1e−05
MR Test
Inverse variance weighted (fixed effects)
Inverse variance weighted (multiplicative random effects)
MR Egger
MR RAPS
Weighted median
Weighted mode
0.1
0.0
0.1
0.2
SNP effect on Ectonucleoside triphosphate diphosphohydrolase 6
SNP effect on Gut microbiota abundance (genus Parasutterella id.2892)

Batch 2−1909 : Gut microbiota abundance (genus Catenibacterium id.2153) on Ephrin type−A receptor 10
with a threshold of 1e−05

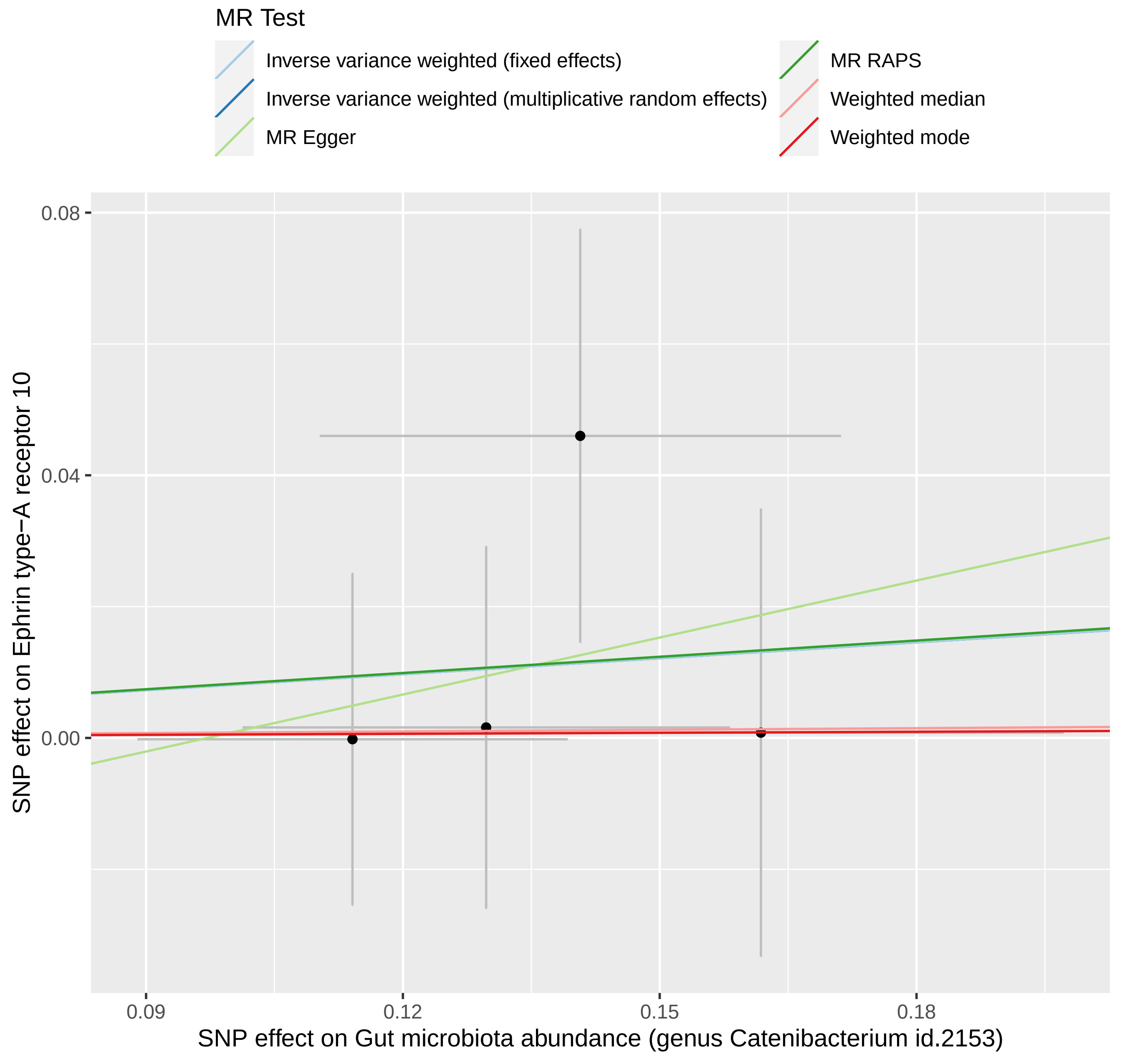

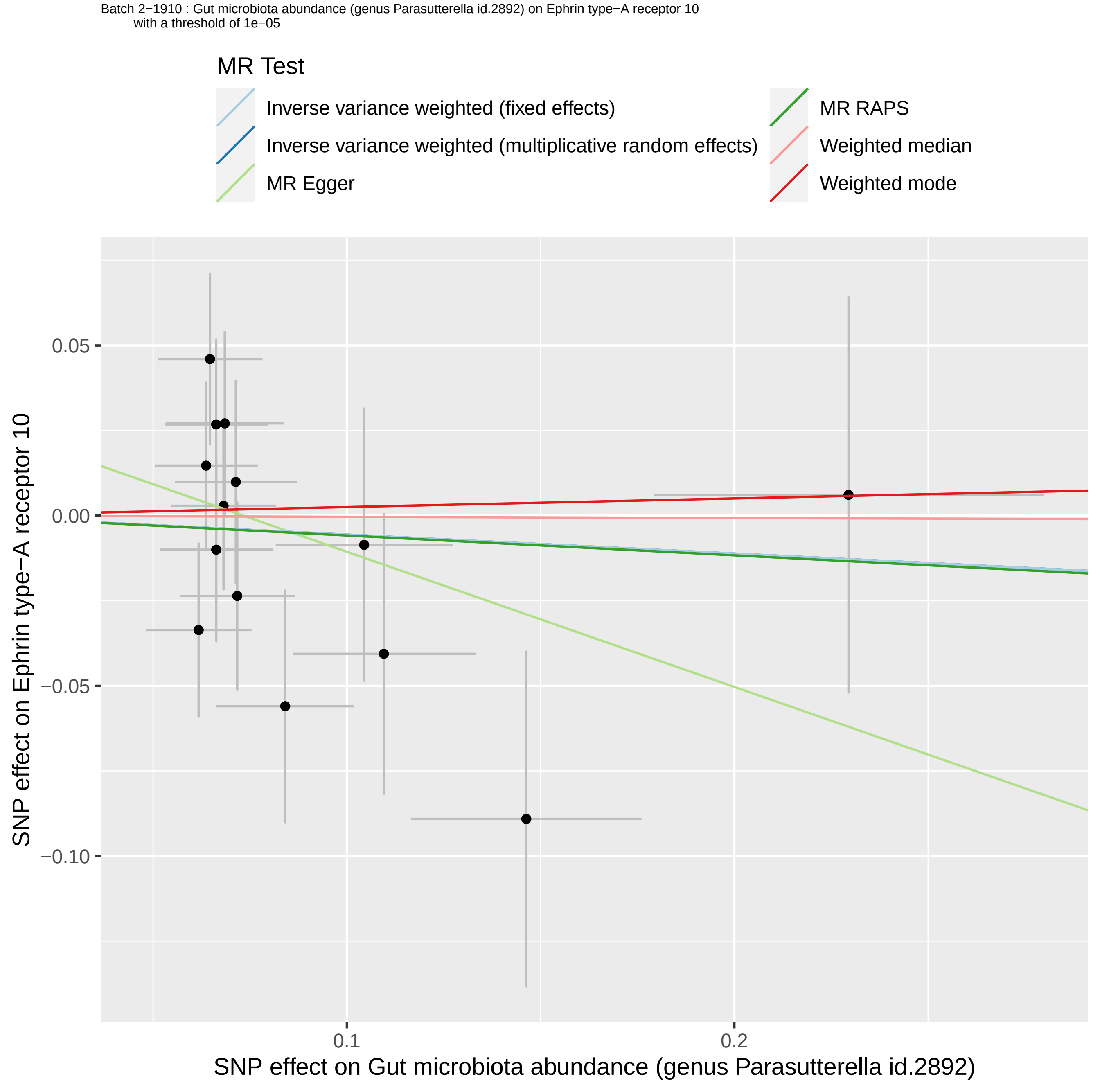
Batch 2−1910 : Gut microbiota abundance (genus Parasutterella id.2892) on Ephrin type−A receptor 10
with a threshold of 1e−05
MR Test
Inverse variance weighted (fixed effects)
Inverse variance weighted (multiplicative random effects)
MR Egger
MR RAPS
Weighted median
Weighted mode
0.05
0.00
−0.05
−0.10
0.1
0.2
SNP effect on Ephrin type−A receptor 10
SNP effect on Gut microbiota abundance (genus Parasutterella id.2892)

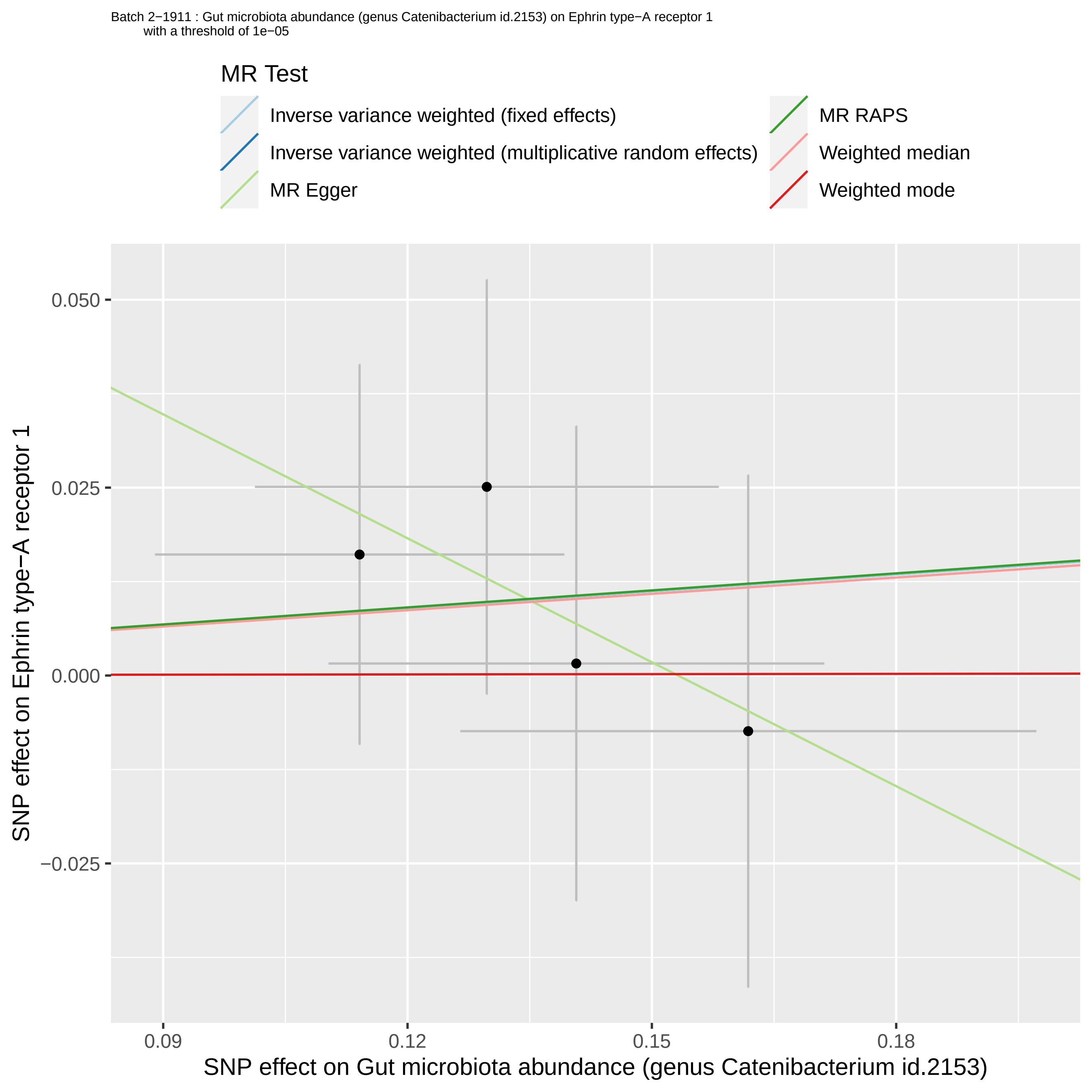

Batch 2−1911 : Gut microbiota abundance (genus Catenibacterium id.2153) on Ephrin type−A receptor 1
with a threshold of 1e−05
MR Test
Inverse variance weighted (fixed effects)
Inverse variance weighted (multiplicative random effects)
MR Egger
MR RAPS
Weighted median
Weighted mode
0.050
0.025
0.000
−0.025
0.09
0.12
0.15
0.18
SNP effect on Ephrin type−A receptor 1
SNP effect on Gut microbiota abundance (genus Catenibacterium id.2153)

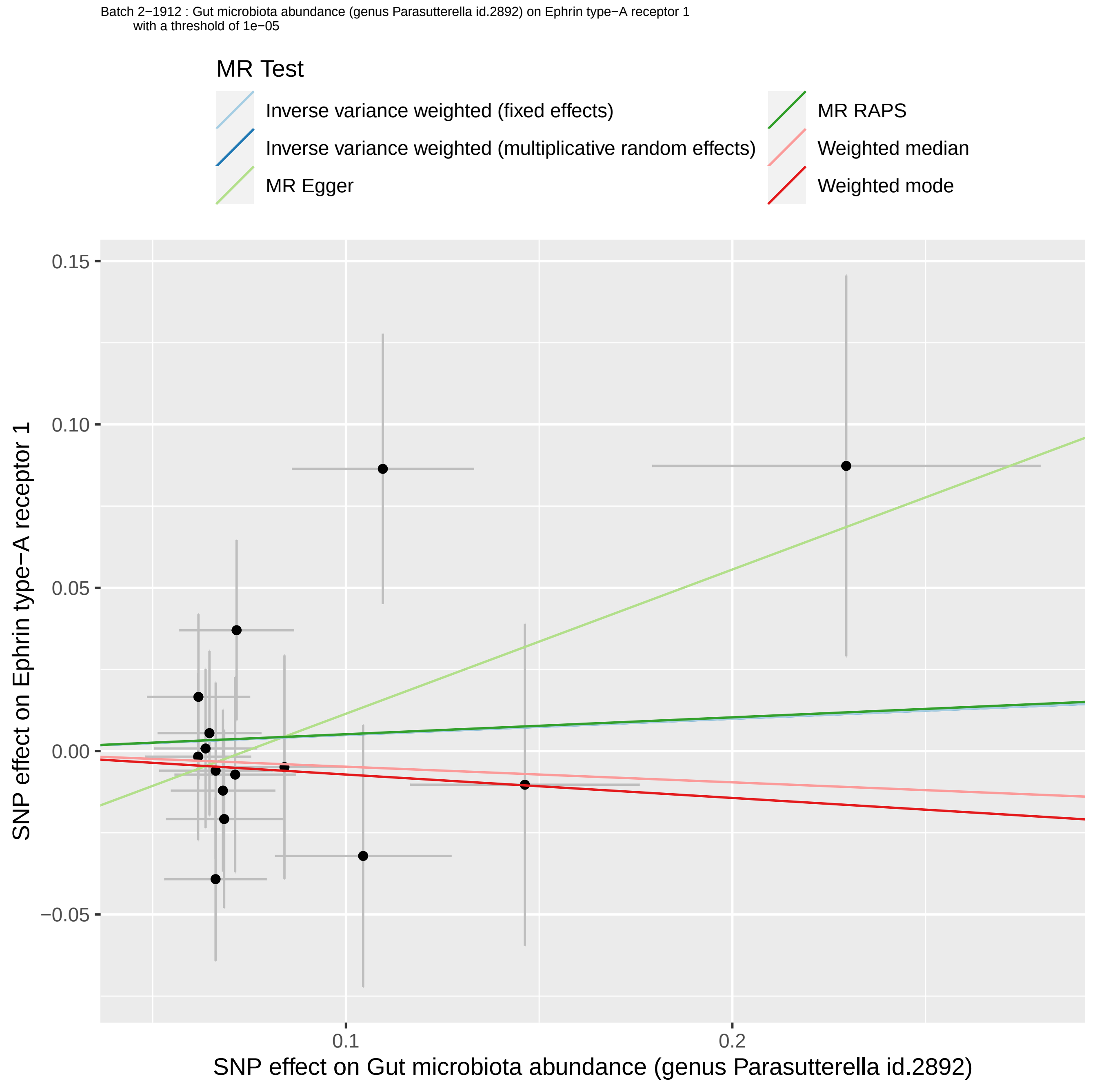
Batch 2−1912 : Gut microbiota abundance (genus Parasutterella id.2892) on Ephrin type−A receptor 1
with a threshold of 1e−05
MR Test
Inverse variance weighted (fixed effects)
Inverse variance weighted (multiplicative random effects)
MR Egger
MR RAPS
Weighted median
Weighted mode
0.15
0.10
0.05
0.00
−0.05
0.1
0.2
SNP effect on Ephrin type−A receptor 1
SNP effect on Gut microbiota abundance (genus Parasutterella id.2892)

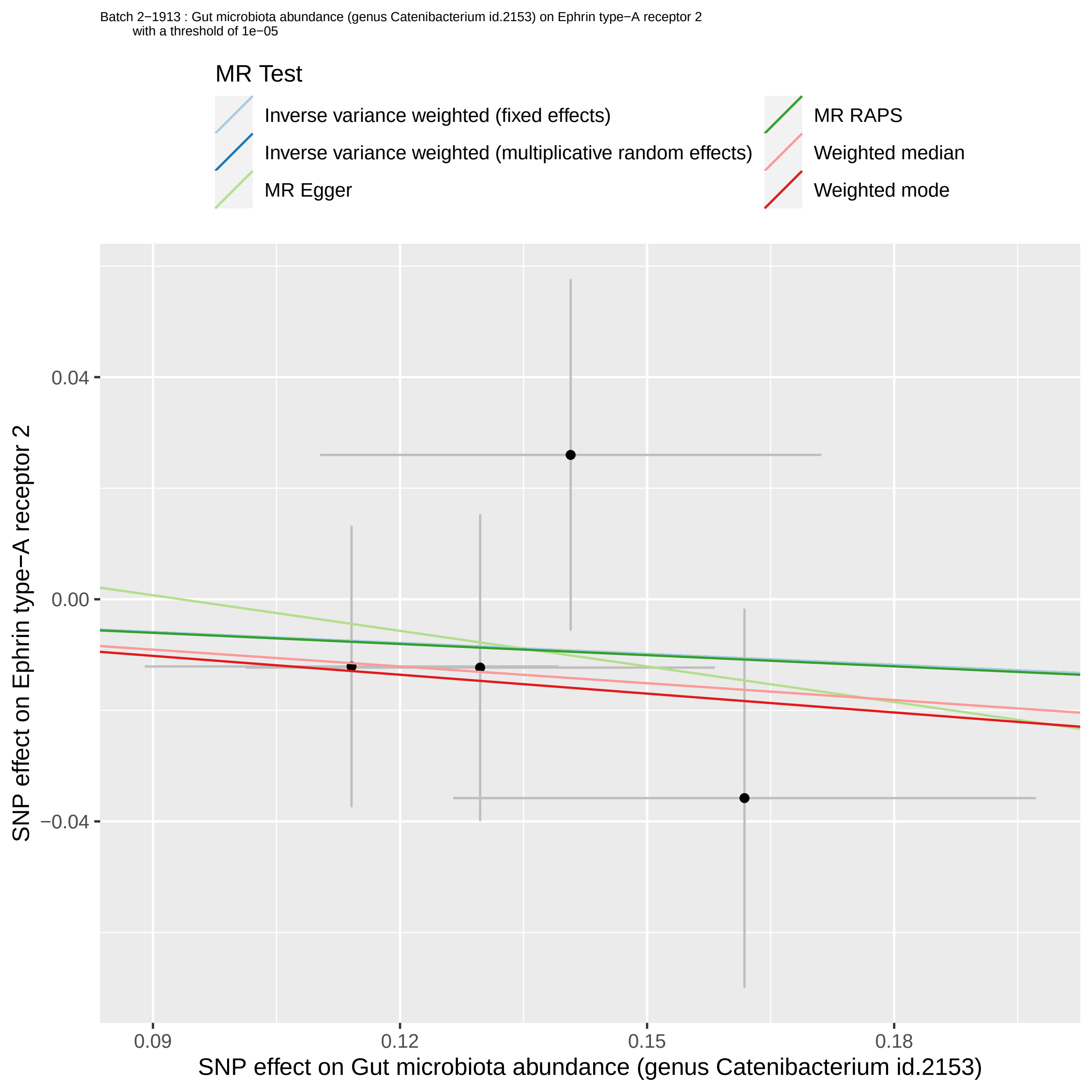

Batch 2−1913 : Gut microbiota abundance (genus Catenibacterium id.2153) on Ephrin type−A receptor 2
with a threshold of 1e−05
MR Test
Inverse variance weighted (fixed effects)
Inverse variance weighted (multiplicative random effects)
MR Egger
MR RAPS
Weighted median
Weighted mode
0.04
0.00
−0.04
0.09
0.12
0.15
0.18
SNP effect on Ephrin type−A receptor 2
SNP effect on Gut microbiota abundance (genus Catenibacterium id.2153)

Batch 2−1914 : Gut microbiota abundance (genus Parasutterella id.2892) on Ephrin type−A receptor 2
with a threshold of 1e−05

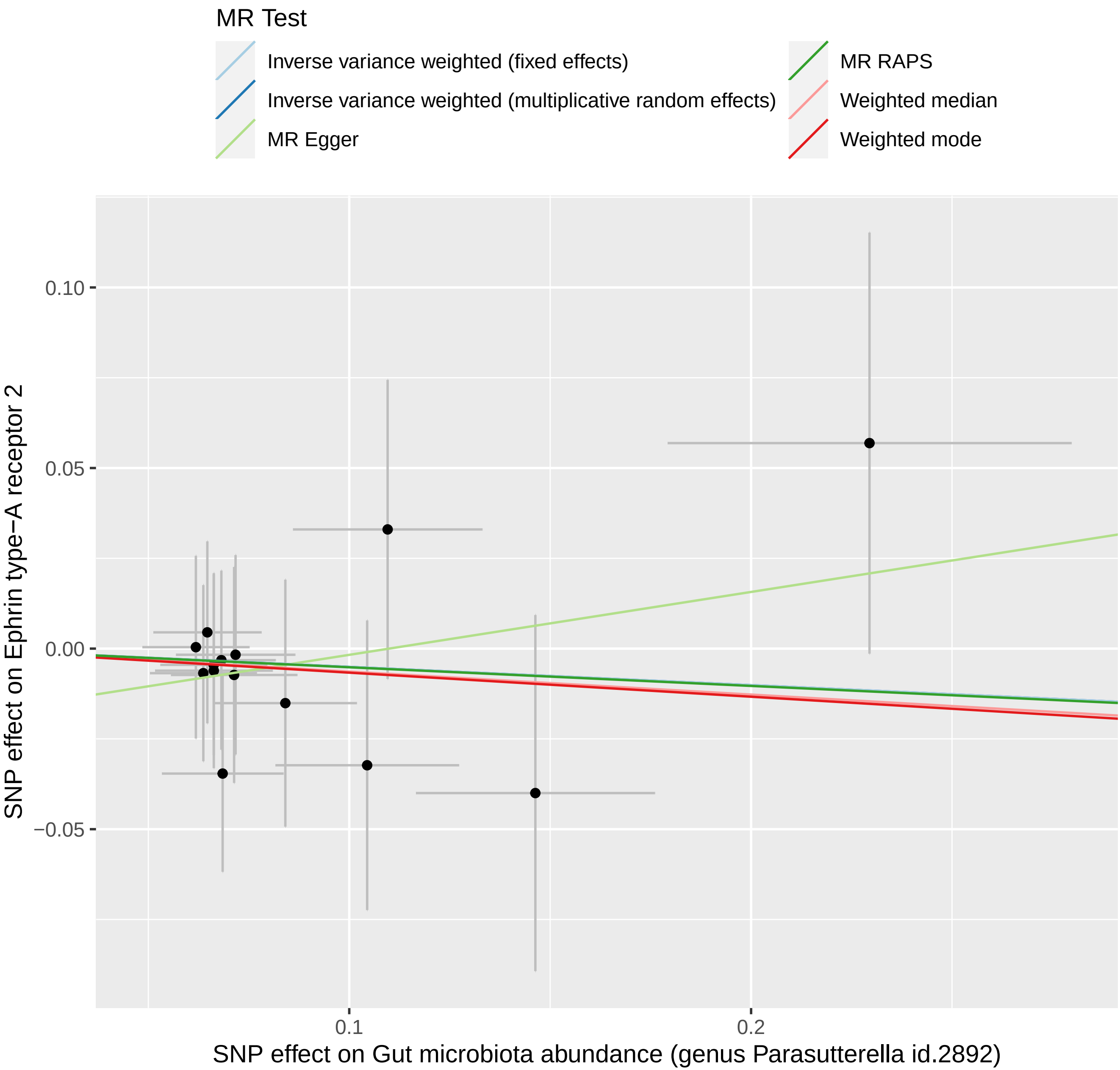

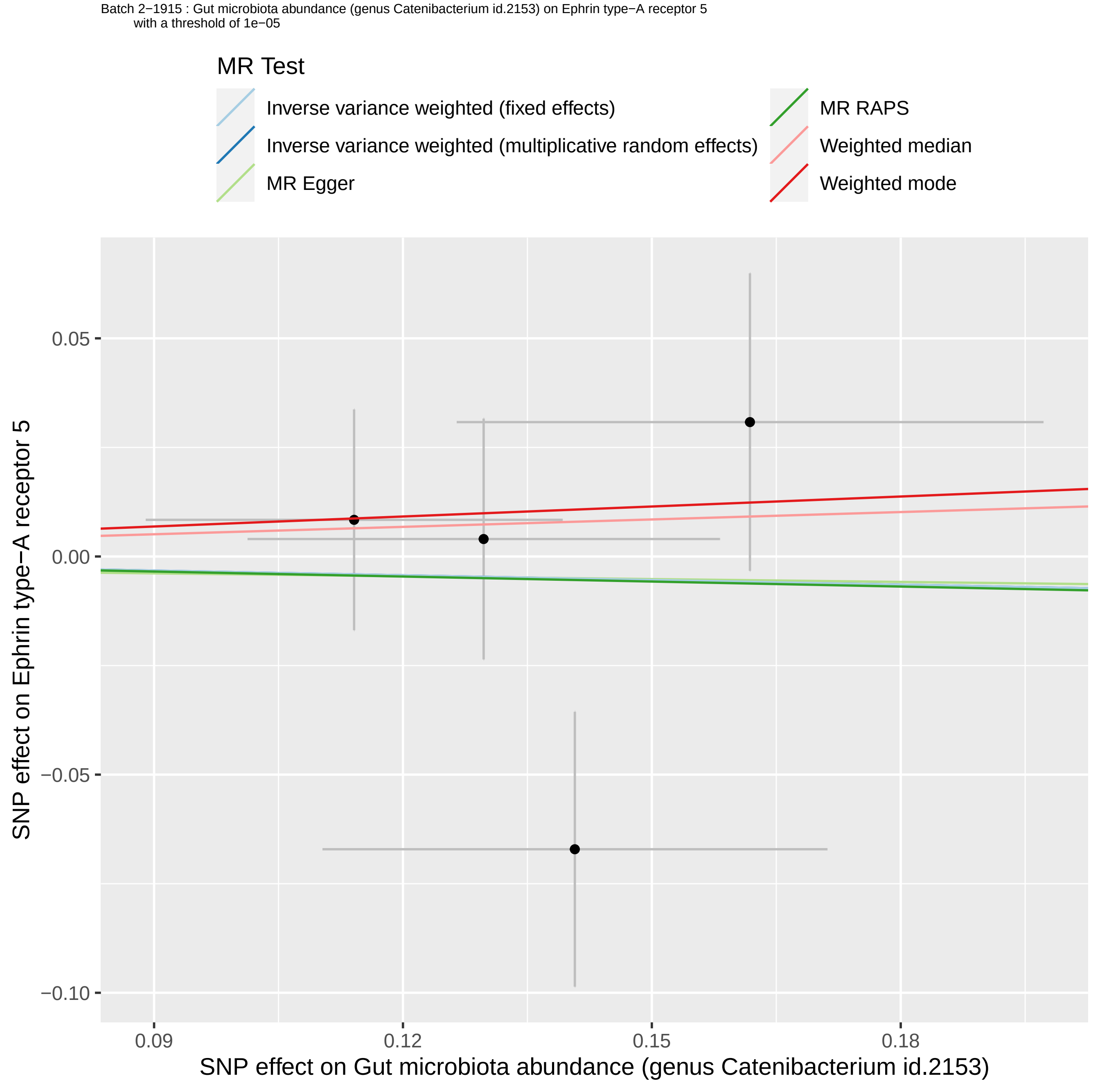

Batch 2−1915 : Gut microbiota abundance (genus Catenibacterium id.2153) on Ephrin type−A receptor 5
with a threshold of 1e−05
MR Test
Inverse variance weighted (fixed effects)
Inverse variance weighted (multiplicative random effects)
MR Egger
MR RAPS
Weighted median
Weighted mode
0.05
0.00
−0.05
−0.10
0.09
0.12
0.15
0.18
SNP effect on Ephrin type−A receptor 5
SNP effect on Gut microbiota abundance (genus Catenibacterium id.2153)

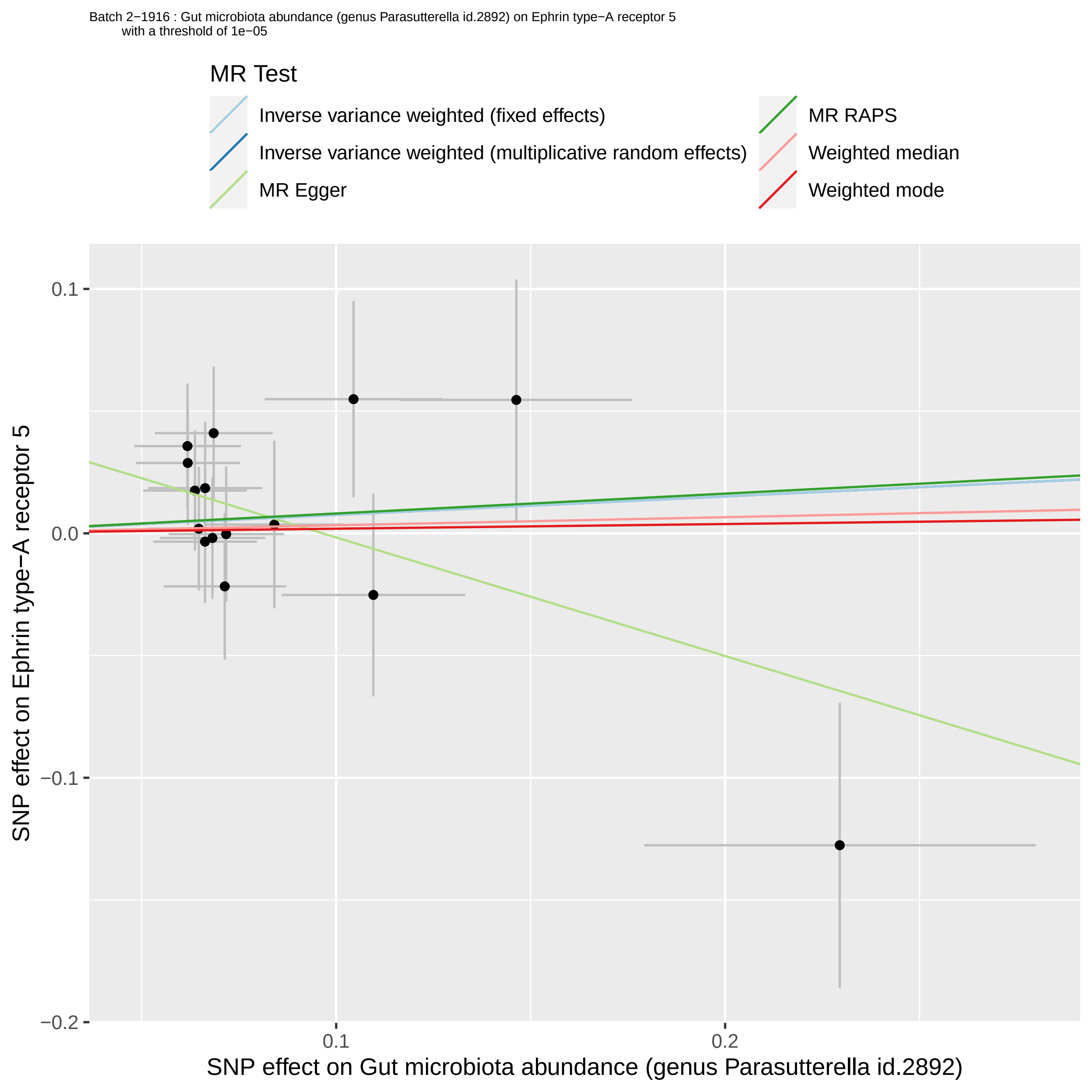

Batch 2−1916 : Gut microbiota abundance (genus Parasutterella id.2892) on Ephrin type−A receptor 5
with a threshold of 1e−05
MR Test
Inverse variance weighted (fixed effects)
Inverse variance weighted (multiplicative random effects)
MR Egger
MR RAPS
Weighted median
Weighted mode
0.1
0.0
−0.1
−0.2
0.1
0.2
SNP effect on Ephrin type−A receptor 5
SNP effect on Gut microbiota abundance (genus Parasutterella id.2892)

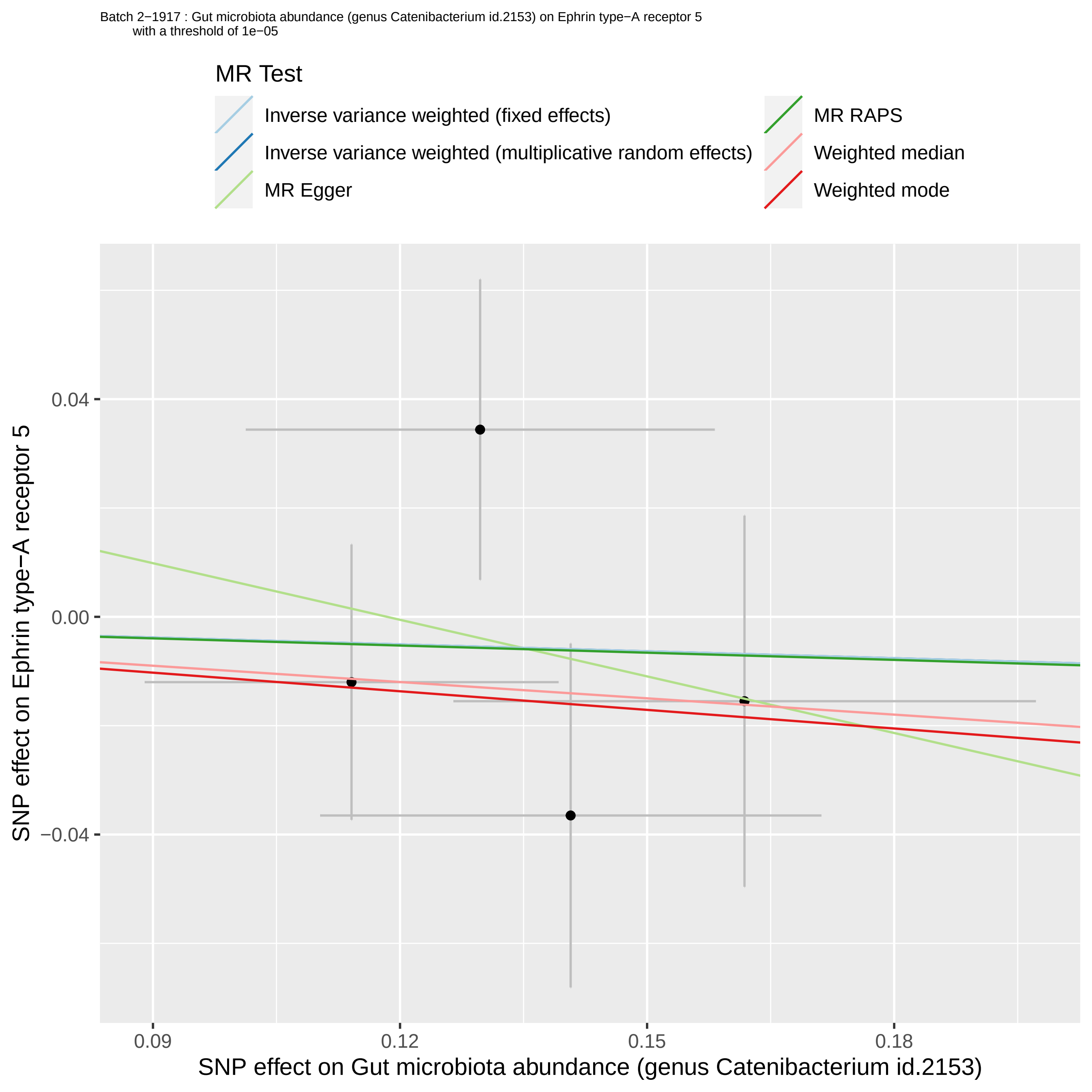
Batch 2−1917 : Gut microbiota abundance (genus Catenibacterium id.2153) on Ephrin type−A receptor 5
with a threshold of 1e−05
MR Test
Inverse variance weighted (fixed effects)
Inverse variance weighted (multiplicative random effects)
MR Egger
MR RAPS
Weighted median
Weighted mode
0.04
0.00
−0.04
0.09
0.12
0.15
0.18
SNP effect on Ephrin type−A receptor 5
SNP effect on Gut microbiota abundance (genus Catenibacterium id.2153)

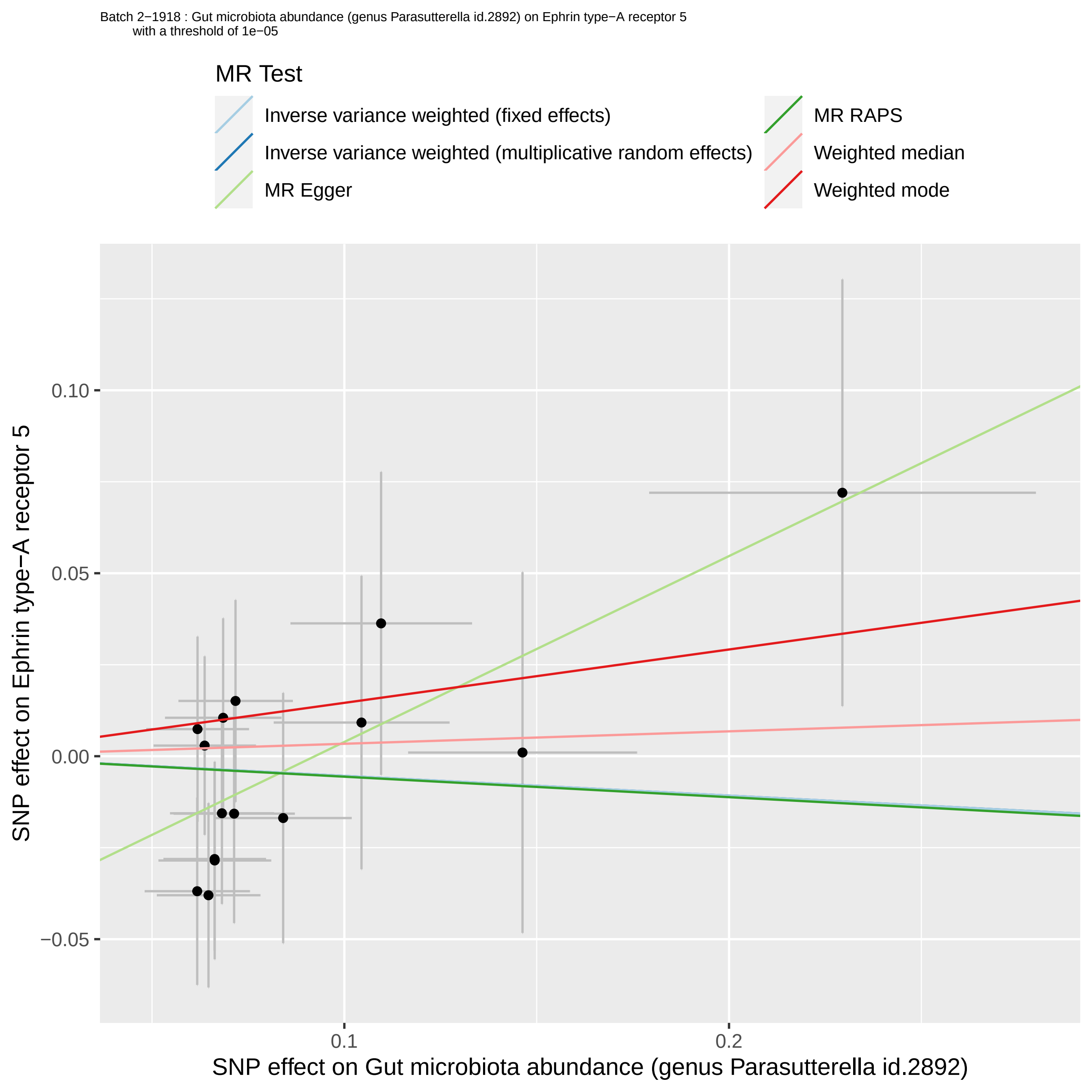
Batch 2−1918 : Gut microbiota abundance (genus Parasutterella id.2892) on Ephrin type−A receptor 5
with a threshold of 1e−05
MR Test
Inverse variance weighted (fixed effects)
Inverse variance weighted (multiplicative random effects)
MR Egger
MR RAPS
Weighted median
Weighted mode
0.10
0.05
0.00
−0.05
0.1
0.2
SNP effect on Ephrin type−A receptor 5
SNP effect on Gut microbiota abundance (genus Parasutterella id.2892)

Batch 2−1919 : Gut microbiota abundance (genus Catenibacterium id.2153) on Ephrin type−B receptor 2
with a threshold of 1e−05

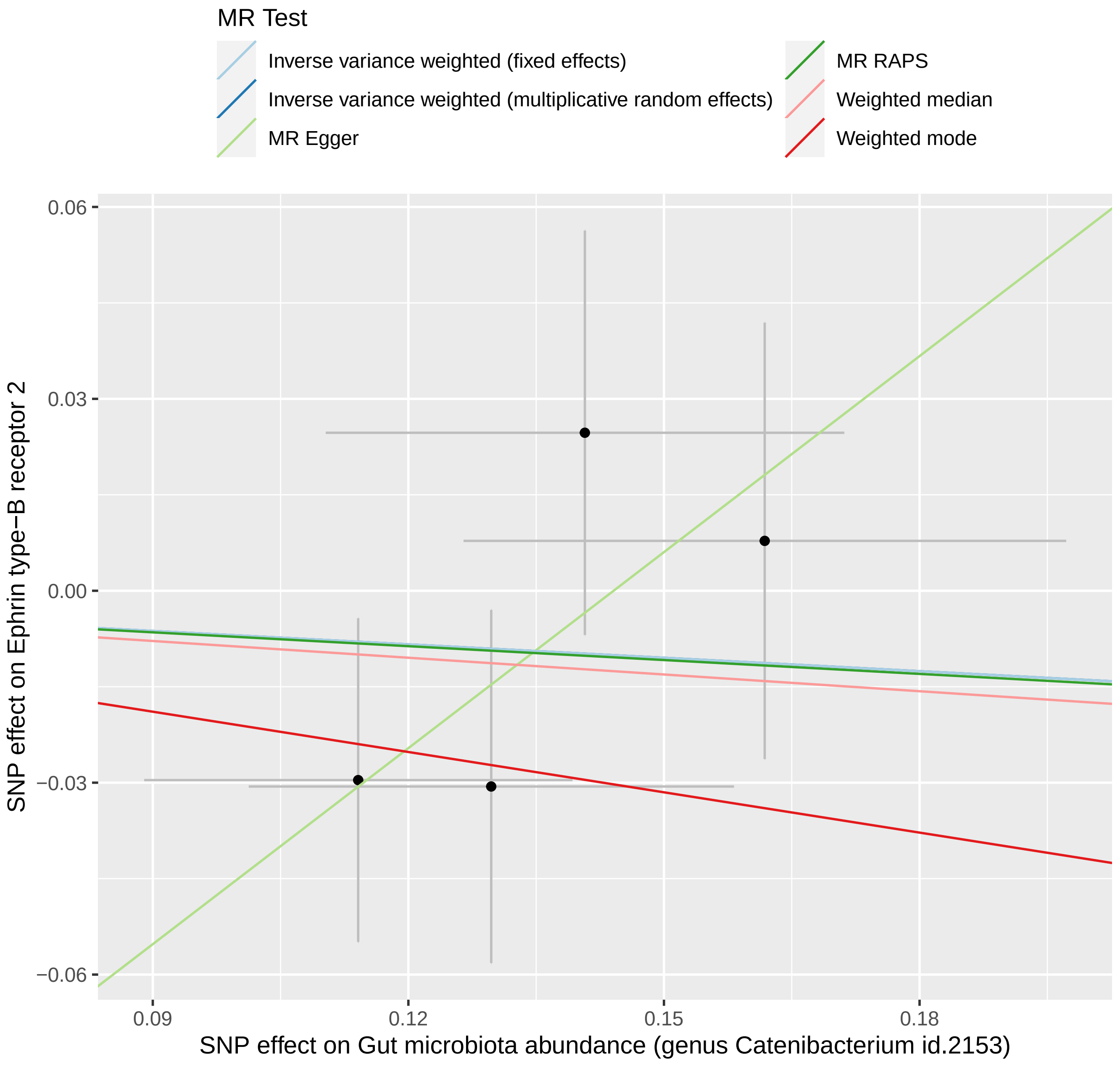

Batch 2−1920 : Gut microbiota abundance (genus Parasutterella id.2892) on Ephrin type−B receptor 2
with a threshold of 1e−05

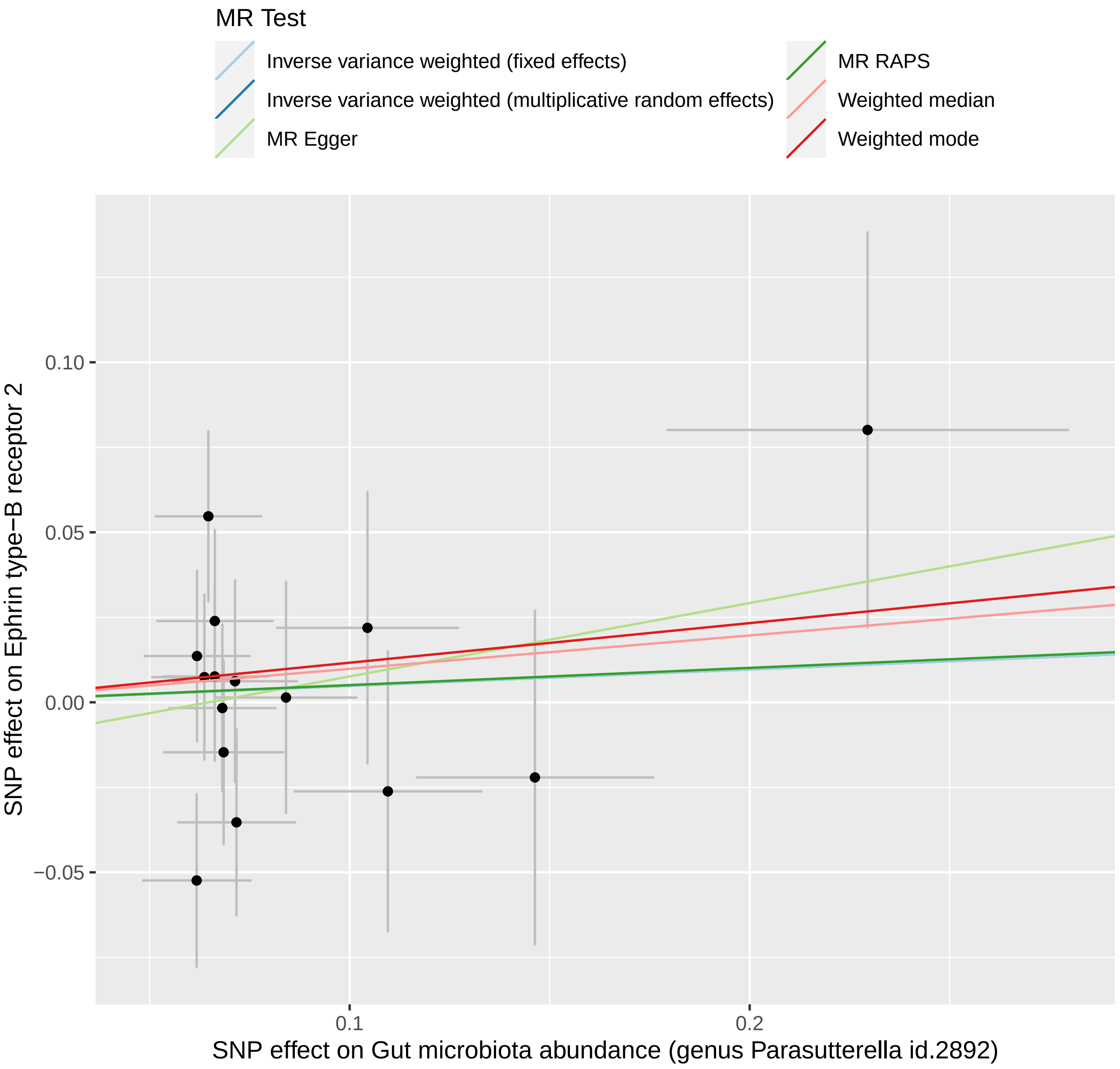

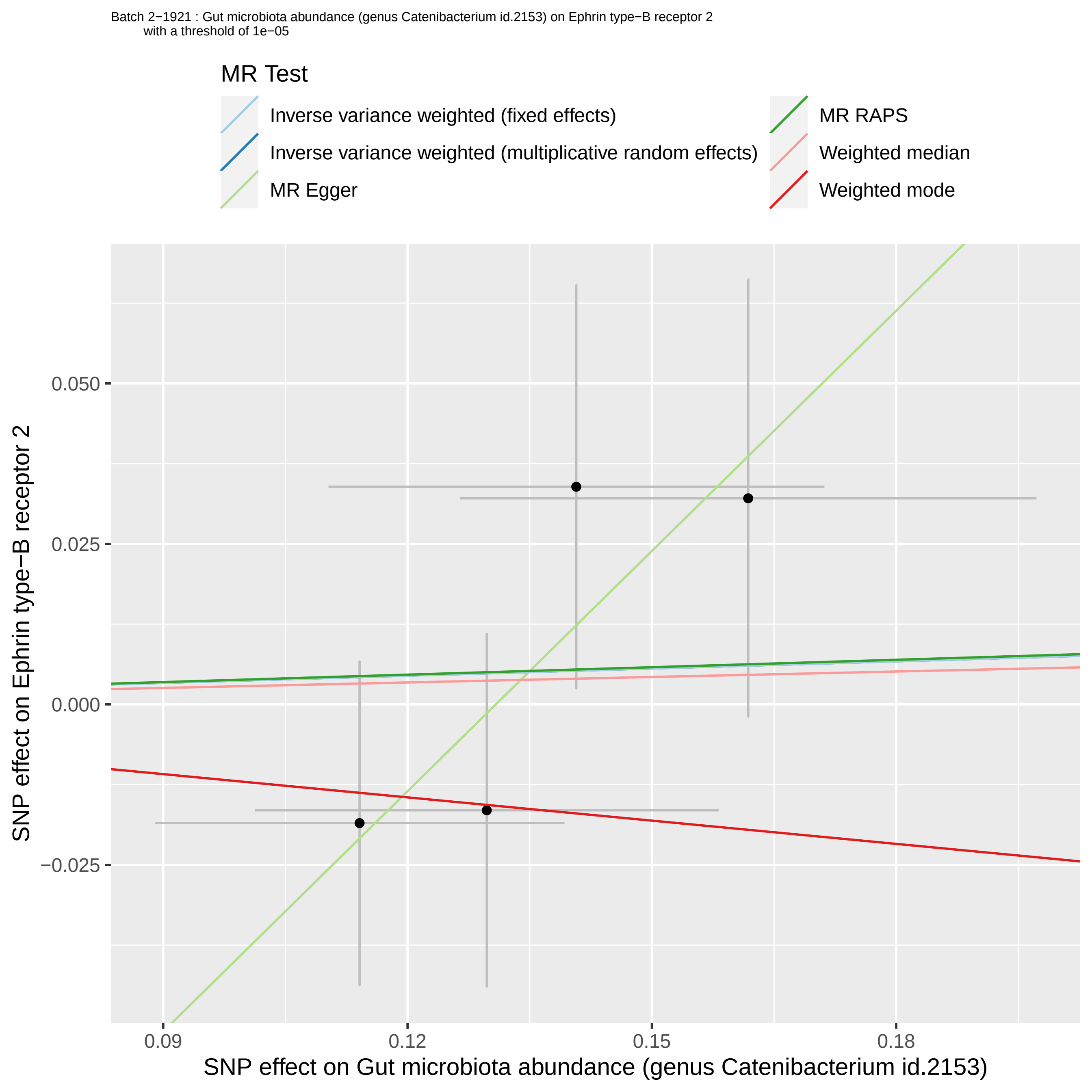
Batch 2−1921 : Gut microbiota abundance (genus Catenibacterium id.2153) on Ephrin type−B receptor 2
with a threshold of 1e−05
MR Test
Inverse variance weighted (fixed effects)
Inverse variance weighted (multiplicative random effects)
MR Egger
MR RAPS
Weighted median
Weighted mode
0.050
0.025
0.000
−0.025
0.09
0.12
0.15
0.18
SNP effect on Ephrin type−B receptor 2
SNP effect on Gut microbiota abundance (genus Catenibacterium id.2153)

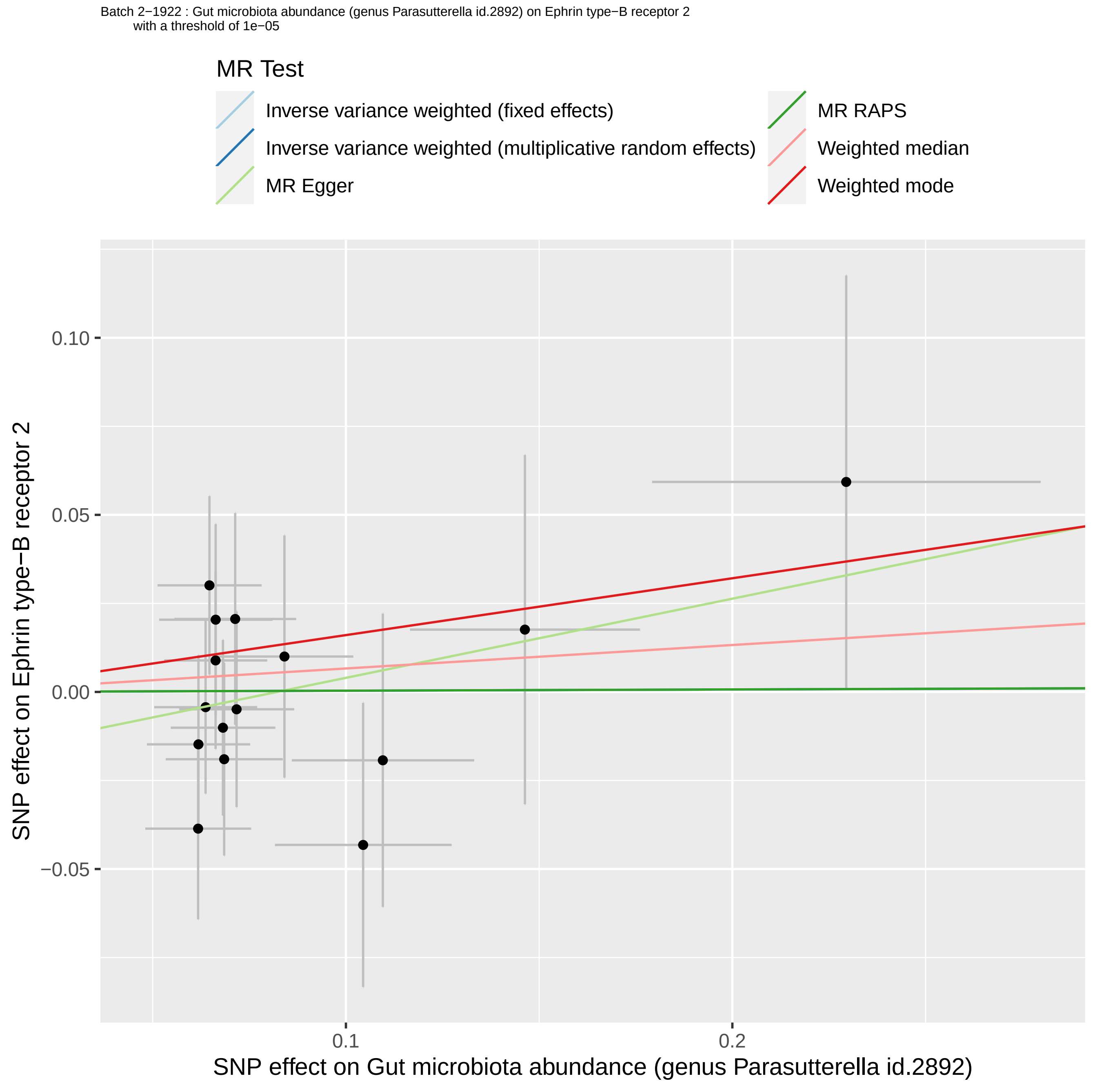

Batch 2−1922 : Gut microbiota abundance (genus Parasutterella id.2892) on Ephrin type−B receptor 2
with a threshold of 1e−05
MR Test
Inverse variance weighted (fixed effects)
Inverse variance weighted (multiplicative random effects)
MR Egger
MR RAPS
Weighted median
Weighted mode
0.10
0.05
0.00
−0.05
0.1
0.2
SNP effect on Ephrin type−B receptor 2
SNP effect on Gut microbiota abundance (genus Parasutterella id.2892)

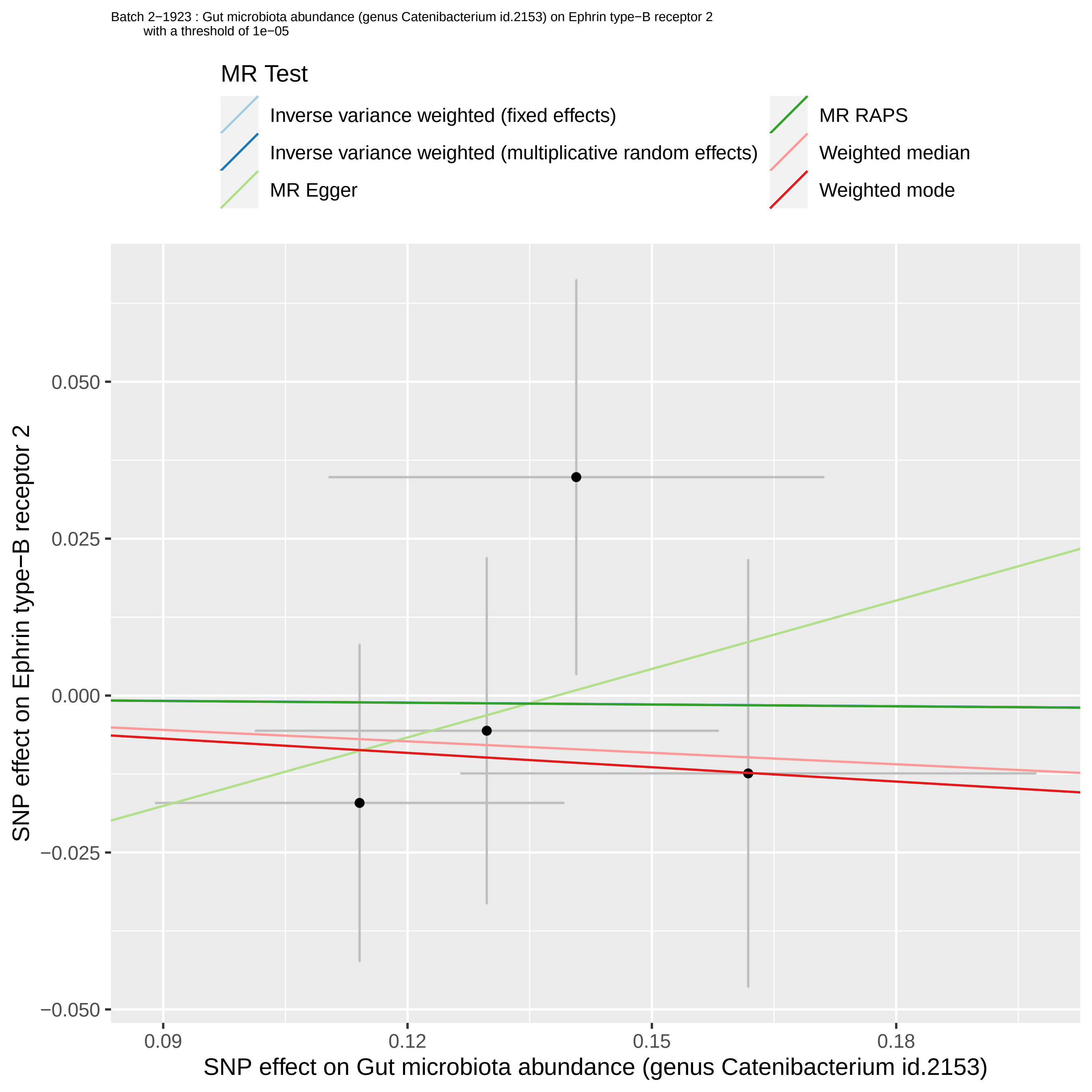
Batch 2−1923 : Gut microbiota abundance (genus Catenibacterium id.2153) on Ephrin type−B receptor 2
with a threshold of 1e−05
MR Test
Inverse variance weighted (fixed effects)
Inverse variance weighted (multiplicative random effects)
MR Egger
MR RAPS
Weighted median
Weighted mode
0.050
0.025
0.000
−0.025
−0.050
0.09
0.12
0.15
0.18
SNP effect on Ephrin type−B receptor 2
SNP effect on Gut microbiota abundance (genus Catenibacterium id.2153)

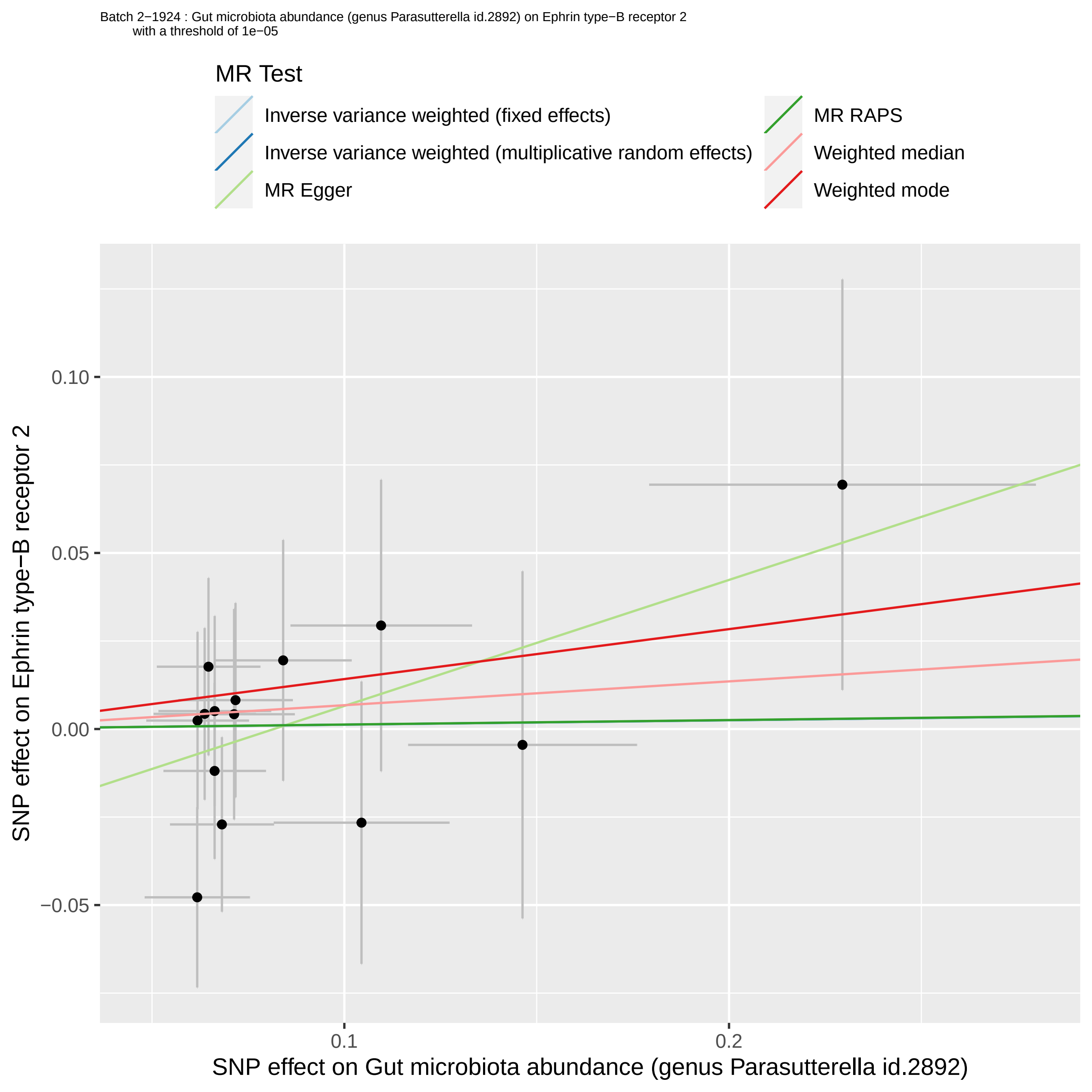

Batch 2−1924 : Gut microbiota abundance (genus Parasutterella id.2892) on Ephrin type−B receptor 2
with a threshold of 1e−05
MR Test
Inverse variance weighted (fixed effects)
Inverse variance weighted (multiplicative random effects)
MR Egger
MR RAPS
Weighted median
Weighted mode
0.10
0.05
0.00
−0.05
0.1
0.2
SNP effect on Ephrin type−B receptor 2
SNP effect on Gut microbiota abundance (genus Parasutterella id.2892)

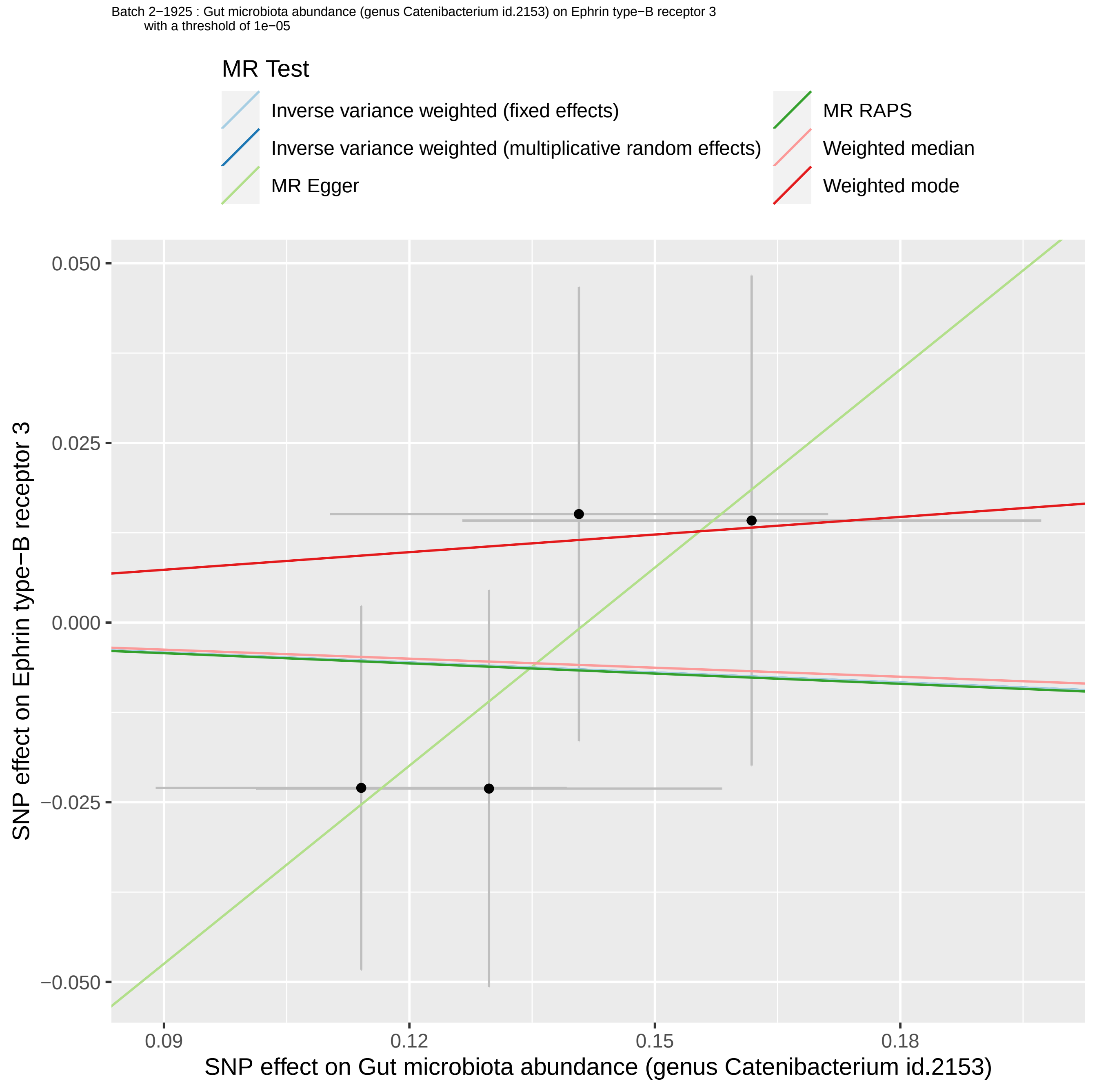
Batch 2−1925 : Gut microbiota abundance (genus Catenibacterium id.2153) on Ephrin type−B receptor 3
with a threshold of 1e−05
MR Test
Inverse variance weighted (fixed effects)
Inverse variance weighted (multiplicative random effects)
MR Egger
MR RAPS
Weighted median
Weighted mode
0.050
0.025
0.000
−0.025
−0.050
0.09
0.12
0.15
0.18
SNP effect on Ephrin type−B receptor 3
SNP effect on Gut microbiota abundance (genus Catenibacterium id.2153)

Batch 2−1926 : Gut microbiota abundance (genus Parasutterella id.2892) on Ephrin type−B receptor 3
with a threshold of 1e−05

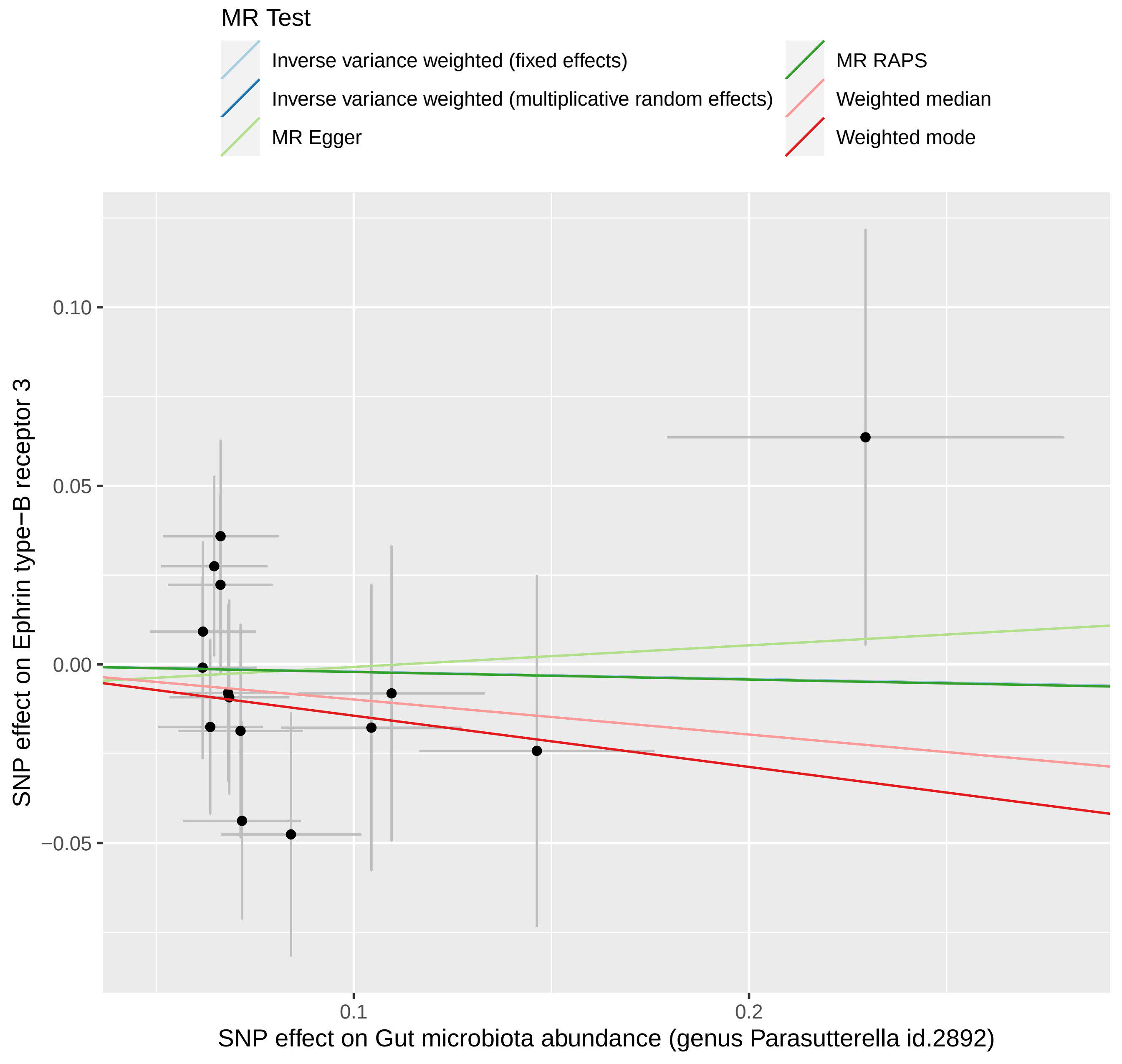

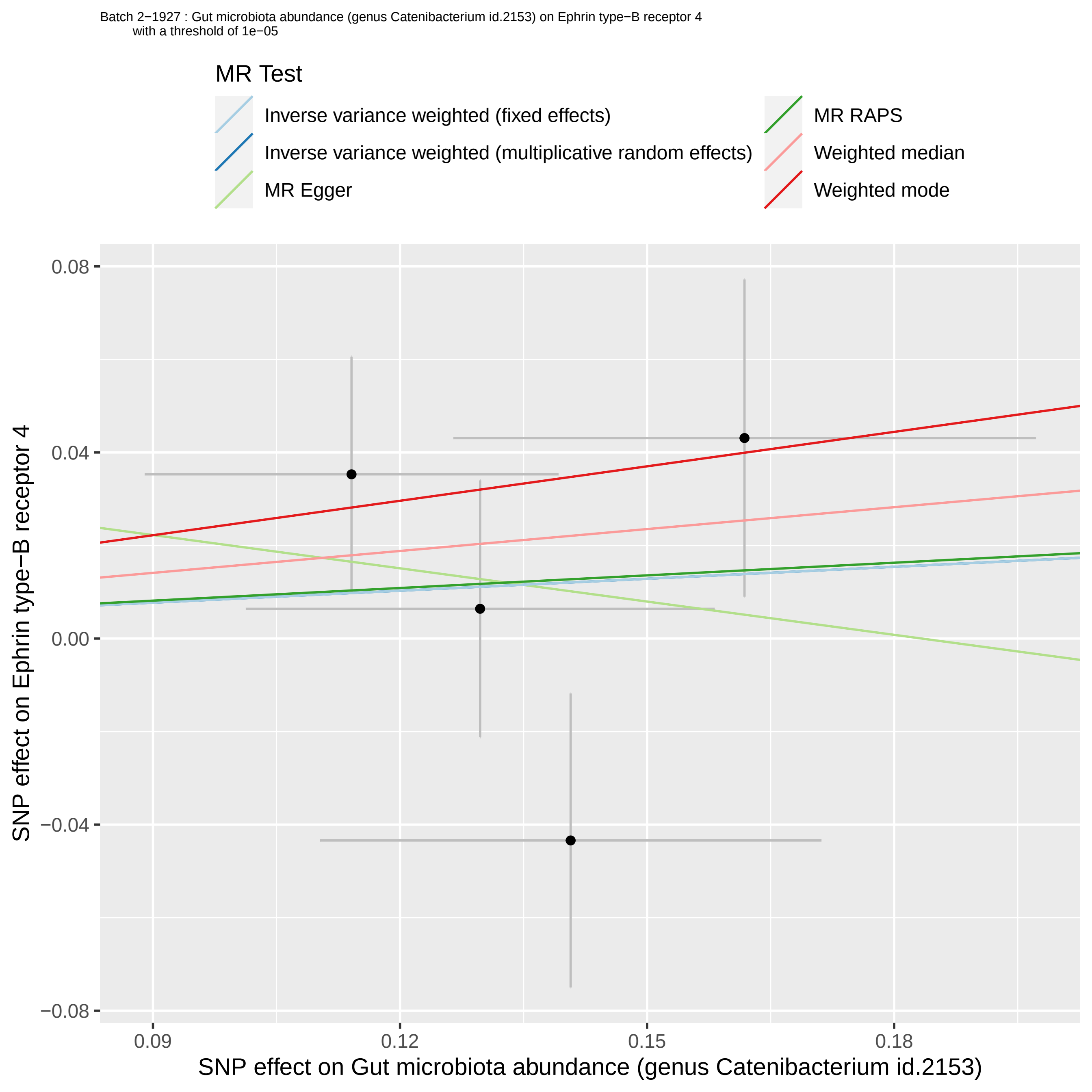
Batch 2−1927 : Gut microbiota abundance (genus Catenibacterium id.2153) on Ephrin type−B receptor 4
with a threshold of 1e−05
MR Test
Inverse variance weighted (fixed effects)
Inverse variance weighted (multiplicative random effects)
MR Egger
MR RAPS
Weighted median
Weighted mode
0.08
0.04
0.00
−0.04
−0.08
0.09
0.12
0.15
0.18
SNP effect on Ephrin type−B receptor 4
SNP effect on Gut microbiota abundance (genus Catenibacterium id.2153)

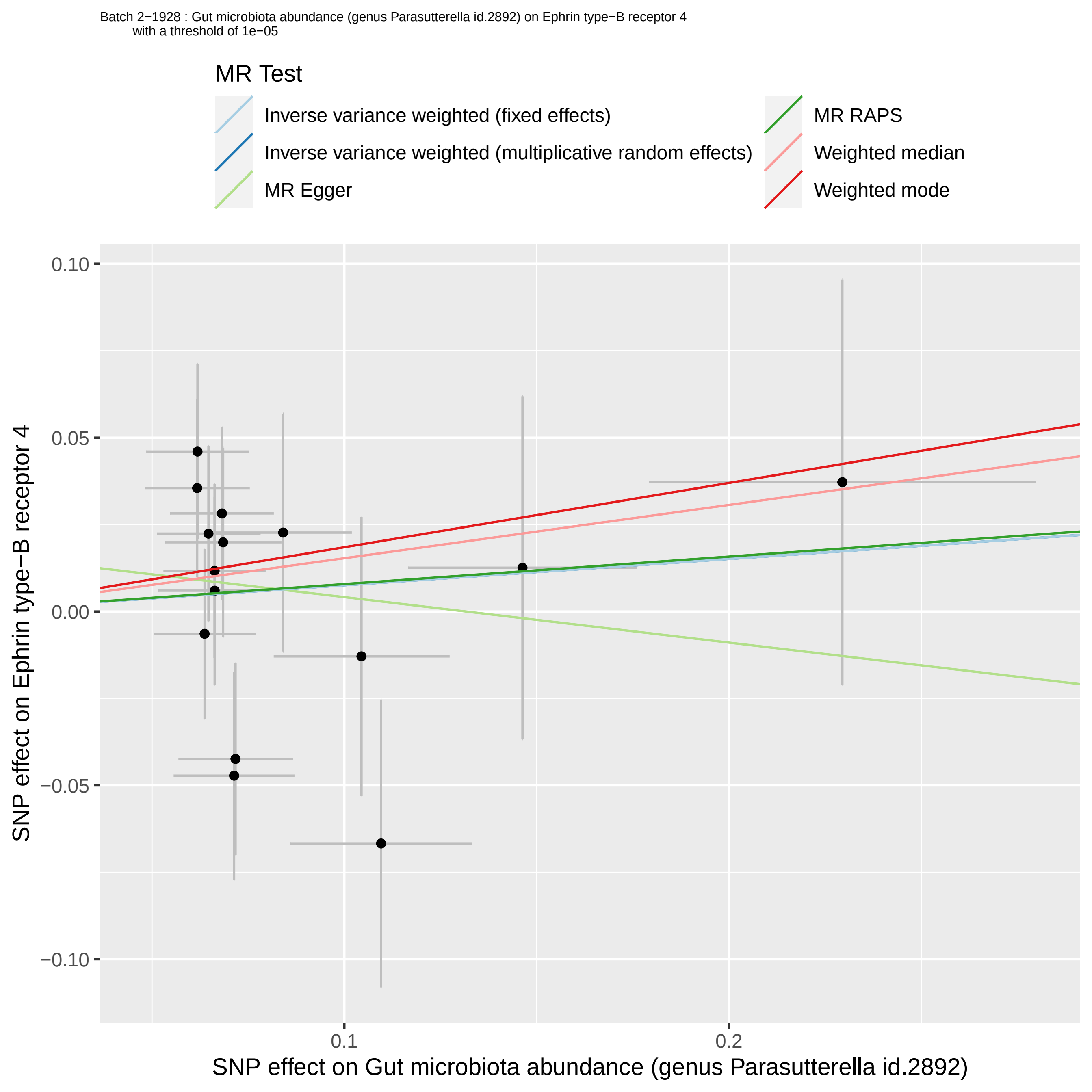
Batch 2−1928 : Gut microbiota abundance (genus Parasutterella id.2892) on Ephrin type−B receptor 4
with a threshold of 1e−05
MR Test
Inverse variance weighted (fixed effects)
Inverse variance weighted (multiplicative random effects)
MR Egger
MR RAPS
Weighted median
Weighted mode
0.10
0.05
0.00
−0.05
−0.10
0.1
0.2
SNP effect on Ephrin type−B receptor 4
SNP effect on Gut microbiota abundance (genus Parasutterella id.2892)

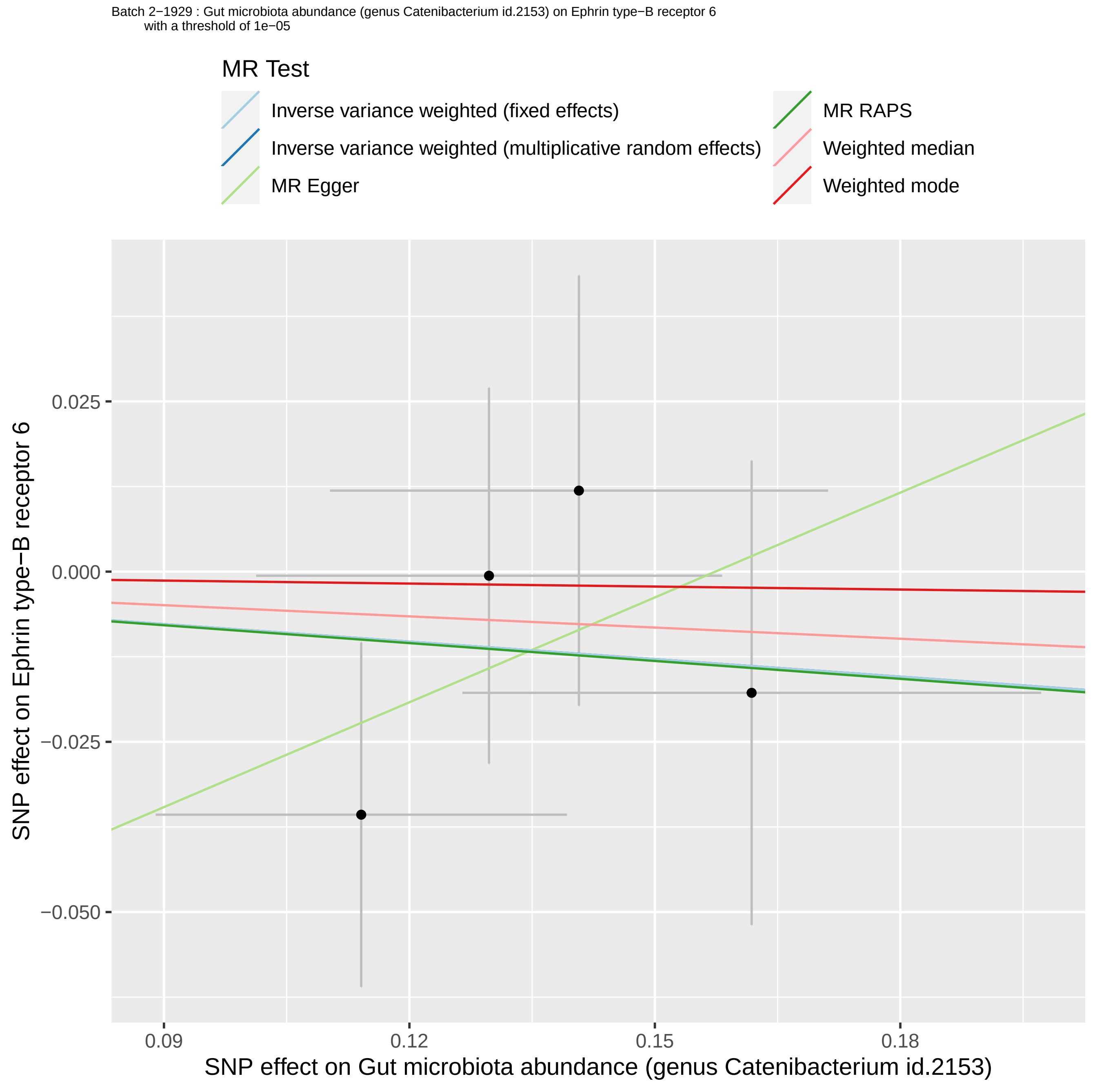
Batch 2−1929 : Gut microbiota abundance (genus Catenibacterium id.2153) on Ephrin type−B receptor 6
with a threshold of 1e−05
MR Test
Inverse variance weighted (fixed effects)
Inverse variance weighted (multiplicative random effects)
MR Egger
MR RAPS
Weighted median
Weighted mode
0.025
0.000
−0.025
−0.050
0.09
0.12
0.15
0.18
SNP effect on Ephrin type−B receptor 6
SNP effect on Gut microbiota abundance (genus Catenibacterium id.2153)

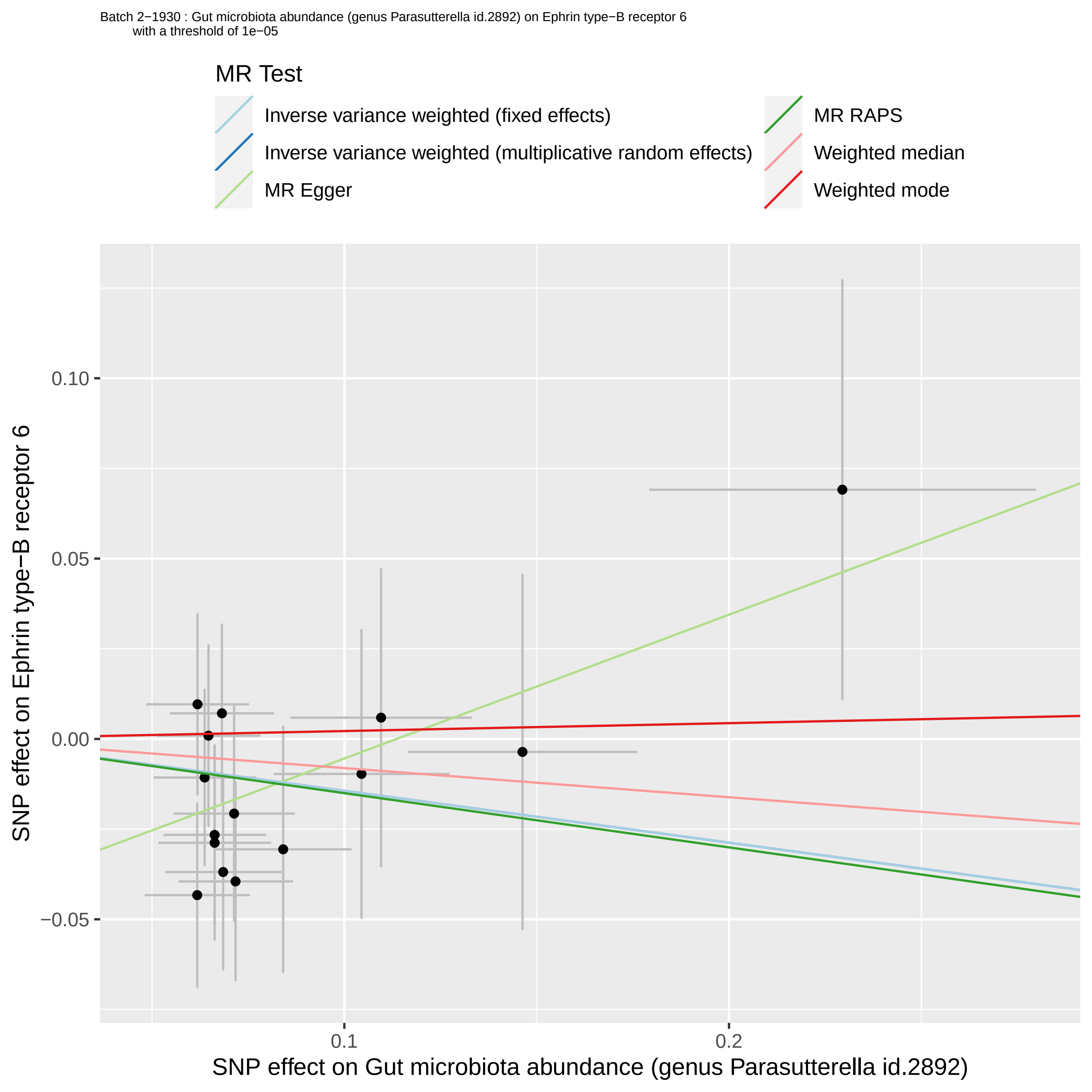
Batch 2−1930 : Gut microbiota abundance (genus Parasutterella id.2892) on Ephrin type−B receptor 6
with a threshold of 1e−05
MR Test
Inverse variance weighted (fixed effects)
Inverse variance weighted (multiplicative random effects)
MR Egger
MR RAPS
Weighted median
Weighted mode
0.10
0.05
0.00
−0.05
0.1
0.2
SNP effect on Ephrin type−B receptor 6
SNP effect on Gut microbiota abundance (genus Parasutterella id.2892)

Batch 2−1931 : Gut microbiota abundance (genus Catenibacterium id.2153) on Ephrin type−B receptor 6
with a threshold of 1e−05

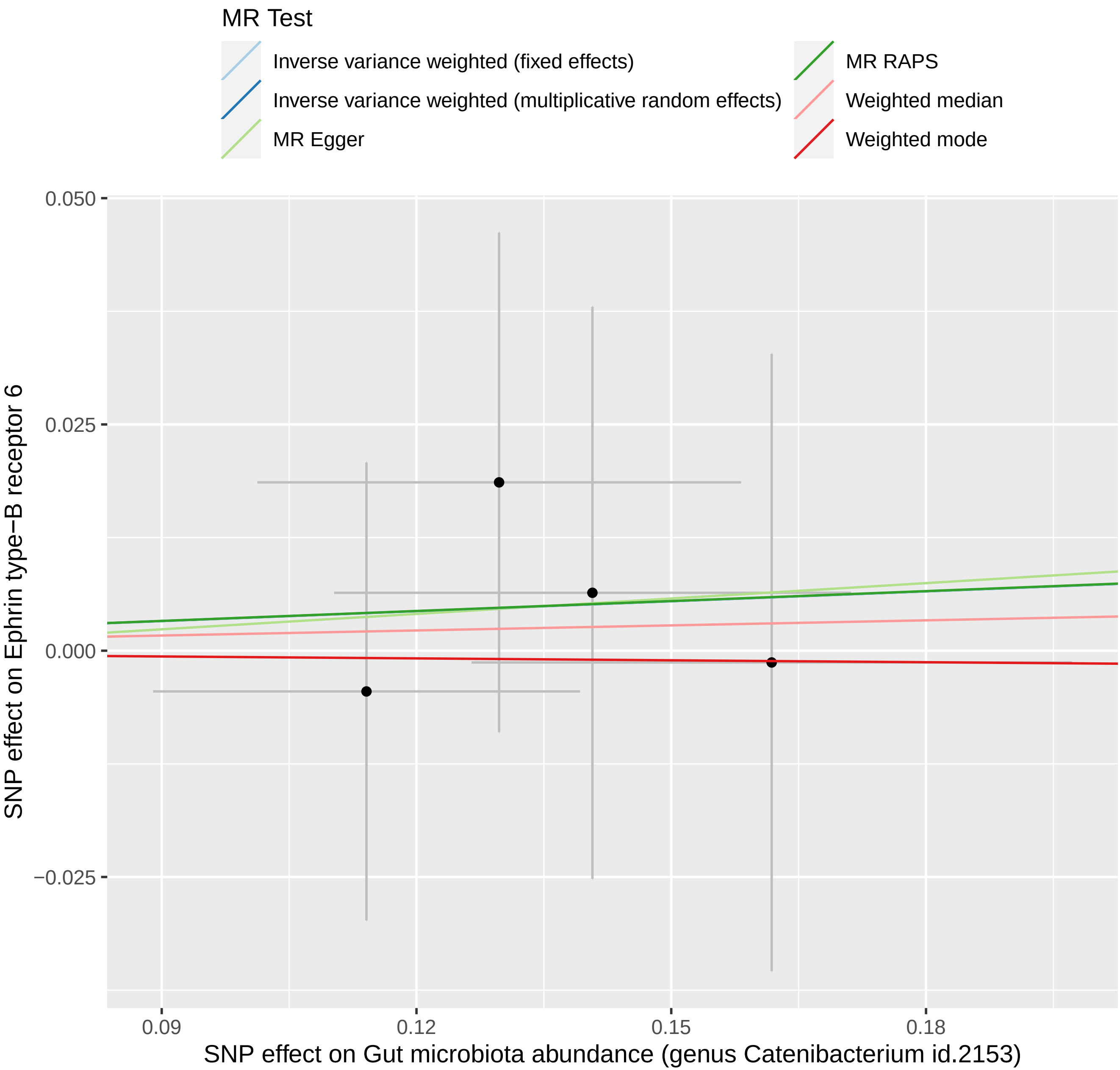

Batch 2−1932 : Gut microbiota abundance (genus Parasutterella id.2892) on Ephrin type−B receptor 6
with a threshold of 1e−05

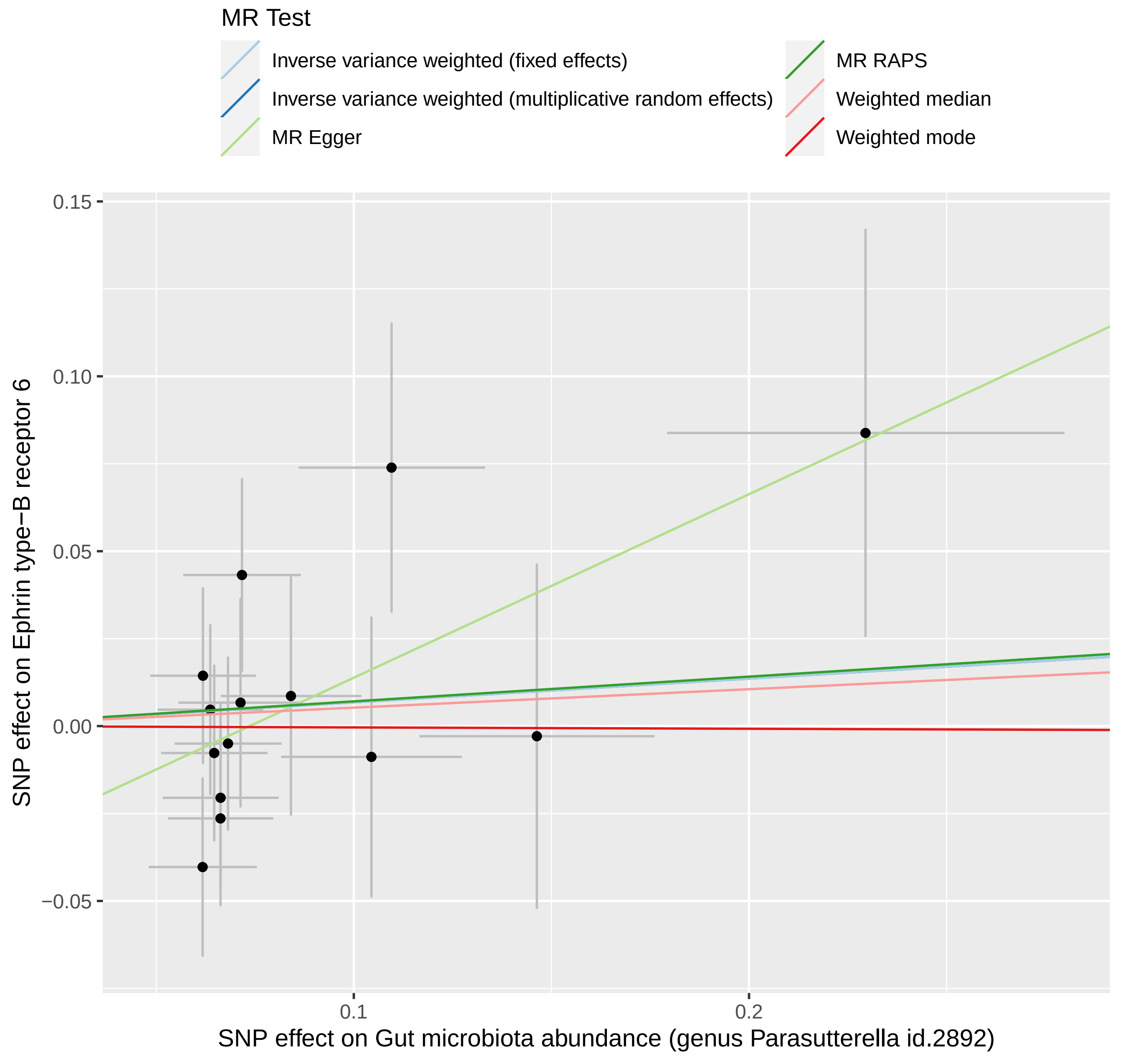

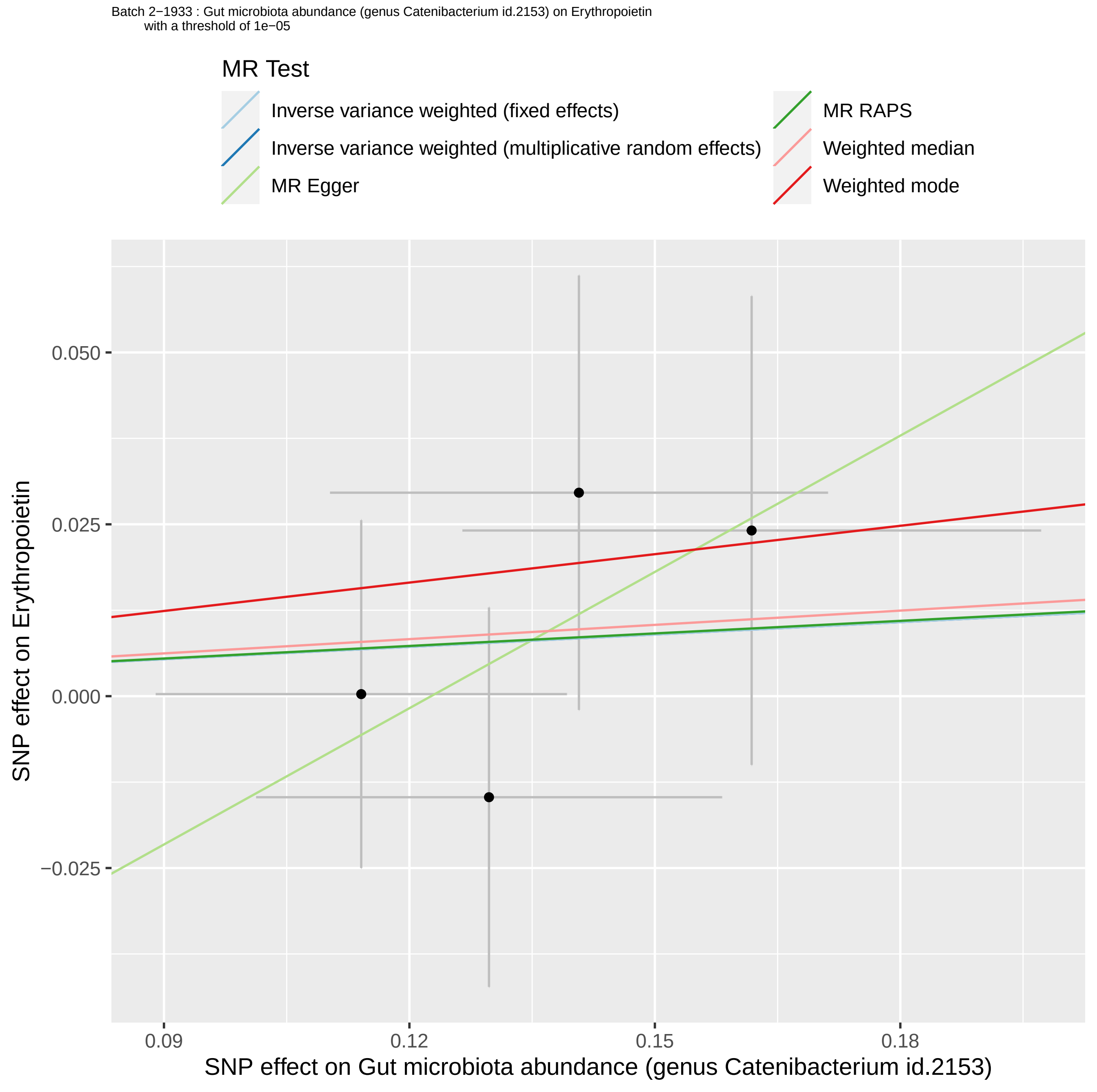

Batch 2−1933 : Gut microbiota abundance (genus Catenibacterium id.2153) on Erythropoietin
with a threshold of 1e−05
MR Test
Inverse variance weighted (fixed effects)
Inverse variance weighted (multiplicative random effects)
MR Egger
MR RAPS
Weighted median
Weighted mode
0.050
0.025
0.000
−0.025
0.09
0.12
0.15
0.18
SNP effect on Erythropoietin
SNP effect on Gut microbiota abundance (genus Catenibacterium id.2153)

Batch 2−1934 : Gut microbiota abundance (genus Parasutterella id.2892) on Erythropoietin
with a threshold of 1e−05

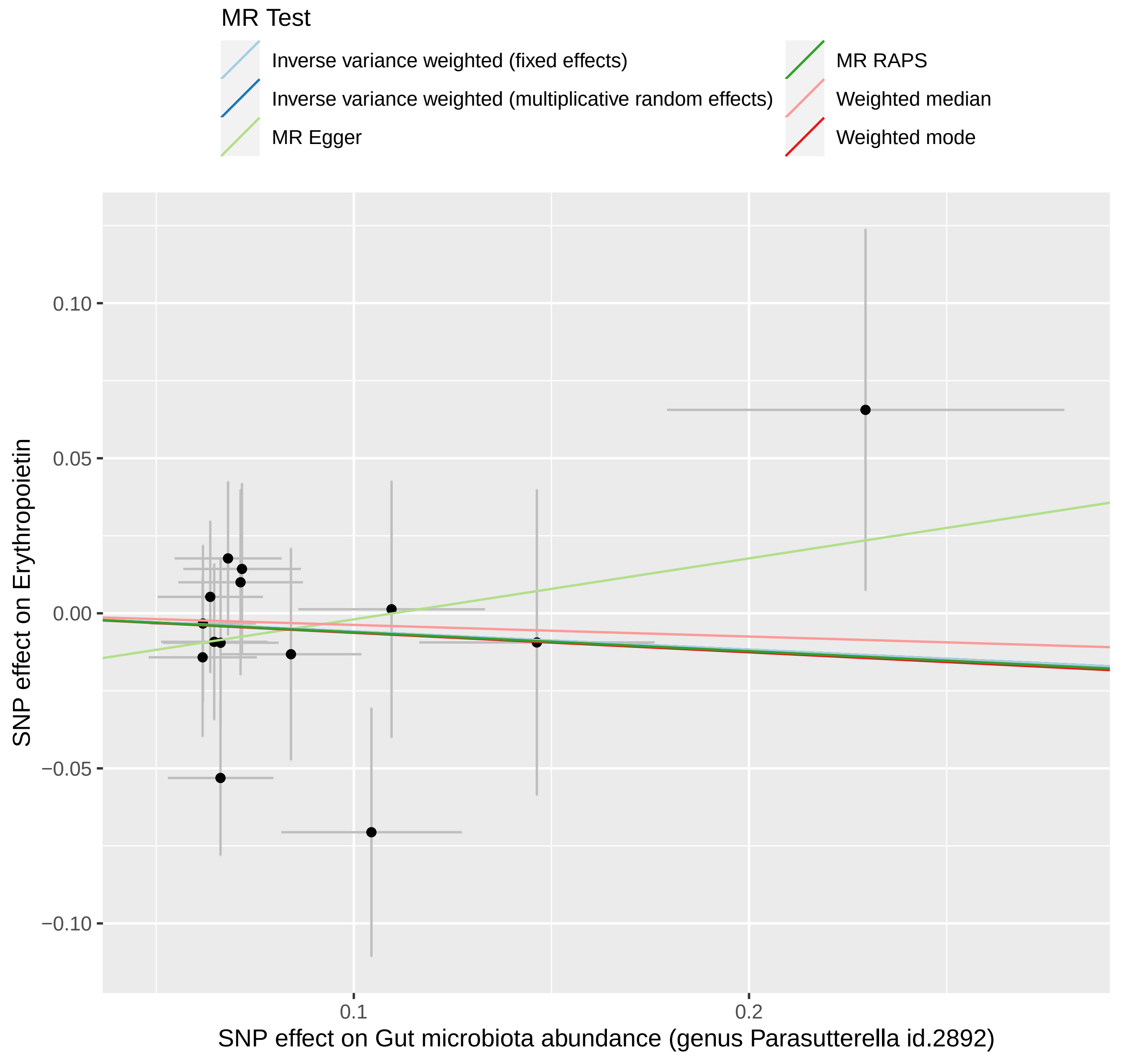

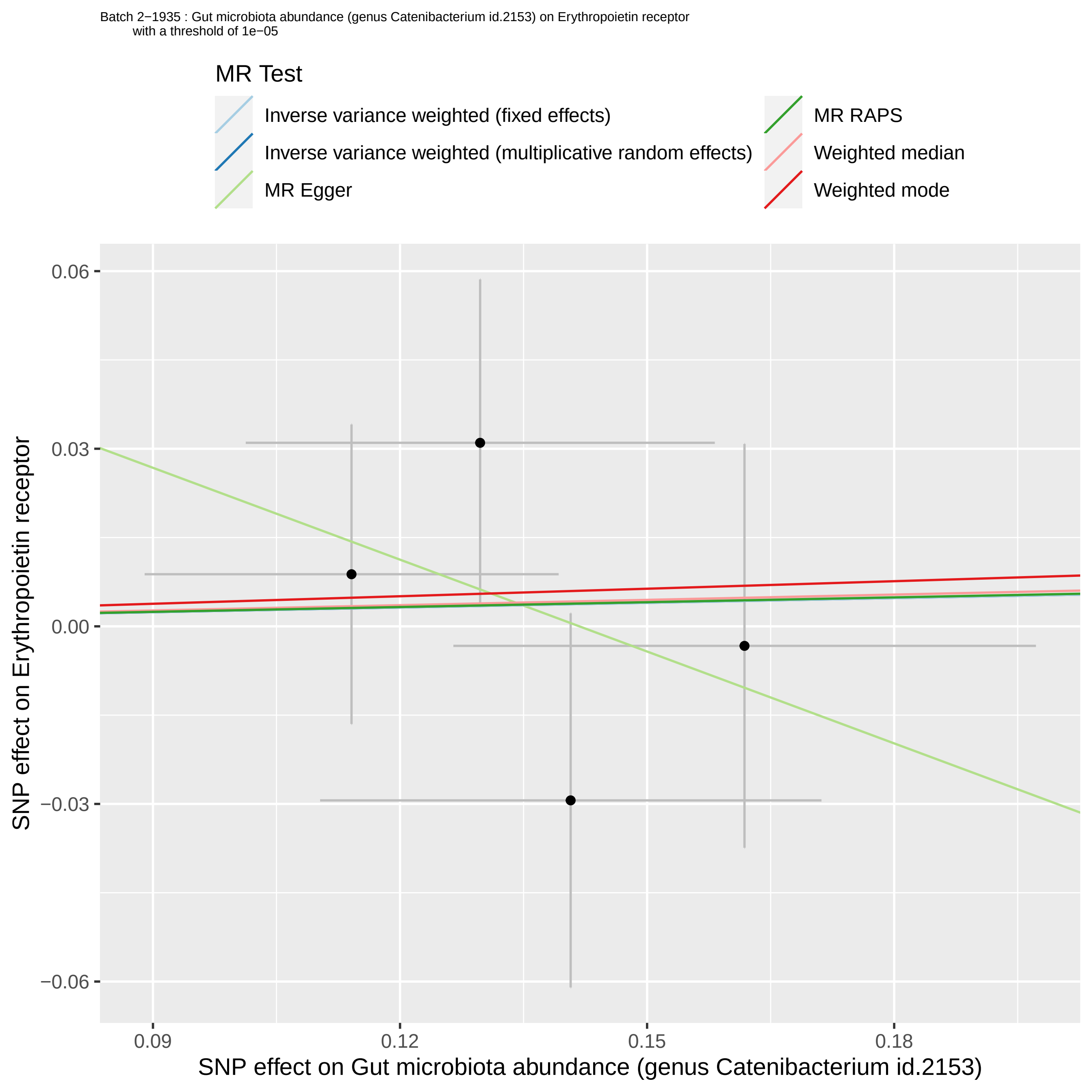
Batch 2−1935 : Gut microbiota abundance (genus Catenibacterium id.2153) on Erythropoietin receptor
with a threshold of 1e−05
MR Test
Inverse variance weighted (fixed effects)
Inverse variance weighted (multiplicative random effects)
MR Egger
MR RAPS
Weighted median
Weighted mode
0.06
0.03
0.00
−0.03
−0.06
0.09
0.12
0.15
0.18
SNP effect on Erythropoietin receptor
SNP effect on Gut microbiota abundance (genus Catenibacterium id.2153)

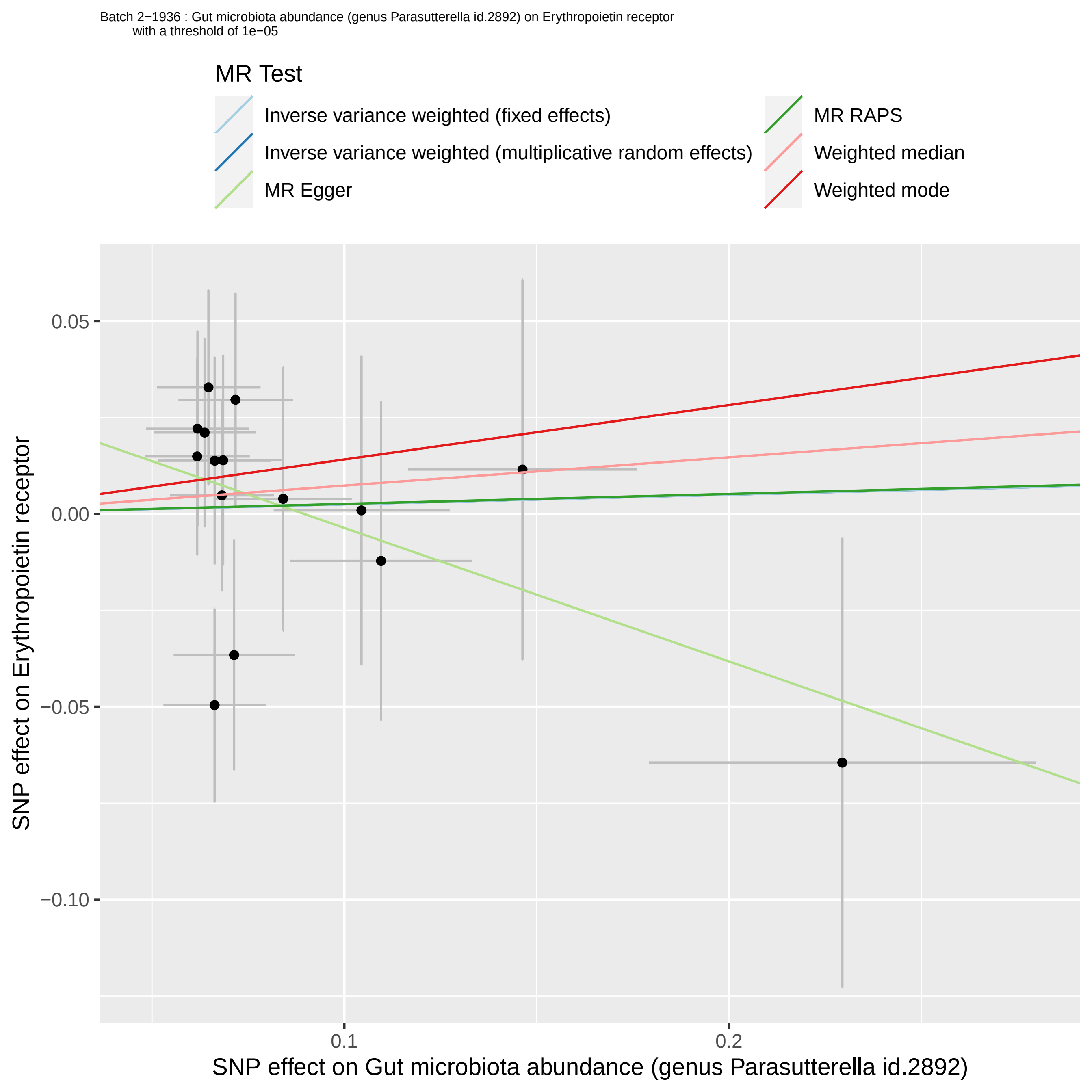
Batch 2−1936 : Gut microbiota abundance (genus Parasutterella id.2892) on Erythropoietin receptor
with a threshold of 1e−05
MR Test
Inverse variance weighted (fixed effects)
Inverse variance weighted (multiplicative random effects)
MR Egger
MR RAPS
Weighted median
Weighted mode
0.05
0.00
−0.05
−0.10
0.1
0.2
SNP effect on Erythropoietin receptor
SNP effect on Gut microbiota abundance (genus Parasutterella id.2892)

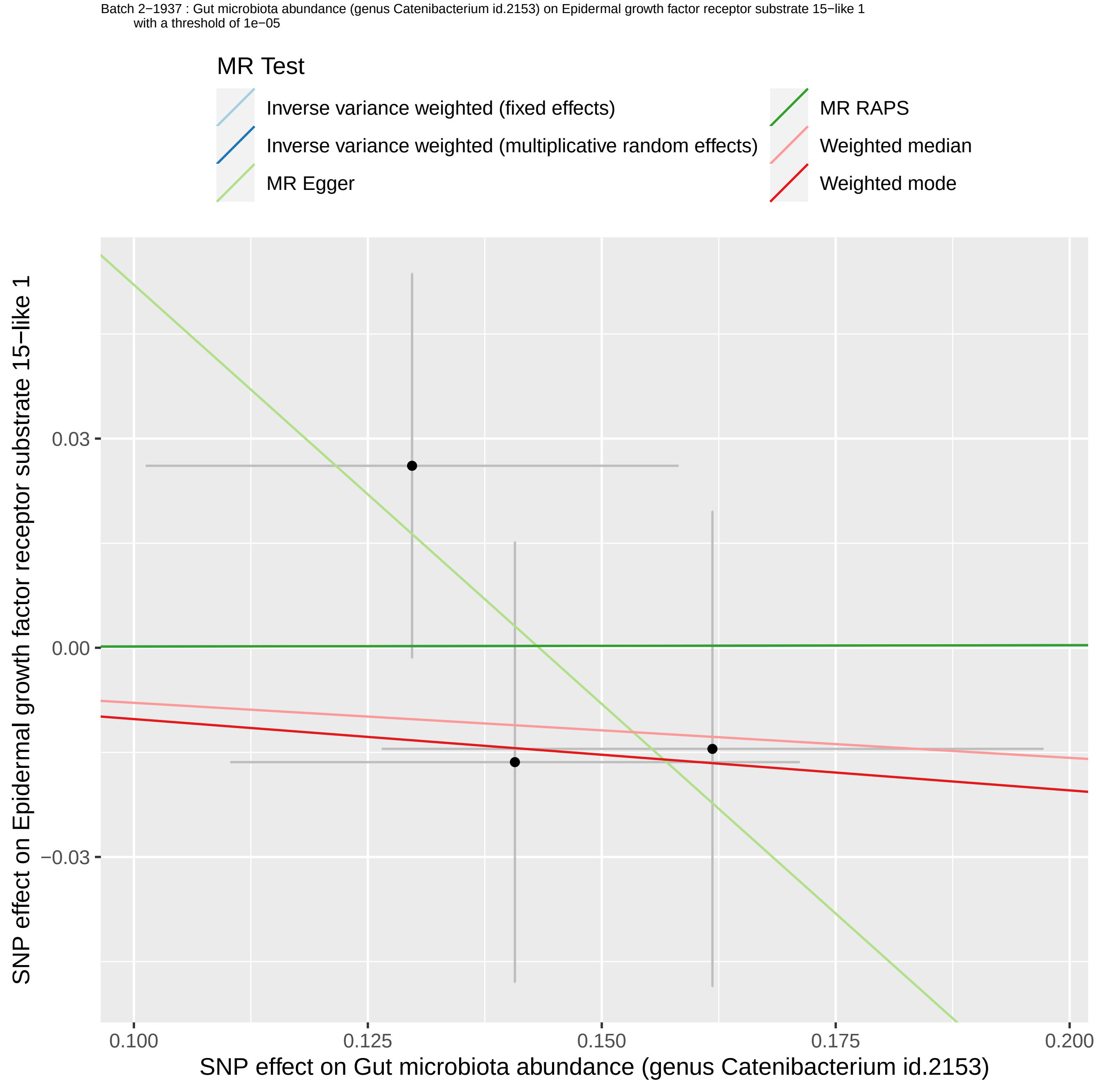

Batch 2−1937 : Gut microbiota abundance (genus Catenibacterium id.2153) on Epidermal growth factor receptor substrate 15−like 1
with a threshold of 1e−05
MR Test
Inverse variance weighted (fixed effects)
Inverse variance weighted (multiplicative random effects)
MR Egger
MR RAPS
Weighted median
Weighted mode
0.03
0.00
−0.03
0.100
0.125
0.150
0.175
0.200
SNP effect on Epidermal growth factor receptor substrate 15−like 1
SNP effect on Gut microbiota abundance (genus Catenibacterium id.2153)

Batch 2−1938 : Gut microbiota abundance (genus Parasutterella id.2892) on Epidermal growth factor receptor substrate 15−like 1
with a threshold of 1e−05

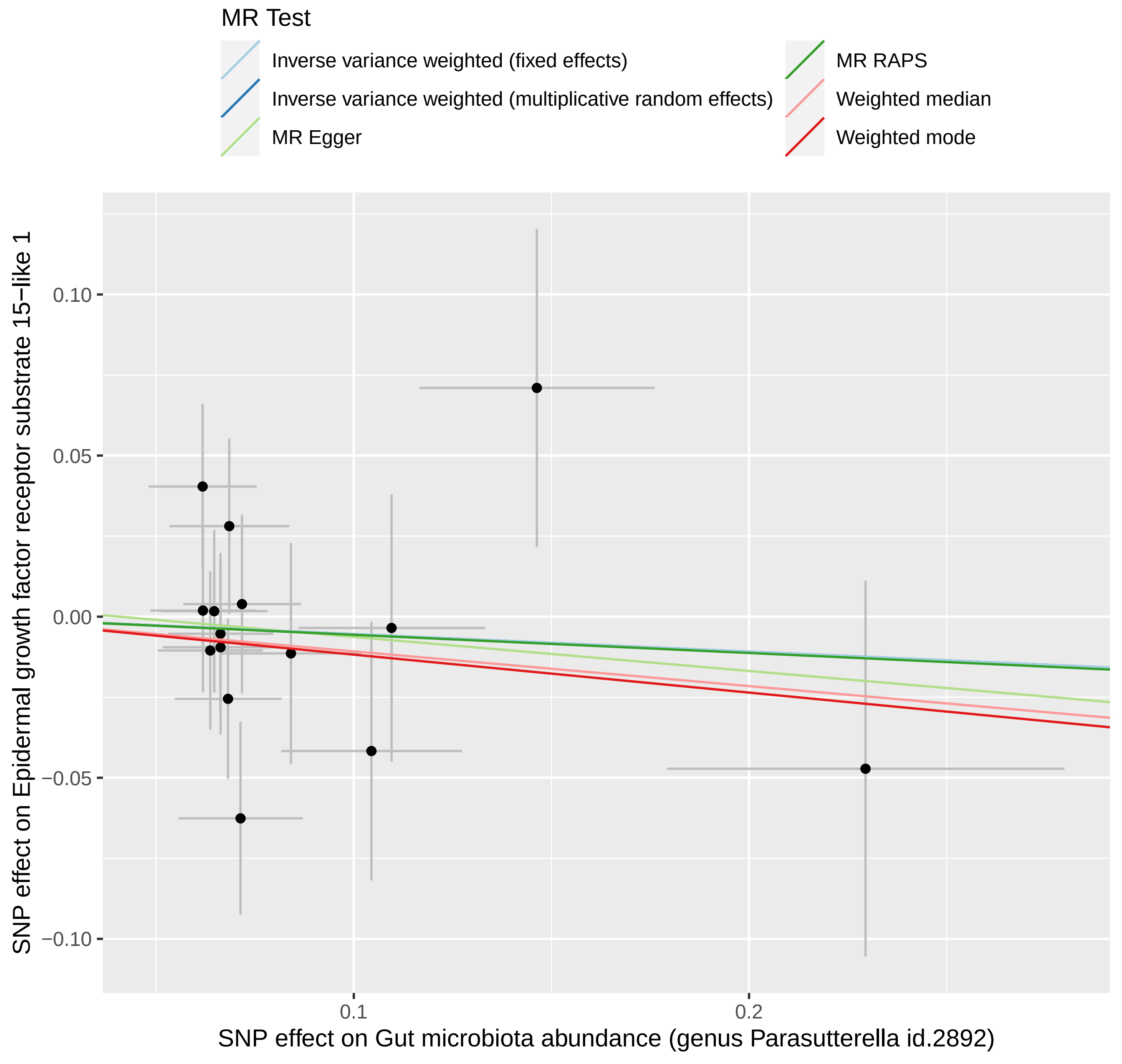

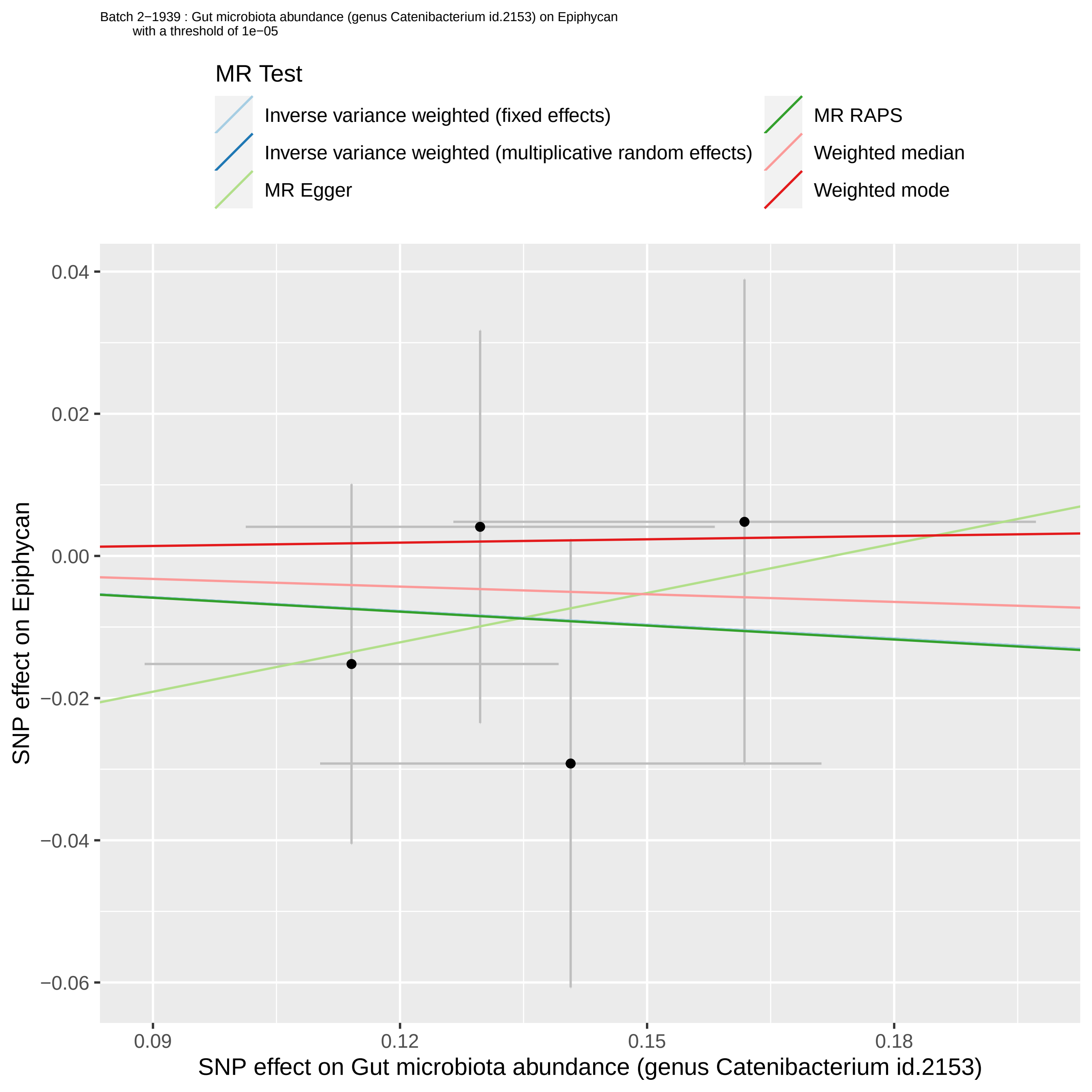
Batch 2−1939 : Gut microbiota abundance (genus Catenibacterium id.2153) on Epiphycan
with a threshold of 1e−05
MR Test
Inverse variance weighted (fixed effects)
Inverse variance weighted (multiplicative random effects)
MR Egger
MR RAPS
Weighted median
Weighted mode
SNP effect on Epiphycan
0.04
0.02
0.00
−0.02
−0.04
−0.06
0.09
0.12
0.15
0.18
SNP effect on Gut microbiota abundance (genus Catenibacterium id.2153)

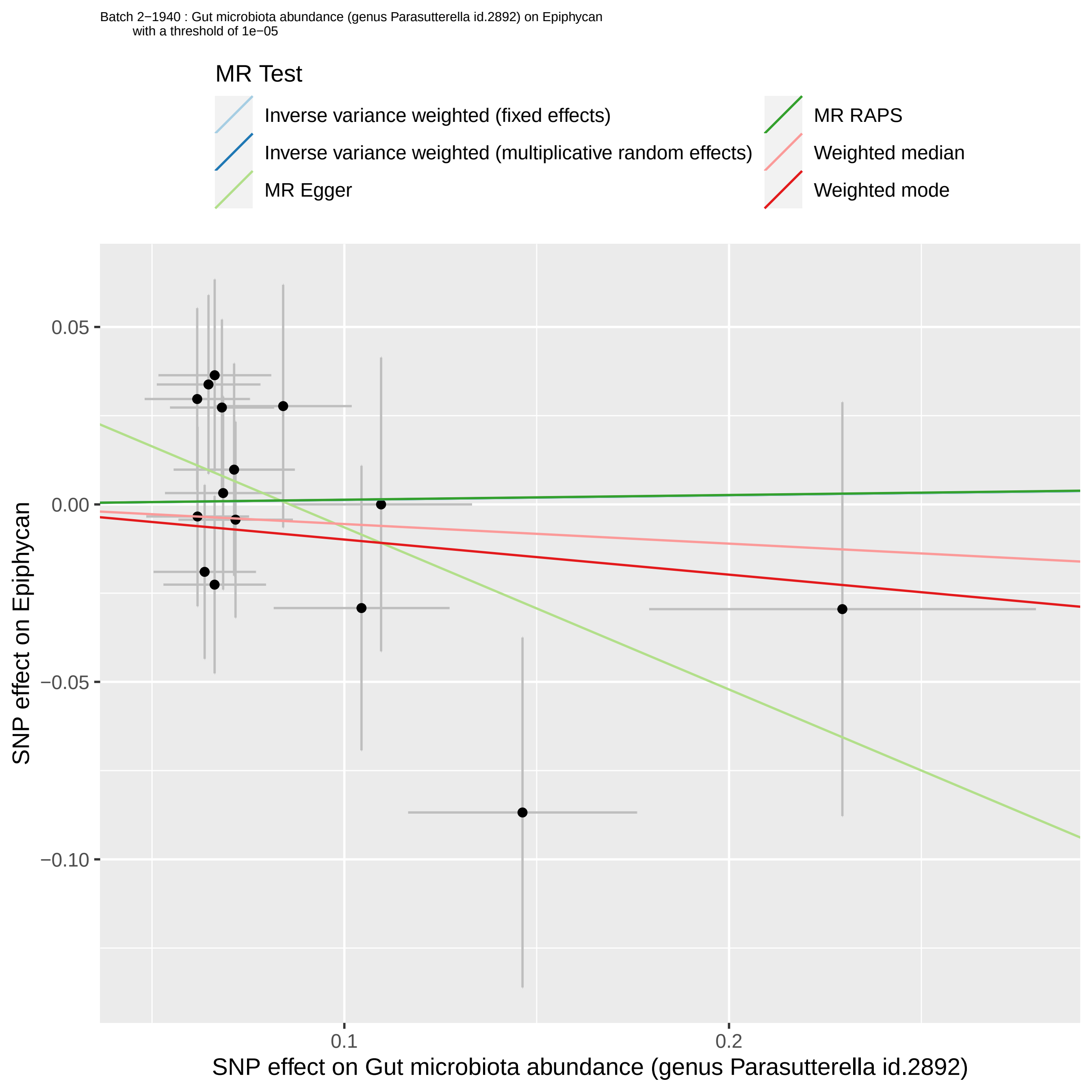
Batch 2−1940 : Gut microbiota abundance (genus Parasutterella id.2892) on Epiphycan
with a threshold of 1e−05
MR Test
Inverse variance weighted (fixed effects)
Inverse variance weighted (multiplicative random effects)
MR Egger
MR RAPS
Weighted median
Weighted mode
0.05
0.00
−0.05
−0.10
0.1
0.2
SNP effect on Epiphycan
SNP effect on Gut microbiota abundance (genus Parasutterella id.2892)

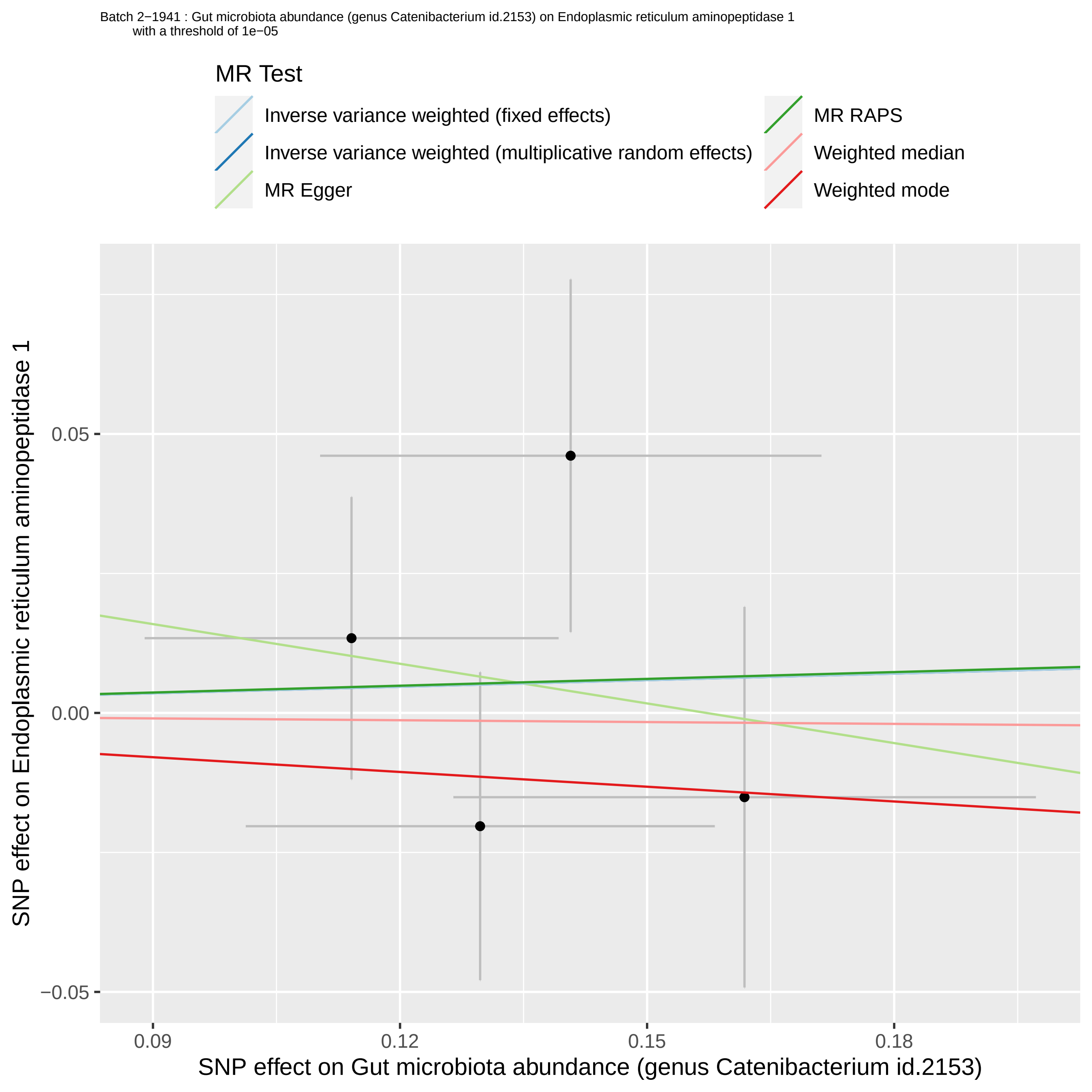
Batch 2−1941 : Gut microbiota abundance (genus Catenibacterium id.2153) on Endoplasmic reticulum aminopeptidase 1
with a threshold of 1e−05
MR Test
Inverse variance weighted (fixed effects)
Inverse variance weighted (multiplicative random effects)
MR Egger
MR RAPS
Weighted median
Weighted mode
0.05
0.00
−0.05
0.09
0.12
0.15
0.18
SNP effect on Endoplasmic reticulum aminopeptidase 1
SNP effect on Gut microbiota abundance (genus Catenibacterium id.2153)

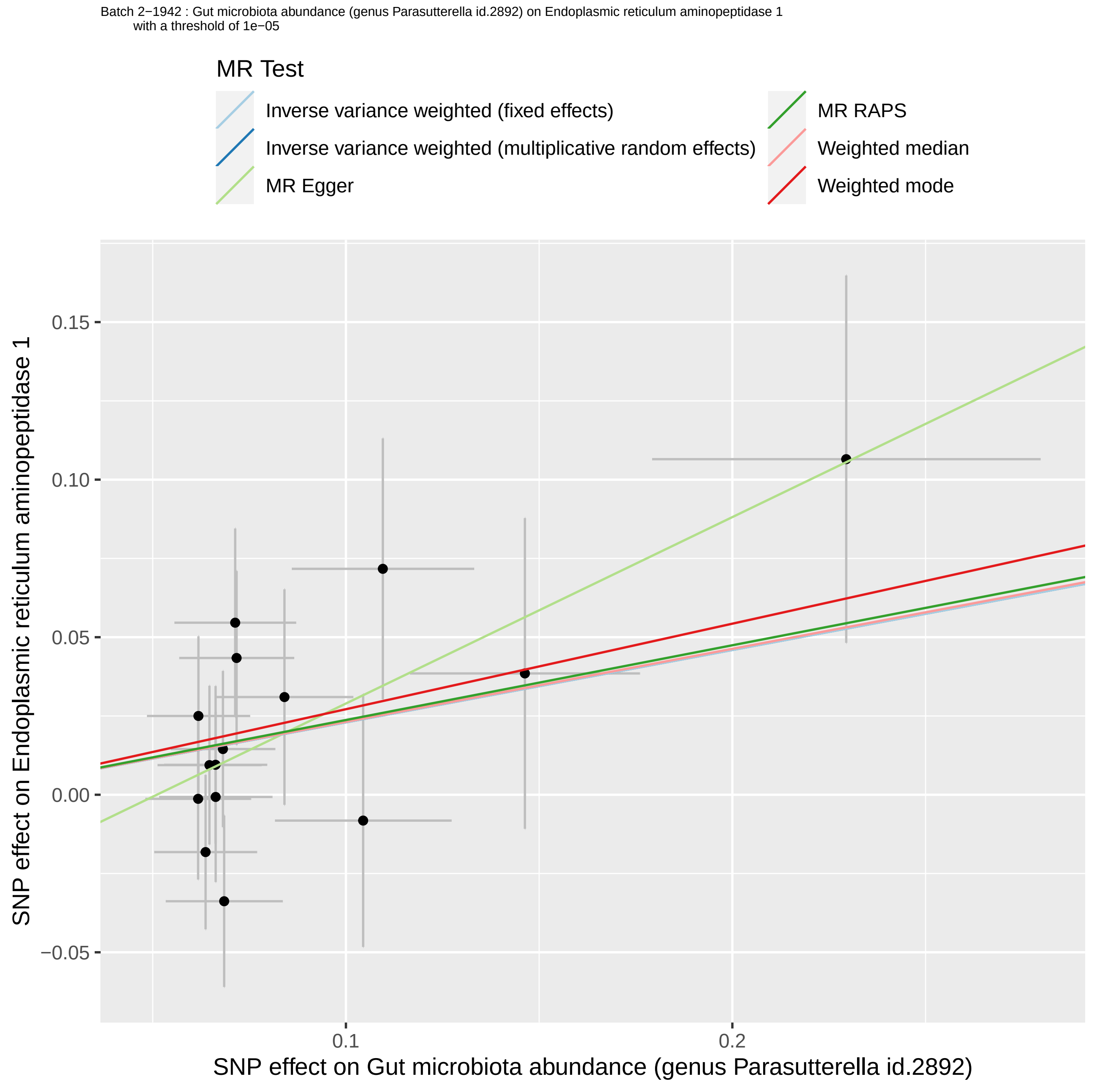
Batch 2−1942 : Gut microbiota abundance (genus Parasutterella id.2892) on Endoplasmic reticulum aminopeptidase 1
with a threshold of 1e−05
MR Test
Inverse variance weighted (fixed effects)
Inverse variance weighted (multiplicative random effects)
MR Egger
MR RAPS
Weighted median
Weighted mode
0.15
0.10
0.05
0.00
−0.05
0.1
0.2
SNP effect on Endoplasmic reticulum aminopeptidase 1
SNP effect on Gut microbiota abundance (genus Parasutterella id.2892)

Batch 2−1943 : Gut microbiota abundance (genus Catenibacterium id.2153) on Endoplasmic reticulum aminopeptidase 2
with a threshold of 1e−05

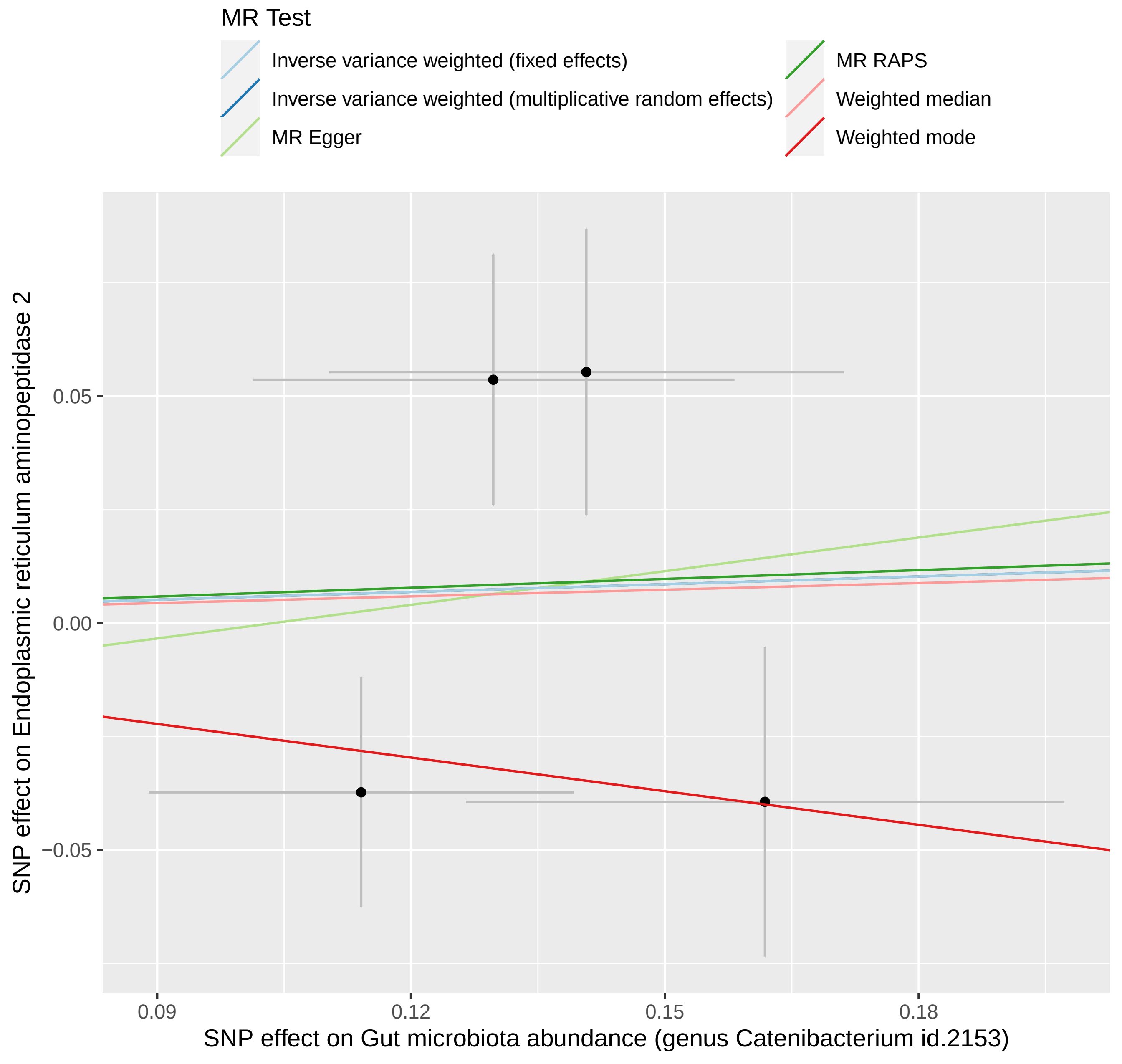

Batch 2−1944 : Gut microbiota abundance (genus Parasutterella id.2892) on Endoplasmic reticulum aminopeptidase 2
with a threshold of 1e−05

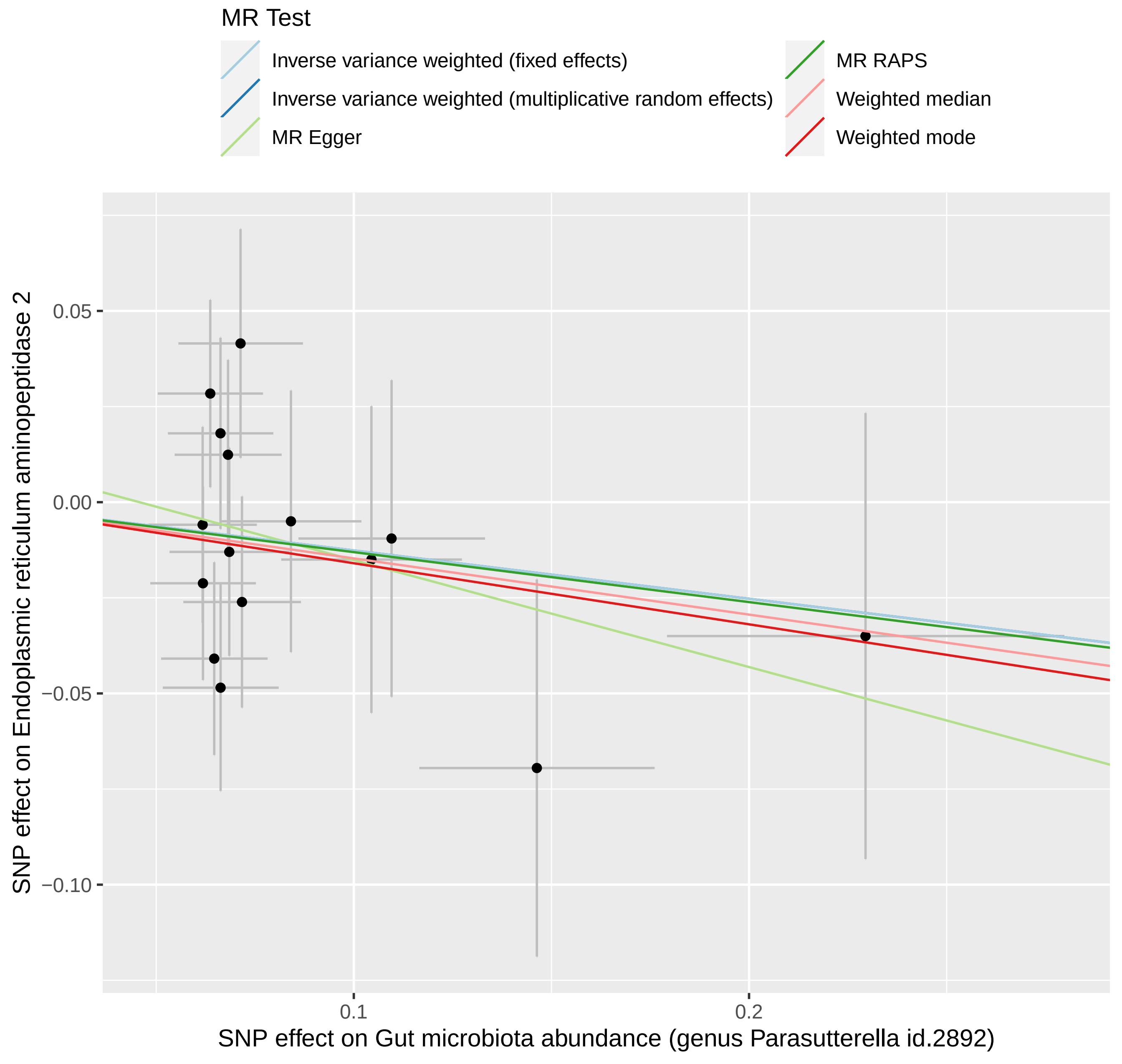

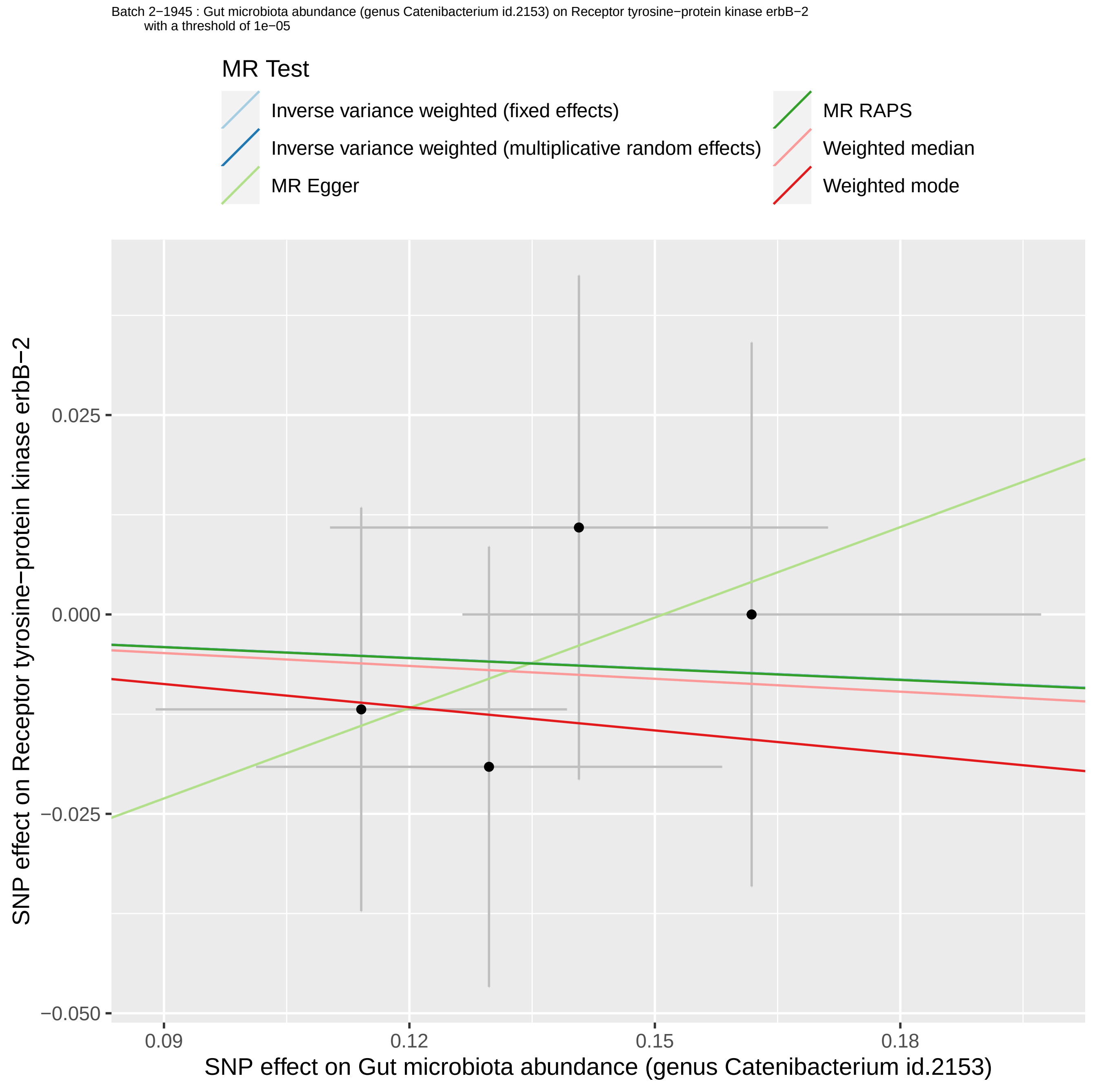
Batch 2−1945 : Gut microbiota abundance (genus Catenibacterium id.2153) on Receptor tyrosine−protein kinase erbB−2
with a threshold of 1e−05
MR Test
Inverse variance weighted (fixed effects)
Inverse variance weighted (multiplicative random effects)
MR Egger
MR RAPS
Weighted median
Weighted mode
0.025
0.000
−0.025
−0.050
0.09
0.12
0.15
0.18
SNP effect on Receptor tyrosine−protein kinase erbB−2
SNP effect on Gut microbiota abundance (genus Catenibacterium id.2153)

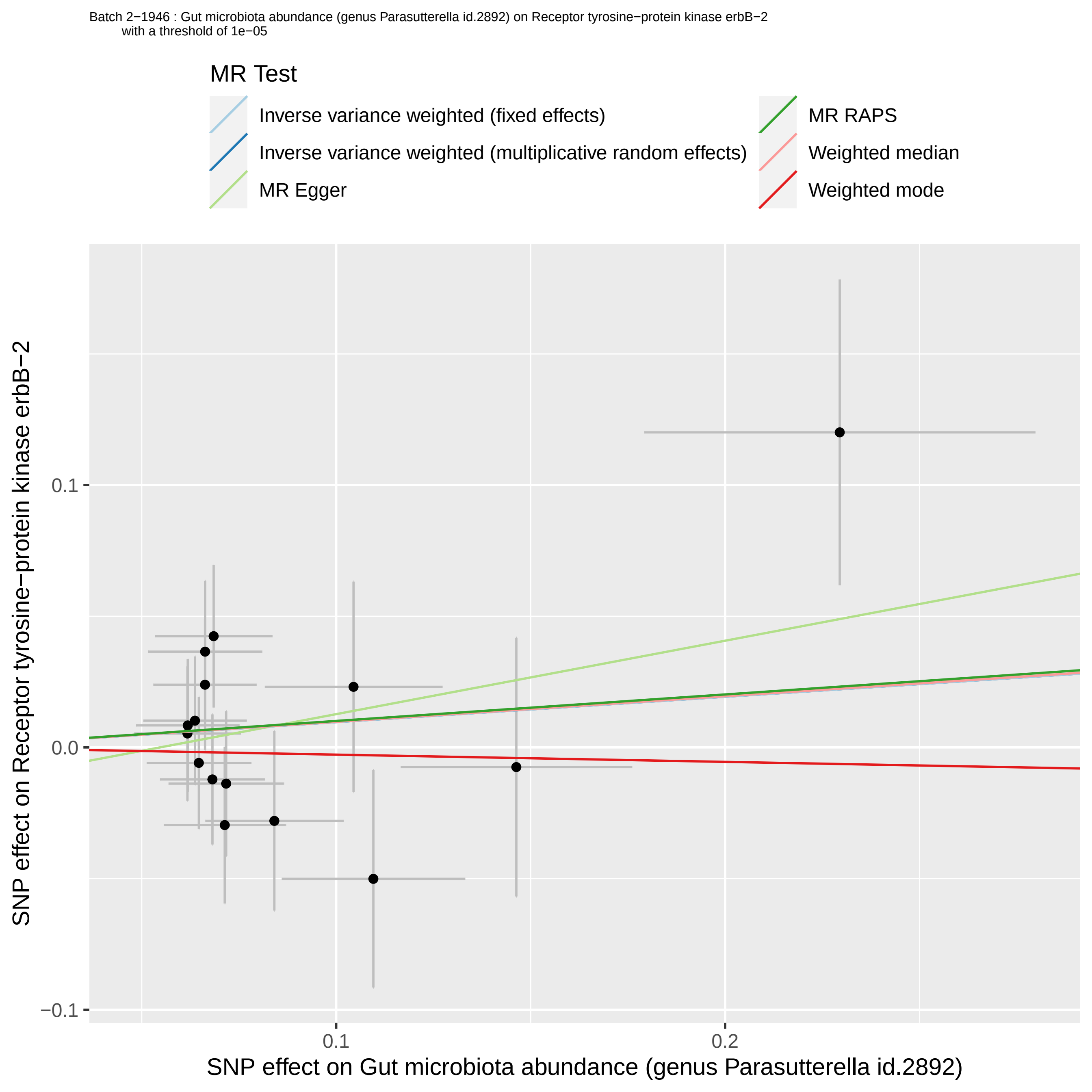
Batch 2−1946 : Gut microbiota abundance (genus Parasutterella id.2892) on Receptor tyrosine−protein kinase erbB−2
with a threshold of 1e−05
MR Test
Inverse variance weighted (fixed effects)
Inverse variance weighted (multiplicative random effects)
MR Egger
MR RAPS
Weighted median
Weighted mode
0.1
0.0
−0.1
0.1
0.2
SNP effect on Receptor tyrosine−protein kinase erbB−2
SNP effect on Gut microbiota abundance (genus Parasutterella id.2892)

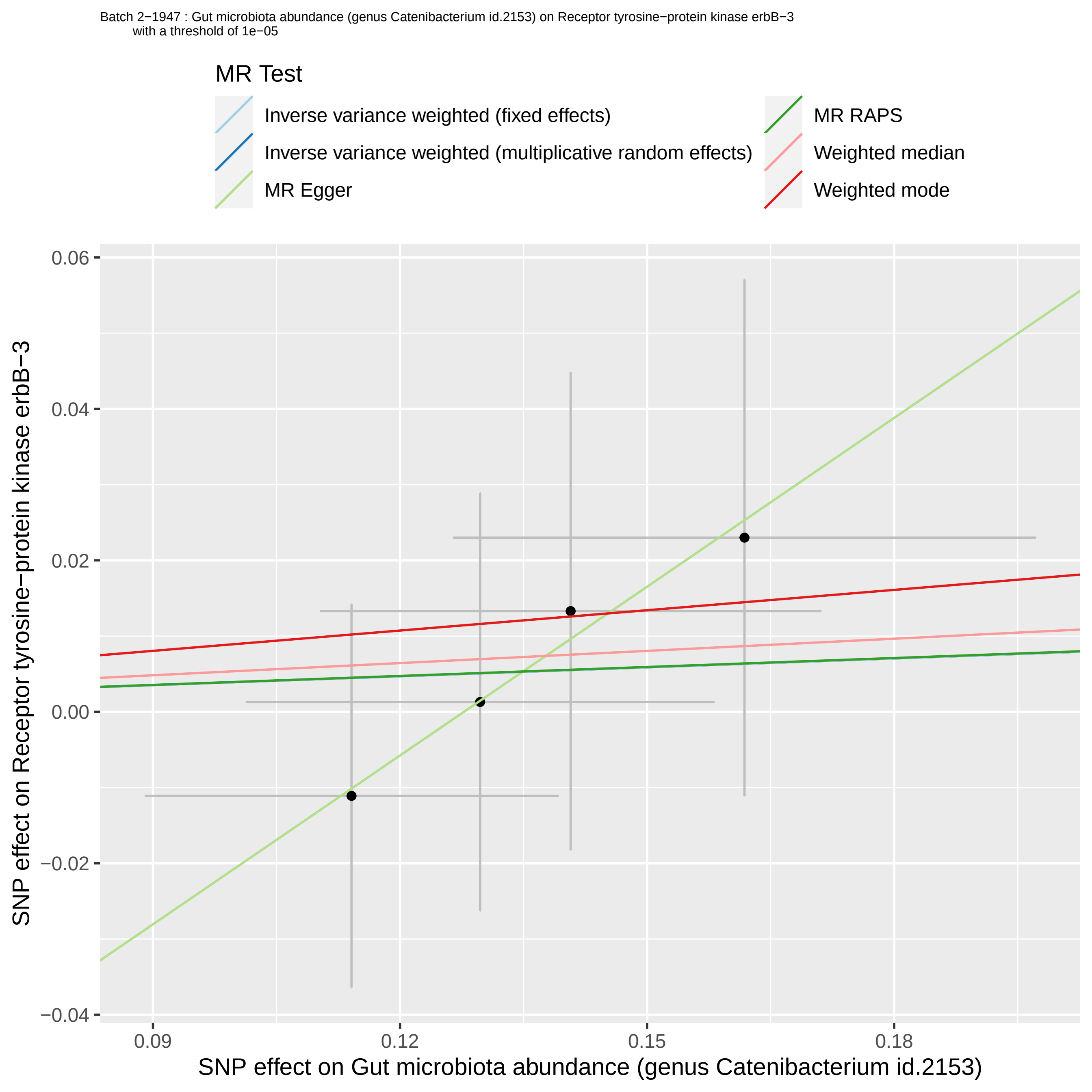

Batch 2−1947 : Gut microbiota abundance (genus Catenibacterium id.2153) on Receptor tyrosine−protein kinase erbB−3
with a threshold of 1e−05
MR Test
Inverse variance weighted (fixed effects)
Inverse variance weighted (multiplicative random effects)
MR Egger
MR RAPS
Weighted median
Weighted mode
SNP effect on Receptor tyrosine−protein kinase erbB−3
SNP effect on Gut microbiota abundance (genus Catenibacterium id.2153)
0.06
0.04
0.02
0.00
−0.02
−0.04
0.09
0.12
0.15
0.18

Batch 2−1948 : Gut microbiota abundance (genus Parasutterella id.2892) on Receptor tyrosine−protein kinase erbB−3
with a threshold of 1e−05

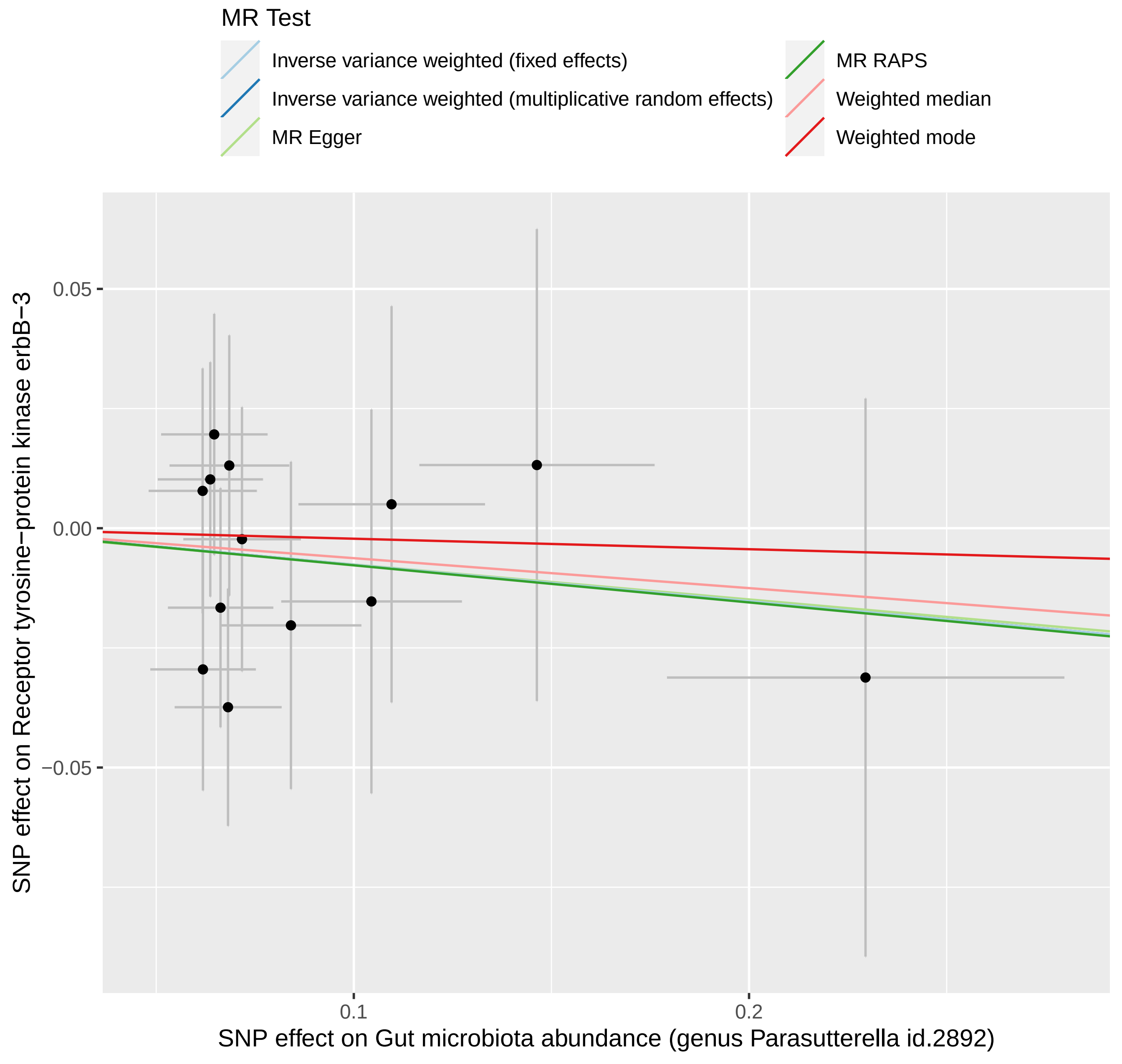

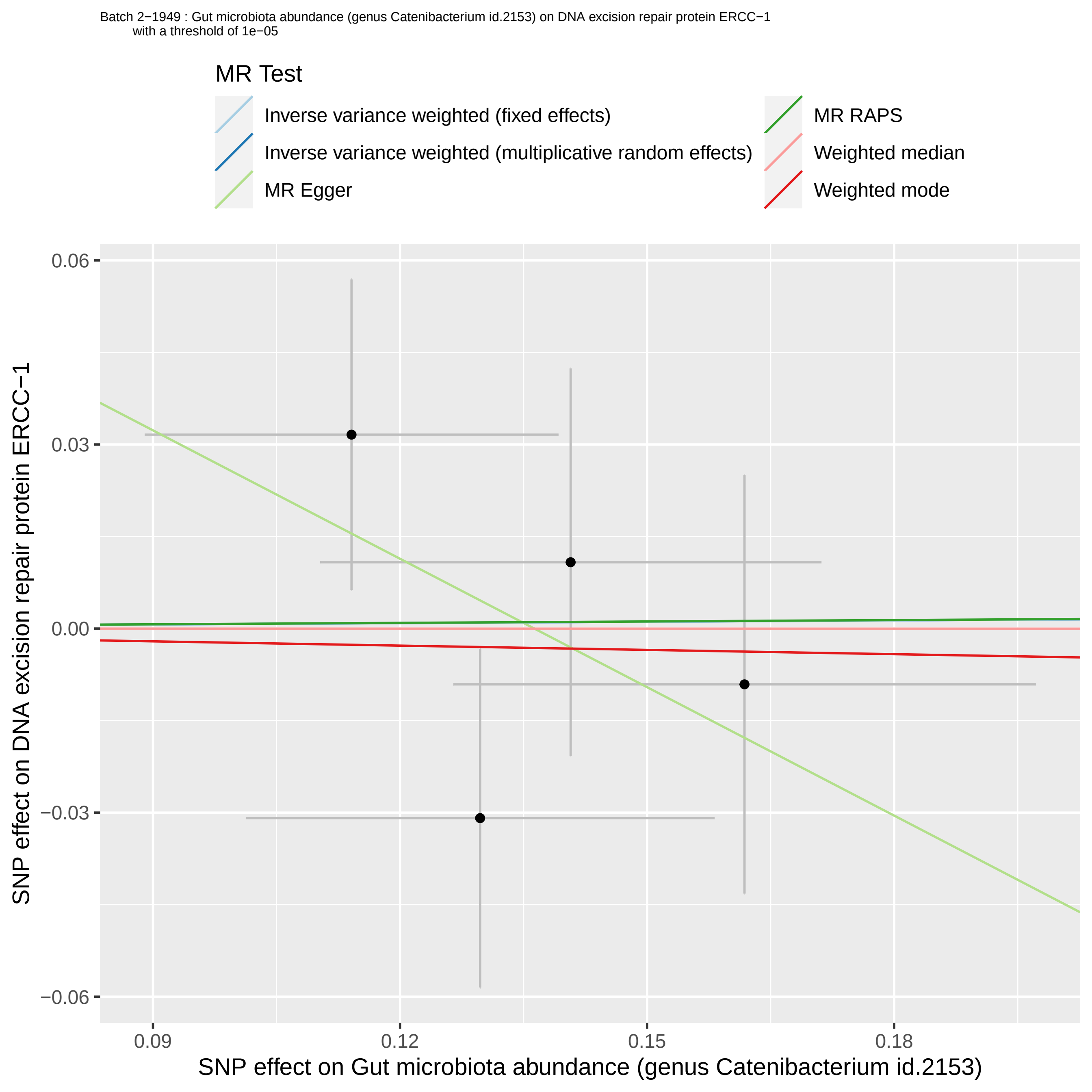

Batch 2−1949 : Gut microbiota abundance (genus Catenibacterium id.2153) on DNA excision repair protein ERCC−1
with a threshold of 1e−05
MR Test
Inverse variance weighted (fixed effects)
Inverse variance weighted (multiplicative random effects)
MR Egger
MR RAPS
Weighted median
Weighted mode
0.06
0.03
0.00
−0.03
−0.06
0.09
0.12
0.15
0.18
SNP effect on DNA excision repair protein ERCC−1
SNP effect on Gut microbiota abundance (genus Catenibacterium id.2153)

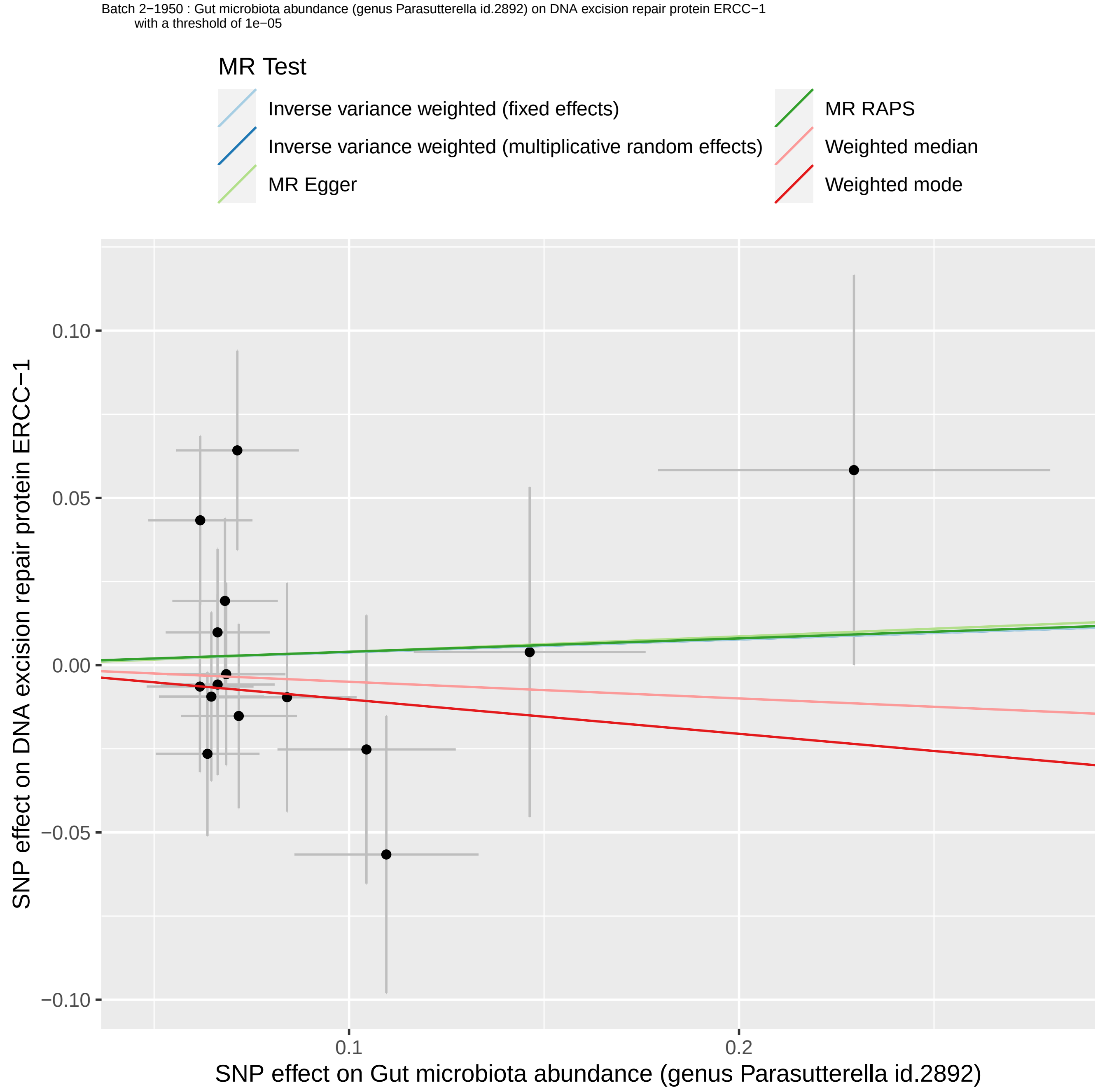
Batch 2−1950 : Gut microbiota abundance (genus Parasutterella id.2892) on DNA excision repair protein ERCC−1
with a threshold of 1e−05
MR Test
Inverse variance weighted (fixed effects)
Inverse variance weighted (multiplicative random effects)
MR Egger
MR RAPS
Weighted median
Weighted mode
0.10
0.05
0.00
−0.05
−0.10
0.1
0.2
SNP effect on DNA excision repair protein ERCC−1
SNP effect on Gut microbiota abundance (genus Parasutterella id.2892)

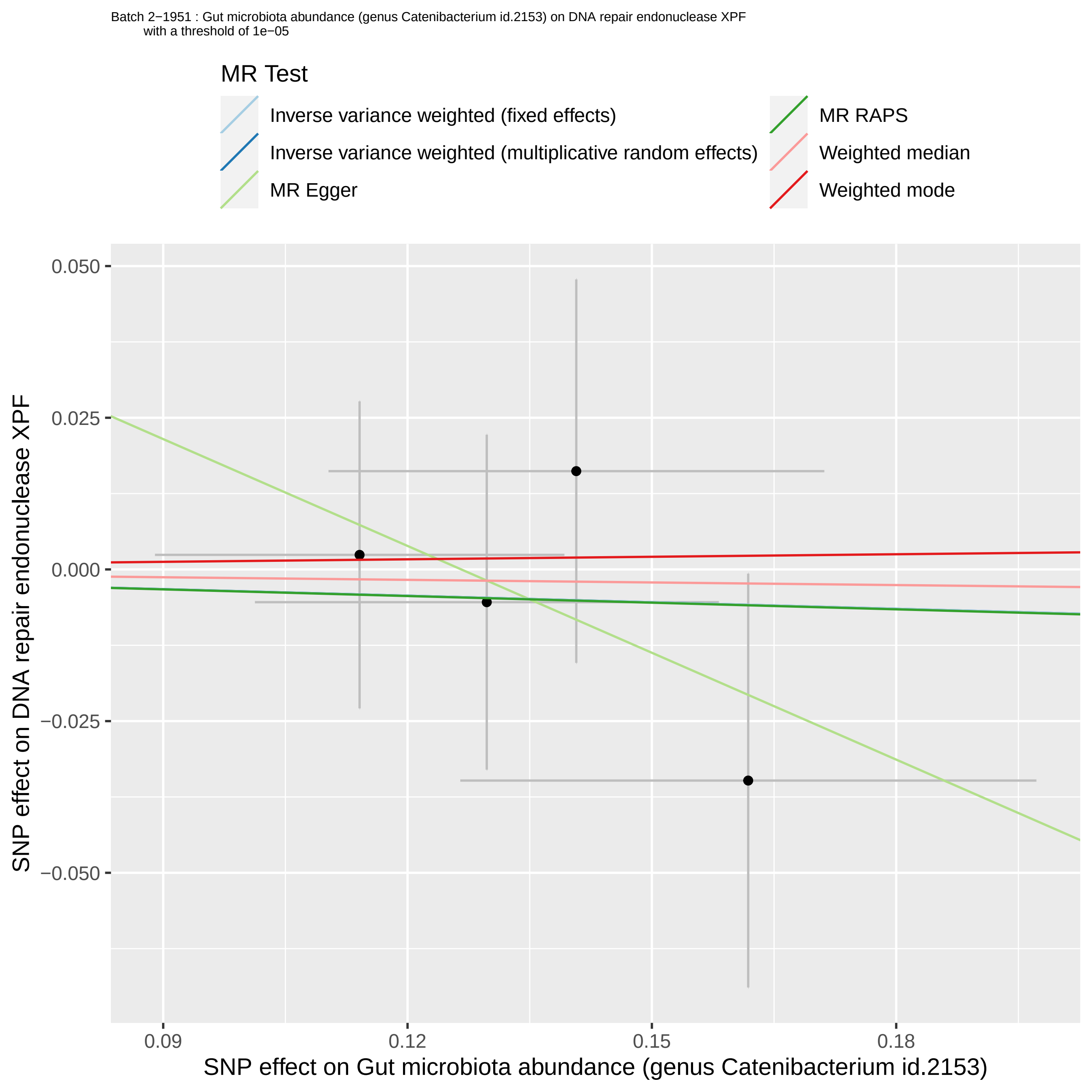
Batch 2−1951 : Gut microbiota abundance (genus Catenibacterium id.2153) on DNA repair endonuclease XPF
with a threshold of 1e−05
MR Test
Inverse variance weighted (fixed effects)
Inverse variance weighted (multiplicative random effects)
MR Egger
MR RAPS
Weighted median
Weighted mode
0.050
0.025
0.000
−0.025
−0.050
0.09
0.12
0.15
0.18
SNP effect on DNA repair endonuclease XPF
SNP effect on Gut microbiota abundance (genus Catenibacterium id.2153)

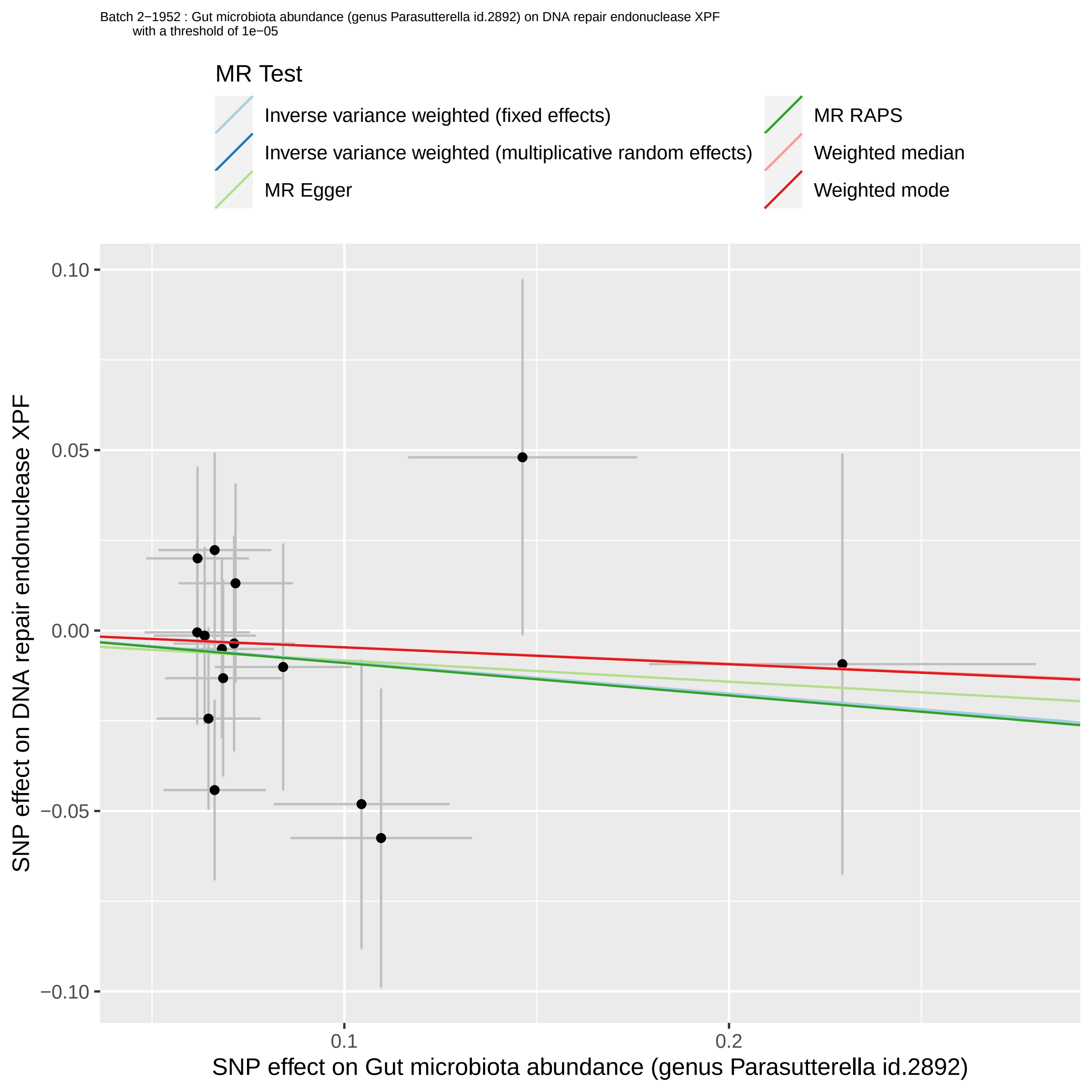

Batch 2−1952 : Gut microbiota abundance (genus Parasutterella id.2892) on DNA repair endonuclease XPF
with a threshold of 1e−05
MR Test
Inverse variance weighted (fixed effects)
Inverse variance weighted (multiplicative random effects)
MR Egger
MR RAPS
Weighted median
Weighted mode
0.10
0.05
0.00
−0.05
−0.10
0.1
0.2
SNP effect on DNA repair endonuclease XPF
SNP effect on Gut microbiota abundance (genus Parasutterella id.2892)

Batch 2−1953 : Gut microbiota abundance (genus Catenibacterium id.2153) on Epiregulin with a threshold of 1e−05

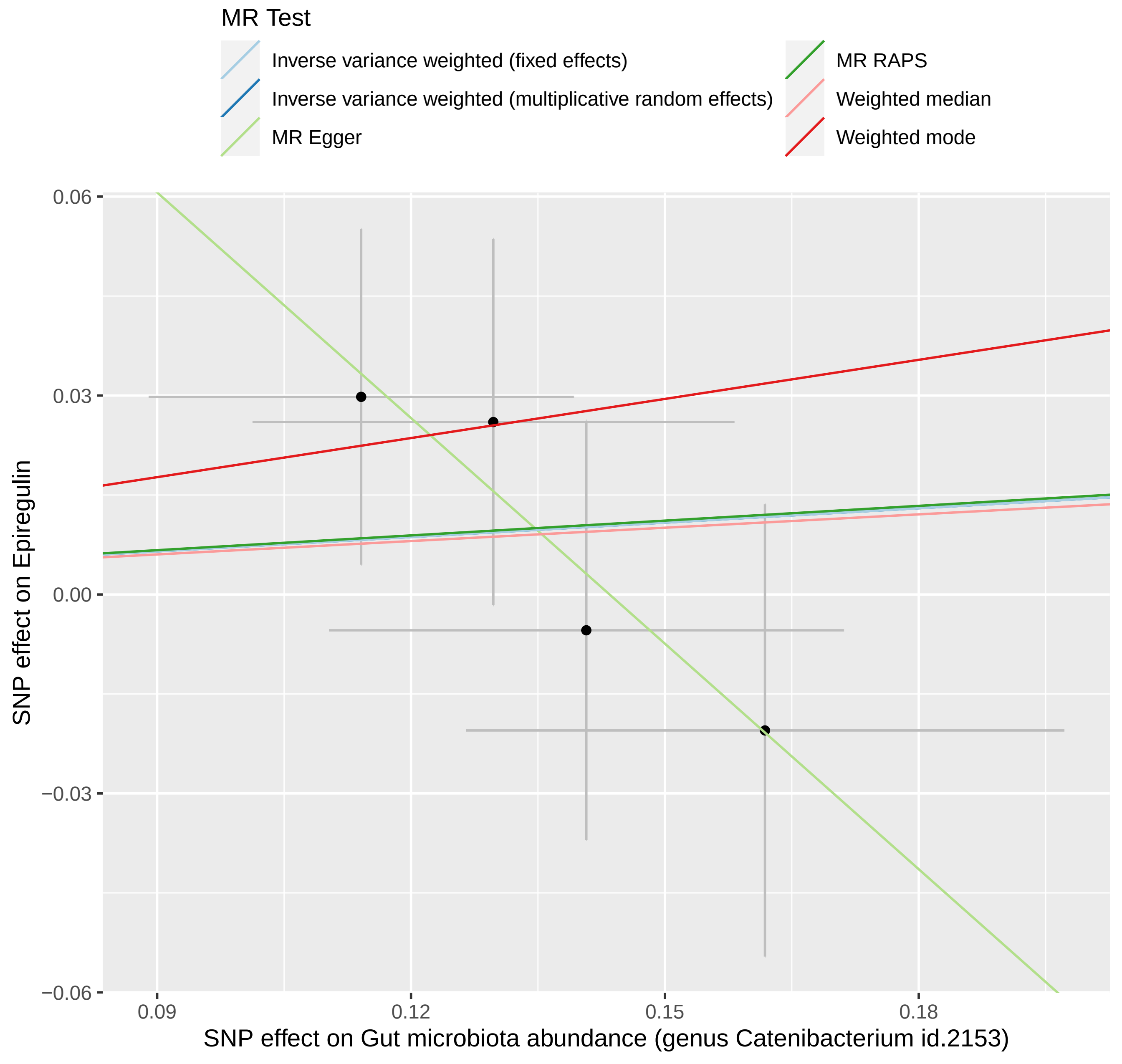

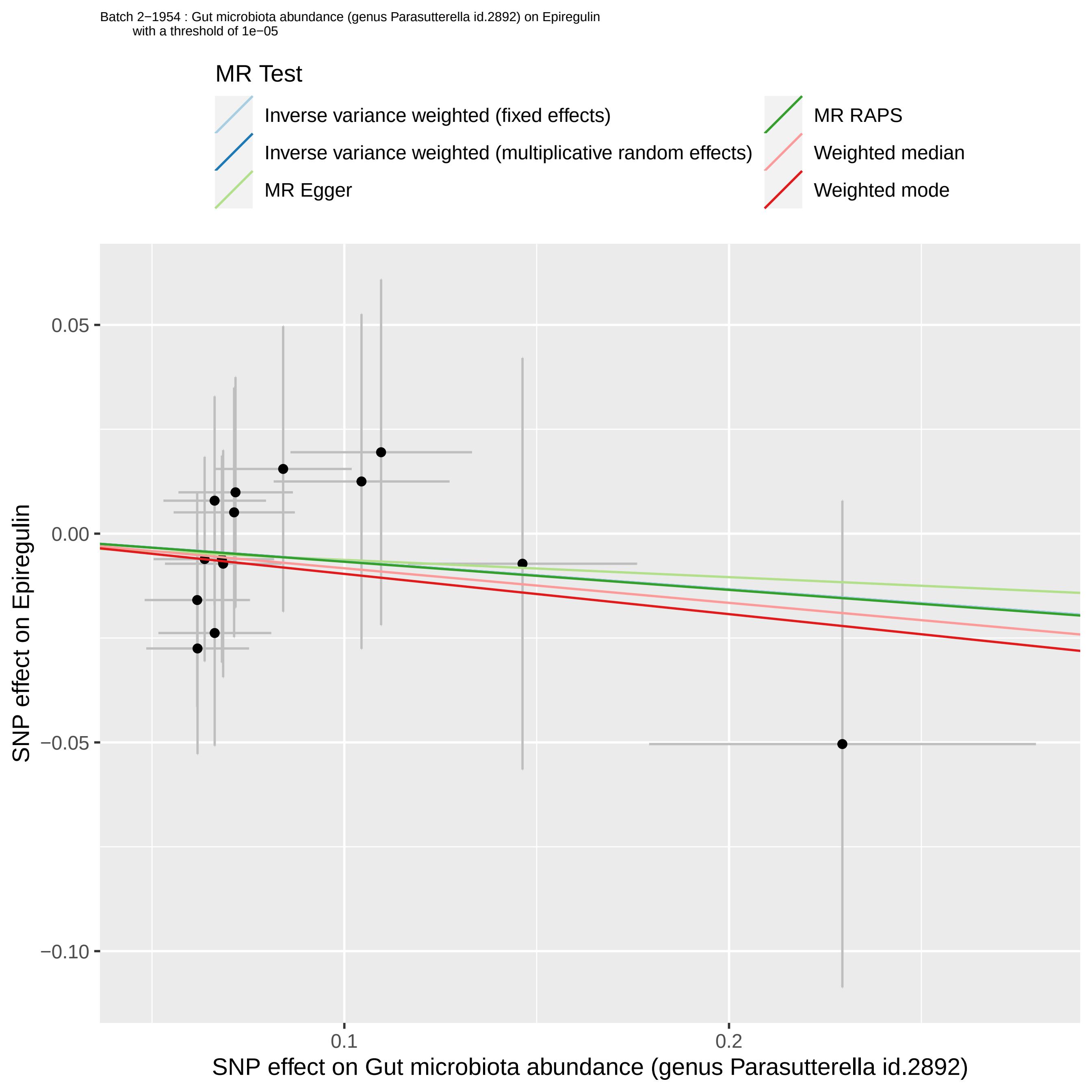
Batch 2−1954 : Gut microbiota abundance (genus Parasutterella id.2892) on Epiregulin
with a threshold of 1e−05
MR Test
Inverse variance weighted (fixed effects)
Inverse variance weighted (multiplicative random effects)
MR Egger
MR RAPS
Weighted median
Weighted mode
0.05
0.00
−0.05
−0.10
0.1
0.2
SNP effect on Epiregulin
SNP effect on Gut microbiota abundance (genus Parasutterella id.2892)

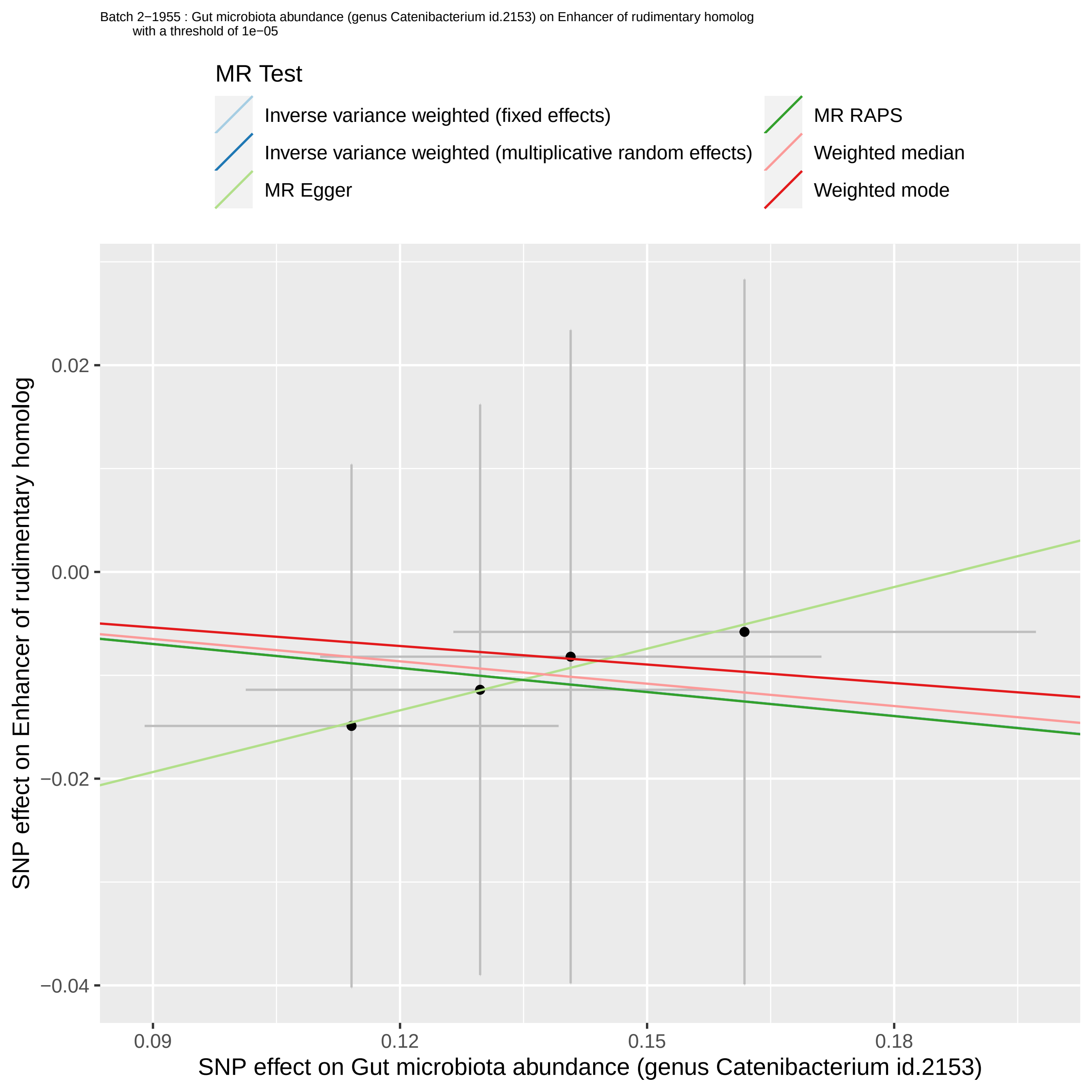

Batch 2−1955 : Gut microbiota abundance (genus Catenibacterium id.2153) on Enhancer of rudimentary homolog
with a threshold of 1e−05
MR Test
Inverse variance weighted (fixed effects)
Inverse variance weighted (multiplicative random effects)
MR Egger
MR RAPS
Weighted median
Weighted mode
0.02
0.00
−0.02
−0.04
0.09
0.12
0.15
0.18
SNP effect on Enhancer of rudimentary homolog
SNP effect on Gut microbiota abundance (genus Catenibacterium id.2153)

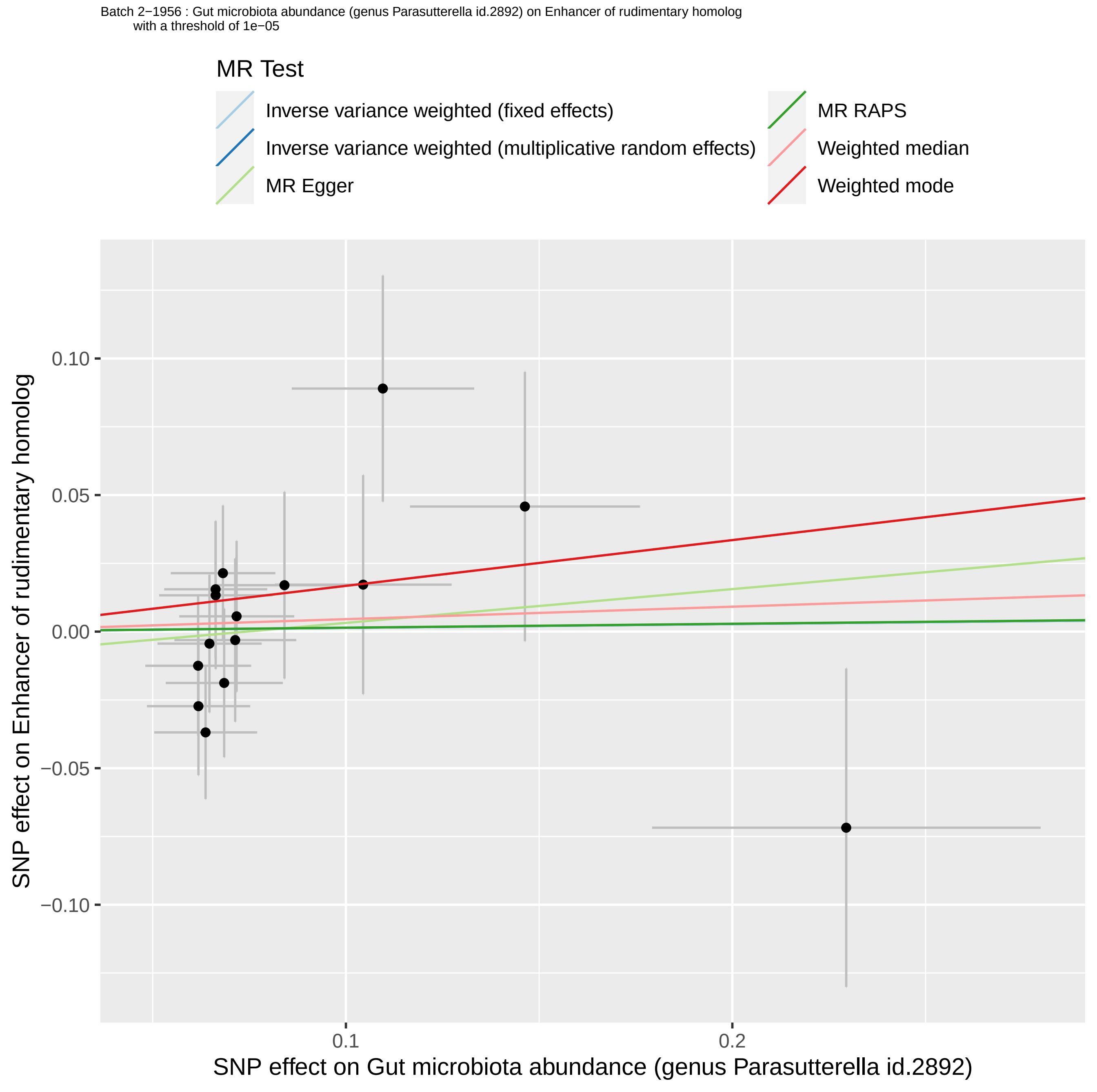

Batch 2−1956 : Gut microbiota abundance (genus Parasutterella id.2892) on Enhancer of rudimentary homolog
with a threshold of 1e−05
MR Test
Inverse variance weighted (fixed effects)
Inverse variance weighted (multiplicative random effects)
MR Egger
MR RAPS
Weighted median
Weighted mode
0.10
0.05
0.00
−0.05
−0.10
0.1
0.2
SNP effect on Enhancer of rudimentary homolog
SNP effect on Gut microbiota abundance (genus Parasutterella id.2892)

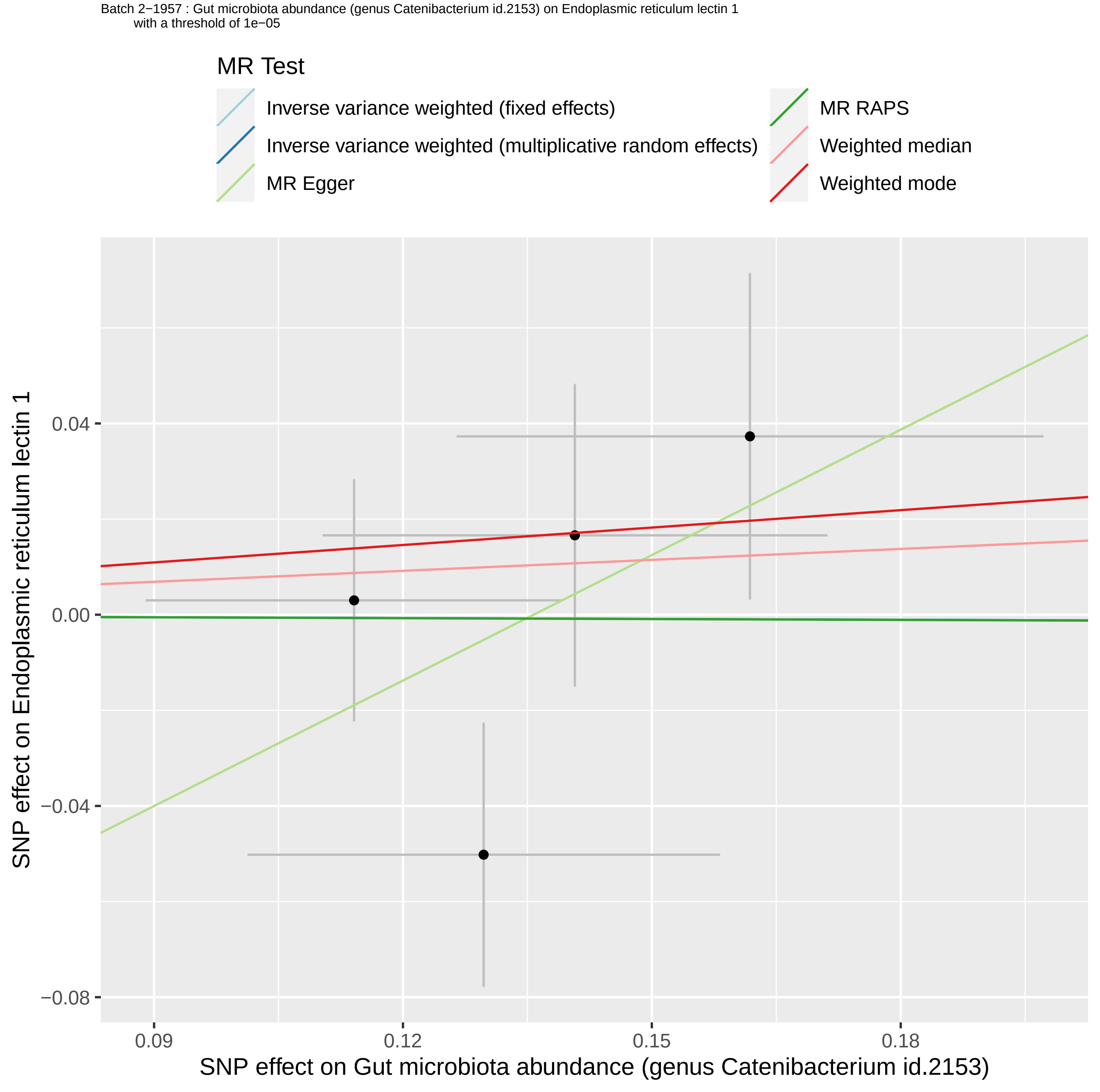

Batch 2−1957 : Gut microbiota abundance (genus Catenibacterium id.2153) on Endoplasmic reticulum lectin 1
with a threshold of 1e−05
MR Test
Inverse variance weighted (fixed effects)
Inverse variance weighted (multiplicative random effects)
MR Egger
MR RAPS
Weighted median
Weighted mode
0.04
0.00
−0.04
−0.08
0.09
0.12
0.15
0.18
SNP effect on Endoplasmic reticulum lectin 1
SNP effect on Gut microbiota abundance (genus Catenibacterium id.2153)

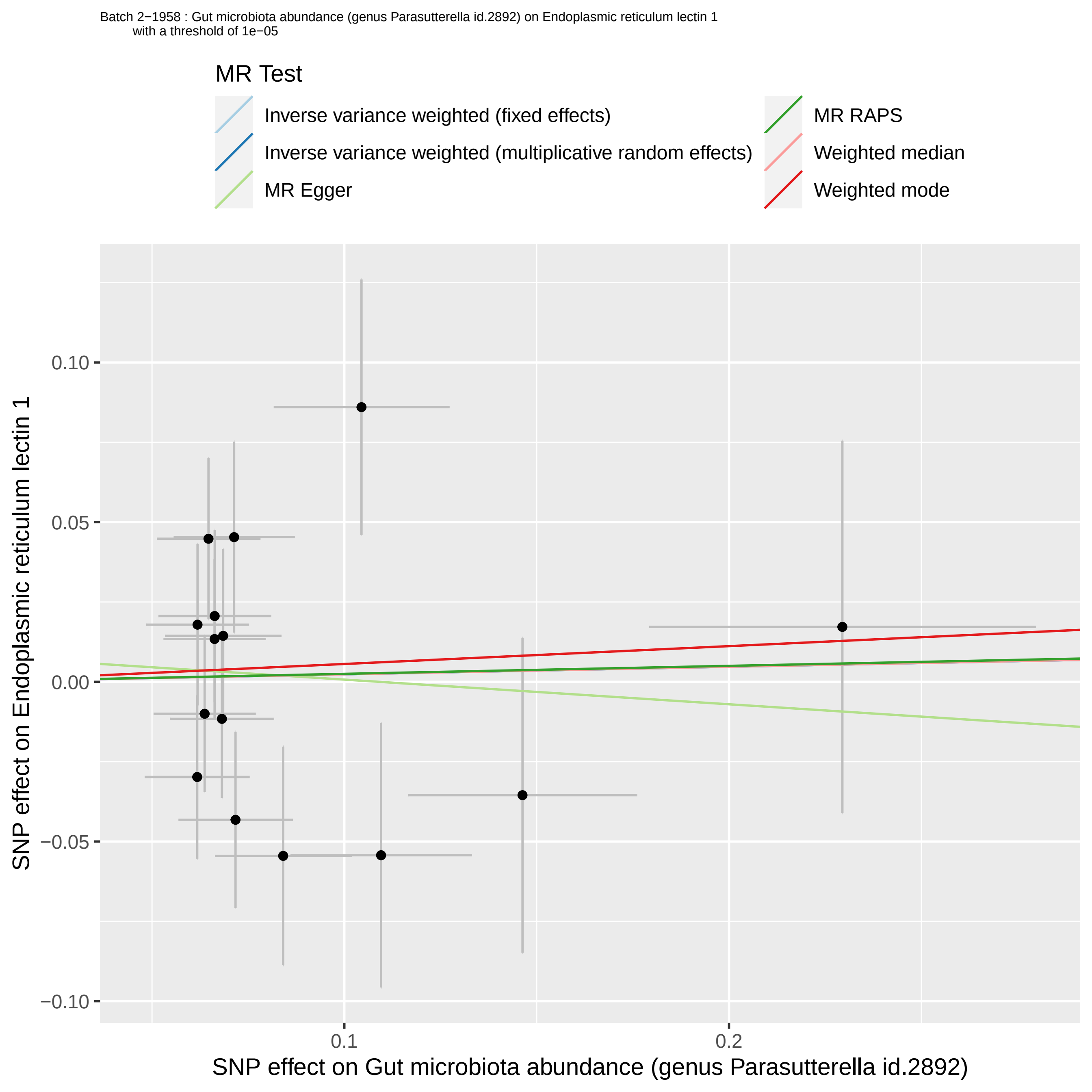
Batch 2−1958 : Gut microbiota abundance (genus Parasutterella id.2892) on Endoplasmic reticulum lectin 1
with a threshold of 1e−05
MR Test
Inverse variance weighted (fixed effects)
Inverse variance weighted (multiplicative random effects)
MR Egger
MR RAPS
Weighted median
Weighted mode
0.10
0.05
0.00
−0.05
−0.10
0.1
0.2
SNP effect on Endoplasmic reticulum lectin 1
SNP effect on Gut microbiota abundance (genus Parasutterella id.2892)

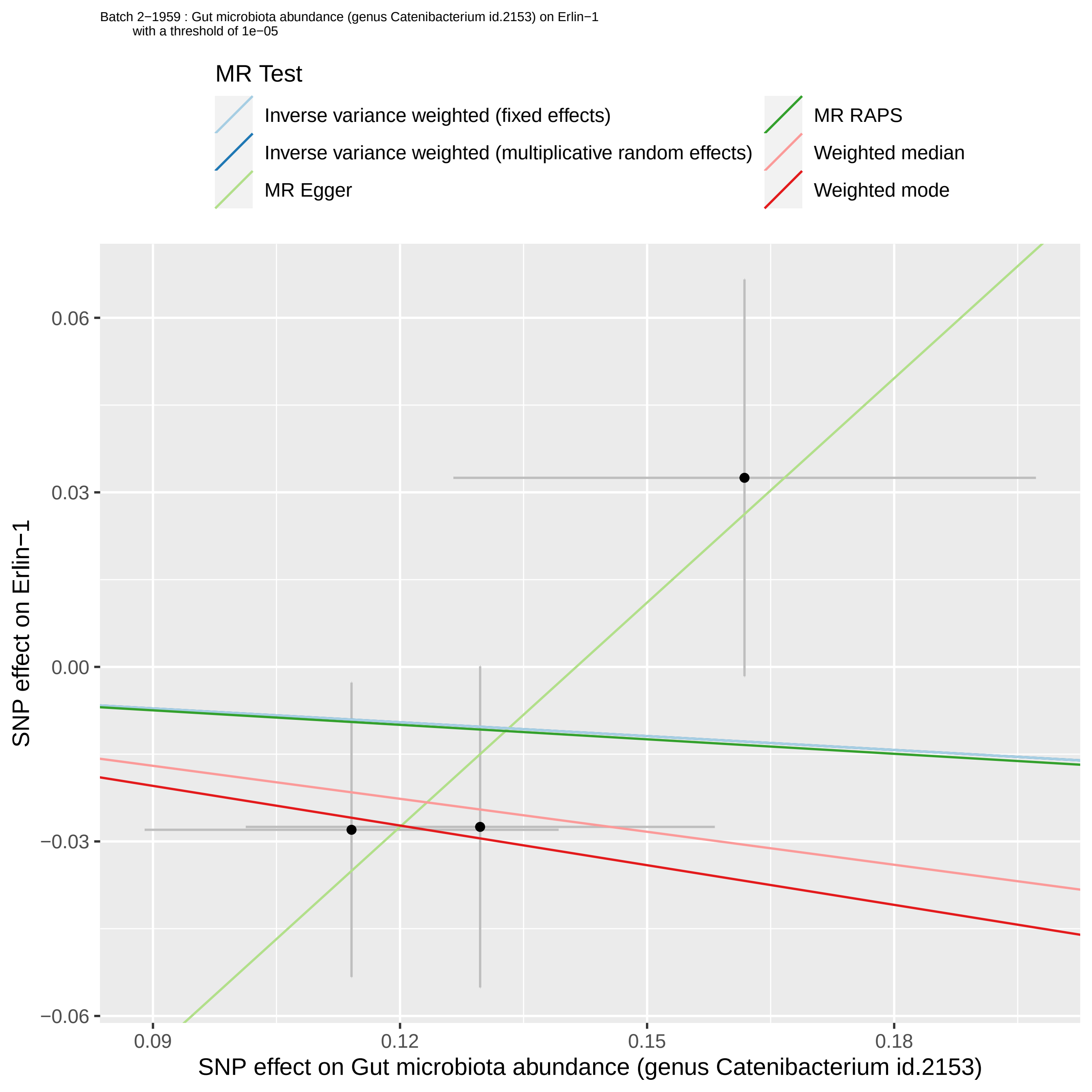
Batch 2−1959 : Gut microbiota abundance (genus Catenibacterium id.2153) on Erlin−1
with a threshold of 1e−05
MR Test
Inverse variance weighted (fixed effects)
Inverse variance weighted (multiplicative random effects)
MR Egger
MR RAPS
Weighted median
Weighted mode
0.06
0.03
0.00
−0.03
−0.06
SNP effect on Erlin−1
0.09
0.12
0.15
0.18
SNP effect on Gut microbiota abundance (genus Catenibacterium id.2153)

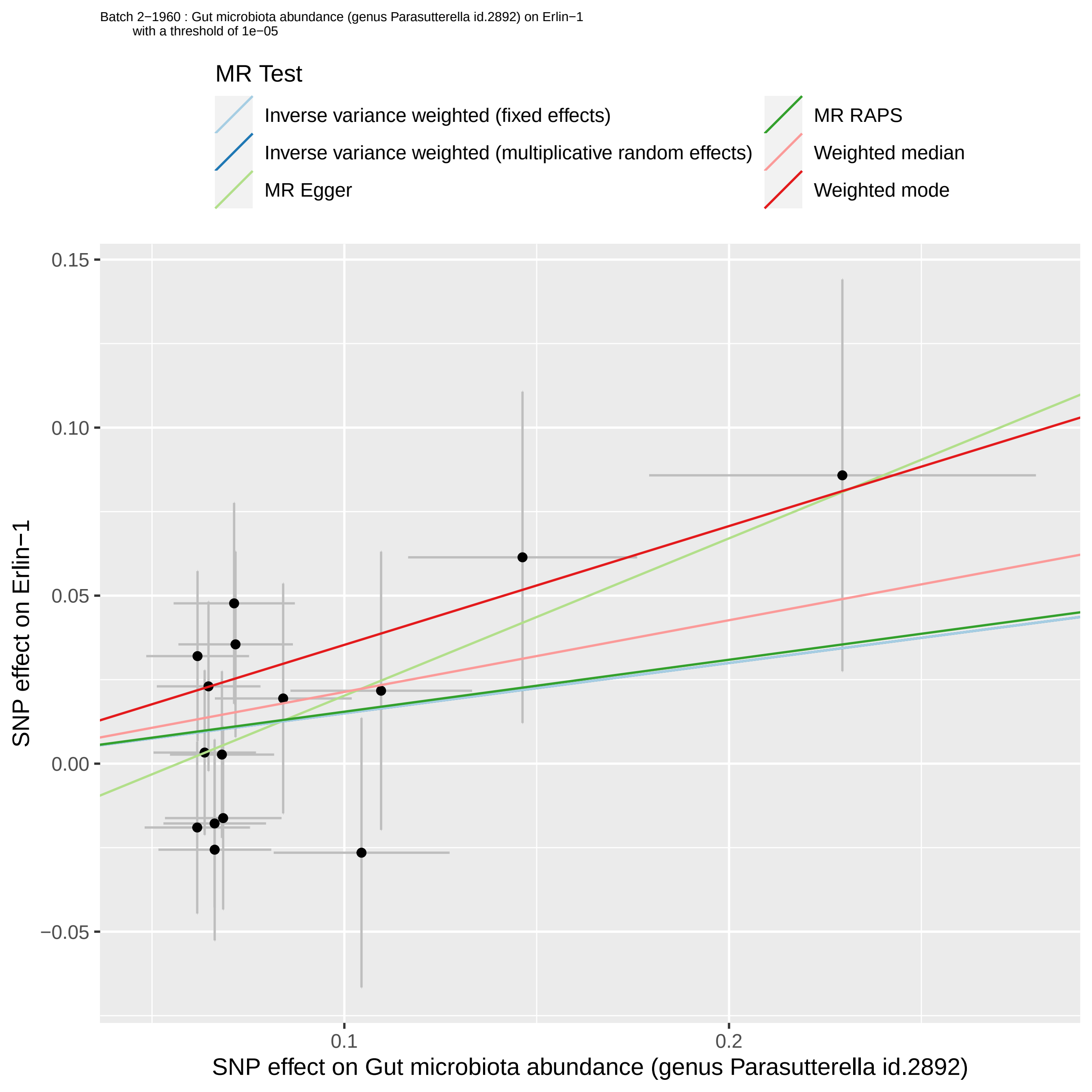
Batch 2−1960 : Gut microbiota abundance (genus Parasutterella id.2892) on Erlin−1
with a threshold of 1e−05
MR Test
Inverse variance weighted (fixed effects)
Inverse variance weighted (multiplicative random effects)
MR Egger
MR RAPS
Weighted median
Weighted mode
0.15
0.10
0.05
0.00
−0.05
0.1
0.2
SNP effect on Erlin−1
SNP effect on Gut microbiota abundance (genus Parasutterella id.2892)

Batch 2−1961 : Gut microbiota abundance (genus Catenibacterium id.2153) on Erythroid membrane−associated protein with a threshold of 1e−05

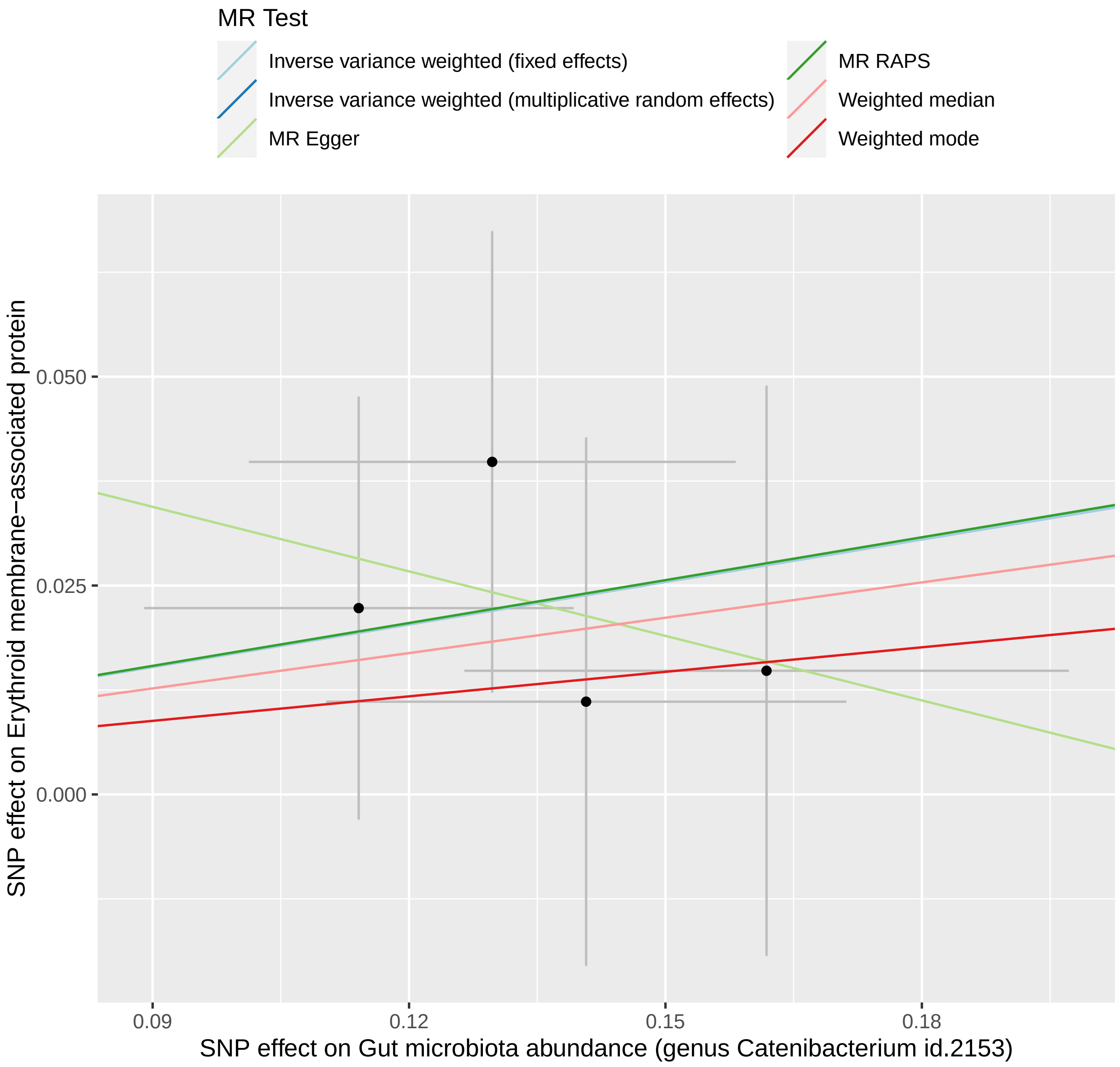

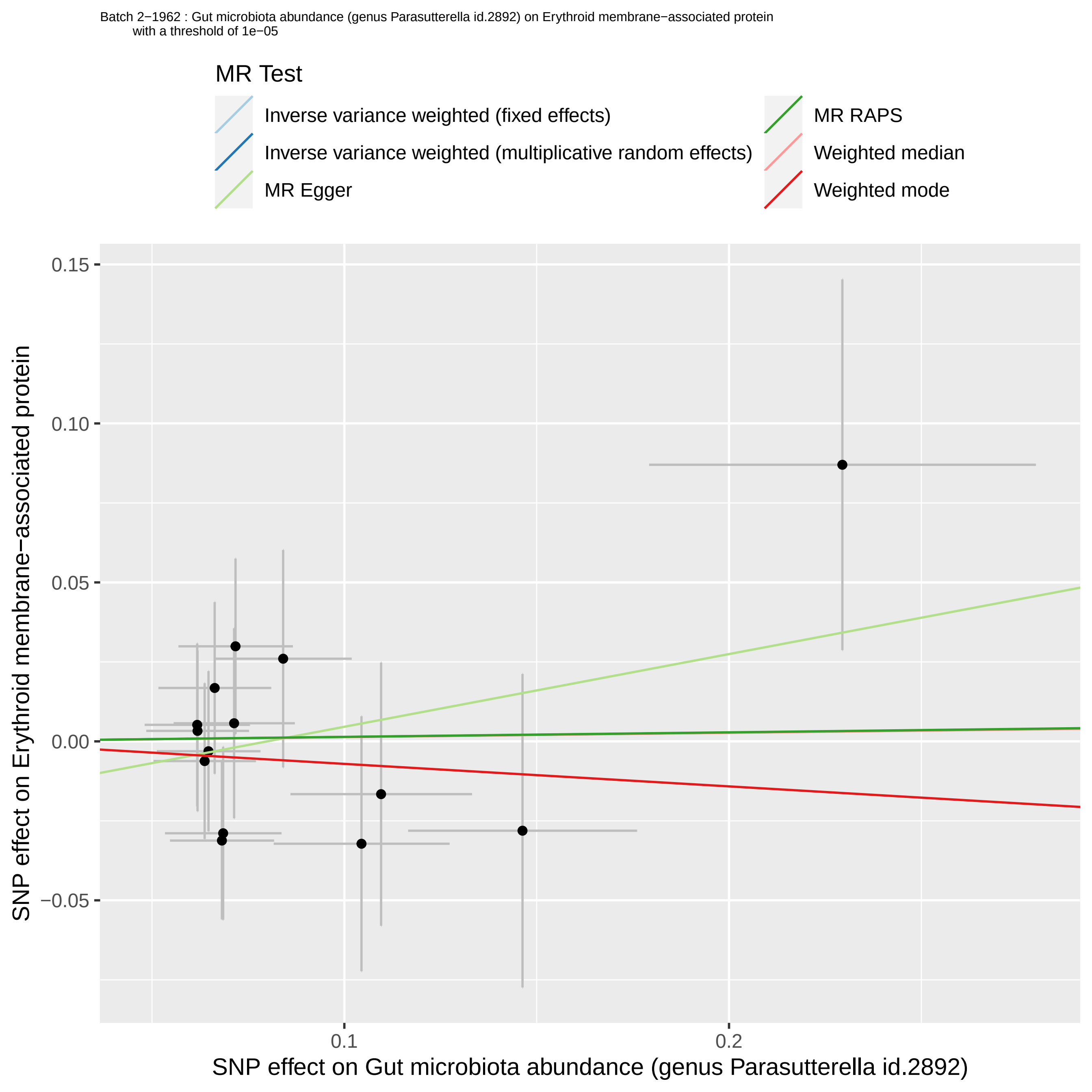
Batch 2−1962 : Gut microbiota abundance (genus Parasutterella id.2892) on Erythroid membrane−associated protein
with a threshold of 1e−05
MR Test
Inverse variance weighted (fixed effects)
Inverse variance weighted (multiplicative random effects)
MR Egger
MR RAPS
Weighted median
Weighted mode
0.15
0.10
0.05
0.00
−0.05
0.1
0.2
SNP effect on Erythroid membrane−associated protein
SNP effect on Gut microbiota abundance (genus Parasutterella id.2892)

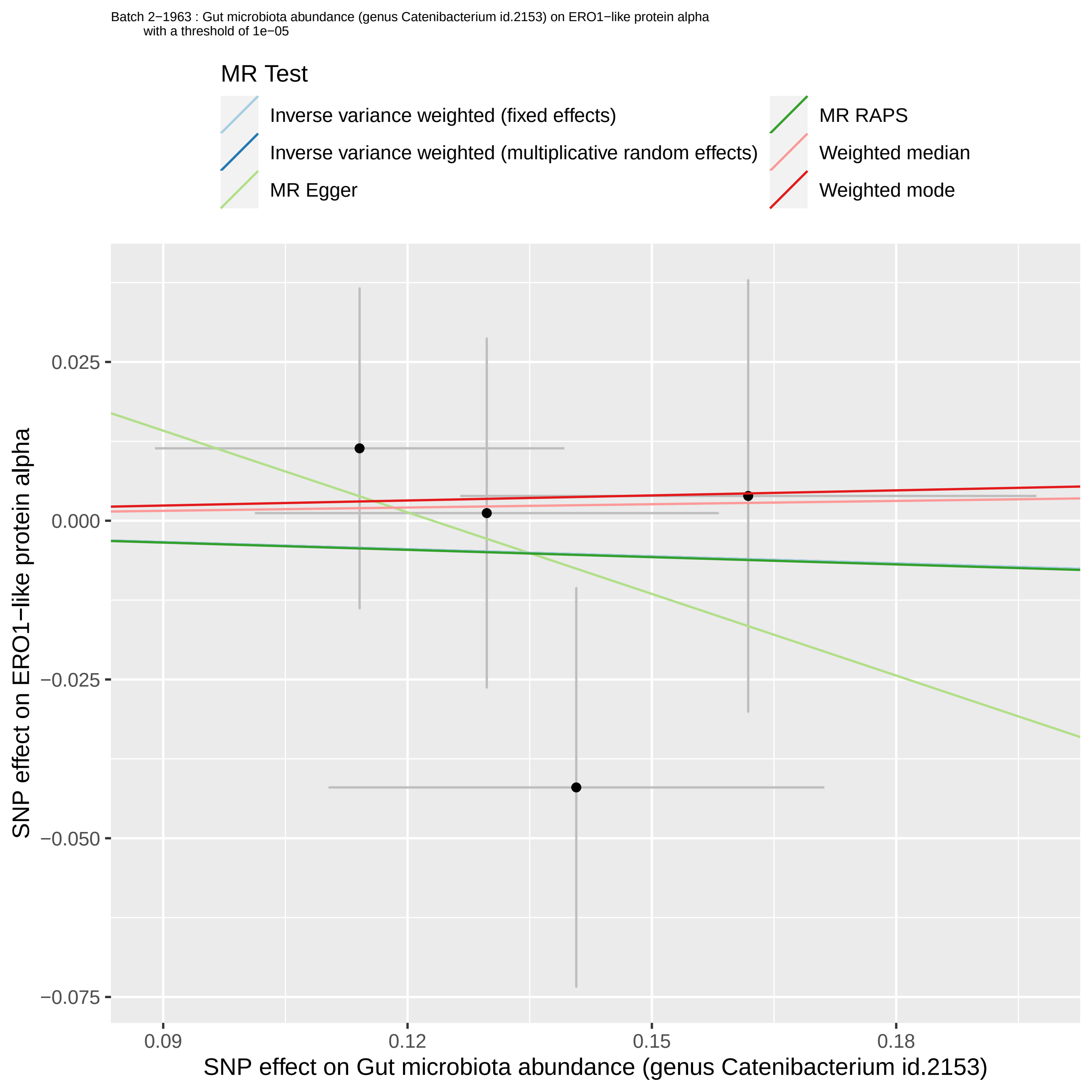
Batch 2−1963 : Gut microbiota abundance (genus Catenibacterium id.2153) on ERO1−like protein alpha
with a threshold of 1e−05
MR Test
Inverse variance weighted (fixed effects)
Inverse variance weighted (multiplicative random effects)
MR Egger
MR RAPS
Weighted median
Weighted mode
0.025
0.000
−0.025
−0.050
−0.075
0.09
0.12
0.15
0.18
SNP effect on ERO1−like protein alpha
SNP effect on Gut microbiota abundance (genus Catenibacterium id.2153)

Batch 2−1964 : Gut microbiota abundance (genus Parasutterella id.2892) on ERO1−like protein alpha
with a threshold of 1e−05

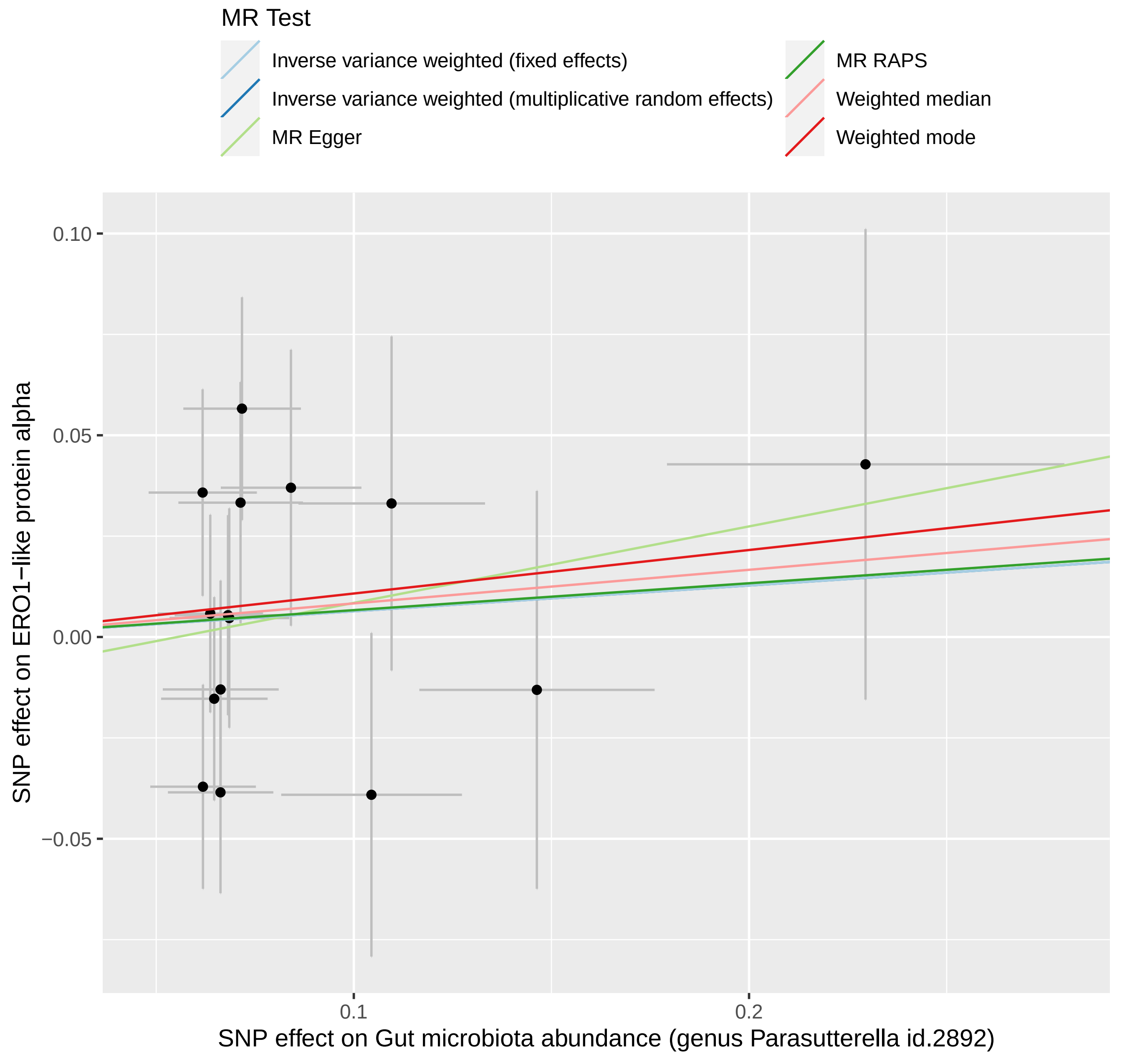

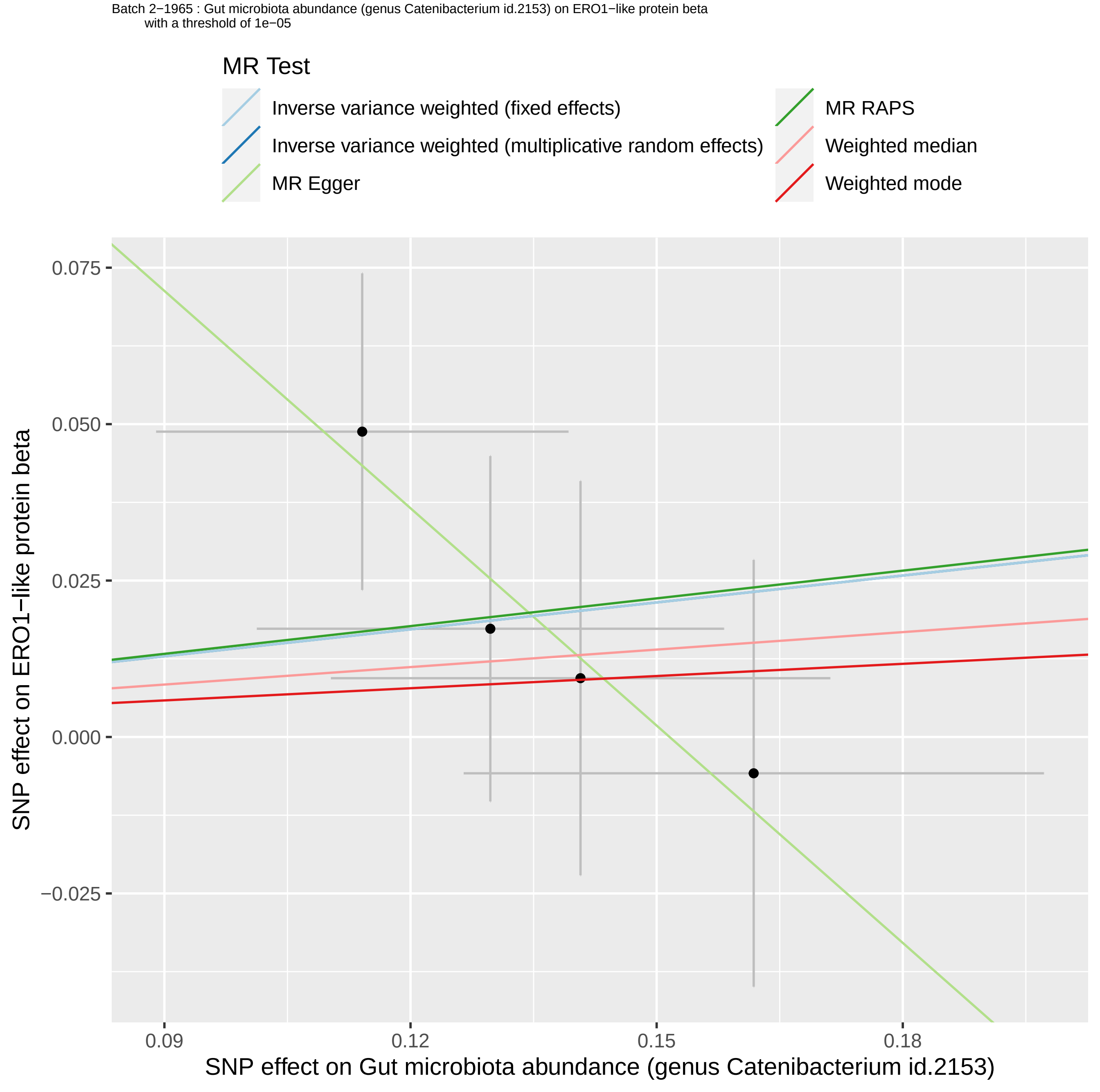
Batch 2−1965 : Gut microbiota abundance (genus Catenibacterium id.2153) on ERO1−like protein beta
with a threshold of 1e−05
MR Test
Inverse variance weighted (fixed effects)
Inverse variance weighted (multiplicative random effects)
MR Egger
MR RAPS
Weighted median
Weighted mode
0.075
0.050
0.025
0.000
−0.025
SNP effect on ERO1−like protein beta
0.09
0.12
0.15
0.18
SNP effect on Gut microbiota abundance (genus Catenibacterium id.2153)

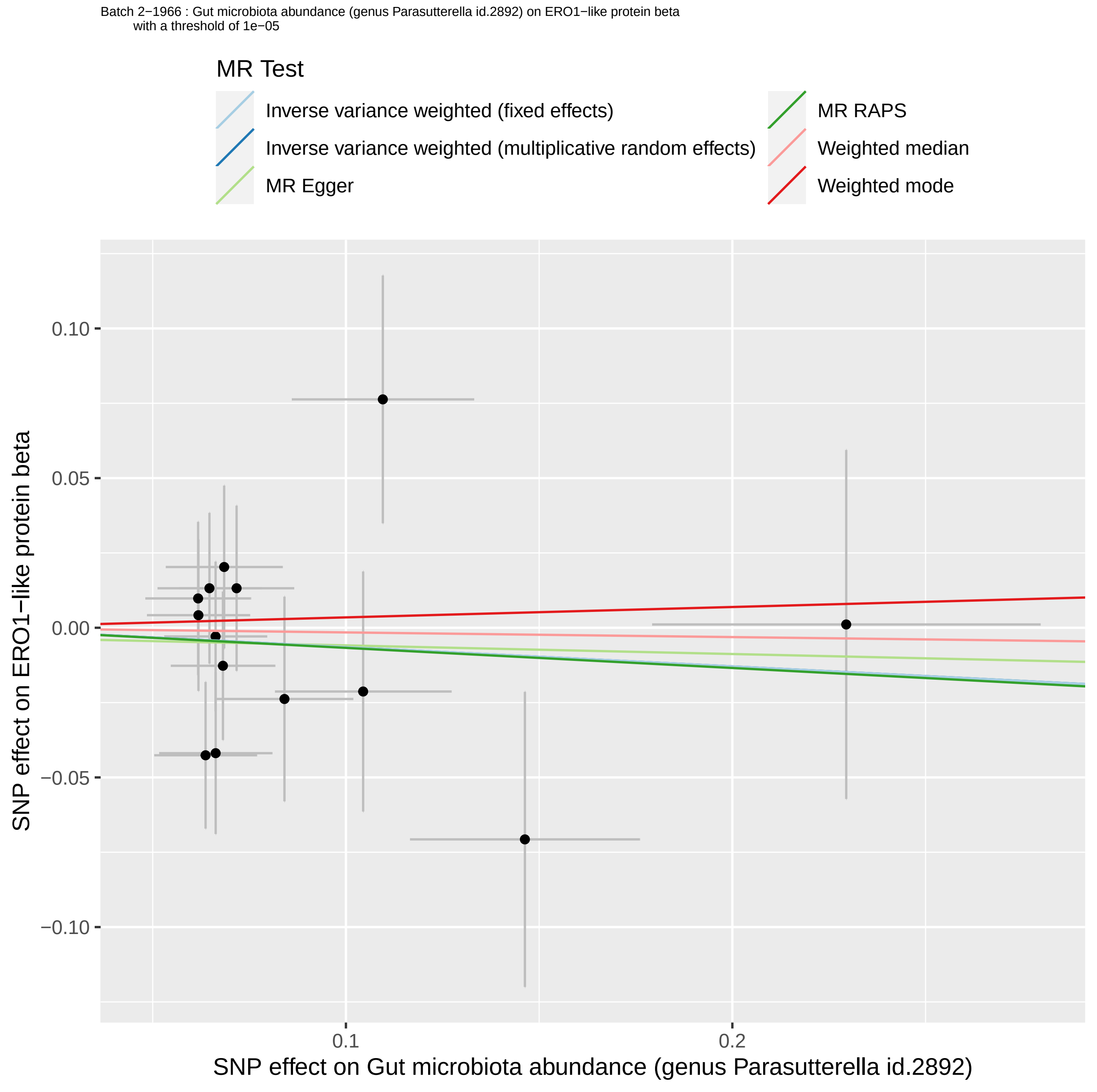
Batch 2−1966 : Gut microbiota abundance (genus Parasutterella id.2892) on ERO1−like protein beta
with a threshold of 1e−05
MR Test
Inverse variance weighted (fixed effects)
Inverse variance weighted (multiplicative random effects)
MR Egger
MR RAPS
Weighted median
Weighted mode
0.10
0.05
0.00
−0.05
−0.10
0.1
0.2
SNP effect on ERO1−like protein beta
SNP effect on Gut microbiota abundance (genus Parasutterella id.2892)

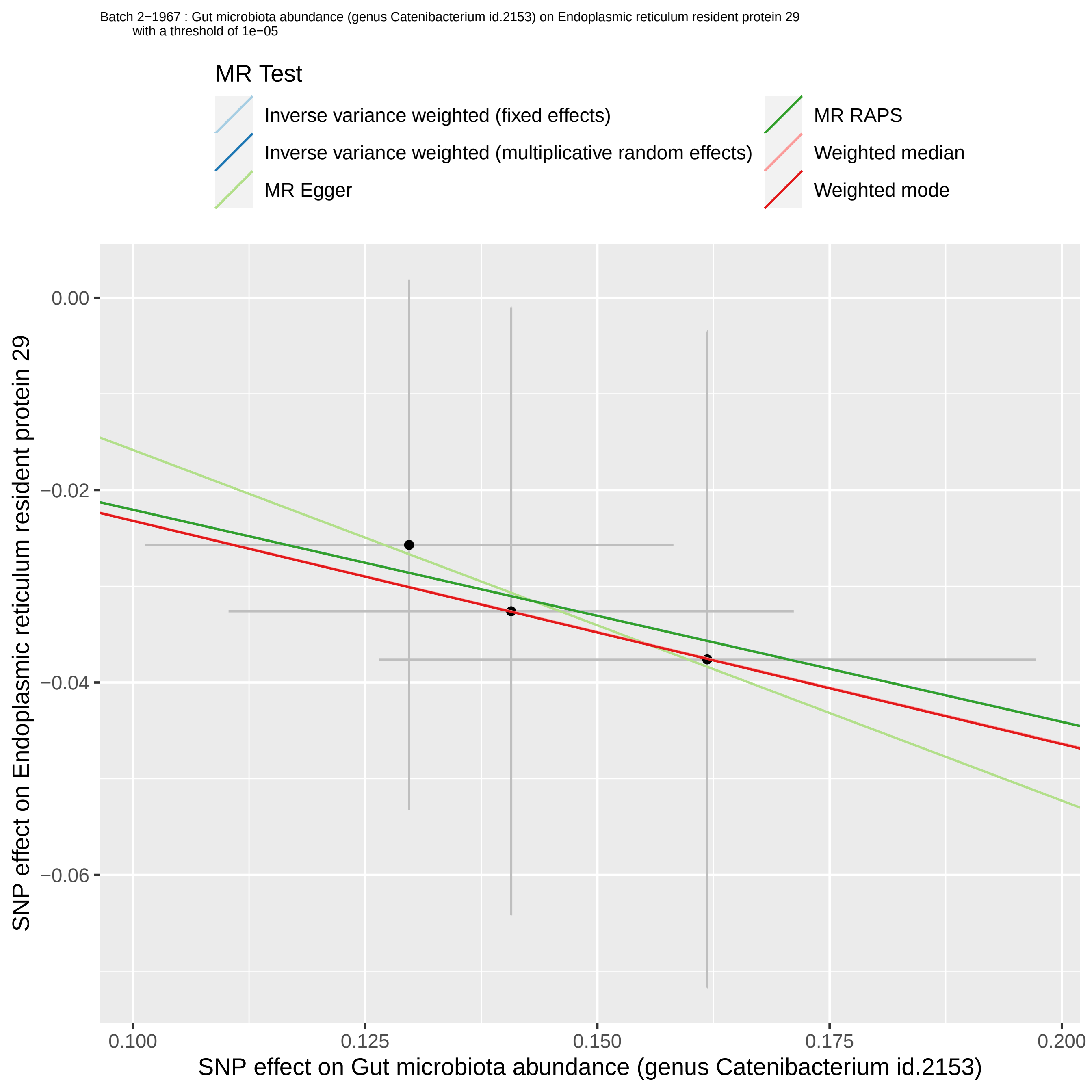
Batch 2−1967 : Gut microbiota abundance (genus Catenibacterium id.2153) on Endoplasmic reticulum resident protein 29
with a threshold of 1e−05
MR Test
Inverse variance weighted (fixed effects)
Inverse variance weighted (multiplicative random effects)
MR Egger
MR RAPS
Weighted median
Weighted mode
0.00
−0.02
−0.04
−0.06
0.100
0.125
0.150
0.175
0.200
SNP effect on Endoplasmic reticulum resident protein 29
SNP effect on Gut microbiota abundance (genus Catenibacterium id.2153)

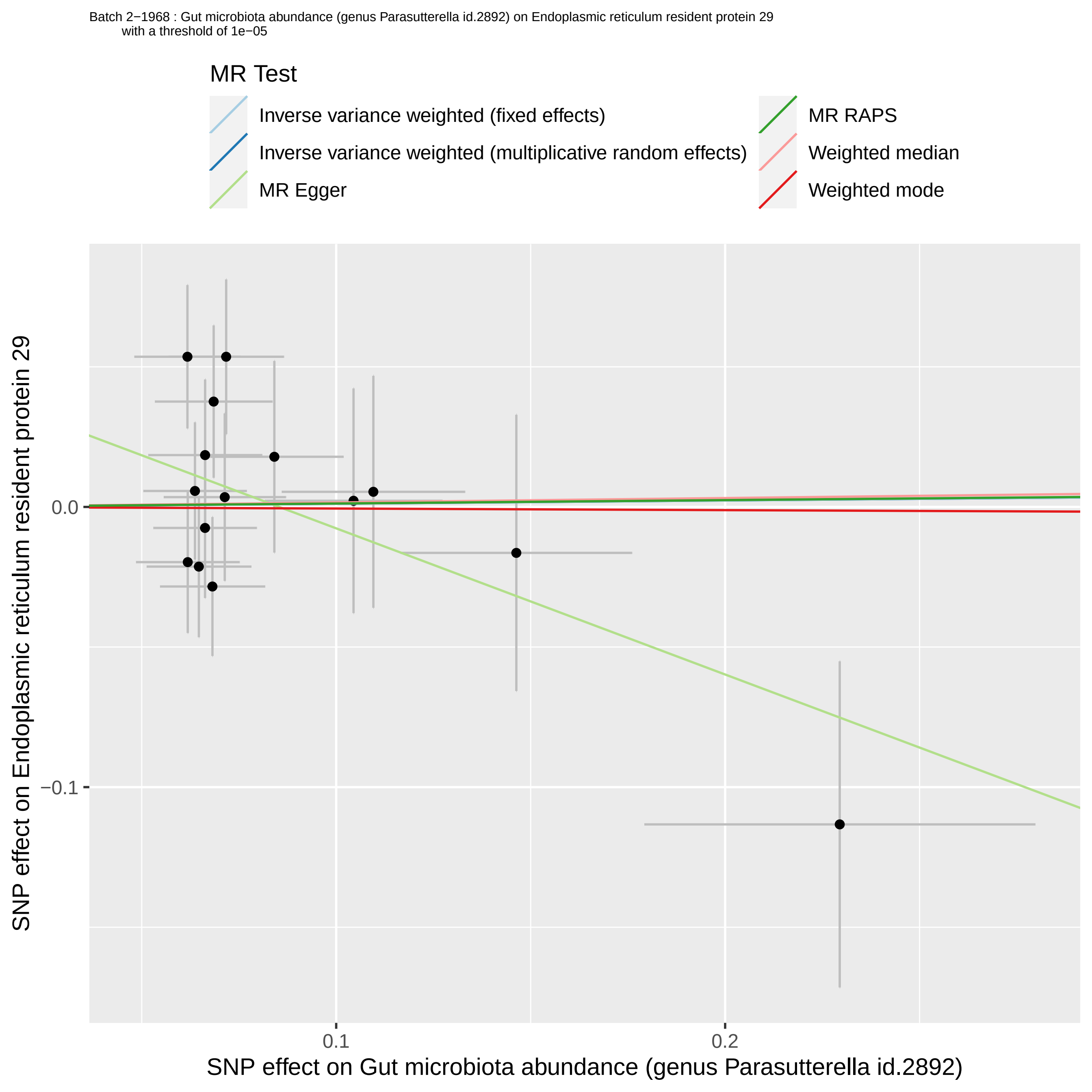
Batch 2−1968 : Gut microbiota abundance (genus Parasutterella id.2892) on Endoplasmic reticulum resident protein 29
with a threshold of 1e−05
MR Test
Inverse variance weighted (fixed effects)
Inverse variance weighted (multiplicative random effects)
MR Egger
MR RAPS
Weighted median
Weighted mode
SNP effect on Endoplasmic reticulum resident protein 29
0.0
−0.1
0.1
0.2
SNP effect on Gut microbiota abundance (genus Parasutterella id.2892)

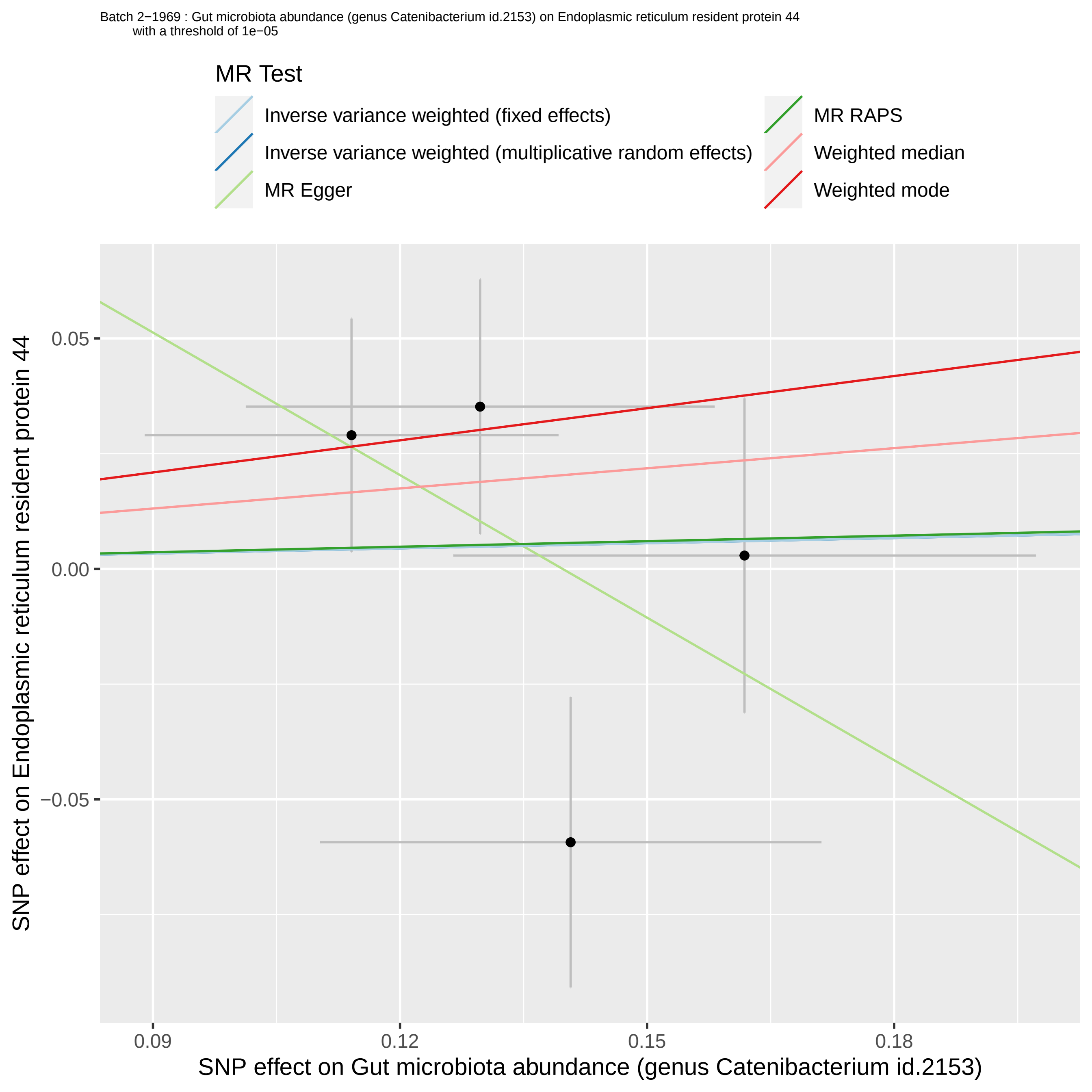

Batch 2−1969 : Gut microbiota abundance (genus Catenibacterium id.2153) on Endoplasmic reticulum resident protein 44
with a threshold of 1e−05
MR Test
Inverse variance weighted (fixed effects)
Inverse variance weighted (multiplicative random effects)
MR Egger
MR RAPS
Weighted median
Weighted mode
0.05
0.00
−0.05
0.09
0.12
0.15
0.18
SNP effect on Endoplasmic reticulum resident protein 44
SNP effect on Gut microbiota abundance (genus Catenibacterium id.2153)

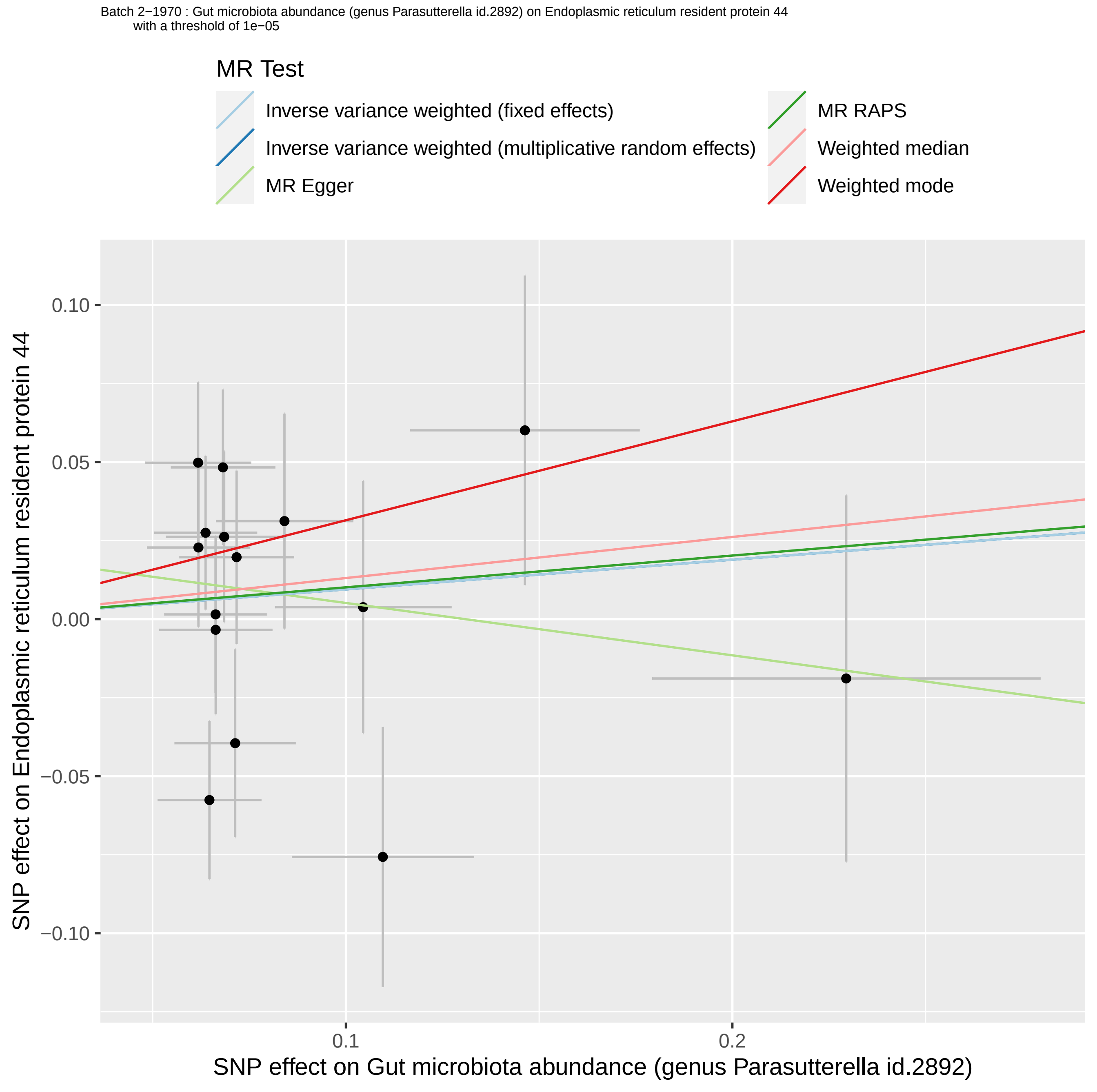

Batch 2−1970 : Gut microbiota abundance (genus Parasutterella id.2892) on Endoplasmic reticulum resident protein 44
with a threshold of 1e−05
MR Test
Inverse variance weighted (fixed effects)
Inverse variance weighted (multiplicative random effects)
MR Egger
MR RAPS
Weighted median
Weighted mode
SNP effect on Endoplasmic reticulum resident protein 44
0.10
0.05
0.00
−0.05
−0.10
0.1
0.2
SNP effect on Gut microbiota abundance (genus Parasutterella id.2892)

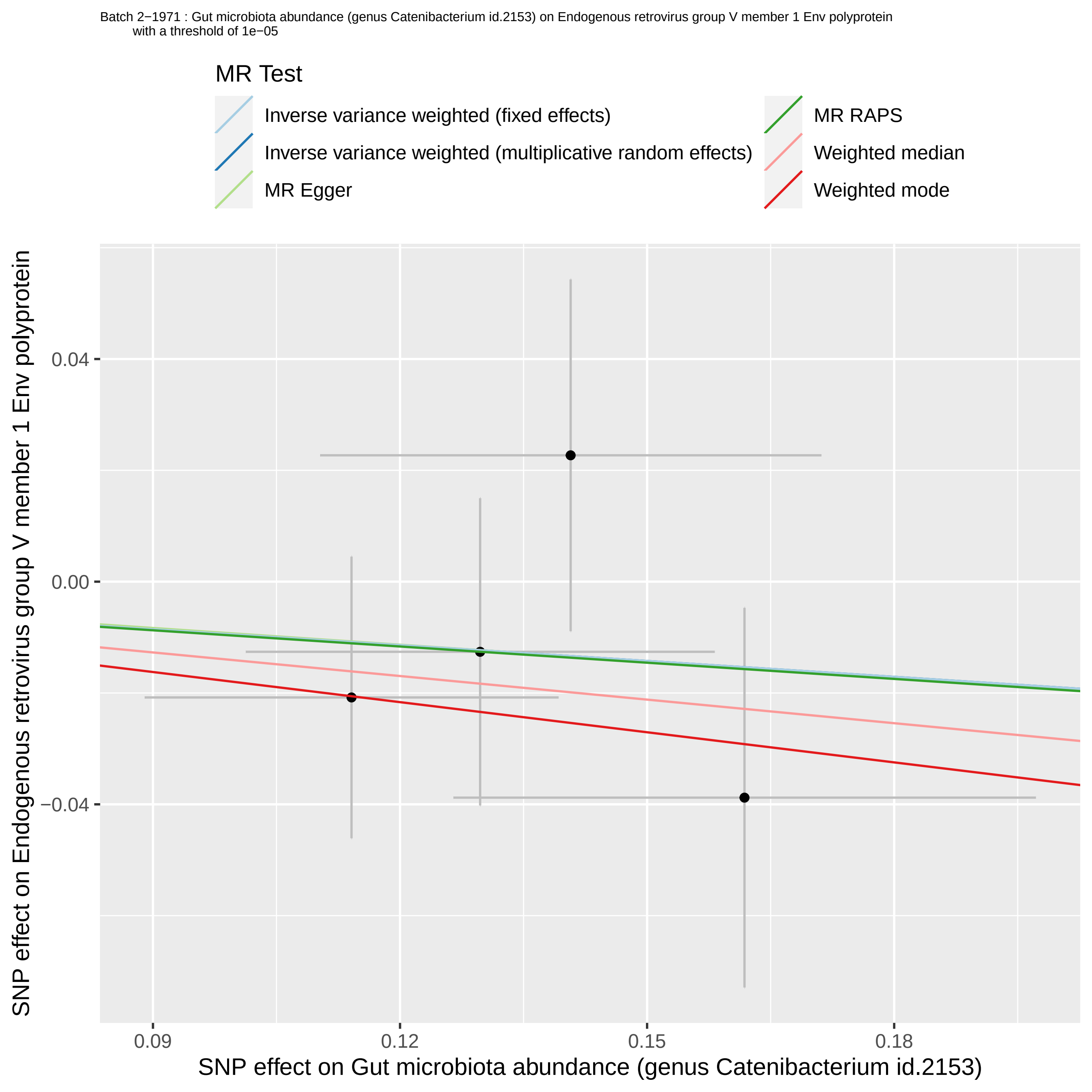
Batch 2−1971 : Gut microbiota abundance (genus Catenibacterium id.2153) on Endogenous retrovirus group V member 1 Env polyprotein
with a threshold of 1e−05
MR Test
Inverse variance weighted (fixed effects)
Inverse variance weighted (multiplicative random effects)
MR Egger
MR RAPS
Weighted median
Weighted mode
0.04
0.00
−0.04
0.09
0.12
0.15
0.18
SNP effect on Endogenous retrovirus group V member 1 Env polyprotein
SNP effect on Gut microbiota abundance (genus Catenibacterium id.2153)

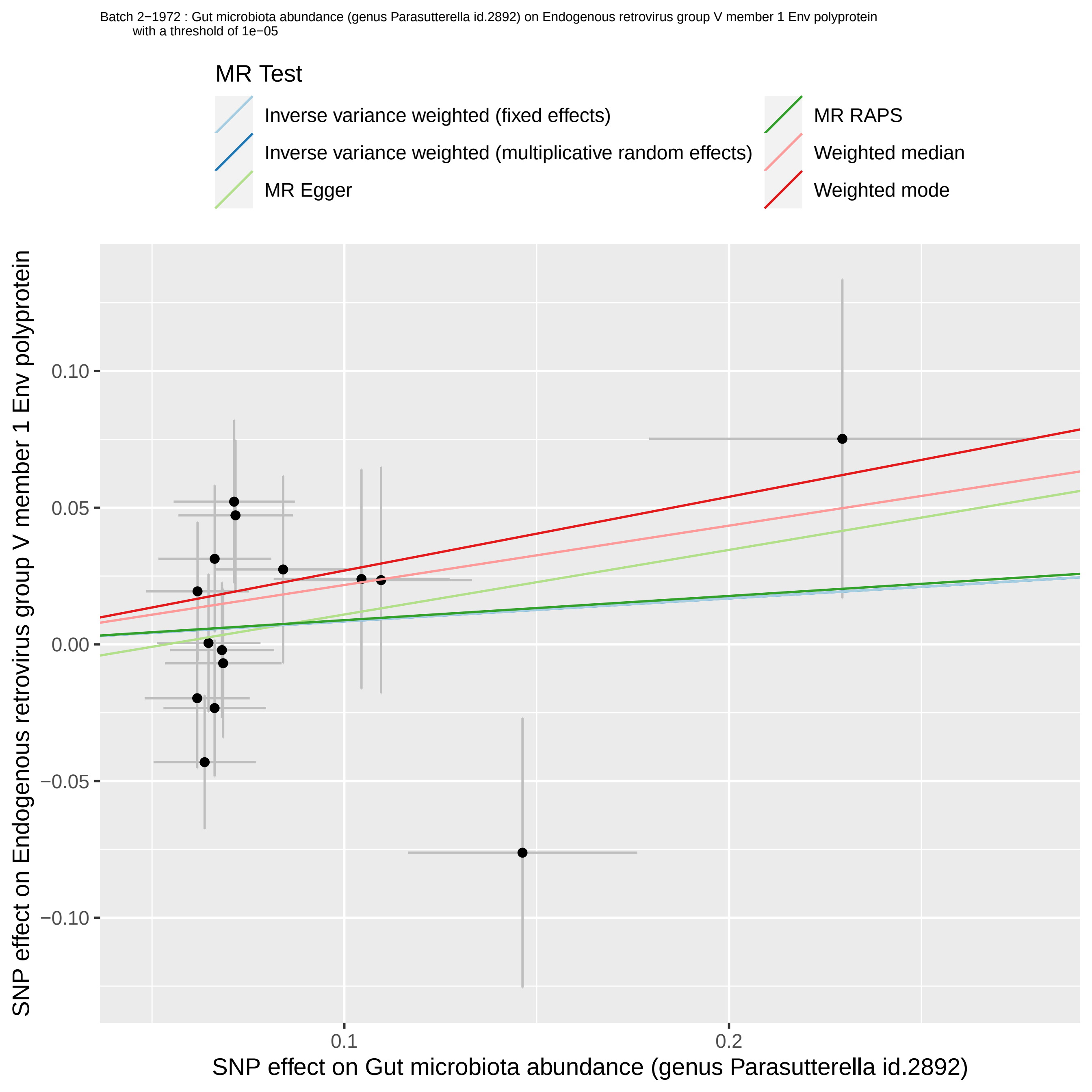
Batch 2−1972 : Gut microbiota abundance (genus Parasutterella id.2892) on Endogenous retrovirus group V member 1 Env polyprotein
with a threshold of 1e−05
MR Test
Inverse variance weighted (fixed effects)
Inverse variance weighted (multiplicative random effects)
MR Egger
MR RAPS
Weighted median
Weighted mode
SNP effect on Endogenous retrovirus group V member 1 Env polyprotein
0.10
0.05
0.00
−0.05
−0.10
0.1
0.2
SNP effect on Gut microbiota abundance (genus Parasutterella id.2892)

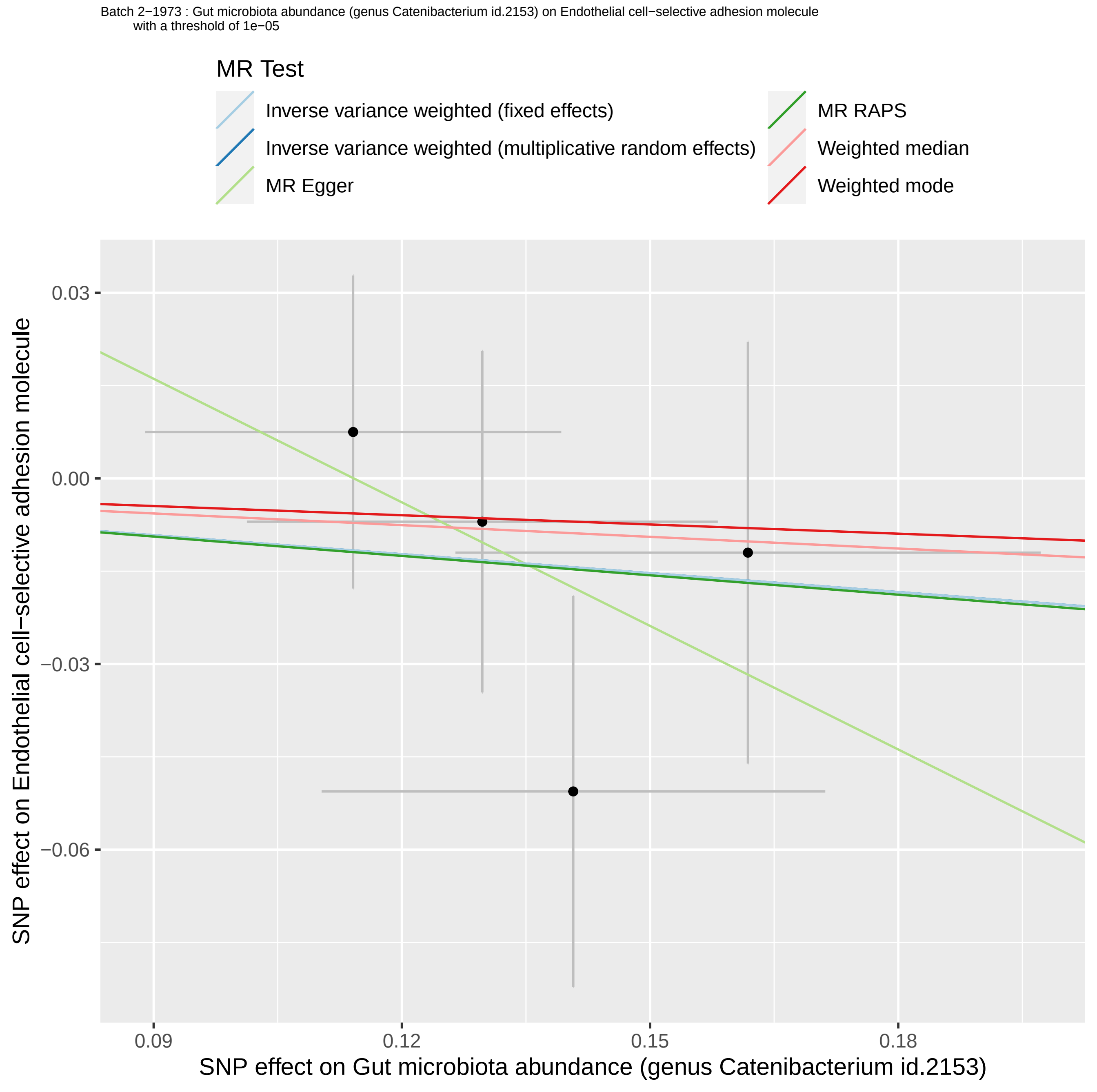

Batch 2−1973 : Gut microbiota abundance (genus Catenibacterium id.2153) on Endothelial cell−selective adhesion molecule with a threshold of 1e−05
MR Test
Inverse variance weighted (fixed effects)
Inverse variance weighted (multiplicative random effects)
MR Egger
MR RAPS
Weighted median
Weighted mode
0.03
0.00
−0.03
−0.06
0.09
0.12
0.15
0.18
SNP effect on Endothelial cell−selective adhesion molecule
SNP effect on Gut microbiota abundance (genus Catenibacterium id.2153)

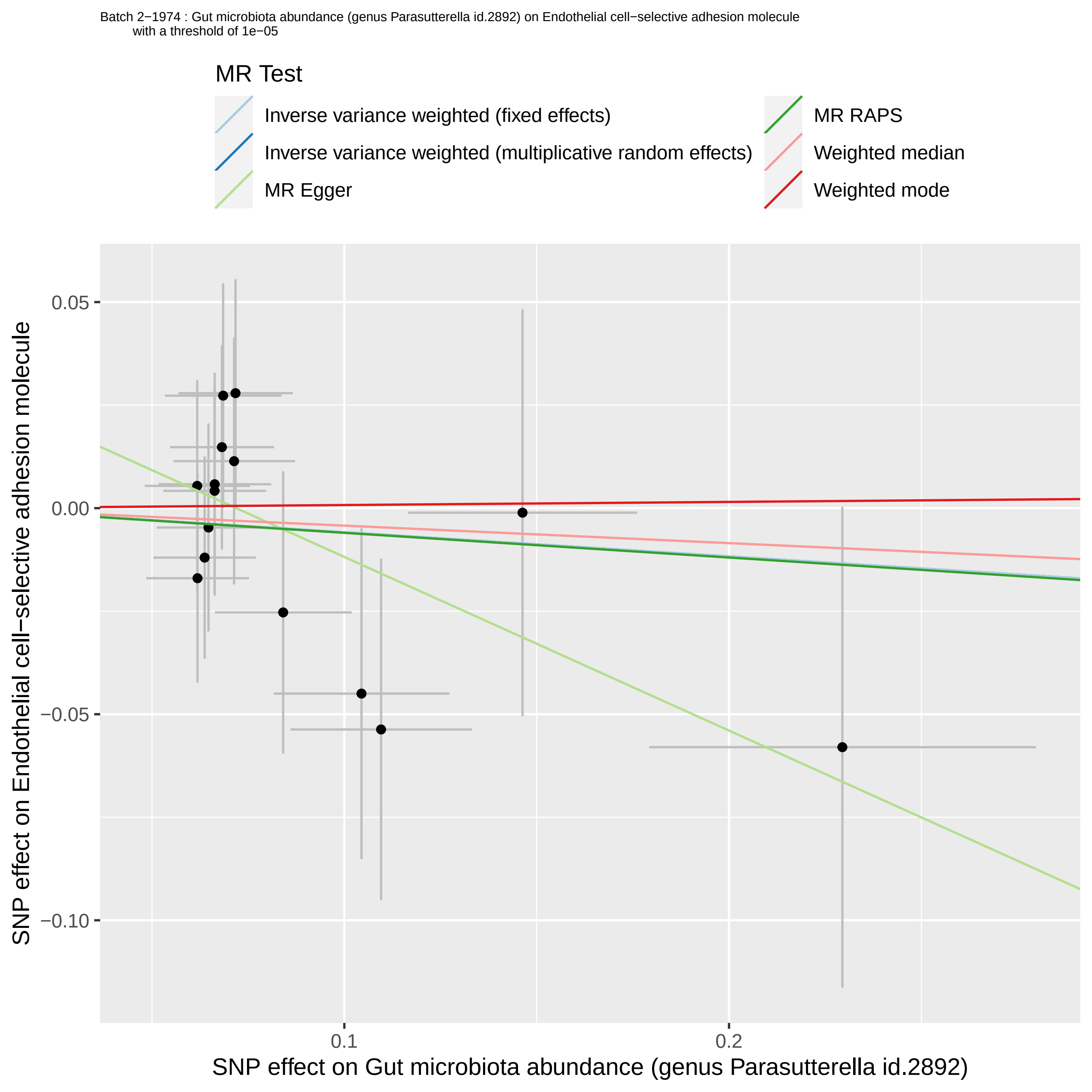

Batch 2−1974 : Gut microbiota abundance (genus Parasutterella id.2892) on Endothelial cell−selective adhesion molecule
with a threshold of 1e−05
MR Test
Inverse variance weighted (fixed effects)
Inverse variance weighted (multiplicative random effects)
MR Egger
MR RAPS
Weighted median
Weighted mode
0.05
0.00
−0.05
−0.10
0.1
0.2
SNP effect on Endothelial cell−selective adhesion molecule
SNP effect on Gut microbiota abundance (genus Parasutterella id.2892)

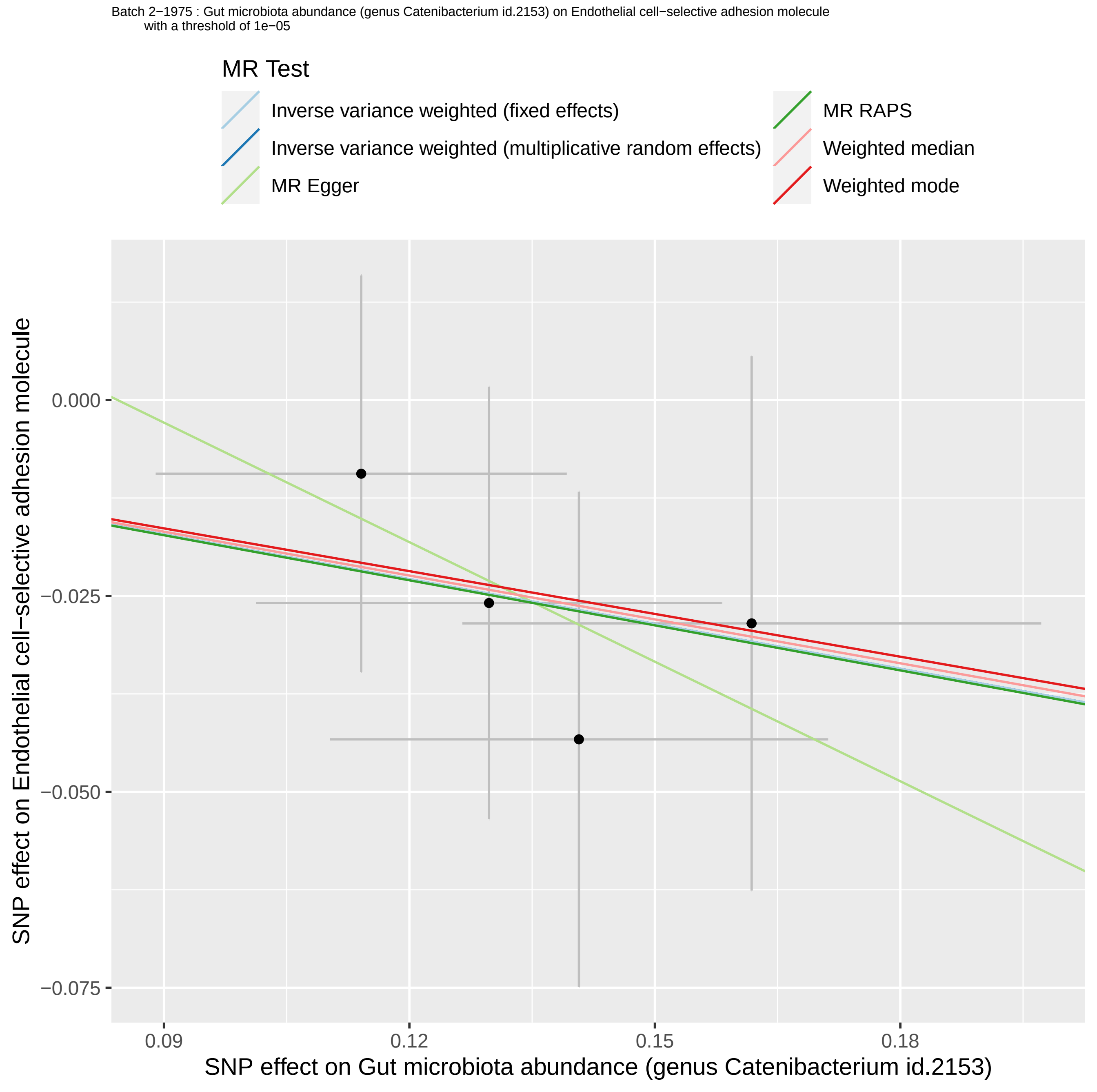

Batch 2−1975 : Gut microbiota abundance (genus Catenibacterium id.2153) on Endothelial cell−selective adhesion molecule
with a threshold of 1e−05
MR Test
Inverse variance weighted (fixed effects)
Inverse variance weighted (multiplicative random effects)
MR Egger
MR RAPS
Weighted median
Weighted mode
0.000
−0.025
−0.050
−0.075
0.09
0.12
0.15
0.18
SNP effect on Endothelial cell−selective adhesion molecule
SNP effect on Gut microbiota abundance (genus Catenibacterium id.2153)

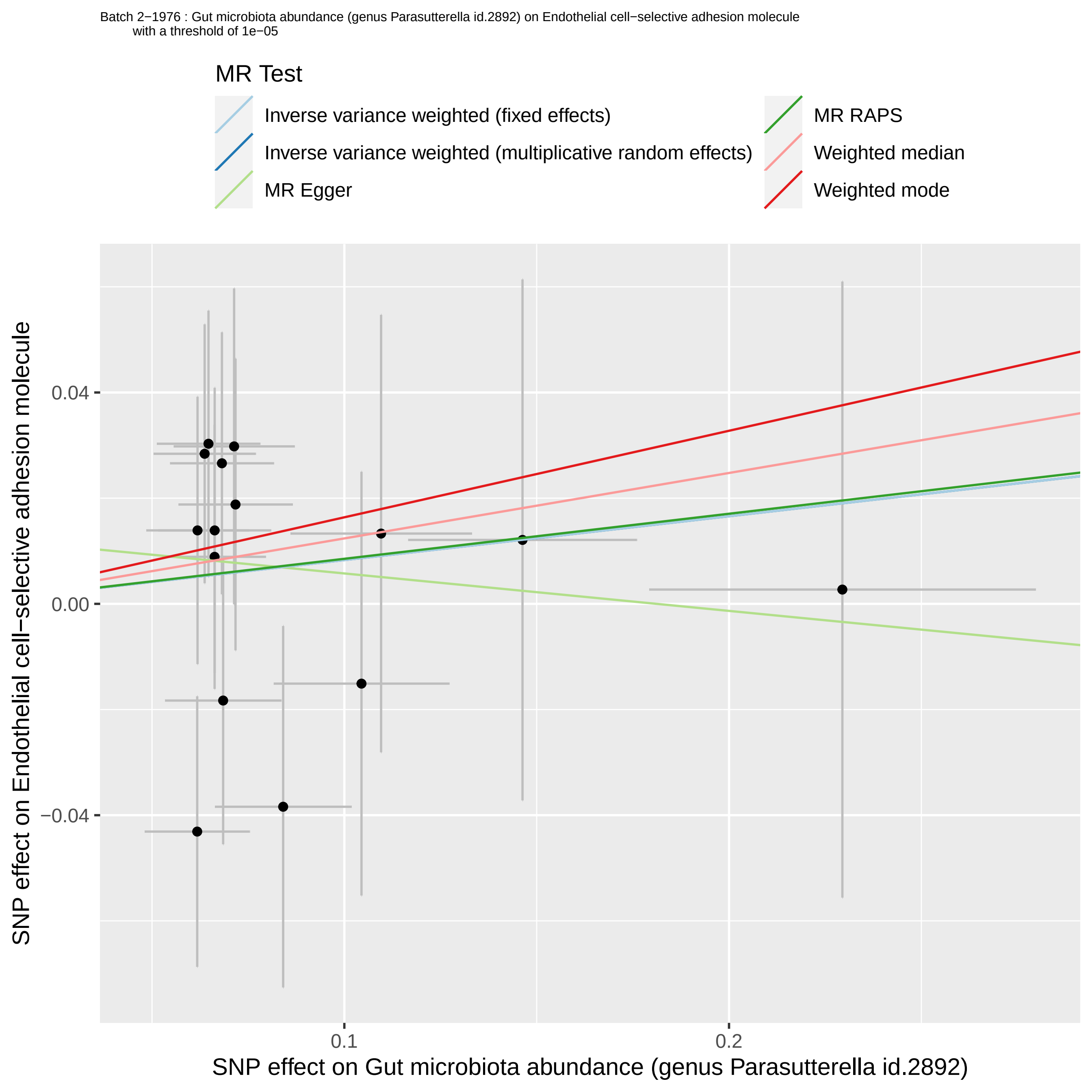
Batch 2−1976 : Gut microbiota abundance (genus Parasutterella id.2892) on Endothelial cell−selective adhesion molecule
with a threshold of 1e−05
MR Test
Inverse variance weighted (fixed effects)
Inverse variance weighted (multiplicative random effects)
MR Egger
MR RAPS
Weighted median
Weighted mode
0.04
0.00
−0.04
0.1
0.2
SNP effect on Endothelial cell−selective adhesion molecule
SNP effect on Gut microbiota abundance (genus Parasutterella id.2892)

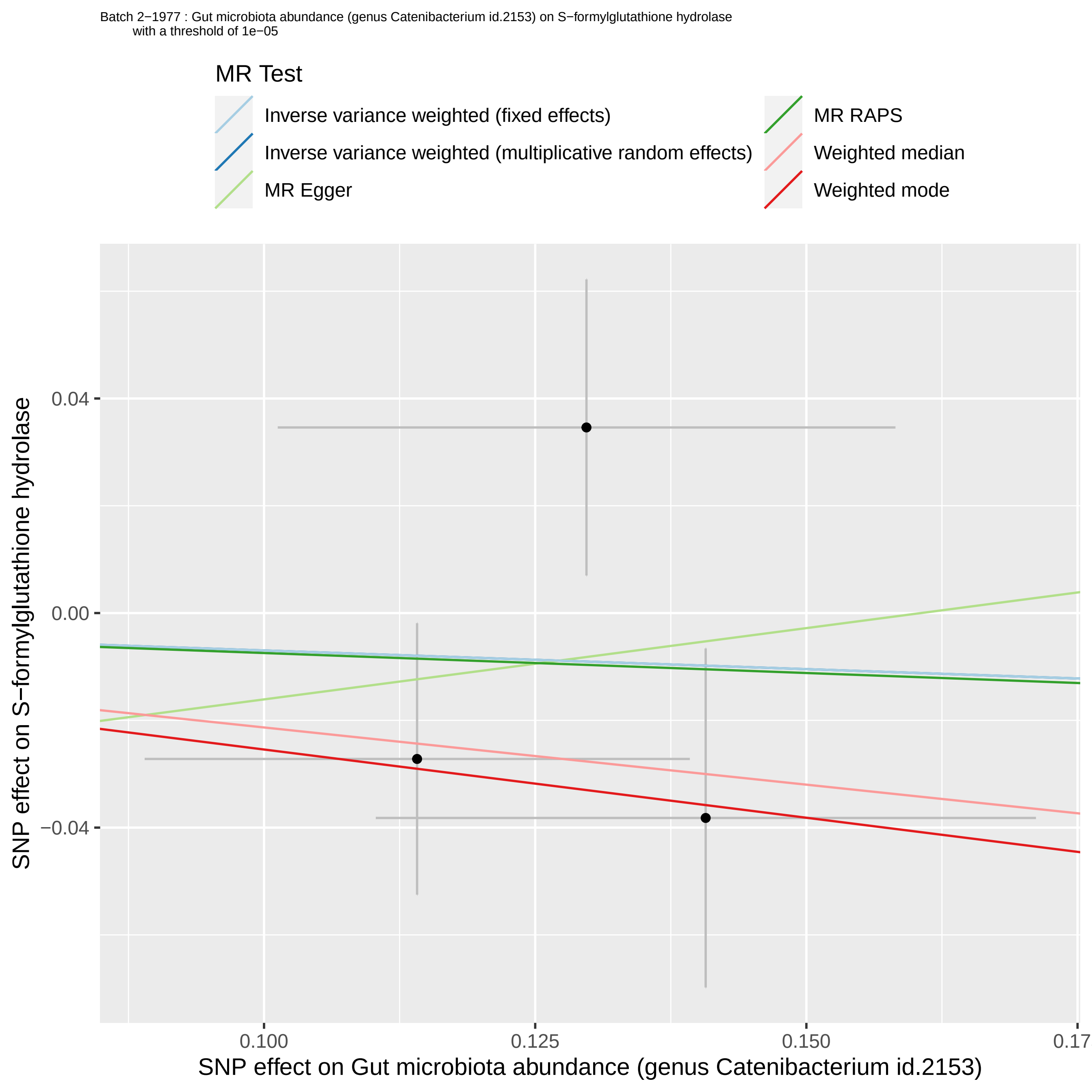

Batch 2−1977 : Gut microbiota abundance (genus Catenibacterium id.2153) on S−formylglutathione hydrolase
with a threshold of 1e−05
MR Test
Inverse variance weighted (fixed effects)
Inverse variance weighted (multiplicative random effects)
MR Egger
MR RAPS
Weighted median
Weighted mode
0.04
0.00
−0.04
0.100
0.125
0.150
0.17
SNP effect on S−formylglutathione hydrolase
SNP effect on Gut microbiota abundance (genus Catenibacterium id.2153)

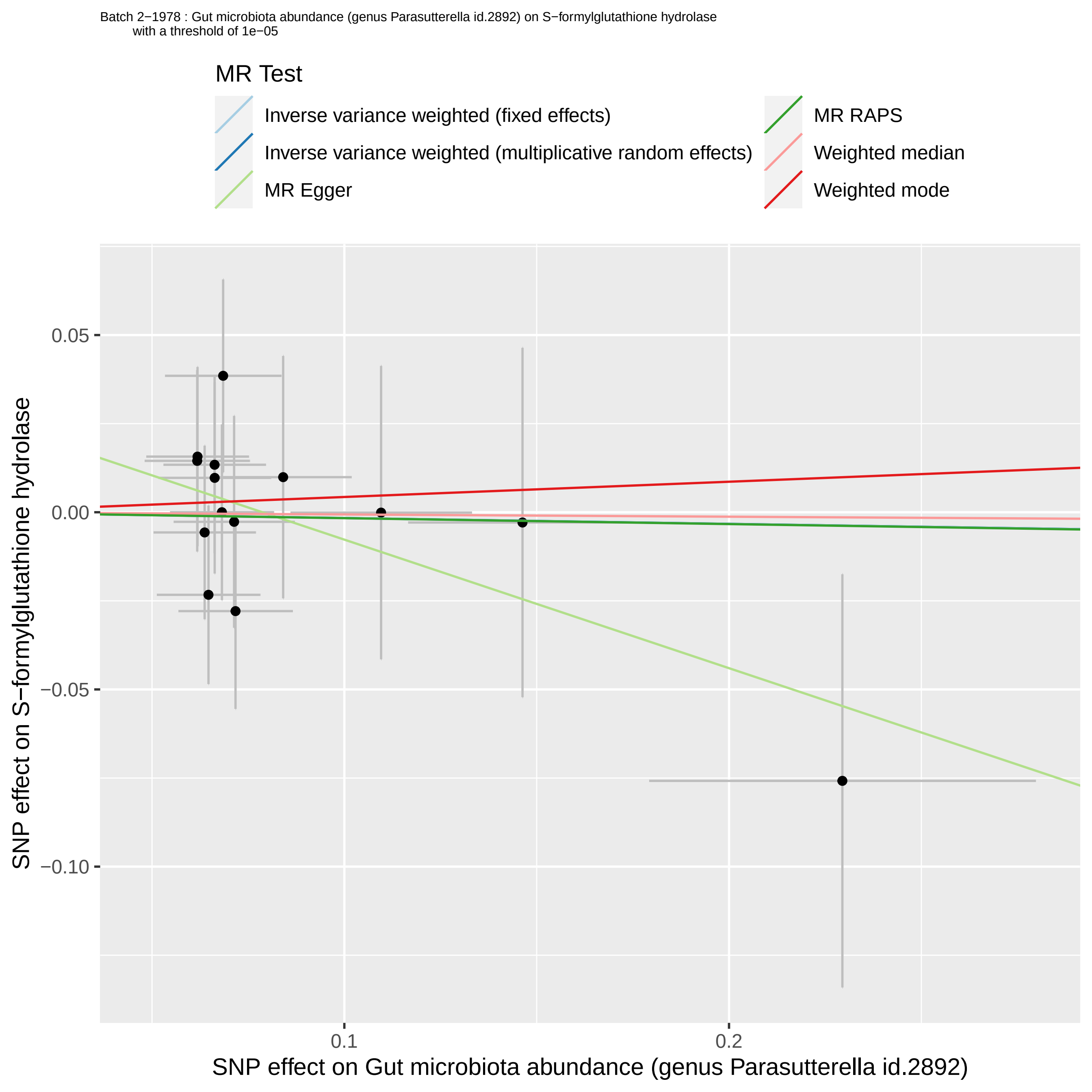
Batch 2−1978 : Gut microbiota abundance (genus Parasutterella id.2892) on S−formylglutathione hydrolase
with a threshold of 1e−05
MR Test
Inverse variance weighted (fixed effects)
Inverse variance weighted (multiplicative random effects)
MR Egger
MR RAPS
Weighted median
Weighted mode
0.05
0.00
−0.05
−0.10
0.1
0.2
SNP effect on S−formylglutathione hydrolase
SNP effect on Gut microbiota abundance (genus Parasutterella id.2892)

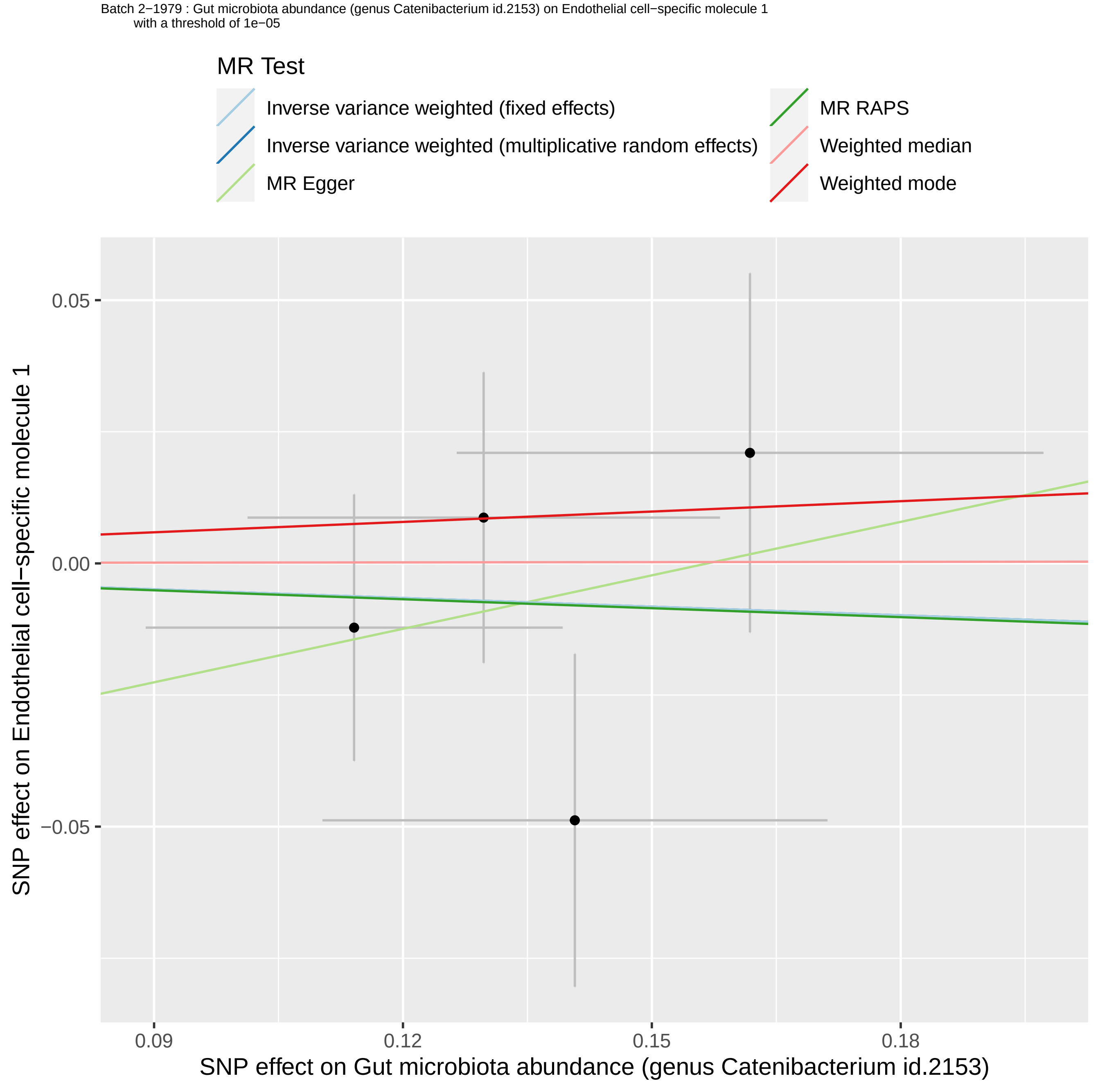

Batch 2−1979 : Gut microbiota abundance (genus Catenibacterium id.2153) on Endothelial cell−specific molecule 1
with a threshold of 1e−05
MR Test
Inverse variance weighted (fixed effects)
Inverse variance weighted (multiplicative random effects)
MR Egger
MR RAPS
Weighted median
Weighted mode
0.05
0.00
−0.05
0.09
0.12
0.15
0.18
SNP effect on Endothelial cell−specific molecule 1
SNP effect on Gut microbiota abundance (genus Catenibacterium id.2153)

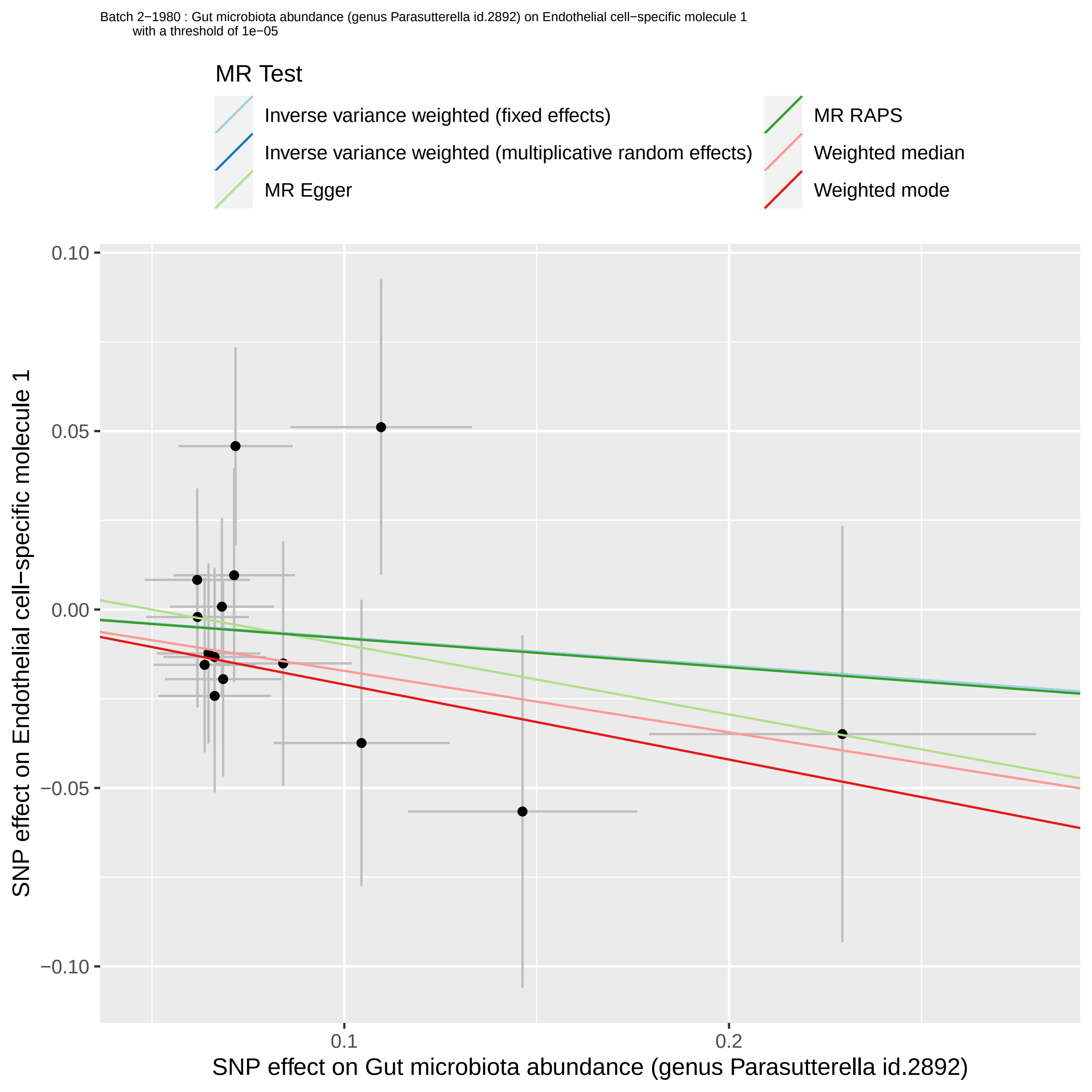
Batch 2−1980 : Gut microbiota abundance (genus Parasutterella id.2892) on Endothelial cell−specific molecule 1
with a threshold of 1e−05
MR Test
Inverse variance weighted (fixed effects)
Inverse variance weighted (multiplicative random effects)
MR Egger
MR RAPS
Weighted median
Weighted mode
0.10
0.05
0.00
−0.05
−0.10
0.1
0.2
SNP effect on Endothelial cell−specific molecule 1
SNP effect on Gut microbiota abundance (genus Parasutterella id.2892)

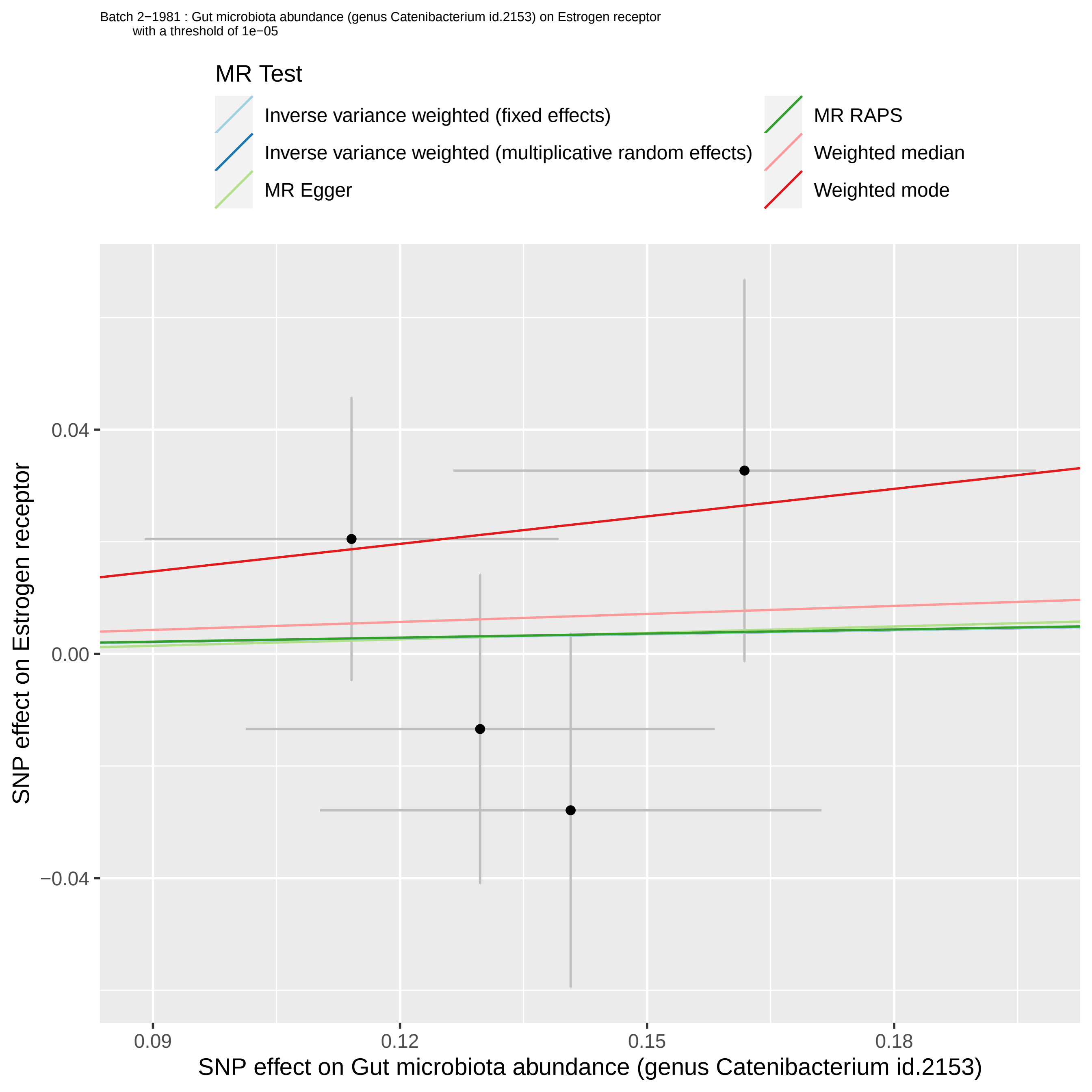

Batch 2−1981 : Gut microbiota abundance (genus Catenibacterium id.2153) on Estrogen receptor
with a threshold of 1e−05
MR Test
Inverse variance weighted (fixed effects)
Inverse variance weighted (multiplicative random effects)
MR Egger
MR RAPS
Weighted median
Weighted mode
0.04
0.00
−0.04
0.09
0.12
0.15
0.18
SNP effect on Estrogen receptor
SNP effect on Gut microbiota abundance (genus Catenibacterium id.2153)

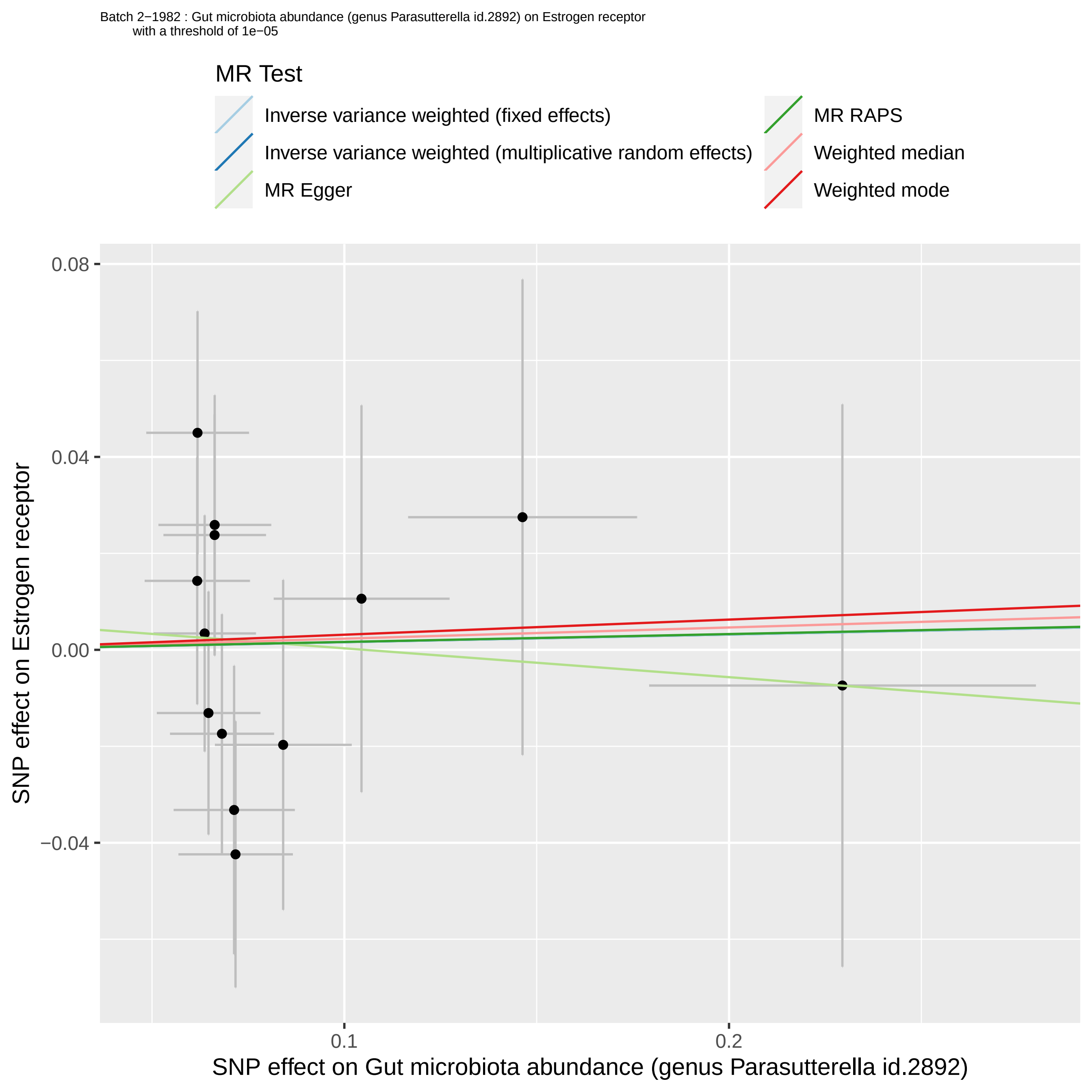

Batch 2−1982 : Gut microbiota abundance (genus Parasutterella id.2892) on Estrogen receptor
with a threshold of 1e−05
MR Test
Inverse variance weighted (fixed effects)
Inverse variance weighted (multiplicative random effects)
MR Egger
MR RAPS
Weighted median
Weighted mode
0.08
0.04
0.00
−0.04
0.1
0.2
SNP effect on Estrogen receptor
SNP effect on Gut microbiota abundance (genus Parasutterella id.2892)

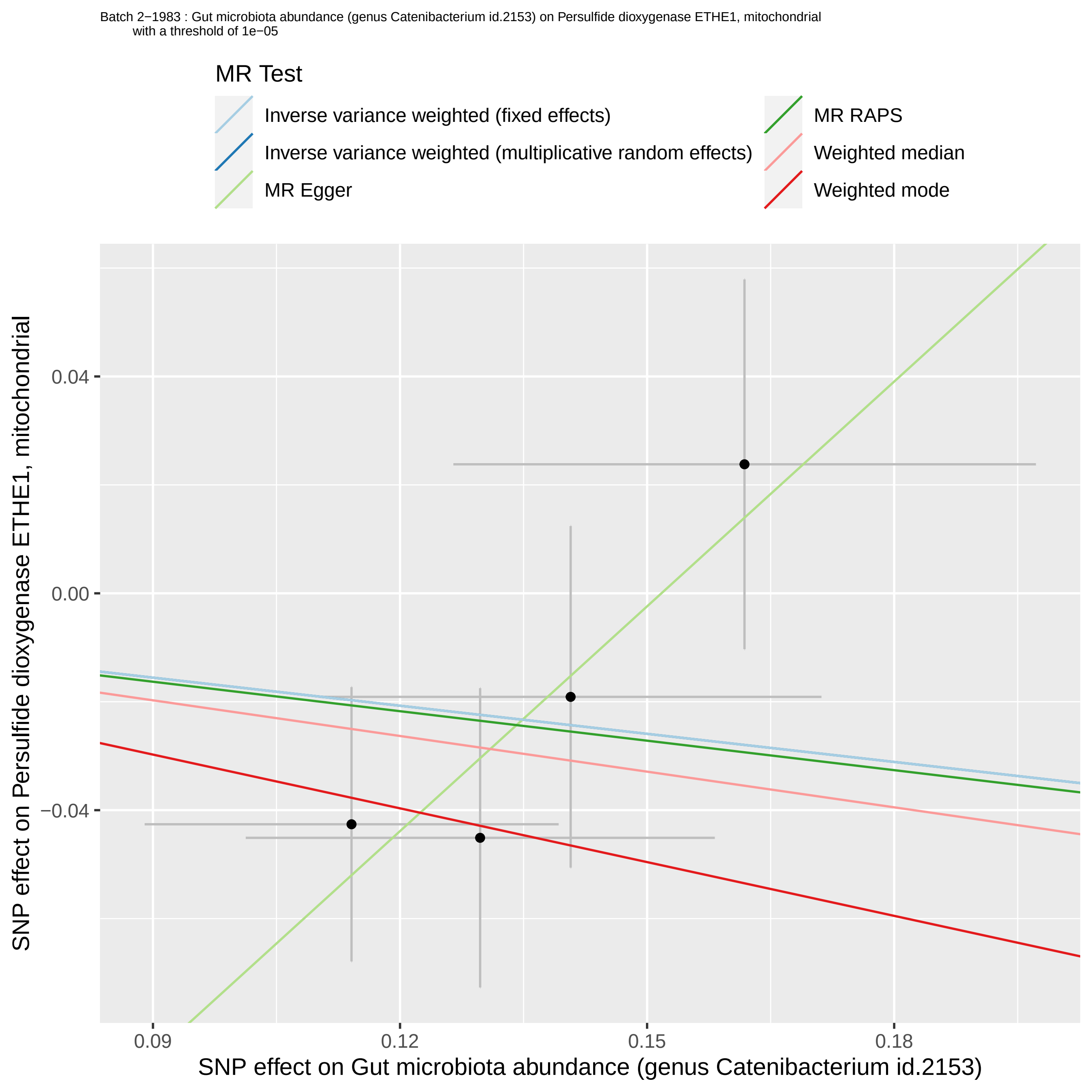
Batch 2−1983 : Gut microbiota abundance (genus Catenibacterium id.2153) on Persulfide dioxygenase ETHE1, mitochondrial
with a threshold of 1e−05
MR Test
Inverse variance weighted (fixed effects)
Inverse variance weighted (multiplicative random effects)
MR Egger
MR RAPS
Weighted median
Weighted mode
0.04
0.00
−0.04
0.09
0.12
0.15
0.18
SNP effect on Persulfide dioxygenase ETHE1, mitochondrial
SNP effect on Gut microbiota abundance (genus Catenibacterium id.2153)

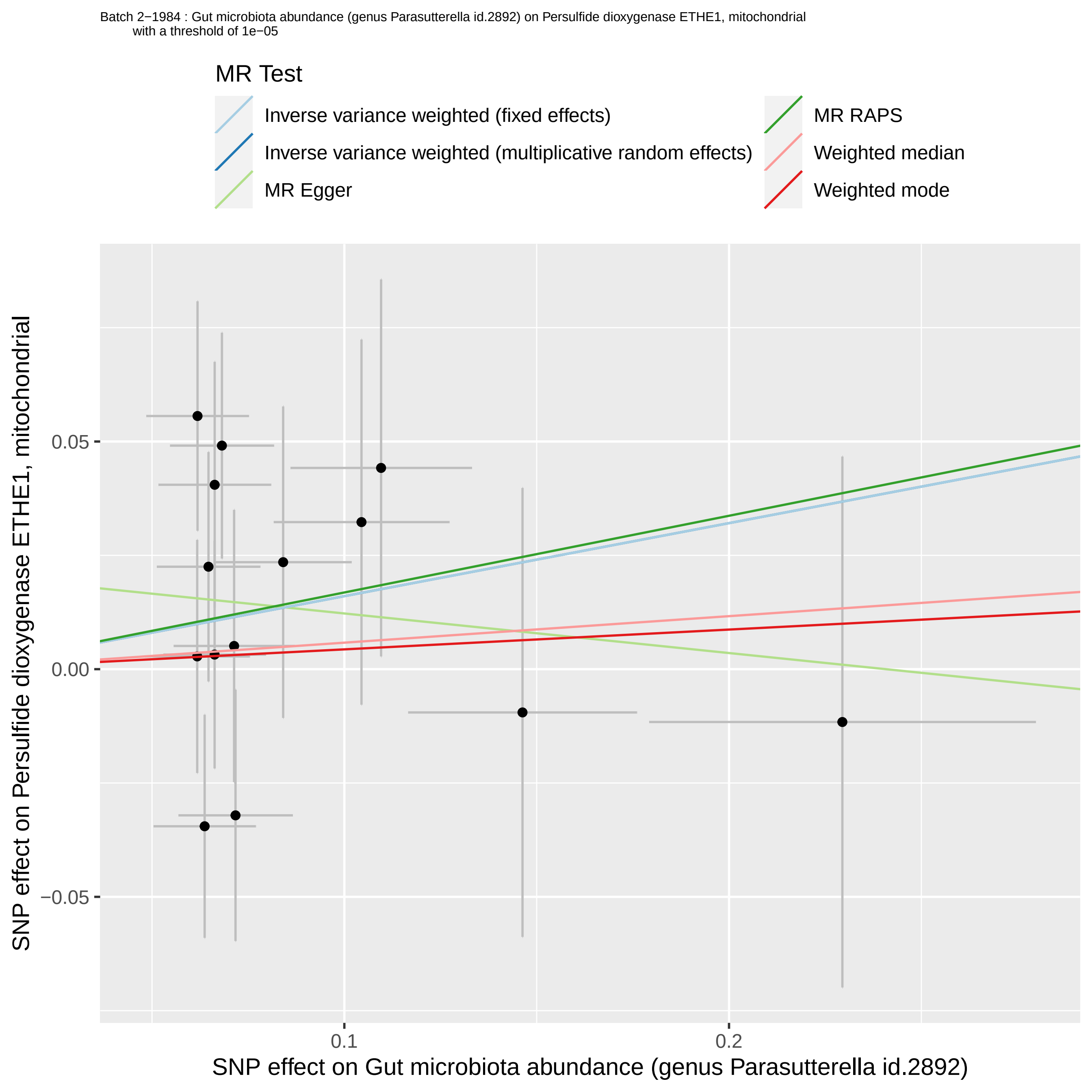

Batch 2−1984 : Gut microbiota abundance (genus Parasutterella id.2892) on Persulfide dioxygenase ETHE1, mitochondrial
with a threshold of 1e−05
MR Test
Inverse variance weighted (fixed effects)
Inverse variance weighted (multiplicative random effects)
MR Egger
MR RAPS
Weighted median
Weighted mode
0.05
0.00
−0.05
0.1
0.2
SNP effect on Persulfide dioxygenase ETHE1, mitochondrial
SNP effect on Gut microbiota abundance (genus Parasutterella id.2892)

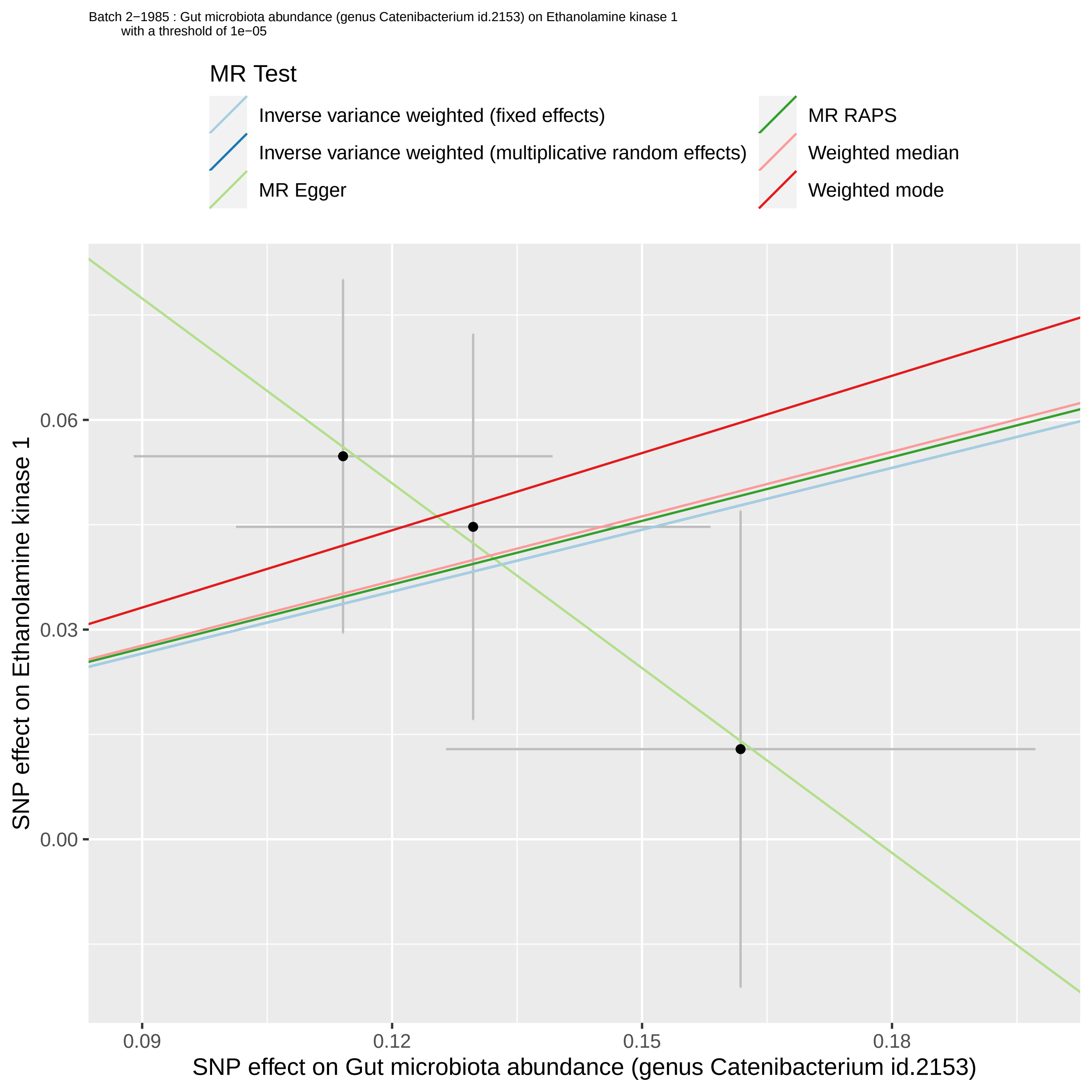

Batch 2−1985 : Gut microbiota abundance (genus Catenibacterium id.2153) on Ethanolamine kinase 1
with a threshold of 1e−05
MR Test
Inverse variance weighted (fixed effects)
Inverse variance weighted (multiplicative random effects)
MR Egger
MR RAPS
Weighted median
Weighted mode
0.06
0.03
0.00
0.09
0.12
0.15
0.18
SNP effect on Ethanolamine kinase 1
SNP effect on Gut microbiota abundance (genus Catenibacterium id.2153)

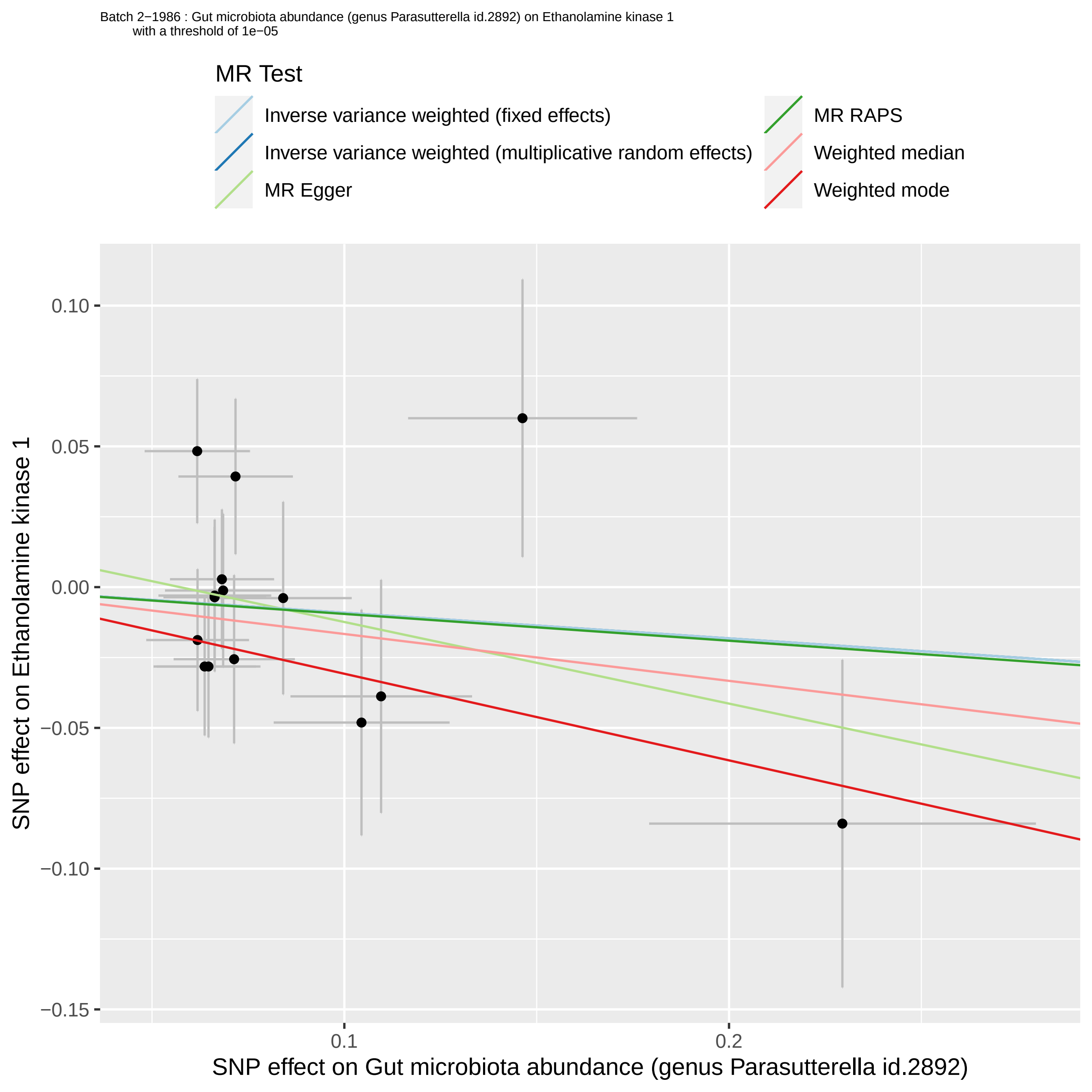
Batch 2−1986 : Gut microbiota abundance (genus Parasutterella id.2892) on Ethanolamine kinase 1
with a threshold of 1e−05
MR Test
Inverse variance weighted (fixed effects)
Inverse variance weighted (multiplicative random effects)
MR Egger
MR RAPS
Weighted median
Weighted mode
0.10
0.05
0.00
−0.05
−0.10
−0.15
0.1
0.2
SNP effect on Ethanolamine kinase 1
SNP effect on Gut microbiota abundance (genus Parasutterella id.2892)

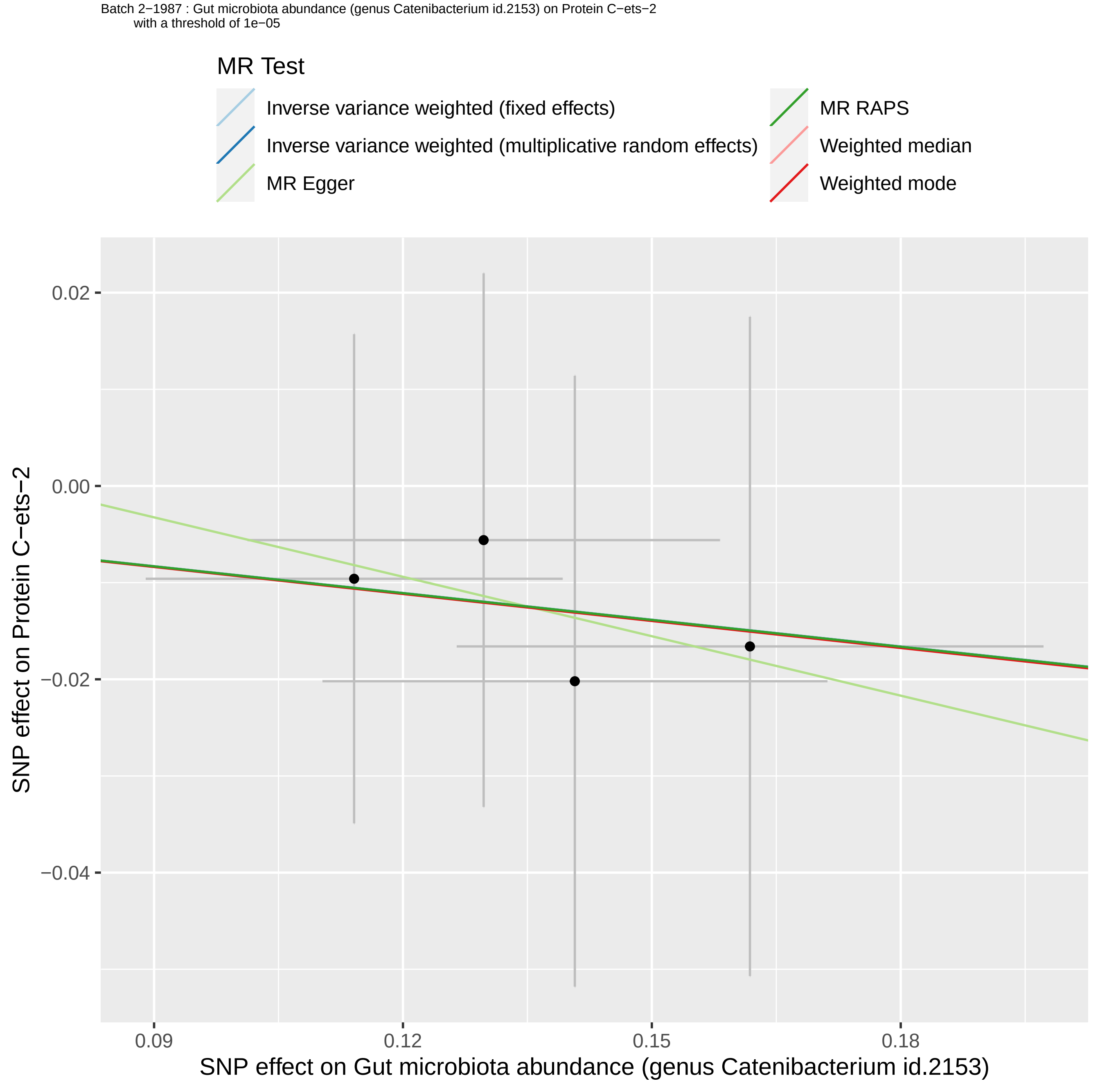

Batch 2−1987 : Gut microbiota abundance (genus Catenibacterium id.2153) on Protein C−ets−2
with a threshold of 1e−05
MR Test
Inverse variance weighted (fixed effects)
Inverse variance weighted (multiplicative random effects)
MR Egger
MR RAPS
Weighted median
Weighted mode
0.02
0.00
−0.02
−0.04
SNP effect on Protein C−ets−2
0.09
0.12
0.15
0.18
SNP effect on Gut microbiota abundance (genus Catenibacterium id.2153)

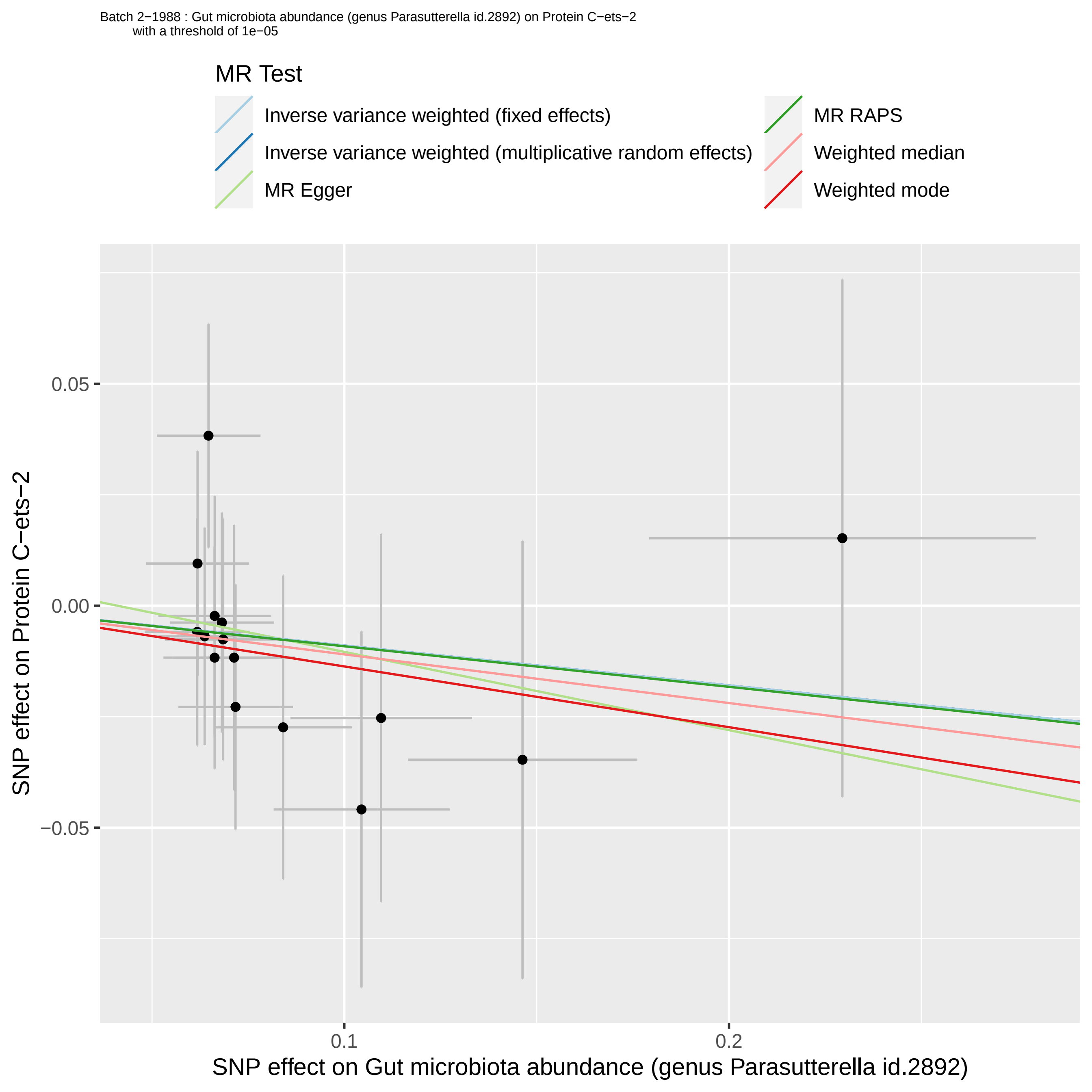
Batch 2−1988 : Gut microbiota abundance (genus Parasutterella id.2892) on Protein C−ets−2
with a threshold of 1e−05
MR Test
Inverse variance weighted (fixed effects)
Inverse variance weighted (multiplicative random effects)
MR Egger
MR RAPS
Weighted median
Weighted mode
0.05
0.00
−0.05
0.1
0.2
SNP effect on Protein C−ets−2
SNP effect on Gut microbiota abundance (genus Parasutterella id.2892)

Batch 2−1989 : Gut microbiota abundance (genus Catenibacterium id.2153) on Protein eva−1 homolog C
with a threshold of 1e−05

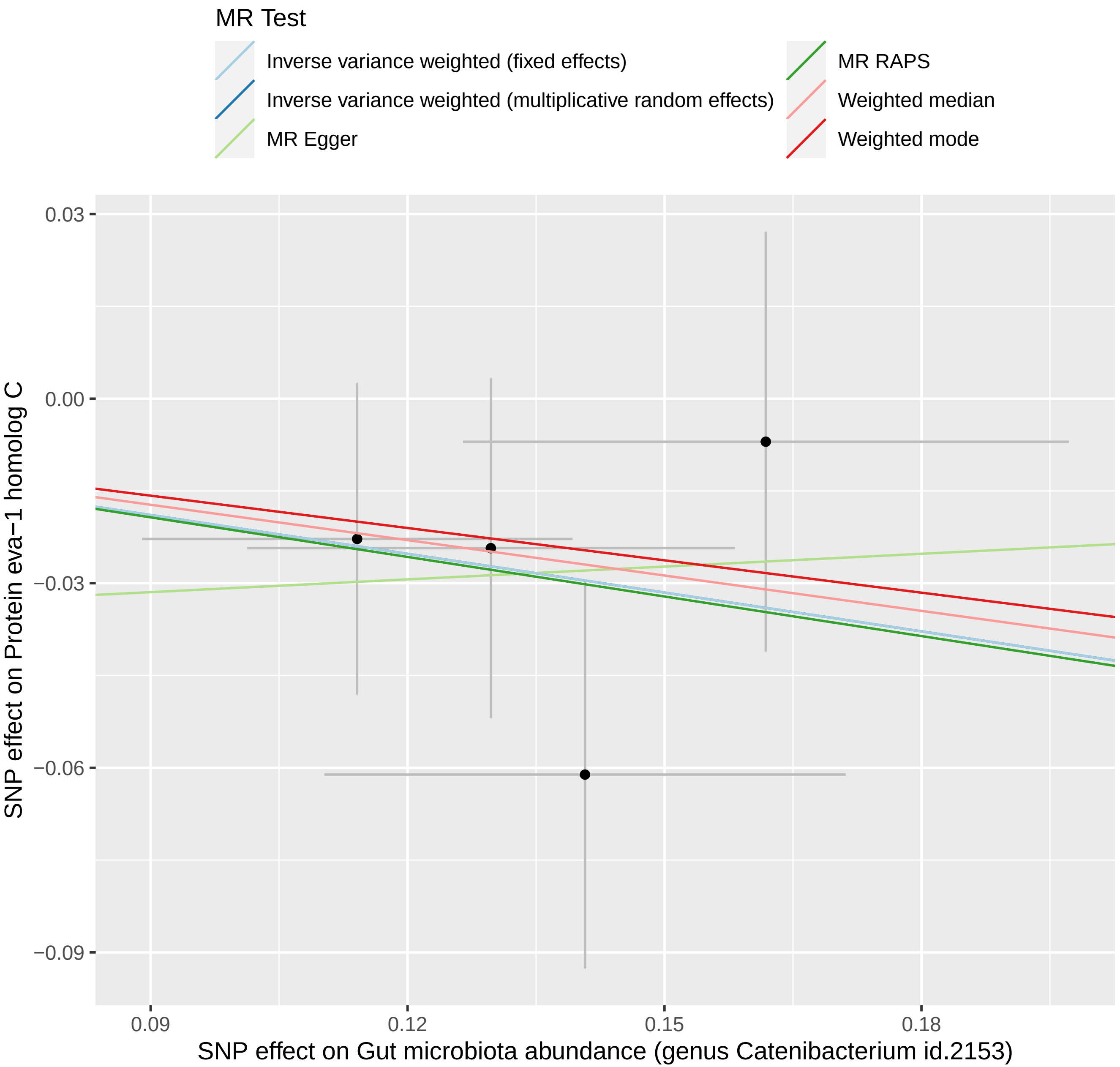

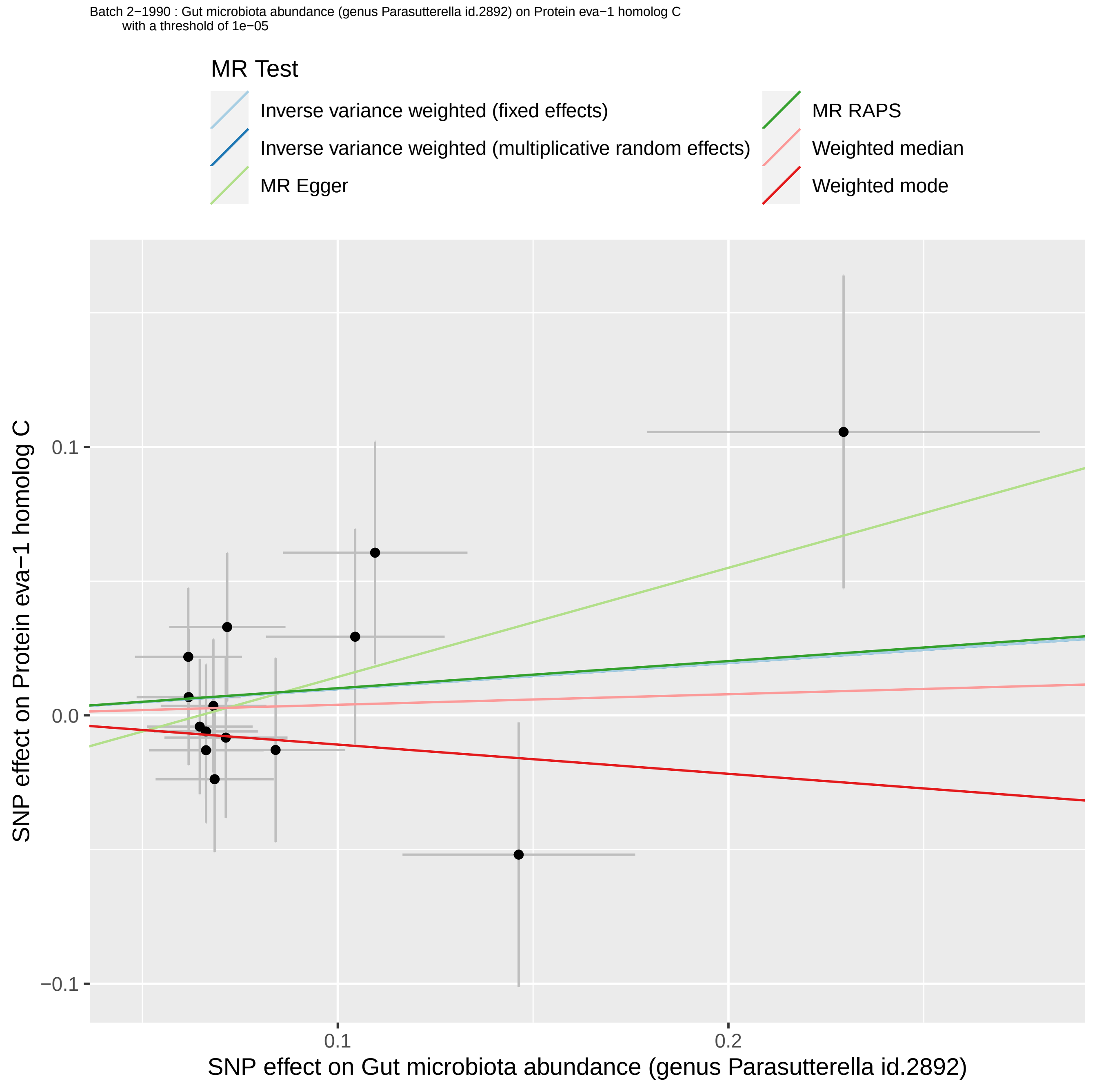
Batch 2−1990 : Gut microbiota abundance (genus Parasutterella id.2892) on Protein eva−1 homolog C
with a threshold of 1e−05
MR Test
Inverse variance weighted (fixed effects)
Inverse variance weighted (multiplicative random effects)
MR Egger
MR RAPS
Weighted median
Weighted mode
SNP effect on Protein eva−1 homolog C
0.1
0.0
−0.1
0.1
0.2
SNP effect on Gut microbiota abundance (genus Parasutterella id.2892)

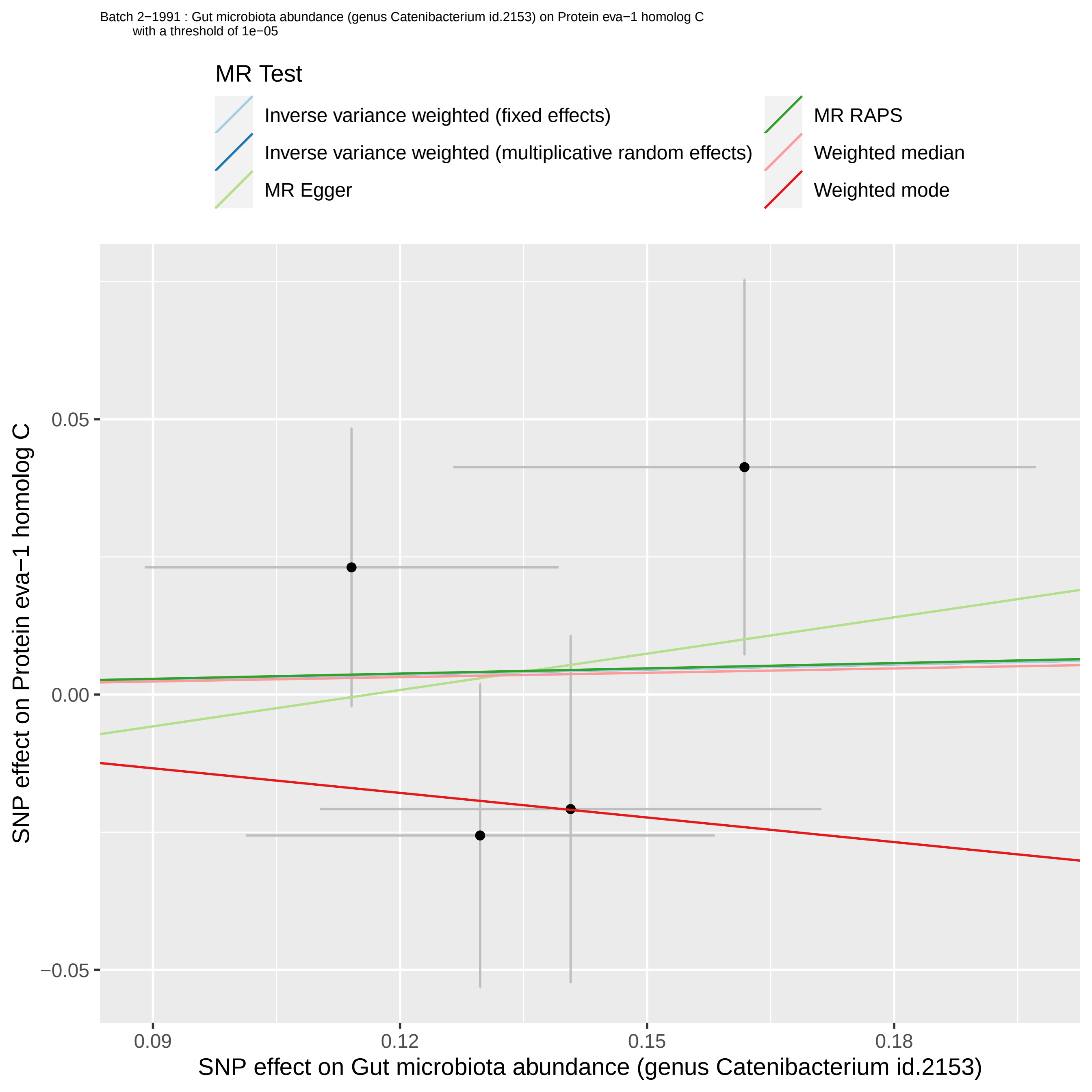

Batch 2−1991 : Gut microbiota abundance (genus Catenibacterium id.2153) on Protein eva−1 homolog C
with a threshold of 1e−05
MR Test
Inverse variance weighted (fixed effects)
Inverse variance weighted (multiplicative random effects)
MR Egger
MR RAPS
Weighted median
Weighted mode
0.05
0.00
−0.05
0.09
0.12
0.15
0.18
SNP effect on Protein eva−1 homolog C
SNP effect on Gut microbiota abundance (genus Catenibacterium id.2153)

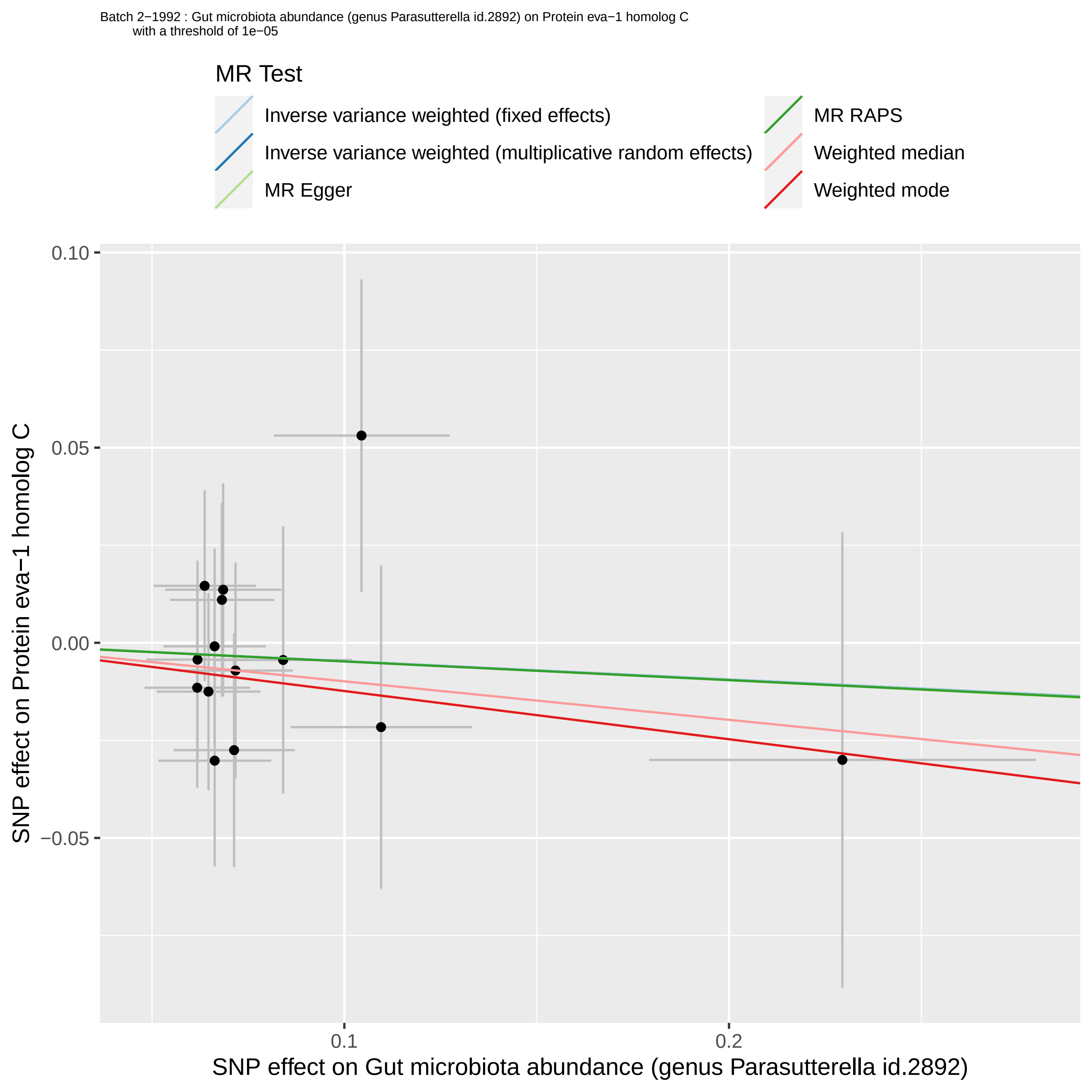
Batch 2−1992 : Gut microbiota abundance (genus Parasutterella id.2892) on Protein eva−1 homolog C
with a threshold of 1e−05
MR Test
Inverse variance weighted (fixed effects)
Inverse variance weighted (multiplicative random effects)
MR Egger
MR RAPS
Weighted median
Weighted mode
0.10
0.05
0.00
−0.05
0.1
0.2
SNP effect on Protein eva−1 homolog C
SNP effect on Gut microbiota abundance (genus Parasutterella id.2892)

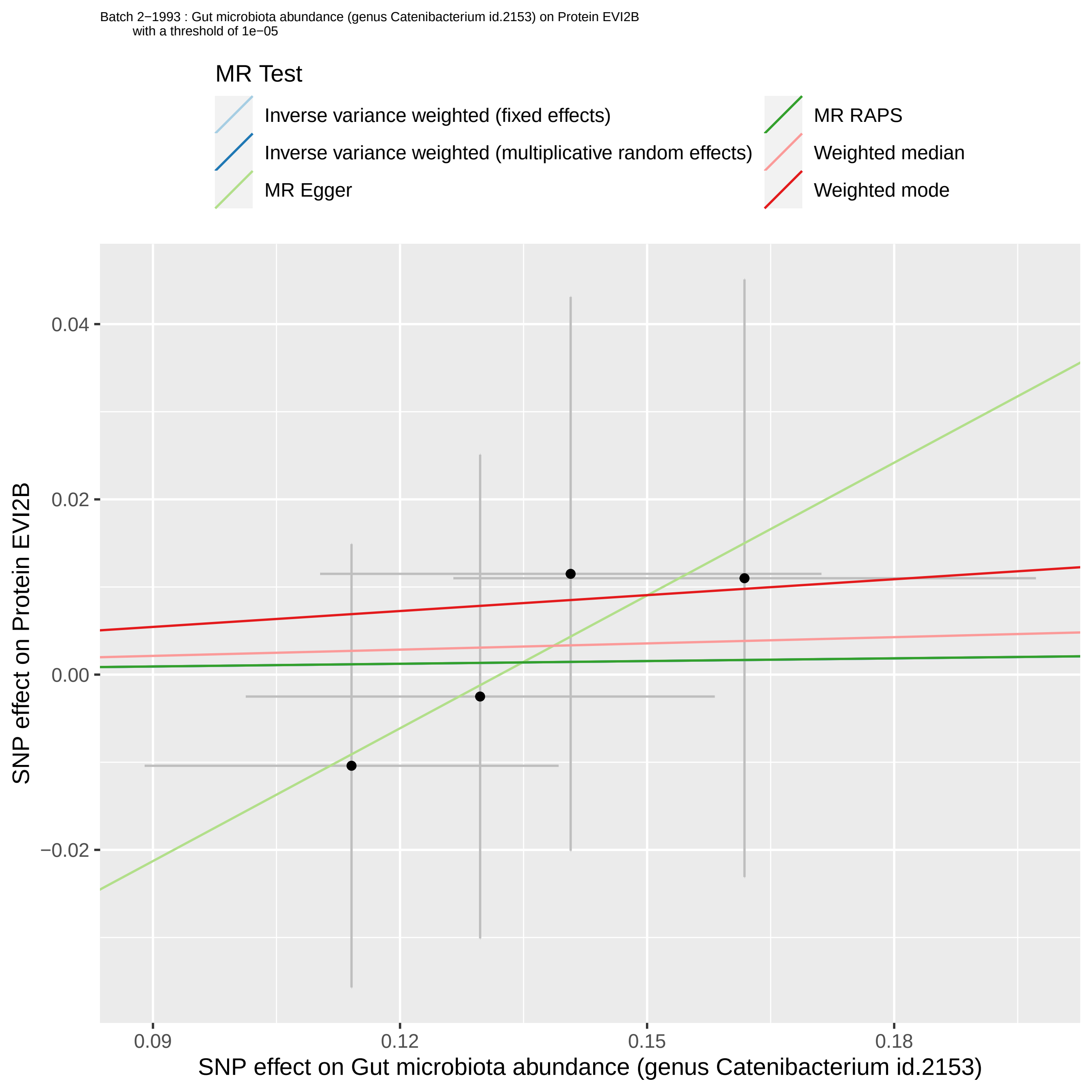
Batch 2−1993 : Gut microbiota abundance (genus Catenibacterium id.2153) on Protein EVI2B
with a threshold of 1e−05
MR Test
Inverse variance weighted (fixed effects)
Inverse variance weighted (multiplicative random effects)
MR Egger
MR RAPS
Weighted median
Weighted mode
SNP effect on Protein EVI2B
0.04
0.02
0.00
−0.02
0.09
0.12
0.15
0.18
SNP effect on Gut microbiota abundance (genus Catenibacterium id.2153)

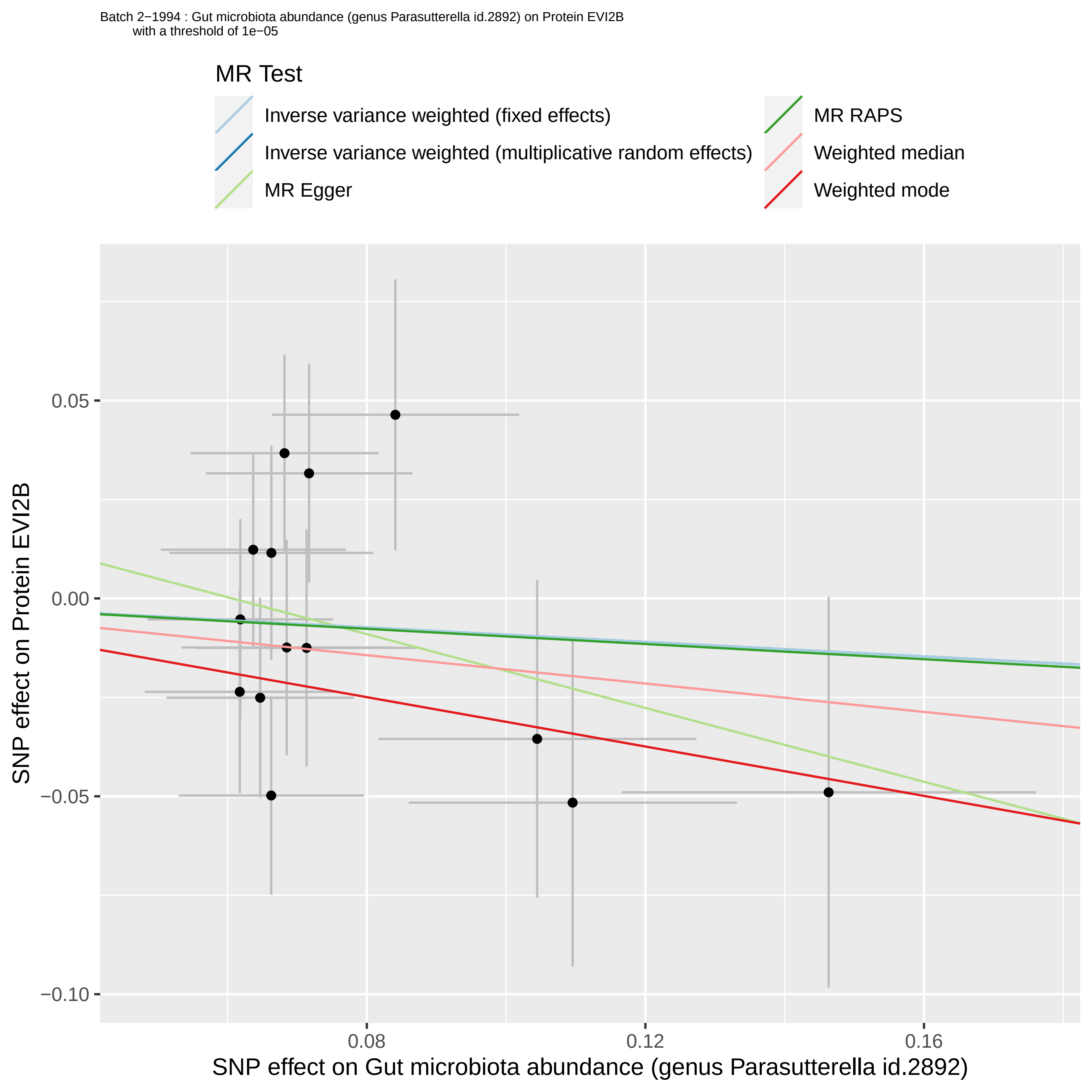
Batch 2−1994 : Gut microbiota abundance (genus Parasutterella id.2892) on Protein EVI2B
with a threshold of 1e−05
MR Test
Inverse variance weighted (fixed effects)
Inverse variance weighted (multiplicative random effects)
MR Egger
MR RAPS
Weighted median
Weighted mode
0.05
0.00
−0.05
−0.10
0.08
0.12
0.16
SNP effect on Protein EVI2B
SNP effect on Gut microbiota abundance (genus Parasutterella id.2892)

Batch 2−1995 : Gut microbiota abundance (genus Catenibacterium id.2153) on Ena/VASP−like protein
with a threshold of 1e−05

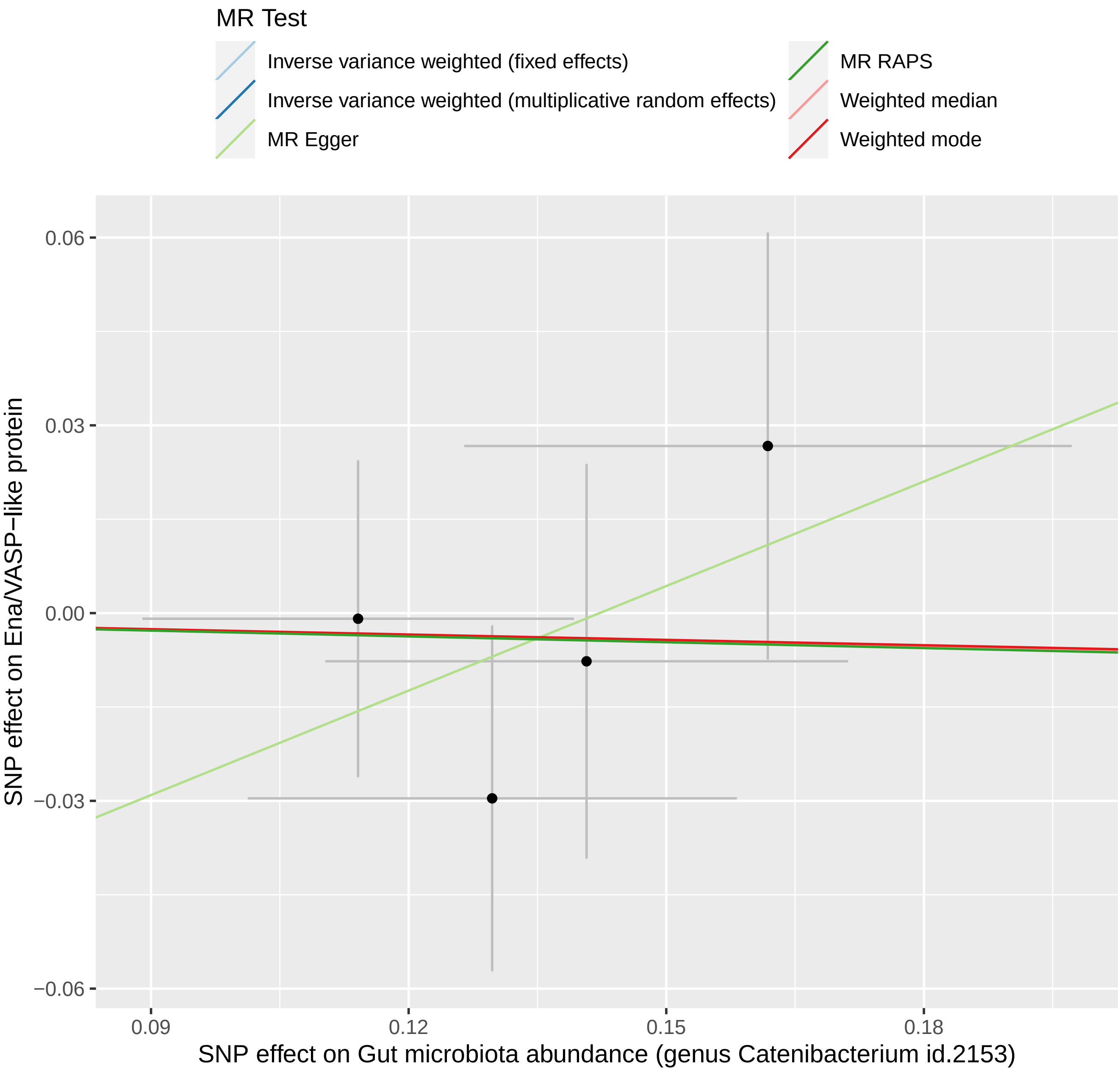

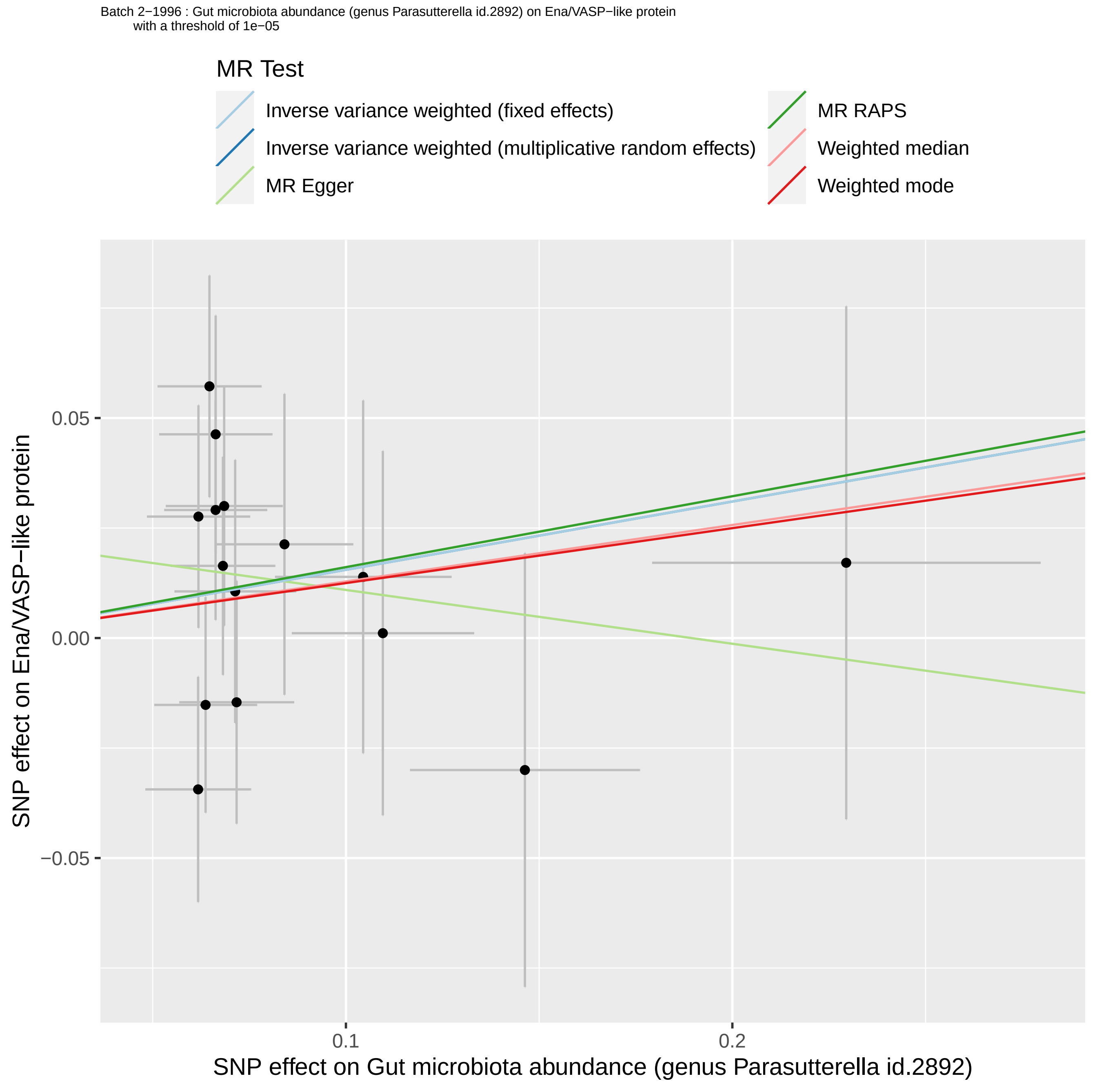
Batch 2−1996 : Gut microbiota abundance (genus Parasutterella id.2892) on Ena/VASP−like protein
with a threshold of 1e−05
MR Test
Inverse variance weighted (fixed effects)
Inverse variance weighted (multiplicative random effects)
MR Egger
MR RAPS
Weighted median
Weighted mode
0.05
0.00
−0.05
0.1
0.2
SNP effect on Ena/VASP−like protein
SNP effect on Gut microbiota abundance (genus Parasutterella id.2892)

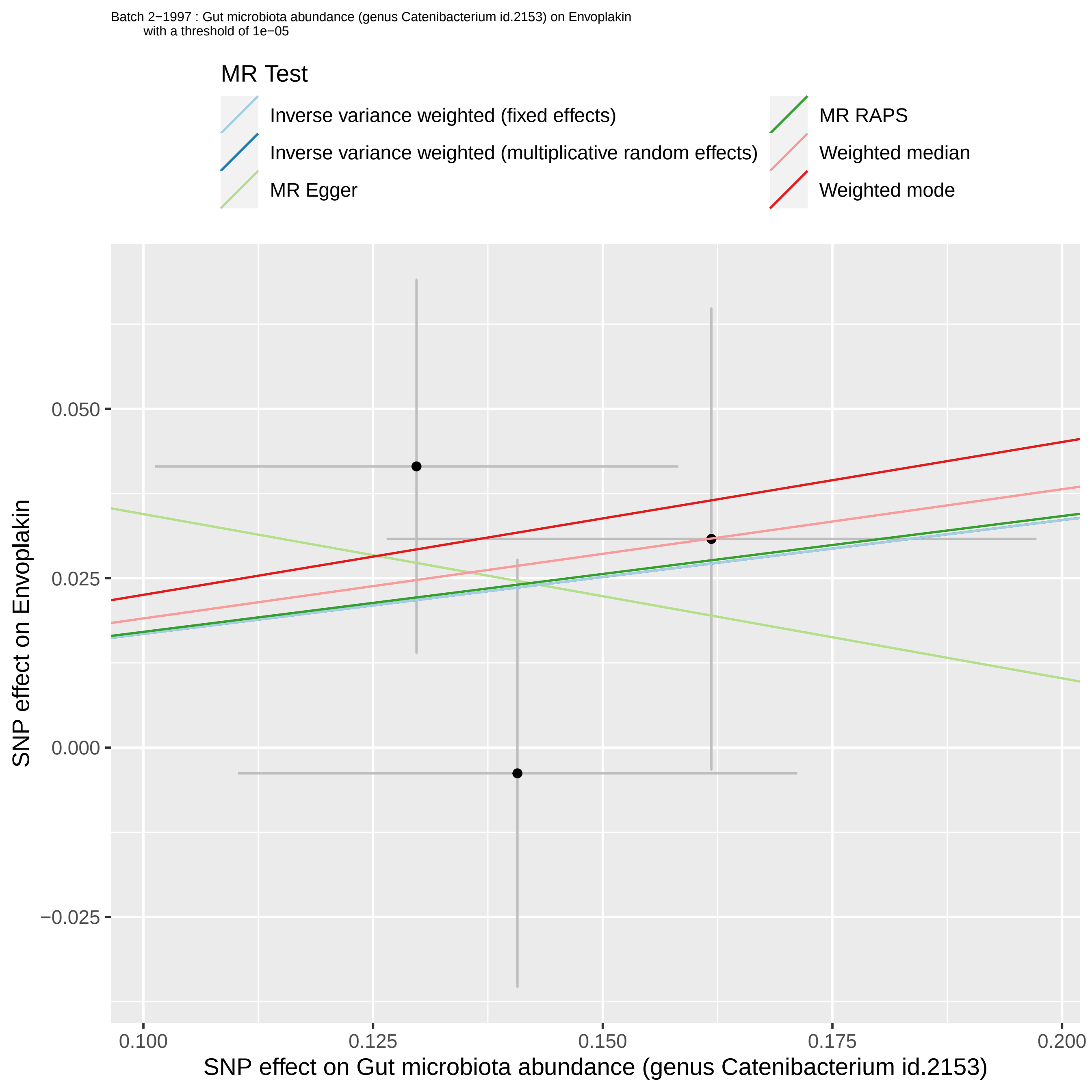
Batch 2−1997 : Gut microbiota abundance (genus Catenibacterium id.2153) on Envoplakin
with a threshold of 1e−05
MR Test
Inverse variance weighted (fixed effects)
Inverse variance weighted (multiplicative random effects)
MR Egger
MR RAPS
Weighted median
Weighted mode
0.050
0.025
0.000
−0.025
SNP effect on Envoplakin
0.100
0.125
0.150
0.175
0.200
SNP effect on Gut microbiota abundance (genus Catenibacterium id.2153)

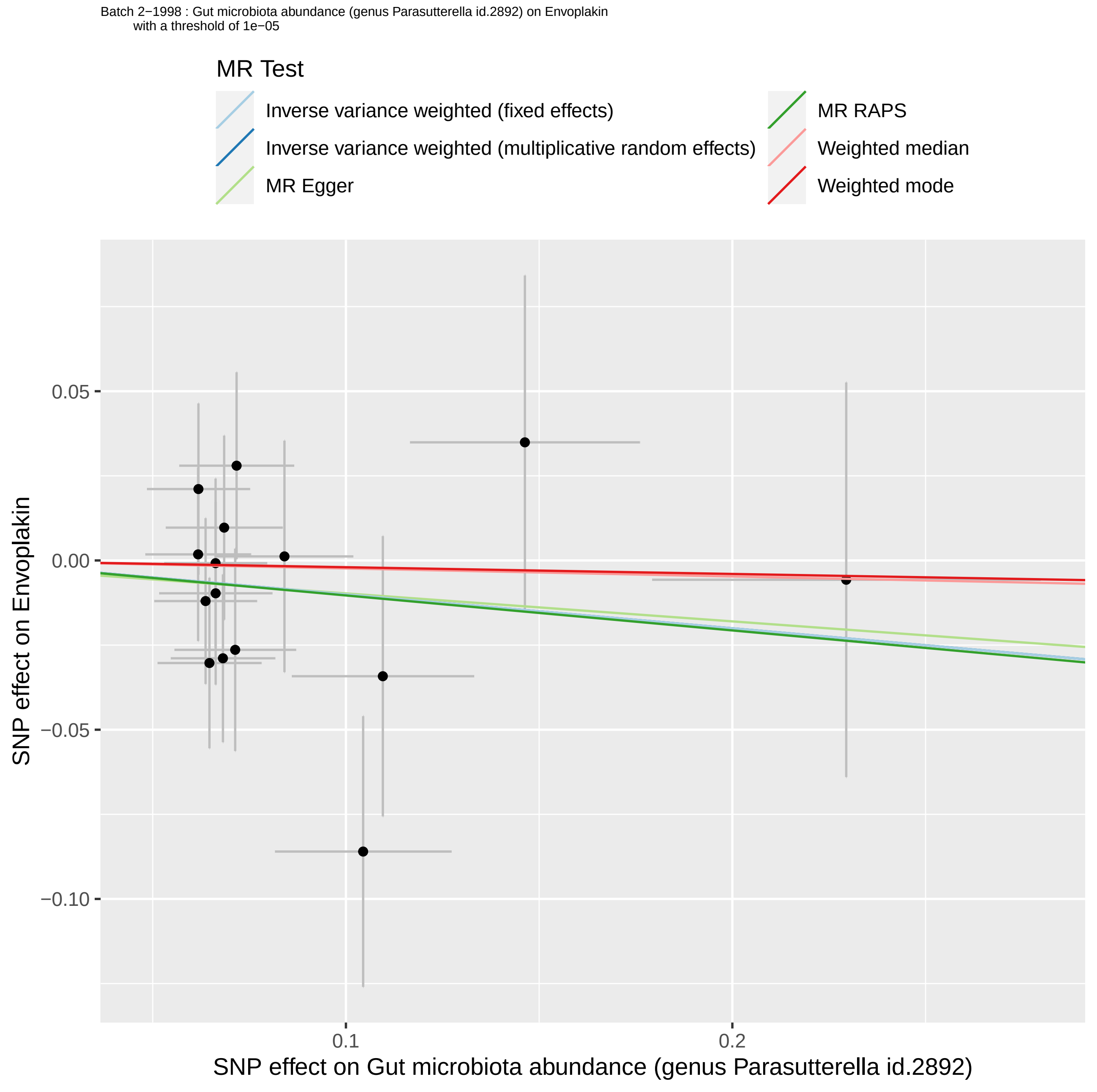
Batch 2−1998 : Gut microbiota abundance (genus Parasutterella id.2892) on Envoplakin
with a threshold of 1e−05
MR Test
Inverse variance weighted (fixed effects)
Inverse variance weighted (multiplicative random effects)
MR Egger
MR RAPS
Weighted median
Weighted mode
0.05
0.00
−0.05
−0.10
0.1
0.2
SNP effect on Envoplakin
SNP effect on Gut microbiota abundance (genus Parasutterella id.2892)

Batch 2−1999 : Gut microbiota abundance (genus Catenibacterium id.2153) on RNA−binding protein EWS
with a threshold of 1e−05

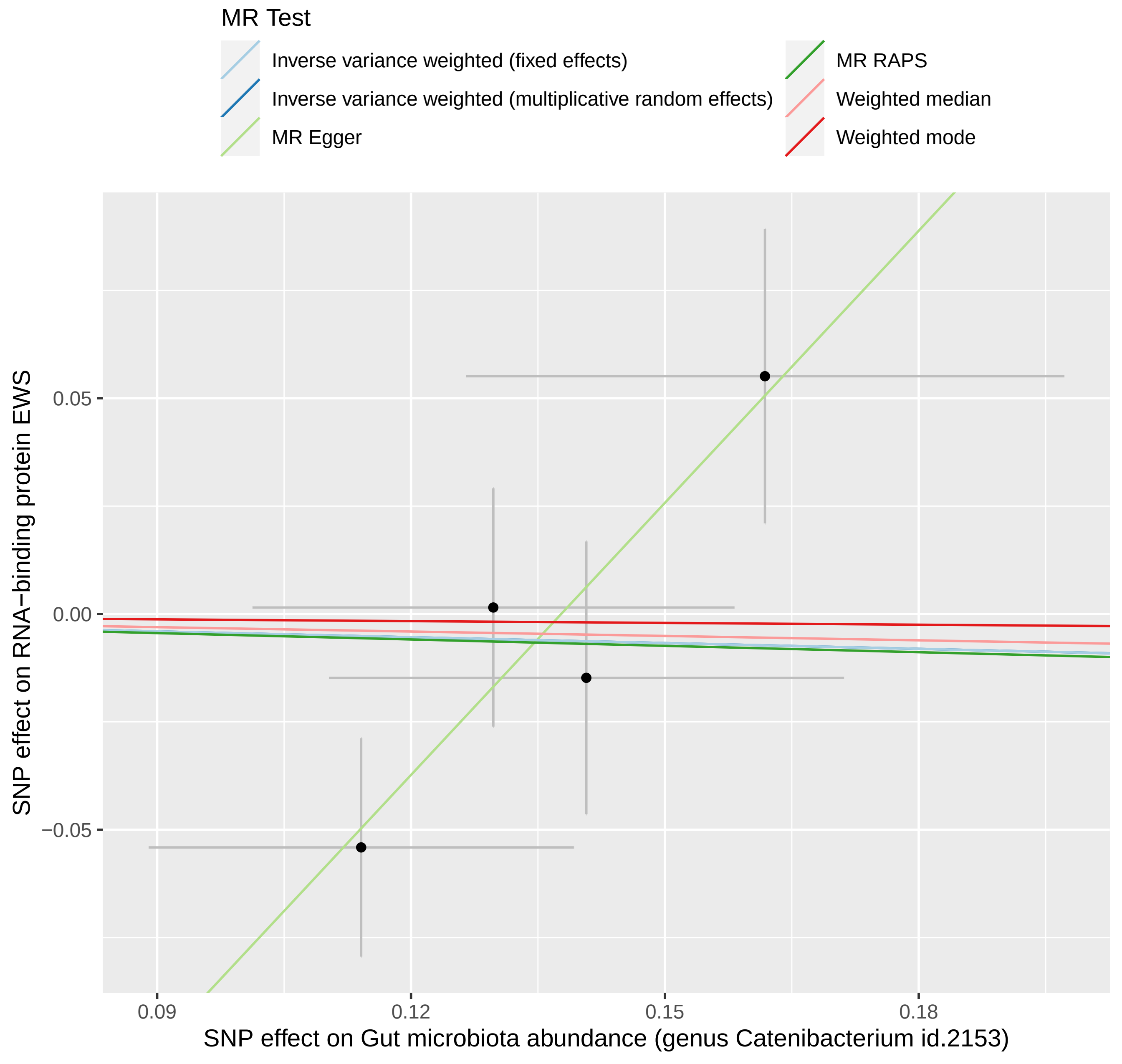

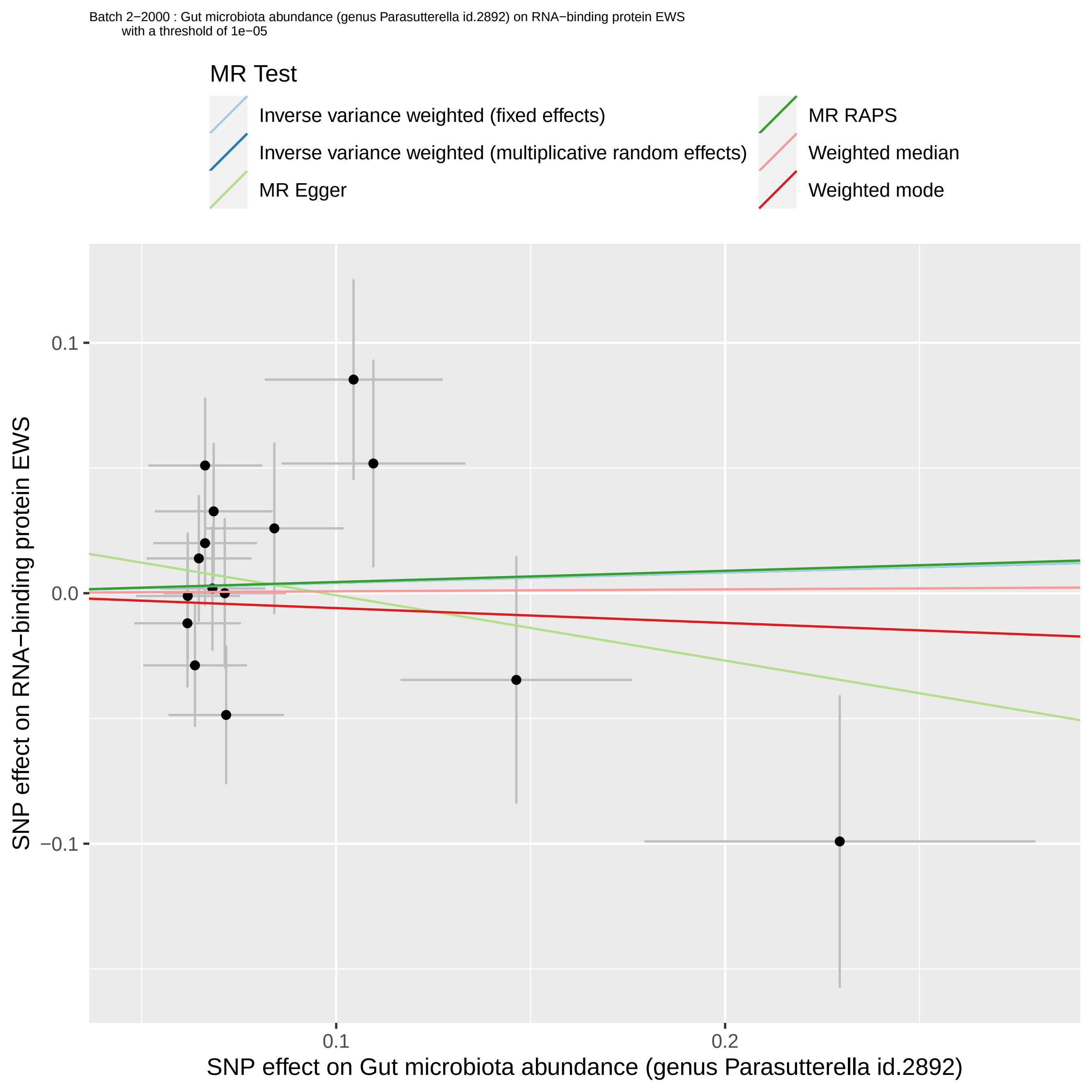

Batch 2−2000 : Gut microbiota abundance (genus Parasutterella id.2892) on RNA−binding protein EWS
with a threshold of 1e−05
MR Test
Inverse variance weighted (fixed effects)
Inverse variance weighted (multiplicative random effects)
MR Egger
MR RAPS
Weighted median
Weighted mode
0.1
0.0
−0.1
0.1
0.2
SNP effect on RNA−binding protein EWS
SNP effect on Gut microbiota abundance (genus Parasutterella id.2892)

Batch 2−2001 : Gut microbiota abundance (genus Catenibacterium id.2153) on Exosome complex component CSL4
with a threshold of 1e−05

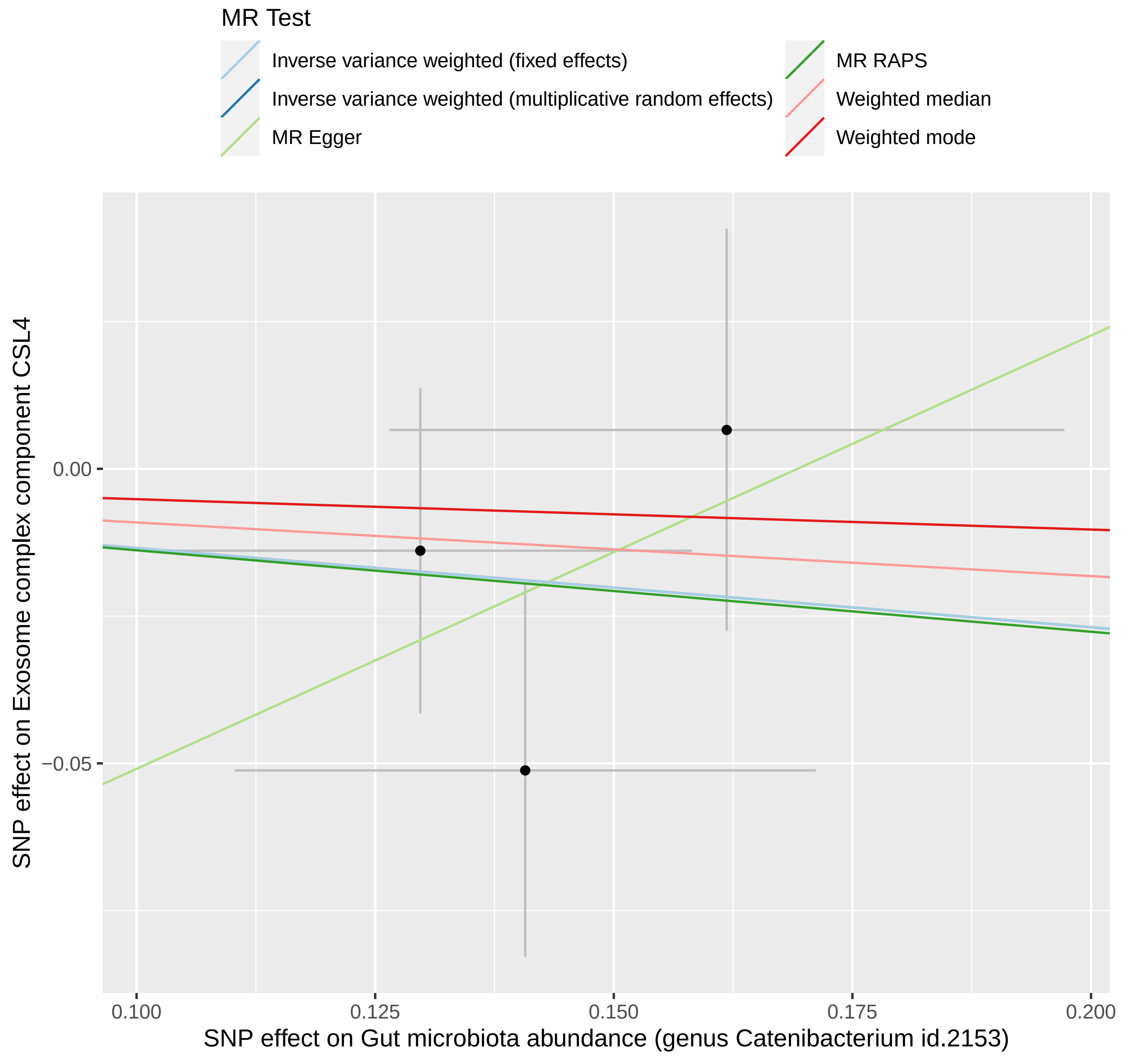

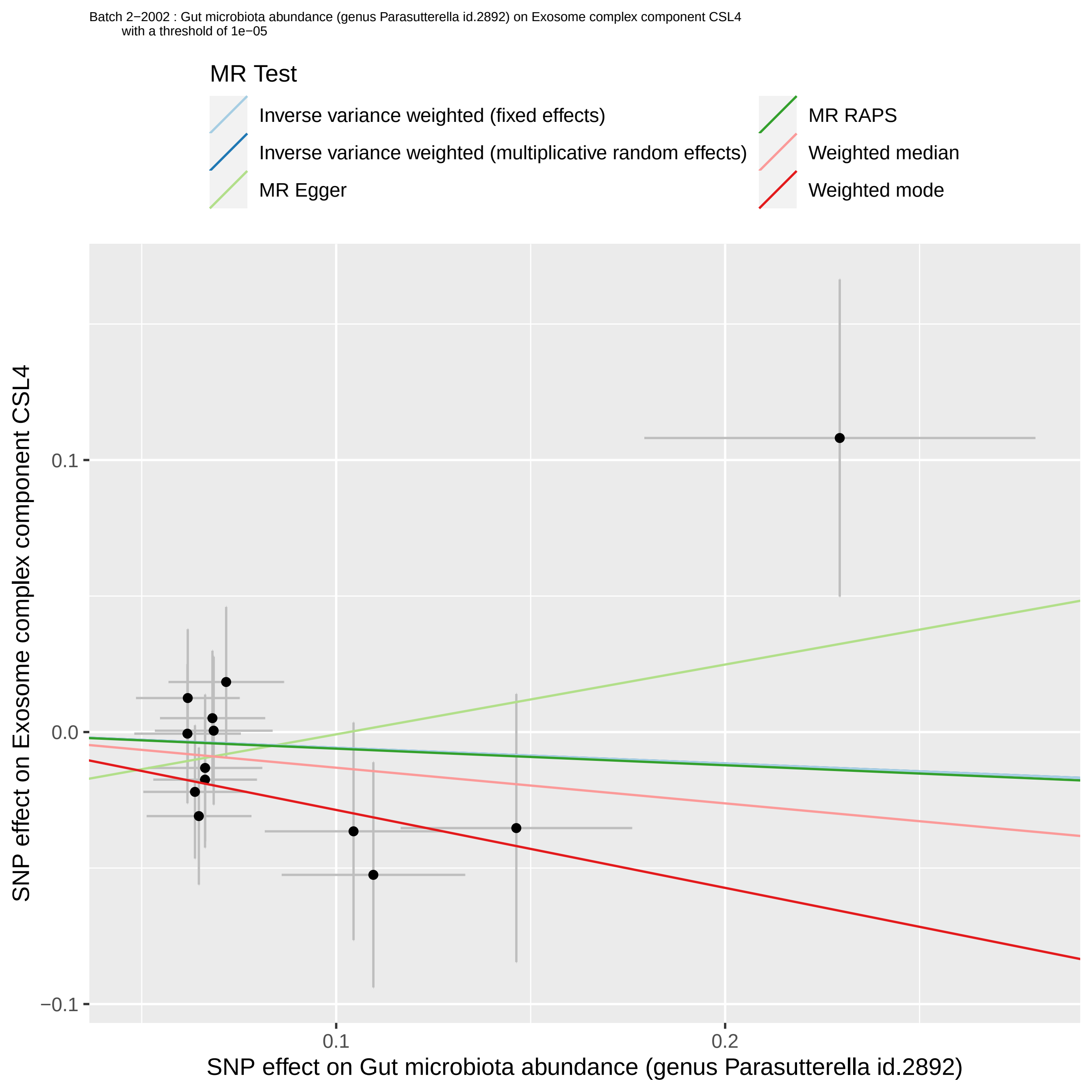
Batch 2−2002 : Gut microbiota abundance (genus Parasutterella id.2892) on Exosome complex component CSL4
with a threshold of 1e−05
MR Test
Inverse variance weighted (fixed effects)
Inverse variance weighted (multiplicative random effects)
MR Egger
MR RAPS
Weighted median
Weighted mode
0.1
0.0
−0.1
0.1
0.2
SNP effect on Exosome complex component CSL4
SNP effect on Gut microbiota abundance (genus Parasutterella id.2892)

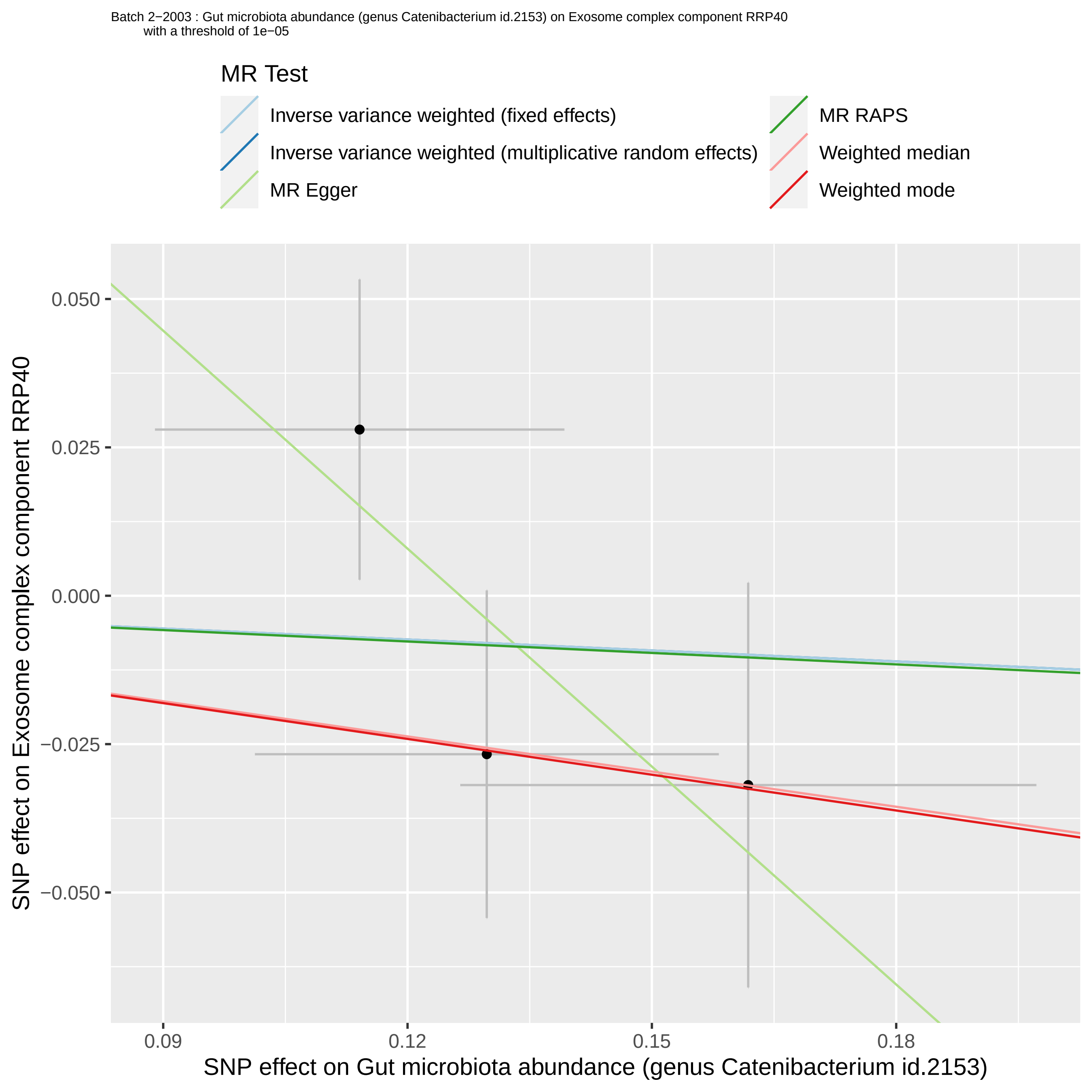
Batch 2−2003 : Gut microbiota abundance (genus Catenibacterium id.2153) on Exosome complex component RRP40
with a threshold of 1e−05
MR Test
Inverse variance weighted (fixed effects)
Inverse variance weighted (multiplicative random effects)
MR Egger
MR RAPS
Weighted median
Weighted mode
0.050
0.025
0.000
−0.025
−0.050
0.09
0.12
0.15
0.18
SNP effect on Exosome complex component RRP40
SNP effect on Gut microbiota abundance (genus Catenibacterium id.2153)

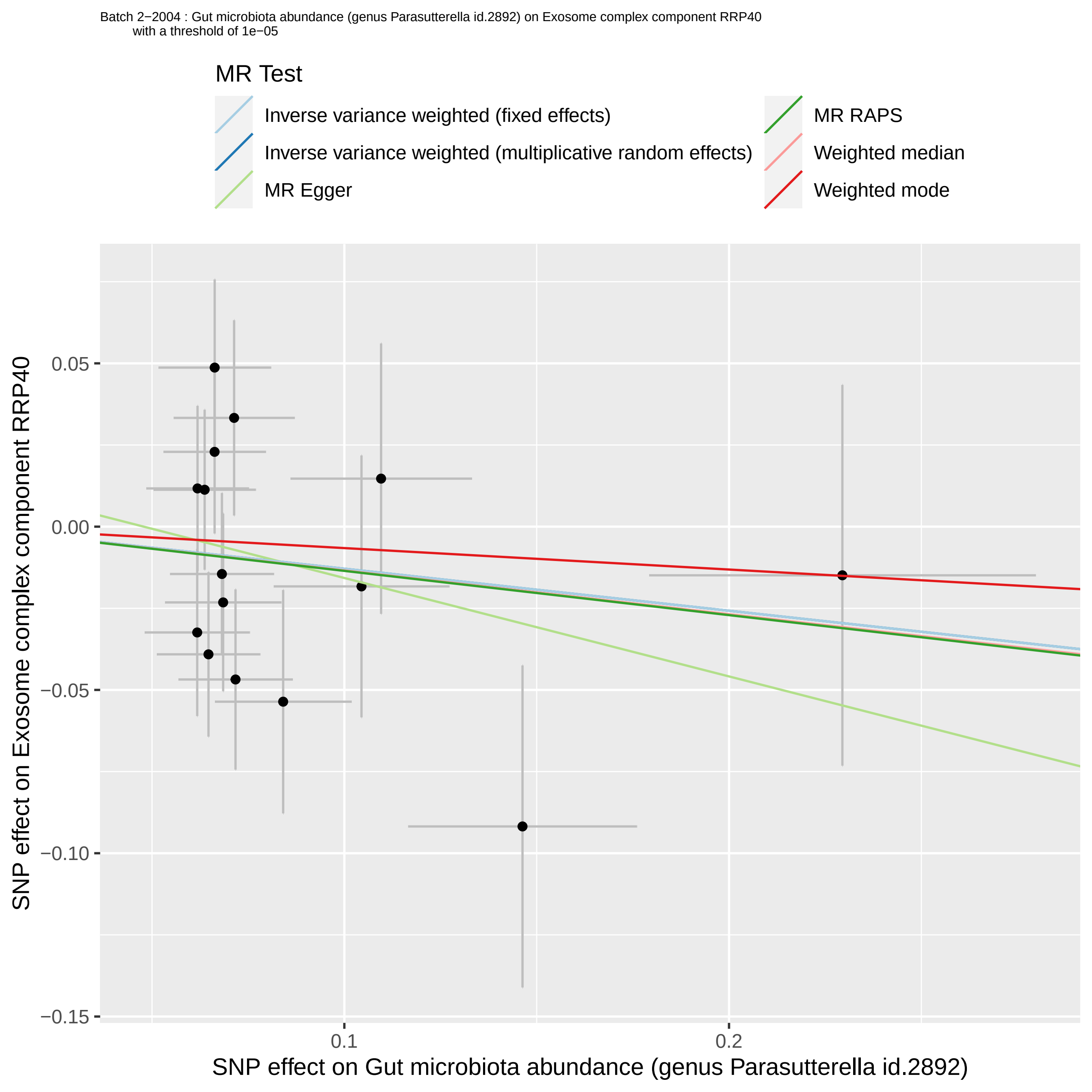
Batch 2−2004 : Gut microbiota abundance (genus Parasutterella id.2892) on Exosome complex component RRP40
with a threshold of 1e−05
MR Test
Inverse variance weighted (fixed effects)
Inverse variance weighted (multiplicative random effects)
MR Egger
MR RAPS
Weighted median
Weighted mode
0.05
0.00
−0.05
−0.10
−0.15
0.1
0.2
SNP effect on Exosome complex component RRP40
SNP effect on Gut microbiota abundance (genus Parasutterella id.2892)

Batch 2−2005 : Gut microbiota abundance (genus Catenibacterium id.2153) on Exostosin−like 2
with a threshold of 1e−05

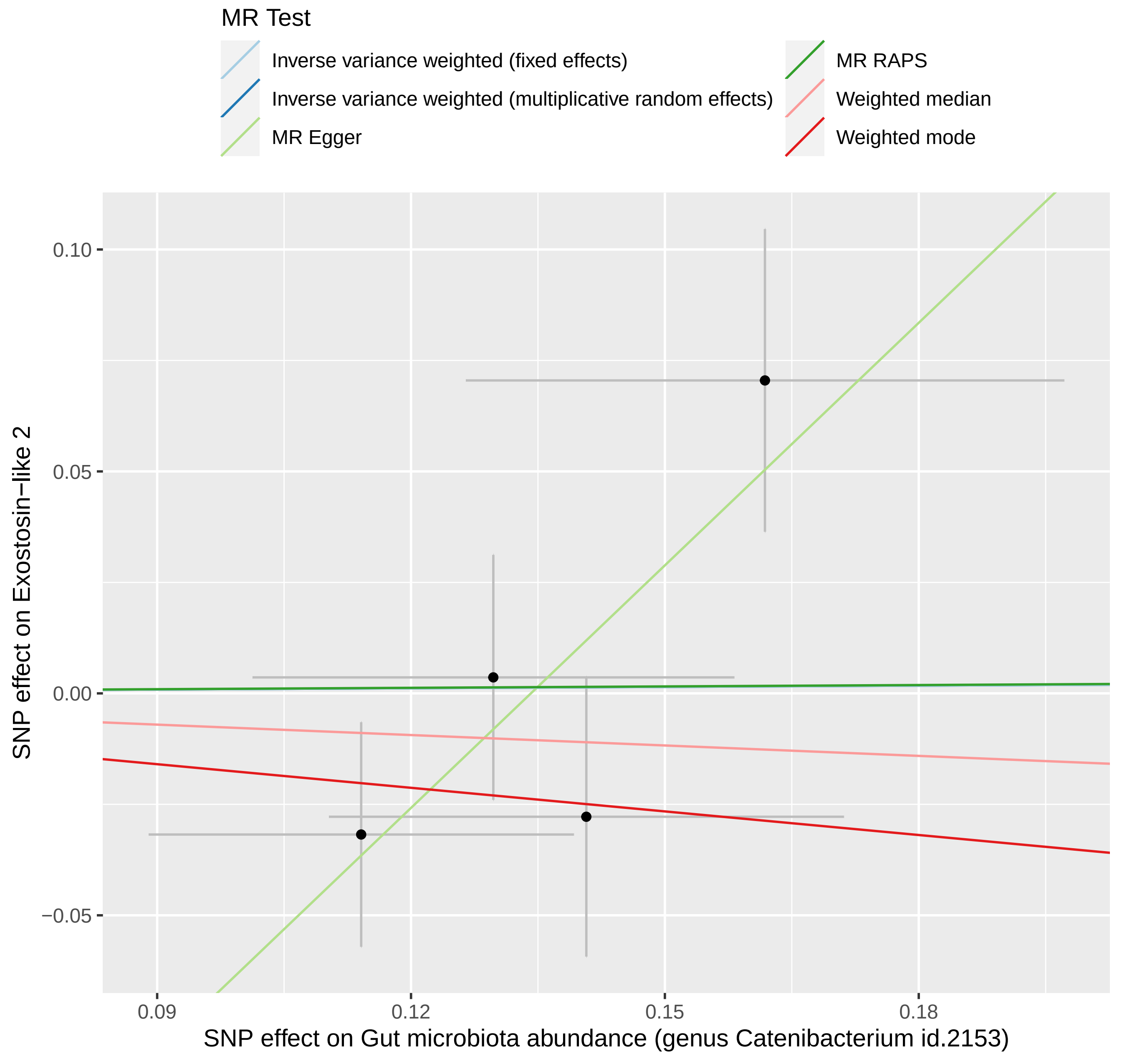

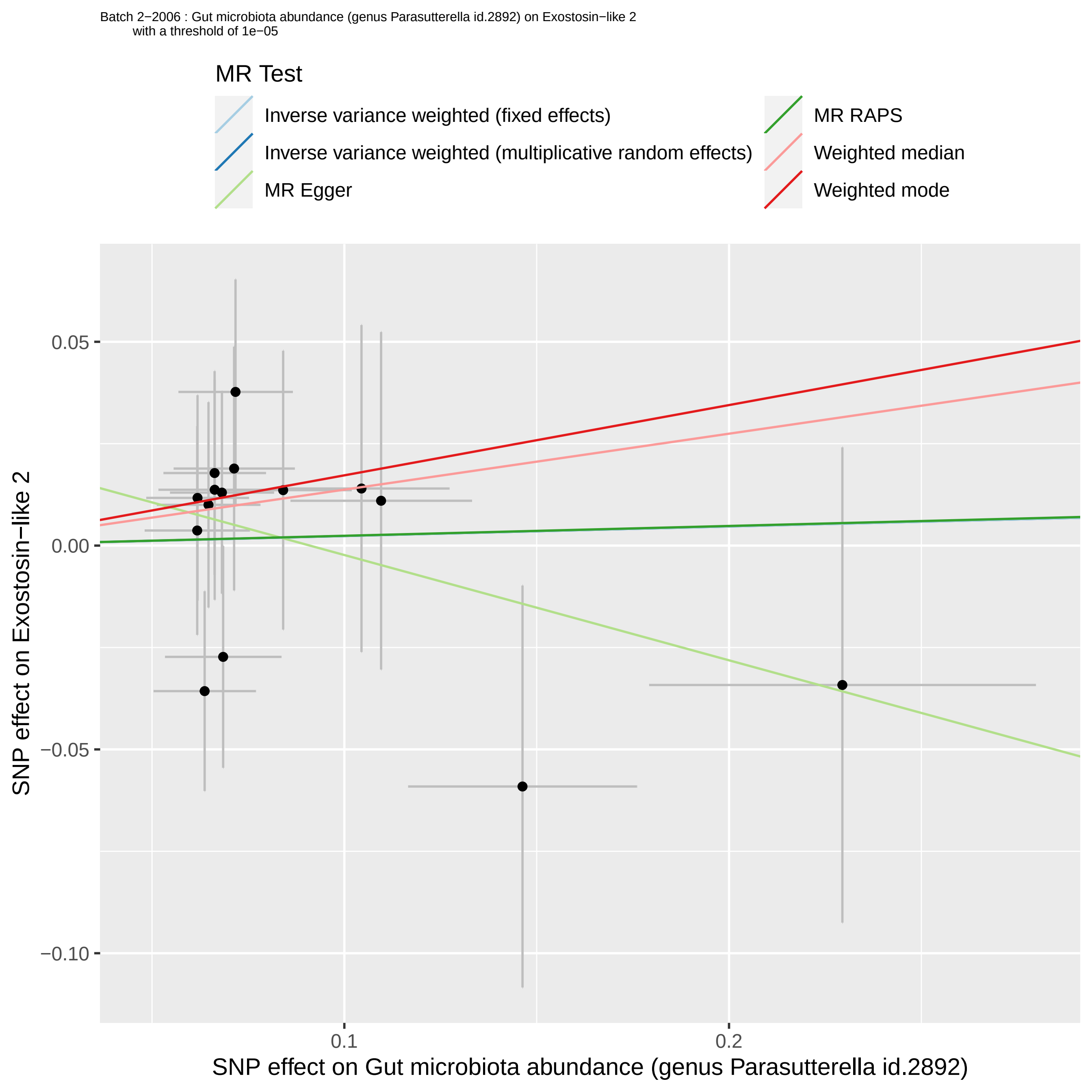
Batch 2−2006 : Gut microbiota abundance (genus Parasutterella id.2892) on Exostosin−like 2
with a threshold of 1e−05
MR Test
Inverse variance weighted (fixed effects)
Inverse variance weighted (multiplicative random effects)
MR Egger
MR RAPS
Weighted median
Weighted mode
0.05
0.00
−0.05
−0.10
0.1
0.2
SNP effect on Exostosin−like 2
SNP effect on Gut microbiota abundance (genus Parasutterella id.2892)

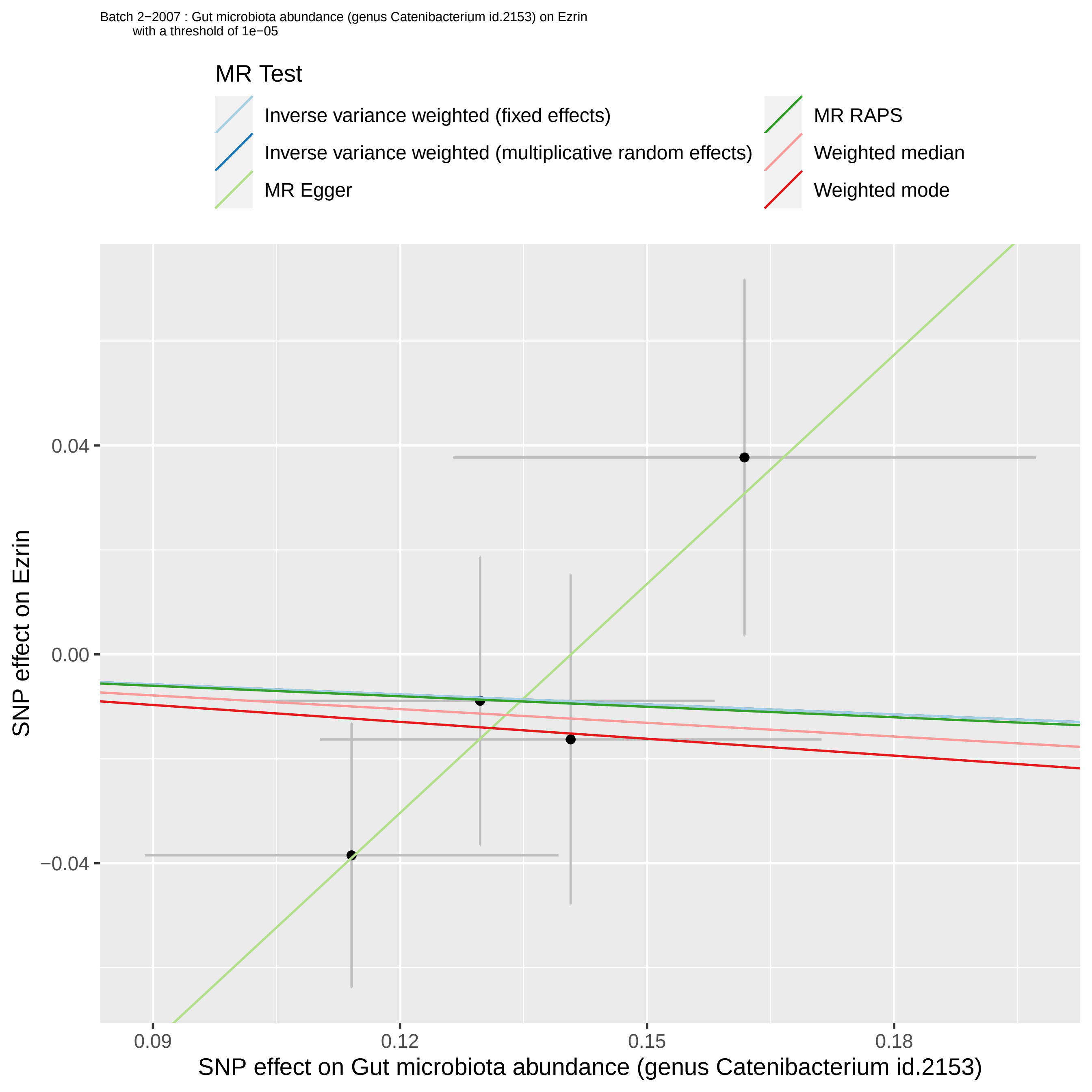

Batch 2−2007 : Gut microbiota abundance (genus Catenibacterium id.2153) on Ezrin
with a threshold of 1e−05
MR Test
Inverse variance weighted (fixed effects)
Inverse variance weighted (multiplicative random effects)
MR Egger
MR RAPS
Weighted median
Weighted mode
0.04
0.00
−0.04
0.09
0.12
0.15
0.18
SNP effect on Ezrin
SNP effect on Gut microbiota abundance (genus Catenibacterium id.2153)

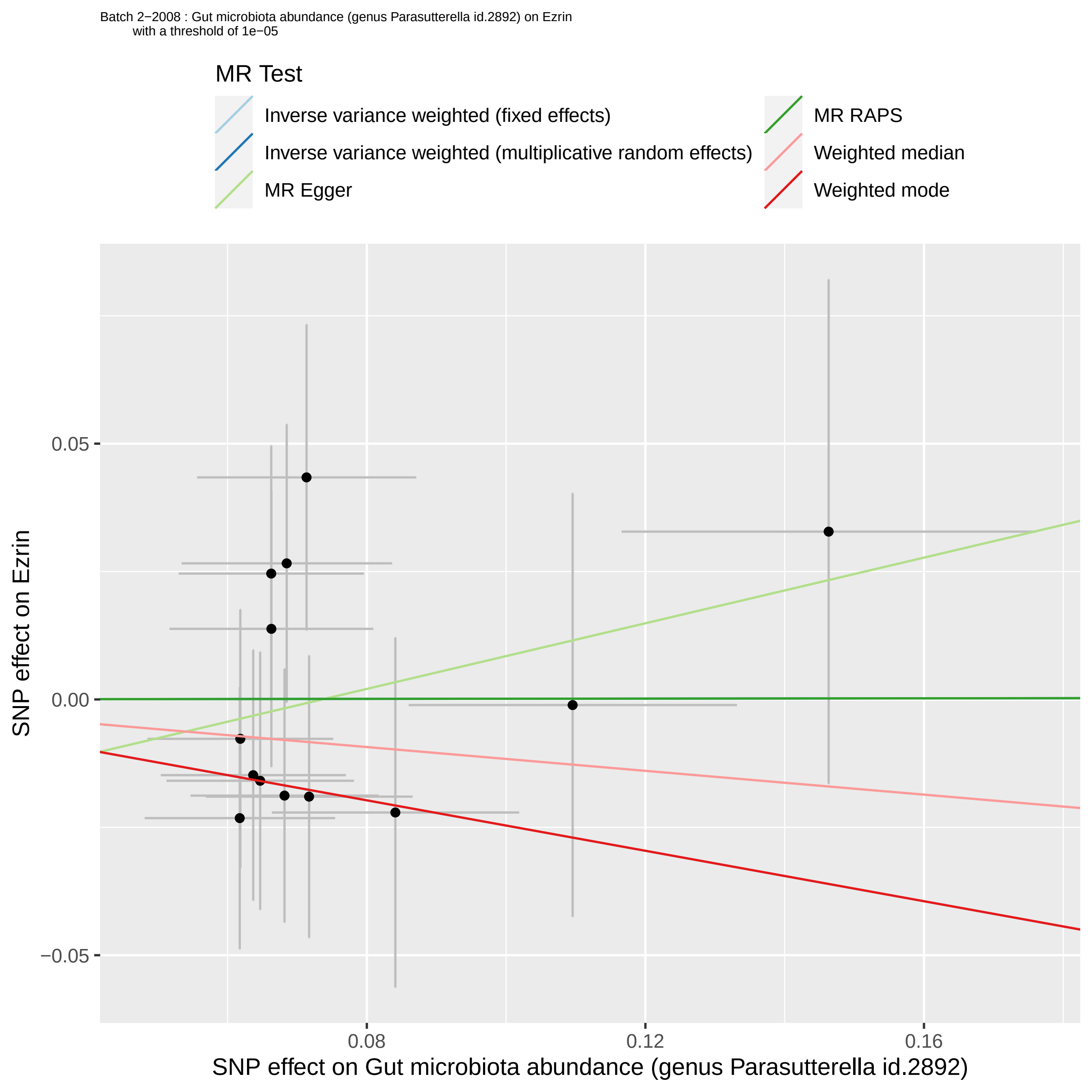
Batch 2−2008 : Gut microbiota abundance (genus Parasutterella id.2892) on Ezrin
with a threshold of 1e−05
MR Test
Inverse variance weighted (fixed effects)
Inverse variance weighted (multiplicative random effects)
MR Egger
MR RAPS
Weighted median
Weighted mode
SNP effect on Ezrin
0.05
0.00
−0.05
0.08
0.12
0.16
SNP effect on Gut microbiota abundance (genus Parasutterella id.2892)

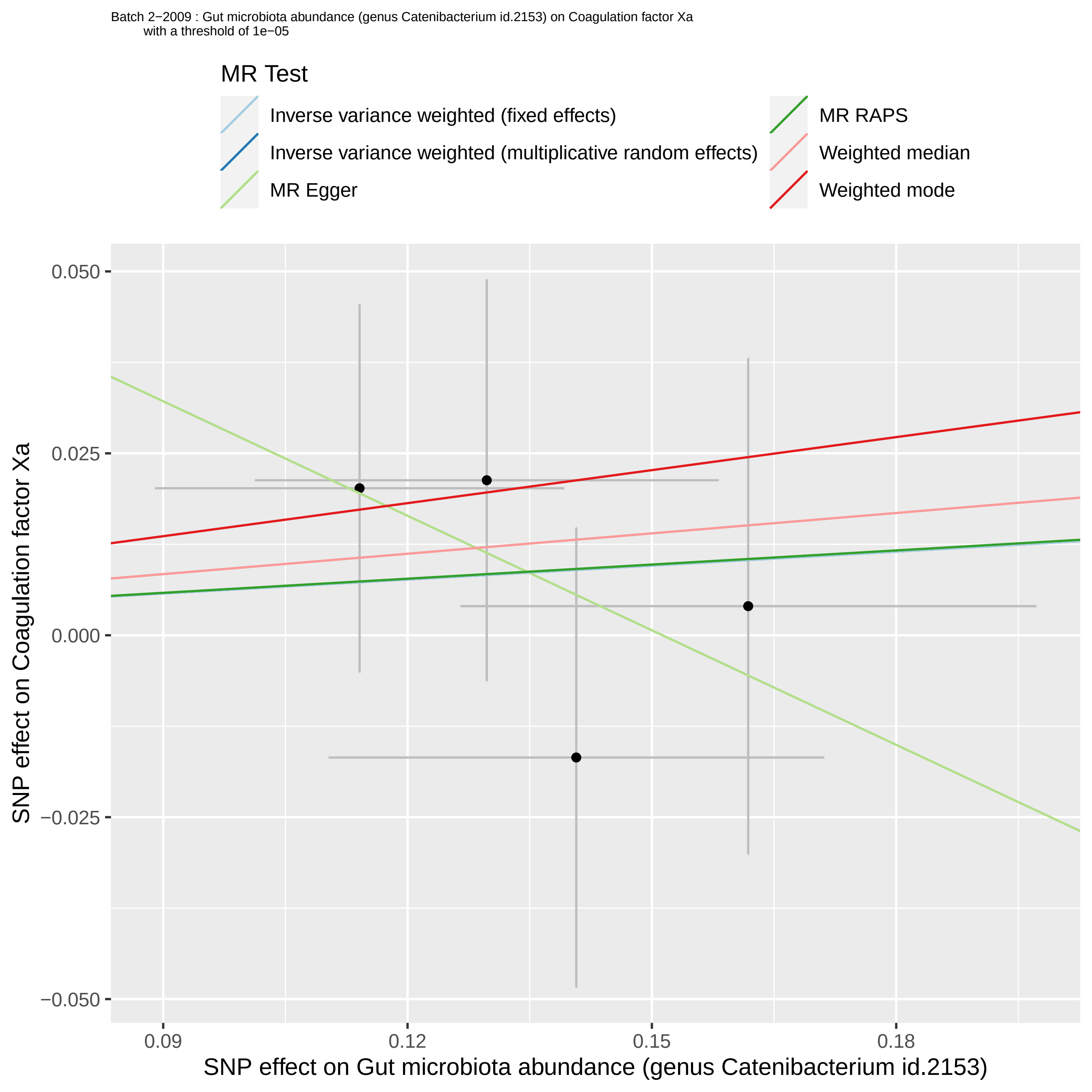

Batch 2−2009 : Gut microbiota abundance (genus Catenibacterium id.2153) on Coagulation factor Xa
with a threshold of 1e−05
MR Test
Inverse variance weighted (fixed effects)
Inverse variance weighted (multiplicative random effects)
MR Egger
MR RAPS
Weighted median
Weighted mode
0.050
0.025
0.000
−0.025
−0.050
0.09
0.12
0.15
0.18
SNP effect on Coagulation factor Xa
SNP effect on Gut microbiota abundance (genus Catenibacterium id.2153)

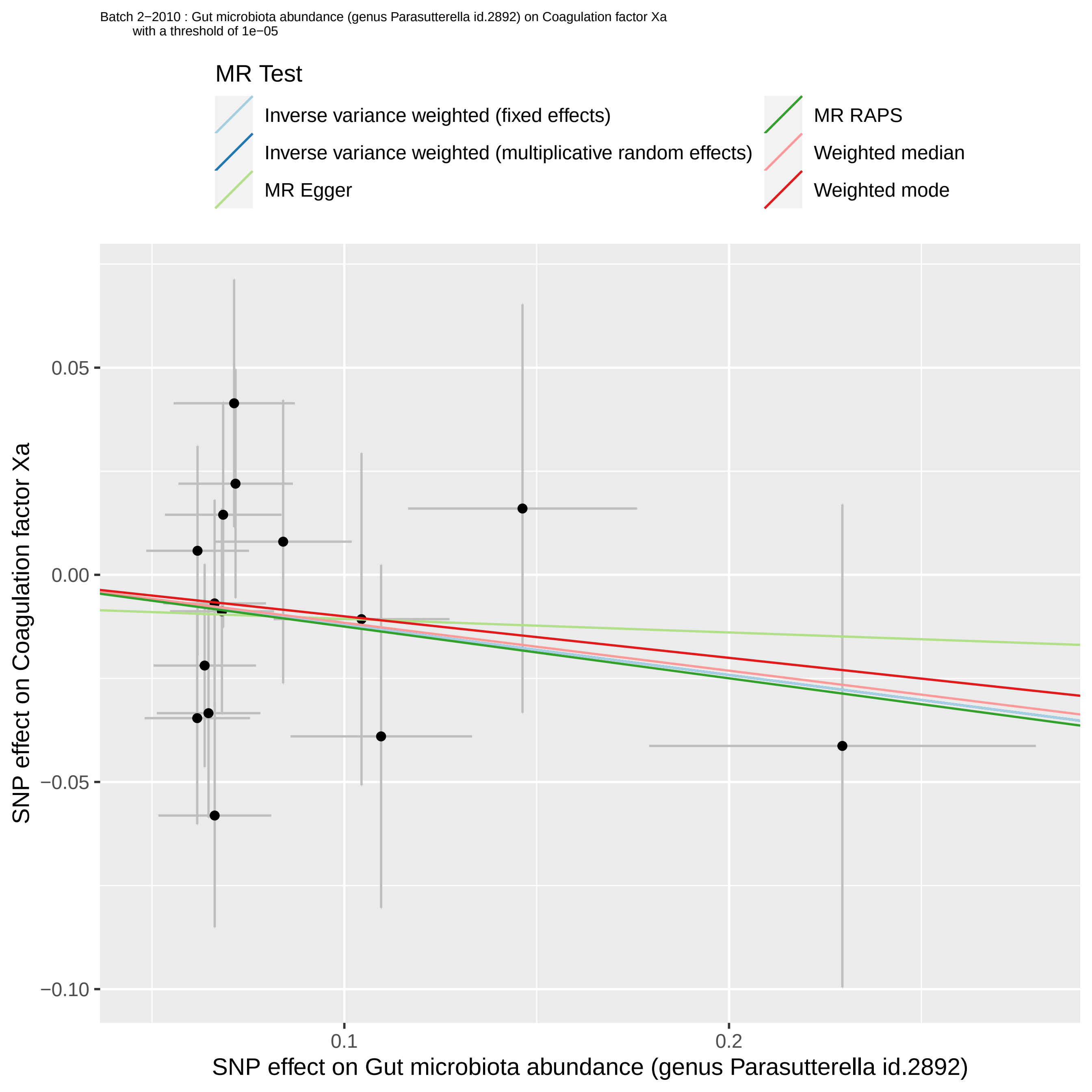
Batch 2−2010 : Gut microbiota abundance (genus Parasutterella id.2892) on Coagulation factor Xa
with a threshold of 1e−05
MR Test
Inverse variance weighted (fixed effects)
Inverse variance weighted (multiplicative random effects)
MR Egger
MR RAPS
Weighted median
Weighted mode
0.05
0.00
−0.05
−0.10
0.1
0.2
SNP effect on Coagulation factor Xa
SNP effect on Gut microbiota abundance (genus Parasutterella id.2892)

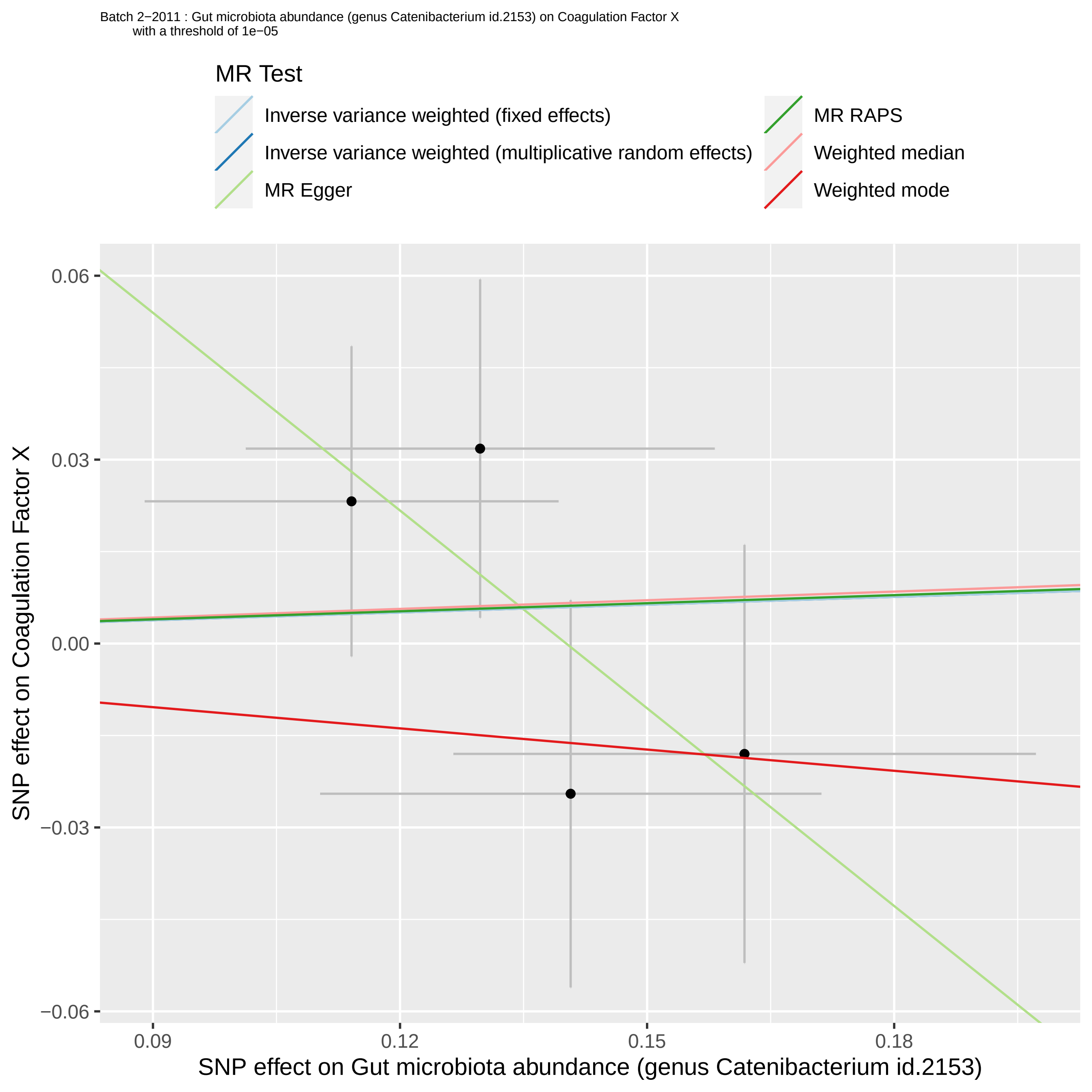

Batch 2−2011 : Gut microbiota abundance (genus Catenibacterium id.2153) on Coagulation Factor X
with a threshold of 1e−05
MR Test
Inverse variance weighted (fixed effects)
Inverse variance weighted (multiplicative random effects)
MR Egger
MR RAPS
Weighted median
Weighted mode
0.06
0.03
0.00
−0.03
−0.06
0.09
0.12
0.15
0.18
SNP effect on Coagulation Factor X
SNP effect on Gut microbiota abundance (genus Catenibacterium id.2153)

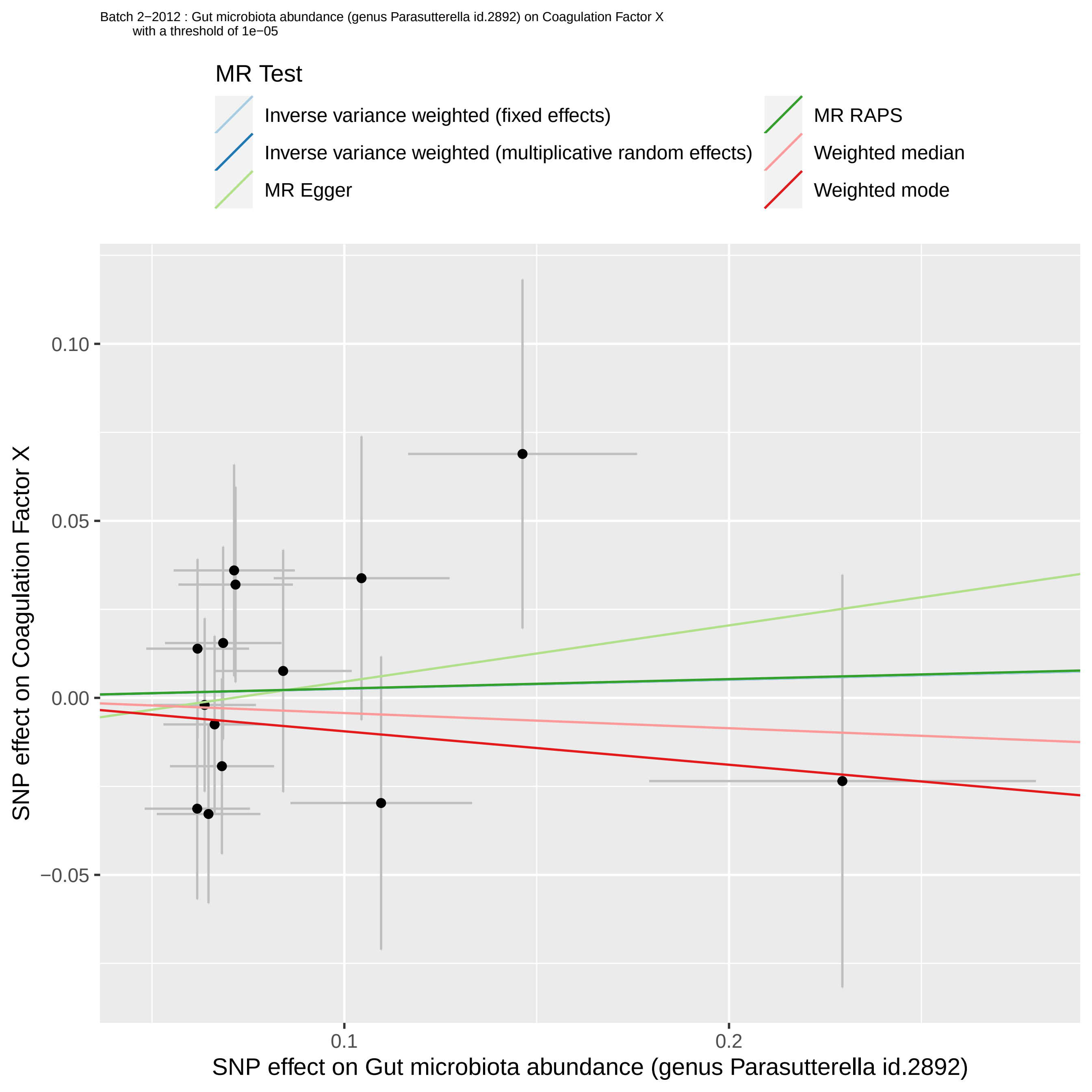
Batch 2−2012 : Gut microbiota abundance (genus Parasutterella id.2892) on Coagulation Factor X
with a threshold of 1e−05
MR Test
Inverse variance weighted (fixed effects)
Inverse variance weighted (multiplicative random effects)
MR Egger
MR RAPS
Weighted median
Weighted mode
0.10
0.05
0.00
−0.05
0.1
0.2
SNP effect on Coagulation Factor X
SNP effect on Gut microbiota abundance (genus Parasutterella id.2892)

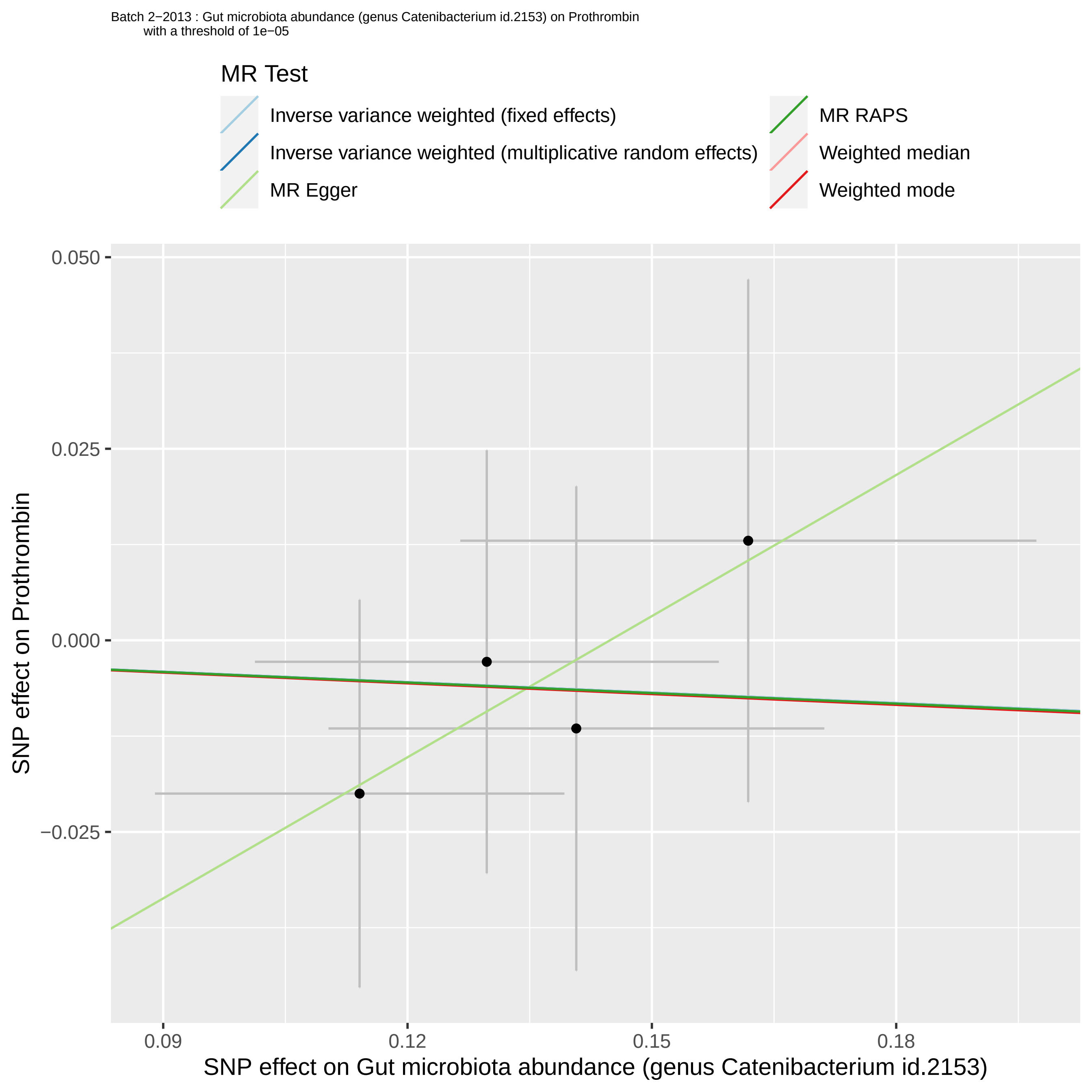
Batch 2−2013 : Gut microbiota abundance (genus Catenibacterium id.2153) on Prothrombin
with a threshold of 1e−05
MR Test
Inverse variance weighted (fixed effects)
Inverse variance weighted (multiplicative random effects)
MR Egger
MR RAPS
Weighted median
Weighted mode
0.050
0.025
0.000
−0.025
0.09
0.12
0.15
0.18
SNP effect on Prothrombin
SNP effect on Gut microbiota abundance (genus Catenibacterium id.2153)

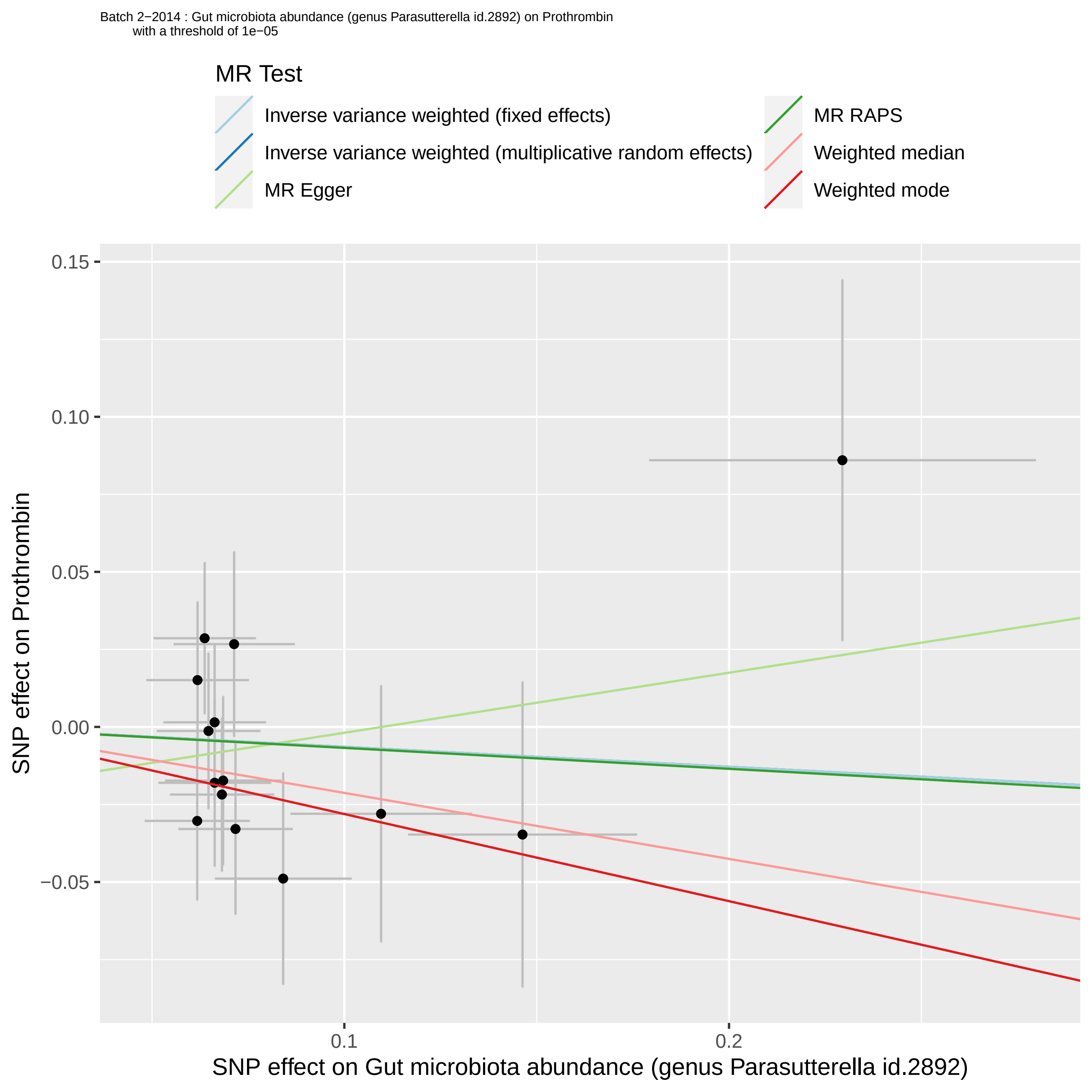
Batch 2−2014 : Gut microbiota abundance (genus Parasutterella id.2892) on Prothrombin
with a threshold of 1e−05
MR Test
Inverse variance weighted (fixed effects)
Inverse variance weighted (multiplicative random effects)
MR Egger
MR RAPS
Weighted median
Weighted mode
0.15
0.10
0.05
0.00
−0.05
0.1
0.2
SNP effect on Prothrombin
SNP effect on Gut microbiota abundance (genus Parasutterella id.2892)

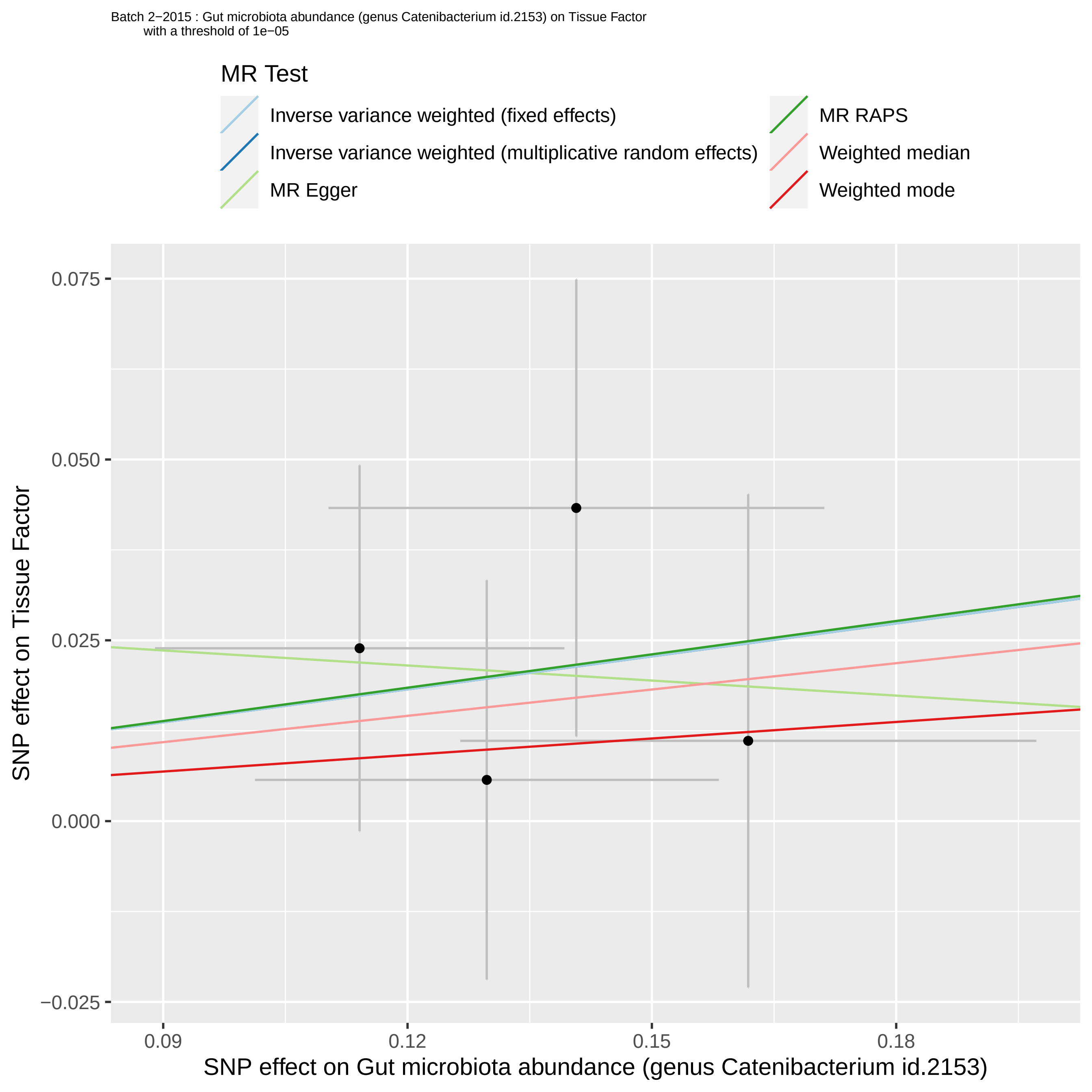

Batch 2−2015 : Gut microbiota abundance (genus Catenibacterium id.2153) on Tissue Factor
with a threshold of 1e−05
MR Test
Inverse variance weighted (fixed effects)
Inverse variance weighted (multiplicative random effects)
MR Egger
MR RAPS
Weighted median
Weighted mode
0.075
0.050
0.025
0.000
−0.025
0.09
0.12
0.15
0.18
SNP effect on Tissue Factor
SNP effect on Gut microbiota abundance (genus Catenibacterium id.2153)

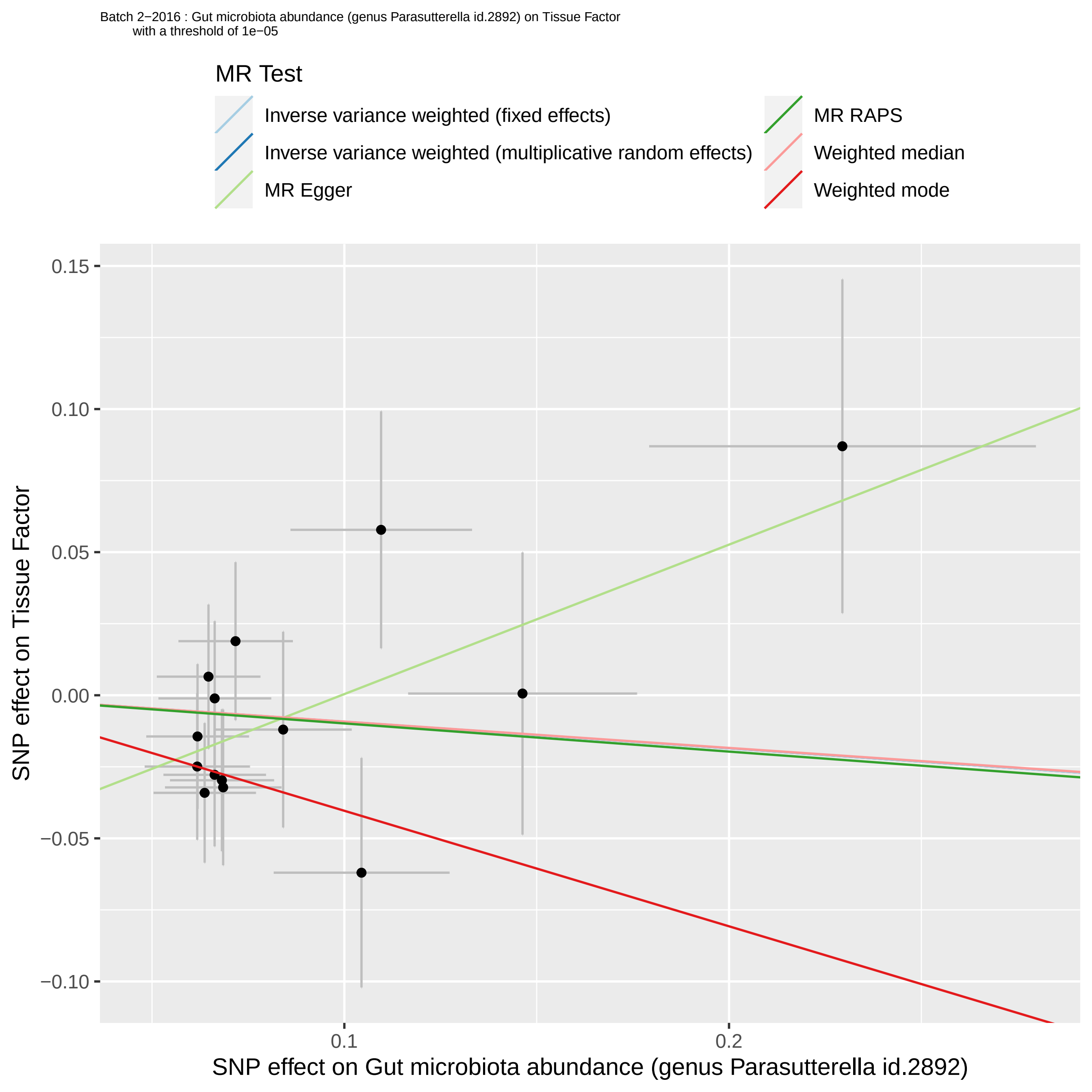
Batch 2−2016 : Gut microbiota abundance (genus Parasutterella id.2892) on Tissue Factor
with a threshold of 1e−05
MR Test
Inverse variance weighted (fixed effects)
Inverse variance weighted (multiplicative random effects)
MR Egger
MR RAPS
Weighted median
Weighted mode
0.15
0.10
0.05
0.00
−0.05
−0.10
0.1
0.2
SNP effect on Tissue Factor
SNP effect on Gut microbiota abundance (genus Parasutterella id.2892)

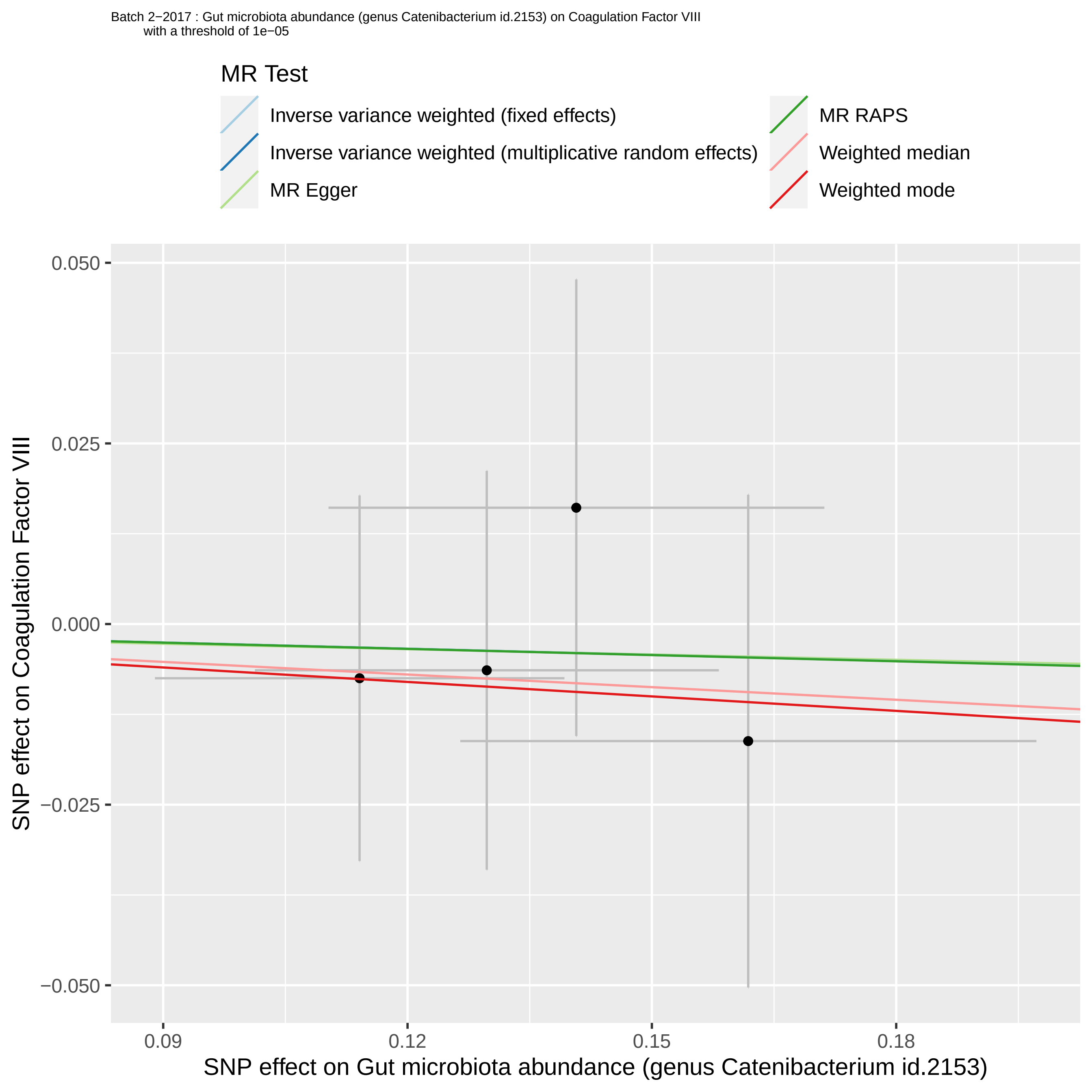

Batch 2−2017 : Gut microbiota abundance (genus Catenibacterium id.2153) on Coagulation Factor VIII
with a threshold of 1e−05
MR Test
Inverse variance weighted (fixed effects)
Inverse variance weighted (multiplicative random effects)
MR Egger
MR RAPS
Weighted median
Weighted mode
0.050
0.025
0.000
−0.025
−0.050
0.09
0.12
0.15
0.18
SNP effect on Coagulation Factor VIII
SNP effect on Gut microbiota abundance (genus Catenibacterium id.2153)

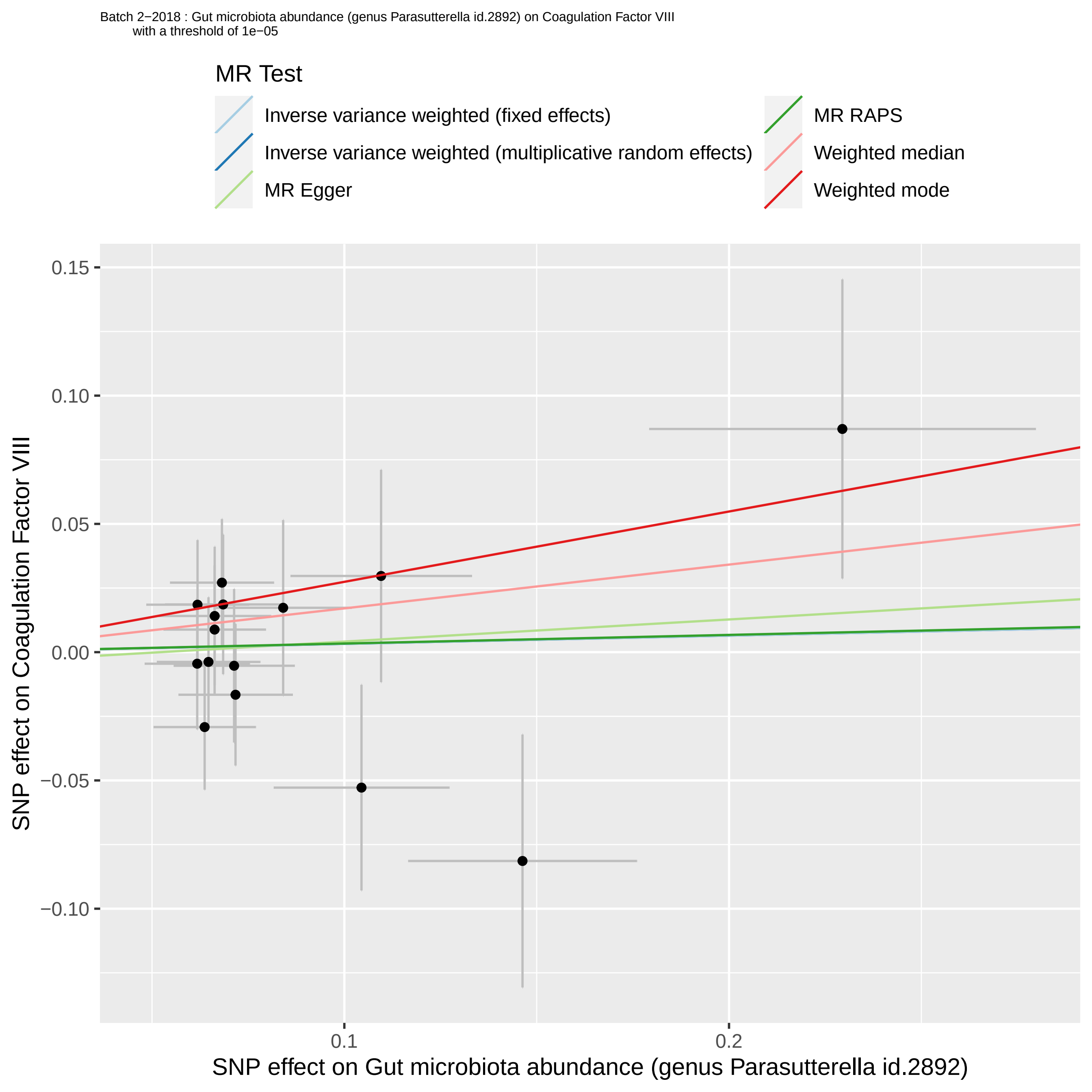
Batch 2−2018 : Gut microbiota abundance (genus Parasutterella id.2892) on Coagulation Factor VIII
with a threshold of 1e−05
MR Test
Inverse variance weighted (fixed effects)
Inverse variance weighted (multiplicative random effects)
MR Egger
MR RAPS
Weighted median
Weighted mode
0.15
0.10
0.05
0.00
−0.05
−0.10
0.1
0.2
SNP effect on Coagulation Factor VIII
SNP effect on Gut microbiota abundance (genus Parasutterella id.2892)

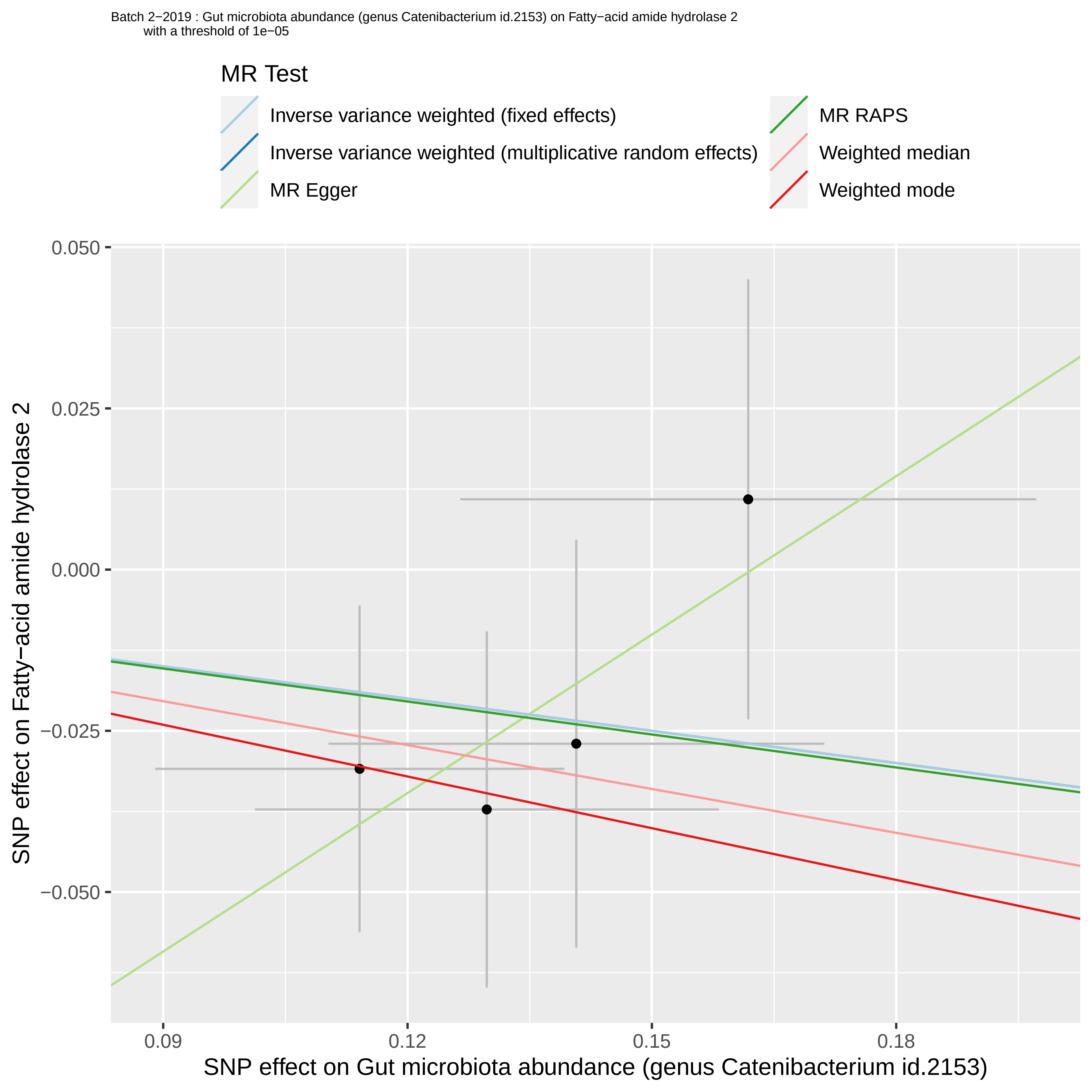
Batch 2−2019 : Gut microbiota abundance (genus Catenibacterium id.2153) on Fatty−acid amide hydrolase 2
with a threshold of 1e−05
MR Test
Inverse variance weighted (fixed effects)
Inverse variance weighted (multiplicative random effects)
MR Egger
MR RAPS
Weighted median
Weighted mode
0.050
0.025
0.000
−0.025
−0.050
0.09
0.12
0.15
0.18
SNP effect on Fatty−acid amide hydrolase 2
SNP effect on Gut microbiota abundance (genus Catenibacterium id.2153)

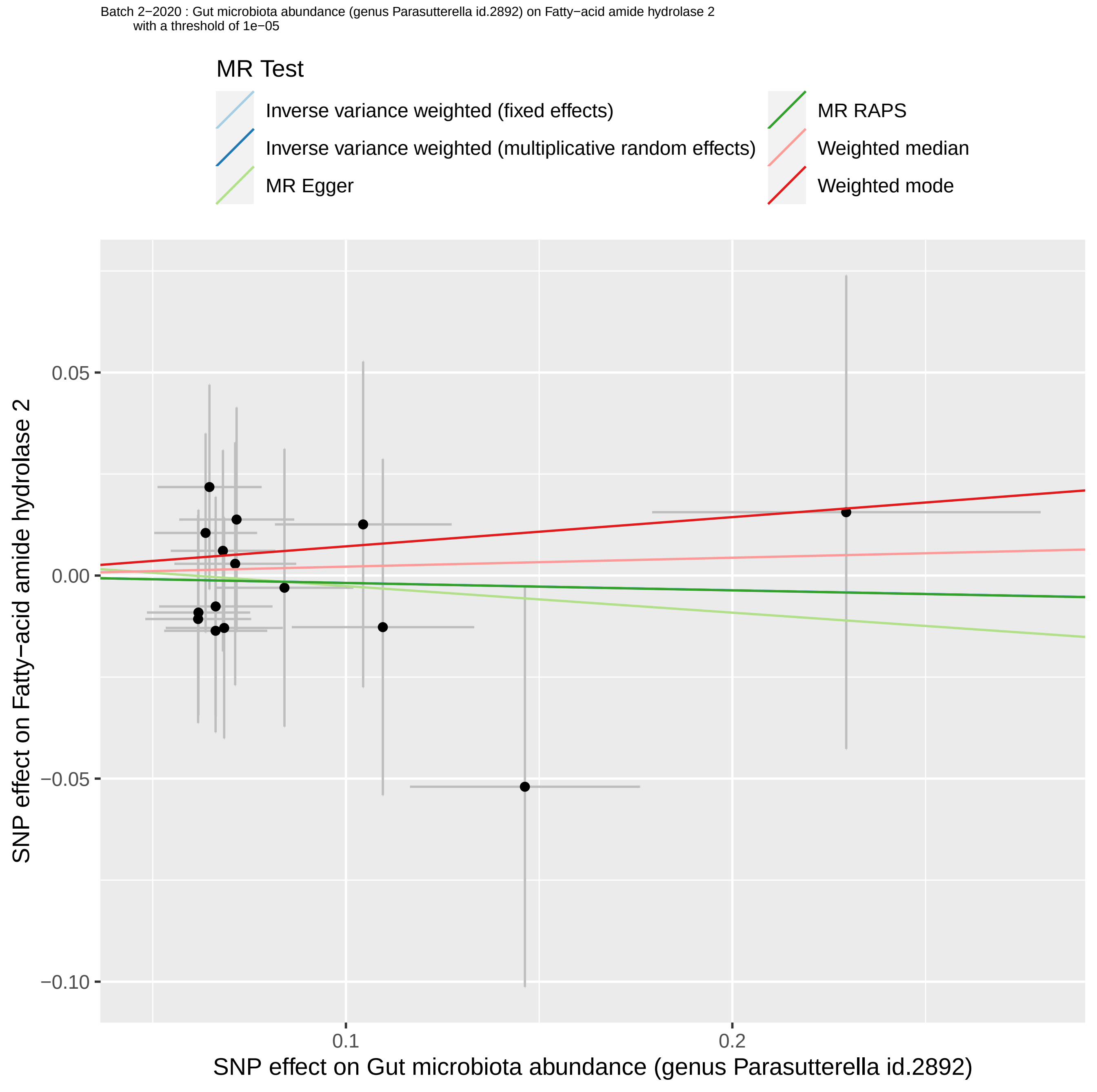
Batch 2−2020 : Gut microbiota abundance (genus Parasutterella id.2892) on Fatty−acid amide hydrolase 2
with a threshold of 1e−05
MR Test
Inverse variance weighted (fixed effects)
Inverse variance weighted (multiplicative random effects)
MR Egger
MR RAPS
Weighted median
Weighted mode
0.05
0.00
−0.05
−0.10
0.1
0.2
SNP effect on Fatty−acid amide hydrolase 2
SNP effect on Gut microbiota abundance (genus Parasutterella id.2892)

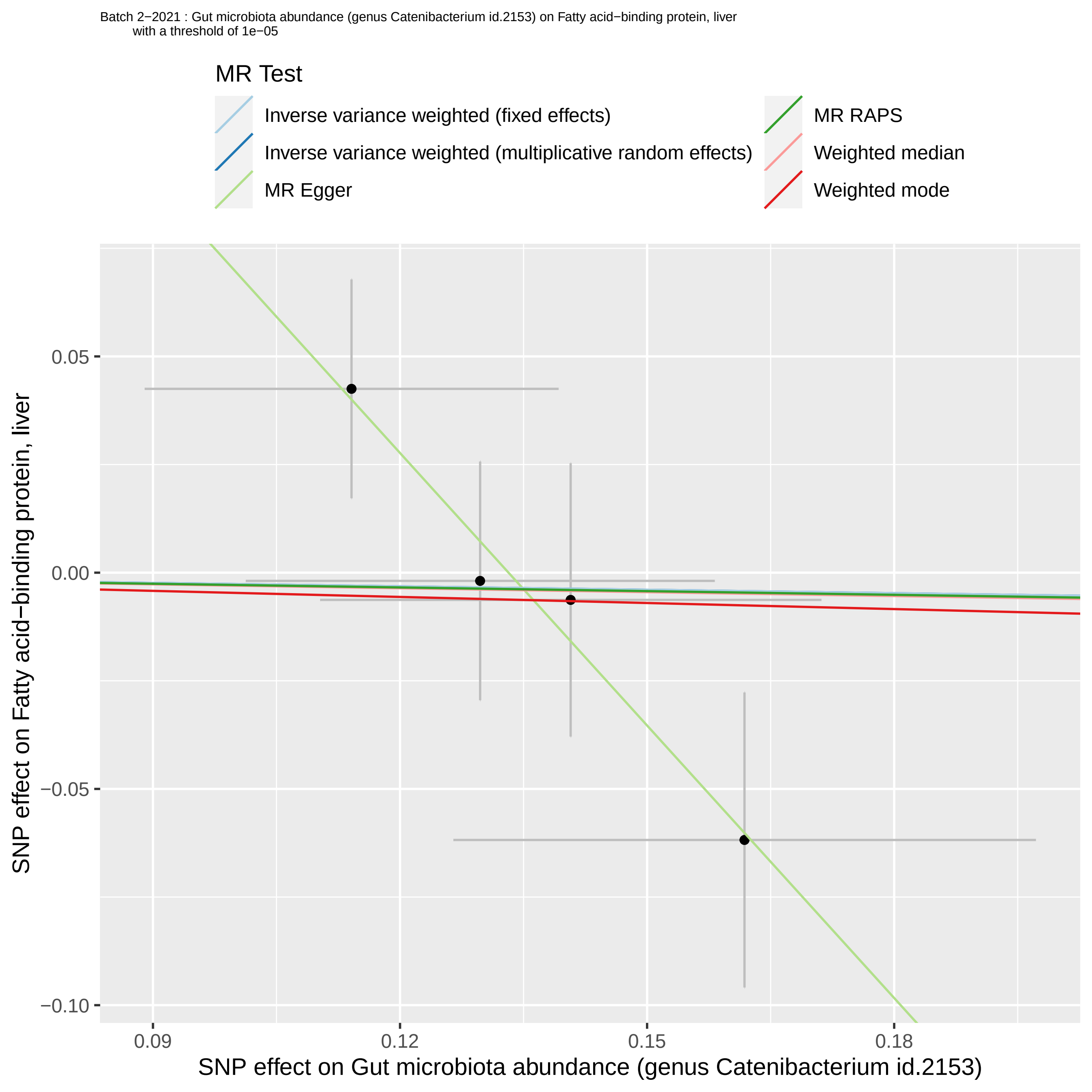

Batch 2−2021 : Gut microbiota abundance (genus Catenibacterium id.2153) on Fatty acid−binding protein, liver
with a threshold of 1e−05
MR Test
Inverse variance weighted (fixed effects)
Inverse variance weighted (multiplicative random effects)
MR Egger
MR RAPS
Weighted median
Weighted mode
0.05
0.00
−0.05
−0.10
0.09
0.12
0.15
0.18
SNP effect on Fatty acid−binding protein, liver
SNP effect on Gut microbiota abundance (genus Catenibacterium id.2153)

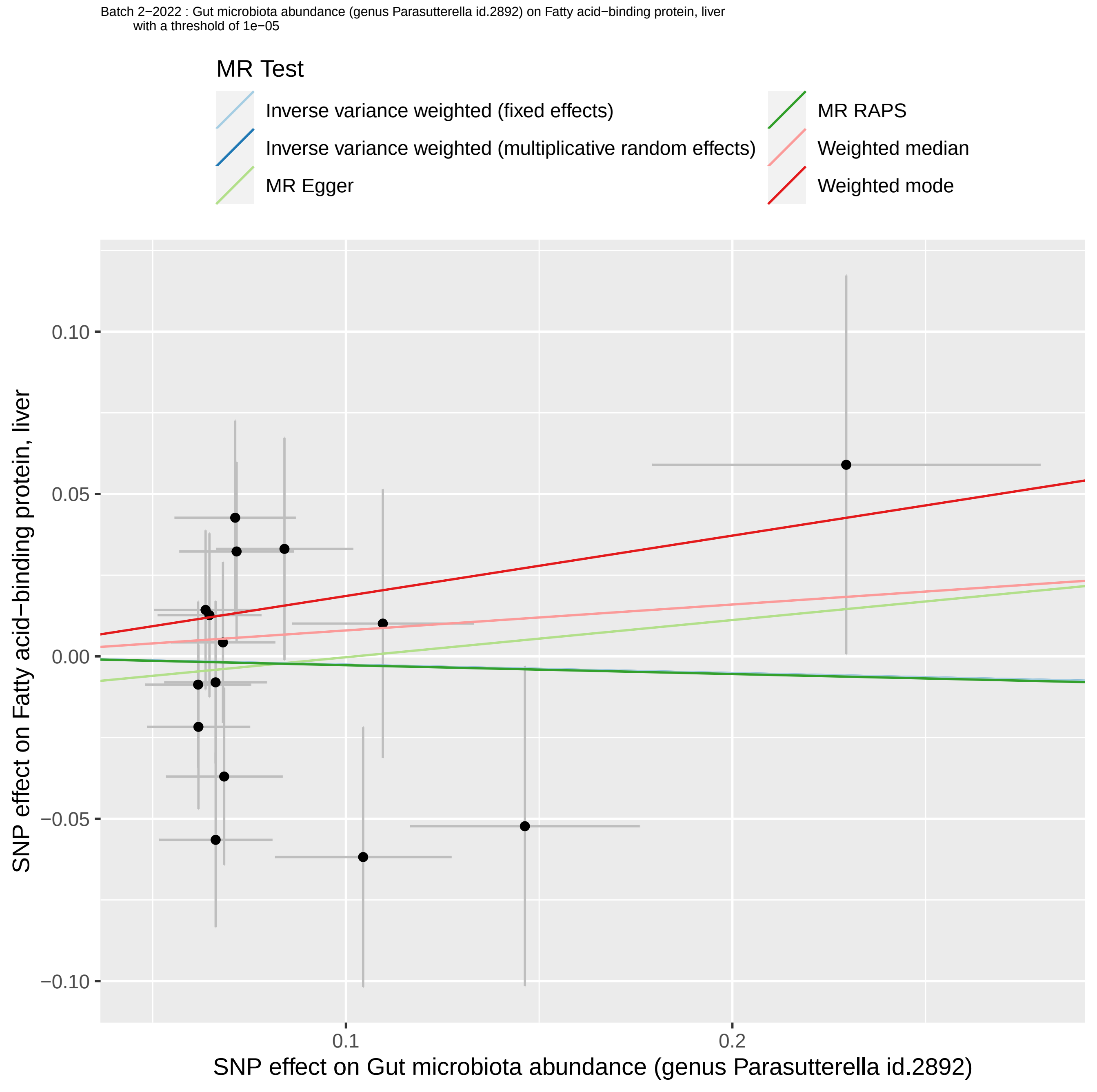
Batch 2−2022 : Gut microbiota abundance (genus Parasutterella id.2892) on Fatty acid−binding protein, liver
with a threshold of 1e−05
MR Test
Inverse variance weighted (fixed effects)
Inverse variance weighted (multiplicative random effects)
MR Egger
MR RAPS
Weighted median
Weighted mode
0.10
0.05
0.00
−0.05
−0.10
0.1
0.2
SNP effect on Gut microbiota abundance (genus Parasutterella id.2892)
SNP effect on Fatty acid−binding protein, liver

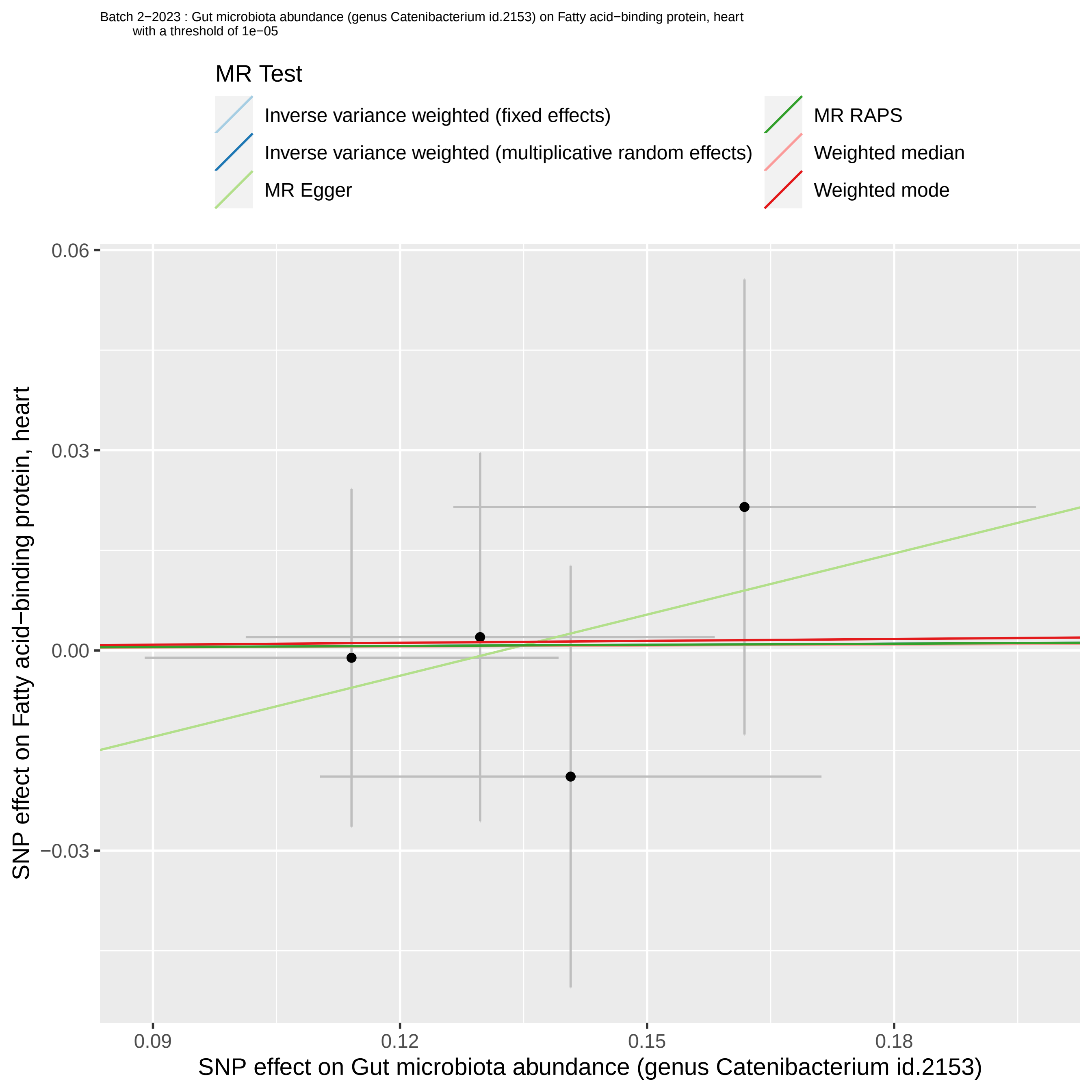

Batch 2−2023 : Gut microbiota abundance (genus Catenibacterium id.2153) on Fatty acid−binding protein, heart
with a threshold of 1e−05
MR Test
Inverse variance weighted (fixed effects)
Inverse variance weighted (multiplicative random effects)
MR Egger
MR RAPS
Weighted median
Weighted mode
0.06
0.03
0.00
−0.03
0.09
0.12
0.15
0.18
SNP effect on Fatty acid−binding protein, heart
SNP effect on Gut microbiota abundance (genus Catenibacterium id.2153)

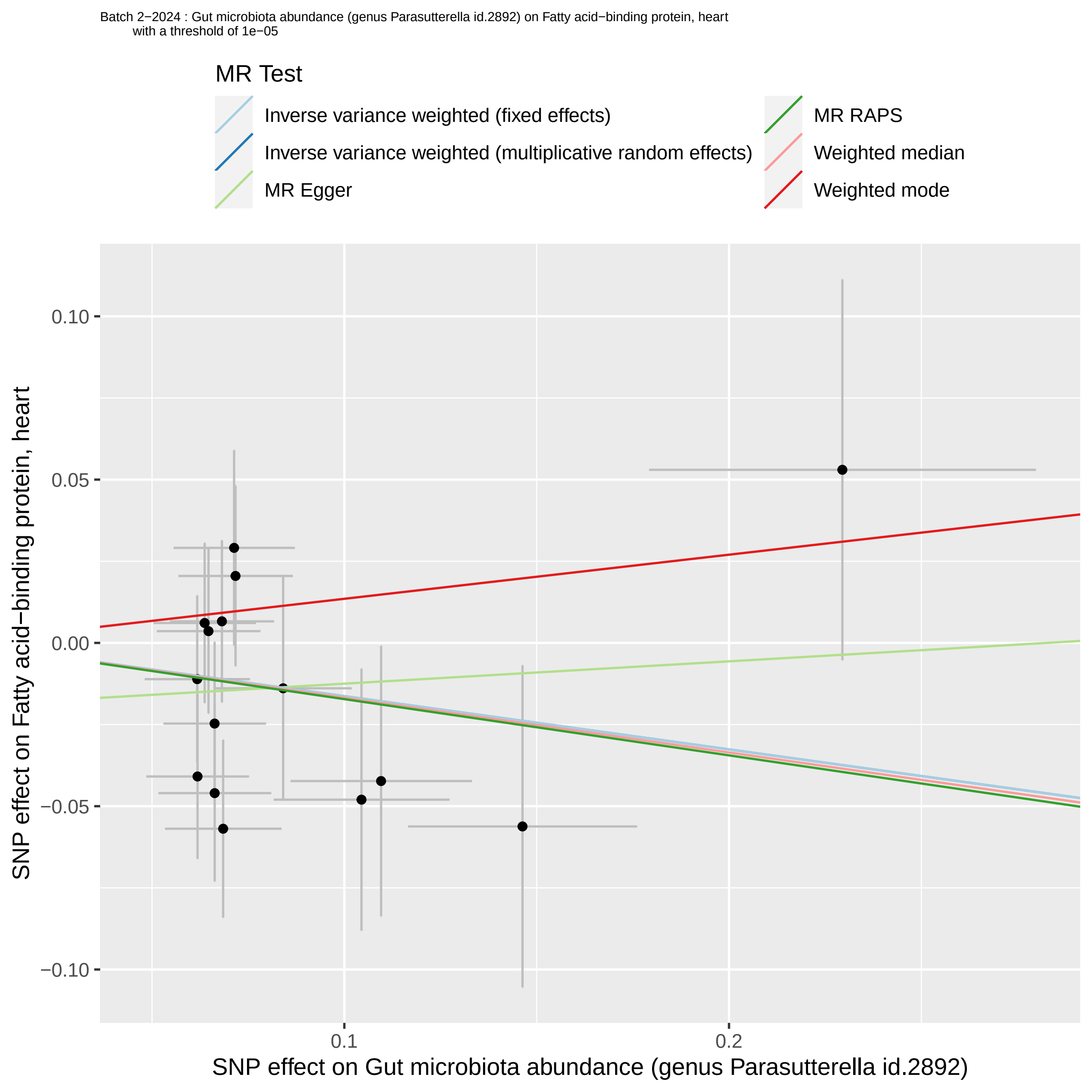

Batch 2−2024 : Gut microbiota abundance (genus Parasutterella id.2892) on Fatty acid−binding protein, heart
with a threshold of 1e−05
MR Test
Inverse variance weighted (fixed effects)
Inverse variance weighted (multiplicative random effects)
MR Egger
MR RAPS
Weighted median
Weighted mode
0.10
0.05
0.00
−0.05
−0.10
0.1
0.2
SNP effect on Fatty acid−binding protein, heart
SNP effect on Gut microbiota abundance (genus Parasutterella id.2892)

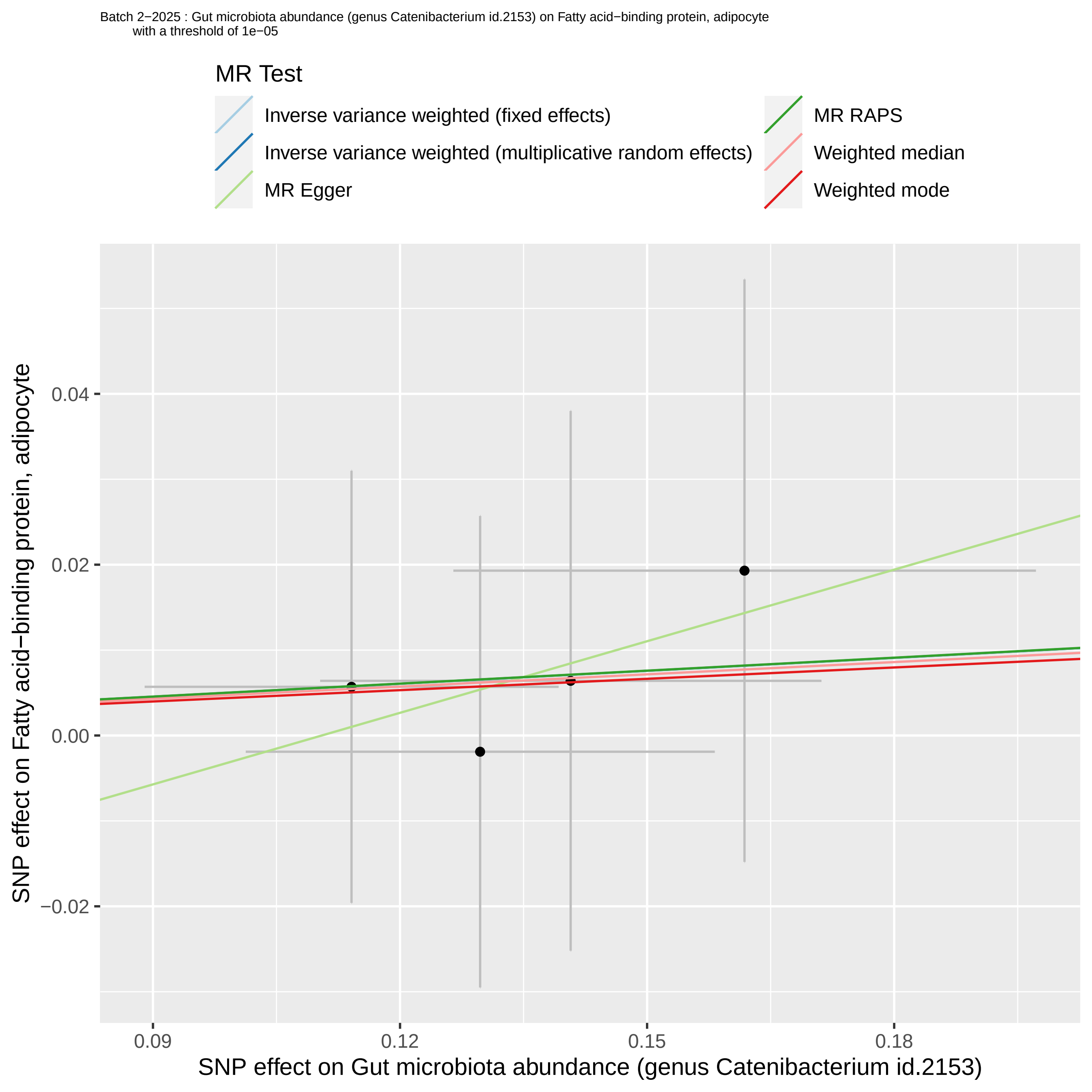

Batch 2−2025 : Gut microbiota abundance (genus Catenibacterium id.2153) on Fatty acid−binding protein, adipocyte
with a threshold of 1e−05
MR Test
Inverse variance weighted (fixed effects)
Inverse variance weighted (multiplicative random effects)
MR Egger
MR RAPS
Weighted median
Weighted mode
SNP effect on Fatty acid−binding protein, adipocyte
0.04
0.02
0.00
−0.02
0.09
0.12
0.15
0.18
SNP effect on Gut microbiota abundance (genus Catenibacterium id.2153)

Batch 2−2026 : Gut microbiota abundance (genus Parasutterella id.2892) on Fatty acid−binding protein, adipocyte
with a threshold of 1e−05

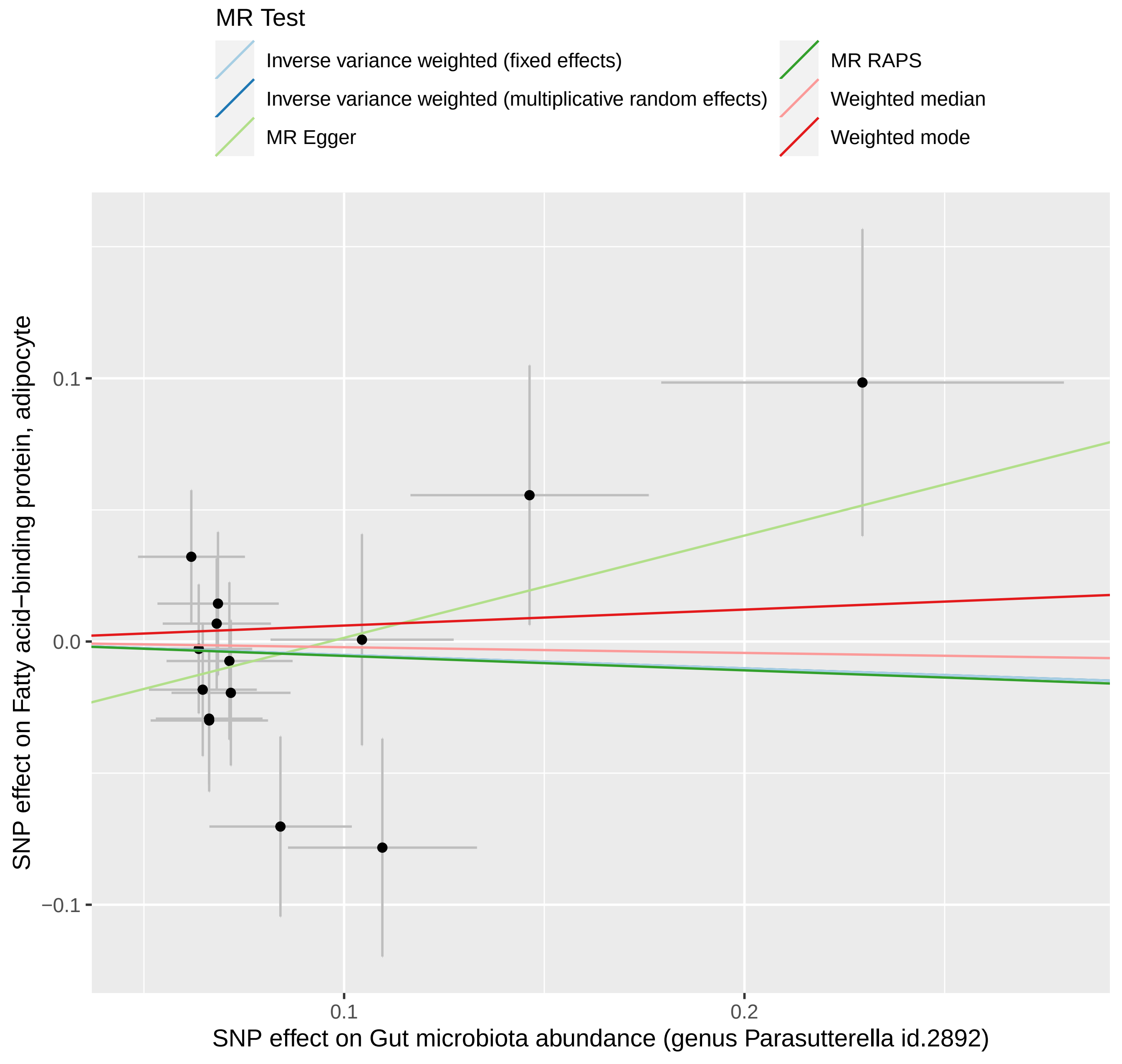

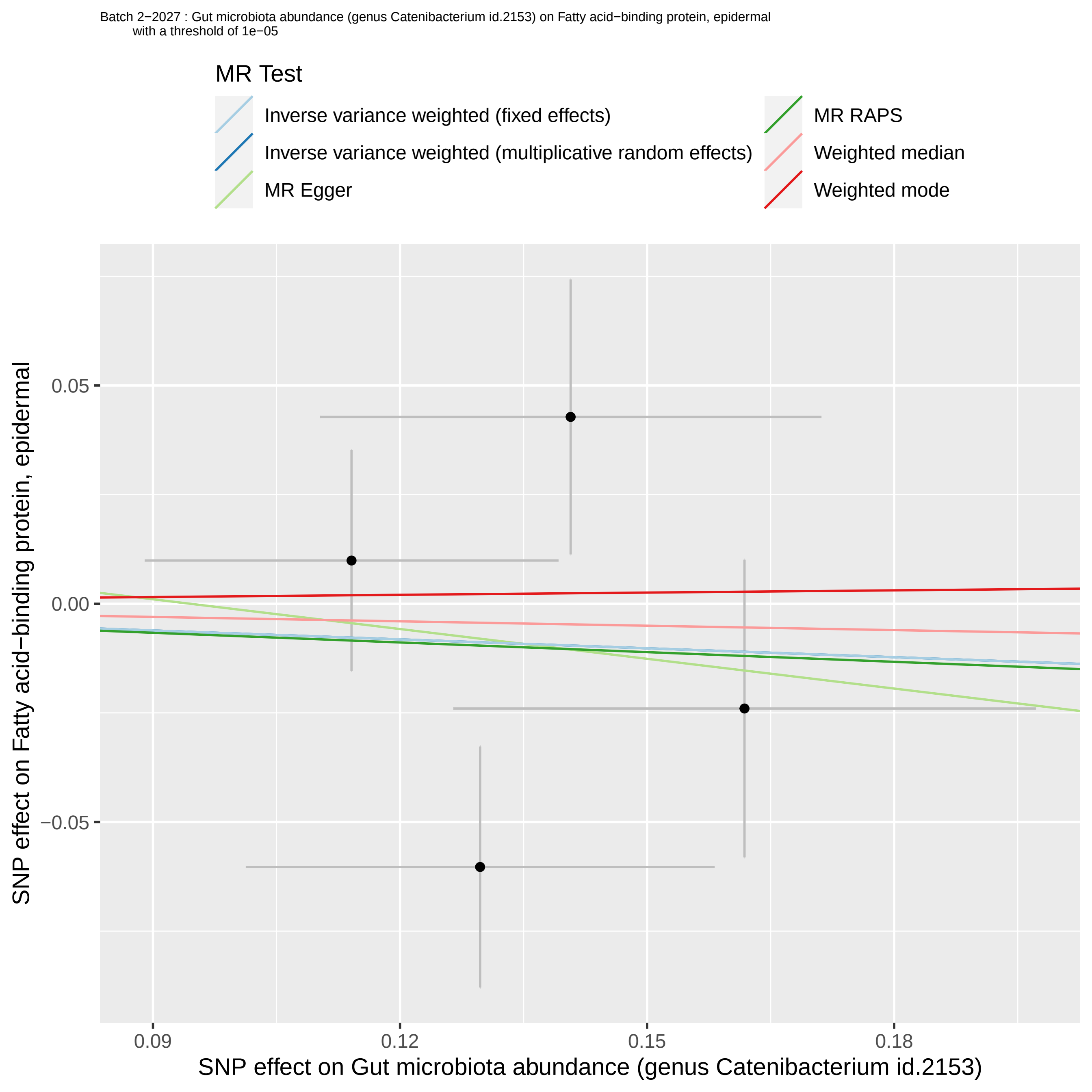

Batch 2−2027 : Gut microbiota abundance (genus Catenibacterium id.2153) on Fatty acid−binding protein, epidermal
with a threshold of 1e−05
MR Test
Inverse variance weighted (fixed effects)
Inverse variance weighted (multiplicative random effects)
MR Egger
MR RAPS
Weighted median
Weighted mode
0.05
0.00
−0.05
0.09
0.12
0.15
0.18
SNP effect on Fatty acid−binding protein, epidermal
SNP effect on Gut microbiota abundance (genus Catenibacterium id.2153)

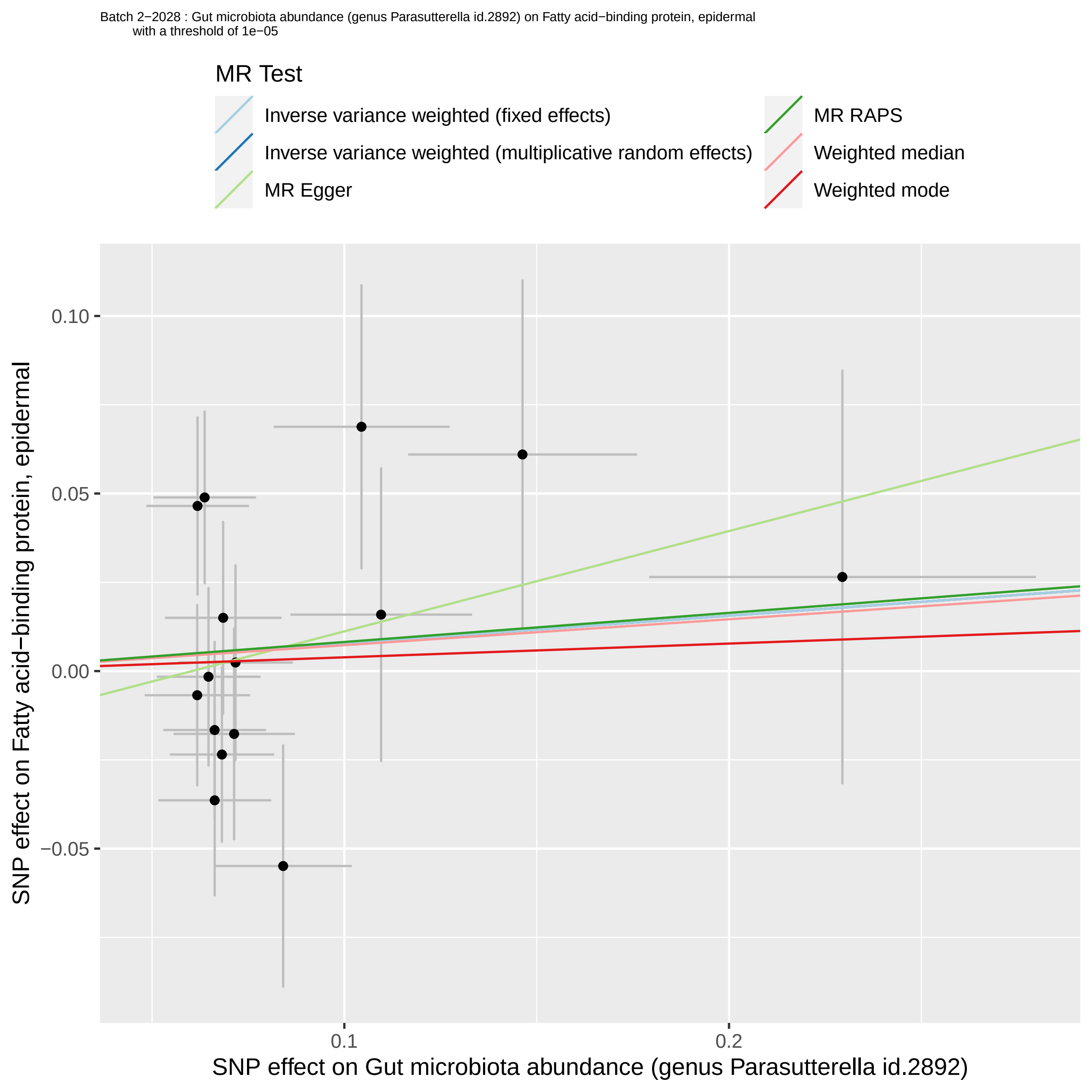
Batch 2−2028 : Gut microbiota abundance (genus Parasutterella id.2892) on Fatty acid−binding protein, epidermal
with a threshold of 1e−05
MR Test
Inverse variance weighted (fixed effects)
Inverse variance weighted (multiplicative random effects)
MR Egger
MR RAPS
Weighted median
Weighted mode
0.10
0.05
0.00
−0.05
0.1
0.2
SNP effect on Fatty acid−binding protein, epidermal
SNP effect on Gut microbiota abundance (genus Parasutterella id.2892)

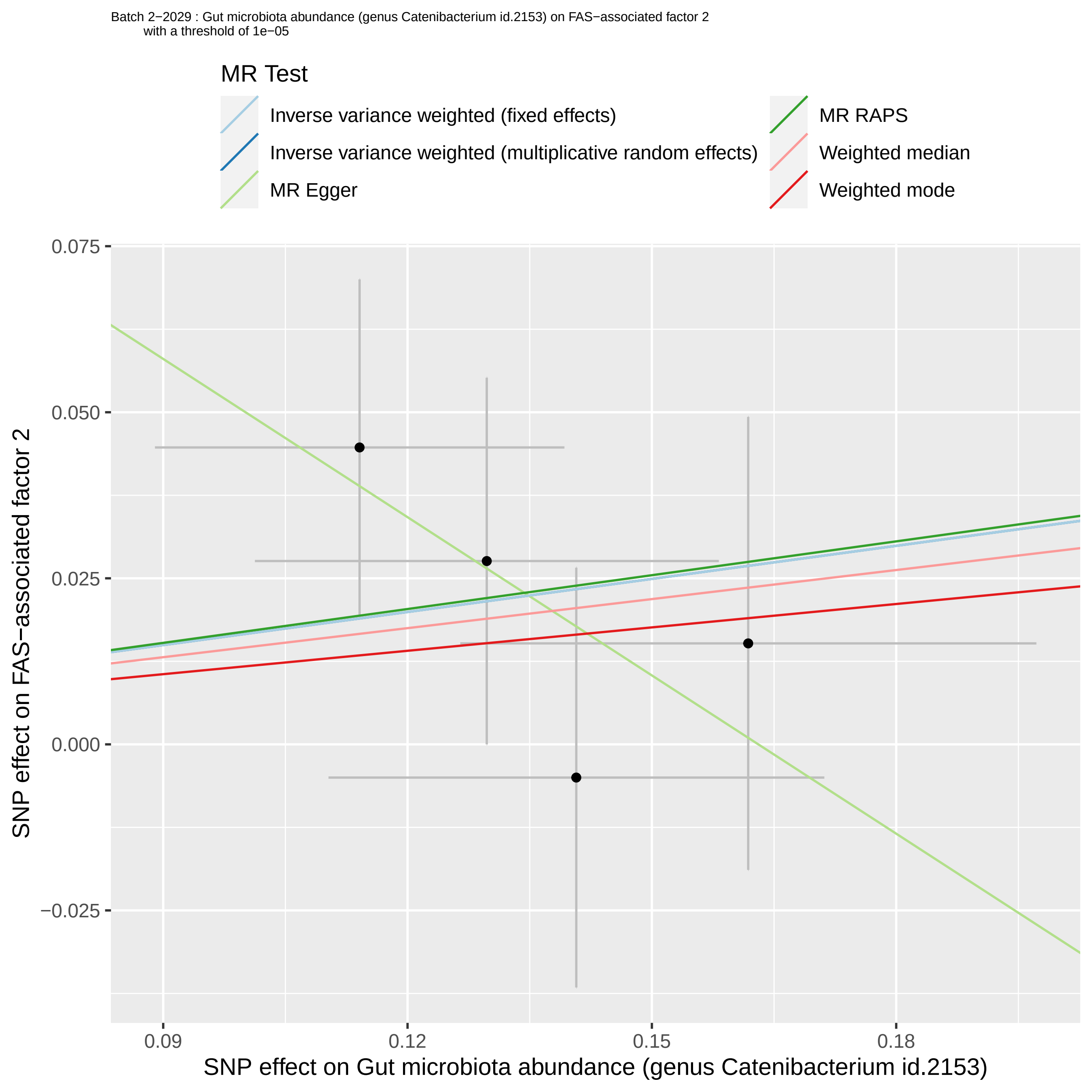
Batch 2−2029 : Gut microbiota abundance (genus Catenibacterium id.2153) on FAS−associated factor 2
with a threshold of 1e−05
MR Test
Inverse variance weighted (fixed effects)
Inverse variance weighted (multiplicative random effects)
MR Egger
MR RAPS
Weighted median
Weighted mode
0.075
0.050
0.025
0.000
−0.025
0.09
0.12
0.15
0.18
SNP effect on FAS−associated factor 2
SNP effect on Gut microbiota abundance (genus Catenibacterium id.2153)

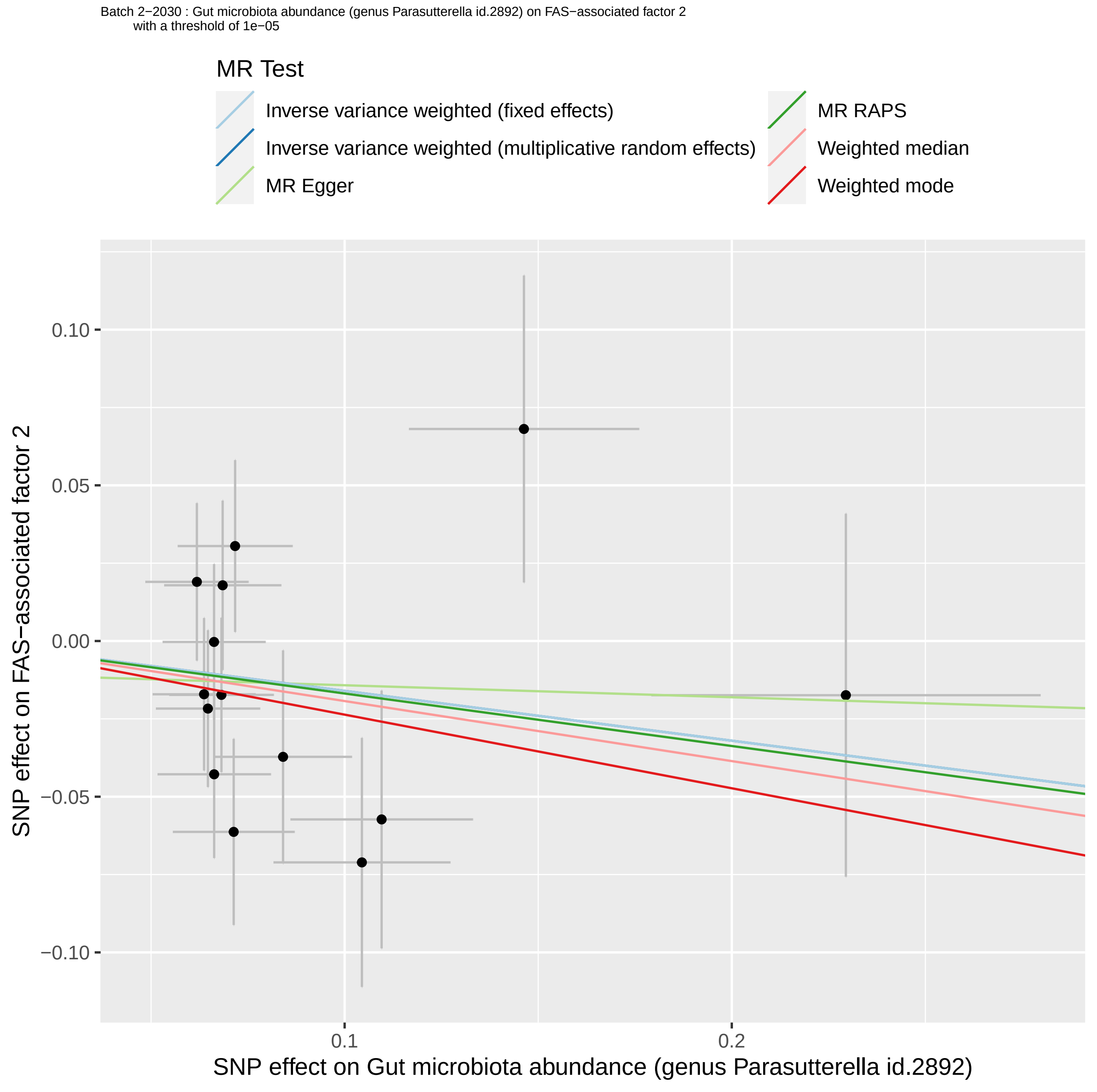
Batch 2−2030 : Gut microbiota abundance (genus Parasutterella id.2892) on FAS−associated factor 2
with a threshold of 1e−05
MR Test
Inverse variance weighted (fixed effects)
Inverse variance weighted (multiplicative random effects)
MR Egger
MR RAPS
Weighted median
Weighted mode
0.10
0.05
0.00
−0.05
−0.10
0.1
0.2
SNP effect on FAS−associated factor 2
SNP effect on Gut microbiota abundance (genus Parasutterella id.2892)

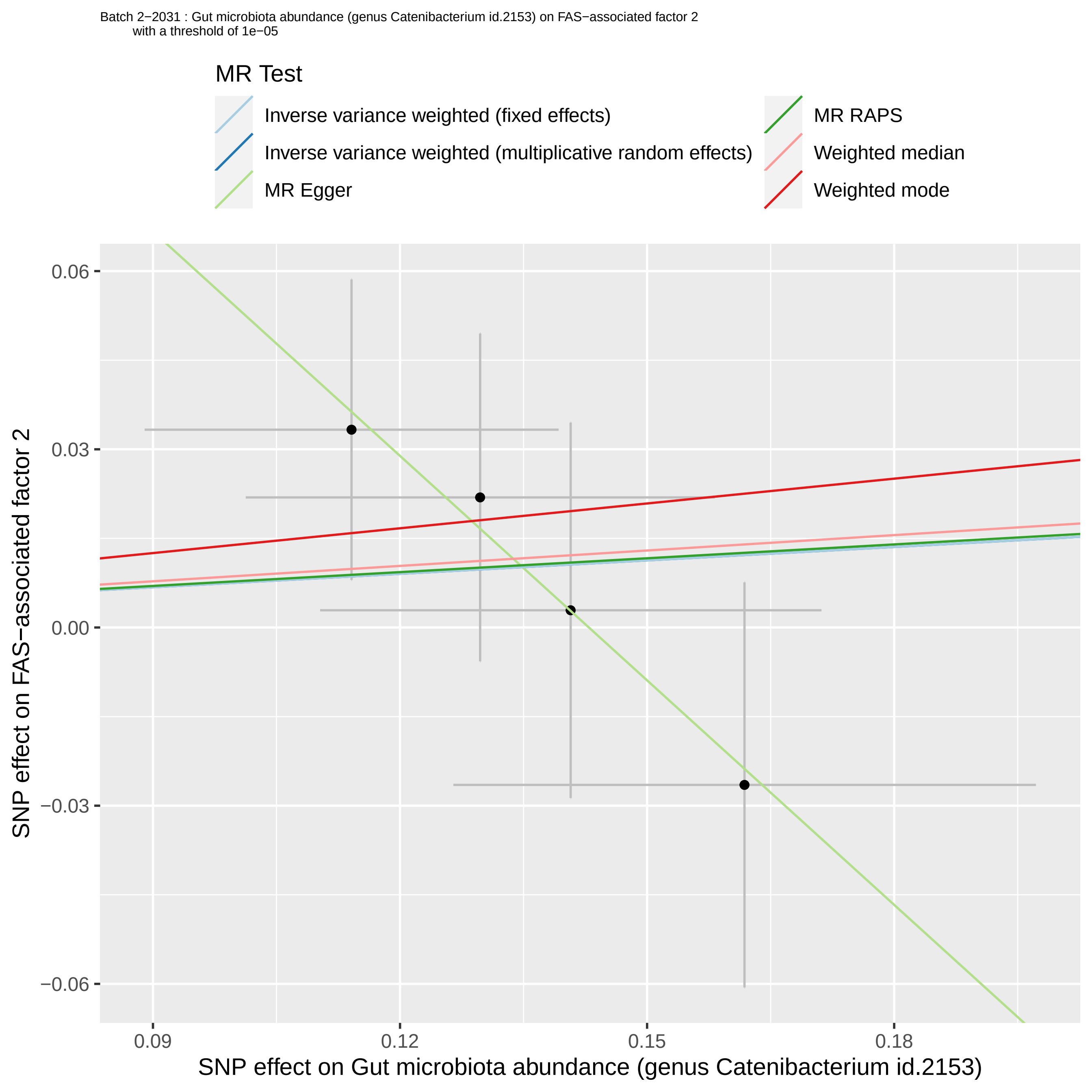

Batch 2−2031 : Gut microbiota abundance (genus Catenibacterium id.2153) on FAS−associated factor 2
with a threshold of 1e−05
MR Test
Inverse variance weighted (fixed effects)
Inverse variance weighted (multiplicative random effects)
MR Egger
MR RAPS
Weighted median
Weighted mode
0.06
0.03
0.00
−0.03
−0.06
SNP effect on FAS−associated factor 2
0.09
0.12
0.15
0.18
SNP effect on Gut microbiota abundance (genus Catenibacterium id.2153)

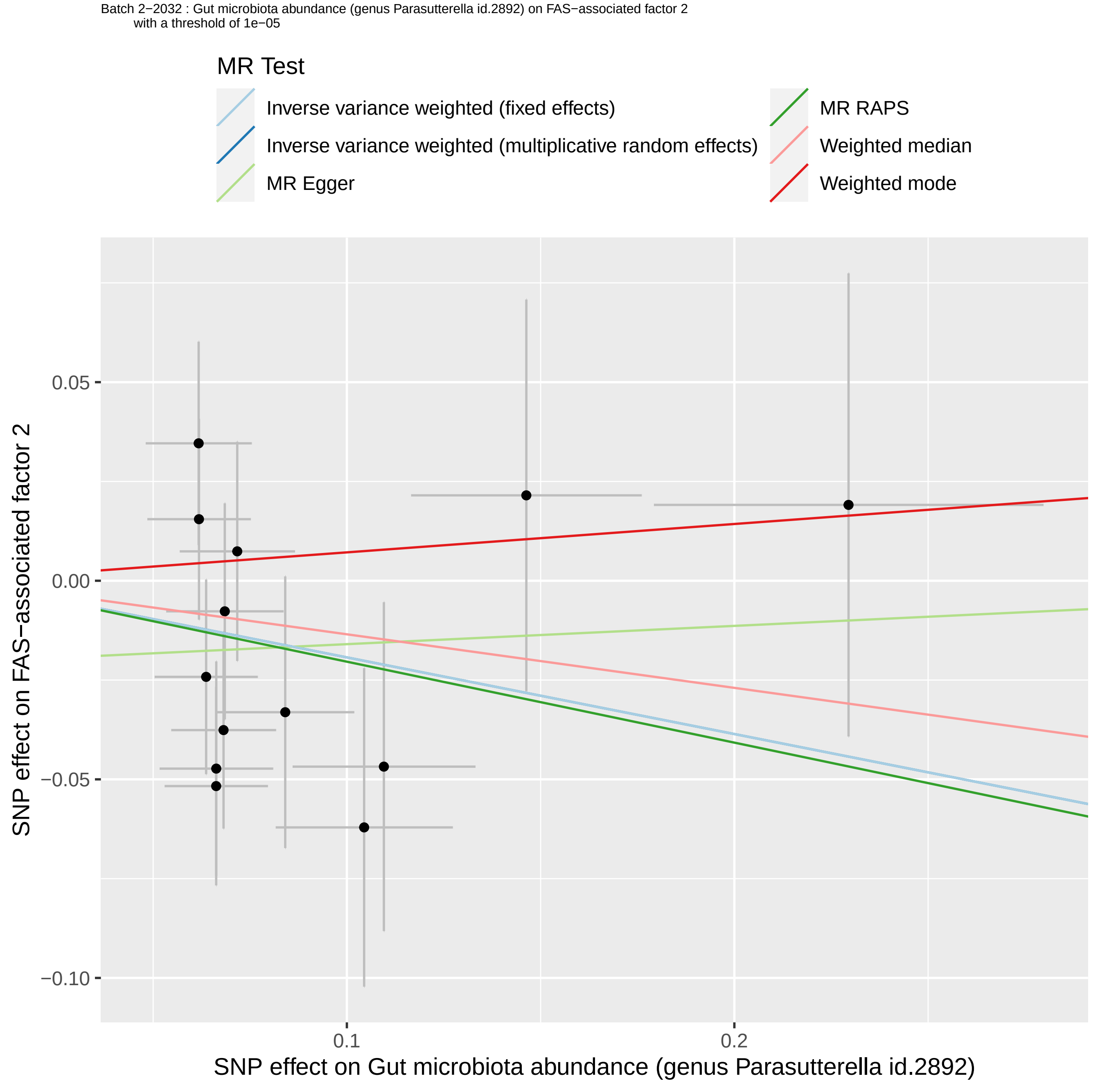
Batch 2−2032 : Gut microbiota abundance (genus Parasutterella id.2892) on FAS−associated factor 2
with a threshold of 1e−05
MR Test
Inverse variance weighted (fixed effects)
Inverse variance weighted (multiplicative random effects)
MR Egger
MR RAPS
Weighted median
Weighted mode
0.05
0.00
−0.05
−0.10
0.1
0.2
SNP effect on FAS−associated factor 2
SNP effect on Gut microbiota abundance (genus Parasutterella id.2892)

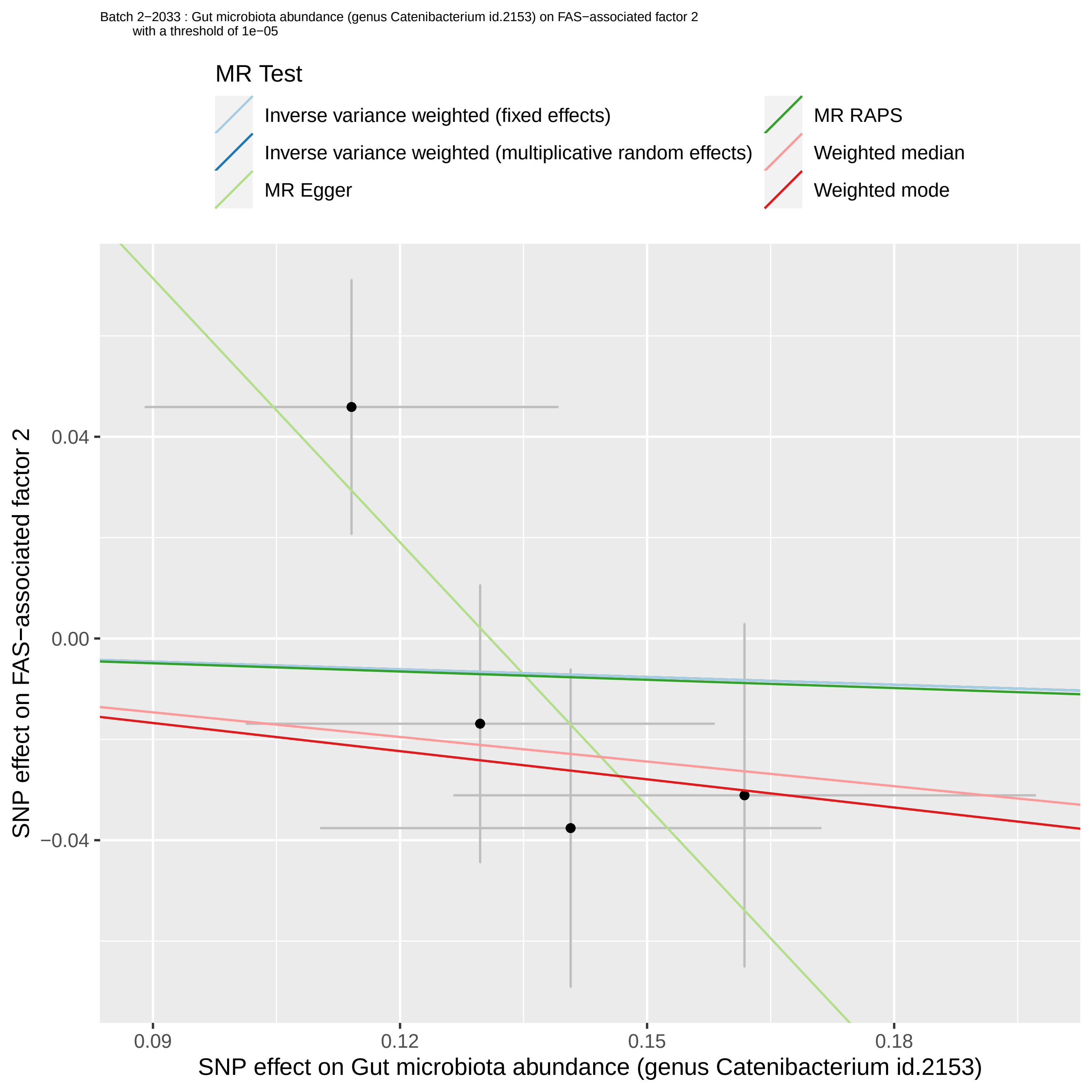

Batch 2−2033 : Gut microbiota abundance (genus Catenibacterium id.2153) on FAS−associated factor 2
with a threshold of 1e−05
MR Test
Inverse variance weighted (fixed effects)
Inverse variance weighted (multiplicative random effects)
MR Egger
MR RAPS
Weighted median
Weighted mode
SNP effect on FAS−associated factor 2
0.04
0.00
−0.04
0.09
0.12
0.15
0.18
SNP effect on Gut microbiota abundance (genus Catenibacterium id.2153)

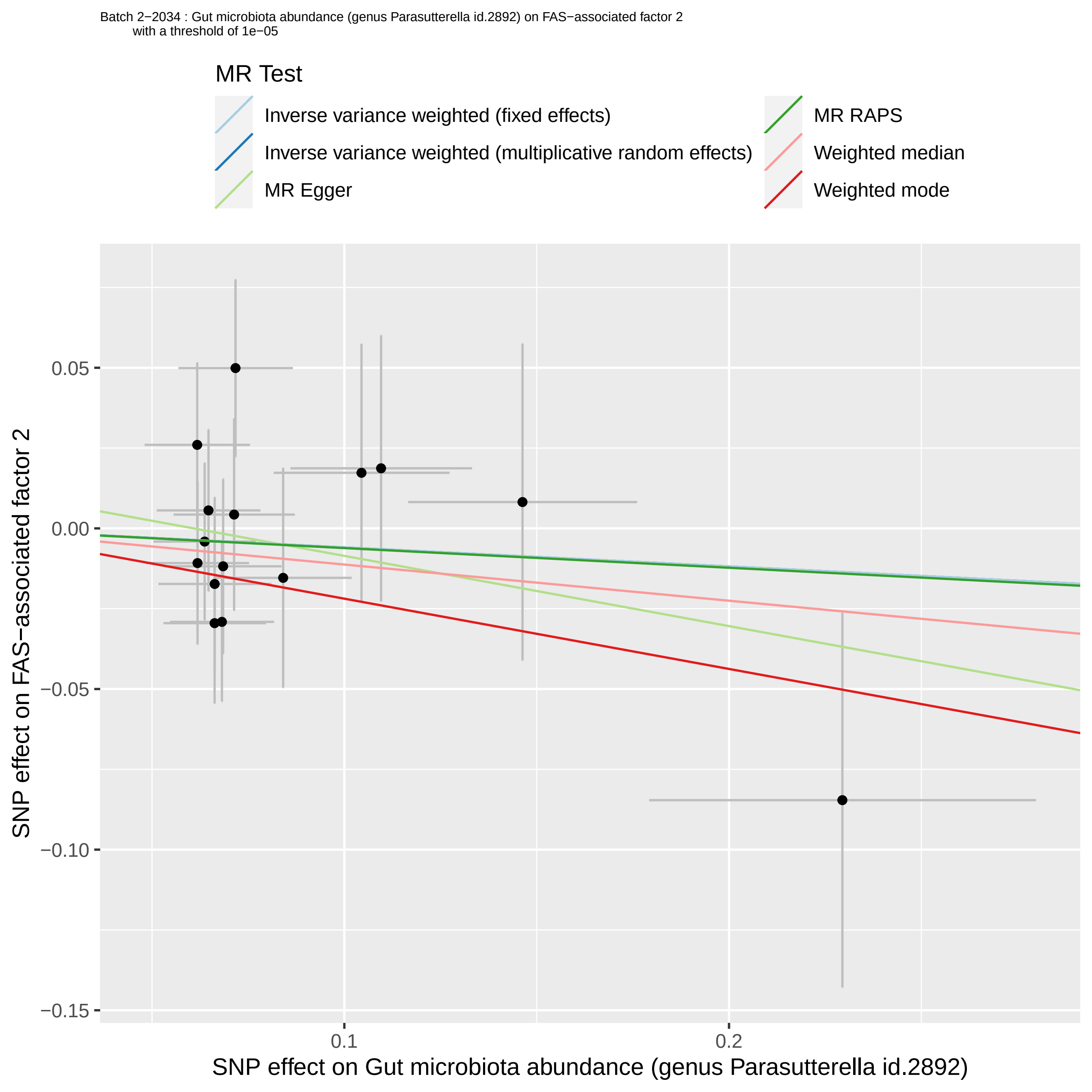
Batch 2−2034 : Gut microbiota abundance (genus Parasutterella id.2892) on FAS−associated factor 2
with a threshold of 1e−05
MR Test
Inverse variance weighted (fixed effects)
Inverse variance weighted (multiplicative random effects)
MR Egger
MR RAPS
Weighted median
Weighted mode
SNP effect on FAS−associated factor 2
0.05
0.00
−0.05
−0.10
−0.15
0.1
0.2
SNP effect on Gut microbiota abundance (genus Parasutterella id.2892)

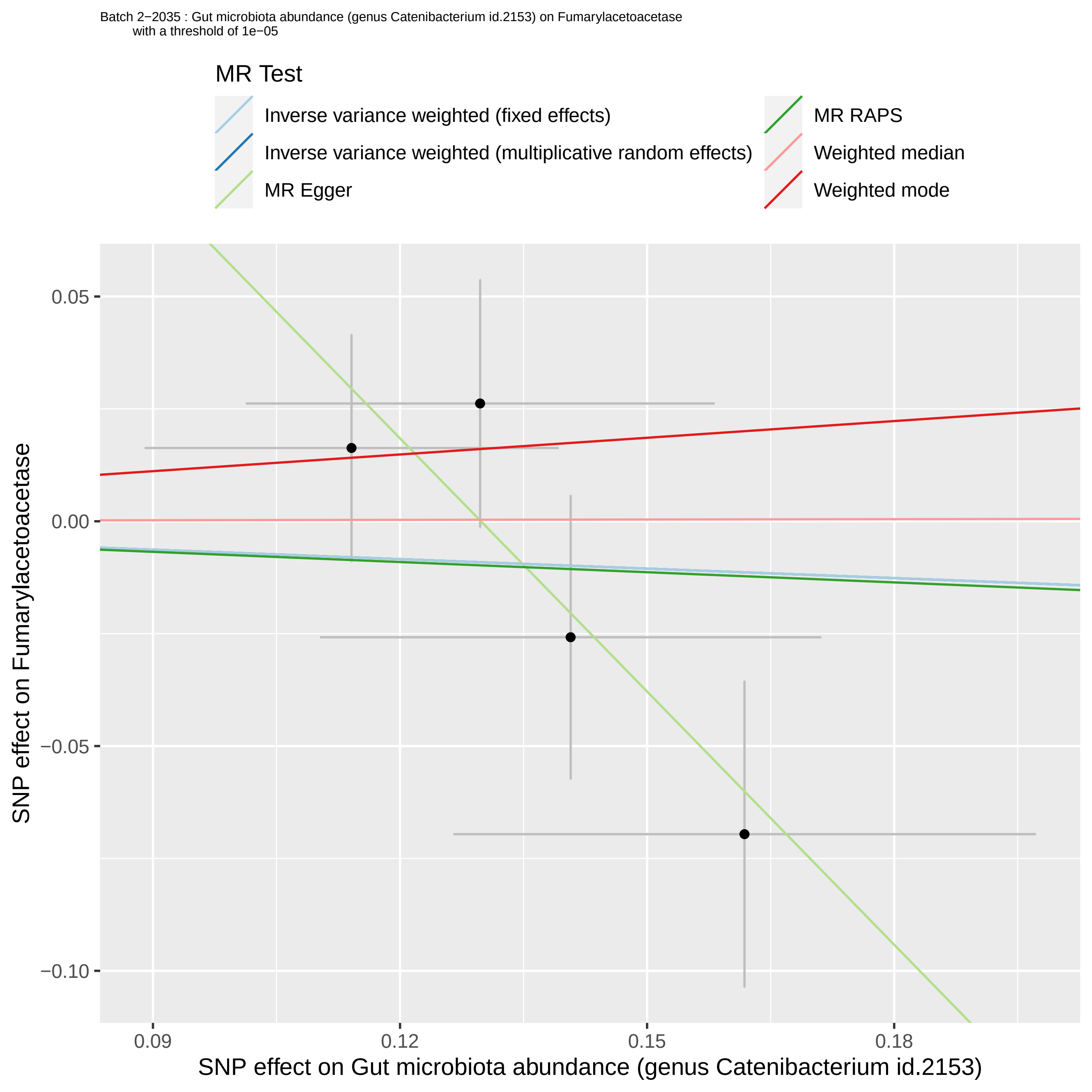

Batch 2−2035 : Gut microbiota abundance (genus Catenibacterium id.2153) on Fumarylacetoacetase
with a threshold of 1e−05
MR Test
Inverse variance weighted (fixed effects)
Inverse variance weighted (multiplicative random effects)
MR Egger
MR RAPS
Weighted median
Weighted mode
0.05
0.00
−0.05
−0.10
0.09
0.12
0.15
0.18
SNP effect on Fumarylacetoacetase
SNP effect on Gut microbiota abundance (genus Catenibacterium id.2153)

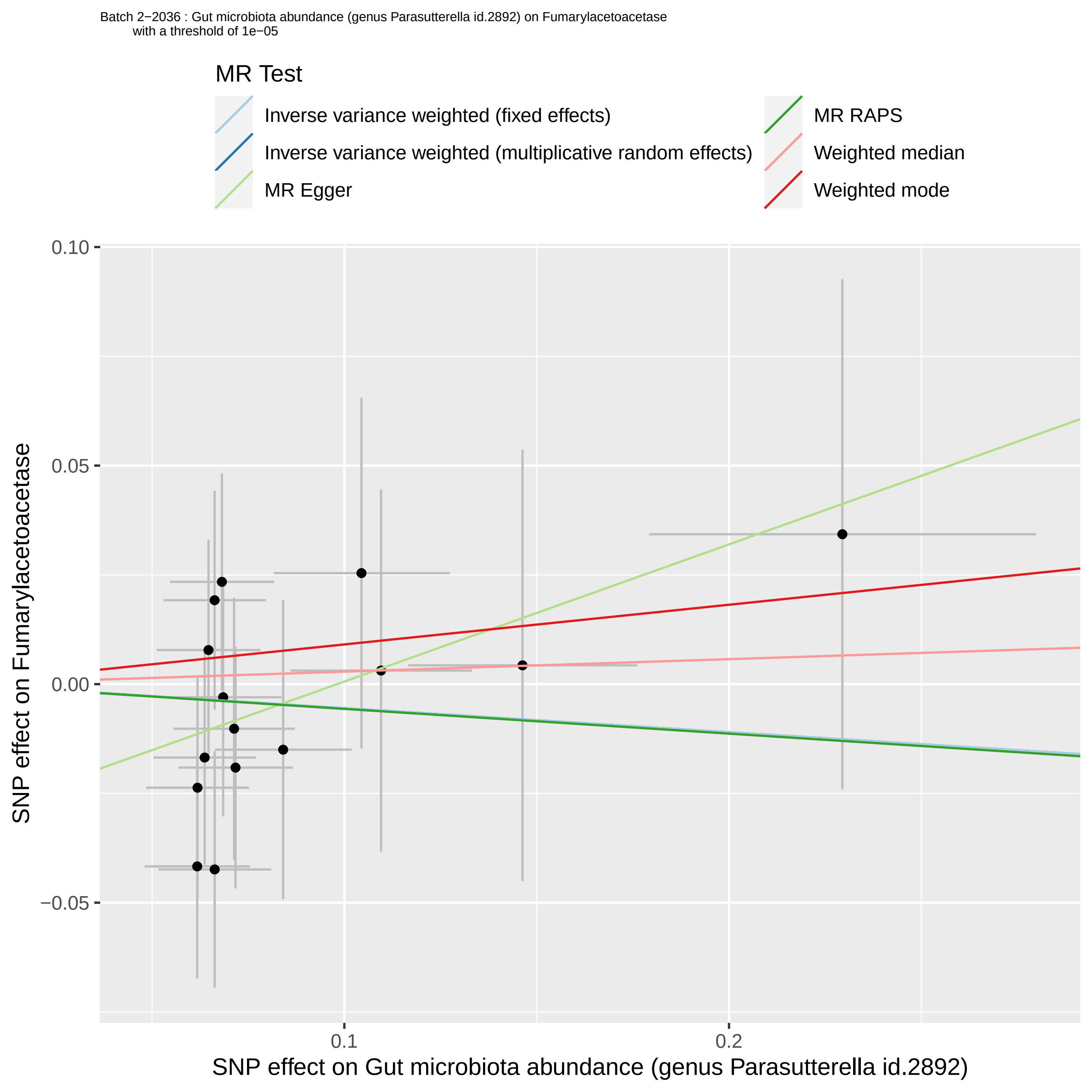

Batch 2−2036 : Gut microbiota abundance (genus Parasutterella id.2892) on Fumarylacetoacetase
with a threshold of 1e−05
MR Test
Inverse variance weighted (fixed effects)
Inverse variance weighted (multiplicative random effects)
MR Egger
MR RAPS
Weighted median
Weighted mode
0.10
0.05
0.00
−0.05
0.1
0.2
SNP effect on Fumarylacetoacetase
SNP effect on Gut microbiota abundance (genus Parasutterella id.2892)

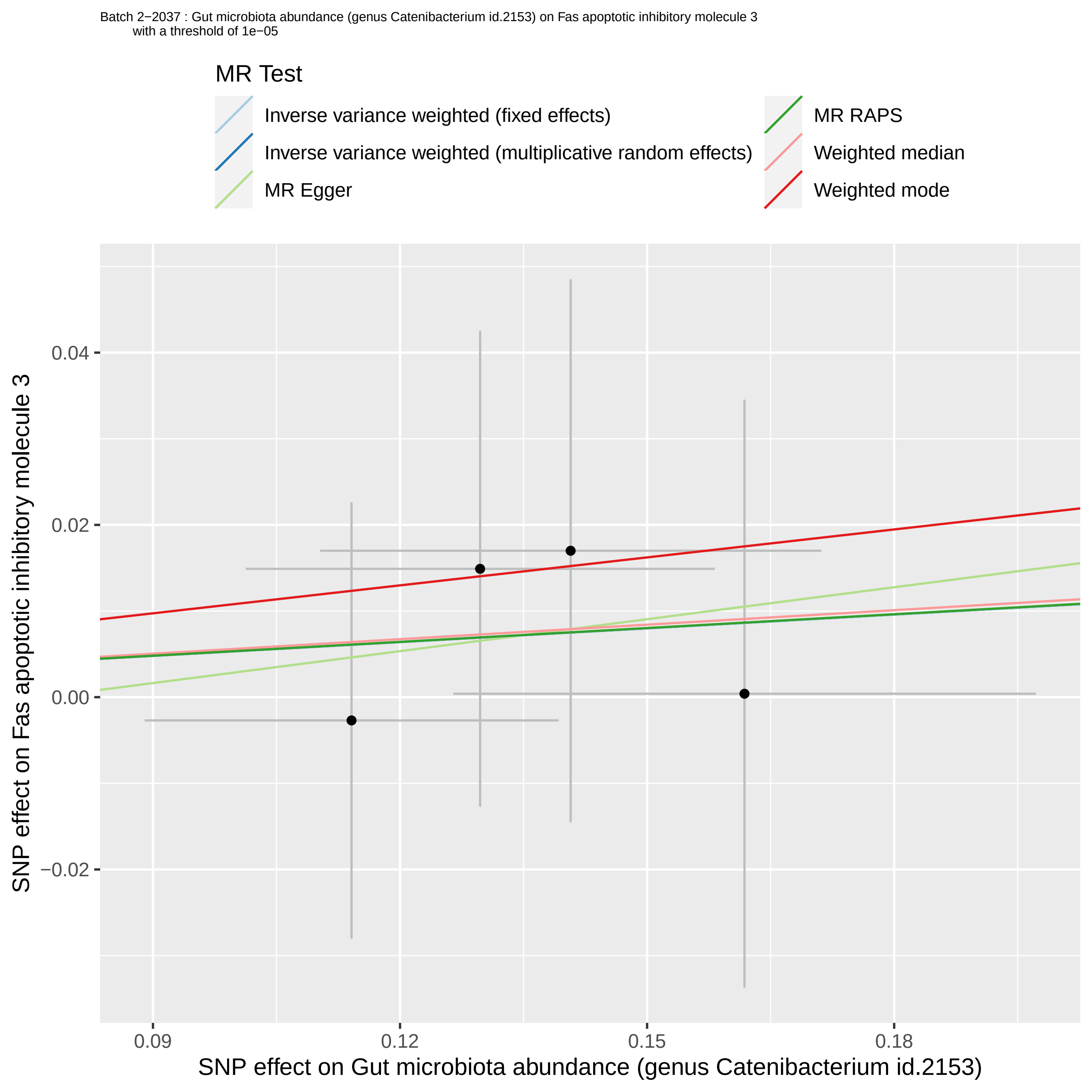

Batch 2−2037 : Gut microbiota abundance (genus Catenibacterium id.2153) on Fas apoptotic inhibitory molecule 3
with a threshold of 1e−05
MR Test
Inverse variance weighted (fixed effects)
Inverse variance weighted (multiplicative random effects)
MR Egger
MR RAPS
Weighted median
Weighted mode
SNP effect on Fas apoptotic inhibitory molecule 3
0.04
0.02
0.00
−0.02
0.09
0.12
0.15
0.18
SNP effect on Gut microbiota abundance (genus Catenibacterium id.2153)

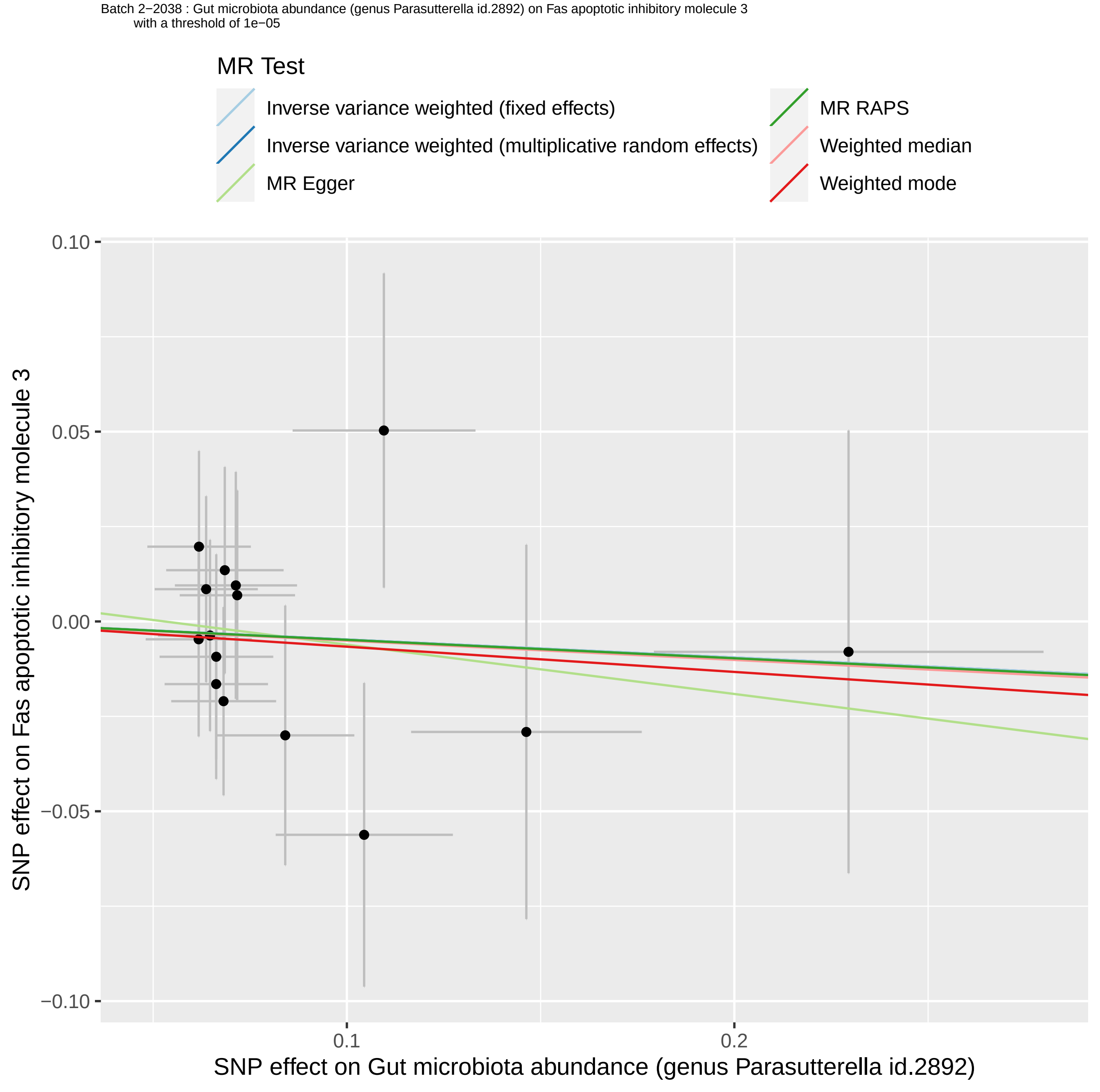

Batch 2−2038 : Gut microbiota abundance (genus Parasutterella id.2892) on Fas apoptotic inhibitory molecule 3
with a threshold of 1e−05
MR Test
Inverse variance weighted (fixed effects)
Inverse variance weighted (multiplicative random effects)
MR Egger
MR RAPS
Weighted median
Weighted mode
0.10
0.05
0.00
−0.05
−0.10
0.1
0.2
SNP effect on Fas apoptotic inhibitory molecule 3
SNP effect on Gut microbiota abundance (genus Parasutterella id.2892)

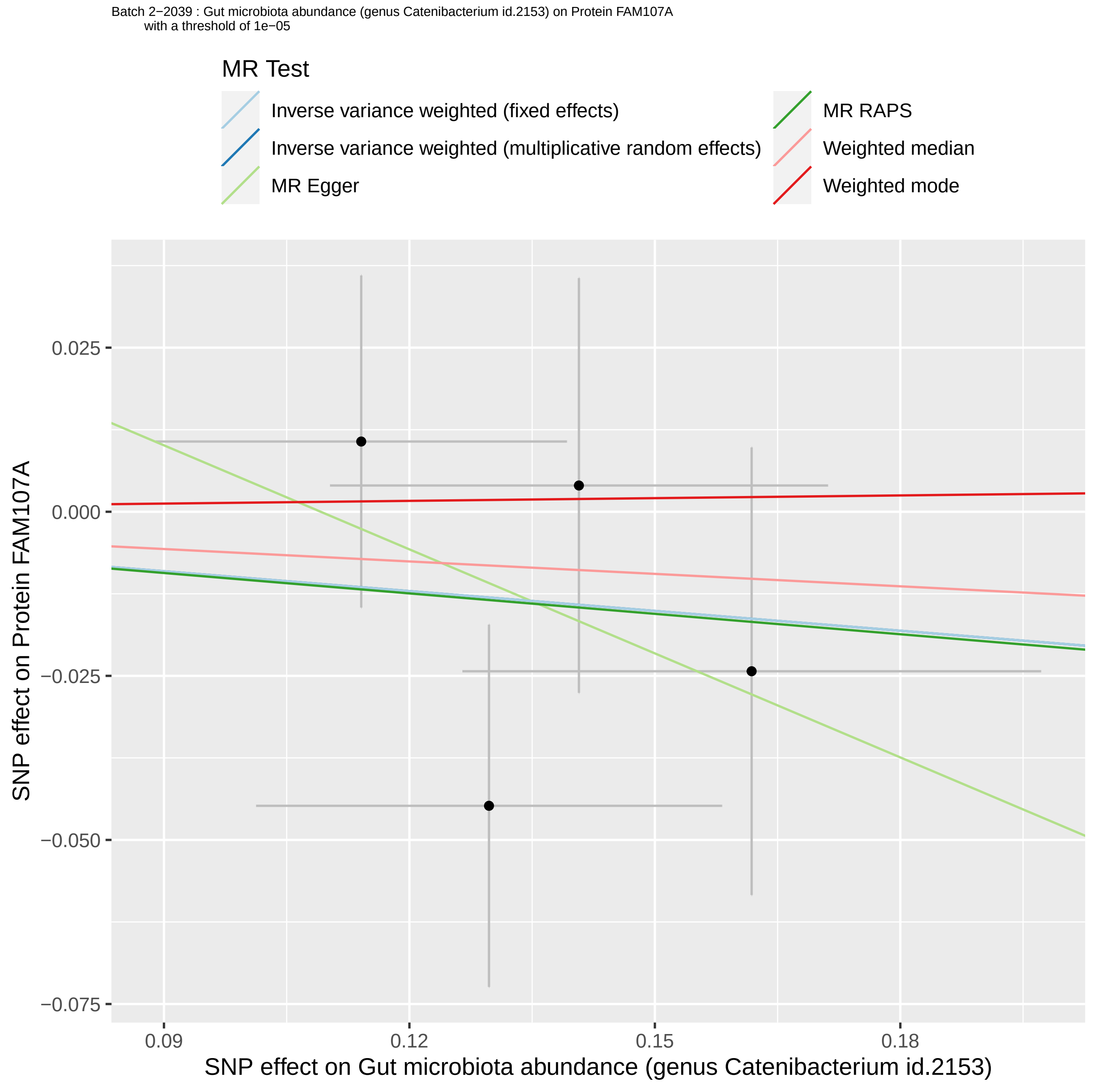
Batch 2−2039 : Gut microbiota abundance (genus Catenibacterium id.2153) on Protein FAM107A
with a threshold of 1e−05
MR Test
Inverse variance weighted (fixed effects)
Inverse variance weighted (multiplicative random effects)
MR Egger
MR RAPS
Weighted median
Weighted mode
0.025
0.000
−0.025
−0.050
−0.075
0.09
0.12
0.15
0.18
SNP effect on Protein FAM107A
SNP effect on Gut microbiota abundance (genus Catenibacterium id.2153)

Batch 2−2040 : Gut microbiota abundance (genus Parasutterella id.2892) on Protein FAM107A
with a threshold of 1e−05

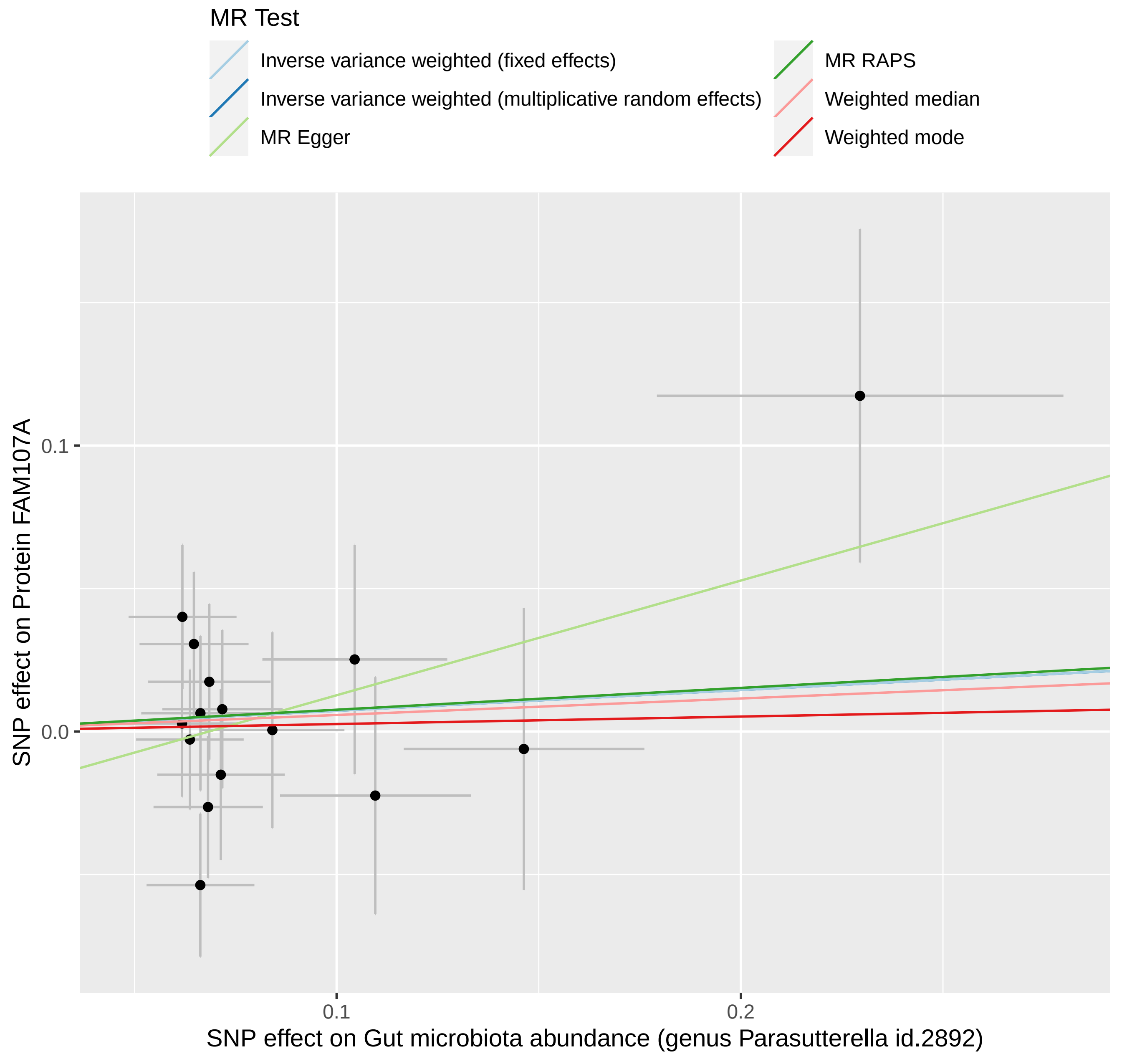

Batch 2−2041 : Gut microbiota abundance (genus Catenibacterium id.2153) on Protein FAM107B
with a threshold of 1e−05

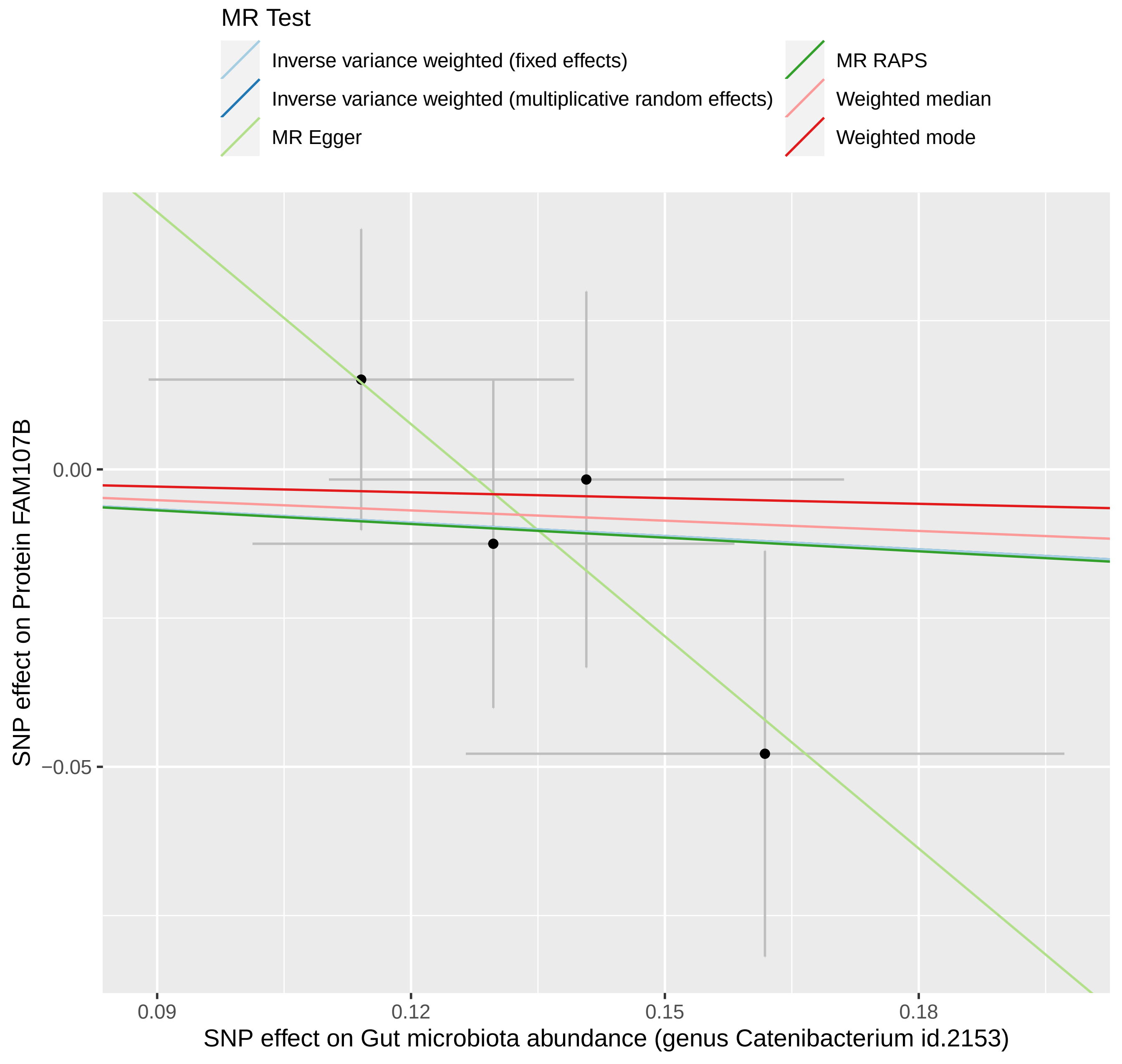

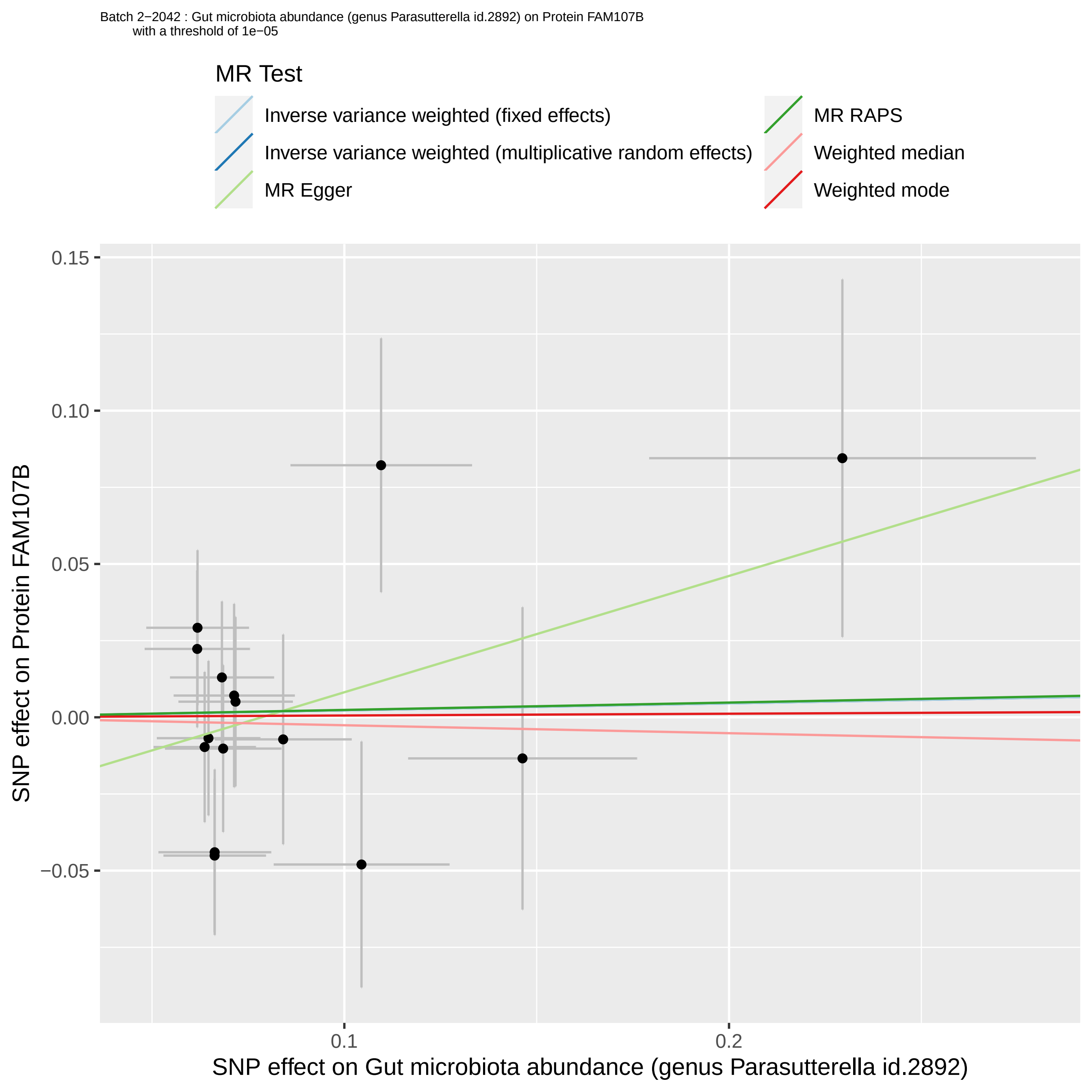

Batch 2−2042 : Gut microbiota abundance (genus Parasutterella id.2892) on Protein FAM107B
with a threshold of 1e−05
MR Test
Inverse variance weighted (fixed effects)
Inverse variance weighted (multiplicative random effects)
MR Egger
MR RAPS
Weighted median
Weighted mode
0.15
0.10
0.05
0.00
−0.05
0.1
0.2
SNP effect on Protein FAM107B
SNP effect on Gut microbiota abundance (genus Parasutterella id.2892)

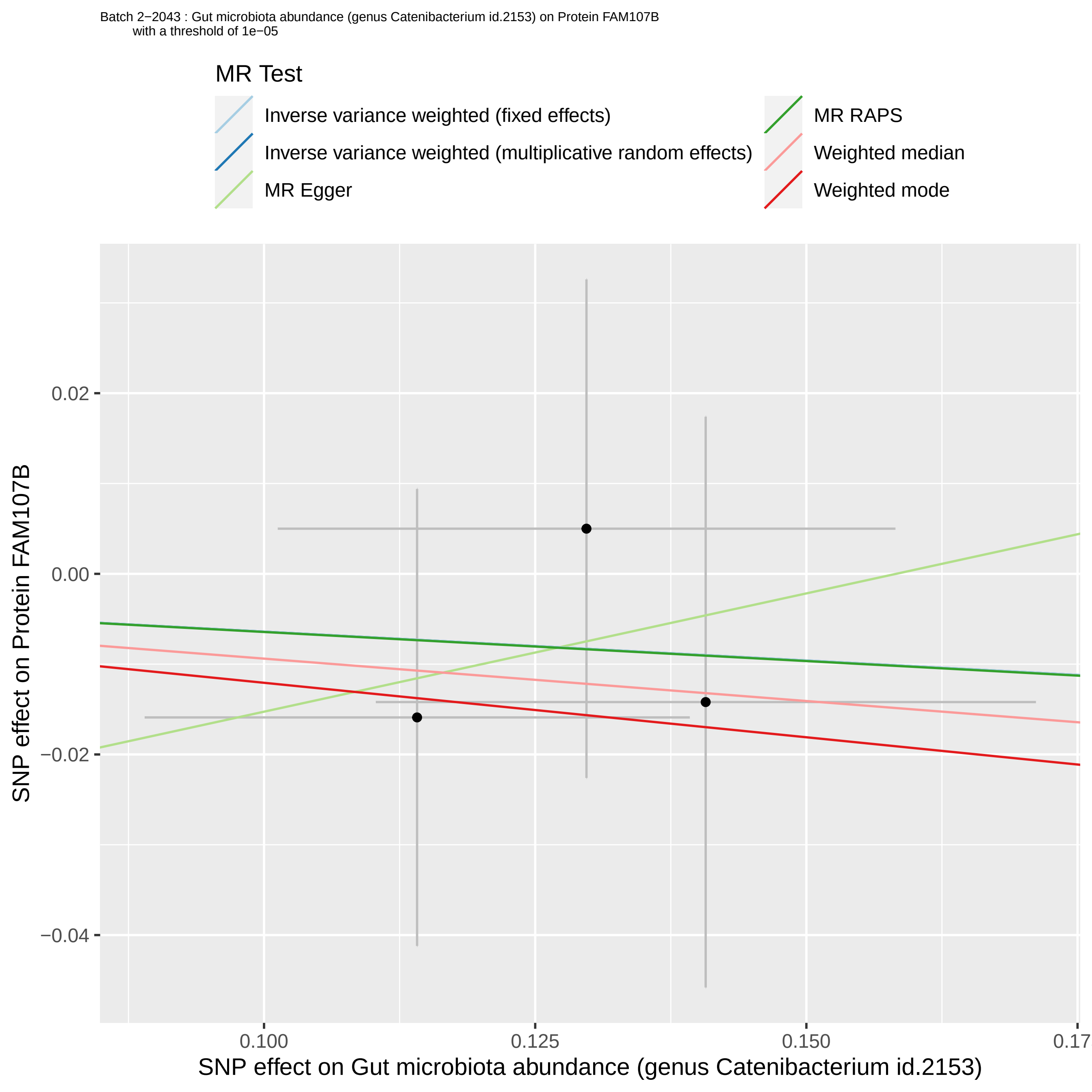

Batch 2−2043 : Gut microbiota abundance (genus Catenibacterium id.2153) on Protein FAM107B
with a threshold of 1e−05
MR Test
Inverse variance weighted (fixed effects)
Inverse variance weighted (multiplicative random effects)
MR Egger
MR RAPS
Weighted median
Weighted mode
0.02
0.00
−0.02
−0.04
0.100
0.125
0.150
0.17
SNP effect on Protein FAM107B
SNP effect on Gut microbiota abundance (genus Catenibacterium id.2153)

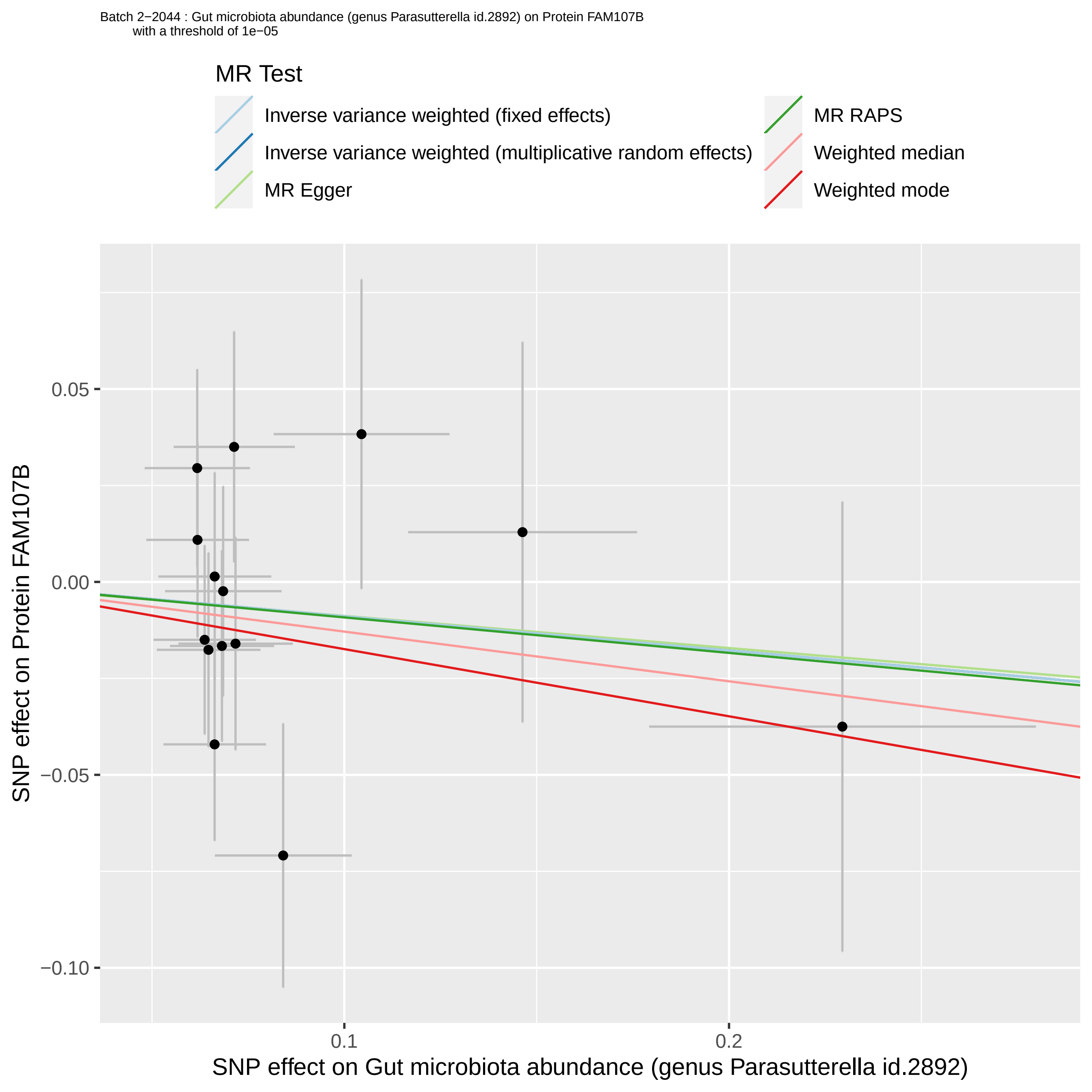

Batch 2−2044 : Gut microbiota abundance (genus Parasutterella id.2892) on Protein FAM107B
with a threshold of 1e−05
MR Test
Inverse variance weighted (fixed effects)
Inverse variance weighted (multiplicative random effects)
MR Egger
MR RAPS
Weighted median
Weighted mode
0.05
0.00
−0.05
−0.10
0.1
0.2
SNP effect on Protein FAM107B
SNP effect on Gut microbiota abundance (genus Parasutterella id.2892)

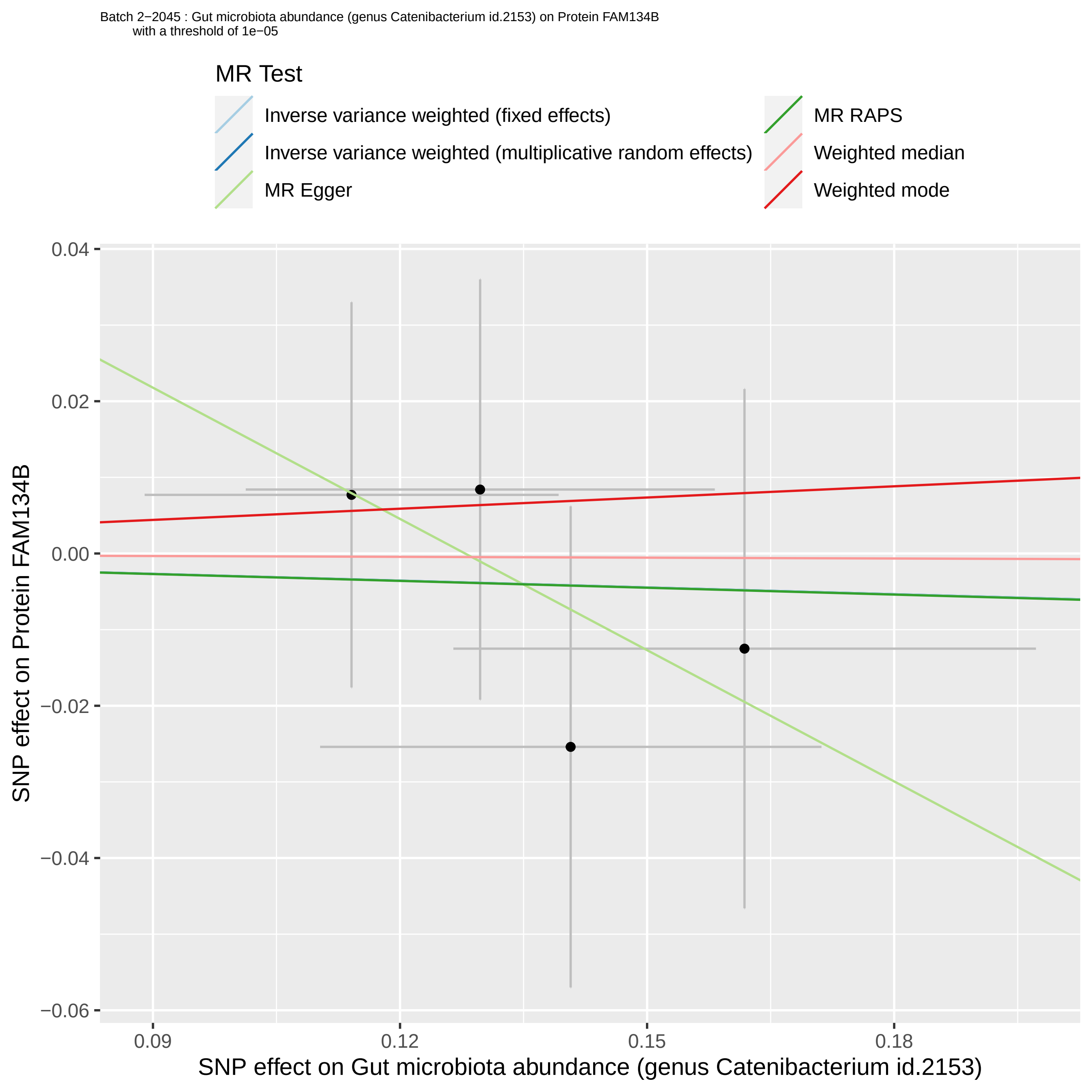

Batch 2−2045 : Gut microbiota abundance (genus Catenibacterium id.2153) on Protein FAM134B
with a threshold of 1e−05
MR Test
Inverse variance weighted (fixed effects)
Inverse variance weighted (multiplicative random effects)
MR Egger
MR RAPS
Weighted median
Weighted mode
0.04
0.02
0.00
−0.02
−0.04
−0.06
0.09
0.12
0.15
0.18
SNP effect on Protein FAM134B
SNP effect on Gut microbiota abundance (genus Catenibacterium id.2153)

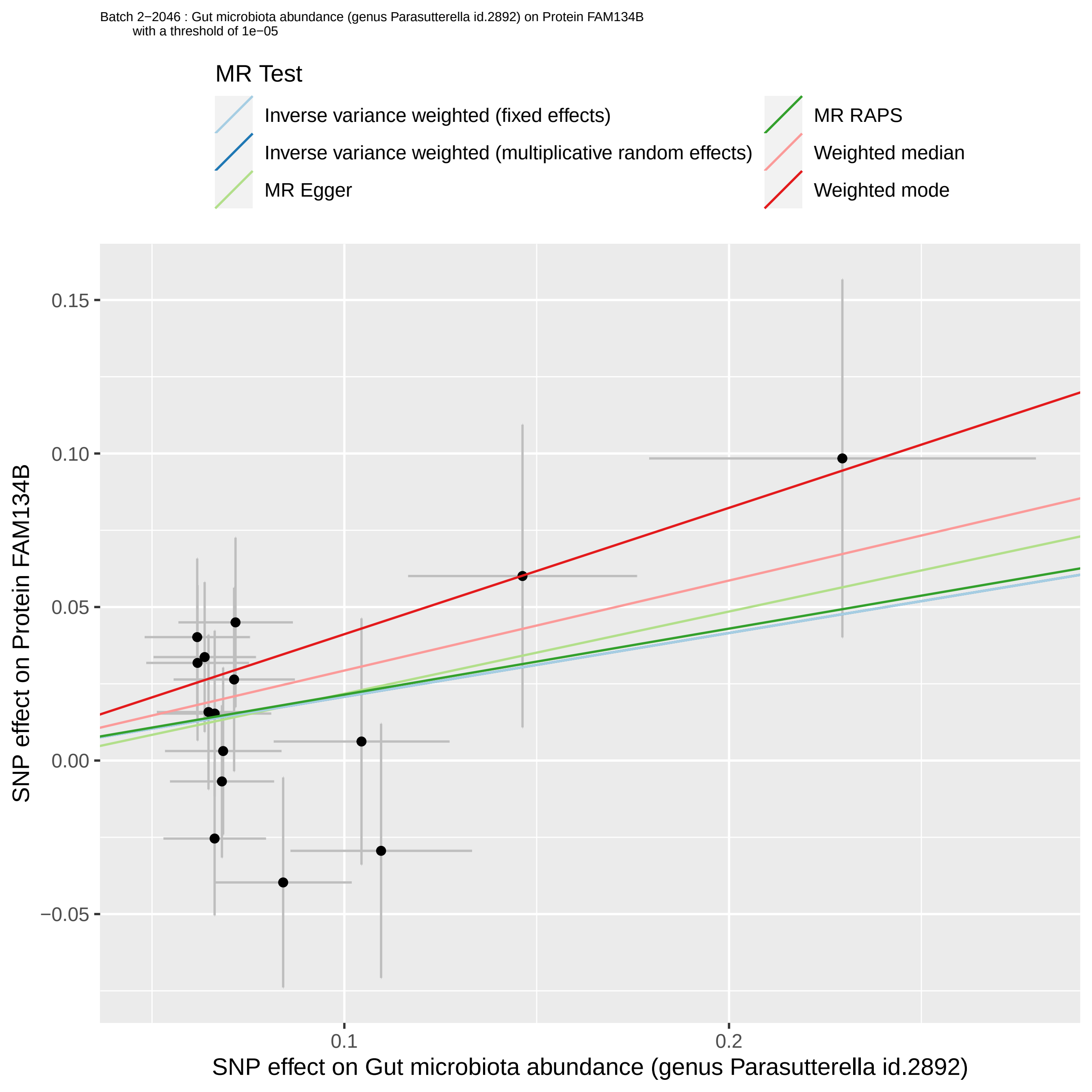

Batch 2−2046 : Gut microbiota abundance (genus Parasutterella id.2892) on Protein FAM134B
with a threshold of 1e−05
MR Test
Inverse variance weighted (fixed effects)
Inverse variance weighted (multiplicative random effects)
MR Egger
MR RAPS
Weighted median
Weighted mode
0.15
0.10
0.05
0.00
−0.05
0.1
0.2
SNP effect on Protein FAM134B
SNP effect on Gut microbiota abundance (genus Parasutterella id.2892)

Batch 2−2047 : Gut microbiota abundance (genus Catenibacterium id.2153) on Protein FAM150B
with a threshold of 1e−05

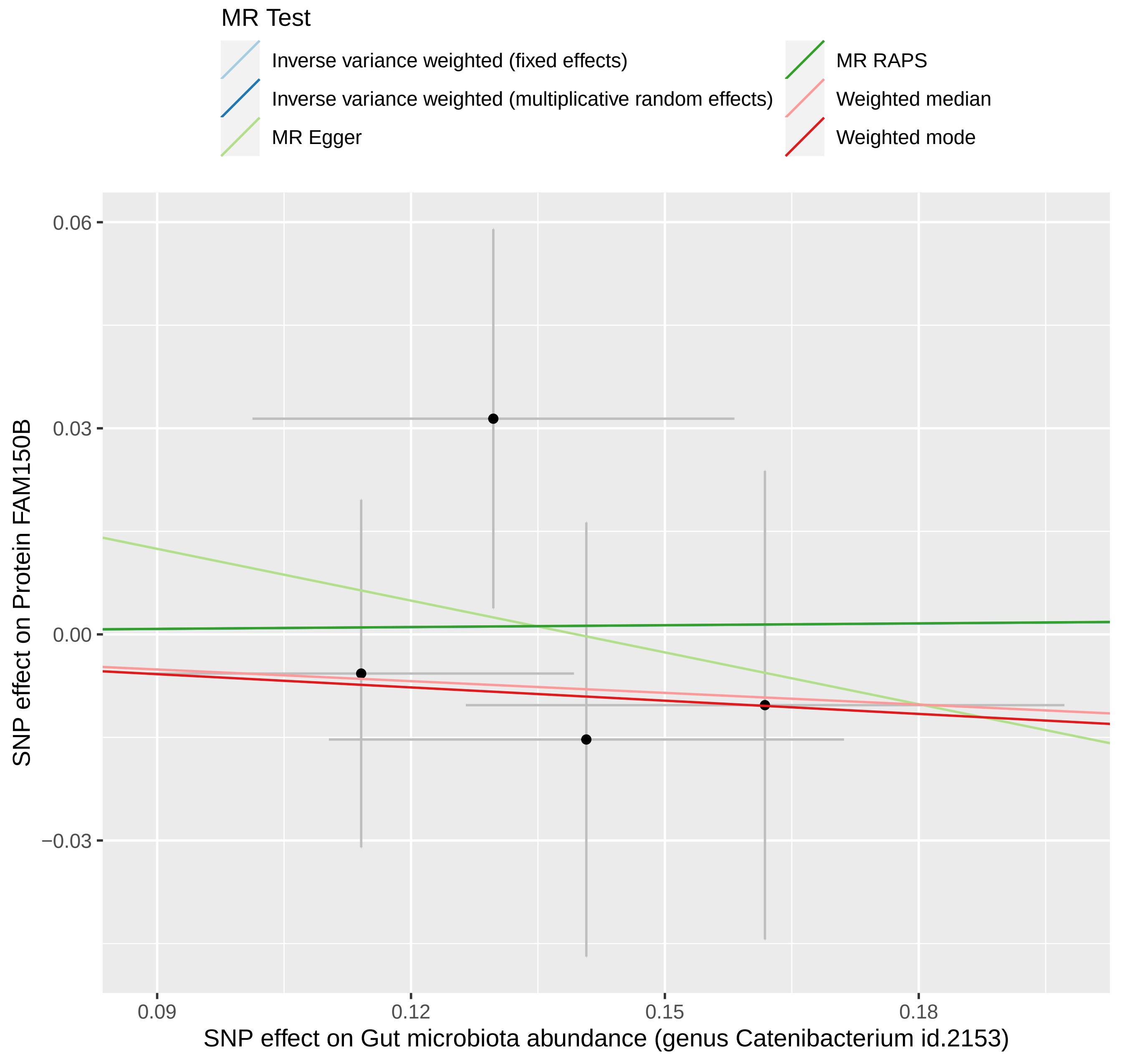

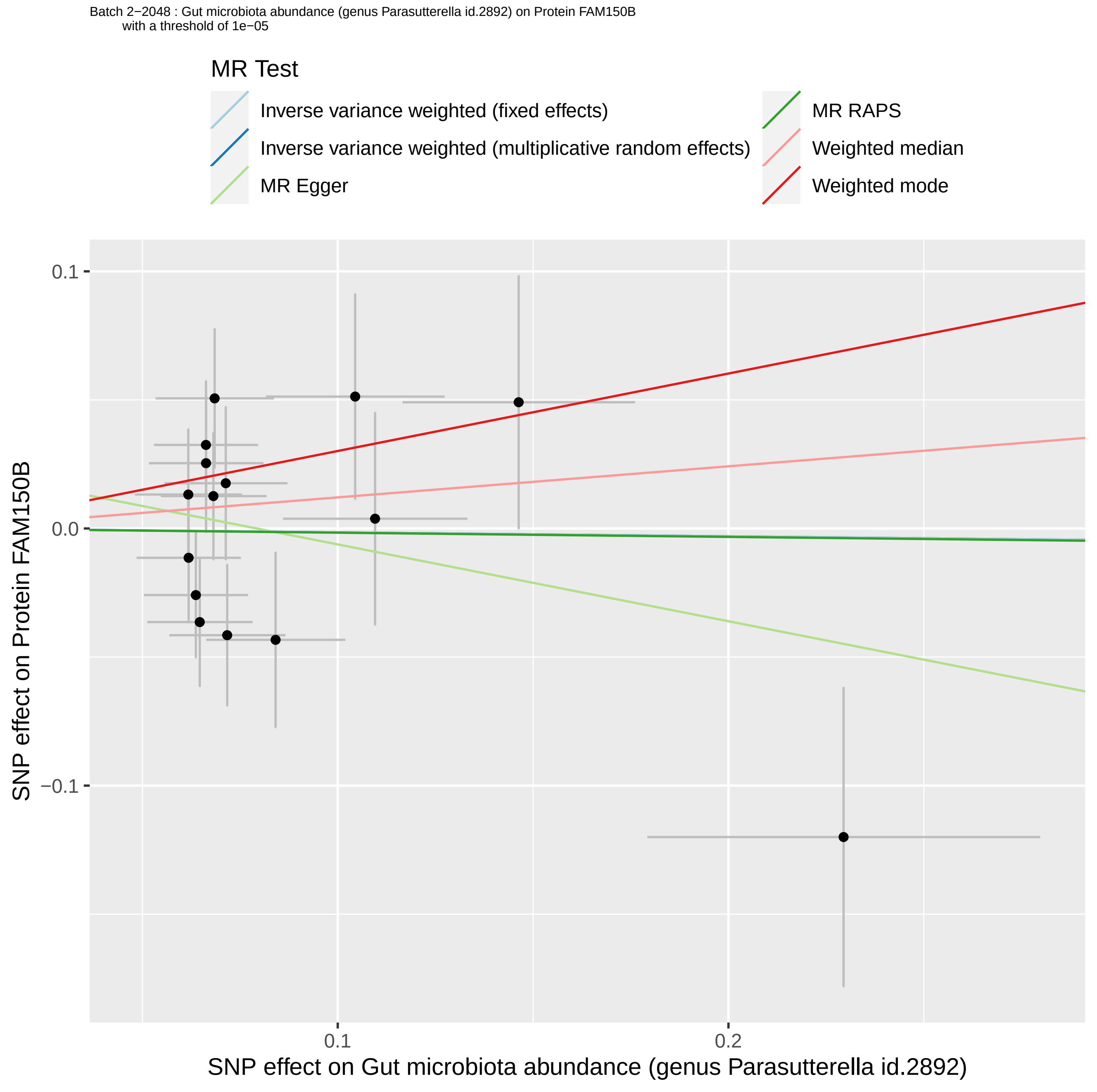

Batch 2−2048 : Gut microbiota abundance (genus Parasutterella id.2892) on Protein FAM150B
with a threshold of 1e−05
MR Test
Inverse variance weighted (fixed effects)
Inverse variance weighted (multiplicative random effects)
MR Egger
MR RAPS
Weighted median
Weighted mode
0.1
0.0
−0.1
0.1
0.2
SNP effect on Protein FAM150B
SNP effect on Gut microbiota abundance (genus Parasutterella id.2892)

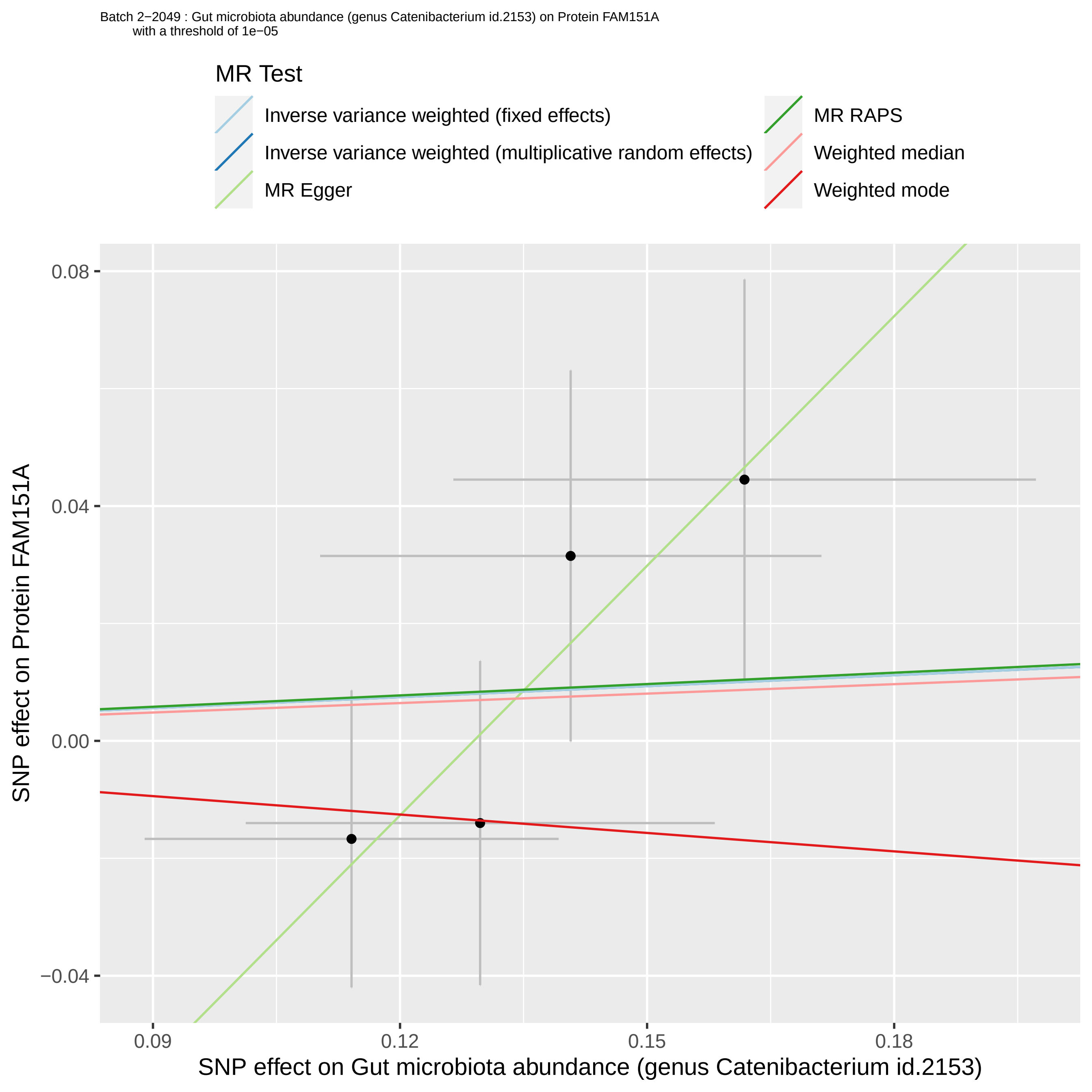
Batch 2−2049 : Gut microbiota abundance (genus Catenibacterium id.2153) on Protein FAM151A
with a threshold of 1e−05
MR Test
Inverse variance weighted (fixed effects)
Inverse variance weighted (multiplicative random effects)
MR Egger
MR RAPS
Weighted median
Weighted mode
0.08
0.04
0.00
−0.04
0.09
0.12
0.15
0.18
SNP effect on Protein FAM151A
SNP effect on Gut microbiota abundance (genus Catenibacterium id.2153)

Batch 2−2050 : Gut microbiota abundance (genus Parasutterella id.2892) on Protein FAM151A
with a threshold of 1e−05

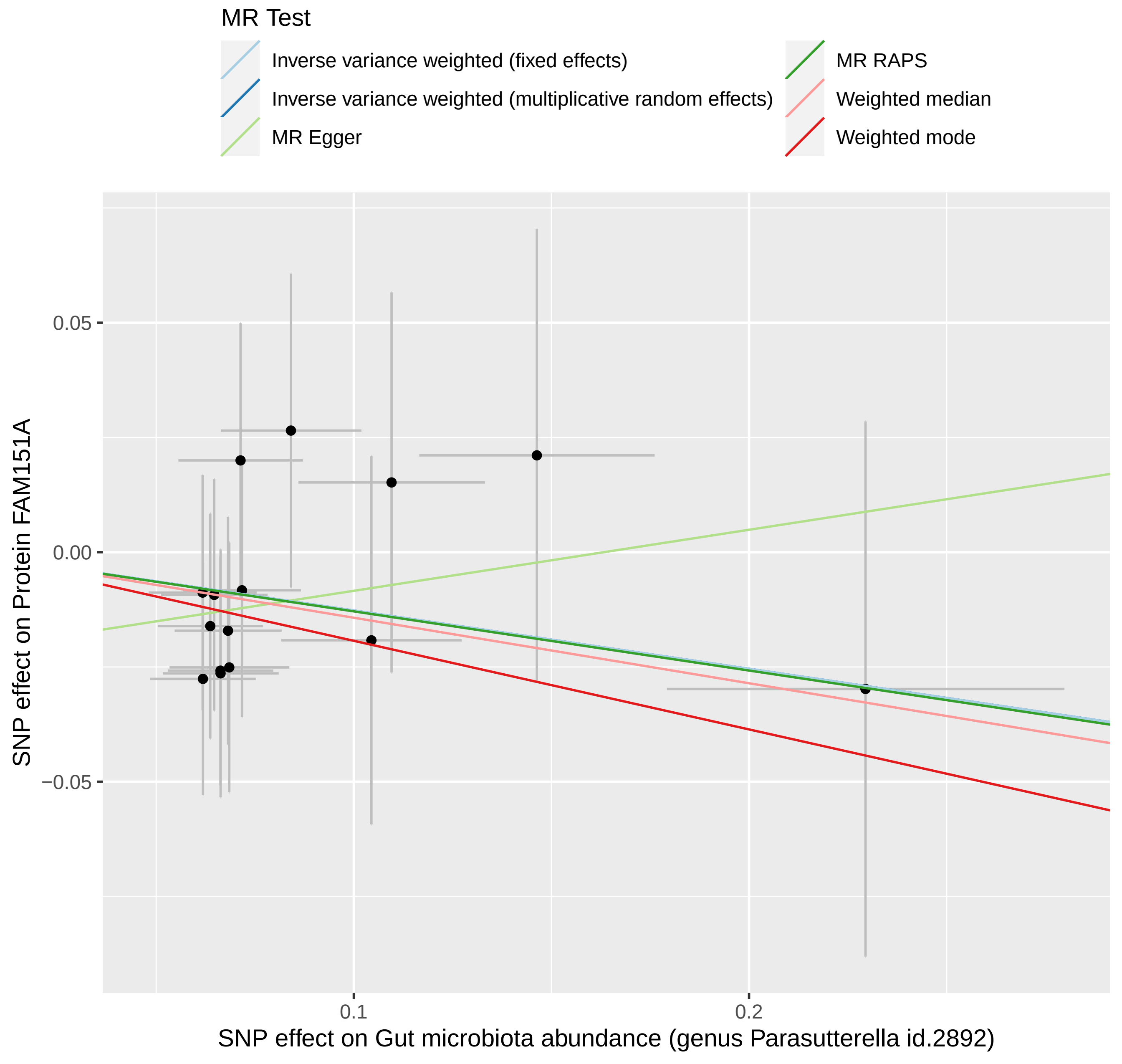

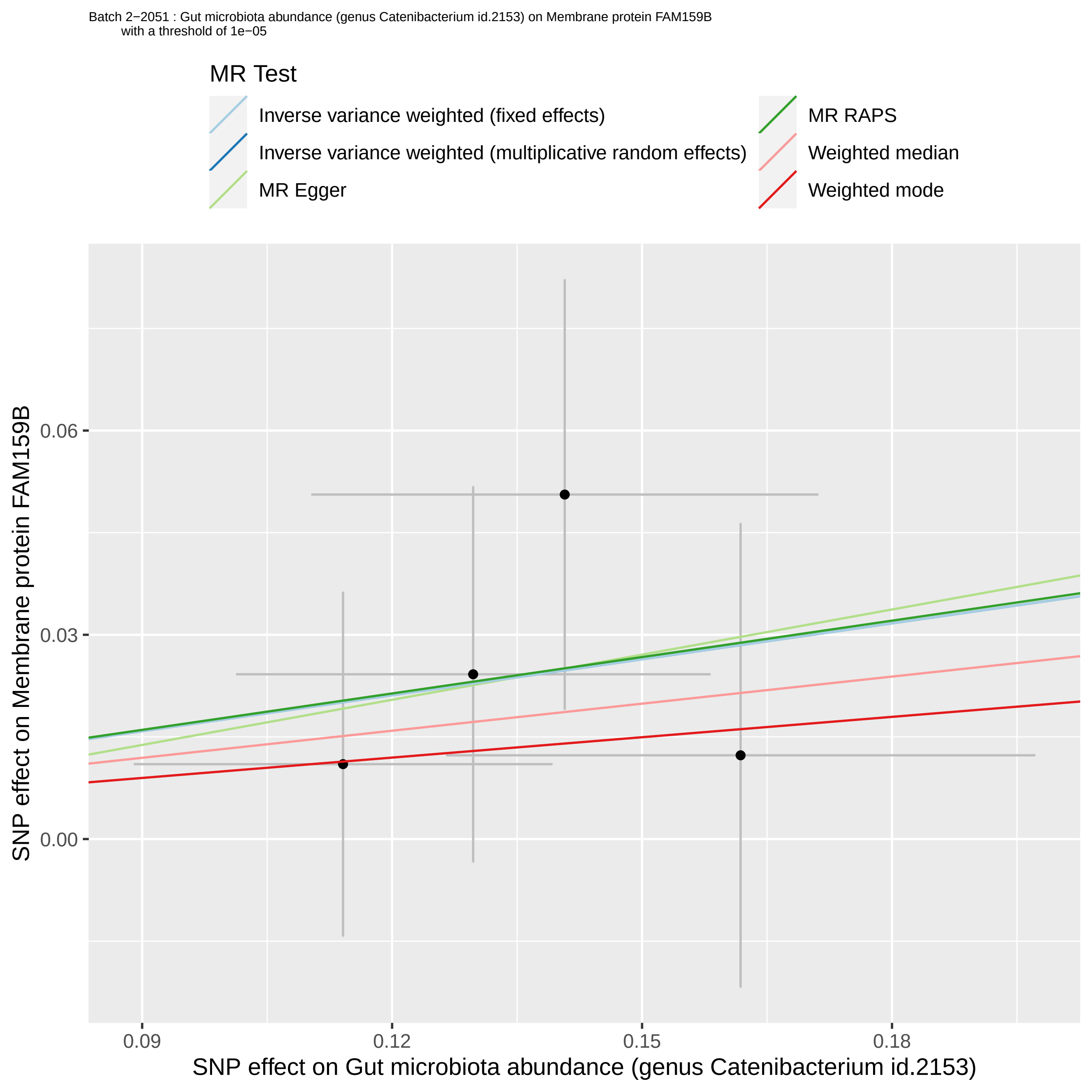

Batch 2−2051 : Gut microbiota abundance (genus Catenibacterium id.2153) on Membrane protein FAM159B
with a threshold of 1e−05
MR Test
Inverse variance weighted (fixed effects)
Inverse variance weighted (multiplicative random effects)
MR Egger
MR RAPS
Weighted median
Weighted mode
SNP effect on Membrane protein FAM159B
0.06
0.03
0.00
0.09
0.12
0.15
0.18
SNP effect on Gut microbiota abundance (genus Catenibacterium id.2153)

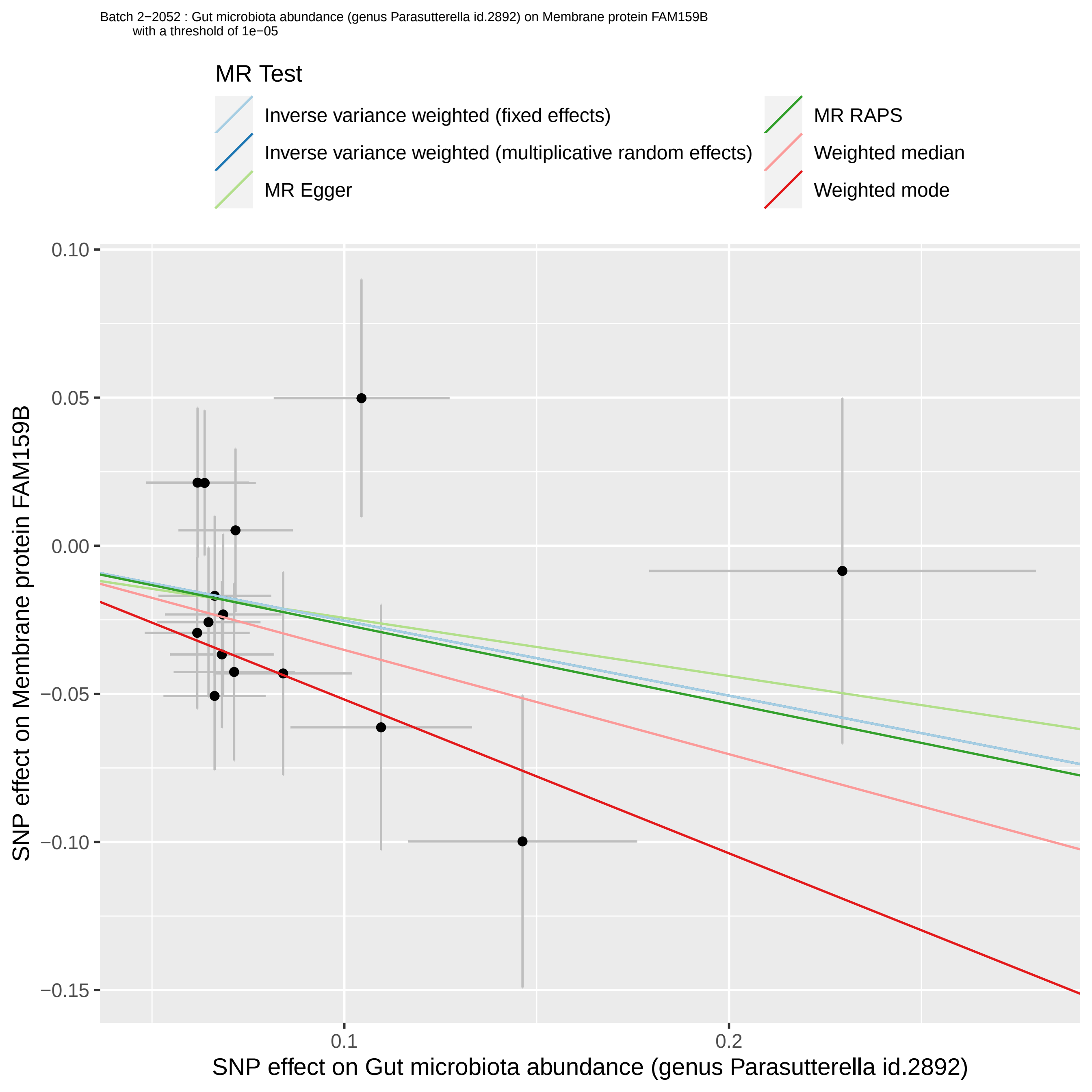
Batch 2−2052 : Gut microbiota abundance (genus Parasutterella id.2892) on Membrane protein FAM159B
with a threshold of 1e−05
MR Test
Inverse variance weighted (fixed effects)
Inverse variance weighted (multiplicative random effects)
MR Egger
MR RAPS
Weighted median
Weighted mode
0.10
0.05
0.00
−0.05
−0.10
−0.15
0.1
0.2
SNP effect on Membrane protein FAM159B
SNP effect on Gut microbiota abundance (genus Parasutterella id.2892)

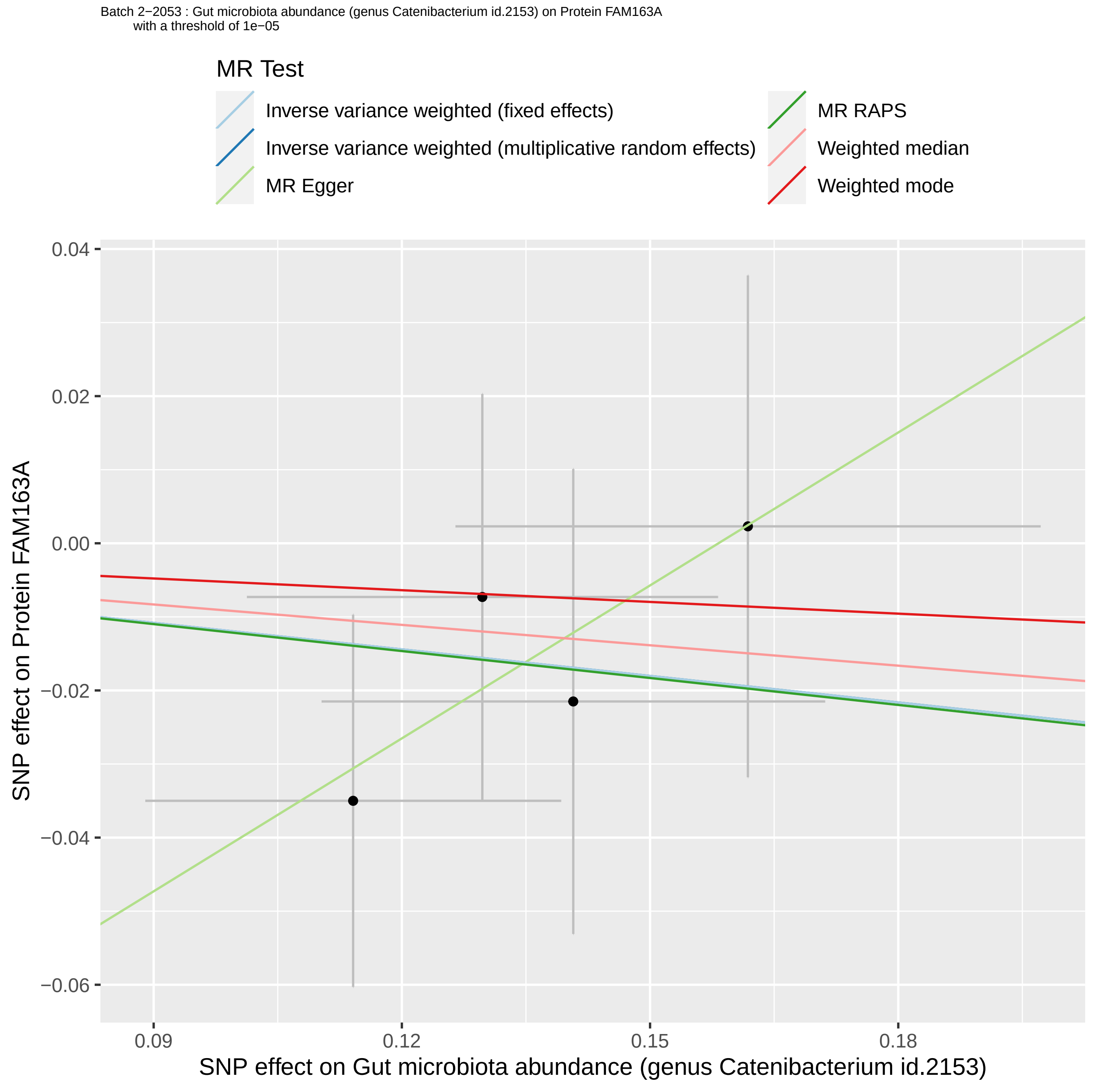

Batch 2−2053 : Gut microbiota abundance (genus Catenibacterium id.2153) on Protein FAM163A
with a threshold of 1e−05
MR Test
Inverse variance weighted (fixed effects)
Inverse variance weighted (multiplicative random effects)
MR Egger
MR RAPS
Weighted median
Weighted mode
0.04
0.02
0.00
−0.02
−0.04
−0.06
SNP effect on Protein FAM163A
0.09
0.12
0.15
0.18
SNP effect on Gut microbiota abundance (genus Catenibacterium id.2153)

Batch 2−2054 : Gut microbiota abundance (genus Parasutterella id.2892) on Protein FAM163A
with a threshold of 1e−05

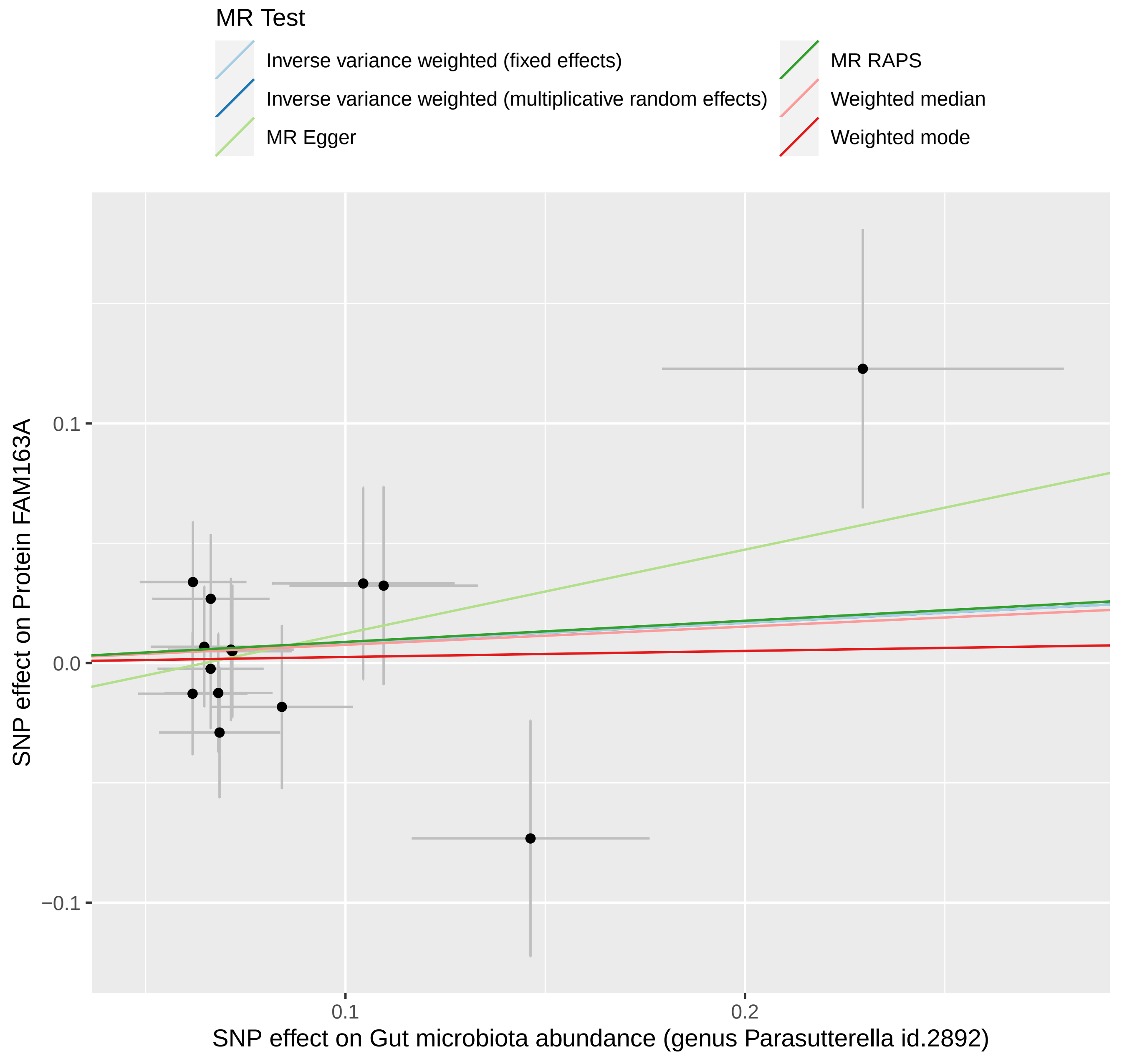

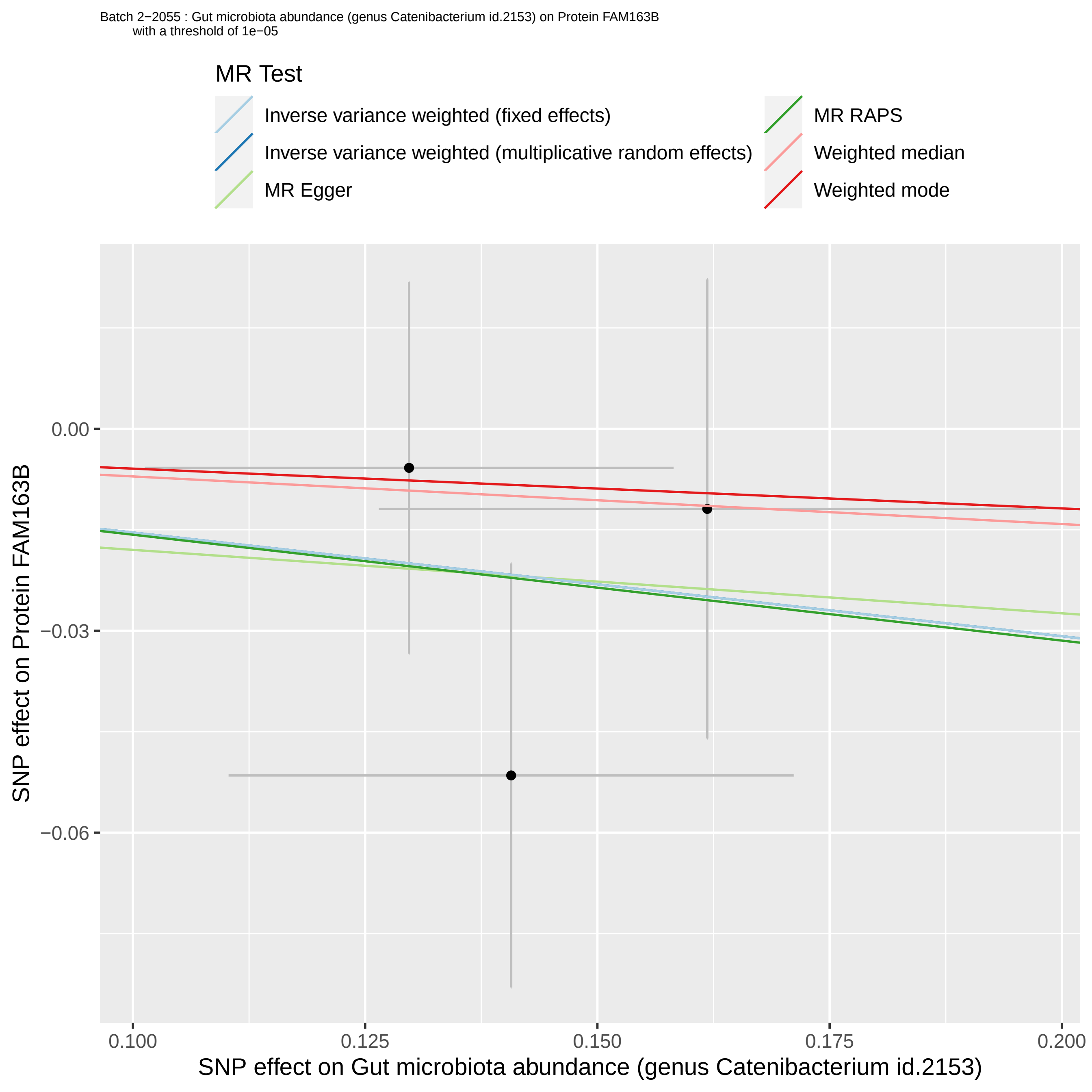

Batch 2−2055 : Gut microbiota abundance (genus Catenibacterium id.2153) on Protein FAM163B
with a threshold of 1e−05
MR Test
Inverse variance weighted (fixed effects)
Inverse variance weighted (multiplicative random effects)
MR Egger
MR RAPS
Weighted median
Weighted mode
0.00
−0.03
−0.06
SNP effect on Protein FAM163B
0.100
0.125
0.150
0.175
0.200
SNP effect on Gut microbiota abundance (genus Catenibacterium id.2153)

Batch 2−2056 : Gut microbiota abundance (genus Parasutterella id.2892) on Protein FAM163B
with a threshold of 1e−05

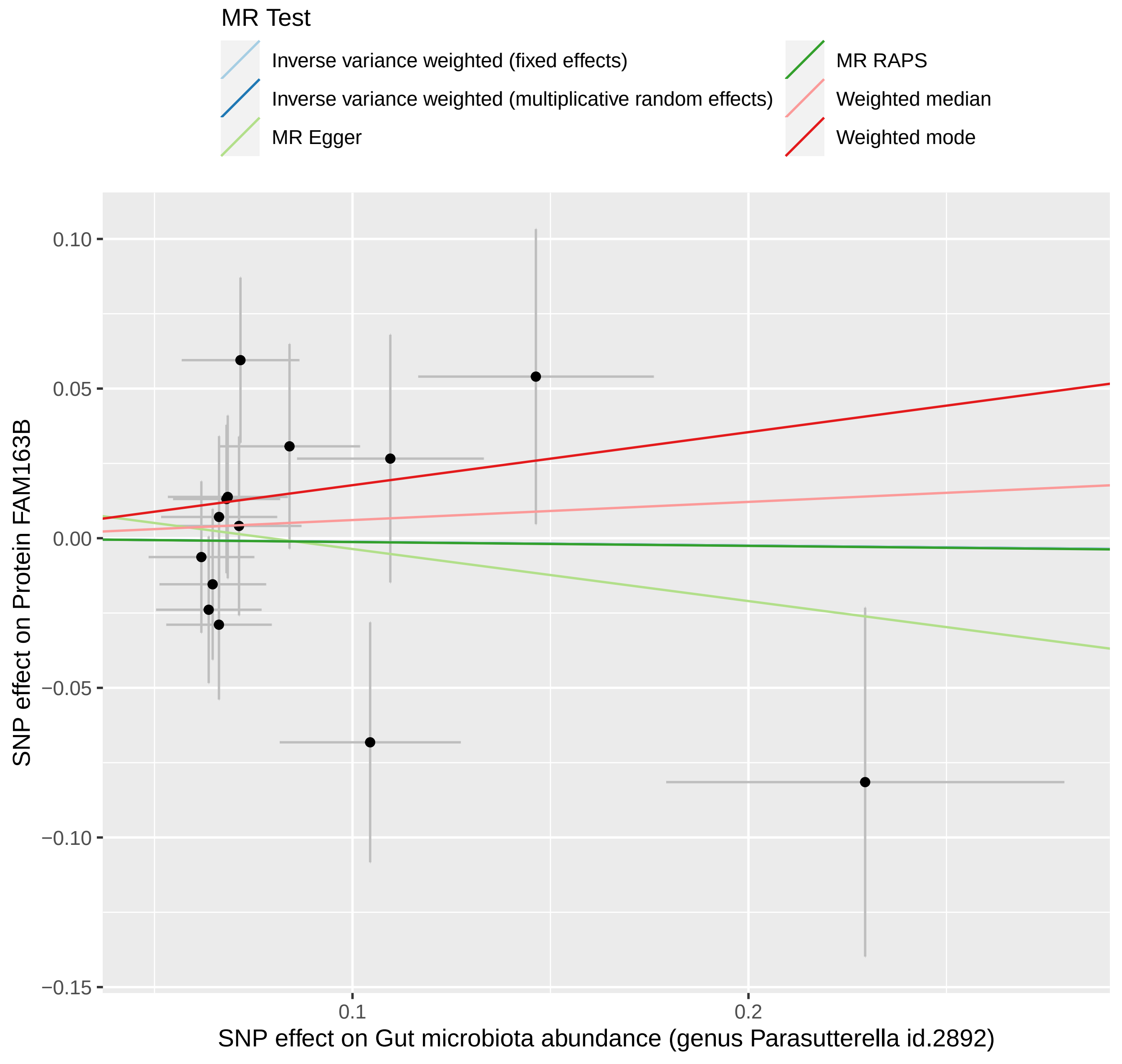

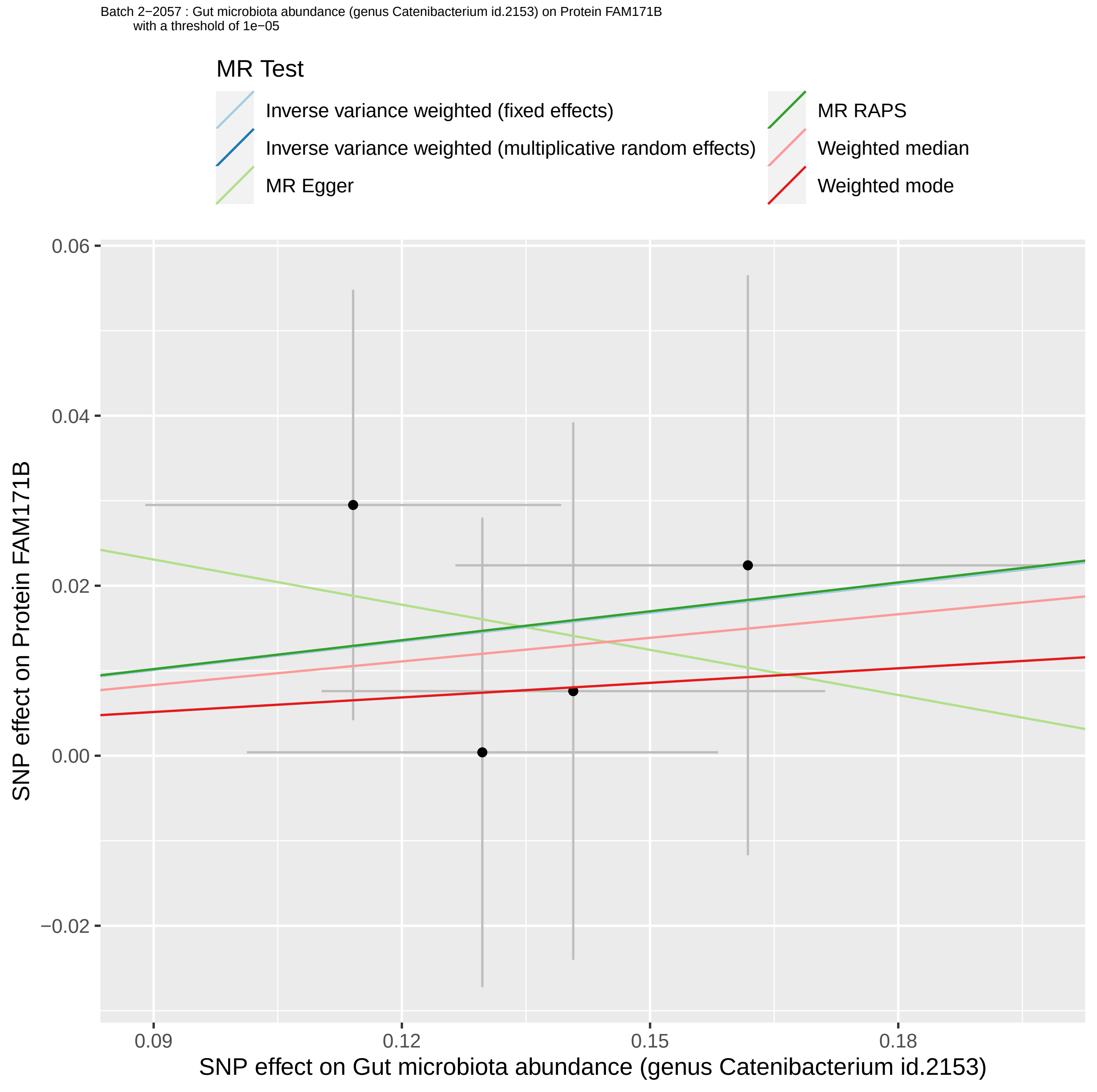
Batch 2−2057 : Gut microbiota abundance (genus Catenibacterium id.2153) on Protein FAM171B
with a threshold of 1e−05
MR Test
Inverse variance weighted (fixed effects)
Inverse variance weighted (multiplicative random effects)
MR Egger
MR RAPS
Weighted median
Weighted mode
0.06
0.04
0.02
0.00
−0.02
0.09
0.12
0.15
0.18
SNP effect on Protein FAM171B
SNP effect on Gut microbiota abundance (genus Catenibacterium id.2153)

Batch 2−2058 : Gut microbiota abundance (genus Parasutterella id.2892) on Protein FAM171B
with a threshold of 1e−05

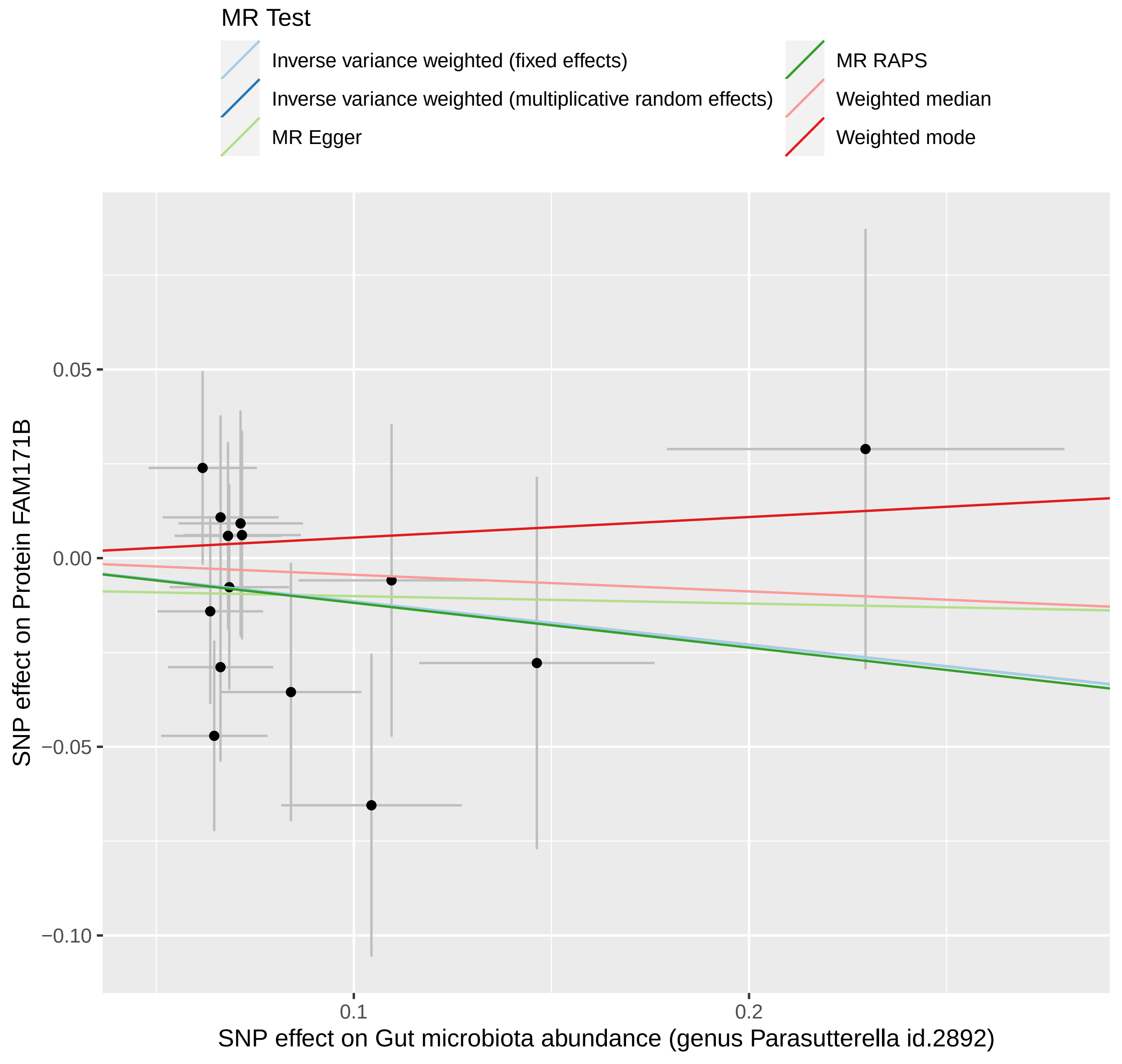

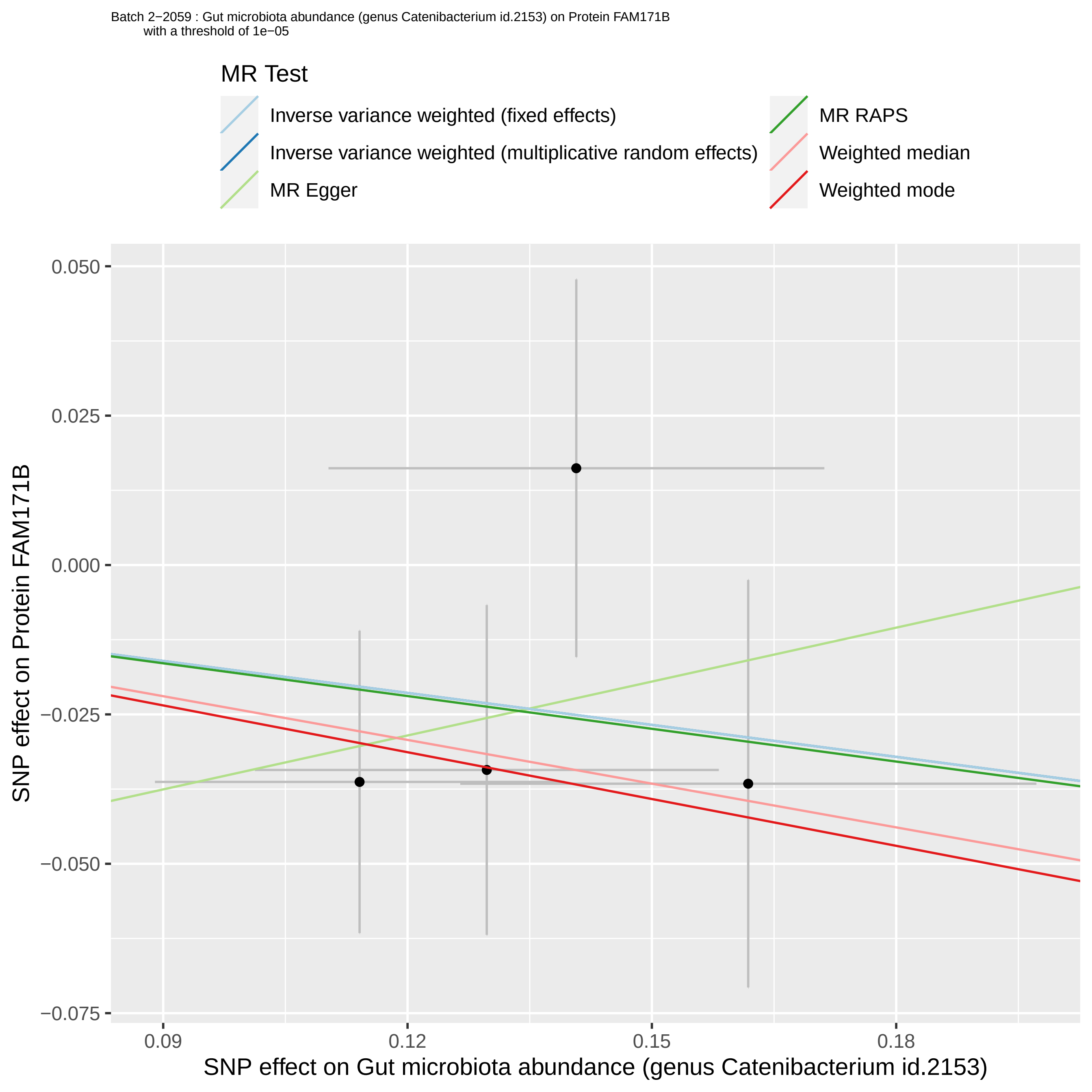
Batch 2−2059 : Gut microbiota abundance (genus Catenibacterium id.2153) on Protein FAM171B
with a threshold of 1e−05
MR Test
Inverse variance weighted (fixed effects)
Inverse variance weighted (multiplicative random effects)
MR Egger
MR RAPS
Weighted median
Weighted mode
0.050
0.025
0.000
−0.025
−0.050
−0.075
0.09
0.12
0.15
0.18
SNP effect on Protein FAM171B
SNP effect on Gut microbiota abundance (genus Catenibacterium id.2153)

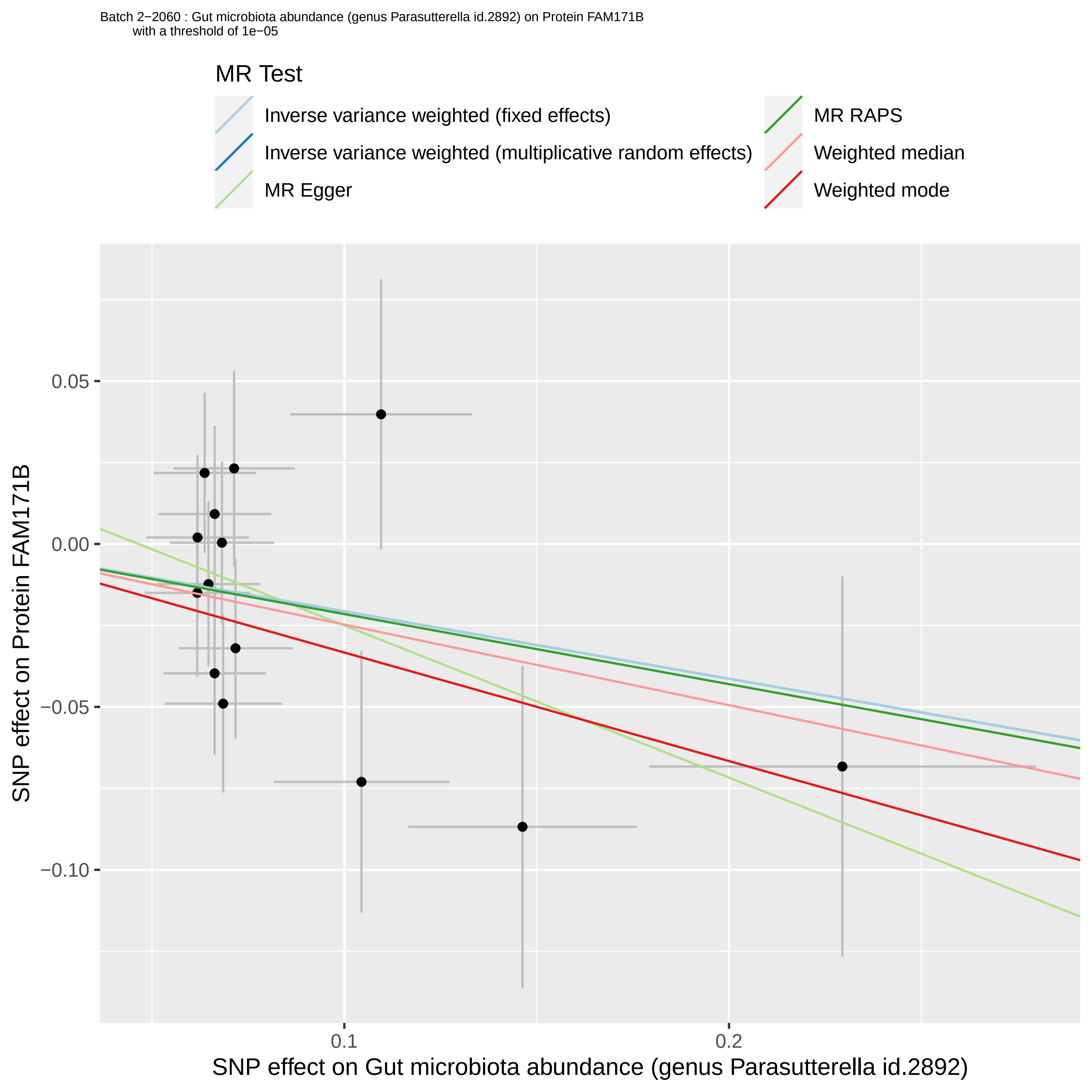

Batch 2−2060 : Gut microbiota abundance (genus Parasutterella id.2892) on Protein FAM171B
with a threshold of 1e−05
MR Test
Inverse variance weighted (fixed effects)
Inverse variance weighted (multiplicative random effects)
MR Egger
MR RAPS
Weighted median
Weighted mode
0.05
0.00
−0.05
−0.10
0.1
0.2
SNP effect on Protein FAM171B
SNP effect on Gut microbiota abundance (genus Parasutterella id.2892)

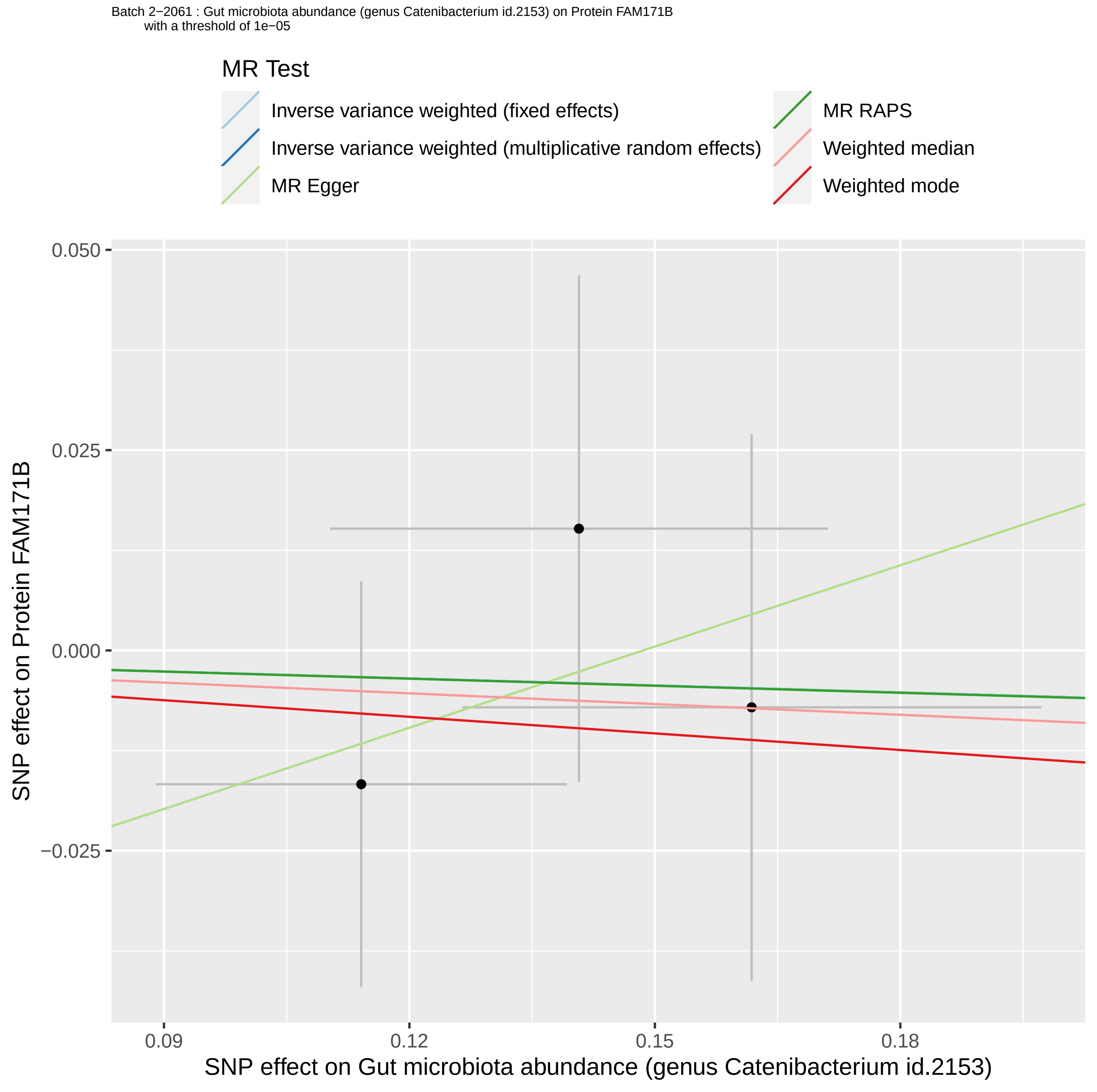

Batch 2−2061 : Gut microbiota abundance (genus Catenibacterium id.2153) on Protein FAM171B
with a threshold of 1e−05
MR Test
Inverse variance weighted (fixed effects)
Inverse variance weighted (multiplicative random effects)
MR Egger
MR RAPS
Weighted median
Weighted mode
0.050
0.025
0.000
−0.025
0.09
0.12
0.15
0.18
SNP effect on Protein FAM171B
SNP effect on Gut microbiota abundance (genus Catenibacterium id.2153)

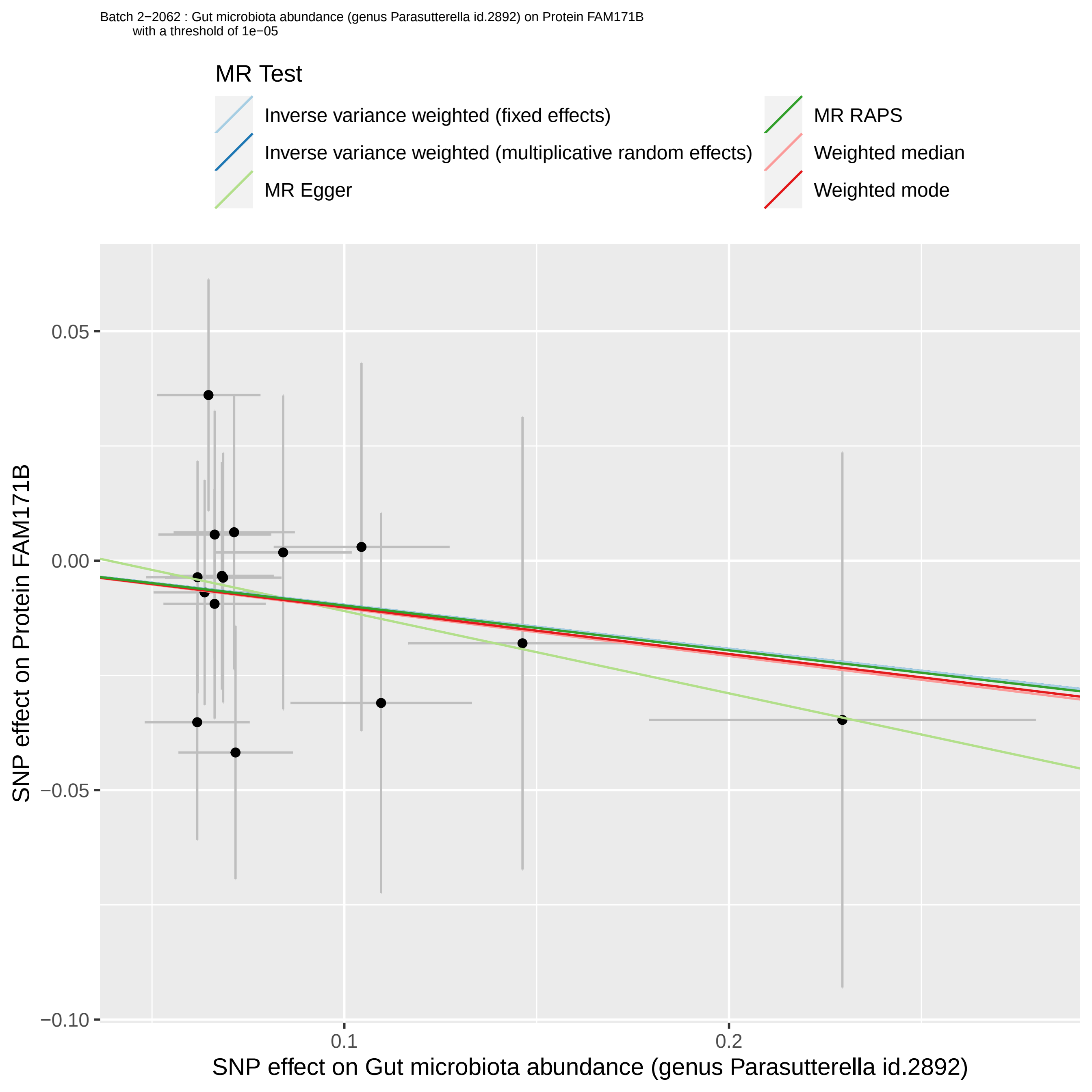

Batch 2−2062 : Gut microbiota abundance (genus Parasutterella id.2892) on Protein FAM171B
with a threshold of 1e−05
MR Test
Inverse variance weighted (fixed effects)
Inverse variance weighted (multiplicative random effects)
MR Egger
MR RAPS
Weighted median
Weighted mode
0.05
0.00
−0.05
−0.10
0.1
0.2
SNP effect on Protein FAM171B
SNP effect on Gut microbiota abundance (genus Parasutterella id.2892)

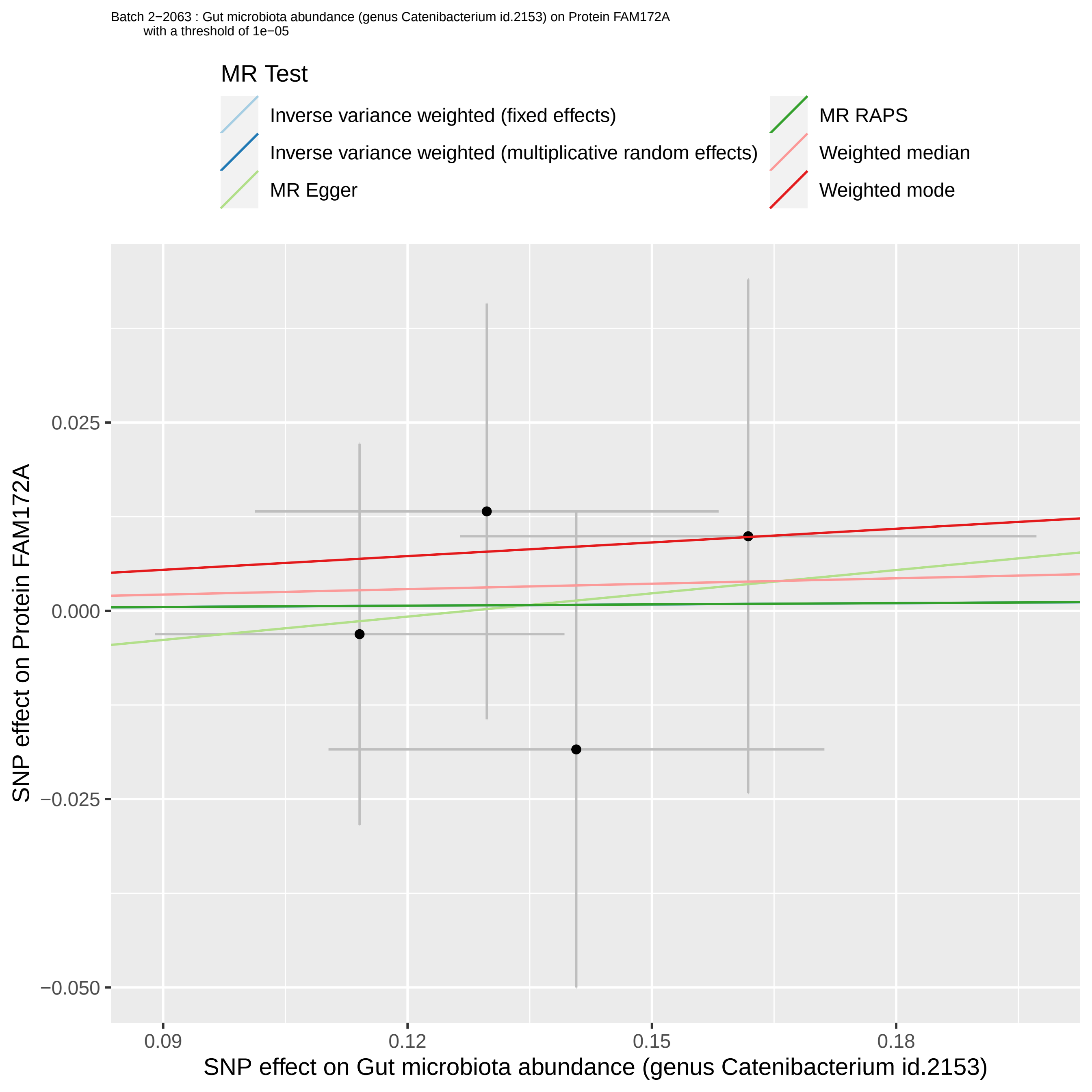
Batch 2−2063 : Gut microbiota abundance (genus Catenibacterium id.2153) on Protein FAM172A
with a threshold of 1e−05
MR Test
Inverse variance weighted (fixed effects)
Inverse variance weighted (multiplicative random effects)
MR Egger
MR RAPS
Weighted median
Weighted mode
SNP effect on Protein FAM172A
0.025
0.000
−0.025
−0.050
0.09
0.12
0.15
0.18
SNP effect on Gut microbiota abundance (genus Catenibacterium id.2153)

Batch 2−2064 : Gut microbiota abundance (genus Parasutterella id.2892) on Protein FAM172A
with a threshold of 1e−05

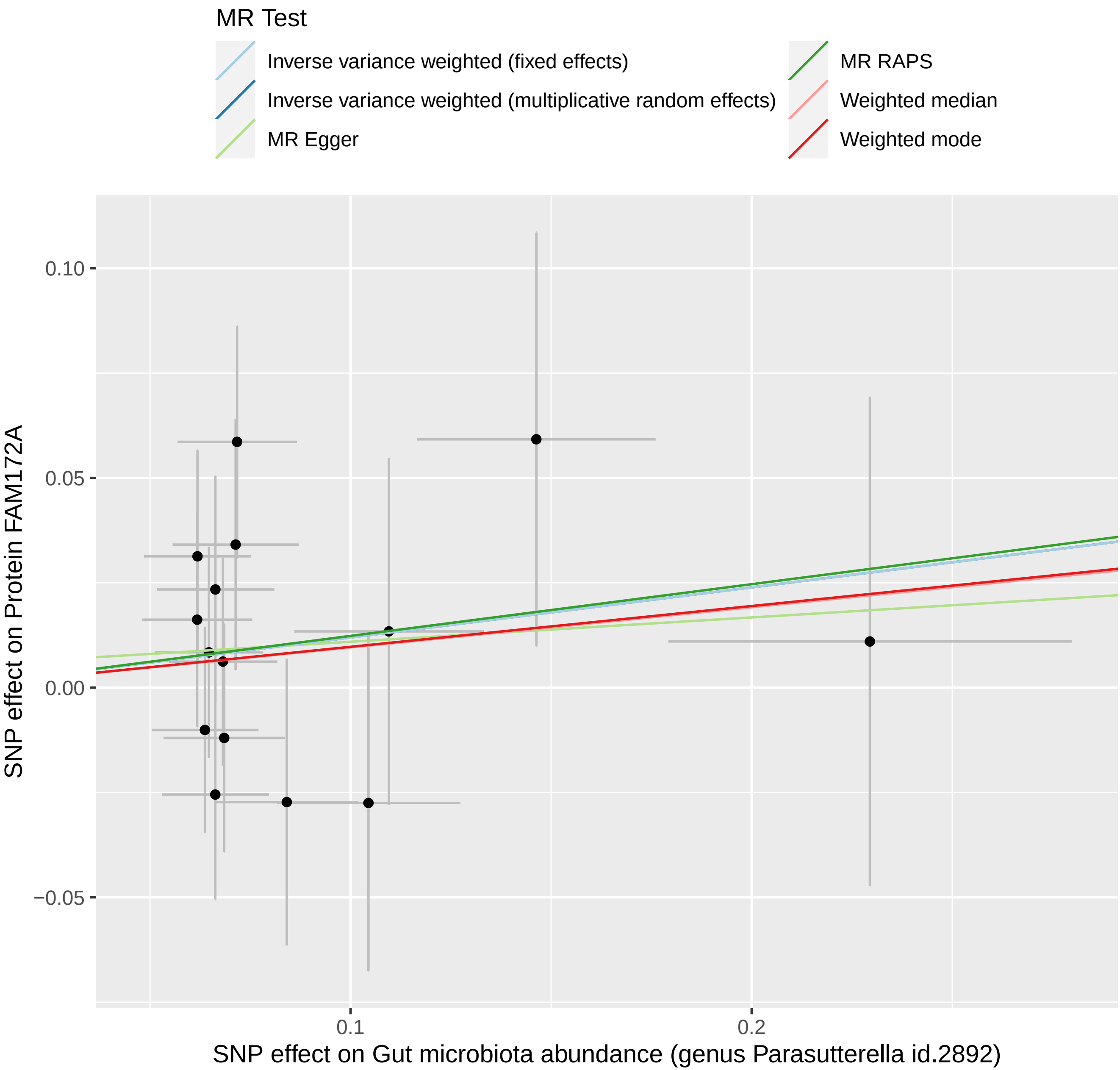

Batch 2−2065 : Gut microbiota abundance (genus Catenibacterium id.2153) on Protein FAM173A
with a threshold of 1e−05

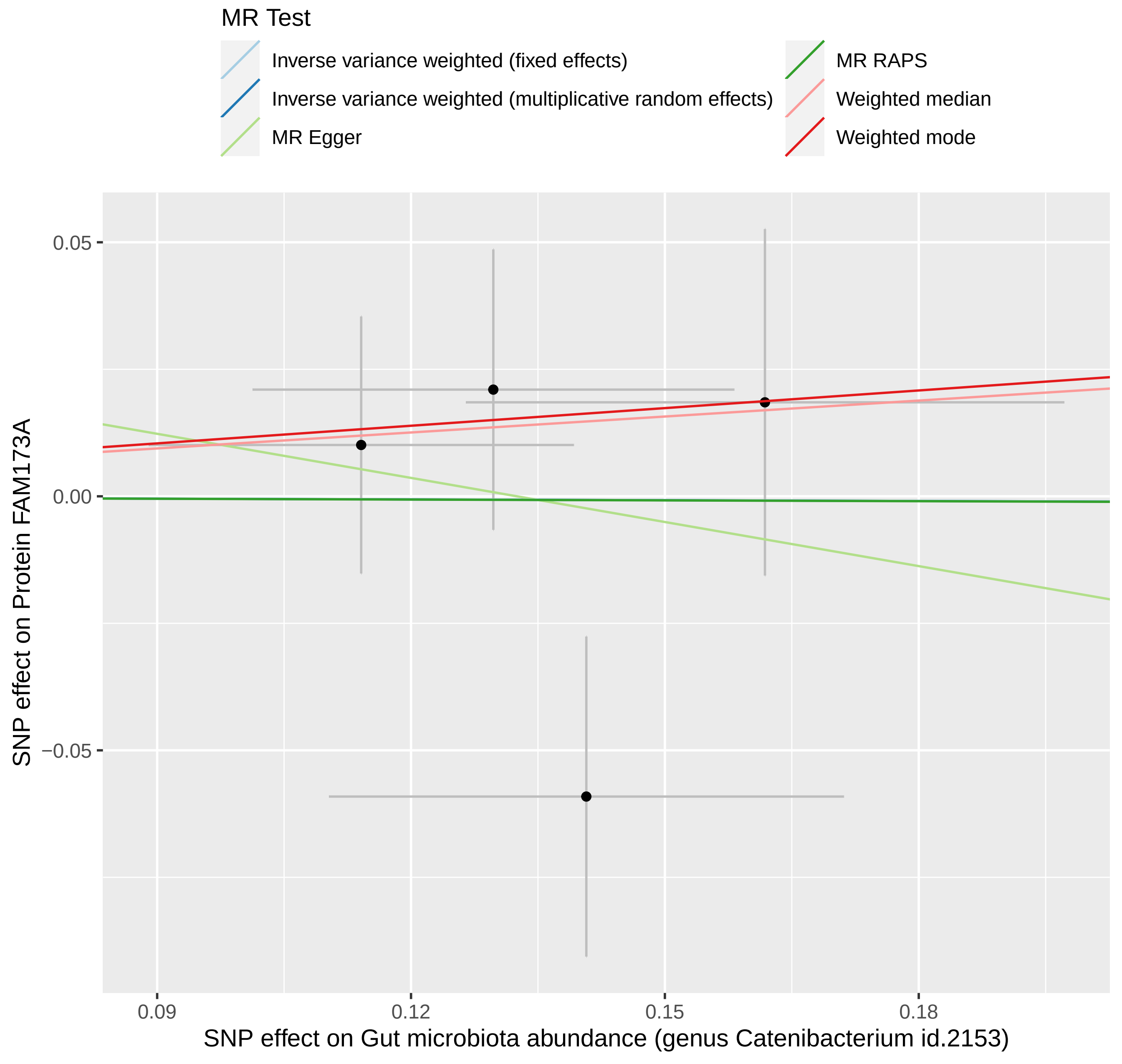

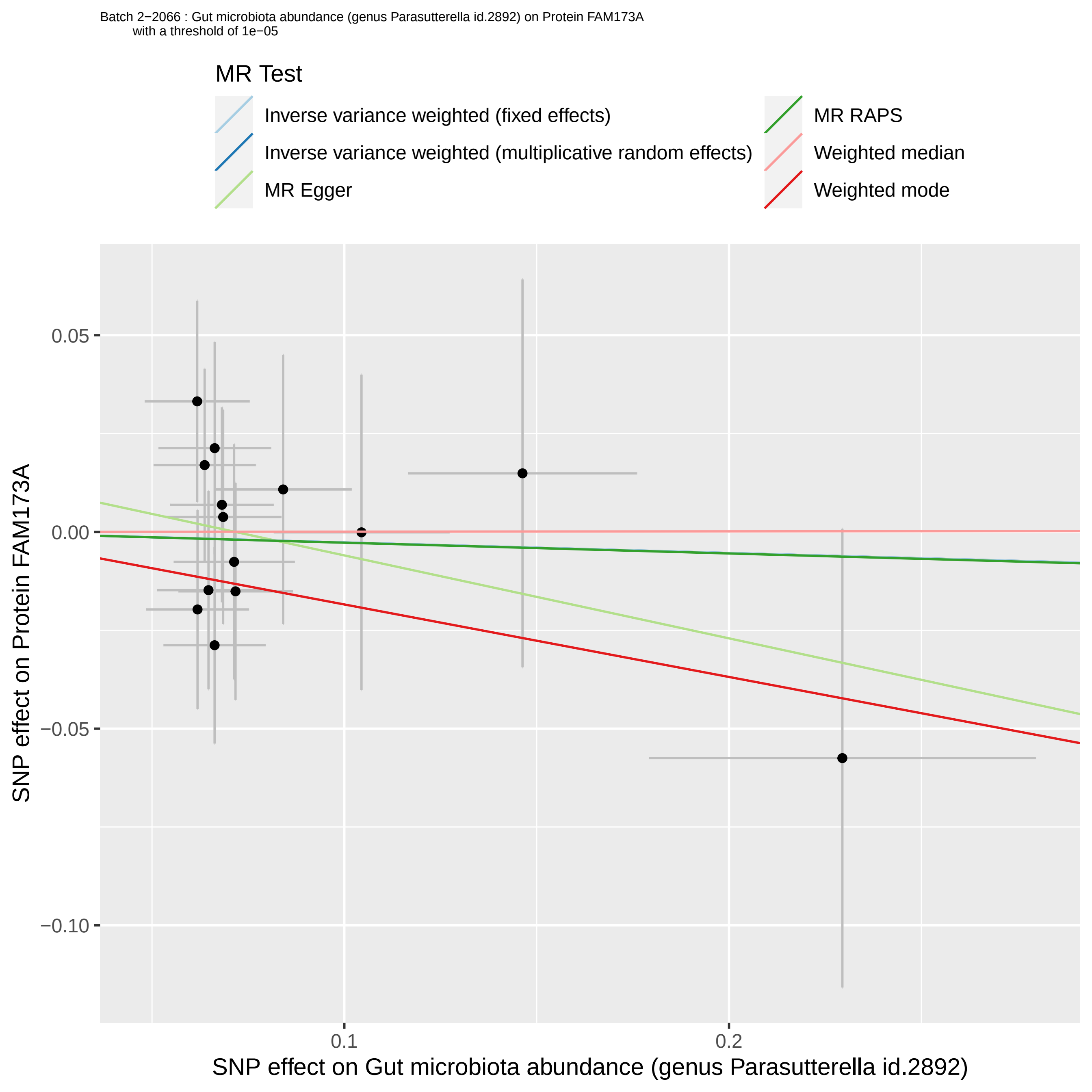

Batch 2−2066 : Gut microbiota abundance (genus Parasutterella id.2892) on Protein FAM173A
with a threshold of 1e−05
MR Test
Inverse variance weighted (fixed effects)
Inverse variance weighted (multiplicative random effects)
MR Egger
MR RAPS
Weighted median
Weighted mode
0.05
0.00
−0.05
−0.10
0.1
0.2
SNP effect on Protein FAM173A
SNP effect on Gut microbiota abundance (genus Parasutterella id.2892)

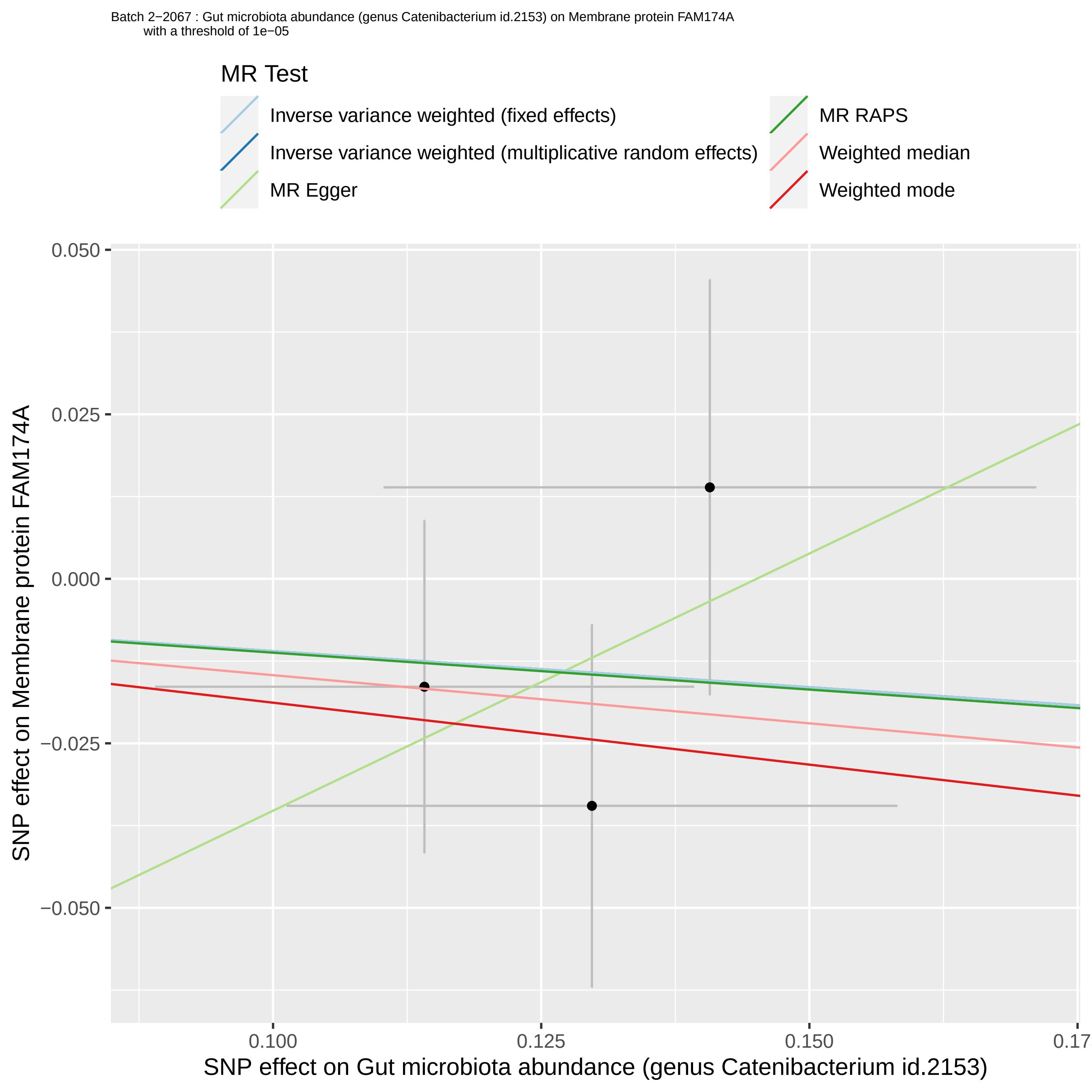
Batch 2−2067 : Gut microbiota abundance (genus Catenibacterium id.2153) on Membrane protein FAM174A
with a threshold of 1e−05
MR Test
Inverse variance weighted (fixed effects)
Inverse variance weighted (multiplicative random effects)
MR Egger
MR RAPS
Weighted median
Weighted mode
0.050
0.025
0.000
−0.025
−0.050
0.100
0.125
0.150
0.17
SNP effect on Membrane protein FAM174A
SNP effect on Gut microbiota abundance (genus Catenibacterium id.2153)

Batch 2−2068 : Gut microbiota abundance (genus Parasutterella id.2892) on Membrane protein FAM174A
with a threshold of 1e−05

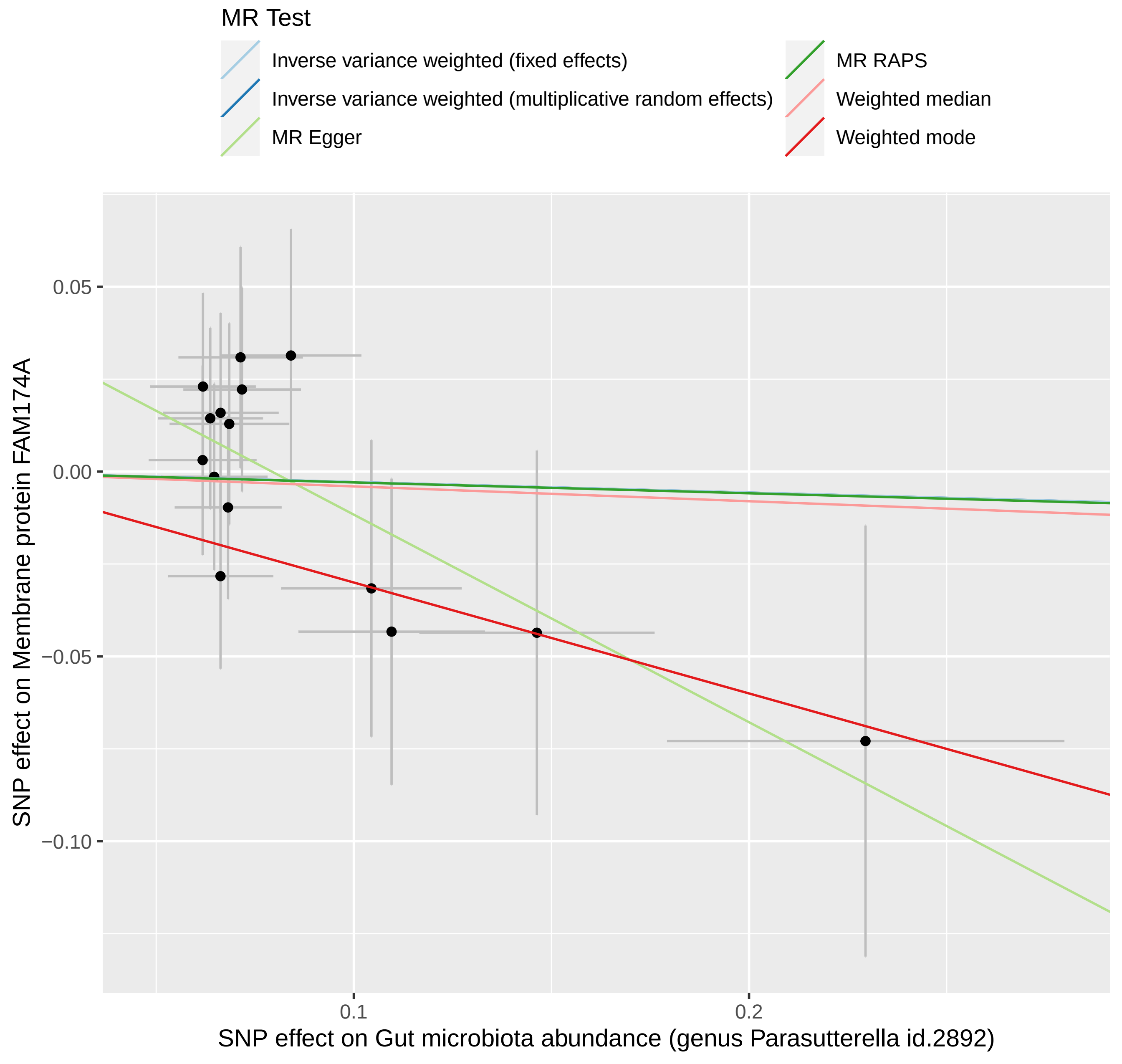

Batch 2−2069 : Gut microbiota abundance (genus Catenibacterium id.2153) on Membrane protein FAM174B
with a threshold of 1e−05

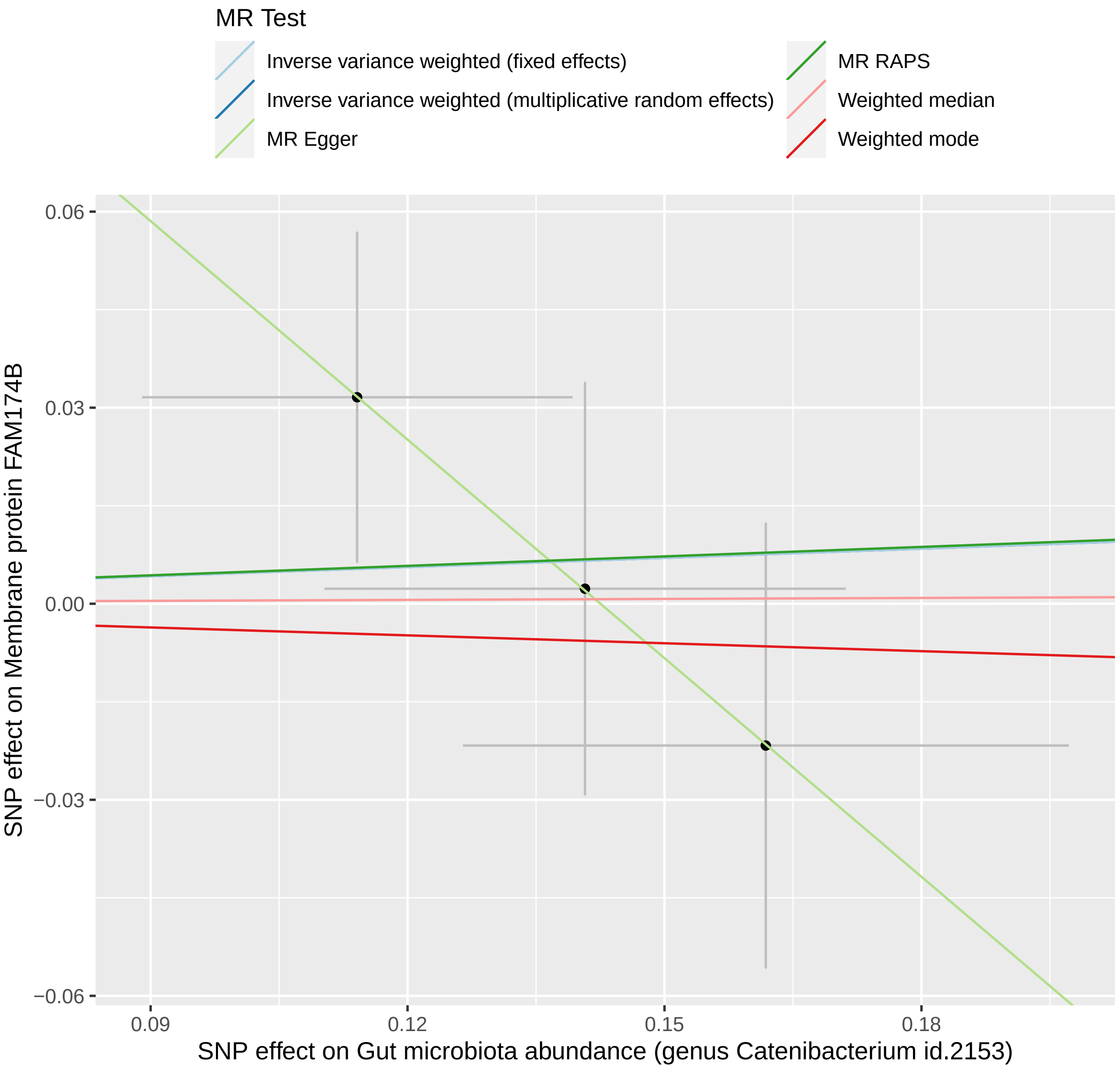

Batch 2−2070 : Gut microbiota abundance (genus Parasutterella id.2892) on Membrane protein FAM174B
with a threshold of 1e−05

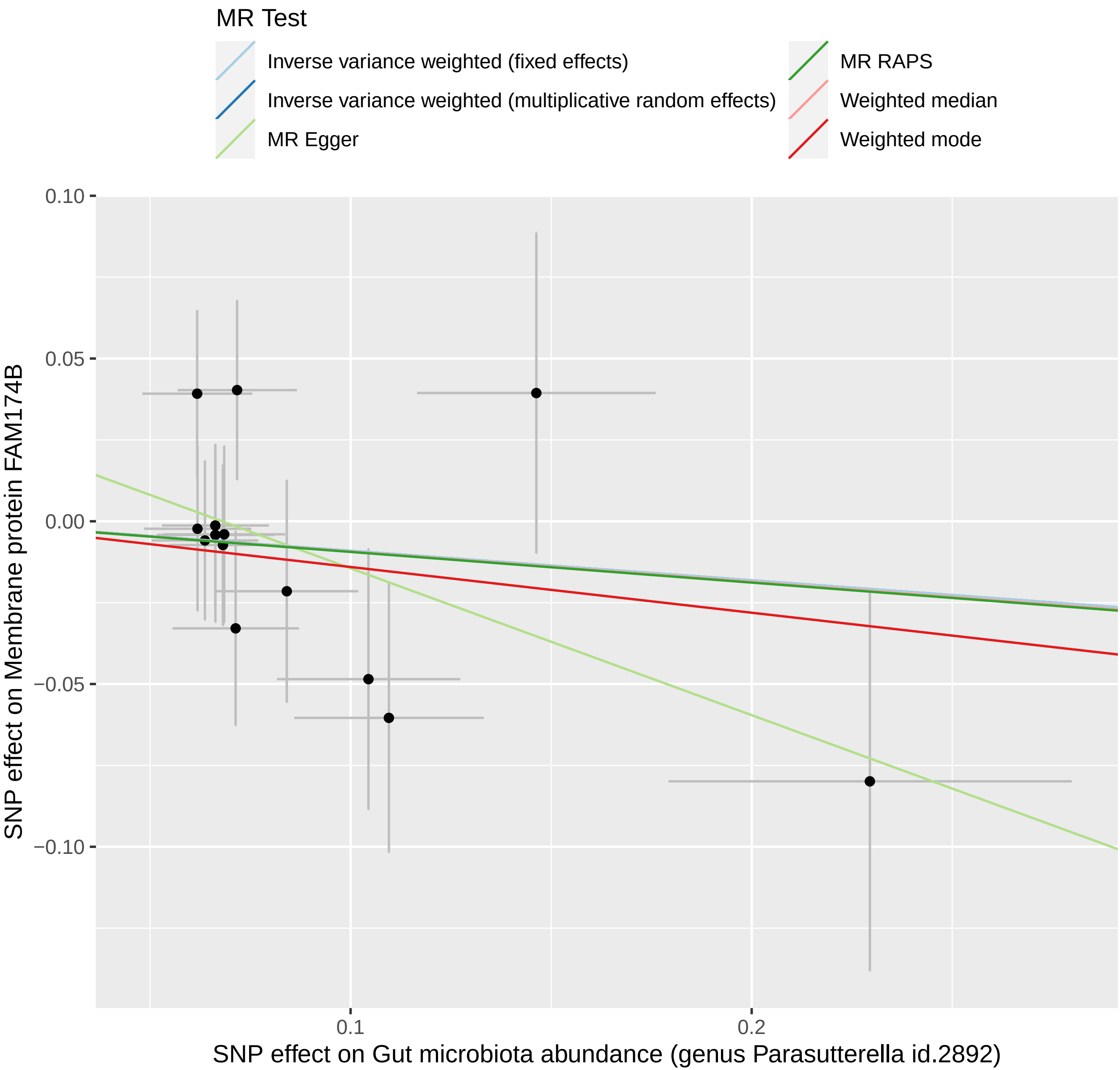

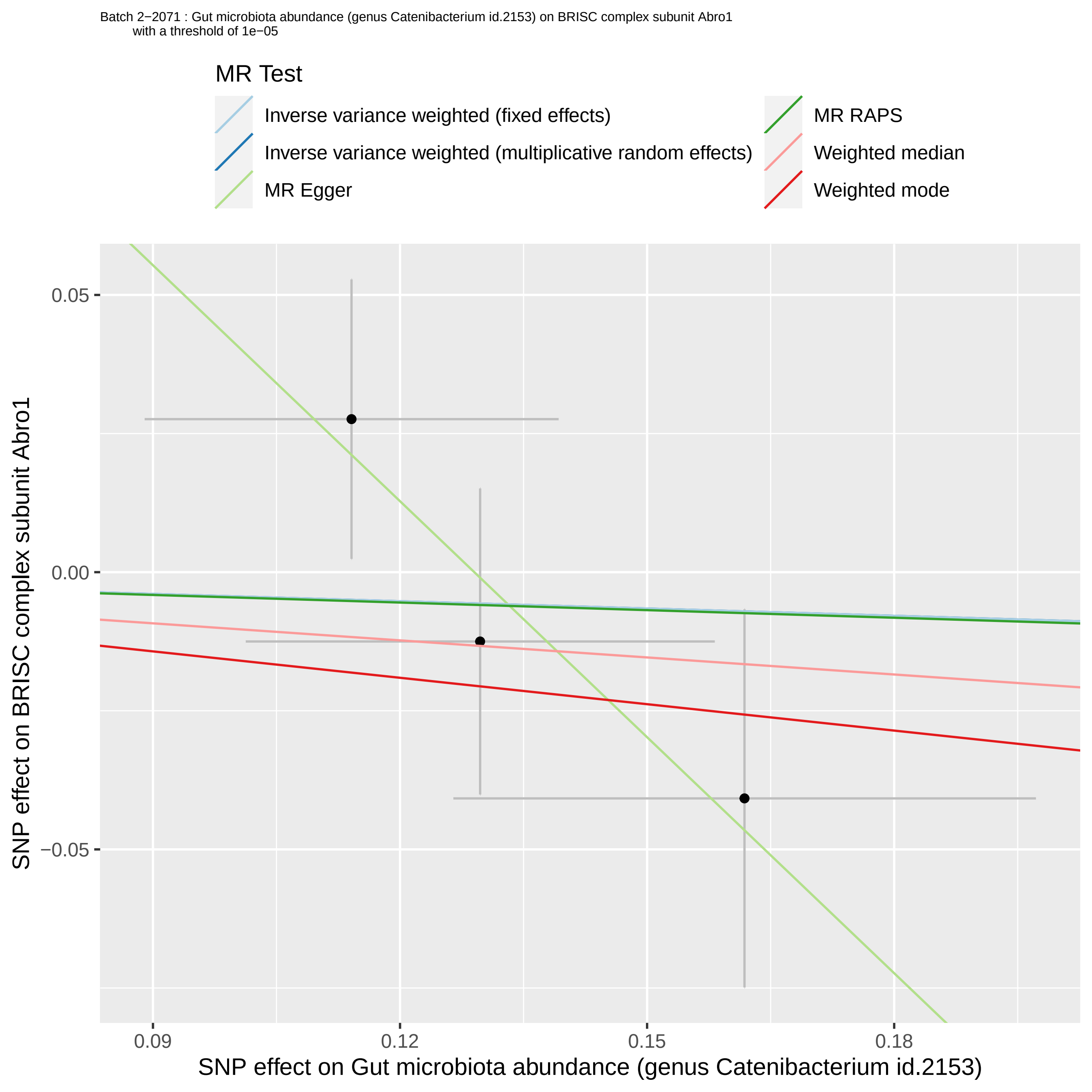
Batch 2−2071 : Gut microbiota abundance (genus Catenibacterium id.2153) on BRISC complex subunit Abro1
with a threshold of 1e−05
MR Test
Inverse variance weighted (fixed effects)
Inverse variance weighted (multiplicative random effects)
MR Egger
MR RAPS
Weighted median
Weighted mode
SNP effect on BRISC complex subunit Abro1
0.05
0.00
−0.05
0.09
0.12
0.15
0.18
SNP effect on Gut microbiota abundance (genus Catenibacterium id.2153)

Batch 2−2072 : Gut microbiota abundance (genus Parasutterella id.2892) on BRISC complex subunit Abro1
with a threshold of 1e−05

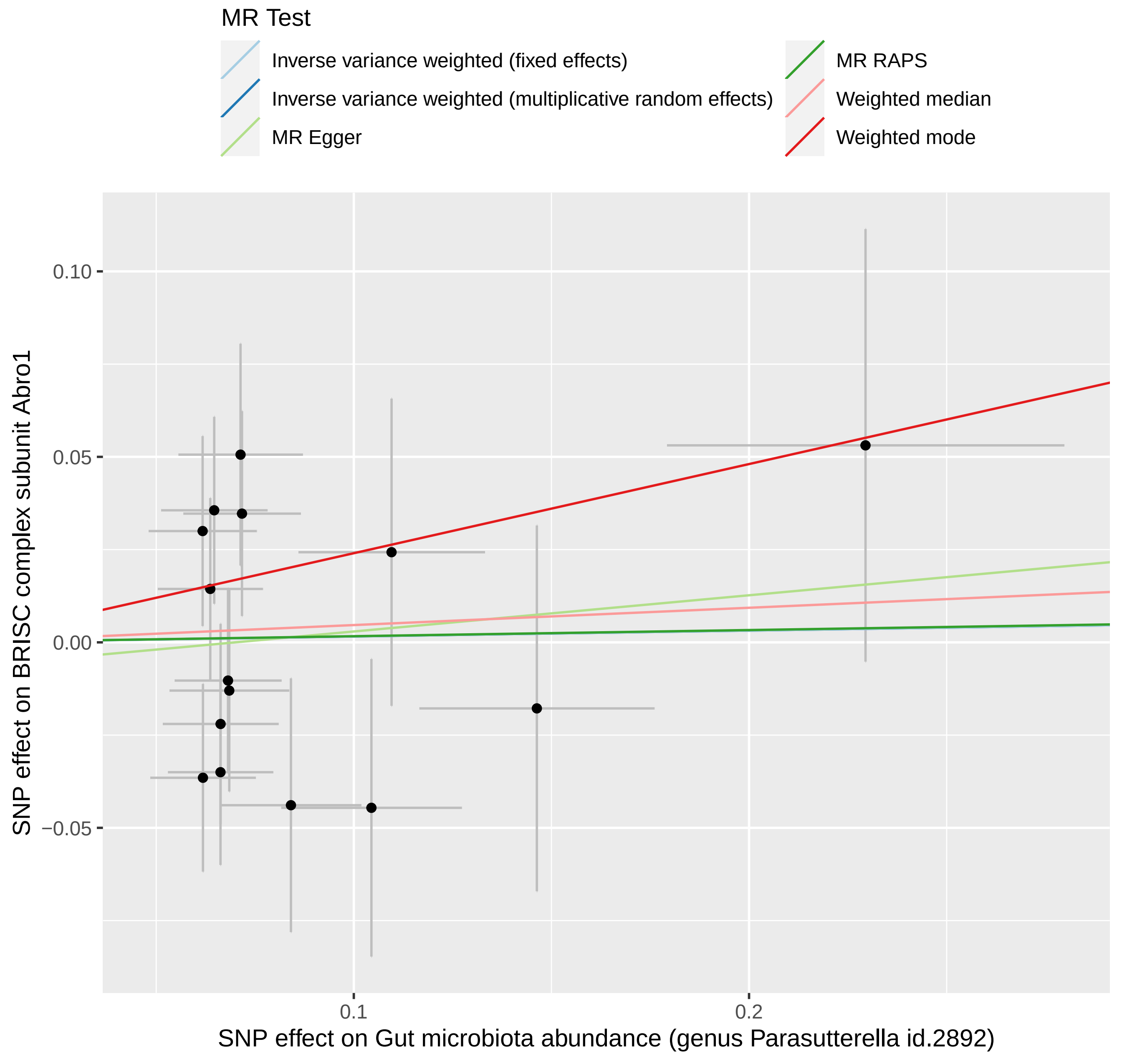

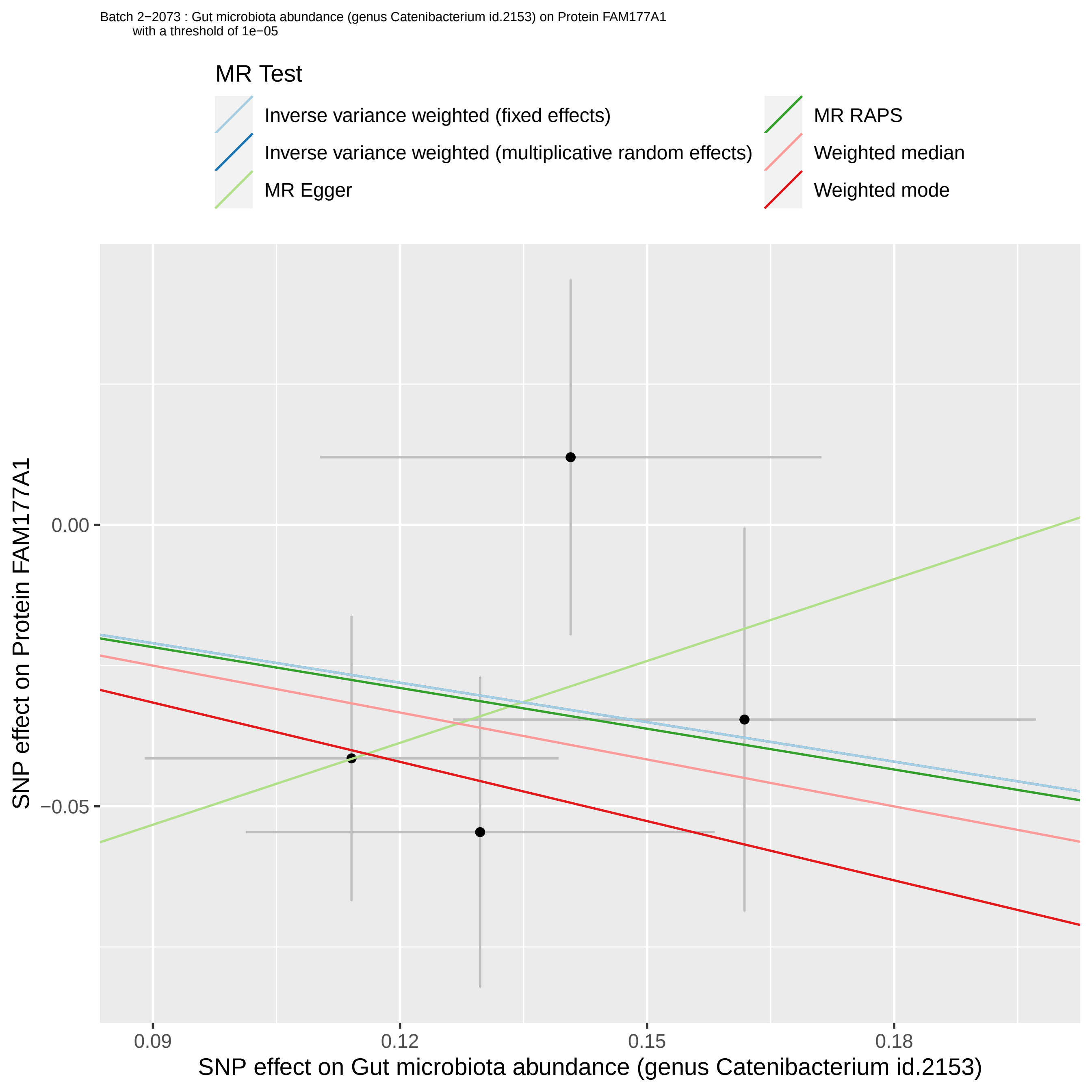

Batch 2−2073 : Gut microbiota abundance (genus Catenibacterium id.2153) on Protein FAM177A1
with a threshold of 1e−05
MR Test
Inverse variance weighted (fixed effects)
Inverse variance weighted (multiplicative random effects)
MR Egger
MR RAPS
Weighted median
Weighted mode
0.00
−0.05
0.09
0.12
0.15
0.18
SNP effect on Protein FAM177A1
SNP effect on Gut microbiota abundance (genus Catenibacterium id.2153)

Batch 2−2074 : Gut microbiota abundance (genus Parasutterella id.2892) on Protein FAM177A1
with a threshold of 1e−05

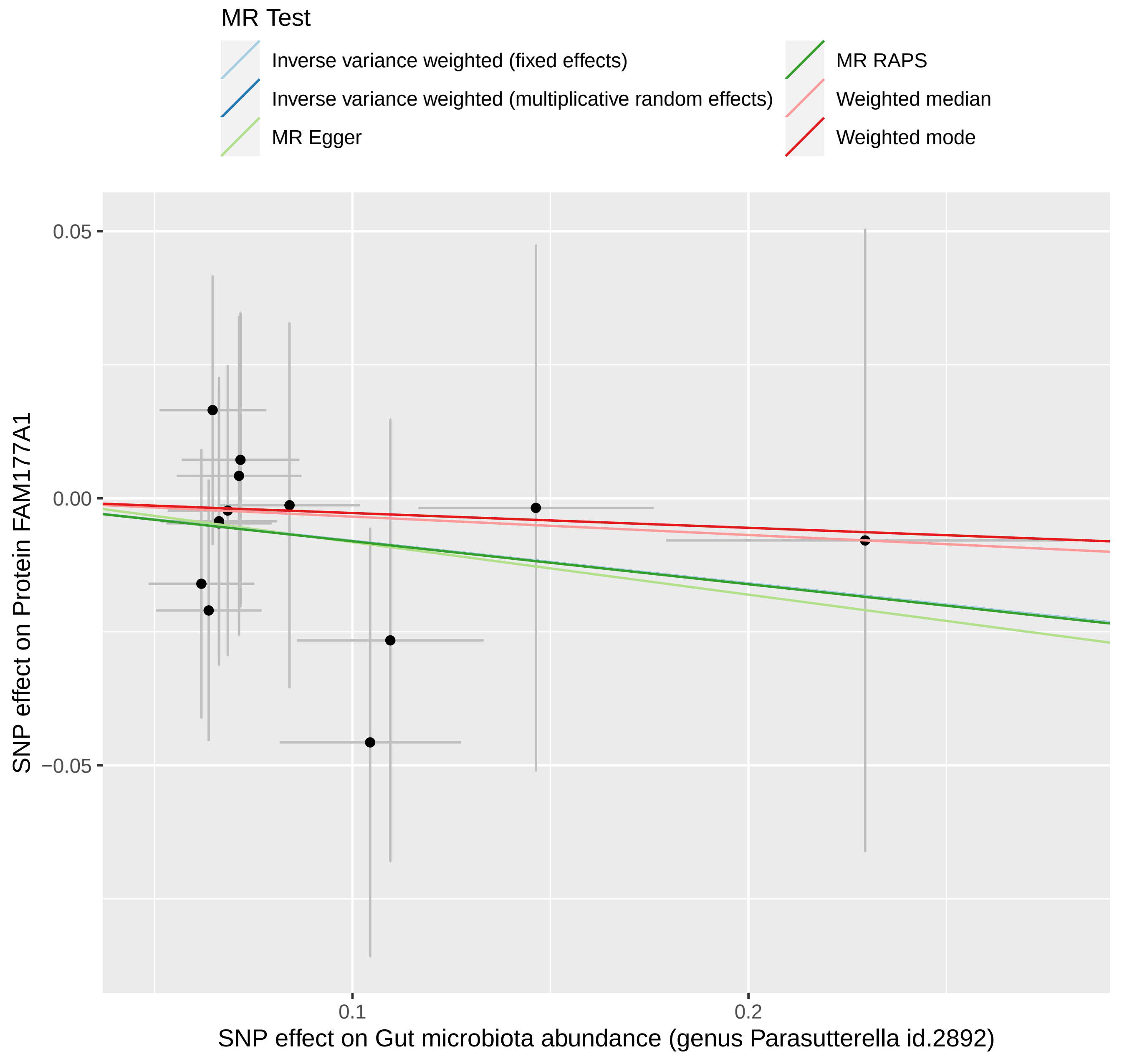

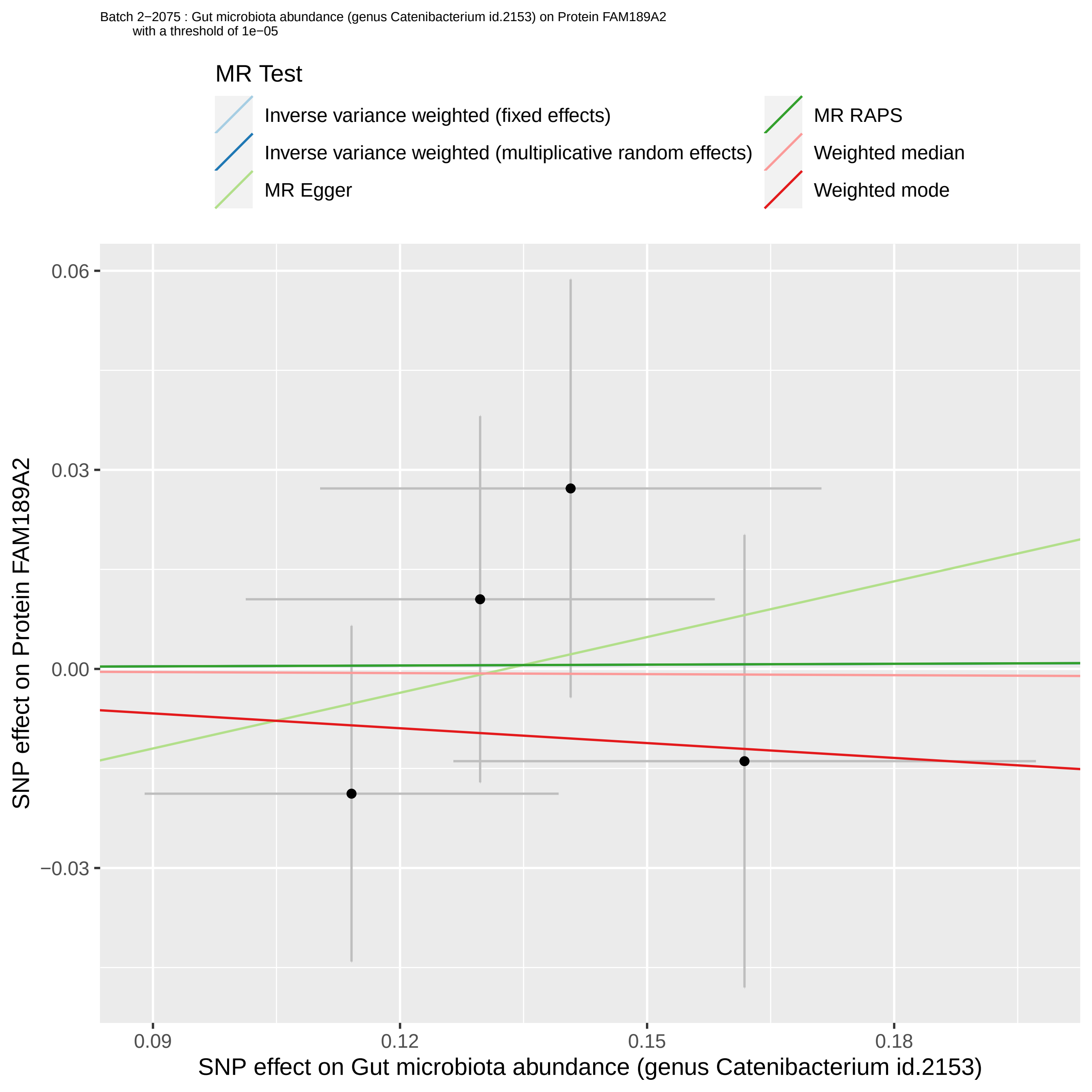
Batch 2−2075 : Gut microbiota abundance (genus Catenibacterium id.2153) on Protein FAM189A2
with a threshold of 1e−05
MR Test
Inverse variance weighted (fixed effects)
Inverse variance weighted (multiplicative random effects)
MR Egger
MR RAPS
Weighted median
Weighted mode
0.06
0.03
0.00
−0.03
0.09
0.12
0.15
0.18
SNP effect on Protein FAM189A2
SNP effect on Gut microbiota abundance (genus Catenibacterium id.2153)

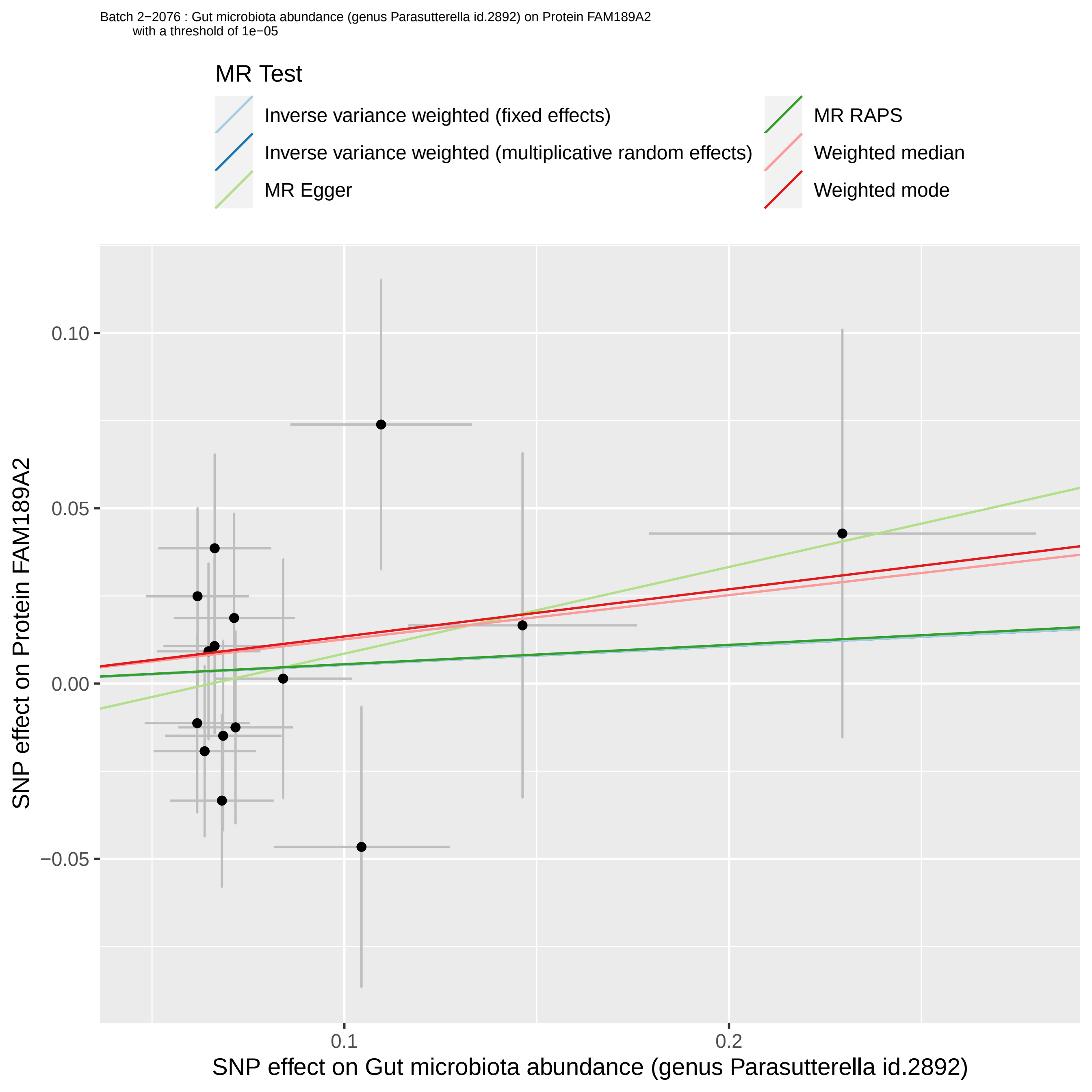

Batch 2−2076 : Gut microbiota abundance (genus Parasutterella id.2892) on Protein FAM189A2
with a threshold of 1e−05
MR Test
Inverse variance weighted (fixed effects)
Inverse variance weighted (multiplicative random effects)
MR Egger
MR RAPS
Weighted median
Weighted mode
0.10
0.05
0.00
−0.05
0.1
0.2
SNP effect on Protein FAM189A2
SNP effect on Gut microbiota abundance (genus Parasutterella id.2892)

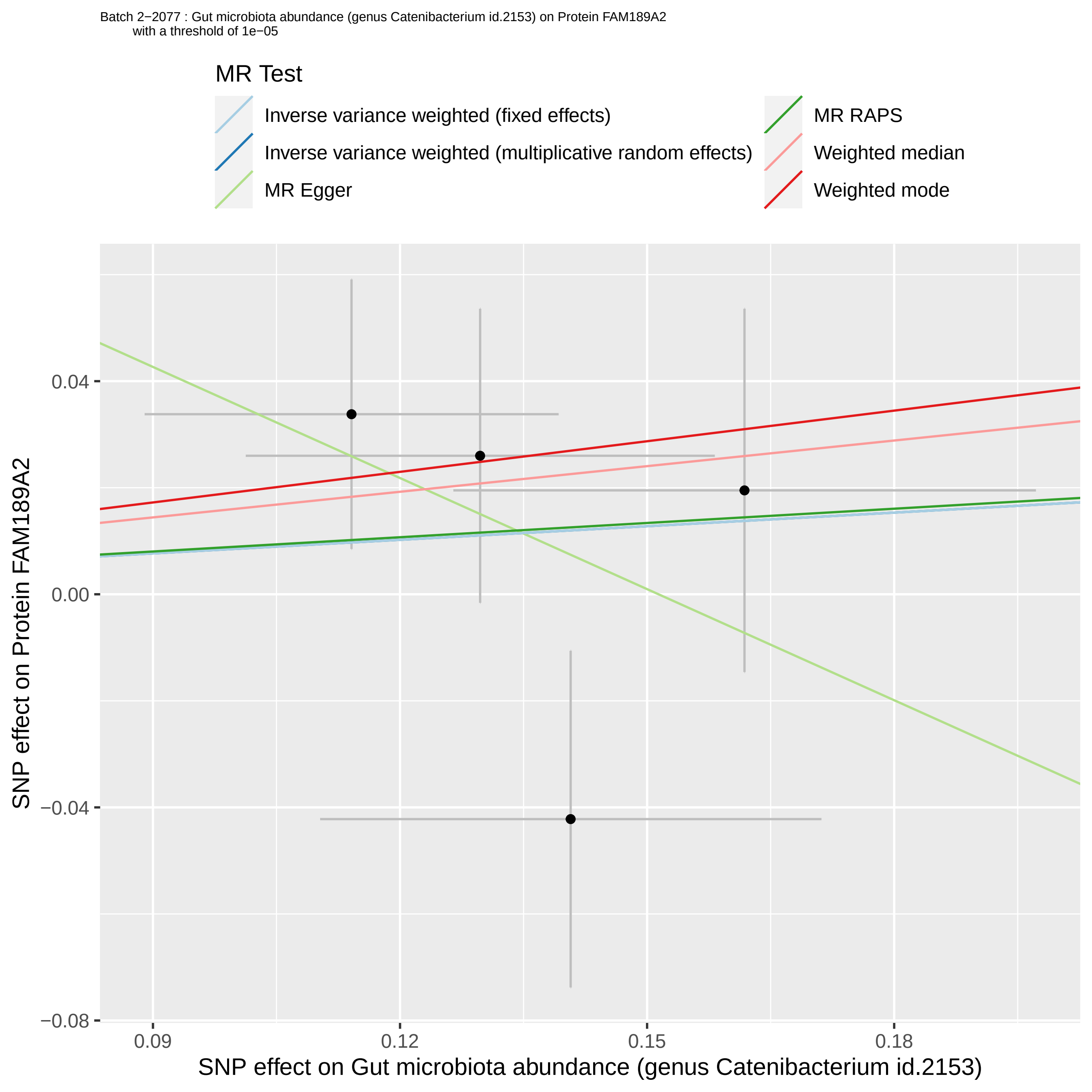
Batch 2−2077 : Gut microbiota abundance (genus Catenibacterium id.2153) on Protein FAM189A2
with a threshold of 1e−05
MR Test
Inverse variance weighted (fixed effects)
Inverse variance weighted (multiplicative random effects)
MR Egger
MR RAPS
Weighted median
Weighted mode
0.04
0.00
−0.04
−0.08
0.09
0.12
0.15
0.18
SNP effect on Protein FAM189A2
SNP effect on Gut microbiota abundance (genus Catenibacterium id.2153)

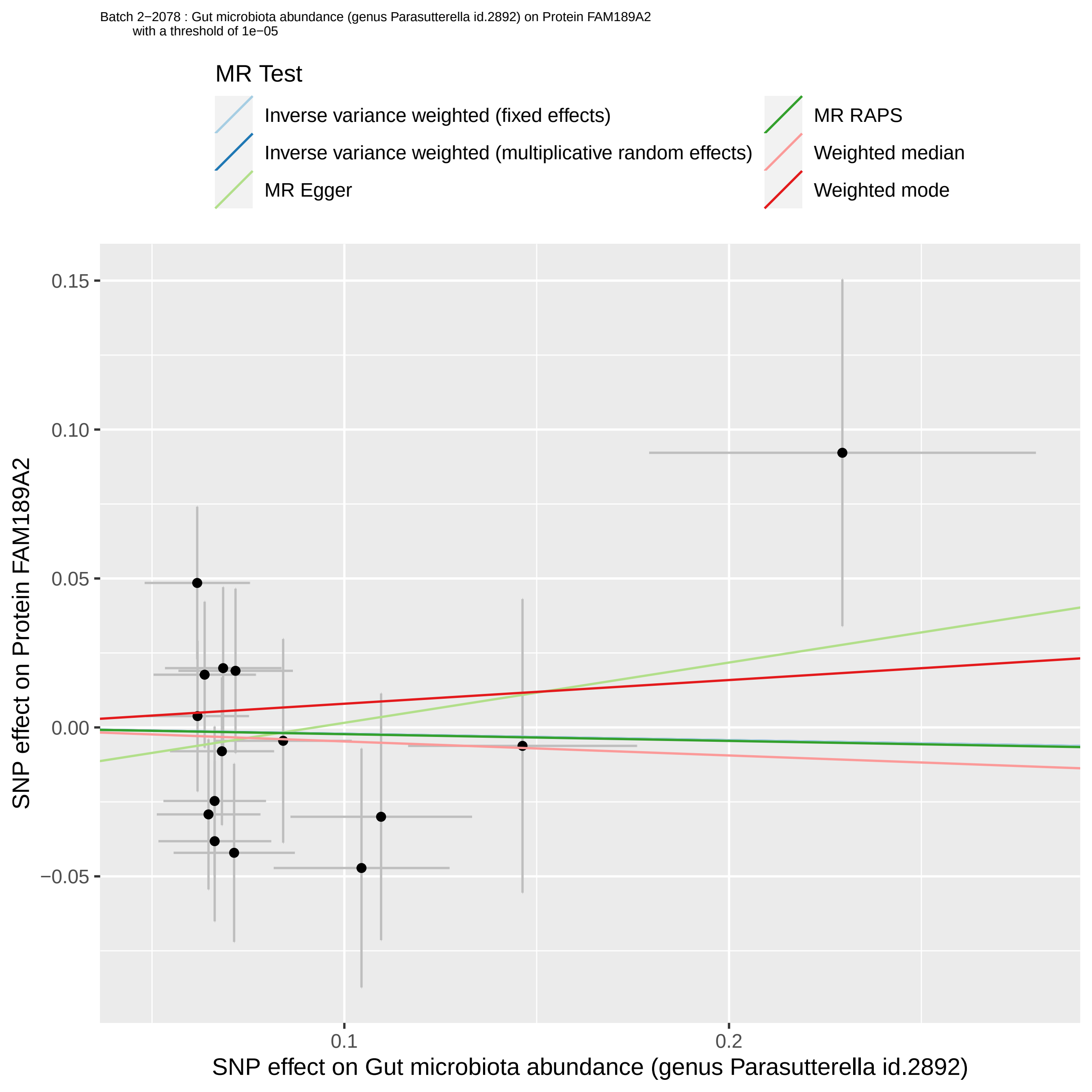

Batch 2−2078 : Gut microbiota abundance (genus Parasutterella id.2892) on Protein FAM189A2
with a threshold of 1e−05
MR Test
Inverse variance weighted (fixed effects)
Inverse variance weighted (multiplicative random effects)
MR Egger
MR RAPS
Weighted median
Weighted mode
0.15
0.10
0.05
0.00
−0.05
0.1
0.2
SNP effect on Protein FAM189A2
SNP effect on Gut microbiota abundance (genus Parasutterella id.2892)

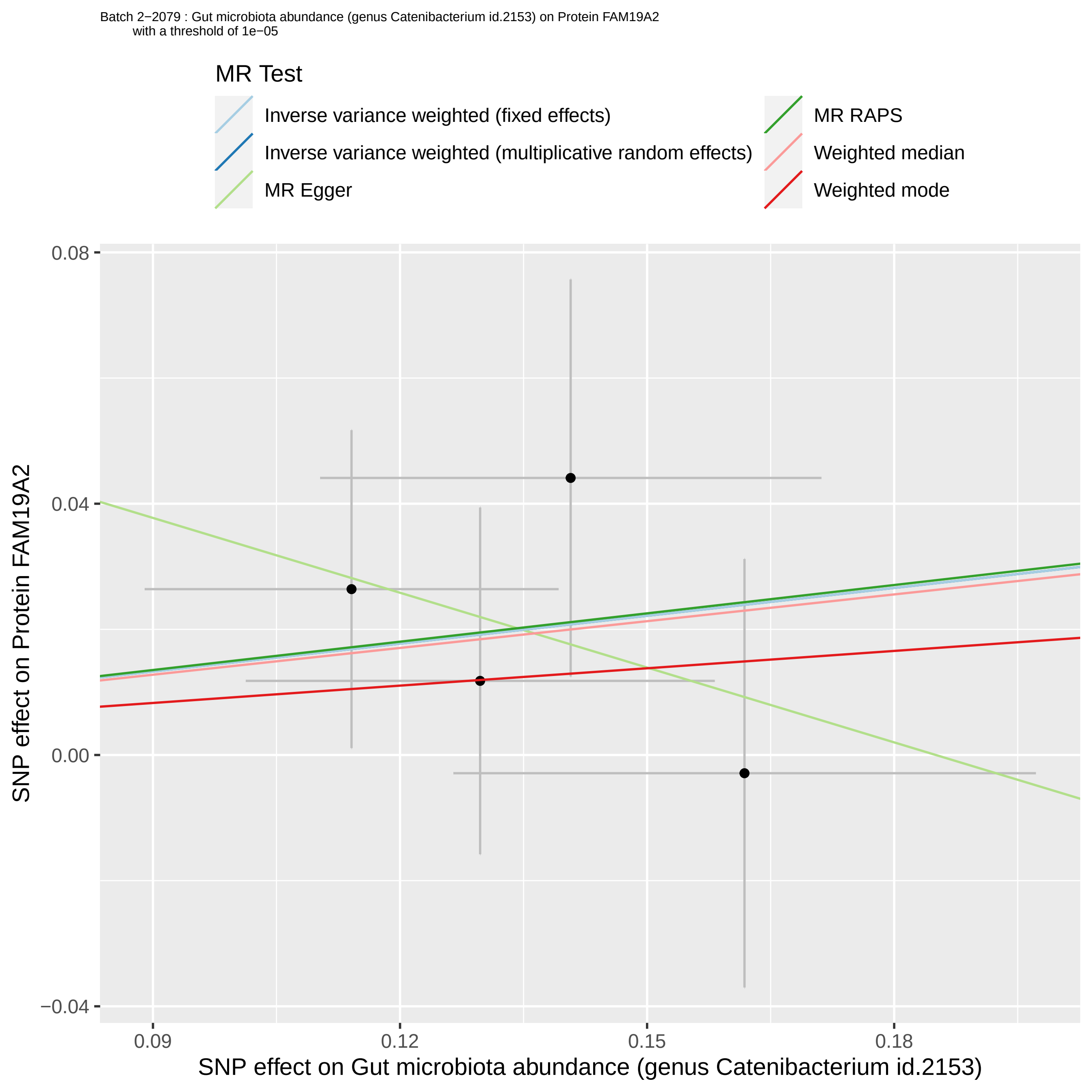

Batch 2−2079 : Gut microbiota abundance (genus Catenibacterium id.2153) on Protein FAM19A2
with a threshold of 1e−05
MR Test
Inverse variance weighted (fixed effects)
Inverse variance weighted (multiplicative random effects)
MR Egger
MR RAPS
Weighted median
Weighted mode
0.08
0.04
0.00
−0.04
0.09
0.12
0.15
0.18
SNP effect on Protein FAM19A2
SNP effect on Gut microbiota abundance (genus Catenibacterium id.2153)

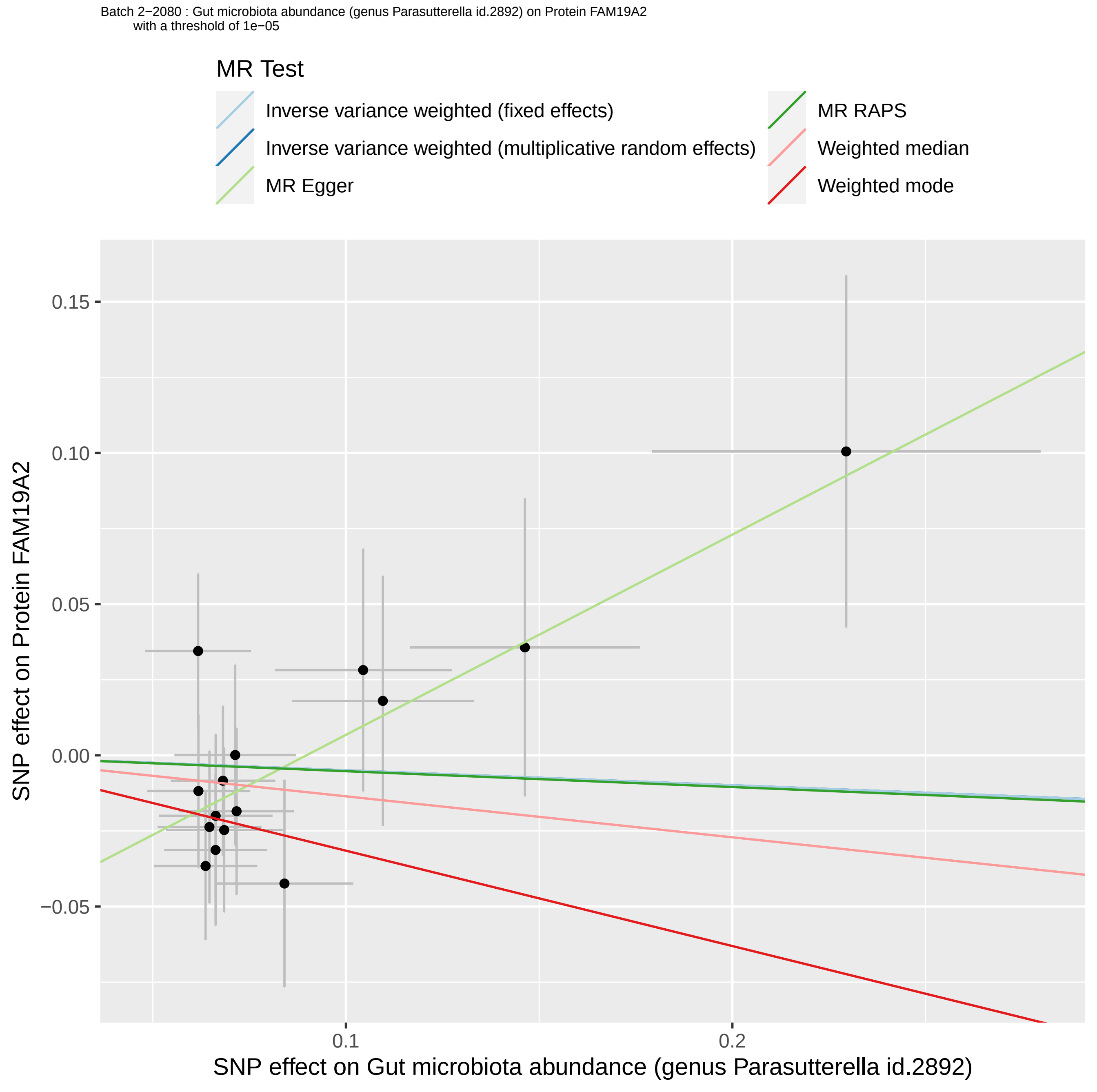

Batch 2−2080 : Gut microbiota abundance (genus Parasutterella id.2892) on Protein FAM19A2
with a threshold of 1e−05
MR Test
Inverse variance weighted (fixed effects)
Inverse variance weighted (multiplicative random effects)
MR Egger
MR RAPS
Weighted median
Weighted mode
SNP effect on Protein FAM19A2
0.15
0.10
0.05
0.00
−0.05
0.1
0.2
SNP effect on Gut microbiota abundance (genus Parasutterella id.2892)

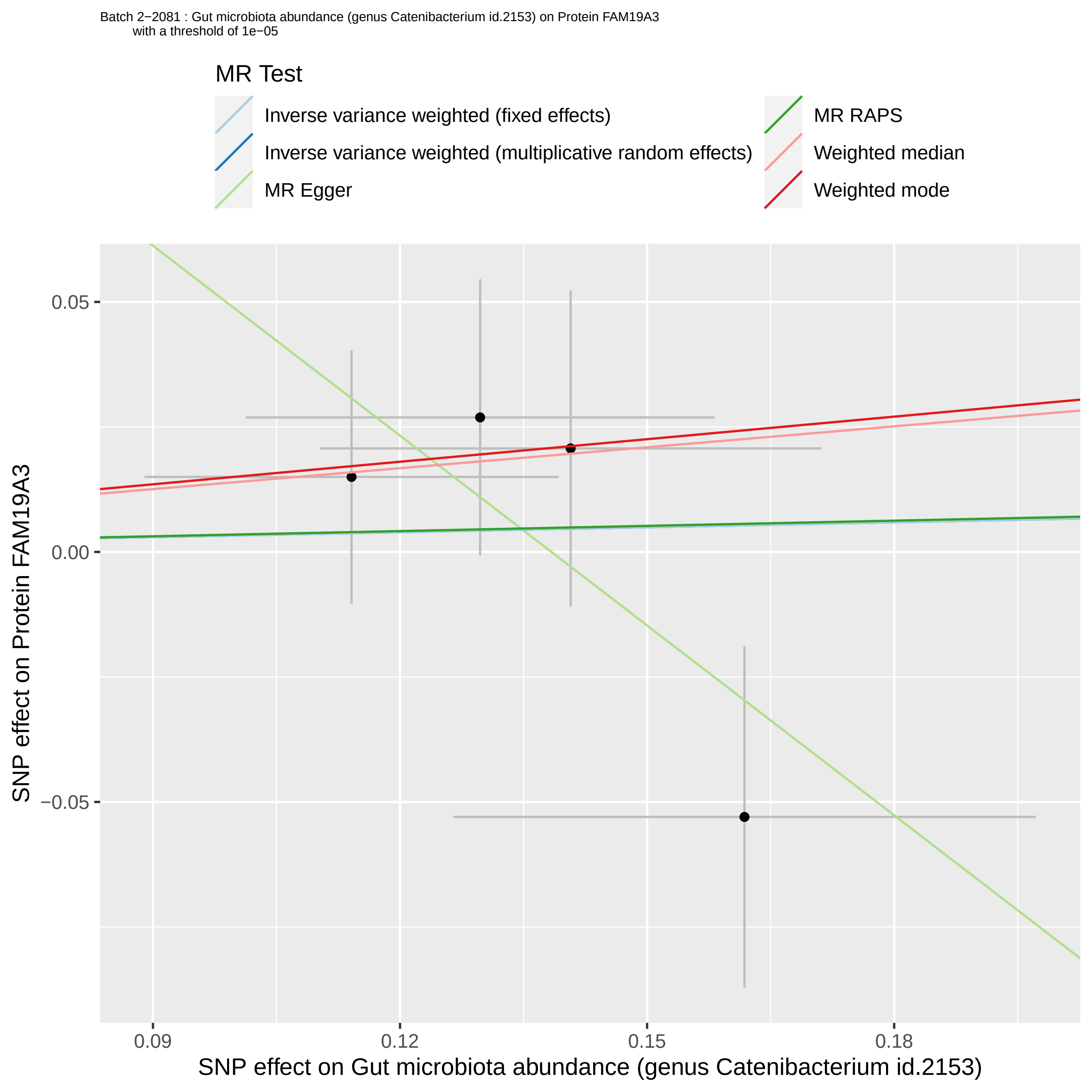
Batch 2−2081 : Gut microbiota abundance (genus Catenibacterium id.2153) on Protein FAM19A3
with a threshold of 1e−05
MR Test
Inverse variance weighted (fixed effects)
Inverse variance weighted (multiplicative random effects)
MR Egger
MR RAPS
Weighted median
Weighted mode
0.05
0.00
−0.05
0.09
0.12
0.15
0.18
SNP effect on Protein FAM19A3
SNP effect on Gut microbiota abundance (genus Catenibacterium id.2153)

Batch 2−2082 : Gut microbiota abundance (genus Parasutterella id.2892) on Protein FAM19A3
with a threshold of 1e−05

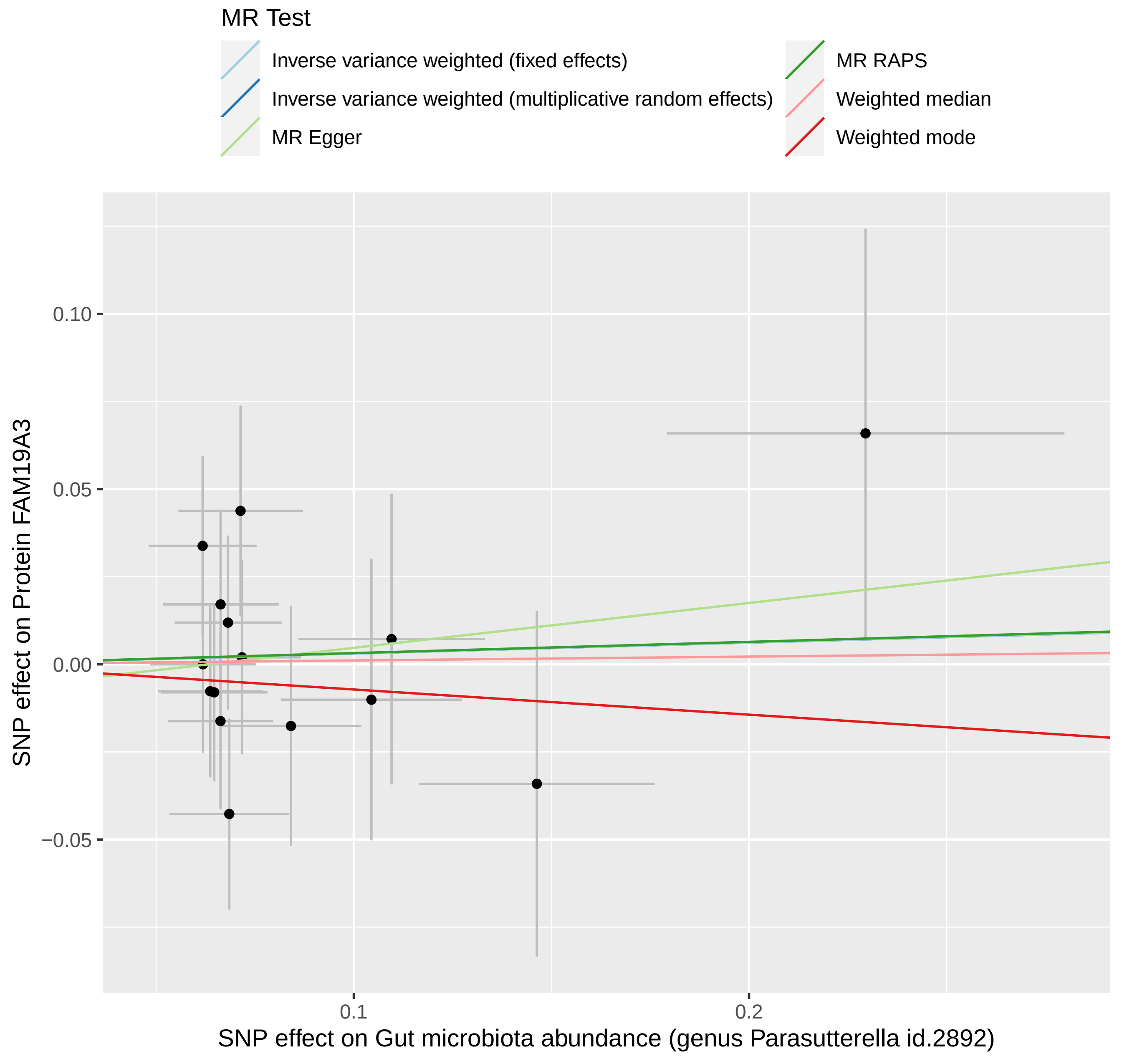

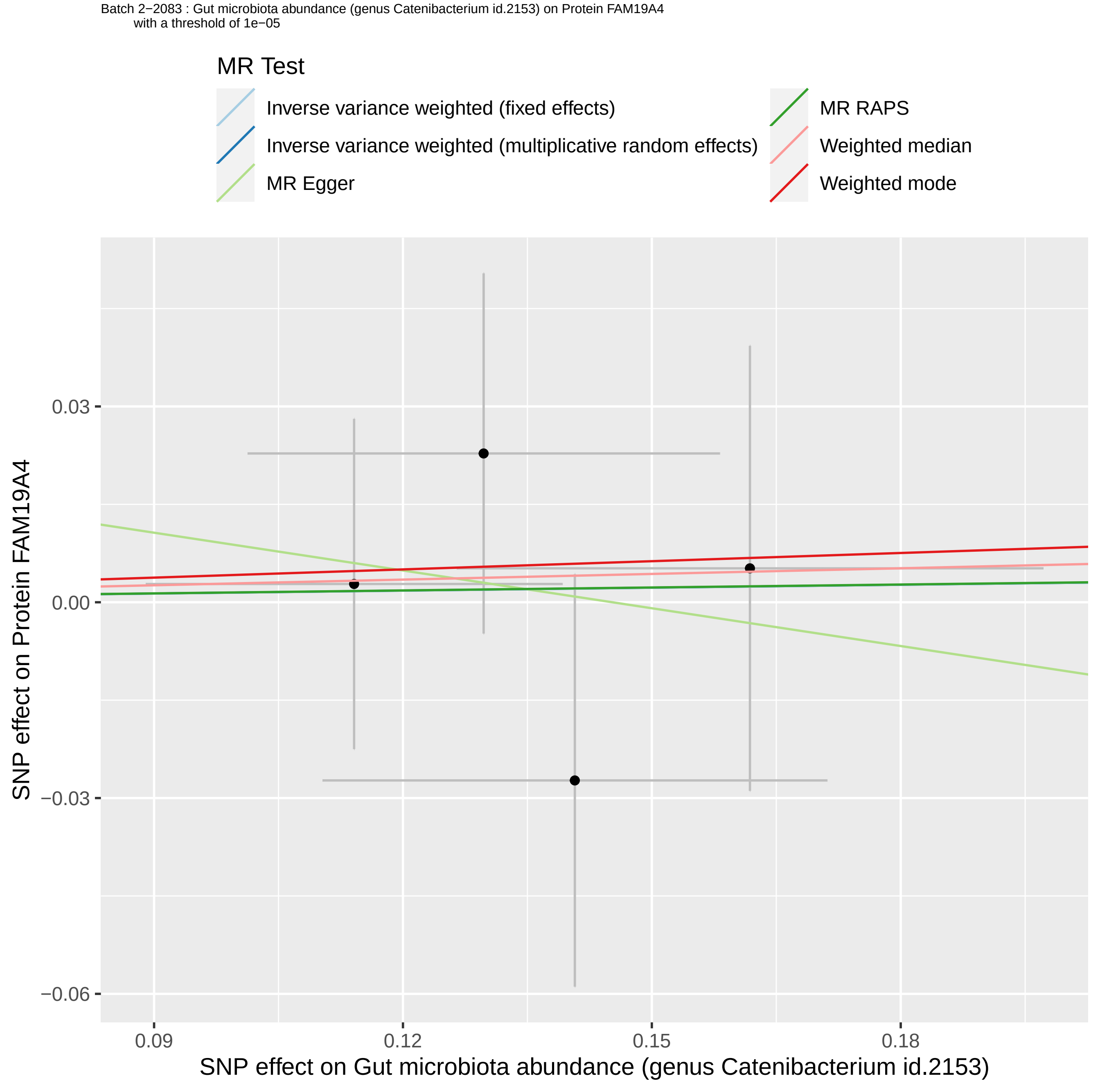
Batch 2−2083 : Gut microbiota abundance (genus Catenibacterium id.2153) on Protein FAM19A4
with a threshold of 1e−05
MR Test
Inverse variance weighted (fixed effects)
Inverse variance weighted (multiplicative random effects)
MR Egger
MR RAPS
Weighted median
Weighted mode
0.03
0.00
−0.03
−0.06
0.09
0.12
0.15
0.18
SNP effect on Protein FAM19A4
SNP effect on Gut microbiota abundance (genus Catenibacterium id.2153)

Batch 2−2084 : Gut microbiota abundance (genus Parasutterella id.2892) on Protein FAM19A4
with a threshold of 1e−05

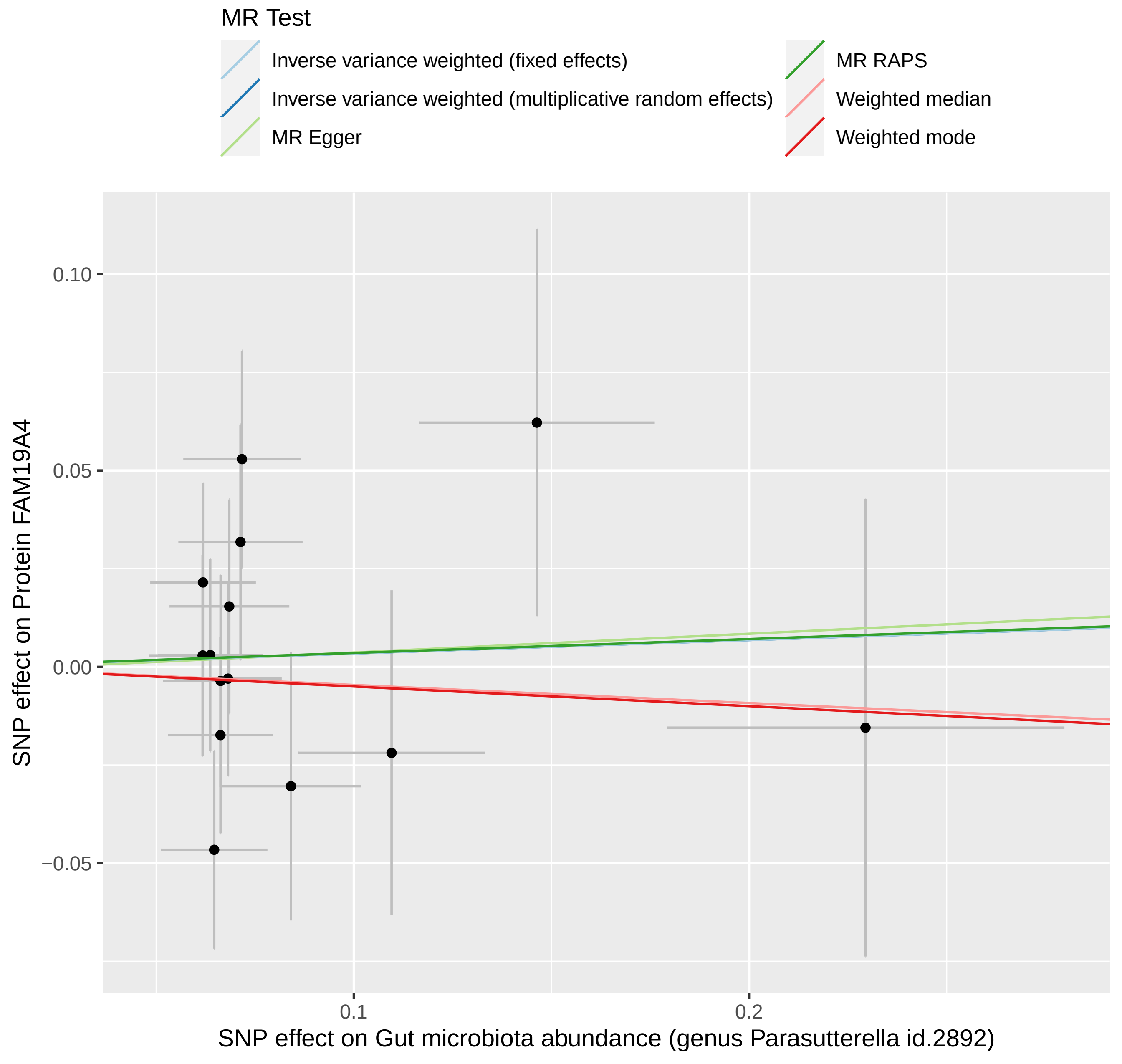

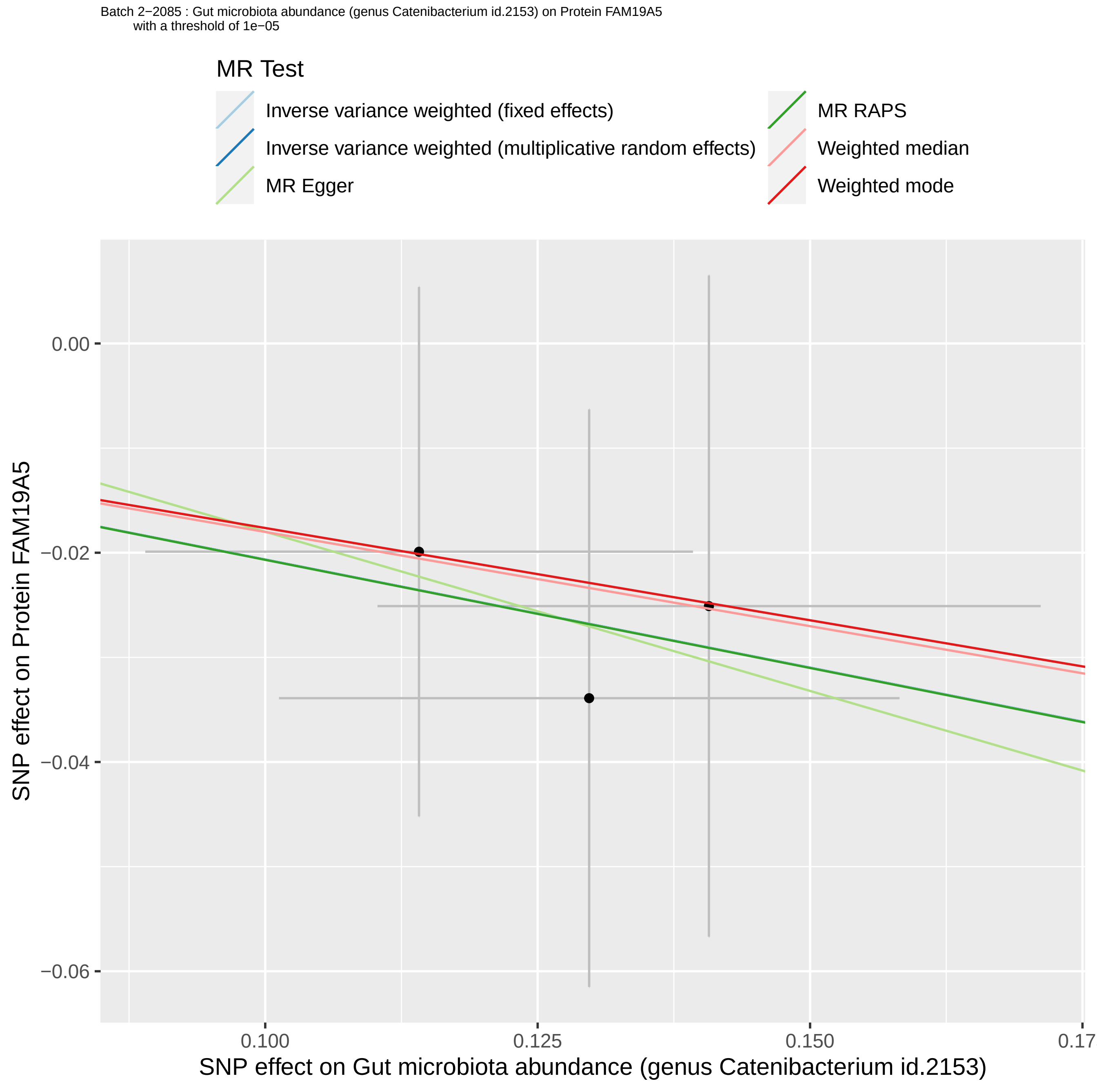
Batch 2−2085 : Gut microbiota abundance (genus Catenibacterium id.2153) on Protein FAM19A5
with a threshold of 1e−05
MR Test
Inverse variance weighted (fixed effects)
Inverse variance weighted (multiplicative random effects)
MR Egger
MR RAPS
Weighted median
Weighted mode
0.00
−0.02
−0.04
−0.06
0.100
0.125
0.150
0.17
SNP effect on Protein FAM19A5
SNP effect on Gut microbiota abundance (genus Catenibacterium id.2153)

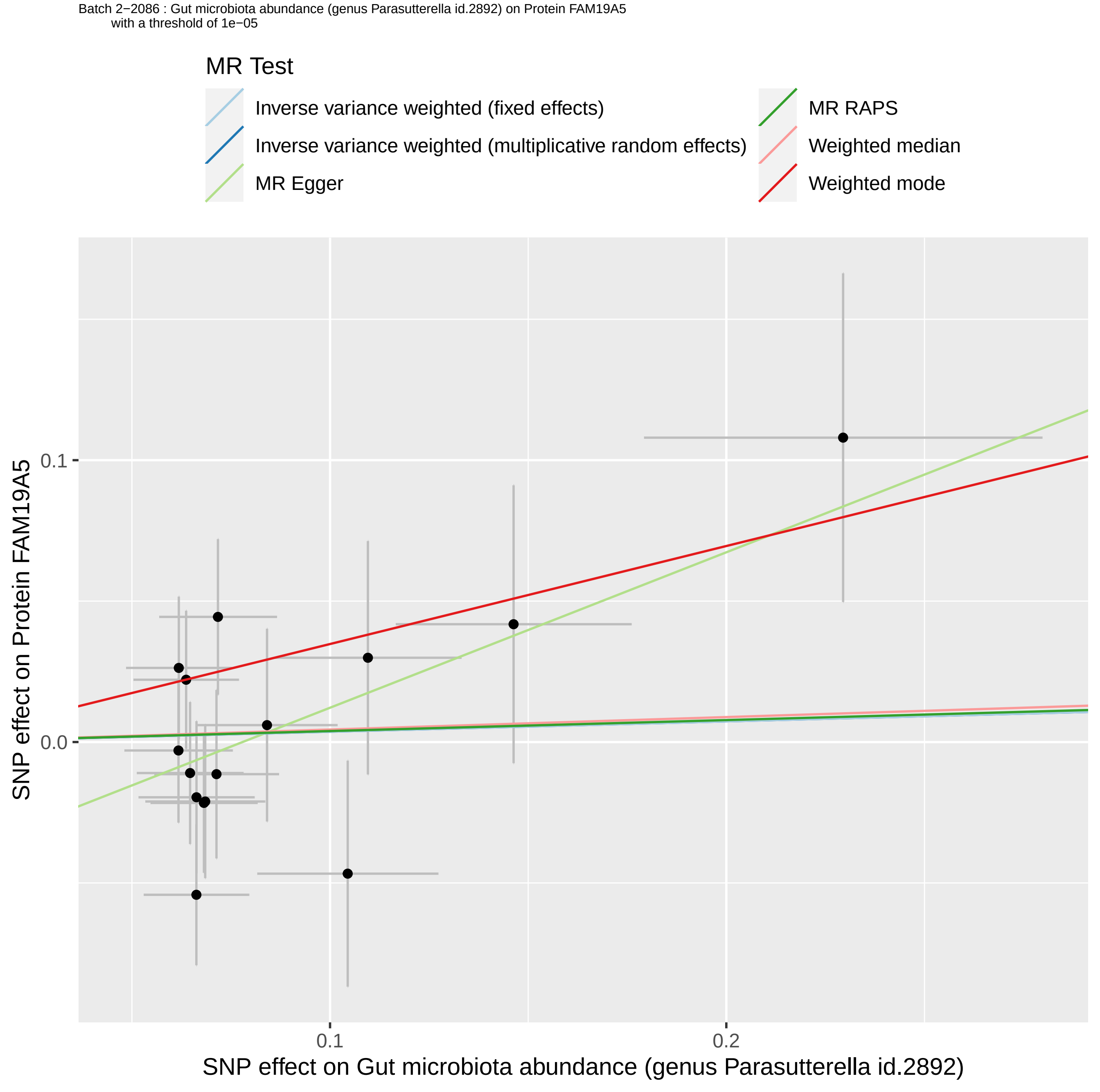

Batch 2−2086 : Gut microbiota abundance (genus Parasutterella id.2892) on Protein FAM19A5
with a threshold of 1e−05
MR Test
Inverse variance weighted (fixed effects)
Inverse variance weighted (multiplicative random effects)
MR Egger
MR RAPS
Weighted median
Weighted mode
0.1
0.0
0.1
0.2
SNP effect on Protein FAM19A5
SNP effect on Gut microbiota abundance (genus Parasutterella id.2892)

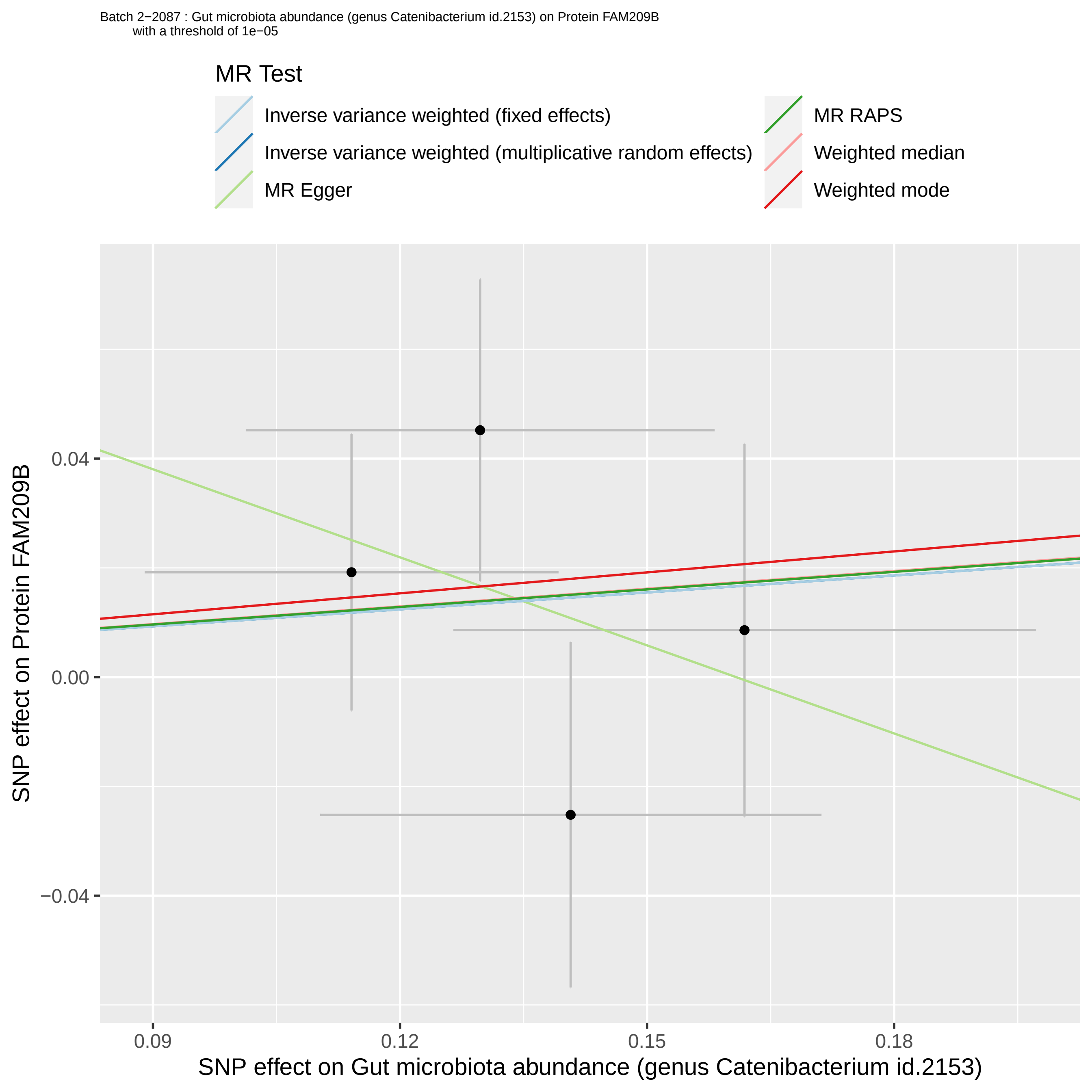

Batch 2−2087 : Gut microbiota abundance (genus Catenibacterium id.2153) on Protein FAM209B
with a threshold of 1e−05
MR Test
Inverse variance weighted (fixed effects)
Inverse variance weighted (multiplicative random effects)
MR Egger
MR RAPS
Weighted median
Weighted mode
0.04
0.00
−0.04
0.09
0.12
0.15
0.18
SNP effect on Protein FAM209B
SNP effect on Gut microbiota abundance (genus Catenibacterium id.2153)

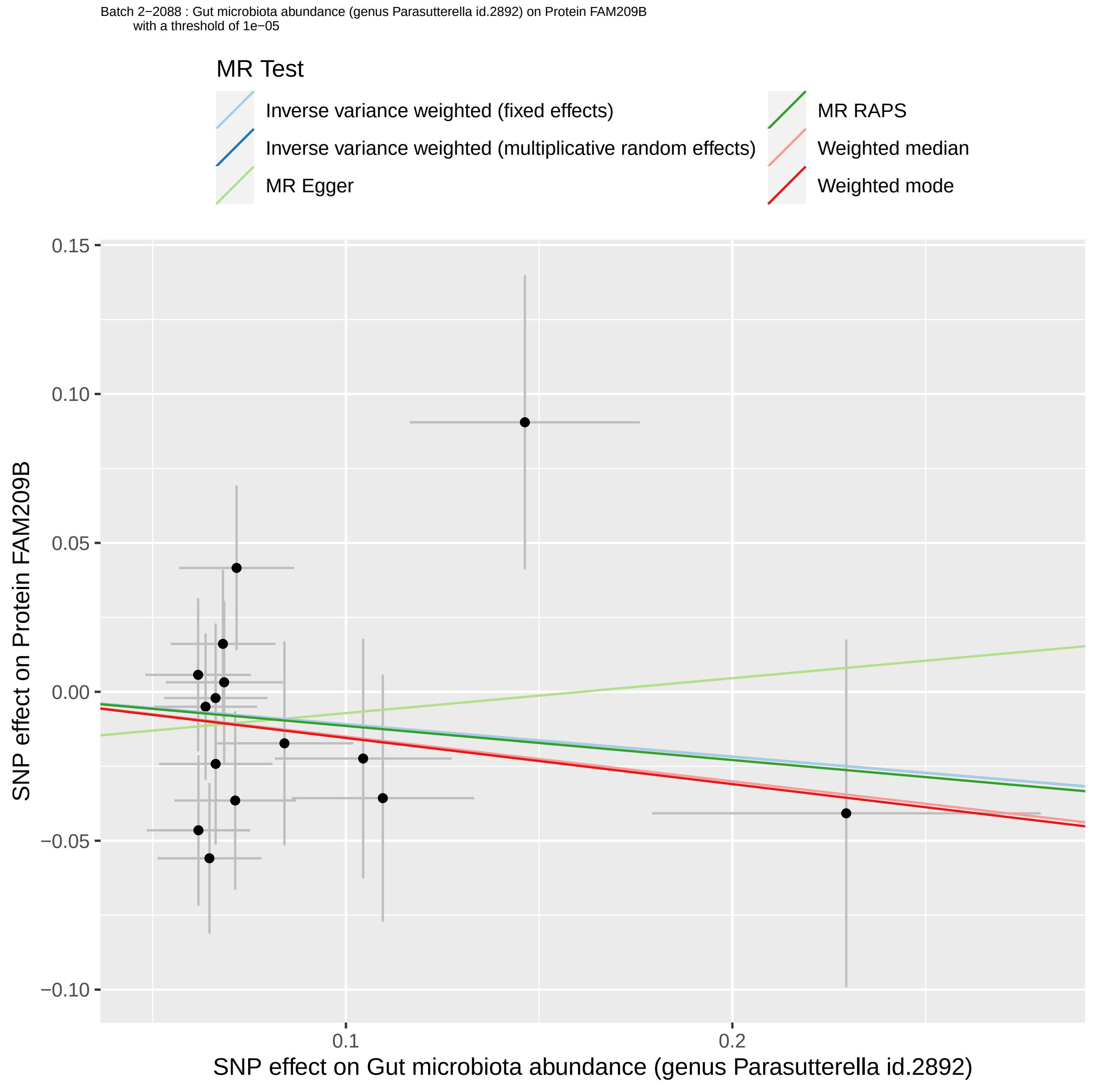

Batch 2−2088 : Gut microbiota abundance (genus Parasutterella id.2892) on Protein FAM209B
with a threshold of 1e−05
MR Test
Inverse variance weighted (fixed effects)
Inverse variance weighted (multiplicative random effects)
MR Egger
MR RAPS
Weighted median
Weighted mode
0.15
0.10
0.05
0.00
−0.05
−0.10
0.1
0.2
SNP effect on Protein FAM209B
SNP effect on Gut microbiota abundance (genus Parasutterella id.2892)

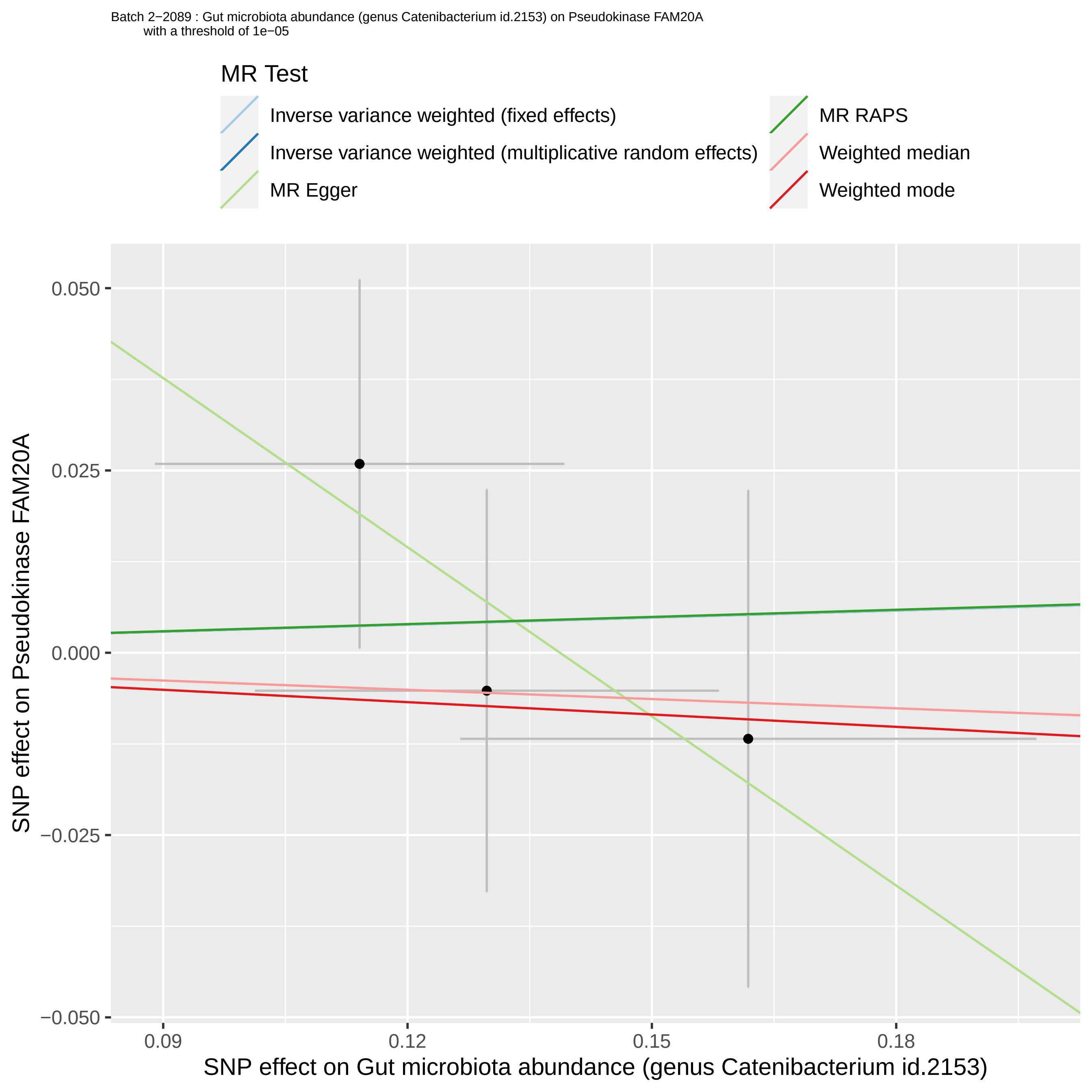

Batch 2−2089 : Gut microbiota abundance (genus Catenibacterium id.2153) on Pseudokinase FAM20A
with a threshold of 1e−05
MR Test
Inverse variance weighted (fixed effects)
Inverse variance weighted (multiplicative random effects)
MR Egger
MR RAPS
Weighted median
Weighted mode
0.050
0.025
0.000
−0.025
−0.050
0.09
0.12
0.15
0.18
SNP effect on Pseudokinase FAM20A
SNP effect on Gut microbiota abundance (genus Catenibacterium id.2153)

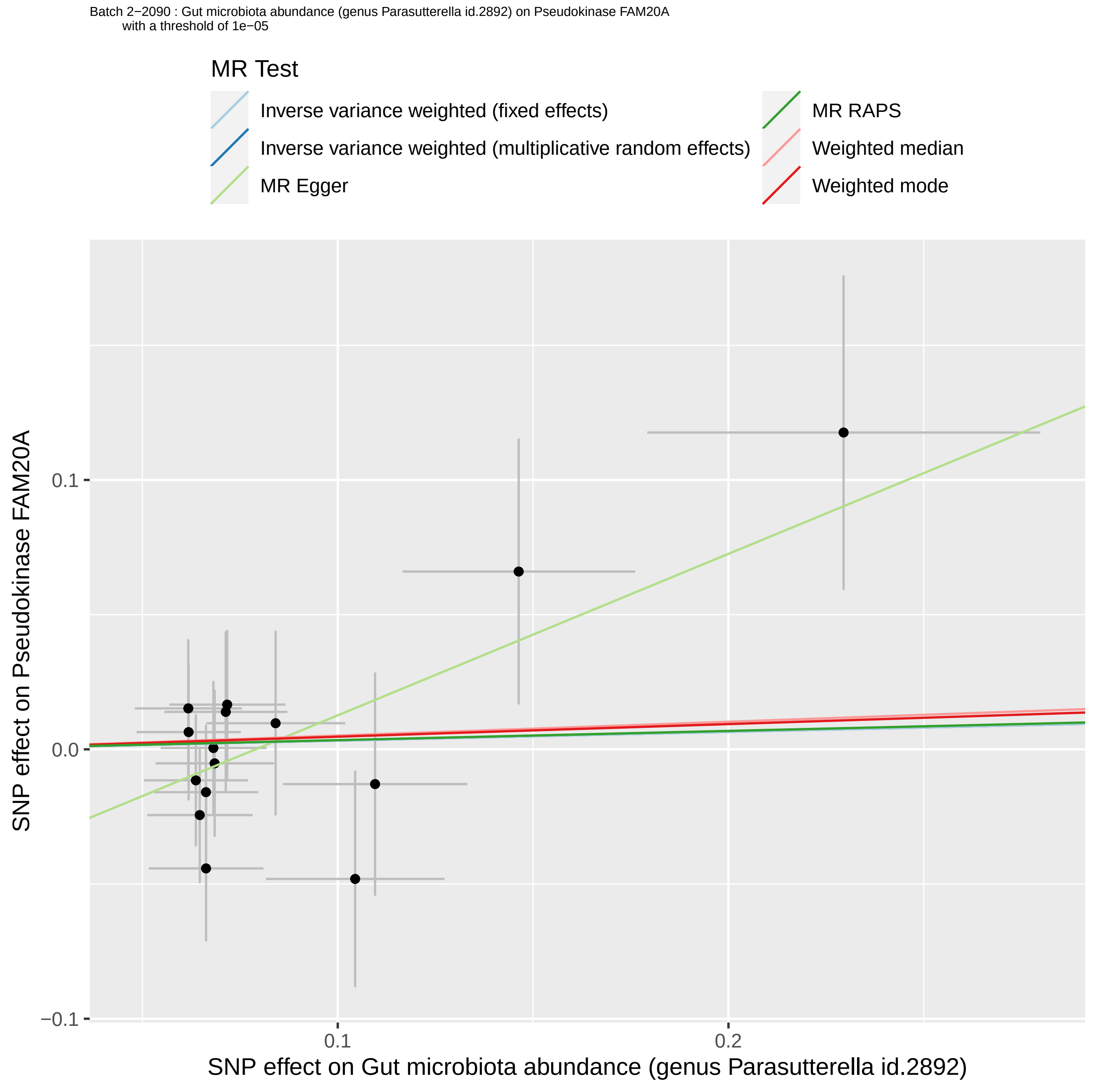
Batch 2−2090 : Gut microbiota abundance (genus Parasutterella id.2892) on Pseudokinase FAM20A
with a threshold of 1e−05
MR Test
Inverse variance weighted (fixed effects)
Inverse variance weighted (multiplicative random effects)
MR Egger
MR RAPS
Weighted median
Weighted mode
0.1
0.0
−0.1
0.1
0.2
SNP effect on Pseudokinase FAM20A
SNP effect on Gut microbiota abundance (genus Parasutterella id.2892)

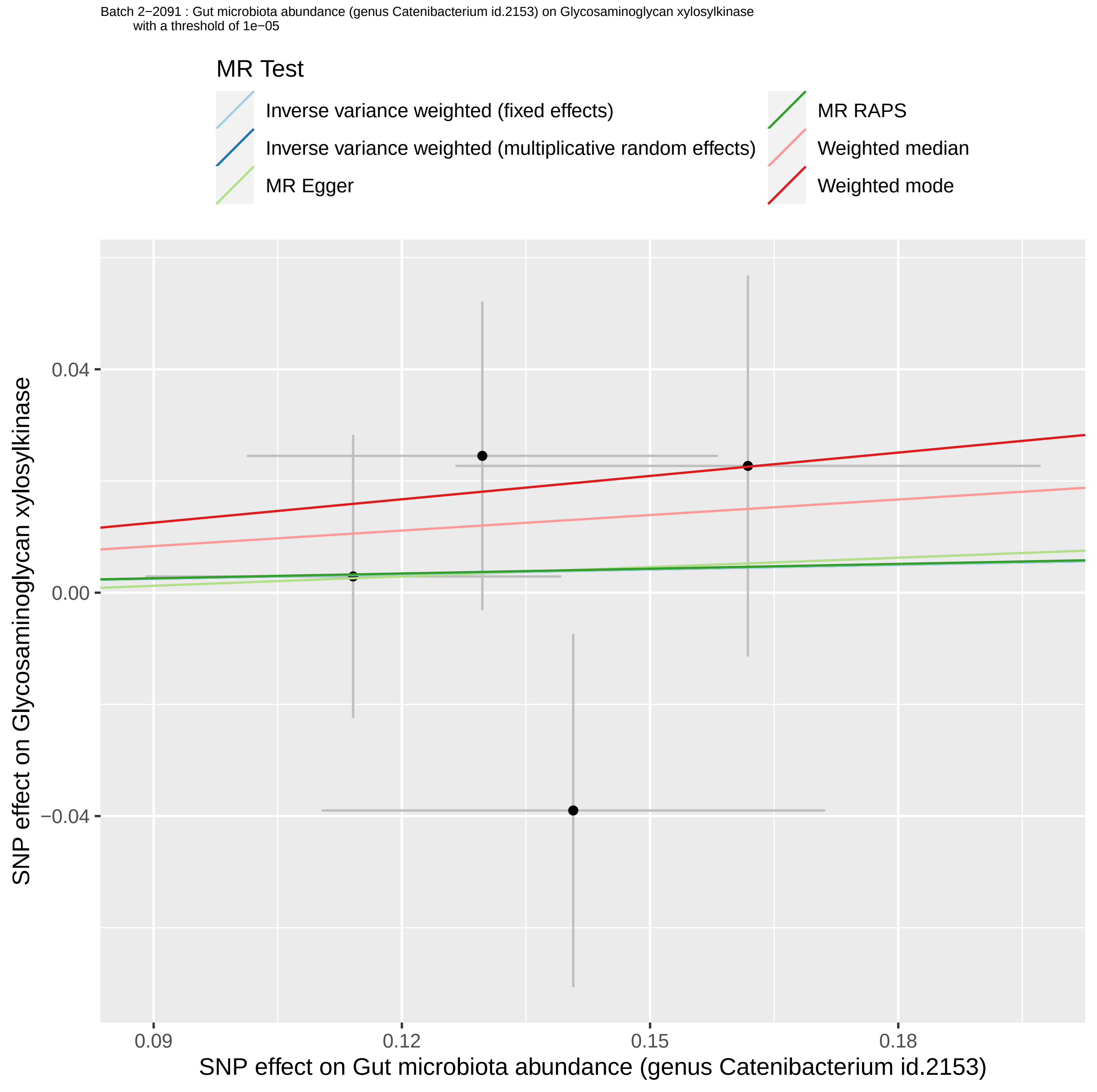
Batch 2−2091 : Gut microbiota abundance (genus Catenibacterium id.2153) on Glycosaminoglycan xylosylkinase
with a threshold of 1e−05
MR Test
Inverse variance weighted (fixed effects)
Inverse variance weighted (multiplicative random effects)
MR Egger
MR RAPS
Weighted median
Weighted mode
0.04
0.00
−0.04
0.09
0.12
0.15
0.18
SNP effect on Glycosaminoglycan xylosylkinase
SNP effect on Gut microbiota abundance (genus Catenibacterium id.2153)

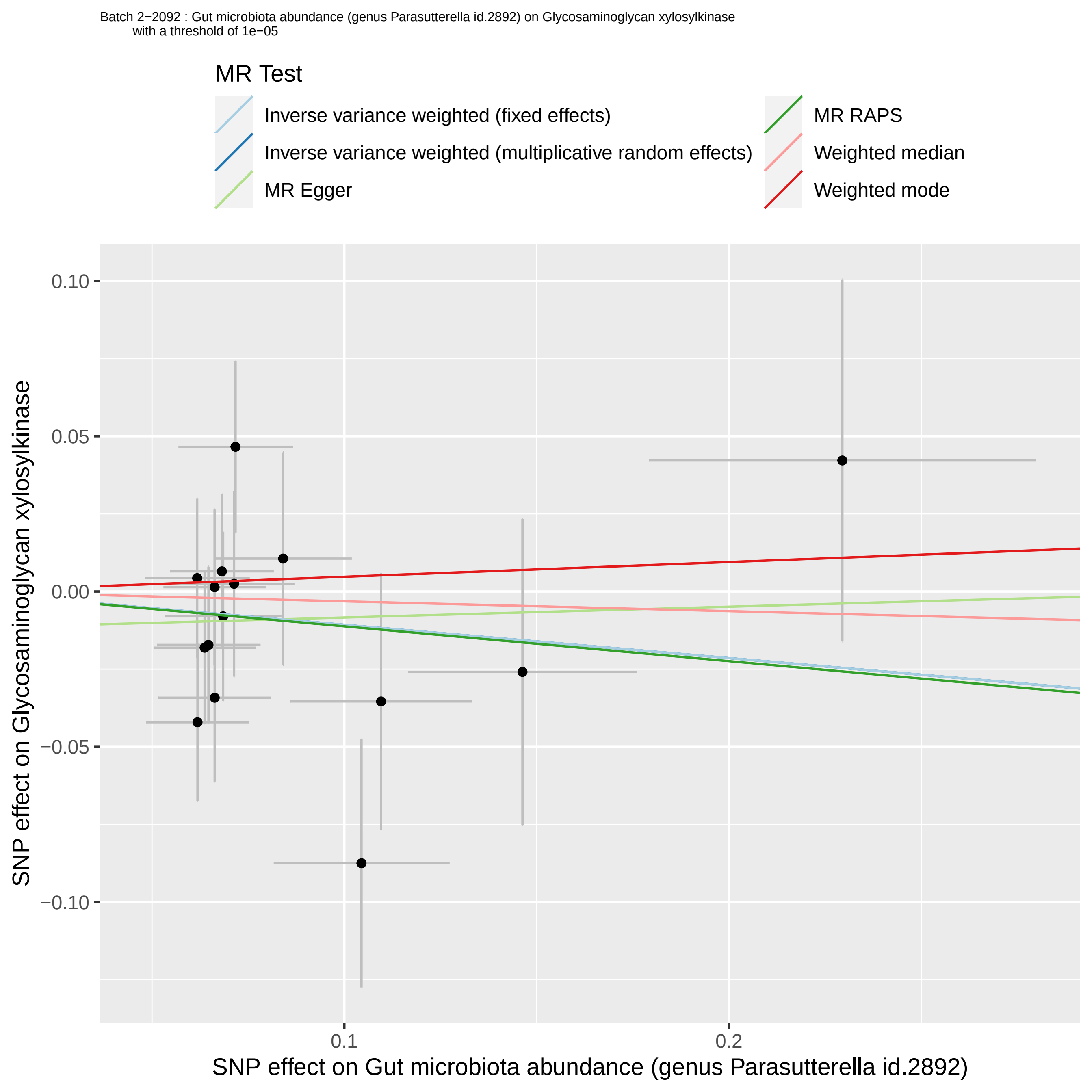
Batch 2−2092 : Gut microbiota abundance (genus Parasutterella id.2892) on Glycosaminoglycan xylosylkinase
with a threshold of 1e−05
MR Test
Inverse variance weighted (fixed effects)
Inverse variance weighted (multiplicative random effects)
MR Egger
MR RAPS
Weighted median
Weighted mode
0.10
0.05
0.00
−0.05
−0.10
0.1
0.2
SNP effect on Glycosaminoglycan xylosylkinase
SNP effect on Gut microbiota abundance (genus Parasutterella id.2892)

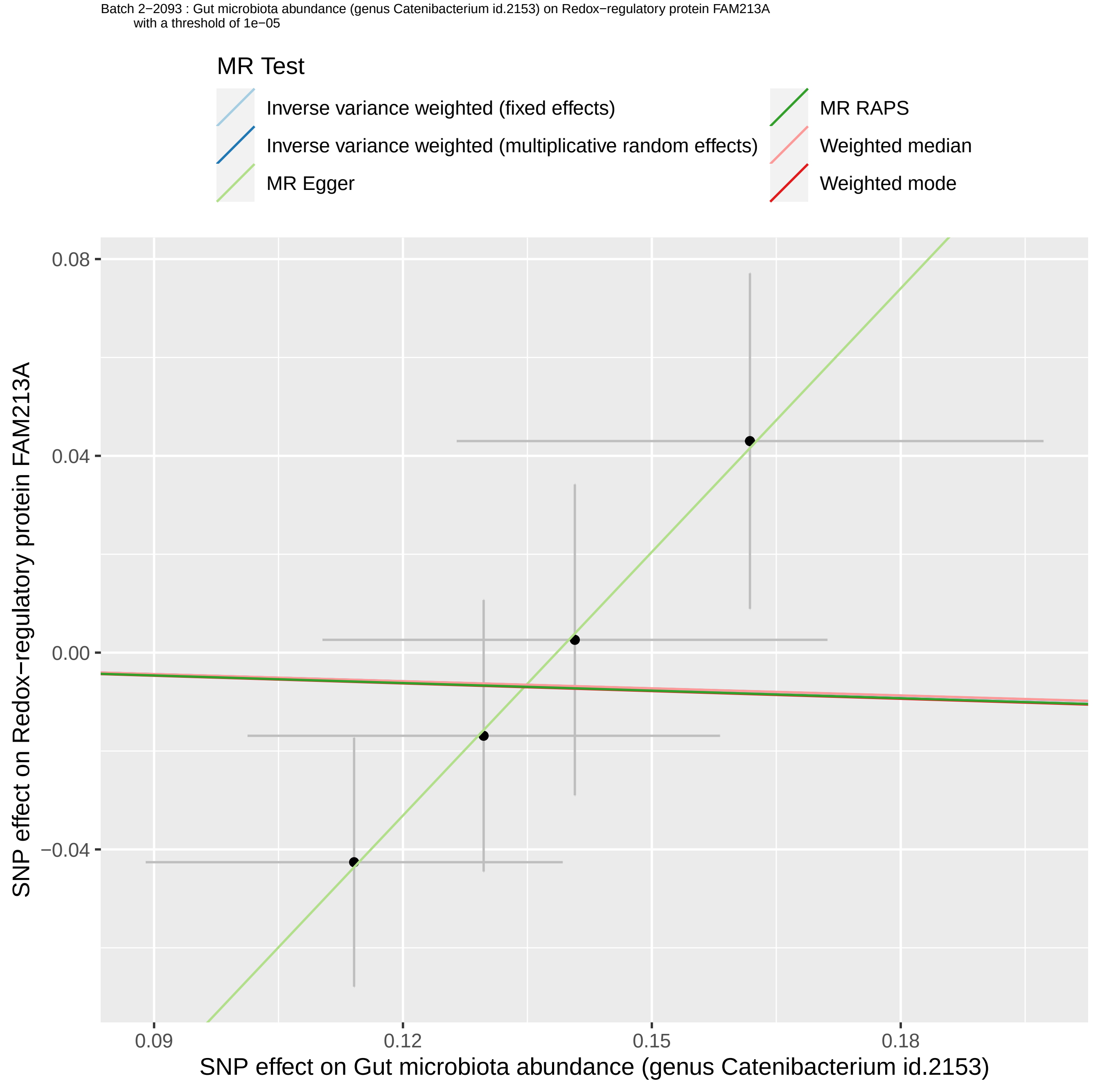

Batch 2−2093 : Gut microbiota abundance (genus Catenibacterium id.2153) on Redox−regulatory protein FAM213A
with a threshold of 1e−05
MR Test
Inverse variance weighted (fixed effects)
Inverse variance weighted (multiplicative random effects)
MR Egger
MR RAPS
Weighted median
Weighted mode
0.08
0.04
0.00
−0.04
0.09
0.12
0.15
0.18
SNP effect on Redox−regulatory protein FAM213A
SNP effect on Gut microbiota abundance (genus Catenibacterium id.2153)

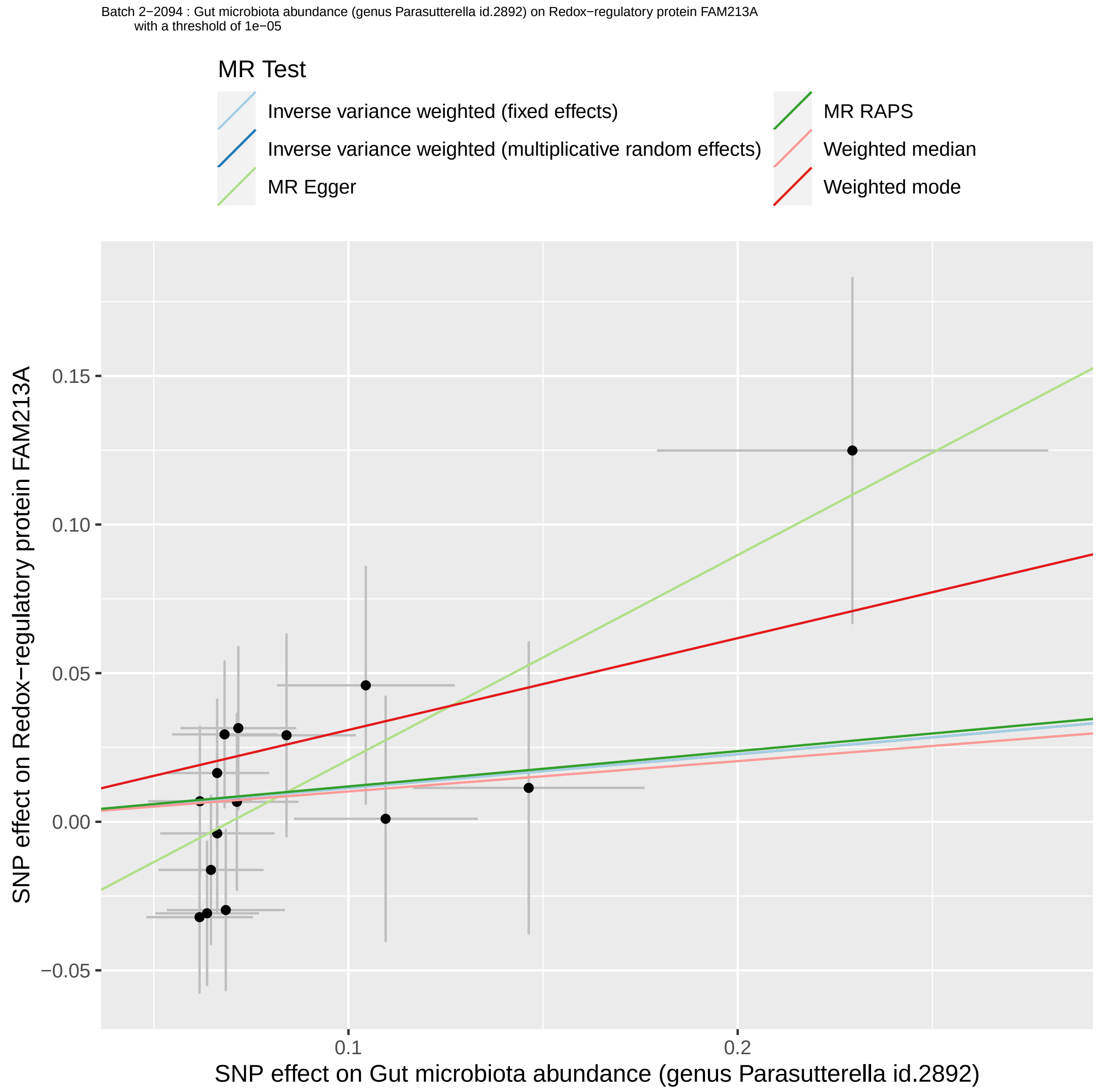

Batch 2−2094 : Gut microbiota abundance (genus Parasutterella id.2892) on Redox−regulatory protein FAM213A
with a threshold of 1e−05
MR Test
Inverse variance weighted (fixed effects)
Inverse variance weighted (multiplicative random effects)
MR Egger
MR RAPS
Weighted median
Weighted mode
0.15
0.10
0.05
0.00
−0.05
0.1
0.2
SNP effect on Redox−regulatory protein FAM213A
SNP effect on Gut microbiota abundance (genus Parasutterella id.2892)

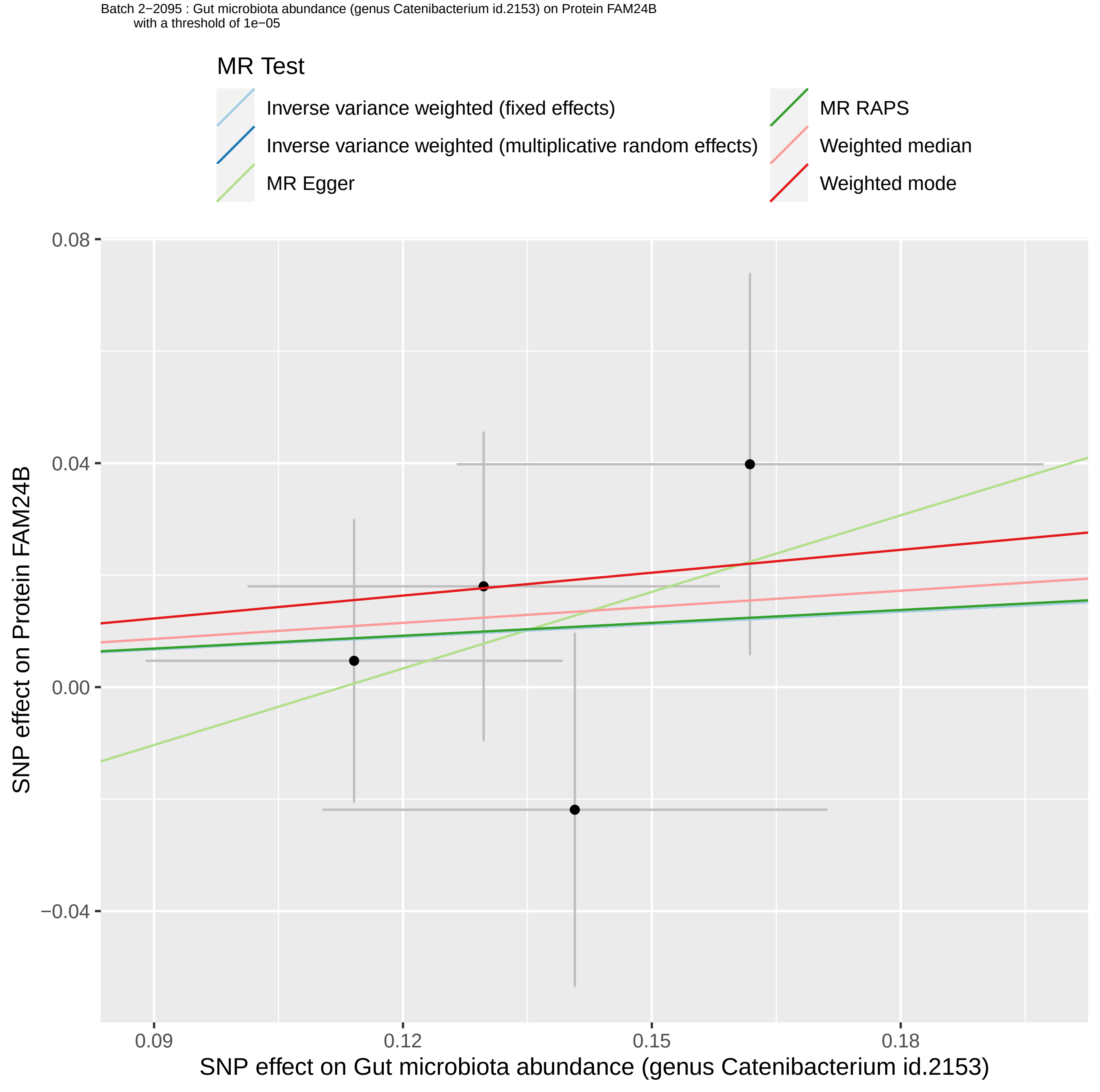

Batch 2−2095 : Gut microbiota abundance (genus Catenibacterium id.2153) on Protein FAM24B
with a threshold of 1e−05
MR Test
Inverse variance weighted (fixed effects)
Inverse variance weighted (multiplicative random effects)
MR Egger
MR RAPS
Weighted median
Weighted mode
0.08
0.04
0.00
−0.04
0.09
0.12
0.15
0.18
SNP effect on Protein FAM24B
SNP effect on Gut microbiota abundance (genus Catenibacterium id.2153)

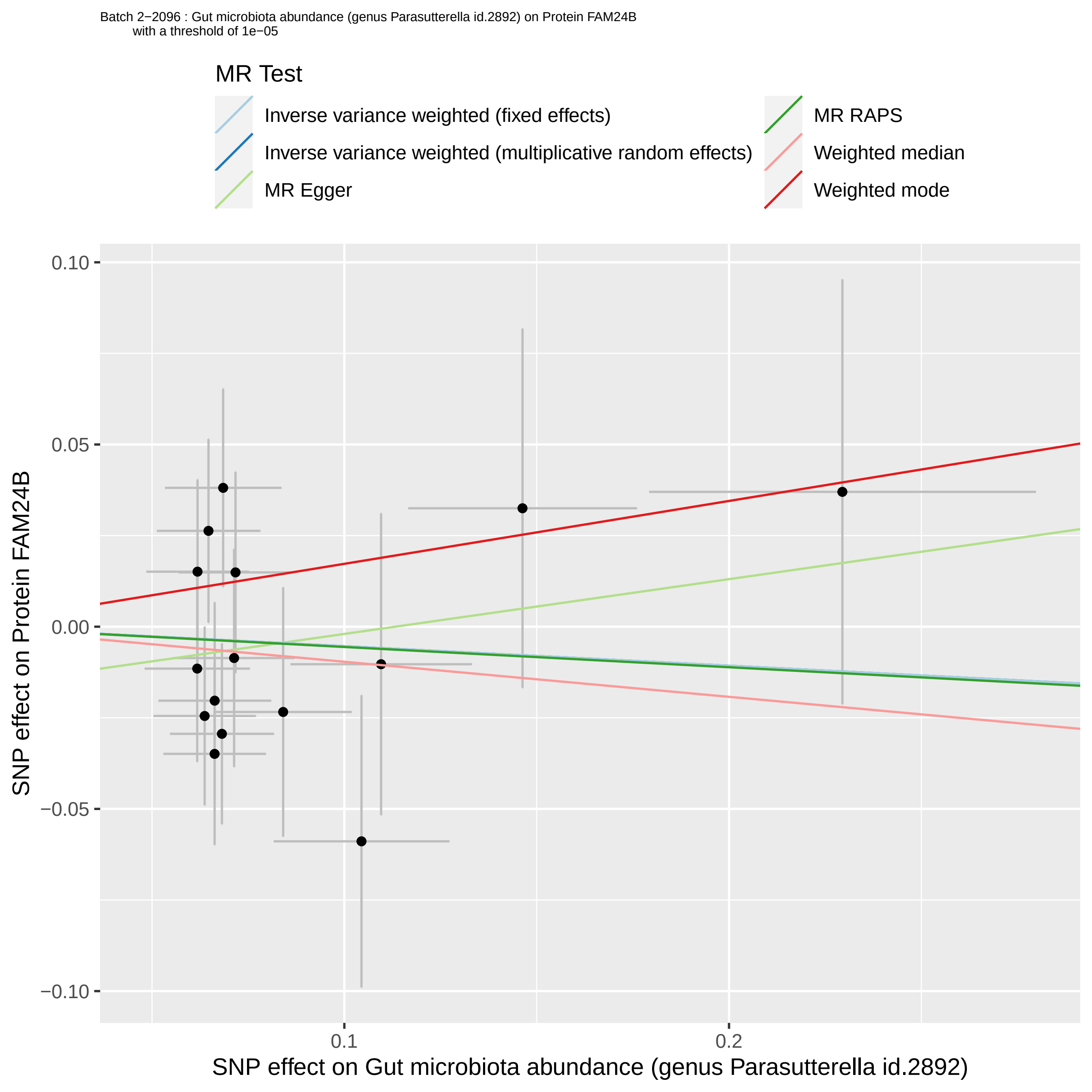

Batch 2−2096 : Gut microbiota abundance (genus Parasutterella id.2892) on Protein FAM24B
with a threshold of 1e−05
MR Test
Inverse variance weighted (fixed effects)
Inverse variance weighted (multiplicative random effects)
MR Egger
MR RAPS
Weighted median
Weighted mode
0.10
0.05
0.00
−0.05
−0.10
SNP effect on Protein FAM24B
0.1
0.2
SNP effect on Gut microbiota abundance (genus Parasutterella id.2892)

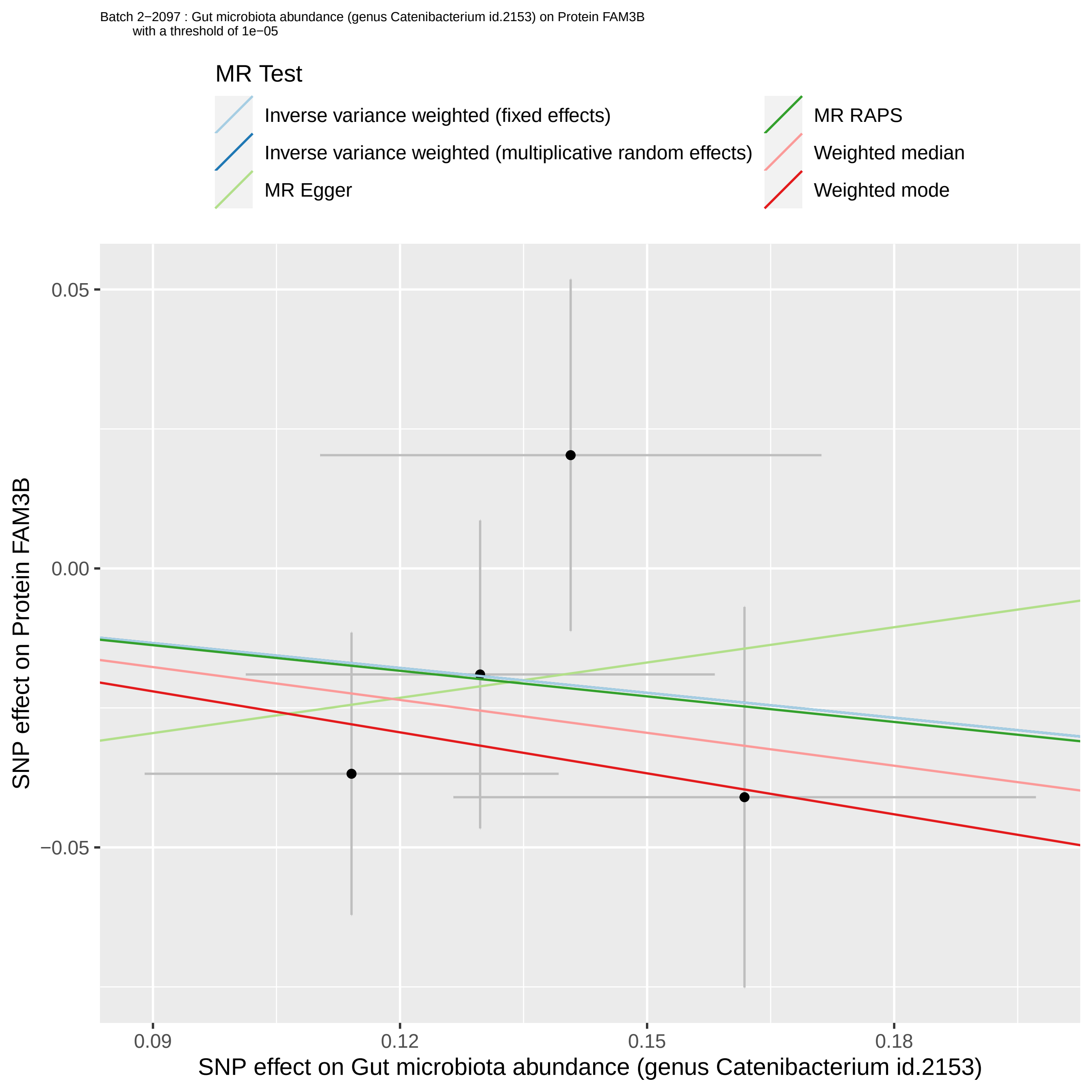
Batch 2−2097 : Gut microbiota abundance (genus Catenibacterium id.2153) on Protein FAM3B
with a threshold of 1e−05
MR Test
Inverse variance weighted (fixed effects)
Inverse variance weighted (multiplicative random effects)
MR Egger
MR RAPS
Weighted median
Weighted mode
0.05
0.00
−0.05
SNP effect on Protein FAM3B
0.09
0.12
0.15
0.18
SNP effect on Gut microbiota abundance (genus Catenibacterium id.2153)

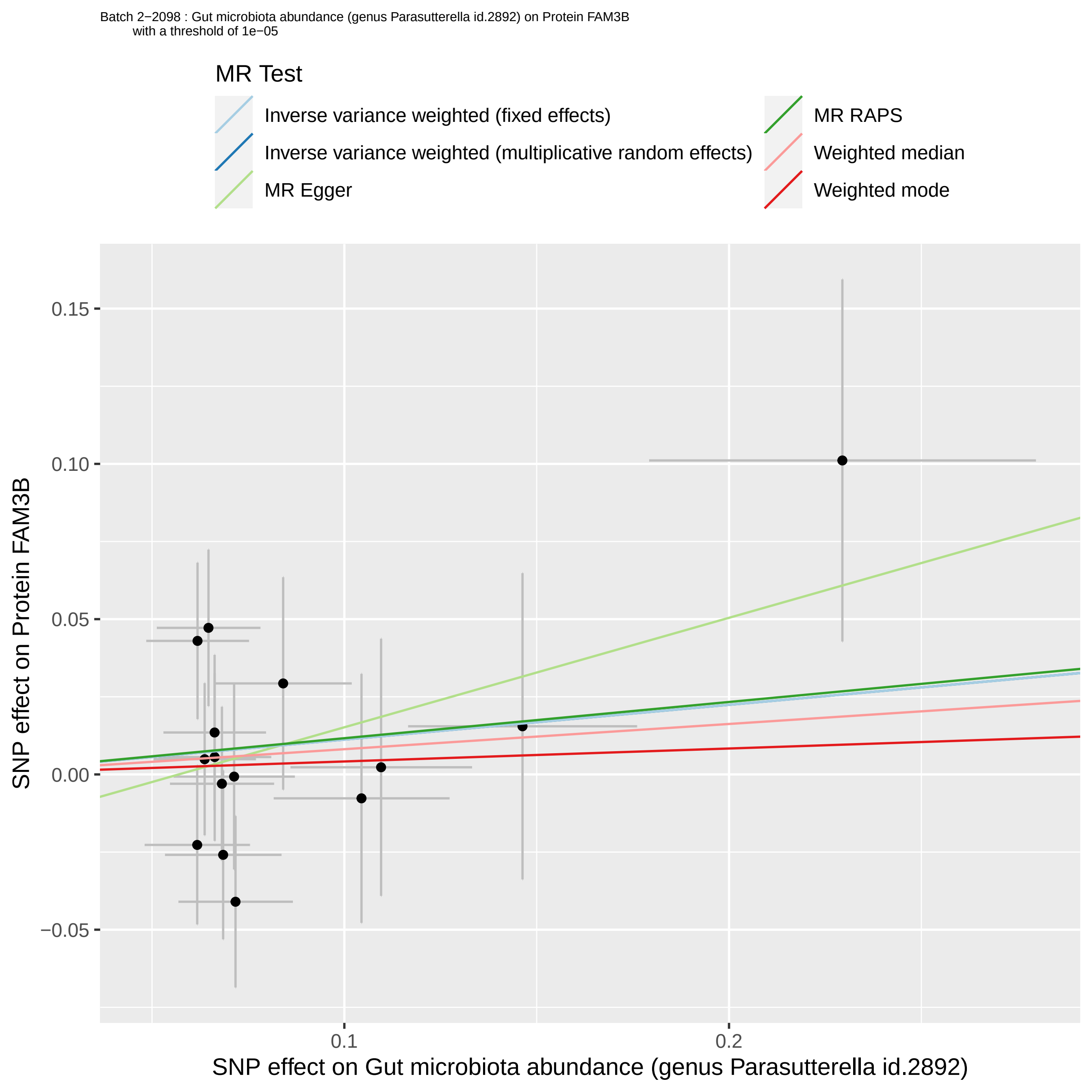

Batch 2−2098 : Gut microbiota abundance (genus Parasutterella id.2892) on Protein FAM3B
with a threshold of 1e−05
MR Test
Inverse variance weighted (fixed effects)
Inverse variance weighted (multiplicative random effects)
MR Egger
MR RAPS
Weighted median
Weighted mode
SNP effect on Protein FAM3B
0.15
0.10
0.05
0.00
−0.05
0.1
0.2
SNP effect on Gut microbiota abundance (genus Parasutterella id.2892)

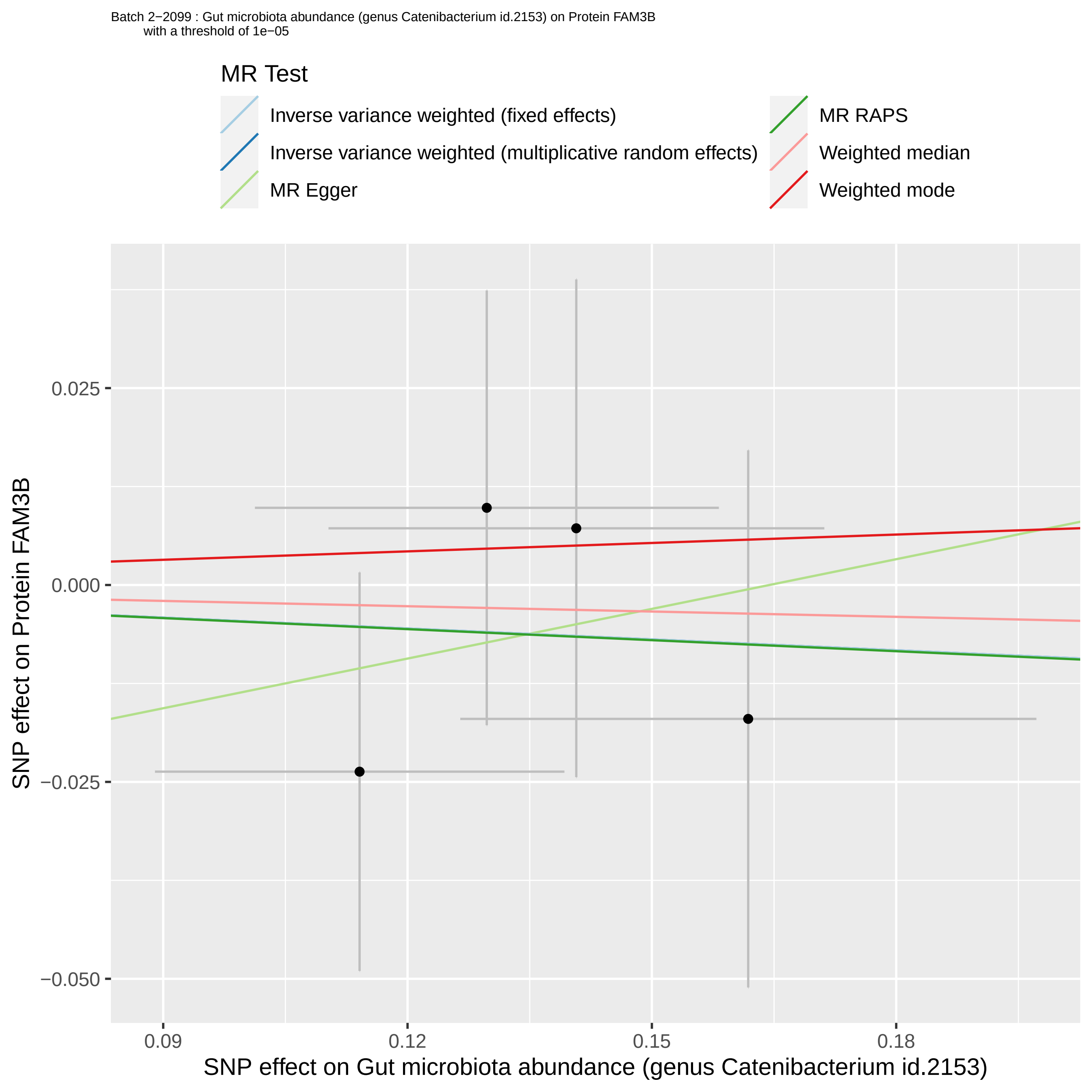

Batch 2−2099 : Gut microbiota abundance (genus Catenibacterium id.2153) on Protein FAM3B
with a threshold of 1e−05
MR Test
Inverse variance weighted (fixed effects)
Inverse variance weighted (multiplicative random effects)
MR Egger
MR RAPS
Weighted median
Weighted mode
0.025
0.000
−0.025
−0.050
0.09
0.12
0.15
0.18
SNP effect on Protein FAM3B
SNP effect on Gut microbiota abundance (genus Catenibacterium id.2153)

Batch 2−2100 : Gut microbiota abundance (genus Parasutterella id.2892) on Protein FAM3B
with a threshold of 1e−05

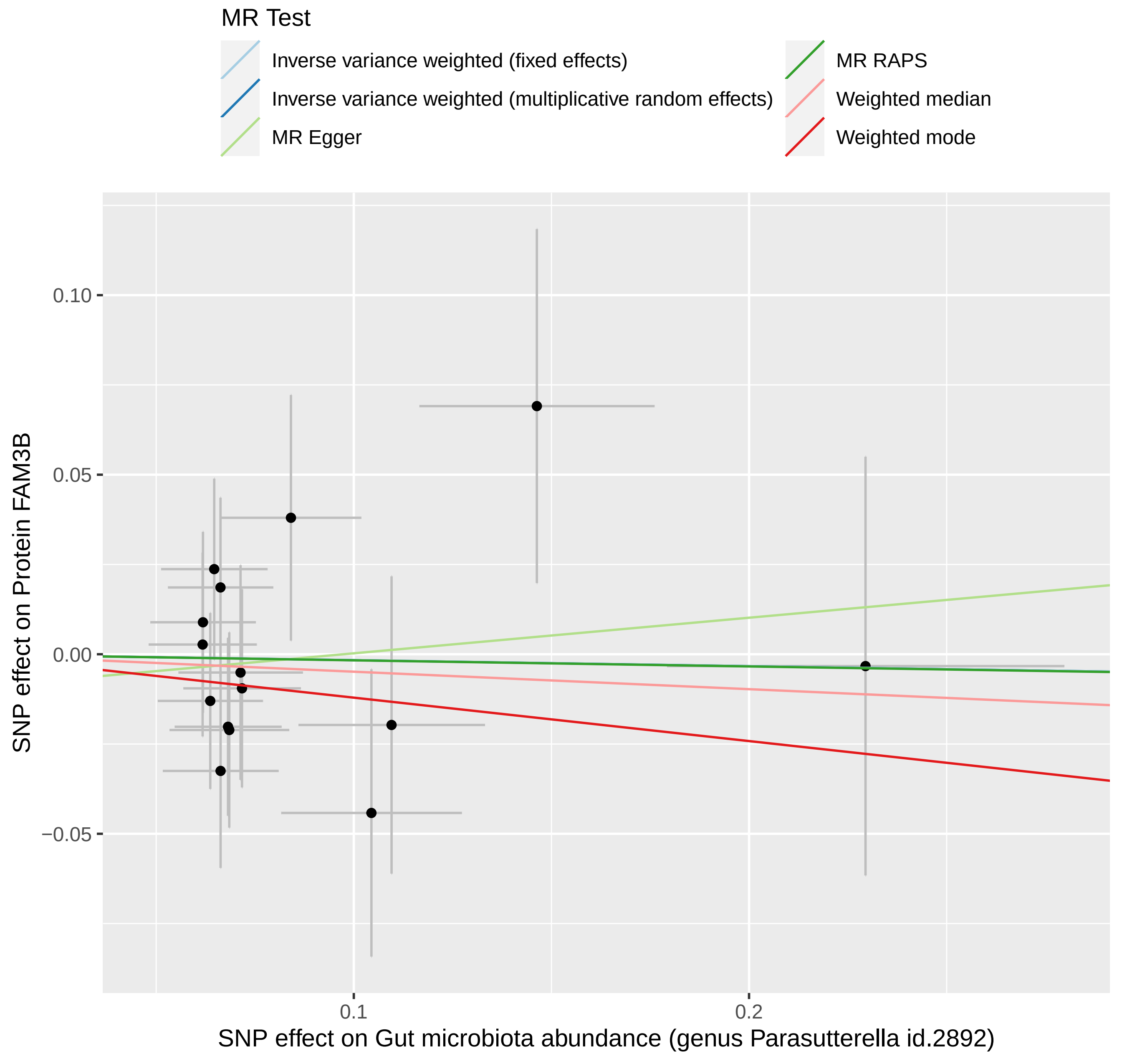

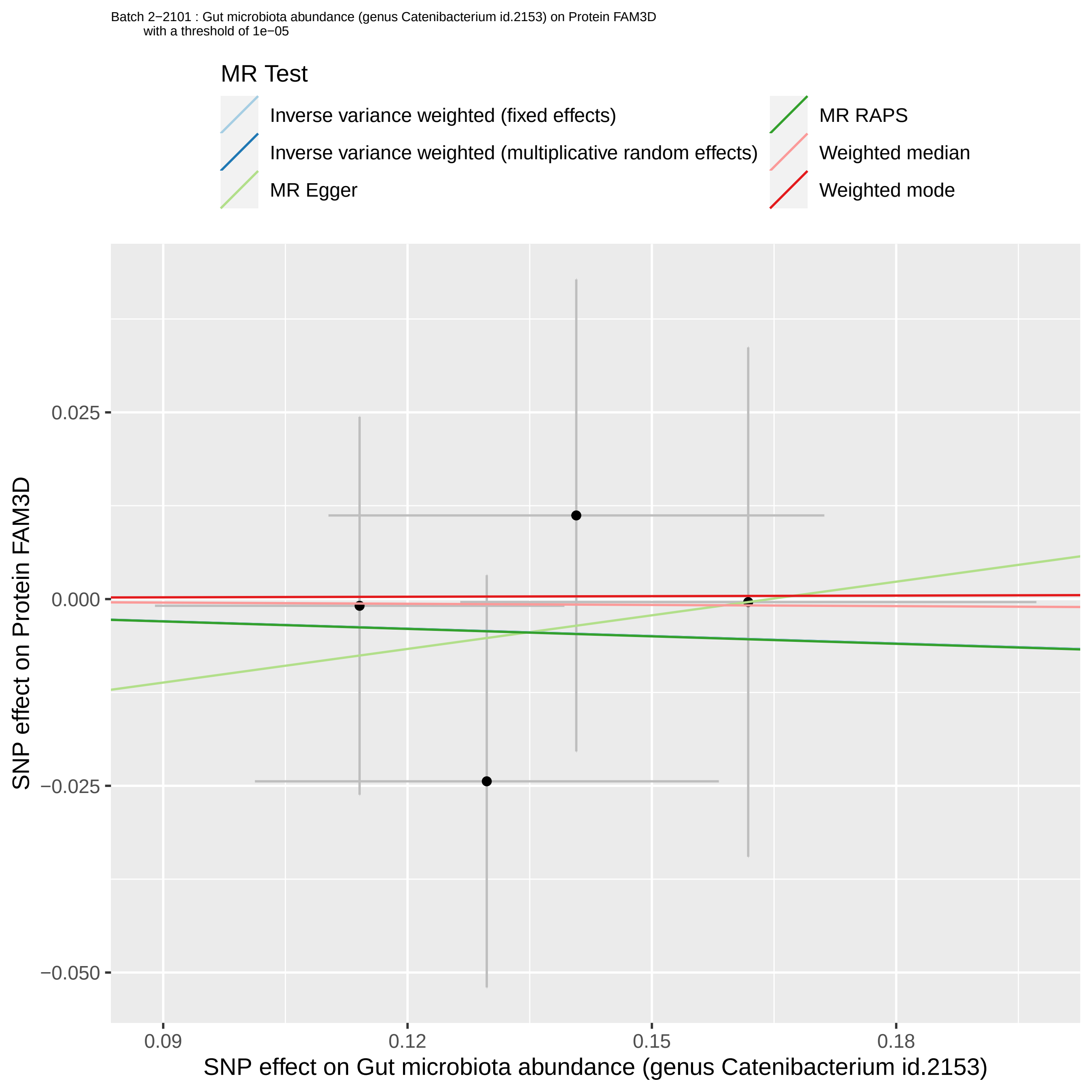
Batch 2−2101 : Gut microbiota abundance (genus Catenibacterium id.2153) on Protein FAM3D
with a threshold of 1e−05
MR Test
Inverse variance weighted (fixed effects)
Inverse variance weighted (multiplicative random effects)
MR Egger
MR RAPS
Weighted median
Weighted mode
0.025
0.000
−0.025
−0.050
0.09
0.12
0.15
0.18
SNP effect on Protein FAM3D
SNP effect on Gut microbiota abundance (genus Catenibacterium id.2153)

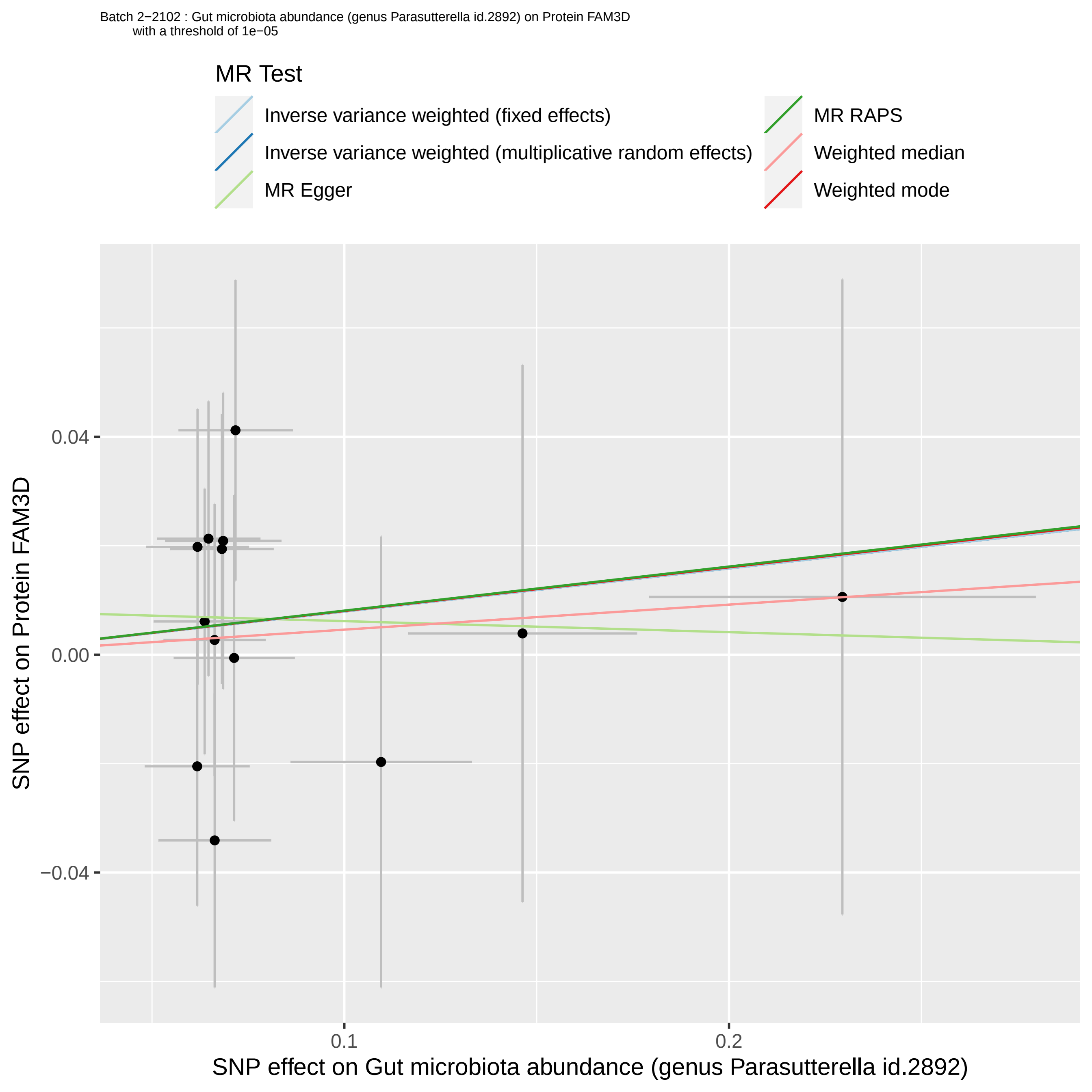

Batch 2−2102 : Gut microbiota abundance (genus Parasutterella id.2892) on Protein FAM3D
with a threshold of 1e−05
MR Test
Inverse variance weighted (fixed effects)
Inverse variance weighted (multiplicative random effects)
MR Egger
MR RAPS
Weighted median
Weighted mode
0.04
0.00
−0.04
0.1
0.2
SNP effect on Protein FAM3D
SNP effect on Gut microbiota abundance (genus Parasutterella id.2892)

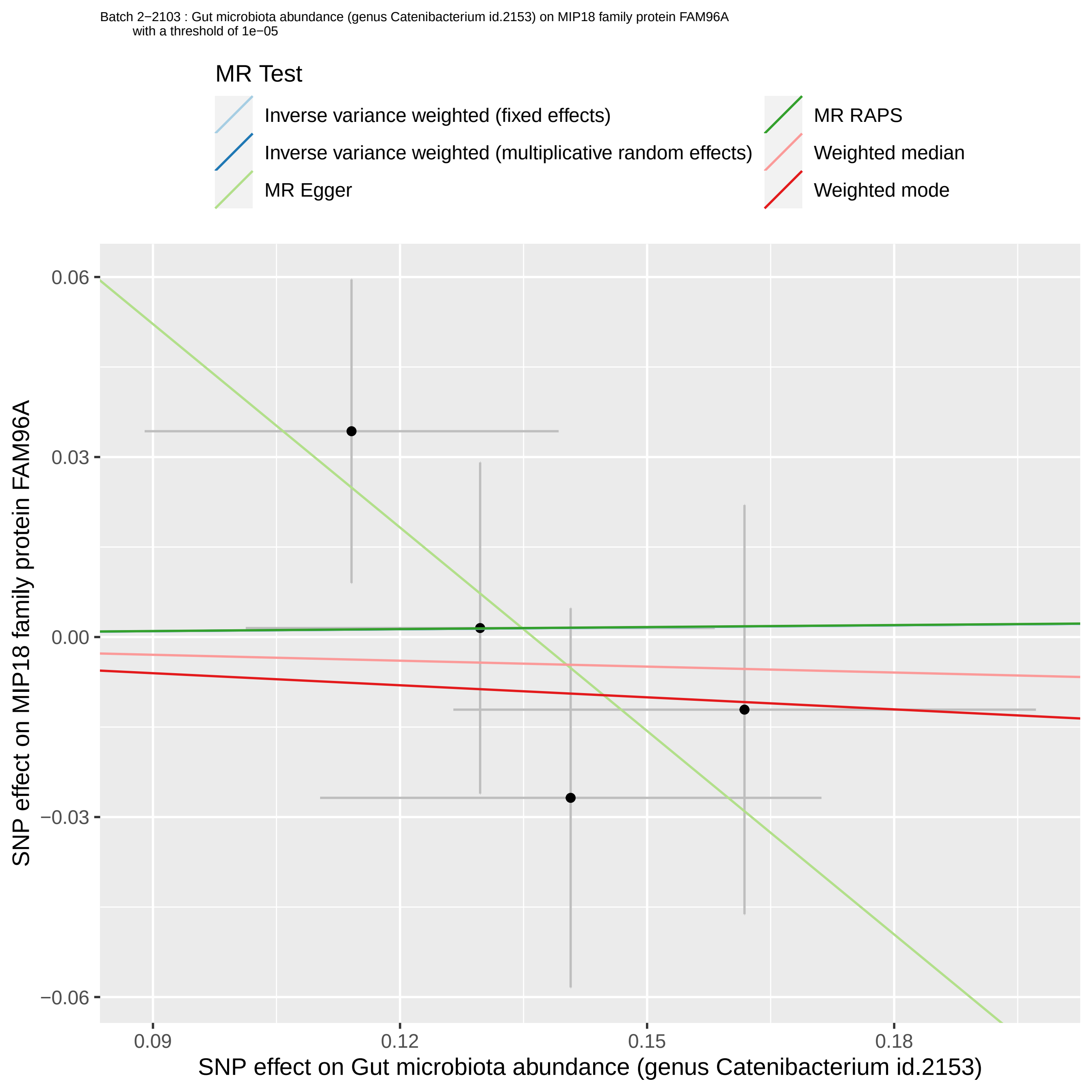
Batch 2−2103 : Gut microbiota abundance (genus Catenibacterium id.2153) on MIP18 family protein FAM96A
with a threshold of 1e−05
MR Test
Inverse variance weighted (fixed effects)
Inverse variance weighted (multiplicative random effects)
MR Egger
MR RAPS
Weighted median
Weighted mode
0.06
0.03
0.00
−0.03
−0.06
0.09
0.12
0.15
0.18
SNP effect on MIP18 family protein FAM96A
SNP effect on Gut microbiota abundance (genus Catenibacterium id.2153)

Batch 2−2104 : Gut microbiota abundance (genus Parasutterella id.2892) on MIP18 family protein FAM96A
with a threshold of 1e−05

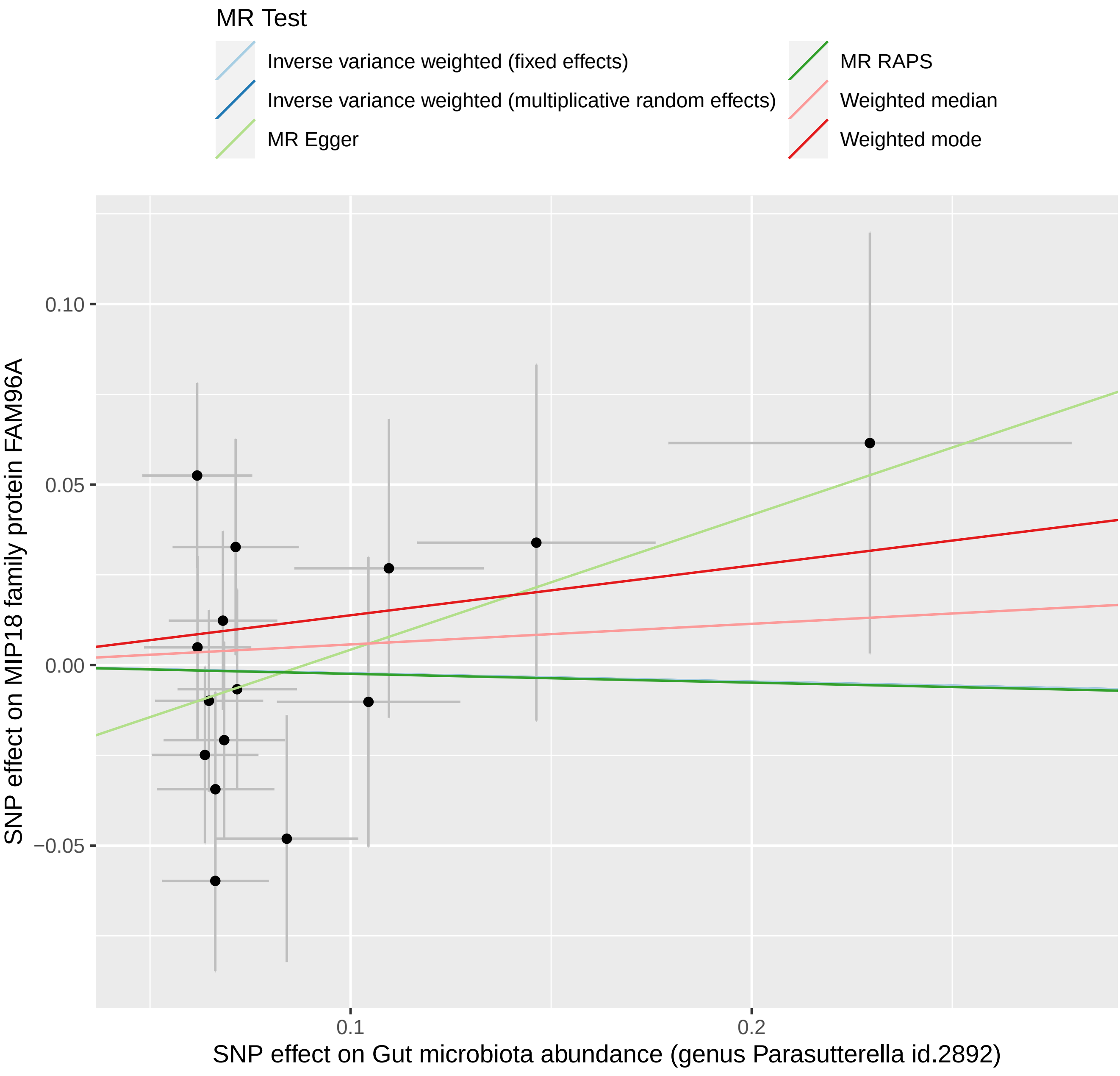

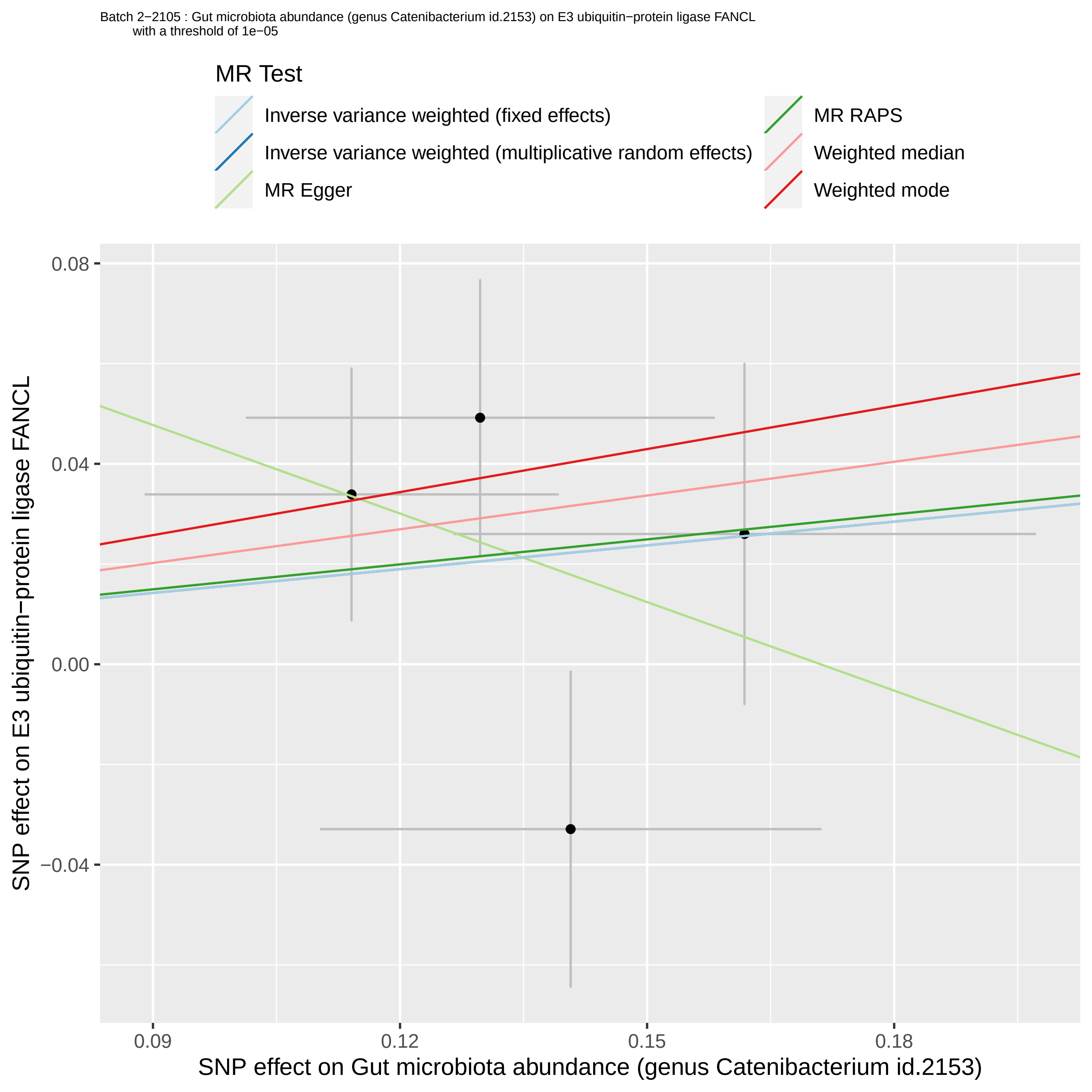
Batch 2−2105 : Gut microbiota abundance (genus Catenibacterium id.2153) on E3 ubiquitin−protein ligase FANCL
with a threshold of 1e−05
MR Test
Inverse variance weighted (fixed effects)
Inverse variance weighted (multiplicative random effects)
MR Egger
MR RAPS
Weighted median
Weighted mode
0.08
0.04
0.00
−0.04
0.09
0.12
0.15
0.18
SNP effect on E3 ubiquitin−protein ligase FANCL
SNP effect on Gut microbiota abundance (genus Catenibacterium id.2153)

Batch 2−2106 : Gut microbiota abundance (genus Parasutterella id.2892) on E3 ubiquitin−protein ligase FANCL with a threshold of 1e−05

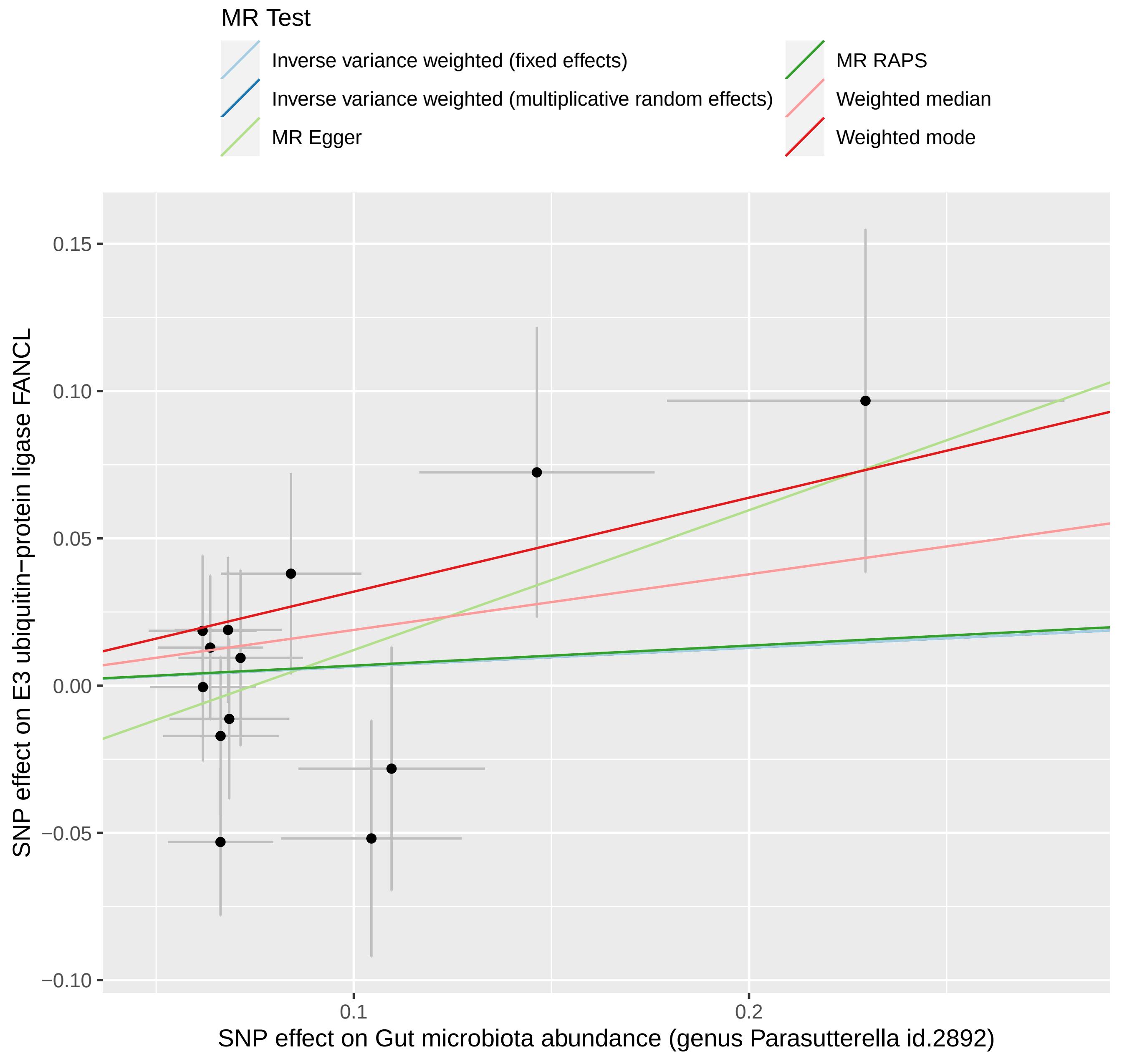

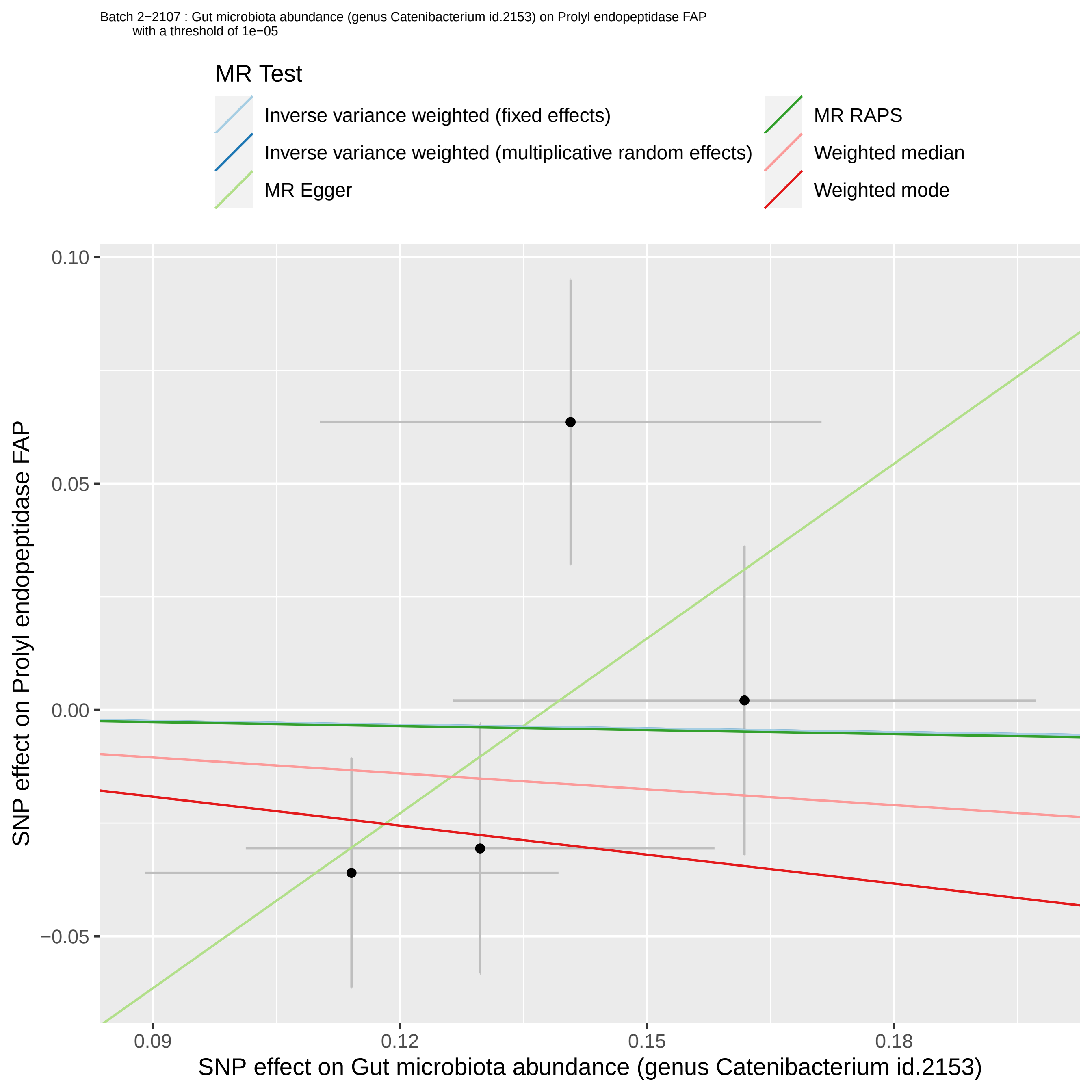
Batch 2−2107 : Gut microbiota abundance (genus Catenibacterium id.2153) on Prolyl endopeptidase FAP
with a threshold of 1e−05
MR Test
Inverse variance weighted (fixed effects)
Inverse variance weighted (multiplicative random effects)
MR Egger
MR RAPS
Weighted median
Weighted mode
0.10
0.05
0.00
−0.05
0.09
0.12
0.15
0.18
SNP effect on Prolyl endopeptidase FAP
SNP effect on Gut microbiota abundance (genus Catenibacterium id.2153)

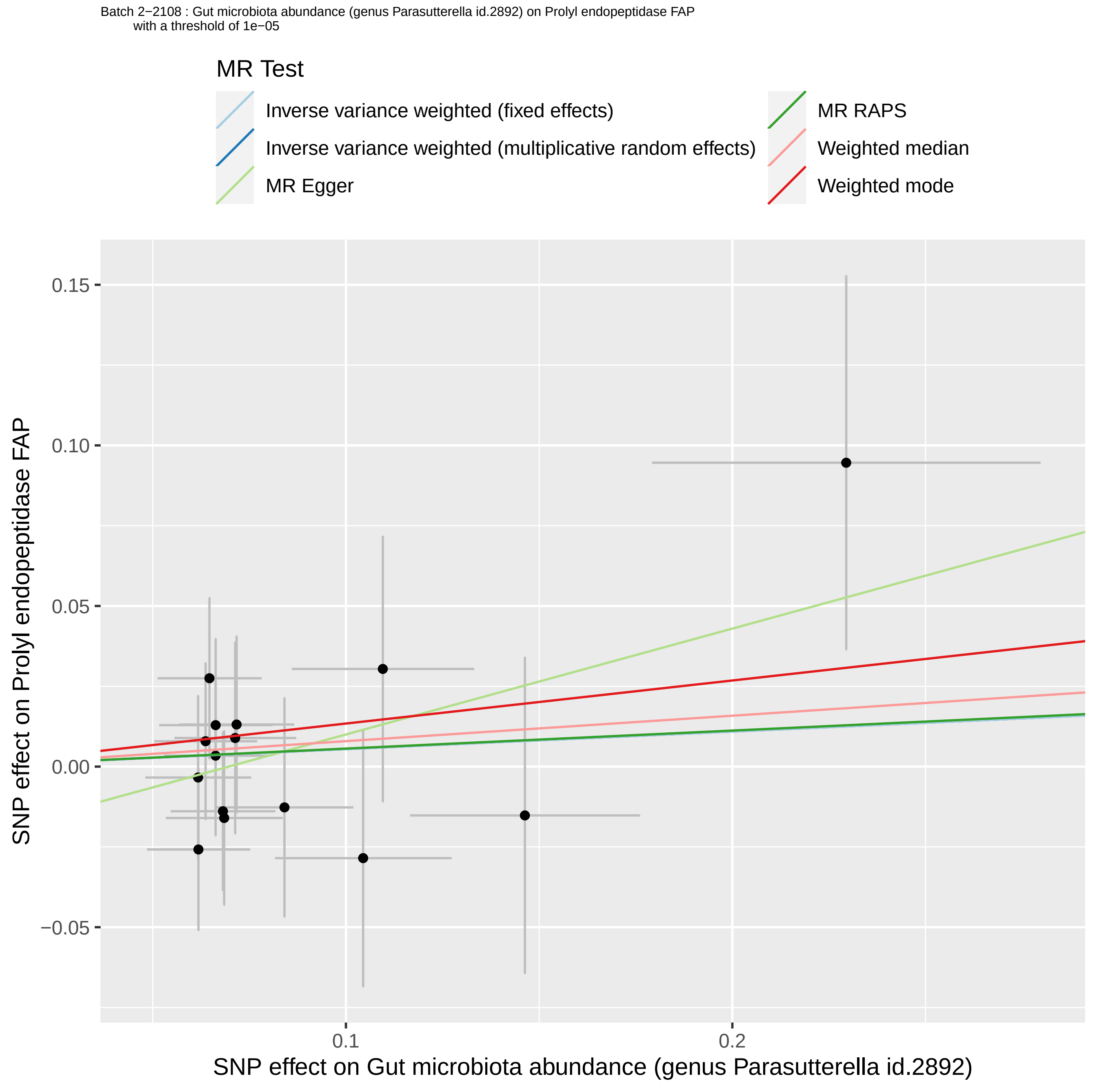
Batch 2−2108 : Gut microbiota abundance (genus Parasutterella id.2892) on Prolyl endopeptidase FAP
with a threshold of 1e−05
MR Test
Inverse variance weighted (fixed effects)
Inverse variance weighted (multiplicative random effects)
MR Egger
MR RAPS
Weighted median
Weighted mode
0.15
0.10
0.05
0.00
−0.05
0.1
0.2
SNP effect on Prolyl endopeptidase FAP
SNP effect on Gut microbiota abundance (genus Parasutterella id.2892)

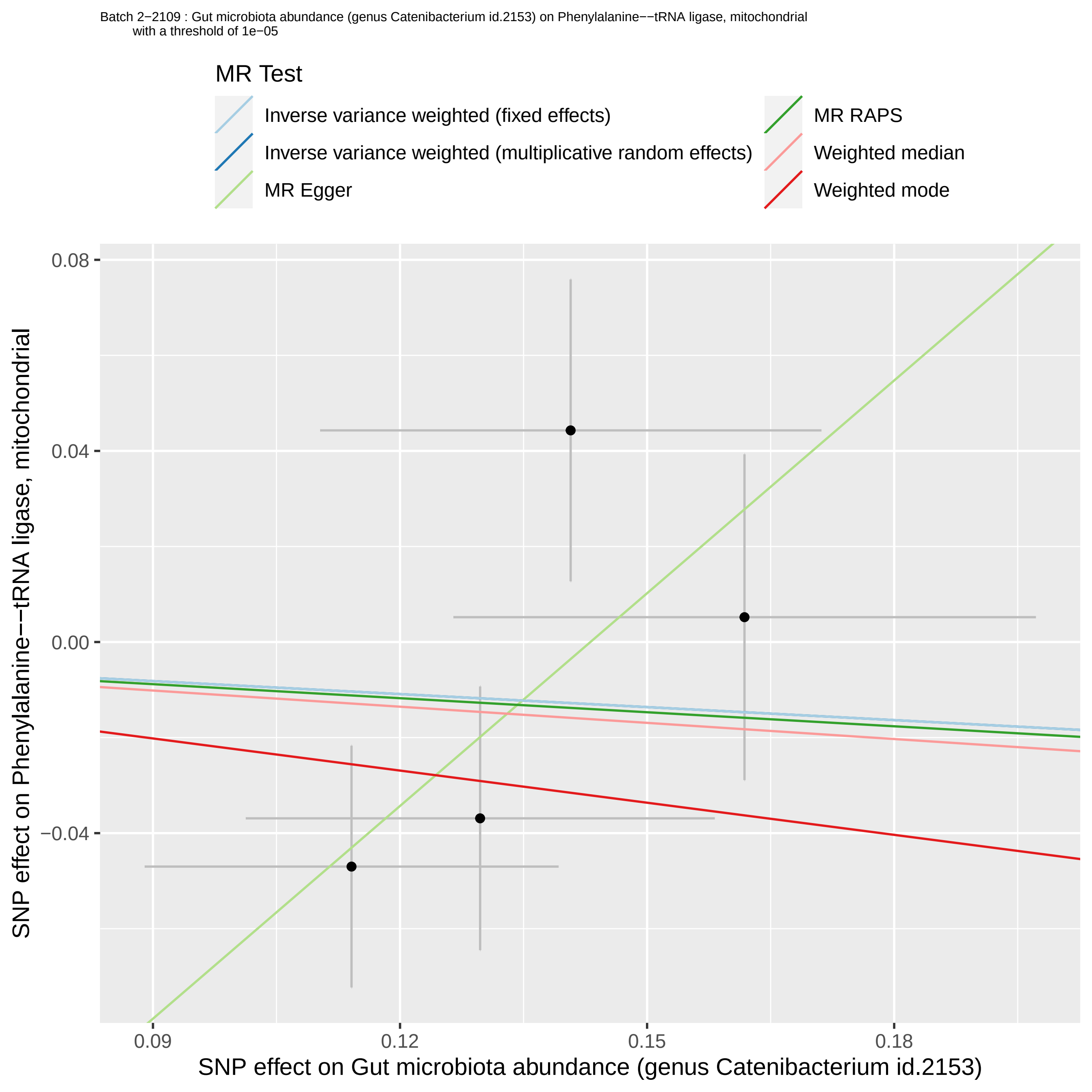

Batch 2−2109 : Gut microbiota abundance (genus Catenibacterium id.2153) on Phenylalanine−−tRNA ligase, mitochondrial
with a threshold of 1e−05
MR Test
Inverse variance weighted (fixed effects)
Inverse variance weighted (multiplicative random effects)
MR Egger
MR RAPS
Weighted median
Weighted mode
0.08
0.04
0.00
−0.04
0.09
0.12
0.15
0.18
SNP effect on Phenylalanine−−tRNA ligase, mitochondrial
SNP effect on Gut microbiota abundance (genus Catenibacterium id.2153)

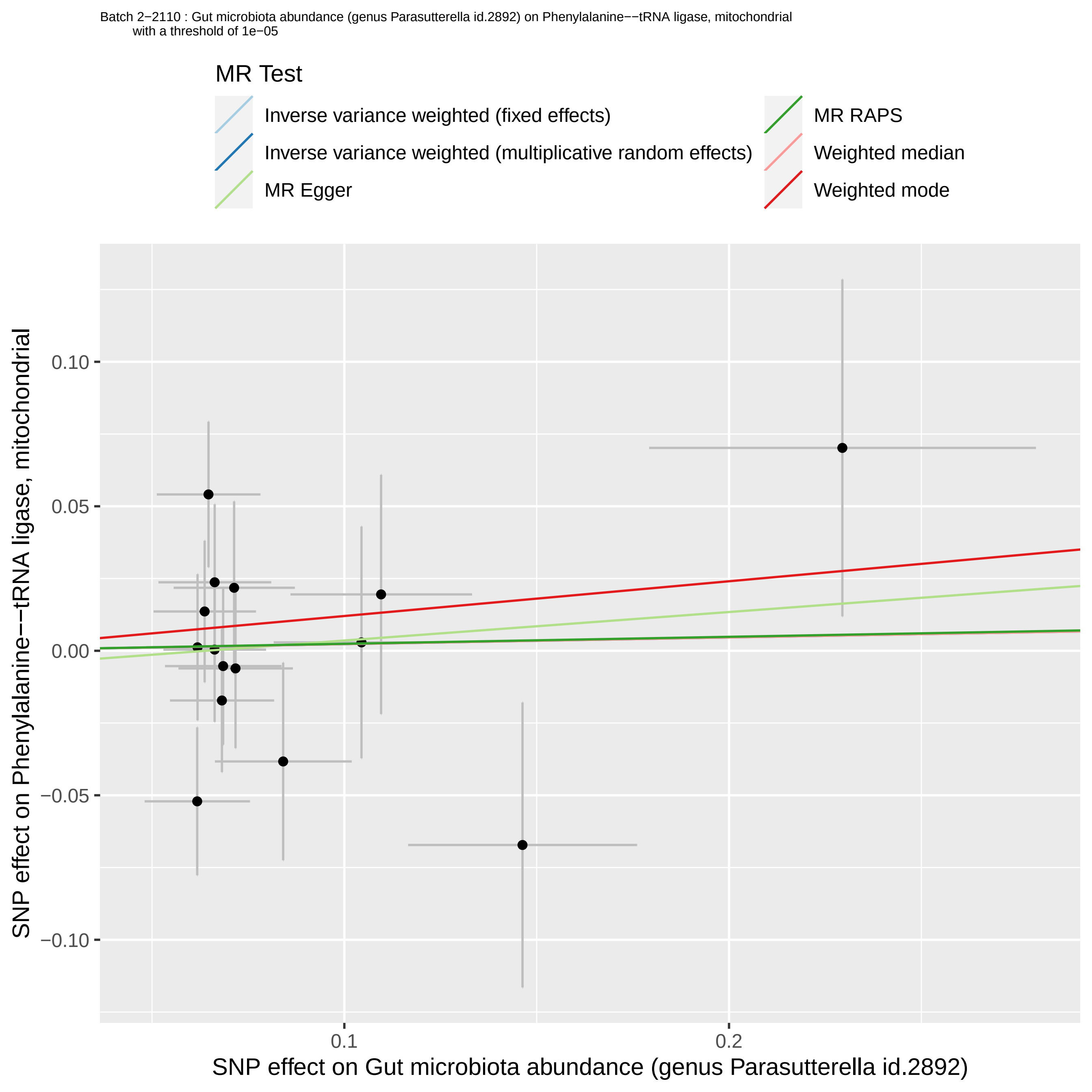
Batch 2−2110 : Gut microbiota abundance (genus Parasutterella id.2892) on Phenylalanine−−tRNA ligase, mitochondrial
with a threshold of 1e−05
MR Test
Inverse variance weighted (fixed effects)
Inverse variance weighted (multiplicative random effects)
MR Egger
MR RAPS
Weighted median
Weighted mode
SNP effect on Phenylalanine−−tRNA ligase, mitochondrial
0.10
0.05
0.00
−0.05
−0.10
0.1
0.2
SNP effect on Gut microbiota abundance (genus Parasutterella id.2892)

Batch 2−2111 : Gut microbiota abundance (genus Catenibacterium id.2153) on Tumor necrosis factor receptor superfamily member 6
with a threshold of 1e−05

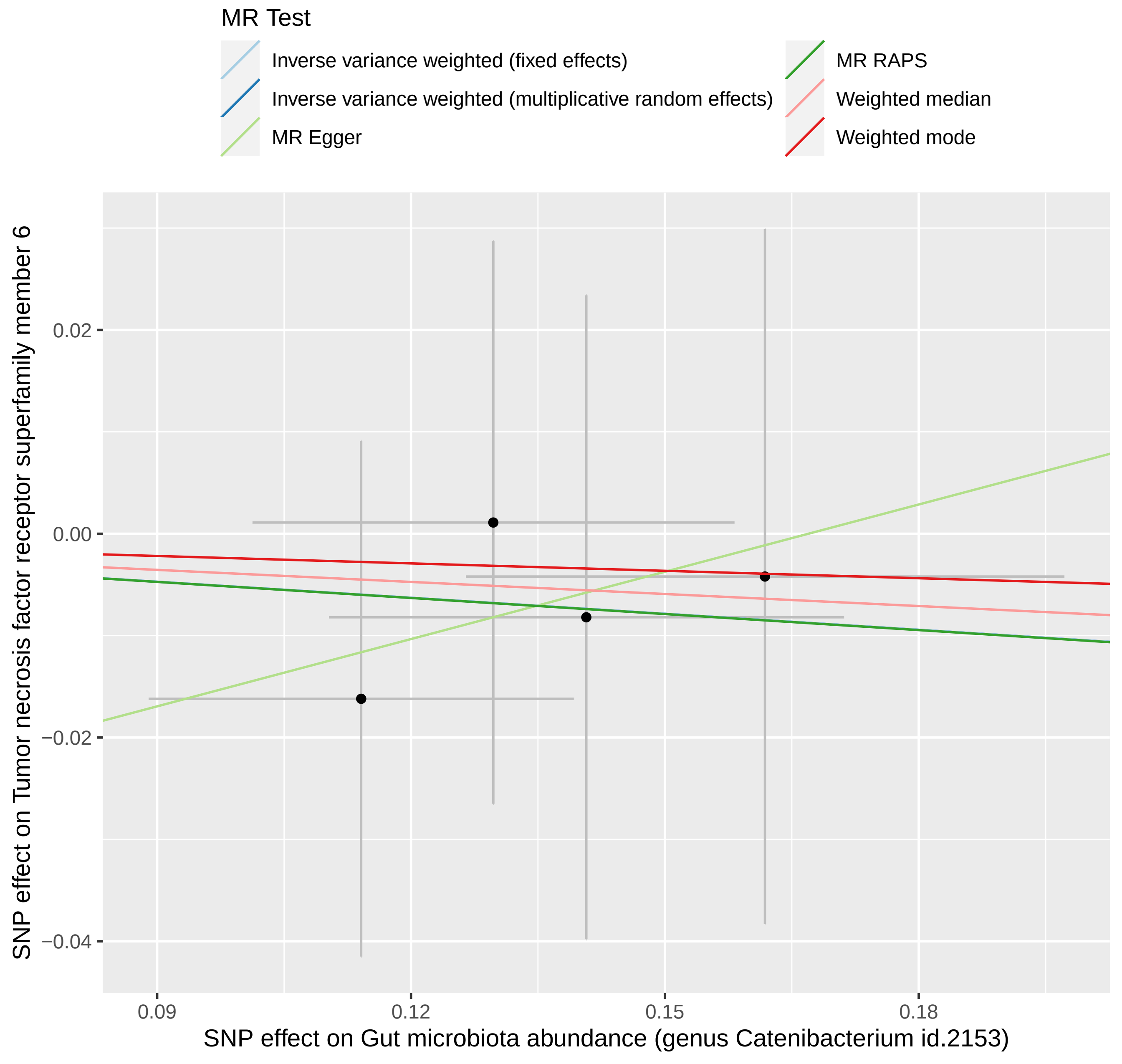

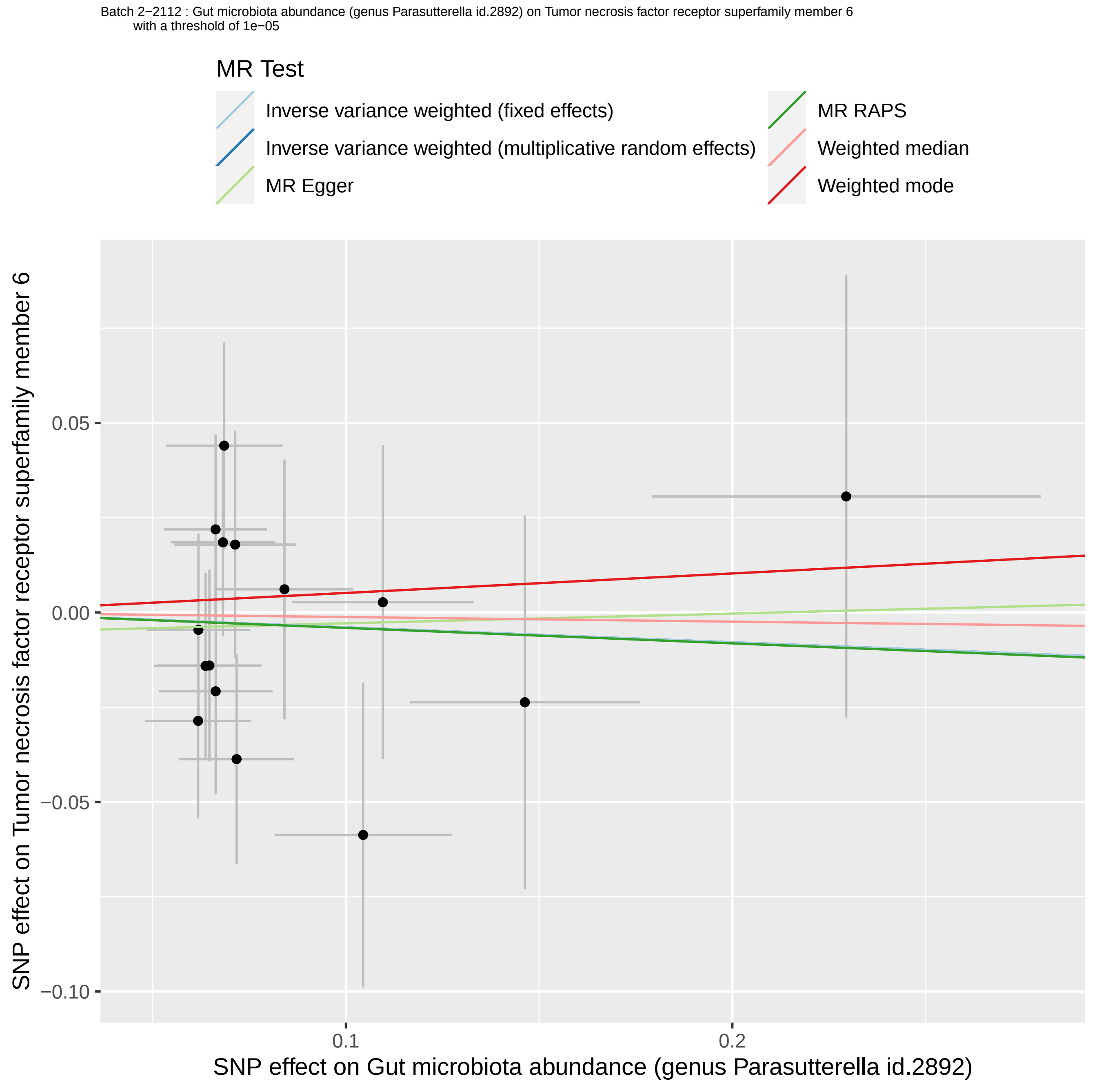

Batch 2−2112 : Gut microbiota abundance (genus Parasutterella id.2892) on Tumor necrosis factor receptor superfamily member 6
with a threshold of 1e−05
MR Test
Inverse variance weighted (fixed effects)
Inverse variance weighted (multiplicative random effects)
MR Egger
MR RAPS
Weighted median
Weighted mode
SNP effect on Tumor necrosis factor receptor superfamily member 6
0.05
0.00
−0.05
−0.10
0.1
0.2
SNP effect on Gut microbiota abundance (genus Parasutterella id.2892)

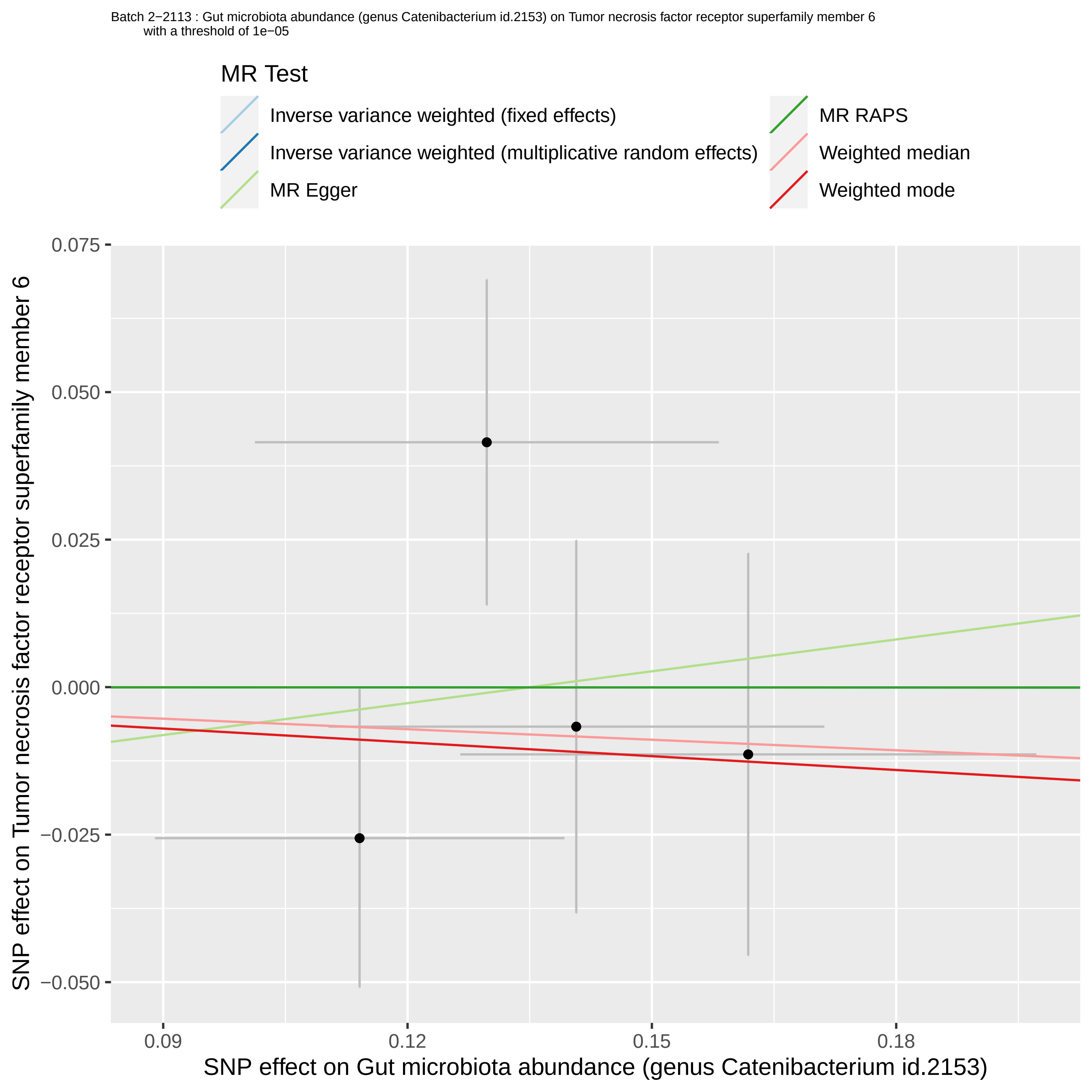

Batch 2−2113 : Gut microbiota abundance (genus Catenibacterium id.2153) on Tumor necrosis factor receptor superfamily member 6
with a threshold of 1e−05
MR Test
Inverse variance weighted (fixed effects)
Inverse variance weighted (multiplicative random effects)
MR Egger
MR RAPS
Weighted median
Weighted mode
0.075
0.050
0.025
0.000
−0.025
−0.050
0.09
0.12
0.15
0.18
SNP effect on Tumor necrosis factor receptor superfamily member 6
SNP effect on Gut microbiota abundance (genus Catenibacterium id.2153)

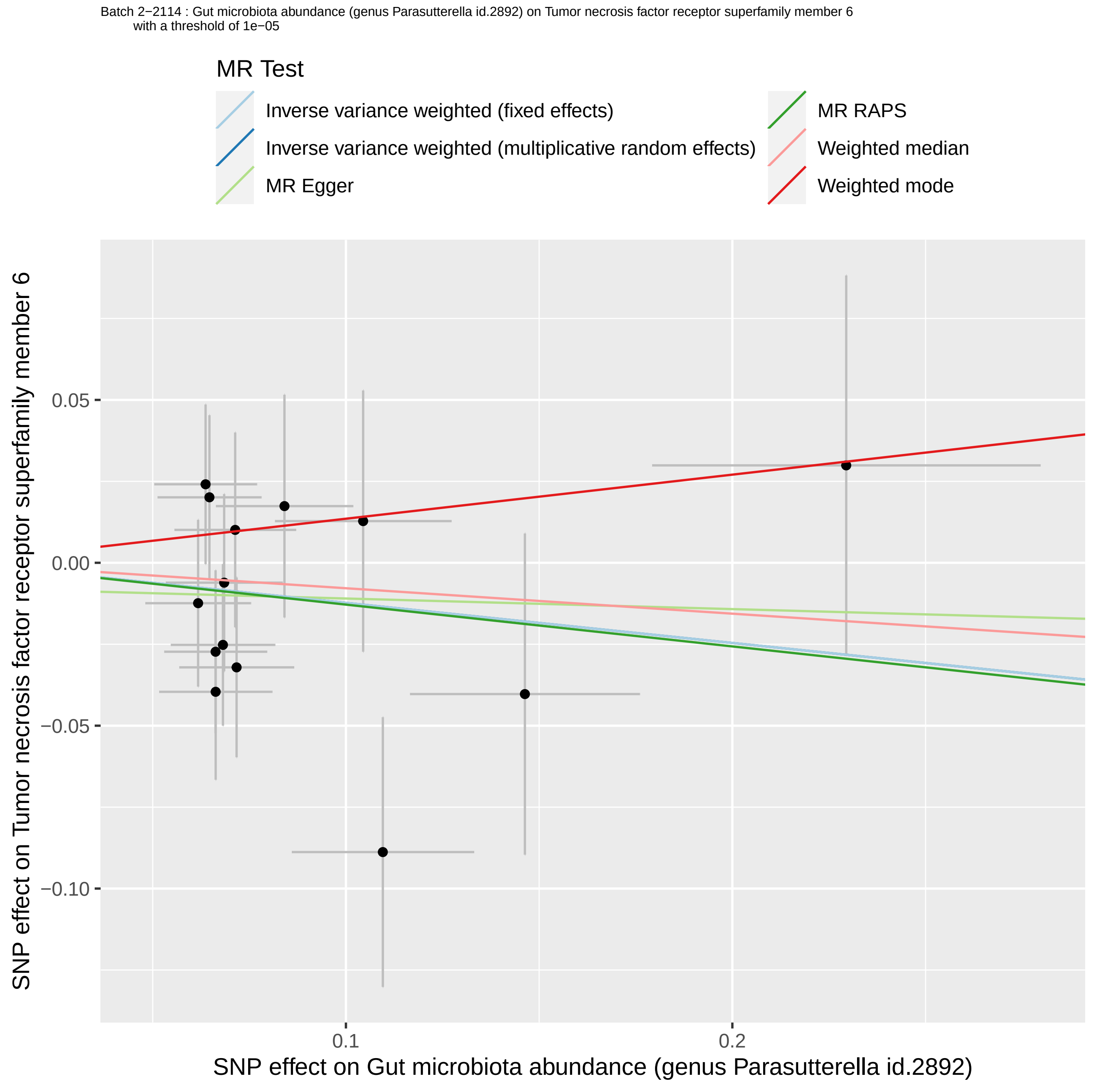
Batch 2−2114 : Gut microbiota abundance (genus Parasutterella id.2892) on Tumor necrosis factor receptor superfamily member 6
with a threshold of 1e−05
MR Test
Inverse variance weighted (fixed effects)
Inverse variance weighted (multiplicative random effects)
MR Egger
MR RAPS
Weighted median
Weighted mode
0.05
0.00
−0.05
−0.10
0.1
0.2
SNP effect on Tumor necrosis factor receptor superfamily member 6
SNP effect on Gut microbiota abundance (genus Parasutterella id.2892)

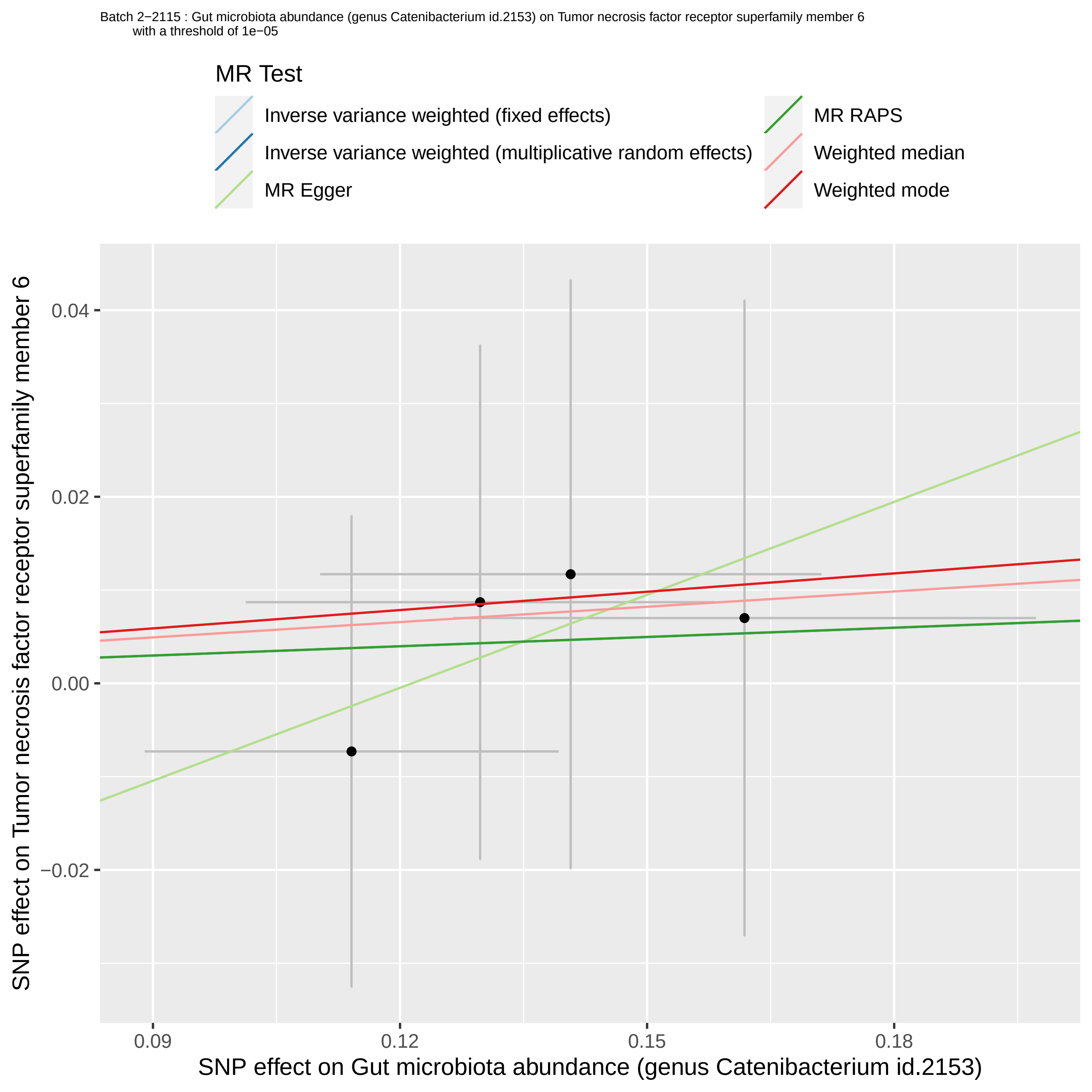

Batch 2−2115 : Gut microbiota abundance (genus Catenibacterium id.2153) on Tumor necrosis factor receptor superfamily member 6
with a threshold of 1e−05
MR Test
Inverse variance weighted (fixed effects)
Inverse variance weighted (multiplicative random effects)
MR Egger
MR RAPS
Weighted median
Weighted mode
SNP effect on Tumor necrosis factor receptor superfamily member 6
0.04
0.02
0.00
−0.02
0.09
0.12
0.15
0.18
SNP effect on Gut microbiota abundance (genus Catenibacterium id.2153)

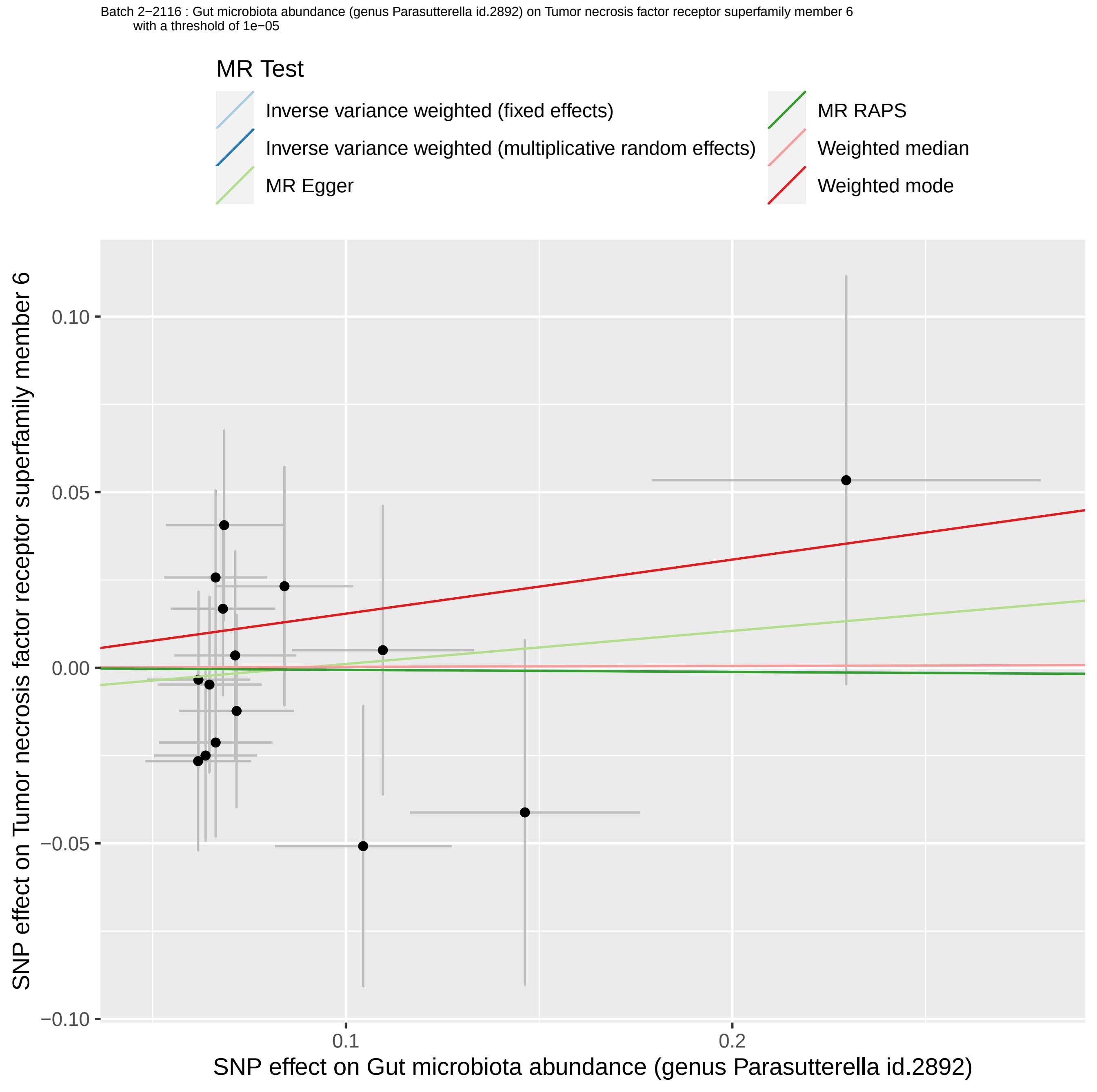

Batch 2−2116 : Gut microbiota abundance (genus Parasutterella id.2892) on Tumor necrosis factor receptor superfamily member 6
with a threshold of 1e−05
MR Test
Inverse variance weighted (fixed effects)
Inverse variance weighted (multiplicative random effects)
MR Egger
MR RAPS
Weighted median
Weighted mode
0.10
0.05
0.00
−0.05
−0.10
0.1
0.2
SNP effect on Tumor necrosis factor receptor superfamily member 6
SNP effect on Gut microbiota abundance (genus Parasutterella id.2892)

Batch 2−2117 : Gut microbiota abundance (genus Catenibacterium id.2153) on Tumor necrosis factor ligand superfamily member 6, soluble form with a threshold of 1e−05

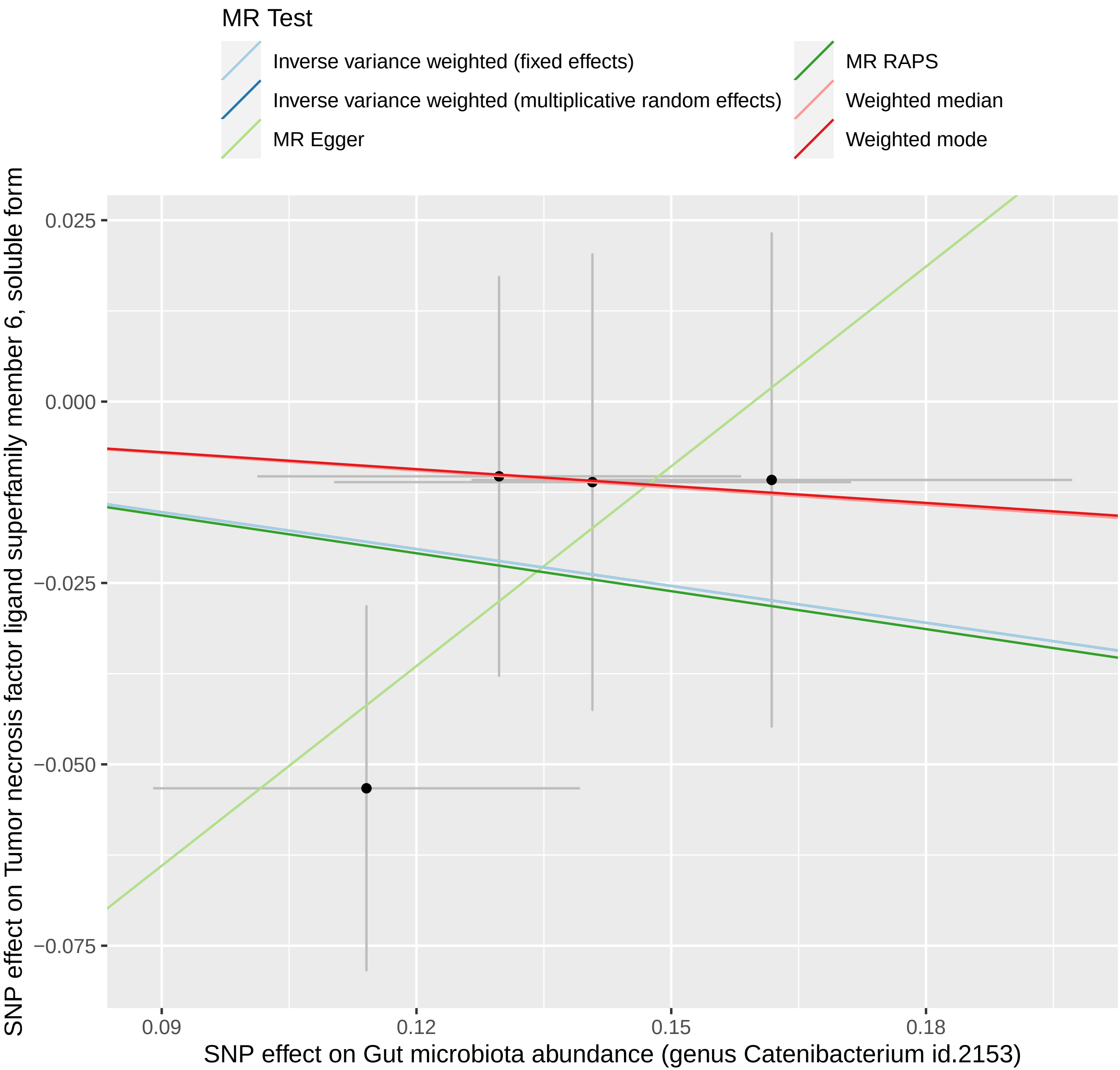

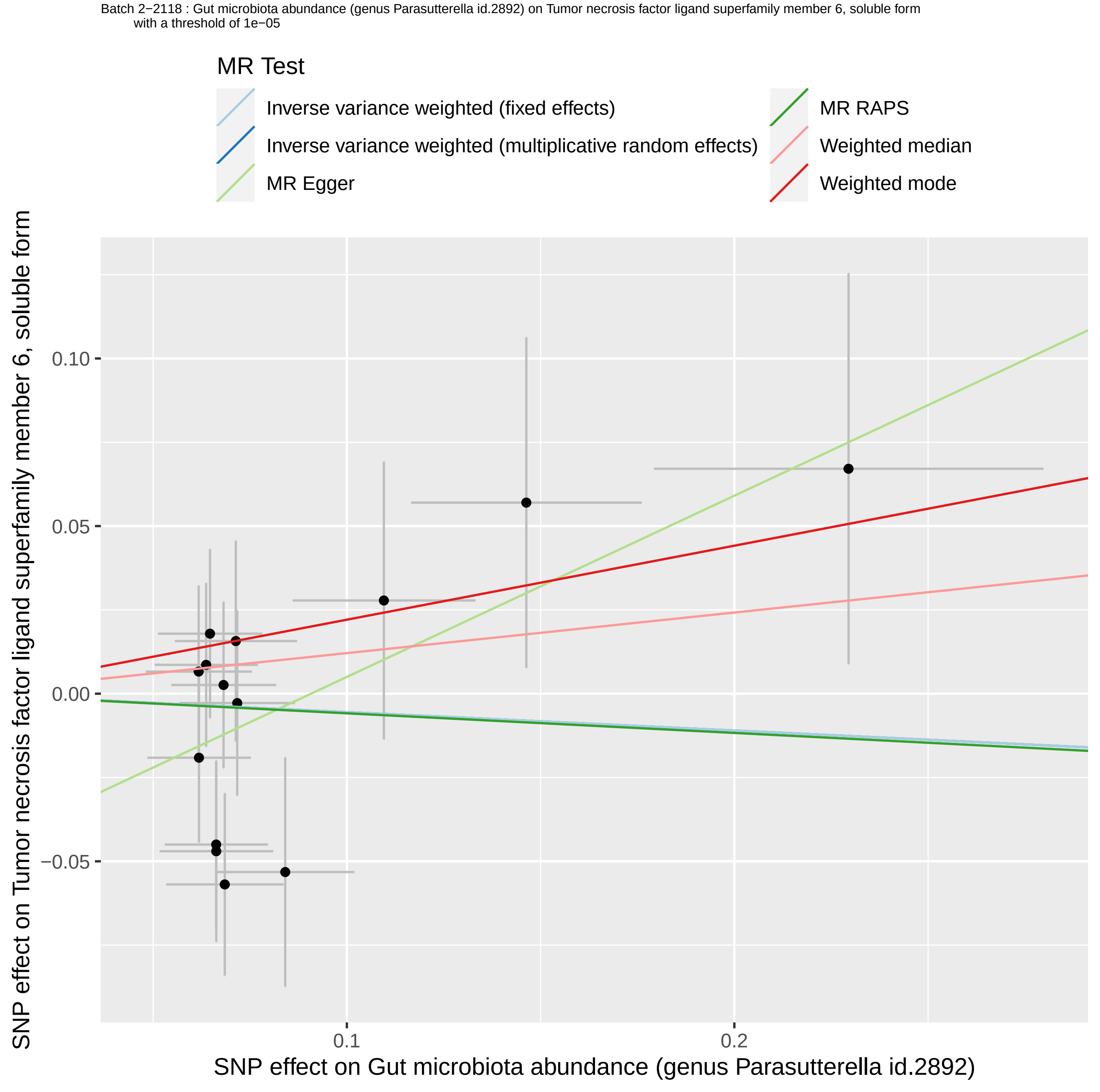

Batch 2−2118 : Gut microbiota abundance (genus Parasutterella id.2892) on Tumor necrosis factor ligand superfamily member 6, soluble form
with a threshold of 1e−05
MR Test
Inverse variance weighted (fixed effects)
Inverse variance weighted (multiplicative random effects)
MR Egger
MR RAPS
Weighted median
Weighted mode
0.10
0.05
0.00
−0.05
0.1
0.2
SNP effect on Tumor necrosis factor ligand superfamily member 6, soluble form
SNP effect on Gut microbiota abundance (genus Parasutterella id.2892)

Batch 2−2119 : Gut microbiota abundance (genus Catenibacterium id.2153) on Fetal and adult testis−expressed transcript protein with a threshold of 1e−05

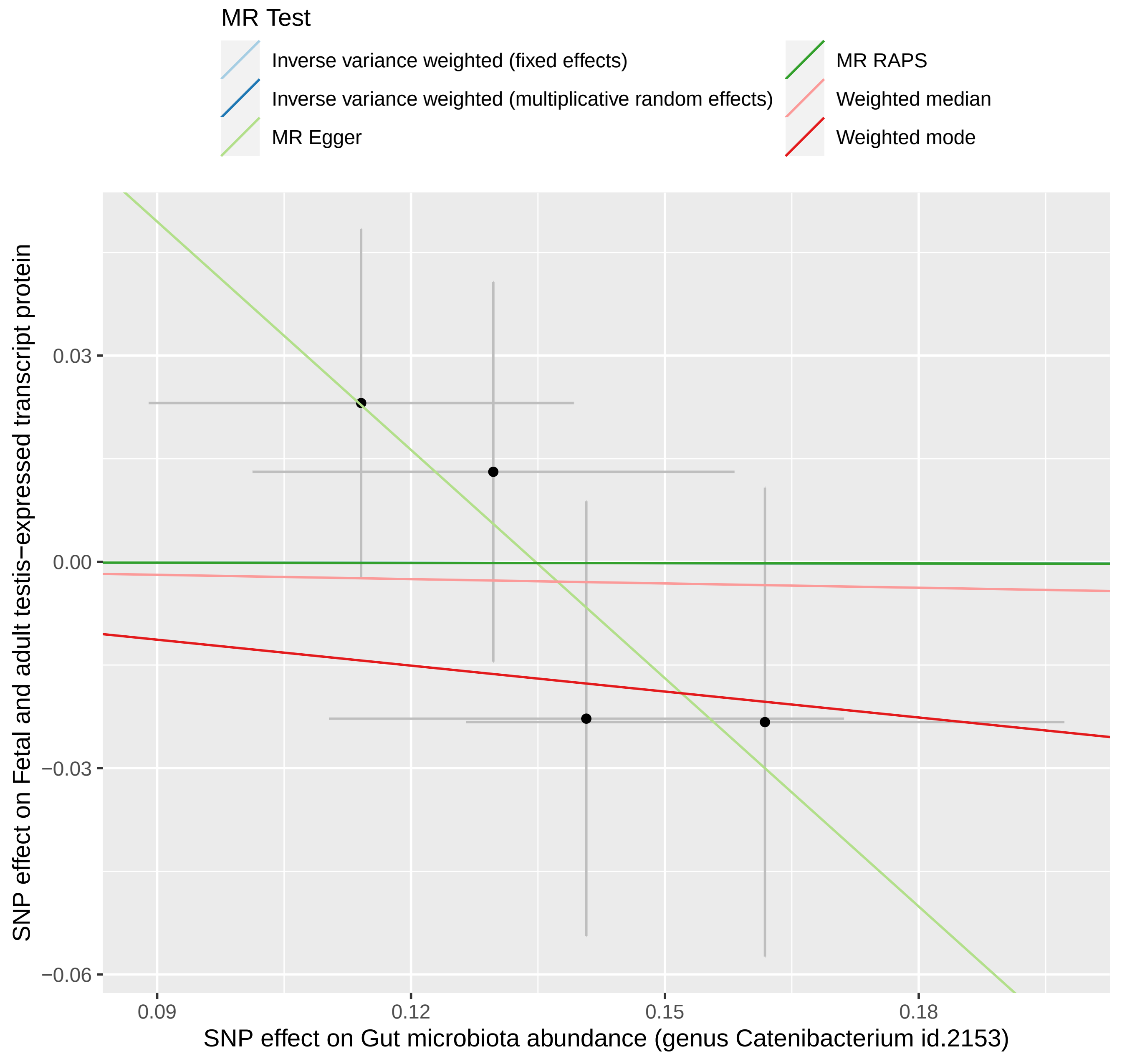

Batch 2−2120 : Gut microbiota abundance (genus Parasutterella id.2892) on Fetal and adult testis−expressed transcript protein
with a threshold of 1e−05

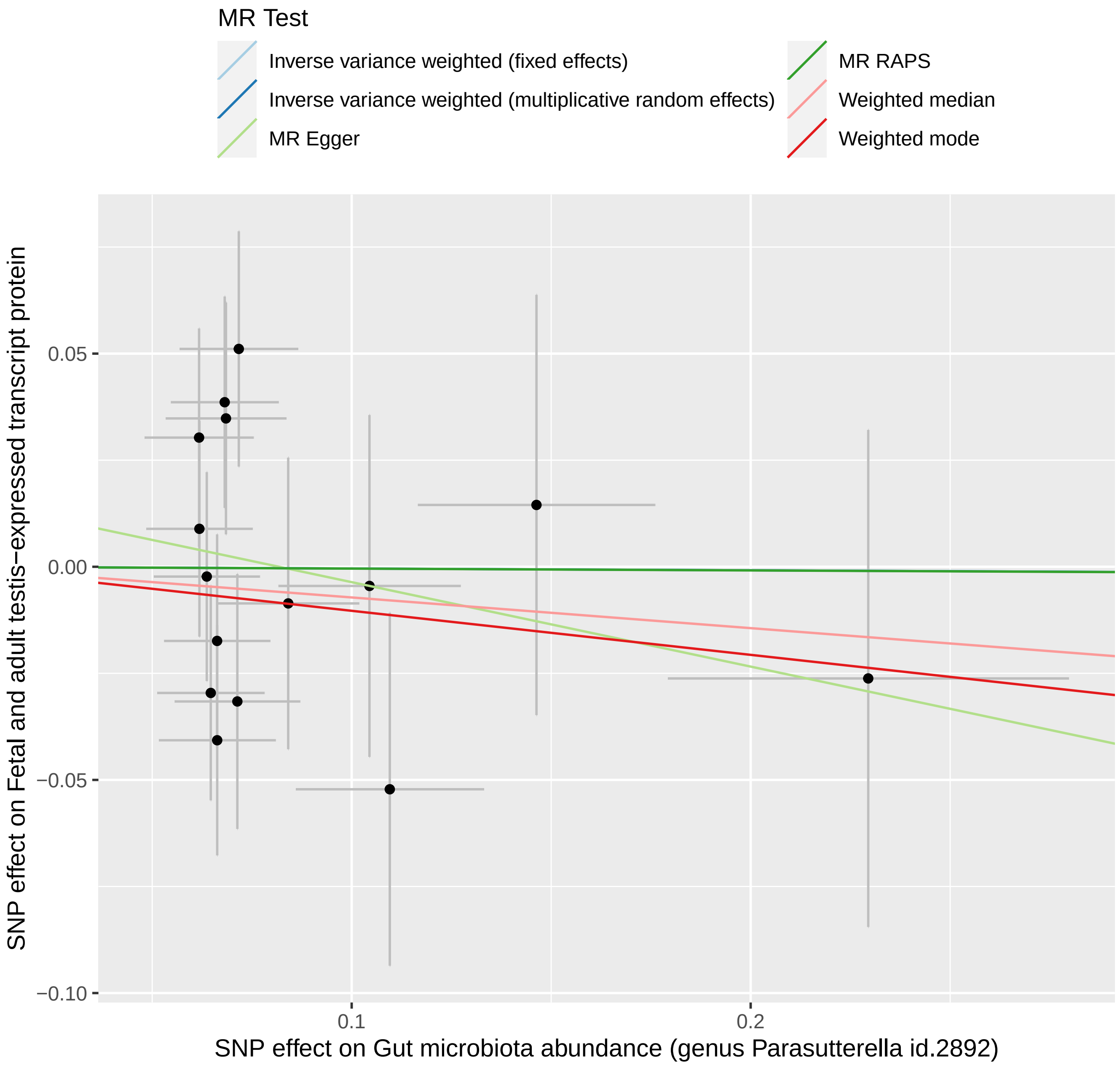

Batch 2−2121 : Gut microbiota abundance (genus Catenibacterium id.2153) on Fatty acid hydroxylase domain−containing protein 2
with a threshold of 1e−05

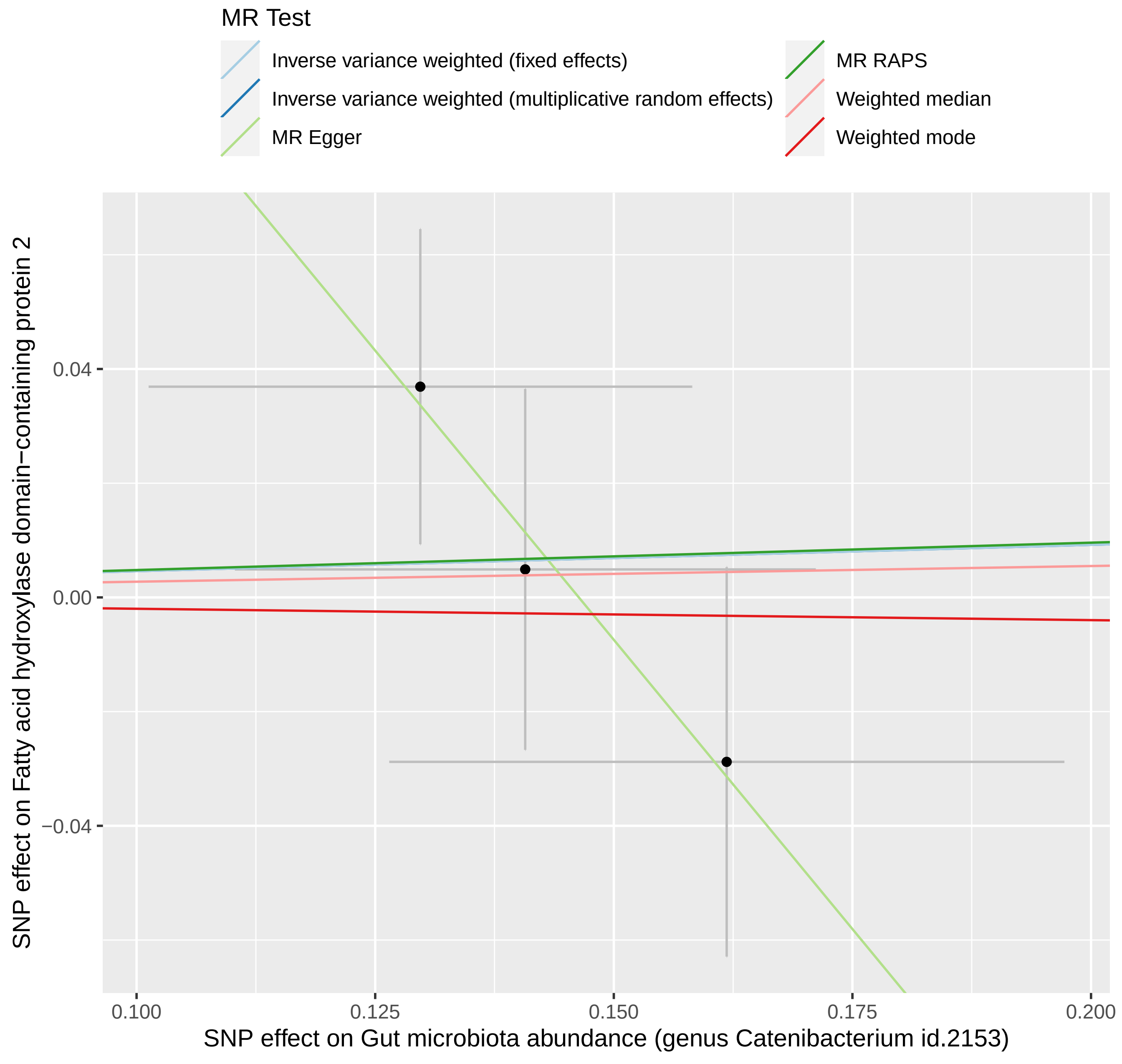

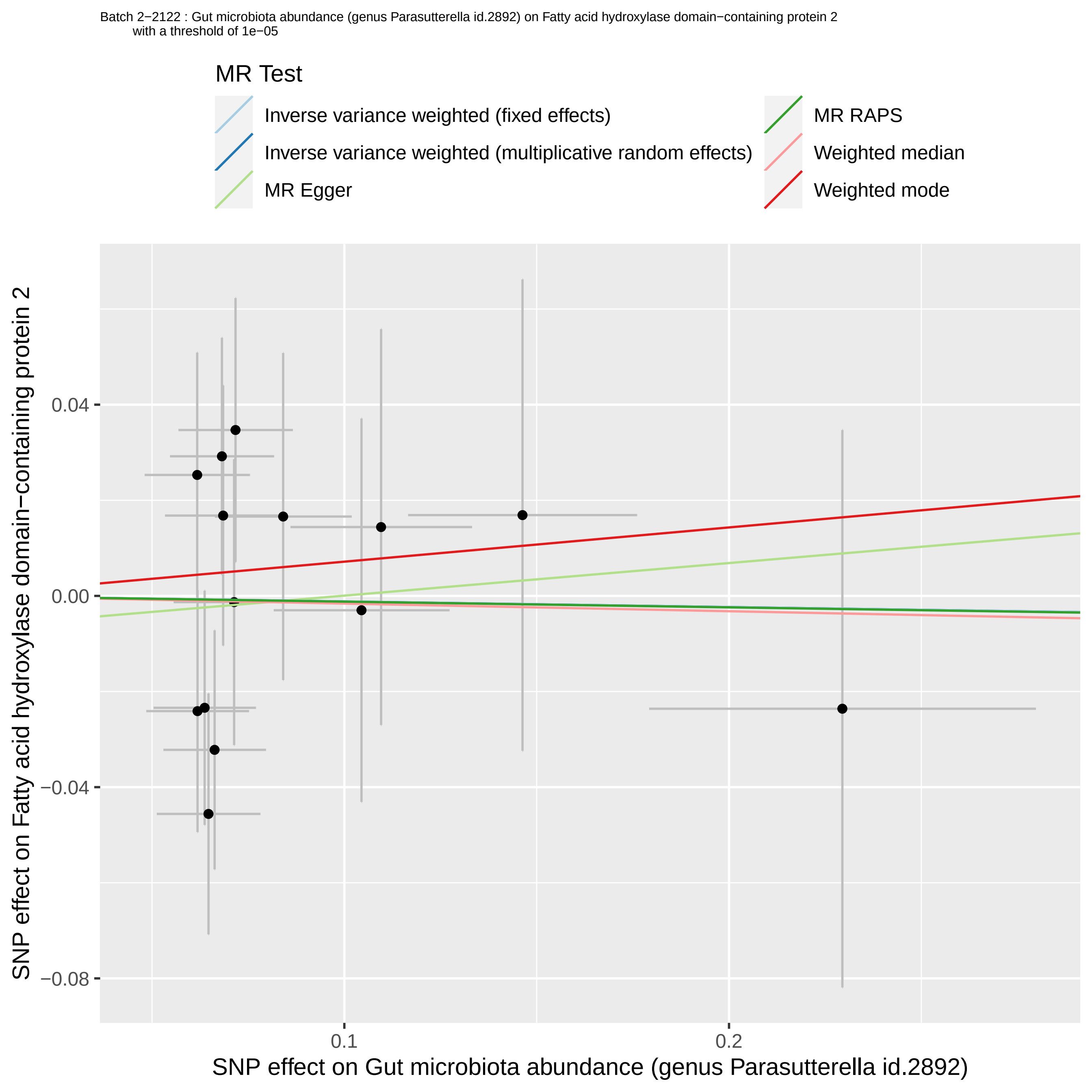
Batch 2−2122 : Gut microbiota abundance (genus Parasutterella id.2892) on Fatty acid hydroxylase domain−containing protein 2
with a threshold of 1e−05
MR Test
Inverse variance weighted (fixed effects)
Inverse variance weighted (multiplicative random effects)
MR Egger
MR RAPS
Weighted median
Weighted mode
0.04
0.00
−0.04
−0.08
0.1
0.2
SNP effect on Fatty acid hydroxylase domain−containing protein 2
SNP effect on Gut microbiota abundance (genus Parasutterella id.2892)

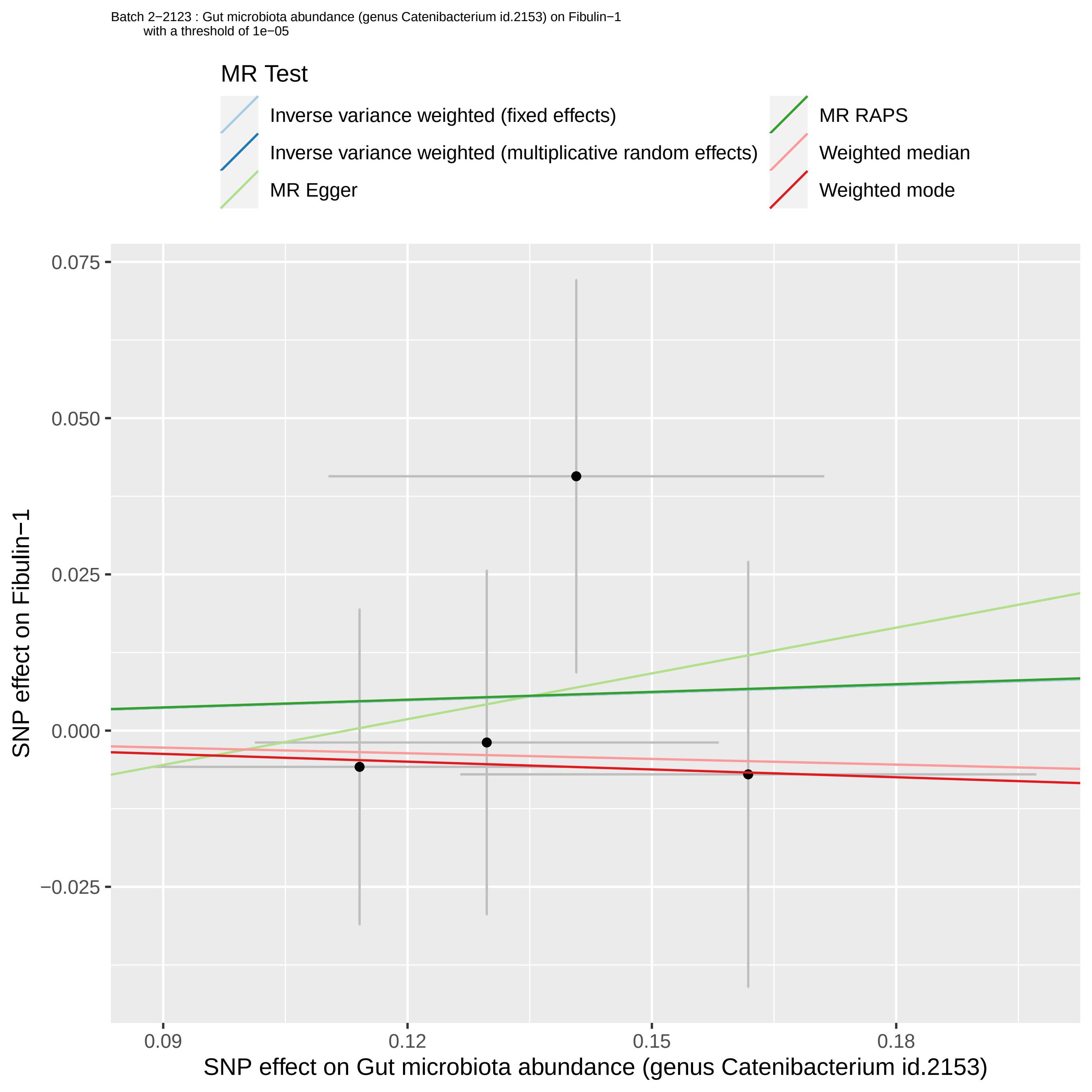
Batch 2−2123 : Gut microbiota abundance (genus Catenibacterium id.2153) on Fibulin−1
with a threshold of 1e−05
MR Test
Inverse variance weighted (fixed effects)
Inverse variance weighted (multiplicative random effects)
MR Egger
MR RAPS
Weighted median
Weighted mode
0.075
0.050
0.025
0.000
−0.025
0.09
0.12
0.15
0.18
SNP effect on Fibulin−1
SNP effect on Gut microbiota abundance (genus Catenibacterium id.2153)

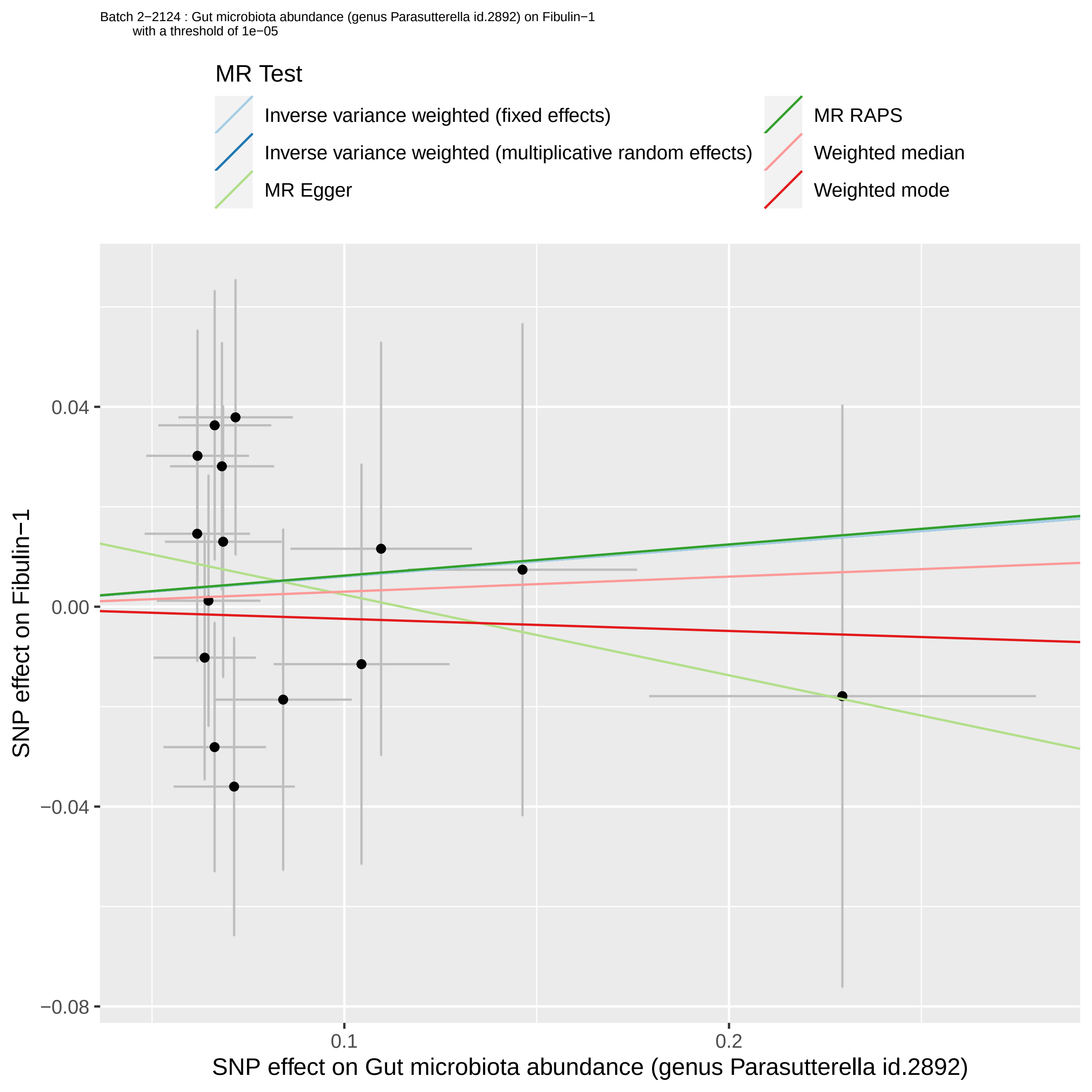
Batch 2−2124 : Gut microbiota abundance (genus Parasutterella id.2892) on Fibulin−1
with a threshold of 1e−05
MR Test
Inverse variance weighted (fixed effects)
Inverse variance weighted (multiplicative random effects)
MR Egger
MR RAPS
Weighted median
Weighted mode
SNP effect on Fibulin−1
0.04
0.00
−0.04
−0.08
0.1
0.2
SNP effect on Gut microbiota abundance (genus Parasutterella id.2892)

Batch 2−2125 : Gut microbiota abundance (genus Catenibacterium id.2153) on Fructose−1,6−bisphosphatase 1
with a threshold of 1e−05

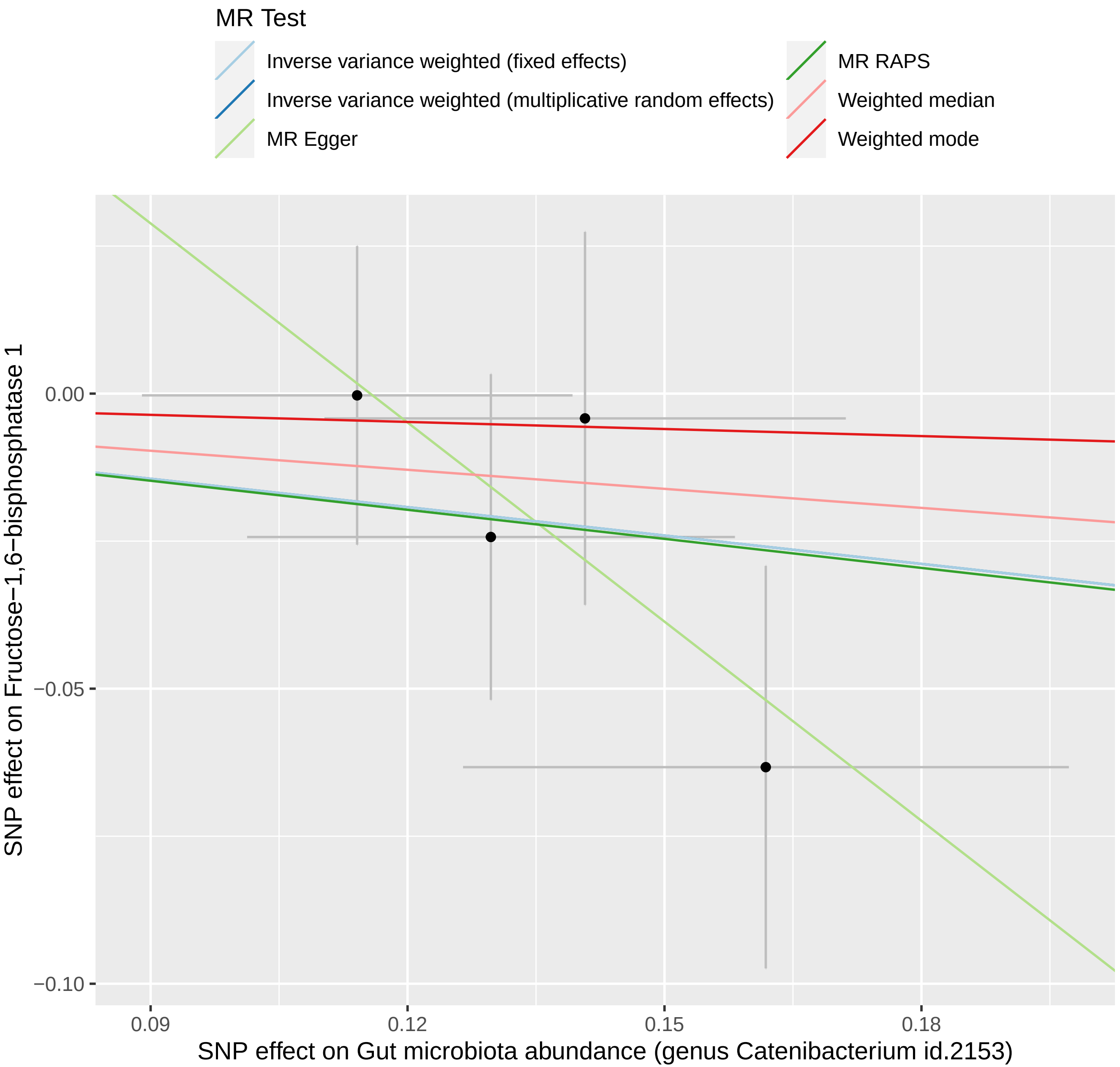

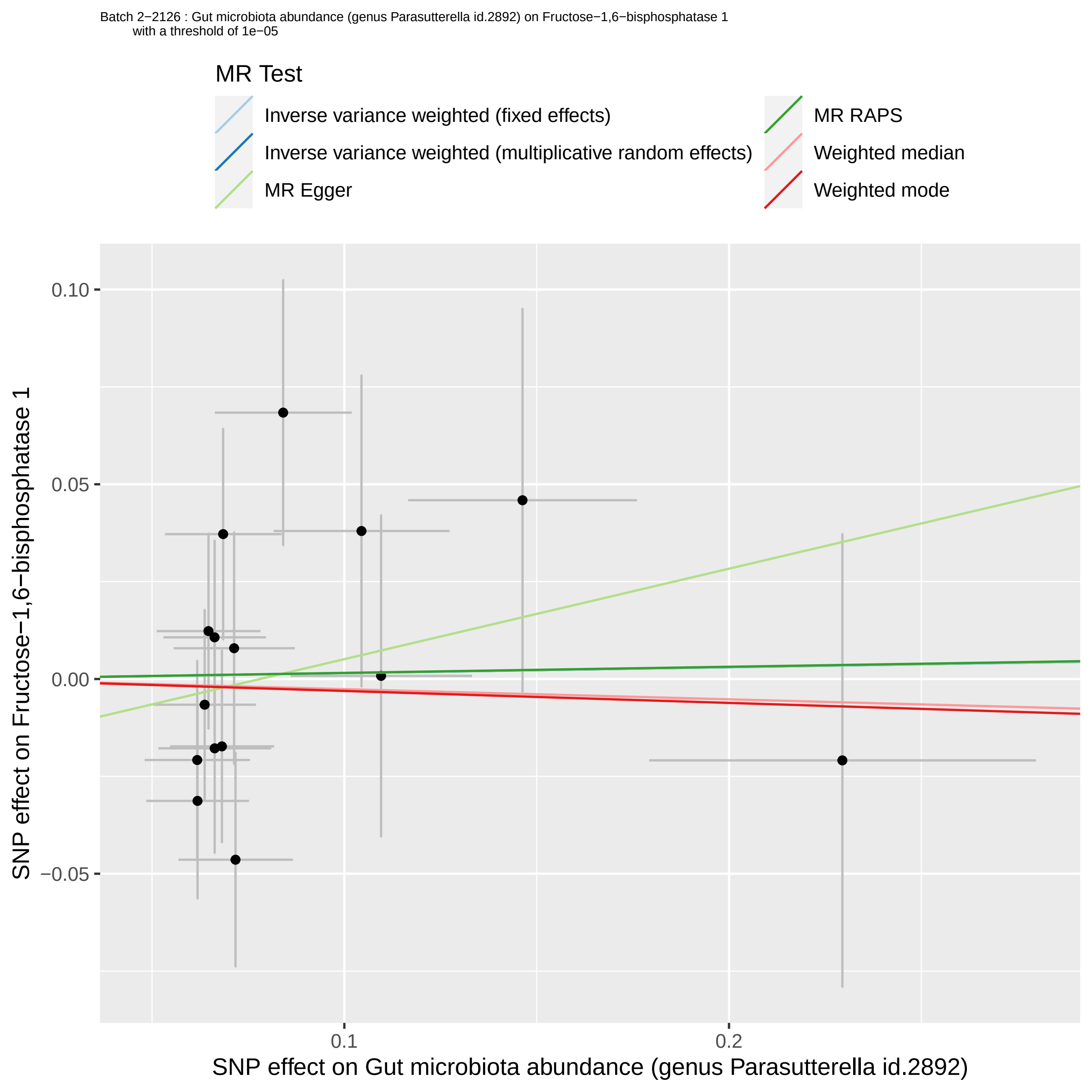

Batch 2−2126 : Gut microbiota abundance (genus Parasutterella id.2892) on Fructose−1,6−bisphosphatase 1
with a threshold of 1e−05
MR Test
Inverse variance weighted (fixed effects)
Inverse variance weighted (multiplicative random effects)
MR Egger
MR RAPS
Weighted median
Weighted mode
0.10
0.05
0.00
−0.05
0.1
0.2
SNP effect on Fructose−1,6−bisphosphatase 1
SNP effect on Gut microbiota abundance (genus Parasutterella id.2892)

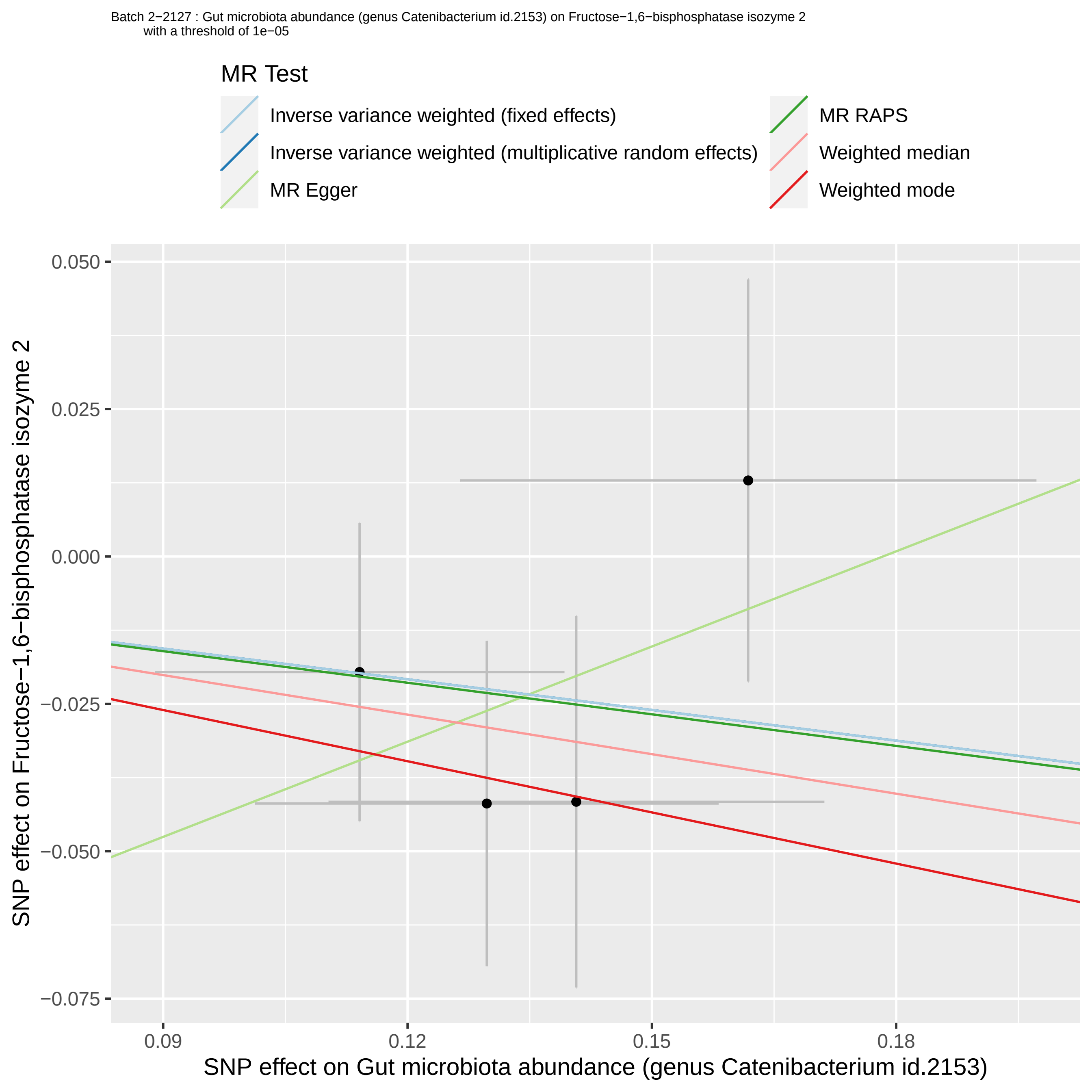
Batch 2−2127 : Gut microbiota abundance (genus Catenibacterium id.2153) on Fructose−1,6−bisphosphatase isozyme 2
with a threshold of 1e−05
MR Test
Inverse variance weighted (fixed effects)
Inverse variance weighted (multiplicative random effects)
MR Egger
MR RAPS
Weighted median
Weighted mode
0.050
0.025
0.000
−0.025
−0.050
−0.075
0.09
0.12
0.15
0.18
SNP effect on Fructose−1,6−bisphosphatase isozyme 2
SNP effect on Gut microbiota abundance (genus Catenibacterium id.2153)

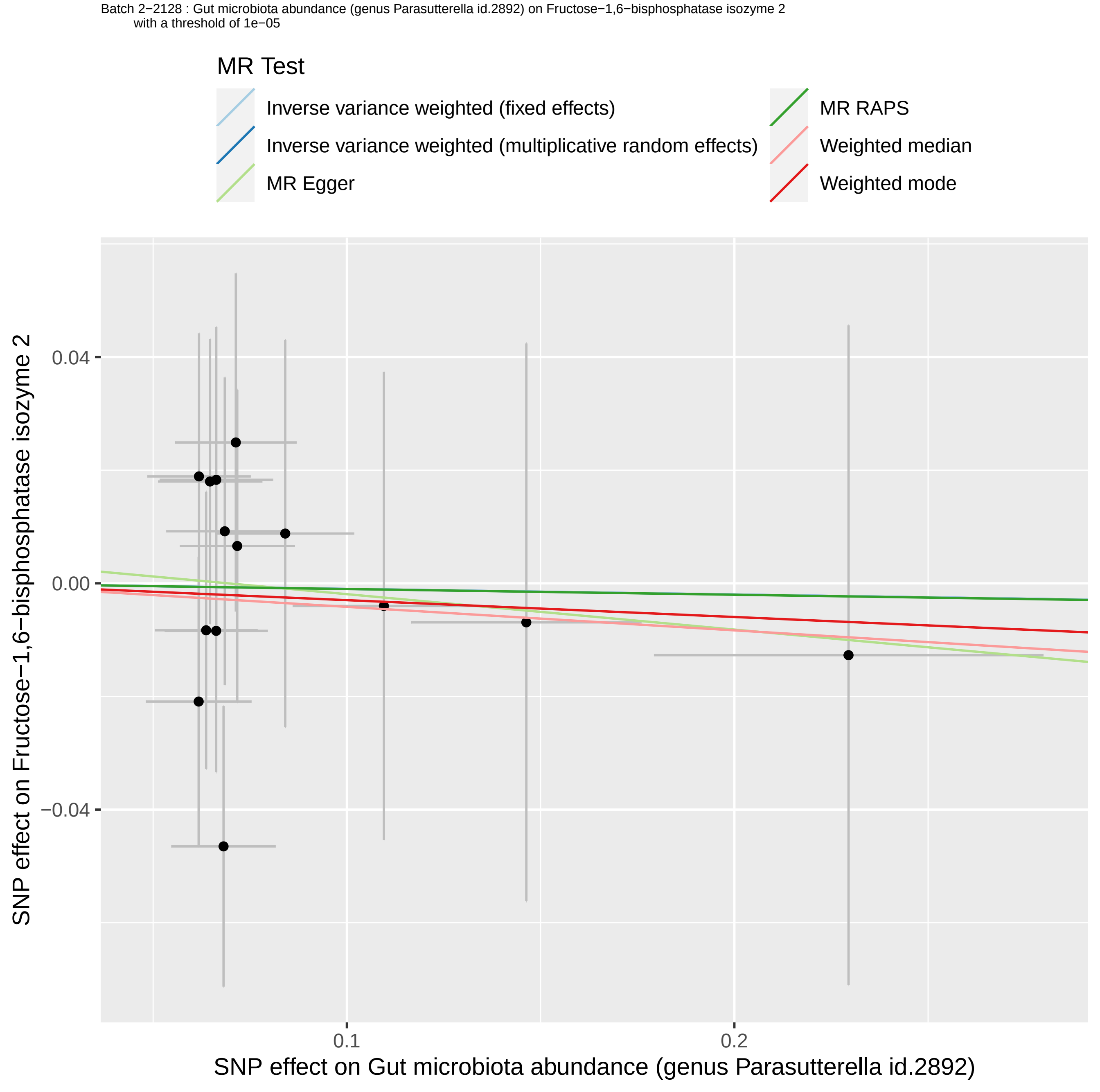

Batch 2−2128 : Gut microbiota abundance (genus Parasutterella id.2892) on Fructose−1,6−bisphosphatase isozyme 2
with a threshold of 1e−05
MR Test
Inverse variance weighted (fixed effects)
Inverse variance weighted (multiplicative random effects)
MR Egger
MR RAPS
Weighted median
Weighted mode
0.04
0.00
−0.04
0.1
0.2
SNP effect on Fructose−1,6−bisphosphatase isozyme 2
SNP effect on Gut microbiota abundance (genus Parasutterella id.2892)

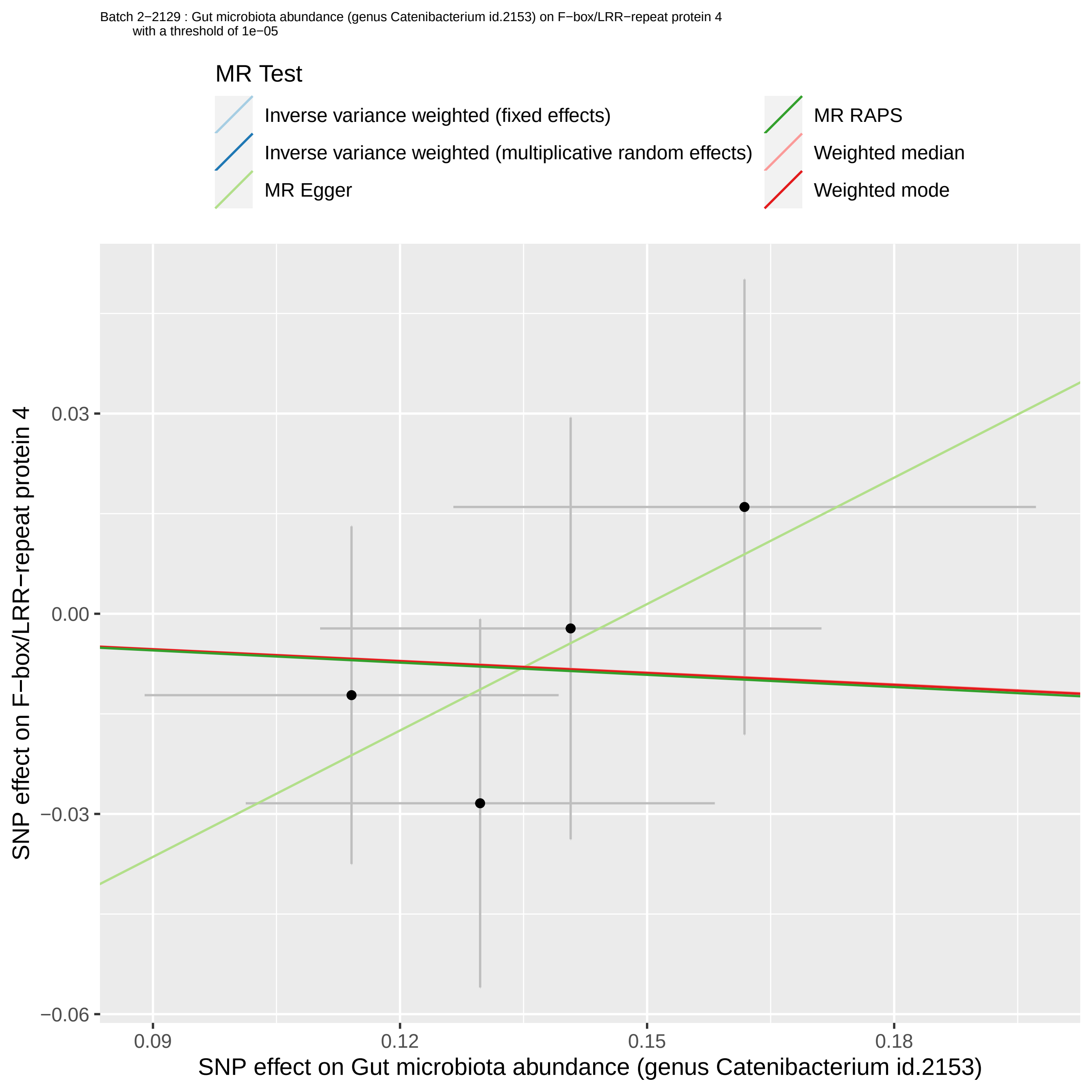

Batch 2−2129 : Gut microbiota abundance (genus Catenibacterium id.2153) on F−box/LRR−repeat protein 4
with a threshold of 1e−05
MR Test
Inverse variance weighted (fixed effects)
Inverse variance weighted (multiplicative random effects)
MR Egger
MR RAPS
Weighted median
Weighted mode
0.03
0.00
−0.03
−0.06
0.09
0.12
0.15
0.18
SNP effect on F−box/LRR−repeat protein 4
SNP effect on Gut microbiota abundance (genus Catenibacterium id.2153)

Batch 2−2130 : Gut microbiota abundance (genus Parasutterella id.2892) on F−box/LRR−repeat protein 4
with a threshold of 1e−05

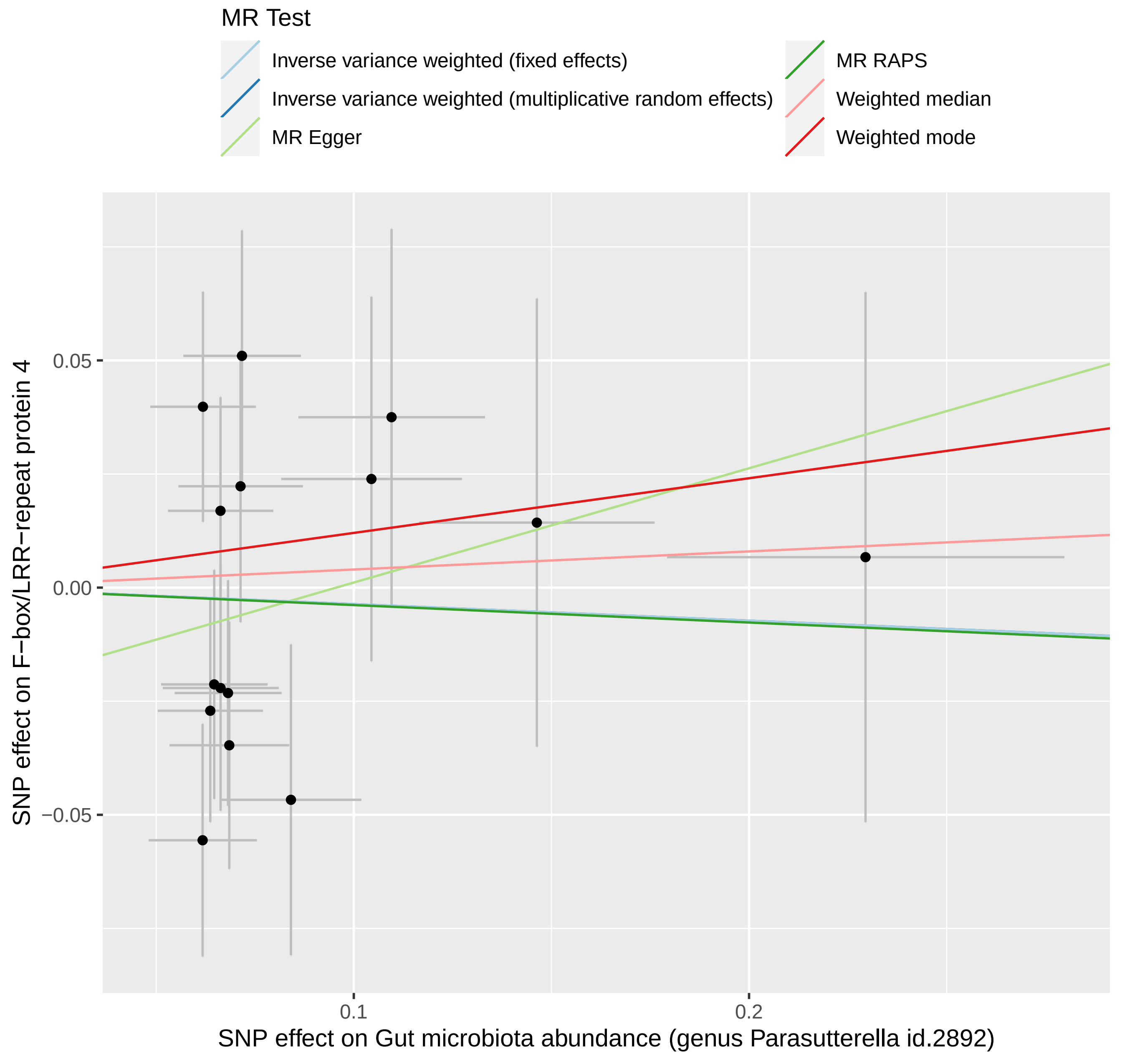

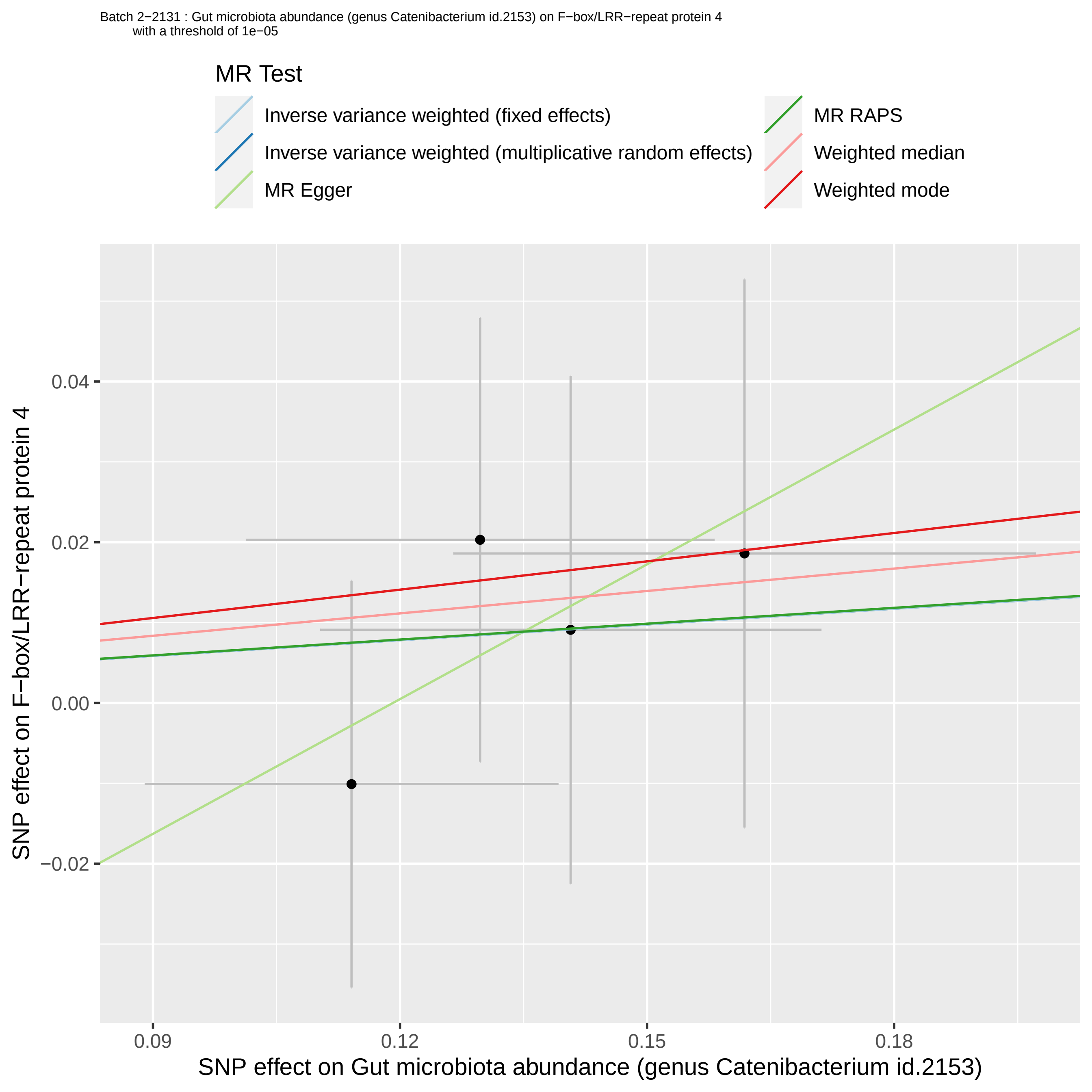

Batch 2−2131 : Gut microbiota abundance (genus Catenibacterium id.2153) on F−box/LRR−repeat protein 4
with a threshold of 1e−05
MR Test
Inverse variance weighted (fixed effects)
Inverse variance weighted (multiplicative random effects)
MR Egger
MR RAPS
Weighted median
Weighted mode
0.04
0.02
0.00
−0.02
0.09
0.12
0.15
0.18
SNP effect on F−box/LRR−repeat protein 4
SNP effect on Gut microbiota abundance (genus Catenibacterium id.2153)

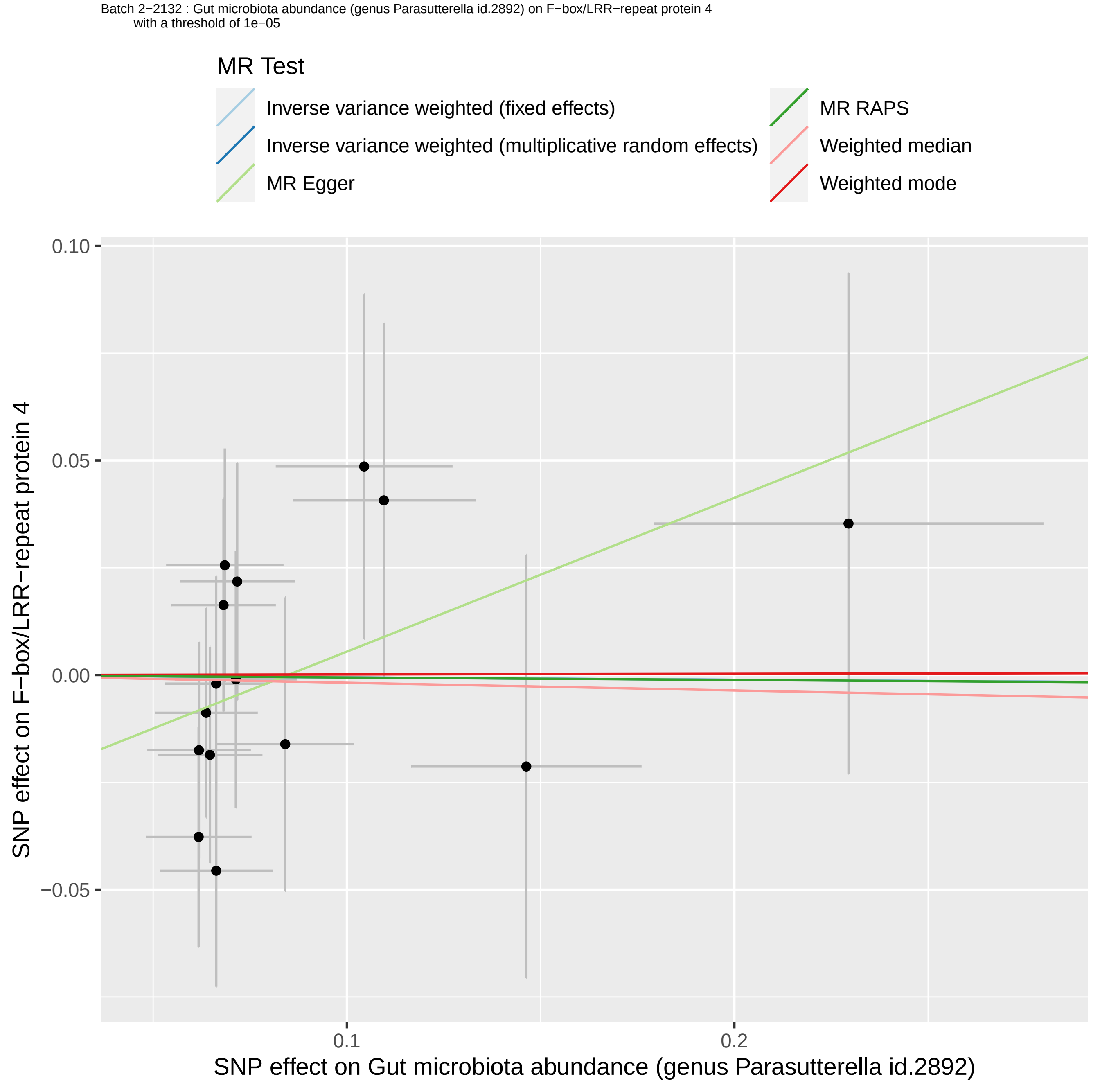
Batch 2−2132 : Gut microbiota abundance (genus Parasutterella id.2892) on F−box/LRR−repeat protein 4
with a threshold of 1e−05
MR Test
Inverse variance weighted (fixed effects)
Inverse variance weighted (multiplicative random effects)
MR Egger
MR RAPS
Weighted median
Weighted mode
0.10
0.05
0.00
−0.05
0.1
0.2
SNP effect on F−box/LRR−repeat protein 4
SNP effect on Gut microbiota abundance (genus Parasutterella id.2892)

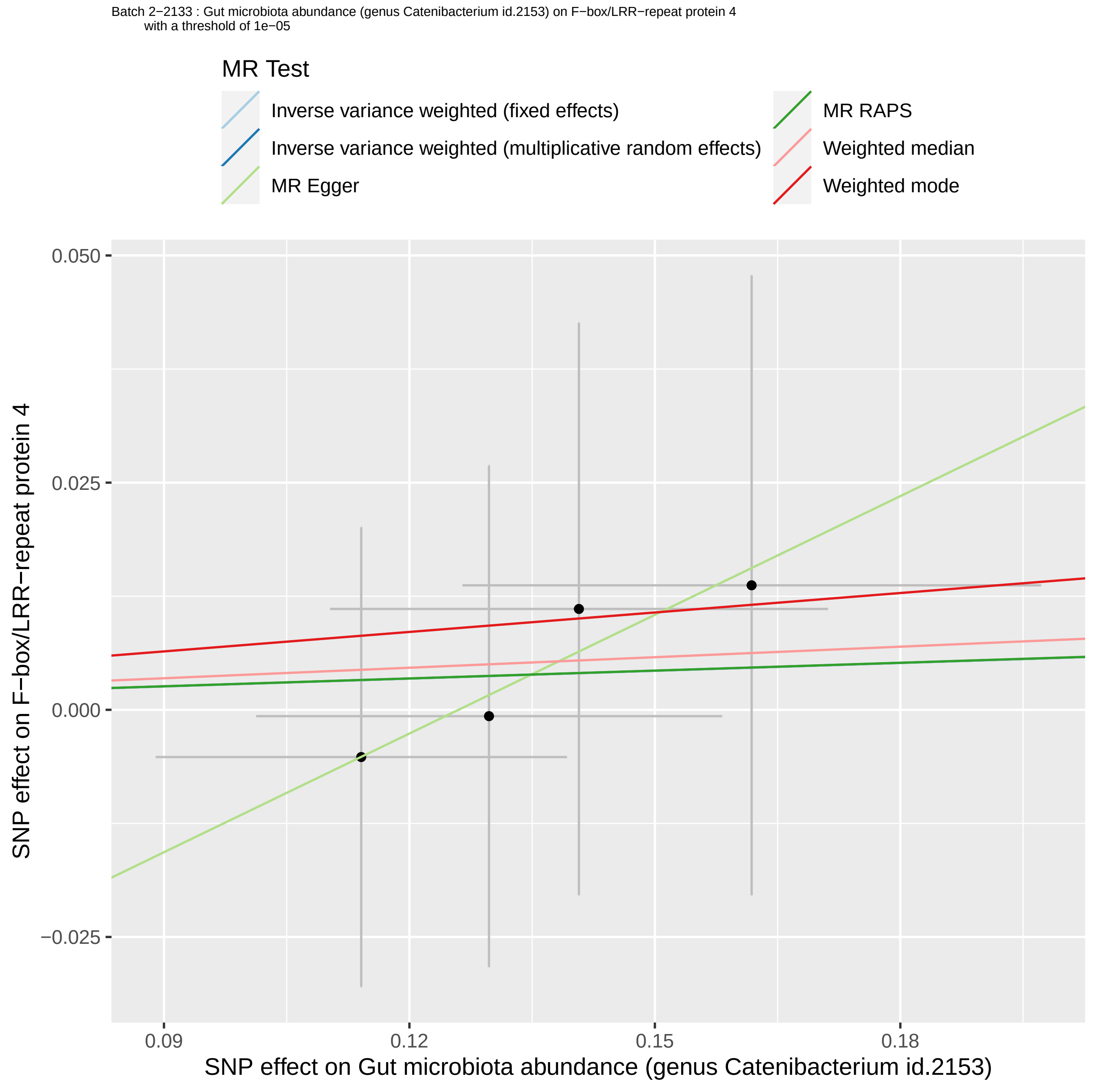
Batch 2−2133 : Gut microbiota abundance (genus Catenibacterium id.2153) on F−box/LRR−repeat protein 4
with a threshold of 1e−05
MR Test
Inverse variance weighted (fixed effects)
Inverse variance weighted (multiplicative random effects)
MR Egger
MR RAPS
Weighted median
Weighted mode
0.050
0.025
0.000
−0.025
0.09
0.12
0.15
0.18
SNP effect on F−box/LRR−repeat protein 4
SNP effect on Gut microbiota abundance (genus Catenibacterium id.2153)

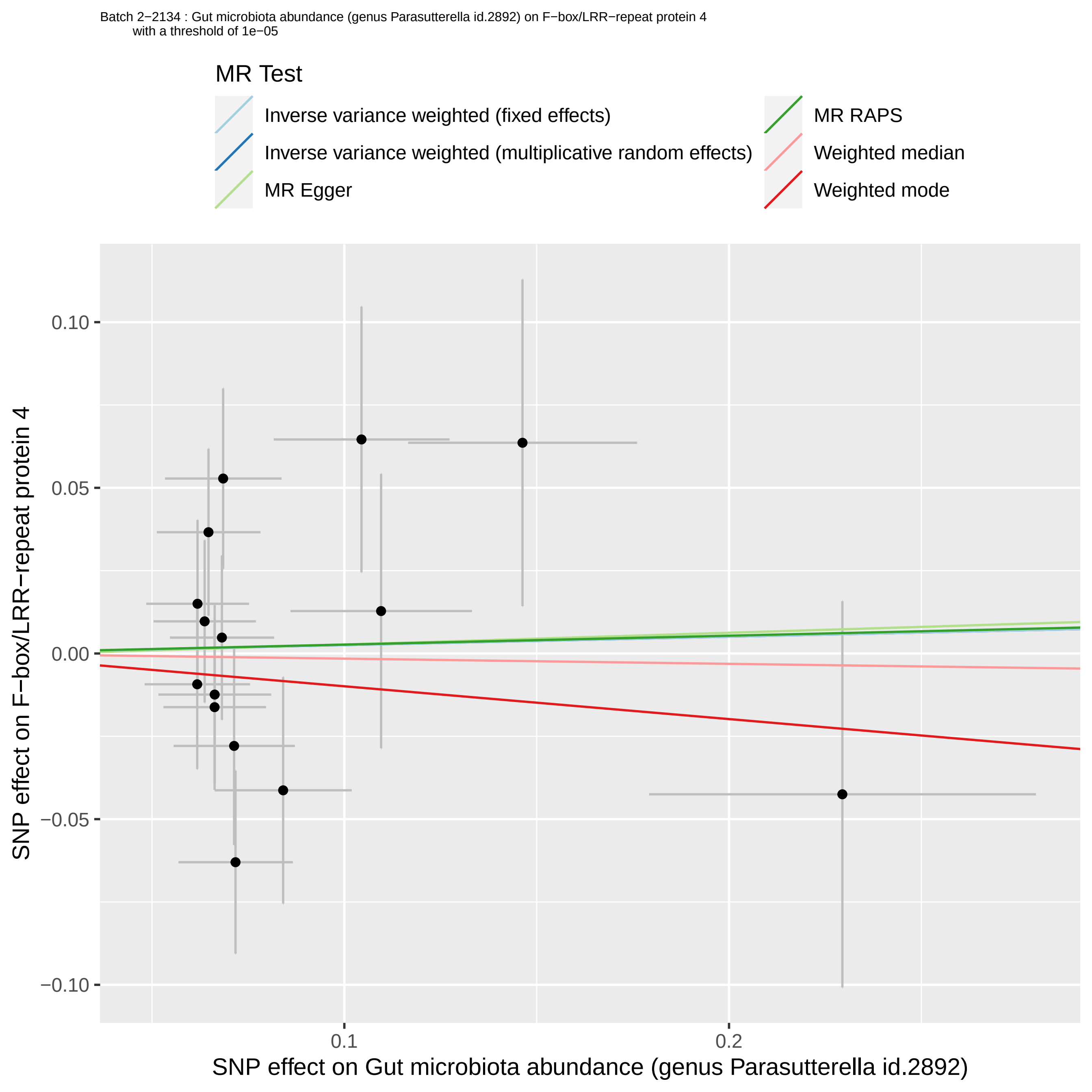

Batch 2−2134 : Gut microbiota abundance (genus Parasutterella id.2892) on F−box/LRR−repeat protein 4
with a threshold of 1e−05
MR Test
Inverse variance weighted (fixed effects)
Inverse variance weighted (multiplicative random effects)
MR Egger
MR RAPS
Weighted median
Weighted mode
0.10
0.05
0.00
−0.05
−0.10
0.1
0.2
SNP effect on F−box/LRR−repeat protein 4
SNP effect on Gut microbiota abundance (genus Parasutterella id.2892)

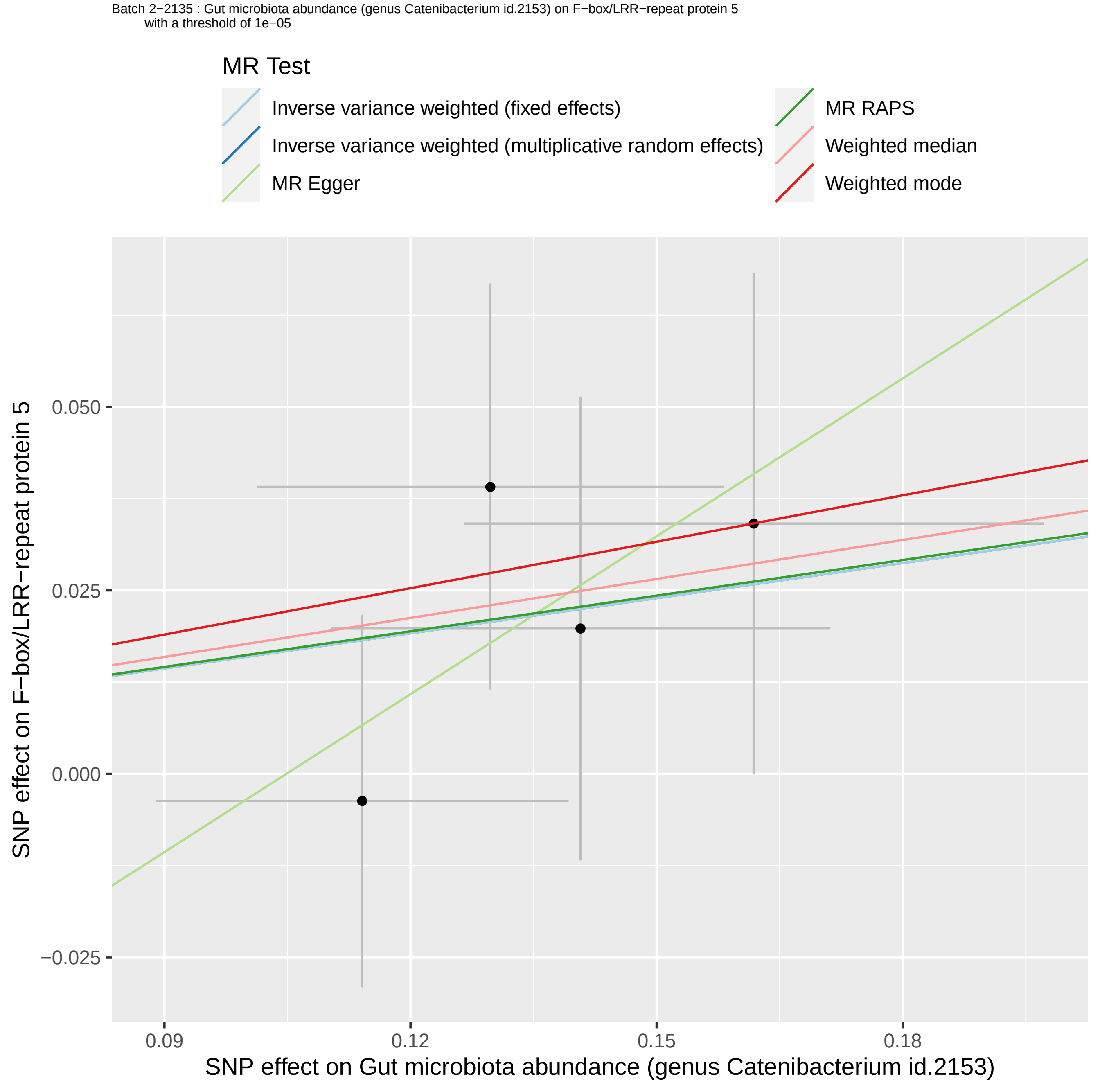

Batch 2−2135 : Gut microbiota abundance (genus Catenibacterium id.2153) on F−box/LRR−repeat protein 5
with a threshold of 1e−05
MR Test
Inverse variance weighted (fixed effects)
Inverse variance weighted (multiplicative random effects)
MR Egger
MR RAPS
Weighted median
Weighted mode
SNP effect on F−box/LRR−repeat protein 5
0.050
0.025
0.000
−0.025
0.09
0.12
0.15
0.18
SNP effect on Gut microbiota abundance (genus Catenibacterium id.2153)

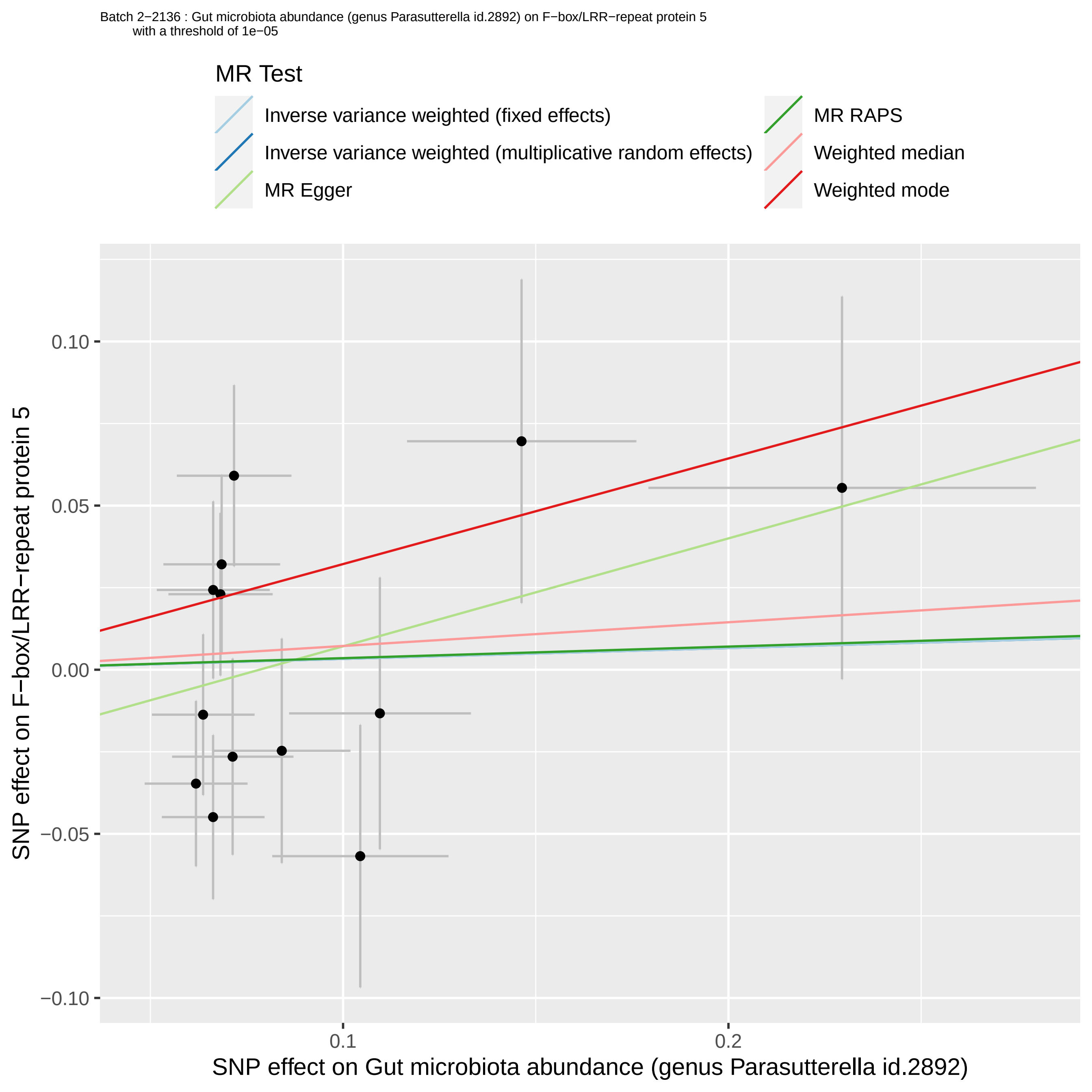

Batch 2−2136 : Gut microbiota abundance (genus Parasutterella id.2892) on F−box/LRR−repeat protein 5
with a threshold of 1e−05
MR Test
Inverse variance weighted (fixed effects)
Inverse variance weighted (multiplicative random effects)
MR Egger
MR RAPS
Weighted median
Weighted mode
0.10
0.05
0.00
−0.05
−0.10
0.1
0.2
SNP effect on F−box/LRR−repeat protein 5
SNP effect on Gut microbiota abundance (genus Parasutterella id.2892)

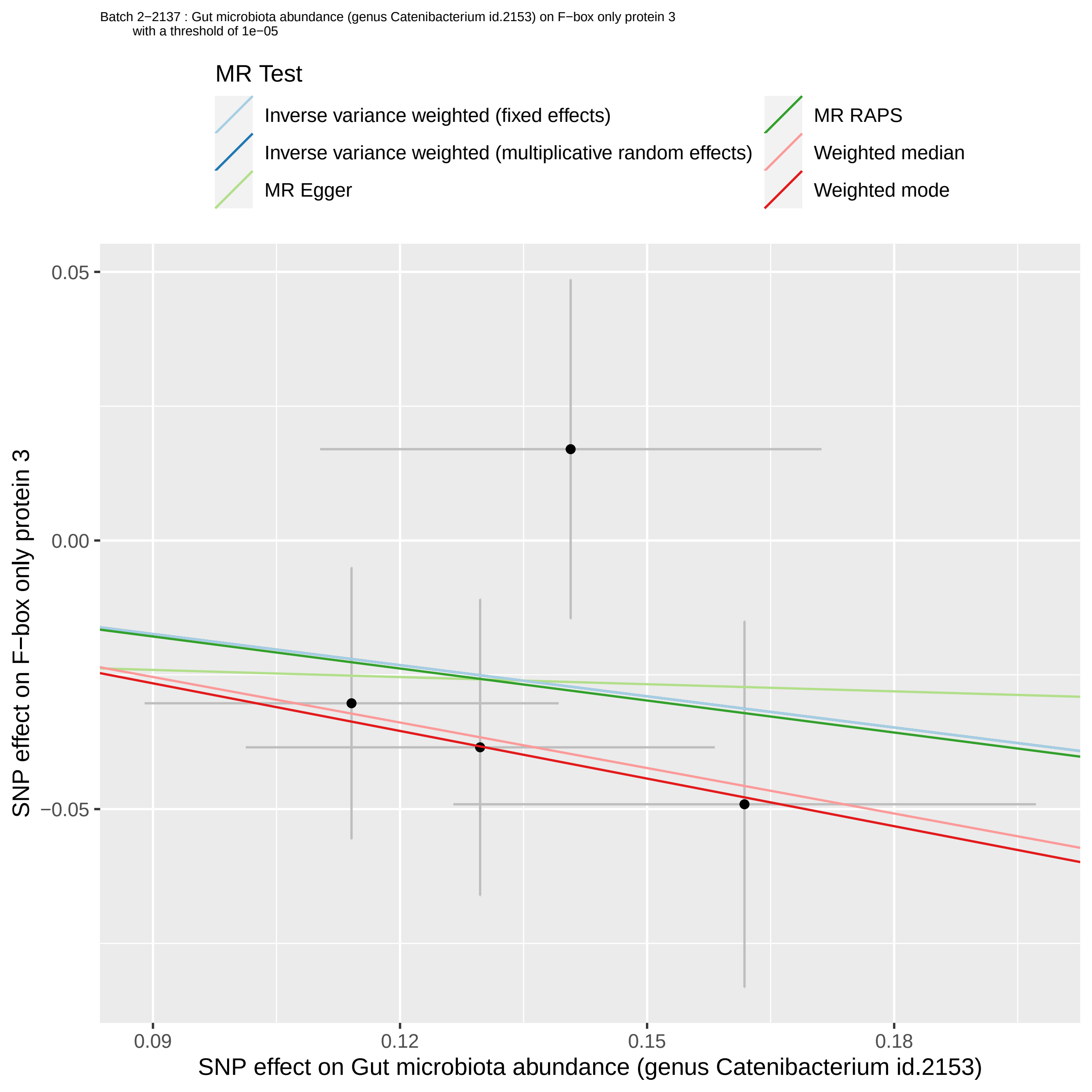

Batch 2−2137 : Gut microbiota abundance (genus Catenibacterium id.2153) on F−box only protein 3
with a threshold of 1e−05
MR Test
Inverse variance weighted (fixed effects)
Inverse variance weighted (multiplicative random effects)
MR Egger
MR RAPS
Weighted median
Weighted mode
0.05
0.00
−0.05
0.09
0.12
0.15
0.18
SNP effect on F−box only protein 3
SNP effect on Gut microbiota abundance (genus Catenibacterium id.2153)

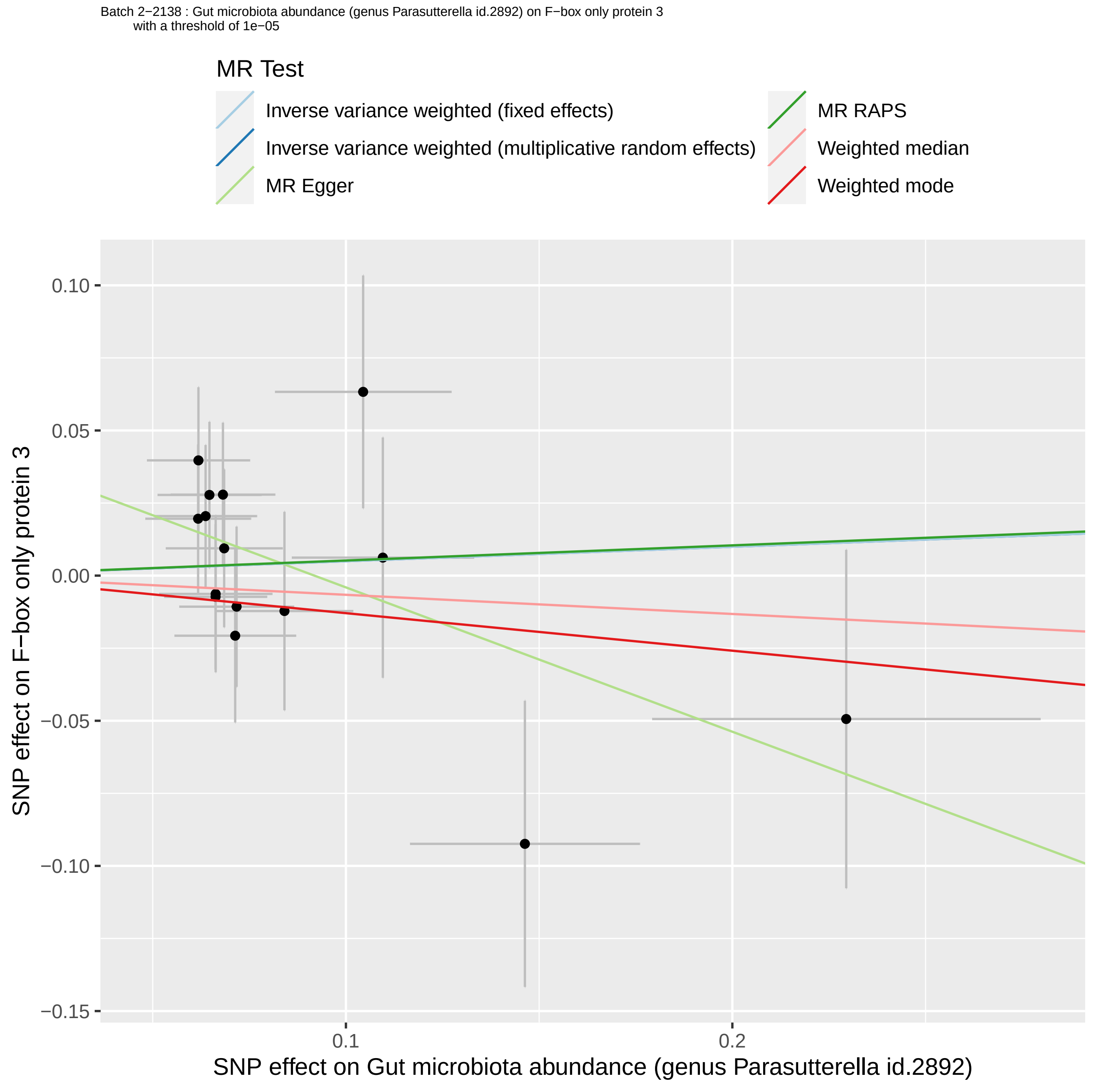
Batch 2−2138 : Gut microbiota abundance (genus Parasutterella id.2892) on F−box only protein 3
with a threshold of 1e−05
MR Test
Inverse variance weighted (fixed effects)
Inverse variance weighted (multiplicative random effects)
MR Egger
MR RAPS
Weighted median
Weighted mode
0.10
0.05
0.00
−0.05
−0.10
−0.15
0.1
0.2
SNP effect on F−box only protein 3
SNP effect on Gut microbiota abundance (genus Parasutterella id.2892)

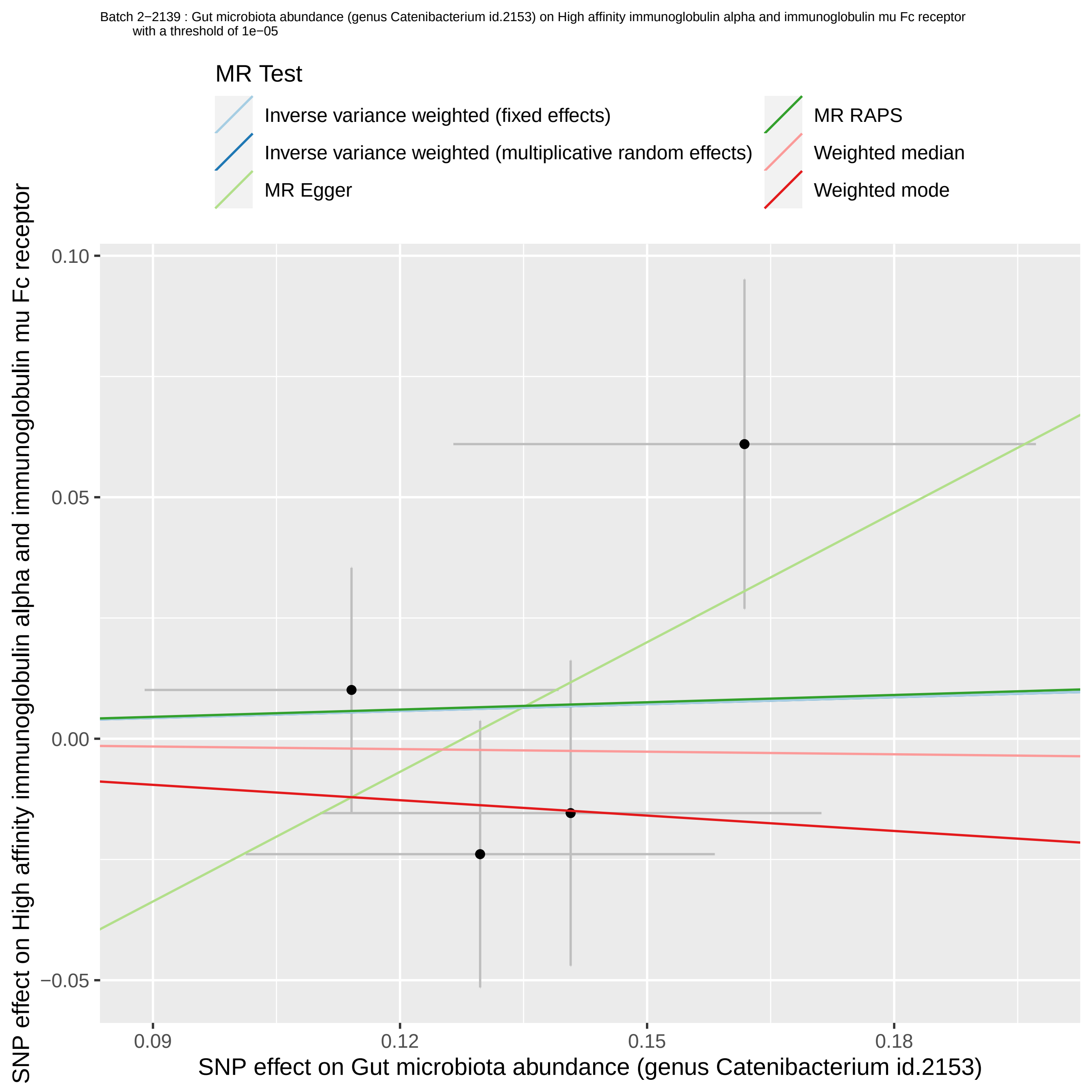
Batch 2−2139 : Gut microbiota abundance (genus Catenibacterium id.2153) on High affinity immunoglobulin alpha and immunoglobulin mu Fc receptor with a threshold of 1e−05
MR Test
Inverse variance weighted (fixed effects)
Inverse variance weighted (multiplicative random effects)
MR Egger
MR RAPS
Weighted median
Weighted mode
0.10
0.05
0.00
−0.05
0.09
0.12
0.15
0.18
SNP effect on High affinity immunoglobulin alpha and immunoglobulin mu Fc receptor
SNP effect on Gut microbiota abundance (genus Catenibacterium id.2153)

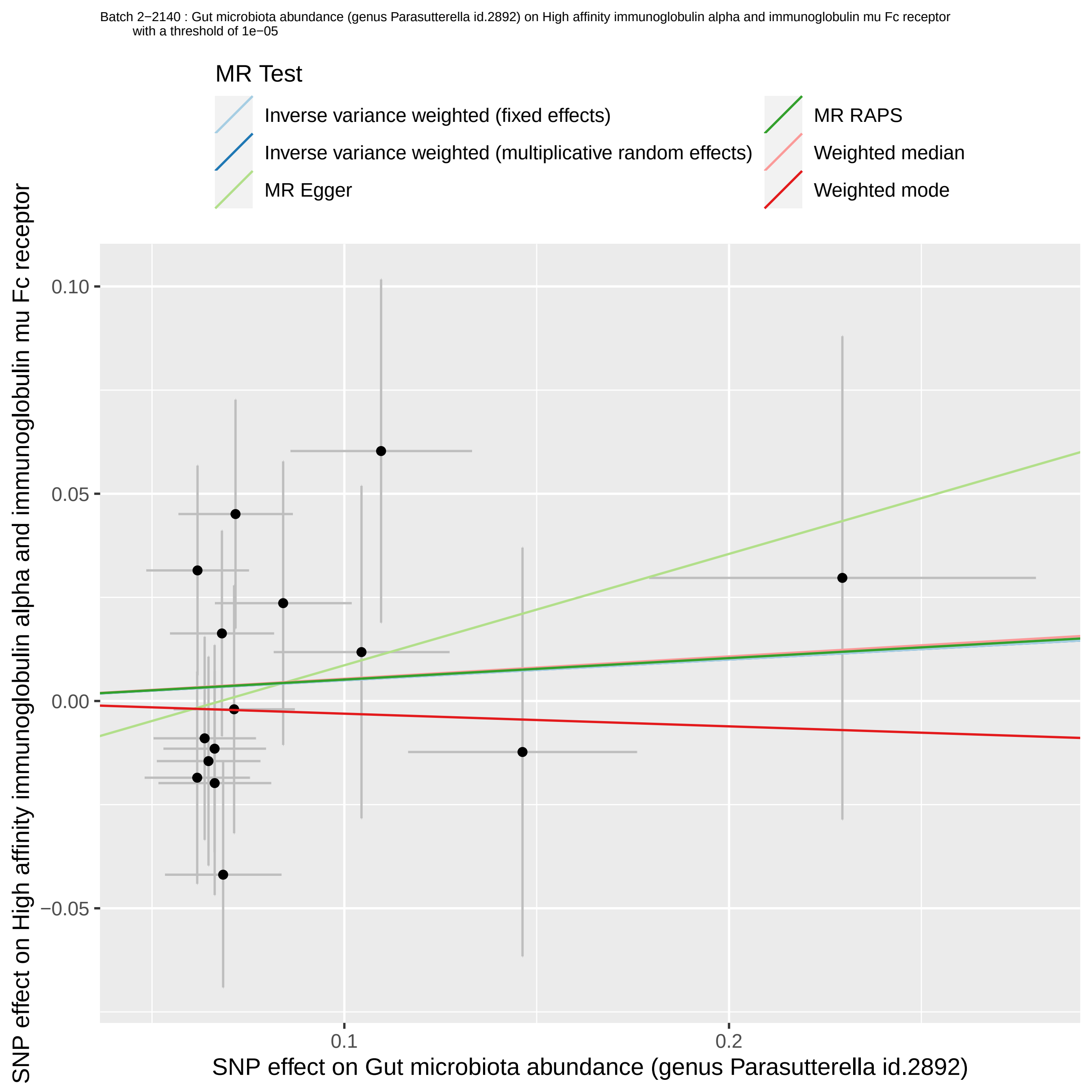

Batch 2−2140 : Gut microbiota abundance (genus Parasutterella id.2892) on High affinity immunoglobulin alpha and immunoglobulin mu Fc receptor with a threshold of 1e−05
MR Test
Inverse variance weighted (fixed effects)
Inverse variance weighted (multiplicative random effects)
MR Egger
MR RAPS
Weighted median
Weighted mode
0.10
0.05
0.00
−0.05
0.1
0.2
SNP effect on High affinity immunoglobulin alpha and immunoglobulin mu Fc receptor
SNP effect on Gut microbiota abundance (genus Parasutterella id.2892)

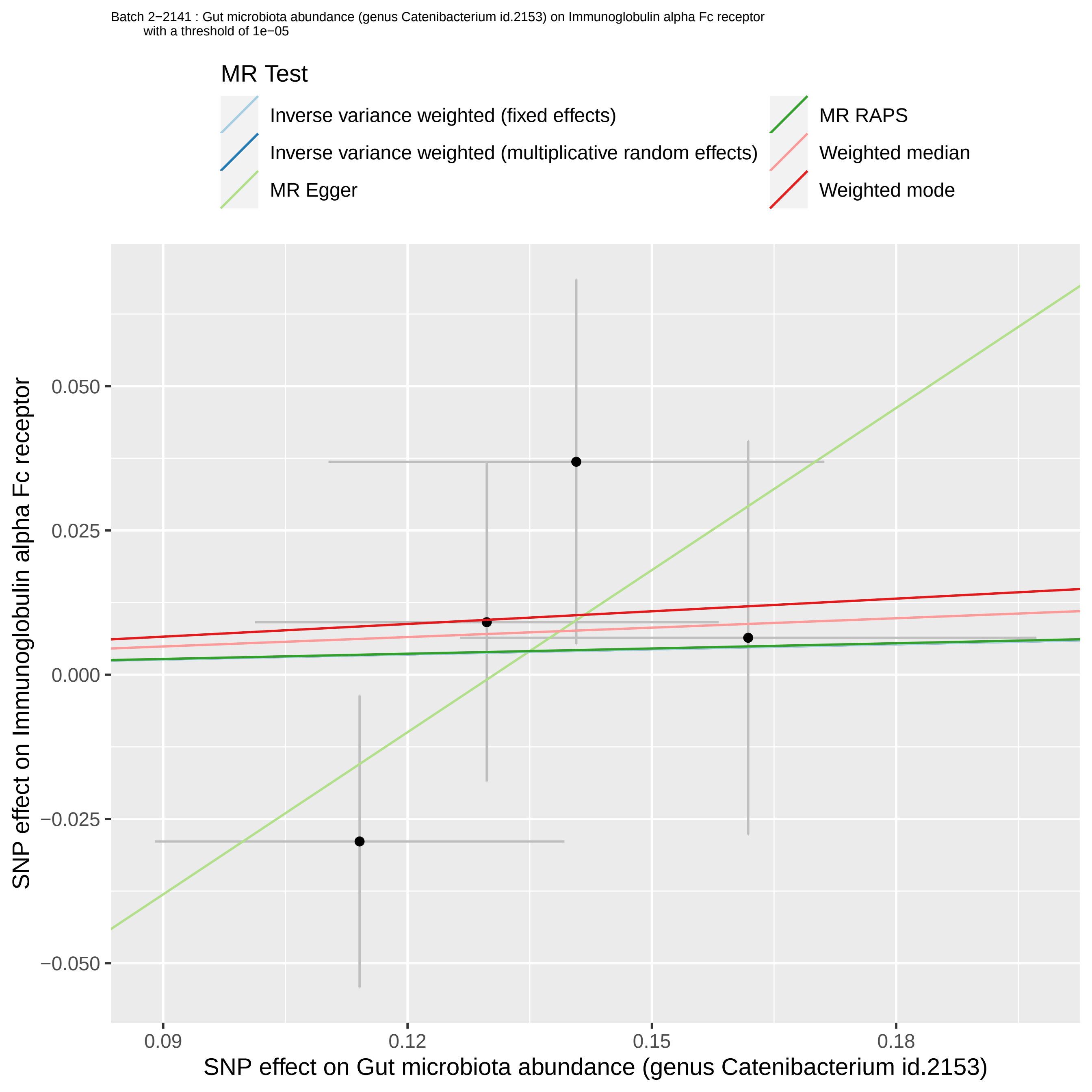
Batch 2−2141 : Gut microbiota abundance (genus Catenibacterium id.2153) on Immunoglobulin alpha Fc receptor
with a threshold of 1e−05
MR Test
Inverse variance weighted (fixed effects)
Inverse variance weighted (multiplicative random effects)
MR Egger
MR RAPS
Weighted median
Weighted mode
0.050
0.025
0.000
−0.025
−0.050
0.09
0.12
0.15
0.18
SNP effect on Immunoglobulin alpha Fc receptor
SNP effect on Gut microbiota abundance (genus Catenibacterium id.2153)

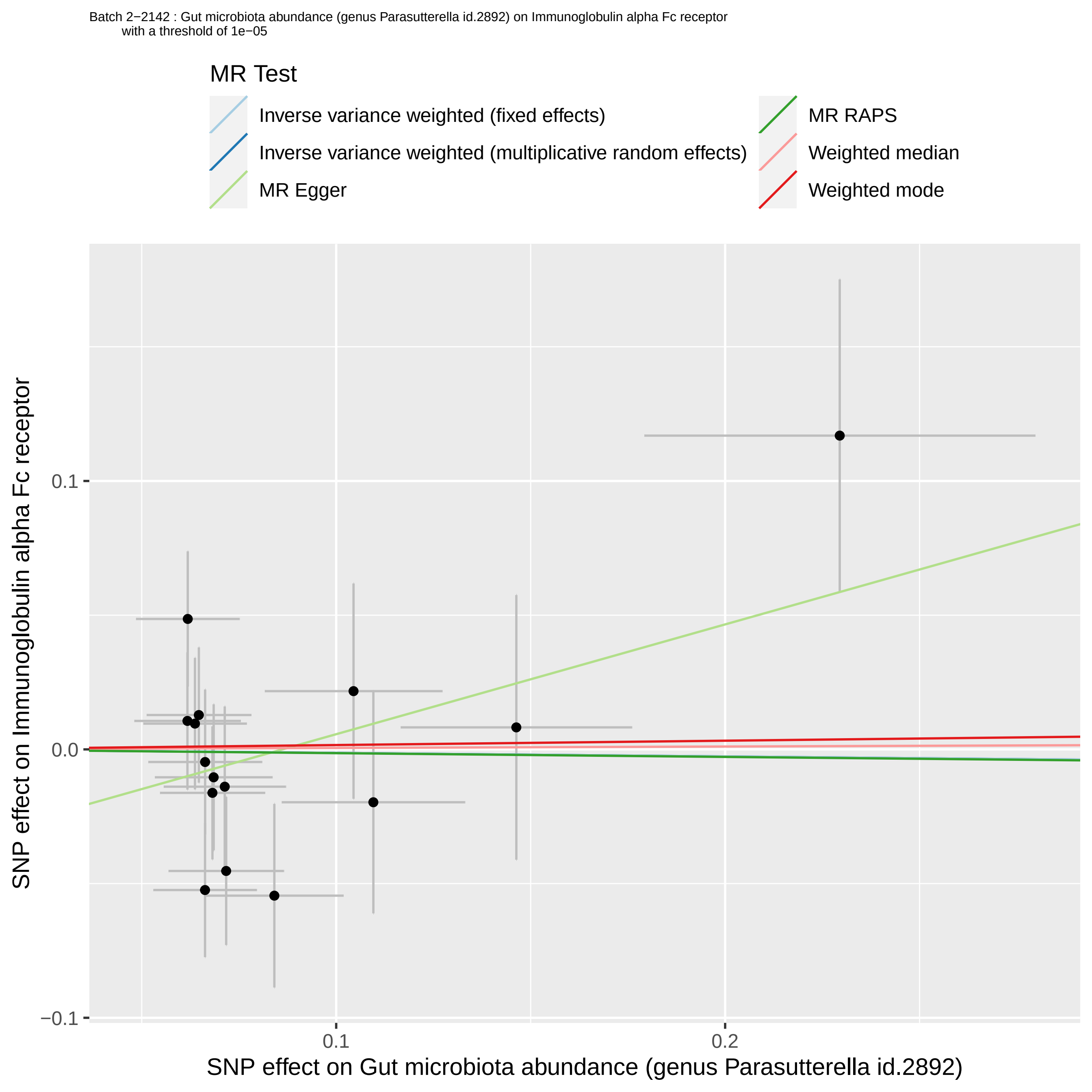
Batch 2−2142 : Gut microbiota abundance (genus Parasutterella id.2892) on Immunoglobulin alpha Fc receptor
with a threshold of 1e−05
MR Test
Inverse variance weighted (fixed effects)
Inverse variance weighted (multiplicative random effects)
MR Egger
MR RAPS
Weighted median
Weighted mode
0.1
0.0
−0.1
0.1
0.2
SNP effect on Immunoglobulin alpha Fc receptor
SNP effect on Gut microbiota abundance (genus Parasutterella id.2892)

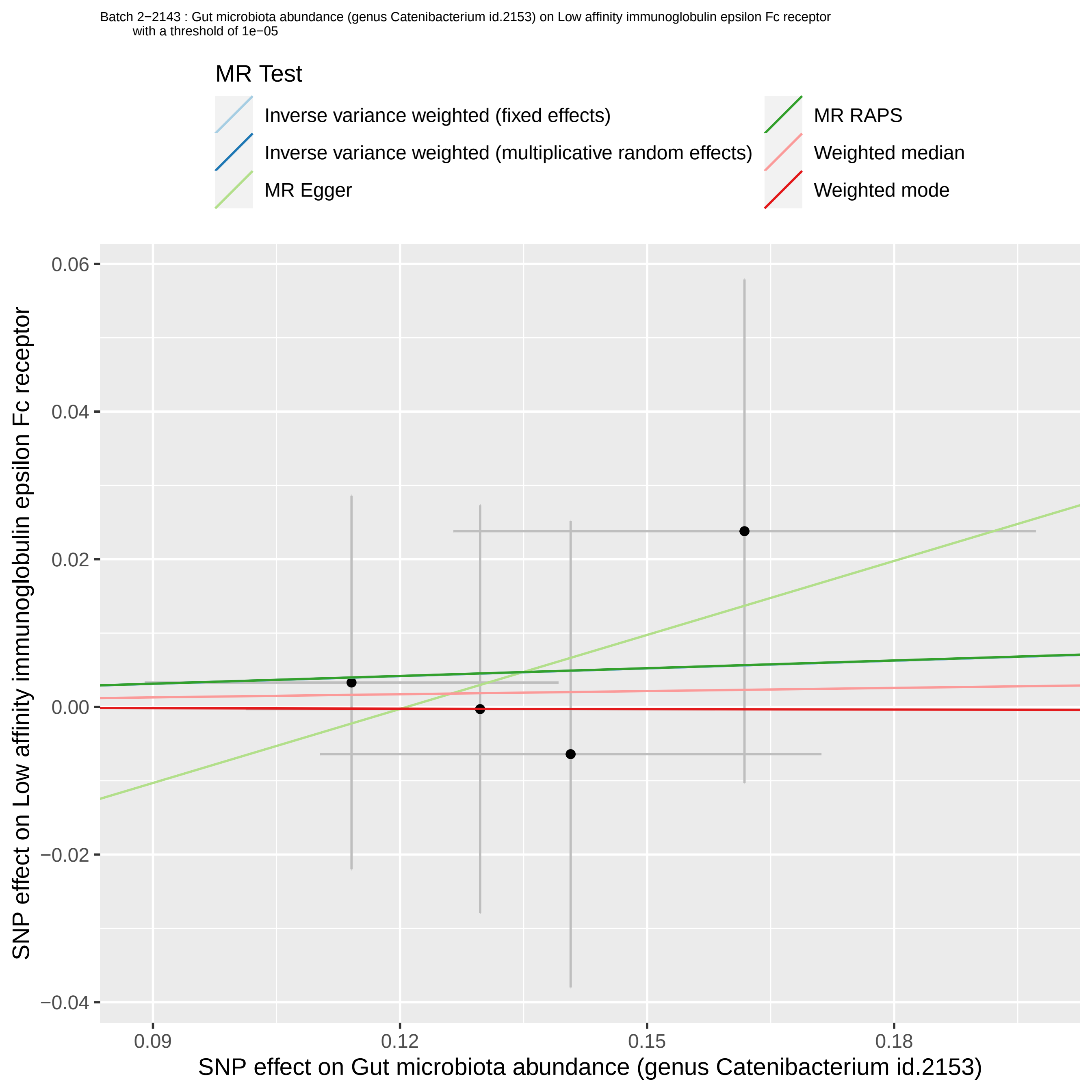
Batch 2−2143 : Gut microbiota abundance (genus Catenibacterium id.2153) on Low affinity immunoglobulin epsilon Fc receptor with a threshold of 1e−05
MR Test
Inverse variance weighted (fixed effects)
Inverse variance weighted (multiplicative random effects)
MR Egger
MR RAPS
Weighted median
Weighted mode
0.06
0.04
0.02
0.00
−0.02
−0.04
0.09
0.12
0.15
0.18
SNP effect on Low affinity immunoglobulin epsilon Fc receptor
SNP effect on Gut microbiota abundance (genus Catenibacterium id.2153)

Batch 2−2144 : Gut microbiota abundance (genus Parasutterella id.2892) on Low affinity immunoglobulin epsilon Fc receptor
with a threshold of 1e−05

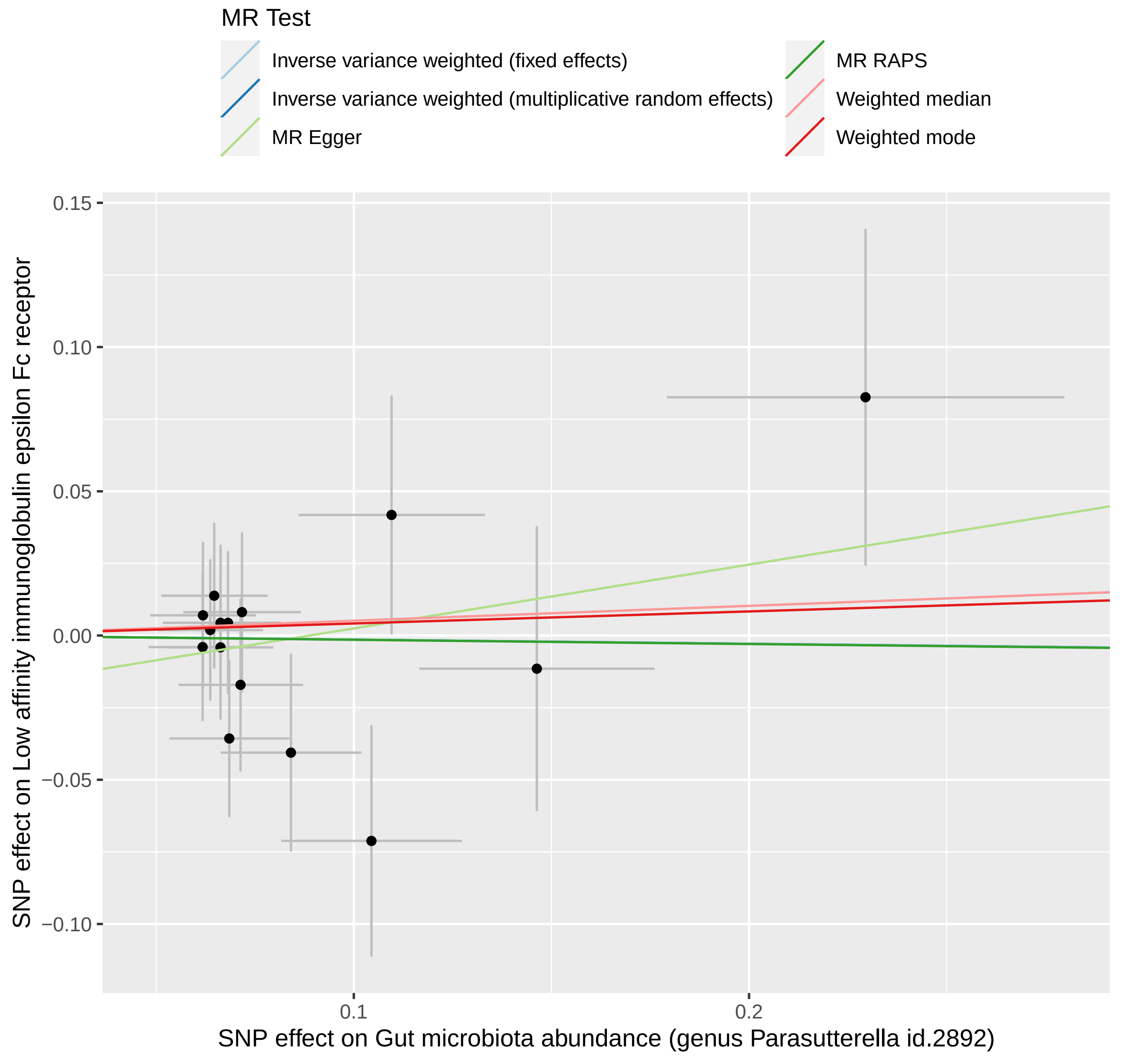

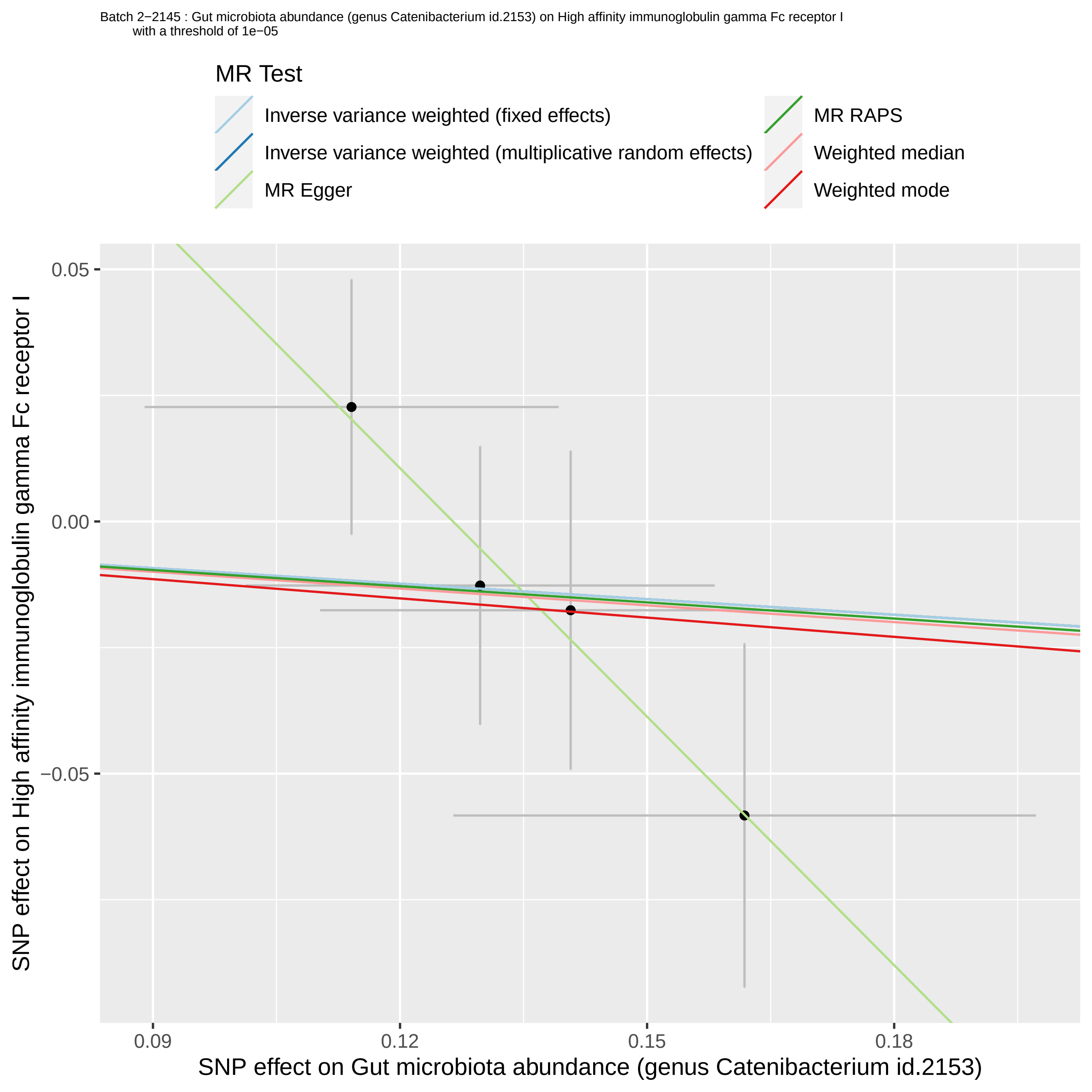
Batch 2−2145 : Gut microbiota abundance (genus Catenibacterium id.2153) on High affinity immunoglobulin gamma Fc receptor I
with a threshold of 1e−05
MR Test
Inverse variance weighted (fixed effects)
Inverse variance weighted (multiplicative random effects)
MR Egger
MR RAPS
Weighted median
Weighted mode
0.05
0.00
−0.05
0.09
0.12
0.15
0.18
SNP effect on High affinity immunoglobulin gamma Fc receptor I
SNP effect on Gut microbiota abundance (genus Catenibacterium id.2153)

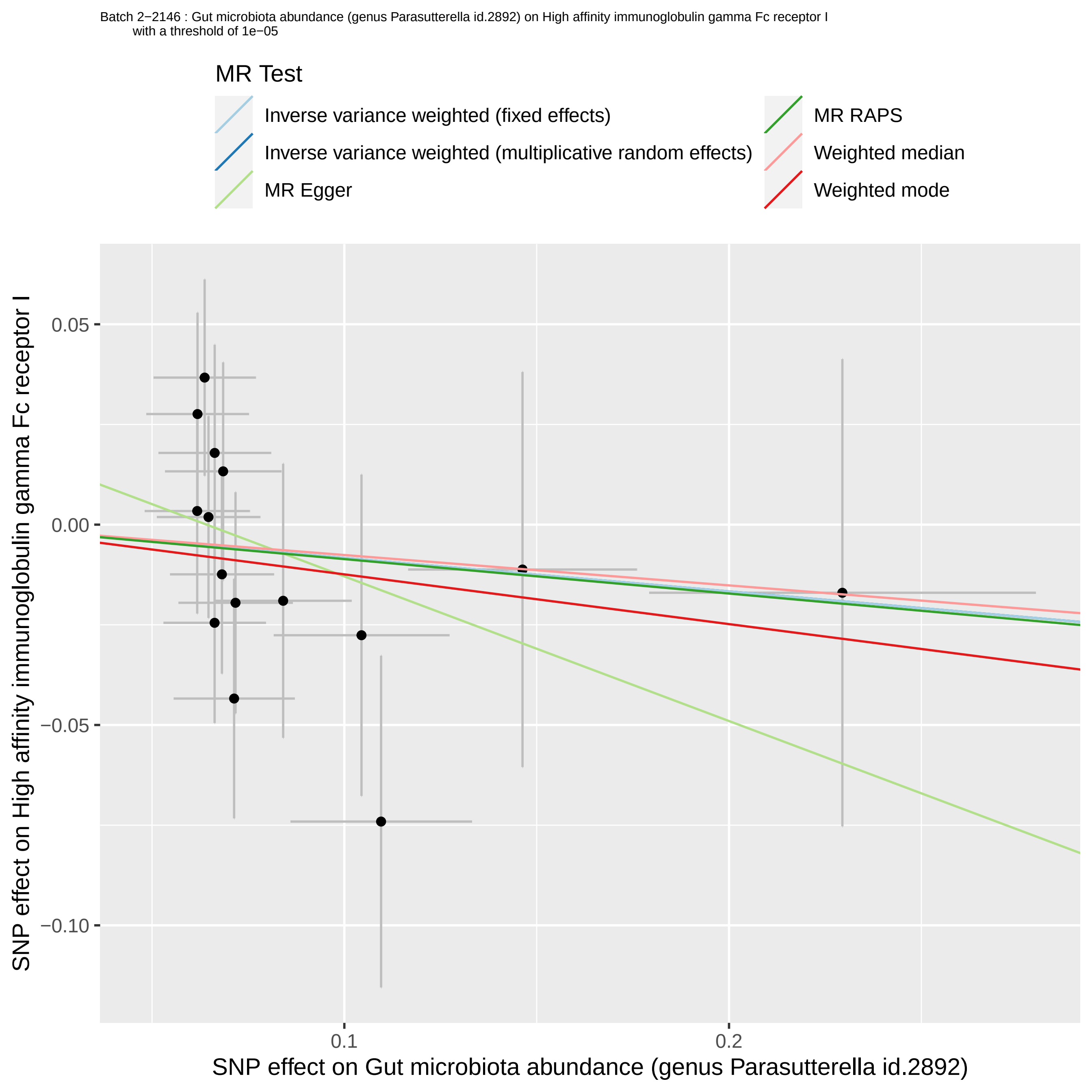
Batch 2−2146 : Gut microbiota abundance (genus Parasutterella id.2892) on High affinity immunoglobulin gamma Fc receptor I
with a threshold of 1e−05
MR Test
Inverse variance weighted (fixed effects)
Inverse variance weighted (multiplicative random effects)
MR Egger
MR RAPS
Weighted median
Weighted mode
0.05
0.00
−0.05
−0.10
0.1
0.2
SNP effect on High affinity immunoglobulin gamma Fc receptor I
SNP effect on Gut microbiota abundance (genus Parasutterella id.2892)

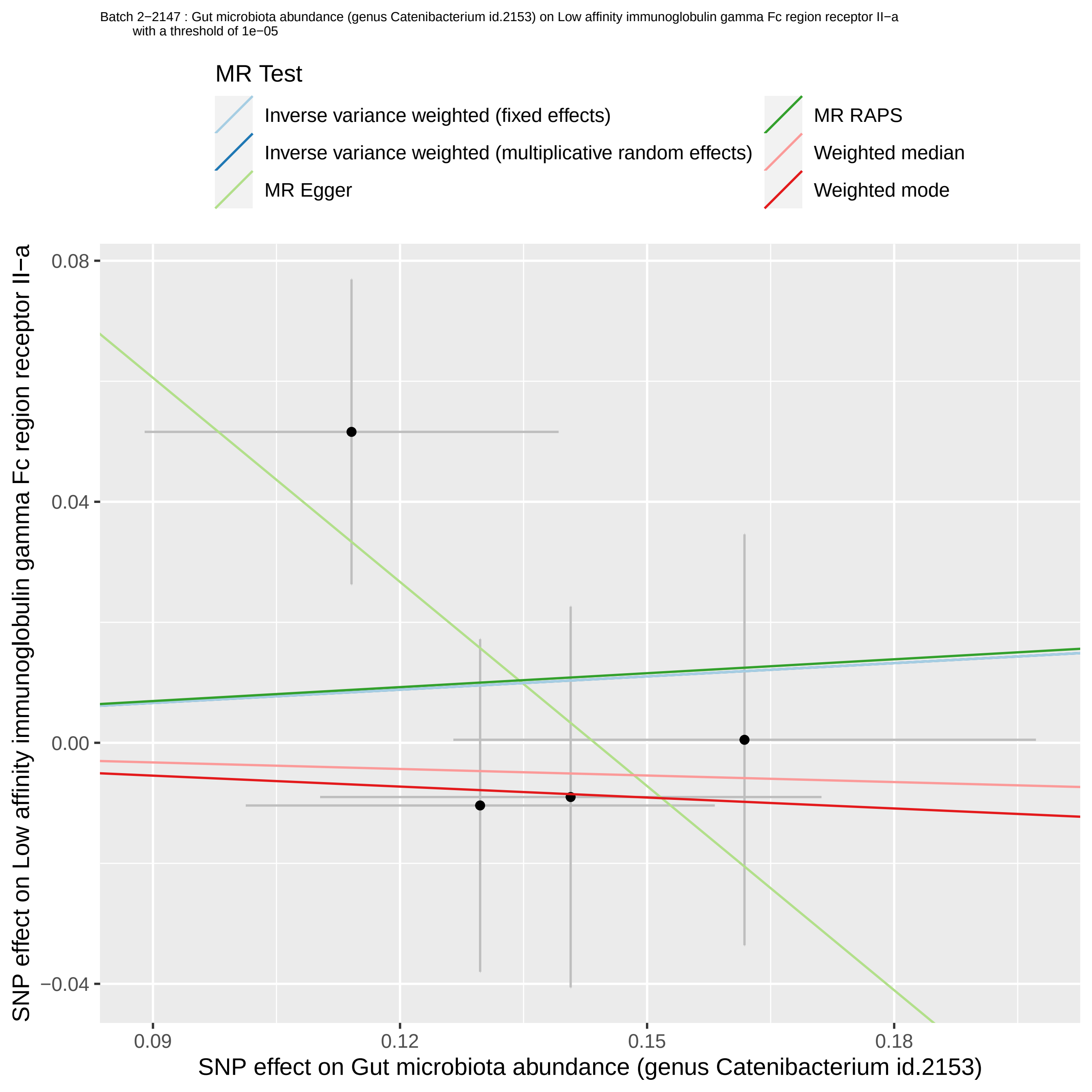
Batch 2−2147 : Gut microbiota abundance (genus Catenibacterium id.2153) on Low affinity immunoglobulin gamma Fc region receptor II−a
with a threshold of 1e−05
MR Test
Inverse variance weighted (fixed effects)
Inverse variance weighted (multiplicative random effects)
MR Egger
MR RAPS
Weighted median
Weighted mode
0.08
0.04
0.00
−0.04
0.09
0.12
0.15
0.18
SNP effect on Low affinity immunoglobulin gamma Fc region receptor II−a
SNP effect on Gut microbiota abundance (genus Catenibacterium id.2153)

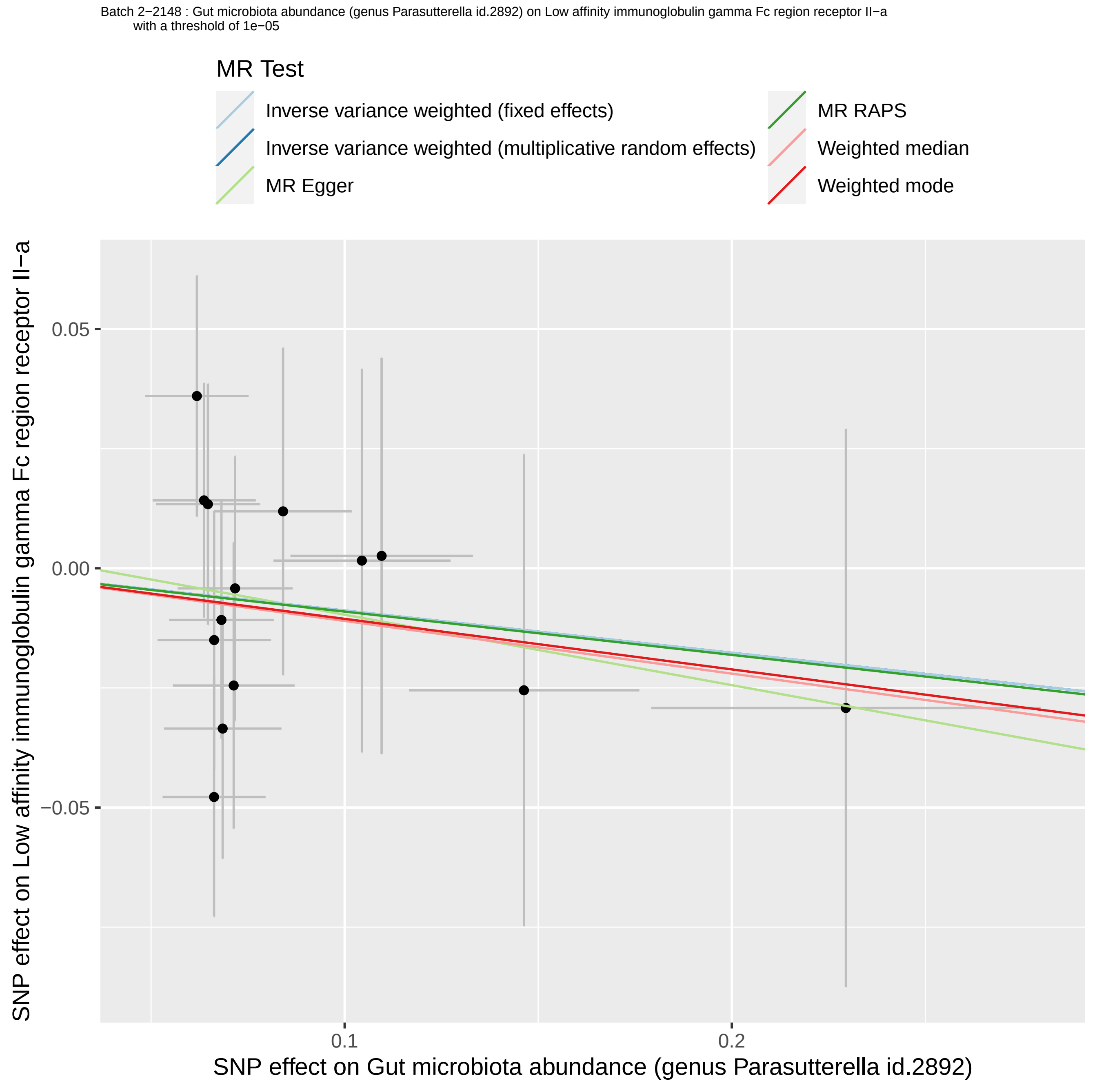
Batch 2−2148 : Gut microbiota abundance (genus Parasutterella id.2892) on Low affinity immunoglobulin gamma Fc region receptor II−a
with a threshold of 1e−05
MR Test
Inverse variance weighted (fixed effects)
Inverse variance weighted (multiplicative random effects)
MR Egger
MR RAPS
Weighted median
Weighted mode
0.05
0.00
−0.05
0.1
0.2
SNP effect on Low affinity immunoglobulin gamma Fc region receptor II−a
SNP effect on Gut microbiota abundance (genus Parasutterella id.2892)

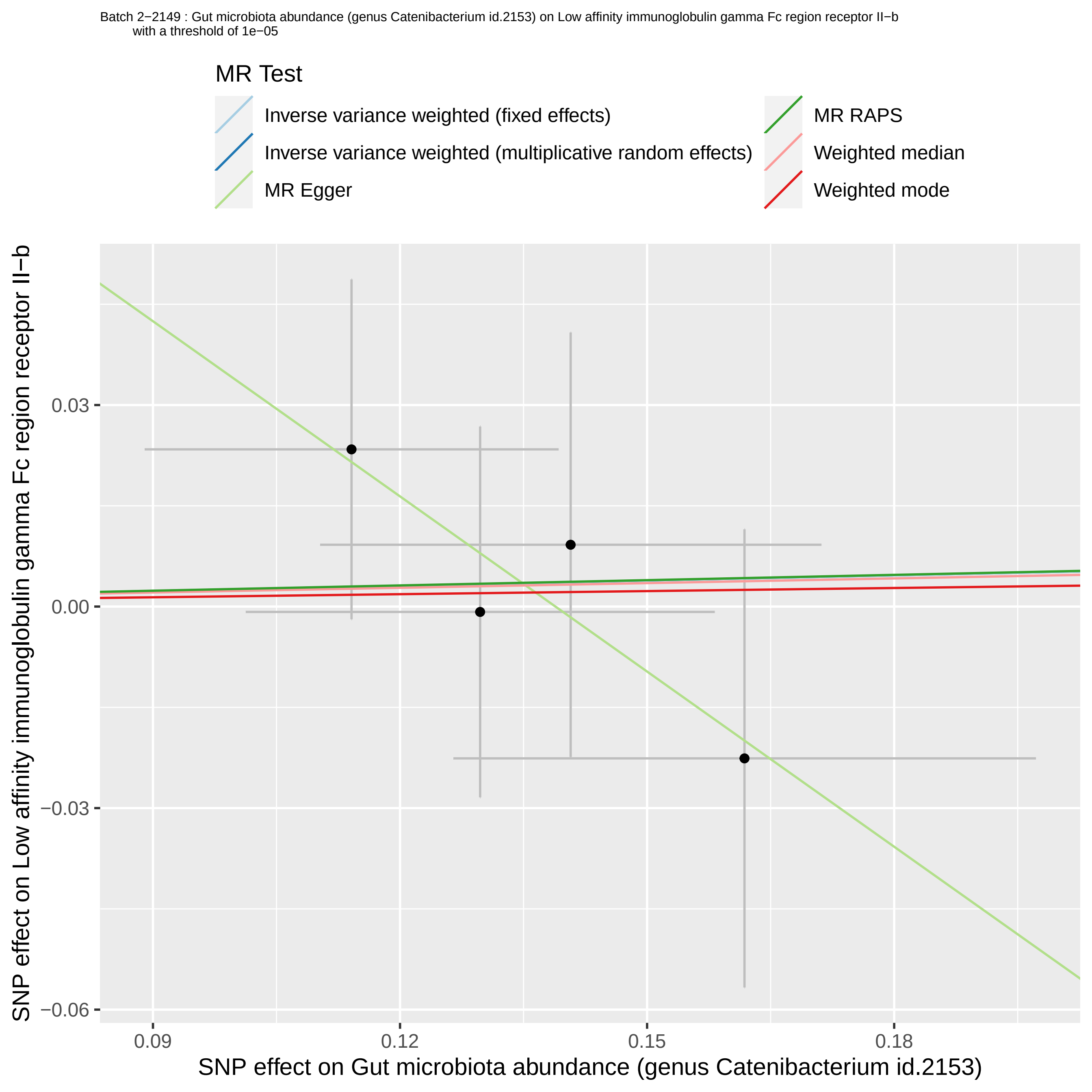
Batch 2−2149 : Gut microbiota abundance (genus Catenibacterium id.2153) on Low affinity immunoglobulin gamma Fc region receptor II−b
with a threshold of 1e−05
MR Test
Inverse variance weighted (fixed effects)
Inverse variance weighted (multiplicative random effects)
MR Egger
MR RAPS
Weighted median
Weighted mode
0.03
0.00
−0.03
−0.06
0.09
0.12
0.15
0.18
SNP effect on Low affinity immunoglobulin gamma Fc region receptor II−b
SNP effect on Gut microbiota abundance (genus Catenibacterium id.2153)

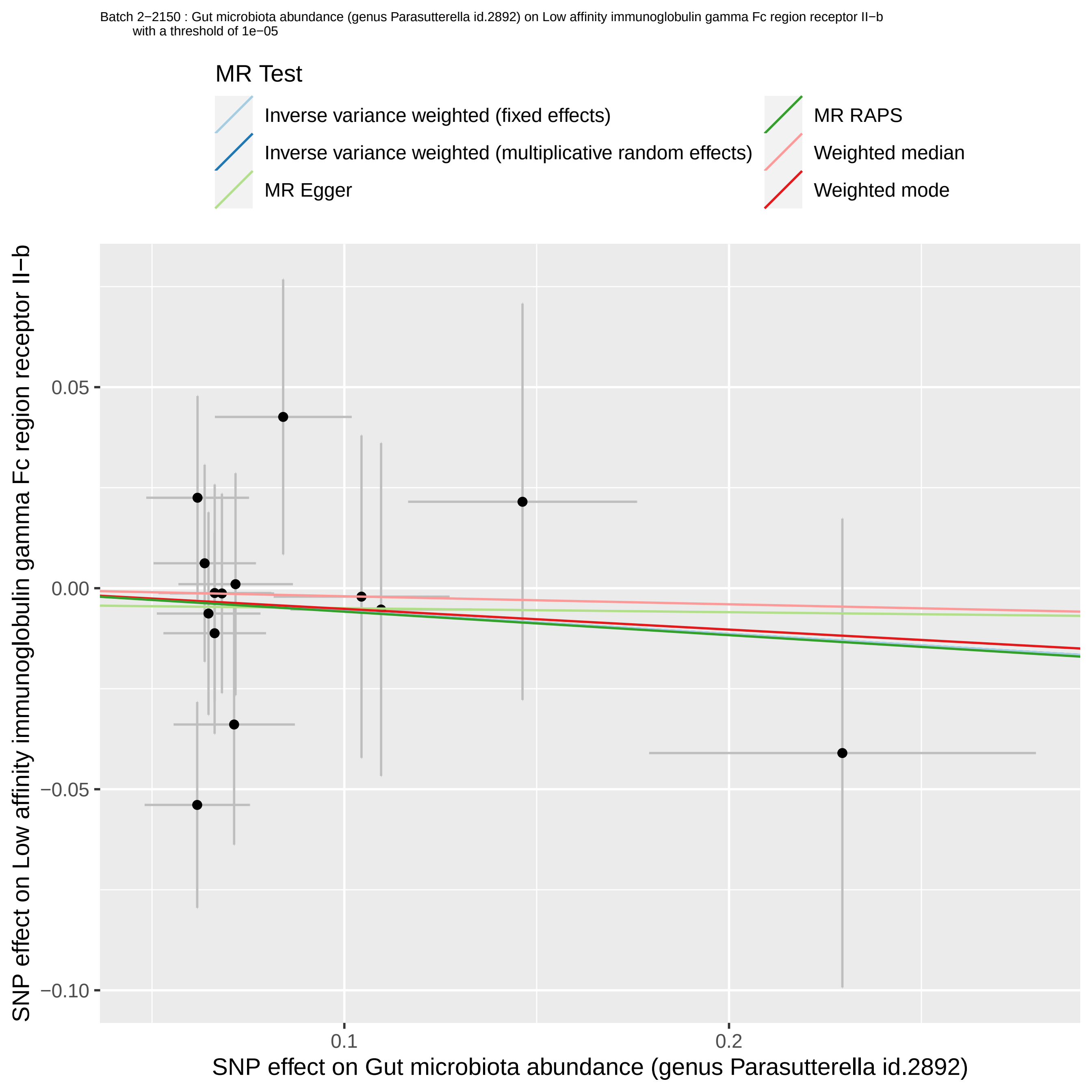
Batch 2−2150 : Gut microbiota abundance (genus Parasutterella id.2892) on Low affinity immunoglobulin gamma Fc region receptor II−b
with a threshold of 1e−05
MR Test
Inverse variance weighted (fixed effects)
Inverse variance weighted (multiplicative random effects)
MR Egger
MR RAPS
Weighted median
Weighted mode
SNP effect on Low affinity immunoglobulin gamma Fc region receptor II−b
0.05
0.00
−0.05
−0.10
0.1
0.2
SNP effect on Gut microbiota abundance (genus Parasutterella id.2892)

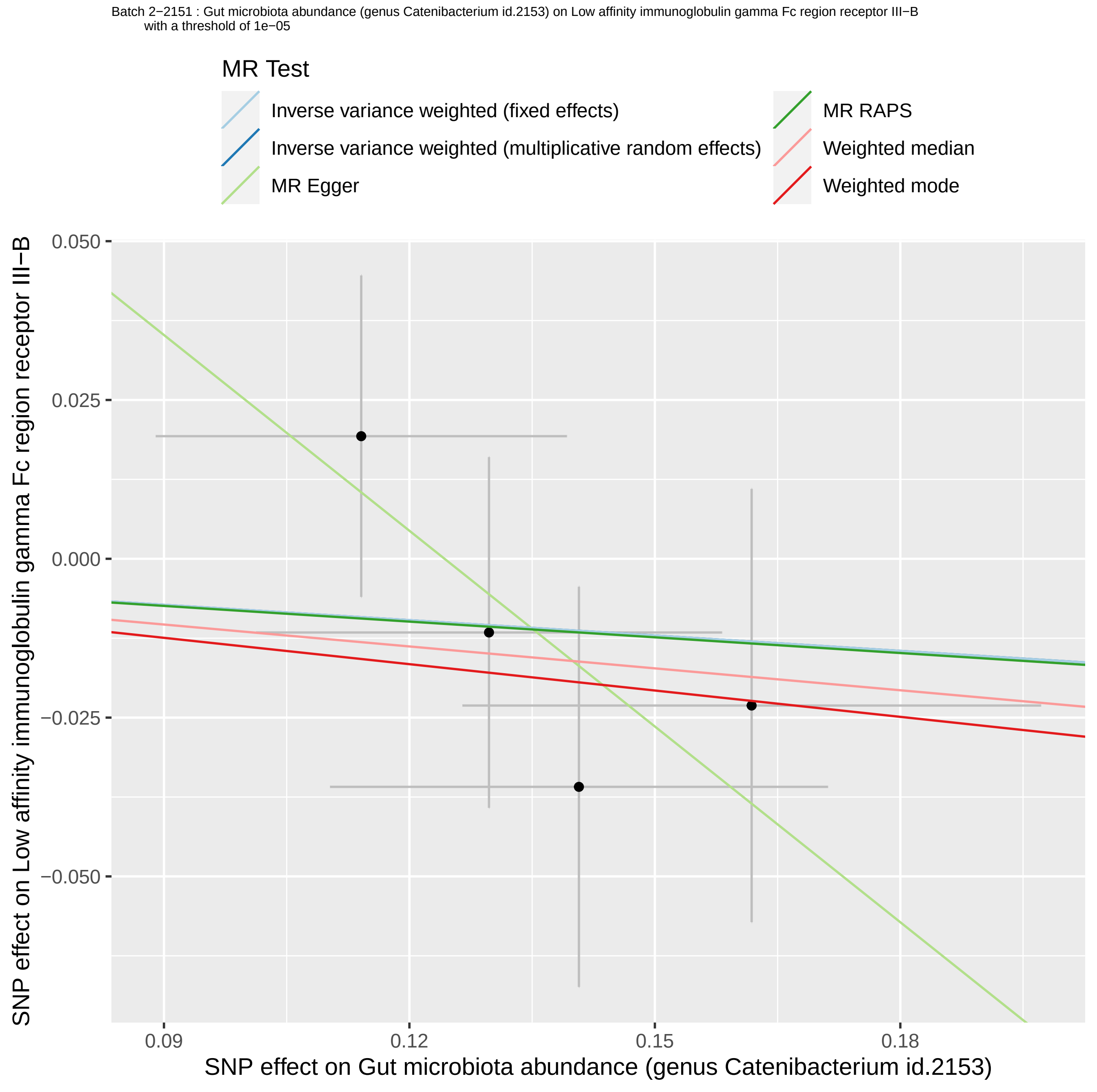

Batch 2−2151 : Gut microbiota abundance (genus Catenibacterium id.2153) on Low affinity immunoglobulin gamma Fc region receptor III−B
with a threshold of 1e−05
MR Test
Inverse variance weighted (fixed effects)
Inverse variance weighted (multiplicative random effects)
MR Egger
MR RAPS
Weighted median
Weighted mode
0.050
0.025
0.000
−0.025
−0.050
0.09
0.12
0.15
0.18
SNP effect on Low affinity immunoglobulin gamma Fc region receptor III−B
SNP effect on Gut microbiota abundance (genus Catenibacterium id.2153)

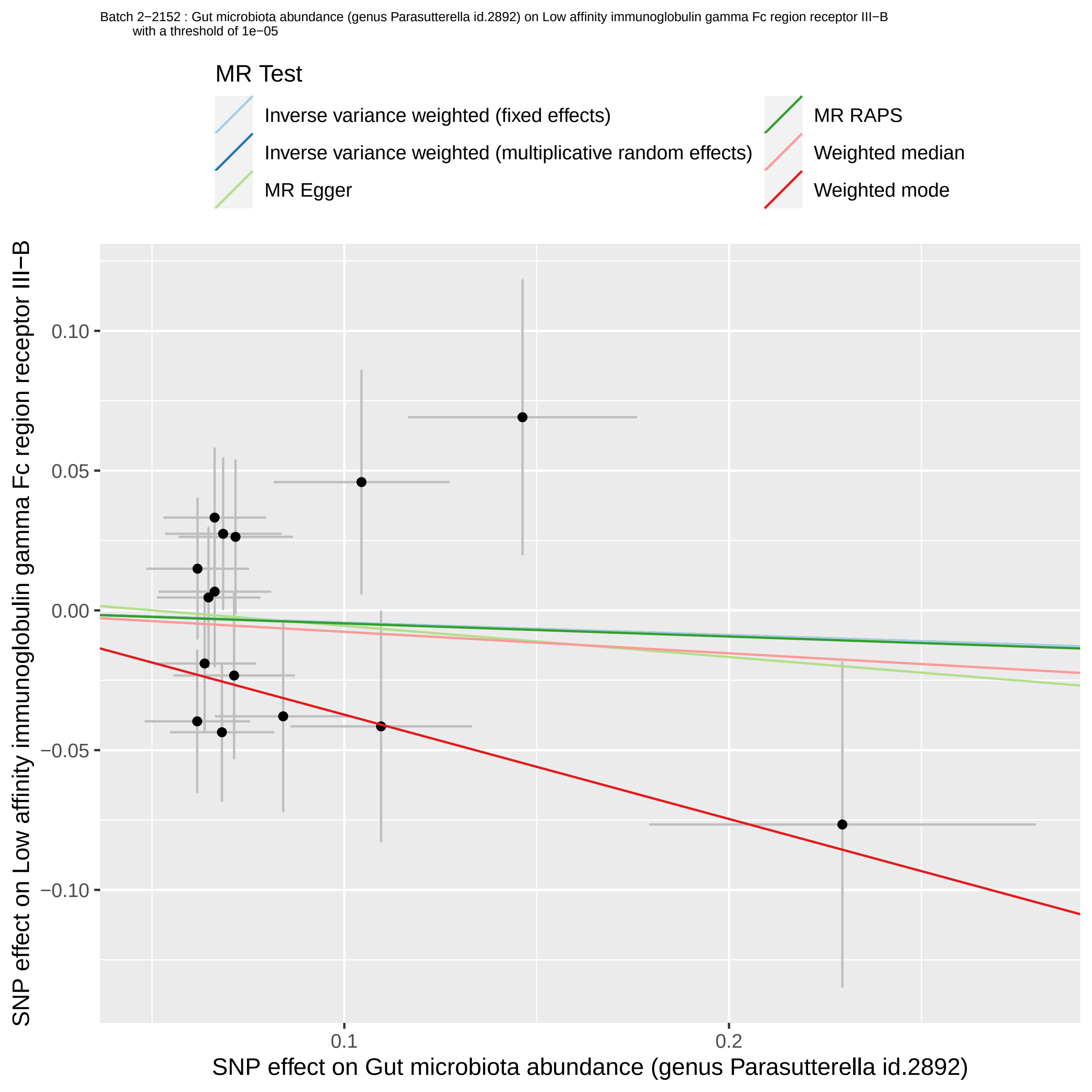
Batch 2−2152 : Gut microbiota abundance (genus Parasutterella id.2892) on Low affinity immunoglobulin gamma Fc region receptor III−B
with a threshold of 1e−05
MR Test
Inverse variance weighted (fixed effects)
Inverse variance weighted (multiplicative random effects)
MR Egger
MR RAPS
Weighted median
Weighted mode
0.10
0.05
0.00
−0.05
−0.10
0.1
0.2
SNP effect on Low affinity immunoglobulin gamma Fc region receptor III−B
SNP effect on Gut microbiota abundance (genus Parasutterella id.2892)

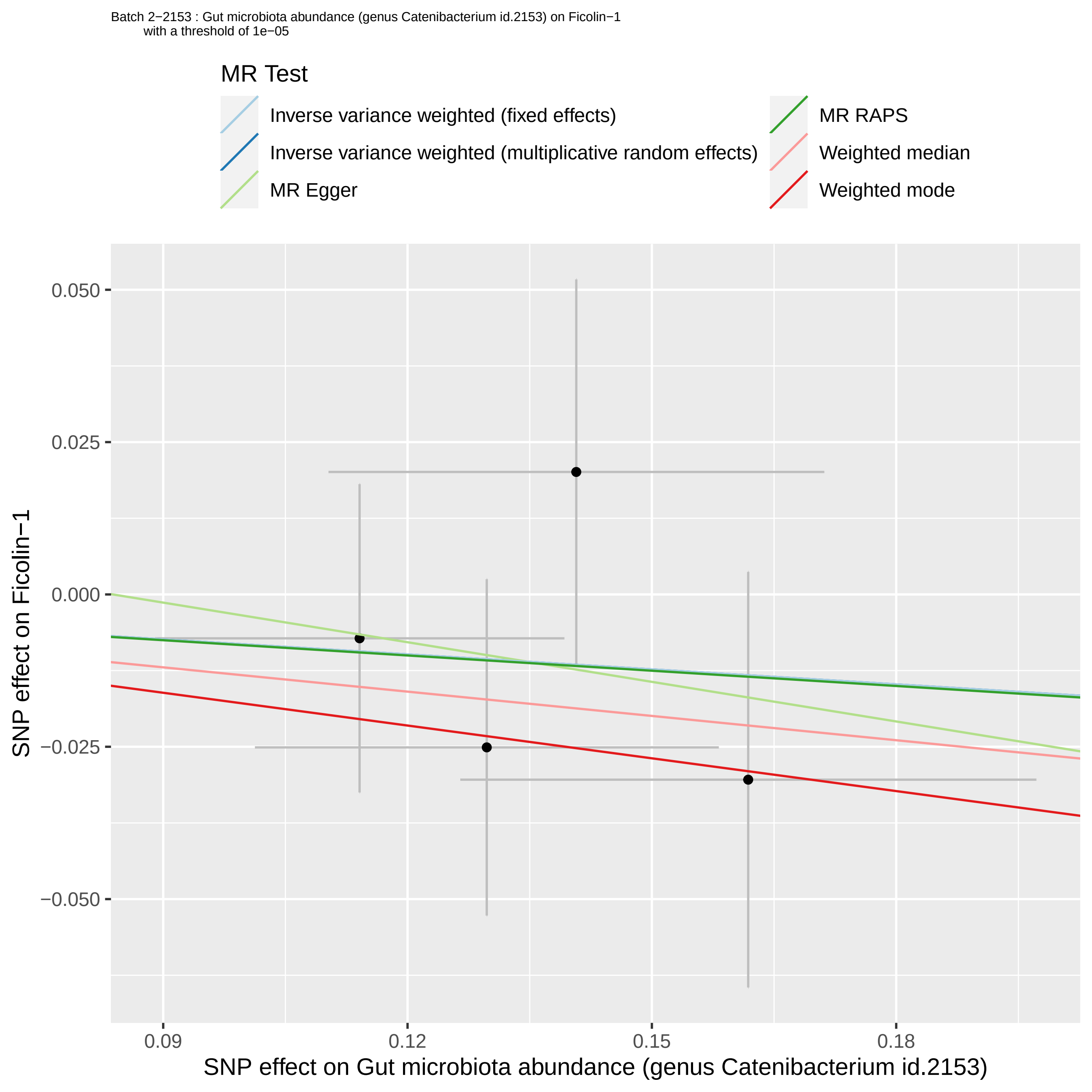

Batch 2−2153 : Gut microbiota abundance (genus Catenibacterium id.2153) on Ficolin−1
with a threshold of 1e−05
MR Test
Inverse variance weighted (fixed effects)
Inverse variance weighted (multiplicative random effects)
MR Egger
MR RAPS
Weighted median
Weighted mode
0.050
0.025
0.000
−0.025
−0.050
0.09
0.12
0.15
0.18
SNP effect on Ficolin−1
SNP effect on Gut microbiota abundance (genus Catenibacterium id.2153)

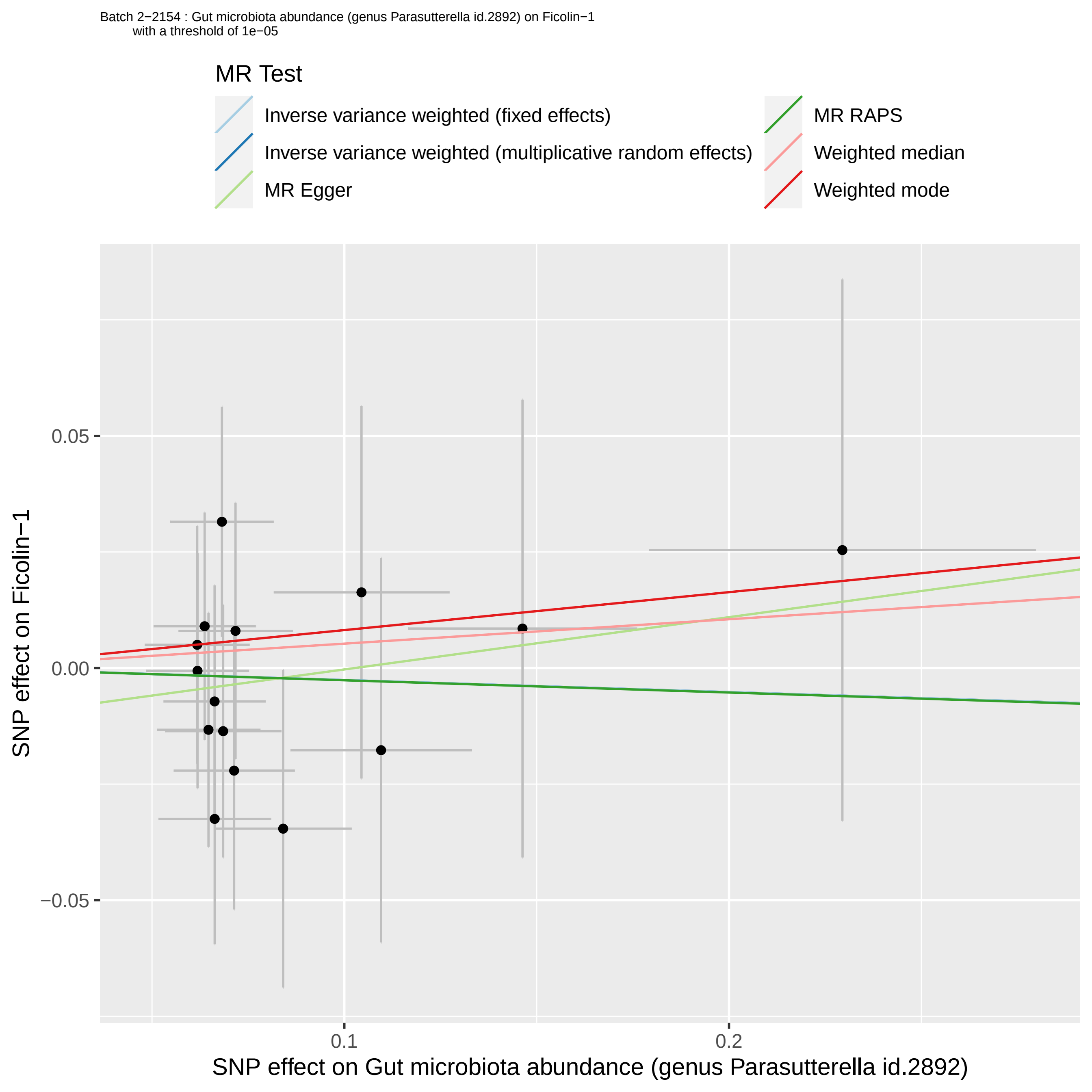

Batch 2−2154 : Gut microbiota abundance (genus Parasutterella id.2892) on Ficolin−1
with a threshold of 1e−05
MR Test
Inverse variance weighted (fixed effects)
Inverse variance weighted (multiplicative random effects)
MR Egger
MR RAPS
Weighted median
Weighted mode
0.05
0.00
−0.05
0.1
0.2
SNP effect on Ficolin−1
SNP effect on Gut microbiota abundance (genus Parasutterella id.2892)

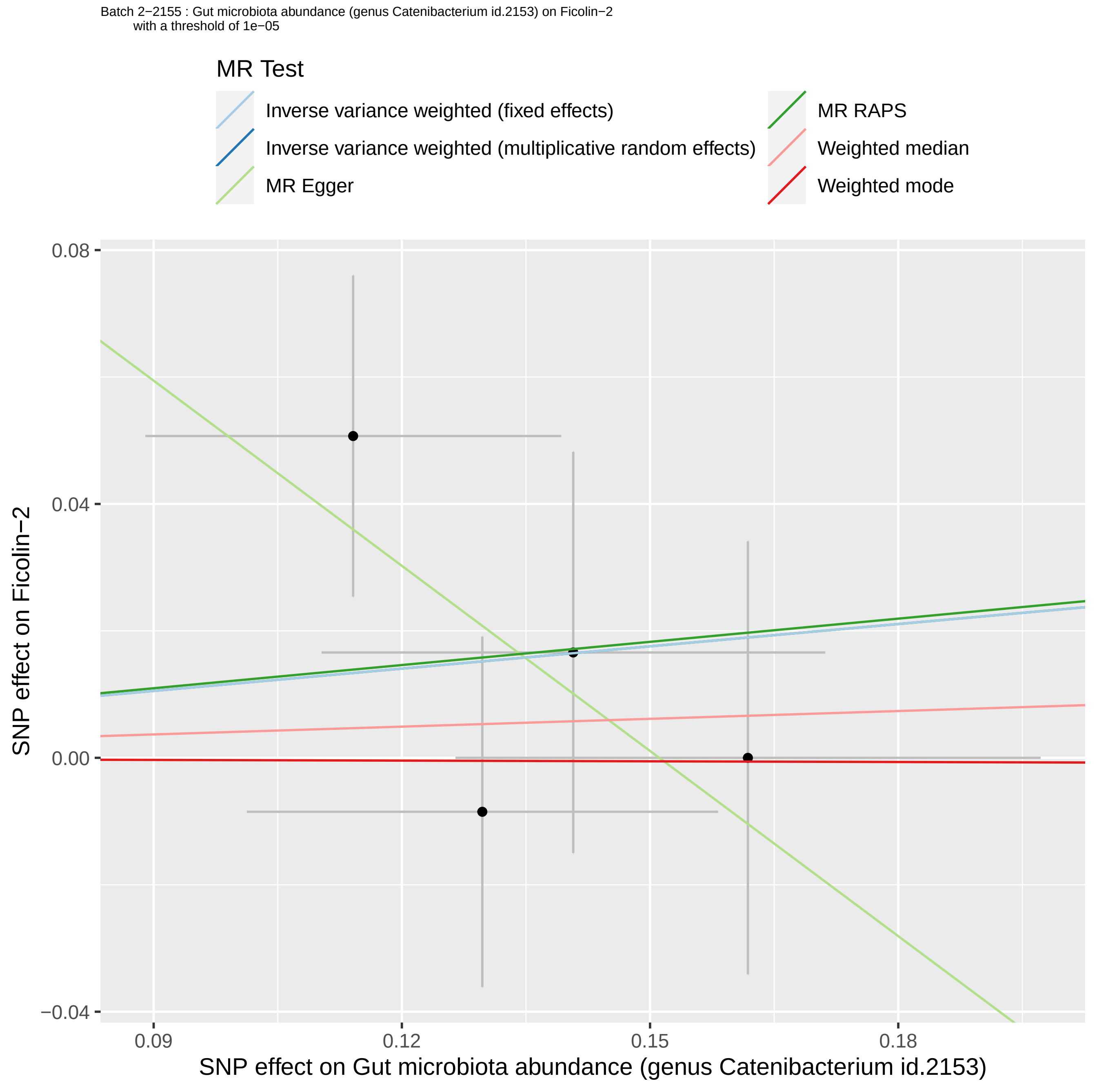

Batch 2−2155 : Gut microbiota abundance (genus Catenibacterium id.2153) on Ficolin−2
with a threshold of 1e−05
MR Test
Inverse variance weighted (fixed effects)
Inverse variance weighted (multiplicative random effects)
MR Egger
MR RAPS
Weighted median
Weighted mode
SNP effect on Ficolin−2
0.08
0.04
0.00
−0.04
0.09
0.12
0.15
0.18
SNP effect on Gut microbiota abundance (genus Catenibacterium id.2153)

Batch 2−2156 : Gut microbiota abundance (genus Parasutterella id.2892) on Ficolin−2
with a threshold of 1e−05

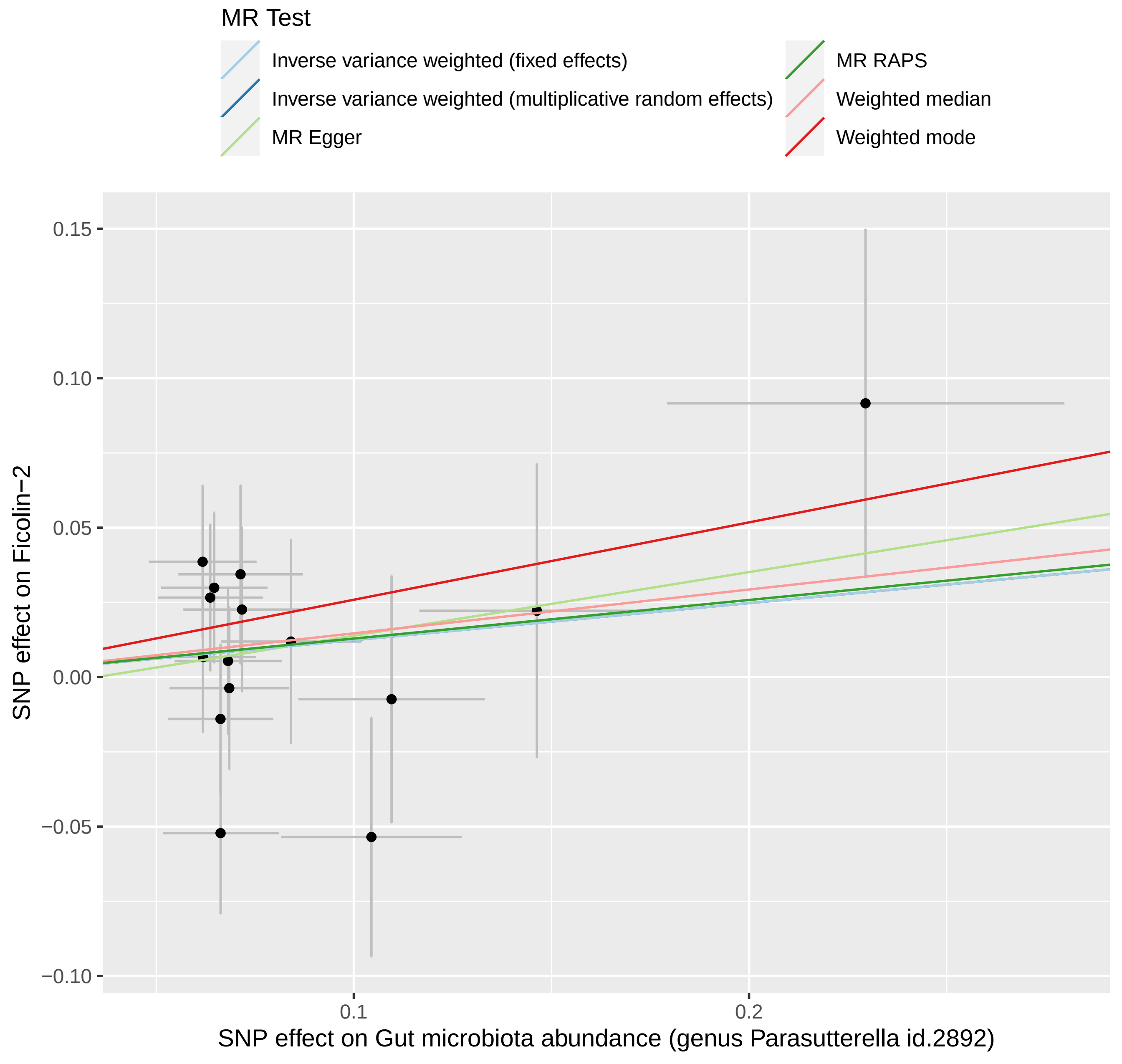

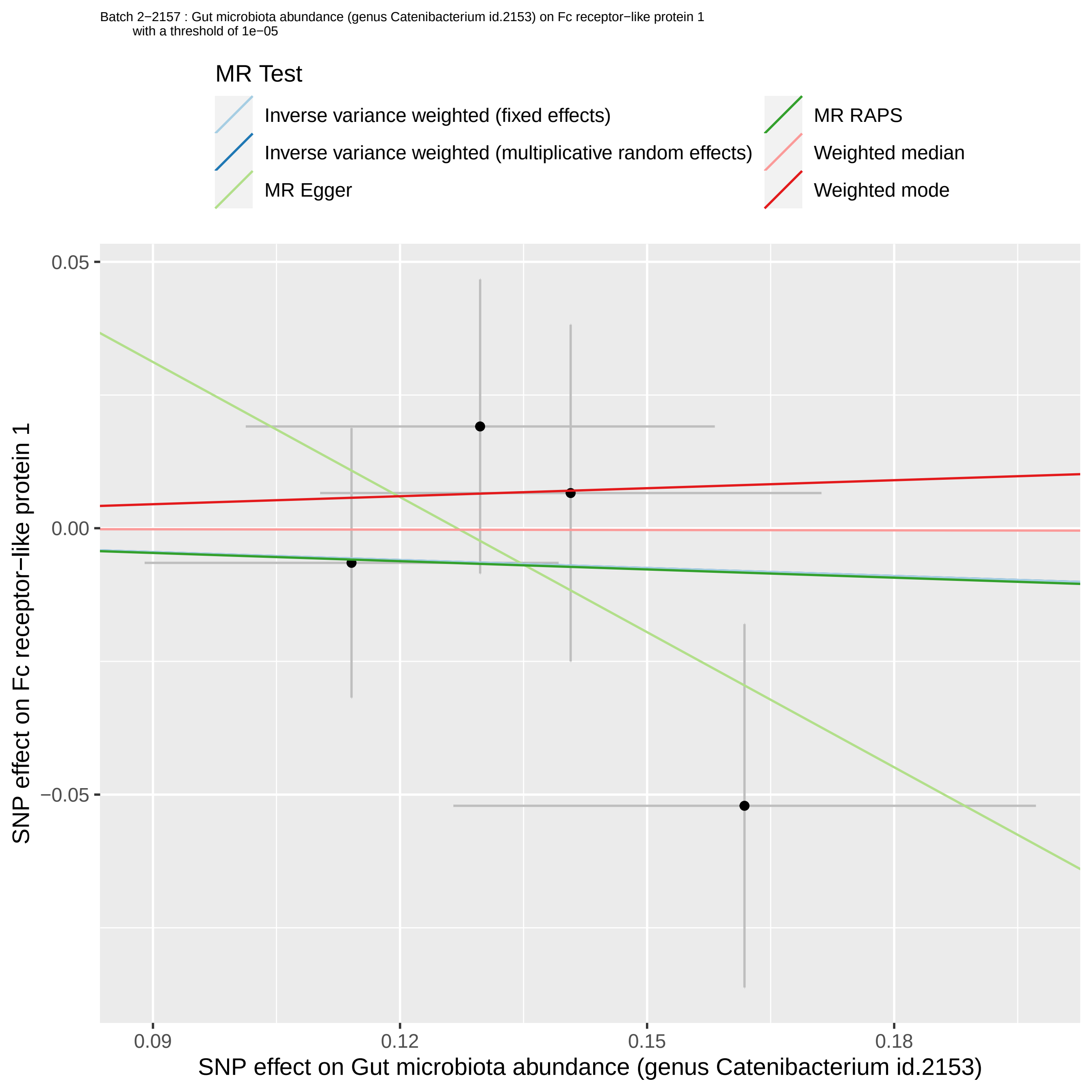
Batch 2−2157 : Gut microbiota abundance (genus Catenibacterium id.2153) on Fc receptor−like protein 1
with a threshold of 1e−05
MR Test
Inverse variance weighted (fixed effects)
Inverse variance weighted (multiplicative random effects)
MR Egger
MR RAPS
Weighted median
Weighted mode
0.05
0.00
−0.05
0.09
0.12
0.15
0.18
SNP effect on Fc receptor−like protein 1
SNP effect on Gut microbiota abundance (genus Catenibacterium id.2153)

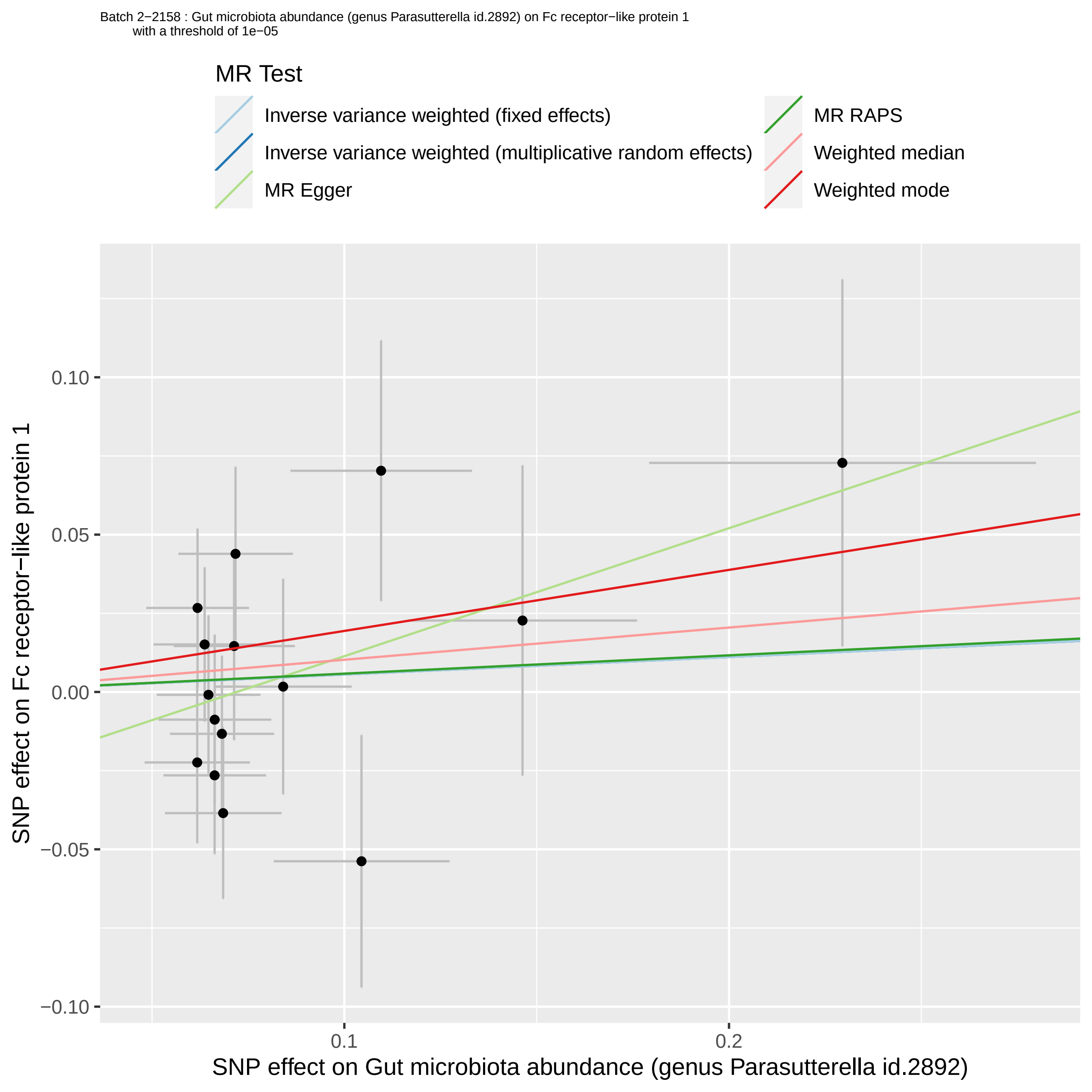

Batch 2−2158 : Gut microbiota abundance (genus Parasutterella id.2892) on Fc receptor−like protein 1
with a threshold of 1e−05
MR Test
Inverse variance weighted (fixed effects)
Inverse variance weighted (multiplicative random effects)
MR Egger
MR RAPS
Weighted median
Weighted mode
0.10
0.05
0.00
−0.05
−0.10
0.1
0.2
SNP effect on Fc receptor−like protein 1
SNP effect on Gut microbiota abundance (genus Parasutterella id.2892)

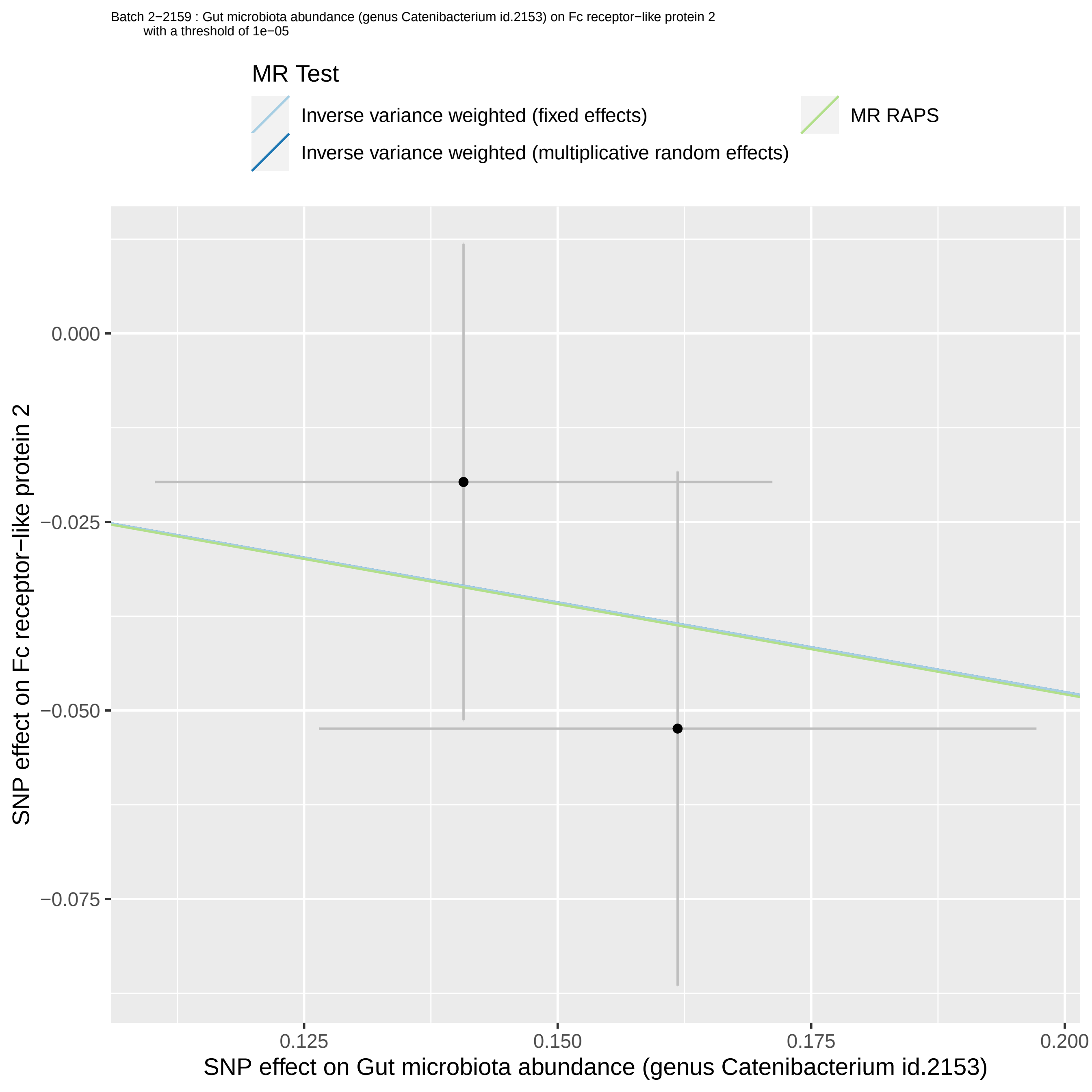
Batch 2−2159 : Gut microbiota abundance (genus Catenibacterium id.2153) on Fc receptor−like protein 2
with a threshold of 1e−05
MR Test
Inverse variance weighted (fixed effects)
Inverse variance weighted (multiplicative random effects)
MR RAPS
0.000
−0.025
−0.050
−0.075
0.125
0.150
0.175
0.200
SNP effect on Fc receptor−like protein 2
SNP effect on Gut microbiota abundance (genus Catenibacterium id.2153)

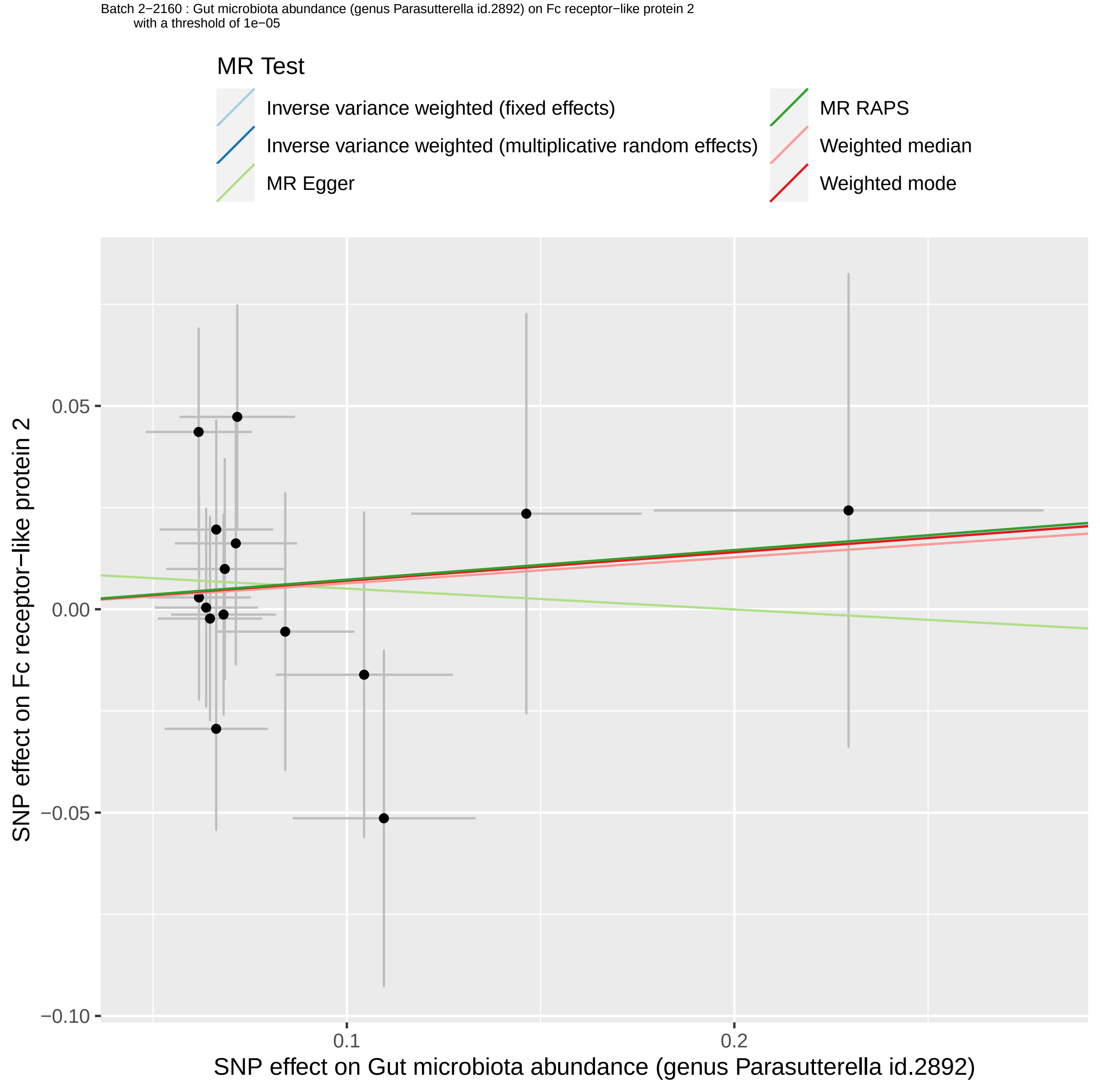

Batch 2−2160 : Gut microbiota abundance (genus Parasutterella id.2892) on Fc receptor−like protein 2
with a threshold of 1e−05
MR Test
Inverse variance weighted (fixed effects)
Inverse variance weighted (multiplicative random effects)
MR Egger
MR RAPS
Weighted median
Weighted mode
0.05
0.00
−0.05
−0.10
0.1
0.2
SNP effect on Fc receptor−like protein 2
SNP effect on Gut microbiota abundance (genus Parasutterella id.2892)

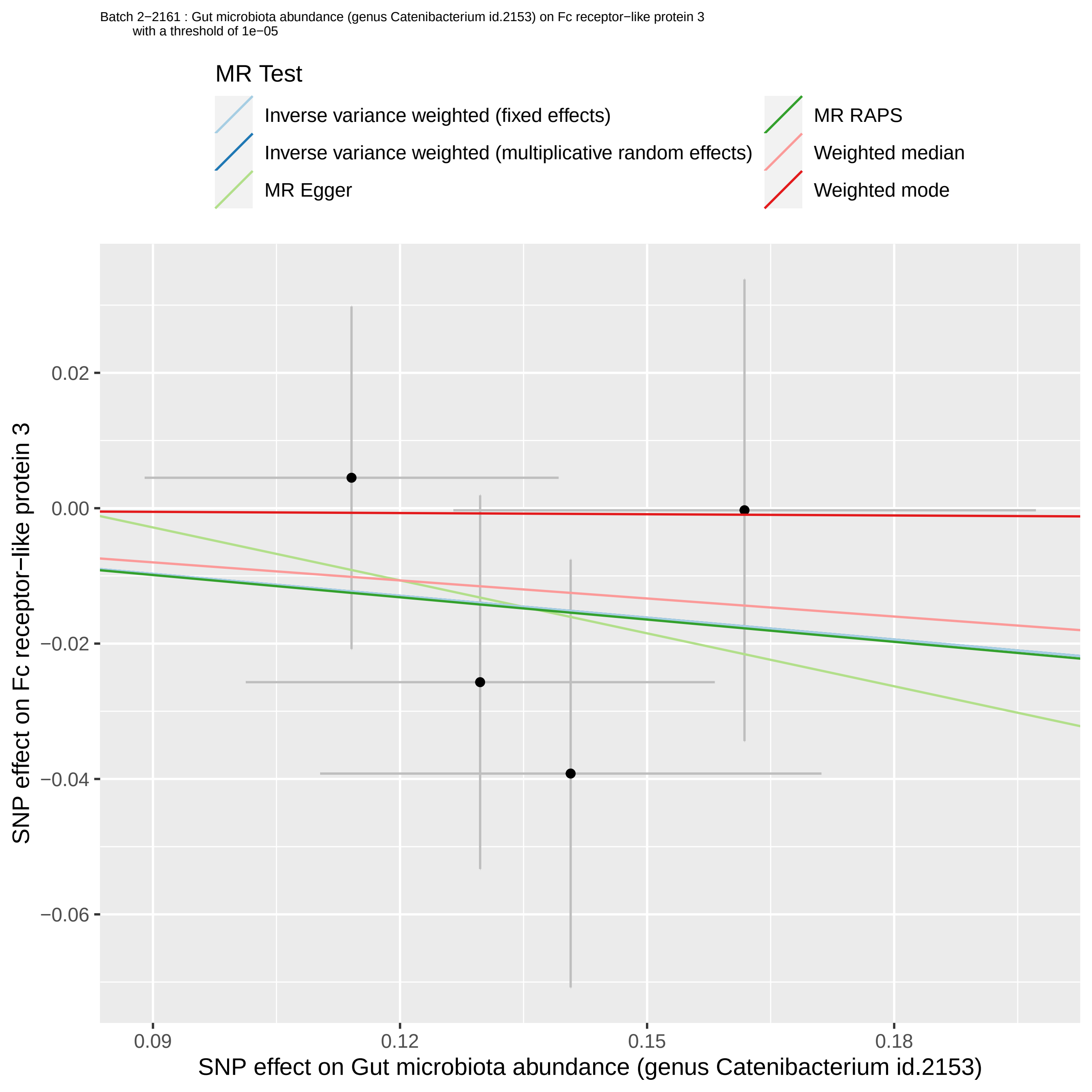
Batch 2−2161 : Gut microbiota abundance (genus Catenibacterium id.2153) on Fc receptor−like protein 3
with a threshold of 1e−05
MR Test
Inverse variance weighted (fixed effects)
Inverse variance weighted (multiplicative random effects)
MR Egger
MR RAPS
Weighted median
Weighted mode
SNP effect on Fc receptor−like protein 3
0.02
0.00
−0.02
−0.04
−0.06
0.09
0.12
0.15
0.18
SNP effect on Gut microbiota abundance (genus Catenibacterium id.2153)

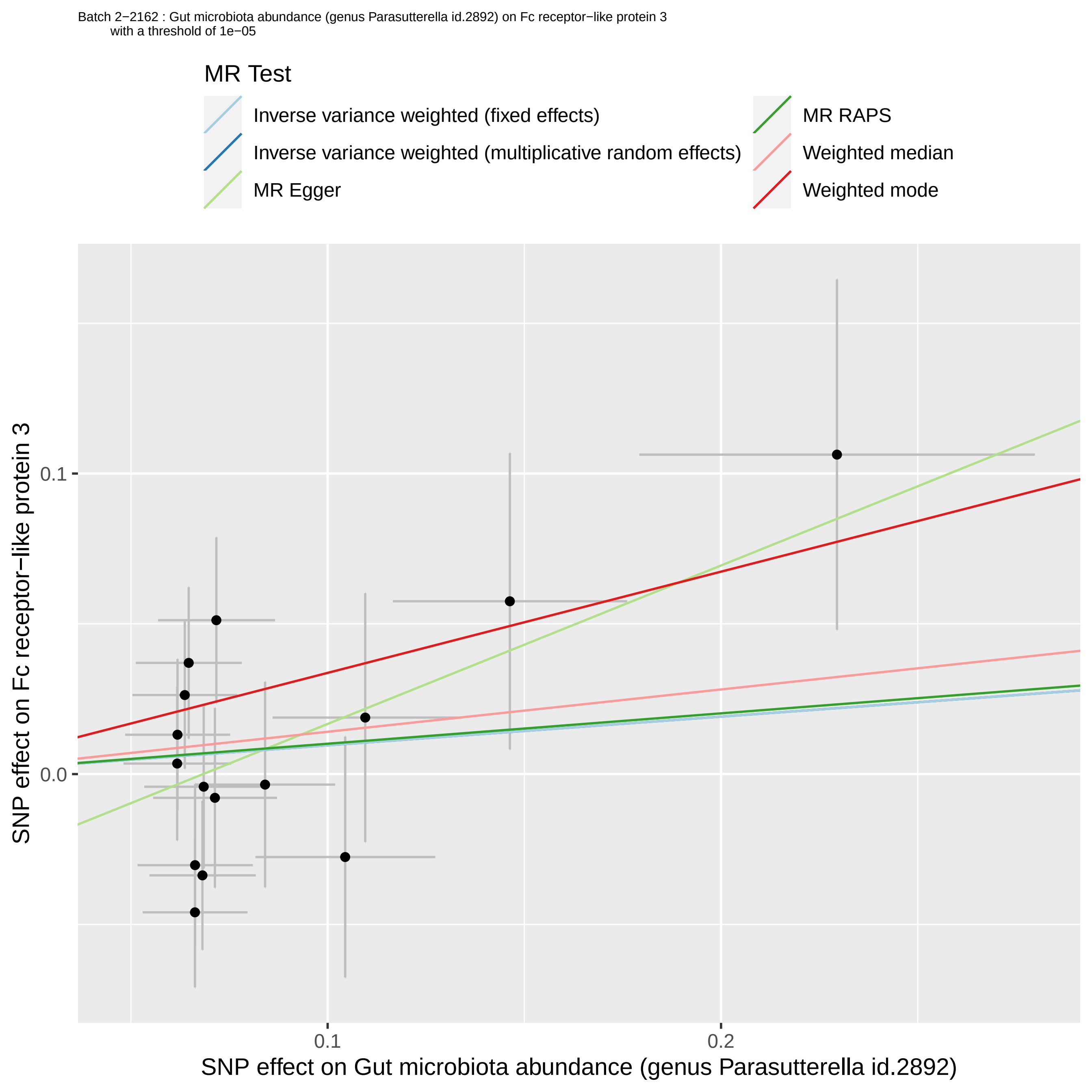
Batch 2−2162 : Gut microbiota abundance (genus Parasutterella id.2892) on Fc receptor−like protein 3
with a threshold of 1e−05
MR Test
Inverse variance weighted (fixed effects)
Inverse variance weighted (multiplicative random effects)
MR Egger
MR RAPS
Weighted median
Weighted mode
0.1
0.0
0.1
0.2
SNP effect on Fc receptor−like protein 3
SNP effect on Gut microbiota abundance (genus Parasutterella id.2892)

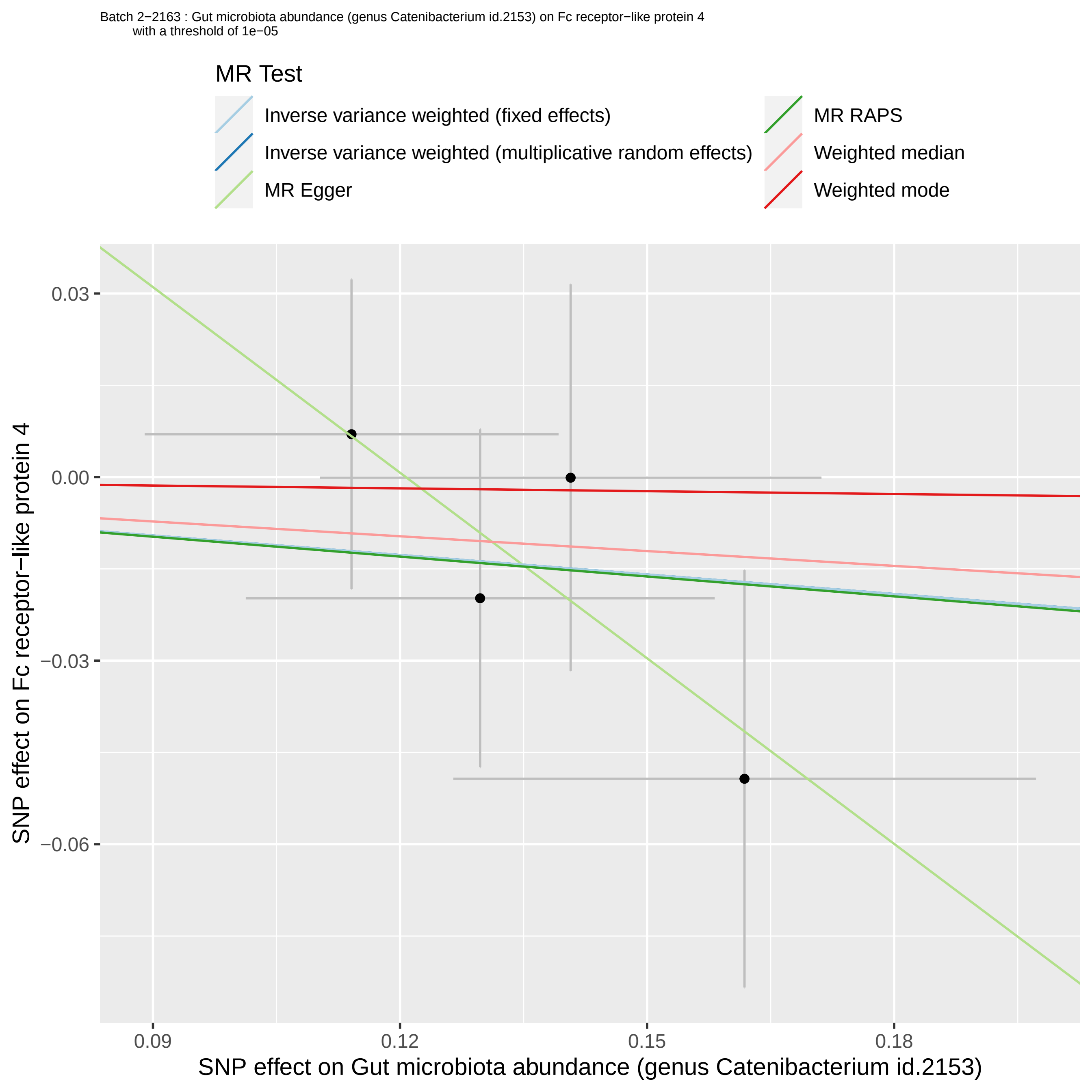

Batch 2−2163 : Gut microbiota abundance (genus Catenibacterium id.2153) on Fc receptor−like protein 4
with a threshold of 1e−05
MR Test
Inverse variance weighted (fixed effects)
Inverse variance weighted (multiplicative random effects)
MR Egger
MR RAPS
Weighted median
Weighted mode
0.03
0.00
−0.03
−0.06
0.09
0.12
0.15
0.18
SNP effect on Fc receptor−like protein 4
SNP effect on Gut microbiota abundance (genus Catenibacterium id.2153)

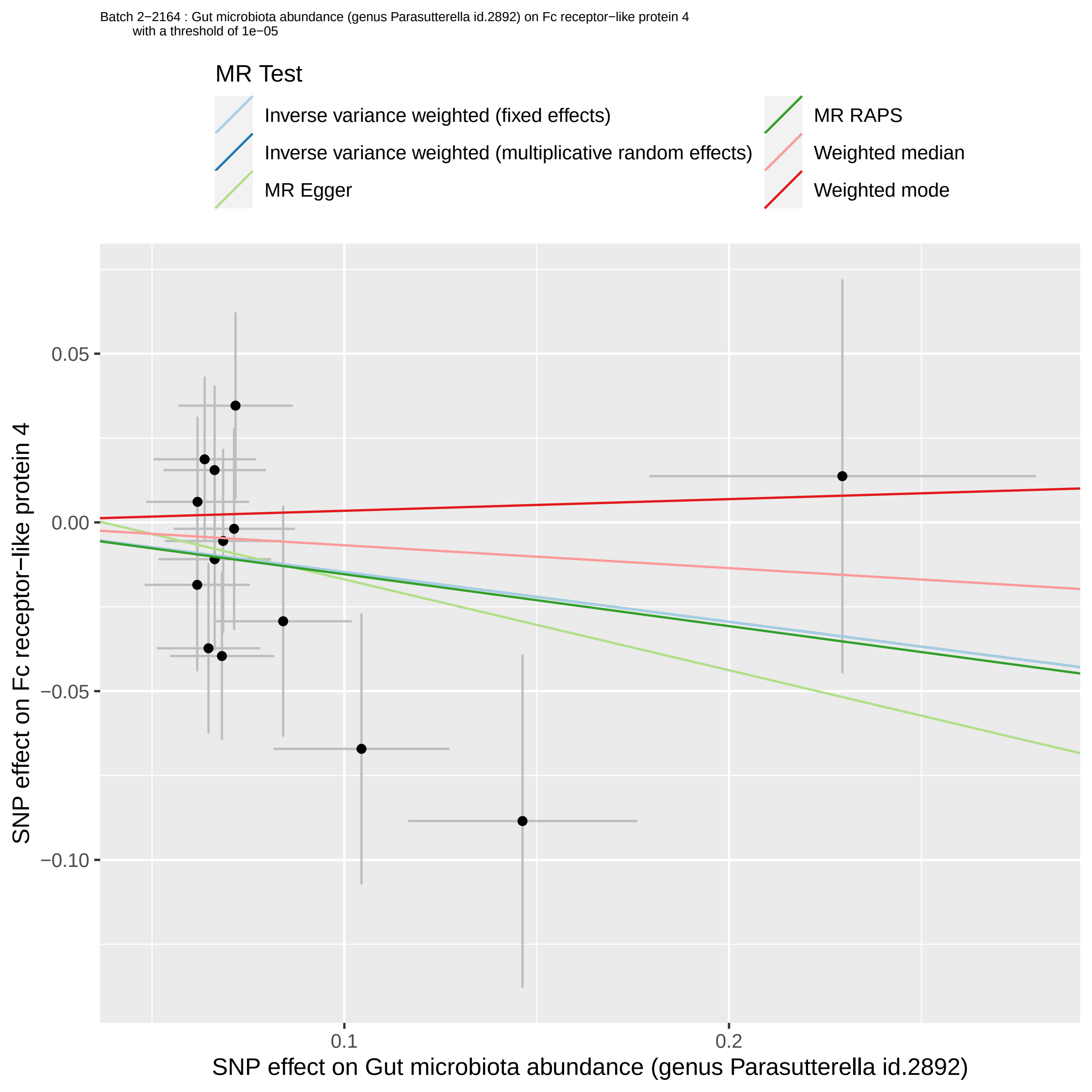
Batch 2−2164 : Gut microbiota abundance (genus Parasutterella id.2892) on Fc receptor−like protein 4
with a threshold of 1e−05
MR Test
Inverse variance weighted (fixed effects)
Inverse variance weighted (multiplicative random effects)
MR Egger
MR RAPS
Weighted median
Weighted mode
0.05
0.00
−0.05
−0.10
0.1
0.2
SNP effect on Fc receptor−like protein 4
SNP effect on Gut microbiota abundance (genus Parasutterella id.2892)

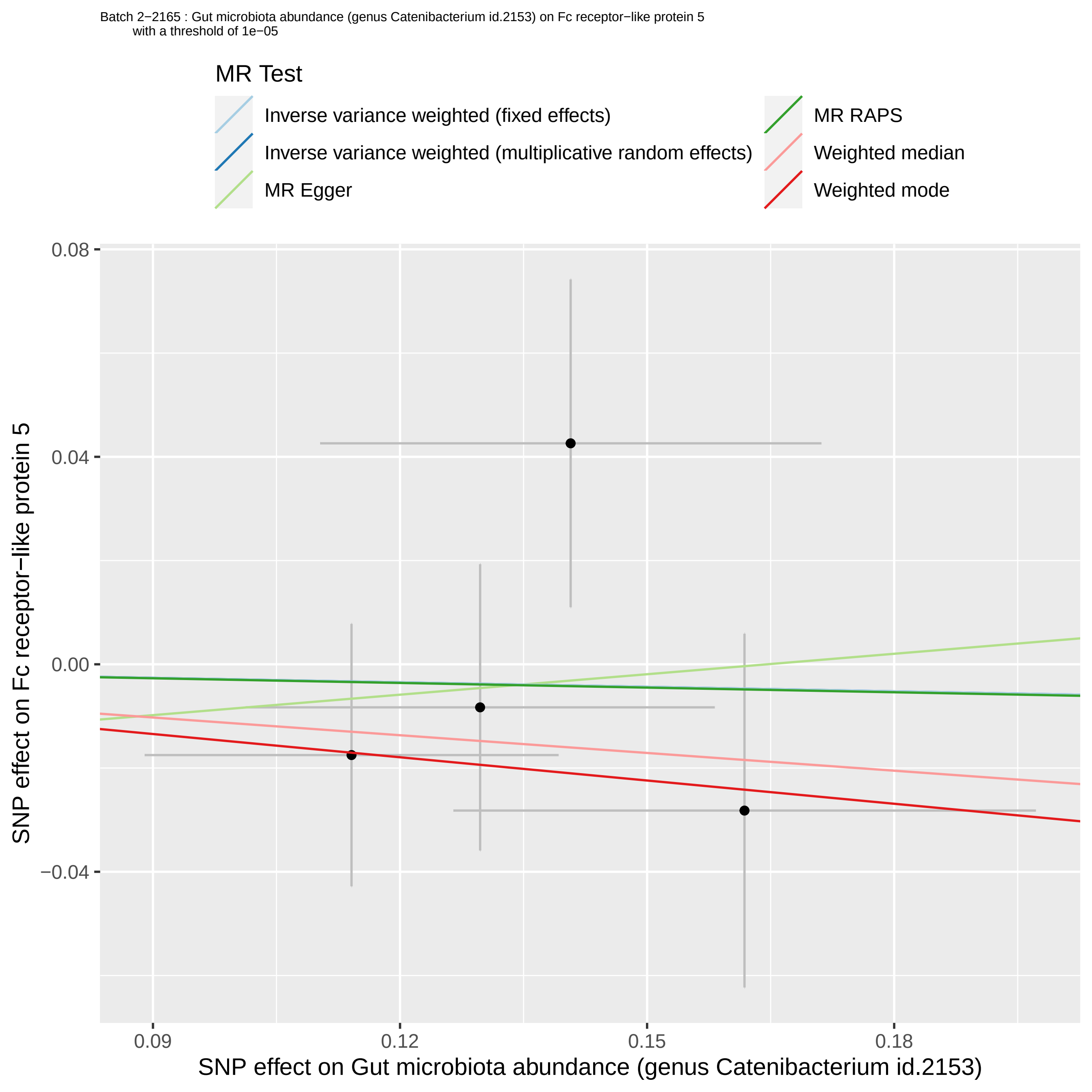
Batch 2−2165 : Gut microbiota abundance (genus Catenibacterium id.2153) on Fc receptor−like protein 5
with a threshold of 1e−05
MR Test
Inverse variance weighted (fixed effects)
Inverse variance weighted (multiplicative random effects)
MR Egger
MR RAPS
Weighted median
Weighted mode
0.08
0.04
0.00
−0.04
0.09
0.12
0.15
0.18
SNP effect on Fc receptor−like protein 5
SNP effect on Gut microbiota abundance (genus Catenibacterium id.2153)

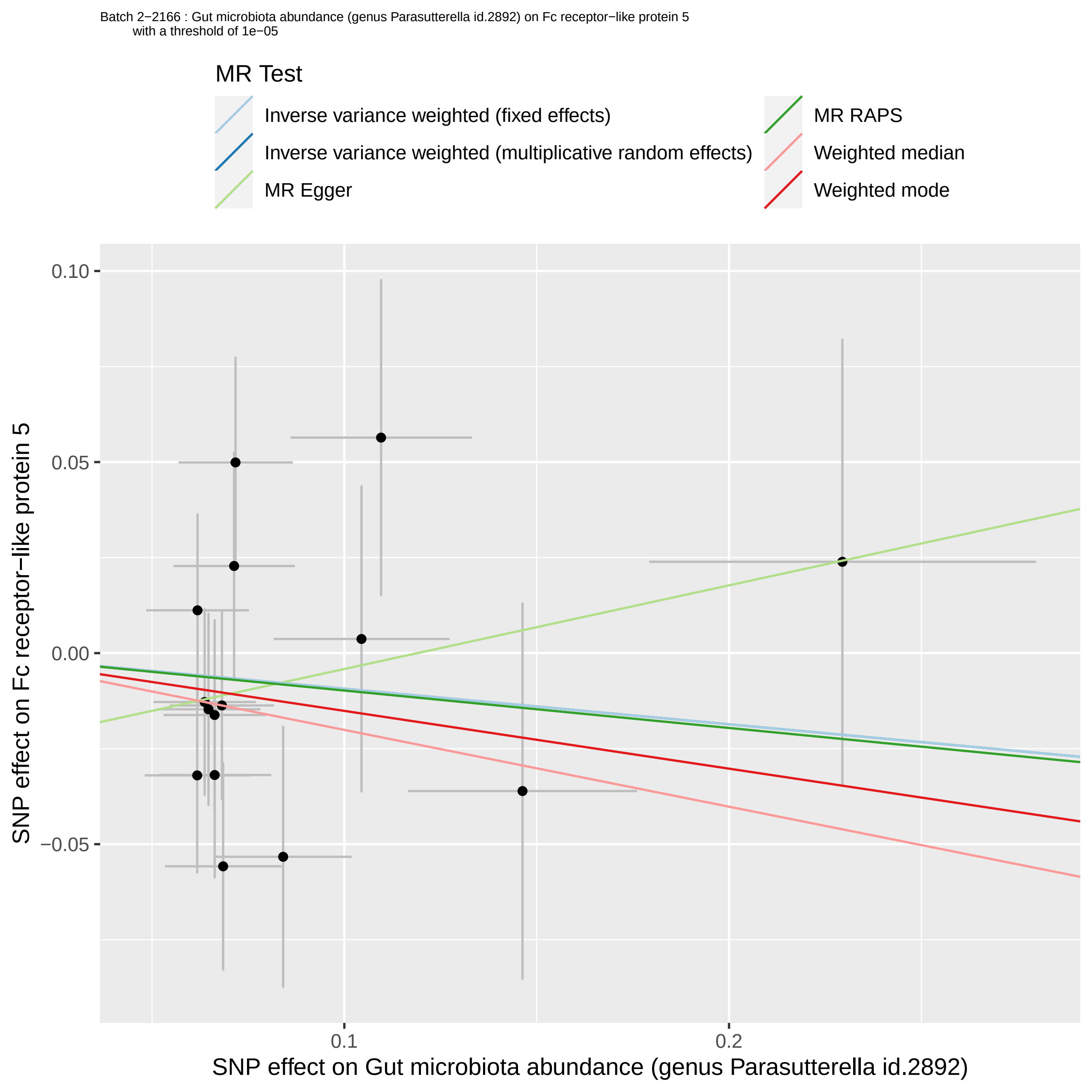
Batch 2−2166 : Gut microbiota abundance (genus Parasutterella id.2892) on Fc receptor−like protein 5
with a threshold of 1e−05
MR Test
Inverse variance weighted (fixed effects)
Inverse variance weighted (multiplicative random effects)
MR Egger
MR RAPS
Weighted median
Weighted mode
0.10
0.05
0.00
−0.05
0.1
0.2
SNP effect on Fc receptor−like protein 5
SNP effect on Gut microbiota abundance (genus Parasutterella id.2892)

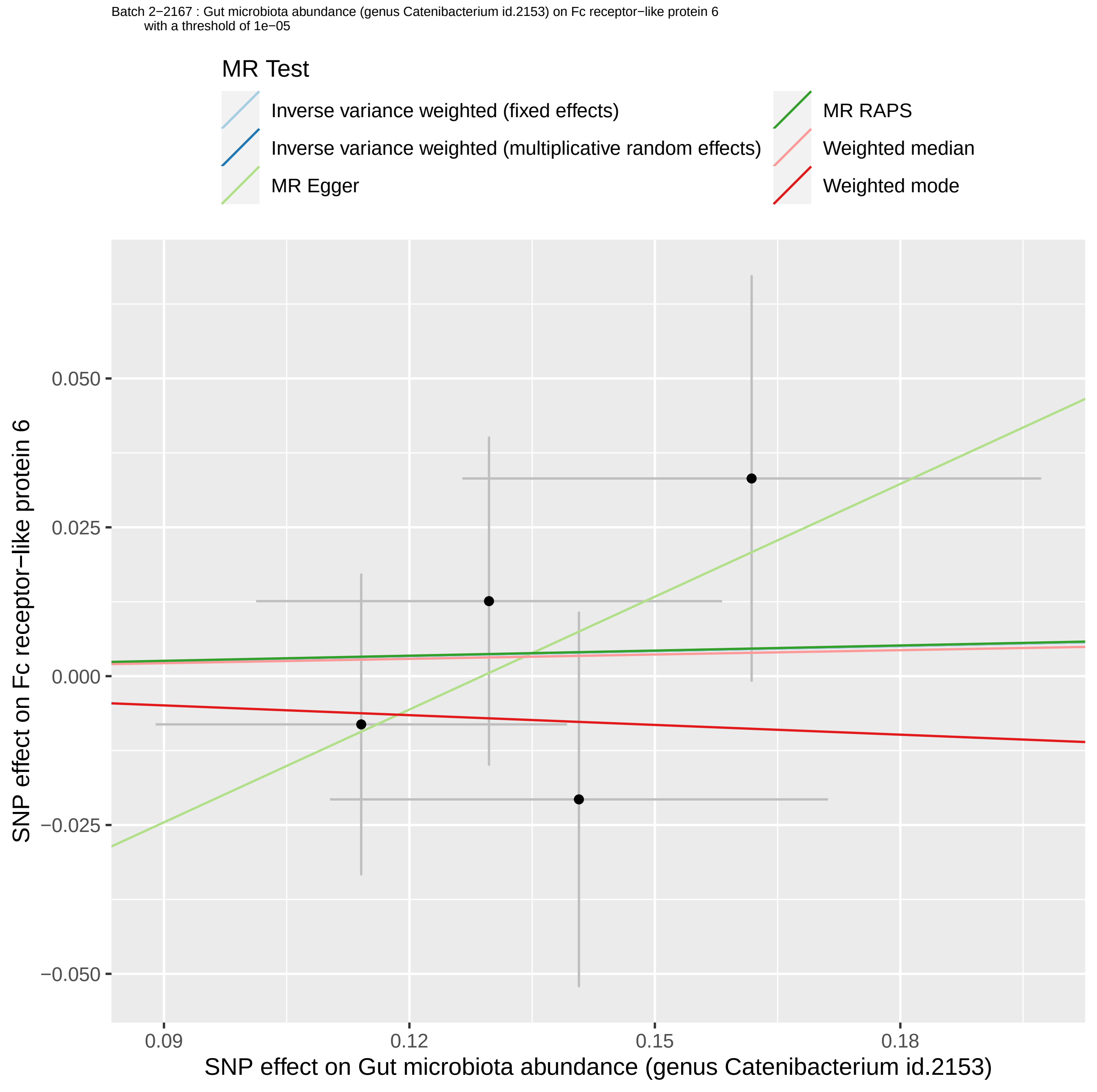
Batch 2−2167 : Gut microbiota abundance (genus Catenibacterium id.2153) on Fc receptor−like protein 6
with a threshold of 1e−05
MR Test
Inverse variance weighted (fixed effects)
Inverse variance weighted (multiplicative random effects)
MR Egger
MR RAPS
Weighted median
Weighted mode
0.050
0.025
0.000
−0.025
−0.050
0.09
0.12
0.15
0.18
SNP effect on Fc receptor−like protein 6
SNP effect on Gut microbiota abundance (genus Catenibacterium id.2153)

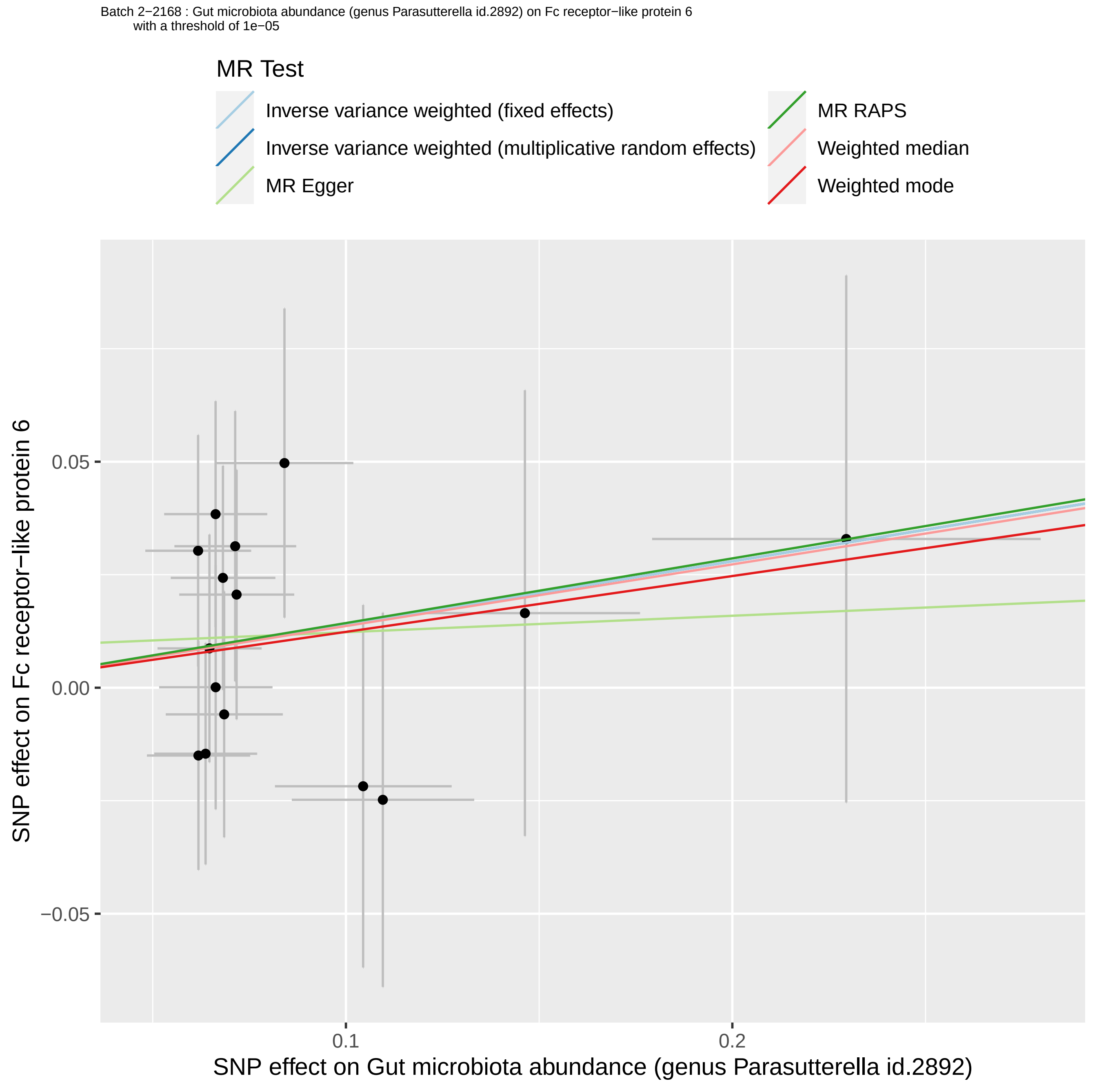

Batch 2−2168 : Gut microbiota abundance (genus Parasutterella id.2892) on Fc receptor−like protein 6
with a threshold of 1e−05
MR Test
Inverse variance weighted (fixed effects)
Inverse variance weighted (multiplicative random effects)
MR Egger
MR RAPS
Weighted median
Weighted mode
SNP effect on Fc receptor−like protein 6
0.05
0.00
−0.05
0.1
0.2
SNP effect on Gut microbiota abundance (genus Parasutterella id.2892)

Batch 2−2169 : Gut microbiota abundance (genus Catenibacterium id.2153) on Flap endonuclease 1
with a threshold of 1e−05

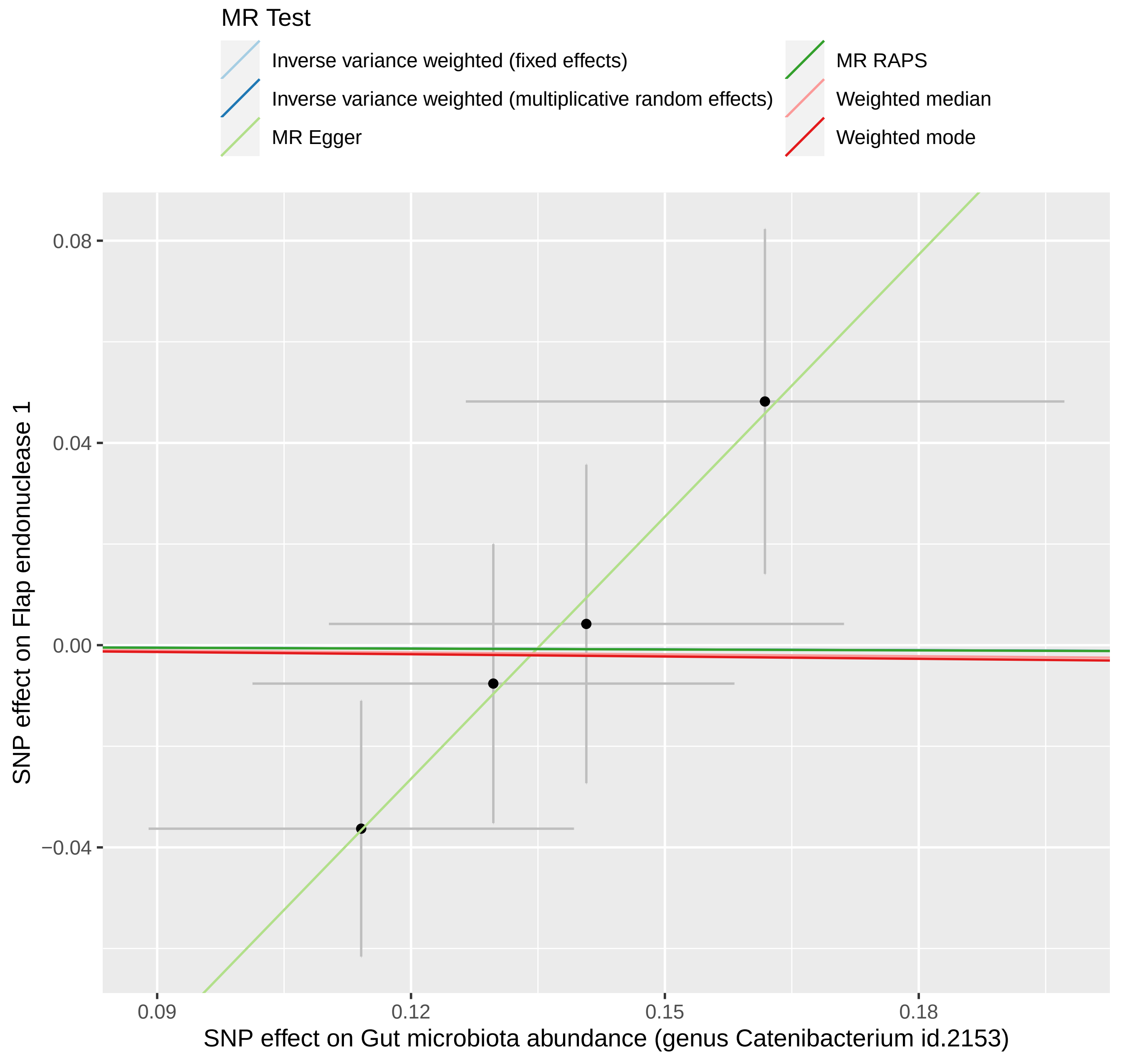

Batch 2−2170 : Gut microbiota abundance (genus Parasutterella id.2892) on Flap endonuclease 1
with a threshold of 1e−05

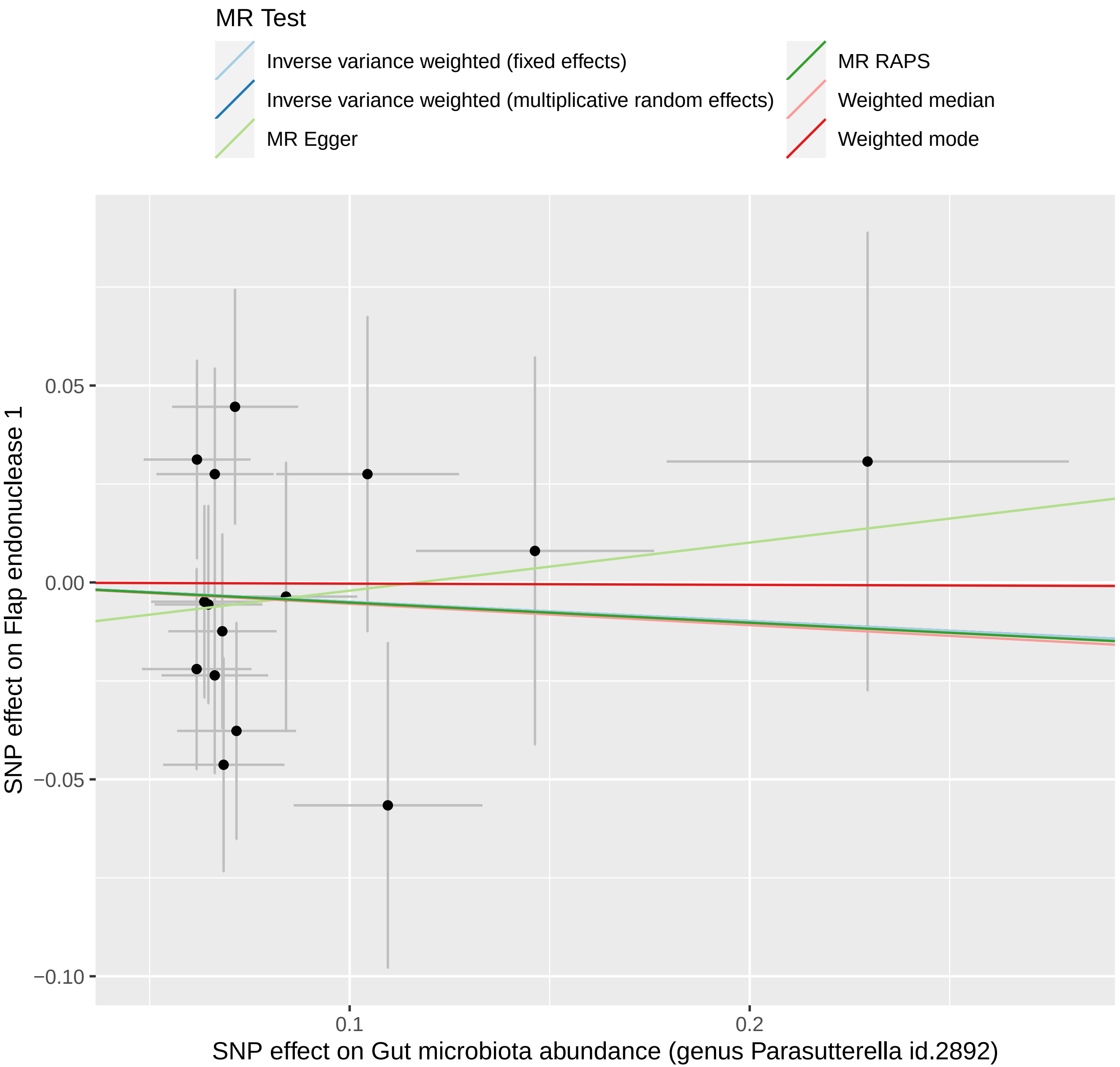

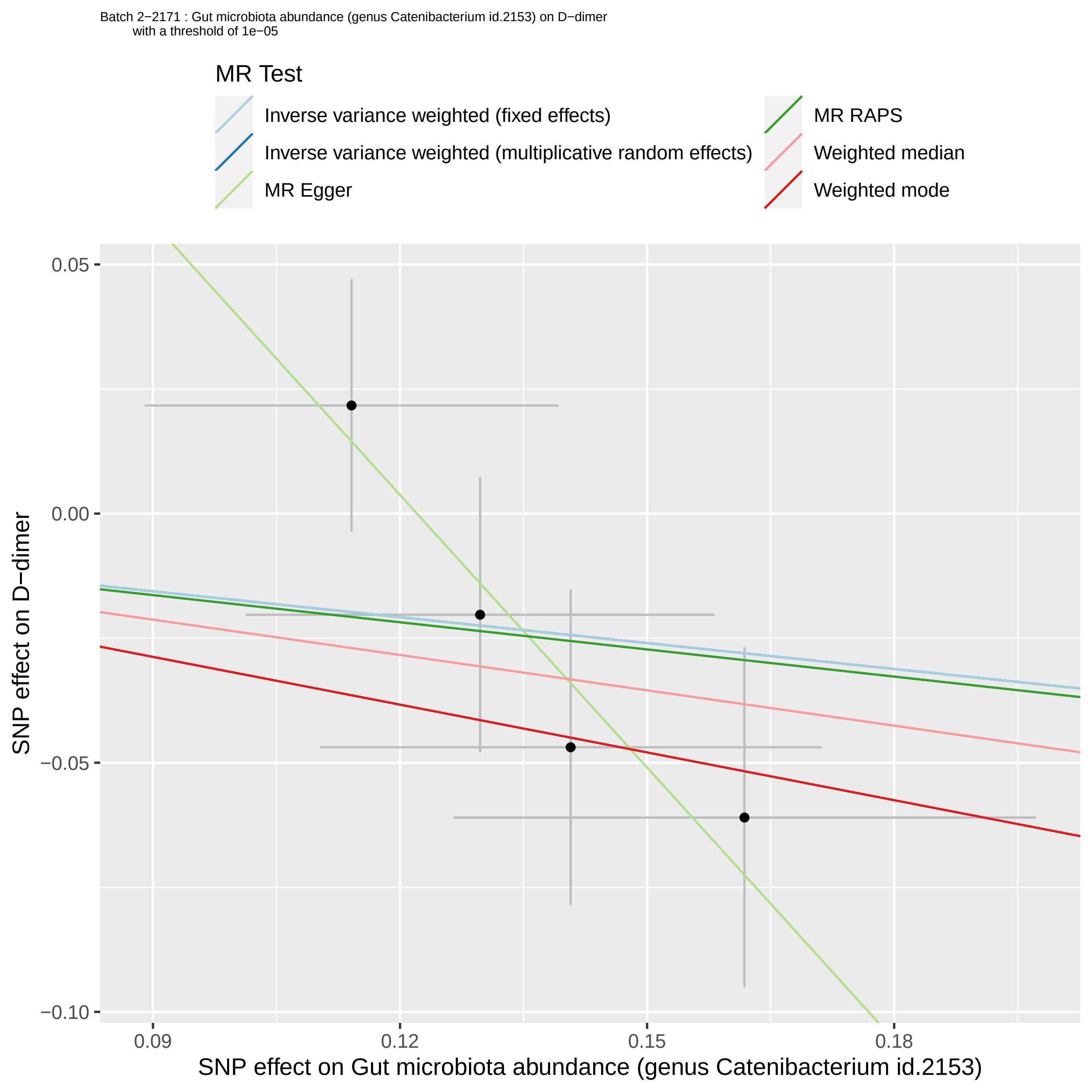
Batch 2−2171 : Gut microbiota abundance (genus Catenibacterium id.2153) on D−dimer
with a threshold of 1e−05
MR Test
Inverse variance weighted (fixed effects)
Inverse variance weighted (multiplicative random effects)
MR Egger
MR RAPS
Weighted median
Weighted mode
0.05
0.00
−0.05
−0.10
0.09
0.12
0.15
0.18
SNP effect on D−dimer
SNP effect on Gut microbiota abundance (genus Catenibacterium id.2153)

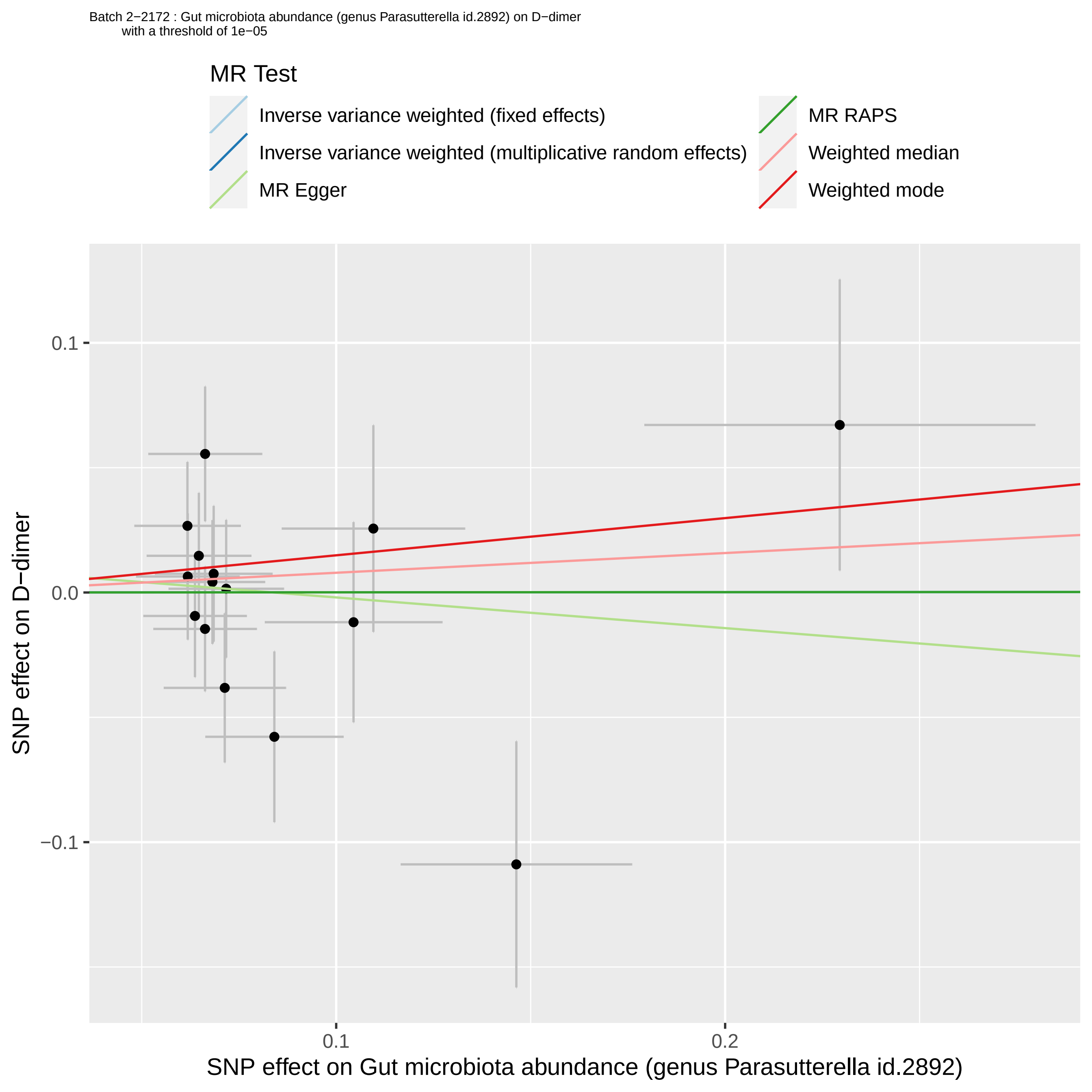
Batch 2−2172 : Gut microbiota abundance (genus Parasutterella id.2892) on D−dimer
with a threshold of 1e−05
MR Test
Inverse variance weighted (fixed effects)
Inverse variance weighted (multiplicative random effects)
MR Egger
MR RAPS
Weighted median
Weighted mode
0.1
0.0
−0.1
0.1
0.2
SNP effect on D−dimer
SNP effect on Gut microbiota abundance (genus Parasutterella id.2892)

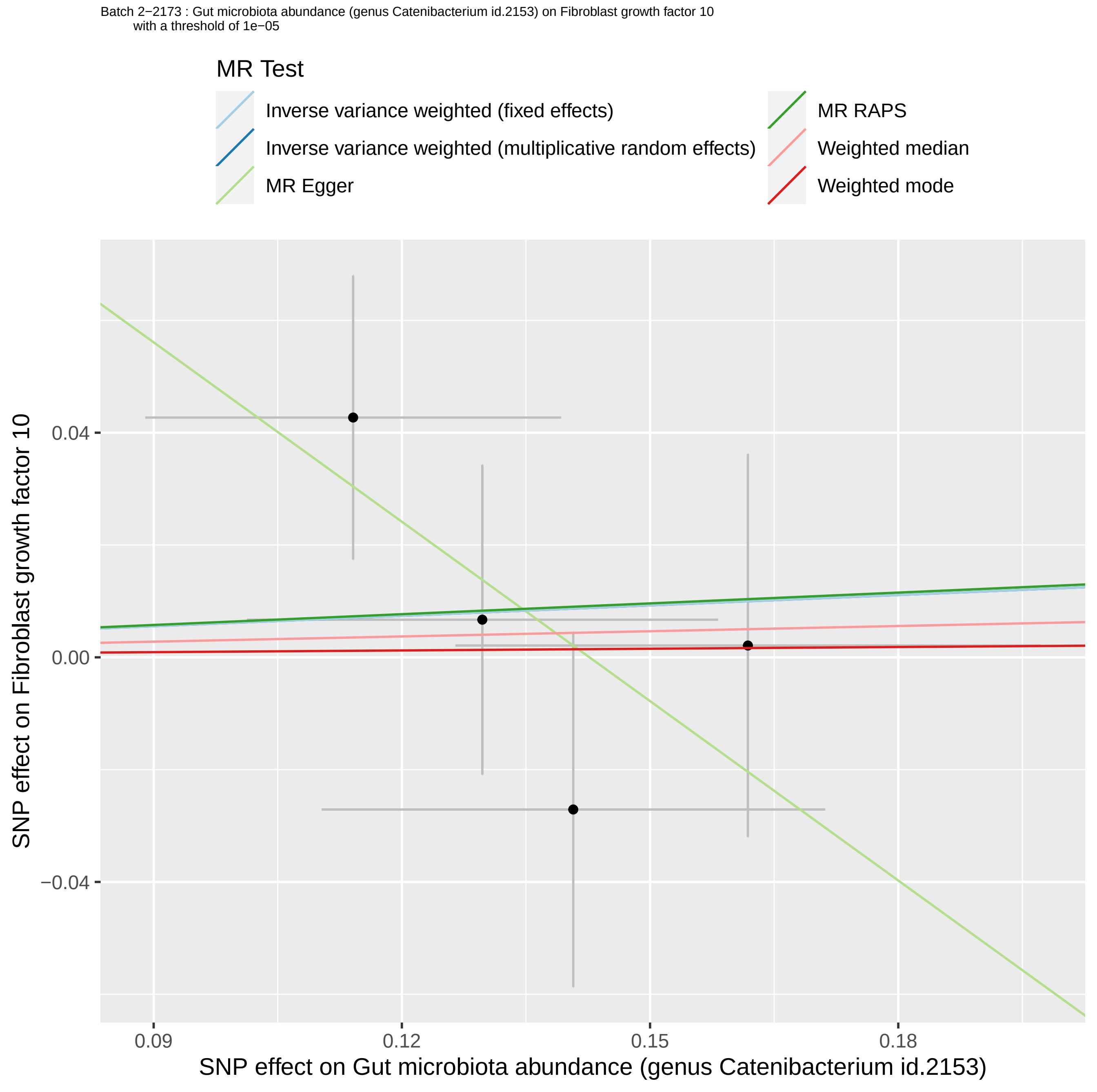

Batch 2−2173 : Gut microbiota abundance (genus Catenibacterium id.2153) on Fibroblast growth factor 10
with a threshold of 1e−05
MR Test
Inverse variance weighted (fixed effects)
Inverse variance weighted (multiplicative random effects)
MR Egger
MR RAPS
Weighted median
Weighted mode
0.04
0.00
−0.04
0.09
0.12
0.15
0.18
SNP effect on Fibroblast growth factor 10
SNP effect on Gut microbiota abundance (genus Catenibacterium id.2153)

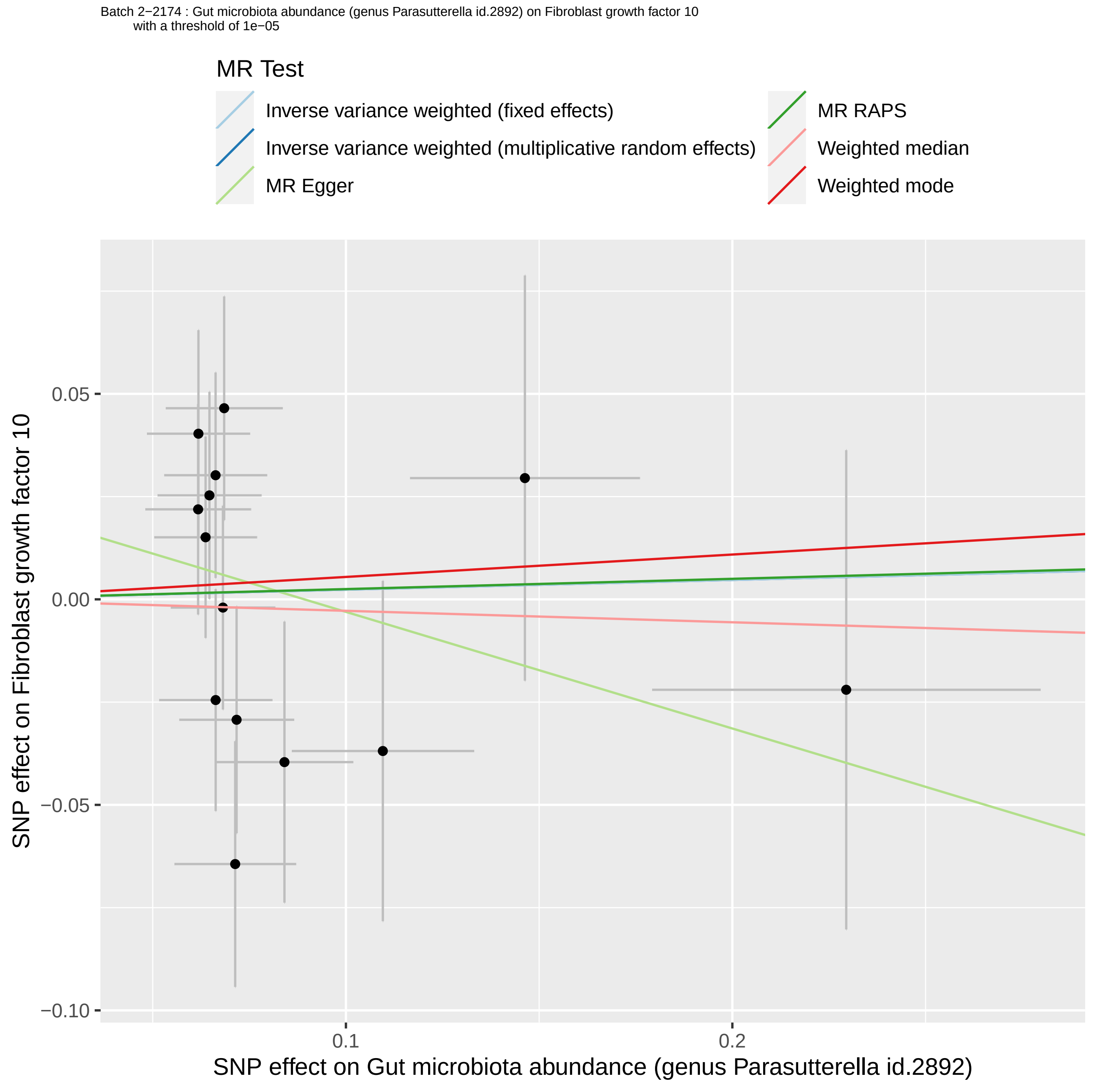
Batch 2−2174 : Gut microbiota abundance (genus Parasutterella id.2892) on Fibroblast growth factor 10
with a threshold of 1e−05
MR Test
Inverse variance weighted (fixed effects)
Inverse variance weighted (multiplicative random effects)
MR Egger
MR RAPS
Weighted median
Weighted mode
0.05
0.00
−0.05
−0.10
0.1
0.2
SNP effect on Fibroblast growth factor 10
SNP effect on Gut microbiota abundance (genus Parasutterella id.2892)

Batch 2−2175 : Gut microbiota abundance (genus Catenibacterium id.2153) on Fibroblast growth factor 1
with a threshold of 1e−05

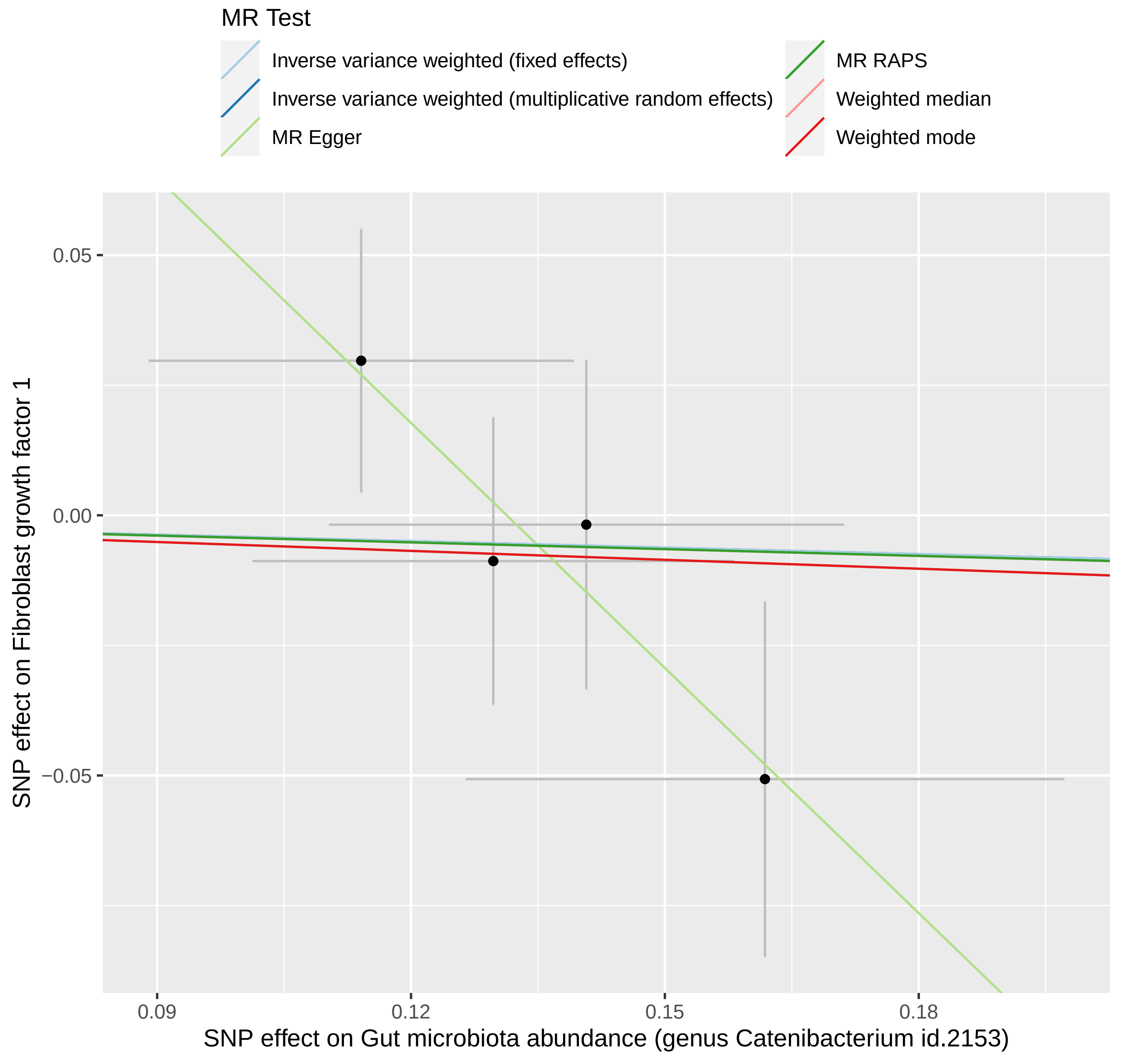

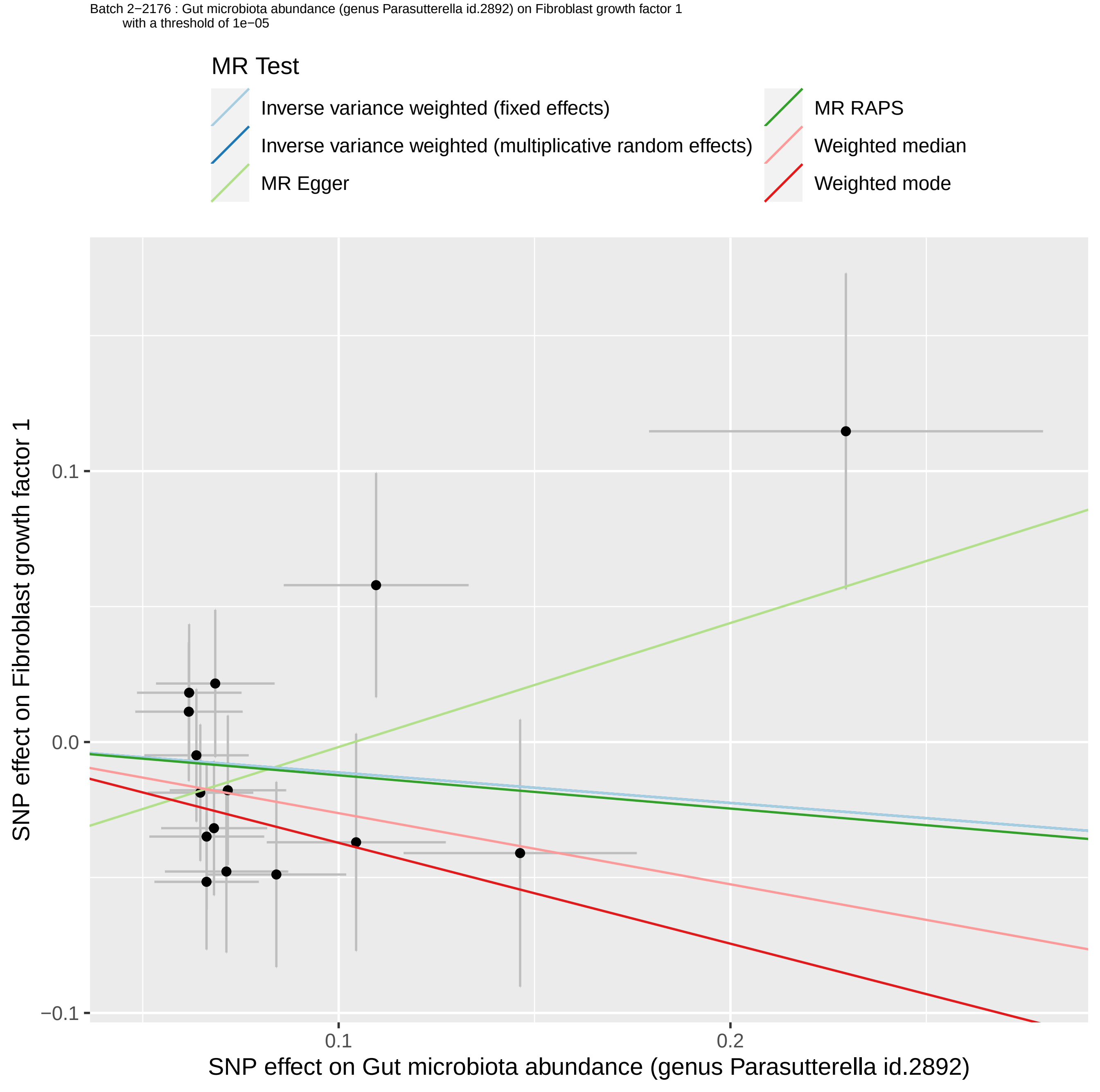
Batch 2−2176 : Gut microbiota abundance (genus Parasutterella id.2892) on Fibroblast growth factor 1
with a threshold of 1e−05
MR Test
Inverse variance weighted (fixed effects)
Inverse variance weighted (multiplicative random effects)
MR Egger
MR RAPS
Weighted median
Weighted mode
0.1
0.0
−0.1
0.1
0.2
SNP effect on Fibroblast growth factor 1
SNP effect on Gut microbiota abundance (genus Parasutterella id.2892)

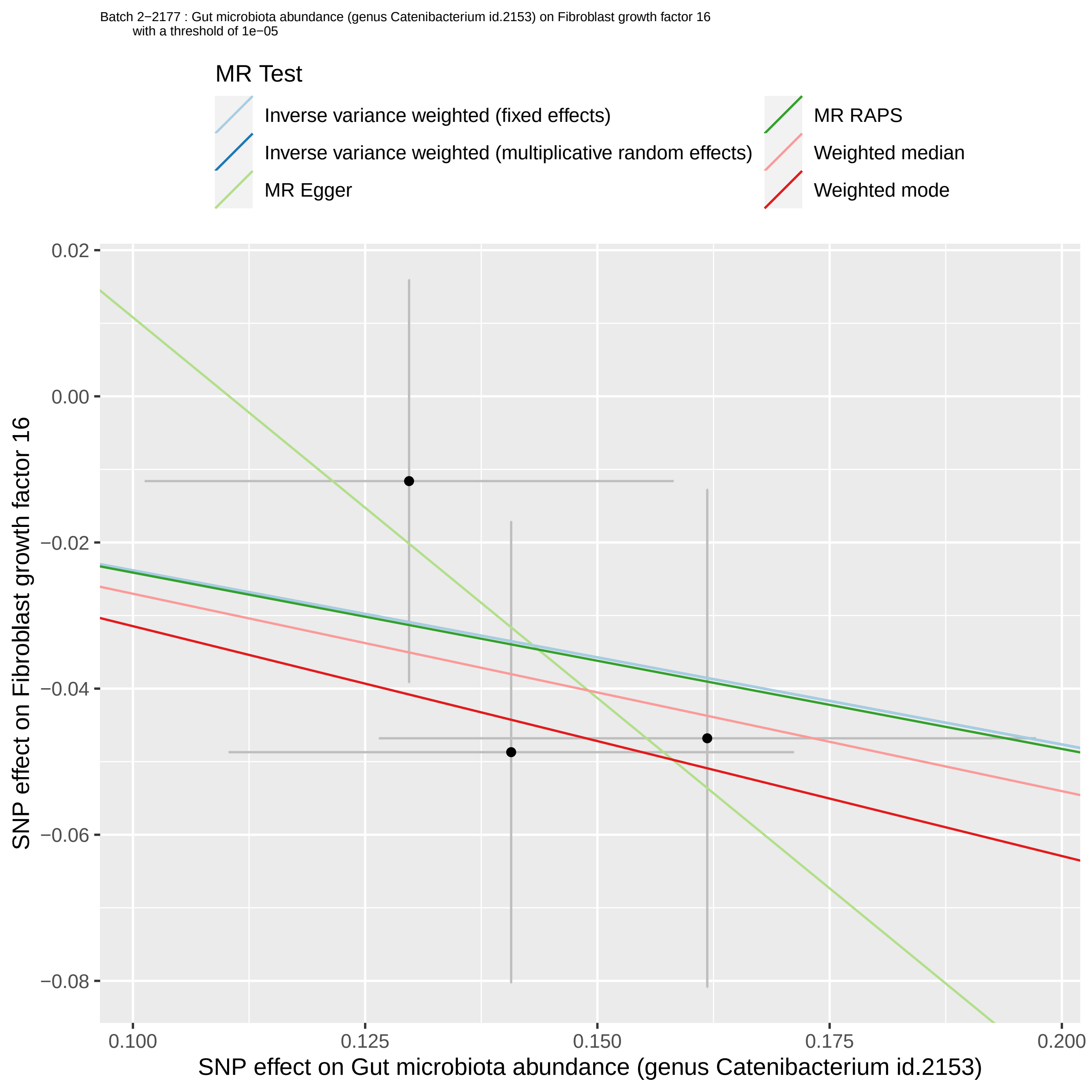
Batch 2−2177 : Gut microbiota abundance (genus Catenibacterium id.2153) on Fibroblast growth factor 16
with a threshold of 1e−05
MR Test
Inverse variance weighted (fixed effects)
Inverse variance weighted (multiplicative random effects)
MR Egger
MR RAPS
Weighted median
Weighted mode
0.02
0.00
−0.02
−0.04
−0.06
−0.08
0.100
0.125
0.150
0.175
0.200
SNP effect on Fibroblast growth factor 16
SNP effect on Gut microbiota abundance (genus Catenibacterium id.2153)

Batch 2−2178 : Gut microbiota abundance (genus Parasutterella id.2892) on Fibroblast growth factor 16
with a threshold of 1e−05

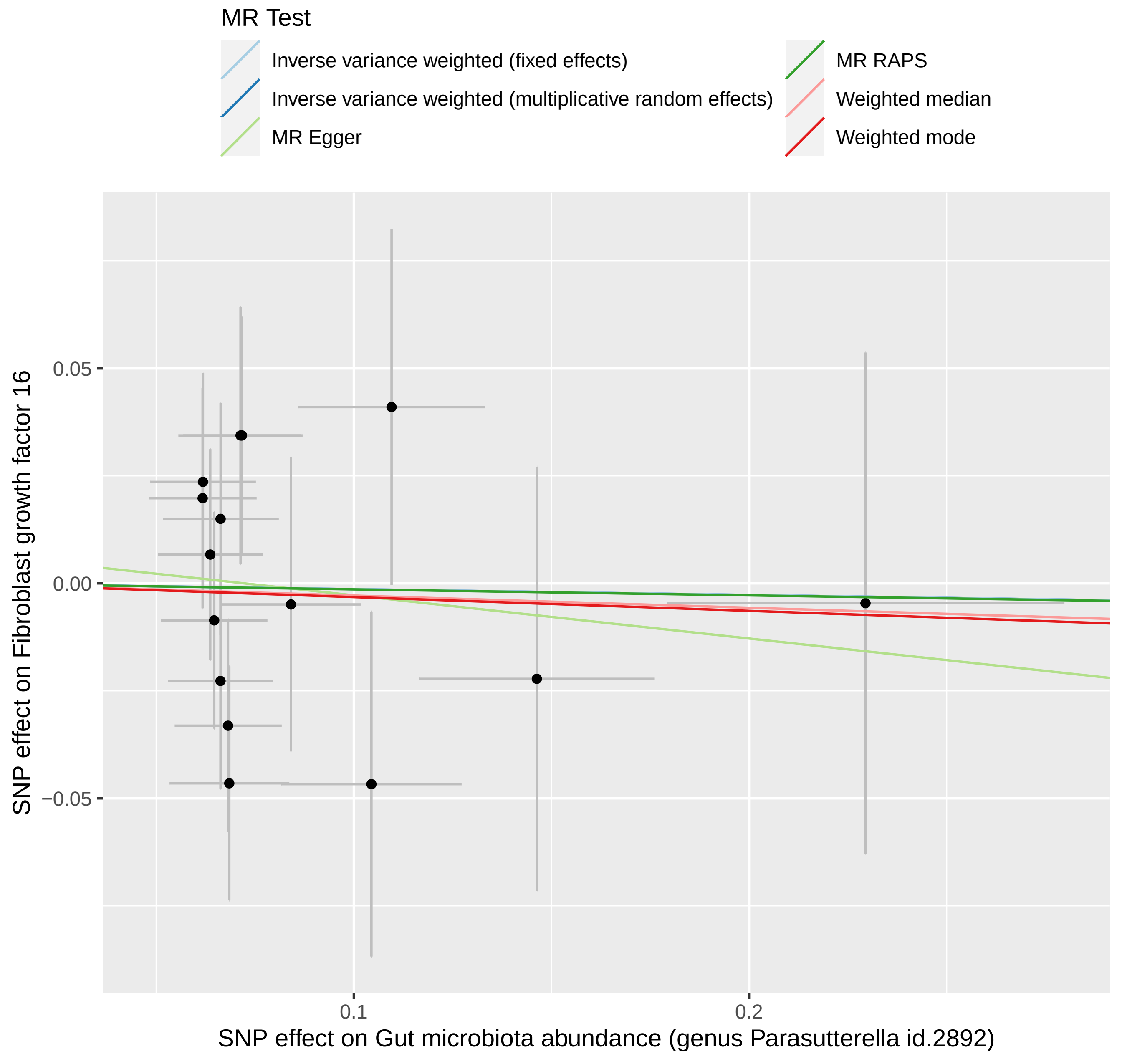

Batch 2−2179 : Gut microbiota abundance (genus Catenibacterium id.2153) on Fibroblast growth factor 16
with a threshold of 1e−05

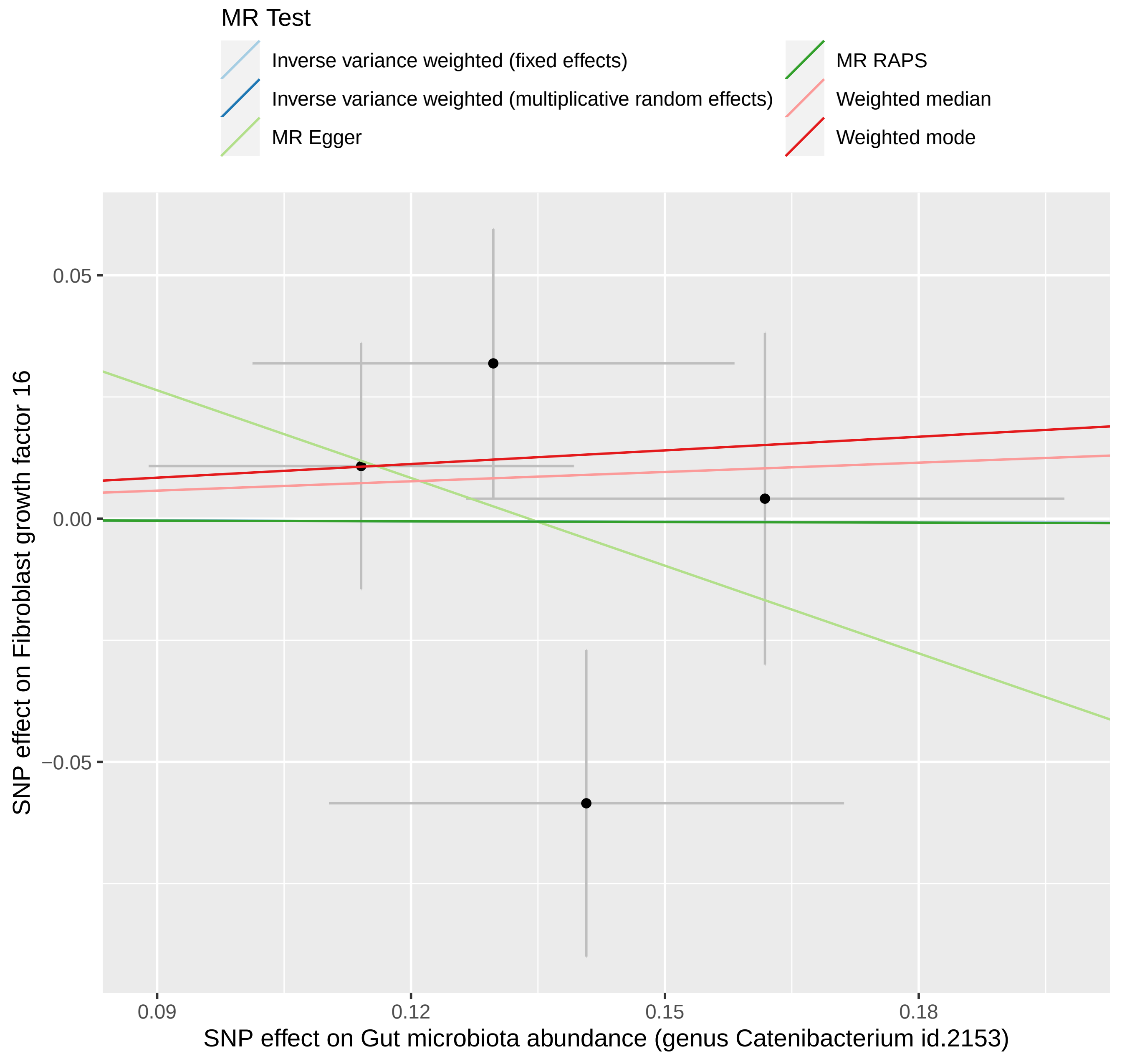

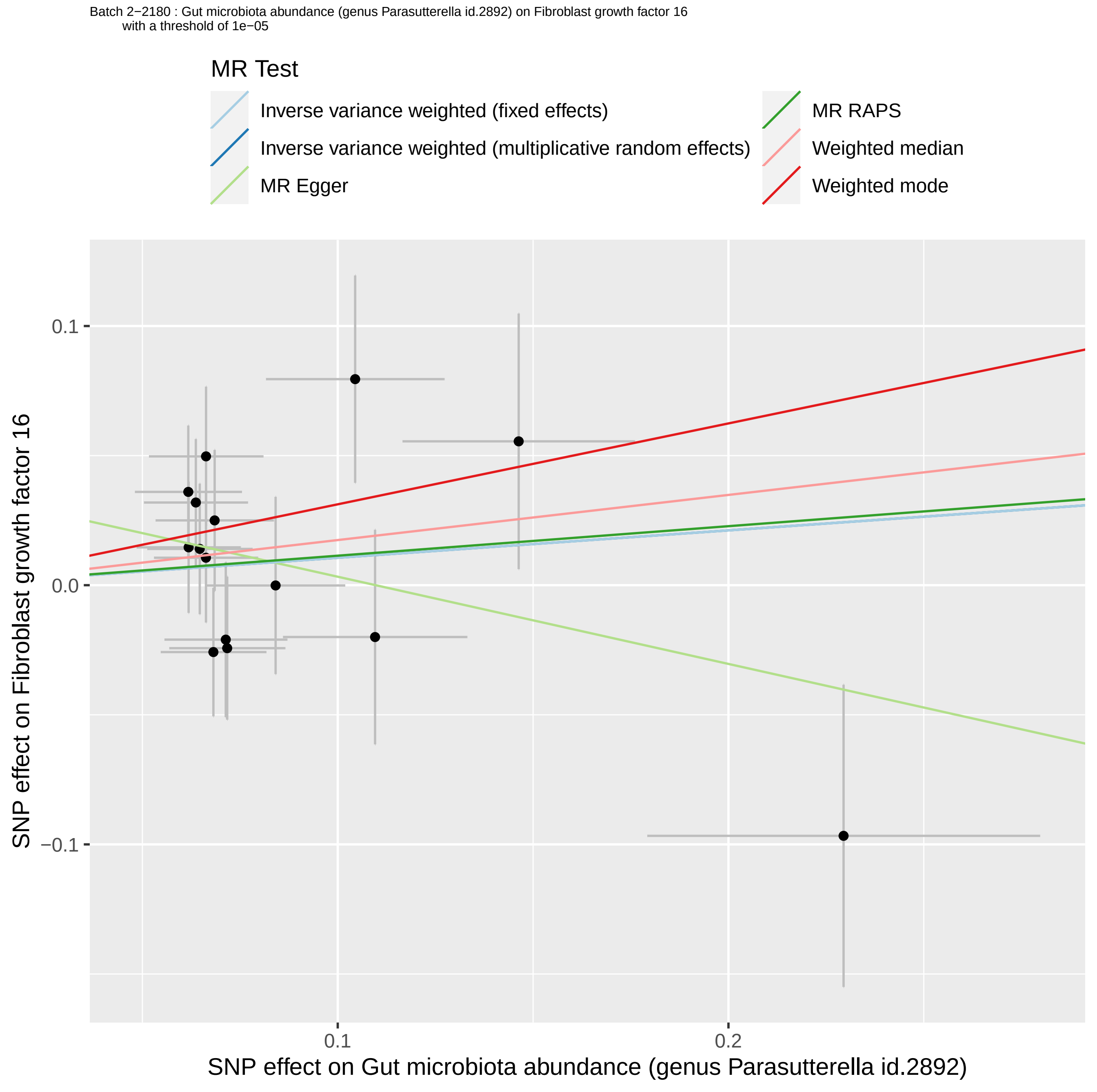

Batch 2−2180 : Gut microbiota abundance (genus Parasutterella id.2892) on Fibroblast growth factor 16
with a threshold of 1e−05
MR Test
Inverse variance weighted (fixed effects)
Inverse variance weighted (multiplicative random effects)
MR Egger
MR RAPS
Weighted median
Weighted mode
0.1
0.0
−0.1
0.1
0.2
SNP effect on Fibroblast growth factor 16
SNP effect on Gut microbiota abundance (genus Parasutterella id.2892)

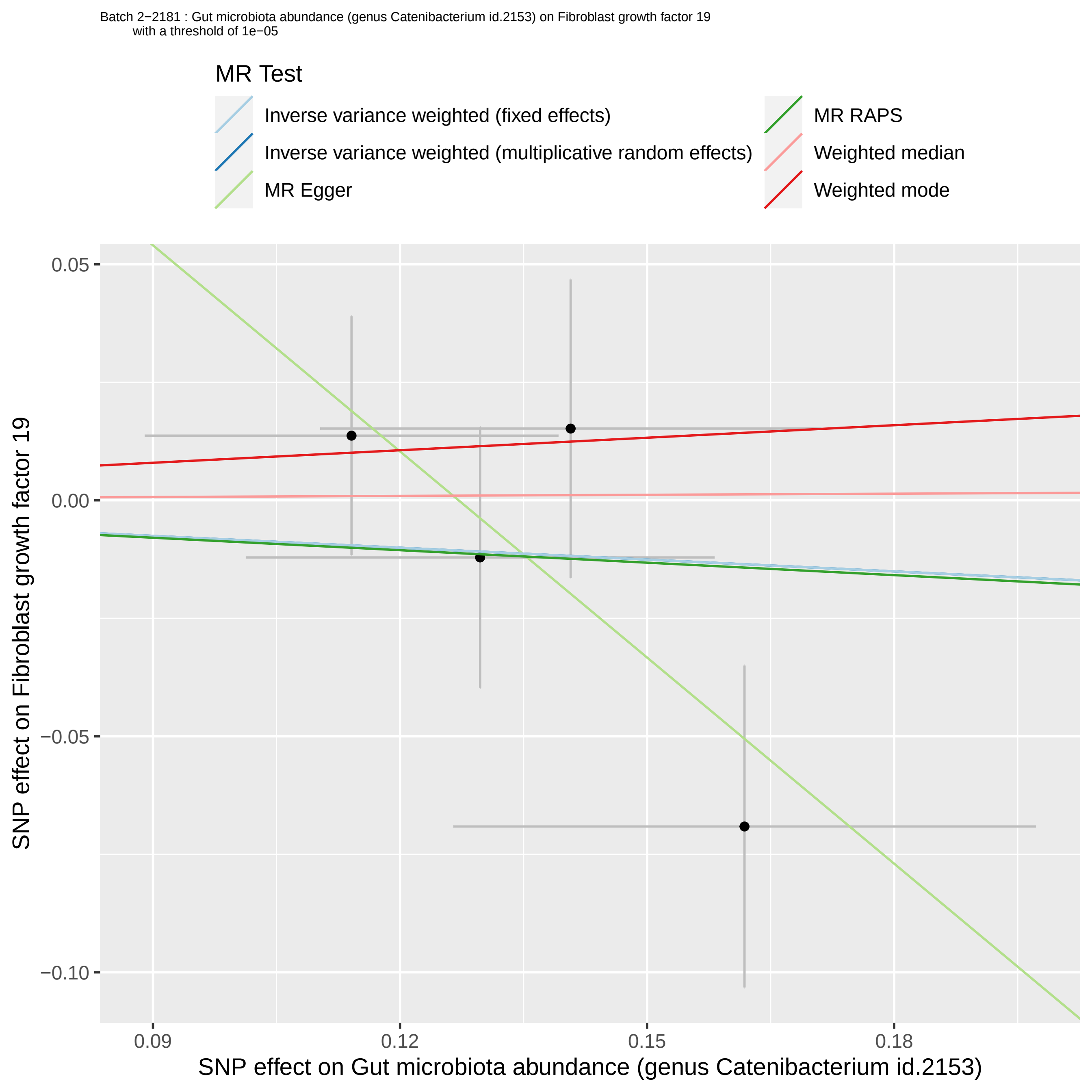
Batch 2−2181 : Gut microbiota abundance (genus Catenibacterium id.2153) on Fibroblast growth factor 19
with a threshold of 1e−05
MR Test
Inverse variance weighted (fixed effects)
Inverse variance weighted (multiplicative random effects)
MR Egger
MR RAPS
Weighted median
Weighted mode
0.05
0.00
−0.05
−0.10
0.09
0.12
0.15
0.18
SNP effect on Gut microbiota abundance (genus Catenibacterium id.2153)
SNP effect on Fibroblast growth factor 19

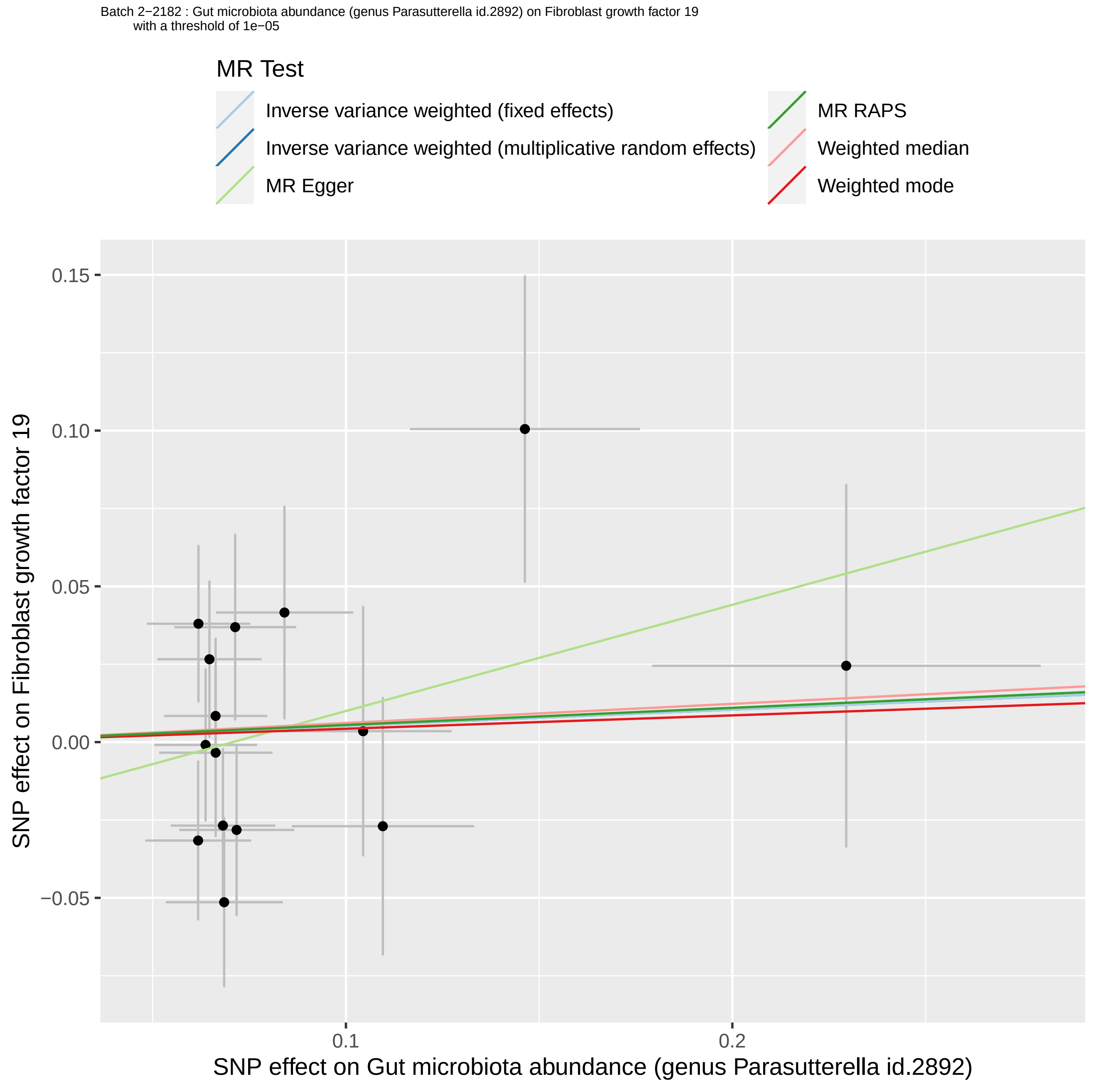
Batch 2−2182 : Gut microbiota abundance (genus Parasutterella id.2892) on Fibroblast growth factor 19
with a threshold of 1e−05
MR Test
Inverse variance weighted (fixed effects)
Inverse variance weighted (multiplicative random effects)
MR Egger
MR RAPS
Weighted median
Weighted mode
0.15
0.10
0.05
0.00
−0.05
0.1
0.2
SNP effect on Fibroblast growth factor 19
SNP effect on Gut microbiota abundance (genus Parasutterella id.2892)

Batch 2−2183 : Gut microbiota abundance (genus Catenibacterium id.2153) on Fibroblast growth factor 20
with a threshold of 1e−05

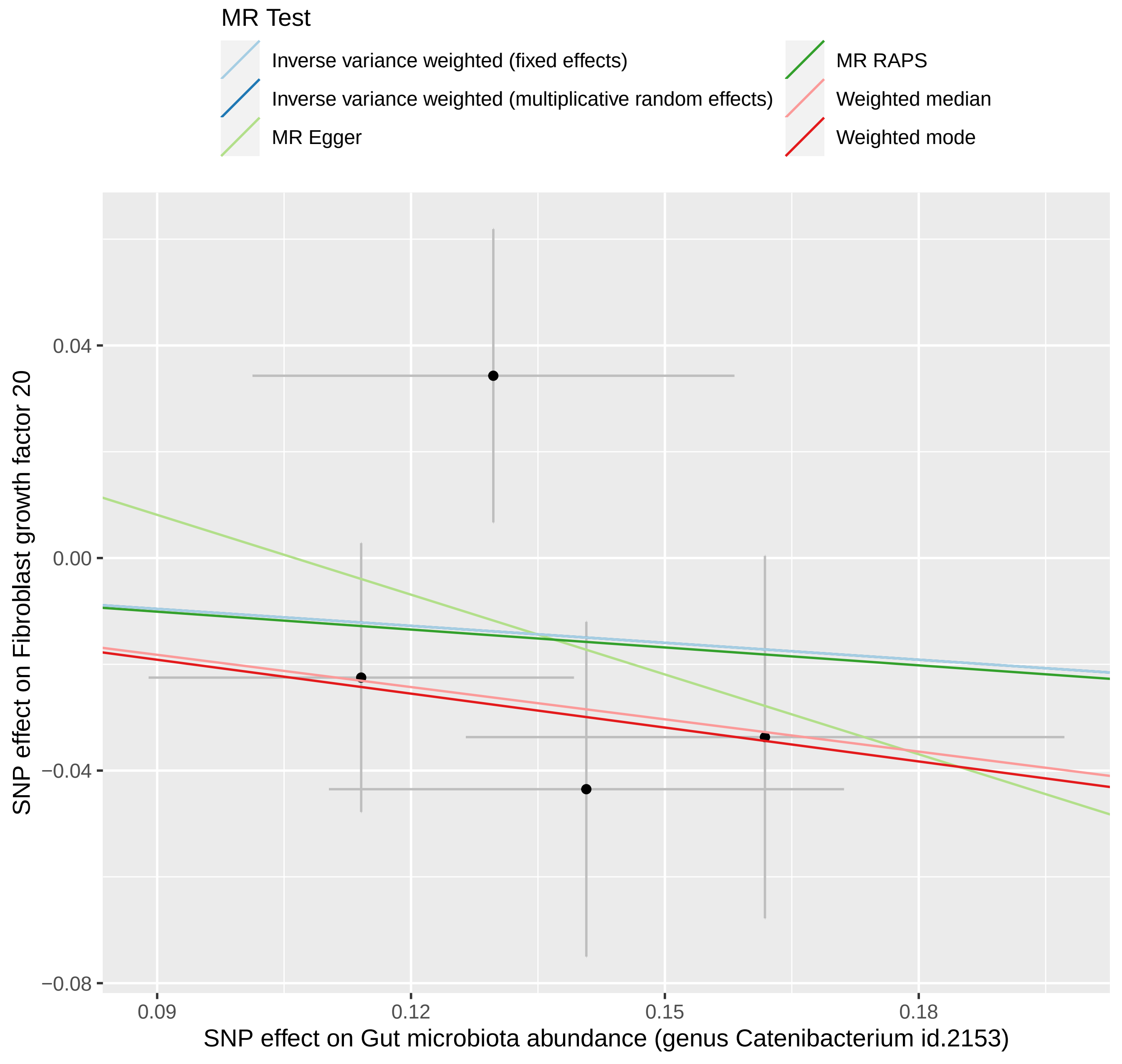

Batch 2−2184 : Gut microbiota abundance (genus Parasutterella id.2892) on Fibroblast growth factor 20
with a threshold of 1e−05

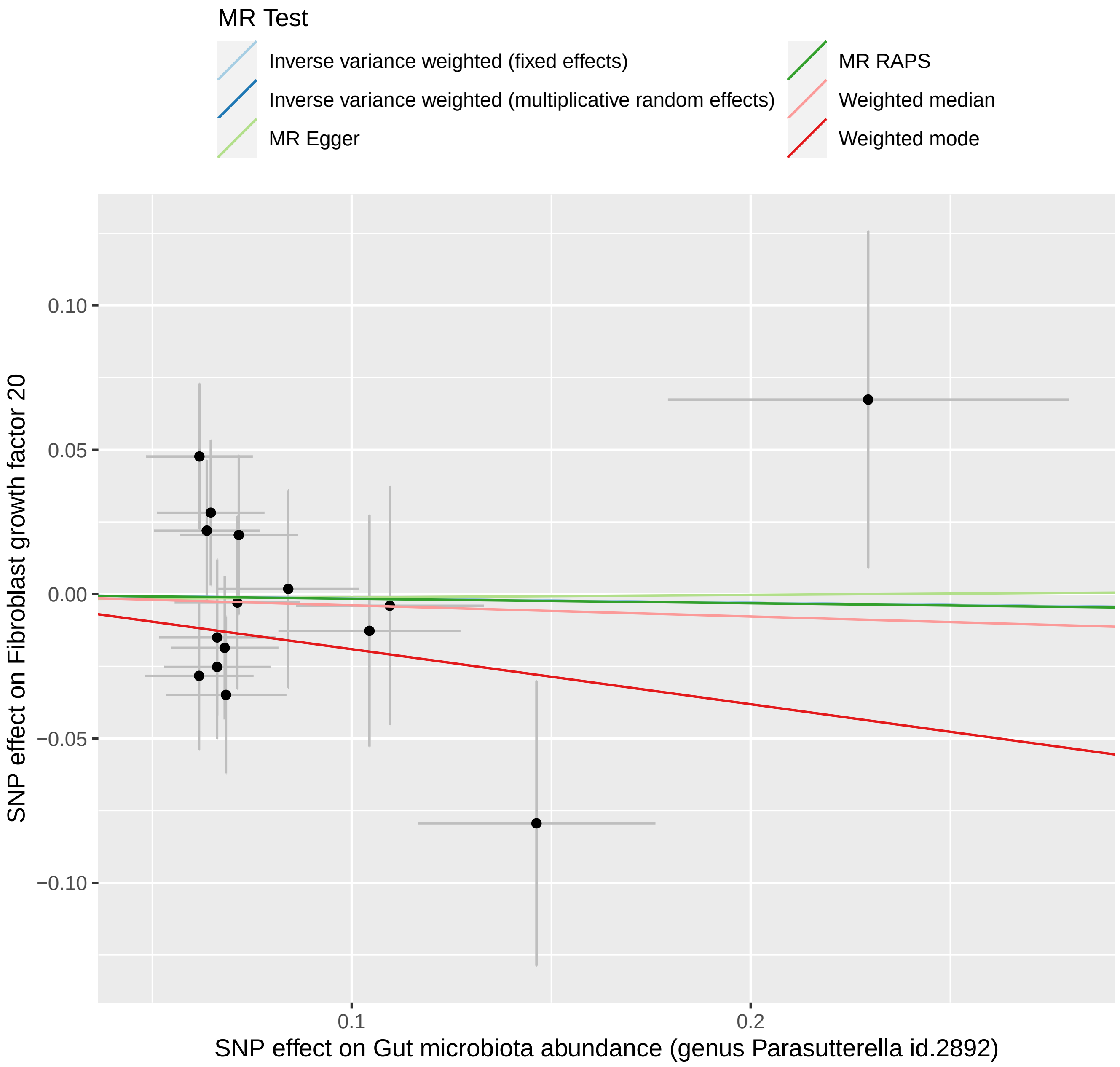

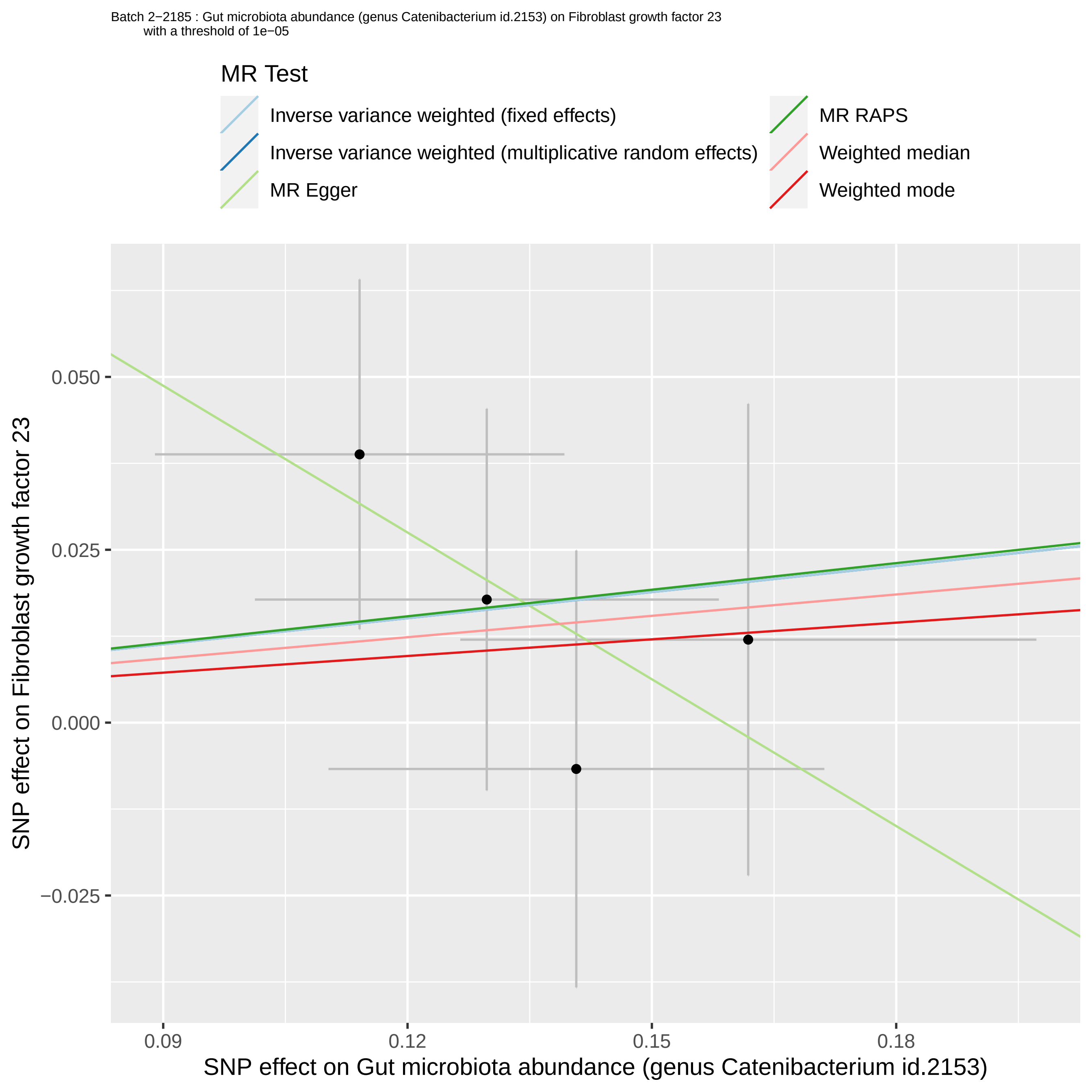
Batch 2−2185 : Gut microbiota abundance (genus Catenibacterium id.2153) on Fibroblast growth factor 23
with a threshold of 1e−05
MR Test
Inverse variance weighted (fixed effects)
Inverse variance weighted (multiplicative random effects)
MR Egger
MR RAPS
Weighted median
Weighted mode
0.050
0.025
0.000
−0.025
0.09
0.12
0.15
0.18
SNP effect on Fibroblast growth factor 23
SNP effect on Gut microbiota abundance (genus Catenibacterium id.2153)

Batch 2−2186 : Gut microbiota abundance (genus Parasutterella id.2892) on Fibroblast growth factor 23
with a threshold of 1e−05

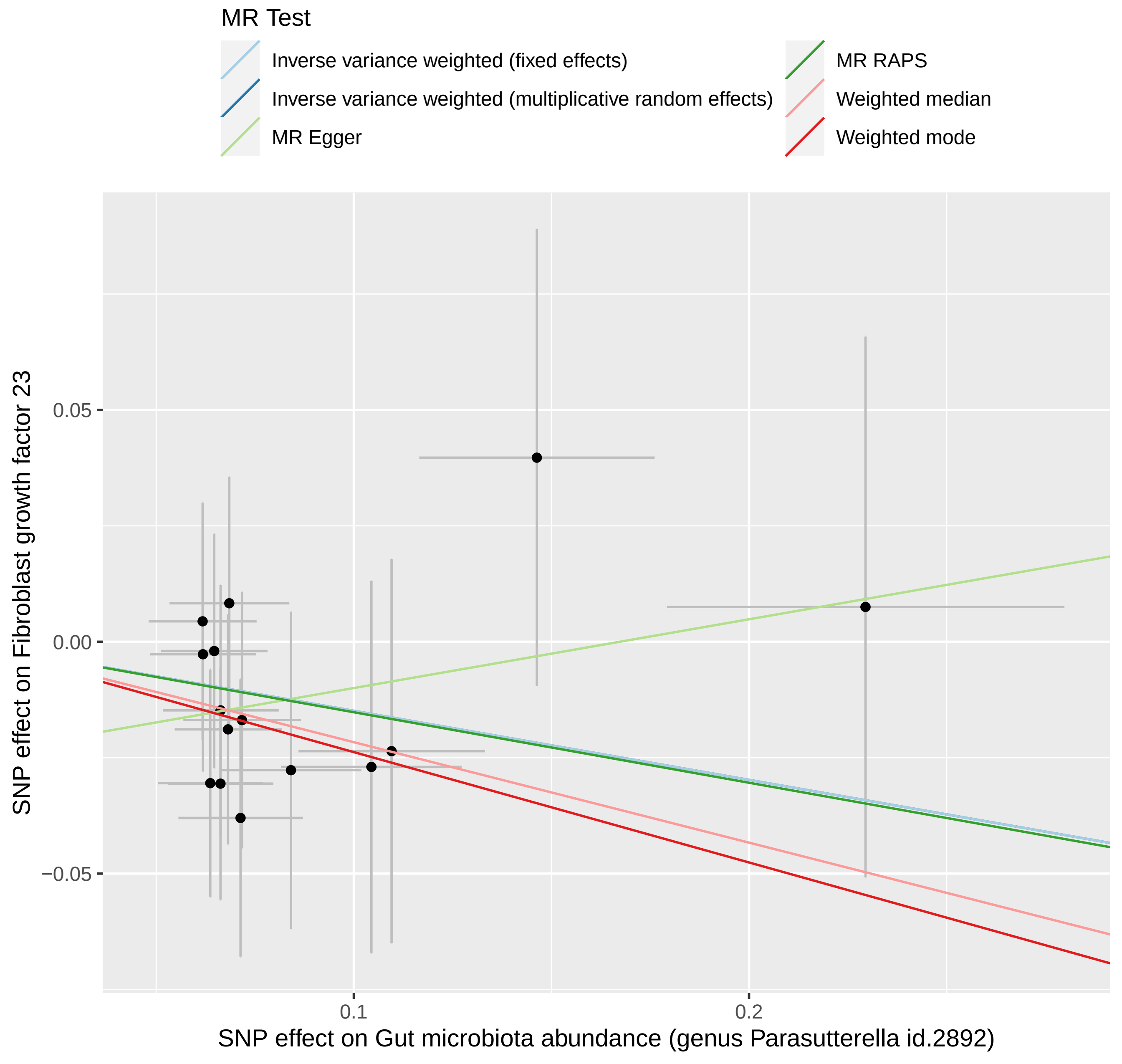

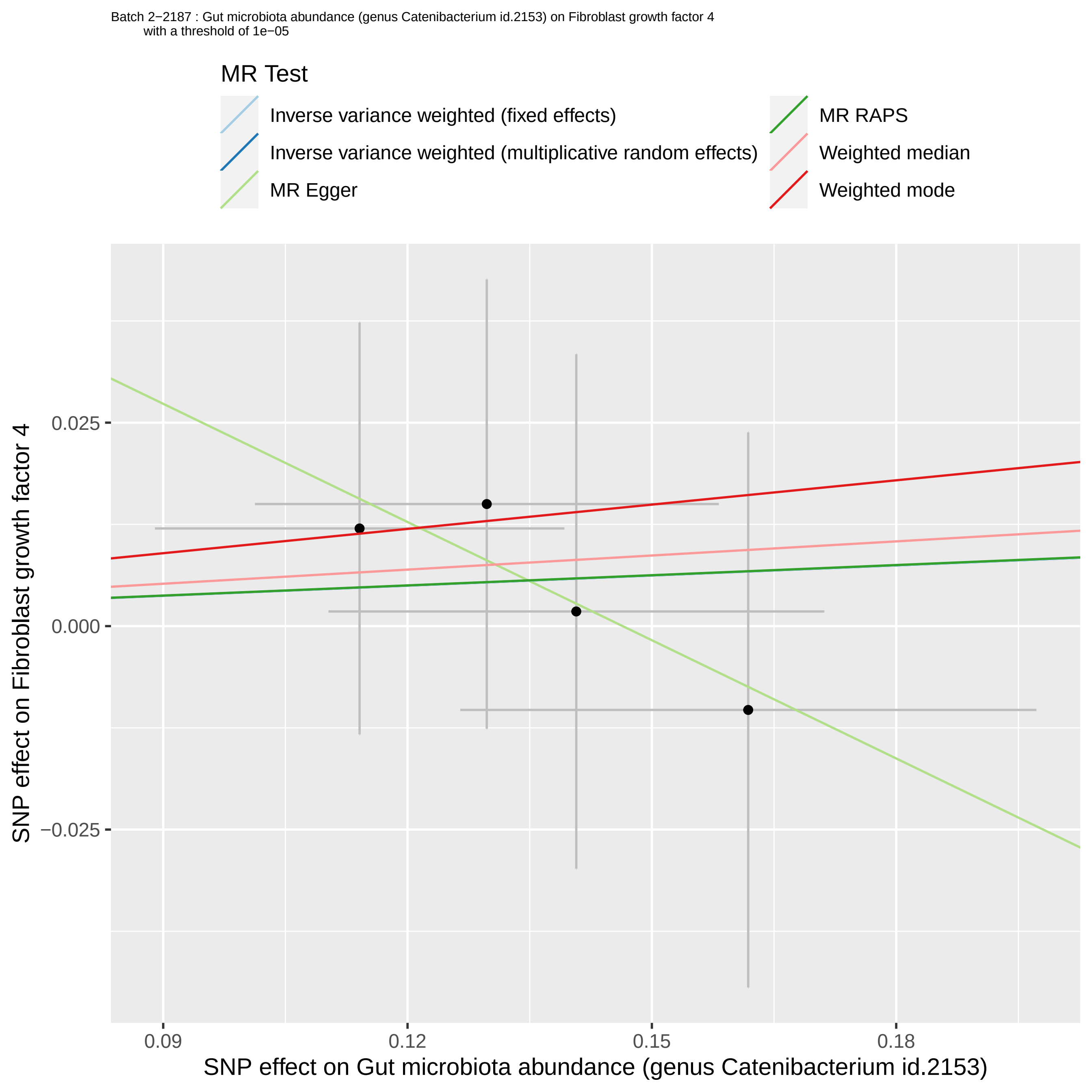

Batch 2−2187 : Gut microbiota abundance (genus Catenibacterium id.2153) on Fibroblast growth factor 4
with a threshold of 1e−05
MR Test
Inverse variance weighted (fixed effects)
Inverse variance weighted (multiplicative random effects)
MR Egger
MR RAPS
Weighted median
Weighted mode
0.025
0.000
−0.025
0.09
0.12
0.15
0.18
SNP effect on Fibroblast growth factor 4
SNP effect on Gut microbiota abundance (genus Catenibacterium id.2153)

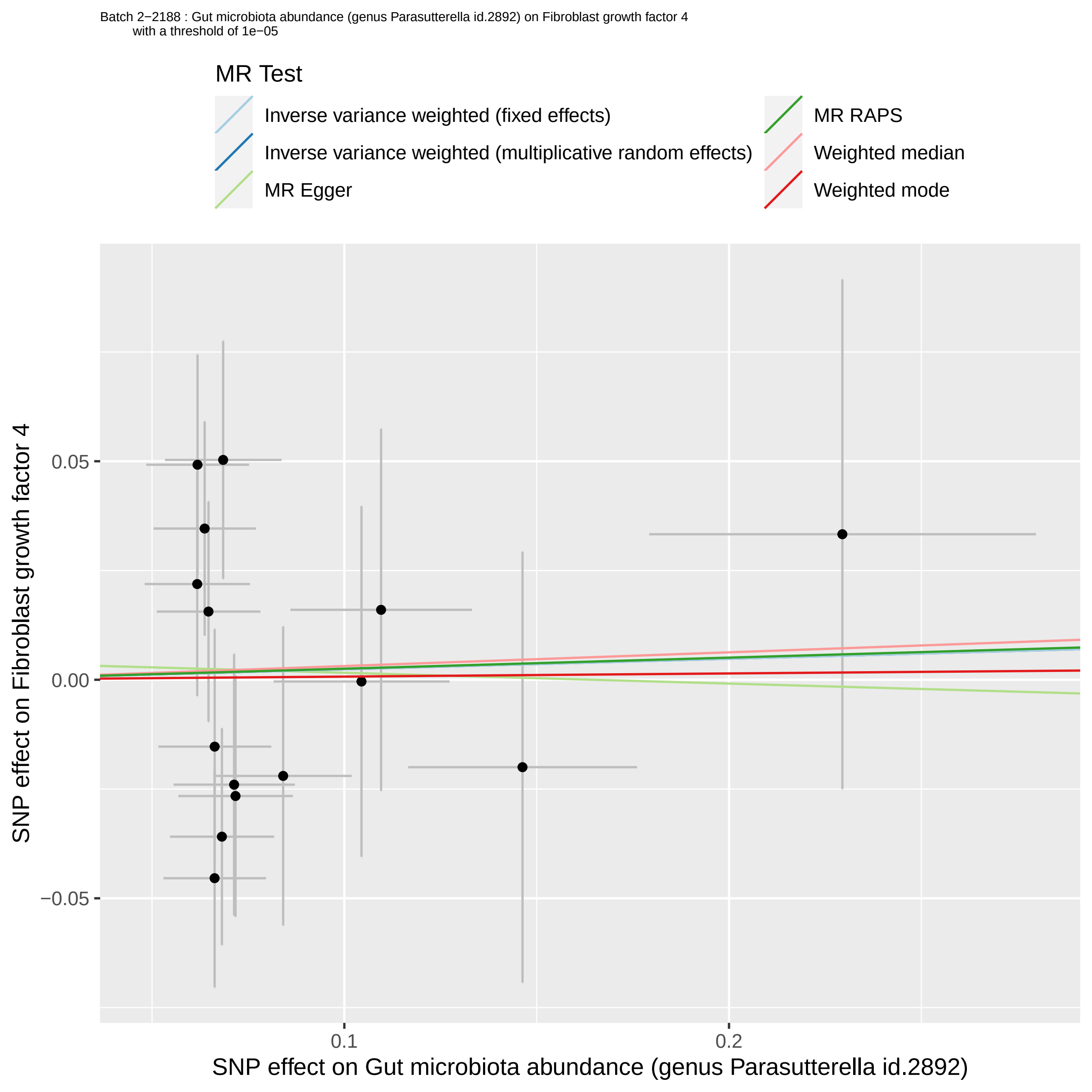
Batch 2−2188 : Gut microbiota abundance (genus Parasutterella id.2892) on Fibroblast growth factor 4
with a threshold of 1e−05
MR Test
Inverse variance weighted (fixed effects)
Inverse variance weighted (multiplicative random effects)
MR Egger
MR RAPS
Weighted median
Weighted mode
0.05
0.00
−0.05
0.1
0.2
SNP effect on Fibroblast growth factor 4
SNP effect on Gut microbiota abundance (genus Parasutterella id.2892)

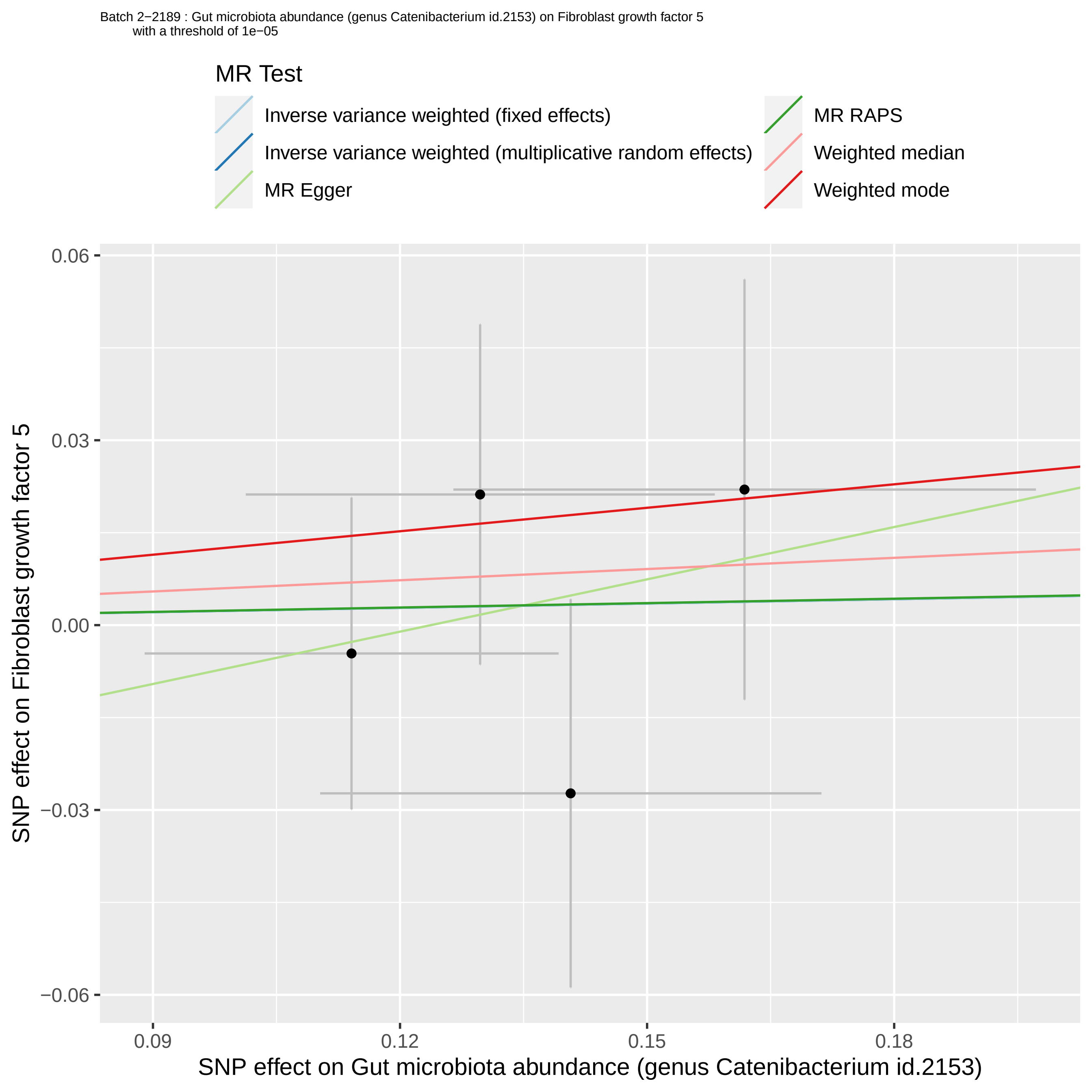

Batch 2−2189 : Gut microbiota abundance (genus Catenibacterium id.2153) on Fibroblast growth factor 5
with a threshold of 1e−05
MR Test
Inverse variance weighted (fixed effects)
Inverse variance weighted (multiplicative random effects)
MR Egger
MR RAPS
Weighted median
Weighted mode
0.06
0.03
0.00
−0.03
−0.06
0.09
0.12
0.15
0.18
SNP effect on Fibroblast growth factor 5
SNP effect on Gut microbiota abundance (genus Catenibacterium id.2153)

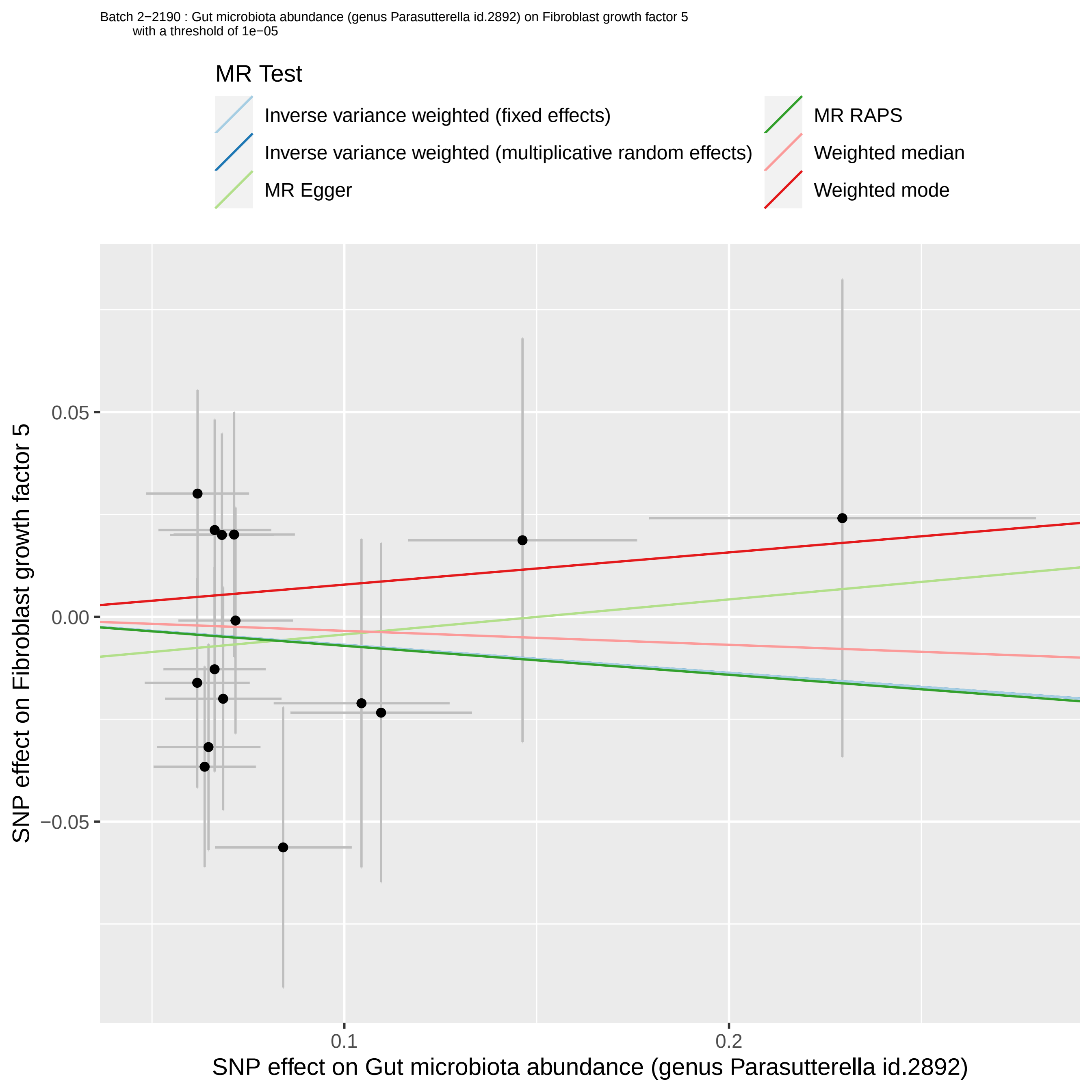

Batch 2−2190 : Gut microbiota abundance (genus Parasutterella id.2892) on Fibroblast growth factor 5
with a threshold of 1e−05
MR Test
Inverse variance weighted (fixed effects)
Inverse variance weighted (multiplicative random effects)
MR Egger
MR RAPS
Weighted median
Weighted mode
0.05
0.00
−0.05
0.1
0.2
SNP effect on Fibroblast growth factor 5
SNP effect on Gut microbiota abundance (genus Parasutterella id.2892)

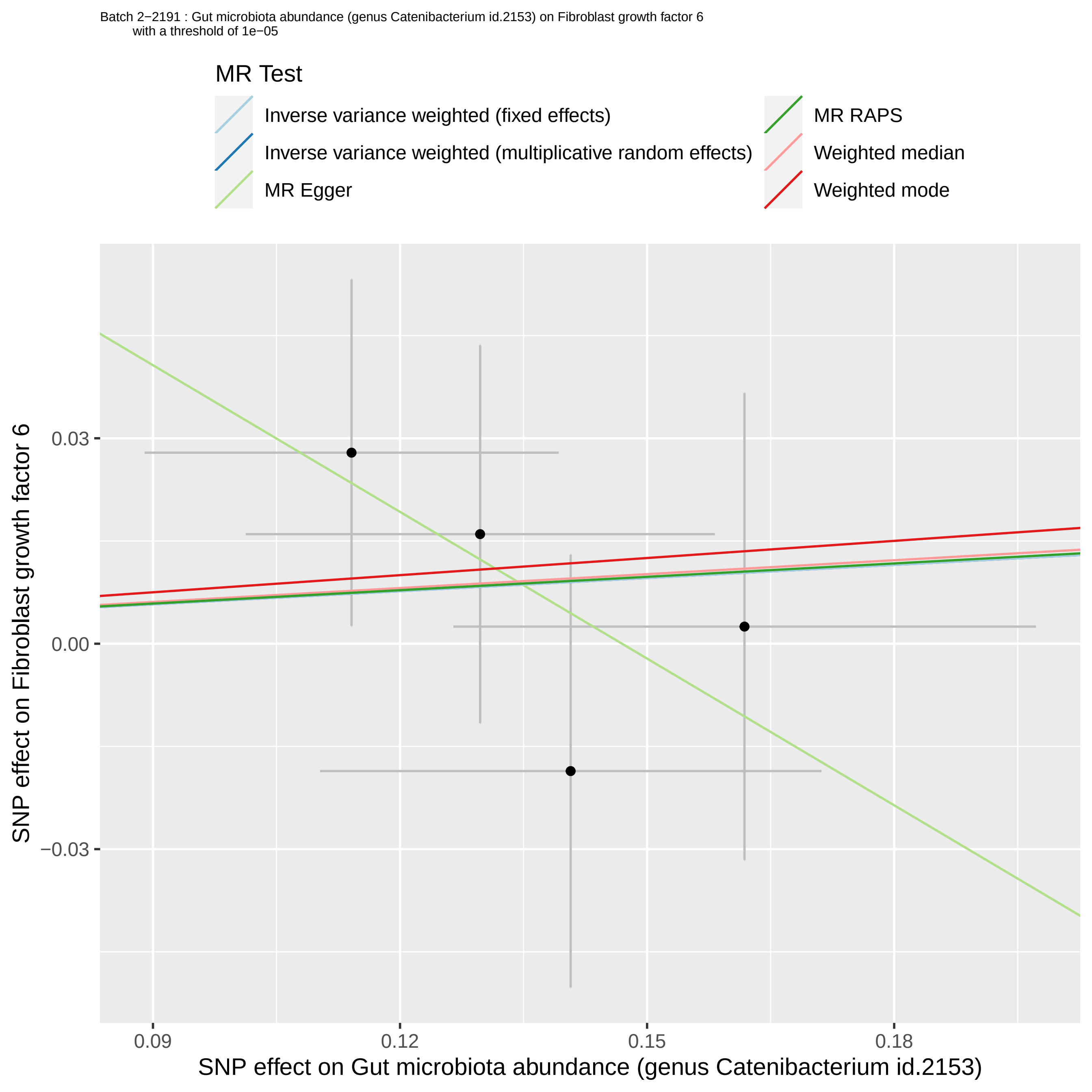

Batch 2−2191 : Gut microbiota abundance (genus Catenibacterium id.2153) on Fibroblast growth factor 6
with a threshold of 1e−05
MR Test
Inverse variance weighted (fixed effects)
Inverse variance weighted (multiplicative random effects)
MR Egger
MR RAPS
Weighted median
Weighted mode
SNP effect on Fibroblast growth factor 6
0.03
0.00
−0.03
0.09
0.12
0.15
0.18
SNP effect on Gut microbiota abundance (genus Catenibacterium id.2153)

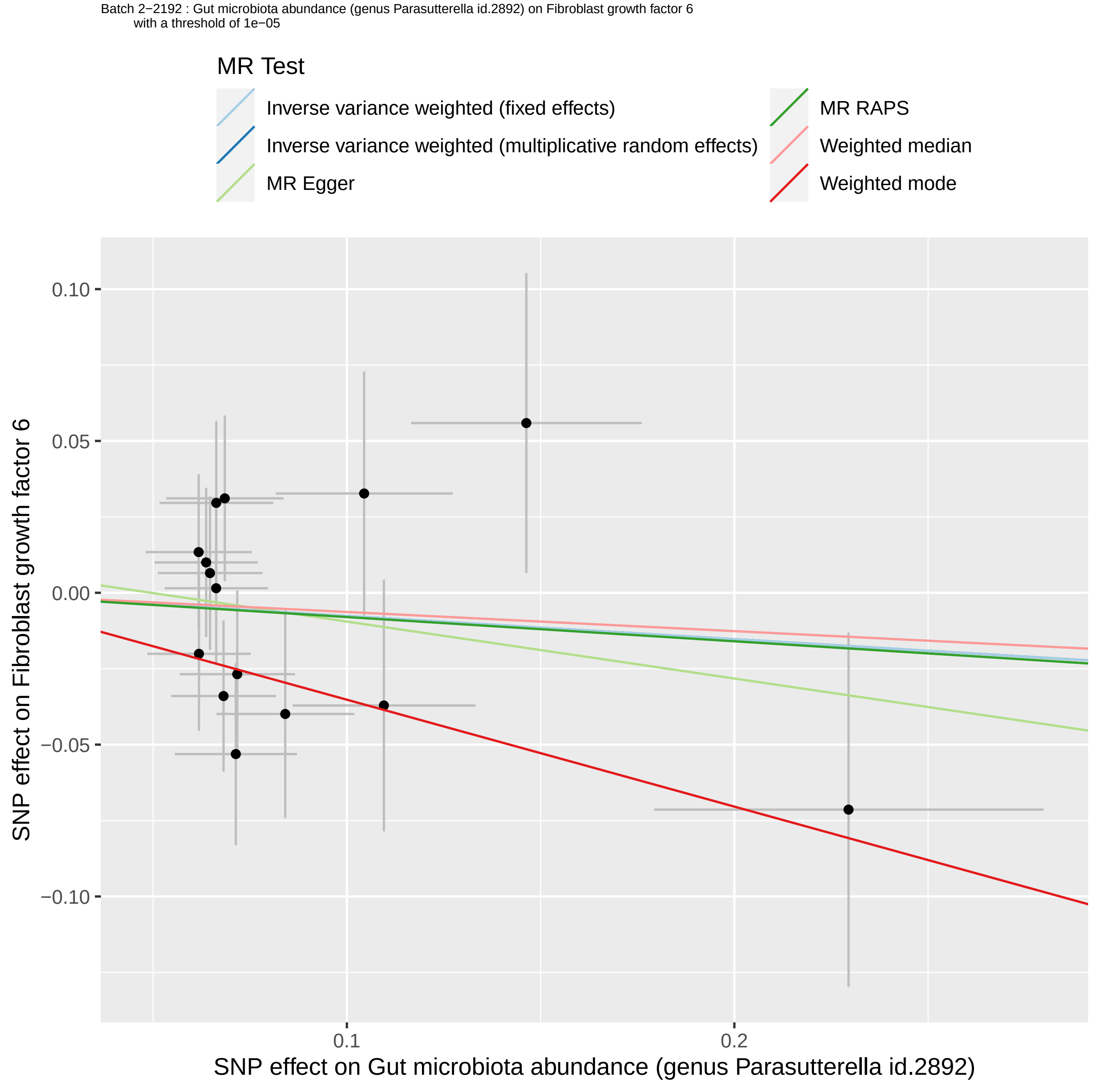
Batch 2−2192 : Gut microbiota abundance (genus Parasutterella id.2892) on Fibroblast growth factor 6
with a threshold of 1e−05
MR Test
Inverse variance weighted (fixed effects)
Inverse variance weighted (multiplicative random effects)
MR Egger
MR RAPS
Weighted median
Weighted mode
0.10
0.05
0.00
−0.05
−0.10
0.1
0.2
SNP effect on Fibroblast growth factor 6
SNP effect on Gut microbiota abundance (genus Parasutterella id.2892)

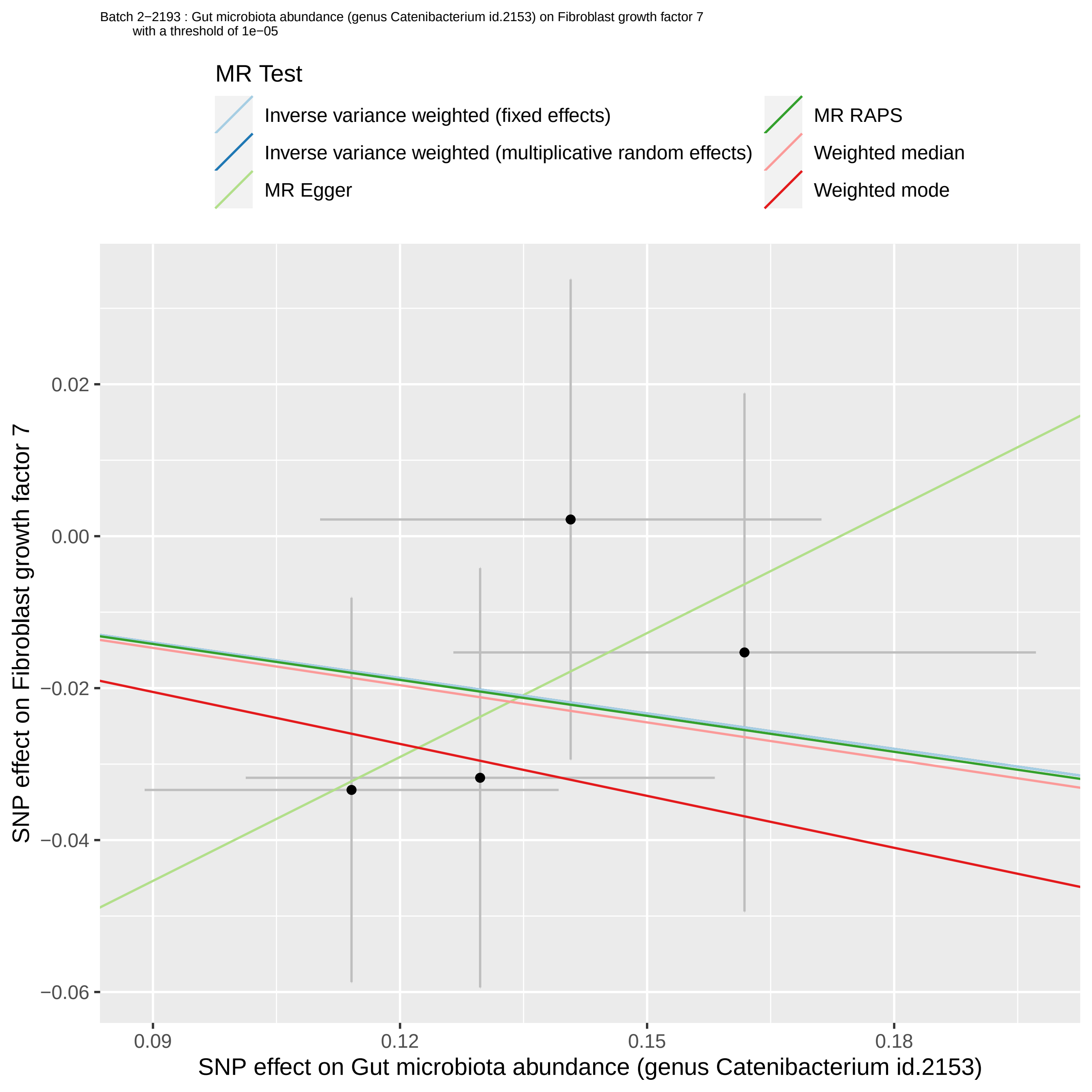
Batch 2−2193 : Gut microbiota abundance (genus Catenibacterium id.2153) on Fibroblast growth factor 7
with a threshold of 1e−05
MR Test
Inverse variance weighted (fixed effects)
Inverse variance weighted (multiplicative random effects)
MR Egger
MR RAPS
Weighted median
Weighted mode
0.02
0.00
−0.02
−0.04
−0.06
0.09
0.12
0.15
0.18
SNP effect on Fibroblast growth factor 7
SNP effect on Gut microbiota abundance (genus Catenibacterium id.2153)

Batch 2−2194 : Gut microbiota abundance (genus Parasutterella id.2892) on Fibroblast growth factor 7
with a threshold of 1e−05

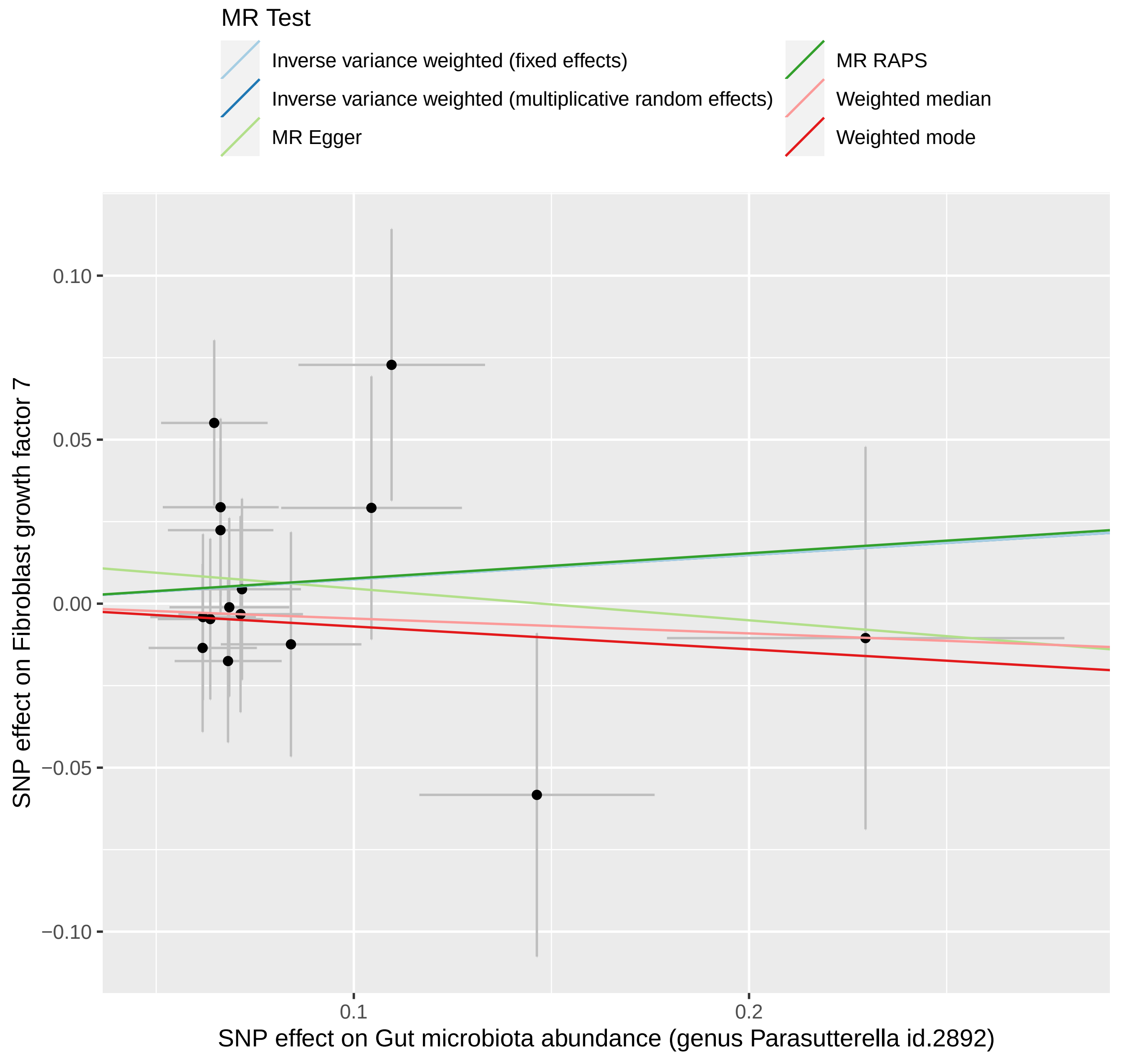

Batch 2−2195 : Gut microbiota abundance (genus Catenibacterium id.2153) on Fibroblast growth factor 7
with a threshold of 1e−05

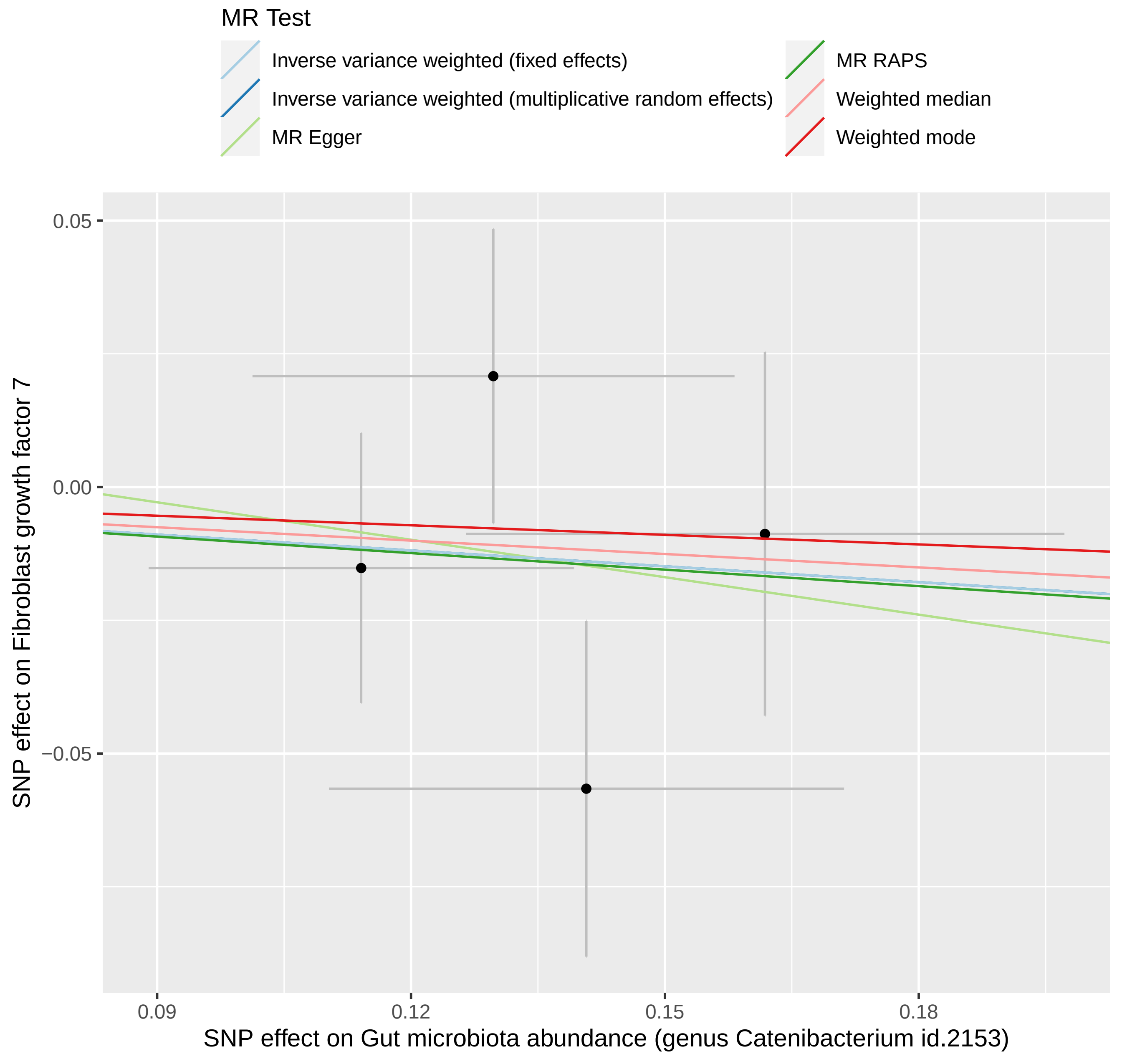

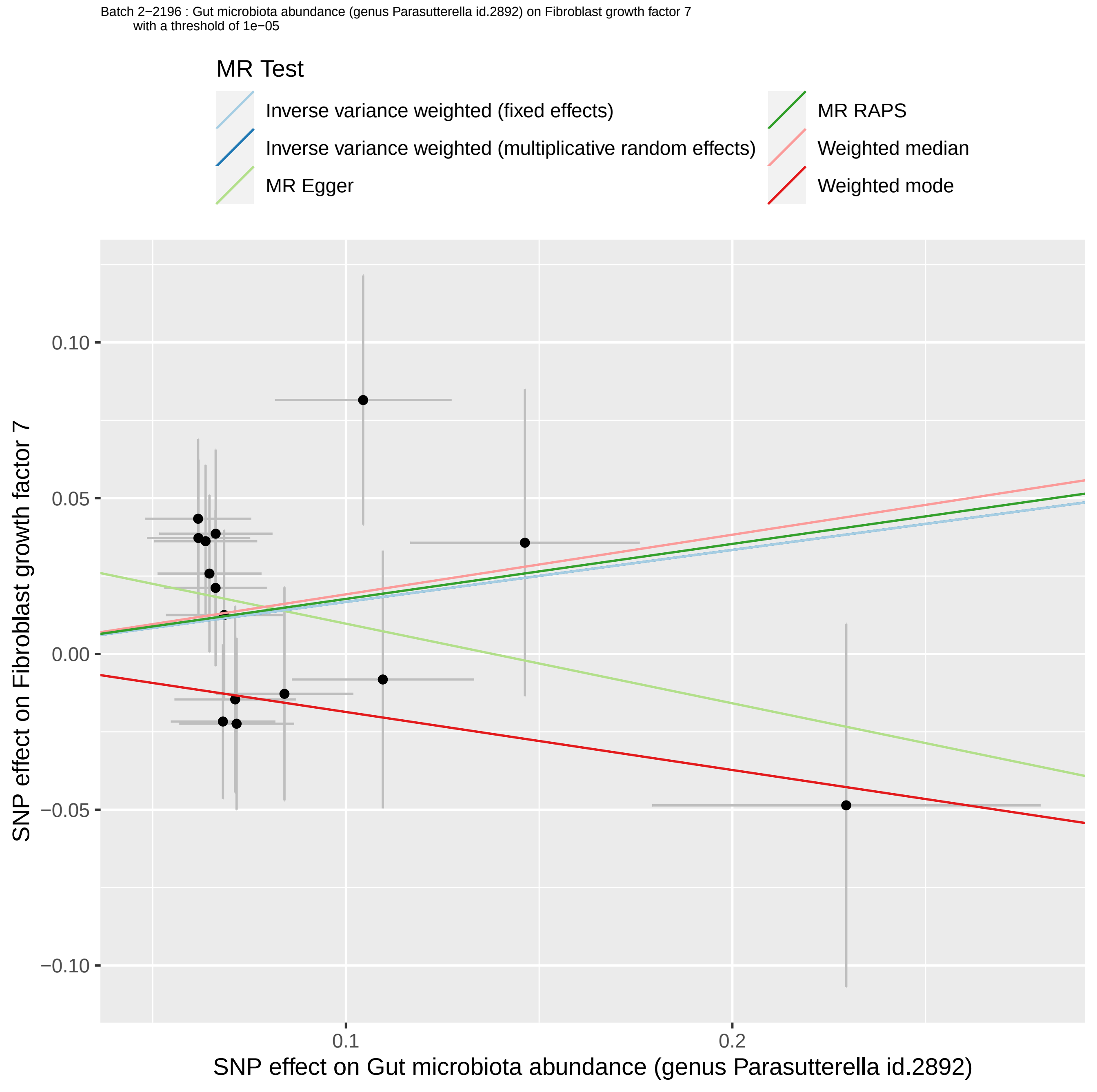

Batch 2−2196 : Gut microbiota abundance (genus Parasutterella id.2892) on Fibroblast growth factor 7
with a threshold of 1e−05
MR Test
Inverse variance weighted (fixed effects)
Inverse variance weighted (multiplicative random effects)
MR Egger
MR RAPS
Weighted median
Weighted mode
0.10
0.05
0.00
−0.05
−0.10
0.1
0.2
SNP effect on Fibroblast growth factor 7
SNP effect on Gut microbiota abundance (genus Parasutterella id.2892)

Batch 2−2197 : Gut microbiota abundance (genus Catenibacterium id.2153) on Fibroblast growth factor 8 isoform B
with a threshold of 1e−05

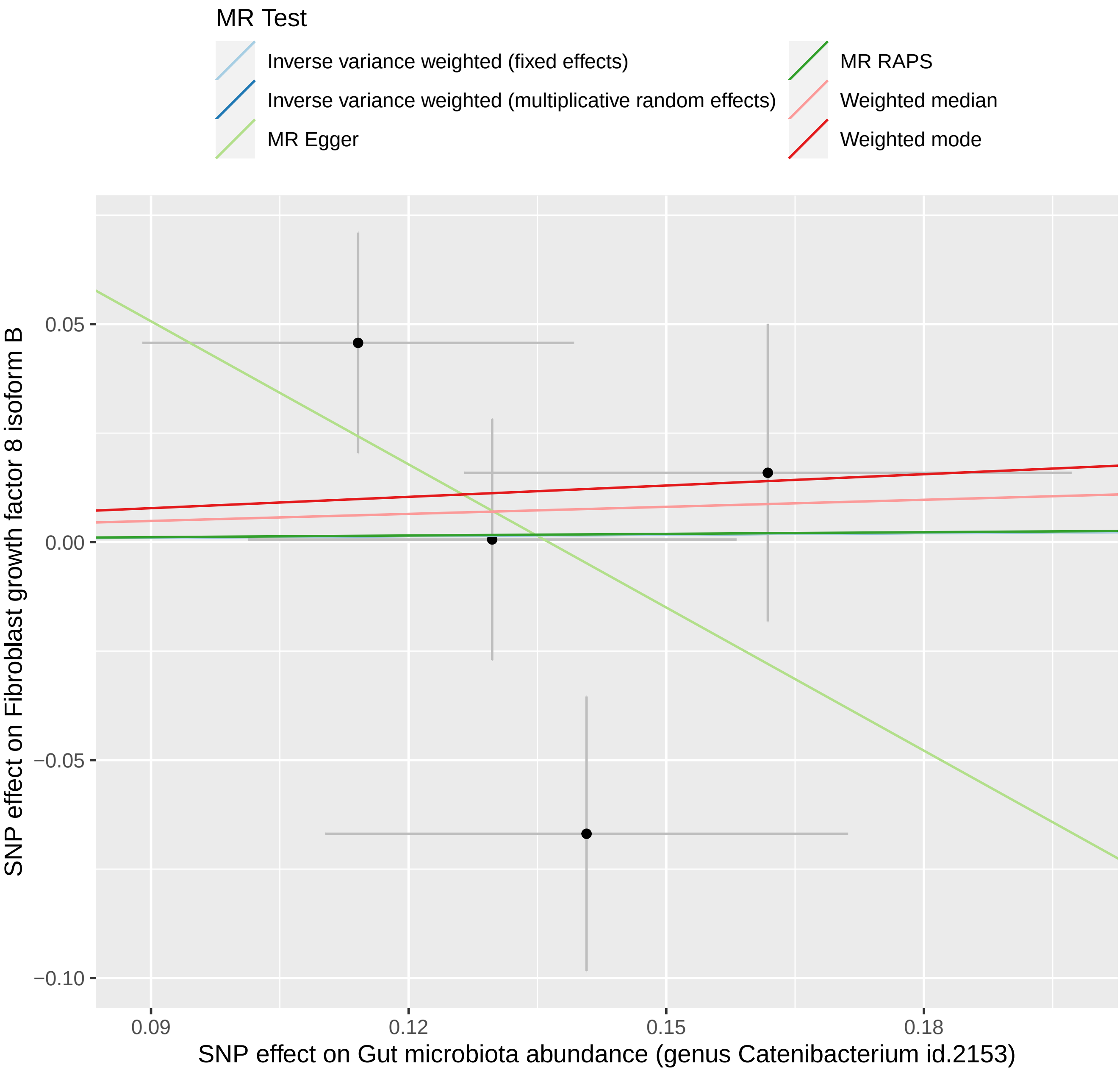

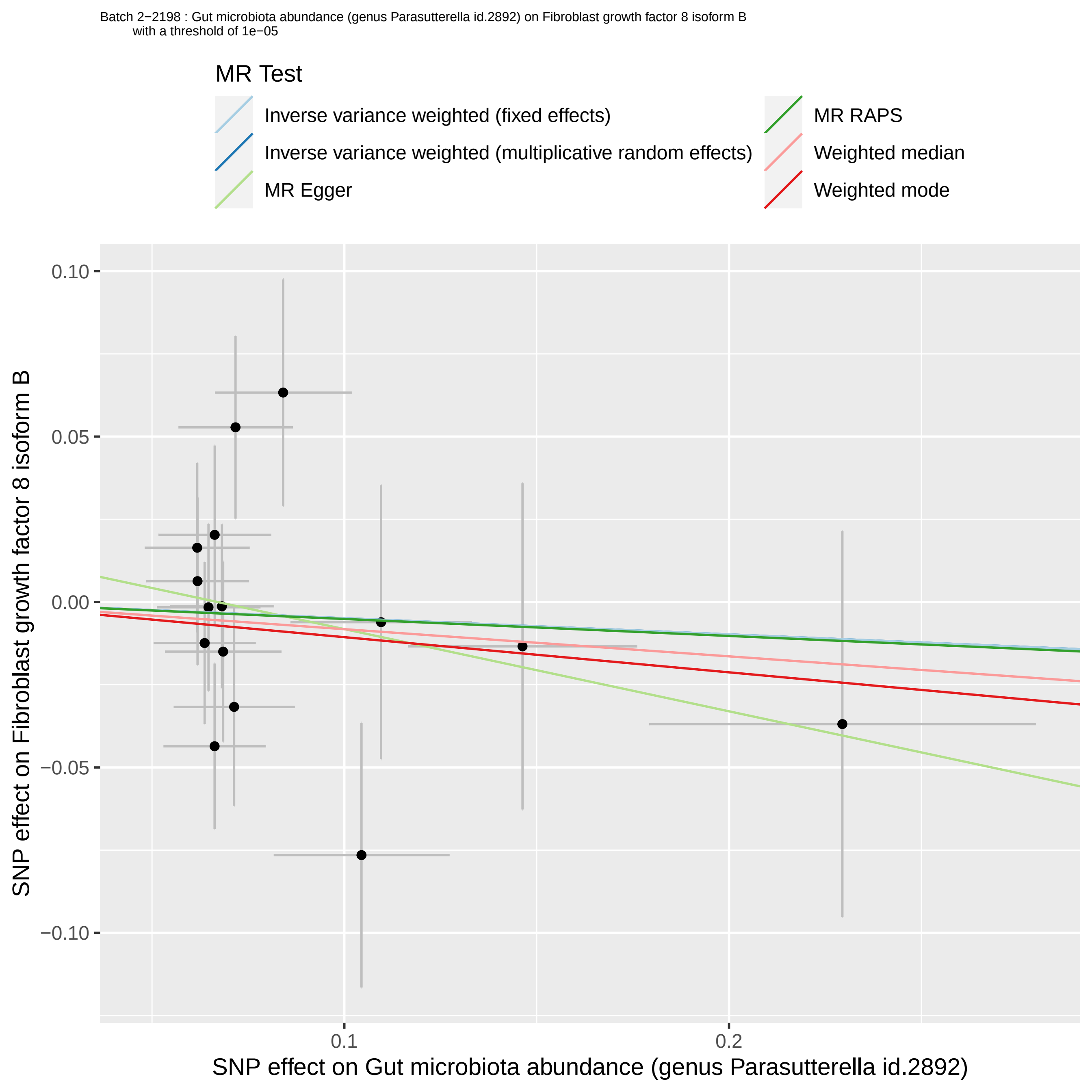
Batch 2−2198 : Gut microbiota abundance (genus Parasutterella id.2892) on Fibroblast growth factor 8 isoform B
with a threshold of 1e−05
MR Test
Inverse variance weighted (fixed effects)
Inverse variance weighted (multiplicative random effects)
MR Egger
MR RAPS
Weighted median
Weighted mode
0.10
0.05
0.00
−0.05
−0.10
0.1
0.2
SNP effect on Fibroblast growth factor 8 isoform B
SNP effect on Gut microbiota abundance (genus Parasutterella id.2892)

Batch 2−2199 : Gut microbiota abundance (genus Catenibacterium id.2153) on Fibroblast growth factor 8 isoform A
with a threshold of 1e−05

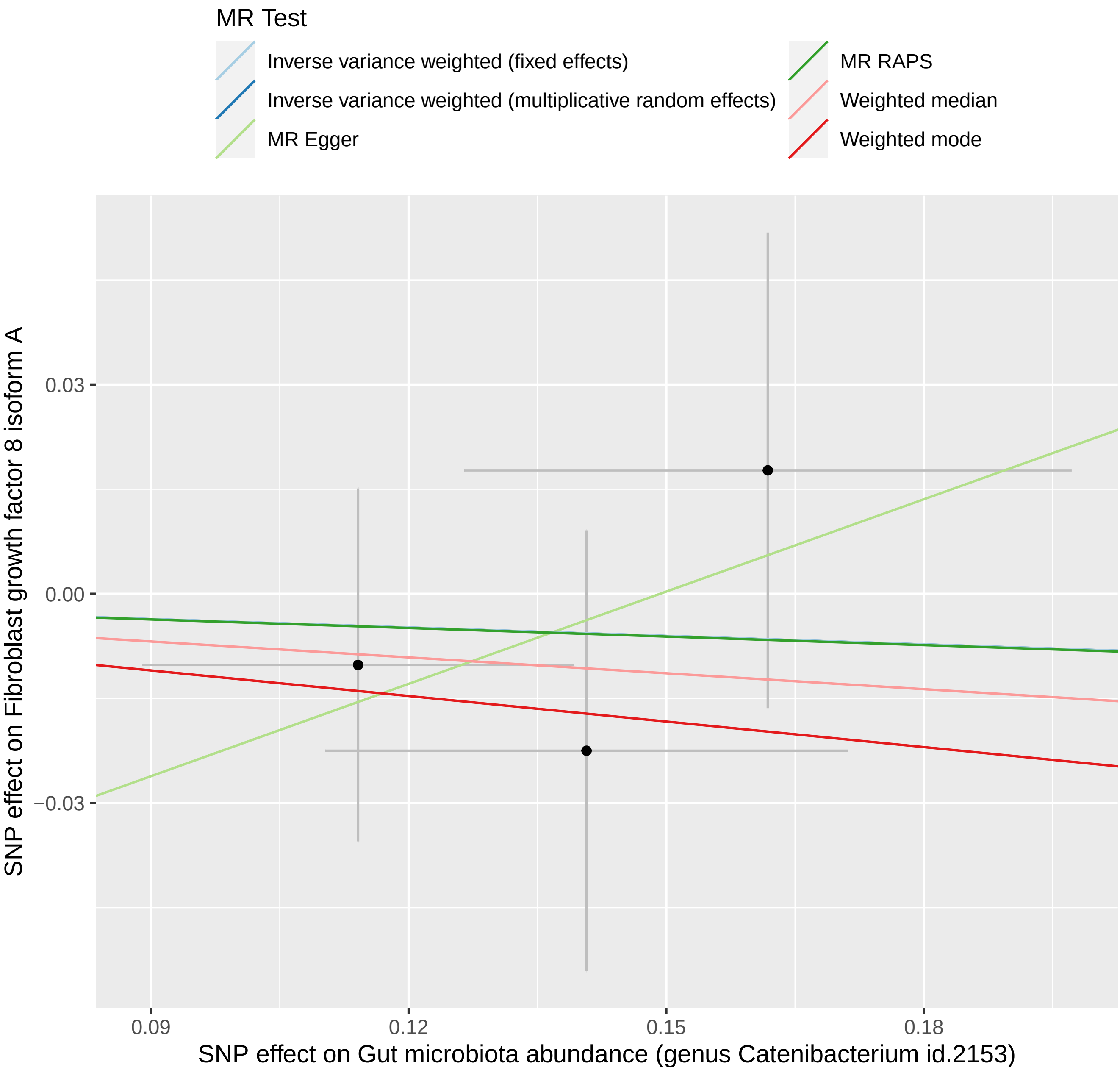

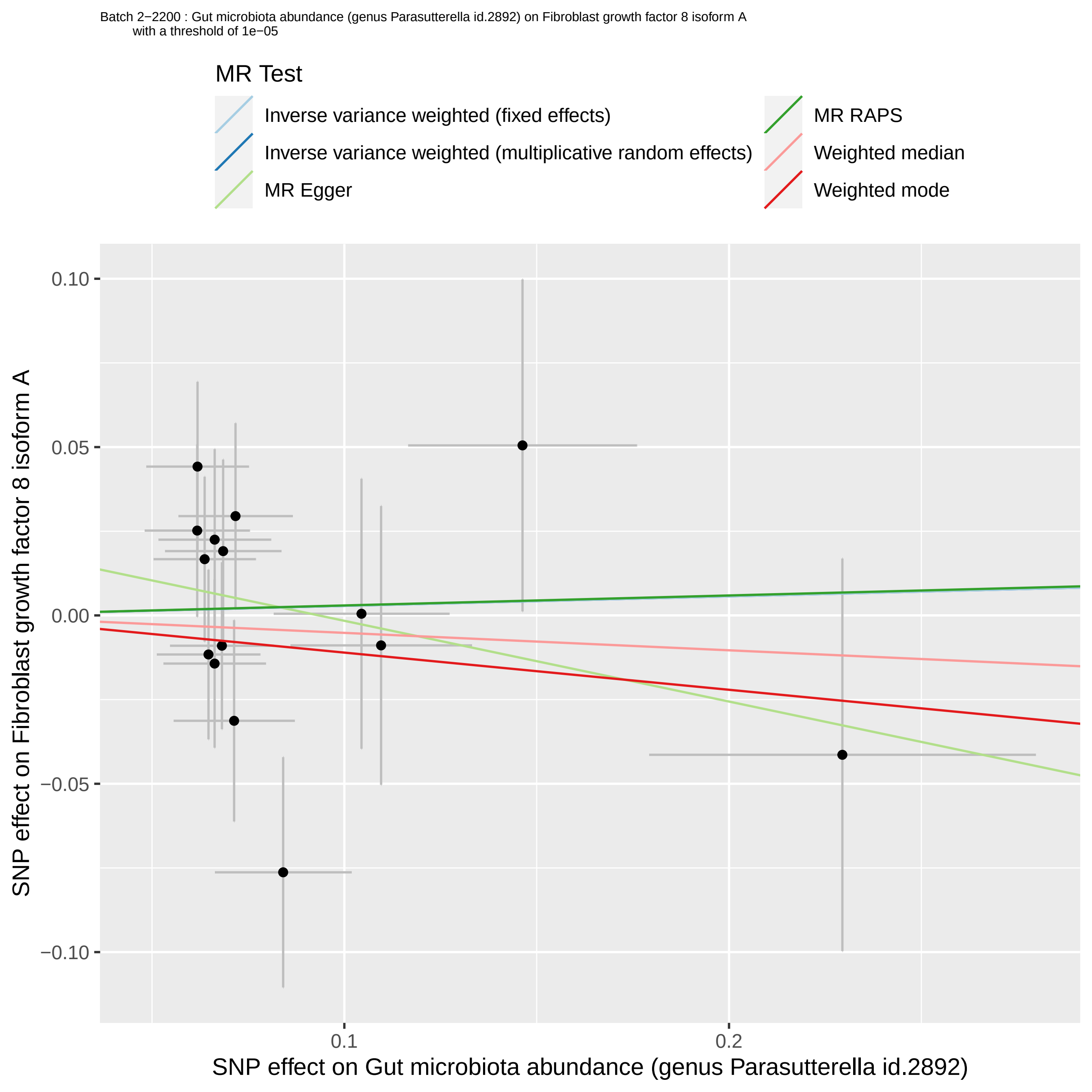

Batch 2−2200 : Gut microbiota abundance (genus Parasutterella id.2892) on Fibroblast growth factor 8 isoform A
with a threshold of 1e−05
MR Test
Inverse variance weighted (fixed effects)
Inverse variance weighted (multiplicative random effects)
MR Egger
MR RAPS
Weighted median
Weighted mode
0.10
0.05
0.00
−0.05
−0.10
0.1
0.2
SNP effect on Fibroblast growth factor 8 isoform A
SNP effect on Gut microbiota abundance (genus Parasutterella id.2892)

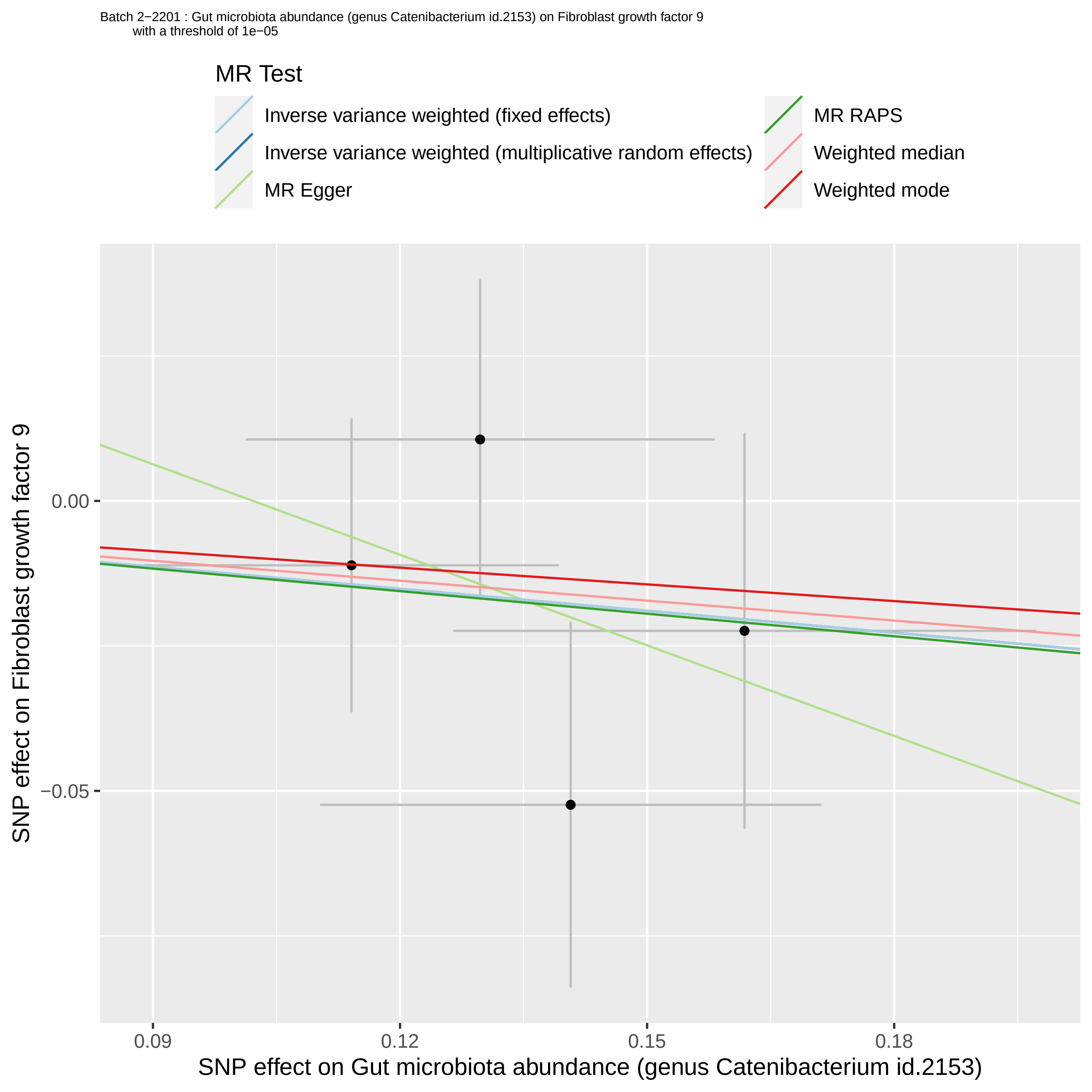
Batch 2−2201 : Gut microbiota abundance (genus Catenibacterium id.2153) on Fibroblast growth factor 9
with a threshold of 1e−05
MR Test
Inverse variance weighted (fixed effects)
Inverse variance weighted (multiplicative random effects)
MR Egger
MR RAPS
Weighted median
Weighted mode
0.00
−0.05
0.09
0.12
0.15
0.18
SNP effect on Fibroblast growth factor 9
SNP effect on Gut microbiota abundance (genus Catenibacterium id.2153)

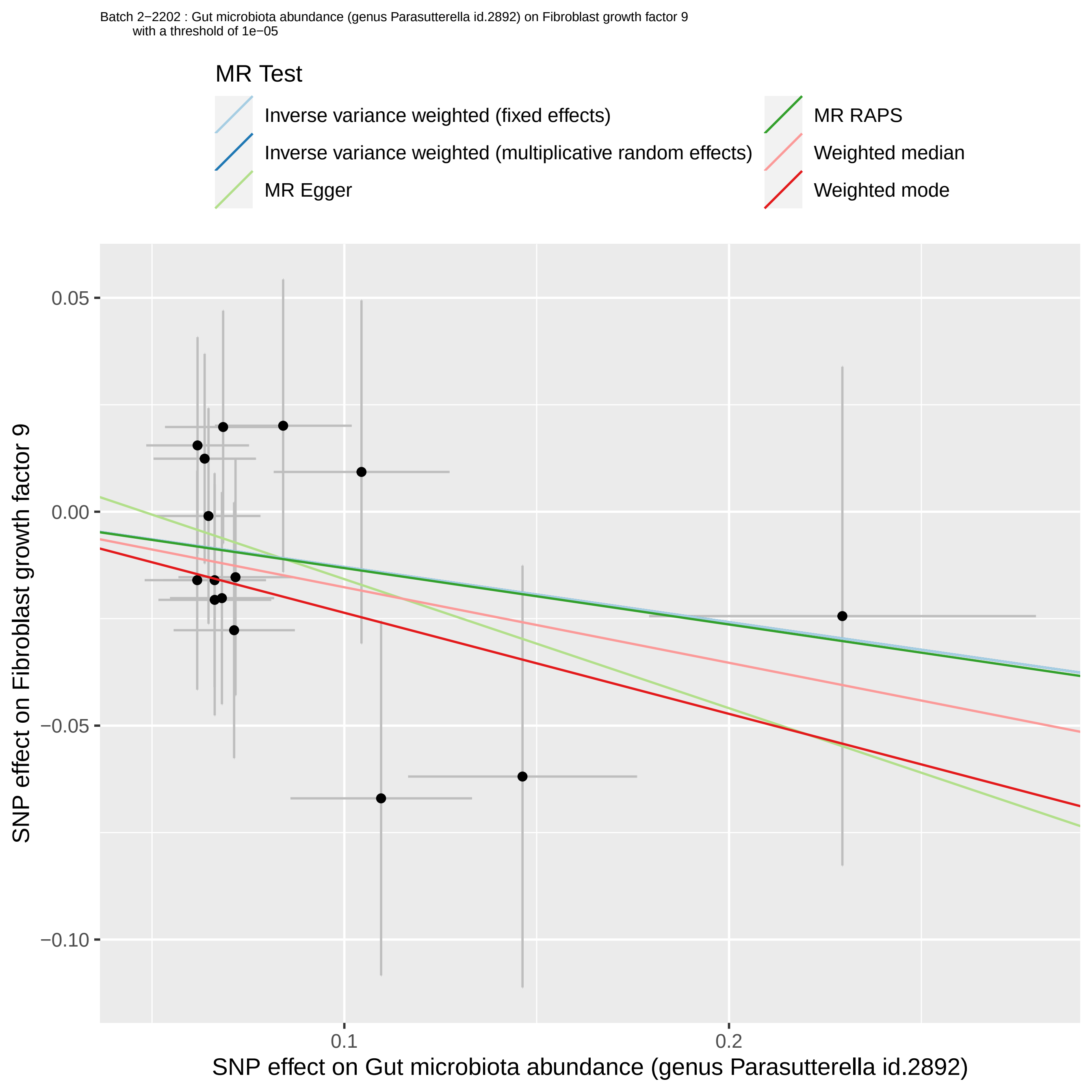
Batch 2−2202 : Gut microbiota abundance (genus Parasutterella id.2892) on Fibroblast growth factor 9
with a threshold of 1e−05
MR Test
Inverse variance weighted (fixed effects)
Inverse variance weighted (multiplicative random effects)
MR Egger
MR RAPS
Weighted median
Weighted mode
0.05
0.00
−0.05
−0.10
0.1
0.2
SNP effect on Fibroblast growth factor 9
SNP effect on Gut microbiota abundance (genus Parasutterella id.2892)

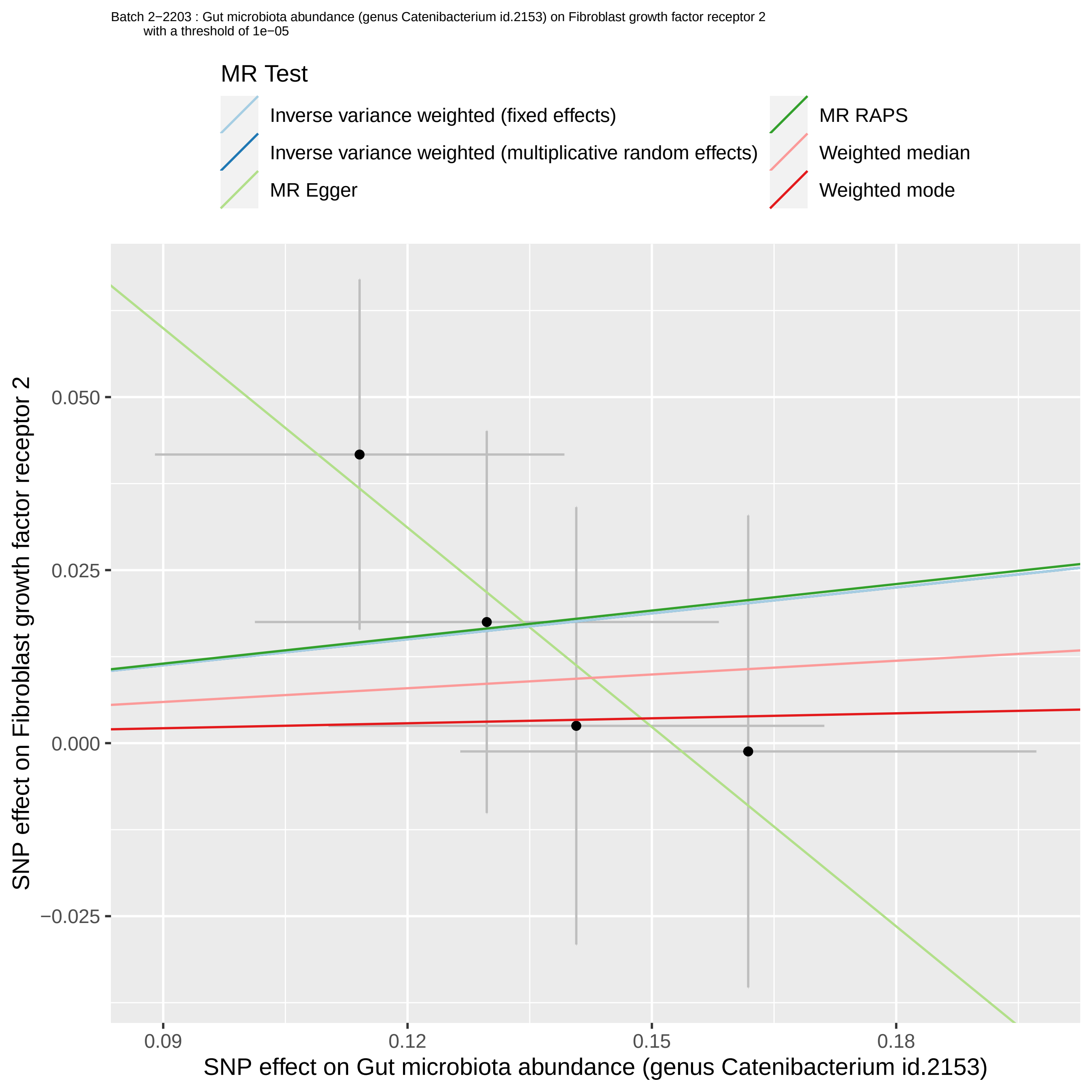

Batch 2−2203 : Gut microbiota abundance (genus Catenibacterium id.2153) on Fibroblast growth factor receptor 2
with a threshold of 1e−05
MR Test
Inverse variance weighted (fixed effects)
Inverse variance weighted (multiplicative random effects)
MR Egger
MR RAPS
Weighted median
Weighted mode
0.050
0.025
0.000
−0.025
0.09
0.12
0.15
0.18
SNP effect on Fibroblast growth factor receptor 2
SNP effect on Gut microbiota abundance (genus Catenibacterium id.2153)

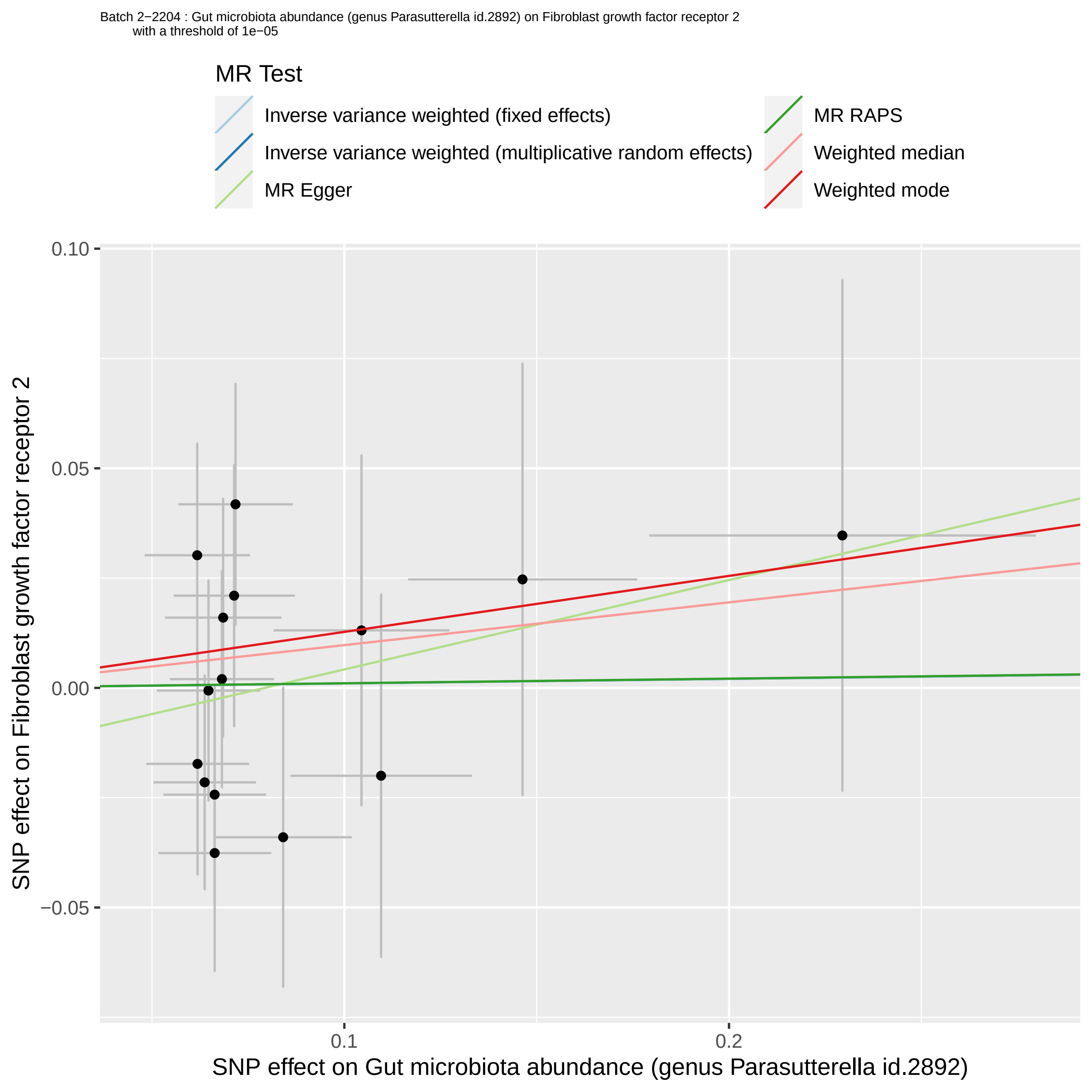
Batch 2−2204 : Gut microbiota abundance (genus Parasutterella id.2892) on Fibroblast growth factor receptor 2
with a threshold of 1e−05
MR Test
Inverse variance weighted (fixed effects)
Inverse variance weighted (multiplicative random effects)
MR Egger
MR RAPS
Weighted median
Weighted mode
0.10
0.05
0.00
−0.05
0.1
0.2
SNP effect on Fibroblast growth factor receptor 2
SNP effect on Gut microbiota abundance (genus Parasutterella id.2892)

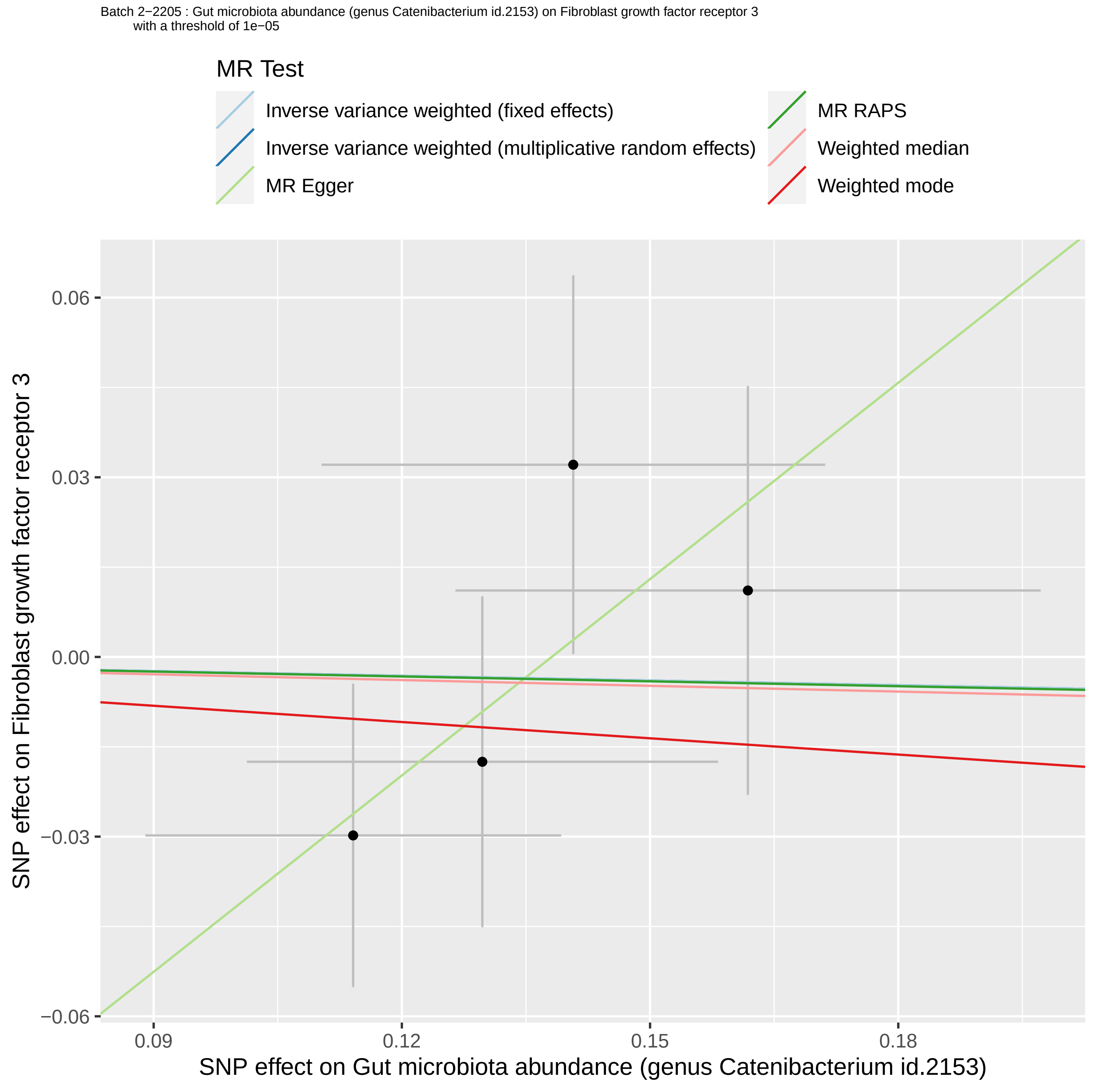

Batch 2−2205 : Gut microbiota abundance (genus Catenibacterium id.2153) on Fibroblast growth factor receptor 3
with a threshold of 1e−05
MR Test
Inverse variance weighted (fixed effects)
Inverse variance weighted (multiplicative random effects)
MR Egger
MR RAPS
Weighted median
Weighted mode
0.06
0.03
0.00
−0.03
−0.06
0.09
0.12
0.15
0.18
SNP effect on Fibroblast growth factor receptor 3
SNP effect on Gut microbiota abundance (genus Catenibacterium id.2153)

Batch 2−2206 : Gut microbiota abundance (genus Parasutterella id.2892) on Fibroblast growth factor receptor 3
with a threshold of 1e−05

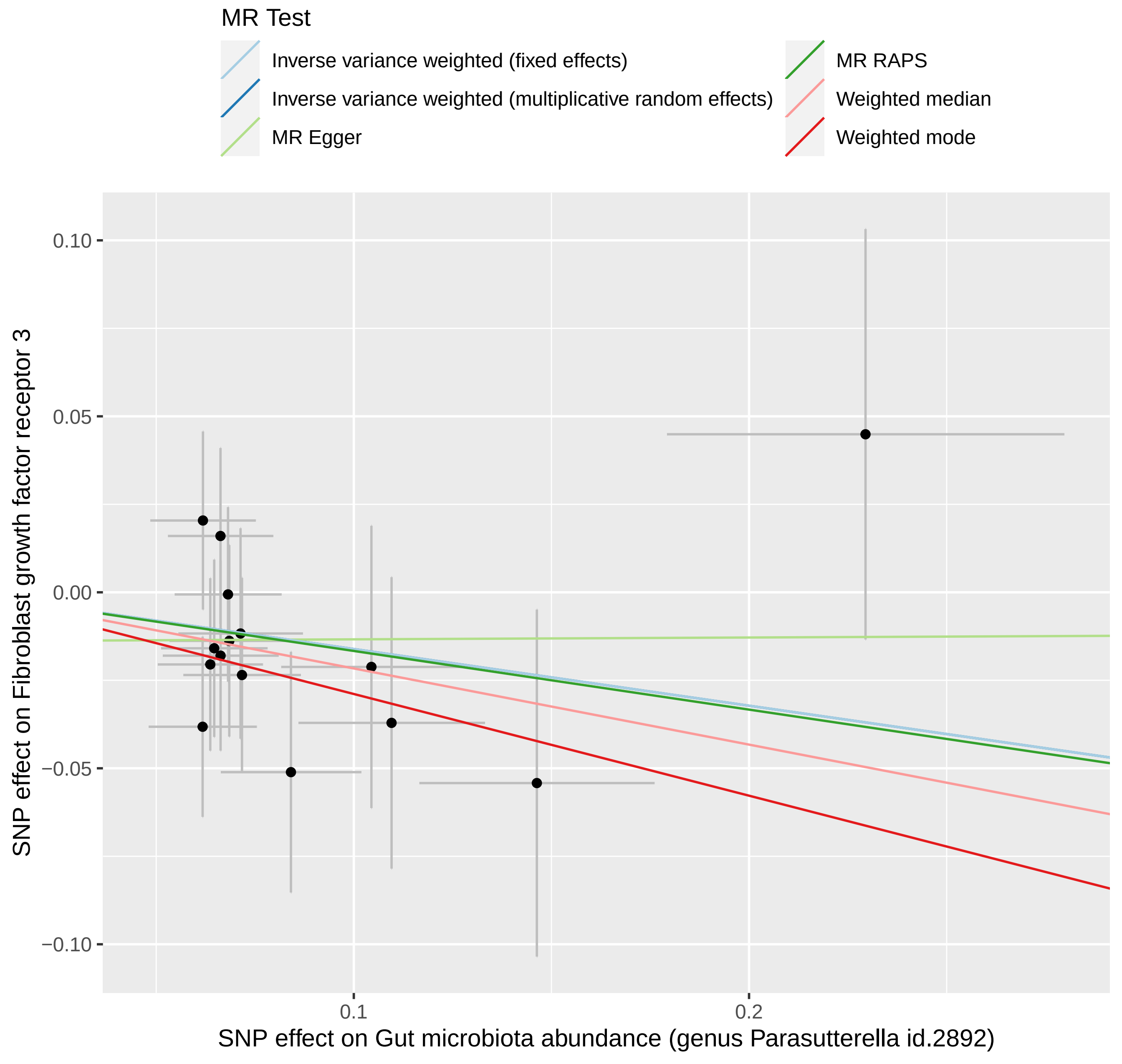

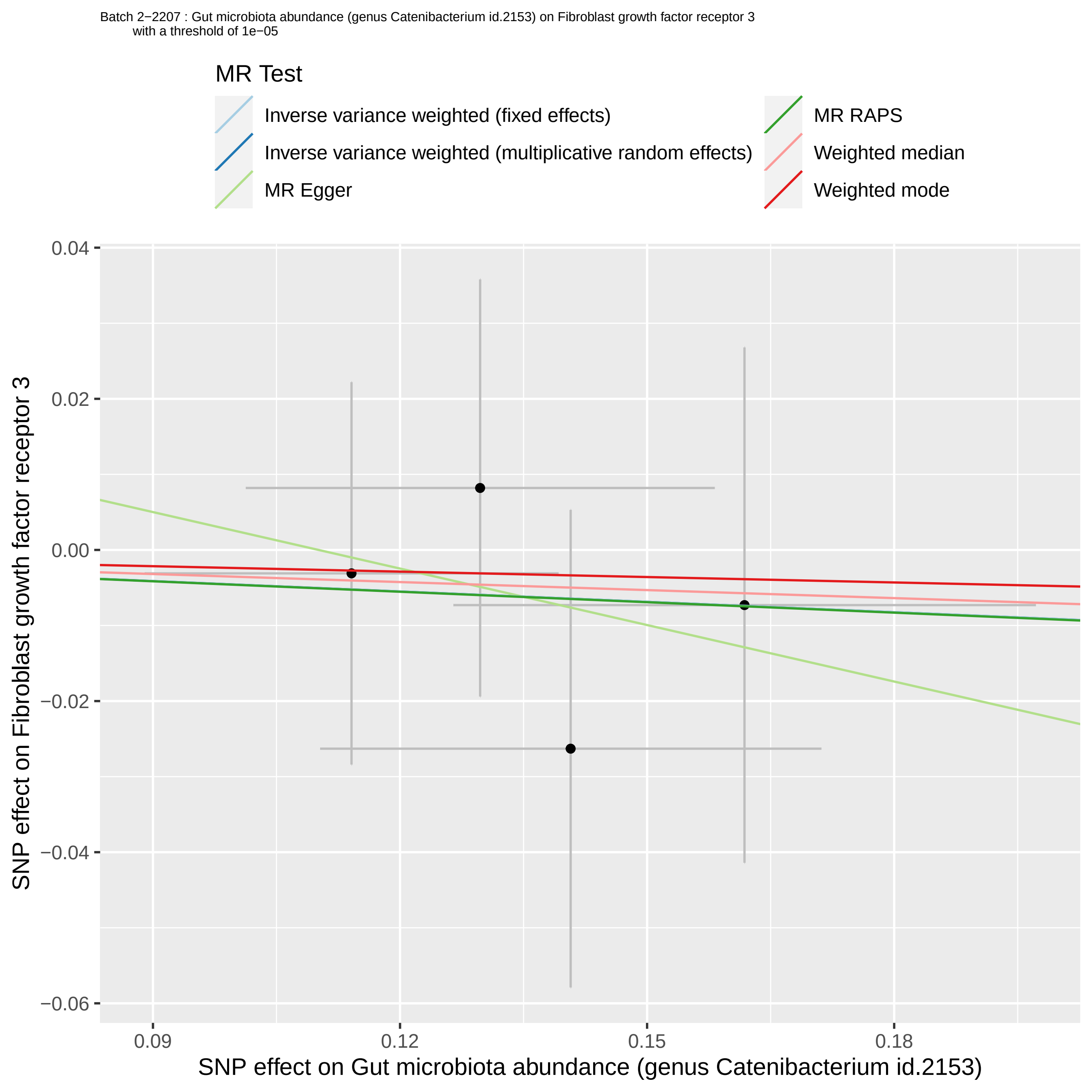
Batch 2−2207 : Gut microbiota abundance (genus Catenibacterium id.2153) on Fibroblast growth factor receptor 3
with a threshold of 1e−05
MR Test
Inverse variance weighted (fixed effects)
Inverse variance weighted (multiplicative random effects)
MR Egger
MR RAPS
Weighted median
Weighted mode
0.04
0.02
0.00
−0.02
−0.04
−0.06
0.09
0.12
0.15
0.18
SNP effect on Fibroblast growth factor receptor 3
SNP effect on Gut microbiota abundance (genus Catenibacterium id.2153)

Batch 2−2208 : Gut microbiota abundance (genus Parasutterella id.2892) on Fibroblast growth factor receptor 3
with a threshold of 1e−05

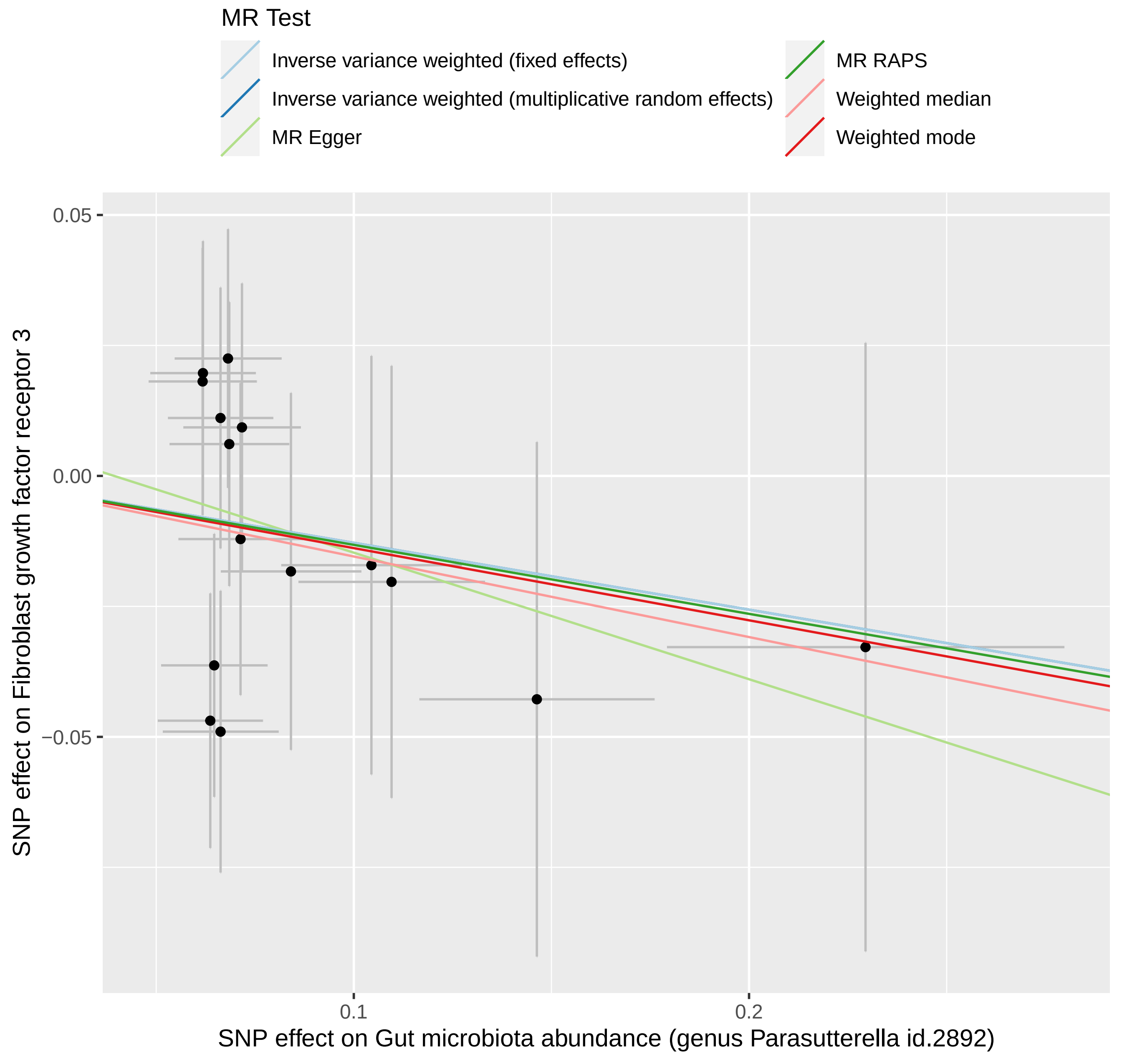

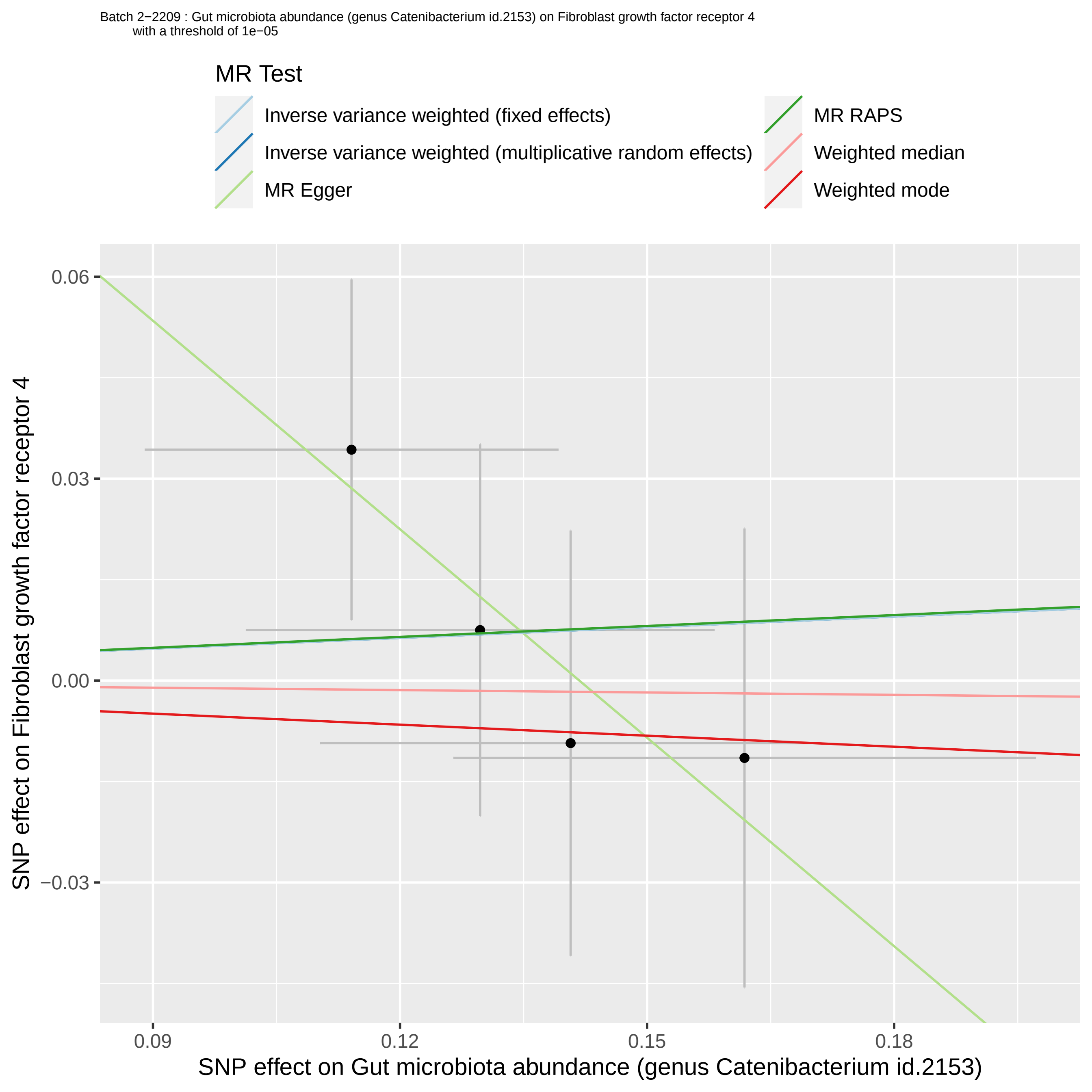
Batch 2−2209 : Gut microbiota abundance (genus Catenibacterium id.2153) on Fibroblast growth factor receptor 4
with a threshold of 1e−05
MR Test
Inverse variance weighted (fixed effects)
Inverse variance weighted (multiplicative random effects)
MR Egger
MR RAPS
Weighted median
Weighted mode
0.06
0.03
0.00
−0.03
0.09
0.12
0.15
0.18
SNP effect on Fibroblast growth factor receptor 4
SNP effect on Gut microbiota abundance (genus Catenibacterium id.2153)

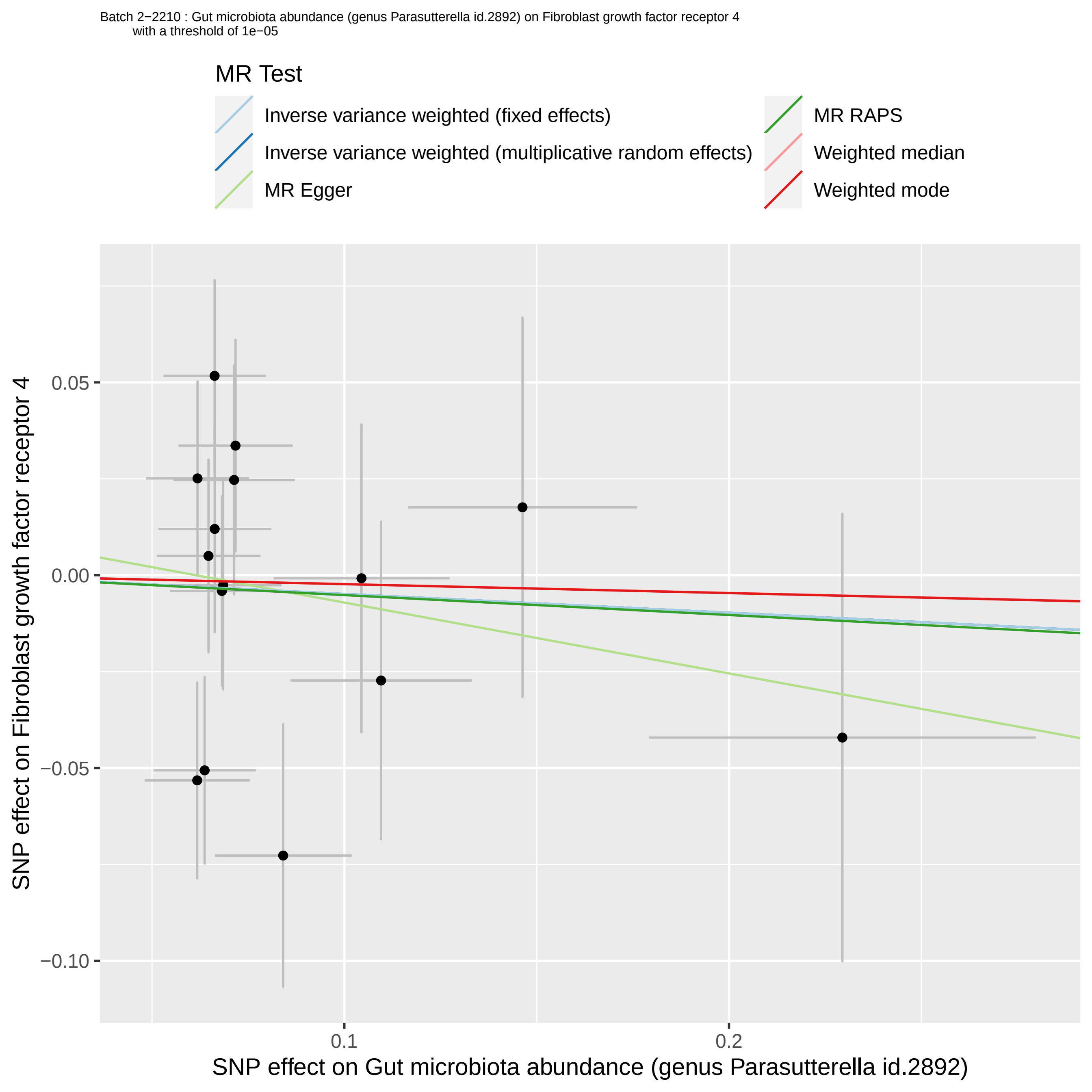
Batch 2−2210 : Gut microbiota abundance (genus Parasutterella id.2892) on Fibroblast growth factor receptor 4
with a threshold of 1e−05
MR Test
Inverse variance weighted (fixed effects)
Inverse variance weighted (multiplicative random effects)
MR Egger
MR RAPS
Weighted median
Weighted mode
SNP effect on Fibroblast growth factor receptor 4
0.05
0.00
−0.05
−0.10
0.1
0.2
SNP effect on Gut microbiota abundance (genus Parasutterella id.2892)

Batch 2−2211 : Gut microbiota abundance (genus Catenibacterium id.2153) on Tyrosine−protein kinase Fgr
with a threshold of 1e−05

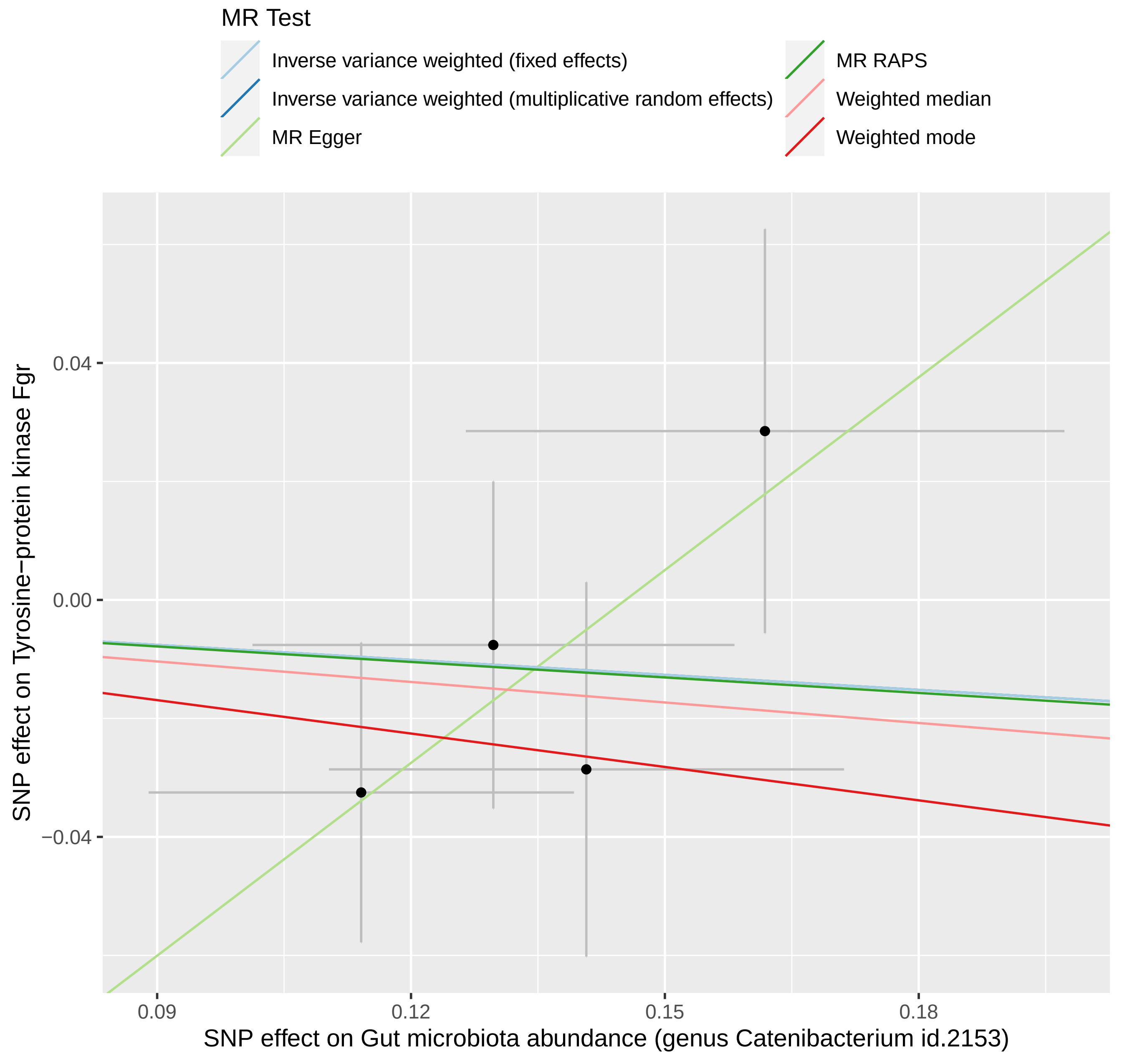

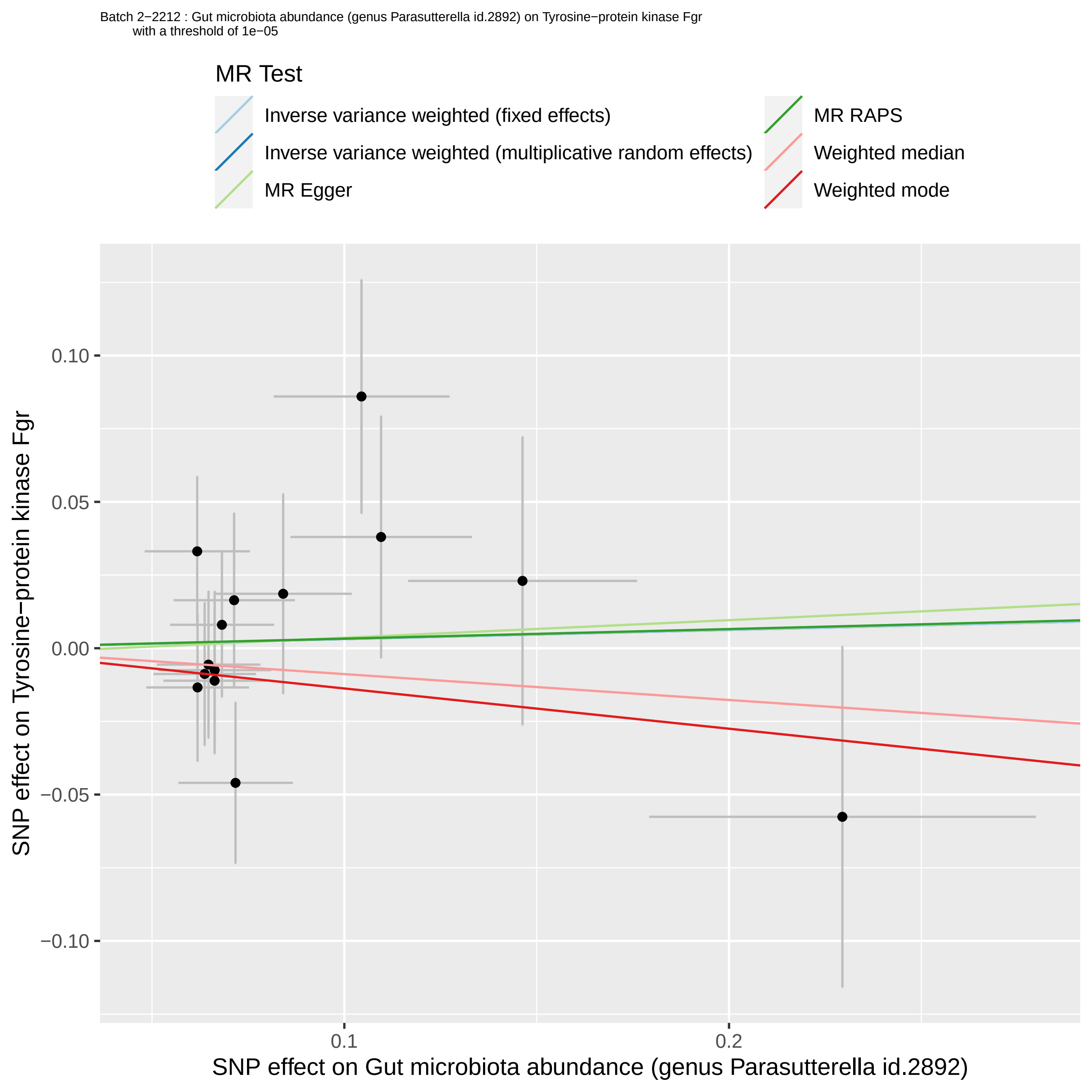

Batch 2−2212 : Gut microbiota abundance (genus Parasutterella id.2892) on Tyrosine−protein kinase Fgr
with a threshold of 1e−05
MR Test
Inverse variance weighted (fixed effects)
Inverse variance weighted (multiplicative random effects)
MR Egger
MR RAPS
Weighted median
Weighted mode
0.10
0.05
0.00
−0.05
−0.10
0.1
0.2
SNP effect on Tyrosine−protein kinase Fgr
SNP effect on Gut microbiota abundance (genus Parasutterella id.2892)

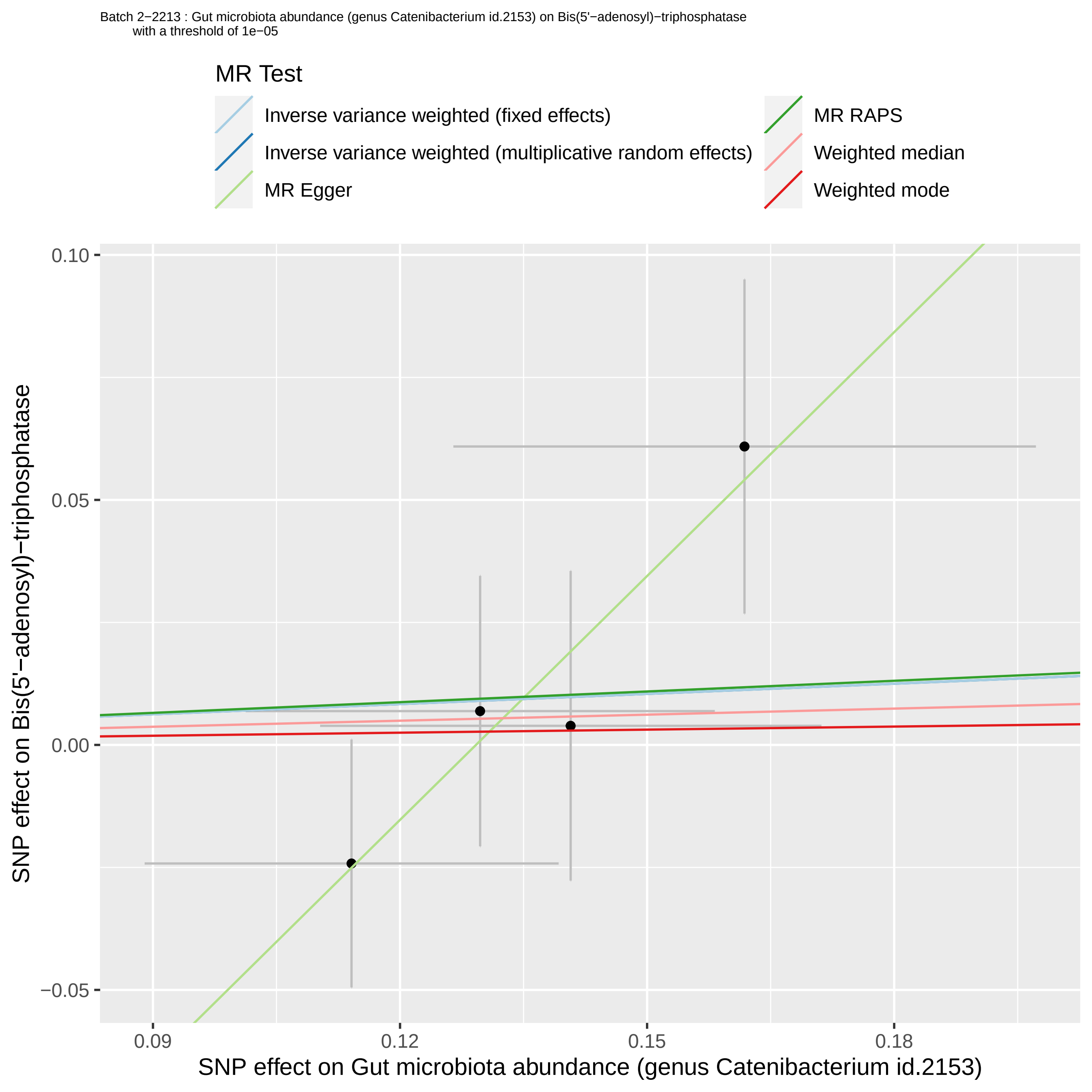

Batch 2−2213 : Gut microbiota abundance (genus Catenibacterium id.2153) on Bis(5'−adenosyl)−triphosphatase
with a threshold of 1e−05
MR Test
Inverse variance weighted (fixed effects)
Inverse variance weighted (multiplicative random effects)
MR Egger
MR RAPS
Weighted median
Weighted mode
0.10
0.05
0.00
−0.05
0.09
0.12
0.15
0.18
SNP effect on Bis(5'−adenosyl)−triphosphatase
SNP effect on Gut microbiota abundance (genus Catenibacterium id.2153)

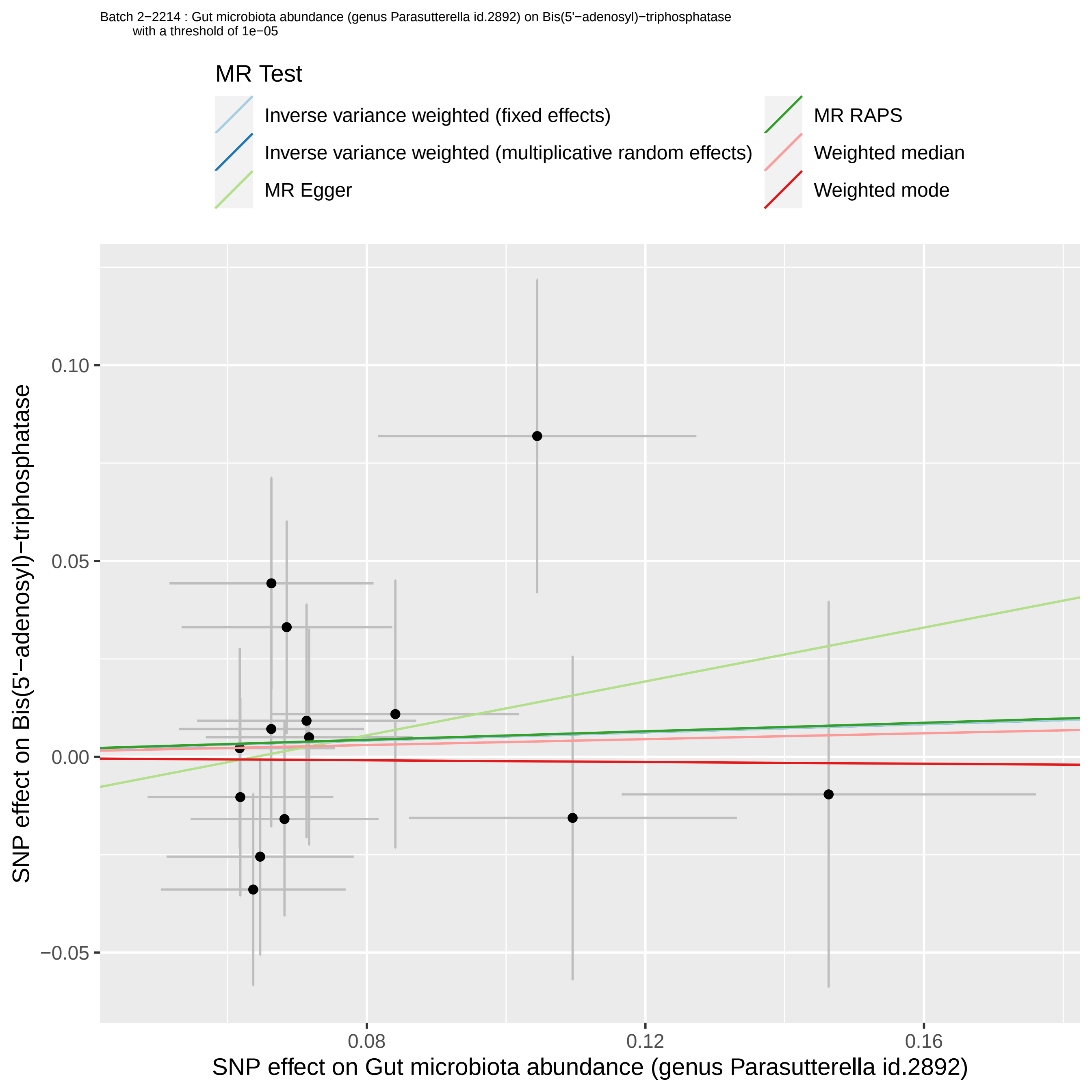
Batch 2−2214 : Gut microbiota abundance (genus Parasutterella id.2892) on Bis(5'−adenosyl)−triphosphatase
with a threshold of 1e−05
MR Test
Inverse variance weighted (fixed effects)
Inverse variance weighted (multiplicative random effects)
MR Egger
MR RAPS
Weighted median
Weighted mode
0.10
0.05
0.00
−0.05
0.08
0.12
0.16
SNP effect on Bis(5'−adenosyl)−triphosphatase
SNP effect on Gut microbiota abundance (genus Parasutterella id.2892)

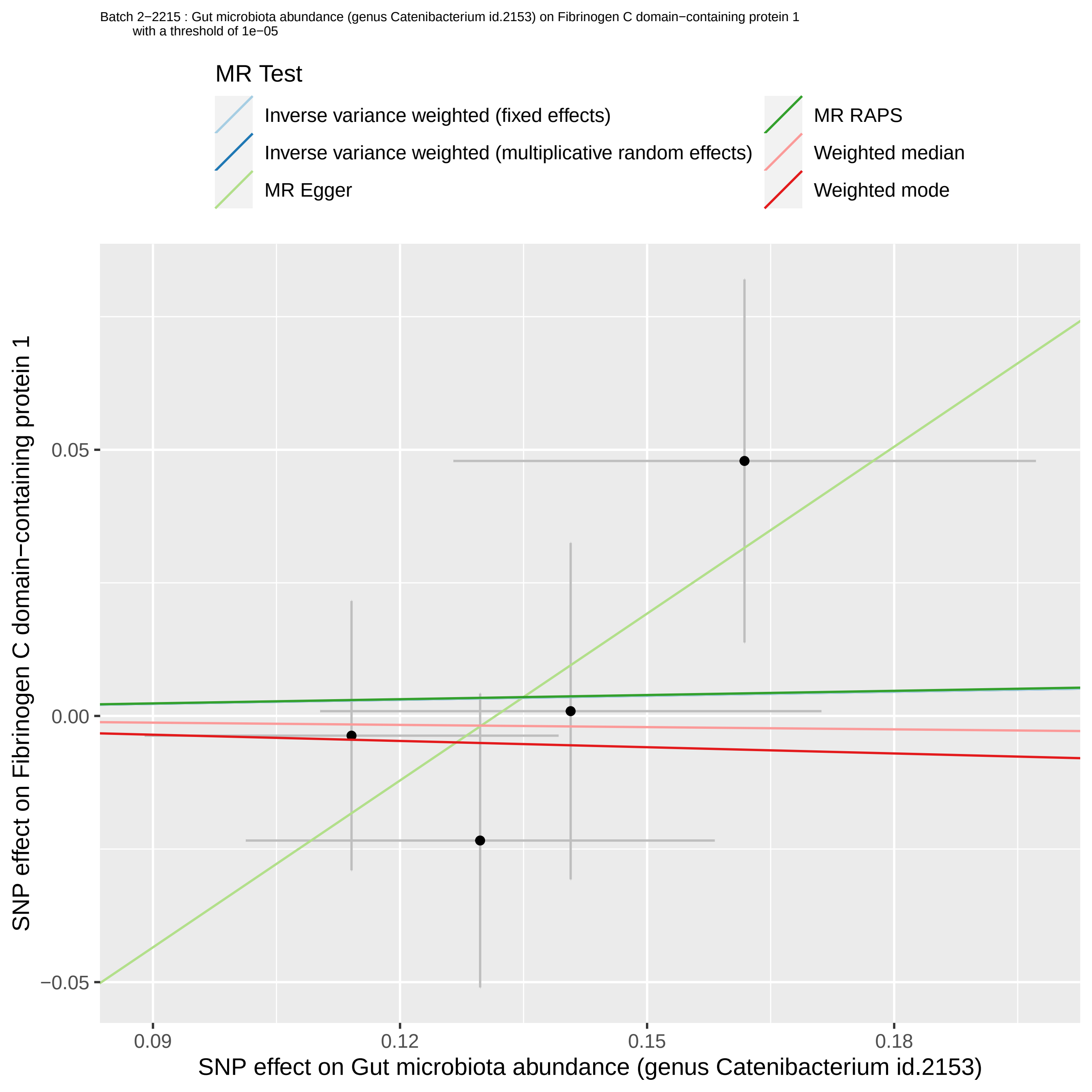

Batch 2−2215 : Gut microbiota abundance (genus Catenibacterium id.2153) on Fibrinogen C domain−containing protein 1 with a threshold of 1e−05
MR Test
Inverse variance weighted (fixed effects)
Inverse variance weighted (multiplicative random effects)
MR Egger
MR RAPS
Weighted median
Weighted mode
0.05
0.00
−0.05
0.09
0.12
0.15
0.18
SNP effect on Fibrinogen C domain−containing protein 1
SNP effect on Gut microbiota abundance (genus Catenibacterium id.2153)

Batch 2−2216 : Gut microbiota abundance (genus Parasutterella id.2892) on Fibrinogen C domain−containing protein 1
with a threshold of 1e−05

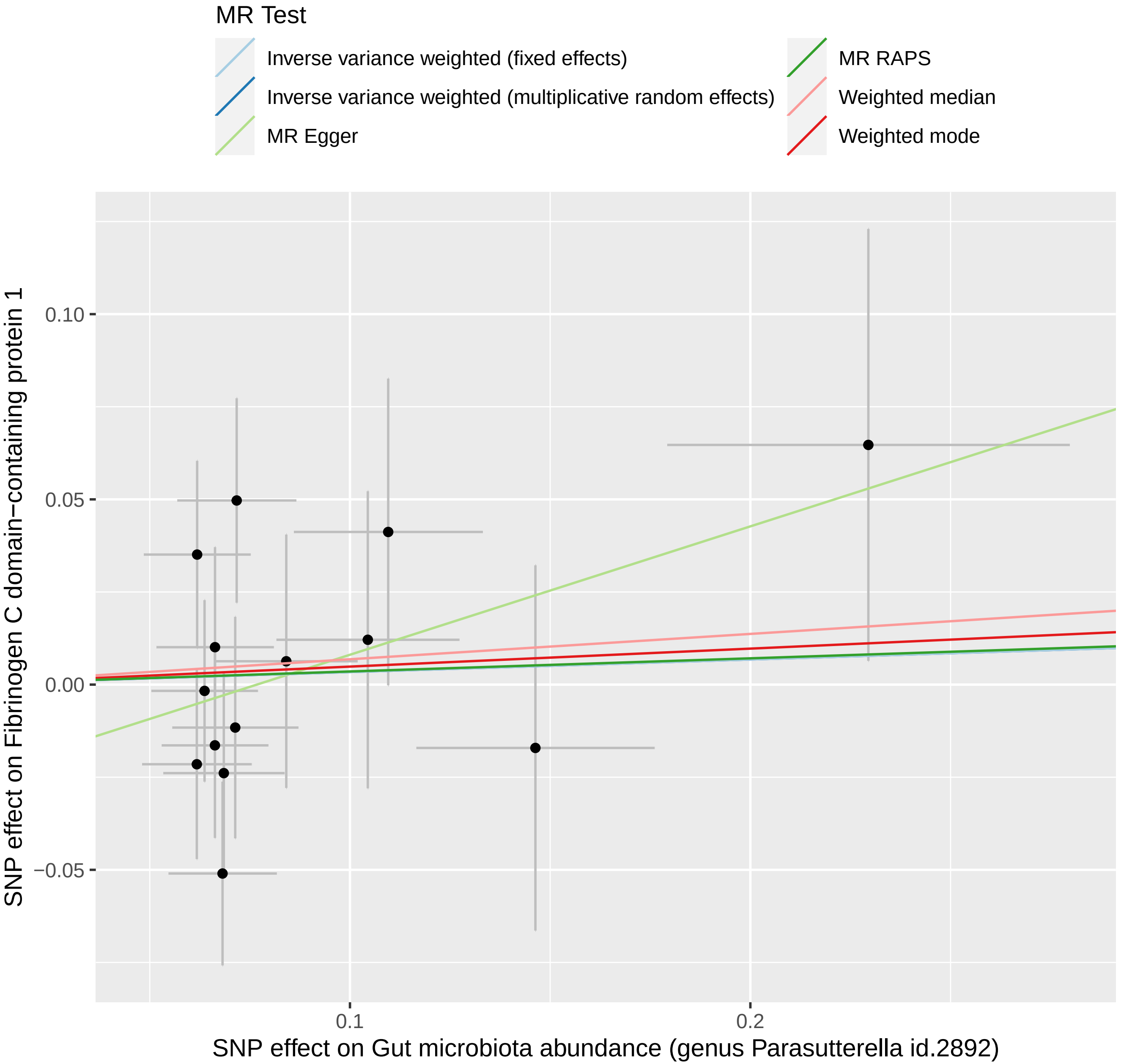

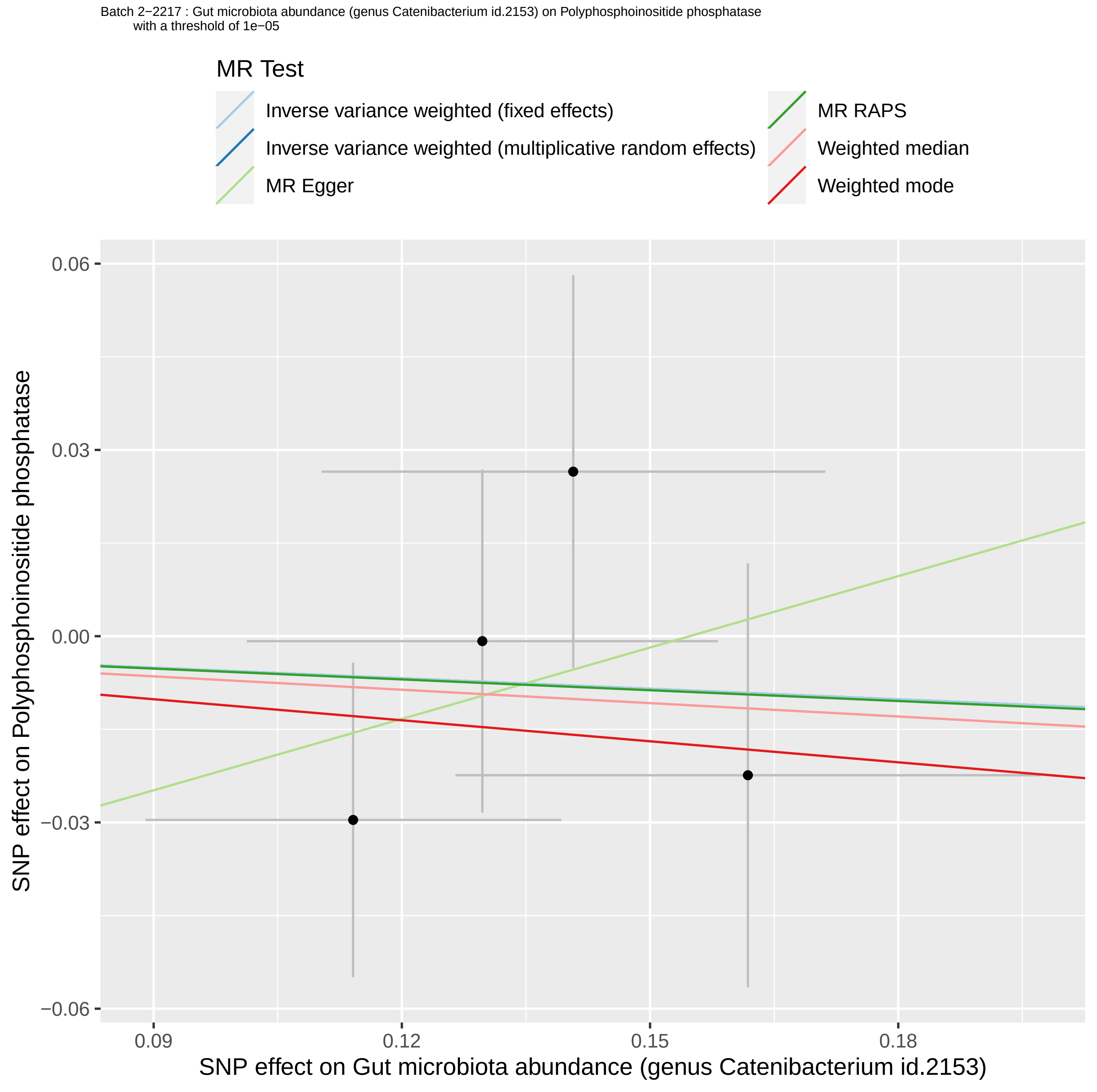

Batch 2−2217 : Gut microbiota abundance (genus Catenibacterium id.2153) on Polyphosphoinositide phosphatase
with a threshold of 1e−05
MR Test
Inverse variance weighted (fixed effects)
Inverse variance weighted (multiplicative random effects)
MR Egger
MR RAPS
Weighted median
Weighted mode
0.06
0.03
0.00
−0.03
−0.06
0.09
0.12
0.15
0.18
SNP effect on Polyphosphoinositide phosphatase
SNP effect on Gut microbiota abundance (genus Catenibacterium id.2153)

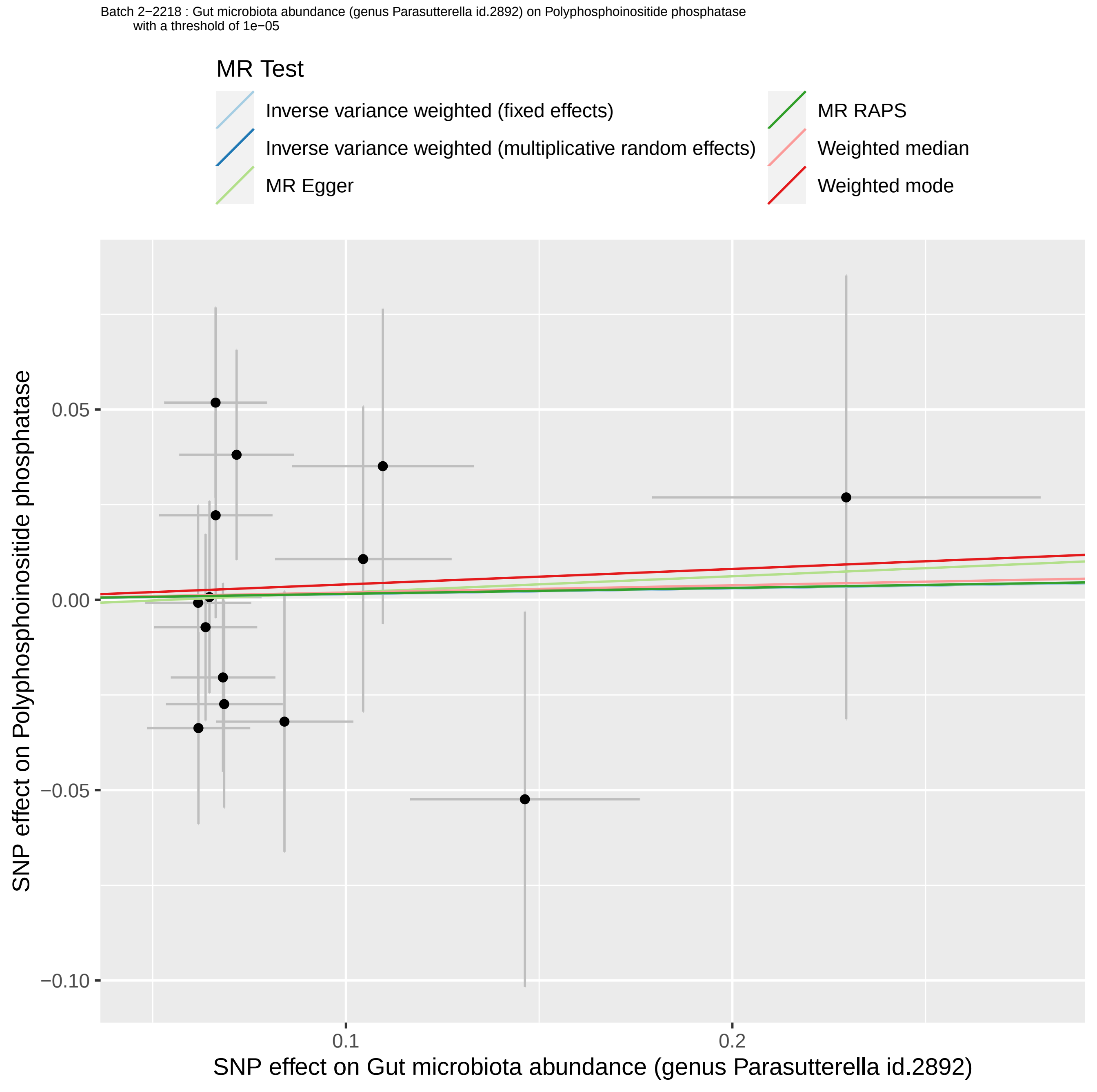
Batch 2−2218 : Gut microbiota abundance (genus Parasutterella id.2892) on Polyphosphoinositide phosphatase
with a threshold of 1e−05
MR Test
Inverse variance weighted (fixed effects)
Inverse variance weighted (multiplicative random effects)
MR Egger
MR RAPS
Weighted median
Weighted mode
SNP effect on Polyphosphoinositide phosphatase
0.05
0.00
−0.05
−0.10
0.1
0.2
SNP effect on Gut microbiota abundance (genus Parasutterella id.2892)

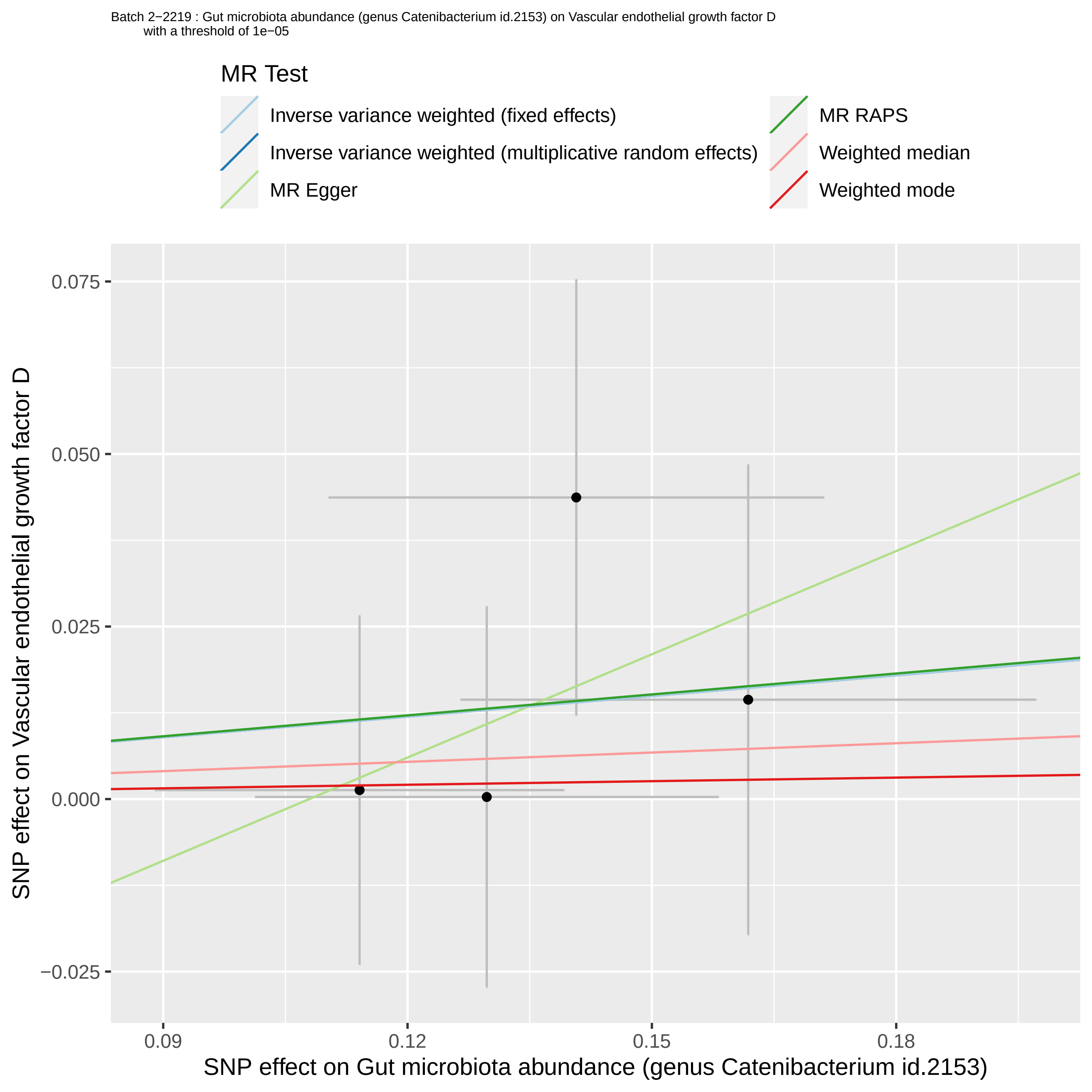
Batch 2−2219 : Gut microbiota abundance (genus Catenibacterium id.2153) on Vascular endothelial growth factor D
with a threshold of 1e−05
MR Test
Inverse variance weighted (fixed effects)
Inverse variance weighted (multiplicative random effects)
MR Egger
MR RAPS
Weighted median
Weighted mode
SNP effect on Vascular endothelial growth factor D
0.075
0.050
0.025
0.000
−0.025
0.09
0.12
0.15
0.18
SNP effect on Gut microbiota abundance (genus Catenibacterium id.2153)

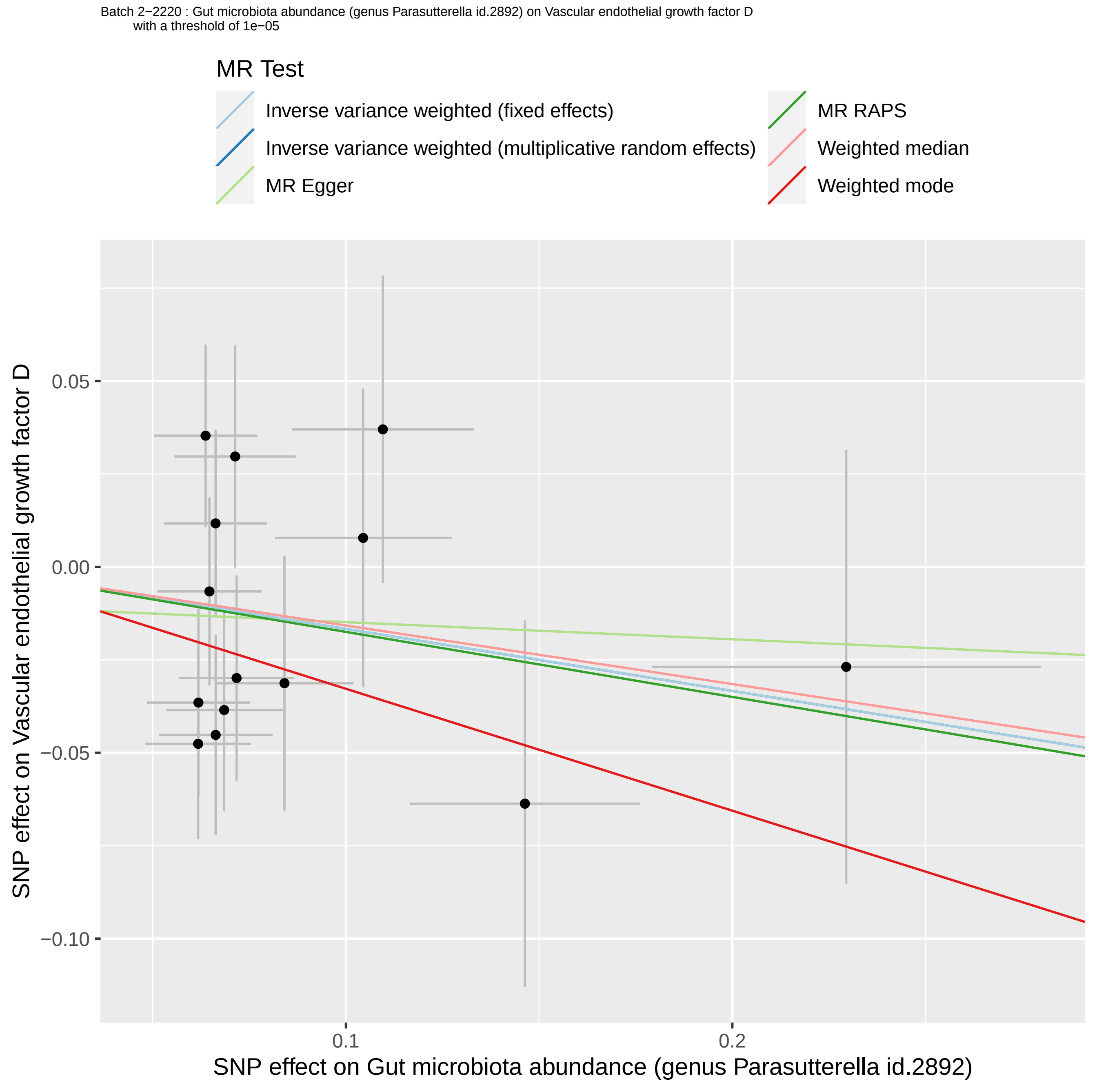
Batch 2−2220 : Gut microbiota abundance (genus Parasutterella id.2892) on Vascular endothelial growth factor D
with a threshold of 1e−05
MR Test
Inverse variance weighted (fixed effects)
Inverse variance weighted (multiplicative random effects)
MR Egger
MR RAPS
Weighted median
Weighted mode
0.05
0.00
−0.05
−0.10
0.1
0.2
SNP effect on Vascular endothelial growth factor D
SNP effect on Gut microbiota abundance (genus Parasutterella id.2892)

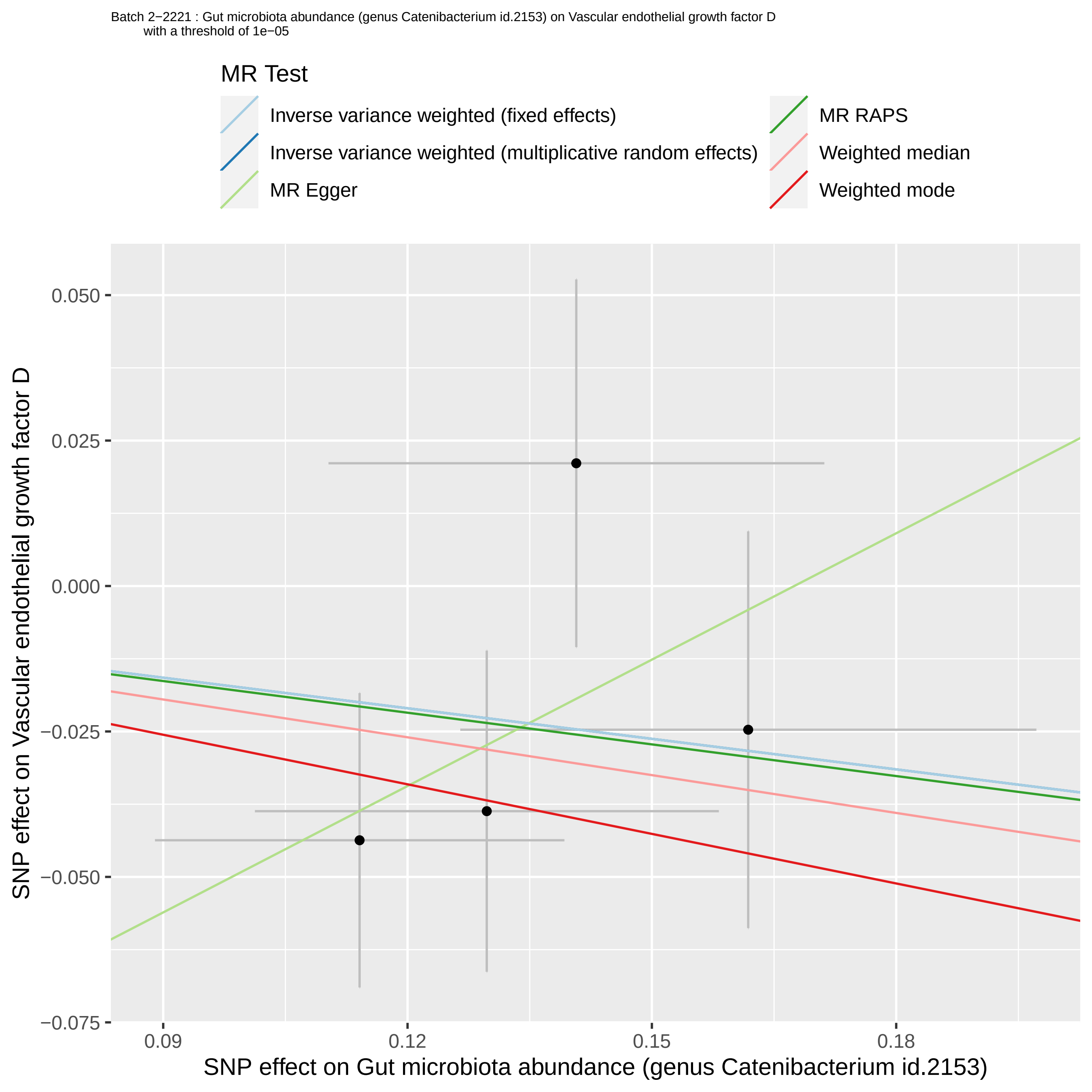
Batch 2−2221 : Gut microbiota abundance (genus Catenibacterium id.2153) on Vascular endothelial growth factor D
with a threshold of 1e−05
MR Test
Inverse variance weighted (fixed effects)
Inverse variance weighted (multiplicative random effects)
MR Egger
MR RAPS
Weighted median
Weighted mode
0.050
0.025
0.000
−0.025
−0.050
−0.075
0.09
0.12
0.15
0.18
SNP effect on Vascular endothelial growth factor D
SNP effect on Gut microbiota abundance (genus Catenibacterium id.2153)

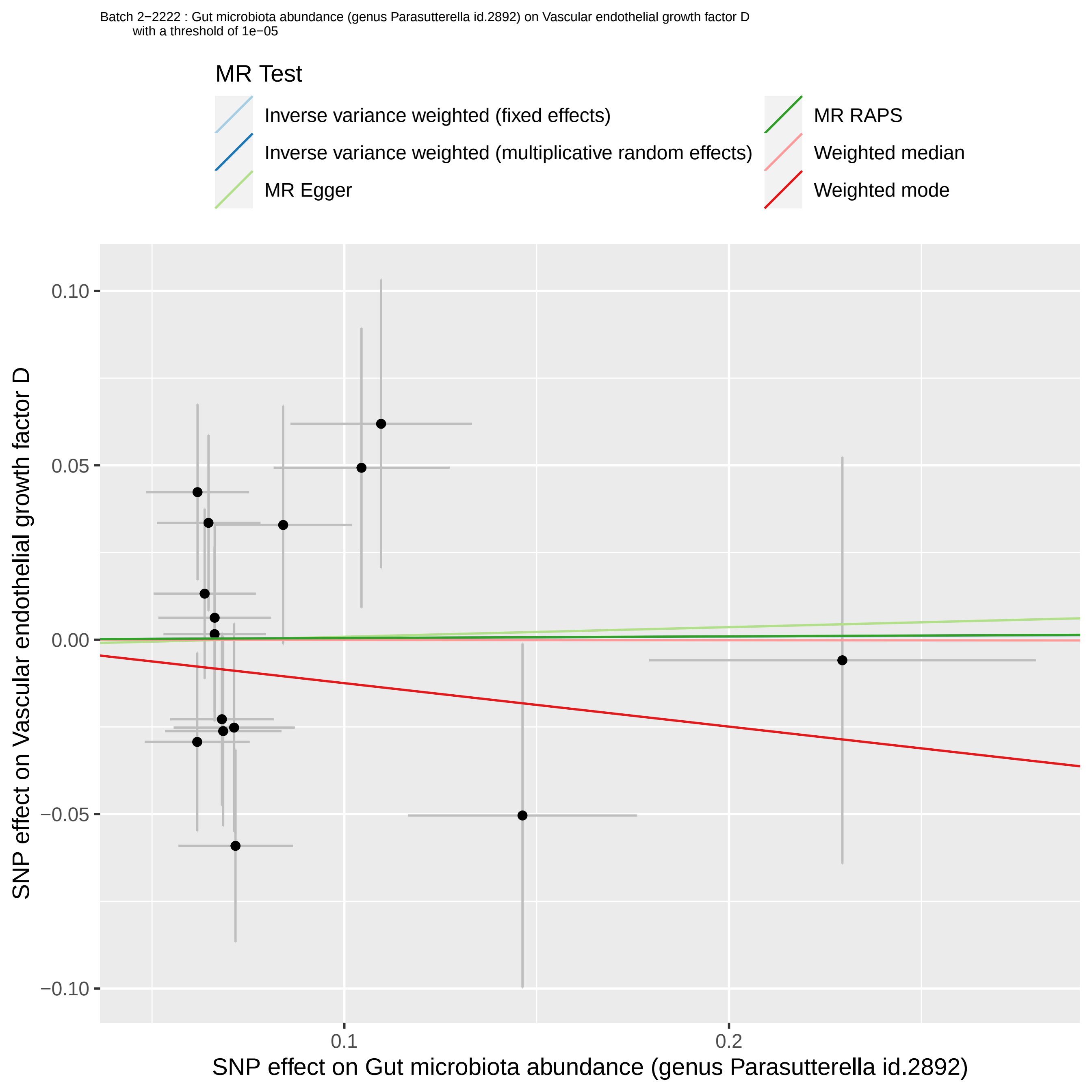
Batch 2−2222 : Gut microbiota abundance (genus Parasutterella id.2892) on Vascular endothelial growth factor D
with a threshold of 1e−05
MR Test
Inverse variance weighted (fixed effects)
Inverse variance weighted (multiplicative random effects)
MR Egger
MR RAPS
Weighted median
Weighted mode
0.10
0.05
0.00
−0.05
−0.10
0.1
0.2
SNP effect on Vascular endothelial growth factor D
SNP effect on Gut microbiota abundance (genus Parasutterella id.2892)

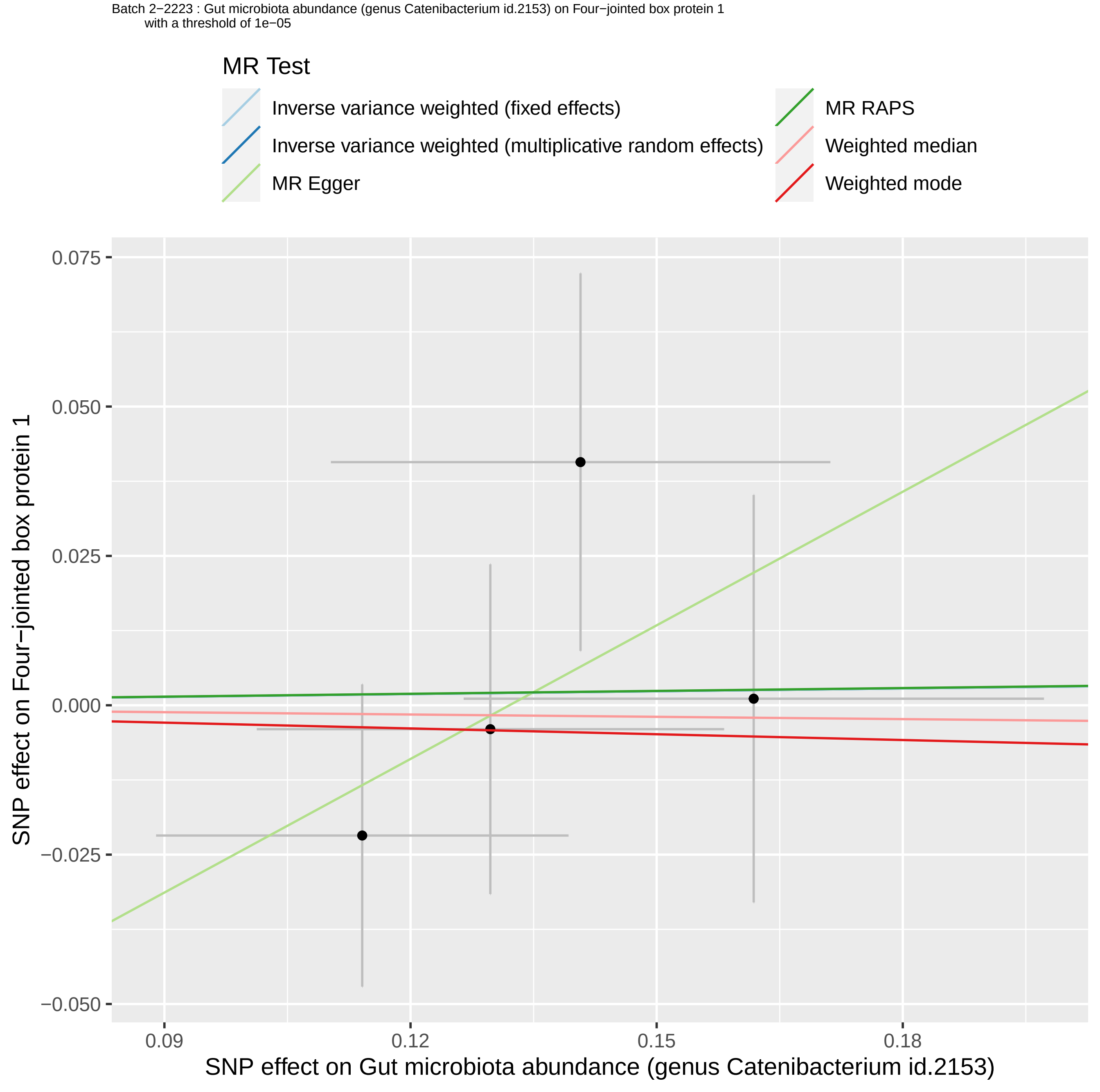

Batch 2−2223 : Gut microbiota abundance (genus Catenibacterium id.2153) on Four−jointed box protein 1
with a threshold of 1e−05
MR Test
Inverse variance weighted (fixed effects)
Inverse variance weighted (multiplicative random effects)
MR Egger
MR RAPS
Weighted median
Weighted mode
0.075
0.050
0.025
0.000
−0.025
−0.050
0.09
0.12
0.15
0.18
SNP effect on Four−jointed box protein 1
SNP effect on Gut microbiota abundance (genus Catenibacterium id.2153)

Batch 2−2224 : Gut microbiota abundance (genus Parasutterella id.2892) on Four−jointed box protein 1
with a threshold of 1e−05

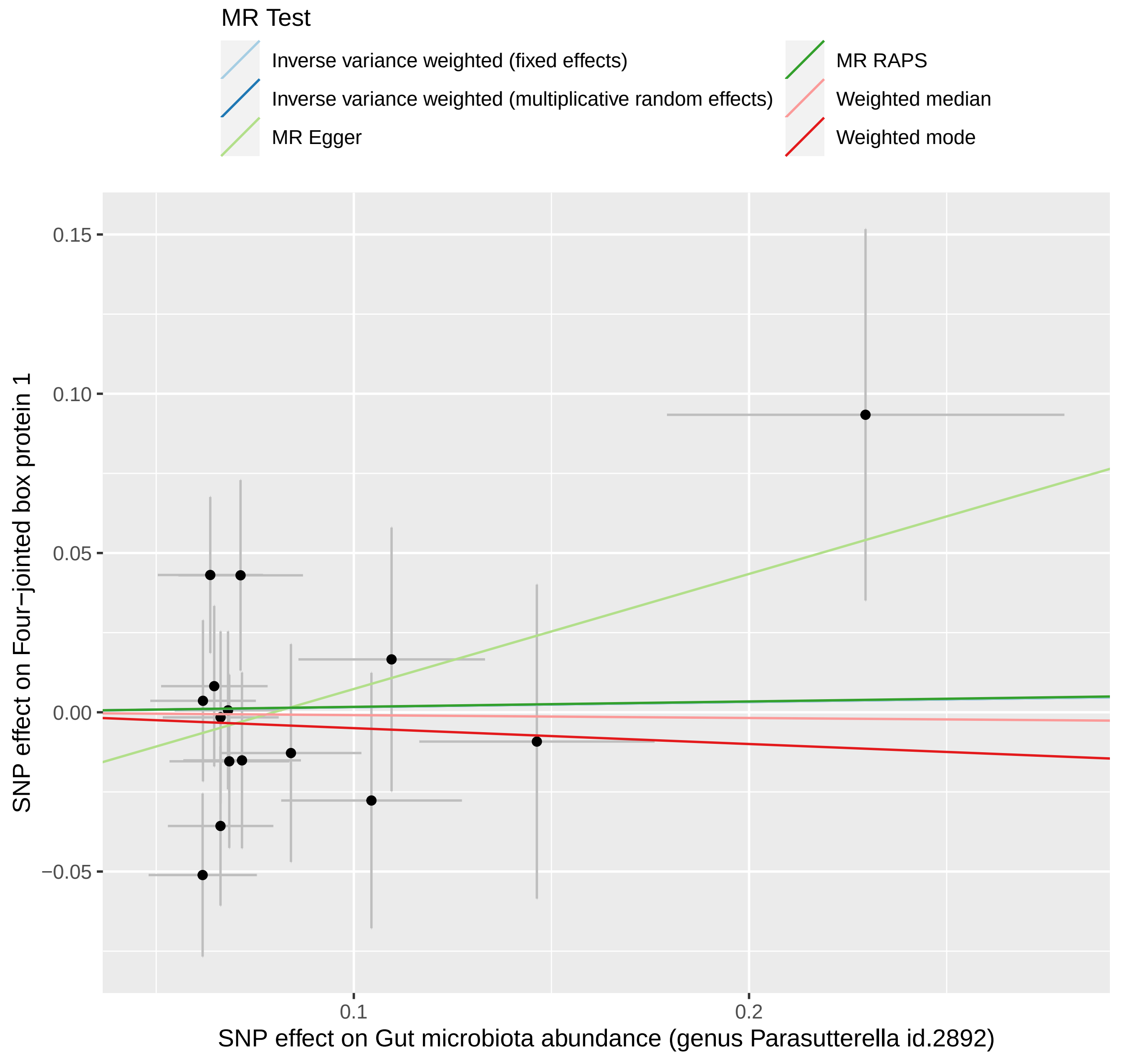

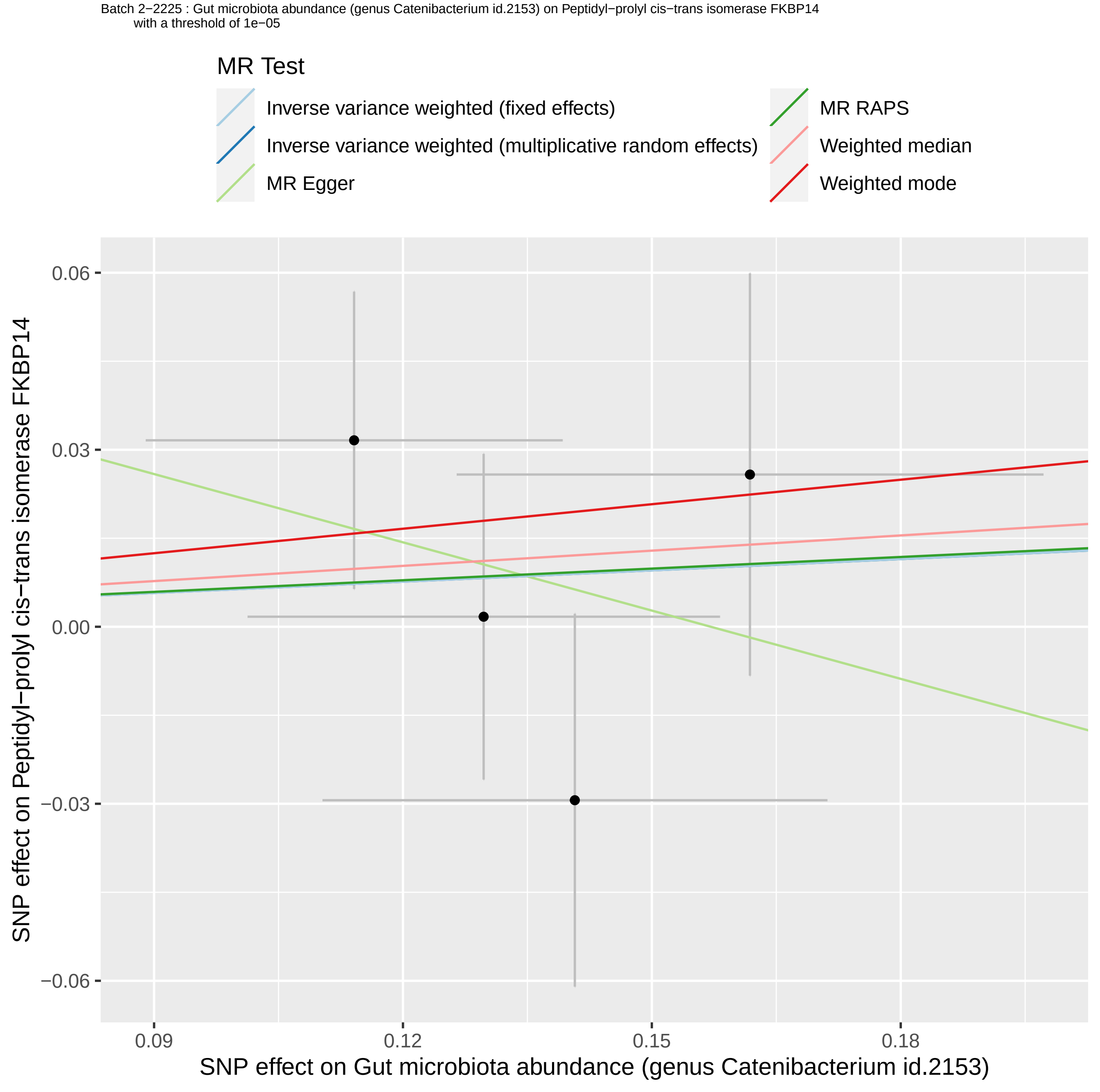

Batch 2−2225 : Gut microbiota abundance (genus Catenibacterium id.2153) on Peptidyl−prolyl cis−trans isomerase FKBP14
with a threshold of 1e−05
MR Test
Inverse variance weighted (fixed effects)
Inverse variance weighted (multiplicative random effects)
MR Egger
MR RAPS
Weighted median
Weighted mode
0.06
0.03
0.00
−0.03
−0.06
0.09
0.12
0.15
0.18
SNP effect on Peptidyl−prolyl cis−trans isomerase FKBP14
SNP effect on Gut microbiota abundance (genus Catenibacterium id.2153)

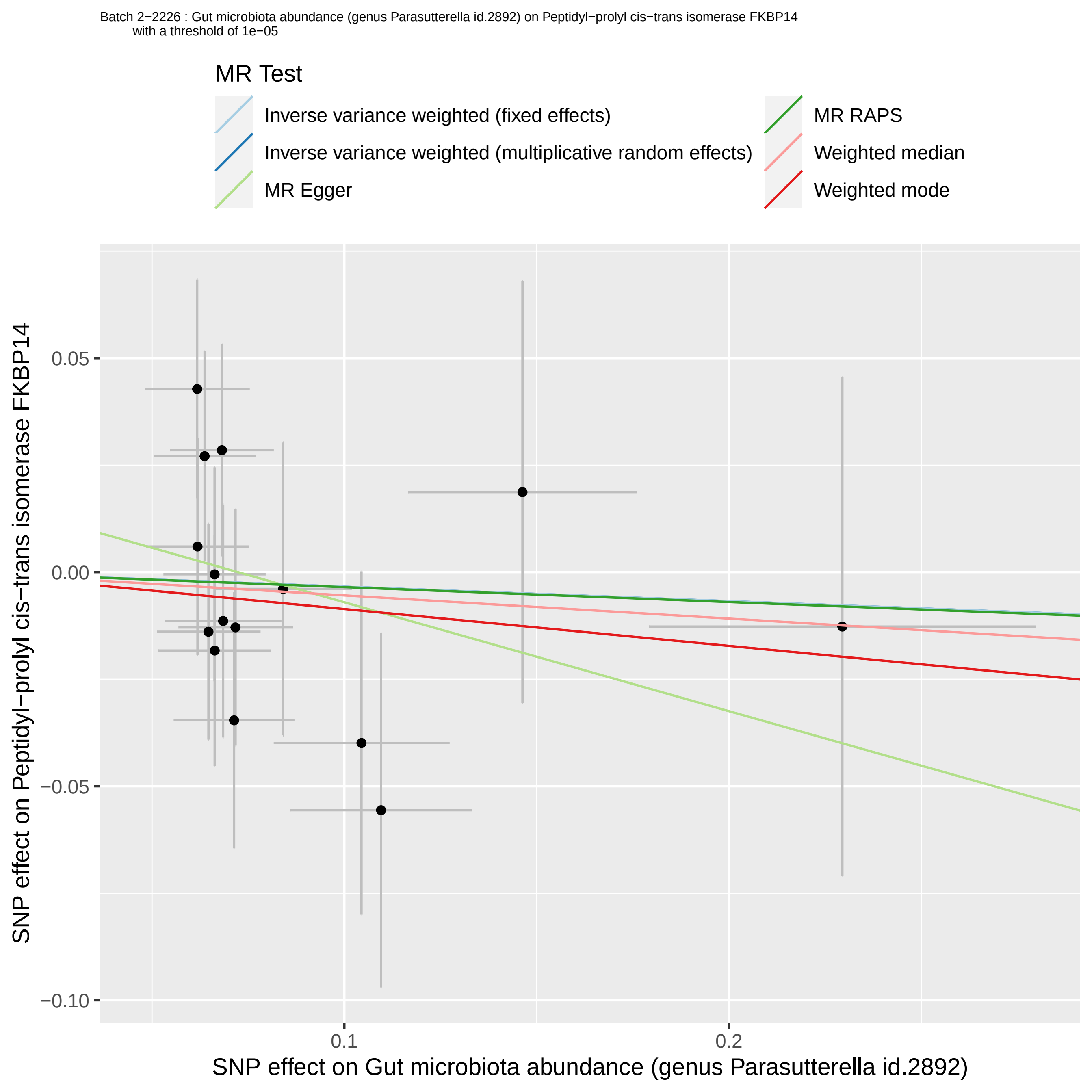
Batch 2−2226 : Gut microbiota abundance (genus Parasutterella id.2892) on Peptidyl−prolyl cis−trans isomerase FKBP14
with a threshold of 1e−05
MR Test
Inverse variance weighted (fixed effects)
Inverse variance weighted (multiplicative random effects)
MR Egger
MR RAPS
Weighted median
Weighted mode
0.05
0.00
−0.05
−0.10
0.1
0.2
SNP effect on Peptidyl−prolyl cis−trans isomerase FKBP14
SNP effect on Gut microbiota abundance (genus Parasutterella id.2892)

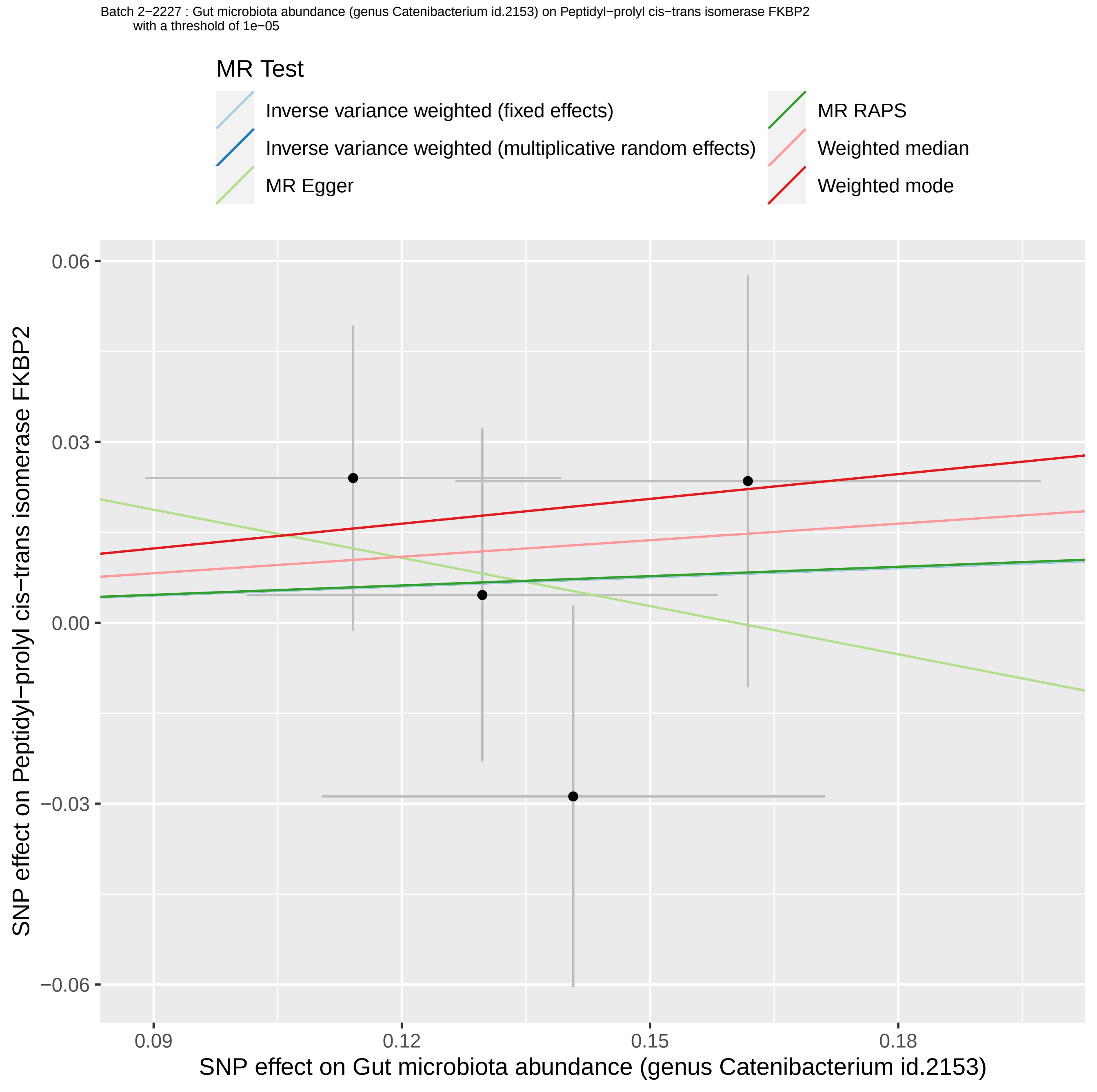
Batch 2−2227 : Gut microbiota abundance (genus Catenibacterium id.2153) on Peptidyl−prolyl cis−trans isomerase FKBP2
with a threshold of 1e−05
MR Test
Inverse variance weighted (fixed effects)
Inverse variance weighted (multiplicative random effects)
MR Egger
MR RAPS
Weighted median
Weighted mode
0.06
0.03
0.00
−0.03
−0.06
0.09
0.12
0.15
0.18
SNP effect on Peptidyl−prolyl cis−trans isomerase FKBP2
SNP effect on Gut microbiota abundance (genus Catenibacterium id.2153)

Batch 2−2228 : Gut microbiota abundance (genus Parasutterella id.2892) on Peptidyl−prolyl cis−trans isomerase FKBP2
with a threshold of 1e−05

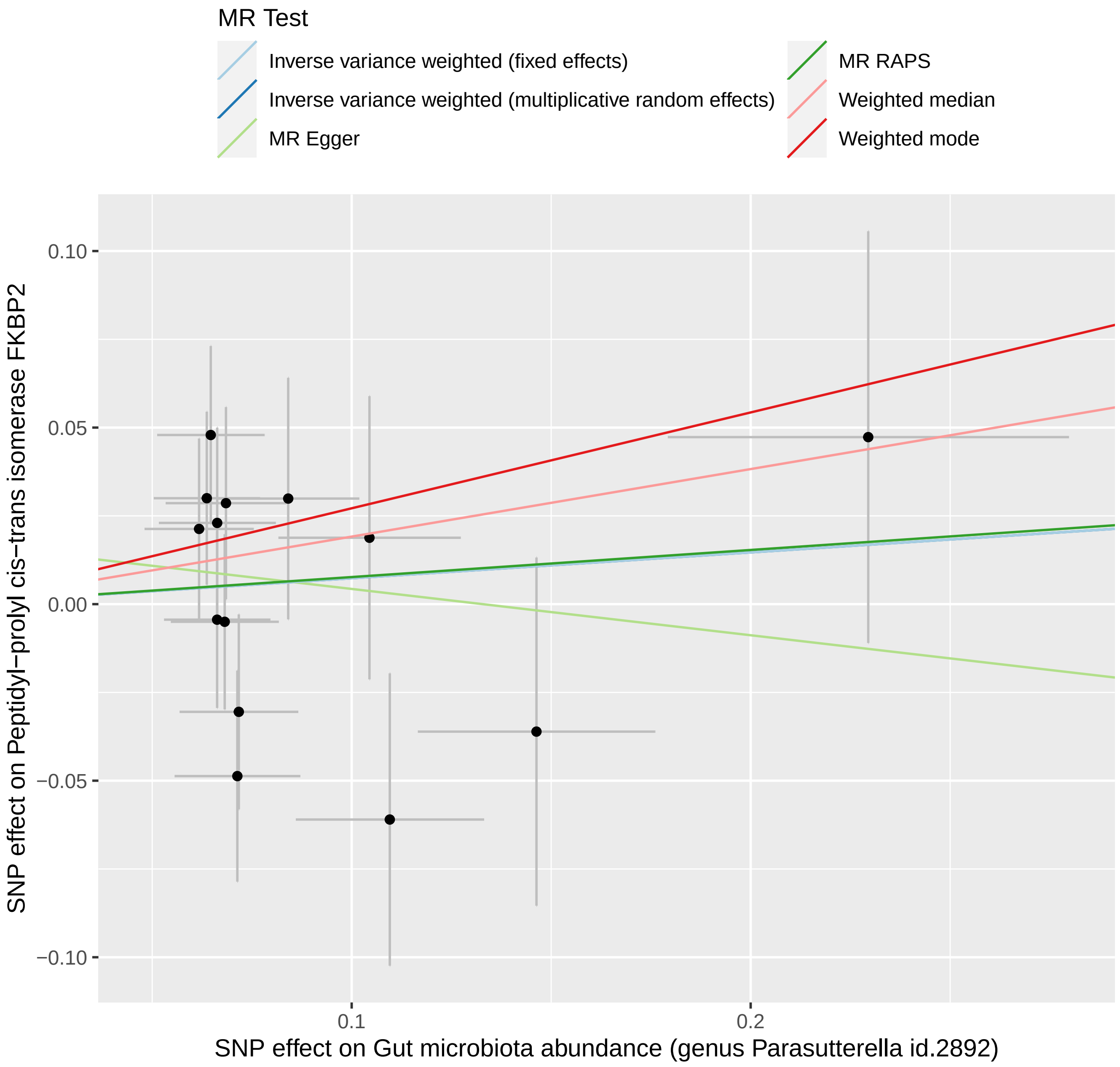

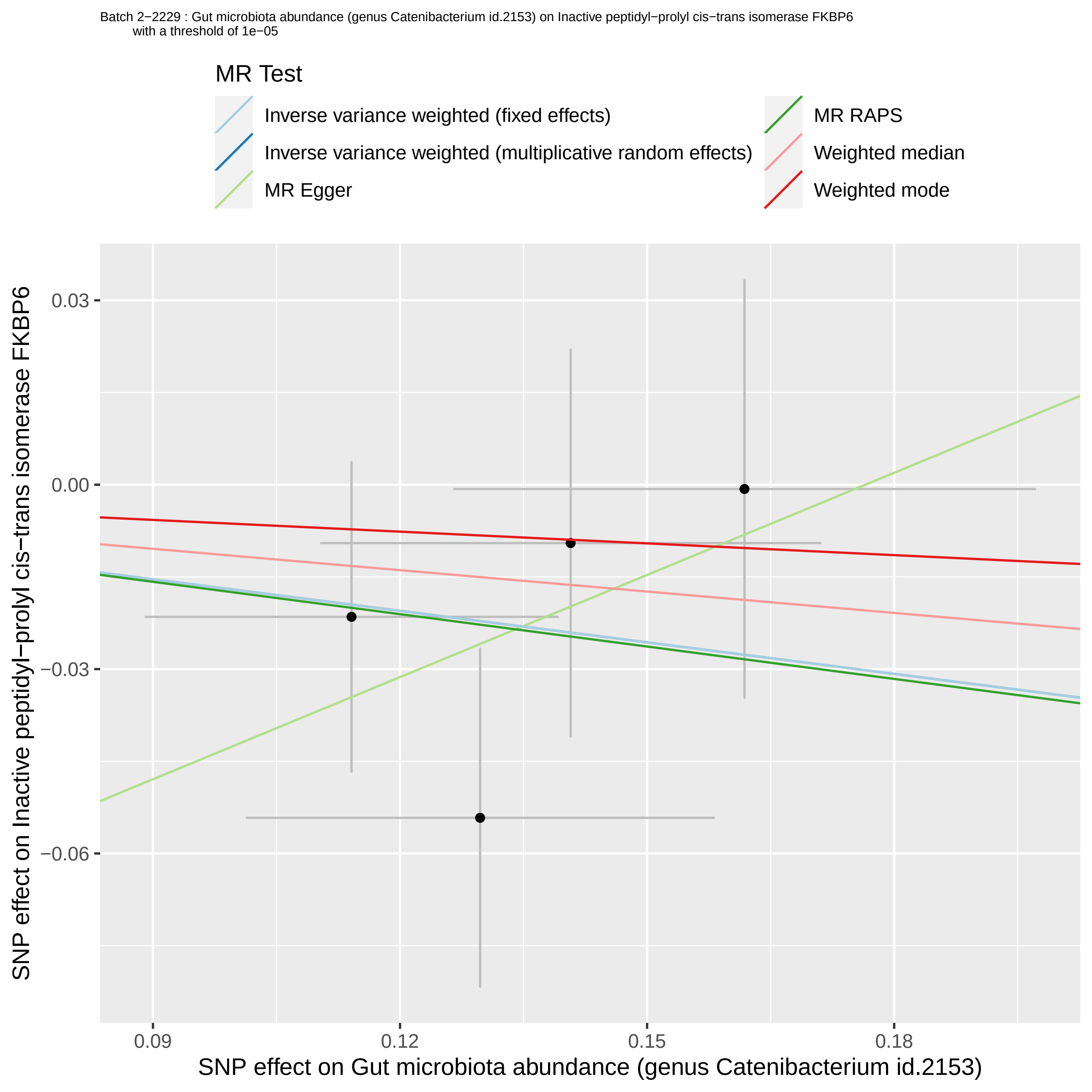

Batch 2−2229 : Gut microbiota abundance (genus Catenibacterium id.2153) on Inactive peptidyl−prolyl cis−trans isomerase FKBP6
with a threshold of 1e−05
MR Test
Inverse variance weighted (fixed effects)
Inverse variance weighted (multiplicative random effects)
MR Egger
MR RAPS
Weighted median
Weighted mode
0.03
0.00
−0.03
−0.06
0.09
0.12
0.15
0.18
SNP effect on Gut microbiota abundance (genus Catenibacterium id.2153)
SNP effect on Inactive peptidyl−prolyl cis−trans isomerase FKBP6

Batch 2−2230 : Gut microbiota abundance (genus Parasutterella id.2892) on Inactive peptidyl−prolyl cis−trans isomerase FKBP6
with a threshold of 1e−05

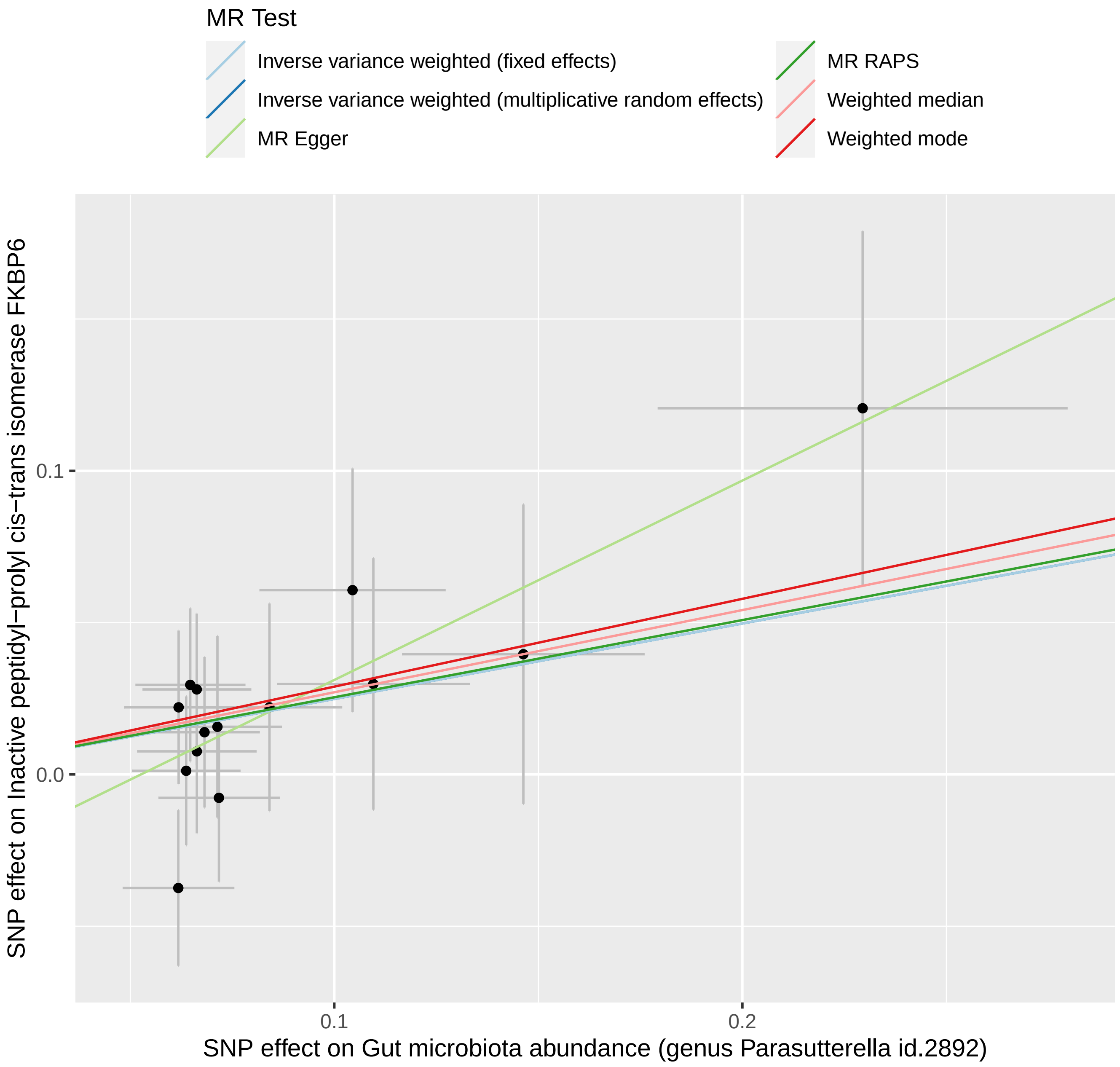

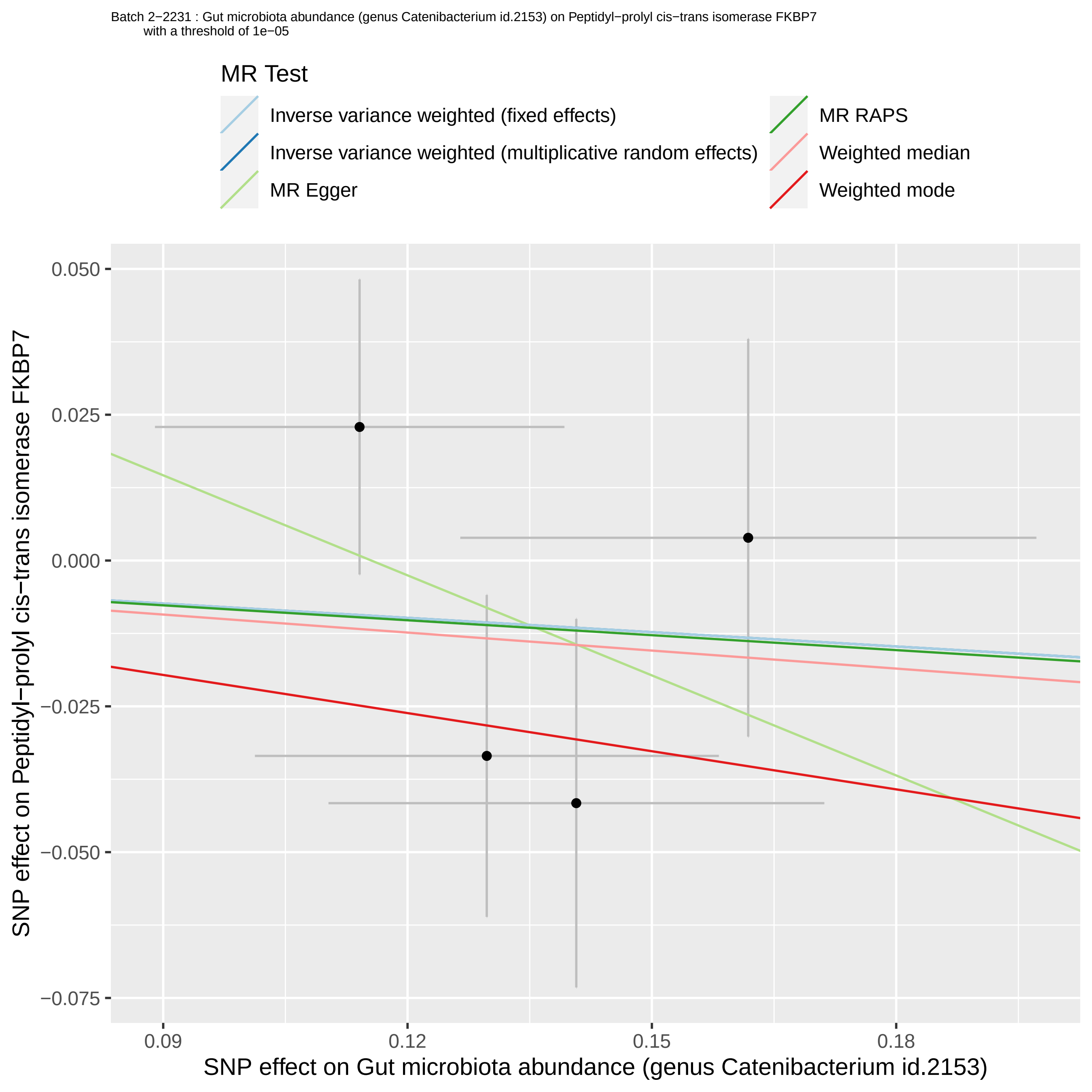
Batch 2−2231 : Gut microbiota abundance (genus Catenibacterium id.2153) on Peptidyl−prolyl cis−trans isomerase FKBP7
with a threshold of 1e−05
MR Test
Inverse variance weighted (fixed effects)
Inverse variance weighted (multiplicative random effects)
MR Egger
MR RAPS
Weighted median
Weighted mode
0.050
0.025
0.000
−0.025
−0.050
−0.075
0.09
0.12
0.15
0.18
SNP effect on Peptidyl−prolyl cis−trans isomerase FKBP7
SNP effect on Gut microbiota abundance (genus Catenibacterium id.2153)

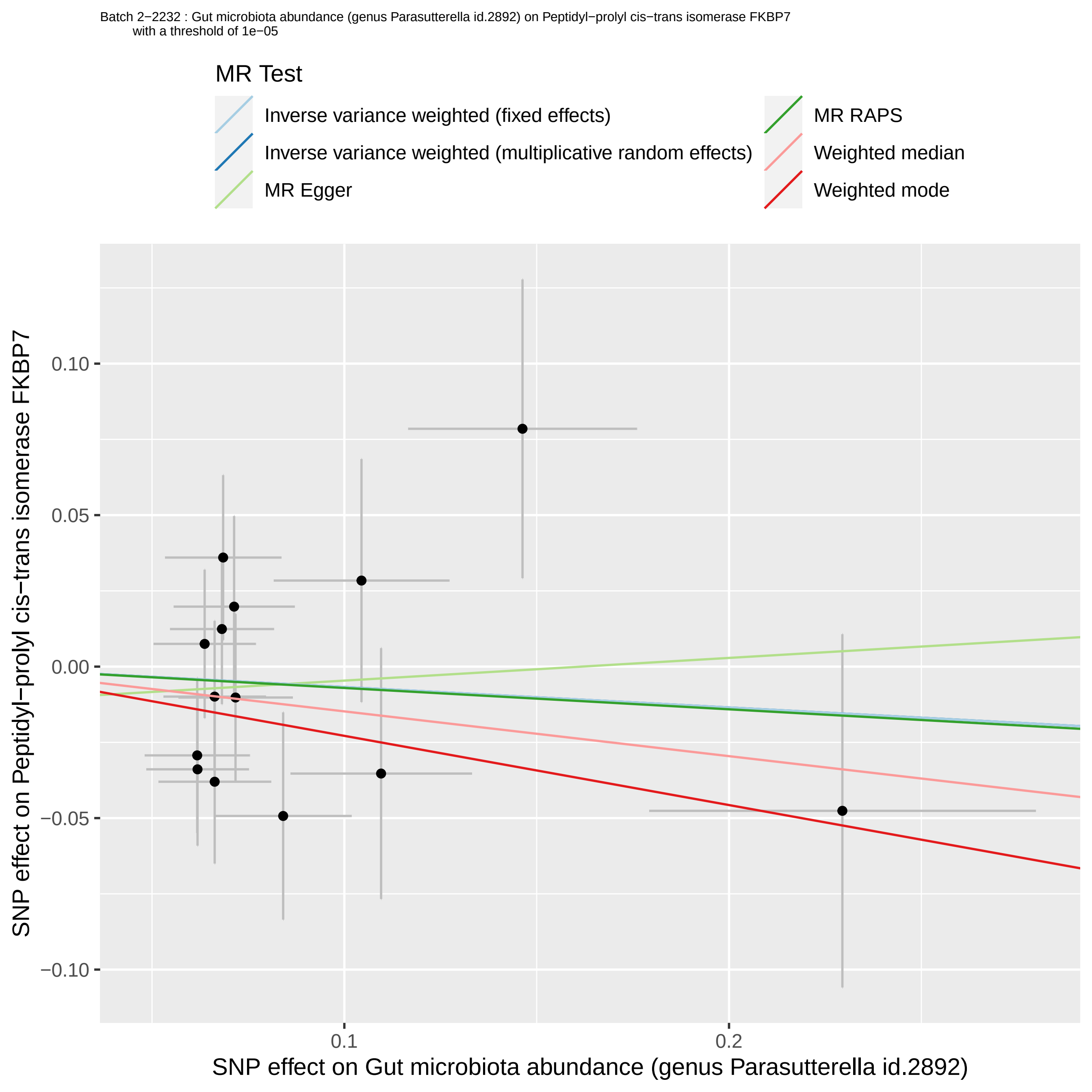
Batch 2−2232 : Gut microbiota abundance (genus Parasutterella id.2892) on Peptidyl−prolyl cis−trans isomerase FKBP7
with a threshold of 1e−05
MR Test
Inverse variance weighted (fixed effects)
Inverse variance weighted (multiplicative random effects)
MR Egger
MR RAPS
Weighted median
Weighted mode
0.10
0.05
0.00
−0.05
−0.10
0.1
0.2
SNP effect on Peptidyl−prolyl cis−trans isomerase FKBP7
SNP effect on Gut microbiota abundance (genus Parasutterella id.2892)

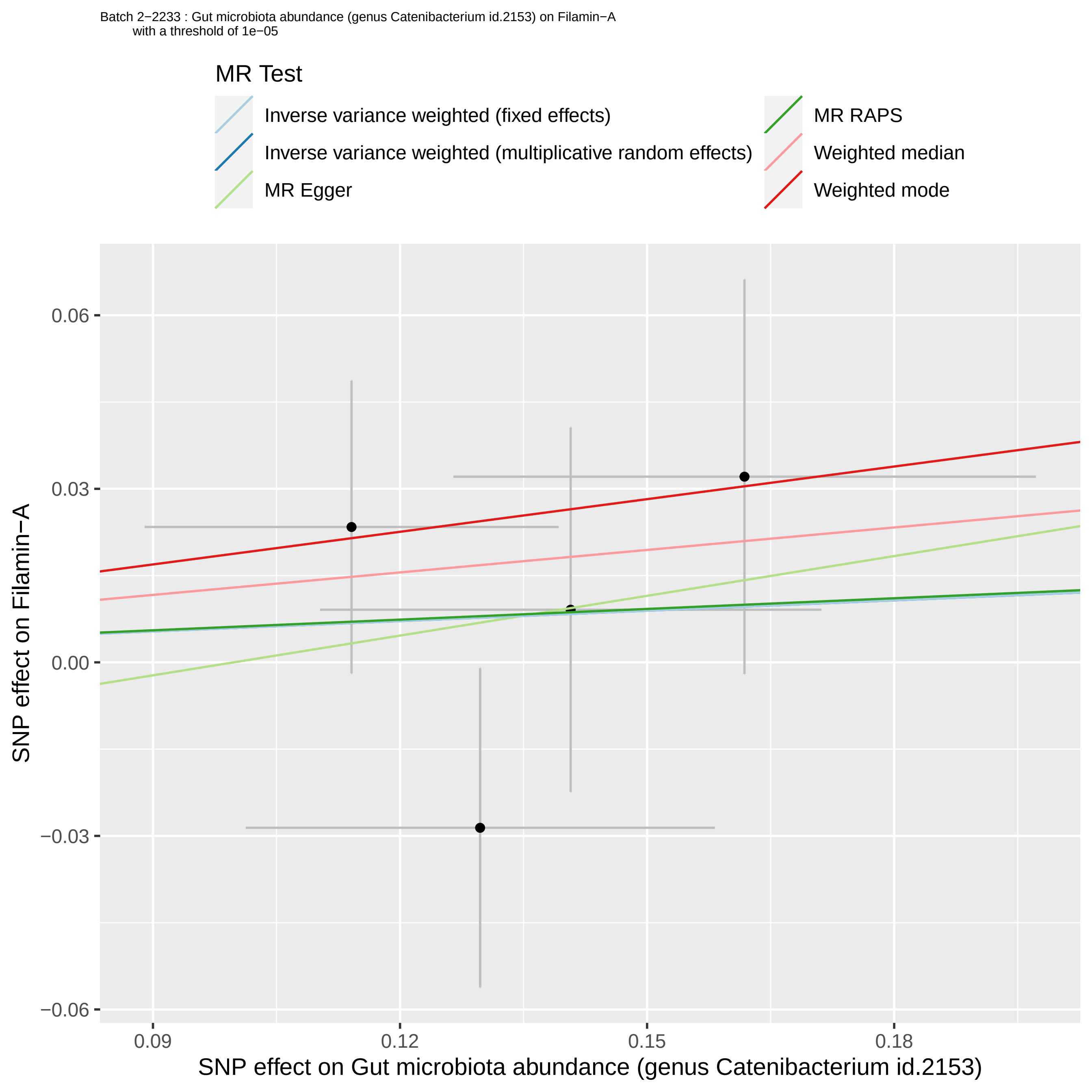
Batch 2−2233 : Gut microbiota abundance (genus Catenibacterium id.2153) on Filamin−A
with a threshold of 1e−05
MR Test
Inverse variance weighted (fixed effects)
Inverse variance weighted (multiplicative random effects)
MR Egger
MR RAPS
Weighted median
Weighted mode
0.06
0.03
0.00
−0.03
−0.06
0.09
0.12
0.15
0.18
SNP effect on Filamin−A
SNP effect on Gut microbiota abundance (genus Catenibacterium id.2153)

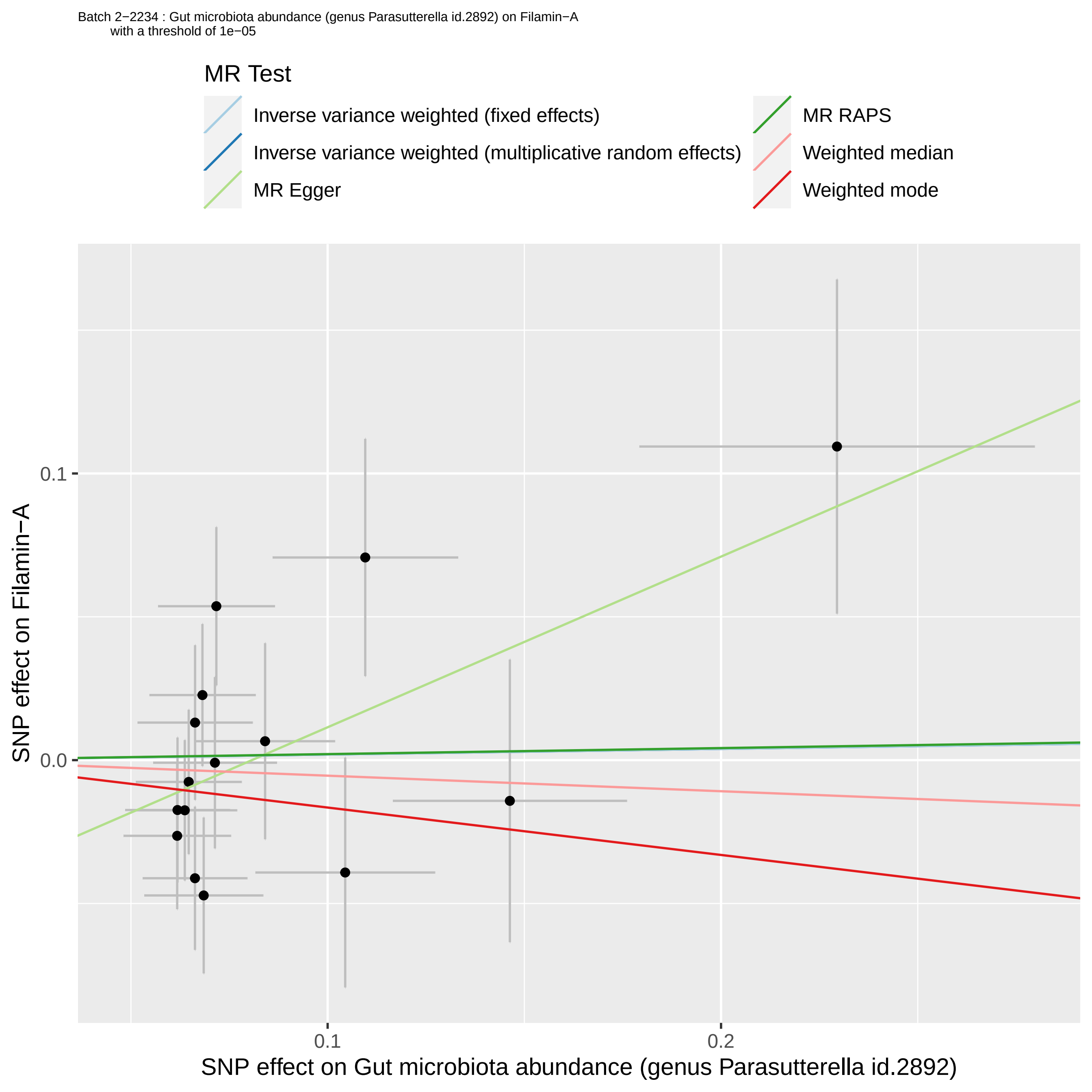

Batch 2−2234 : Gut microbiota abundance (genus Parasutterella id.2892) on Filamin−A
with a threshold of 1e−05
MR Test
Inverse variance weighted (fixed effects)
Inverse variance weighted (multiplicative random effects)
MR Egger
MR RAPS
Weighted median
Weighted mode
SNP effect on Filamin−A
0.1
0.0
0.1
0.2
SNP effect on Gut microbiota abundance (genus Parasutterella id.2892)

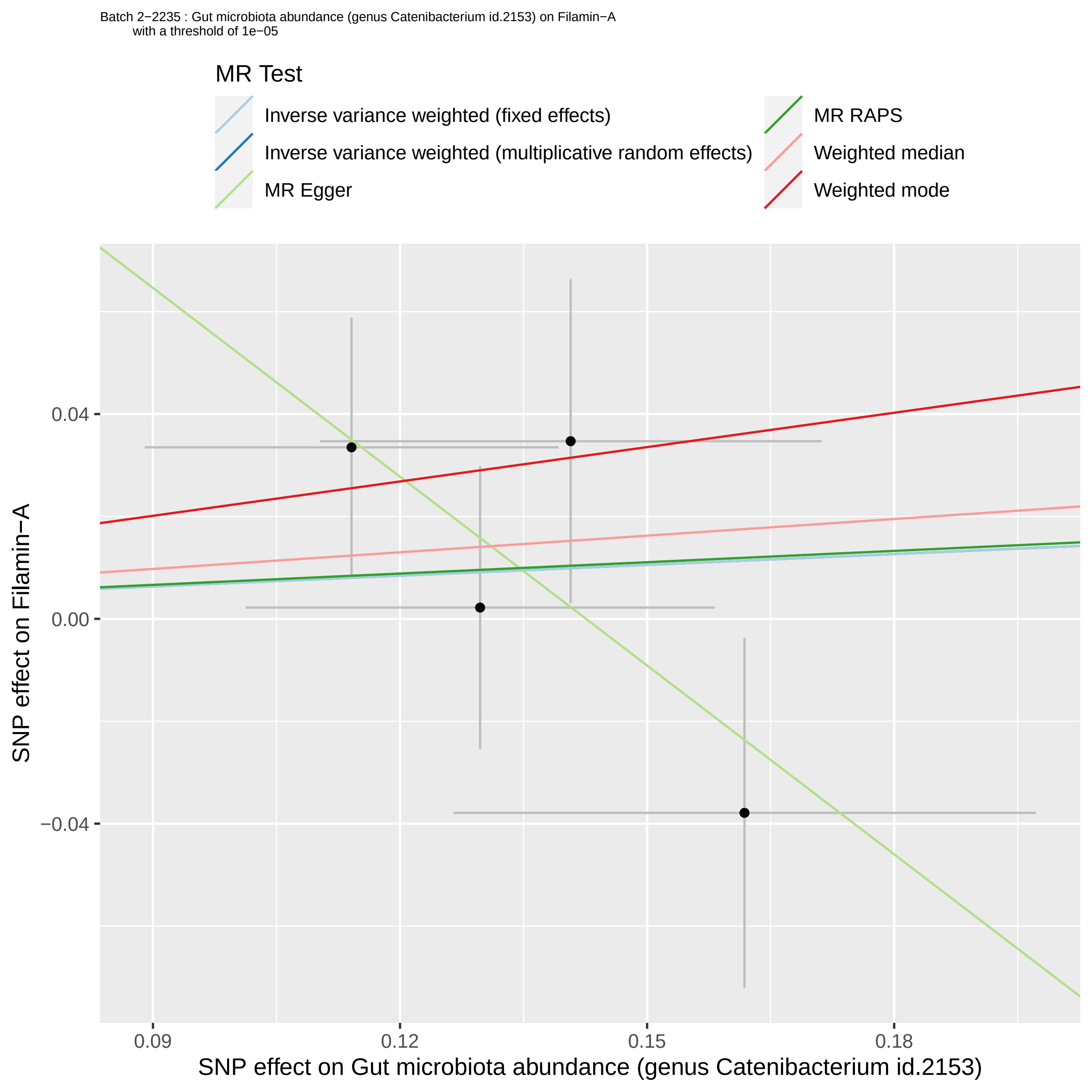
Batch 2−2235 : Gut microbiota abundance (genus Catenibacterium id.2153) on Filamin−A
with a threshold of 1e−05
MR Test
Inverse variance weighted (fixed effects)
Inverse variance weighted (multiplicative random effects)
MR Egger
MR RAPS
Weighted median
Weighted mode
SNP effect on Filamin−A
0.04
0.00
−0.04
0.09
0.12
0.15
0.18
SNP effect on Gut microbiota abundance (genus Catenibacterium id.2153)

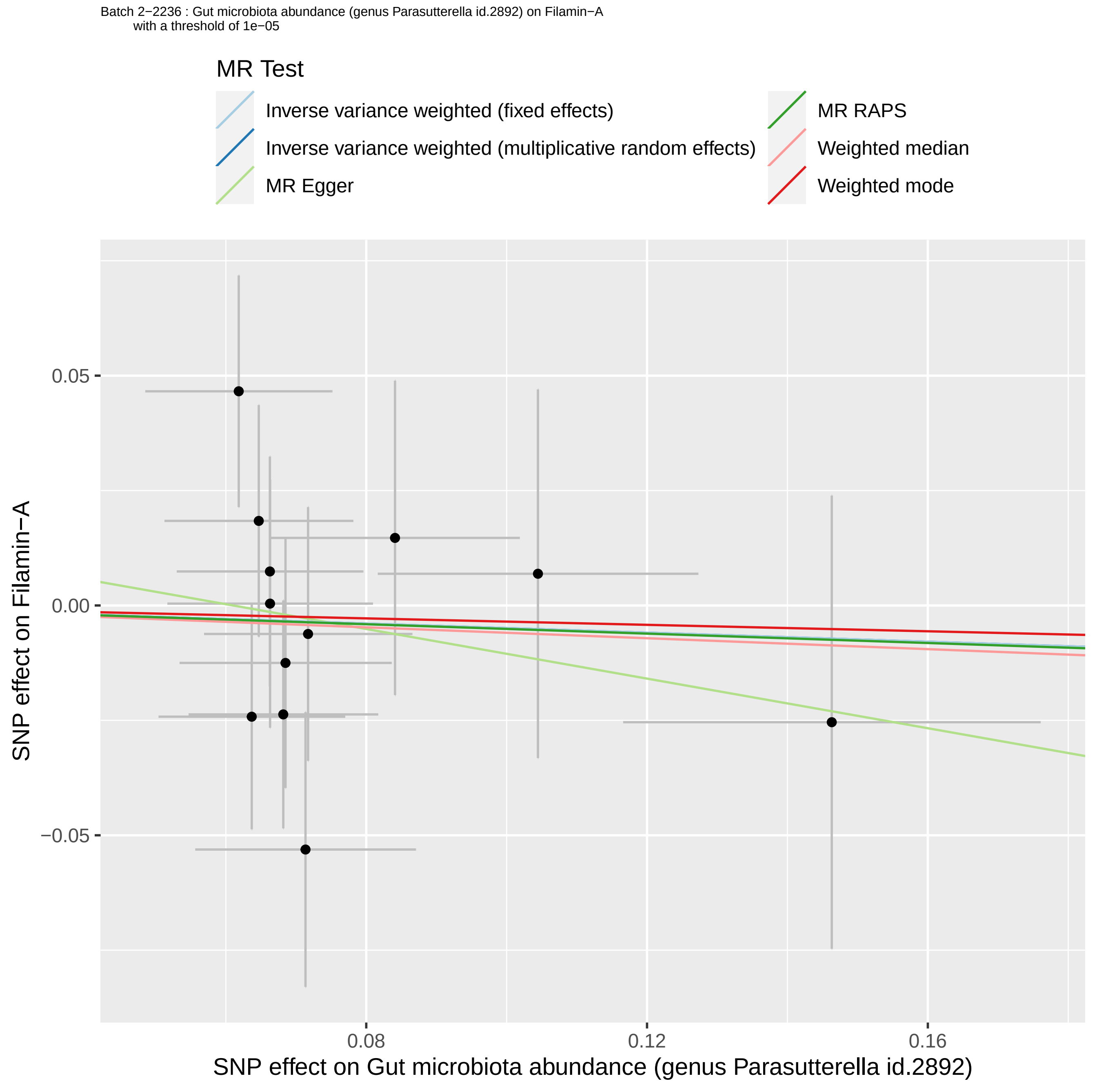
Batch 2−2236 : Gut microbiota abundance (genus Parasutterella id.2892) on Filamin−A
with a threshold of 1e−05
MR Test
Inverse variance weighted (fixed effects)
Inverse variance weighted (multiplicative random effects)
MR Egger
MR RAPS
Weighted median
Weighted mode
0.05
0.00
−0.05
0.08
0.12
0.16
SNP effect on Filamin−A
SNP effect on Gut microbiota abundance (genus Parasutterella id.2892)

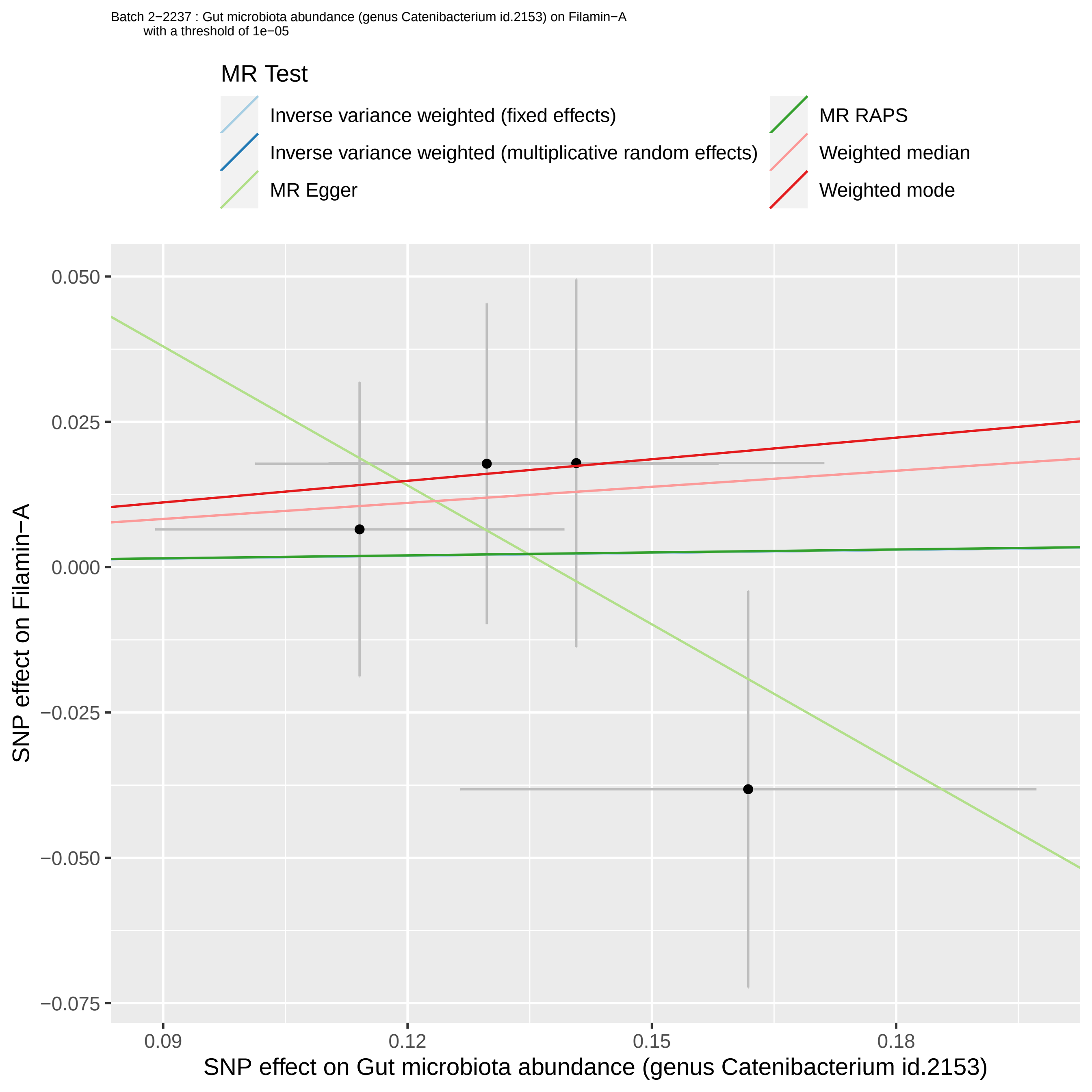
Batch 2−2237 : Gut microbiota abundance (genus Catenibacterium id.2153) on Filamin−A
with a threshold of 1e−05
MR Test
Inverse variance weighted (fixed effects)
Inverse variance weighted (multiplicative random effects)
MR Egger
MR RAPS
Weighted median
Weighted mode
0.050
0.025
0.000
−0.025
−0.050
−0.075
0.09
0.12
0.15
0.18
SNP effect on Filamin−A
SNP effect on Gut microbiota abundance (genus Catenibacterium id.2153)

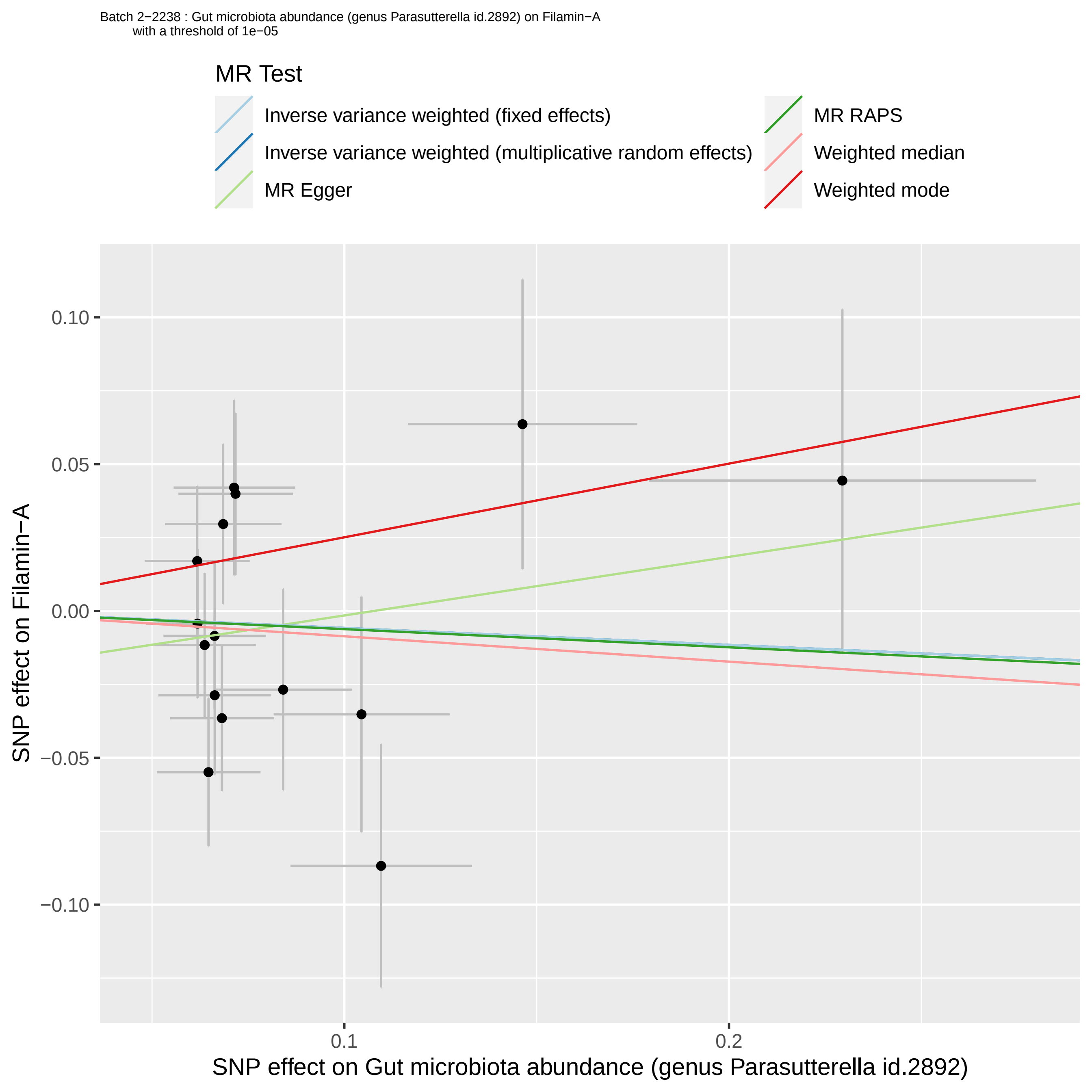

Batch 2−2238 : Gut microbiota abundance (genus Parasutterella id.2892) on Filamin−A
with a threshold of 1e−05
MR Test
Inverse variance weighted (fixed effects)
Inverse variance weighted (multiplicative random effects)
MR Egger
MR RAPS
Weighted median
Weighted mode
0.10
0.05
0.00
−0.05
−0.10
0.1
0.2
SNP effect on Filamin−A
SNP effect on Gut microbiota abundance (genus Parasutterella id.2892)

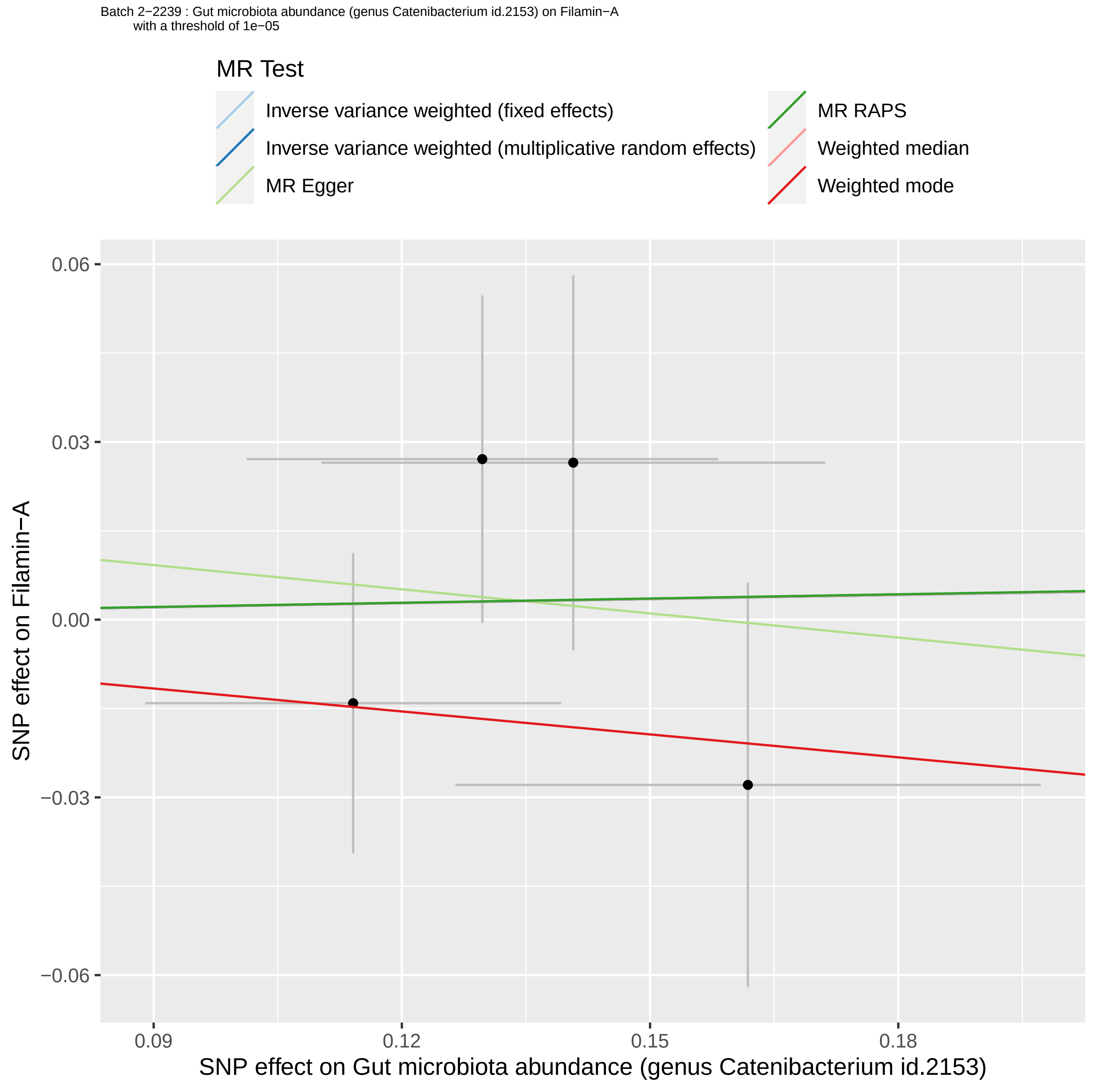
Batch 2−2239 : Gut microbiota abundance (genus Catenibacterium id.2153) on Filamin−A
with a threshold of 1e−05
MR Test
Inverse variance weighted (fixed effects)
Inverse variance weighted (multiplicative random effects)
MR Egger
MR RAPS
Weighted median
Weighted mode
0.06
0.03
0.00
−0.03
−0.06
0.09
0.12
0.15
0.18
SNP effect on Filamin−A
SNP effect on Gut microbiota abundance (genus Catenibacterium id.2153)

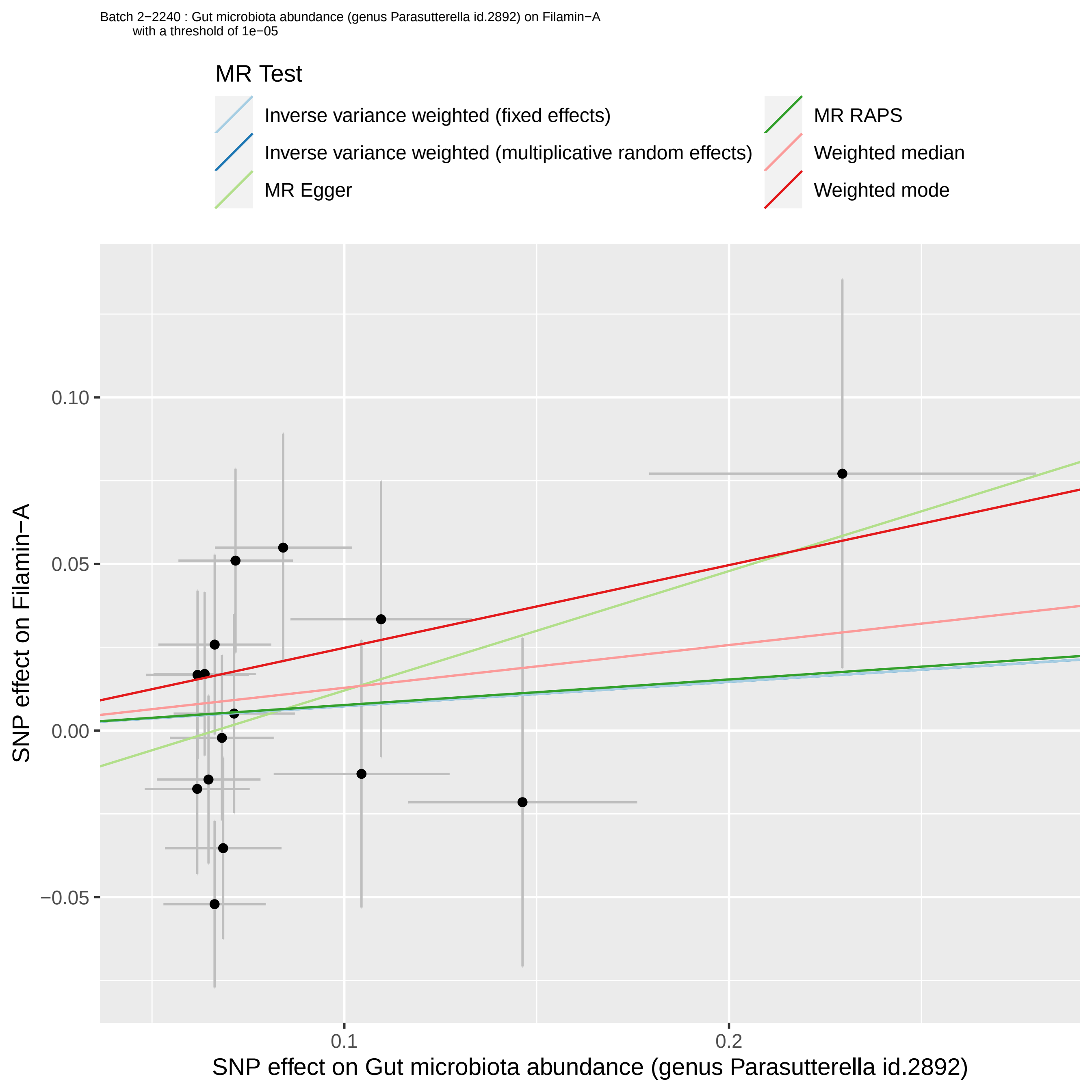

Batch 2−2240 : Gut microbiota abundance (genus Parasutterella id.2892) on Filamin−A
with a threshold of 1e−05
MR Test
Inverse variance weighted (fixed effects)
Inverse variance weighted (multiplicative random effects)
MR Egger
MR RAPS
Weighted median
Weighted mode
0.10
0.05
0.00
−0.05
0.1
0.2
SNP effect on Filamin−A
SNP effect on Gut microbiota abundance (genus Parasutterella id.2892)

Batch 2−2241 : Gut microbiota abundance (genus Catenibacterium id.2153) on Leucine−rich repeat transmembrane protein FLRT1
with a threshold of 1e−05

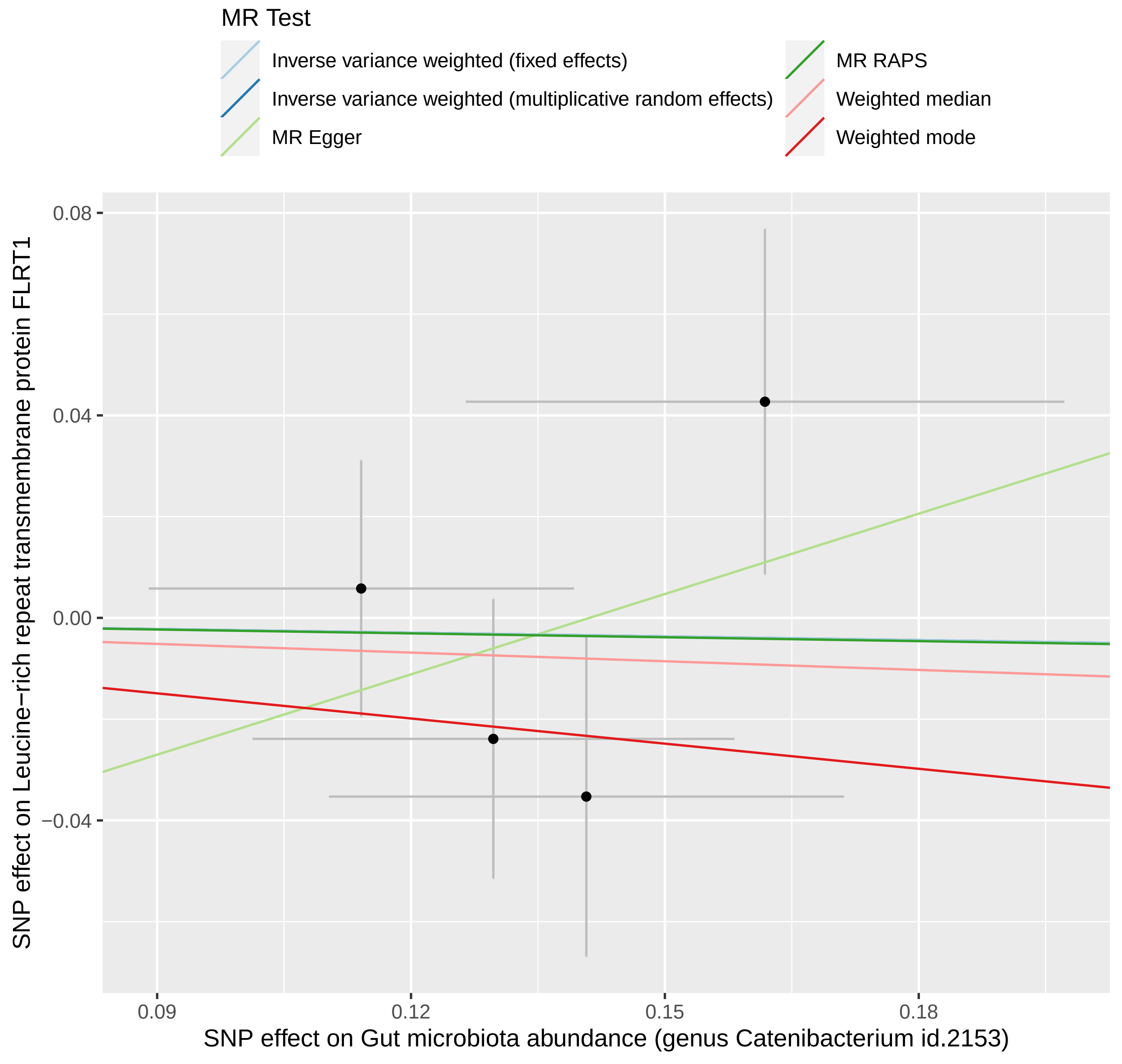

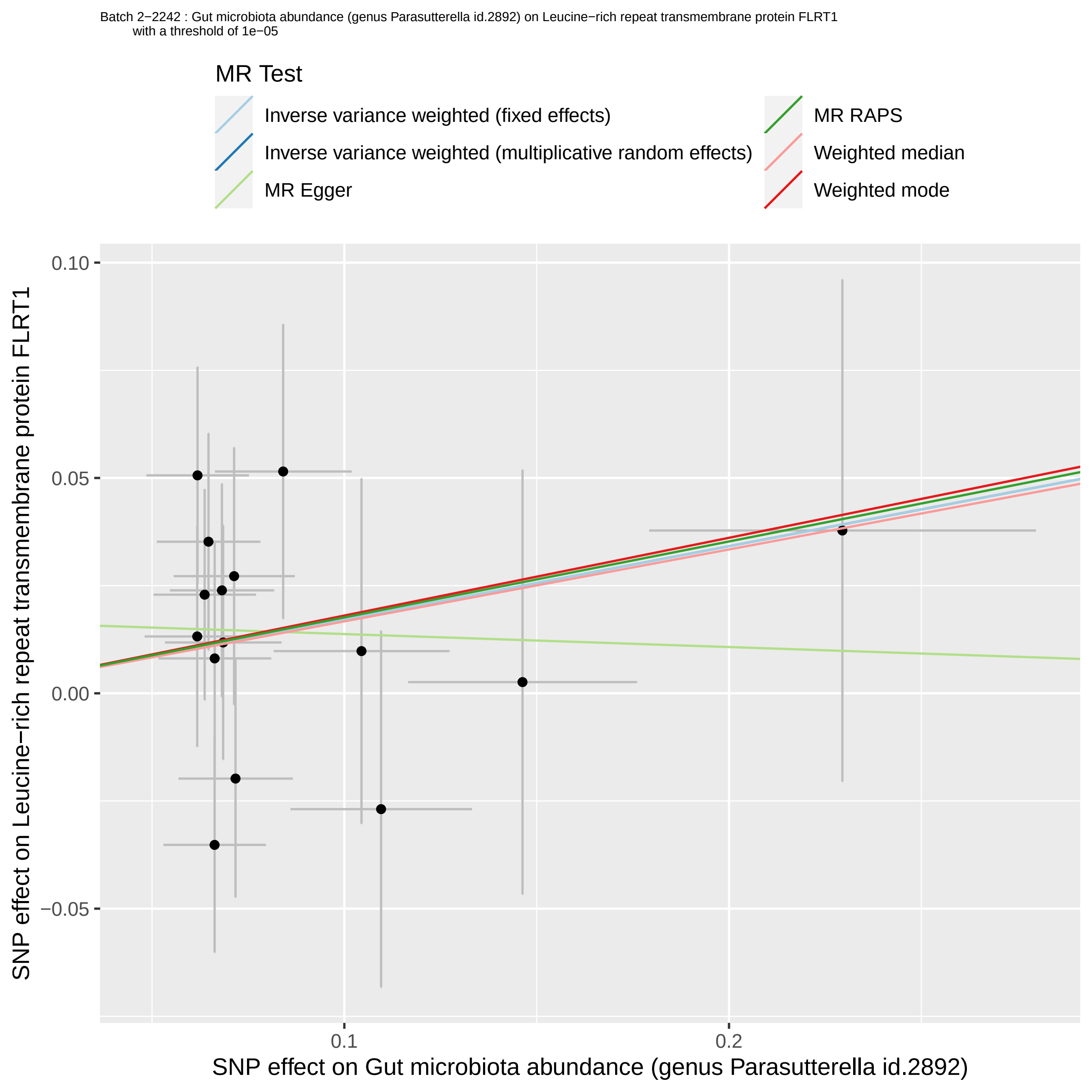

Batch 2−2242 : Gut microbiota abundance (genus Parasutterella id.2892) on Leucine−rich repeat transmembrane protein FLRT1
with a threshold of 1e−05
MR Test
Inverse variance weighted (fixed effects)
Inverse variance weighted (multiplicative random effects)
MR Egger
MR RAPS
Weighted median
Weighted mode
0.10
0.05
0.00
−0.05
0.1
0.2
SNP effect on Leucine−rich repeat transmembrane protein FLRT1
SNP effect on Gut microbiota abundance (genus Parasutterella id.2892)

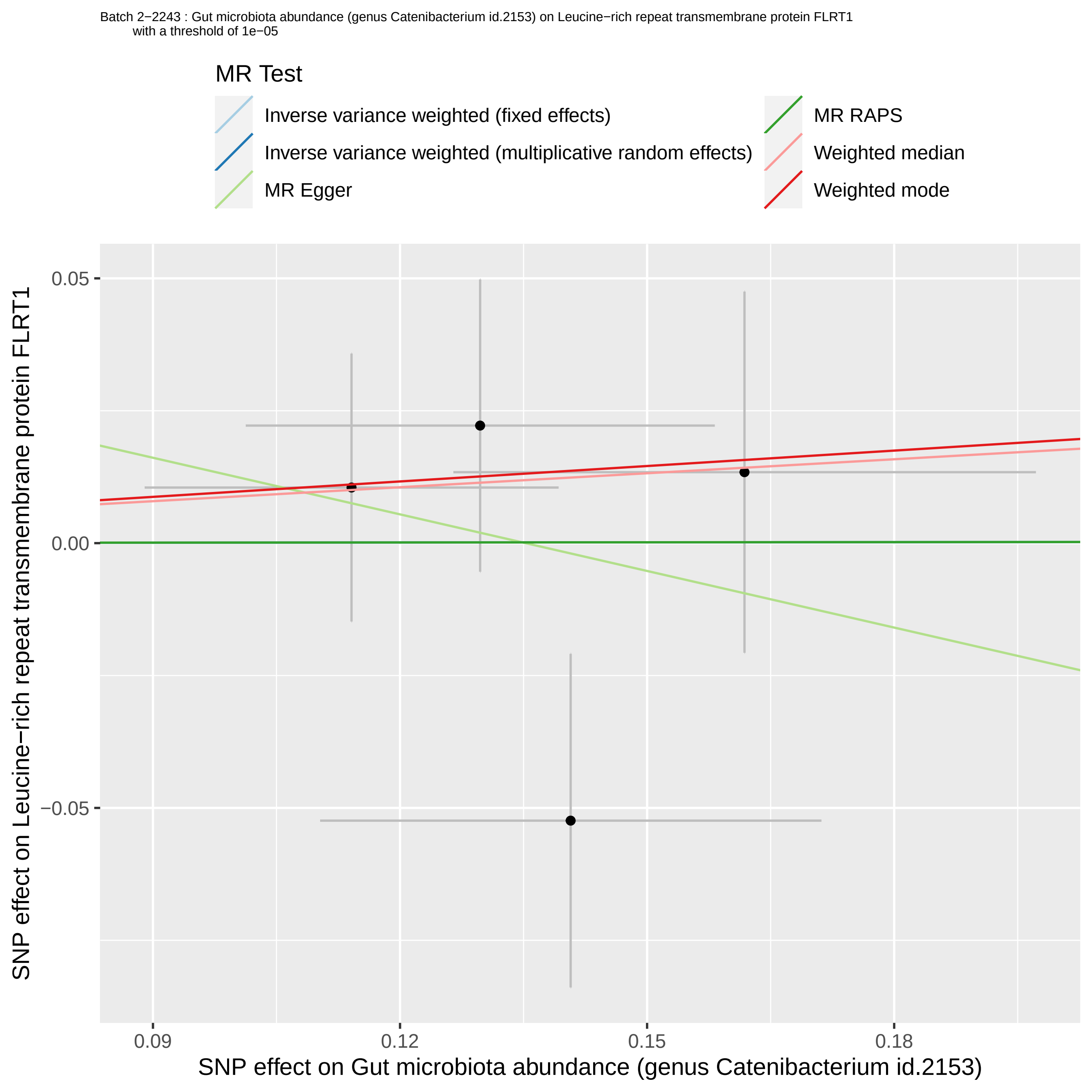
Batch 2−2243 : Gut microbiota abundance (genus Catenibacterium id.2153) on Leucine−rich repeat transmembrane protein FLRT1
with a threshold of 1e−05
MR Test
Inverse variance weighted (fixed effects)
Inverse variance weighted (multiplicative random effects)
MR Egger
MR RAPS
Weighted median
Weighted mode
0.05
0.00
−0.05
0.09
0.12
0.15
0.18
SNP effect on Leucine−rich repeat transmembrane protein FLRT1
SNP effect on Gut microbiota abundance (genus Catenibacterium id.2153)

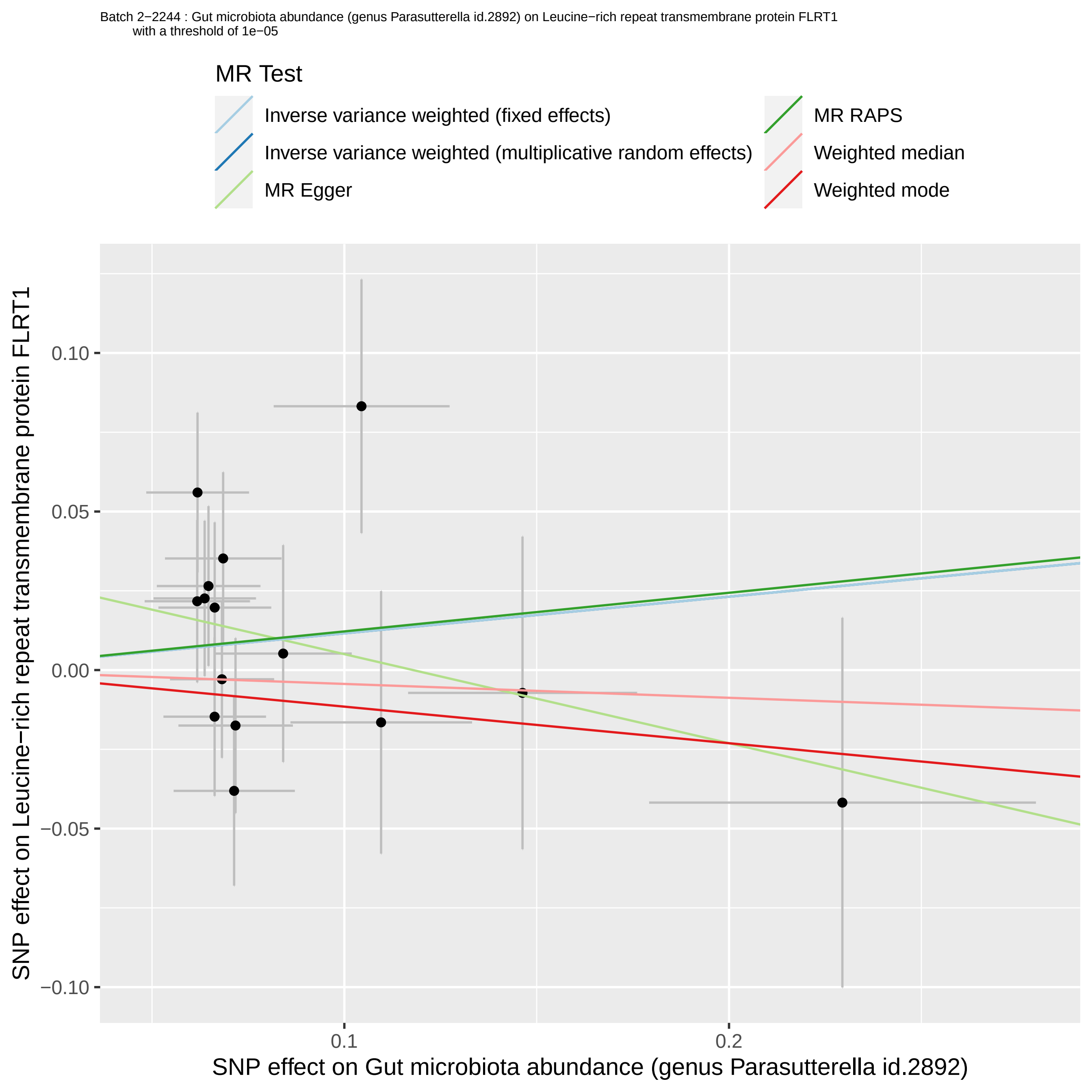
Batch 2−2244 : Gut microbiota abundance (genus Parasutterella id.2892) on Leucine−rich repeat transmembrane protein FLRT1
with a threshold of 1e−05
MR Test
Inverse variance weighted (fixed effects)
Inverse variance weighted (multiplicative random effects)
MR Egger
MR RAPS
Weighted median
Weighted mode
SNP effect on Leucine−rich repeat transmembrane protein FLRT1
0.10
0.05
0.00
−0.05
−0.10
0.1
0.2
SNP effect on Gut microbiota abundance (genus Parasutterella id.2892)

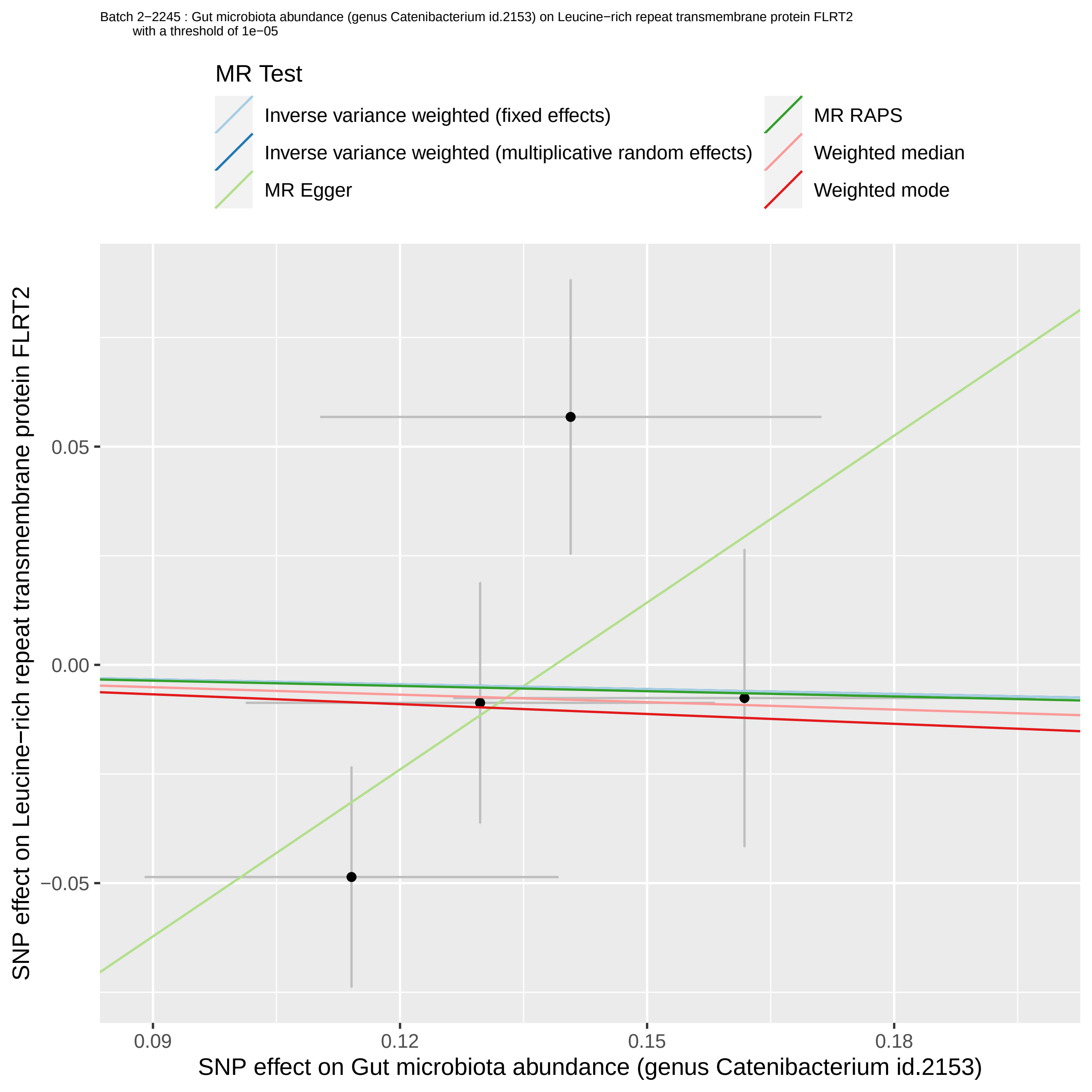

Batch 2−2245 : Gut microbiota abundance (genus Catenibacterium id.2153) on Leucine−rich repeat transmembrane protein FLRT2
with a threshold of 1e−05
MR Test
Inverse variance weighted (fixed effects)
Inverse variance weighted (multiplicative random effects)
MR Egger
MR RAPS
Weighted median
Weighted mode
0.05
0.00
−0.05
0.09
0.12
0.15
0.18
SNP effect on Leucine−rich repeat transmembrane protein FLRT2
SNP effect on Gut microbiota abundance (genus Catenibacterium id.2153)

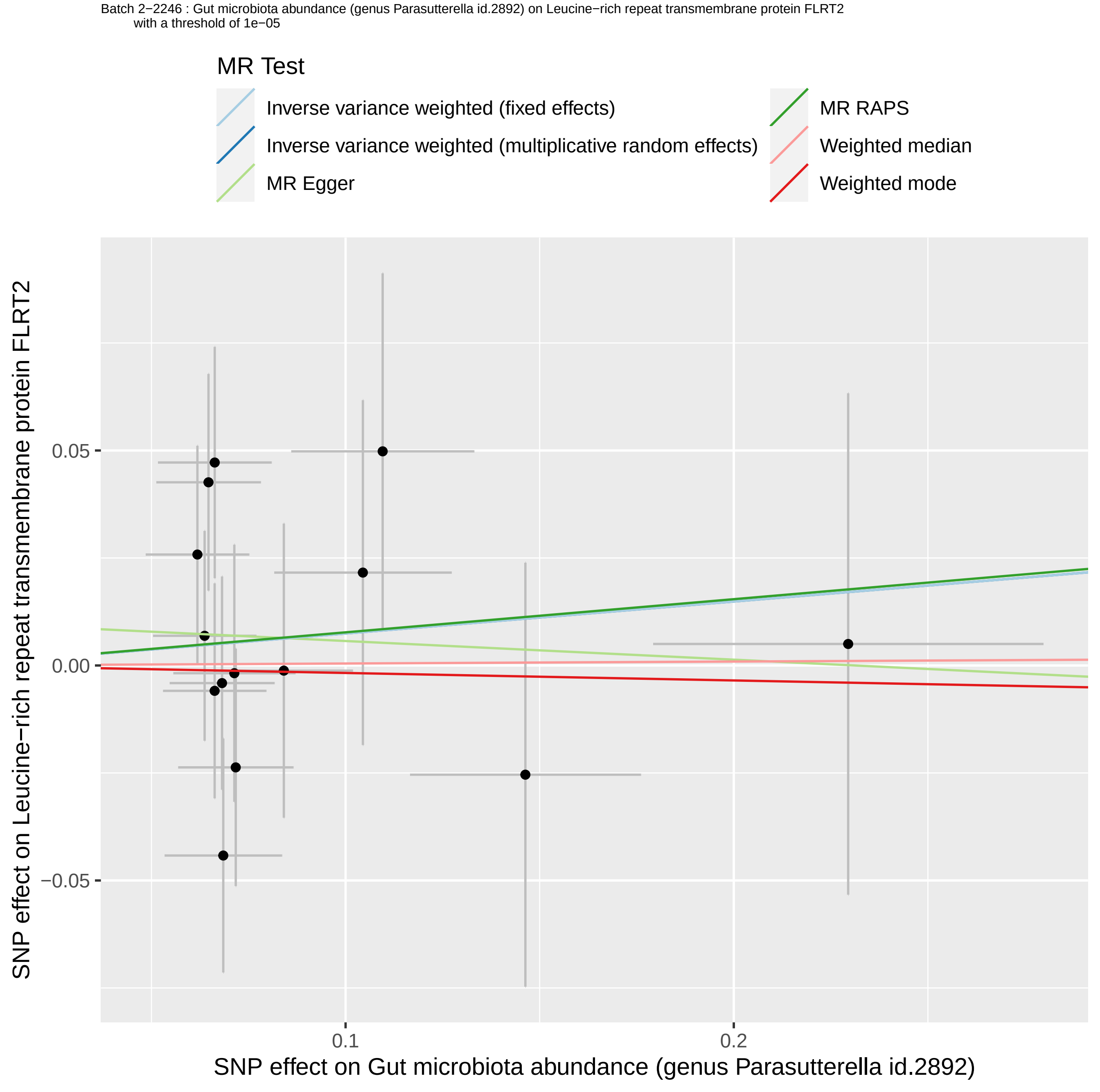

Batch 2−2246 : Gut microbiota abundance (genus Parasutterella id.2892) on Leucine−rich repeat transmembrane protein FLRT2
with a threshold of 1e−05
MR Test
Inverse variance weighted (fixed effects)
Inverse variance weighted (multiplicative random effects)
MR Egger
MR RAPS
Weighted median
Weighted mode
SNP effect on Leucine−rich repeat transmembrane protein FLRT2
0.05
0.00
−0.05
0.1
0.2
SNP effect on Gut microbiota abundance (genus Parasutterella id.2892)

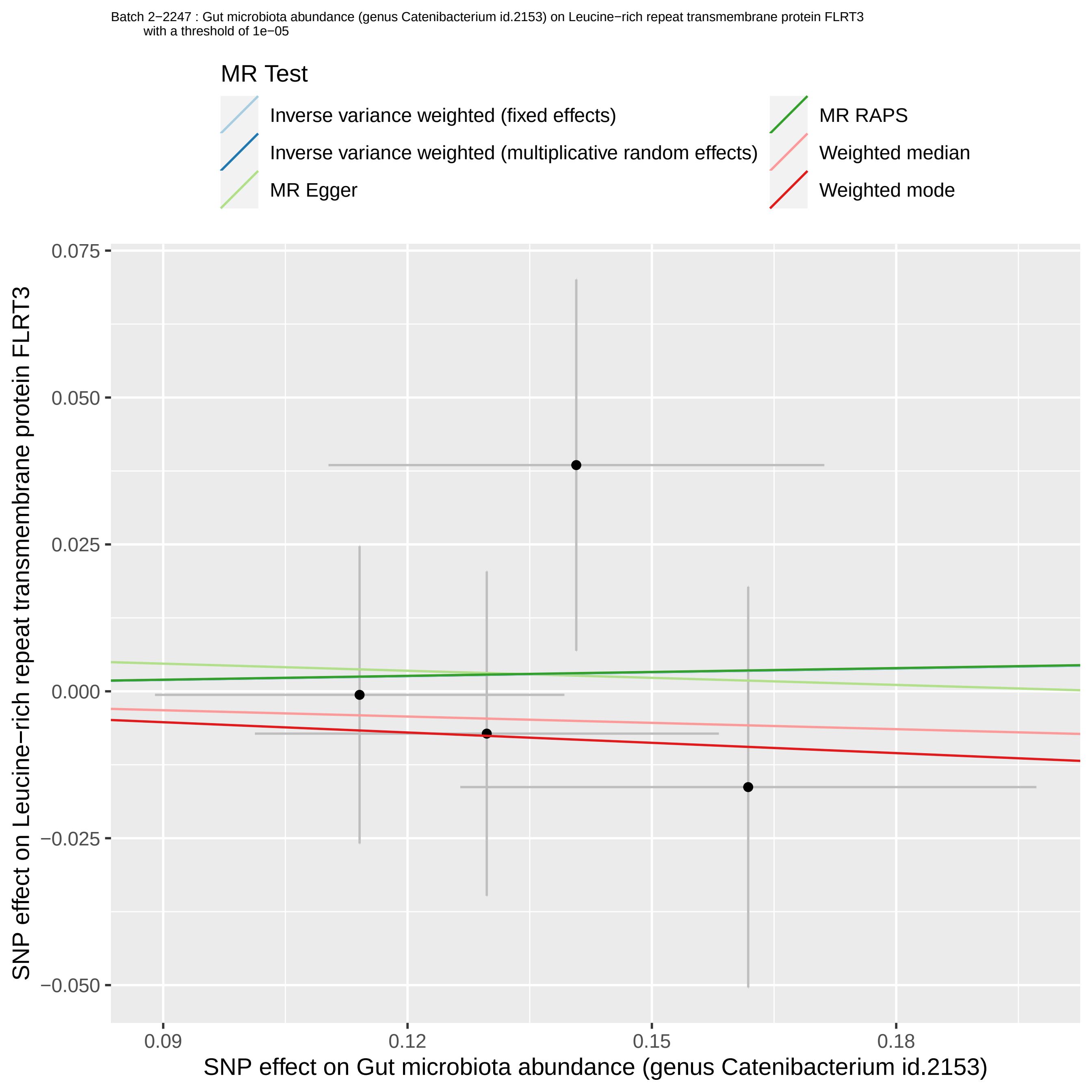
Batch 2−2247 : Gut microbiota abundance (genus Catenibacterium id.2153) on Leucine−rich repeat transmembrane protein FLRT3
with a threshold of 1e−05
MR Test
Inverse variance weighted (fixed effects)
Inverse variance weighted (multiplicative random effects)
MR Egger
MR RAPS
Weighted median
Weighted mode
0.075
0.050
0.025
0.000
−0.025
−0.050
0.09
0.12
0.15
0.18
SNP effect on Leucine−rich repeat transmembrane protein FLRT3
SNP effect on Gut microbiota abundance (genus Catenibacterium id.2153)

Batch 2−2248 : Gut microbiota abundance (genus Parasutterella id.2892) on Leucine−rich repeat transmembrane protein FLRT3
with a threshold of 1e−05

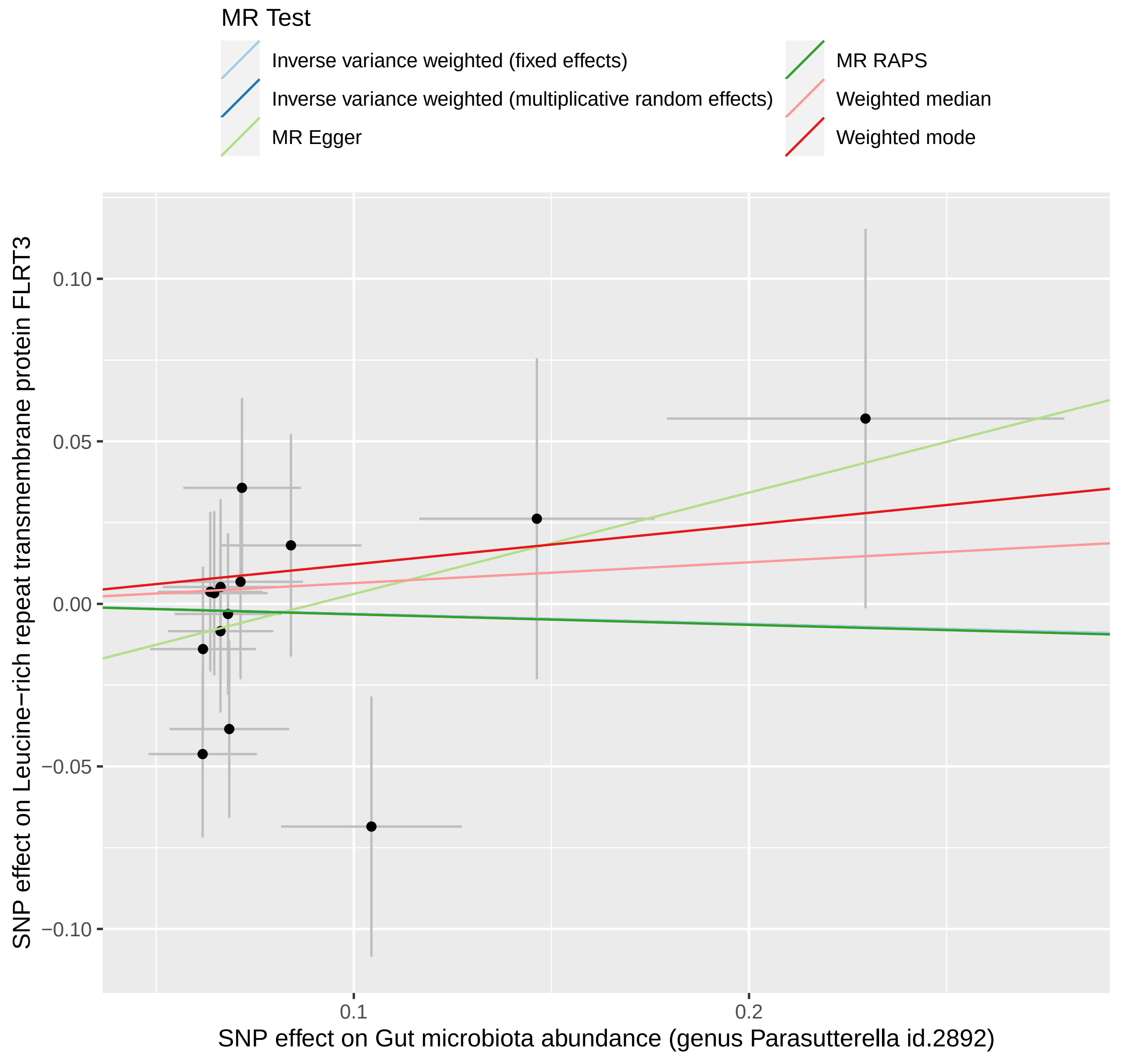

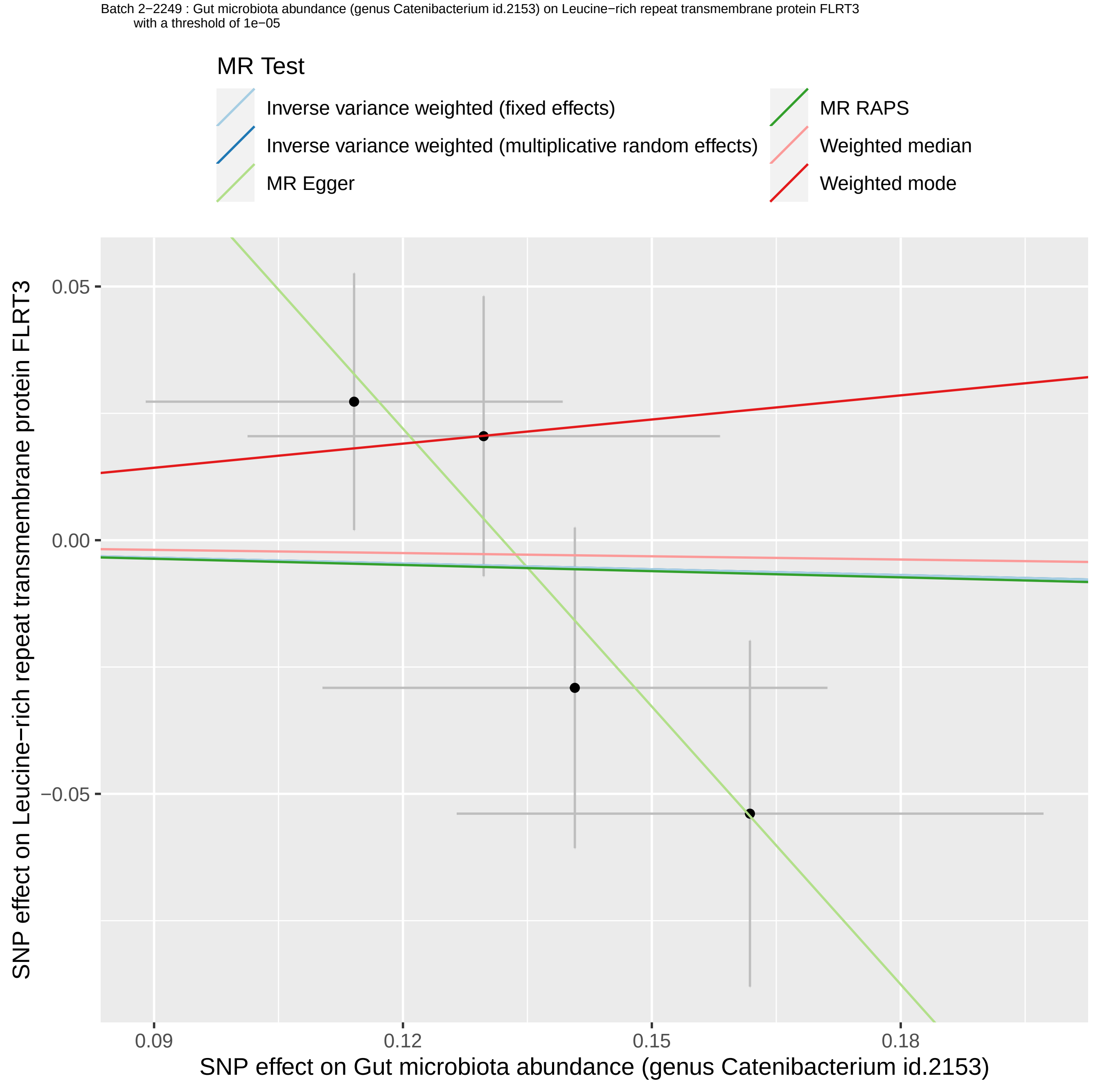
Batch 2−2249 : Gut microbiota abundance (genus Catenibacterium id.2153) on Leucine−rich repeat transmembrane protein FLRT3
with a threshold of 1e−05
MR Test
Inverse variance weighted (fixed effects)
Inverse variance weighted (multiplicative random effects)
MR Egger
MR RAPS
Weighted median
Weighted mode
0.05
0.00
−0.05
0.09
0.12
0.15
0.18
SNP effect on Leucine−rich repeat transmembrane protein FLRT3
SNP effect on Gut microbiota abundance (genus Catenibacterium id.2153)

Batch 2−2250 : Gut microbiota abundance (genus Parasutterella id.2892) on Leucine−rich repeat transmembrane protein FLRT3
with a threshold of 1e−05

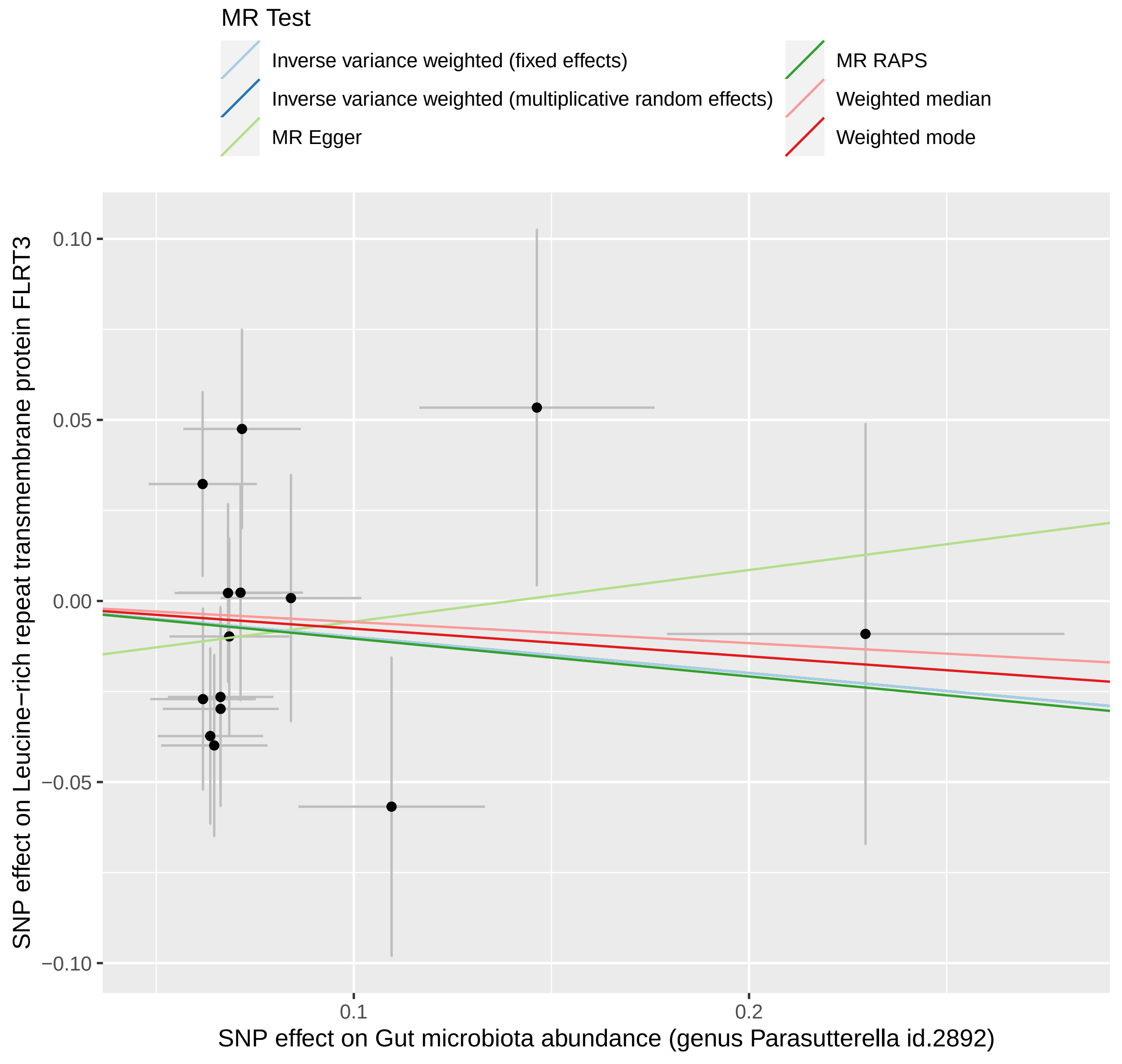

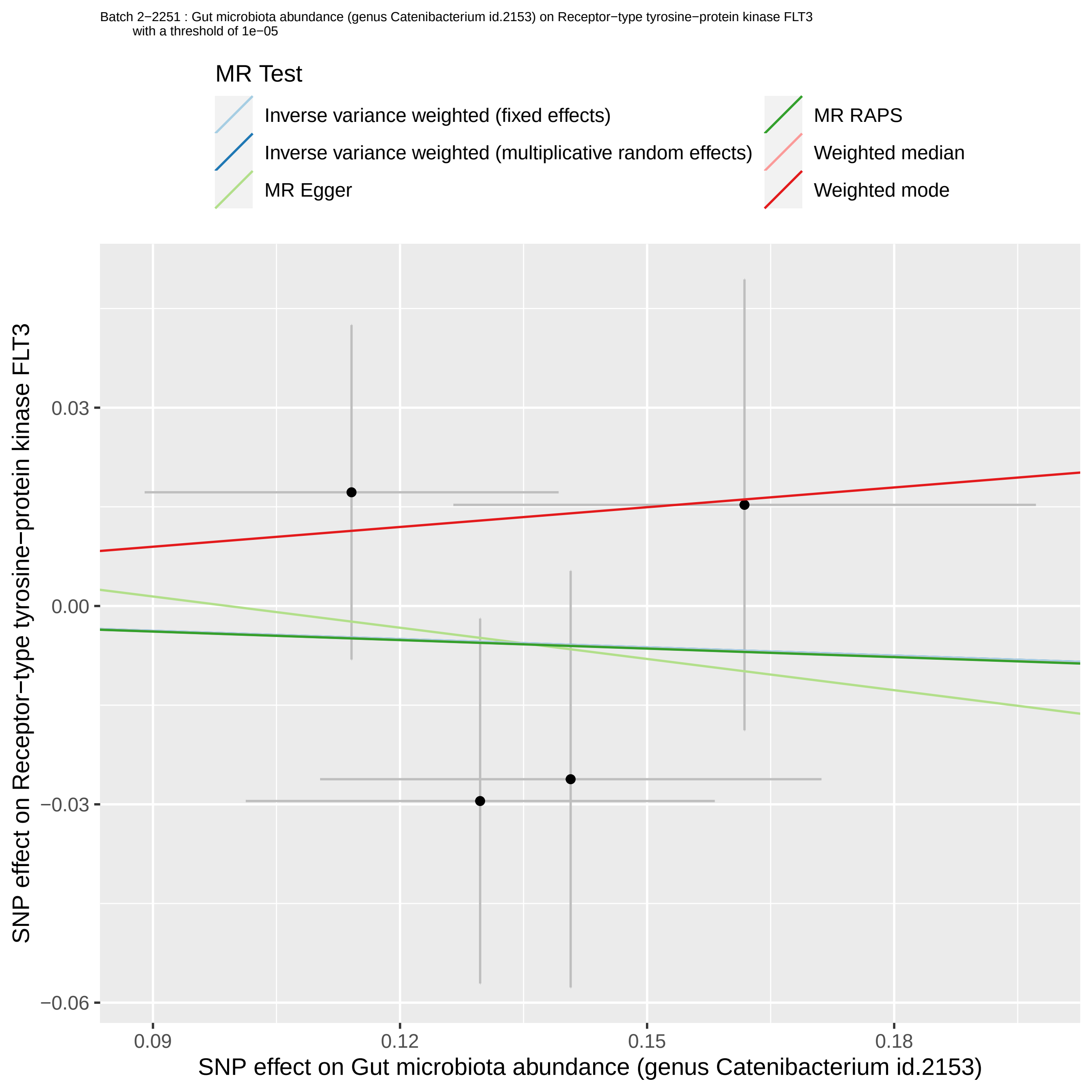

Batch 2−2251 : Gut microbiota abundance (genus Catenibacterium id.2153) on Receptor−type tyrosine−protein kinase FLT3
with a threshold of 1e−05
MR Test
Inverse variance weighted (fixed effects)
Inverse variance weighted (multiplicative random effects)
MR Egger
MR RAPS
Weighted median
Weighted mode
SNP effect on Receptor−type tyrosine−protein kinase FLT3
0.03
0.00
−0.03
−0.06
0.09
0.12
0.15
0.18
SNP effect on Gut microbiota abundance (genus Catenibacterium id.2153)

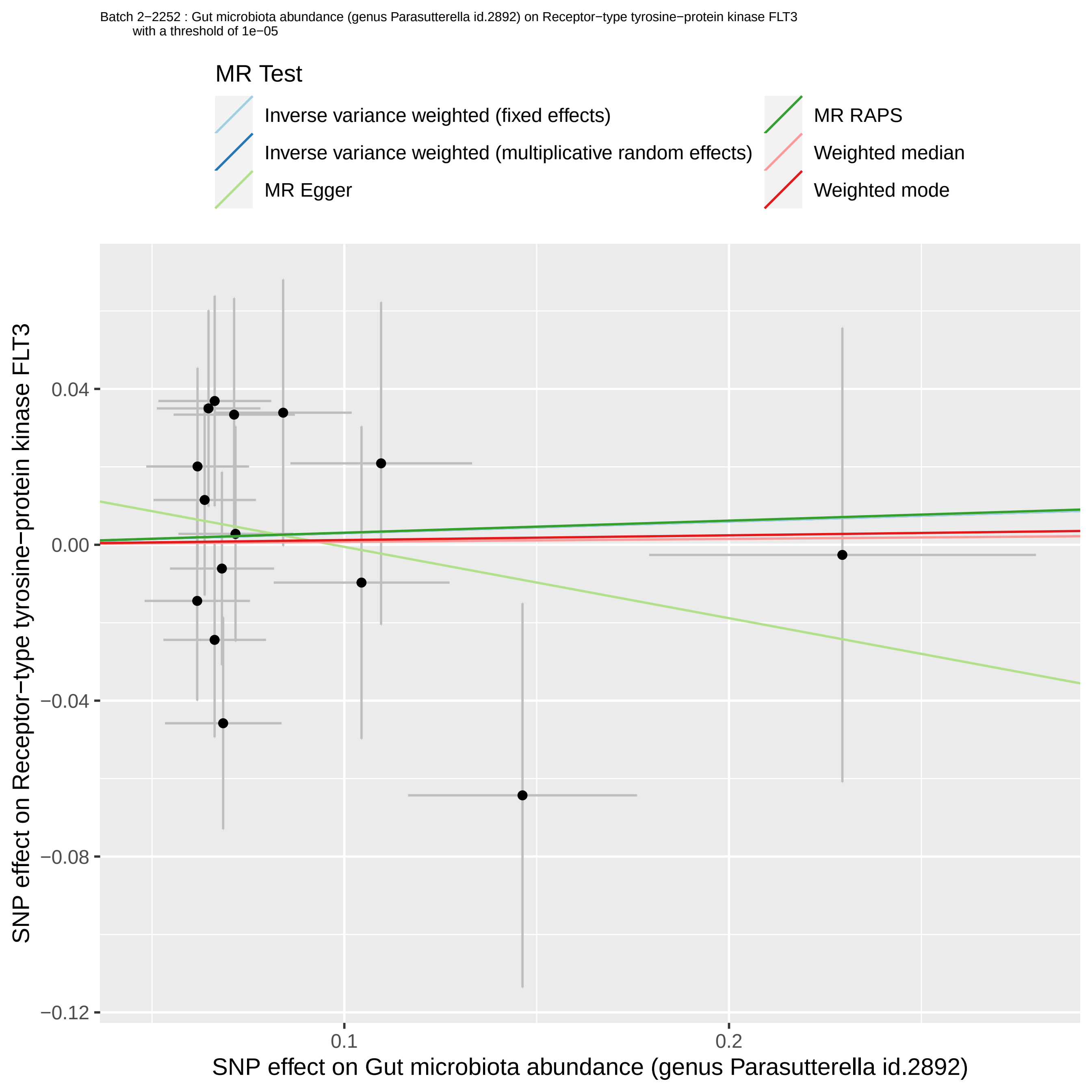
Batch 2−2252 : Gut microbiota abundance (genus Parasutterella id.2892) on Receptor−type tyrosine−protein kinase FLT3
with a threshold of 1e−05
MR Test
Inverse variance weighted (fixed effects)
Inverse variance weighted (multiplicative random effects)
MR Egger
MR RAPS
Weighted median
Weighted mode
0.04
0.00
−0.04
−0.08
−0.12
0.1
0.2
SNP effect on Receptor−type tyrosine−protein kinase FLT3
SNP effect on Gut microbiota abundance (genus Parasutterella id.2892)

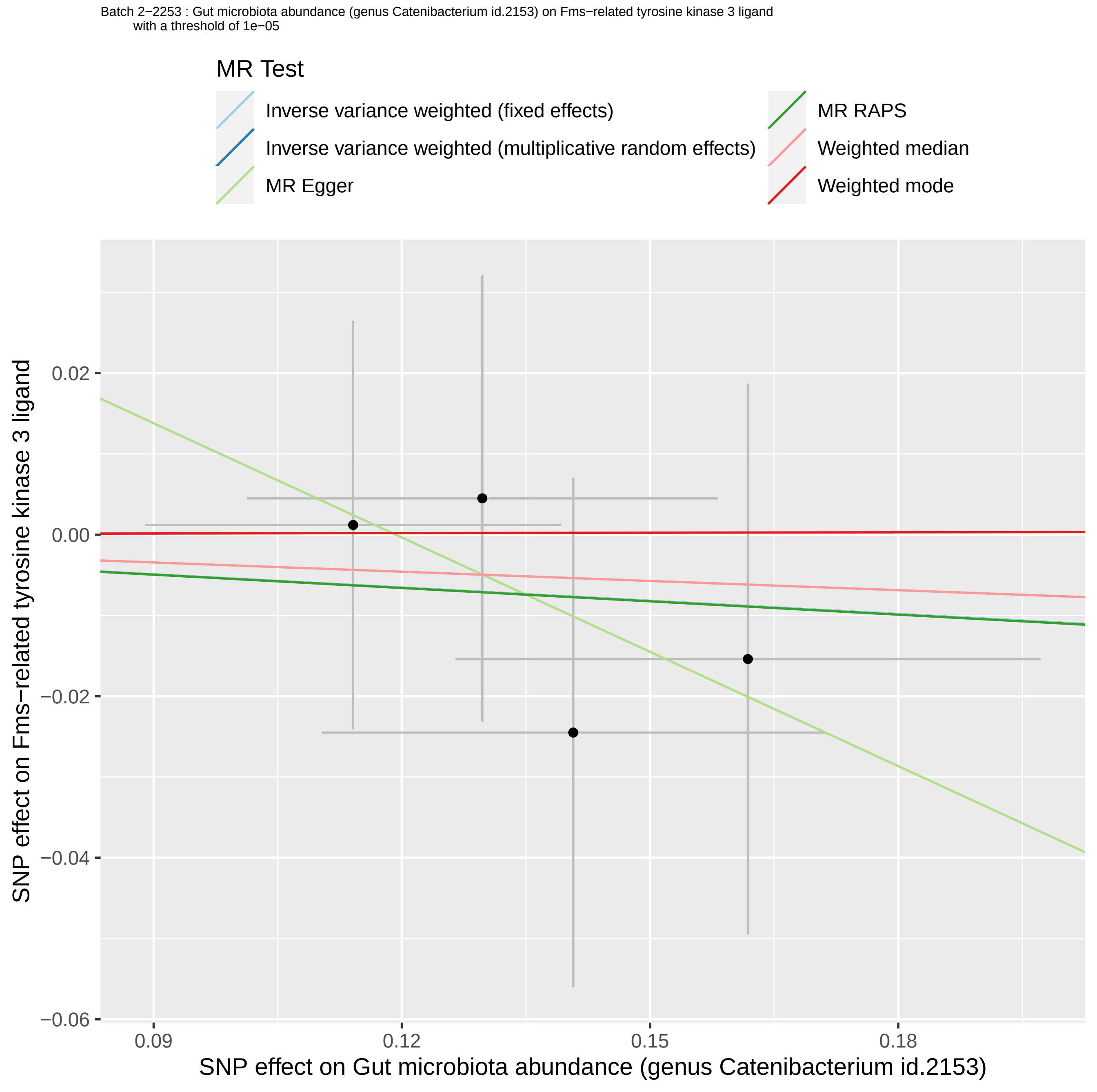
Batch 2−2253 : Gut microbiota abundance (genus Catenibacterium id.2153) on Fms−related tyrosine kinase 3 ligand
with a threshold of 1e−05
MR Test
Inverse variance weighted (fixed effects)
Inverse variance weighted (multiplicative random effects)
MR Egger
MR RAPS
Weighted median
Weighted mode
0.02
0.00
−0.02
−0.04
−0.06
0.09
0.12
0.15
0.18
SNP effect on Fms−related tyrosine kinase 3 ligand
SNP effect on Gut microbiota abundance (genus Catenibacterium id.2153)

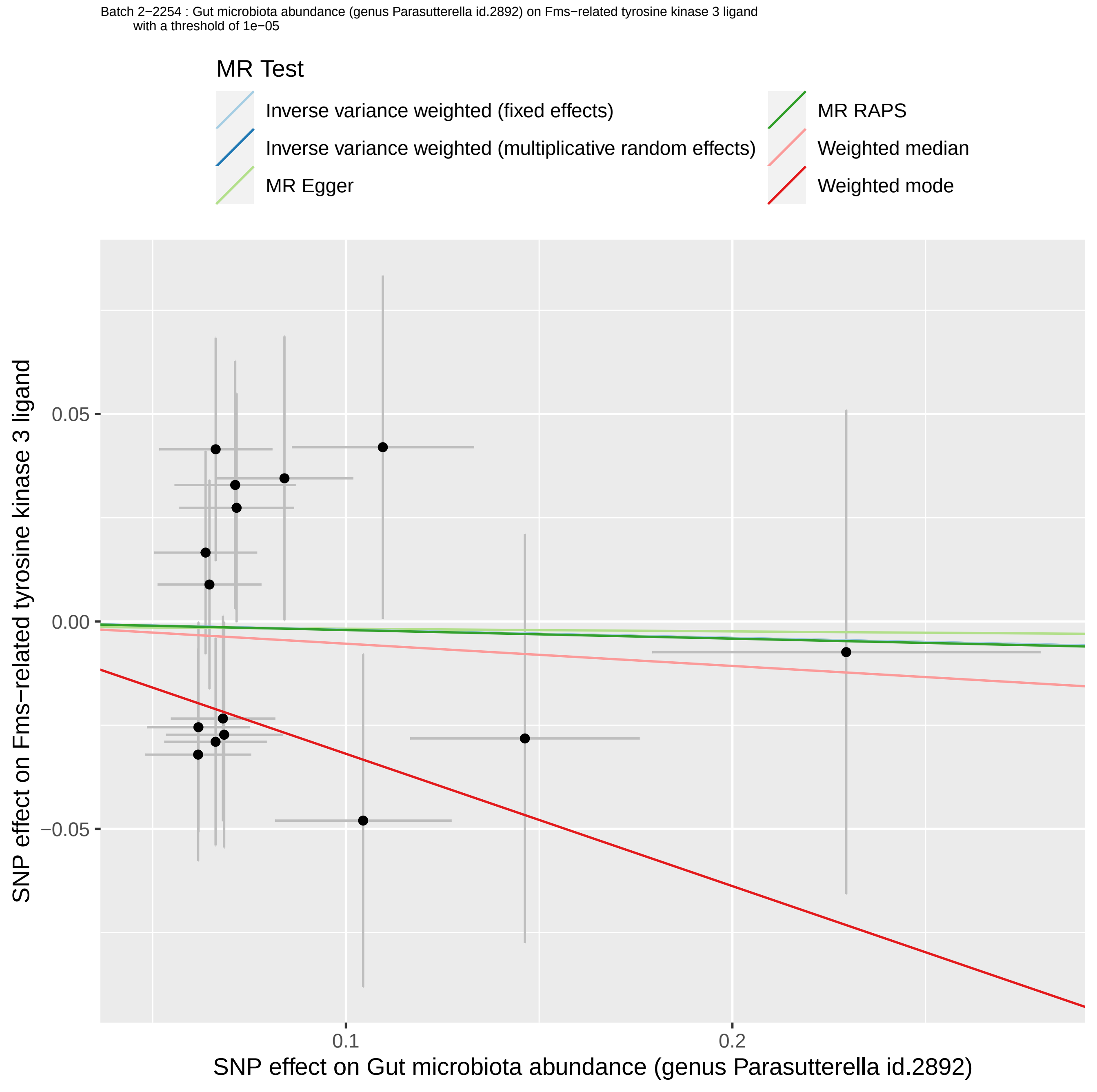
Batch 2−2254 : Gut microbiota abundance (genus Parasutterella id.2892) on Fms−related tyrosine kinase 3 ligand
with a threshold of 1e−05
MR Test
Inverse variance weighted (fixed effects)
Inverse variance weighted (multiplicative random effects)
MR Egger
MR RAPS
Weighted median
Weighted mode
SNP effect on Fms−related tyrosine kinase 3 ligand
0.05
0.00
−0.05
0.1
0.2
SNP effect on Gut microbiota abundance (genus Parasutterella id.2892)

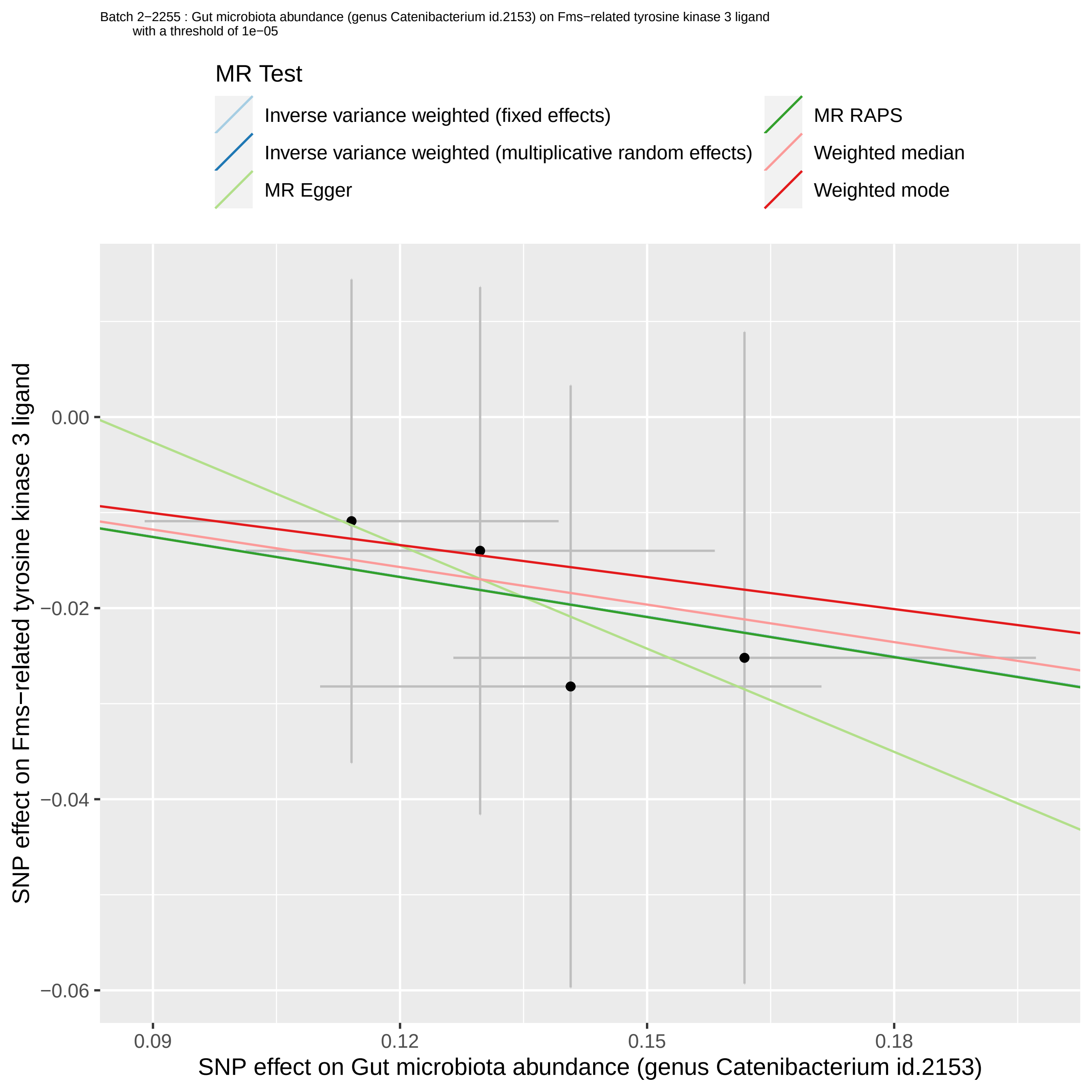

Batch 2−2255 : Gut microbiota abundance (genus Catenibacterium id.2153) on Fms−related tyrosine kinase 3 ligand
with a threshold of 1e−05
MR Test
Inverse variance weighted (fixed effects)
Inverse variance weighted (multiplicative random effects)
MR Egger
MR RAPS
Weighted median
Weighted mode
0.00
−0.02
−0.04
−0.06
0.09
0.12
0.15
0.18
SNP effect on Fms−related tyrosine kinase 3 ligand
SNP effect on Gut microbiota abundance (genus Catenibacterium id.2153)

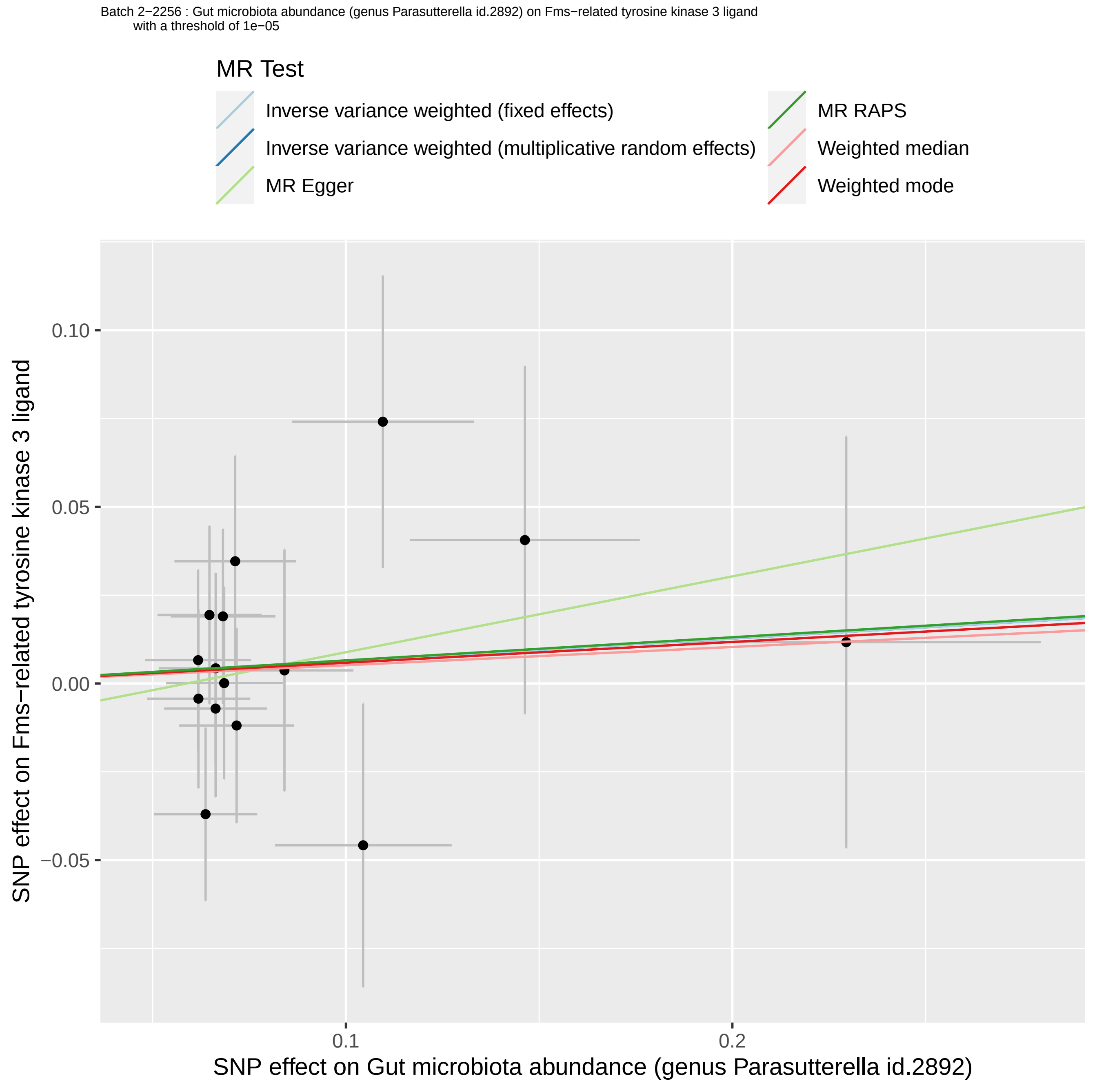
Batch 2−2256 : Gut microbiota abundance (genus Parasutterella id.2892) on Fms−related tyrosine kinase 3 ligand
with a threshold of 1e−05
MR Test
Inverse variance weighted (fixed effects)
Inverse variance weighted (multiplicative random effects)
MR Egger
MR RAPS
Weighted median
Weighted mode
0.10
0.05
0.00
−0.05
0.1
0.2
SNP effect on Fms−related tyrosine kinase 3 ligand
SNP effect on Gut microbiota abundance (genus Parasutterella id.2892)

Batch 2−2257 : Gut microbiota abundance (genus Catenibacterium id.2153) on Vascular endothelial growth factor receptor 3
with a threshold of 1e−05

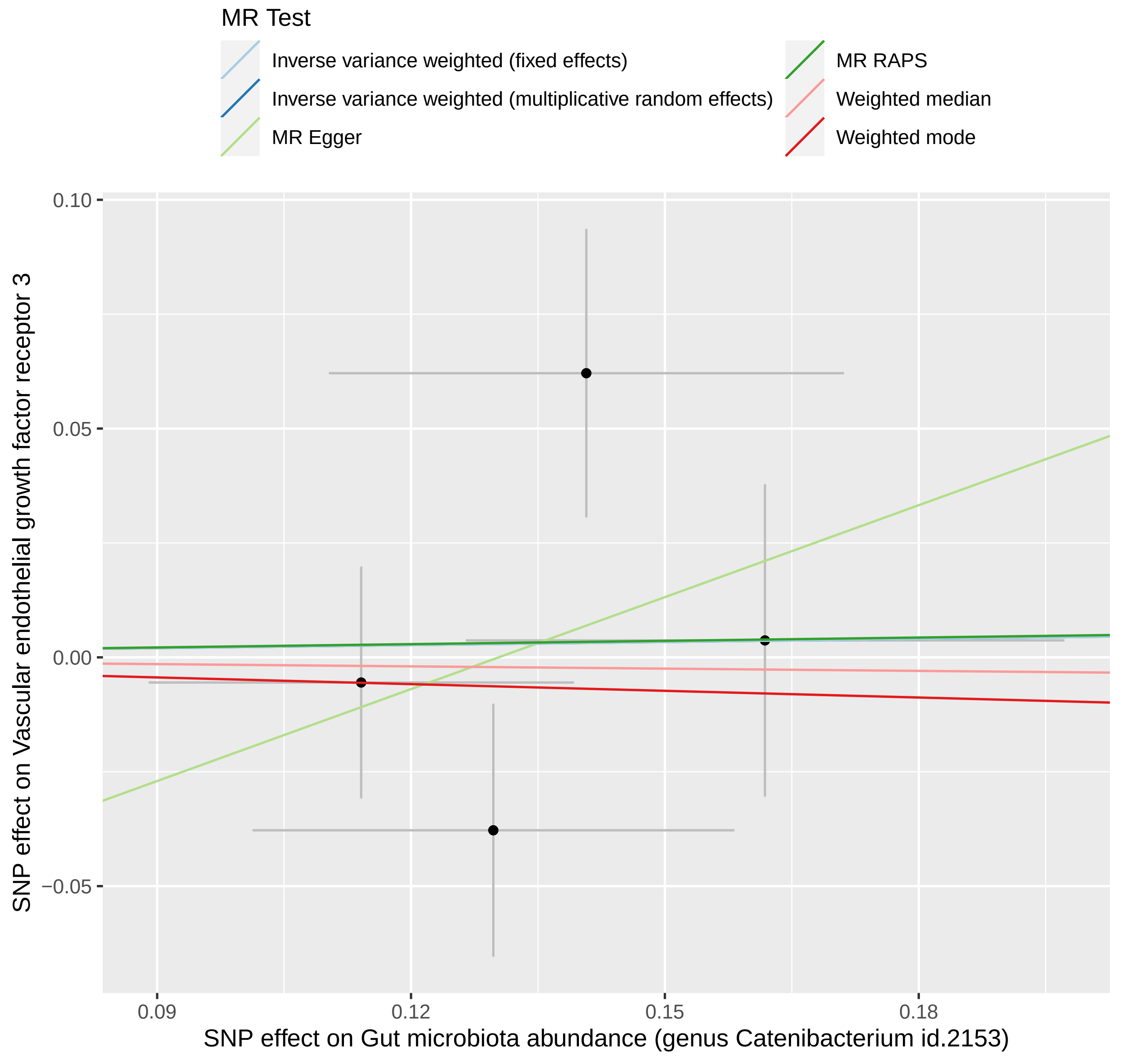

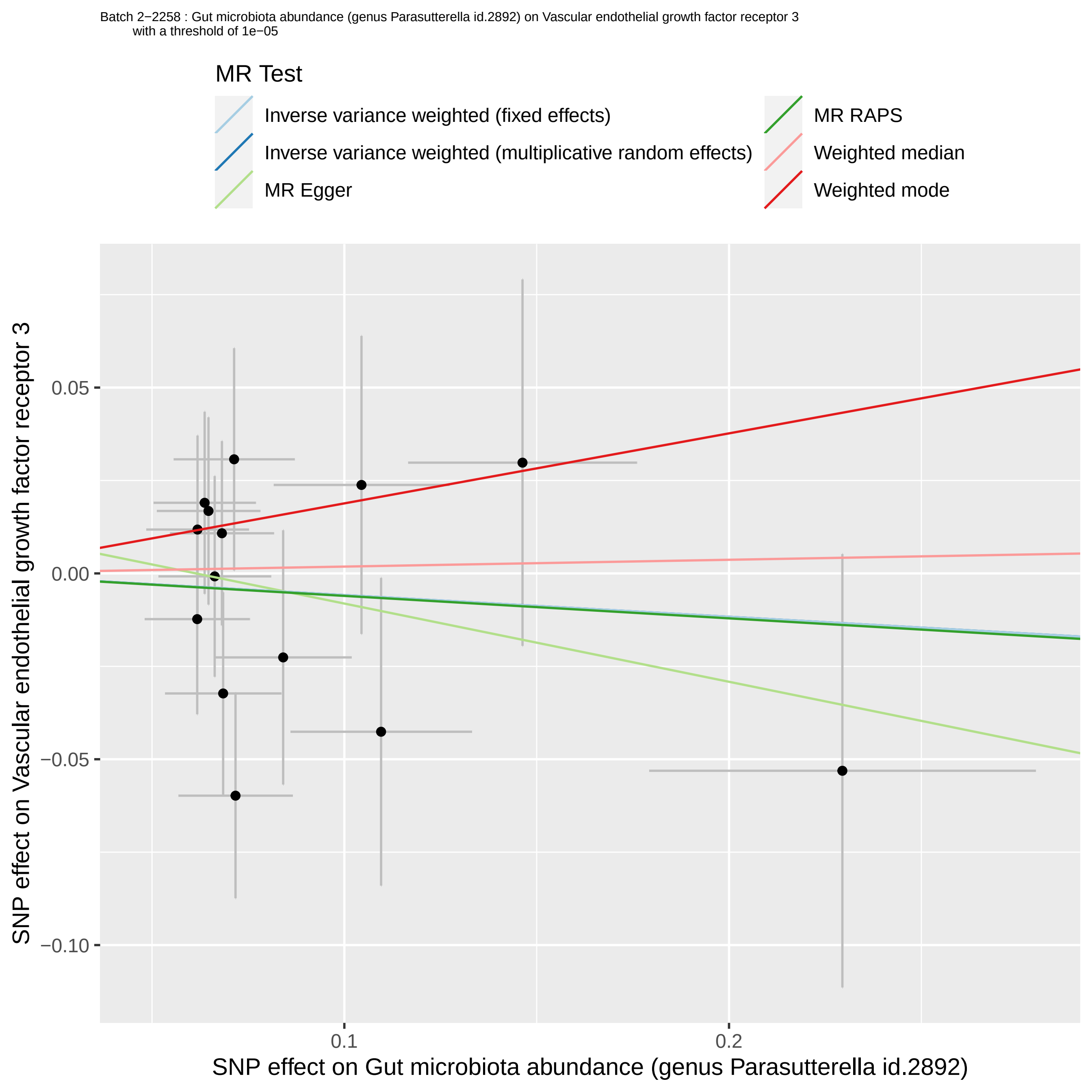

Batch 2−2258 : Gut microbiota abundance (genus Parasutterella id.2892) on Vascular endothelial growth factor receptor 3
with a threshold of 1e−05
MR Test
Inverse variance weighted (fixed effects)
Inverse variance weighted (multiplicative random effects)
MR Egger
MR RAPS
Weighted median
Weighted mode
0.05
0.00
−0.05
−0.10
0.1
0.2
SNP effect on Vascular endothelial growth factor receptor 3
SNP effect on Gut microbiota abundance (genus Parasutterella id.2892)

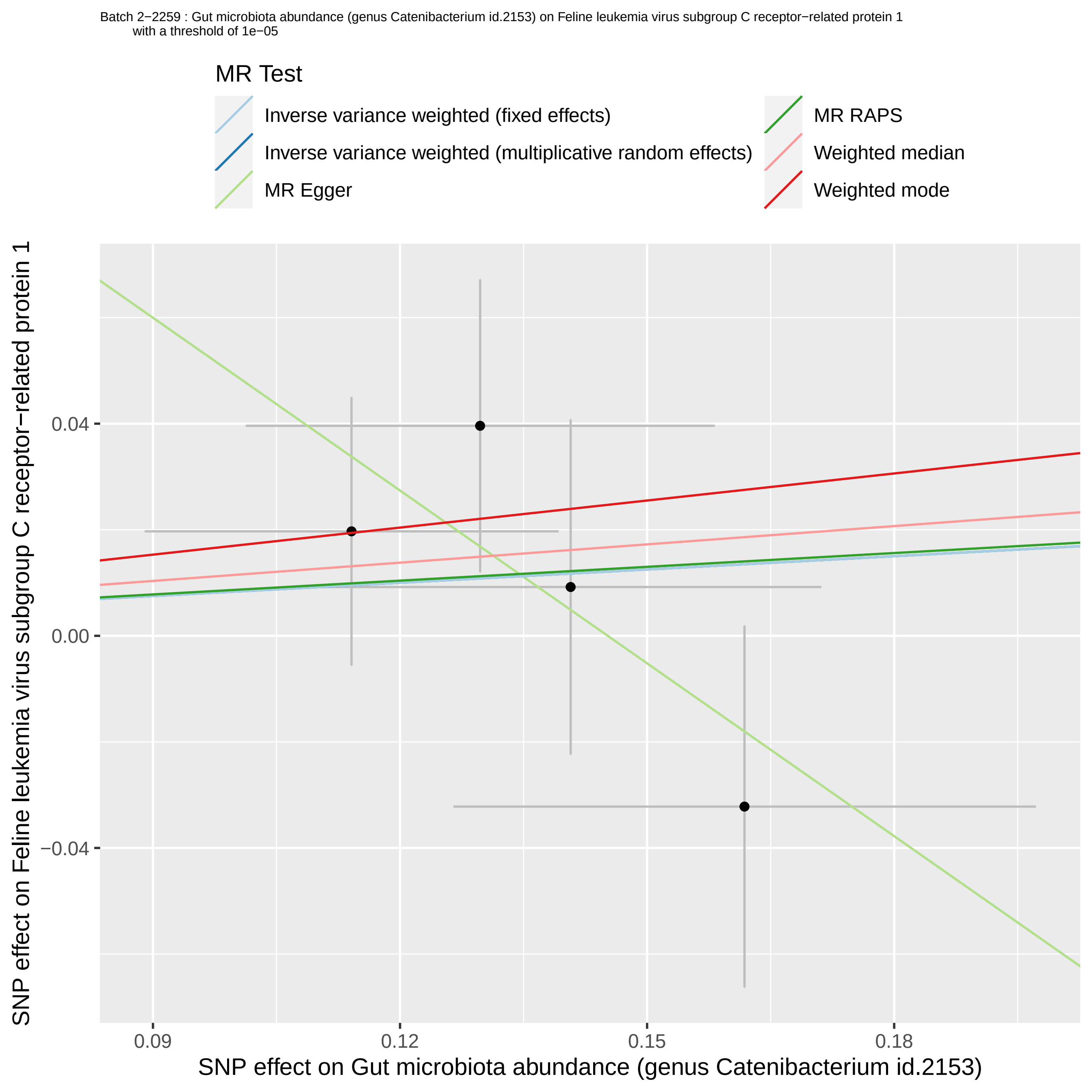
Batch 2−2259 : Gut microbiota abundance (genus Catenibacterium id.2153) on Feline leukemia virus subgroup C receptor−related protein 1 with a threshold of 1e−05
MR Test
Inverse variance weighted (fixed effects)
Inverse variance weighted (multiplicative random effects)
MR Egger
MR RAPS
Weighted median
Weighted mode
SNP effect on Feline leukemia virus subgroup C receptor−related protein 1
0.04
0.00
−0.04
0.09
0.12
0.15
0.18
SNP effect on Gut microbiota abundance (genus Catenibacterium id.2153)

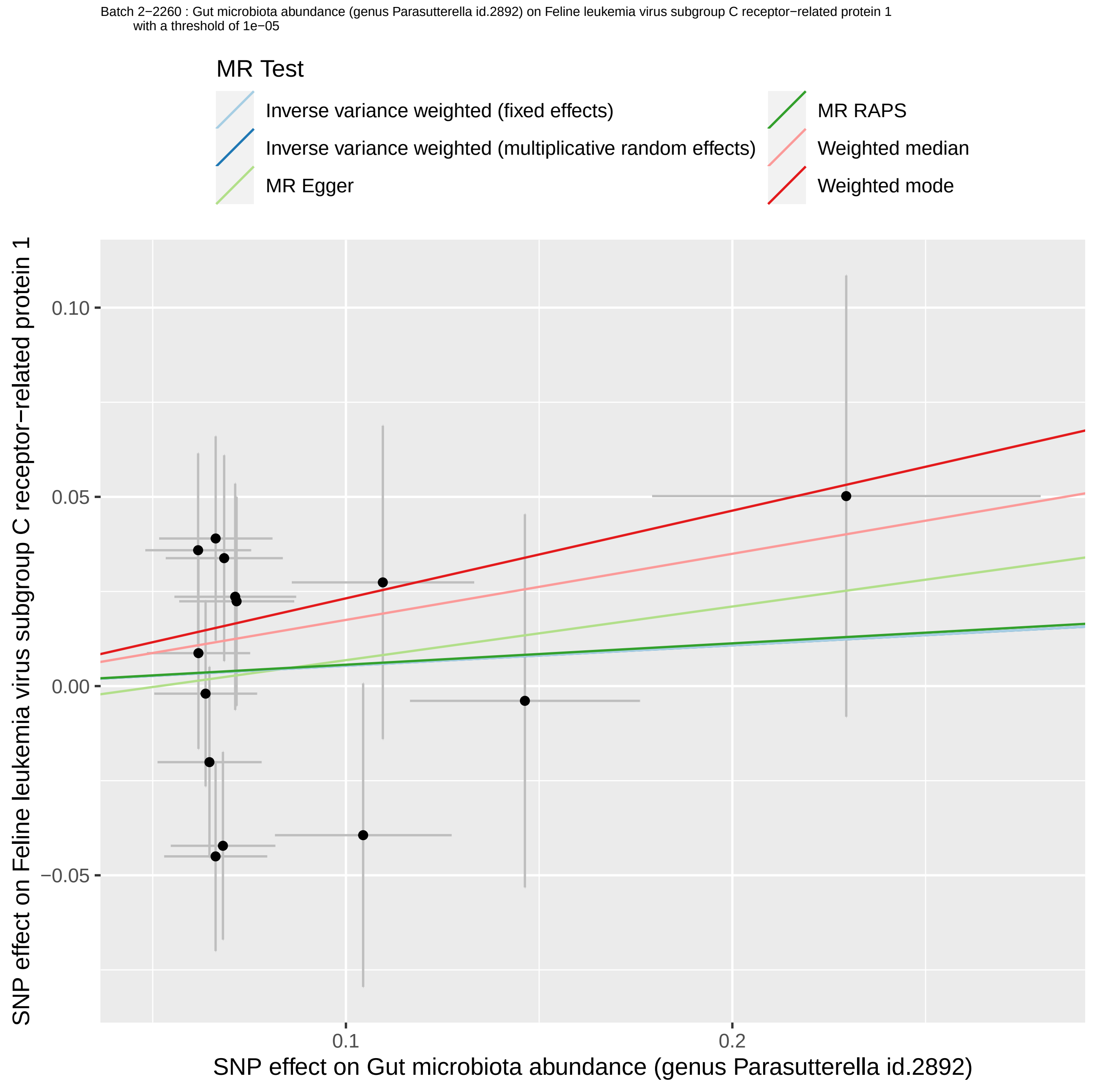
Batch 2−2260 : Gut microbiota abundance (genus Parasutterella id.2892) on Feline leukemia virus subgroup C receptor−related protein 1
with a threshold of 1e−05
MR Test
Inverse variance weighted (fixed effects)
Inverse variance weighted (multiplicative random effects)
MR Egger
MR RAPS
Weighted median
Weighted mode
SNP effect on Feline leukemia virus subgroup C receptor−related protein 1
0.10
0.05
0.00
−0.05
0.1
0.2
SNP effect on Gut microbiota abundance (genus Parasutterella id.2892)

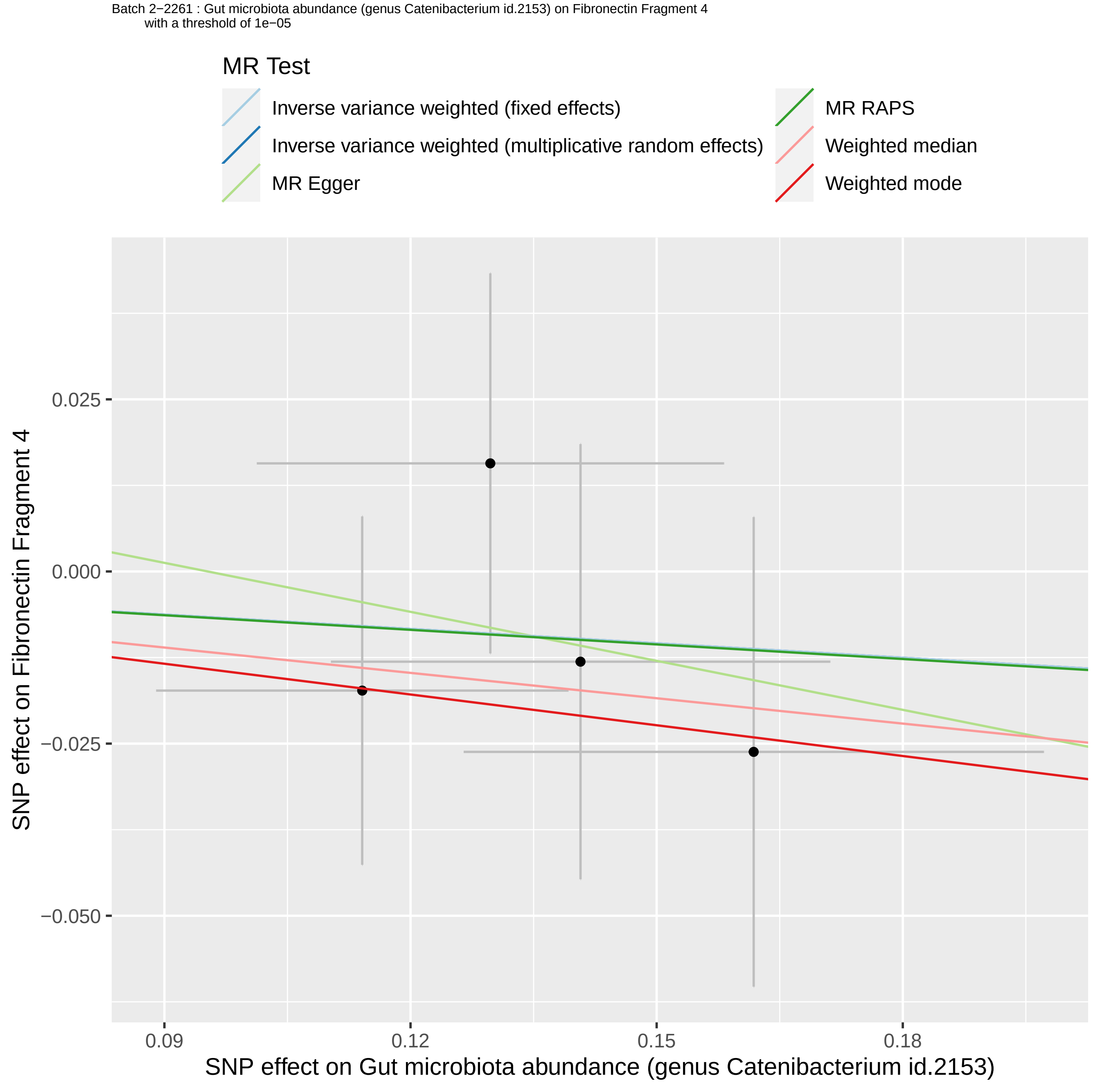

Batch 2−2261 : Gut microbiota abundance (genus Catenibacterium id.2153) on Fibronectin Fragment 4
with a threshold of 1e−05
MR Test
Inverse variance weighted (fixed effects)
Inverse variance weighted (multiplicative random effects)
MR Egger
MR RAPS
Weighted median
Weighted mode
0.025
0.000
−0.025
−0.050
SNP effect on Fibronectin Fragment 4
0.09
0.12
0.15
0.18
SNP effect on Gut microbiota abundance (genus Catenibacterium id.2153)

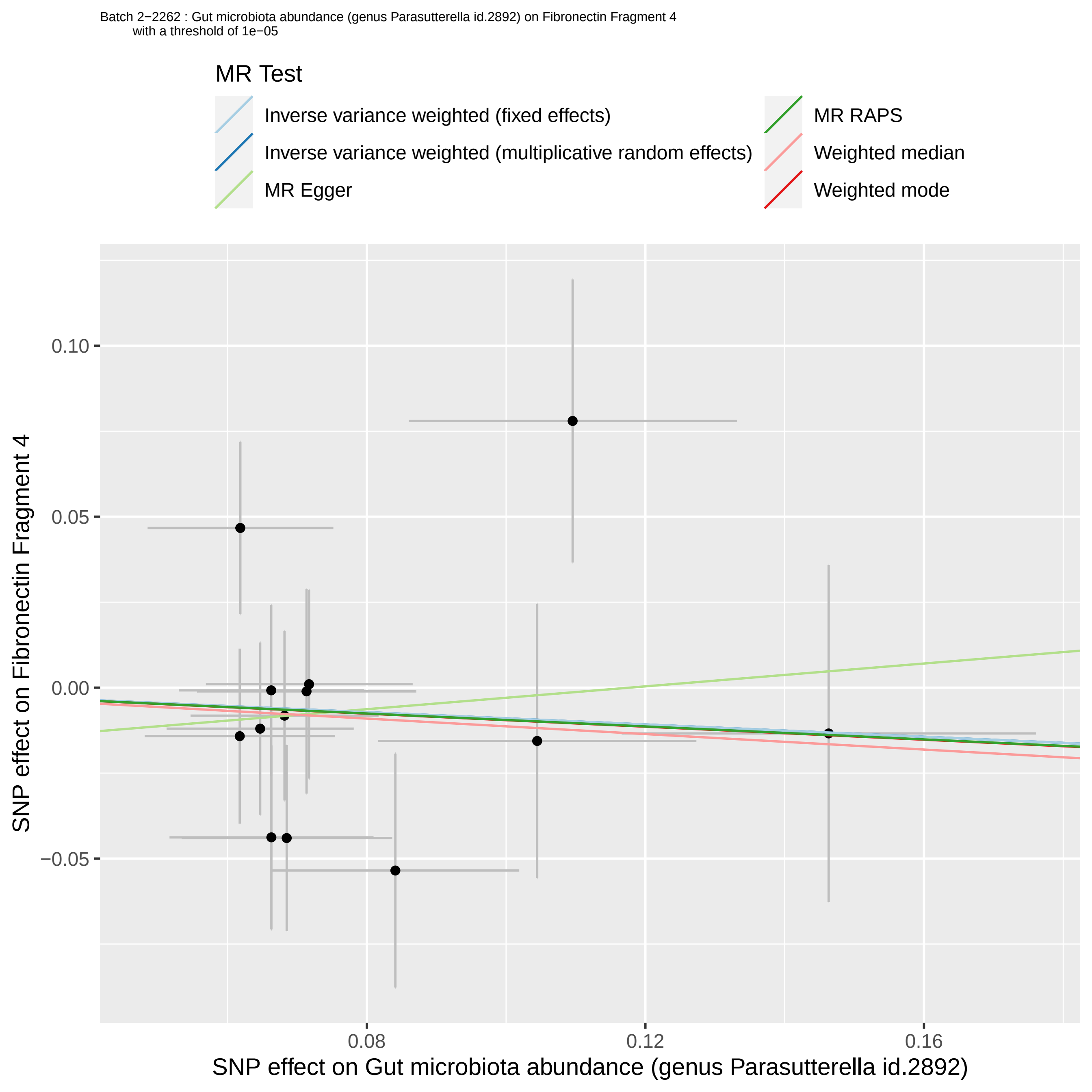
Batch 2−2262 : Gut microbiota abundance (genus Parasutterella id.2892) on Fibronectin Fragment 4
with a threshold of 1e−05
MR Test
Inverse variance weighted (fixed effects)
Inverse variance weighted (multiplicative random effects)
MR Egger
MR RAPS
Weighted median
Weighted mode
0.10
0.05
0.00
−0.05
0.08
0.12
0.16
SNP effect on Fibronectin Fragment 4
SNP effect on Gut microbiota abundance (genus Parasutterella id.2892)

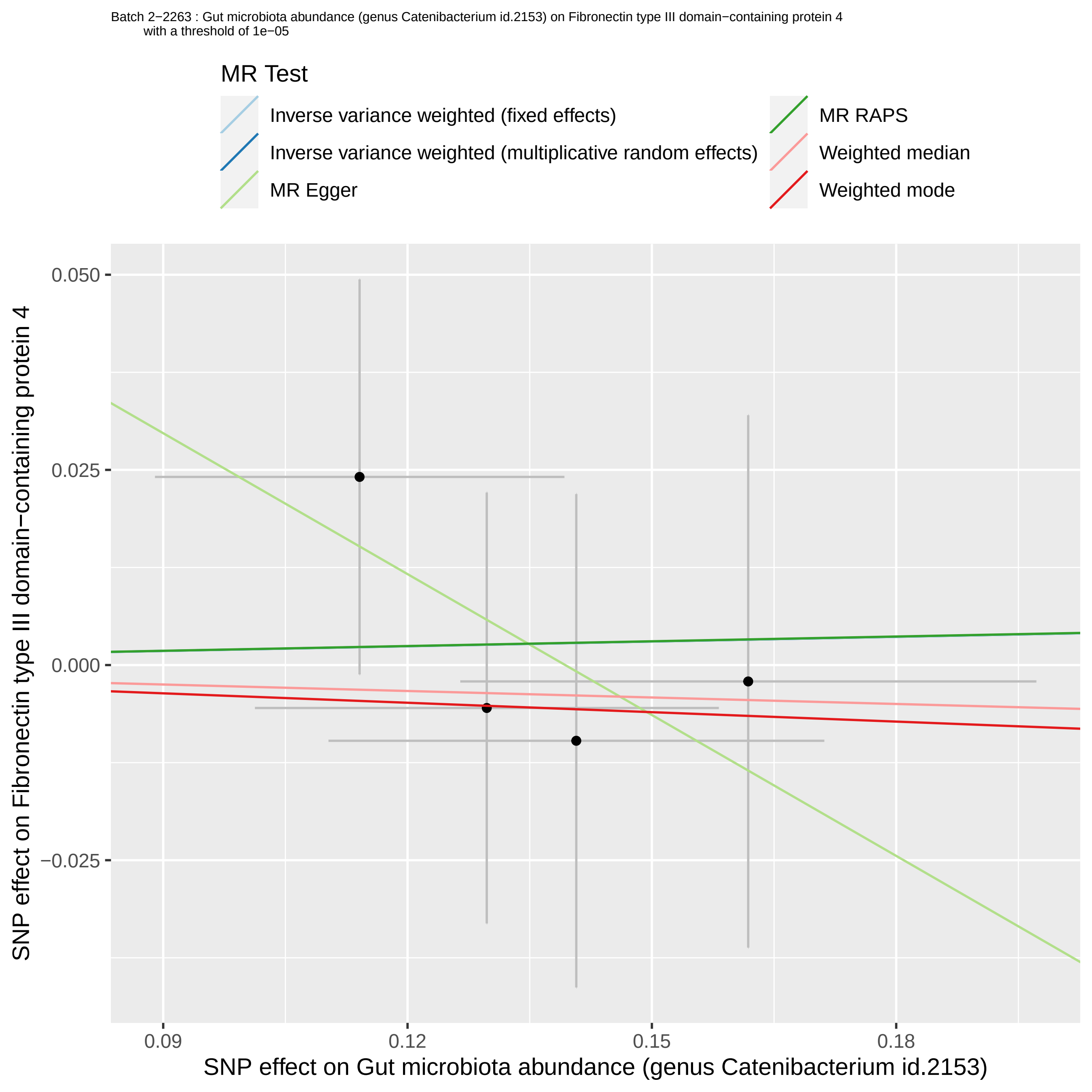

Batch 2−2263 : Gut microbiota abundance (genus Catenibacterium id.2153) on Fibronectin type III domain−containing protein 4
with a threshold of 1e−05
MR Test
Inverse variance weighted (fixed effects)
Inverse variance weighted (multiplicative random effects)
MR Egger
MR RAPS
Weighted median
Weighted mode
0.050
0.025
0.000
−0.025
0.09
0.12
0.15
0.18
SNP effect on Gut microbiota abundance (genus Catenibacterium id.2153)
SNP effect on Fibronectin type III domain−containing protein 4

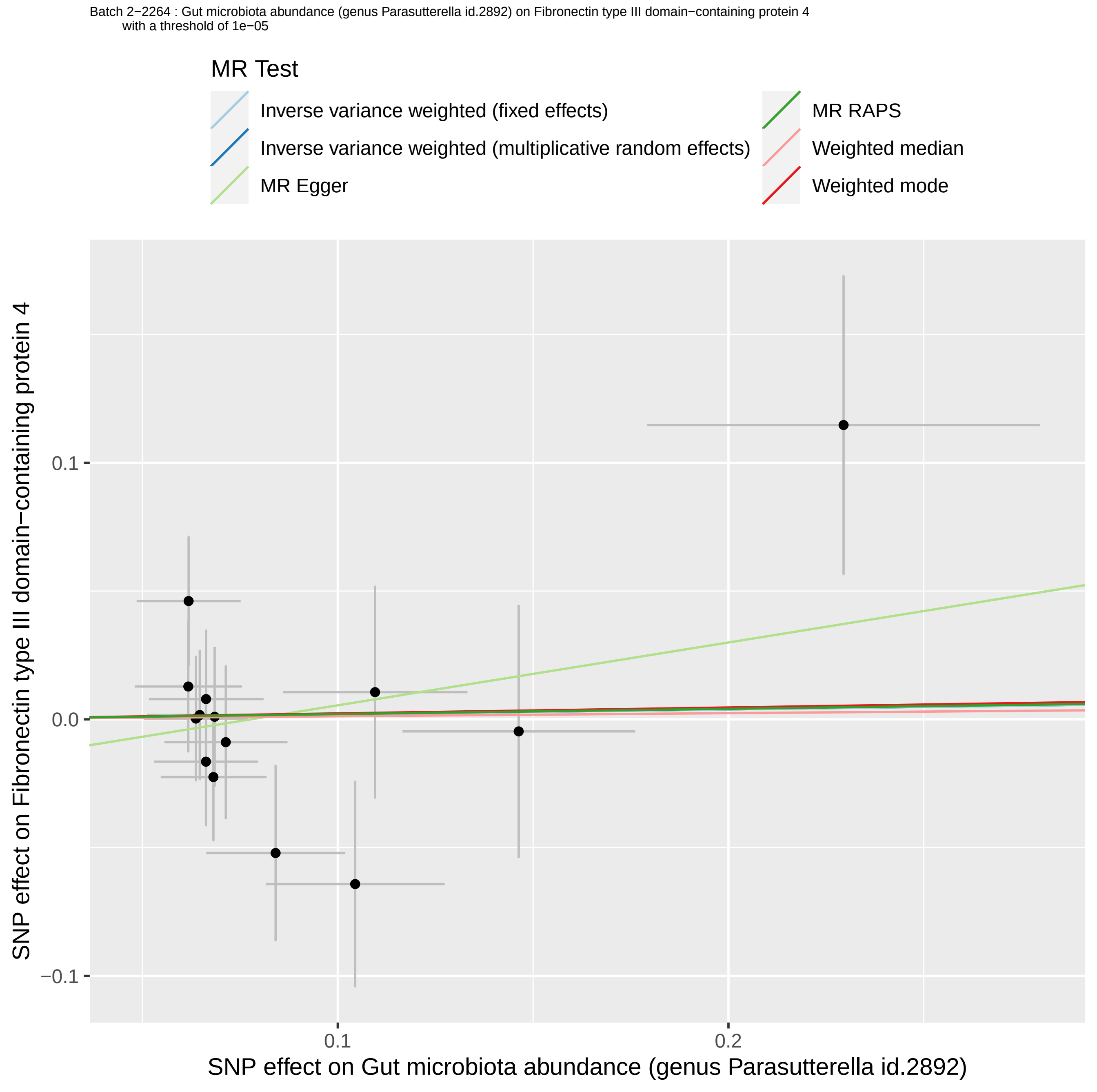

Batch 2−2264 : Gut microbiota abundance (genus Parasutterella id.2892) on Fibronectin type III domain−containing protein 4
with a threshold of 1e−05
MR Test
Inverse variance weighted (fixed effects)
Inverse variance weighted (multiplicative random effects)
MR Egger
MR RAPS
Weighted median
Weighted mode
SNP effect on Fibronectin type III domain−containing protein 4
0.1
0.0
−0.1
0.1
0.2
SNP effect on Gut microbiota abundance (genus Parasutterella id.2892)

Batch 2−2265 : Gut microbiota abundance (genus Catenibacterium id.2153) on Fibronectin type III domain−containing protein 5 with a threshold of 1e−05

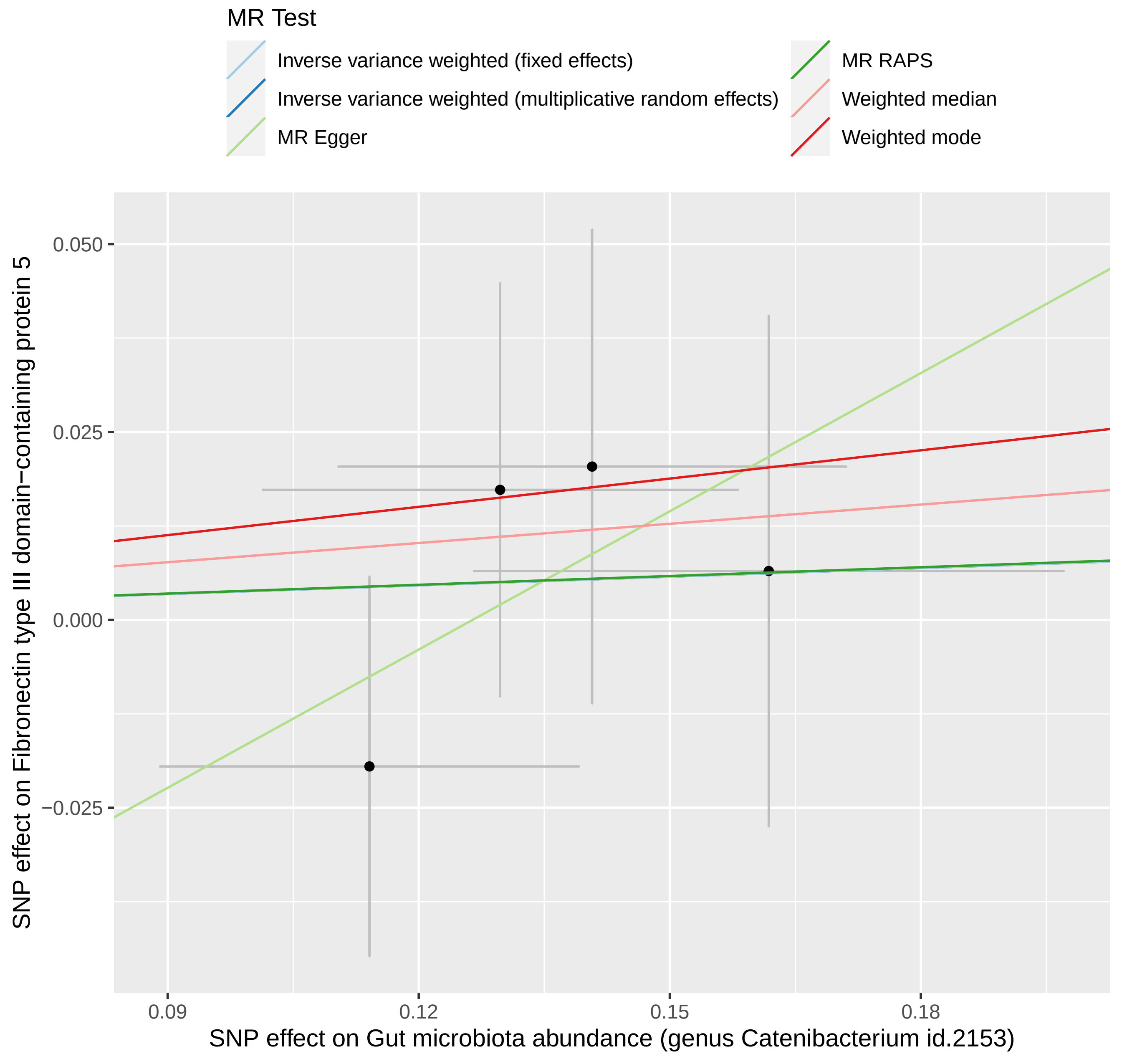

Batch 2−2266 : Gut microbiota abundance (genus Parasutterella id.2892) on Fibronectin type III domain−containing protein 5
with a threshold of 1e−05

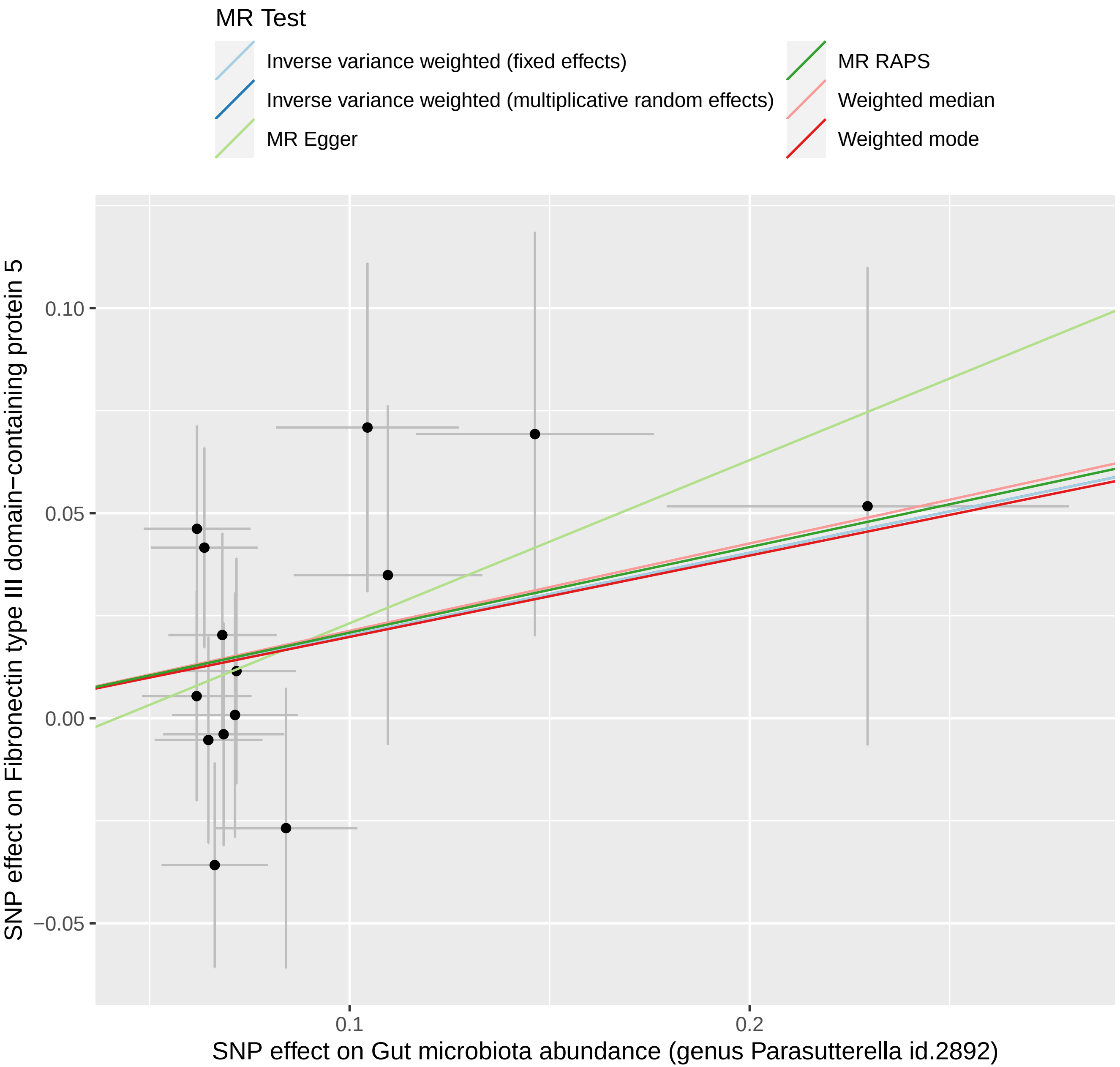

Batch 2−2267 : Gut microbiota abundance (genus Catenibacterium id.2153) on Glutamate carboxypeptidase 2
with a threshold of 1e−05

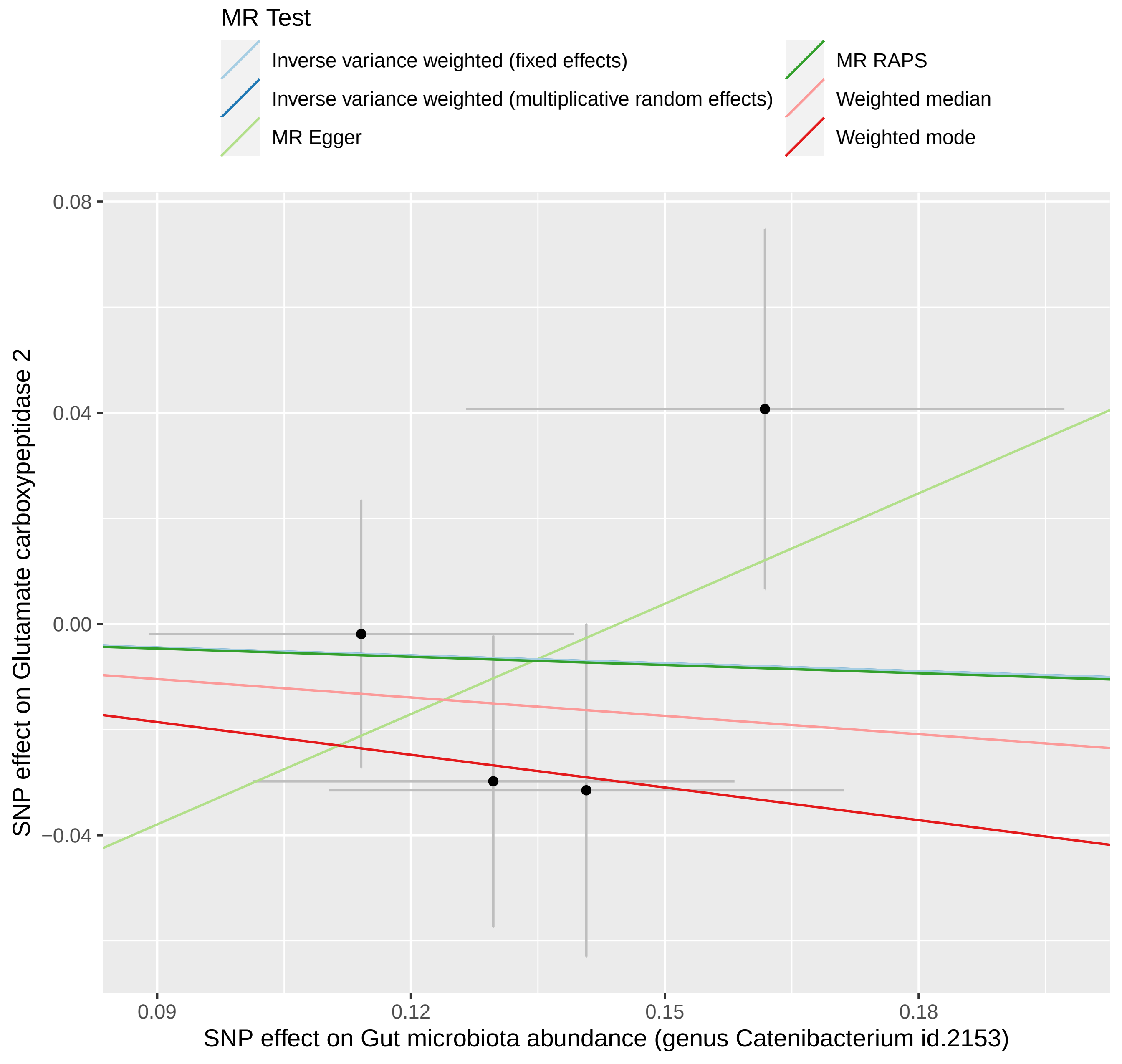

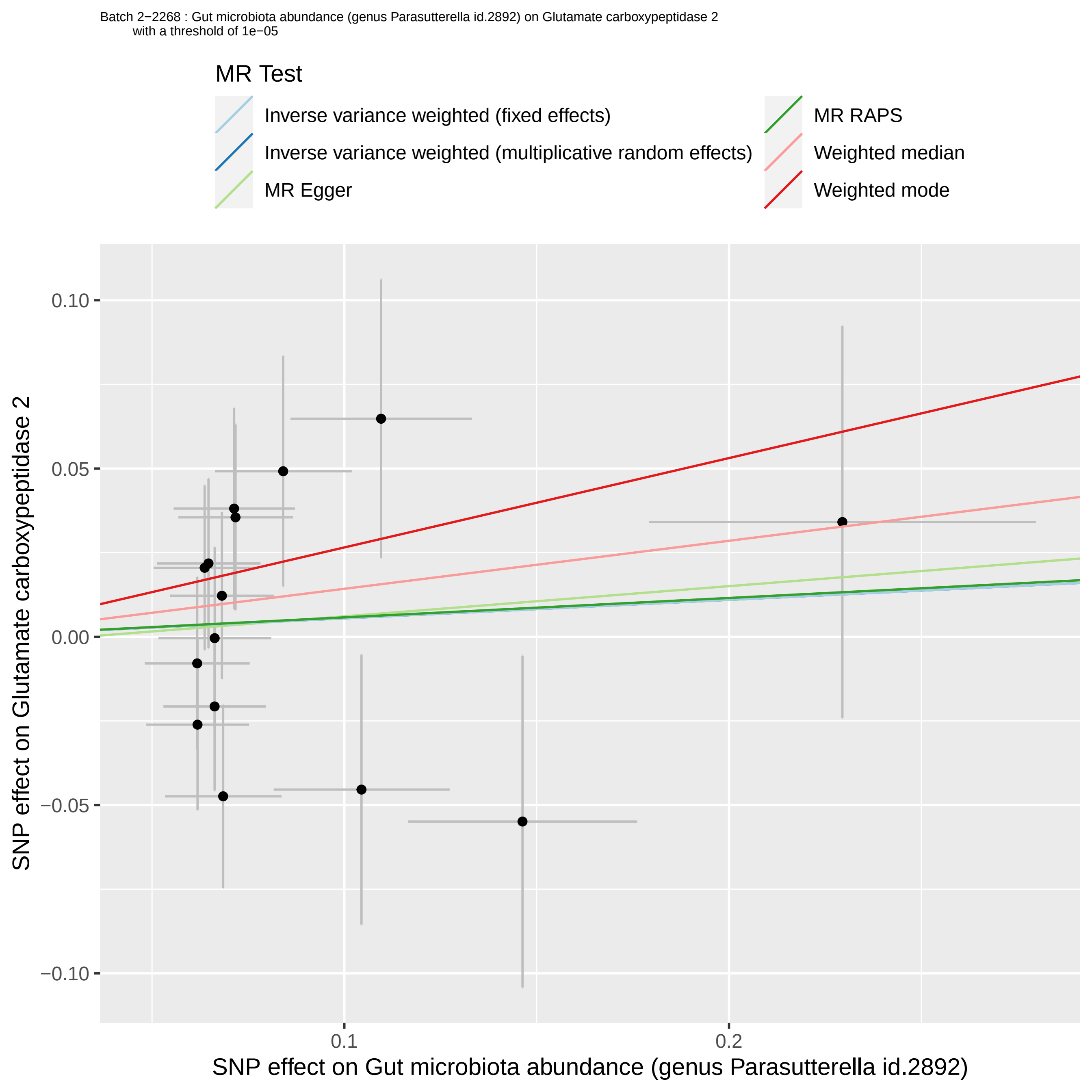
Batch 2−2268 : Gut microbiota abundance (genus Parasutterella id.2892) on Glutamate carboxypeptidase 2
with a threshold of 1e−05
MR Test
Inverse variance weighted (fixed effects)
Inverse variance weighted (multiplicative random effects)
MR Egger
MR RAPS
Weighted median
Weighted mode
0.10
0.05
0.00
−0.05
−0.10
0.1
0.2
SNP effect on Glutamate carboxypeptidase 2
SNP effect on Gut microbiota abundance (genus Parasutterella id.2892)

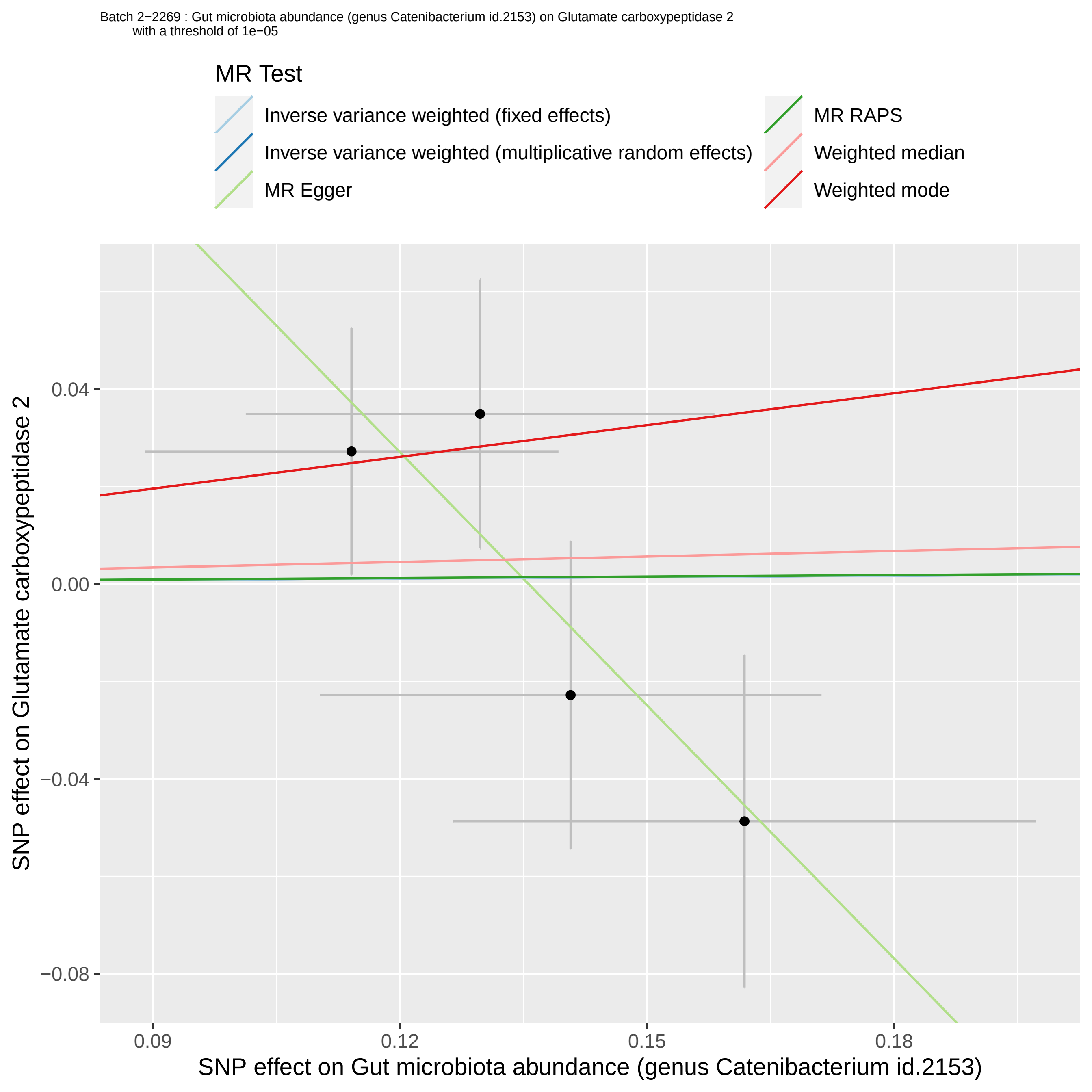
Batch 2−2269 : Gut microbiota abundance (genus Catenibacterium id.2153) on Glutamate carboxypeptidase 2
with a threshold of 1e−05
MR Test
Inverse variance weighted (fixed effects)
Inverse variance weighted (multiplicative random effects)
MR Egger
MR RAPS
Weighted median
Weighted mode
0.04
0.00
−0.04
−0.08
0.09
0.12
0.15
0.18
SNP effect on Glutamate carboxypeptidase 2
SNP effect on Gut microbiota abundance (genus Catenibacterium id.2153)

Batch 2−2270 : Gut microbiota abundance (genus Parasutterella id.2892) on Glutamate carboxypeptidase 2
with a threshold of 1e−05

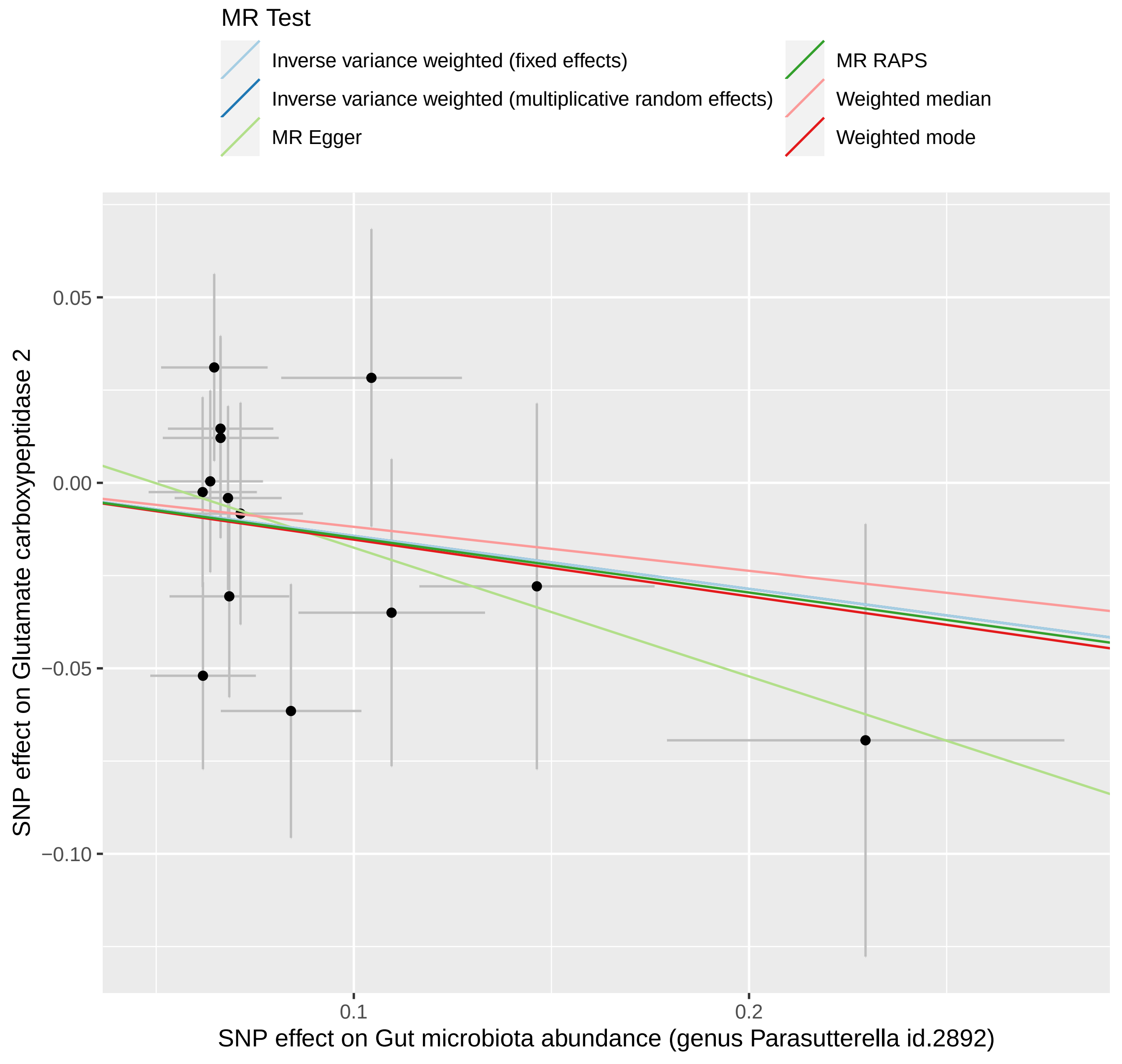

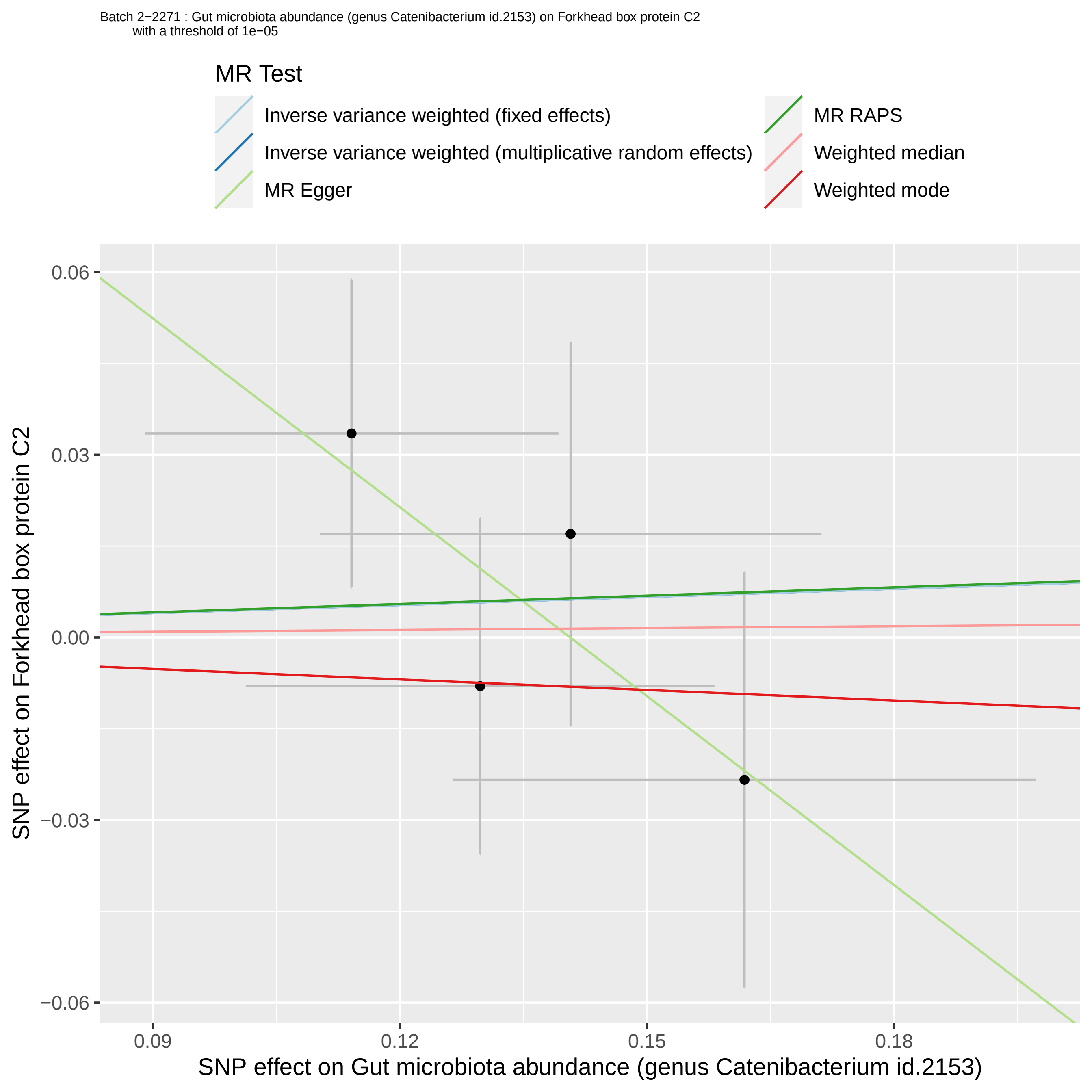
Batch 2−2271 : Gut microbiota abundance (genus Catenibacterium id.2153) on Forkhead box protein C2
with a threshold of 1e−05
MR Test
Inverse variance weighted (fixed effects)
Inverse variance weighted (multiplicative random effects)
MR Egger
MR RAPS
Weighted median
Weighted mode
0.06
0.03
0.00
−0.03
−0.06
0.09
0.12
0.15
0.18
SNP effect on Forkhead box protein C2
SNP effect on Gut microbiota abundance (genus Catenibacterium id.2153)

Batch 2−2272 : Gut microbiota abundance (genus Parasutterella id.2892) on Forkhead box protein C2
with a threshold of 1e−05

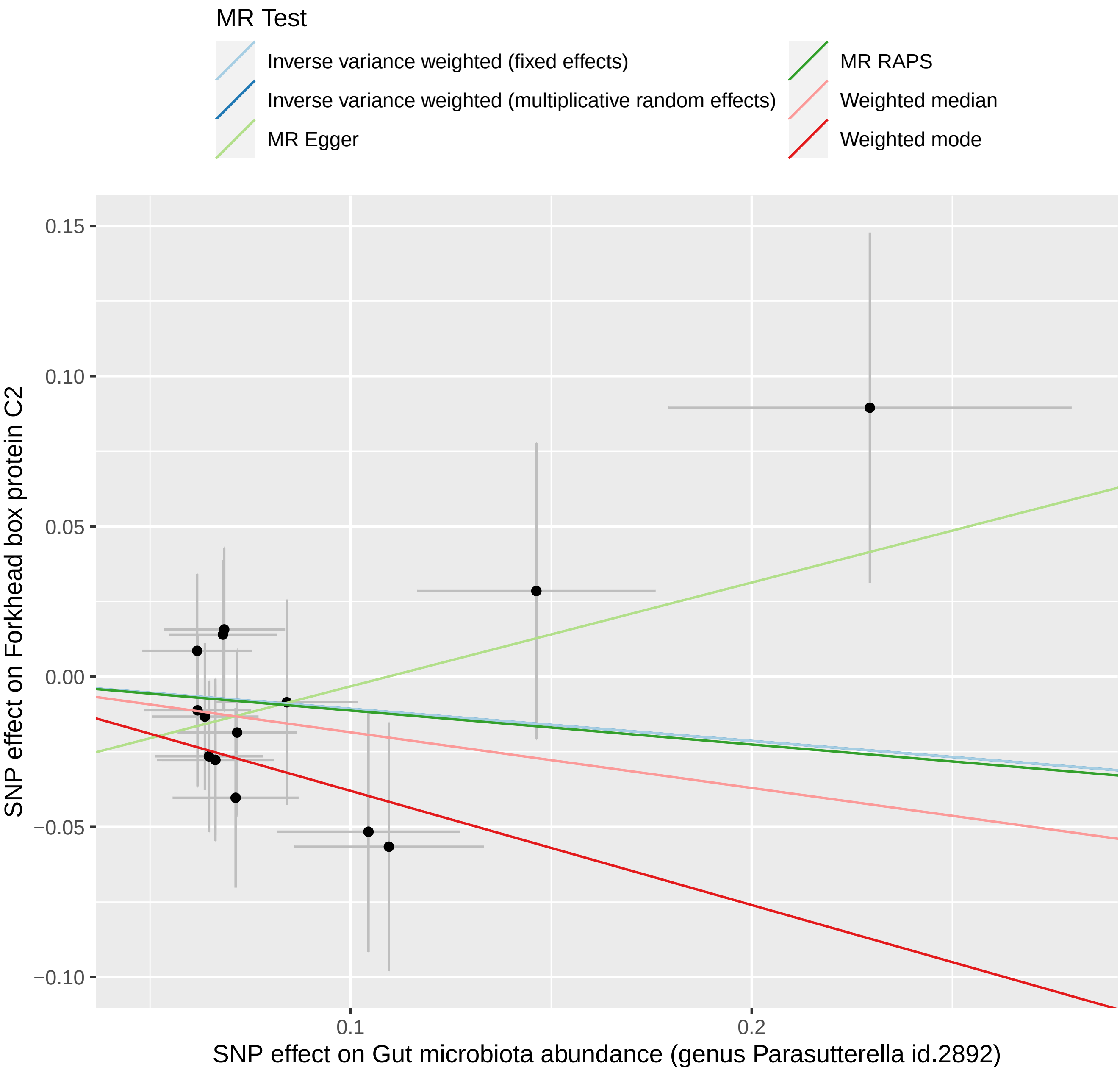

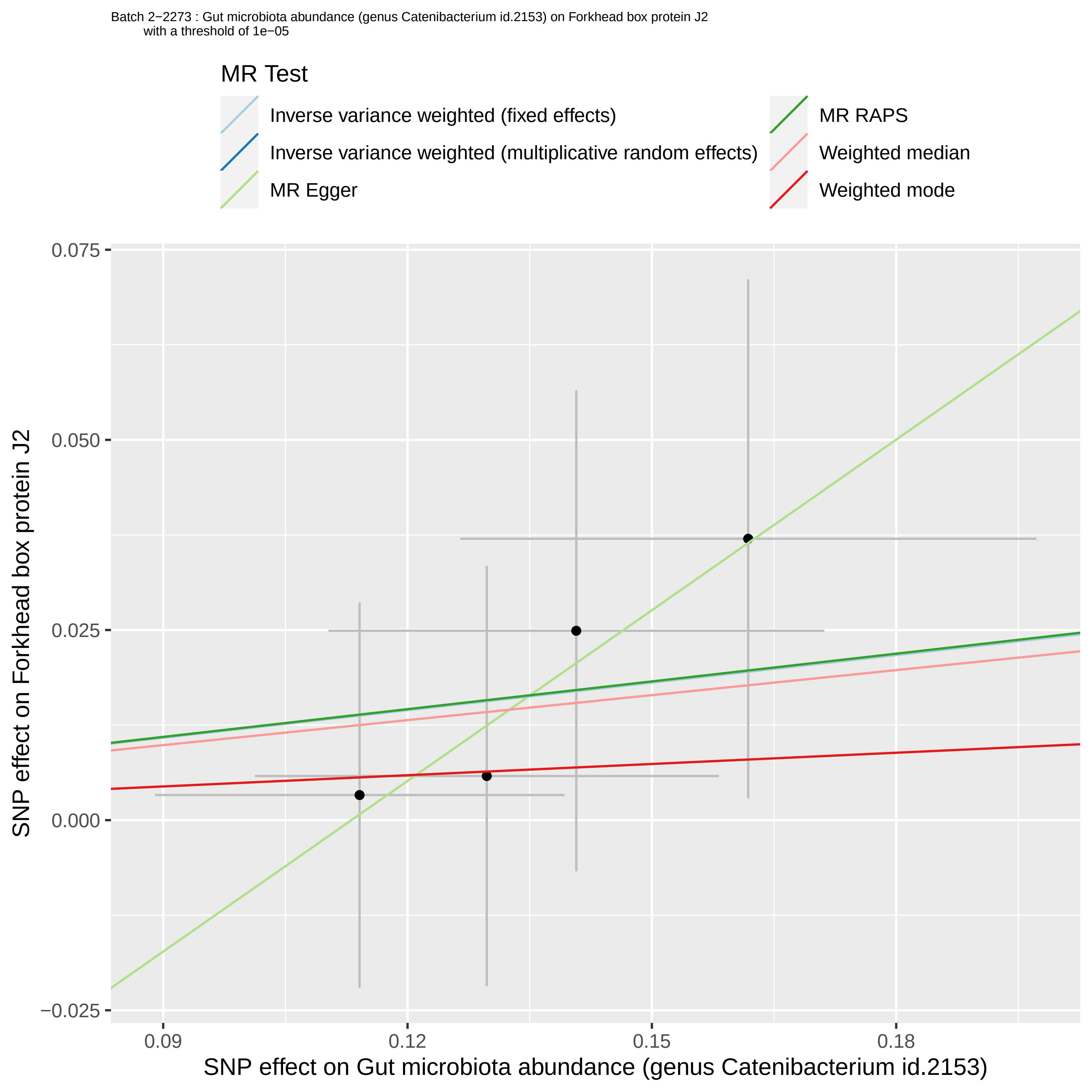
Batch 2−2273 : Gut microbiota abundance (genus Catenibacterium id.2153) on Forkhead box protein J2
with a threshold of 1e−05
MR Test
Inverse variance weighted (fixed effects)
Inverse variance weighted (multiplicative random effects)
MR Egger
MR RAPS
Weighted median
Weighted mode
0.075
0.050
0.025
0.000
−0.025
0.09
0.12
0.15
0.18
SNP effect on Forkhead box protein J2
SNP effect on Gut microbiota abundance (genus Catenibacterium id.2153)

Batch 2−2274 : Gut microbiota abundance (genus Parasutterella id.2892) on Forkhead box protein J2
with a threshold of 1e−05

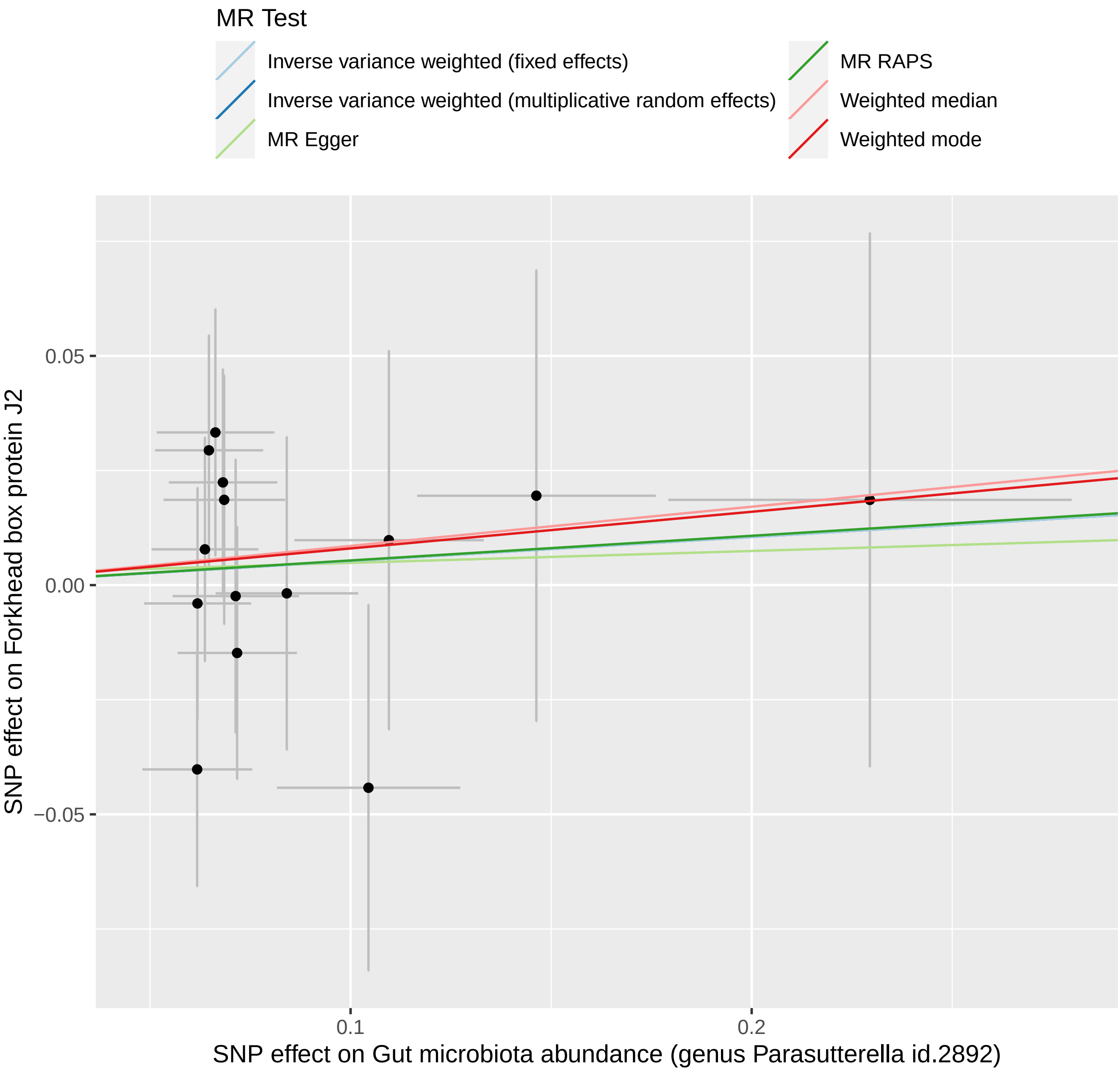

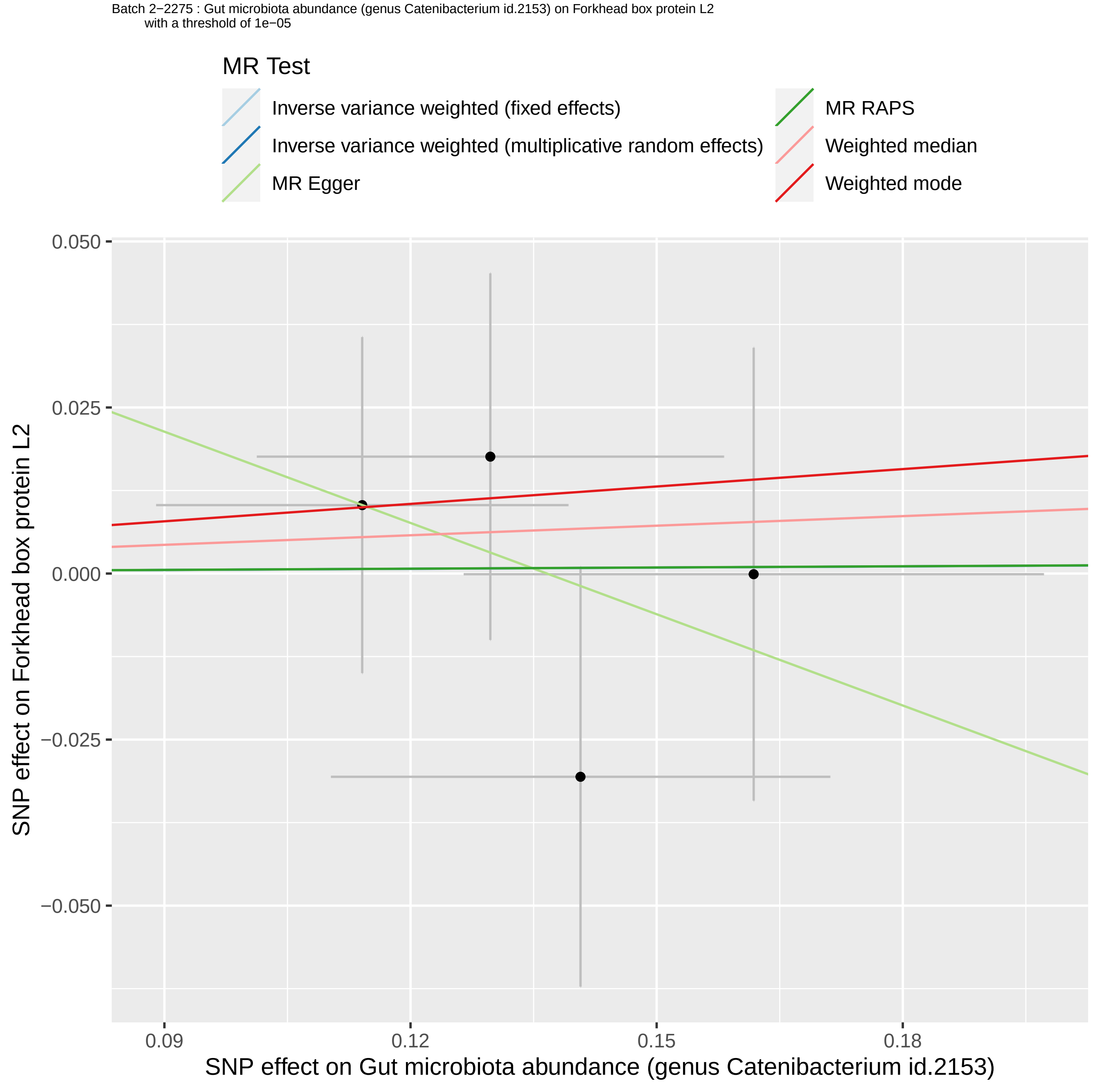
Batch 2−2275 : Gut microbiota abundance (genus Catenibacterium id.2153) on Forkhead box protein L2
with a threshold of 1e−05
MR Test
Inverse variance weighted (fixed effects)
Inverse variance weighted (multiplicative random effects)
MR Egger
MR RAPS
Weighted median
Weighted mode
0.050
0.025
0.000
−0.025
−0.050
SNP effect on Forkhead box protein L2
0.09
0.12
0.15
0.18
SNP effect on Gut microbiota abundance (genus Catenibacterium id.2153)

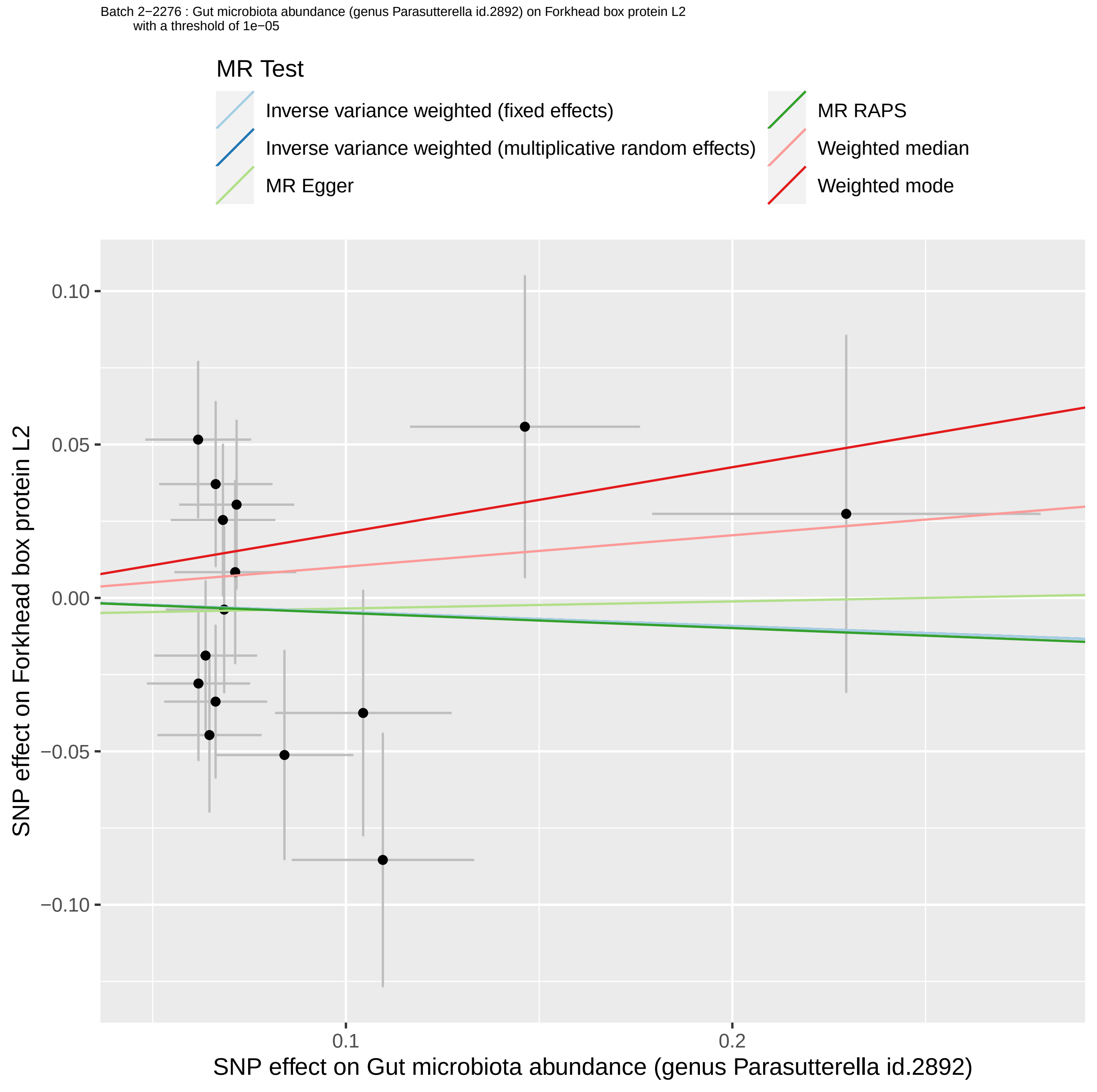

Batch 2−2276 : Gut microbiota abundance (genus Parasutterella id.2892) on Forkhead box protein L2
with a threshold of 1e−05
MR Test
Inverse variance weighted (fixed effects)
Inverse variance weighted (multiplicative random effects)
MR Egger
MR RAPS
Weighted median
Weighted mode
0.10
0.05
0.00
−0.05
−0.10
0.1
0.2
SNP effect on Forkhead box protein L2
SNP effect on Gut microbiota abundance (genus Parasutterella id.2892)

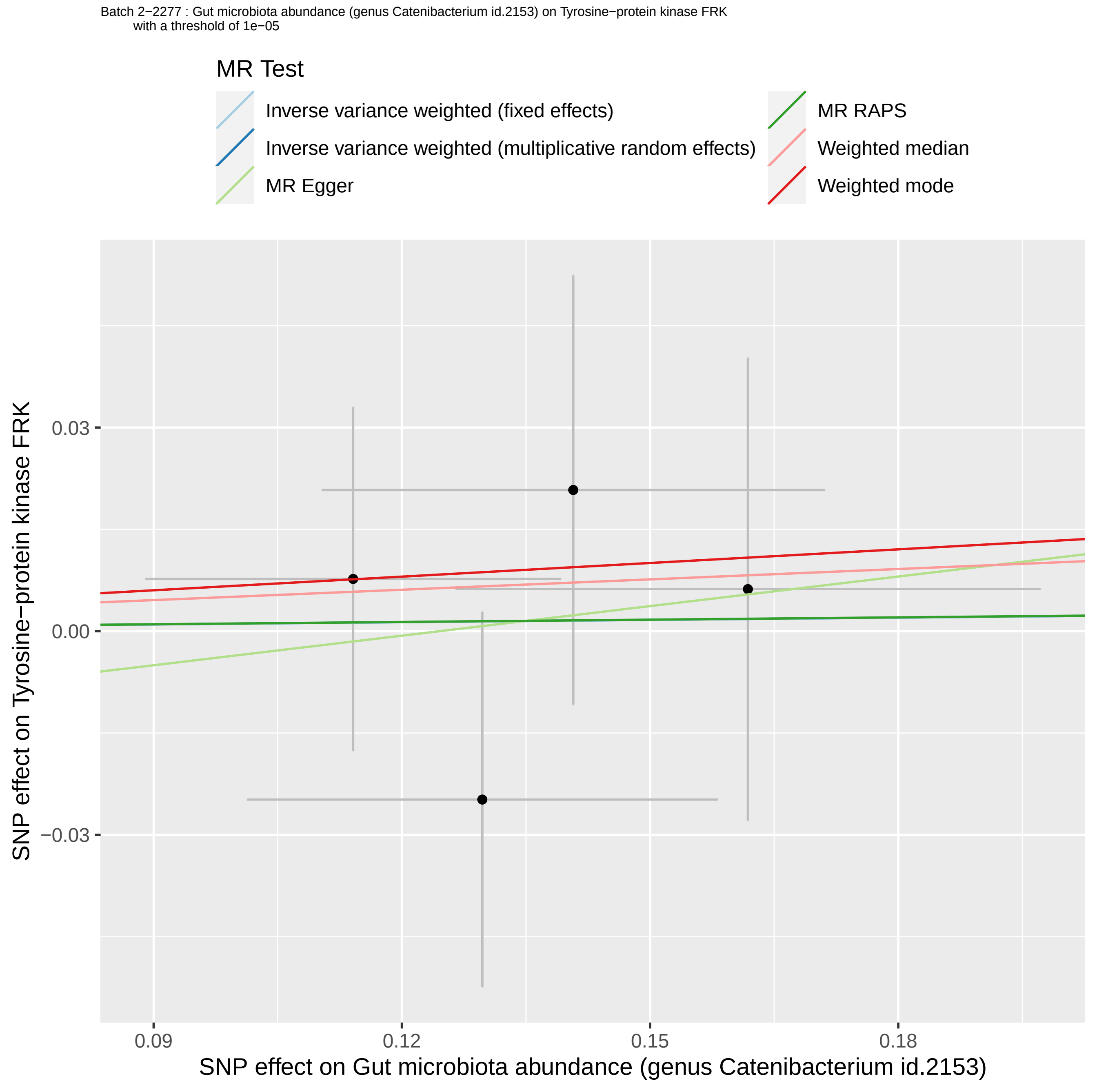
Batch 2−2277 : Gut microbiota abundance (genus Catenibacterium id.2153) on Tyrosine−protein kinase FRK
with a threshold of 1e−05
MR Test
Inverse variance weighted (fixed effects)
Inverse variance weighted (multiplicative random effects)
MR Egger
MR RAPS
Weighted median
Weighted mode
0.03
0.00
−0.03
0.09
0.12
0.15
0.18
SNP effect on Tyrosine−protein kinase FRK
SNP effect on Gut microbiota abundance (genus Catenibacterium id.2153)

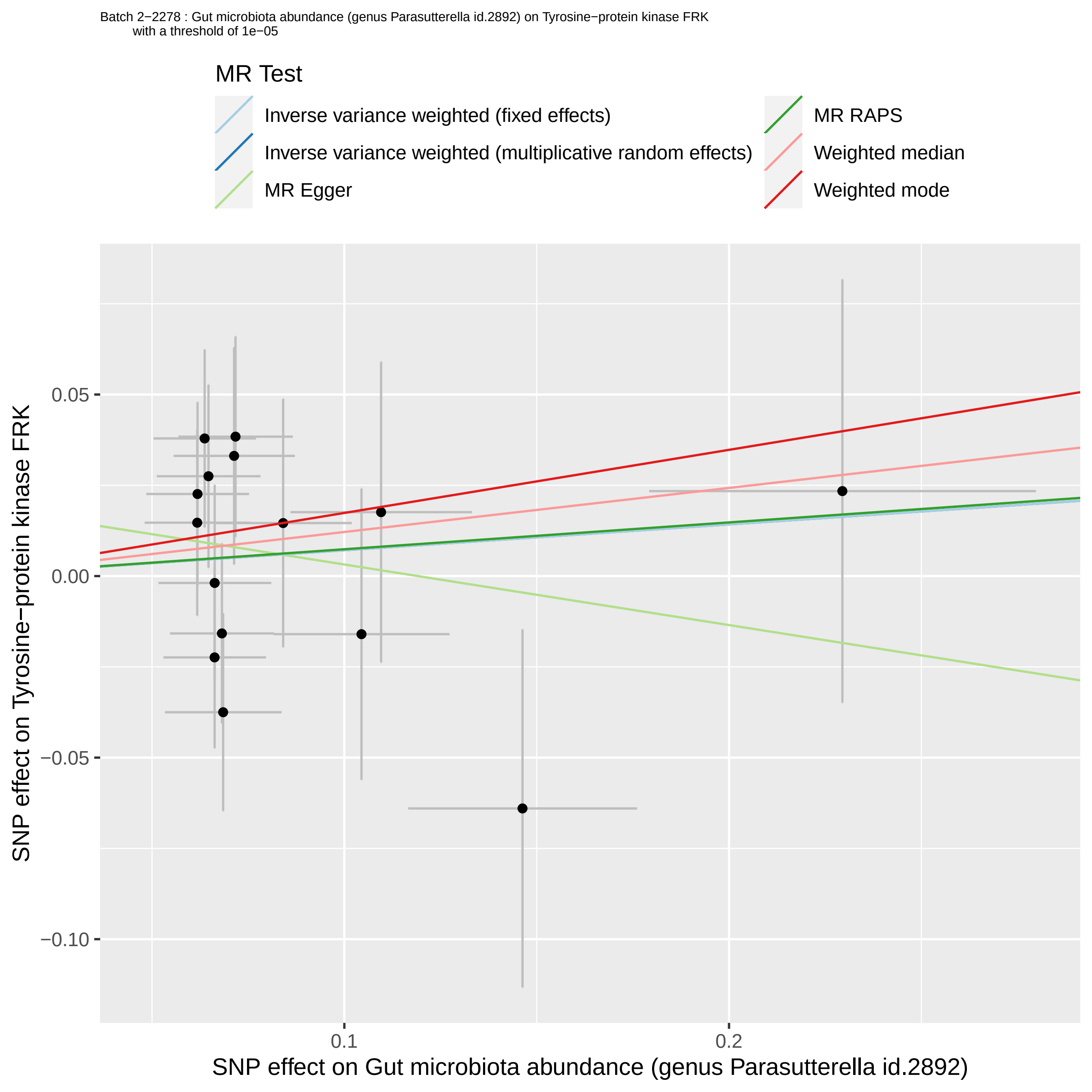

Batch 2−2278 : Gut microbiota abundance (genus Parasutterella id.2892) on Tyrosine−protein kinase FRK
with a threshold of 1e−05
MR Test
Inverse variance weighted (fixed effects)
Inverse variance weighted (multiplicative random effects)
MR Egger
MR RAPS
Weighted median
Weighted mode
0.05
0.00
−0.05
−0.10
0.1
0.2
SNP effect on Tyrosine−protein kinase FRK
SNP effect on Gut microbiota abundance (genus Parasutterella id.2892)

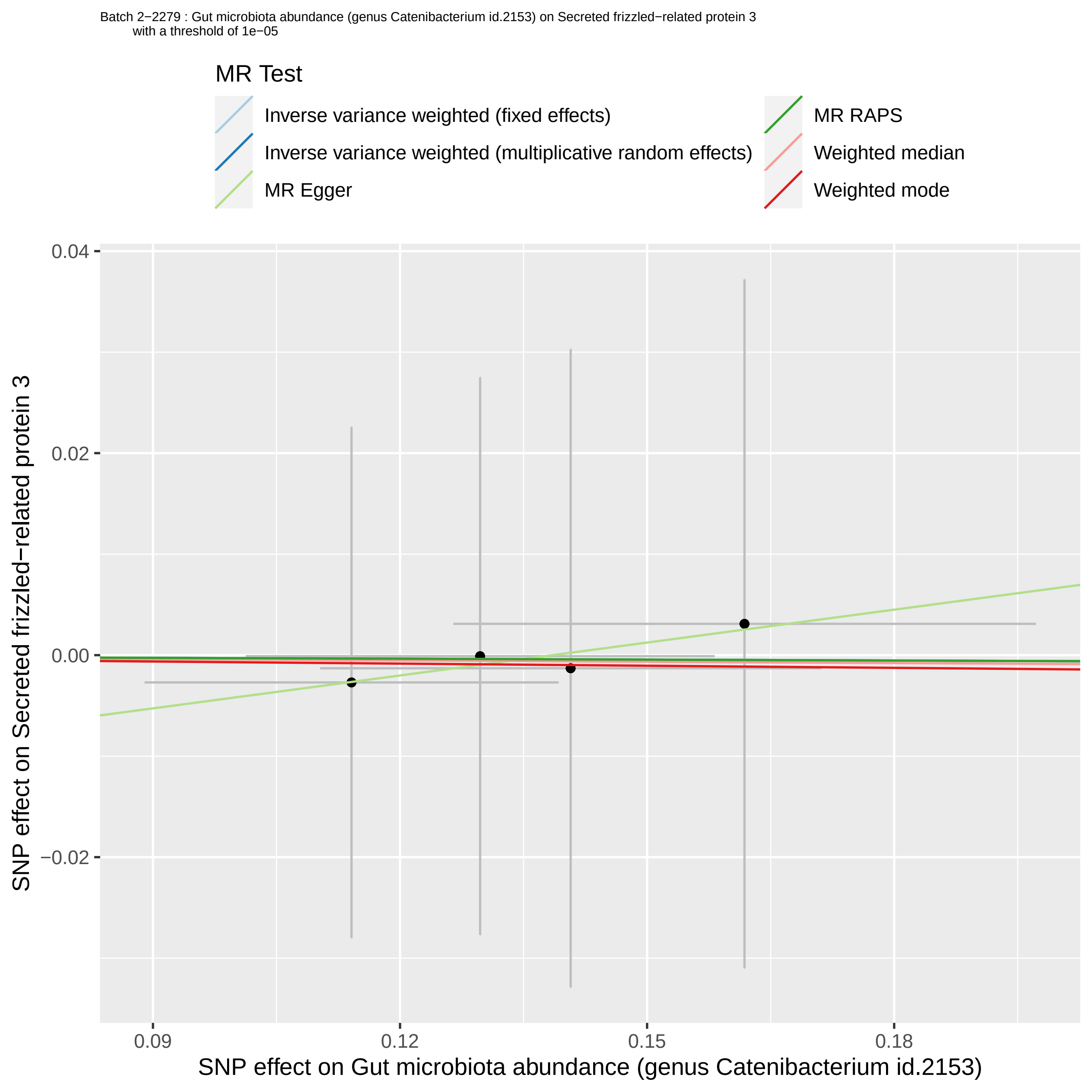
Batch 2−2279 : Gut microbiota abundance (genus Catenibacterium id.2153) on Secreted frizzled−related protein 3
with a threshold of 1e−05
MR Test
Inverse variance weighted (fixed effects)
Inverse variance weighted (multiplicative random effects)
MR Egger
MR RAPS
Weighted median
Weighted mode
0.04
0.02
0.00
−0.02
0.09
0.12
0.15
0.18
SNP effect on Secreted frizzled−related protein 3
SNP effect on Gut microbiota abundance (genus Catenibacterium id.2153)

Batch 2−2280 : Gut microbiota abundance (genus Parasutterella id.2892) on Secreted frizzled−related protein 3 with a threshold of 1e−05

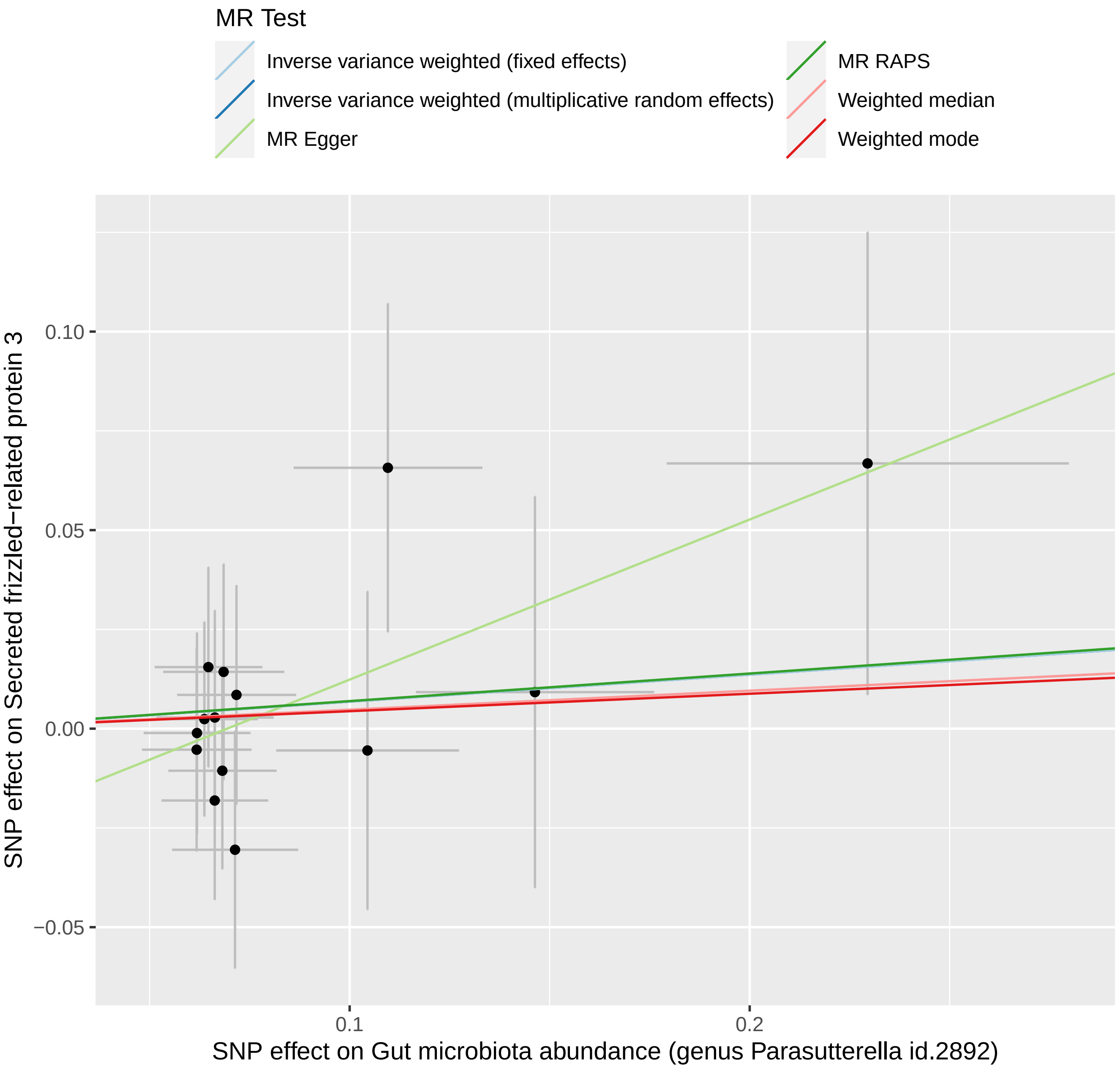

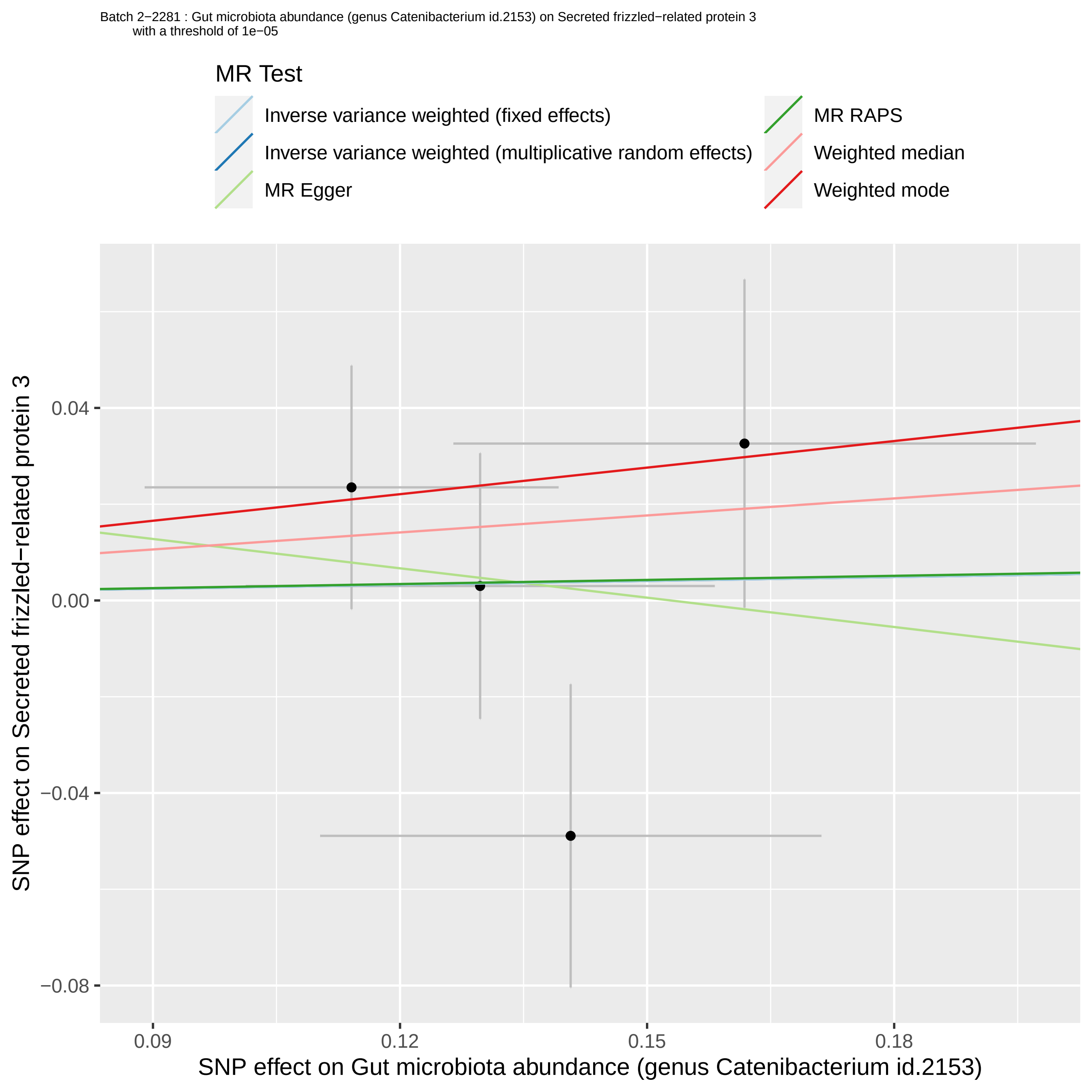
Batch 2−2281 : Gut microbiota abundance (genus Catenibacterium id.2153) on Secreted frizzled−related protein 3
with a threshold of 1e−05
MR Test
Inverse variance weighted (fixed effects)
Inverse variance weighted (multiplicative random effects)
MR Egger
MR RAPS
Weighted median
Weighted mode
0.04
0.00
−0.04
−0.08
0.09
0.12
0.15
0.18
SNP effect on Secreted frizzled−related protein 3
SNP effect on Gut microbiota abundance (genus Catenibacterium id.2153)

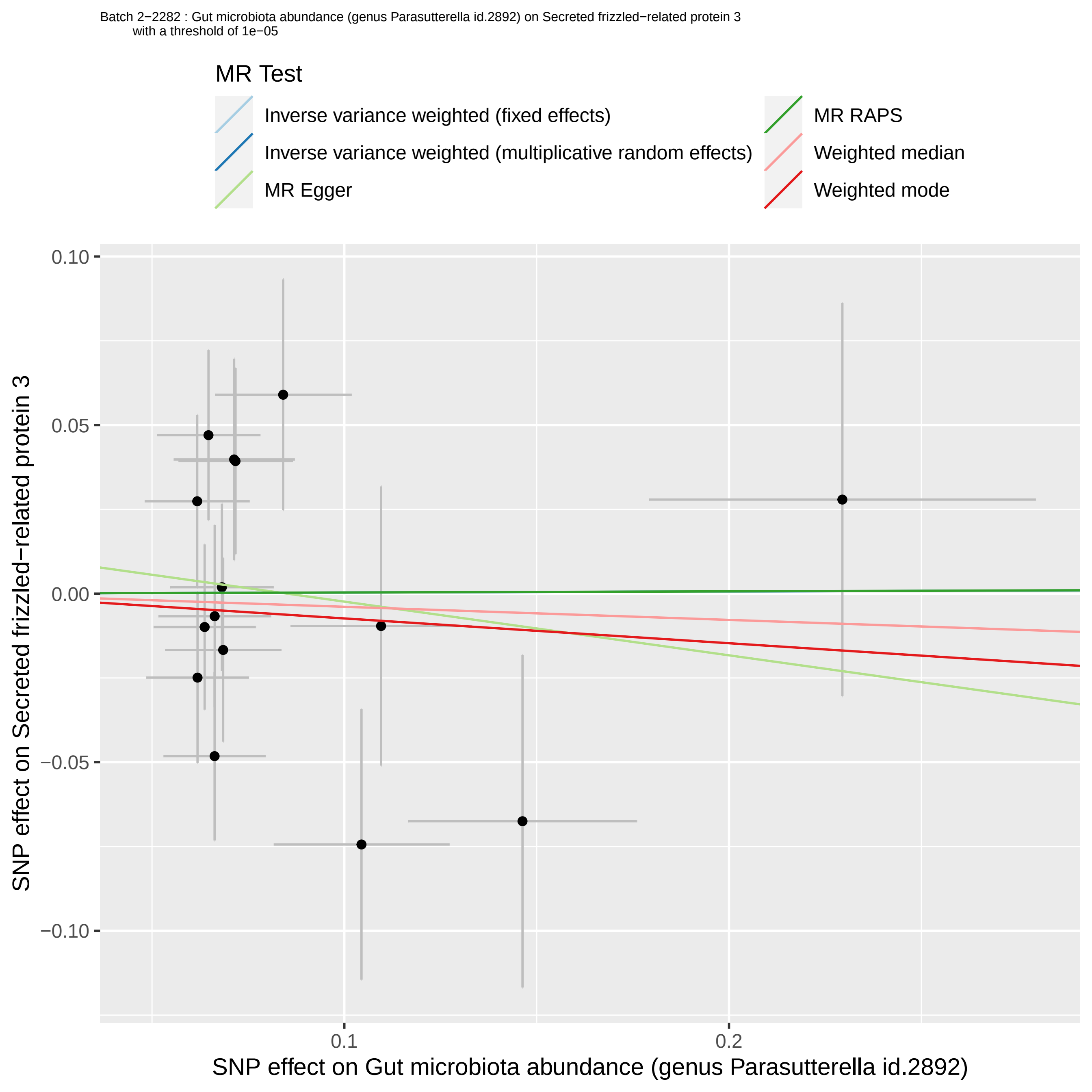
Batch 2−2282 : Gut microbiota abundance (genus Parasutterella id.2892) on Secreted frizzled−related protein 3
with a threshold of 1e−05
MR Test
Inverse variance weighted (fixed effects)
Inverse variance weighted (multiplicative random effects)
MR Egger
MR RAPS
Weighted median
Weighted mode
0.10
0.05
0.00
−0.05
−0.10
0.1
0.2
SNP effect on Secreted frizzled−related protein 3
SNP effect on Gut microbiota abundance (genus Parasutterella id.2892)

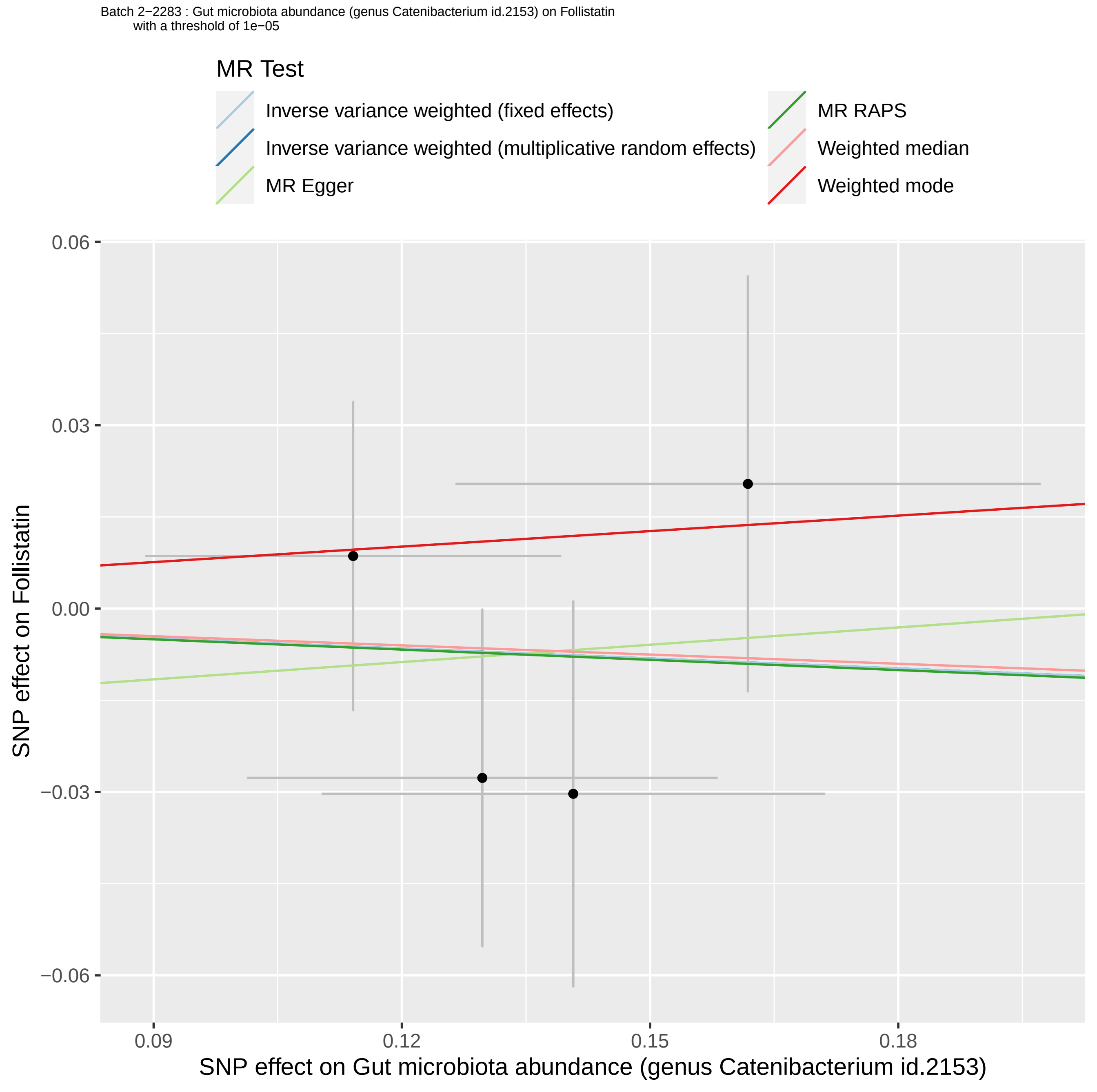
Batch 2−2283 : Gut microbiota abundance (genus Catenibacterium id.2153) on Follistatin
with a threshold of 1e−05
MR Test
Inverse variance weighted (fixed effects)
Inverse variance weighted (multiplicative random effects)
MR Egger
MR RAPS
Weighted median
Weighted mode
0.06
0.03
0.00
−0.03
−0.06
0.09
0.12
0.15
0.18
SNP effect on Follistatin
SNP effect on Gut microbiota abundance (genus Catenibacterium id.2153)

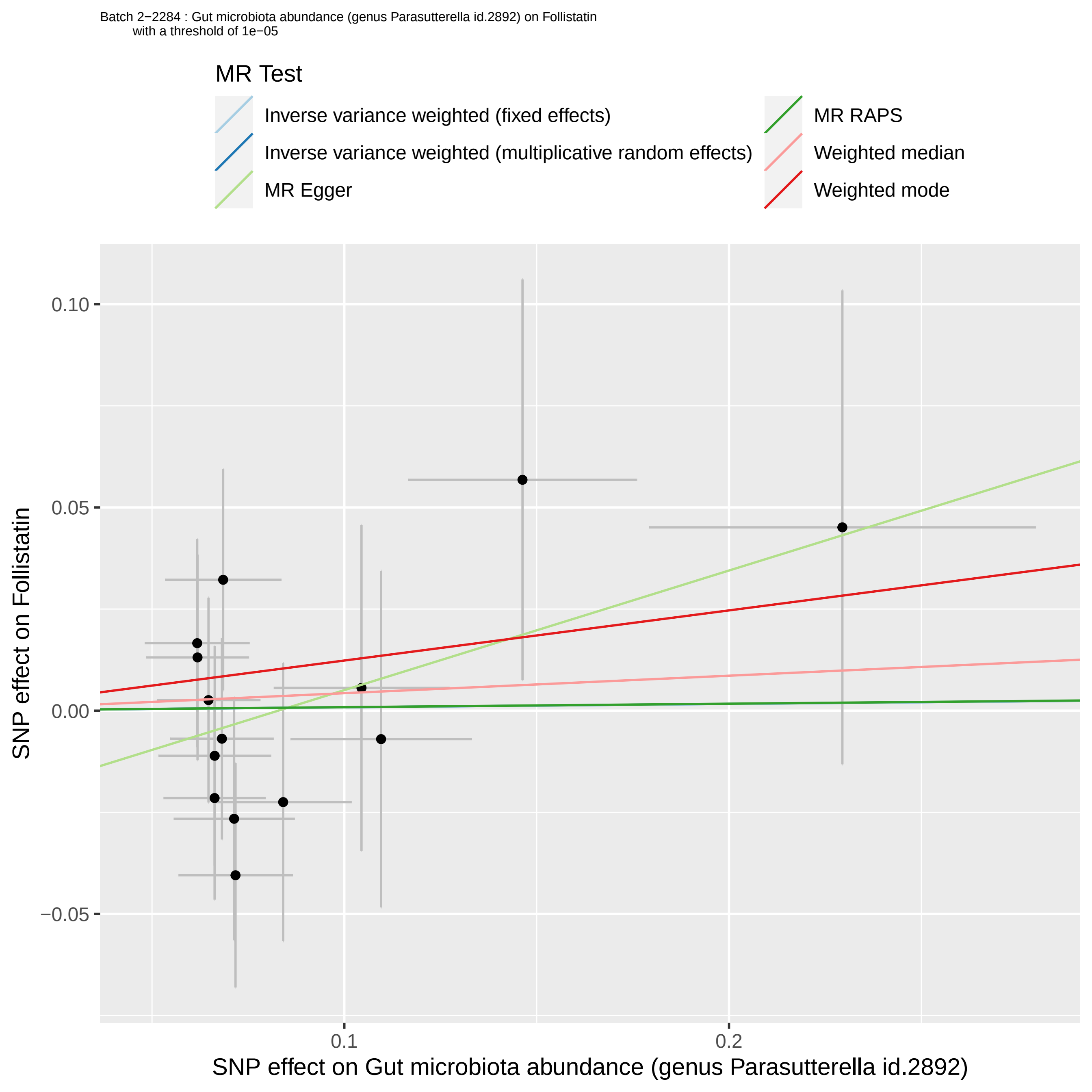

Batch 2−2284 : Gut microbiota abundance (genus Parasutterella id.2892) on Follistatin
with a threshold of 1e−05
MR Test
Inverse variance weighted (fixed effects)
Inverse variance weighted (multiplicative random effects)
MR Egger
MR RAPS
Weighted median
Weighted mode
0.10
0.05
0.00
−0.05
0.1
0.2
SNP effect on Follistatin
SNP effect on Gut microbiota abundance (genus Parasutterella id.2892)

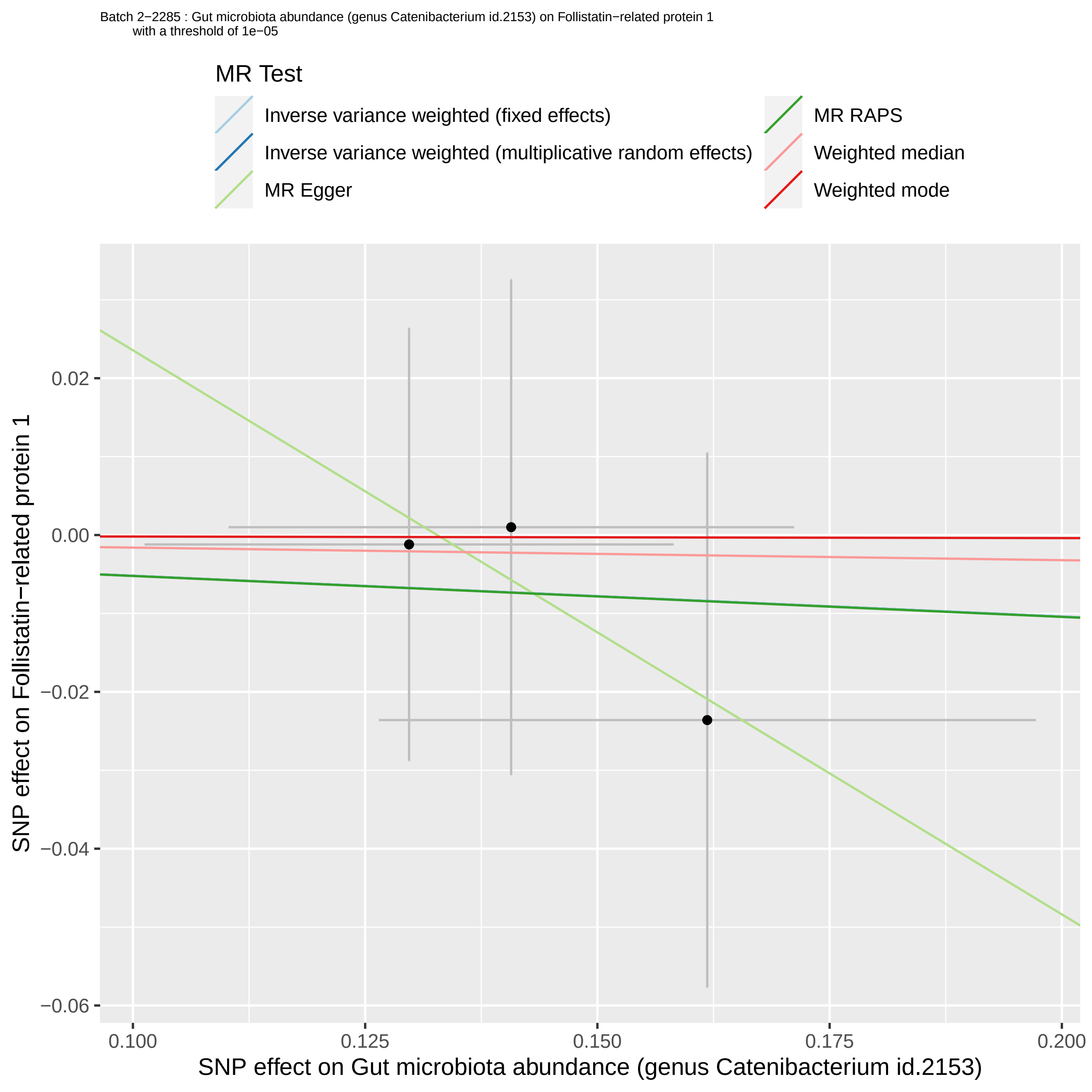

Batch 2−2285 : Gut microbiota abundance (genus Catenibacterium id.2153) on Follistatin−related protein 1
with a threshold of 1e−05
MR Test
Inverse variance weighted (fixed effects)
Inverse variance weighted (multiplicative random effects)
MR Egger
MR RAPS
Weighted median
Weighted mode
0.02
0.00
−0.02
−0.04
−0.06
0.100
0.125
0.150
0.175
0.200
SNP effect on Follistatin−related protein 1
SNP effect on Gut microbiota abundance (genus Catenibacterium id.2153)

Batch 2−2286 : Gut microbiota abundance (genus Parasutterella id.2892) on Follistatin−related protein 1
with a threshold of 1e−05

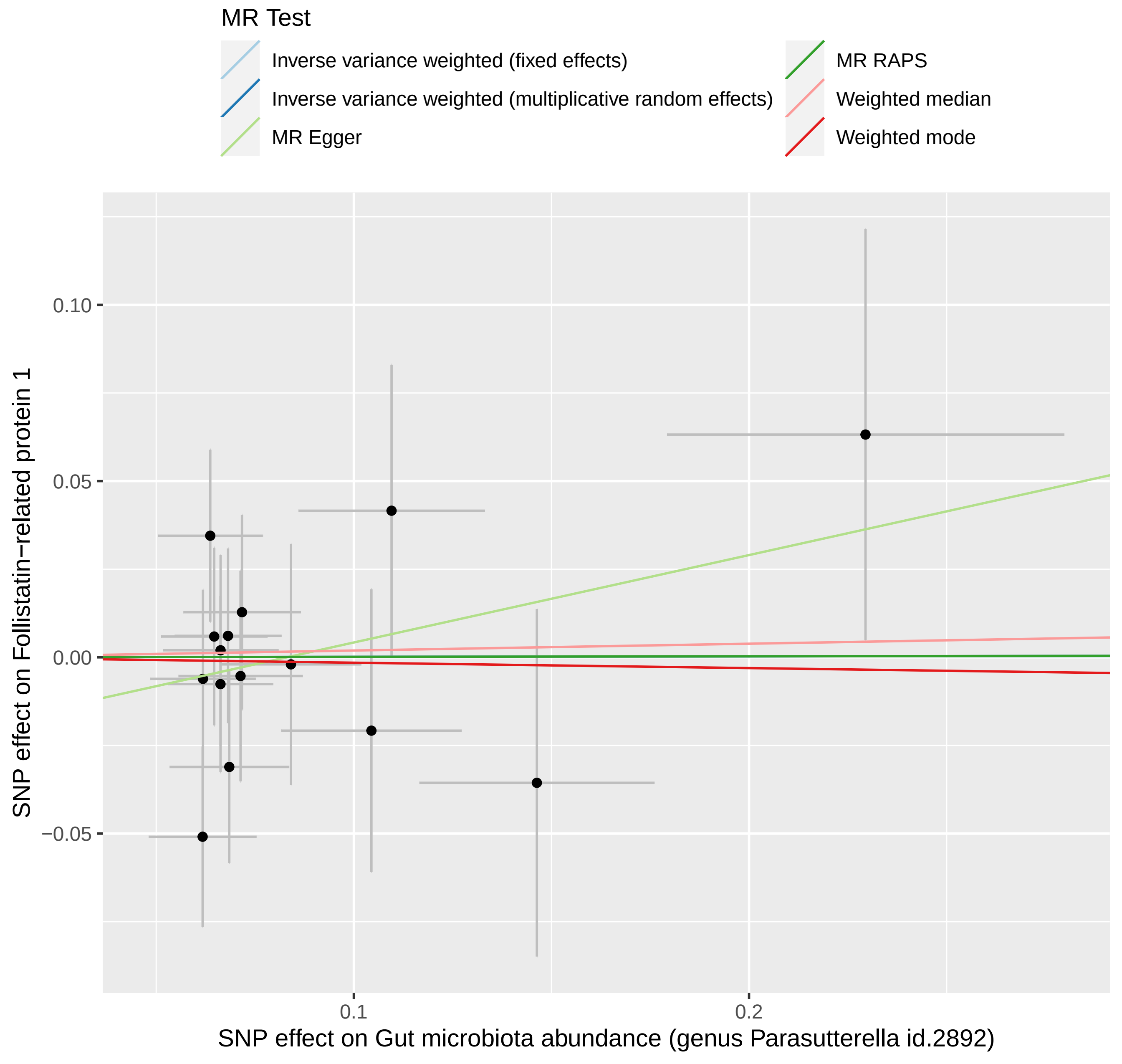

Batch 2−2287 : Gut microbiota abundance (genus Catenibacterium id.2153) on Follistatin−related protein 1
with a threshold of 1e−05

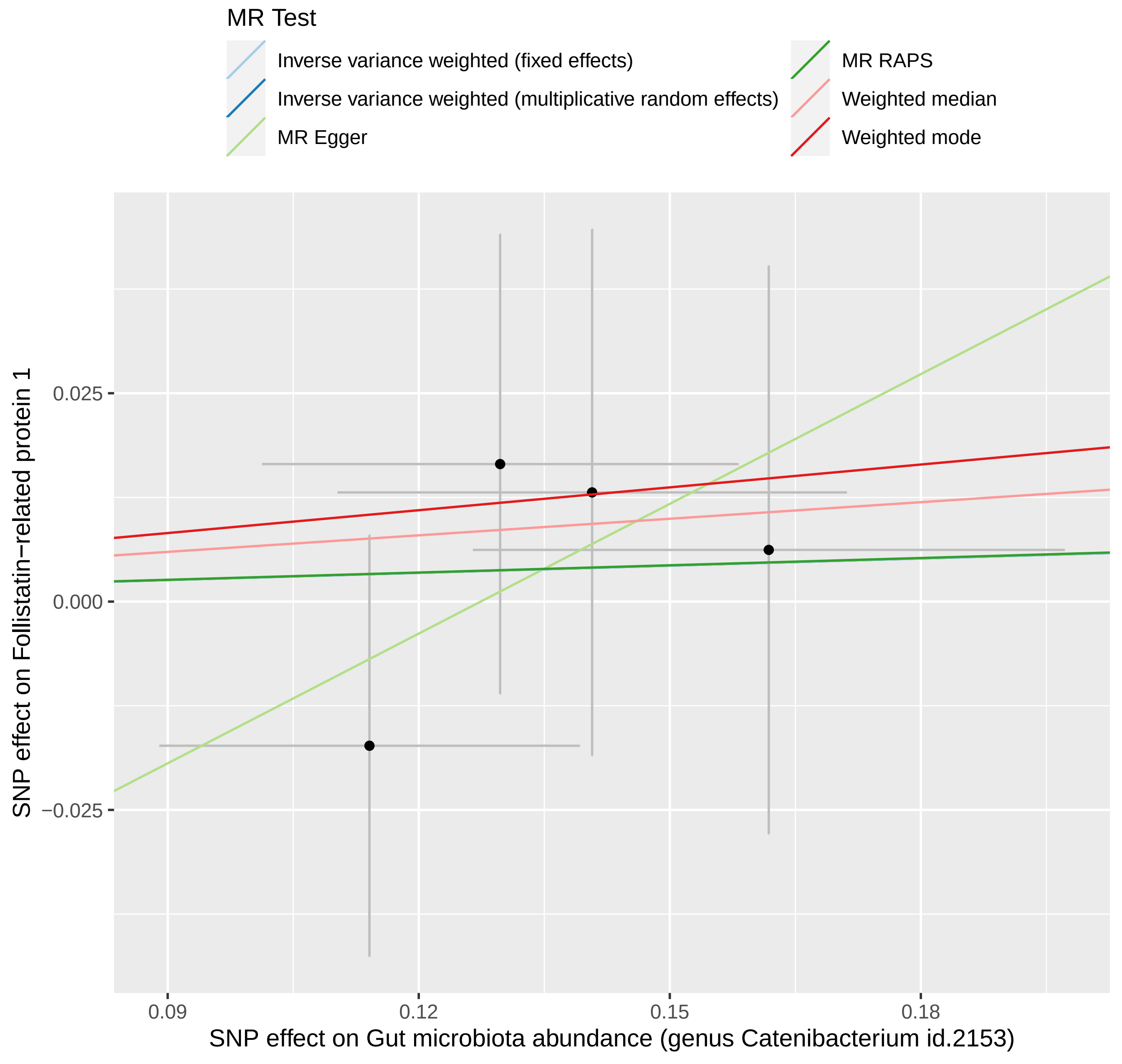

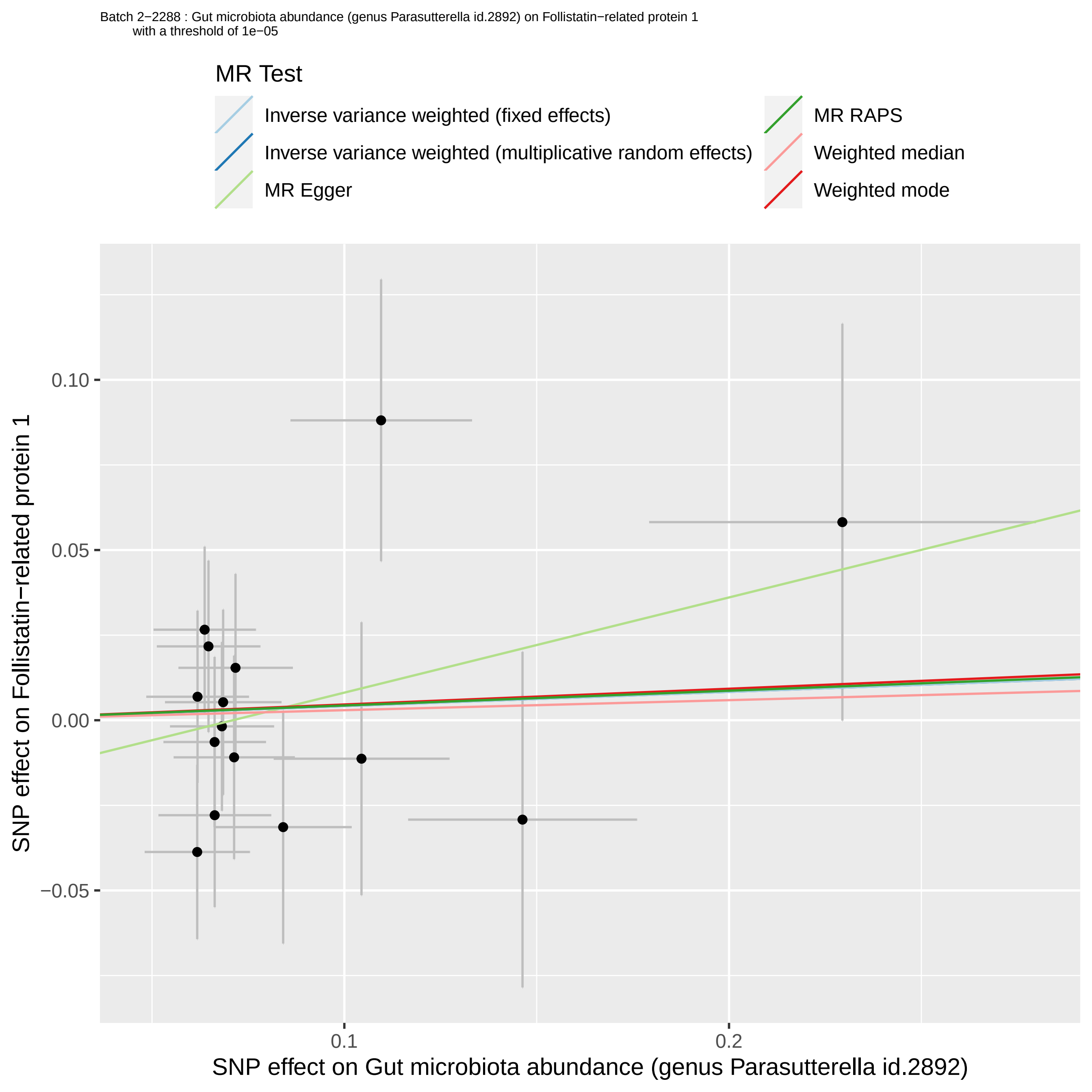

Batch 2−2288 : Gut microbiota abundance (genus Parasutterella id.2892) on Follistatin−related protein 1
with a threshold of 1e−05
MR Test
Inverse variance weighted (fixed effects)
Inverse variance weighted (multiplicative random effects)
MR Egger
MR RAPS
Weighted median
Weighted mode
0.10
0.05
0.00
−0.05
0.1
0.2
SNP effect on Follistatin−related protein 1
SNP effect on Gut microbiota abundance (genus Parasutterella id.2892)

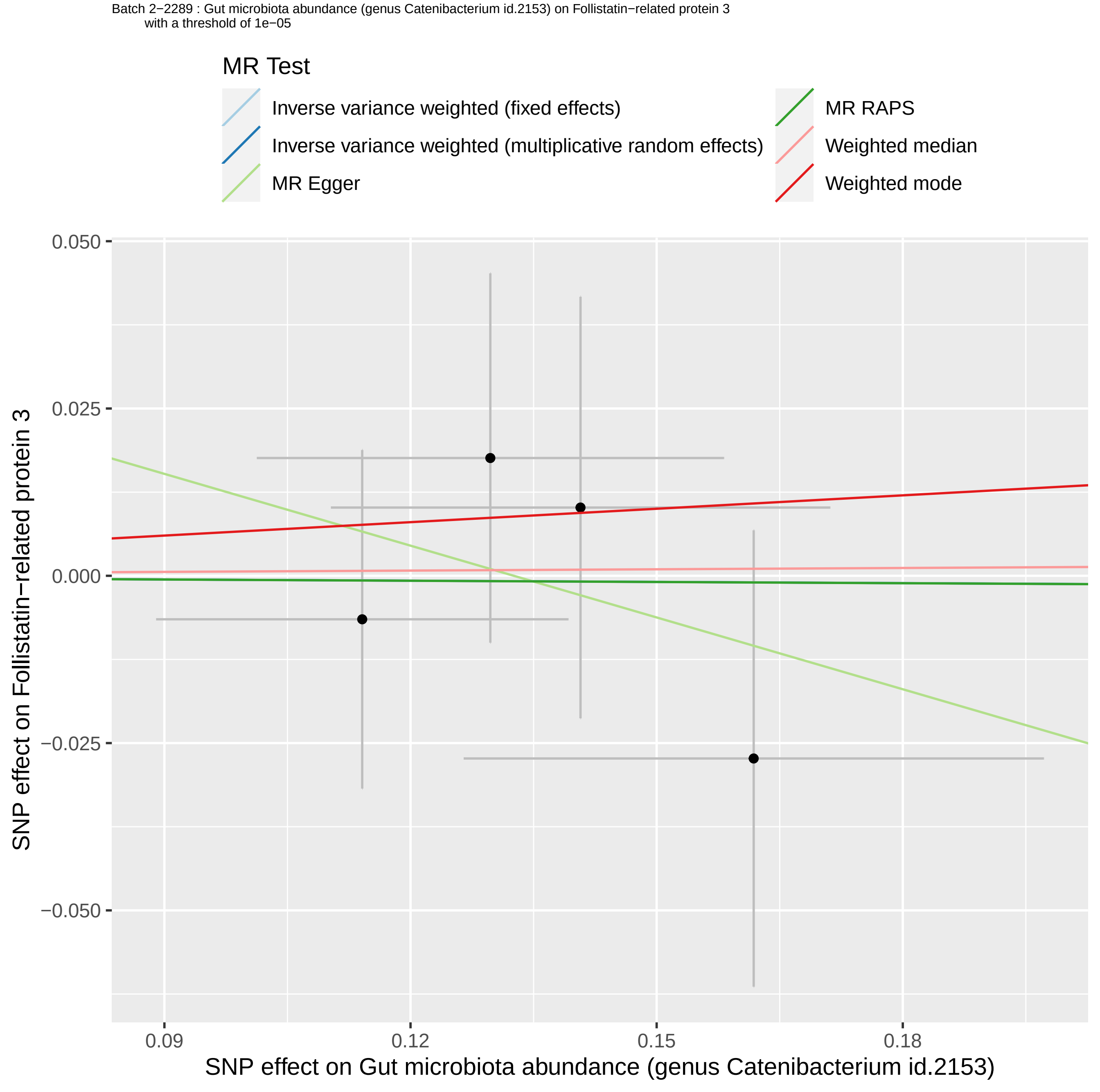
Batch 2−2289 : Gut microbiota abundance (genus Catenibacterium id.2153) on Follistatin−related protein 3
with a threshold of 1e−05
MR Test
Inverse variance weighted (fixed effects)
Inverse variance weighted (multiplicative random effects)
MR Egger
MR RAPS
Weighted median
Weighted mode
0.050
0.025
0.000
−0.025
−0.050
0.09
0.12
0.15
0.18
SNP effect on Follistatin−related protein 3
SNP effect on Gut microbiota abundance (genus Catenibacterium id.2153)

Batch 2−2290 : Gut microbiota abundance (genus Parasutterella id.2892) on Follistatin−related protein 3
with a threshold of 1e−05

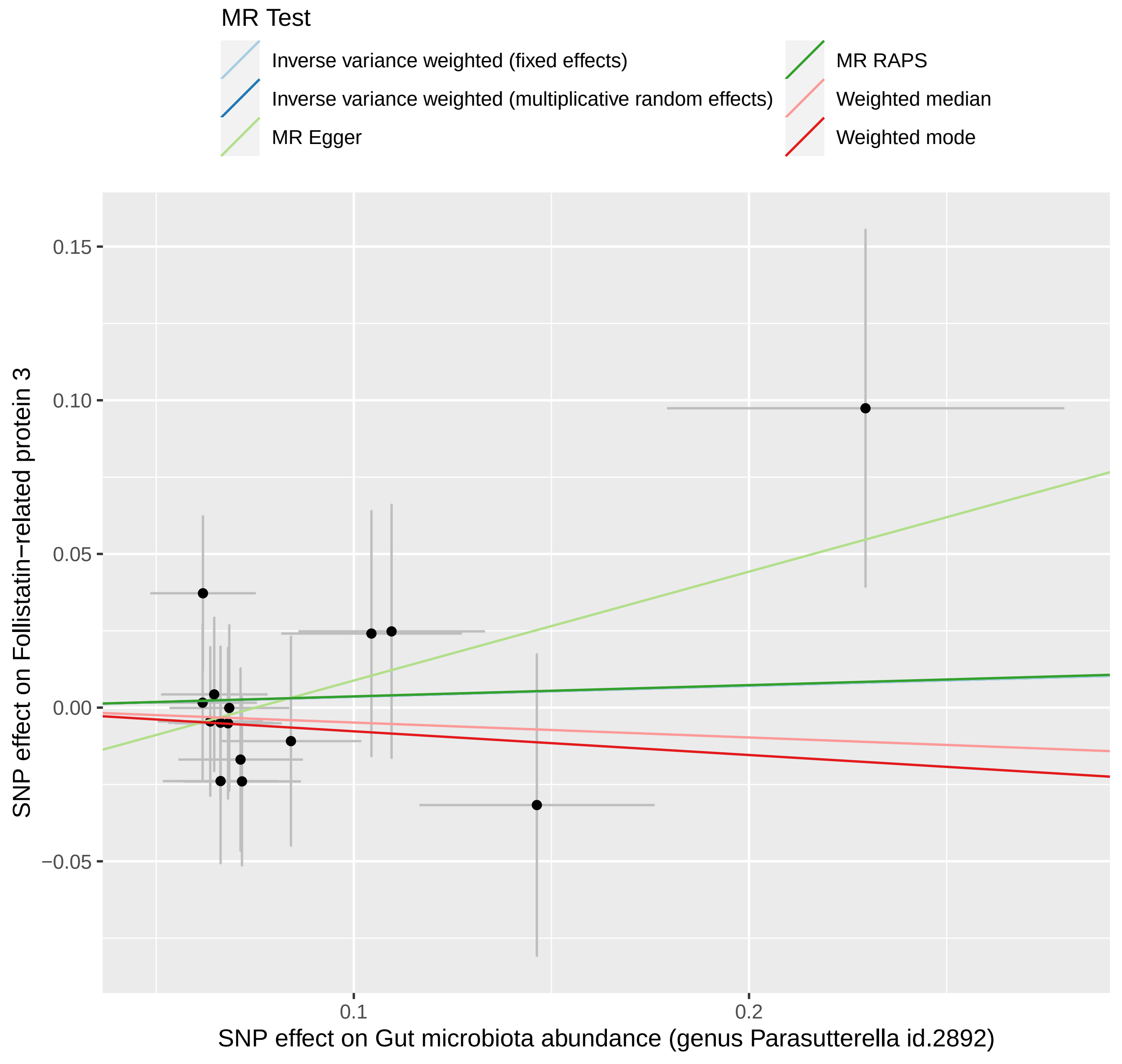

Batch 2−2291 : Gut microbiota abundance (genus Catenibacterium id.2153) on Follistatin−related protein 4
with a threshold of 1e−05

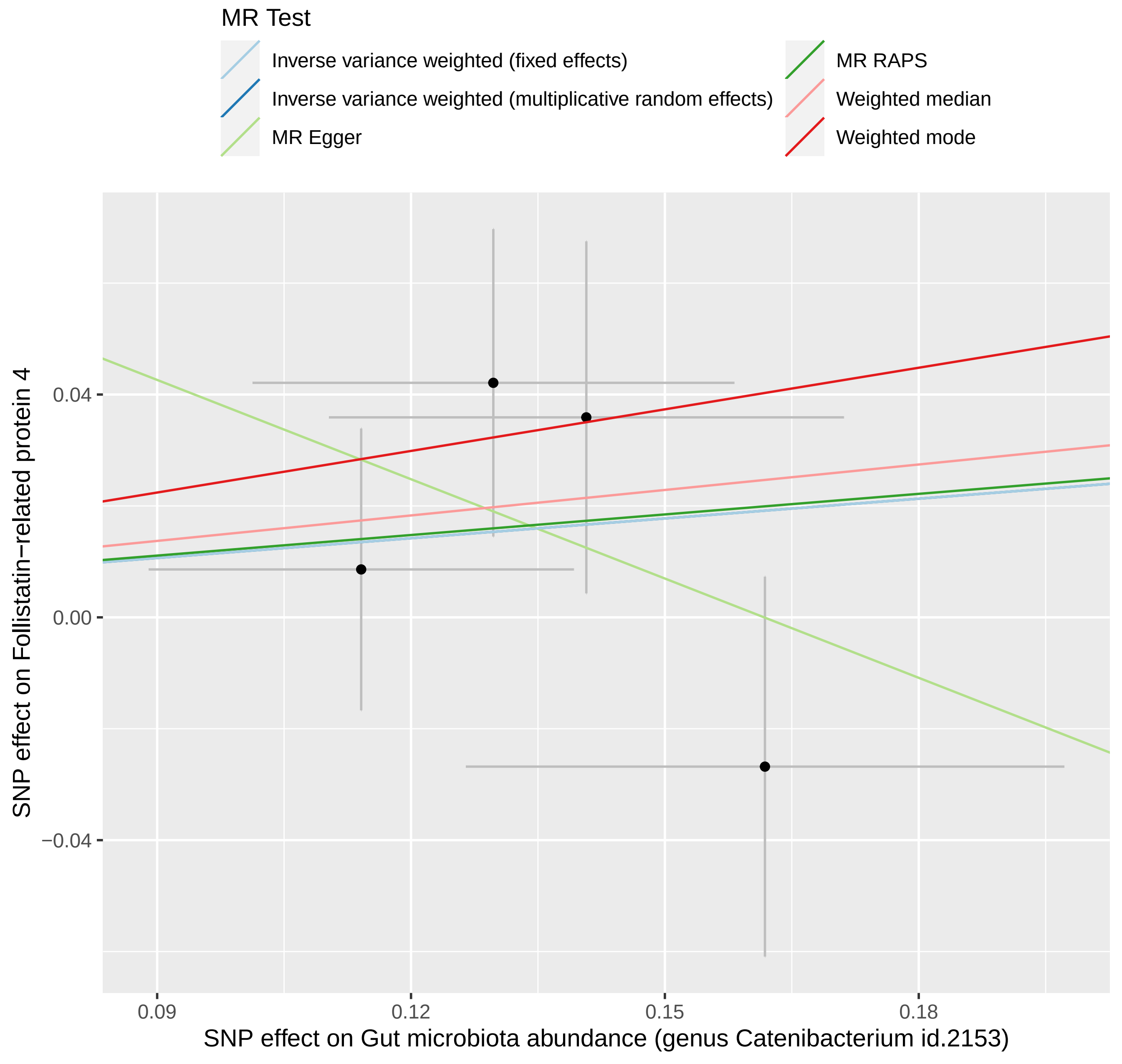

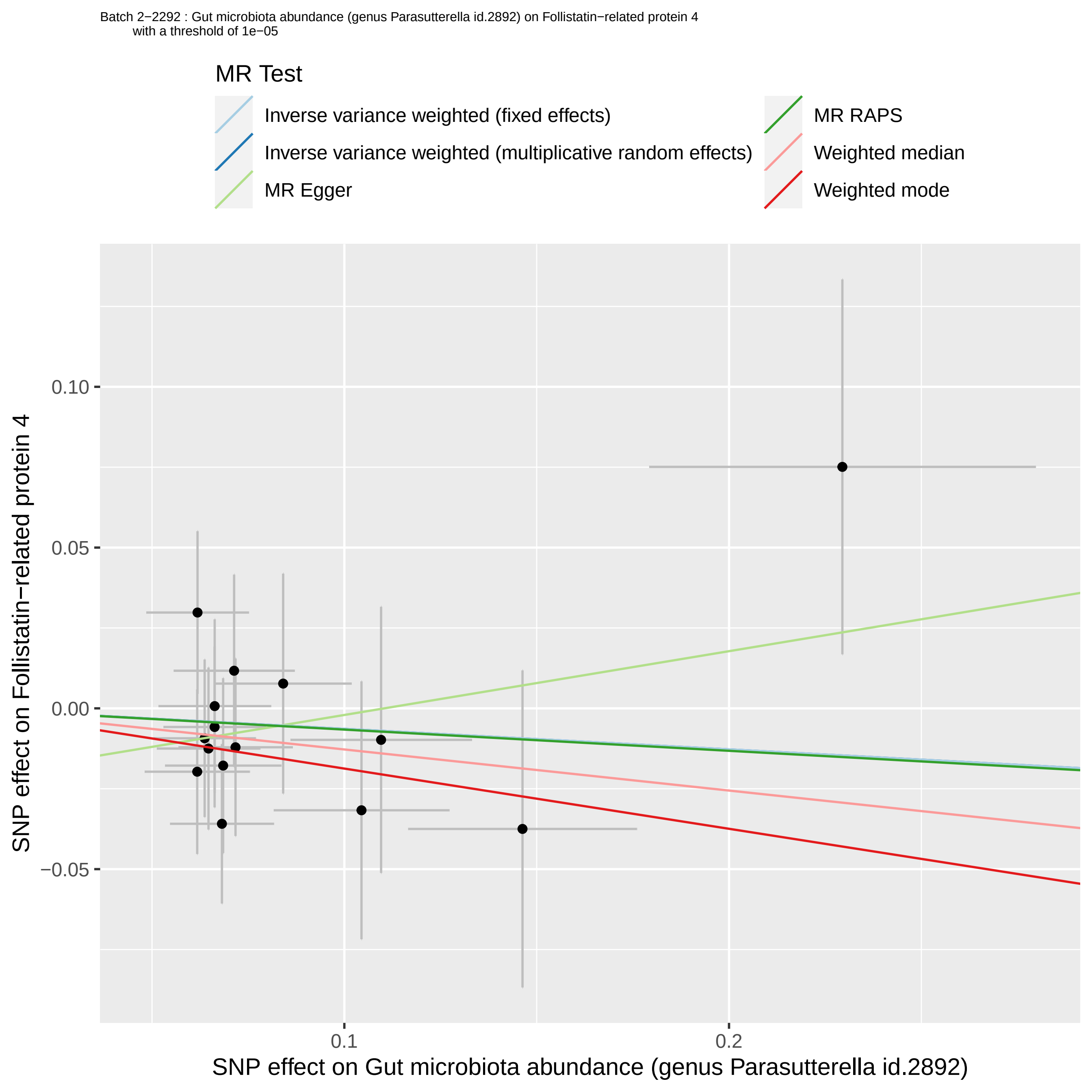

Batch 2−2292 : Gut microbiota abundance (genus Parasutterella id.2892) on Follistatin−related protein 4
with a threshold of 1e−05
MR Test
Inverse variance weighted (fixed effects)
Inverse variance weighted (multiplicative random effects)
MR Egger
MR RAPS
Weighted median
Weighted mode
0.10
0.05
0.00
−0.05
0.1
0.2
SNP effect on Follistatin−related protein 4
SNP effect on Gut microbiota abundance (genus Parasutterella id.2892)

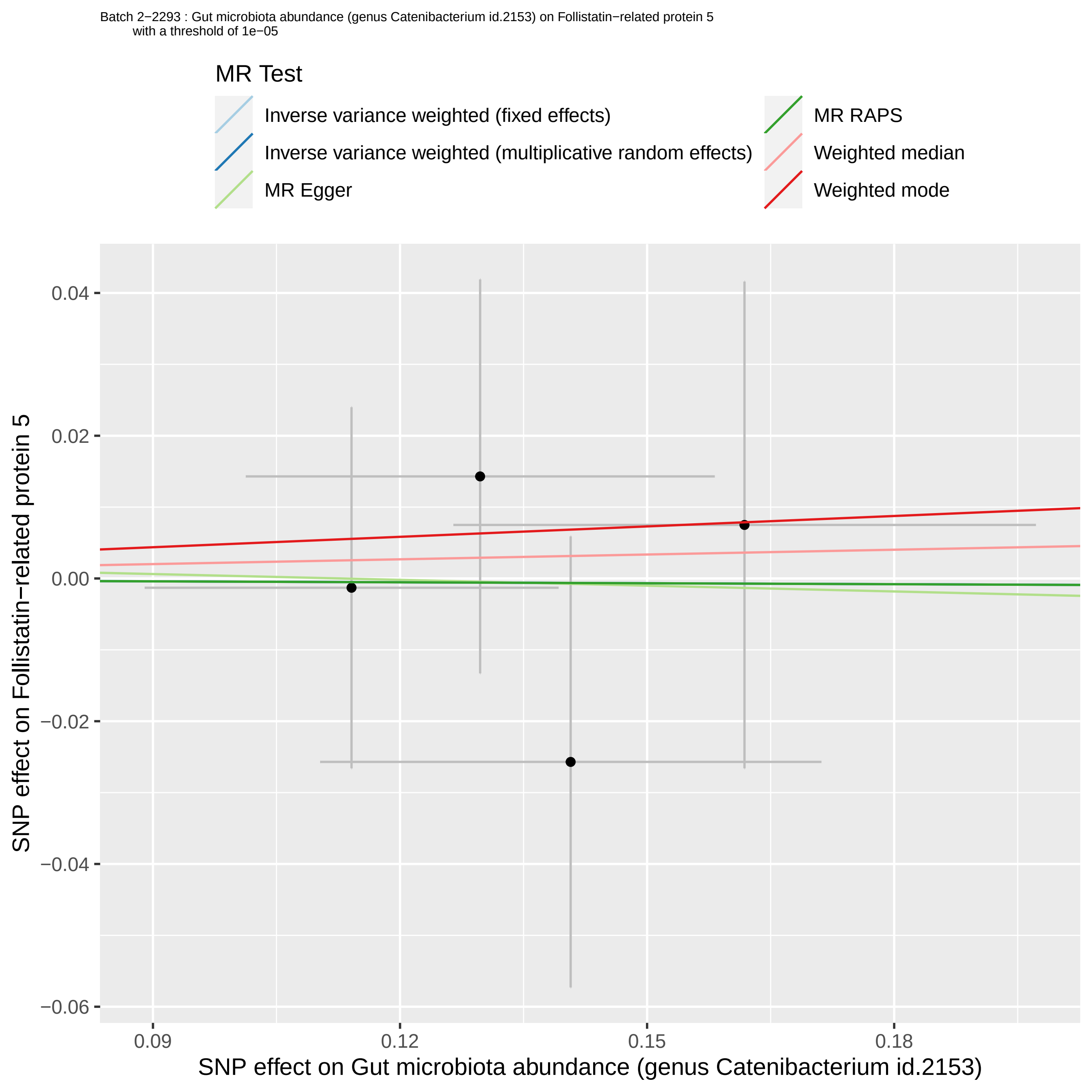

Batch 2−2293 : Gut microbiota abundance (genus Catenibacterium id.2153) on Follistatin−related protein 5
with a threshold of 1e−05
MR Test
Inverse variance weighted (fixed effects)
Inverse variance weighted (multiplicative random effects)
MR Egger
MR RAPS
Weighted median
Weighted mode
0.04
0.02
0.00
−0.02
−0.04
−0.06
0.09
0.12
0.15
0.18
SNP effect on Follistatin−related protein 5
SNP effect on Gut microbiota abundance (genus Catenibacterium id.2153)

Batch 2−2294 : Gut microbiota abundance (genus Parasutterella id.2892) on Follistatin−related protein 5
with a threshold of 1e−05

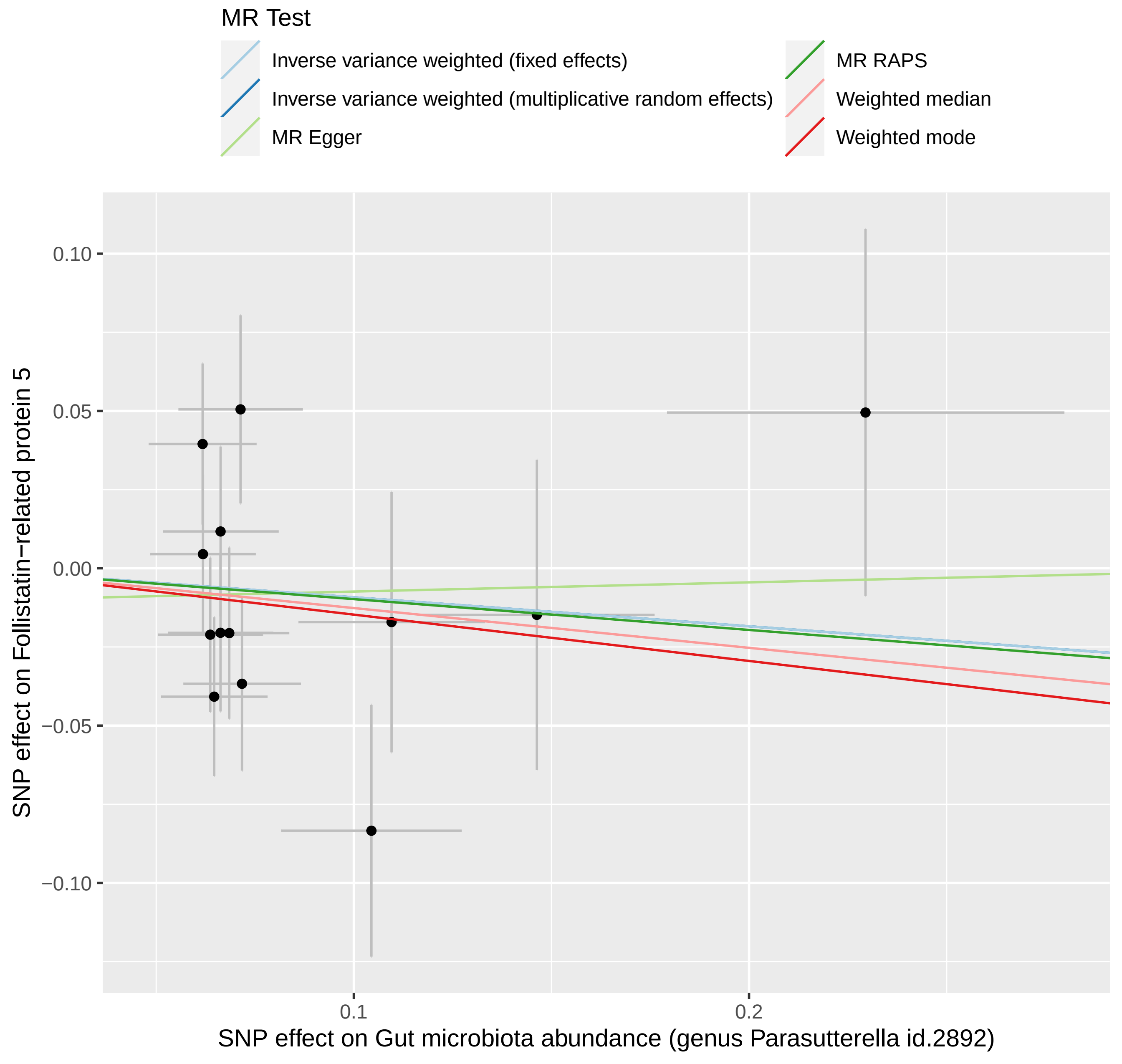

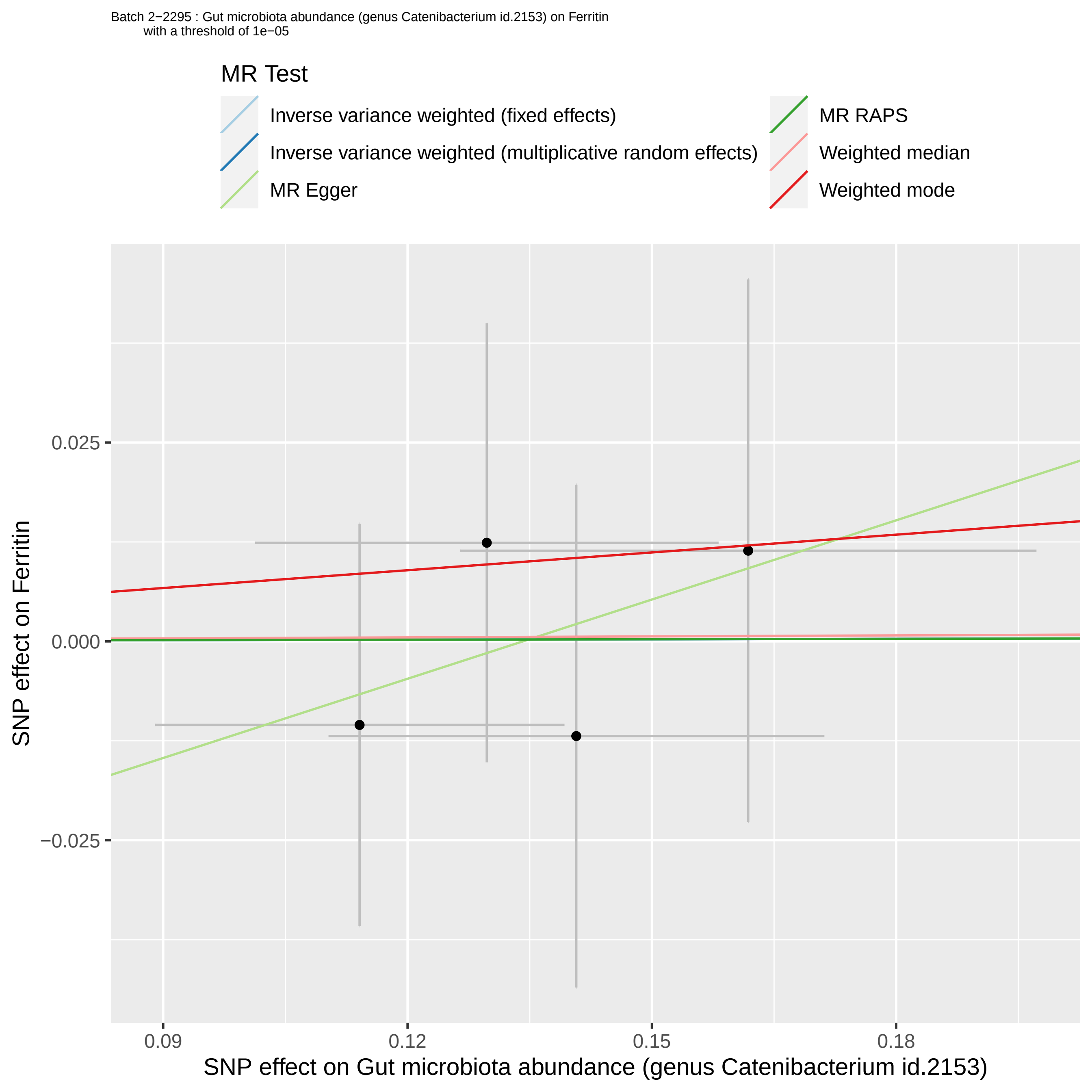
Batch 2−2295 : Gut microbiota abundance (genus Catenibacterium id.2153) on Ferritin
with a threshold of 1e−05
MR Test
Inverse variance weighted (fixed effects)
Inverse variance weighted (multiplicative random effects)
MR Egger
MR RAPS
Weighted median
Weighted mode
0.025
0.000
−0.025
0.09
0.12
0.15
0.18
SNP effect on Ferritin
SNP effect on Gut microbiota abundance (genus Catenibacterium id.2153)

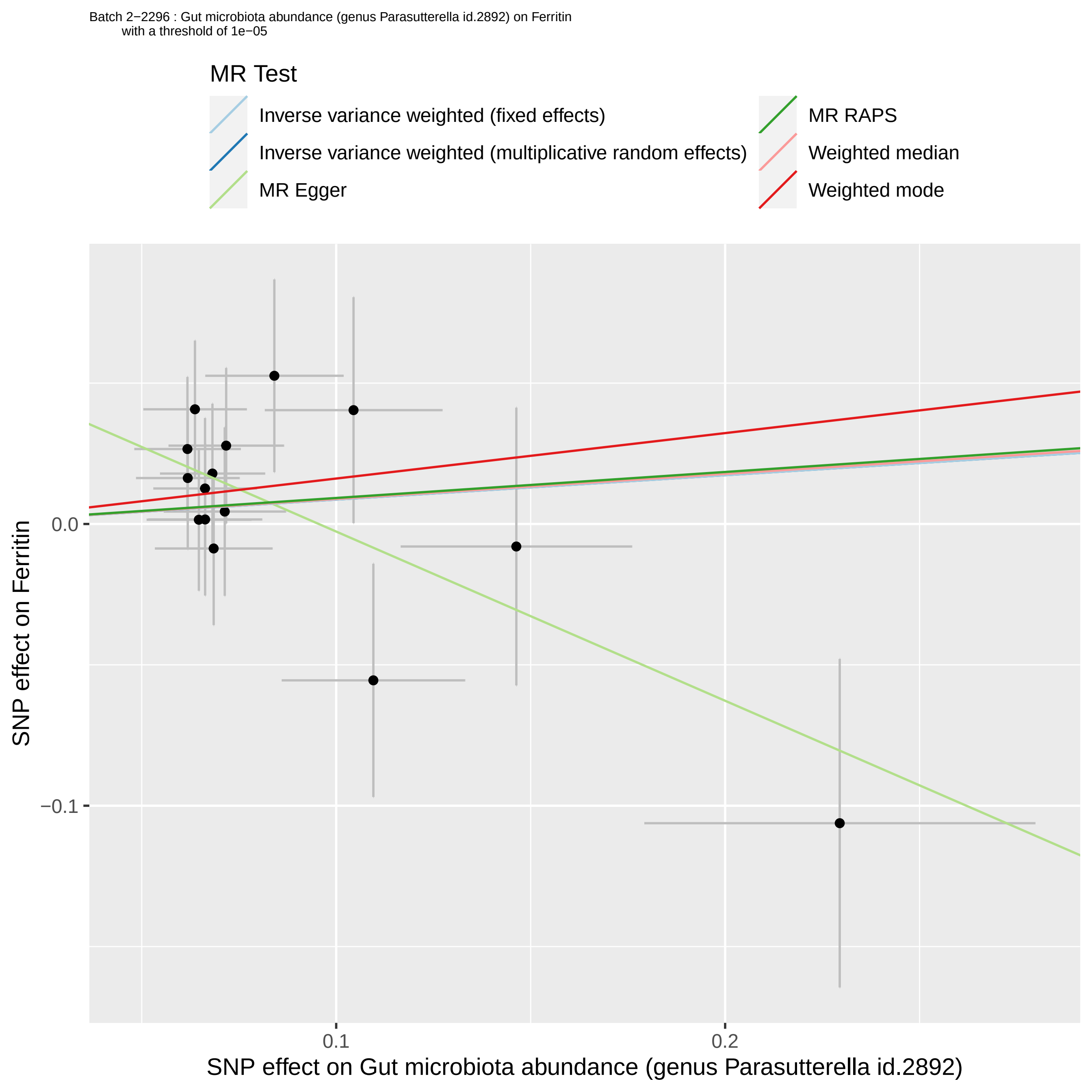
Batch 2−2296 : Gut microbiota abundance (genus Parasutterella id.2892) on Ferritin
with a threshold of 1e−05
MR Test
Inverse variance weighted (fixed effects)
Inverse variance weighted (multiplicative random effects)
MR Egger
MR RAPS
Weighted median
Weighted mode
SNP effect on Ferritin
0.0
−0.1
0.1
0.2
SNP effect on Gut microbiota abundance (genus Parasutterella id.2892)

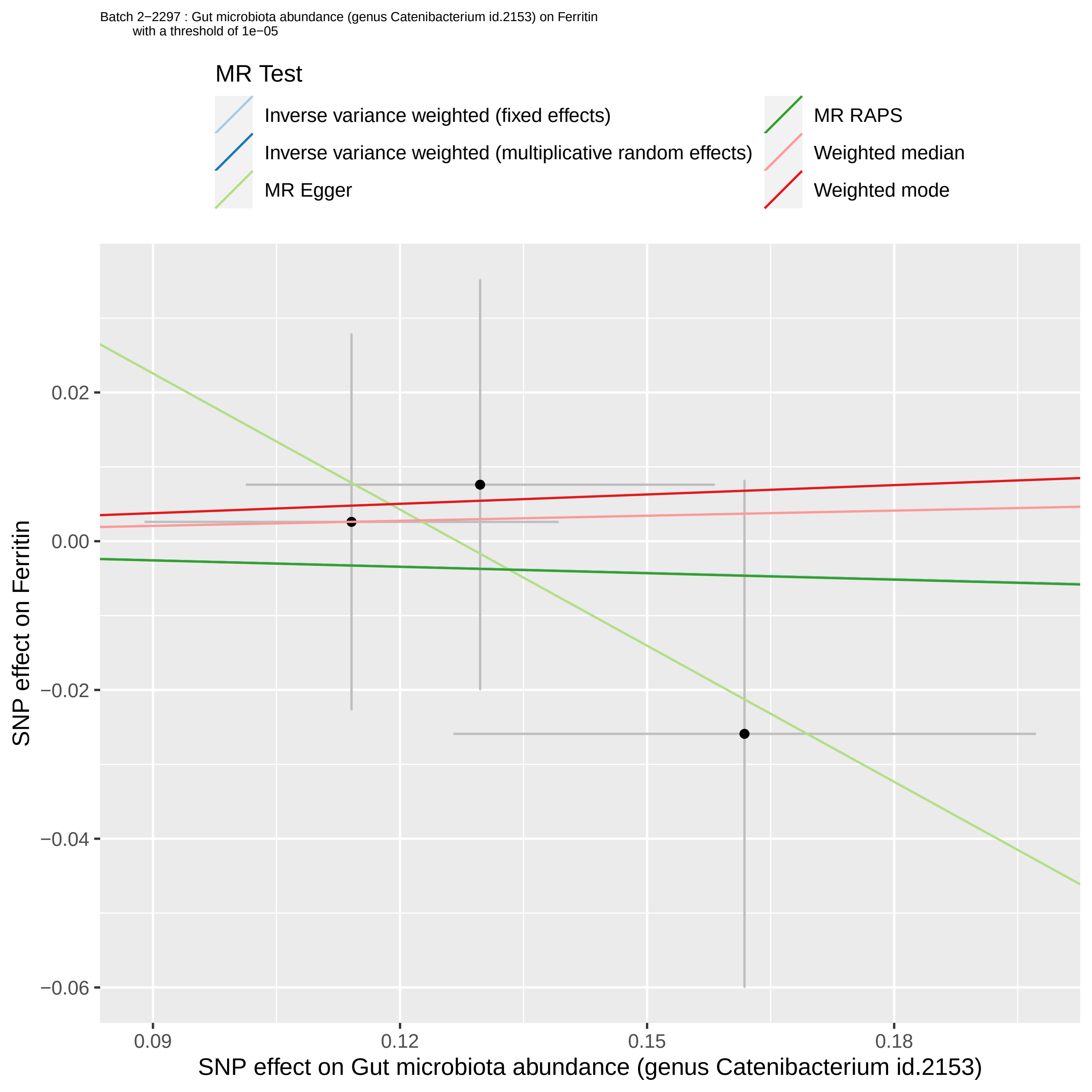

Batch 2−2297 : Gut microbiota abundance (genus Catenibacterium id.2153) on Ferritin
with a threshold of 1e−05
MR Test
Inverse variance weighted (fixed effects)
Inverse variance weighted (multiplicative random effects)
MR Egger
MR RAPS
Weighted median
Weighted mode
0.02
0.00
−0.02
−0.04
−0.06
0.09
0.12
0.15
0.18
SNP effect on Ferritin
SNP effect on Gut microbiota abundance (genus Catenibacterium id.2153)

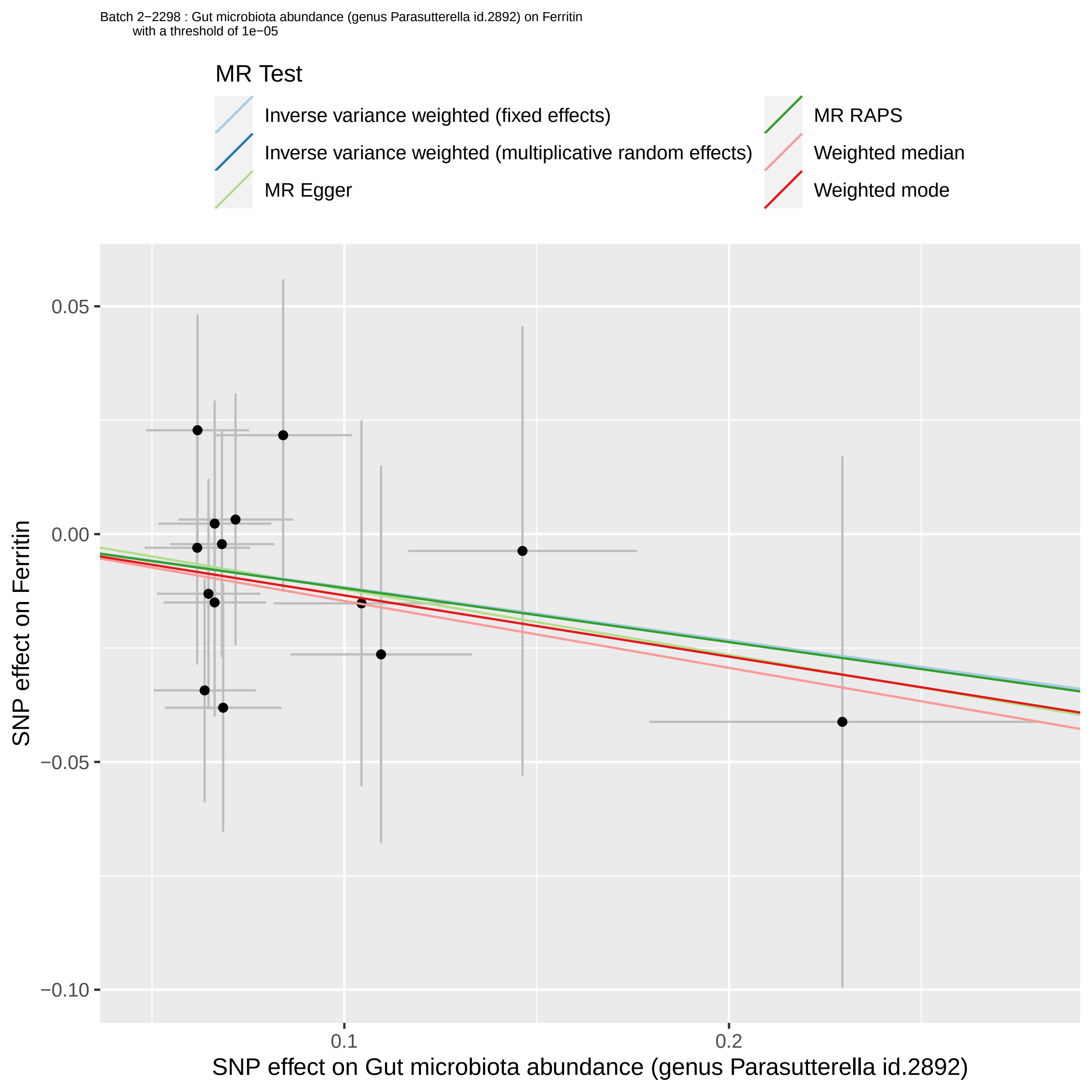

Batch 2−2298 : Gut microbiota abundance (genus Parasutterella id.2892) on Ferritin
with a threshold of 1e−05
MR Test
Inverse variance weighted (fixed effects)
Inverse variance weighted (multiplicative random effects)
MR Egger
MR RAPS
Weighted median
Weighted mode
0.05
0.00
−0.05
−0.10
0.1
0.2
SNP effect on Ferritin
SNP effect on Gut microbiota abundance (genus Parasutterella id.2892)

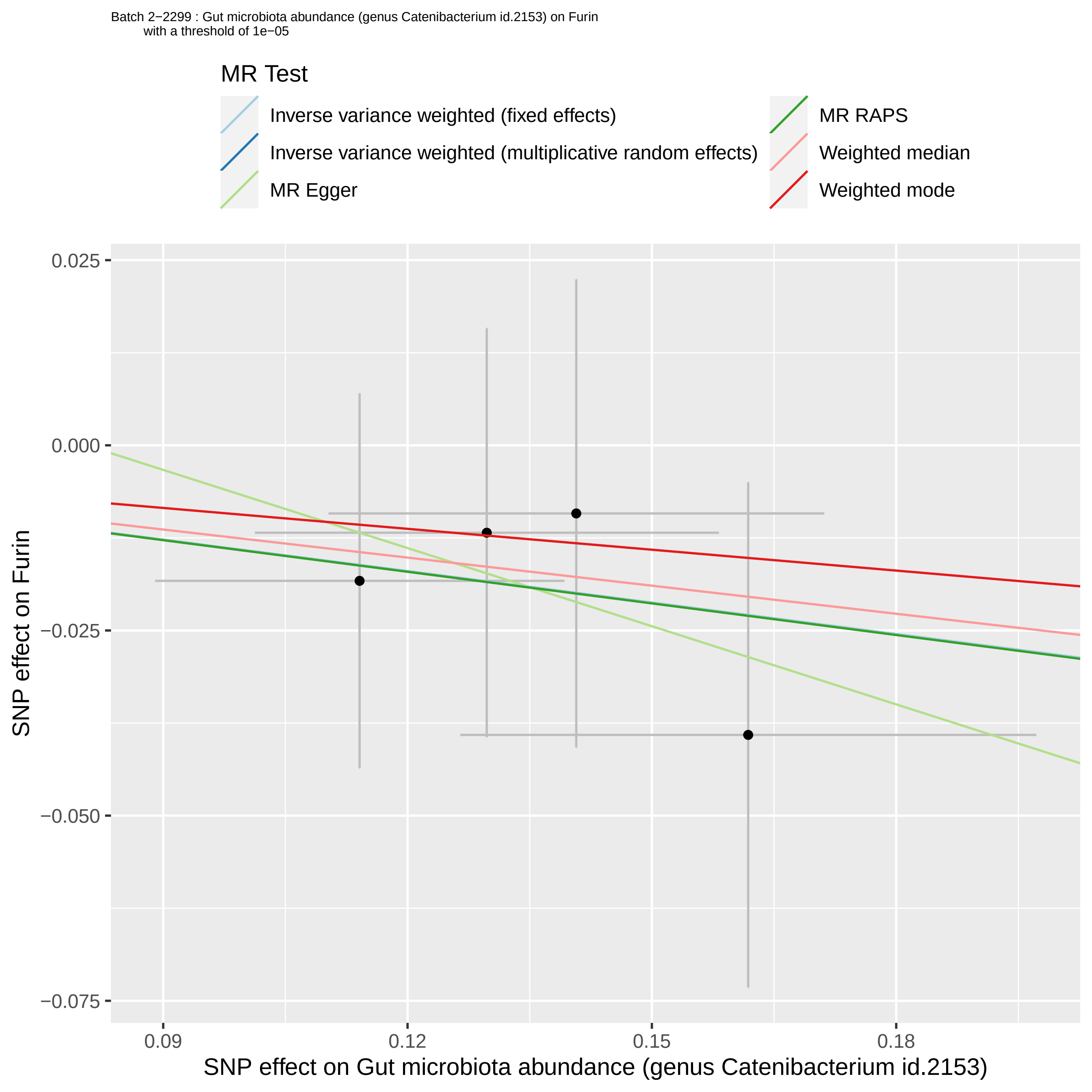

Batch 2−2299 : Gut microbiota abundance (genus Catenibacterium id.2153) on Furin
with a threshold of 1e−05
MR Test
Inverse variance weighted (fixed effects)
Inverse variance weighted (multiplicative random effects)
MR Egger
MR RAPS
Weighted median
Weighted mode
0.025
0.000
−0.025
−0.050
−0.075
SNP effect on Furin
0.09
0.12
0.15
0.18
SNP effect on Gut microbiota abundance (genus Catenibacterium id.2153)

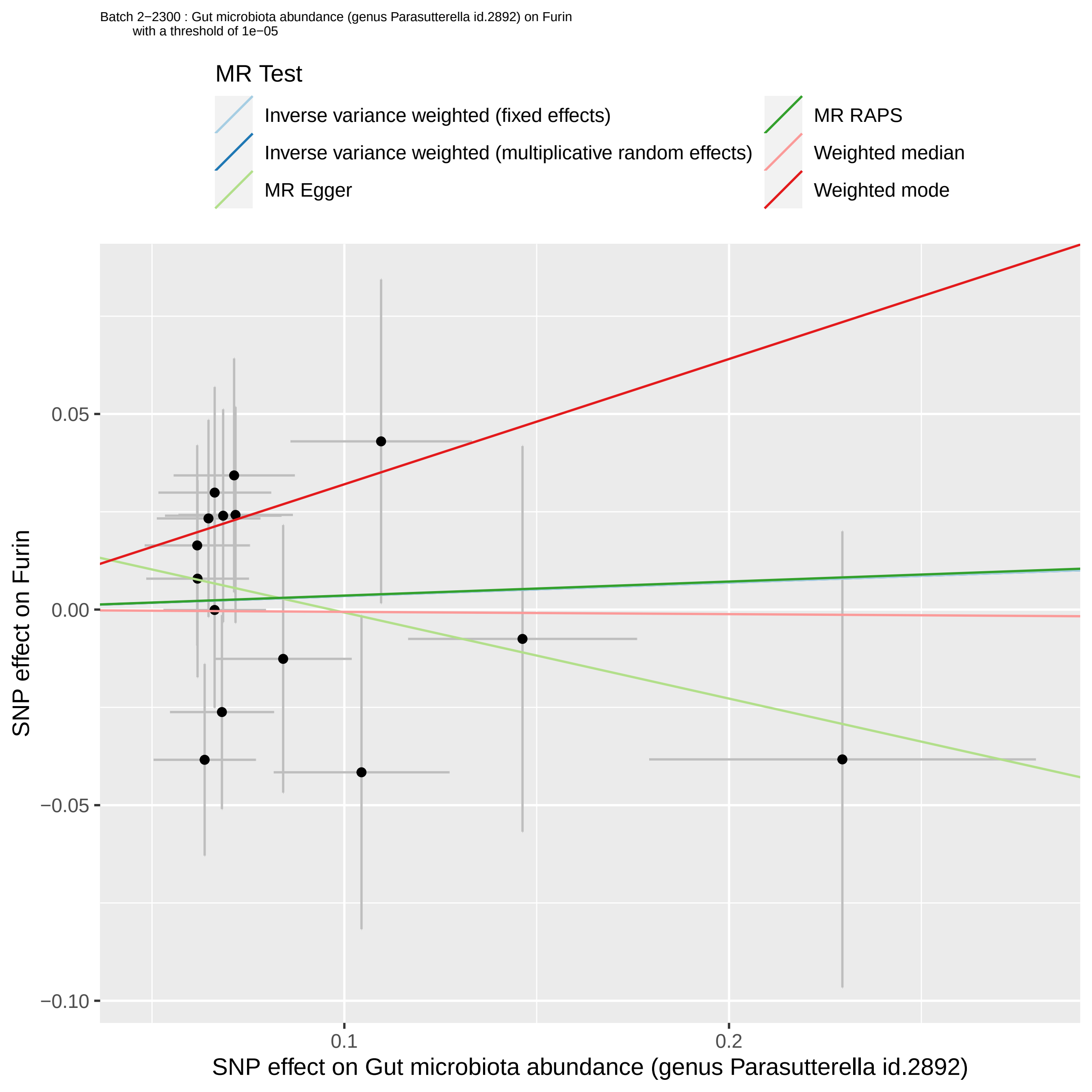

Batch 2−2300 : Gut microbiota abundance (genus Parasutterella id.2892) on Furin
with a threshold of 1e−05
MR Test
Inverse variance weighted (fixed effects)
Inverse variance weighted (multiplicative random effects)
MR Egger
MR RAPS
Weighted median
Weighted mode
0.05
0.00
−0.05
−0.10
0.1
0.2
SNP effect on Furin
SNP effect on Gut microbiota abundance (genus Parasutterella id.2892)

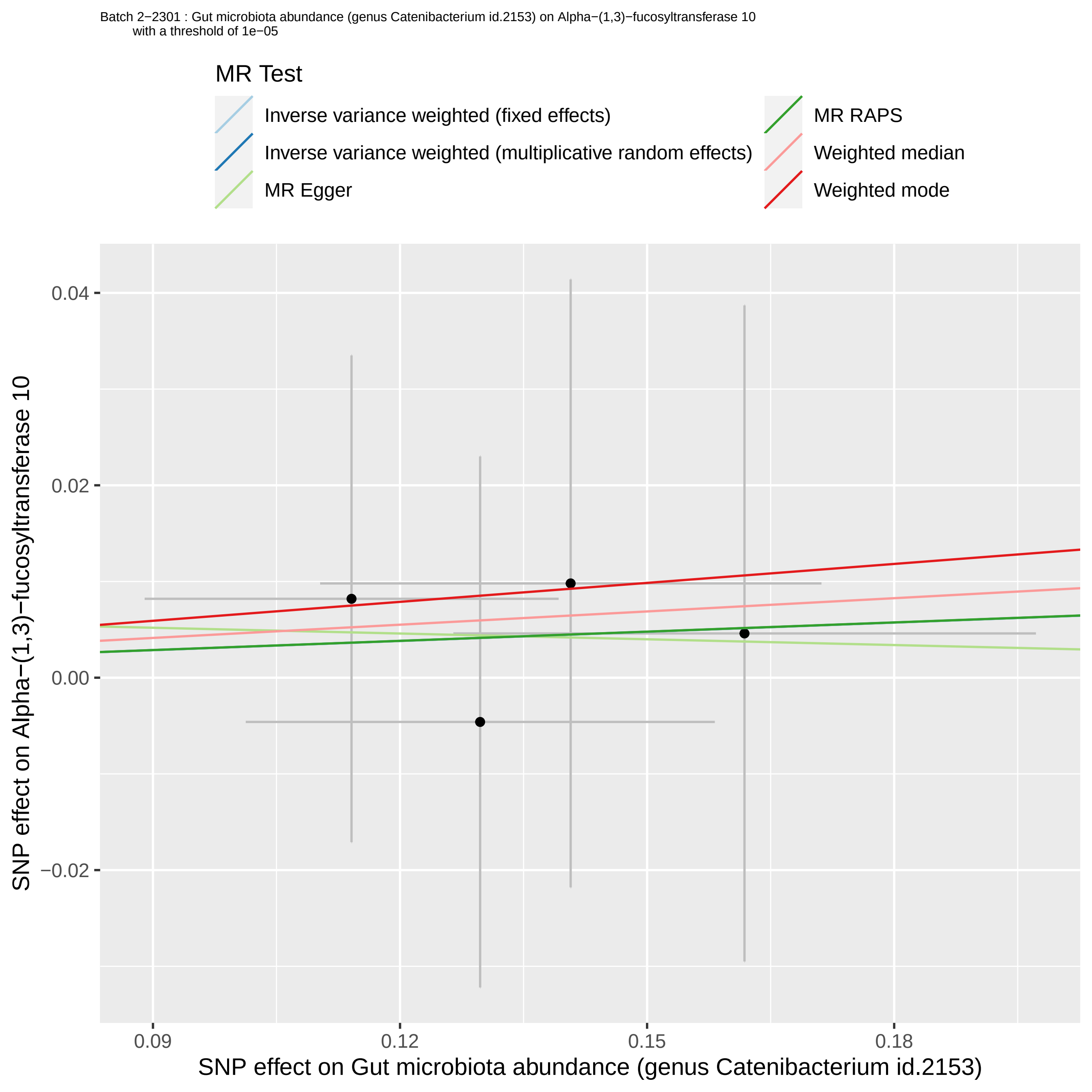

Batch 2−2301 : Gut microbiota abundance (genus Catenibacterium id.2153) on Alpha−(1,3)−fucosyltransferase 10
with a threshold of 1e−05
MR Test
Inverse variance weighted (fixed effects)
Inverse variance weighted (multiplicative random effects)
MR Egger
MR RAPS
Weighted median
Weighted mode
0.04
0.02
0.00
−0.02
0.09
0.12
0.15
0.18
SNP effect on Alpha−(1,3)−fucosyltransferase 10
SNP effect on Gut microbiota abundance (genus Catenibacterium id.2153)

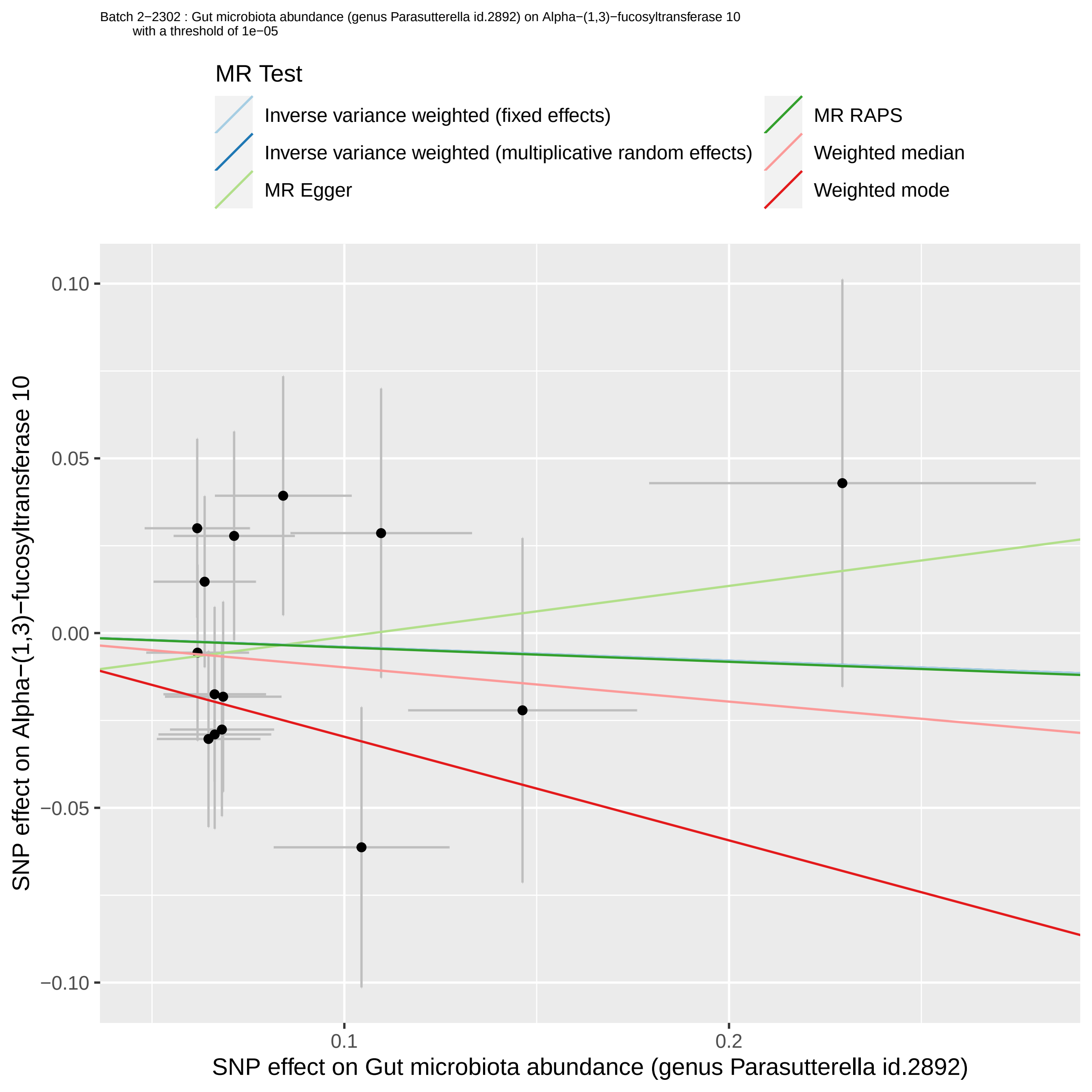
Batch 2−2302 : Gut microbiota abundance (genus Parasutterella id.2892) on Alpha−(1,3)−fucosyltransferase 10
with a threshold of 1e−05
MR Test
Inverse variance weighted (fixed effects)
Inverse variance weighted (multiplicative random effects)
MR Egger
MR RAPS
Weighted median
Weighted mode
0.10
0.05
0.00
−0.05
−0.10
0.1
0.2
SNP effect on Alpha−(1,3)−fucosyltransferase 10
SNP effect on Gut microbiota abundance (genus Parasutterella id.2892)

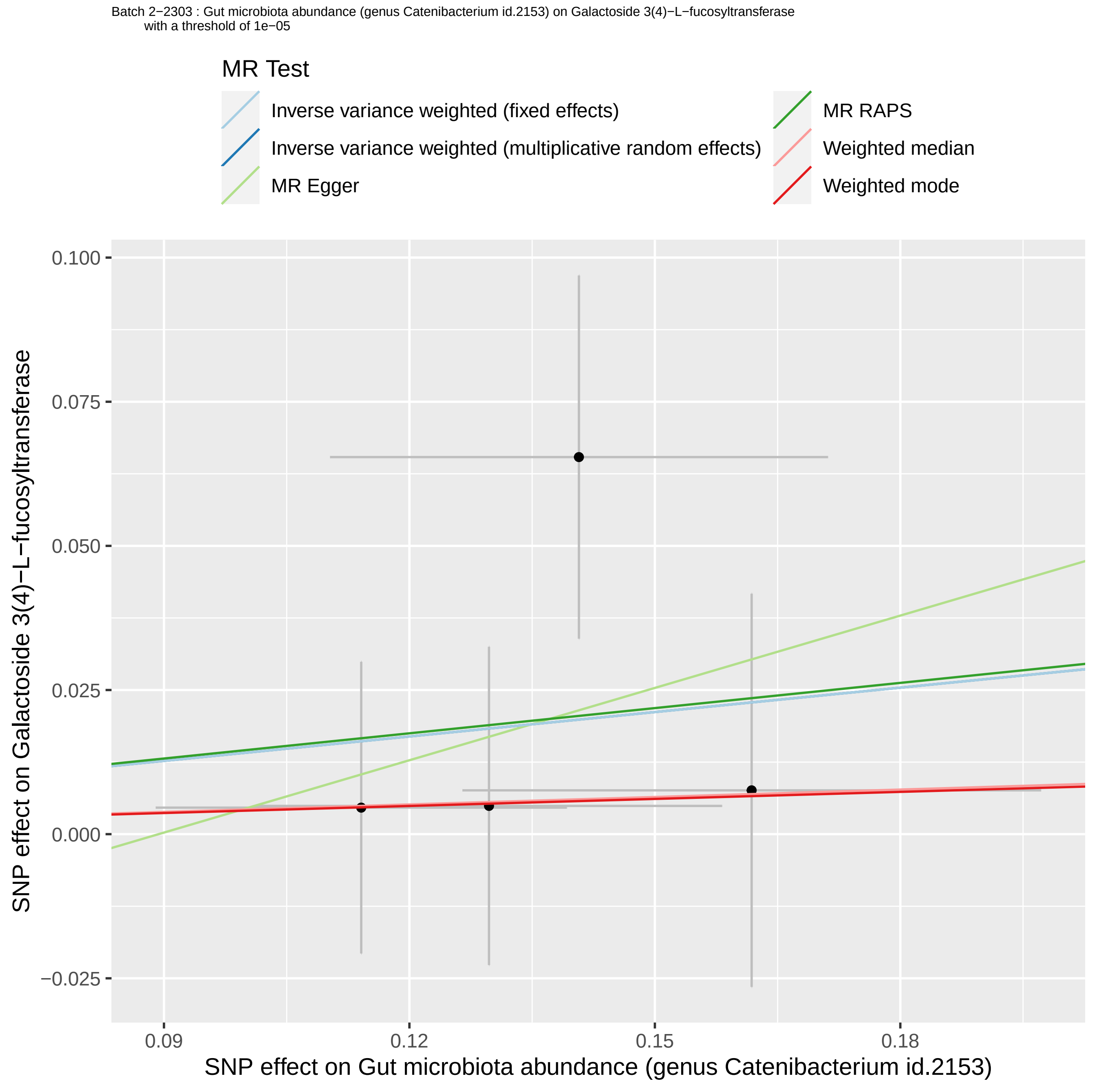

Batch 2−2303 : Gut microbiota abundance (genus Catenibacterium id.2153) on Galactoside 3(4)−L−fucosyltransferase
with a threshold of 1e−05
MR Test
Inverse variance weighted (fixed effects)
Inverse variance weighted (multiplicative random effects)
MR Egger
MR RAPS
Weighted median
Weighted mode
0.100
0.075
0.050
0.025
0.000
−0.025
0.09
0.12
0.15
0.18
SNP effect on Galactoside 3(4)−L−fucosyltransferase
SNP effect on Gut microbiota abundance (genus Catenibacterium id.2153)

Batch 2−2304 : Gut microbiota abundance (genus Parasutterella id.2892) on Galactoside 3(4)−L−fucosyltransferase
with a threshold of 1e−05

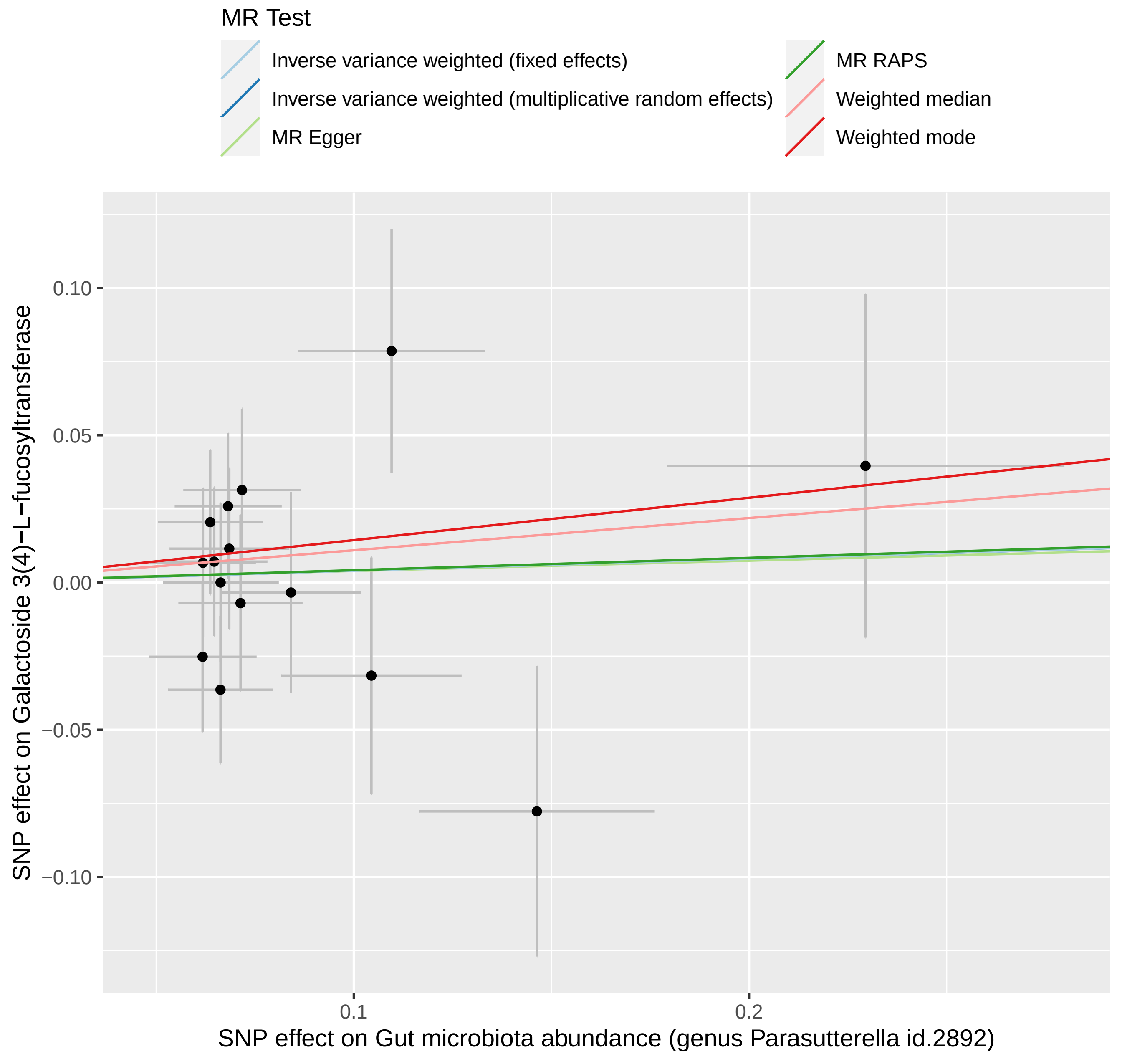

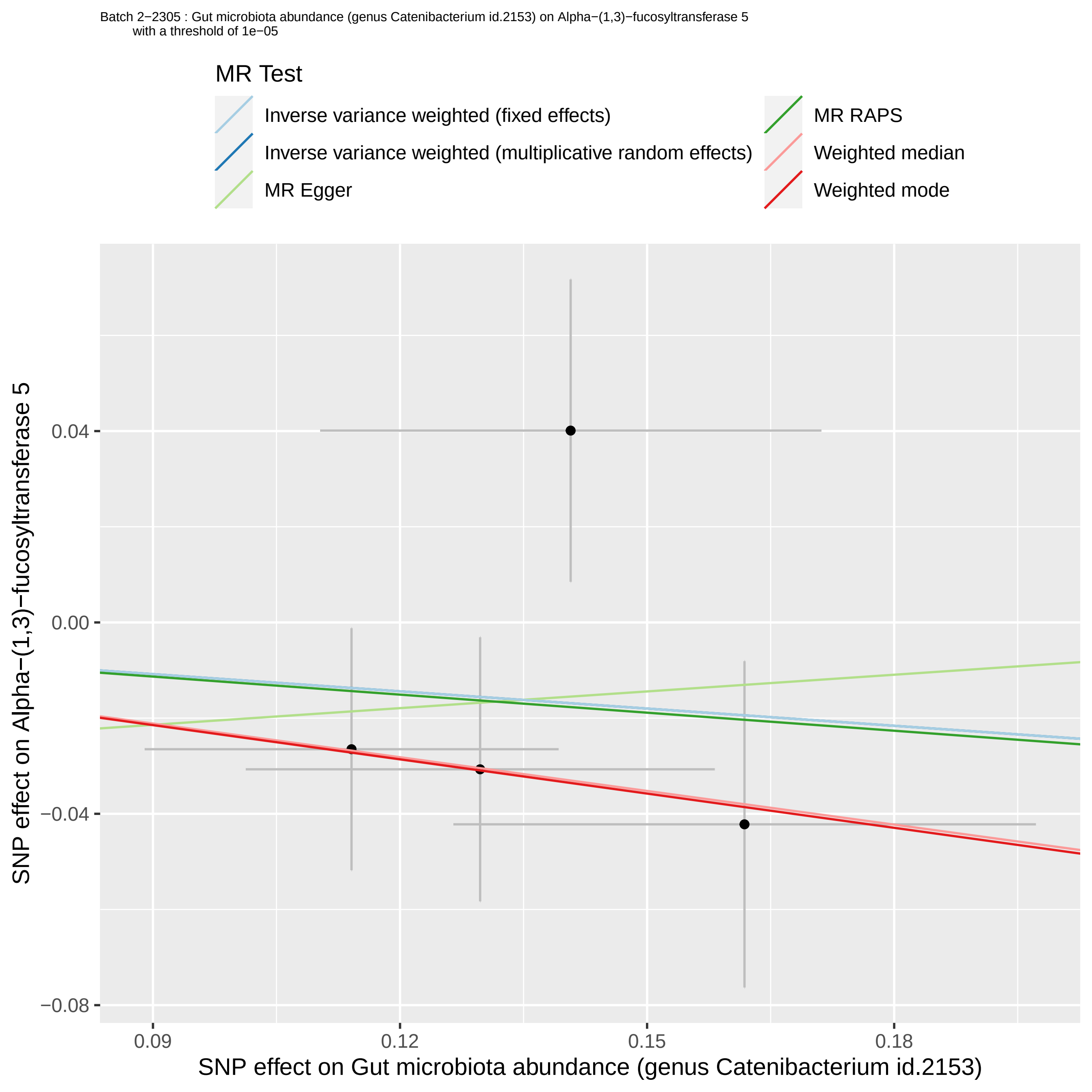
Batch 2−2305 : Gut microbiota abundance (genus Catenibacterium id.2153) on Alpha−(1,3)−fucosyltransferase 5
with a threshold of 1e−05
MR Test
Inverse variance weighted (fixed effects)
Inverse variance weighted (multiplicative random effects)
MR Egger
MR RAPS
Weighted median
Weighted mode
0.04
0.00
−0.04
−0.08
0.09
0.12
0.15
0.18
SNP effect on Gut microbiota abundance (genus Catenibacterium id.2153)
SNP effect on Alpha−(1,3)−fucosyltransferase 5

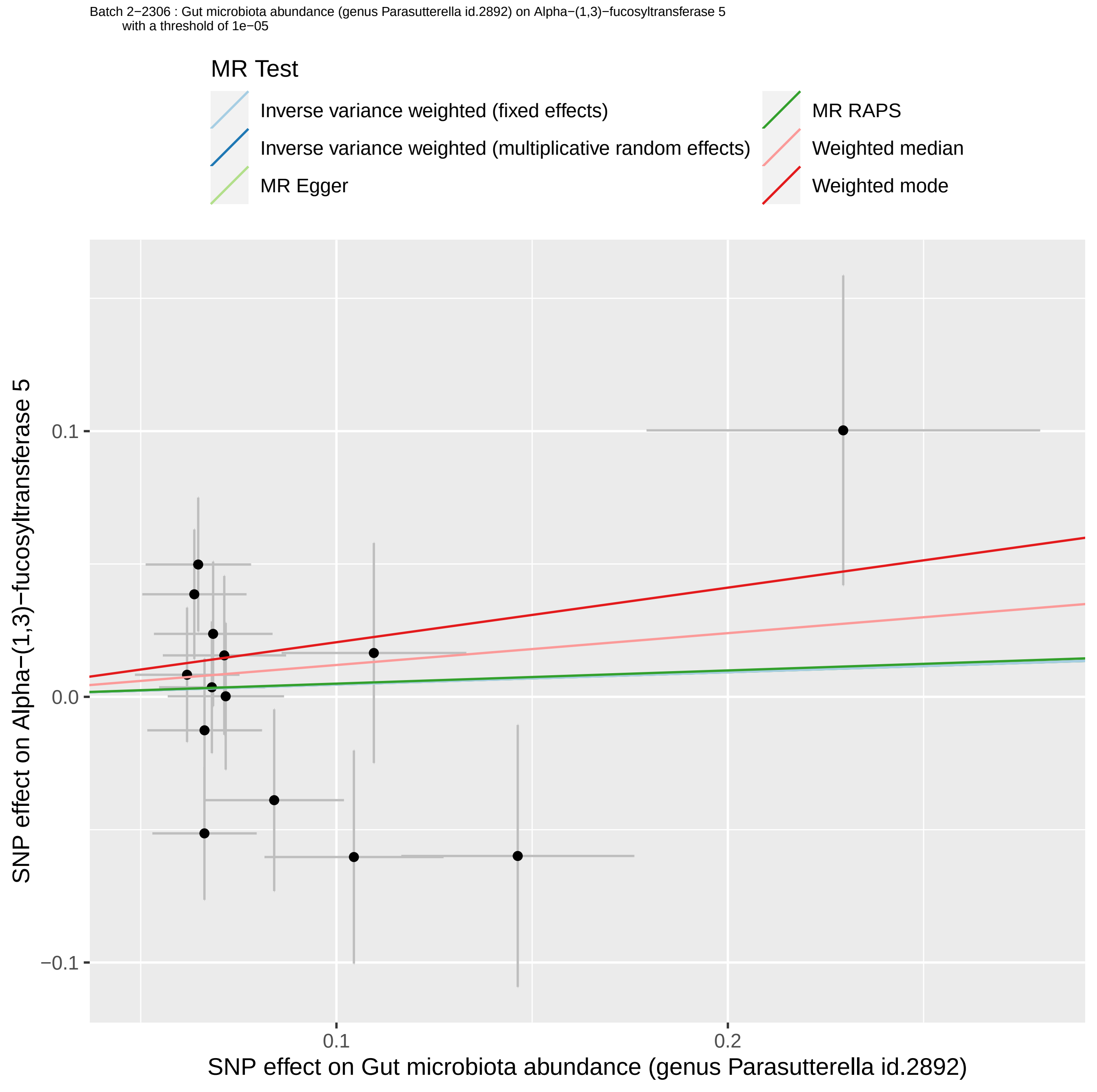

Batch 2−2306 : Gut microbiota abundance (genus Parasutterella id.2892) on Alpha−(1,3)−fucosyltransferase 5
with a threshold of 1e−05
MR Test
Inverse variance weighted (fixed effects)
Inverse variance weighted (multiplicative random effects)
MR Egger
MR RAPS
Weighted median
Weighted mode
SNP effect on Alpha−(1,3)−fucosyltransferase 5
0.1
0.0
−0.1
0.1
0.2
SNP effect on Gut microbiota abundance (genus Parasutterella id.2892)

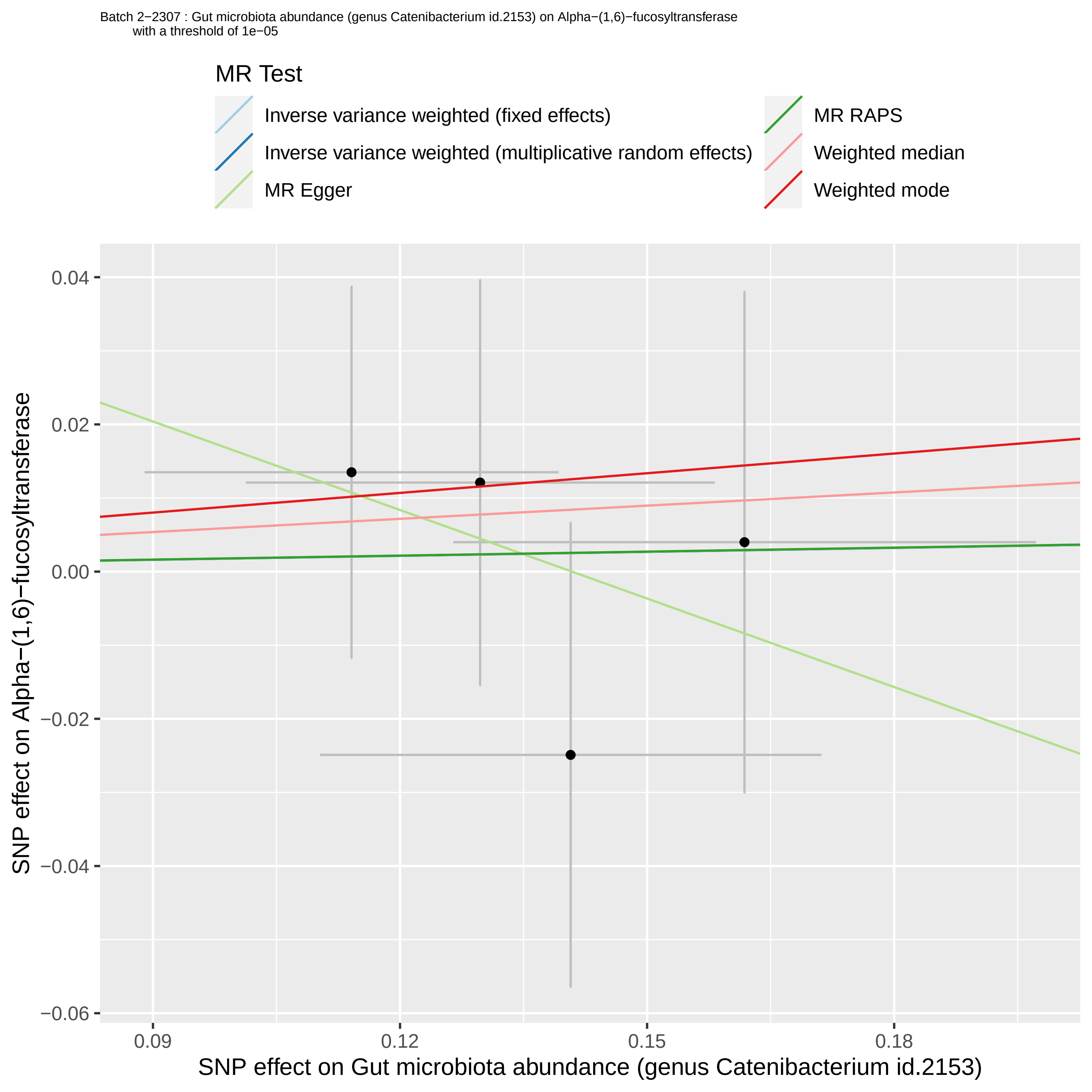
Batch 2−2307 : Gut microbiota abundance (genus Catenibacterium id.2153) on Alpha−(1,6)−fucosyltransferase
with a threshold of 1e−05
MR Test
Inverse variance weighted (fixed effects)
Inverse variance weighted (multiplicative random effects)
MR Egger
MR RAPS
Weighted median
Weighted mode
0.04
0.02
0.00
−0.02
−0.04
−0.06
0.09
0.12
0.15
0.18
SNP effect on Alpha−(1,6)−fucosyltransferase
SNP effect on Gut microbiota abundance (genus Catenibacterium id.2153)

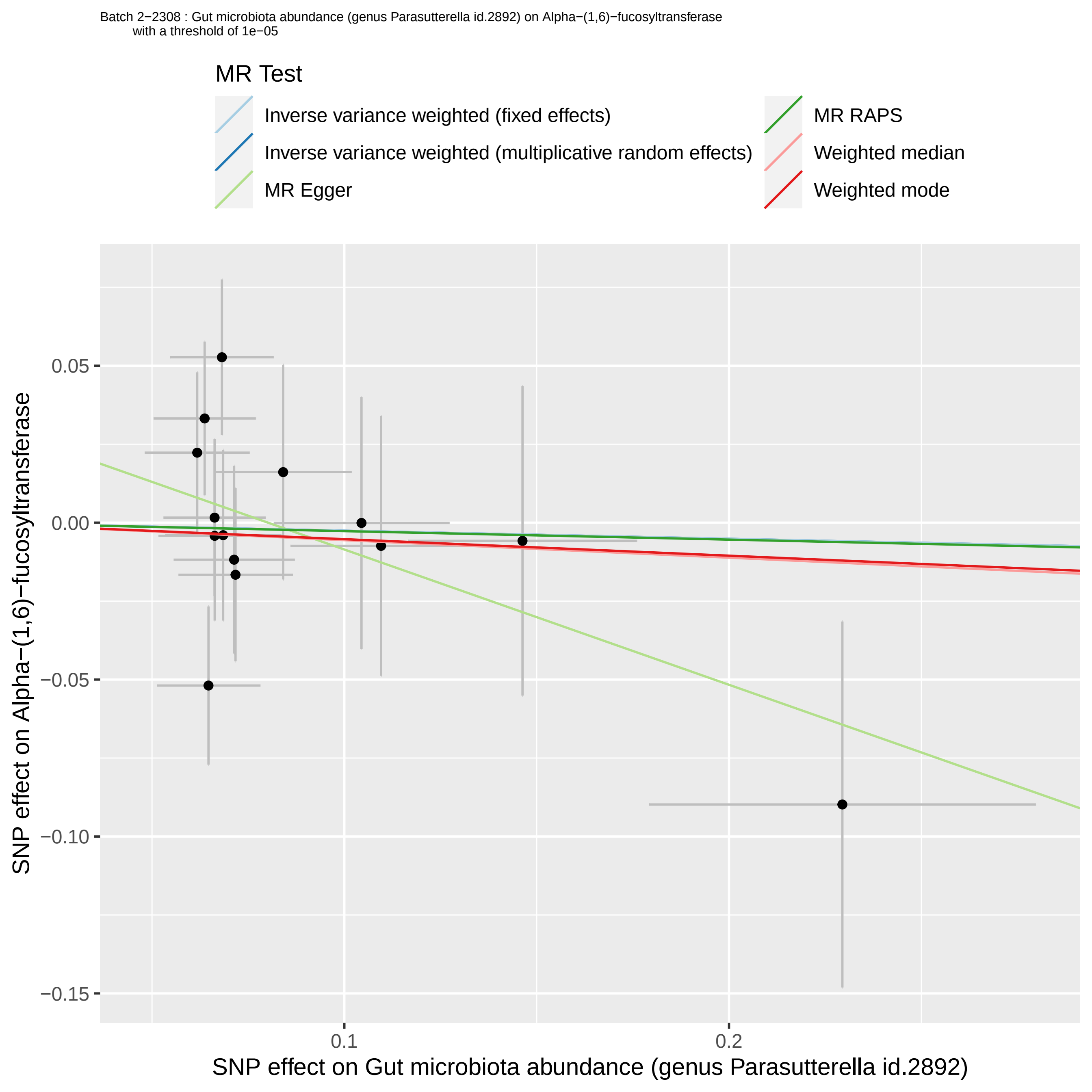
Batch 2−2308 : Gut microbiota abundance (genus Parasutterella id.2892) on Alpha−(1,6)−fucosyltransferase
with a threshold of 1e−05
MR Test
Inverse variance weighted (fixed effects)
Inverse variance weighted (multiplicative random effects)
MR Egger
MR RAPS
Weighted median
Weighted mode
0.05
0.00
−0.05
−0.10
−0.15
0.1
0.2
SNP effect on Alpha−(1,6)−fucosyltransferase
SNP effect on Gut microbiota abundance (genus Parasutterella id.2892)

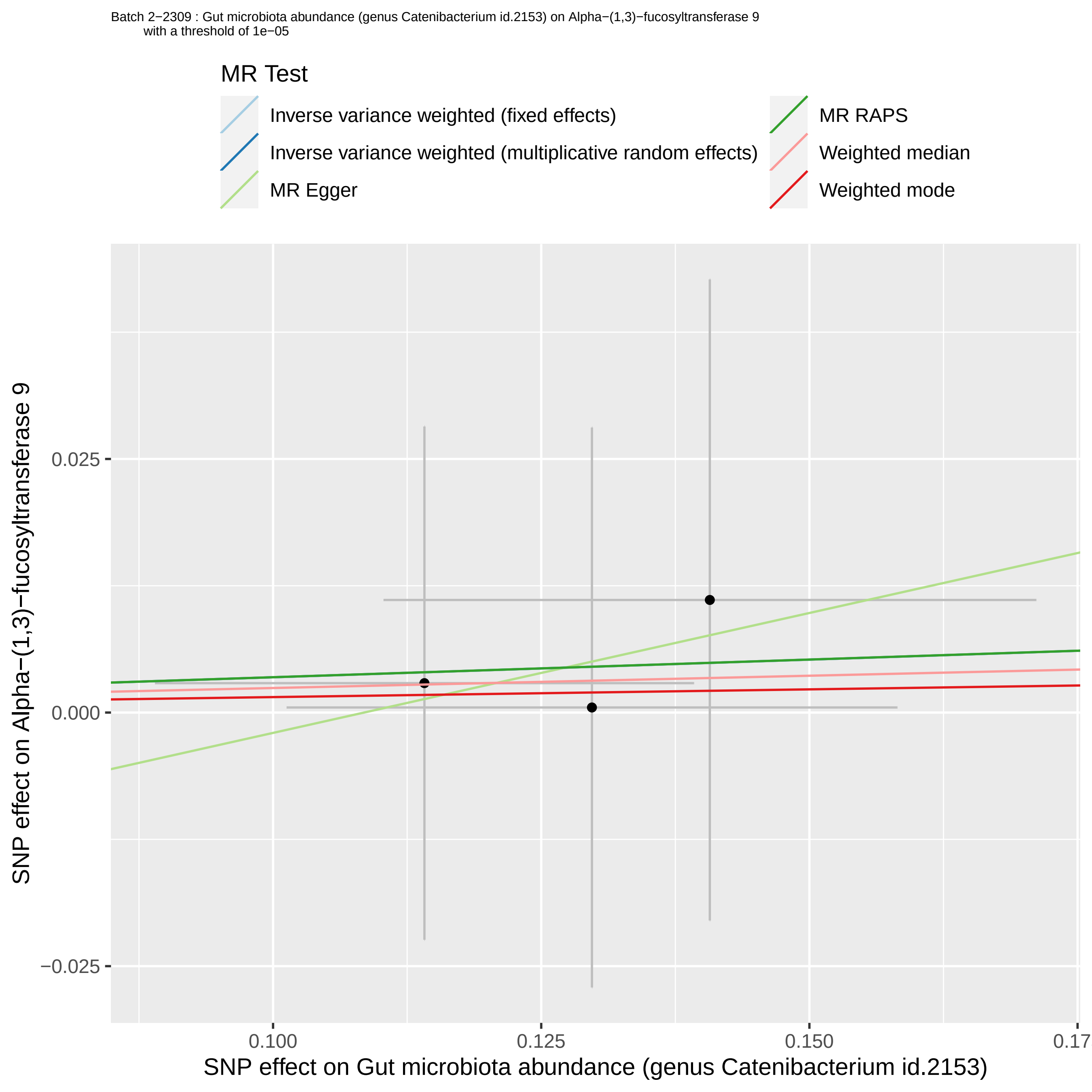
Batch 2−2309 : Gut microbiota abundance (genus Catenibacterium id.2153) on Alpha−(1,3)−fucosyltransferase 9
with a threshold of 1e−05
MR Test
Inverse variance weighted (fixed effects)
Inverse variance weighted (multiplicative random effects)
MR Egger
MR RAPS
Weighted median
Weighted mode
SNP effect on Alpha−(1,3)−fucosyltransferase 9
0.025
0.000
−0.025
0.100
0.125
0.150
0.17
SNP effect on Gut microbiota abundance (genus Catenibacterium id.2153)

Batch 2−2310 : Gut microbiota abundance (genus Parasutterella id.2892) on Alpha−(1,3)−fucosyltransferase 9
with a threshold of 1e−05

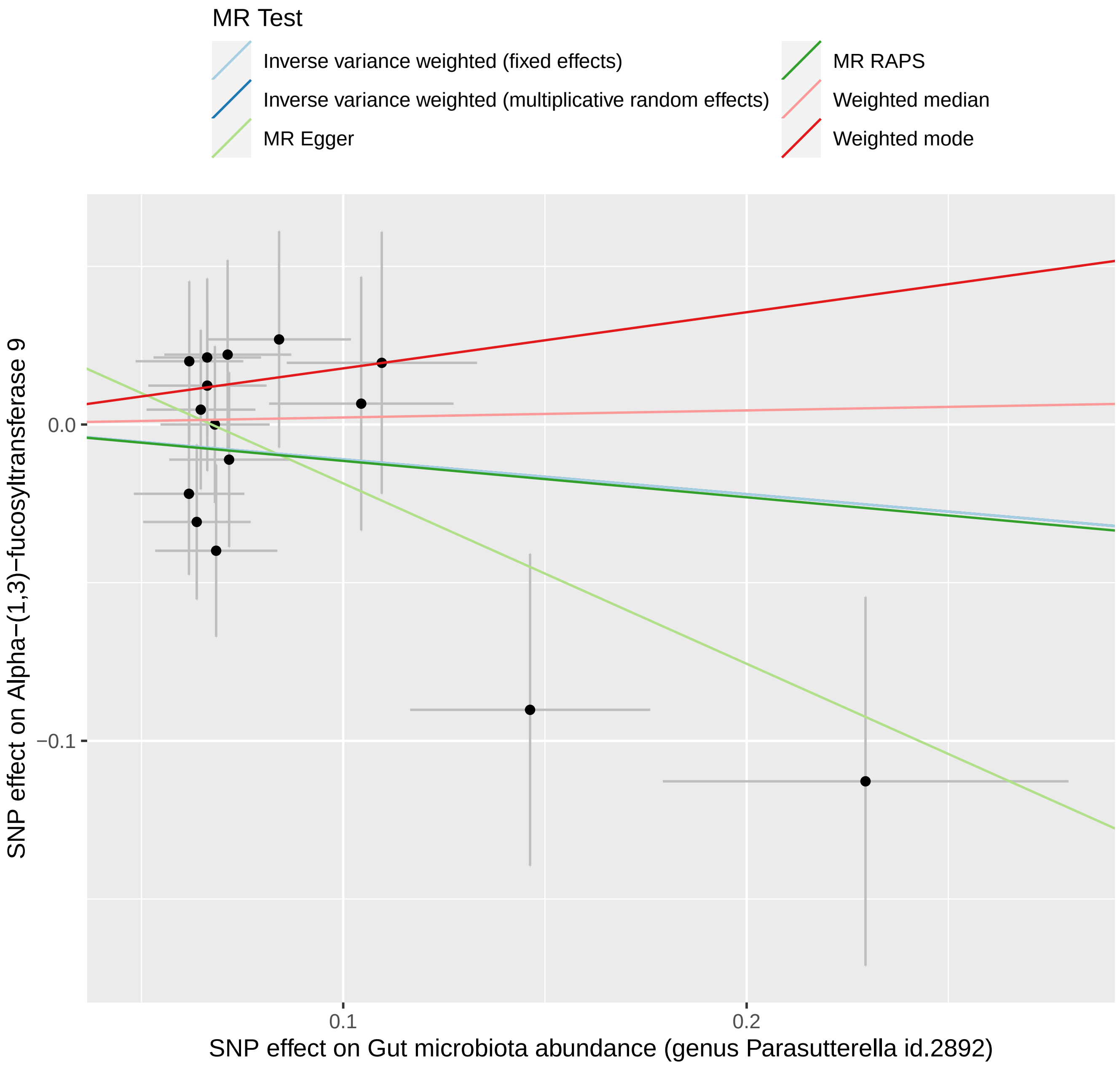

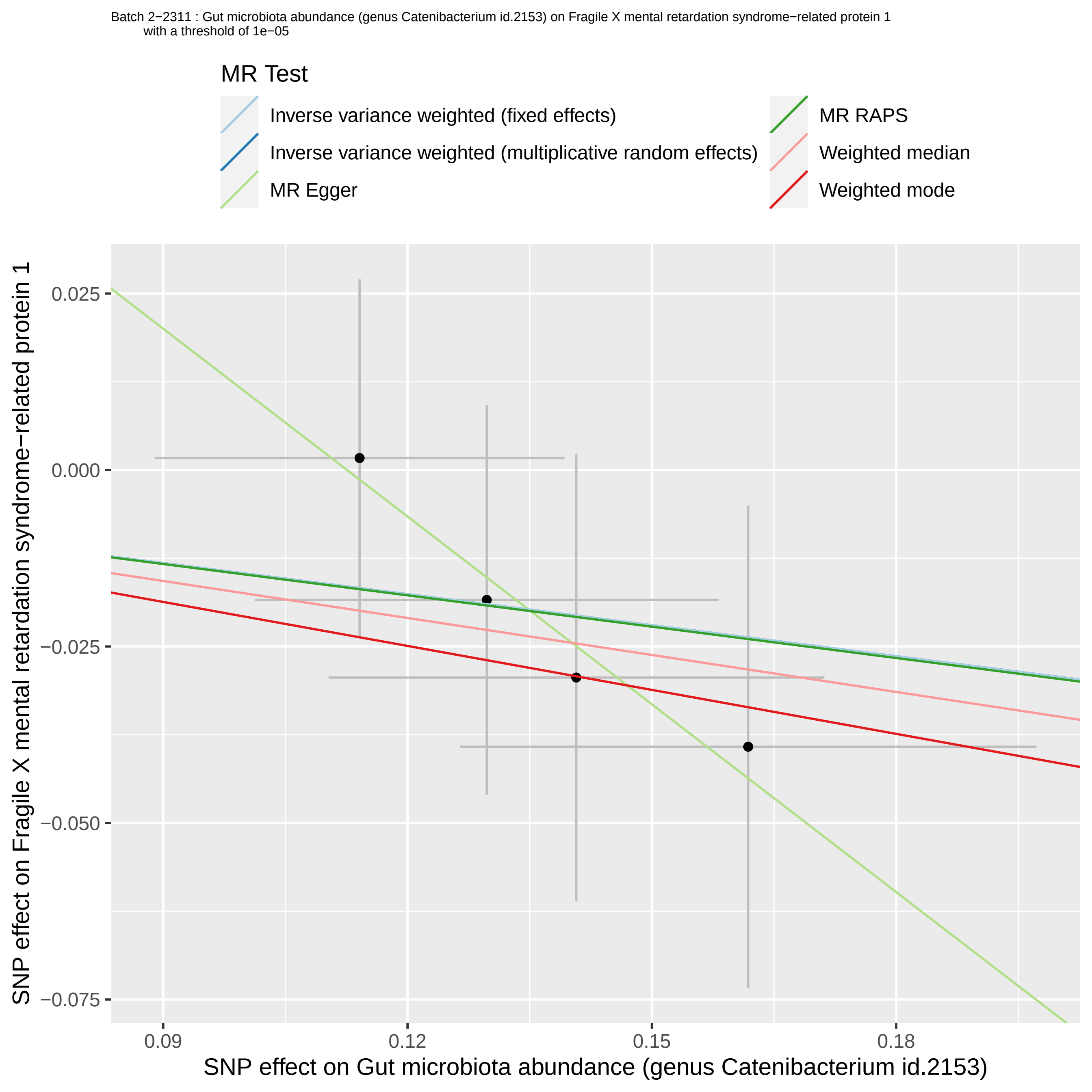
Batch 2−2311 : Gut microbiota abundance (genus Catenibacterium id.2153) on Fragile X mental retardation syndrome−related protein 1 with a threshold of 1e−05
MR Test
Inverse variance weighted (fixed effects)
Inverse variance weighted (multiplicative random effects)
MR Egger
MR RAPS
Weighted median
Weighted mode
SNP effect on Fragile X mental retardation syndrome−related protein 1
0.025
0.000
−0.025
−0.050
−0.075
0.09
0.12
0.15
0.18
SNP effect on Gut microbiota abundance (genus Catenibacterium id.2153)

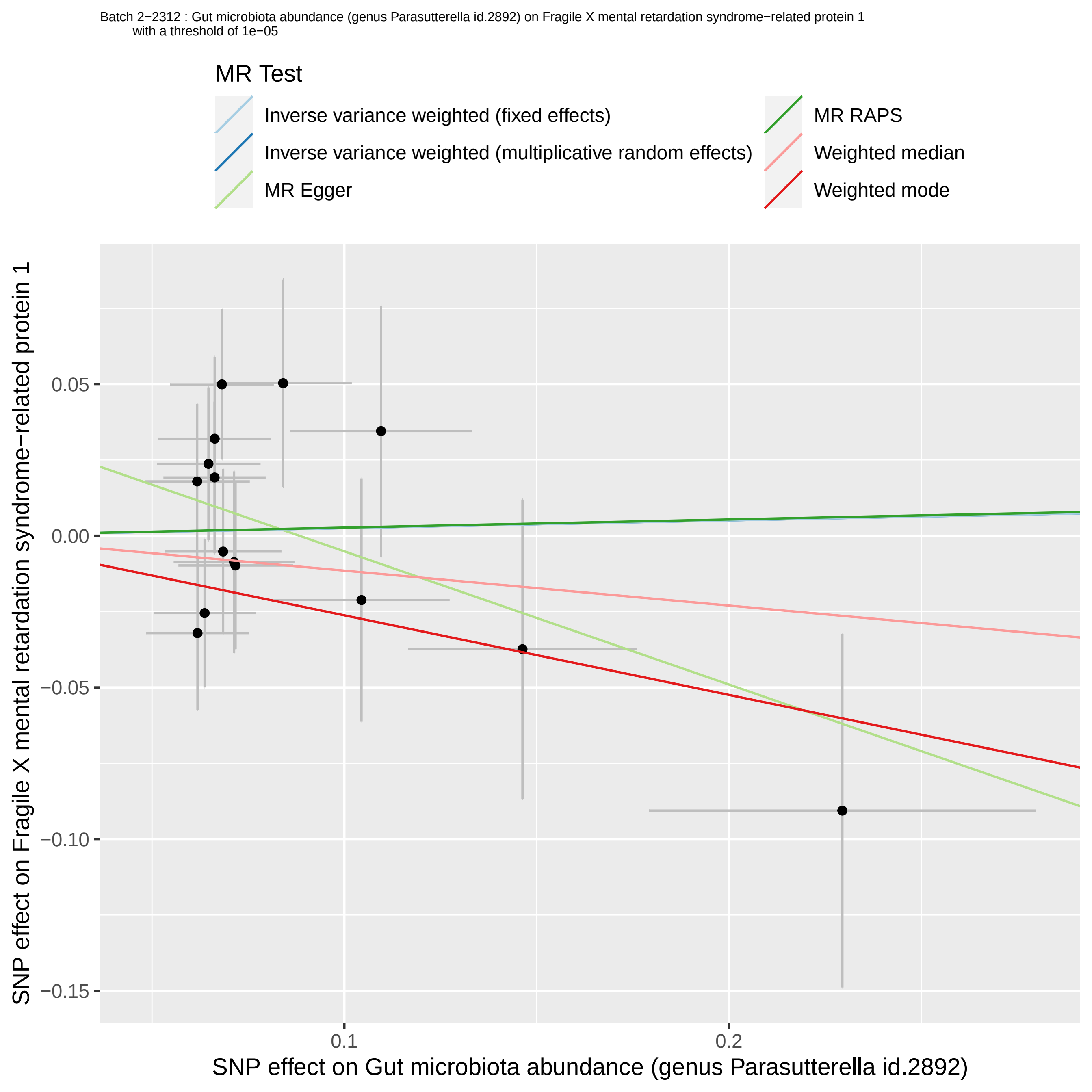
Batch 2−2312 : Gut microbiota abundance (genus Parasutterella id.2892) on Fragile X mental retardation syndrome−related protein 1
with a threshold of 1e−05
MR Test
Inverse variance weighted (fixed effects)
Inverse variance weighted (multiplicative random effects)
MR Egger
MR RAPS
Weighted median
Weighted mode
0.05
0.00
−0.05
−0.10
−0.15
0.1
0.2
SNP effect on Fragile X mental retardation syndrome−related protein 1
SNP effect on Gut microbiota abundance (genus Parasutterella id.2892)

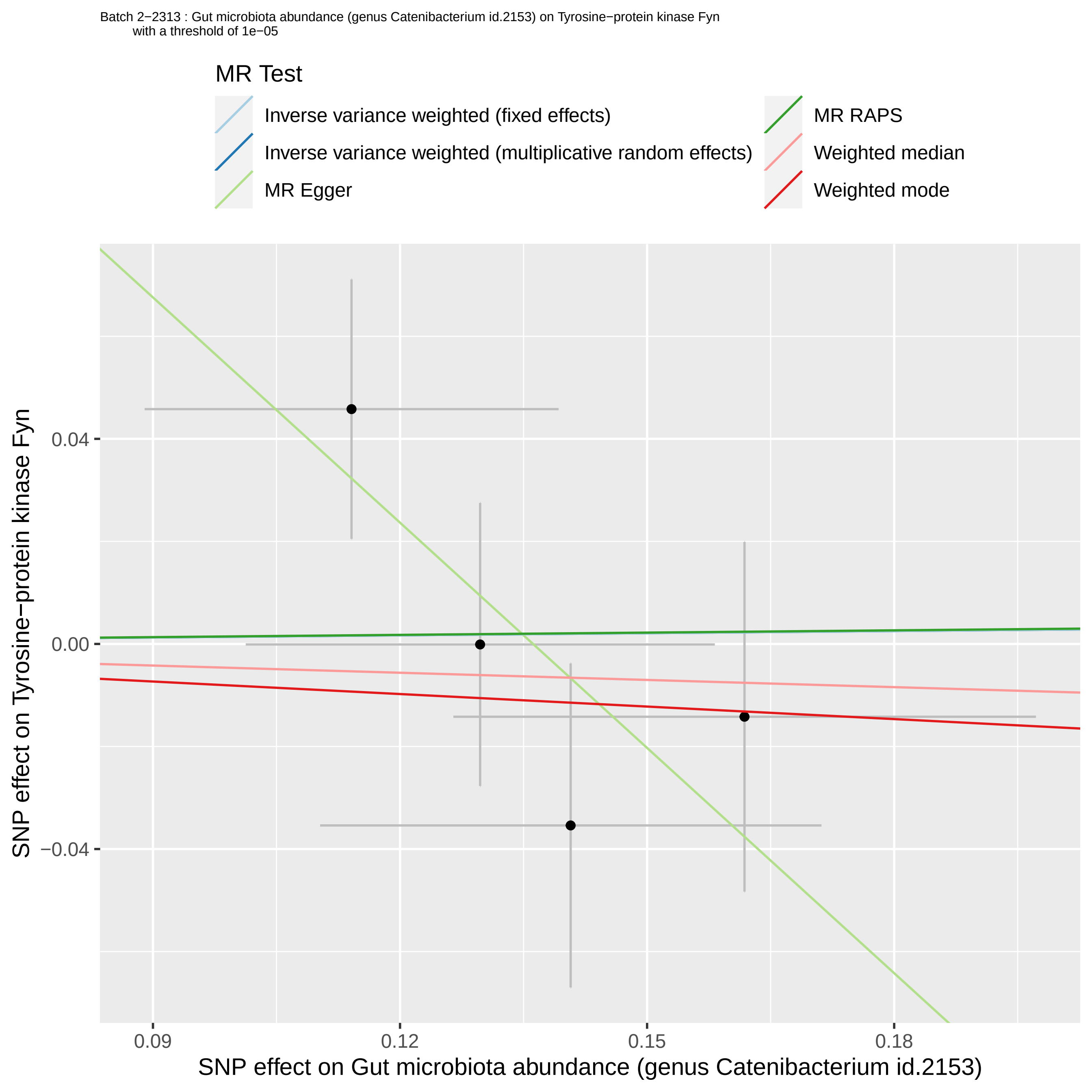
Batch 2−2313 : Gut microbiota abundance (genus Catenibacterium id.2153) on Tyrosine−protein kinase Fyn
with a threshold of 1e−05
MR Test
Inverse variance weighted (fixed effects)
Inverse variance weighted (multiplicative random effects)
MR Egger
MR RAPS
Weighted median
Weighted mode
0.04
0.00
−0.04
0.09
0.12
0.15
0.18
SNP effect on Tyrosine−protein kinase Fyn
SNP effect on Gut microbiota abundance (genus Catenibacterium id.2153)

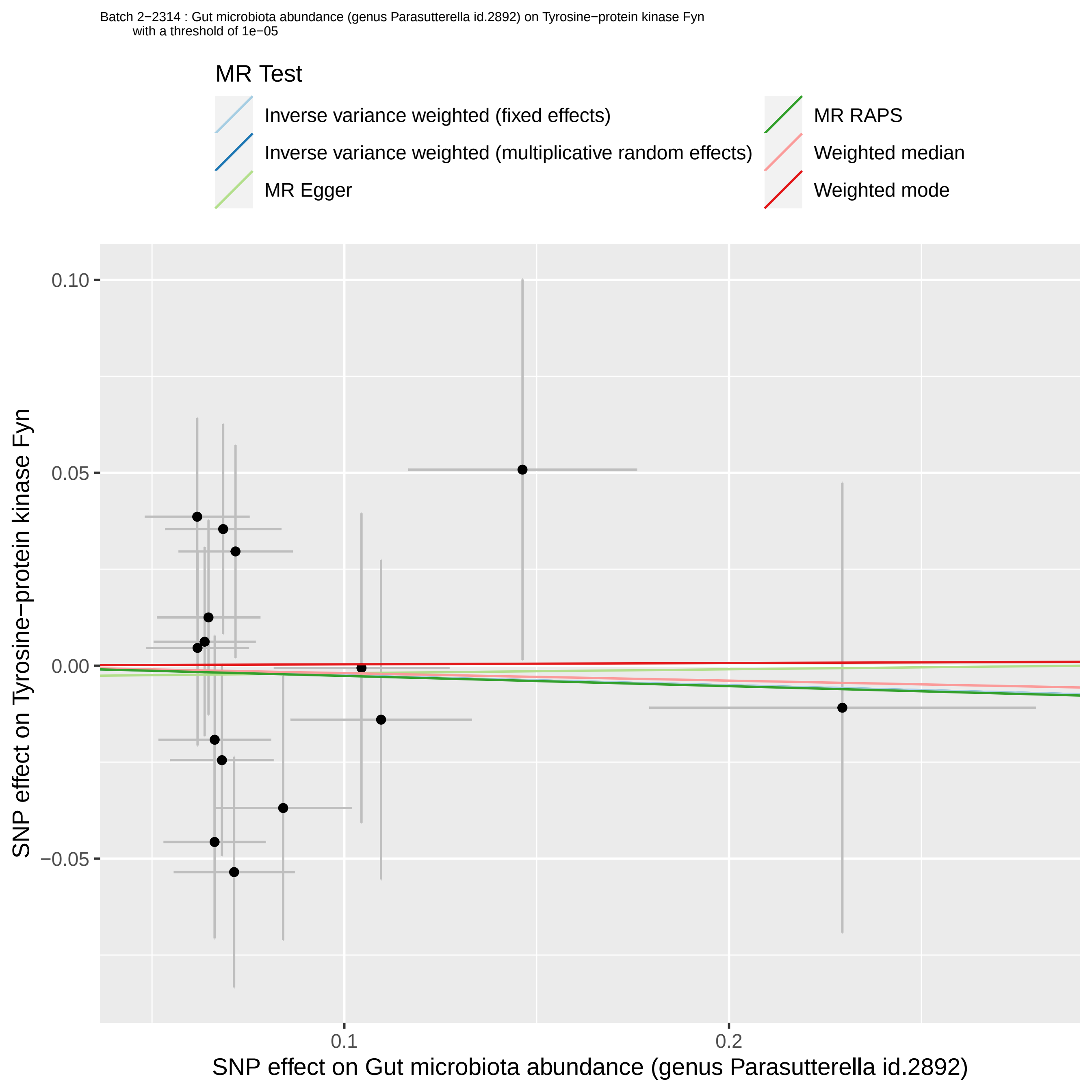

Batch 2−2314 : Gut microbiota abundance (genus Parasutterella id.2892) on Tyrosine−protein kinase Fyn
with a threshold of 1e−05
MR Test
Inverse variance weighted (fixed effects)
Inverse variance weighted (multiplicative random effects)
MR Egger
MR RAPS
Weighted median
Weighted mode
0.10
0.05
0.00
−0.05
0.1
0.2
SNP effect on Tyrosine−protein kinase Fyn
SNP effect on Gut microbiota abundance (genus Parasutterella id.2892)

Batch 2−2315 : Gut microbiota abundance (genus Catenibacterium id.2153) on G0/G1 switch protein 2
with a threshold of 1e−05

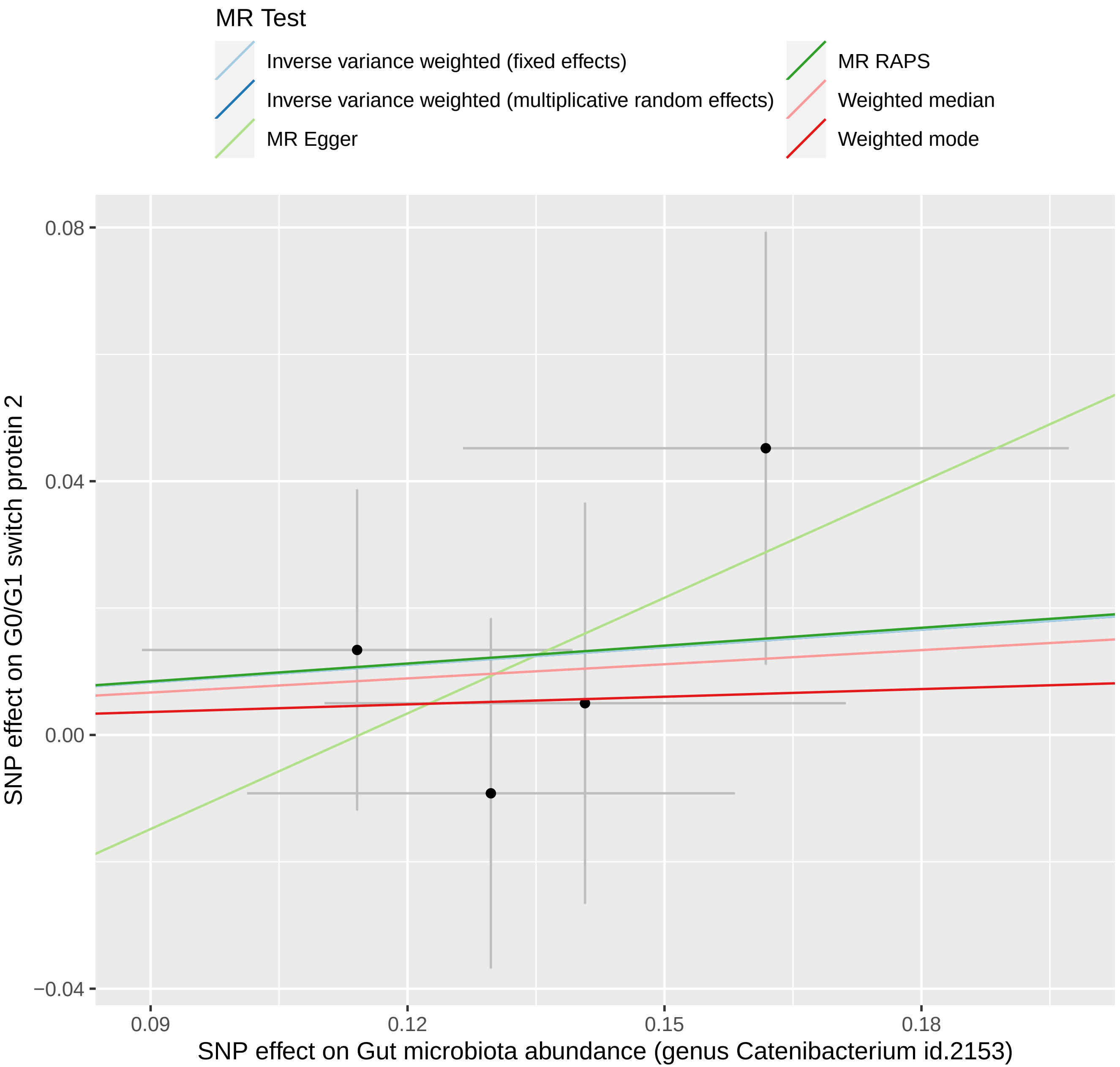

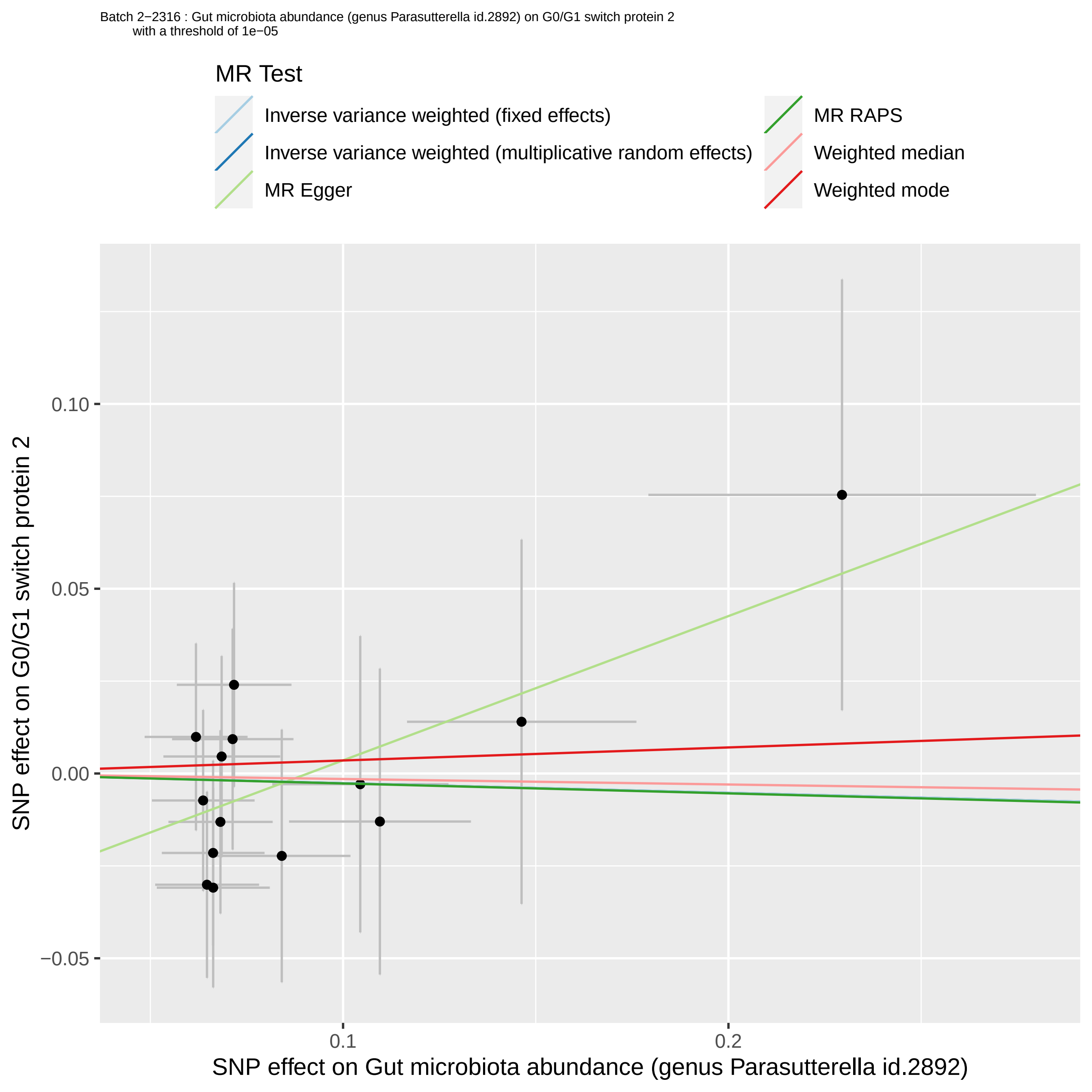

Batch 2−2316 : Gut microbiota abundance (genus Parasutterella id.2892) on G0/G1 switch protein 2
with a threshold of 1e−05
MR Test
Inverse variance weighted (fixed effects)
Inverse variance weighted (multiplicative random effects)
MR Egger
MR RAPS
Weighted median
Weighted mode
SNP effect on G0/G1 switch protein 2
0.10
0.05
0.00
−0.05
0.1
0.2
SNP effect on Gut microbiota abundance (genus Parasutterella id.2892)

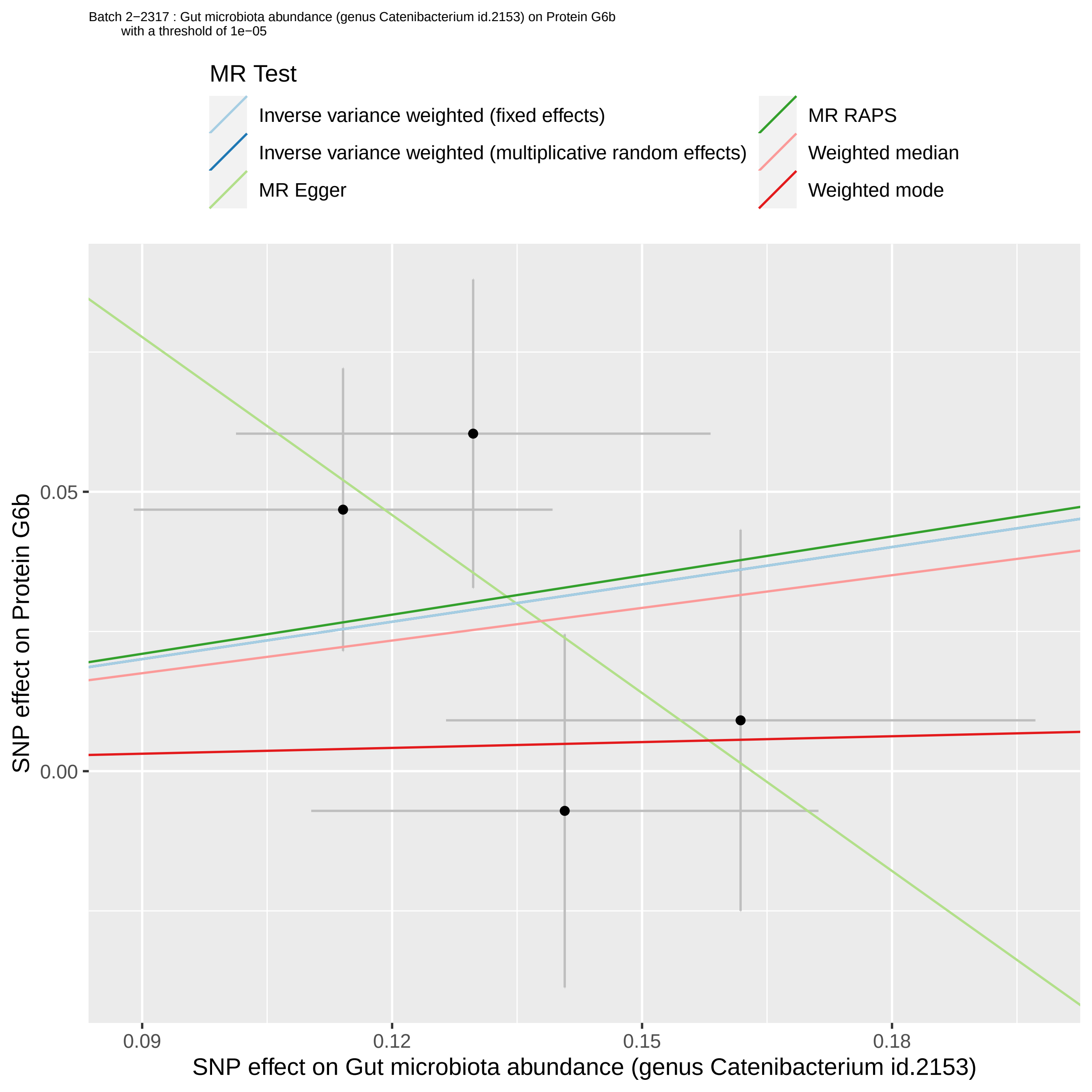
Batch 2−2317 : Gut microbiota abundance (genus Catenibacterium id.2153) on Protein G6b
with a threshold of 1e−05
MR Test
Inverse variance weighted (fixed effects)
Inverse variance weighted (multiplicative random effects)
MR Egger
MR RAPS
Weighted median
Weighted mode
SNP effect on Protein G6b
0.05
0.00
0.09
0.12
0.15
0.18
SNP effect on Gut microbiota abundance (genus Catenibacterium id.2153)

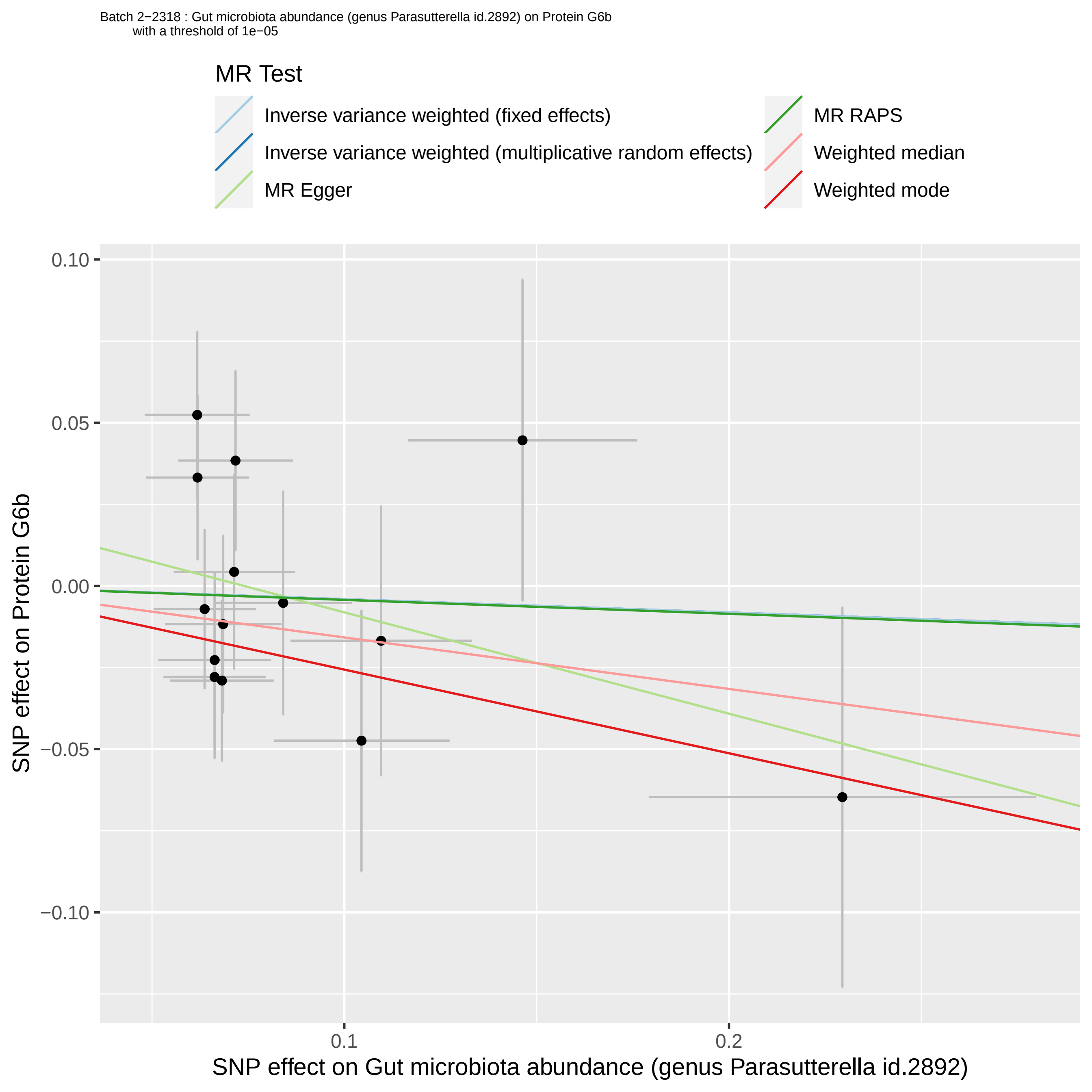

Batch 2−2318 : Gut microbiota abundance (genus Parasutterella id.2892) on Protein G6b
with a threshold of 1e−05
MR Test
Inverse variance weighted (fixed effects)
Inverse variance weighted (multiplicative random effects)
MR Egger
MR RAPS
Weighted median
Weighted mode
0.10
0.05
0.00
−0.05
−0.10
0.1
0.2
SNP effect on Protein G6b
SNP effect on Gut microbiota abundance (genus Parasutterella id.2892)

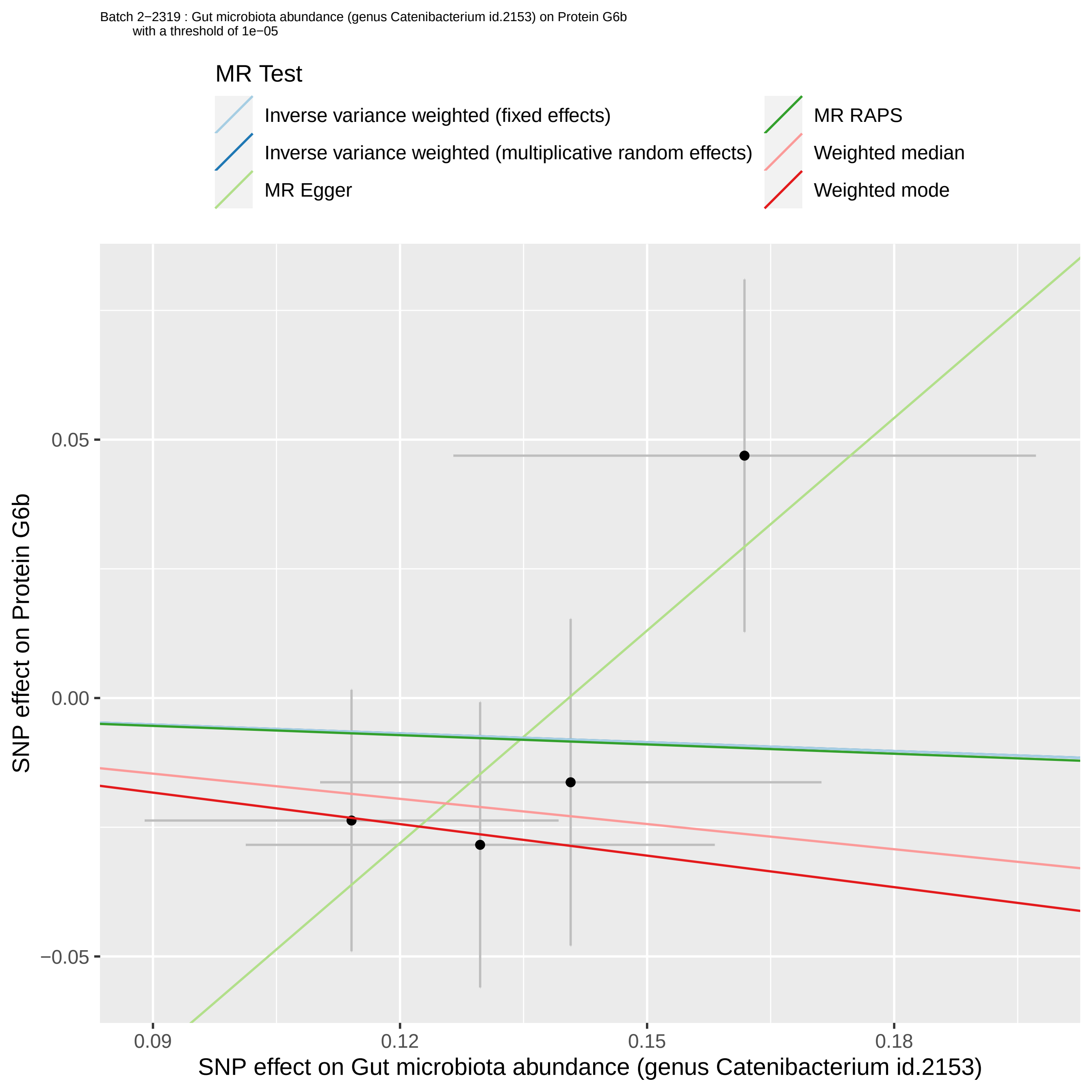
Batch 2−2319 : Gut microbiota abundance (genus Catenibacterium id.2153) on Protein G6b
with a threshold of 1e−05
MR Test
Inverse variance weighted (fixed effects)
Inverse variance weighted (multiplicative random effects)
MR Egger
MR RAPS
Weighted median
Weighted mode
0.05
0.00
−0.05
0.09
0.12
0.15
0.18
SNP effect on Protein G6b
SNP effect on Gut microbiota abundance (genus Catenibacterium id.2153)

Batch 2−2320 : Gut microbiota abundance (genus Parasutterella id.2892) on Protein G6b
with a threshold of 1e−05

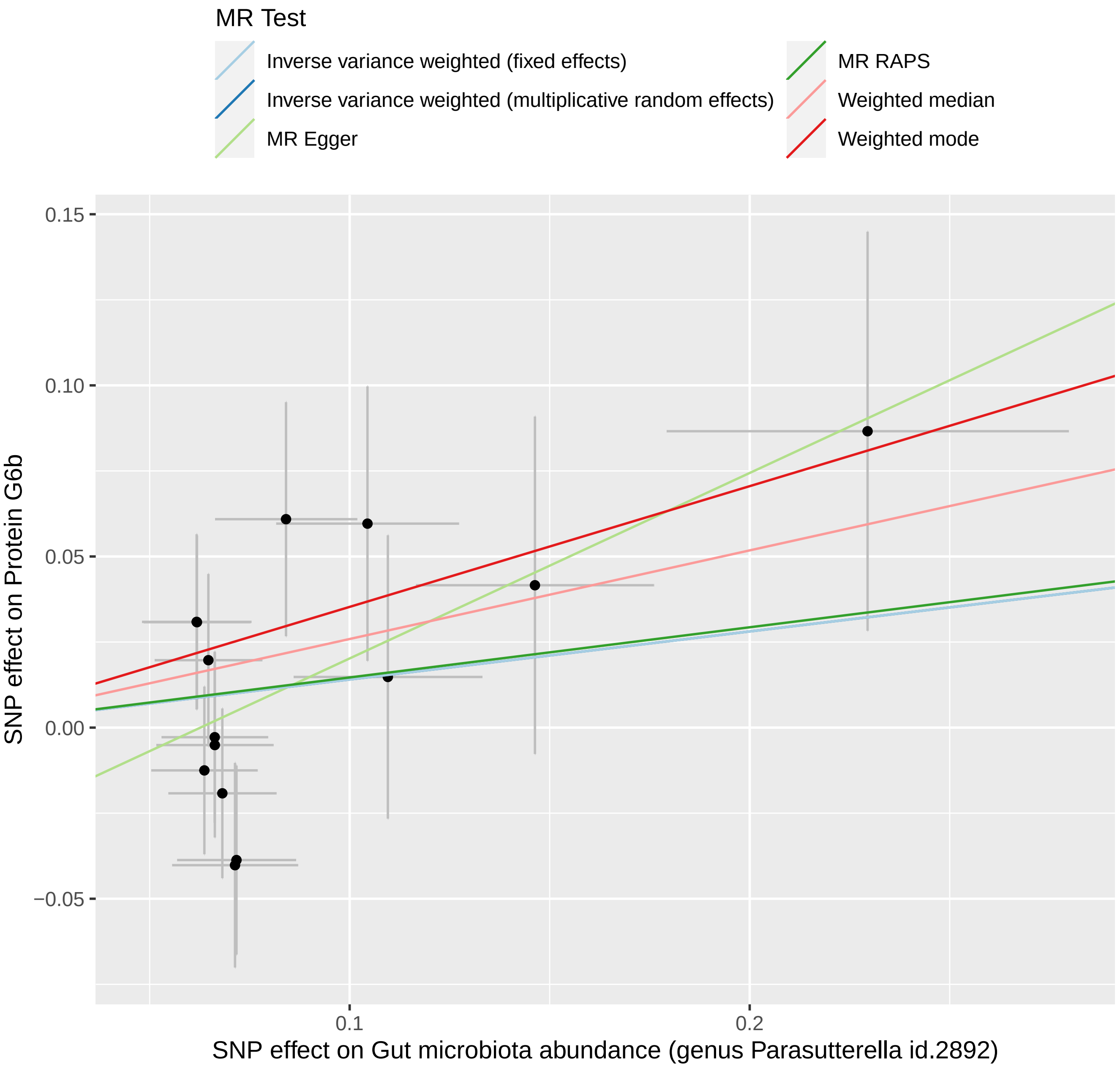

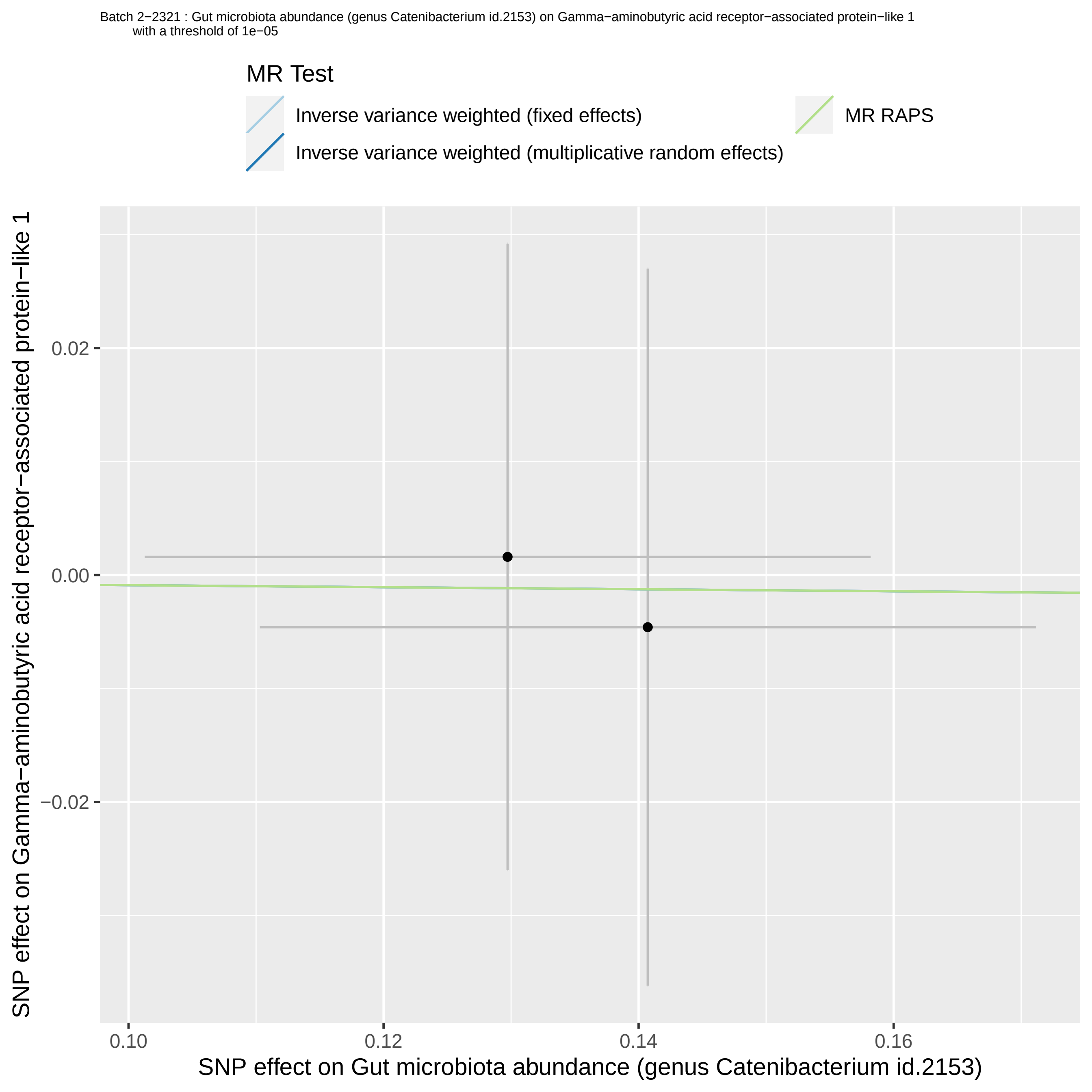
Batch 2−2321 : Gut microbiota abundance (genus Catenibacterium id.2153) on Gamma−aminobutyric acid receptor−associated protein−like 1
with a threshold of 1e−05
MR Test
Inverse variance weighted (fixed effects)
Inverse variance weighted (multiplicative random effects)
MR RAPS
SNP effect on Gamma−aminobutyric acid receptor−associated protein−like 1
0.02
0.00
−0.02
0.10
0.12
0.14
0.16
SNP effect on Gut microbiota abundance (genus Catenibacterium id.2153)

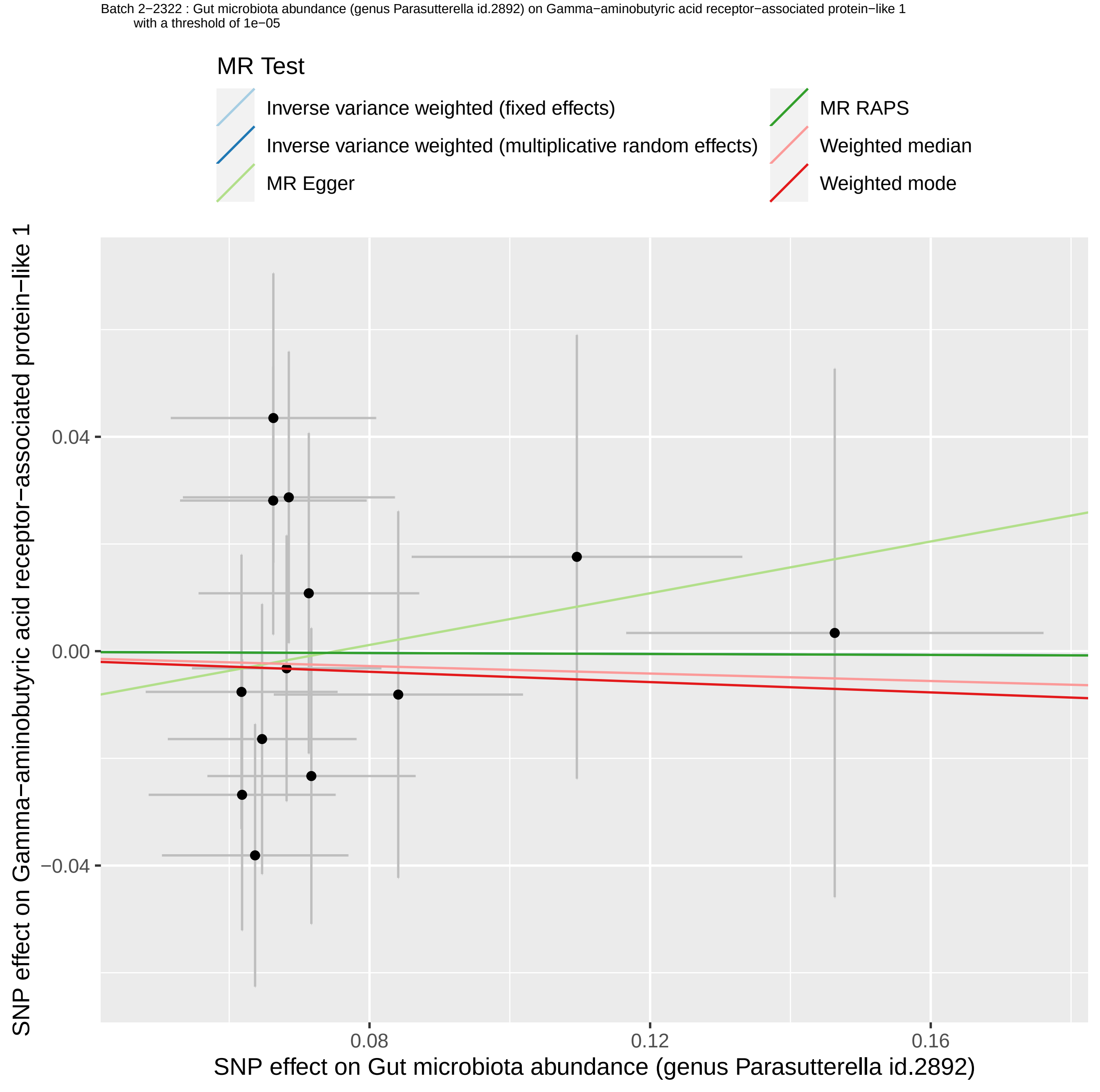

Batch 2−2322 : Gut microbiota abundance (genus Parasutterella id.2892) on Gamma−aminobutyric acid receptor−associated protein−like 1
with a threshold of 1e−05
MR Test
Inverse variance weighted (fixed effects)
Inverse variance weighted (multiplicative random effects)
MR Egger
MR RAPS
Weighted median
Weighted mode
0.04
0.00
−0.04
0.08
0.12
0.16
SNP effect on Gamma−aminobutyric acid receptor−associated protein−like 1
SNP effect on Gut microbiota abundance (genus Parasutterella id.2892)

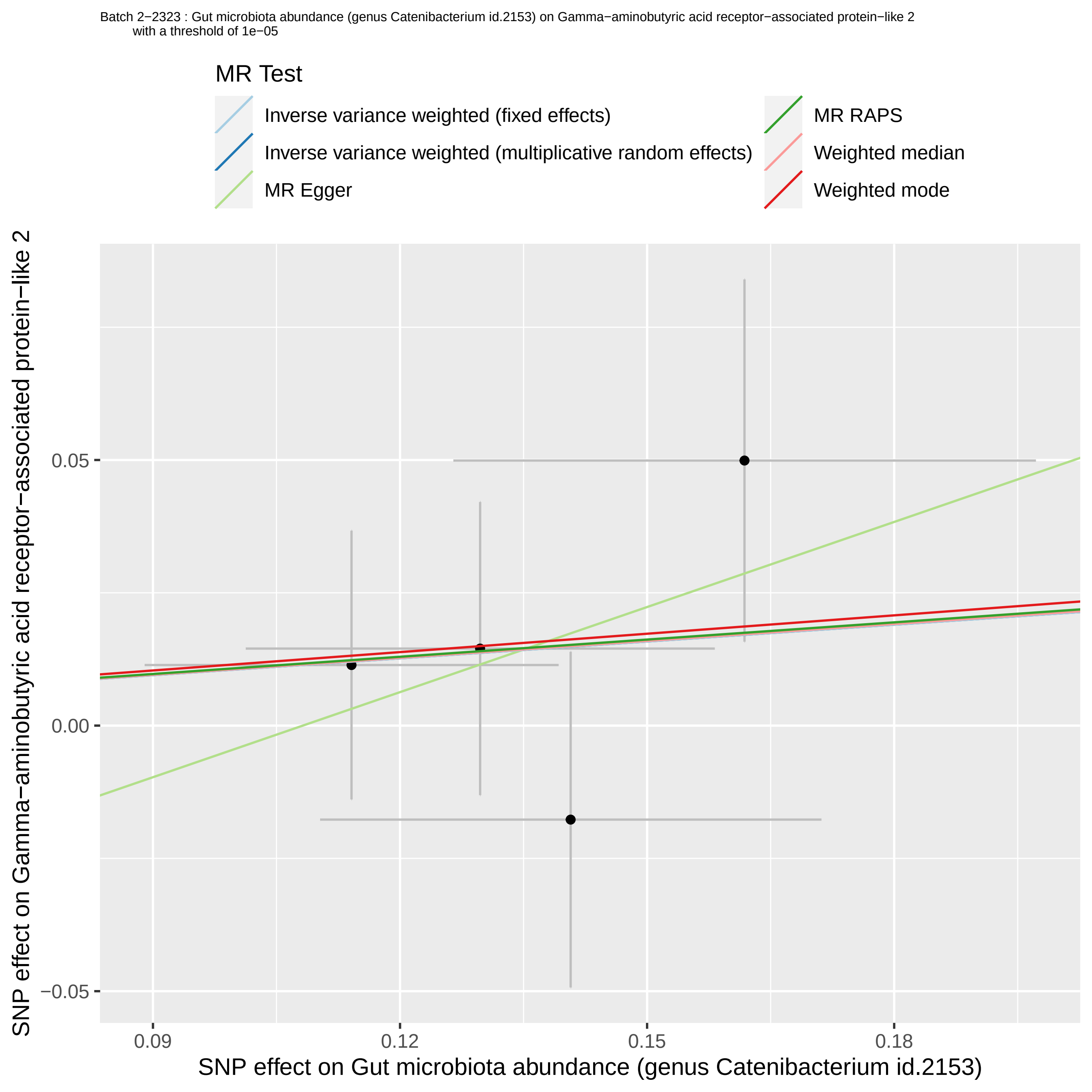
Batch 2−2323 : Gut microbiota abundance (genus Catenibacterium id.2153) on Gamma−aminobutyric acid receptor−associated protein−like 2
with a threshold of 1e−05
MR Test
Inverse variance weighted (fixed effects)
Inverse variance weighted (multiplicative random effects)
MR Egger
MR RAPS
Weighted median
Weighted mode
0.05
0.00
−0.05
0.09
0.12
0.15
0.18
SNP effect on Gamma−aminobutyric acid receptor−associated protein−like 2
SNP effect on Gut microbiota abundance (genus Catenibacterium id.2153)

Batch 2−2324 : Gut microbiota abundance (genus Parasutterella id.2892) on Gamma−aminobutyric acid receptor−associated protein−like 2 with a threshold of 1e−05

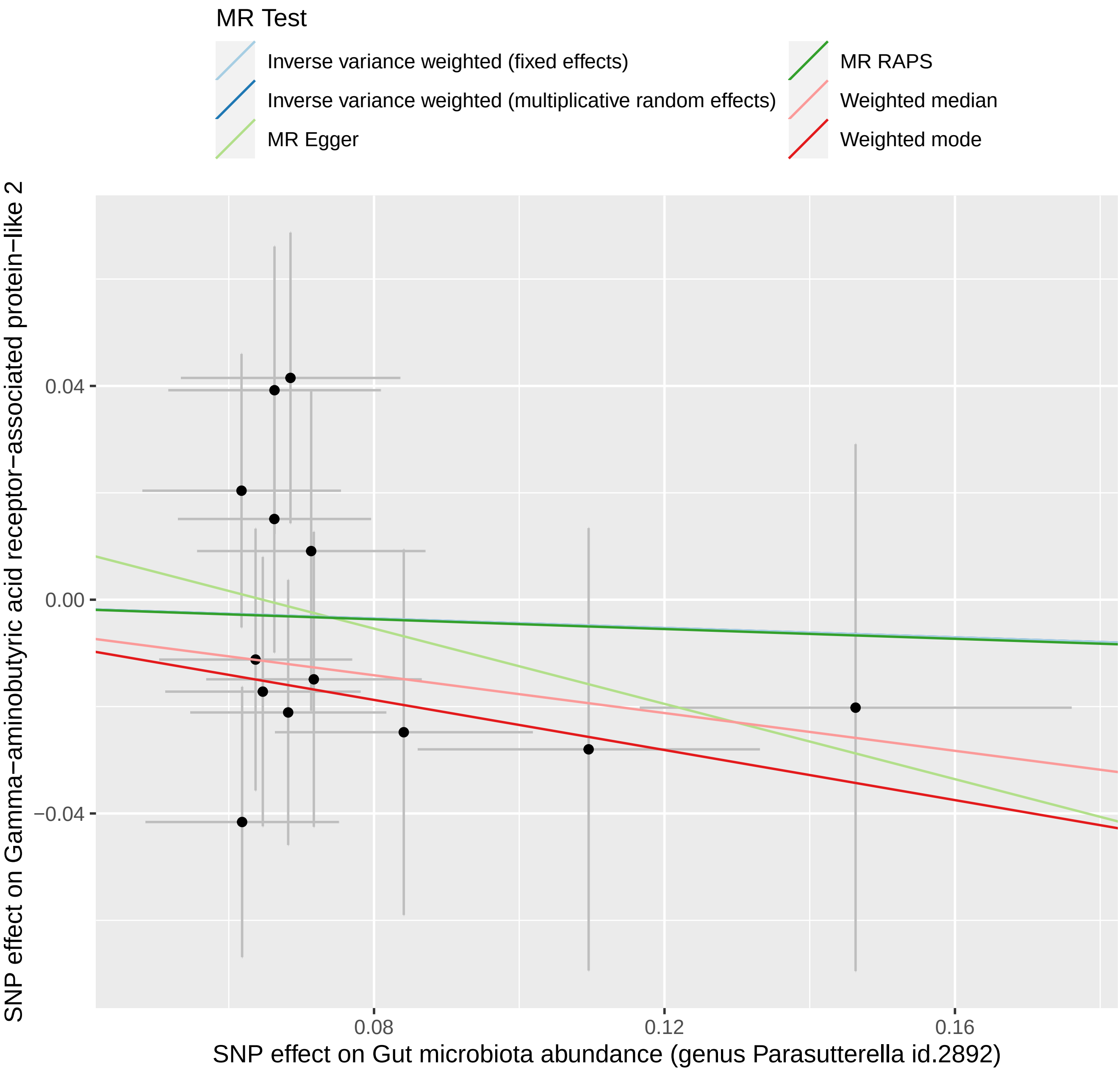

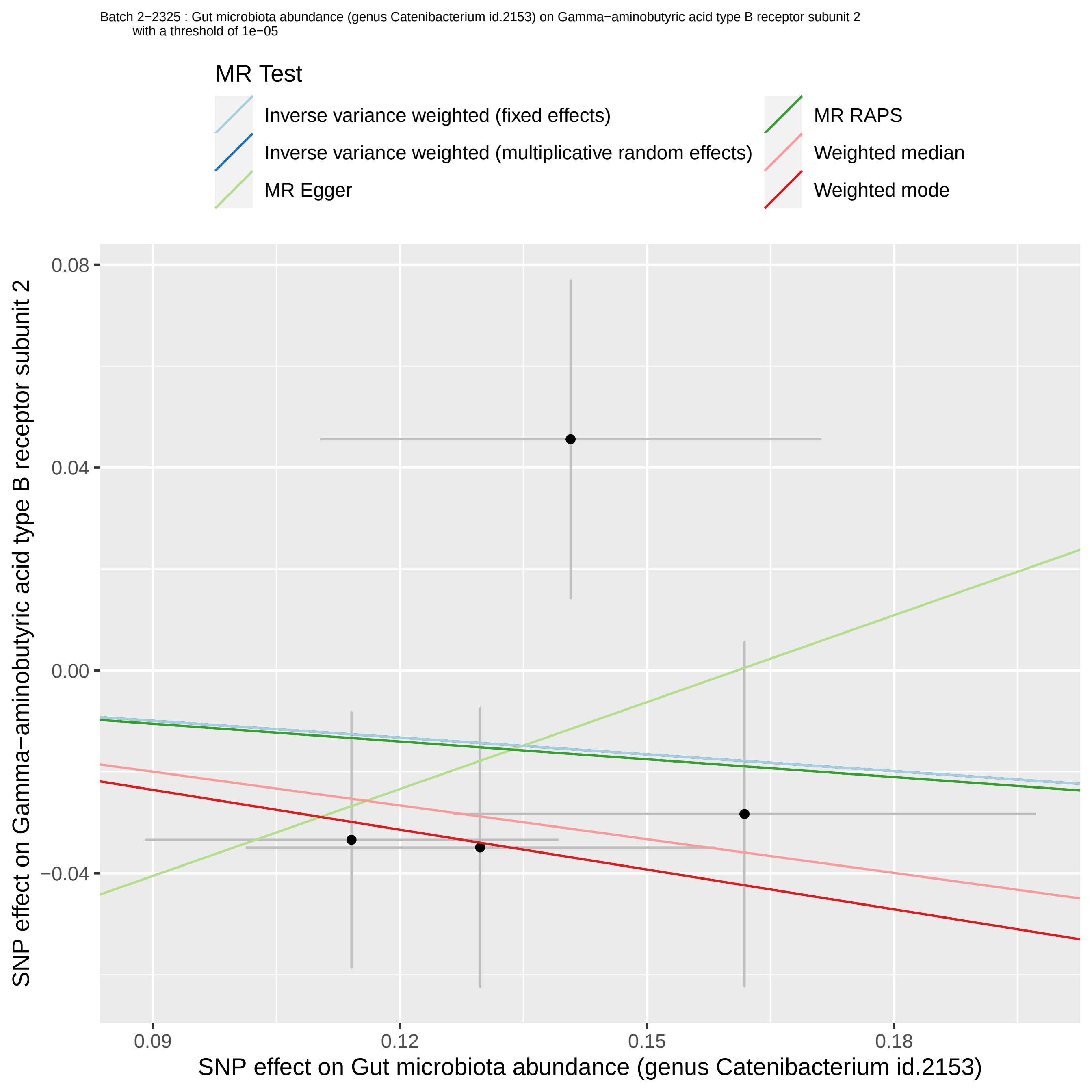
Batch 2−2325 : Gut microbiota abundance (genus Catenibacterium id.2153) on Gamma−aminobutyric acid type B receptor subunit 2
with a threshold of 1e−05
MR Test
Inverse variance weighted (fixed effects)
Inverse variance weighted (multiplicative random effects)
MR Egger
MR RAPS
Weighted median
Weighted mode
0.08
0.04
0.00
−0.04
0.09
0.12
0.15
0.18
SNP effect on Gamma−aminobutyric acid type B receptor subunit 2
SNP effect on Gut microbiota abundance (genus Catenibacterium id.2153)

Batch 2−2326 : Gut microbiota abundance (genus Parasutterella id.2892) on Gamma−aminobutyric acid type B receptor subunit 2
with a threshold of 1e−05

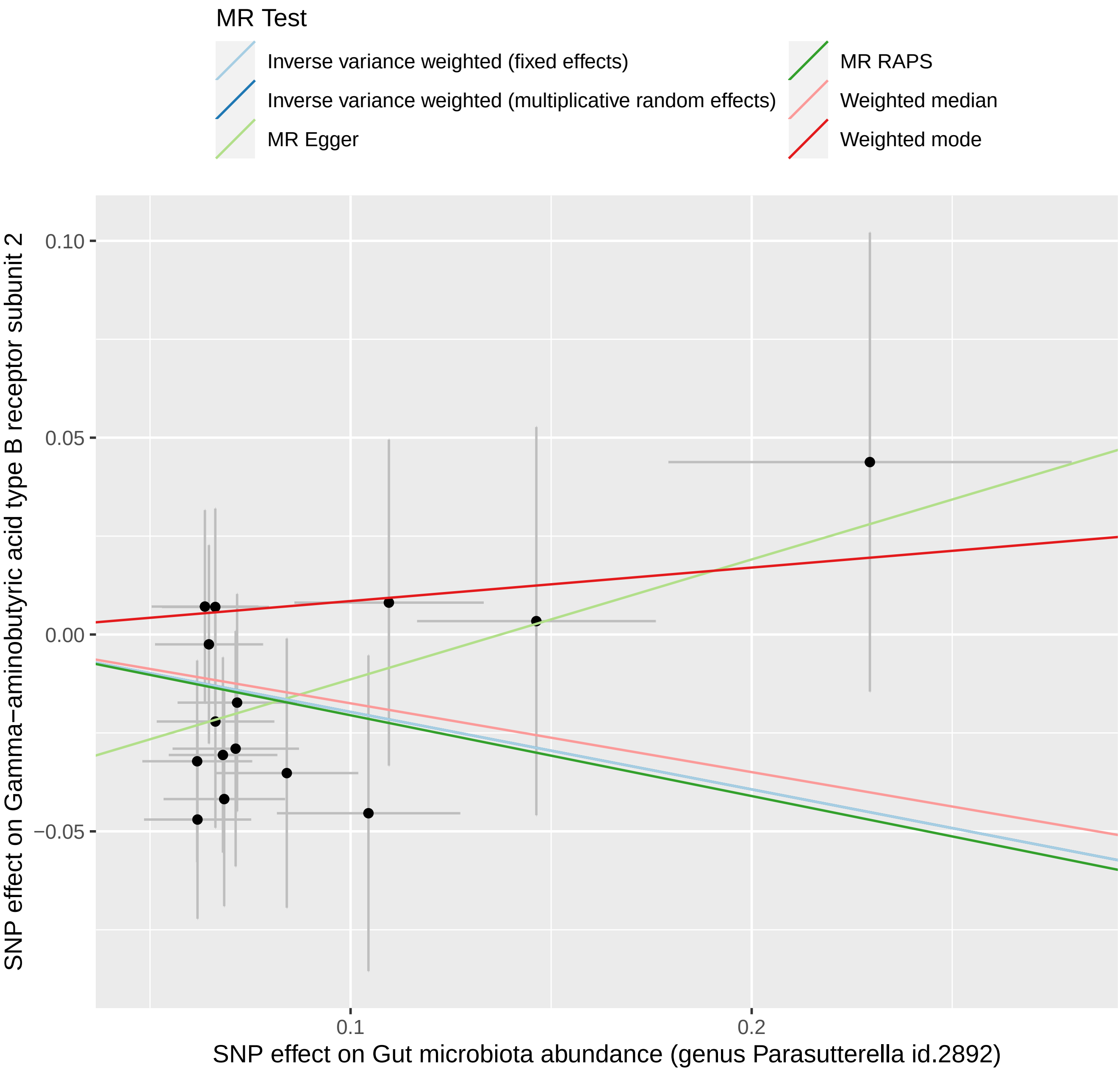

Batch 2−2327 : Gut microbiota abundance (genus Catenibacterium id.2153) on Gamma−aminobutyric acid type B receptor subunit 2 with a threshold of 1e−05

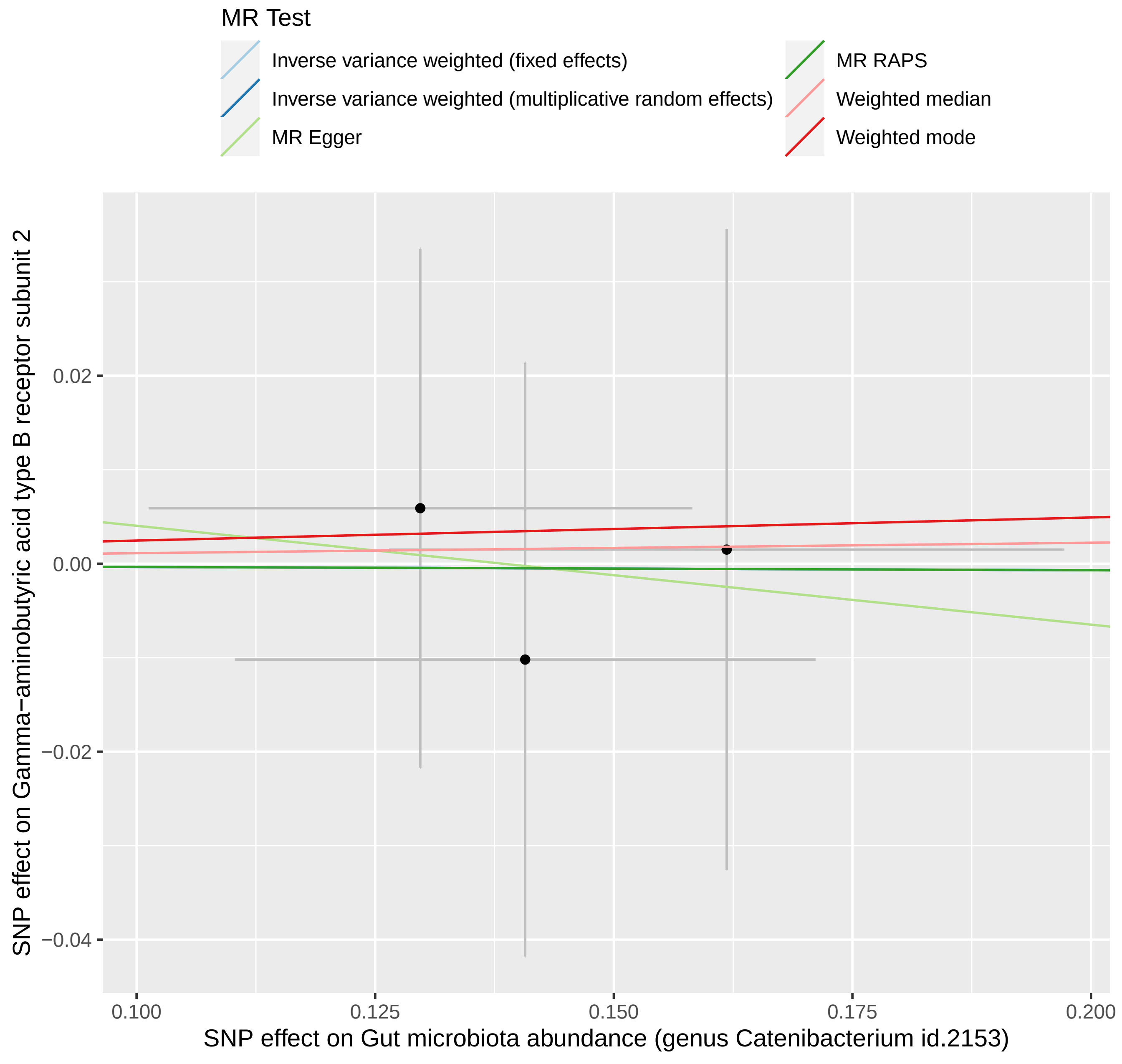

Batch 2−2328 : Gut microbiota abundance (genus Parasutterella id.2892) on Gamma−aminobutyric acid type B receptor subunit 2
with a threshold of 1e−05

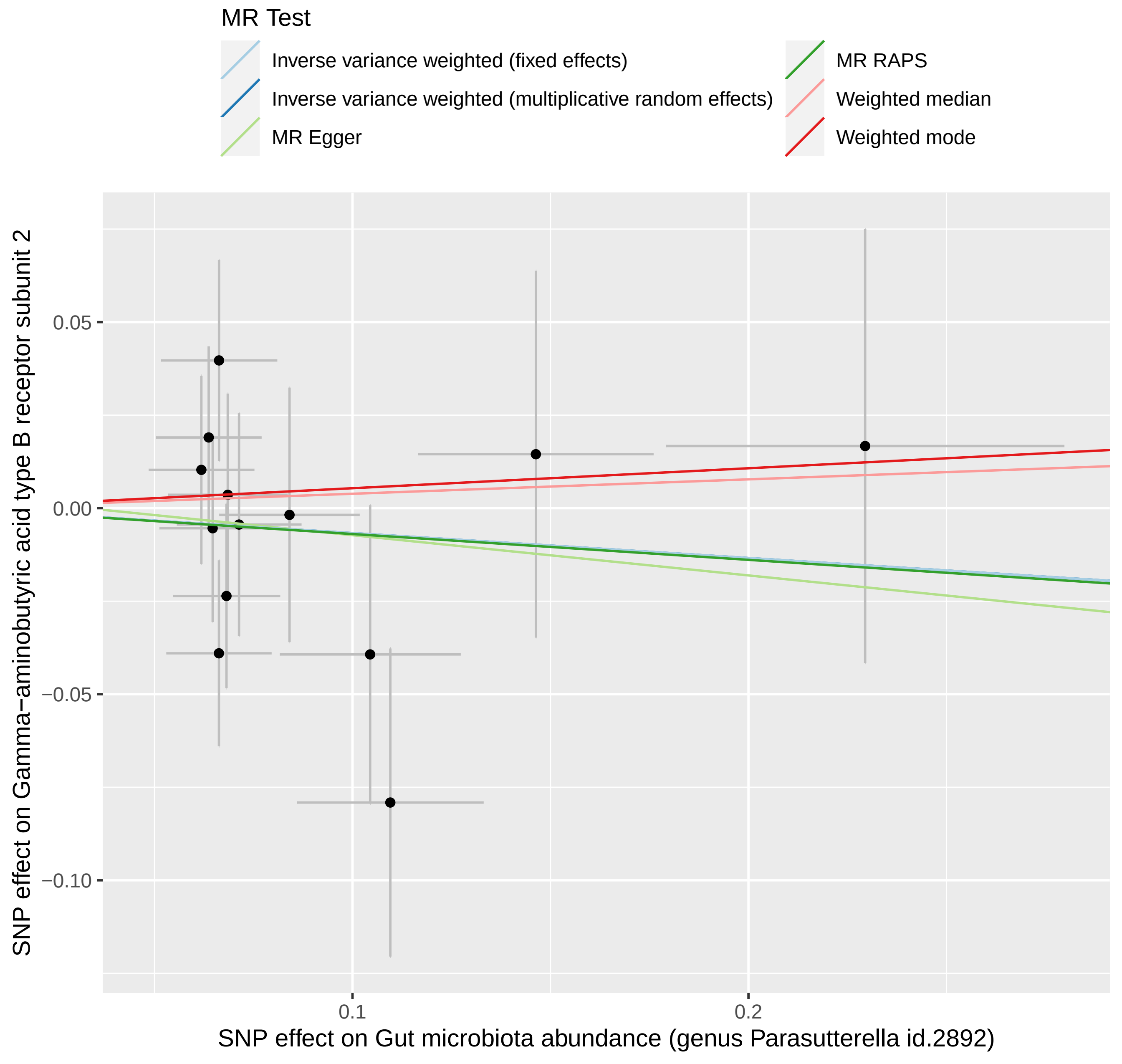

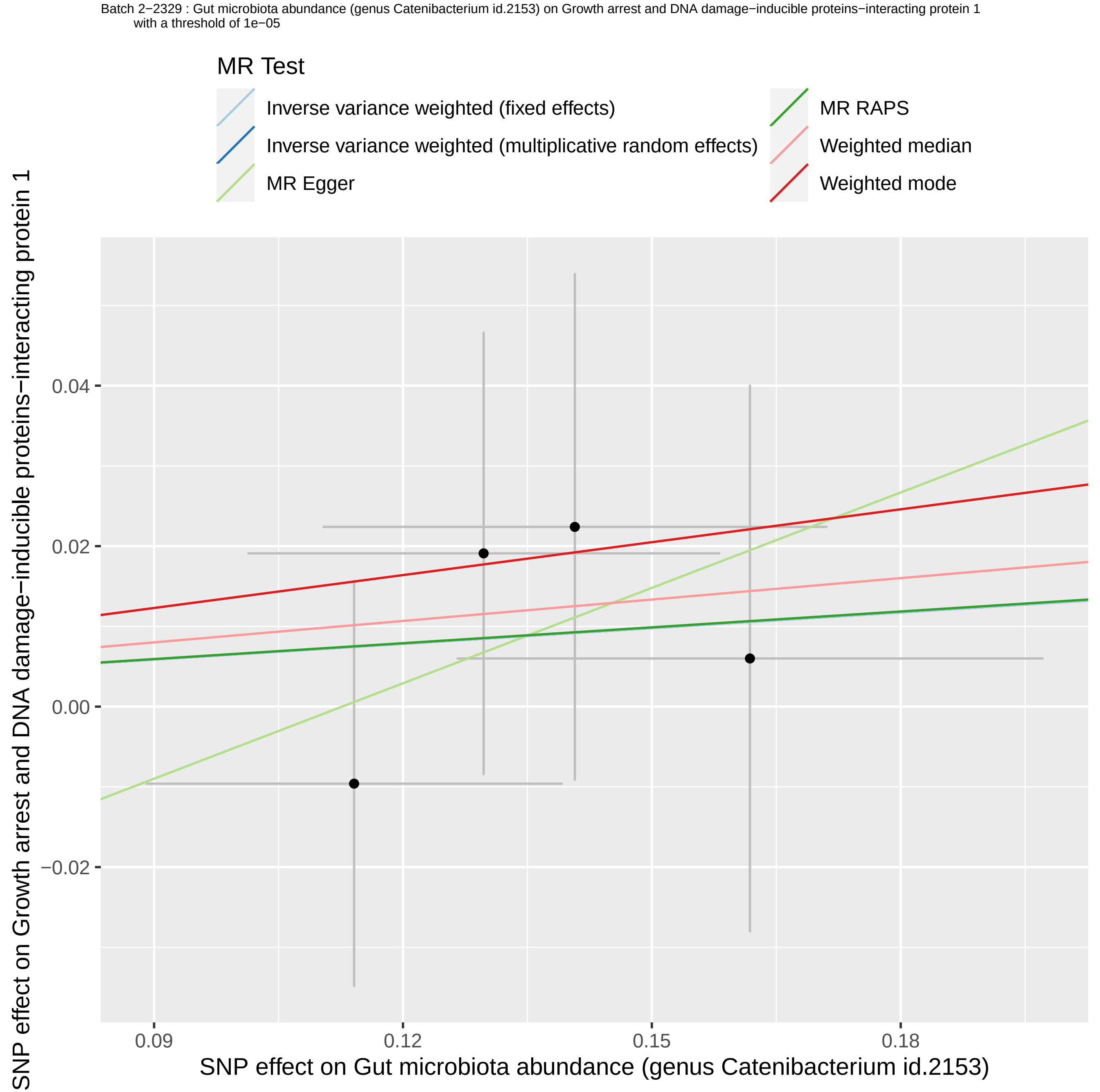

Batch 2−2329 : Gut microbiota abundance (genus Catenibacterium id.2153) on Growth arrest and DNA damage−inducible proteins−interacting protein 1
with a threshold of 1e−05
MR Test
Inverse variance weighted (fixed effects)
Inverse variance weighted (multiplicative random effects)
MR Egger
MR RAPS
Weighted median
Weighted mode
0.04
0.02
0.00
−0.02
0.09
0.12
0.15
0.18
SNP effect on Growth arrest and DNA damage−inducible proteins−interacting protein 1
SNP effect on Gut microbiota abundance (genus Catenibacterium id.2153)

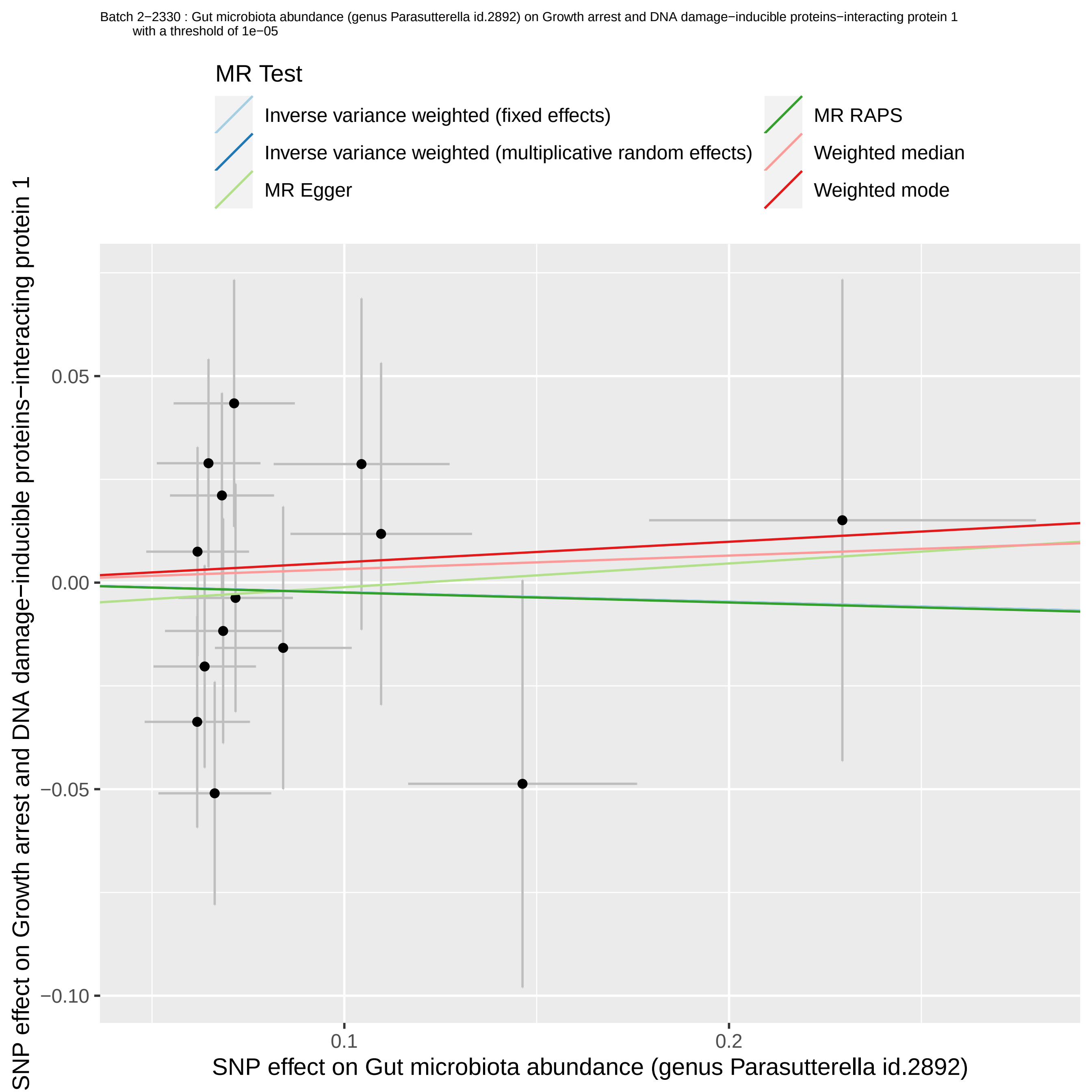
Batch 2−2330 : Gut microbiota abundance (genus Parasutterella id.2892) on Growth arrest and DNA damage−inducible proteins−interacting protein 1 with a threshold of 1e−05
MR Test
Inverse variance weighted (fixed effects)
Inverse variance weighted (multiplicative random effects)
MR Egger
MR RAPS
Weighted median
Weighted mode
SNP effect on Growth arrest and DNA damage−inducible proteins−interacting protein 1
0.05
0.00
−0.05
−0.10
0.1
0.2
SNP effect on Gut microbiota abundance (genus Parasutterella id.2892)

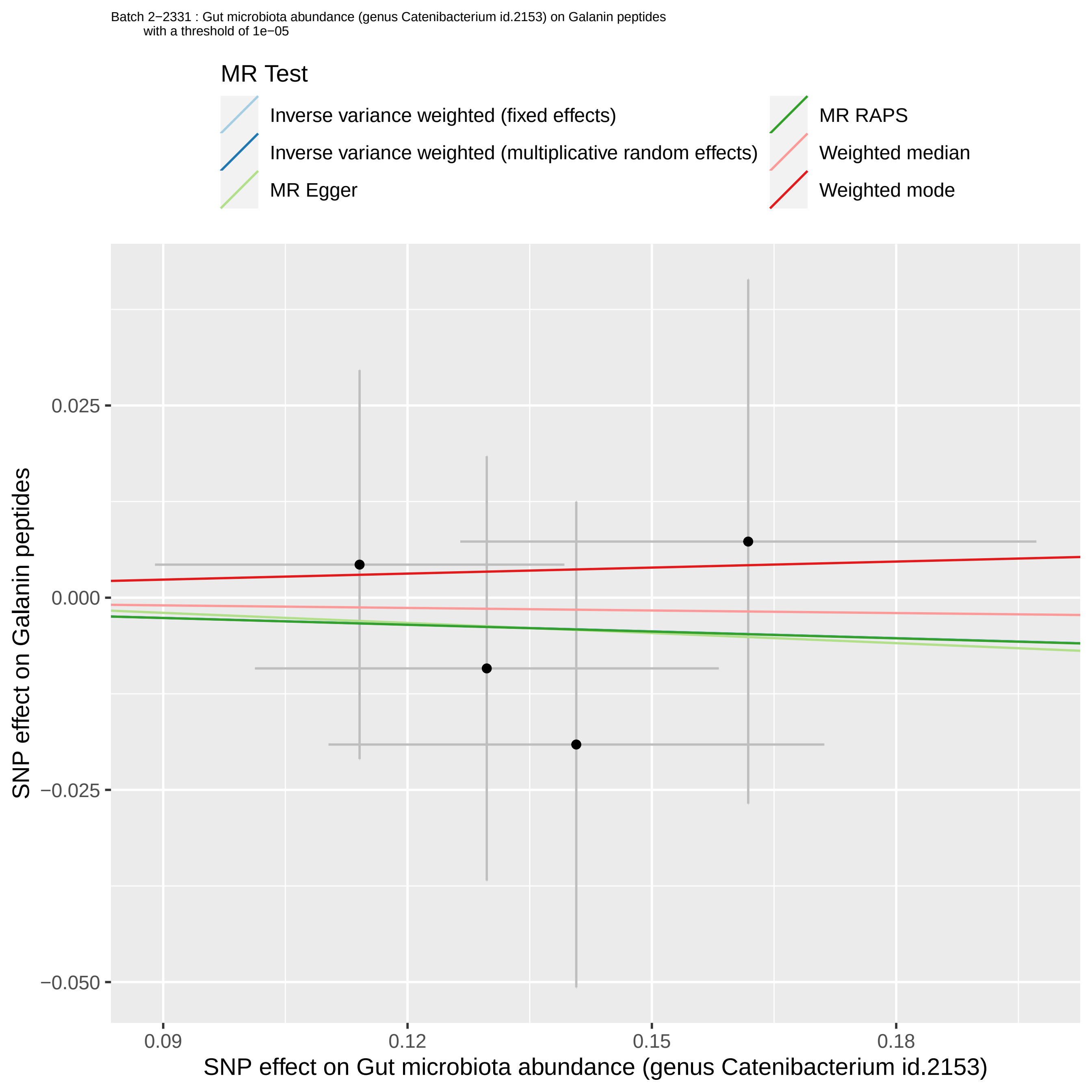
Batch 2−2331 : Gut microbiota abundance (genus Catenibacterium id.2153) on Galanin peptides
with a threshold of 1e−05
MR Test
Inverse variance weighted (fixed effects)
Inverse variance weighted (multiplicative random effects)
MR Egger
MR RAPS
Weighted median
Weighted mode
0.025
0.000
−0.025
−0.050
SNP effect on Galanin peptides
0.09
0.12
0.15
0.18
SNP effect on Gut microbiota abundance (genus Catenibacterium id.2153)

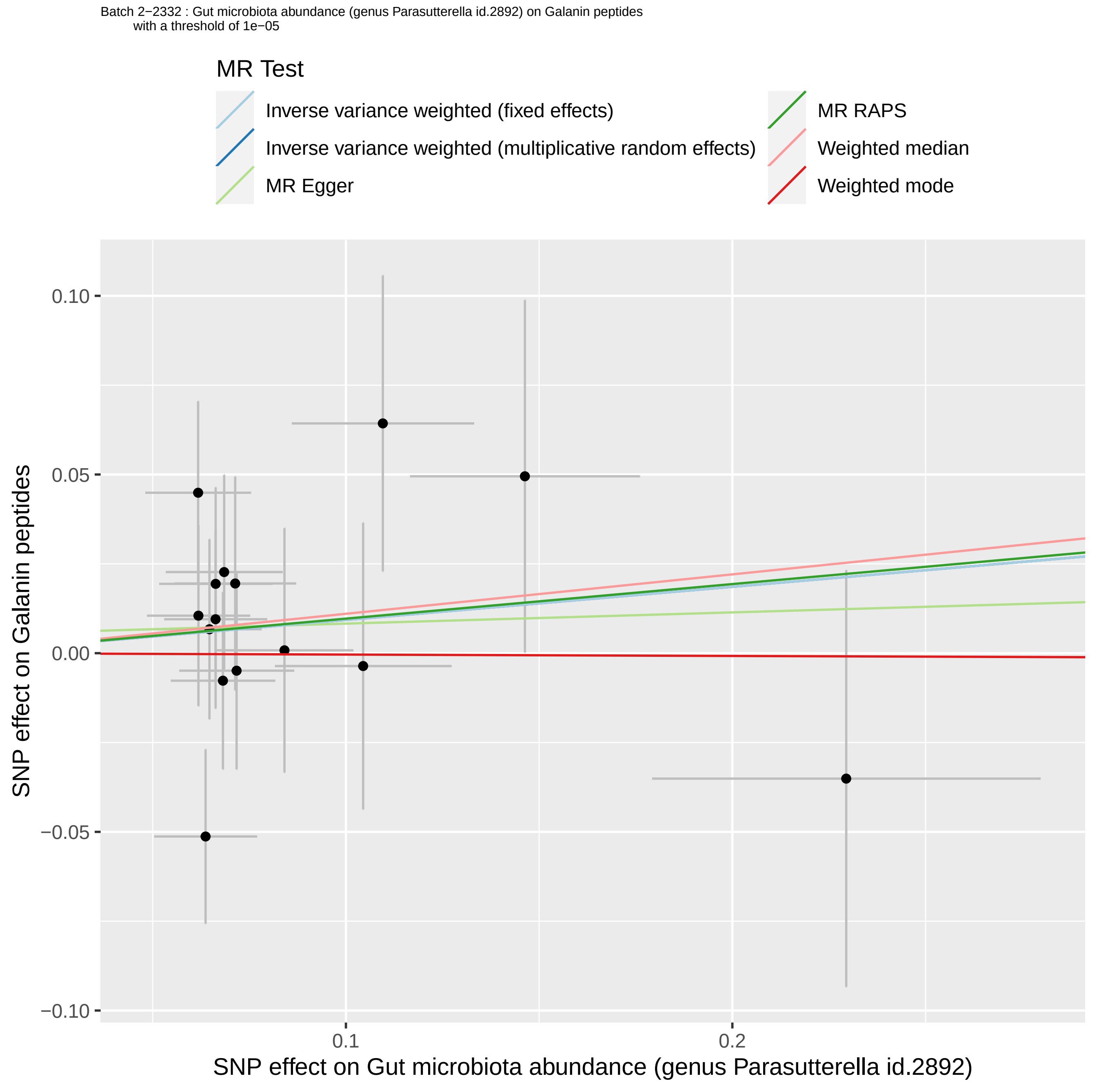

Batch 2−2332 : Gut microbiota abundance (genus Parasutterella id.2892) on Galanin peptides
with a threshold of 1e−05
MR Test
Inverse variance weighted (fixed effects)
Inverse variance weighted (multiplicative random effects)
MR Egger
MR RAPS
Weighted median
Weighted mode
0.10
0.05
0.00
−0.05
−0.10
0.1
0.2
SNP effect on Galanin peptides
SNP effect on Gut microbiota abundance (genus Parasutterella id.2892)

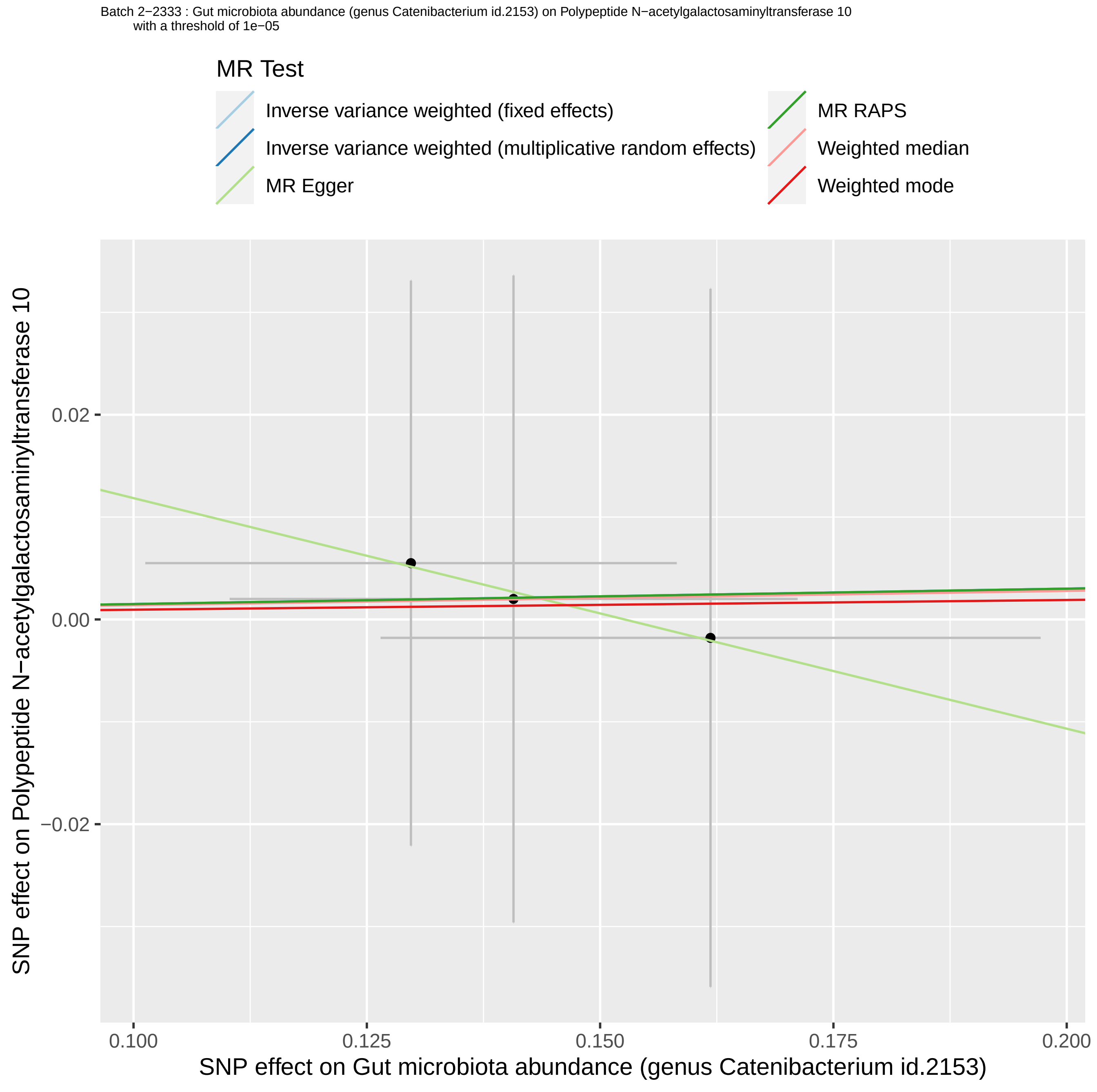

Batch 2−2333 : Gut microbiota abundance (genus Catenibacterium id.2153) on Polypeptide N−acetylgalactosaminyltransferase 10
with a threshold of 1e−05
MR Test
Inverse variance weighted (fixed effects)
Inverse variance weighted (multiplicative random effects)
MR Egger
MR RAPS
Weighted median
Weighted mode
0.02
0.00
−0.02
0.100
0.125
0.150
0.175
0.200
SNP effect on Polypeptide N−acetylgalactosaminyltransferase 10
SNP effect on Gut microbiota abundance (genus Catenibacterium id.2153)

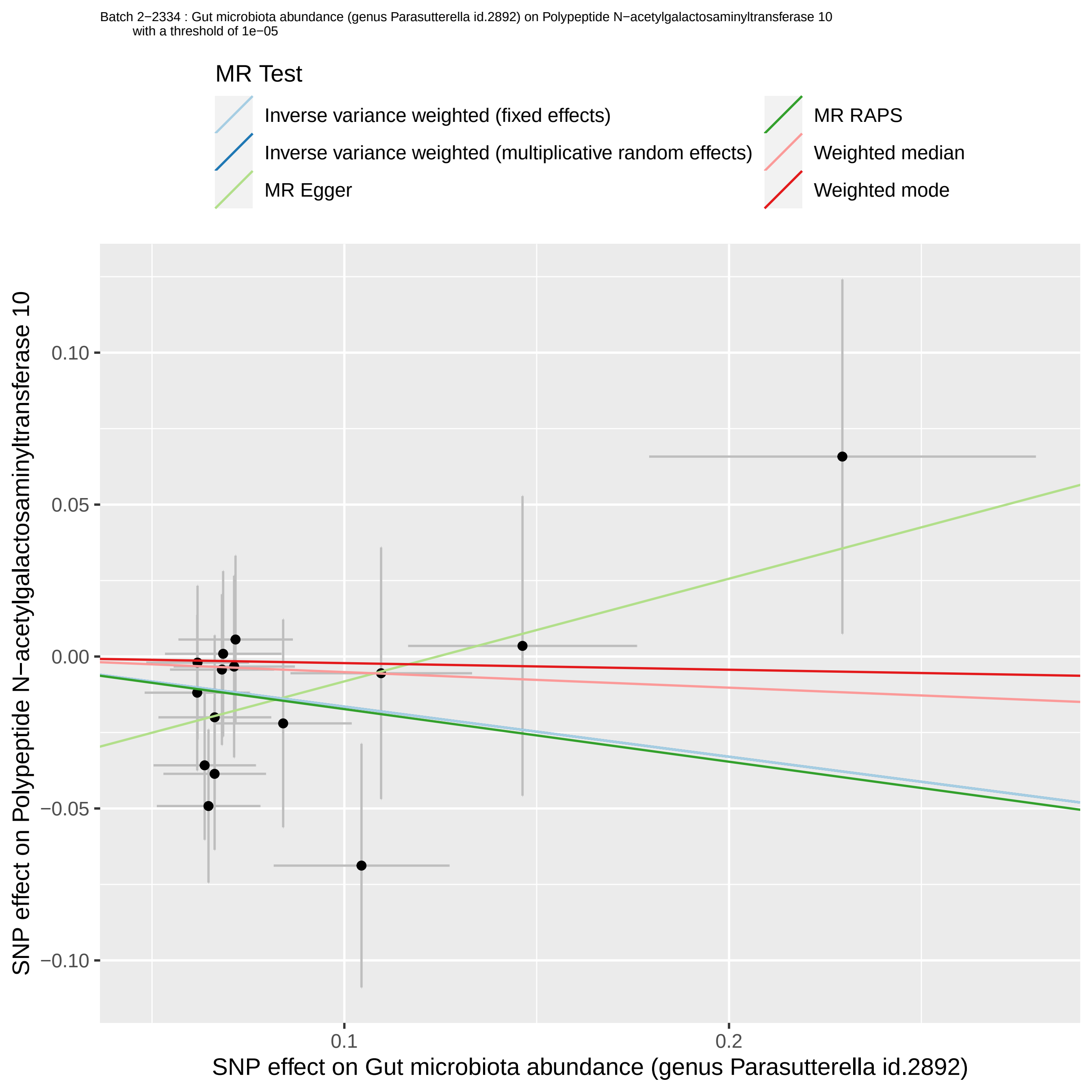
Batch 2−2334 : Gut microbiota abundance (genus Parasutterella id.2892) on Polypeptide N−acetylgalactosaminyltransferase 10
with a threshold of 1e−05
MR Test
Inverse variance weighted (fixed effects)
Inverse variance weighted (multiplicative random effects)
MR Egger
MR RAPS
Weighted median
Weighted mode
SNP effect on Polypeptide N−acetylgalactosaminyltransferase 10
0.10
0.05
0.00
−0.05
−0.10
0.1
0.2
SNP effect on Gut microbiota abundance (genus Parasutterella id.2892)

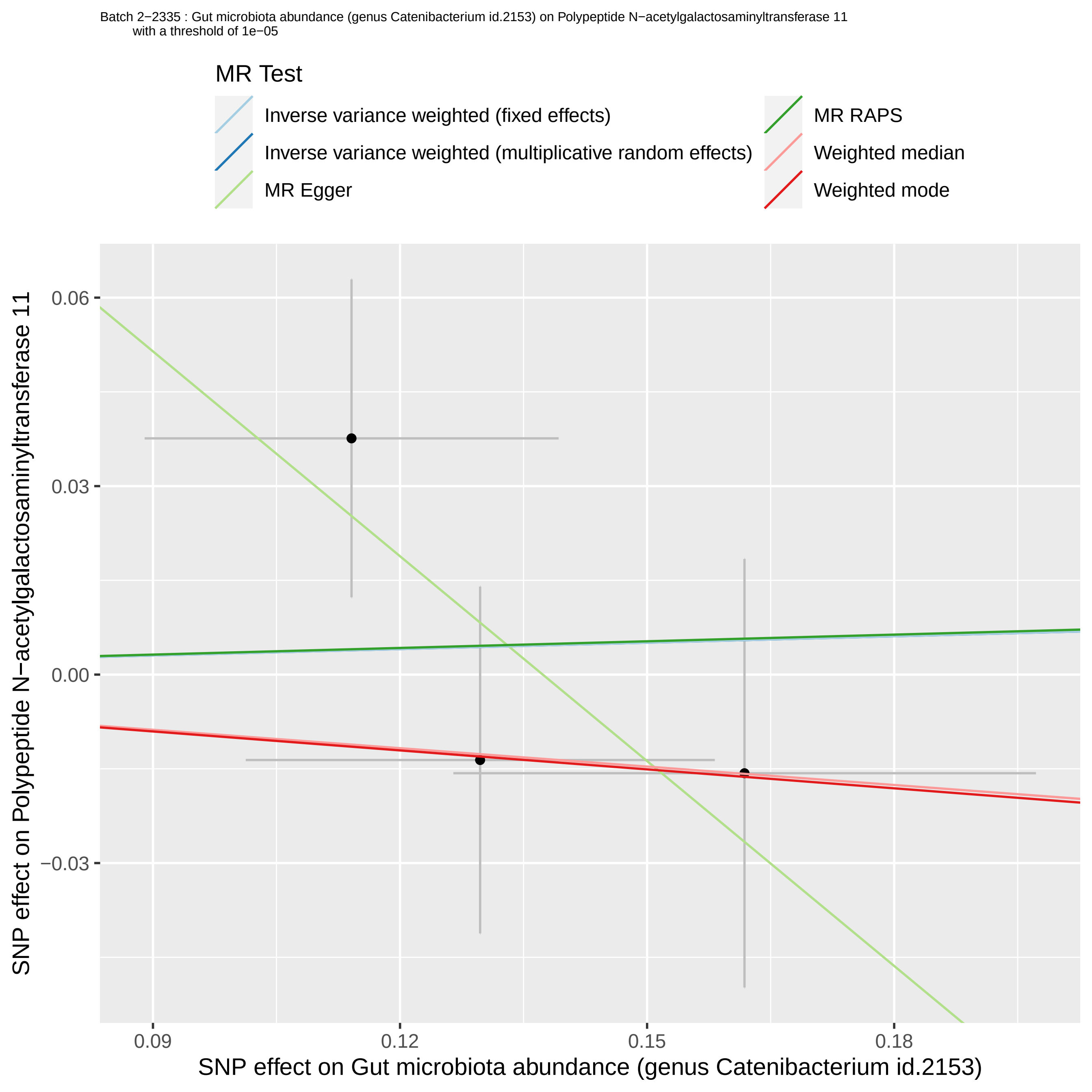
Batch 2−2335 : Gut microbiota abundance (genus Catenibacterium id.2153) on Polypeptide N−acetylgalactosaminyltransferase 11
with a threshold of 1e−05
MR Test
Inverse variance weighted (fixed effects)
Inverse variance weighted (multiplicative random effects)
MR Egger
MR RAPS
Weighted median
Weighted mode
0.06
0.03
0.00
−0.03
0.09
0.12
0.15
0.18
SNP effect on Polypeptide N−acetylgalactosaminyltransferase 11
SNP effect on Gut microbiota abundance (genus Catenibacterium id.2153)

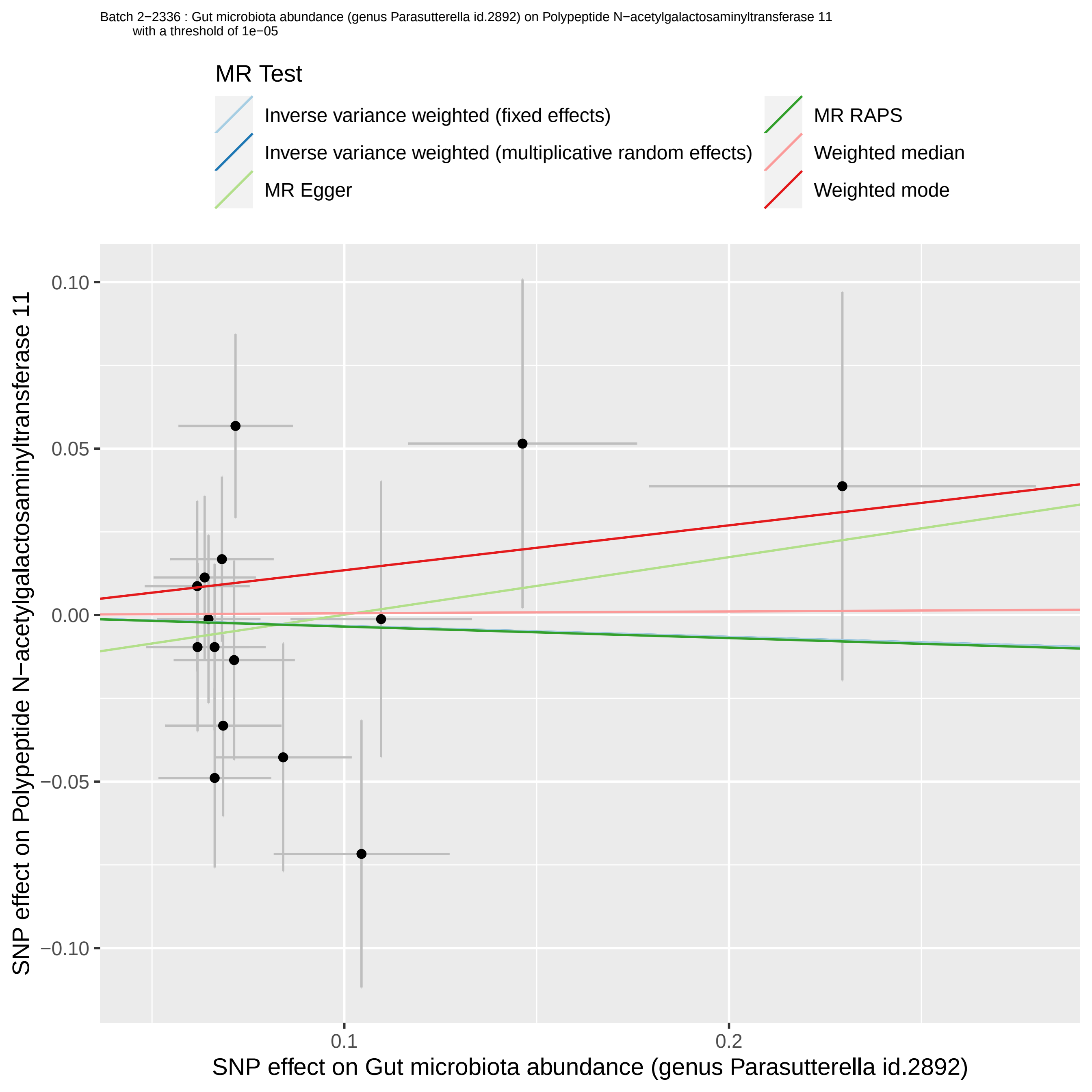
Batch 2−2336 : Gut microbiota abundance (genus Parasutterella id.2892) on Polypeptide N−acetylgalactosaminyltransferase 11
with a threshold of 1e−05
MR Test
Inverse variance weighted (fixed effects)
Inverse variance weighted (multiplicative random effects)
MR Egger
MR RAPS
Weighted median
Weighted mode
0.10
0.05
0.00
−0.05
−0.10
0.1
0.2
SNP effect on Polypeptide N−acetylgalactosaminyltransferase 11
SNP effect on Gut microbiota abundance (genus Parasutterella id.2892)

Batch 2−2337 : Gut microbiota abundance (genus Catenibacterium id.2153) on Polypeptide N−acetylgalactosaminyltransferase 16
with a threshold of 1e−05

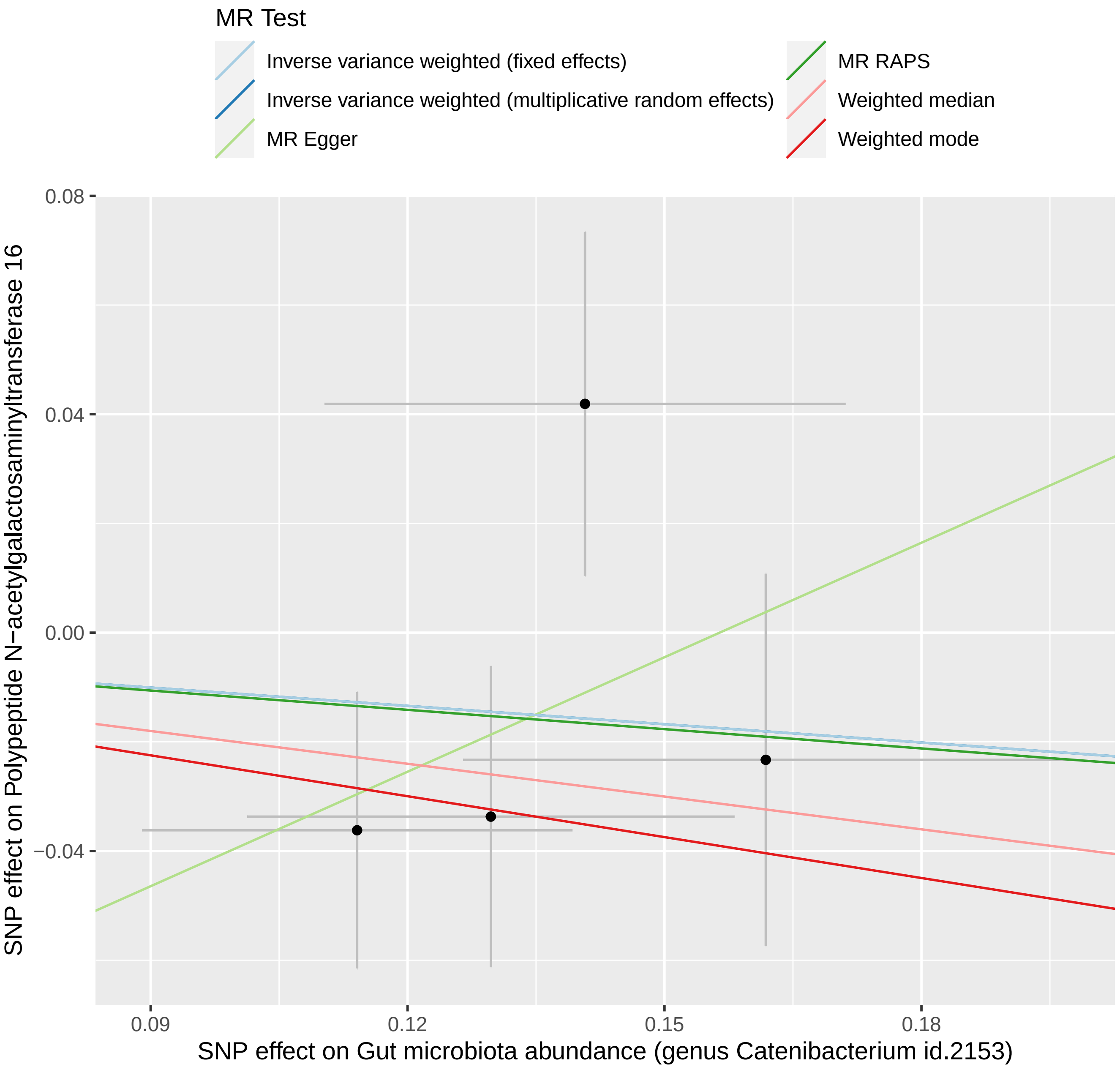

Batch 2−2338 : Gut microbiota abundance (genus Parasutterella id.2892) on Polypeptide N−acetylgalactosaminyltransferase 16
with a threshold of 1e−05

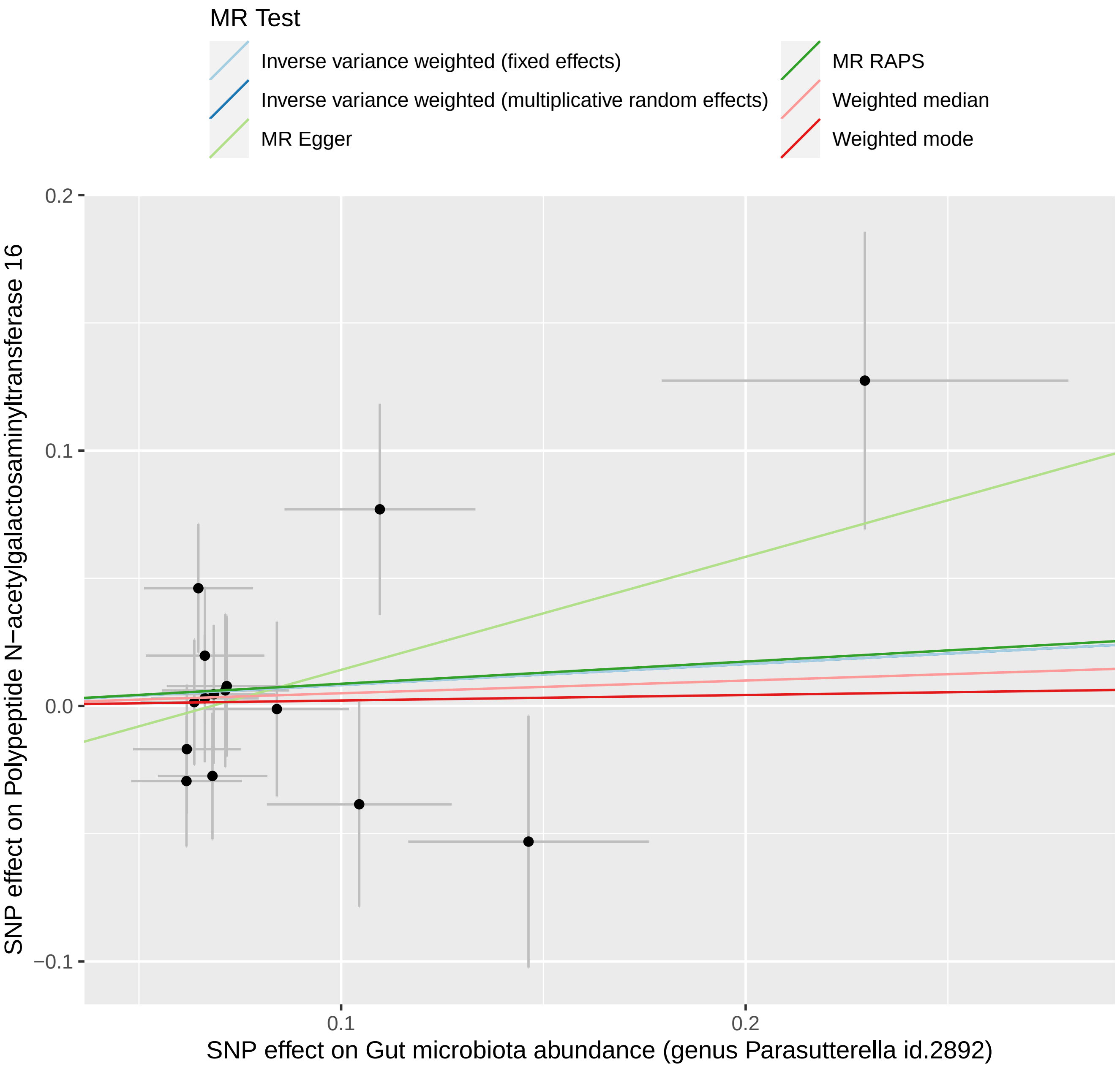

Batch 2−2339 : Gut microbiota abundance (genus Catenibacterium id.2153) on Polypeptide N−acetylgalactosaminyltransferase 1
with a threshold of 1e−05

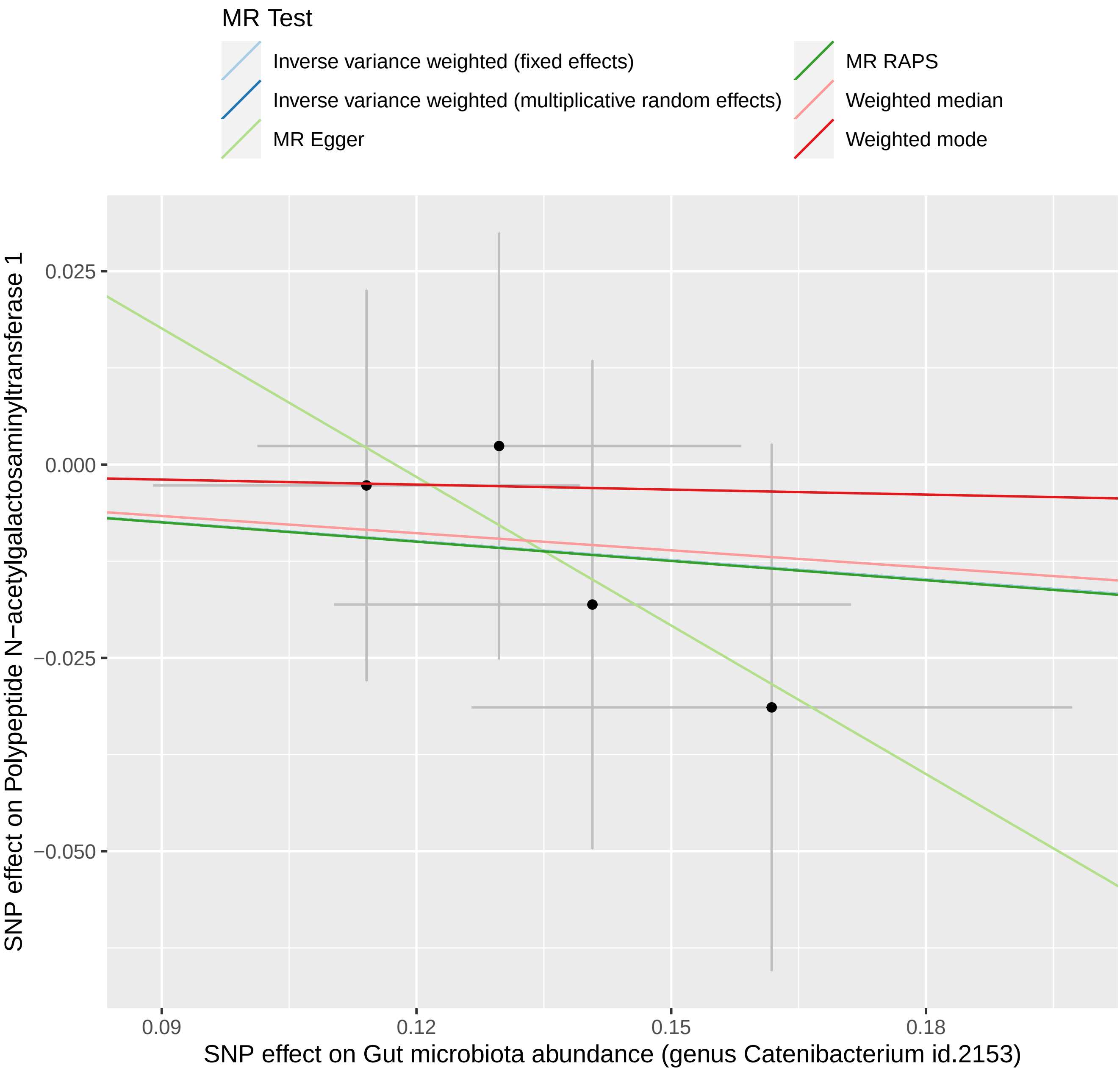

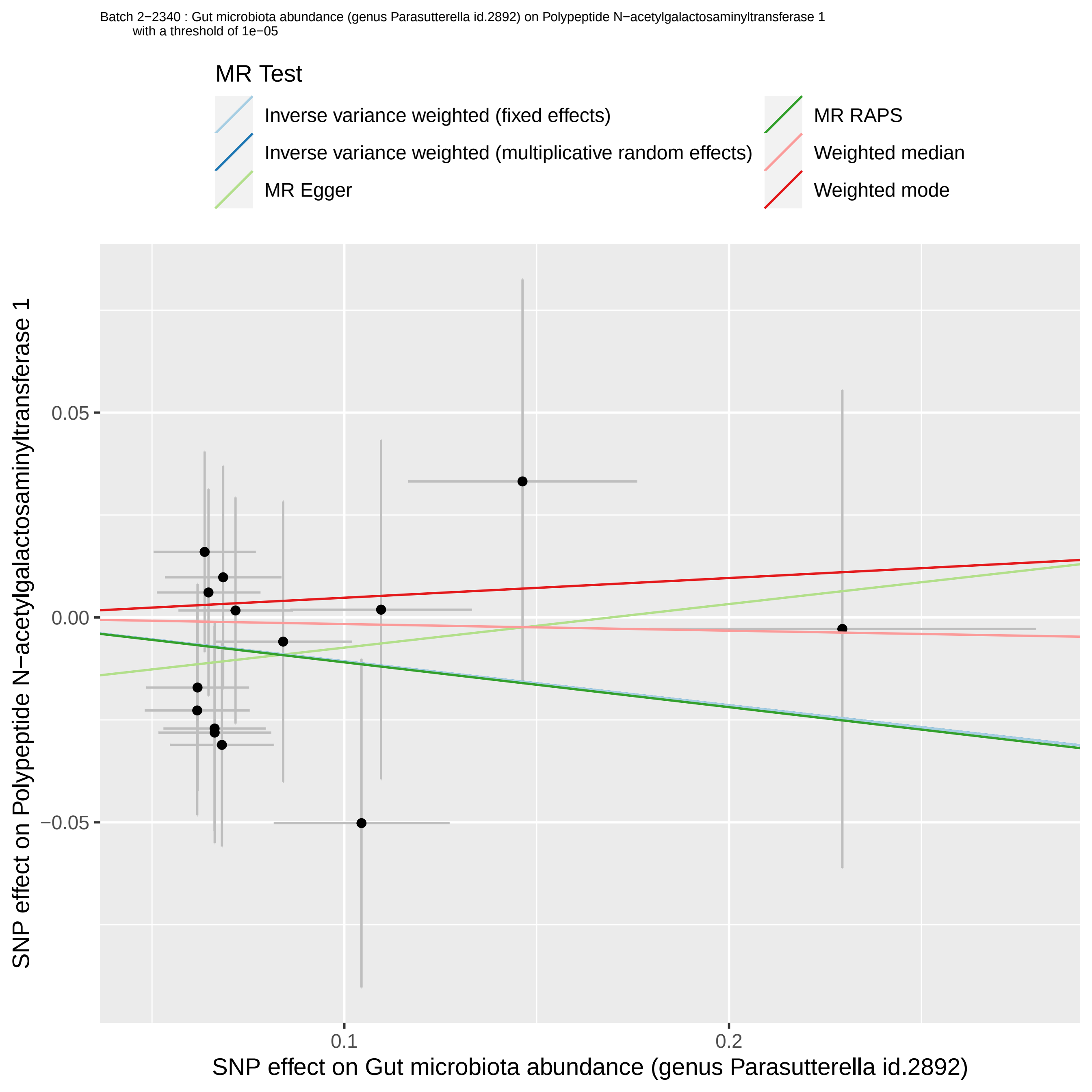
Batch 2−2340 : Gut microbiota abundance (genus Parasutterella id.2892) on Polypeptide N−acetylgalactosaminyltransferase 1
with a threshold of 1e−05
MR Test
Inverse variance weighted (fixed effects)
Inverse variance weighted (multiplicative random effects)
MR Egger
MR RAPS
Weighted median
Weighted mode
0.05
0.00
−0.05
0.1
0.2
SNP effect on Polypeptide N−acetylgalactosaminyltransferase 1
SNP effect on Gut microbiota abundance (genus Parasutterella id.2892)

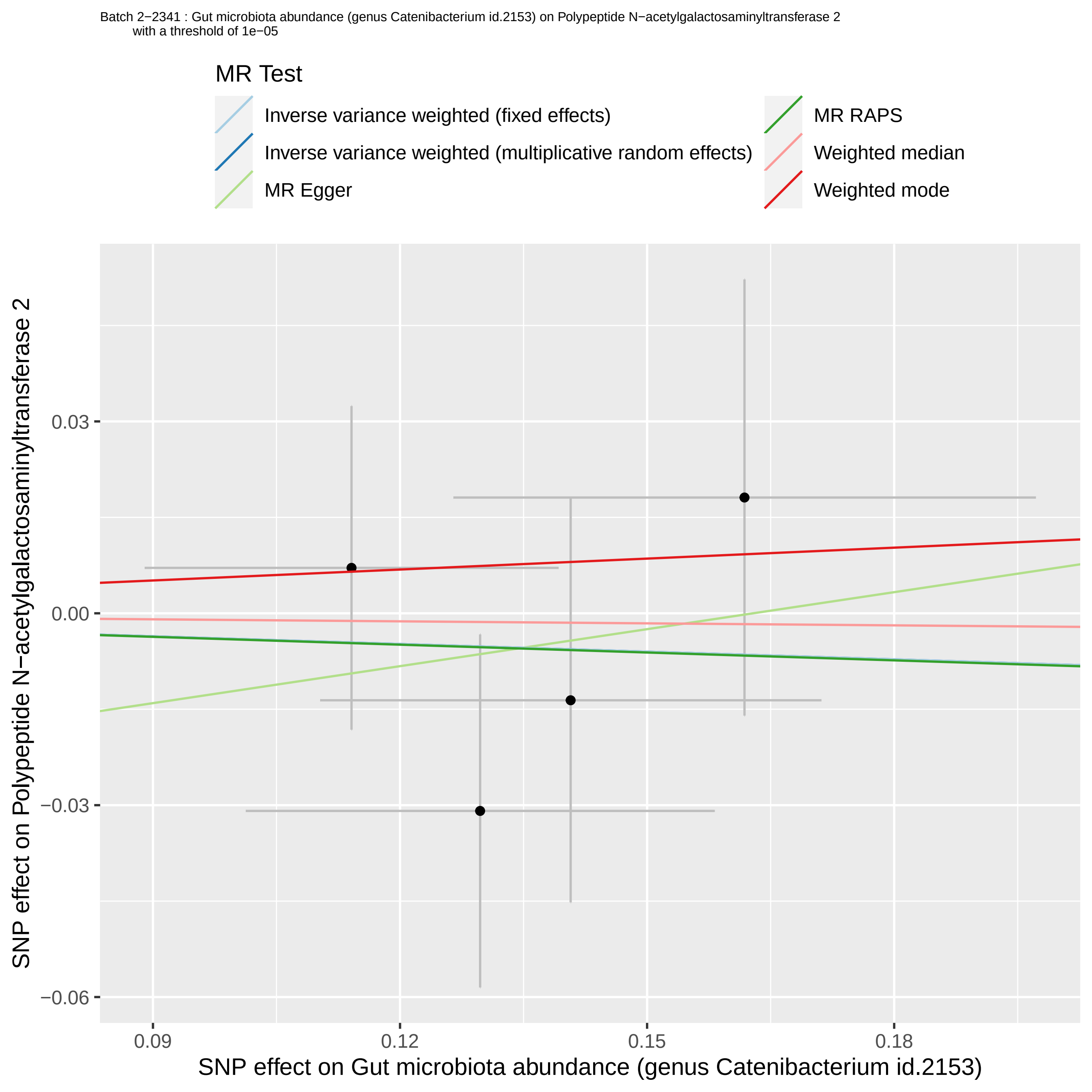

Batch 2−2341 : Gut microbiota abundance (genus Catenibacterium id.2153) on Polypeptide N−acetylgalactosaminyltransferase 2
with a threshold of 1e−05
MR Test
Inverse variance weighted (fixed effects)
Inverse variance weighted (multiplicative random effects)
MR Egger
MR RAPS
Weighted median
Weighted mode
0.03
0.00
−0.03
−0.06
0.09
0.12
0.15
0.18
SNP effect on Polypeptide N−acetylgalactosaminyltransferase 2
SNP effect on Gut microbiota abundance (genus Catenibacterium id.2153)

Batch 2−2342 : Gut microbiota abundance (genus Parasutterella id.2892) on Polypeptide N−acetylgalactosaminyltransferase 2 with a threshold of 1e−05

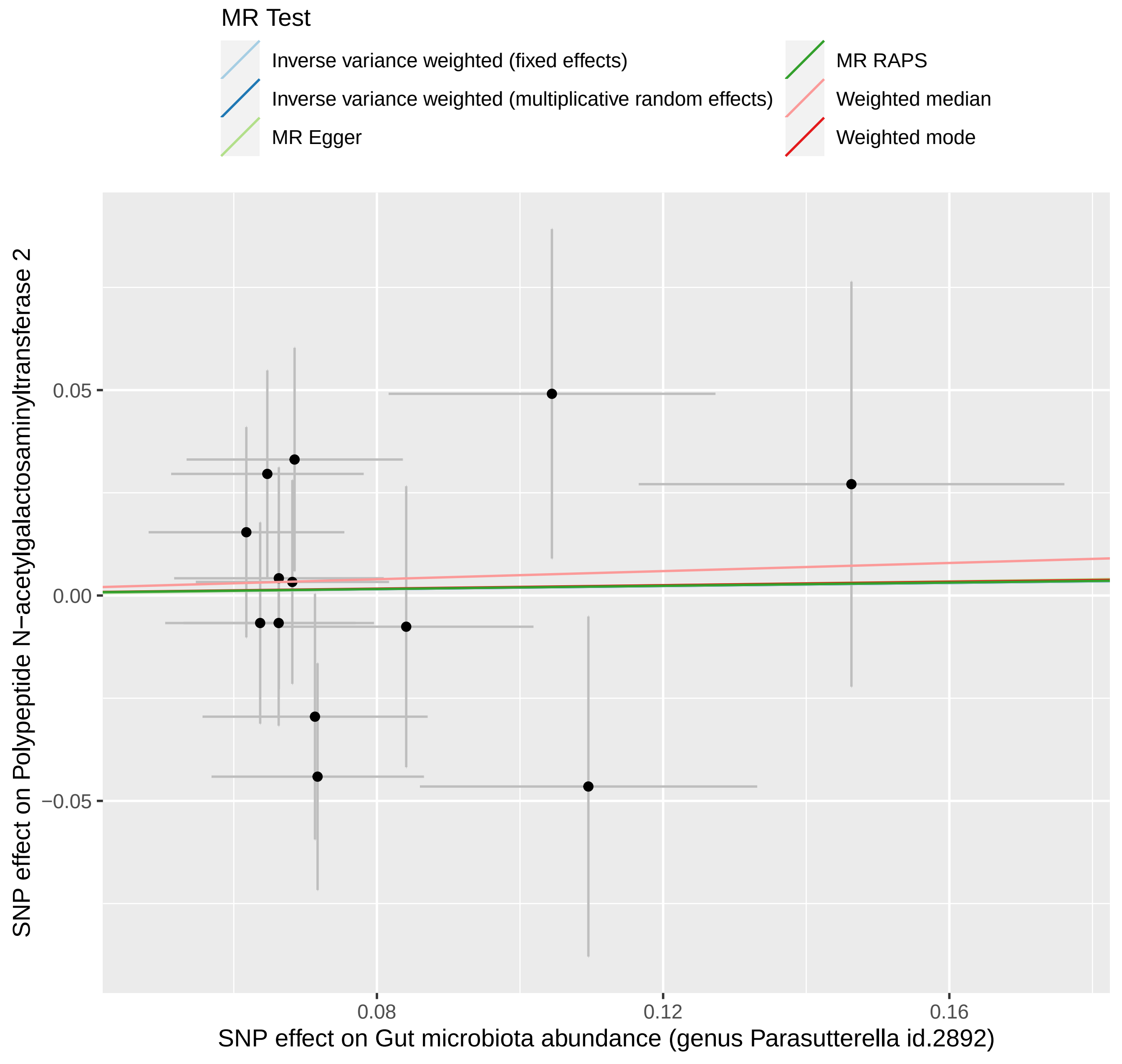

Batch 2−2343 : Gut microbiota abundance (genus Catenibacterium id.2153) on Polypeptide N−acetylgalactosaminyltransferase 3
with a threshold of 1e−05

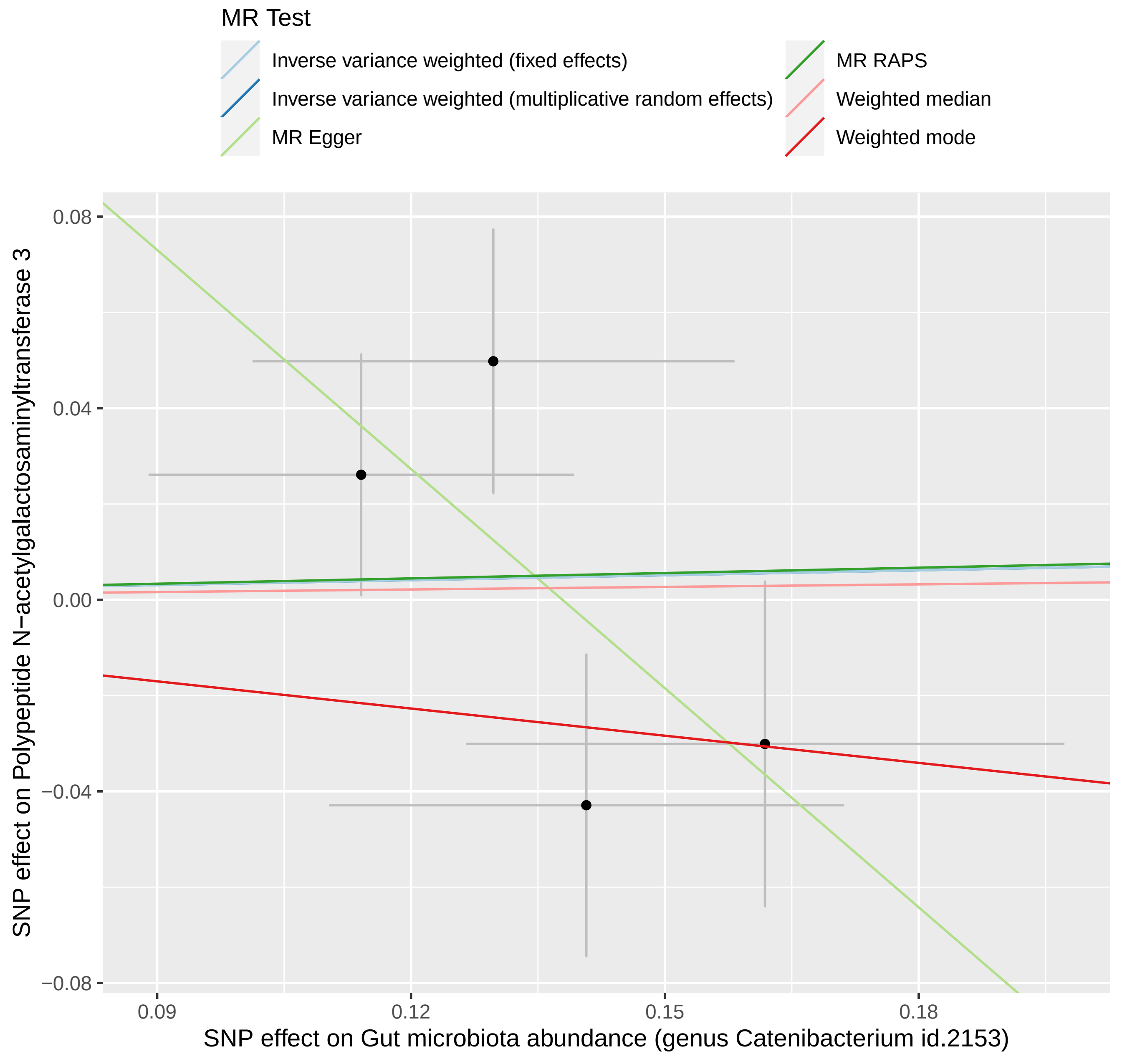

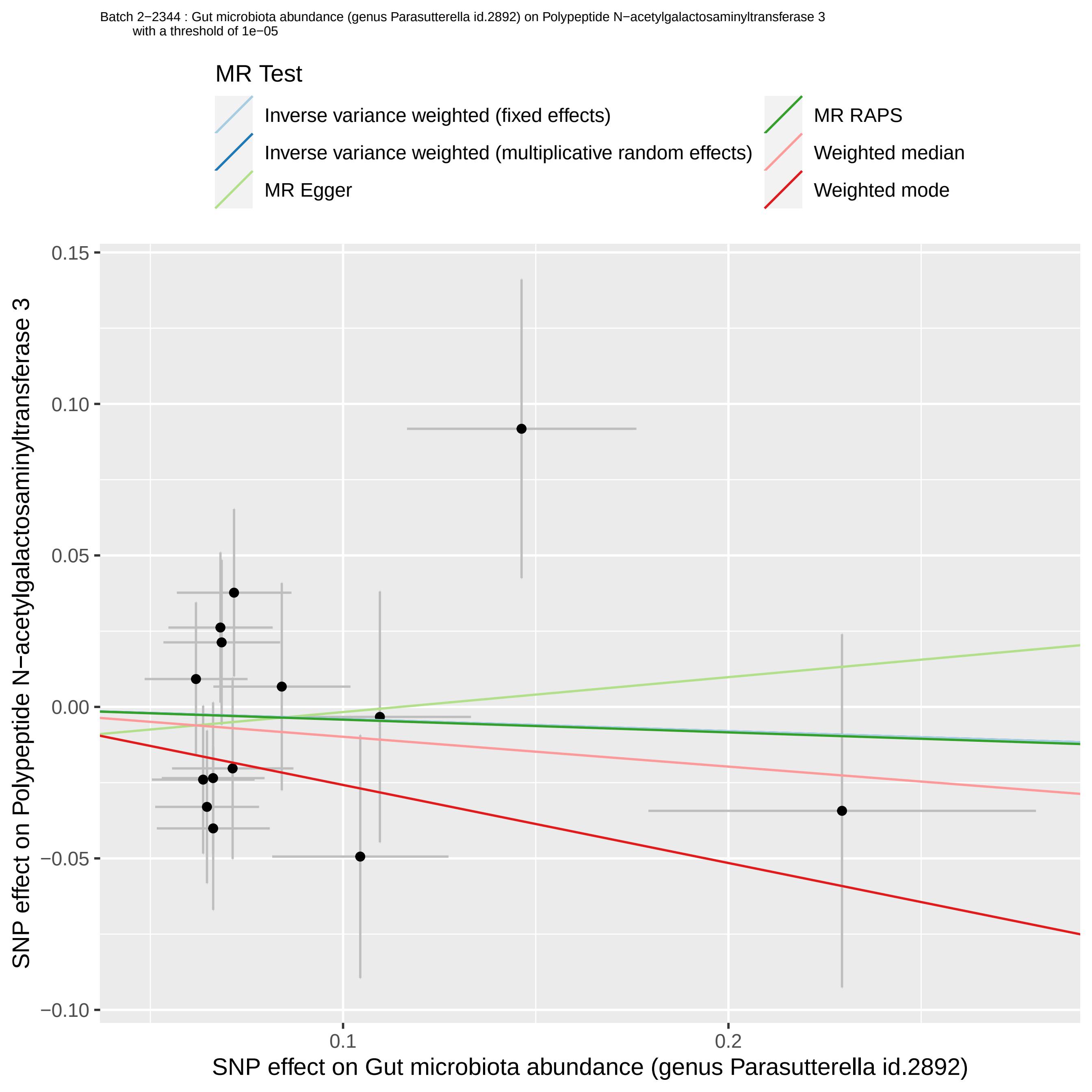

Batch 2−2344 : Gut microbiota abundance (genus Parasutterella id.2892) on Polypeptide N−acetylgalactosaminyltransferase 3
with a threshold of 1e−05
MR Test
Inverse variance weighted (fixed effects)
Inverse variance weighted (multiplicative random effects)
MR Egger
MR RAPS
Weighted median
Weighted mode
0.15
0.10
0.05
0.00
−0.05
−0.10
0.1
0.2
SNP effect on Polypeptide N−acetylgalactosaminyltransferase 3
SNP effect on Gut microbiota abundance (genus Parasutterella id.2892)

Batch 2−2345 : Gut microbiota abundance (genus Catenibacterium id.2153) on N−acetylgalactosaminyltransferase 7
with a threshold of 1e−05

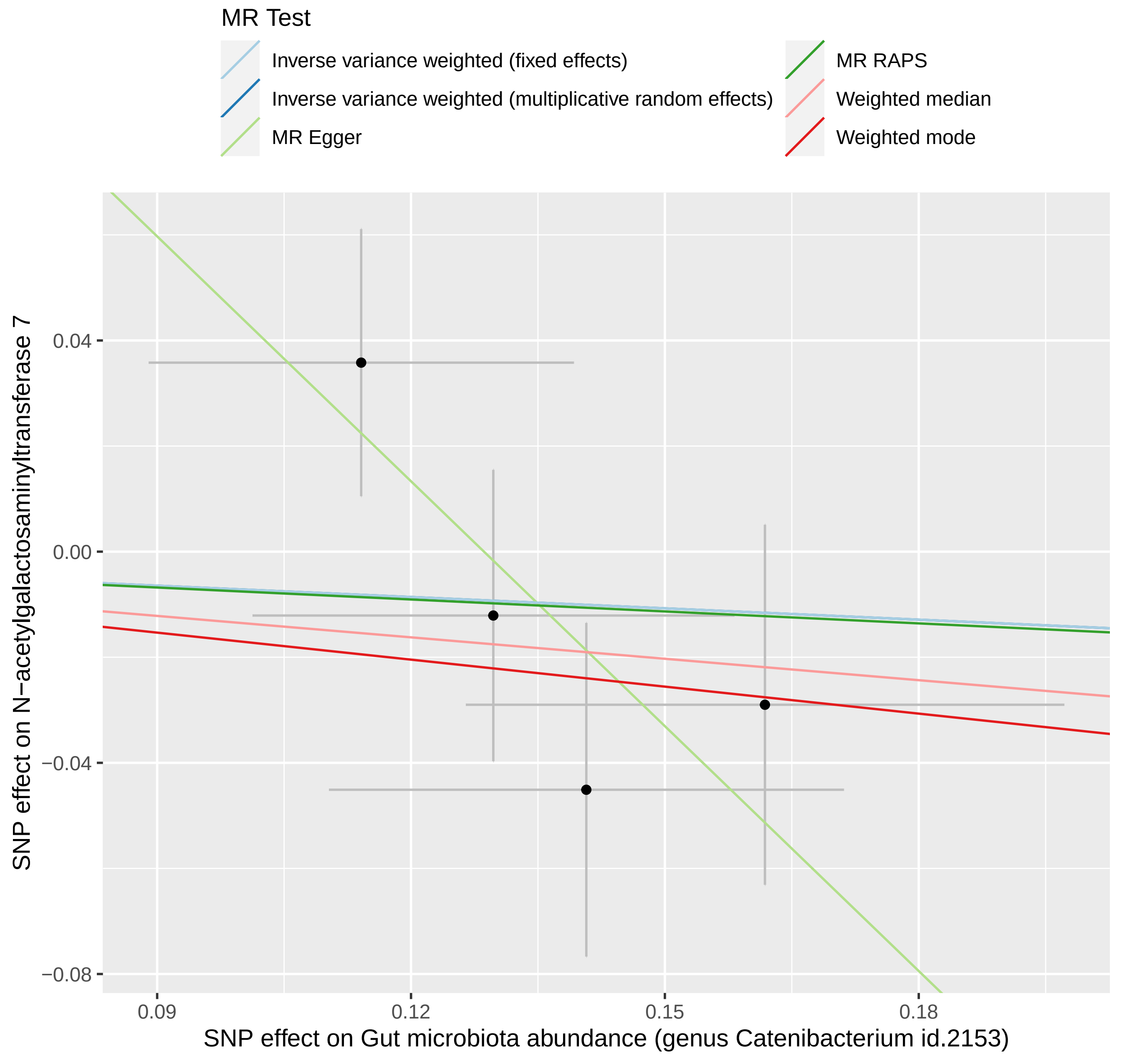

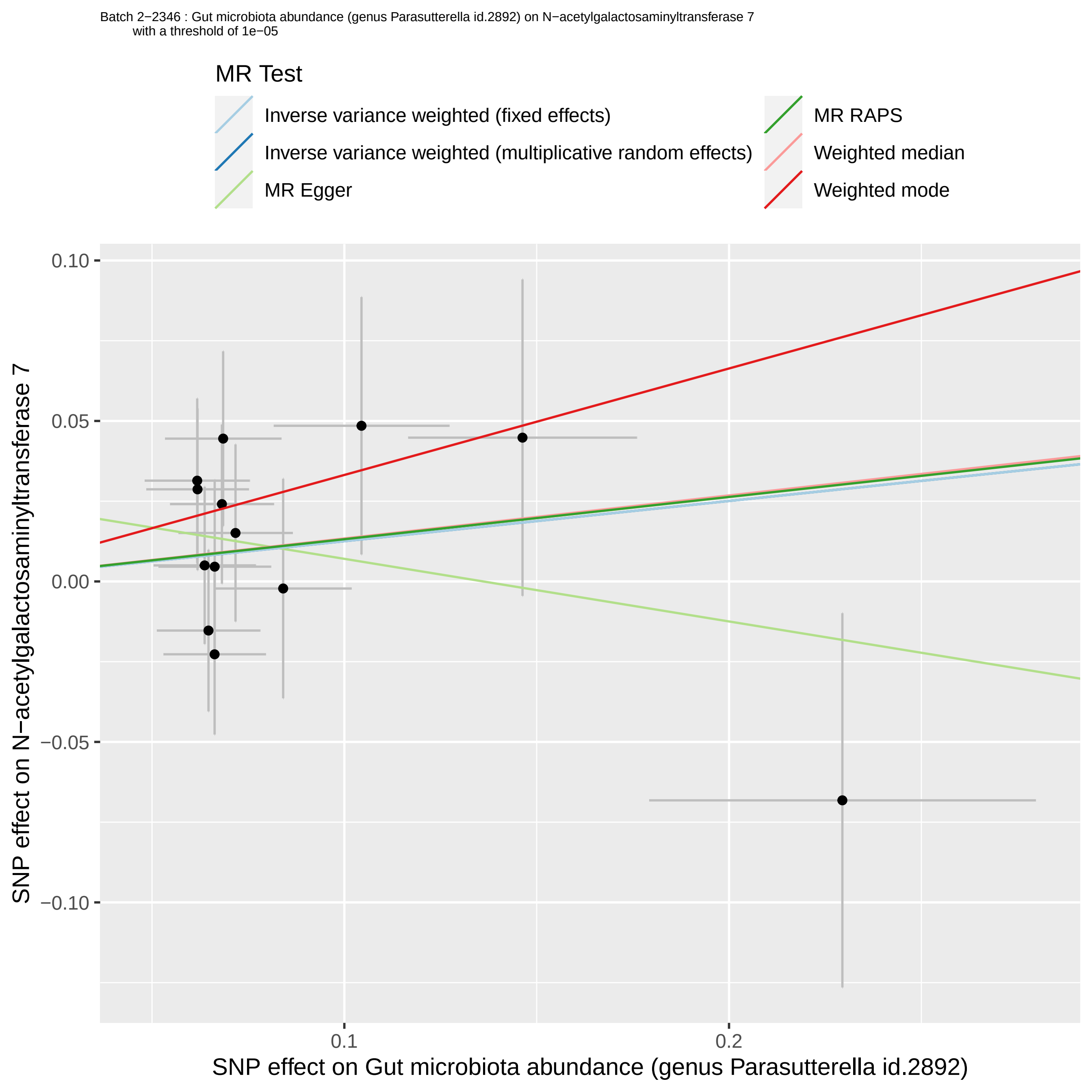

Batch 2−2346 : Gut microbiota abundance (genus Parasutterella id.2892) on N−acetylgalactosaminyltransferase 7
with a threshold of 1e−05
MR Test
Inverse variance weighted (fixed effects)
Inverse variance weighted (multiplicative random effects)
MR Egger
MR RAPS
Weighted median
Weighted mode
0.10
0.05
0.00
−0.05
−0.10
0.1
0.2
SNP effect on N−acetylgalactosaminyltransferase 7
SNP effect on Gut microbiota abundance (genus Parasutterella id.2892)

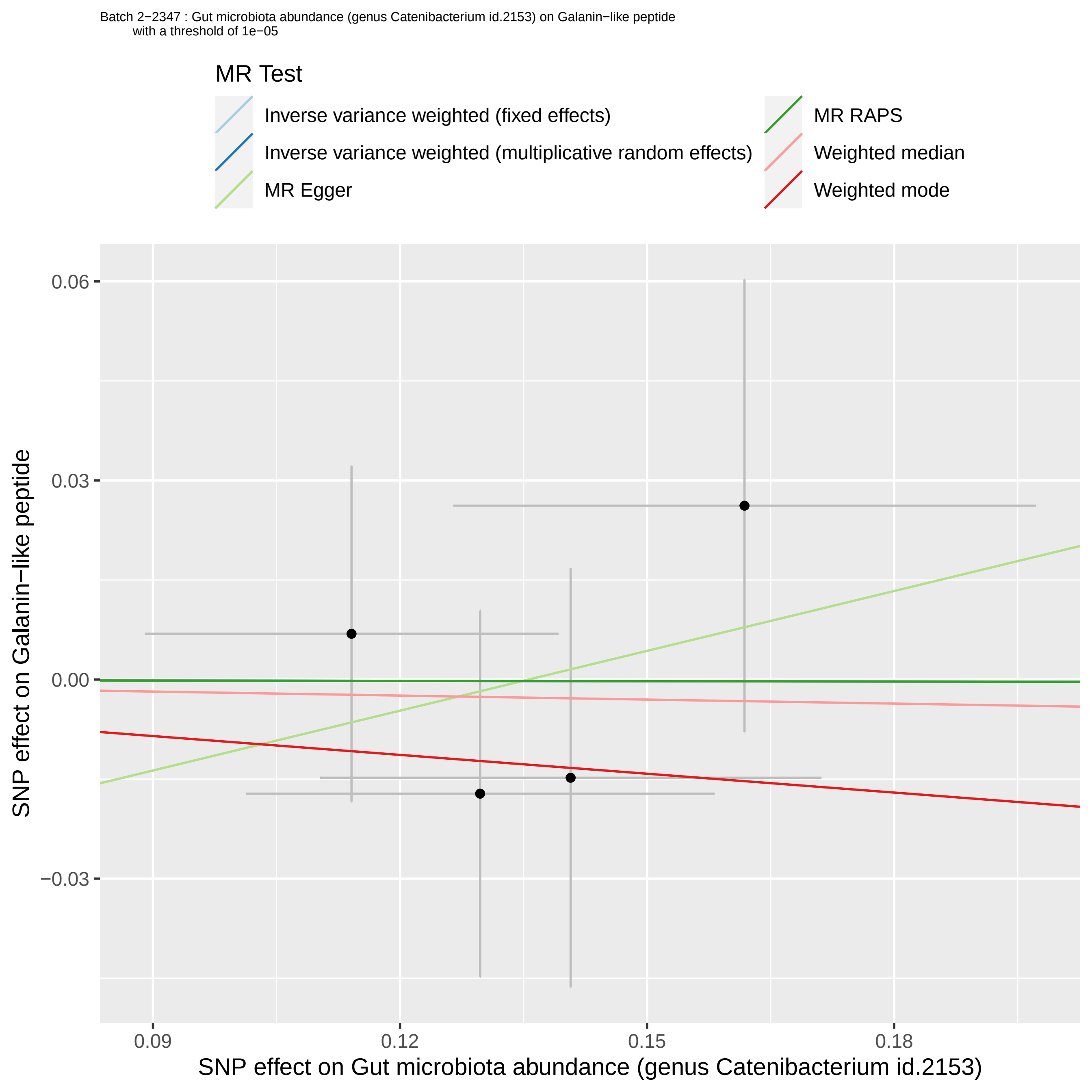

Batch 2−2347 : Gut microbiota abundance (genus Catenibacterium id.2153) on Galanin−like peptide
with a threshold of 1e−05
MR Test
Inverse variance weighted (fixed effects)
Inverse variance weighted (multiplicative random effects)
MR Egger
MR RAPS
Weighted median
Weighted mode
0.06
0.03
0.00
−0.03
0.09
0.12
0.15
0.18
SNP effect on Galanin−like peptide
SNP effect on Gut microbiota abundance (genus Catenibacterium id.2153)

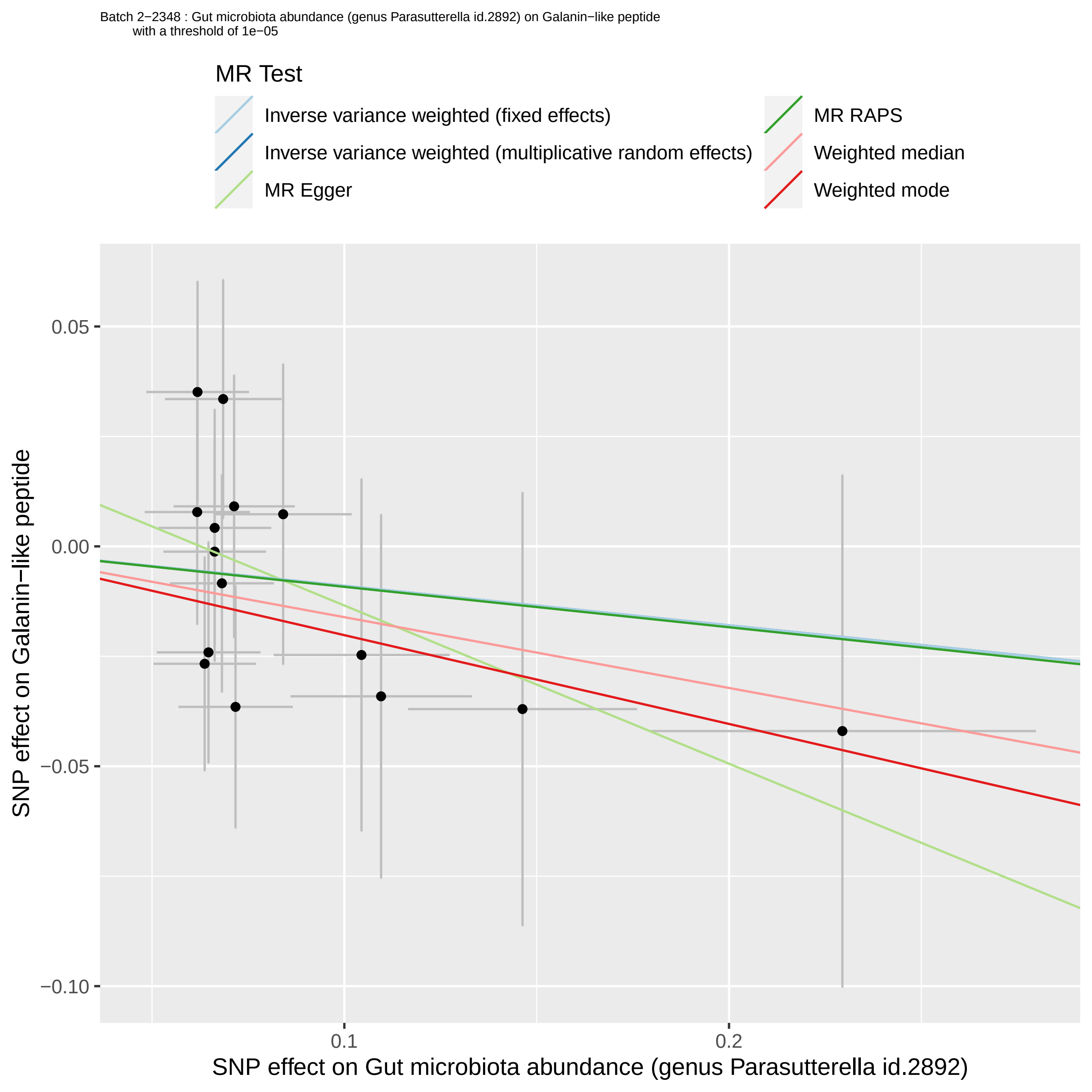
Batch 2−2348 : Gut microbiota abundance (genus Parasutterella id.2892) on Galanin−like peptide
with a threshold of 1e−05
MR Test
Inverse variance weighted (fixed effects)
Inverse variance weighted (multiplicative random effects)
MR Egger
MR RAPS
Weighted median
Weighted mode
SNP effect on Galanin−like peptide
0.05
0.00
−0.05
−0.10
0.1
0.2
SNP effect on Gut microbiota abundance (genus Parasutterella id.2892)

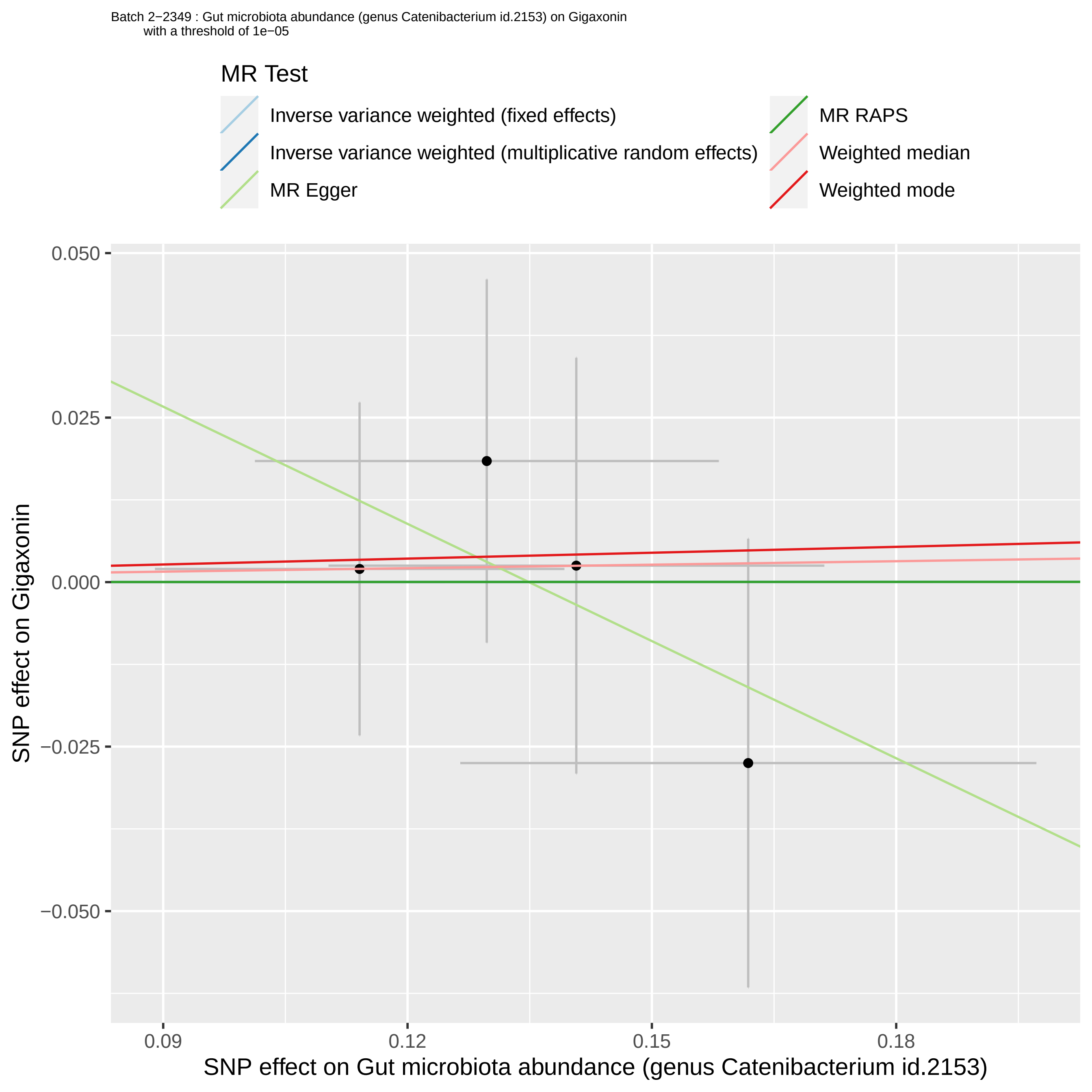

Batch 2−2349 : Gut microbiota abundance (genus Catenibacterium id.2153) on Gigaxonin
with a threshold of 1e−05
MR Test
Inverse variance weighted (fixed effects)
Inverse variance weighted (multiplicative random effects)
MR Egger
MR RAPS
Weighted median
Weighted mode
0.050
0.025
0.000
−0.025
−0.050
0.09
0.12
0.15
0.18
SNP effect on Gigaxonin
SNP effect on Gut microbiota abundance (genus Catenibacterium id.2153)

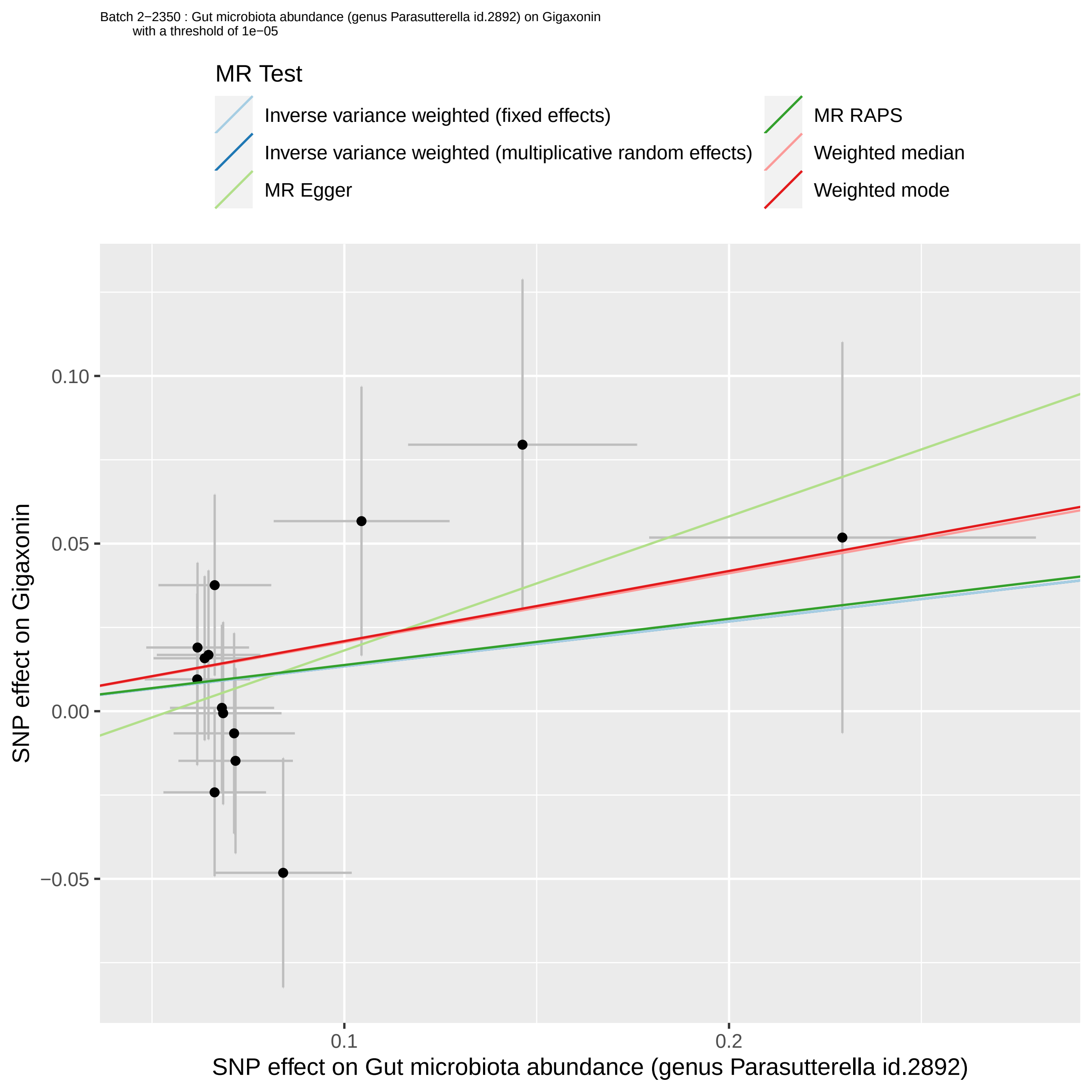
Batch 2−2350 : Gut microbiota abundance (genus Parasutterella id.2892) on Gigaxonin
with a threshold of 1e−05
MR Test
Inverse variance weighted (fixed effects)
Inverse variance weighted (multiplicative random effects)
MR Egger
MR RAPS
Weighted median
Weighted mode
SNP effect on Gigaxonin
0.10
0.05
0.00
−0.05
0.1
0.2
SNP effect on Gut microbiota abundance (genus Parasutterella id.2892)

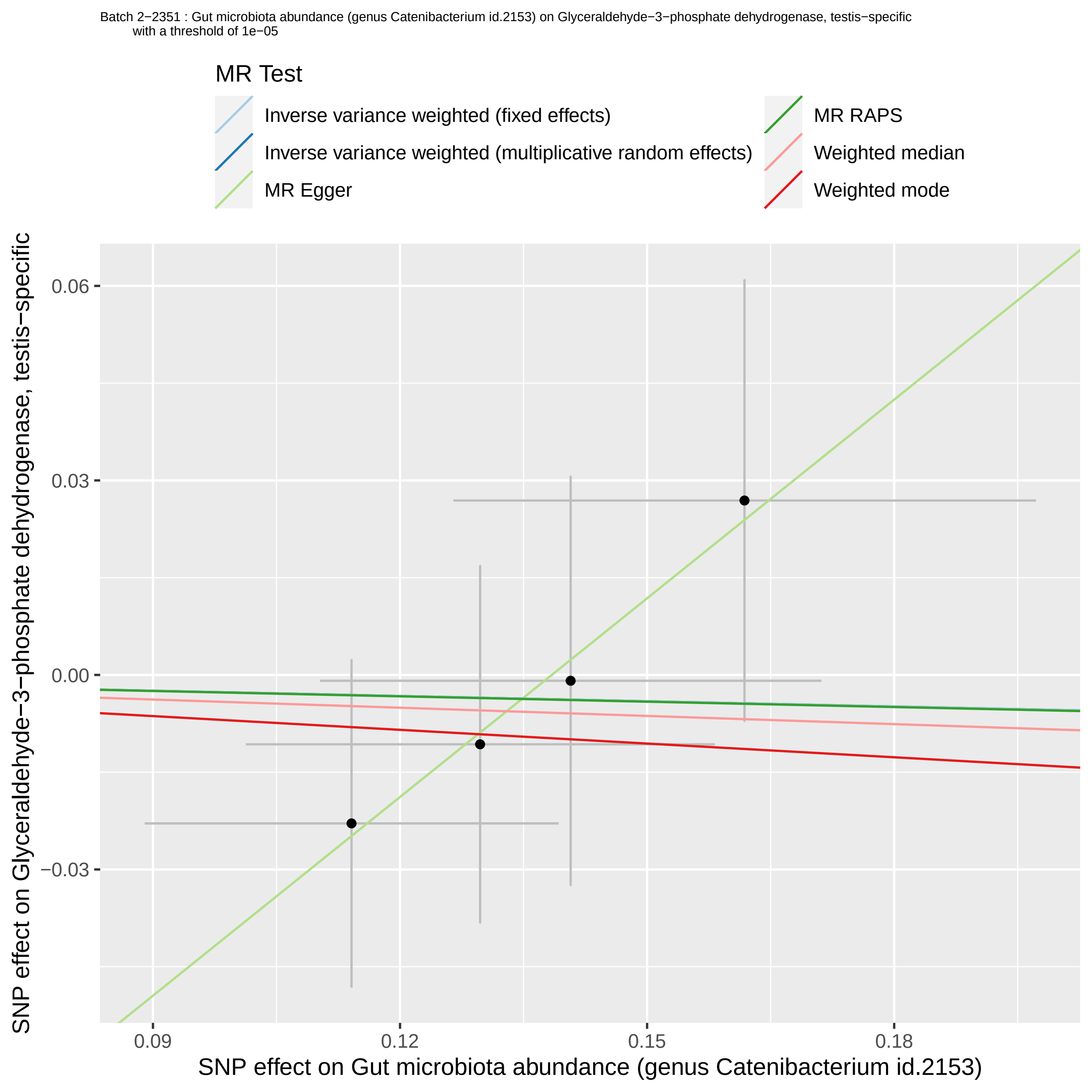
Batch 2−2351 : Gut microbiota abundance (genus Catenibacterium id.2153) on Glyceraldehyde−3−phosphate dehydrogenase, testis−specific
with a threshold of 1e−05
MR Test
Inverse variance weighted (fixed effects)
Inverse variance weighted (multiplicative random effects)
MR Egger
MR RAPS
Weighted median
Weighted mode
0.06
0.03
0.00
−0.03
0.09
0.12
0.15
0.18
SNP effect on Glyceraldehyde−3−phosphate dehydrogenase, testis−specific
SNP effect on Gut microbiota abundance (genus Catenibacterium id.2153)

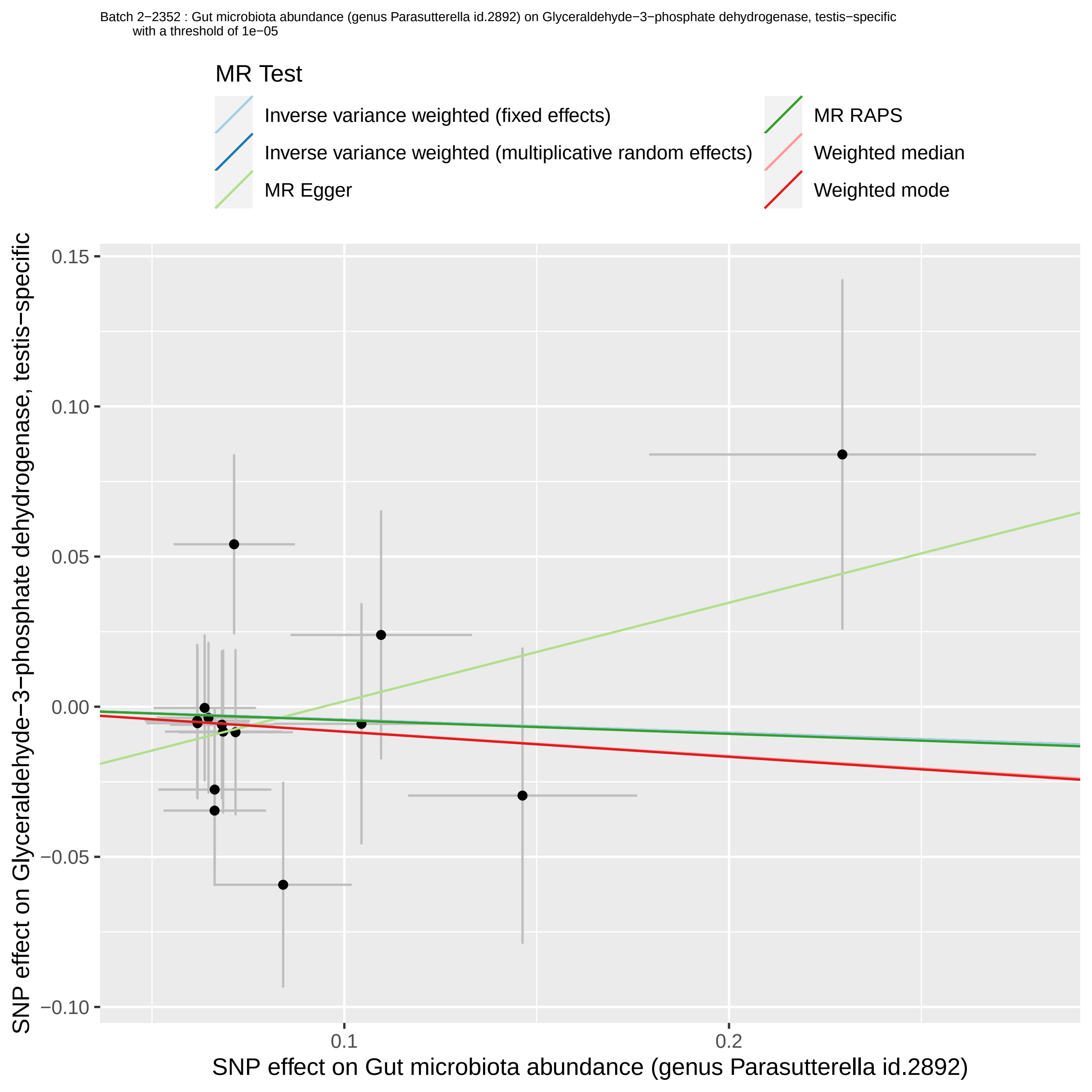

Batch 2−2352 : Gut microbiota abundance (genus Parasutterella id.2892) on Glyceraldehyde−3−phosphate dehydrogenase, testis−specific
with a threshold of 1e−05
MR Test
Inverse variance weighted (fixed effects)
Inverse variance weighted (multiplicative random effects)
MR Egger
MR RAPS
Weighted median
Weighted mode
0.15
0.10
0.05
0.00
−0.05
−0.10
0.1
0.2
SNP effect on Glyceraldehyde−3−phosphate dehydrogenase, testis−specific
SNP effect on Gut microbiota abundance (genus Parasutterella id.2892)

Batch 2−2353 : Gut microbiota abundance (genus Catenibacterium id.2153) on Growth arrest−specific protein 1 with a threshold of 1e−05

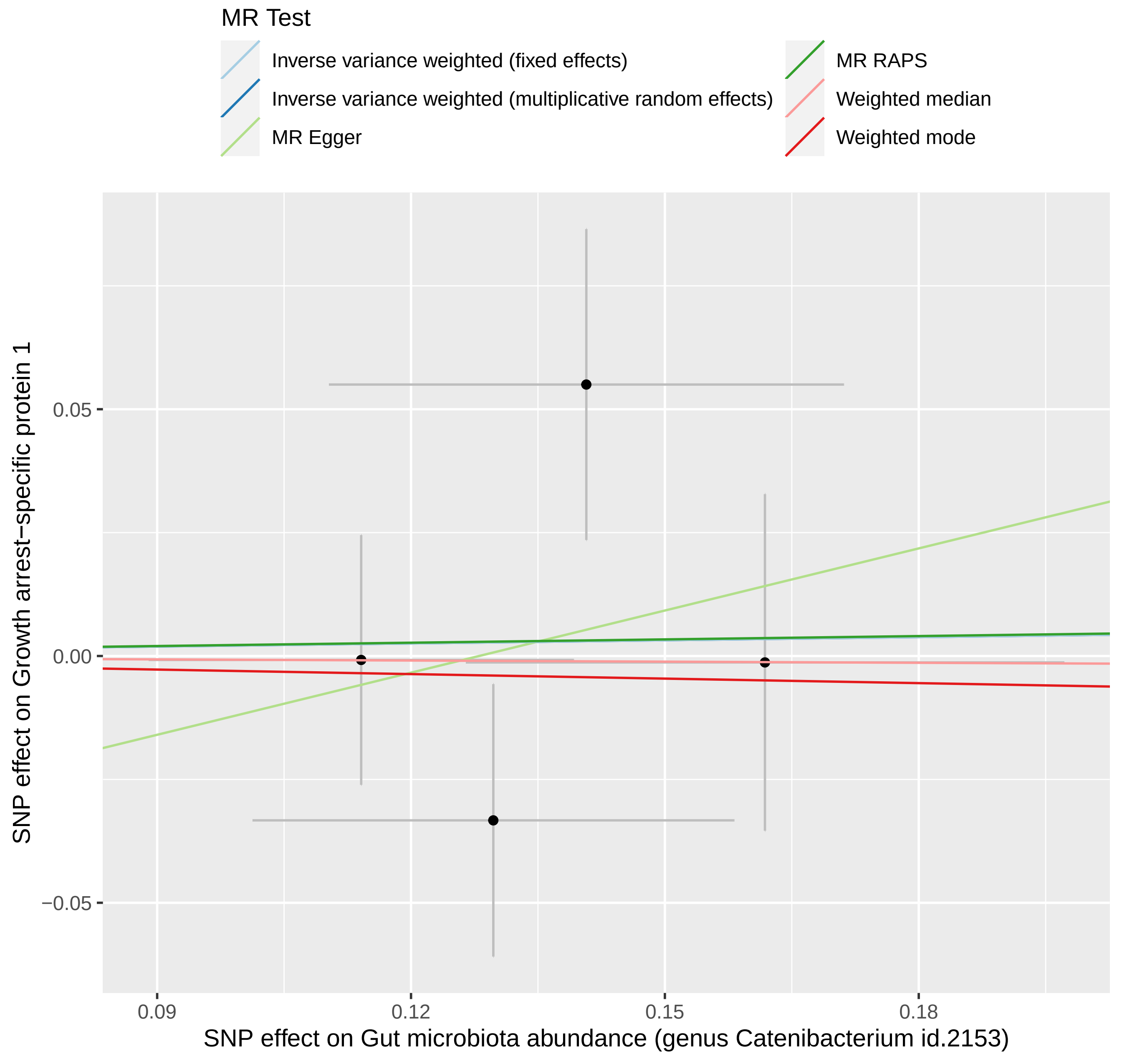

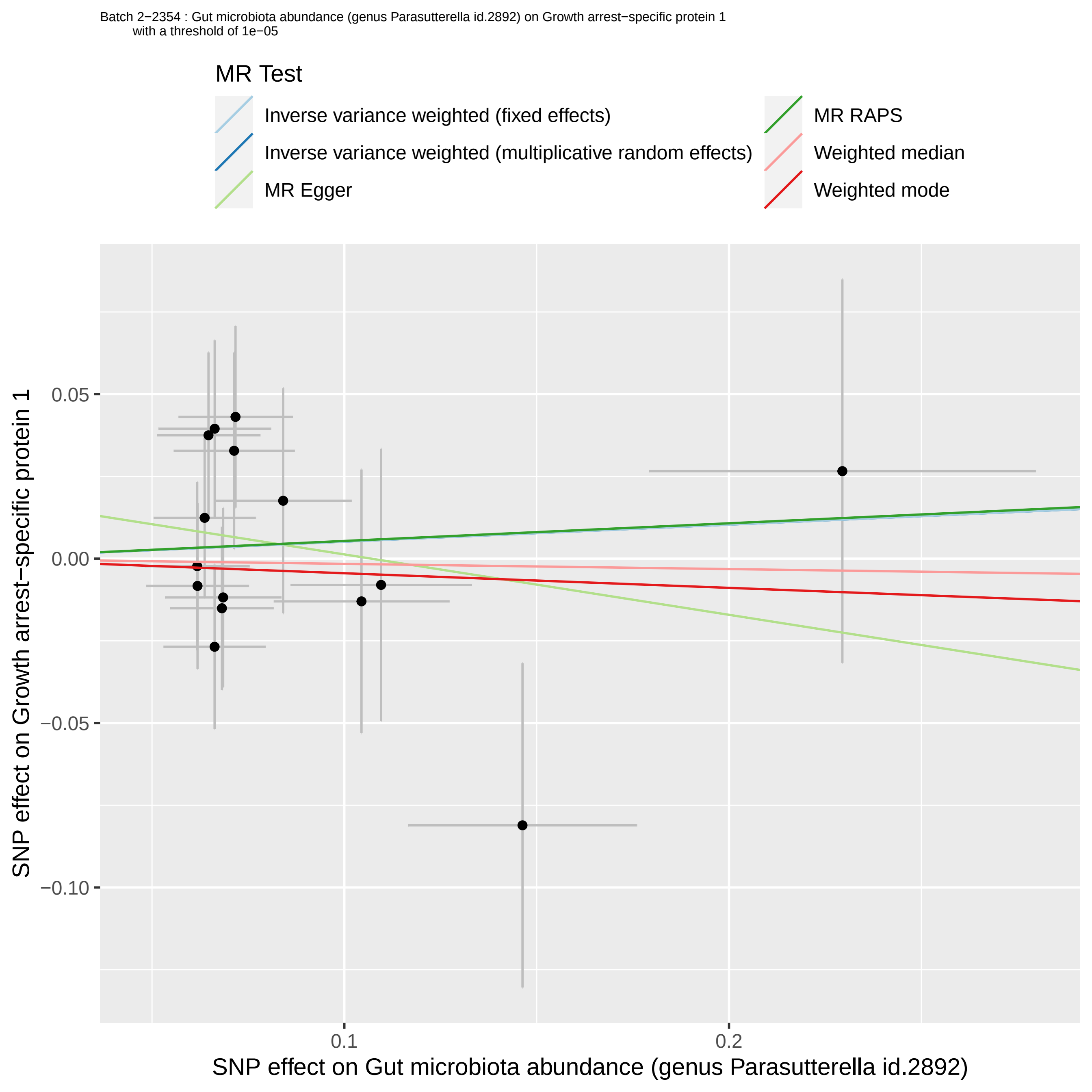
Batch 2−2354 : Gut microbiota abundance (genus Parasutterella id.2892) on Growth arrest−specific protein 1
with a threshold of 1e−05
MR Test
Inverse variance weighted (fixed effects)
Inverse variance weighted (multiplicative random effects)
MR Egger
MR RAPS
Weighted median
Weighted mode
0.05
0.00
−0.05
−0.10
0.1
0.2
SNP effect on Growth arrest−specific protein 1
SNP effect on Gut microbiota abundance (genus Parasutterella id.2892)

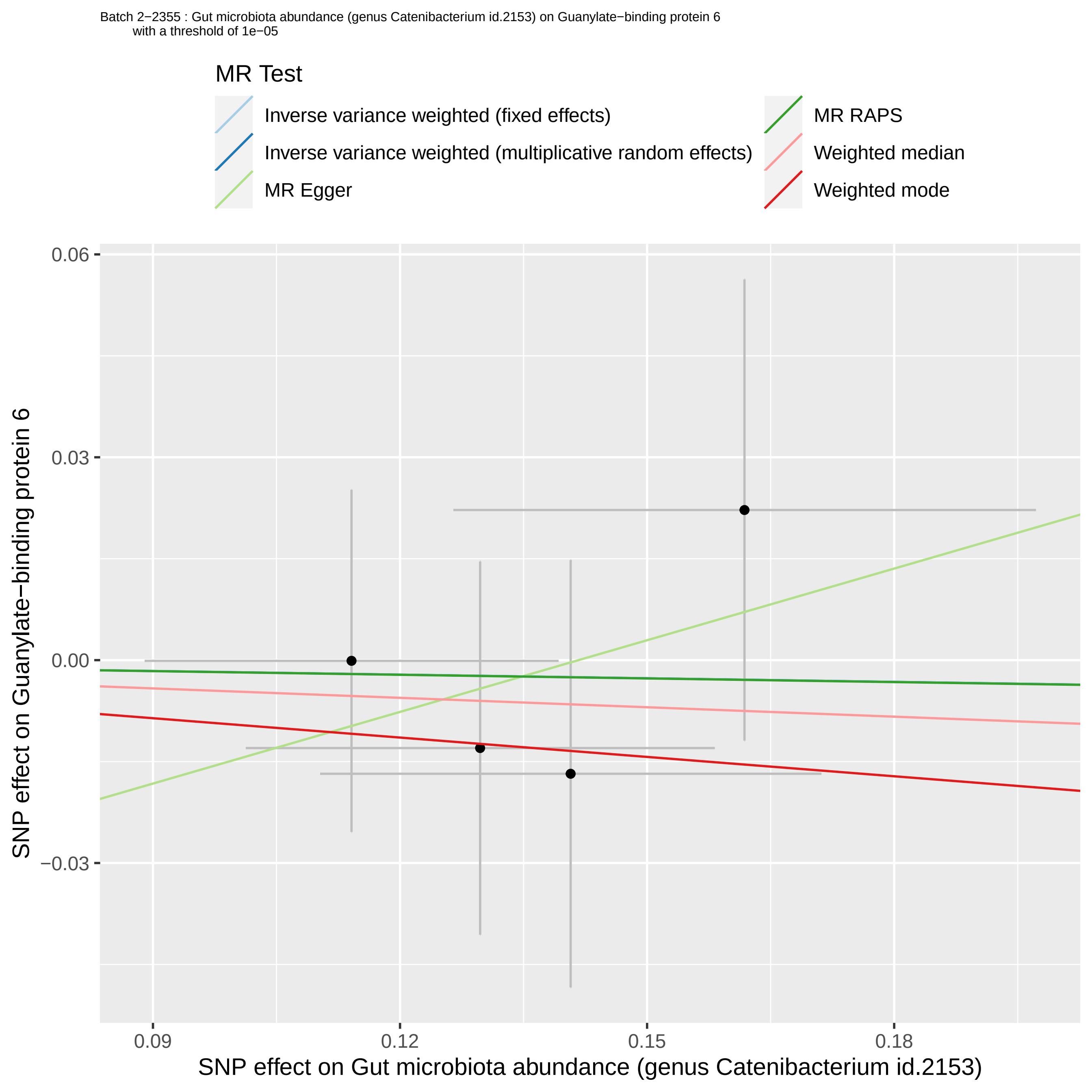
Batch 2−2355 : Gut microbiota abundance (genus Catenibacterium id.2153) on Guanylate−binding protein 6
with a threshold of 1e−05
MR Test
Inverse variance weighted (fixed effects)
Inverse variance weighted (multiplicative random effects)
MR Egger
MR RAPS
Weighted median
Weighted mode
0.06
0.03
0.00
−0.03
0.09
0.12
0.15
0.18
SNP effect on Guanylate−binding protein 6
SNP effect on Gut microbiota abundance (genus Catenibacterium id.2153)

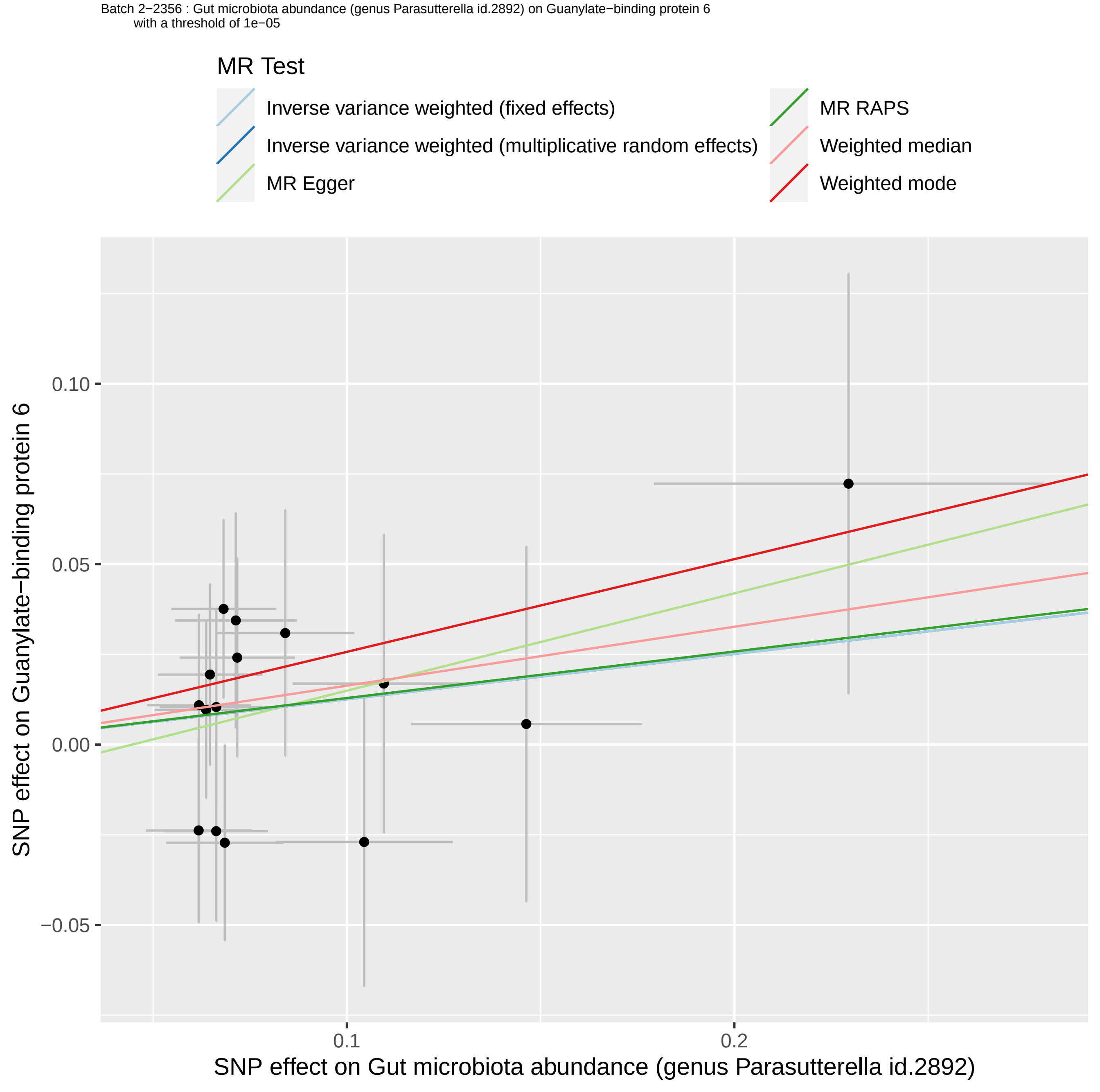

Batch 2−2356 : Gut microbiota abundance (genus Parasutterella id.2892) on Guanylate−binding protein 6
with a threshold of 1e−05
MR Test
Inverse variance weighted (fixed effects)
Inverse variance weighted (multiplicative random effects)
MR Egger
MR RAPS
Weighted median
Weighted mode
0.10
0.05
0.00
−0.05
0.1
0.2
SNP effect on Guanylate−binding protein 6
SNP effect on Gut microbiota abundance (genus Parasutterella id.2892)

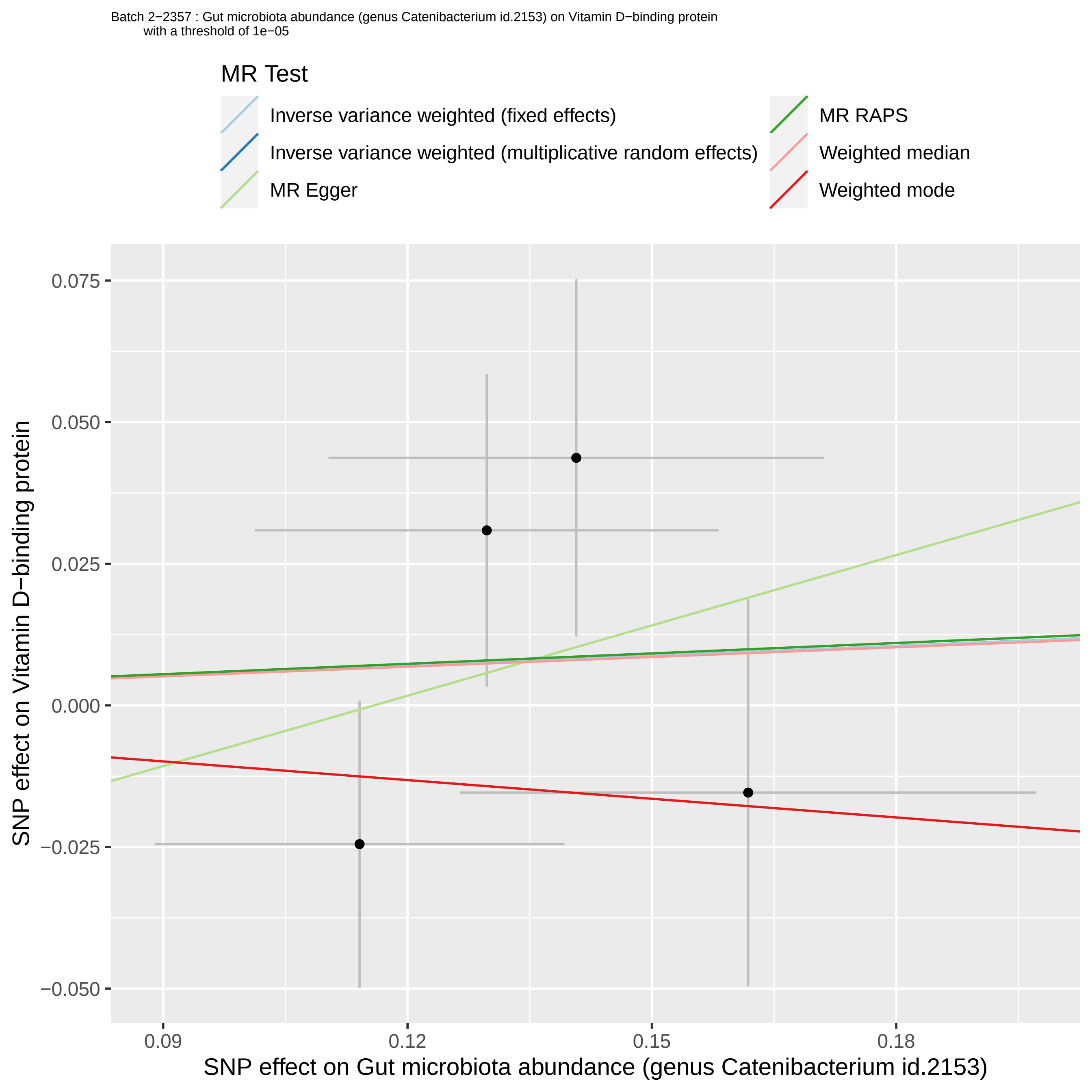

Batch 2−2357 : Gut microbiota abundance (genus Catenibacterium id.2153) on Vitamin D−binding protein
with a threshold of 1e−05
MR Test
Inverse variance weighted (fixed effects)
Inverse variance weighted (multiplicative random effects)
MR Egger
MR RAPS
Weighted median
Weighted mode
0.075
0.050
0.025
0.000
−0.025
−0.050
0.09
0.12
0.15
0.18
SNP effect on Vitamin D−binding protein
SNP effect on Gut microbiota abundance (genus Catenibacterium id.2153)

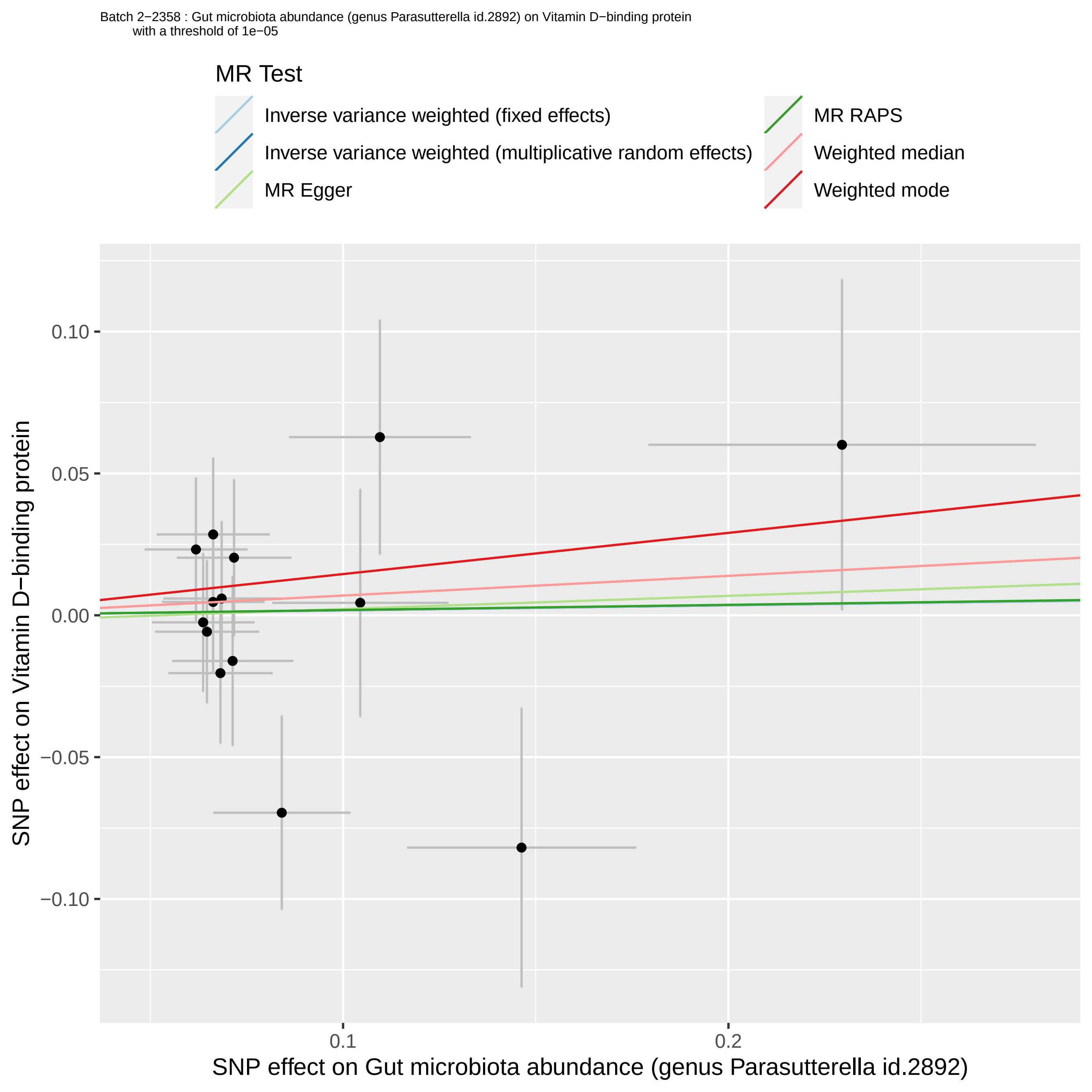

Batch 2−2358 : Gut microbiota abundance (genus Parasutterella id.2892) on Vitamin D−binding protein
with a threshold of 1e−05
MR Test
Inverse variance weighted (fixed effects)
Inverse variance weighted (multiplicative random effects)
MR Egger
MR RAPS
Weighted median
Weighted mode
0.10
0.05
0.00
−0.05
−0.10
0.1
0.2
SNP effect on Vitamin D−binding protein
SNP effect on Gut microbiota abundance (genus Parasutterella id.2892)

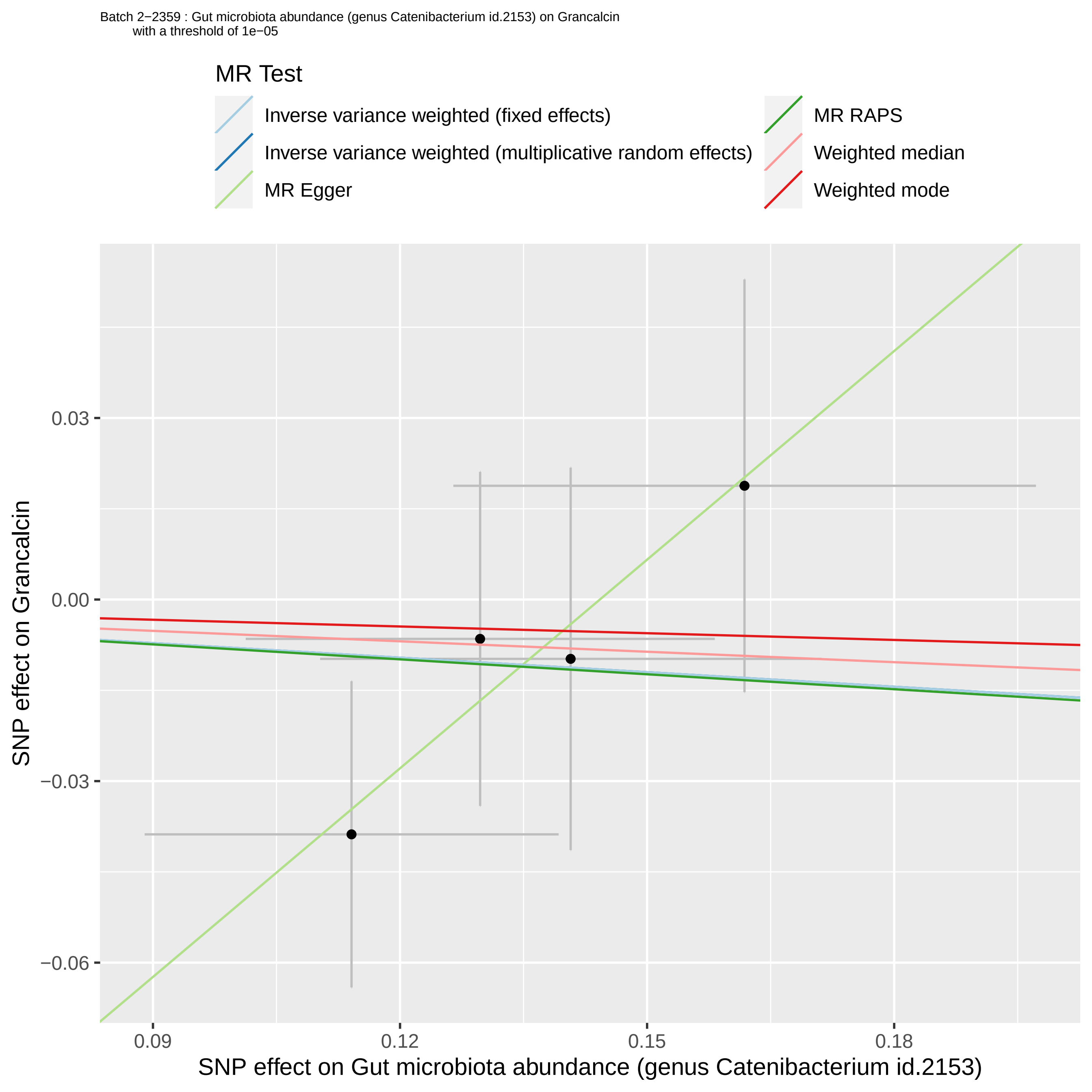
Batch 2−2359 : Gut microbiota abundance (genus Catenibacterium id.2153) on Grancalcin
with a threshold of 1e−05
MR Test
Inverse variance weighted (fixed effects)
Inverse variance weighted (multiplicative random effects)
MR Egger
MR RAPS
Weighted median
Weighted mode
0.03
0.00
−0.03
−0.06
0.09
0.12
0.15
0.18
SNP effect on Grancalcin
SNP effect on Gut microbiota abundance (genus Catenibacterium id.2153)

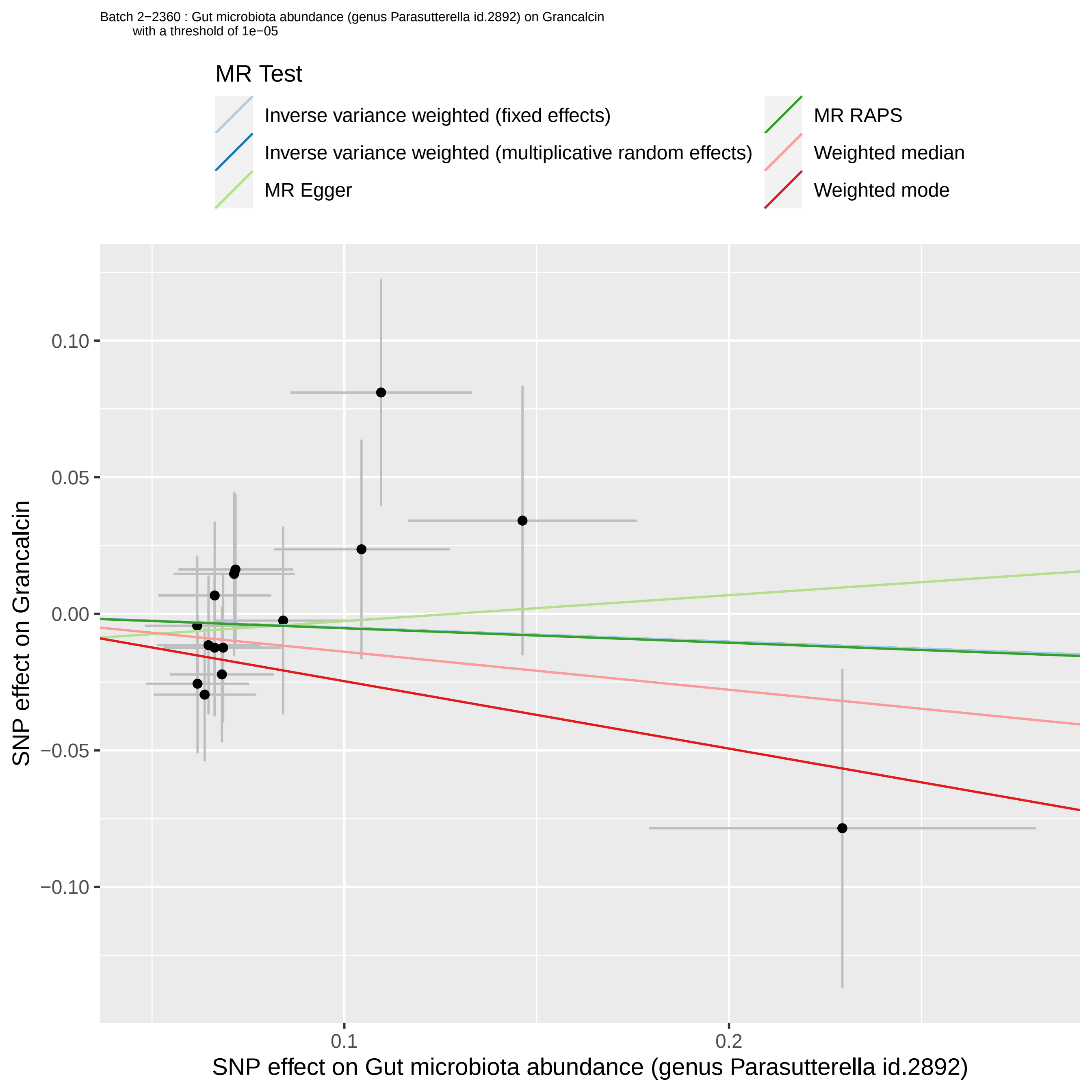

Batch 2−2360 : Gut microbiota abundance (genus Parasutterella id.2892) on Grancalcin
with a threshold of 1e−05
MR Test
Inverse variance weighted (fixed effects)
Inverse variance weighted (multiplicative random effects)
MR Egger
MR RAPS
Weighted median
Weighted mode
0.10
0.05
0.00
−0.05
−0.10
SNP effect on Grancalcin
0.1
0.2
SNP effect on Gut microbiota abundance (genus Parasutterella id.2892)

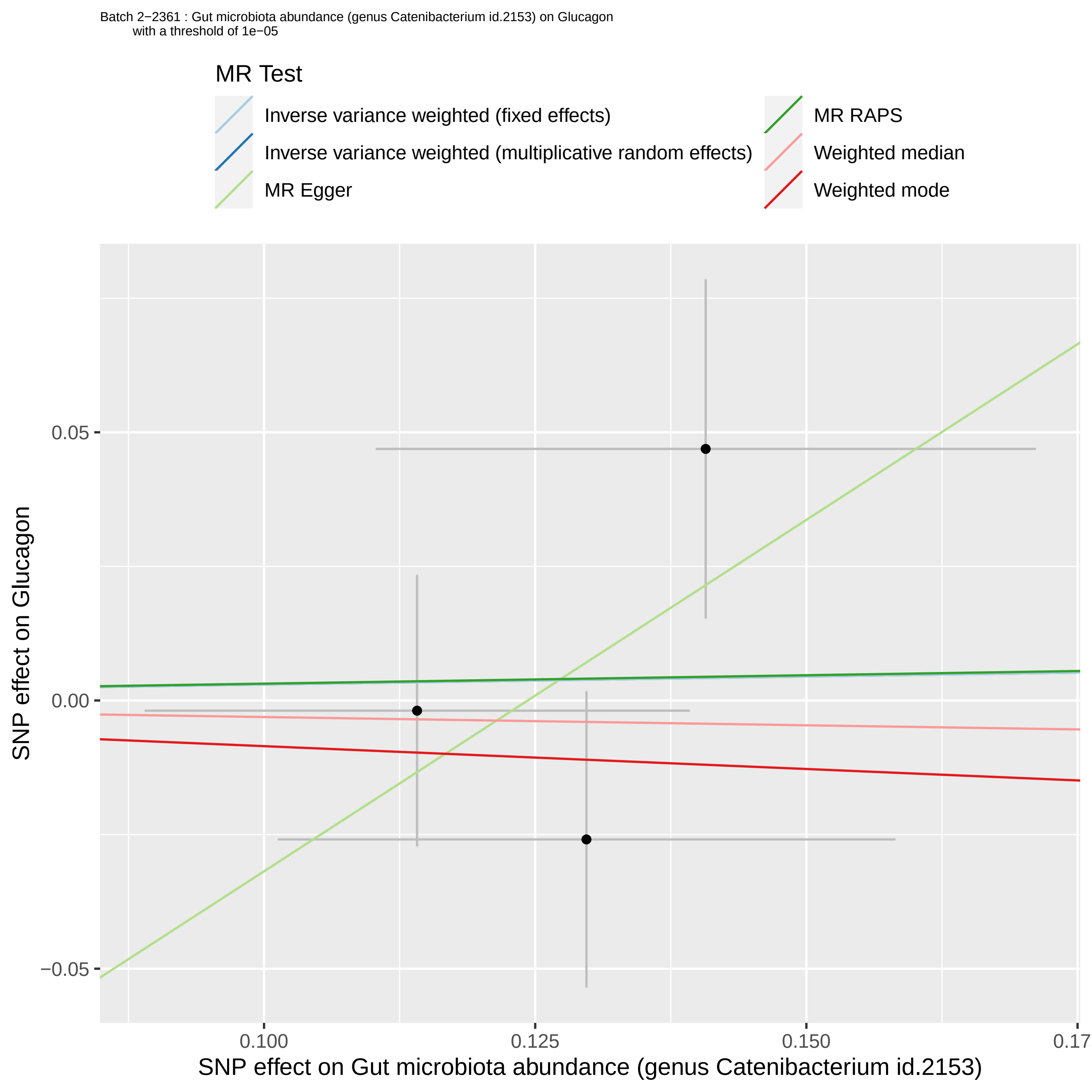
Batch 2−2361 : Gut microbiota abundance (genus Catenibacterium id.2153) on Glucagon
with a threshold of 1e−05
MR Test
Inverse variance weighted (fixed effects)
Inverse variance weighted (multiplicative random effects)
MR Egger
MR RAPS
Weighted median
Weighted mode
0.05
0.00
−0.05
0.100
0.125
0.150
0.17
SNP effect on Glucagon
SNP effect on Gut microbiota abundance (genus Catenibacterium id.2153)

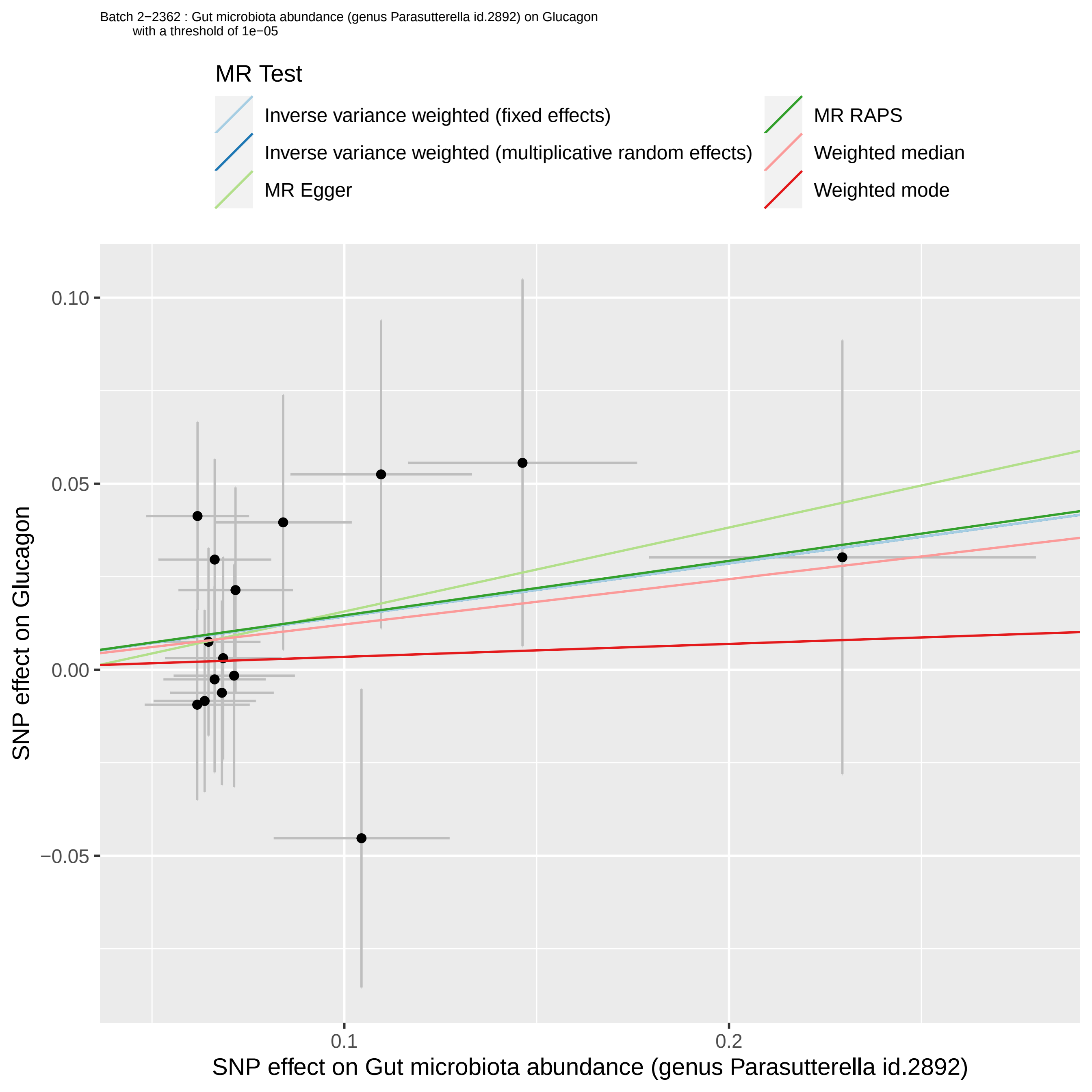
Batch 2−2362 : Gut microbiota abundance (genus Parasutterella id.2892) on Glucagon
with a threshold of 1e−05
MR Test
Inverse variance weighted (fixed effects)
Inverse variance weighted (multiplicative random effects)
MR Egger
MR RAPS
Weighted median
Weighted mode
0.10
0.05
0.00
−0.05
0.1
0.2
SNP effect on Glucagon
SNP effect on Gut microbiota abundance (genus Parasutterella id.2892)

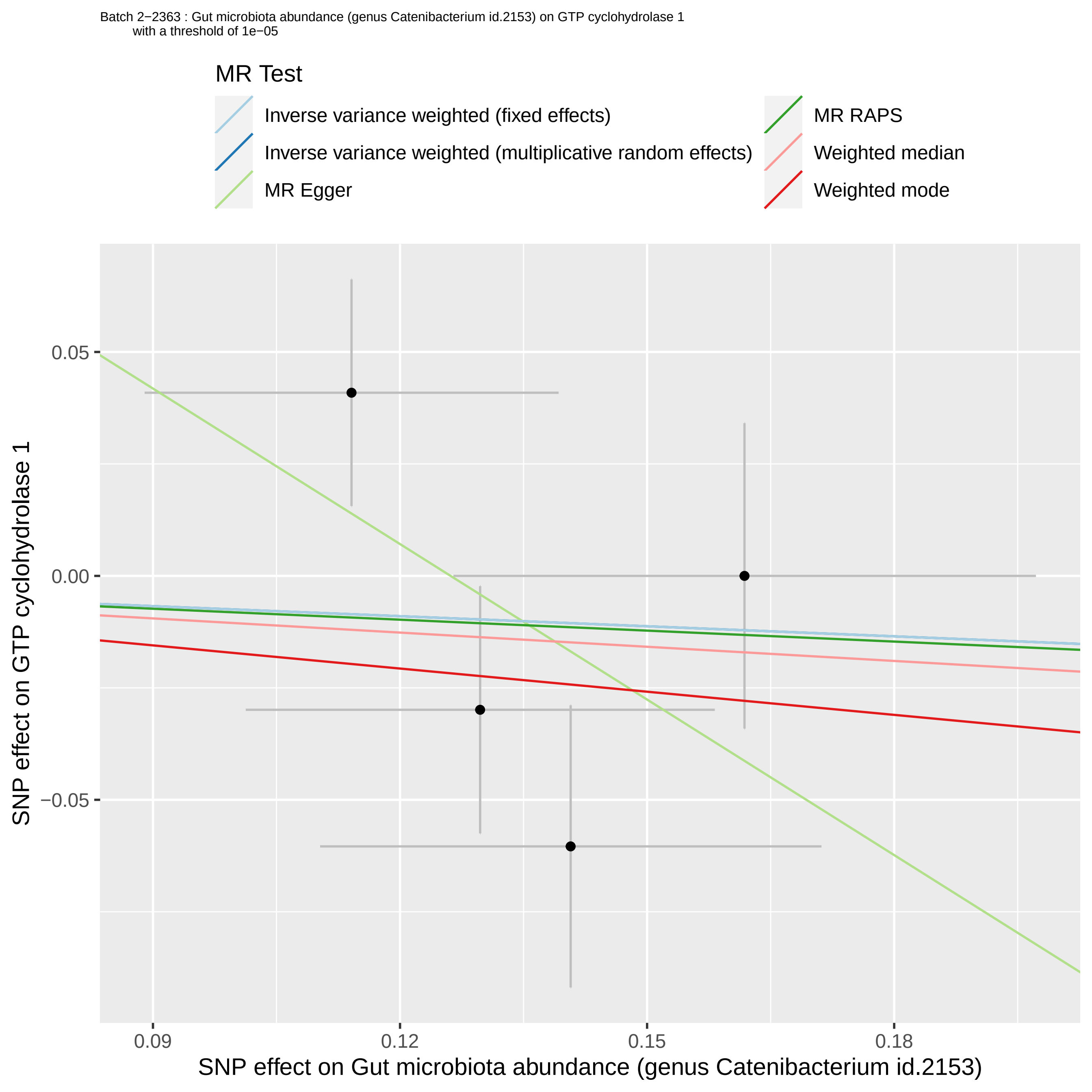

Batch 2−2363 : Gut microbiota abundance (genus Catenibacterium id.2153) on GTP cyclohydrolase 1
with a threshold of 1e−05
MR Test
Inverse variance weighted (fixed effects)
Inverse variance weighted (multiplicative random effects)
MR Egger
MR RAPS
Weighted median
Weighted mode
0.05
0.00
−0.05
0.09
0.12
0.15
0.18
SNP effect on GTP cyclohydrolase 1
SNP effect on Gut microbiota abundance (genus Catenibacterium id.2153)

Batch 2−2364 : Gut microbiota abundance (genus Parasutterella id.2892) on GTP cyclohydrolase 1
with a threshold of 1e−05

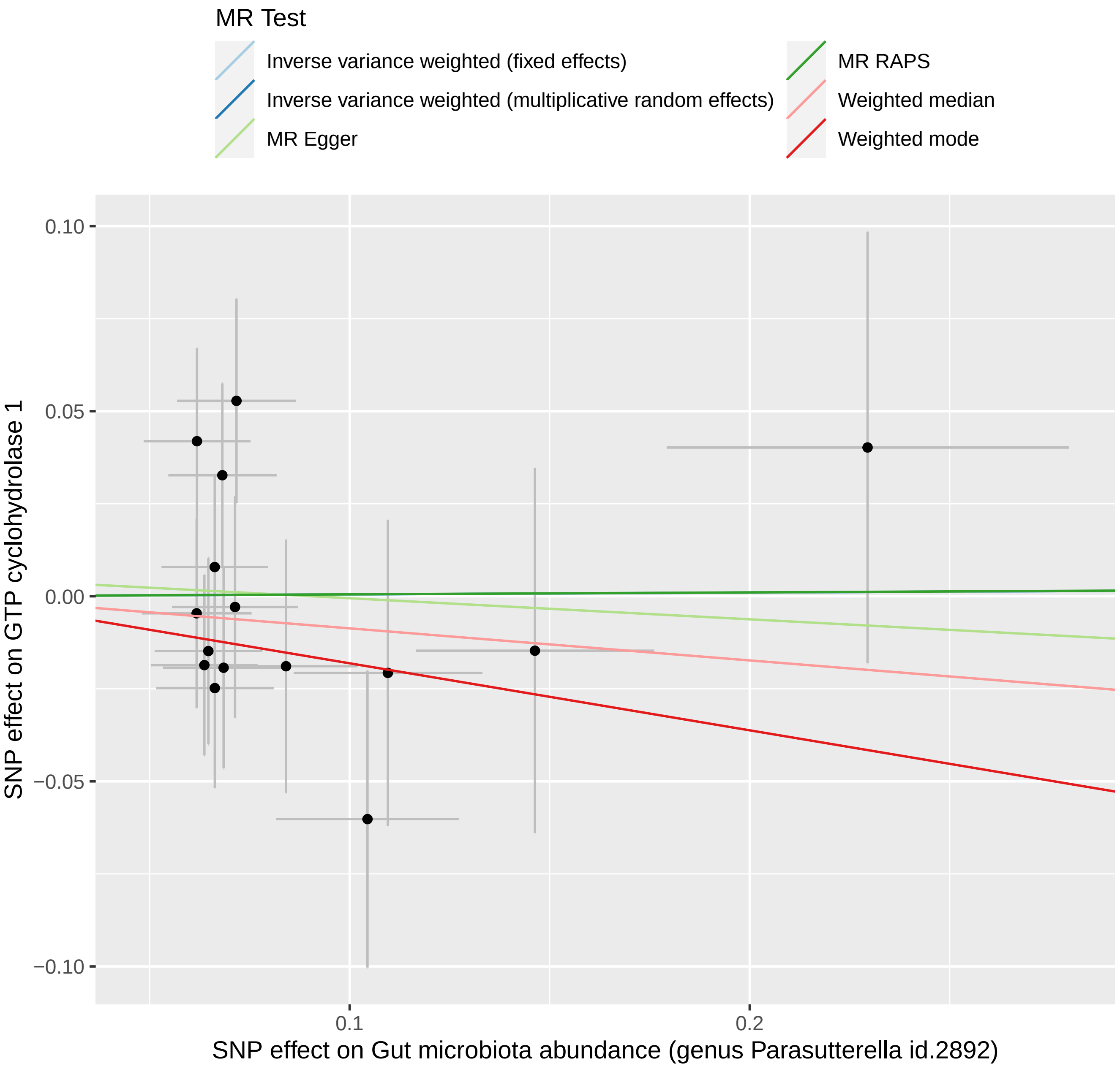

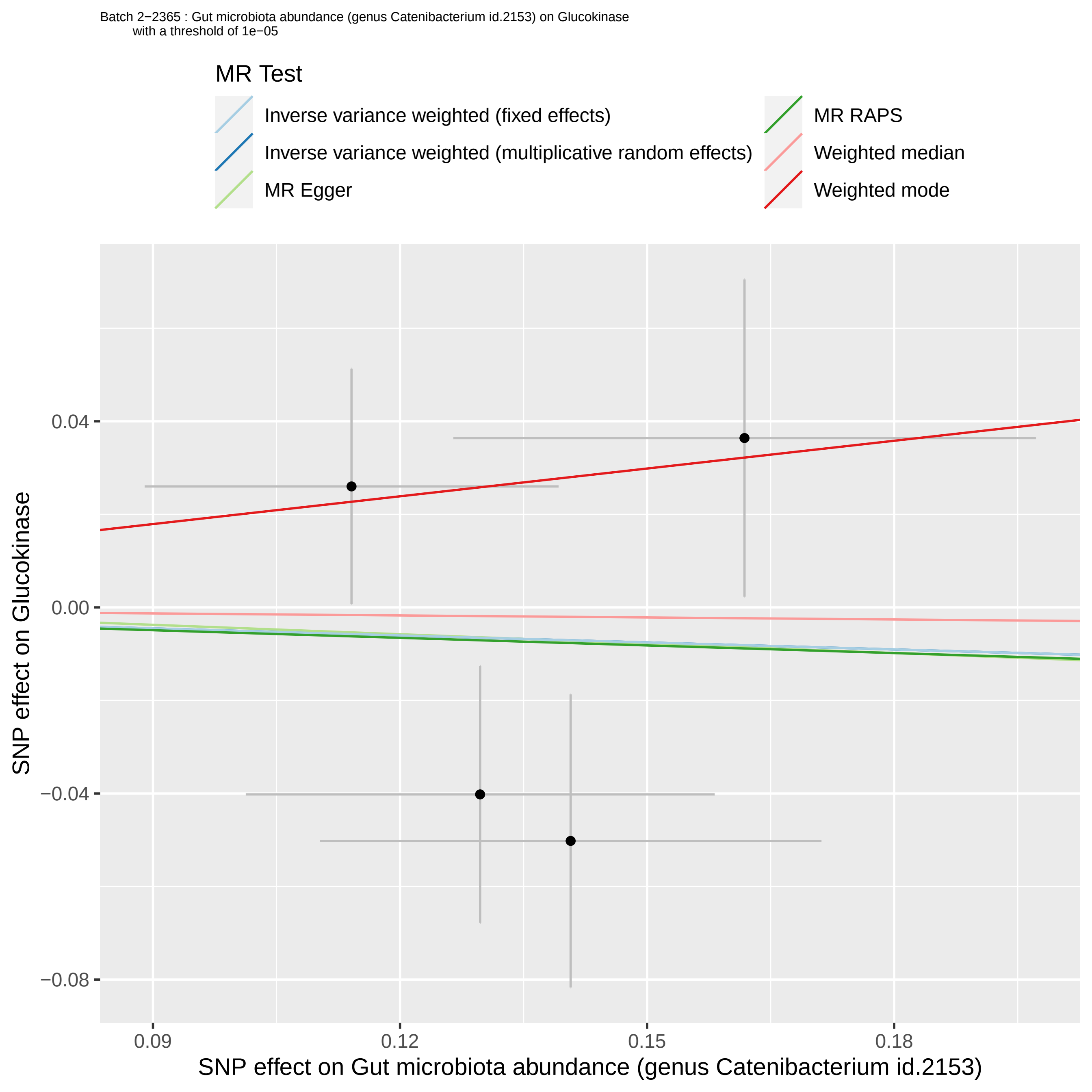
Batch 2−2365 : Gut microbiota abundance (genus Catenibacterium id.2153) on Glucokinase
with a threshold of 1e−05
MR Test
Inverse variance weighted (fixed effects)
Inverse variance weighted (multiplicative random effects)
MR Egger
MR RAPS
Weighted median
Weighted mode
SNP effect on Glucokinase
0.04
0.00
−0.04
−0.08
0.09
0.12
0.15
0.18
SNP effect on Gut microbiota abundance (genus Catenibacterium id.2153)

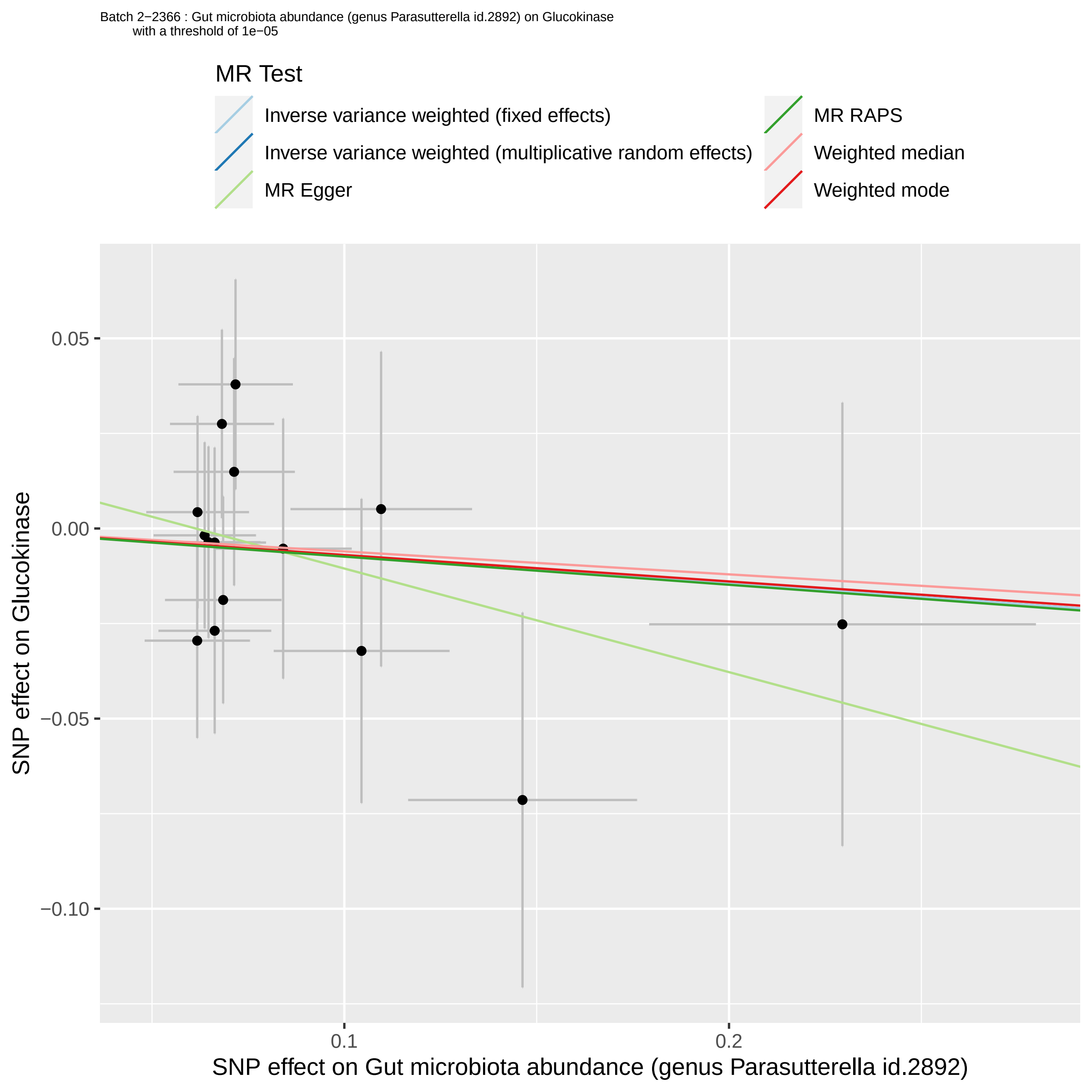
Batch 2−2366 : Gut microbiota abundance (genus Parasutterella id.2892) on Glucokinase
with a threshold of 1e−05
MR Test
Inverse variance weighted (fixed effects)
Inverse variance weighted (multiplicative random effects)
MR Egger
MR RAPS
Weighted median
Weighted mode
0.05
0.00
−0.05
−0.10
0.1
0.2
SNP effect on Glucokinase
SNP effect on Gut microbiota abundance (genus Parasutterella id.2892)

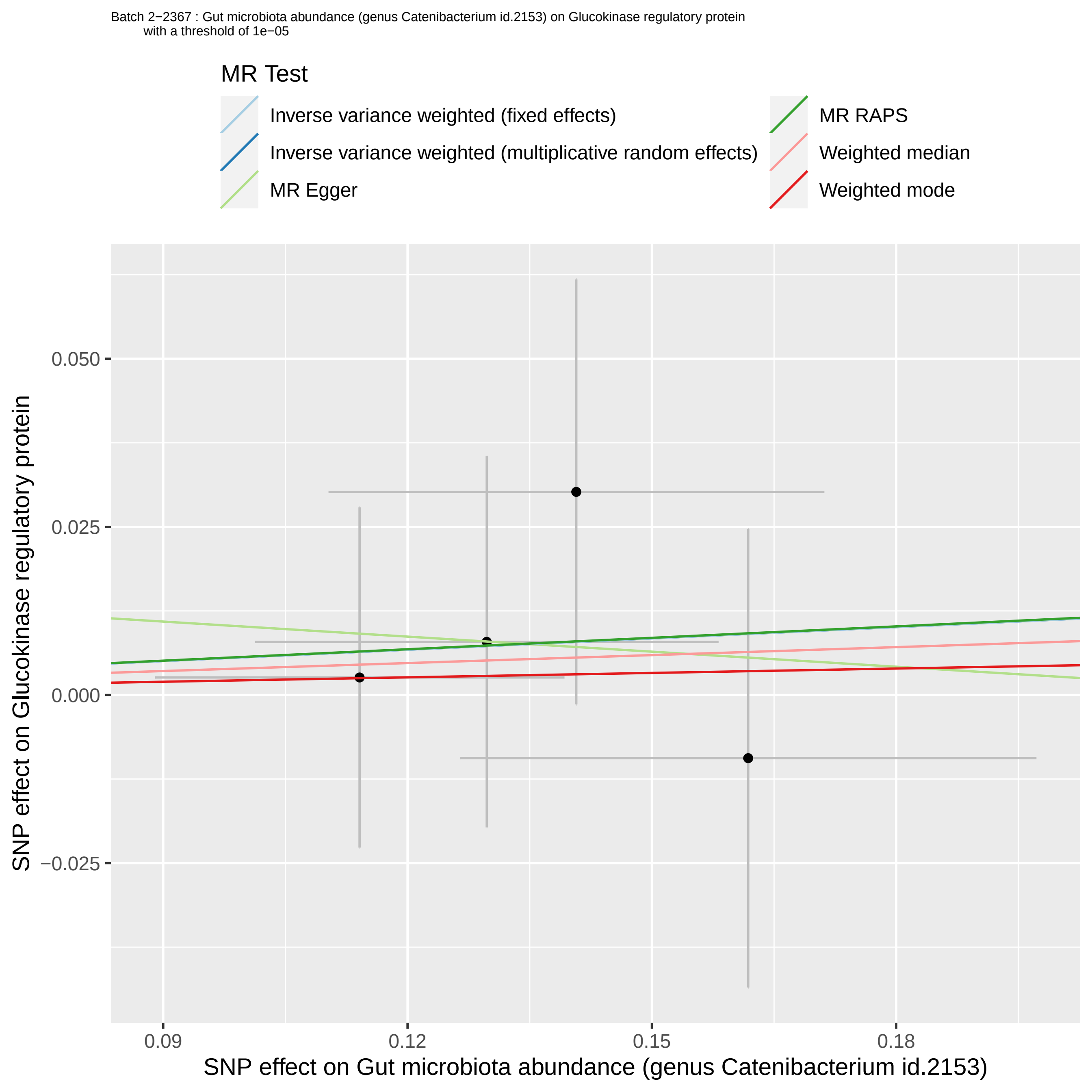
Batch 2−2367 : Gut microbiota abundance (genus Catenibacterium id.2153) on Glucokinase regulatory protein
with a threshold of 1e−05
MR Test
Inverse variance weighted (fixed effects)
Inverse variance weighted (multiplicative random effects)
MR Egger
MR RAPS
Weighted median
Weighted mode
0.050
0.025
0.000
−0.025
0.09
0.12
0.15
0.18
SNP effect on Glucokinase regulatory protein
SNP effect on Gut microbiota abundance (genus Catenibacterium id.2153)

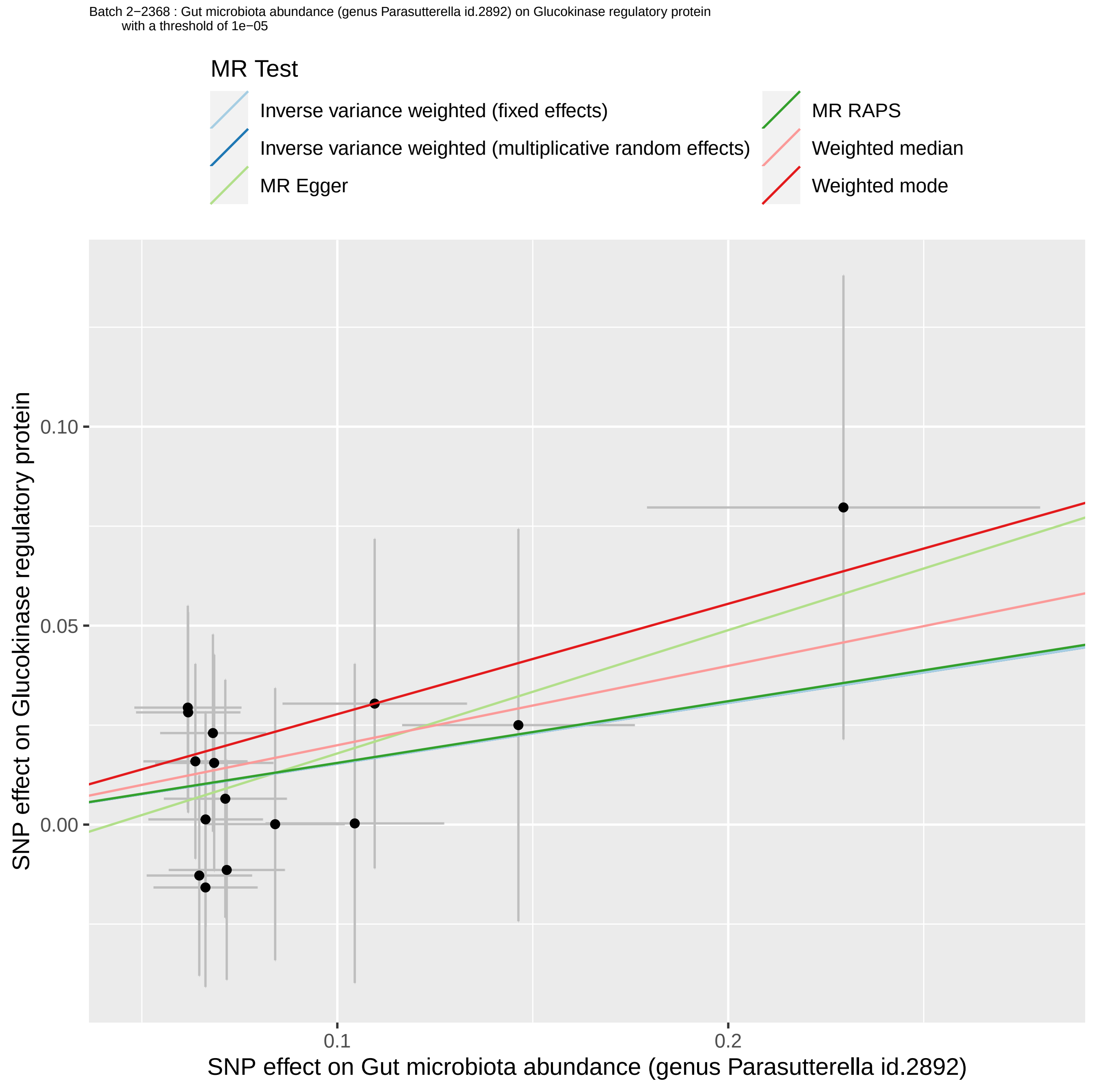
Batch 2−2368 : Gut microbiota abundance (genus Parasutterella id.2892) on Glucokinase regulatory protein
with a threshold of 1e−05
MR Test
Inverse variance weighted (fixed effects)
Inverse variance weighted (multiplicative random effects)
MR Egger
MR RAPS
Weighted median
Weighted mode
SNP effect on Glucokinase regulatory protein
0.10
0.05
0.00
0.1
0.2
SNP effect on Gut microbiota abundance (genus Parasutterella id.2892)

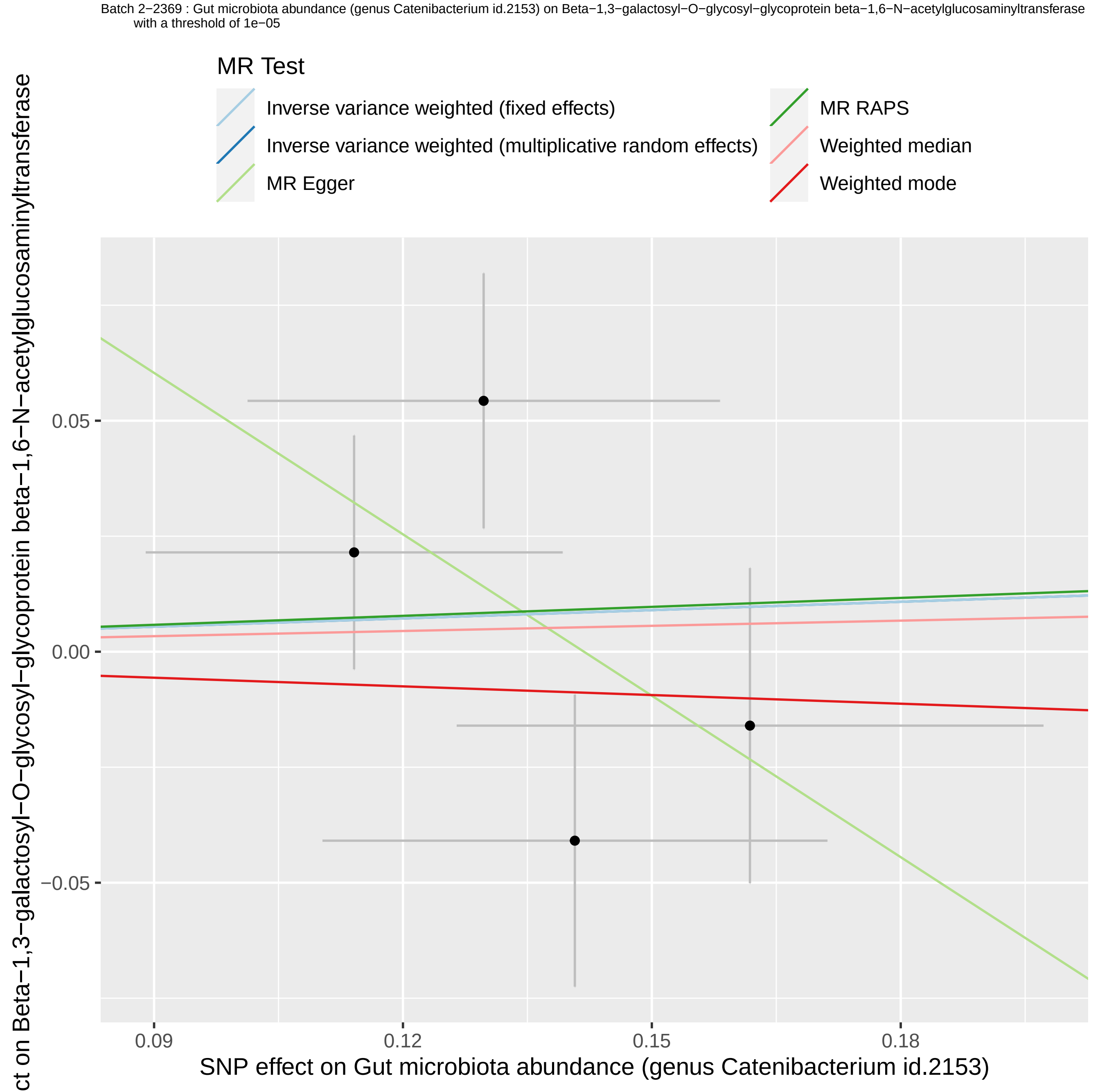

Batch 2−2369 : Gut microbiota abundance (genus Catenibacterium id.2153) on Beta−1,3−galactosyl−O−glycosyl−glycoprotein beta−1,6−N−acetylglucosaminyltransferase with a threshold of 1e−05
MR Test
Inverse variance weighted (fixed effects)
Inverse variance weighted (multiplicative random effects)
MR Egger
MR RAPS
Weighted median
Weighted mode
ct on Beta−1,3−galactosyl−O−glycosyl−glycoprotein beta−1,6−N−acetylglucosaminyltransferase
0.05
0.00
−0.05
0.09
0.12
0.15
0.18
SNP effect on Gut microbiota abundance (genus Catenibacterium id.2153)

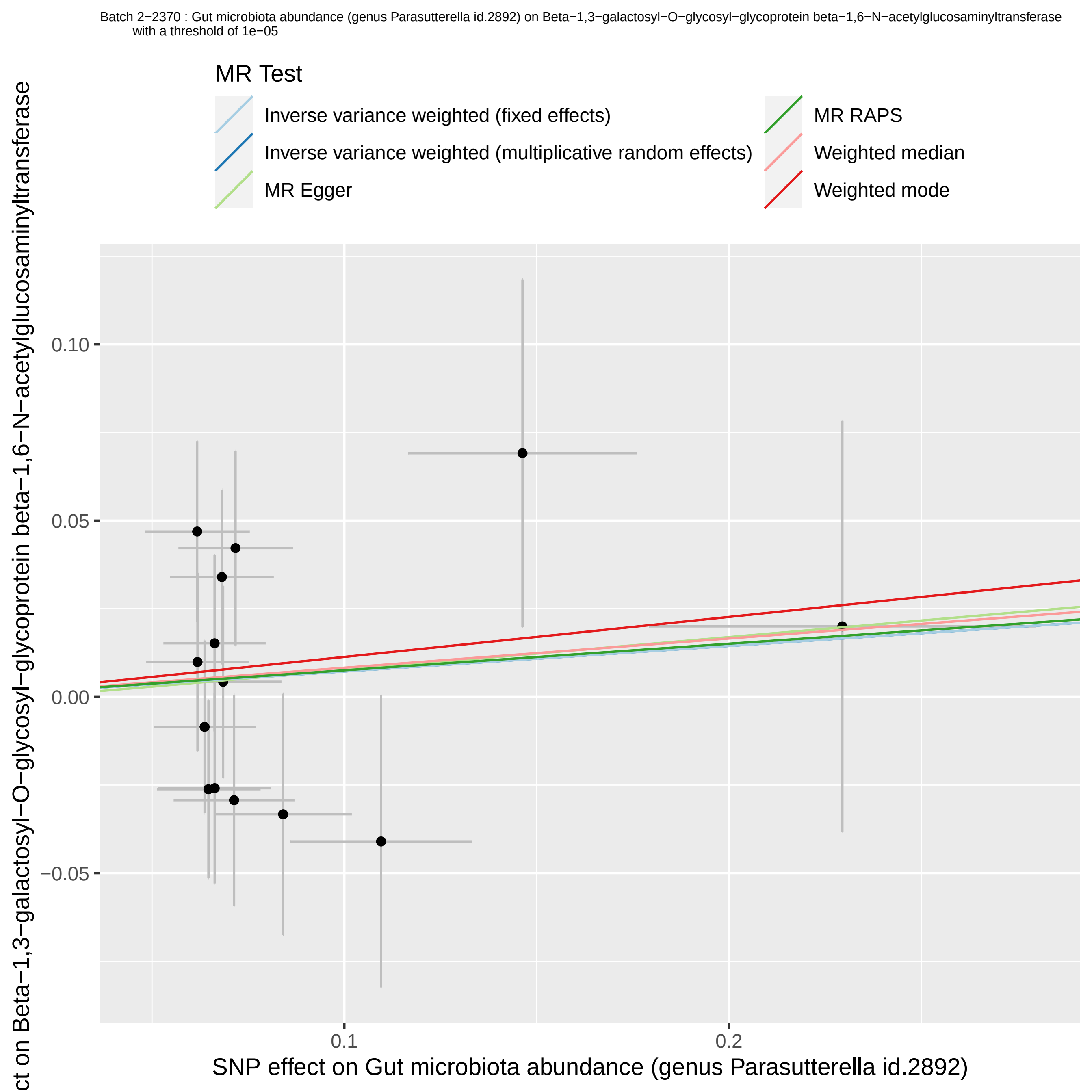
Batch 2−2370 : Gut microbiota abundance (genus Parasutterella id.2892) on Beta−1,3−galactosyl−O−glycosyl−glycoprotein beta−1,6−N−acetylglucosaminyltransferase with a threshold of 1e−05
MR Test
Inverse variance weighted (fixed effects)
Inverse variance weighted (multiplicative random effects)
MR Egger
MR RAPS
Weighted median
Weighted mode
ct on Beta−1,3−galactosyl−O−glycosyl−glycoprotein beta−1,6−N−acetylglucosaminyltransferase
0.10
0.05
0.00
−0.05
0.1
0.2
SNP effect on Gut microbiota abundance (genus Parasutterella id.2892)

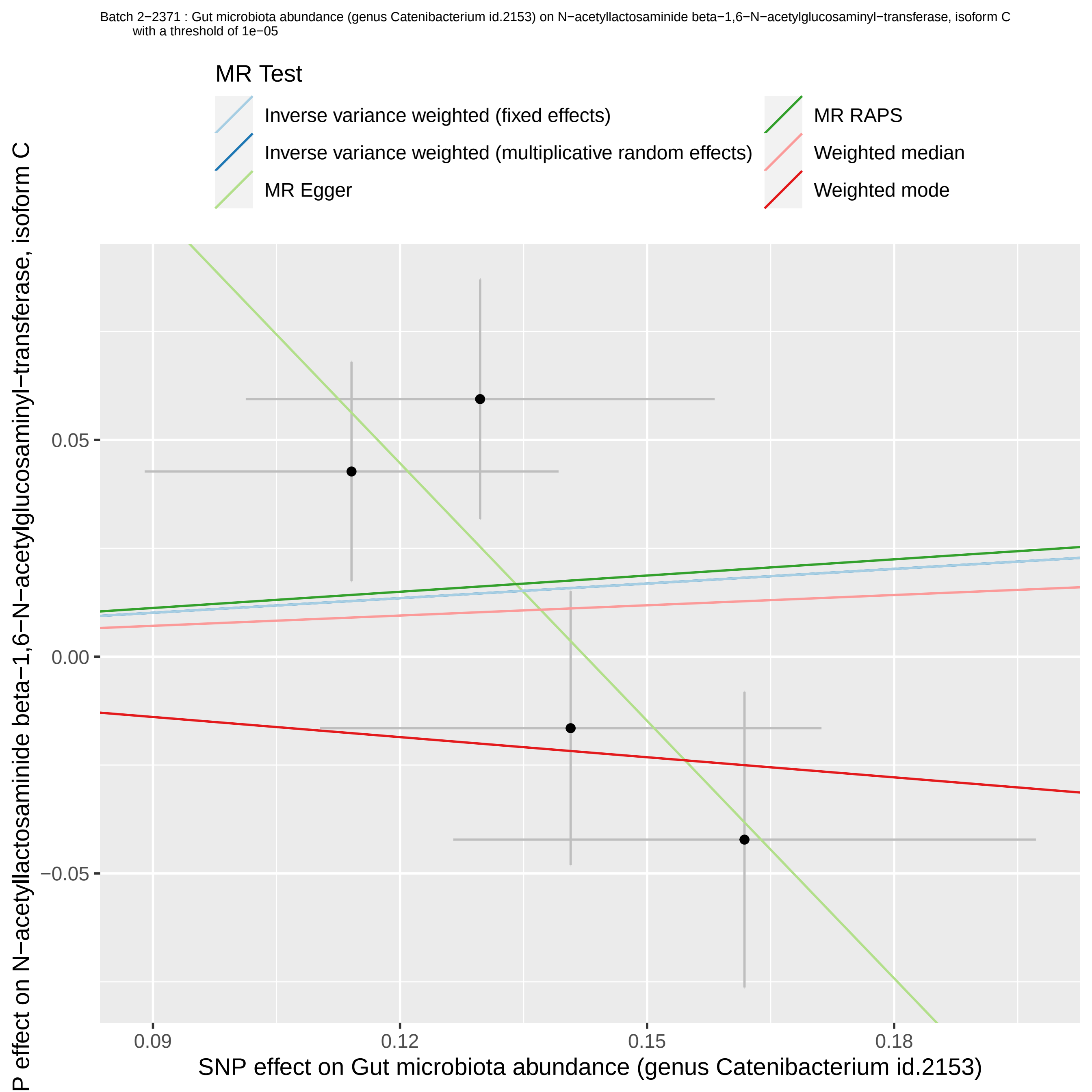

Batch 2−2371 : Gut microbiota abundance (genus Catenibacterium id.2153) on N−acetyllactosaminide beta−1,6−N−acetylglucosaminyl−transferase, isoform C
with a threshold of 1e−05
MR Test
Inverse variance weighted (fixed effects)
Inverse variance weighted (multiplicative random effects)
MR Egger
MR RAPS
Weighted median
Weighted mode
0.05
0.00
−0.05
0.09
0.12
0.15
0.18
SNP effect on Gut microbiota abundance (genus Catenibacterium id.2153)
P effect on N−acetyllactosaminide beta−1,6−N−acetylglucosaminyl−transferase, isoform C

Batch 2−2372 : Gut microbiota abundance (genus Parasutterella id.2892) on N−acetyllactosaminide beta−1,6−N−acetylglucosaminyl−transferase, isoform C
with a threshold of 1e−05

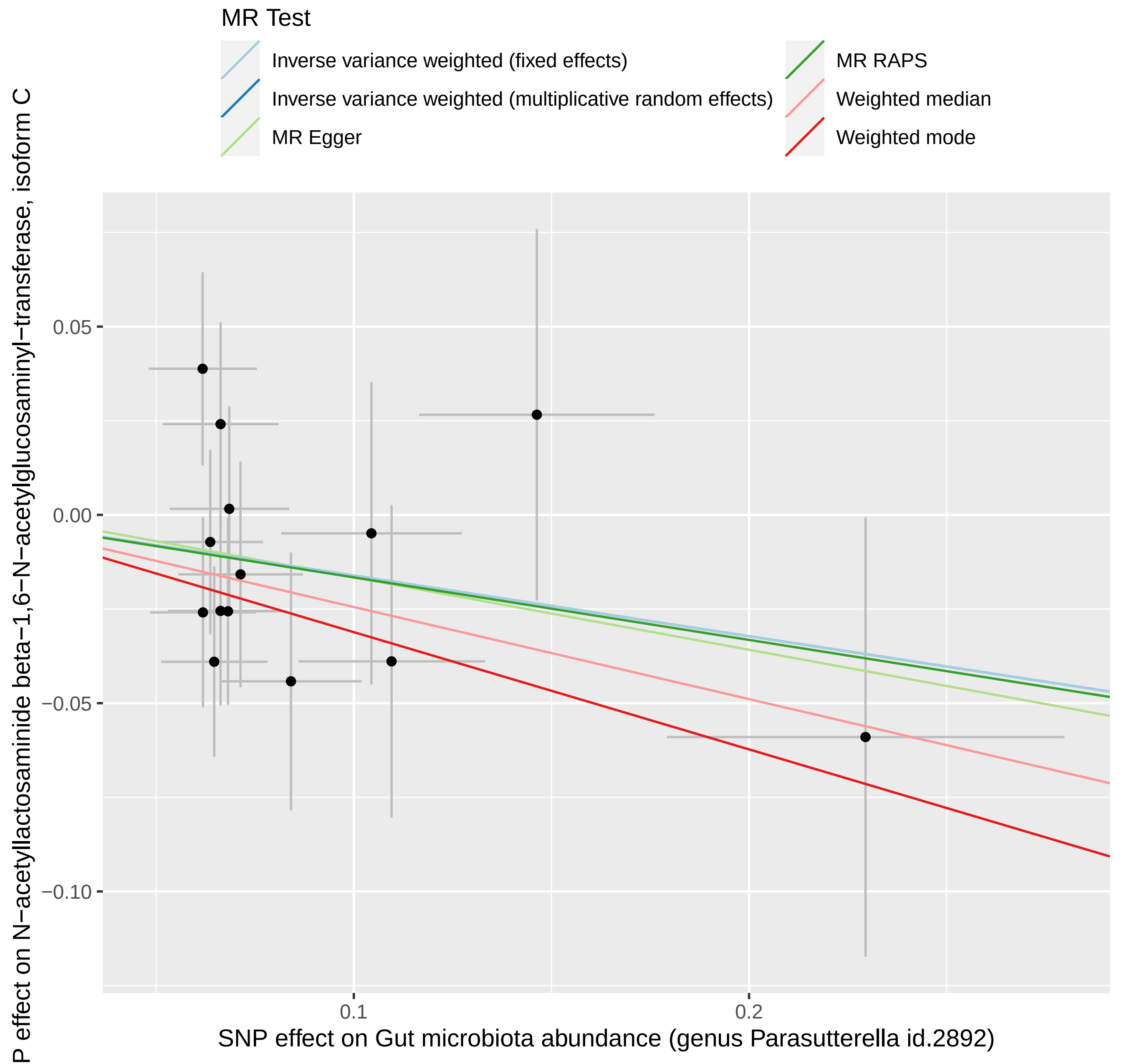

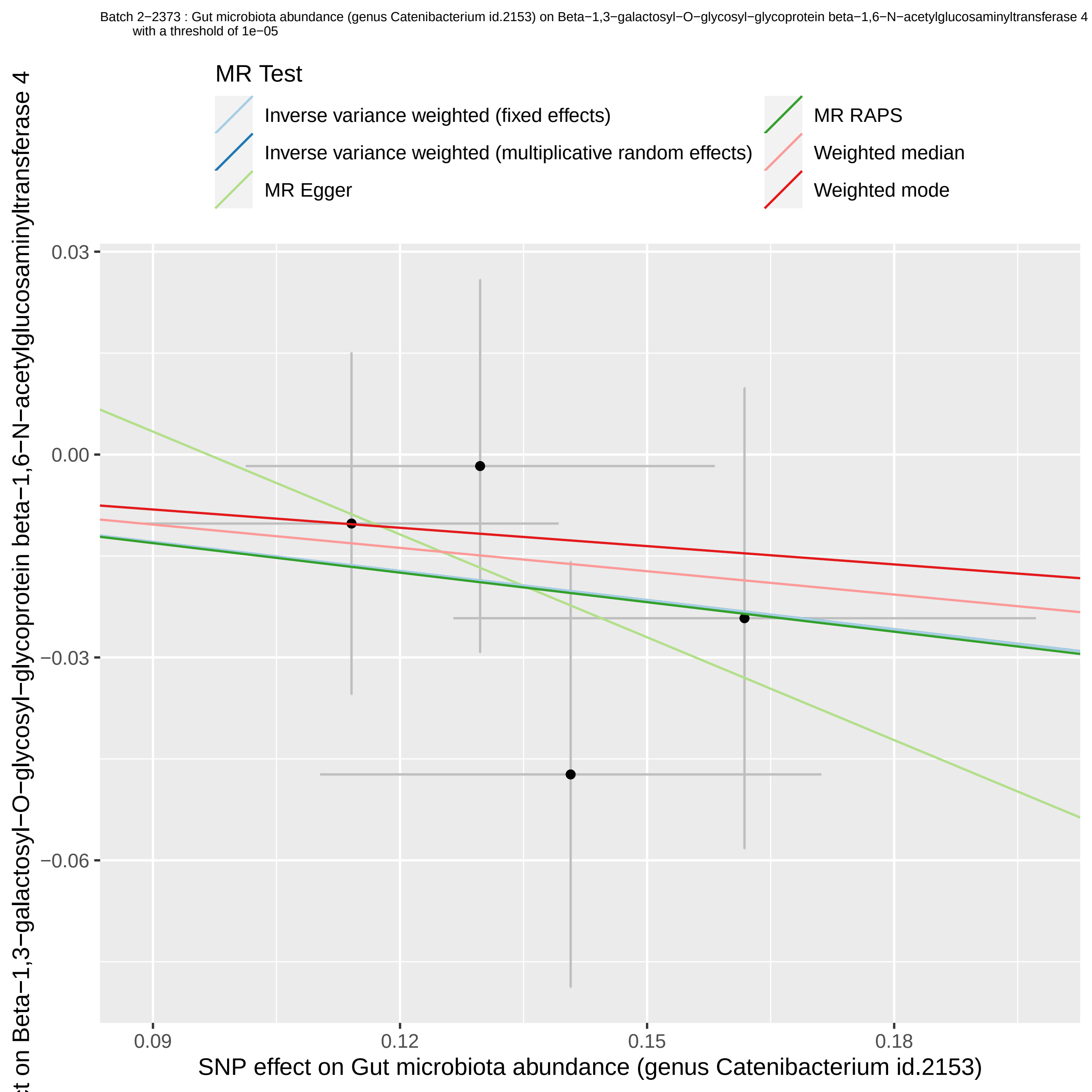
Batch 2−2373 : Gut microbiota abundance (genus Catenibacterium id.2153) on Beta−1,3−galactosyl−O−glycosyl−glycoprotein beta−1,6−N−acetylglucosaminyltransferase 4
with a threshold of 1e−05
MR Test
Inverse variance weighted (fixed effects)
Inverse variance weighted (multiplicative random effects)
MR Egger
MR RAPS
Weighted median
Weighted mode
0.03
0.00
−0.03
−0.06
0.09
0.12
0.15
0.18
SNP effect on Gut microbiota abundance (genus Catenibacterium id.2153)
t on Beta−1,3−galactosyl−O−glycosyl−glycoprotein beta−1,6−N−acetylglucosaminyltransferase 4

Batch 2−2374 : Gut microbiota abundance (genus Parasutterella id.2892) on Beta−1,3−galactosyl−O−glycosyl−glycoprotein beta−1,6−N−acetylglucosaminyltransferase 4 with a threshold of 1e−05

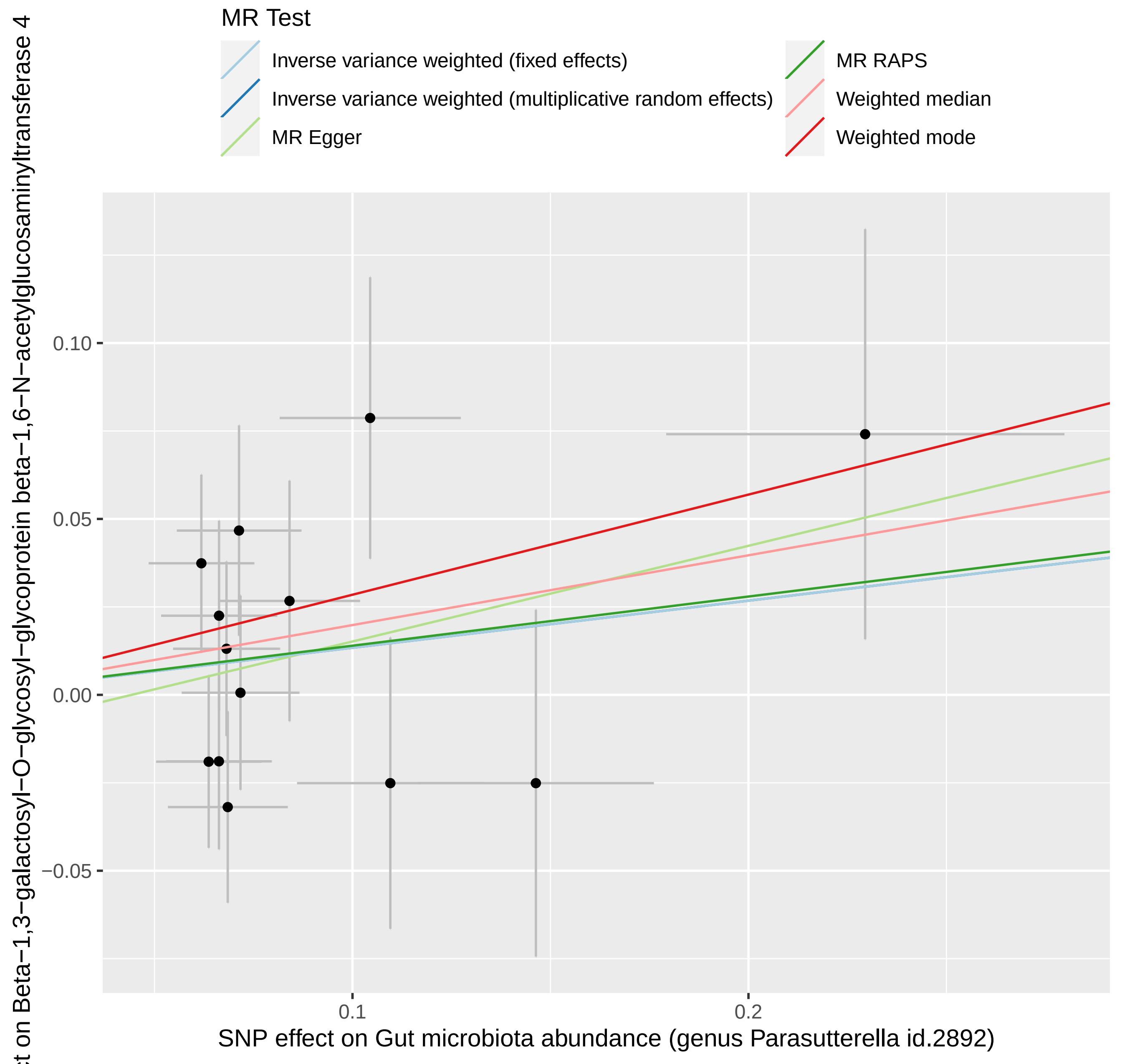

Batch 2−2375 : Gut microbiota abundance (genus Catenibacterium id.2153) on Beta−1,3−galactosyl−O−glycosyl−glycoprotein beta−1,6−N−acetylglucosaminyltransferase 4
with a threshold of 1e−05

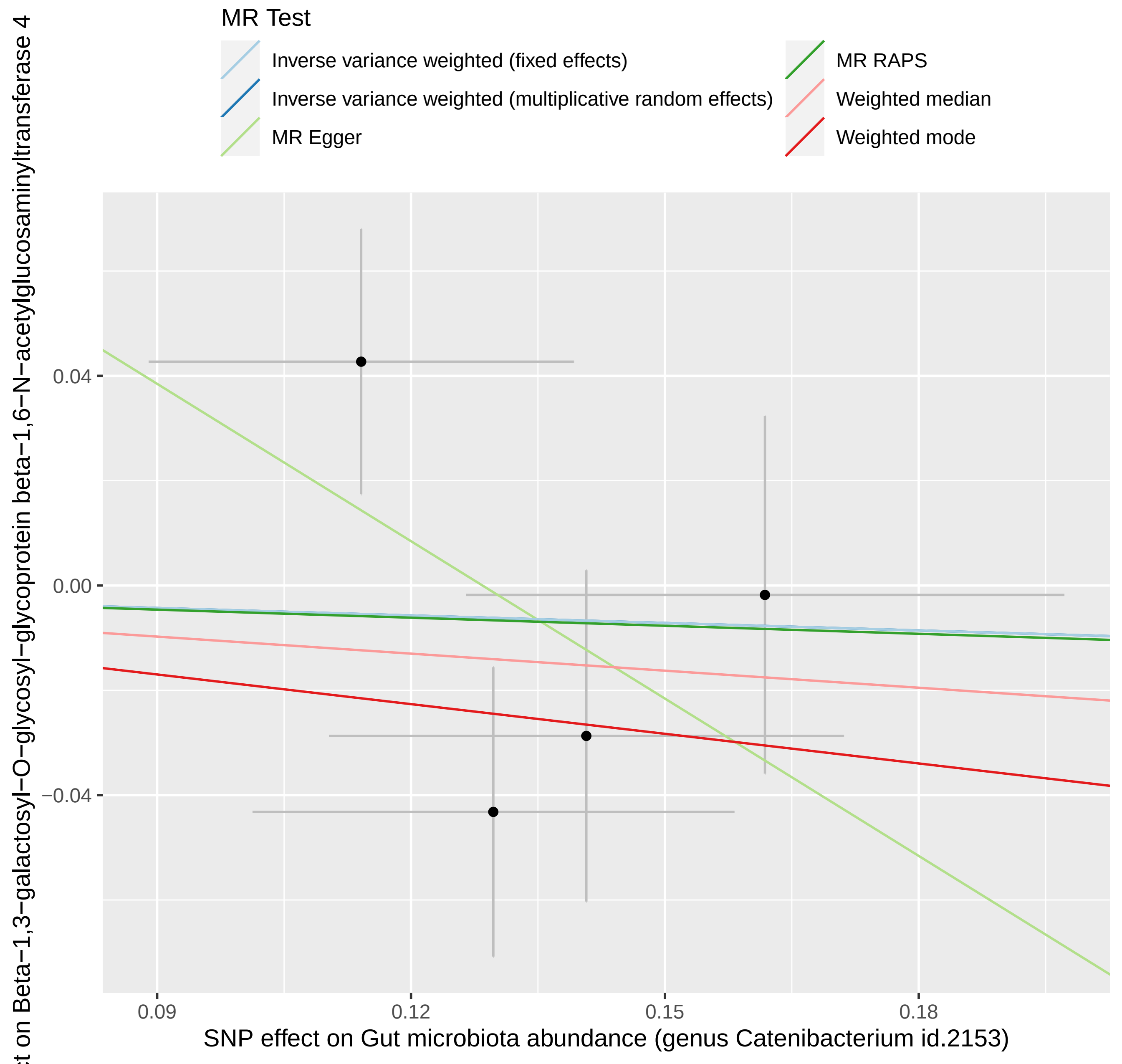

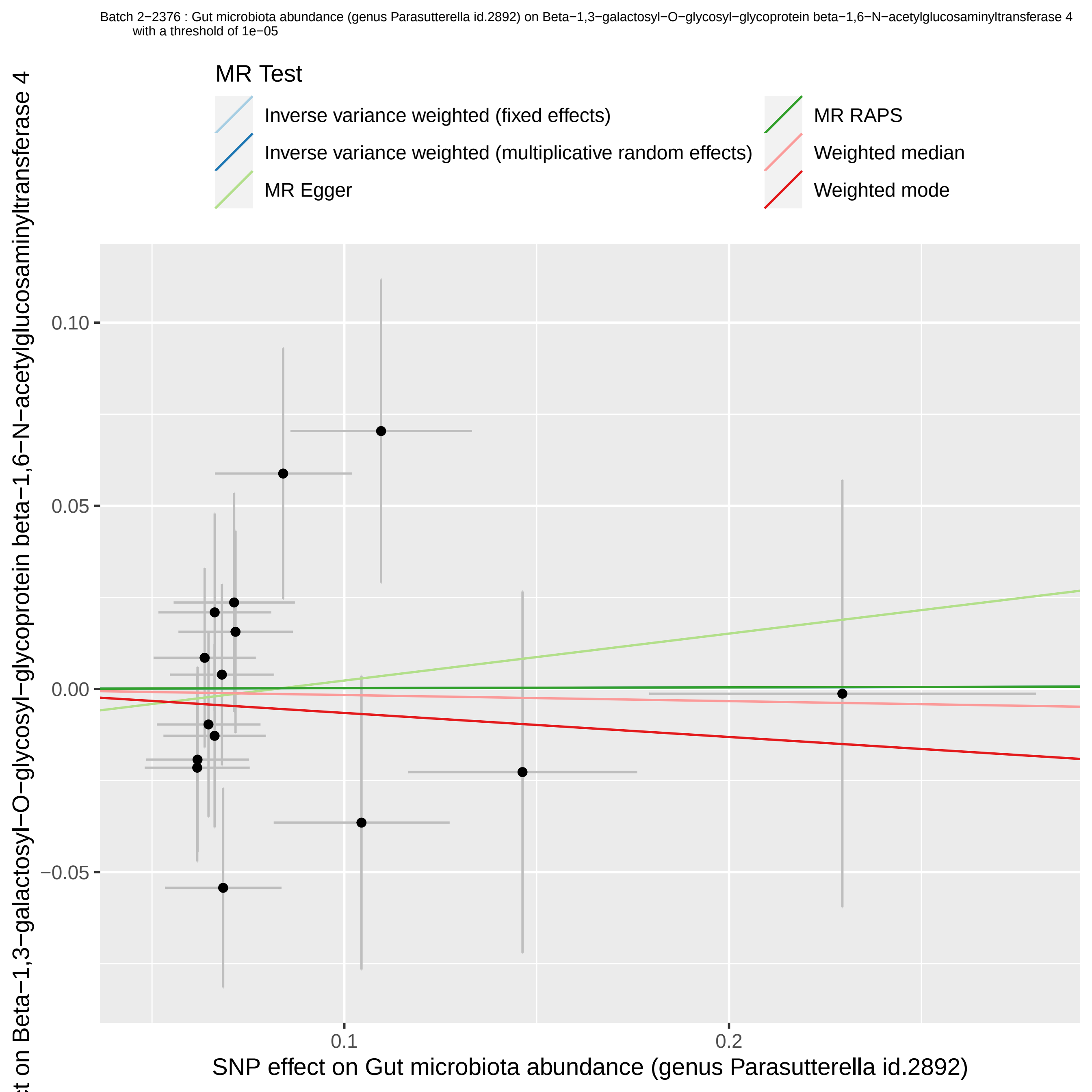
Batch 2−2376 : Gut microbiota abundance (genus Parasutterella id.2892) on Beta−1,3−galactosyl−O−glycosyl−glycoprotein beta−1,6−N−acetylglucosaminyltransferase 4 with a threshold of 1e−05
MR Test
Inverse variance weighted (fixed effects)
Inverse variance weighted (multiplicative random effects)
MR Egger
MR RAPS
Weighted median
Weighted mode
0.10
0.05
0.00
−0.05
0.1
0.2
SNP effect on Gut microbiota abundance (genus Parasutterella id.2892)
t on Beta−1,3−galactosyl−O−glycosyl−glycoprotein beta−1,6−N−acetylglucosaminyltransferase 4

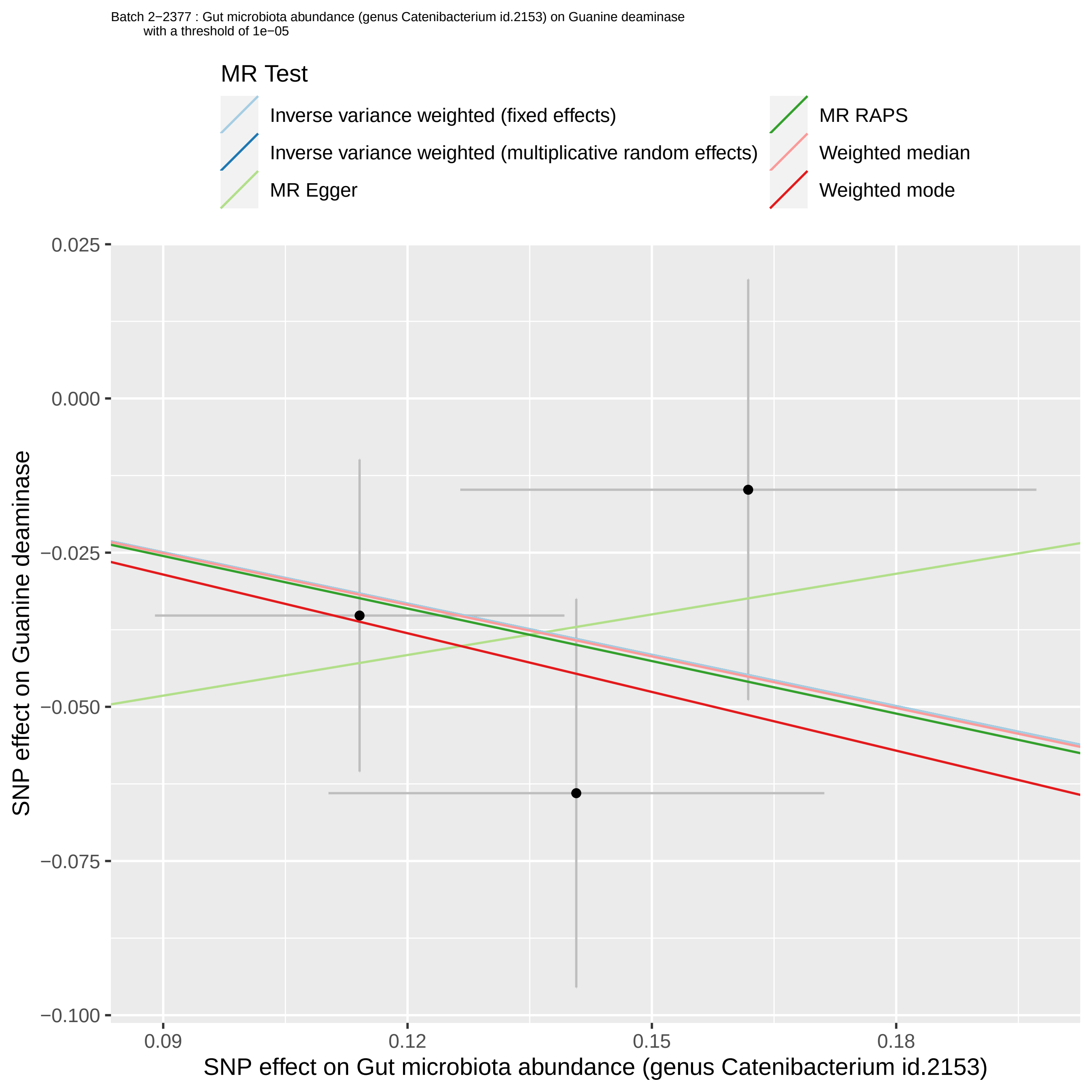

Batch 2−2377 : Gut microbiota abundance (genus Catenibacterium id.2153) on Guanine deaminase
with a threshold of 1e−05
MR Test
Inverse variance weighted (fixed effects)
Inverse variance weighted (multiplicative random effects)
MR Egger
MR RAPS
Weighted median
Weighted mode
0.025
0.000
−0.025
−0.050
−0.075
−0.100
0.09
0.12
0.15
0.18
SNP effect on Guanine deaminase
SNP effect on Gut microbiota abundance (genus Catenibacterium id.2153)

Batch 2−2378 : Gut microbiota abundance (genus Parasutterella id.2892) on Guanine deaminase
with a threshold of 1e−05

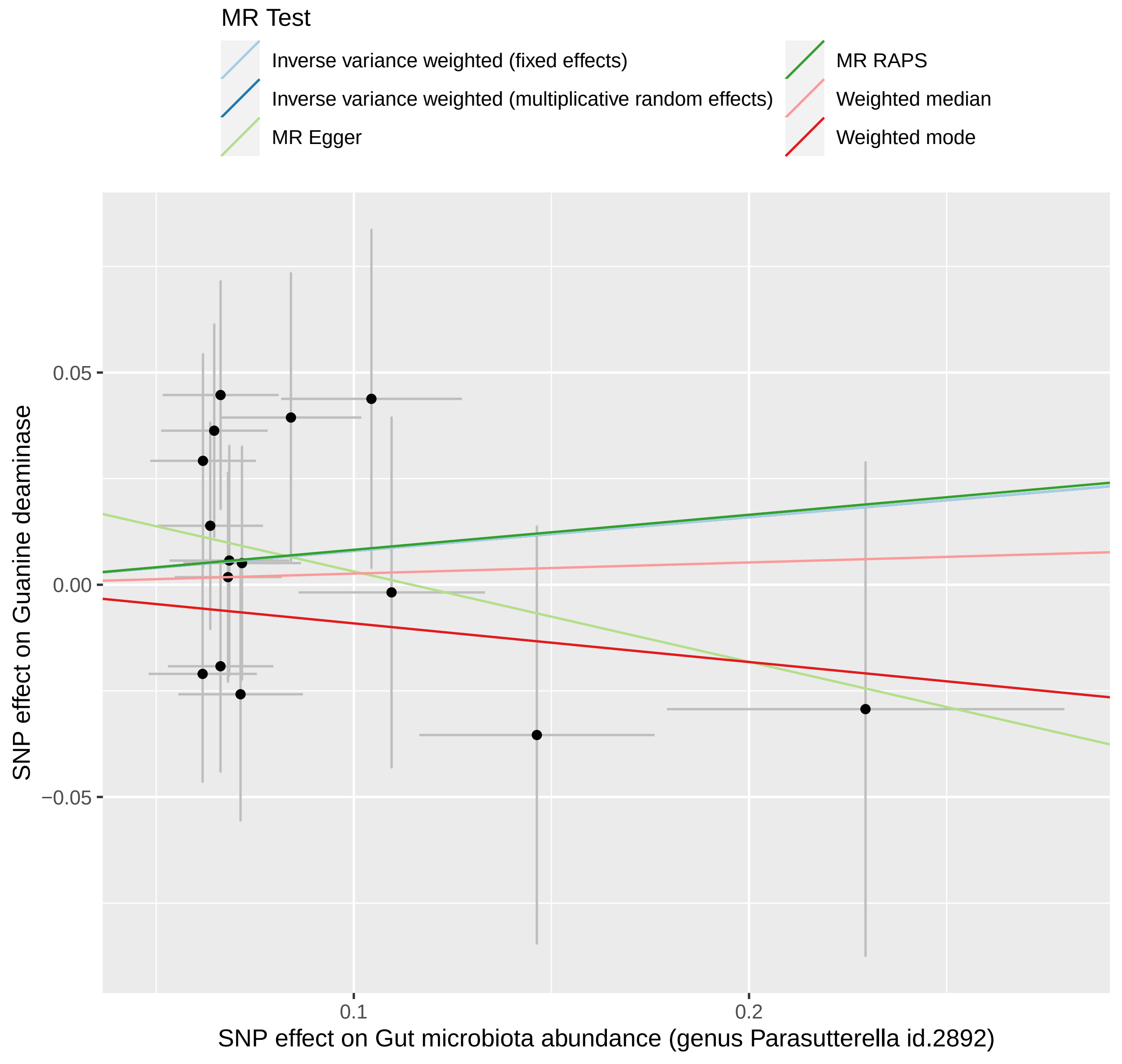

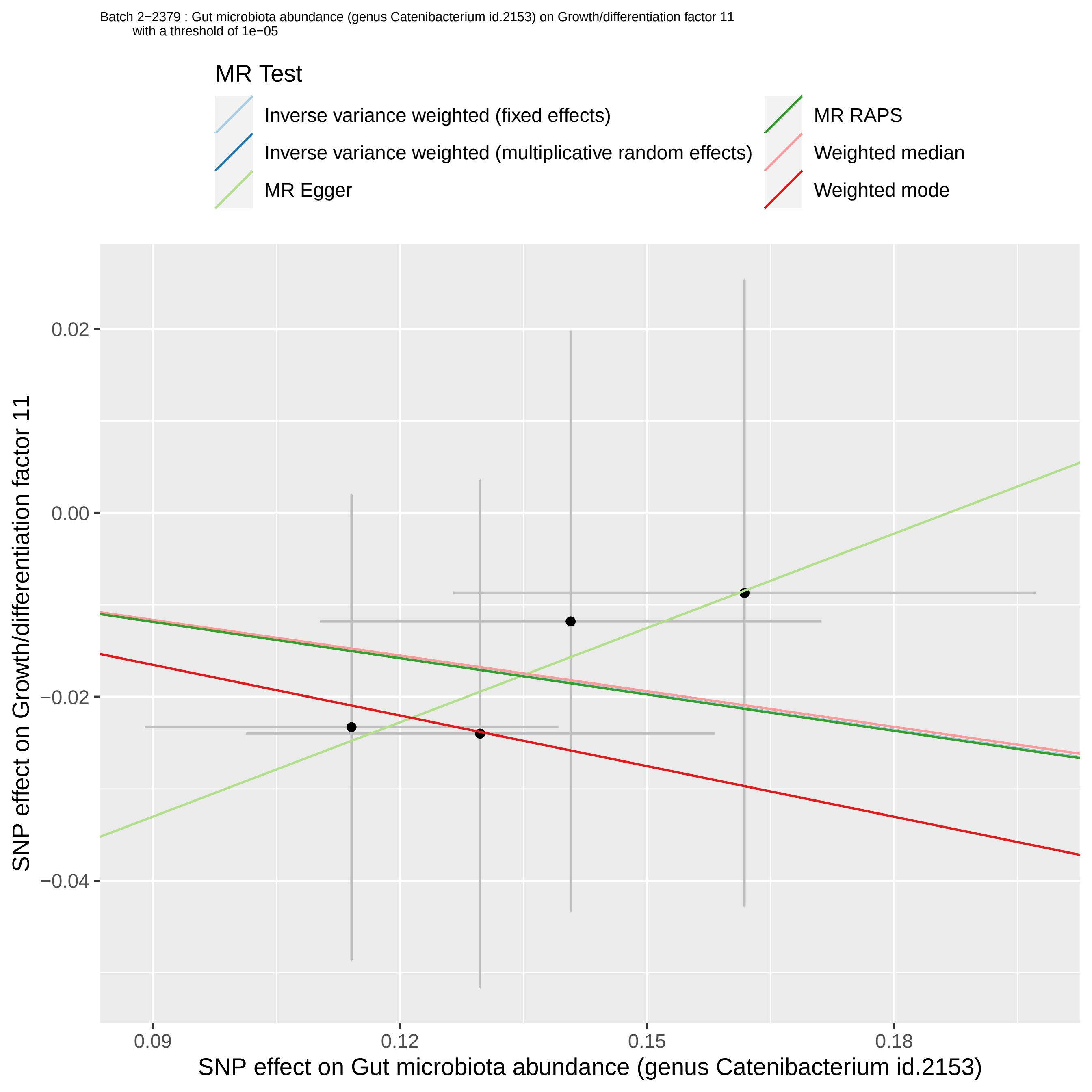
Batch 2−2379 : Gut microbiota abundance (genus Catenibacterium id.2153) on Growth/differentiation factor 11
with a threshold of 1e−05
MR Test
Inverse variance weighted (fixed effects)
Inverse variance weighted (multiplicative random effects)
MR Egger
MR RAPS
Weighted median
Weighted mode
0.02
0.00
−0.02
−0.04
0.09
0.12
0.15
0.18
SNP effect on Growth/differentiation factor 11
SNP effect on Gut microbiota abundance (genus Catenibacterium id.2153)

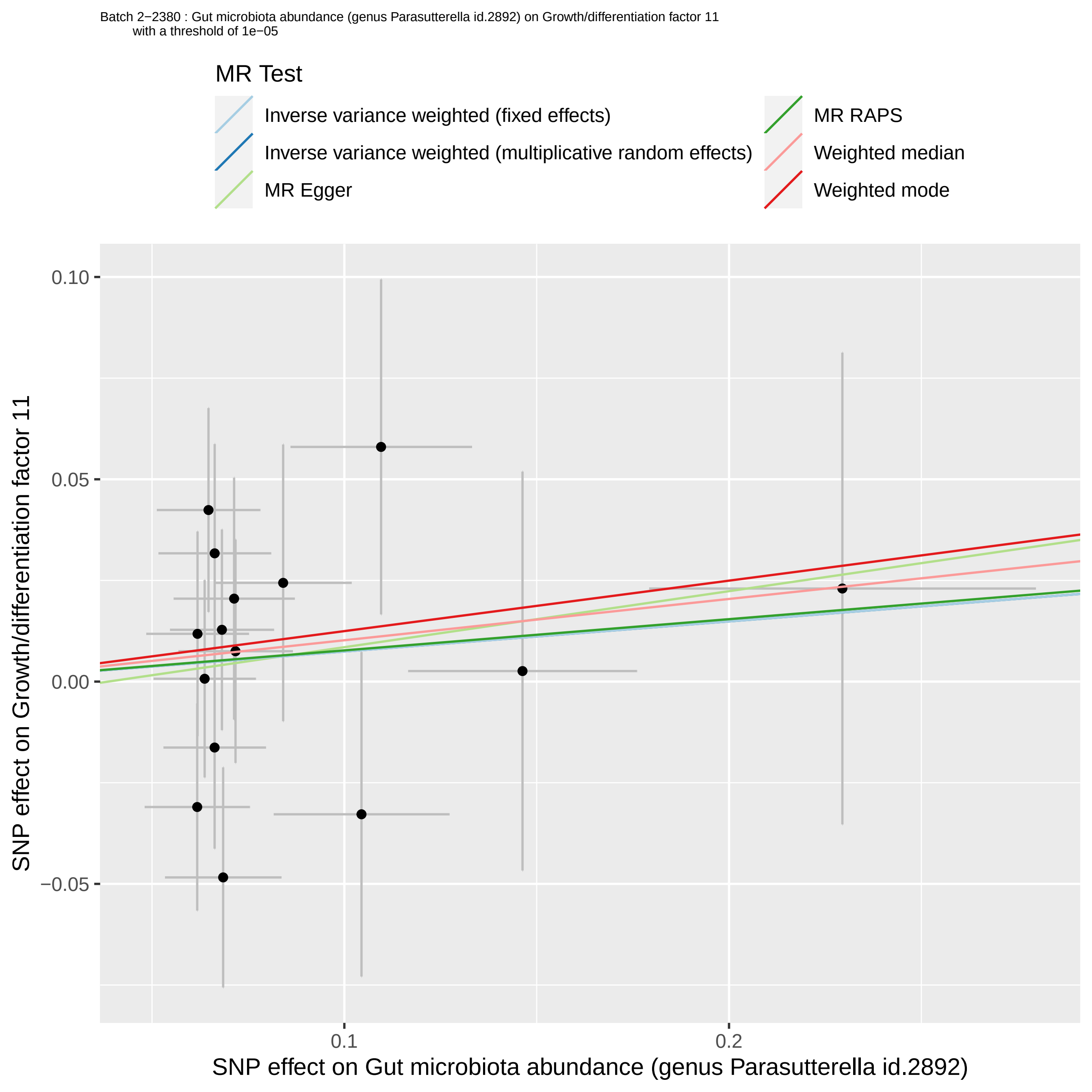

Batch 2−2380 : Gut microbiota abundance (genus Parasutterella id.2892) on Growth/differentiation factor 11
with a threshold of 1e−05
MR Test
Inverse variance weighted (fixed effects)
Inverse variance weighted (multiplicative random effects)
MR Egger
MR RAPS
Weighted median
Weighted mode
0.10
0.05
0.00
−0.05
0.1
0.2
SNP effect on Growth/differentiation factor 11
SNP effect on Gut microbiota abundance (genus Parasutterella id.2892)

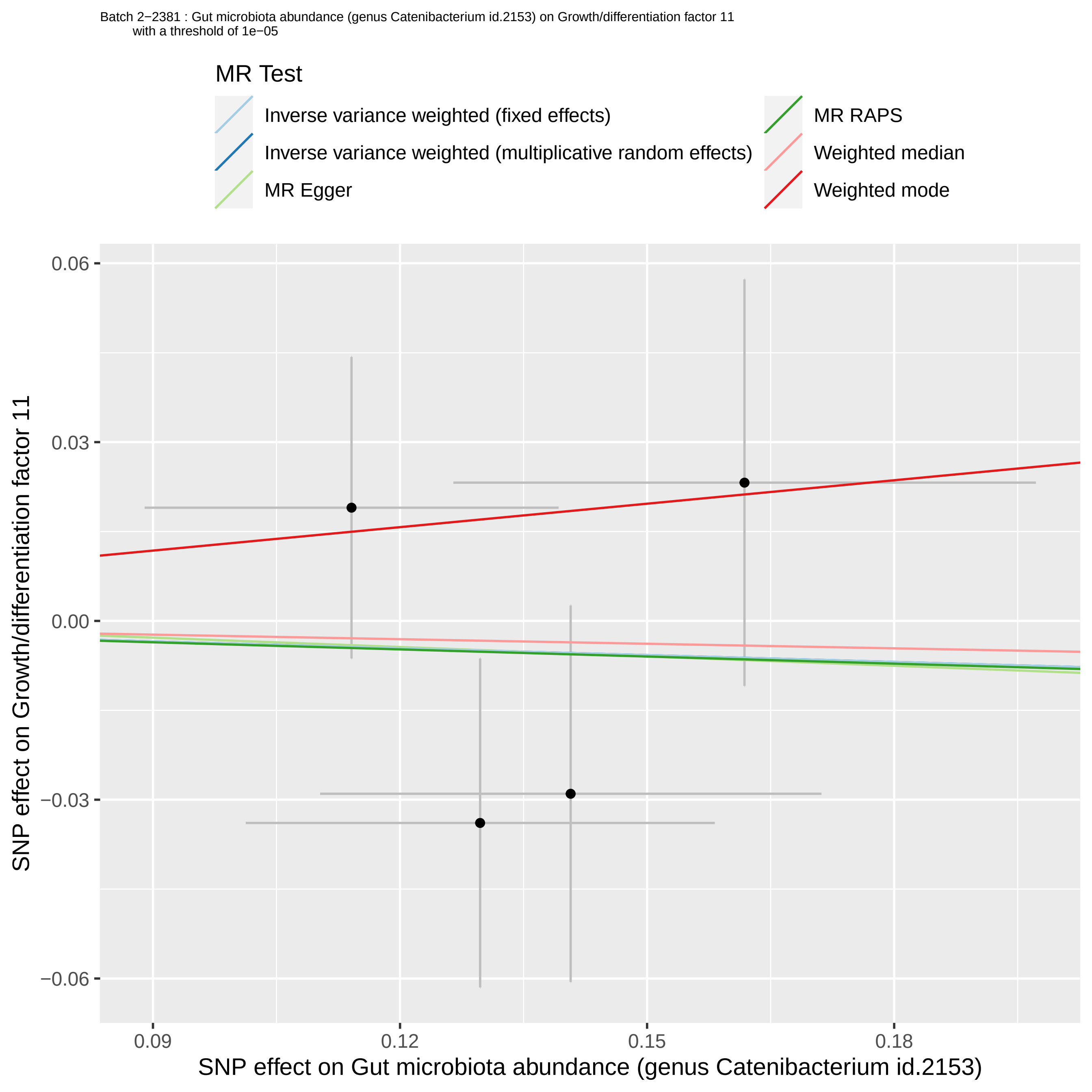

Batch 2−2381 : Gut microbiota abundance (genus Catenibacterium id.2153) on Growth/differentiation factor 11
with a threshold of 1e−05
MR Test
Inverse variance weighted (fixed effects)
Inverse variance weighted (multiplicative random effects)
MR Egger
MR RAPS
Weighted median
Weighted mode
0.06
0.03
0.00
−0.03
−0.06
0.09
0.12
0.15
0.18
SNP effect on Growth/differentiation factor 11
SNP effect on Gut microbiota abundance (genus Catenibacterium id.2153)

Batch 2−2382 : Gut microbiota abundance (genus Parasutterella id.2892) on Growth/differentiation factor 11
with a threshold of 1e−05

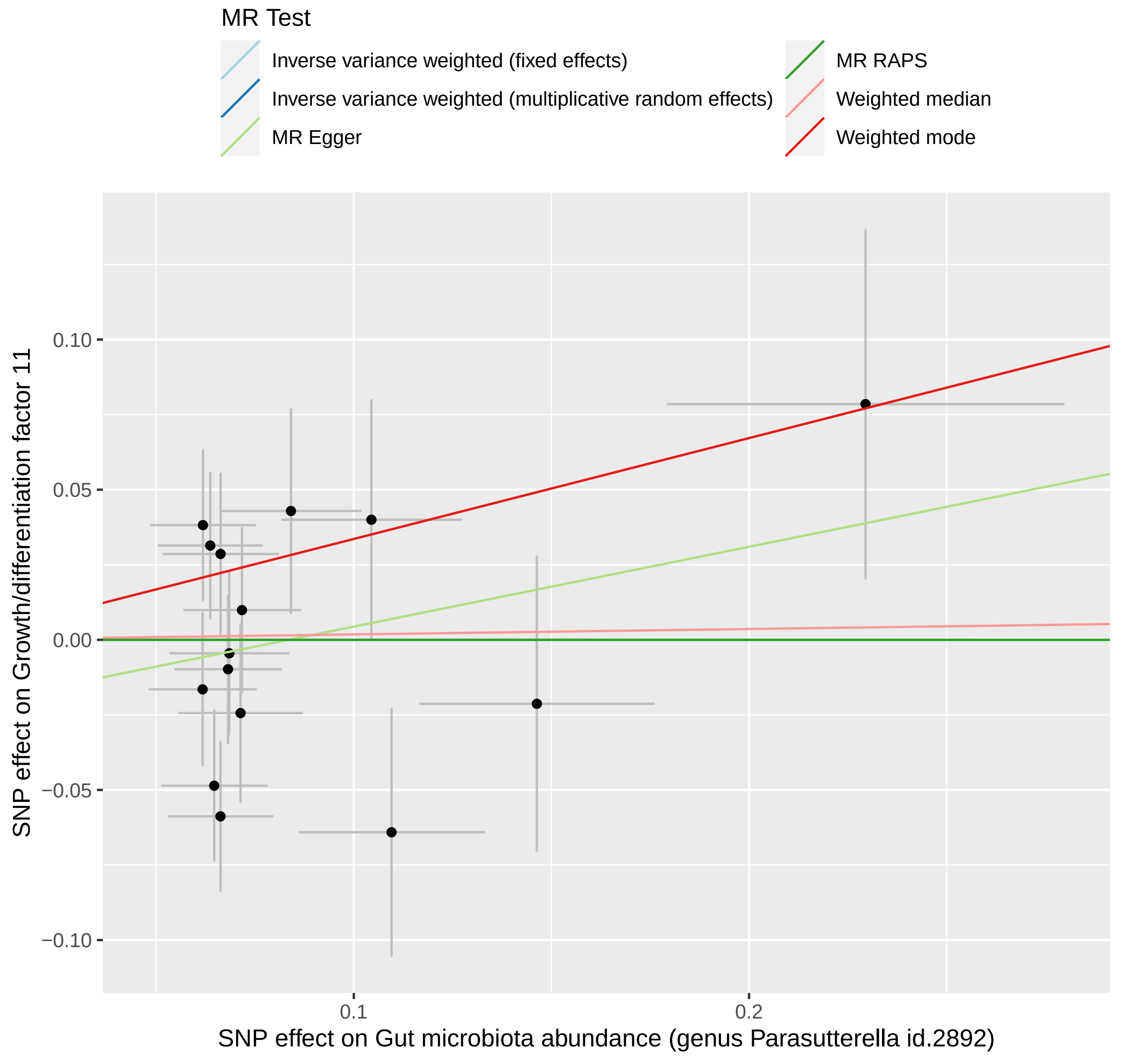

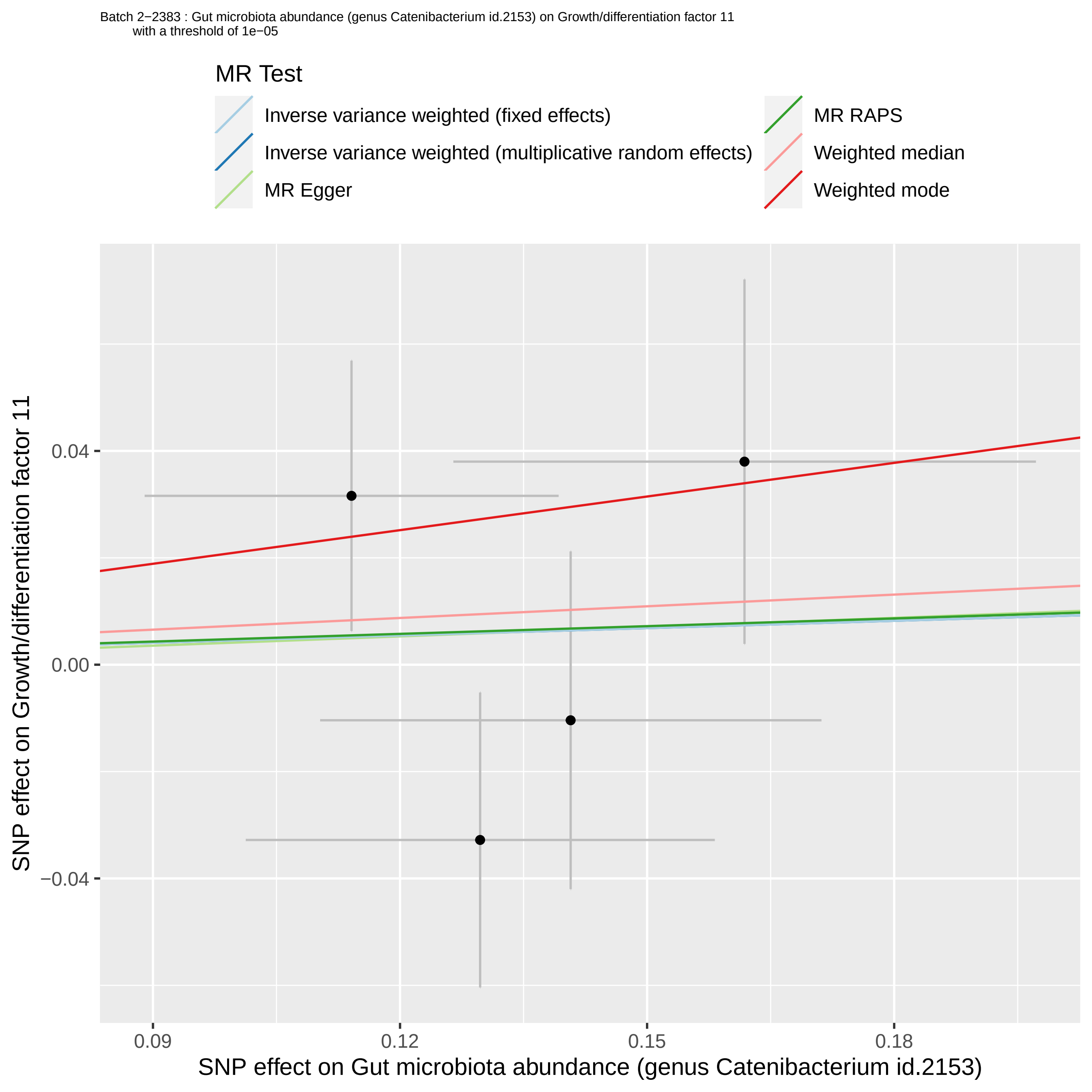

Batch 2−2383 : Gut microbiota abundance (genus Catenibacterium id.2153) on Growth/differentiation factor 11
with a threshold of 1e−05
MR Test
Inverse variance weighted (fixed effects)
Inverse variance weighted (multiplicative random effects)
MR Egger
MR RAPS
Weighted median
Weighted mode
0.04
0.00
−0.04
0.09
0.12
0.15
0.18
SNP effect on Growth/differentiation factor 11
SNP effect on Gut microbiota abundance (genus Catenibacterium id.2153)

Batch 2−2384 : Gut microbiota abundance (genus Parasutterella id.2892) on Growth/differentiation factor 11
with a threshold of 1e−05

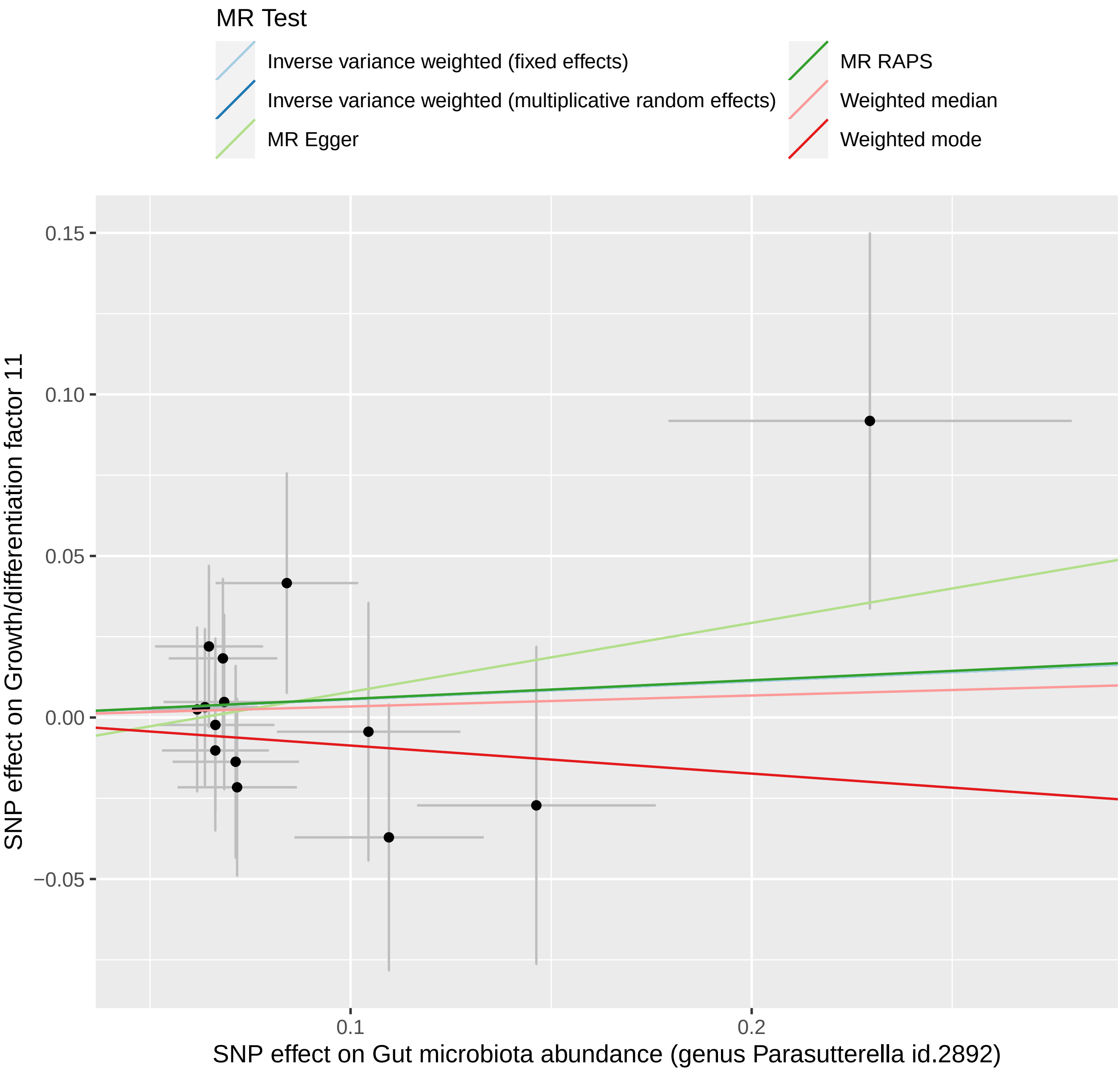

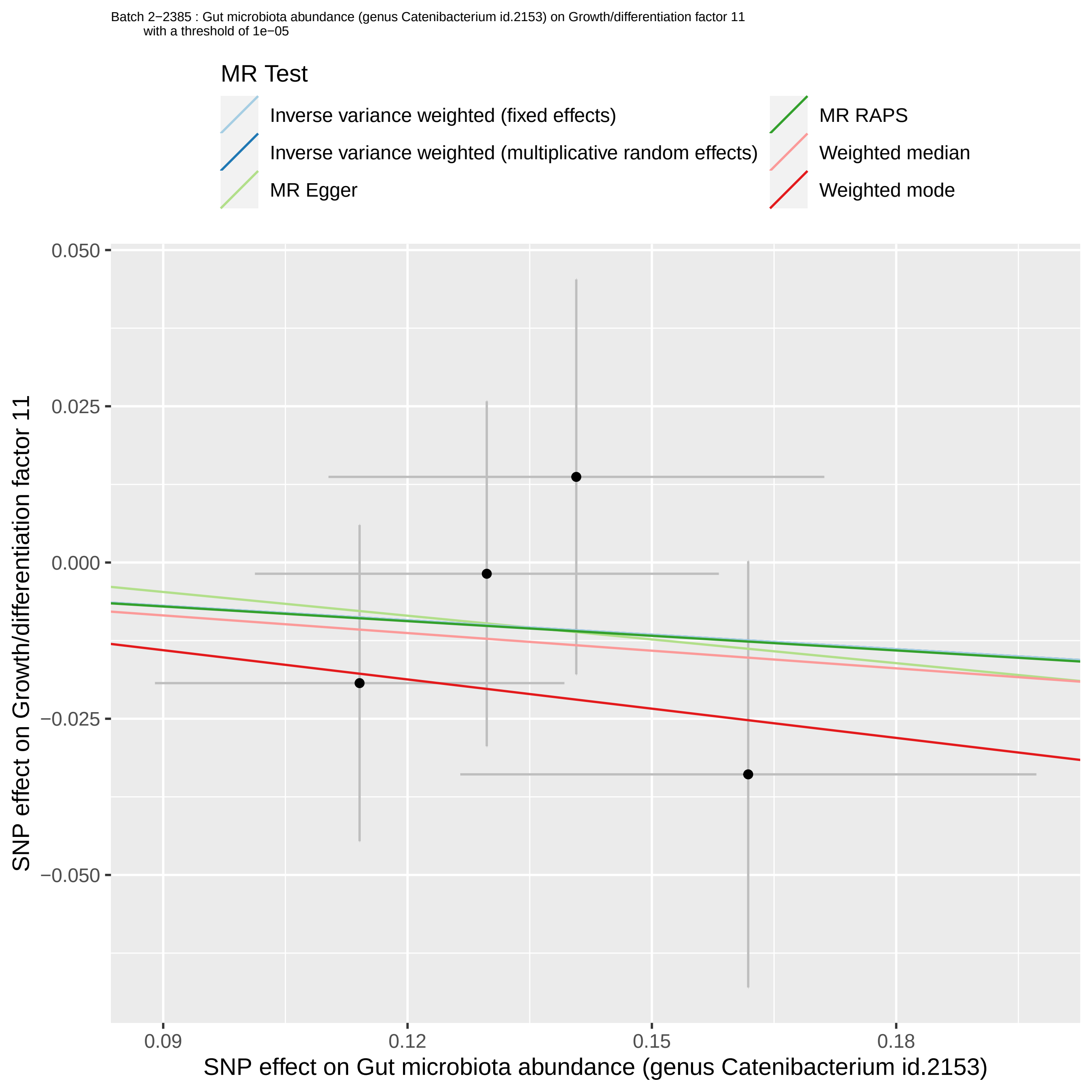
Batch 2−2385 : Gut microbiota abundance (genus Catenibacterium id.2153) on Growth/differentiation factor 11
with a threshold of 1e−05
MR Test
Inverse variance weighted (fixed effects)
Inverse variance weighted (multiplicative random effects)
MR Egger
MR RAPS
Weighted median
Weighted mode
0.050
0.025
0.000
−0.025
−0.050
0.09
0.12
0.15
0.18
SNP effect on Growth/differentiation factor 11
SNP effect on Gut microbiota abundance (genus Catenibacterium id.2153)

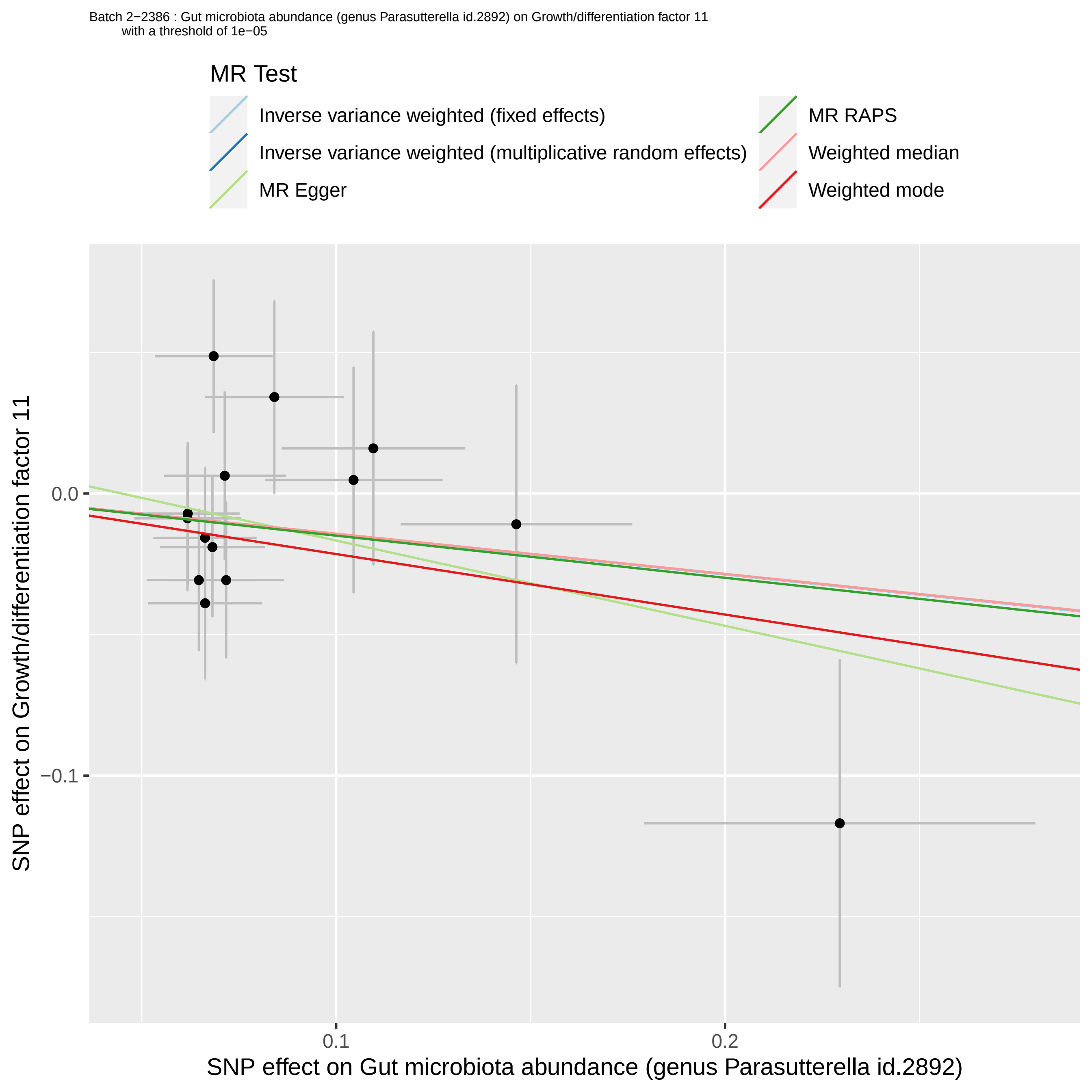
Batch 2−2386 : Gut microbiota abundance (genus Parasutterella id.2892) on Growth/differentiation factor 11
with a threshold of 1e−05
MR Test
Inverse variance weighted (fixed effects)
Inverse variance weighted (multiplicative random effects)
MR Egger
MR RAPS
Weighted median
Weighted mode
0.0
−0.1
0.1
0.2
SNP effect on Gut microbiota abundance (genus Parasutterella id.2892)
SNP effect on Growth/differentiation factor 11

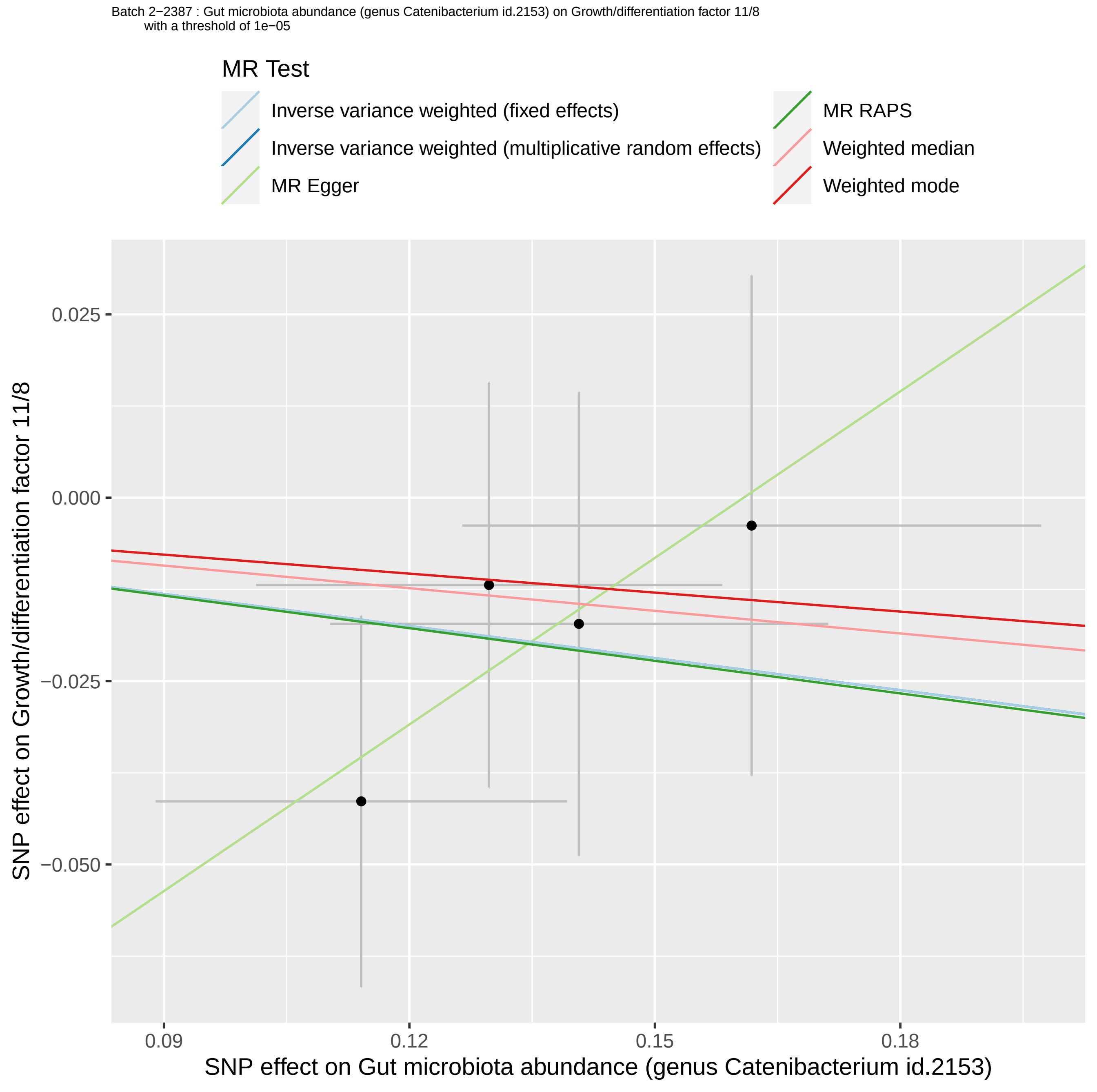
Batch 2−2387 : Gut microbiota abundance (genus Catenibacterium id.2153) on Growth/differentiation factor 11/8
with a threshold of 1e−05
MR Test
Inverse variance weighted (fixed effects)
Inverse variance weighted (multiplicative random effects)
MR Egger
MR RAPS
Weighted median
Weighted mode
0.025
0.000
−0.025
−0.050
0.09
0.12
0.15
0.18
SNP effect on Growth/differentiation factor 11/8
SNP effect on Gut microbiota abundance (genus Catenibacterium id.2153)

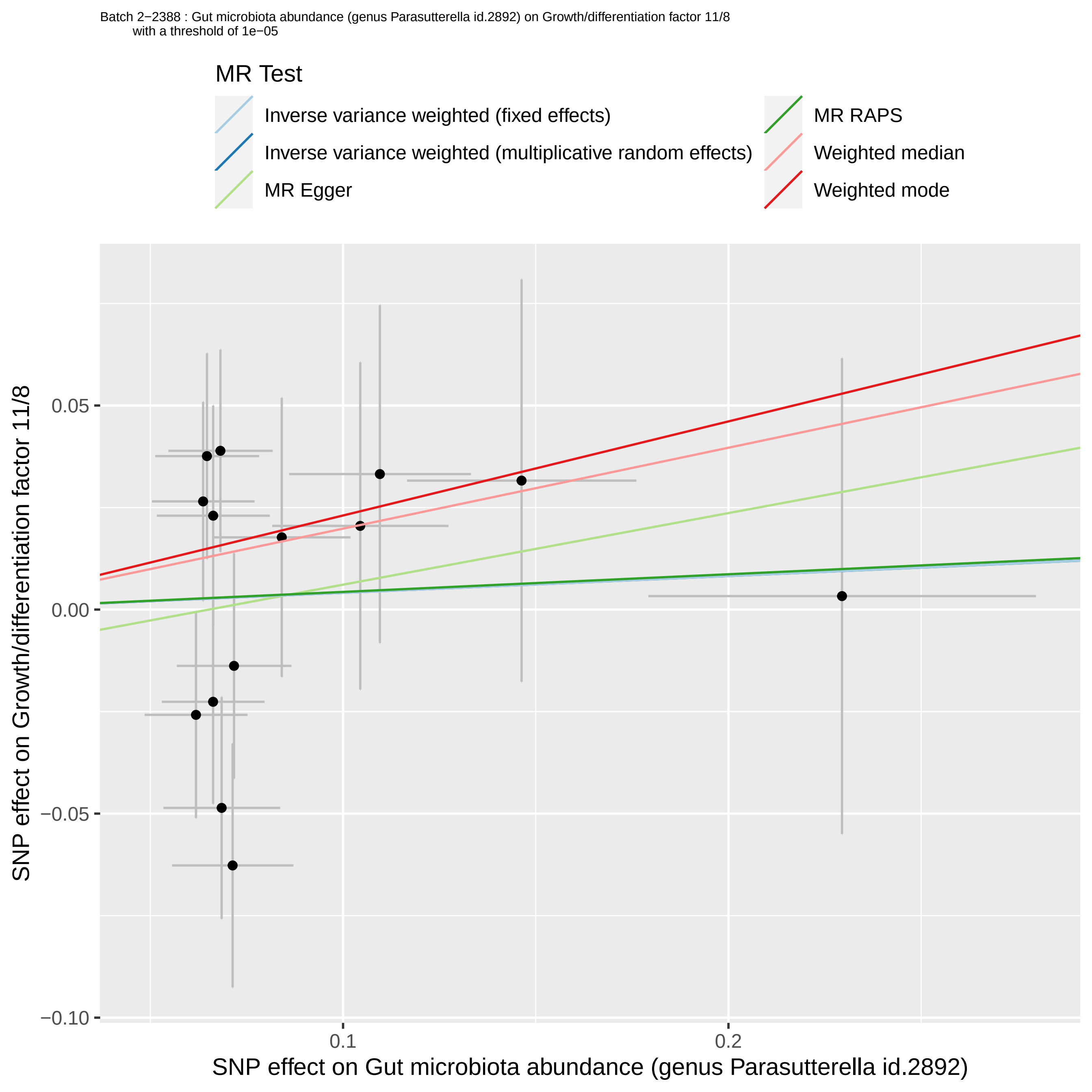
Batch 2−2388 : Gut microbiota abundance (genus Parasutterella id.2892) on Growth/differentiation factor 11/8
with a threshold of 1e−05
MR Test
Inverse variance weighted (fixed effects)
Inverse variance weighted (multiplicative random effects)
MR Egger
MR RAPS
Weighted median
Weighted mode
0.05
0.00
−0.05
−0.10
0.1
0.2
SNP effect on Growth/differentiation factor 11/8
SNP effect on Gut microbiota abundance (genus Parasutterella id.2892)

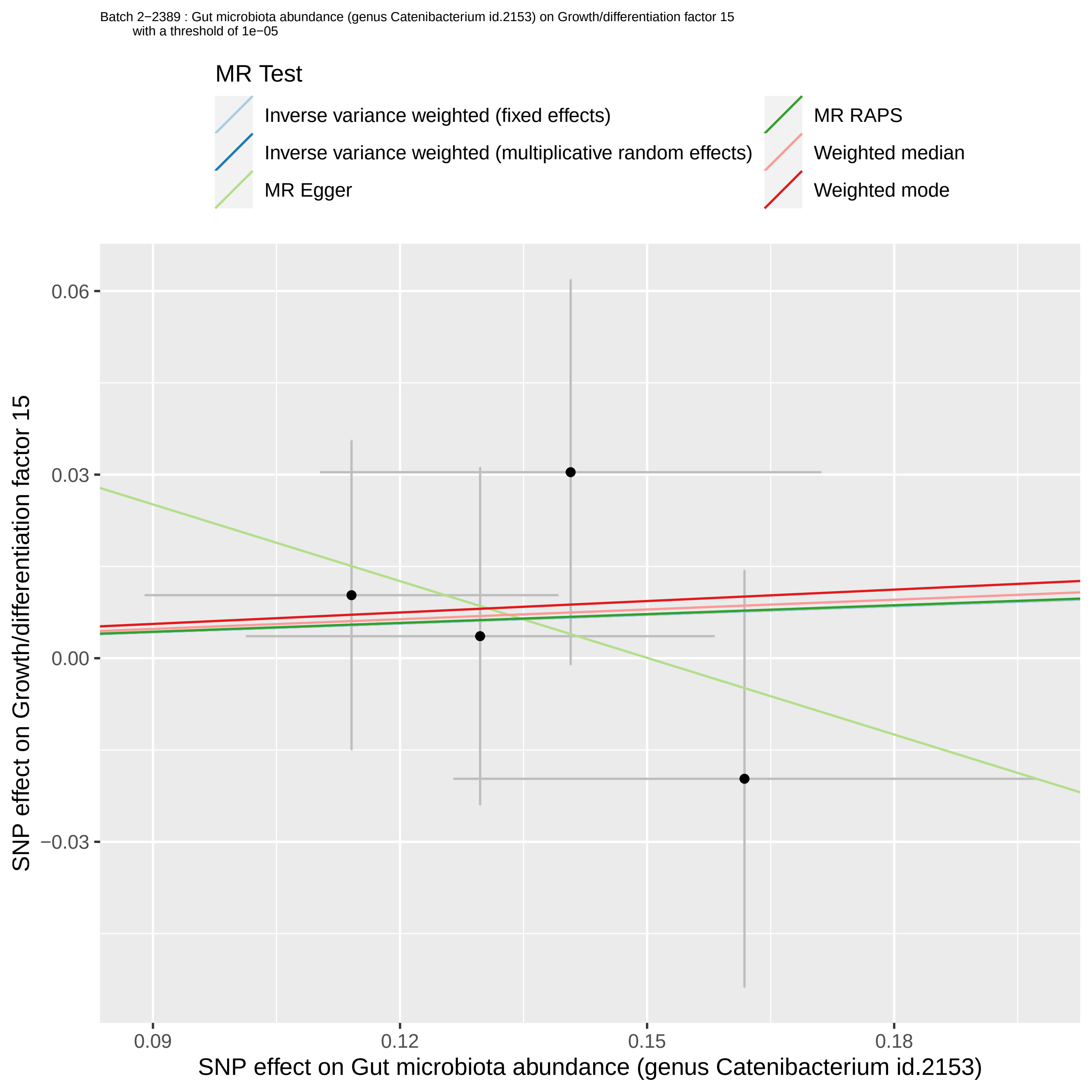
Batch 2−2389 : Gut microbiota abundance (genus Catenibacterium id.2153) on Growth/differentiation factor 15
with a threshold of 1e−05
MR Test
Inverse variance weighted (fixed effects)
Inverse variance weighted (multiplicative random effects)
MR Egger
MR RAPS
Weighted median
Weighted mode
SNP effect on Growth/differentiation factor 15
0.06
0.03
0.00
−0.03
0.09
0.12
0.15
0.18
SNP effect on Gut microbiota abundance (genus Catenibacterium id.2153)

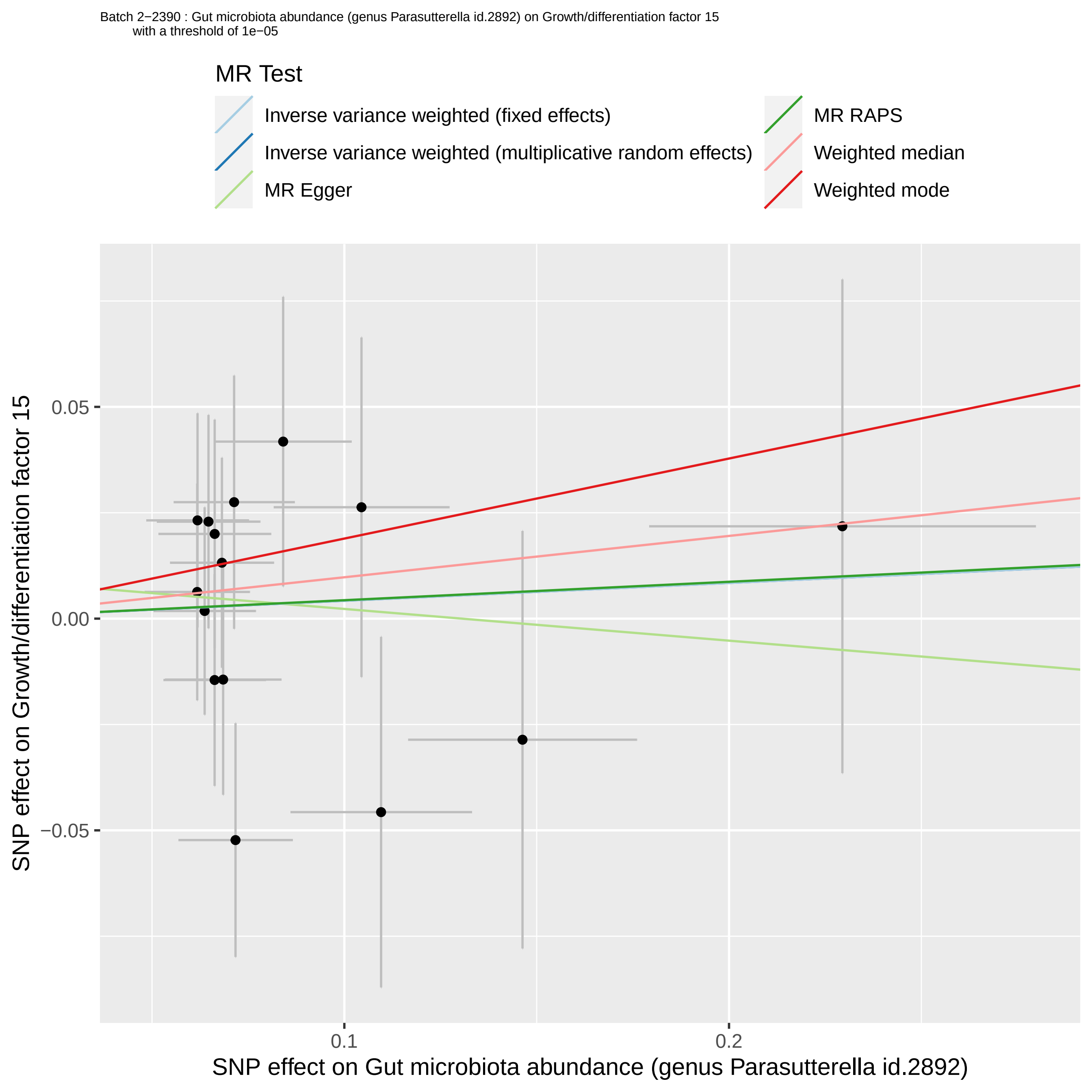

Batch 2−2390 : Gut microbiota abundance (genus Parasutterella id.2892) on Growth/differentiation factor 15
with a threshold of 1e−05
MR Test
Inverse variance weighted (fixed effects)
Inverse variance weighted (multiplicative random effects)
MR Egger
MR RAPS
Weighted median
Weighted mode
0.05
0.00
−0.05
0.1
0.2
SNP effect on Growth/differentiation factor 15
SNP effect on Gut microbiota abundance (genus Parasutterella id.2892)

Batch 2−2391 : Gut microbiota abundance (genus Catenibacterium id.2153) on Growth/differentiation factor 5
with a threshold of 1e−05

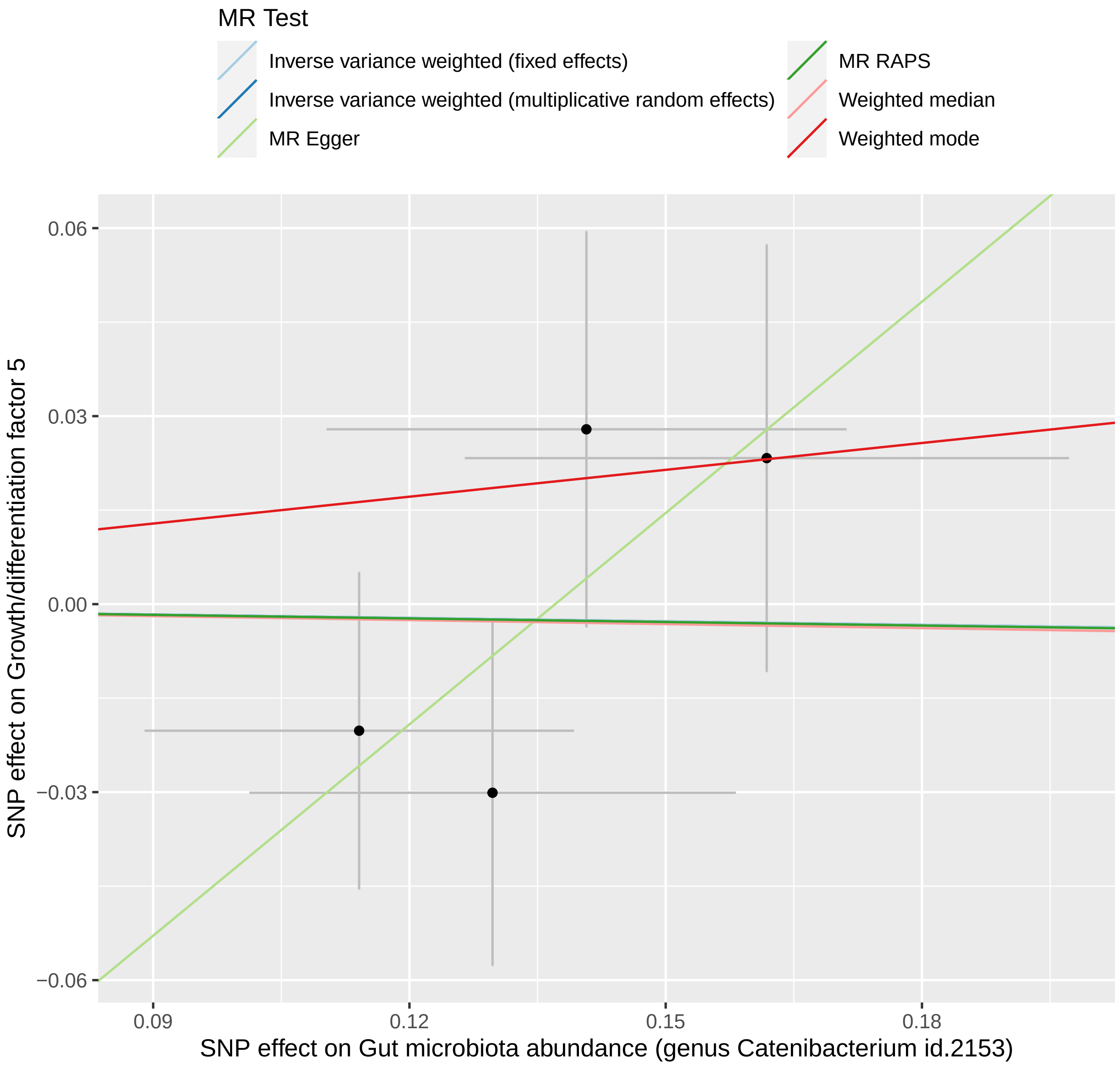

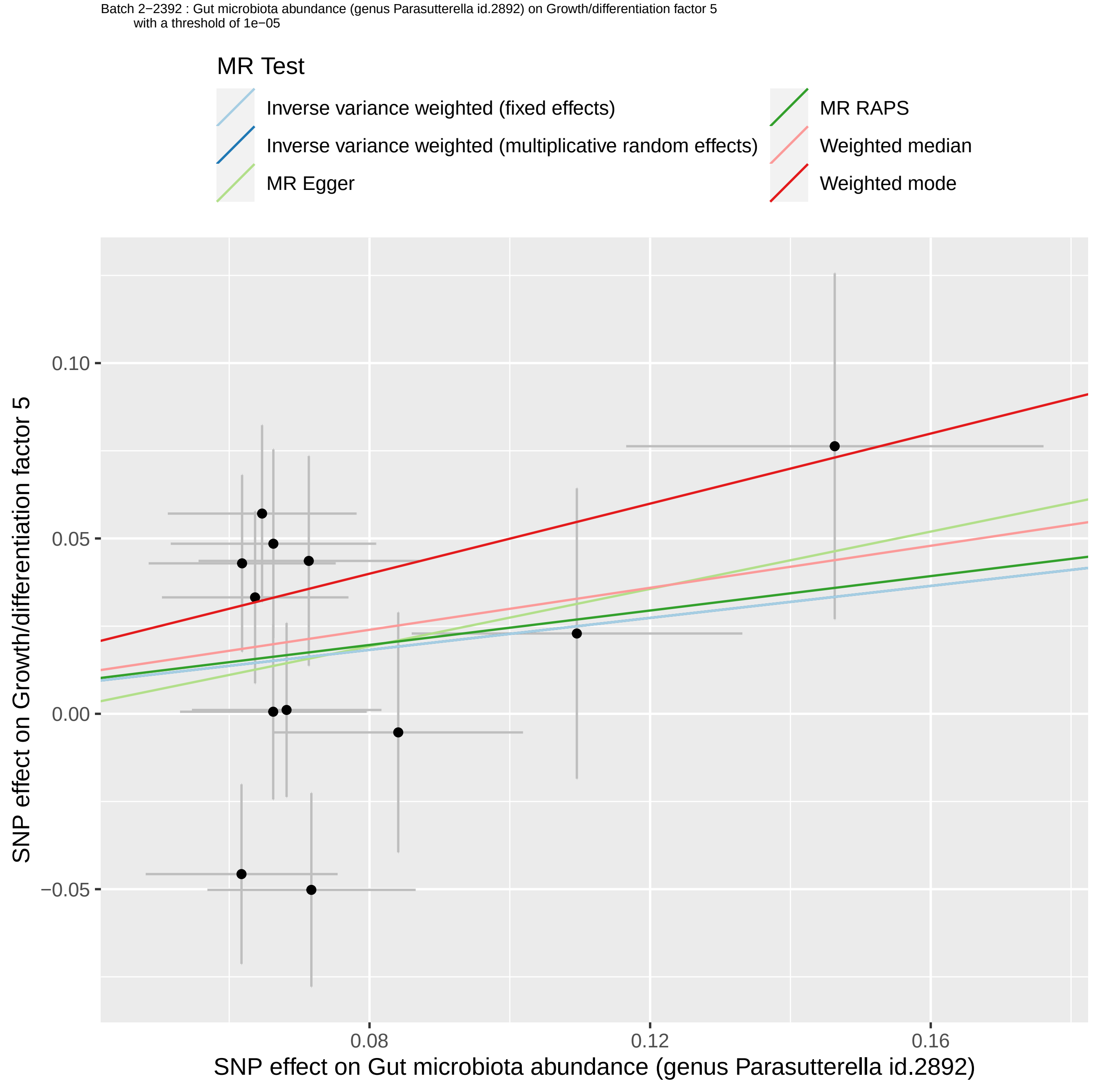

Batch 2−2392 : Gut microbiota abundance (genus Parasutterella id.2892) on Growth/differentiation factor 5
with a threshold of 1e−05
MR Test
Inverse variance weighted (fixed effects)
Inverse variance weighted (multiplicative random effects)
MR Egger
MR RAPS
Weighted median
Weighted mode
0.10
0.05
0.00
−0.05
0.08
0.12
0.16
SNP effect on Growth/differentiation factor 5
SNP effect on Gut microbiota abundance (genus Parasutterella id.2892)

Batch 2−2393 : Gut microbiota abundance (genus Catenibacterium id.2153) on Growth/differentiation factor 9
with a threshold of 1e−05

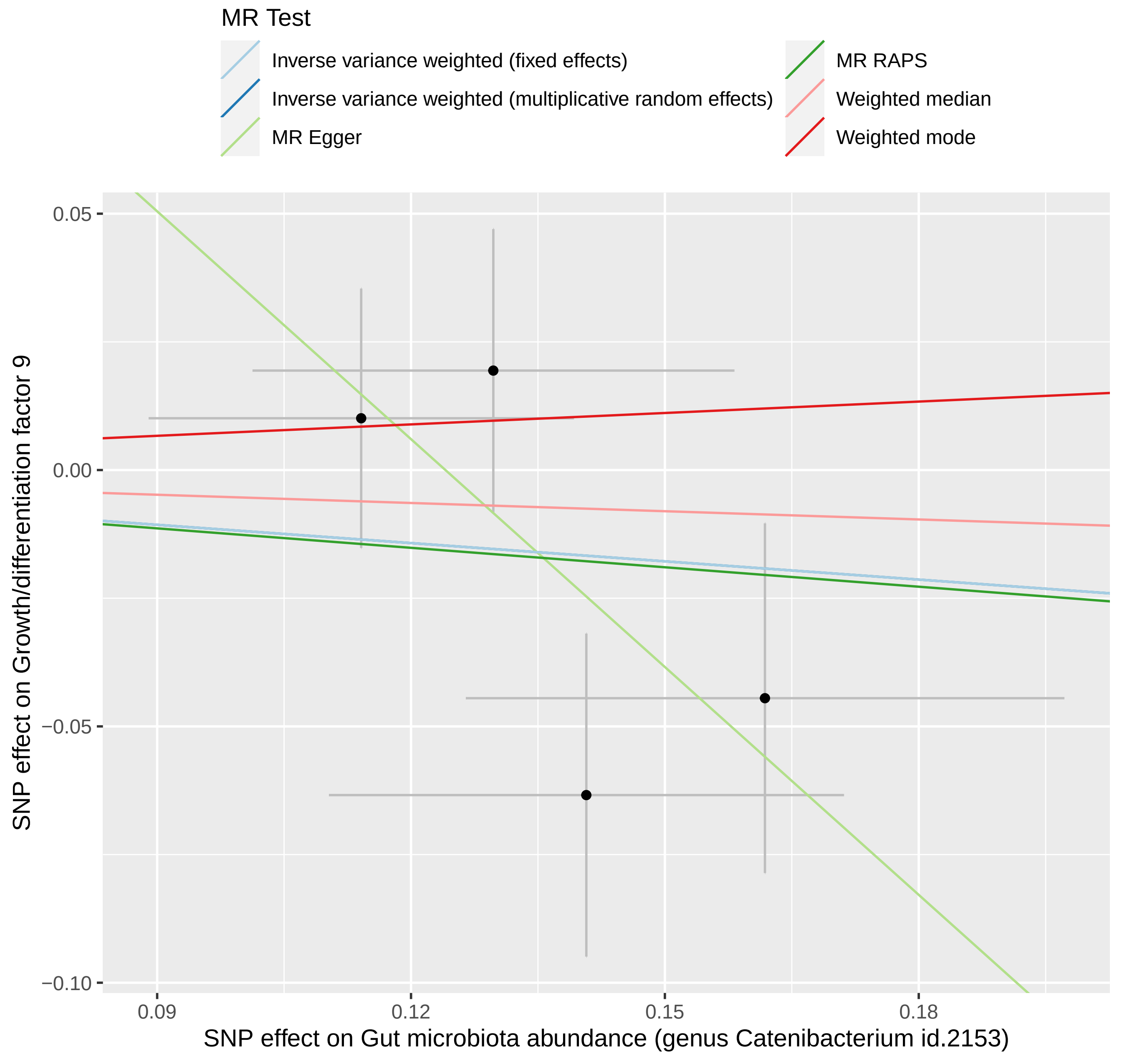

Batch 2−2394 : Gut microbiota abundance (genus Parasutterella id.2892) on Growth/differentiation factor 9
with a threshold of 1e−05

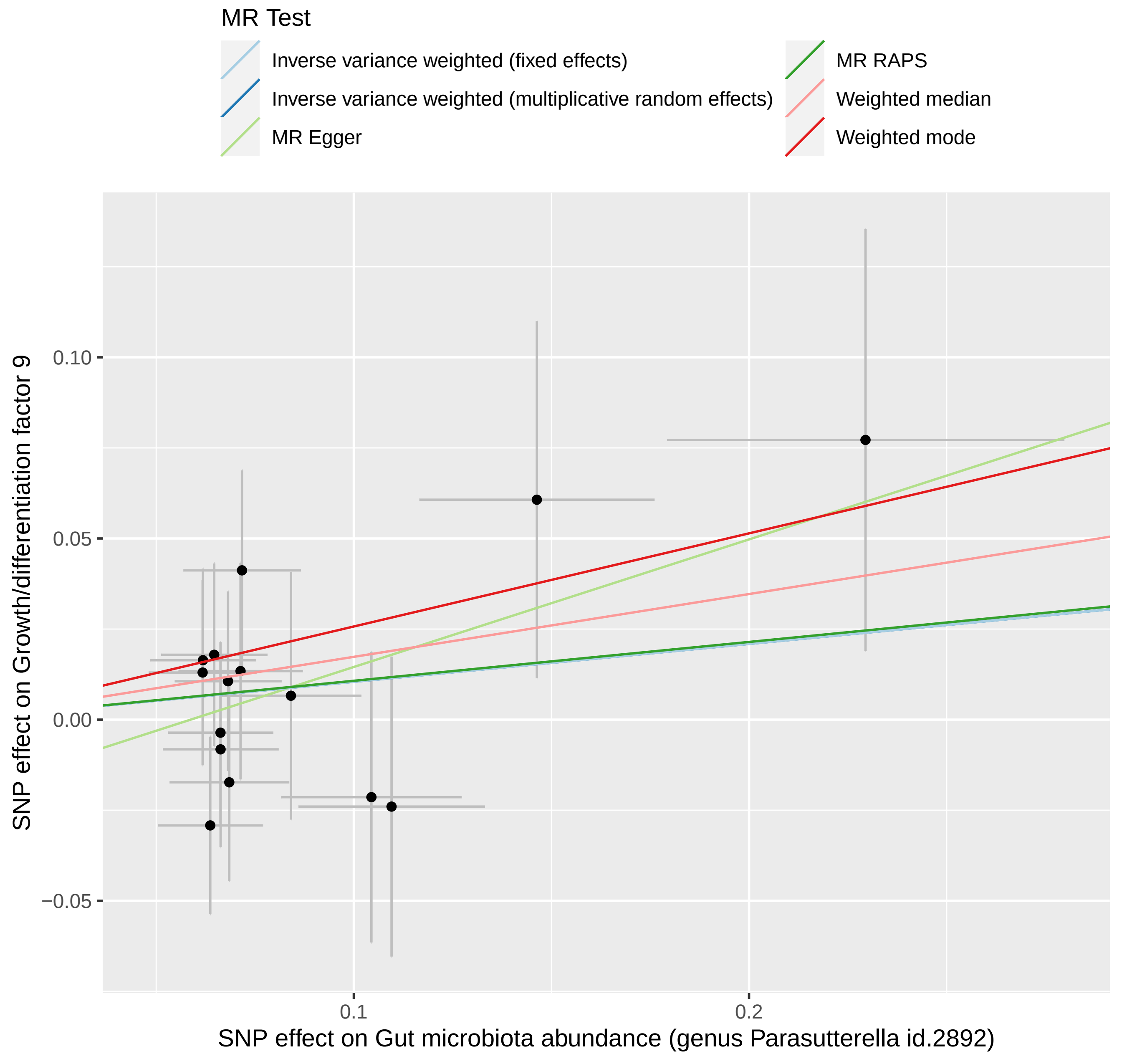

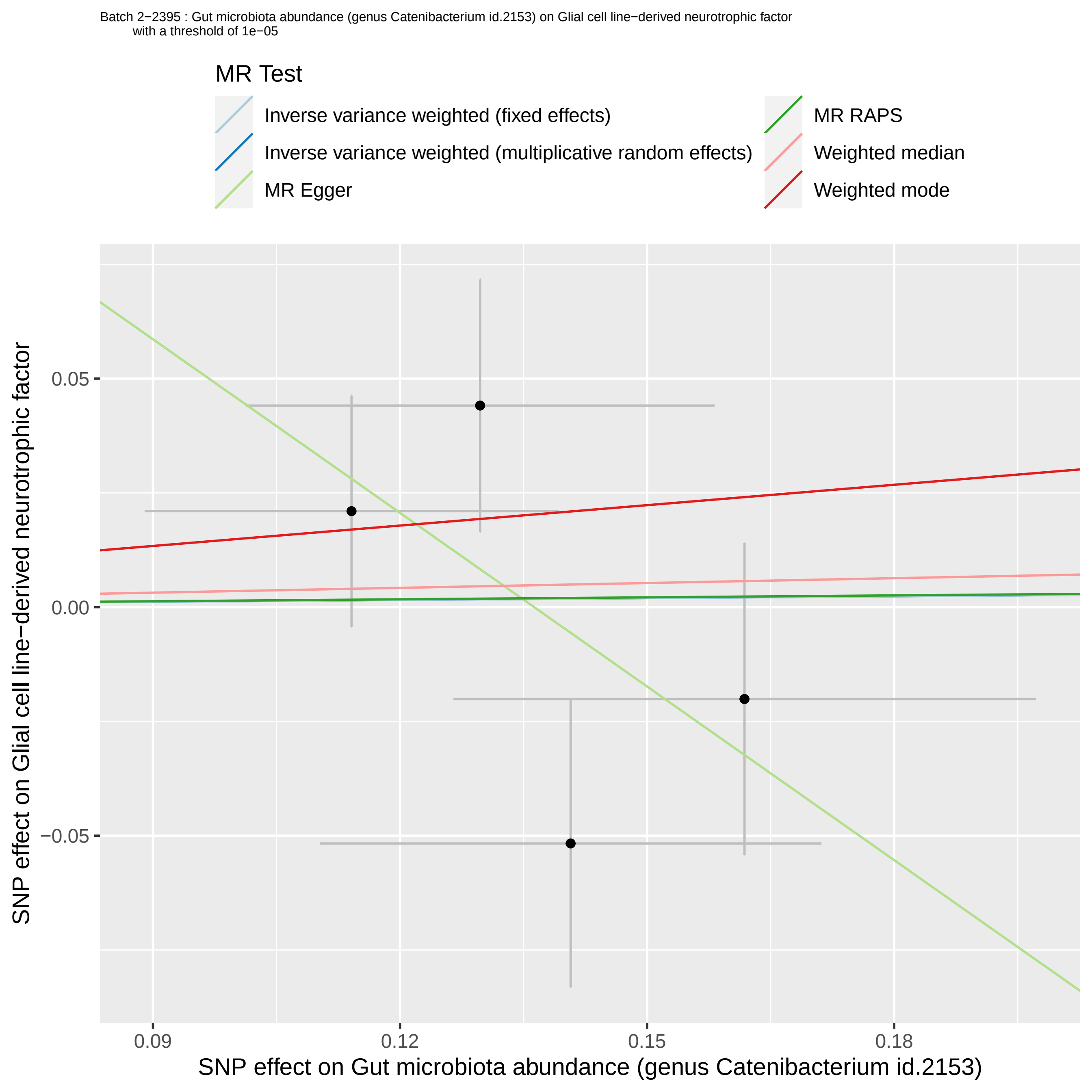
Batch 2−2395 : Gut microbiota abundance (genus Catenibacterium id.2153) on Glial cell line−derived neurotrophic factor
with a threshold of 1e−05
MR Test
Inverse variance weighted (fixed effects)
Inverse variance weighted (multiplicative random effects)
MR Egger
MR RAPS
Weighted median
Weighted mode
0.05
0.00
−0.05
0.09
0.12
0.15
0.18
SNP effect on Glial cell line−derived neurotrophic factor
SNP effect on Gut microbiota abundance (genus Catenibacterium id.2153)

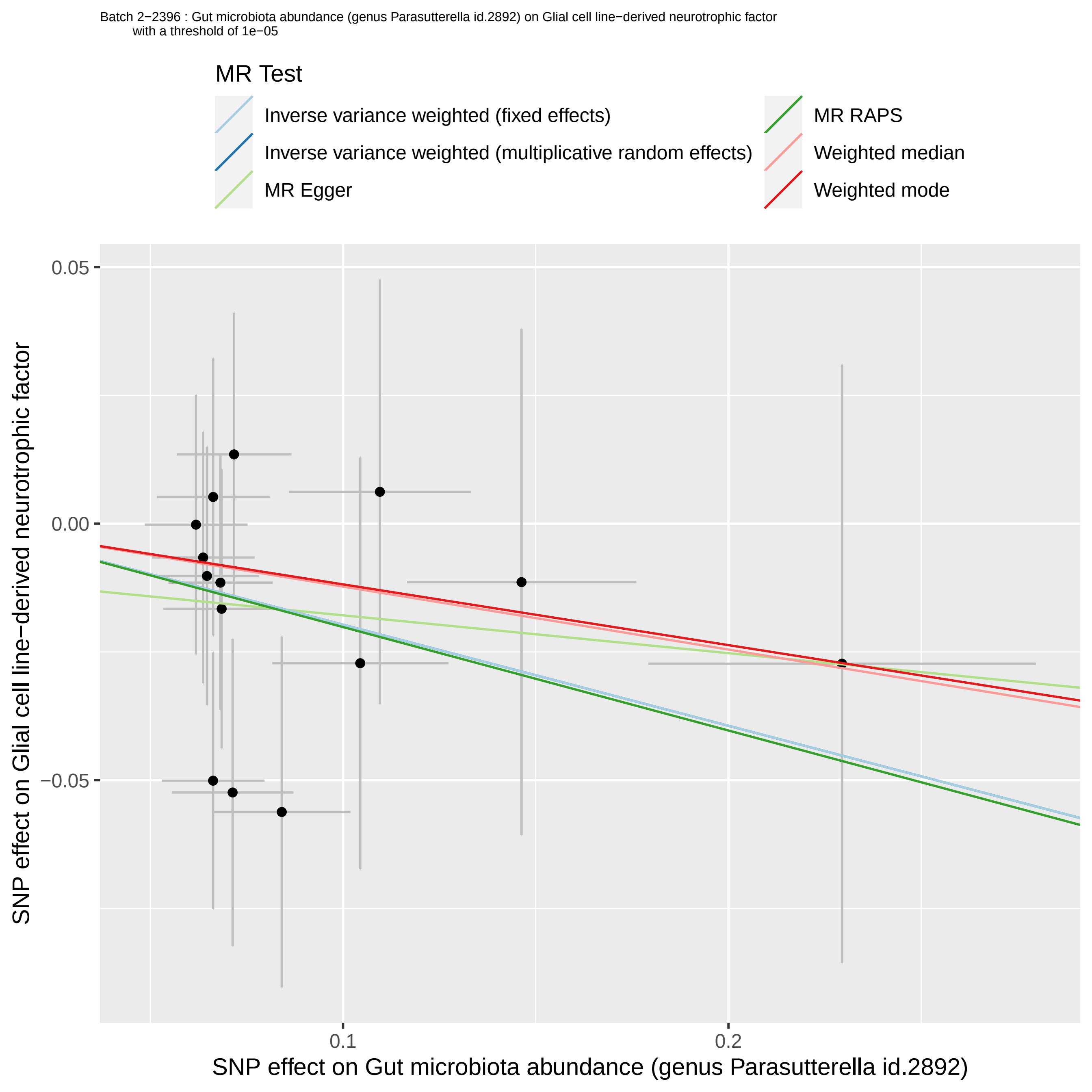

Batch 2−2396 : Gut microbiota abundance (genus Parasutterella id.2892) on Glial cell line−derived neurotrophic factor
with a threshold of 1e−05
MR Test
Inverse variance weighted (fixed effects)
Inverse variance weighted (multiplicative random effects)
MR Egger
MR RAPS
Weighted median
Weighted mode
0.05
0.00
−0.05
0.1
0.2
SNP effect on Glial cell line−derived neurotrophic factor
SNP effect on Gut microbiota abundance (genus Parasutterella id.2892)

Batch 2−2397 : Gut microbiota abundance (genus Catenibacterium id.2153) on GTP−binding protein GEM
with a threshold of 1e−05

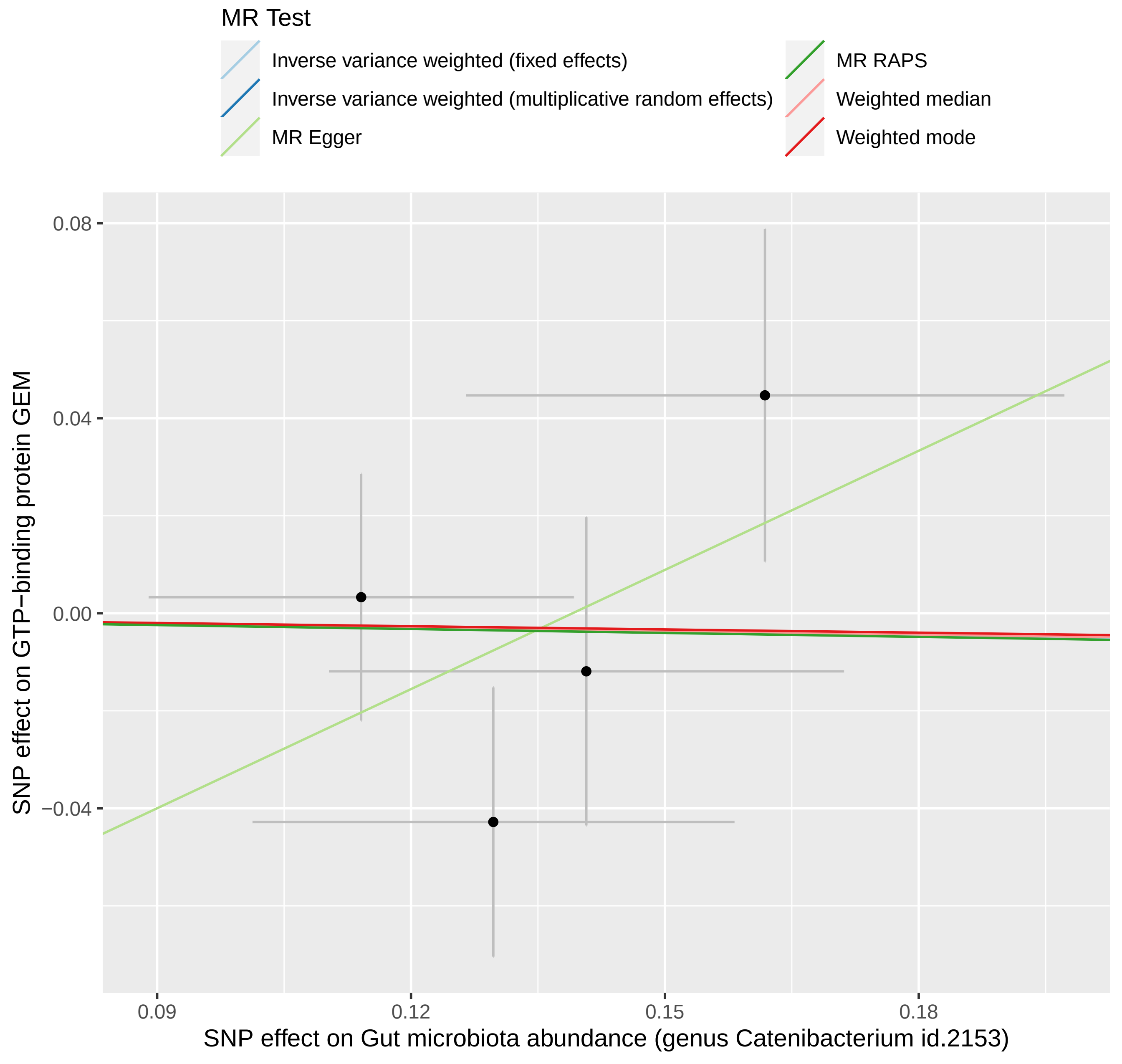

Batch 2−2398 : Gut microbiota abundance (genus Parasutterella id.2892) on GTP−binding protein GEM
with a threshold of 1e−05

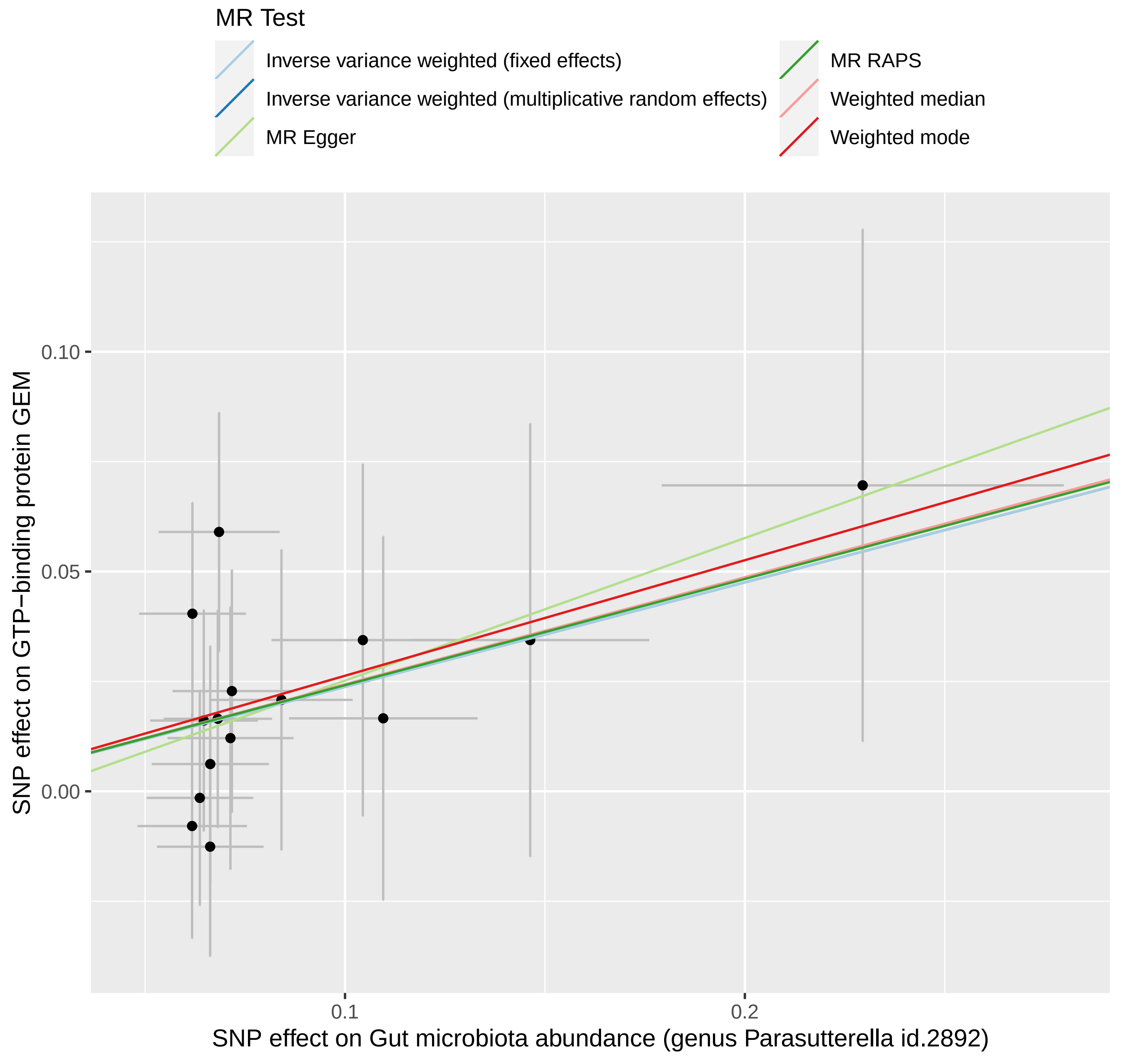

Batch 2−2399 : Gut microbiota abundance (genus Catenibacterium id.2153) on Glial fibrillary acidic protein
with a threshold of 1e−05

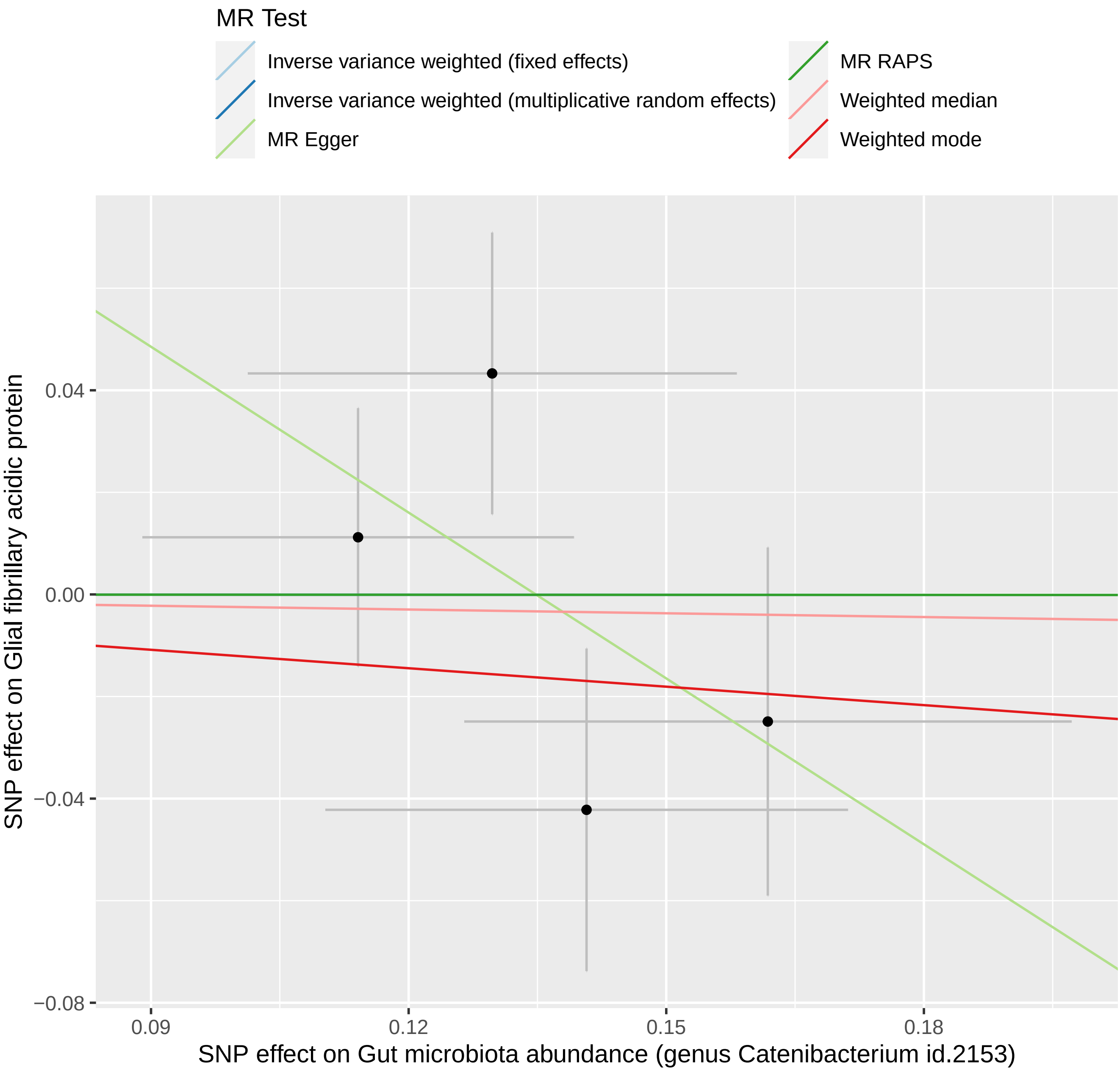

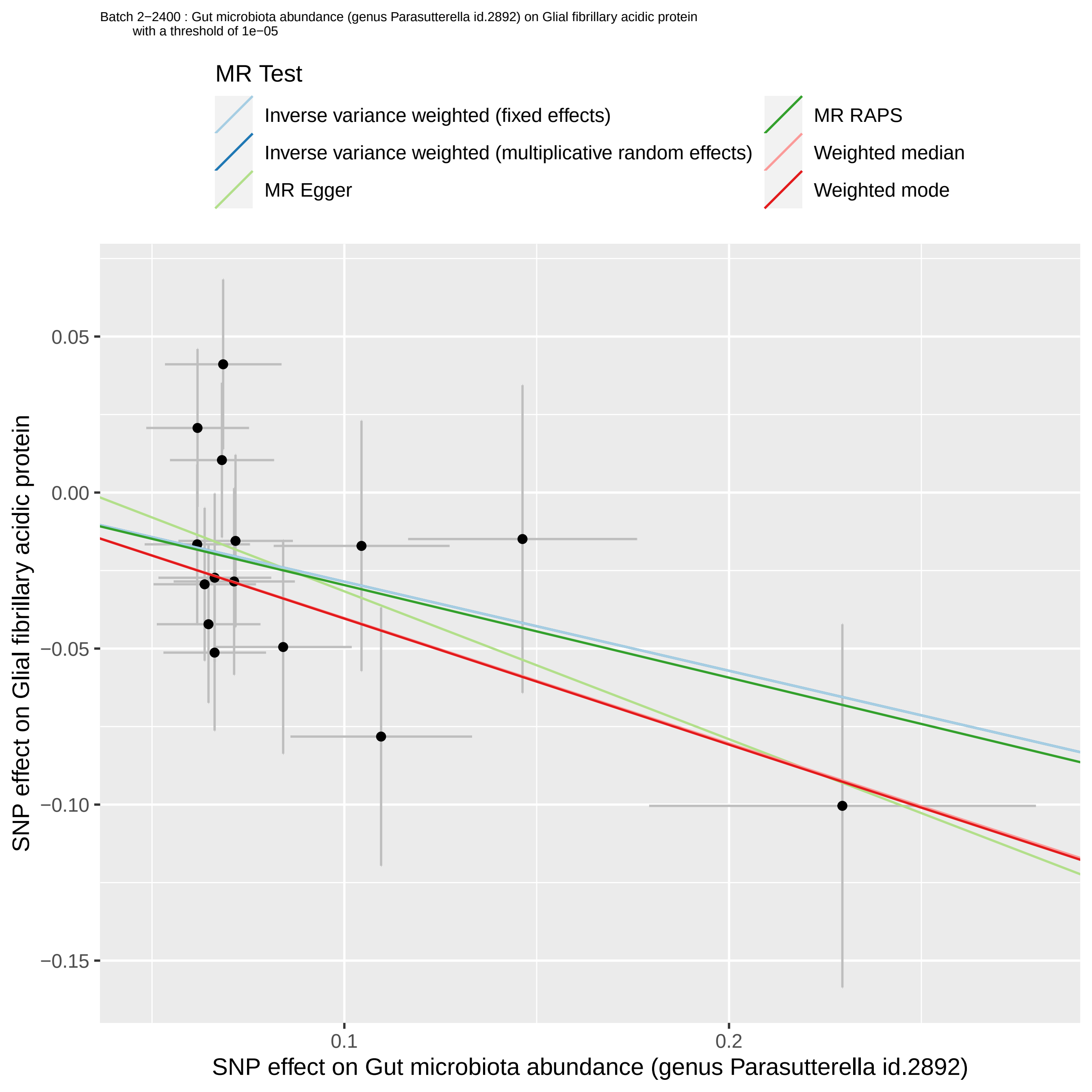
Batch 2−2400 : Gut microbiota abundance (genus Parasutterella id.2892) on Glial fibrillary acidic protein
with a threshold of 1e−05
MR Test
Inverse variance weighted (fixed effects)
Inverse variance weighted (multiplicative random effects)
MR Egger
MR RAPS
Weighted median
Weighted mode
0.05
0.00
−0.05
−0.10
−0.15
0.1
0.2
SNP effect on Glial fibrillary acidic protein
SNP effect on Gut microbiota abundance (genus Parasutterella id.2892)

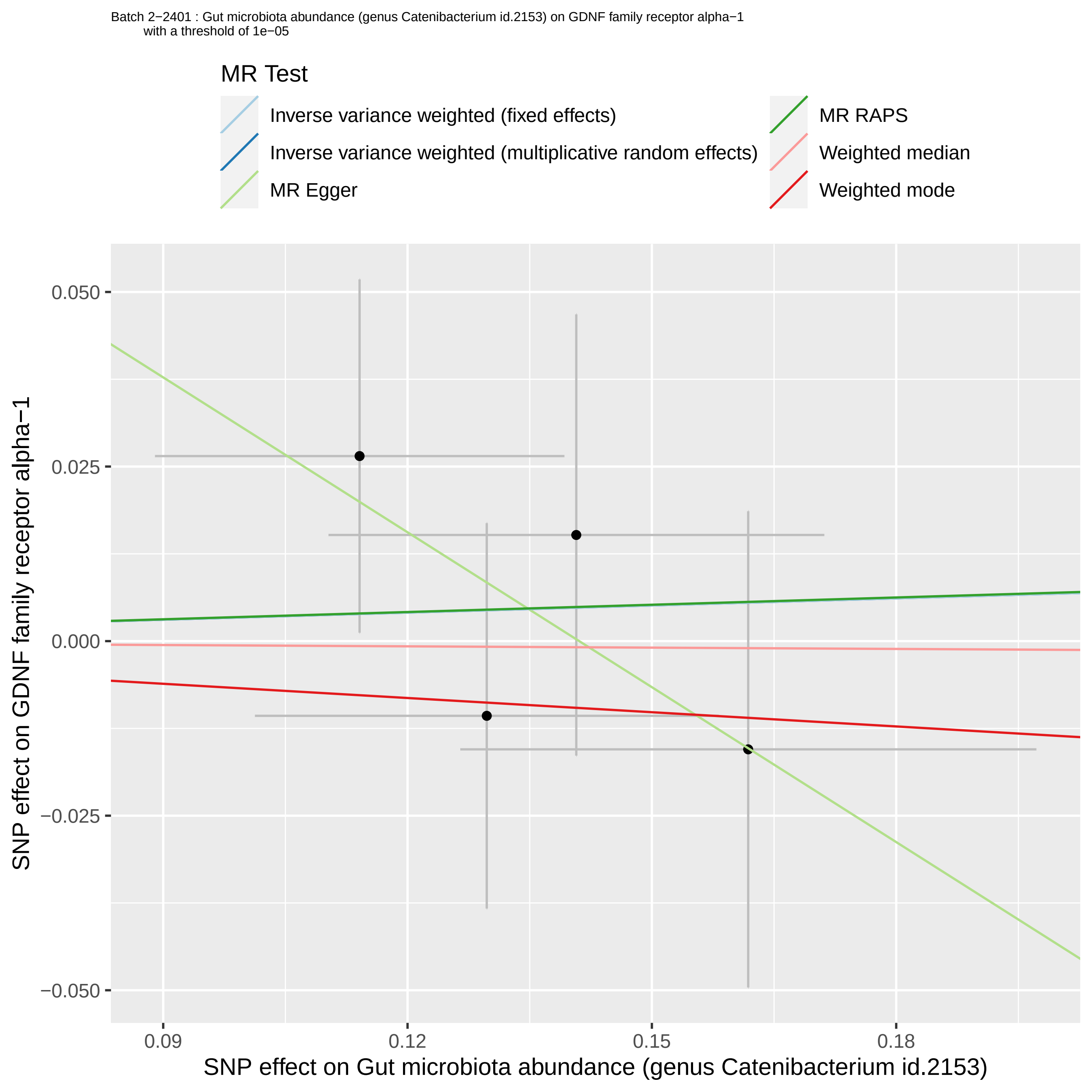

Batch 2−2401 : Gut microbiota abundance (genus Catenibacterium id.2153) on GDNF family receptor alpha−1
with a threshold of 1e−05
MR Test
Inverse variance weighted (fixed effects)
Inverse variance weighted (multiplicative random effects)
MR Egger
MR RAPS
Weighted median
Weighted mode
0.050
0.025
0.000
−0.025
−0.050
0.09
0.12
0.15
0.18
SNP effect on GDNF family receptor alpha−1
SNP effect on Gut microbiota abundance (genus Catenibacterium id.2153)

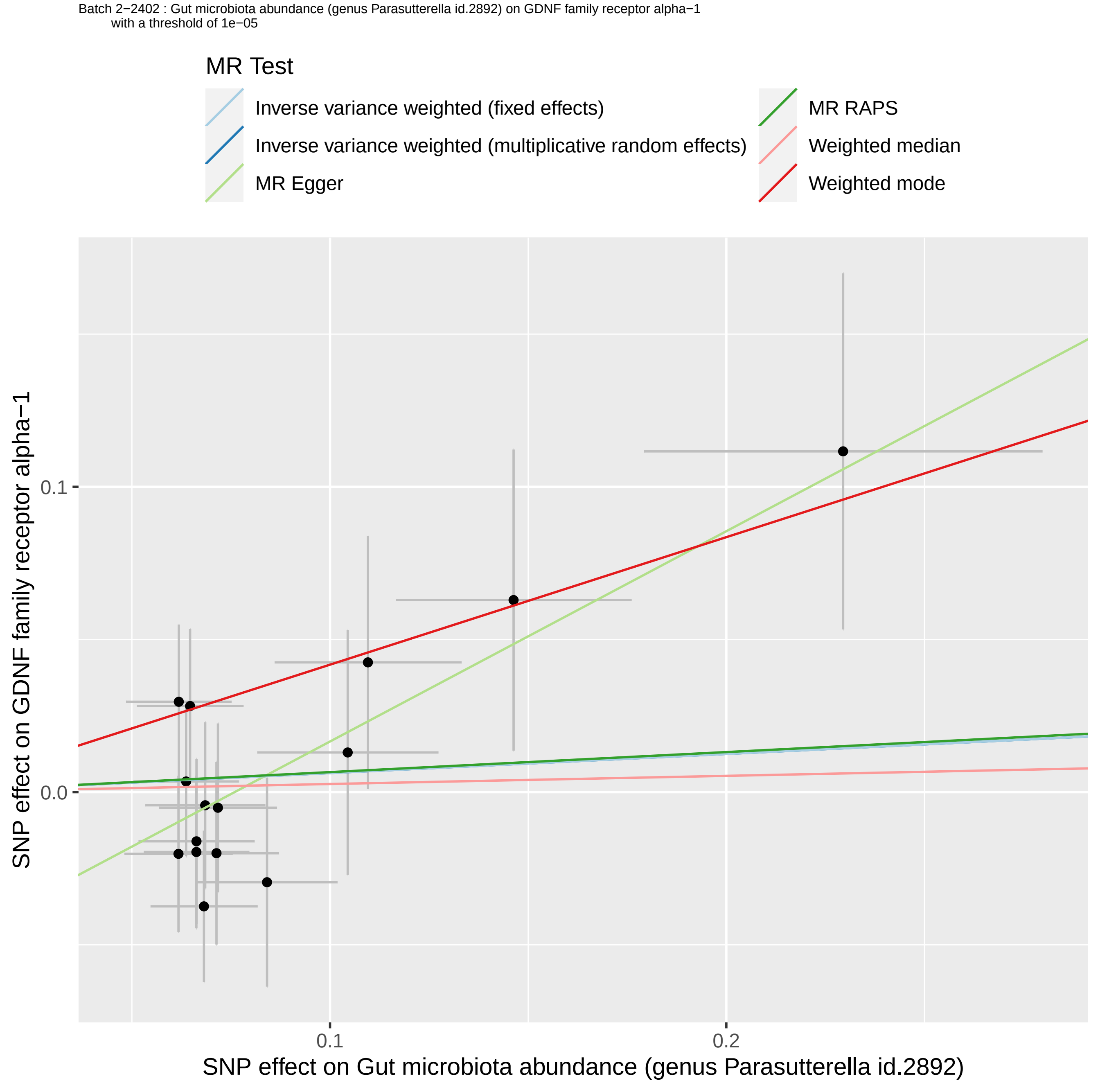

Batch 2−2402 : Gut microbiota abundance (genus Parasutterella id.2892) on GDNF family receptor alpha−1
with a threshold of 1e−05
MR Test
Inverse variance weighted (fixed effects)
Inverse variance weighted (multiplicative random effects)
MR Egger
MR RAPS
Weighted median
Weighted mode
SNP effect on GDNF family receptor alpha−1
0.1
0.0
0.1
0.2
SNP effect on Gut microbiota abundance (genus Parasutterella id.2892)

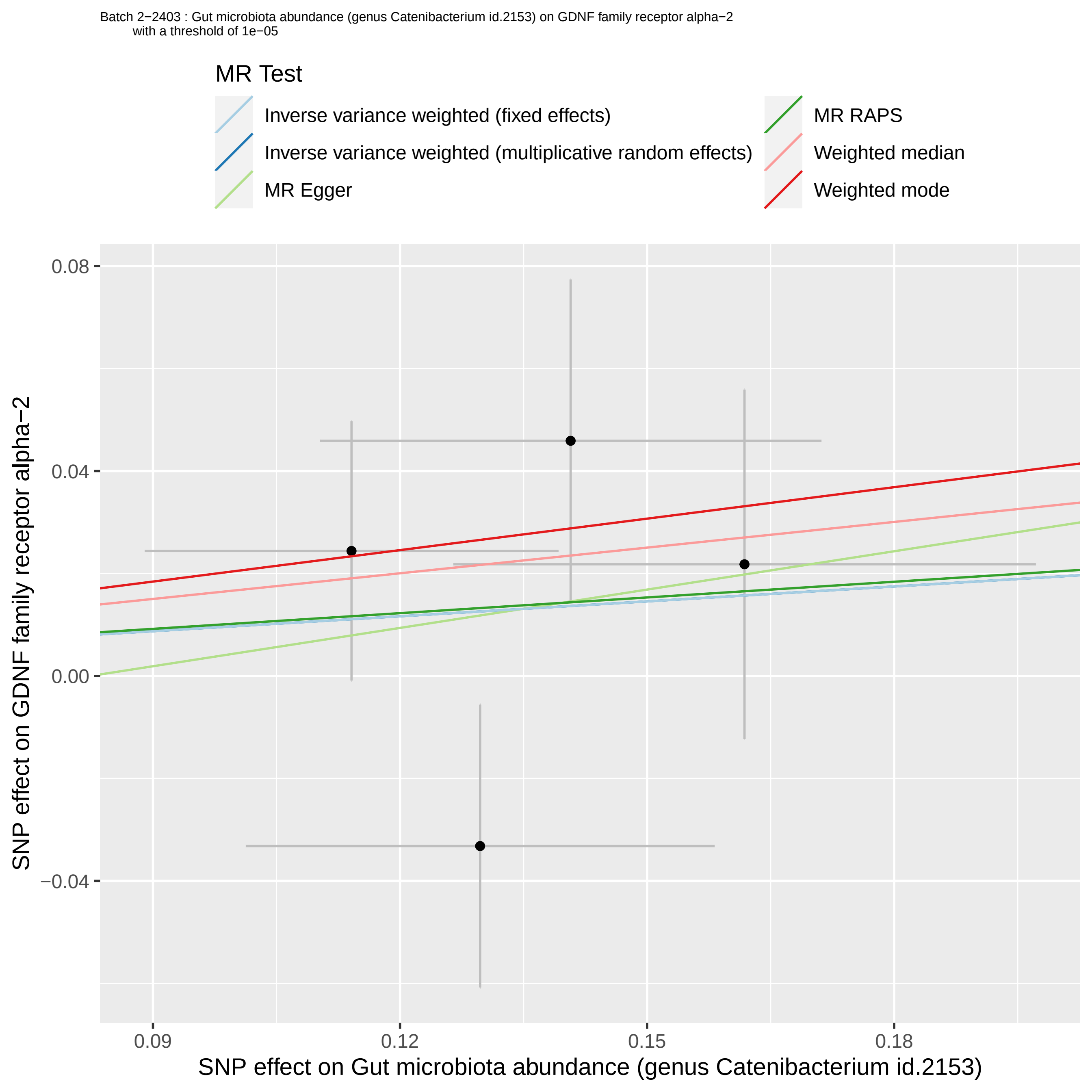

Batch 2−2403 : Gut microbiota abundance (genus Catenibacterium id.2153) on GDNF family receptor alpha−2
with a threshold of 1e−05
MR Test
Inverse variance weighted (fixed effects)
Inverse variance weighted (multiplicative random effects)
MR Egger
MR RAPS
Weighted median
Weighted mode
0.08
0.04
0.00
−0.04
0.09
0.12
0.15
0.18
SNP effect on GDNF family receptor alpha−2
SNP effect on Gut microbiota abundance (genus Catenibacterium id.2153)

Batch 2−2404 : Gut microbiota abundance (genus Parasutterella id.2892) on GDNF family receptor alpha−2
with a threshold of 1e−05

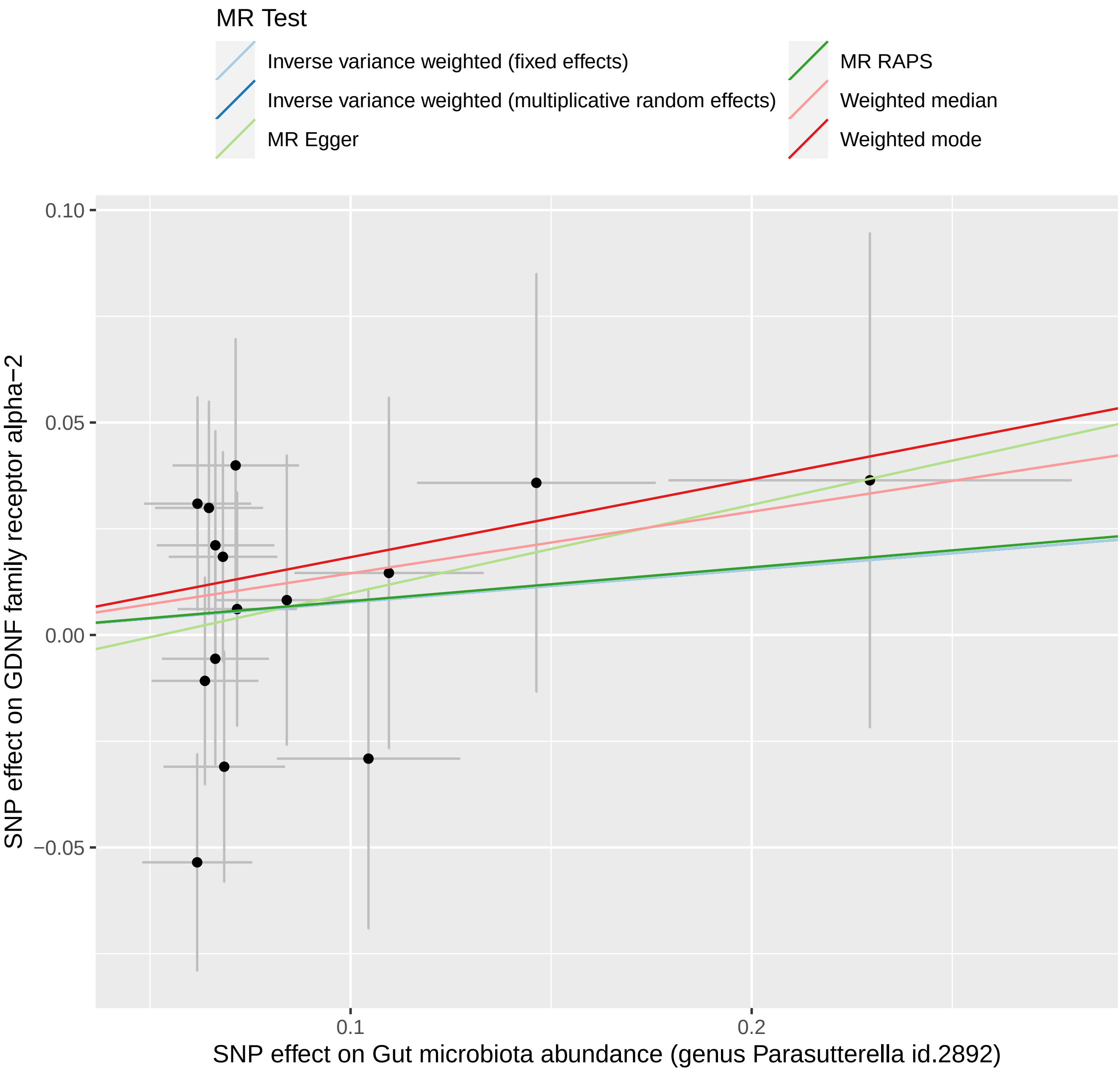

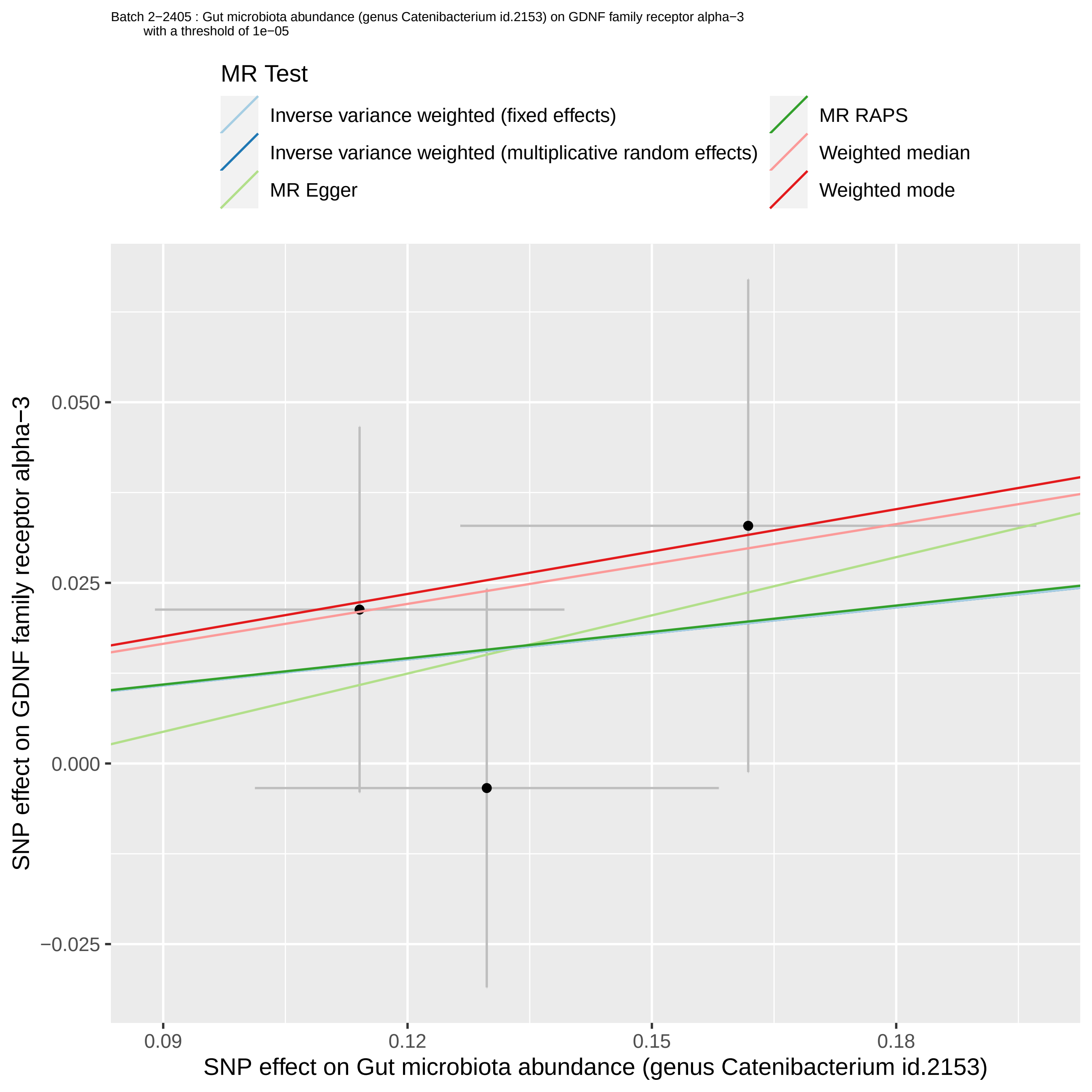
Batch 2−2405 : Gut microbiota abundance (genus Catenibacterium id.2153) on GDNF family receptor alpha−3
with a threshold of 1e−05
MR Test
Inverse variance weighted (fixed effects)
Inverse variance weighted (multiplicative random effects)
MR Egger
MR RAPS
Weighted median
Weighted mode
0.050
0.025
0.000
−0.025
0.09
0.12
0.15
0.18
SNP effect on GDNF family receptor alpha−3
SNP effect on Gut microbiota abundance (genus Catenibacterium id.2153)

Batch 2−2406 : Gut microbiota abundance (genus Parasutterella id.2892) on GDNF family receptor alpha−3
with a threshold of 1e−05

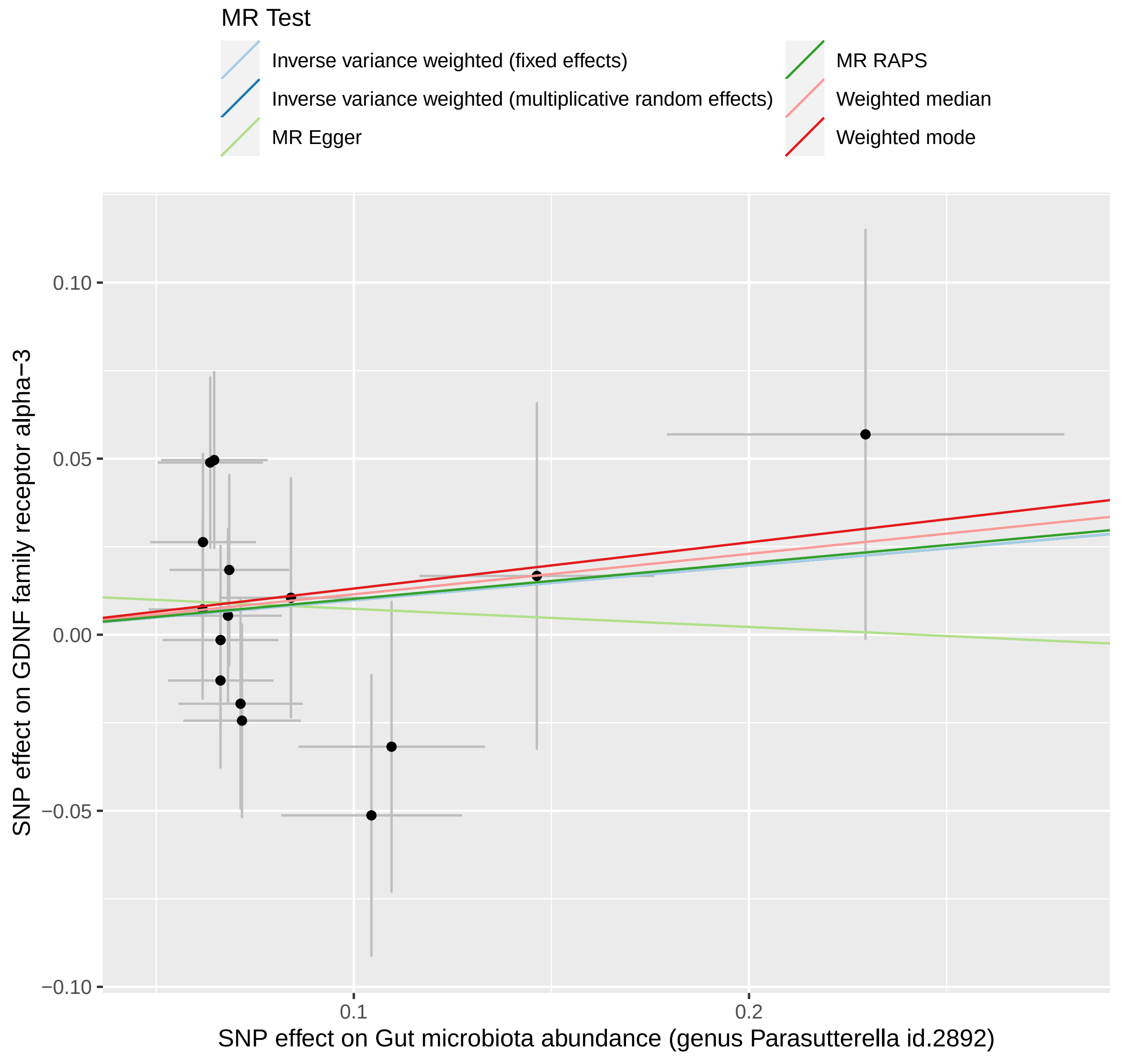

Batch 2−2407 : Gut microbiota abundance (genus Catenibacterium id.2153) on GDNF family receptor alpha−like
with a threshold of 1e−05

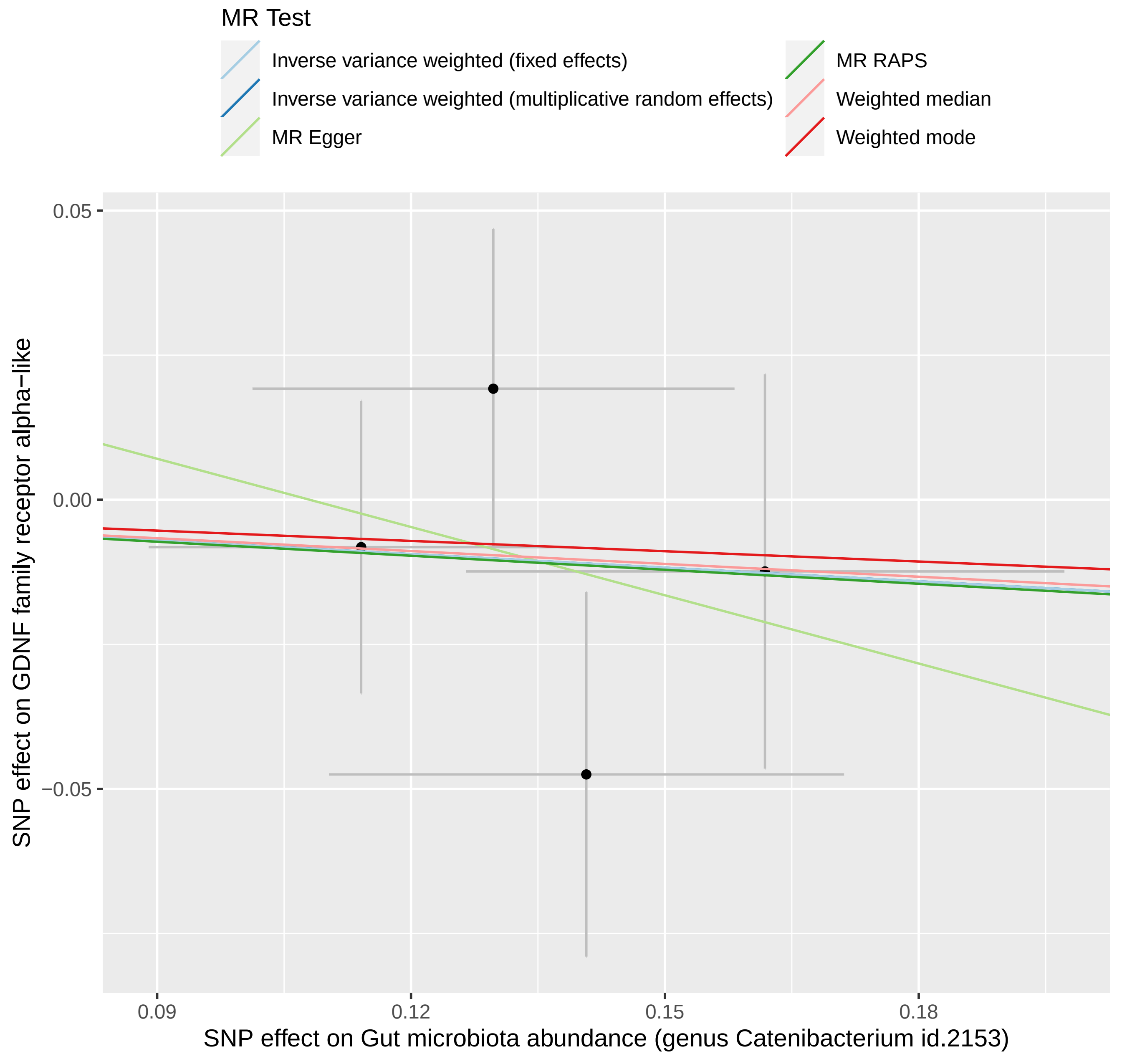

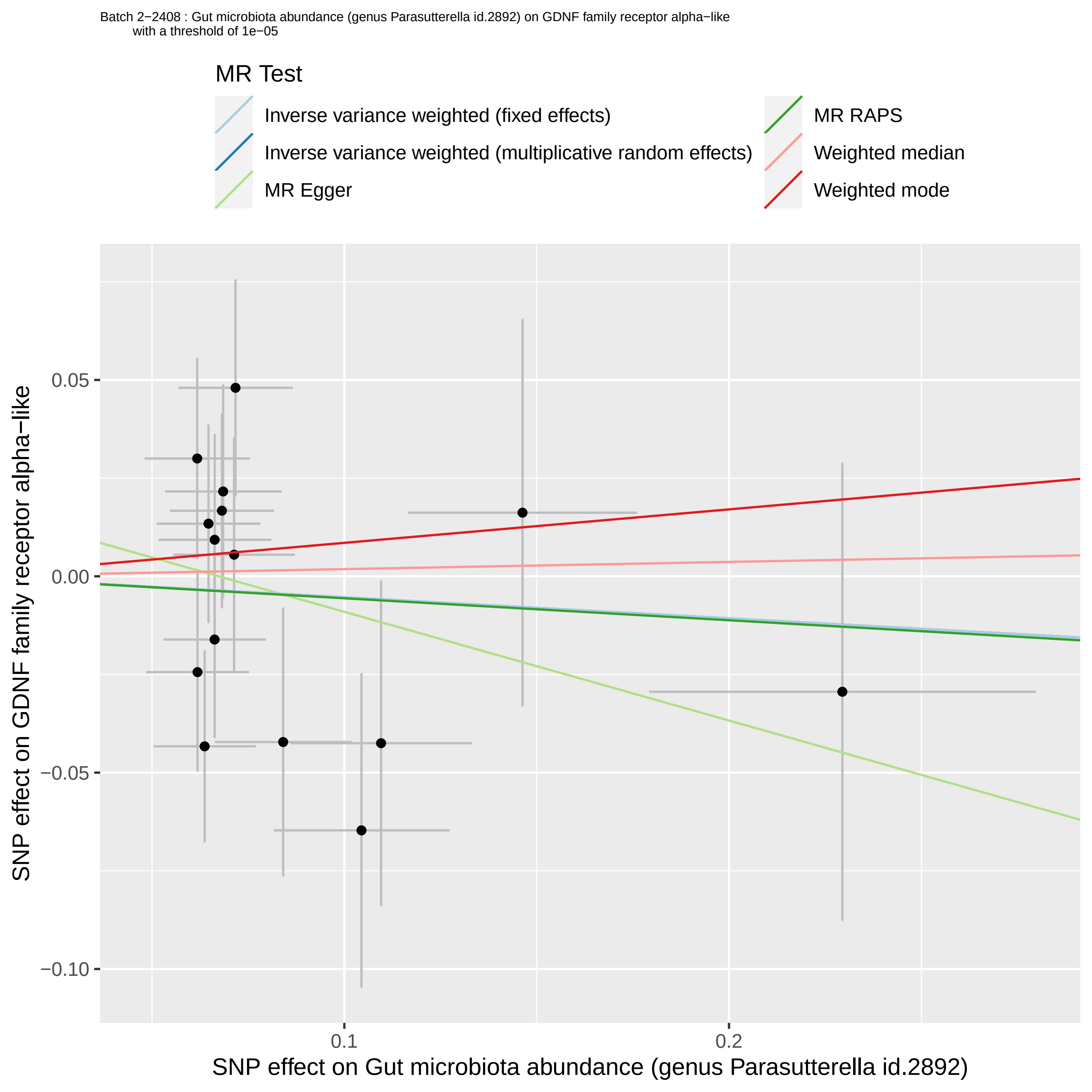
Batch 2−2408 : Gut microbiota abundance (genus Parasutterella id.2892) on GDNF family receptor alpha−like
with a threshold of 1e−05
MR Test
Inverse variance weighted (fixed effects)
Inverse variance weighted (multiplicative random effects)
MR Egger
MR RAPS
Weighted median
Weighted mode
0.05
0.00
−0.05
−0.10
0.1
0.2
SNP effect on GDNF family receptor alpha−like
SNP effect on Gut microbiota abundance (genus Parasutterella id.2892)

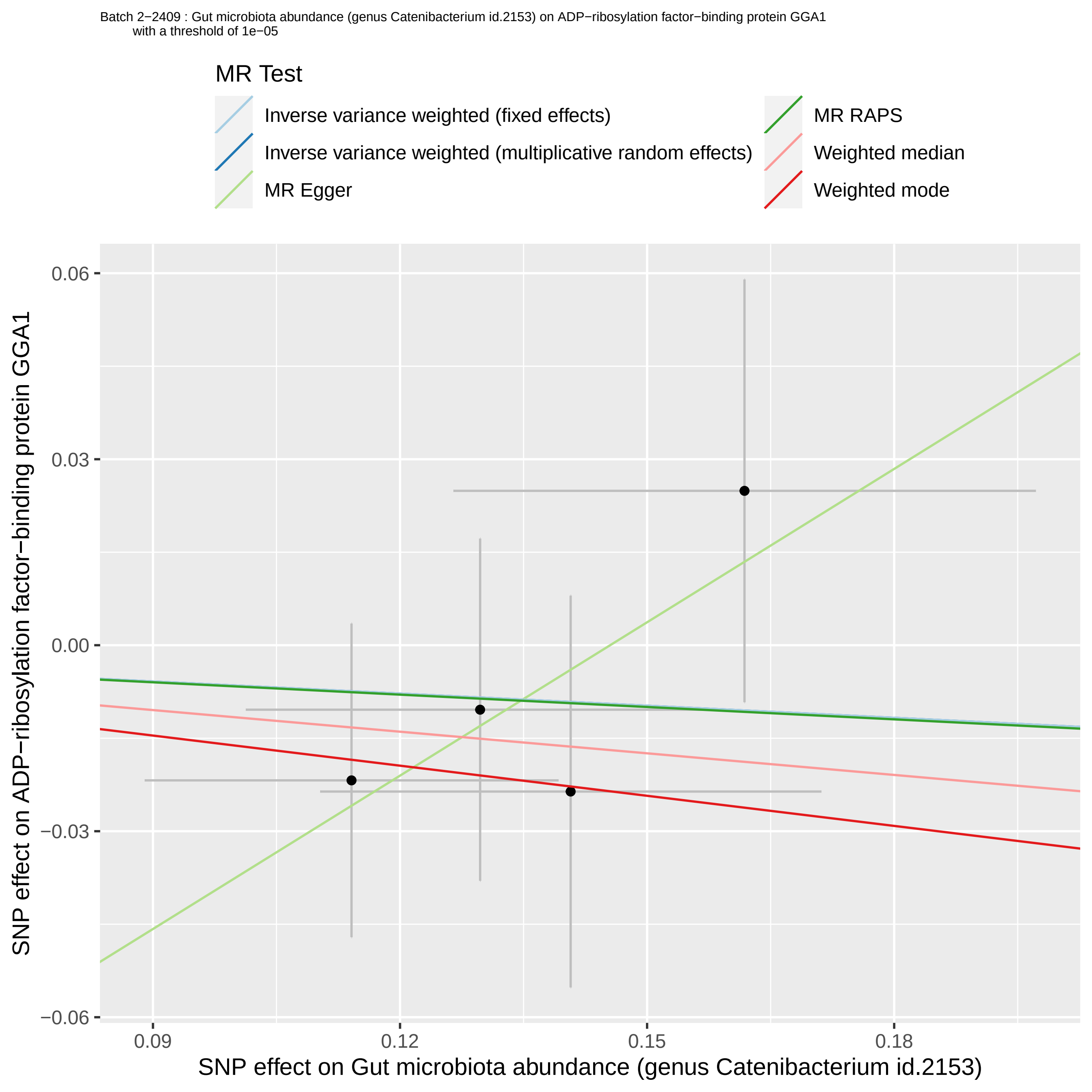

Batch 2−2409 : Gut microbiota abundance (genus Catenibacterium id.2153) on ADP−ribosylation factor−binding protein GGA1
with a threshold of 1e−05
MR Test
Inverse variance weighted (fixed effects)
Inverse variance weighted (multiplicative random effects)
MR Egger
MR RAPS
Weighted median
Weighted mode
0.06
0.03
0.00
−0.03
−0.06
0.09
0.12
0.15
0.18
SNP effect on ADP−ribosylation factor−binding protein GGA1
SNP effect on Gut microbiota abundance (genus Catenibacterium id.2153)

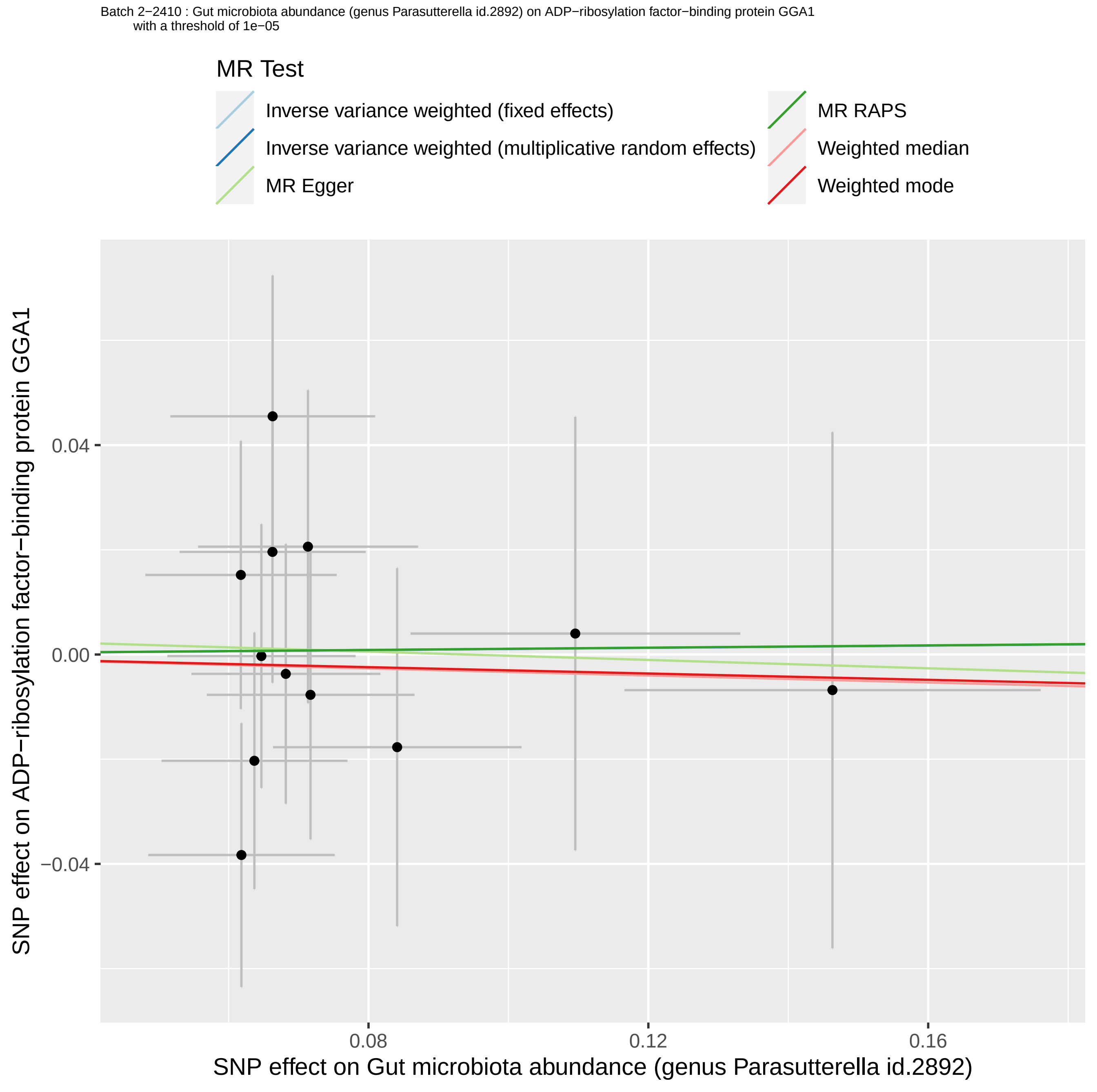

Batch 2−2410 : Gut microbiota abundance (genus Parasutterella id.2892) on ADP−ribosylation factor−binding protein GGA1
with a threshold of 1e−05
MR Test
Inverse variance weighted (fixed effects)
Inverse variance weighted (multiplicative random effects)
MR Egger
MR RAPS
Weighted median
Weighted mode
SNP effect on ADP−ribosylation factor−binding protein GGA1
0.04
0.00
−0.04
0.08
0.12
0.16
SNP effect on Gut microbiota abundance (genus Parasutterella id.2892)

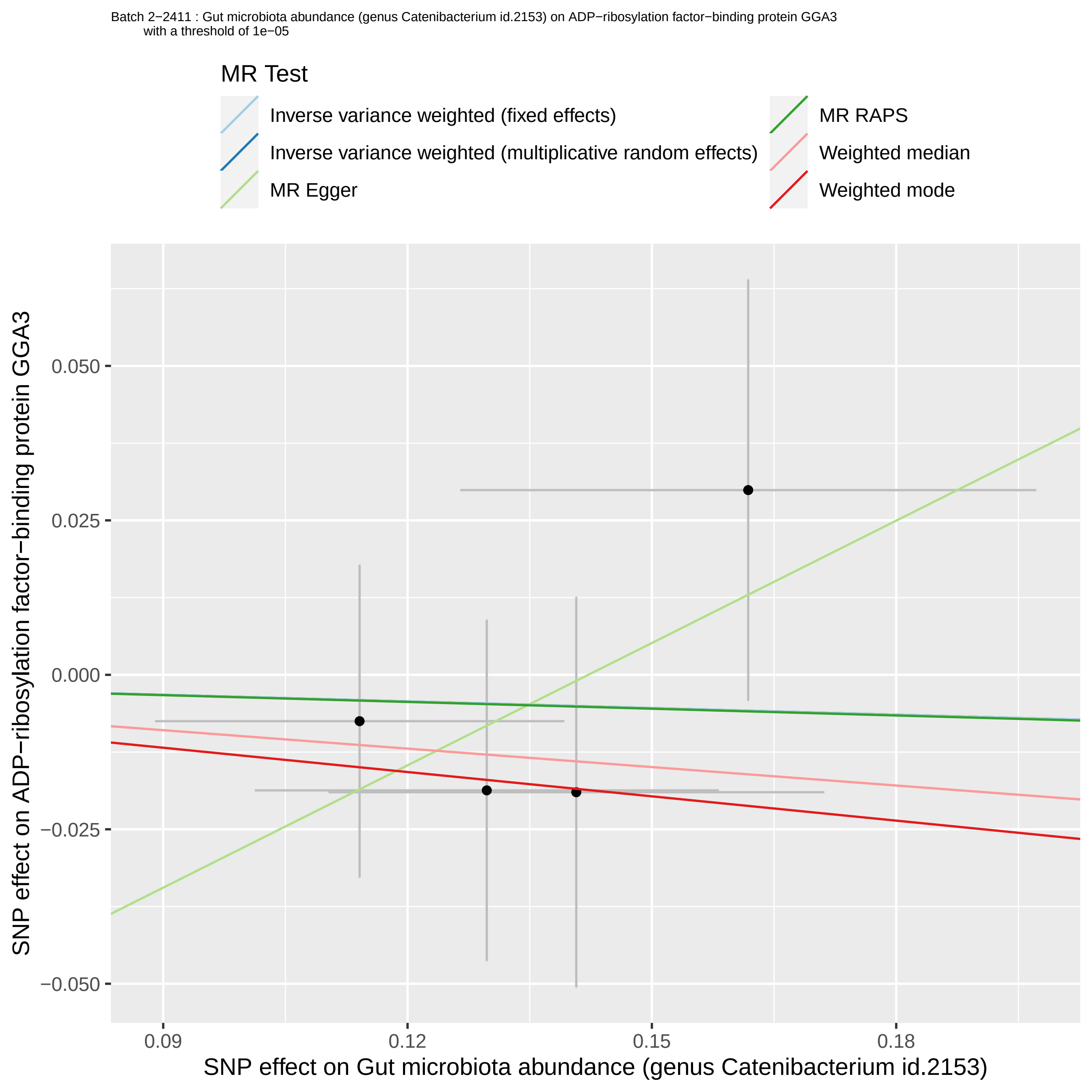
Batch 2−2411 : Gut microbiota abundance (genus Catenibacterium id.2153) on ADP−ribosylation factor−binding protein GGA3
with a threshold of 1e−05
MR Test
Inverse variance weighted (fixed effects)
Inverse variance weighted (multiplicative random effects)
MR Egger
MR RAPS
Weighted median
Weighted mode
SNP effect on ADP−ribosylation factor−binding protein GGA3
0.050
0.025
0.000
−0.025
−0.050
0.09
0.12
0.15
0.18
SNP effect on Gut microbiota abundance (genus Catenibacterium id.2153)

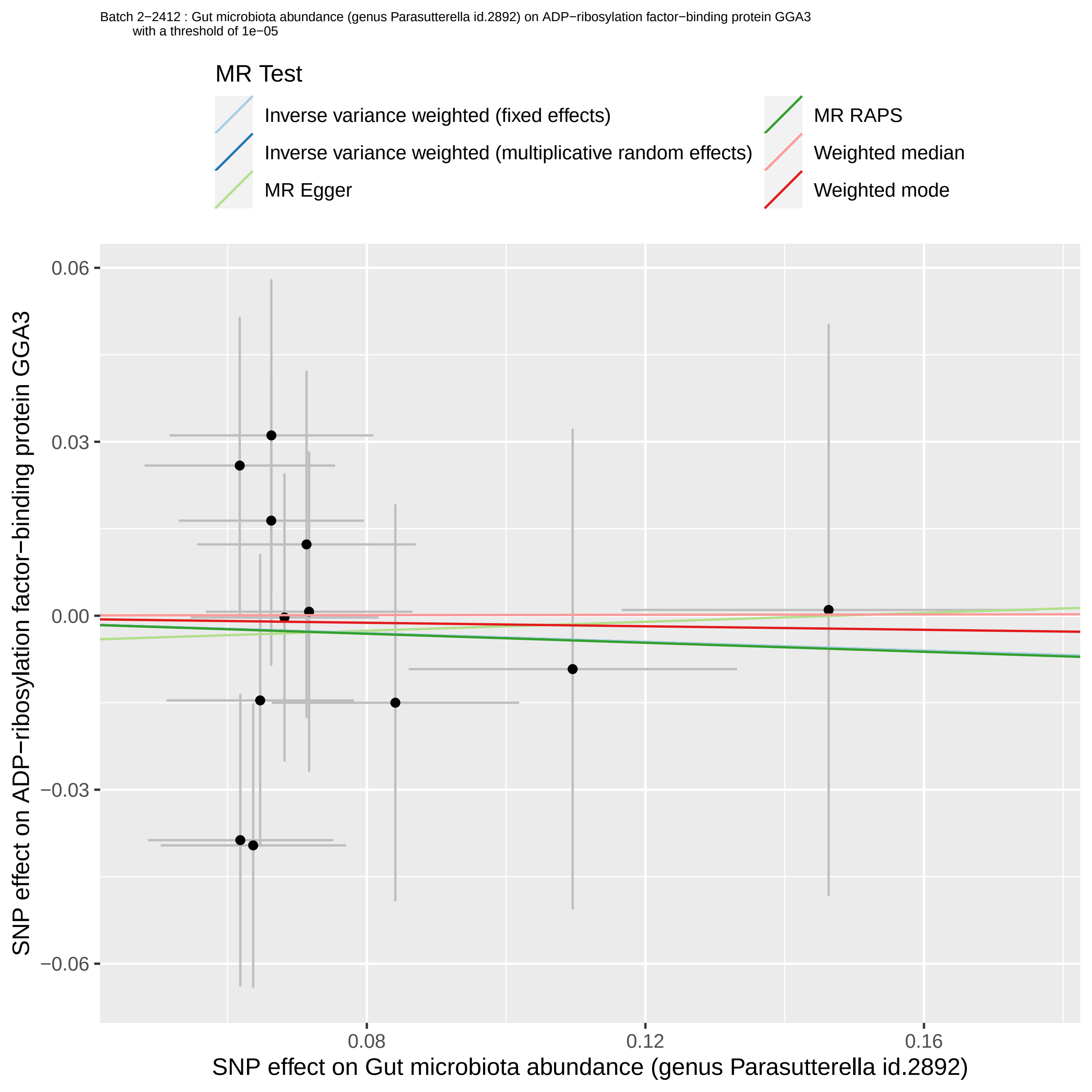

Batch 2−2412 : Gut microbiota abundance (genus Parasutterella id.2892) on ADP−ribosylation factor−binding protein GGA3
with a threshold of 1e−05
MR Test
Inverse variance weighted (fixed effects)
Inverse variance weighted (multiplicative random effects)
MR Egger
MR RAPS
Weighted median
Weighted mode
0.06
0.03
0.00
−0.03
−0.06
0.08
0.12
0.16
SNP effect on ADP−ribosylation factor−binding protein GGA3
SNP effect on Gut microbiota abundance (genus Parasutterella id.2892)

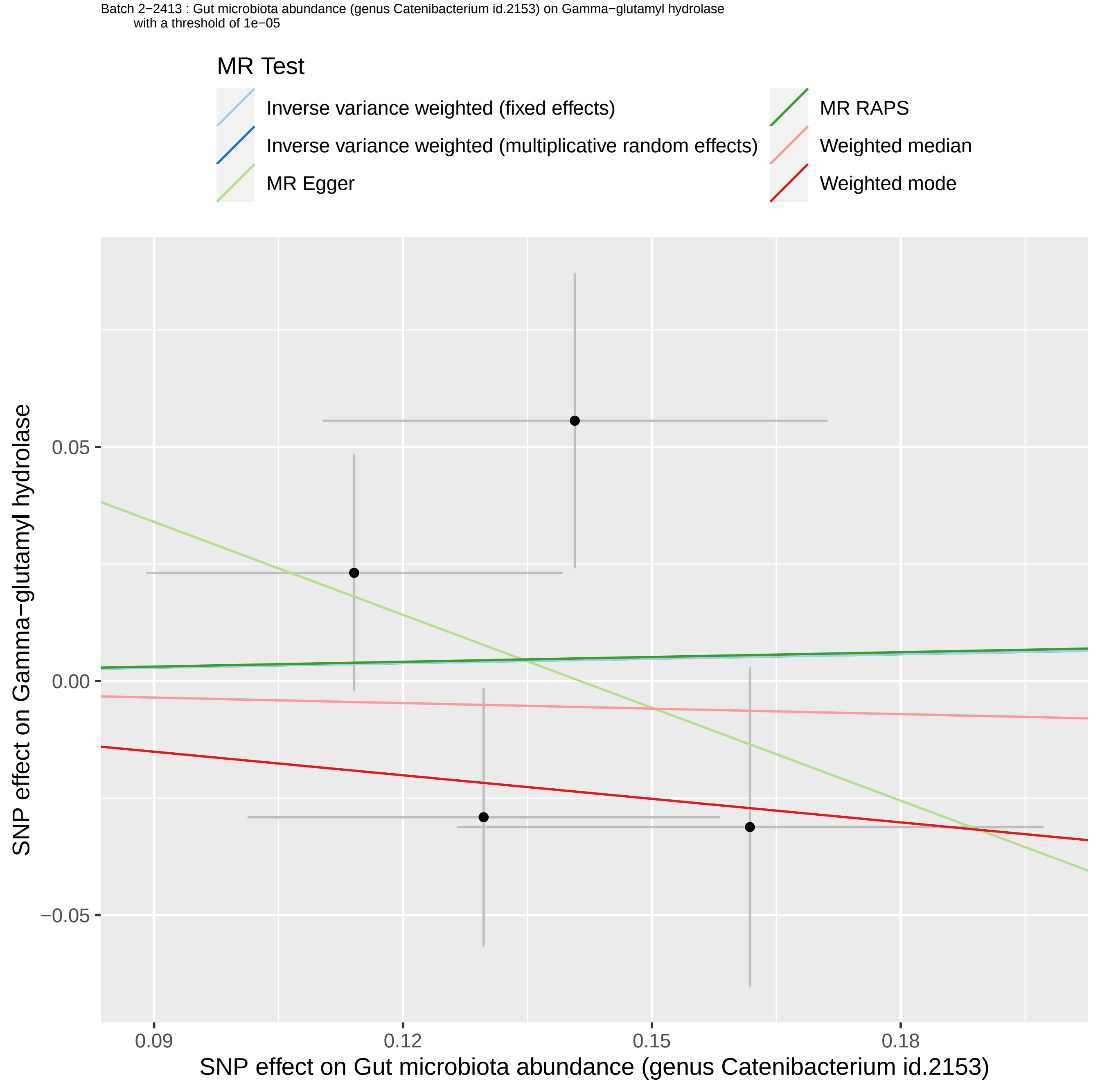
Batch 2−2413 : Gut microbiota abundance (genus Catenibacterium id.2153) on Gamma−glutamyl hydrolase
with a threshold of 1e−05
MR Test
Inverse variance weighted (fixed effects)
Inverse variance weighted (multiplicative random effects)
MR Egger
MR RAPS
Weighted median
Weighted mode
0.05
0.00
−0.05
0.09
0.12
0.15
0.18
SNP effect on Gamma−glutamyl hydrolase
SNP effect on Gut microbiota abundance (genus Catenibacterium id.2153)

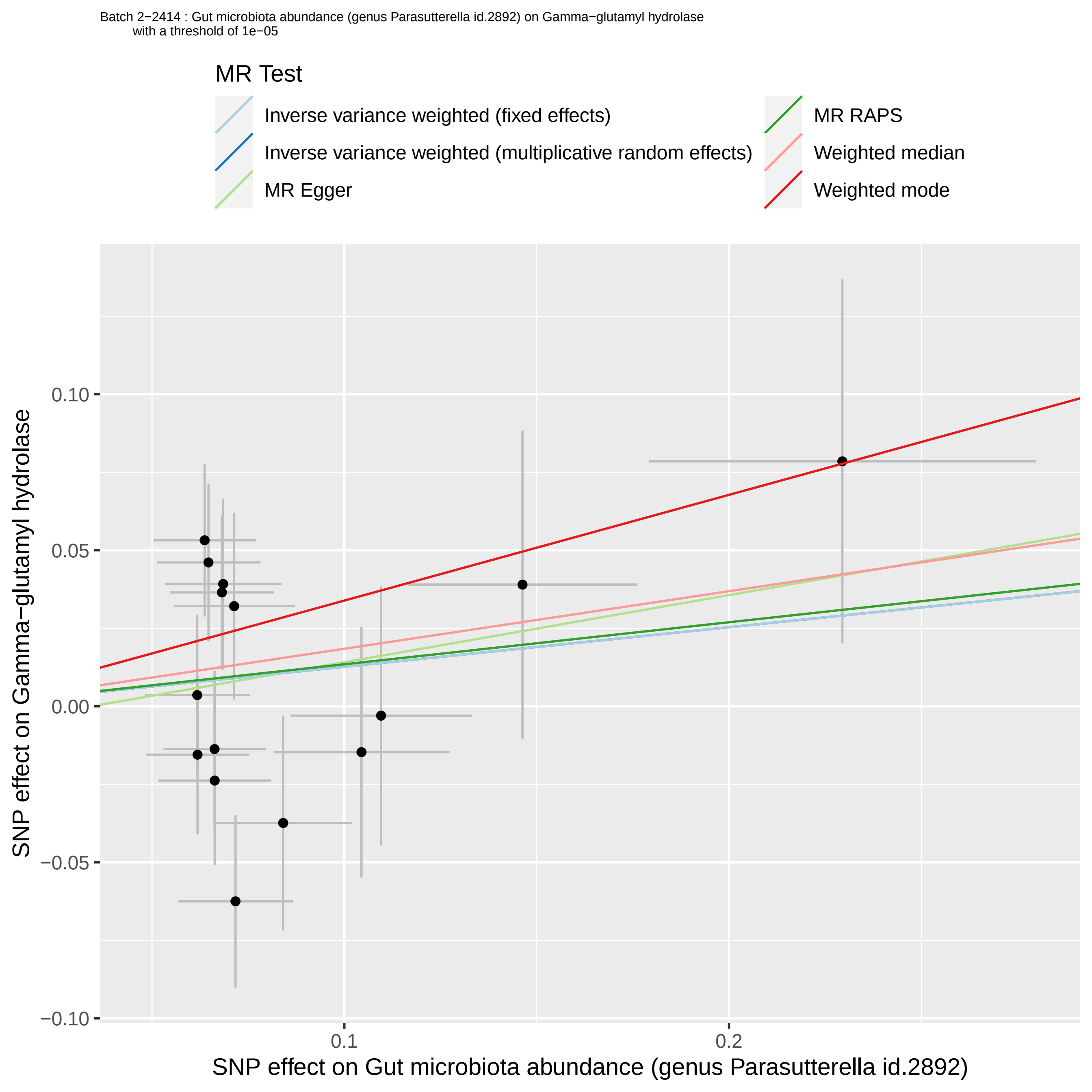

Batch 2−2414 : Gut microbiota abundance (genus Parasutterella id.2892) on Gamma−glutamyl hydrolase
with a threshold of 1e−05
MR Test
Inverse variance weighted (fixed effects)
Inverse variance weighted (multiplicative random effects)
MR Egger
MR RAPS
Weighted median
Weighted mode
0.10
0.05
0.00
−0.05
−0.10
0.1
0.2
SNP effect on Gamma−glutamyl hydrolase
SNP effect on Gut microbiota abundance (genus Parasutterella id.2892)

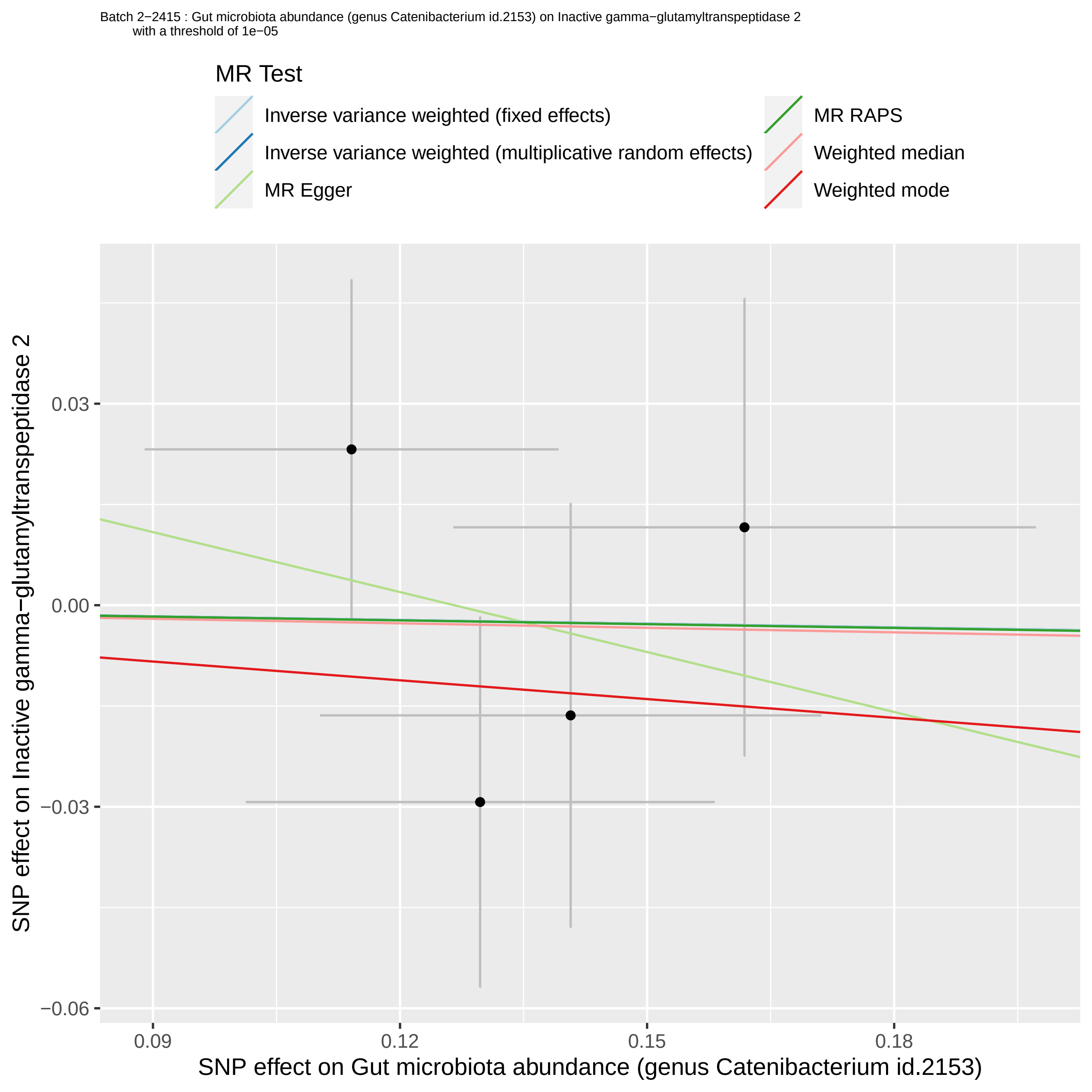

Batch 2−2415 : Gut microbiota abundance (genus Catenibacterium id.2153) on Inactive gamma−glutamyltranspeptidase 2
with a threshold of 1e−05
MR Test
Inverse variance weighted (fixed effects)
Inverse variance weighted (multiplicative random effects)
MR Egger
MR RAPS
Weighted median
Weighted mode
SNP effect on Inactive gamma−glutamyltranspeptidase 2
0.03
0.00
−0.03
−0.06
0.09
0.12
0.15
0.18
SNP effect on Gut microbiota abundance (genus Catenibacterium id.2153)

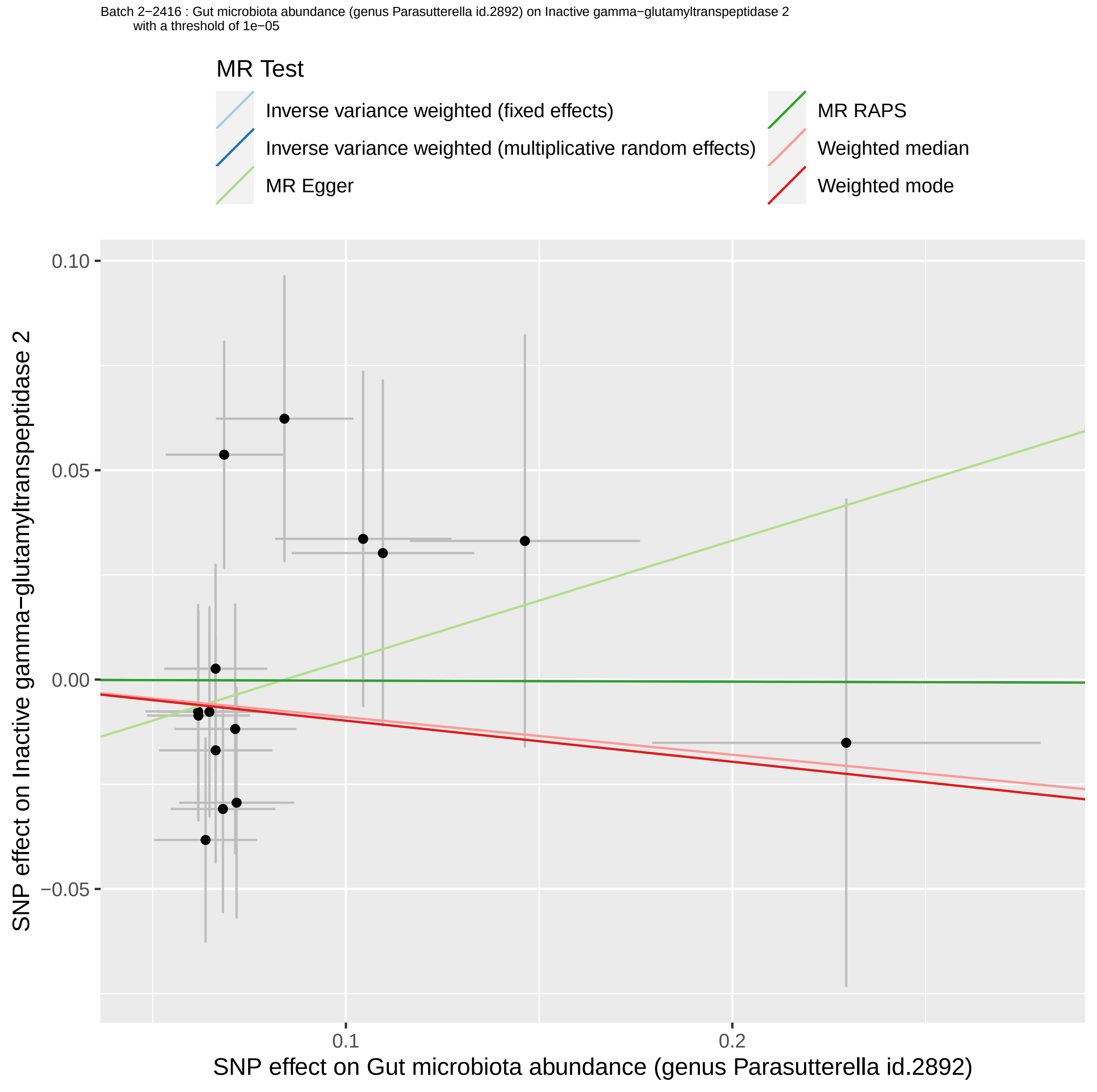
Batch 2−2416 : Gut microbiota abundance (genus Parasutterella id.2892) on Inactive gamma−glutamyltranspeptidase 2
with a threshold of 1e−05
MR Test
Inverse variance weighted (fixed effects)
Inverse variance weighted (multiplicative random effects)
MR Egger
MR RAPS
Weighted median
Weighted mode
0.10
0.05
0.00
−0.05
0.1
0.2
SNP effect on Inactive gamma−glutamyltranspeptidase 2
SNP effect on Gut microbiota abundance (genus Parasutterella id.2892)

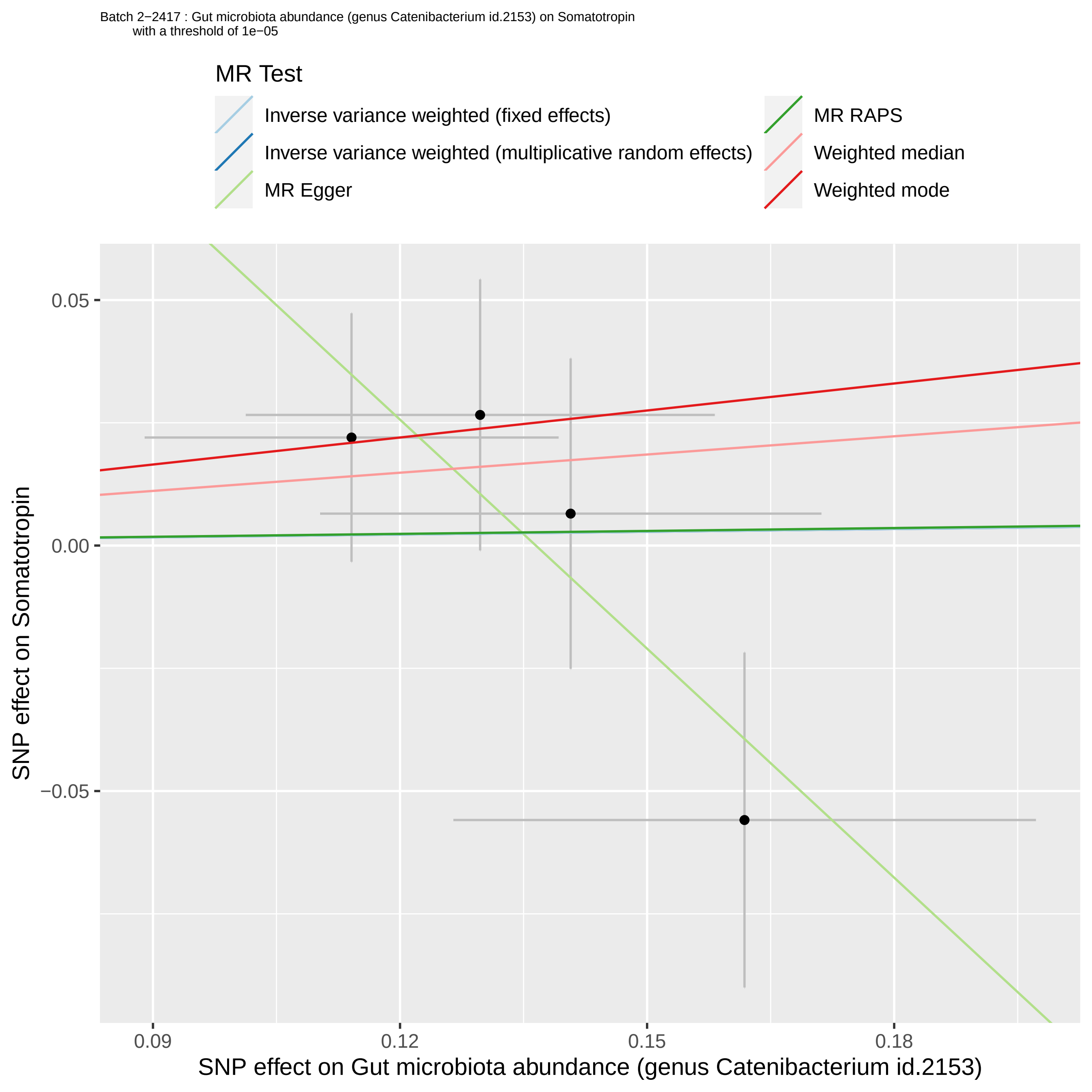
Batch 2−2417 : Gut microbiota abundance (genus Catenibacterium id.2153) on Somatotropin
with a threshold of 1e−05
MR Test
Inverse variance weighted (fixed effects)
Inverse variance weighted (multiplicative random effects)
MR Egger
MR RAPS
Weighted median
Weighted mode
0.05
0.00
−0.05
0.09
0.12
0.15
0.18
SNP effect on Somatotropin
SNP effect on Gut microbiota abundance (genus Catenibacterium id.2153)

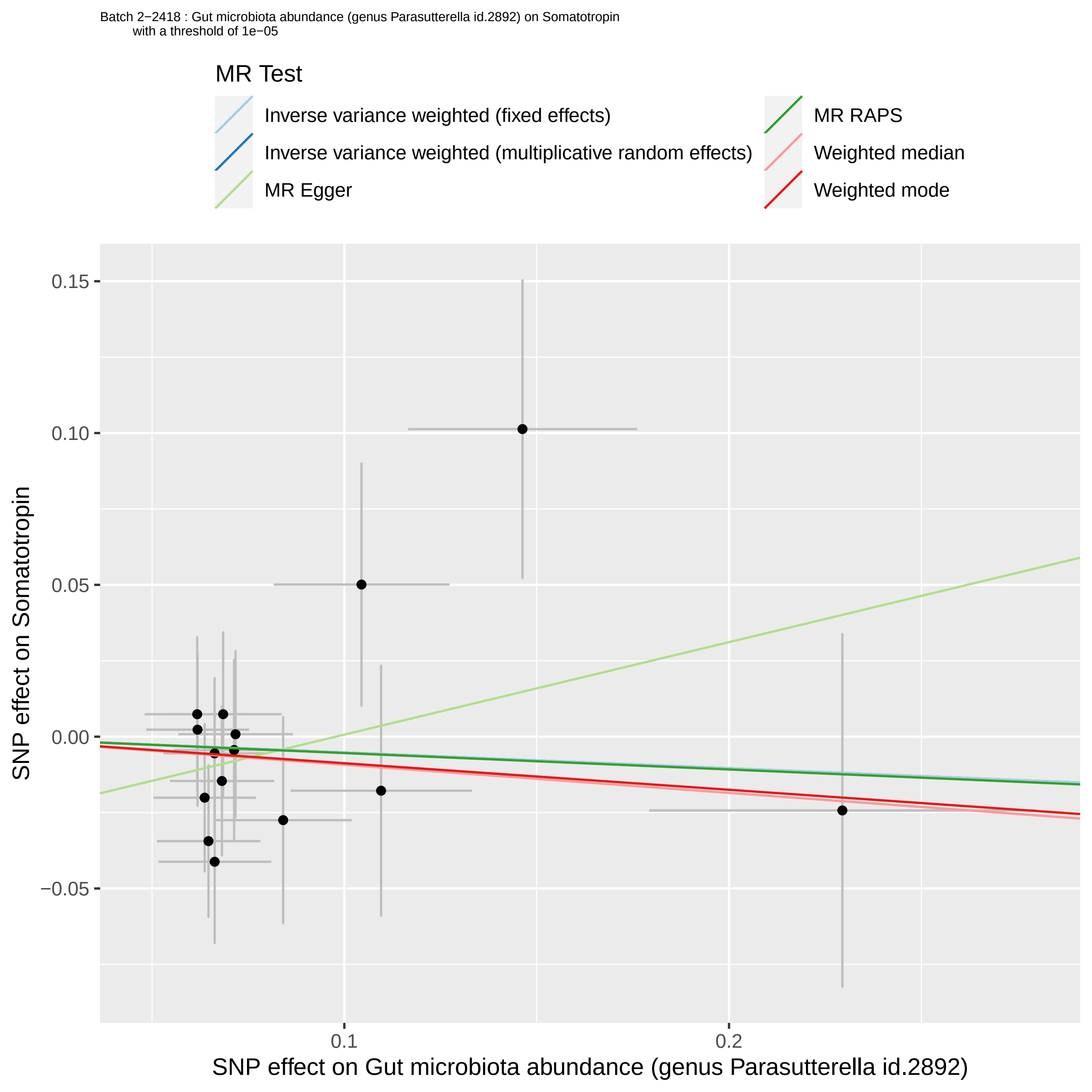

Batch 2−2418 : Gut microbiota abundance (genus Parasutterella id.2892) on Somatotropin
with a threshold of 1e−05
MR Test
Inverse variance weighted (fixed effects)
Inverse variance weighted (multiplicative random effects)
MR Egger
MR RAPS
Weighted median
Weighted mode
0.15
0.10
0.05
0.00
−0.05
0.1
0.2
SNP effect on Somatotropin
SNP effect on Gut microbiota abundance (genus Parasutterella id.2892)

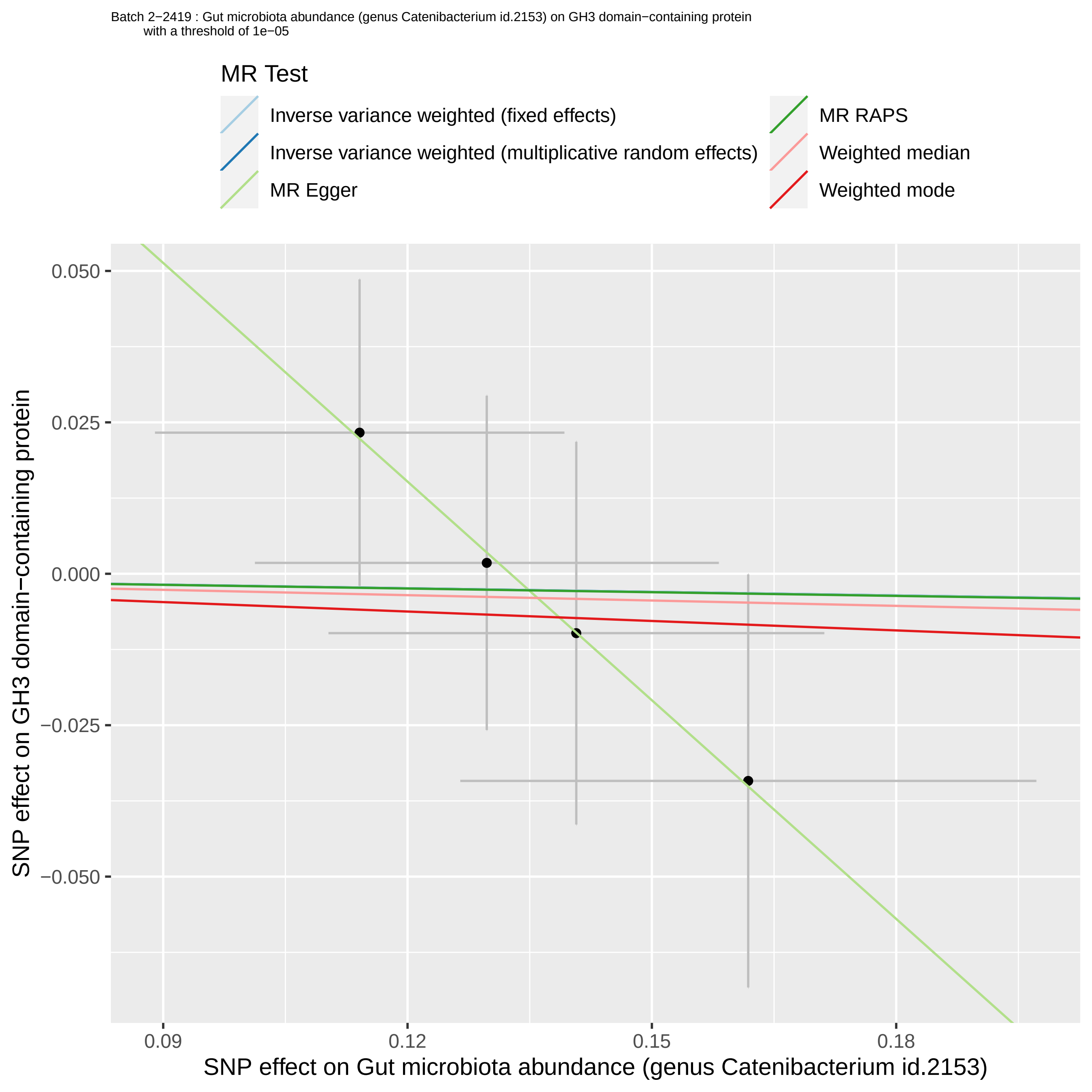

Batch 2−2419 : Gut microbiota abundance (genus Catenibacterium id.2153) on GH3 domain−containing protein
with a threshold of 1e−05
MR Test
Inverse variance weighted (fixed effects)
Inverse variance weighted (multiplicative random effects)
MR Egger
MR RAPS
Weighted median
Weighted mode
SNP effect on GH3 domain−containing protein
0.050
0.025
0.000
−0.025
−0.050
0.09
0.12
0.15
0.18
SNP effect on Gut microbiota abundance (genus Catenibacterium id.2153)

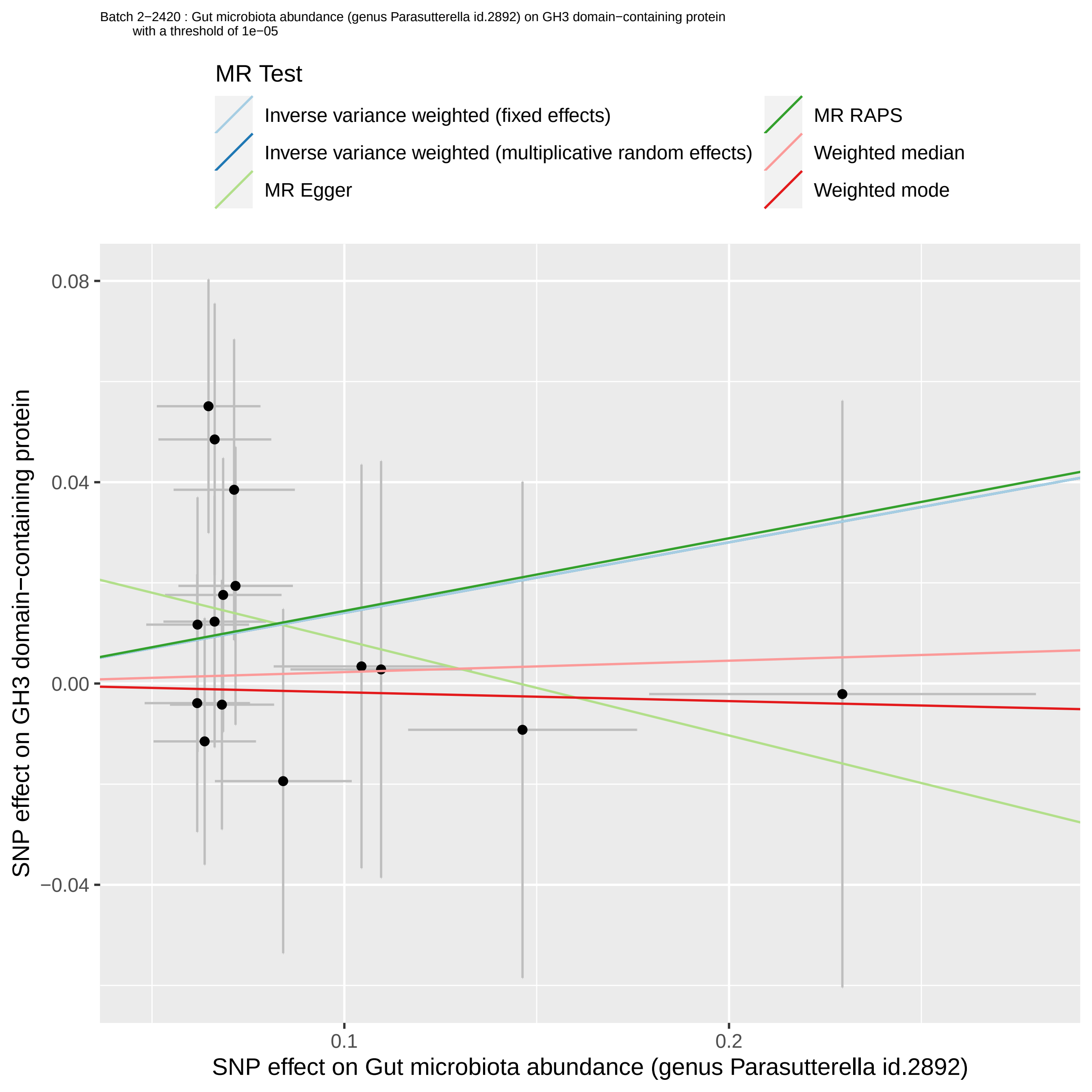
Batch 2−2420 : Gut microbiota abundance (genus Parasutterella id.2892) on GH3 domain−containing protein
with a threshold of 1e−05
MR Test
Inverse variance weighted (fixed effects)
Inverse variance weighted (multiplicative random effects)
MR Egger
MR RAPS
Weighted median
Weighted mode
0.08
0.04
0.00
−0.04
0.1
0.2
SNP effect on GH3 domain−containing protein
SNP effect on Gut microbiota abundance (genus Parasutterella id.2892)

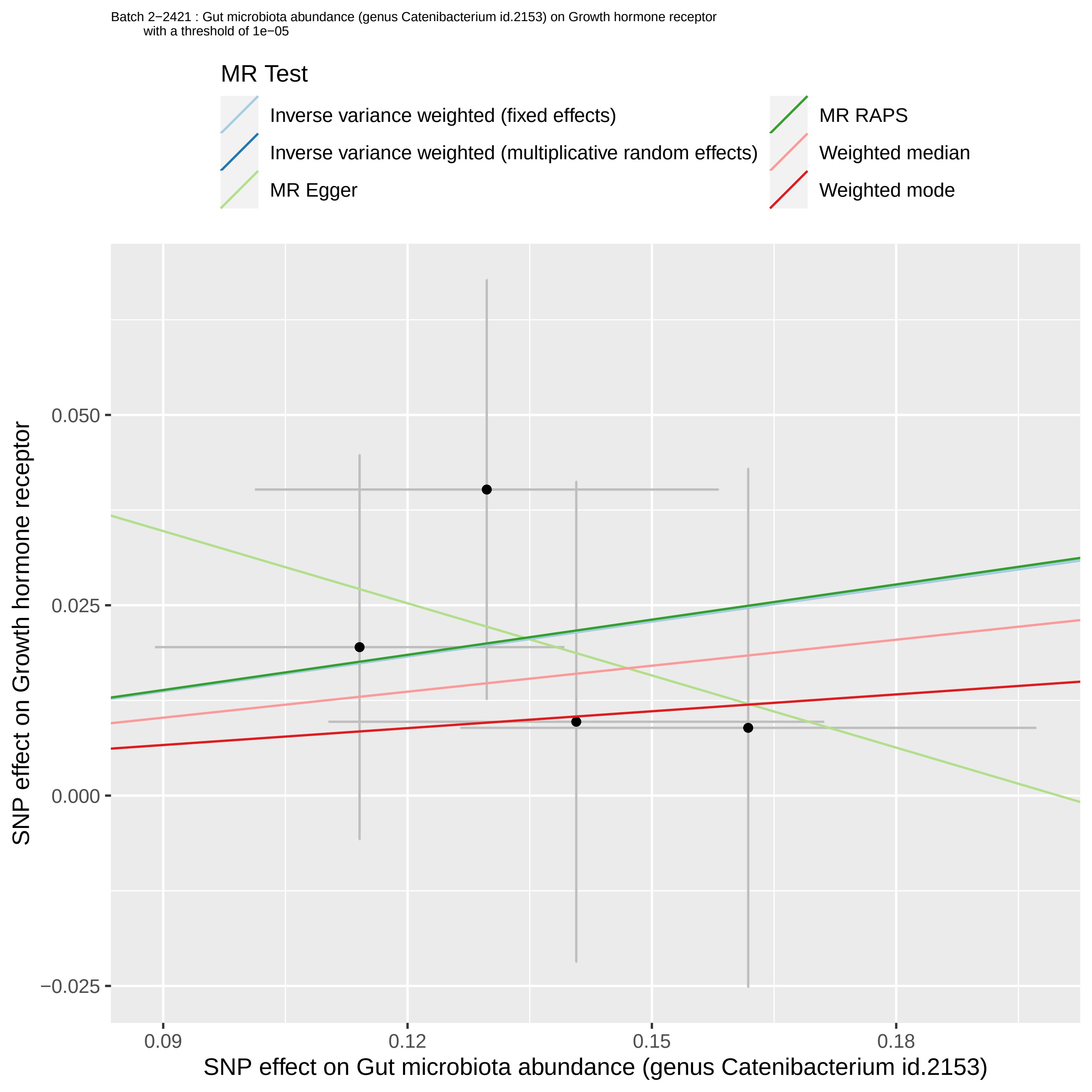
Batch 2−2421 : Gut microbiota abundance (genus Catenibacterium id.2153) on Growth hormone receptor
with a threshold of 1e−05
MR Test
Inverse variance weighted (fixed effects)
Inverse variance weighted (multiplicative random effects)
MR Egger
MR RAPS
Weighted median
Weighted mode
0.050
0.025
0.000
−0.025
0.09
0.12
0.15
0.18
SNP effect on Growth hormone receptor
SNP effect on Gut microbiota abundance (genus Catenibacterium id.2153)

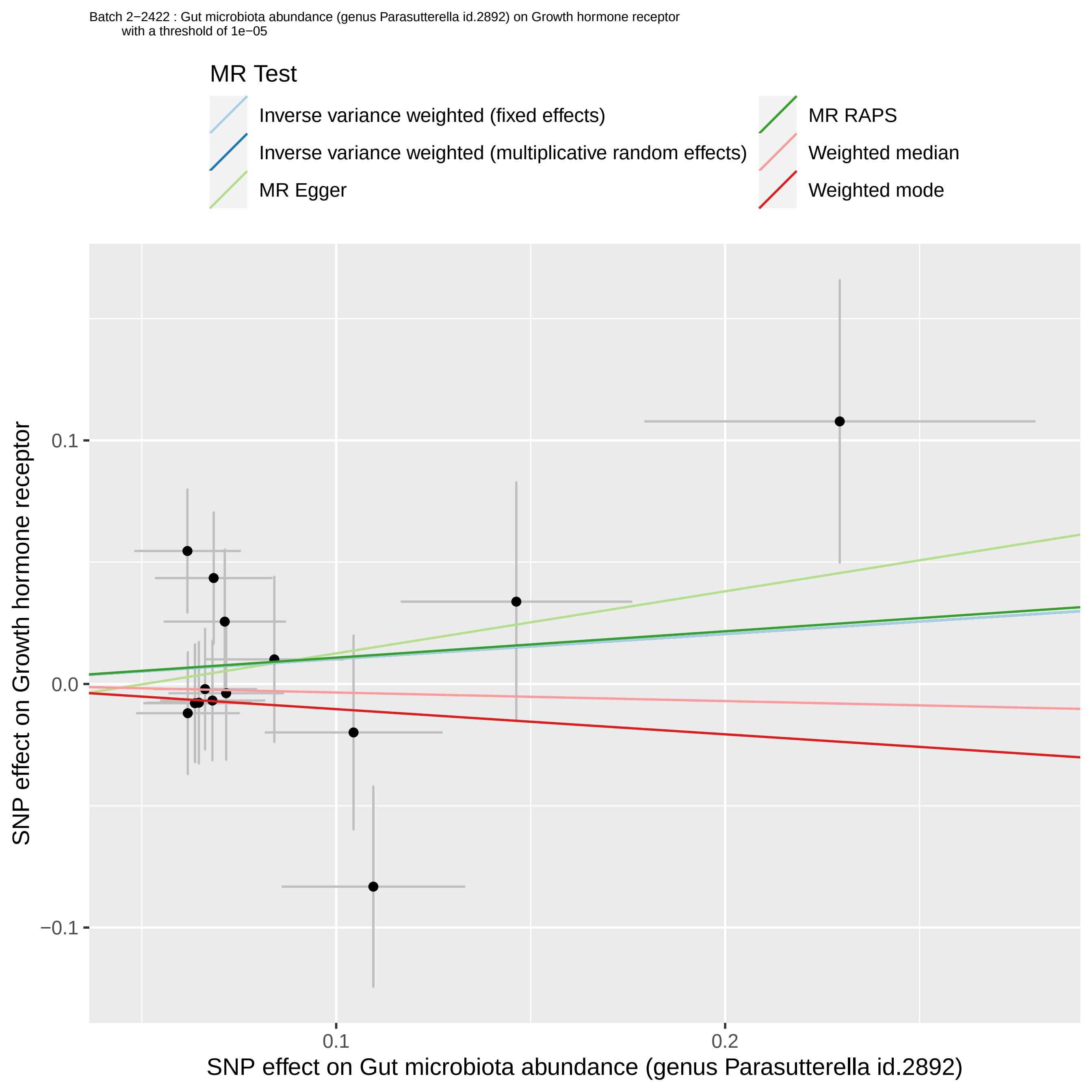

Batch 2−2422 : Gut microbiota abundance (genus Parasutterella id.2892) on Growth hormone receptor
with a threshold of 1e−05
MR Test
Inverse variance weighted (fixed effects)
Inverse variance weighted (multiplicative random effects)
MR Egger
MR RAPS
Weighted median
Weighted mode
0.1
0.0
−0.1
0.1
0.2
SNP effect on Growth hormone receptor
SNP effect on Gut microbiota abundance (genus Parasutterella id.2892)

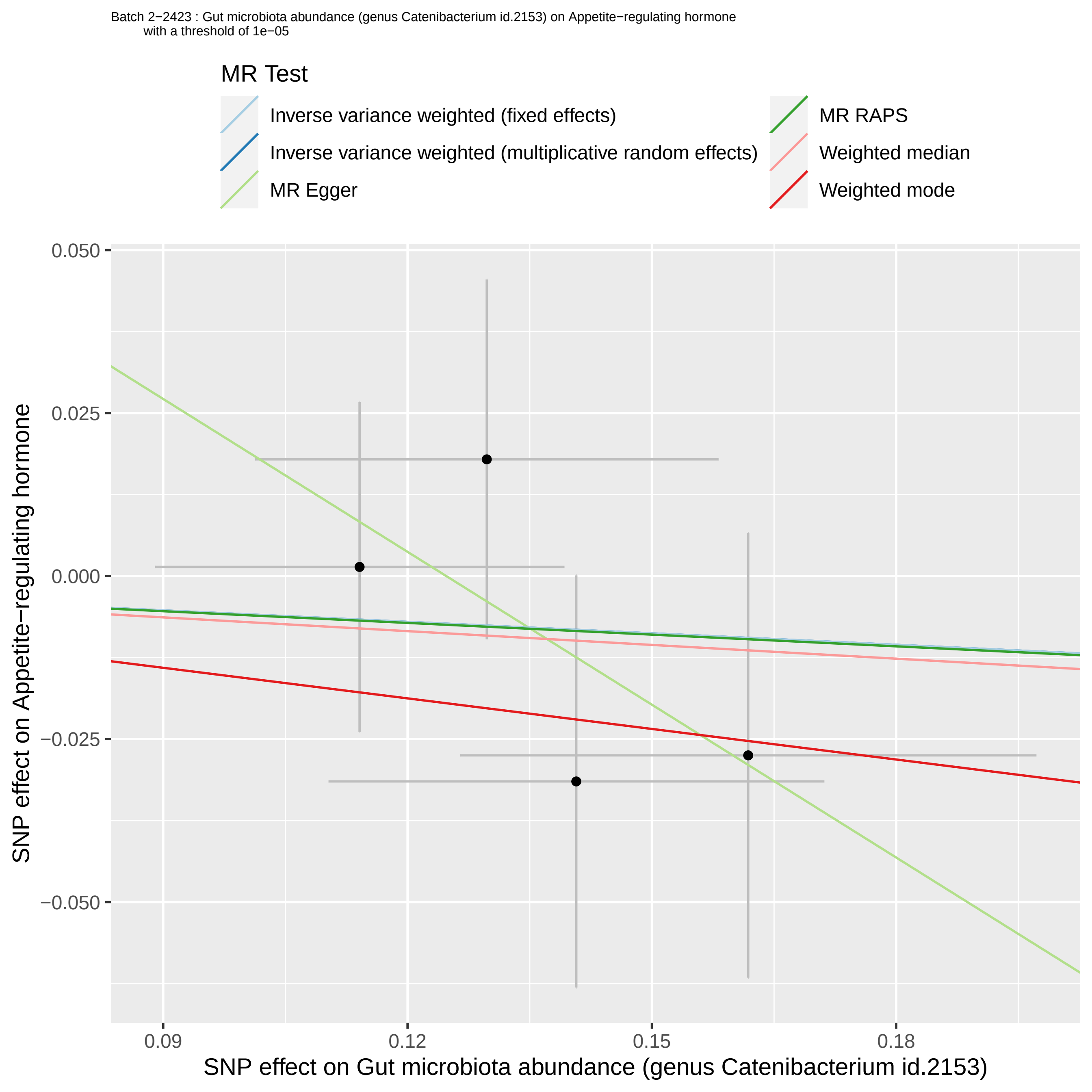
Batch 2−2423 : Gut microbiota abundance (genus Catenibacterium id.2153) on Appetite−regulating hormone with a threshold of 1e−05
MR Test
Inverse variance weighted (fixed effects)
Inverse variance weighted (multiplicative random effects)
MR Egger
MR RAPS
Weighted median
Weighted mode
0.050
0.025
0.000
−0.025
−0.050
0.09
0.12
0.15
0.18
SNP effect on Appetite−regulating hormone
SNP effect on Gut microbiota abundance (genus Catenibacterium id.2153)

Batch 2−2424 : Gut microbiota abundance (genus Parasutterella id.2892) on Appetite−regulating hormone
with a threshold of 1e−05

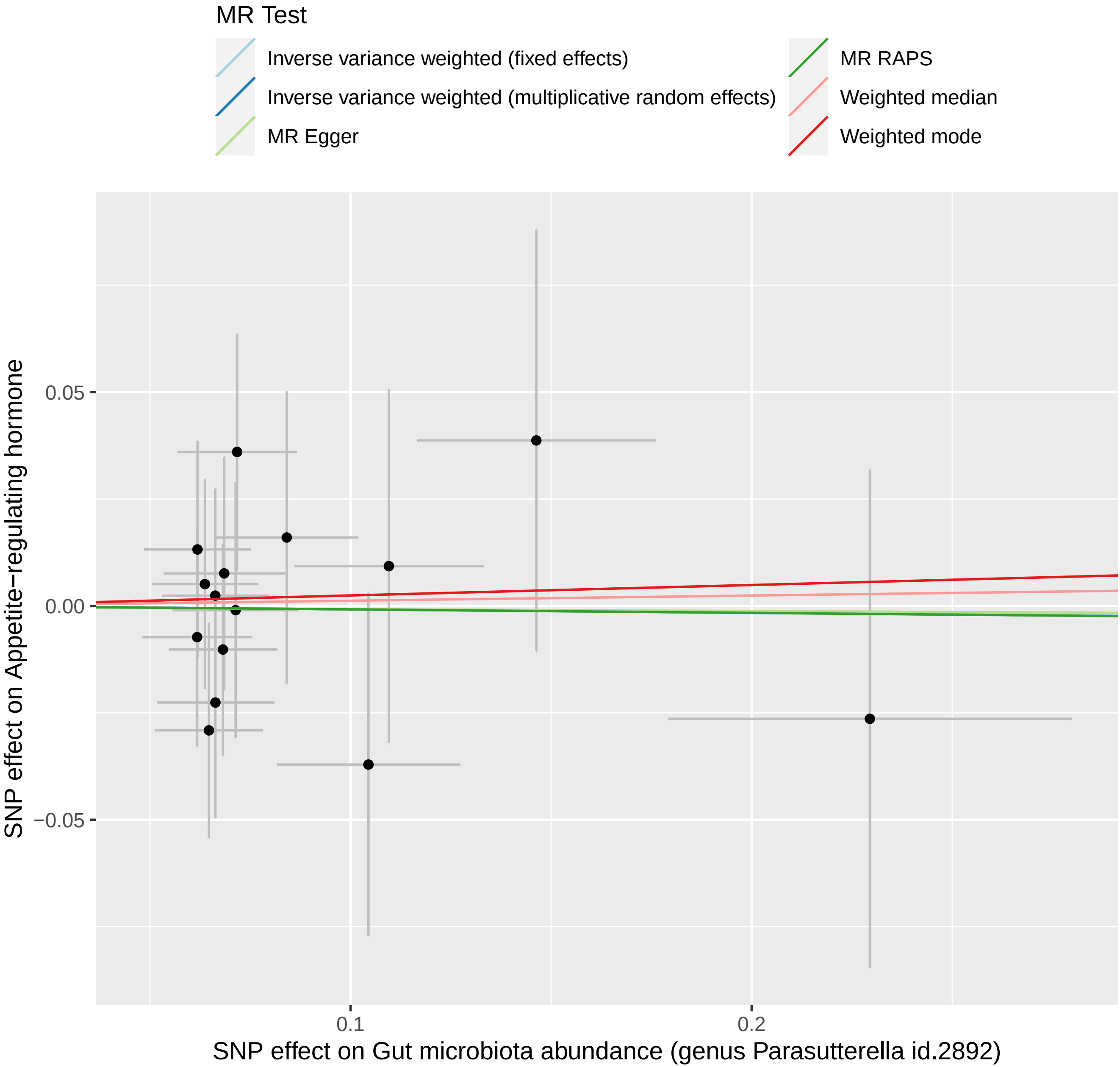

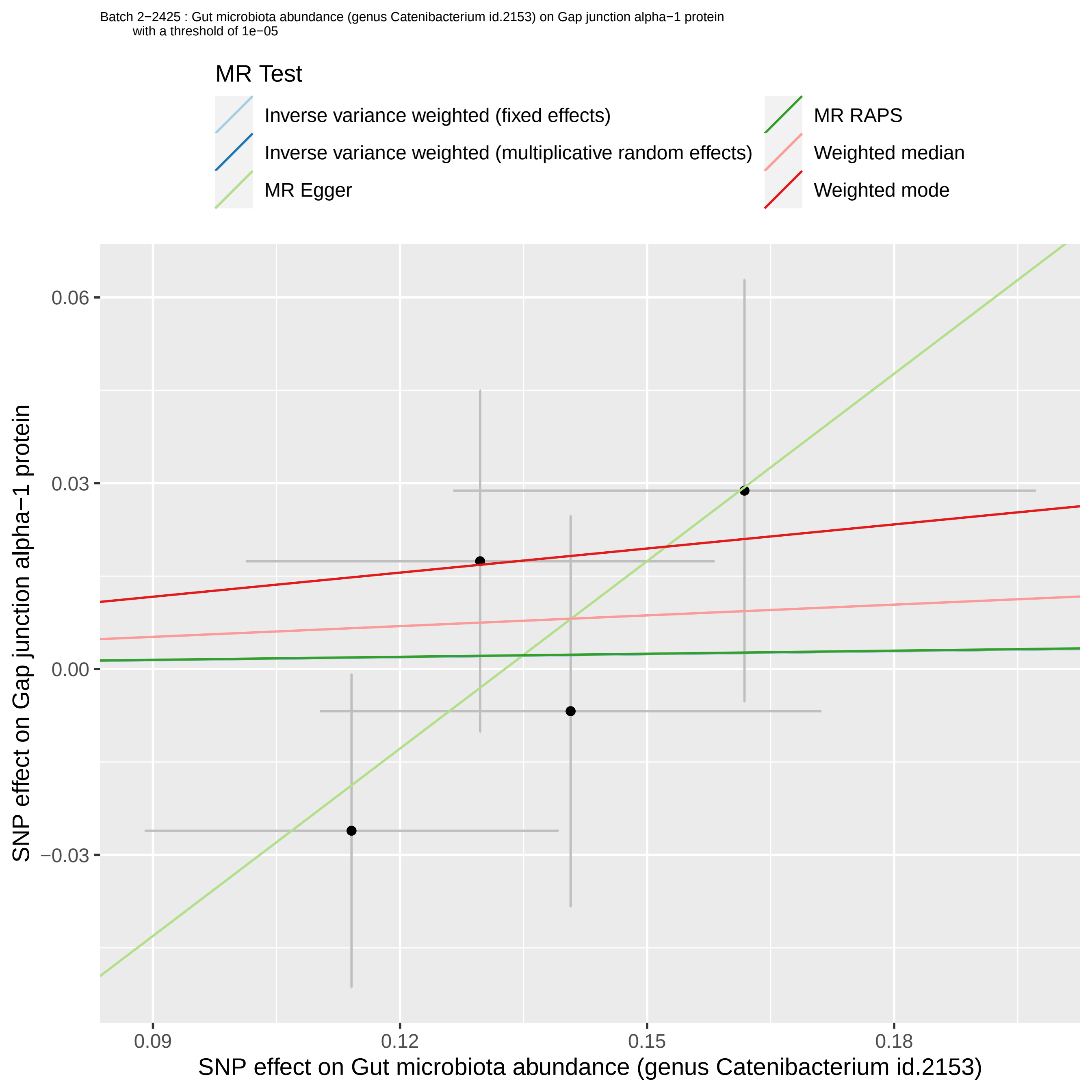

Batch 2−2425 : Gut microbiota abundance (genus Catenibacterium id.2153) on Gap junction alpha−1 protein
with a threshold of 1e−05
MR Test
Inverse variance weighted (fixed effects)
Inverse variance weighted (multiplicative random effects)
MR Egger
MR RAPS
Weighted median
Weighted mode
0.06
0.03
0.00
−0.03
0.09
0.12
0.15
0.18
SNP effect on Gap junction alpha−1 protein
SNP effect on Gut microbiota abundance (genus Catenibacterium id.2153)

Batch 2−2426 : Gut microbiota abundance (genus Parasutterella id.2892) on Gap junction alpha−1 protein
with a threshold of 1e−05

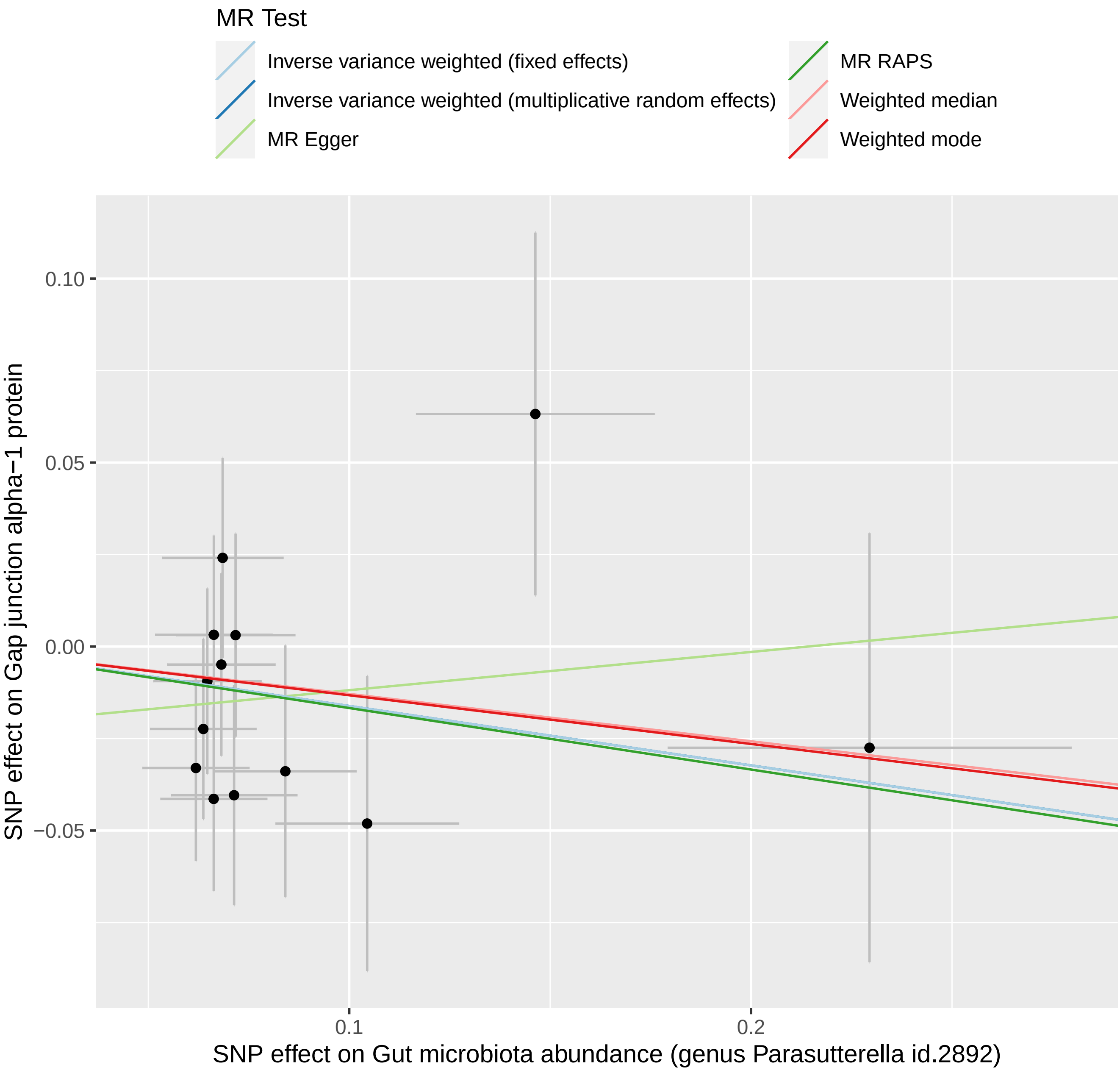

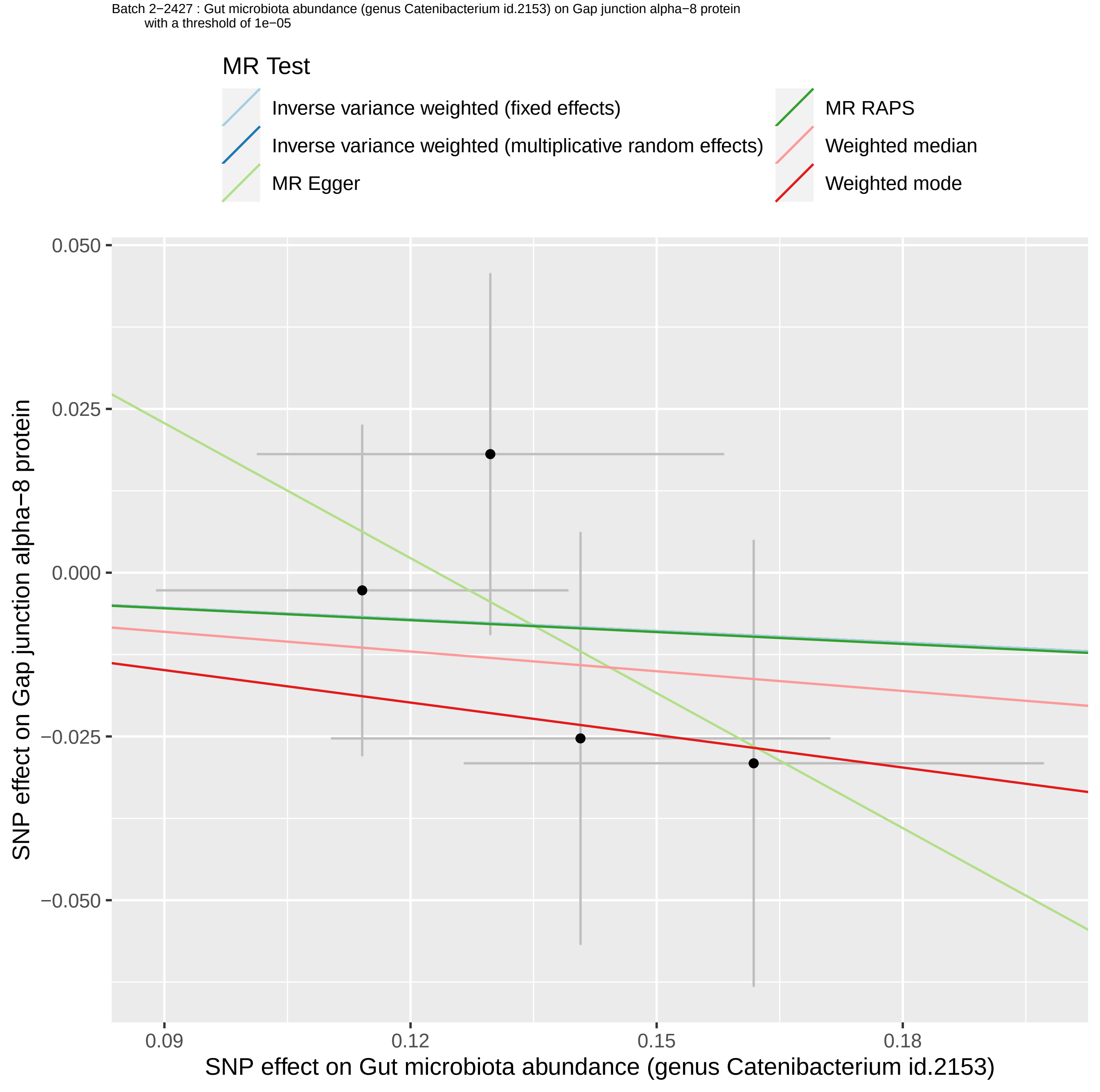

Batch 2−2427 : Gut microbiota abundance (genus Catenibacterium id.2153) on Gap junction alpha−8 protein
with a threshold of 1e−05
MR Test
Inverse variance weighted (fixed effects)
Inverse variance weighted (multiplicative random effects)
MR Egger
MR RAPS
Weighted median
Weighted mode
0.050
0.025
0.000
−0.025
−0.050
0.09
0.12
0.15
0.18
SNP effect on Gap junction alpha−8 protein
SNP effect on Gut microbiota abundance (genus Catenibacterium id.2153)

Batch 2−2428 : Gut microbiota abundance (genus Parasutterella id.2892) on Gap junction alpha−8 protein with a threshold of 1e−05

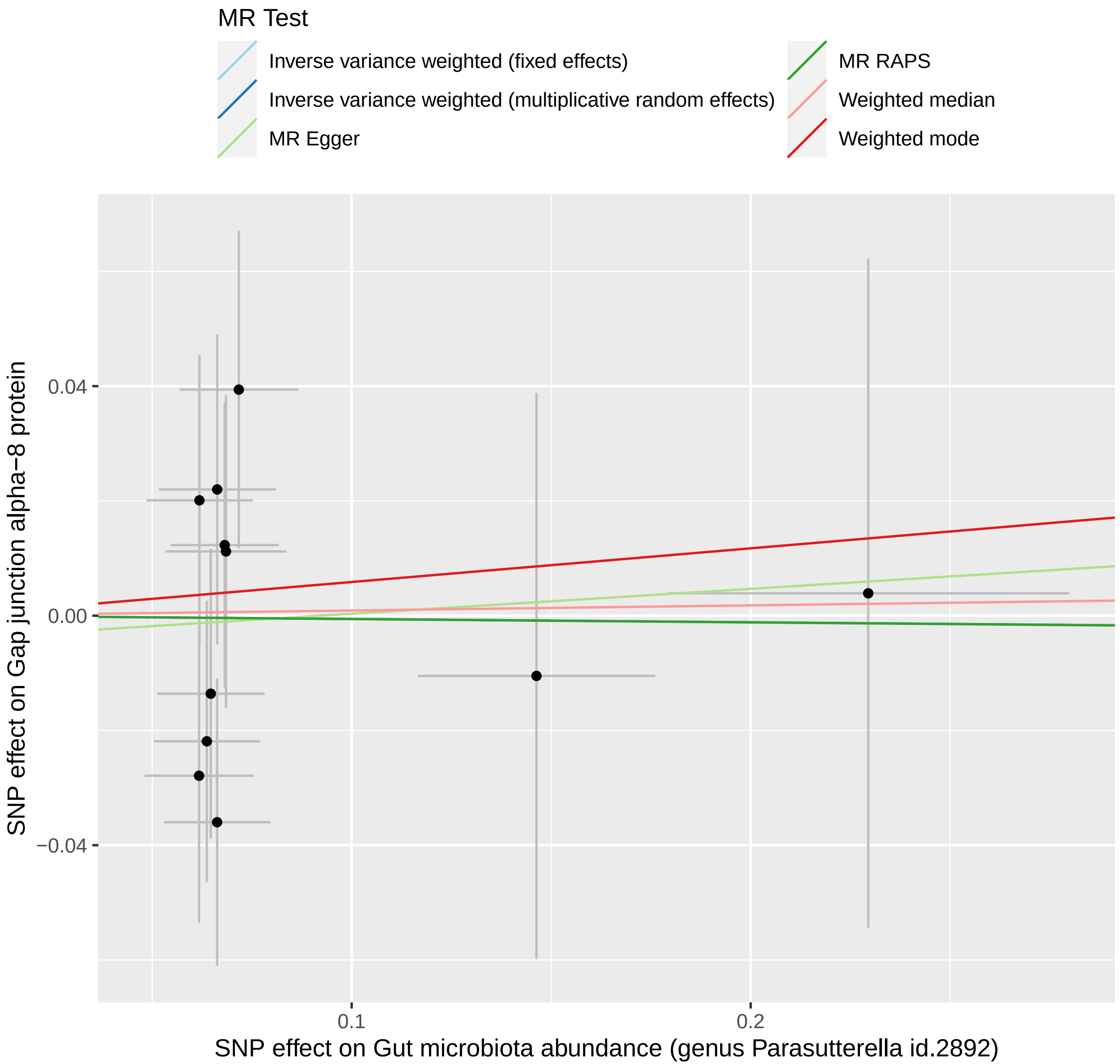

Batch 2−2429 : Gut microbiota abundance (genus Catenibacterium id.2153) on Gap junction delta−2 protein
with a threshold of 1e−05

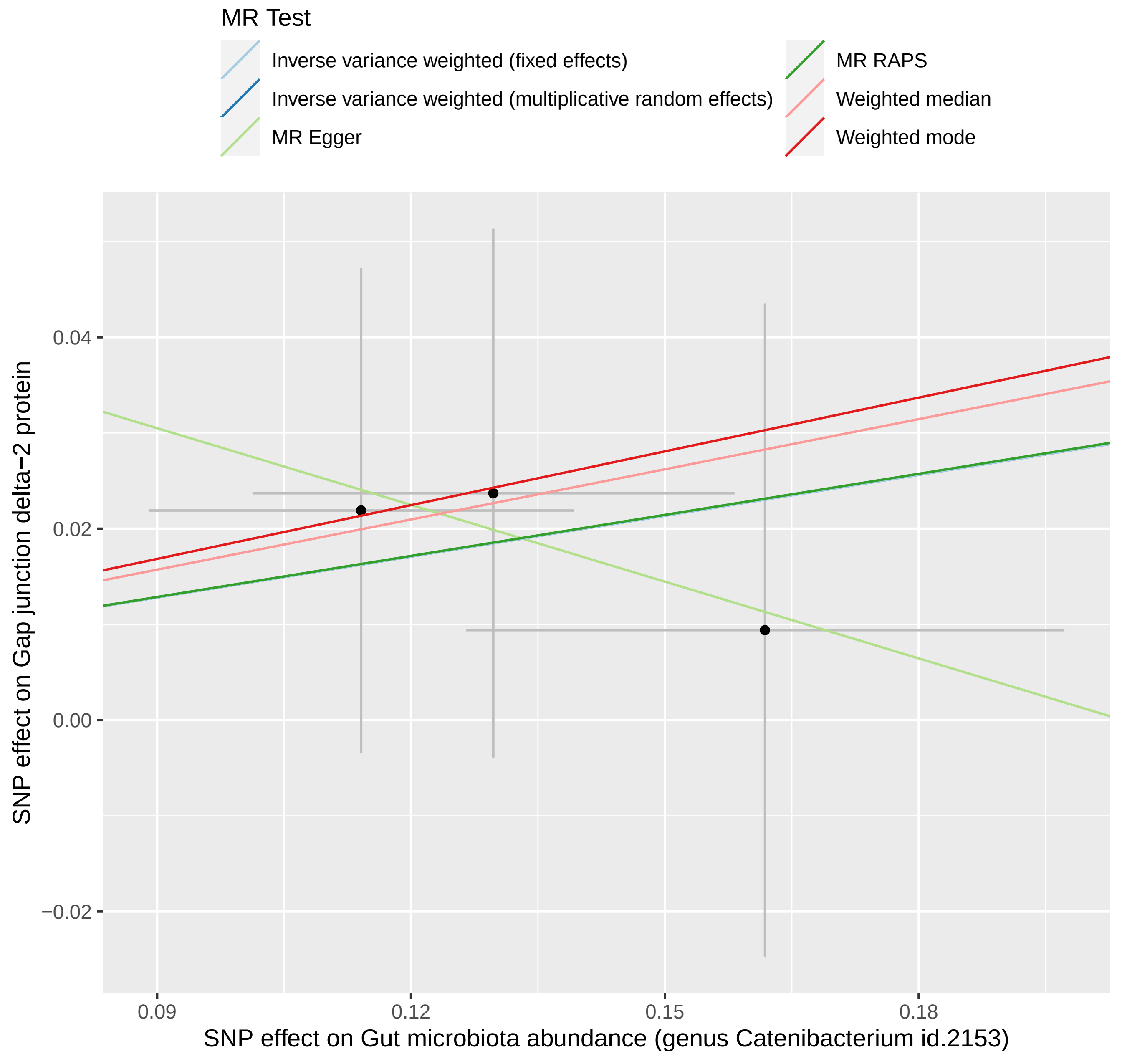

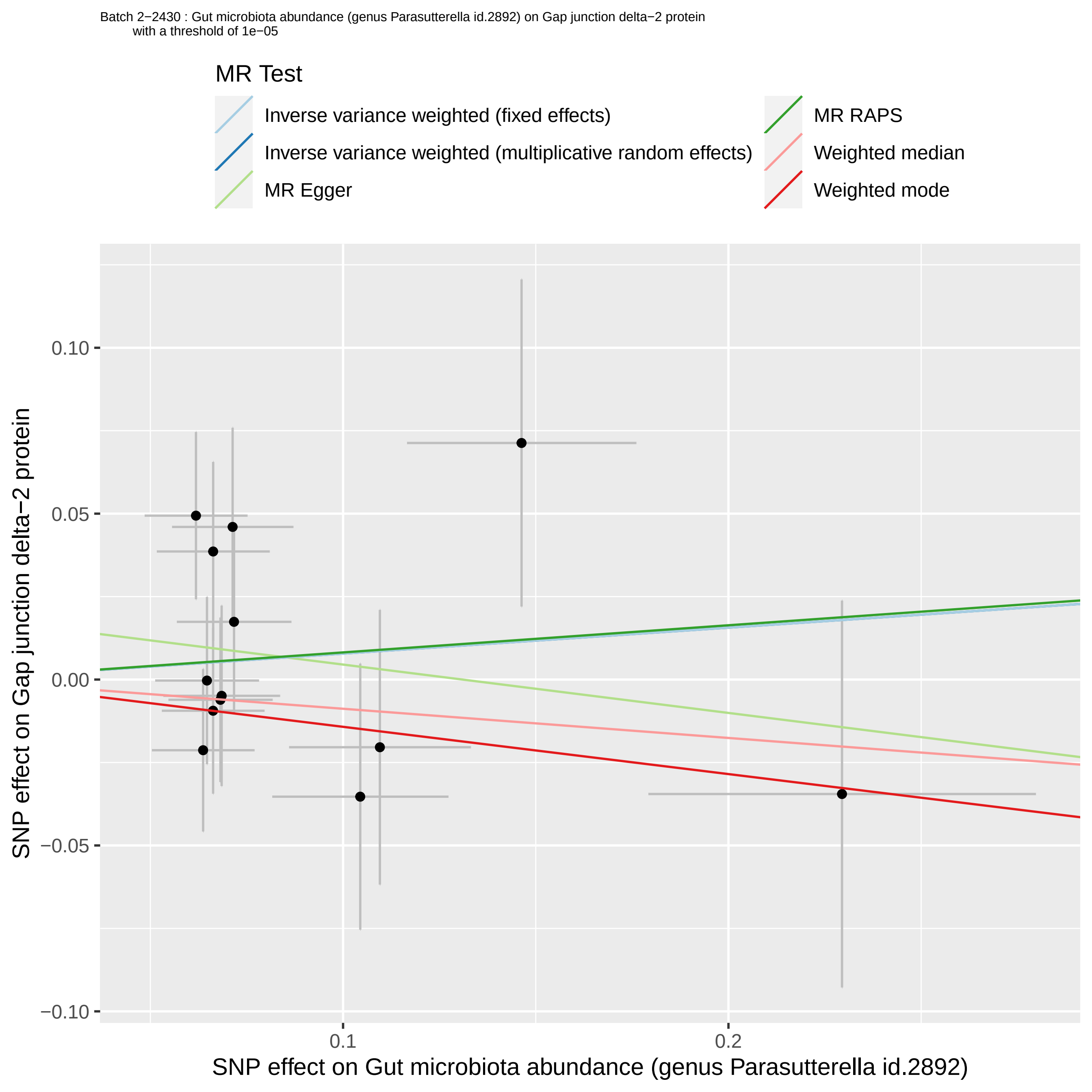
Batch 2−2430 : Gut microbiota abundance (genus Parasutterella id.2892) on Gap junction delta−2 protein
with a threshold of 1e−05
MR Test
Inverse variance weighted (fixed effects)
Inverse variance weighted (multiplicative random effects)
MR Egger
MR RAPS
Weighted median
Weighted mode
0.10
0.05
0.00
−0.05
−0.10
0.1
0.2
SNP effect on Gap junction delta−2 protein
SNP effect on Gut microbiota abundance (genus Parasutterella id.2892)

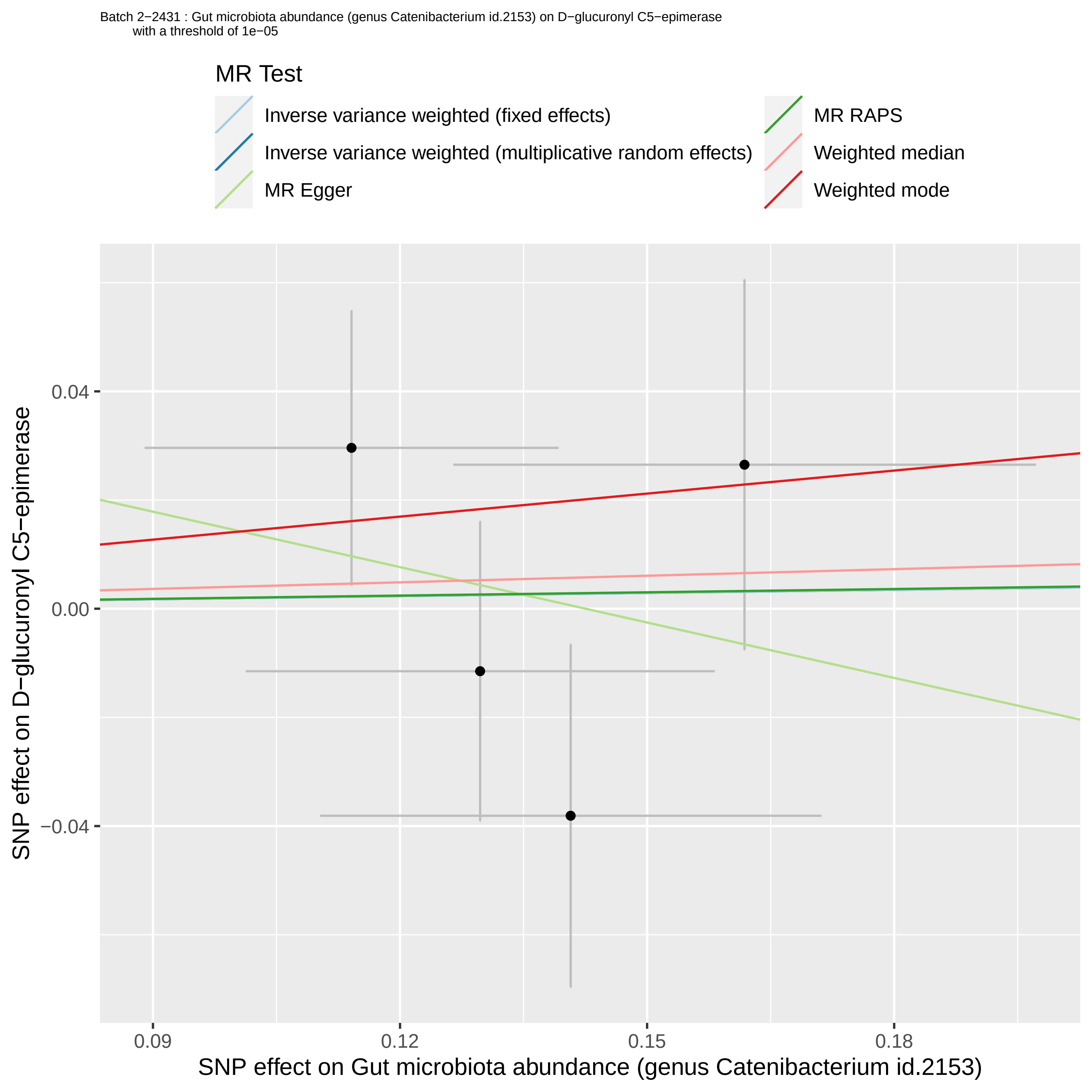

Batch 2−2431 : Gut microbiota abundance (genus Catenibacterium id.2153) on D−glucuronyl C5−epimerase
with a threshold of 1e−05
MR Test
Inverse variance weighted (fixed effects)
Inverse variance weighted (multiplicative random effects)
MR Egger
MR RAPS
Weighted median
Weighted mode
0.04
0.00
−0.04
0.09
0.12
0.15
0.18
SNP effect on D−glucuronyl C5−epimerase
SNP effect on Gut microbiota abundance (genus Catenibacterium id.2153)

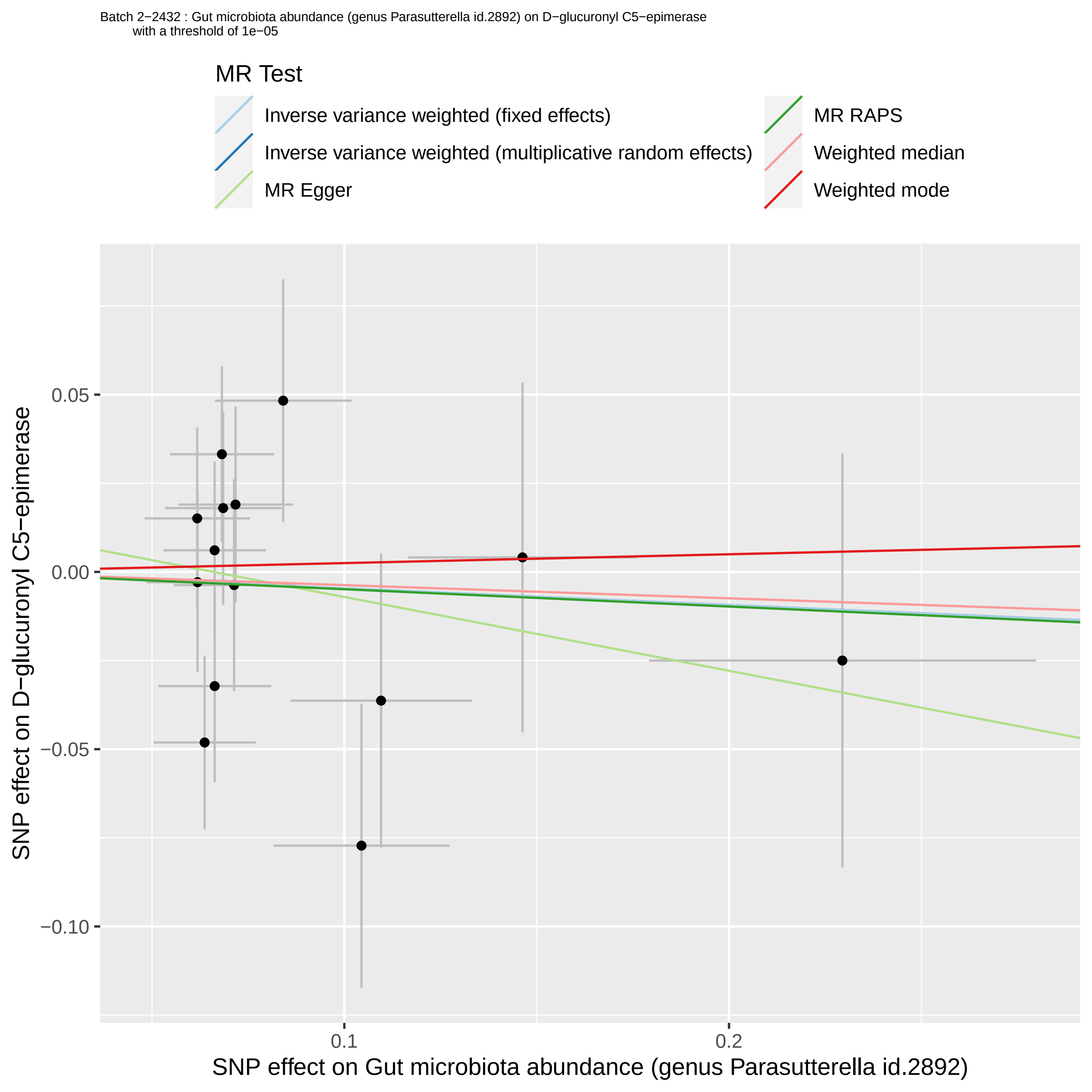

Batch 2−2432 : Gut microbiota abundance (genus Parasutterella id.2892) on D−glucuronyl C5−epimerase
with a threshold of 1e−05
MR Test
Inverse variance weighted (fixed effects)
Inverse variance weighted (multiplicative random effects)
MR Egger
MR RAPS
Weighted median
Weighted mode
0.05
0.00
−0.05
−0.10
0.1
0.2
SNP effect on D−glucuronyl C5−epimerase
SNP effect on Gut microbiota abundance (genus Parasutterella id.2892)

Batch 2−2433 : Gut microbiota abundance (genus Catenibacterium id.2153) on Glioma pathogenesis−related protein 1
with a threshold of 1e−05

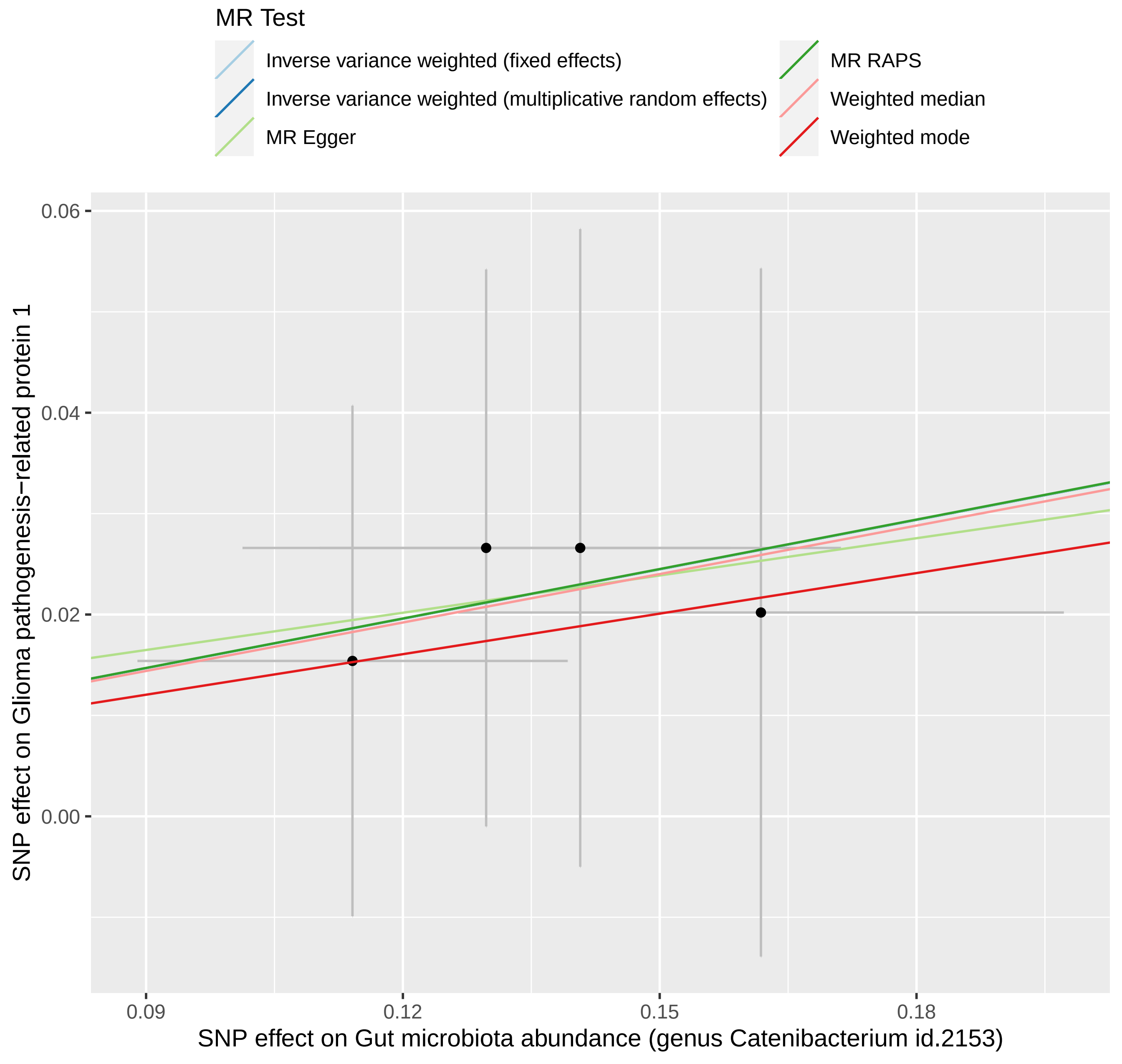

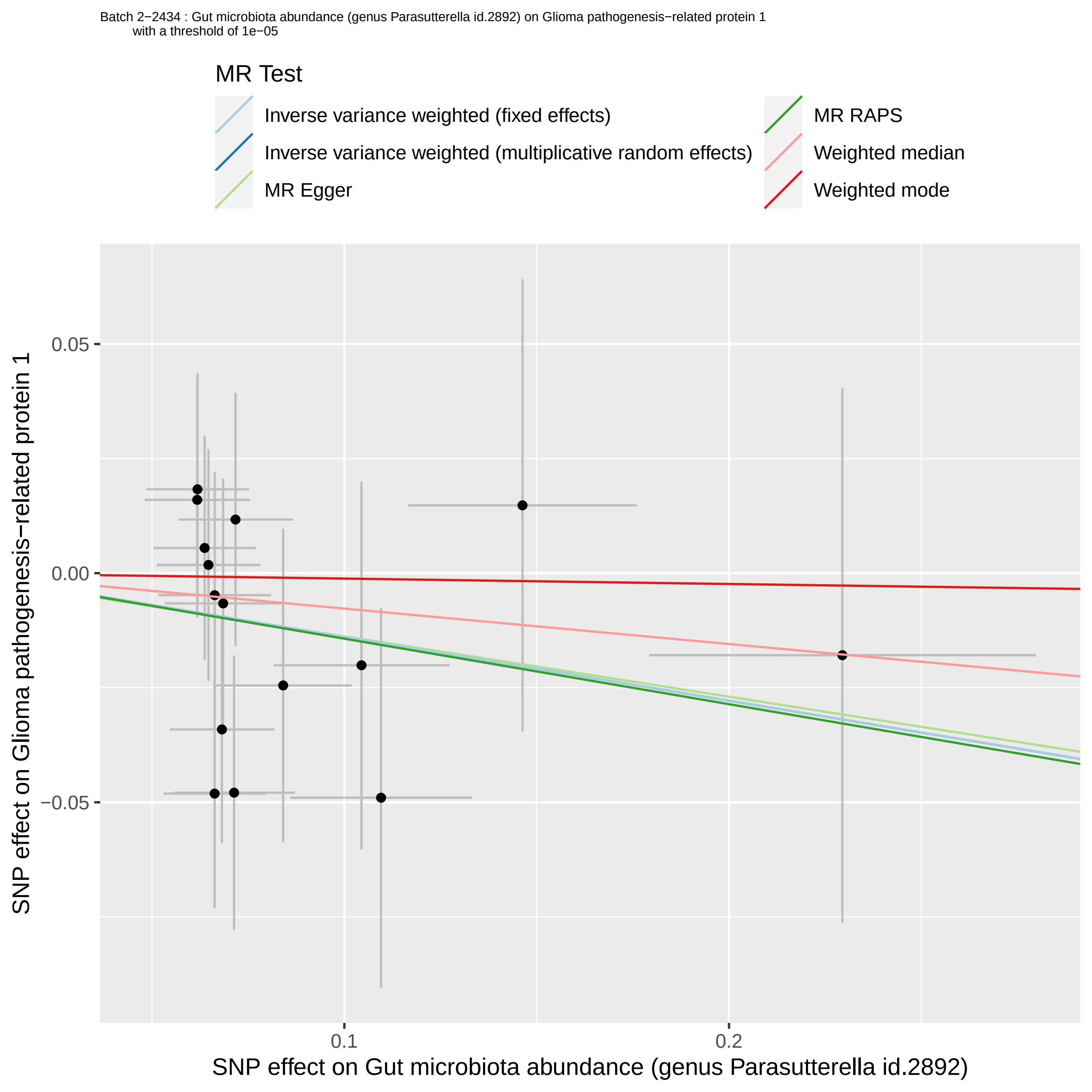
Batch 2−2434 : Gut microbiota abundance (genus Parasutterella id.2892) on Glioma pathogenesis−related protein 1
with a threshold of 1e−05
MR Test
Inverse variance weighted (fixed effects)
Inverse variance weighted (multiplicative random effects)
MR Egger
MR RAPS
Weighted median
Weighted mode
0.05
0.00
−0.05
0.1
0.2
SNP effect on Glioma pathogenesis−related protein 1
SNP effect on Gut microbiota abundance (genus Parasutterella id.2892)

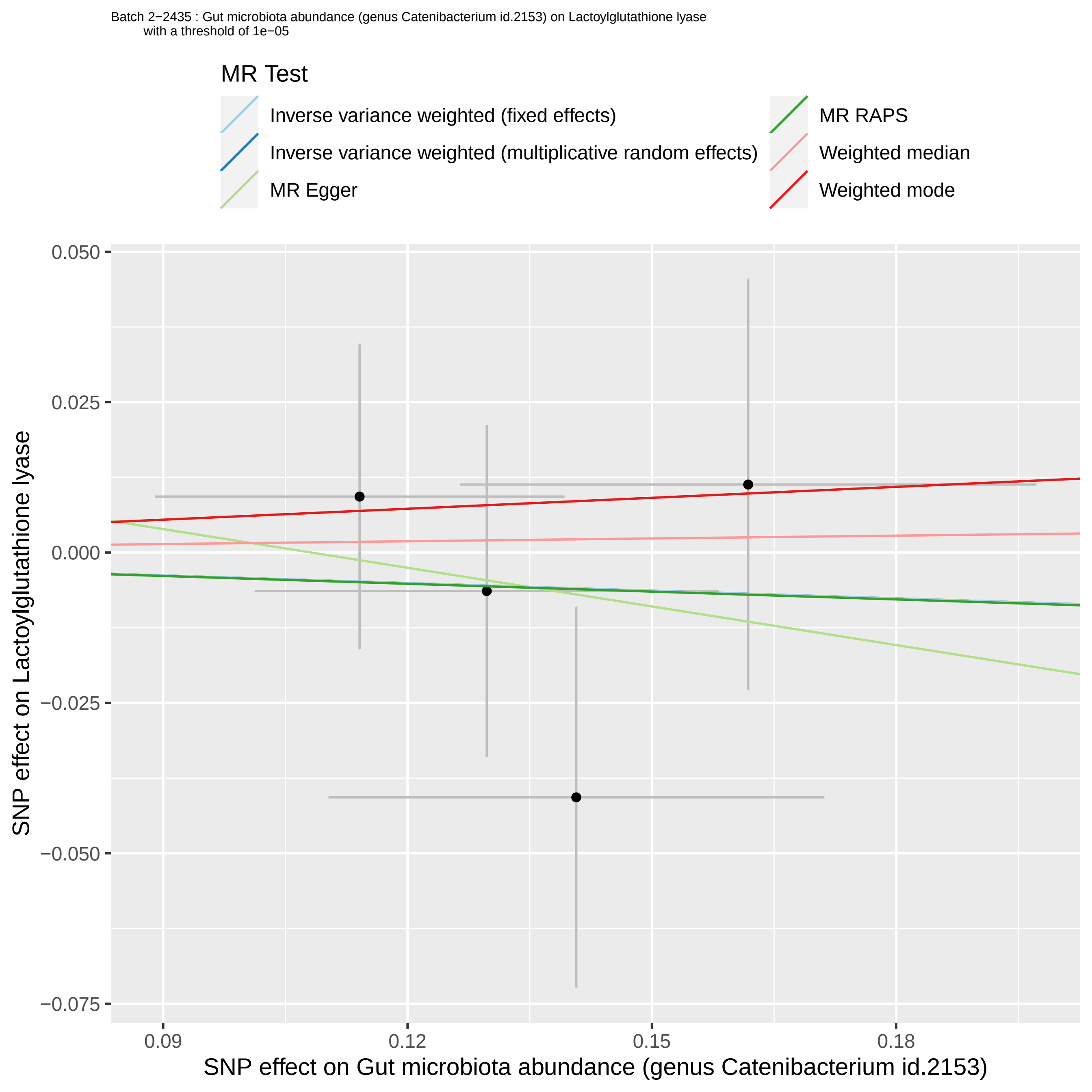

Batch 2−2435 : Gut microbiota abundance (genus Catenibacterium id.2153) on Lactoylglutathione lyase
with a threshold of 1e−05
MR Test
Inverse variance weighted (fixed effects)
Inverse variance weighted (multiplicative random effects)
MR Egger
MR RAPS
Weighted median
Weighted mode
0.050
0.025
0.000
−0.025
−0.050
−0.075
0.09
0.12
0.15
0.18
SNP effect on Lactoylglutathione lyase
SNP effect on Gut microbiota abundance (genus Catenibacterium id.2153)

Batch 2−2436 : Gut microbiota abundance (genus Parasutterella id.2892) on Lactoylglutathione lyase
with a threshold of 1e−05

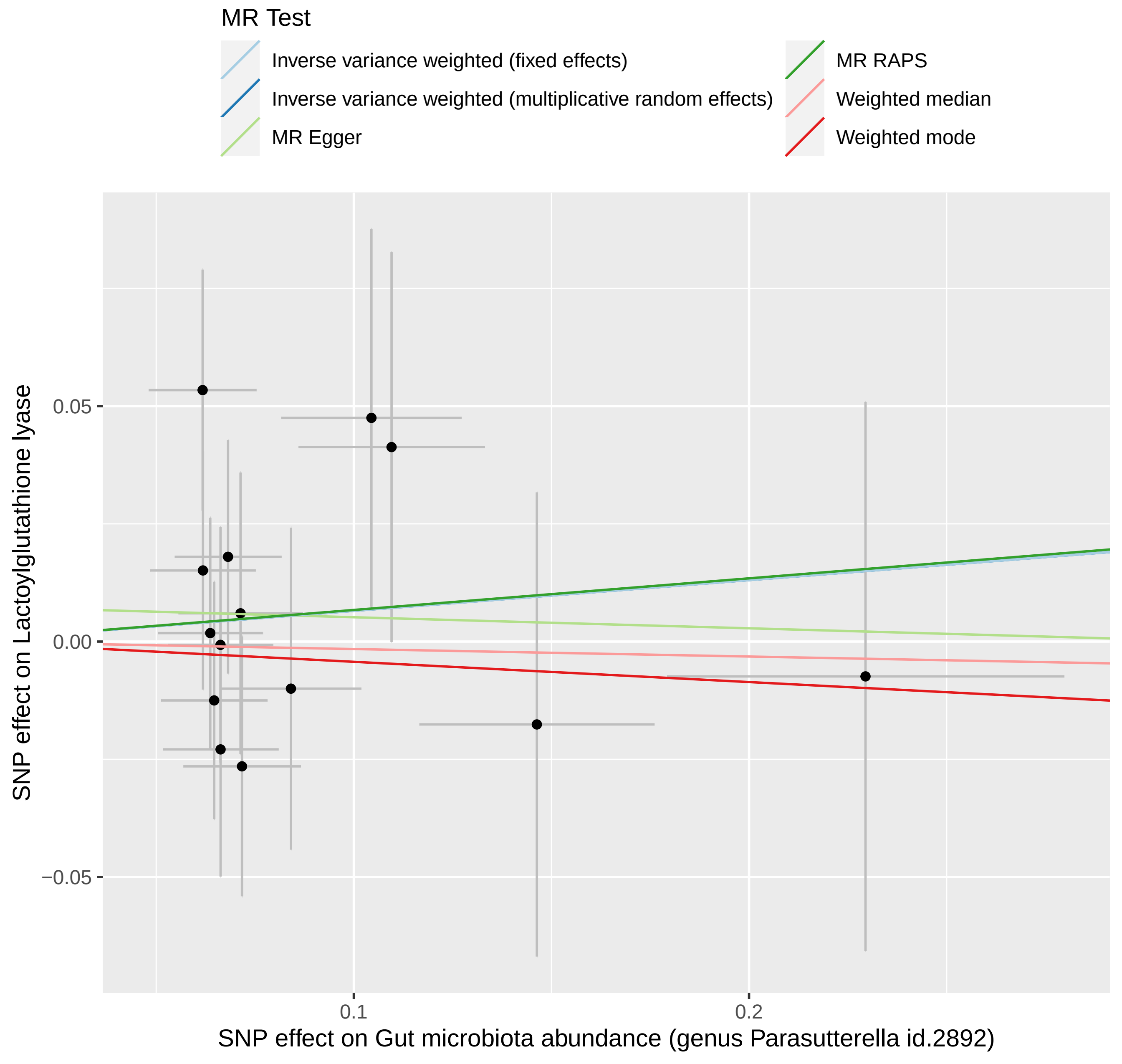

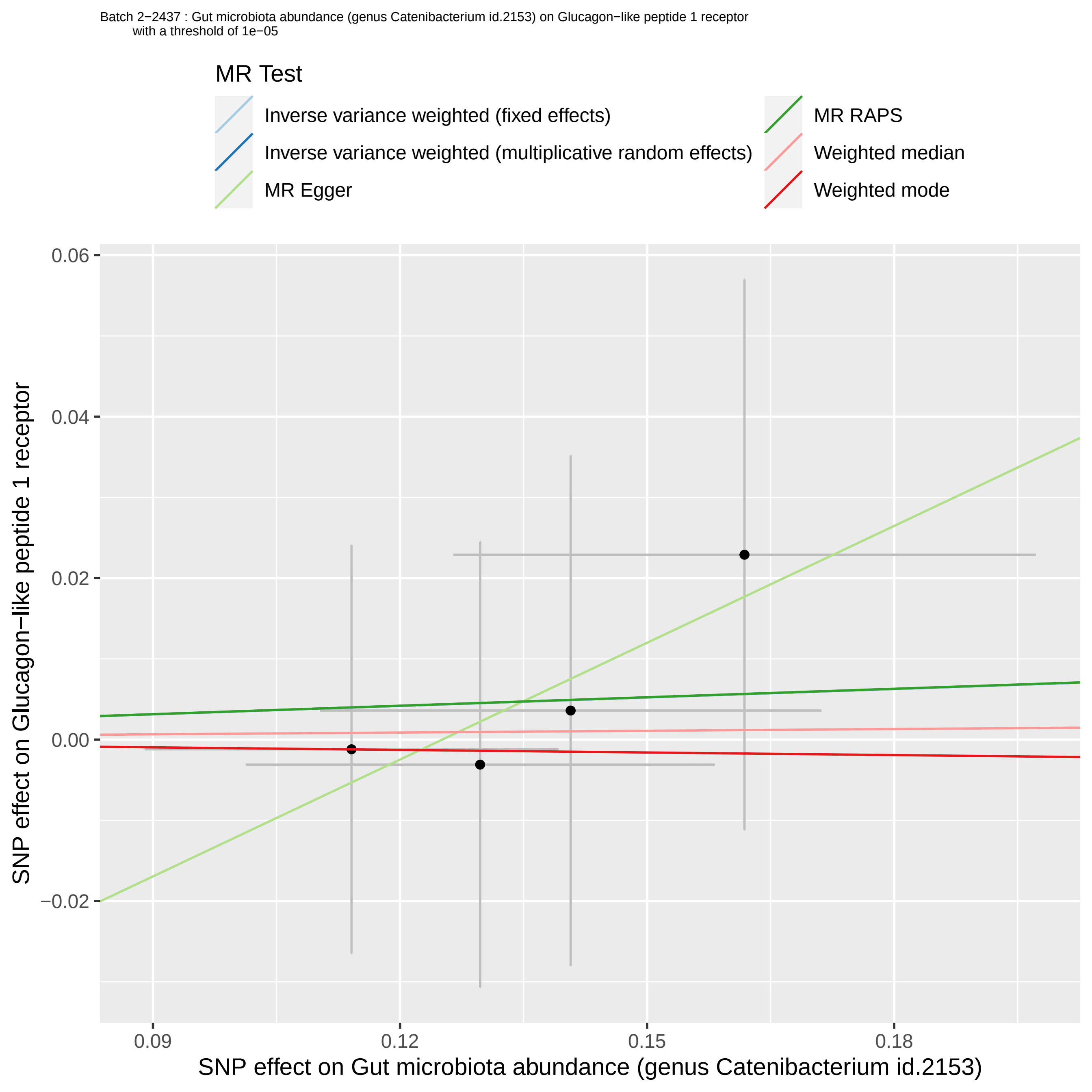
Batch 2−2437 : Gut microbiota abundance (genus Catenibacterium id.2153) on Glucagon−like peptide 1 receptor
with a threshold of 1e−05
MR Test
Inverse variance weighted (fixed effects)
Inverse variance weighted (multiplicative random effects)
MR Egger
MR RAPS
Weighted median
Weighted mode
0.06
0.04
0.02
0.00
−0.02
SNP effect on Glucagon−like peptide 1 receptor
0.09
0.12
0.15
0.18
SNP effect on Gut microbiota abundance (genus Catenibacterium id.2153)

Batch 2−2438 : Gut microbiota abundance (genus Parasutterella id.2892) on Glucagon−like peptide 1 receptor
with a threshold of 1e−05

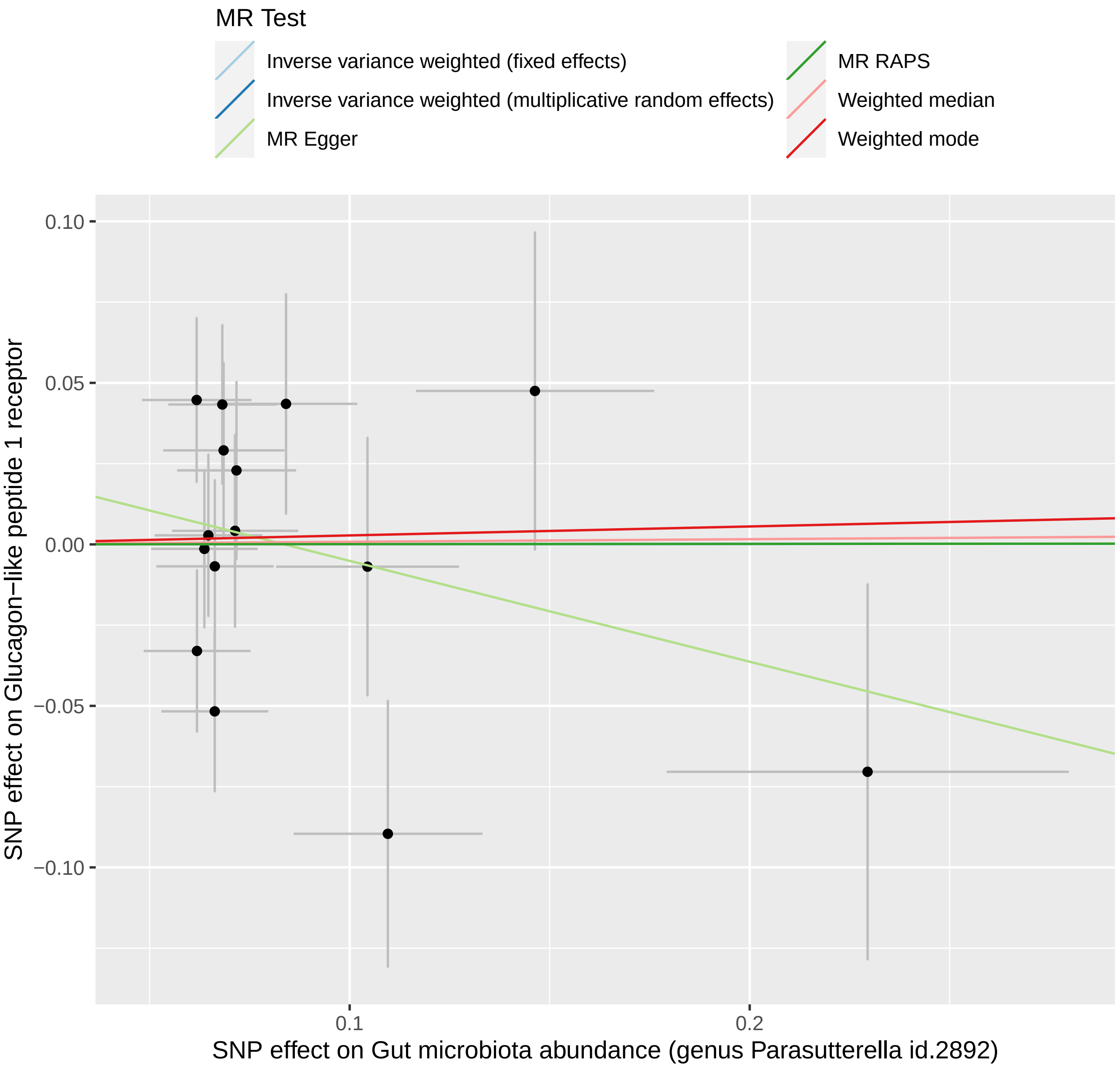

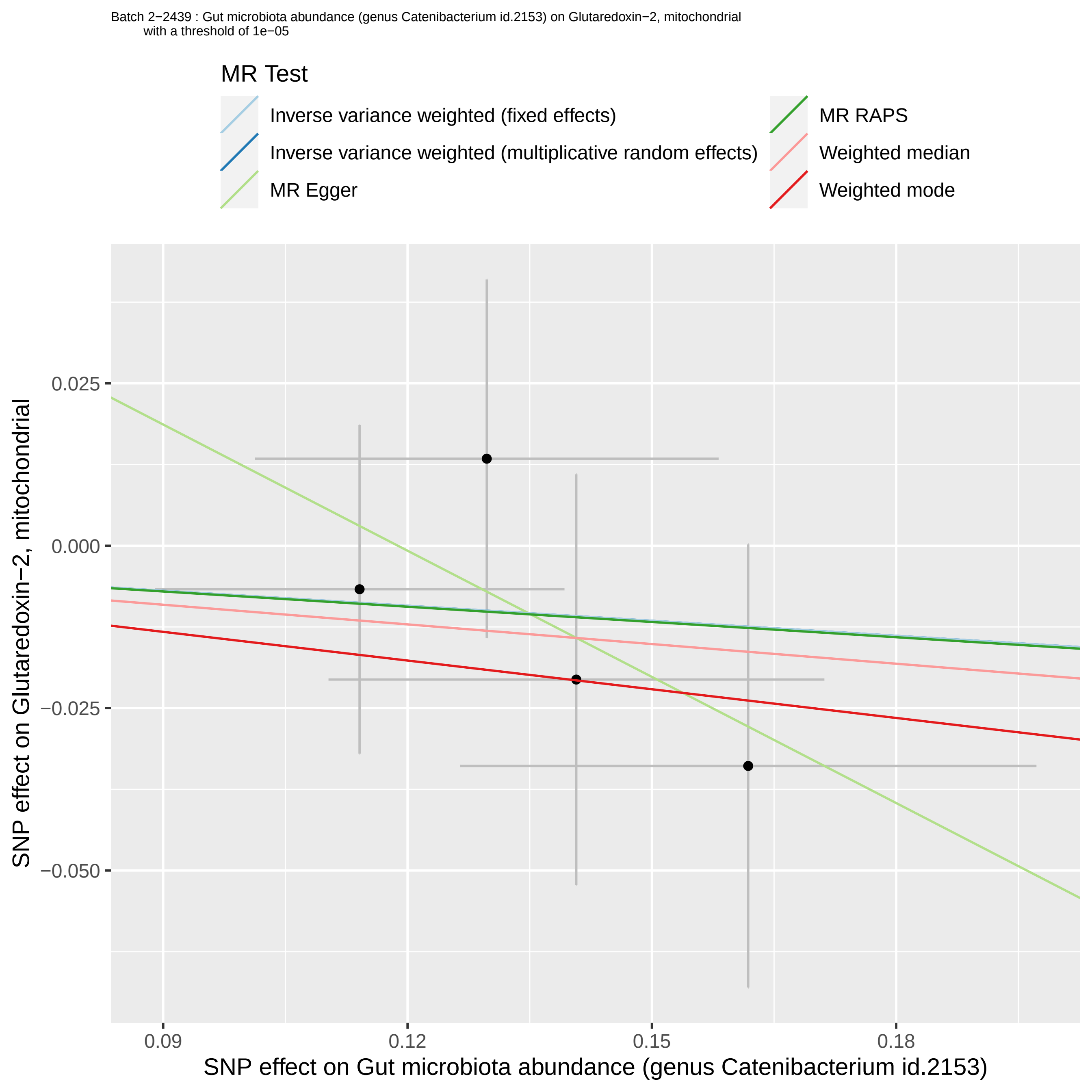

Batch 2−2439 : Gut microbiota abundance (genus Catenibacterium id.2153) on Glutaredoxin−2, mitochondrial
with a threshold of 1e−05
MR Test
Inverse variance weighted (fixed effects)
Inverse variance weighted (multiplicative random effects)
MR Egger
MR RAPS
Weighted median
Weighted mode
0.025
0.000
−0.025
−0.050
0.09
0.12
0.15
0.18
SNP effect on Glutaredoxin−2, mitochondrial
SNP effect on Gut microbiota abundance (genus Catenibacterium id.2153)

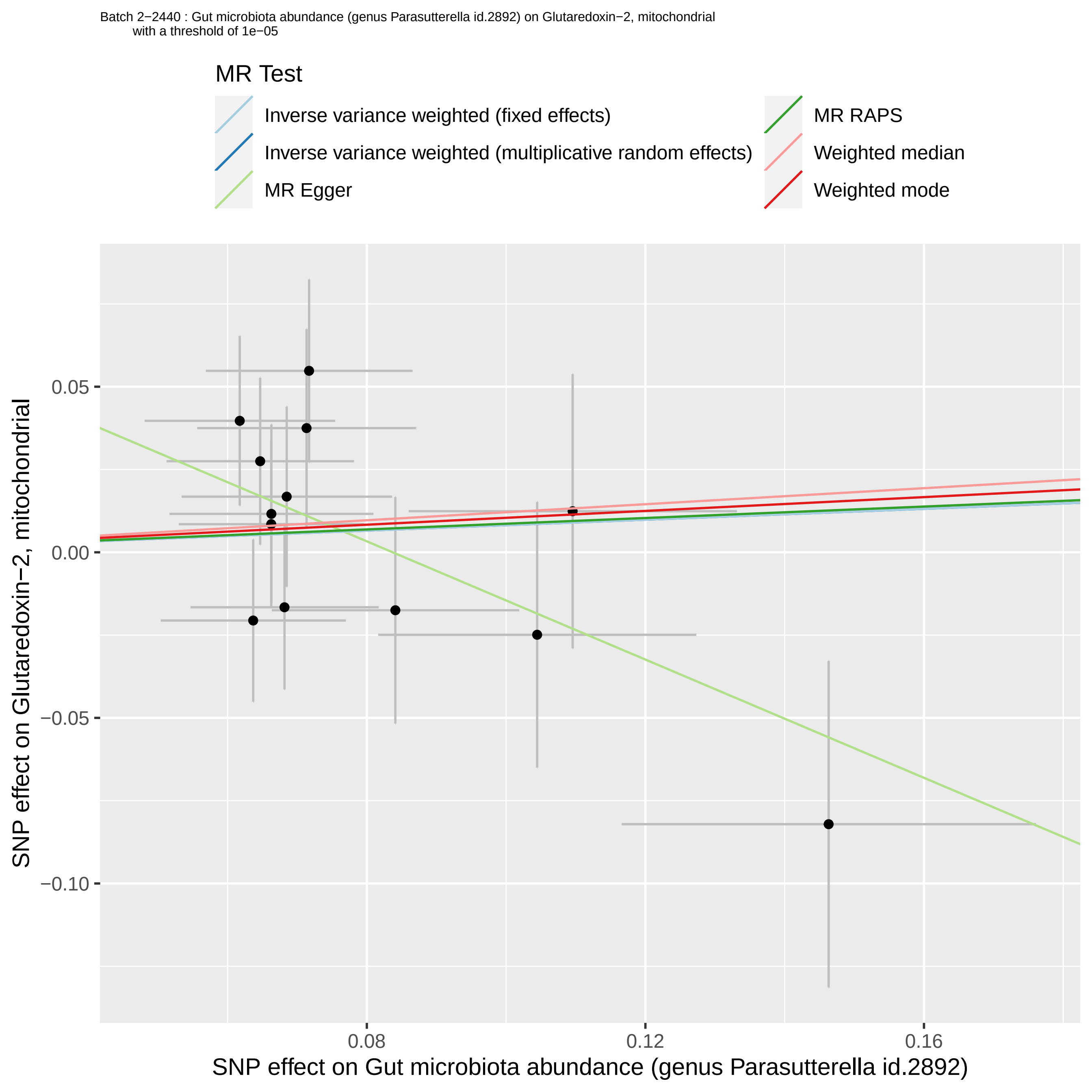
Batch 2−2440 : Gut microbiota abundance (genus Parasutterella id.2892) on Glutaredoxin−2, mitochondrial
with a threshold of 1e−05
MR Test
Inverse variance weighted (fixed effects)
Inverse variance weighted (multiplicative random effects)
MR Egger
MR RAPS
Weighted median
Weighted mode
0.05
0.00
−0.05
−0.10
0.08
0.12
0.16
SNP effect on Glutaredoxin−2, mitochondrial
SNP effect on Gut microbiota abundance (genus Parasutterella id.2892)

Batch 2−2441 : Gut microbiota abundance (genus Catenibacterium id.2153) on Glycosyltransferase 8 domain−containing protein 1 with a threshold of 1e−05

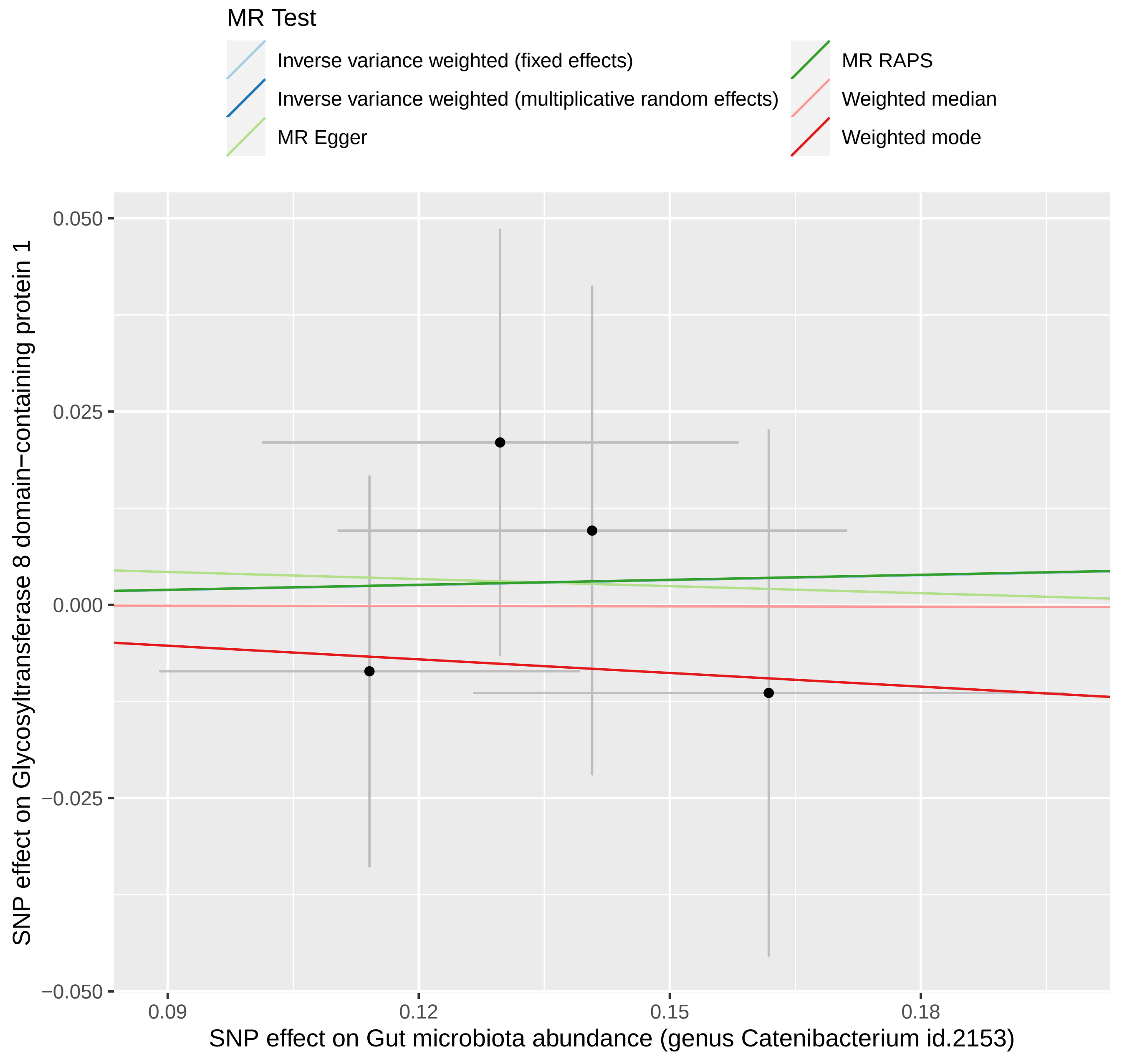

Batch 2−2442 : Gut microbiota abundance (genus Parasutterella id.2892) on Glycosyltransferase 8 domain−containing protein 1
with a threshold of 1e−05

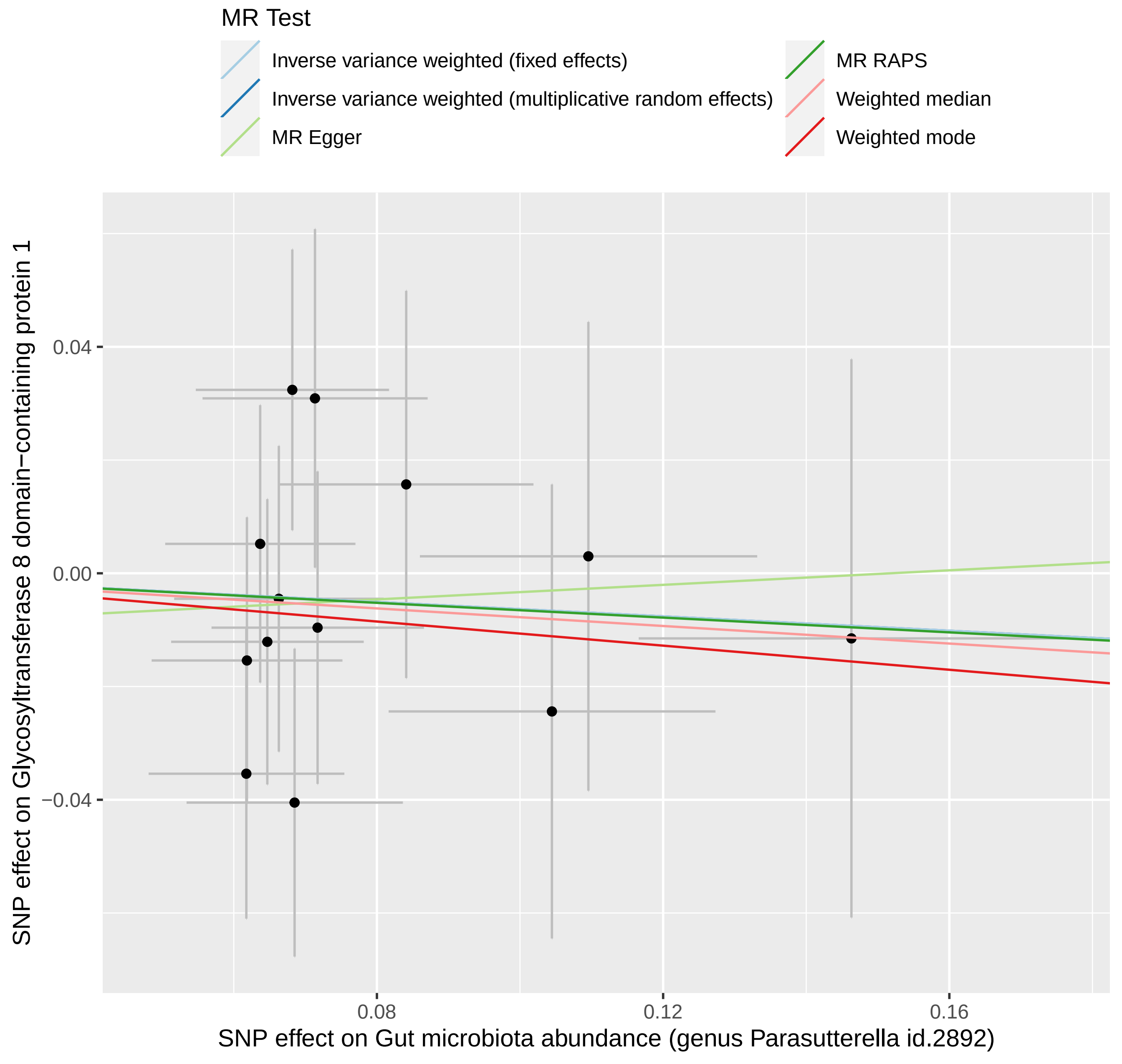

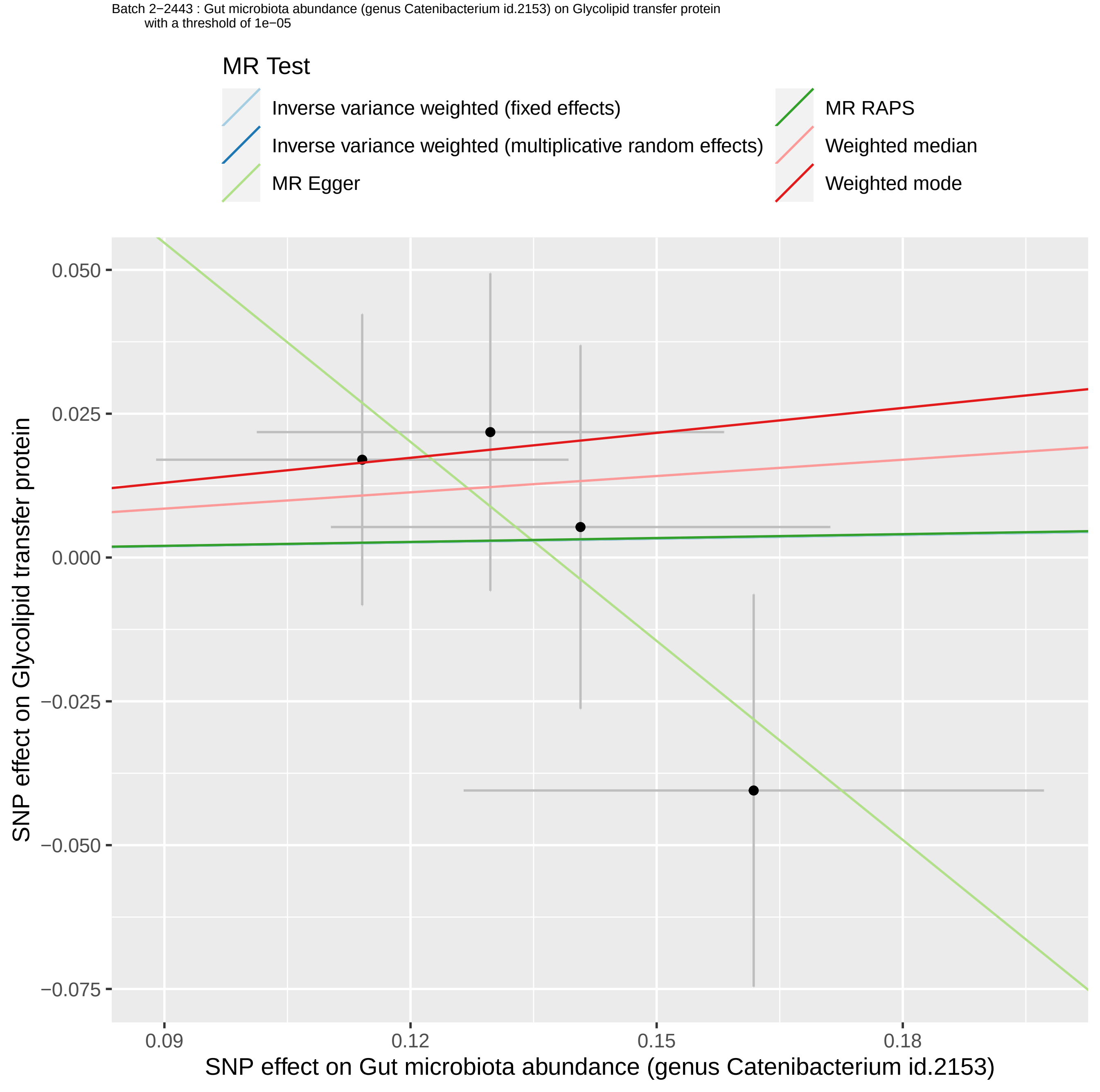

Batch 2−2443 : Gut microbiota abundance (genus Catenibacterium id.2153) on Glycolipid transfer protein
with a threshold of 1e−05
MR Test
Inverse variance weighted (fixed effects)
Inverse variance weighted (multiplicative random effects)
MR Egger
MR RAPS
Weighted median
Weighted mode
0.050
0.025
0.000
−0.025
−0.050
−0.075
0.09
0.12
0.15
0.18
SNP effect on Gut microbiota abundance (genus Catenibacterium id.2153)
SNP effect on Glycolipid transfer protein

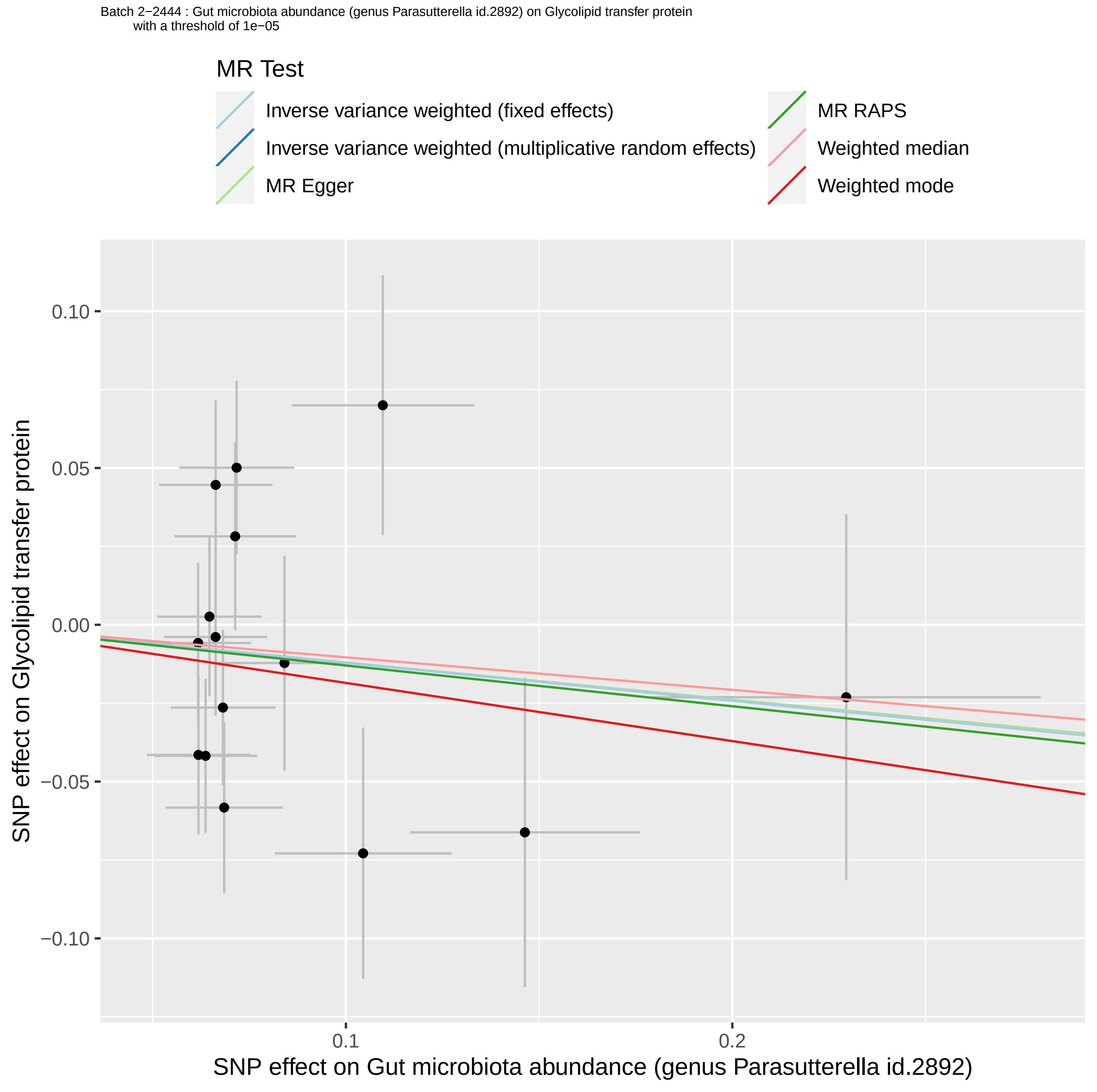
Batch 2−2444 : Gut microbiota abundance (genus Parasutterella id.2892) on Glycolipid transfer protein
with a threshold of 1e−05
MR Test
Inverse variance weighted (fixed effects)
Inverse variance weighted (multiplicative random effects)
MR Egger
MR RAPS
Weighted median
Weighted mode
0.10
0.05
0.00
−0.05
−0.10
0.1
0.2
SNP effect on Glycolipid transfer protein
SNP effect on Gut microbiota abundance (genus Parasutterella id.2892)

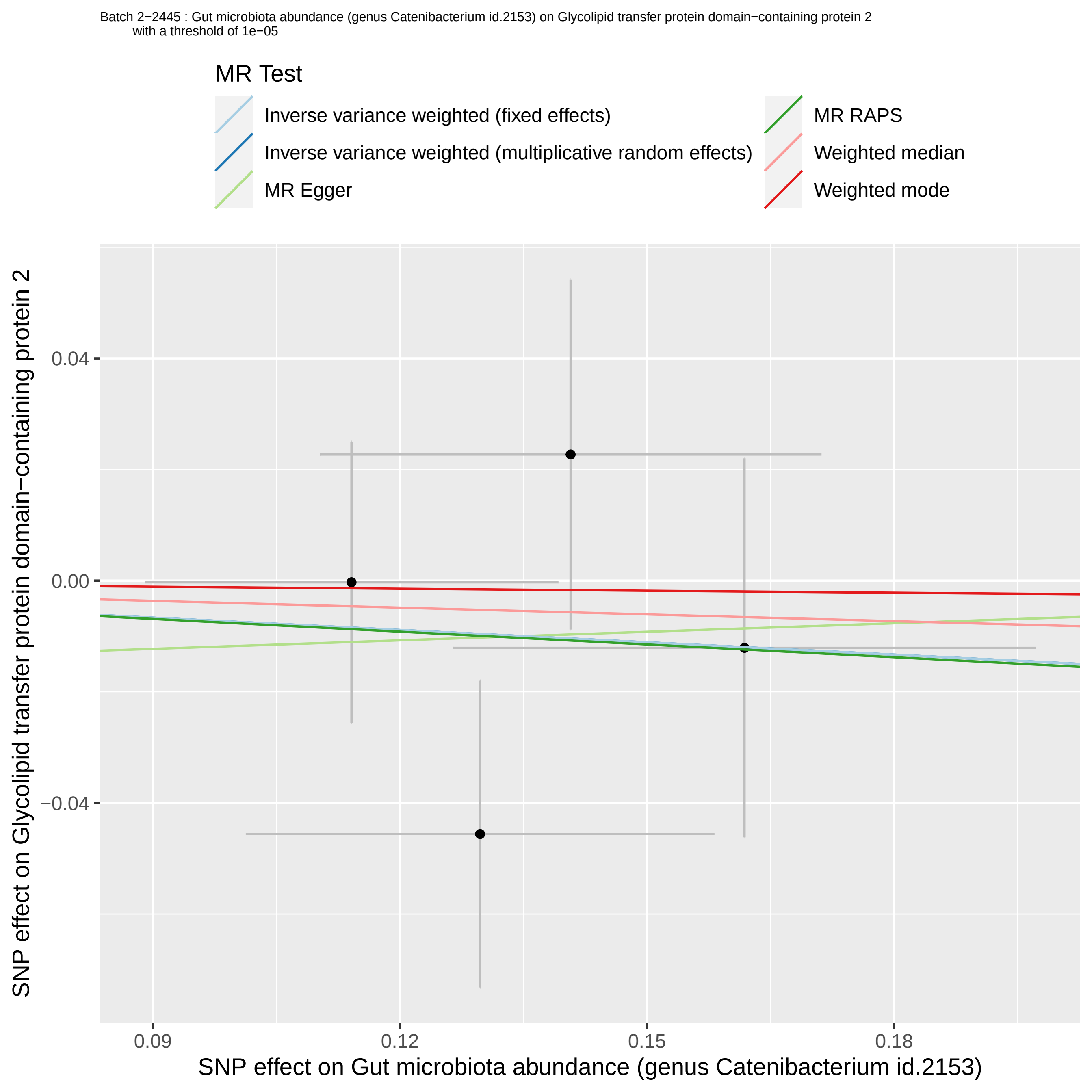
Batch 2−2445 : Gut microbiota abundance (genus Catenibacterium id.2153) on Glycolipid transfer protein domain−containing protein 2
with a threshold of 1e−05
MR Test
Inverse variance weighted (fixed effects)
Inverse variance weighted (multiplicative random effects)
MR Egger
MR RAPS
Weighted median
Weighted mode
0.04
0.00
−0.04
0.09
0.12
0.15
0.18
SNP effect on Glycolipid transfer protein domain−containing protein 2
SNP effect on Gut microbiota abundance (genus Catenibacterium id.2153)

Batch 2−2446 : Gut microbiota abundance (genus Parasutterella id.2892) on Glycolipid transfer protein domain−containing protein 2
with a threshold of 1e−05

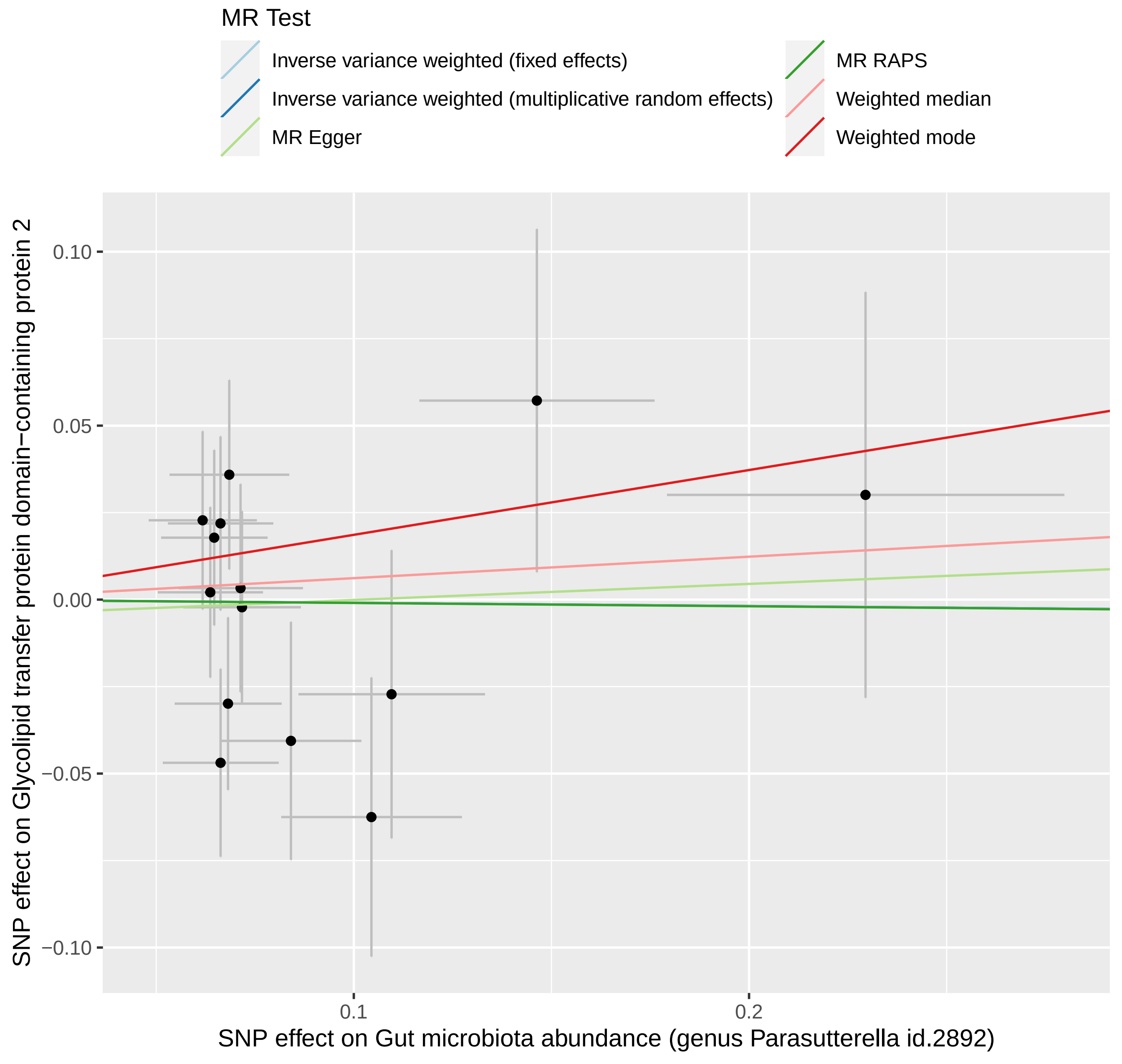

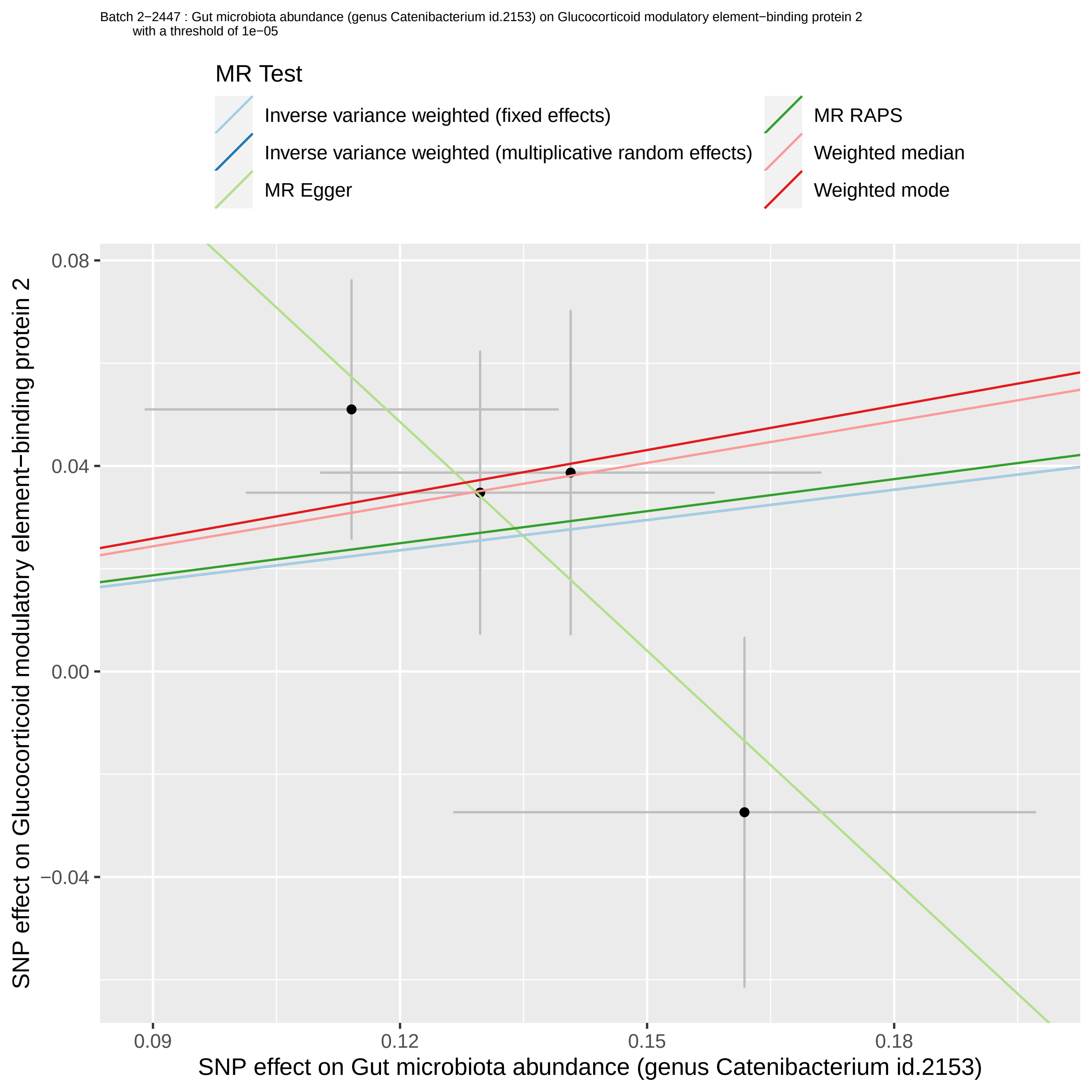
Batch 2−2447 : Gut microbiota abundance (genus Catenibacterium id.2153) on Glucocorticoid modulatory element−binding protein 2
with a threshold of 1e−05
MR Test
Inverse variance weighted (fixed effects)
Inverse variance weighted (multiplicative random effects)
MR Egger
MR RAPS
Weighted median
Weighted mode
0.08
0.04
0.00
−0.04
0.09
0.12
0.15
0.18
SNP effect on Glucocorticoid modulatory element−binding protein 2
SNP effect on Gut microbiota abundance (genus Catenibacterium id.2153)

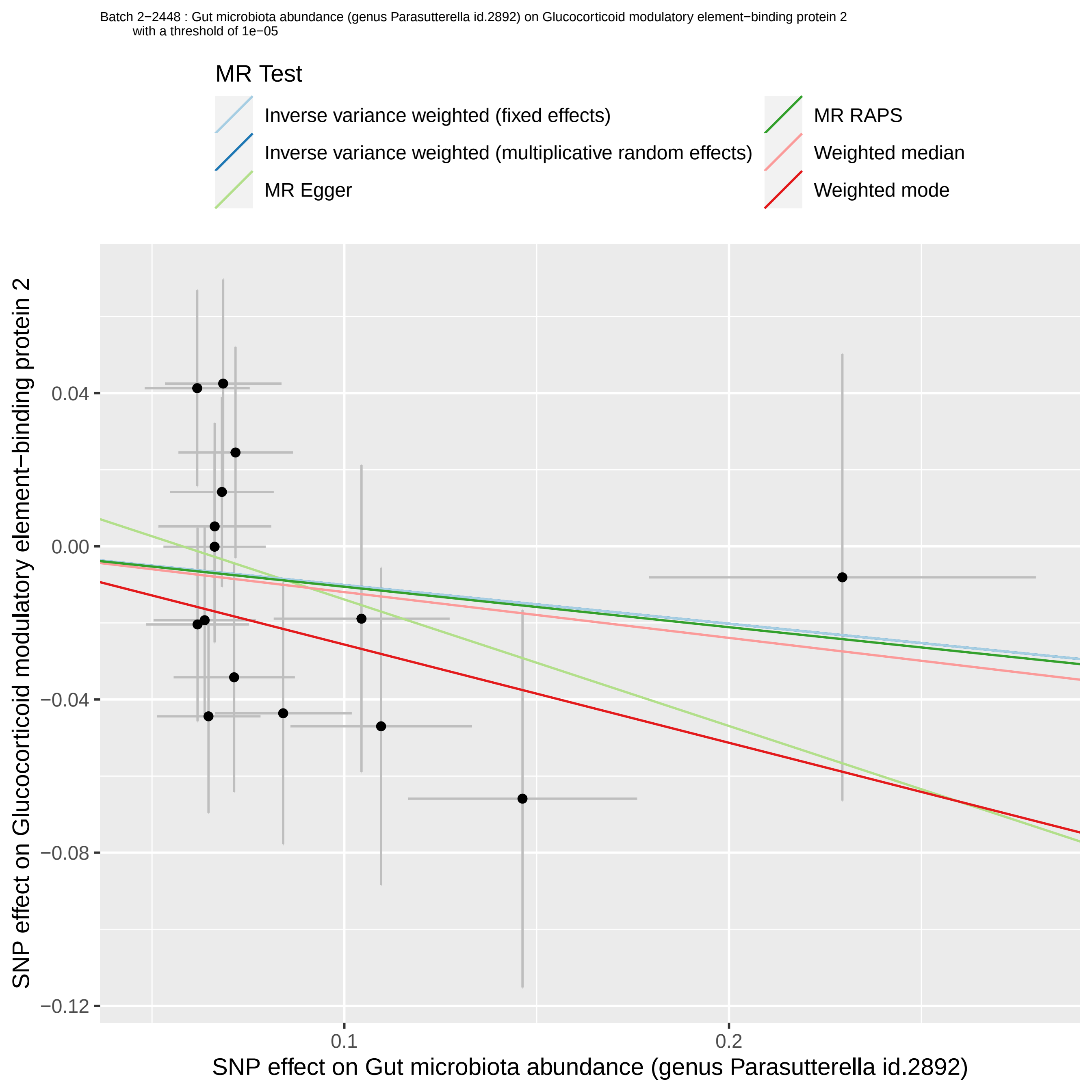
Batch 2−2448 : Gut microbiota abundance (genus Parasutterella id.2892) on Glucocorticoid modulatory element−binding protein 2
with a threshold of 1e−05
MR Test
Inverse variance weighted (fixed effects)
Inverse variance weighted (multiplicative random effects)
MR Egger
MR RAPS
Weighted median
Weighted mode
SNP effect on Glucocorticoid modulatory element−binding protein 2
0.04
0.00
−0.04
−0.08
−0.12
0.1
0.2
SNP effect on Gut microbiota abundance (genus Parasutterella id.2892)

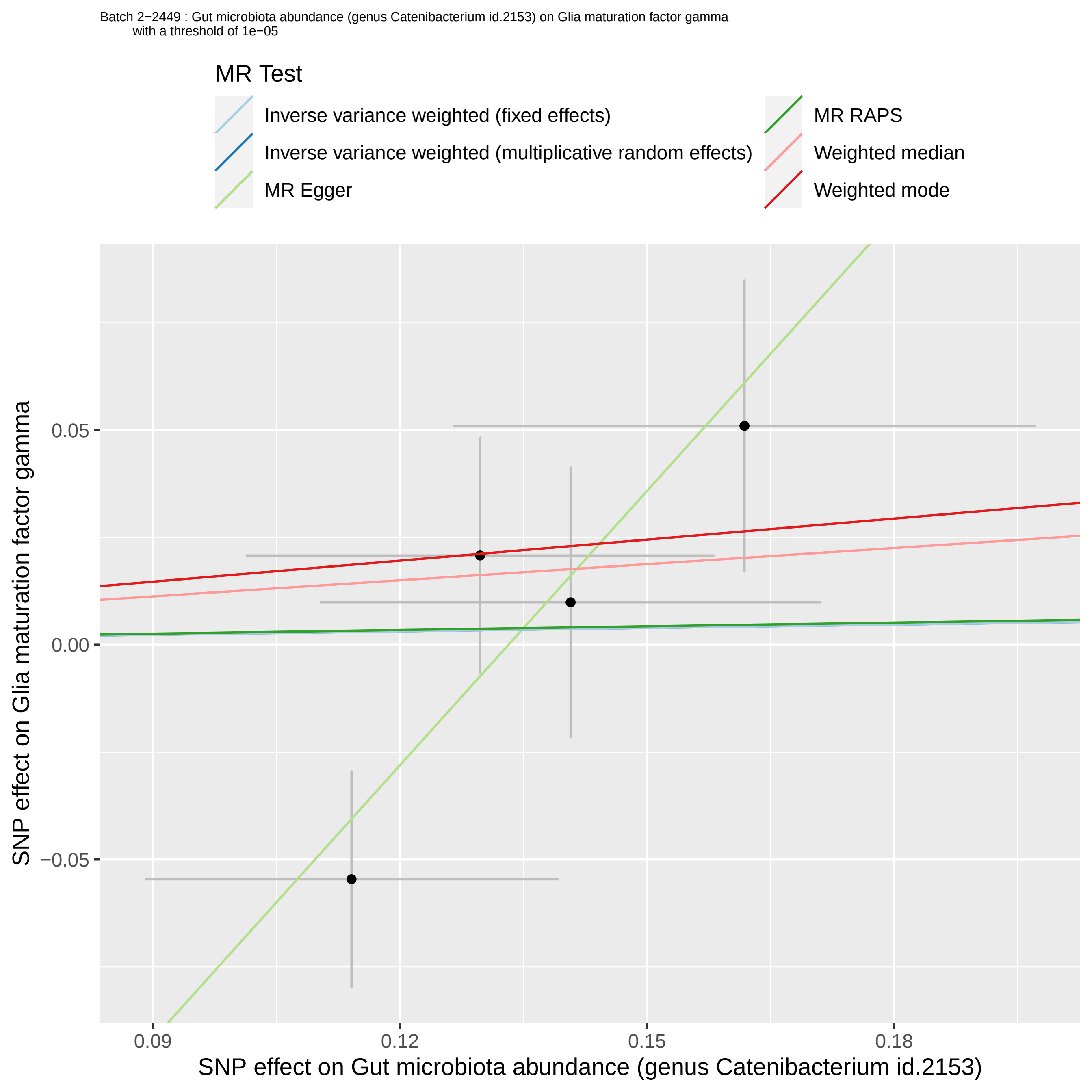

Batch 2−2449 : Gut microbiota abundance (genus Catenibacterium id.2153) on Glia maturation factor gamma
with a threshold of 1e−05
MR Test
Inverse variance weighted (fixed effects)
Inverse variance weighted (multiplicative random effects)
MR Egger
MR RAPS
Weighted median
Weighted mode
0.05
0.00
−0.05
0.09
0.12
0.15
0.18
SNP effect on Glia maturation factor gamma
SNP effect on Gut microbiota abundance (genus Catenibacterium id.2153)

Batch 2−2450 : Gut microbiota abundance (genus Parasutterella id.2892) on Glia maturation factor gamma
with a threshold of 1e−05

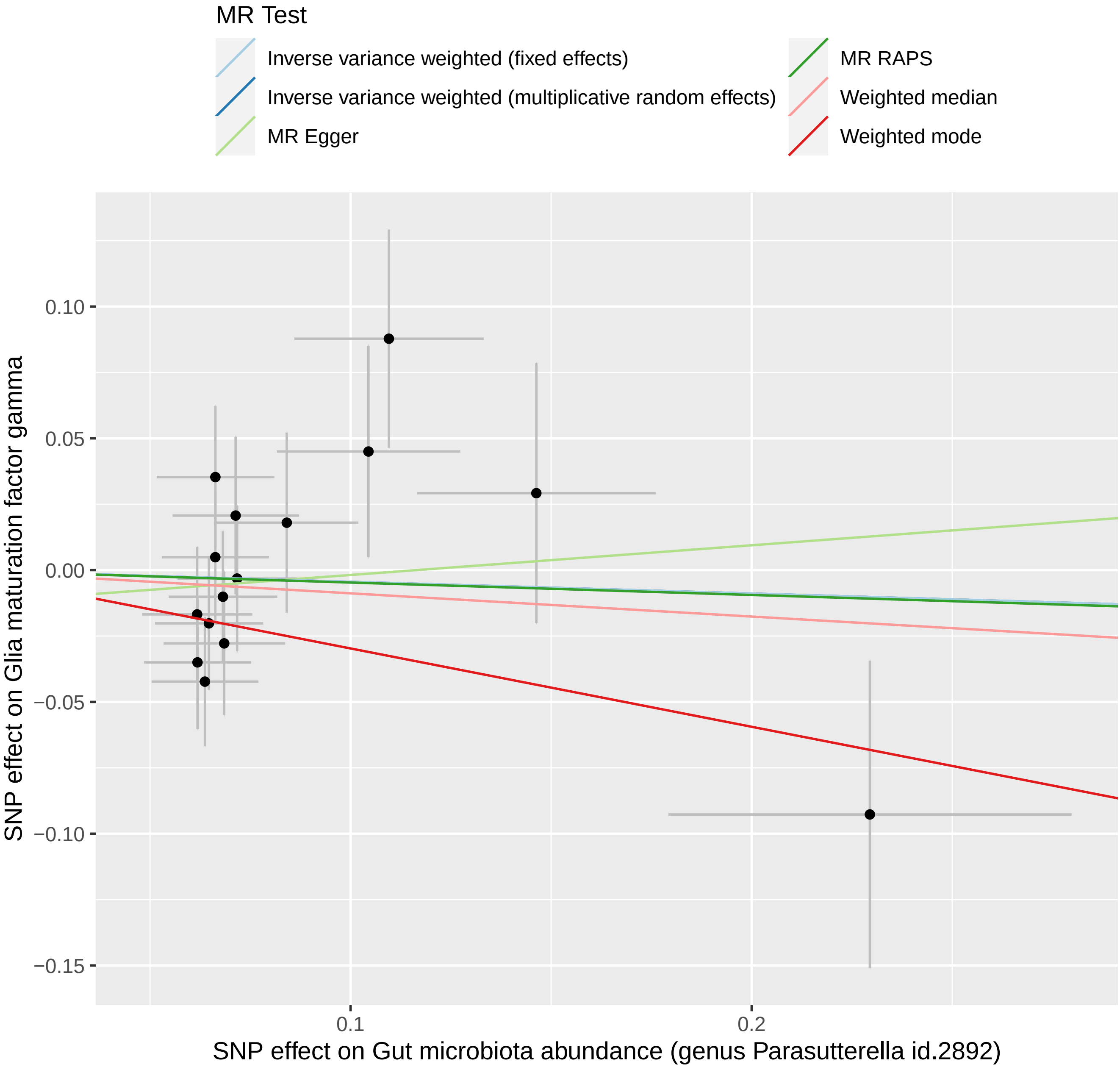

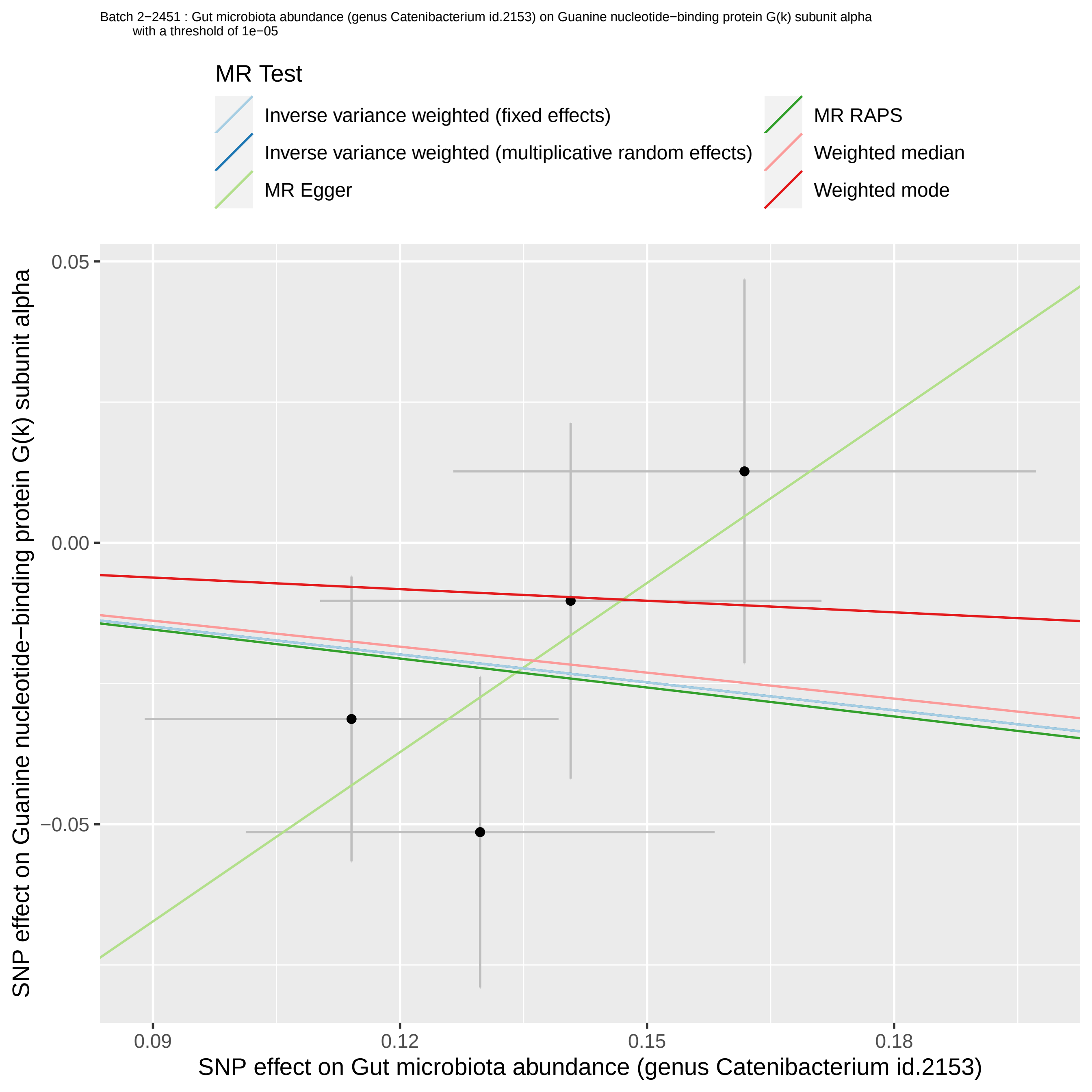

Batch 2−2451 : Gut microbiota abundance (genus Catenibacterium id.2153) on Guanine nucleotide−binding protein G(k) subunit alpha
with a threshold of 1e−05
MR Test
Inverse variance weighted (fixed effects)
Inverse variance weighted (multiplicative random effects)
MR Egger
MR RAPS
Weighted median
Weighted mode
0.05
0.00
−0.05
0.09
0.12
0.15
0.18
SNP effect on Guanine nucleotide−binding protein G(k) subunit alpha
SNP effect on Gut microbiota abundance (genus Catenibacterium id.2153)

Batch 2−2452 : Gut microbiota abundance (genus Parasutterella id.2892) on Guanine nucleotide−binding protein G(k) subunit alpha
with a threshold of 1e−05

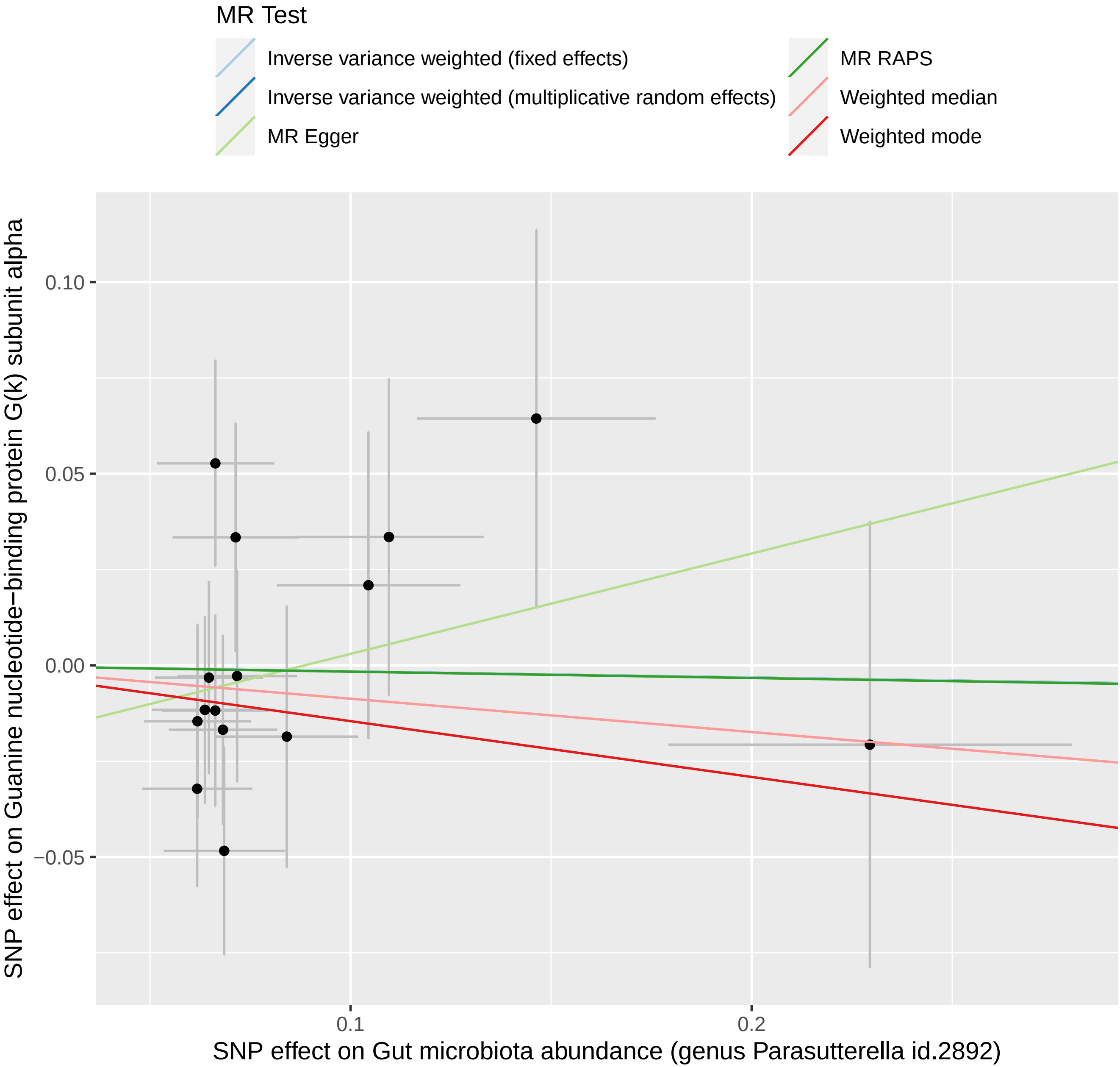

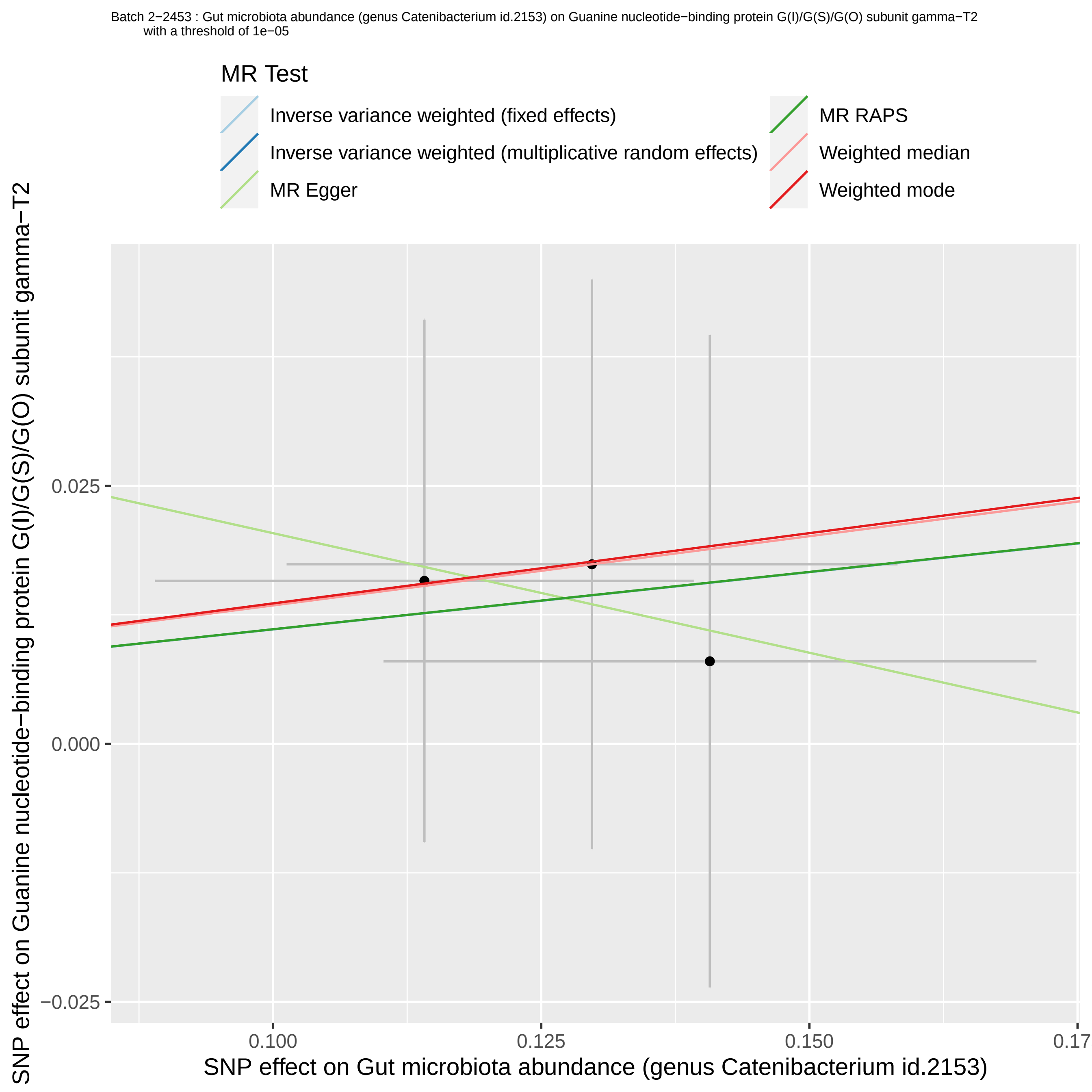
Batch 2−2453 : Gut microbiota abundance (genus Catenibacterium id.2153) on Guanine nucleotide−binding protein G(I)/G(S)/G(O) subunit gamma−T2
with a threshold of 1e−05
MR Test
Inverse variance weighted (fixed effects)
Inverse variance weighted (multiplicative random effects)
MR Egger
MR RAPS
Weighted median
Weighted mode
SNP effect on Guanine nucleotide−binding protein G(I)/G(S)/G(O) subunit gamma−T2
0.025
0.000
−0.025
0.100
0.125
0.150
0.17
SNP effect on Gut microbiota abundance (genus Catenibacterium id.2153)

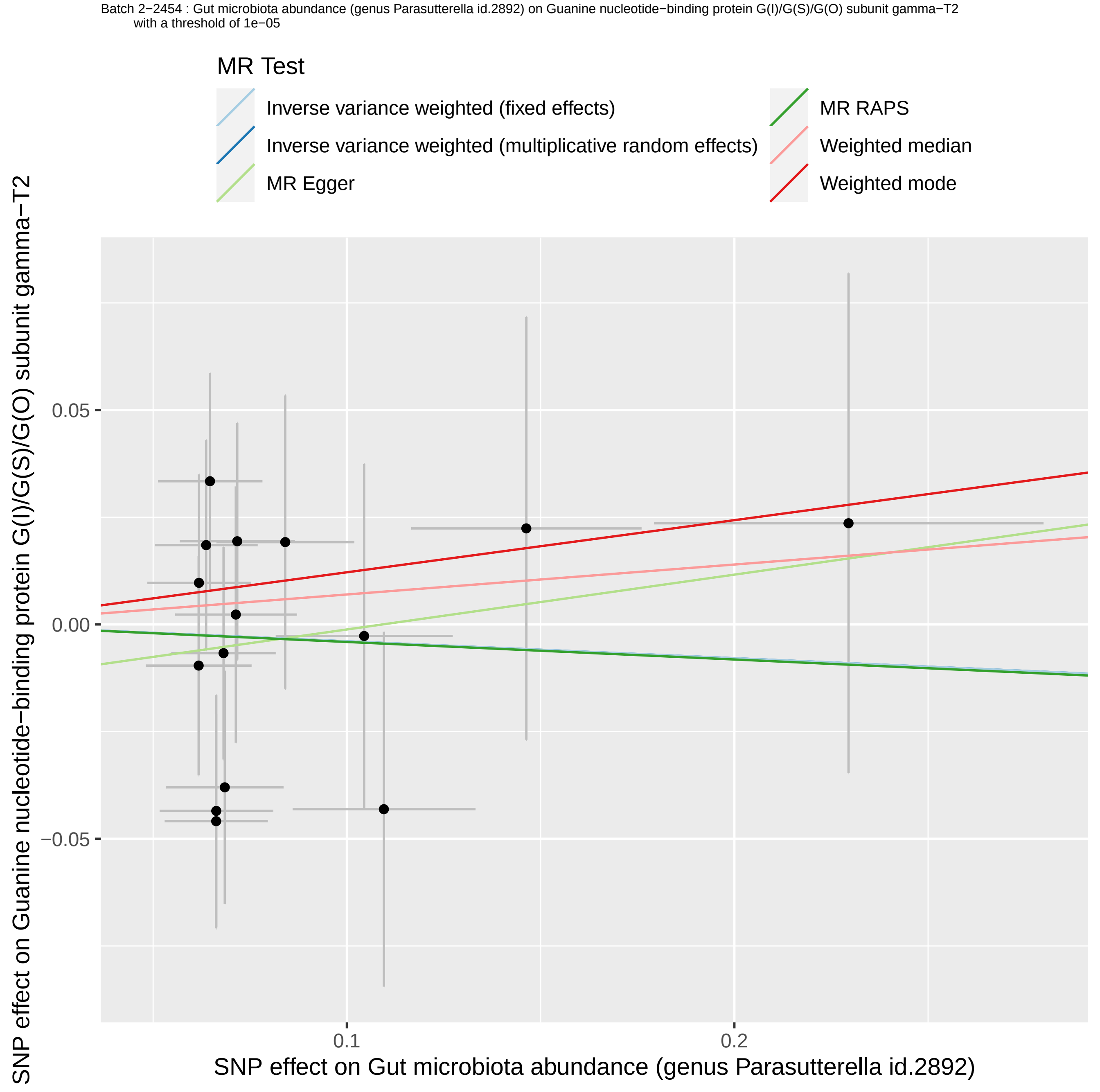
Batch 2−2454 : Gut microbiota abundance (genus Parasutterella id.2892) on Guanine nucleotide−binding protein G(I)/G(S)/G(O) subunit gamma−T2
with a threshold of 1e−05
MR Test
Inverse variance weighted (fixed effects)
Inverse variance weighted (multiplicative random effects)
MR Egger
MR RAPS
Weighted median
Weighted mode
0.05
0.00
−0.05
0.1
0.2
SNP effect on Gut microbiota abundance (genus Parasutterella id.2892)
SNP effect on Guanine nucleotide−binding protein G(I)/G(S)/G(O) subunit gamma−T2

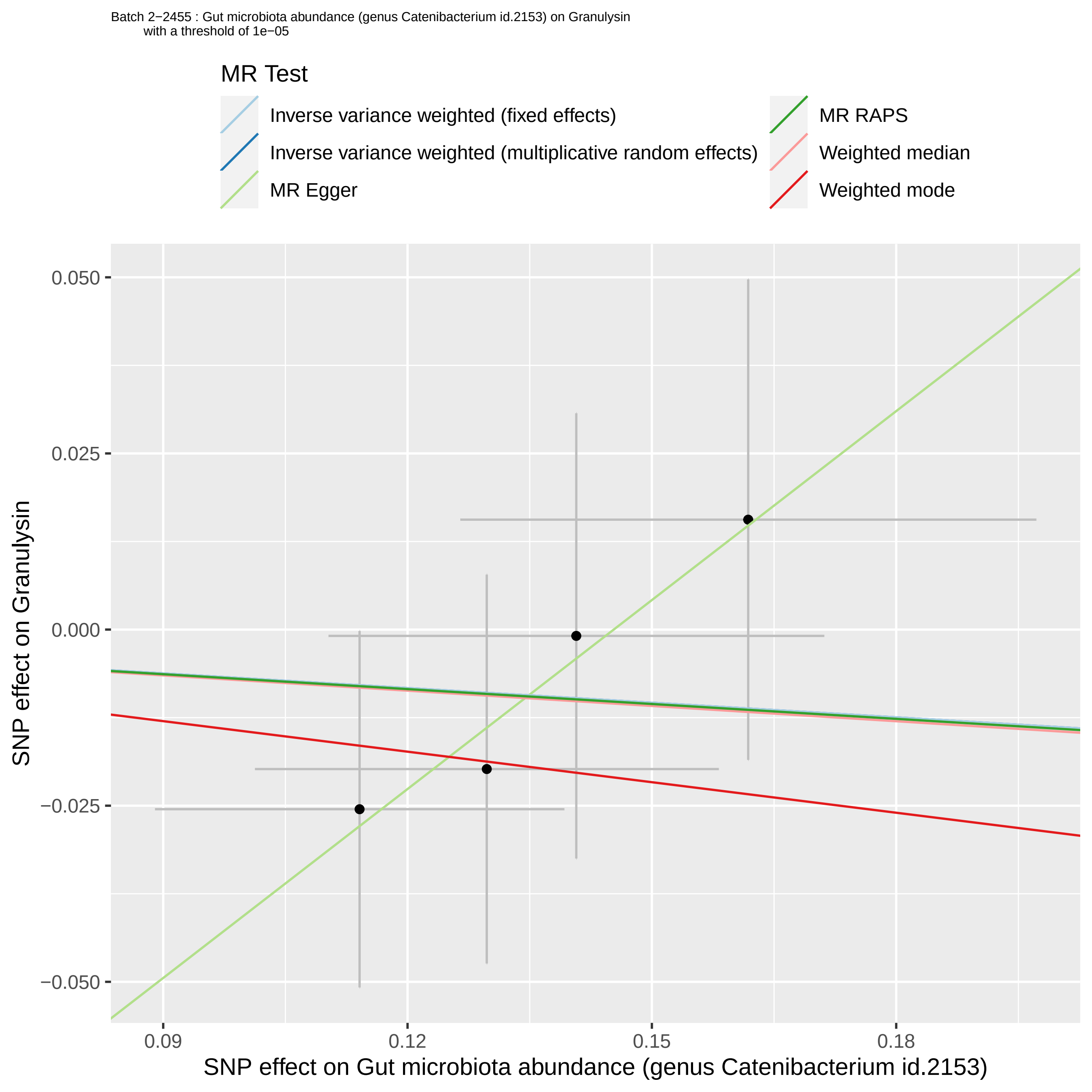

Batch 2−2455 : Gut microbiota abundance (genus Catenibacterium id.2153) on Granulysin
with a threshold of 1e−05
MR Test
Inverse variance weighted (fixed effects)
Inverse variance weighted (multiplicative random effects)
MR Egger
MR RAPS
Weighted median
Weighted mode
0.050
0.025
0.000
−0.025
−0.050
SNP effect on Granulysin
0.09
0.12
0.15
0.18
SNP effect on Gut microbiota abundance (genus Catenibacterium id.2153)

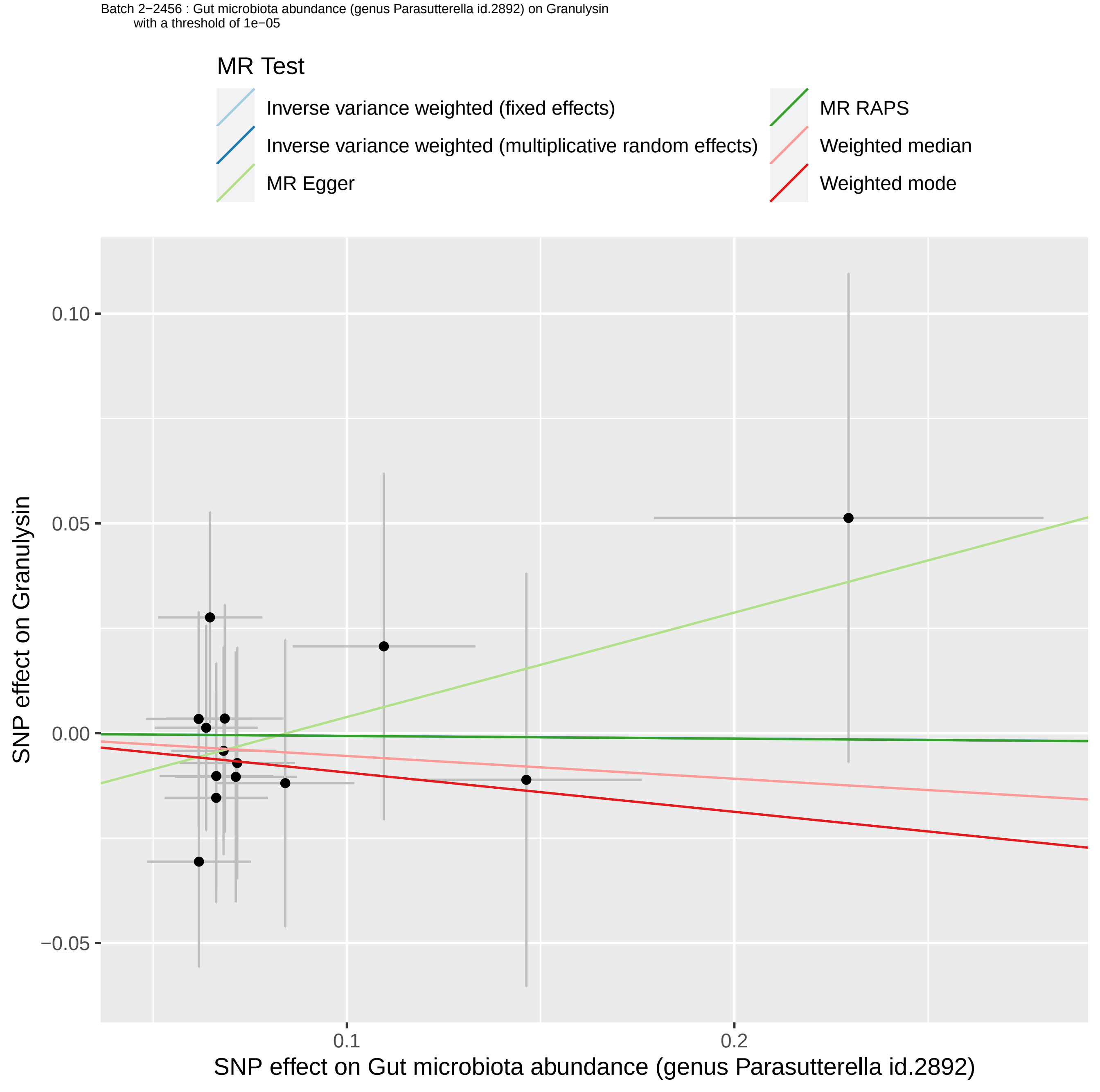
Batch 2−2456 : Gut microbiota abundance (genus Parasutterella id.2892) on Granulysin
with a threshold of 1e−05
MR Test
Inverse variance weighted (fixed effects)
Inverse variance weighted (multiplicative random effects)
MR Egger
MR RAPS
Weighted median
Weighted mode
0.10
0.05
0.00
−0.05
0.1
0.2
SNP effect on Granulysin
SNP effect on Gut microbiota abundance (genus Parasutterella id.2892)

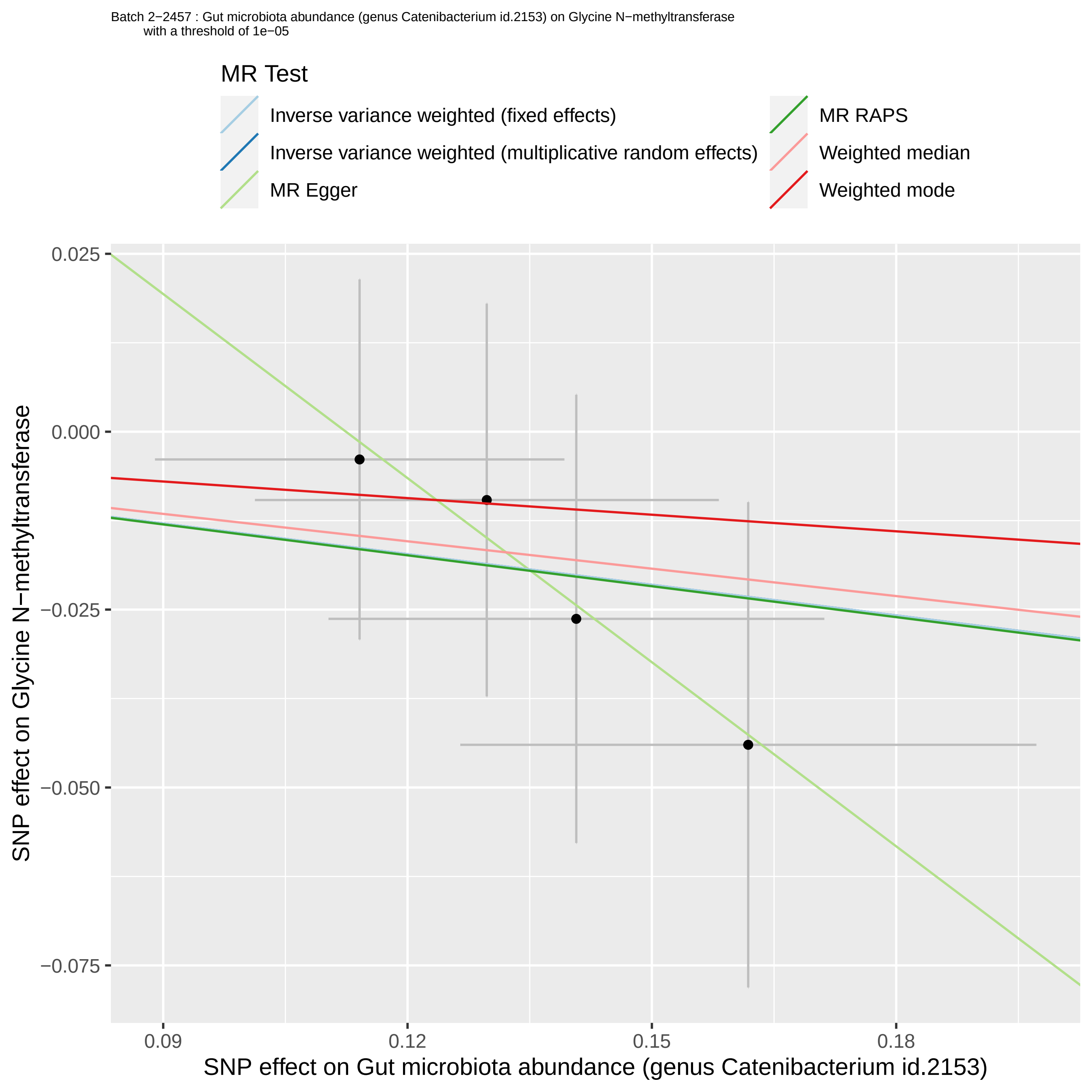
Batch 2−2457 : Gut microbiota abundance (genus Catenibacterium id.2153) on Glycine N−methyltransferase
with a threshold of 1e−05
MR Test
Inverse variance weighted (fixed effects)
Inverse variance weighted (multiplicative random effects)
MR Egger
MR RAPS
Weighted median
Weighted mode
0.025
0.000
−0.025
−0.050
−0.075
0.09
0.12
0.15
0.18
SNP effect on Glycine N−methyltransferase
SNP effect on Gut microbiota abundance (genus Catenibacterium id.2153)

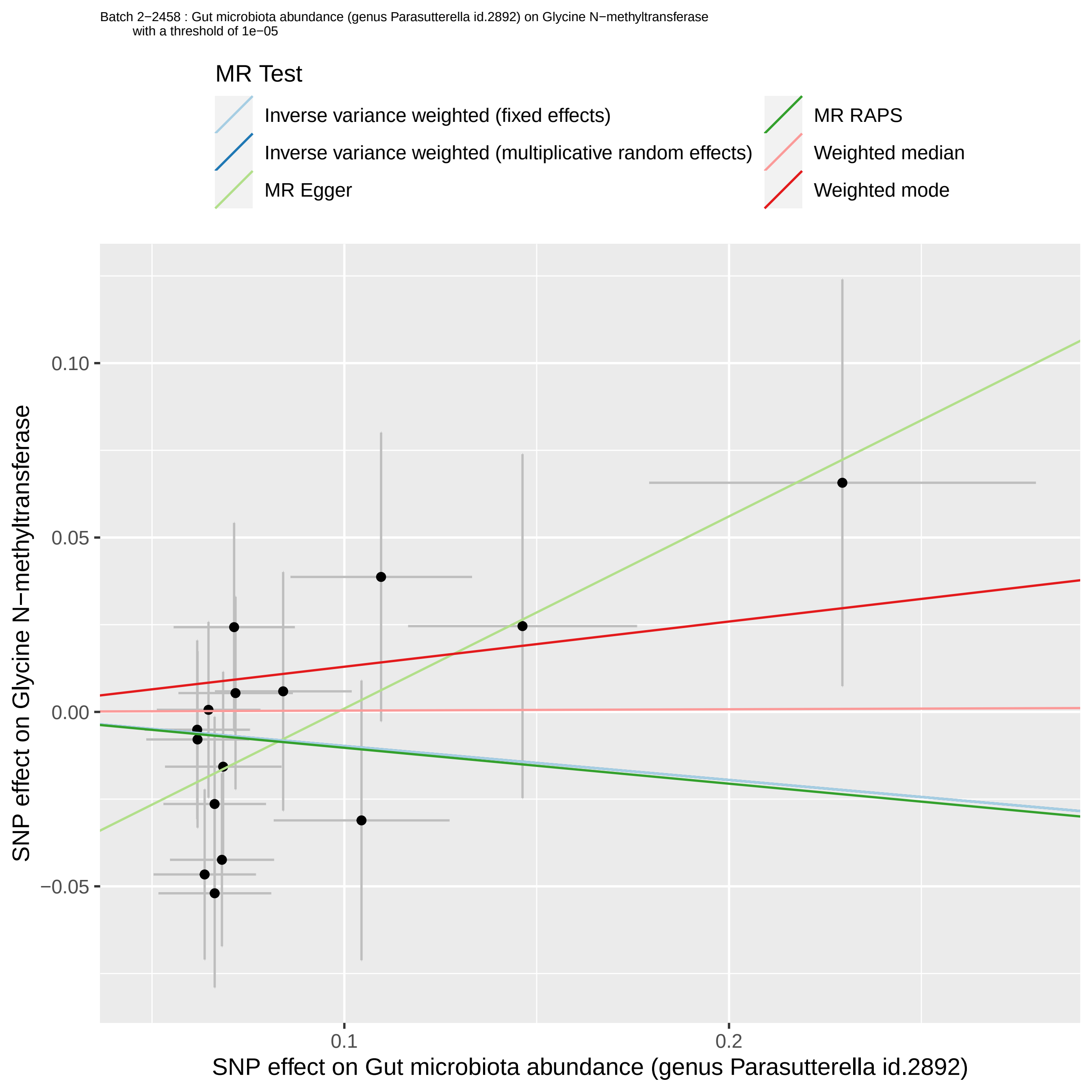

Batch 2−2458 : Gut microbiota abundance (genus Parasutterella id.2892) on Glycine N−methyltransferase
with a threshold of 1e−05
MR Test
Inverse variance weighted (fixed effects)
Inverse variance weighted (multiplicative random effects)
MR Egger
MR RAPS
Weighted median
Weighted mode
0.10
0.05
0.00
−0.05
0.1
0.2
SNP effect on Glycine N−methyltransferase
SNP effect on Gut microbiota abundance (genus Parasutterella id.2892)

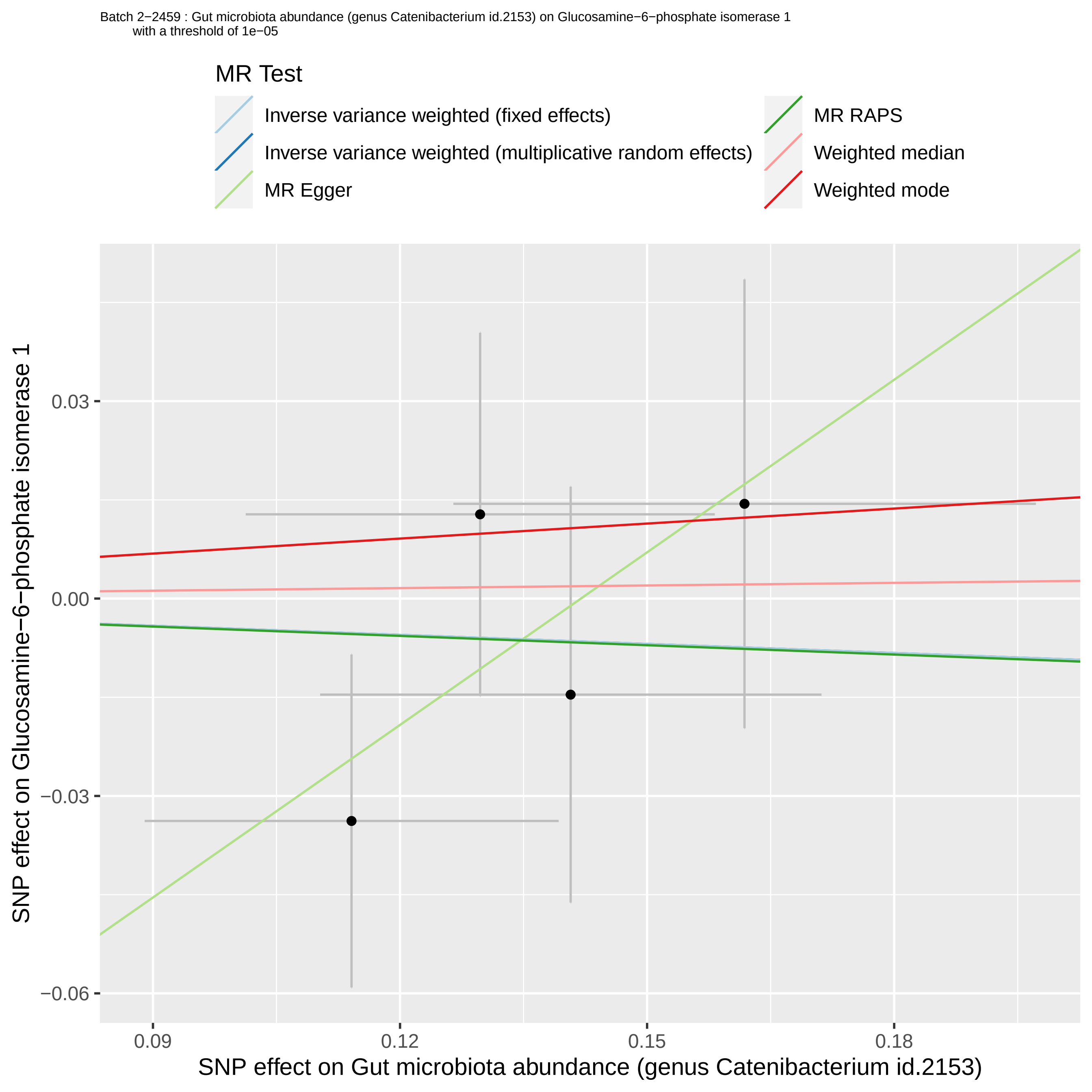
Batch 2−2459 : Gut microbiota abundance (genus Catenibacterium id.2153) on Glucosamine−6−phosphate isomerase 1
with a threshold of 1e−05
MR Test
Inverse variance weighted (fixed effects)
Inverse variance weighted (multiplicative random effects)
MR Egger
MR RAPS
Weighted median
Weighted mode
SNP effect on Glucosamine−6−phosphate isomerase 1
0.03
0.00
−0.03
−0.06
0.09
0.12
0.15
0.18
SNP effect on Gut microbiota abundance (genus Catenibacterium id.2153)

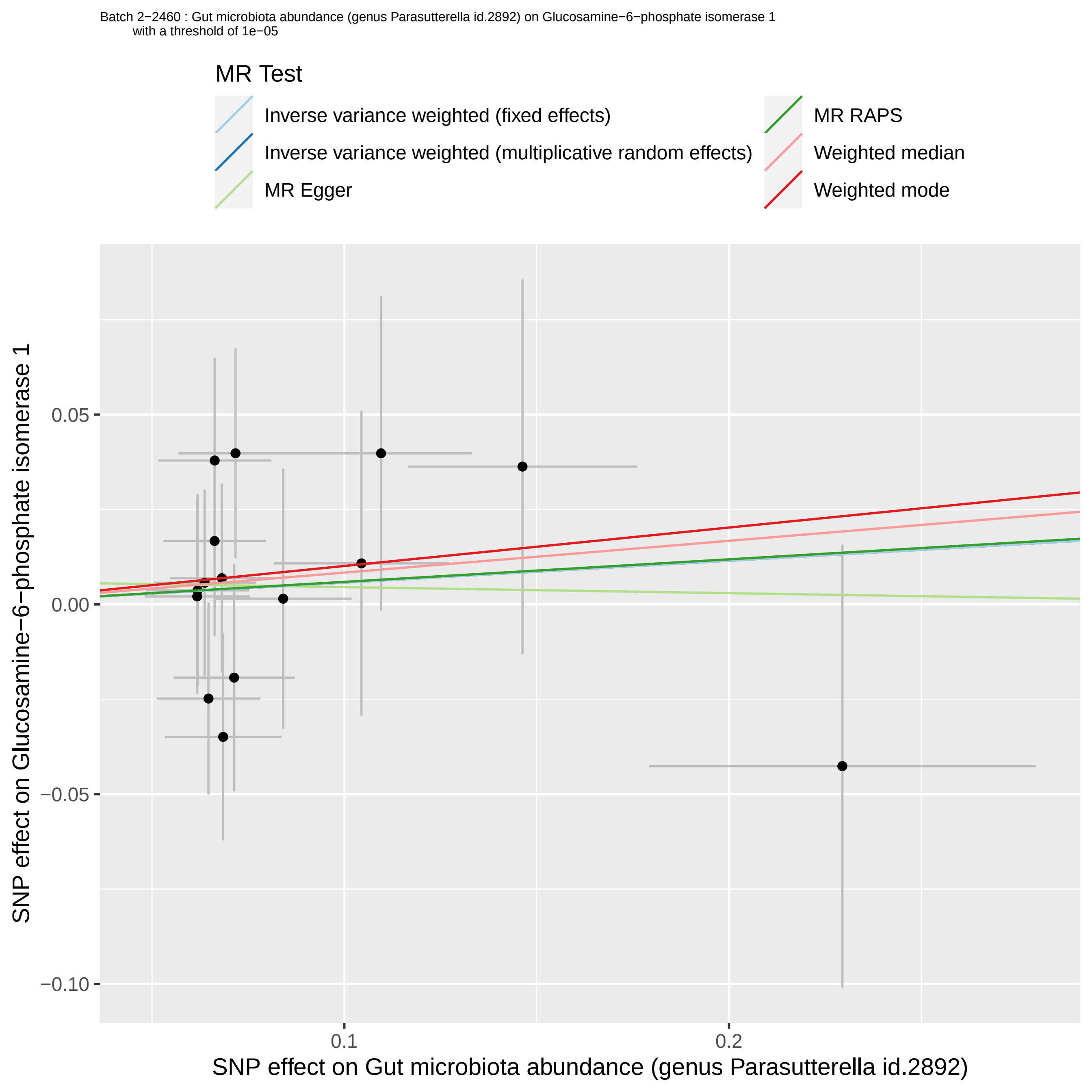

Batch 2−2460 : Gut microbiota abundance (genus Parasutterella id.2892) on Glucosamine−6−phosphate isomerase 1
with a threshold of 1e−05
MR Test
Inverse variance weighted (fixed effects)
Inverse variance weighted (multiplicative random effects)
MR Egger
MR RAPS
Weighted median
Weighted mode
0.05
0.00
−0.05
−0.10
0.1
0.2
SNP effect on Glucosamine−6−phosphate isomerase 1
SNP effect on Gut microbiota abundance (genus Parasutterella id.2892)

Batch 2−2461 : Gut microbiota abundance (genus Catenibacterium id.2153) on Glucosamine 6−phosphate N−acetyltransferase
with a threshold of 1e−05

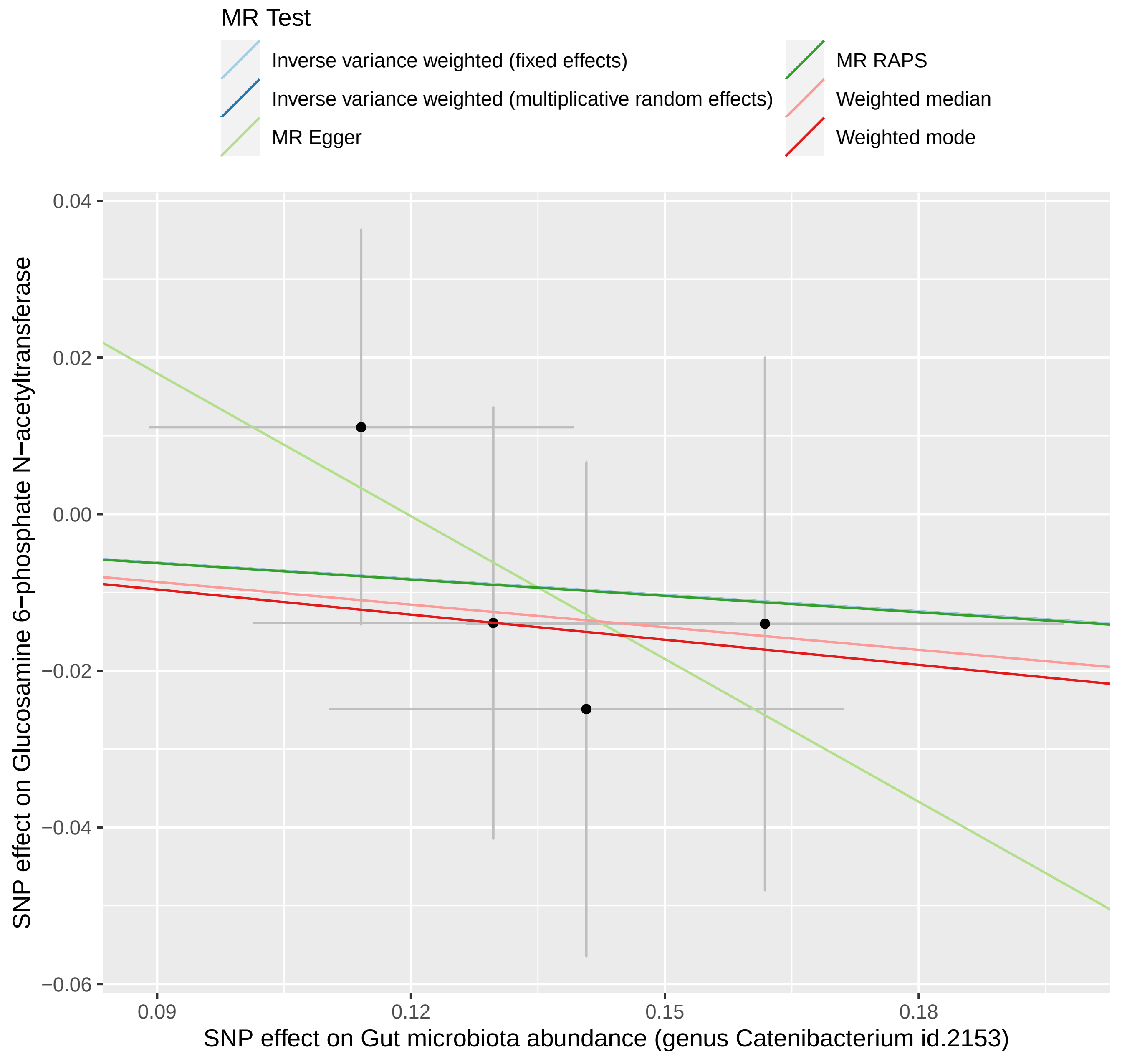

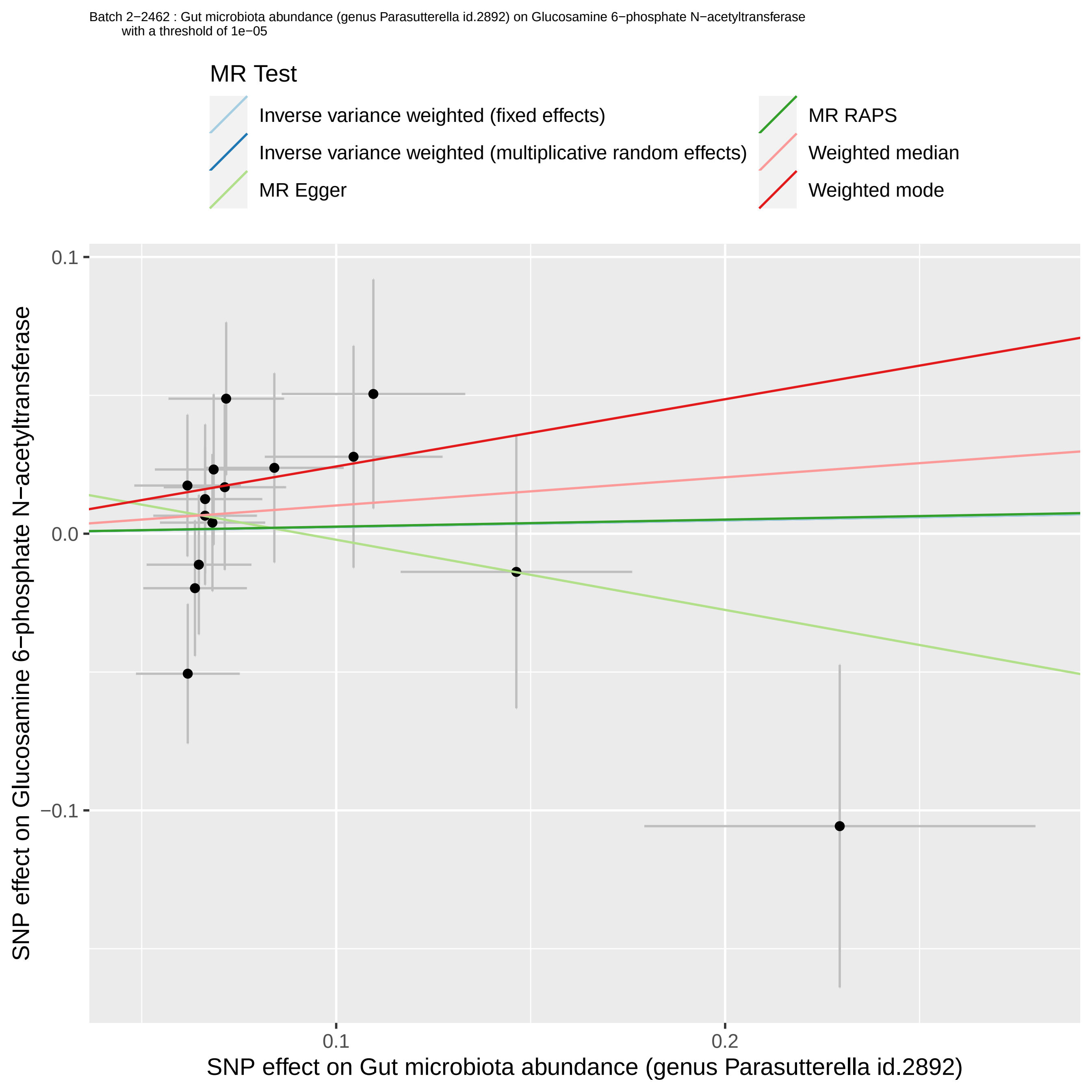

Batch 2−2462 : Gut microbiota abundance (genus Parasutterella id.2892) on Glucosamine 6−phosphate N−acetyltransferase
with a threshold of 1e−05
MR Test
Inverse variance weighted (fixed effects)
Inverse variance weighted (multiplicative random effects)
MR Egger
MR RAPS
Weighted median
Weighted mode
0.1
0.0
−0.1
0.1
0.2
SNP effect on Glucosamine 6−phosphate N−acetyltransferase
SNP effect on Gut microbiota abundance (genus Parasutterella id.2892)

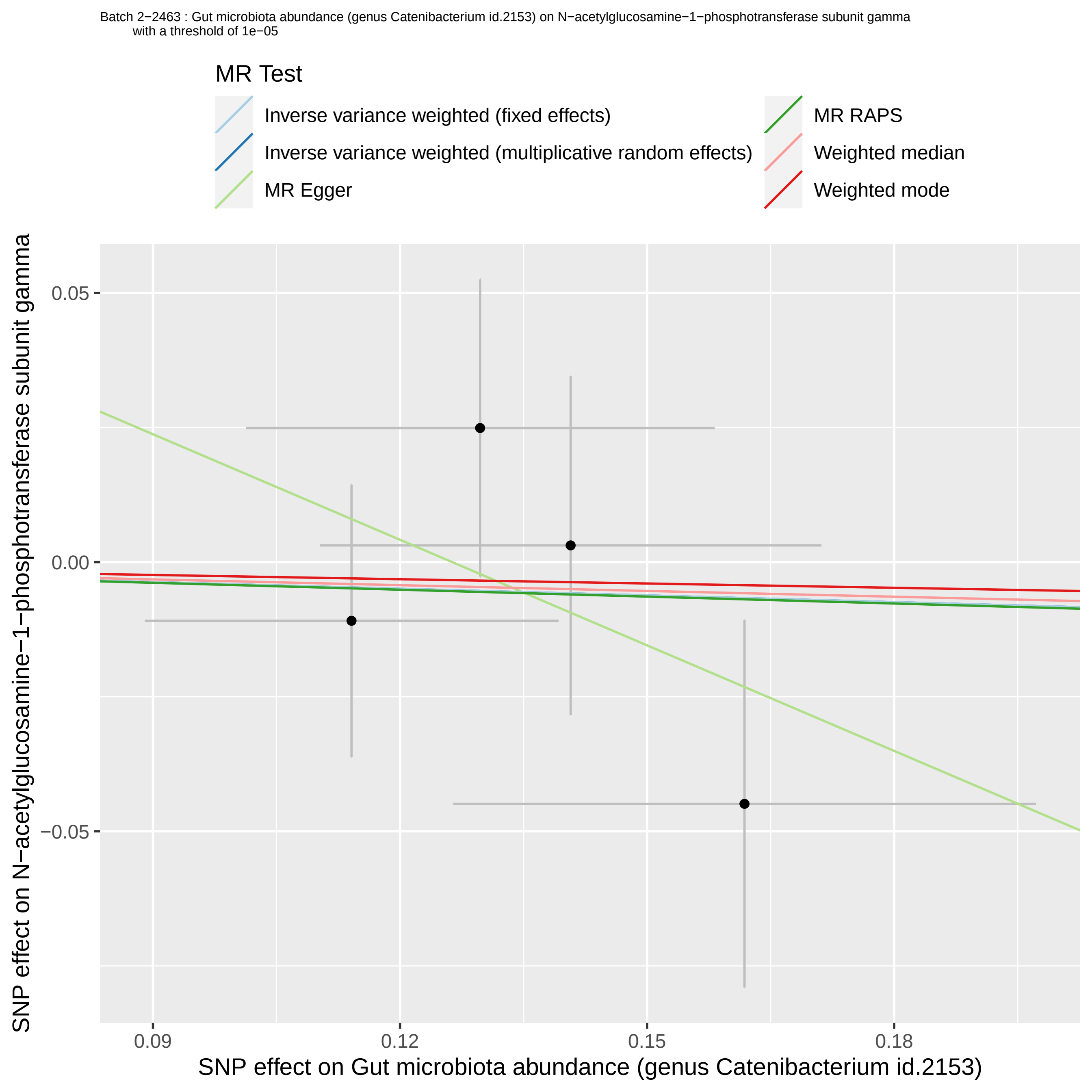
Batch 2−2463 : Gut microbiota abundance (genus Catenibacterium id.2153) on N−acetylglucosamine−1−phosphotransferase subunit gamma
with a threshold of 1e−05
MR Test
Inverse variance weighted (fixed effects)
Inverse variance weighted (multiplicative random effects)
MR Egger
MR RAPS
Weighted median
Weighted mode
SNP effect on N−acetylglucosamine−1−phosphotransferase subunit gamma
0.05
0.00
−0.05
0.09
0.12
0.15
0.18
SNP effect on Gut microbiota abundance (genus Catenibacterium id.2153)

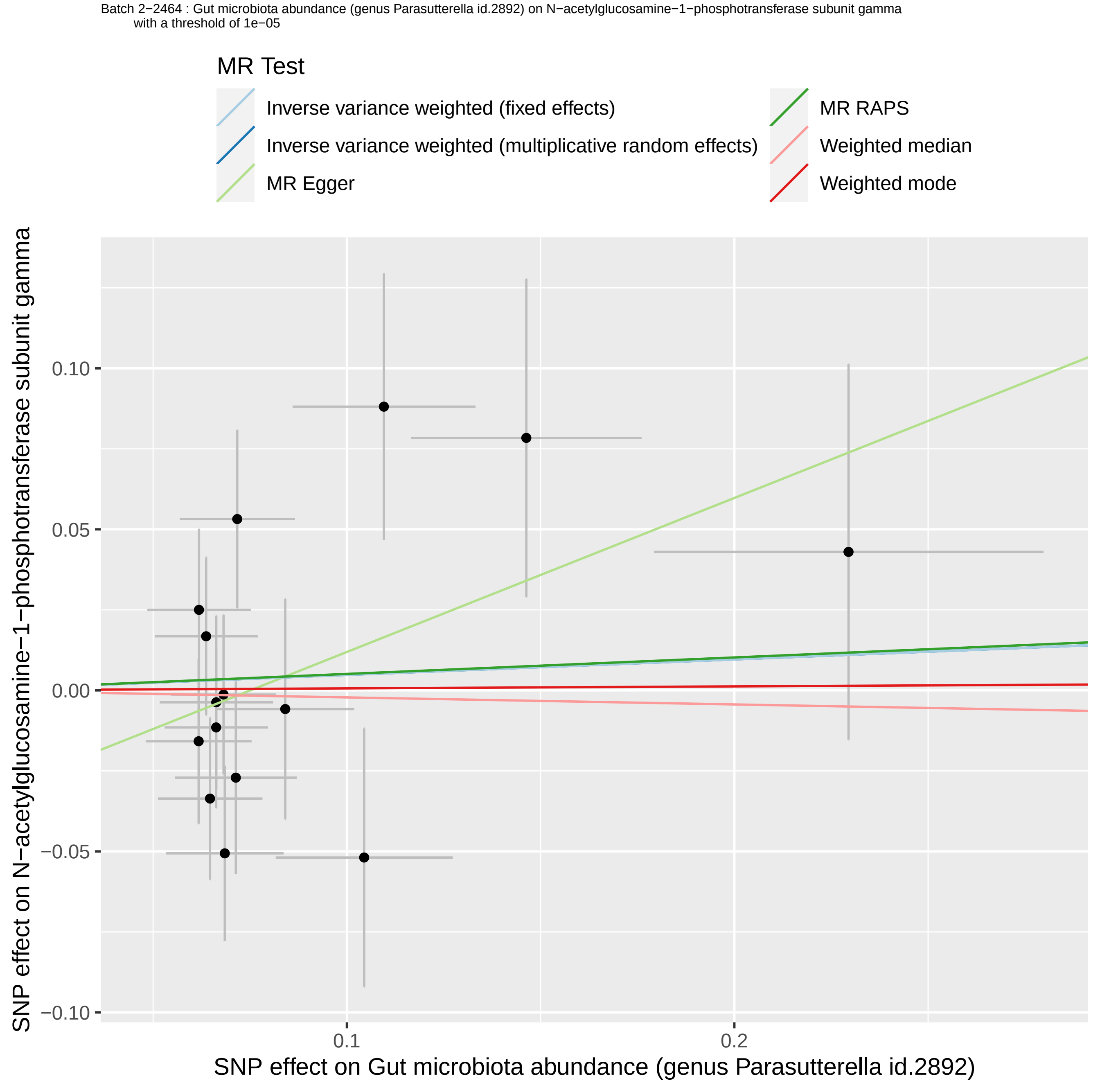

Batch 2−2464 : Gut microbiota abundance (genus Parasutterella id.2892) on N−acetylglucosamine−1−phosphotransferase subunit gamma
with a threshold of 1e−05
MR Test
Inverse variance weighted (fixed effects)
Inverse variance weighted (multiplicative random effects)
MR Egger
MR RAPS
Weighted median
Weighted mode
0.10
0.05
0.00
−0.05
−0.10
0.1
0.2
SNP effect on N−acetylglucosamine−1−phosphotransferase subunit gamma
SNP effect on Gut microbiota abundance (genus Parasutterella id.2892)

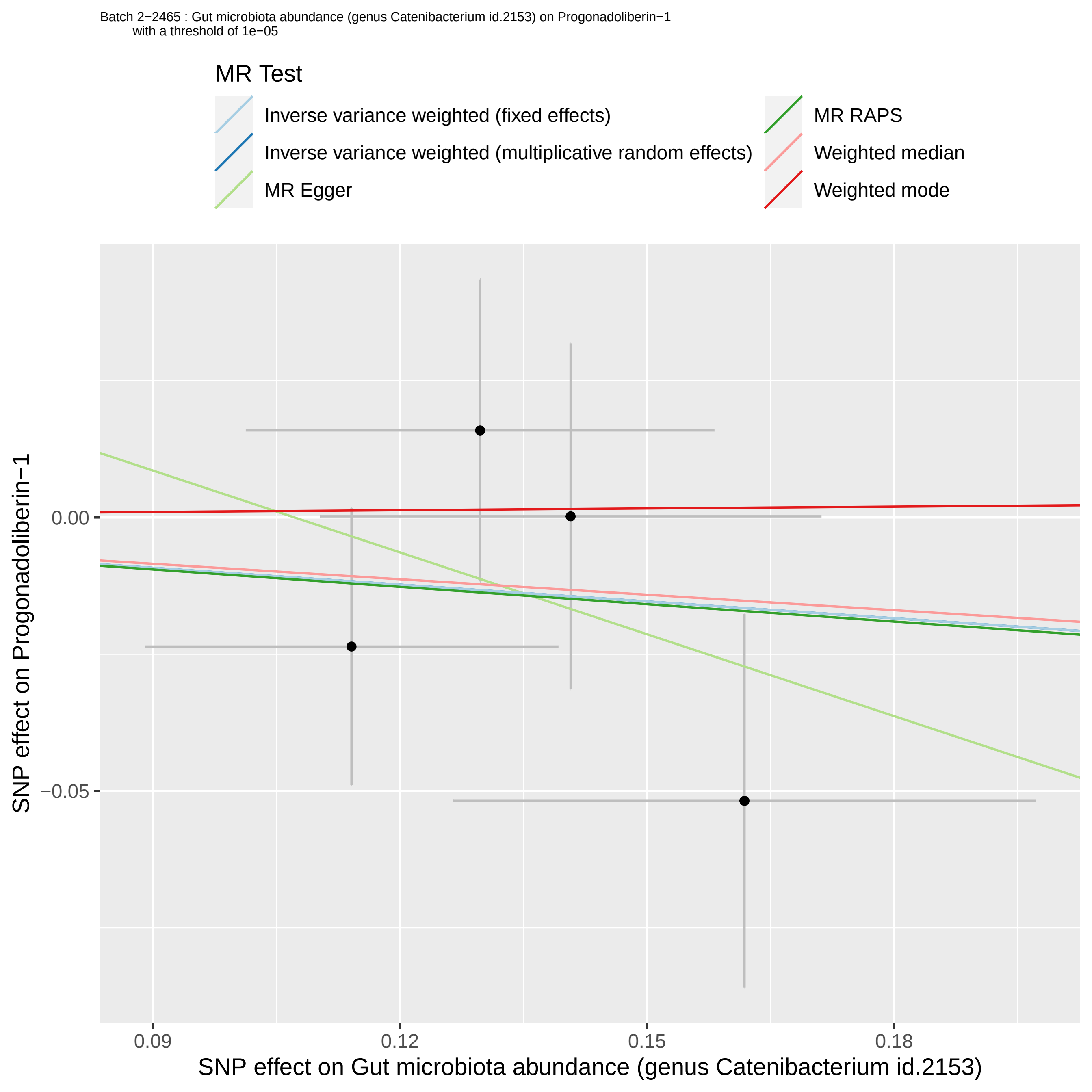

Batch 2−2465 : Gut microbiota abundance (genus Catenibacterium id.2153) on Progonadoliberin−1
with a threshold of 1e−05
MR Test
Inverse variance weighted (fixed effects)
Inverse variance weighted (multiplicative random effects)
MR Egger
MR RAPS
Weighted median
Weighted mode
0.00
−0.05
0.09
0.12
0.15
0.18
SNP effect on Progonadoliberin−1
SNP effect on Gut microbiota abundance (genus Catenibacterium id.2153)

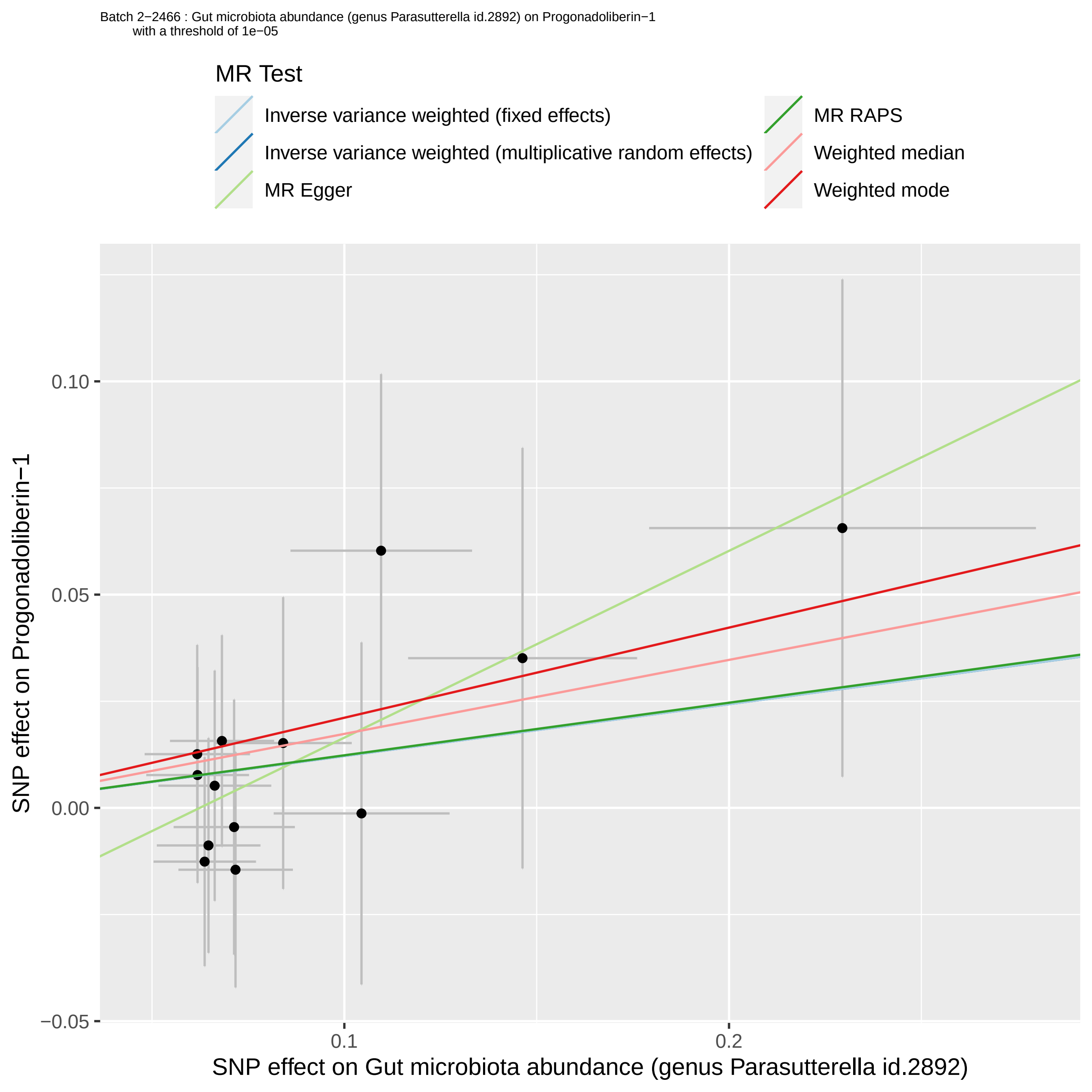
Batch 2−2466 : Gut microbiota abundance (genus Parasutterella id.2892) on Progonadoliberin−1
with a threshold of 1e−05
MR Test
Inverse variance weighted (fixed effects)
Inverse variance weighted (multiplicative random effects)
MR Egger
MR RAPS
Weighted median
Weighted mode
SNP effect on Progonadoliberin−1
0.10
0.05
0.00
−0.05
0.1
0.2
SNP effect on Gut microbiota abundance (genus Parasutterella id.2892)

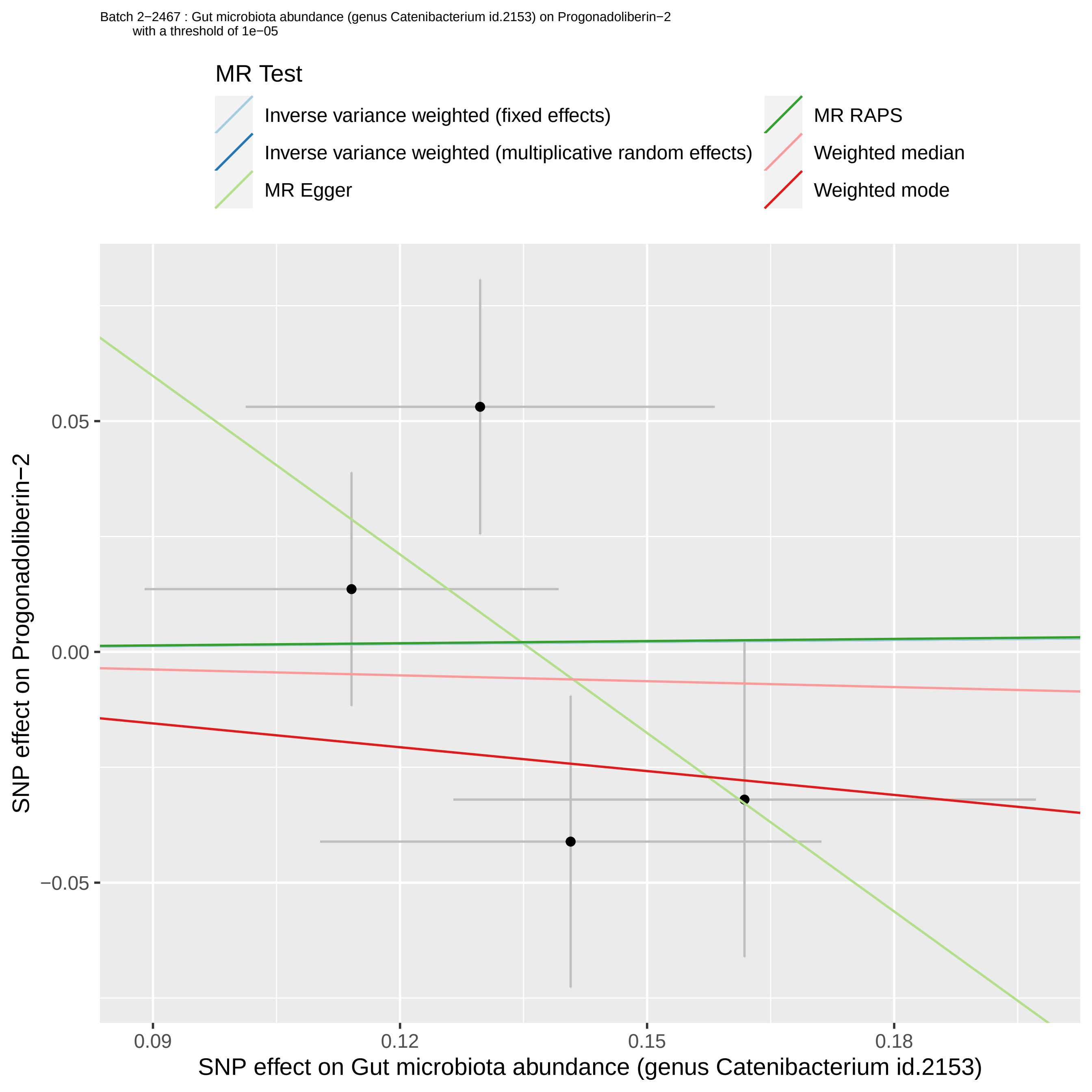

Batch 2−2467 : Gut microbiota abundance (genus Catenibacterium id.2153) on Progonadoliberin−2
with a threshold of 1e−05
MR Test
Inverse variance weighted (fixed effects)
Inverse variance weighted (multiplicative random effects)
MR Egger
MR RAPS
Weighted median
Weighted mode
0.05
0.00
−0.05
0.09
0.12
0.15
0.18
SNP effect on Progonadoliberin−2
SNP effect on Gut microbiota abundance (genus Catenibacterium id.2153)

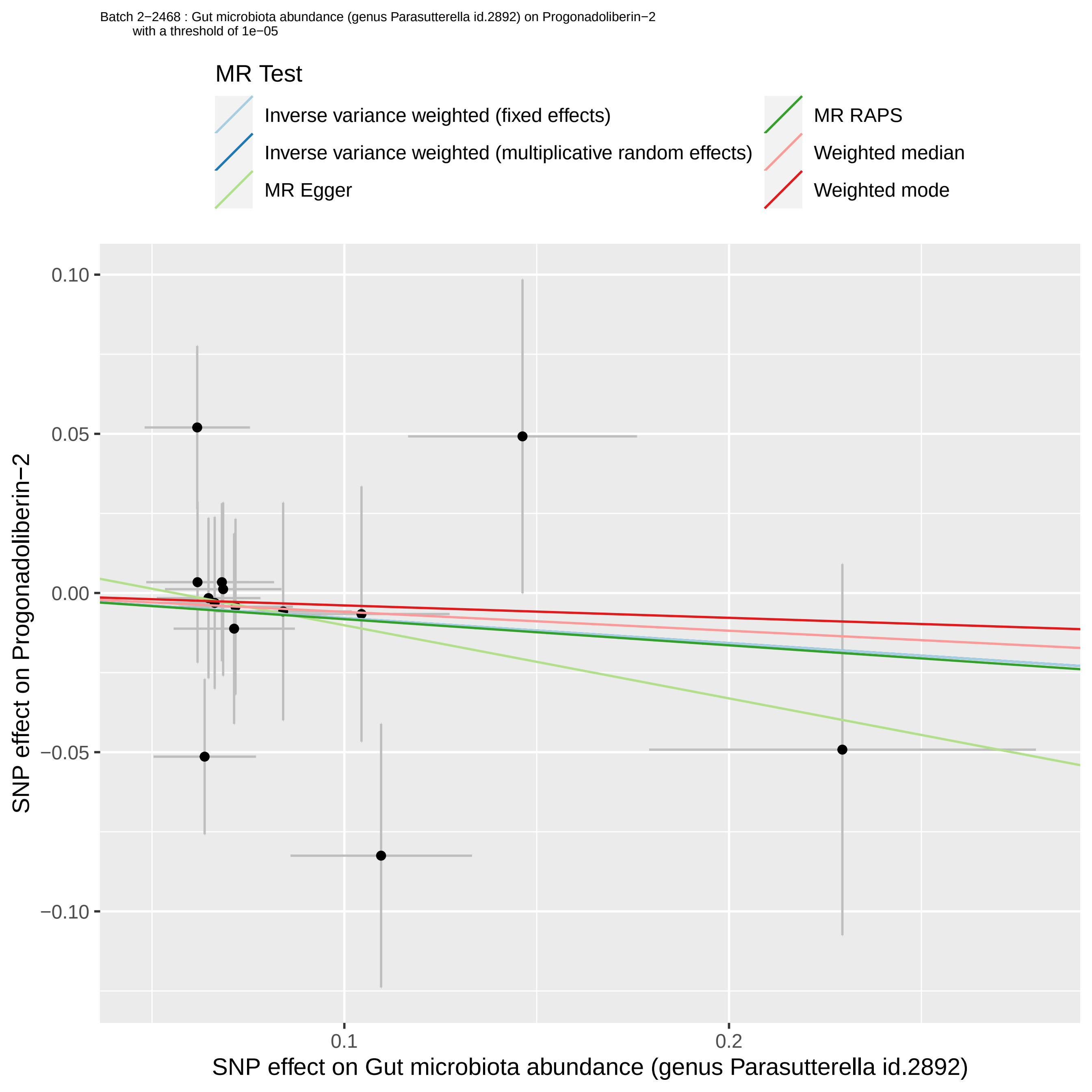
Batch 2−2468 : Gut microbiota abundance (genus Parasutterella id.2892) on Progonadoliberin−2
with a threshold of 1e−05
MR Test
Inverse variance weighted (fixed effects)
Inverse variance weighted (multiplicative random effects)
MR Egger
MR RAPS
Weighted median
Weighted mode
0.10
0.05
0.00
−0.05
−0.10
0.1
0.2
SNP effect on Progonadoliberin−2
SNP effect on Gut microbiota abundance (genus Parasutterella id.2892)

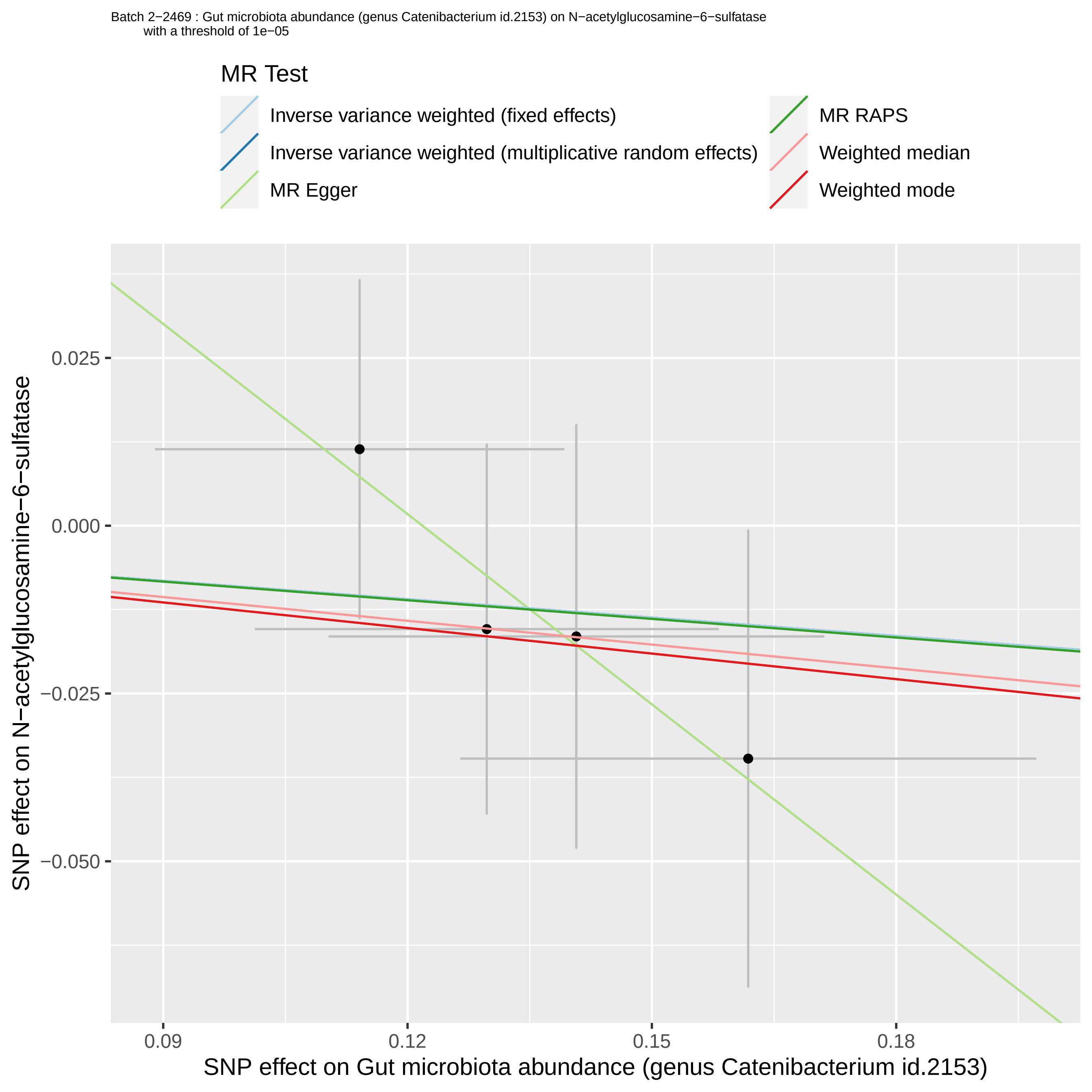

Batch 2−2469 : Gut microbiota abundance (genus Catenibacterium id.2153) on N−acetylglucosamine−6−sulfatase
with a threshold of 1e−05
MR Test
Inverse variance weighted (fixed effects)
Inverse variance weighted (multiplicative random effects)
MR Egger
MR RAPS
Weighted median
Weighted mode
0.025
0.000
−0.025
−0.050
0.09
0.12
0.15
0.18
SNP effect on N−acetylglucosamine−6−sulfatase
SNP effect on Gut microbiota abundance (genus Catenibacterium id.2153)

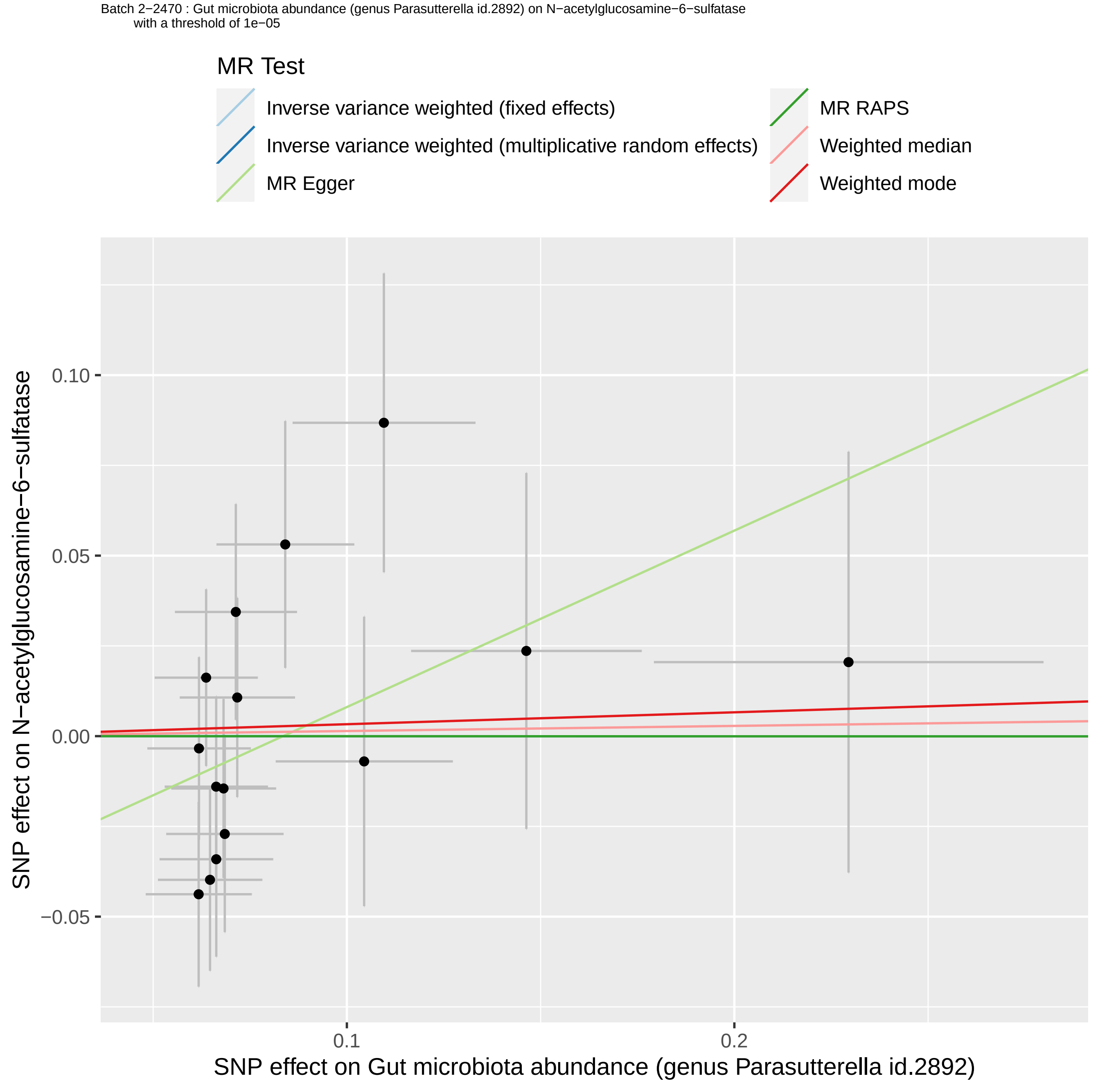

Batch 2−2470 : Gut microbiota abundance (genus Parasutterella id.2892) on N−acetylglucosamine−6−sulfatase
with a threshold of 1e−05
MR Test
Inverse variance weighted (fixed effects)
Inverse variance weighted (multiplicative random effects)
MR Egger
MR RAPS
Weighted median
Weighted mode
SNP effect on N−acetylglucosamine−6−sulfatase
0.10
0.05
0.00
−0.05
0.1
0.2
SNP effect on Gut microbiota abundance (genus Parasutterella id.2892)

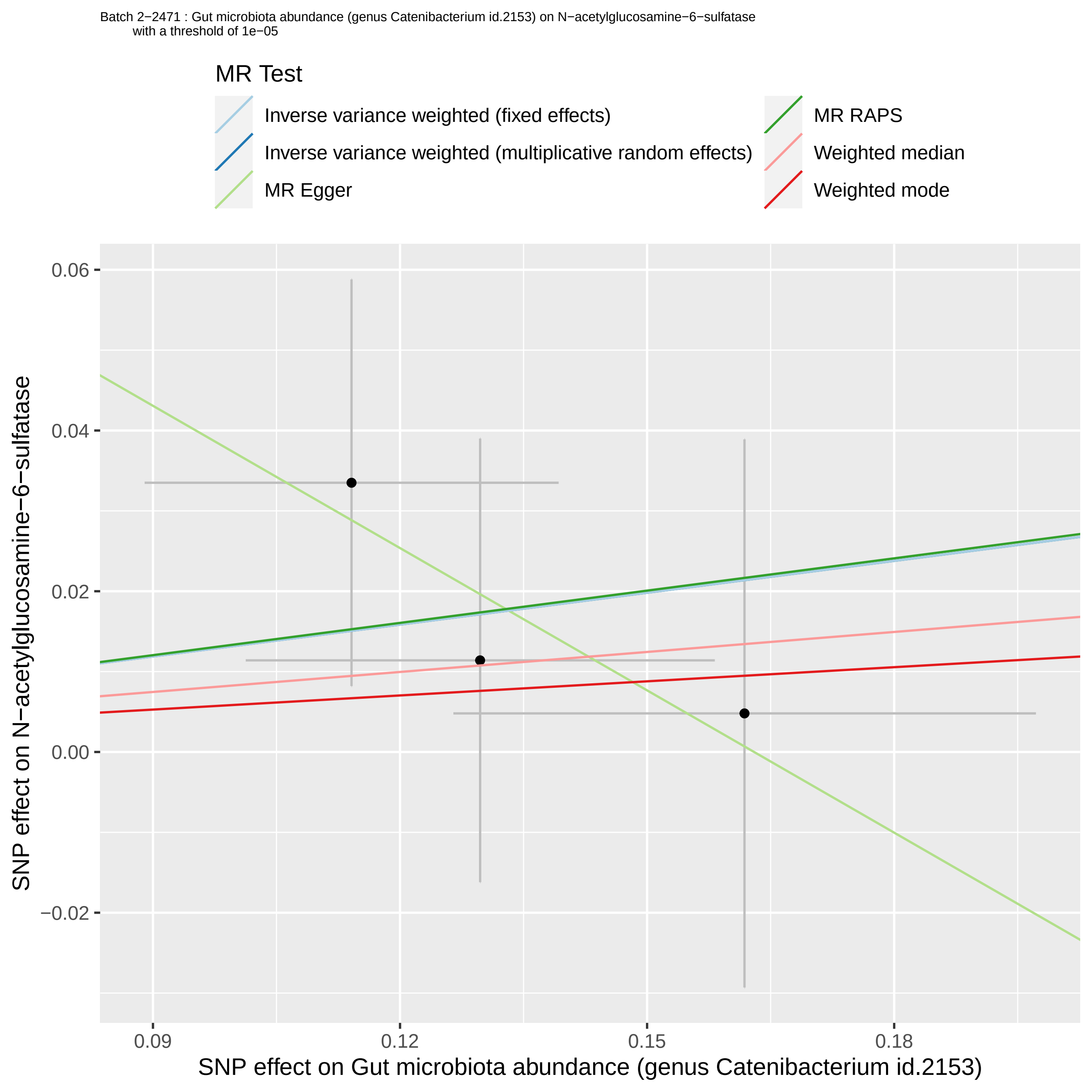
Batch 2−2471 : Gut microbiota abundance (genus Catenibacterium id.2153) on N−acetylglucosamine−6−sulfatase
with a threshold of 1e−05
MR Test
Inverse variance weighted (fixed effects)
Inverse variance weighted (multiplicative random effects)
MR Egger
MR RAPS
Weighted median
Weighted mode
0.06
0.04
0.02
0.00
−0.02
SNP effect on N−acetylglucosamine−6−sulfatase
0.09
0.12
0.15
0.18
SNP effect on Gut microbiota abundance (genus Catenibacterium id.2153)

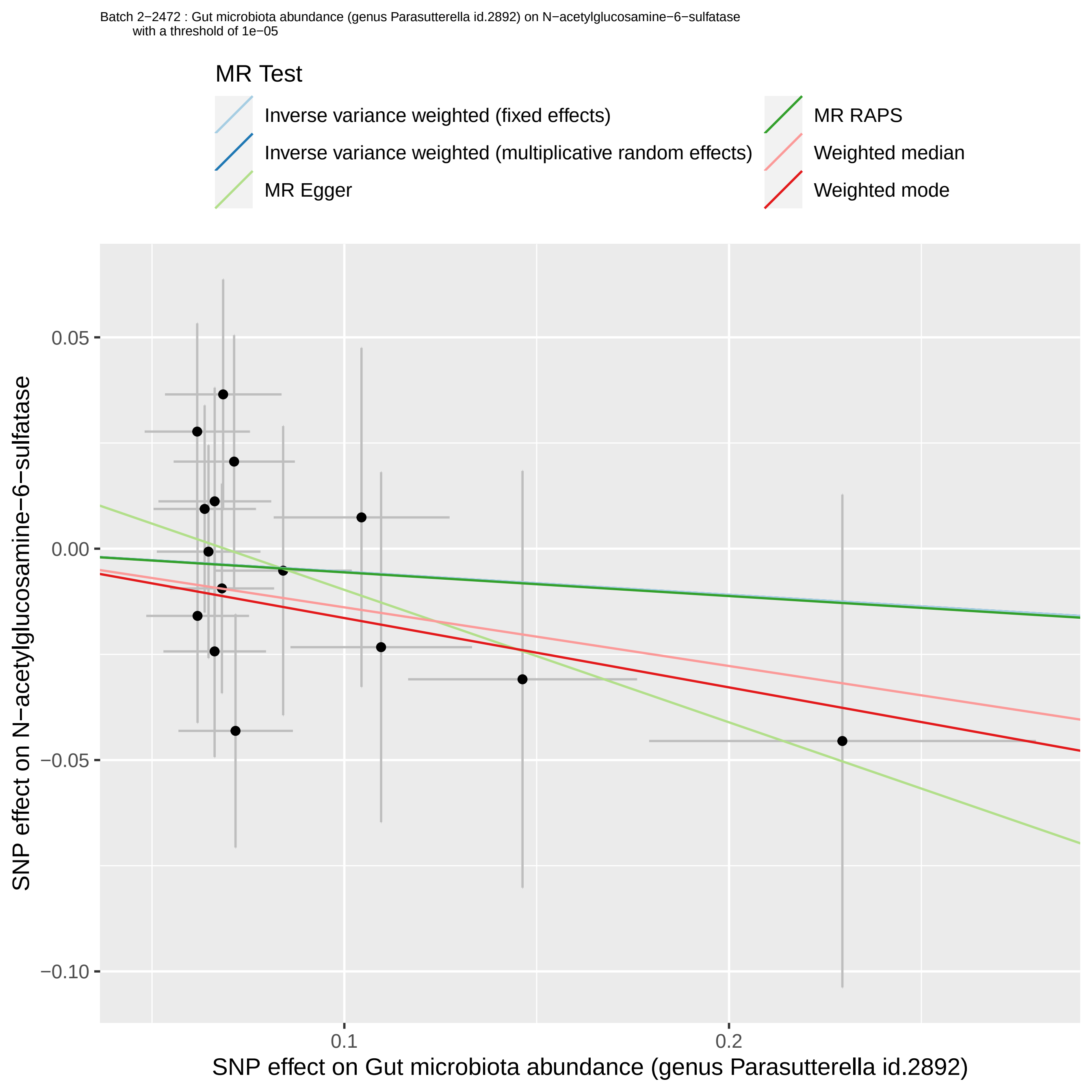
Batch 2−2472 : Gut microbiota abundance (genus Parasutterella id.2892) on N−acetylglucosamine−6−sulfatase
with a threshold of 1e−05
MR Test
Inverse variance weighted (fixed effects)
Inverse variance weighted (multiplicative random effects)
MR Egger
MR RAPS
Weighted median
Weighted mode
0.05
0.00
−0.05
−0.10
0.1
0.2
SNP effect on N−acetylglucosamine−6−sulfatase
SNP effect on Gut microbiota abundance (genus Parasutterella id.2892)

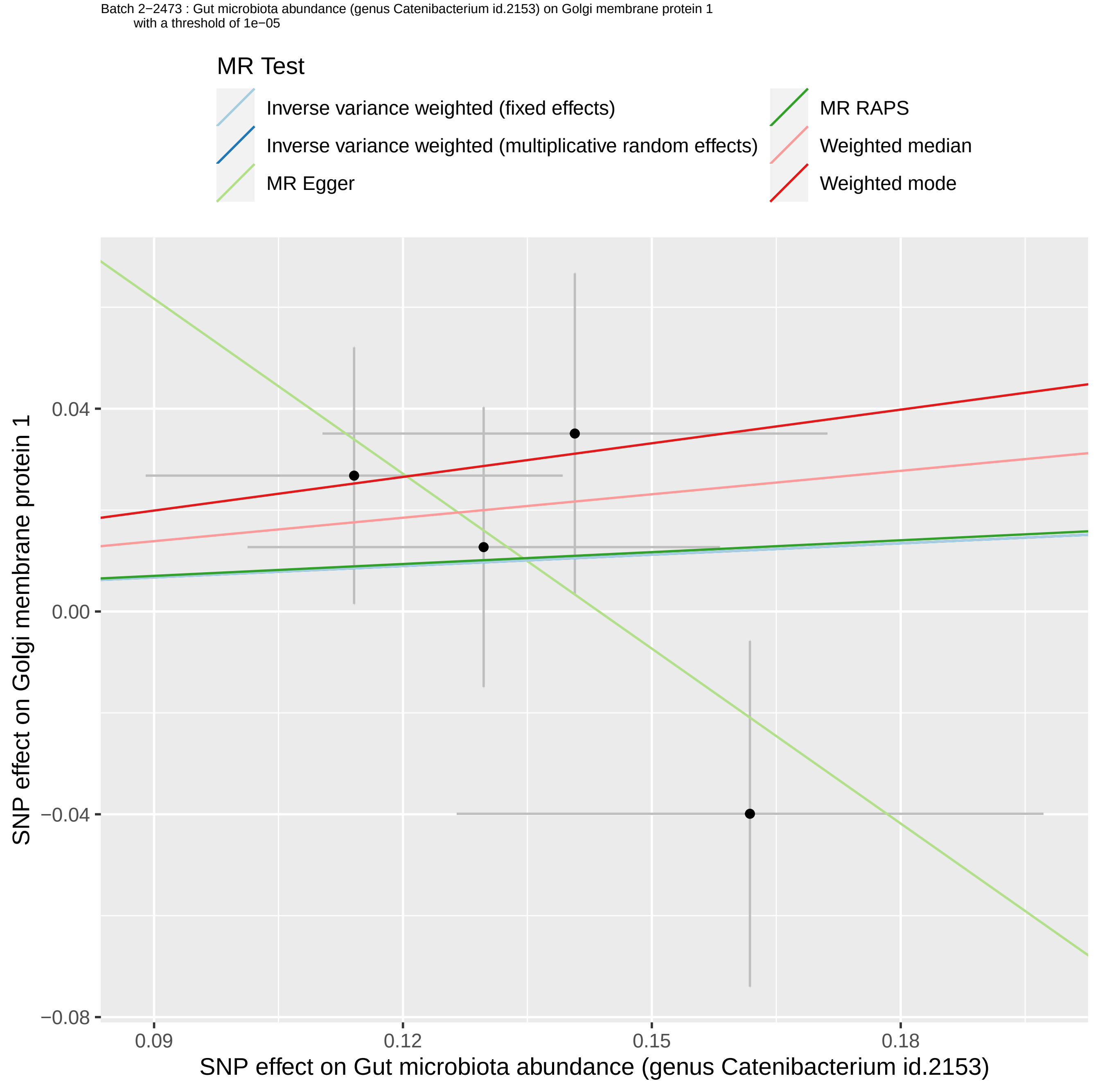
Batch 2−2473 : Gut microbiota abundance (genus Catenibacterium id.2153) on Golgi membrane protein 1
with a threshold of 1e−05
MR Test
Inverse variance weighted (fixed effects)
Inverse variance weighted (multiplicative random effects)
MR Egger
MR RAPS
Weighted median
Weighted mode
0.04
0.00
−0.04
−0.08
0.09
0.12
0.15
0.18
SNP effect on Golgi membrane protein 1
SNP effect on Gut microbiota abundance (genus Catenibacterium id.2153)

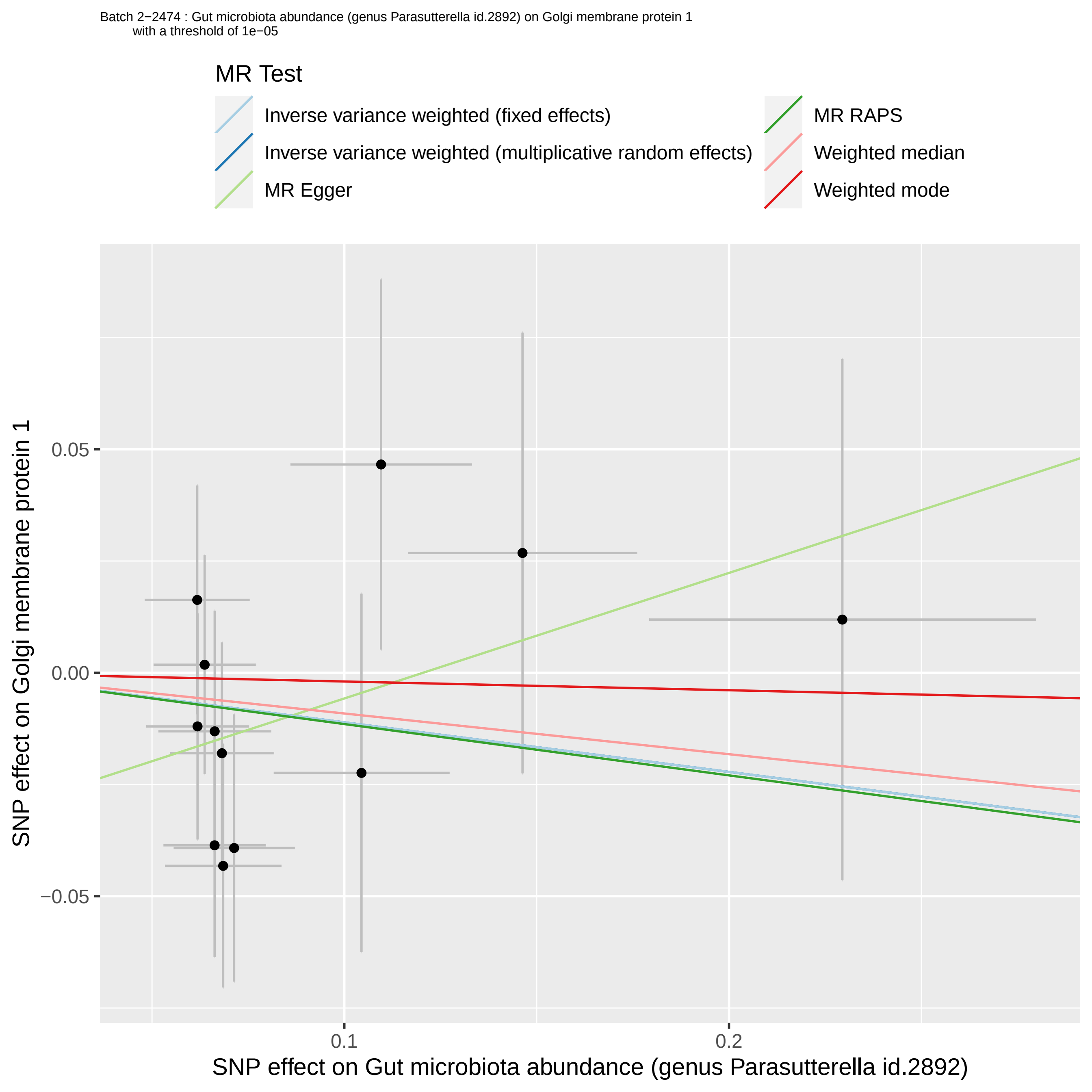
Batch 2−2474 : Gut microbiota abundance (genus Parasutterella id.2892) on Golgi membrane protein 1
with a threshold of 1e−05
MR Test
Inverse variance weighted (fixed effects)
Inverse variance weighted (multiplicative random effects)
MR Egger
MR RAPS
Weighted median
Weighted mode
0.05
0.00
−0.05
0.1
0.2
SNP effect on Golgi membrane protein 1
SNP effect on Gut microbiota abundance (genus Parasutterella id.2892)

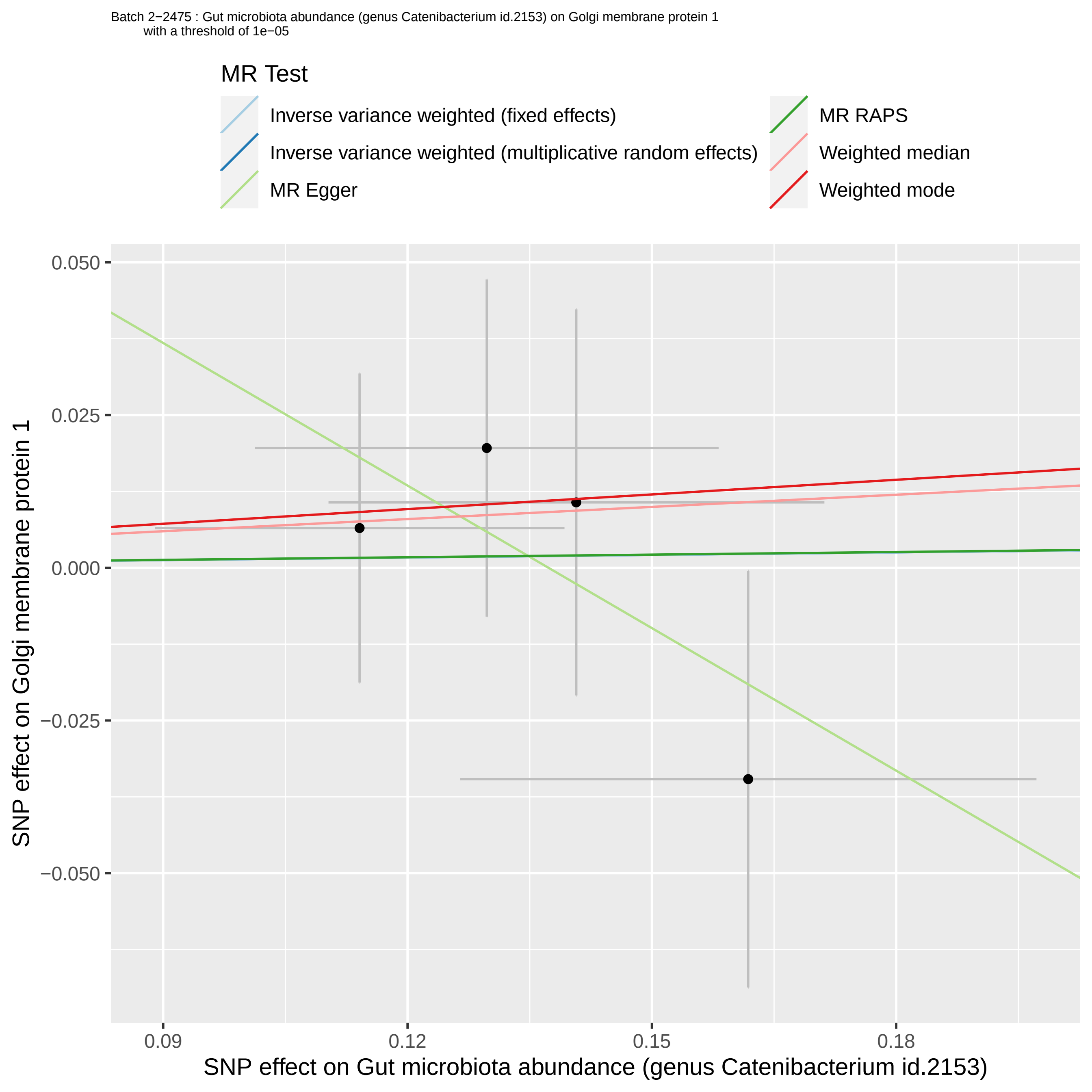
Batch 2−2475 : Gut microbiota abundance (genus Catenibacterium id.2153) on Golgi membrane protein 1
with a threshold of 1e−05
MR Test
Inverse variance weighted (fixed effects)
Inverse variance weighted (multiplicative random effects)
MR Egger
MR RAPS
Weighted median
Weighted mode
0.050
0.025
0.000
−0.025
−0.050
0.09
0.12
0.15
0.18
SNP effect on Golgi membrane protein 1
SNP effect on Gut microbiota abundance (genus Catenibacterium id.2153)

Batch 2−2476 : Gut microbiota abundance (genus Parasutterella id.2892) on Golgi membrane protein 1
with a threshold of 1e−05

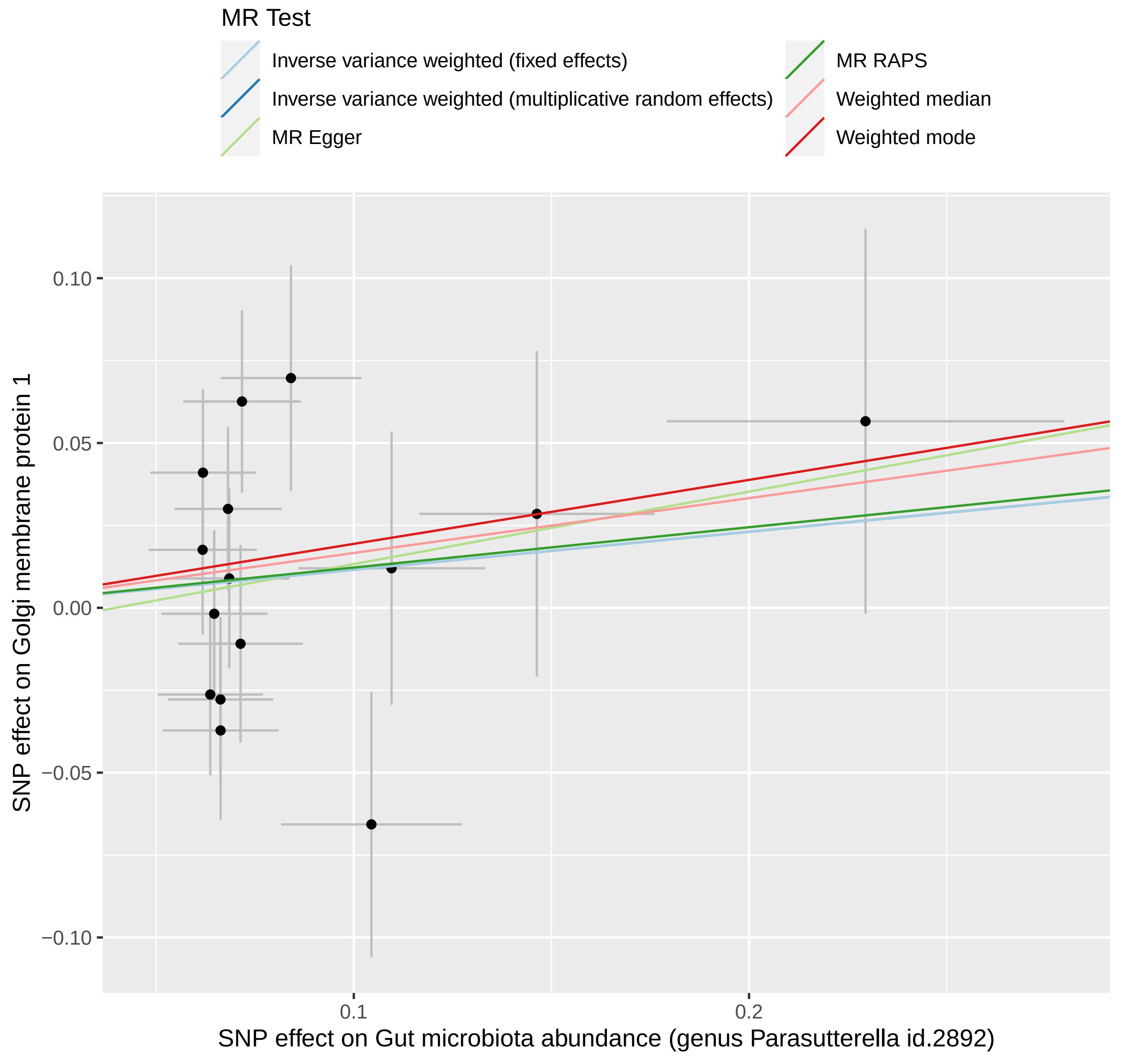

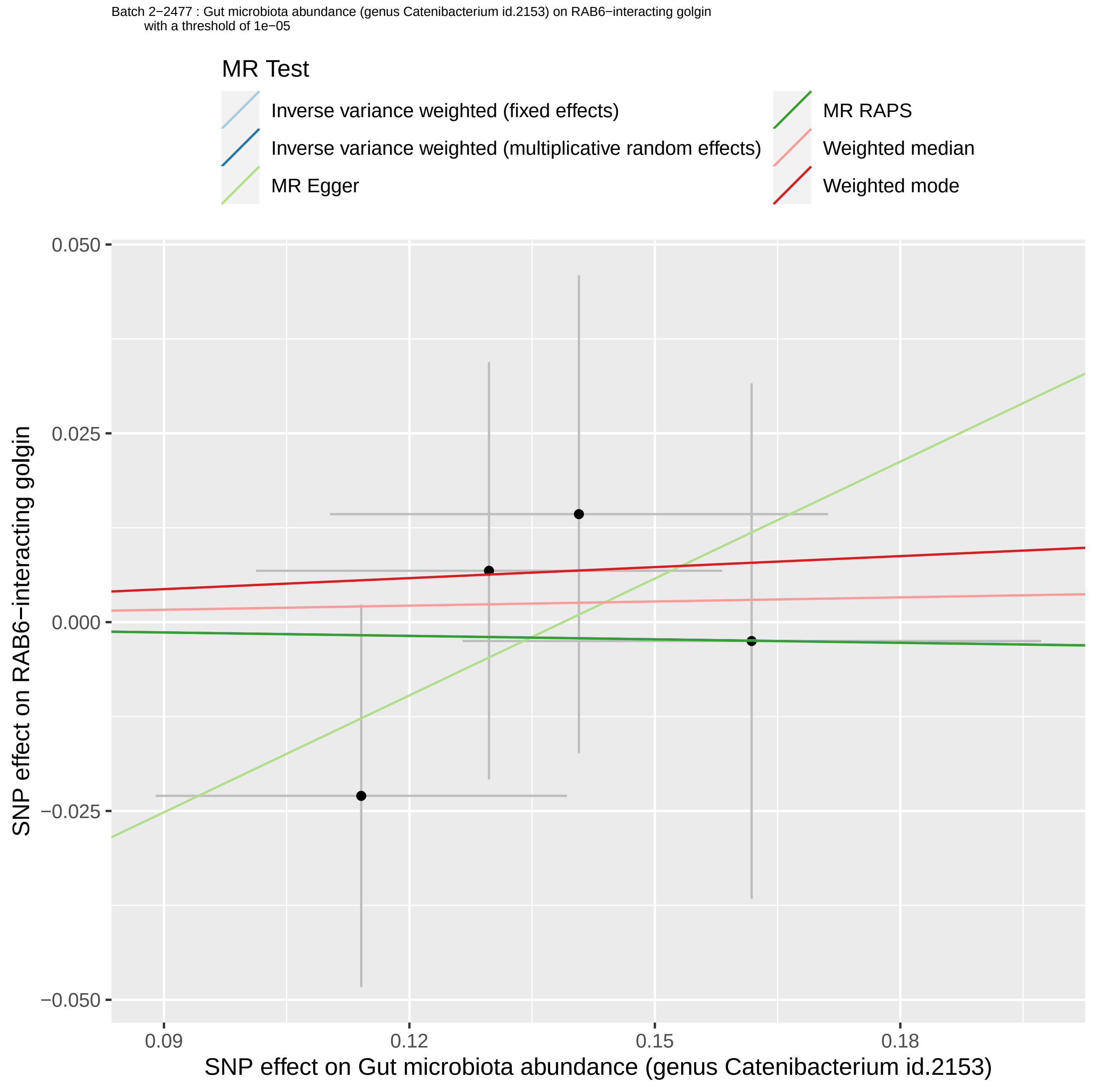

Batch 2−2477 : Gut microbiota abundance (genus Catenibacterium id.2153) on RAB6−interacting golgin
with a threshold of 1e−05
MR Test
Inverse variance weighted (fixed effects)
Inverse variance weighted (multiplicative random effects)
MR Egger
MR RAPS
Weighted median
Weighted mode
0.050
0.025
0.000
−0.025
−0.050
SNP effect on RAB6−interacting golgin
0.09
0.12
0.15
0.18
SNP effect on Gut microbiota abundance (genus Catenibacterium id.2153)

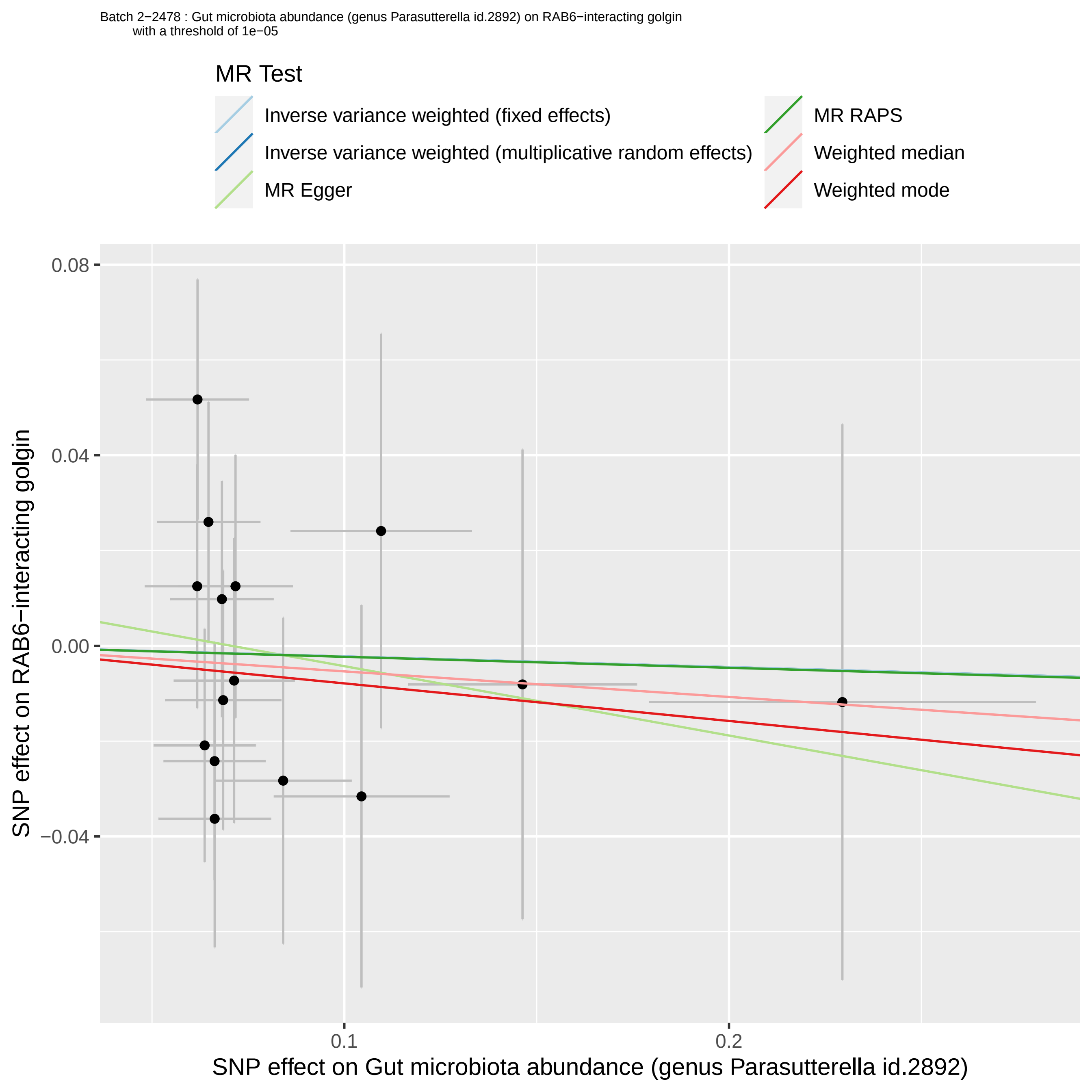

Batch 2−2478 : Gut microbiota abundance (genus Parasutterella id.2892) on RAB6−interacting golgin
with a threshold of 1e−05
MR Test
Inverse variance weighted (fixed effects)
Inverse variance weighted (multiplicative random effects)
MR Egger
MR RAPS
Weighted median
Weighted mode
0.08
0.04
0.00
−0.04
0.1
0.2
SNP effect on RAB6−interacting golgin
SNP effect on Gut microbiota abundance (genus Parasutterella id.2892)

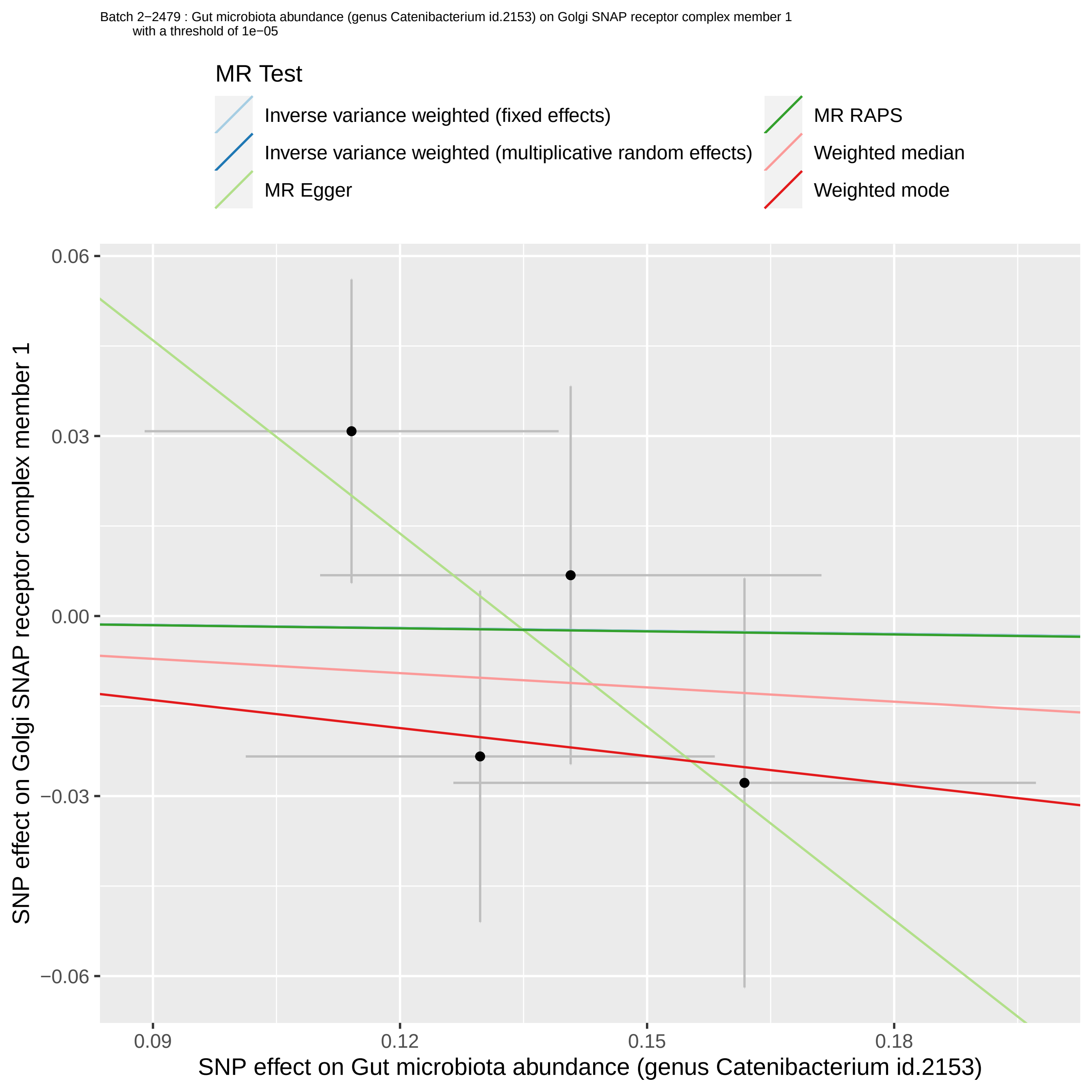

Batch 2−2479 : Gut microbiota abundance (genus Catenibacterium id.2153) on Golgi SNAP receptor complex member 1
with a threshold of 1e−05
MR Test
Inverse variance weighted (fixed effects)
Inverse variance weighted (multiplicative random effects)
MR Egger
MR RAPS
Weighted median
Weighted mode
0.06
0.03
0.00
−0.03
−0.06
0.09
0.12
0.15
0.18
SNP effect on Golgi SNAP receptor complex member 1
SNP effect on Gut microbiota abundance (genus Catenibacterium id.2153)

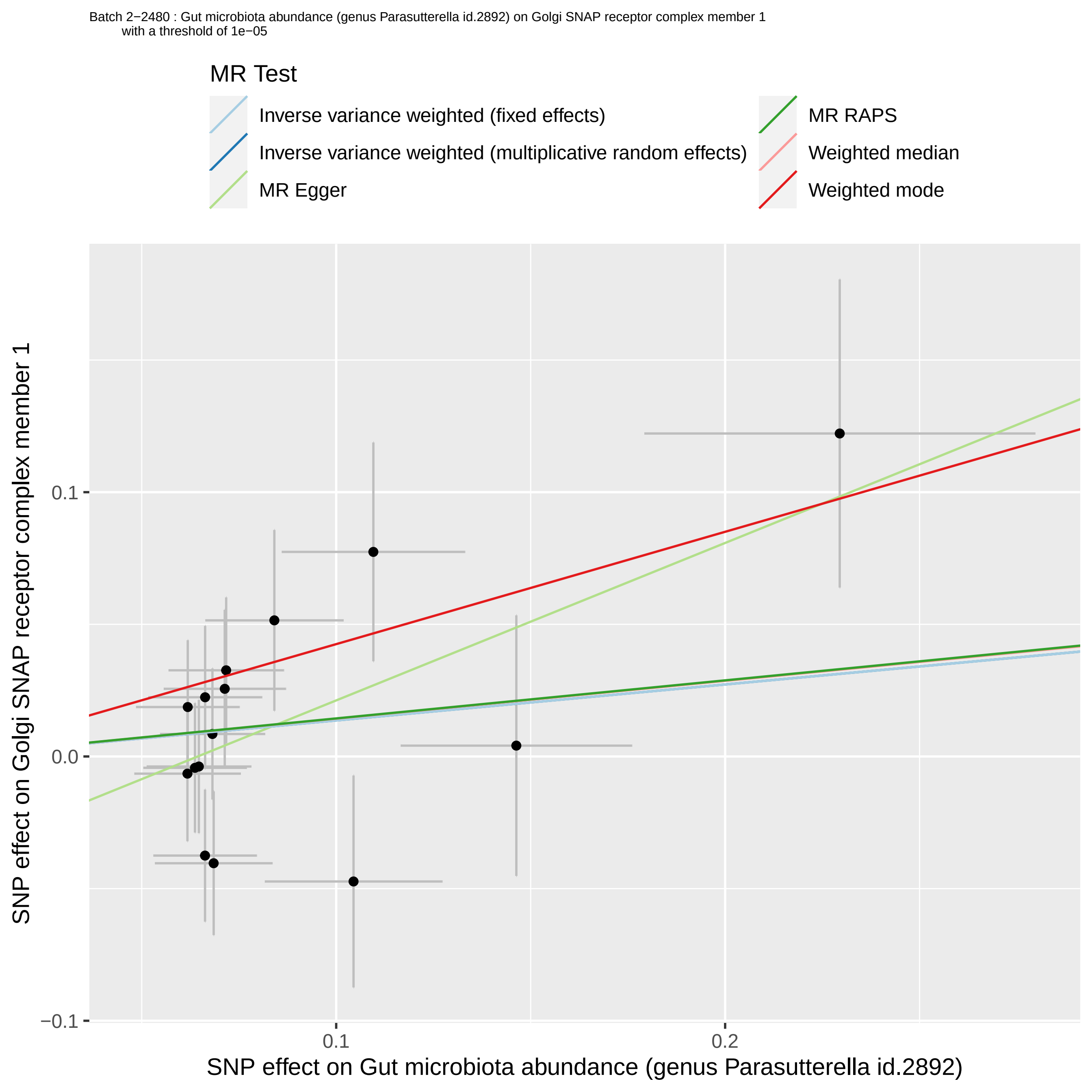
Batch 2−2480 : Gut microbiota abundance (genus Parasutterella id.2892) on Golgi SNAP receptor complex member 1
with a threshold of 1e−05
MR Test
Inverse variance weighted (fixed effects)
Inverse variance weighted (multiplicative random effects)
MR Egger
MR RAPS
Weighted median
Weighted mode
SNP effect on Golgi SNAP receptor complex member 1
0.1
0.0
−0.1
0.1
0.2
SNP effect on Gut microbiota abundance (genus Parasutterella id.2892)

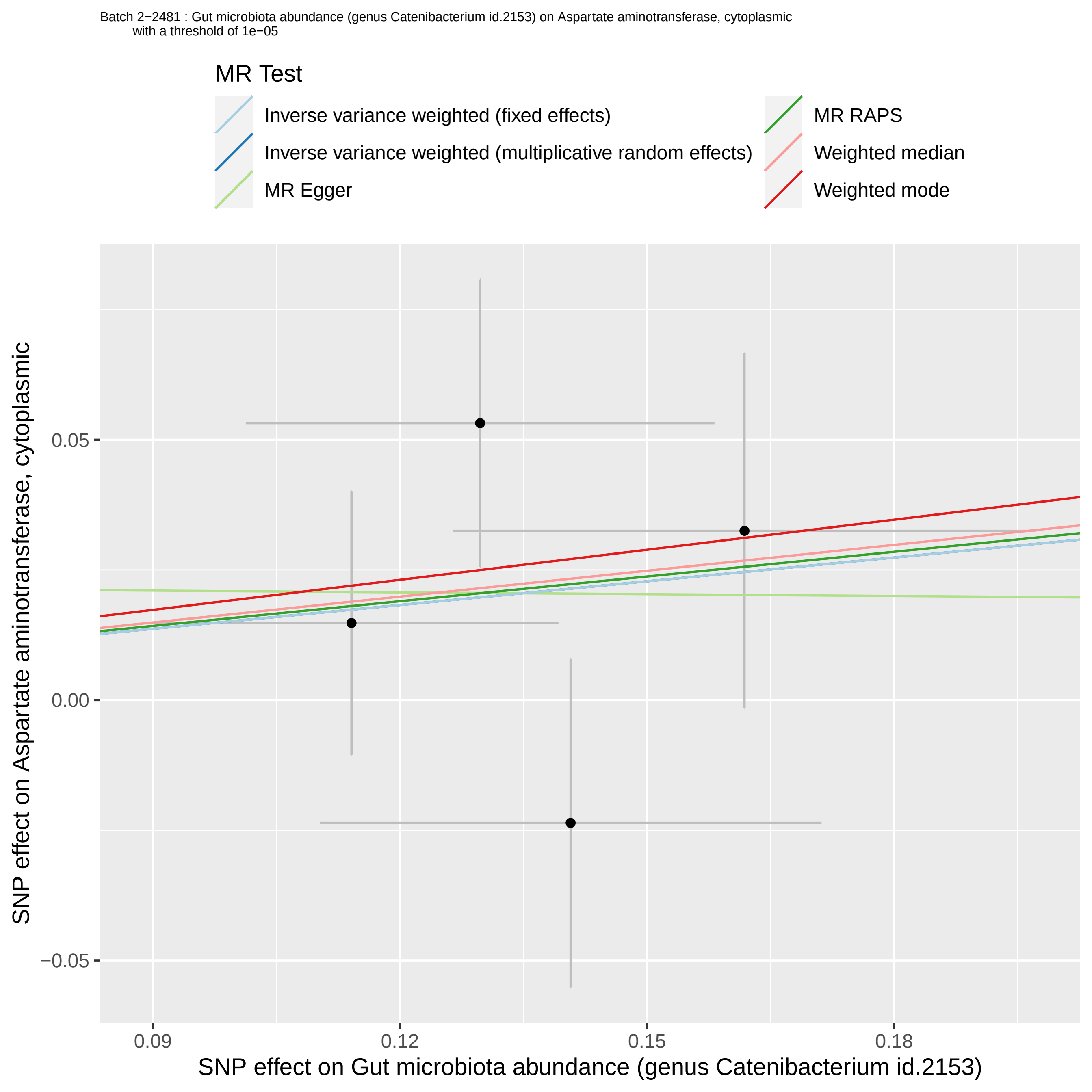
Batch 2−2481 : Gut microbiota abundance (genus Catenibacterium id.2153) on Aspartate aminotransferase, cytoplasmic
with a threshold of 1e−05
MR Test
Inverse variance weighted (fixed effects)
Inverse variance weighted (multiplicative random effects)
MR Egger
MR RAPS
Weighted median
Weighted mode
SNP effect on Aspartate aminotransferase, cytoplasmic
0.05
0.00
−0.05
0.09
0.12
0.15
0.18
SNP effect on Gut microbiota abundance (genus Catenibacterium id.2153)

Batch 2−2482 : Gut microbiota abundance (genus Parasutterella id.2892) on Aspartate aminotransferase, cytoplasmic
with a threshold of 1e−05

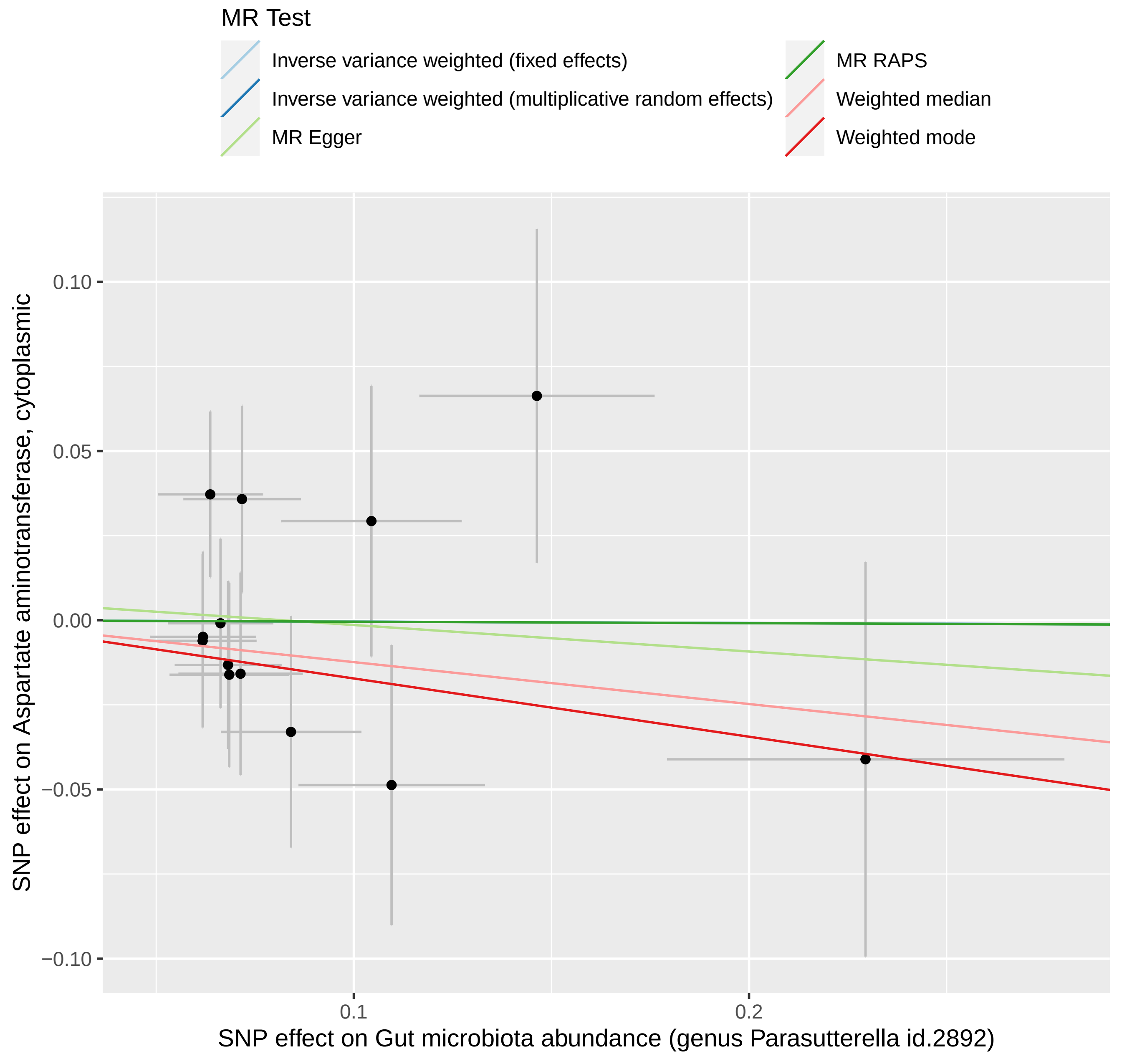

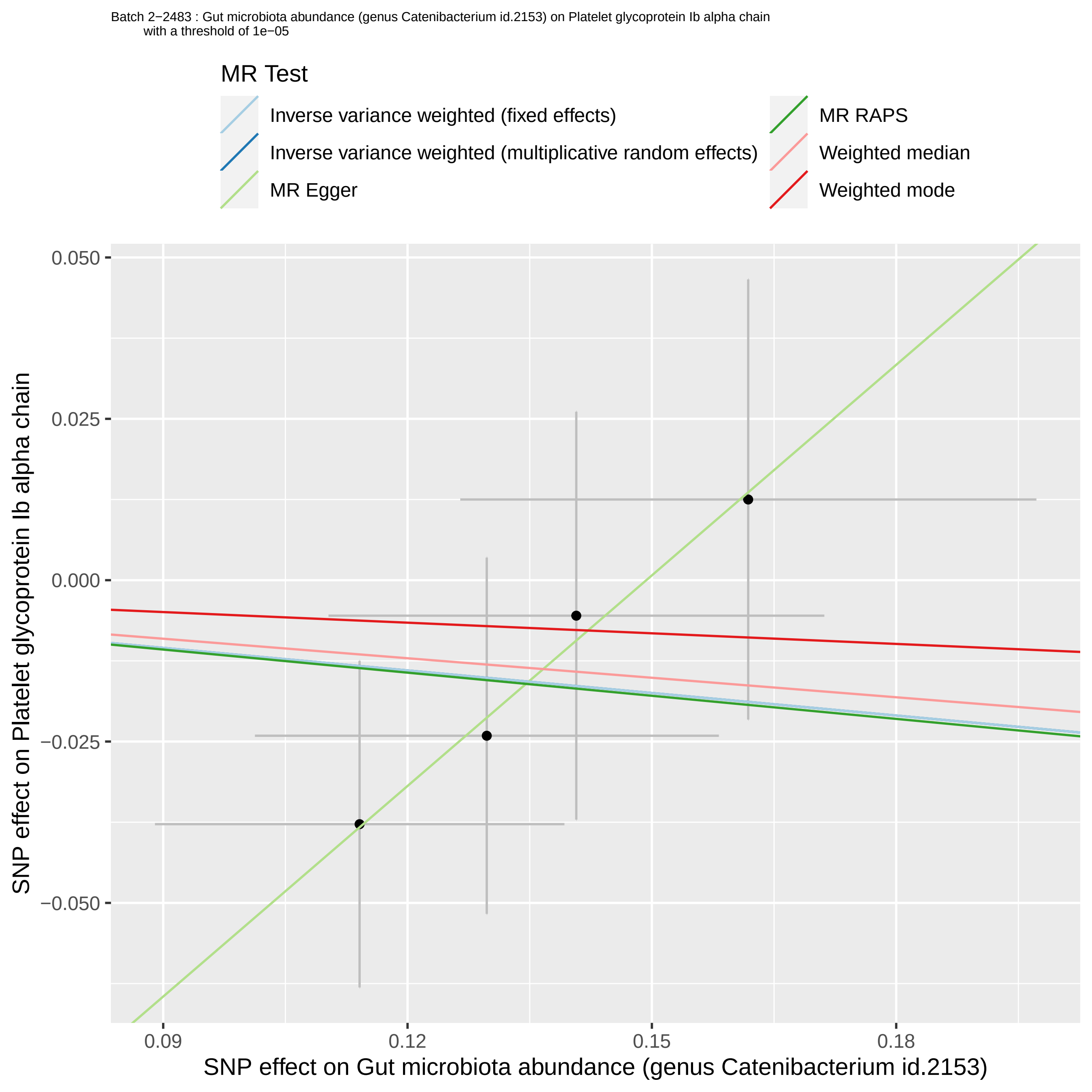

Batch 2−2483 : Gut microbiota abundance (genus Catenibacterium id.2153) on Platelet glycoprotein Ib alpha chain
with a threshold of 1e−05
MR Test
Inverse variance weighted (fixed effects)
Inverse variance weighted (multiplicative random effects)
MR Egger
MR RAPS
Weighted median
Weighted mode
0.050
0.025
0.000
−0.025
−0.050
0.09
0.12
0.15
0.18
SNP effect on Platelet glycoprotein Ib alpha chain
SNP effect on Gut microbiota abundance (genus Catenibacterium id.2153)

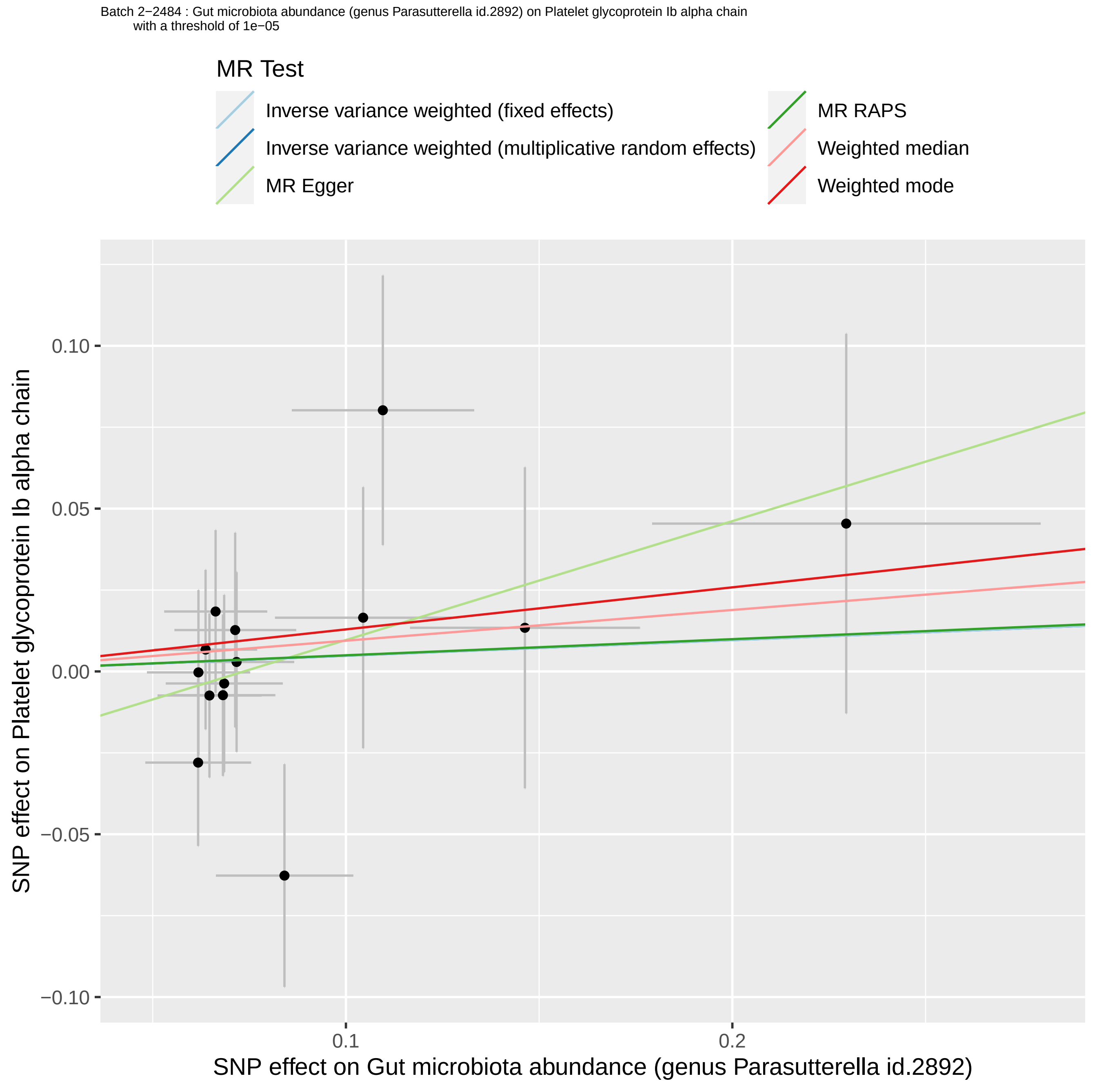
Batch 2−2484 : Gut microbiota abundance (genus Parasutterella id.2892) on Platelet glycoprotein Ib alpha chain
with a threshold of 1e−05
MR Test
Inverse variance weighted (fixed effects)
Inverse variance weighted (multiplicative random effects)
MR Egger
MR RAPS
Weighted median
Weighted mode
SNP effect on Platelet glycoprotein Ib alpha chain
SNP effect on Gut microbiota abundance (genus Parasutterella id.2892)
0.10
0.05
0.00
−0.05
−0.10
0.1
0.2

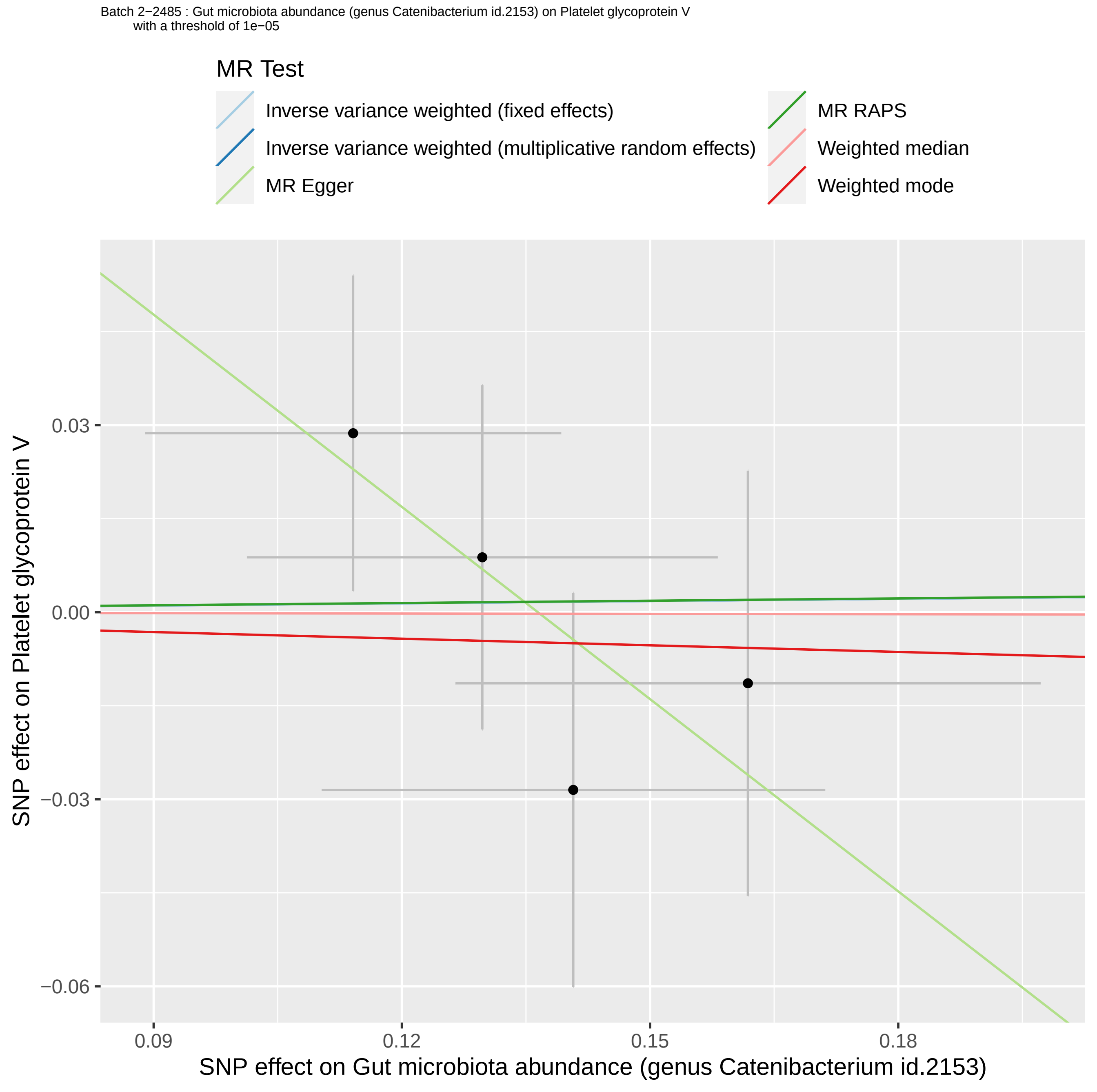
Batch 2−2485 : Gut microbiota abundance (genus Catenibacterium id.2153) on Platelet glycoprotein V
with a threshold of 1e−05
MR Test
Inverse variance weighted (fixed effects)
Inverse variance weighted (multiplicative random effects)
MR Egger
MR RAPS
Weighted median
Weighted mode
0.03
0.00
−0.03
−0.06
0.09
0.12
0.15
0.18
SNP effect on Platelet glycoprotein V
SNP effect on Gut microbiota abundance (genus Catenibacterium id.2153)

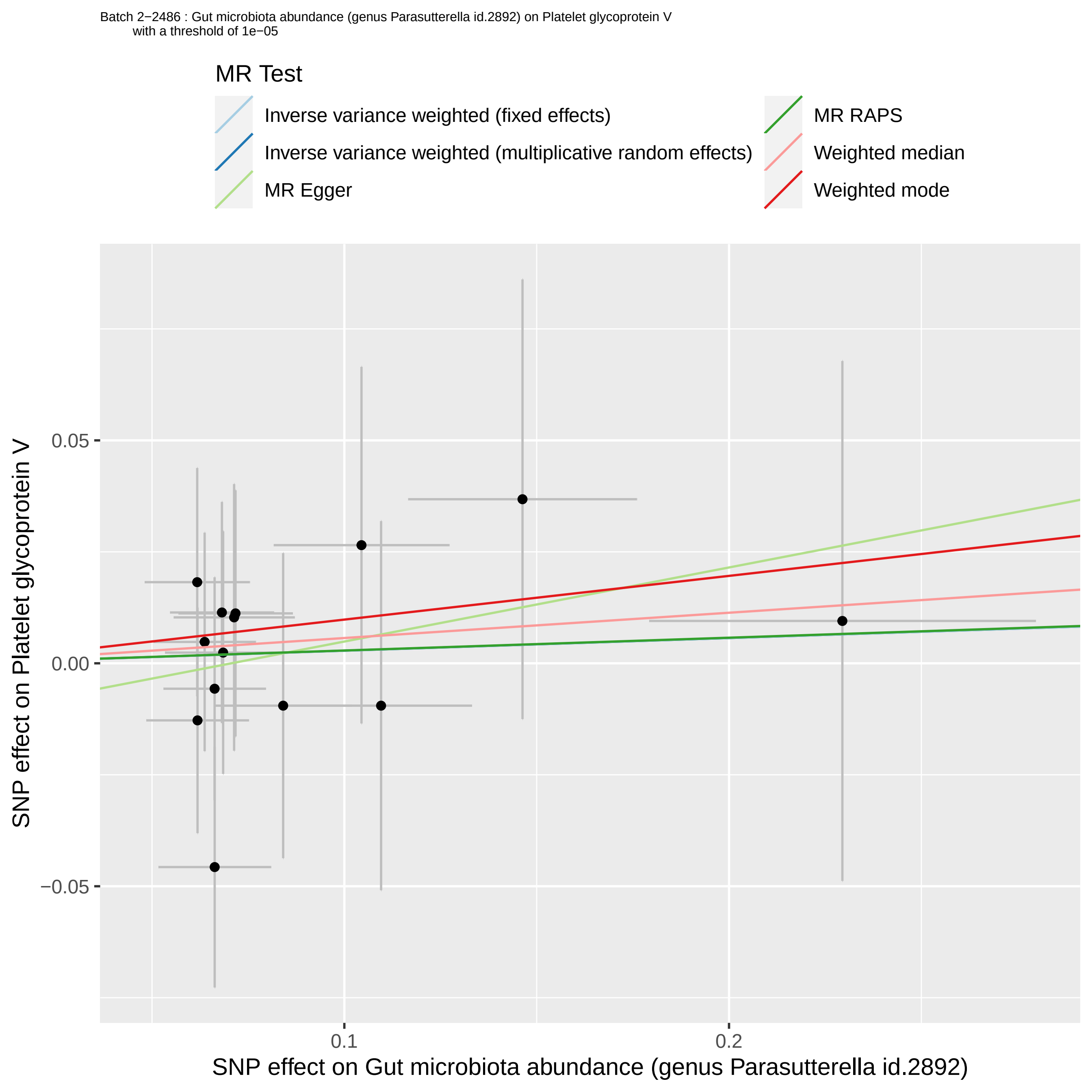
Batch 2−2486 : Gut microbiota abundance (genus Parasutterella id.2892) on Platelet glycoprotein V
with a threshold of 1e−05
MR Test
Inverse variance weighted (fixed effects)
Inverse variance weighted (multiplicative random effects)
MR Egger
MR RAPS
Weighted median
Weighted mode
0.05
0.00
−0.05
0.1
0.2
SNP effect on Platelet glycoprotein V
SNP effect on Gut microbiota abundance (genus Parasutterella id.2892)

Batch 2−2487 : Gut microbiota abundance (genus Catenibacterium id.2153) on Cell surface A33 antigen
with a threshold of 1e−05

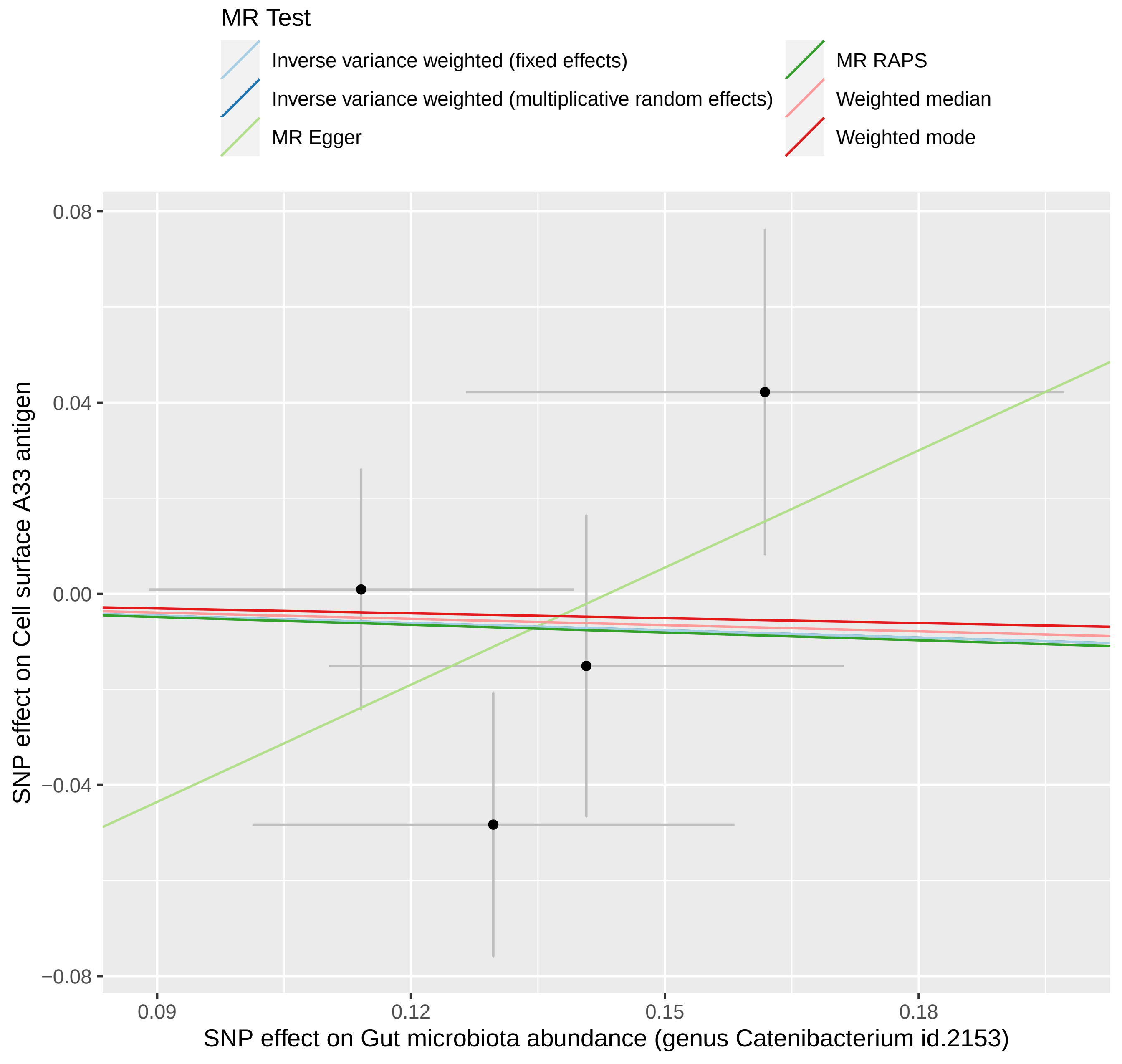

Batch 2−2488 : Gut microbiota abundance (genus Parasutterella id.2892) on Cell surface A33 antigen
with a threshold of 1e−05

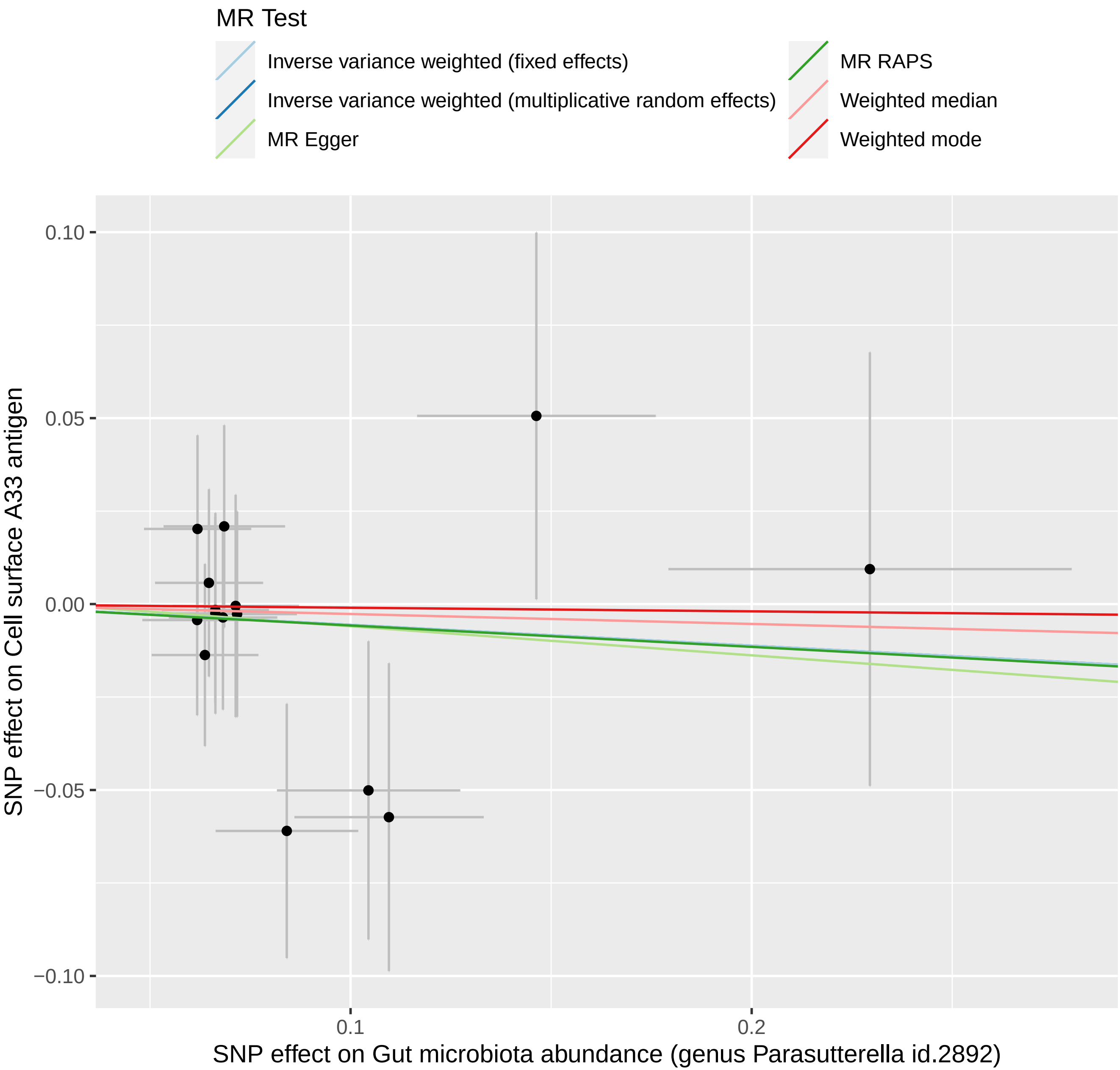

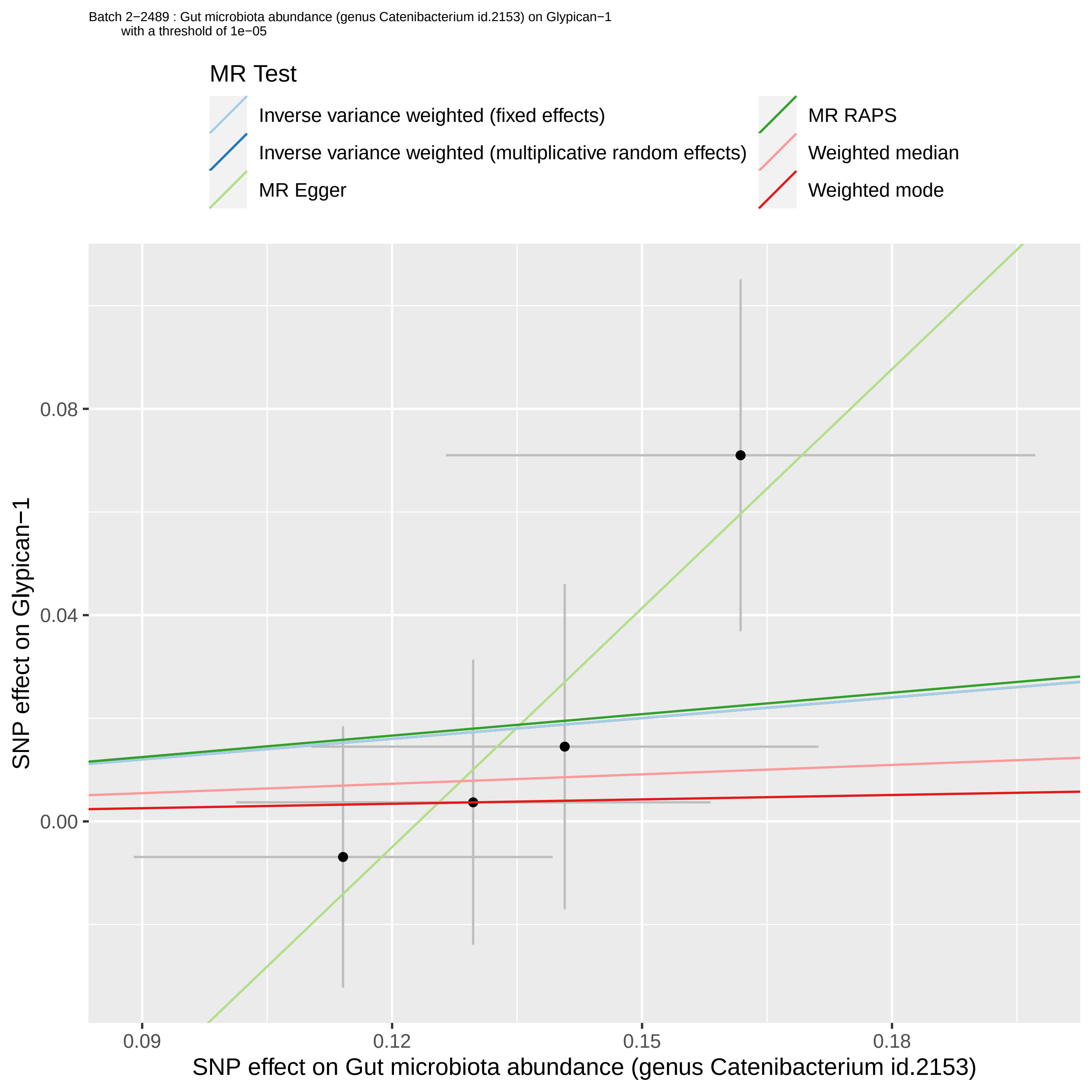

Batch 2−2489 : Gut microbiota abundance (genus Catenibacterium id.2153) on Glypican−1
with a threshold of 1e−05
MR Test
Inverse variance weighted (fixed effects)
Inverse variance weighted (multiplicative random effects)
MR Egger
MR RAPS
Weighted median
Weighted mode
SNP effect on Glypican−1
0.08
0.04
0.00
0.09
0.12
0.15
0.18
SNP effect on Gut microbiota abundance (genus Catenibacterium id.2153)

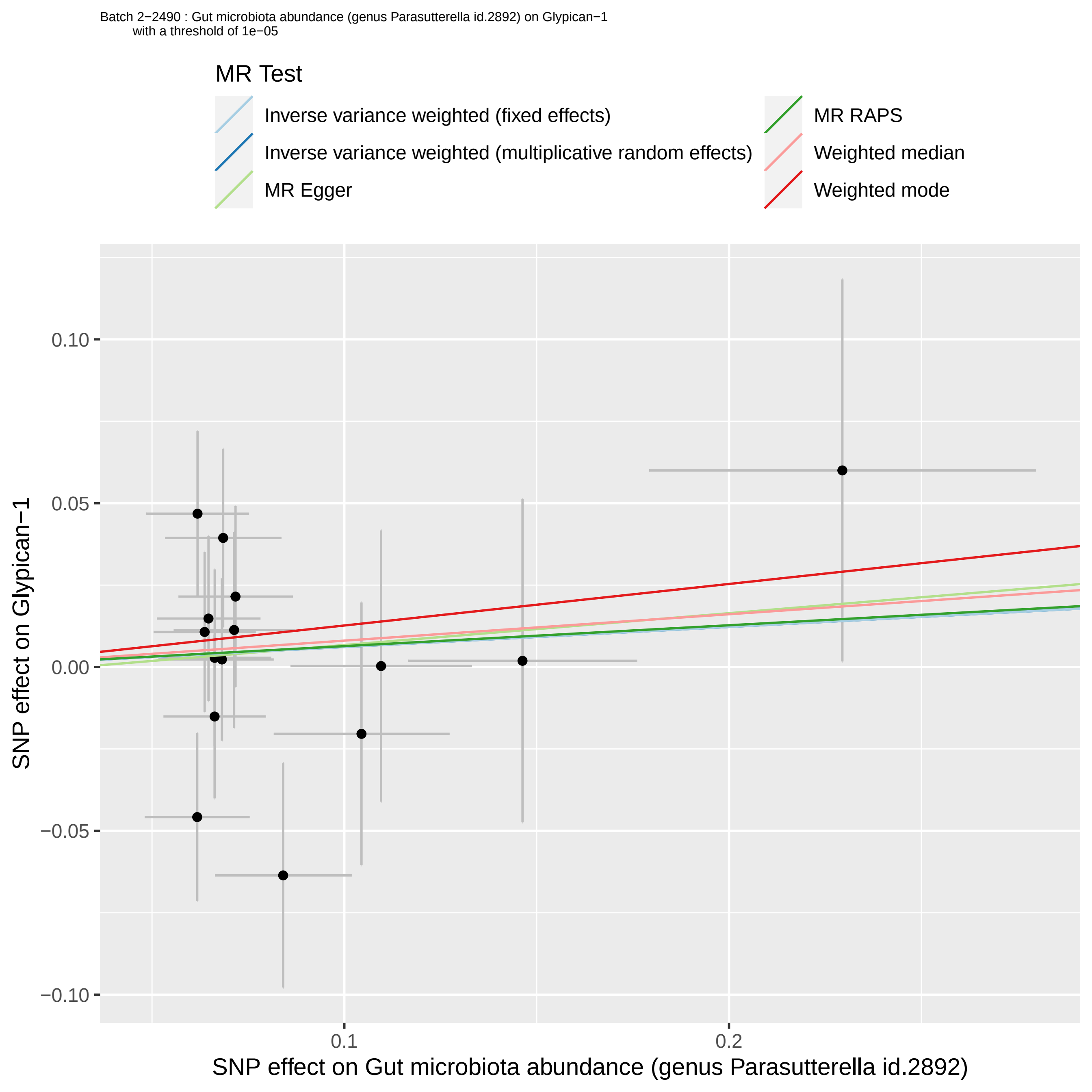

Batch 2−2490 : Gut microbiota abundance (genus Parasutterella id.2892) on Glypican−1
with a threshold of 1e−05
MR Test
Inverse variance weighted (fixed effects)
Inverse variance weighted (multiplicative random effects)
MR Egger
MR RAPS
Weighted median
Weighted mode
0.10
0.05
0.00
−0.05
−0.10
0.1
0.2
SNP effect on Glypican−1
SNP effect on Gut microbiota abundance (genus Parasutterella id.2892)

Batch 2−2491 : Gut microbiota abundance (genus Catenibacterium id.2153) on Glypican−3
with a threshold of 1e−05

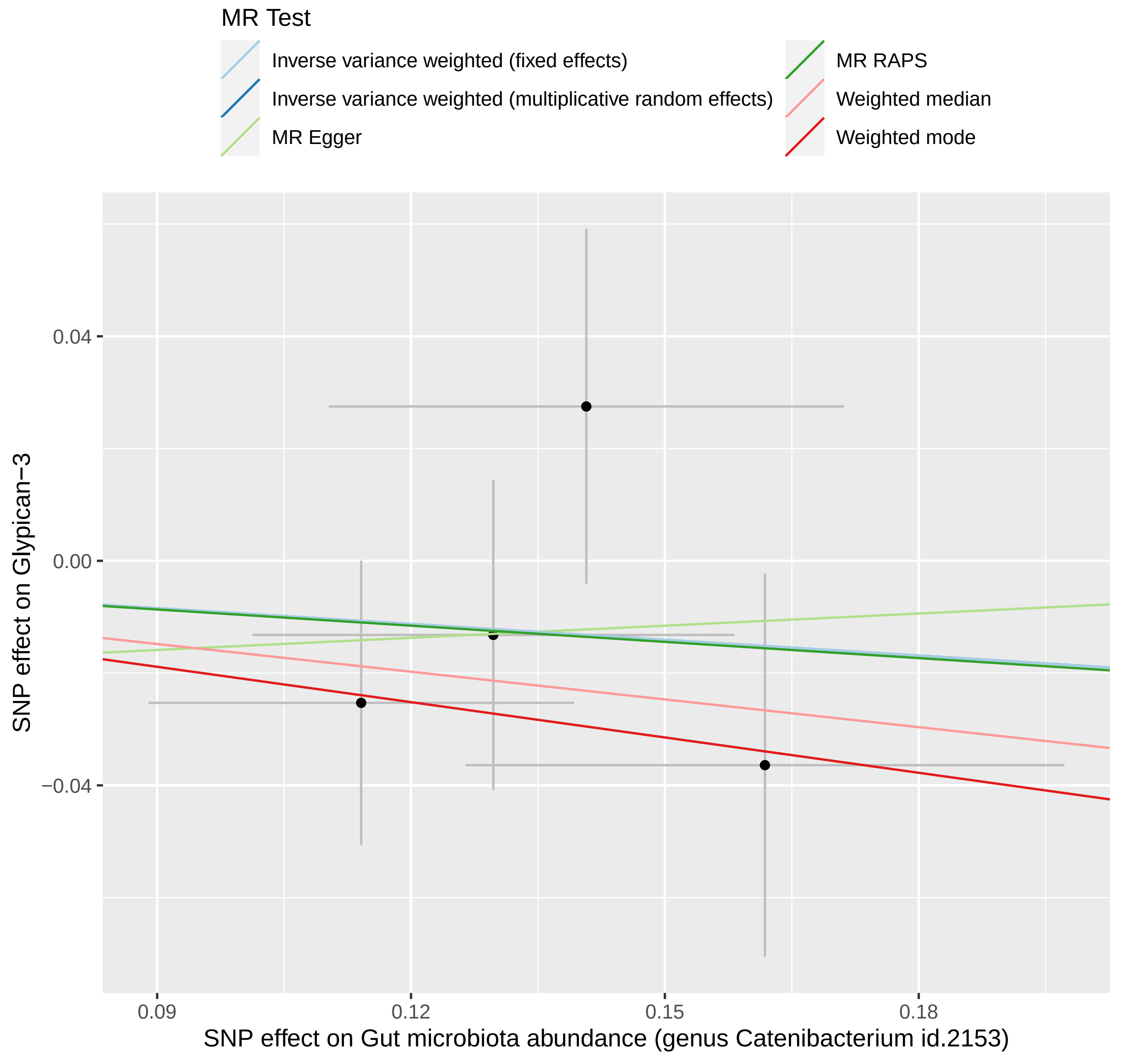

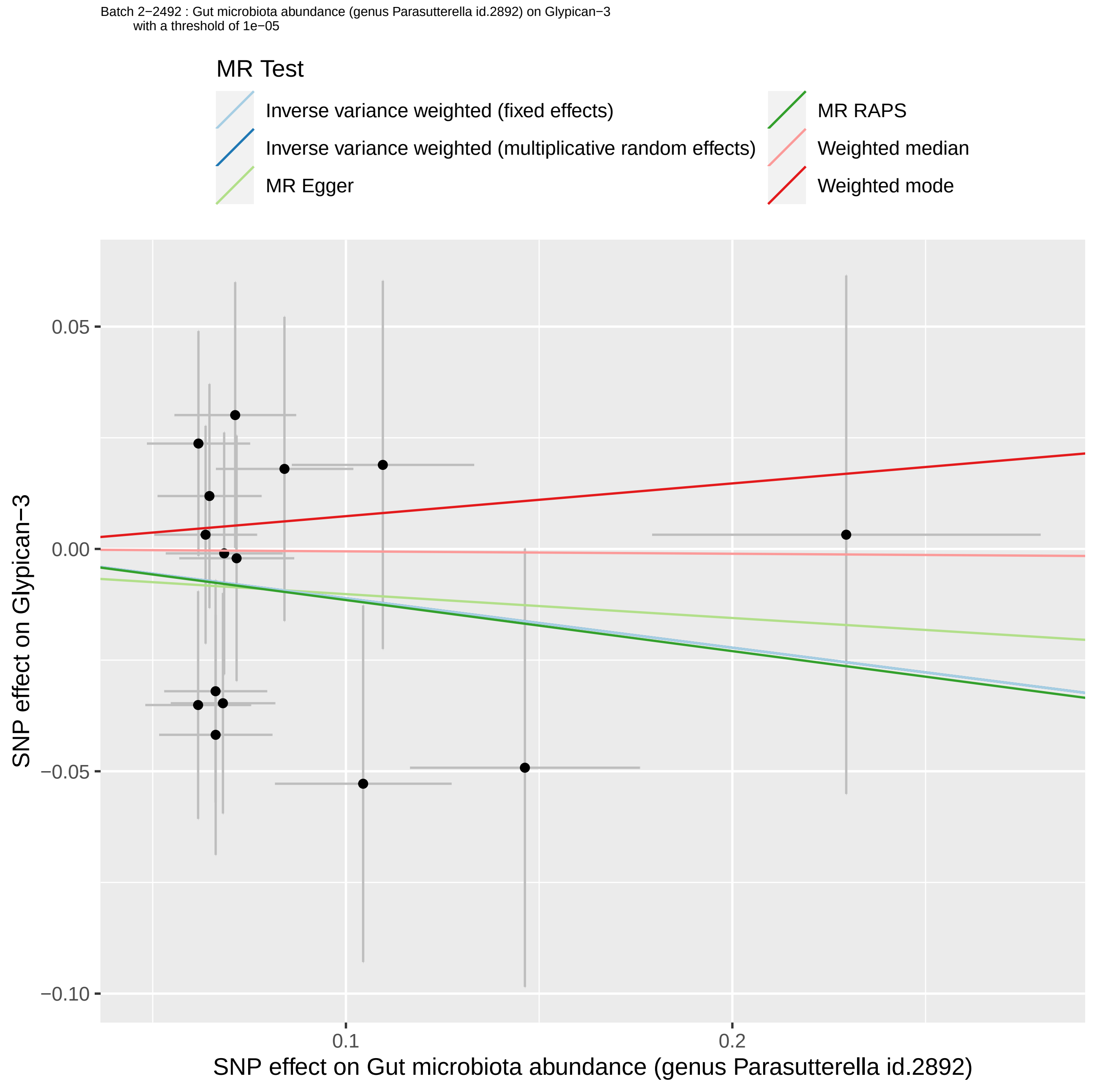

Batch 2−2492 : Gut microbiota abundance (genus Parasutterella id.2892) on Glypican−3
with a threshold of 1e−05
MR Test
Inverse variance weighted (fixed effects)
Inverse variance weighted (multiplicative random effects)
MR Egger
MR RAPS
Weighted median
Weighted mode
0.05
0.00
−0.05
−0.10
0.1
0.2
SNP effect on Glypican−3
SNP effect on Gut microbiota abundance (genus Parasutterella id.2892)

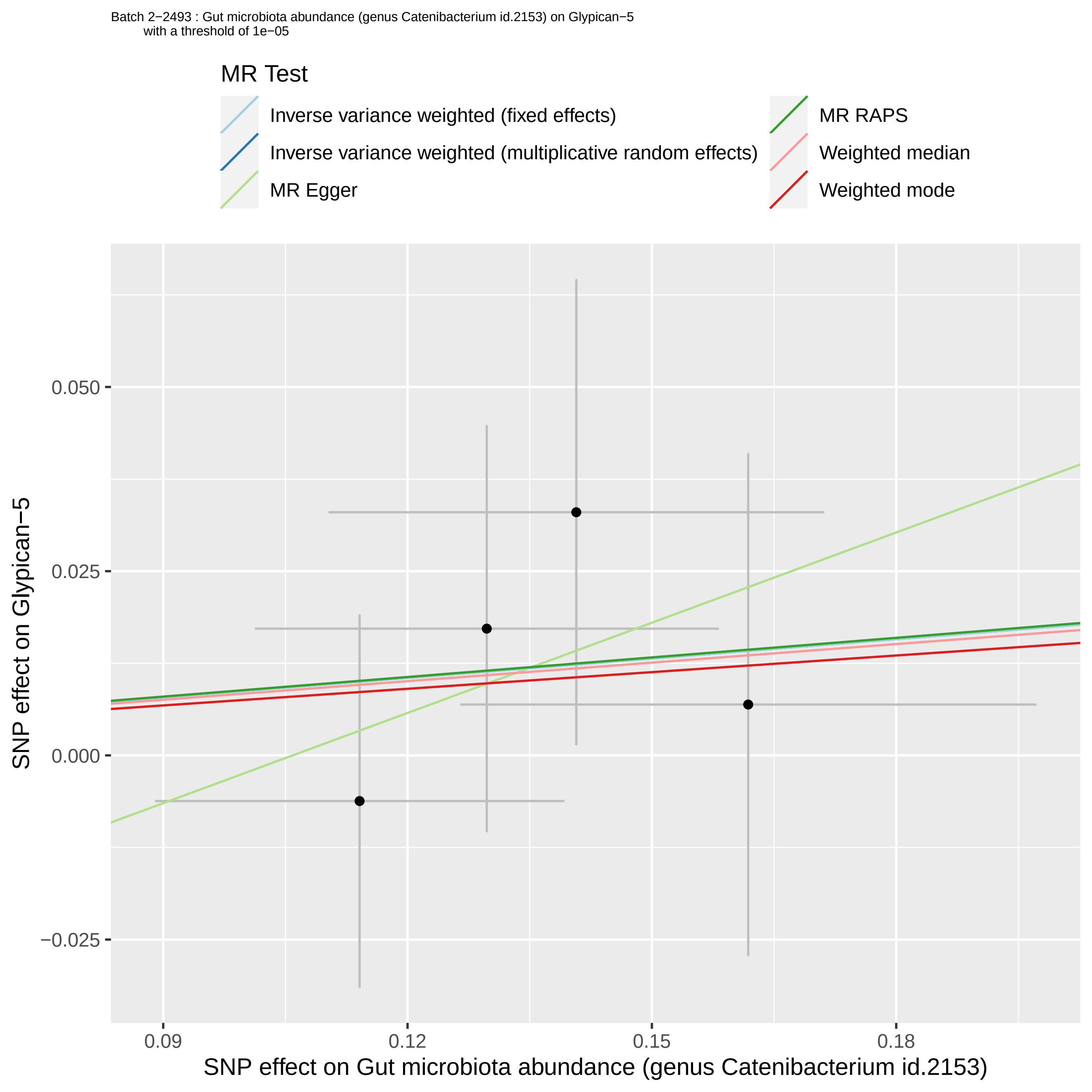

Batch 2−2493 : Gut microbiota abundance (genus Catenibacterium id.2153) on Glypican−5
with a threshold of 1e−05
MR Test
Inverse variance weighted (fixed effects)
Inverse variance weighted (multiplicative random effects)
MR Egger
MR RAPS
Weighted median
Weighted mode
0.050
0.025
0.000
−0.025
0.09
0.12
0.15
0.18
SNP effect on Glypican−5
SNP effect on Gut microbiota abundance (genus Catenibacterium id.2153)

Batch 2−2494 : Gut microbiota abundance (genus Parasutterella id.2892) on Glypican−5
with a threshold of 1e−05

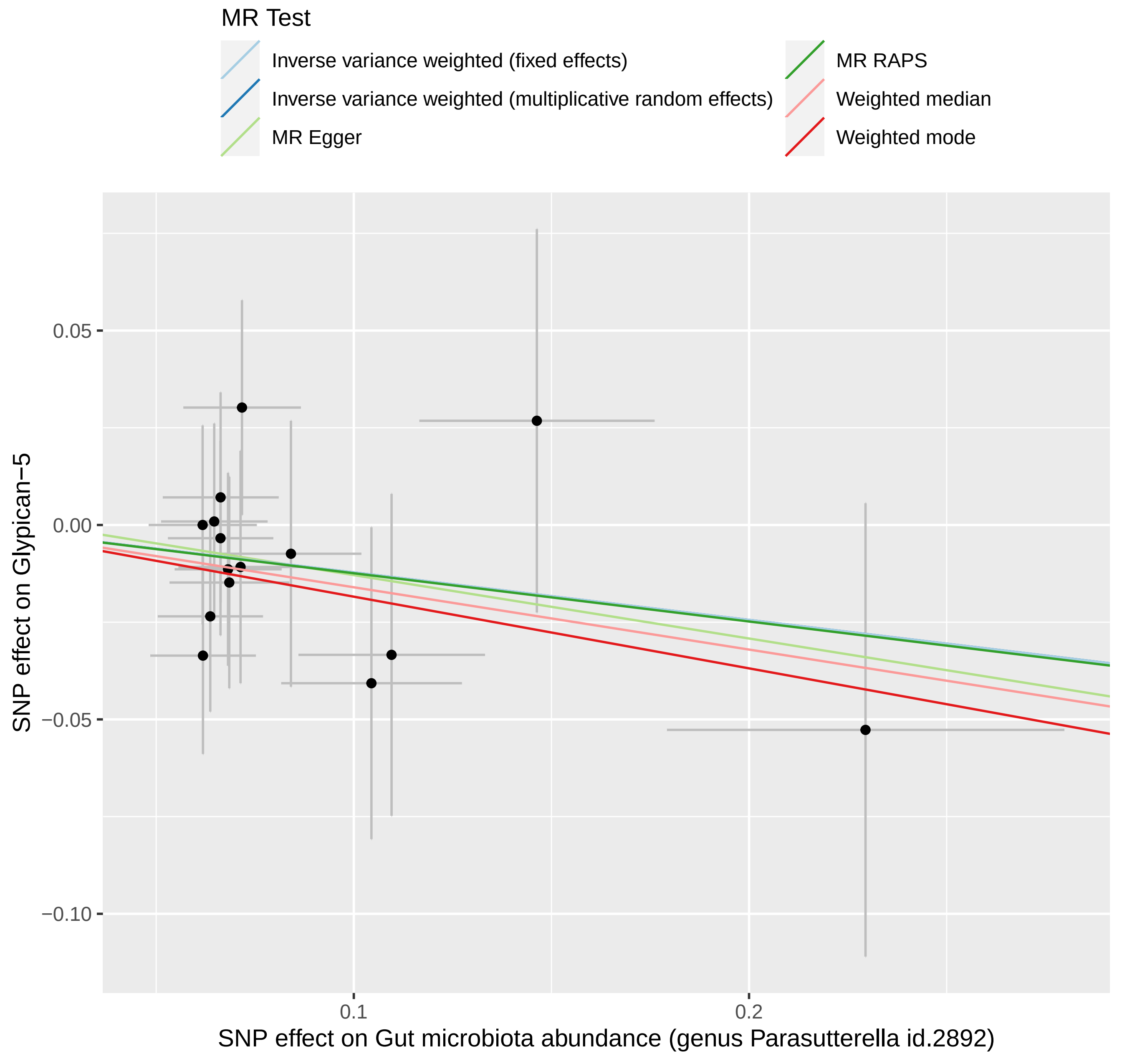

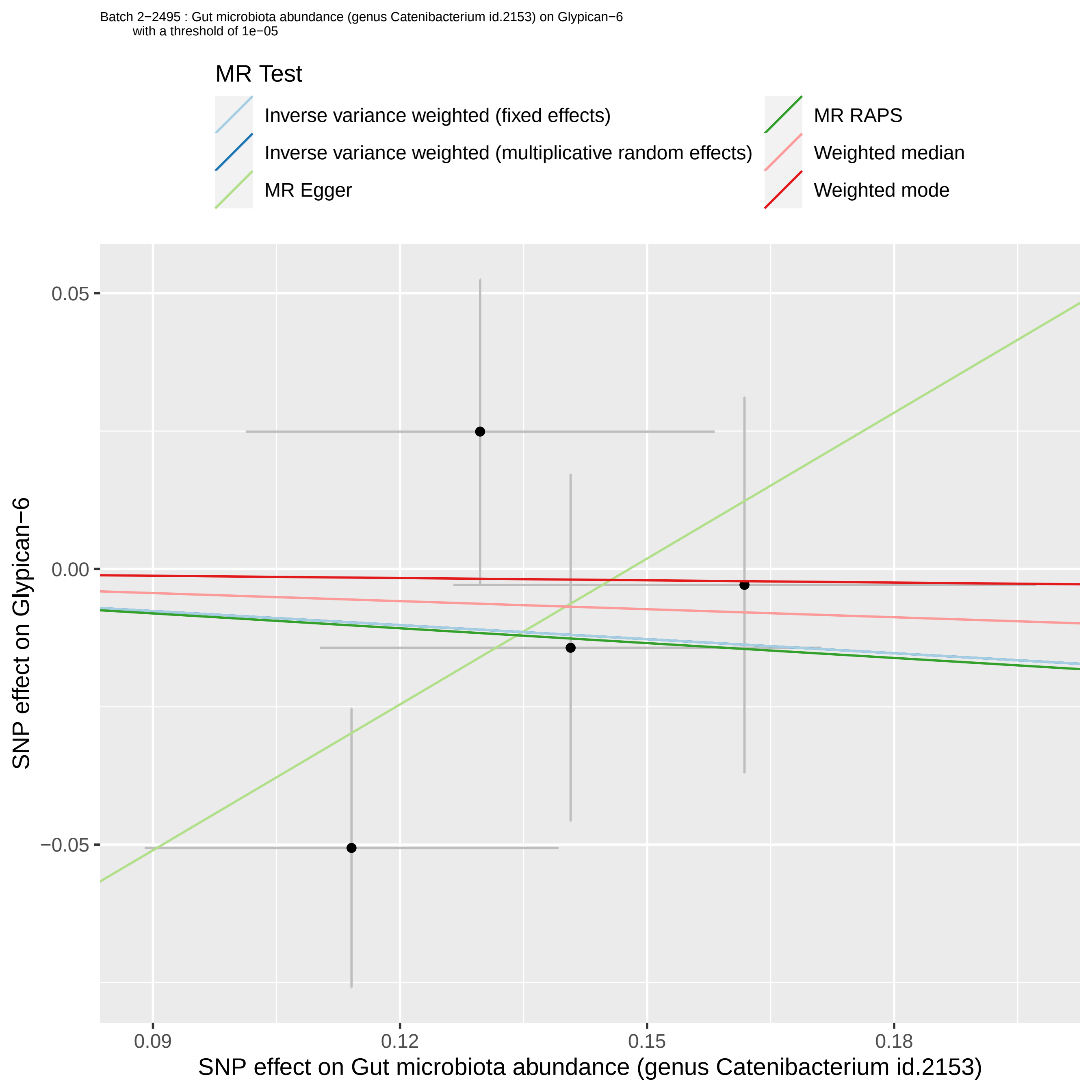
Batch 2−2495 : Gut microbiota abundance (genus Catenibacterium id.2153) on Glypican−6
with a threshold of 1e−05
MR Test
Inverse variance weighted (fixed effects)
Inverse variance weighted (multiplicative random effects)
MR Egger
MR RAPS
Weighted median
Weighted mode
0.05
0.00
−0.05
0.09
0.12
0.15
0.18
SNP effect on Glypican−6
SNP effect on Gut microbiota abundance (genus Catenibacterium id.2153)

Batch 2−2496 : Gut microbiota abundance (genus Parasutterella id.2892) on Glypican−6
with a threshold of 1e−05

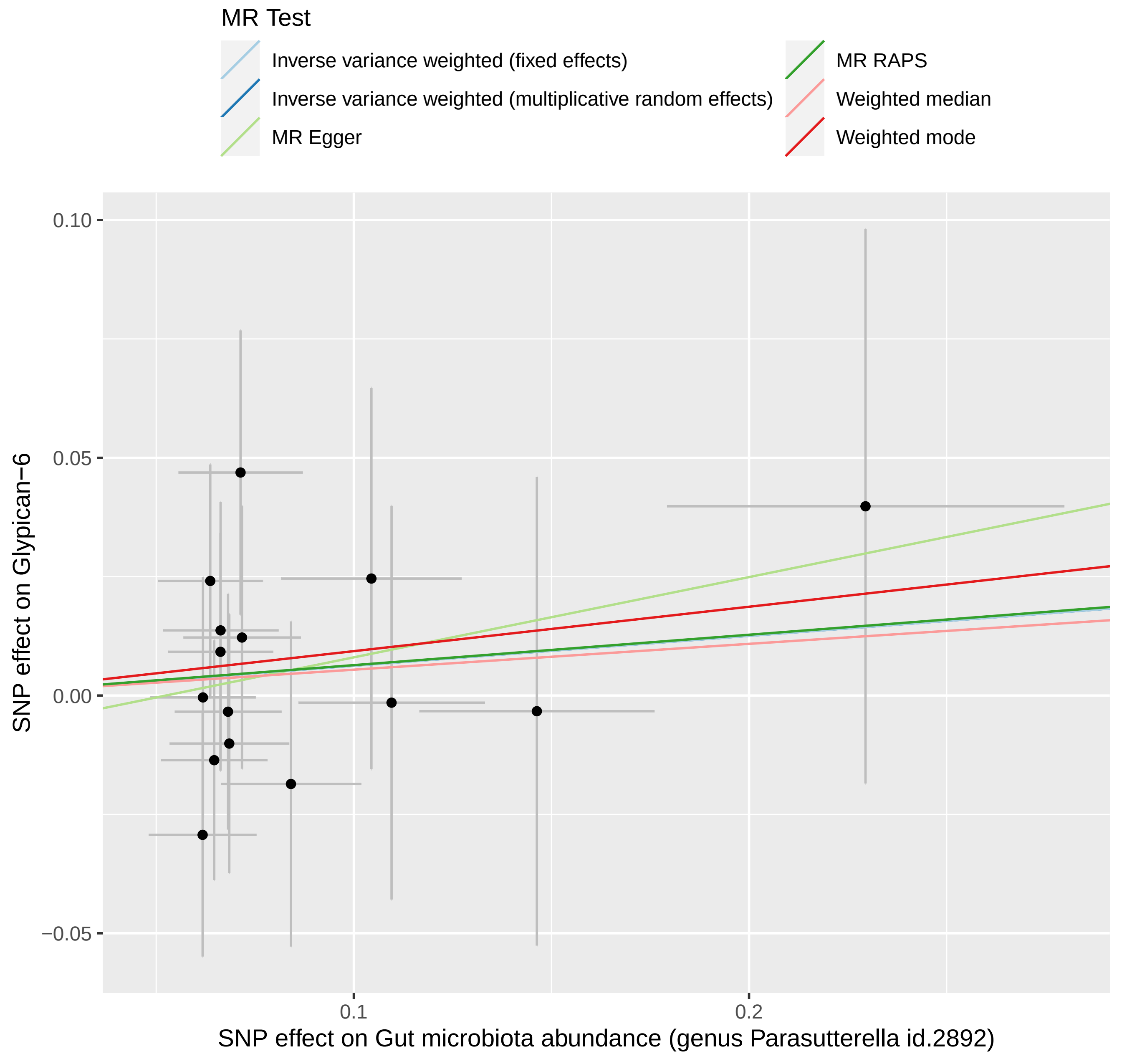

Batch 2−2497 : Gut microbiota abundance (genus Catenibacterium id.2153) on Glycerophosphocholine phosphodiesterase GPCPD1
with a threshold of 1e−05

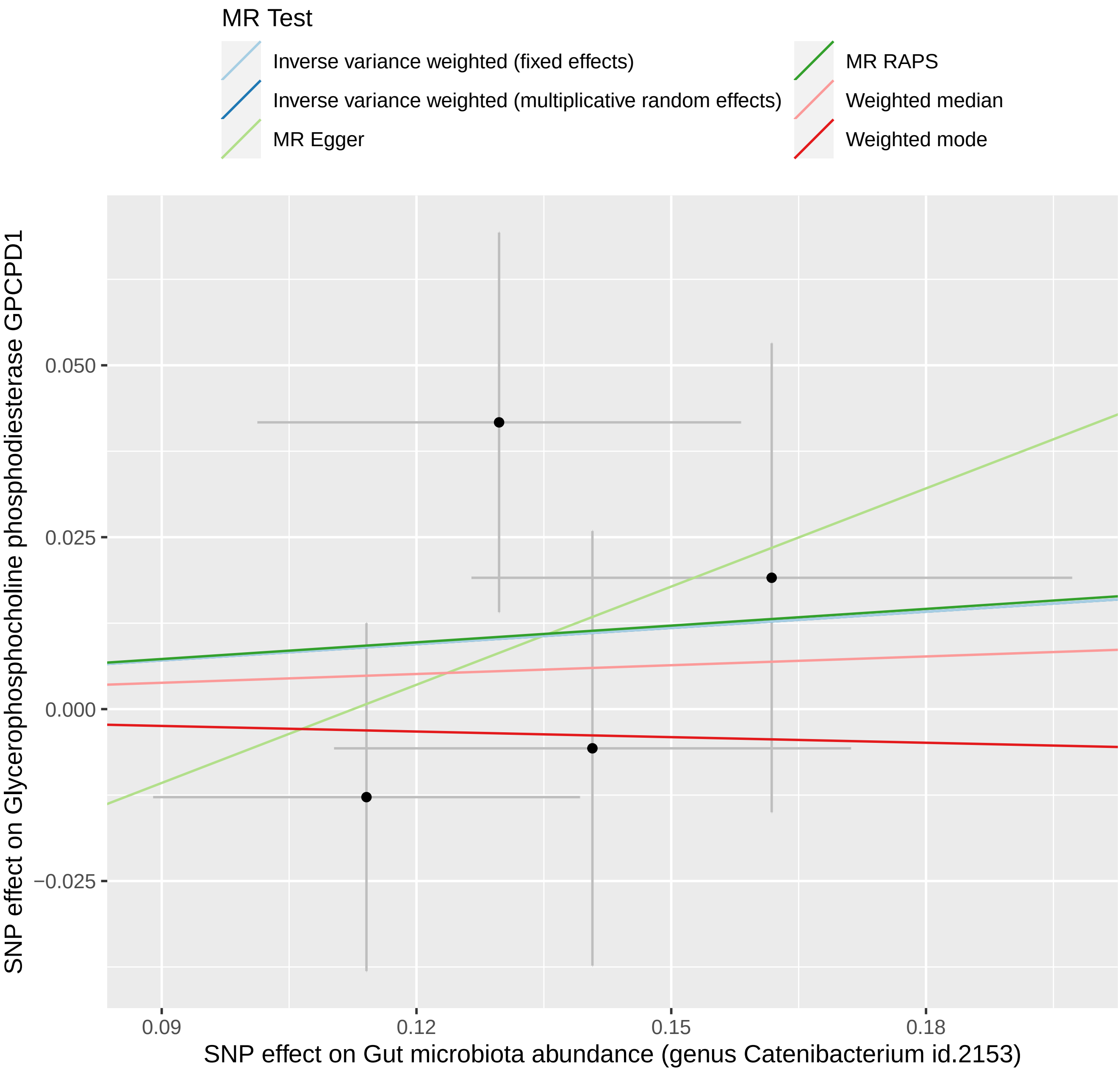

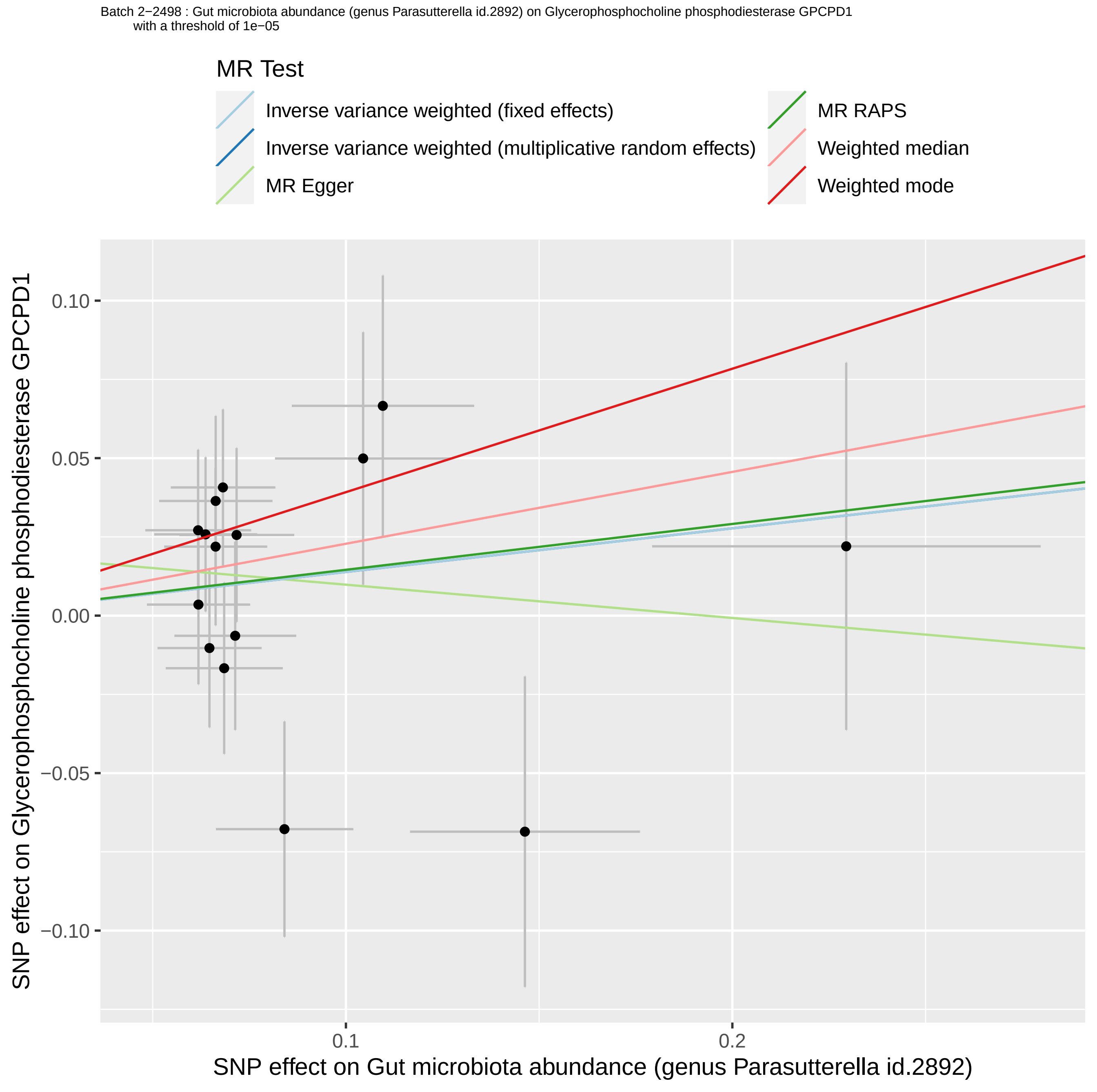
Batch 2−2498 : Gut microbiota abundance (genus Parasutterella id.2892) on Glycerophosphocholine phosphodiesterase GPCPD1
with a threshold of 1e−05
MR Test
Inverse variance weighted (fixed effects)
Inverse variance weighted (multiplicative random effects)
MR Egger
MR RAPS
Weighted median
Weighted mode
0.10
0.05
0.00
−0.05
−0.10
0.1
0.2
SNP effect on Glycerophosphocholine phosphodiesterase GPCPD1
SNP effect on Gut microbiota abundance (genus Parasutterella id.2892)

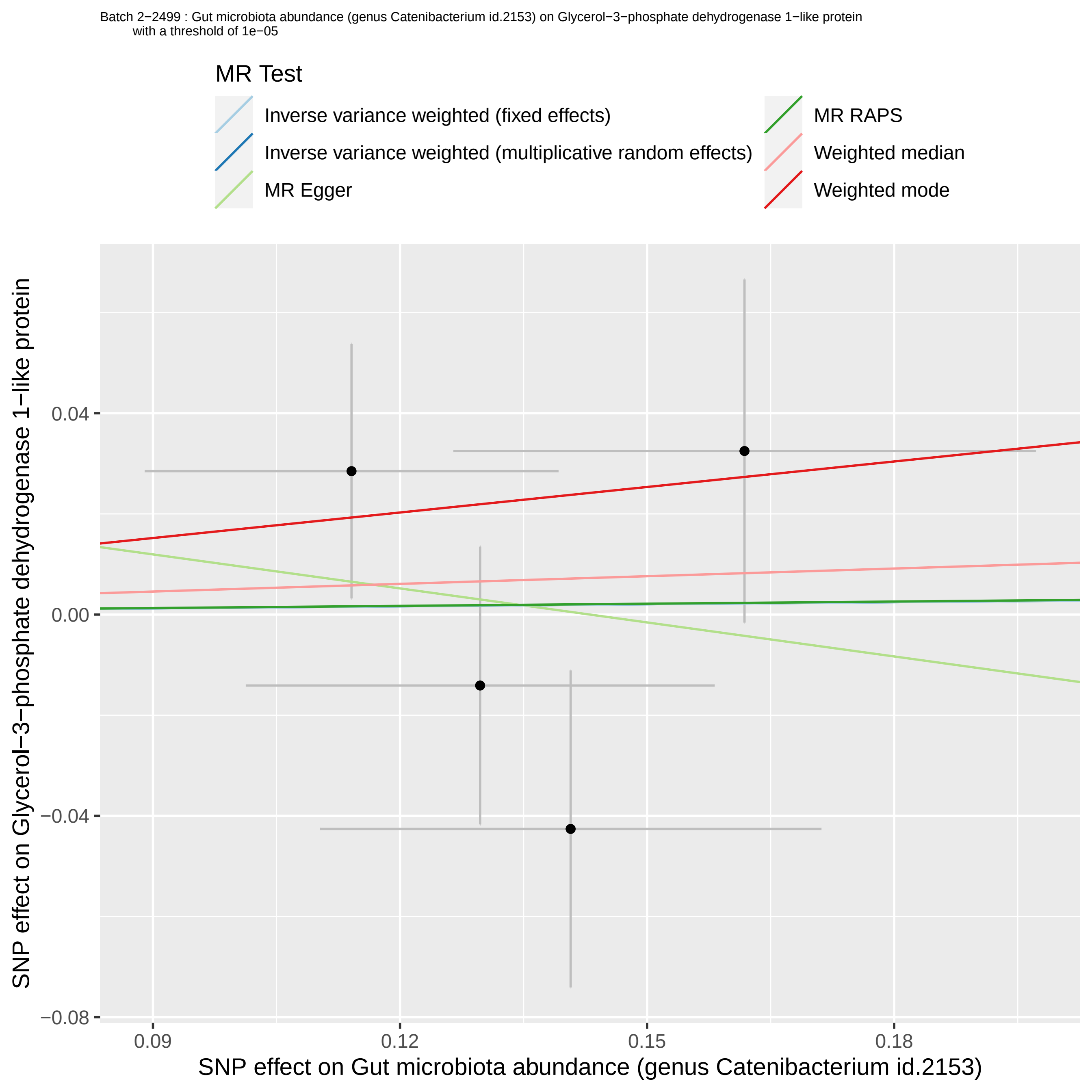

Batch 2−2499 : Gut microbiota abundance (genus Catenibacterium id.2153) on Glycerol−3−phosphate dehydrogenase 1−like protein
with a threshold of 1e−05
MR Test
Inverse variance weighted (fixed effects)
Inverse variance weighted (multiplicative random effects)
MR Egger
MR RAPS
Weighted median
Weighted mode
0.04
0.00
−0.04
−0.08
0.09
0.12
0.15
0.18
SNP effect on Glycerol−3−phosphate dehydrogenase 1−like protein
SNP effect on Gut microbiota abundance (genus Catenibacterium id.2153)

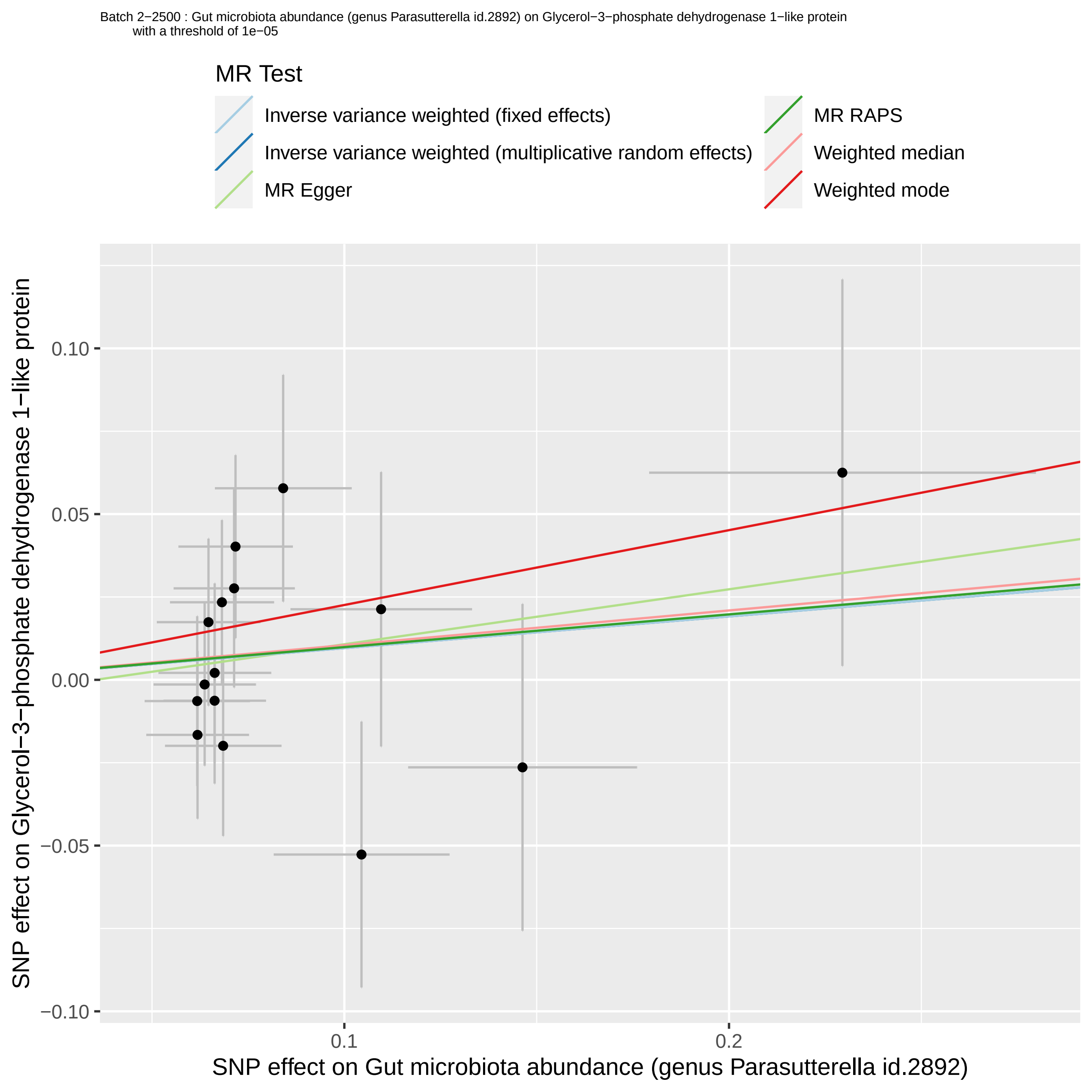

Batch 2−2500 : Gut microbiota abundance (genus Parasutterella id.2892) on Glycerol−3−phosphate dehydrogenase 1−like protein
with a threshold of 1e−05
MR Test
Inverse variance weighted (fixed effects)
Inverse variance weighted (multiplicative random effects)
MR Egger
MR RAPS
Weighted median
Weighted mode
0.10
0.05
0.00
−0.05
−0.10
0.1
0.2
SNP effect on Glycerol−3−phosphate dehydrogenase 1−like protein
SNP effect on Gut microbiota abundance (genus Parasutterella id.2892)

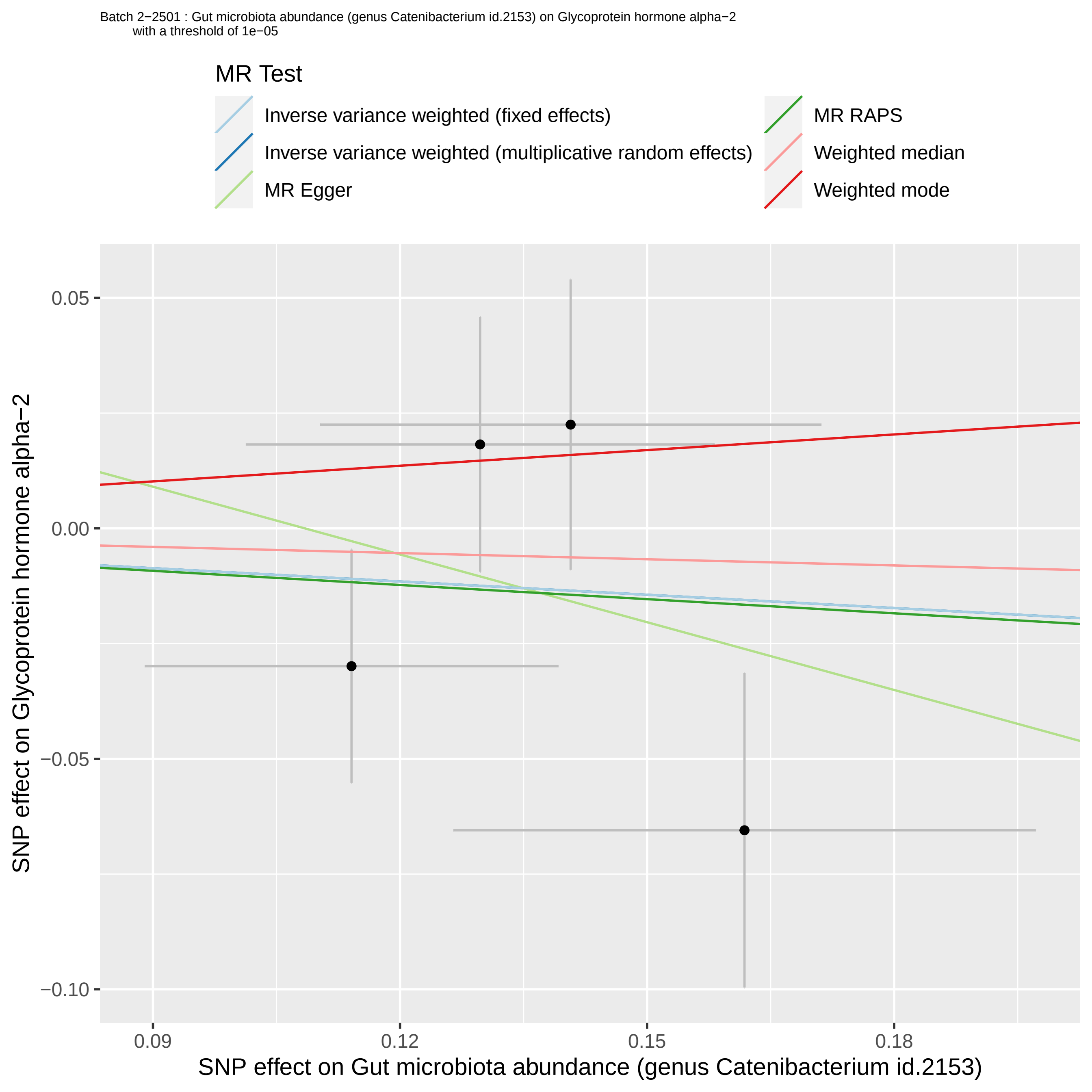

Batch 2−2501 : Gut microbiota abundance (genus Catenibacterium id.2153) on Glycoprotein hormone alpha−2
with a threshold of 1e−05
MR Test
Inverse variance weighted (fixed effects)
Inverse variance weighted (multiplicative random effects)
MR Egger
MR RAPS
Weighted median
Weighted mode
0.05
0.00
−0.05
−0.10
0.09
0.12
0.15
0.18
SNP effect on Glycoprotein hormone alpha−2
SNP effect on Gut microbiota abundance (genus Catenibacterium id.2153)

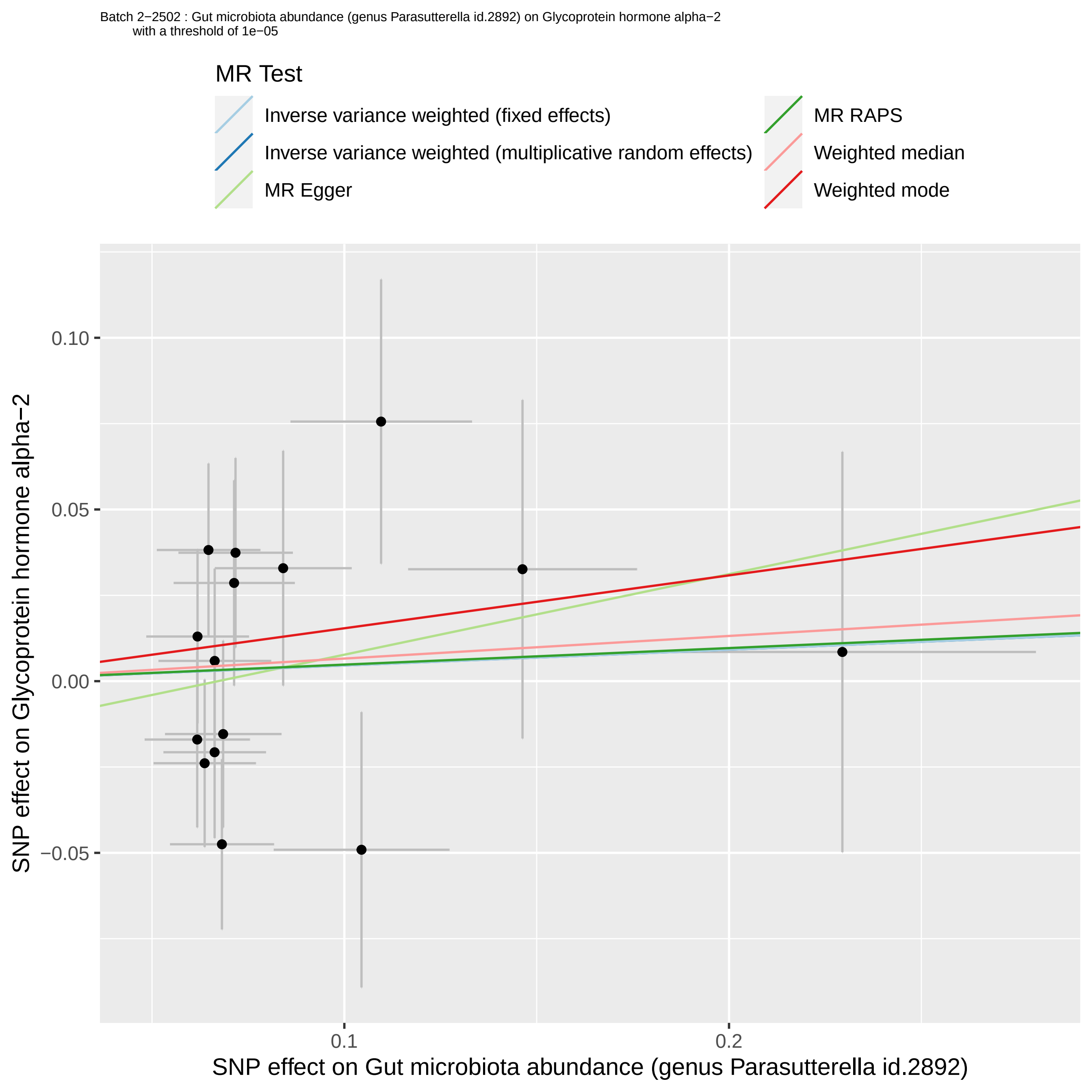

Batch 2−2502 : Gut microbiota abundance (genus Parasutterella id.2892) on Glycoprotein hormone alpha−2
with a threshold of 1e−05
MR Test
Inverse variance weighted (fixed effects)
Inverse variance weighted (multiplicative random effects)
MR Egger
MR RAPS
Weighted median
Weighted mode
0.10
0.05
0.00
−0.05
0.1
0.2
SNP effect on Glycoprotein hormone alpha−2
SNP effect on Gut microbiota abundance (genus Parasutterella id.2892)

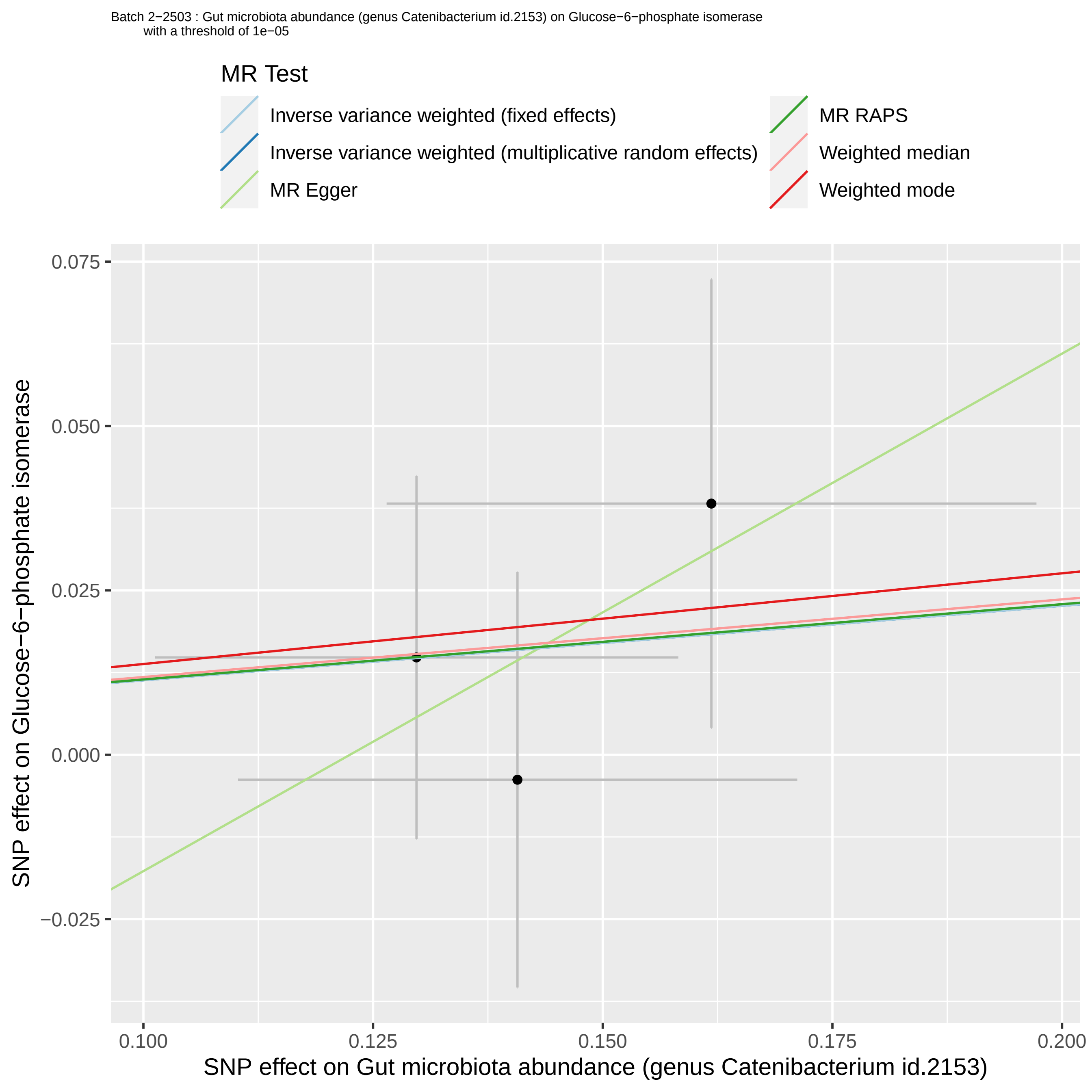
Batch 2−2503 : Gut microbiota abundance (genus Catenibacterium id.2153) on Glucose−6−phosphate isomerase
with a threshold of 1e−05
MR Test
Inverse variance weighted (fixed effects)
Inverse variance weighted (multiplicative random effects)
MR Egger
MR RAPS
Weighted median
Weighted mode
0.075
0.050
0.025
0.000
−0.025
0.100
0.125
0.150
0.175
0.200
SNP effect on Glucose−6−phosphate isomerase
SNP effect on Gut microbiota abundance (genus Catenibacterium id.2153)

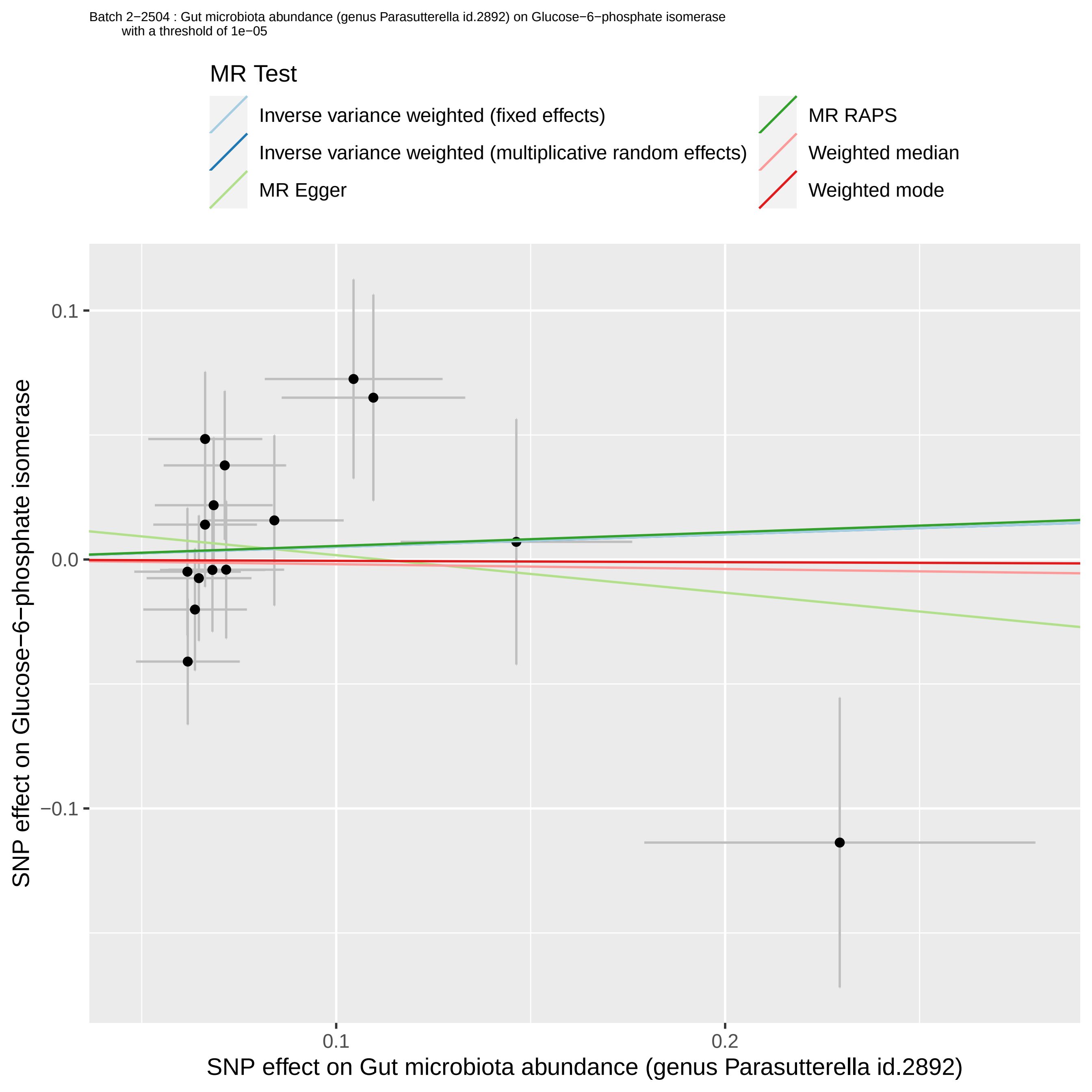

Batch 2−2504 : Gut microbiota abundance (genus Parasutterella id.2892) on Glucose−6−phosphate isomerase
with a threshold of 1e−05
MR Test
Inverse variance weighted (fixed effects)
Inverse variance weighted (multiplicative random effects)
MR Egger
MR RAPS
Weighted median
Weighted mode
0.1
0.0
−0.1
0.1
0.2
SNP effect on Glucose−6−phosphate isomerase
SNP effect on Gut microbiota abundance (genus Parasutterella id.2892)

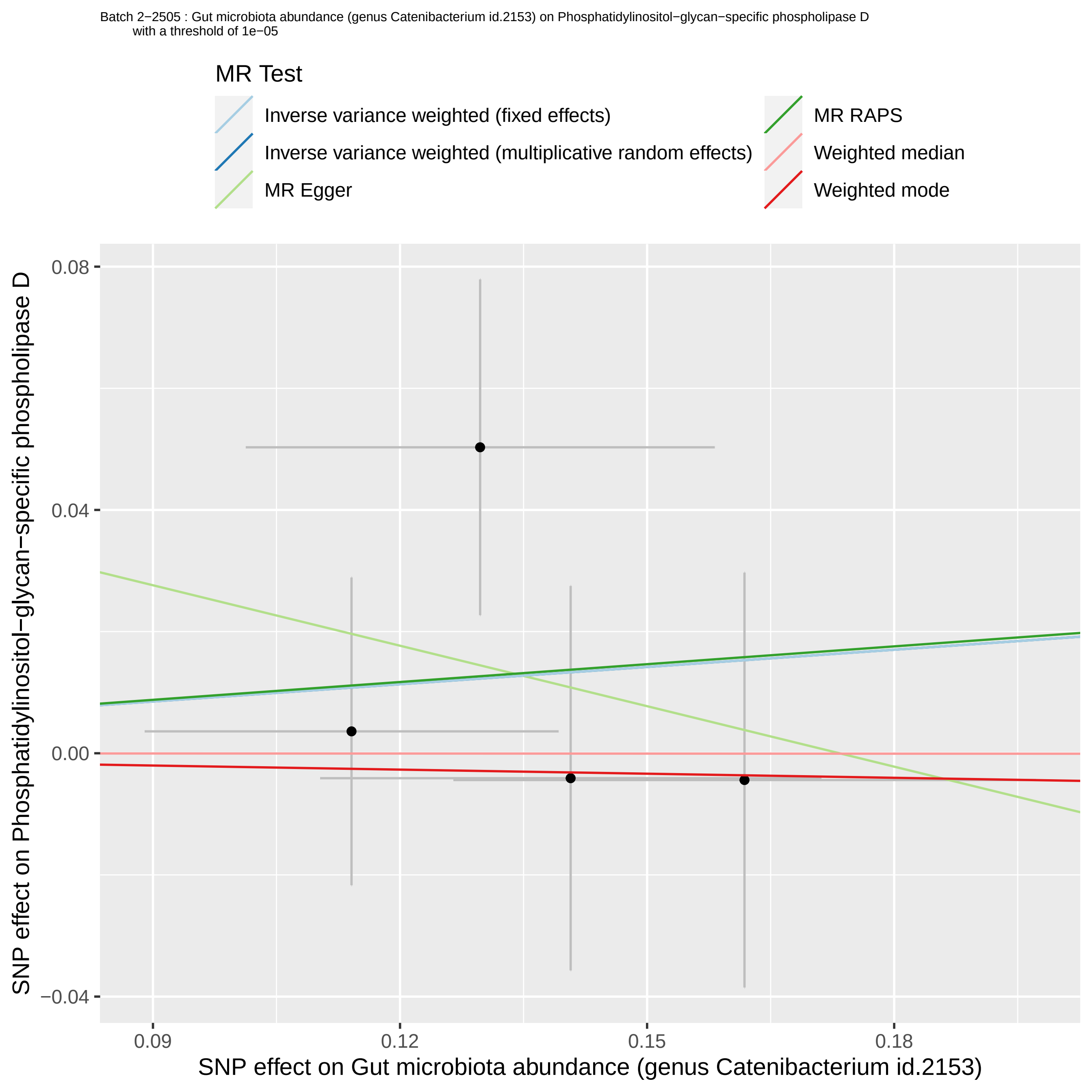
Batch 2−2505 : Gut microbiota abundance (genus Catenibacterium id.2153) on Phosphatidylinositol−glycan−specific phospholipase D
with a threshold of 1e−05
MR Test
Inverse variance weighted (fixed effects)
Inverse variance weighted (multiplicative random effects)
MR Egger
MR RAPS
Weighted median
Weighted mode
0.08
0.04
0.00
−0.04
0.09
0.12
0.15
0.18
SNP effect on Phosphatidylinositol−glycan−specific phospholipase D
SNP effect on Gut microbiota abundance (genus Catenibacterium id.2153)

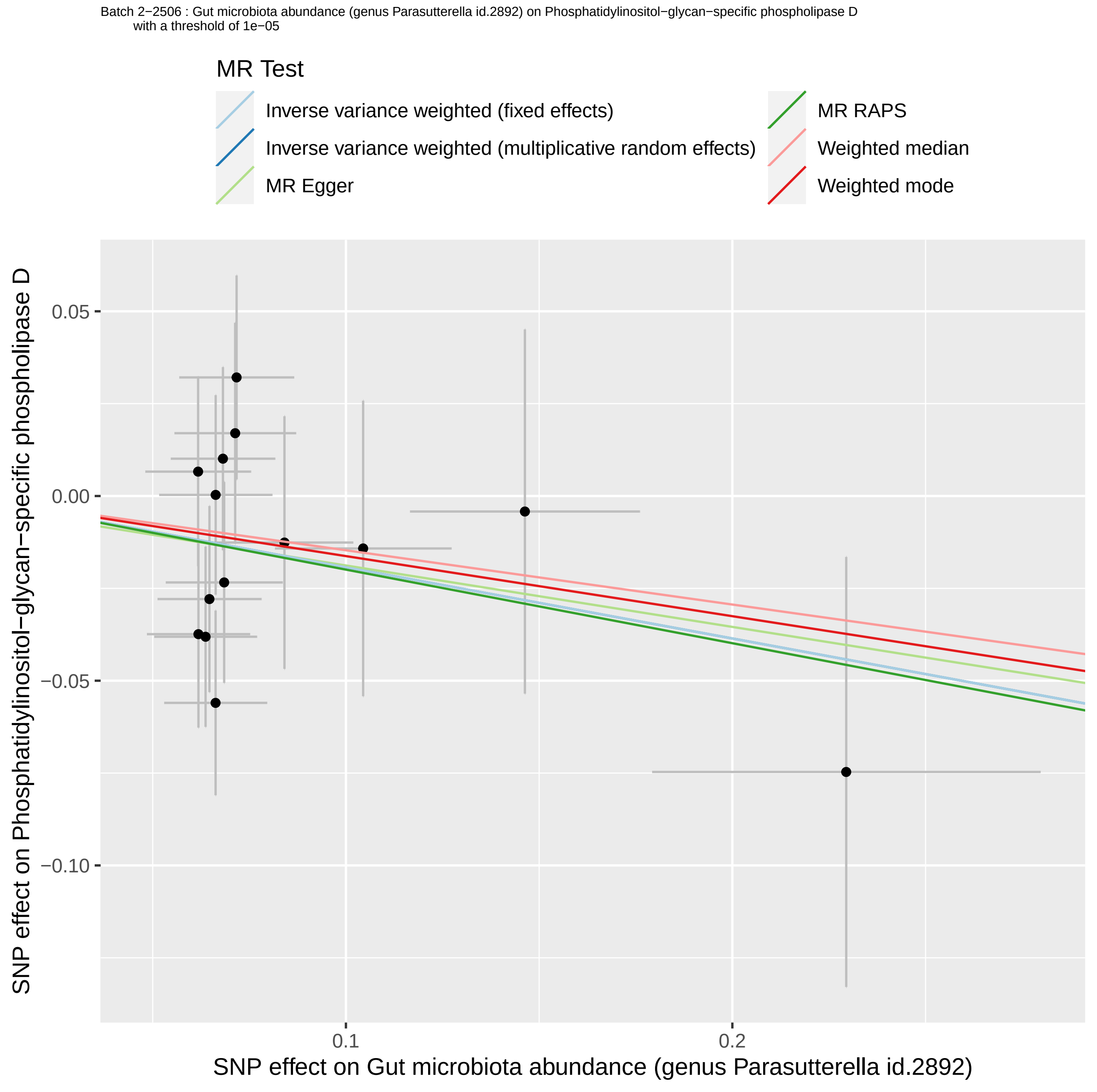

Batch 2−2506 : Gut microbiota abundance (genus Parasutterella id.2892) on Phosphatidylinositol−glycan−specific phospholipase D
with a threshold of 1e−05
MR Test
Inverse variance weighted (fixed effects)
Inverse variance weighted (multiplicative random effects)
MR Egger
MR RAPS
Weighted median
Weighted mode
0.05
0.00
−0.05
−0.10
0.1
0.2
SNP effect on Phosphatidylinositol−glycan−specific phospholipase D
SNP effect on Gut microbiota abundance (genus Parasutterella id.2892)

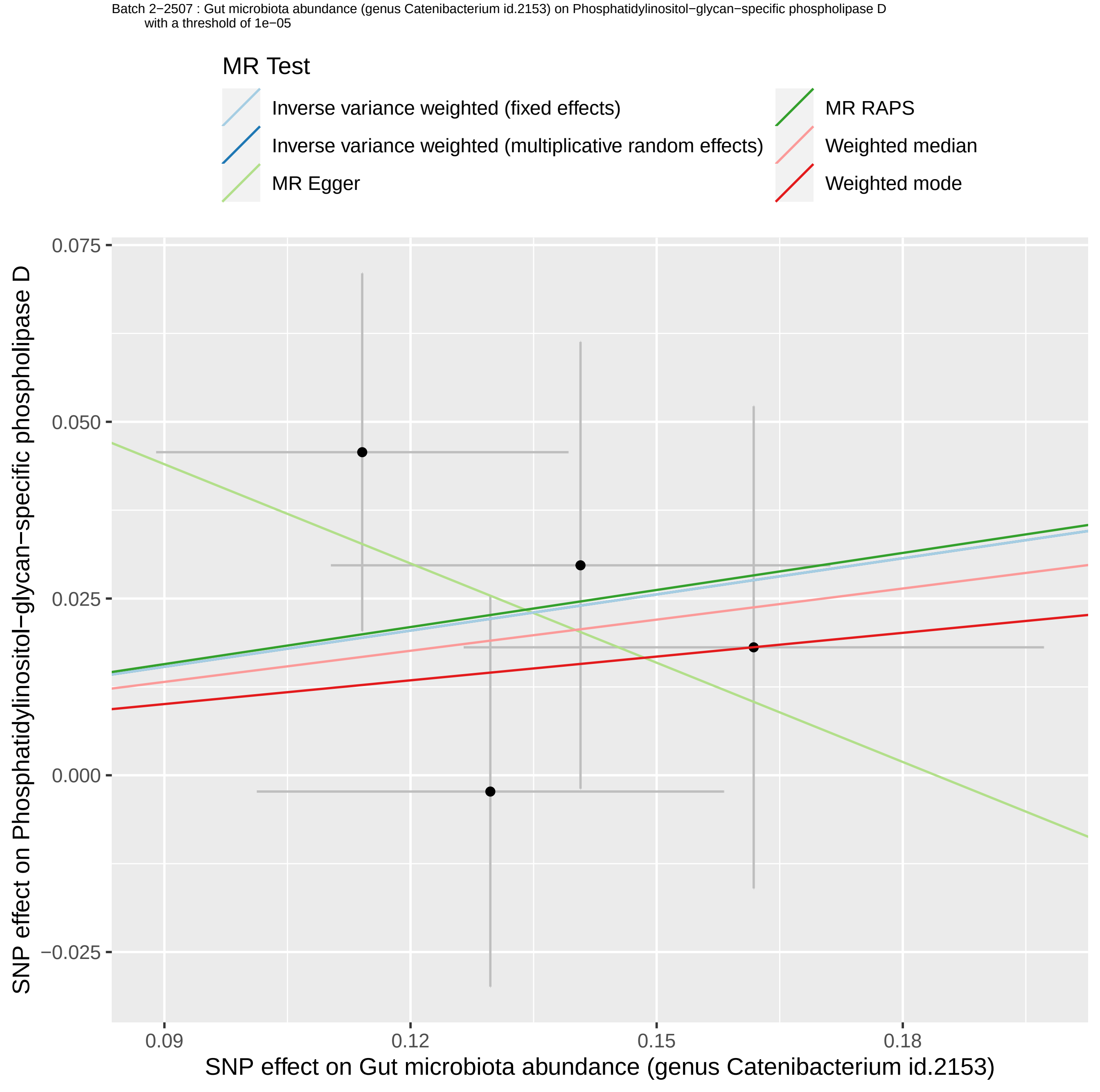

Batch 2−2507 : Gut microbiota abundance (genus Catenibacterium id.2153) on Phosphatidylinositol−glycan−specific phospholipase D
with a threshold of 1e−05
MR Test
Inverse variance weighted (fixed effects)
Inverse variance weighted (multiplicative random effects)
MR Egger
MR RAPS
Weighted median
Weighted mode
0.075
0.050
0.025
0.000
−0.025
0.09
0.12
0.15
0.18
SNP effect on Phosphatidylinositol−glycan−specific phospholipase D
SNP effect on Gut microbiota abundance (genus Catenibacterium id.2153)

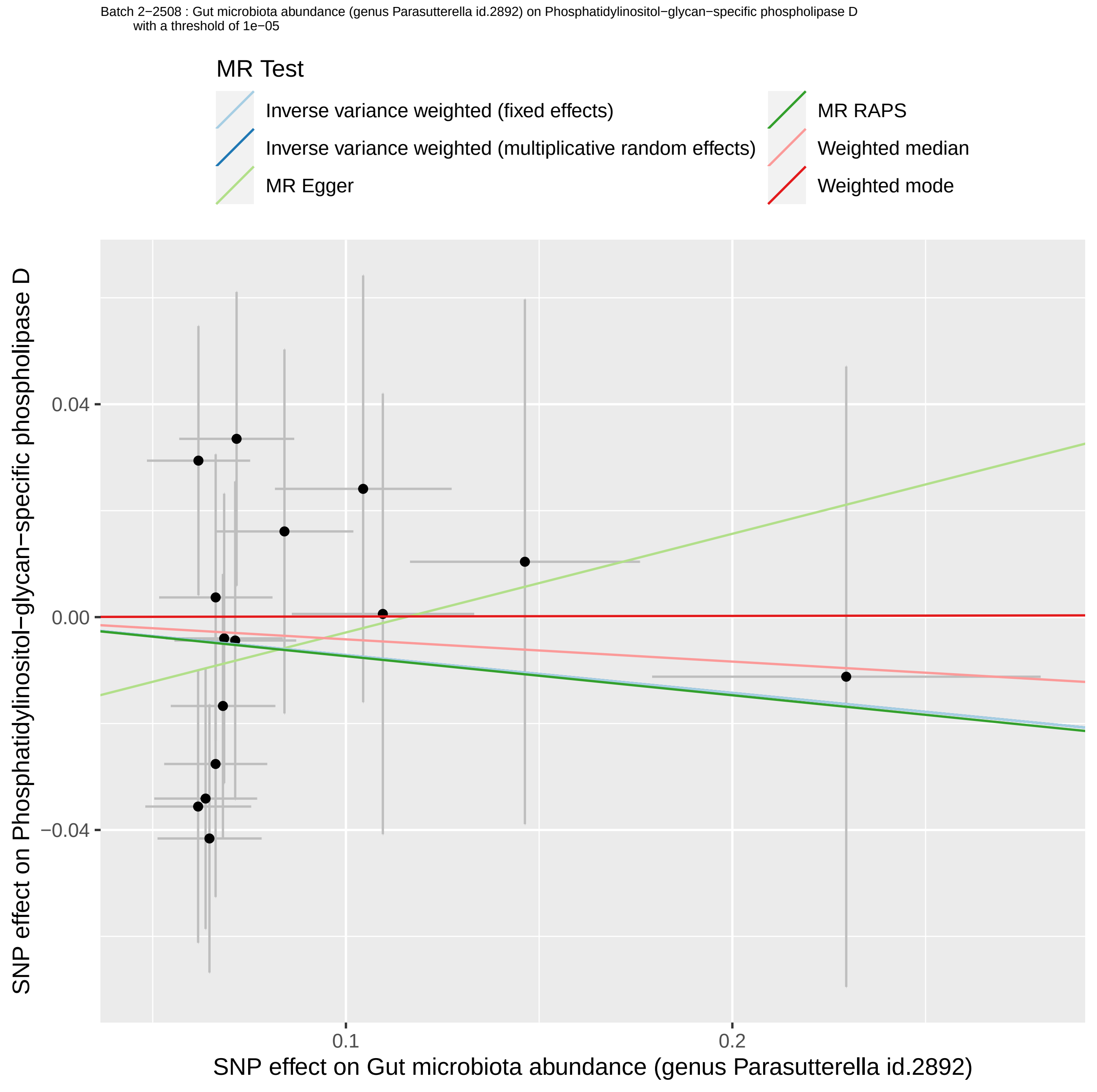
Batch 2−2508 : Gut microbiota abundance (genus Parasutterella id.2892) on Phosphatidylinositol−glycan−specific phospholipase D
with a threshold of 1e−05
MR Test
Inverse variance weighted (fixed effects)
Inverse variance weighted (multiplicative random effects)
MR Egger
MR RAPS
Weighted median
Weighted mode
0.04
0.00
−0.04
0.1
0.2
SNP effect on Phosphatidylinositol−glycan−specific phospholipase D
SNP effect on Gut microbiota abundance (genus Parasutterella id.2892)

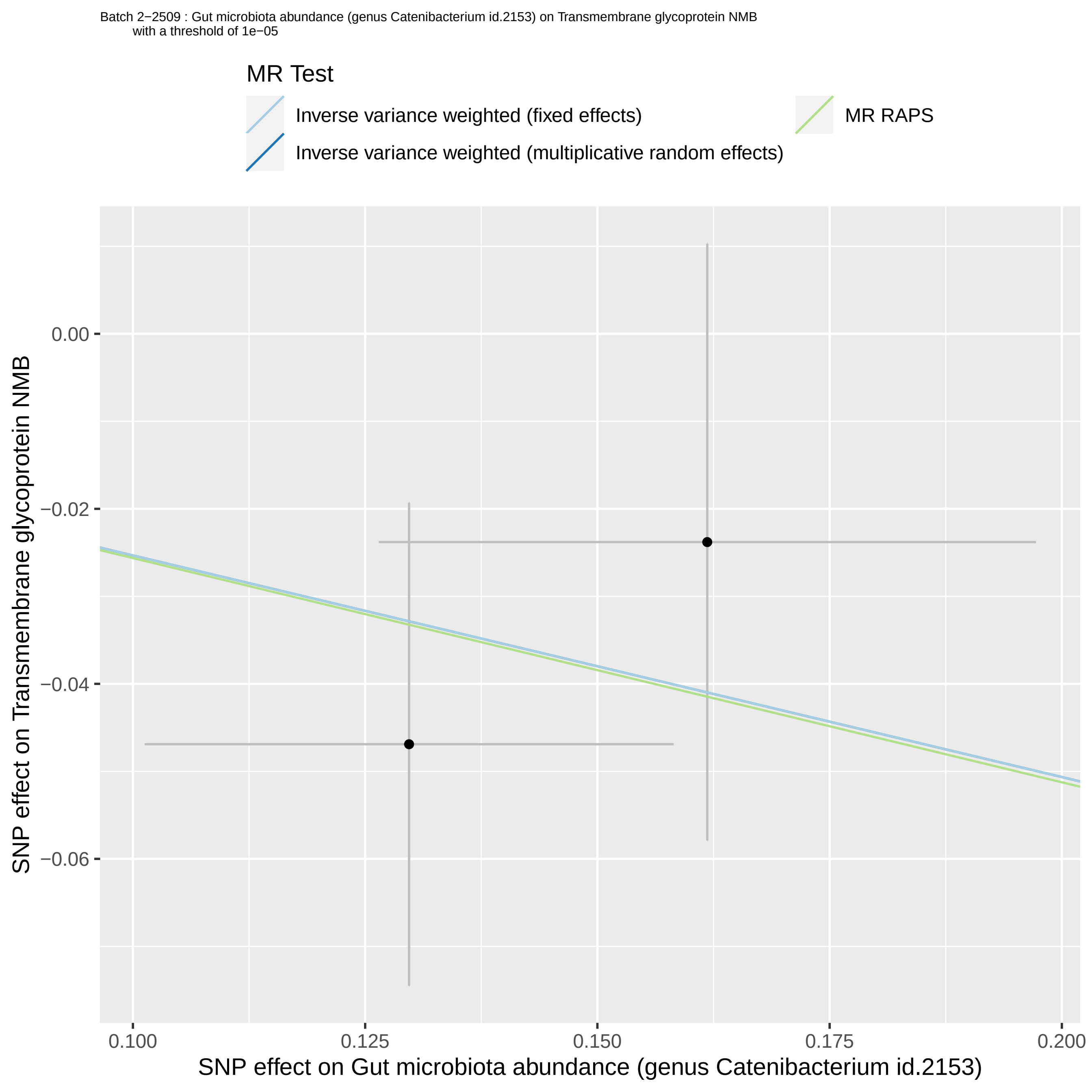
Batch 2−2509 : Gut microbiota abundance (genus Catenibacterium id.2153) on Transmembrane glycoprotein NMB
with a threshold of 1e−05
MR Test
Inverse variance weighted (fixed effects)
Inverse variance weighted (multiplicative random effects)
MR RAPS
0.00
−0.02
−0.04
−0.06
0.100
0.125
0.150
0.175
0.200
SNP effect on Transmembrane glycoprotein NMB
SNP effect on Gut microbiota abundance (genus Catenibacterium id.2153)

Batch 2−2510 : Gut microbiota abundance (genus Parasutterella id.2892) on Transmembrane glycoprotein NMB
with a threshold of 1e−05

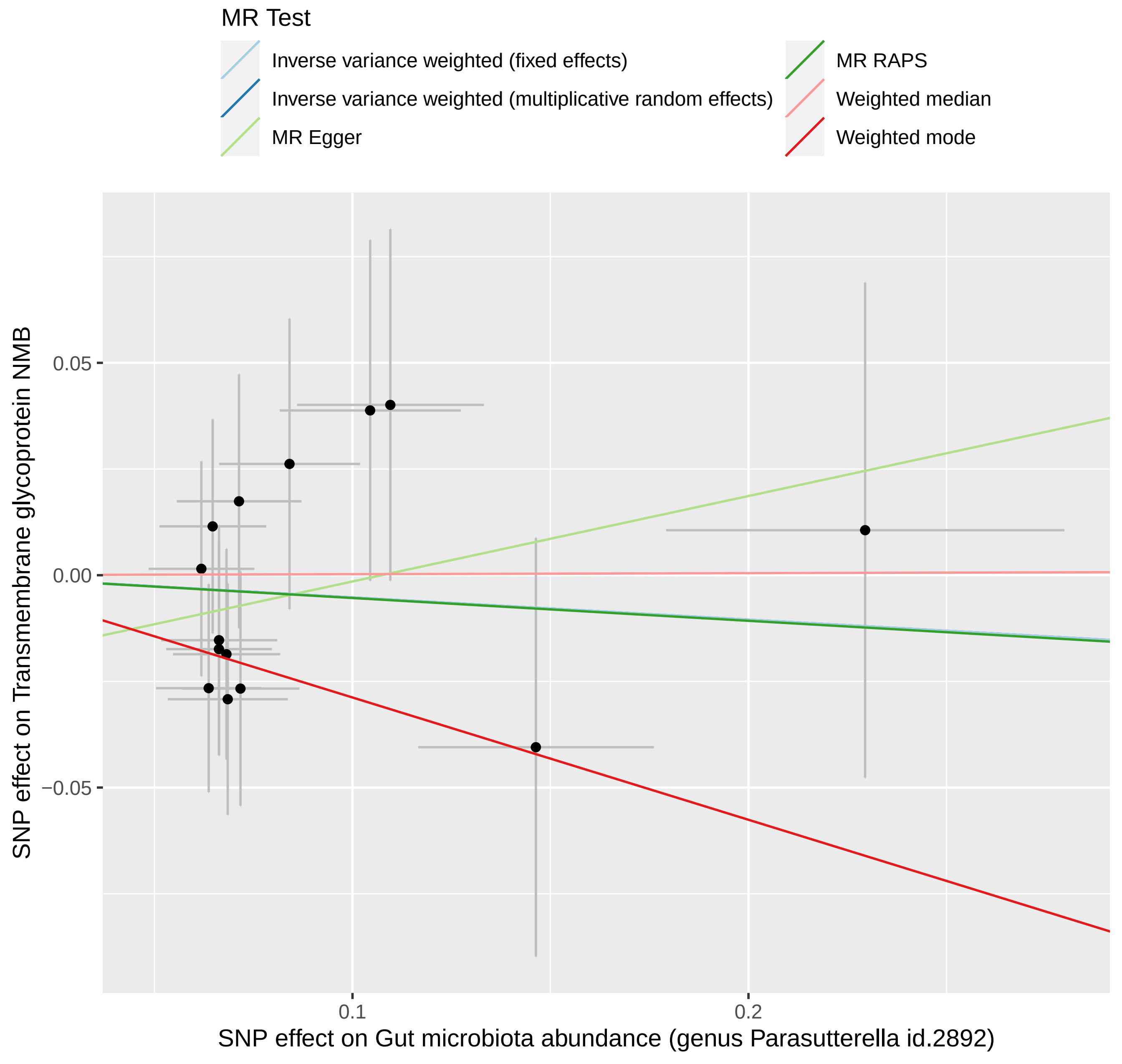

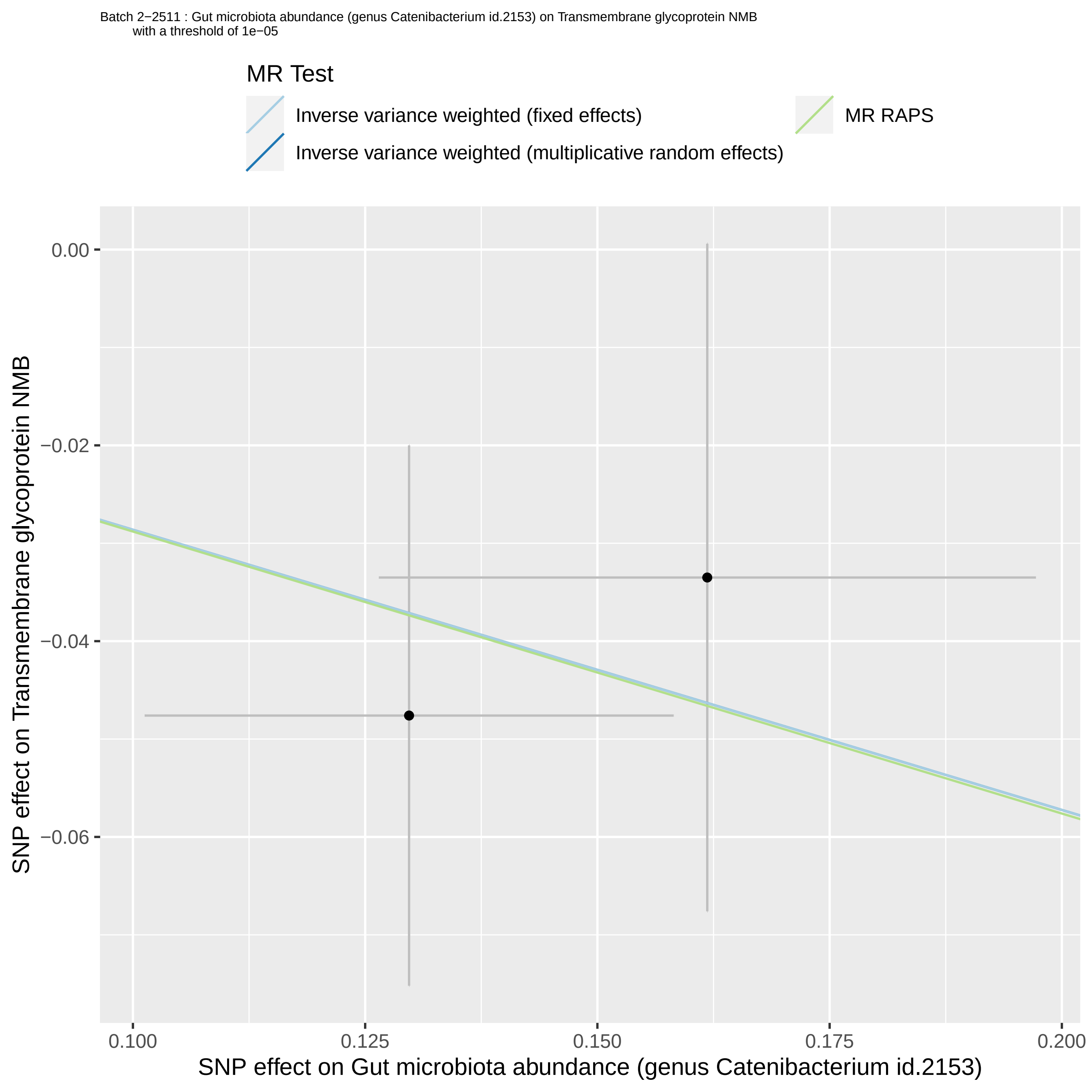

Batch 2−2511 : Gut microbiota abundance (genus Catenibacterium id.2153) on Transmembrane glycoprotein NMB
with a threshold of 1e−05
MR Test
Inverse variance weighted (fixed effects)
Inverse variance weighted (multiplicative random effects)
MR RAPS
0.00
−0.02
−0.04
−0.06
0.100
0.125
0.150
0.175
0.200
SNP effect on Transmembrane glycoprotein NMB
SNP effect on Gut microbiota abundance (genus Catenibacterium id.2153)

Batch 2−2512 : Gut microbiota abundance (genus Parasutterella id.2892) on Transmembrane glycoprotein NMB
with a threshold of 1e−05

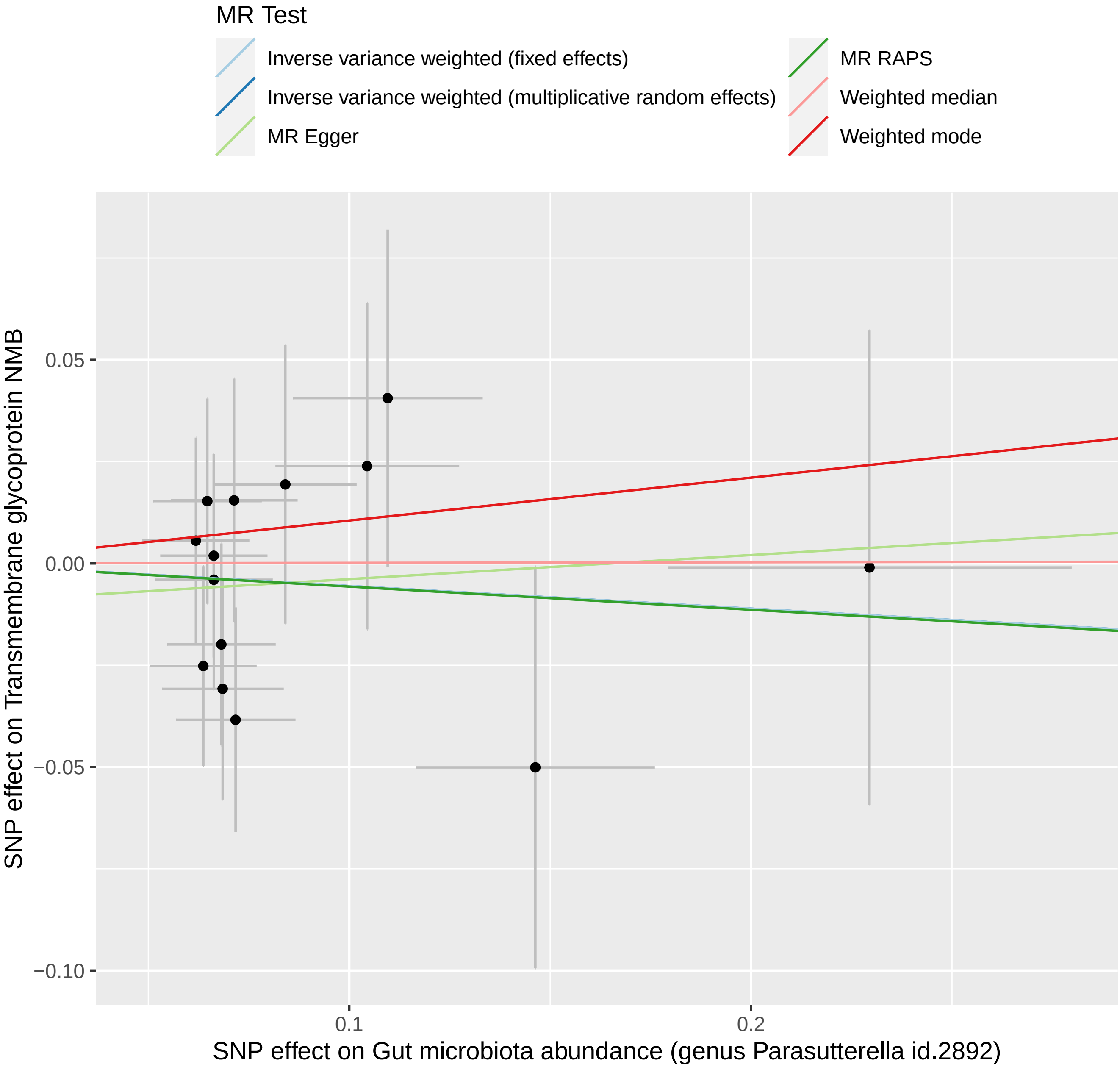

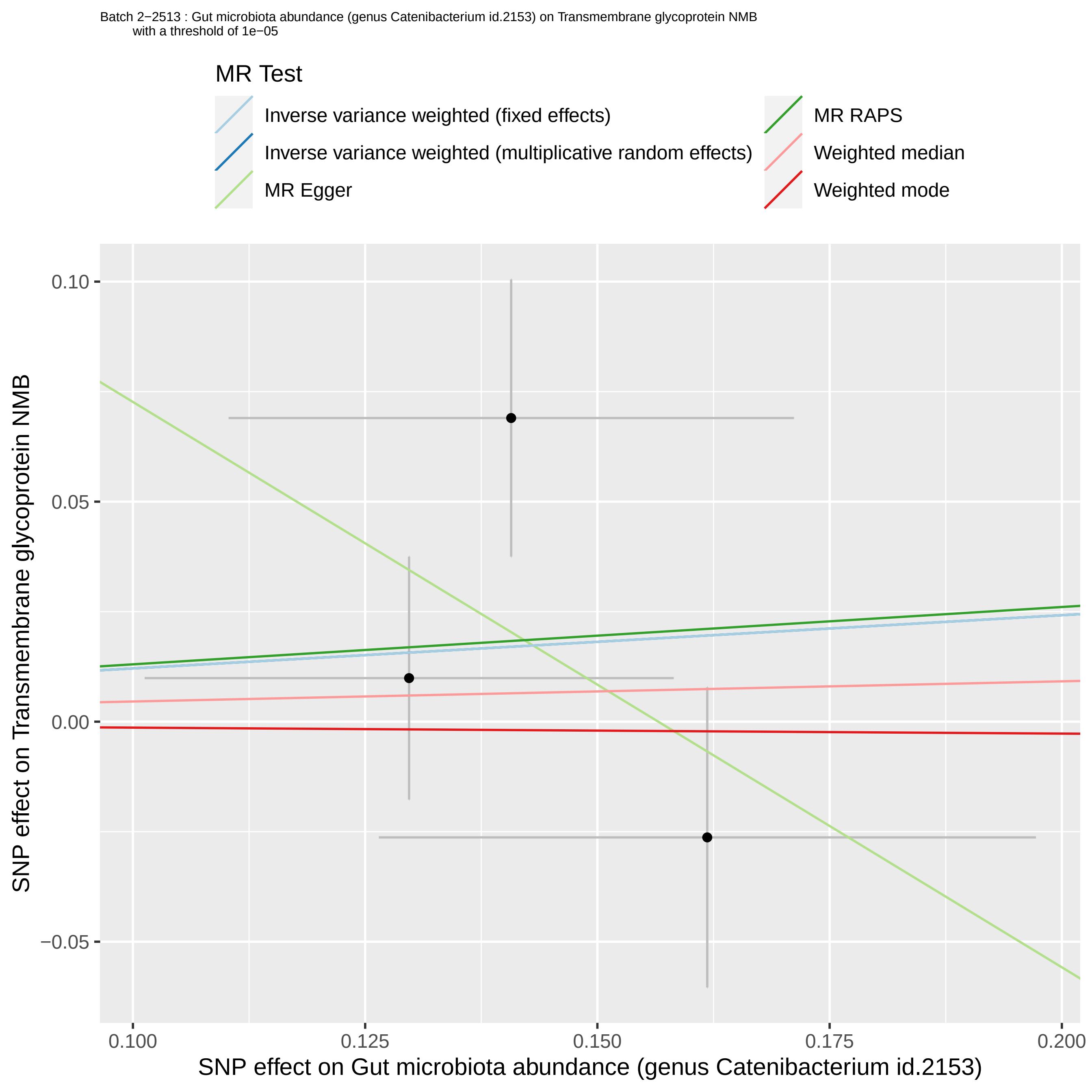
Batch 2−2513 : Gut microbiota abundance (genus Catenibacterium id.2153) on Transmembrane glycoprotein NMB
with a threshold of 1e−05
MR Test
Inverse variance weighted (fixed effects)
Inverse variance weighted (multiplicative random effects)
MR Egger
MR RAPS
Weighted median
Weighted mode
0.10
0.05
0.00
−0.05
0.100
0.125
0.150
0.175
0.200
SNP effect on Transmembrane glycoprotein NMB
SNP effect on Gut microbiota abundance (genus Catenibacterium id.2153)

Batch 2−2514 : Gut microbiota abundance (genus Parasutterella id.2892) on Transmembrane glycoprotein NMB
with a threshold of 1e−05

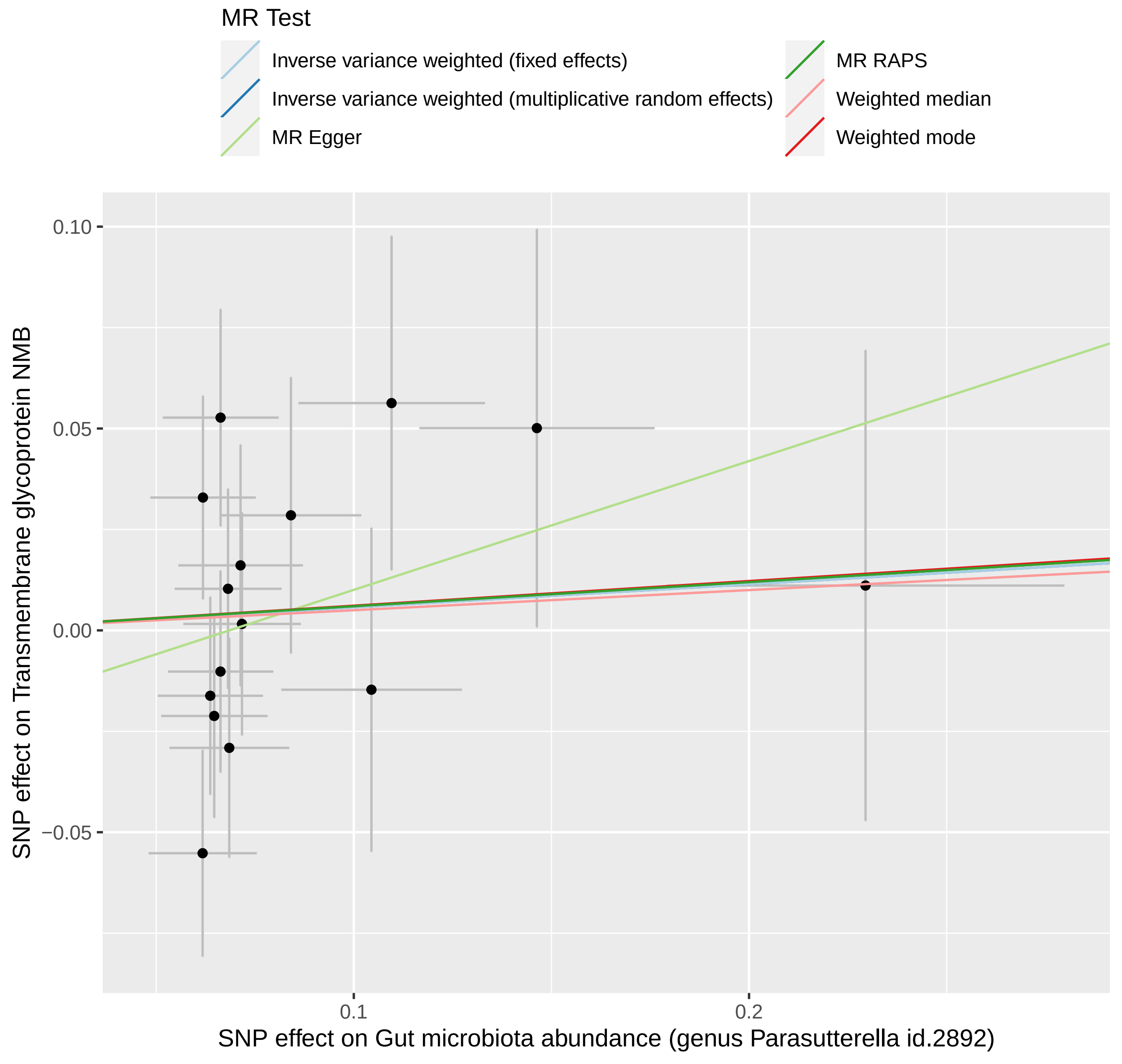

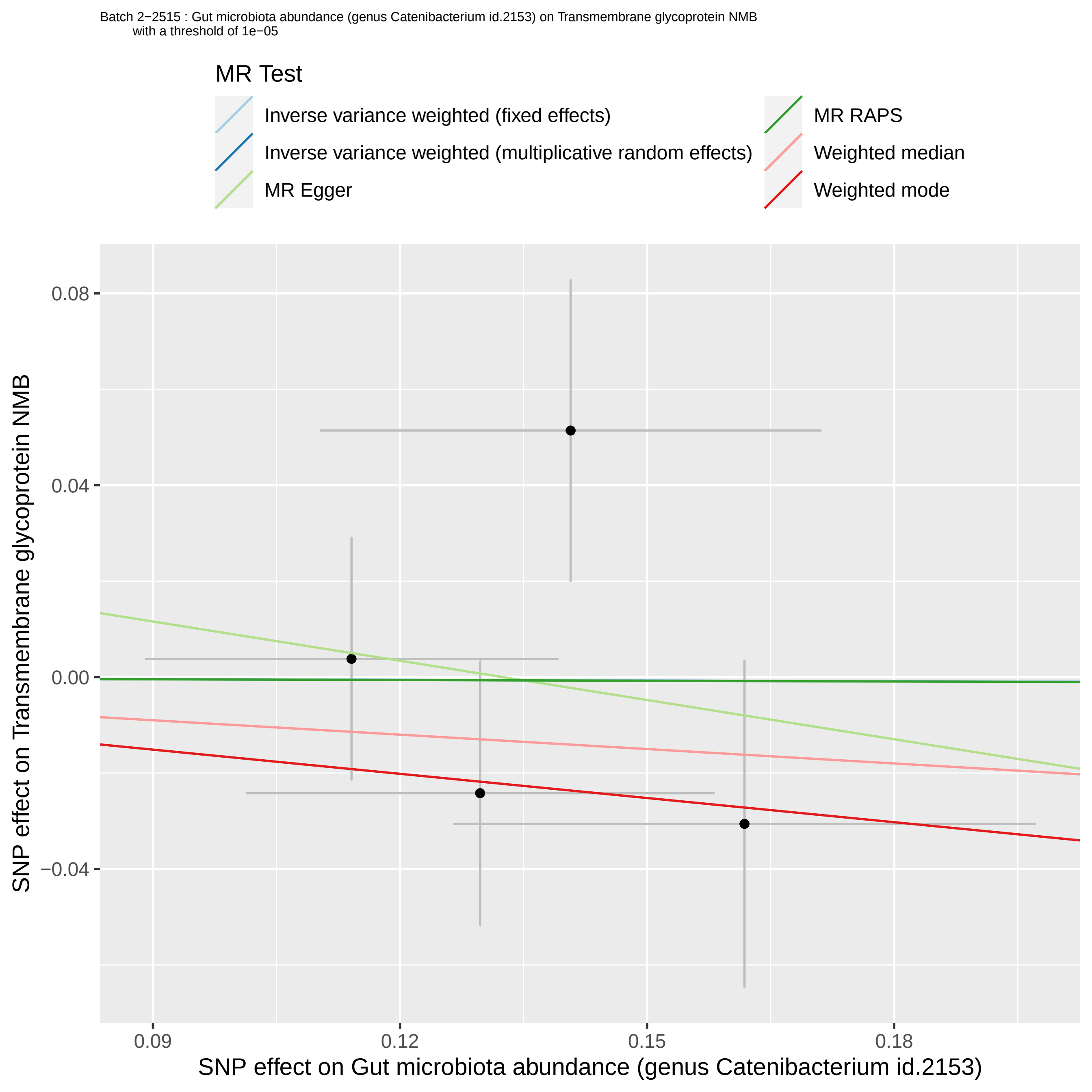

Batch 2−2515 : Gut microbiota abundance (genus Catenibacterium id.2153) on Transmembrane glycoprotein NMB
with a threshold of 1e−05
MR Test
Inverse variance weighted (fixed effects)
Inverse variance weighted (multiplicative random effects)
MR Egger
MR RAPS
Weighted median
Weighted mode
0.08
0.04
0.00
−0.04
0.09
0.12
0.15
0.18
SNP effect on Transmembrane glycoprotein NMB
SNP effect on Gut microbiota abundance (genus Catenibacterium id.2153)

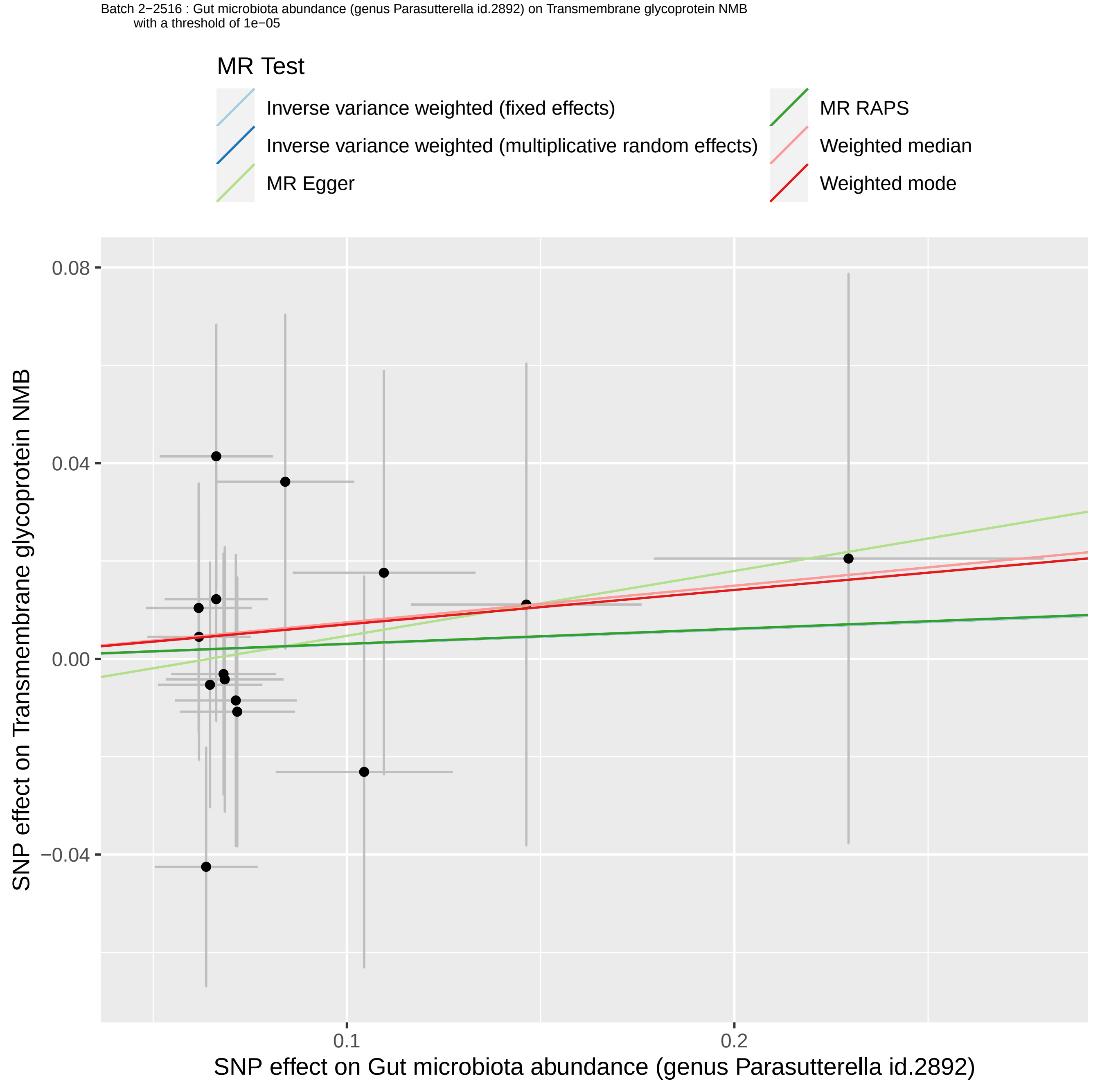
Batch 2−2516 : Gut microbiota abundance (genus Parasutterella id.2892) on Transmembrane glycoprotein NMB
with a threshold of 1e−05
MR Test
Inverse variance weighted (fixed effects)
Inverse variance weighted (multiplicative random effects)
MR Egger
MR RAPS
Weighted median
Weighted mode
0.08
0.04
0.00
−0.04
0.1
0.2
SNP effect on Transmembrane glycoprotein NMB
SNP effect on Gut microbiota abundance (genus Parasutterella id.2892)

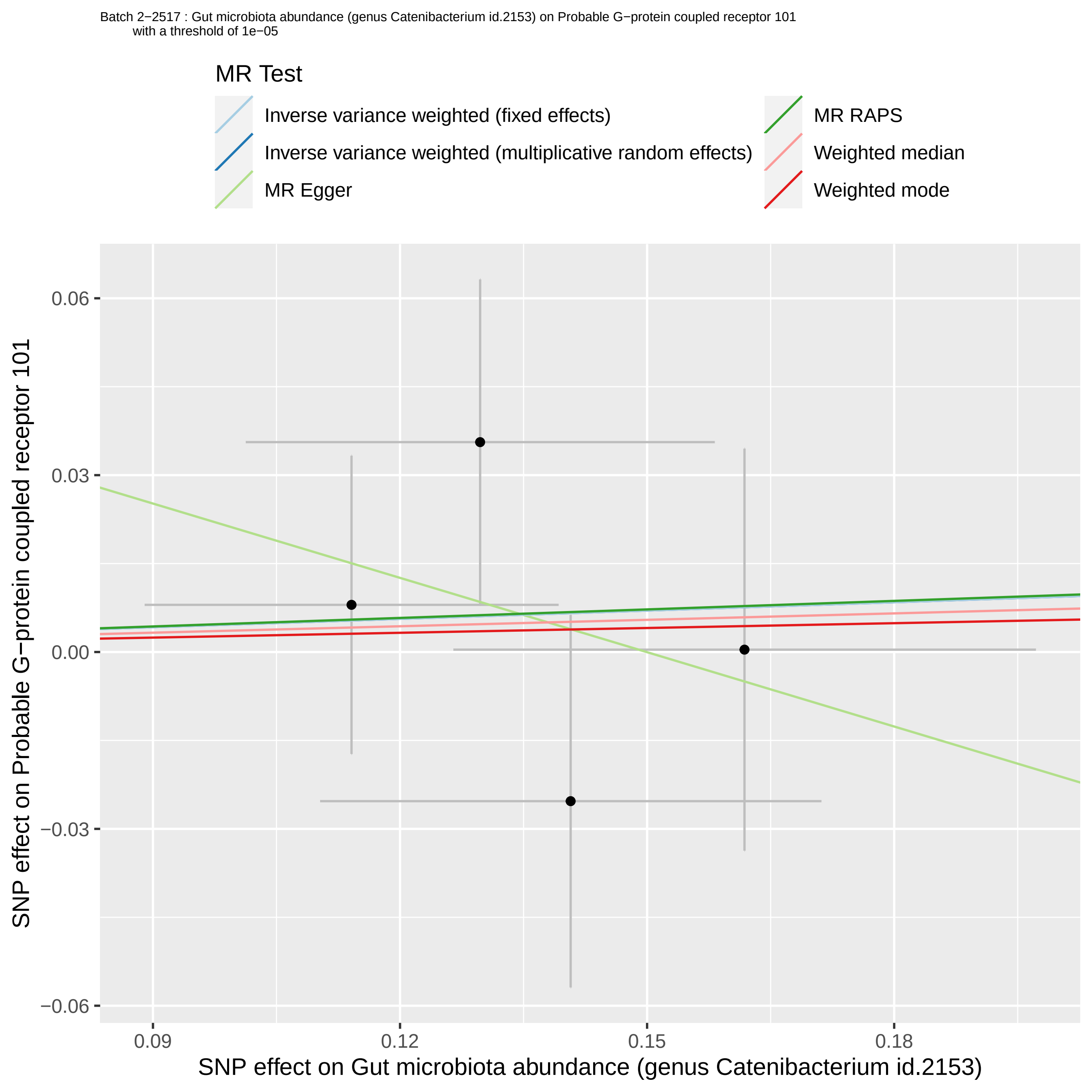
Batch 2−2517 : Gut microbiota abundance (genus Catenibacterium id.2153) on Probable G−protein coupled receptor 101
with a threshold of 1e−05
MR Test
Inverse variance weighted (fixed effects)
Inverse variance weighted (multiplicative random effects)
MR Egger
MR RAPS
Weighted median
Weighted mode
0.06
0.03
0.00
−0.03
−0.06
0.09
0.12
0.15
0.18
SNP effect on Probable G−protein coupled receptor 101
SNP effect on Gut microbiota abundance (genus Catenibacterium id.2153)

Batch 2−2518 : Gut microbiota abundance (genus Parasutterella id.2892) on Probable G−protein coupled receptor 101
with a threshold of 1e−05

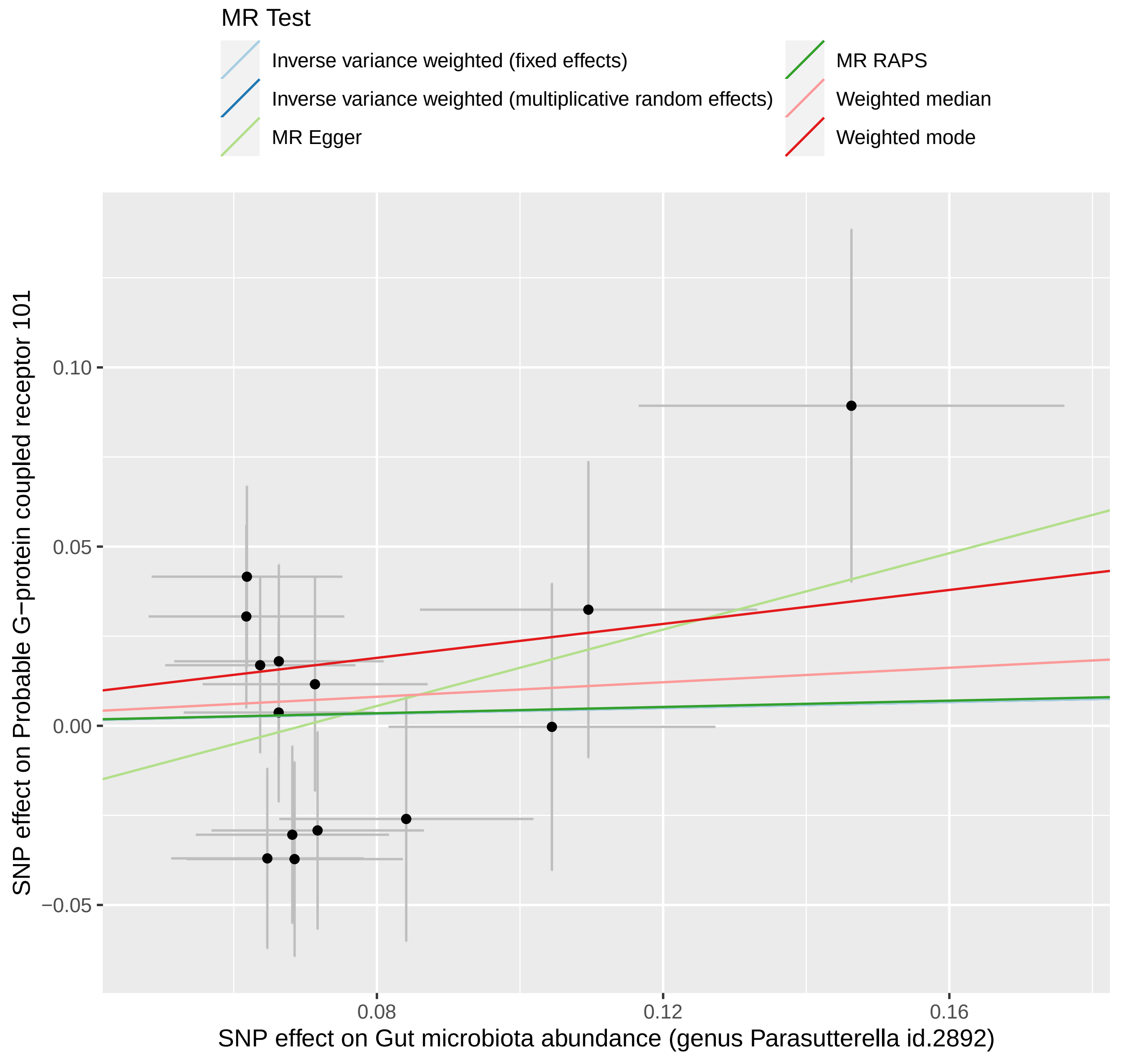

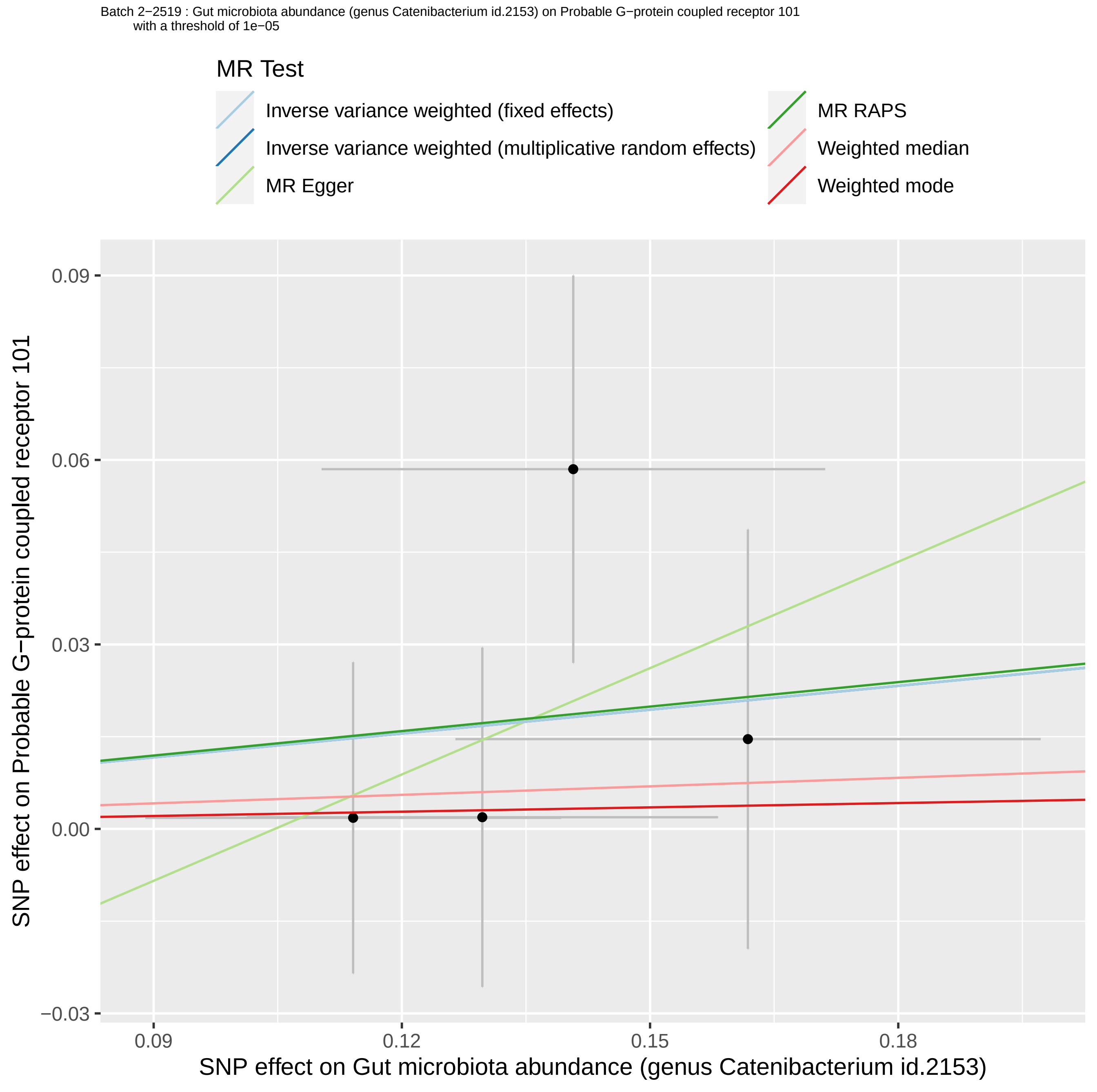
Batch 2−2519 : Gut microbiota abundance (genus Catenibacterium id.2153) on Probable G−protein coupled receptor 101
with a threshold of 1e−05
MR Test
Inverse variance weighted (fixed effects)
Inverse variance weighted (multiplicative random effects)
MR Egger
MR RAPS
Weighted median
Weighted mode
0.09
0.06
0.03
0.00
−0.03
0.09
0.12
0.15
0.18
SNP effect on Probable G−protein coupled receptor 101
SNP effect on Gut microbiota abundance (genus Catenibacterium id.2153)

Batch 2−2520 : Gut microbiota abundance (genus Parasutterella id.2892) on Probable G−protein coupled receptor 101
with a threshold of 1e−05

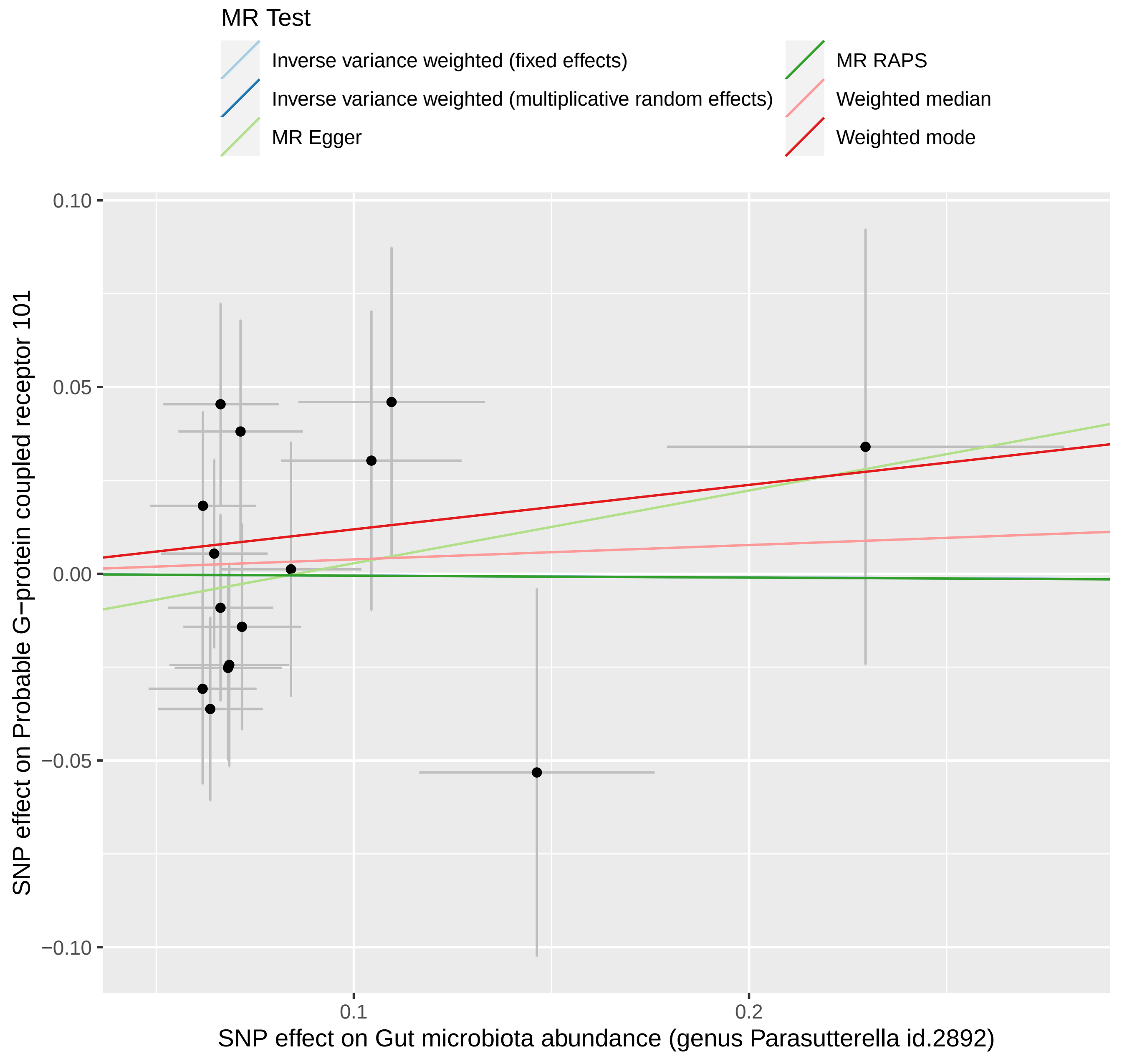

Batch 2−2521 : Gut microbiota abundance (genus Catenibacterium id.2153) on Protein GPR107
with a threshold of 1e−05

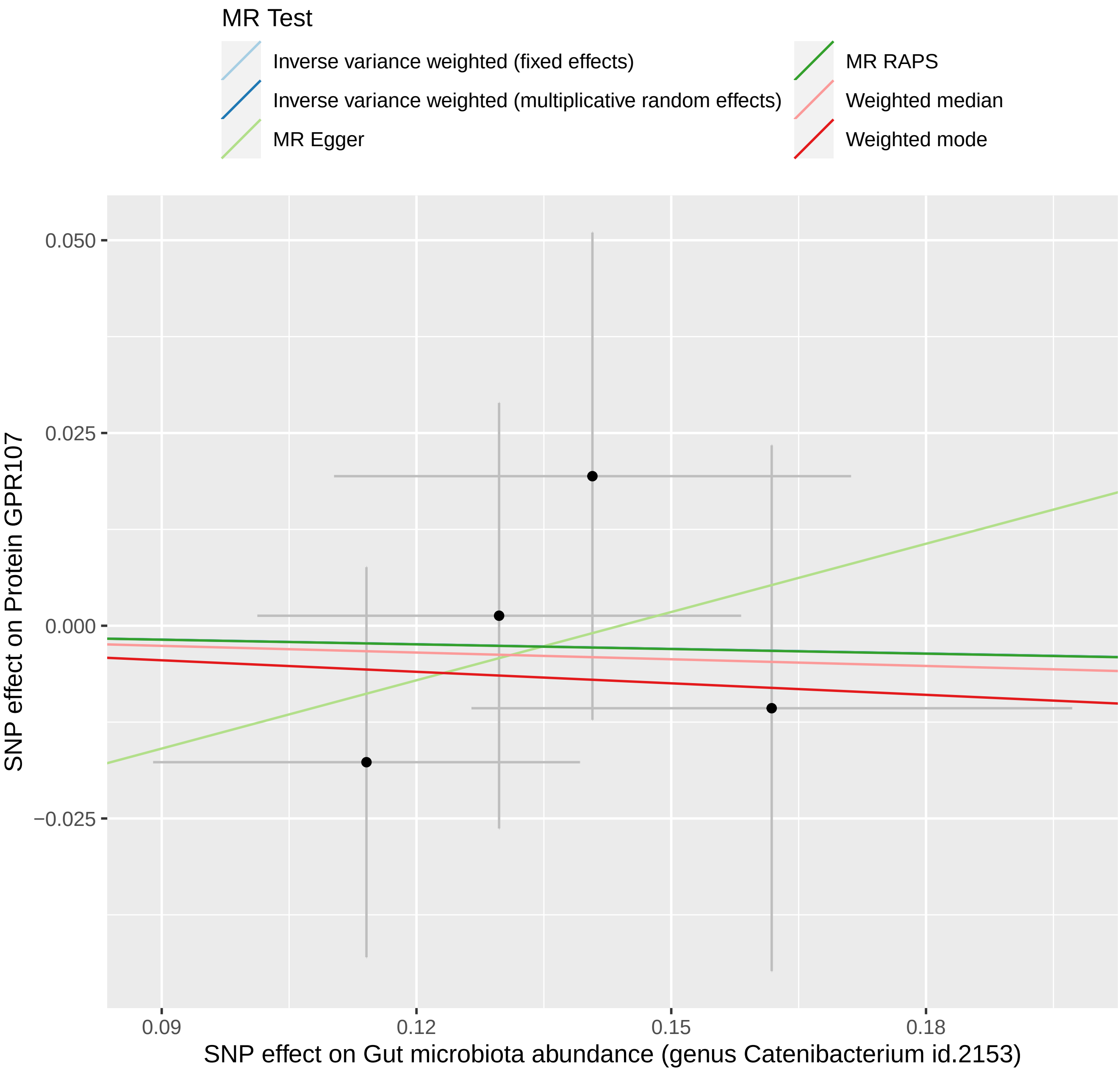

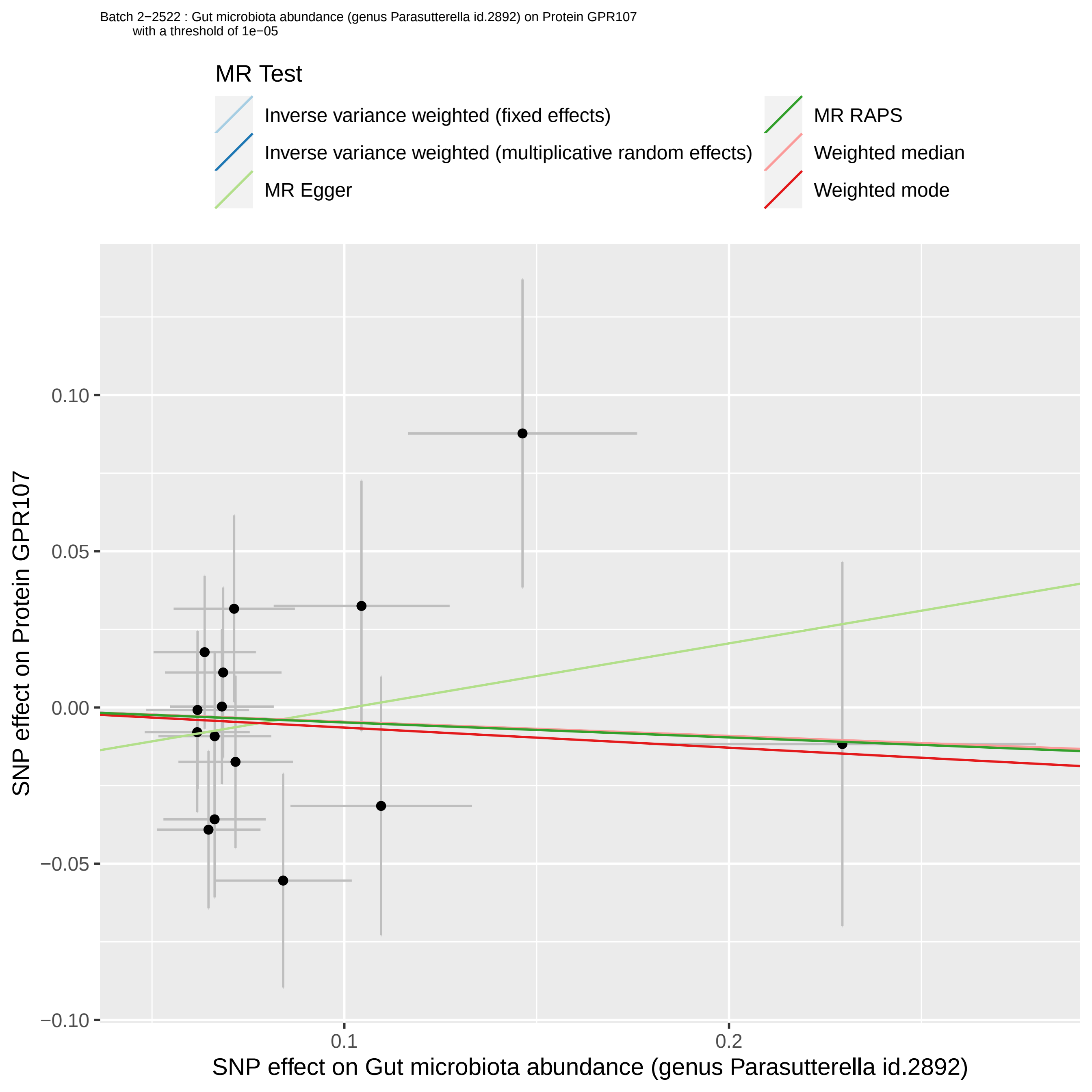

Batch 2−2522 : Gut microbiota abundance (genus Parasutterella id.2892) on Protein GPR107
with a threshold of 1e−05
MR Test
Inverse variance weighted (fixed effects)
Inverse variance weighted (multiplicative random effects)
MR Egger
MR RAPS
Weighted median
Weighted mode
0.10
0.05
0.00
−0.05
−0.10
0.1
0.2
SNP effect on Protein GPR107
SNP effect on Gut microbiota abundance (genus Parasutterella id.2892)

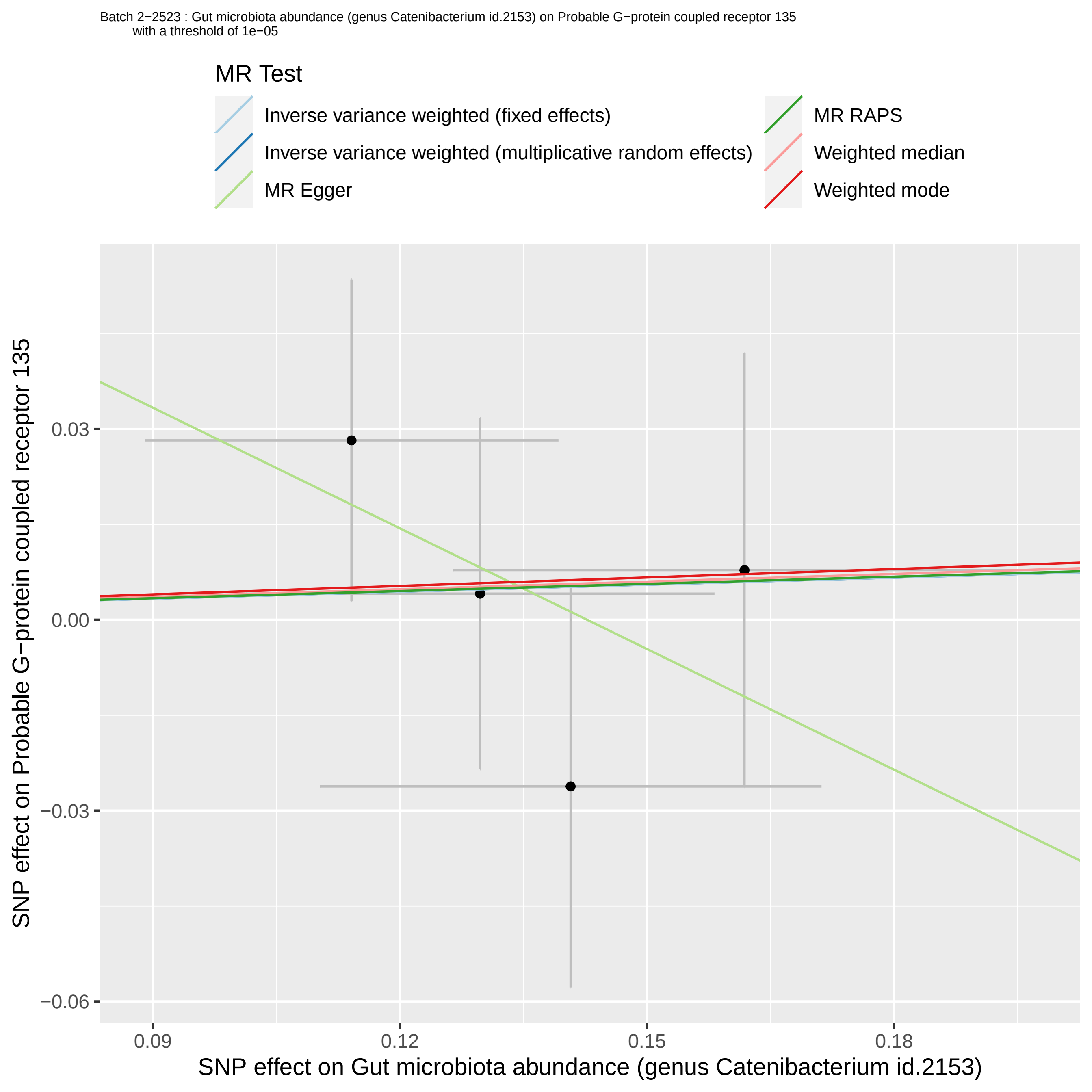

Batch 2−2523 : Gut microbiota abundance (genus Catenibacterium id.2153) on Probable G−protein coupled receptor 135
with a threshold of 1e−05
MR Test
Inverse variance weighted (fixed effects)
Inverse variance weighted (multiplicative random effects)
MR Egger
MR RAPS
Weighted median
Weighted mode
0.03
0.00
−0.03
−0.06
0.09
0.12
0.15
0.18
SNP effect on Probable G−protein coupled receptor 135
SNP effect on Gut microbiota abundance (genus Catenibacterium id.2153)

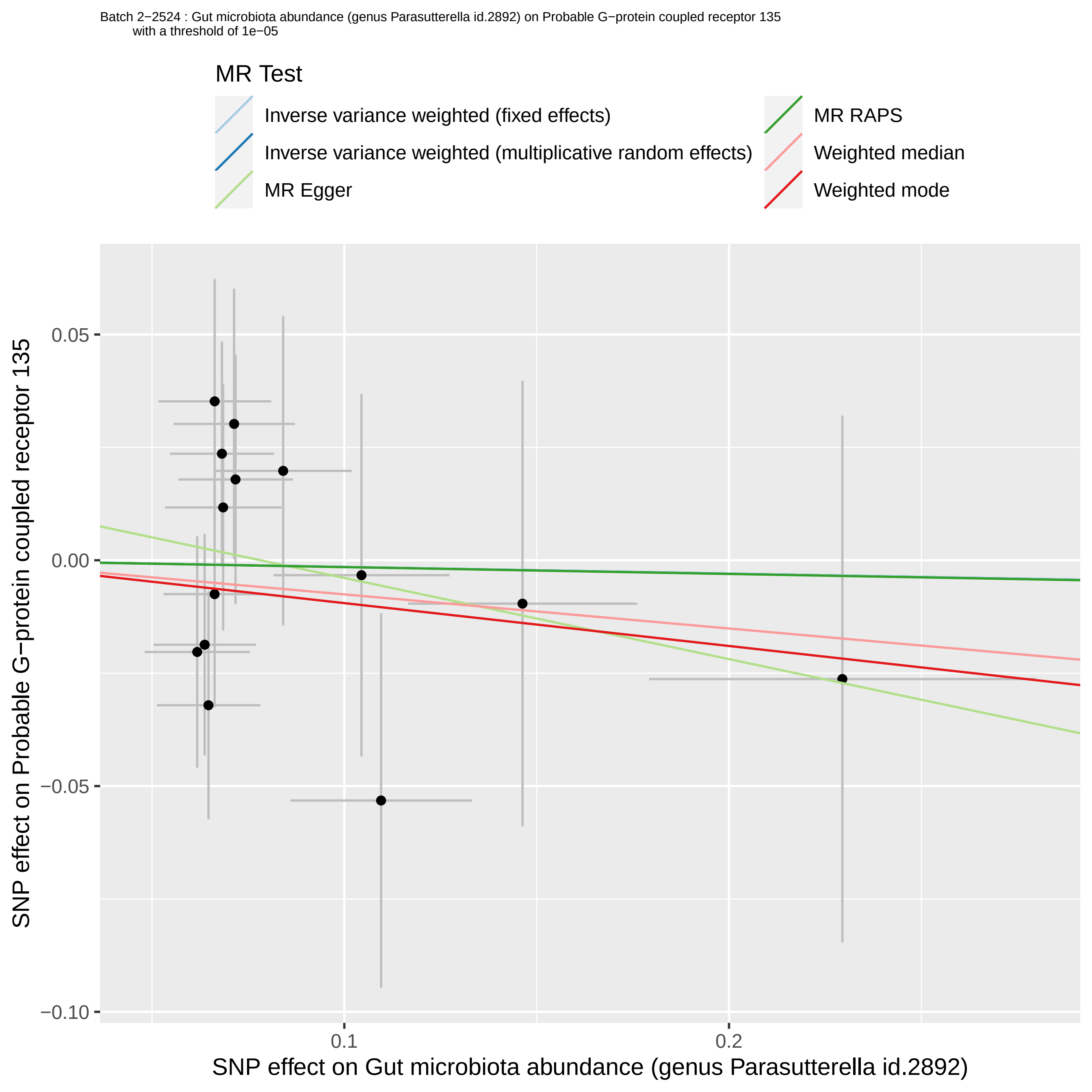
Batch 2−2524 : Gut microbiota abundance (genus Parasutterella id.2892) on Probable G−protein coupled receptor 135
with a threshold of 1e−05
MR Test
Inverse variance weighted (fixed effects)
Inverse variance weighted (multiplicative random effects)
MR Egger
MR RAPS
Weighted median
Weighted mode
0.05
0.00
−0.05
−0.10
0.1
0.2
SNP effect on Probable G−protein coupled receptor 135
SNP effect on Gut microbiota abundance (genus Parasutterella id.2892)

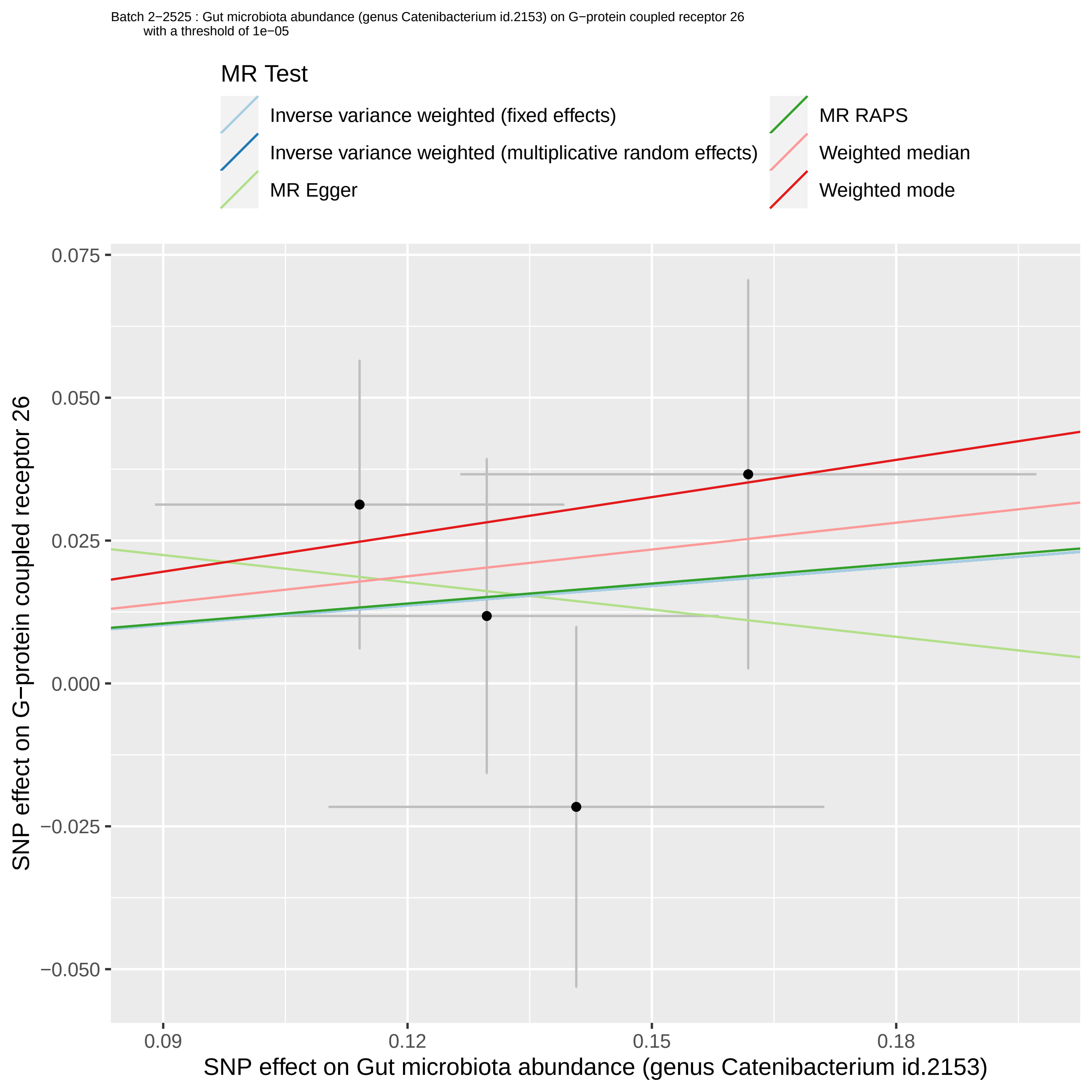
Batch 2−2525 : Gut microbiota abundance (genus Catenibacterium id.2153) on G−protein coupled receptor 26
with a threshold of 1e−05
MR Test
Inverse variance weighted (fixed effects)
Inverse variance weighted (multiplicative random effects)
MR Egger
MR RAPS
Weighted median
Weighted mode
0.075
0.050
0.025
0.000
−0.025
−0.050
0.09
0.12
0.15
0.18
SNP effect on G−protein coupled receptor 26
SNP effect on Gut microbiota abundance (genus Catenibacterium id.2153)

Batch 2−2526 : Gut microbiota abundance (genus Parasutterella id.2892) on G−protein coupled receptor 26
with a threshold of 1e−05

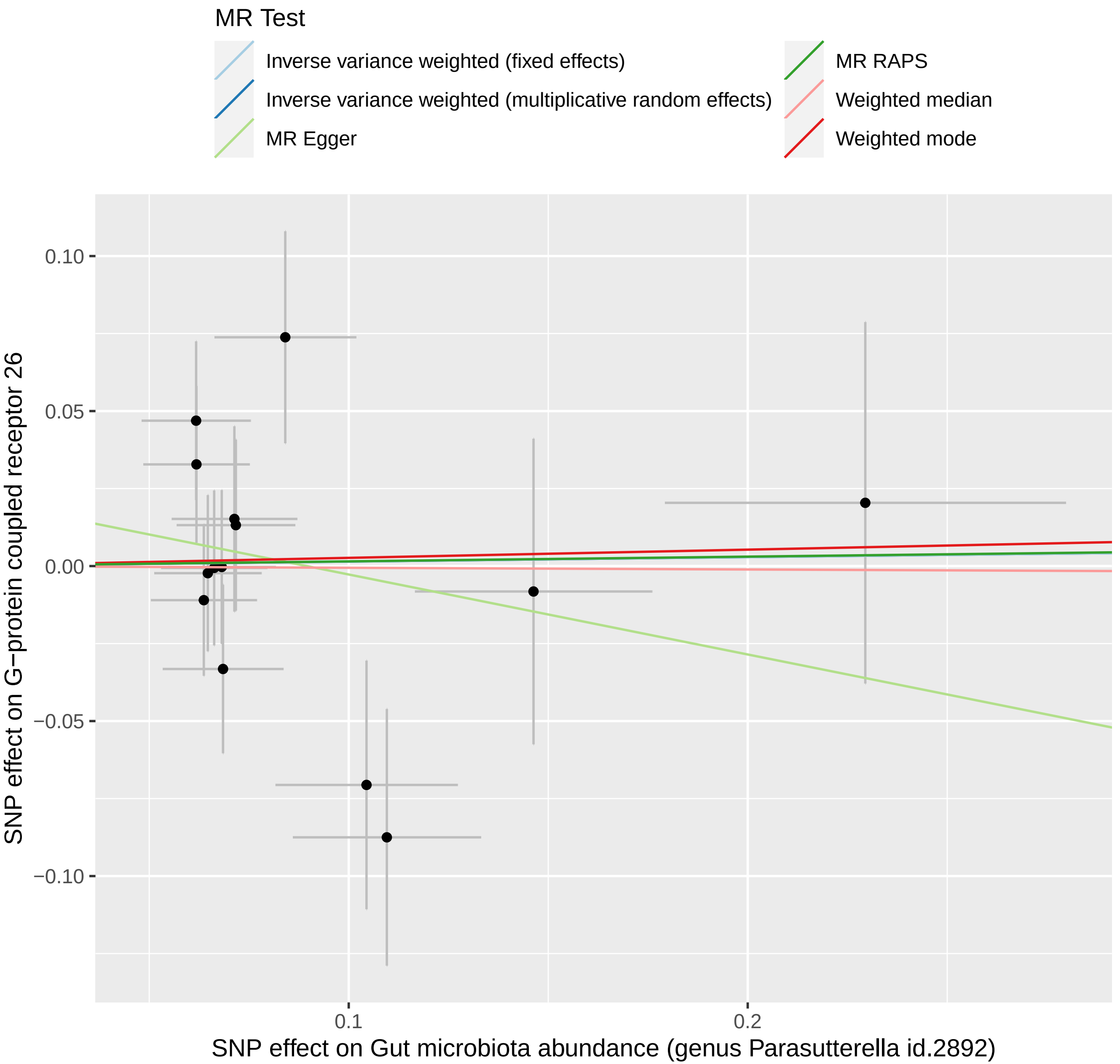

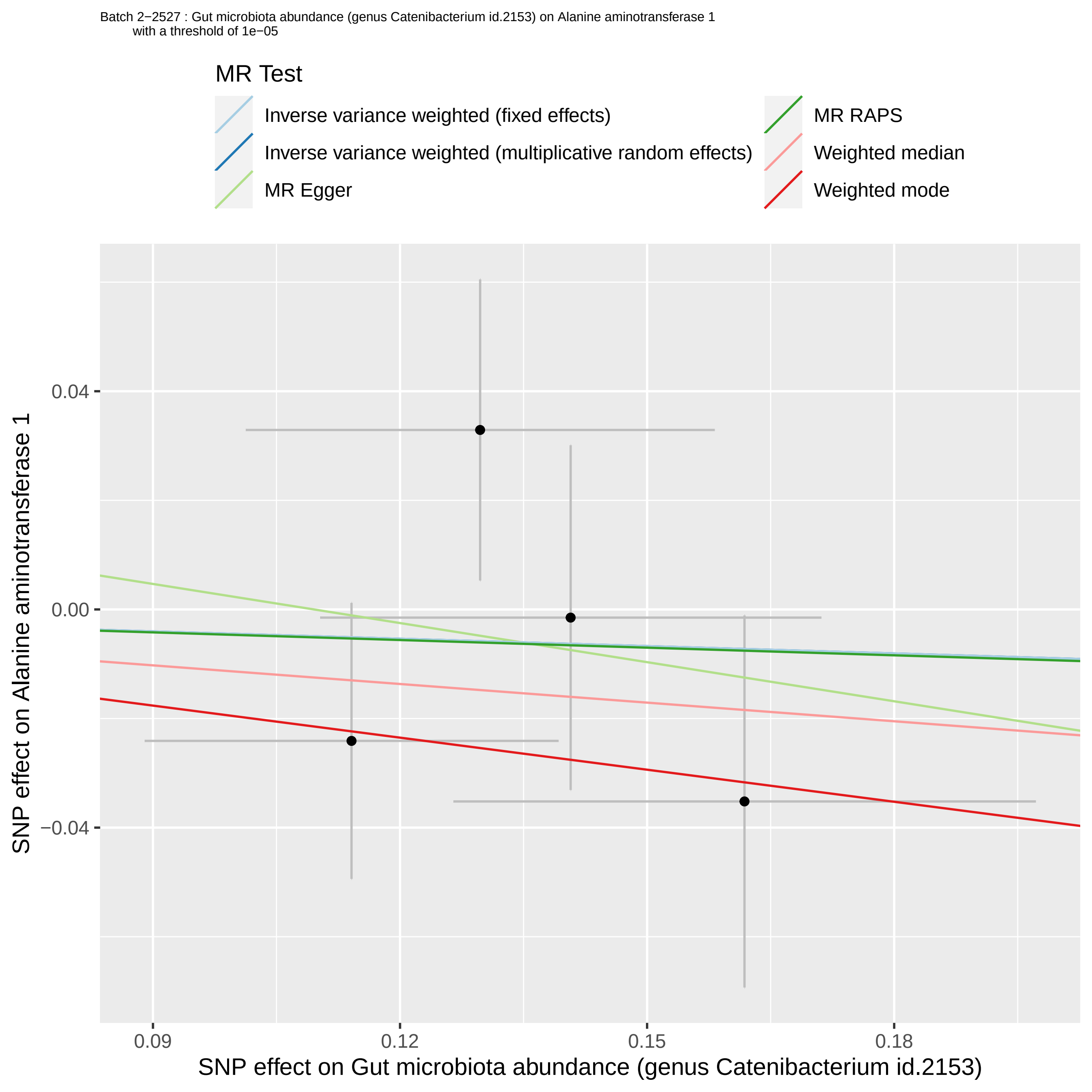

Batch 2−2527 : Gut microbiota abundance (genus Catenibacterium id.2153) on Alanine aminotransferase 1
with a threshold of 1e−05
MR Test
Inverse variance weighted (fixed effects)
Inverse variance weighted (multiplicative random effects)
MR Egger
MR RAPS
Weighted median
Weighted mode
0.04
0.00
−0.04
0.09
0.12
0.15
0.18
SNP effect on Alanine aminotransferase 1
SNP effect on Gut microbiota abundance (genus Catenibacterium id.2153)

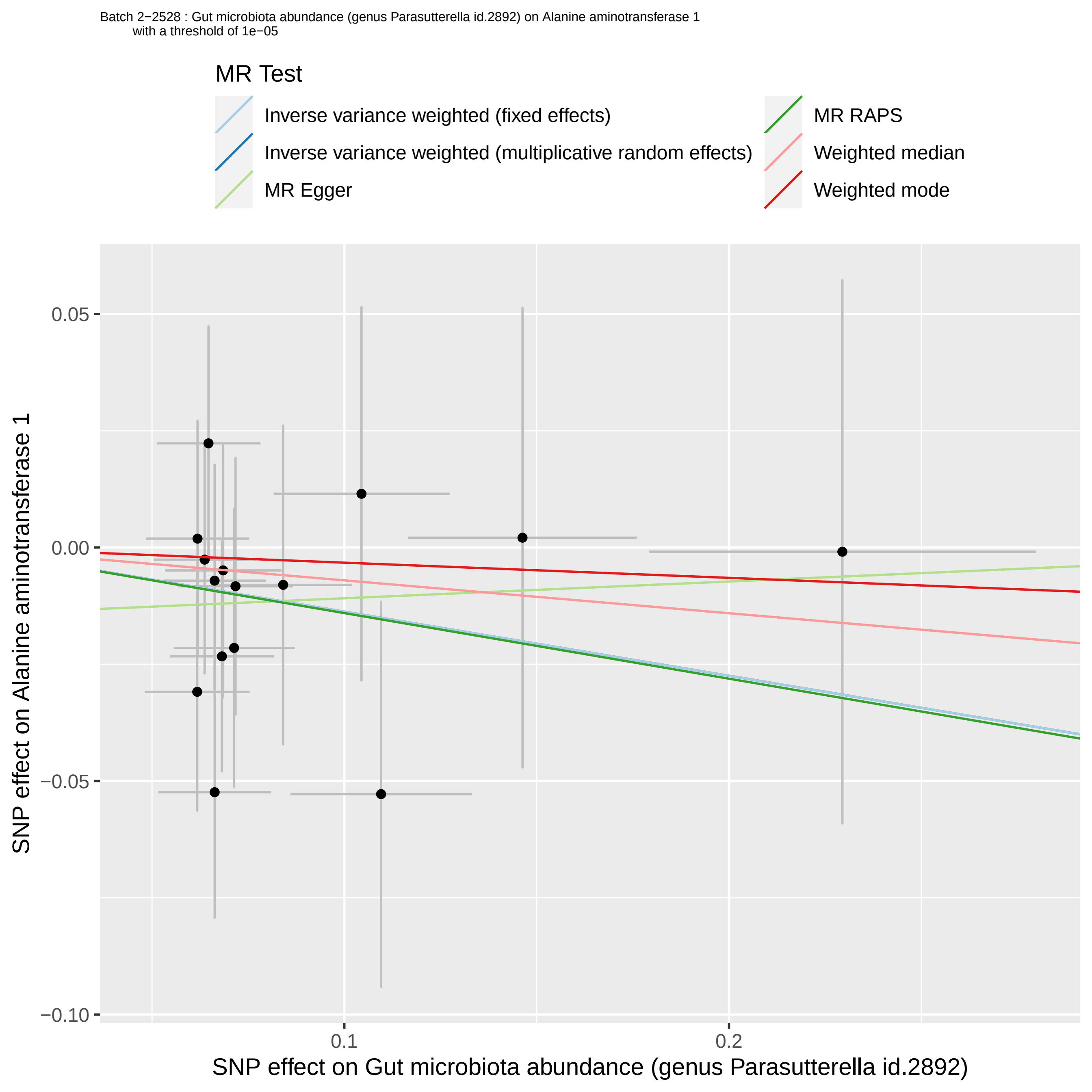
Batch 2−2528 : Gut microbiota abundance (genus Parasutterella id.2892) on Alanine aminotransferase 1
with a threshold of 1e−05
MR Test
Inverse variance weighted (fixed effects)
Inverse variance weighted (multiplicative random effects)
MR Egger
MR RAPS
Weighted median
Weighted mode
0.05
0.00
−0.05
−0.10
0.1
0.2
SNP effect on Alanine aminotransferase 1
SNP effect on Gut microbiota abundance (genus Parasutterella id.2892)

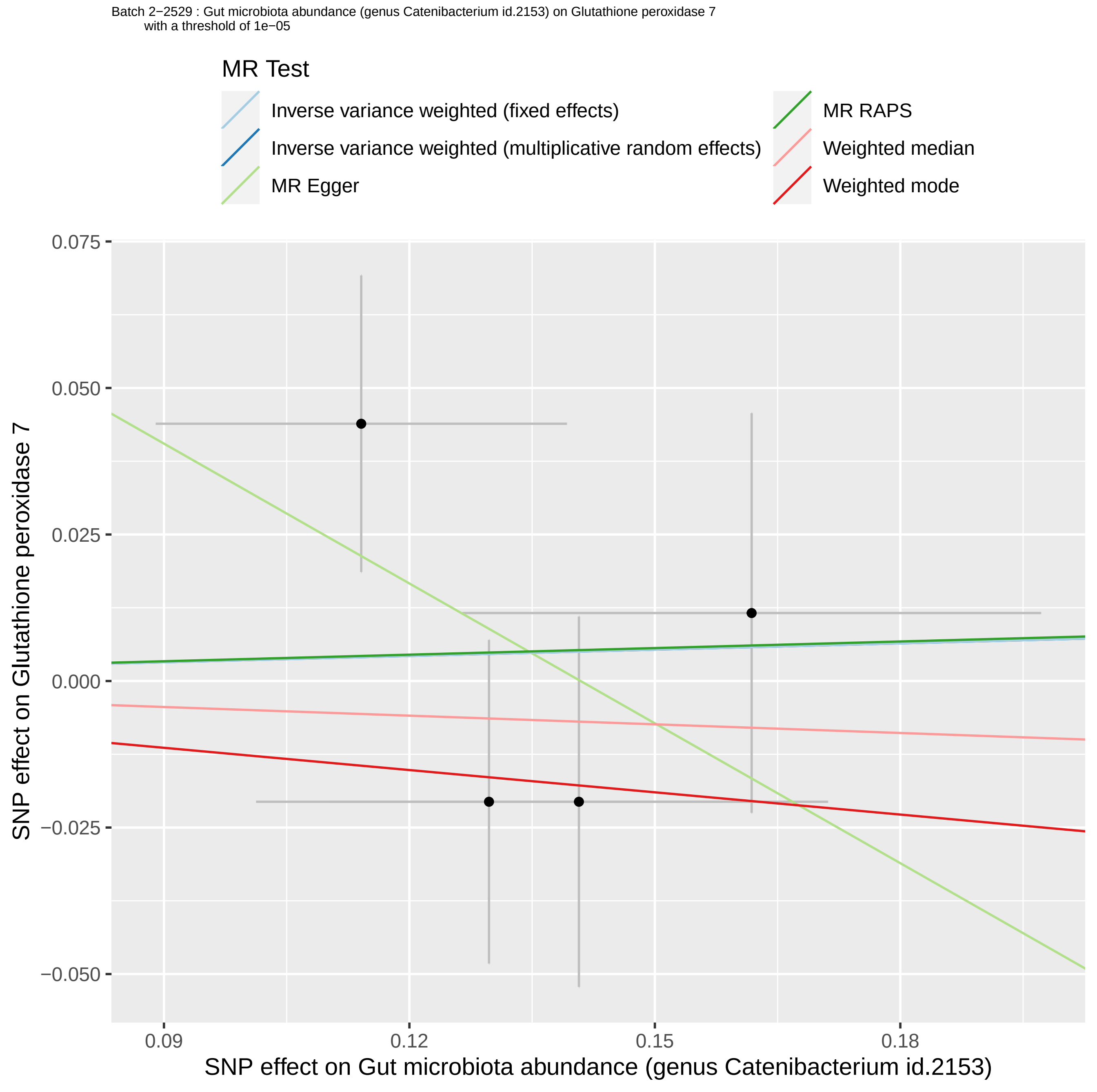

Batch 2−2529 : Gut microbiota abundance (genus Catenibacterium id.2153) on Glutathione peroxidase 7
with a threshold of 1e−05
MR Test
Inverse variance weighted (fixed effects)
Inverse variance weighted (multiplicative random effects)
MR Egger
MR RAPS
Weighted median
Weighted mode
0.075
0.050
0.025
0.000
−0.025
−0.050
0.09
0.12
0.15
0.18
SNP effect on Glutathione peroxidase 7
SNP effect on Gut microbiota abundance (genus Catenibacterium id.2153)

Batch 2−2530 : Gut microbiota abundance (genus Parasutterella id.2892) on Glutathione peroxidase 7
with a threshold of 1e−05

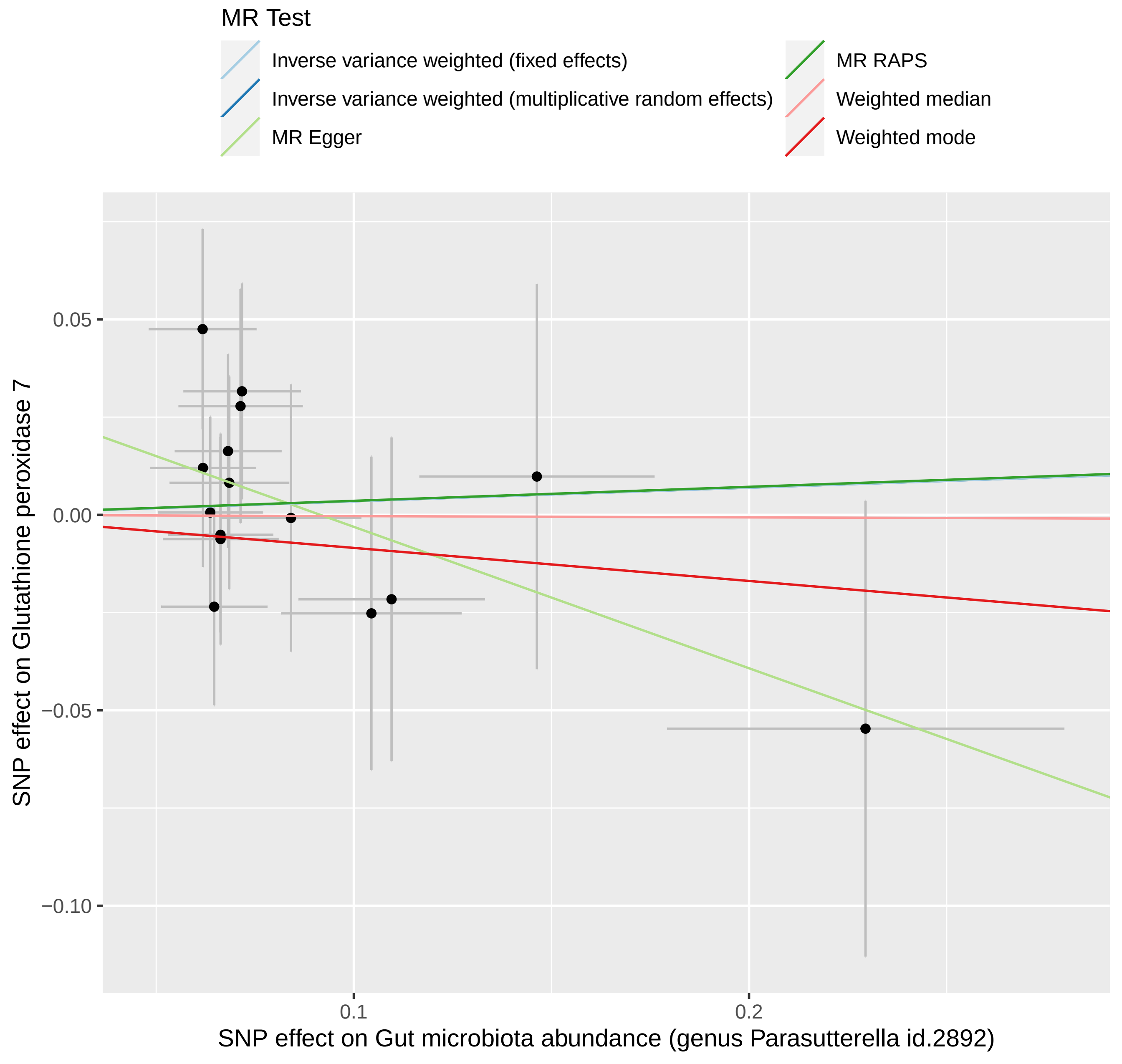

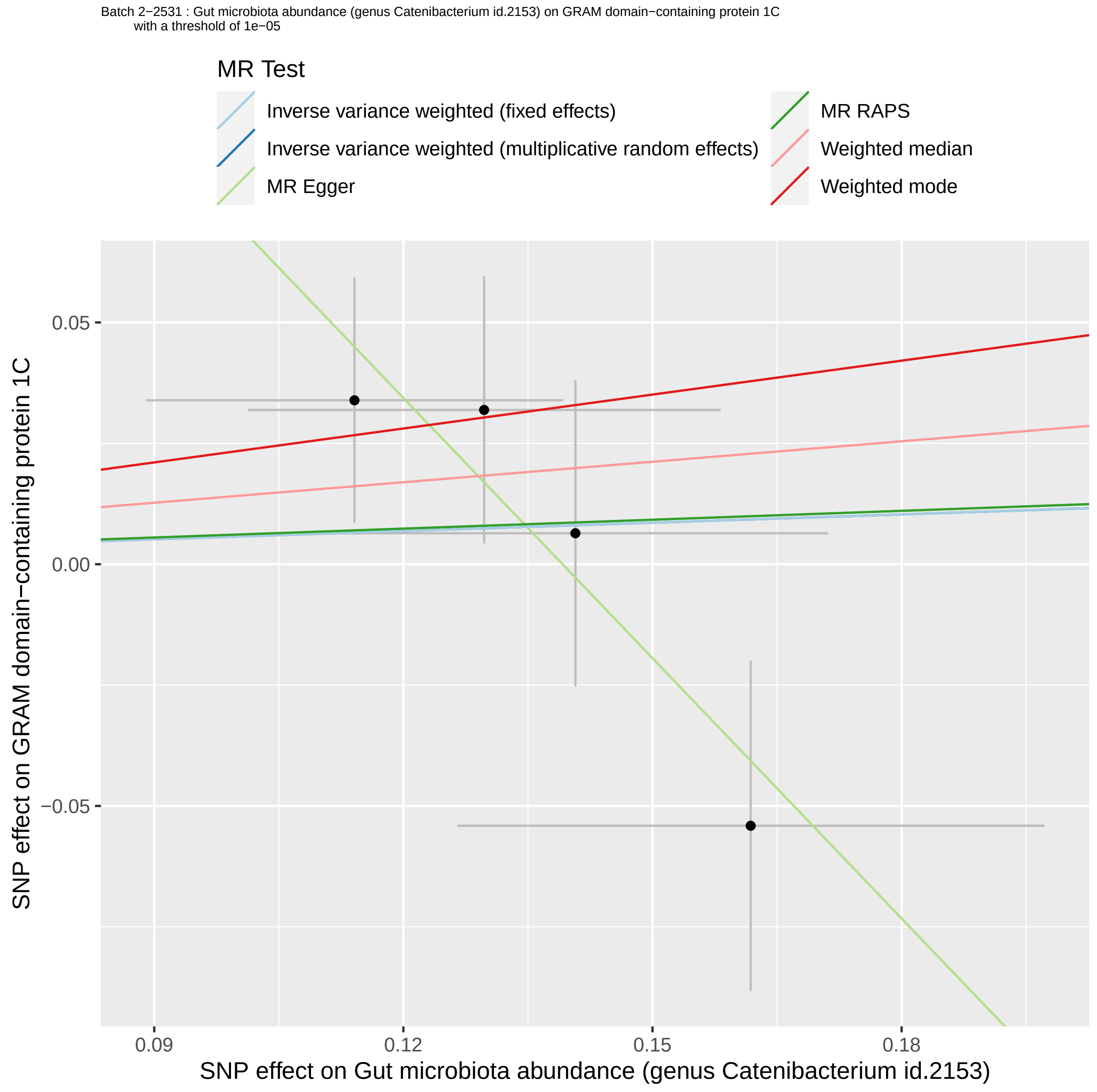
Batch 2−2531 : Gut microbiota abundance (genus Catenibacterium id.2153) on GRAM domain−containing protein 1C
with a threshold of 1e−05
MR Test
Inverse variance weighted (fixed effects)
Inverse variance weighted (multiplicative random effects)
MR Egger
MR RAPS
Weighted median
Weighted mode
0.05
0.00
−0.05
0.09
0.12
0.15
0.18
SNP effect on GRAM domain−containing protein 1C
SNP effect on Gut microbiota abundance (genus Catenibacterium id.2153)

Batch 2−2532 : Gut microbiota abundance (genus Parasutterella id.2892) on GRAM domain−containing protein 1C
with a threshold of 1e−05

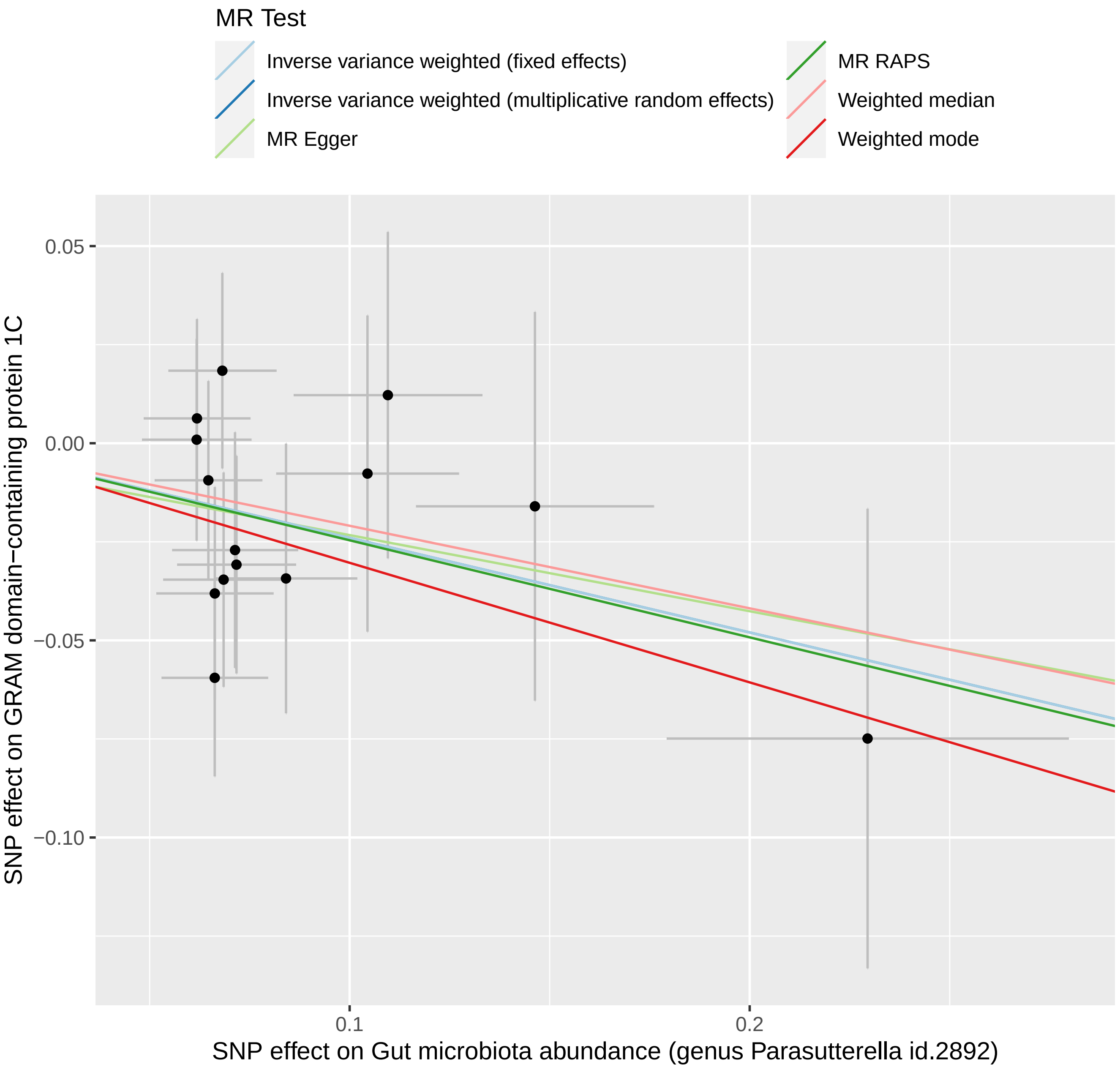

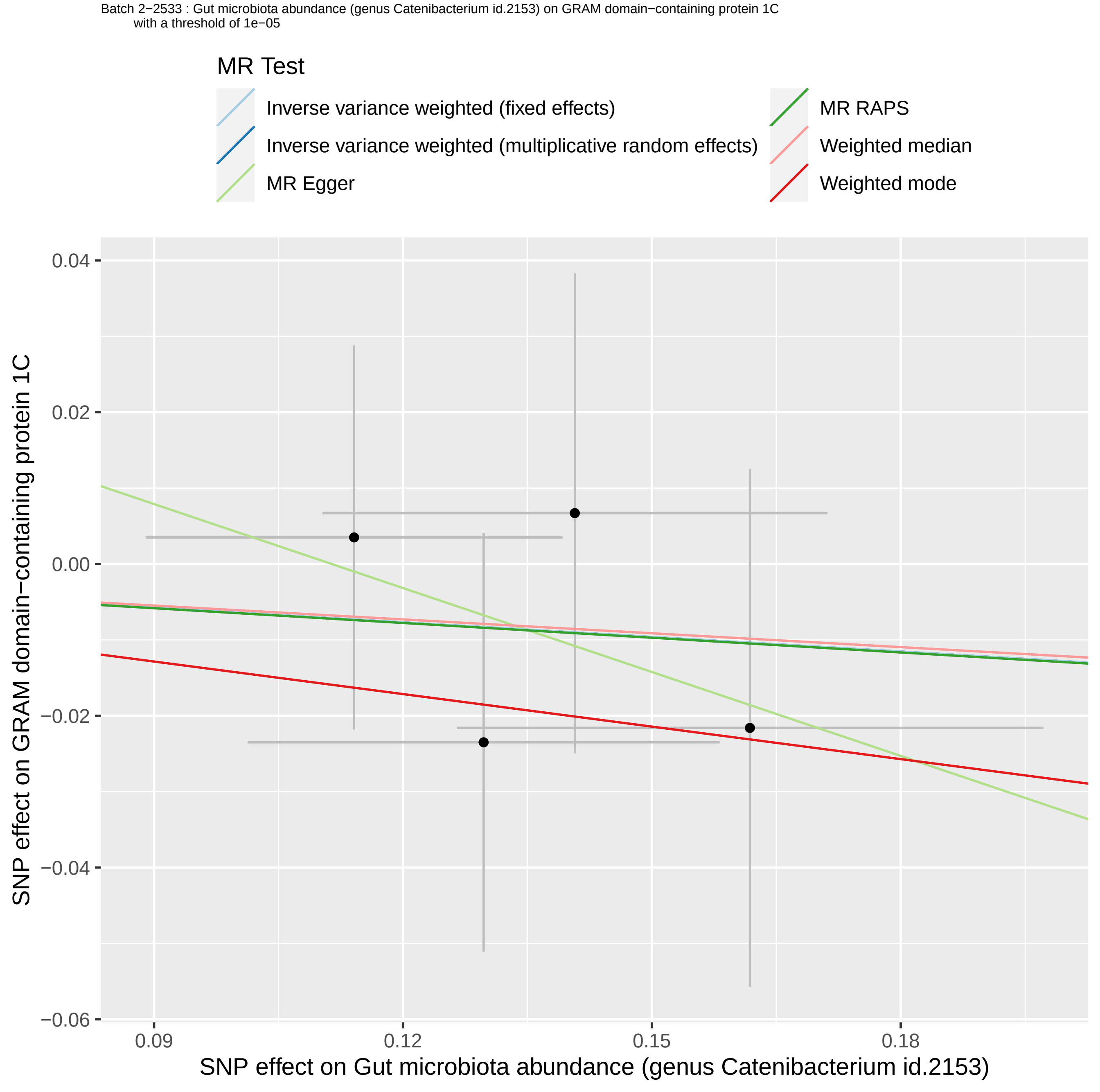

Batch 2−2533 : Gut microbiota abundance (genus Catenibacterium id.2153) on GRAM domain−containing protein 1C
with a threshold of 1e−05
MR Test
Inverse variance weighted (fixed effects)
Inverse variance weighted (multiplicative random effects)
MR Egger
MR RAPS
Weighted median
Weighted mode
0.04
0.02
0.00
−0.02
−0.04
−0.06
0.09
0.12
0.15
0.18
SNP effect on GRAM domain−containing protein 1C
SNP effect on Gut microbiota abundance (genus Catenibacterium id.2153)

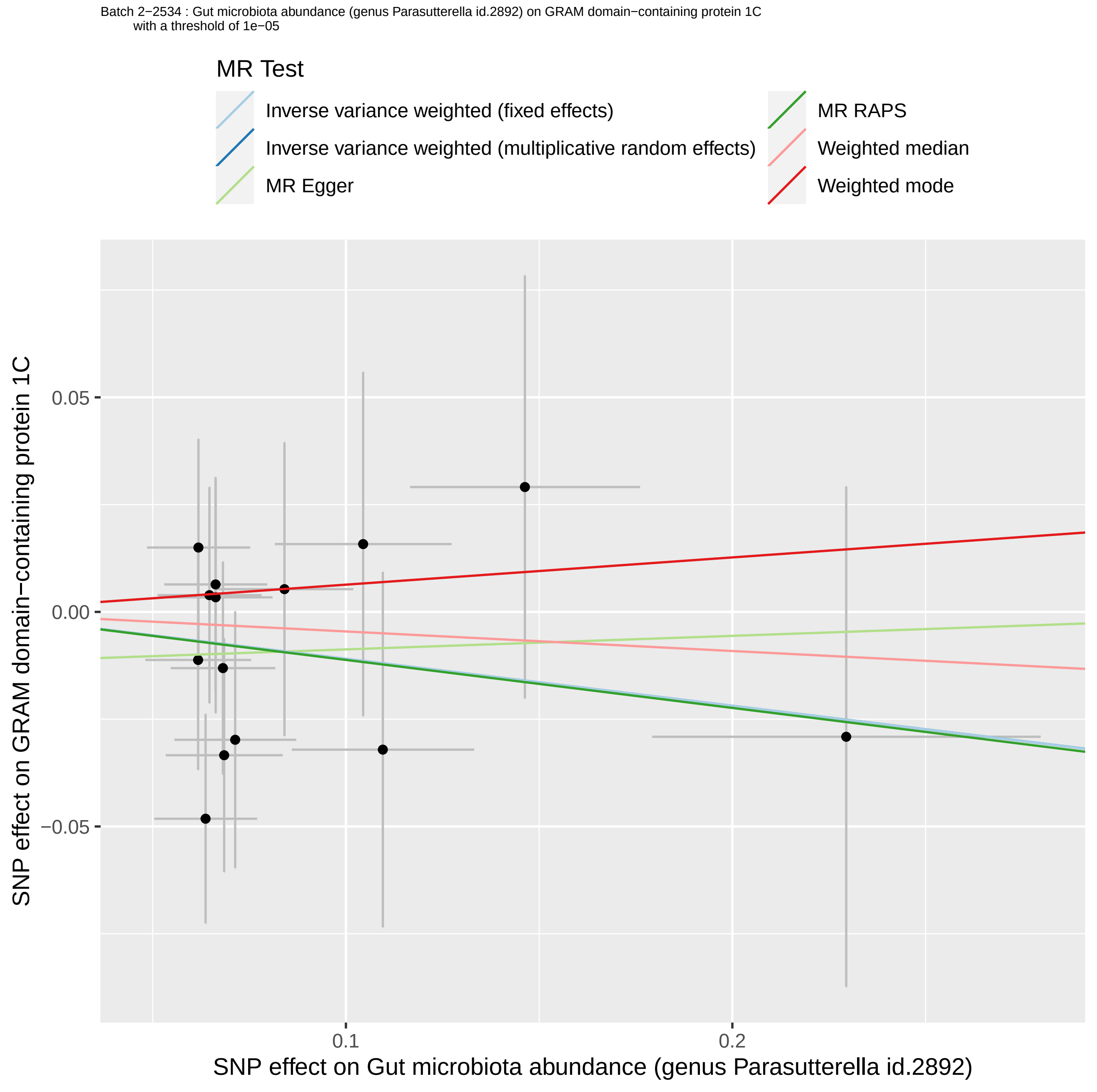

Batch 2−2534 : Gut microbiota abundance (genus Parasutterella id.2892) on GRAM domain−containing protein 1C
with a threshold of 1e−05
MR Test
Inverse variance weighted (fixed effects)
Inverse variance weighted (multiplicative random effects)
MR Egger
MR RAPS
Weighted median
Weighted mode
0.05
0.00
−0.05
0.1
0.2
SNP effect on GRAM domain−containing protein 1C
SNP effect on Gut microbiota abundance (genus Parasutterella id.2892)

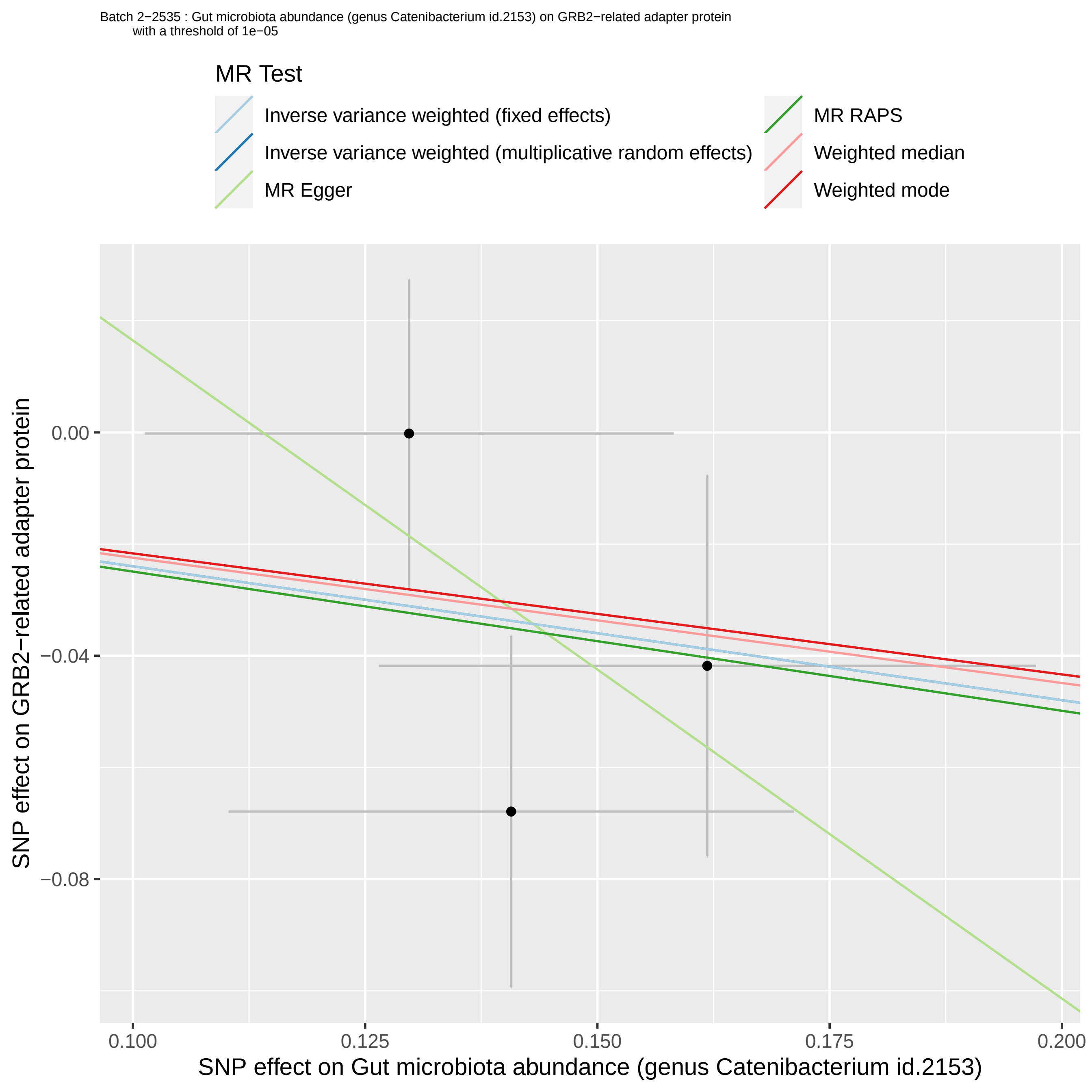
Batch 2−2535 : Gut microbiota abundance (genus Catenibacterium id.2153) on GRB2−related adapter protein
with a threshold of 1e−05
MR Test
Inverse variance weighted (fixed effects)
Inverse variance weighted (multiplicative random effects)
MR Egger
MR RAPS
Weighted median
Weighted mode
0.00
−0.04
−0.08
0.100
0.125
0.150
0.175
0.200
SNP effect on GRB2−related adapter protein
SNP effect on Gut microbiota abundance (genus Catenibacterium id.2153)

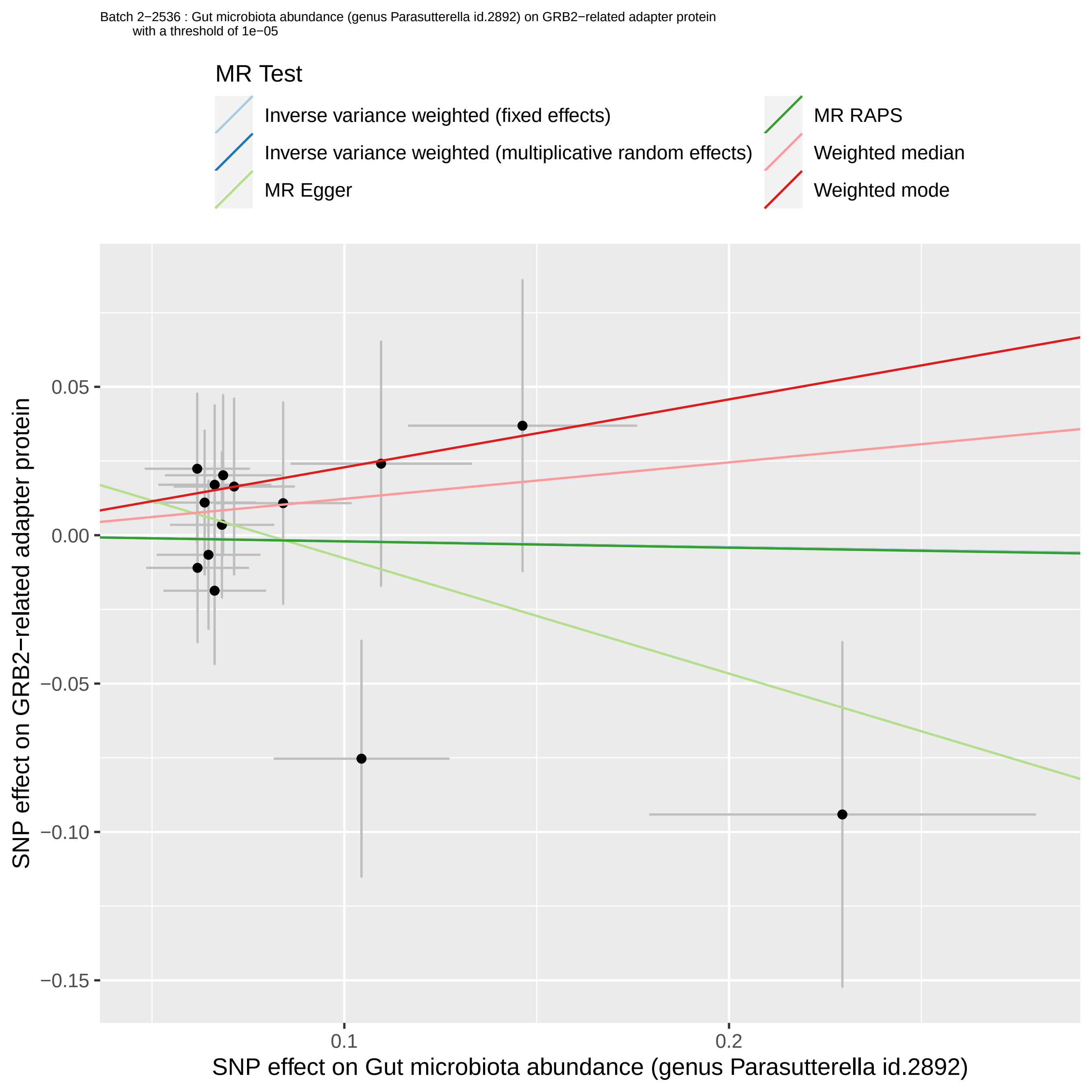
Batch 2−2536 : Gut microbiota abundance (genus Parasutterella id.2892) on GRB2−related adapter protein
with a threshold of 1e−05
MR Test
Inverse variance weighted (fixed effects)
Inverse variance weighted (multiplicative random effects)
MR Egger
MR RAPS
Weighted median
Weighted mode
SNP effect on GRB2−related adapter protein
0.05
0.00
−0.05
−0.10
−0.15
0.1
0.2
SNP effect on Gut microbiota abundance (genus Parasutterella id.2892)

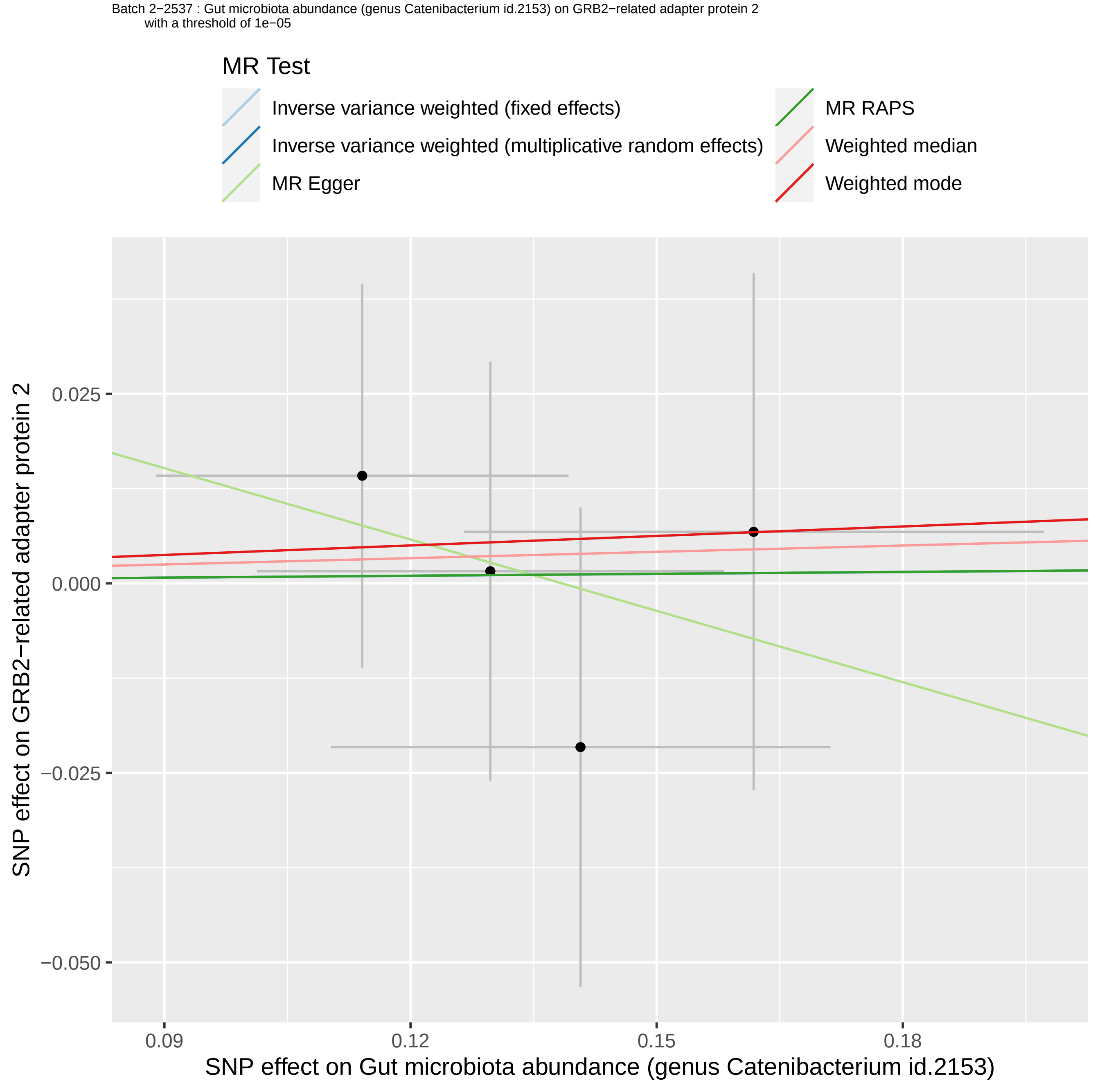
Batch 2−2537 : Gut microbiota abundance (genus Catenibacterium id.2153) on GRB2−related adapter protein 2
with a threshold of 1e−05
MR Test
Inverse variance weighted (fixed effects)
Inverse variance weighted (multiplicative random effects)
MR Egger
MR RAPS
Weighted median
Weighted mode
0.025
0.000
−0.025
−0.050
0.09
0.12
0.15
0.18
SNP effect on GRB2−related adapter protein 2
SNP effect on Gut microbiota abundance (genus Catenibacterium id.2153)

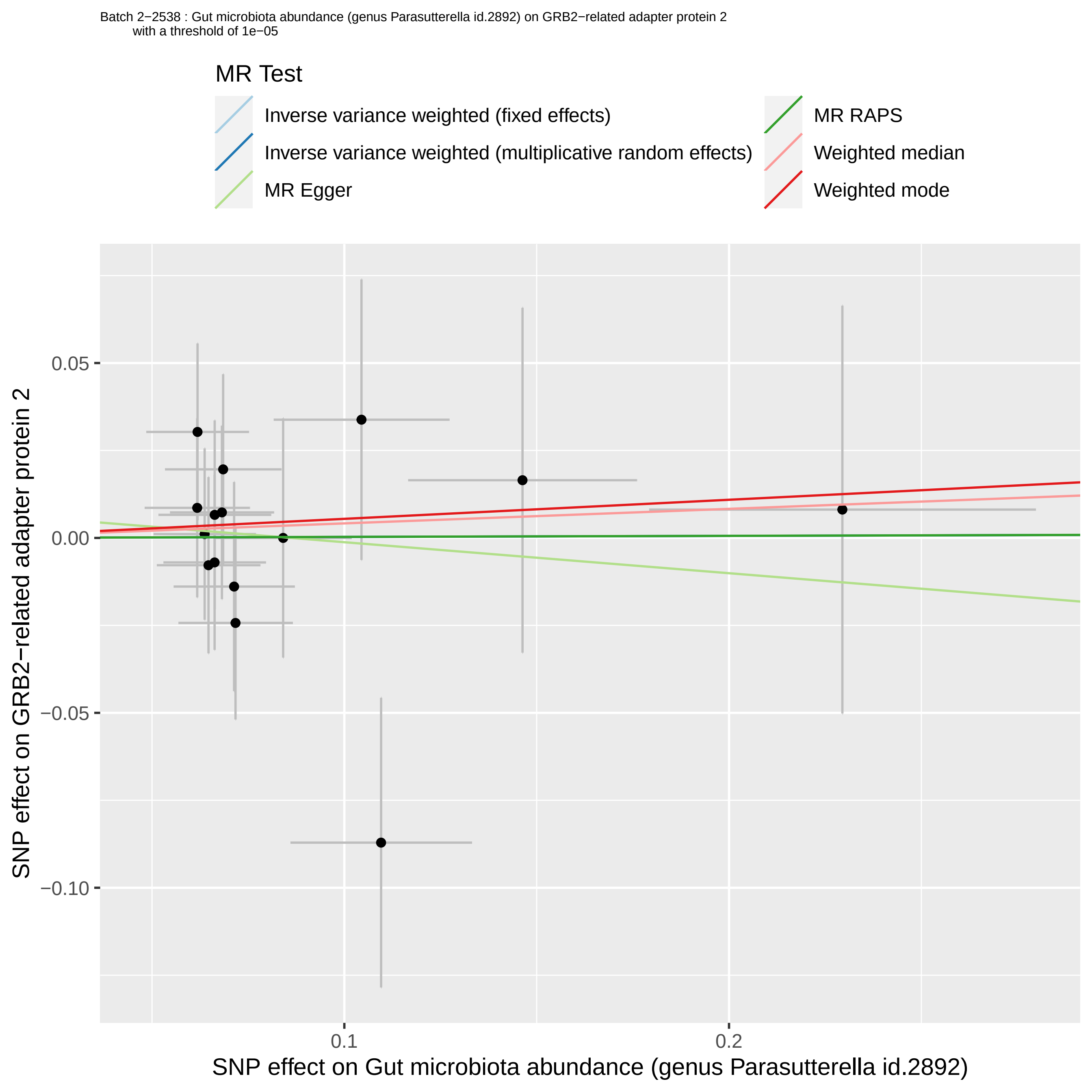
Batch 2−2538 : Gut microbiota abundance (genus Parasutterella id.2892) on GRB2−related adapter protein 2
with a threshold of 1e−05
MR Test
Inverse variance weighted (fixed effects)
Inverse variance weighted (multiplicative random effects)
MR Egger
MR RAPS
Weighted median
Weighted mode
0.05
0.00
−0.05
−0.10
0.1
0.2
SNP effect on GRB2−related adapter protein 2
SNP effect on Gut microbiota abundance (genus Parasutterella id.2892)

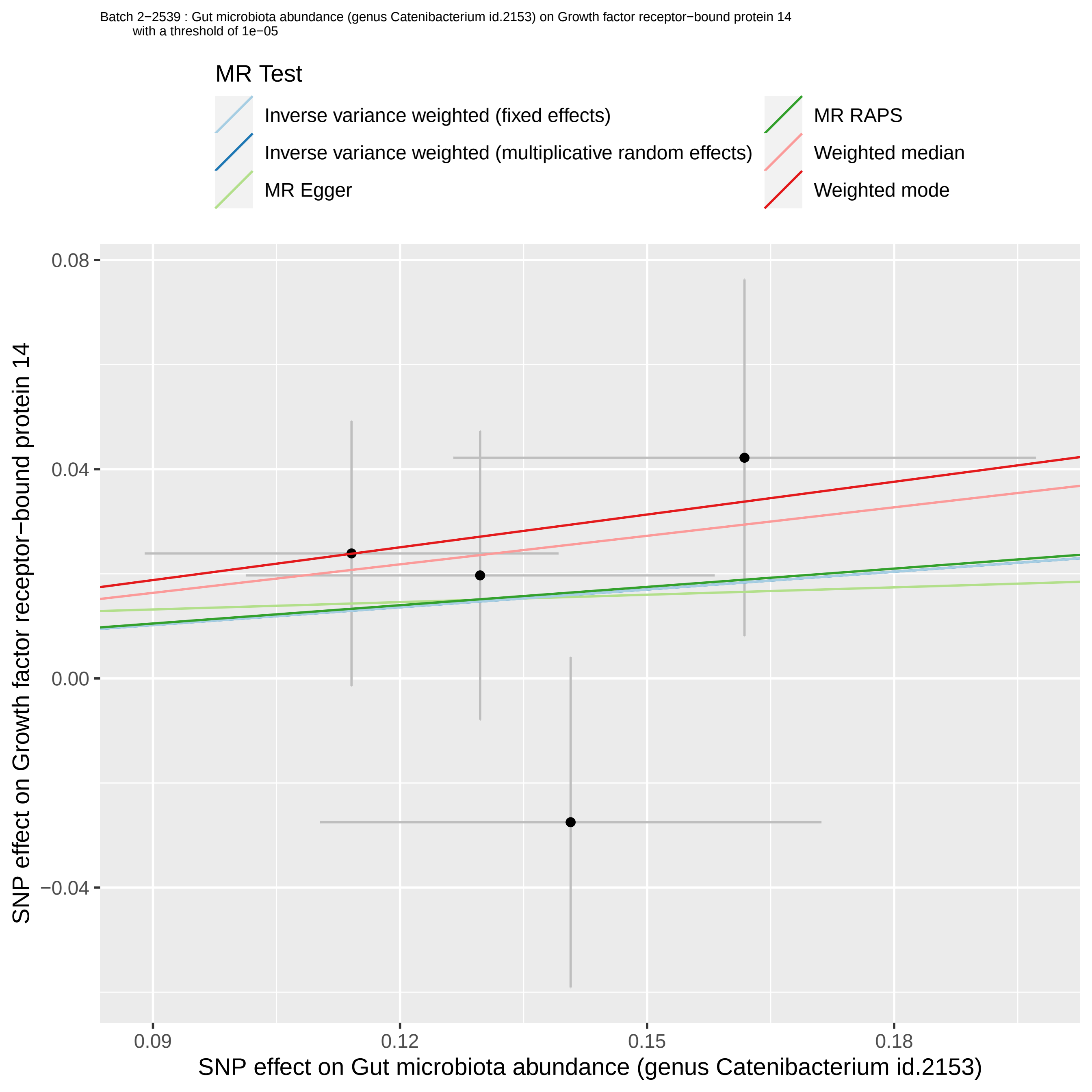
Batch 2−2539 : Gut microbiota abundance (genus Catenibacterium id.2153) on Growth factor receptor−bound protein 14
with a threshold of 1e−05
MR Test
Inverse variance weighted (fixed effects)
Inverse variance weighted (multiplicative random effects)
MR Egger
MR RAPS
Weighted median
Weighted mode
0.08
0.04
0.00
−0.04
0.09
0.12
0.15
0.18
SNP effect on Growth factor receptor−bound protein 14
SNP effect on Gut microbiota abundance (genus Catenibacterium id.2153)

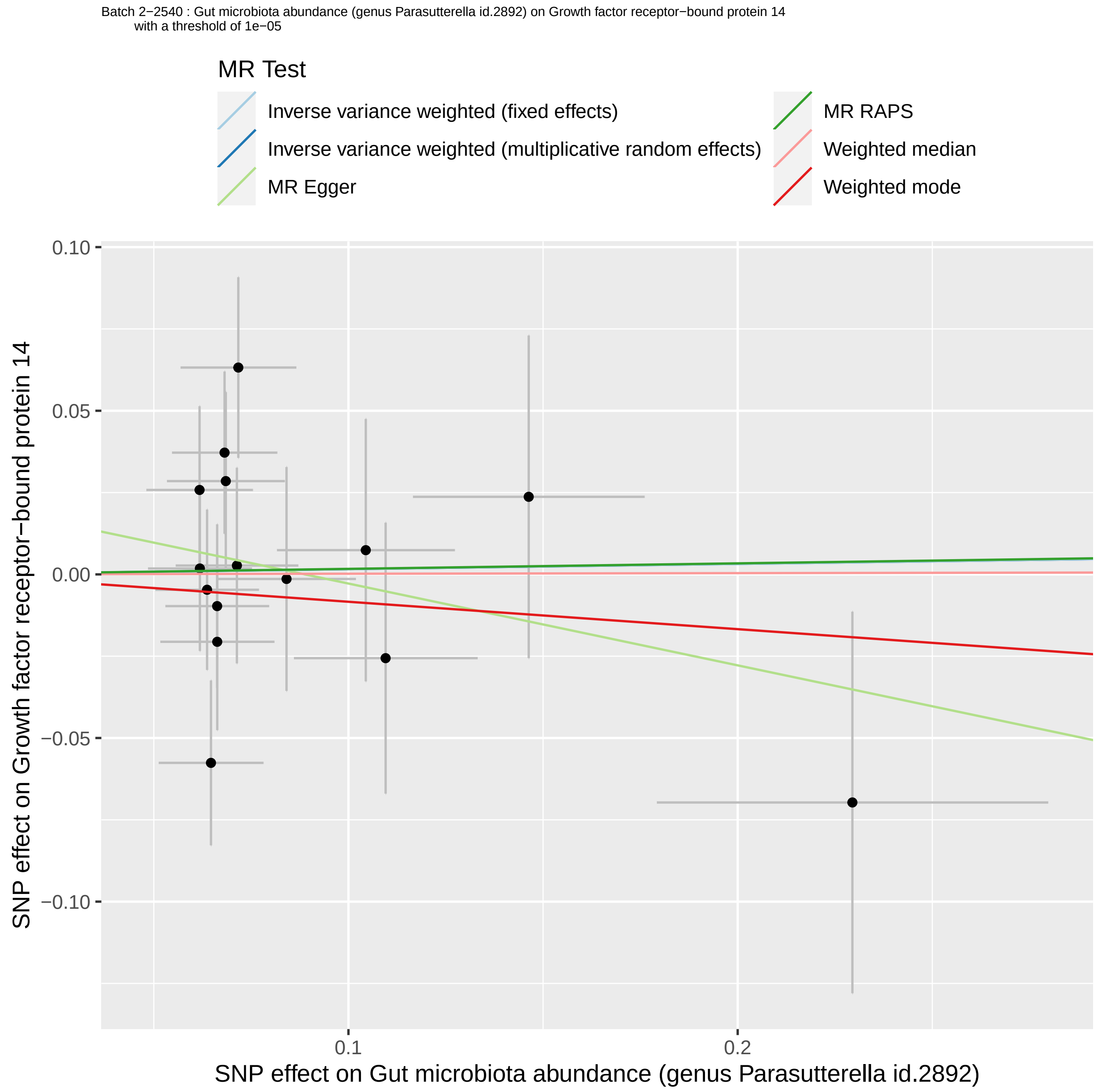
Batch 2−2540 : Gut microbiota abundance (genus Parasutterella id.2892) on Growth factor receptor−bound protein 14
with a threshold of 1e−05
MR Test
Inverse variance weighted (fixed effects)
Inverse variance weighted (multiplicative random effects)
MR Egger
MR RAPS
Weighted median
Weighted mode
0.10
0.05
0.00
−0.05
−0.10
0.1
0.2
SNP effect on Growth factor receptor−bound protein 14
SNP effect on Gut microbiota abundance (genus Parasutterella id.2892)

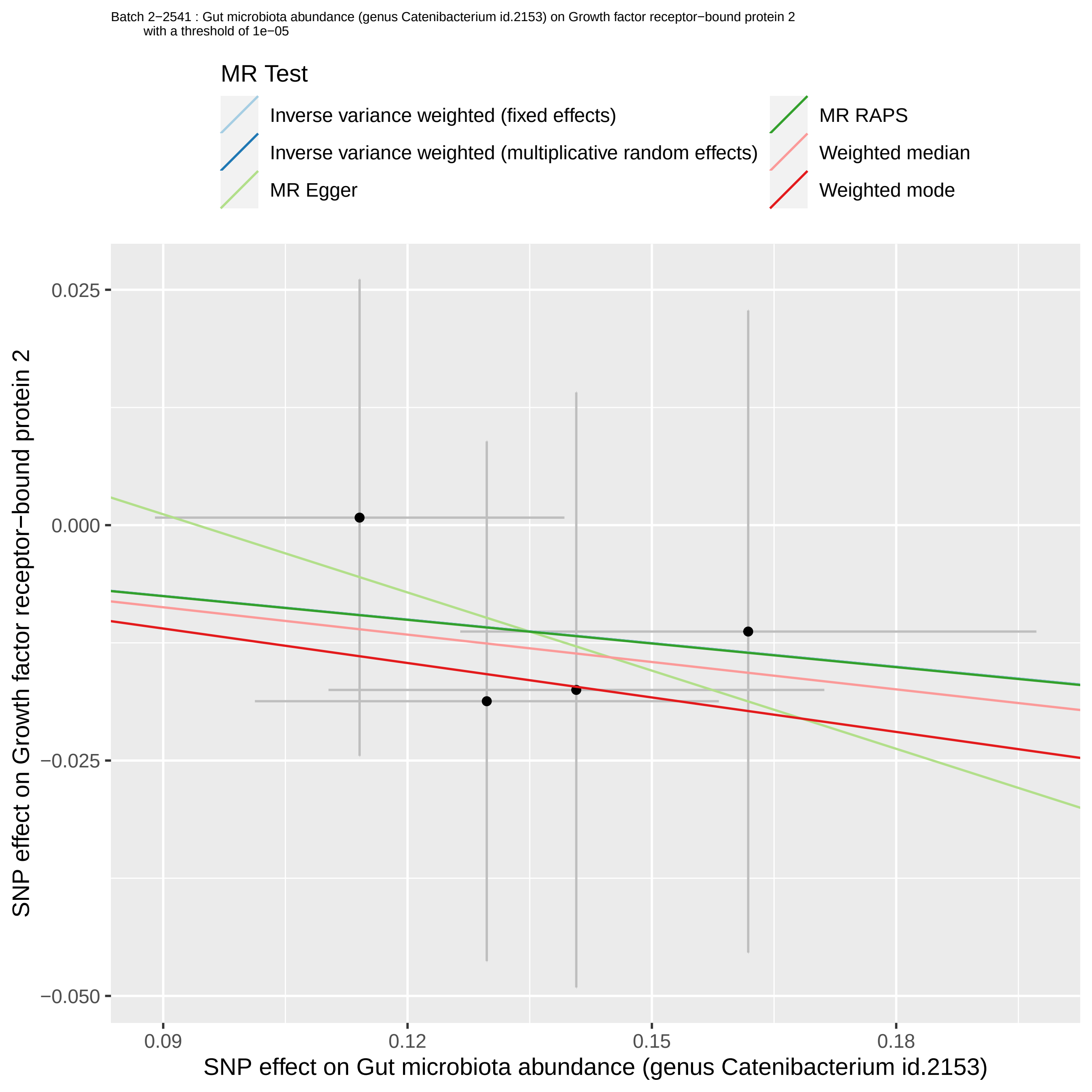

Batch 2−2541 : Gut microbiota abundance (genus Catenibacterium id.2153) on Growth factor receptor−bound protein 2
with a threshold of 1e−05
MR Test
Inverse variance weighted (fixed effects)
Inverse variance weighted (multiplicative random effects)
MR Egger
MR RAPS
Weighted median
Weighted mode
0.025
0.000
−0.025
−0.050
0.09
0.12
0.15
0.18
SNP effect on Gut microbiota abundance (genus Catenibacterium id.2153)
SNP effect on Growth factor receptor−bound protein 2

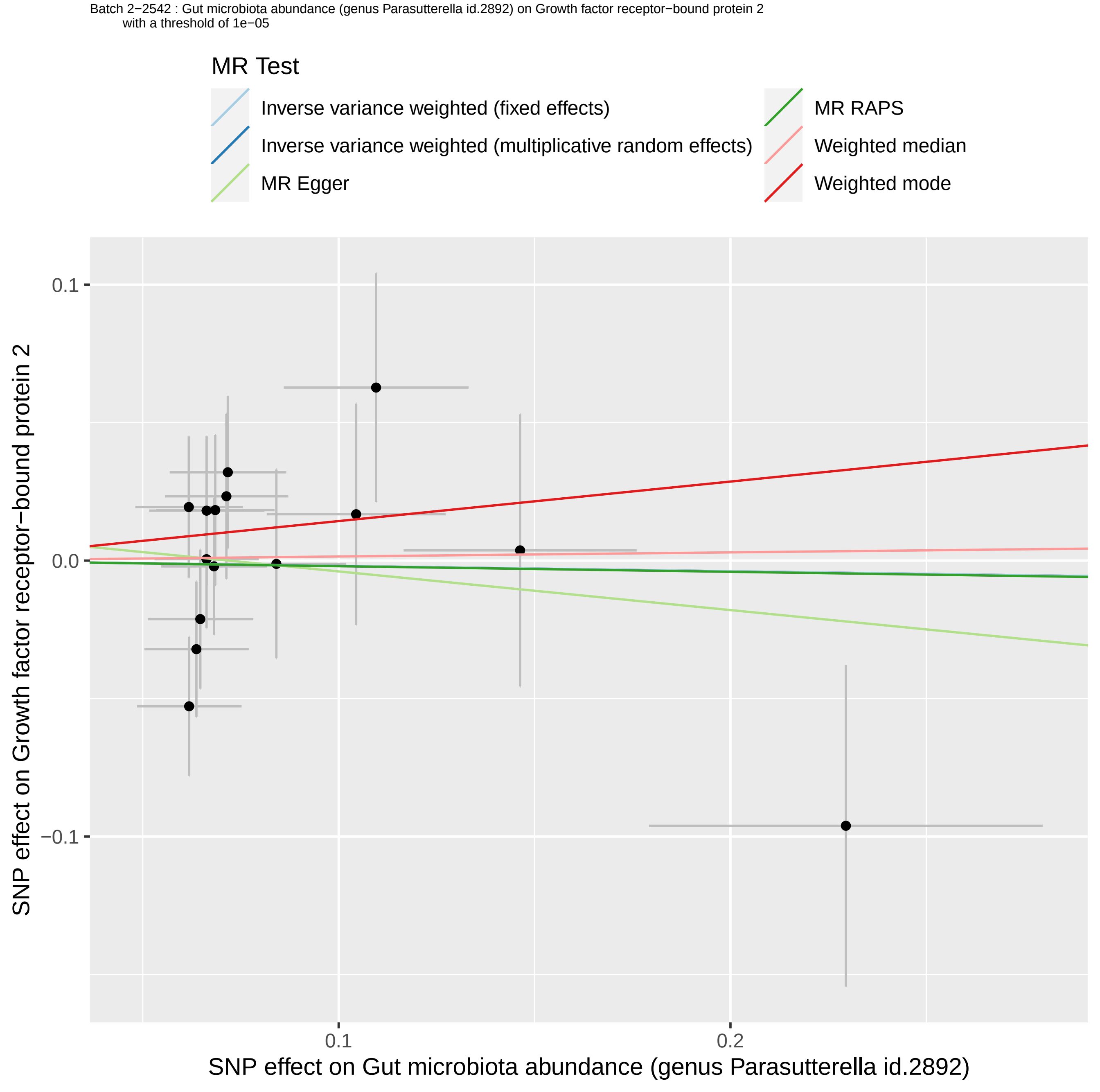
Batch 2−2542 : Gut microbiota abundance (genus Parasutterella id.2892) on Growth factor receptor−bound protein 2
with a threshold of 1e−05
MR Test
Inverse variance weighted (fixed effects)
Inverse variance weighted (multiplicative random effects)
MR Egger
MR RAPS
Weighted median
Weighted mode
0.1
0.0
−0.1
0.1
0.2
SNP effect on Growth factor receptor−bound protein 2
SNP effect on Gut microbiota abundance (genus Parasutterella id.2892)

Batch 2−2543 : Gut microbiota abundance (genus Catenibacterium id.2153) on Growth factor receptor−bound protein 7
with a threshold of 1e−05

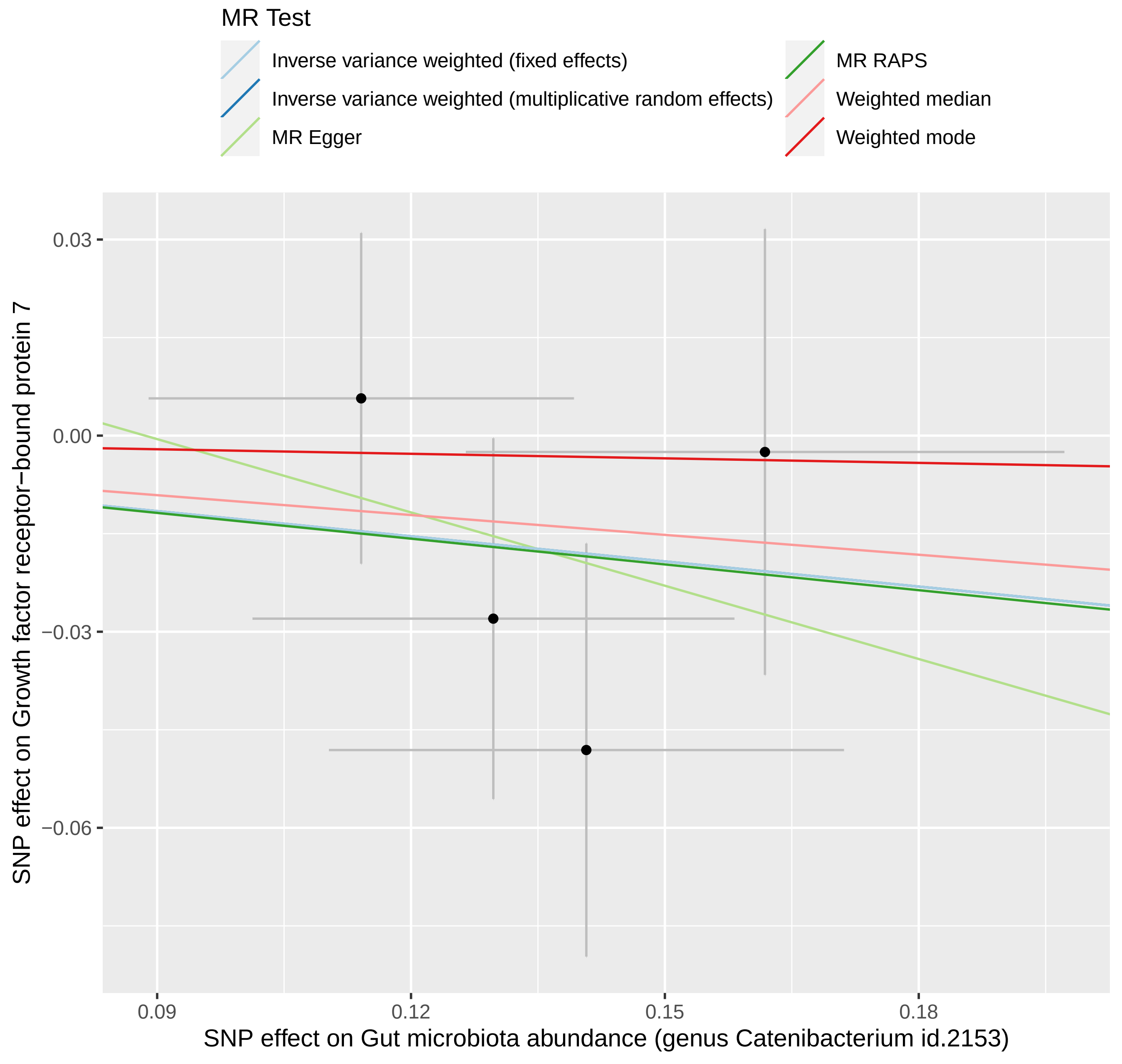

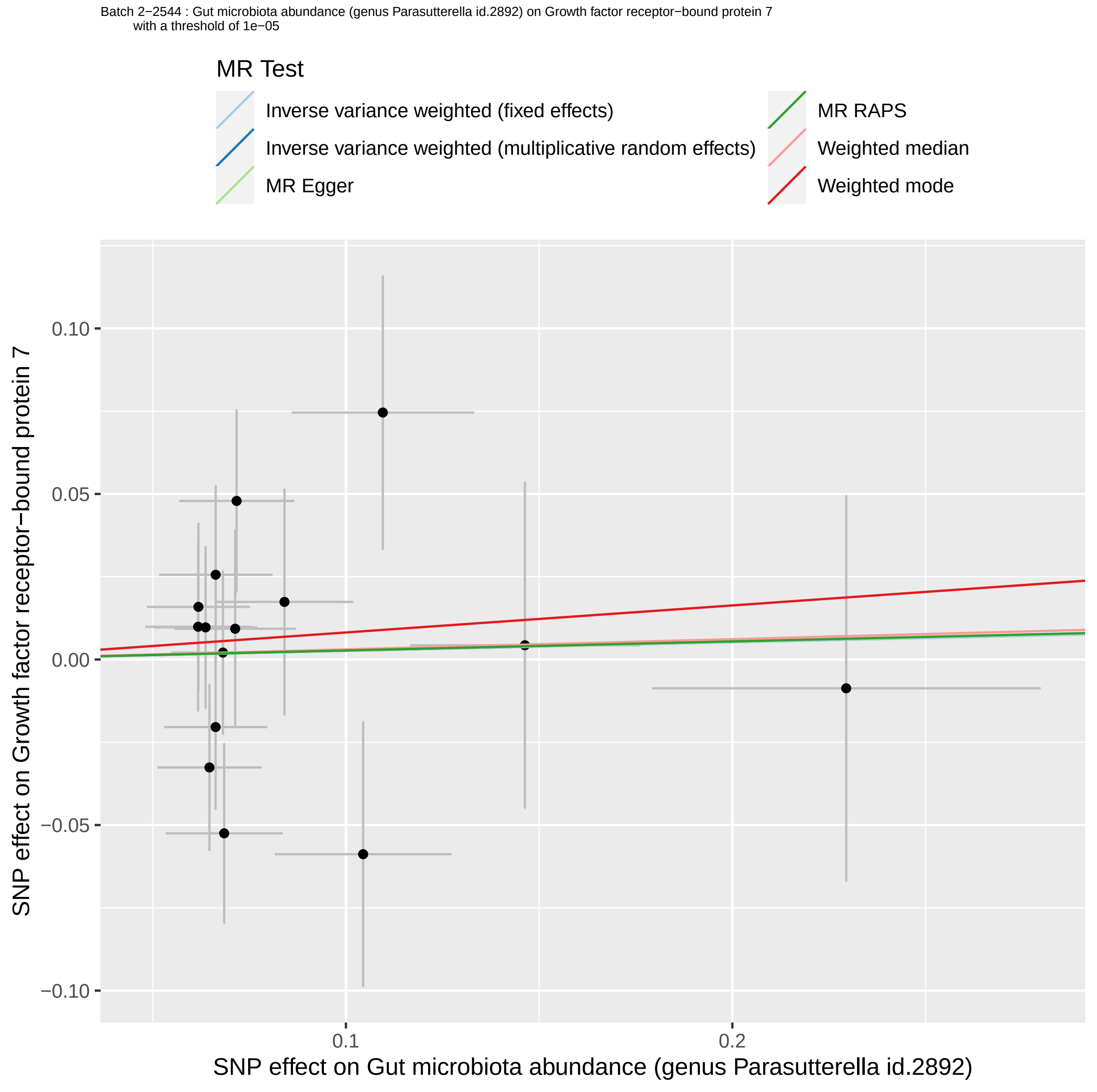

Batch 2−2544 : Gut microbiota abundance (genus Parasutterella id.2892) on Growth factor receptor−bound protein 7
with a threshold of 1e−05
MR Test
Inverse variance weighted (fixed effects)
Inverse variance weighted (multiplicative random effects)
MR Egger
MR RAPS
Weighted median
Weighted mode
0.10
0.05
0.00
−0.05
−0.10
0.1
0.2
SNP effect on Growth factor receptor−bound protein 7
SNP effect on Gut microbiota abundance (genus Parasutterella id.2892)

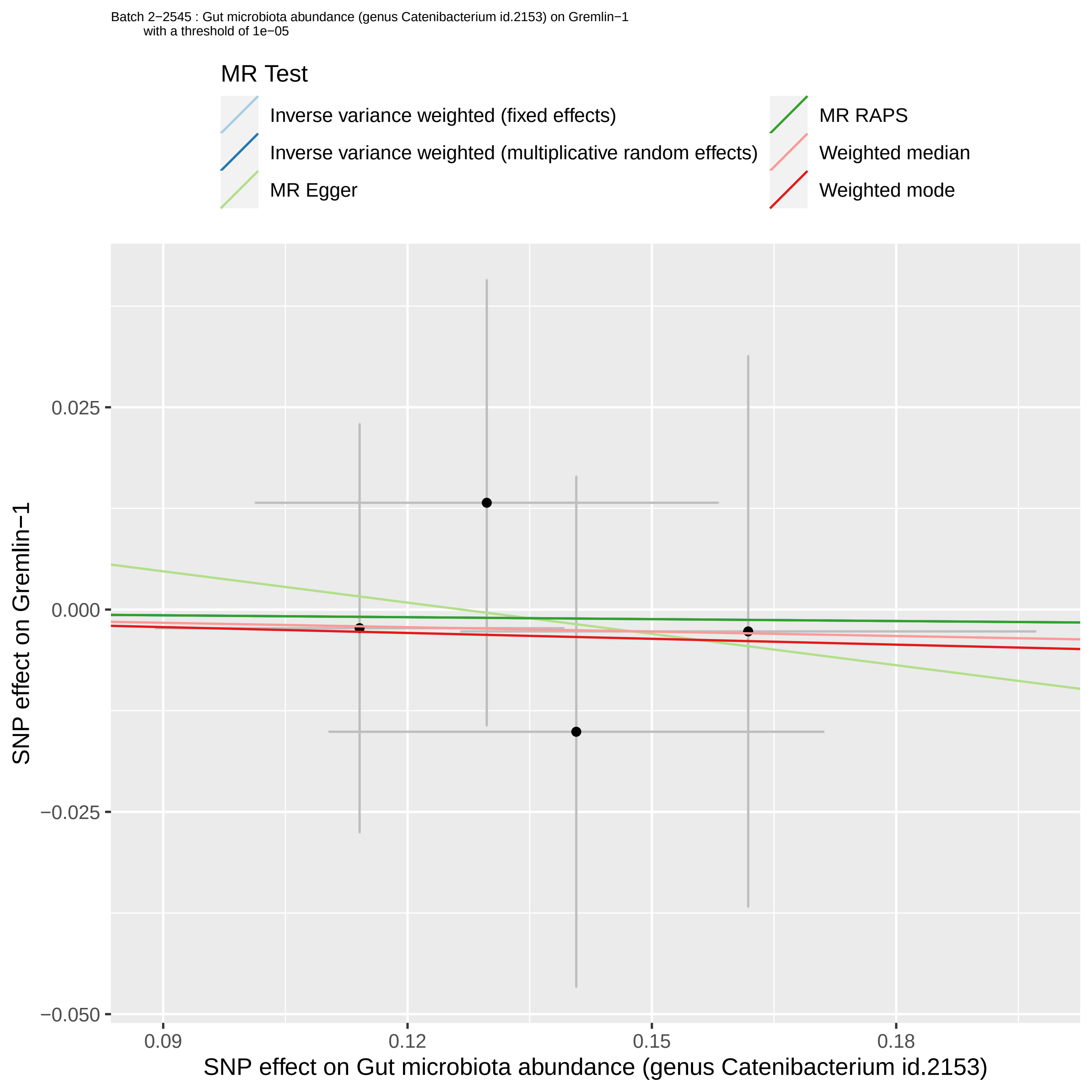
Batch 2−2545 : Gut microbiota abundance (genus Catenibacterium id.2153) on Gremlin−1
with a threshold of 1e−05
MR Test
Inverse variance weighted (fixed effects)
Inverse variance weighted (multiplicative random effects)
MR Egger
MR RAPS
Weighted median
Weighted mode
0.025
0.000
−0.025
−0.050
0.09
0.12
0.15
0.18
SNP effect on Gremlin−1
SNP effect on Gut microbiota abundance (genus Catenibacterium id.2153)

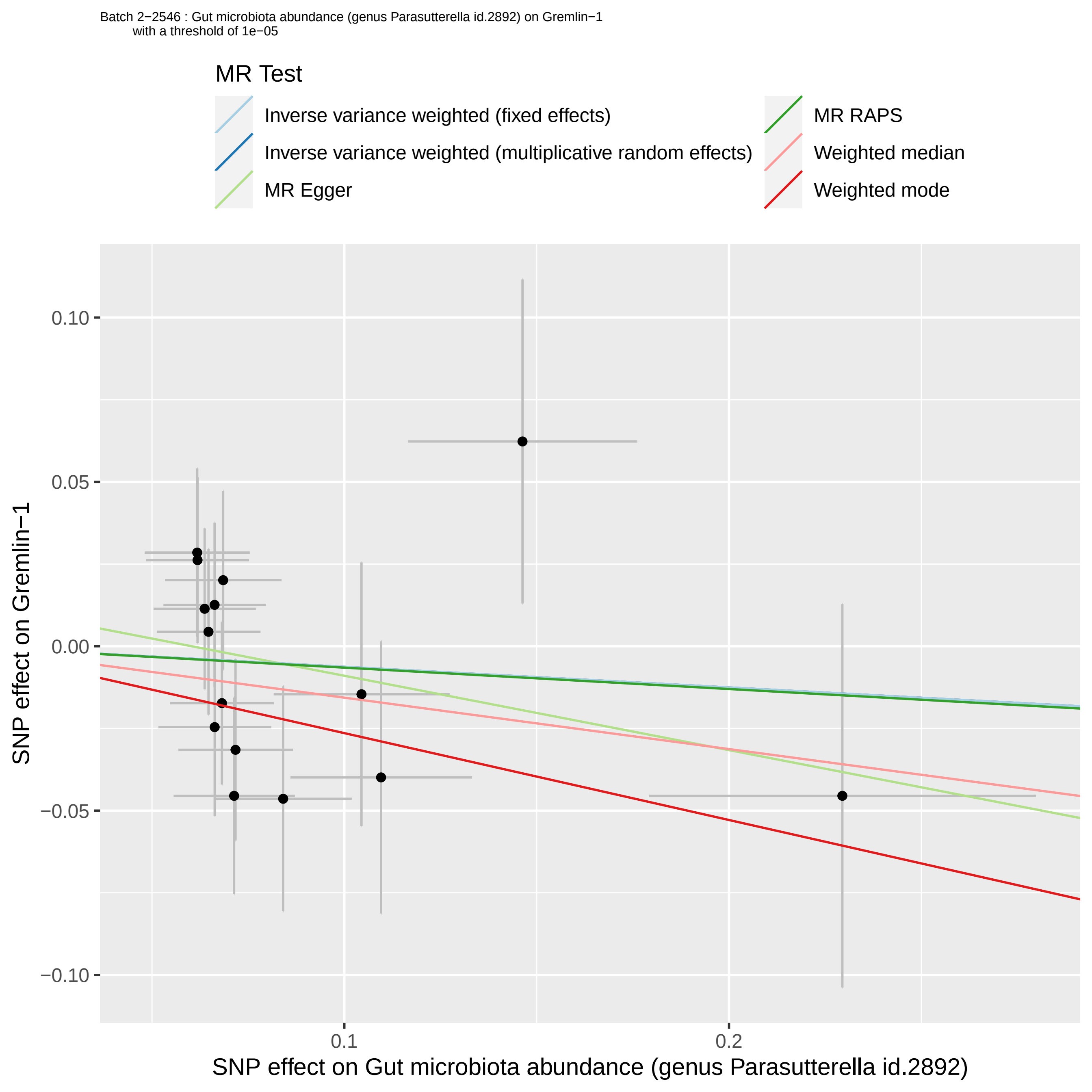
Batch 2−2546 : Gut microbiota abundance (genus Parasutterella id.2892) on Gremlin−1
with a threshold of 1e−05
MR Test
Inverse variance weighted (fixed effects)
Inverse variance weighted (multiplicative random effects)
MR Egger
MR RAPS
Weighted median
Weighted mode
0.10
0.05
0.00
−0.05
−0.10
SNP effect on Gremlin−1
0.1
0.2
SNP effect on Gut microbiota abundance (genus Parasutterella id.2892)

Batch 2−2547 : Gut microbiota abundance (genus Catenibacterium id.2153) on Gremlin−2
with a threshold of 1e−05

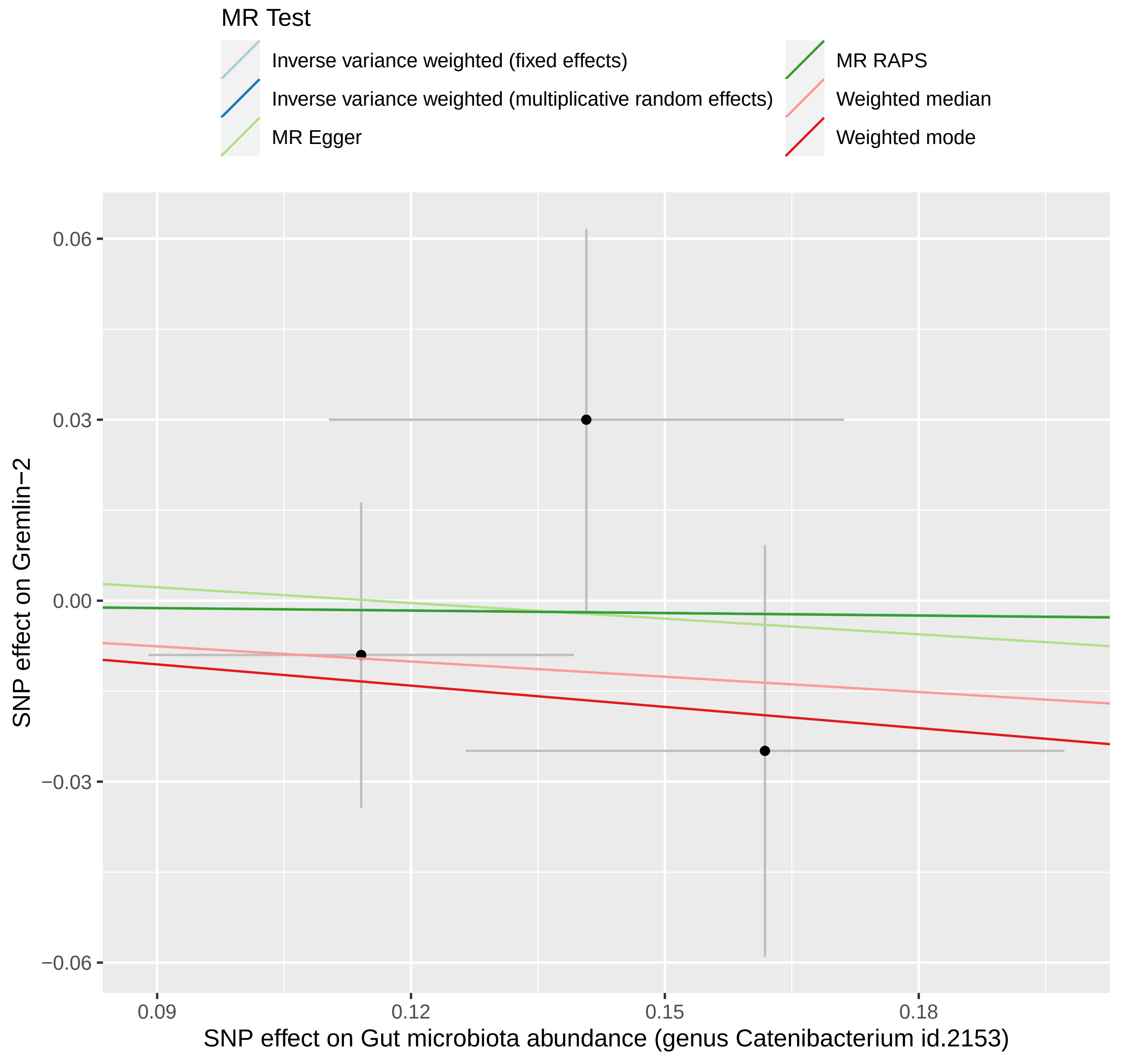

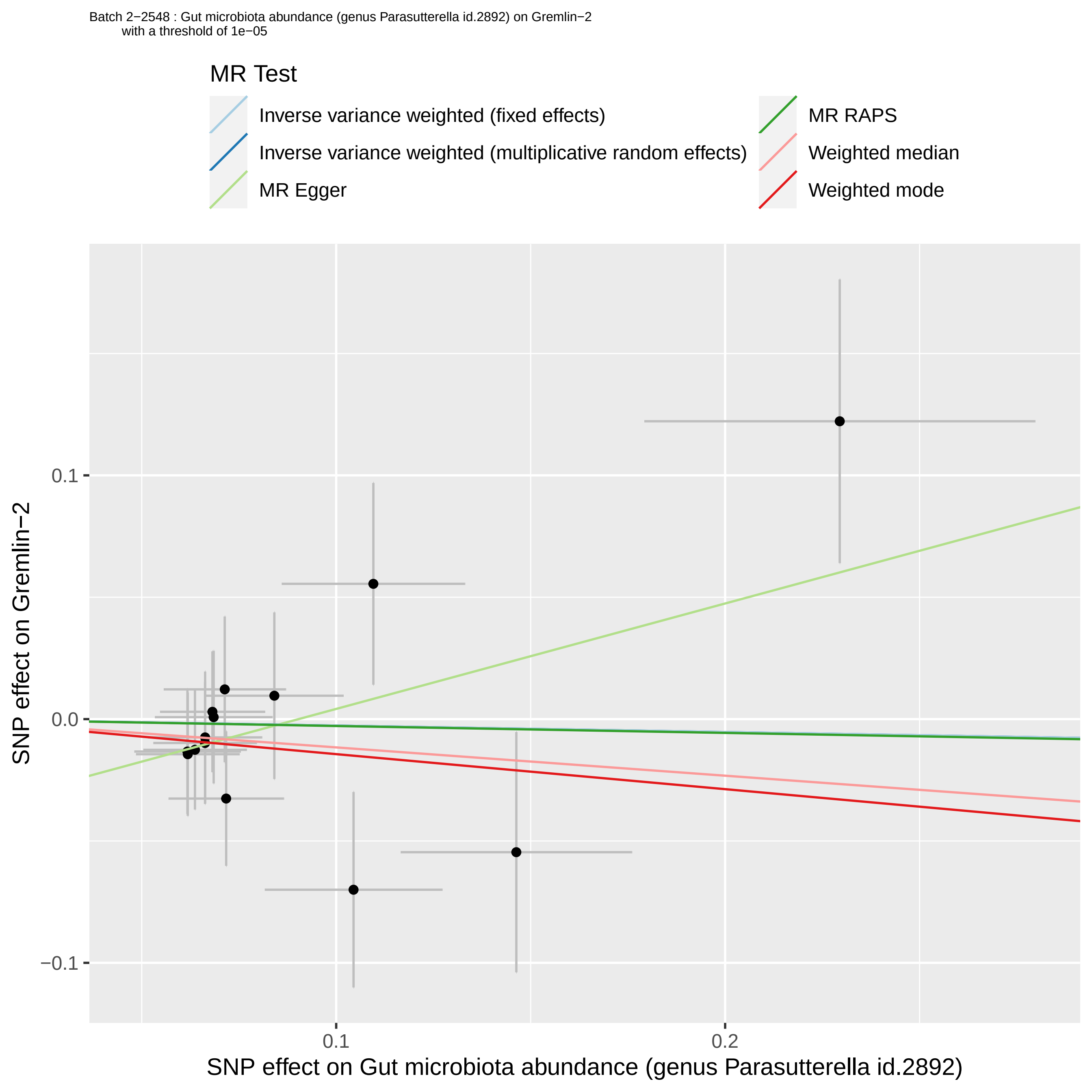
Batch 2−2548 : Gut microbiota abundance (genus Parasutterella id.2892) on Gremlin−2
with a threshold of 1e−05
MR Test
Inverse variance weighted (fixed effects)
Inverse variance weighted (multiplicative random effects)
MR Egger
MR RAPS
Weighted median
Weighted mode
0.1
0.0
−0.1
0.1
0.2
SNP effect on Gremlin−2
SNP effect on Gut microbiota abundance (genus Parasutterella id.2892)

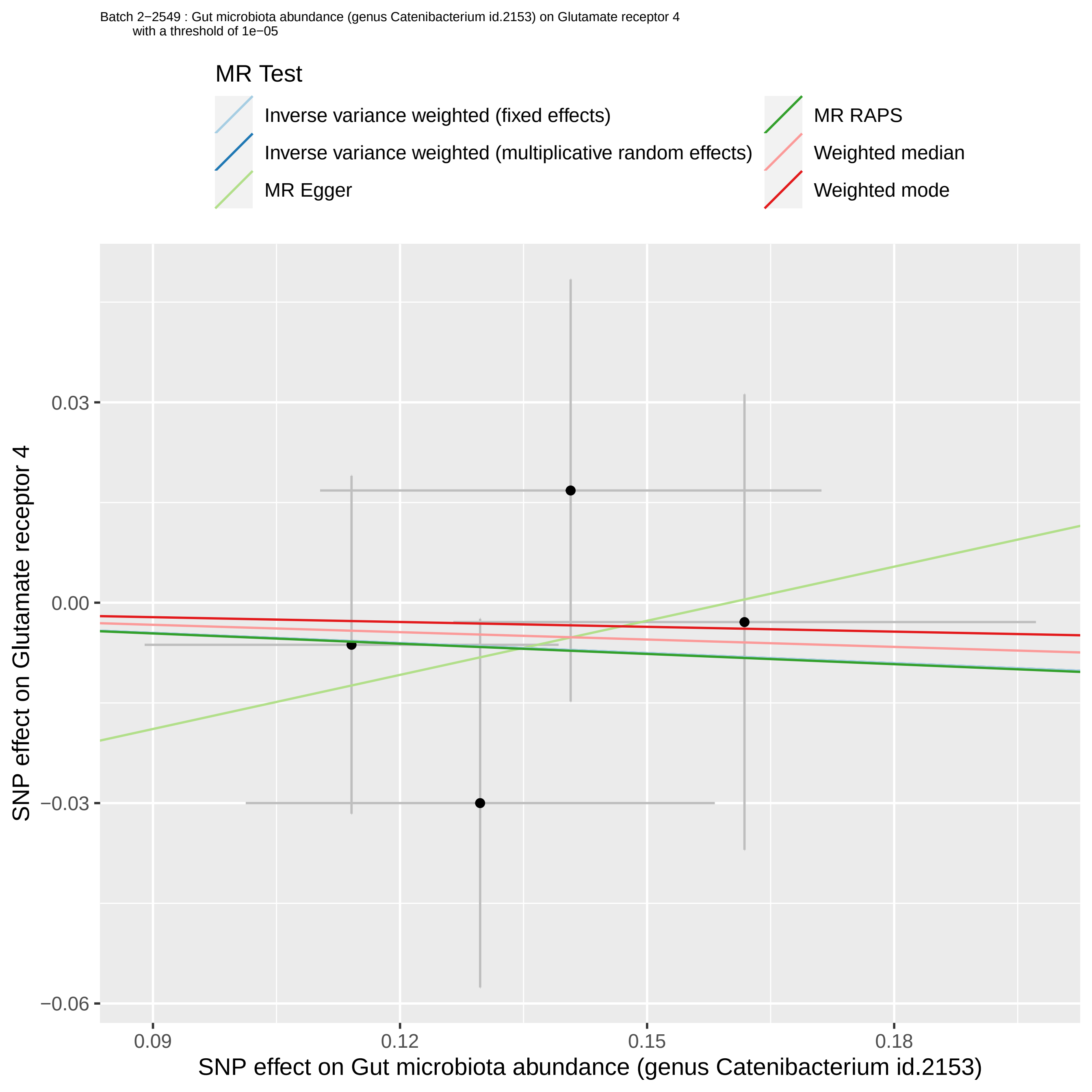
Batch 2−2549 : Gut microbiota abundance (genus Catenibacterium id.2153) on Glutamate receptor 4
with a threshold of 1e−05
MR Test
Inverse variance weighted (fixed effects)
Inverse variance weighted (multiplicative random effects)
MR Egger
MR RAPS
Weighted median
Weighted mode
0.03
0.00
−0.03
−0.06
0.09
0.12
0.15
0.18
SNP effect on Glutamate receptor 4
SNP effect on Gut microbiota abundance (genus Catenibacterium id.2153)

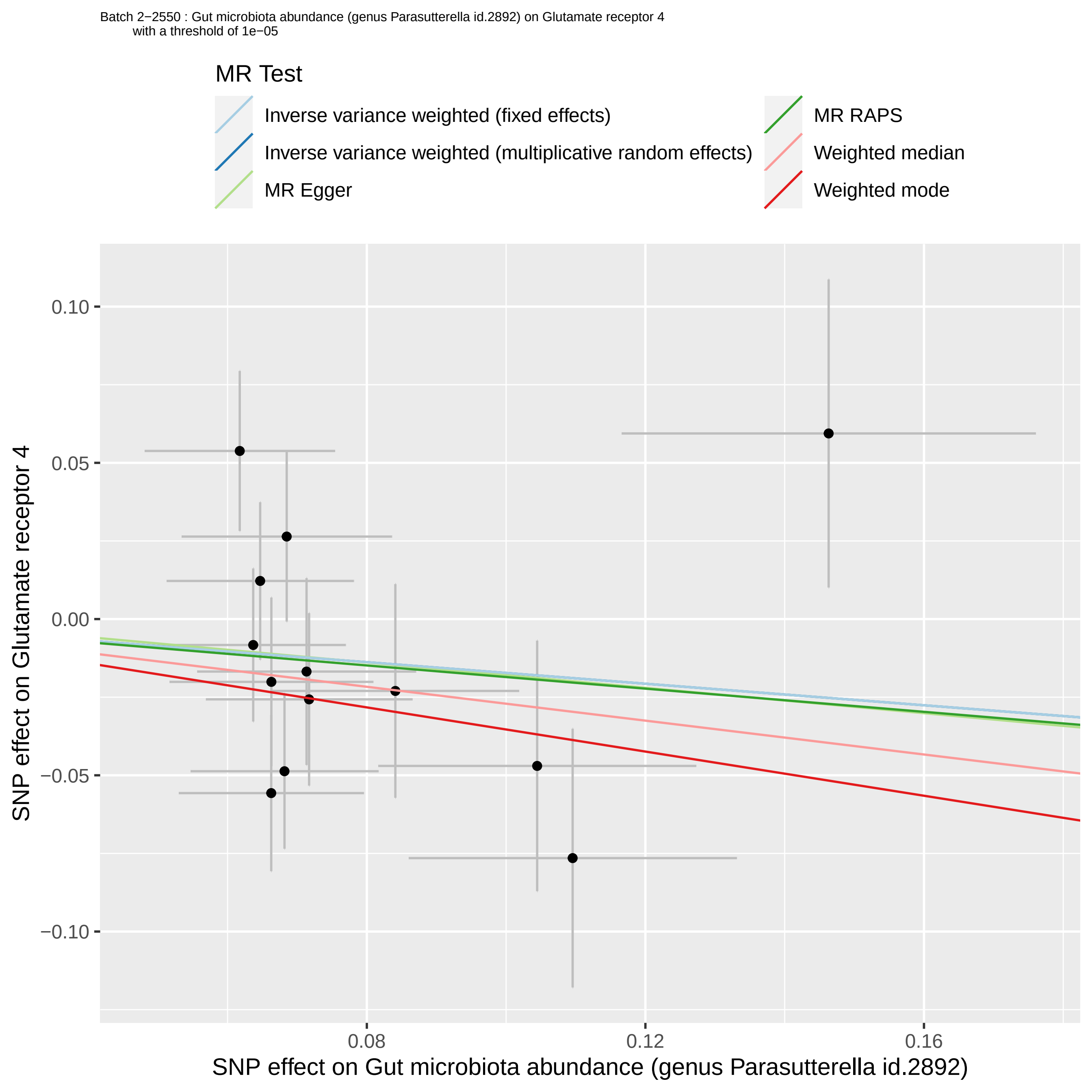
Batch 2−2550 : Gut microbiota abundance (genus Parasutterella id.2892) on Glutamate receptor 4
with a threshold of 1e−05
MR Test
Inverse variance weighted (fixed effects)
Inverse variance weighted (multiplicative random effects)
MR Egger
MR RAPS
Weighted median
Weighted mode
0.10
0.05
0.00
−0.05
−0.10
0.08
0.12
0.16
SNP effect on Glutamate receptor 4
SNP effect on Gut microbiota abundance (genus Parasutterella id.2892)

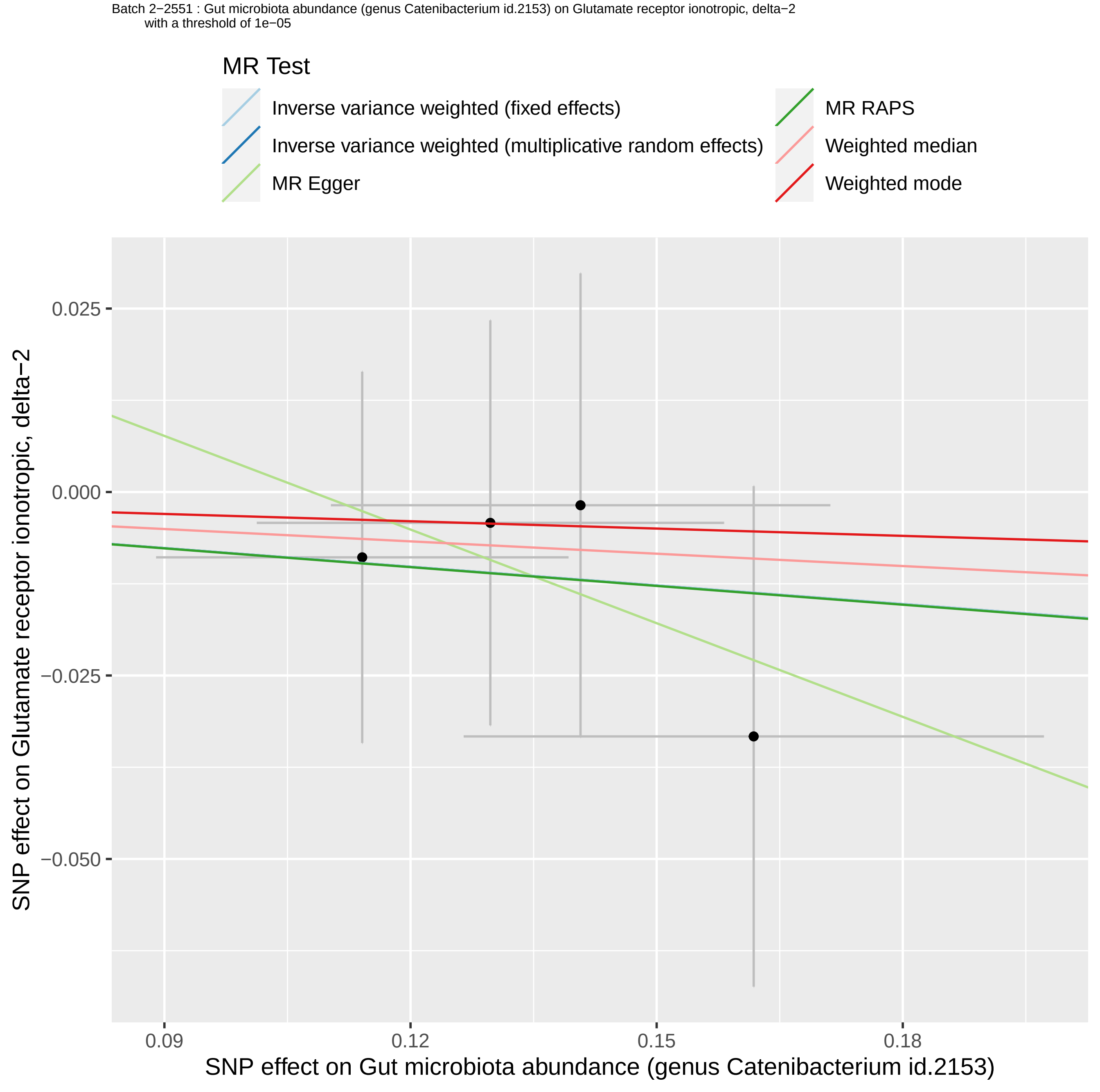

Batch 2−2551 : Gut microbiota abundance (genus Catenibacterium id.2153) on Glutamate receptor ionotropic, delta−2
with a threshold of 1e−05
MR Test
Inverse variance weighted (fixed effects)
Inverse variance weighted (multiplicative random effects)
MR Egger
MR RAPS
Weighted median
Weighted mode
0.025
0.000
−0.025
−0.050
0.09
0.12
0.15
0.18
SNP effect on Glutamate receptor ionotropic, delta−2
SNP effect on Gut microbiota abundance (genus Catenibacterium id.2153)

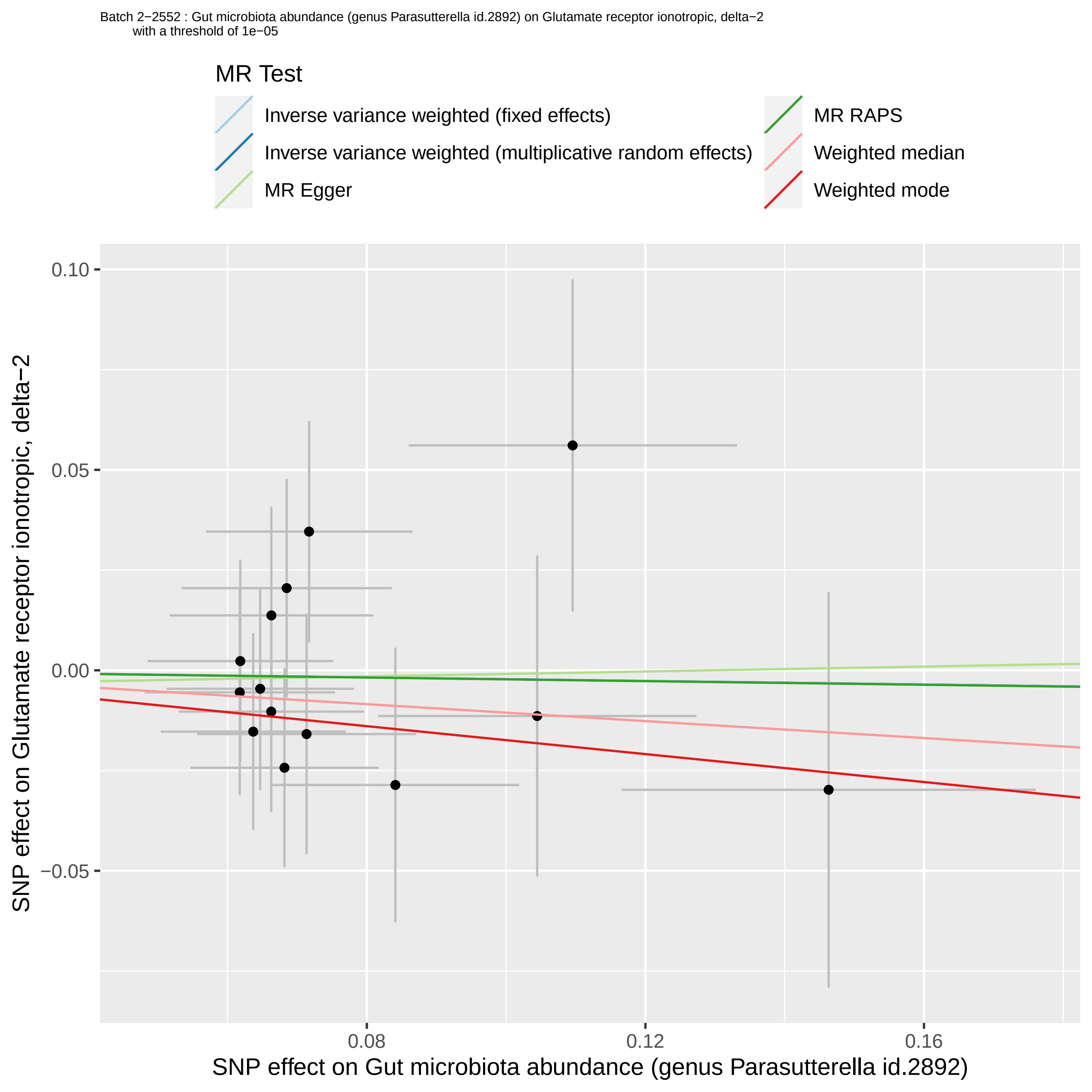
Batch 2−2552 : Gut microbiota abundance (genus Parasutterella id.2892) on Glutamate receptor ionotropic, delta−2
with a threshold of 1e−05
MR Test
Inverse variance weighted (fixed effects)
Inverse variance weighted (multiplicative random effects)
MR Egger
MR RAPS
Weighted median
Weighted mode
0.10
0.05
0.00
−0.05
0.08
0.12
0.16
SNP effect on Gut microbiota abundance (genus Parasutterella id.2892)
SNP effect on Glutamate receptor ionotropic, delta−2

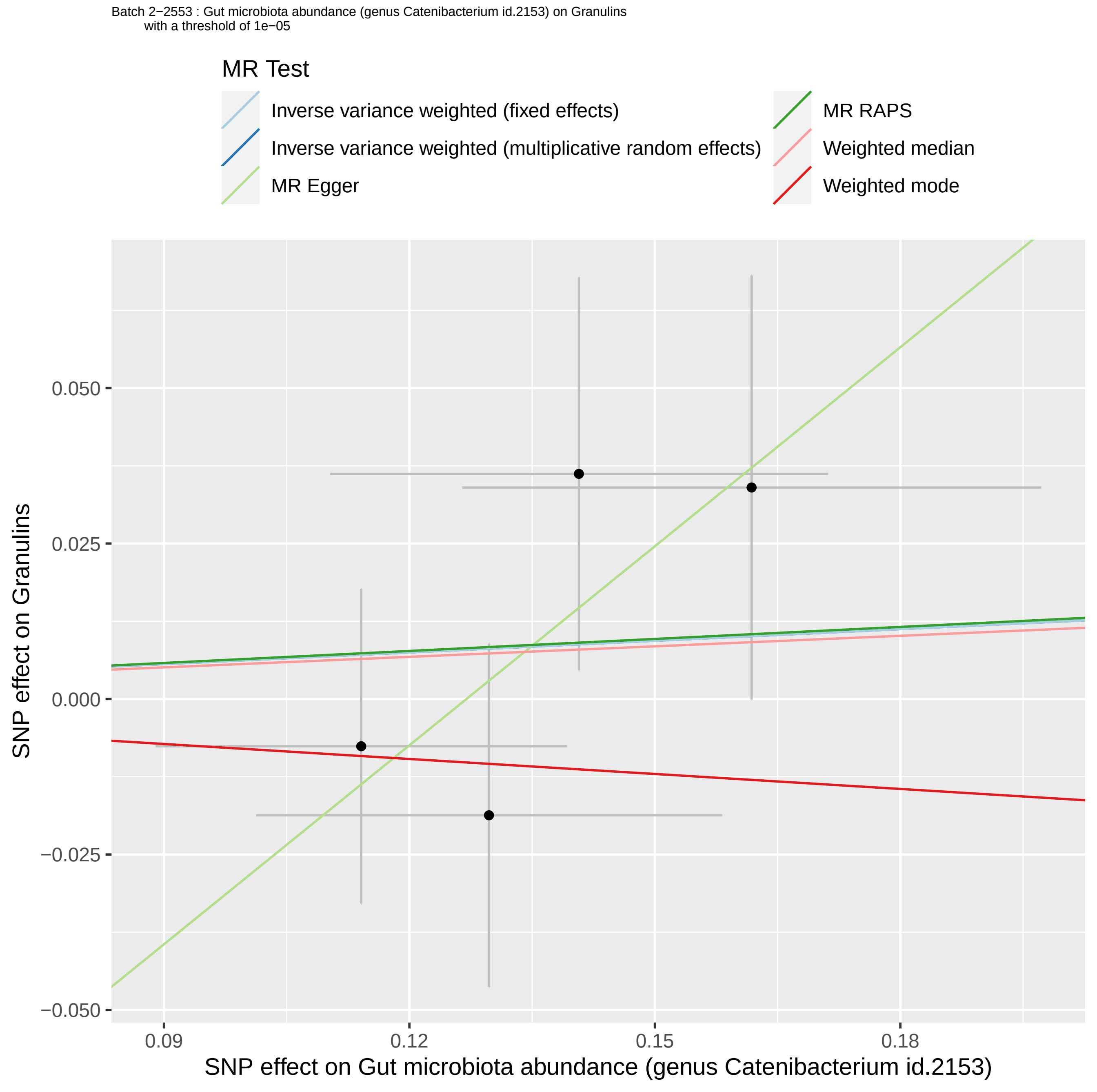

Batch 2−2553 : Gut microbiota abundance (genus Catenibacterium id.2153) on Granulins
with a threshold of 1e−05
MR Test
Inverse variance weighted (fixed effects)
Inverse variance weighted (multiplicative random effects)
MR Egger
MR RAPS
Weighted median
Weighted mode
0.050
0.025
0.000
−0.025
−0.050
SNP effect on Granulins
0.09
0.12
0.15
0.18
SNP effect on Gut microbiota abundance (genus Catenibacterium id.2153)

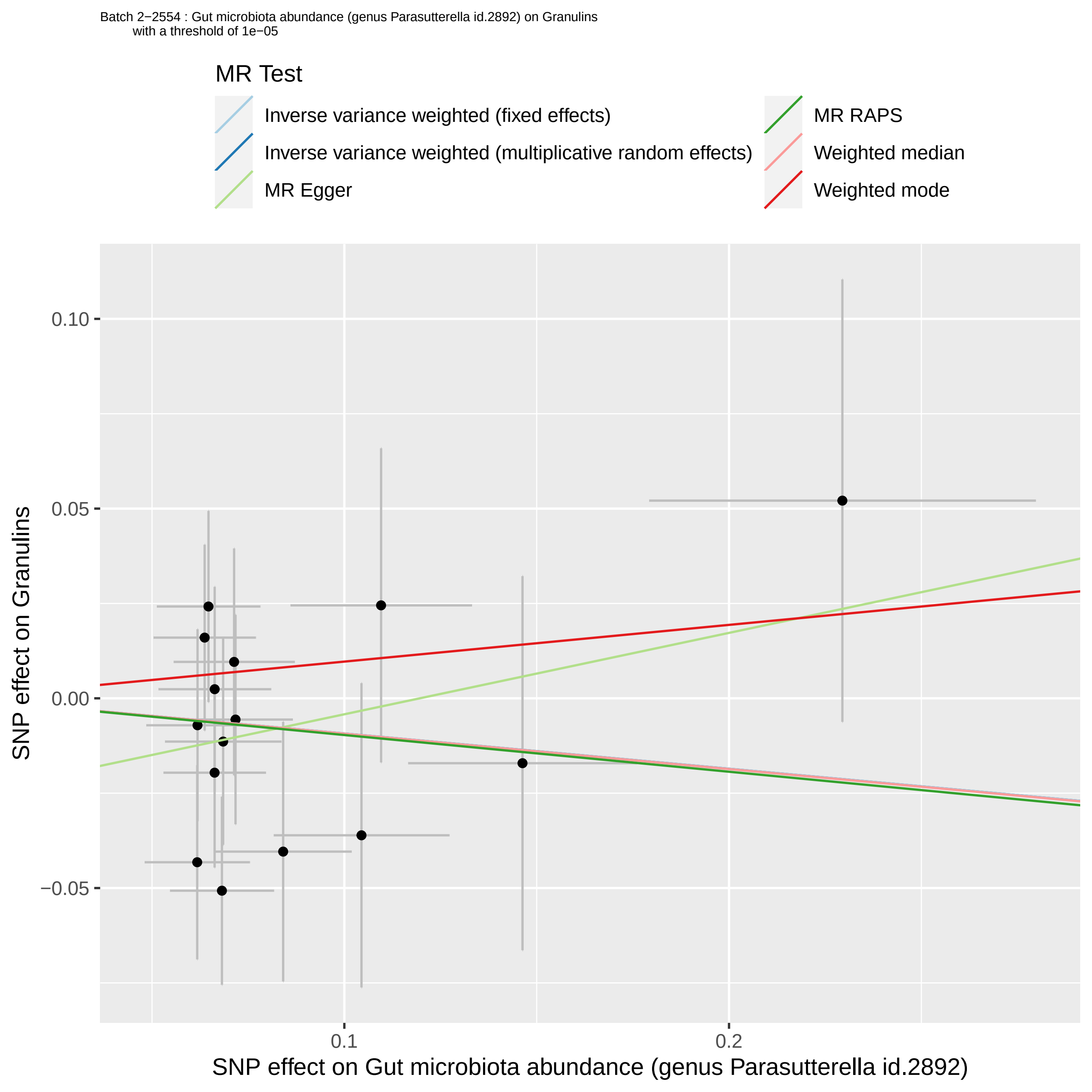

Batch 2−2554 : Gut microbiota abundance (genus Parasutterella id.2892) on Granulins
with a threshold of 1e−05
MR Test
Inverse variance weighted (fixed effects)
Inverse variance weighted (multiplicative random effects)
MR Egger
MR RAPS
Weighted median
Weighted mode
0.10
0.05
0.00
−0.05
0.1
0.2
SNP effect on Granulins
SNP effect on Gut microbiota abundance (genus Parasutterella id.2892)

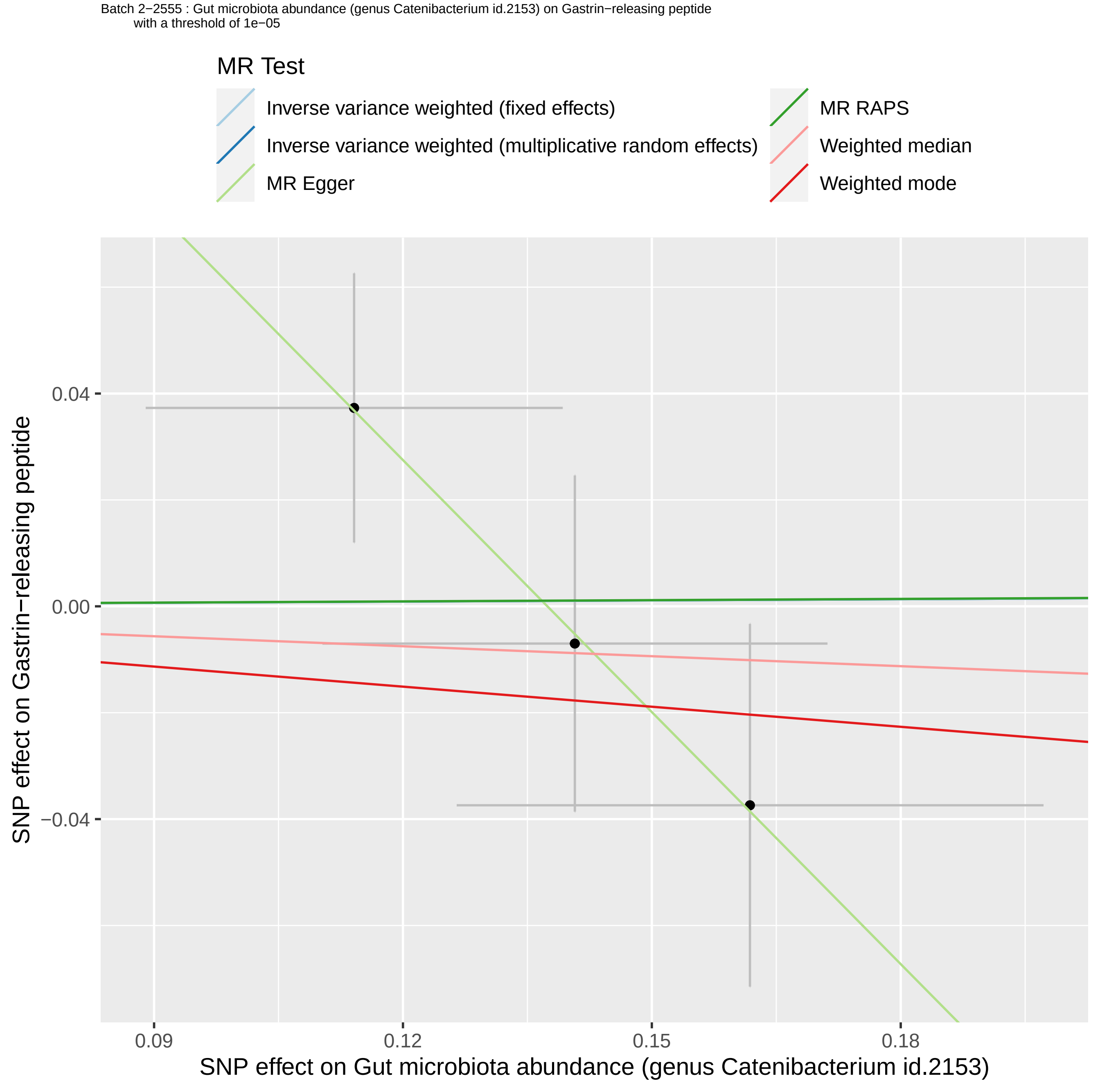

Batch 2−2555 : Gut microbiota abundance (genus Catenibacterium id.2153) on Gastrin−releasing peptide
with a threshold of 1e−05
MR Test
Inverse variance weighted (fixed effects)
Inverse variance weighted (multiplicative random effects)
MR Egger
MR RAPS
Weighted median
Weighted mode
0.04
0.00
−0.04
0.09
0.12
0.15
0.18
SNP effect on Gastrin−releasing peptide
SNP effect on Gut microbiota abundance (genus Catenibacterium id.2153)

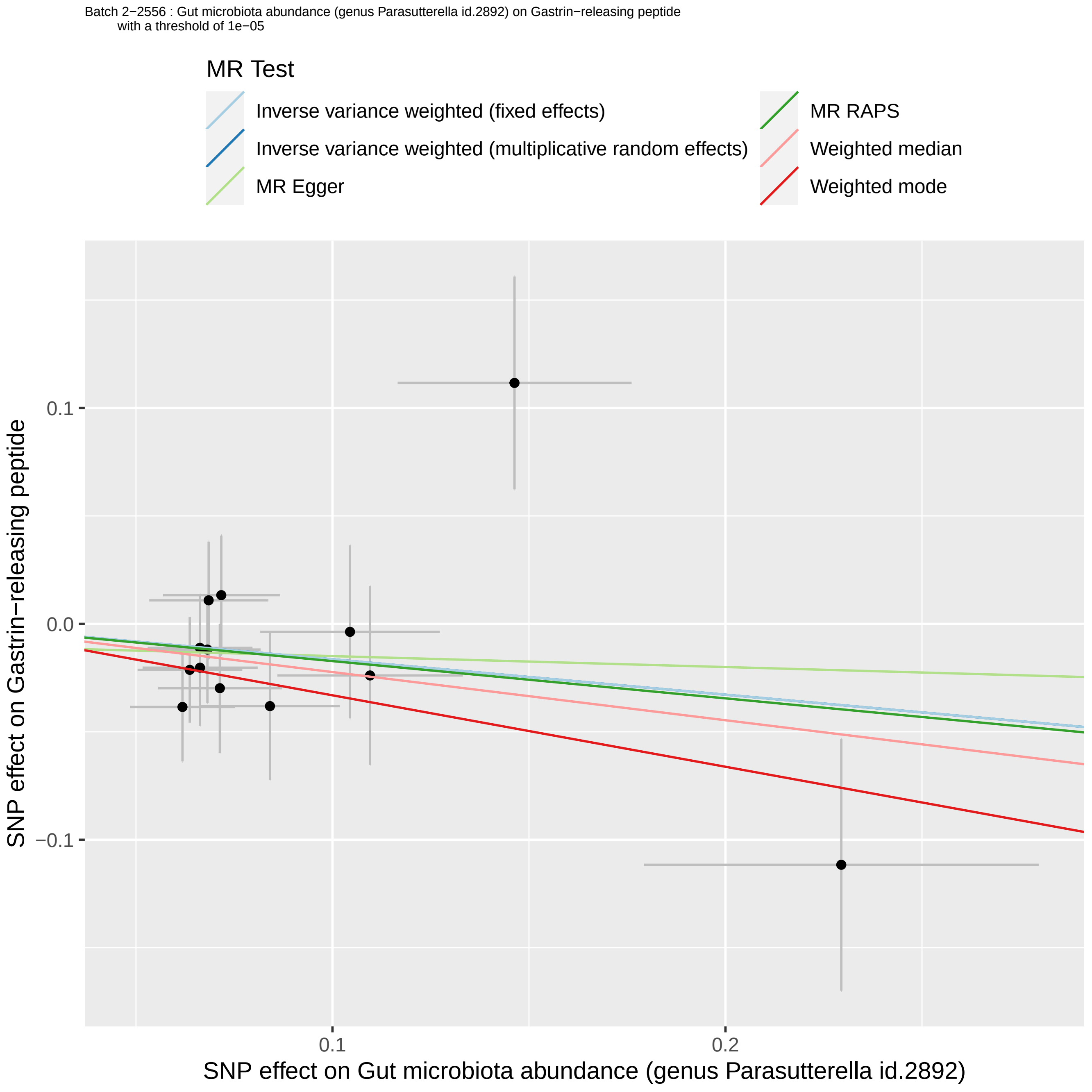

Batch 2−2556 : Gut microbiota abundance (genus Parasutterella id.2892) on Gastrin−releasing peptide
with a threshold of 1e−05
MR Test
Inverse variance weighted (fixed effects)
Inverse variance weighted (multiplicative random effects)
MR Egger
MR RAPS
Weighted median
Weighted mode
0.1
0.0
−0.1
0.1
0.2
SNP effect on Gastrin−releasing peptide
SNP effect on Gut microbiota abundance (genus Parasutterella id.2892)

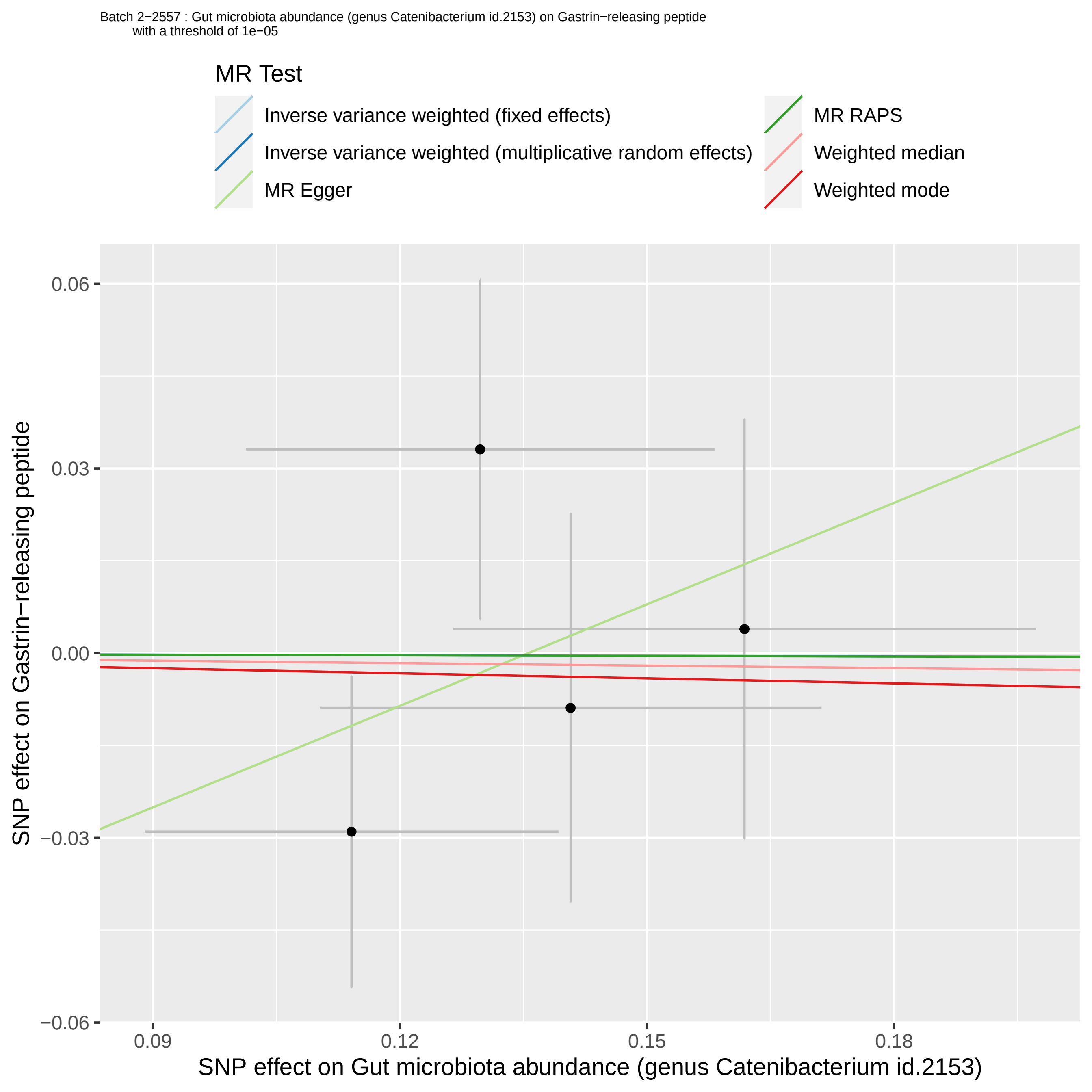

Batch 2−2557 : Gut microbiota abundance (genus Catenibacterium id.2153) on Gastrin−releasing peptide
with a threshold of 1e−05
MR Test
Inverse variance weighted (fixed effects)
Inverse variance weighted (multiplicative random effects)
MR Egger
MR RAPS
Weighted median
Weighted mode
0.06
0.03
0.00
−0.03
−0.06
0.09
0.12
0.15
0.18
SNP effect on Gastrin−releasing peptide
SNP effect on Gut microbiota abundance (genus Catenibacterium id.2153)

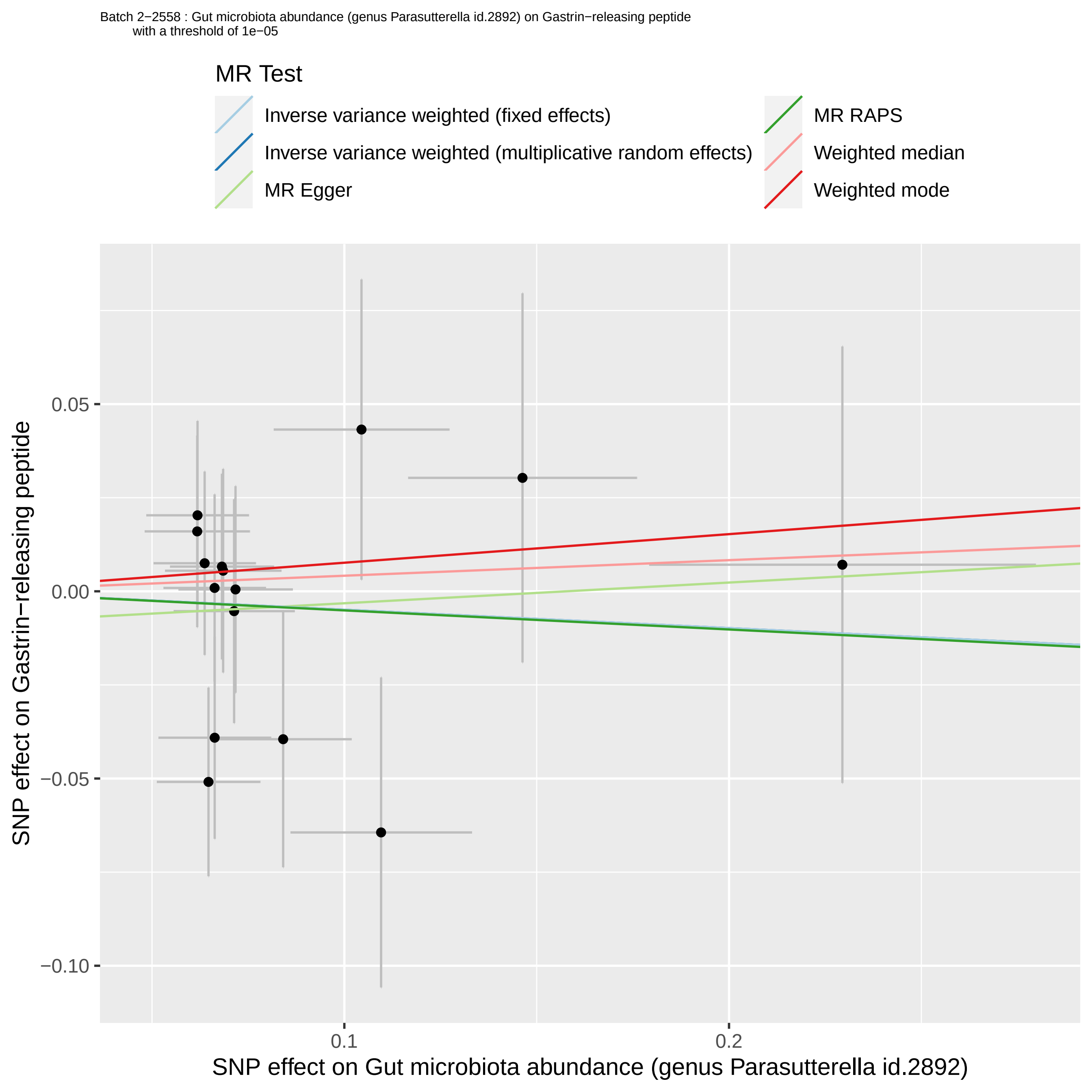
Batch 2−2558 : Gut microbiota abundance (genus Parasutterella id.2892) on Gastrin−releasing peptide
with a threshold of 1e−05
MR Test
Inverse variance weighted (fixed effects)
Inverse variance weighted (multiplicative random effects)
MR Egger
MR RAPS
Weighted median
Weighted mode
0.05
0.00
−0.05
−0.10
0.1
0.2
SNP effect on Gastrin−releasing peptide
SNP effect on Gut microbiota abundance (genus Parasutterella id.2892)

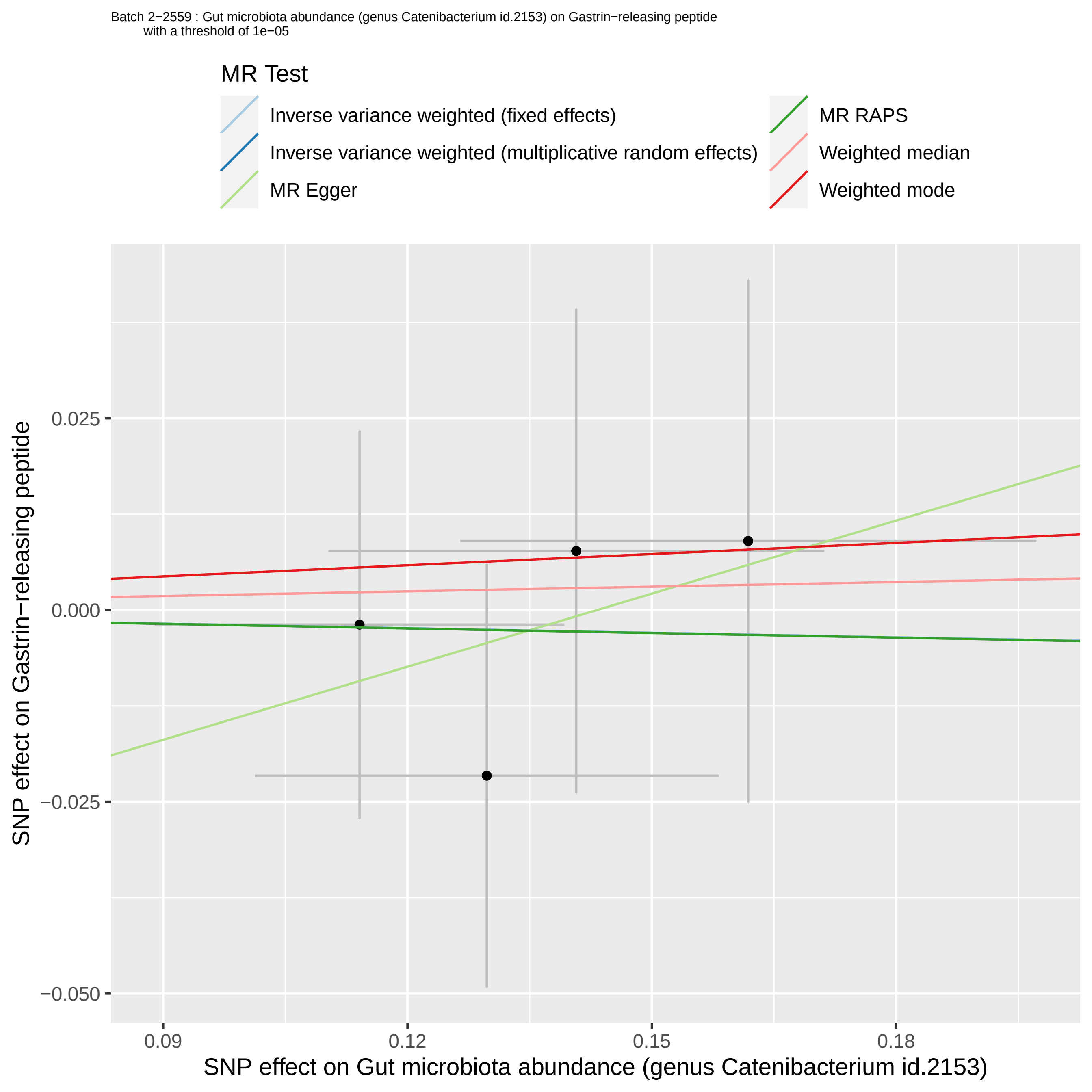
Batch 2−2559 : Gut microbiota abundance (genus Catenibacterium id.2153) on Gastrin−releasing peptide
with a threshold of 1e−05
MR Test
Inverse variance weighted (fixed effects)
Inverse variance weighted (multiplicative random effects)
MR Egger
MR RAPS
Weighted median
Weighted mode
SNP effect on Gastrin−releasing peptide
0.025
0.000
−0.025
−0.050
0.09
0.12
0.15
0.18
SNP effect on Gut microbiota abundance (genus Catenibacterium id.2153)

Batch 2−2560 : Gut microbiota abundance (genus Parasutterella id.2892) on Gastrin−releasing peptide
with a threshold of 1e−05

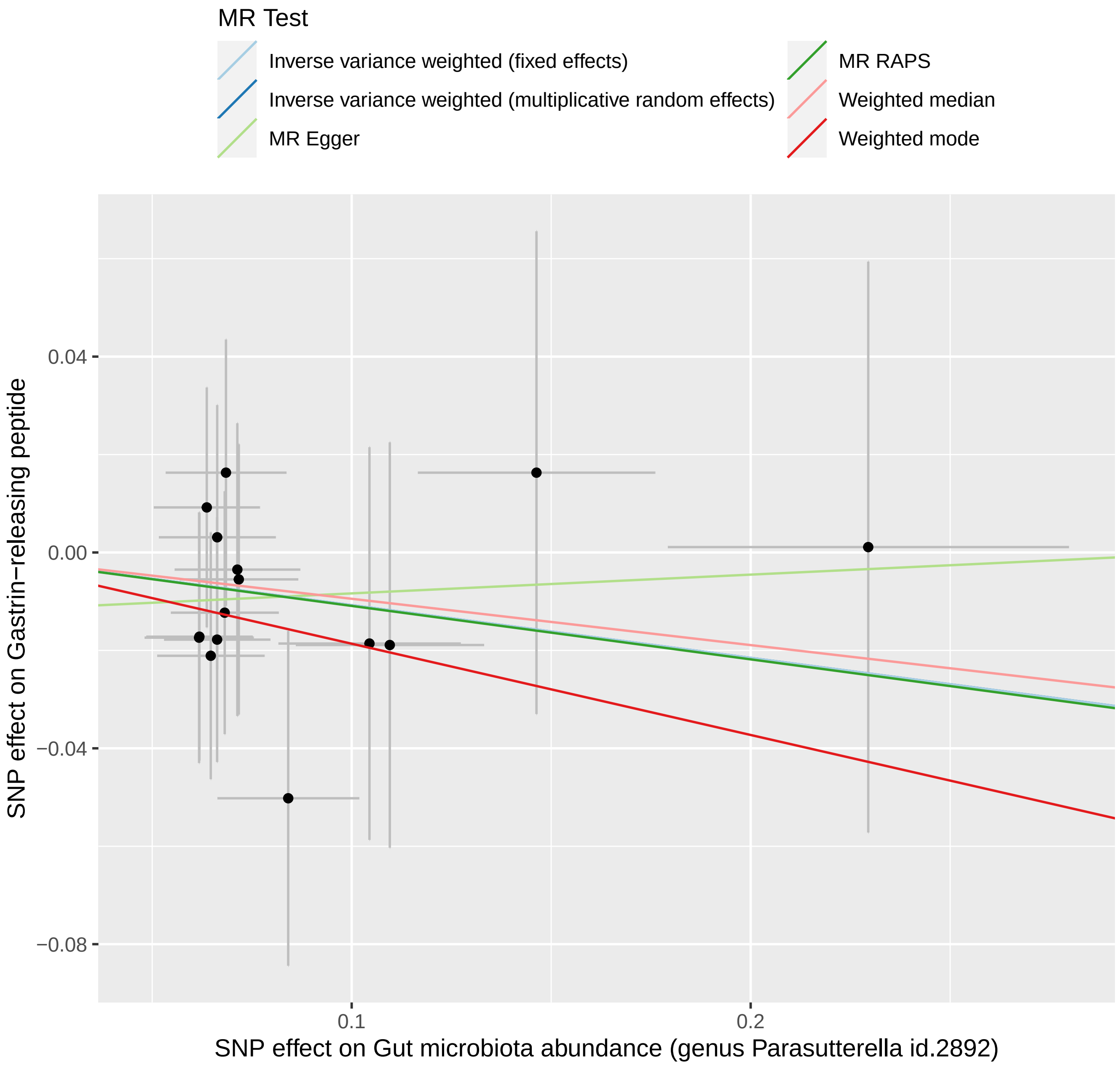

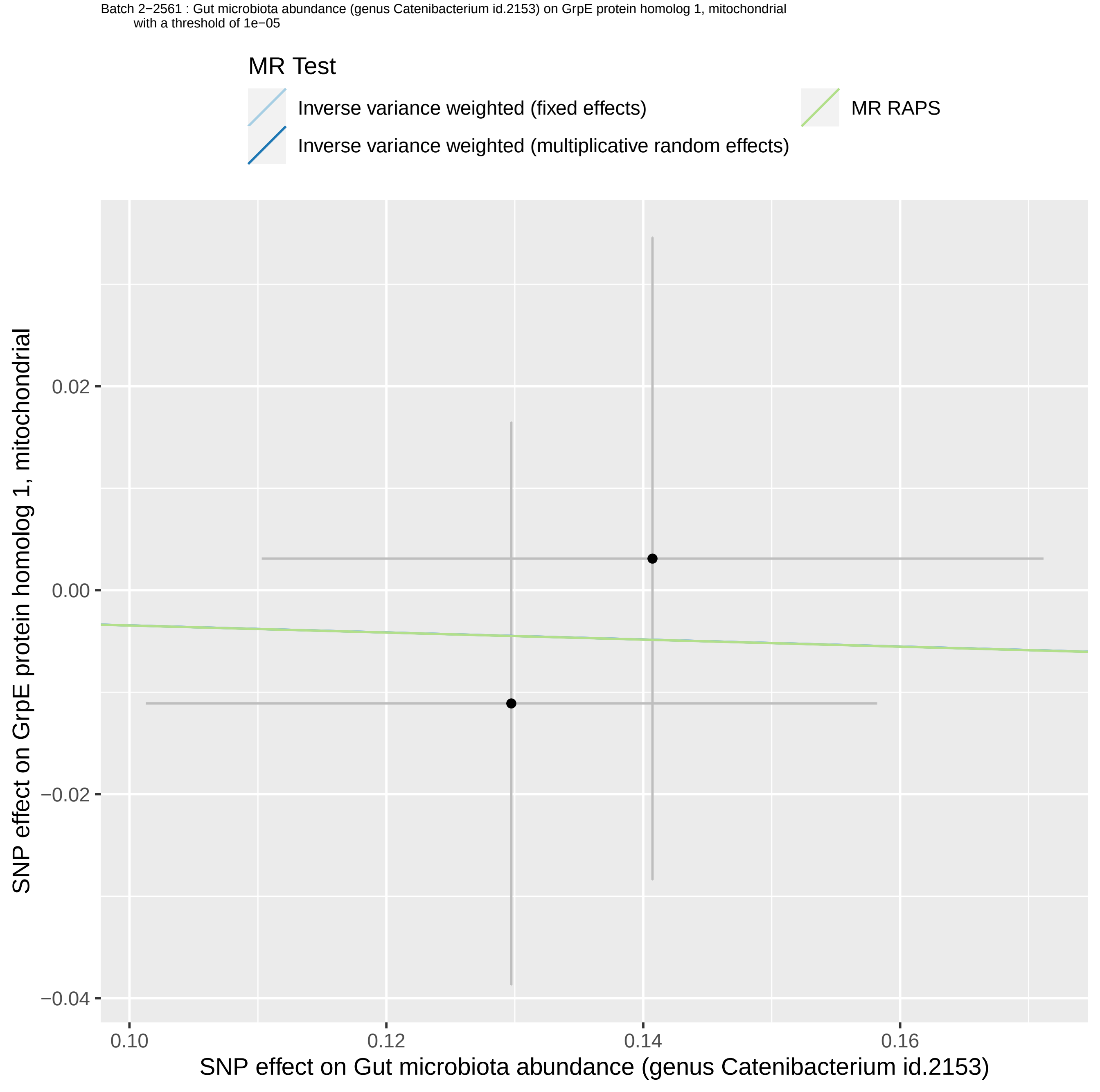

Batch 2−2561 : Gut microbiota abundance (genus Catenibacterium id.2153) on GrpE protein homolog 1, mitochondrial
with a threshold of 1e−05
MR Test
Inverse variance weighted (fixed effects)
Inverse variance weighted (multiplicative random effects)
MR RAPS
SNP effect on GrpE protein homolog 1, mitochondrial
0.02
0.00
−0.02
−0.04
0.10
0.12
0.14
0.16
SNP effect on Gut microbiota abundance (genus Catenibacterium id.2153)

Batch 2−2562 : Gut microbiota abundance (genus Parasutterella id.2892) on GrpE protein homolog 1, mitochondrial
with a threshold of 1e−05

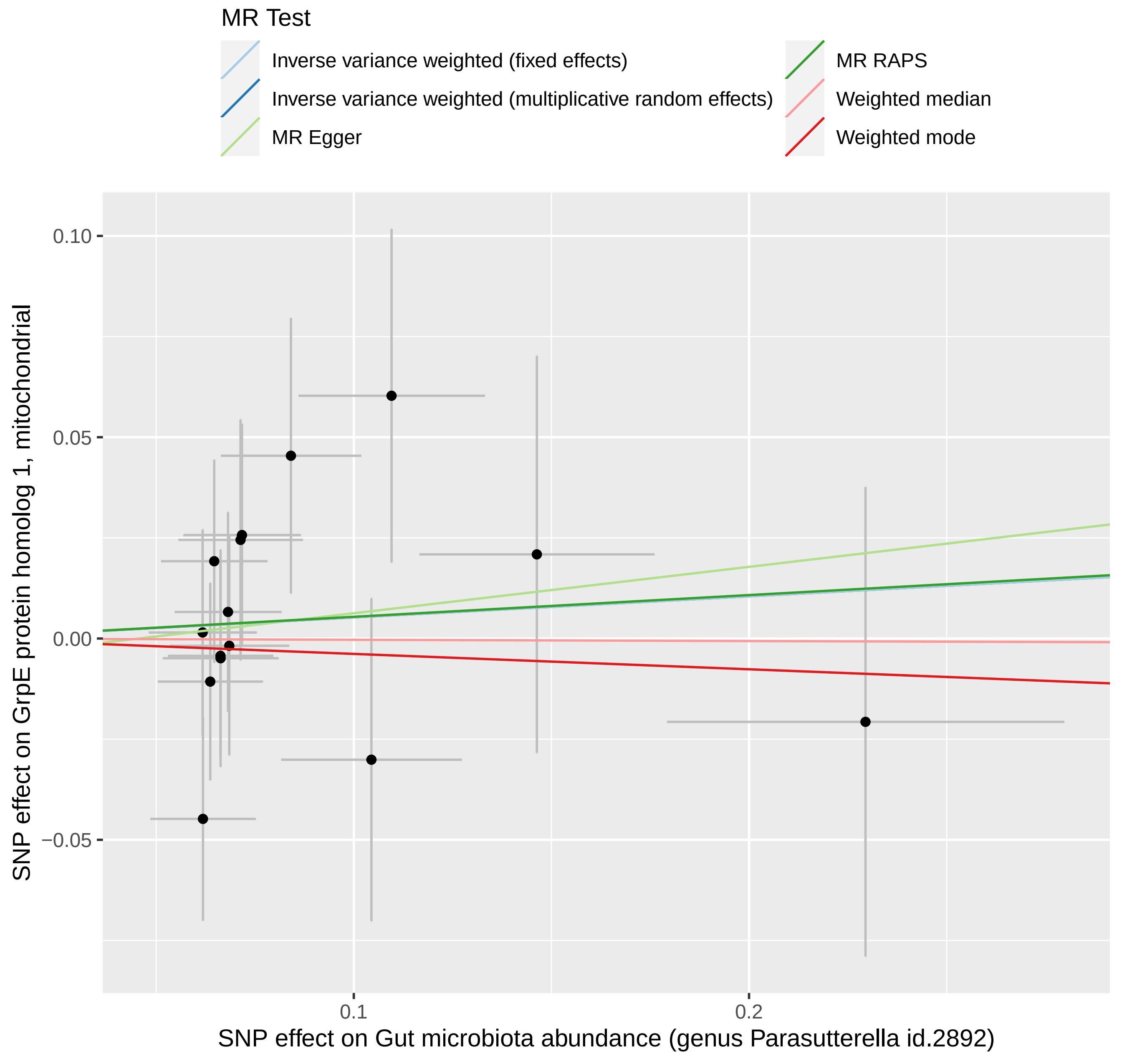

Batch 2−2563 : Gut microbiota abundance (genus Catenibacterium id.2153) on GSK3−beta interaction protein
with a threshold of 1e−05

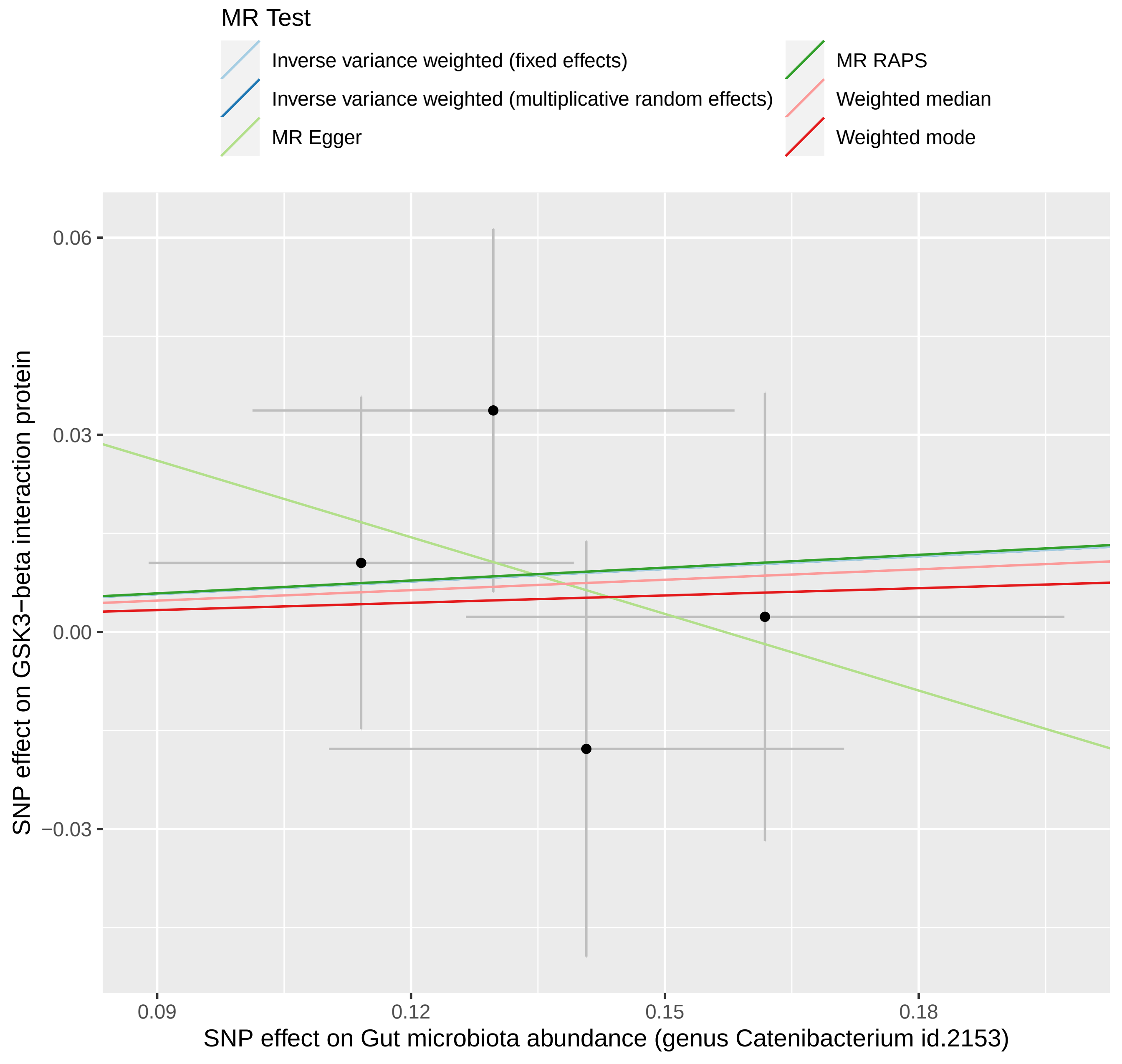

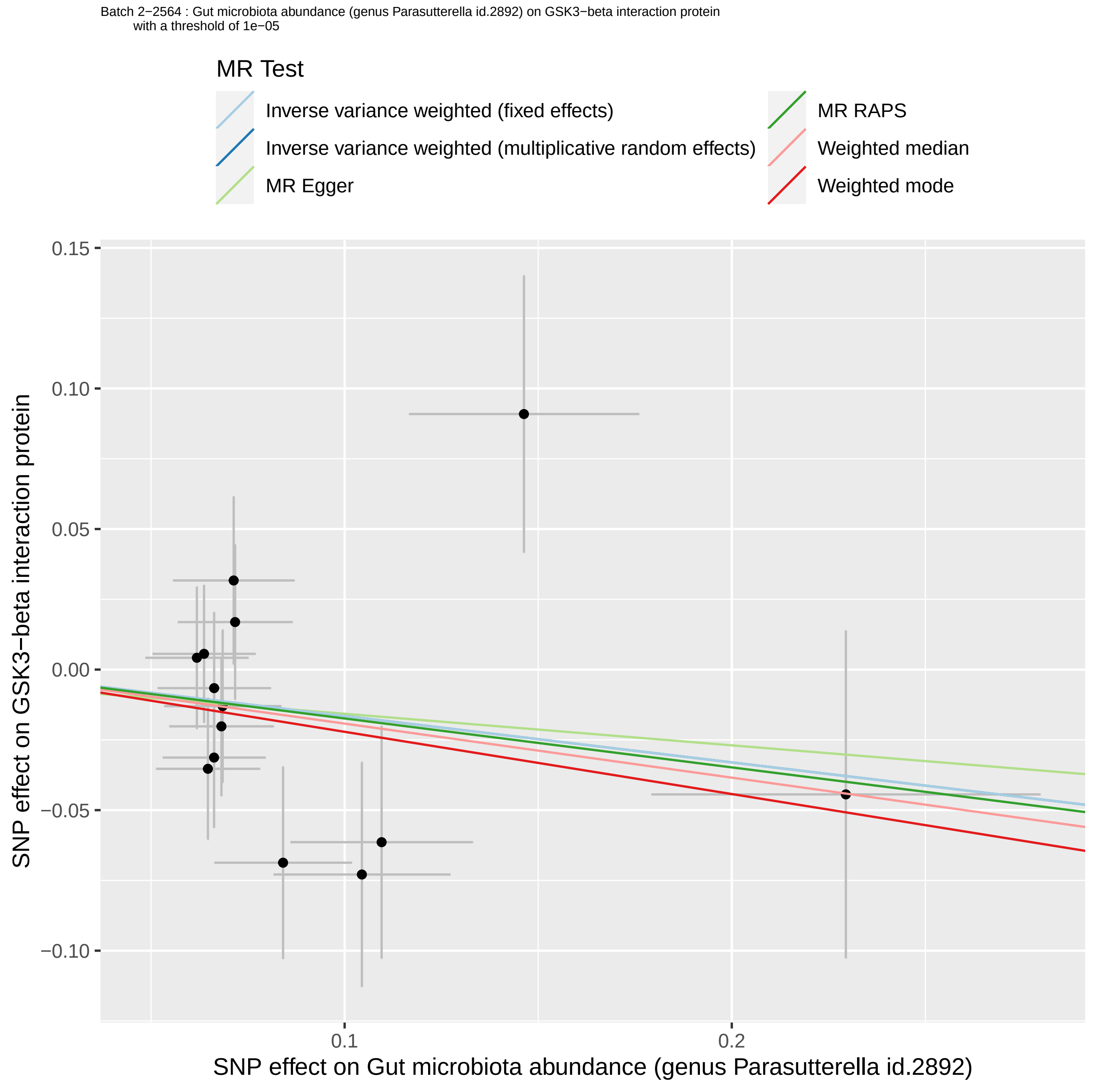

Batch 2−2564 : Gut microbiota abundance (genus Parasutterella id.2892) on GSK3−beta interaction protein
with a threshold of 1e−05
MR Test
Inverse variance weighted (fixed effects)
Inverse variance weighted (multiplicative random effects)
MR Egger
MR RAPS
Weighted median
Weighted mode
0.15
0.10
0.05
0.00
−0.05
−0.10
0.1
0.2
SNP effect on GSK3−beta interaction protein
SNP effect on Gut microbiota abundance (genus Parasutterella id.2892)

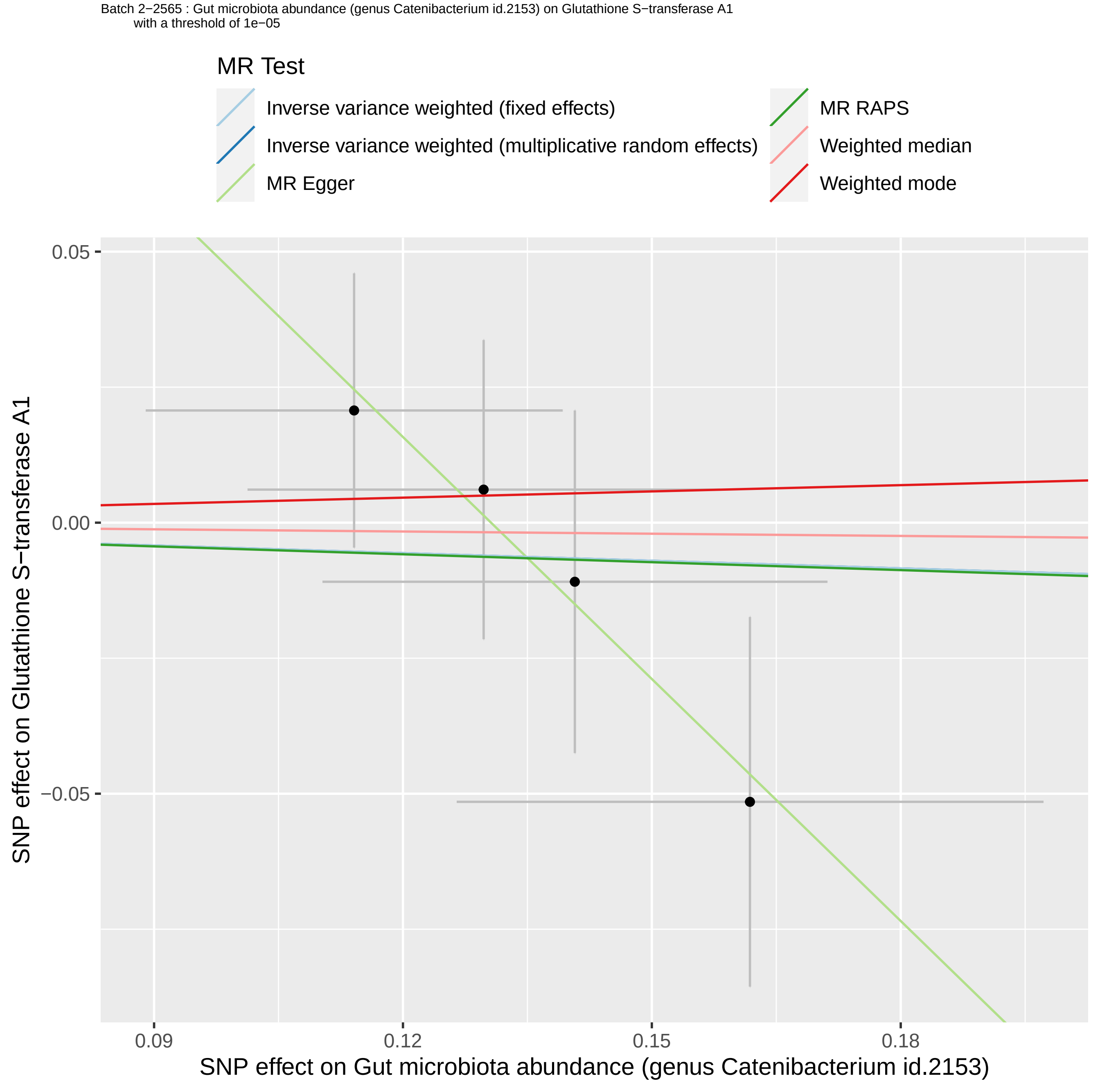
Batch 2−2565 : Gut microbiota abundance (genus Catenibacterium id.2153) on Glutathione S−transferase A1
with a threshold of 1e−05
MR Test
Inverse variance weighted (fixed effects)
Inverse variance weighted (multiplicative random effects)
MR Egger
MR RAPS
Weighted median
Weighted mode
0.05
0.00
−0.05
0.09
0.12
0.15
0.18
SNP effect on Glutathione S−transferase A1
SNP effect on Gut microbiota abundance (genus Catenibacterium id.2153)

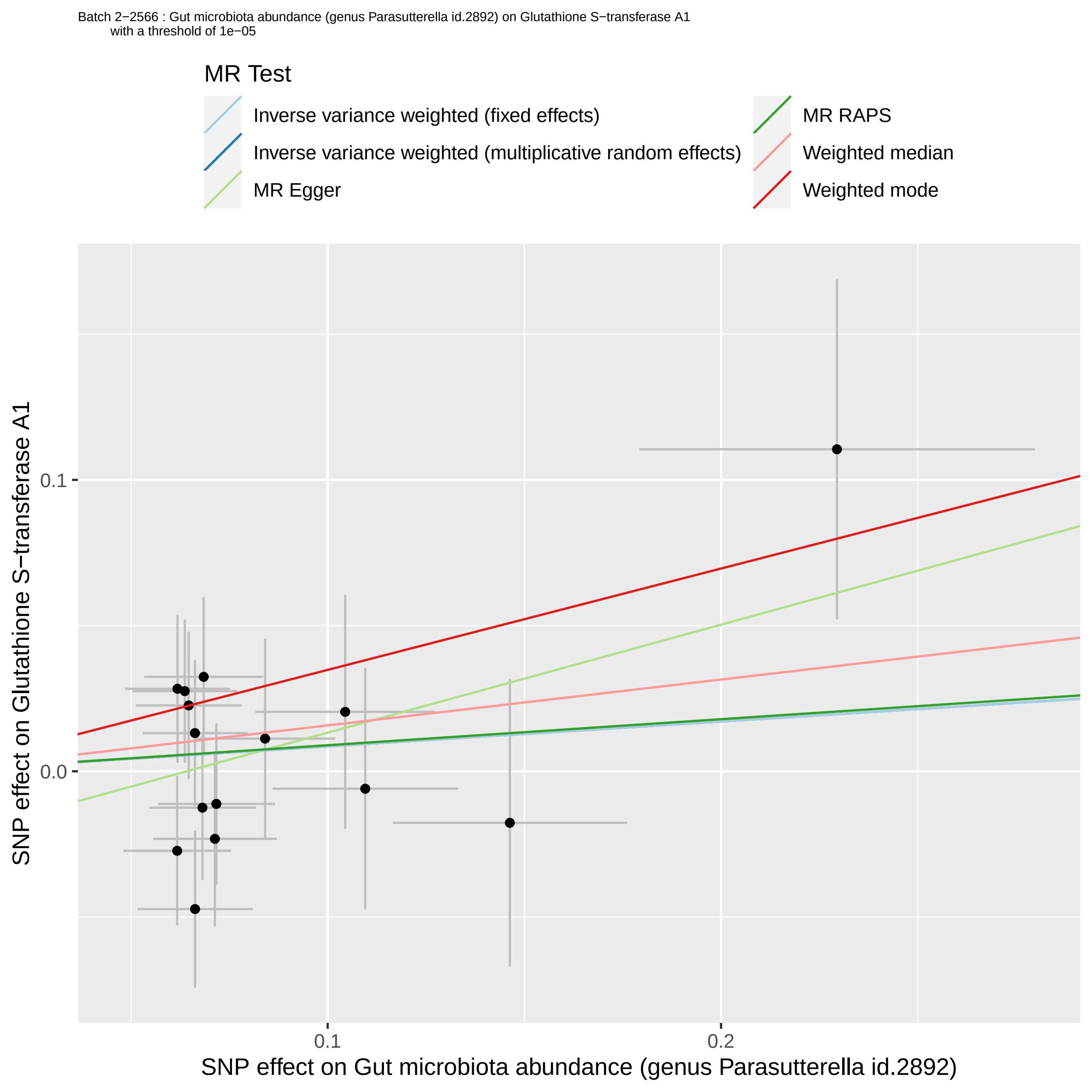
Batch 2−2566 : Gut microbiota abundance (genus Parasutterella id.2892) on Glutathione S−transferase A1
with a threshold of 1e−05
MR Test
Inverse variance weighted (fixed effects)
Inverse variance weighted (multiplicative random effects)
MR Egger
MR RAPS
Weighted median
Weighted mode
SNP effect on Glutathione S−transferase A1
0.1
0.0
0.1
0.2
SNP effect on Gut microbiota abundance (genus Parasutterella id.2892)

Batch 2−2567 : Gut microbiota abundance (genus Catenibacterium id.2153) on Glutathione S−transferase A3
with a threshold of 1e−05

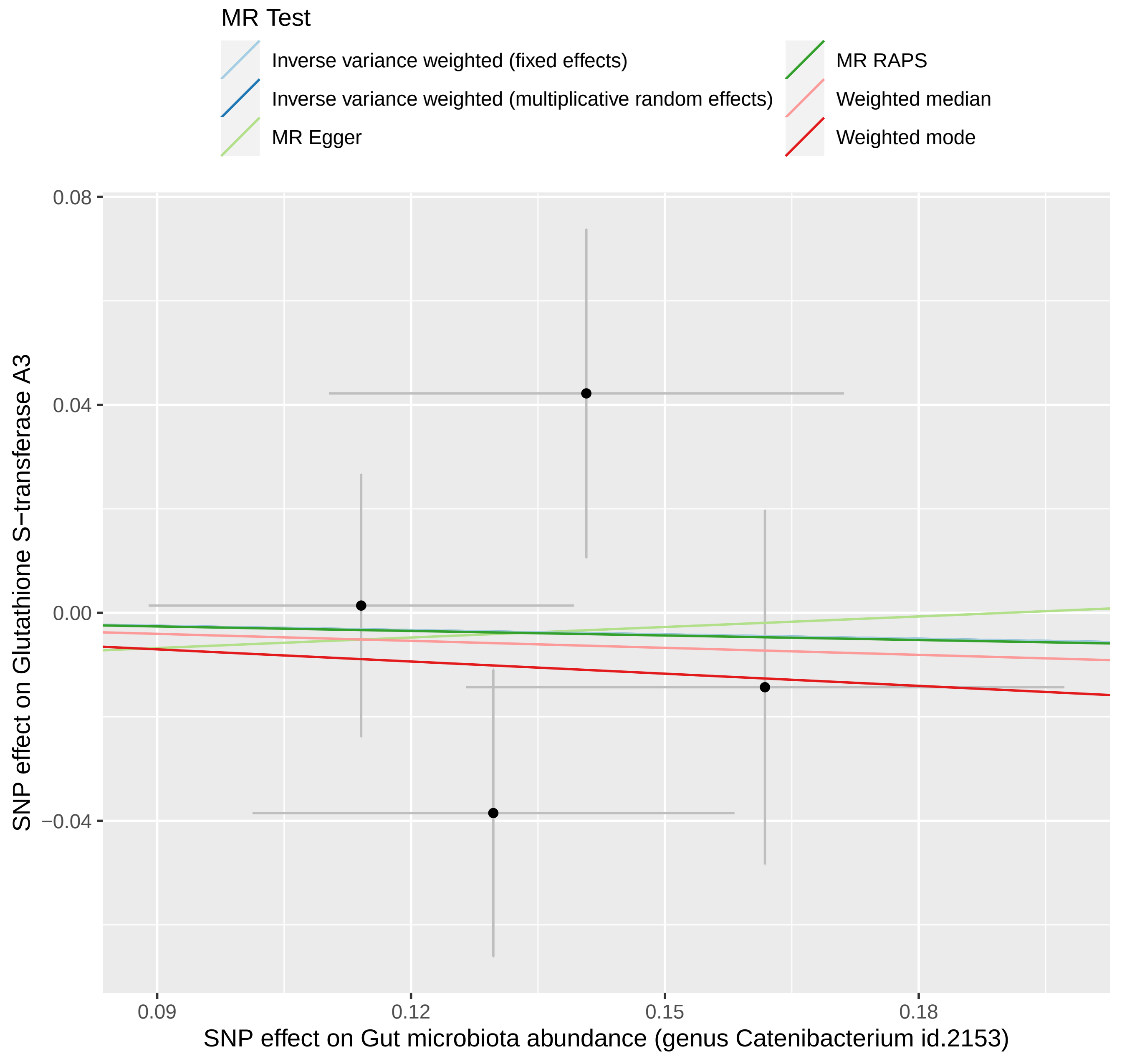

Batch 2−2568 : Gut microbiota abundance (genus Parasutterella id.2892) on Glutathione S−transferase A3
with a threshold of 1e−05

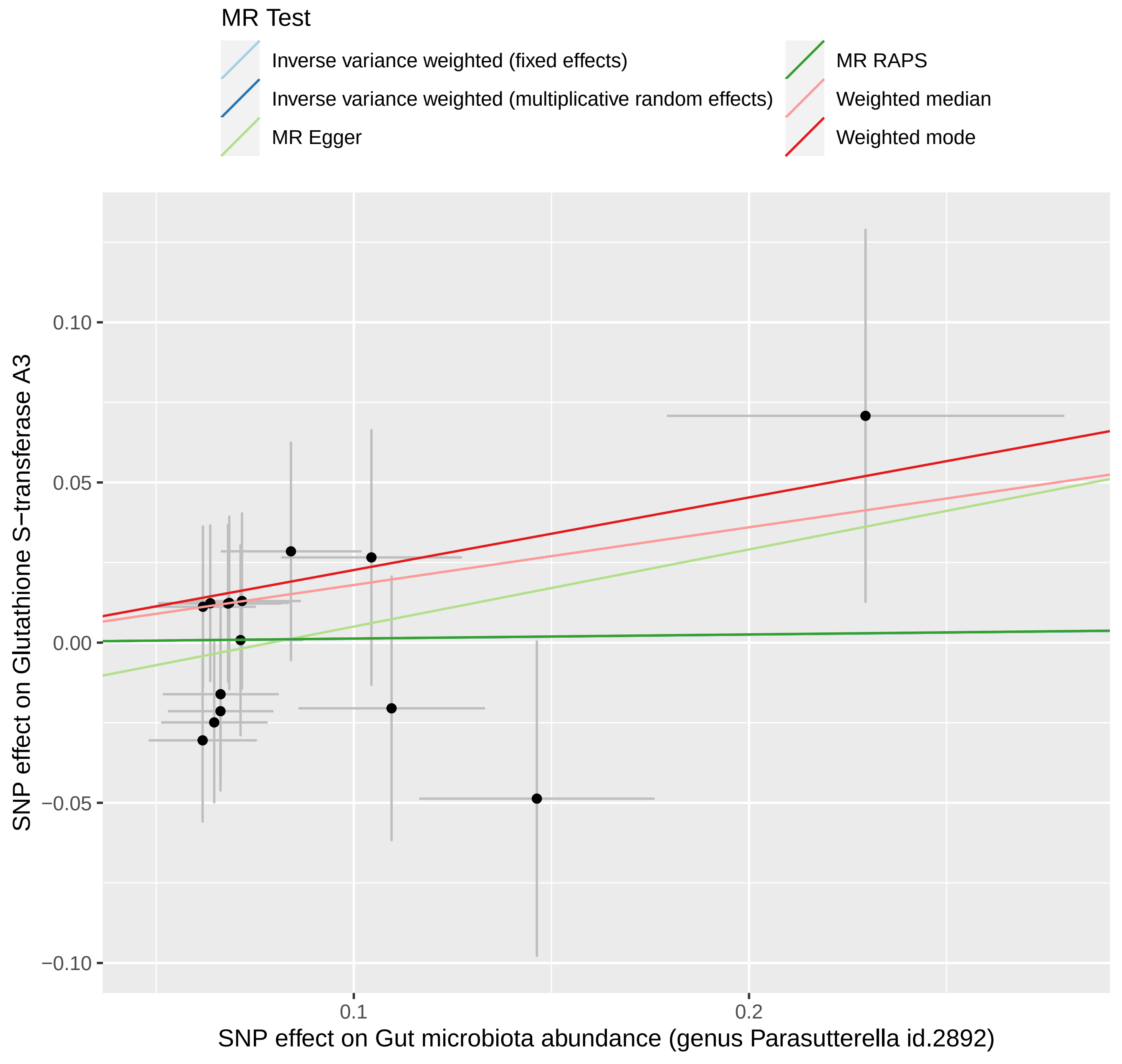

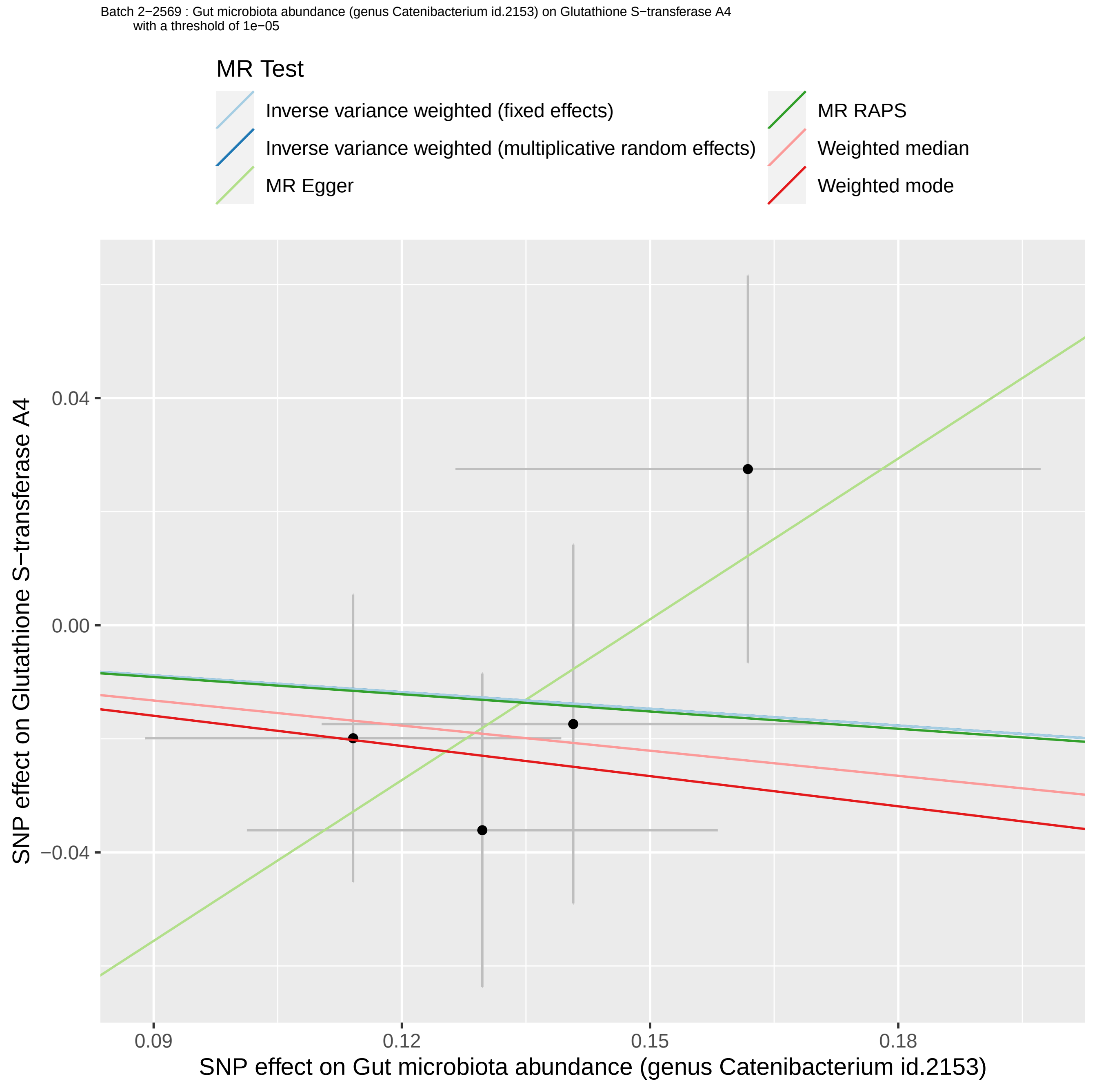

Batch 2−2569 : Gut microbiota abundance (genus Catenibacterium id.2153) on Glutathione S−transferase A4
with a threshold of 1e−05
MR Test
Inverse variance weighted (fixed effects)
Inverse variance weighted (multiplicative random effects)
MR Egger
MR RAPS
Weighted median
Weighted mode
0.04
0.00
−0.04
0.09
0.12
0.15
0.18
SNP effect on Glutathione S−transferase A4
SNP effect on Gut microbiota abundance (genus Catenibacterium id.2153)

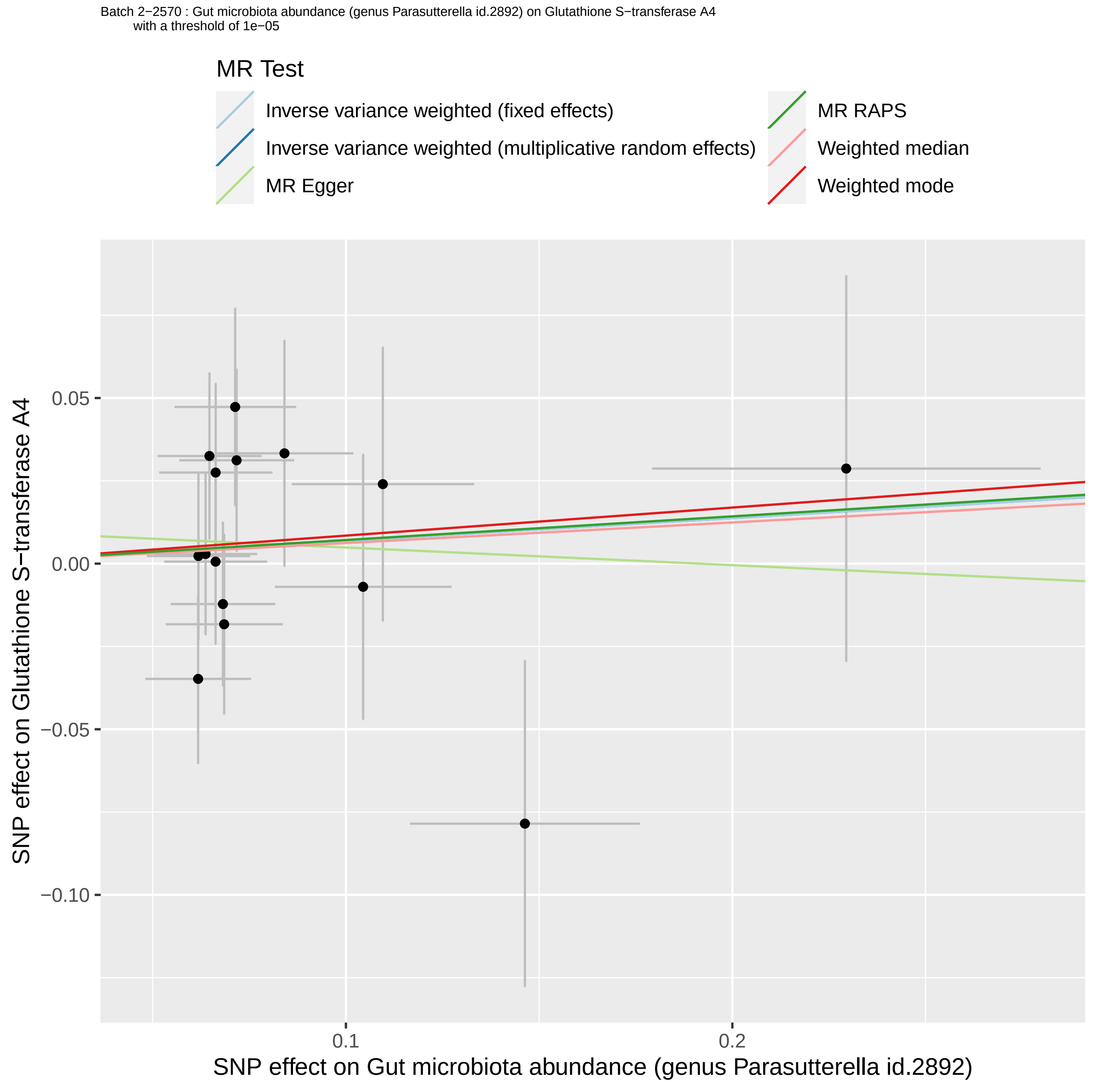

Batch 2−2570 : Gut microbiota abundance (genus Parasutterella id.2892) on Glutathione S−transferase A4
with a threshold of 1e−05
MR Test
Inverse variance weighted (fixed effects)
Inverse variance weighted (multiplicative random effects)
MR Egger
MR RAPS
Weighted median
Weighted mode
0.05
0.00
−0.05
−0.10
0.1
0.2
SNP effect on Glutathione S−transferase A4
SNP effect on Gut microbiota abundance (genus Parasutterella id.2892)

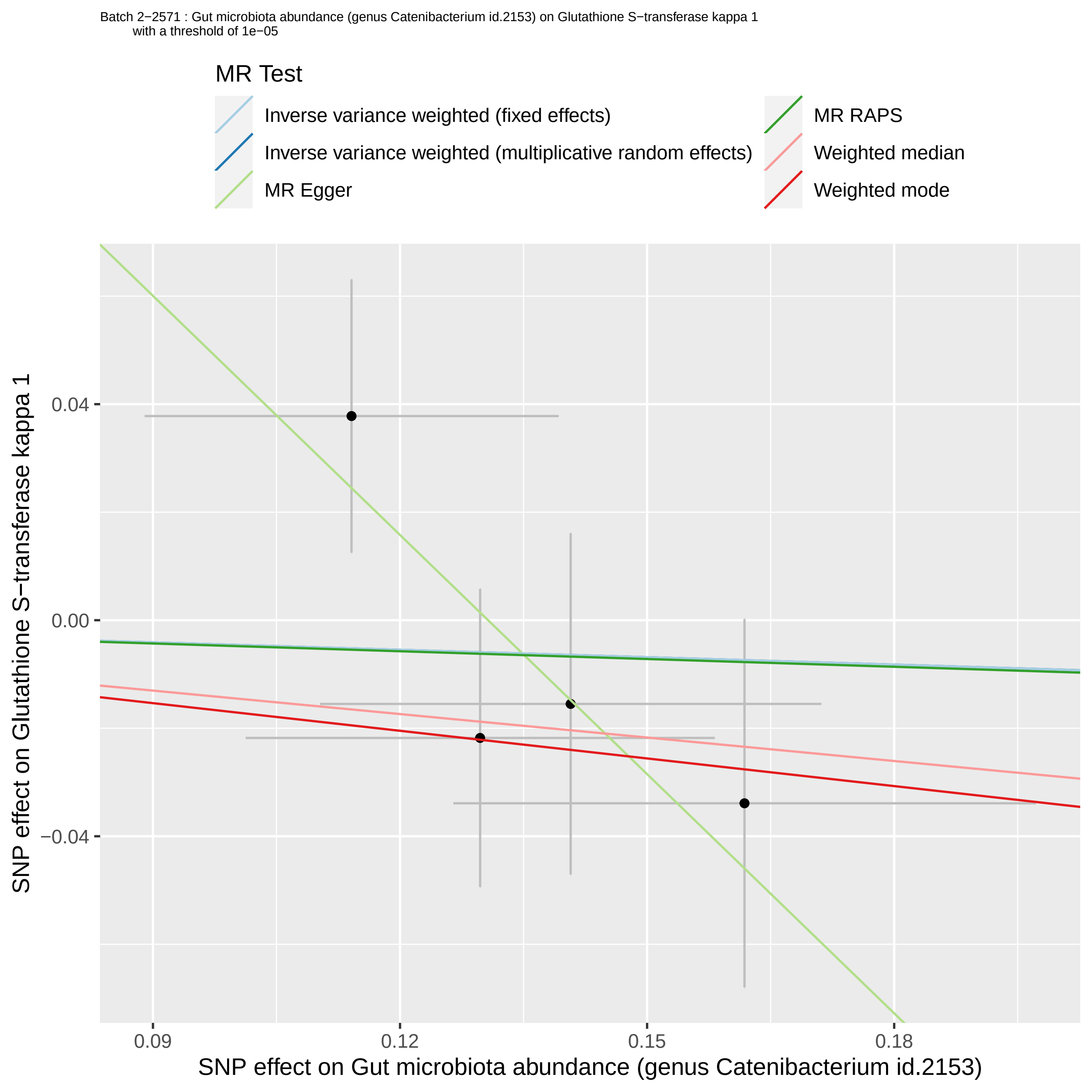
Batch 2−2571 : Gut microbiota abundance (genus Catenibacterium id.2153) on Glutathione S−transferase kappa 1
with a threshold of 1e−05
MR Test
Inverse variance weighted (fixed effects)
Inverse variance weighted (multiplicative random effects)
MR Egger
MR RAPS
Weighted median
Weighted mode
0.04
0.00
−0.04
0.09
0.12
0.15
0.18
SNP effect on Glutathione S−transferase kappa 1
SNP effect on Gut microbiota abundance (genus Catenibacterium id.2153)

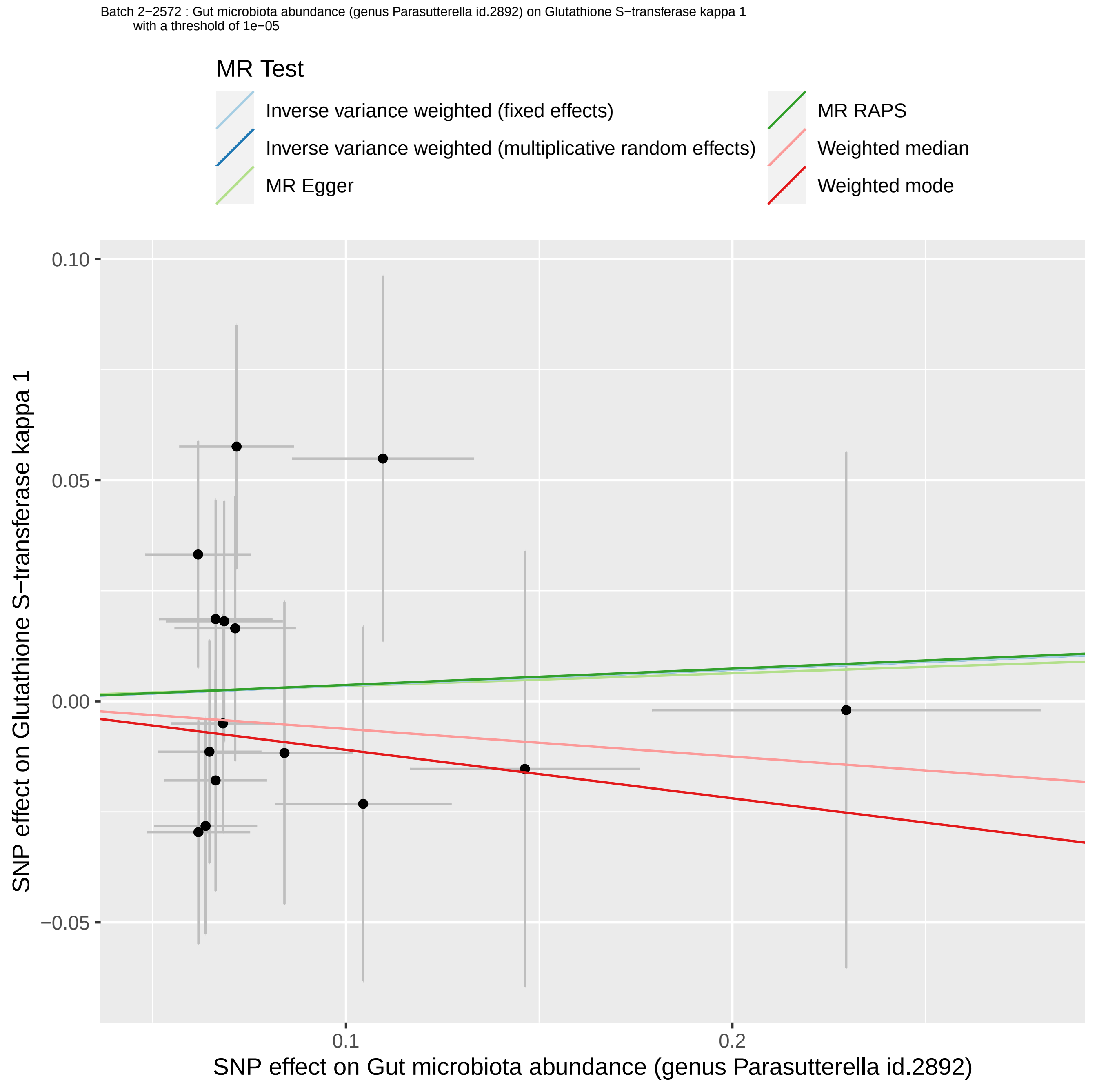
Batch 2−2572 : Gut microbiota abundance (genus Parasutterella id.2892) on Glutathione S−transferase kappa 1
with a threshold of 1e−05
MR Test
Inverse variance weighted (fixed effects)
Inverse variance weighted (multiplicative random effects)
MR Egger
MR RAPS
Weighted median
Weighted mode
0.10
0.05
0.00
−0.05
0.1
0.2
SNP effect on Glutathione S−transferase kappa 1
SNP effect on Gut microbiota abundance (genus Parasutterella id.2892)

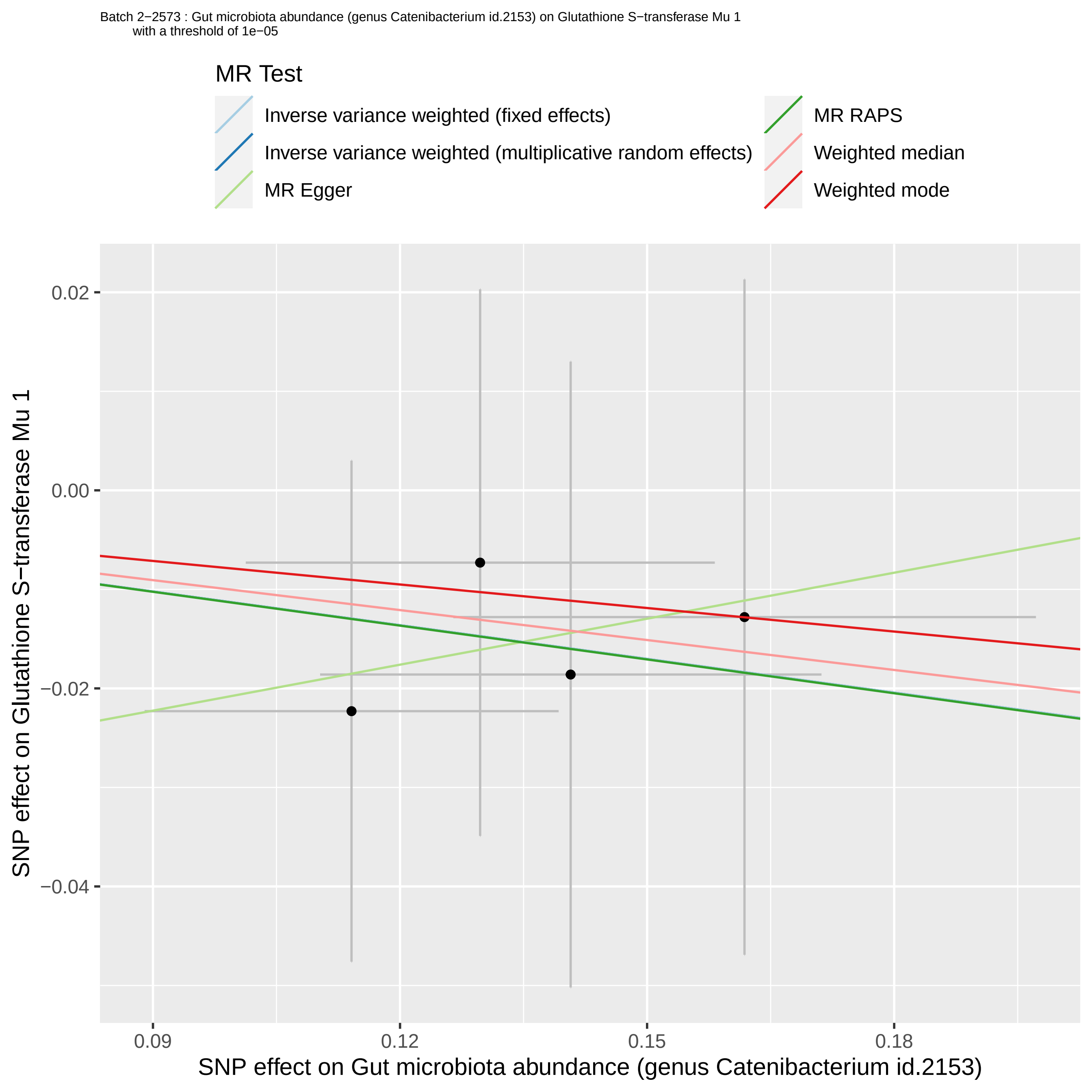

Batch 2−2573 : Gut microbiota abundance (genus Catenibacterium id.2153) on Glutathione S−transferase Mu 1
with a threshold of 1e−05
MR Test
Inverse variance weighted (fixed effects)
Inverse variance weighted (multiplicative random effects)
MR Egger
MR RAPS
Weighted median
Weighted mode
0.02
0.00
−0.02
−0.04
0.09
0.12
0.15
0.18
SNP effect on Glutathione S−transferase Mu 1
SNP effect on Gut microbiota abundance (genus Catenibacterium id.2153)

Batch 2−2574 : Gut microbiota abundance (genus Parasutterella id.2892) on Glutathione S−transferase Mu 1
with a threshold of 1e−05

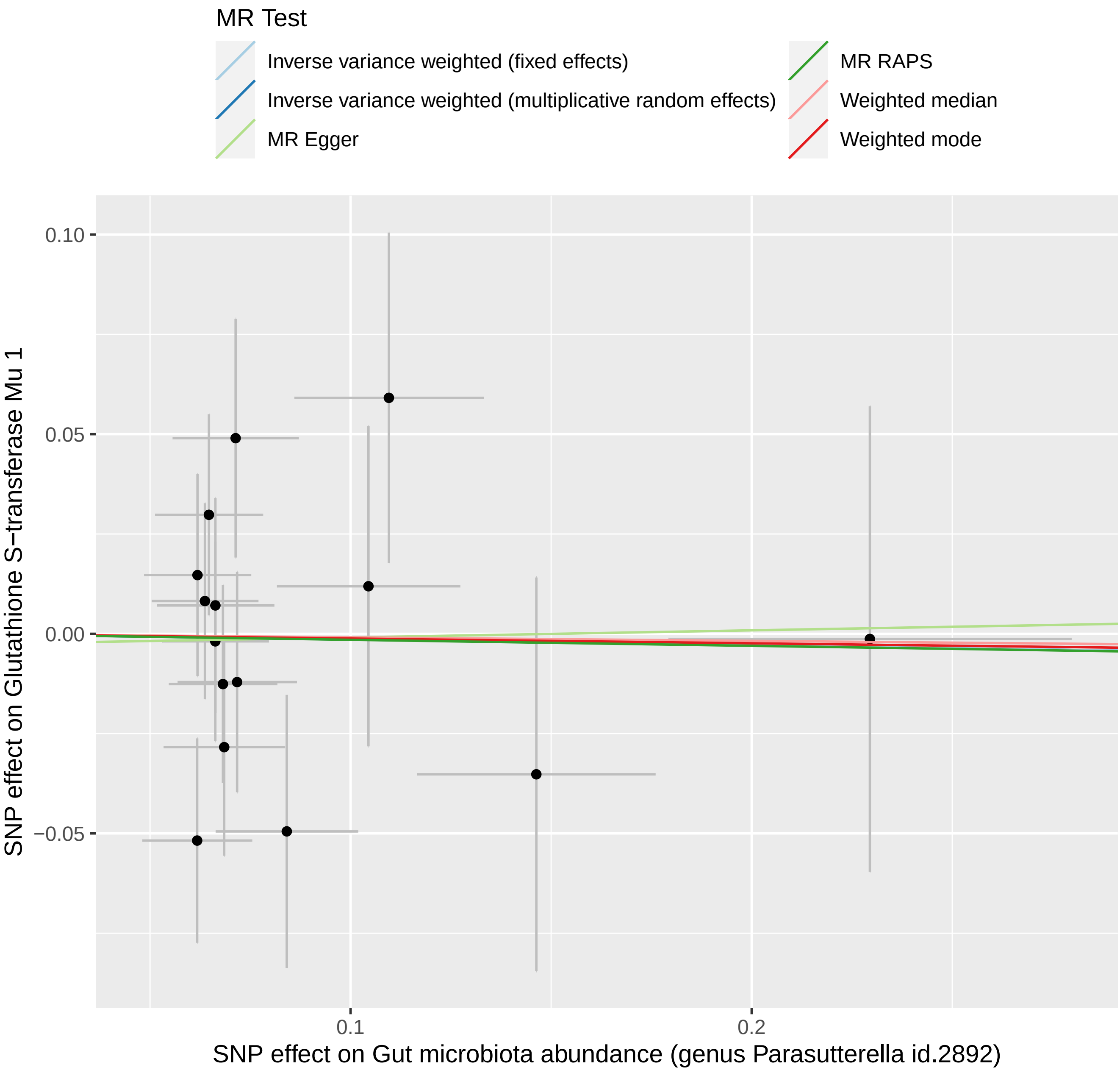

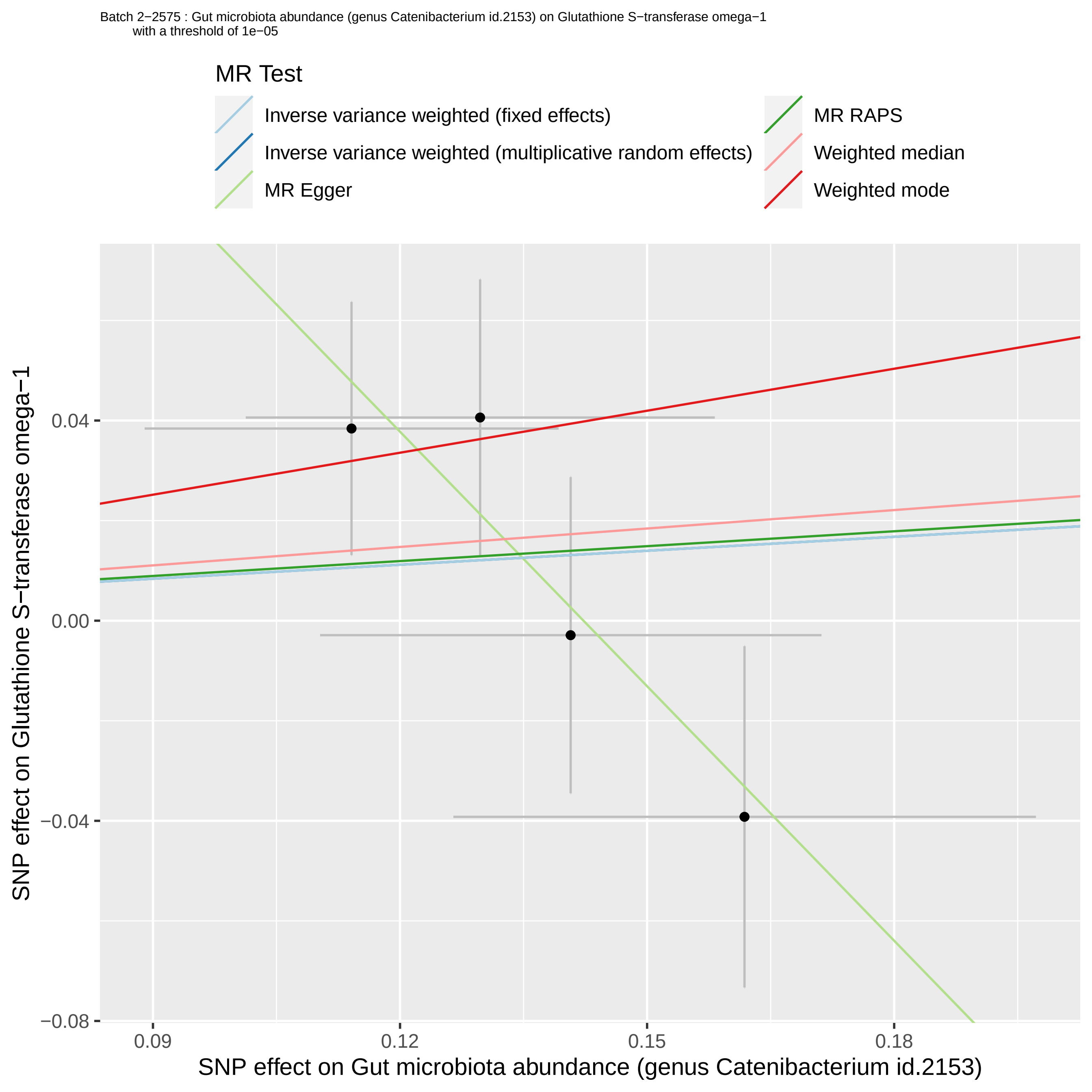
Batch 2−2575 : Gut microbiota abundance (genus Catenibacterium id.2153) on Glutathione S−transferase omega−1
with a threshold of 1e−05
MR Test
Inverse variance weighted (fixed effects)
Inverse variance weighted (multiplicative random effects)
MR Egger
MR RAPS
Weighted median
Weighted mode
SNP effect on Glutathione S−transferase omega−1
0.04
0.00
−0.04
−0.08
0.09
0.12
0.15
0.18
SNP effect on Gut microbiota abundance (genus Catenibacterium id.2153)

Batch 2−2576 : Gut microbiota abundance (genus Parasutterella id.2892) on Glutathione S−transferase omega−1
with a threshold of 1e−05

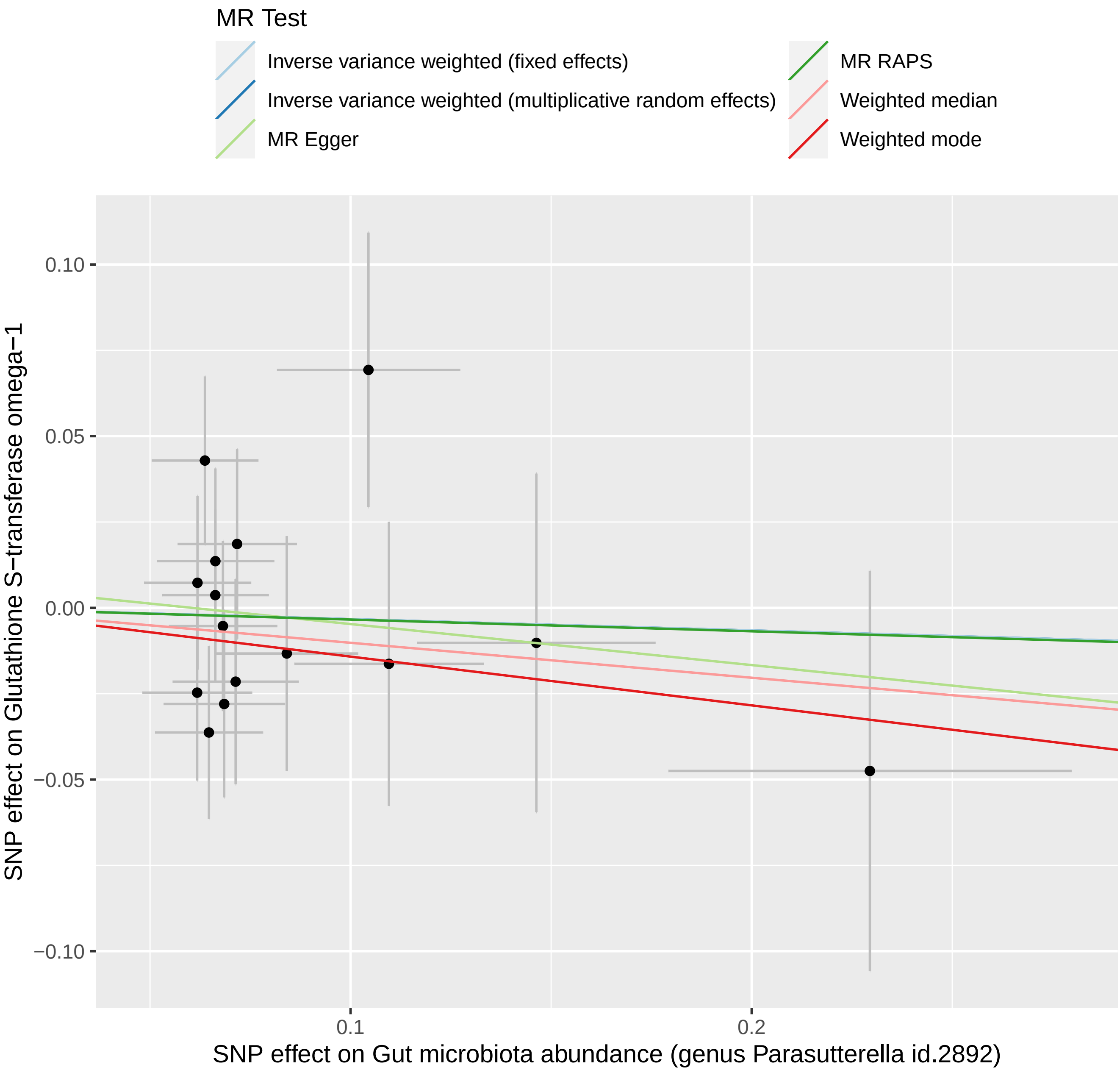

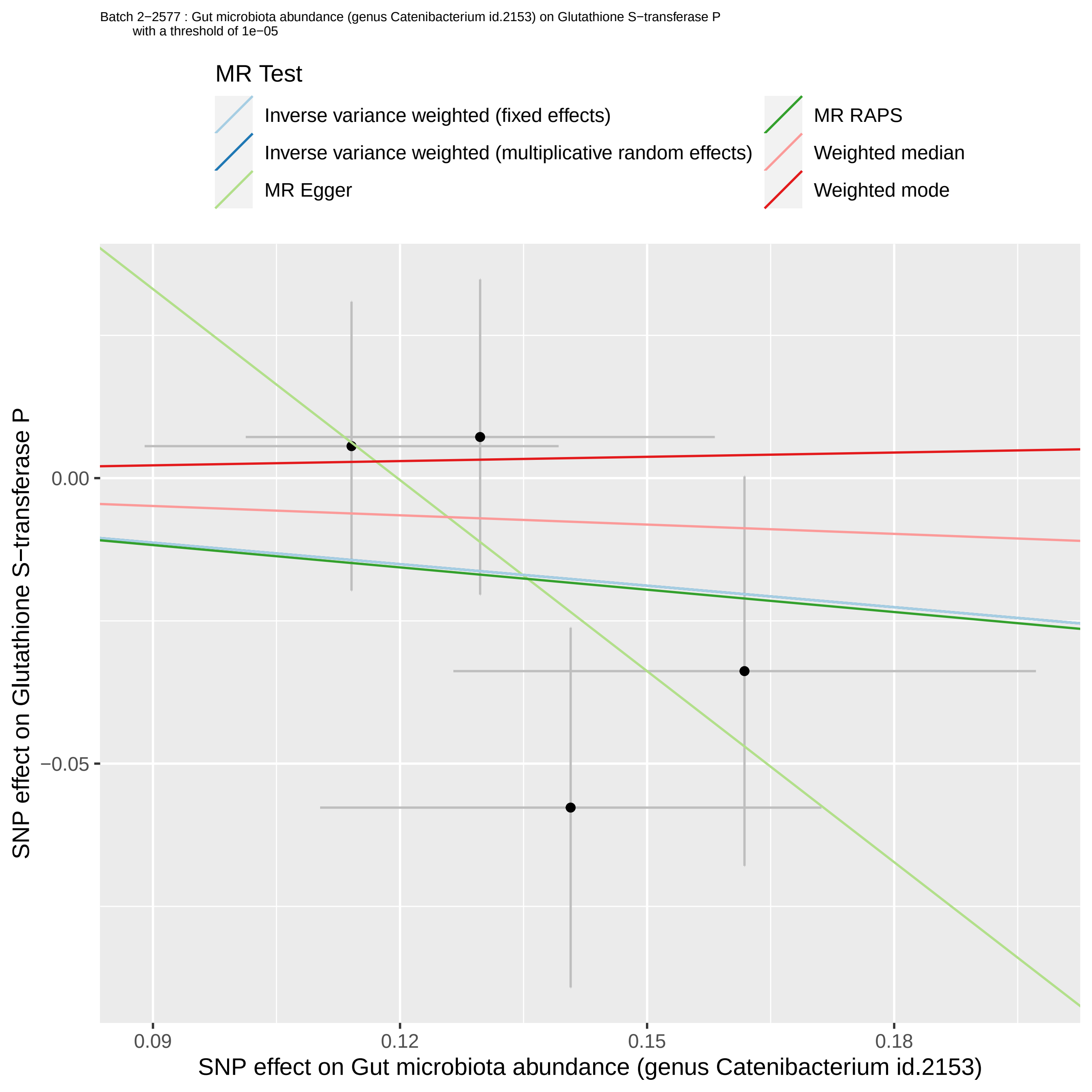
Batch 2−2577 : Gut microbiota abundance (genus Catenibacterium id.2153) on Glutathione S−transferase P
with a threshold of 1e−05
MR Test
Inverse variance weighted (fixed effects)
Inverse variance weighted (multiplicative random effects)
MR Egger
MR RAPS
Weighted median
Weighted mode
0.00
−0.05
0.09
0.12
0.15
0.18
SNP effect on Glutathione S−transferase P
SNP effect on Gut microbiota abundance (genus Catenibacterium id.2153)

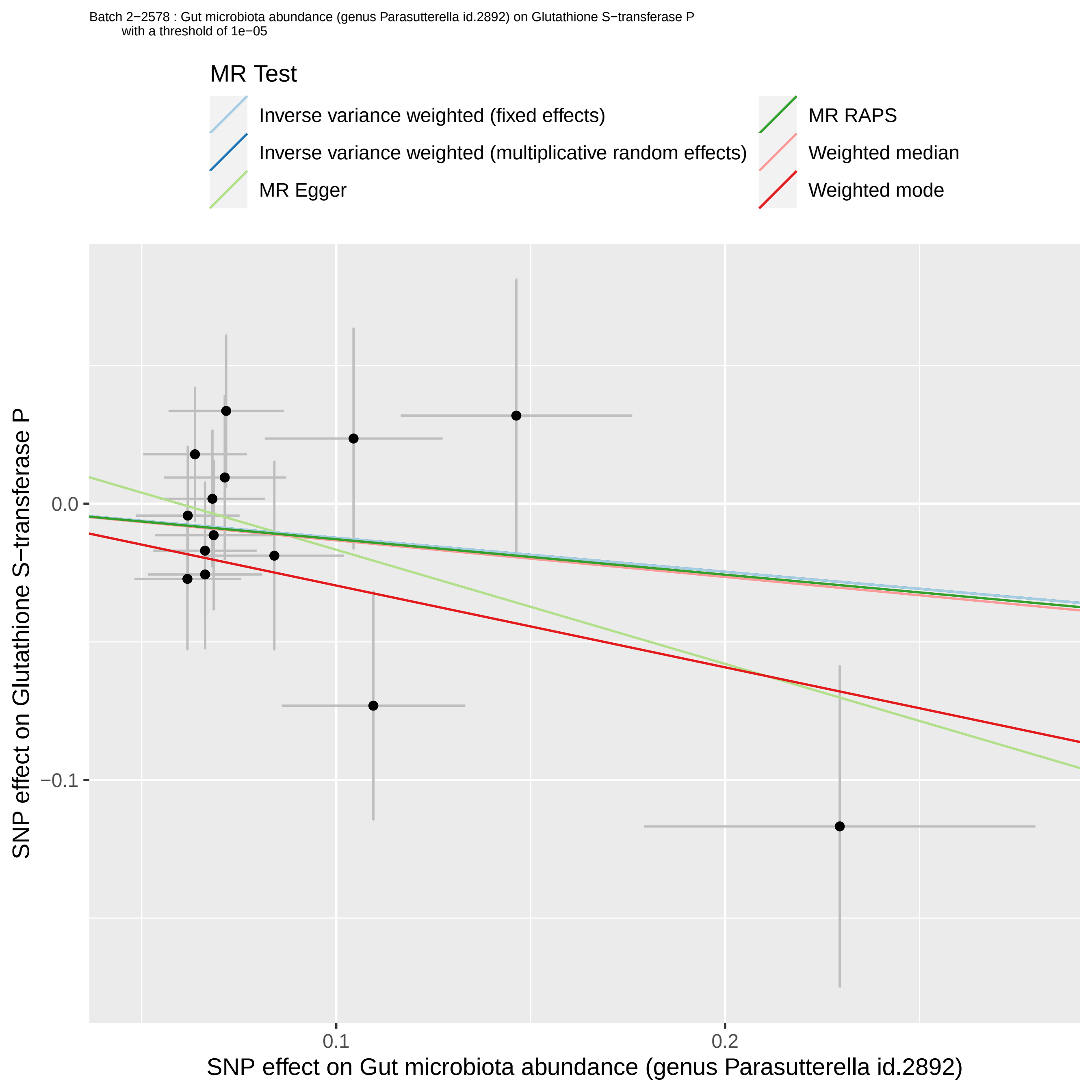
Batch 2−2578 : Gut microbiota abundance (genus Parasutterella id.2892) on Glutathione S−transferase P
with a threshold of 1e−05
MR Test
Inverse variance weighted (fixed effects)
Inverse variance weighted (multiplicative random effects)
MR Egger
MR RAPS
Weighted median
Weighted mode
0.0
−0.1
0.1
0.2
SNP effect on Glutathione S−transferase P
SNP effect on Gut microbiota abundance (genus Parasutterella id.2892)

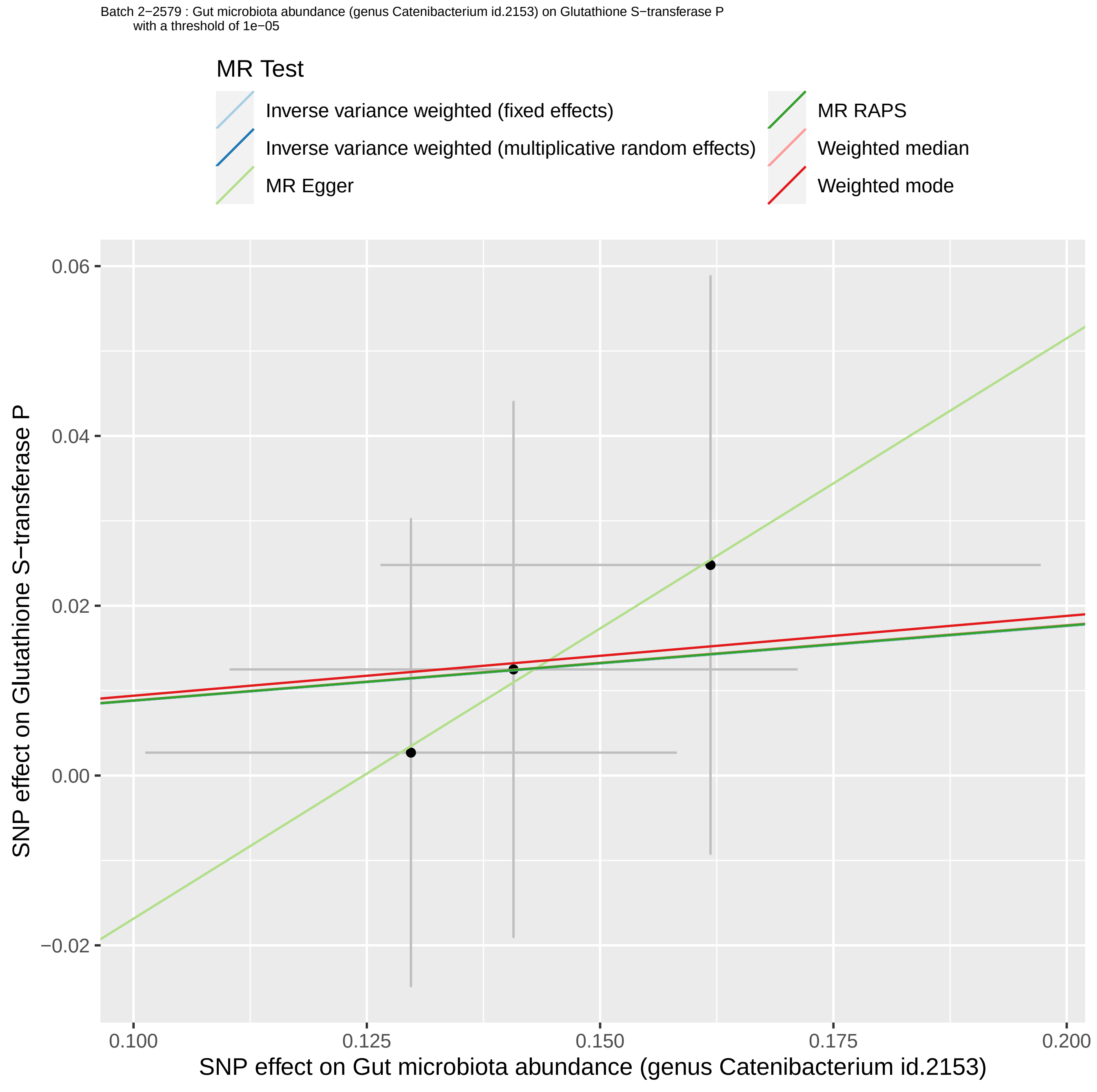
Batch 2−2579 : Gut microbiota abundance (genus Catenibacterium id.2153) on Glutathione S−transferase P
with a threshold of 1e−05
MR Test
Inverse variance weighted (fixed effects)
Inverse variance weighted (multiplicative random effects)
MR Egger
MR RAPS
Weighted median
Weighted mode
0.06
0.04
0.02
0.00
−0.02
0.100
0.125
0.150
0.175
0.200
SNP effect on Glutathione S−transferase P
SNP effect on Gut microbiota abundance (genus Catenibacterium id.2153)

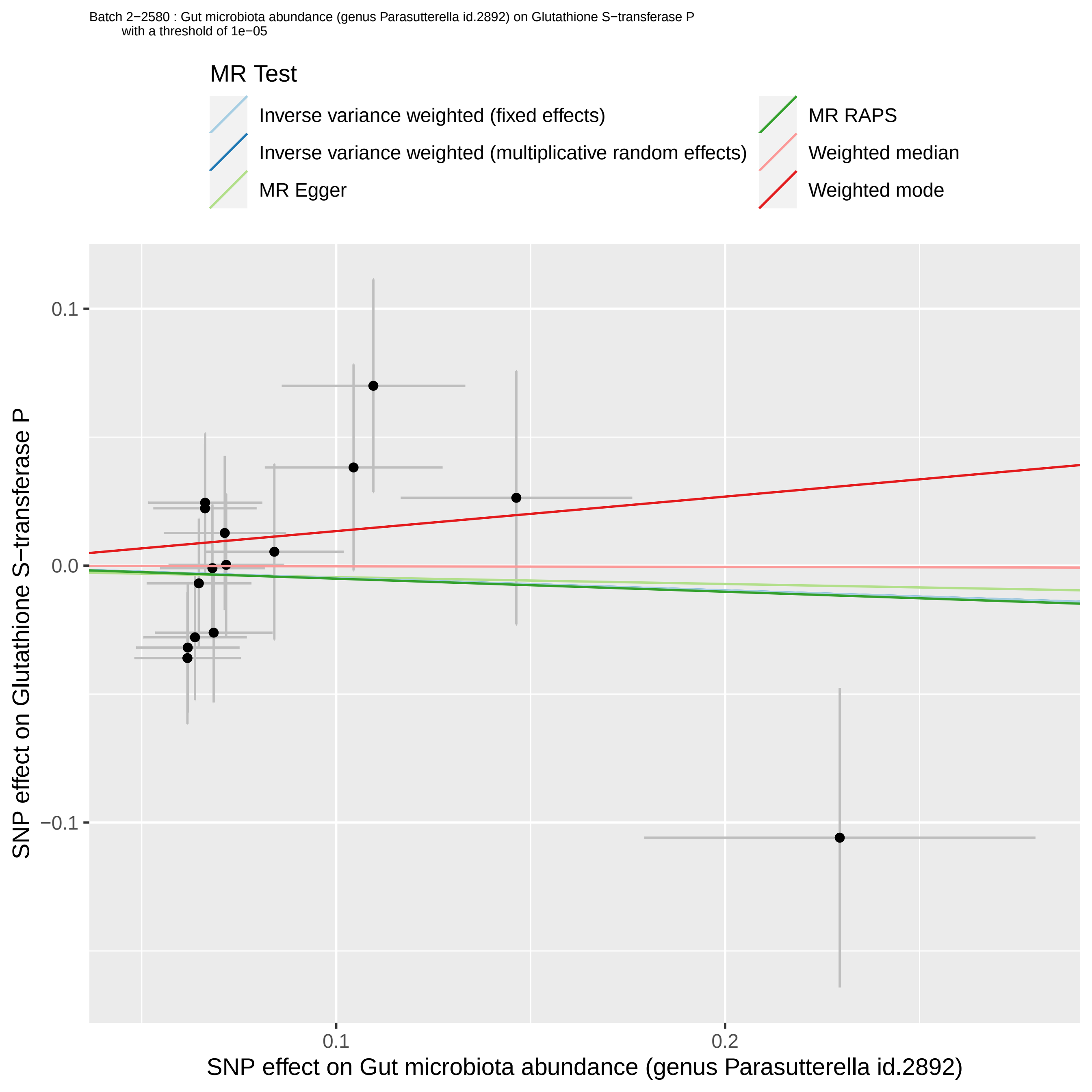

Batch 2−2580 : Gut microbiota abundance (genus Parasutterella id.2892) on Glutathione S−transferase P
with a threshold of 1e−05
MR Test
Inverse variance weighted (fixed effects)
Inverse variance weighted (multiplicative random effects)
MR Egger
MR RAPS
Weighted median
Weighted mode
0.1
0.0
−0.1
0.1
0.2
SNP effect on Glutathione S−transferase P
SNP effect on Gut microbiota abundance (genus Parasutterella id.2892)

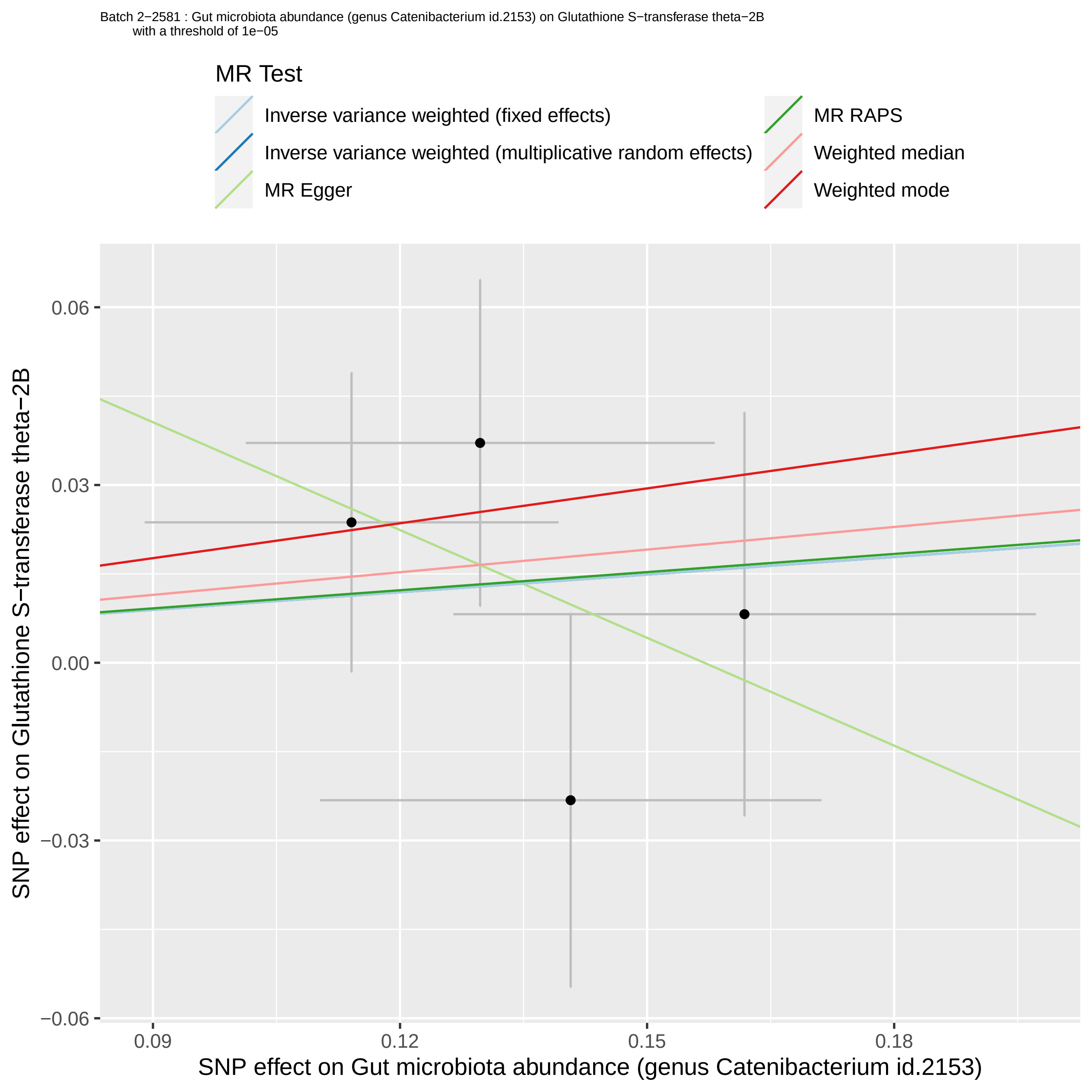

Batch 2−2581 : Gut microbiota abundance (genus Catenibacterium id.2153) on Glutathione S−transferase theta−2B
with a threshold of 1e−05
MR Test
Inverse variance weighted (fixed effects)
Inverse variance weighted (multiplicative random effects)
MR Egger
MR RAPS
Weighted median
Weighted mode
0.06
0.03
0.00
−0.03
−0.06
0.09
0.12
0.15
0.18
SNP effect on Glutathione S−transferase theta−2B
SNP effect on Gut microbiota abundance (genus Catenibacterium id.2153)

Batch 2−2582 : Gut microbiota abundance (genus Parasutterella id.2892) on Glutathione S−transferase theta−2B
with a threshold of 1e−05

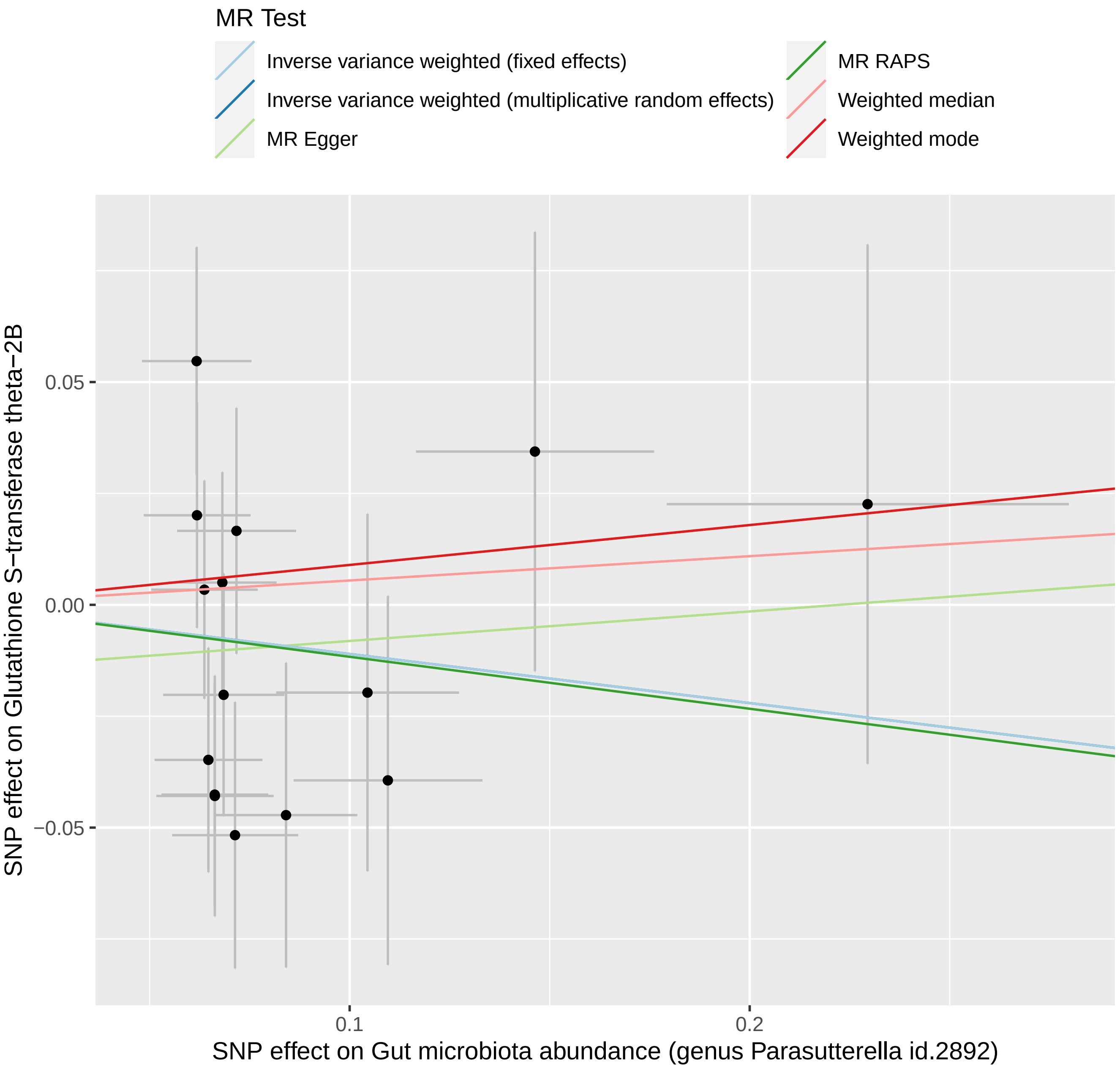

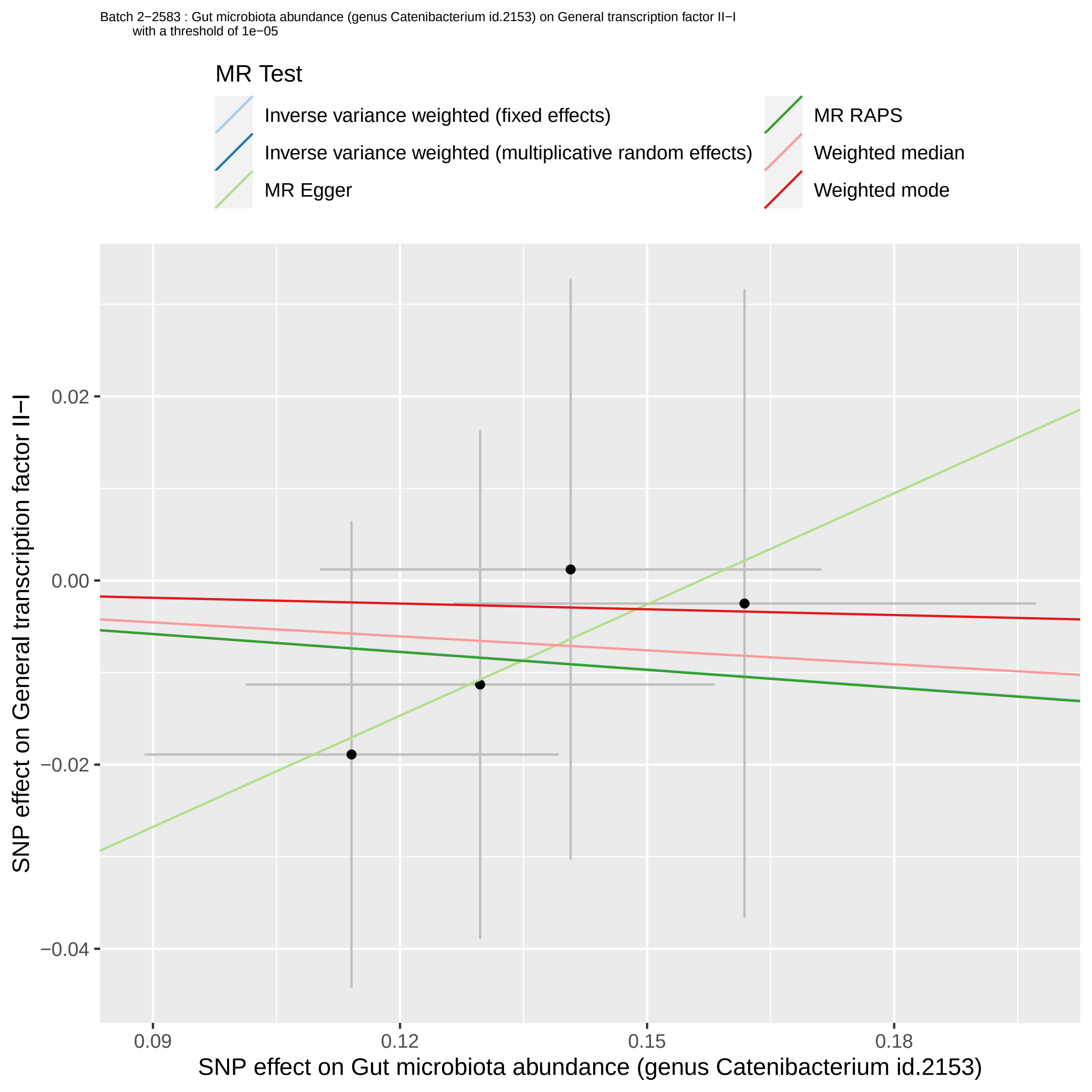
Batch 2−2583 : Gut microbiota abundance (genus Catenibacterium id.2153) on General transcription factor II−I
with a threshold of 1e−05
MR Test
Inverse variance weighted (fixed effects)
Inverse variance weighted (multiplicative random effects)
MR Egger
MR RAPS
Weighted median
Weighted mode
0.02
0.00
−0.02
−0.04
0.09
0.12
0.15
0.18
SNP effect on General transcription factor II−I
SNP effect on Gut microbiota abundance (genus Catenibacterium id.2153)

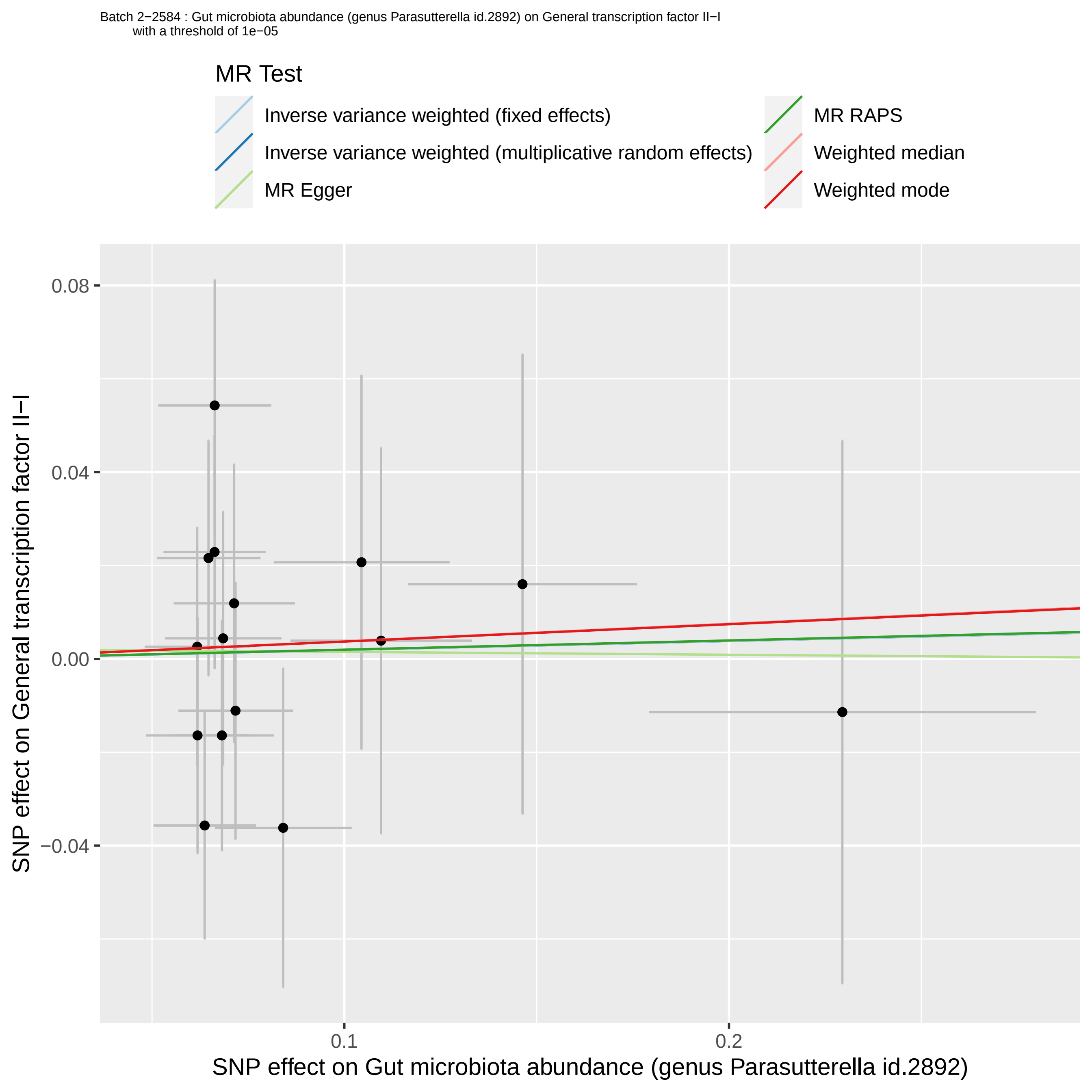
Batch 2−2584 : Gut microbiota abundance (genus Parasutterella id.2892) on General transcription factor II−I
with a threshold of 1e−05
MR Test
Inverse variance weighted (fixed effects)
Inverse variance weighted (multiplicative random effects)
MR Egger
MR RAPS
Weighted median
Weighted mode
0.08
0.04
0.00
−0.04
0.1
0.2
SNP effect on General transcription factor II−I
SNP effect on Gut microbiota abundance (genus Parasutterella id.2892)

Batch 2−2585 : Gut microbiota abundance (genus Catenibacterium id.2153) on Guanylyl cyclase−activating protein 1
with a threshold of 1e−05

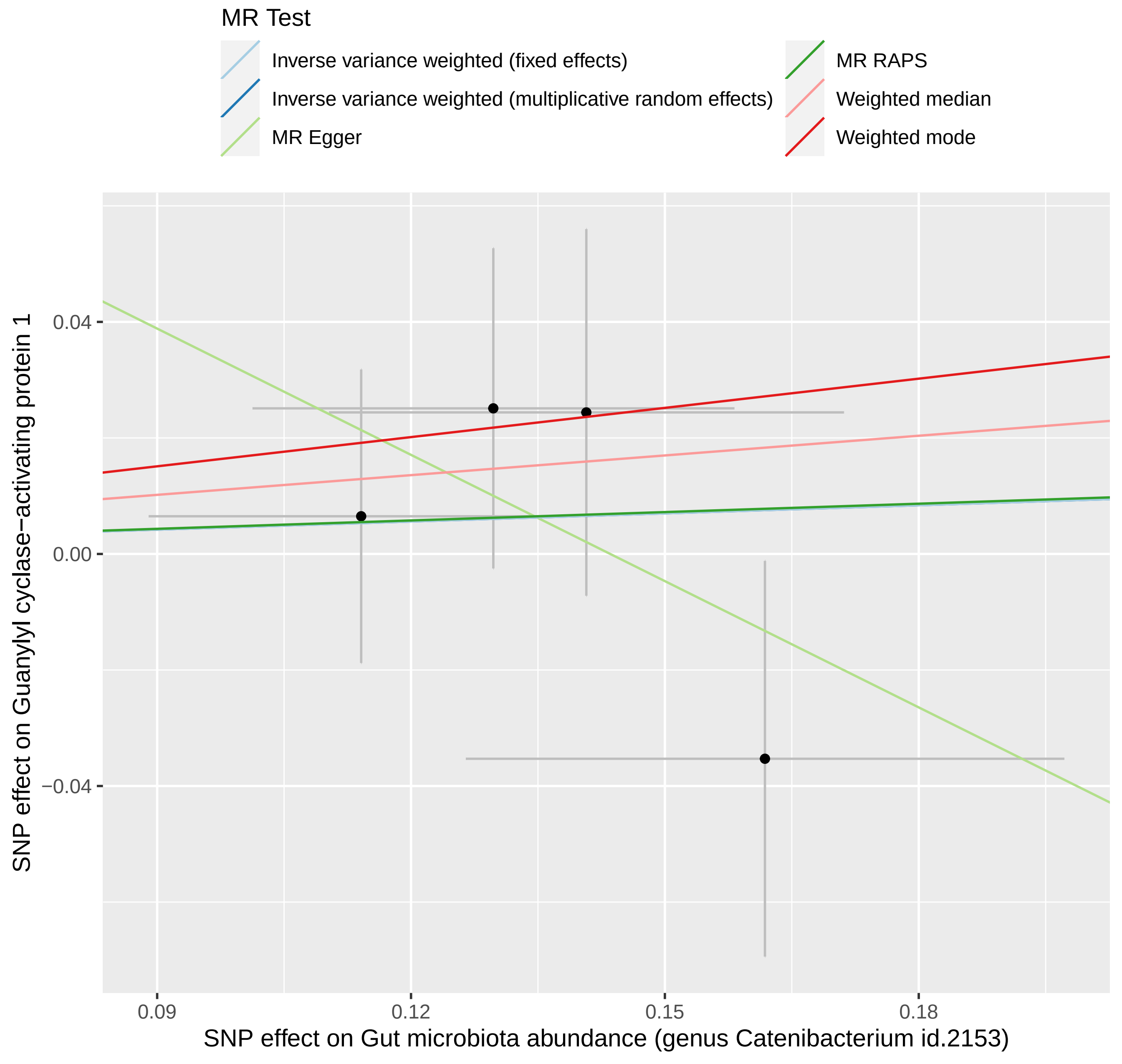

Batch 2−2586 : Gut microbiota abundance (genus Parasutterella id.2892) on Guanylyl cyclase−activating protein 1
with a threshold of 1e−05

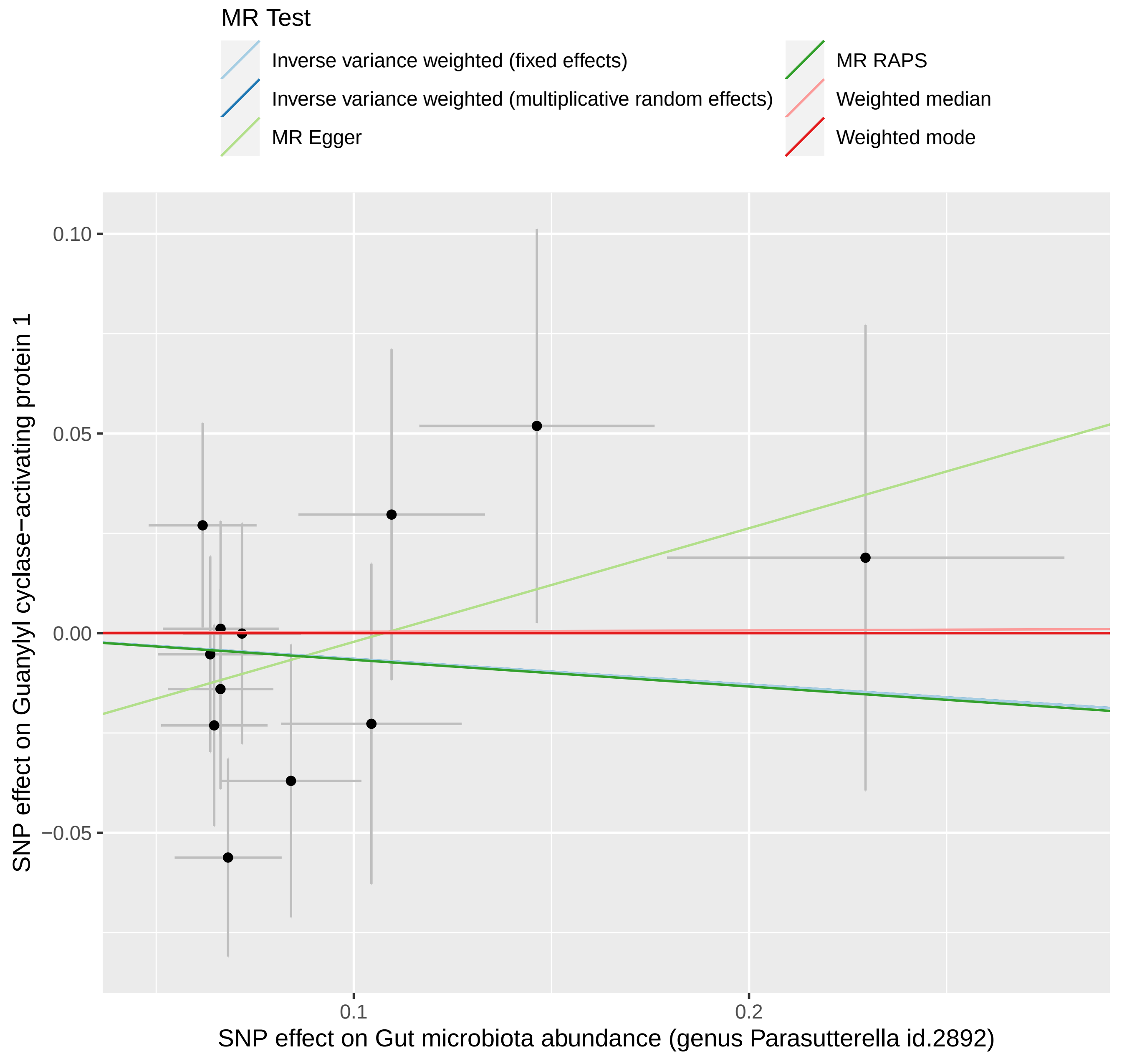

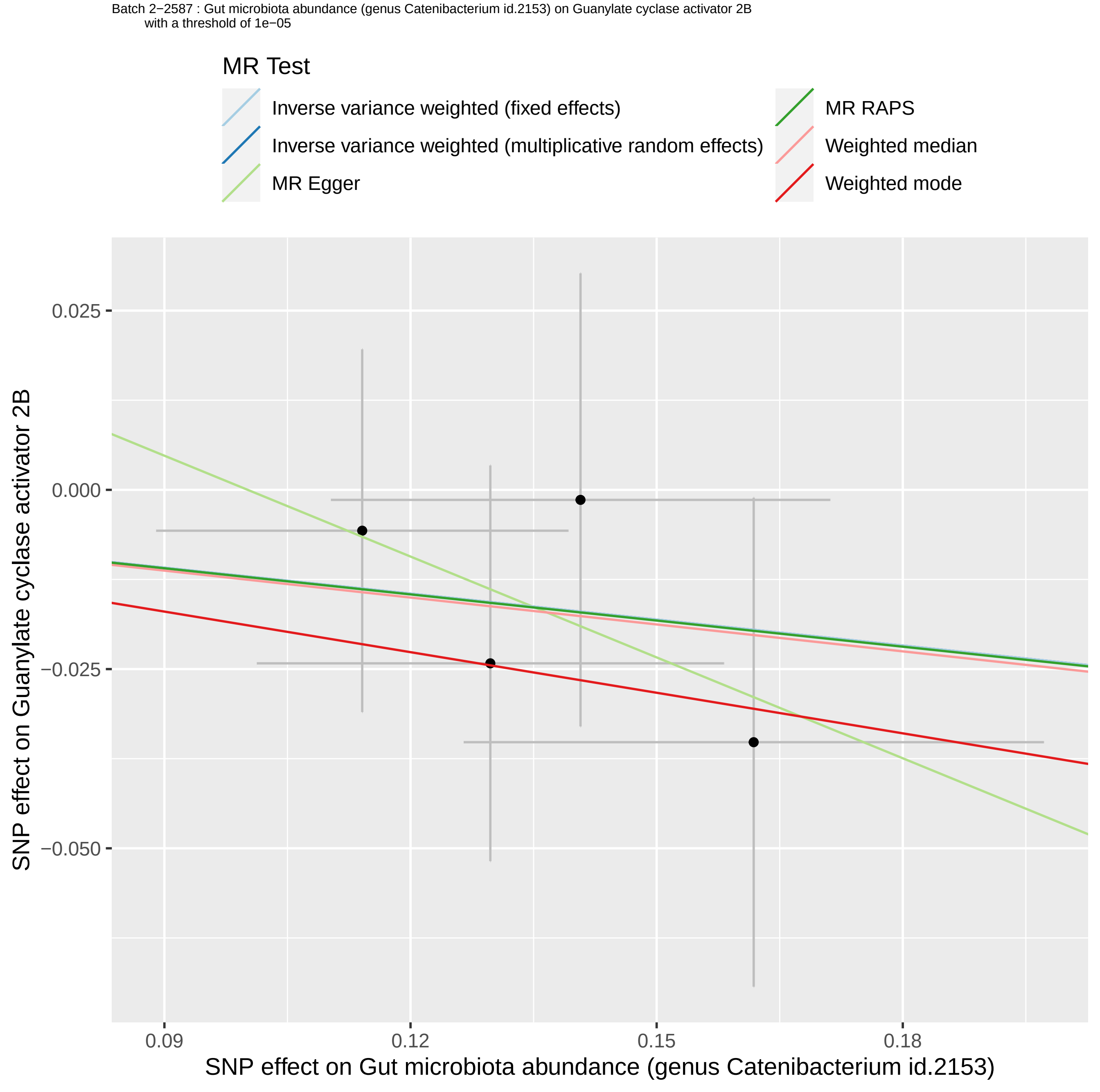
Batch 2−2587 : Gut microbiota abundance (genus Catenibacterium id.2153) on Guanylate cyclase activator 2B
with a threshold of 1e−05
MR Test
Inverse variance weighted (fixed effects)
Inverse variance weighted (multiplicative random effects)
MR Egger
MR RAPS
Weighted median
Weighted mode
0.025
0.000
−0.025
−0.050
0.09
0.12
0.15
0.18
SNP effect on Guanylate cyclase activator 2B
SNP effect on Gut microbiota abundance (genus Catenibacterium id.2153)

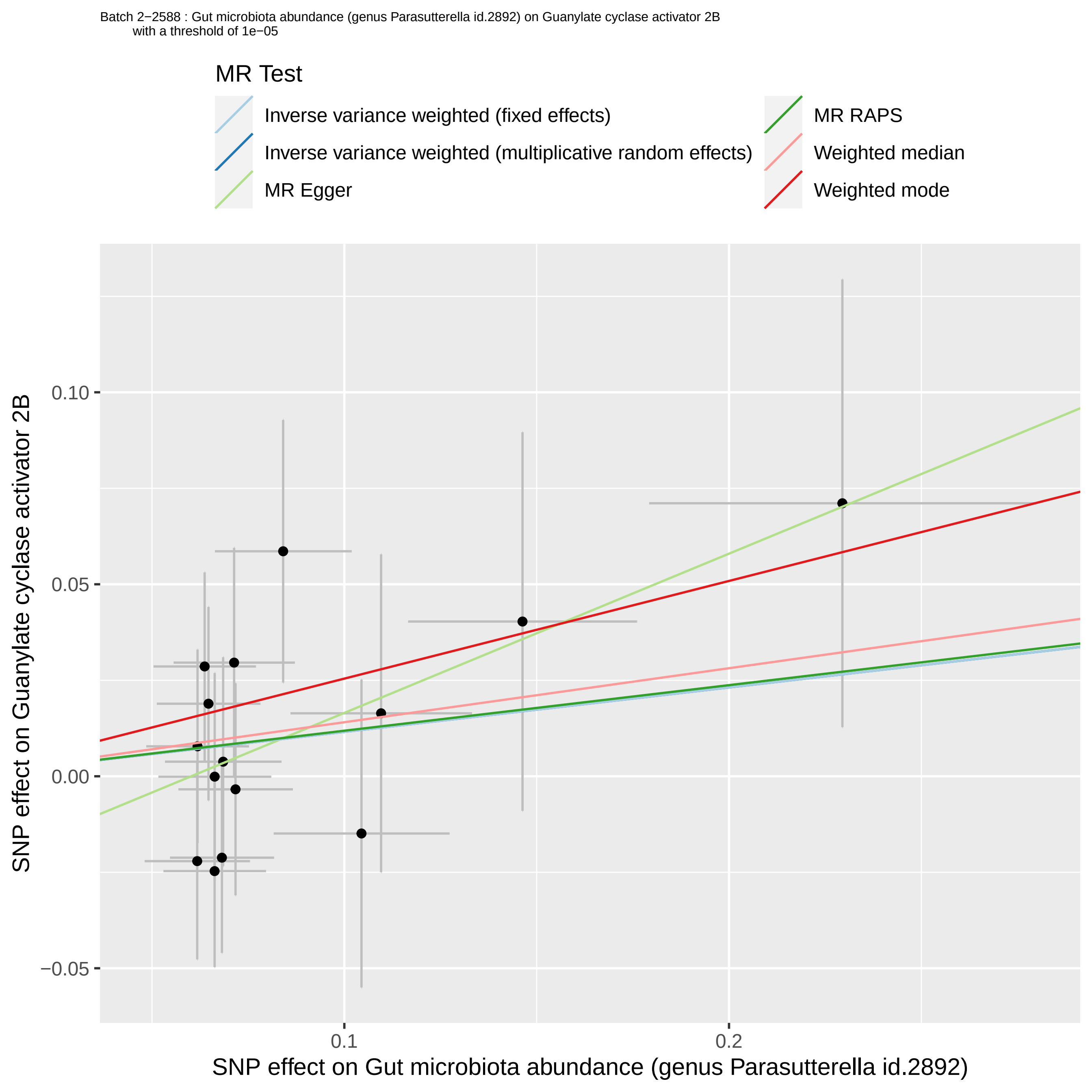

Batch 2−2588 : Gut microbiota abundance (genus Parasutterella id.2892) on Guanylate cyclase activator 2B
with a threshold of 1e−05
MR Test
Inverse variance weighted (fixed effects)
Inverse variance weighted (multiplicative random effects)
MR Egger
MR RAPS
Weighted median
Weighted mode
0.10
0.05
0.00
−0.05
0.1
0.2
SNP effect on Guanylate cyclase activator 2B
SNP effect on Gut microbiota abundance (genus Parasutterella id.2892)

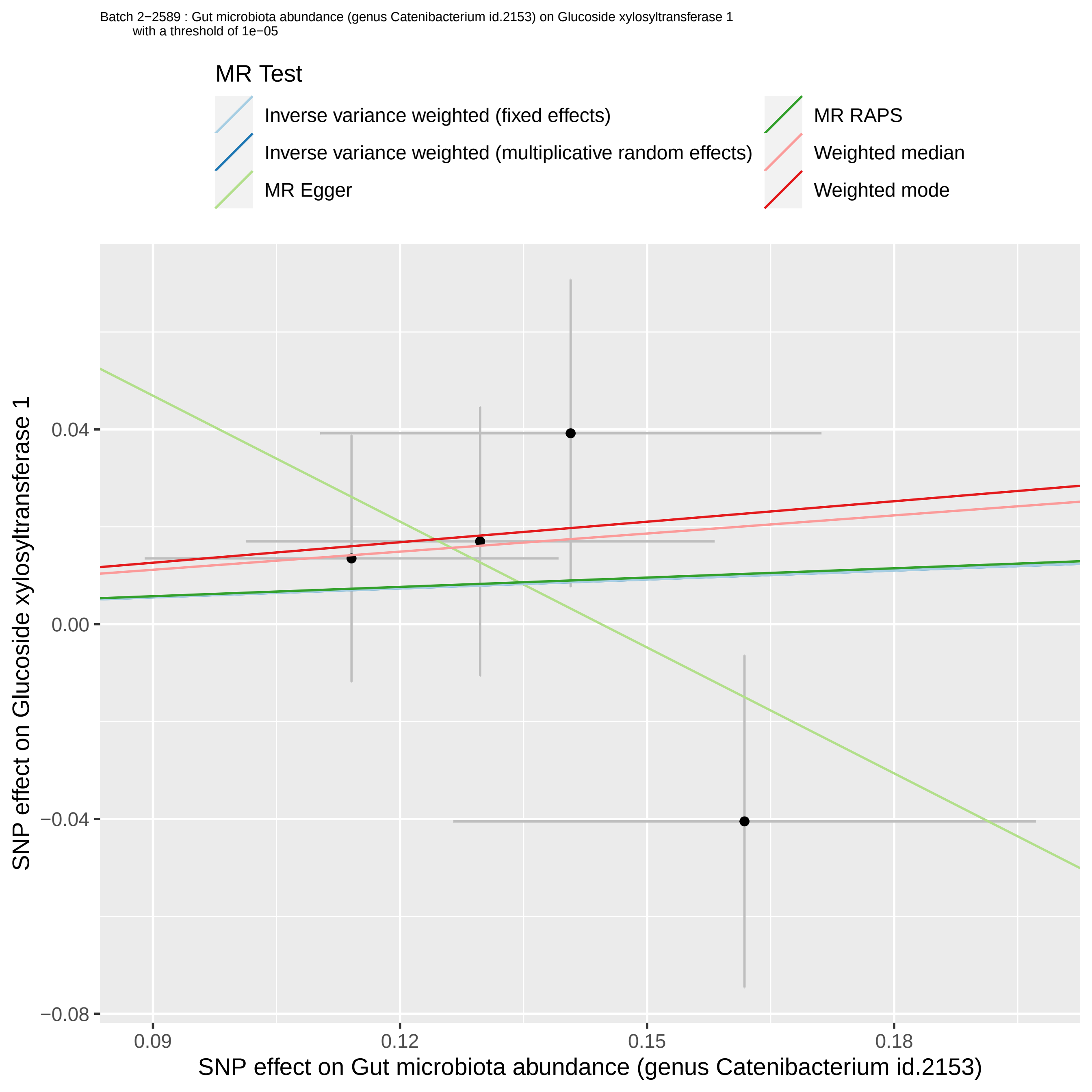
Batch 2−2589 : Gut microbiota abundance (genus Catenibacterium id.2153) on Glucoside xylosyltransferase 1
with a threshold of 1e−05
MR Test
Inverse variance weighted (fixed effects)
Inverse variance weighted (multiplicative random effects)
MR Egger
MR RAPS
Weighted median
Weighted mode
0.04
0.00
−0.04
−0.08
0.09
0.12
0.15
0.18
SNP effect on Glucoside xylosyltransferase 1
SNP effect on Gut microbiota abundance (genus Catenibacterium id.2153)

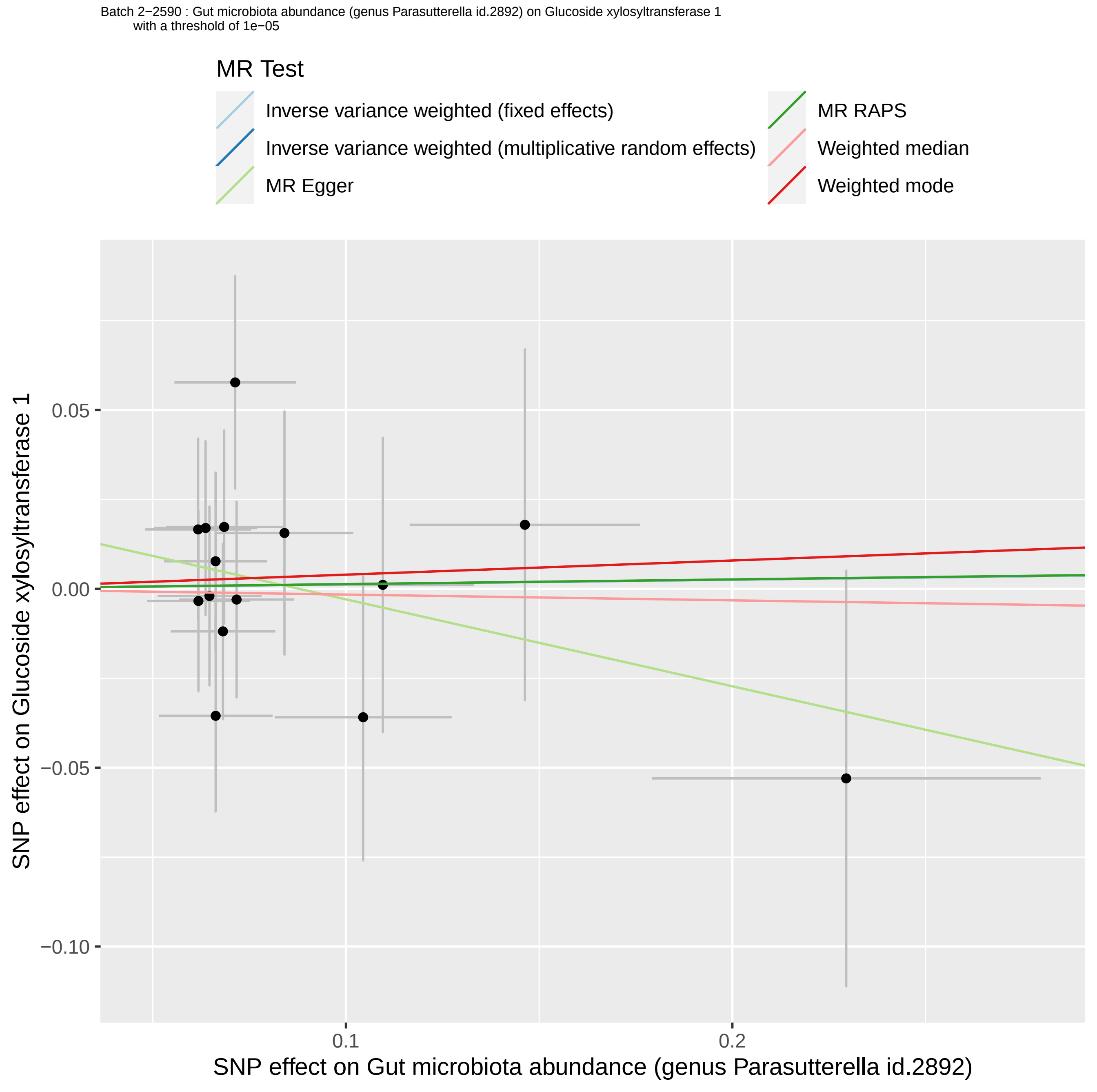

Batch 2−2590 : Gut microbiota abundance (genus Parasutterella id.2892) on Glucoside xylosyltransferase 1
with a threshold of 1e−05
MR Test
Inverse variance weighted (fixed effects)
Inverse variance weighted (multiplicative random effects)
MR Egger
MR RAPS
Weighted median
Weighted mode
0.05
0.00
−0.05
−0.10
0.1
0.2
SNP effect on Glucoside xylosyltransferase 1
SNP effect on Gut microbiota abundance (genus Parasutterella id.2892)

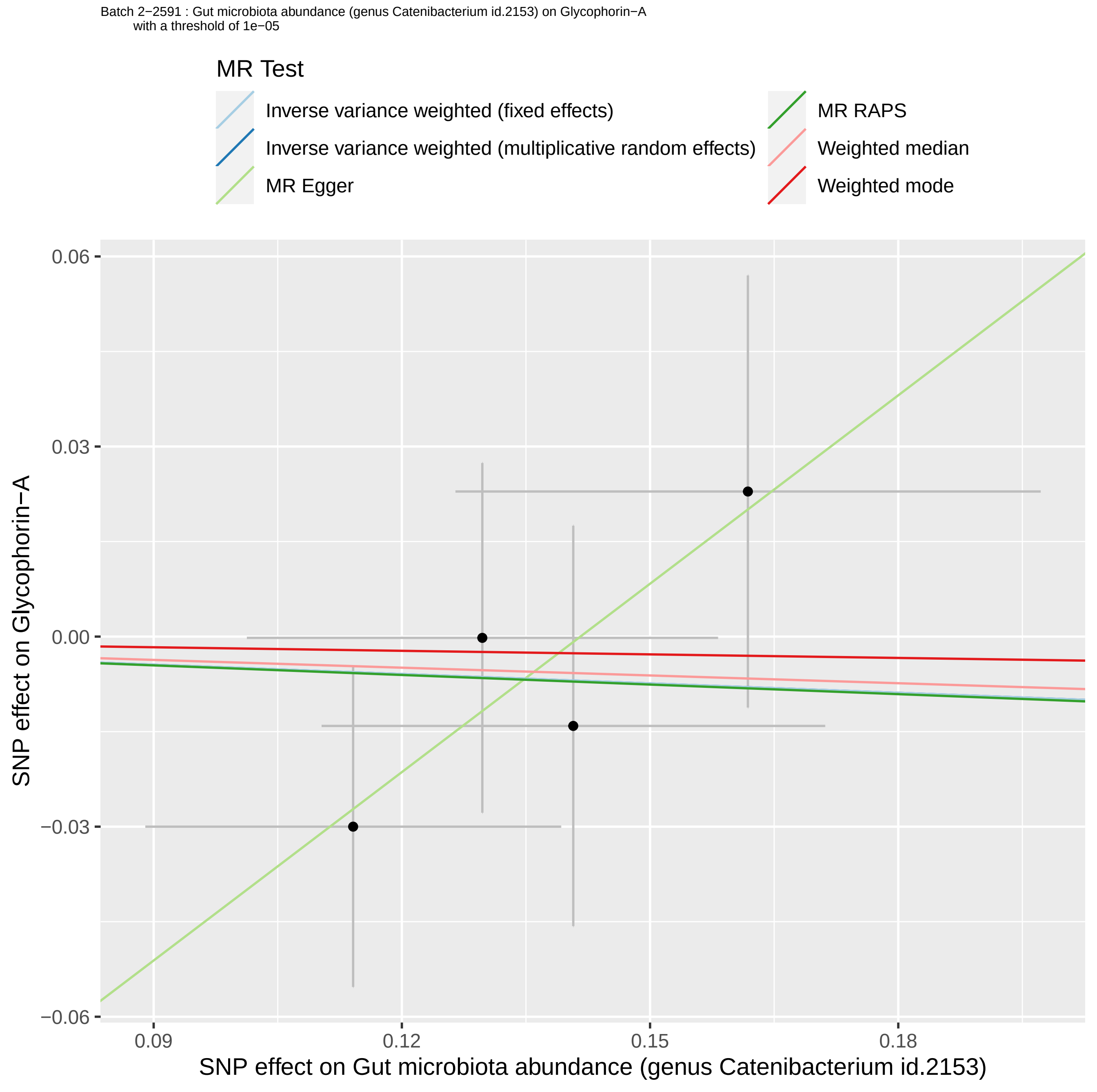

Batch 2−2591 : Gut microbiota abundance (genus Catenibacterium id.2153) on Glycophorin−A
with a threshold of 1e−05
MR Test
Inverse variance weighted (fixed effects)
Inverse variance weighted (multiplicative random effects)
MR Egger
MR RAPS
Weighted median
Weighted mode
0.06
0.03
0.00
−0.03
−0.06
0.09
0.12
0.15
0.18
SNP effect on Glycophorin−A
SNP effect on Gut microbiota abundance (genus Catenibacterium id.2153)

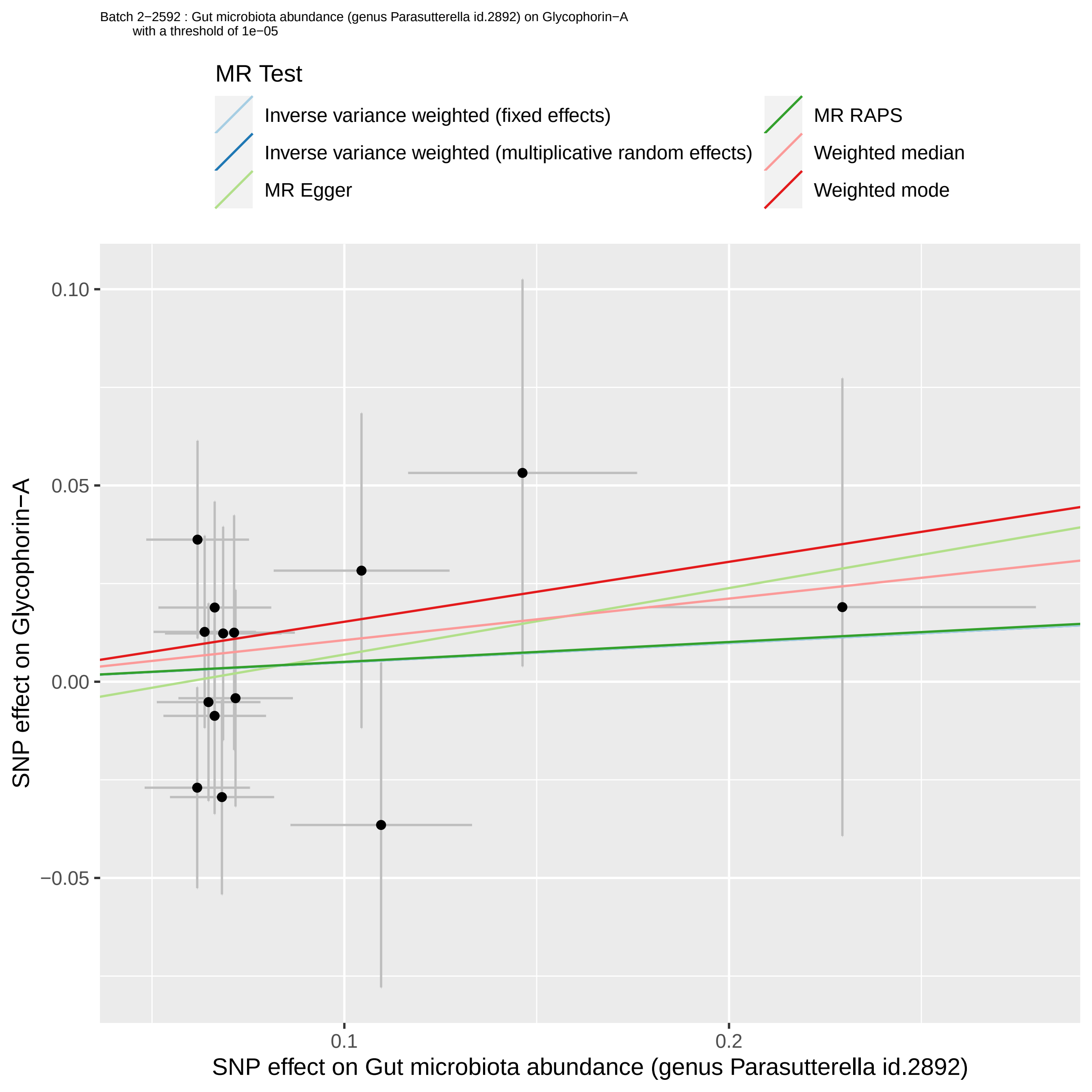
Batch 2−2592 : Gut microbiota abundance (genus Parasutterella id.2892) on Glycophorin−A
with a threshold of 1e−05
MR Test
Inverse variance weighted (fixed effects)
Inverse variance weighted (multiplicative random effects)
MR Egger
MR RAPS
Weighted median
Weighted mode
0.10
0.05
0.00
−0.05
0.1
0.2
SNP effect on Glycophorin−A
SNP effect on Gut microbiota abundance (genus Parasutterella id.2892)

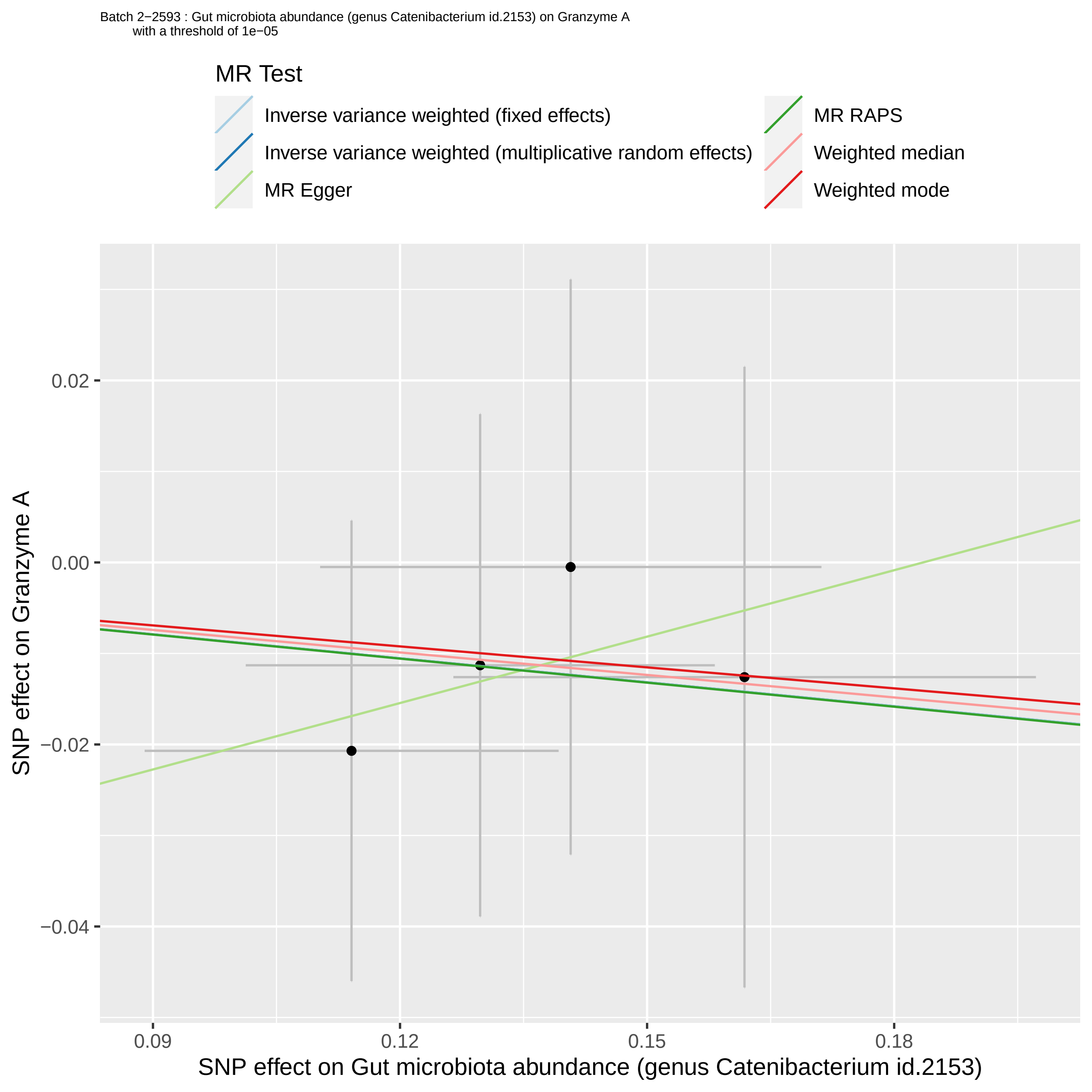

Batch 2−2593 : Gut microbiota abundance (genus Catenibacterium id.2153) on Granzyme A
with a threshold of 1e−05
MR Test
Inverse variance weighted (fixed effects)
Inverse variance weighted (multiplicative random effects)
MR Egger
MR RAPS
Weighted median
Weighted mode
0.02
0.00
−0.02
−0.04
0.09
0.12
0.15
0.18
SNP effect on Granzyme A
SNP effect on Gut microbiota abundance (genus Catenibacterium id.2153)

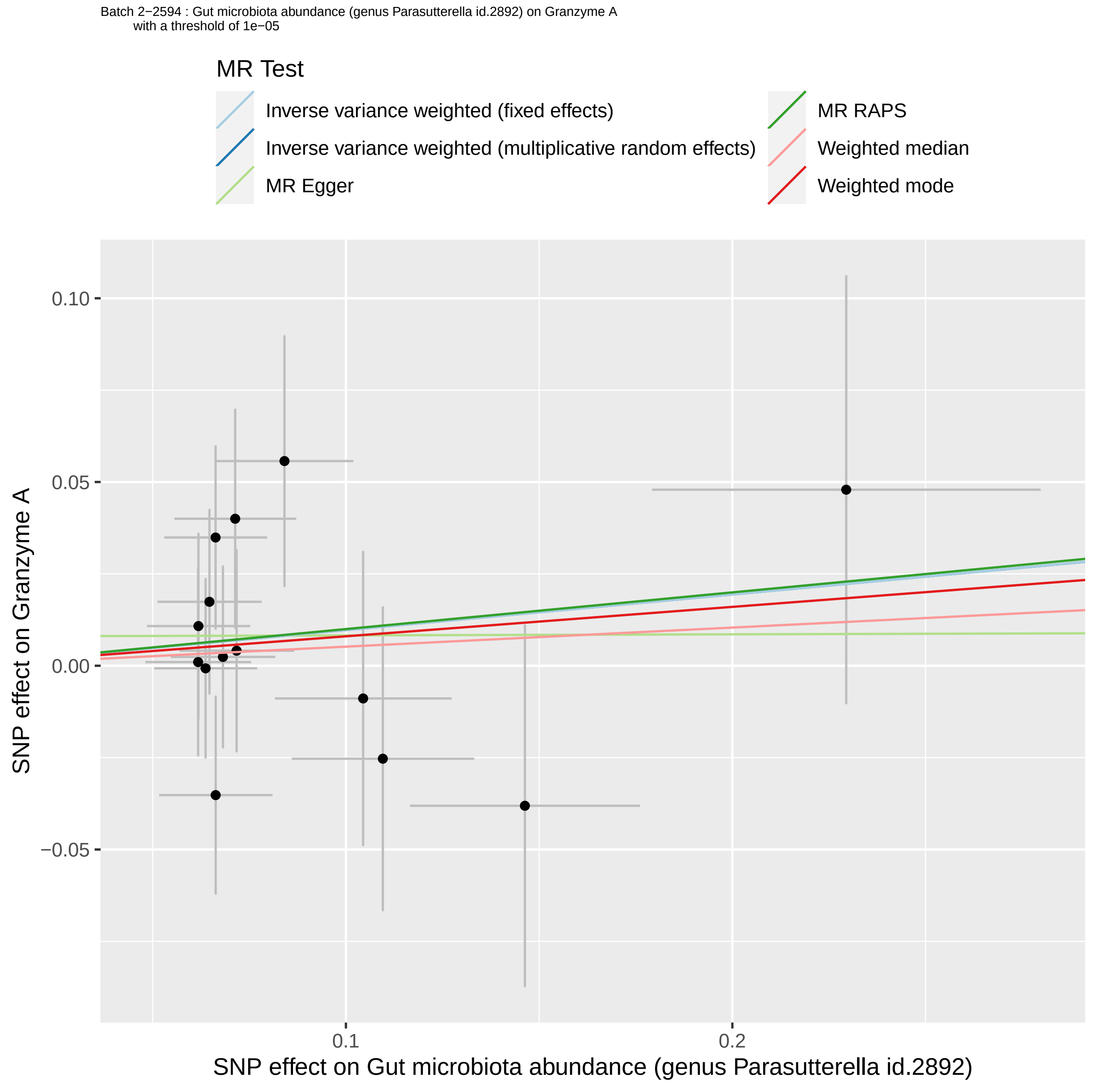
Batch 2−2594 : Gut microbiota abundance (genus Parasutterella id.2892) on Granzyme A
with a threshold of 1e−05
MR Test
Inverse variance weighted (fixed effects)
Inverse variance weighted (multiplicative random effects)
MR Egger
MR RAPS
Weighted median
Weighted mode
0.10
0.05
0.00
−0.05
0.1
0.2
SNP effect on Granzyme A
SNP effect on Gut microbiota abundance (genus Parasutterella id.2892)

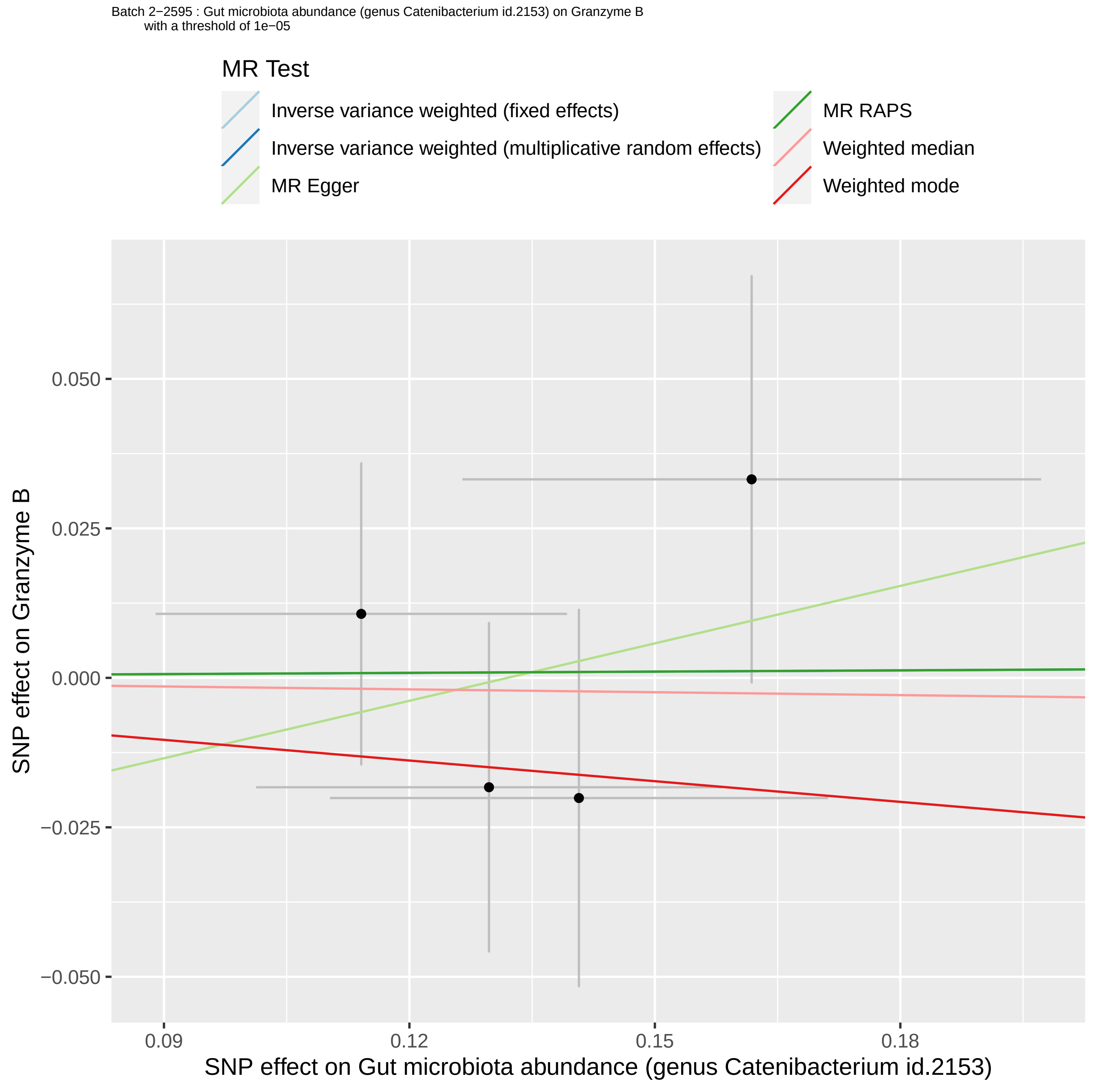
Batch 2−2595 : Gut microbiota abundance (genus Catenibacterium id.2153) on Granzyme B
with a threshold of 1e−05
MR Test
Inverse variance weighted (fixed effects)
Inverse variance weighted (multiplicative random effects)
MR Egger
MR RAPS
Weighted median
Weighted mode
0.050
0.025
0.000
−0.025
−0.050
0.09
0.12
0.15
0.18
SNP effect on Granzyme B
SNP effect on Gut microbiota abundance (genus Catenibacterium id.2153)

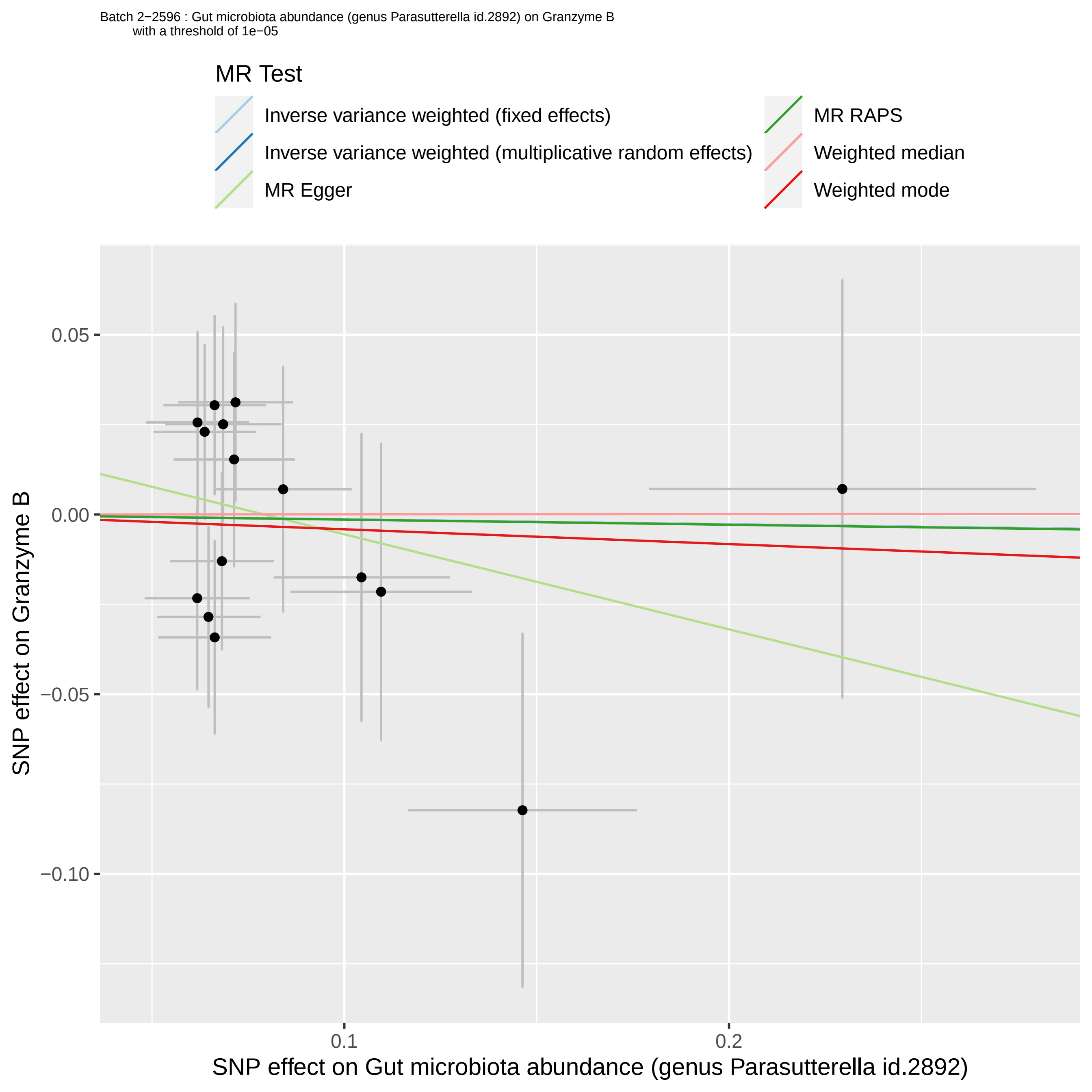
Batch 2−2596 : Gut microbiota abundance (genus Parasutterella id.2892) on Granzyme B
with a threshold of 1e−05
MR Test
Inverse variance weighted (fixed effects)
Inverse variance weighted (multiplicative random effects)
MR Egger
MR RAPS
Weighted median
Weighted mode
SNP effect on Granzyme B
0.05
0.00
−0.05
−0.10
0.1
0.2
SNP effect on Gut microbiota abundance (genus Parasutterella id.2892)

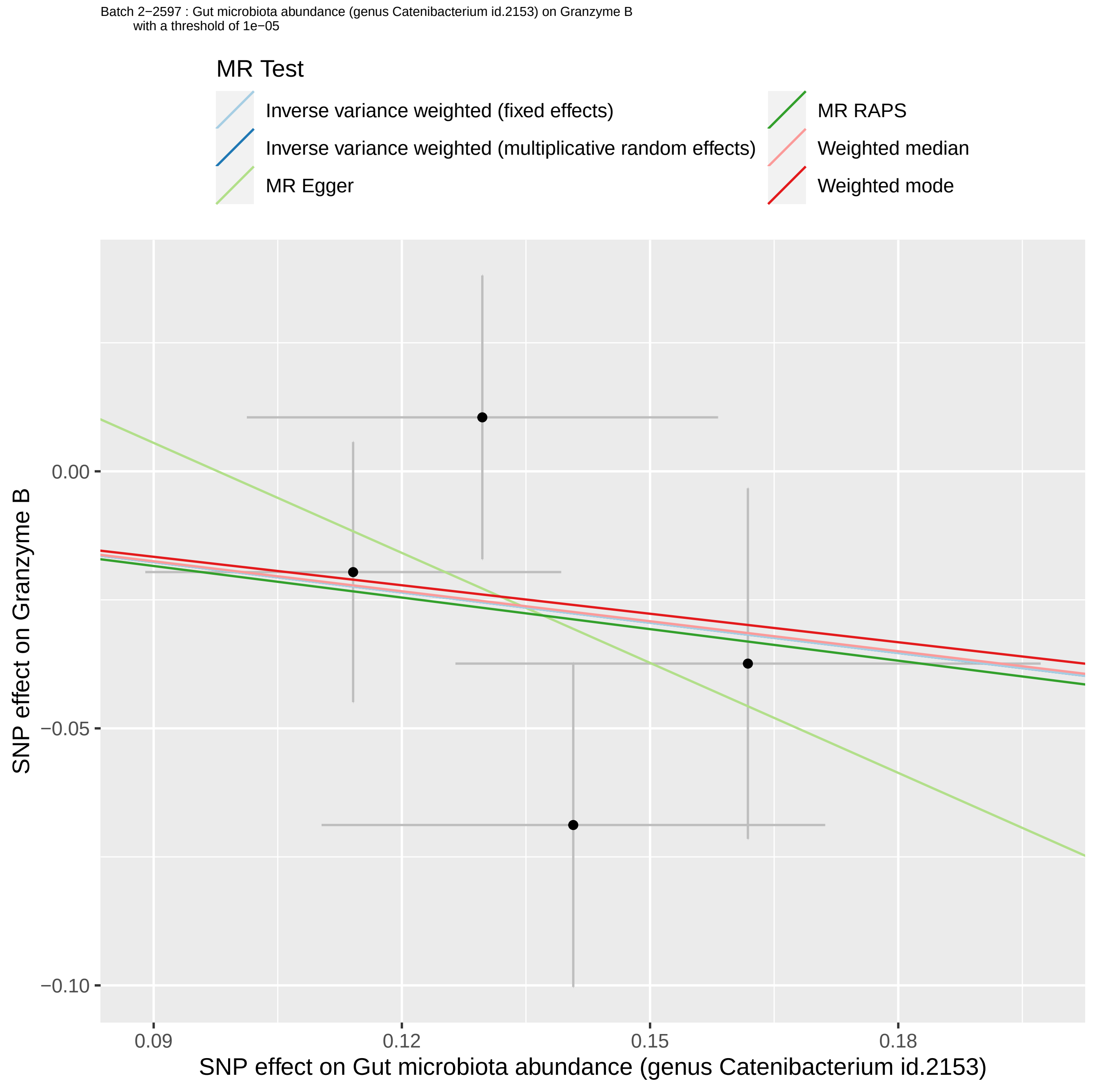
Batch 2−2597 : Gut microbiota abundance (genus Catenibacterium id.2153) on Granzyme B
with a threshold of 1e−05
MR Test
Inverse variance weighted (fixed effects)
Inverse variance weighted (multiplicative random effects)
MR Egger
MR RAPS
Weighted median
Weighted mode
0.00
−0.05
−0.10
0.09
0.12
0.15
0.18
SNP effect on Granzyme B
SNP effect on Gut microbiota abundance (genus Catenibacterium id.2153)

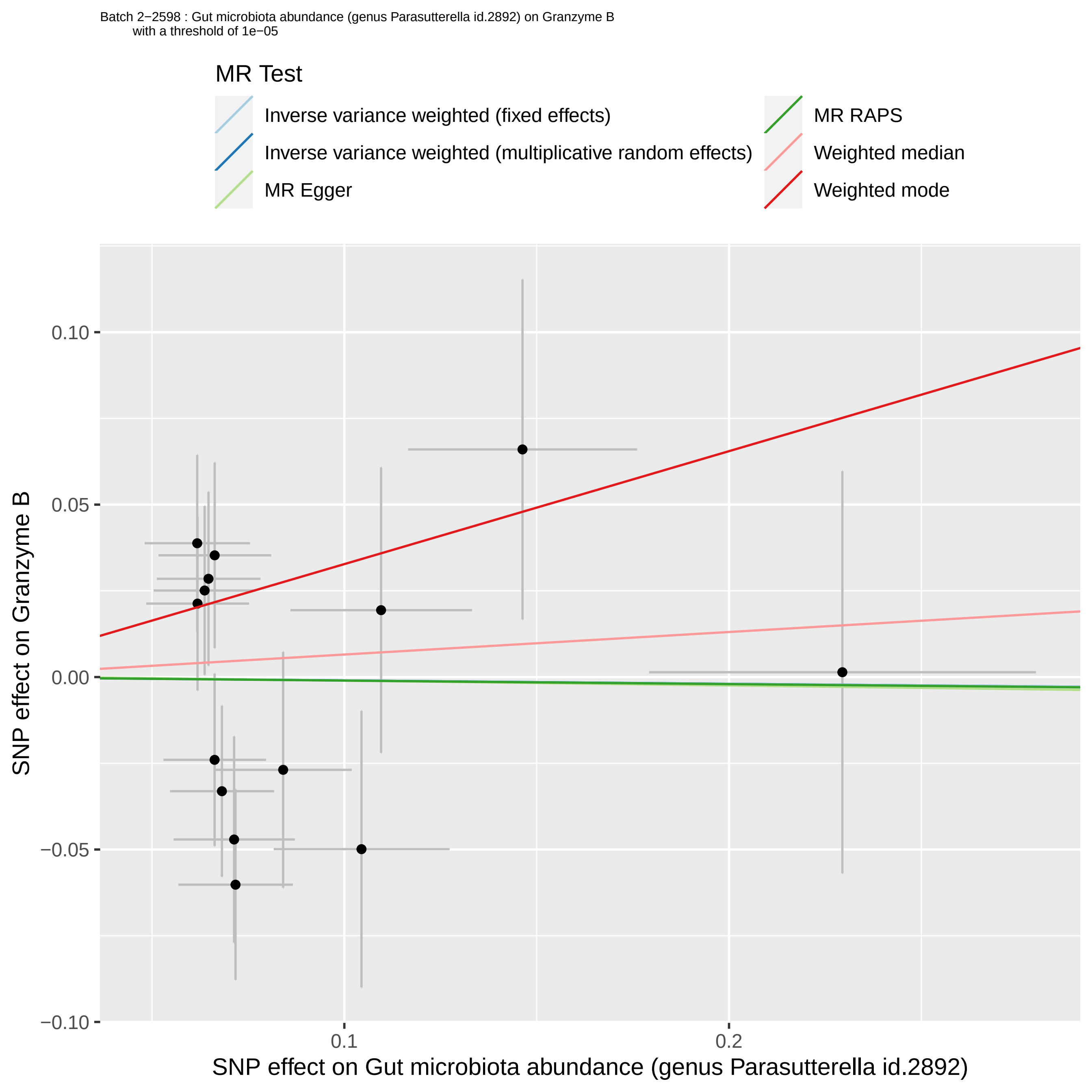
Batch 2−2598 : Gut microbiota abundance (genus Parasutterella id.2892) on Granzyme B
with a threshold of 1e−05
MR Test
Inverse variance weighted (fixed effects)
Inverse variance weighted (multiplicative random effects)
MR Egger
MR RAPS
Weighted median
Weighted mode
0.10
0.05
0.00
−0.05
−0.10
0.1
0.2
SNP effect on Granzyme B
SNP effect on Gut microbiota abundance (genus Parasutterella id.2892)

Batch 2−2599 : Gut microbiota abundance (genus Catenibacterium id.2153) on Granzyme H
with a threshold of 1e−05

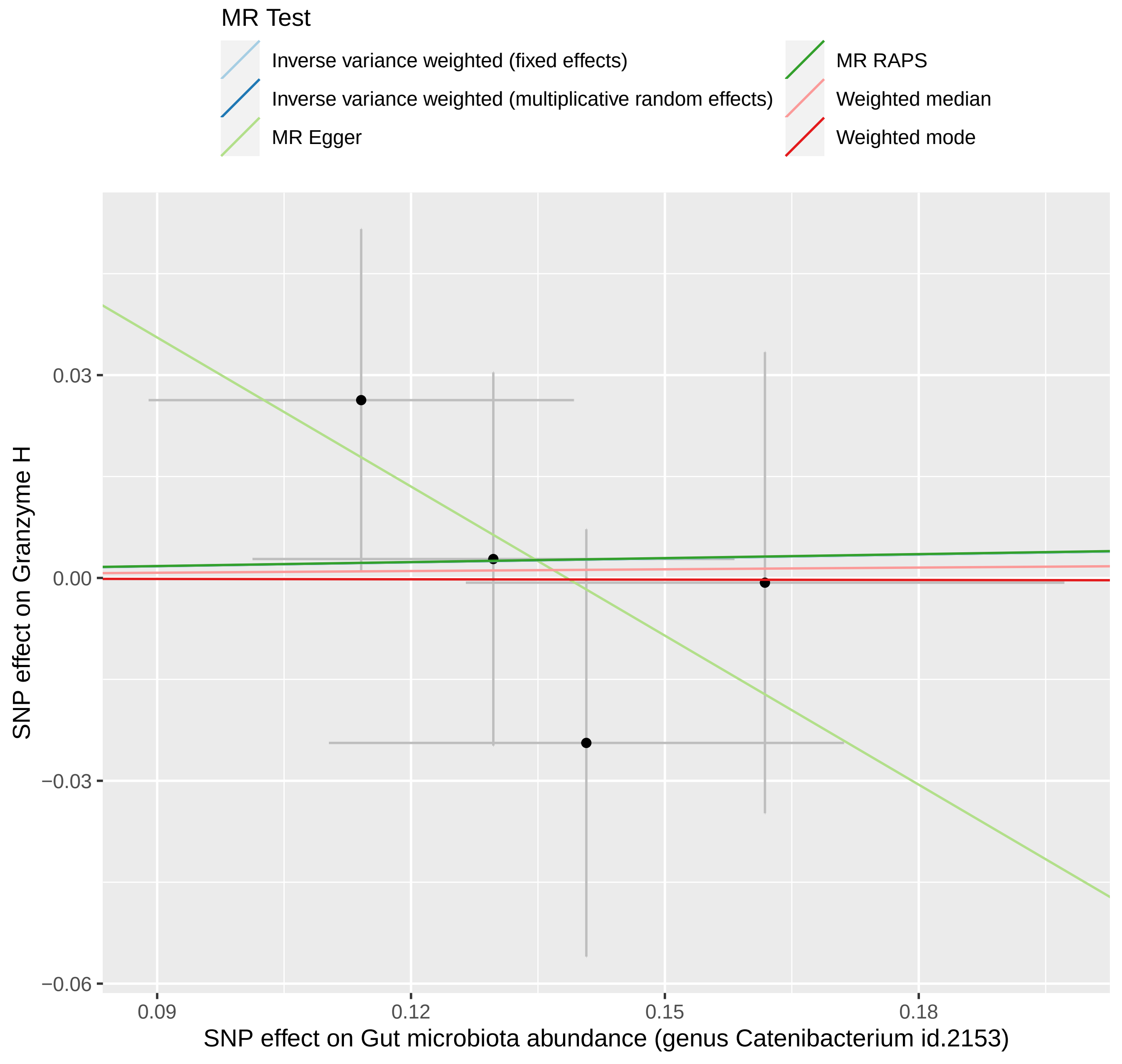

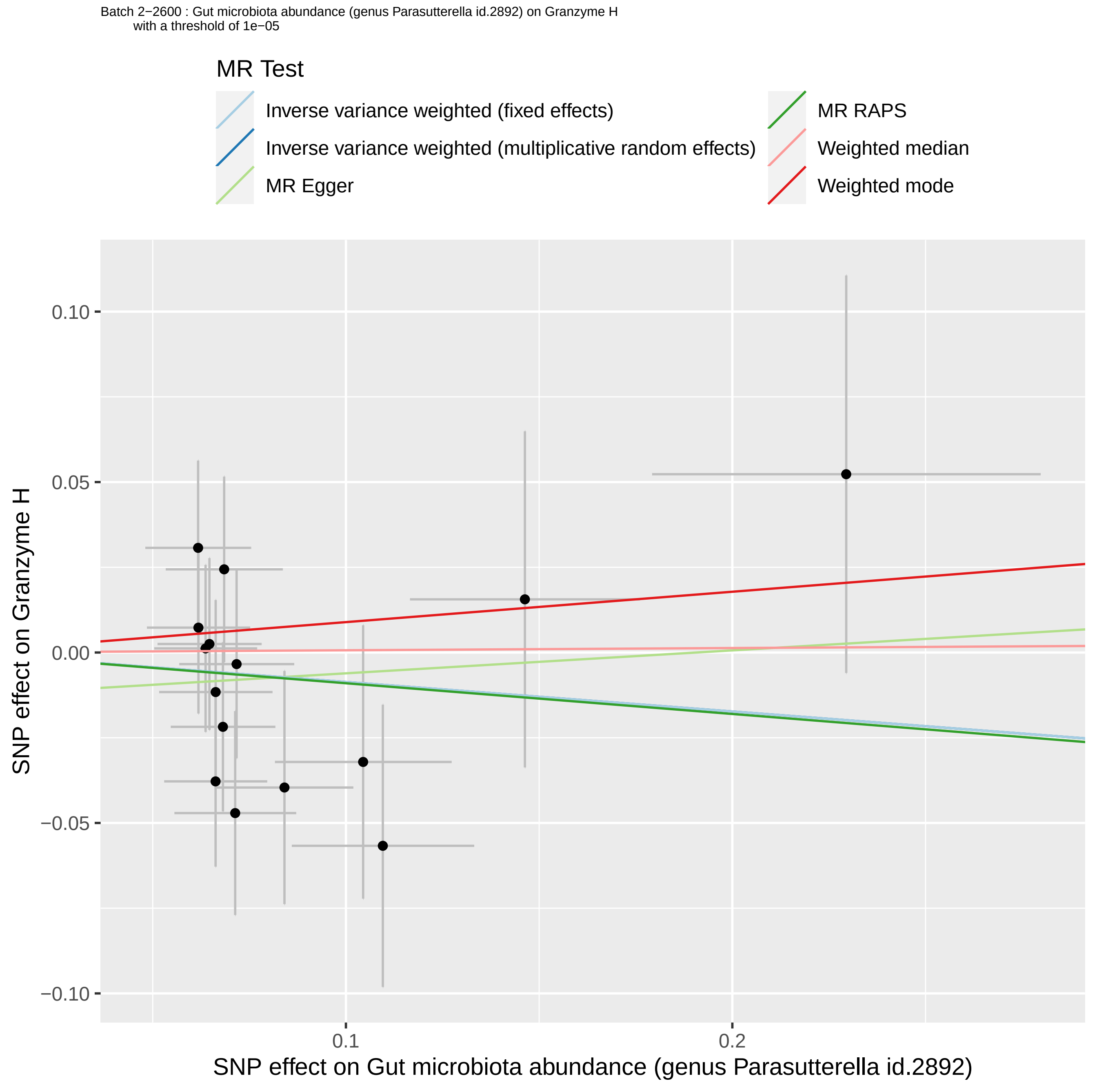
Batch 2−2600 : Gut microbiota abundance (genus Parasutterella id.2892) on Granzyme H
with a threshold of 1e−05
MR Test
Inverse variance weighted (fixed effects)
Inverse variance weighted (multiplicative random effects)
MR Egger
MR RAPS
Weighted median
Weighted mode
0.10
0.05
0.00
−0.05
−0.10
0.1
0.2
SNP effect on Granzyme H
SNP effect on Gut microbiota abundance (genus Parasutterella id.2892)

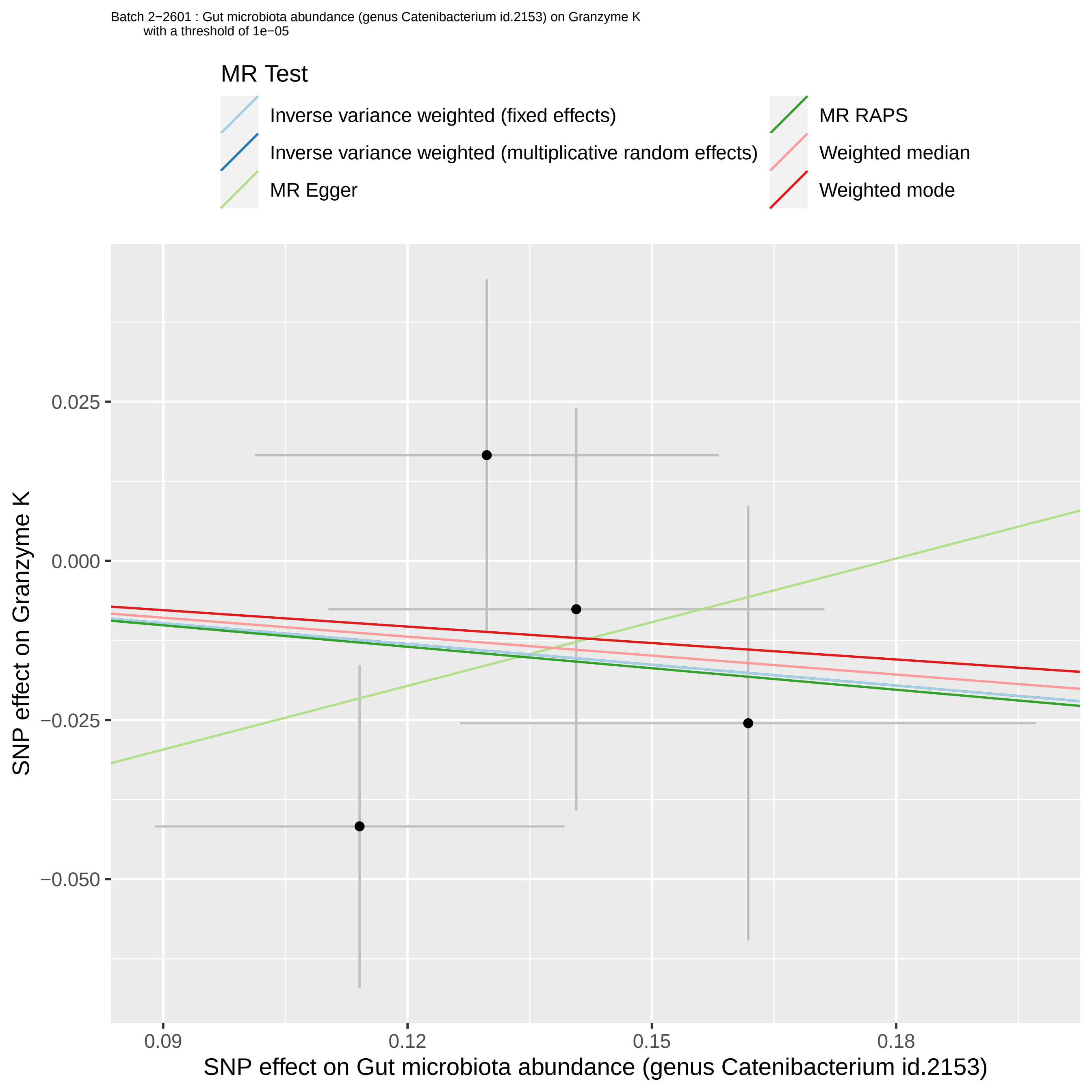

Batch 2−2601 : Gut microbiota abundance (genus Catenibacterium id.2153) on Granzyme K
with a threshold of 1e−05
MR Test
Inverse variance weighted (fixed effects)
Inverse variance weighted (multiplicative random effects)
MR Egger
MR RAPS
Weighted median
Weighted mode
0.025
0.000
−0.025
−0.050
0.09
0.12
0.15
0.18
SNP effect on Granzyme K
SNP effect on Gut microbiota abundance (genus Catenibacterium id.2153)

Batch 2−2602 : Gut microbiota abundance (genus Parasutterella id.2892) on Granzyme K
with a threshold of 1e−05

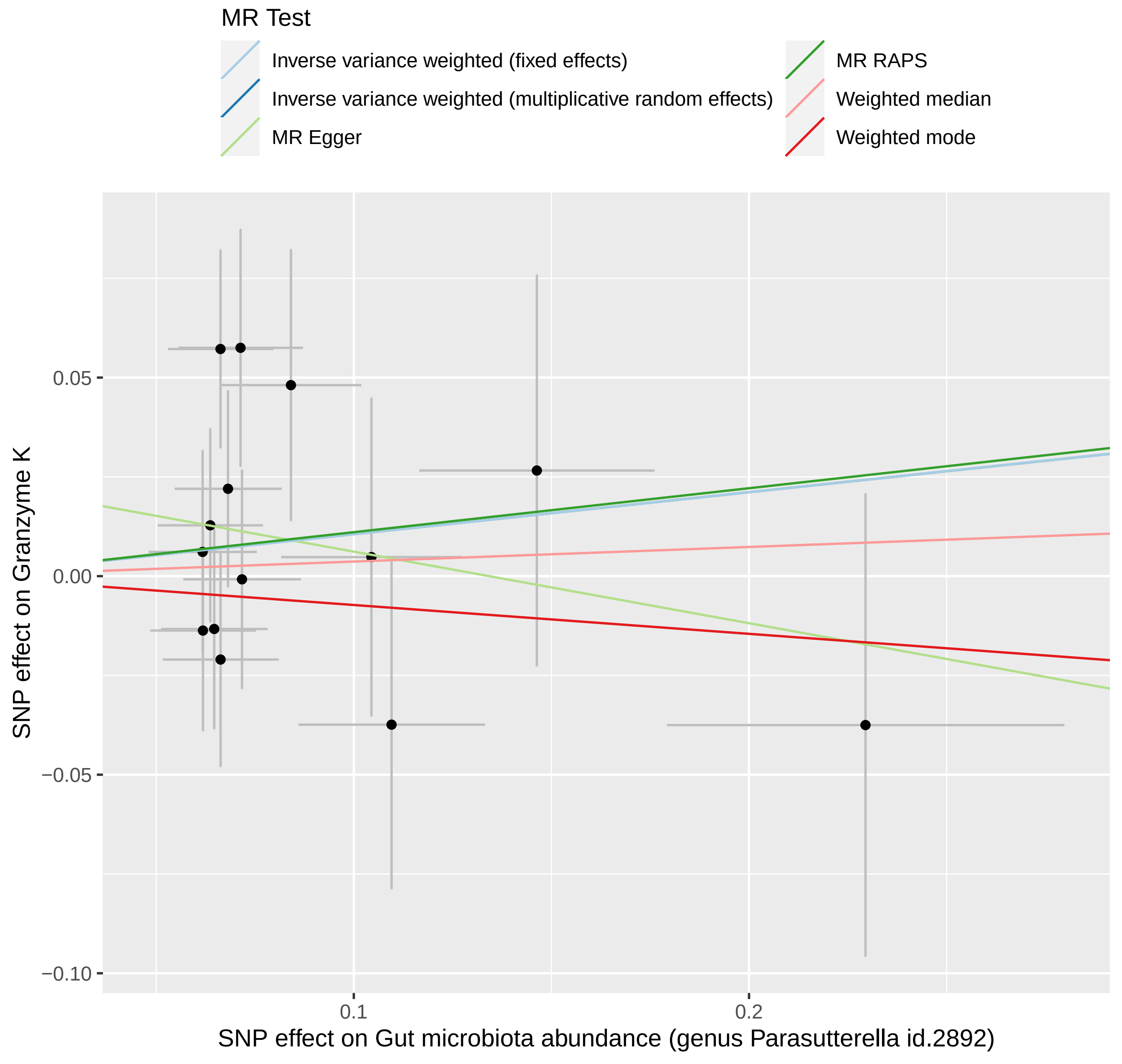

Batch 2−2603 : Gut microbiota abundance (genus Catenibacterium id.2153) on Granzyme M
with a threshold of 1e−05

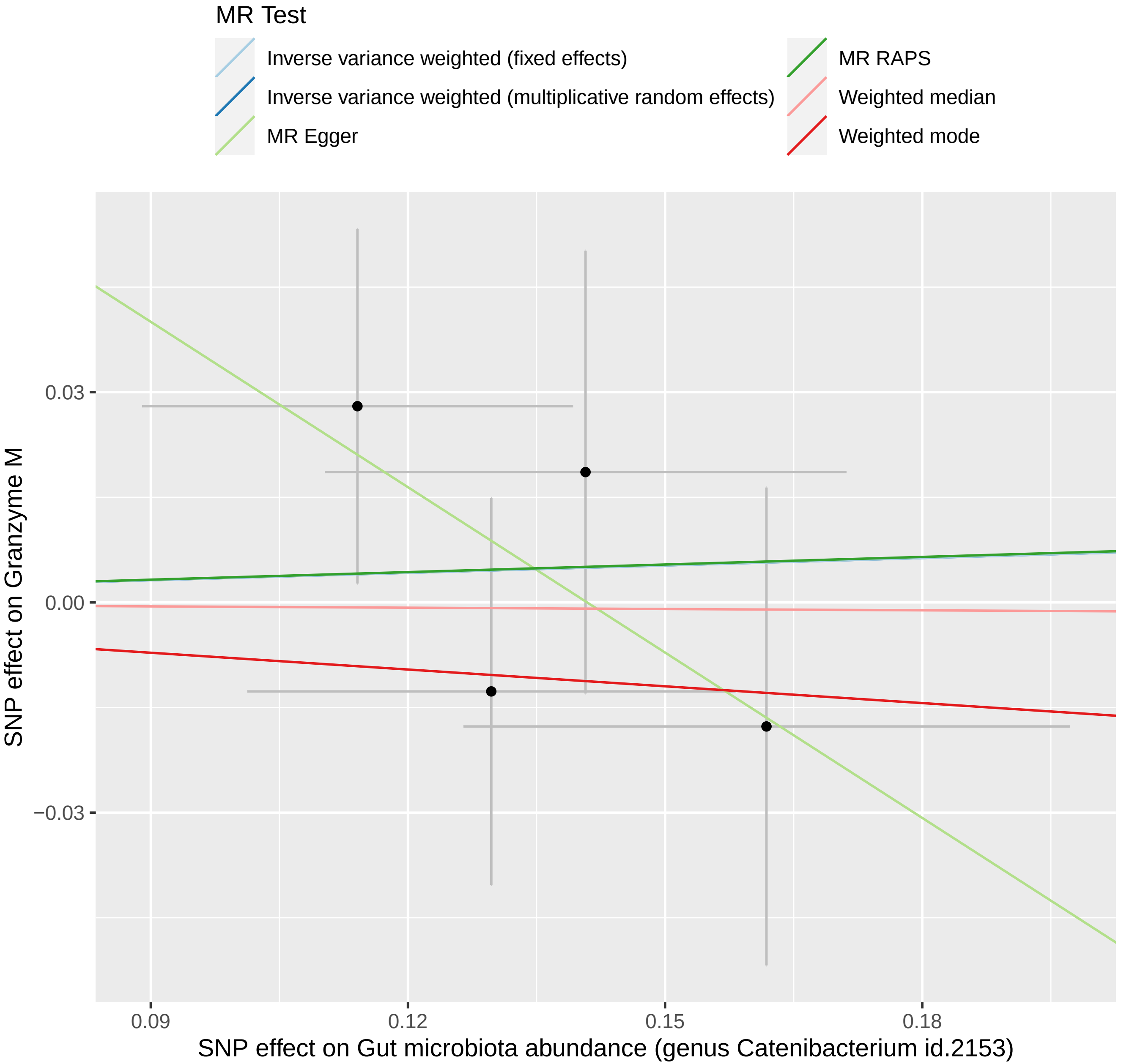

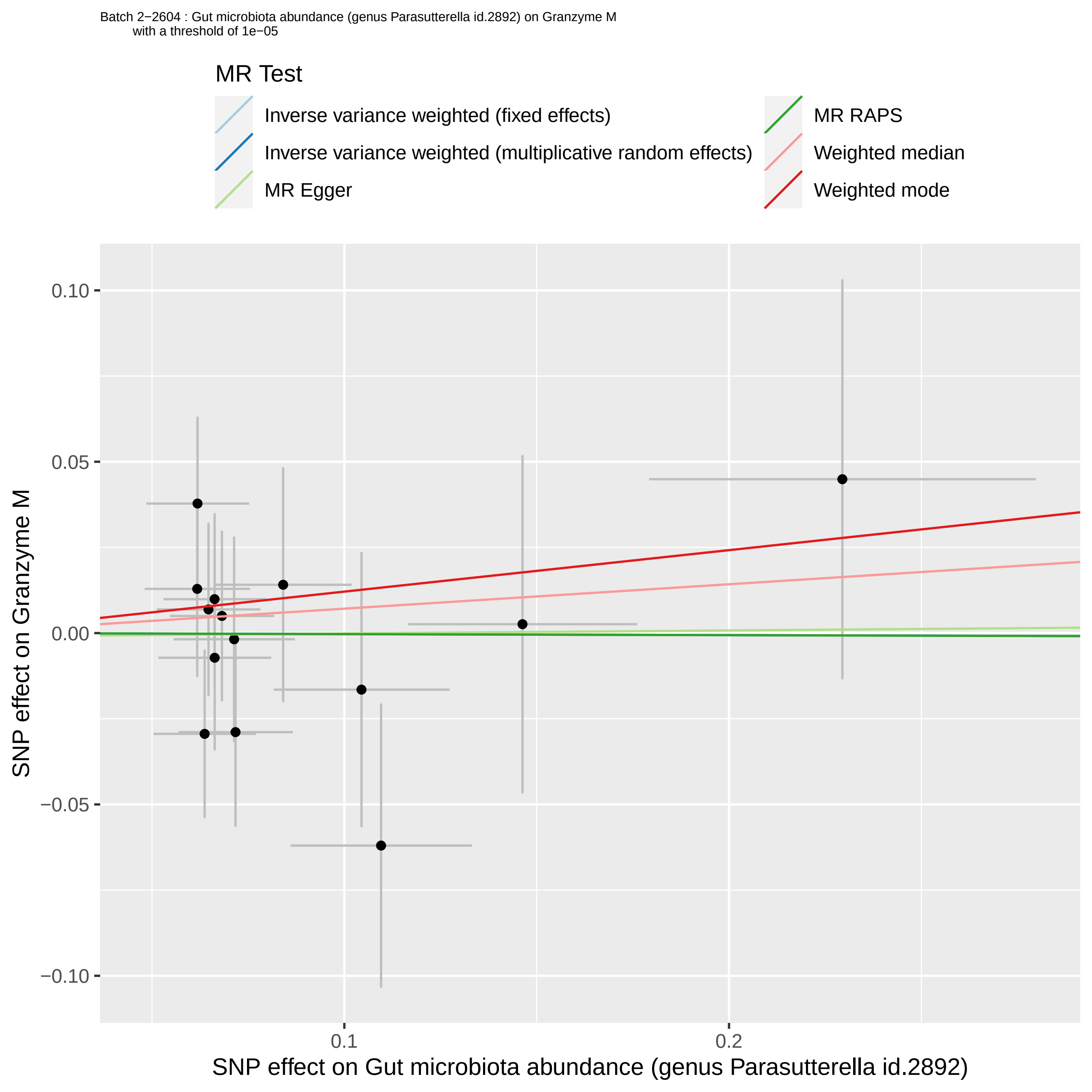
Batch 2−2604 : Gut microbiota abundance (genus Parasutterella id.2892) on Granzyme M
with a threshold of 1e−05
MR Test
Inverse variance weighted (fixed effects)
Inverse variance weighted (multiplicative random effects)
MR Egger
MR RAPS
Weighted median
Weighted mode
0.10
0.05
0.00
−0.05
−0.10
0.1
0.2
SNP effect on Granzyme M
SNP effect on Gut microbiota abundance (genus Parasutterella id.2892)

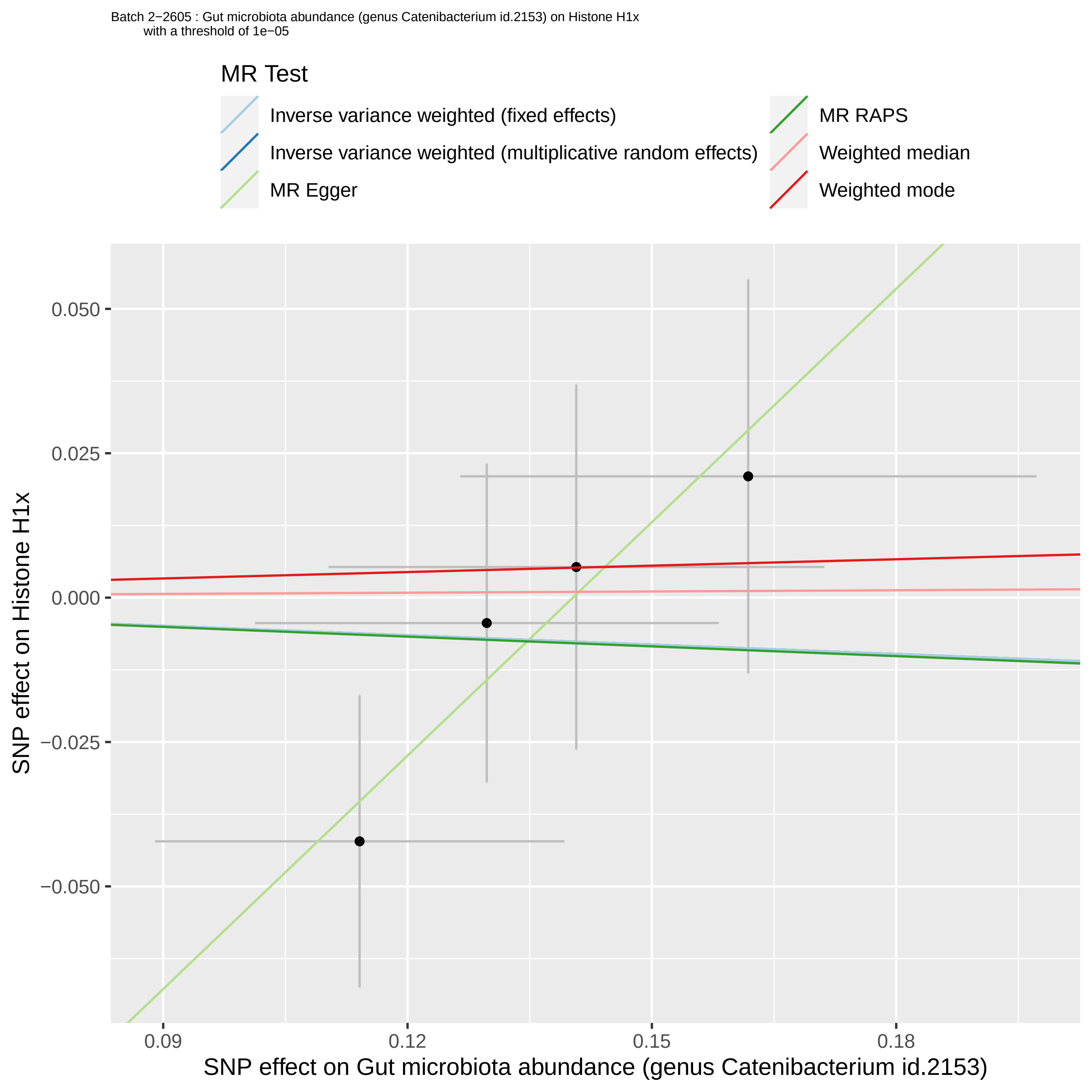
Batch 2−2605 : Gut microbiota abundance (genus Catenibacterium id.2153) on Histone H1x
with a threshold of 1e−05
MR Test
Inverse variance weighted (fixed effects)
Inverse variance weighted (multiplicative random effects)
MR Egger
MR RAPS
Weighted median
Weighted mode
0.050
0.025
0.000
−0.025
−0.050
0.09
0.12
0.15
0.18
SNP effect on Histone H1x
SNP effect on Gut microbiota abundance (genus Catenibacterium id.2153)

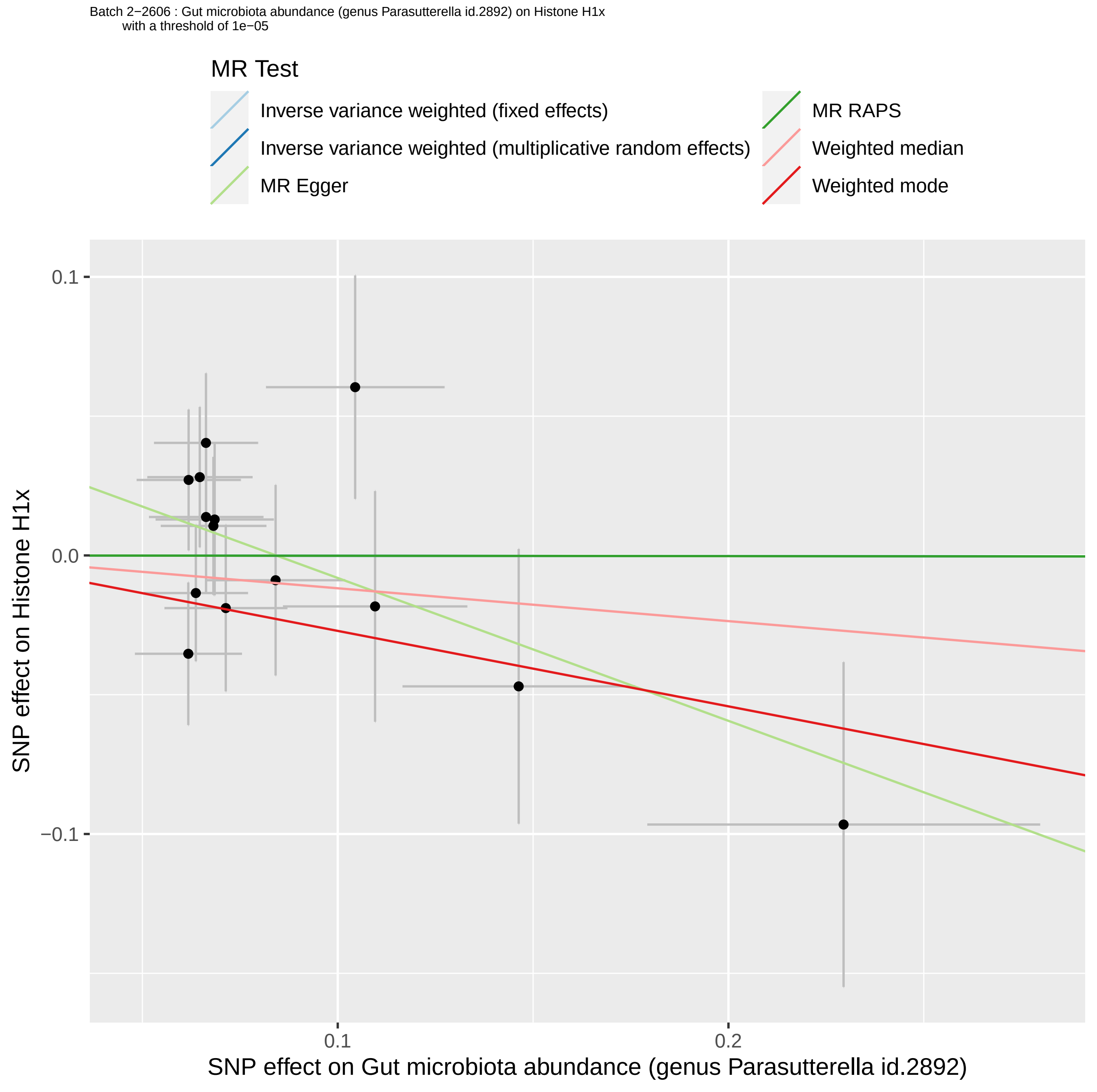
Batch 2−2606 : Gut microbiota abundance (genus Parasutterella id.2892) on Histone H1x
with a threshold of 1e−05
MR Test
Inverse variance weighted (fixed effects)
Inverse variance weighted (multiplicative random effects)
MR Egger
MR RAPS
Weighted median
Weighted mode
0.1
0.0
−0.1
0.1
0.2
SNP effect on Histone H1x
SNP effect on Gut microbiota abundance (genus Parasutterella id.2892)

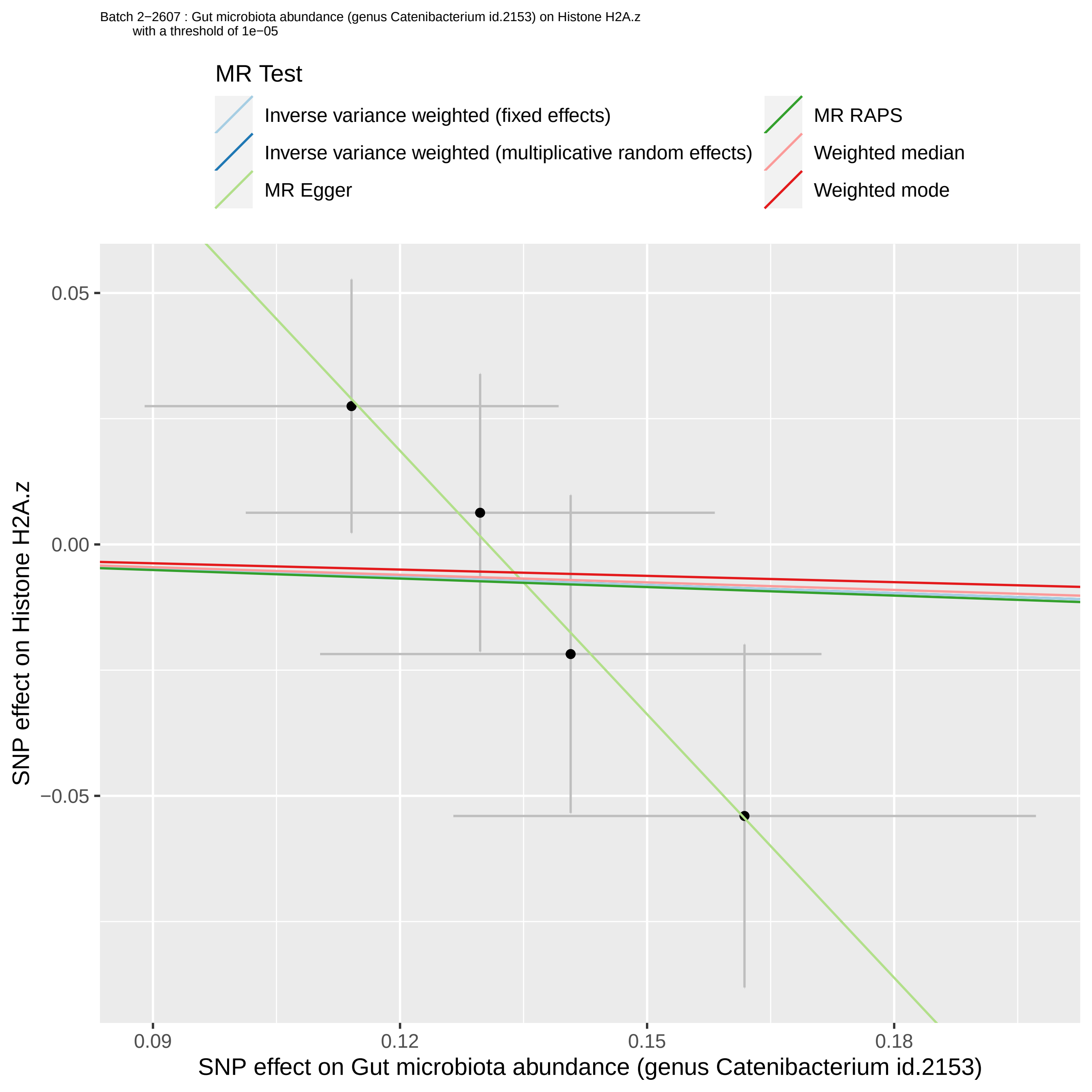

Batch 2−2607 : Gut microbiota abundance (genus Catenibacterium id.2153) on Histone H2A.z
with a threshold of 1e−05
MR Test
Inverse variance weighted (fixed effects)
Inverse variance weighted (multiplicative random effects)
MR Egger
MR RAPS
Weighted median
Weighted mode
0.05
0.00
−0.05
0.09
0.12
0.15
0.18
SNP effect on Histone H2A.z
SNP effect on Gut microbiota abundance (genus Catenibacterium id.2153)

Batch 2−2608 : Gut microbiota abundance (genus Parasutterella id.2892) on Histone H2A.z
with a threshold of 1e−05

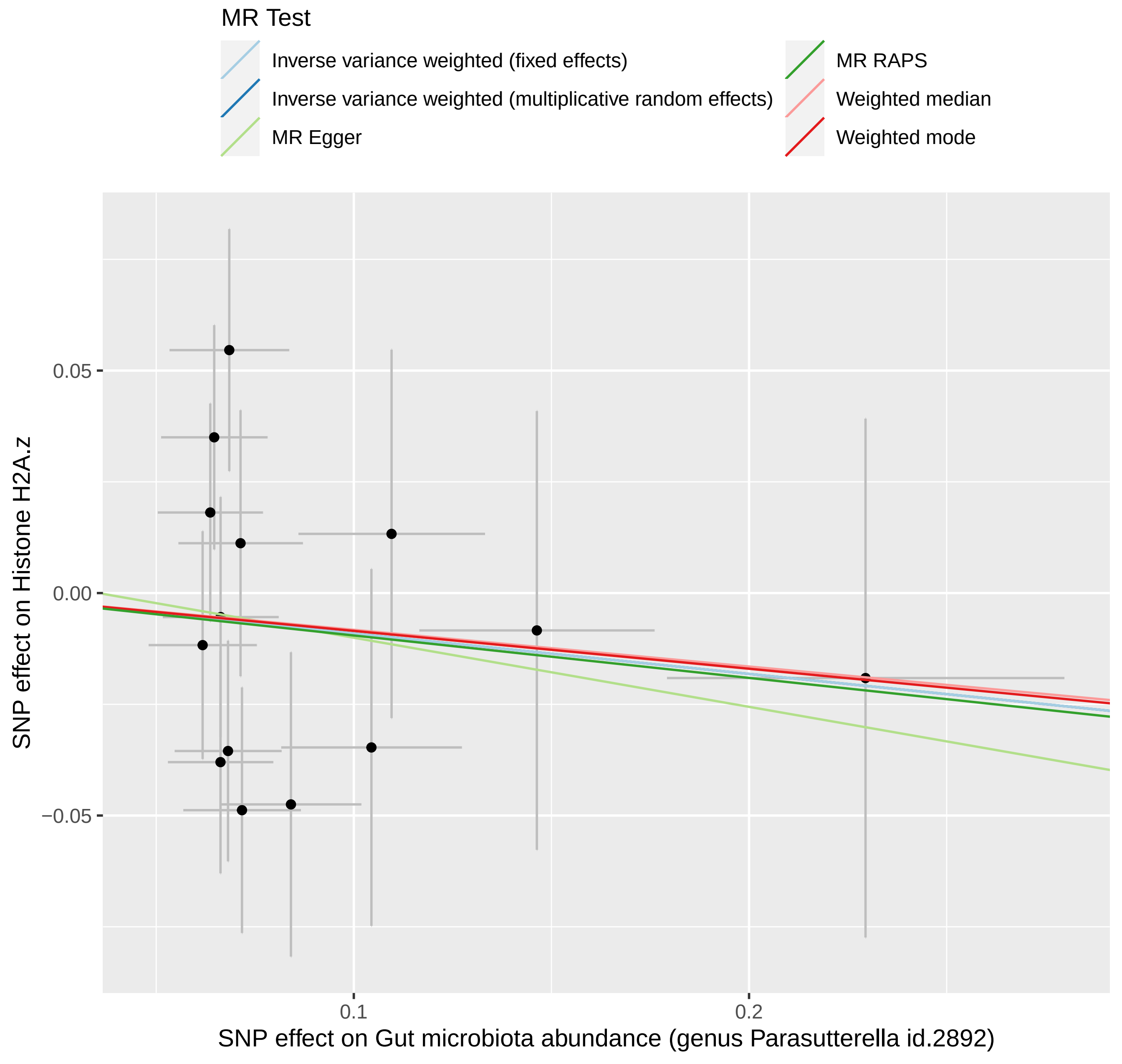

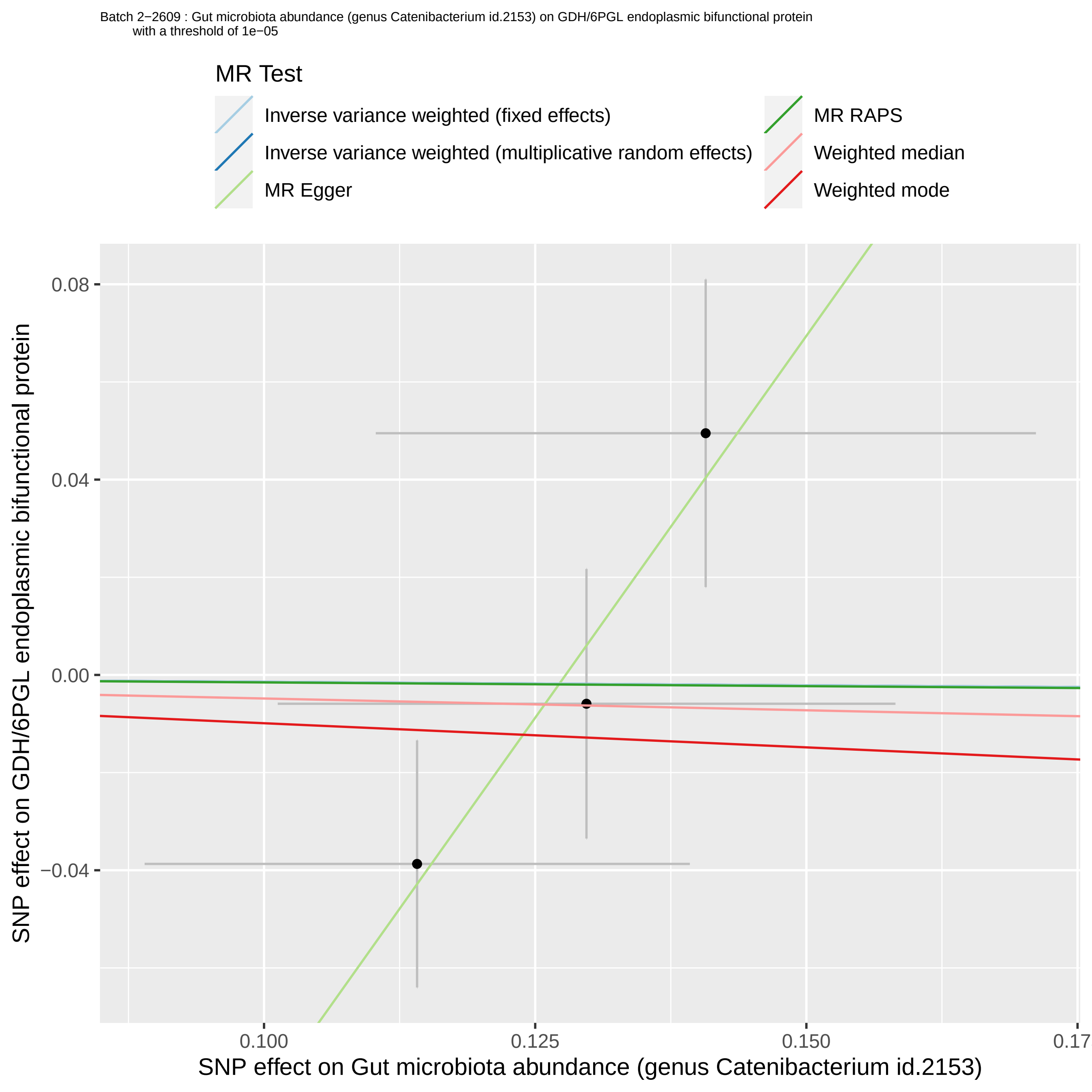
Batch 2−2609 : Gut microbiota abundance (genus Catenibacterium id.2153) on GDH/6PGL endoplasmic bifunctional protein
with a threshold of 1e−05
MR Test
Inverse variance weighted (fixed effects)
Inverse variance weighted (multiplicative random effects)
MR Egger
MR RAPS
Weighted median
Weighted mode
0.08
0.04
0.00
−0.04
0.100
0.125
0.150
0.17
SNP effect on GDH/6PGL endoplasmic bifunctional protein
SNP effect on Gut microbiota abundance (genus Catenibacterium id.2153)

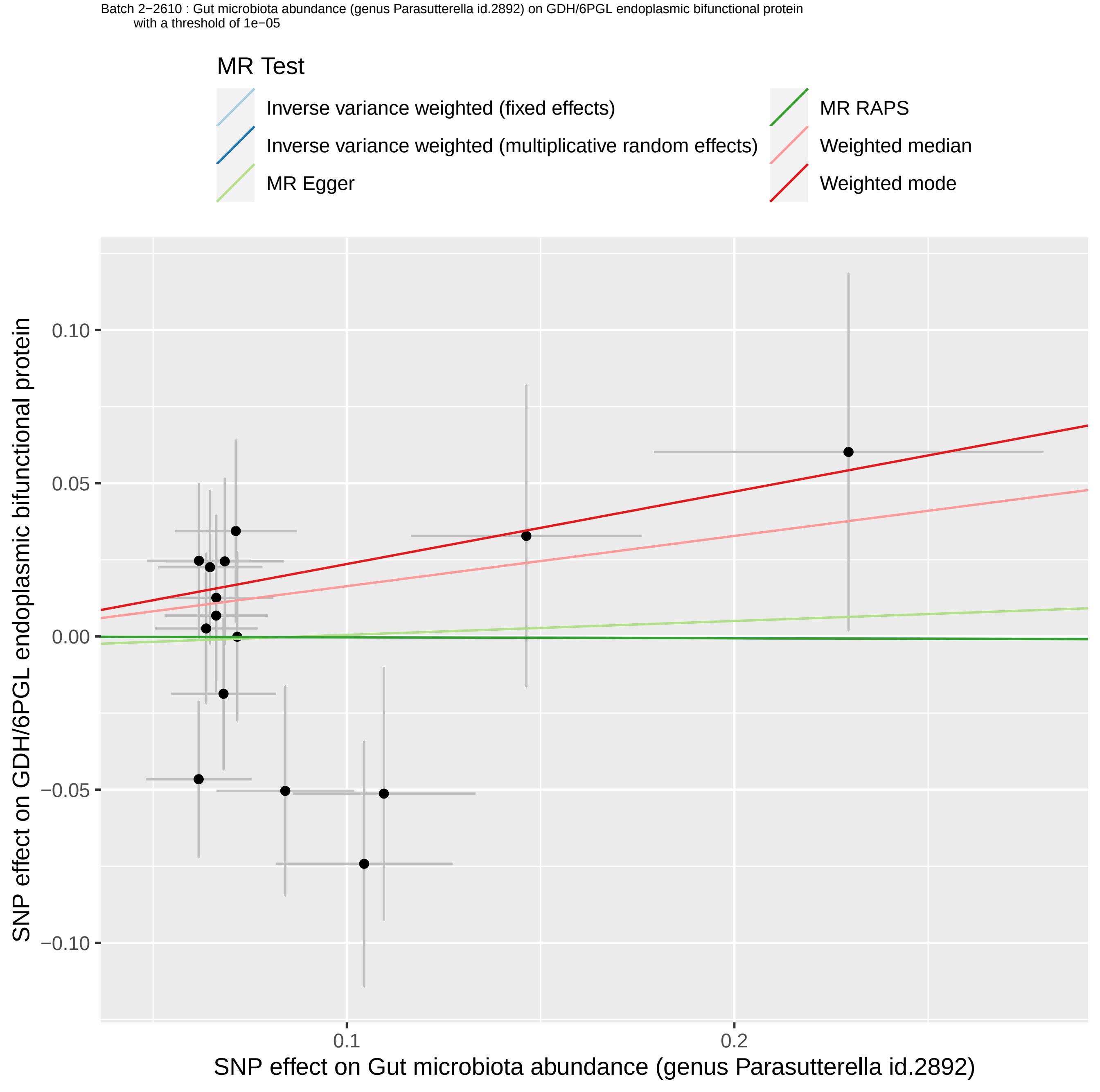

Batch 2−2610 : Gut microbiota abundance (genus Parasutterella id.2892) on GDH/6PGL endoplasmic bifunctional protein
with a threshold of 1e−05
MR Test
Inverse variance weighted (fixed effects)
Inverse variance weighted (multiplicative random effects)
MR Egger
MR RAPS
Weighted median
Weighted mode
0.10
0.05
0.00
−0.05
−0.10
0.1
0.2
SNP effect on GDH/6PGL endoplasmic bifunctional protein
SNP effect on Gut microbiota abundance (genus Parasutterella id.2892)

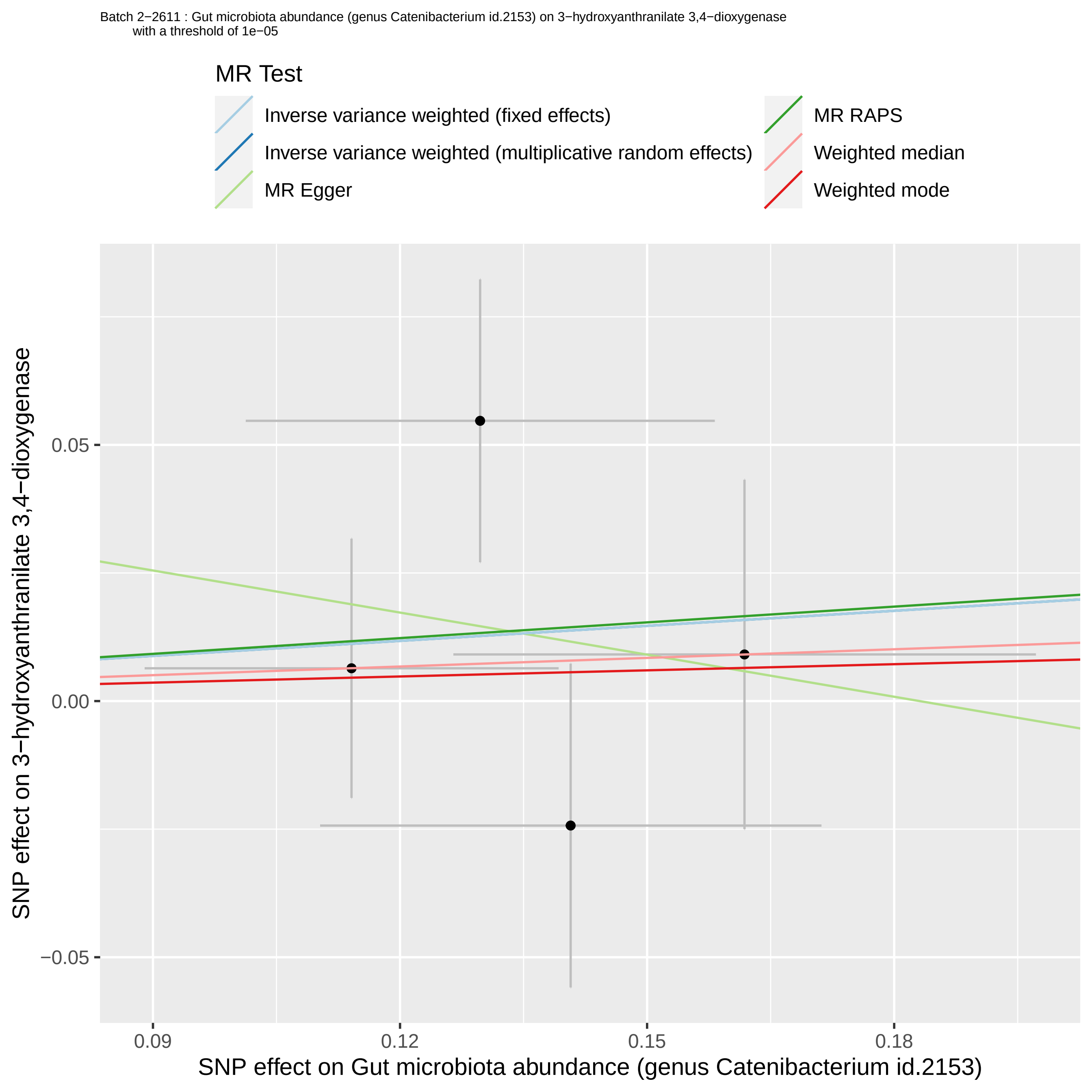

Batch 2−2611 : Gut microbiota abundance (genus Catenibacterium id.2153) on 3−hydroxyanthranilate 3,4−dioxygenase
with a threshold of 1e−05
MR Test
Inverse variance weighted (fixed effects)
Inverse variance weighted (multiplicative random effects)
MR Egger
MR RAPS
Weighted median
Weighted mode
SNP effect on 3−hydroxyanthranilate 3,4−dioxygenase
0.05
0.00
−0.05
0.09
0.12
0.15
0.18
SNP effect on Gut microbiota abundance (genus Catenibacterium id.2153)

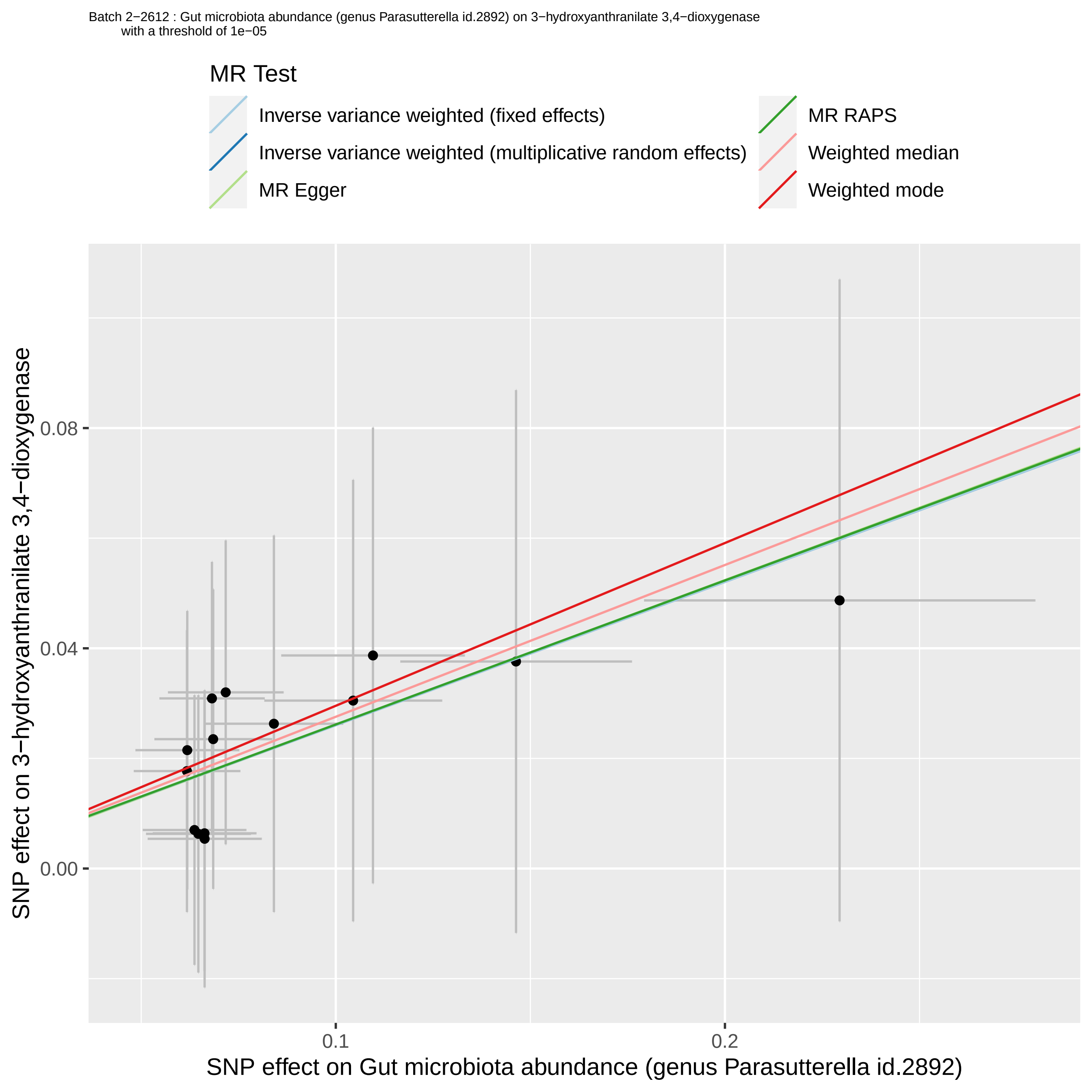
Batch 2−2612 : Gut microbiota abundance (genus Parasutterella id.2892) on 3−hydroxyanthranilate 3,4−dioxygenase
with a threshold of 1e−05
MR Test
Inverse variance weighted (fixed effects)
Inverse variance weighted (multiplicative random effects)
MR Egger
MR RAPS
Weighted median
Weighted mode
0.08
0.04
0.00
0.1
0.2
SNP effect on 3−hydroxyanthranilate 3,4−dioxygenase
SNP effect on Gut microbiota abundance (genus Parasutterella id.2892)

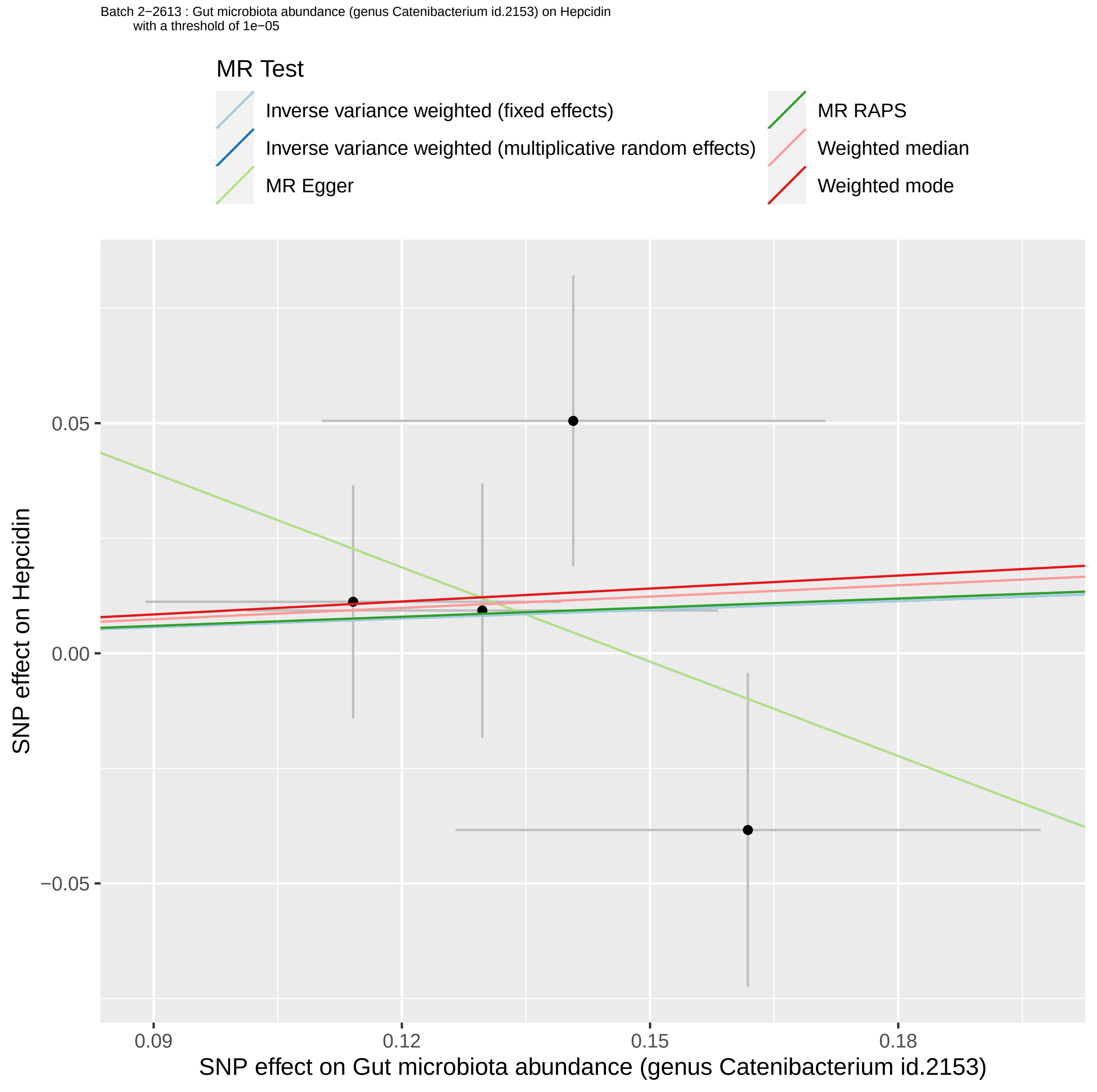

Batch 2−2613 : Gut microbiota abundance (genus Catenibacterium id.2153) on Hepcidin
with a threshold of 1e−05
MR Test
Inverse variance weighted (fixed effects)
Inverse variance weighted (multiplicative random effects)
MR Egger
MR RAPS
Weighted median
Weighted mode
0.05
0.00
−0.05
0.09
0.12
0.15
0.18
SNP effect on Hepcidin
SNP effect on Gut microbiota abundance (genus Catenibacterium id.2153)

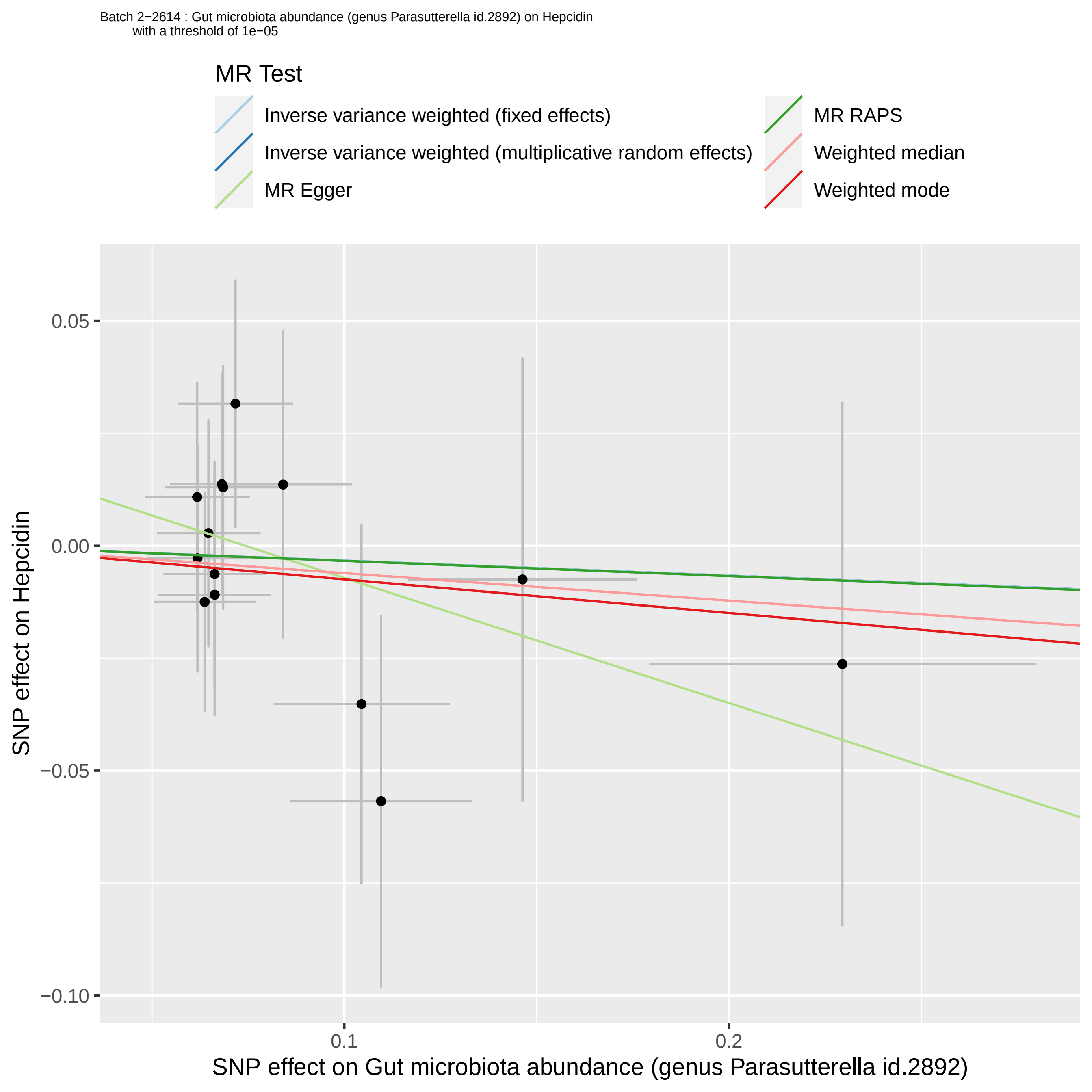
Batch 2−2614 : Gut microbiota abundance (genus Parasutterella id.2892) on Hepcidin
with a threshold of 1e−05
MR Test
Inverse variance weighted (fixed effects)
Inverse variance weighted (multiplicative random effects)
MR Egger
MR RAPS
Weighted median
Weighted mode
SNP effect on Hepcidin
0.05
0.00
−0.05
−0.10
0.1
0.2
SNP effect on Gut microbiota abundance (genus Parasutterella id.2892)

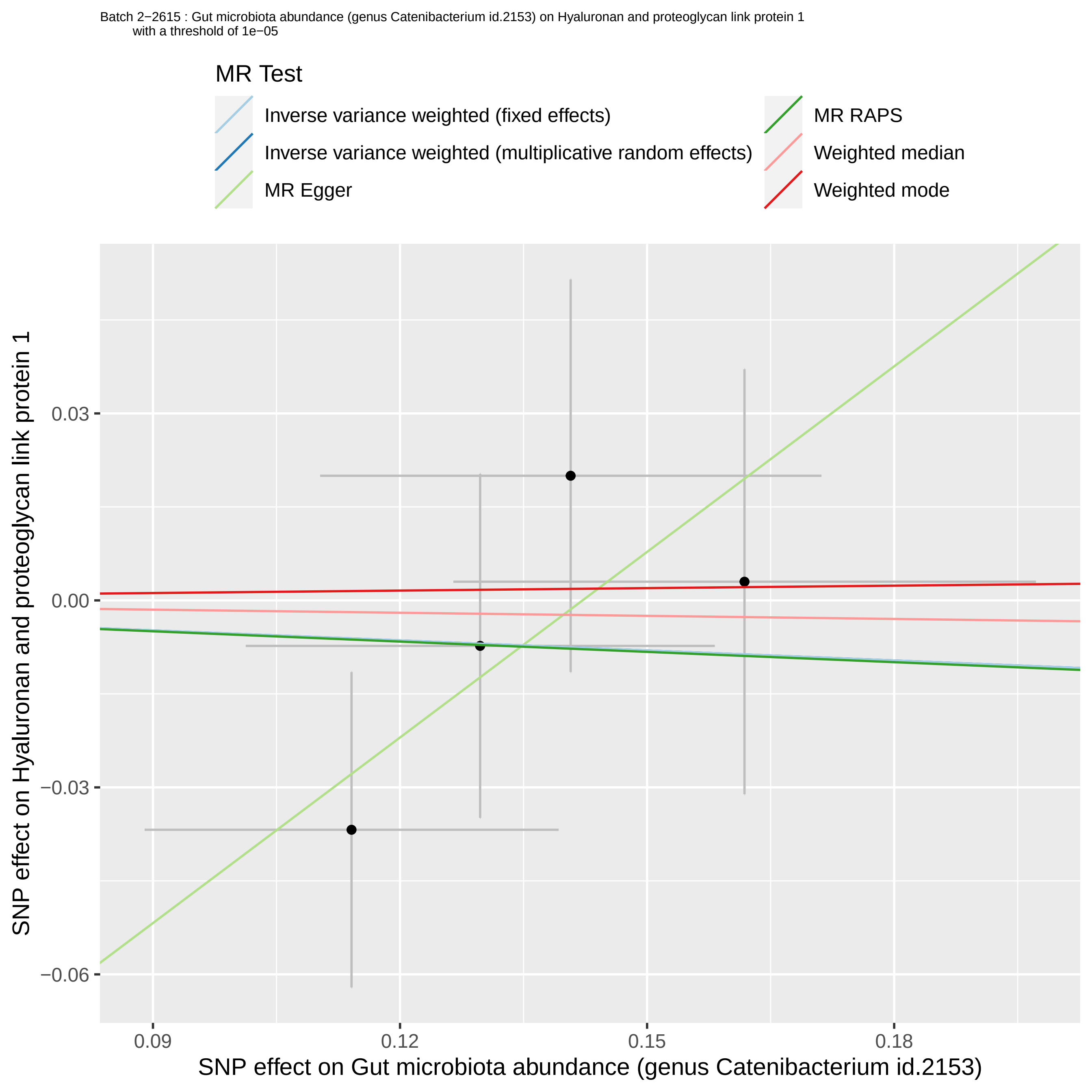
Batch 2−2615 : Gut microbiota abundance (genus Catenibacterium id.2153) on Hyaluronan and proteoglycan link protein 1
with a threshold of 1e−05
MR Test
Inverse variance weighted (fixed effects)
Inverse variance weighted (multiplicative random effects)
MR Egger
MR RAPS
Weighted median
Weighted mode
0.03
0.00
−0.03
−0.06
0.09
0.12
0.15
0.18
SNP effect on Gut microbiota abundance (genus Catenibacterium id.2153)
SNP effect on Hyaluronan and proteoglycan link protein 1

Batch 2−2616 : Gut microbiota abundance (genus Parasutterella id.2892) on Hyaluronan and proteoglycan link protein 1
with a threshold of 1e−05

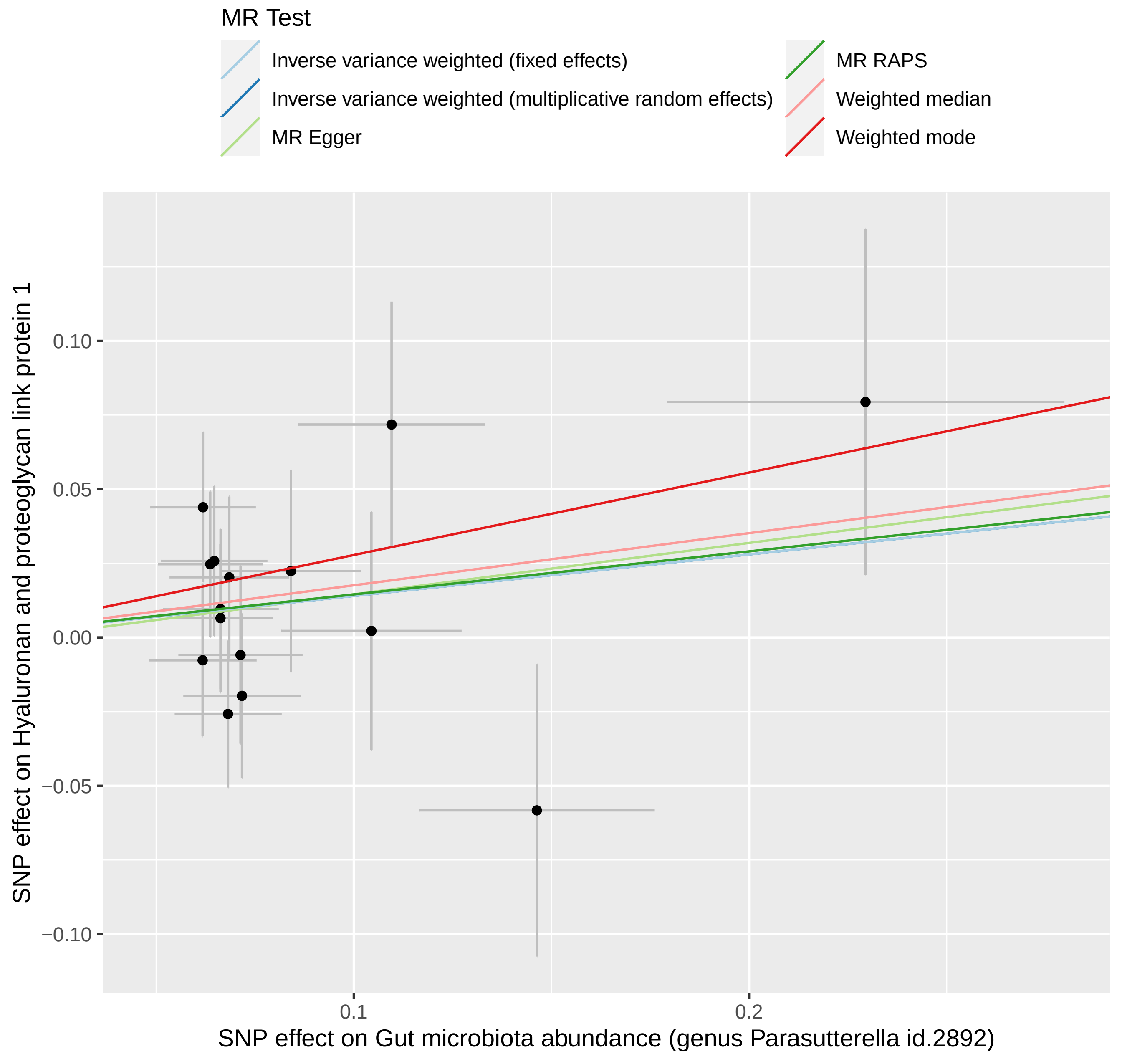

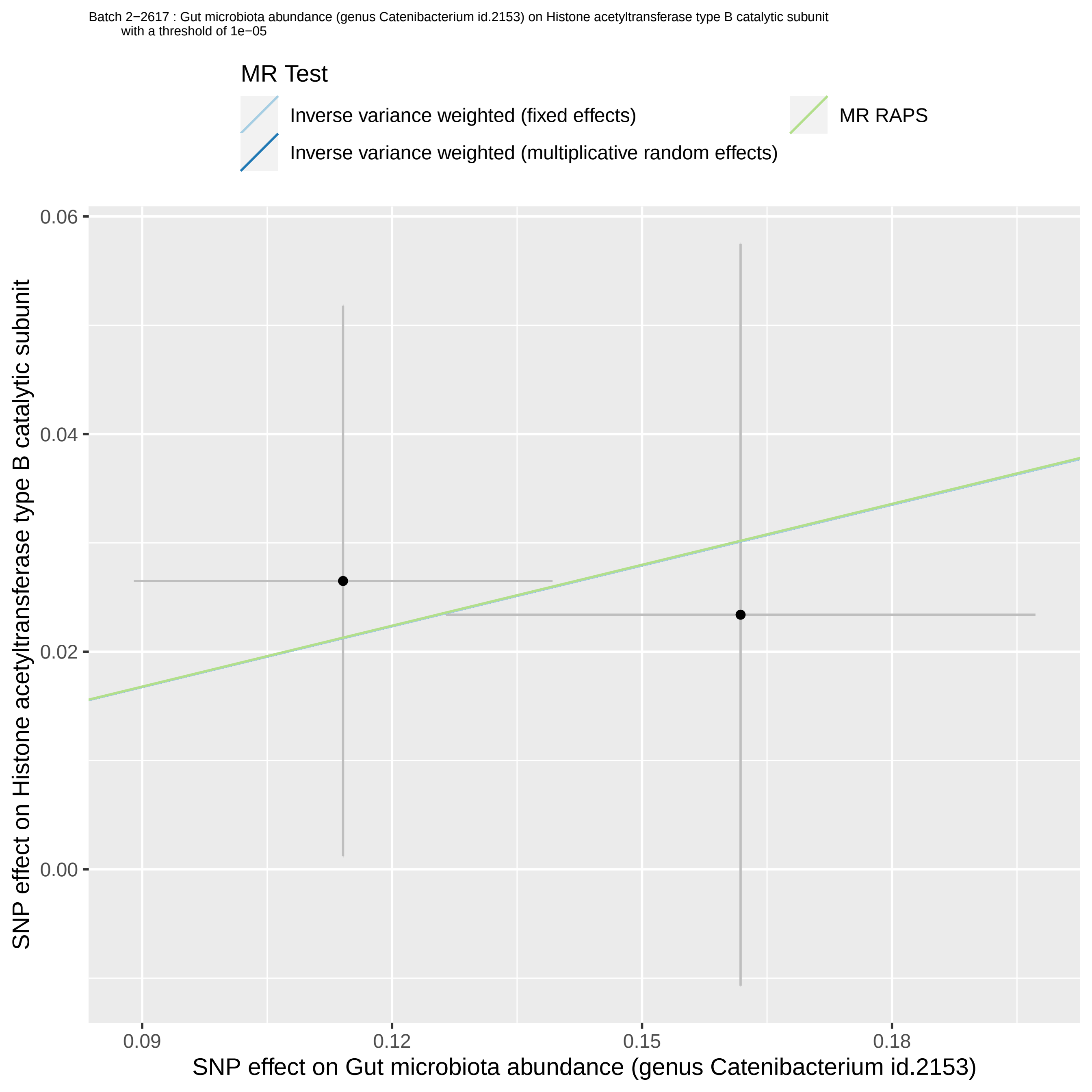

Batch 2−2617 : Gut microbiota abundance (genus Catenibacterium id.2153) on Histone acetyltransferase type B catalytic subunit
with a threshold of 1e−05
MR Test
Inverse variance weighted (fixed effects)
Inverse variance weighted (multiplicative random effects)
MR RAPS
0.06
0.04
0.02
0.00
0.09
0.12
0.15
0.18
SNP effect on Gut microbiota abundance (genus Catenibacterium id.2153)
SNP effect on Histone acetyltransferase type B catalytic subunit

Batch 2−2618 : Gut microbiota abundance (genus Parasutterella id.2892) on Histone acetyltransferase type B catalytic subunit
with a threshold of 1e−05

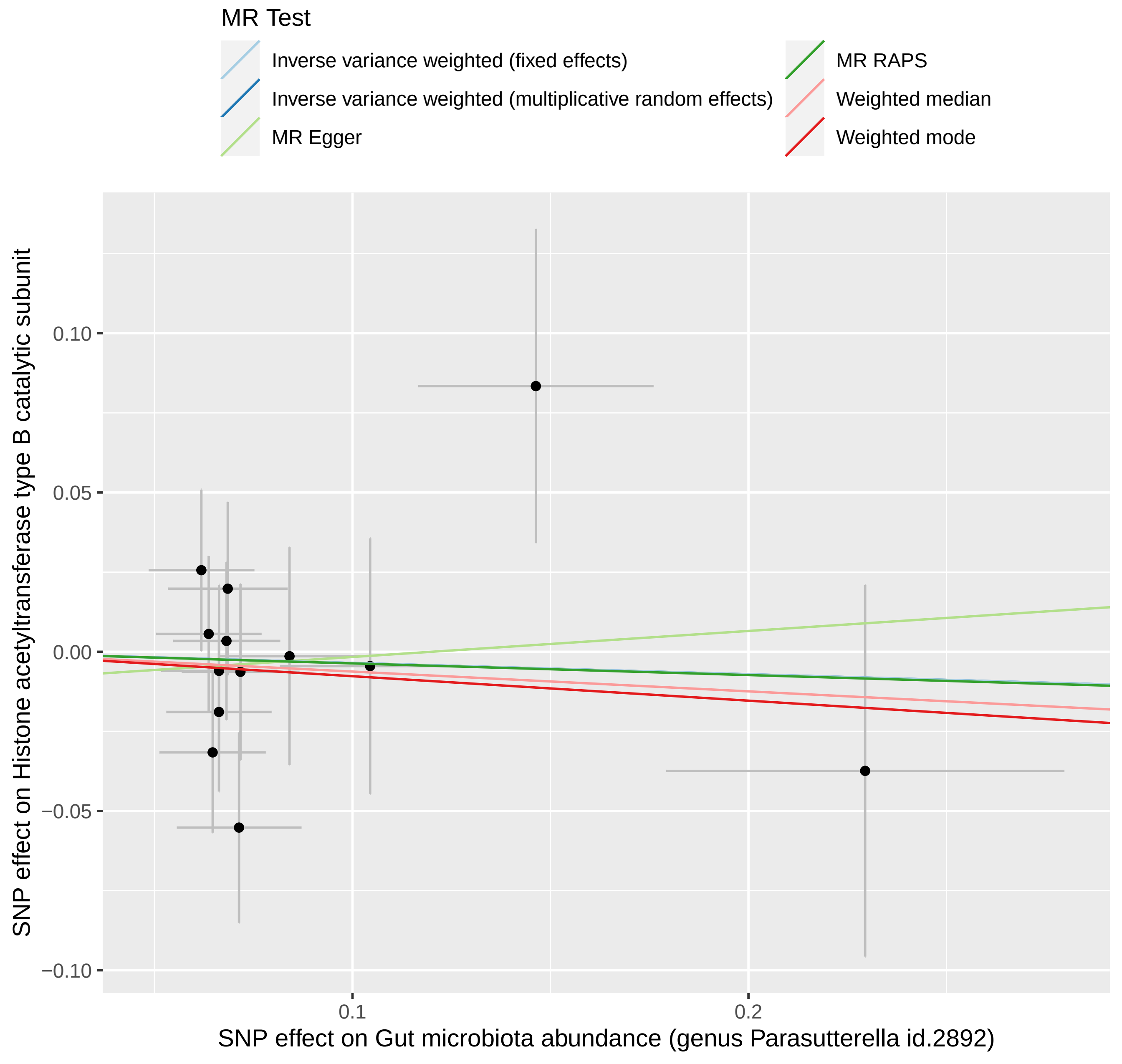

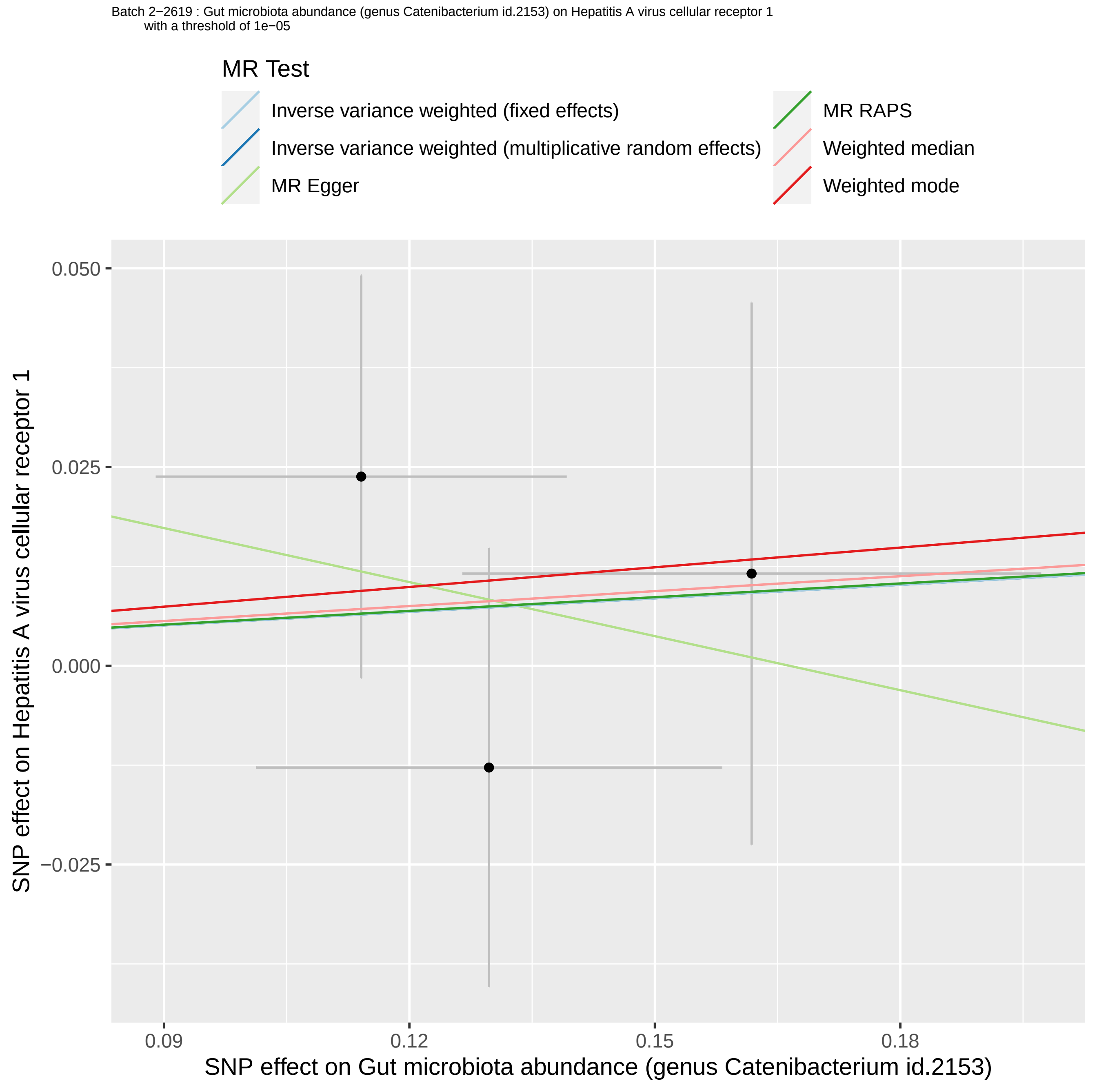

Batch 2−2619 : Gut microbiota abundance (genus Catenibacterium id.2153) on Hepatitis A virus cellular receptor 1
with a threshold of 1e−05
MR Test
Inverse variance weighted (fixed effects)
Inverse variance weighted (multiplicative random effects)
MR Egger
MR RAPS
Weighted median
Weighted mode
0.050
0.025
0.000
−0.025
0.09
0.12
0.15
0.18
SNP effect on Hepatitis A virus cellular receptor 1
SNP effect on Gut microbiota abundance (genus Catenibacterium id.2153)

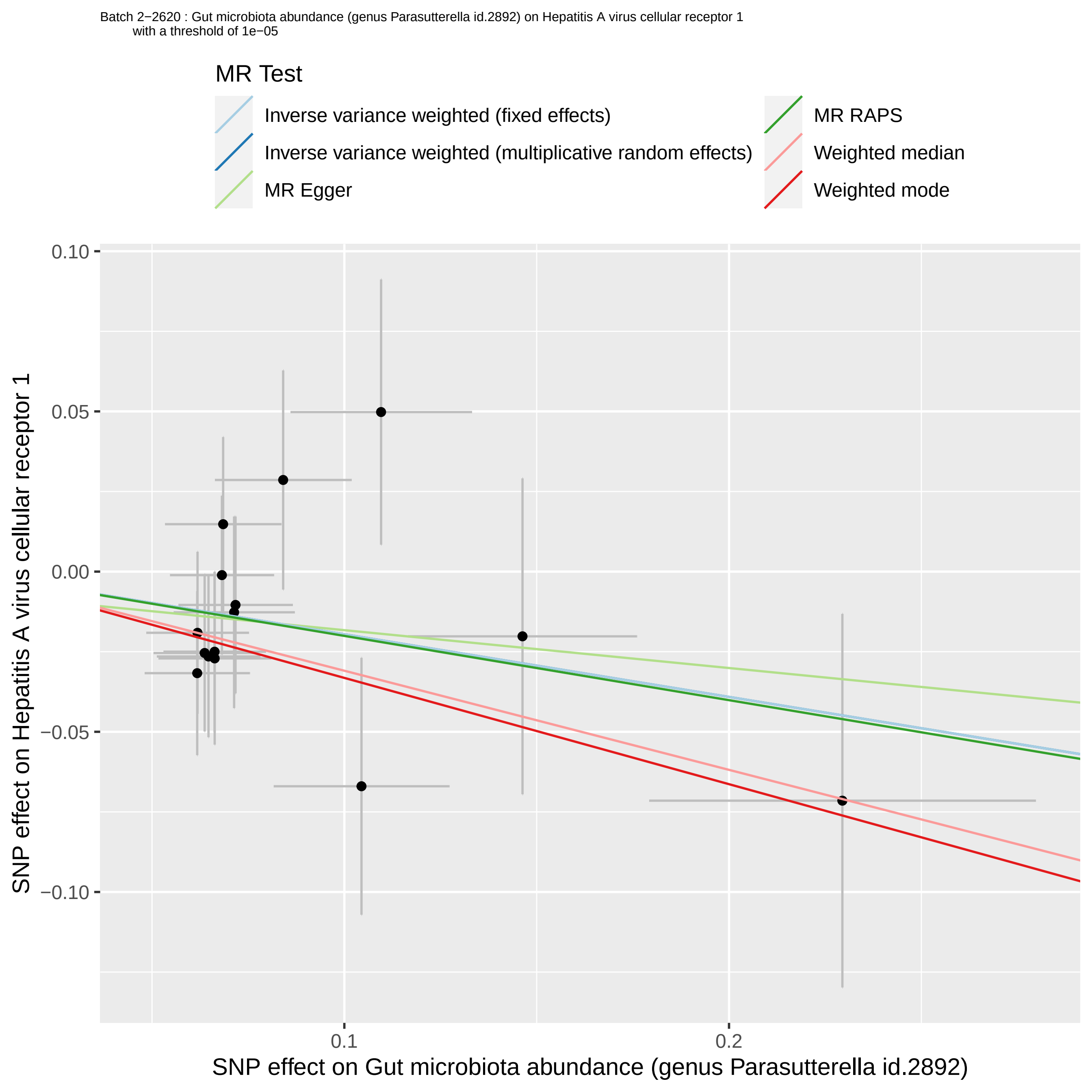
Batch 2−2620 : Gut microbiota abundance (genus Parasutterella id.2892) on Hepatitis A virus cellular receptor 1
with a threshold of 1e−05
MR Test
Inverse variance weighted (fixed effects)
Inverse variance weighted (multiplicative random effects)
MR Egger
MR RAPS
Weighted median
Weighted mode
0.10
0.05
0.00
−0.05
−0.10
0.1
0.2
SNP effect on Hepatitis A virus cellular receptor 1
SNP effect on Gut microbiota abundance (genus Parasutterella id.2892)

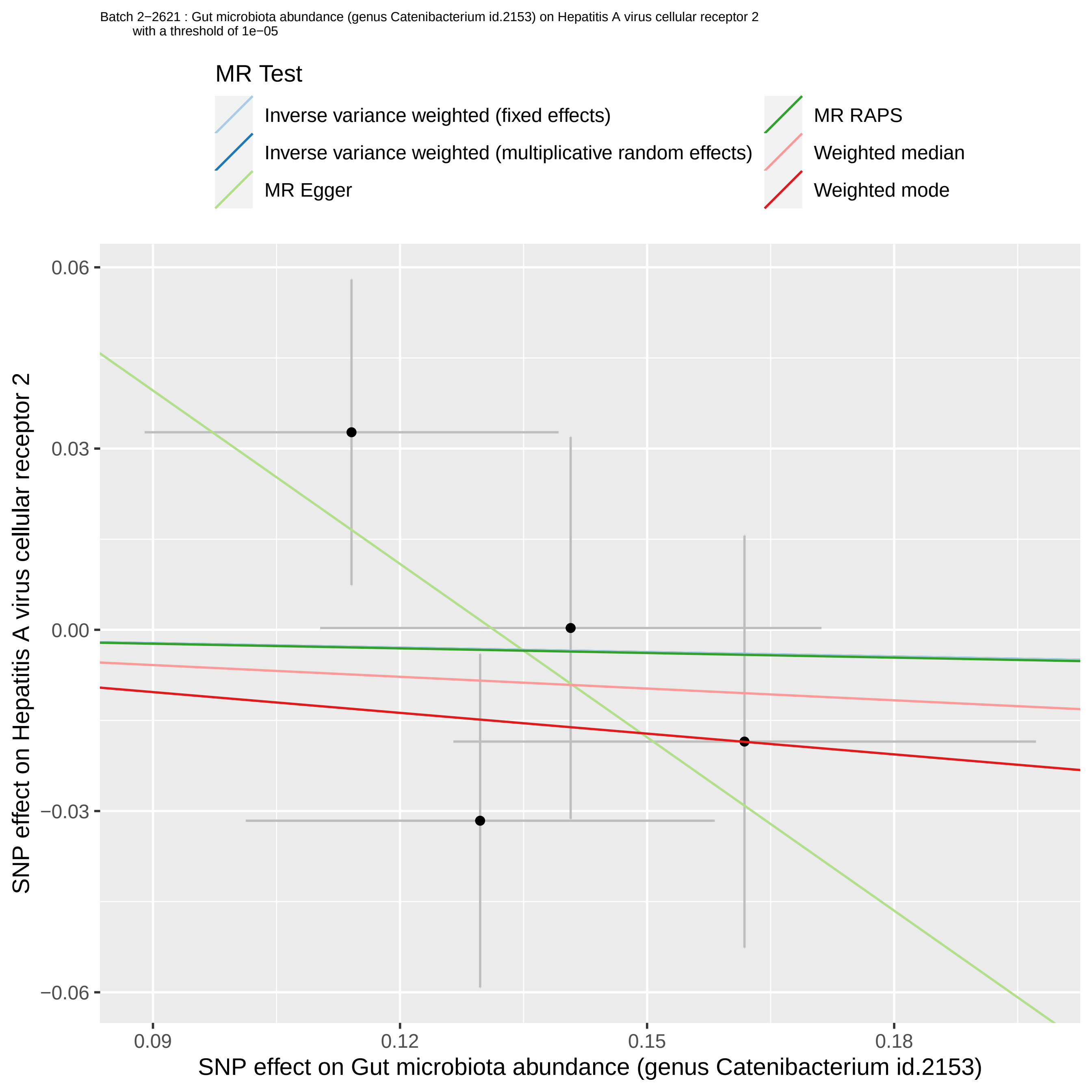

Batch 2−2621 : Gut microbiota abundance (genus Catenibacterium id.2153) on Hepatitis A virus cellular receptor 2
with a threshold of 1e−05
MR Test
Inverse variance weighted (fixed effects)
Inverse variance weighted (multiplicative random effects)
MR Egger
MR RAPS
Weighted median
Weighted mode
0.06
0.03
0.00
−0.03
−0.06
0.09
0.12
0.15
0.18
SNP effect on Hepatitis A virus cellular receptor 2
SNP effect on Gut microbiota abundance (genus Catenibacterium id.2153)

Batch 2−2622 : Gut microbiota abundance (genus Parasutterella id.2892) on Hepatitis A virus cellular receptor 2
with a threshold of 1e−05

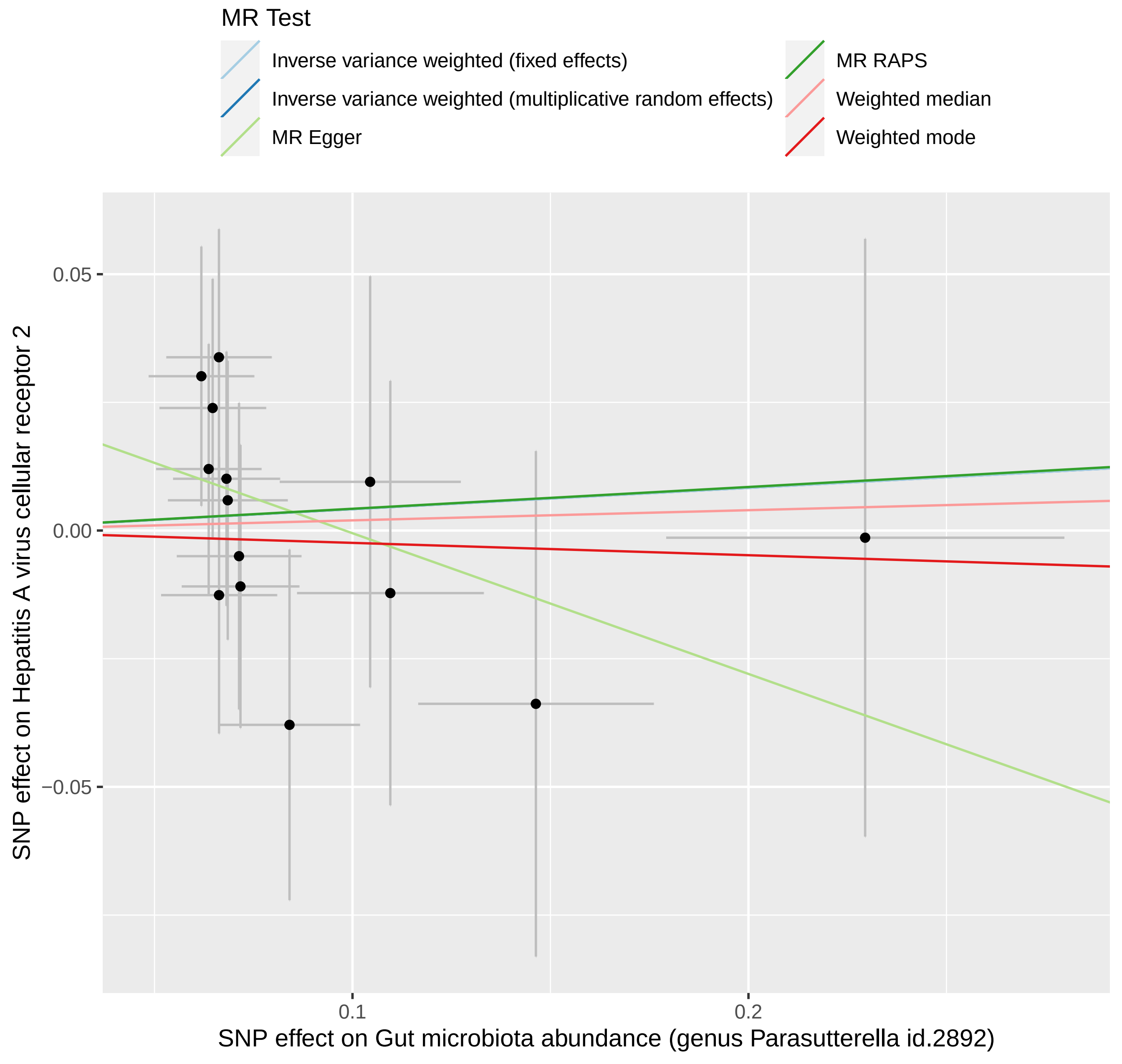

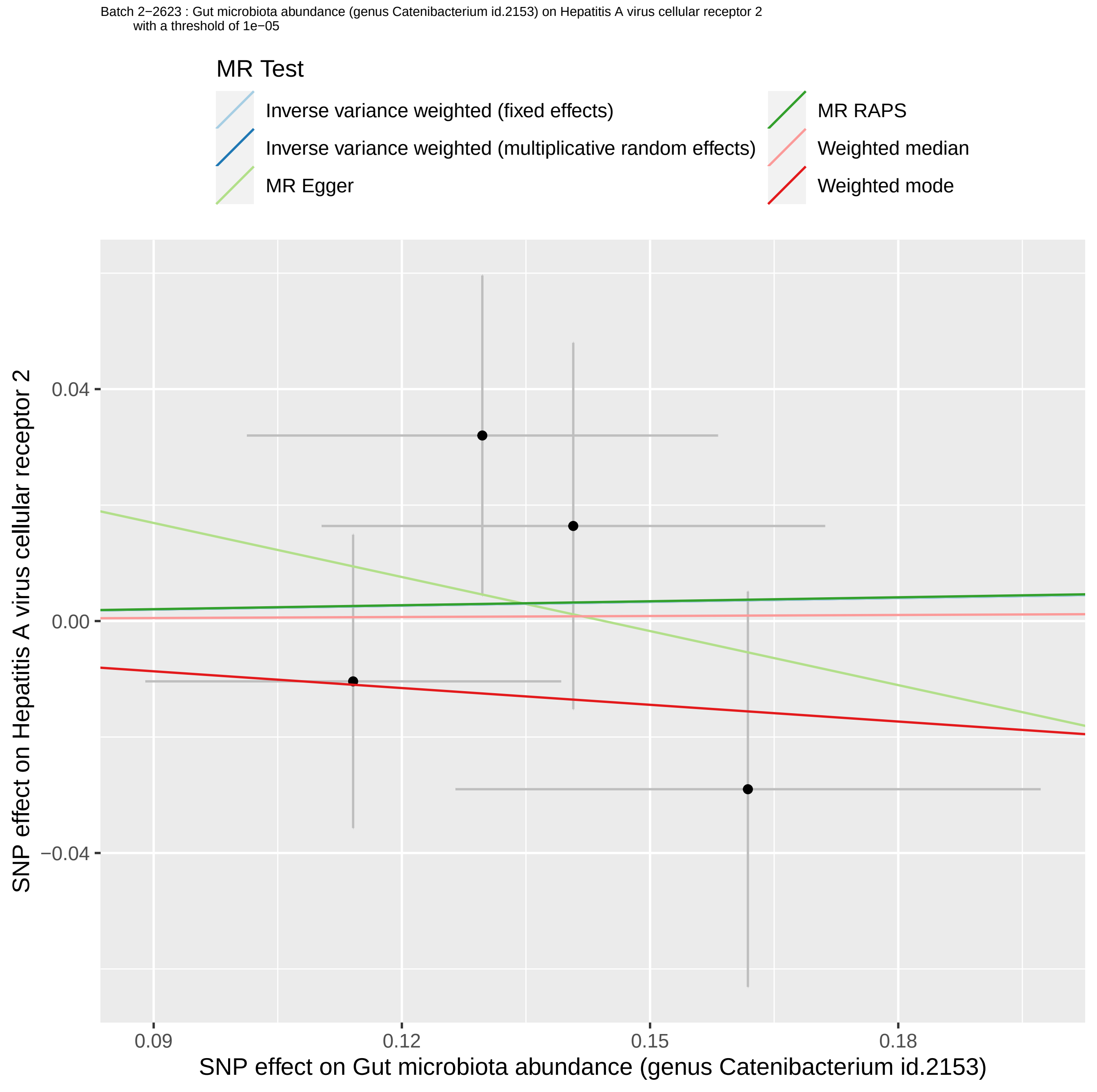

Batch 2−2623 : Gut microbiota abundance (genus Catenibacterium id.2153) on Hepatitis A virus cellular receptor 2
with a threshold of 1e−05
MR Test
Inverse variance weighted (fixed effects)
Inverse variance weighted (multiplicative random effects)
MR Egger
MR RAPS
Weighted median
Weighted mode
0.04
0.00
−0.04
0.09
0.12
0.15
0.18
SNP effect on Gut microbiota abundance (genus Catenibacterium id.2153)
SNP effect on Hepatitis A virus cellular receptor 2

Batch 2−2624 : Gut microbiota abundance (genus Parasutterella id.2892) on Hepatitis A virus cellular receptor 2
with a threshold of 1e−05

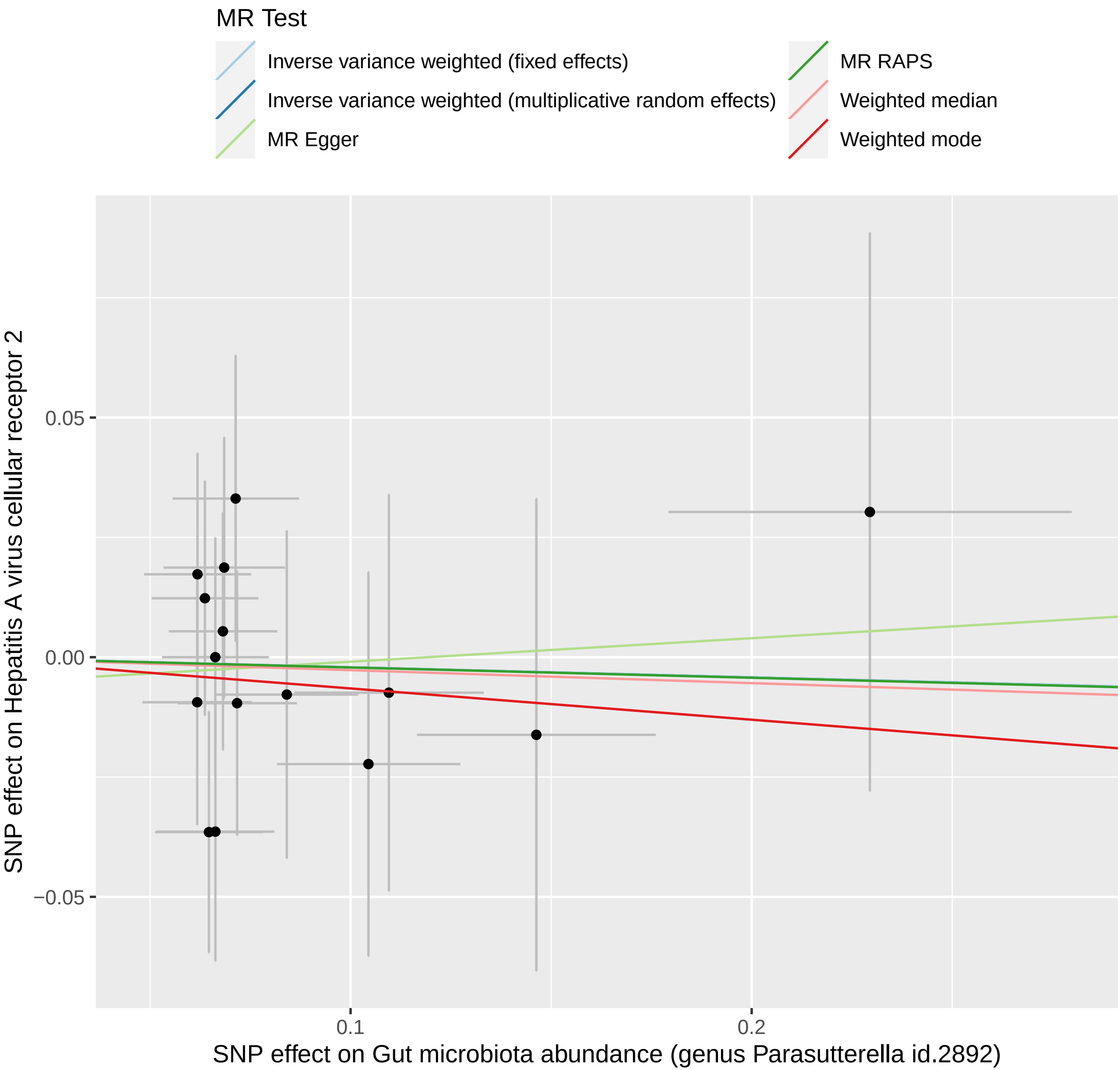

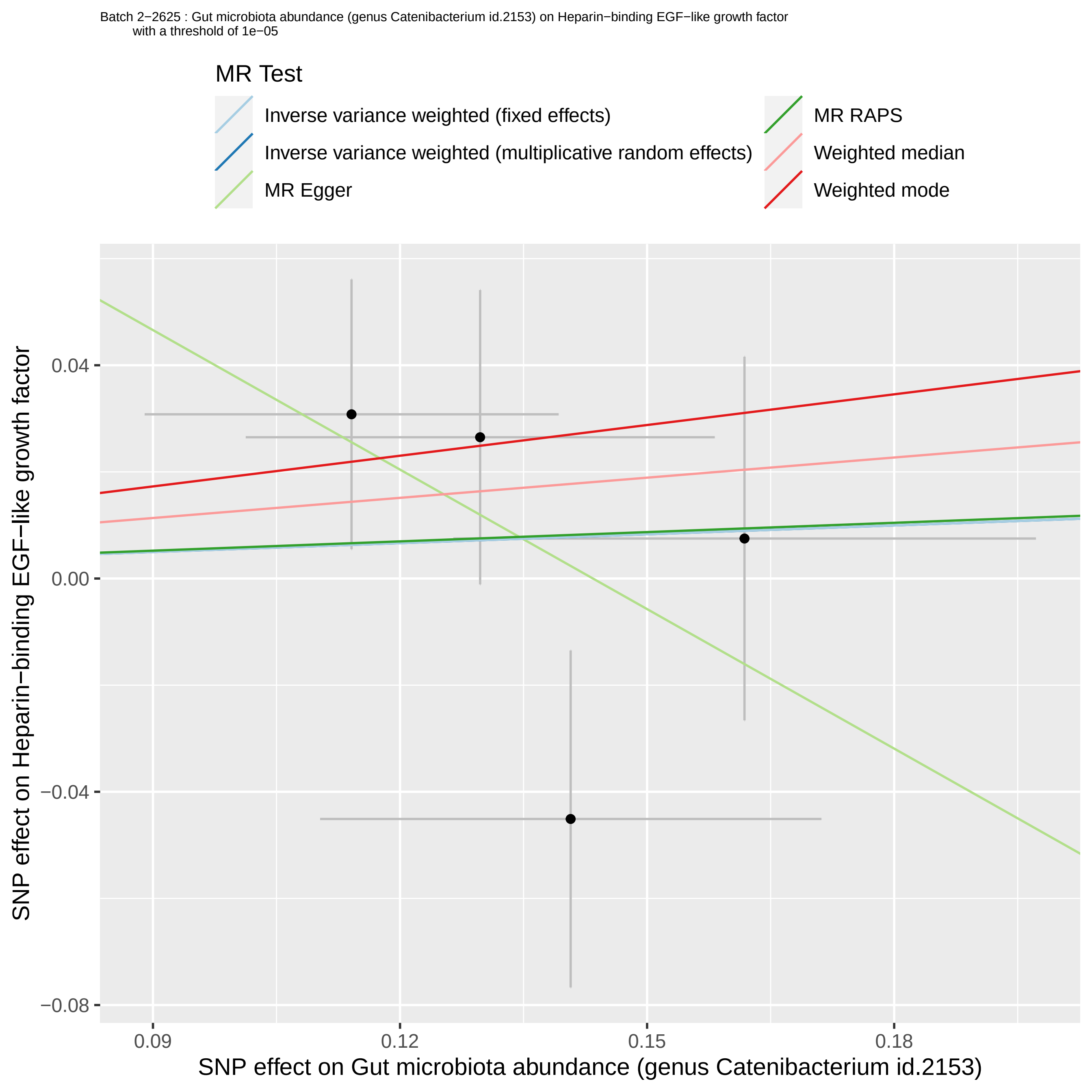
Batch 2−2625 : Gut microbiota abundance (genus Catenibacterium id.2153) on Heparin−binding EGF−like growth factor
with a threshold of 1e−05
MR Test
Inverse variance weighted (fixed effects)
Inverse variance weighted (multiplicative random effects)
MR Egger
MR RAPS
Weighted median
Weighted mode
0.04
0.00
−0.04
−0.08
0.09
0.12
0.15
0.18
SNP effect on Heparin−binding EGF−like growth factor
SNP effect on Gut microbiota abundance (genus Catenibacterium id.2153)

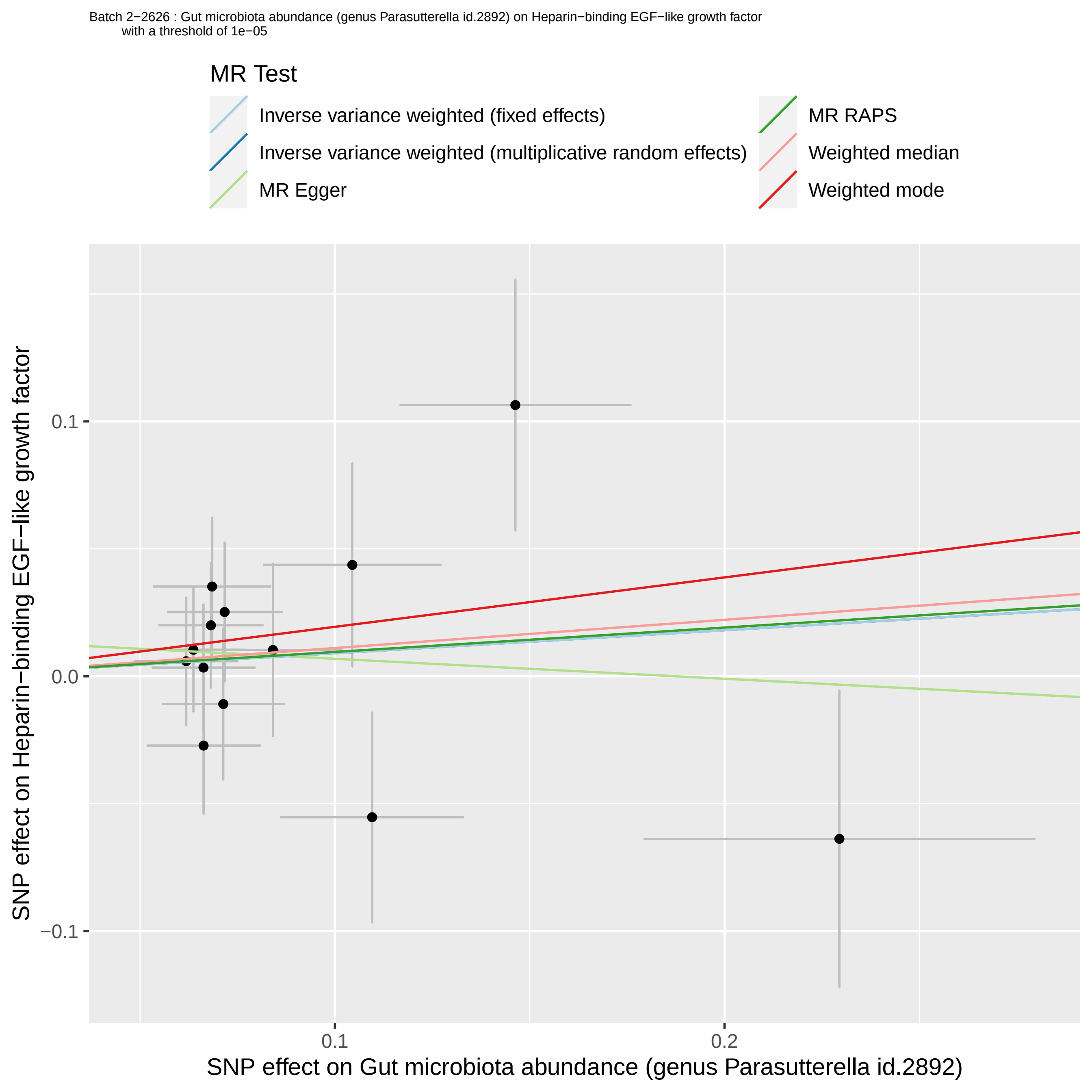

Batch 2−2626 : Gut microbiota abundance (genus Parasutterella id.2892) on Heparin−binding EGF−like growth factor
with a threshold of 1e−05
MR Test
Inverse variance weighted (fixed effects)
Inverse variance weighted (multiplicative random effects)
MR Egger
MR RAPS
Weighted median
Weighted mode
SNP effect on Heparin−binding EGF−like growth factor
0.1
0.0
−0.1
0.1
0.2
SNP effect on Gut microbiota abundance (genus Parasutterella id.2892)

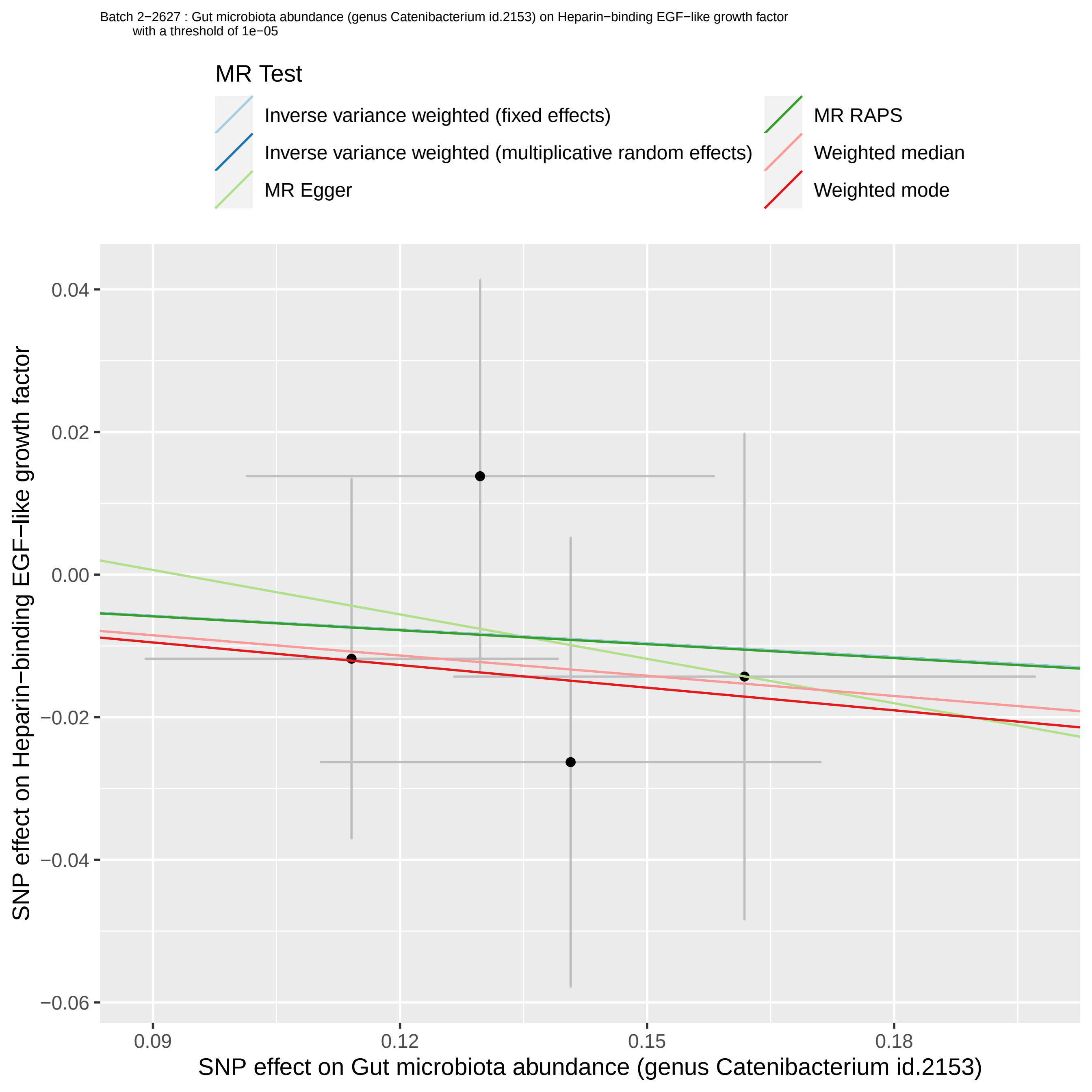
Batch 2−2627 : Gut microbiota abundance (genus Catenibacterium id.2153) on Heparin−binding EGF−like growth factor
with a threshold of 1e−05
MR Test
Inverse variance weighted (fixed effects)
Inverse variance weighted (multiplicative random effects)
MR Egger
MR RAPS
Weighted median
Weighted mode
0.04
0.02
0.00
−0.02
−0.04
−0.06
SNP effect on Heparin−binding EGF−like growth factor
0.09
0.12
0.15
0.18
SNP effect on Gut microbiota abundance (genus Catenibacterium id.2153)

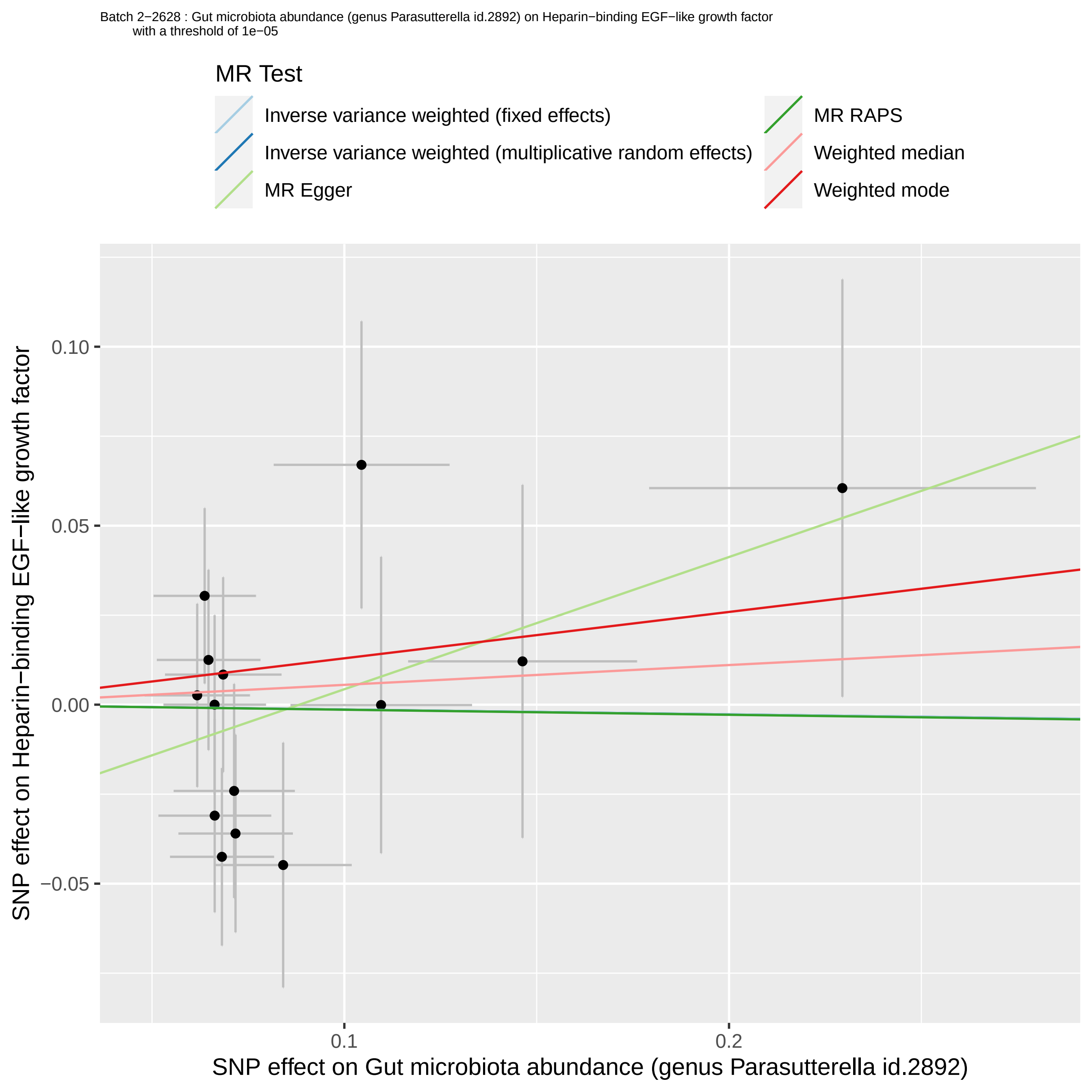

Batch 2−2628 : Gut microbiota abundance (genus Parasutterella id.2892) on Heparin−binding EGF−like growth factor
with a threshold of 1e−05
MR Test
Inverse variance weighted (fixed effects)
Inverse variance weighted (multiplicative random effects)
MR Egger
MR RAPS
Weighted median
Weighted mode
0.10
0.05
0.00
−0.05
0.1
0.2
SNP effect on Heparin−binding EGF−like growth factor
SNP effect on Gut microbiota abundance (genus Parasutterella id.2892)

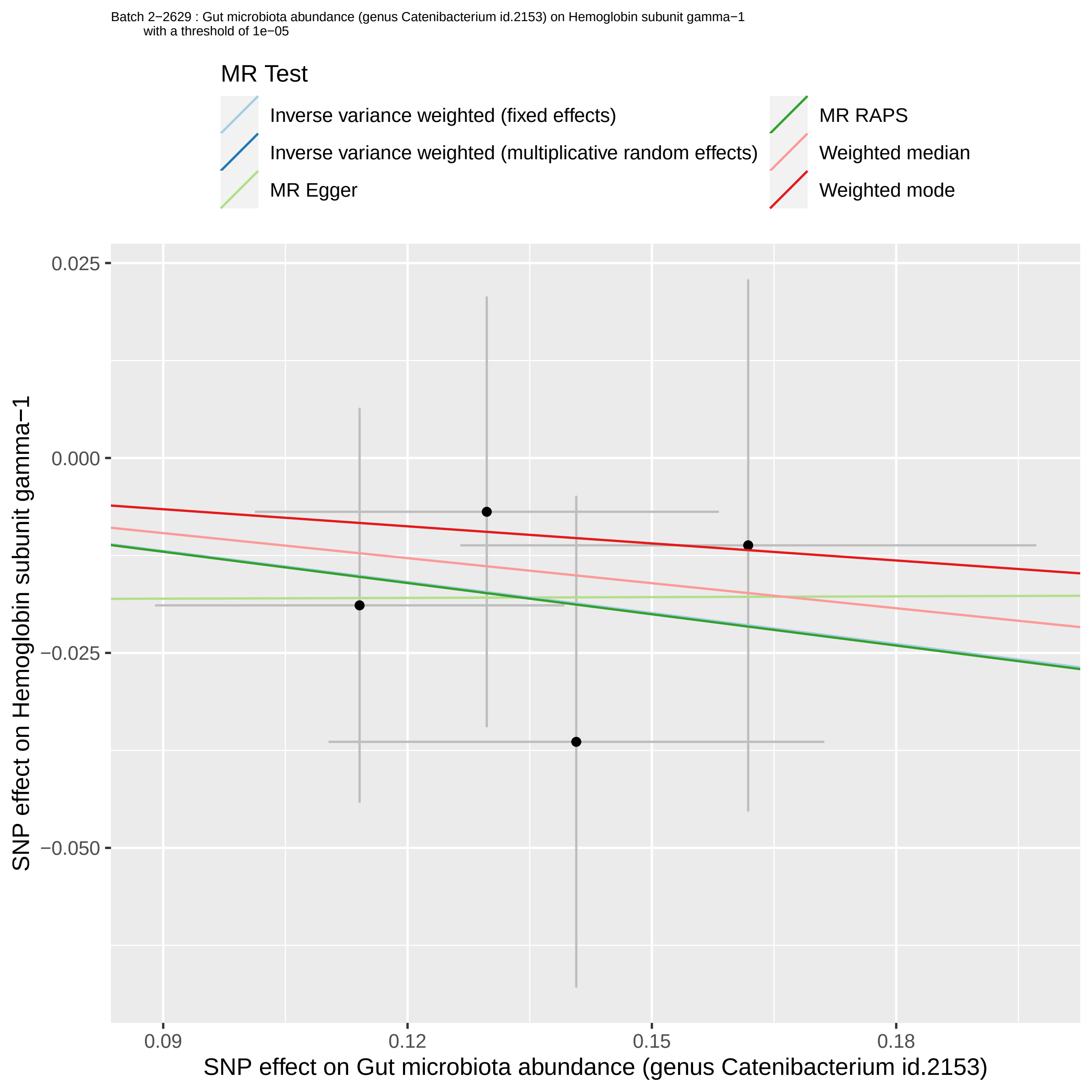

Batch 2−2629 : Gut microbiota abundance (genus Catenibacterium id.2153) on Hemoglobin subunit gamma−1
with a threshold of 1e−05
MR Test
Inverse variance weighted (fixed effects)
Inverse variance weighted (multiplicative random effects)
MR Egger
MR RAPS
Weighted median
Weighted mode
0.025
0.000
−0.025
−0.050
0.09
0.12
0.15
0.18
SNP effect on Hemoglobin subunit gamma−1
SNP effect on Gut microbiota abundance (genus Catenibacterium id.2153)

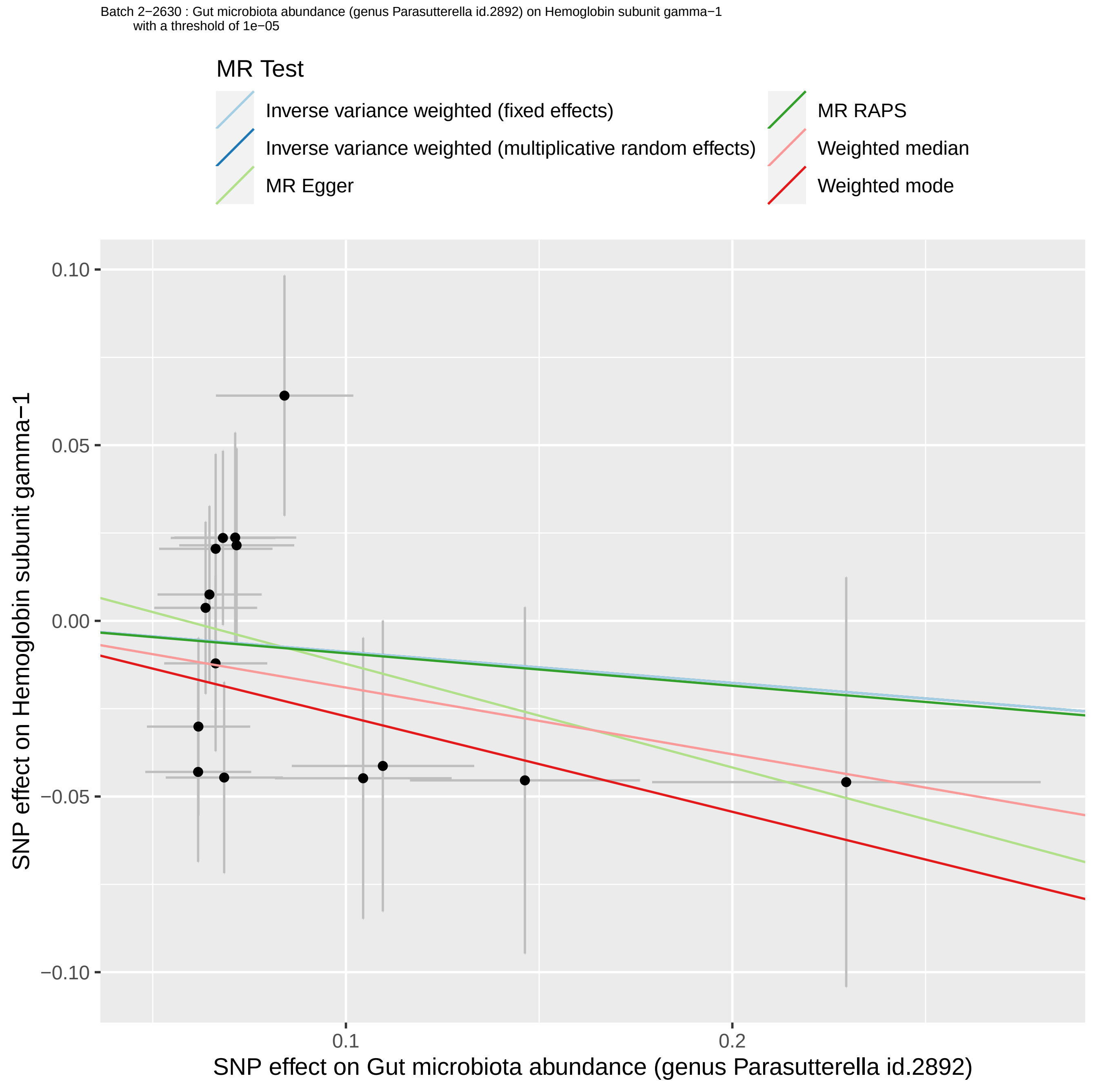

Batch 2−2630 : Gut microbiota abundance (genus Parasutterella id.2892) on Hemoglobin subunit gamma−1
with a threshold of 1e−05
MR Test
Inverse variance weighted (fixed effects)
Inverse variance weighted (multiplicative random effects)
MR Egger
MR RAPS
Weighted median
Weighted mode
0.10
0.05
0.00
−0.05
−0.10
0.1
0.2
SNP effect on Hemoglobin subunit gamma−1
SNP effect on Gut microbiota abundance (genus Parasutterella id.2892)

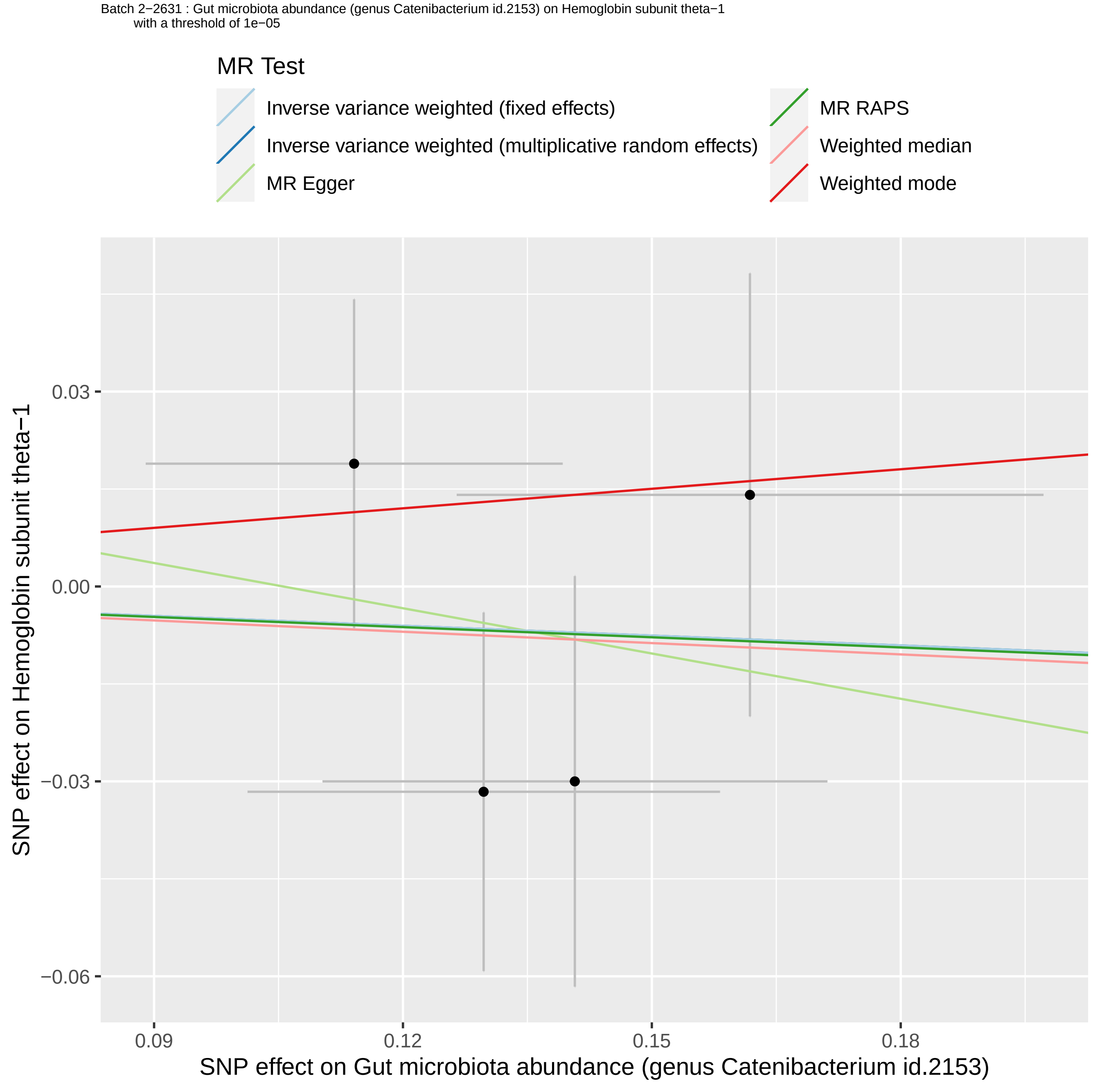
Batch 2−2631 : Gut microbiota abundance (genus Catenibacterium id.2153) on Hemoglobin subunit theta−1
with a threshold of 1e−05
MR Test
Inverse variance weighted (fixed effects)
Inverse variance weighted (multiplicative random effects)
MR Egger
MR RAPS
Weighted median
Weighted mode
0.03
0.00
−0.03
−0.06
0.09
0.12
0.15
0.18
SNP effect on Hemoglobin subunit theta−1
SNP effect on Gut microbiota abundance (genus Catenibacterium id.2153)

Batch 2−2632 : Gut microbiota abundance (genus Parasutterella id.2892) on Hemoglobin subunit theta−1
with a threshold of 1e−05

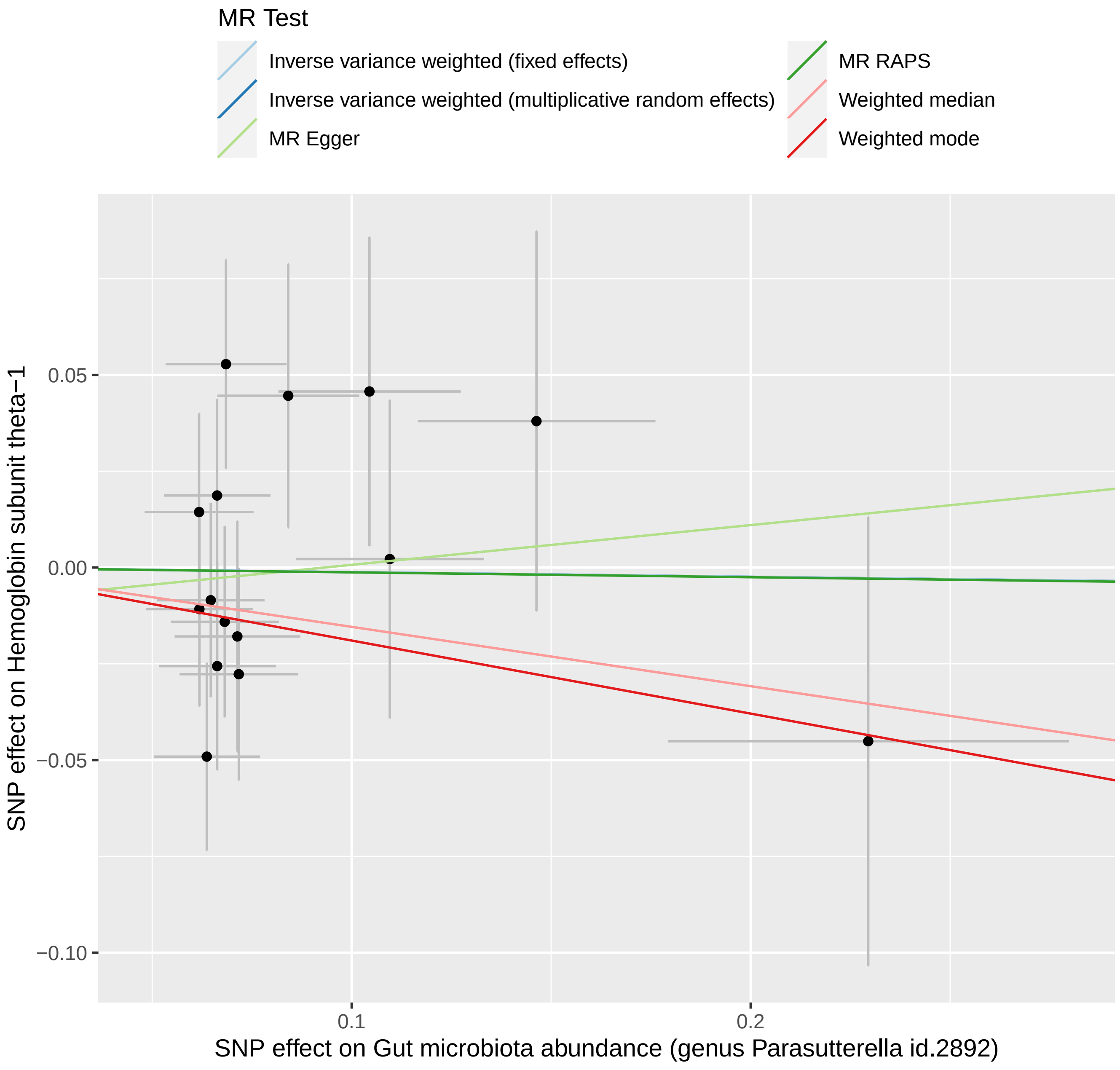

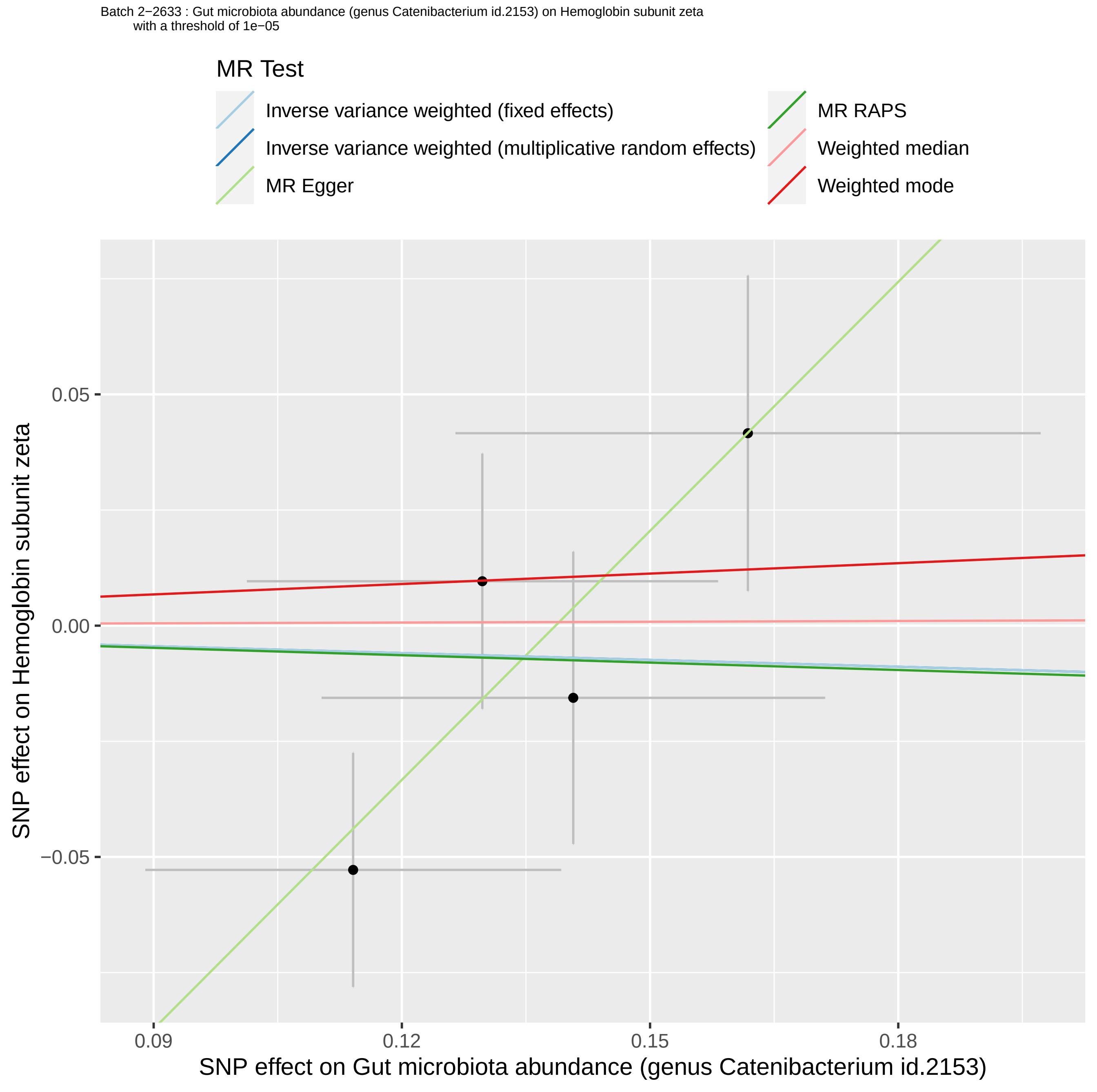

Batch 2−2633 : Gut microbiota abundance (genus Catenibacterium id.2153) on Hemoglobin subunit zeta
with a threshold of 1e−05
MR Test
Inverse variance weighted (fixed effects)
Inverse variance weighted (multiplicative random effects)
MR Egger
MR RAPS
Weighted median
Weighted mode
0.05
0.00
−0.05
0.09
0.12
0.15
0.18
SNP effect on Hemoglobin subunit zeta
SNP effect on Gut microbiota abundance (genus Catenibacterium id.2153)

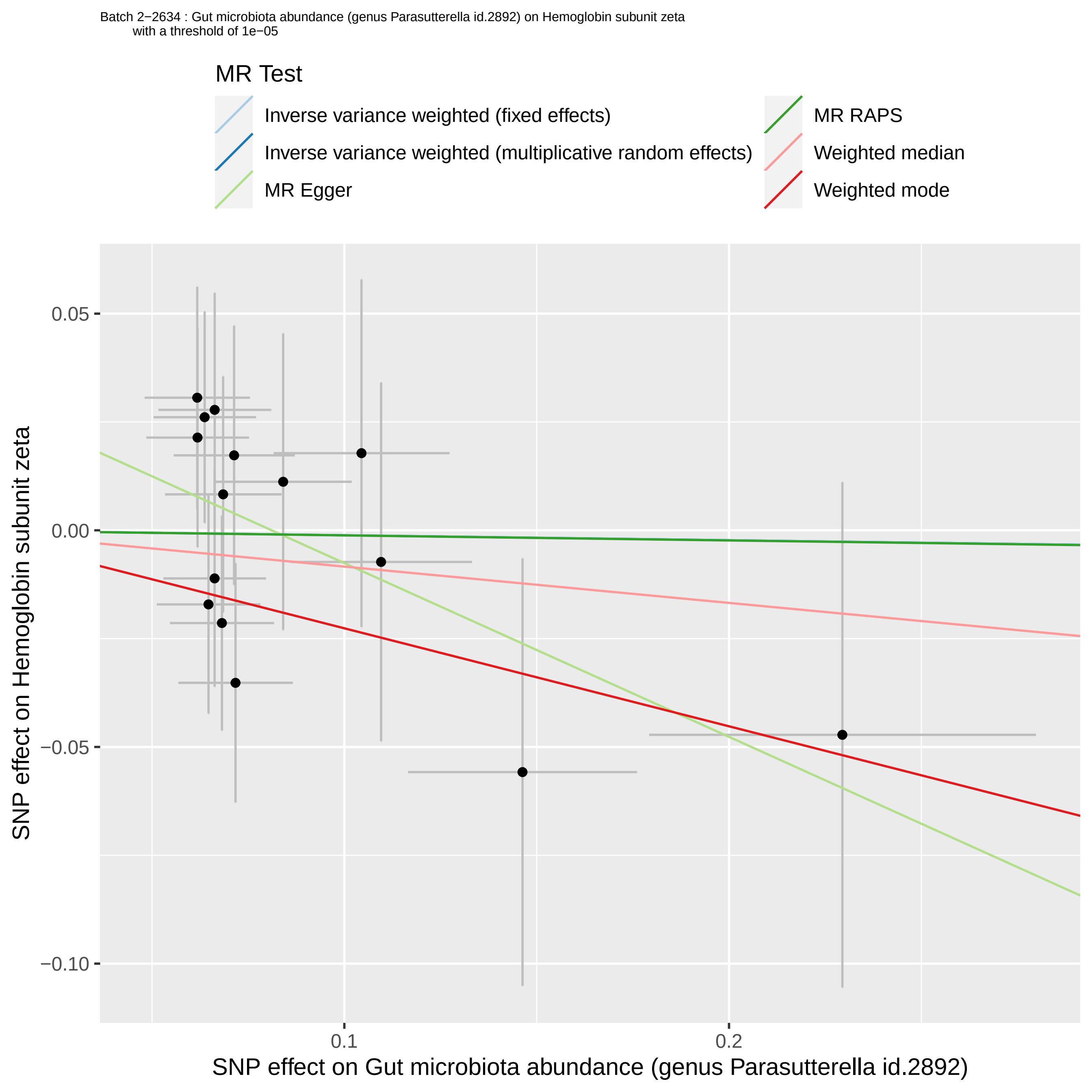

Batch 2−2634 : Gut microbiota abundance (genus Parasutterella id.2892) on Hemoglobin subunit zeta
with a threshold of 1e−05
MR Test
Inverse variance weighted (fixed effects)
Inverse variance weighted (multiplicative random effects)
MR Egger
MR RAPS
Weighted median
Weighted mode
0.05
0.00
−0.05
−0.10
0.1
0.2
SNP effect on Hemoglobin subunit zeta
SNP effect on Gut microbiota abundance (genus Parasutterella id.2892)

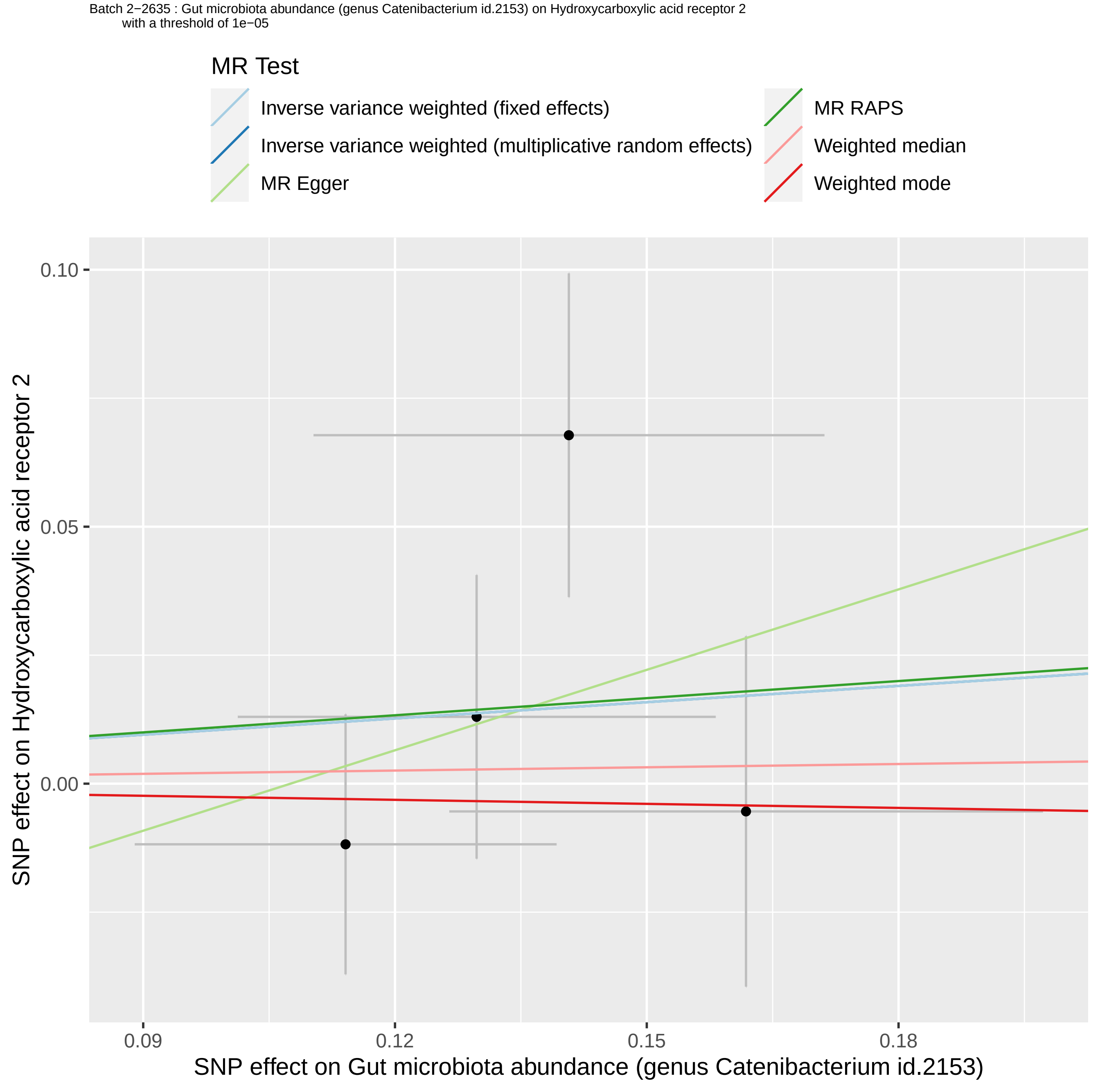

Batch 2−2635 : Gut microbiota abundance (genus Catenibacterium id.2153) on Hydroxycarboxylic acid receptor 2
with a threshold of 1e−05
MR Test
Inverse variance weighted (fixed effects)
Inverse variance weighted (multiplicative random effects)
MR Egger
MR RAPS
Weighted median
Weighted mode
0.10
0.05
0.00
0.09
0.12
0.15
0.18
SNP effect on Hydroxycarboxylic acid receptor 2
SNP effect on Gut microbiota abundance (genus Catenibacterium id.2153)

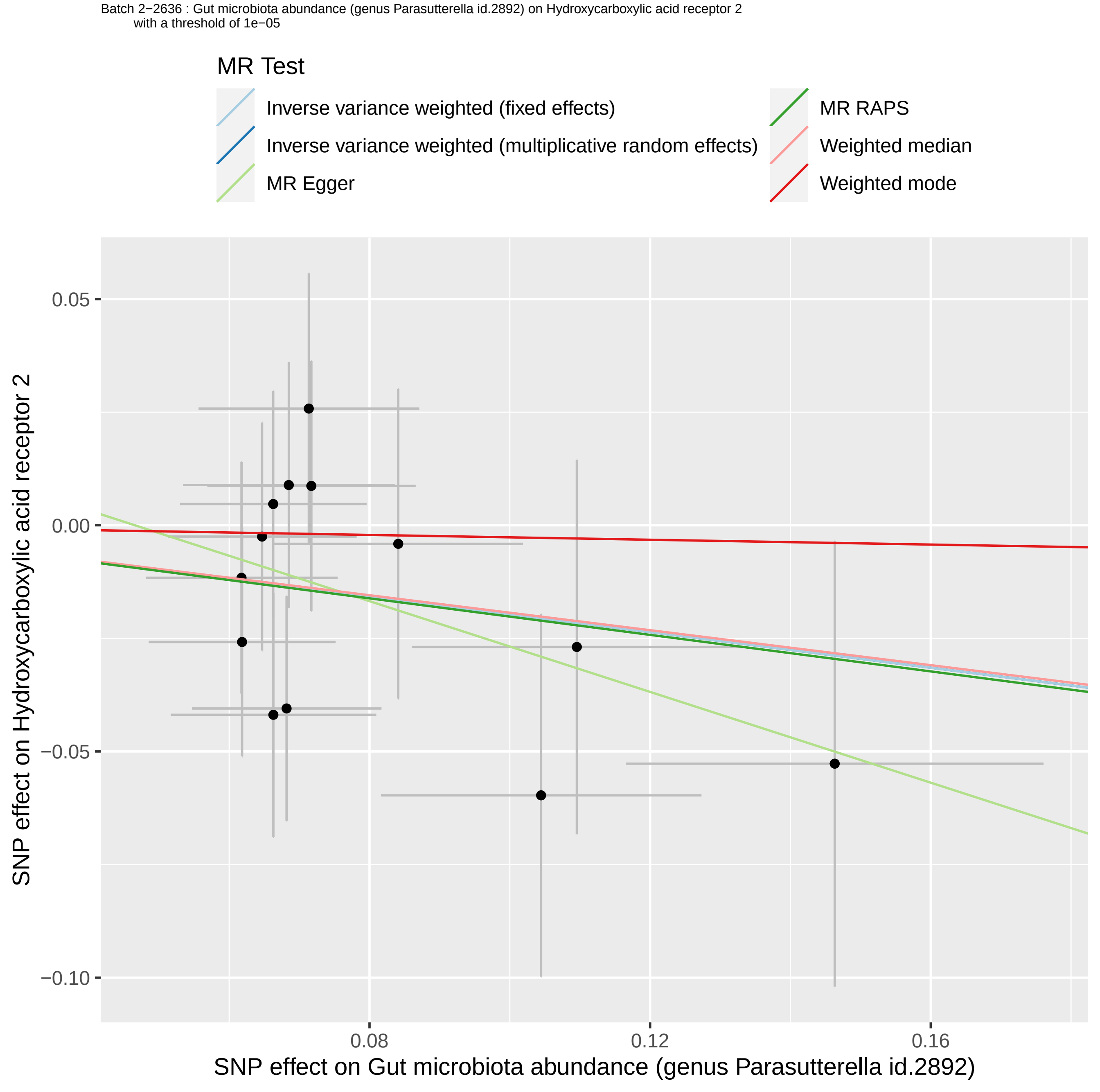

Batch 2−2636 : Gut microbiota abundance (genus Parasutterella id.2892) on Hydroxycarboxylic acid receptor 2
with a threshold of 1e−05
MR Test
Inverse variance weighted (fixed effects)
Inverse variance weighted (multiplicative random effects)
MR Egger
MR RAPS
Weighted median
Weighted mode
0.05
0.00
−0.05
−0.10
0.08
0.12
0.16
SNP effect on Hydroxycarboxylic acid receptor 2
SNP effect on Gut microbiota abundance (genus Parasutterella id.2892)

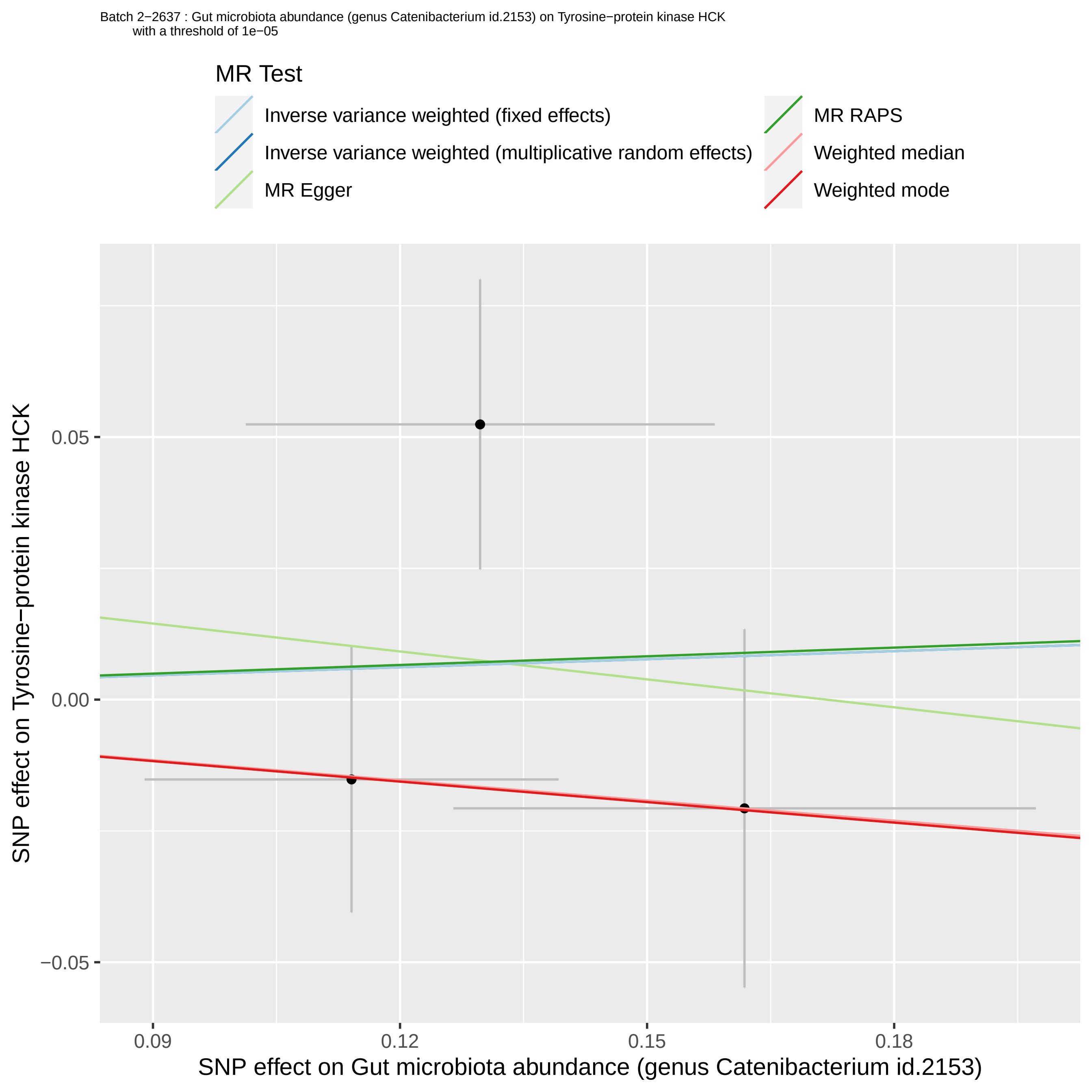
Batch 2−2637 : Gut microbiota abundance (genus Catenibacterium id.2153) on Tyrosine−protein kinase HCK
with a threshold of 1e−05
MR Test
Inverse variance weighted (fixed effects)
Inverse variance weighted (multiplicative random effects)
MR Egger
MR RAPS
Weighted median
Weighted mode
0.05
0.00
−0.05
0.09
0.12
0.15
0.18
SNP effect on Tyrosine−protein kinase HCK
SNP effect on Gut microbiota abundance (genus Catenibacterium id.2153)

Batch 2−2638 : Gut microbiota abundance (genus Parasutterella id.2892) on Tyrosine−protein kinase HCK
with a threshold of 1e−05

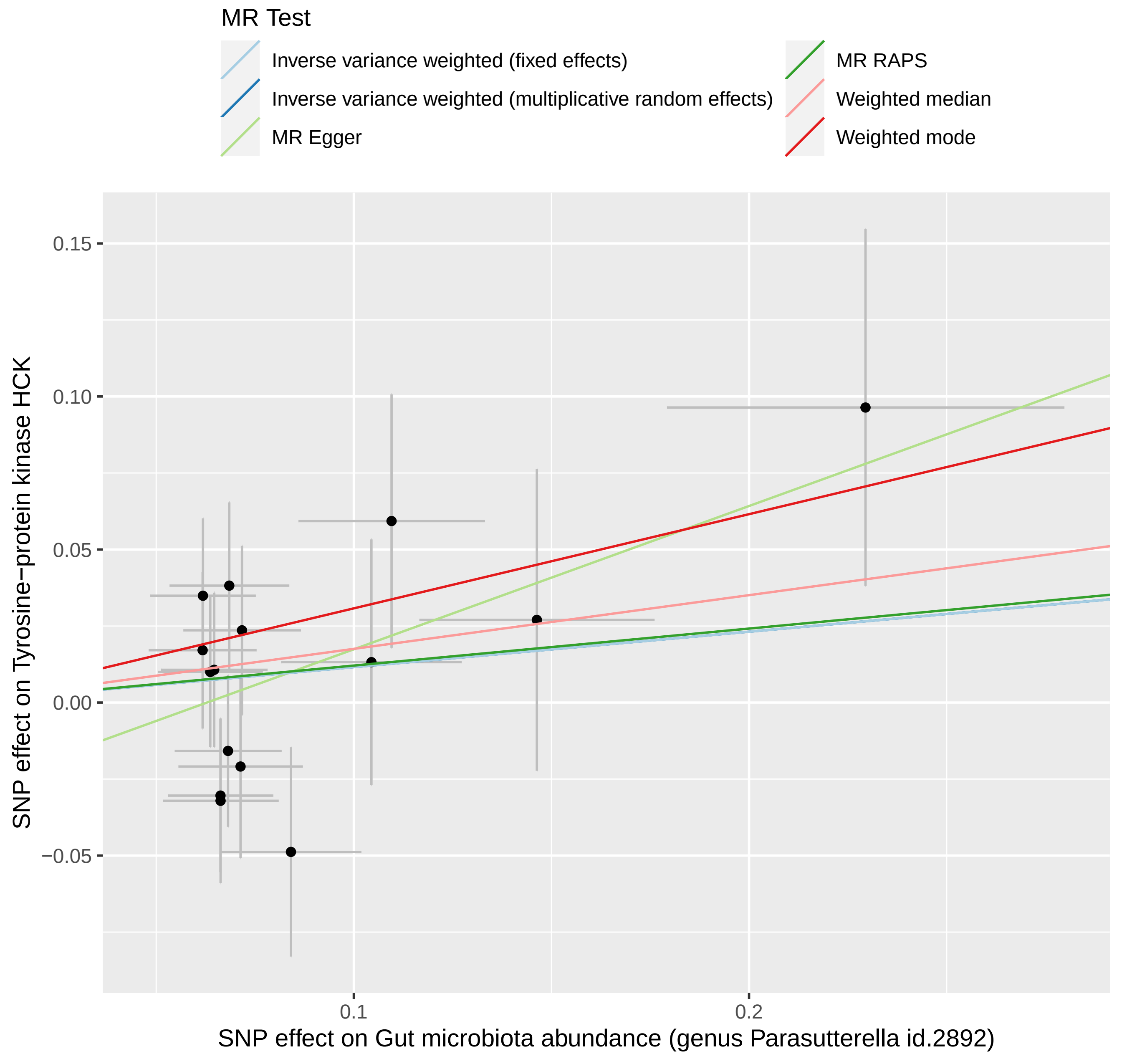

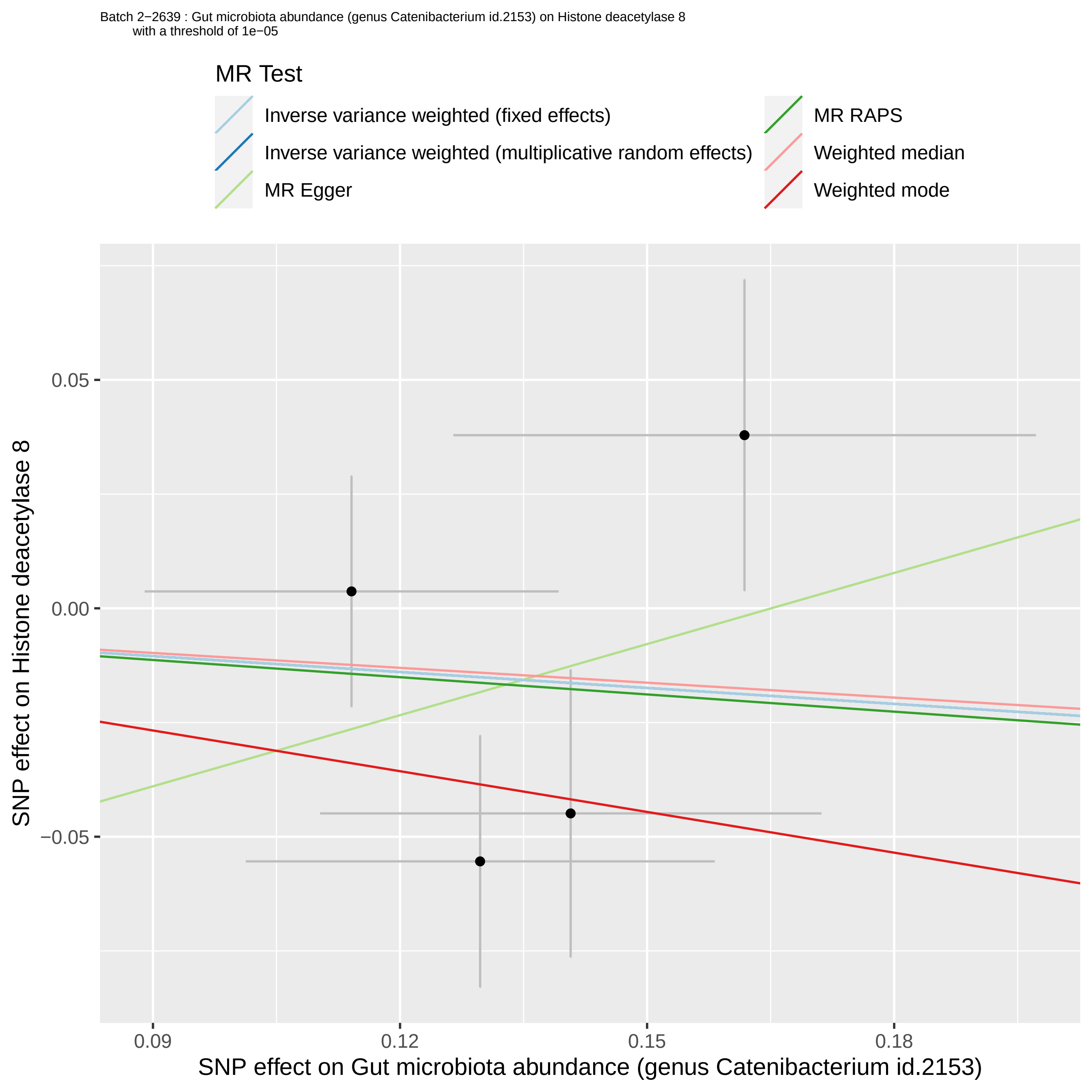

Batch 2−2639 : Gut microbiota abundance (genus Catenibacterium id.2153) on Histone deacetylase 8
with a threshold of 1e−05
MR Test
Inverse variance weighted (fixed effects)
Inverse variance weighted (multiplicative random effects)
MR Egger
MR RAPS
Weighted median
Weighted mode
0.05
0.00
−0.05
SNP effect on Histone deacetylase 8
0.09
0.12
0.15
0.18
SNP effect on Gut microbiota abundance (genus Catenibacterium id.2153)

Batch 2−2640 : Gut microbiota abundance (genus Parasutterella id.2892) on Histone deacetylase 8
with a threshold of 1e−05

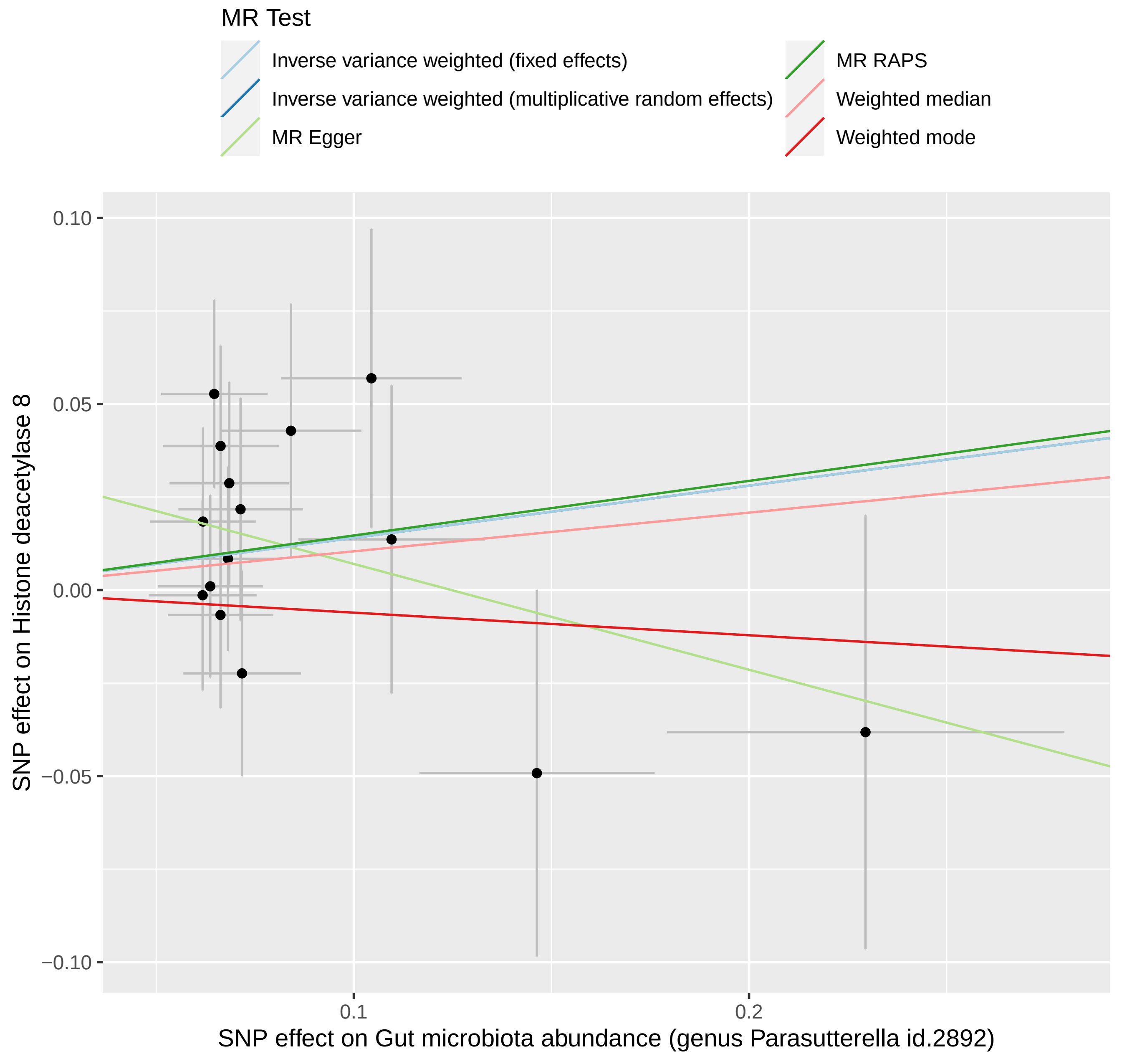

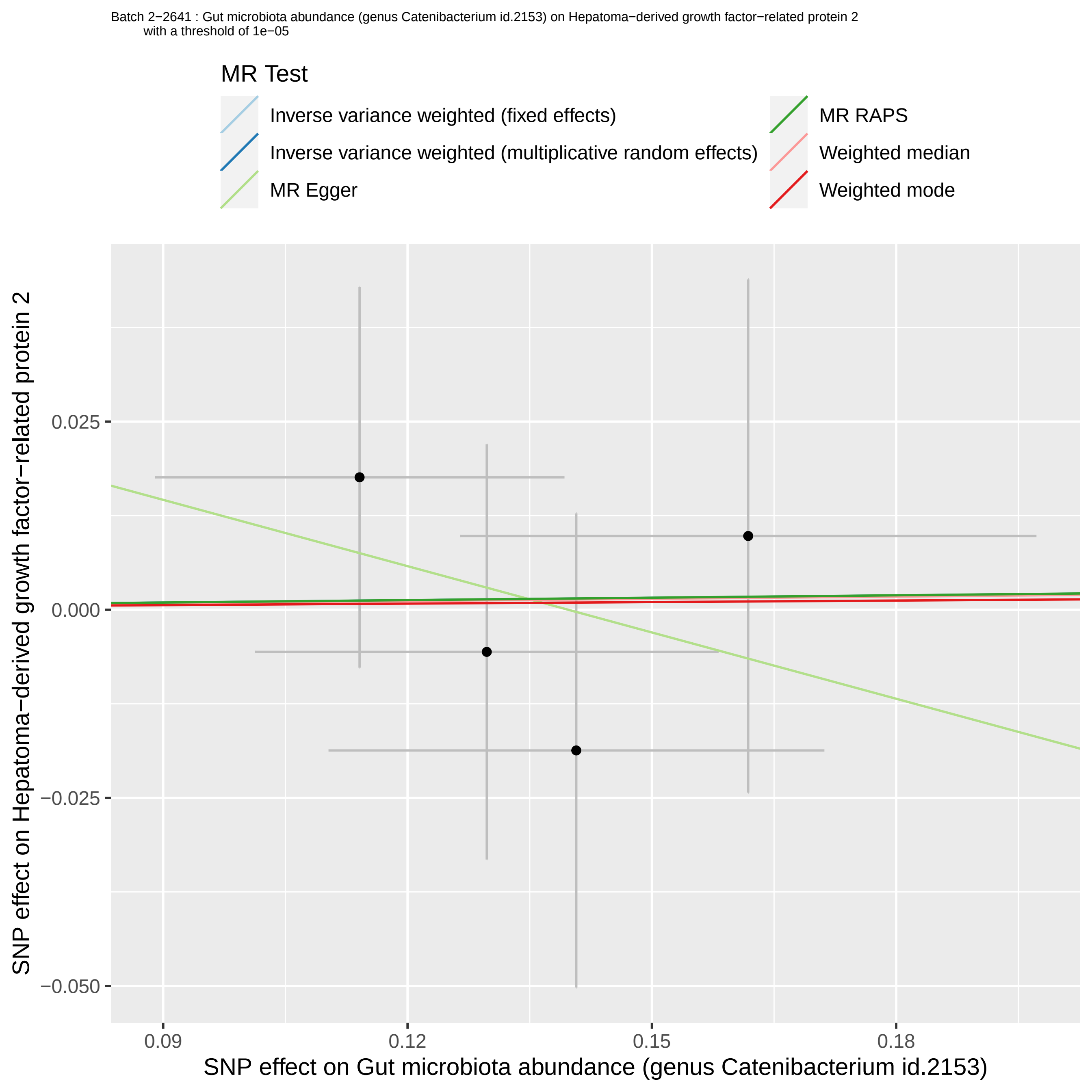
Batch 2−2641 : Gut microbiota abundance (genus Catenibacterium id.2153) on Hepatoma−derived growth factor−related protein 2
with a threshold of 1e−05
MR Test
Inverse variance weighted (fixed effects)
Inverse variance weighted (multiplicative random effects)
MR Egger
MR RAPS
Weighted median
Weighted mode
0.025
0.000
−0.025
−0.050
0.09
0.12
0.15
0.18
SNP effect on Gut microbiota abundance (genus Catenibacterium id.2153)
SNP effect on Hepatoma−derived growth factor−related protein 2

Batch 2−2642 : Gut microbiota abundance (genus Parasutterella id.2892) on Hepatoma−derived growth factor−related protein 2 with a threshold of 1e−05

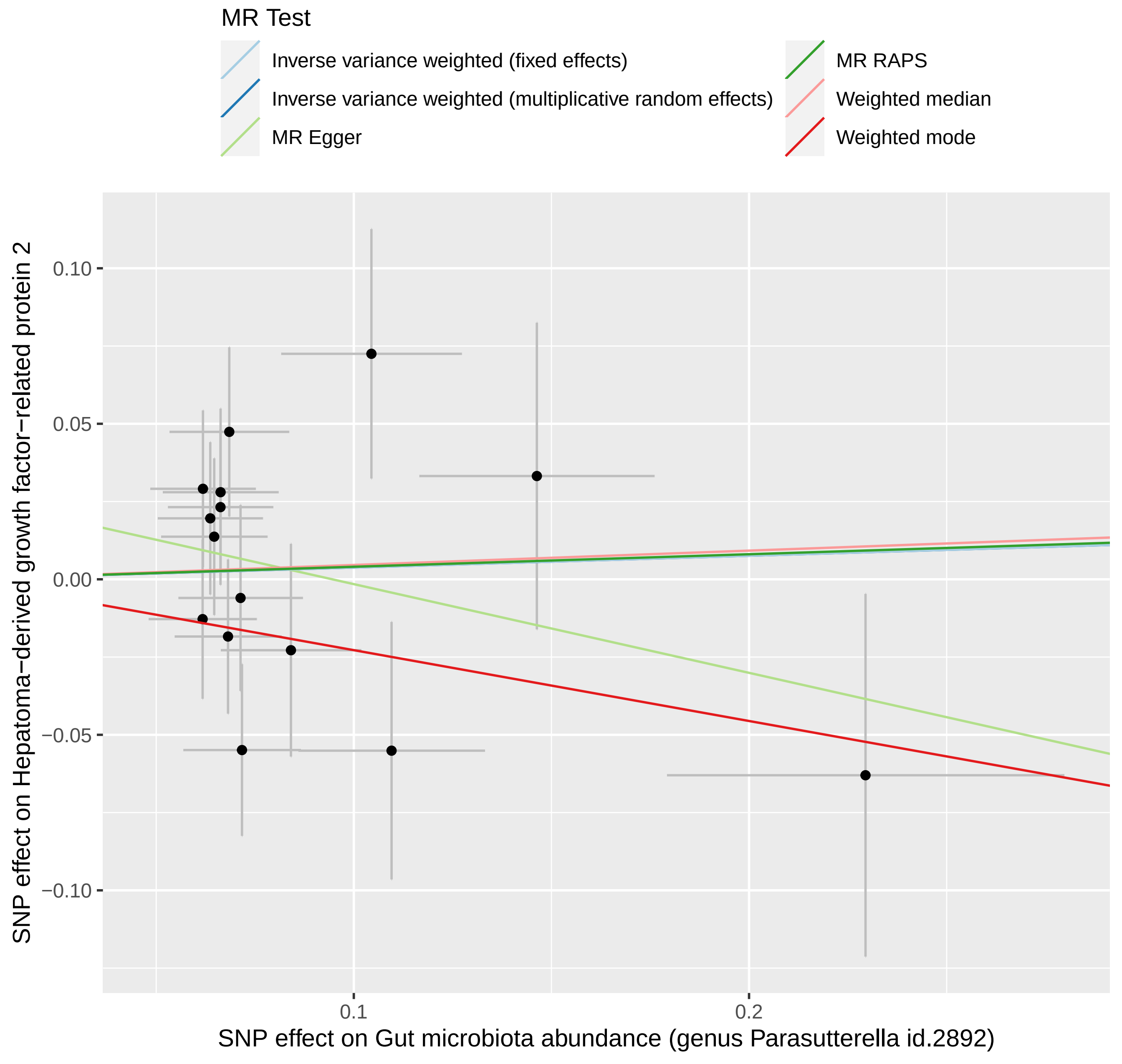

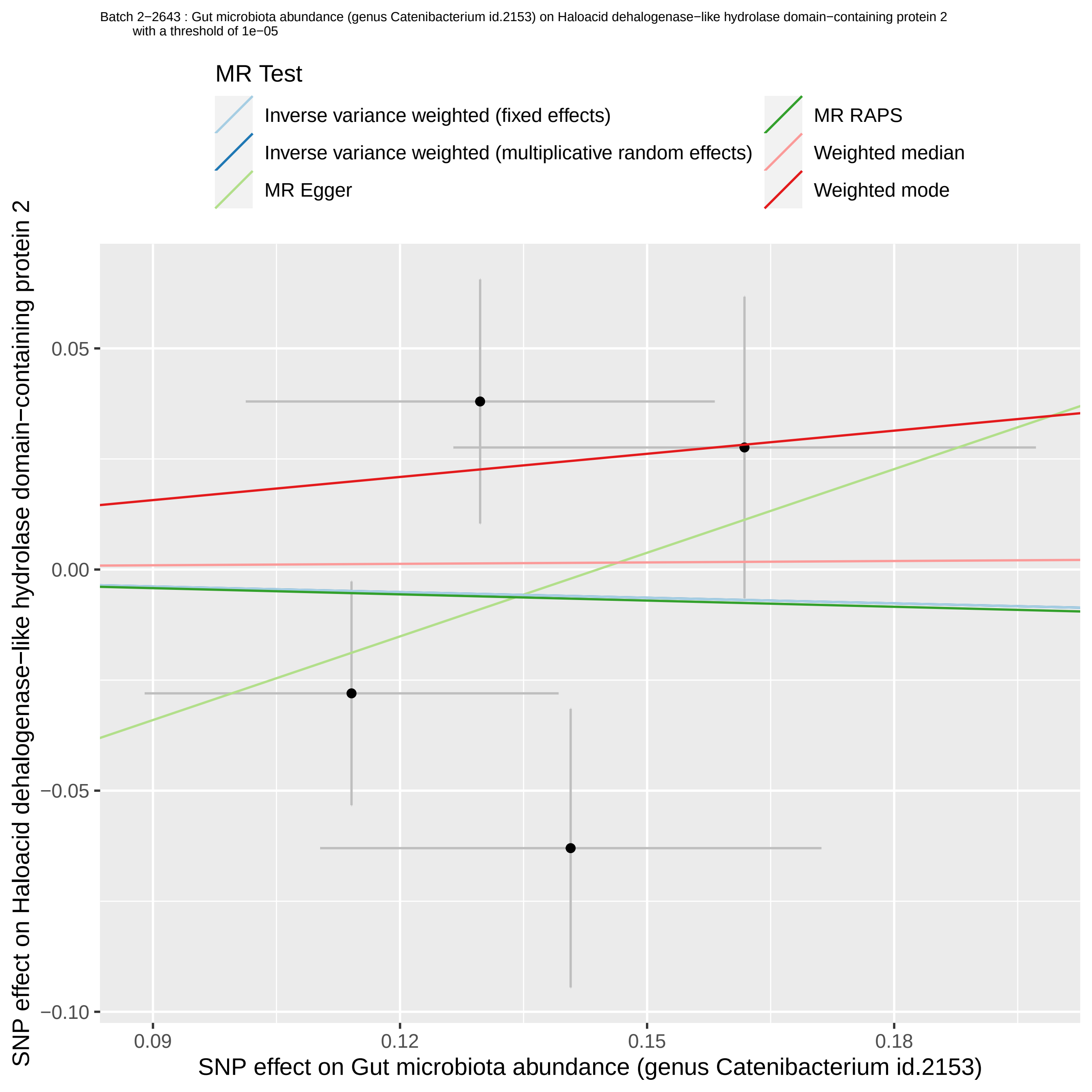

Batch 2−2643 : Gut microbiota abundance (genus Catenibacterium id.2153) on Haloacid dehalogenase−like hydrolase domain−containing protein 2
with a threshold of 1e−05
MR Test
Inverse variance weighted (fixed effects)
Inverse variance weighted (multiplicative random effects)
MR Egger
MR RAPS
Weighted median
Weighted mode
SNP effect on Haloacid dehalogenase−like hydrolase domain−containing protein 2
0.05
0.00
−0.05
−0.10
0.09
0.12
0.15
0.18
SNP effect on Gut microbiota abundance (genus Catenibacterium id.2153)

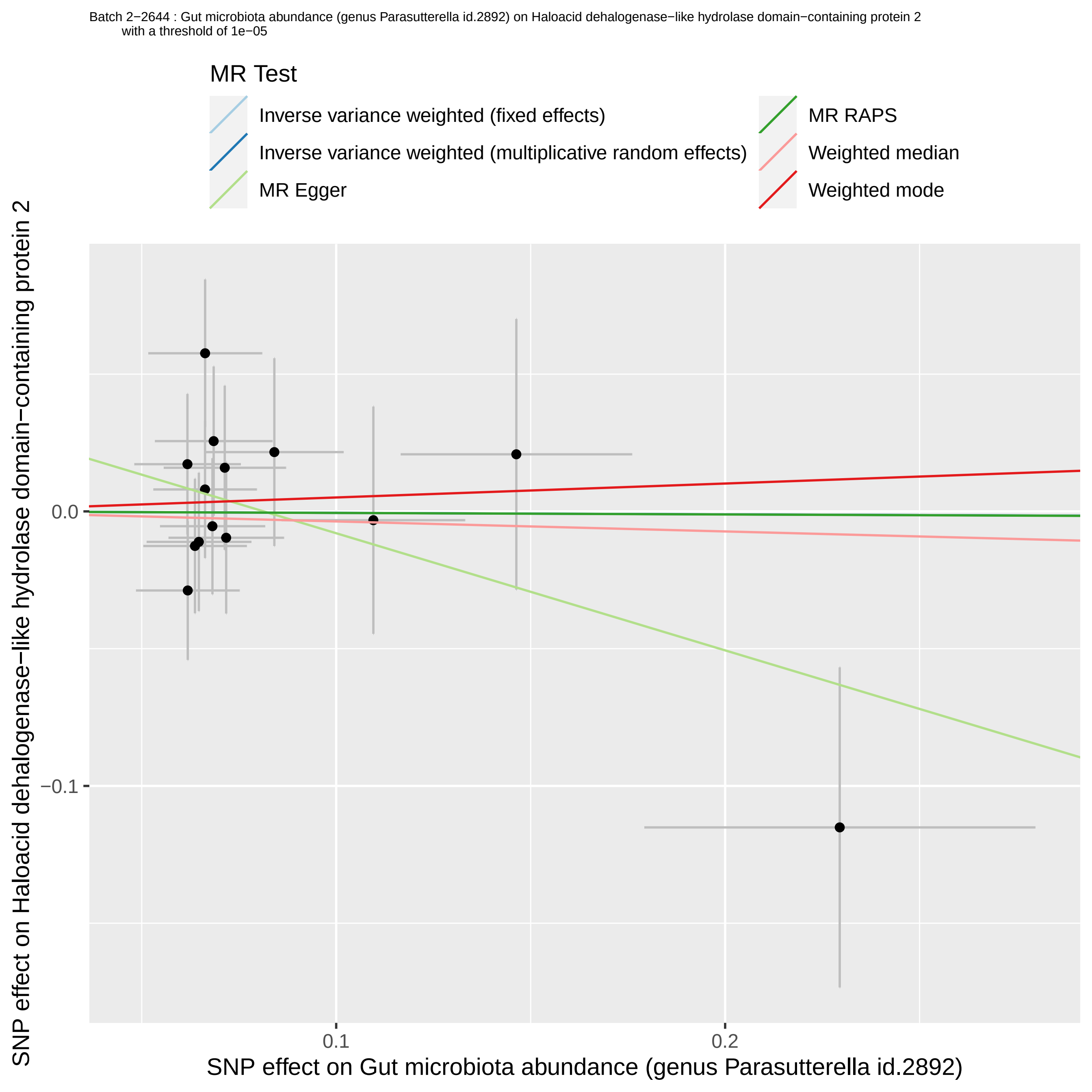

Batch 2−2644 : Gut microbiota abundance (genus Parasutterella id.2892) on Haloacid dehalogenase−like hydrolase domain−containing protein 2
with a threshold of 1e−05
MR Test
Inverse variance weighted (fixed effects)
Inverse variance weighted (multiplicative random effects)
MR Egger
MR RAPS
Weighted median
Weighted mode
SNP effect on Haloacid dehalogenase−like hydrolase domain−containing protein 2
0.0
−0.1
0.1
0.2
SNP effect on Gut microbiota abundance (genus Parasutterella id.2892)

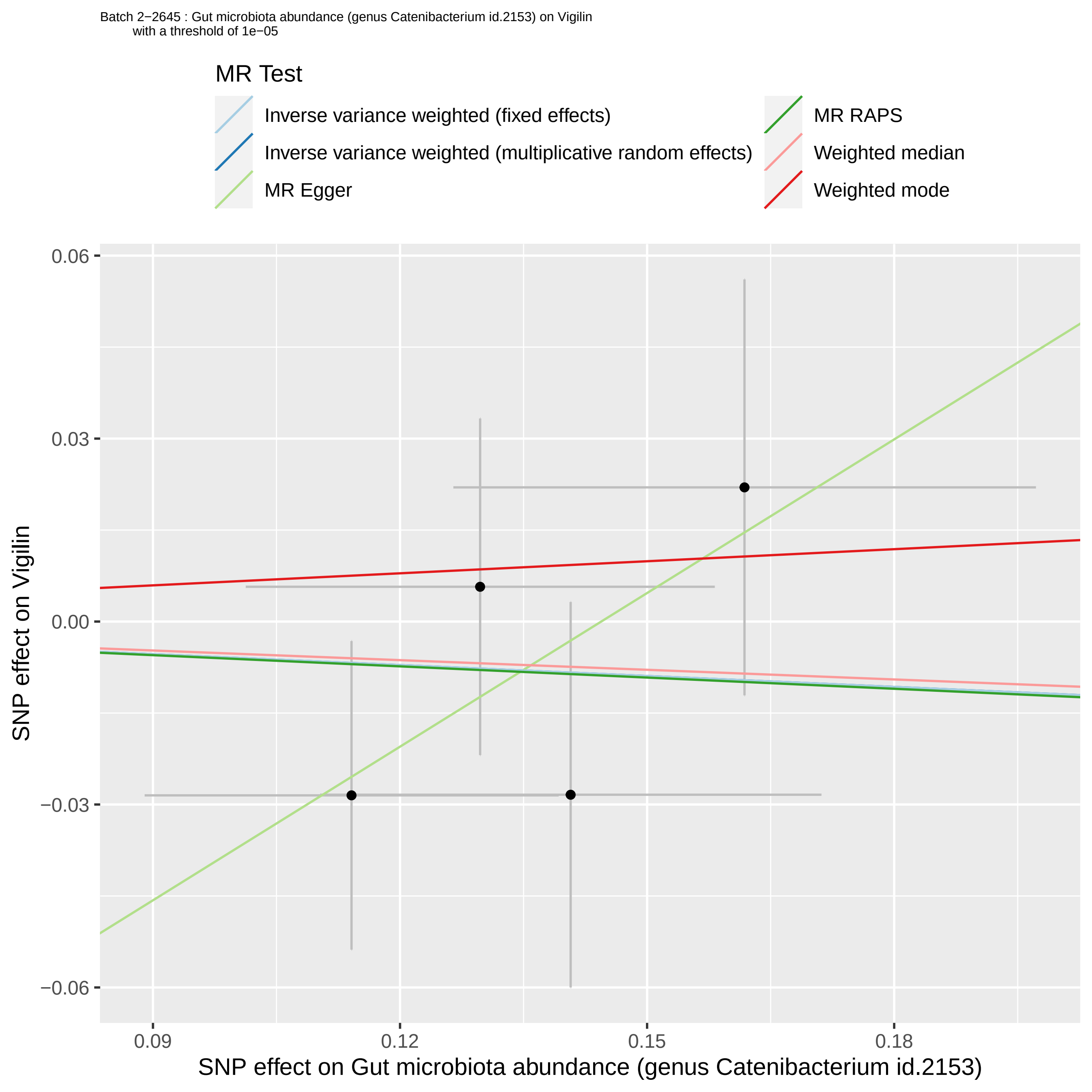
Batch 2−2645 : Gut microbiota abundance (genus Catenibacterium id.2153) on Vigilin
with a threshold of 1e−05
MR Test
Inverse variance weighted (fixed effects)
Inverse variance weighted (multiplicative random effects)
MR Egger
MR RAPS
Weighted median
Weighted mode
0.06
0.03
0.00
−0.03
−0.06
0.09
0.12
0.15
0.18
SNP effect on Vigilin
SNP effect on Gut microbiota abundance (genus Catenibacterium id.2153)

Batch 2−2646 : Gut microbiota abundance (genus Parasutterella id.2892) on Vigilin
with a threshold of 1e−05

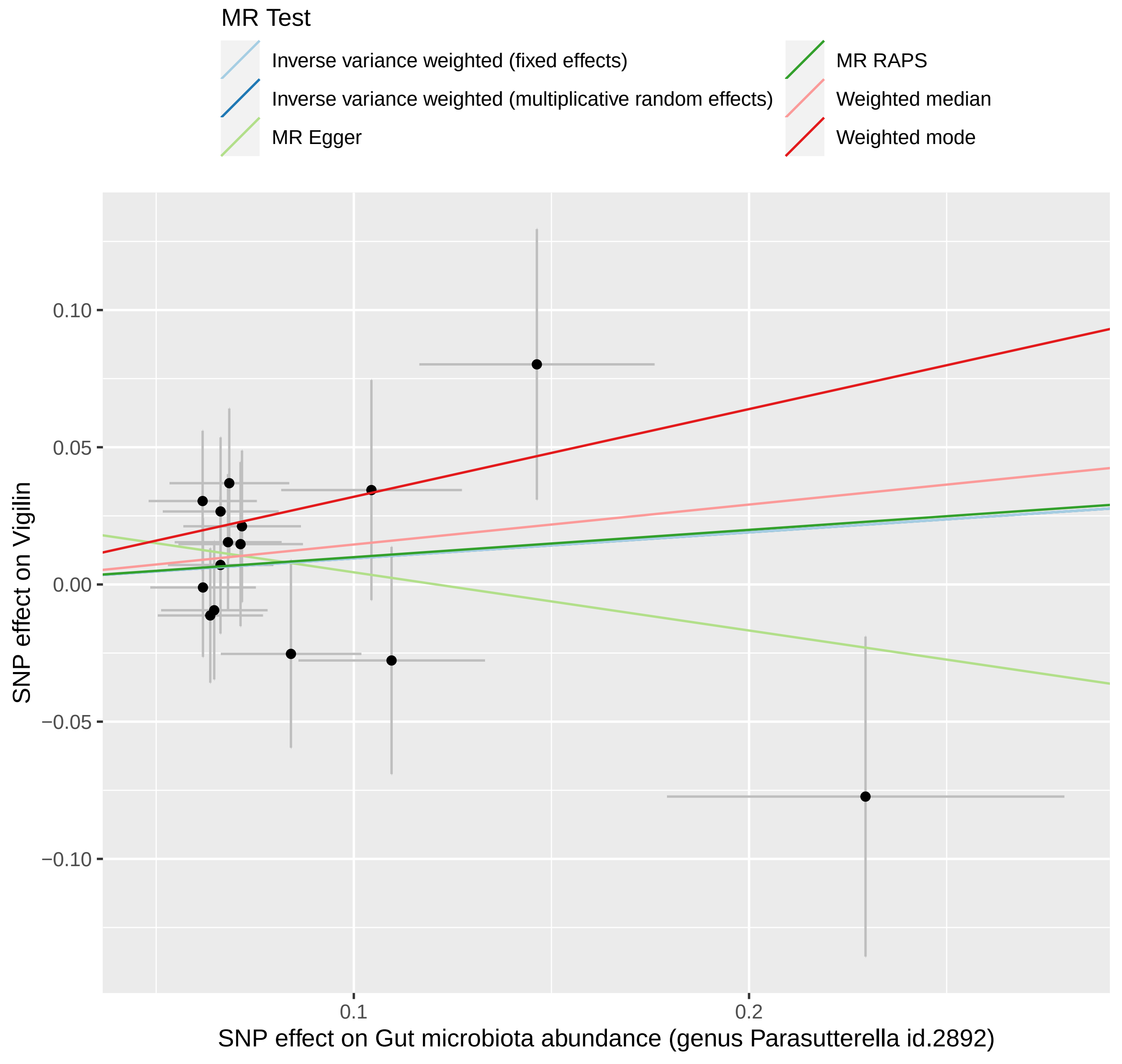

Batch 2−2647 : Gut microbiota abundance (genus Catenibacterium id.2153) on E3 ubiquitin−protein ligase HECW1
with a threshold of 1e−05

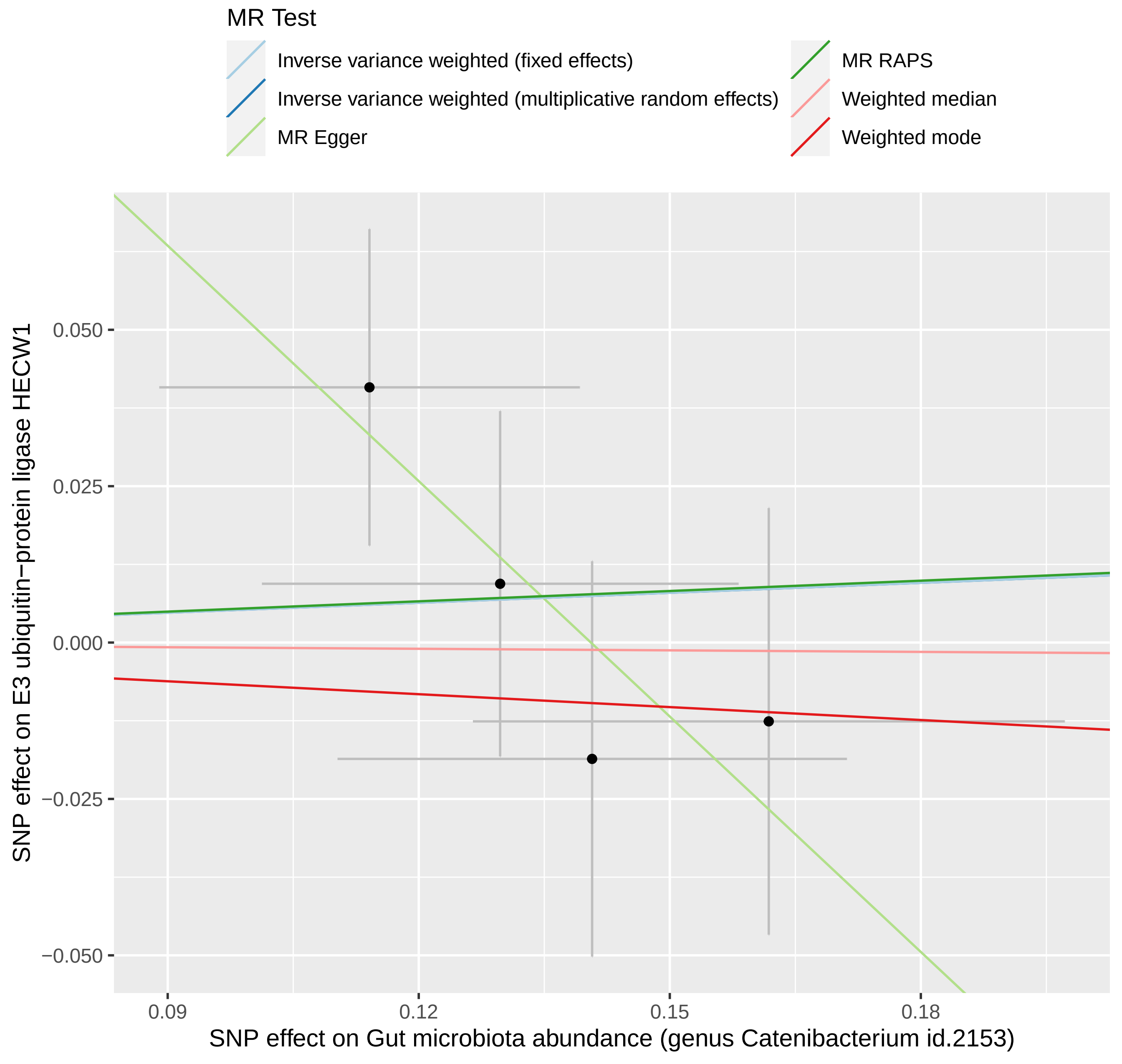

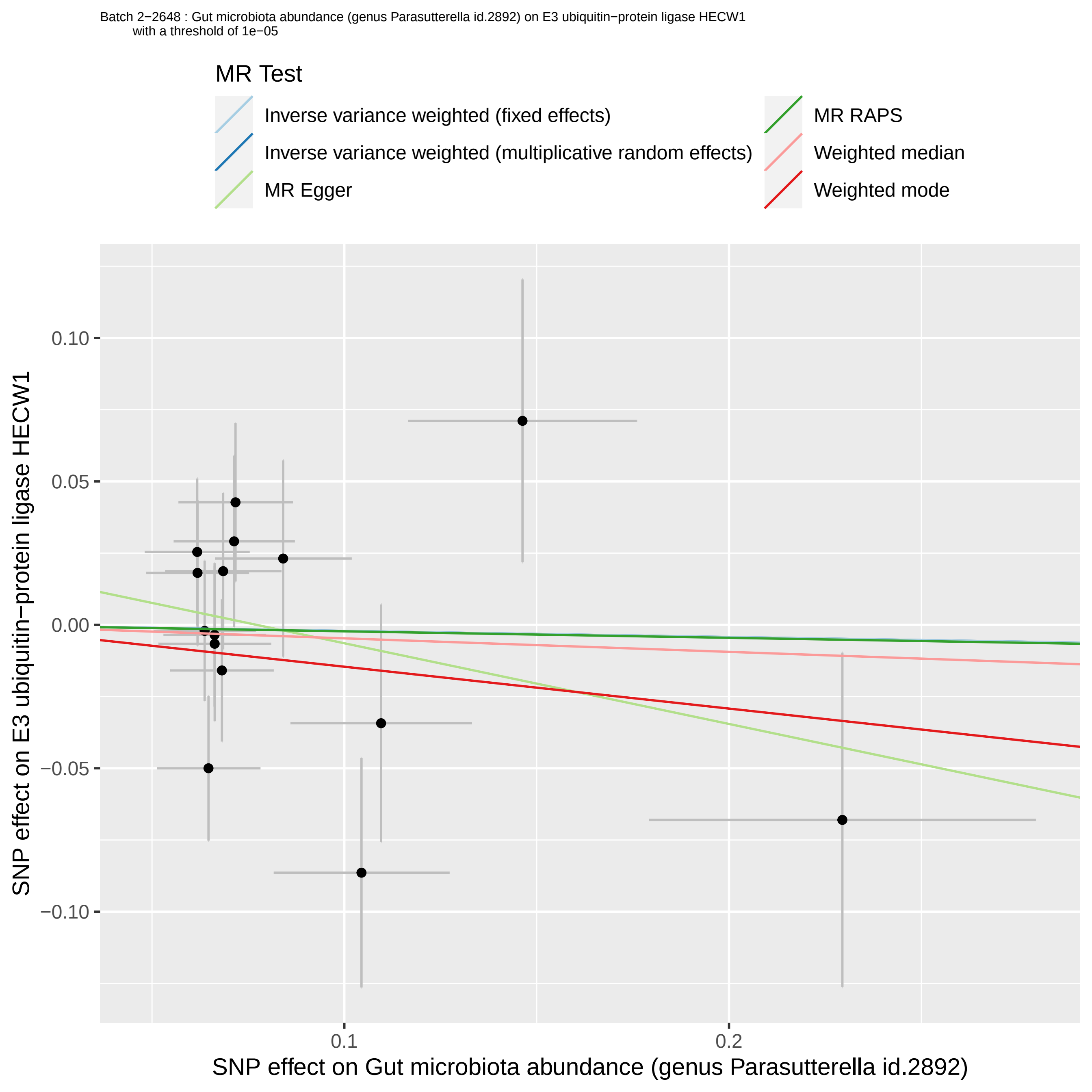
Batch 2−2648 : Gut microbiota abundance (genus Parasutterella id.2892) on E3 ubiquitin−protein ligase HECW1
with a threshold of 1e−05
MR Test
Inverse variance weighted (fixed effects)
Inverse variance weighted (multiplicative random effects)
MR Egger
MR RAPS
Weighted median
Weighted mode
0.10
0.05
0.00
−0.05
−0.10
0.1
0.2
SNP effect on E3 ubiquitin−protein ligase HECW1
SNP effect on Gut microbiota abundance (genus Parasutterella id.2892)

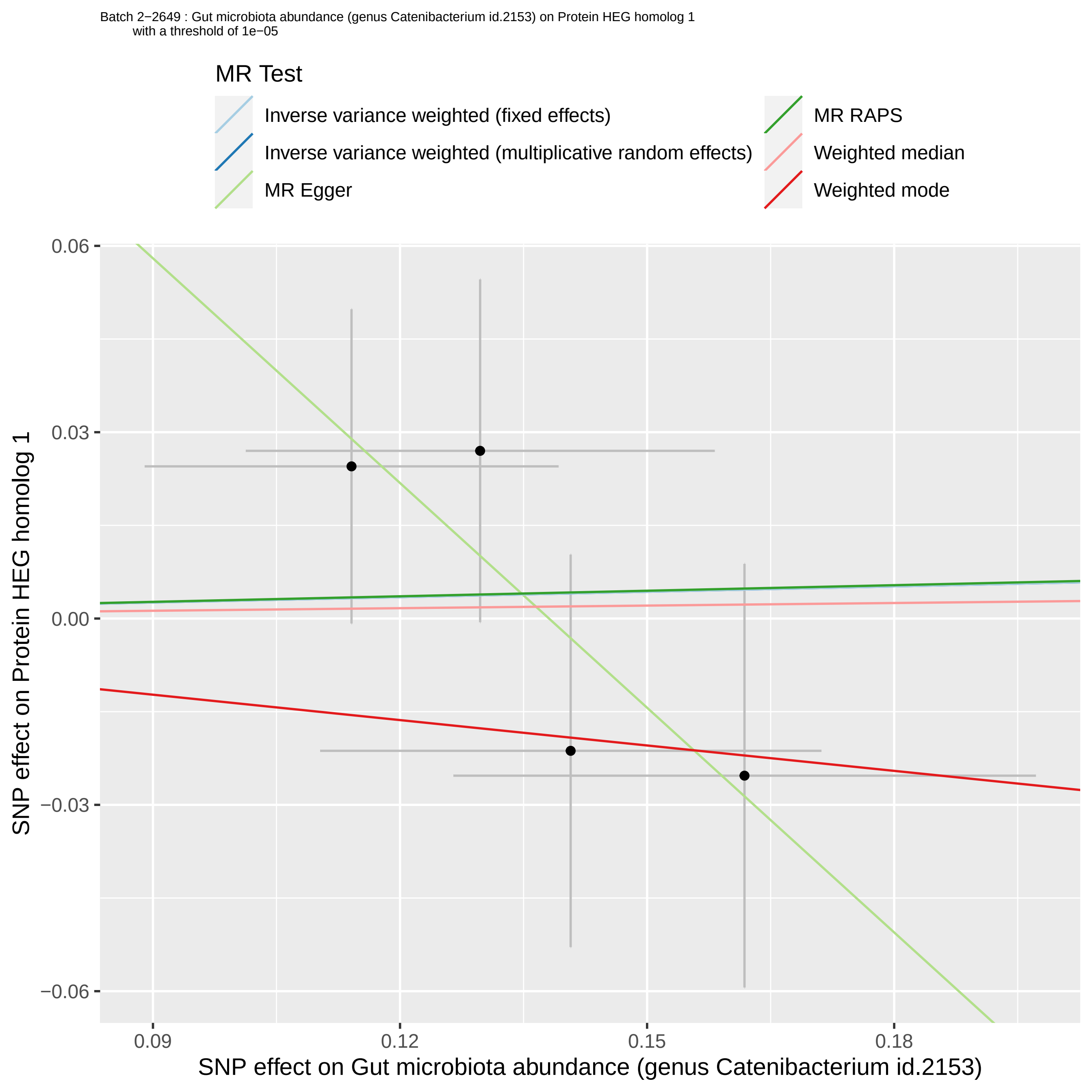
Batch 2−2649 : Gut microbiota abundance (genus Catenibacterium id.2153) on Protein HEG homolog 1
with a threshold of 1e−05
MR Test
Inverse variance weighted (fixed effects)
Inverse variance weighted (multiplicative random effects)
MR Egger
MR RAPS
Weighted median
Weighted mode
0.06
0.03
0.00
−0.03
−0.06
0.09
0.12
0.15
0.18
SNP effect on Protein HEG homolog 1
SNP effect on Gut microbiota abundance (genus Catenibacterium id.2153)

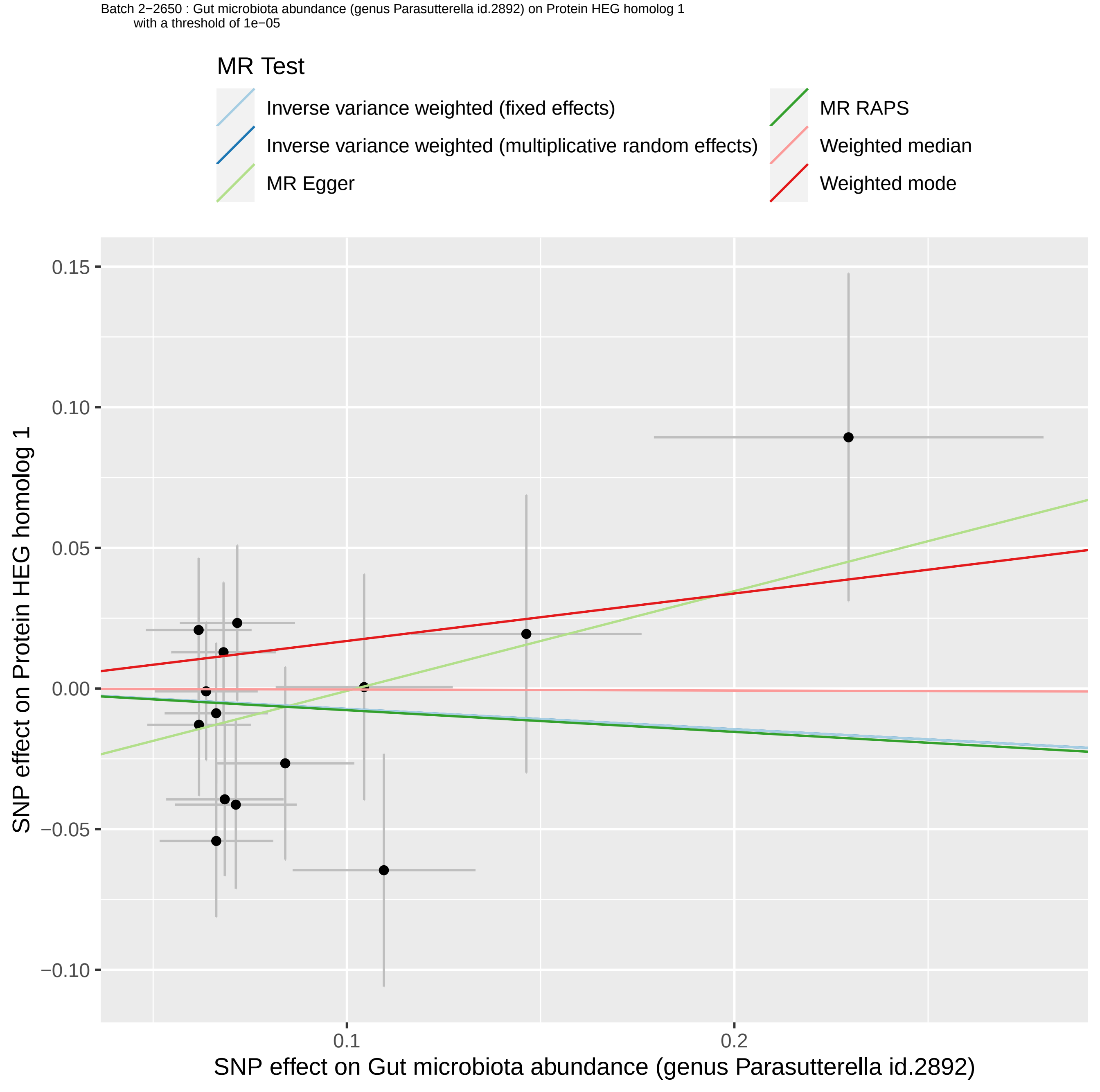
Batch 2−2650 : Gut microbiota abundance (genus Parasutterella id.2892) on Protein HEG homolog 1
with a threshold of 1e−05
MR Test
Inverse variance weighted (fixed effects)
Inverse variance weighted (multiplicative random effects)
MR Egger
MR RAPS
Weighted median
Weighted mode
0.15
0.10
0.05
0.00
−0.05
−0.10
0.1
0.2
SNP effect on Protein HEG homolog 1
SNP effect on Gut microbiota abundance (genus Parasutterella id.2892)

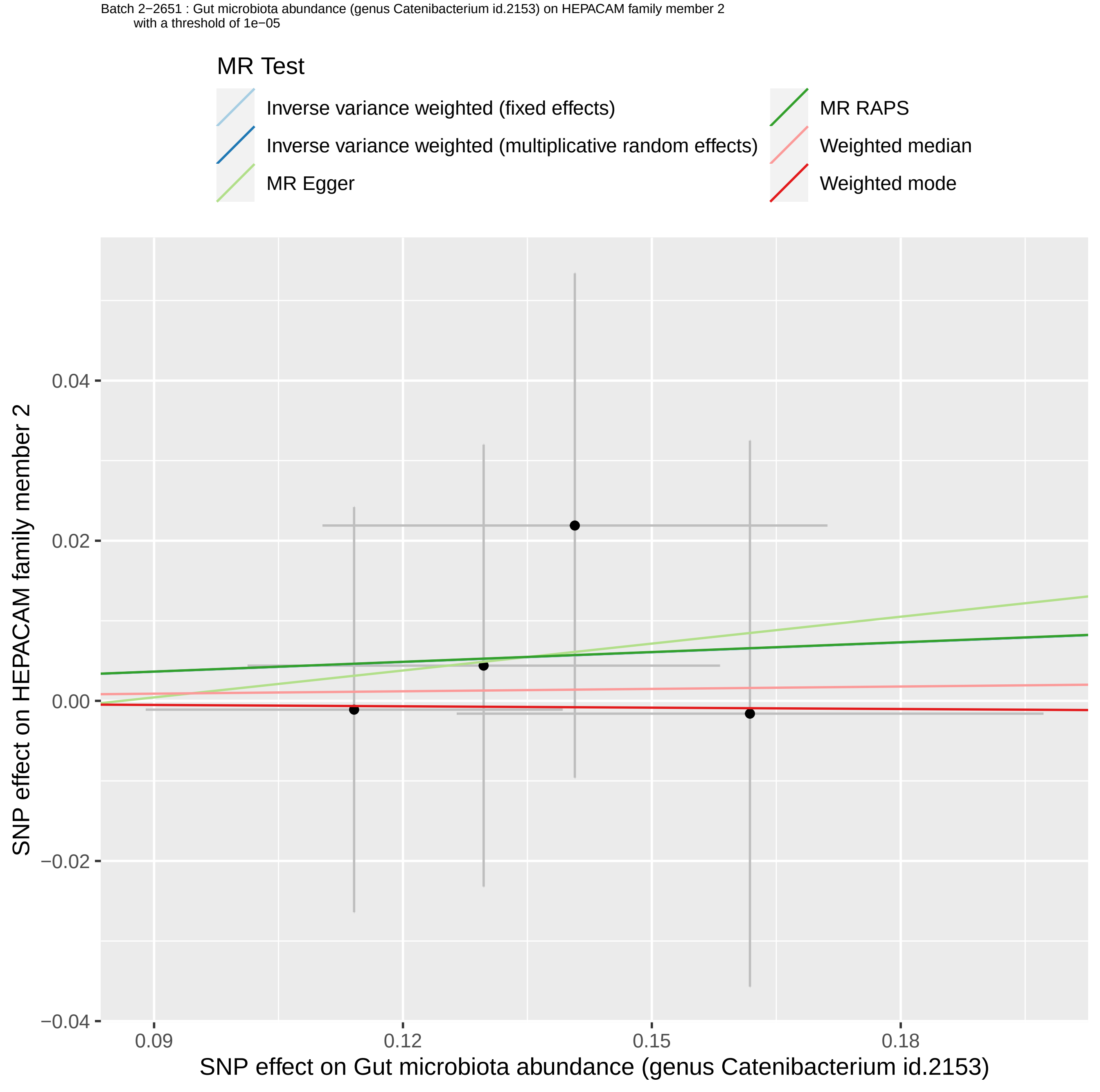
Batch 2−2651 : Gut microbiota abundance (genus Catenibacterium id.2153) on HEPACAM family member 2
with a threshold of 1e−05
MR Test
Inverse variance weighted (fixed effects)
Inverse variance weighted (multiplicative random effects)
MR Egger
MR RAPS
Weighted median
Weighted mode
SNP effect on HEPACAM family member 2
0.04
0.02
0.00
−0.02
−0.04
0.09
0.12
0.15
0.18
SNP effect on Gut microbiota abundance (genus Catenibacterium id.2153)

Batch 2−2652 : Gut microbiota abundance (genus Parasutterella id.2892) on HEPACAM family member 2
with a threshold of 1e−05

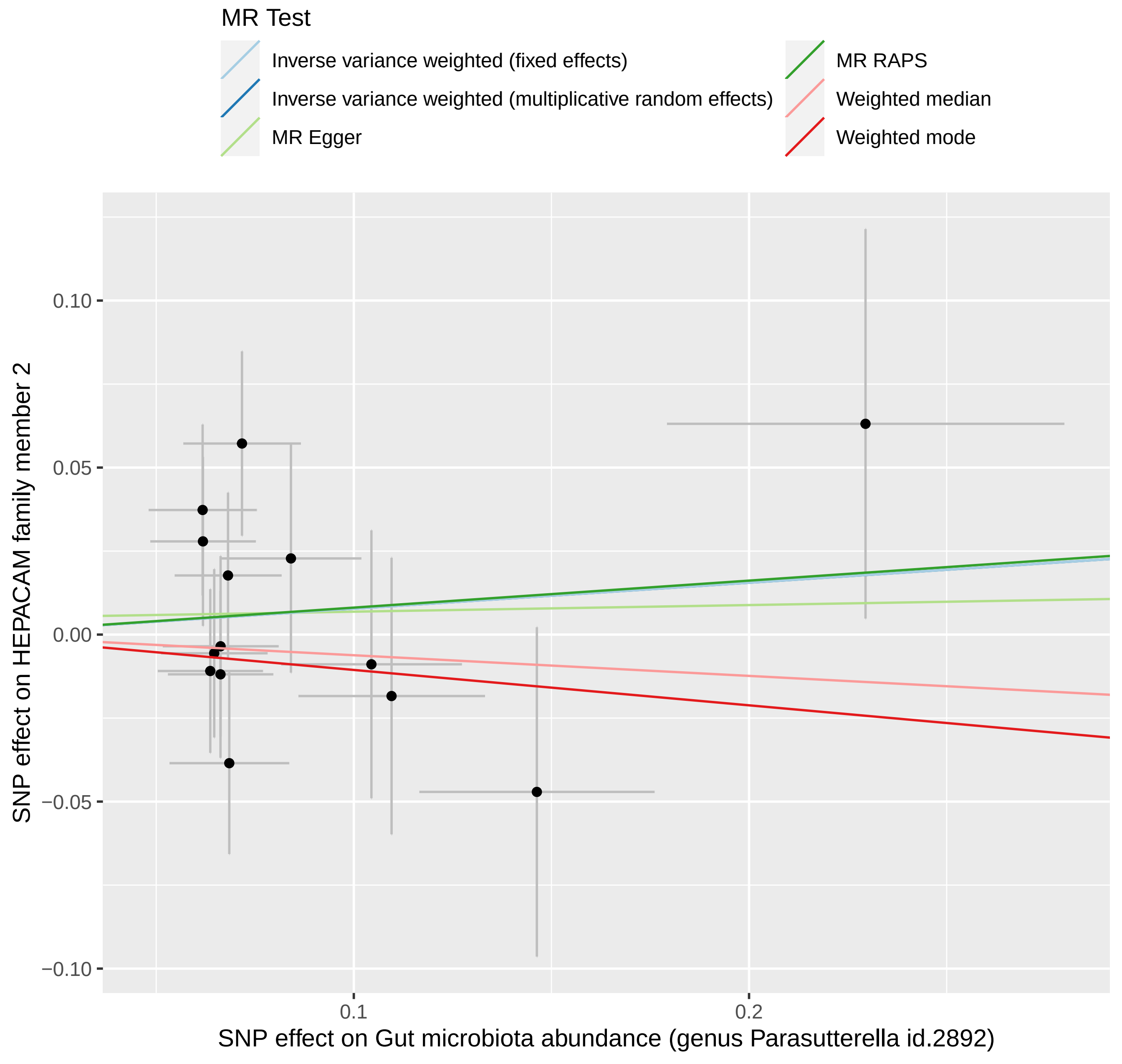

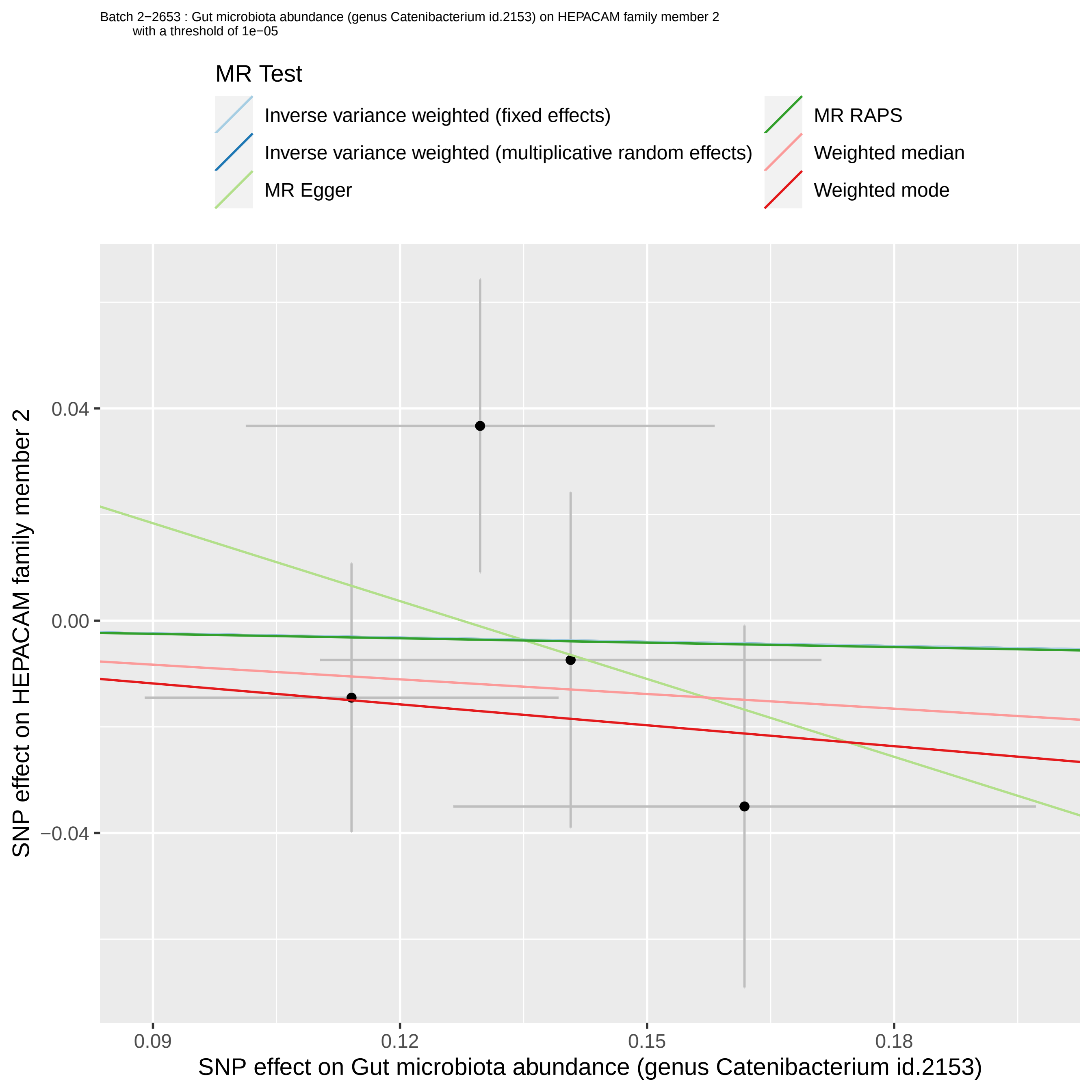

Batch 2−2653 : Gut microbiota abundance (genus Catenibacterium id.2153) on HEPACAM family member 2
with a threshold of 1e−05
MR Test
Inverse variance weighted (fixed effects)
Inverse variance weighted (multiplicative random effects)
MR Egger
MR RAPS
Weighted median
Weighted mode
0.04
0.00
−0.04
0.09
0.12
0.15
0.18
SNP effect on HEPACAM family member 2
SNP effect on Gut microbiota abundance (genus Catenibacterium id.2153)

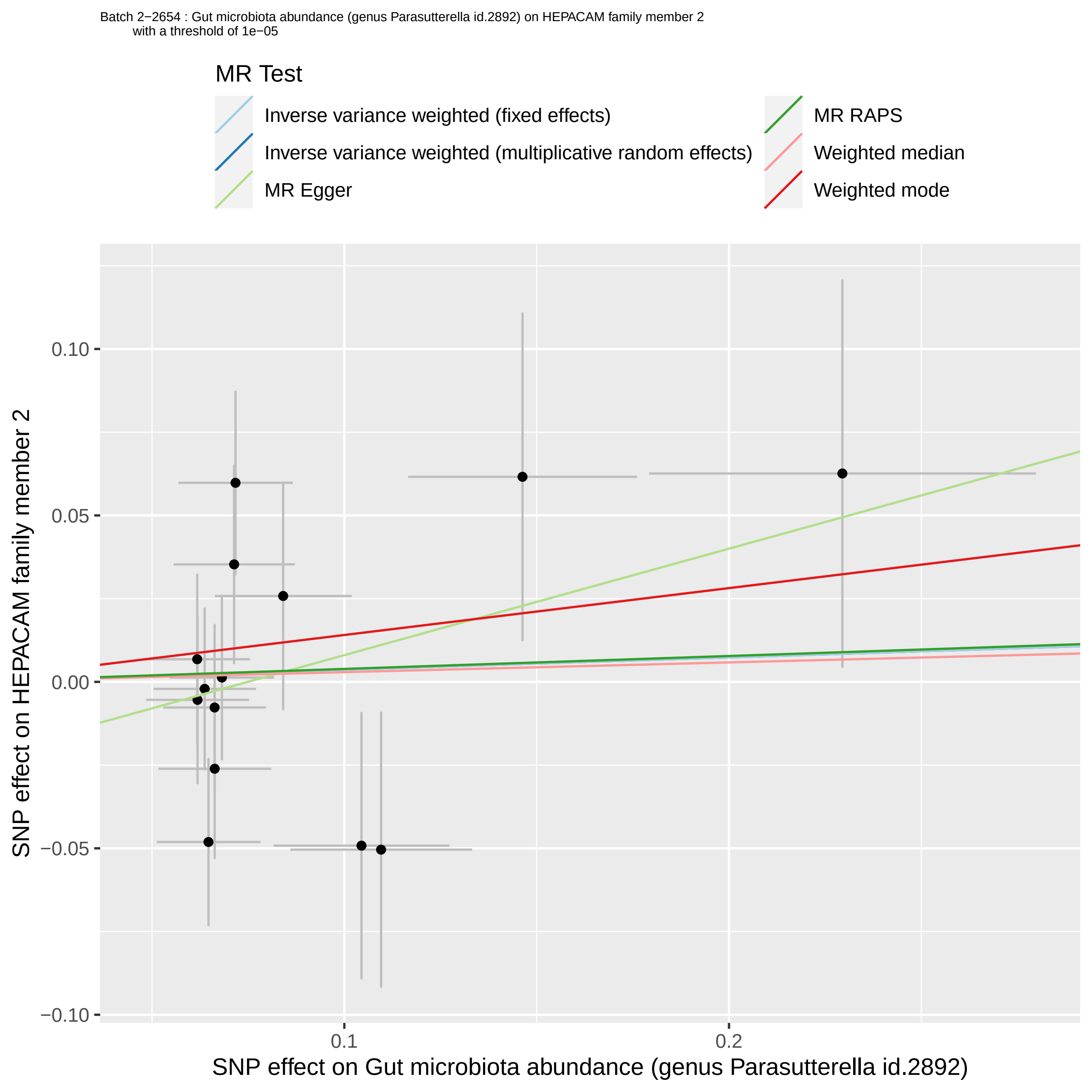
Batch 2−2654 : Gut microbiota abundance (genus Parasutterella id.2892) on HEPACAM family member 2
with a threshold of 1e−05
MR Test
Inverse variance weighted (fixed effects)
Inverse variance weighted (multiplicative random effects)
MR Egger
MR RAPS
Weighted median
Weighted mode
0.10
0.05
0.00
−0.05
−0.10
0.1
0.2
SNP effect on HEPACAM family member 2
SNP effect on Gut microbiota abundance (genus Parasutterella id.2892)

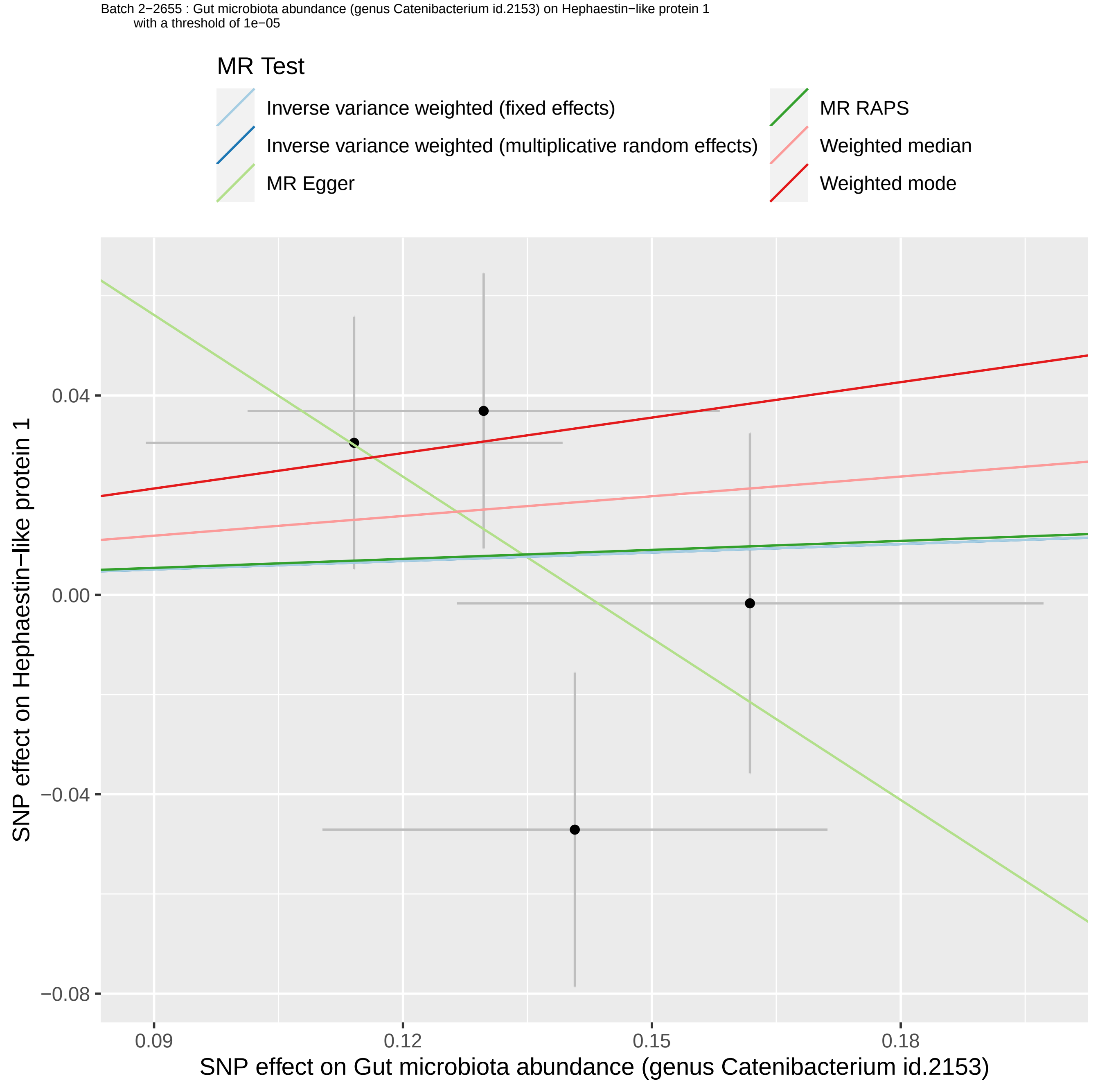

Batch 2−2655 : Gut microbiota abundance (genus Catenibacterium id.2153) on Hephaestin−like protein 1
with a threshold of 1e−05
MR Test
Inverse variance weighted (fixed effects)
Inverse variance weighted (multiplicative random effects)
MR Egger
MR RAPS
Weighted median
Weighted mode
0.04
0.00
−0.04
−0.08
0.09
0.12
0.15
0.18
SNP effect on Hephaestin−like protein 1
SNP effect on Gut microbiota abundance (genus Catenibacterium id.2153)

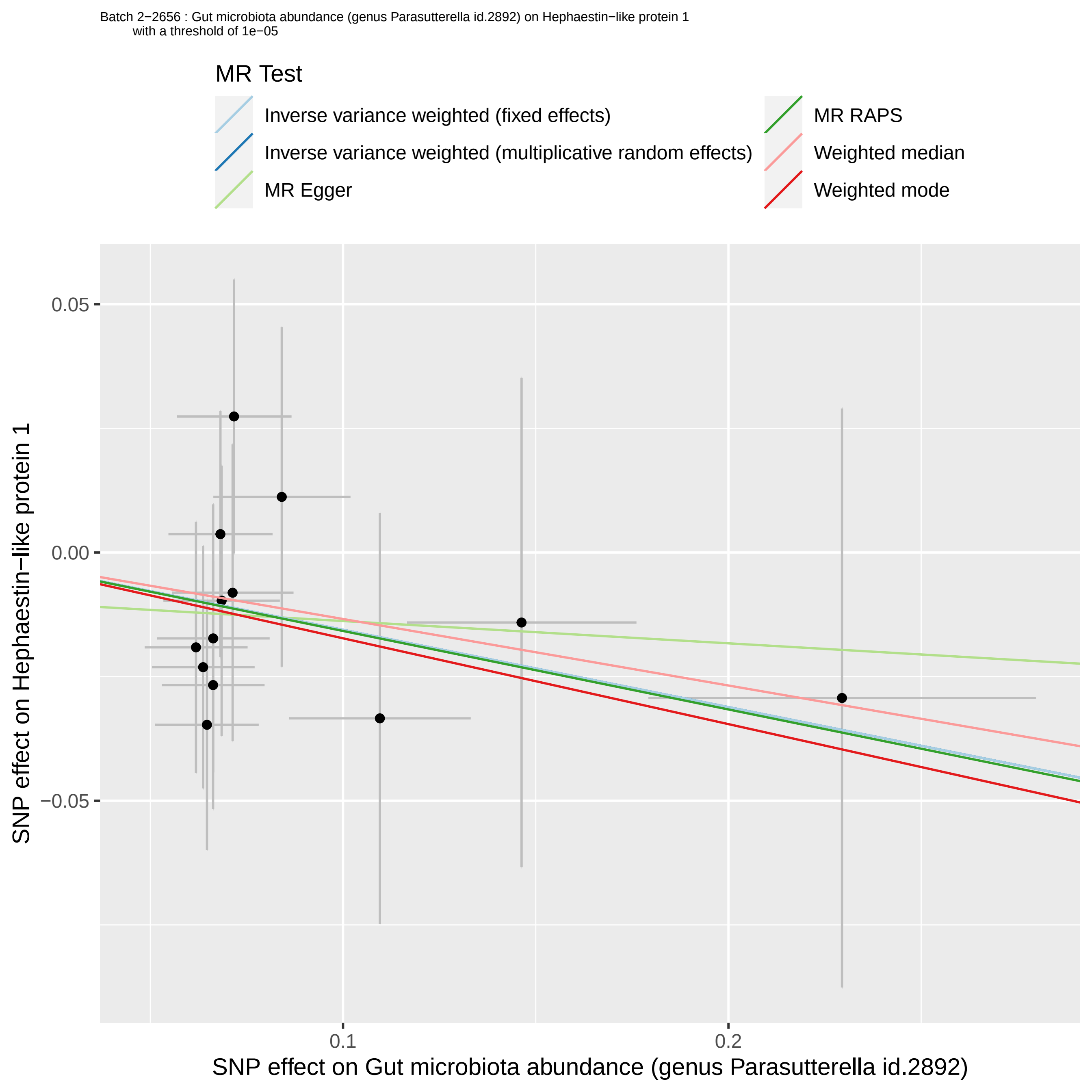

Batch 2−2656 : Gut microbiota abundance (genus Parasutterella id.2892) on Hephaestin−like protein 1
with a threshold of 1e−05
MR Test
Inverse variance weighted (fixed effects)
Inverse variance weighted (multiplicative random effects)
MR Egger
MR RAPS
Weighted median
Weighted mode
0.05
0.00
−0.05
0.1
0.2
SNP effect on Hephaestin−like protein 1
SNP effect on Gut microbiota abundance (genus Parasutterella id.2892)

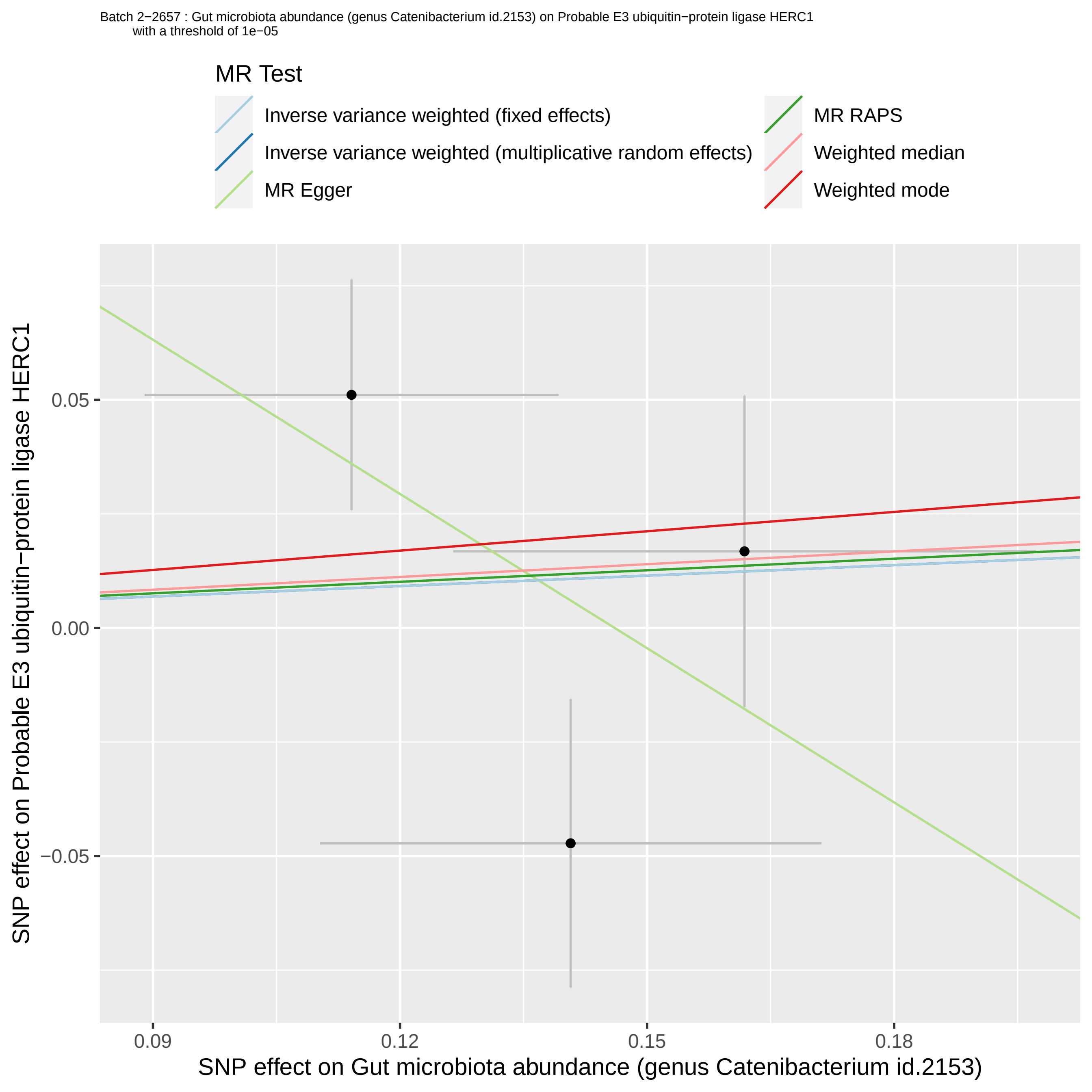
Batch 2−2657 : Gut microbiota abundance (genus Catenibacterium id.2153) on Probable E3 ubiquitin−protein ligase HERC1
with a threshold of 1e−05
MR Test
Inverse variance weighted (fixed effects)
Inverse variance weighted (multiplicative random effects)
MR Egger
MR RAPS
Weighted median
Weighted mode
0.05
0.00
−0.05
0.09
0.12
0.15
0.18
SNP effect on Probable E3 ubiquitin−protein ligase HERC1
SNP effect on Gut microbiota abundance (genus Catenibacterium id.2153)

Batch 2−2658 : Gut microbiota abundance (genus Parasutterella id.2892) on Probable E3 ubiquitin−protein ligase HERC1
with a threshold of 1e−05

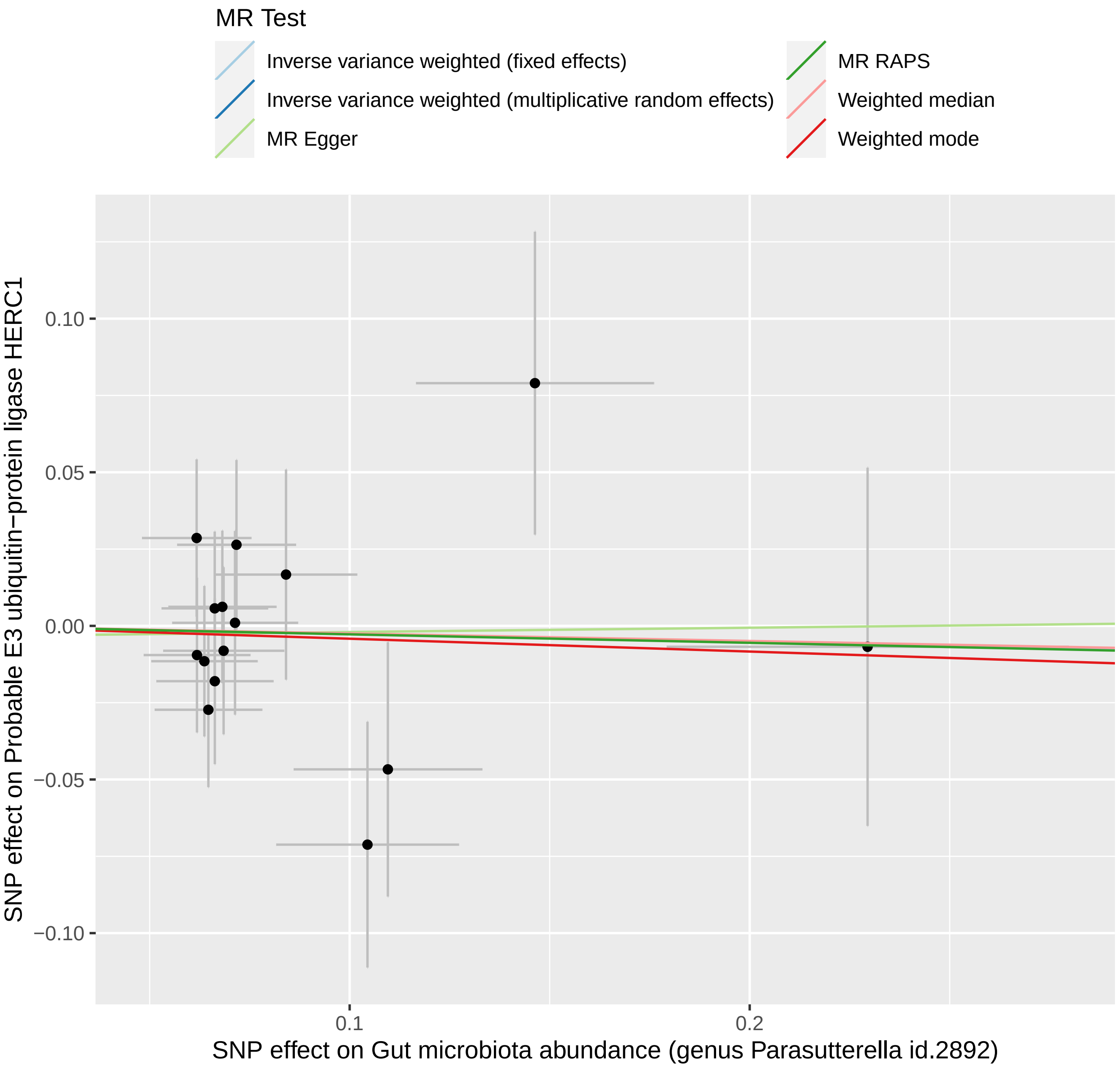

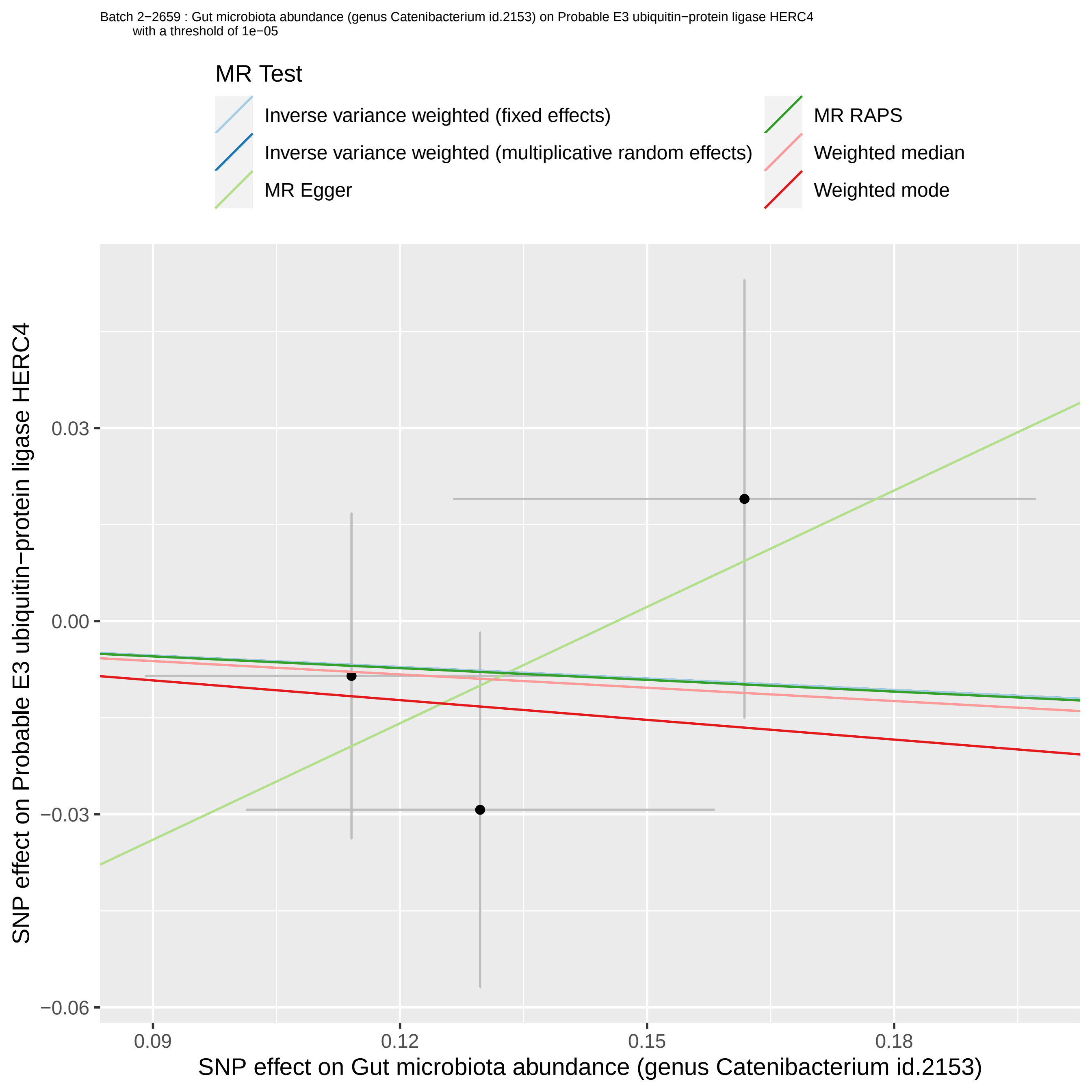
Batch 2−2659 : Gut microbiota abundance (genus Catenibacterium id.2153) on Probable E3 ubiquitin−protein ligase HERC4
with a threshold of 1e−05
MR Test
Inverse variance weighted (fixed effects)
Inverse variance weighted (multiplicative random effects)
MR Egger
MR RAPS
Weighted median
Weighted mode
0.03
0.00
−0.03
−0.06
0.09
0.12
0.15
0.18
SNP effect on Probable E3 ubiquitin−protein ligase HERC4
SNP effect on Gut microbiota abundance (genus Catenibacterium id.2153)

Batch 2−2660 : Gut microbiota abundance (genus Parasutterella id.2892) on Probable E3 ubiquitin−protein ligase HERC4 with a threshold of 1e−05

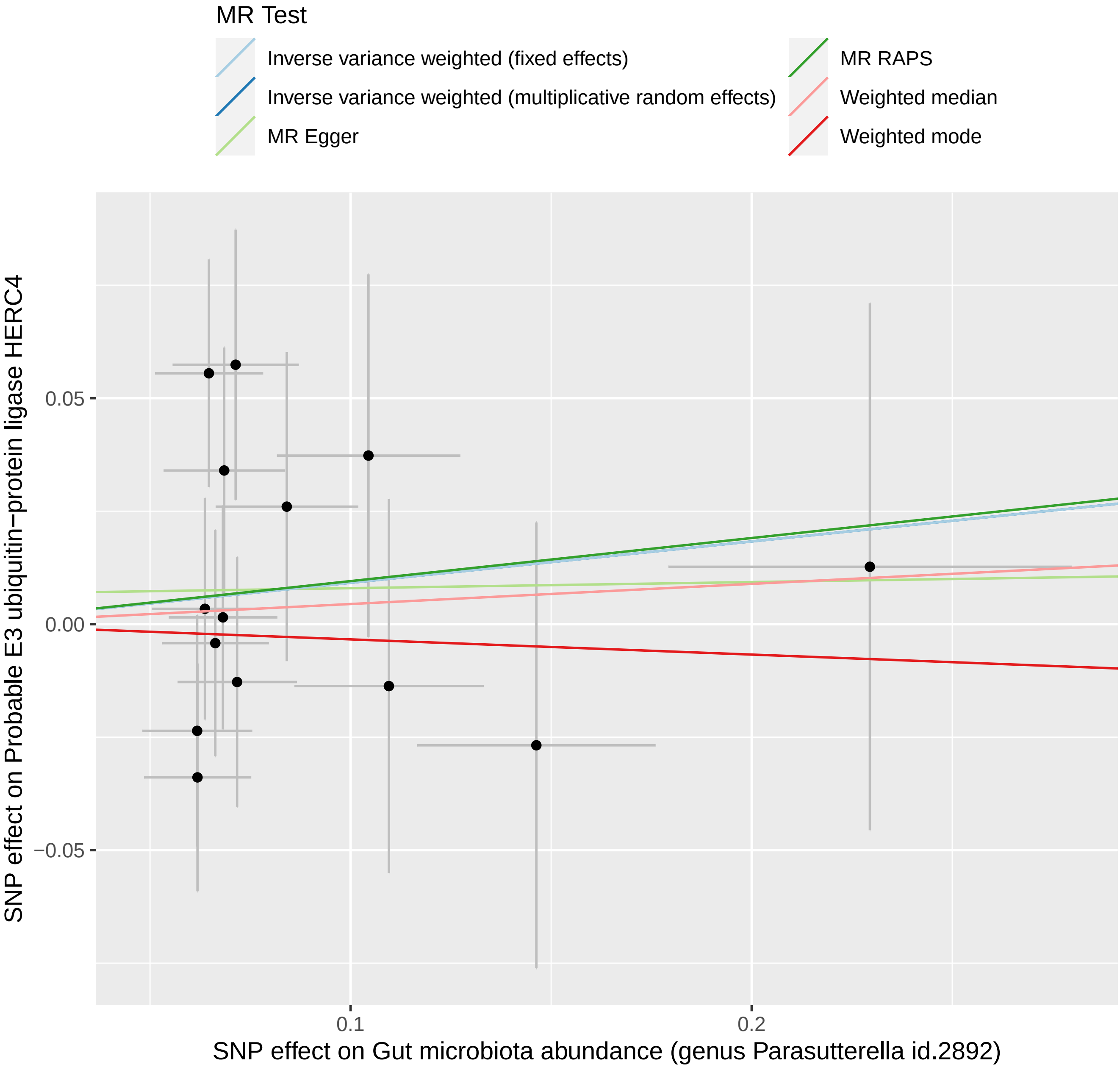

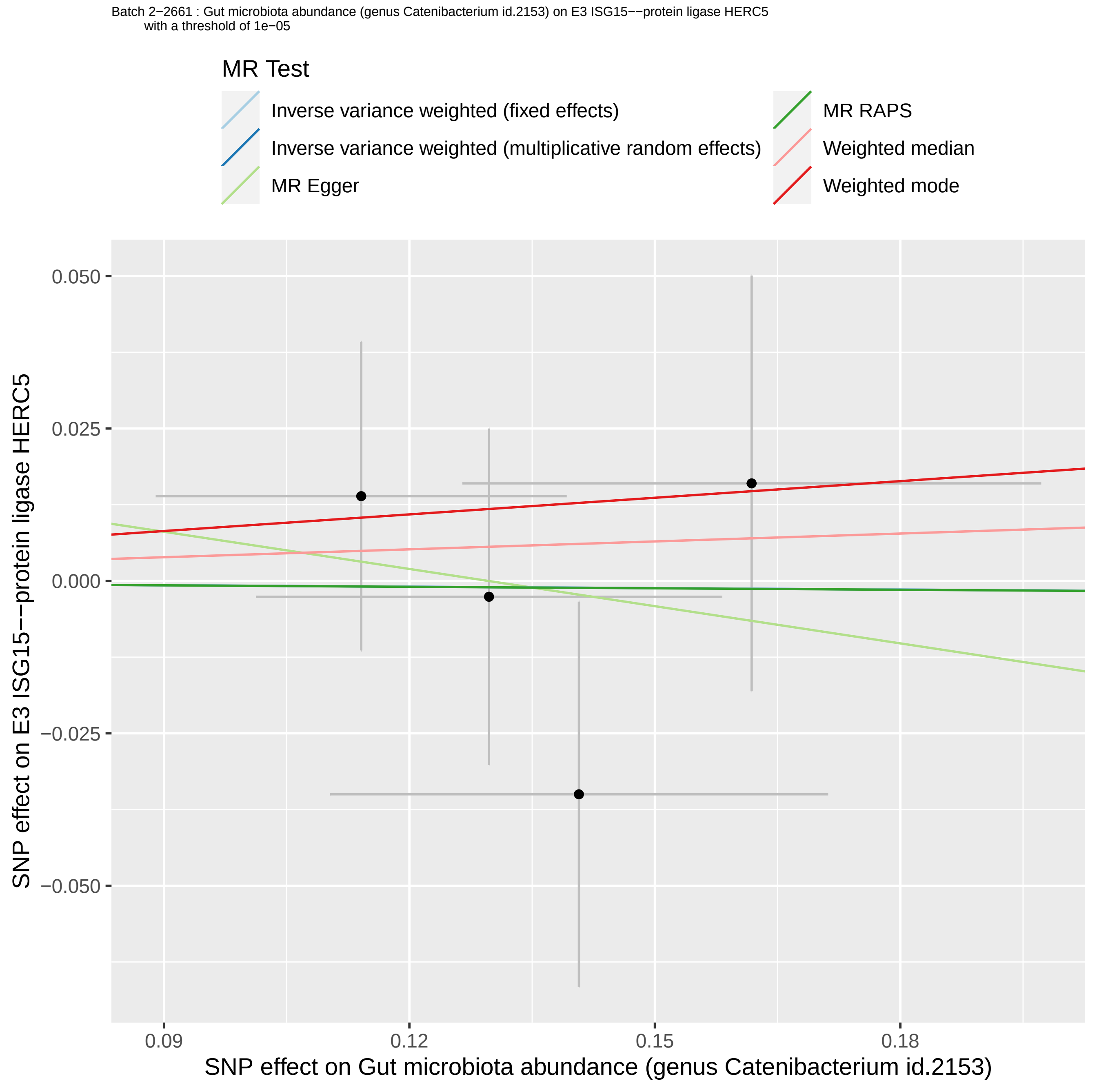
Batch 2−2661 : Gut microbiota abundance (genus Catenibacterium id.2153) on E3 ISG15−−protein ligase HERC5
with a threshold of 1e−05
MR Test
Inverse variance weighted (fixed effects)
Inverse variance weighted (multiplicative random effects)
MR Egger
MR RAPS
Weighted median
Weighted mode
0.050
0.025
0.000
−0.025
−0.050
0.09
0.12
0.15
0.18
SNP effect on E3 ISG15−−protein ligase HERC5
SNP effect on Gut microbiota abundance (genus Catenibacterium id.2153)

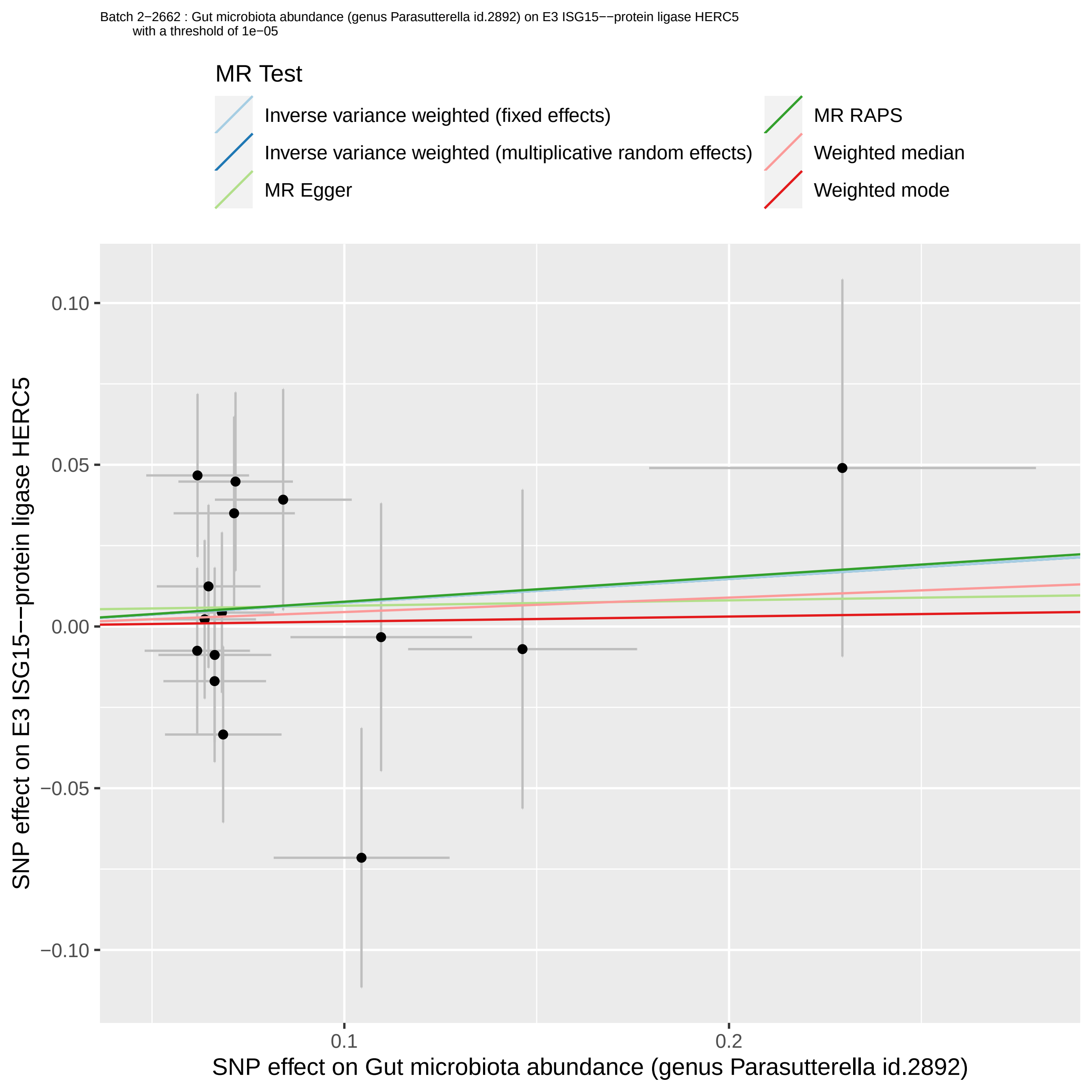

Batch 2−2662 : Gut microbiota abundance (genus Parasutterella id.2892) on E3 ISG15−−protein ligase HERC5
with a threshold of 1e−05
MR Test
Inverse variance weighted (fixed effects)
Inverse variance weighted (multiplicative random effects)
MR Egger
MR RAPS
Weighted median
Weighted mode
0.10
0.05
0.00
−0.05
−0.10
0.1
0.2
SNP effect on E3 ISG15−−protein ligase HERC5
SNP effect on Gut microbiota abundance (genus Parasutterella id.2892)

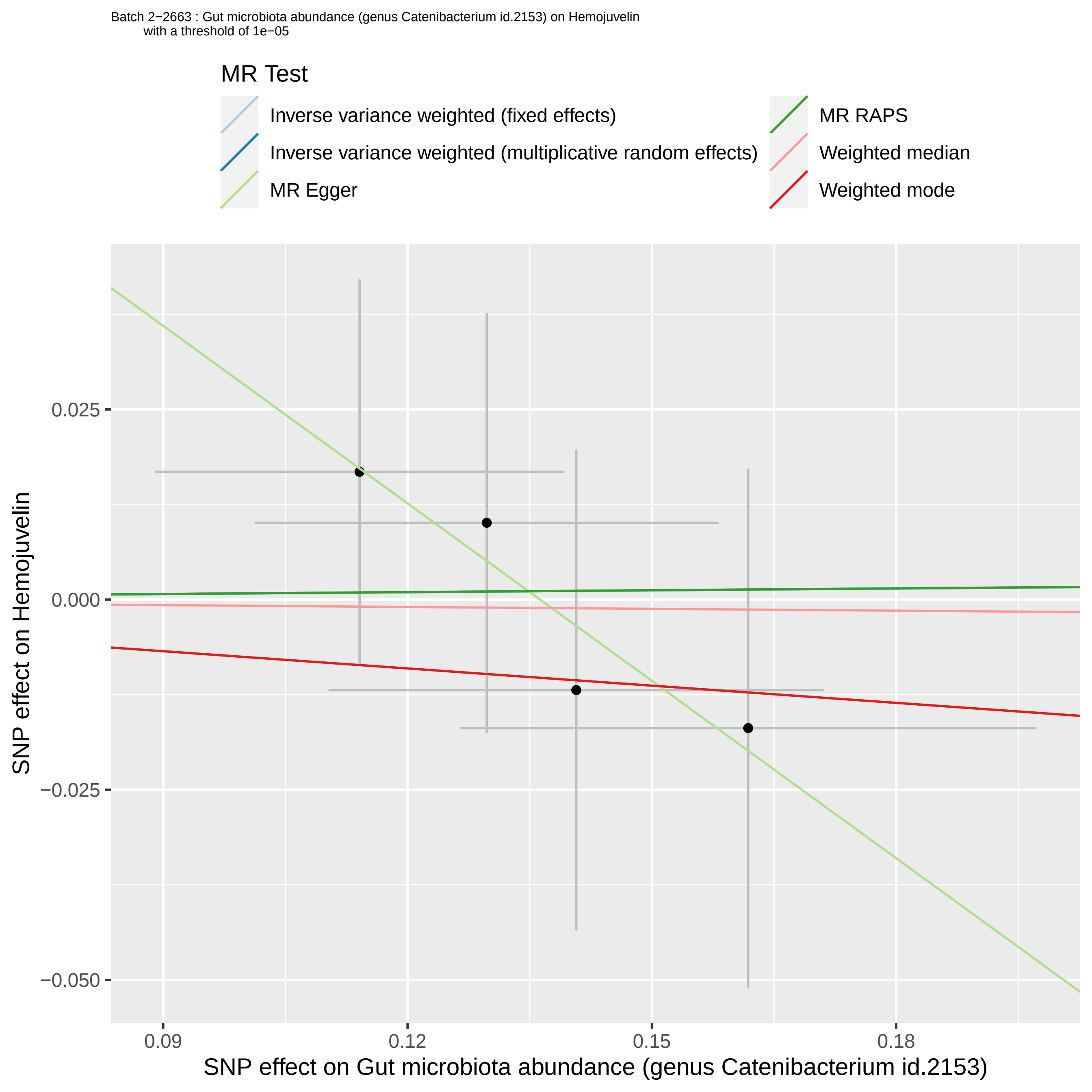

Batch 2−2663 : Gut microbiota abundance (genus Catenibacterium id.2153) on Hemojuvelin
with a threshold of 1e−05
MR Test
Inverse variance weighted (fixed effects)
Inverse variance weighted (multiplicative random effects)
MR Egger
MR RAPS
Weighted median
Weighted mode
0.025
0.000
−0.025
−0.050
0.09
0.12
0.15
0.18
SNP effect on Hemojuvelin
SNP effect on Gut microbiota abundance (genus Catenibacterium id.2153)

Batch 2−2664 : Gut microbiota abundance (genus Parasutterella id.2892) on Hemojuvelin
with a threshold of 1e−05

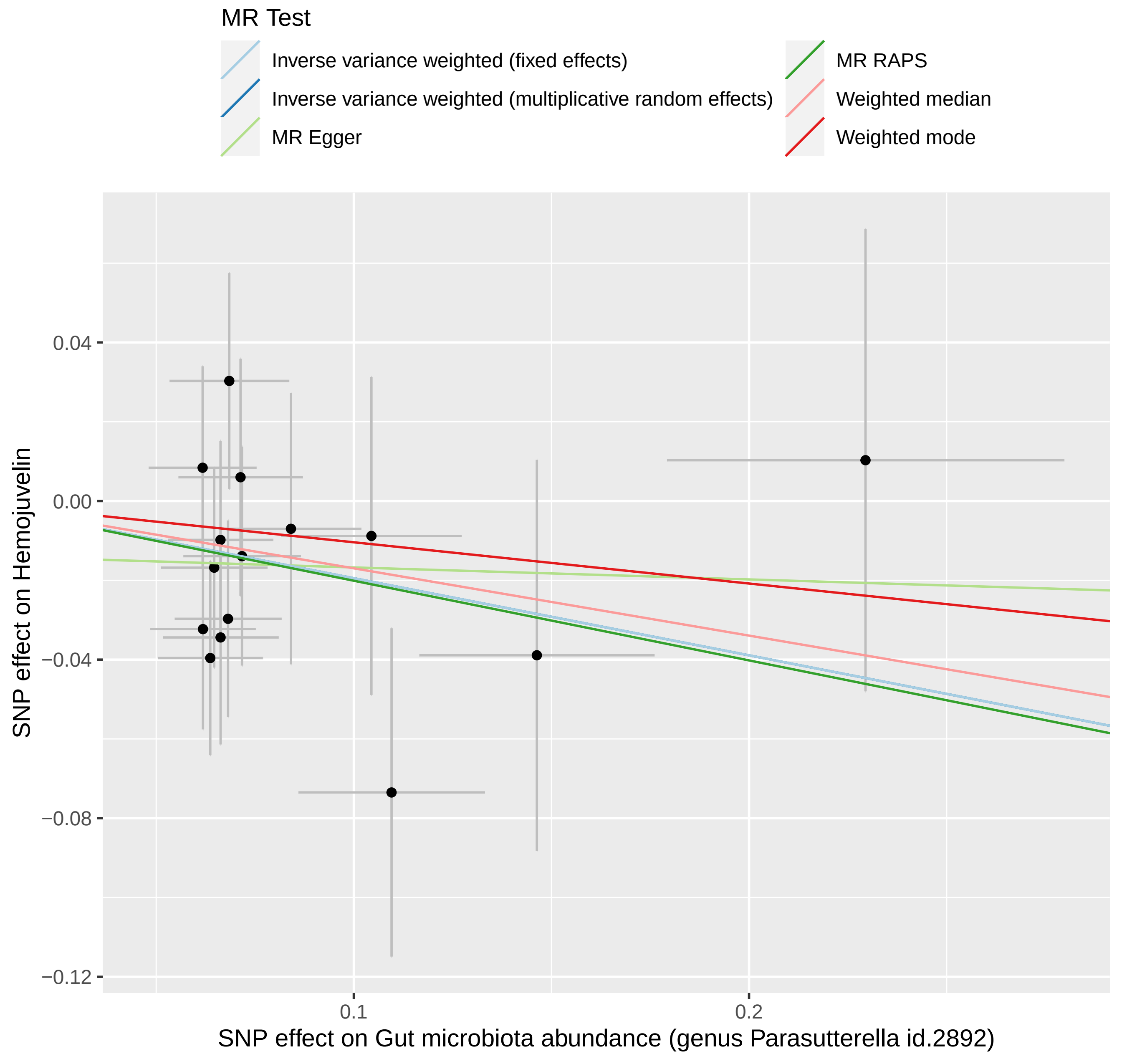

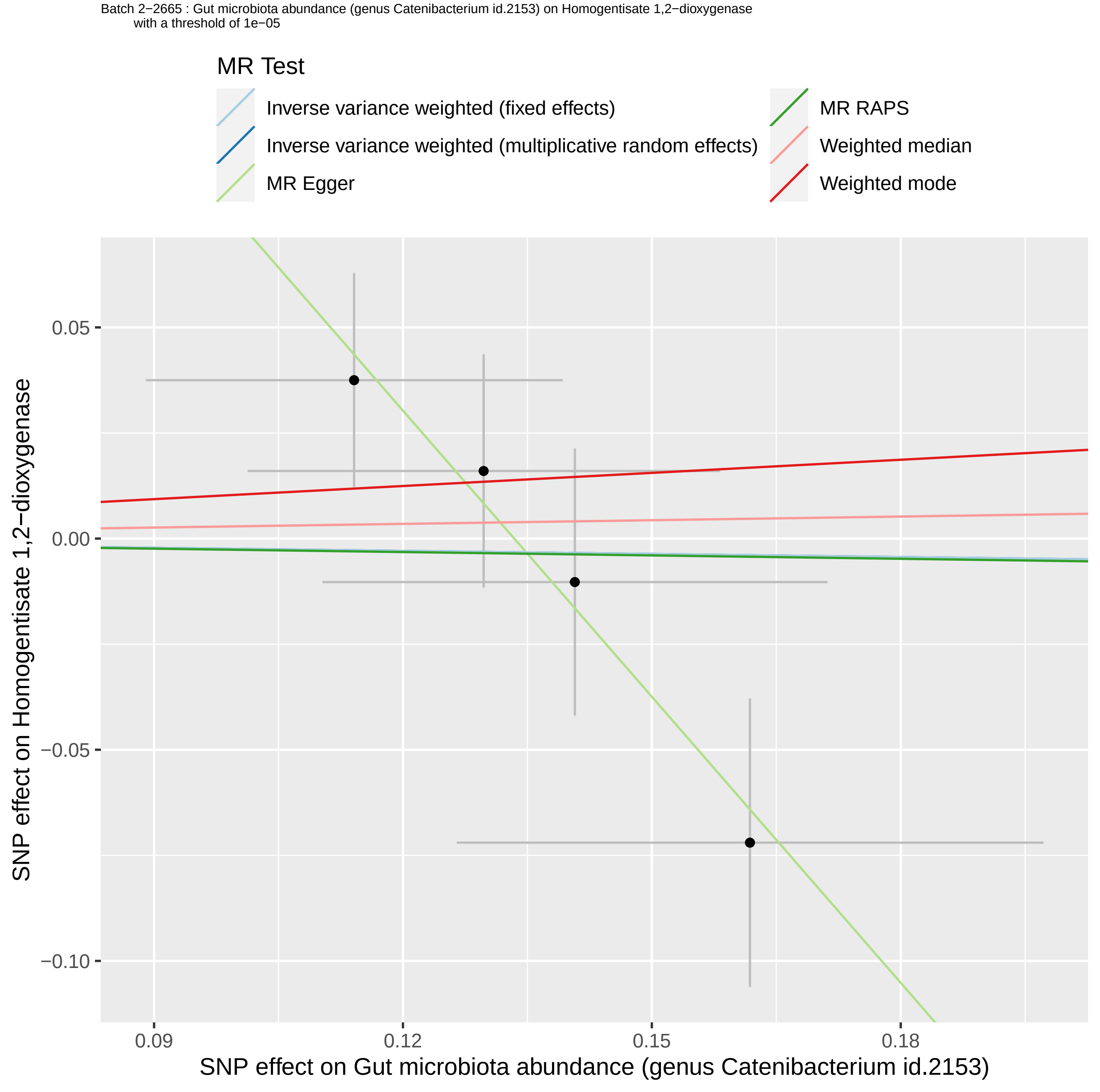
Batch 2−2665 : Gut microbiota abundance (genus Catenibacterium id.2153) on Homogentisate 1,2−dioxygenase
with a threshold of 1e−05
MR Test
Inverse variance weighted (fixed effects)
Inverse variance weighted (multiplicative random effects)
MR Egger
MR RAPS
Weighted median
Weighted mode
0.05
0.00
−0.05
−0.10
0.09
0.12
0.15
0.18
SNP effect on Homogentisate 1,2−dioxygenase
SNP effect on Gut microbiota abundance (genus Catenibacterium id.2153)

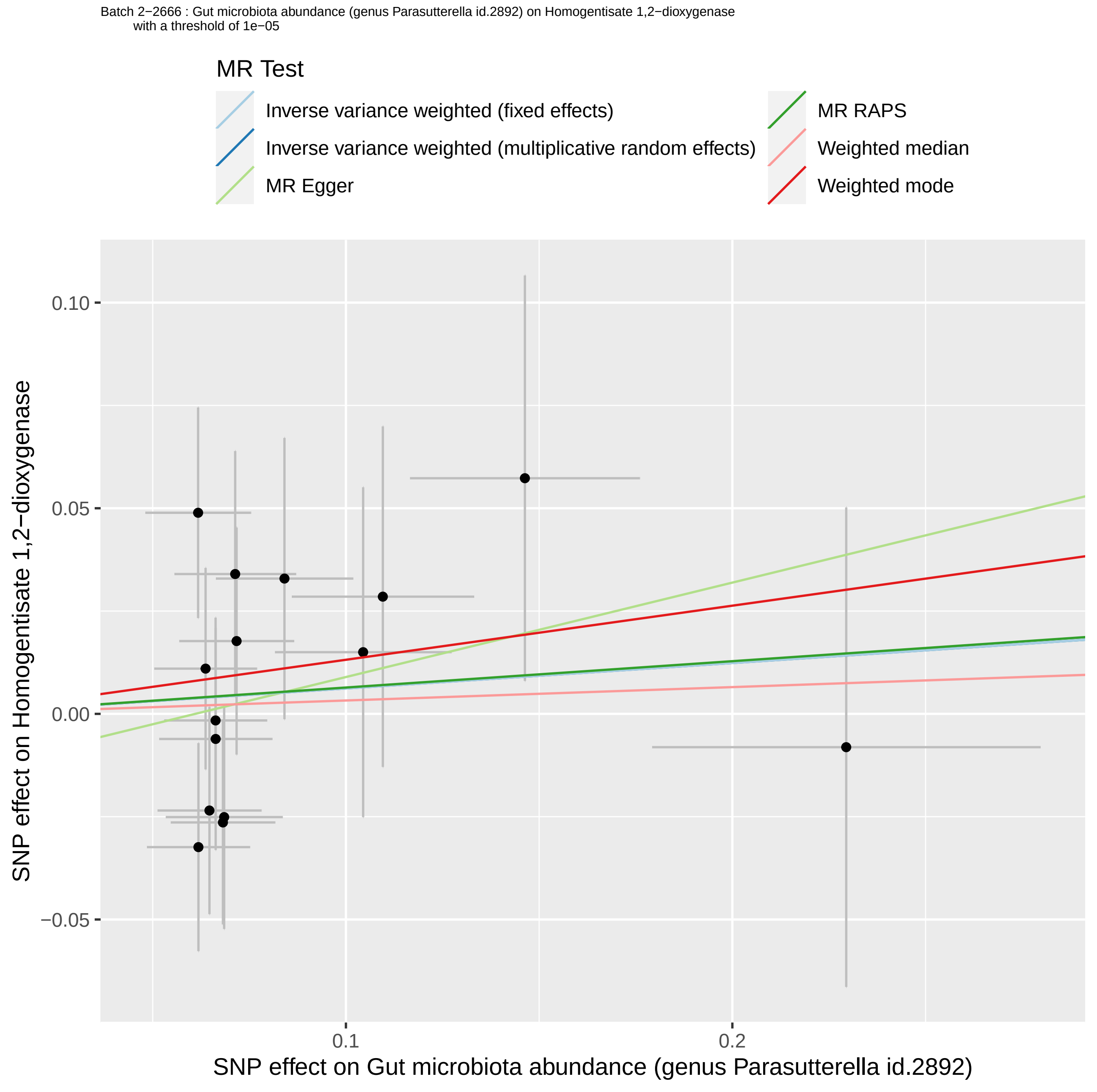
Batch 2−2666 : Gut microbiota abundance (genus Parasutterella id.2892) on Homogentisate 1,2−dioxygenase
with a threshold of 1e−05
MR Test
Inverse variance weighted (fixed effects)
Inverse variance weighted (multiplicative random effects)
MR Egger
MR RAPS
Weighted median
Weighted mode
0.10
0.05
0.00
−0.05
SNP effect on Homogentisate 1,2−dioxygenase
0.1
0.2
SNP effect on Gut microbiota abundance (genus Parasutterella id.2892)

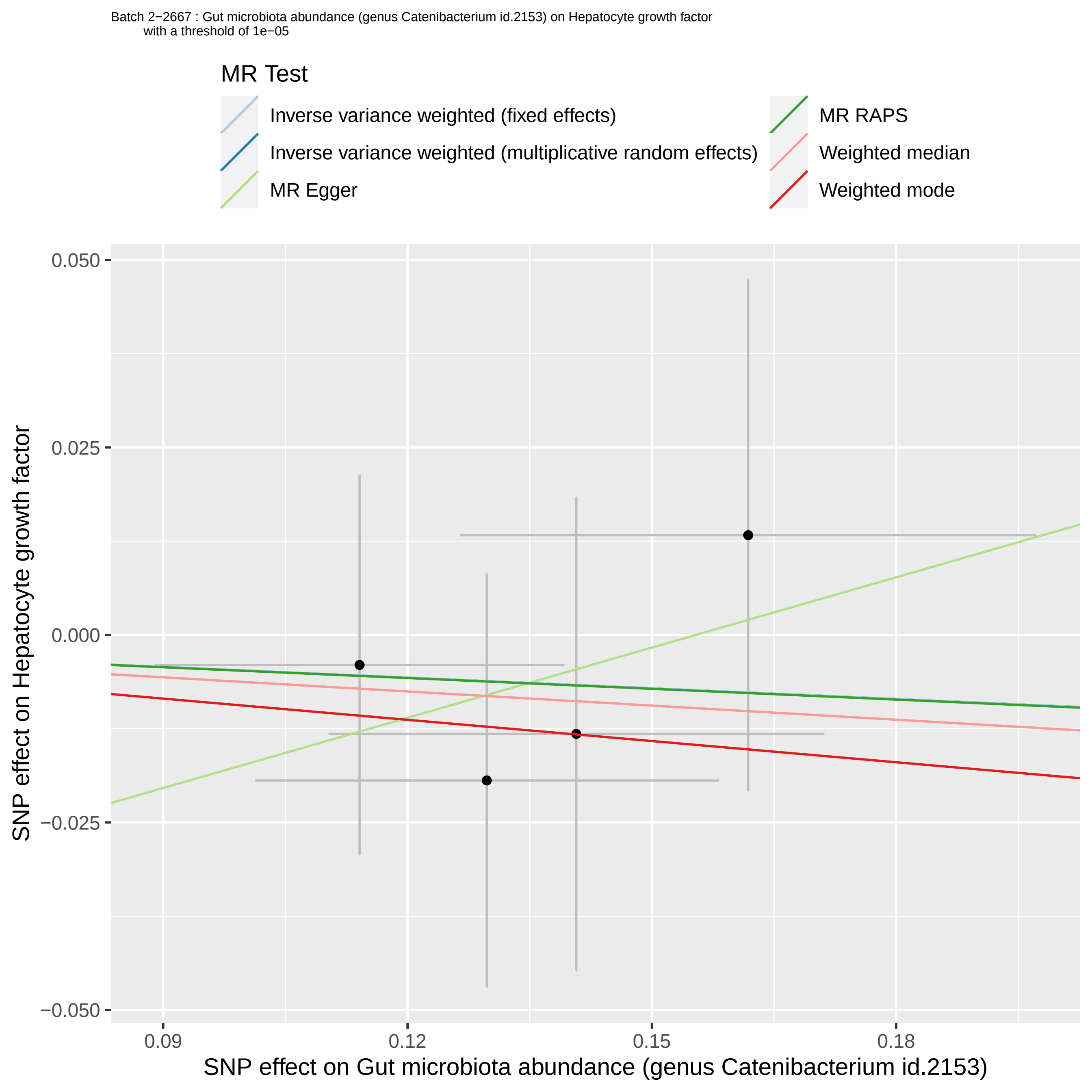
Batch 2−2667 : Gut microbiota abundance (genus Catenibacterium id.2153) on Hepatocyte growth factor
with a threshold of 1e−05
MR Test
Inverse variance weighted (fixed effects)
Inverse variance weighted (multiplicative random effects)
MR Egger
MR RAPS
Weighted median
Weighted mode
0.050
0.025
0.000
−0.025
−0.050
0.09
0.12
0.15
0.18
SNP effect on Hepatocyte growth factor
SNP effect on Gut microbiota abundance (genus Catenibacterium id.2153)

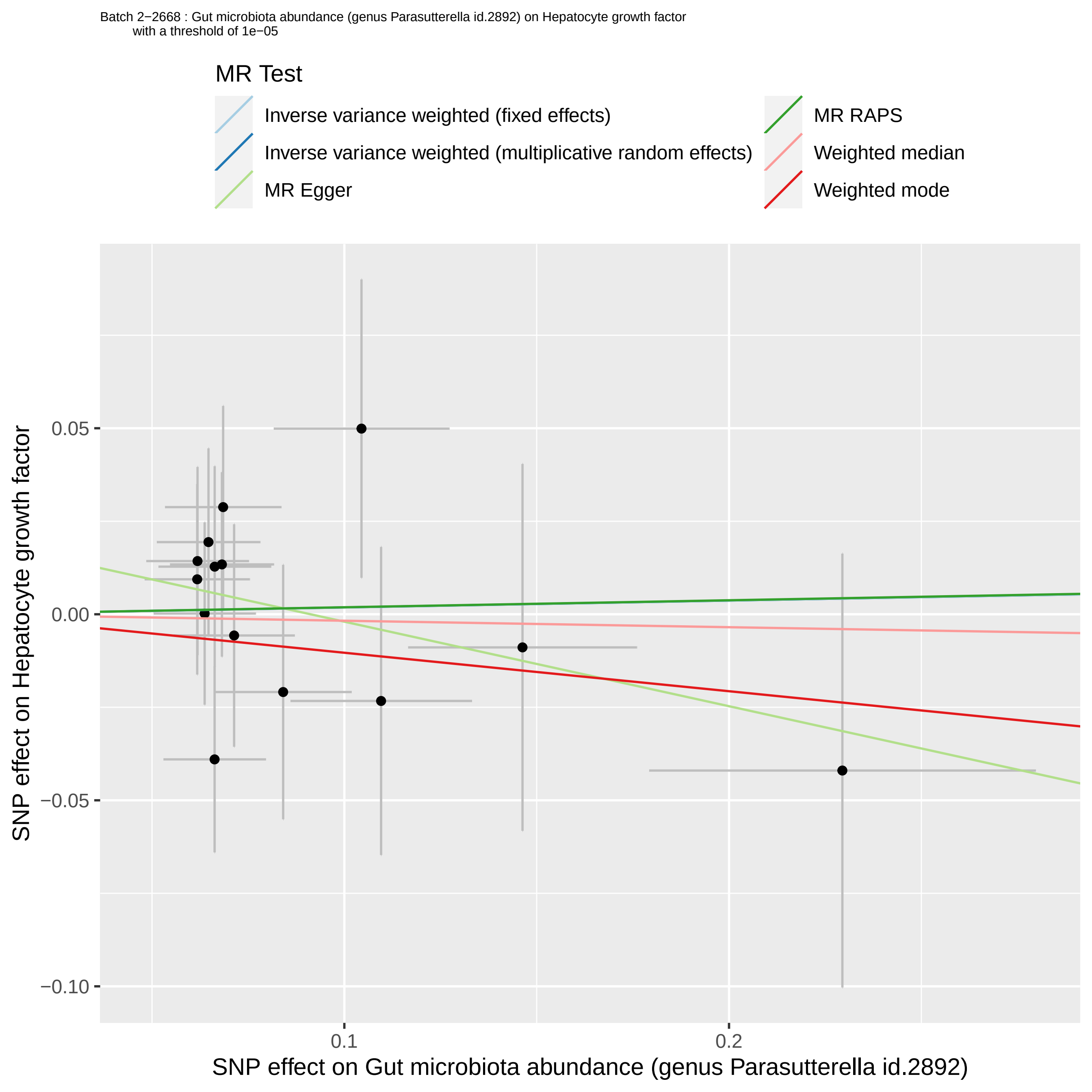

Batch 2−2668 : Gut microbiota abundance (genus Parasutterella id.2892) on Hepatocyte growth factor
with a threshold of 1e−05
MR Test
Inverse variance weighted (fixed effects)
Inverse variance weighted (multiplicative random effects)
MR Egger
MR RAPS
Weighted median
Weighted mode
0.05
0.00
−0.05
−0.10
0.1
0.2
SNP effect on Hepatocyte growth factor
SNP effect on Gut microbiota abundance (genus Parasutterella id.2892)

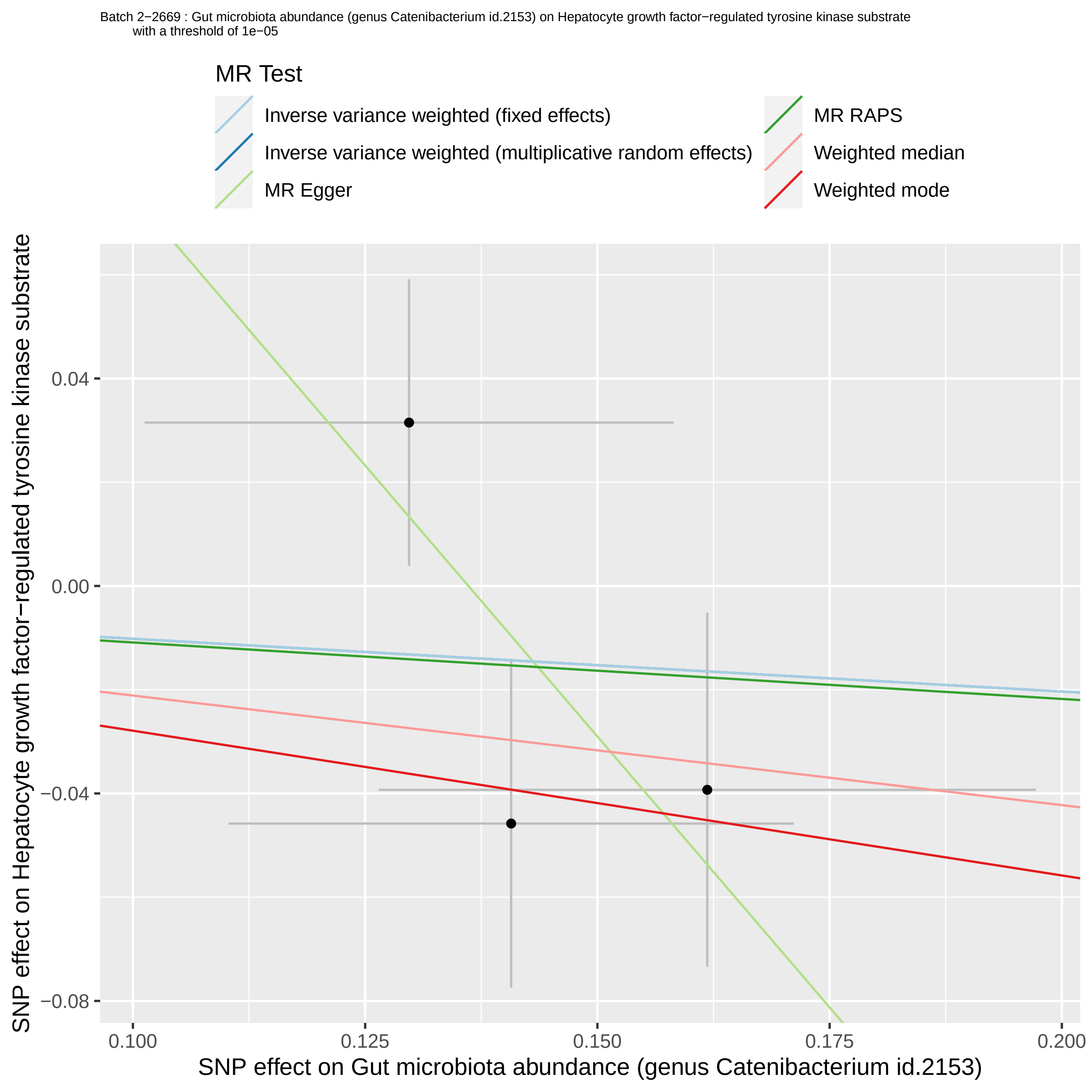

Batch 2−2669 : Gut microbiota abundance (genus Catenibacterium id.2153) on Hepatocyte growth factor−regulated tyrosine kinase substrate
with a threshold of 1e−05
MR Test
Inverse variance weighted (fixed effects)
Inverse variance weighted (multiplicative random effects)
MR Egger
MR RAPS
Weighted median
Weighted mode
0.04
0.00
−0.04
−0.08
SNP effect on Hepatocyte growth factor−regulated tyrosine kinase substrate
0.100
0.125
0.150
0.175
0.200
SNP effect on Gut microbiota abundance (genus Catenibacterium id.2153)

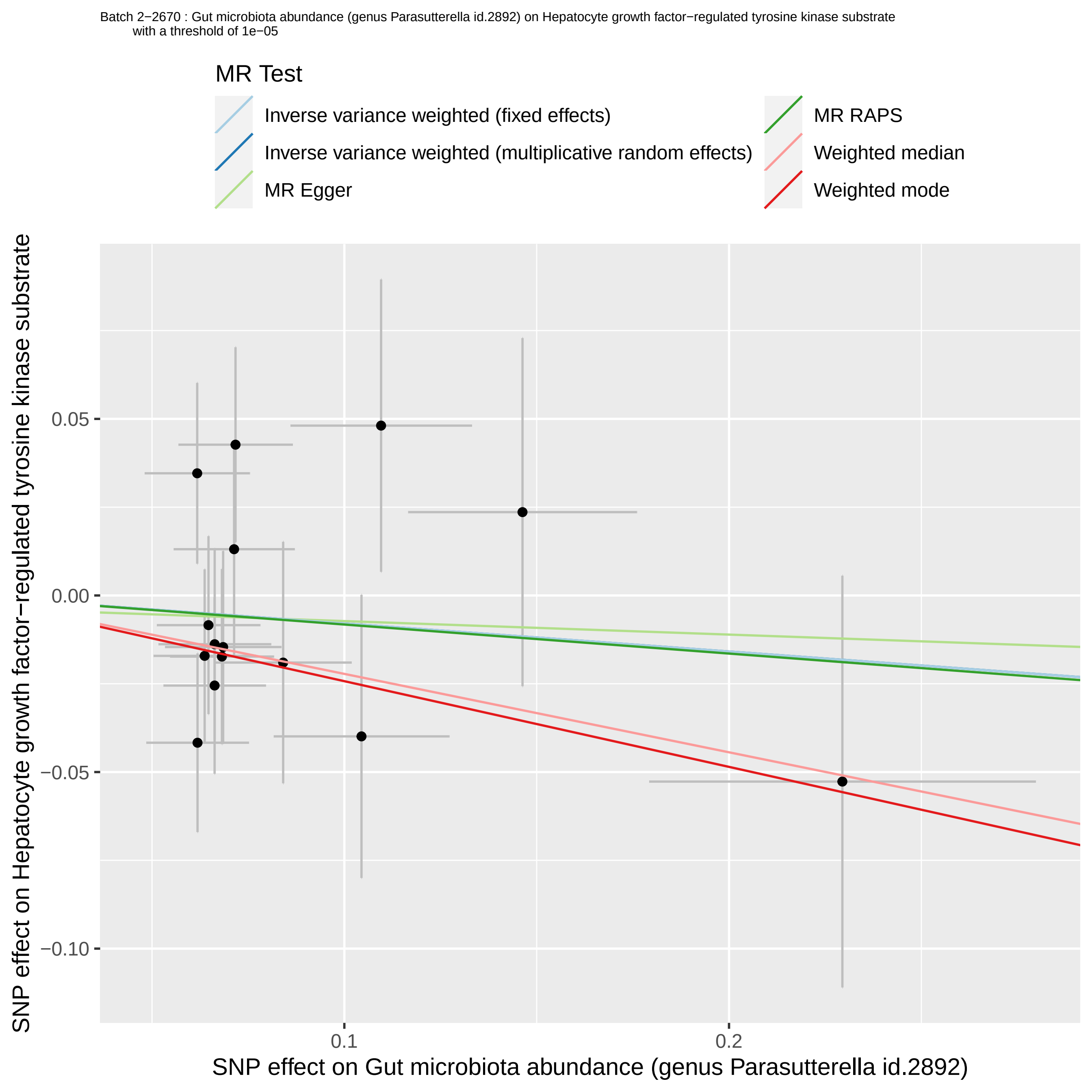
Batch 2−2670 : Gut microbiota abundance (genus Parasutterella id.2892) on Hepatocyte growth factor−regulated tyrosine kinase substrate with a threshold of 1e−05
MR Test
Inverse variance weighted (fixed effects)
Inverse variance weighted (multiplicative random effects)
MR Egger
MR RAPS
Weighted median
Weighted mode
SNP effect on Hepatocyte growth factor−regulated tyrosine kinase substrate
0.05
0.00
−0.05
−0.10
0.1
0.2
SNP effect on Gut microbiota abundance (genus Parasutterella id.2892)

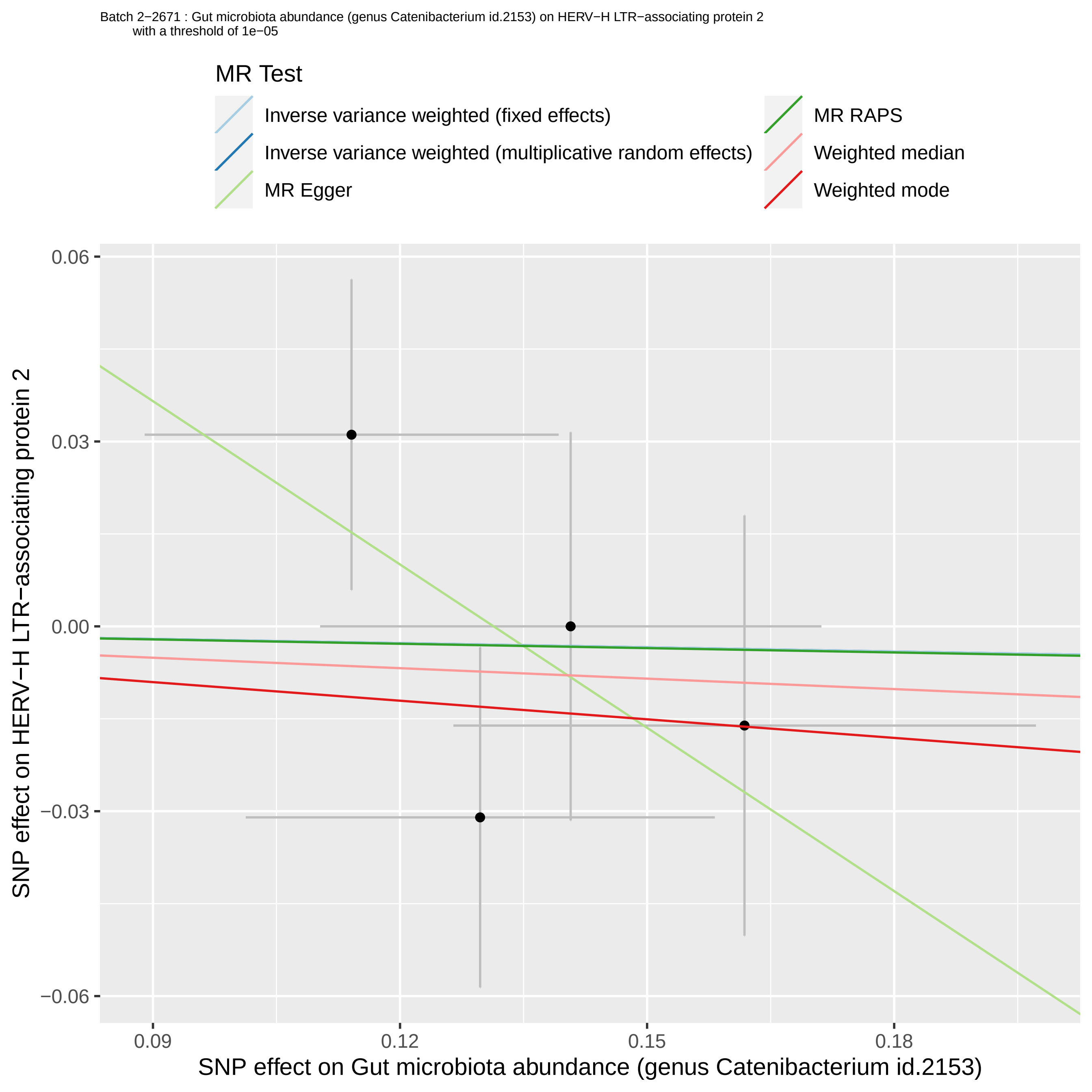
Batch 2−2671 : Gut microbiota abundance (genus Catenibacterium id.2153) on HERV−H LTR−associating protein 2
with a threshold of 1e−05
MR Test
Inverse variance weighted (fixed effects)
Inverse variance weighted (multiplicative random effects)
MR Egger
MR RAPS
Weighted median
Weighted mode
0.06
0.03
0.00
−0.03
−0.06
0.09
0.12
0.15
0.18
SNP effect on HERV−H LTR−associating protein 2
SNP effect on Gut microbiota abundance (genus Catenibacterium id.2153)

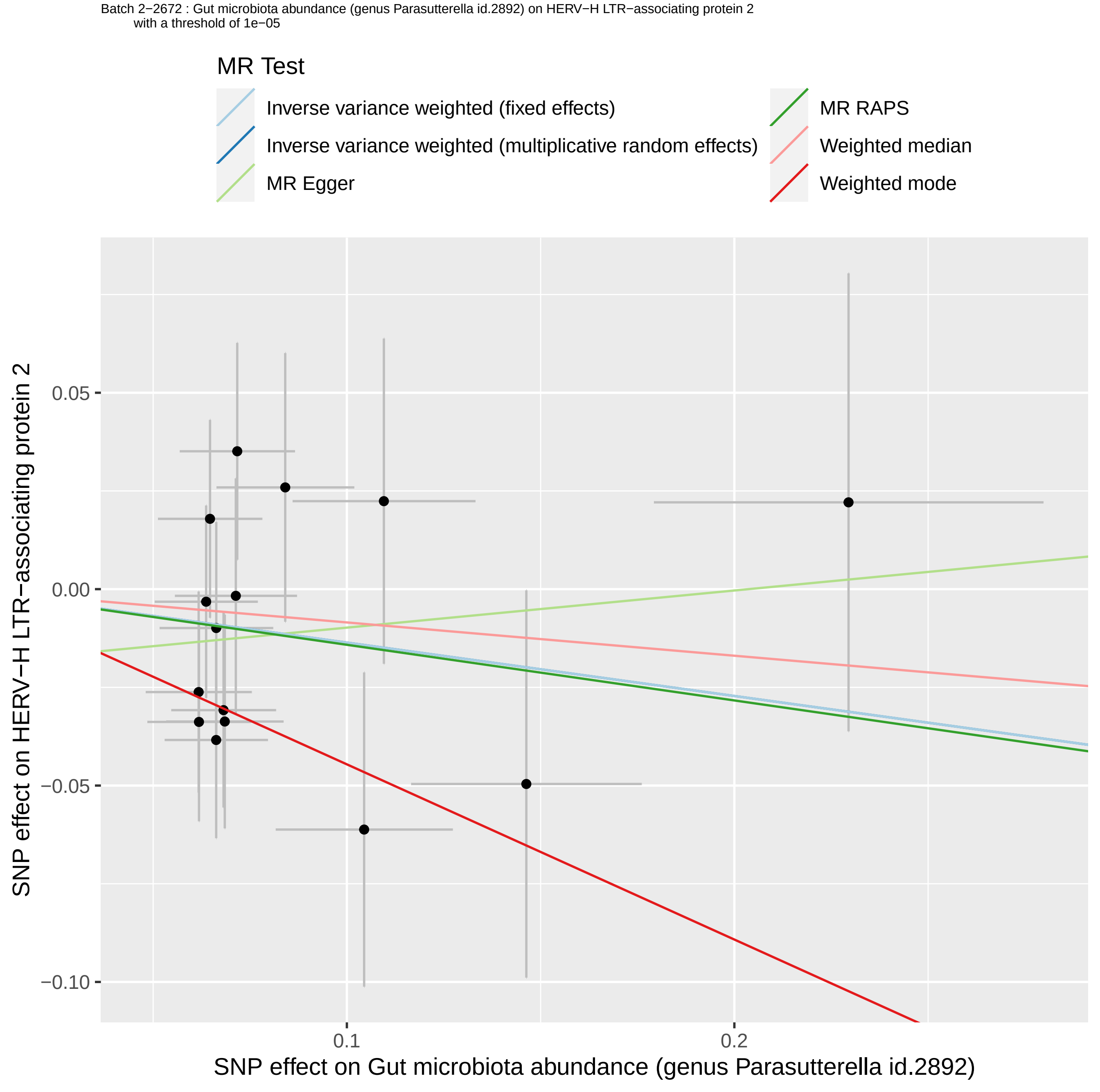

Batch 2−2672 : Gut microbiota abundance (genus Parasutterella id.2892) on HERV−H LTR−associating protein 2
with a threshold of 1e−05
MR Test
Inverse variance weighted (fixed effects)
Inverse variance weighted (multiplicative random effects)
MR Egger
MR RAPS
Weighted median
Weighted mode
0.05
0.00
−0.05
−0.10
0.1
0.2
SNP effect on HERV−H LTR−associating protein 2
SNP effect on Gut microbiota abundance (genus Parasutterella id.2892)

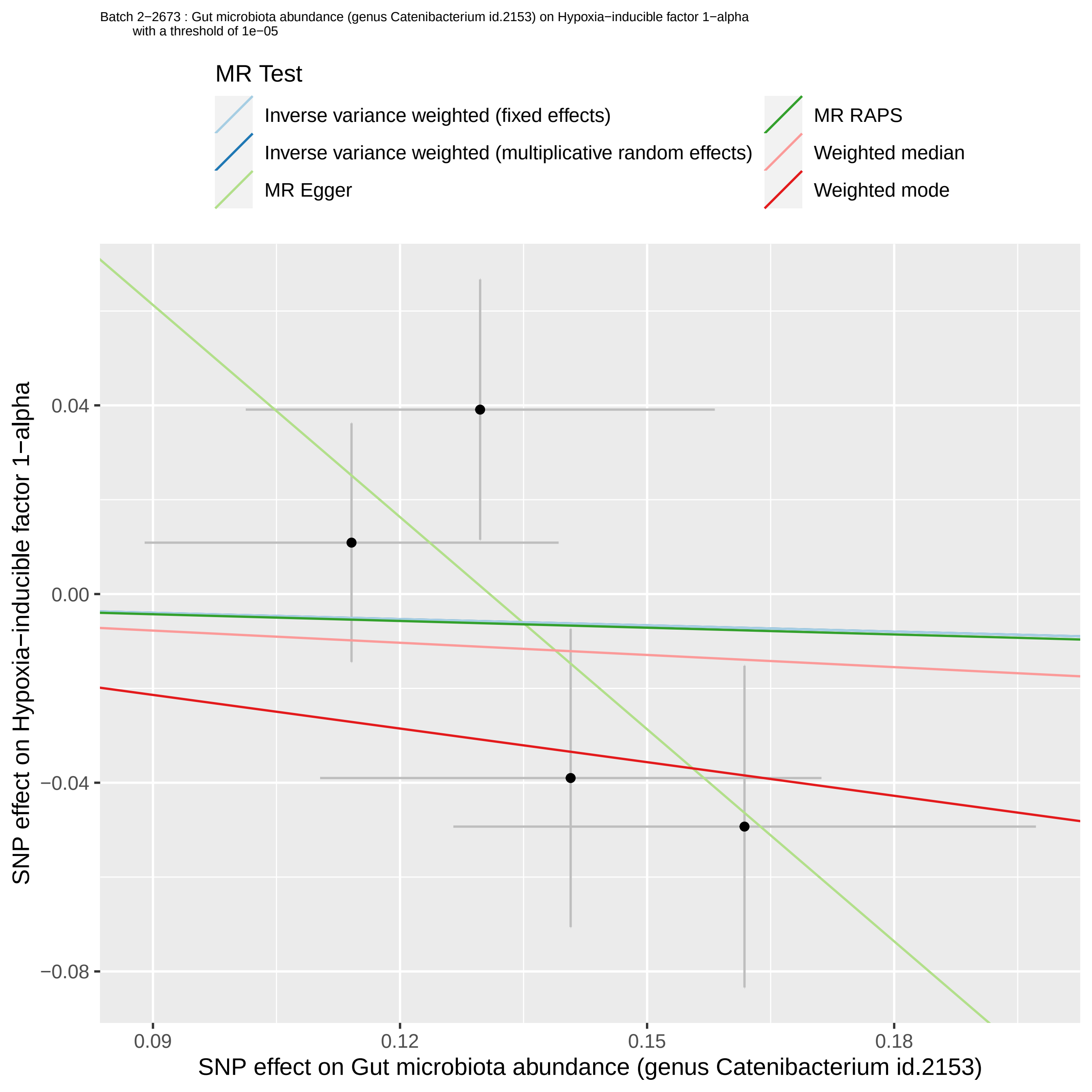

Batch 2−2673 : Gut microbiota abundance (genus Catenibacterium id.2153) on Hypoxia−inducible factor 1−alpha
with a threshold of 1e−05
MR Test
Inverse variance weighted (fixed effects)
Inverse variance weighted (multiplicative random effects)
MR Egger
MR RAPS
Weighted median
Weighted mode
0.04
0.00
−0.04
−0.08
0.09
0.12
0.15
0.18
SNP effect on Hypoxia−inducible factor 1−alpha
SNP effect on Gut microbiota abundance (genus Catenibacterium id.2153)

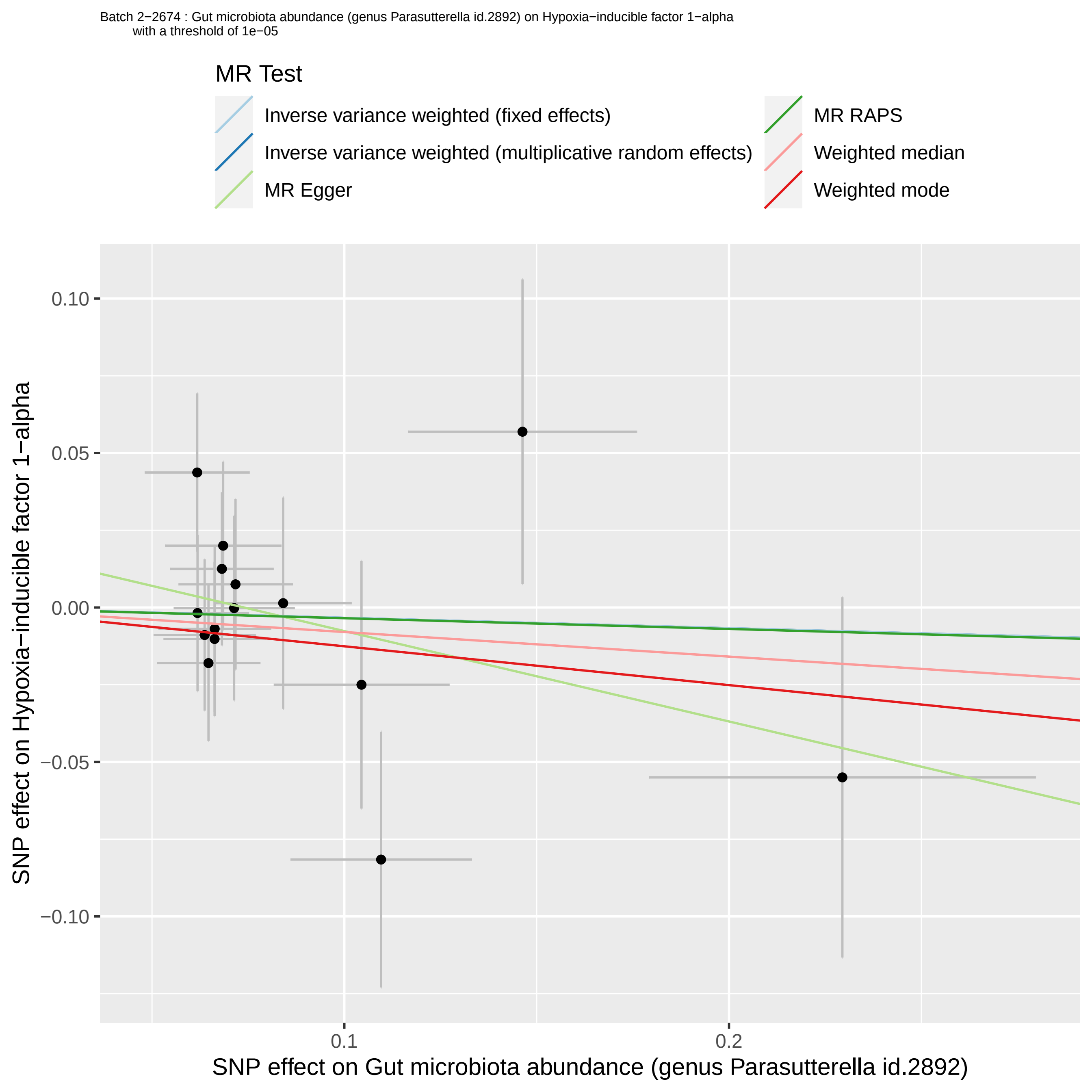
Batch 2−2674 : Gut microbiota abundance (genus Parasutterella id.2892) on Hypoxia−inducible factor 1−alpha
with a threshold of 1e−05
MR Test
Inverse variance weighted (fixed effects)
Inverse variance weighted (multiplicative random effects)
MR Egger
MR RAPS
Weighted median
Weighted mode
0.10
0.05
0.00
−0.05
−0.10
0.1
0.2
SNP effect on Hypoxia−inducible factor 1−alpha
SNP effect on Gut microbiota abundance (genus Parasutterella id.2892)

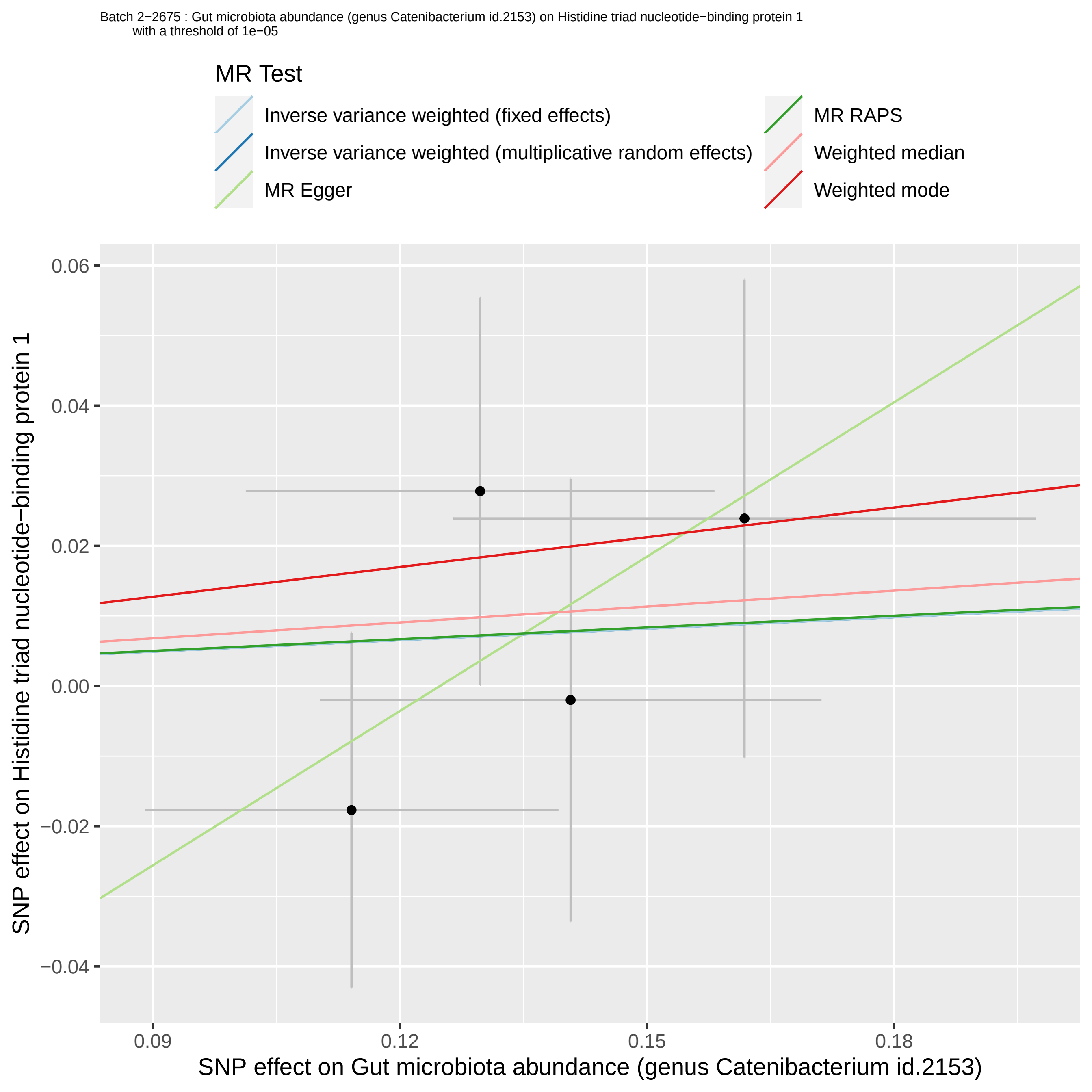
Batch 2−2675 : Gut microbiota abundance (genus Catenibacterium id.2153) on Histidine triad nucleotide−binding protein 1 with a threshold of 1e−05
MR Test
Inverse variance weighted (fixed effects)
Inverse variance weighted (multiplicative random effects)
MR Egger
MR RAPS
Weighted median
Weighted mode
SNP effect on Histidine triad nucleotide−binding protein 1
0.06
0.04
0.02
0.00
−0.02
−0.04
0.09
0.12
0.15
0.18
SNP effect on Gut microbiota abundance (genus Catenibacterium id.2153)

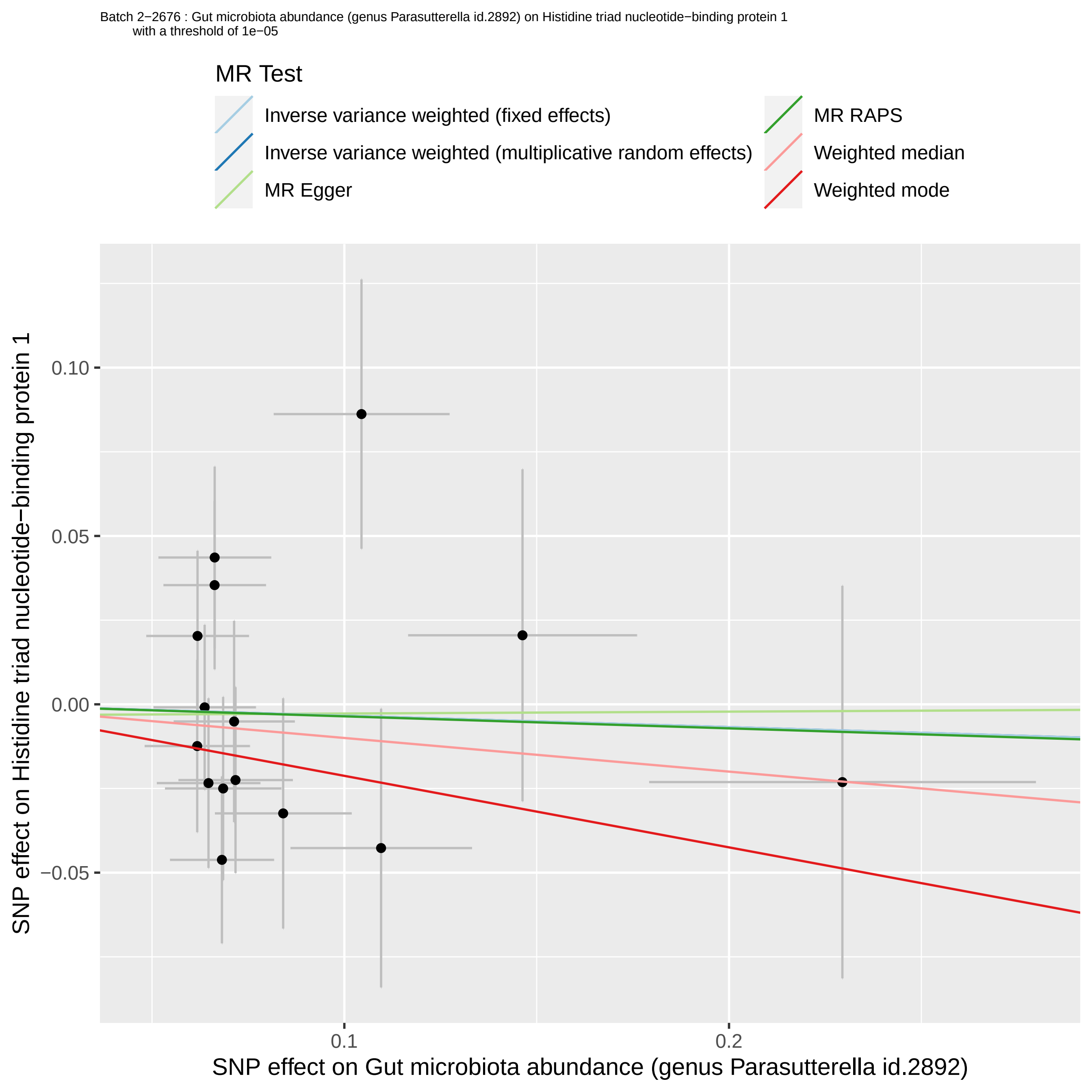
Batch 2−2676 : Gut microbiota abundance (genus Parasutterella id.2892) on Histidine triad nucleotide−binding protein 1
with a threshold of 1e−05
MR Test
Inverse variance weighted (fixed effects)
Inverse variance weighted (multiplicative random effects)
MR Egger
MR RAPS
Weighted median
Weighted mode
0.10
0.05
0.00
−0.05
0.1
0.2
SNP effect on Histidine triad nucleotide−binding protein 1
SNP effect on Gut microbiota abundance (genus Parasutterella id.2892)

Batch 2−2677 : Gut microbiota abundance (genus Catenibacterium id.2153) on Histidine triad nucleotide−binding protein 2, mitochondrial
with a threshold of 1e−05

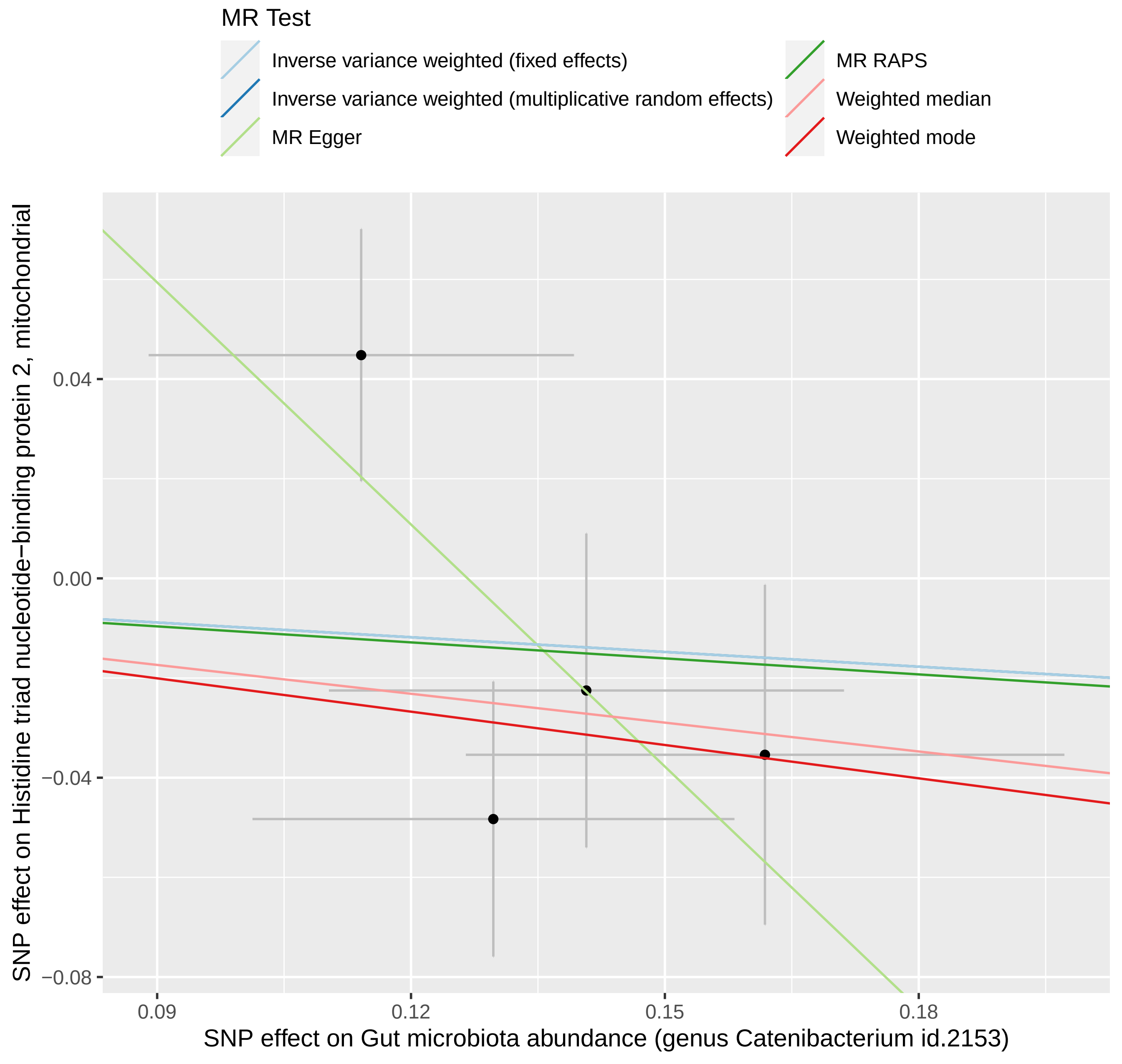

Batch 2−2678 : Gut microbiota abundance (genus Parasutterella id.2892) on Histidine triad nucleotide−binding protein 2, mitochondrial
with a threshold of 1e−05

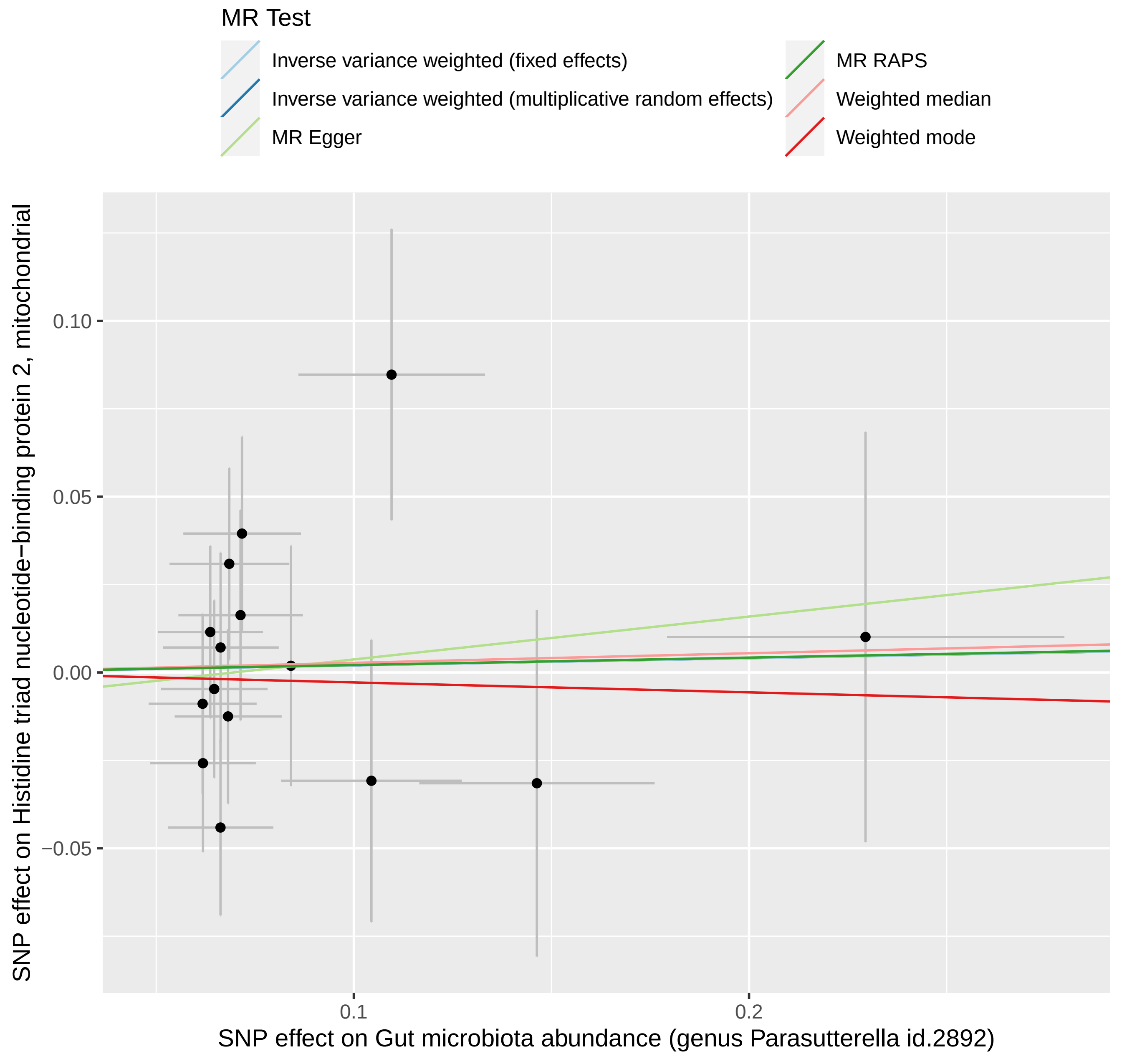

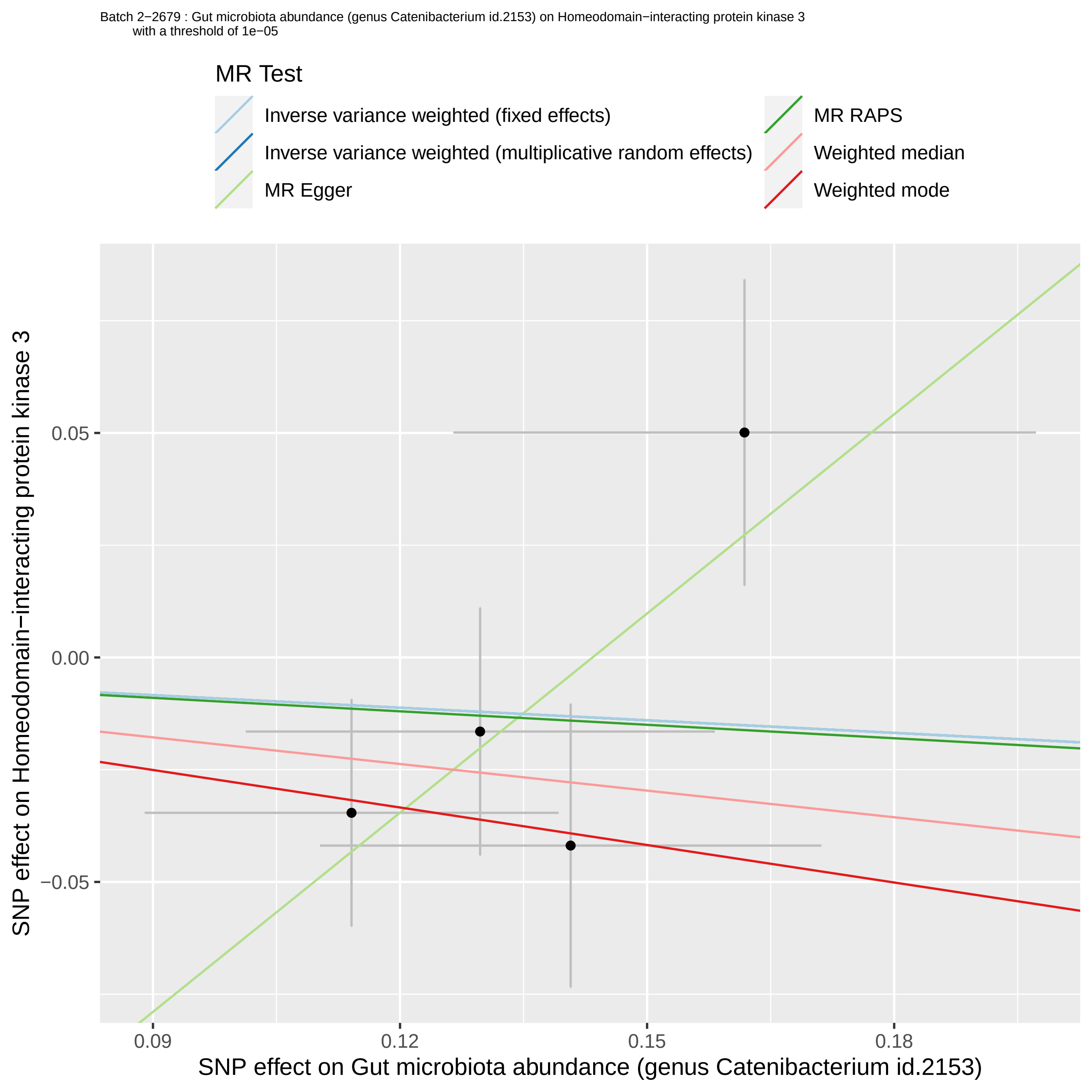
Batch 2−2679 : Gut microbiota abundance (genus Catenibacterium id.2153) on Homeodomain−interacting protein kinase 3
with a threshold of 1e−05
MR Test
Inverse variance weighted (fixed effects)
Inverse variance weighted (multiplicative random effects)
MR Egger
MR RAPS
Weighted median
Weighted mode
0.05
0.00
−0.05
0.09
0.12
0.15
0.18
SNP effect on Homeodomain−interacting protein kinase 3
SNP effect on Gut microbiota abundance (genus Catenibacterium id.2153)

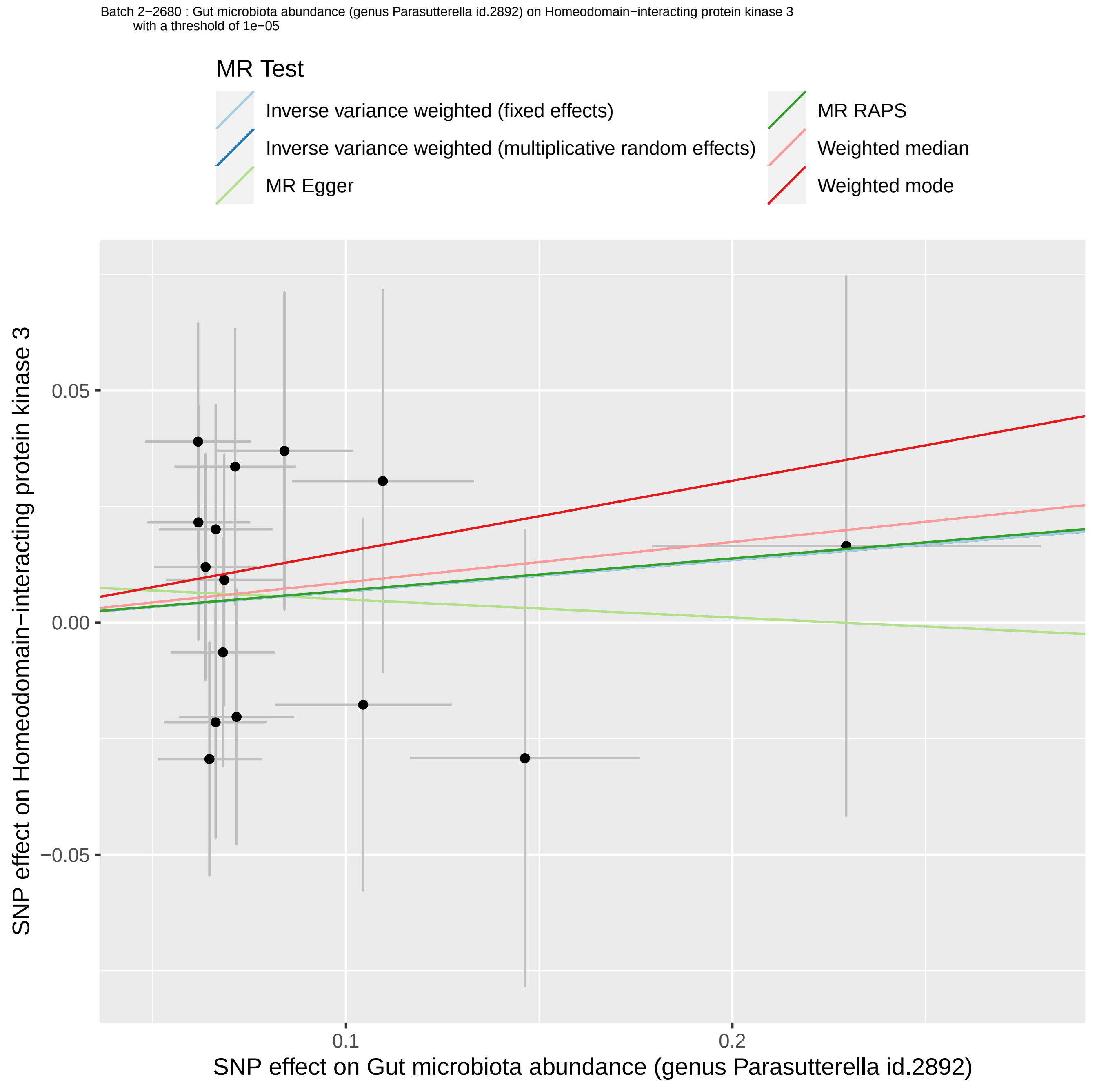
Batch 2−2680 : Gut microbiota abundance (genus Parasutterella id.2892) on Homeodomain−interacting protein kinase 3
with a threshold of 1e−05
MR Test
Inverse variance weighted (fixed effects)
Inverse variance weighted (multiplicative random effects)
MR Egger
MR RAPS
Weighted median
Weighted mode
0.05
0.00
−0.05
0.1
0.2
SNP effect on Homeodomain−interacting protein kinase 3
SNP effect on Gut microbiota abundance (genus Parasutterella id.2892)

Batch 2−2681 : Gut microbiota abundance (genus Catenibacterium id.2153) on Histone H1.2
with a threshold of 1e−05

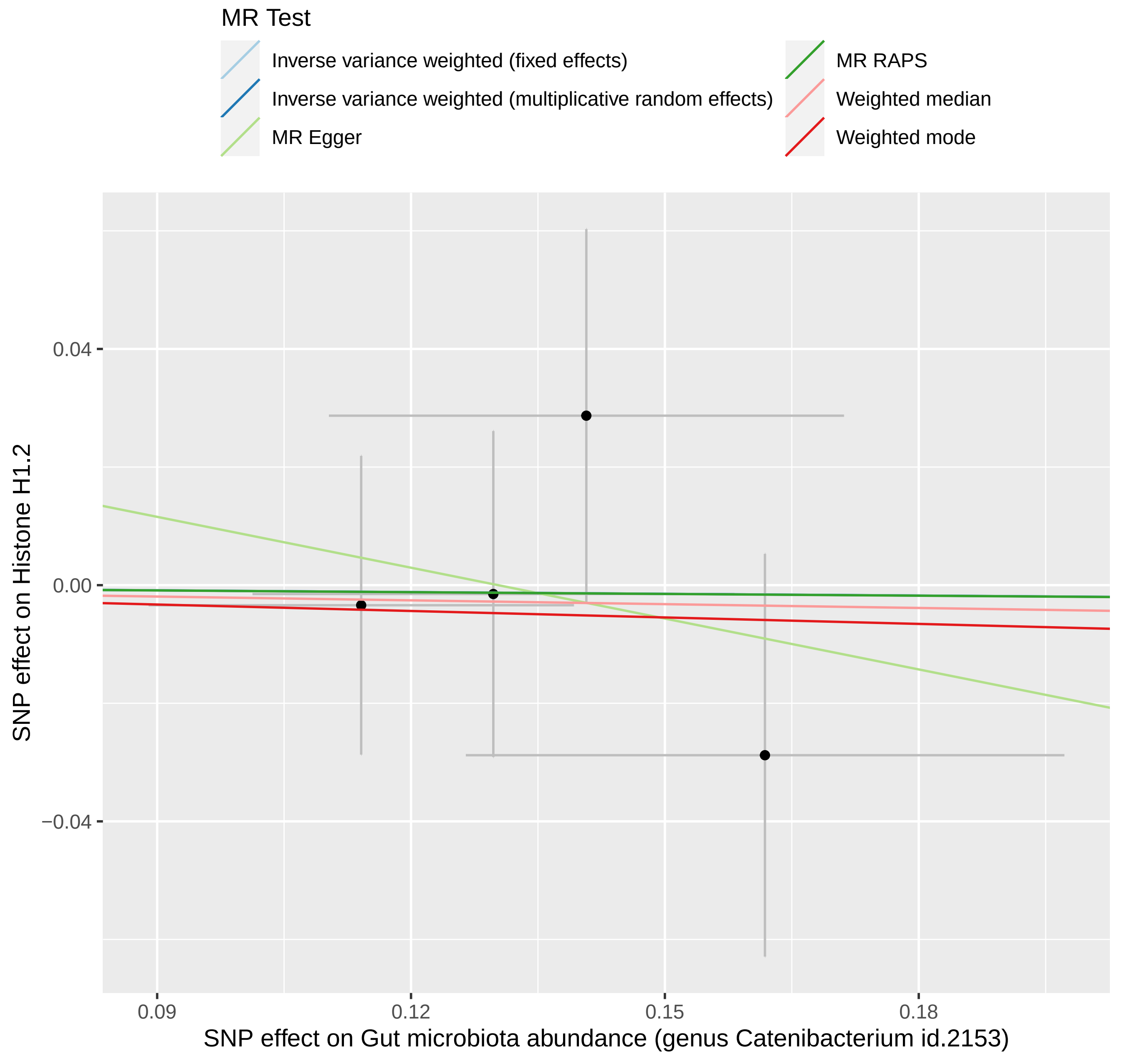

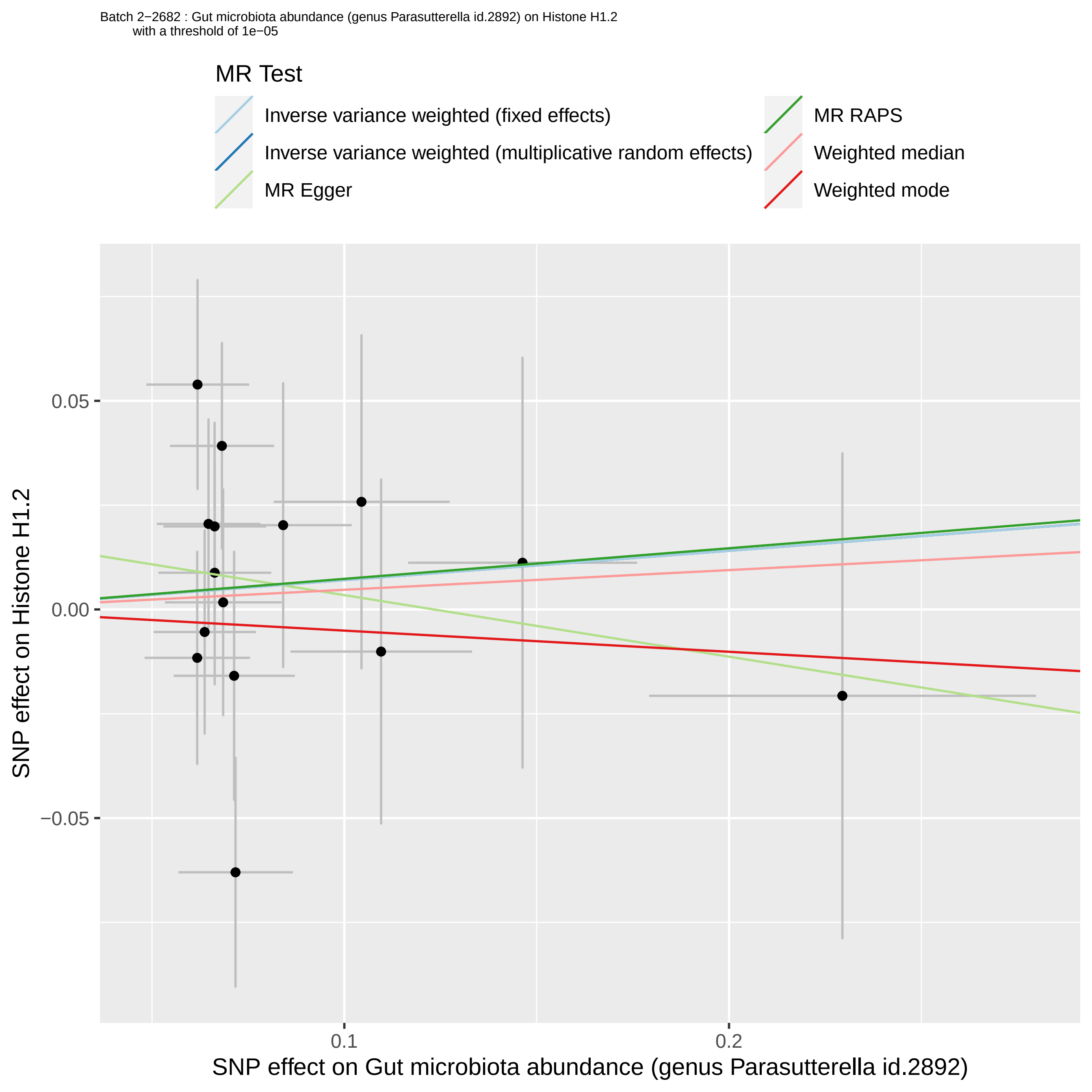

Batch 2−2682 : Gut microbiota abundance (genus Parasutterella id.2892) on Histone H1.2
with a threshold of 1e−05
MR Test
Inverse variance weighted (fixed effects)
Inverse variance weighted (multiplicative random effects)
MR Egger
MR RAPS
Weighted median
Weighted mode
SNP effect on Histone H1.2
0.05
0.00
−0.05
0.1
0.2
SNP effect on Gut microbiota abundance (genus Parasutterella id.2892)

Batch 2−2683 : Gut microbiota abundance (genus Catenibacterium id.2153) on Histone H2A type 3
with a threshold of 1e−05

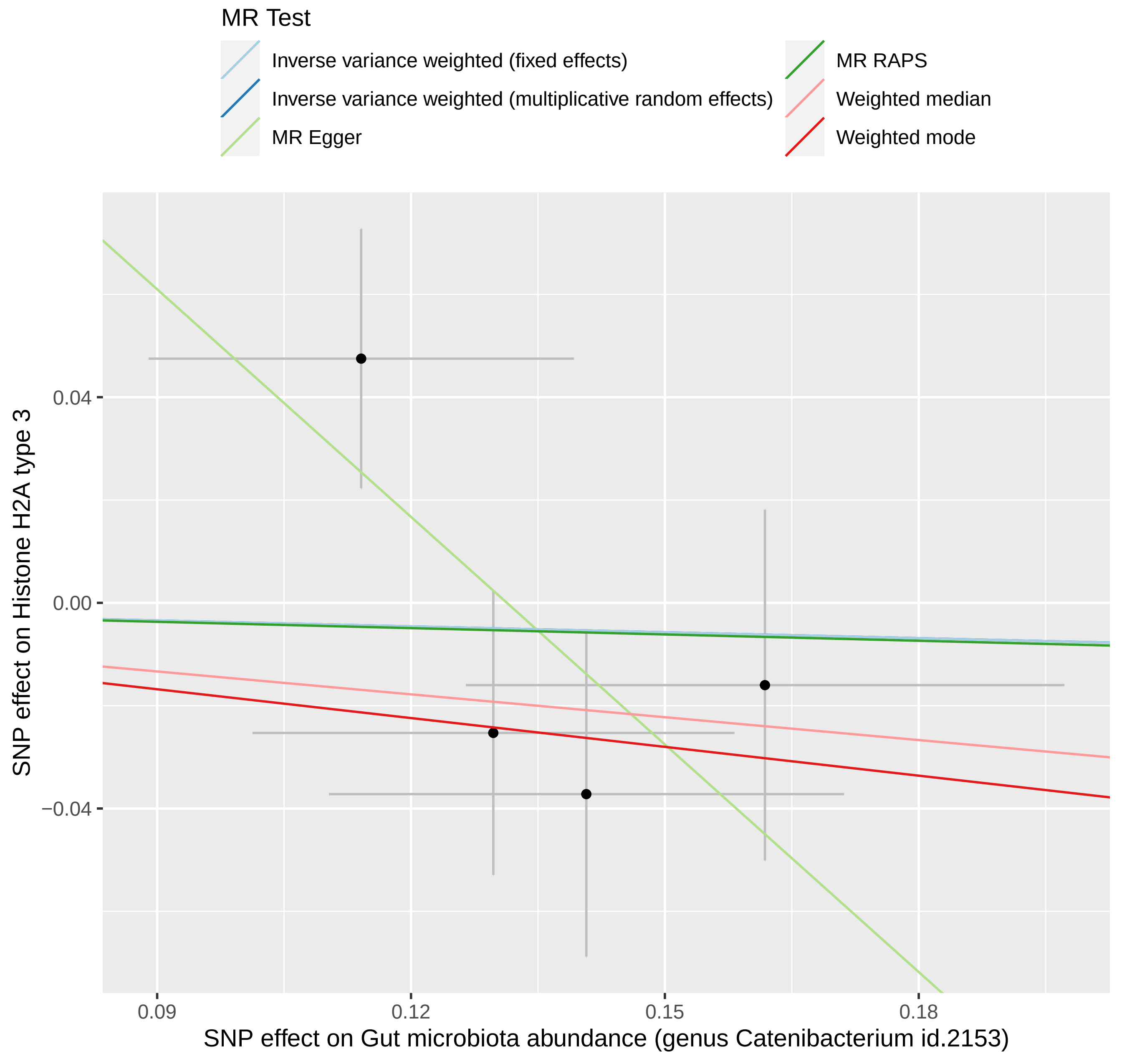

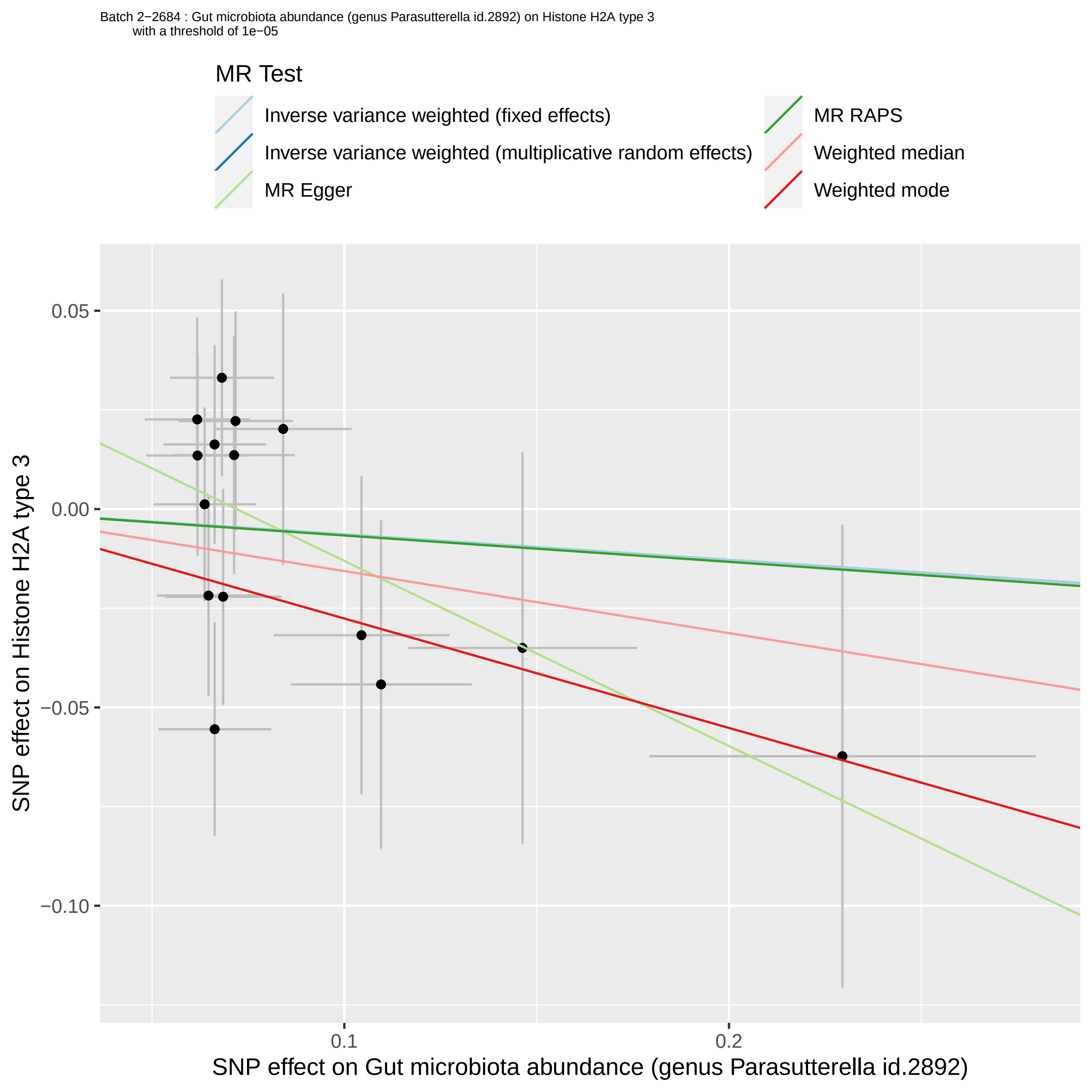
Batch 2−2684 : Gut microbiota abundance (genus Parasutterella id.2892) on Histone H2A type 3
with a threshold of 1e−05
MR Test
Inverse variance weighted (fixed effects)
Inverse variance weighted (multiplicative random effects)
MR Egger
MR RAPS
Weighted median
Weighted mode
0.05
0.00
−0.05
−0.10
0.1
0.2
SNP effect on Histone H2A type 3
SNP effect on Gut microbiota abundance (genus Parasutterella id.2892)

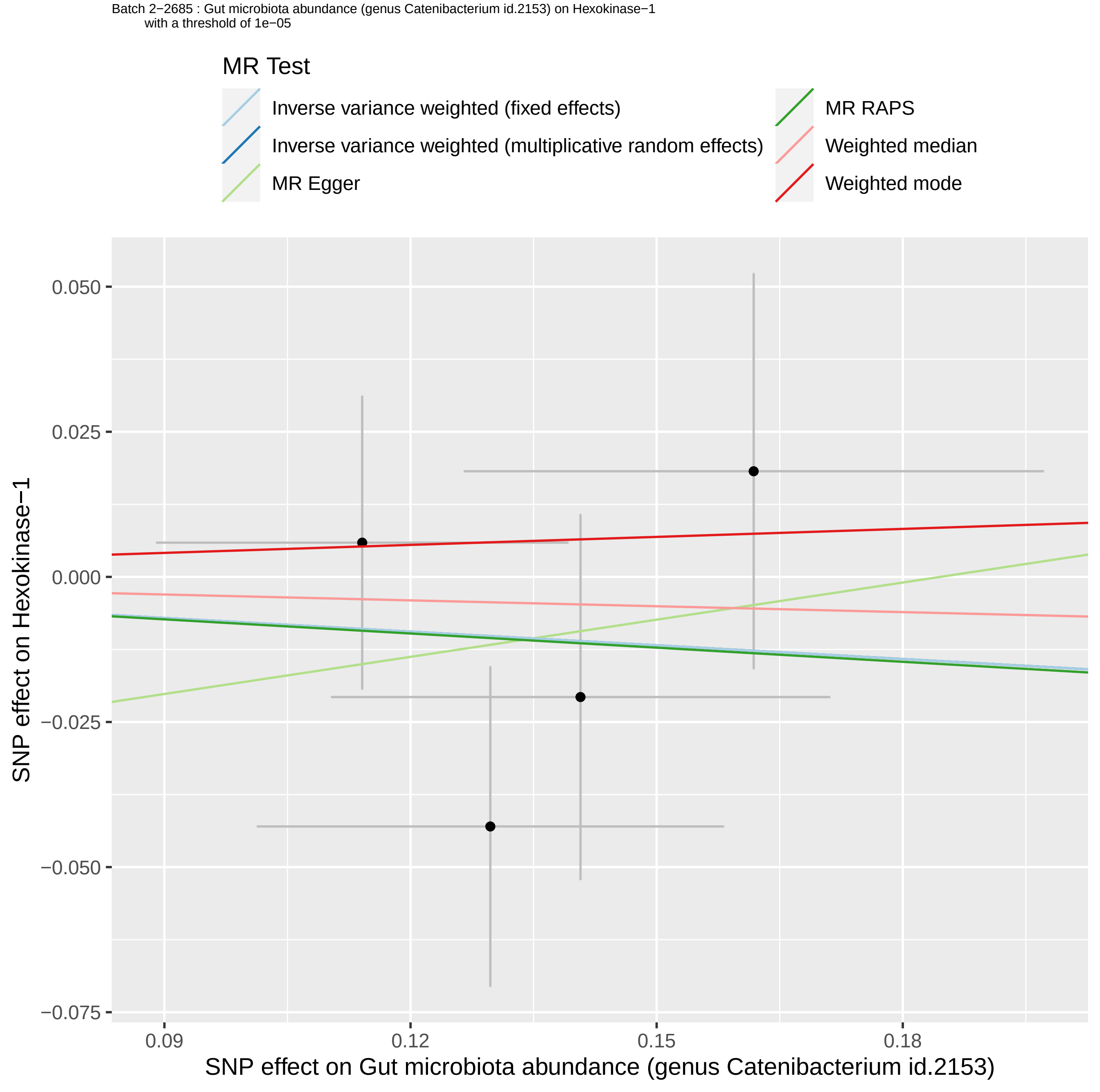
Batch 2−2685 : Gut microbiota abundance (genus Catenibacterium id.2153) on Hexokinase−1
with a threshold of 1e−05
MR Test
Inverse variance weighted (fixed effects)
Inverse variance weighted (multiplicative random effects)
MR Egger
MR RAPS
Weighted median
Weighted mode
0.050
0.025
0.000
−0.025
−0.050
−0.075
0.09
0.12
0.15
0.18
SNP effect on Hexokinase−1
SNP effect on Gut microbiota abundance (genus Catenibacterium id.2153)

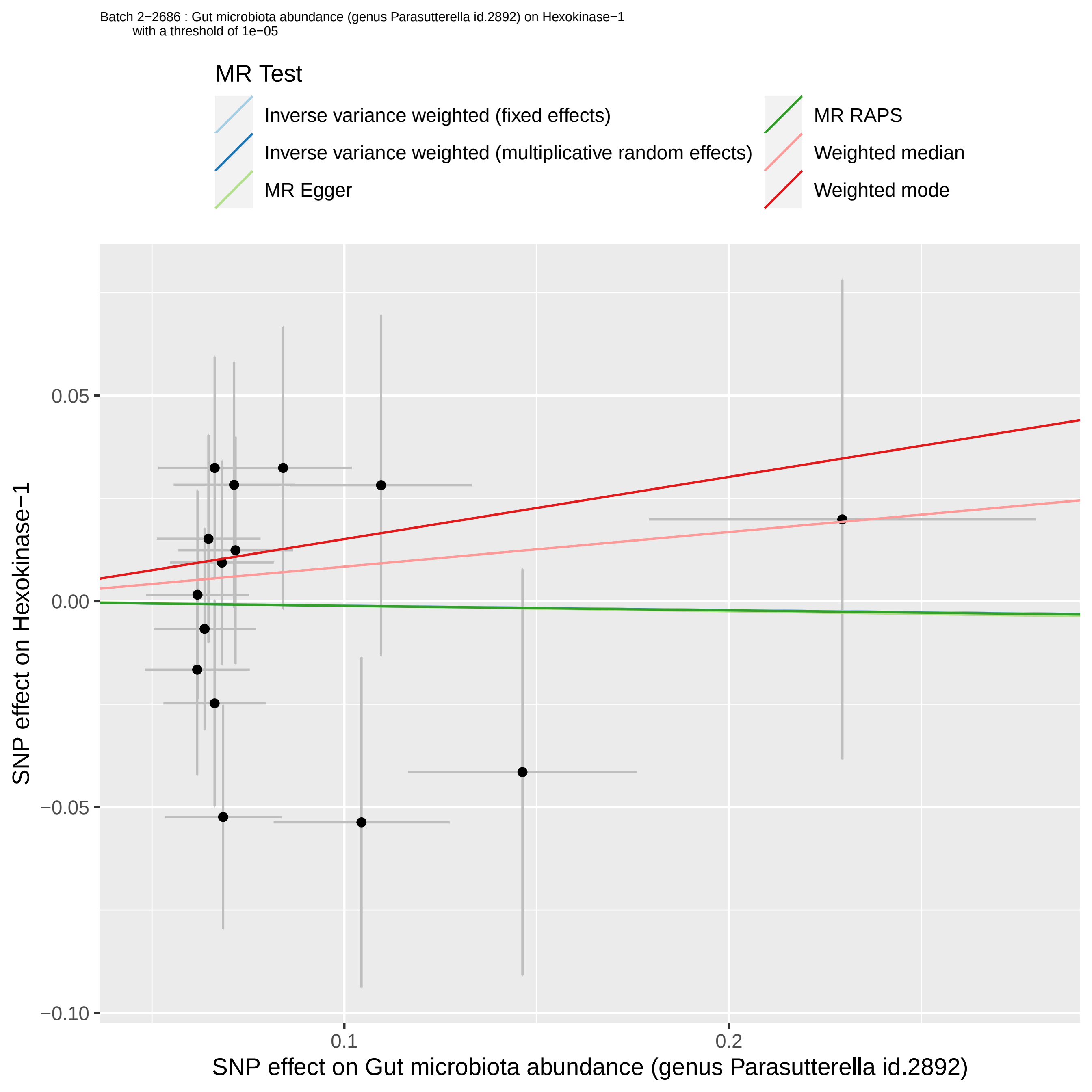
Batch 2−2686 : Gut microbiota abundance (genus Parasutterella id.2892) on Hexokinase−1
with a threshold of 1e−05
MR Test
Inverse variance weighted (fixed effects)
Inverse variance weighted (multiplicative random effects)
MR Egger
MR RAPS
Weighted median
Weighted mode
0.05
0.00
−0.05
−0.10
0.1
0.2
SNP effect on Hexokinase−1
SNP effect on Gut microbiota abundance (genus Parasutterella id.2892)

Batch 2−2687 : Gut microbiota abundance (genus Catenibacterium id.2153) on Hexokinase−2
with a threshold of 1e−05

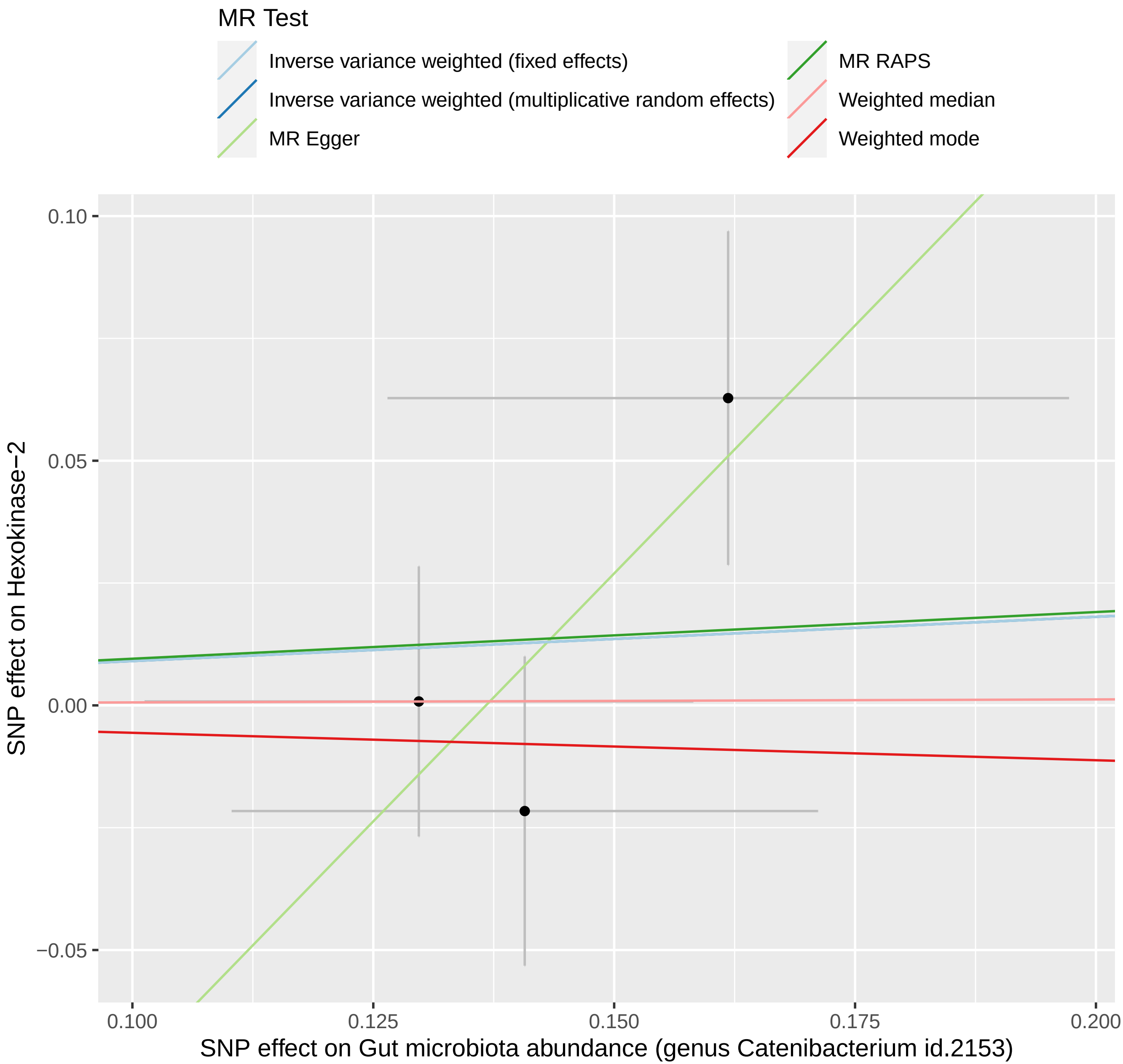

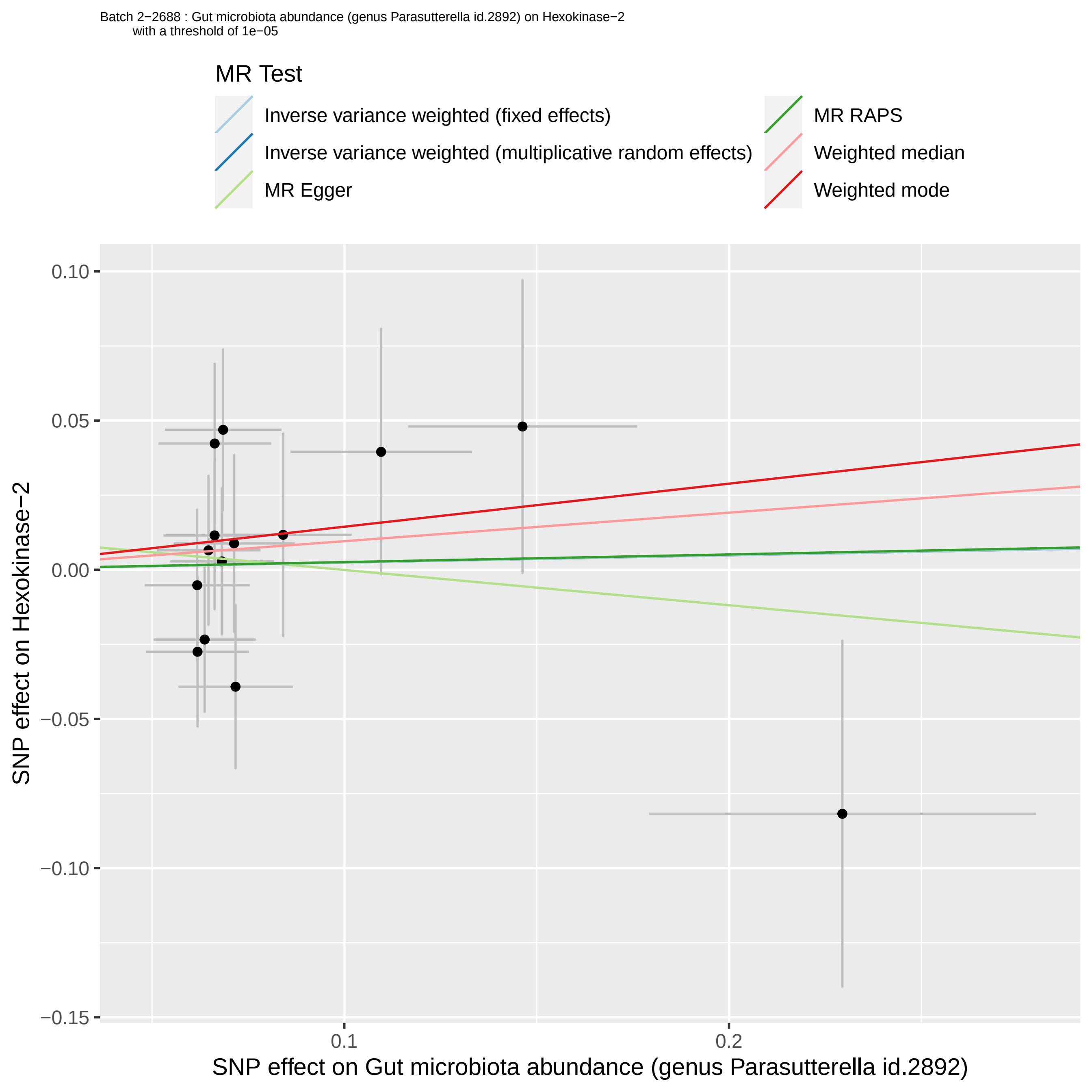
Batch 2−2688 : Gut microbiota abundance (genus Parasutterella id.2892) on Hexokinase−2
with a threshold of 1e−05
MR Test
Inverse variance weighted (fixed effects)
Inverse variance weighted (multiplicative random effects)
MR Egger
MR RAPS
Weighted median
Weighted mode
0.10
0.05
0.00
−0.05
−0.10
−0.15
0.1
0.2
SNP effect on Hexokinase−2
SNP effect on Gut microbiota abundance (genus Parasutterella id.2892)

Batch 2−2689 : Gut microbiota abundance (genus Catenibacterium id.2153) on HLA class II histocompatibility antigen, DM alpha chain with a threshold of 1e−05

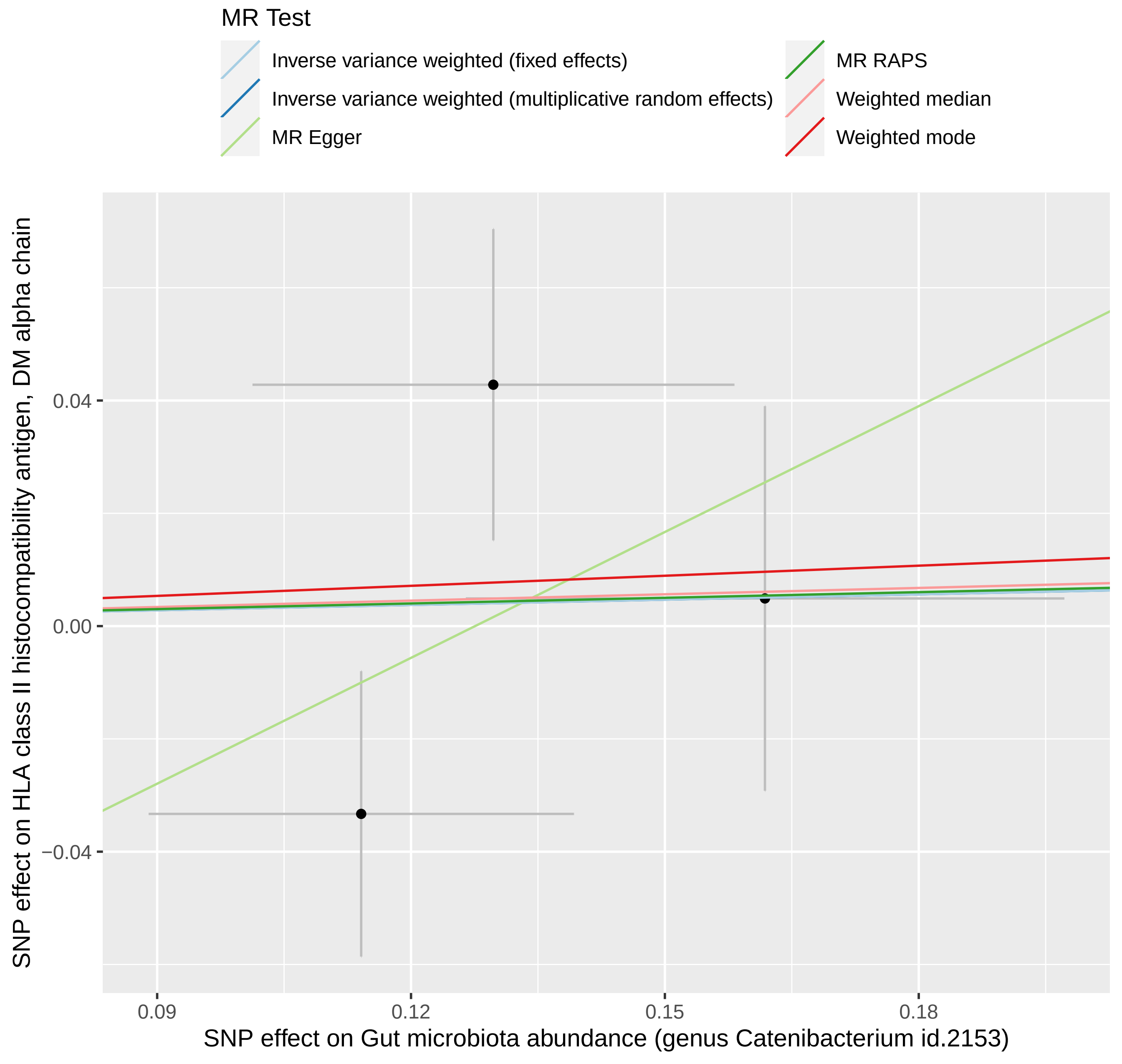

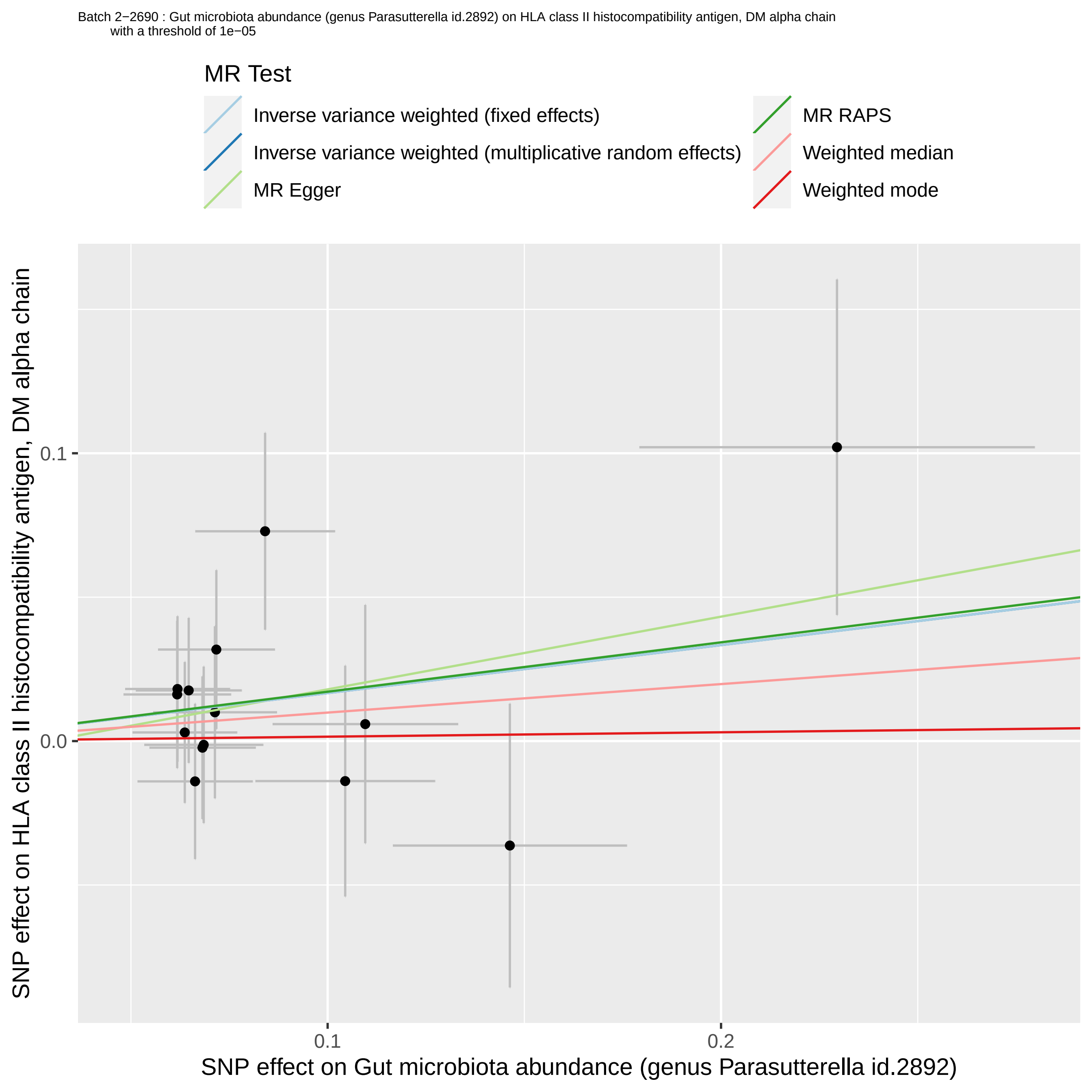

Batch 2−2690 : Gut microbiota abundance (genus Parasutterella id.2892) on HLA class II histocompatibility antigen, DM alpha chain
with a threshold of 1e−05
MR Test
Inverse variance weighted (fixed effects)
Inverse variance weighted (multiplicative random effects)
MR Egger
MR RAPS
Weighted median
Weighted mode
SNP effect on HLA class II histocompatibility antigen, DM alpha chain
0.1
0.0
0.1
0.2
SNP effect on Gut microbiota abundance (genus Parasutterella id.2892)

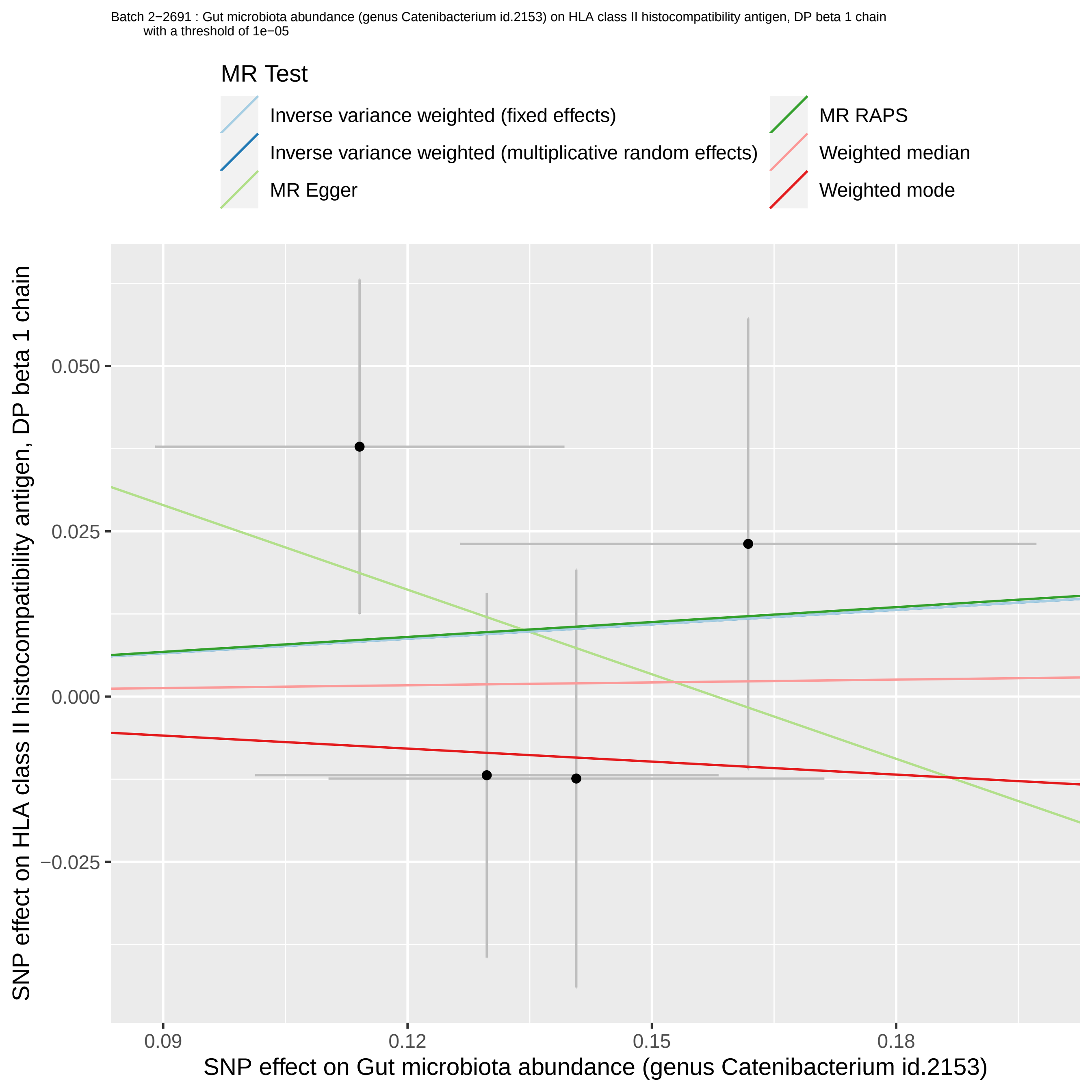

Batch 2−2691 : Gut microbiota abundance (genus Catenibacterium id.2153) on HLA class II histocompatibility antigen, DP beta 1 chain
with a threshold of 1e−05
MR Test
Inverse variance weighted (fixed effects)
Inverse variance weighted (multiplicative random effects)
MR Egger
MR RAPS
Weighted median
Weighted mode
0.050
0.025
0.000
−0.025
0.09
0.12
0.15
0.18
SNP effect on HLA class II histocompatibility antigen, DP beta 1 chain
SNP effect on Gut microbiota abundance (genus Catenibacterium id.2153)

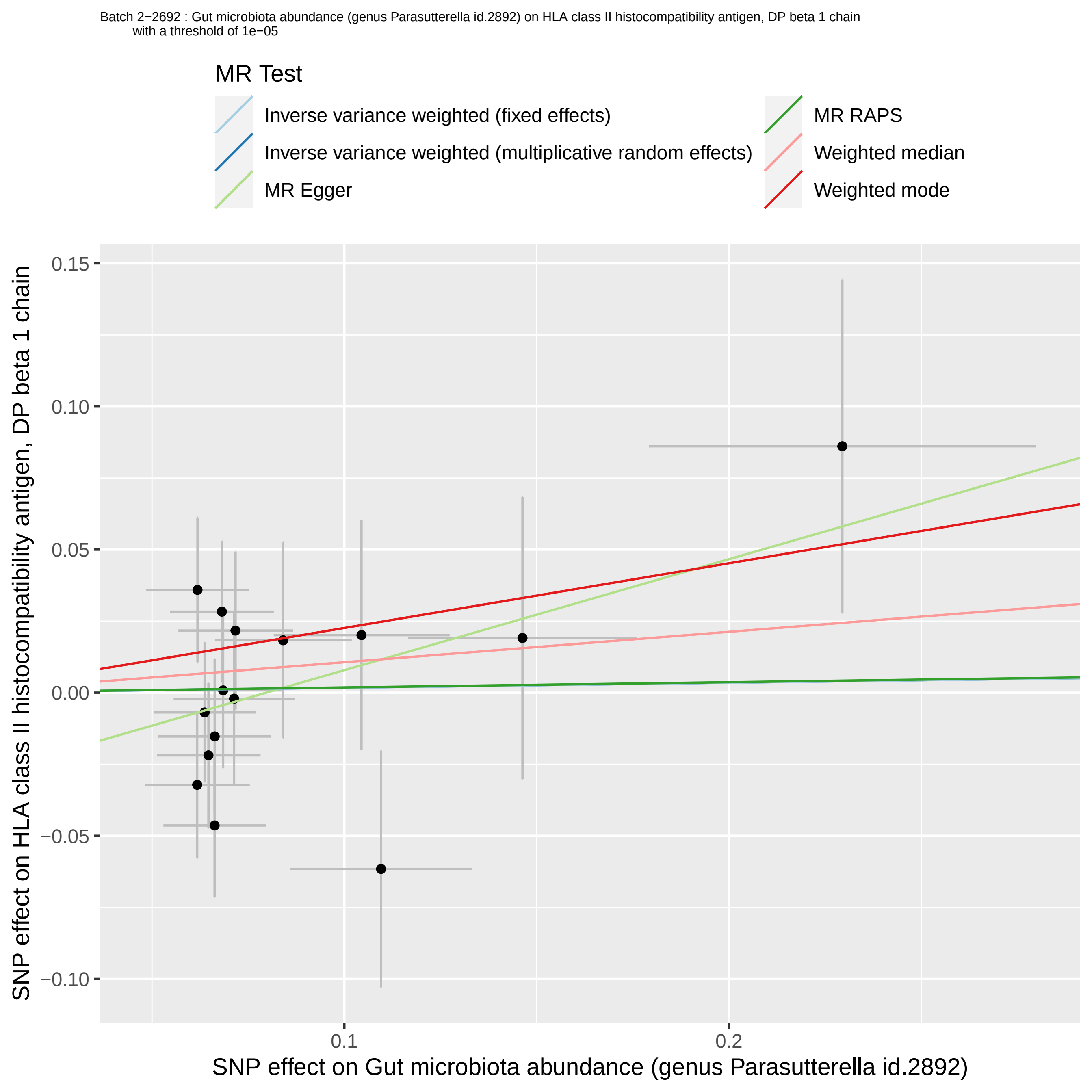
Batch 2−2692 : Gut microbiota abundance (genus Parasutterella id.2892) on HLA class II histocompatibility antigen, DP beta 1 chain
with a threshold of 1e−05
MR Test
Inverse variance weighted (fixed effects)
Inverse variance weighted (multiplicative random effects)
MR Egger
MR RAPS
Weighted median
Weighted mode
0.15
0.10
0.05
0.00
−0.05
−0.10
0.1
0.2
SNP effect on HLA class II histocompatibility antigen, DP beta 1 chain
SNP effect on Gut microbiota abundance (genus Parasutterella id.2892)

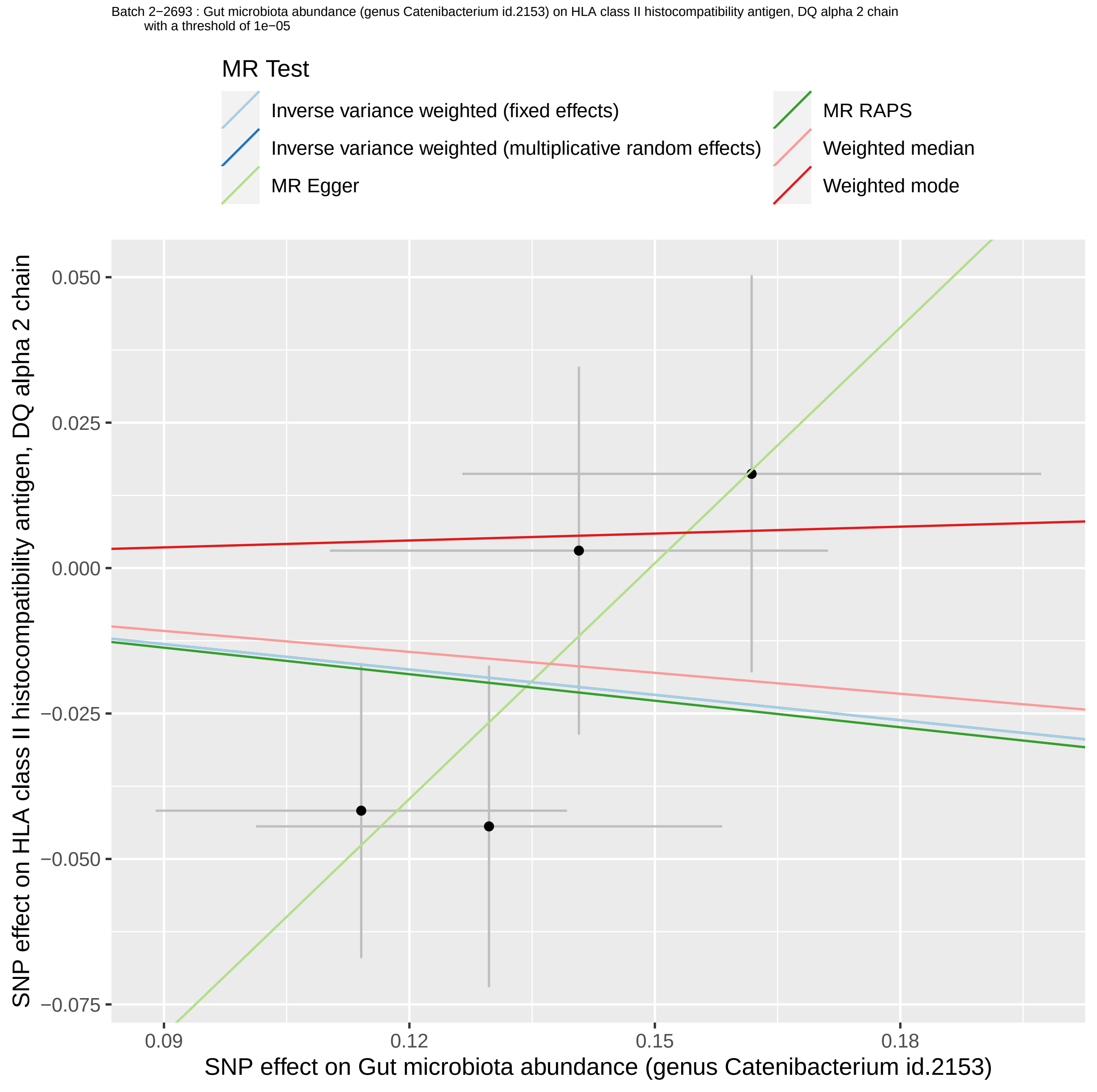
Batch 2−2693 : Gut microbiota abundance (genus Catenibacterium id.2153) on HLA class II histocompatibility antigen, DQ alpha 2 chain
with a threshold of 1e−05
MR Test
Inverse variance weighted (fixed effects)
Inverse variance weighted (multiplicative random effects)
MR Egger
MR RAPS
Weighted median
Weighted mode
SNP effect on HLA class II histocompatibility antigen, DQ alpha 2 chain
0.050
0.025
0.000
−0.025
−0.050
−0.075
0.09
0.12
0.15
0.18
SNP effect on Gut microbiota abundance (genus Catenibacterium id.2153)

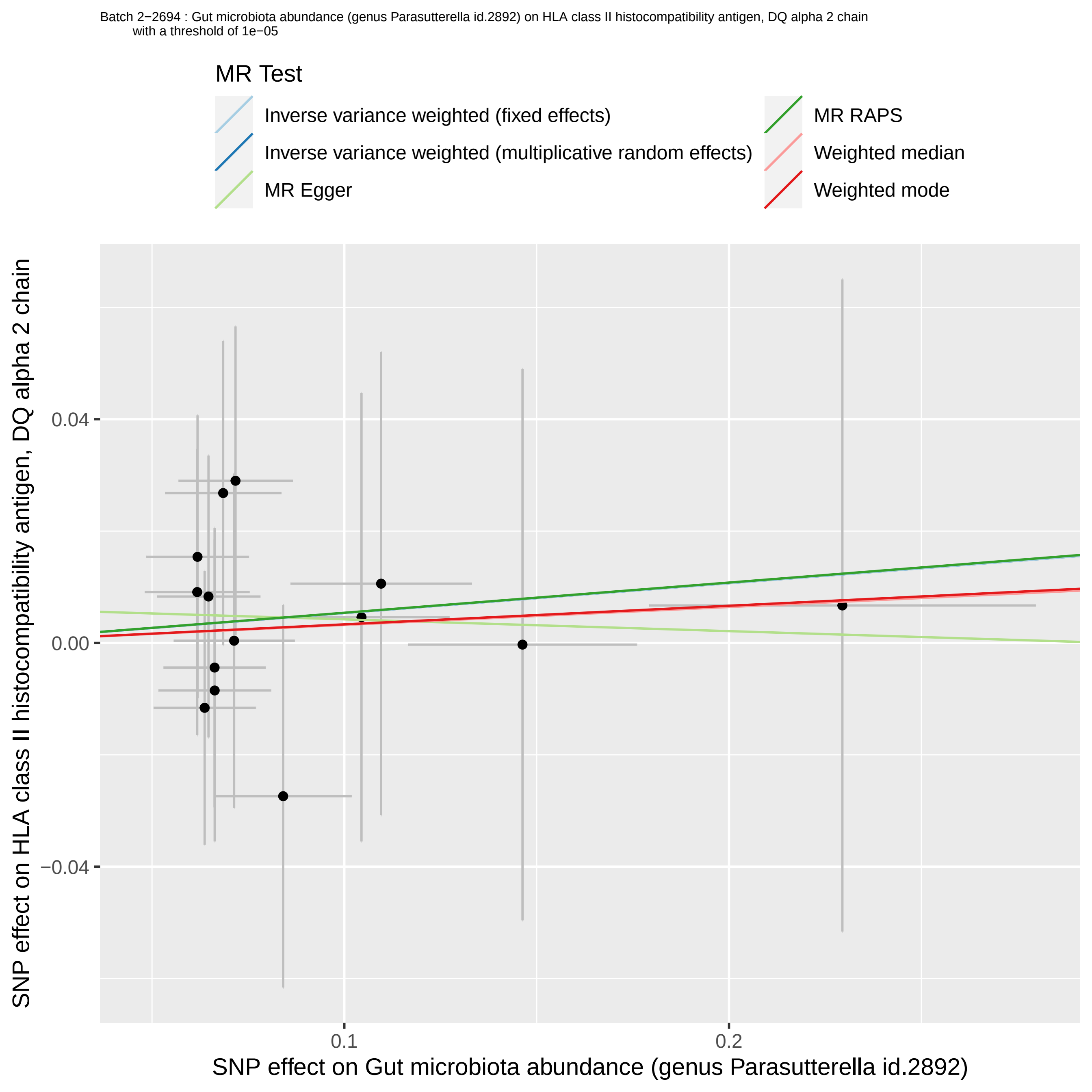
Batch 2−2694 : Gut microbiota abundance (genus Parasutterella id.2892) on HLA class II histocompatibility antigen, DQ alpha 2 chain
with a threshold of 1e−05
MR Test
Inverse variance weighted (fixed effects)
Inverse variance weighted (multiplicative random effects)
MR Egger
MR RAPS
Weighted median
Weighted mode
SNP effect on HLA class II histocompatibility antigen, DQ alpha 2 chain
0.04
0.00
−0.04
0.1
0.2
SNP effect on Gut microbiota abundance (genus Parasutterella id.2892)

Batch 2−2695 : Gut microbiota abundance (genus Catenibacterium id.2153) on Porphobilinogen deaminase
with a threshold of 1e−05

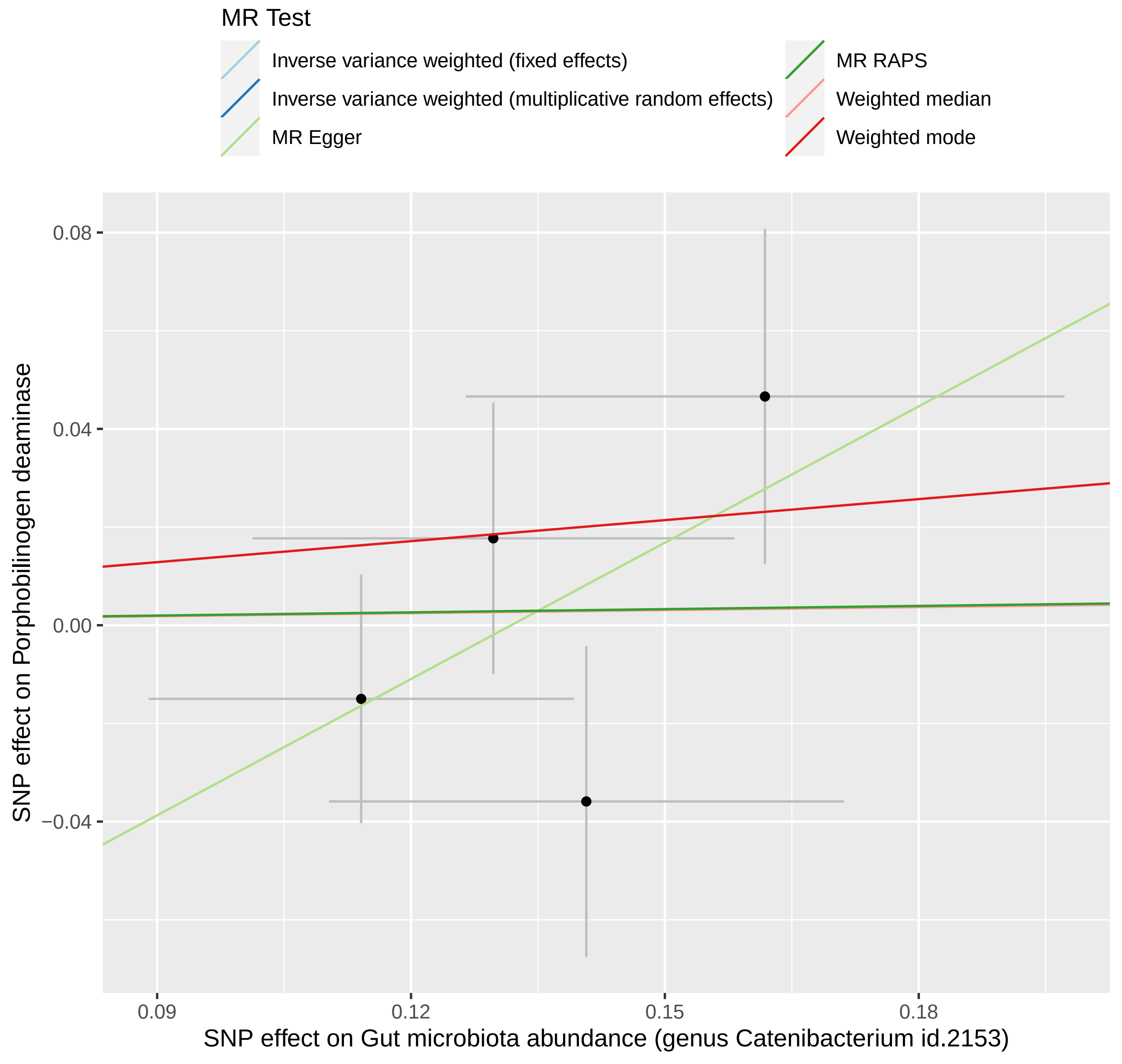

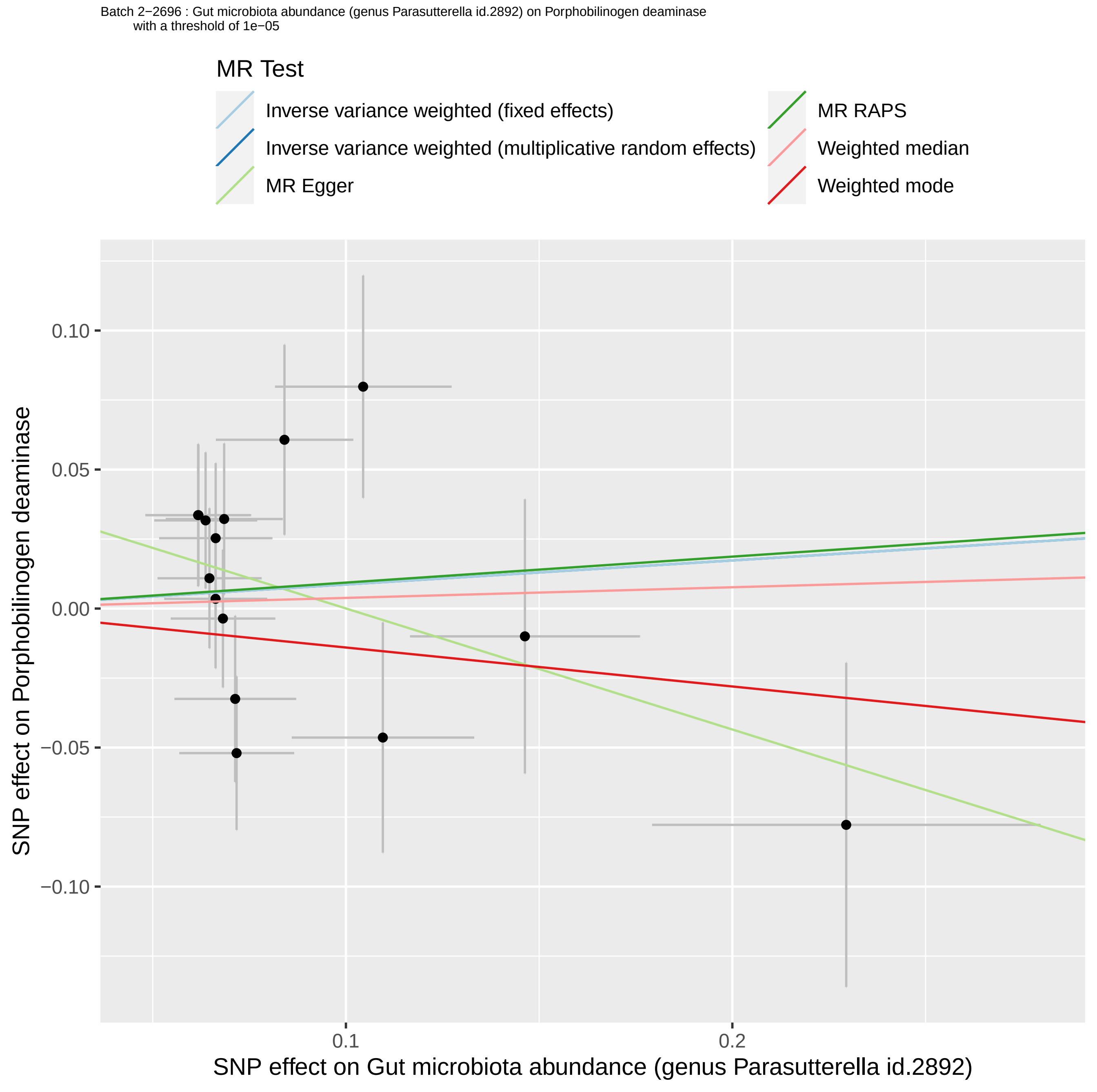

Batch 2−2696 : Gut microbiota abundance (genus Parasutterella id.2892) on Porphobilinogen deaminase
with a threshold of 1e−05
MR Test
Inverse variance weighted (fixed effects)
Inverse variance weighted (multiplicative random effects)
MR Egger
MR RAPS
Weighted median
Weighted mode
0.10
0.05
0.00
−0.05
−0.10
0.1
0.2
SNP effect on Porphobilinogen deaminase
SNP effect on Gut microbiota abundance (genus Parasutterella id.2892)

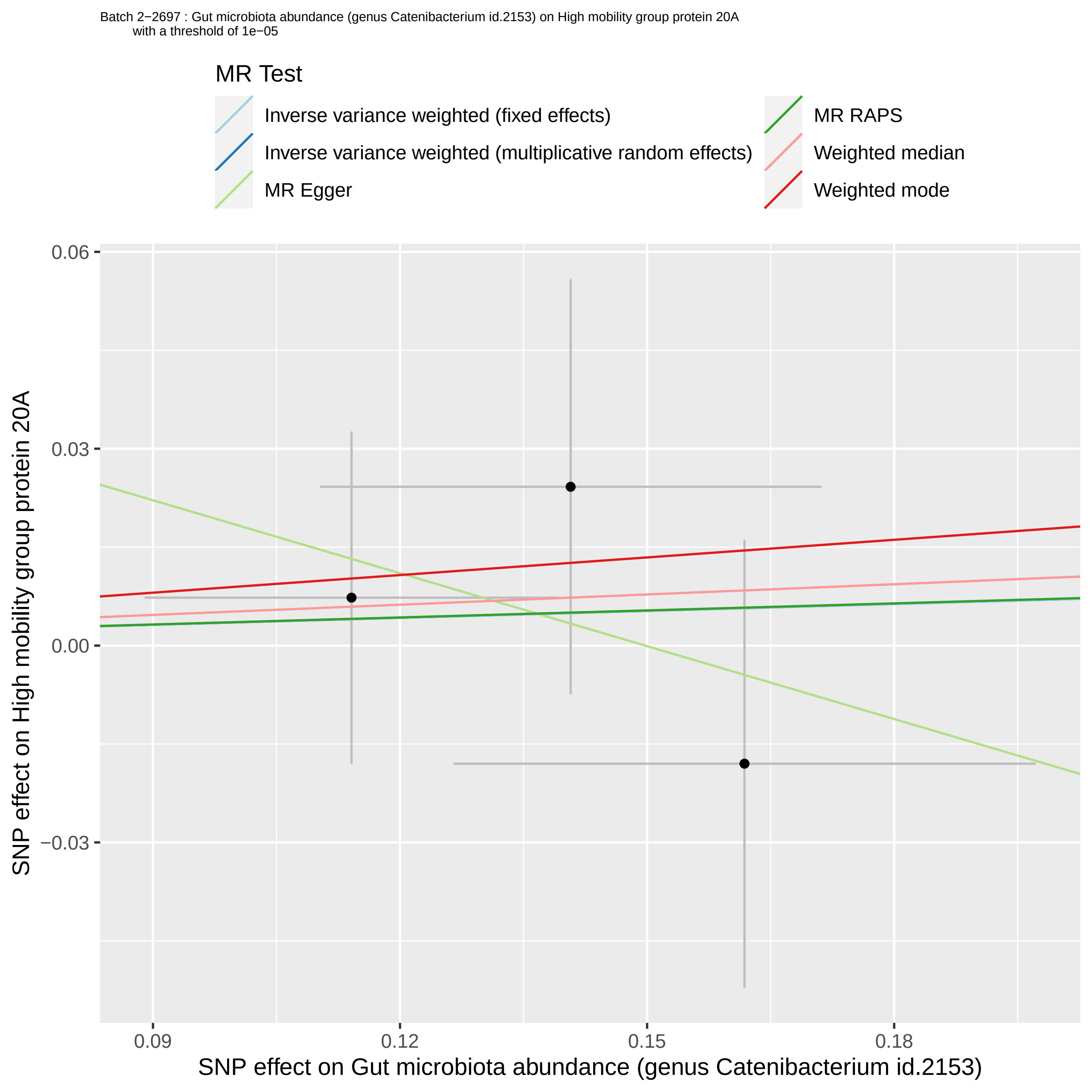
Batch 2−2697 : Gut microbiota abundance (genus Catenibacterium id.2153) on High mobility group protein 20A
with a threshold of 1e−05
MR Test
Inverse variance weighted (fixed effects)
Inverse variance weighted (multiplicative random effects)
MR Egger
MR RAPS
Weighted median
Weighted mode
0.06
0.03
0.00
−0.03
0.09
0.12
0.15
0.18
SNP effect on High mobility group protein 20A
SNP effect on Gut microbiota abundance (genus Catenibacterium id.2153)

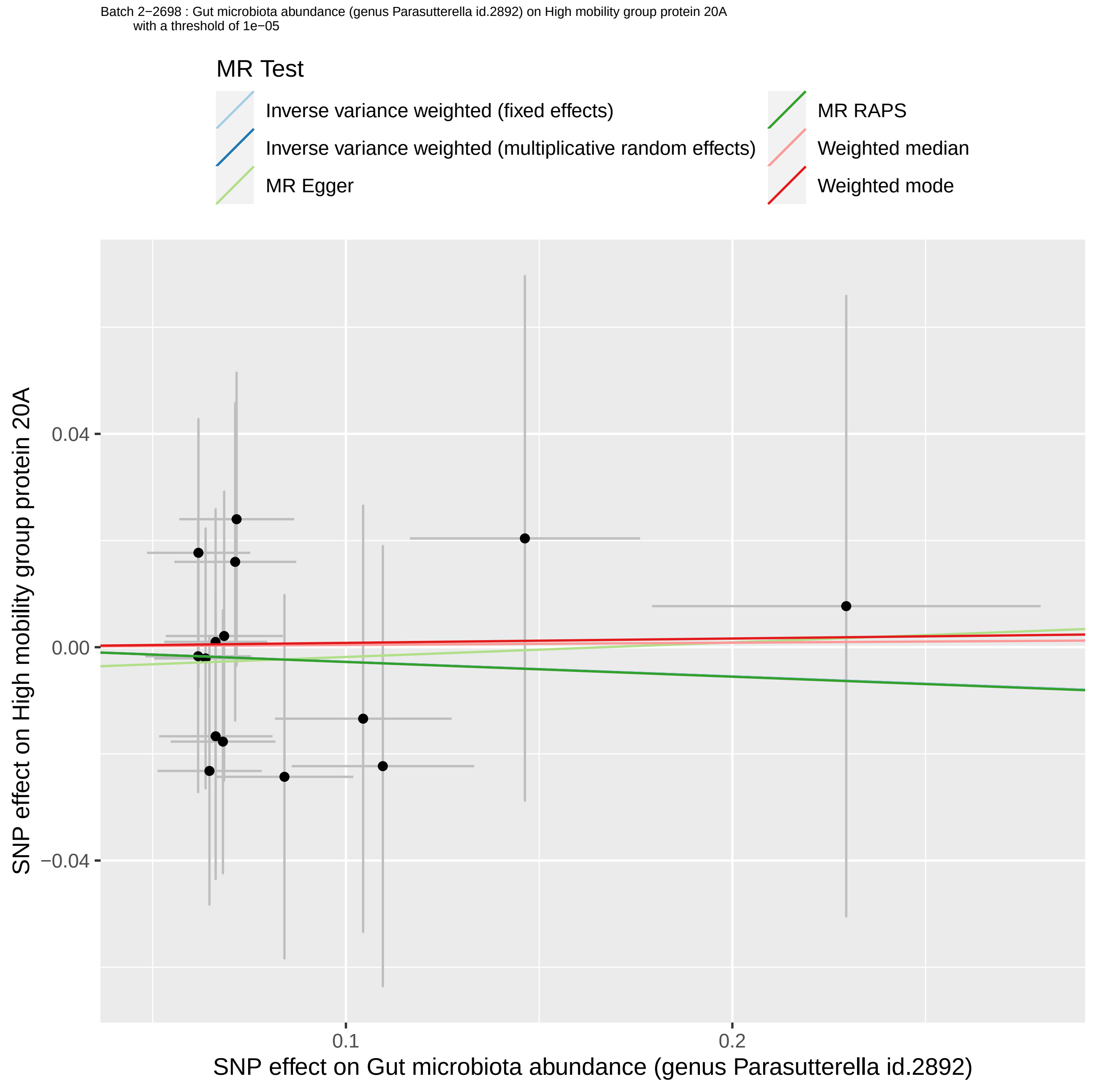
Batch 2−2698 : Gut microbiota abundance (genus Parasutterella id.2892) on High mobility group protein 20A
with a threshold of 1e−05
MR Test
Inverse variance weighted (fixed effects)
Inverse variance weighted (multiplicative random effects)
MR Egger
MR RAPS
Weighted median
Weighted mode
SNP effect on High mobility group protein 20A
0.04
0.00
−0.04
0.1
0.2
SNP effect on Gut microbiota abundance (genus Parasutterella id.2892)

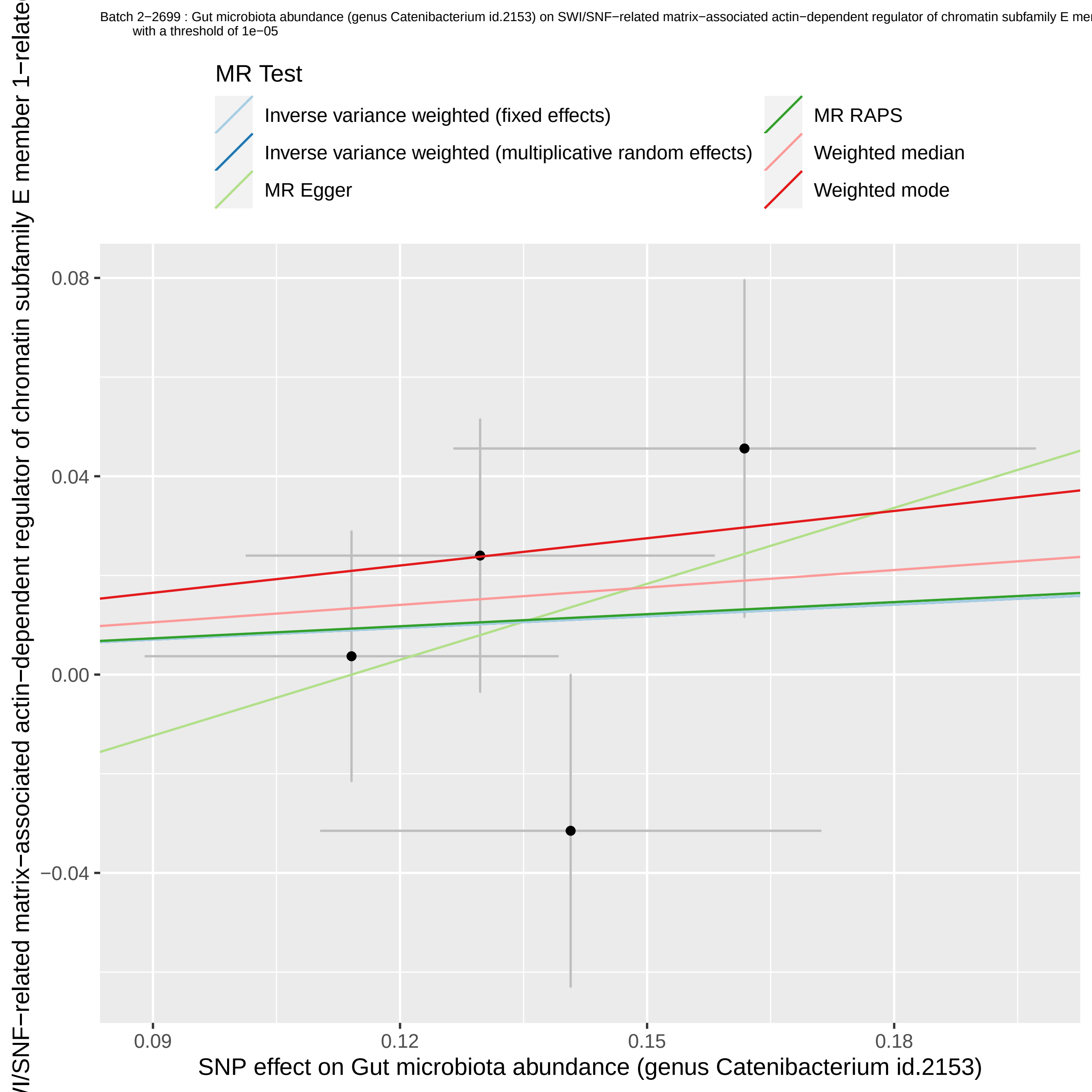

Batch 2−2699 : Gut microbiota abundance (genus Catenibacterium id.2153) on SWI/SNF−related matrix−associated actin−dependent regulator of chromatin subfamily E me
with a threshold of 1e−05
MR Test
Inverse variance weighted (fixed effects)
Inverse variance weighted (multiplicative random effects)
MR Egger
MR RAPS
Weighted median
Weighted mode
0.08
0.04
0.00
−0.04
0.09
0.12
0.15
0.18
SNP effect on Gut microbiota abundance (genus Catenibacterium id.2153)
/I/SNF−related matrix−associated actin−dependent regulator of chromatin subfamily E member 1−relate

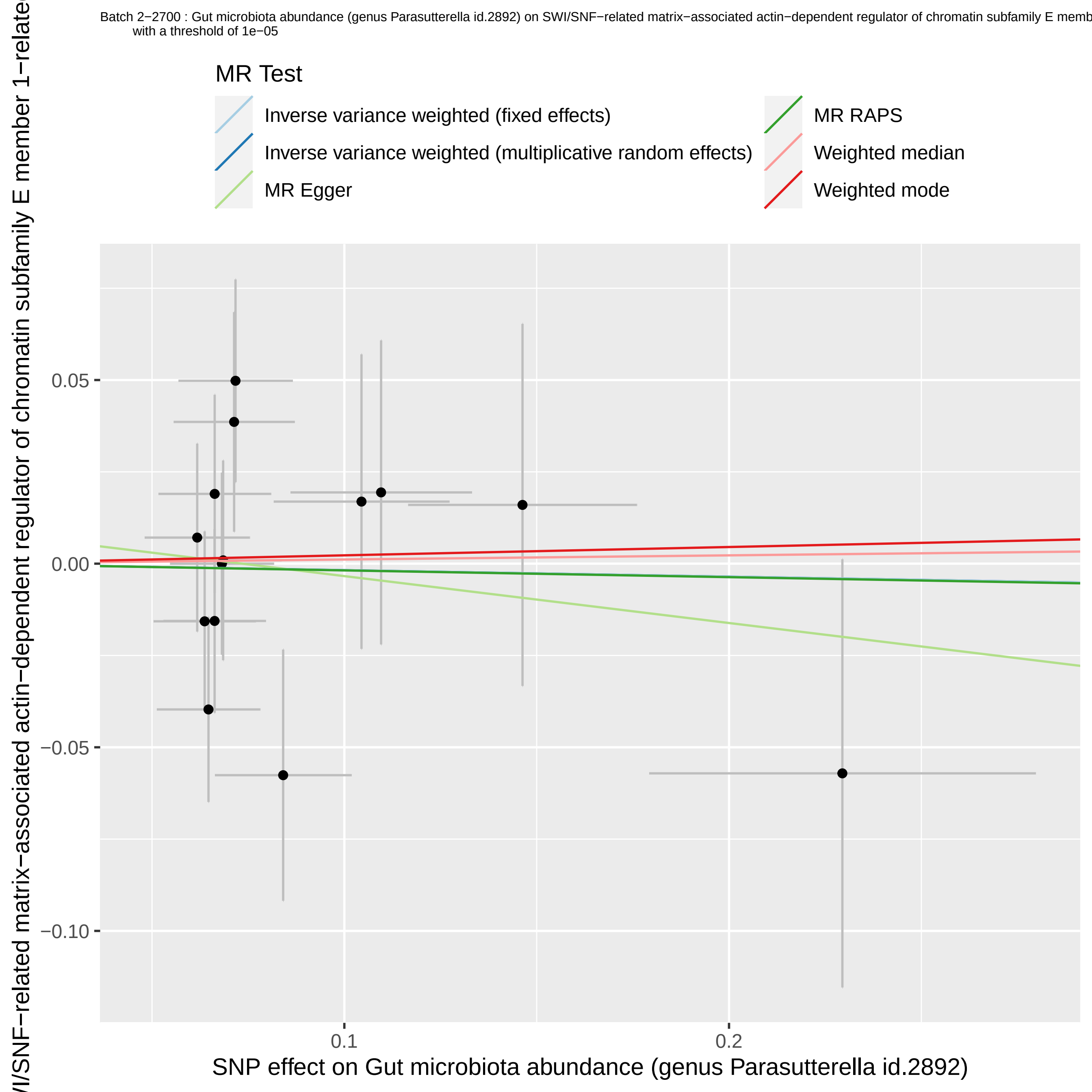

Batch 2−2700 : Gut microbiota abundance (genus Parasutterella id.2892) on SWI/SNF−related matrix−associated actin−dependent regulator of chromatin subfamily E memb
with a threshold of 1e−05
MR Test
Inverse variance weighted (fixed effects)
Inverse variance weighted (multiplicative random effects)
MR Egger
MR RAPS
Weighted median
Weighted mode
0.05
0.00
−0.05
−0.10
0.1
0.2
SNP effect on Gut microbiota abundance (genus Parasutterella id.2892)
I/SNF−related matrix−associated actin−dependent regulator of chromatin subfamily E member 1−relate

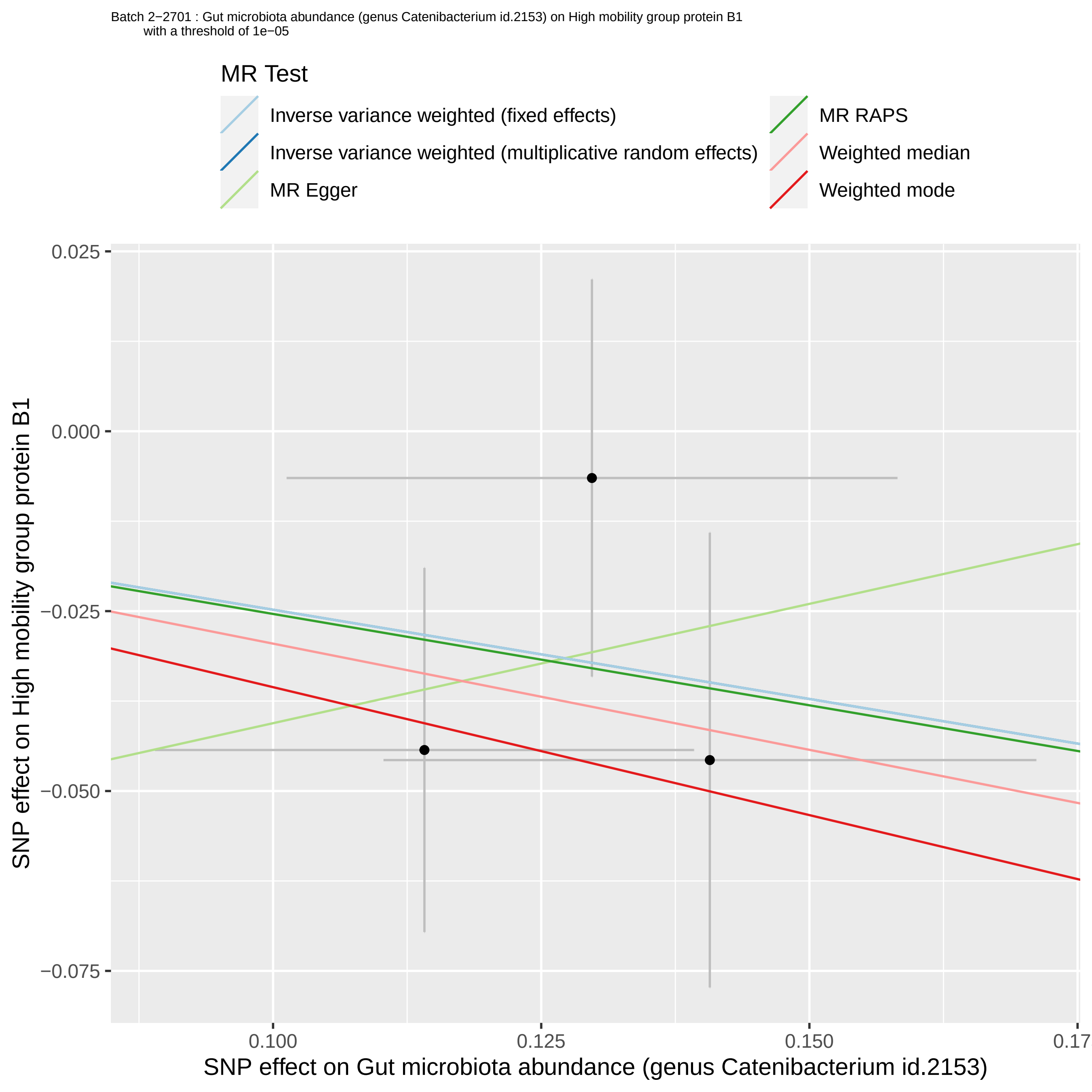
Batch 2−2701 : Gut microbiota abundance (genus Catenibacterium id.2153) on High mobility group protein B1
with a threshold of 1e−05
MR Test
Inverse variance weighted (fixed effects)
Inverse variance weighted (multiplicative random effects)
MR Egger
MR RAPS
Weighted median
Weighted mode
0.025
0.000
−0.025
−0.050
−0.075
0.100
0.125
0.150
0.17
SNP effect on High mobility group protein B1
SNP effect on Gut microbiota abundance (genus Catenibacterium id.2153)

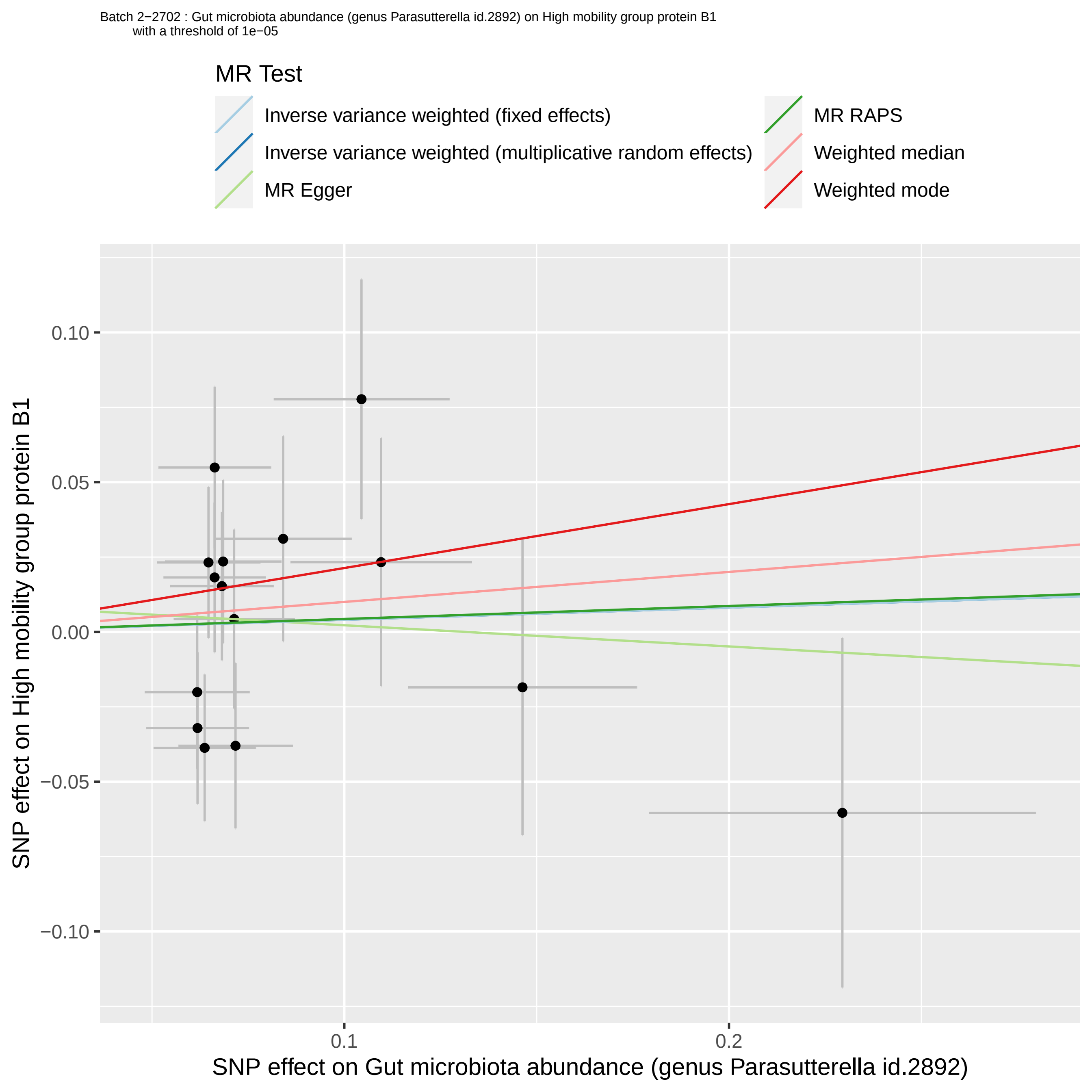

Batch 2−2702 : Gut microbiota abundance (genus Parasutterella id.2892) on High mobility group protein B1
with a threshold of 1e−05
MR Test
Inverse variance weighted (fixed effects)
Inverse variance weighted (multiplicative random effects)
MR Egger
MR RAPS
Weighted median
Weighted mode
0.10
0.05
0.00
−0.05
−0.10
0.1
0.2
SNP effect on High mobility group protein B1
SNP effect on Gut microbiota abundance (genus Parasutterella id.2892)

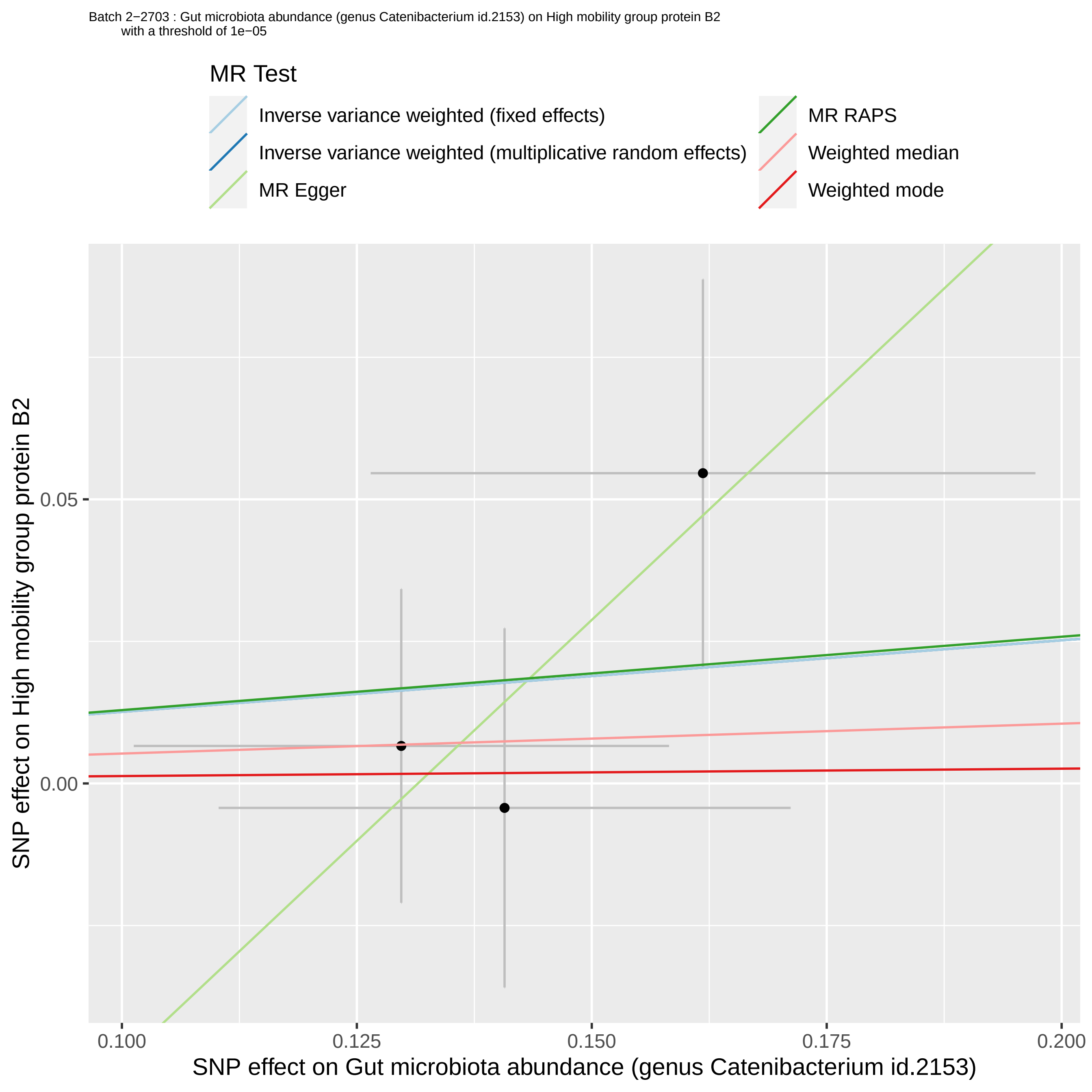

Batch 2−2703 : Gut microbiota abundance (genus Catenibacterium id.2153) on High mobility group protein B2
with a threshold of 1e−05
MR Test
Inverse variance weighted (fixed effects)
Inverse variance weighted (multiplicative random effects)
MR Egger
MR RAPS
Weighted median
Weighted mode
0.05
0.00
0.100
0.125
0.150
0.175
0.200
SNP effect on High mobility group protein B2
SNP effect on Gut microbiota abundance (genus Catenibacterium id.2153)

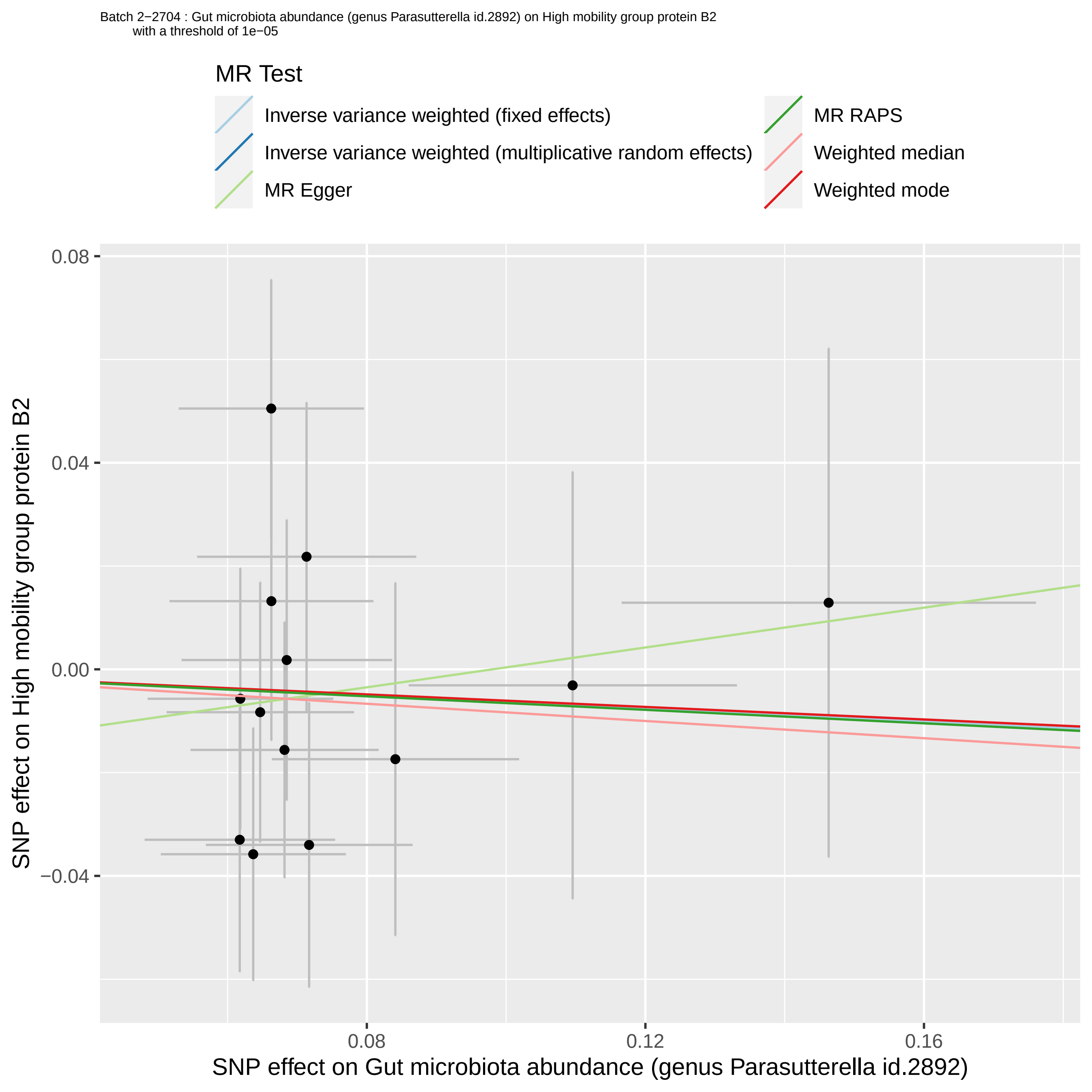
Batch 2−2704 : Gut microbiota abundance (genus Parasutterella id.2892) on High mobility group protein B2
with a threshold of 1e−05
MR Test
Inverse variance weighted (fixed effects)
Inverse variance weighted (multiplicative random effects)
MR Egger
MR RAPS
Weighted median
Weighted mode
0.08
0.04
0.00
−0.04
0.08
0.12
0.16
SNP effect on High mobility group protein B2
SNP effect on Gut microbiota abundance (genus Parasutterella id.2892)

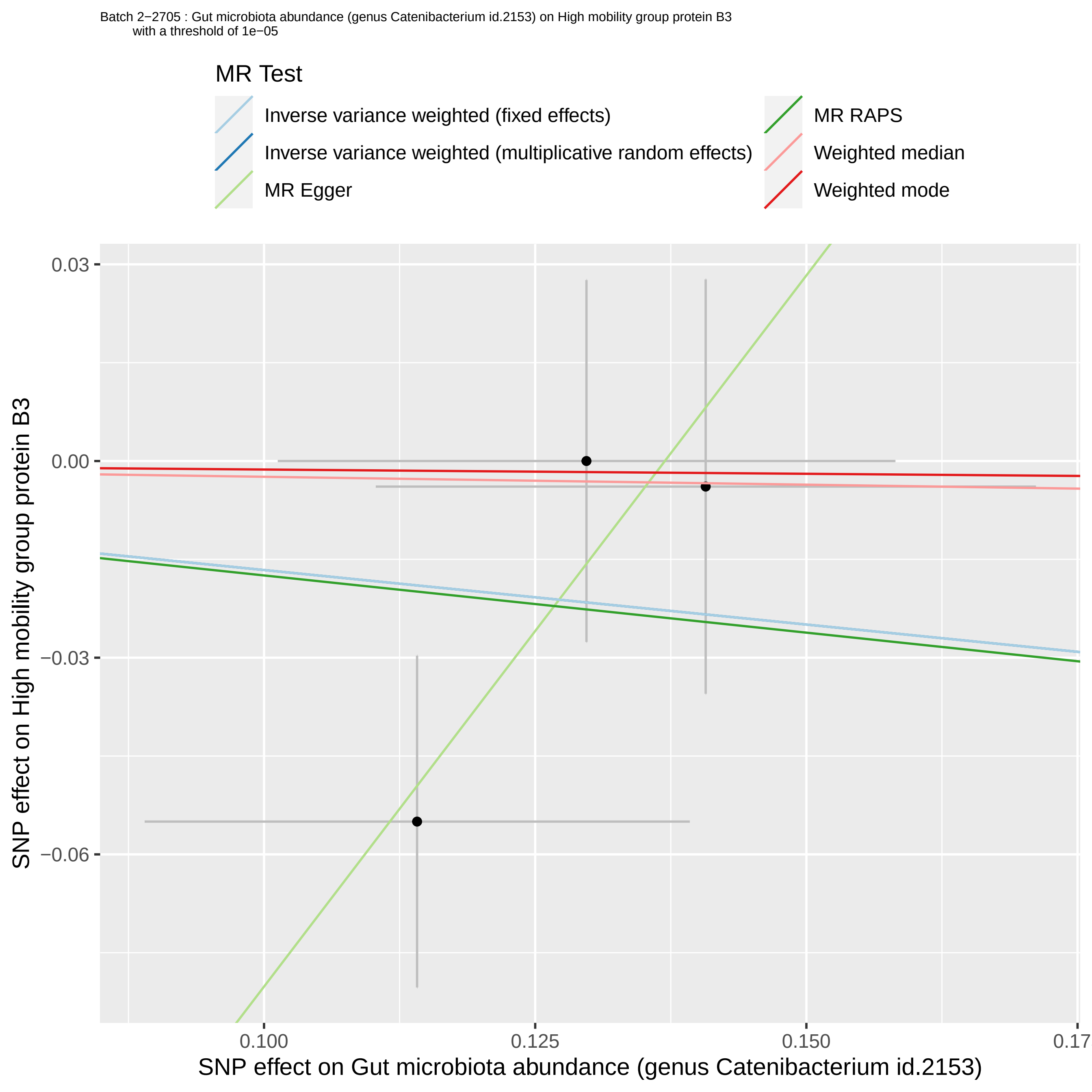

Batch 2−2705 : Gut microbiota abundance (genus Catenibacterium id.2153) on High mobility group protein B3
with a threshold of 1e−05
MR Test
Inverse variance weighted (fixed effects)
Inverse variance weighted (multiplicative random effects)
MR Egger
MR RAPS
Weighted median
Weighted mode
SNP effect on High mobility group protein B3
0.03
0.00
−0.03
−0.06
0.100
0.125
0.150
0.17
SNP effect on Gut microbiota abundance (genus Catenibacterium id.2153)

Batch 2−2706 : Gut microbiota abundance (genus Parasutterella id.2892) on High mobility group protein B3
with a threshold of 1e−05

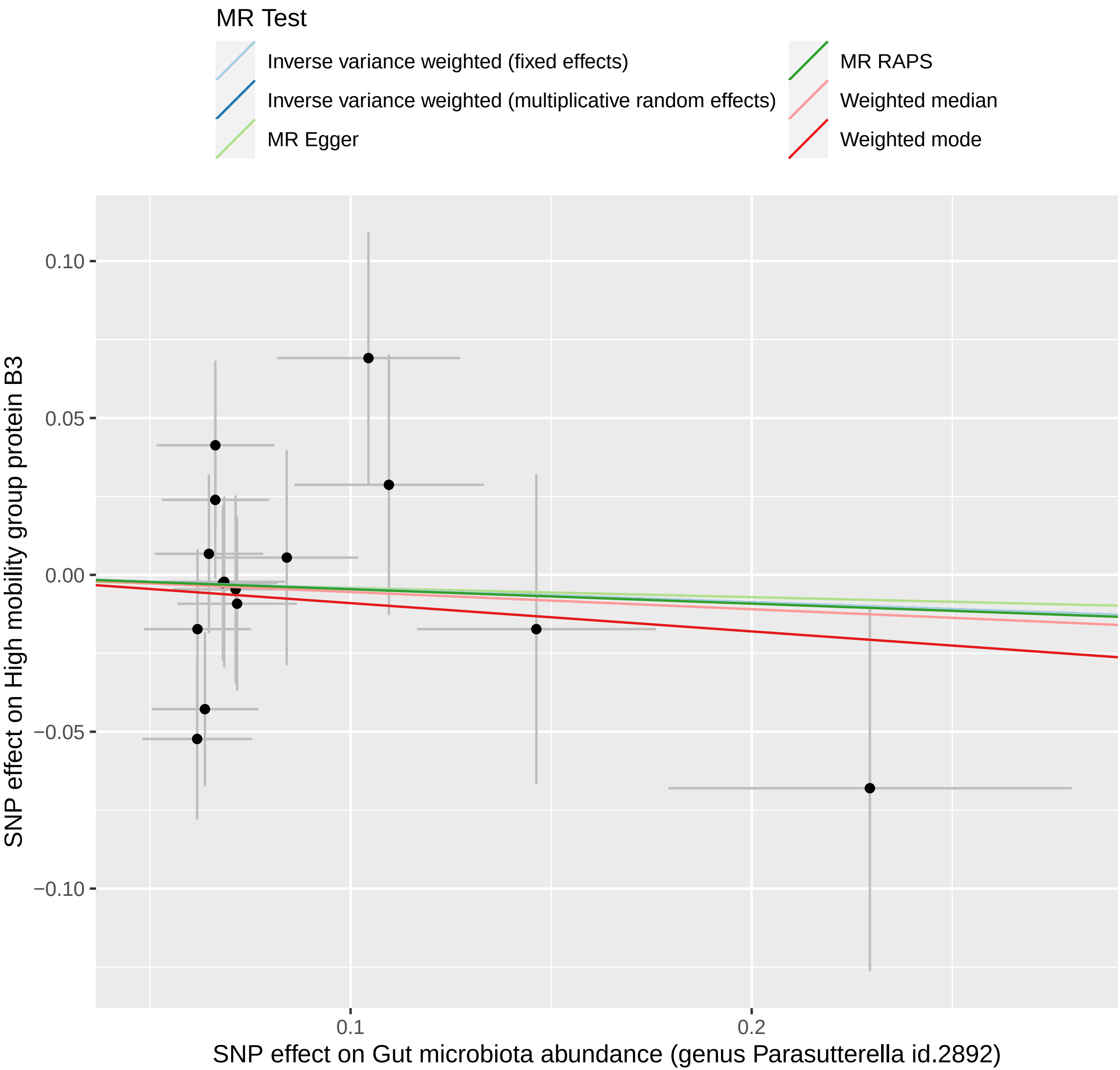

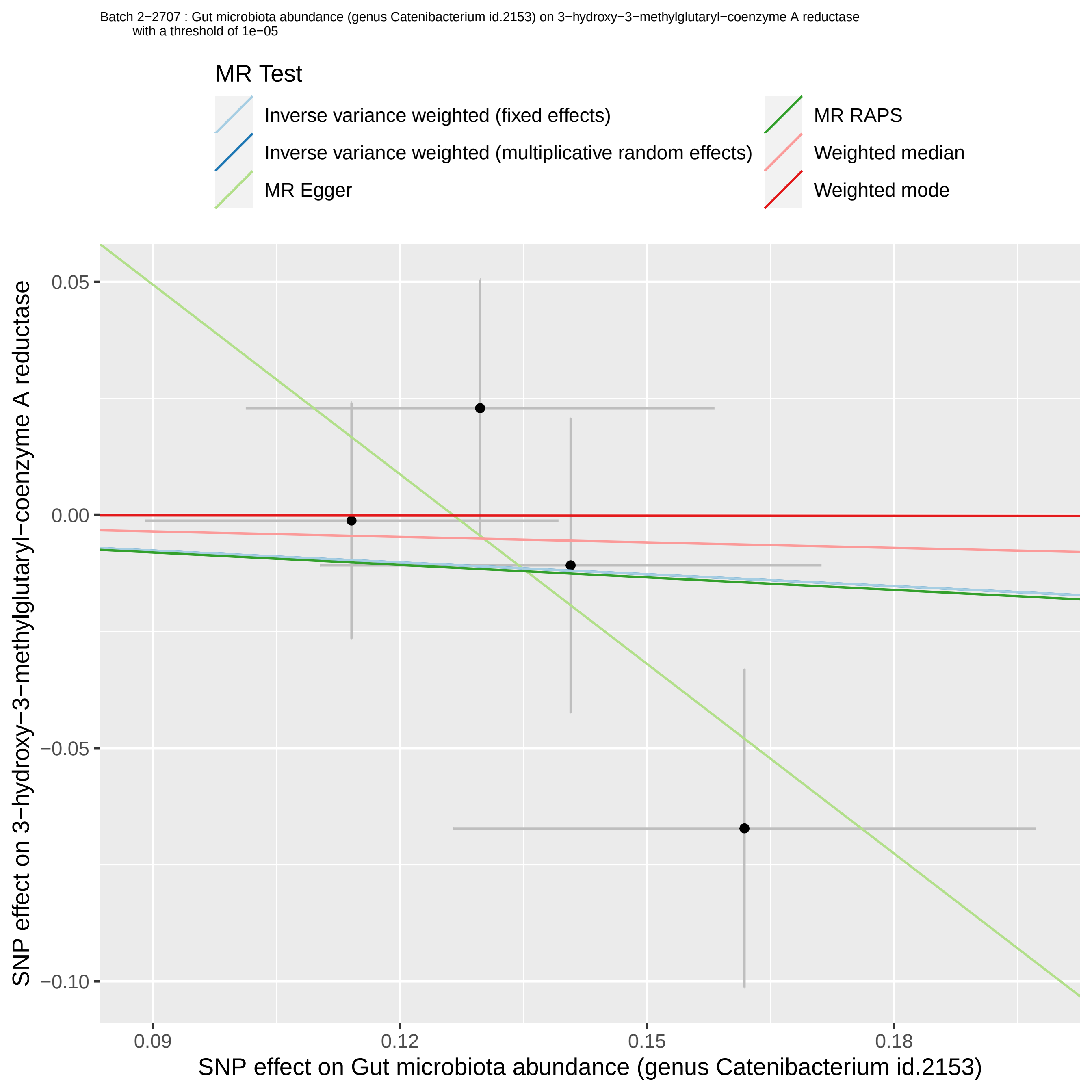
Batch 2−2707 : Gut microbiota abundance (genus Catenibacterium id.2153) on 3−hydroxy−3−methylglutaryl−coenzyme A reductase
with a threshold of 1e−05
MR Test
Inverse variance weighted (fixed effects)
Inverse variance weighted (multiplicative random effects)
MR Egger
MR RAPS
Weighted median
Weighted mode
0.05
0.00
−0.05
−0.10
0.09
0.12
0.15
0.18
SNP effect on 3−hydroxy−3−methylglutaryl−coenzyme A reductase
SNP effect on Gut microbiota abundance (genus Catenibacterium id.2153)

Batch 2−2708 : Gut microbiota abundance (genus Parasutterella id.2892) on 3−hydroxy−3−methylglutaryl−coenzyme A reductase
with a threshold of 1e−05

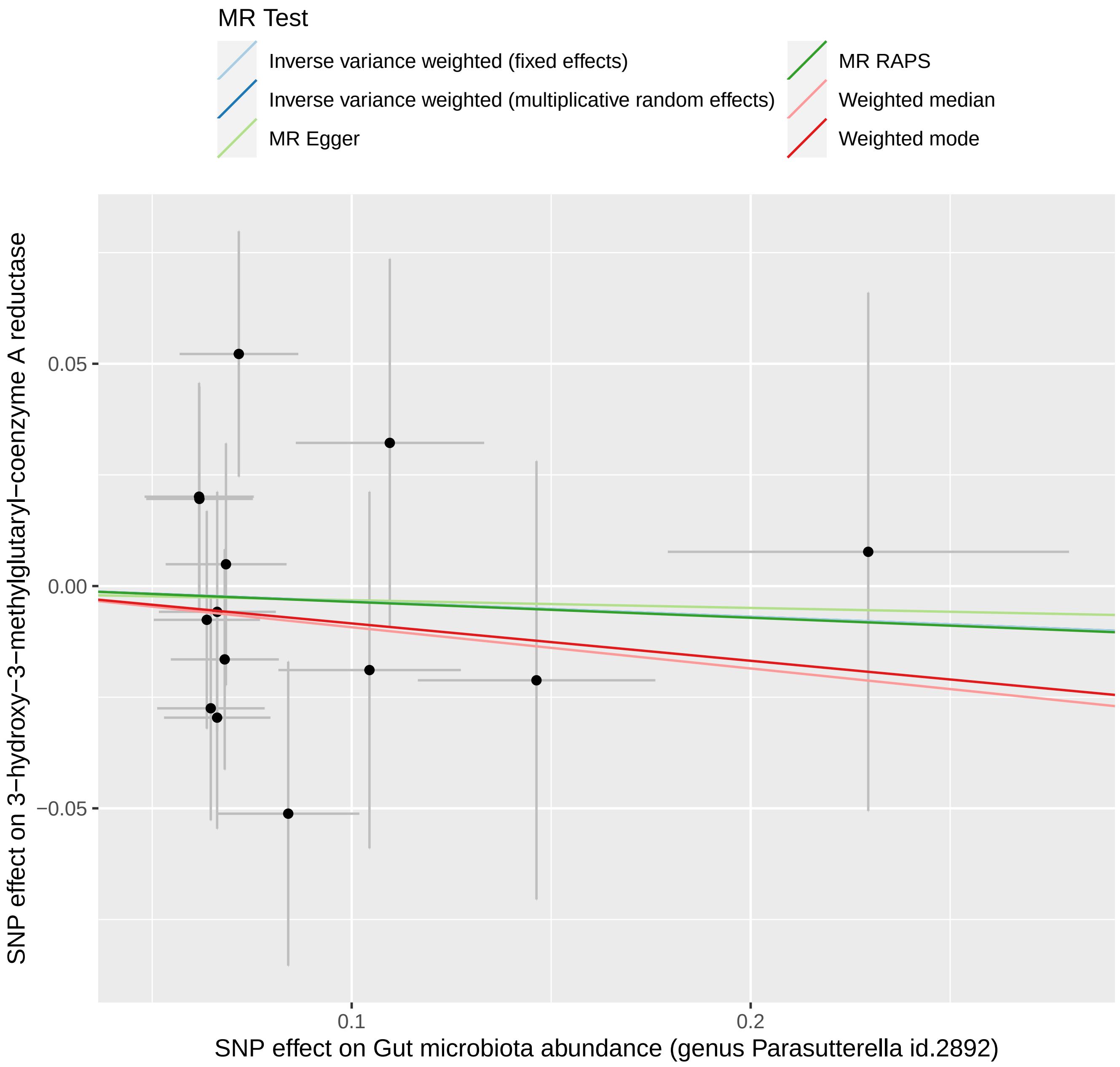

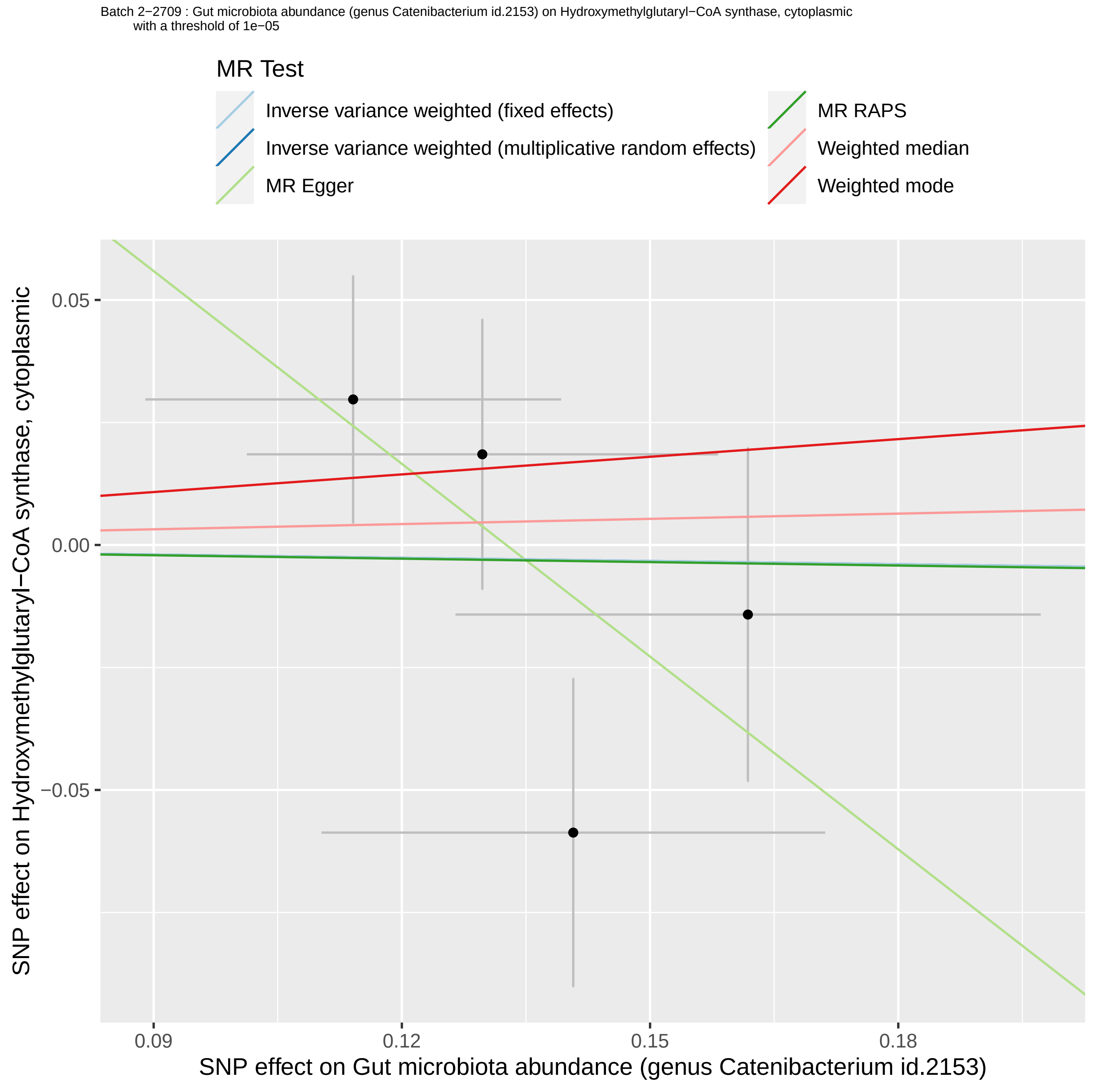

Batch 2−2709 : Gut microbiota abundance (genus Catenibacterium id.2153) on Hydroxymethylglutaryl−CoA synthase, cytoplasmic
with a threshold of 1e−05
MR Test
Inverse variance weighted (fixed effects)
Inverse variance weighted (multiplicative random effects)
MR Egger
MR RAPS
Weighted median
Weighted mode
0.05
0.00
−0.05
0.09
0.12
0.15
0.18
SNP effect on Hydroxymethylglutaryl−CoA synthase, cytoplasmic
SNP effect on Gut microbiota abundance (genus Catenibacterium id.2153)

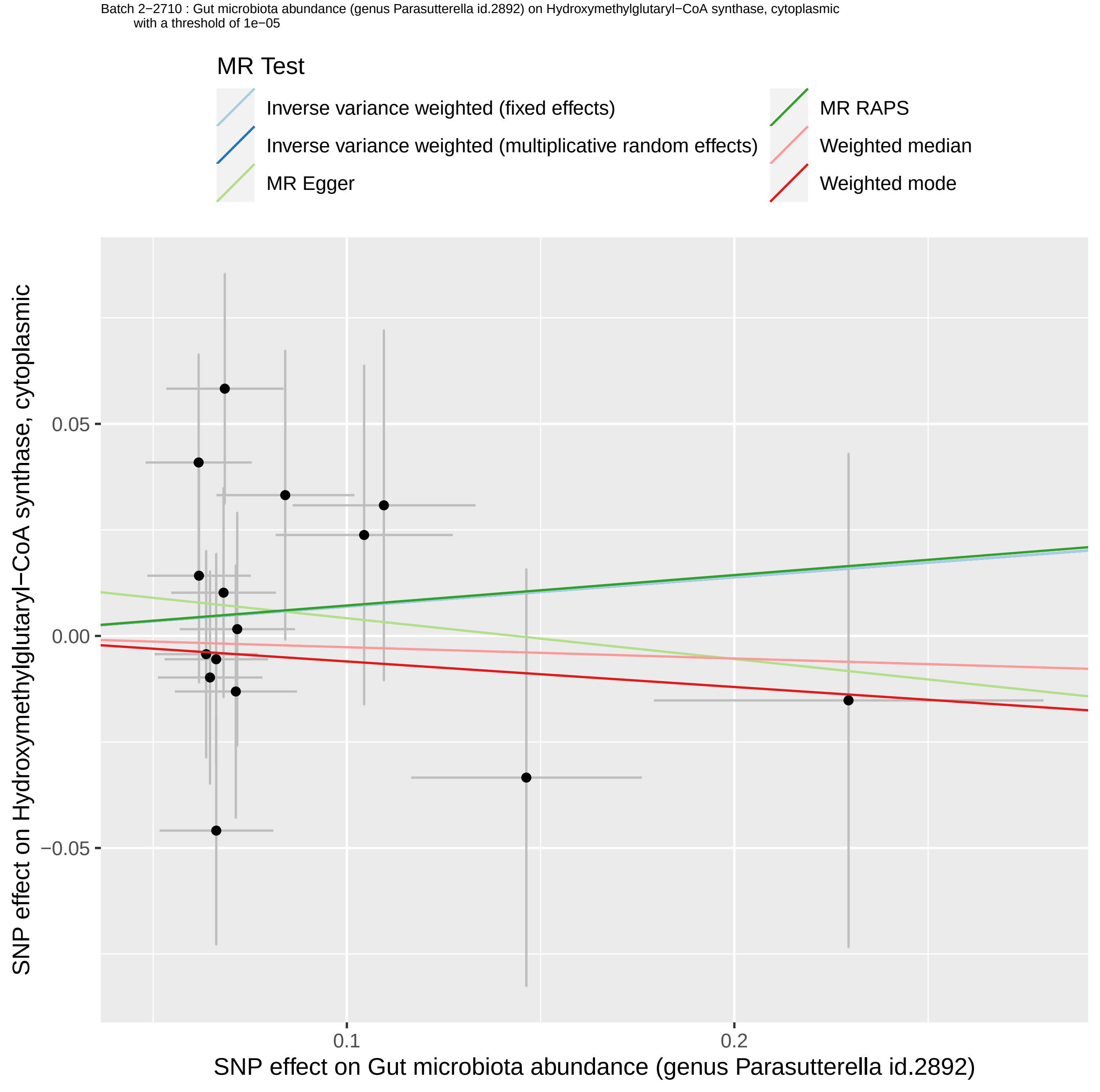

Batch 2−2710 : Gut microbiota abundance (genus Parasutterella id.2892) on Hydroxymethylglutaryl−CoA synthase, cytoplasmic
with a threshold of 1e−05
MR Test
Inverse variance weighted (fixed effects)
Inverse variance weighted (multiplicative random effects)
MR Egger
MR RAPS
Weighted median
Weighted mode
0.05
0.00
−0.05
0.1
0.2
SNP effect on Hydroxymethylglutaryl−CoA synthase, cytoplasmic
SNP effect on Gut microbiota abundance (genus Parasutterella id.2892)

Batch 2−2711 : Gut microbiota abundance (genus Catenibacterium id.2153) on Hydroxymethylglutaryl−CoA synthase, mitochondrial
with a threshold of 1e−05

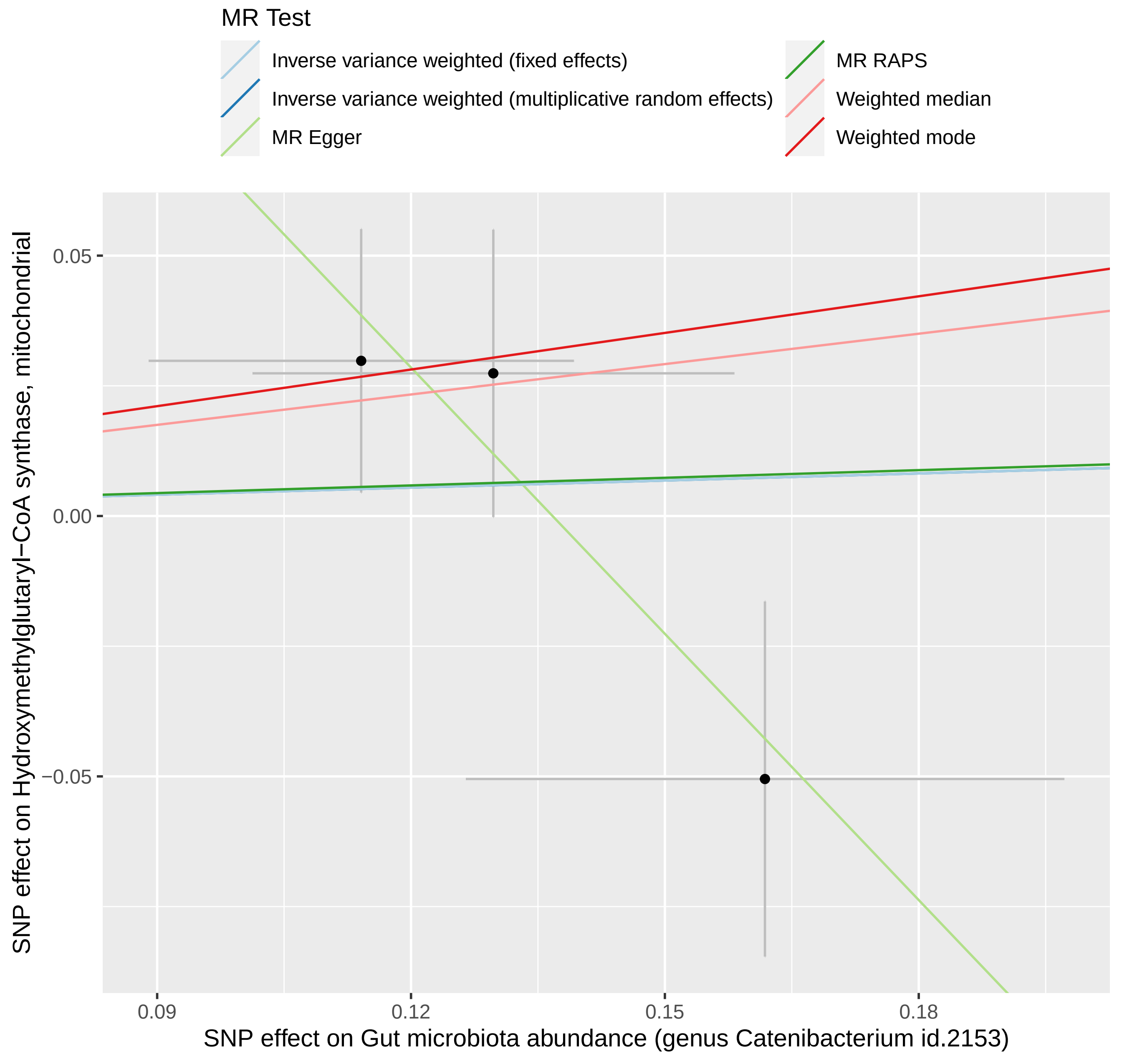

Batch 2−2712 : Gut microbiota abundance (genus Parasutterella id.2892) on Hydroxymethylglutaryl−CoA synthase, mitochondrial
with a threshold of 1e−05

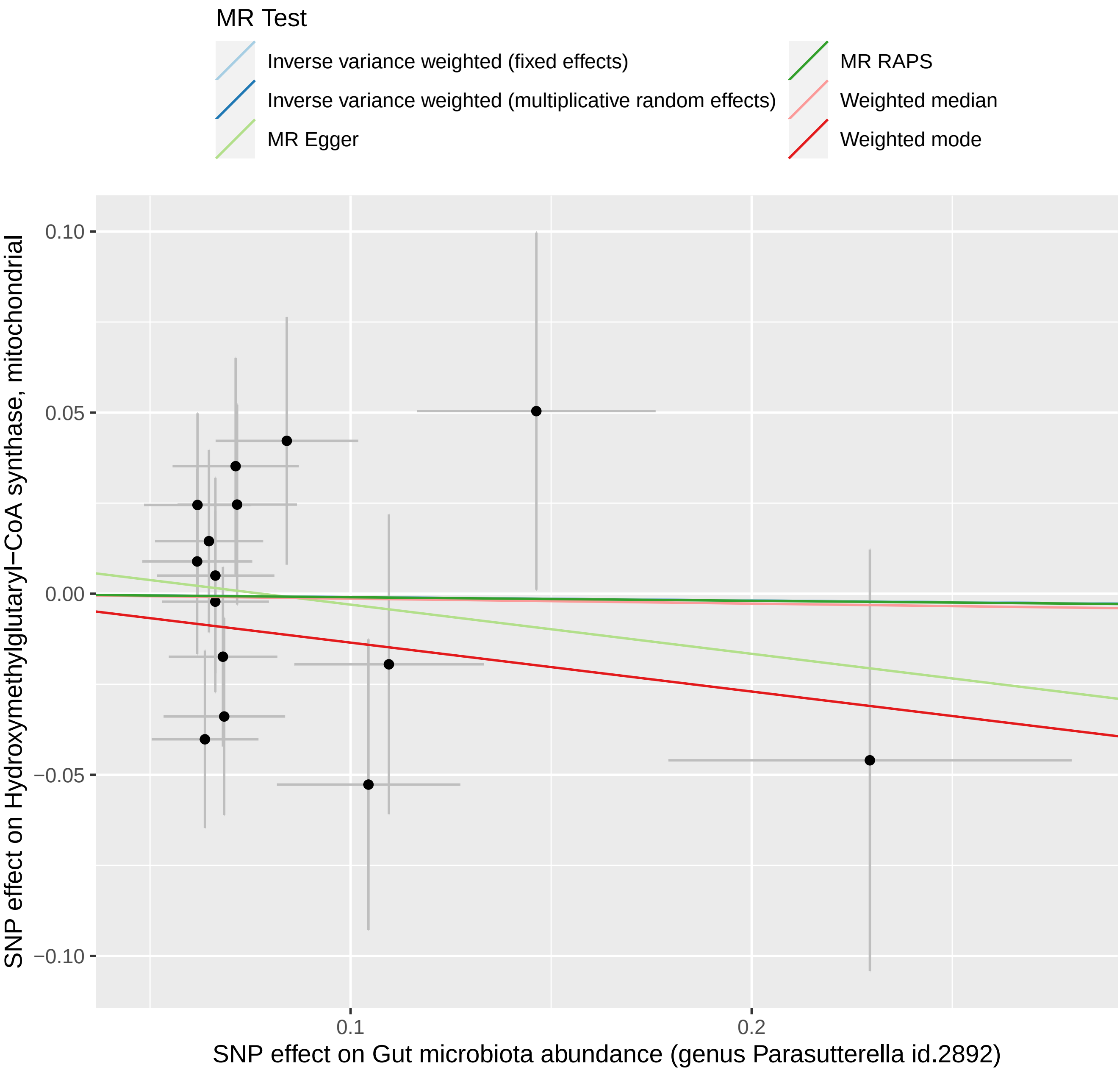

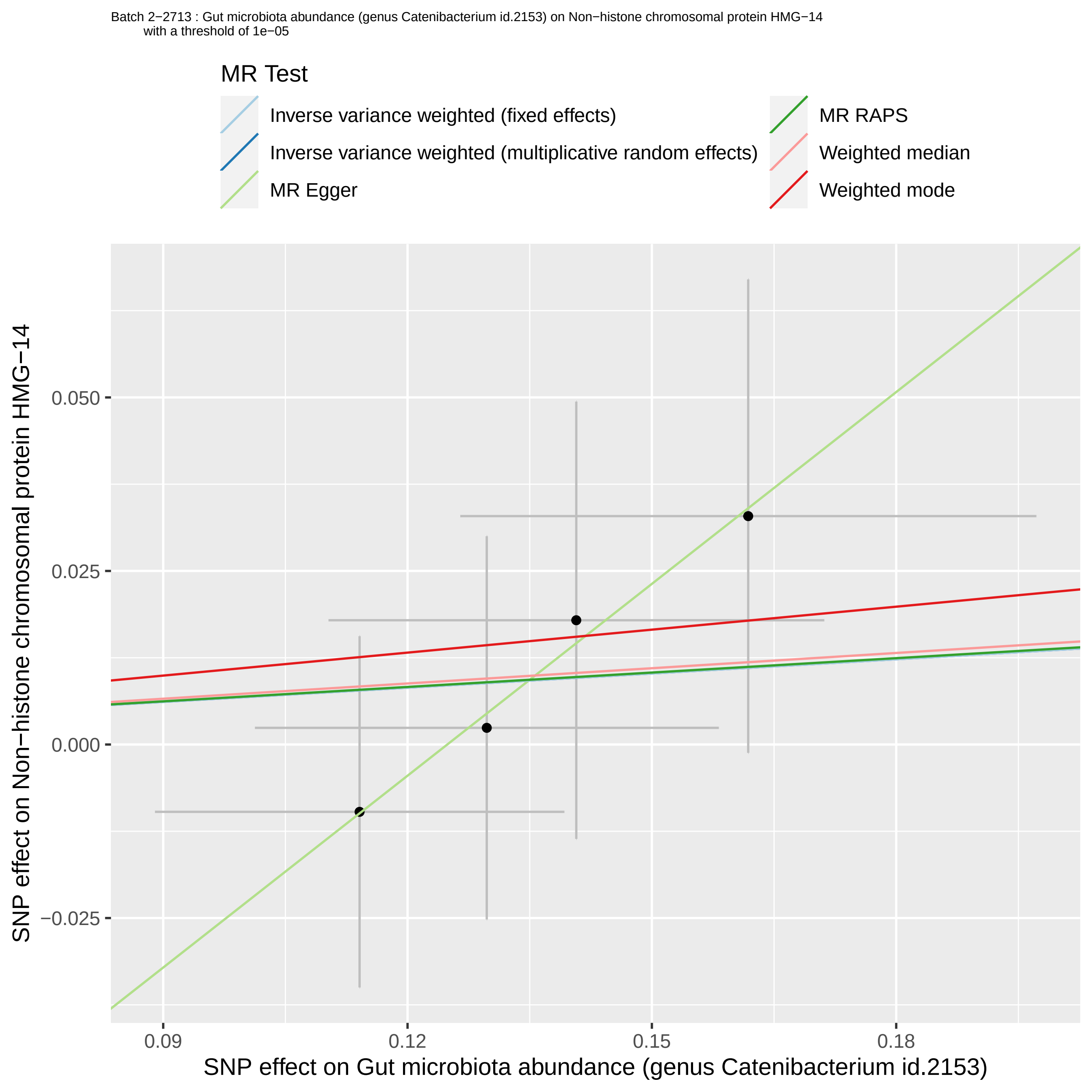

Batch 2−2713 : Gut microbiota abundance (genus Catenibacterium id.2153) on Non−histone chromosomal protein HMG−14
with a threshold of 1e−05
MR Test
Inverse variance weighted (fixed effects)
Inverse variance weighted (multiplicative random effects)
MR Egger
MR RAPS
Weighted median
Weighted mode
SNP effect on Non−histone chromosomal protein HMG−14
0.050
0.025
0.000
−0.025
0.09
0.12
0.15
0.18
SNP effect on Gut microbiota abundance (genus Catenibacterium id.2153)

Batch 2−2714 : Gut microbiota abundance (genus Parasutterella id.2892) on Non−histone chromosomal protein HMG−14
with a threshold of 1e−05

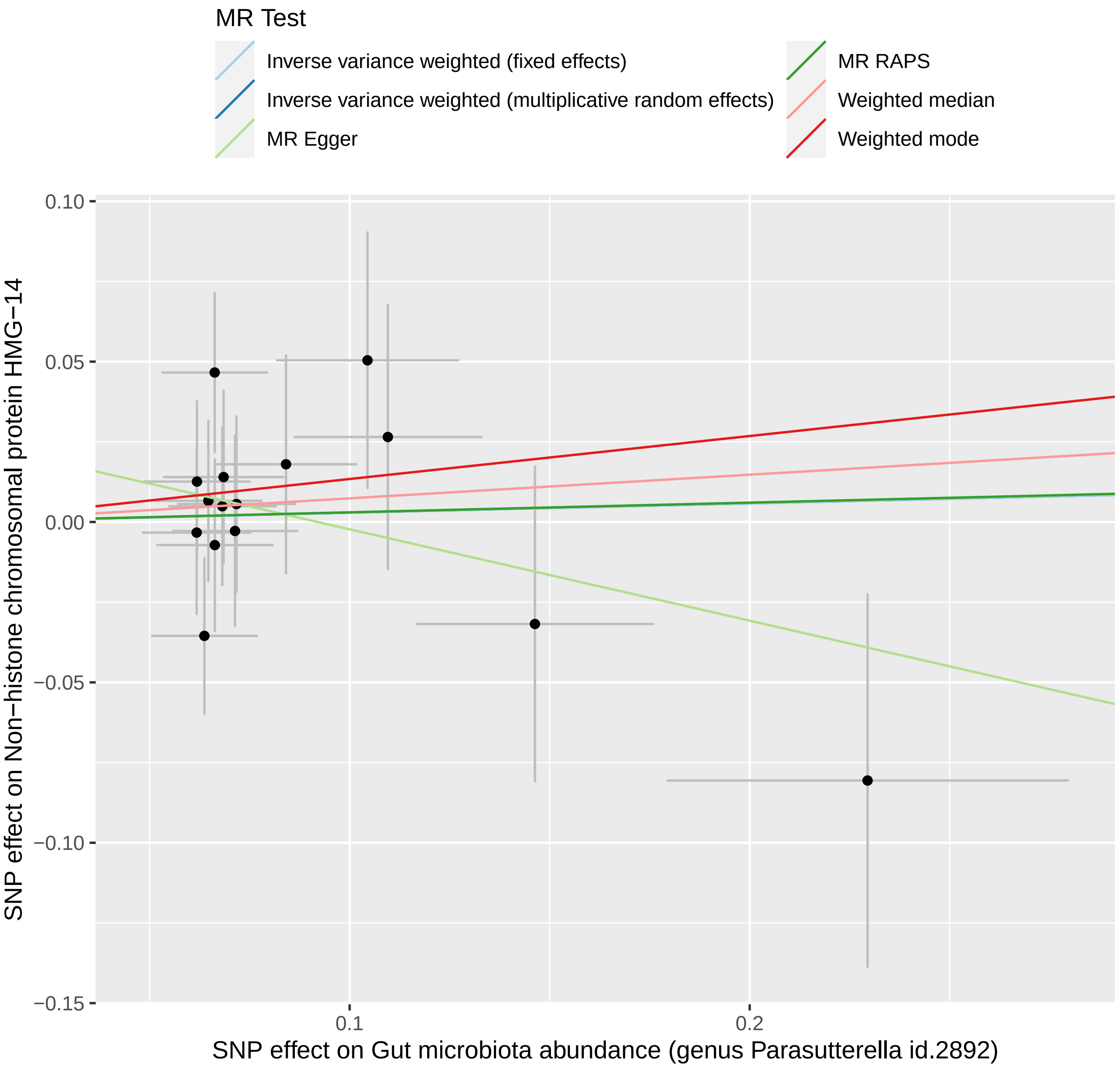

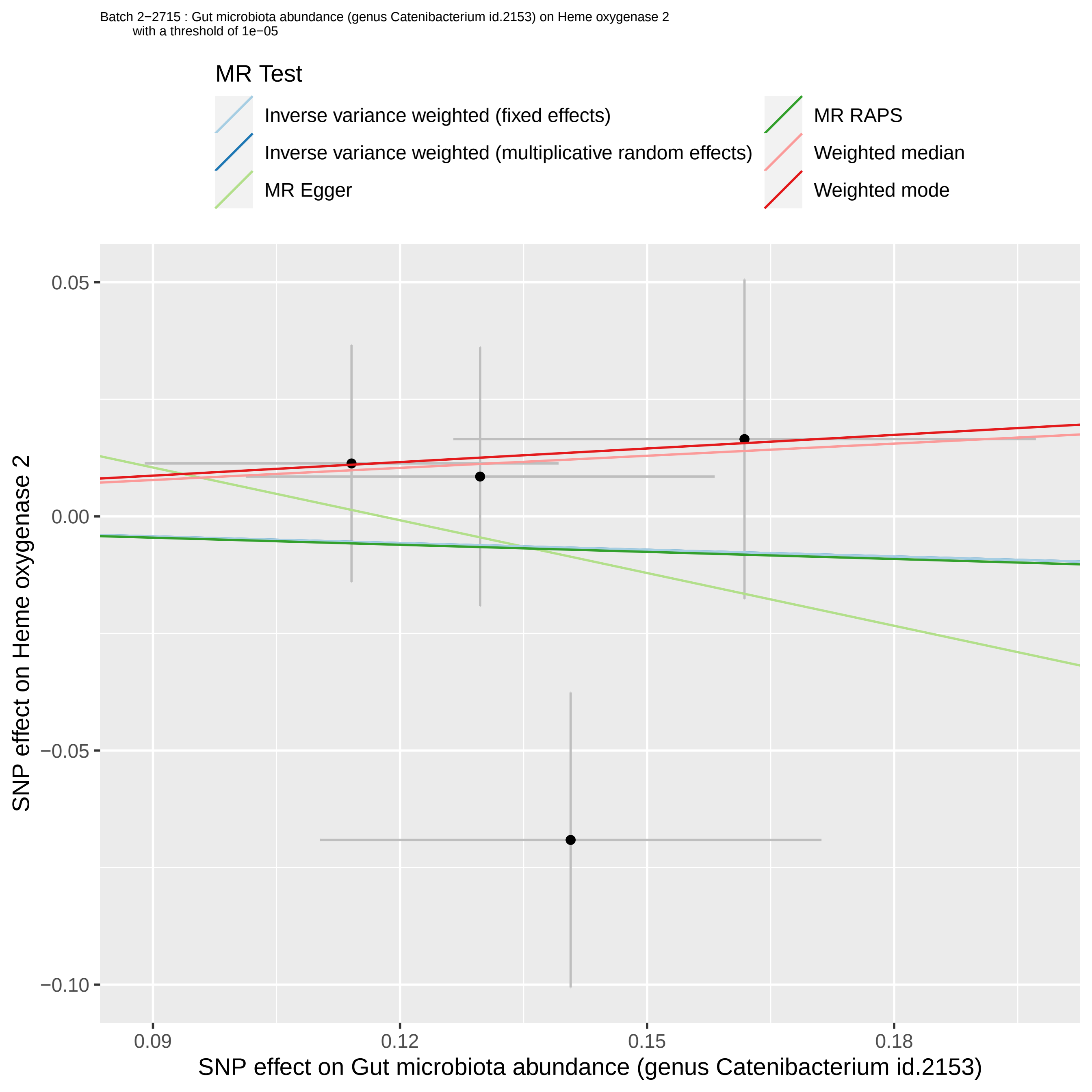
Batch 2−2715 : Gut microbiota abundance (genus Catenibacterium id.2153) on Heme oxygenase 2
with a threshold of 1e−05
MR Test
Inverse variance weighted (fixed effects)
Inverse variance weighted (multiplicative random effects)
MR Egger
MR RAPS
Weighted median
Weighted mode
0.05
0.00
−0.05
−0.10
0.09
0.12
0.15
0.18
SNP effect on Heme oxygenase 2
SNP effect on Gut microbiota abundance (genus Catenibacterium id.2153)

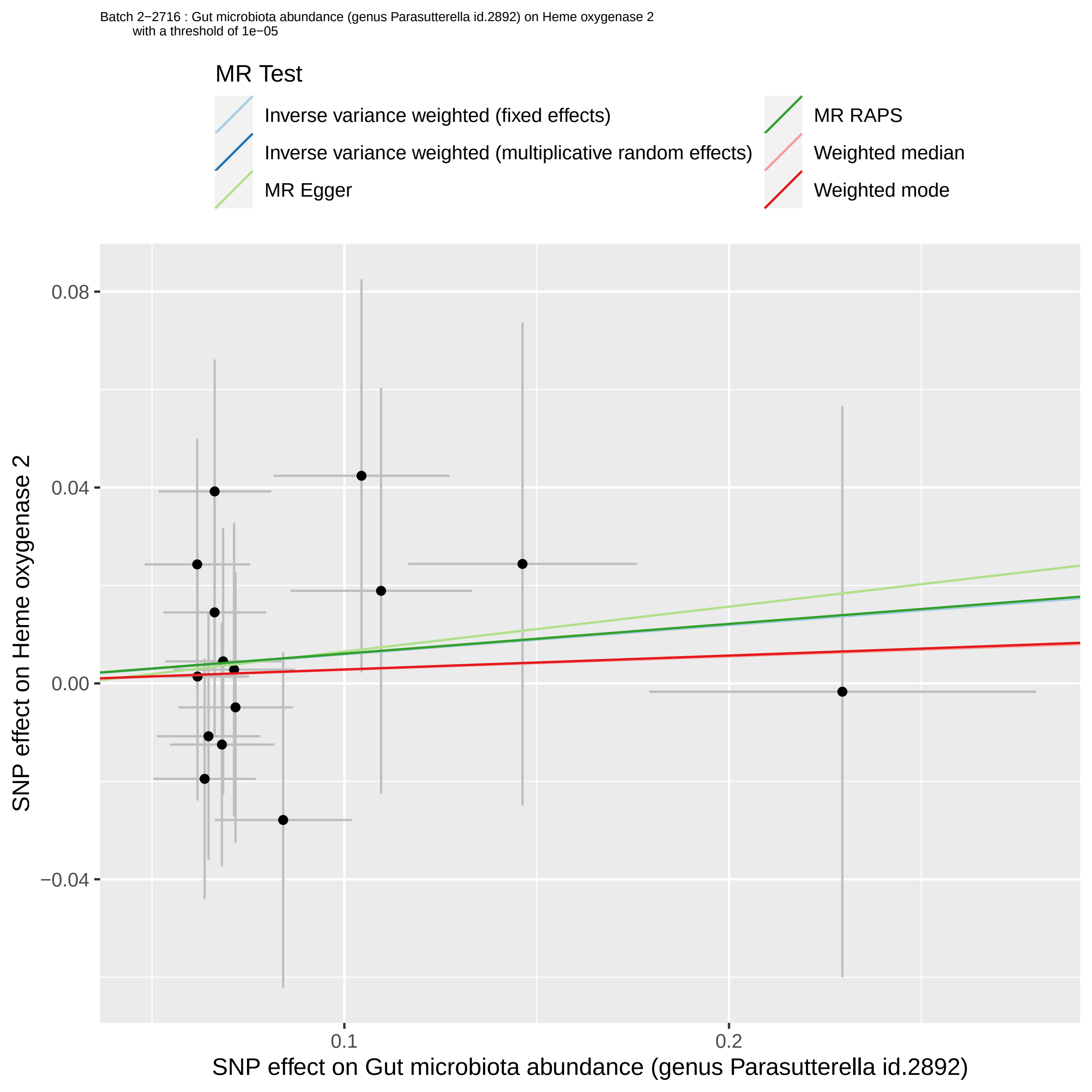

Batch 2−2716 : Gut microbiota abundance (genus Parasutterella id.2892) on Heme oxygenase 2
with a threshold of 1e−05
MR Test
Inverse variance weighted (fixed effects)
Inverse variance weighted (multiplicative random effects)
MR Egger
MR RAPS
Weighted median
Weighted mode
0.08
0.04
0.00
−0.04
0.1
0.2
SNP effect on Heme oxygenase 2
SNP effect on Gut microbiota abundance (genus Parasutterella id.2892)

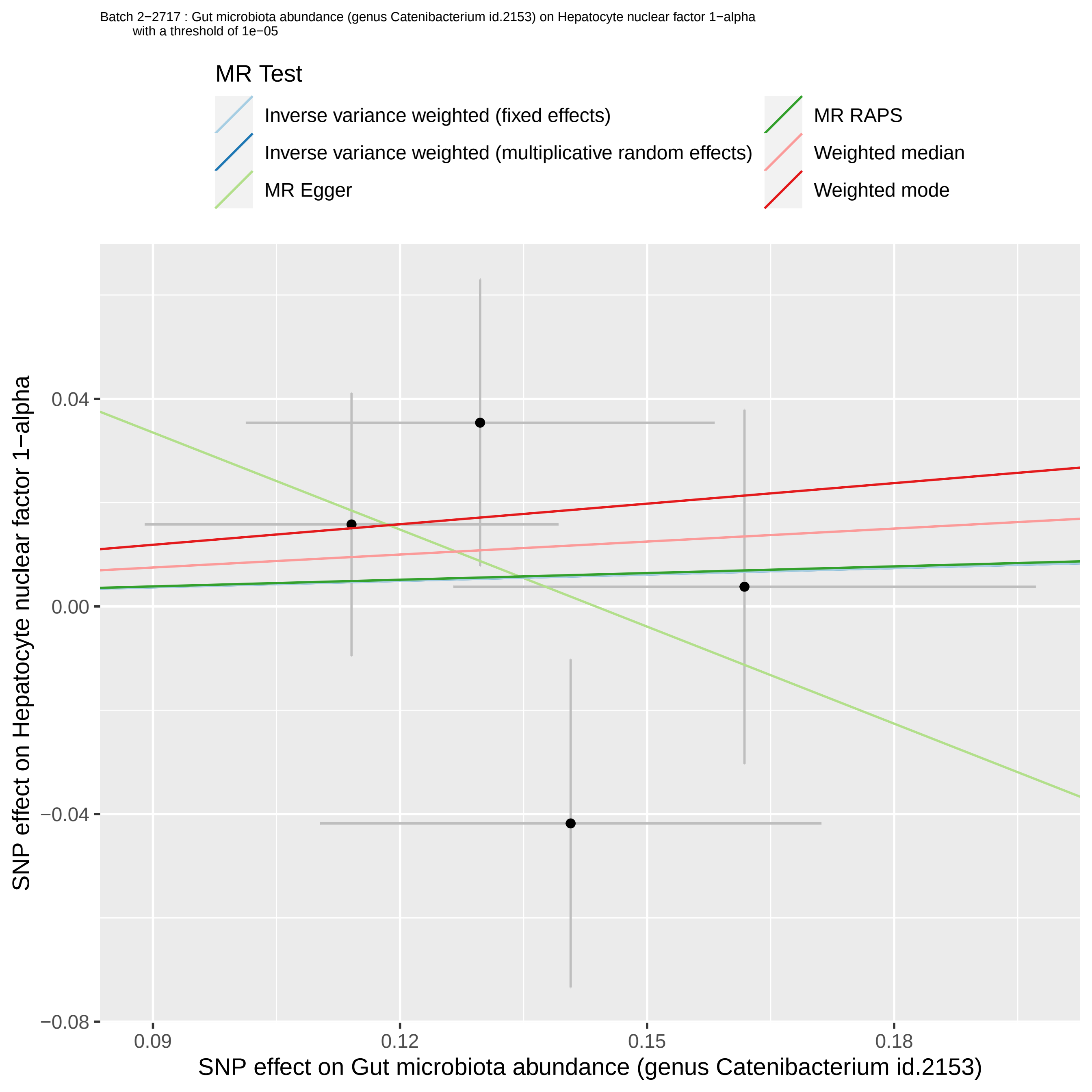

Batch 2−2717 : Gut microbiota abundance (genus Catenibacterium id.2153) on Hepatocyte nuclear factor 1−alpha
with a threshold of 1e−05
MR Test
Inverse variance weighted (fixed effects)
Inverse variance weighted (multiplicative random effects)
MR Egger
MR RAPS
Weighted median
Weighted mode
0.04
0.00
−0.04
−0.08
0.09
0.12
0.15
0.18
SNP effect on Hepatocyte nuclear factor 1−alpha
SNP effect on Gut microbiota abundance (genus Catenibacterium id.2153)

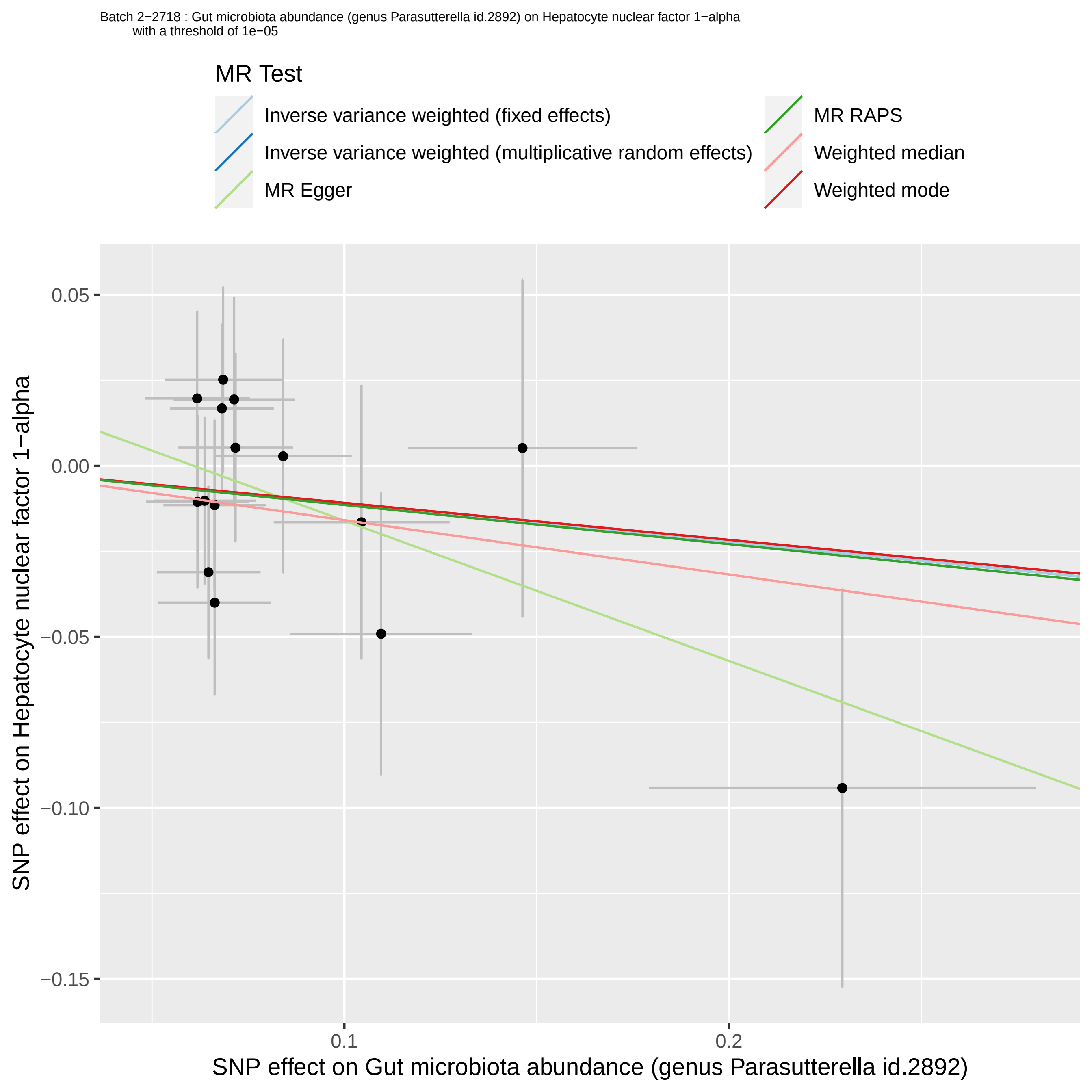

Batch 2−2718 : Gut microbiota abundance (genus Parasutterella id.2892) on Hepatocyte nuclear factor 1−alpha
with a threshold of 1e−05
MR Test
Inverse variance weighted (fixed effects)
Inverse variance weighted (multiplicative random effects)
MR Egger
MR RAPS
Weighted median
Weighted mode
0.05
0.00
−0.05
−0.10
−0.15
0.1
0.2
SNP effect on Hepatocyte nuclear factor 1−alpha
SNP effect on Gut microbiota abundance (genus Parasutterella id.2892)

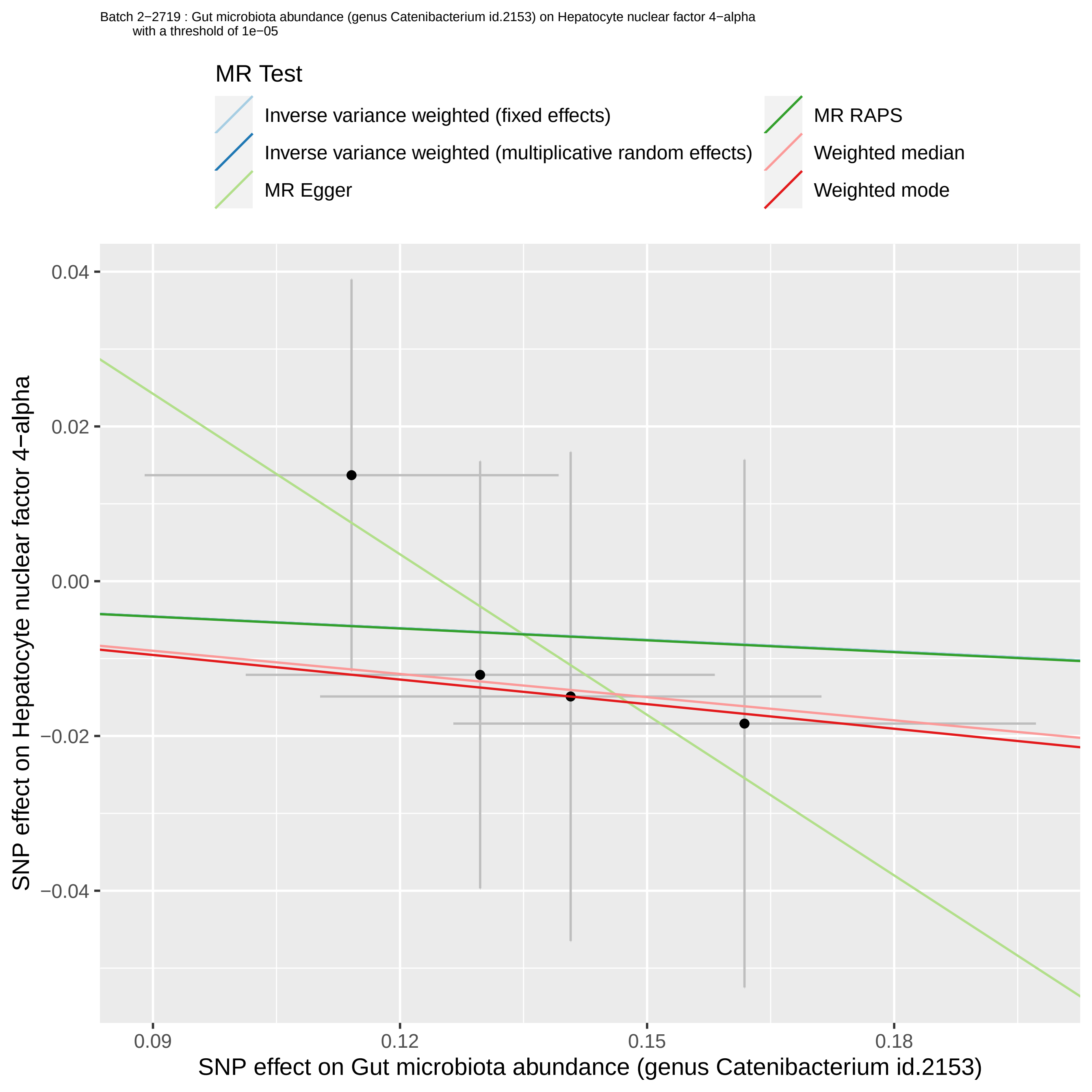

Batch 2−2719 : Gut microbiota abundance (genus Catenibacterium id.2153) on Hepatocyte nuclear factor 4−alpha
with a threshold of 1e−05
MR Test
Inverse variance weighted (fixed effects)
Inverse variance weighted (multiplicative random effects)
MR Egger
MR RAPS
Weighted median
Weighted mode
0.04
0.02
0.00
−0.02
−0.04
0.09
0.12
0.15
0.18
SNP effect on Hepatocyte nuclear factor 4−alpha
SNP effect on Gut microbiota abundance (genus Catenibacterium id.2153)

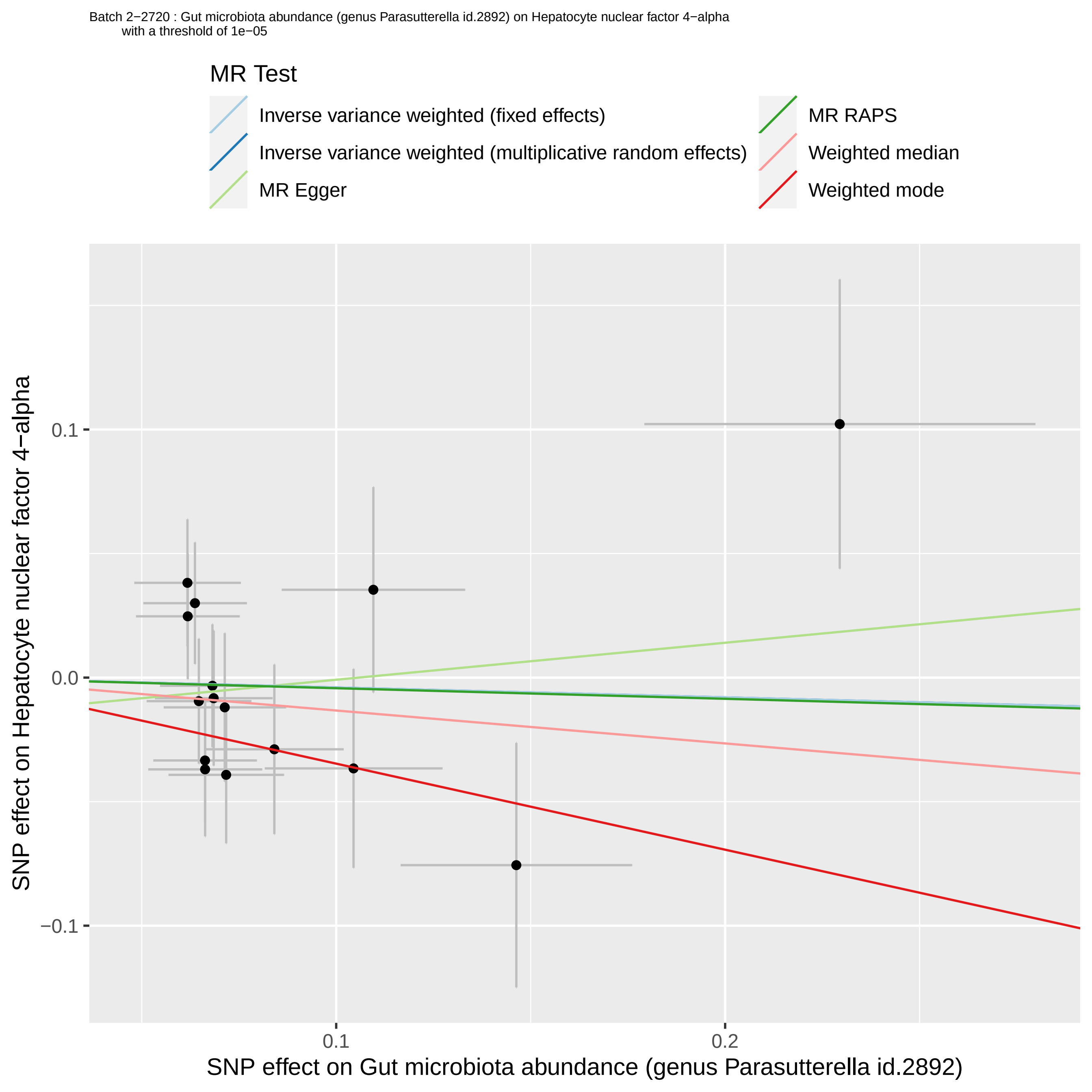

Batch 2−2720 : Gut microbiota abundance (genus Parasutterella id.2892) on Hepatocyte nuclear factor 4−alpha
with a threshold of 1e−05
MR Test
Inverse variance weighted (fixed effects)
Inverse variance weighted (multiplicative random effects)
MR Egger
MR RAPS
Weighted median
Weighted mode
0.1
0.0
−0.1
0.1
0.2
SNP effect on Hepatocyte nuclear factor 4−alpha
SNP effect on Gut microbiota abundance (genus Parasutterella id.2892)

Batch 2−2721 : Gut microbiota abundance (genus Catenibacterium id.2153) on Heterogeneous nuclear ribonucleoproteins A2/B1
with a threshold of 1e−05

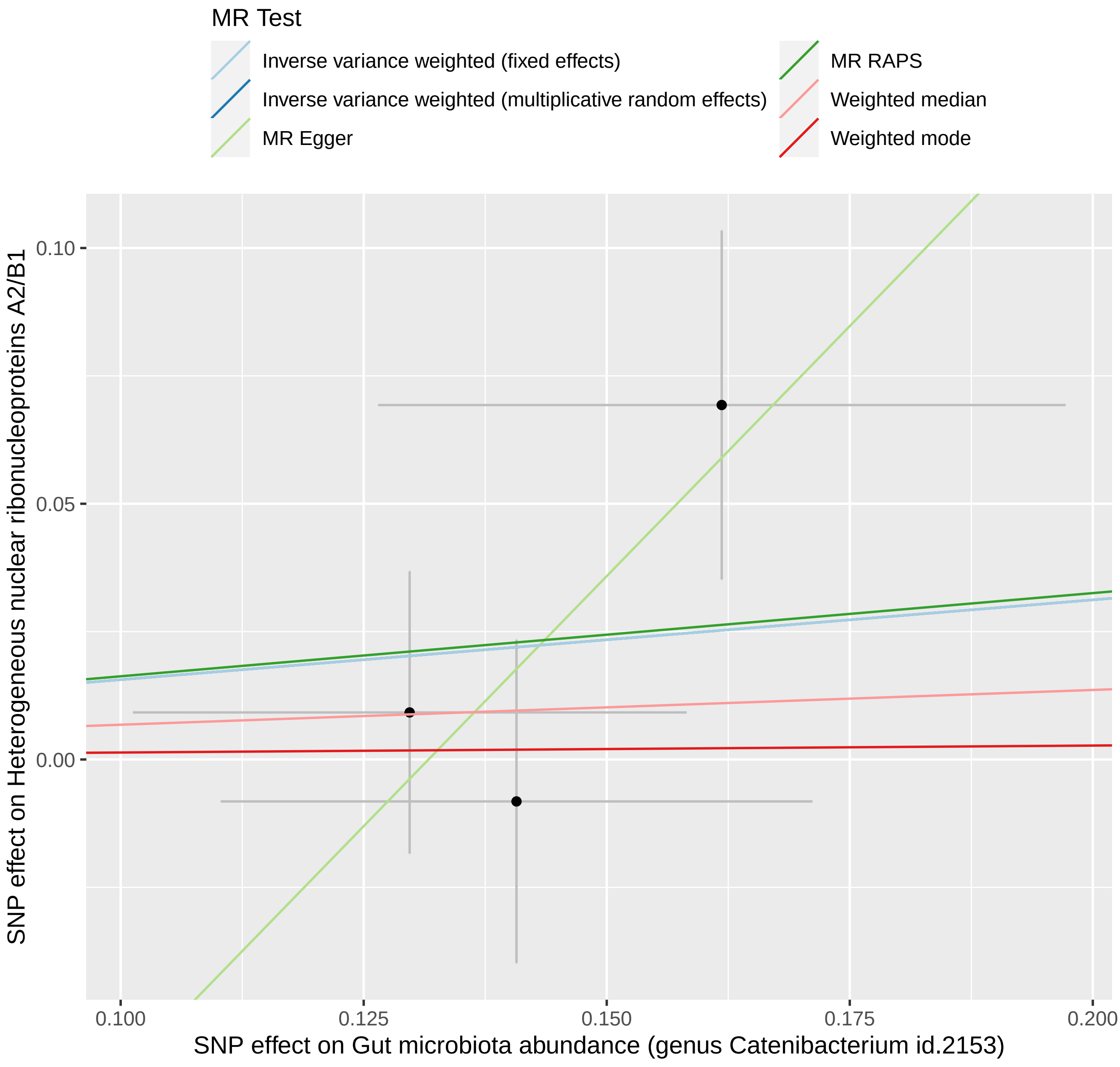

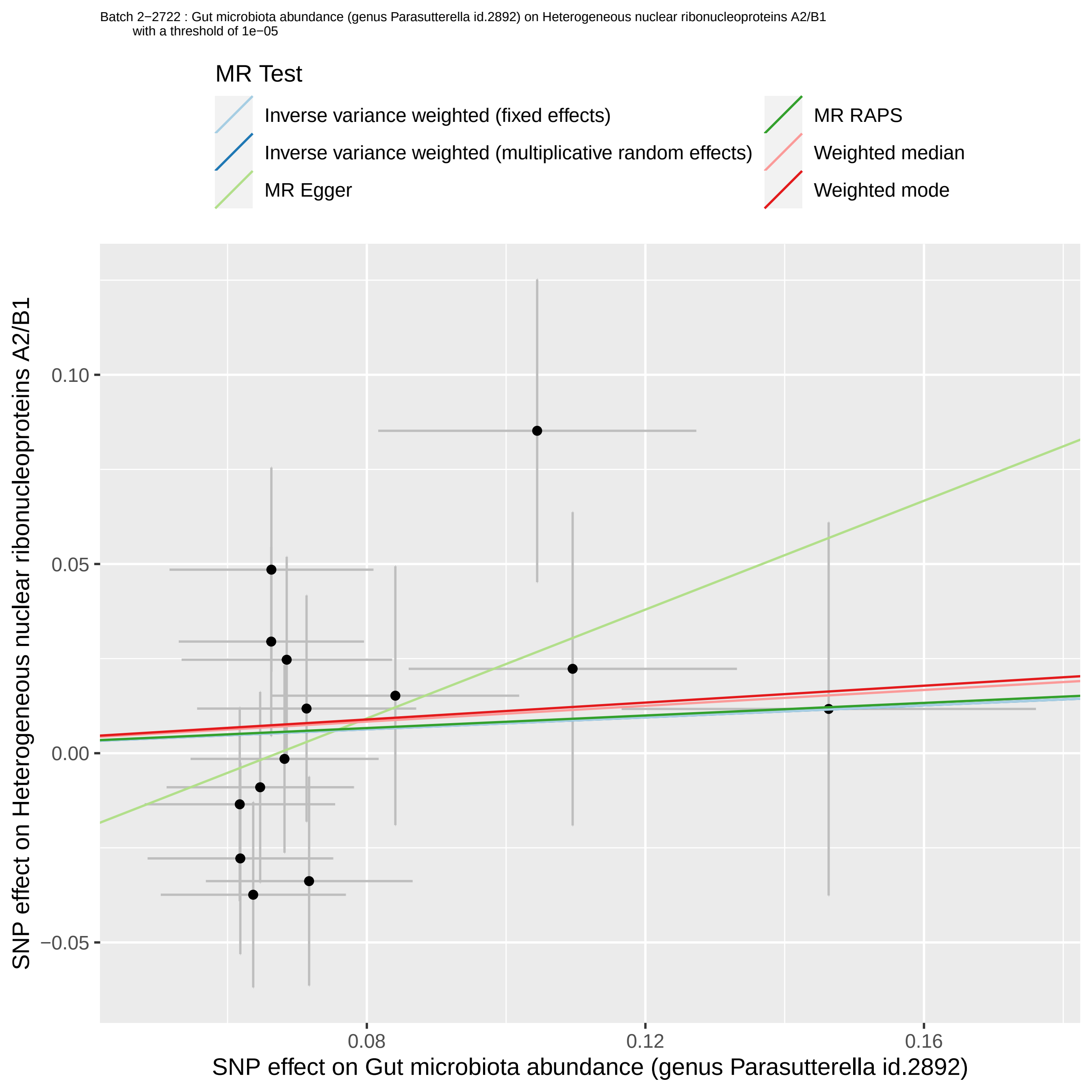
Batch 2−2722 : Gut microbiota abundance (genus Parasutterella id.2892) on Heterogeneous nuclear ribonucleoproteins A2/B1
with a threshold of 1e−05
MR Test
Inverse variance weighted (fixed effects)
Inverse variance weighted (multiplicative random effects)
MR Egger
MR RAPS
Weighted median
Weighted mode
0.10
0.05
0.00
−0.05
0.08
0.12
0.16
SNP effect on Heterogeneous nuclear ribonucleoproteins A2/B1
SNP effect on Gut microbiota abundance (genus Parasutterella id.2892)

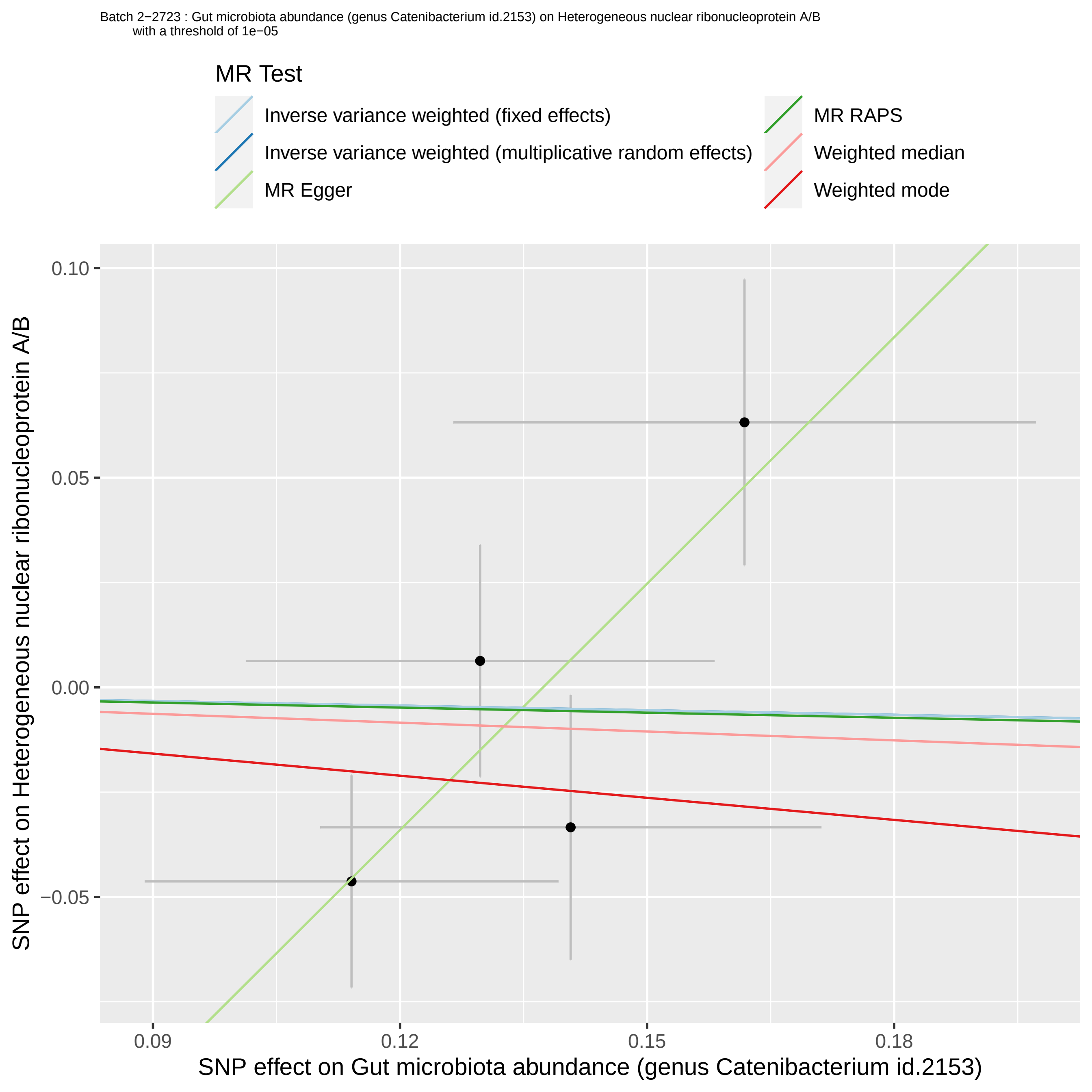

Batch 2−2723 : Gut microbiota abundance (genus Catenibacterium id.2153) on Heterogeneous nuclear ribonucleoprotein A/B
with a threshold of 1e−05
MR Test
Inverse variance weighted (fixed effects)
Inverse variance weighted (multiplicative random effects)
MR Egger
MR RAPS
Weighted median
Weighted mode
0.10
0.05
0.00
−0.05
0.09
0.12
0.15
0.18
SNP effect on Heterogeneous nuclear ribonucleoprotein A/B
SNP effect on Gut microbiota abundance (genus Catenibacterium id.2153)

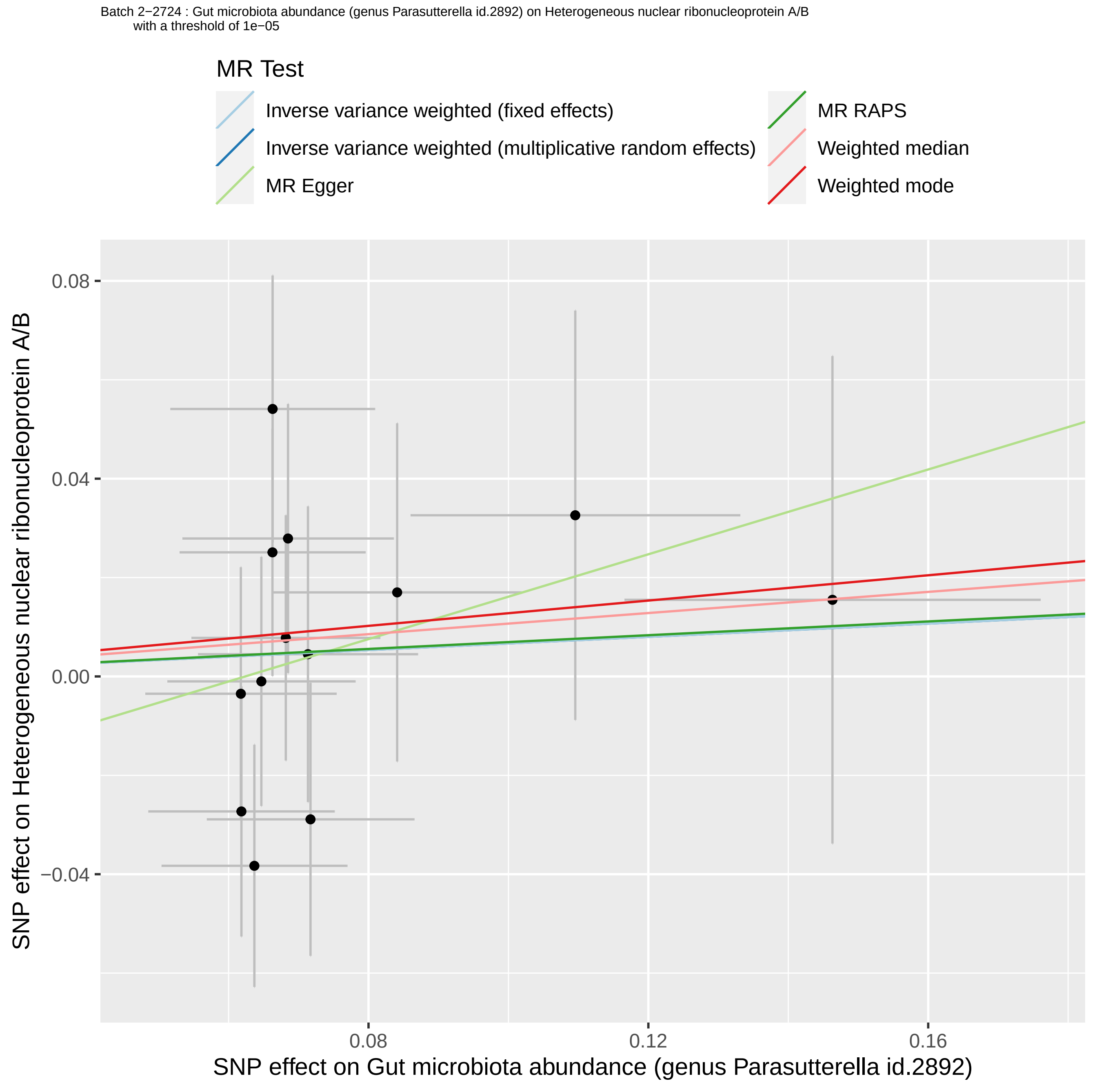

Batch 2−2724 : Gut microbiota abundance (genus Parasutterella id.2892) on Heterogeneous nuclear ribonucleoprotein A/B
with a threshold of 1e−05
MR Test
Inverse variance weighted (fixed effects)
Inverse variance weighted (multiplicative random effects)
MR Egger
MR RAPS
Weighted median
Weighted mode
0.08
0.04
0.00
−0.04
0.08
0.12
0.16
SNP effect on Heterogeneous nuclear ribonucleoprotein A/B
SNP effect on Gut microbiota abundance (genus Parasutterella id.2892)

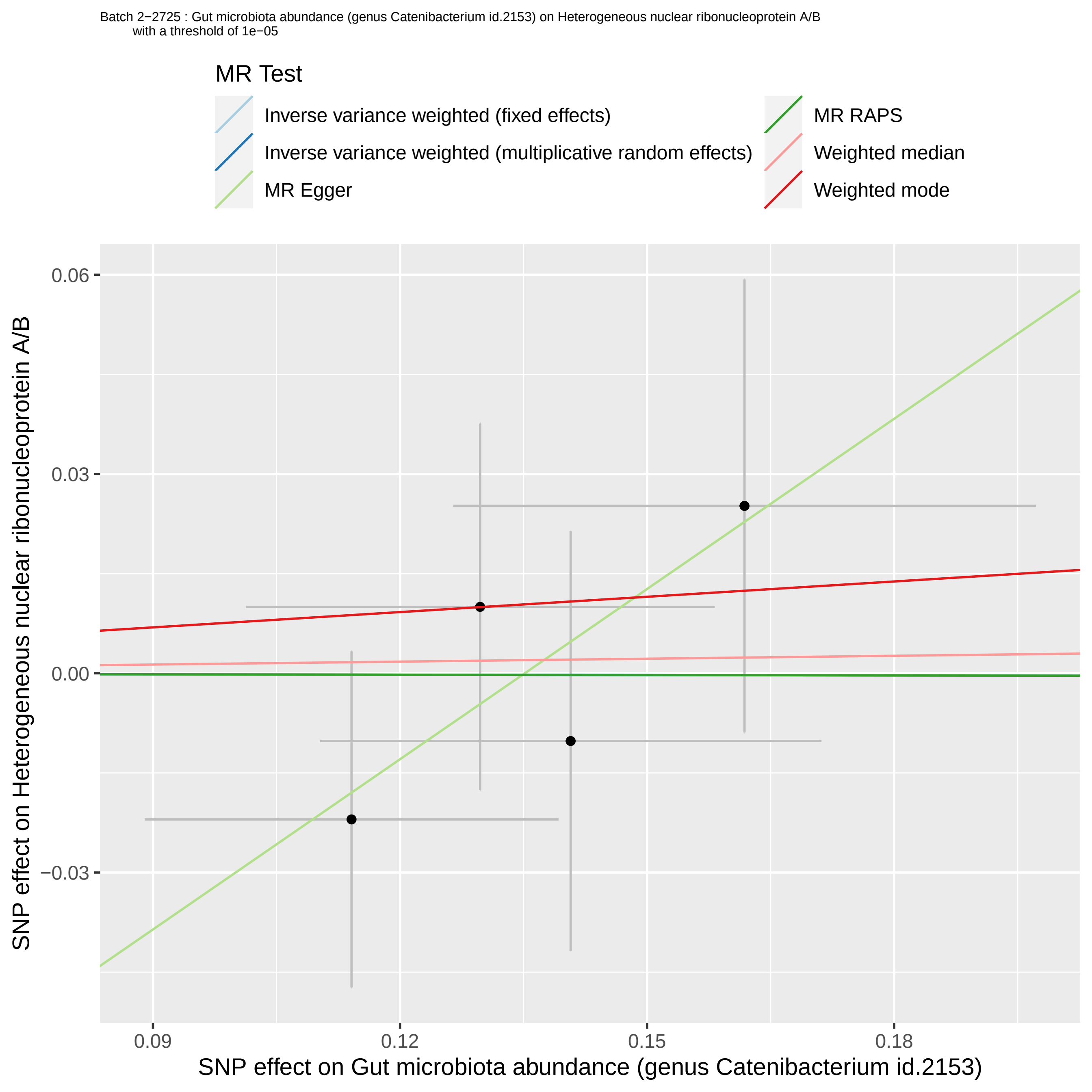
Batch 2−2725 : Gut microbiota abundance (genus Catenibacterium id.2153) on Heterogeneous nuclear ribonucleoprotein A/B
with a threshold of 1e−05
MR Test
Inverse variance weighted (fixed effects)
Inverse variance weighted (multiplicative random effects)
MR Egger
MR RAPS
Weighted median
Weighted mode
0.06
0.03
0.00
−0.03
0.09
0.12
0.15
0.18
SNP effect on Heterogeneous nuclear ribonucleoprotein A/B
SNP effect on Gut microbiota abundance (genus Catenibacterium id.2153)

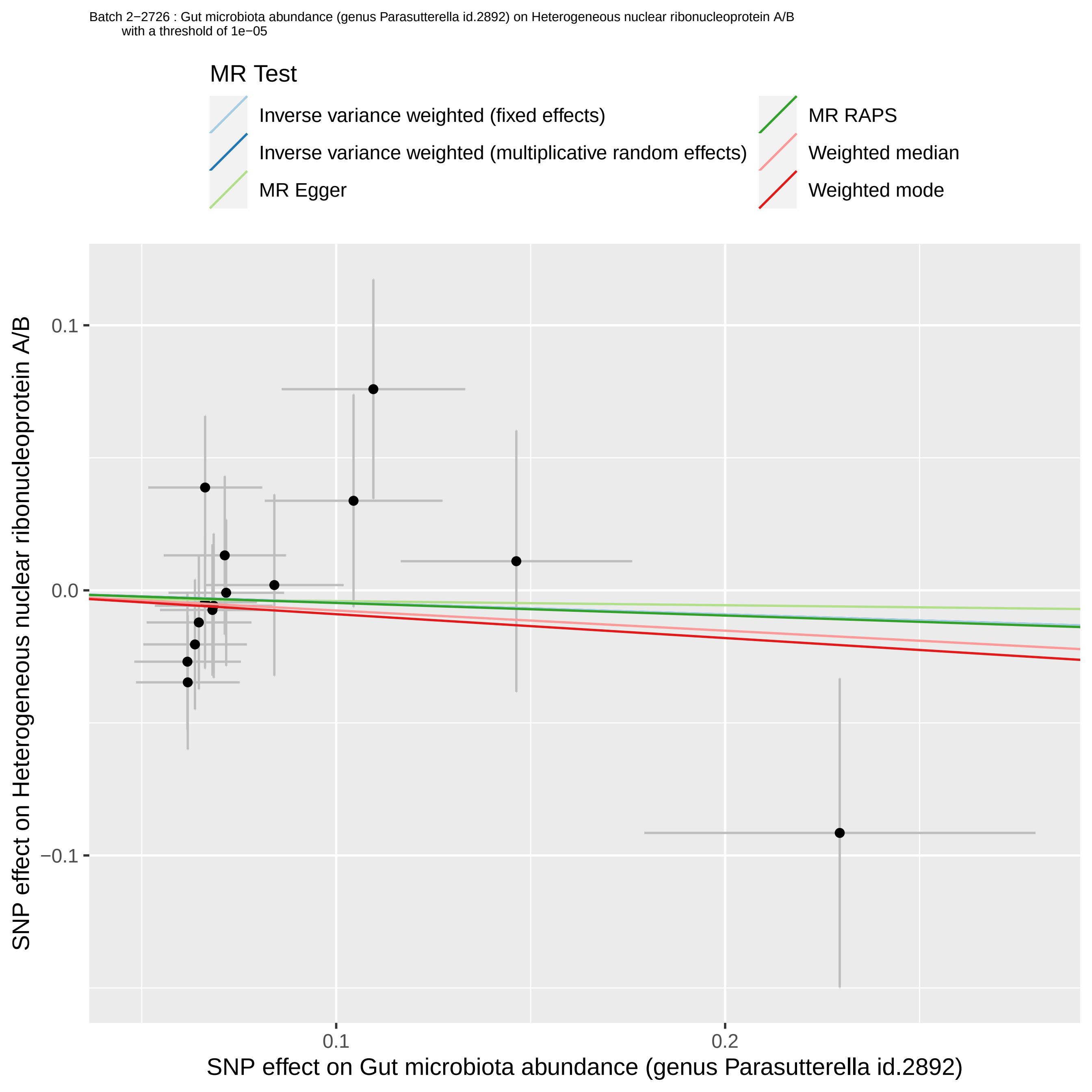
Batch 2−2726 : Gut microbiota abundance (genus Parasutterella id.2892) on Heterogeneous nuclear ribonucleoprotein A/B
with a threshold of 1e−05
MR Test
Inverse variance weighted (fixed effects)
Inverse variance weighted (multiplicative random effects)
MR Egger
MR RAPS
Weighted median
Weighted mode
0.1
0.0
−0.1
0.1
0.2
SNP effect on Heterogeneous nuclear ribonucleoprotein A/B
SNP effect on Gut microbiota abundance (genus Parasutterella id.2892)

Batch 2−2727 : Gut microbiota abundance (genus Catenibacterium id.2153) on Heterogeneous nuclear ribonucleoproteins C1/C2
with a threshold of 1e−05

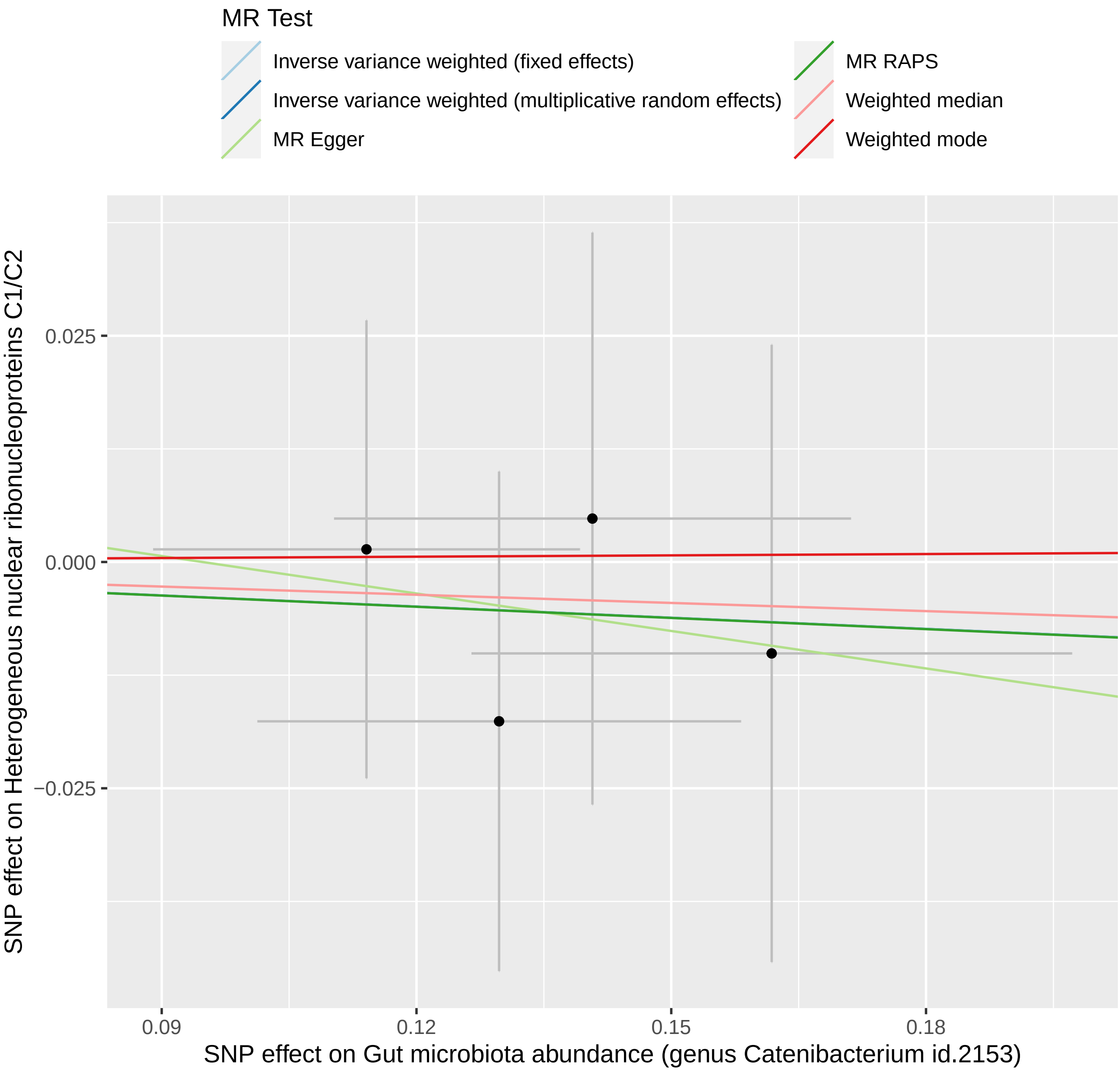

Batch 2−2728 : Gut microbiota abundance (genus Parasutterella id.2892) on Heterogeneous nuclear ribonucleoproteins C1/C2
with a threshold of 1e−05

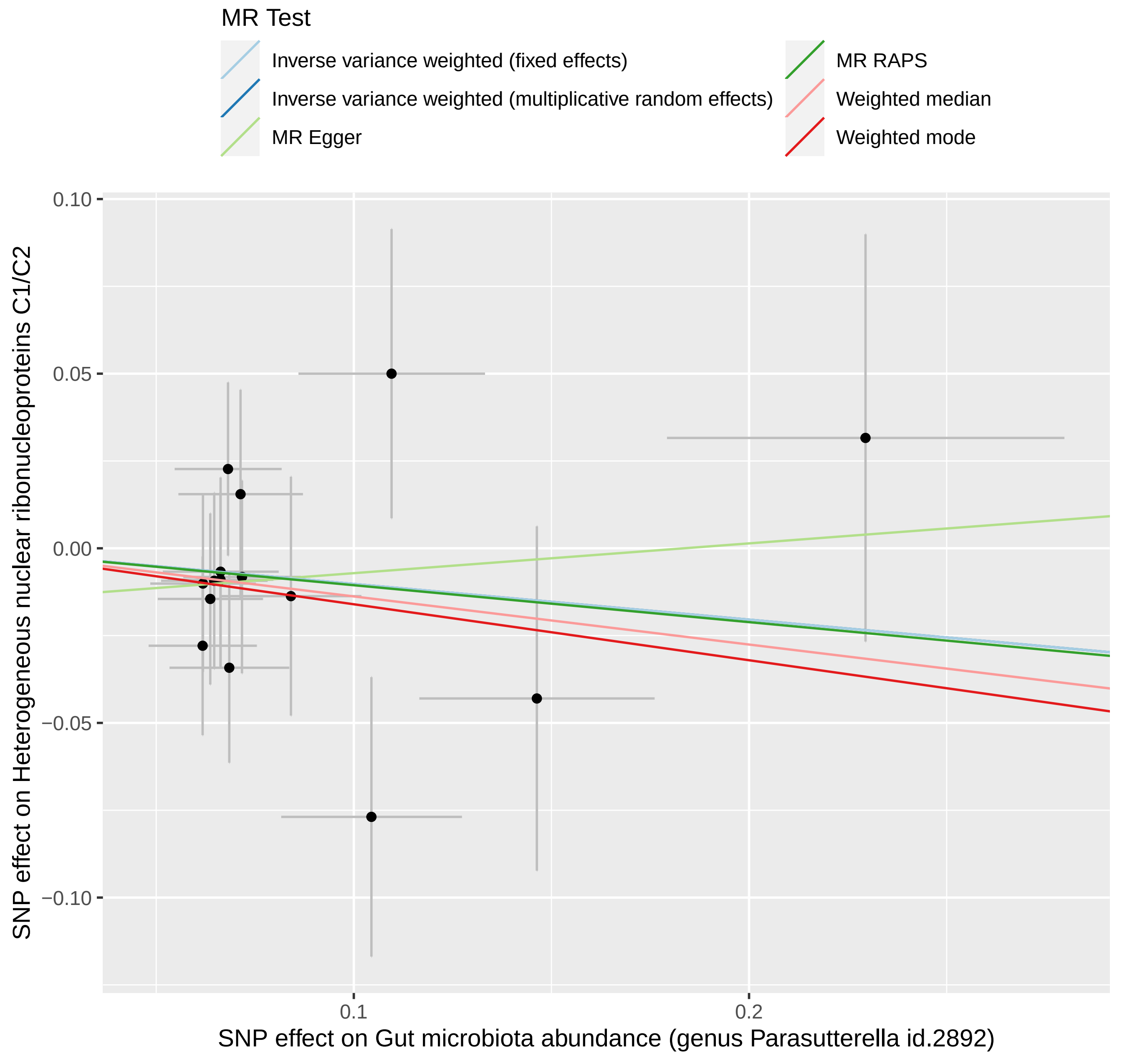

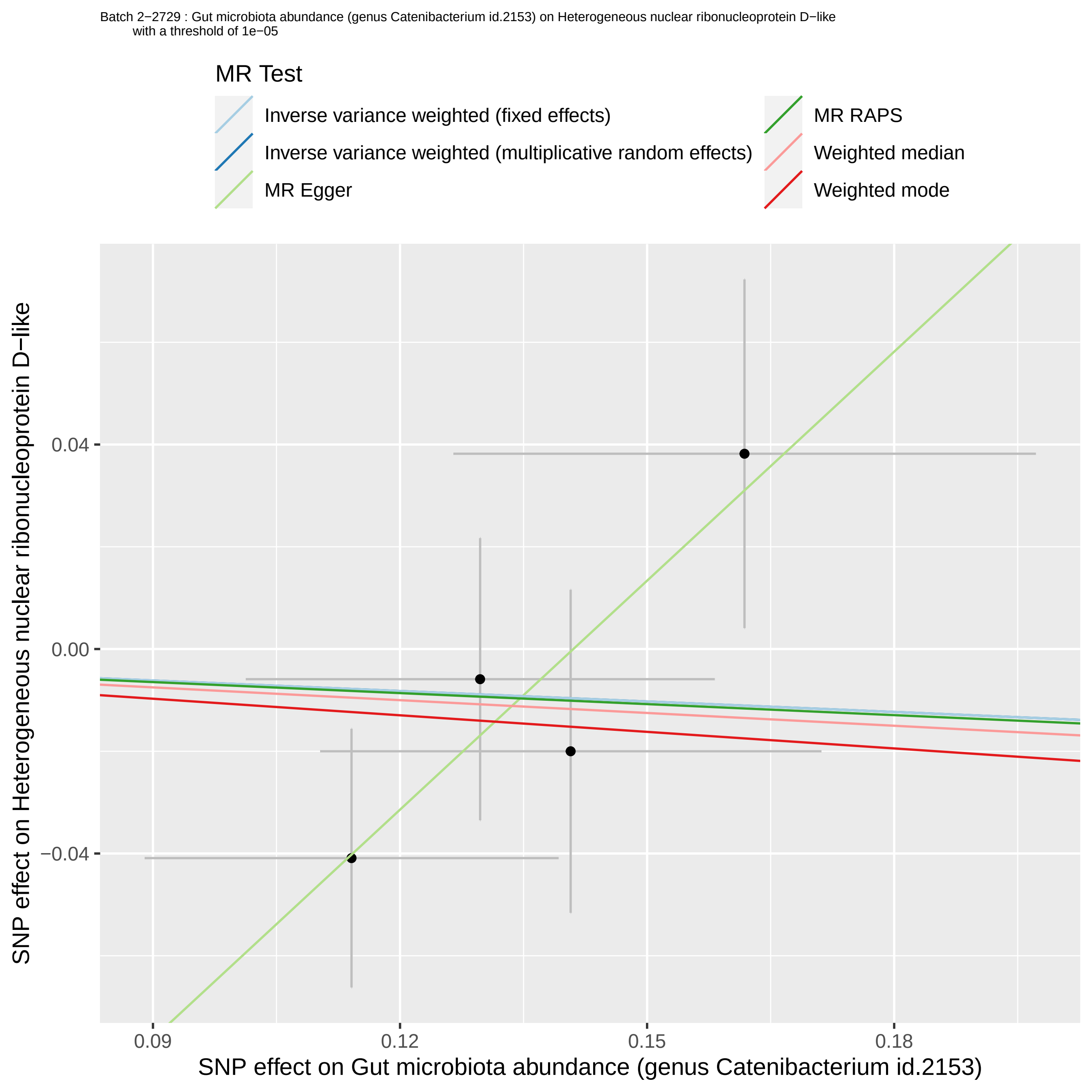
Batch 2−2729 : Gut microbiota abundance (genus Catenibacterium id.2153) on Heterogeneous nuclear ribonucleoprotein D−like
with a threshold of 1e−05
MR Test
Inverse variance weighted (fixed effects)
Inverse variance weighted (multiplicative random effects)
MR Egger
MR RAPS
Weighted median
Weighted mode
0.04
0.00
−0.04
0.09
0.12
0.15
0.18
SNP effect on Heterogeneous nuclear ribonucleoprotein D−like
SNP effect on Gut microbiota abundance (genus Catenibacterium id.2153)

Batch 2−2730 : Gut microbiota abundance (genus Parasutterella id.2892) on Heterogeneous nuclear ribonucleoprotein D−like with a threshold of 1e−05

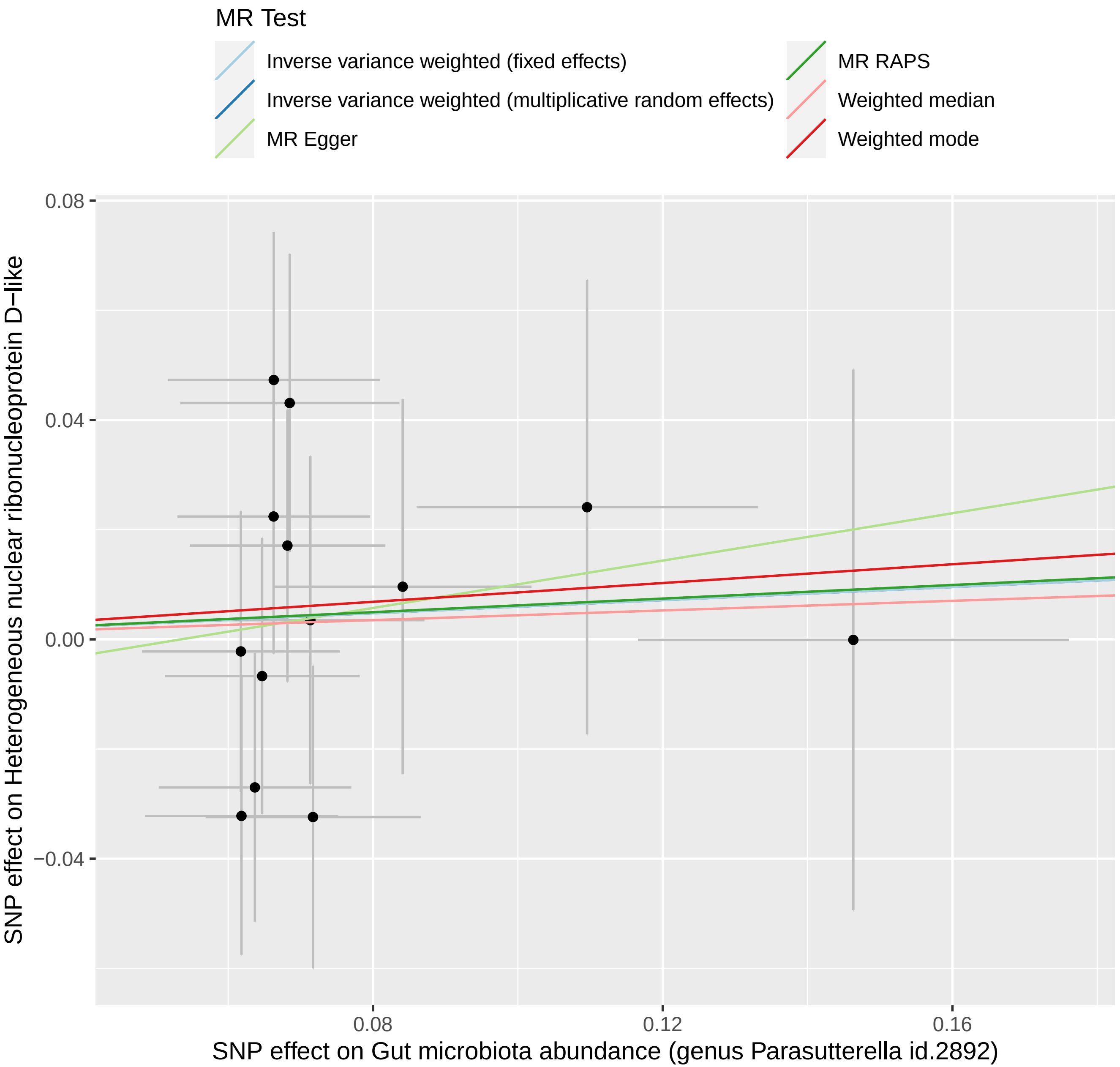

Batch 2−2731 : Gut microbiota abundance (genus Catenibacterium id.2153) on Heterogeneous nuclear ribonucleoprotein K
with a threshold of 1e−05

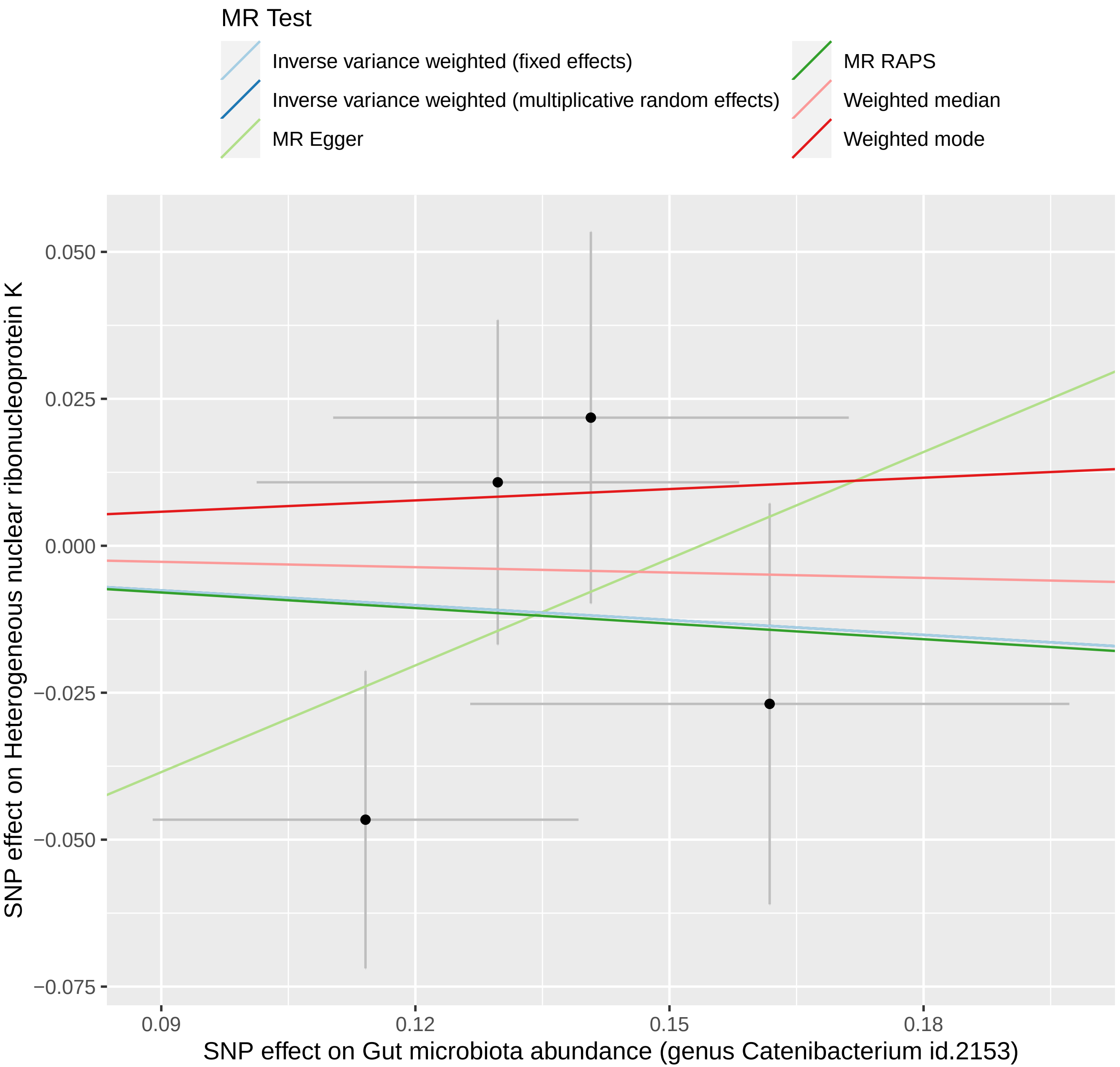

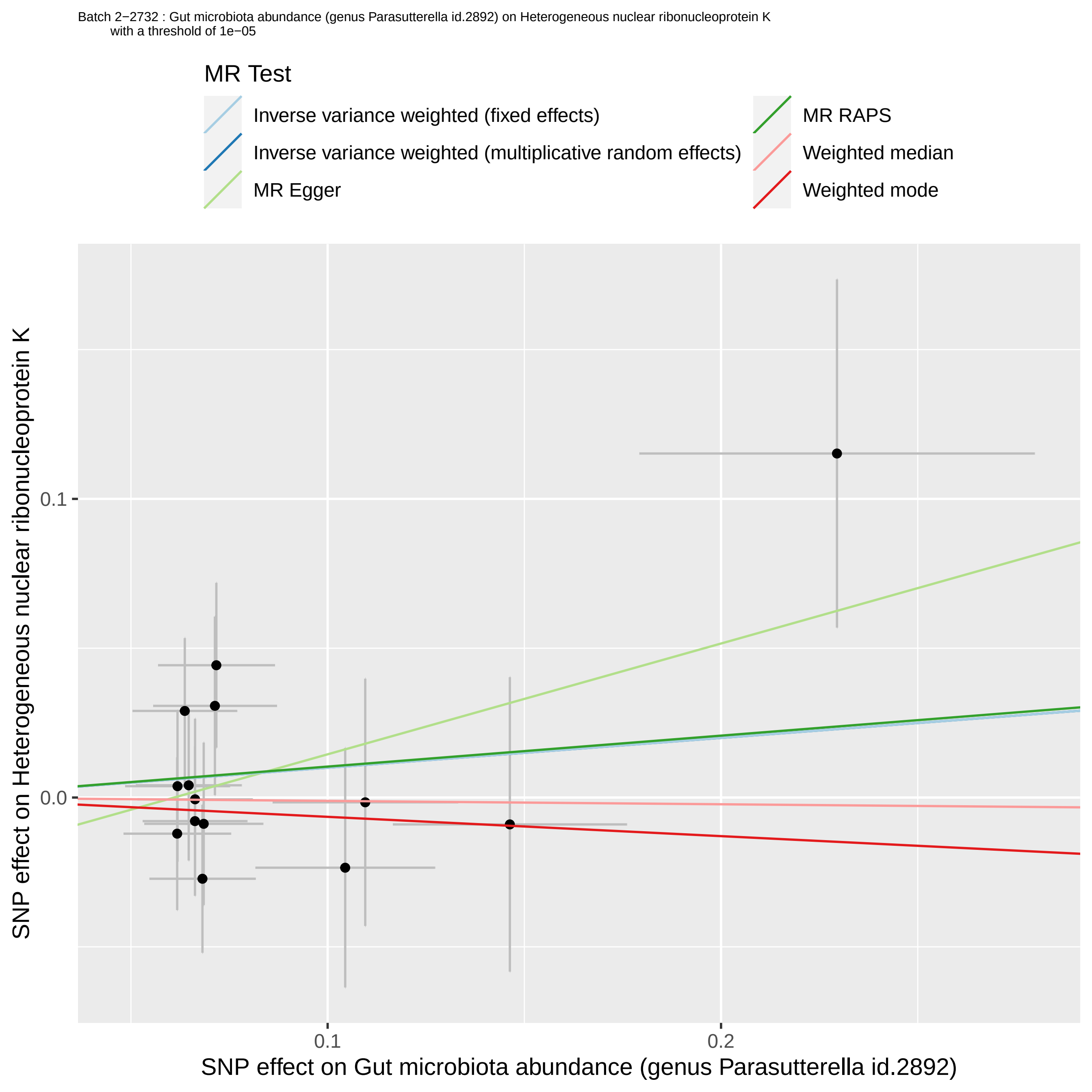

Batch 2−2732 : Gut microbiota abundance (genus Parasutterella id.2892) on Heterogeneous nuclear ribonucleoprotein K
with a threshold of 1e−05
MR Test
Inverse variance weighted (fixed effects)
Inverse variance weighted (multiplicative random effects)
MR Egger
MR RAPS
Weighted median
Weighted mode
0.1
0.0
0.1
0.2
SNP effect on Heterogeneous nuclear ribonucleoprotein K
SNP effect on Gut microbiota abundance (genus Parasutterella id.2892)

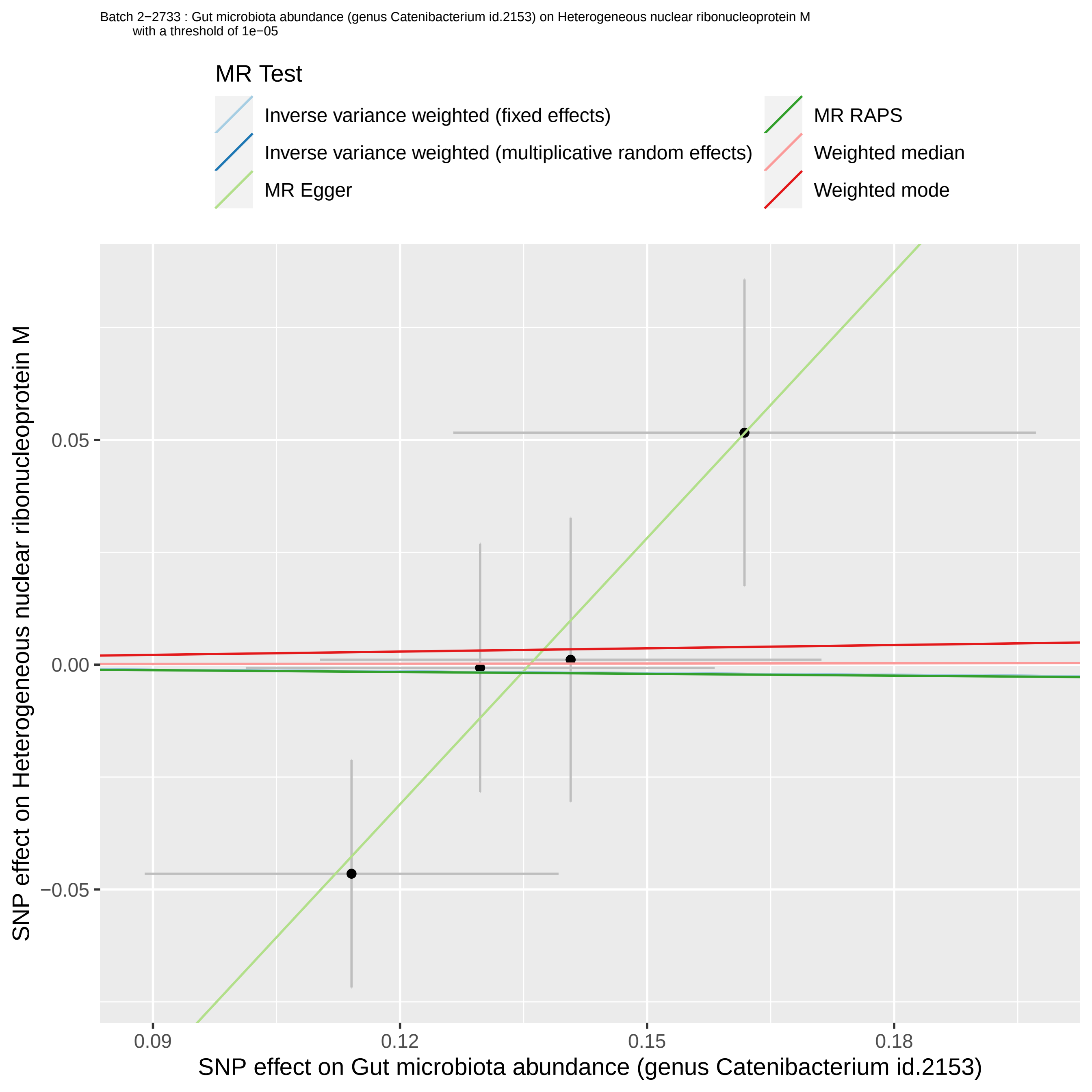
Batch 2−2733 : Gut microbiota abundance (genus Catenibacterium id.2153) on Heterogeneous nuclear ribonucleoprotein M
with a threshold of 1e−05
MR Test
Inverse variance weighted (fixed effects)
Inverse variance weighted (multiplicative random effects)
MR Egger
MR RAPS
Weighted median
Weighted mode
0.05
0.00
−0.05
0.09
0.12
0.15
0.18
SNP effect on Heterogeneous nuclear ribonucleoprotein M
SNP effect on Gut microbiota abundance (genus Catenibacterium id.2153)

Batch 2−2734 : Gut microbiota abundance (genus Parasutterella id.2892) on Heterogeneous nuclear ribonucleoprotein M
with a threshold of 1e−05

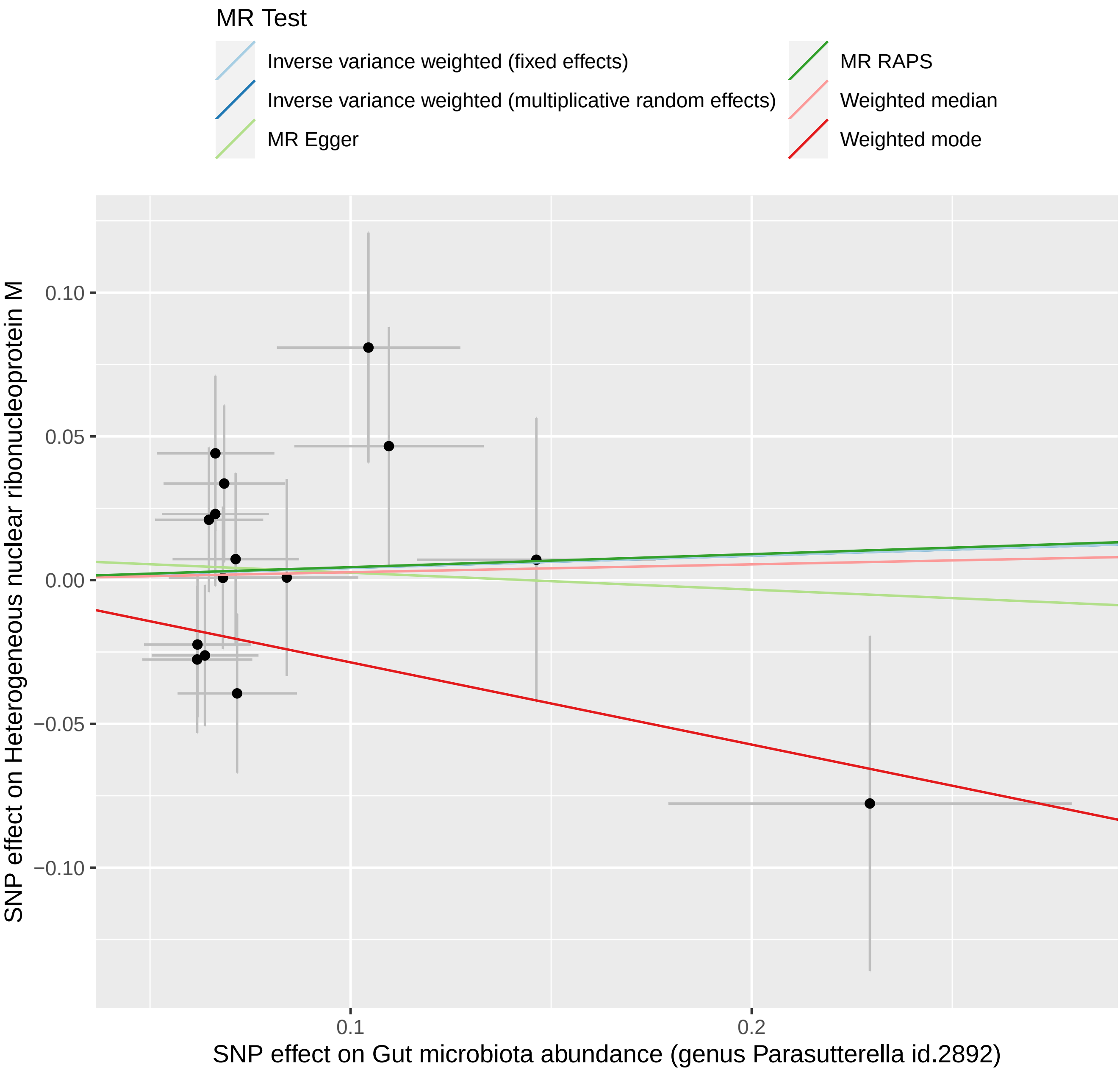

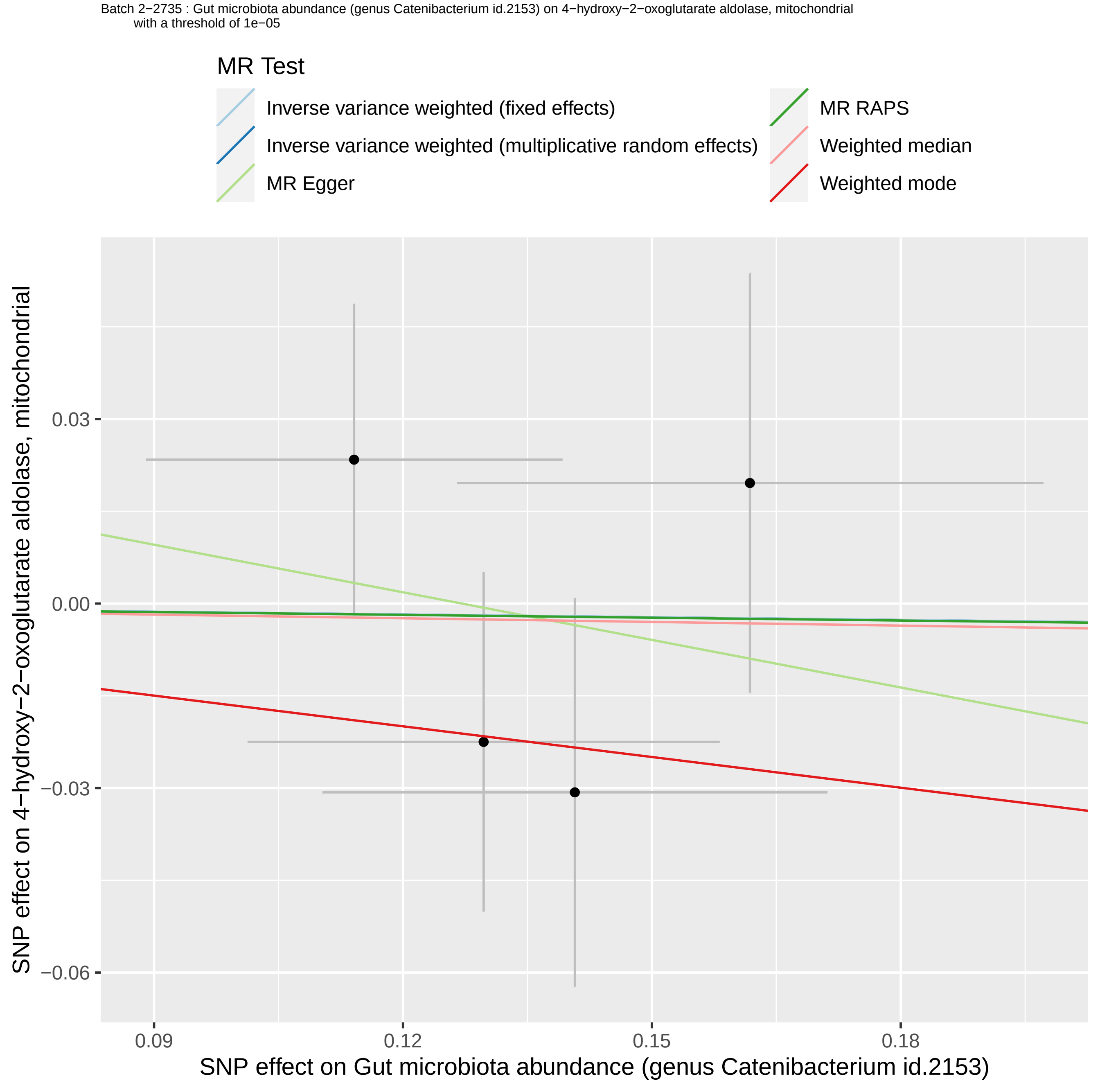
Batch 2−2735 : Gut microbiota abundance (genus Catenibacterium id.2153) on 4−hydroxy−2−oxoglutarate aldolase, mitochondrial
with a threshold of 1e−05
MR Test
Inverse variance weighted (fixed effects)
Inverse variance weighted (multiplicative random effects)
MR Egger
MR RAPS
Weighted median
Weighted mode
0.03
0.00
−0.03
−0.06
0.09
0.12
0.15
0.18
SNP effect on 4−hydroxy−2−oxoglutarate aldolase, mitochondrial
SNP effect on Gut microbiota abundance (genus Catenibacterium id.2153)

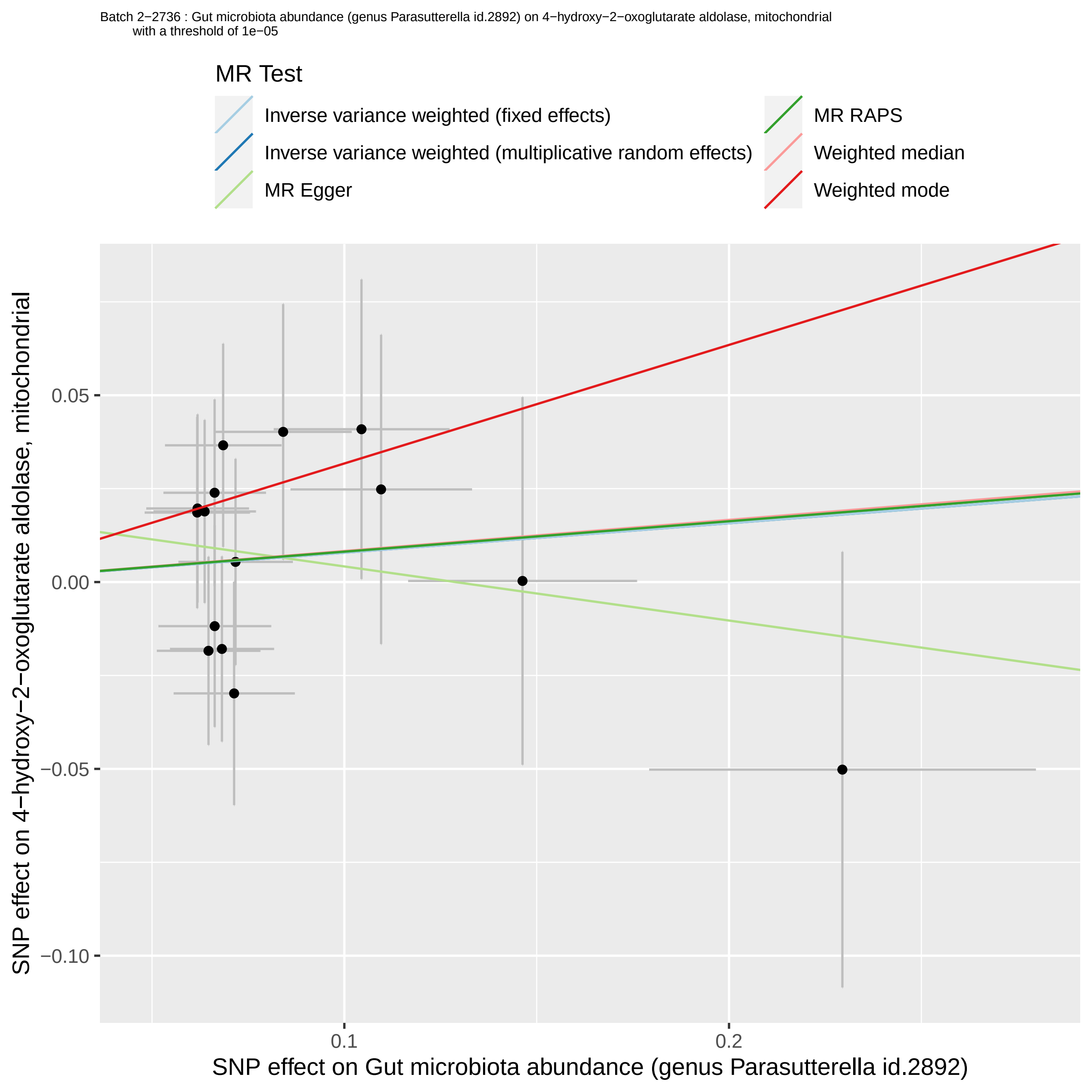
Batch 2−2736 : Gut microbiota abundance (genus Parasutterella id.2892) on 4−hydroxy−2−oxoglutarate aldolase, mitochondrial
with a threshold of 1e−05
MR Test
Inverse variance weighted (fixed effects)
Inverse variance weighted (multiplicative random effects)
MR Egger
MR RAPS
Weighted median
Weighted mode
0.05
0.00
−0.05
−0.10
0.1
0.2
SNP effect on 4−hydroxy−2−oxoglutarate aldolase, mitochondrial
SNP effect on Gut microbiota abundance (genus Parasutterella id.2892)

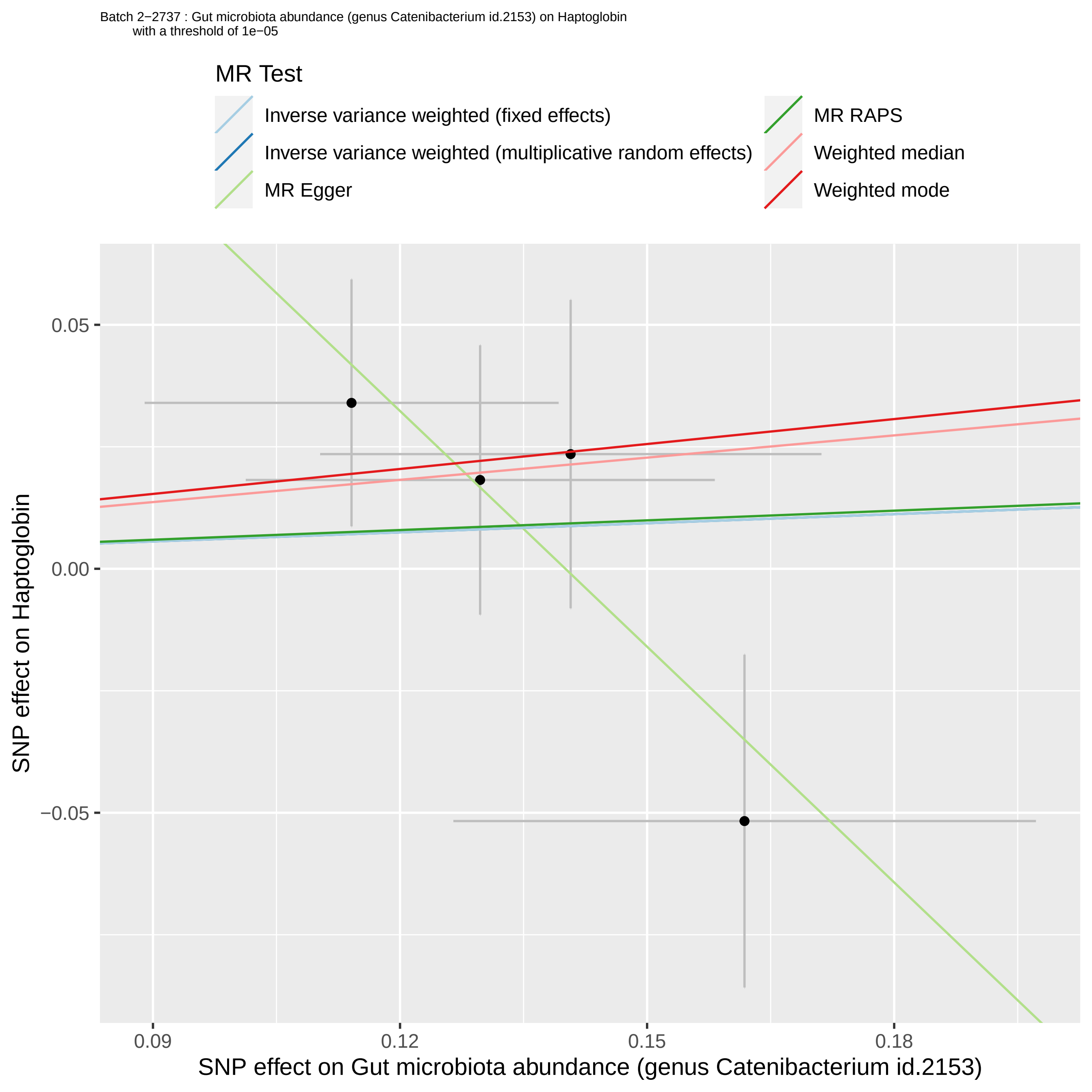

Batch 2−2737 : Gut microbiota abundance (genus Catenibacterium id.2153) on Haptoglobin
with a threshold of 1e−05
MR Test
Inverse variance weighted (fixed effects)
Inverse variance weighted (multiplicative random effects)
MR Egger
MR RAPS
Weighted median
Weighted mode
0.05
0.00
−0.05
0.09
0.12
0.15
0.18
SNP effect on Haptoglobin
SNP effect on Gut microbiota abundance (genus Catenibacterium id.2153)

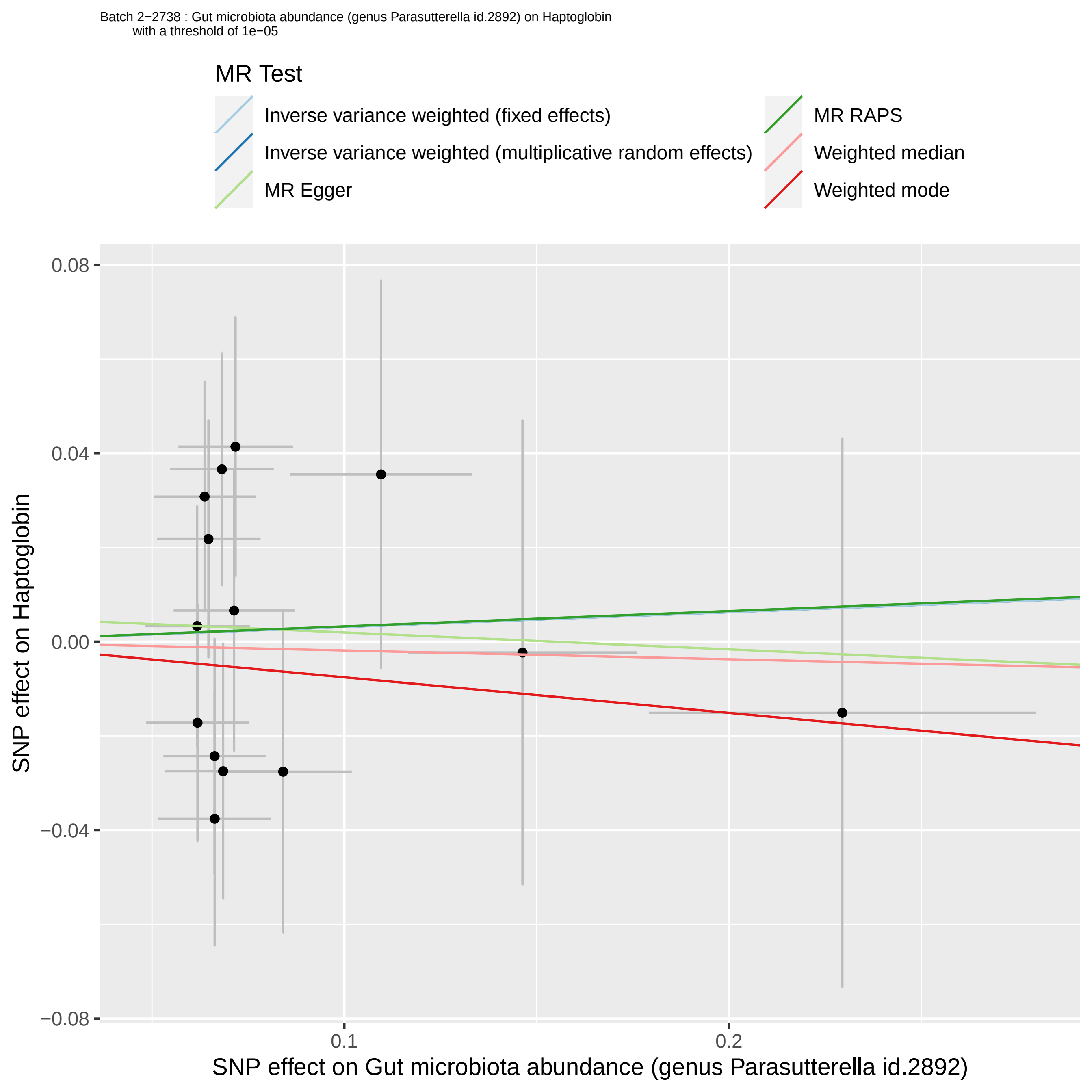
Batch 2−2738 : Gut microbiota abundance (genus Parasutterella id.2892) on Haptoglobin
with a threshold of 1e−05
MR Test
Inverse variance weighted (fixed effects)
Inverse variance weighted (multiplicative random effects)
MR Egger
MR RAPS
Weighted median
Weighted mode
0.08
0.04
0.00
−0.04
−0.08
0.1
0.2
SNP effect on Haptoglobin
SNP effect on Gut microbiota abundance (genus Parasutterella id.2892)

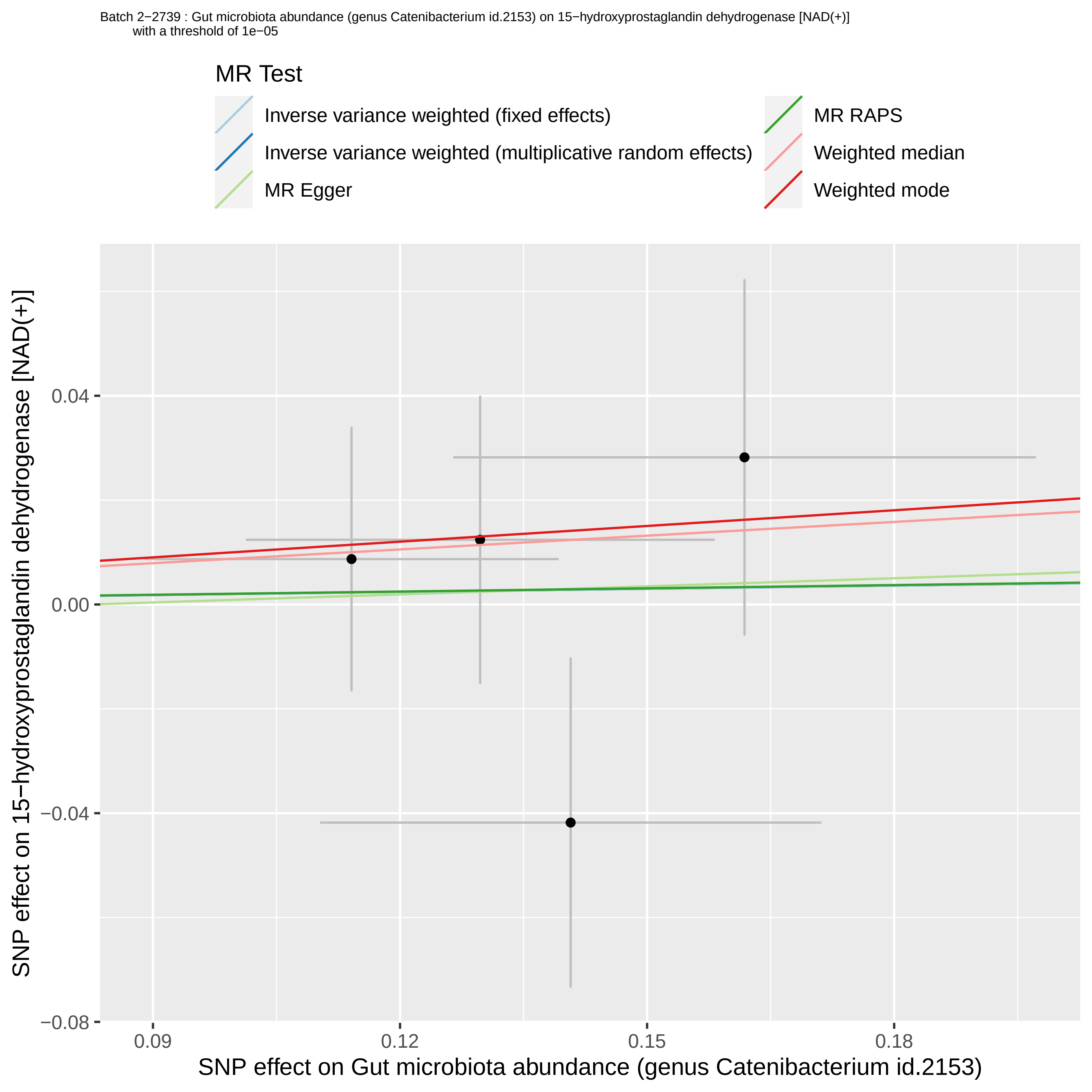
Batch 2−2739 : Gut microbiota abundance (genus Catenibacterium id.2153) on 15−hydroxyprostaglandin dehydrogenase [NAD(+)]
with a threshold of 1e−05
MR Test
Inverse variance weighted (fixed effects)
Inverse variance weighted (multiplicative random effects)
MR Egger
MR RAPS
Weighted median
Weighted mode
0.04
0.00
−0.04
−0.08
0.09
0.12
0.15
0.18
SNP effect on Gut microbiota abundance (genus Catenibacterium id.2153)
SNP effect on 15−hydroxyprostaglandin dehydrogenase [NAD(+)]

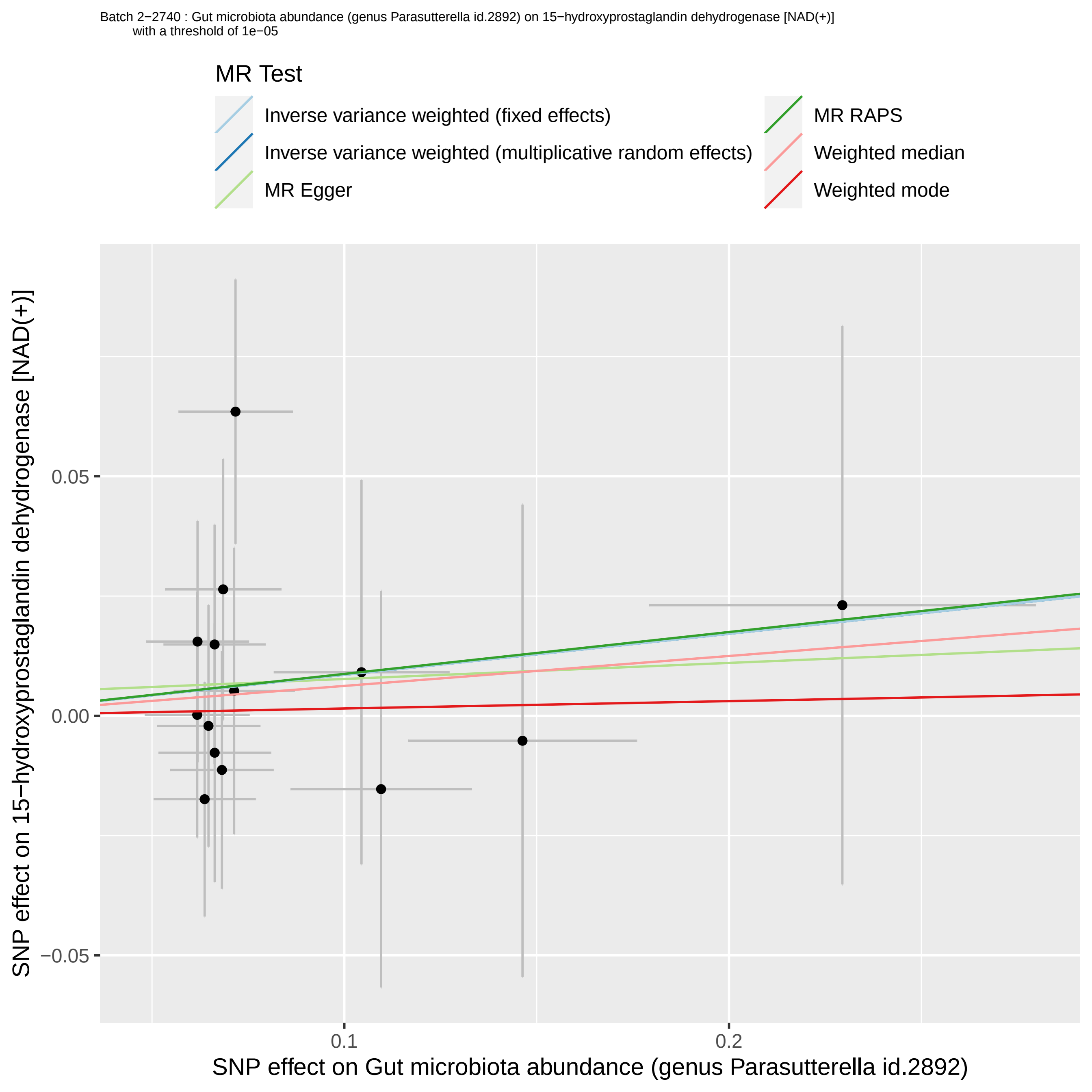
Batch 2−2740 : Gut microbiota abundance (genus Parasutterella id.2892) on 15−hydroxyprostaglandin dehydrogenase [NAD(+)]
with a threshold of 1e−05
MR Test
Inverse variance weighted (fixed effects)
Inverse variance weighted (multiplicative random effects)
MR Egger
MR RAPS
Weighted median
Weighted mode
0.05
0.00
−0.05
0.1
0.2
SNP effect on 15−hydroxyprostaglandin dehydrogenase [NAD(+)]
SNP effect on Gut microbiota abundance (genus Parasutterella id.2892)

Batch 2−2741 : Gut microbiota abundance (genus Catenibacterium id.2153) on Hematopoietic prostaglandin D synthase with a threshold of 1e−05

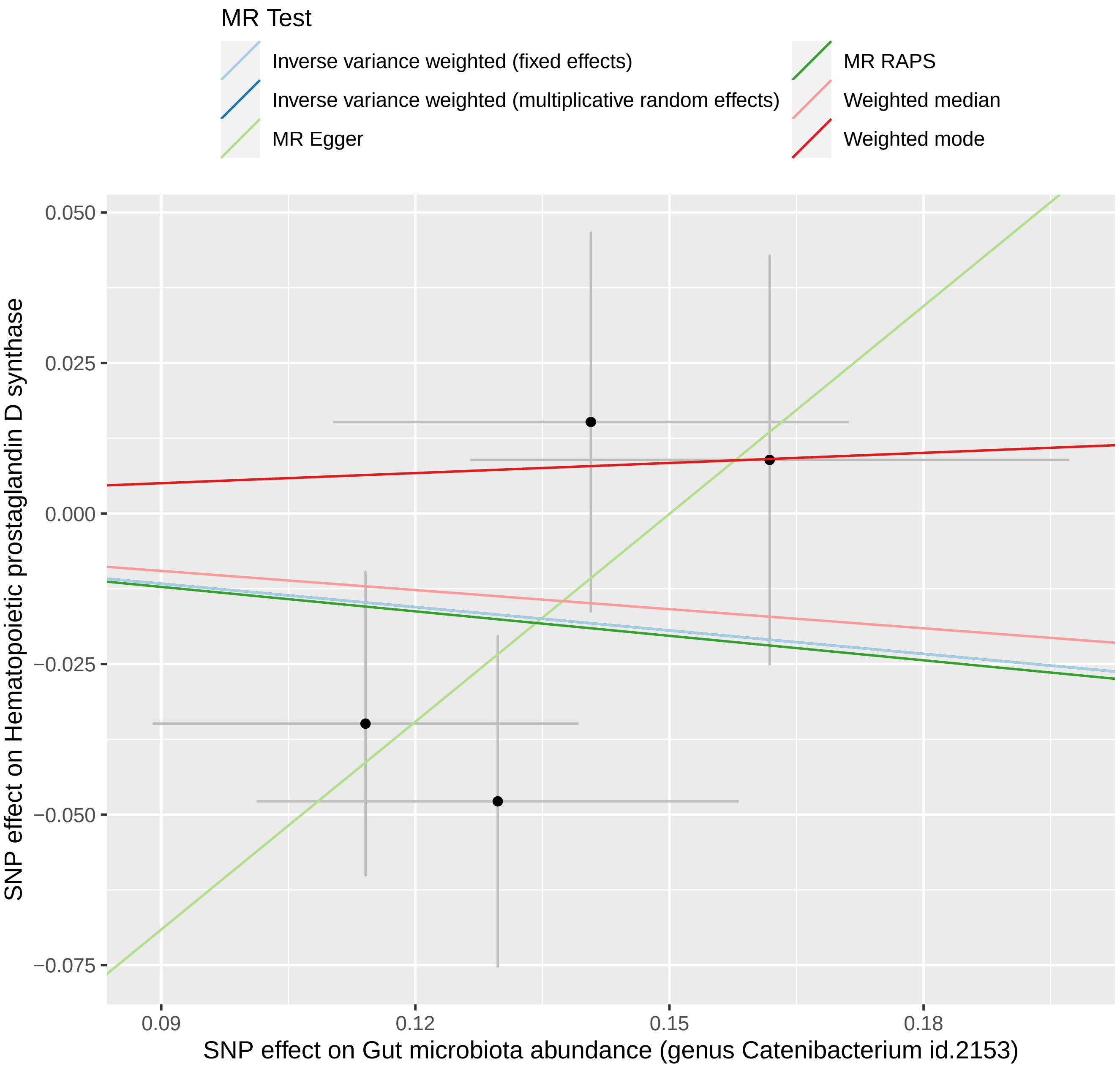

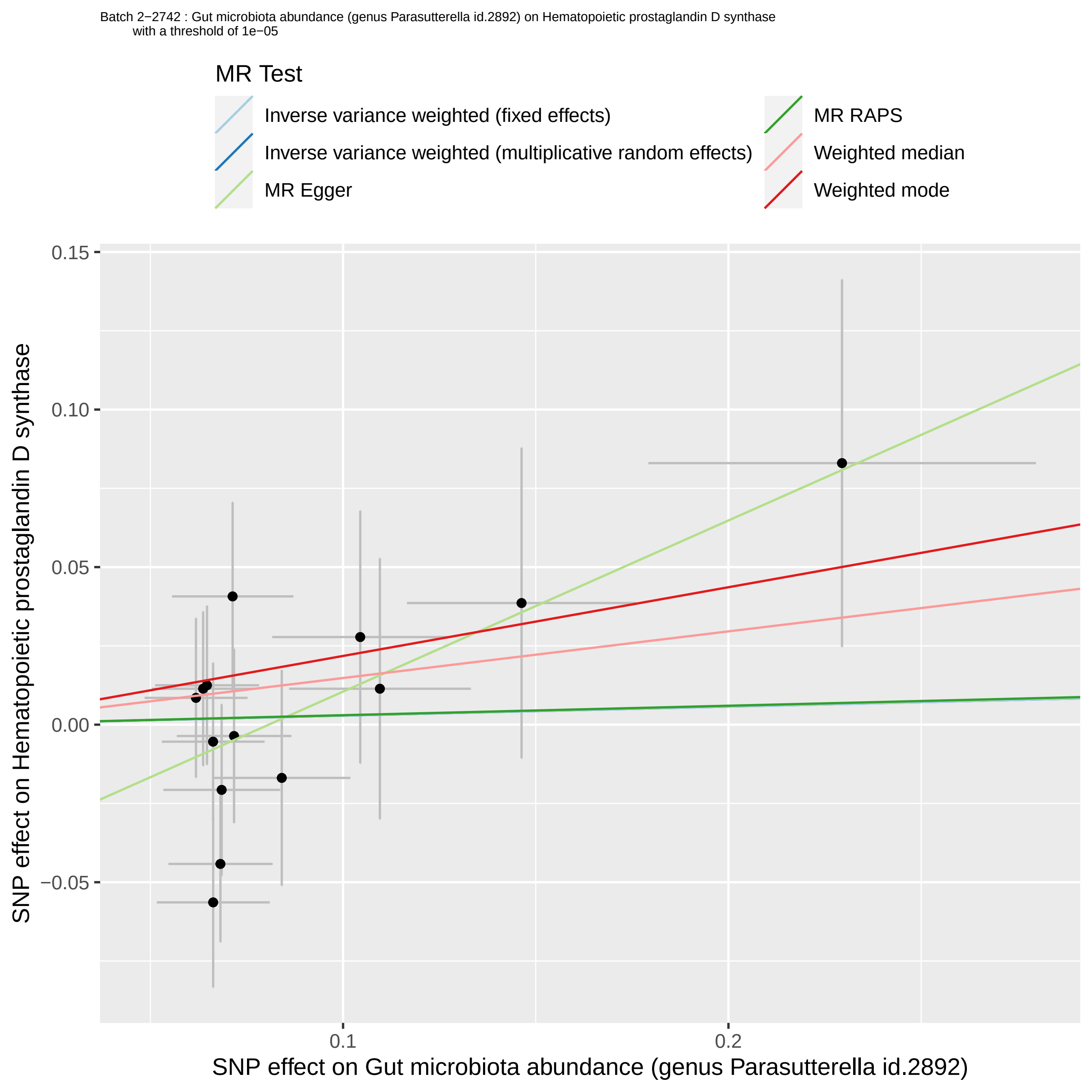

Batch 2−2742 : Gut microbiota abundance (genus Parasutterella id.2892) on Hematopoietic prostaglandin D synthase
with a threshold of 1e−05
MR Test
Inverse variance weighted (fixed effects)
Inverse variance weighted (multiplicative random effects)
MR Egger
MR RAPS
Weighted median
Weighted mode
0.15
0.10
0.05
0.00
−0.05
0.1
0.2
SNP effect on Hematopoietic prostaglandin D synthase
SNP effect on Gut microbiota abundance (genus Parasutterella id.2892)

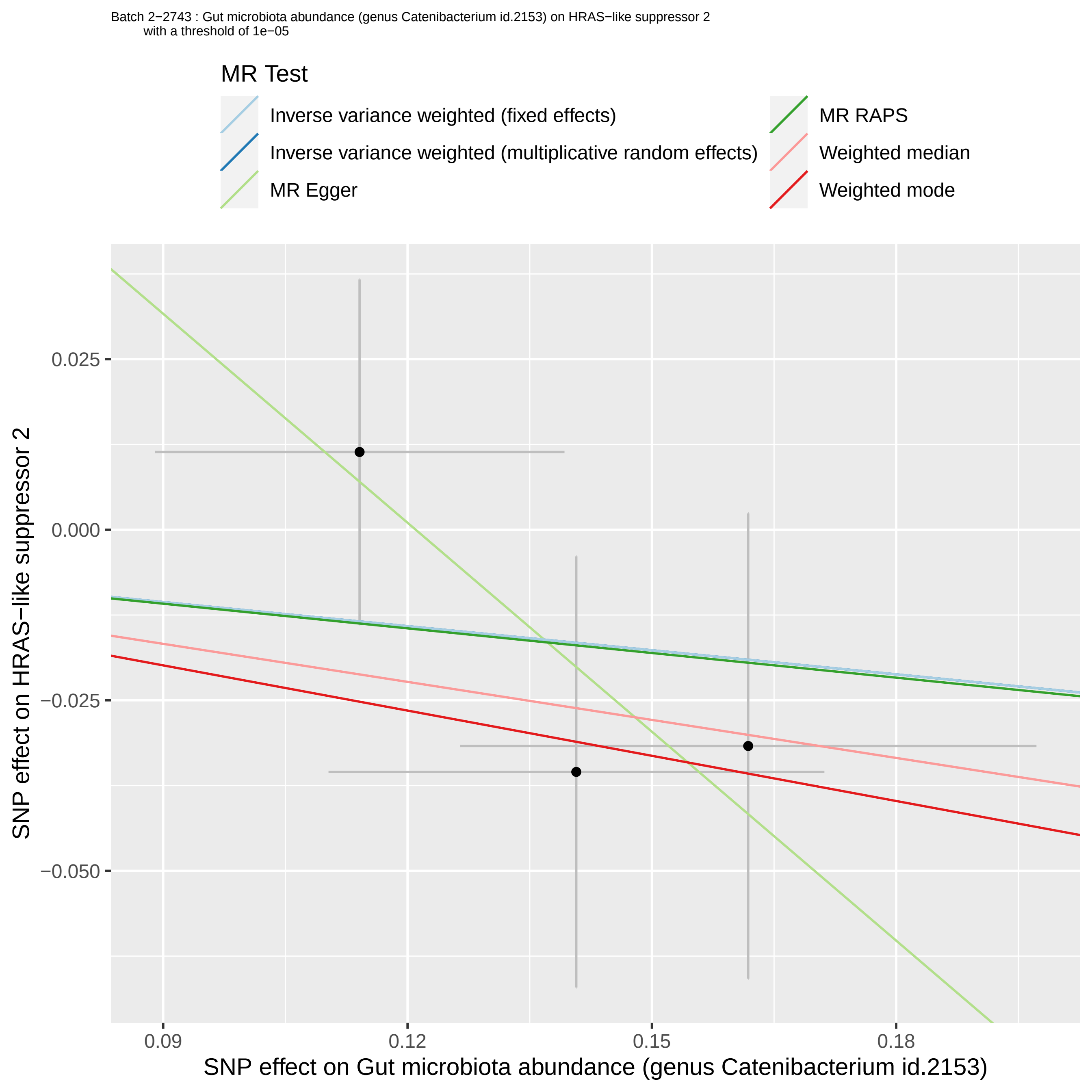

Batch 2−2743 : Gut microbiota abundance (genus Catenibacterium id.2153) on HRAS−like suppressor 2
with a threshold of 1e−05
MR Test
Inverse variance weighted (fixed effects)
Inverse variance weighted (multiplicative random effects)
MR Egger
MR RAPS
Weighted median
Weighted mode
0.025
0.000
−0.025
−0.050
0.09
0.12
0.15
0.18
SNP effect on HRAS−like suppressor 2
SNP effect on Gut microbiota abundance (genus Catenibacterium id.2153)

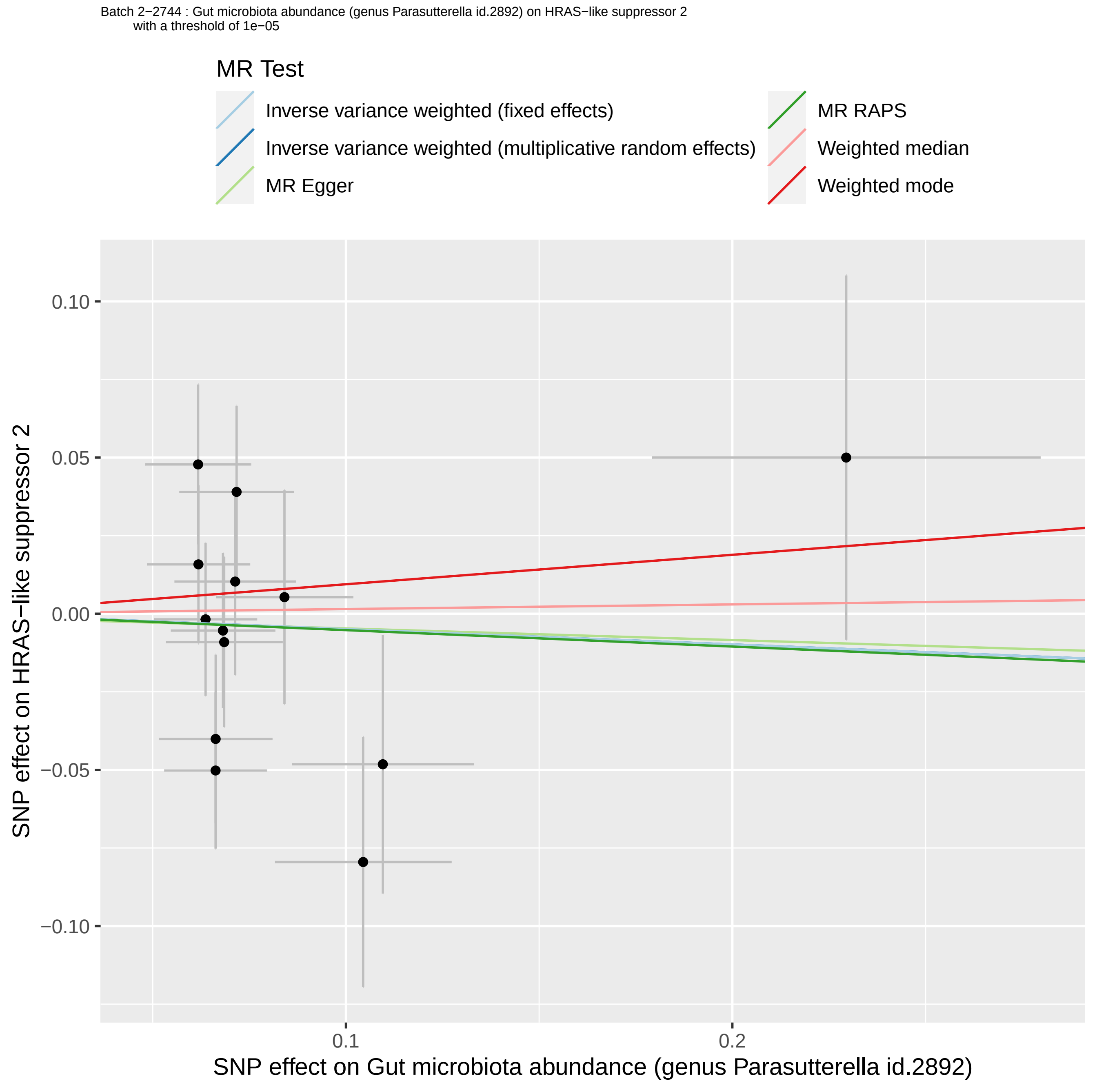

Batch 2−2744 : Gut microbiota abundance (genus Parasutterella id.2892) on HRAS−like suppressor 2
with a threshold of 1e−05
MR Test
Inverse variance weighted (fixed effects)
Inverse variance weighted (multiplicative random effects)
MR Egger
MR RAPS
Weighted median
Weighted mode
0.10
0.05
0.00
−0.05
−0.10
0.1
0.2
SNP effect on HRAS−like suppressor 2
SNP effect on Gut microbiota abundance (genus Parasutterella id.2892)

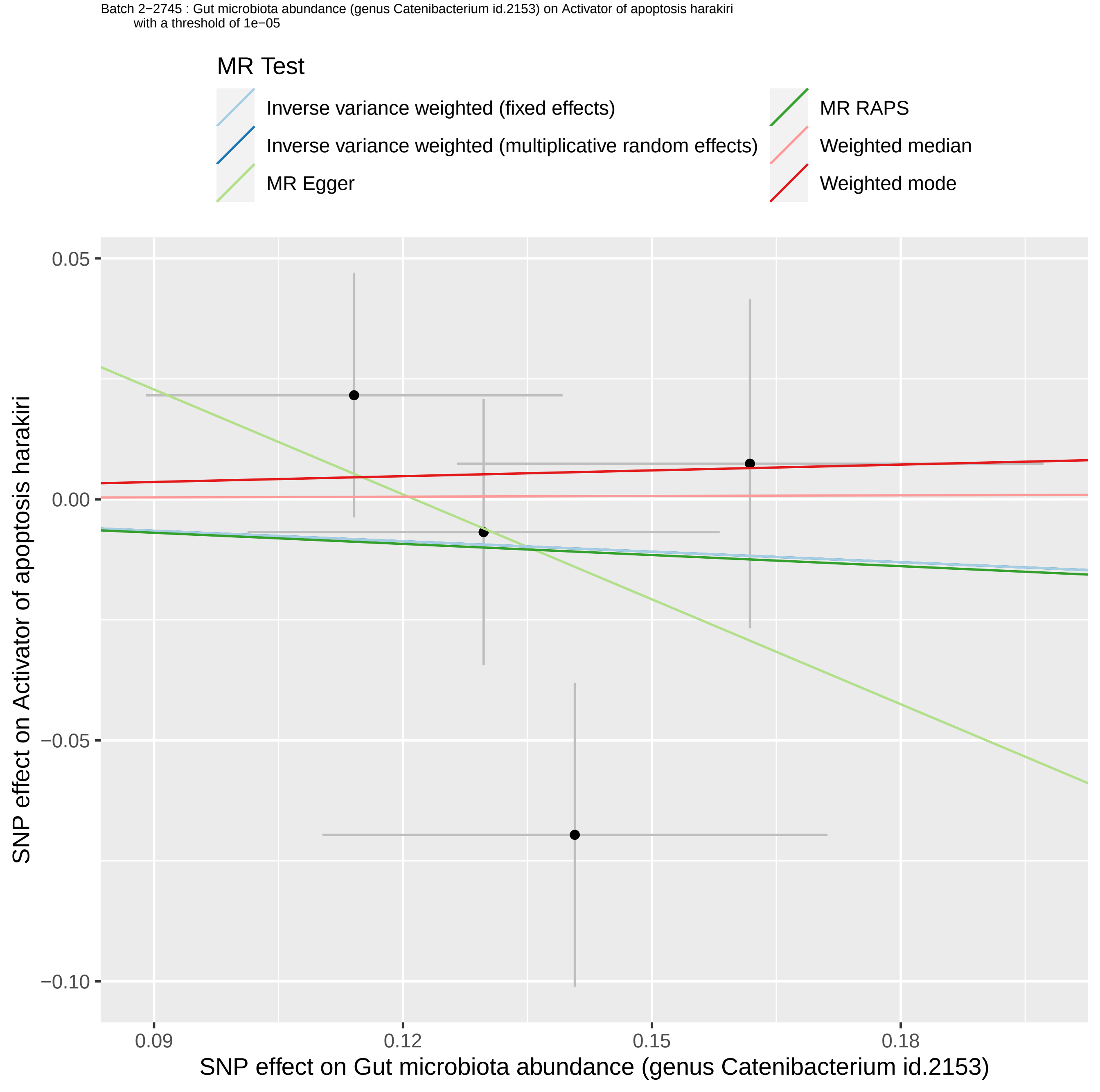

Batch 2−2745 : Gut microbiota abundance (genus Catenibacterium id.2153) on Activator of apoptosis harakiri
with a threshold of 1e−05
MR Test
Inverse variance weighted (fixed effects)
Inverse variance weighted (multiplicative random effects)
MR Egger
MR RAPS
Weighted median
Weighted mode
0.05
0.00
−0.05
−0.10
0.09
0.12
0.15
0.18
SNP effect on Gut microbiota abundance (genus Catenibacterium id.2153)
SNP effect on Activator of apoptosis harakiri

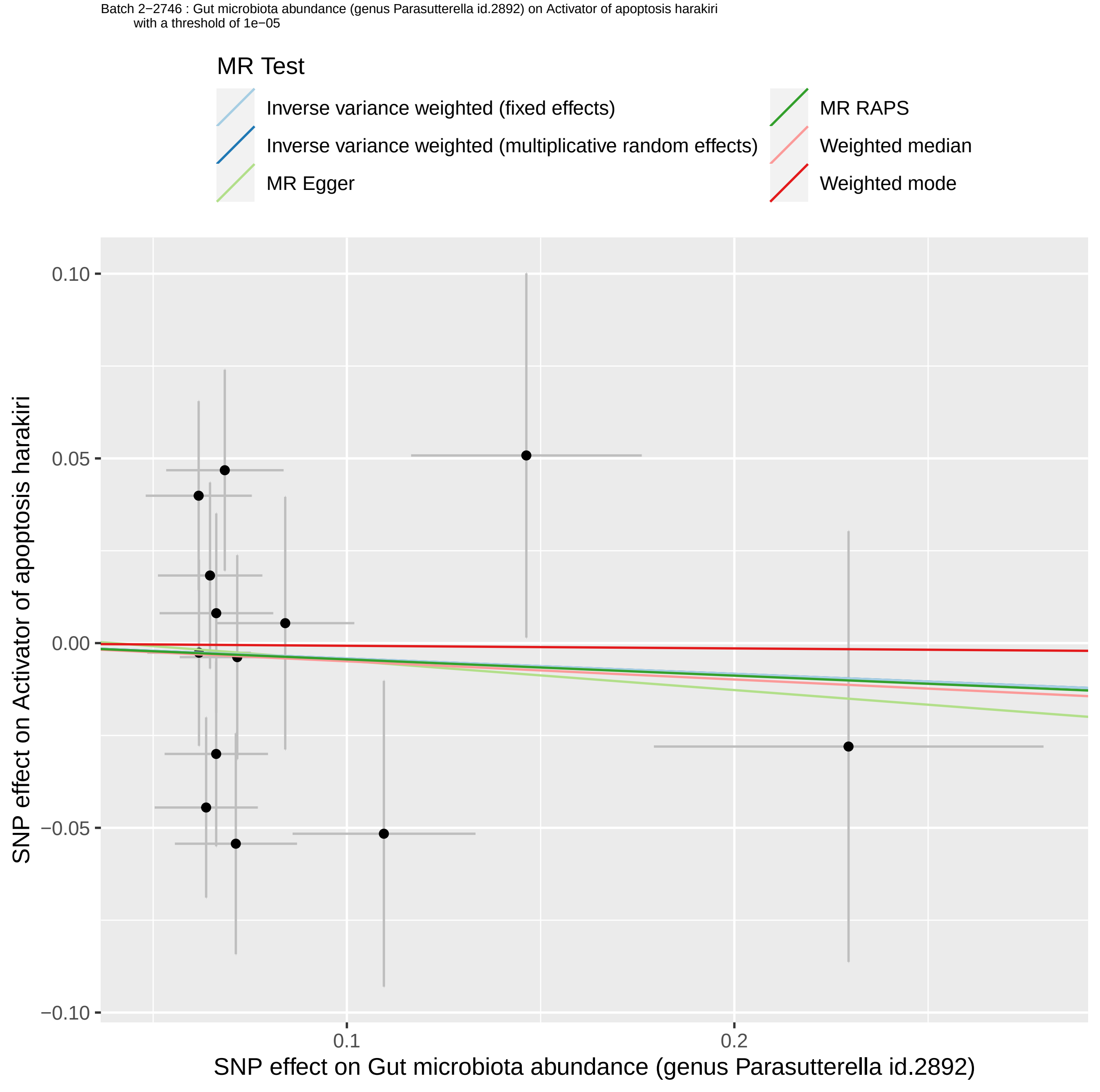
Batch 2−2746 : Gut microbiota abundance (genus Parasutterella id.2892) on Activator of apoptosis harakiri
with a threshold of 1e−05
MR Test
Inverse variance weighted (fixed effects)
Inverse variance weighted (multiplicative random effects)
MR Egger
MR RAPS
Weighted median
Weighted mode
0.10
0.05
0.00
−0.05
−0.10
0.1
0.2
SNP effect on Activator of apoptosis harakiri
SNP effect on Gut microbiota abundance (genus Parasutterella id.2892)

Batch 2−2747 : Gut microbiota abundance (genus Catenibacterium id.2153) on Ribonuclease UK114
with a threshold of 1e−05

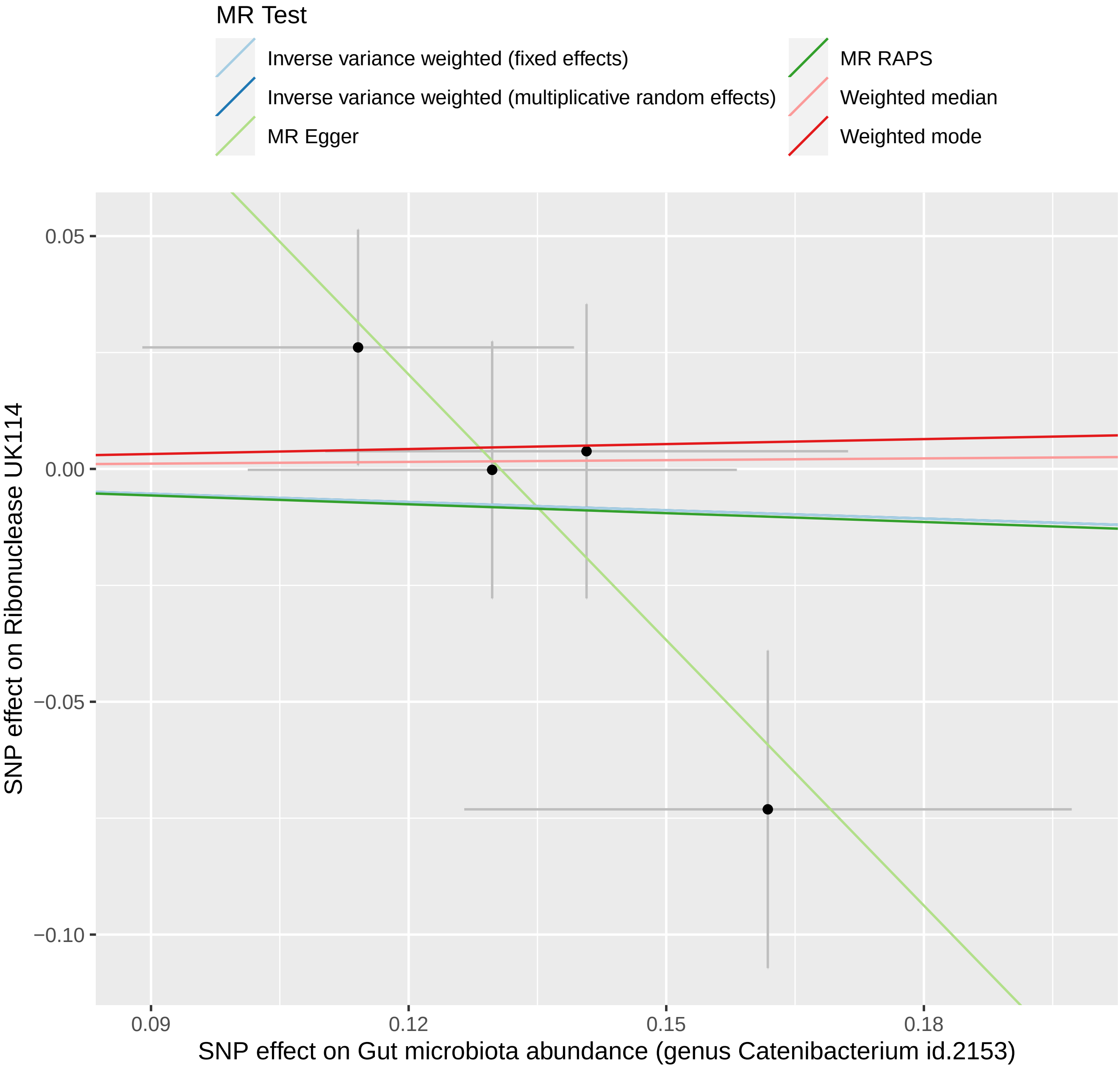

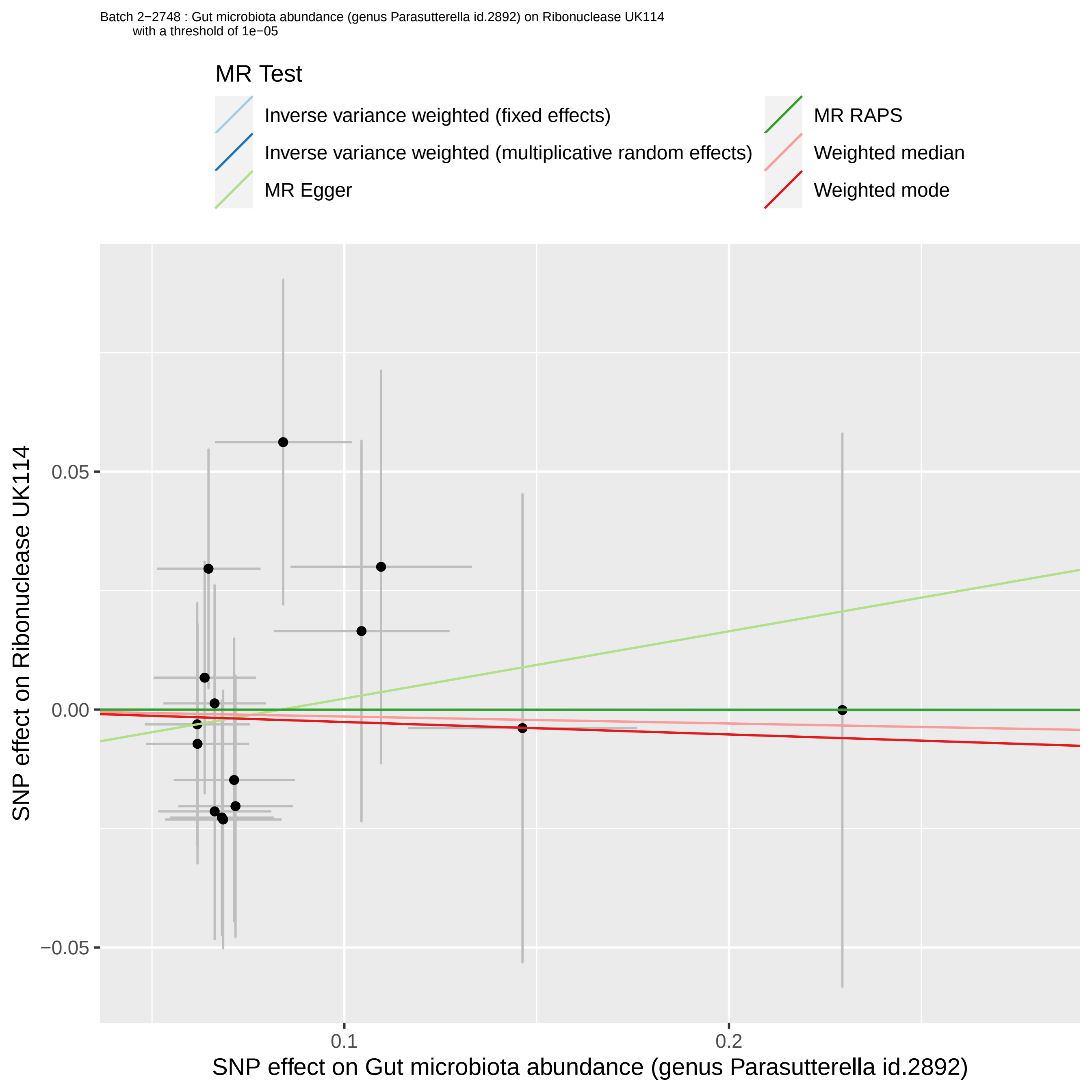
Batch 2−2748 : Gut microbiota abundance (genus Parasutterella id.2892) on Ribonuclease UK114
with a threshold of 1e−05
MR Test
Inverse variance weighted (fixed effects)
Inverse variance weighted (multiplicative random effects)
MR Egger
MR RAPS
Weighted median
Weighted mode
0.05
0.00
−0.05
0.1
0.2
SNP effect on Ribonuclease UK114
SNP effect on Gut microbiota abundance (genus Parasutterella id.2892)

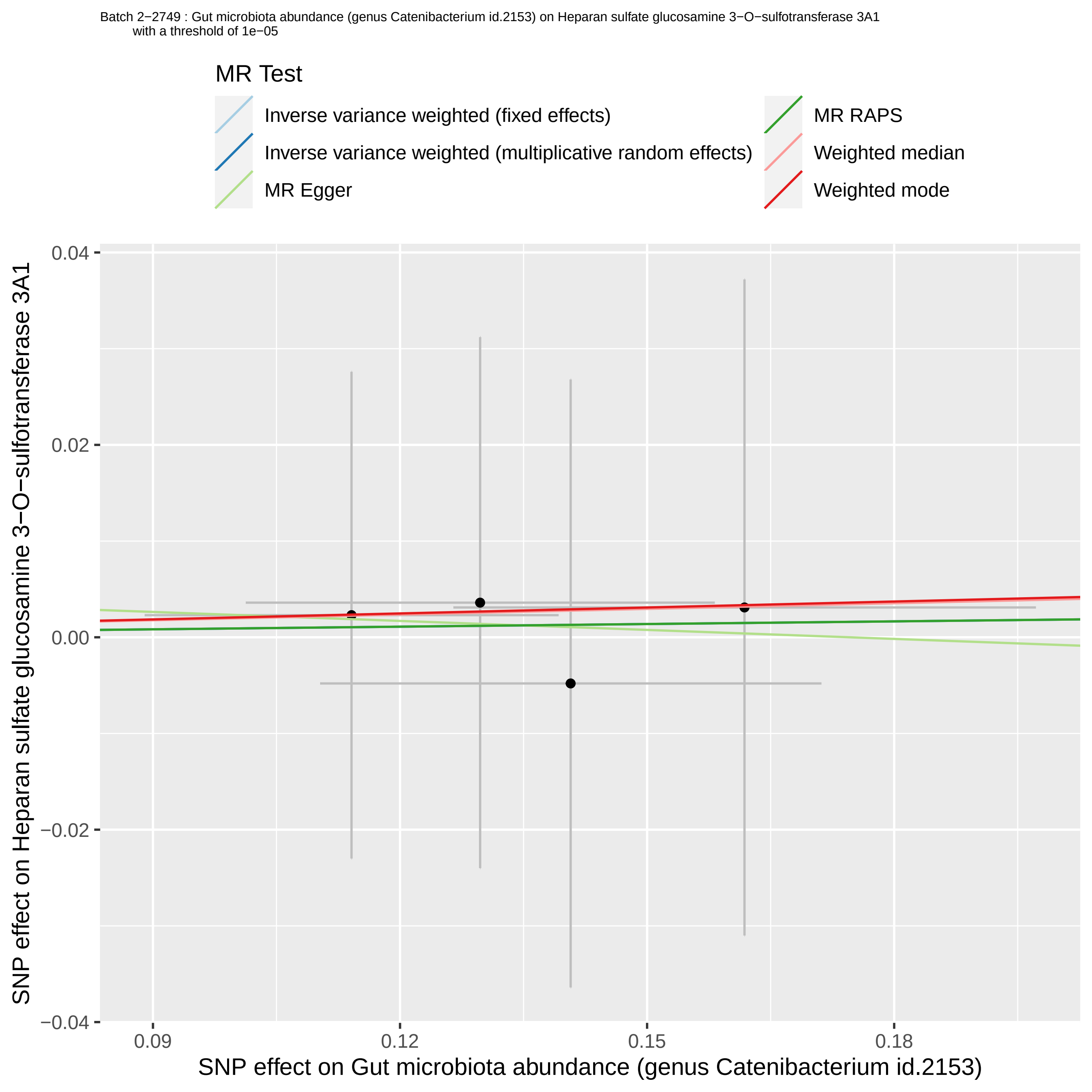
Batch 2−2749 : Gut microbiota abundance (genus Catenibacterium id.2153) on Heparan sulfate glucosamine 3−O−sulfotransferase 3A1
with a threshold of 1e−05
MR Test
Inverse variance weighted (fixed effects)
Inverse variance weighted (multiplicative random effects)
MR Egger
MR RAPS
Weighted median
Weighted mode
0.04
0.02
0.00
−0.02
−0.04
0.09
0.12
0.15
0.18
SNP effect on Heparan sulfate glucosamine 3−O−sulfotransferase 3A1
SNP effect on Gut microbiota abundance (genus Catenibacterium id.2153)

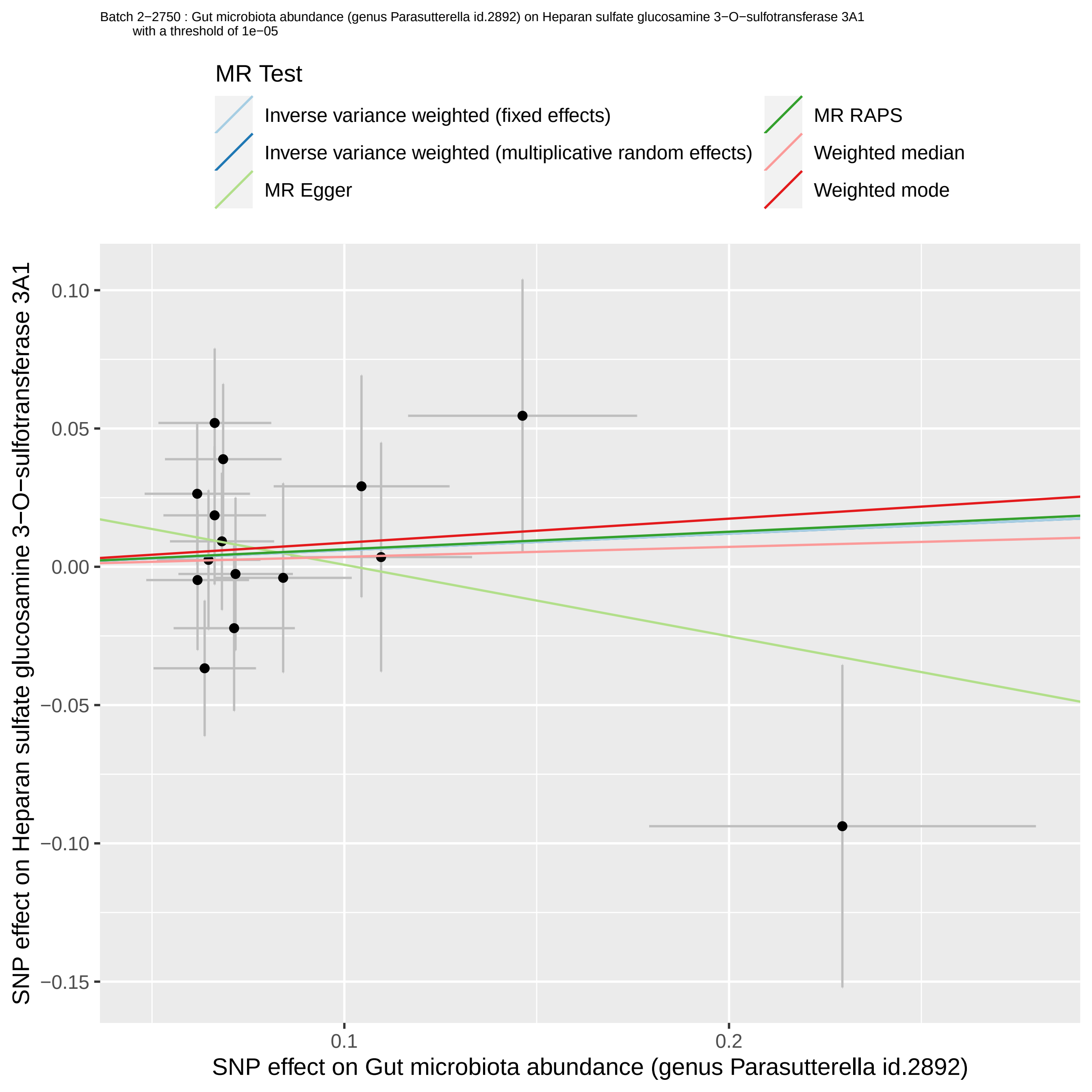

Batch 2−2750 : Gut microbiota abundance (genus Parasutterella id.2892) on Heparan sulfate glucosamine 3−O−sulfotransferase 3A1
with a threshold of 1e−05
MR Test
Inverse variance weighted (fixed effects)
Inverse variance weighted (multiplicative random effects)
MR Egger
MR RAPS
Weighted median
Weighted mode
0.10
0.05
0.00
−0.05
−0.10
−0.15
0.1
0.2
SNP effect on Heparan sulfate glucosamine 3−O−sulfotransferase 3A1
SNP effect on Gut microbiota abundance (genus Parasutterella id.2892)

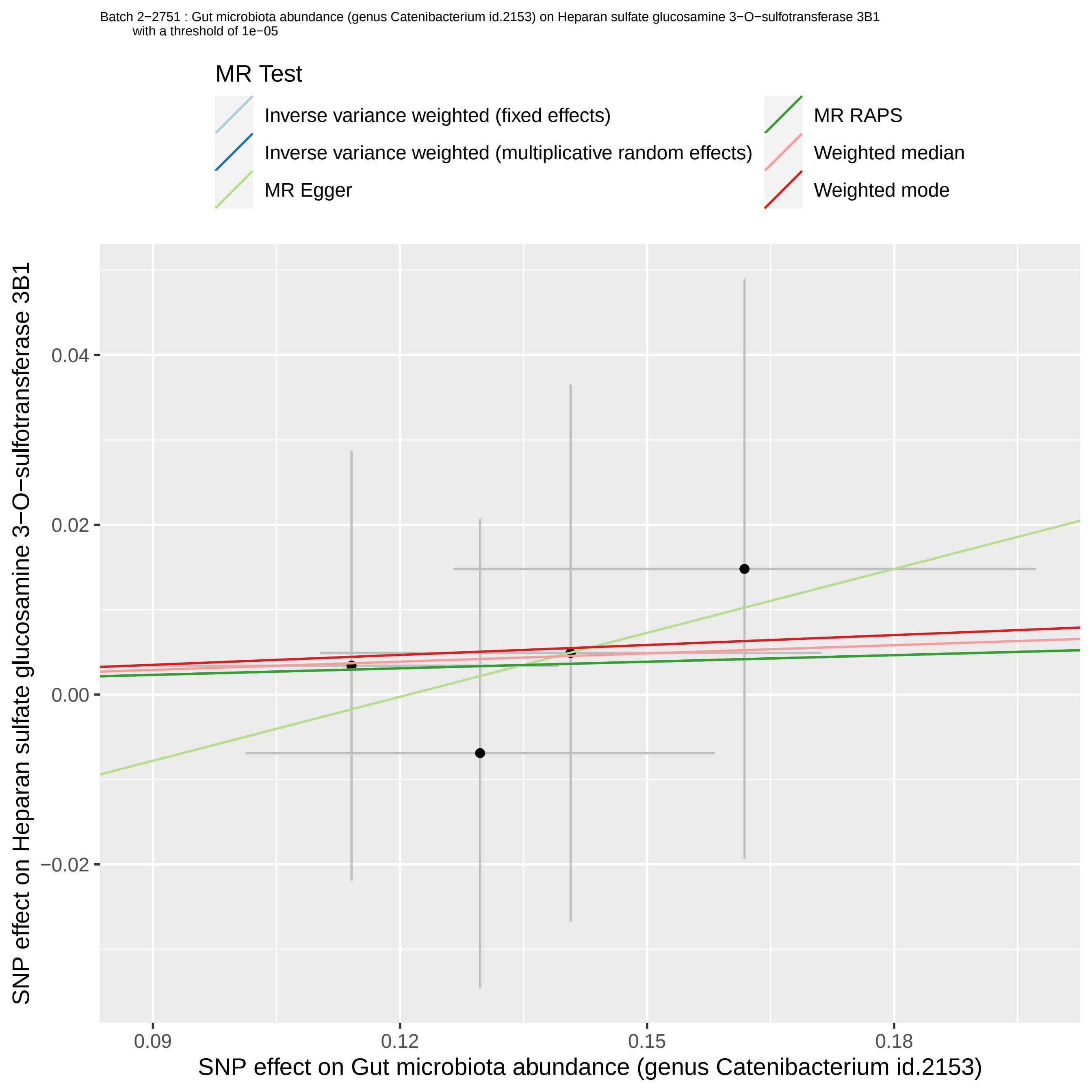
Batch 2−2751 : Gut microbiota abundance (genus Catenibacterium id.2153) on Heparan sulfate glucosamine 3−O−sulfotransferase 3B1 with a threshold of 1e−05
MR Test
Inverse variance weighted (fixed effects)
Inverse variance weighted (multiplicative random effects)
MR Egger
MR RAPS
Weighted median
Weighted mode
0.04
0.02
0.00
−0.02
0.09
0.12
0.15
0.18
SNP effect on Heparan sulfate glucosamine 3−O−sulfotransferase 3B1
SNP effect on Gut microbiota abundance (genus Catenibacterium id.2153)

Batch 2−2752 : Gut microbiota abundance (genus Parasutterella id.2892) on Heparan sulfate glucosamine 3−O−sulfotransferase 3B1 with a threshold of 1e−05

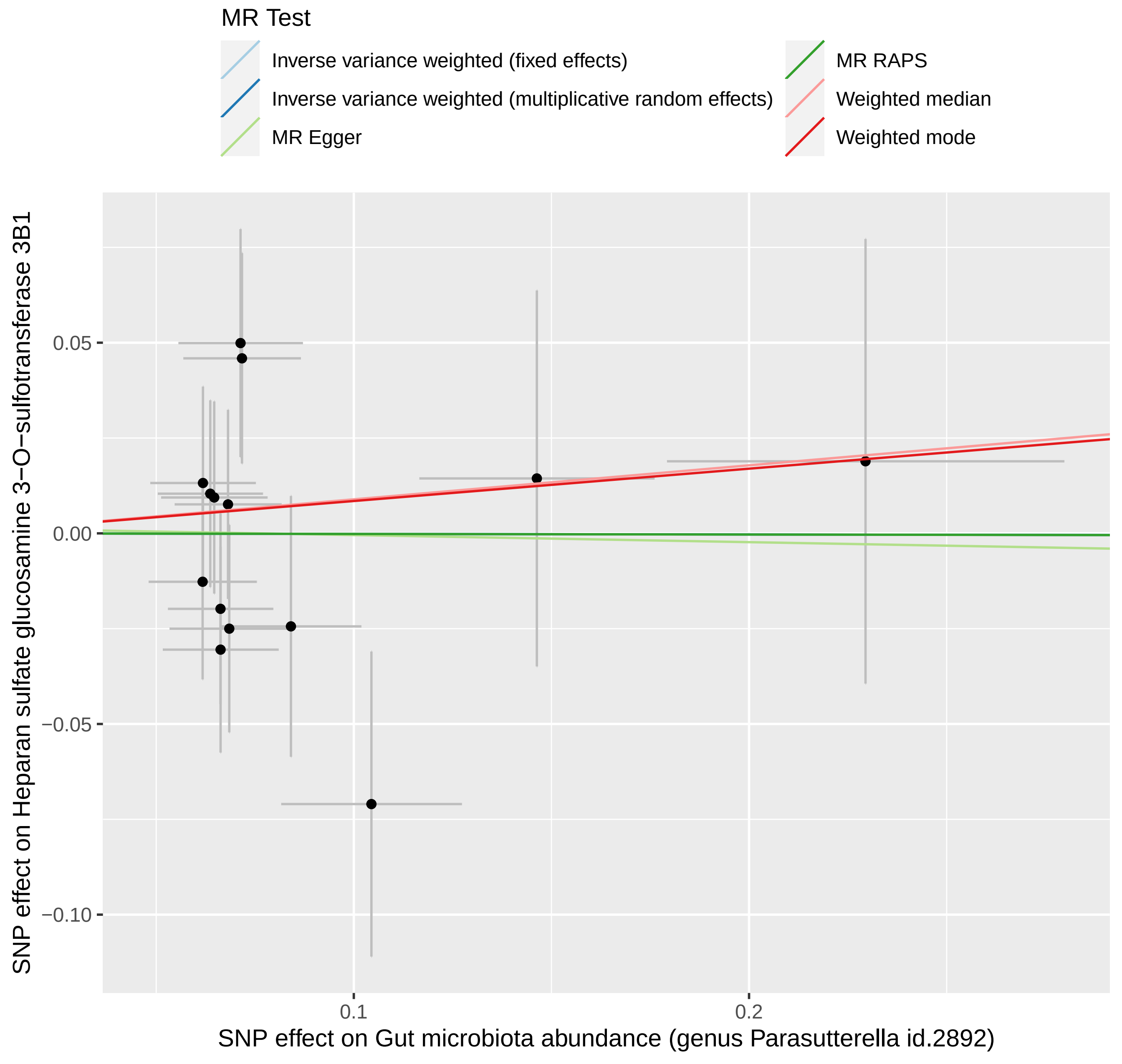

Batch 2−2753 : Gut microbiota abundance (genus Catenibacterium id.2153) on Heparan sulfate glucosamine 3−O−sulfotransferase 4 with a threshold of 1e−05

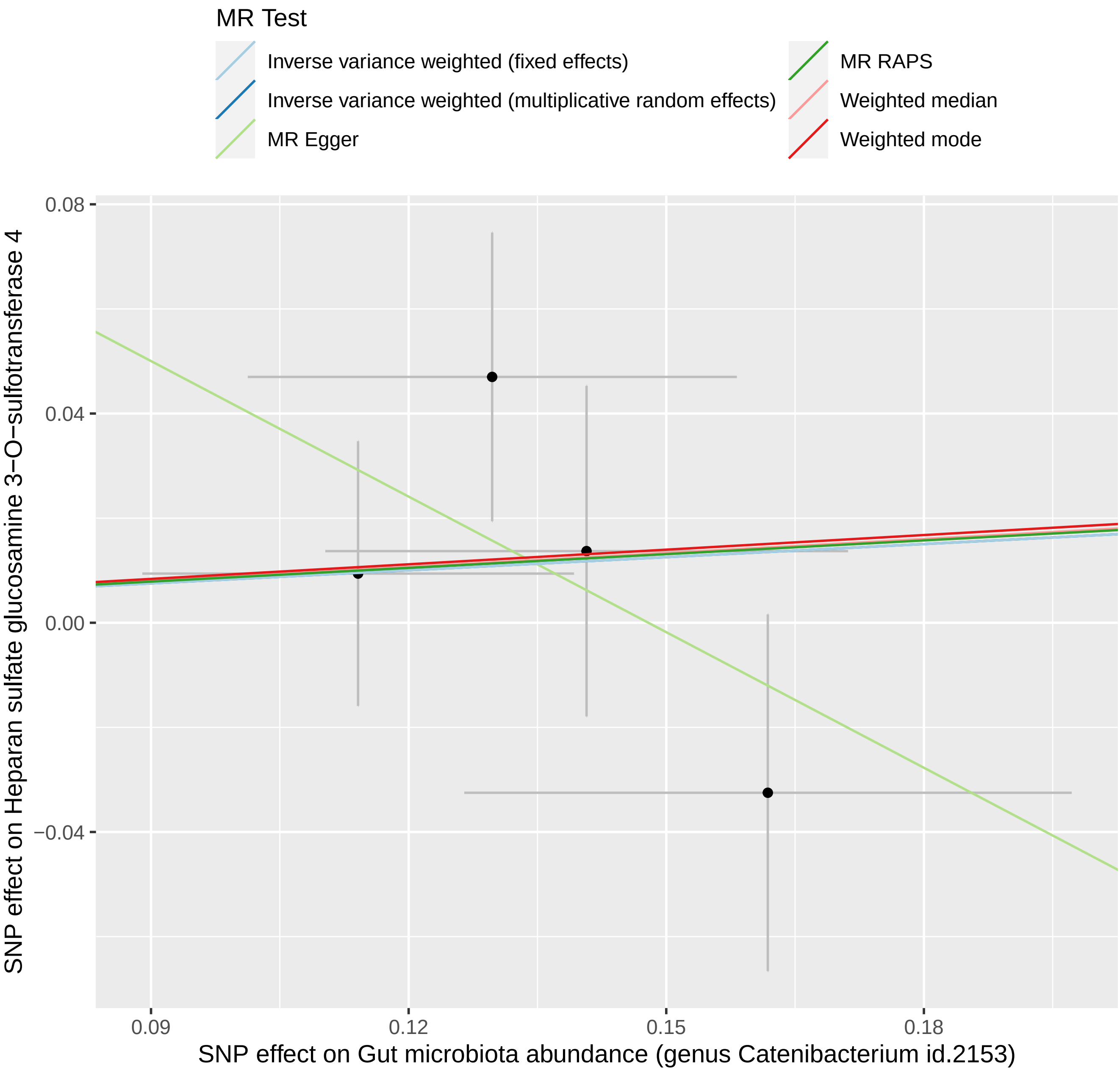

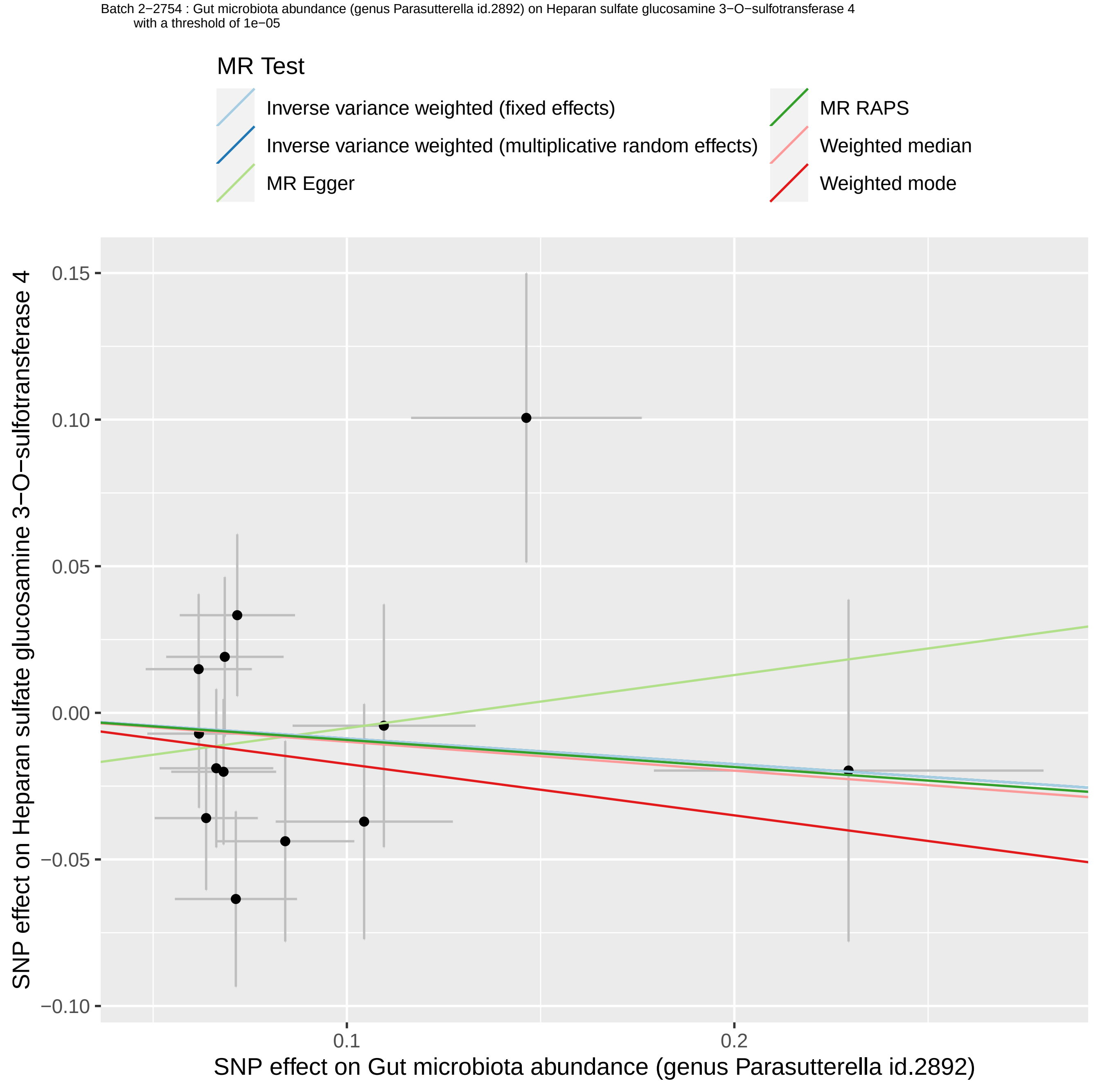

Batch 2−2754 : Gut microbiota abundance (genus Parasutterella id.2892) on Heparan sulfate glucosamine 3−O−sulfotransferase 4
with a threshold of 1e−05
MR Test
Inverse variance weighted (fixed effects)
Inverse variance weighted (multiplicative random effects)
MR Egger
MR RAPS
Weighted median
Weighted mode
0.15
0.10
0.05
0.00
−0.05
−0.10
0.1
0.2
SNP effect on Heparan sulfate glucosamine 3−O−sulfotransferase 4
SNP effect on Gut microbiota abundance (genus Parasutterella id.2892)

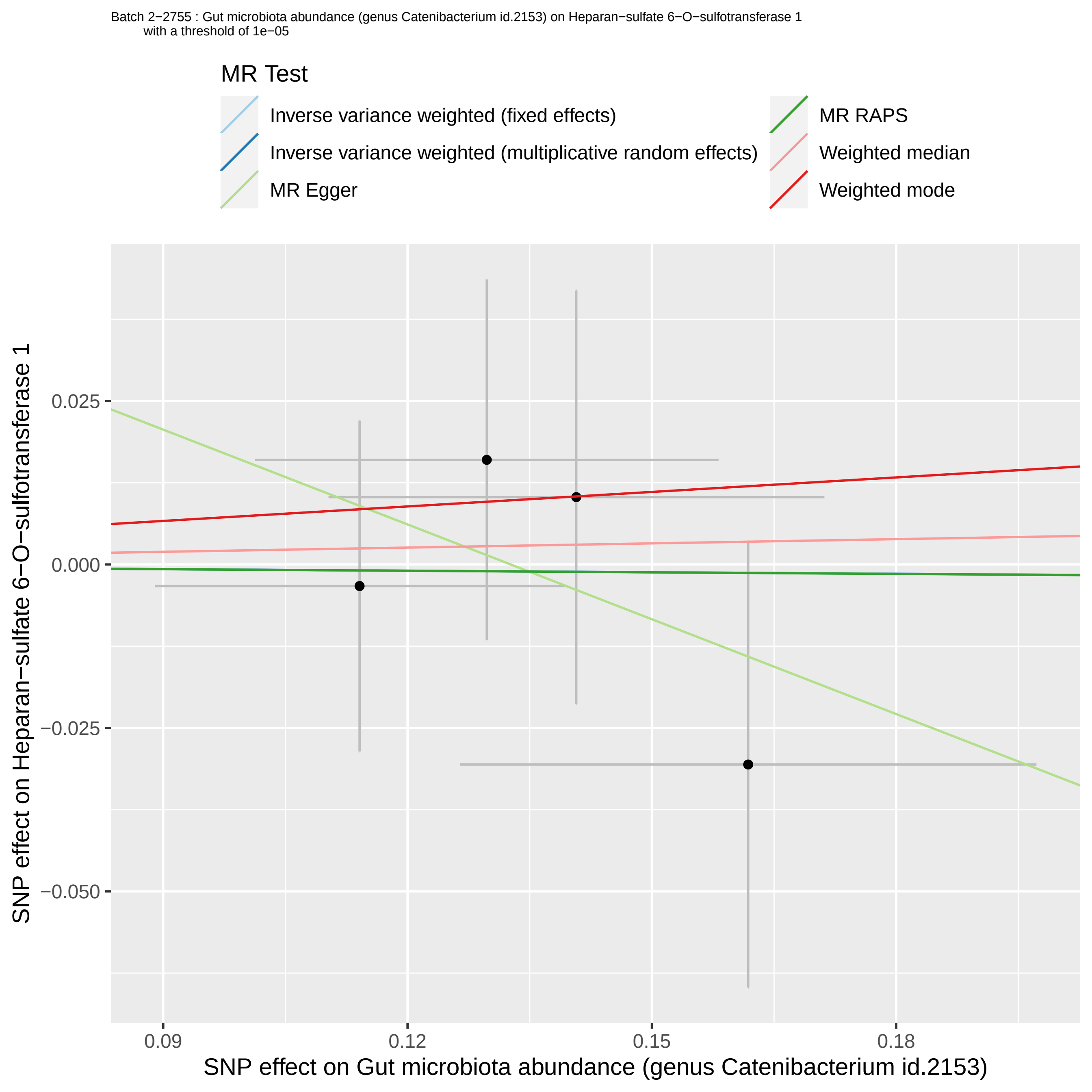
Batch 2−2755 : Gut microbiota abundance (genus Catenibacterium id.2153) on Heparan−sulfate 6−O−sulfotransferase 1
with a threshold of 1e−05
MR Test
Inverse variance weighted (fixed effects)
Inverse variance weighted (multiplicative random effects)
MR Egger
MR RAPS
Weighted median
Weighted mode
0.025
0.000
−0.025
−0.050
0.09
0.12
0.15
0.18
SNP effect on Gut microbiota abundance (genus Catenibacterium id.2153)
SNP effect on Heparan−sulfate 6−O−sulfotransferase 1

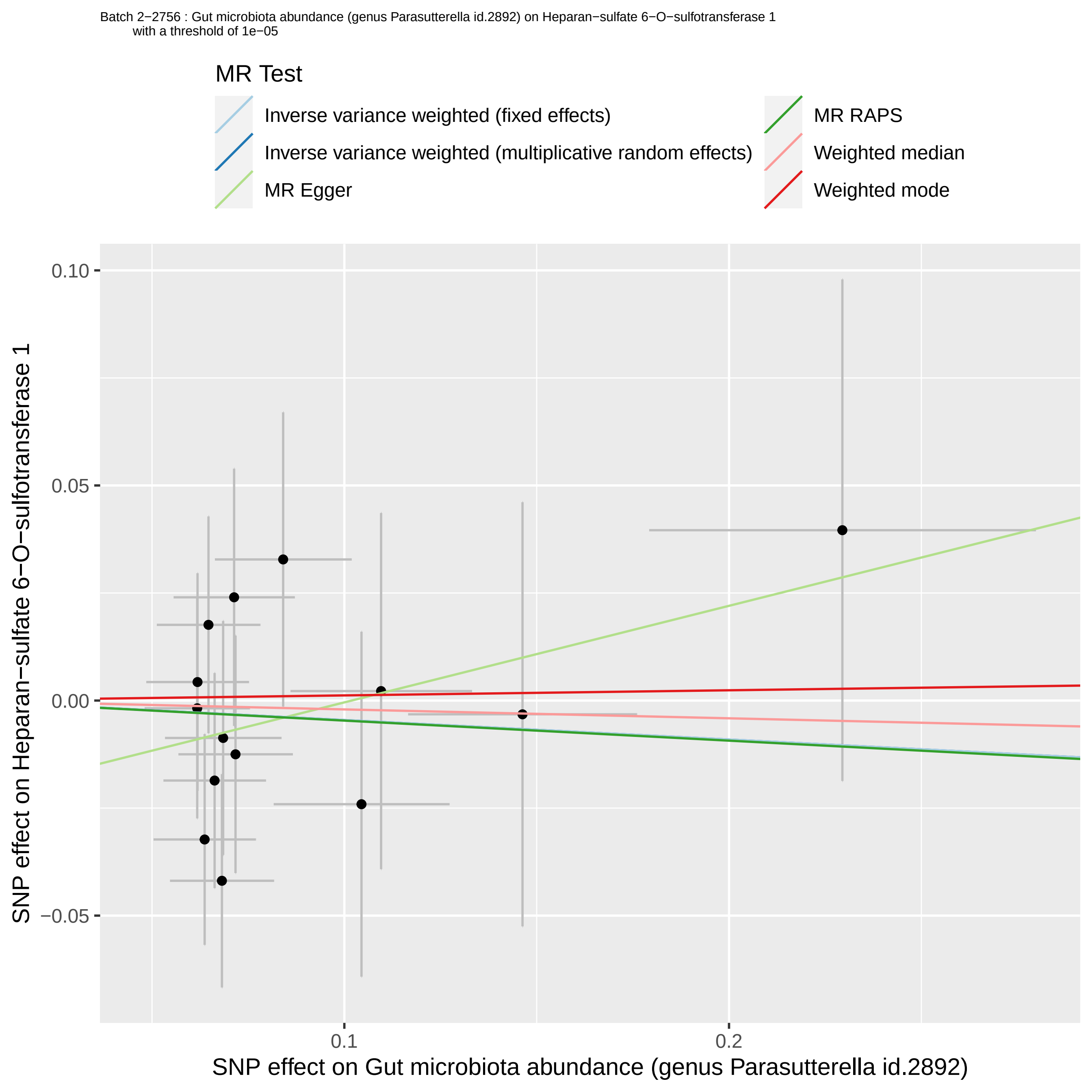

Batch 2−2756 : Gut microbiota abundance (genus Parasutterella id.2892) on Heparan−sulfate 6−O−sulfotransferase 1
with a threshold of 1e−05
MR Test
Inverse variance weighted (fixed effects)
Inverse variance weighted (multiplicative random effects)
MR Egger
MR RAPS
Weighted median
Weighted mode
0.10
0.05
0.00
−0.05
0.1
0.2
SNP effect on Heparan−sulfate 6−O−sulfotransferase 1
SNP effect on Gut microbiota abundance (genus Parasutterella id.2892)

Batch 2−2757 : Gut microbiota abundance (genus Catenibacterium id.2153) on Heparan−sulfate 6−O−sulfotransferase 2 with a threshold of 1e−05

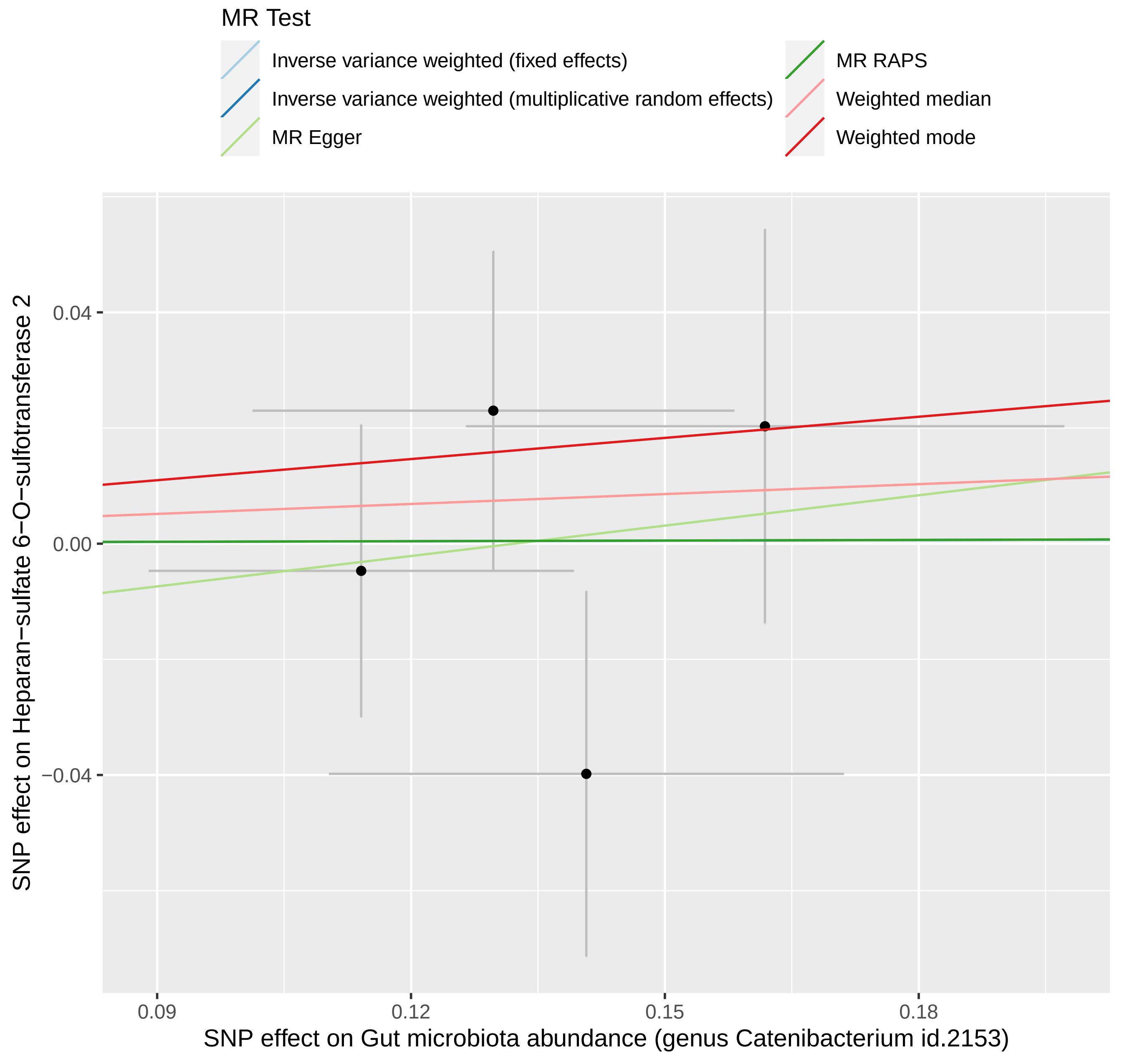

Batch 2−2758 : Gut microbiota abundance (genus Parasutterella id.2892) on Heparan−sulfate 6−O−sulfotransferase 2
with a threshold of 1e−05

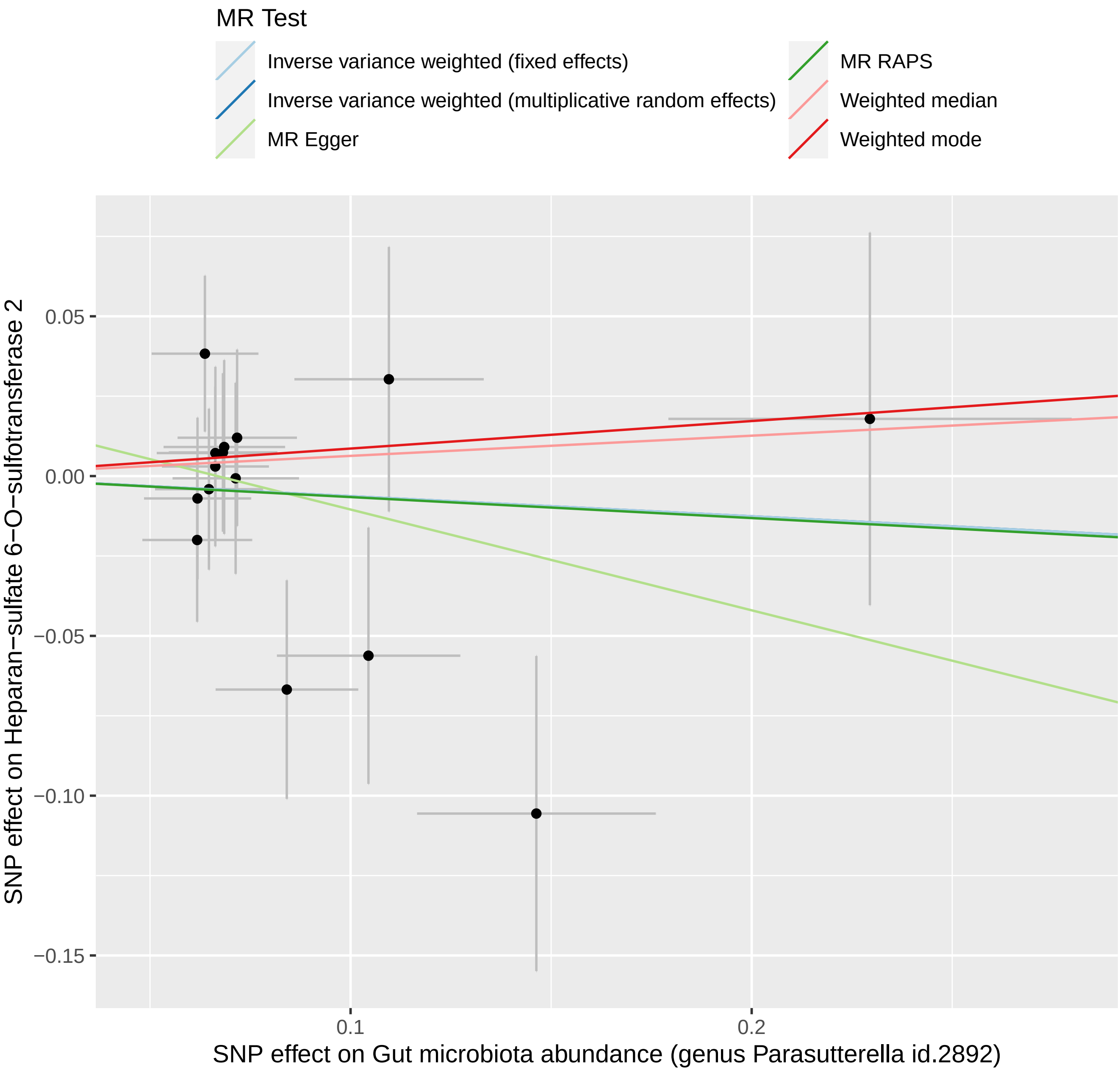

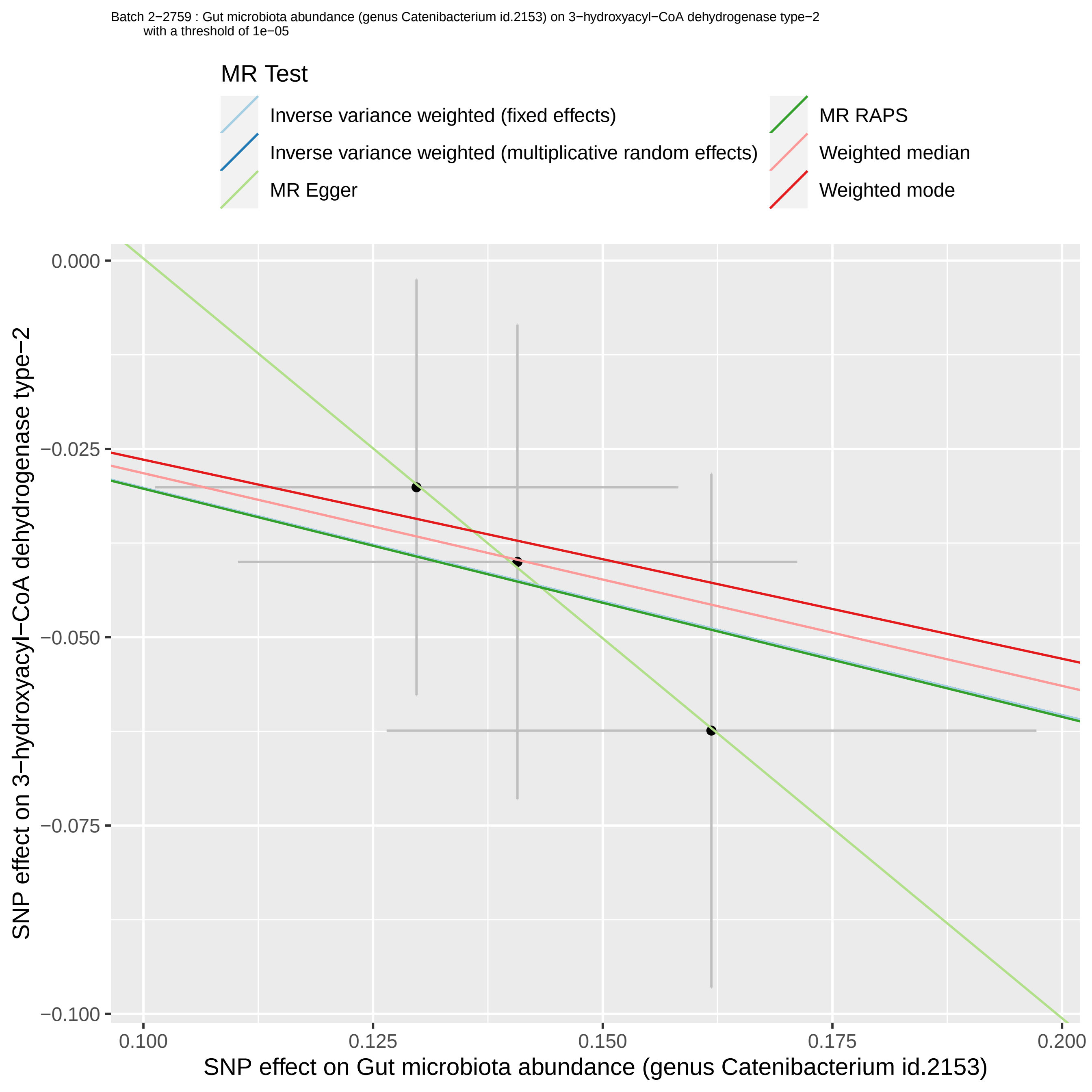

Batch 2−2759 : Gut microbiota abundance (genus Catenibacterium id.2153) on 3−hydroxyacyl−CoA dehydrogenase type−2
with a threshold of 1e−05
MR Test
Inverse variance weighted (fixed effects)
Inverse variance weighted (multiplicative random effects)
MR Egger
MR RAPS
Weighted median
Weighted mode
0.000
−0.025
−0.050
−0.075
−0.100
0.100
0.125
0.150
0.175
0.200
SNP effect on 3−hydroxyacyl−CoA dehydrogenase type−2
SNP effect on Gut microbiota abundance (genus Catenibacterium id.2153)

Batch 2−2760 : Gut microbiota abundance (genus Parasutterella id.2892) on 3−hydroxyacyl−CoA dehydrogenase type−2
with a threshold of 1e−05

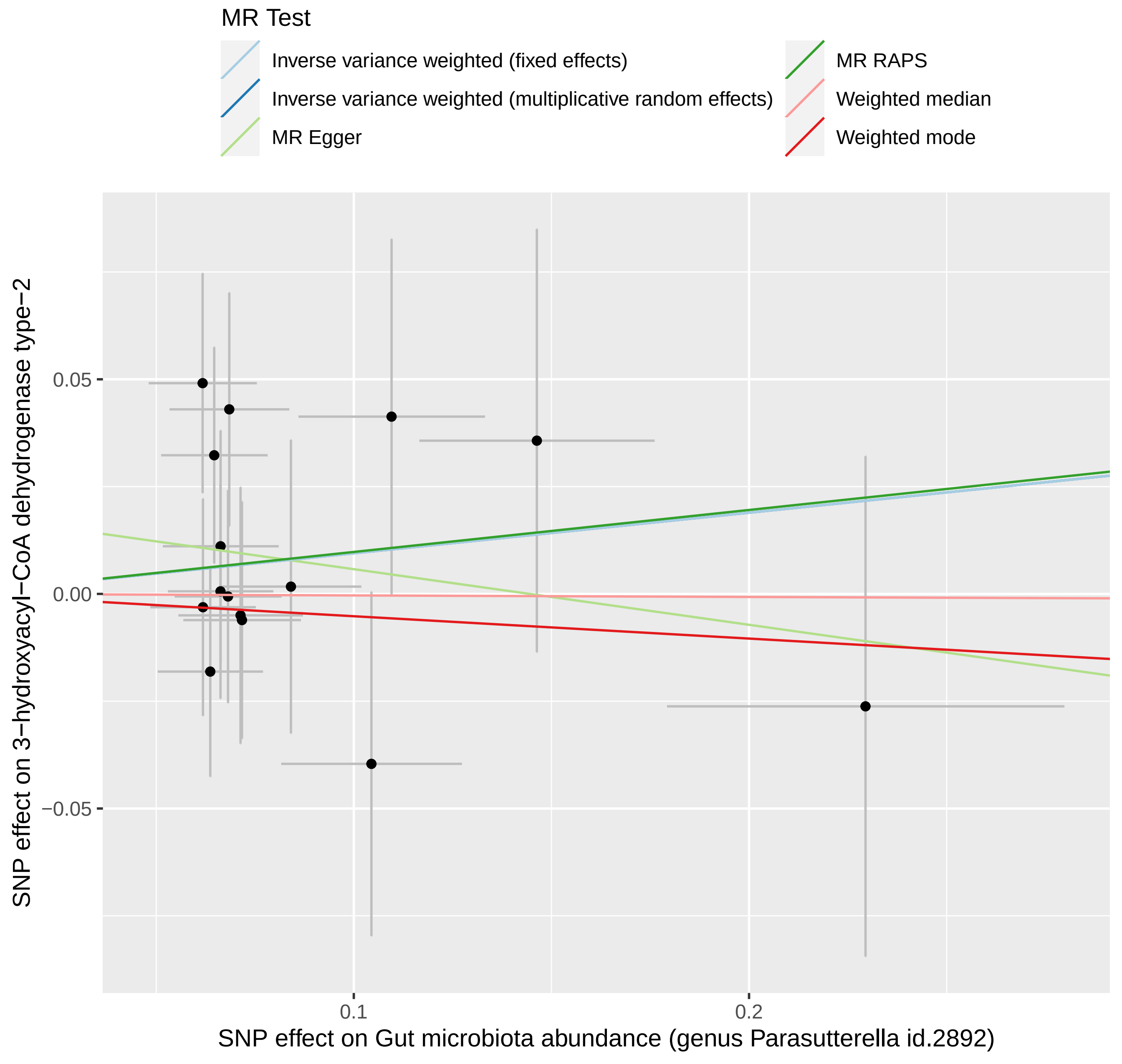

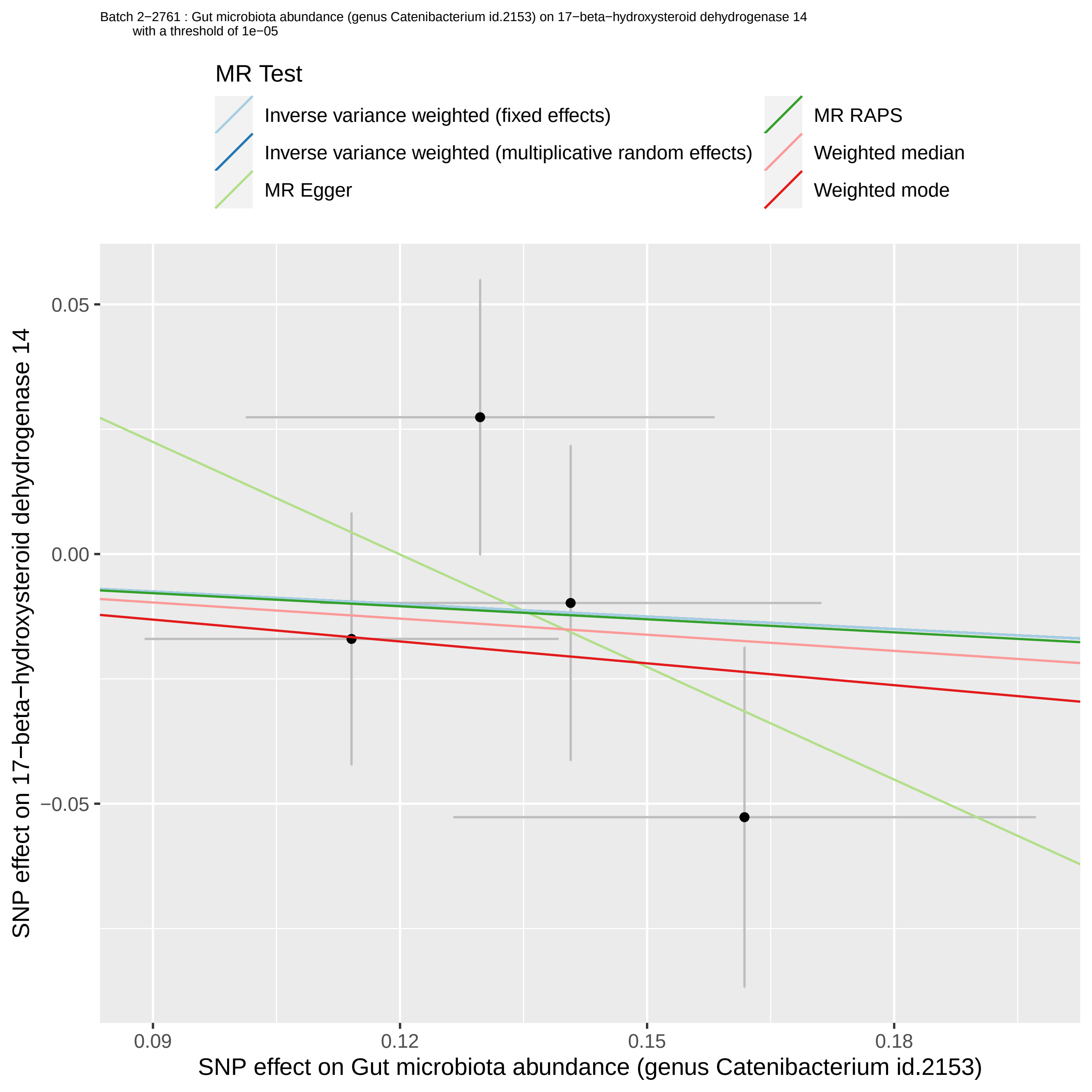

Batch 2−2761 : Gut microbiota abundance (genus Catenibacterium id.2153) on 17−beta−hydroxysteroid dehydrogenase 14
with a threshold of 1e−05
MR Test
Inverse variance weighted (fixed effects)
Inverse variance weighted (multiplicative random effects)
MR Egger
MR RAPS
Weighted median
Weighted mode
0.05
0.00
−0.05
0.09
0.12
0.15
0.18
SNP effect on 17−beta−hydroxysteroid dehydrogenase 14
SNP effect on Gut microbiota abundance (genus Catenibacterium id.2153)

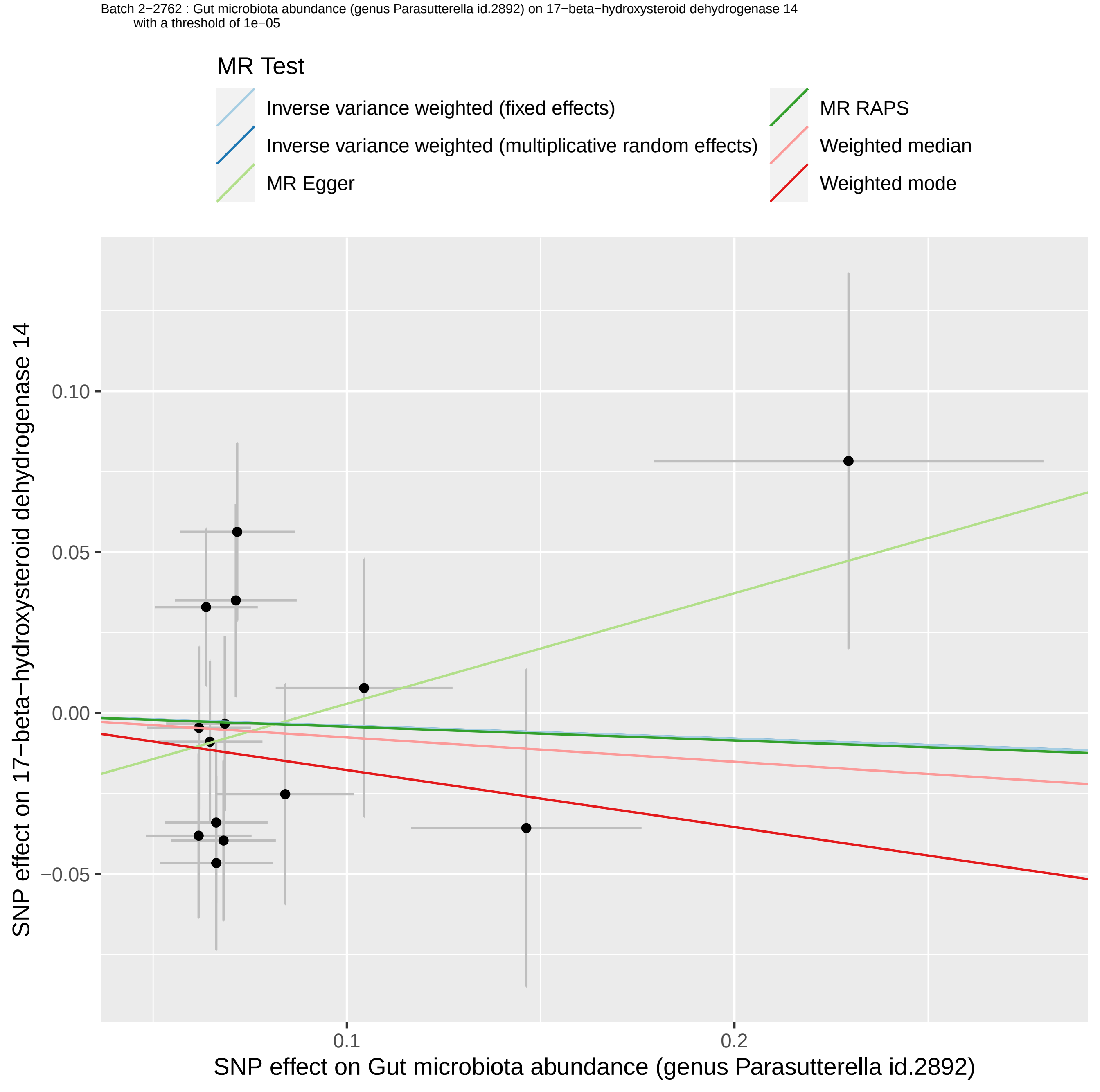
Batch 2−2762 : Gut microbiota abundance (genus Parasutterella id.2892) on 17−beta−hydroxysteroid dehydrogenase 14
with a threshold of 1e−05
MR Test
Inverse variance weighted (fixed effects)
Inverse variance weighted (multiplicative random effects)
MR Egger
MR RAPS
Weighted median
Weighted mode
0.10
0.05
0.00
−0.05
0.1
0.2
SNP effect on 17−beta−hydroxysteroid dehydrogenase 14
SNP effect on Gut microbiota abundance (genus Parasutterella id.2892)

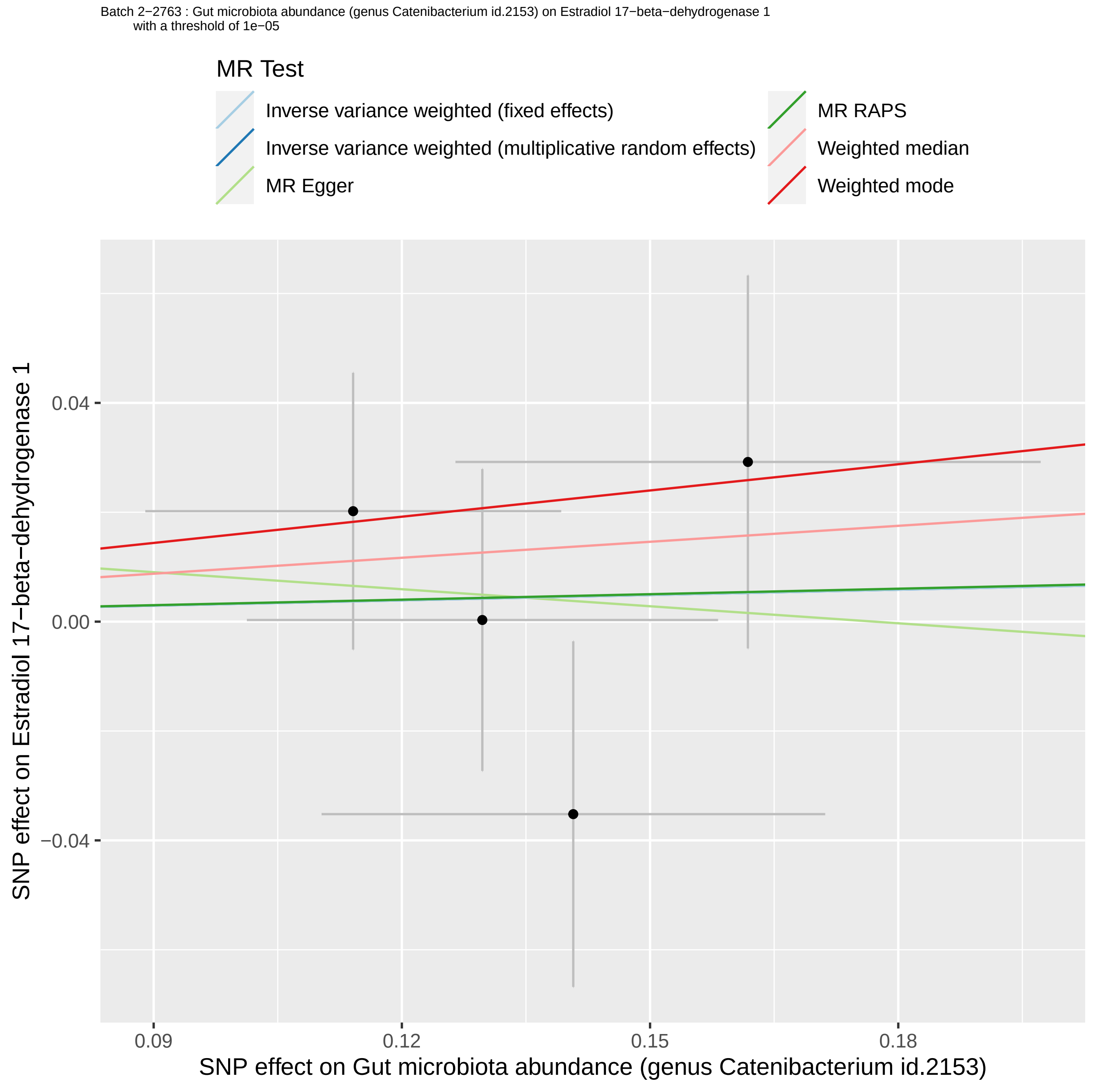

Batch 2−2763 : Gut microbiota abundance (genus Catenibacterium id.2153) on Estradiol 17−beta−dehydrogenase 1
with a threshold of 1e−05
MR Test
Inverse variance weighted (fixed effects)
Inverse variance weighted (multiplicative random effects)
MR Egger
MR RAPS
Weighted median
Weighted mode
0.04
0.00
−0.04
0.09
0.12
0.15
0.18
SNP effect on Estradiol 17−beta−dehydrogenase 1
SNP effect on Gut microbiota abundance (genus Catenibacterium id.2153)

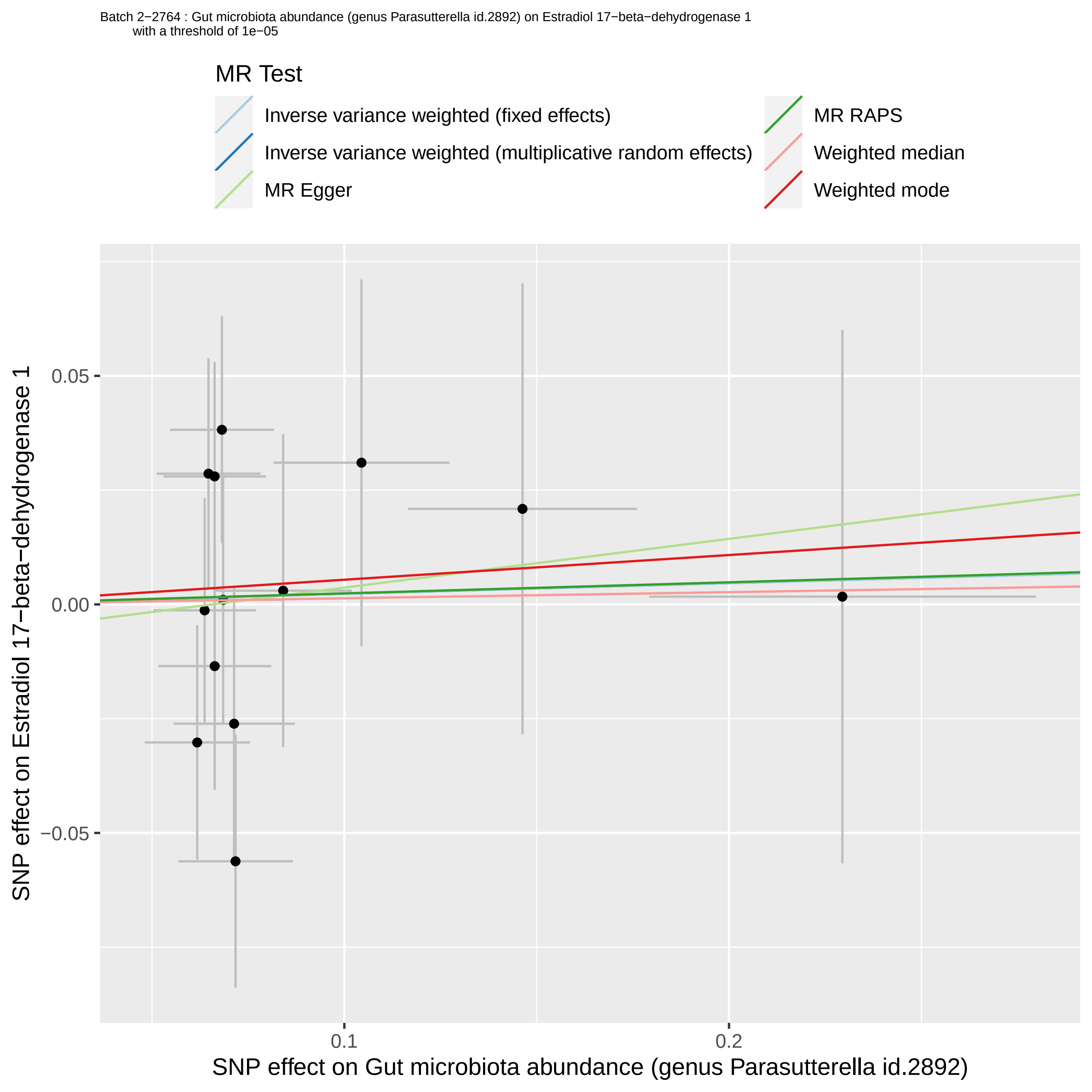
Batch 2−2764 : Gut microbiota abundance (genus Parasutterella id.2892) on Estradiol 17−beta−dehydrogenase 1
with a threshold of 1e−05
MR Test
Inverse variance weighted (fixed effects)
Inverse variance weighted (multiplicative random effects)
MR Egger
MR RAPS
Weighted median
Weighted mode
0.05
0.00
−0.05
0.1
0.2
SNP effect on Estradiol 17−beta−dehydrogenase 1
SNP effect on Gut microbiota abundance (genus Parasutterella id.2892)

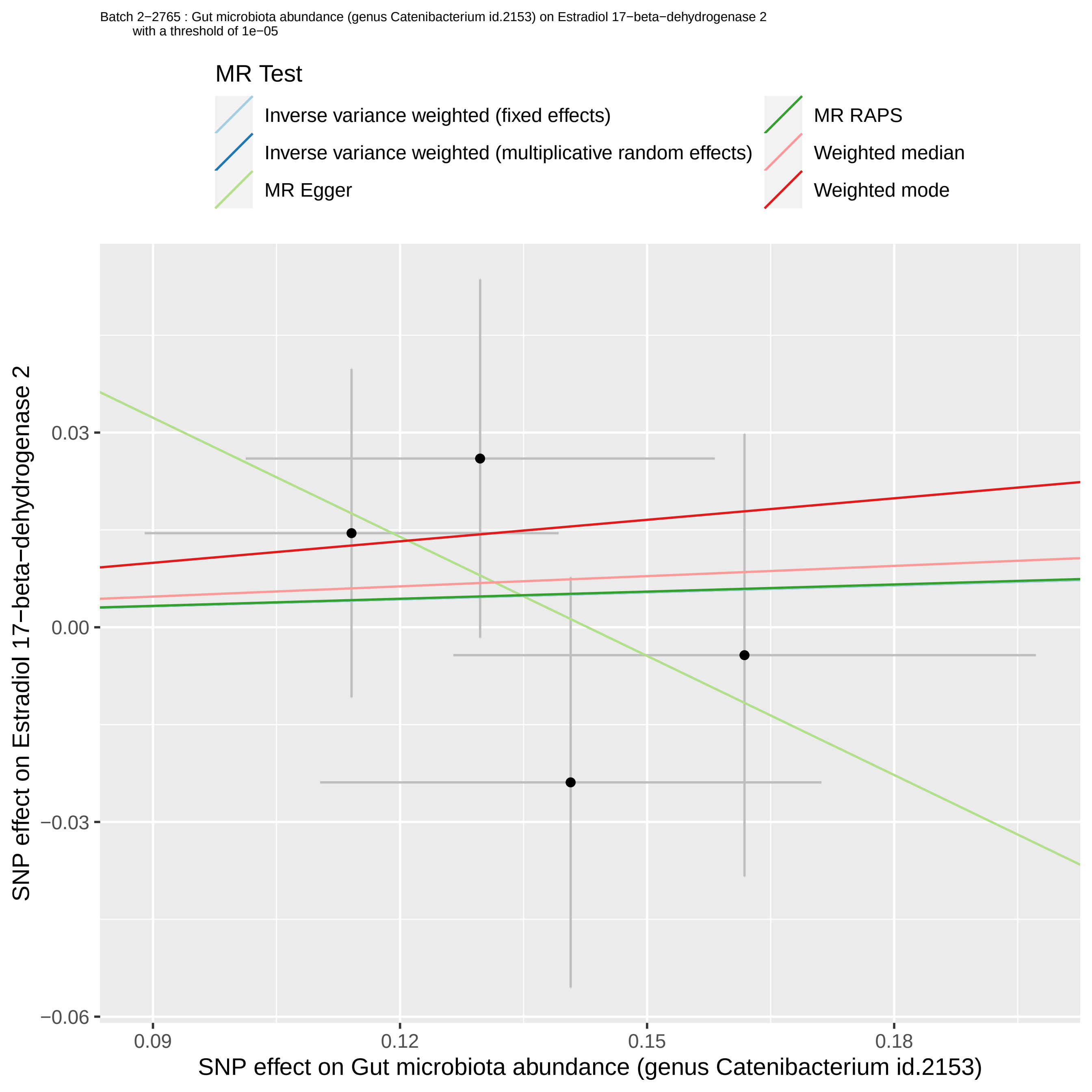

Batch 2−2765 : Gut microbiota abundance (genus Catenibacterium id.2153) on Estradiol 17−beta−dehydrogenase 2
with a threshold of 1e−05
MR Test
Inverse variance weighted (fixed effects)
Inverse variance weighted (multiplicative random effects)
MR Egger
MR RAPS
Weighted median
Weighted mode
SNP effect on Estradiol 17−beta−dehydrogenase 2
0.03
0.00
−0.03
−0.06
0.09
0.12
0.15
0.18
SNP effect on Gut microbiota abundance (genus Catenibacterium id.2153)

Batch 2−2766 : Gut microbiota abundance (genus Parasutterella id.2892) on Estradiol 17−beta−dehydrogenase 2
with a threshold of 1e−05

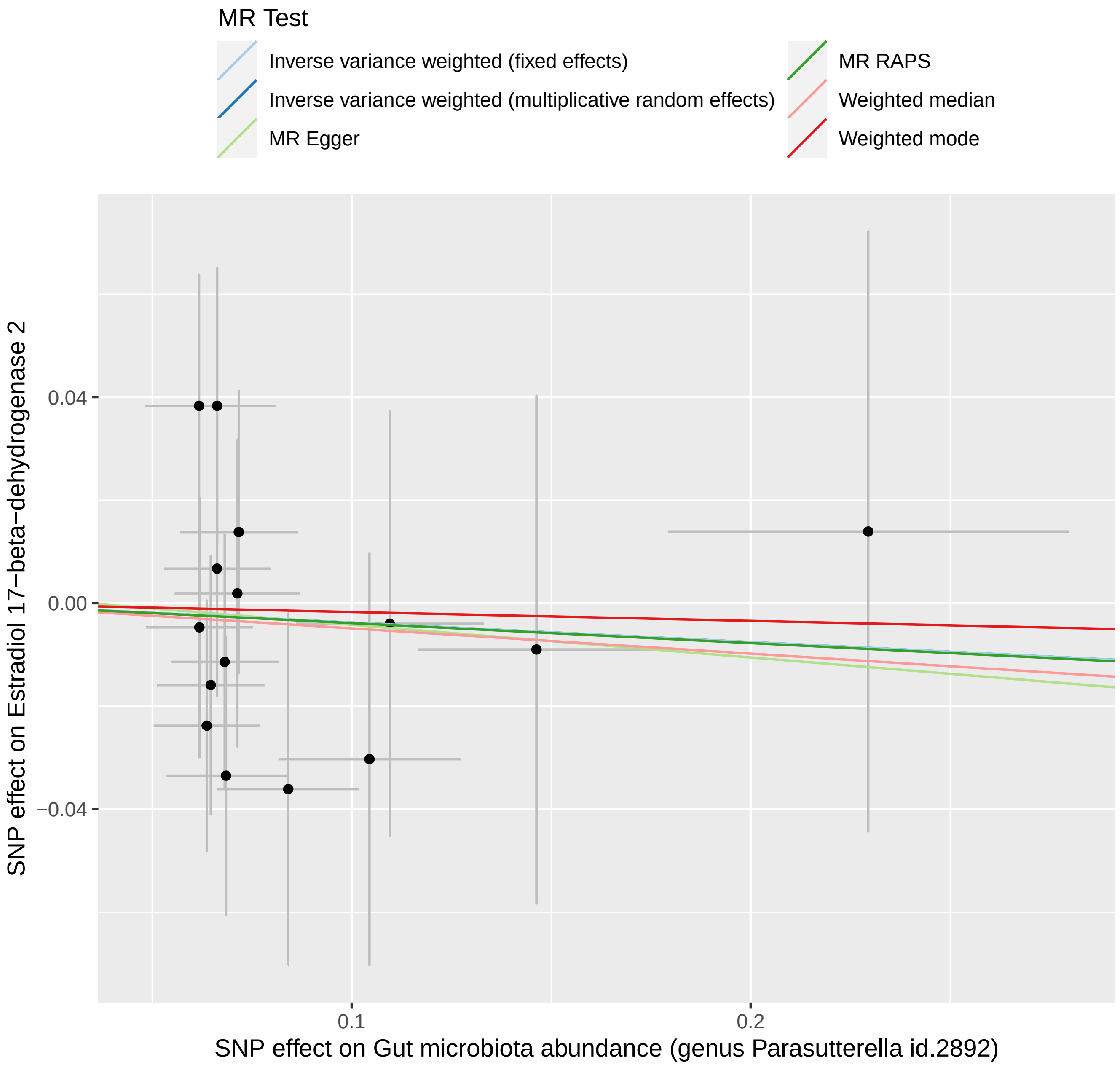

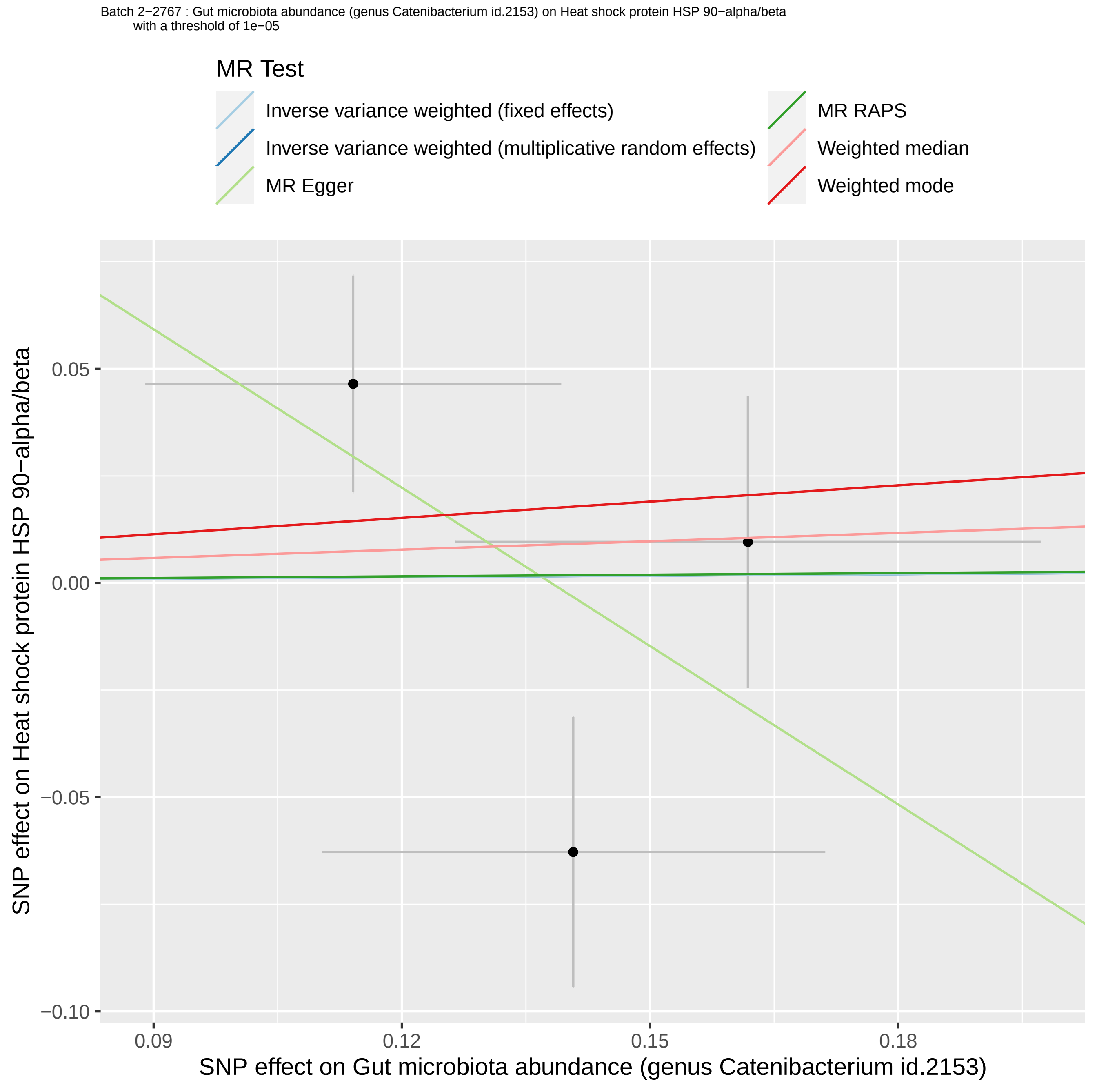
Batch 2−2767 : Gut microbiota abundance (genus Catenibacterium id.2153) on Heat shock protein HSP 90−alpha/beta
with a threshold of 1e−05
MR Test
Inverse variance weighted (fixed effects)
Inverse variance weighted (multiplicative random effects)
MR Egger
MR RAPS
Weighted median
Weighted mode
SNP effect on Heat shock protein HSP 90−alpha/beta
0.05
0.00
−0.05
−0.10
0.09
0.12
0.15
0.18
SNP effect on Gut microbiota abundance (genus Catenibacterium id.2153)

Batch 2−2768 : Gut microbiota abundance (genus Parasutterella id.2892) on Heat shock protein HSP 90−alpha/beta
with a threshold of 1e−05

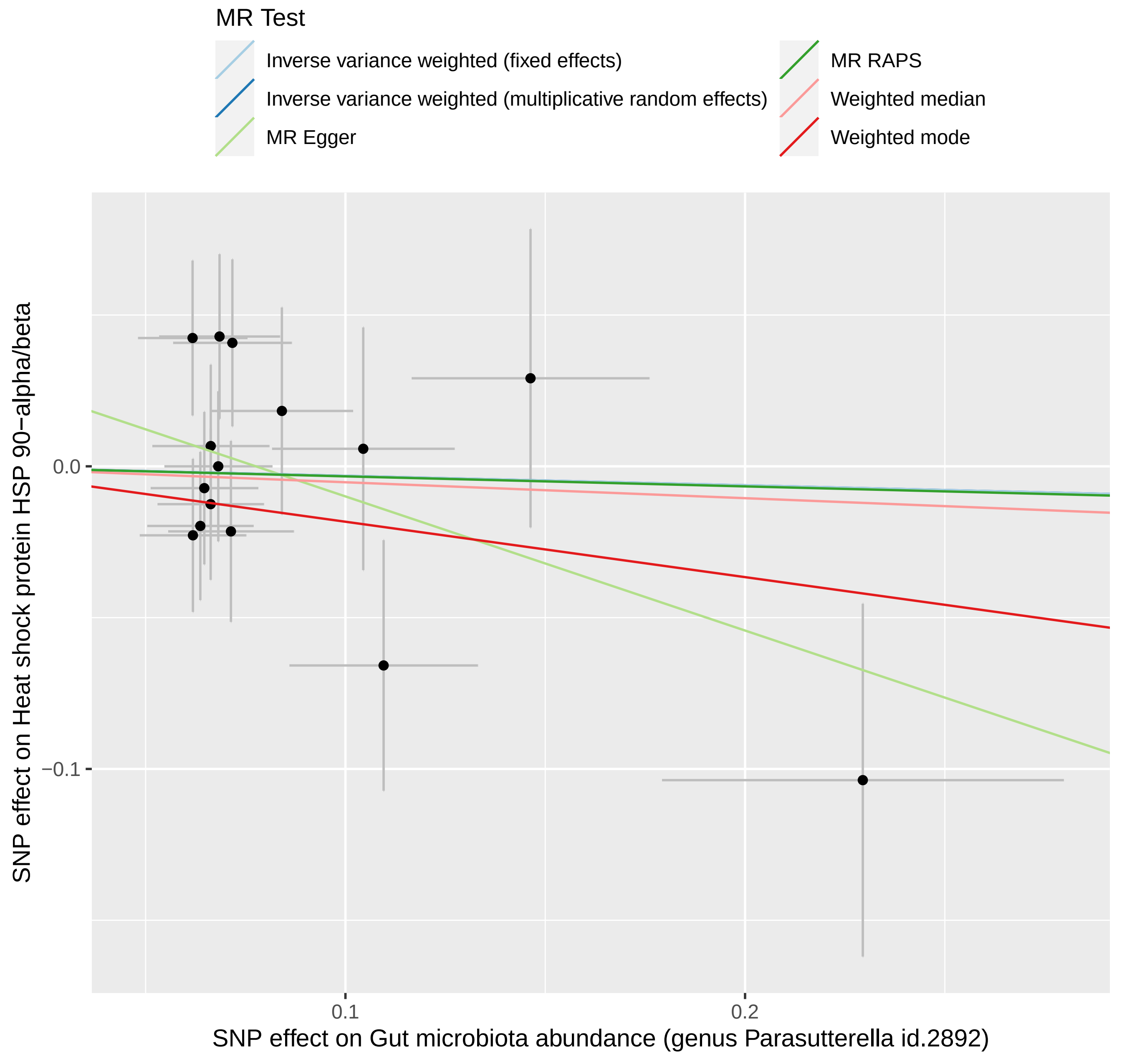

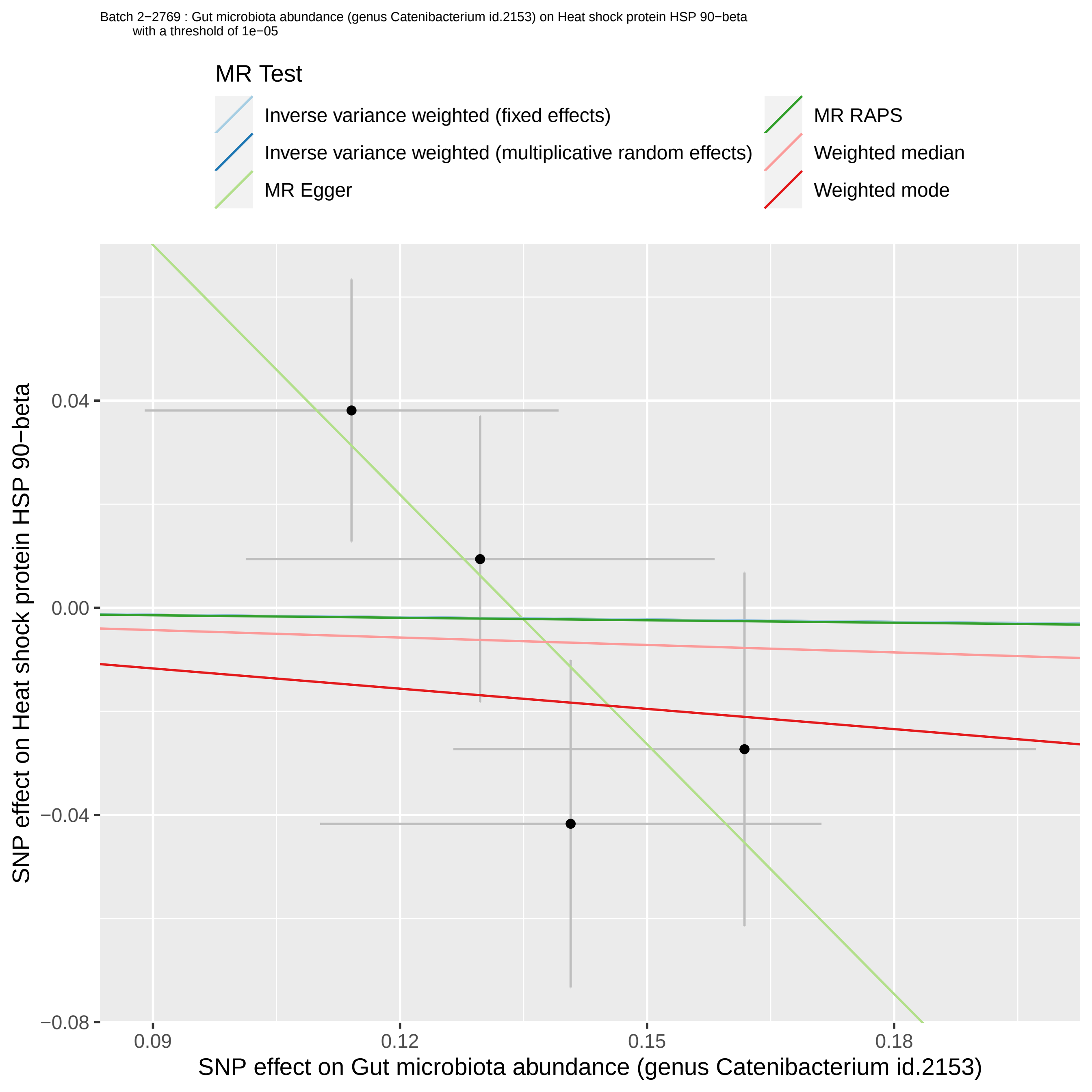
Batch 2−2769 : Gut microbiota abundance (genus Catenibacterium id.2153) on Heat shock protein HSP 90−beta
with a threshold of 1e−05
MR Test
Inverse variance weighted (fixed effects)
Inverse variance weighted (multiplicative random effects)
MR Egger
MR RAPS
Weighted median
Weighted mode
0.04
0.00
−0.04
−0.08
0.09
0.12
0.15
0.18
SNP effect on Heat shock protein HSP 90−beta
SNP effect on Gut microbiota abundance (genus Catenibacterium id.2153)

Batch 2−2770 : Gut microbiota abundance (genus Parasutterella id.2892) on Heat shock protein HSP 90−beta
with a threshold of 1e−05

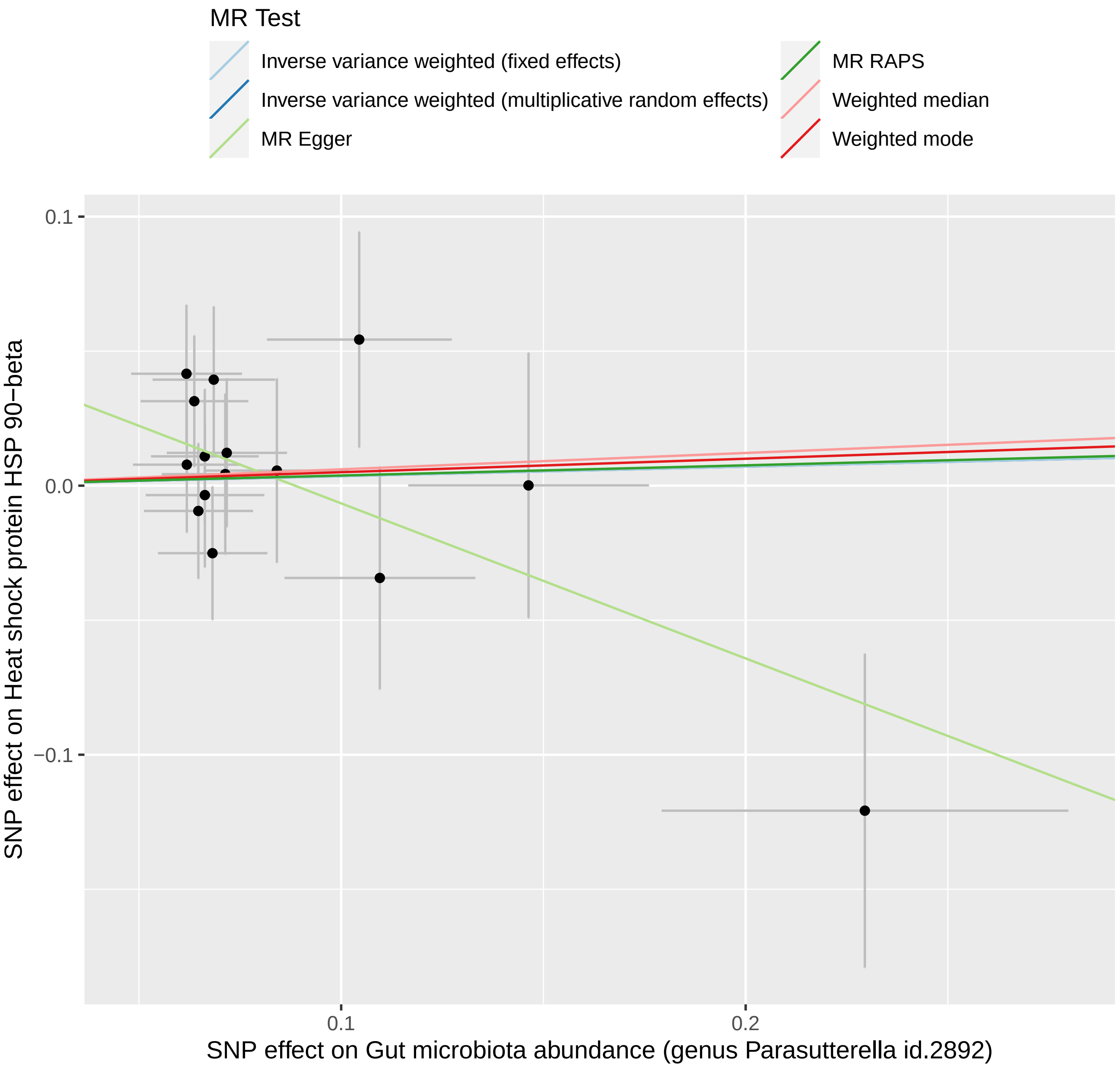

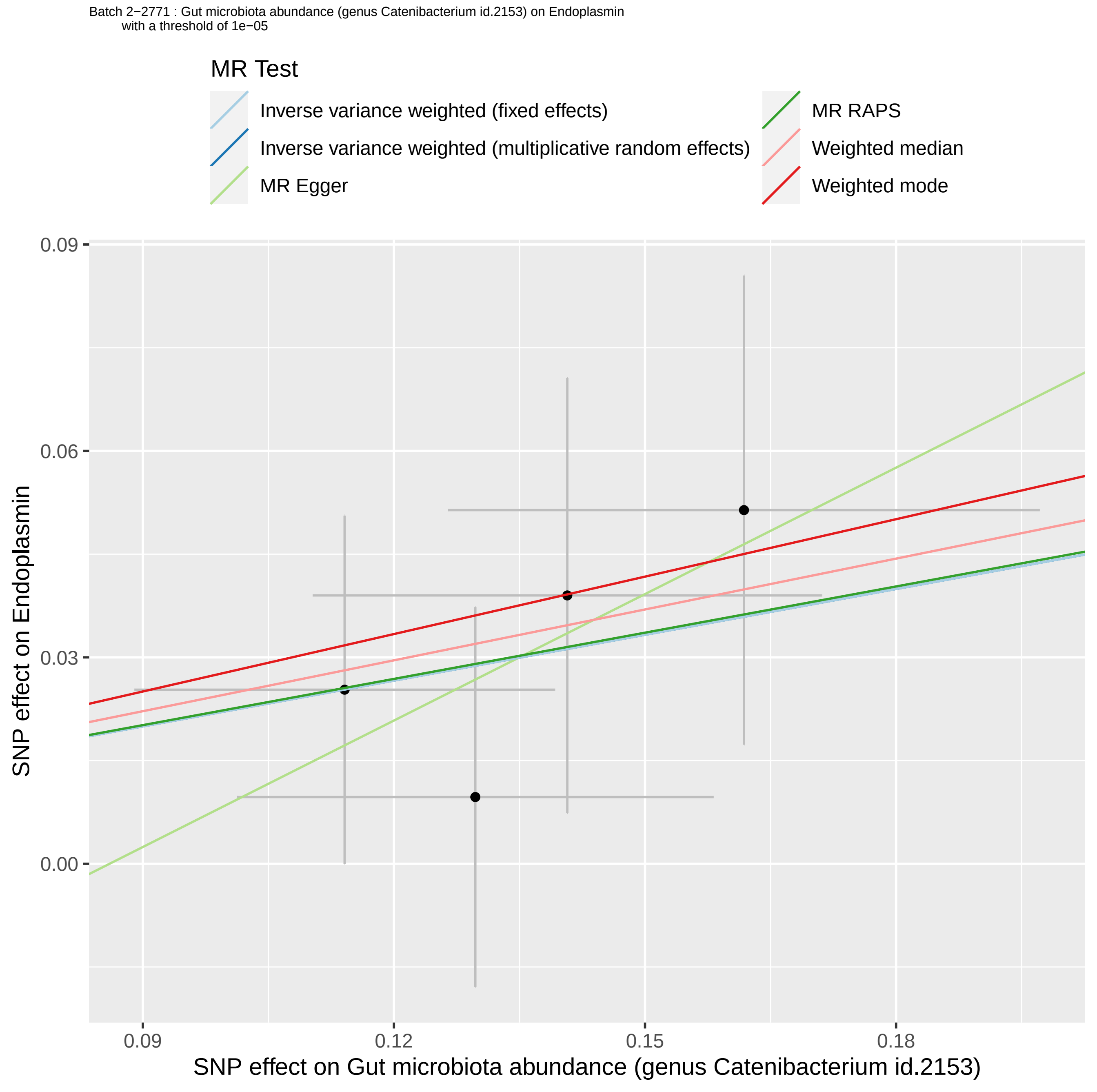

Batch 2−2771 : Gut microbiota abundance (genus Catenibacterium id.2153) on Endoplasmin
with a threshold of 1e−05
MR Test
Inverse variance weighted (fixed effects)
Inverse variance weighted (multiplicative random effects)
MR Egger
MR RAPS
Weighted median
Weighted mode
0.09
0.06
0.03
0.00
SNP effect on Endoplasmin
0.09
0.12
0.15
0.18
SNP effect on Gut microbiota abundance (genus Catenibacterium id.2153)

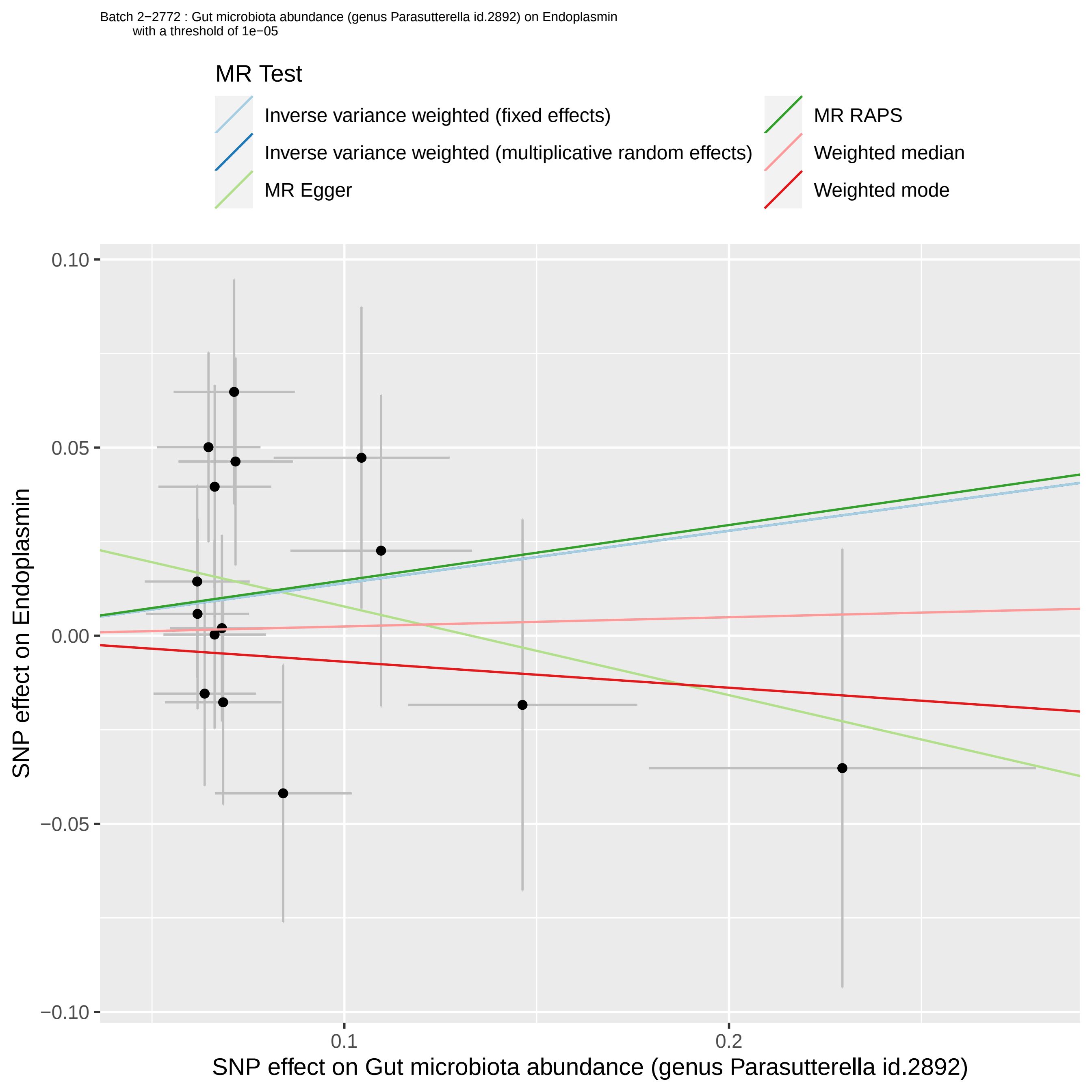
Batch 2−2772 : Gut microbiota abundance (genus Parasutterella id.2892) on Endoplasmin
with a threshold of 1e−05
MR Test
Inverse variance weighted (fixed effects)
Inverse variance weighted (multiplicative random effects)
MR Egger
MR RAPS
Weighted median
Weighted mode
0.10
0.05
0.00
−0.05
−0.10
0.1
0.2
SNP effect on Endoplasmin
SNP effect on Gut microbiota abundance (genus Parasutterella id.2892)

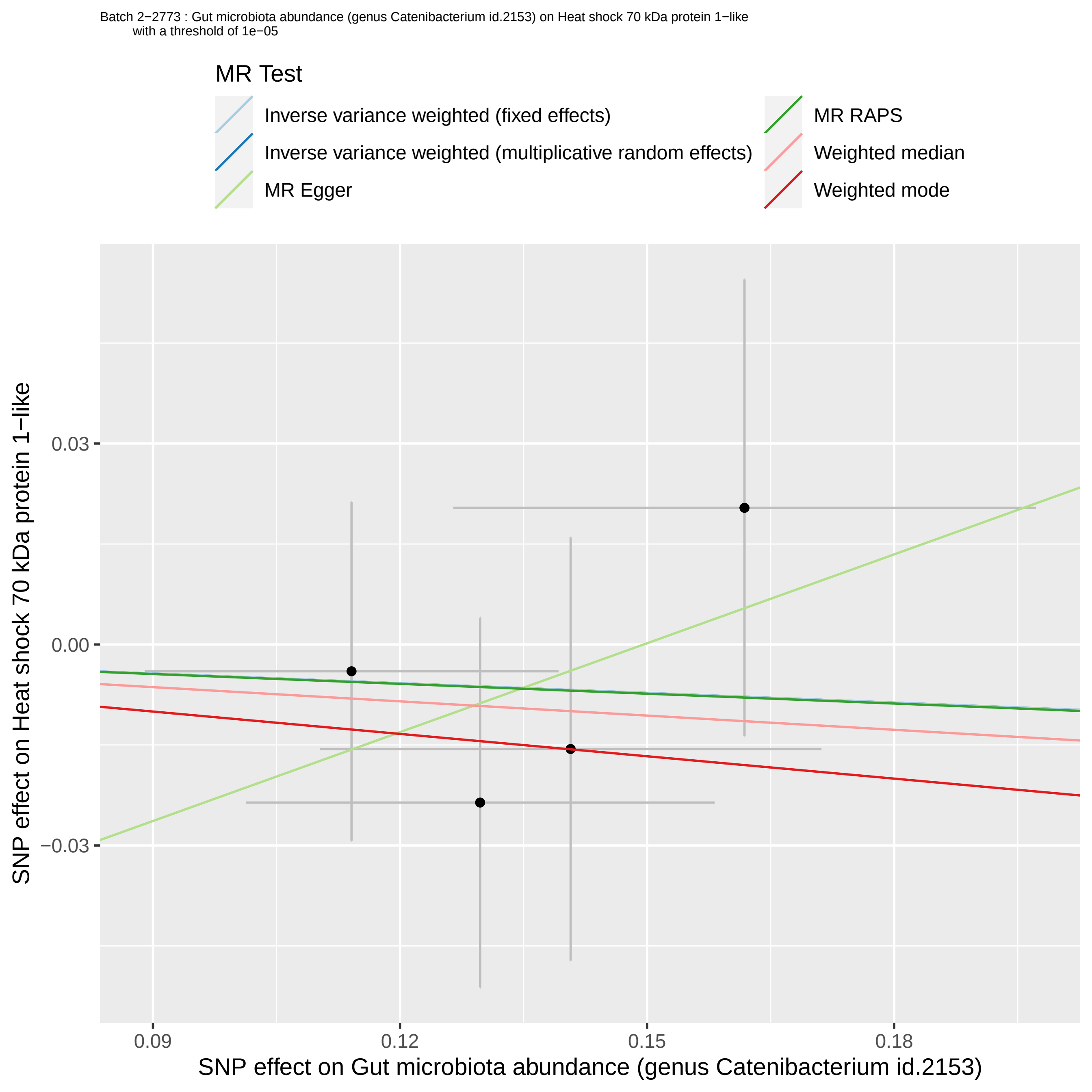

Batch 2−2773 : Gut microbiota abundance (genus Catenibacterium id.2153) on Heat shock 70 kDa protein 1−like
with a threshold of 1e−05
MR Test
Inverse variance weighted (fixed effects)
Inverse variance weighted (multiplicative random effects)
MR Egger
MR RAPS
Weighted median
Weighted mode
0.03
0.00
−0.03
0.09
0.12
0.15
0.18
SNP effect on Heat shock 70 kDa protein 1−like
SNP effect on Gut microbiota abundance (genus Catenibacterium id.2153)

Batch 2−2774 : Gut microbiota abundance (genus Parasutterella id.2892) on Heat shock 70 kDa protein 1−like
with a threshold of 1e−05

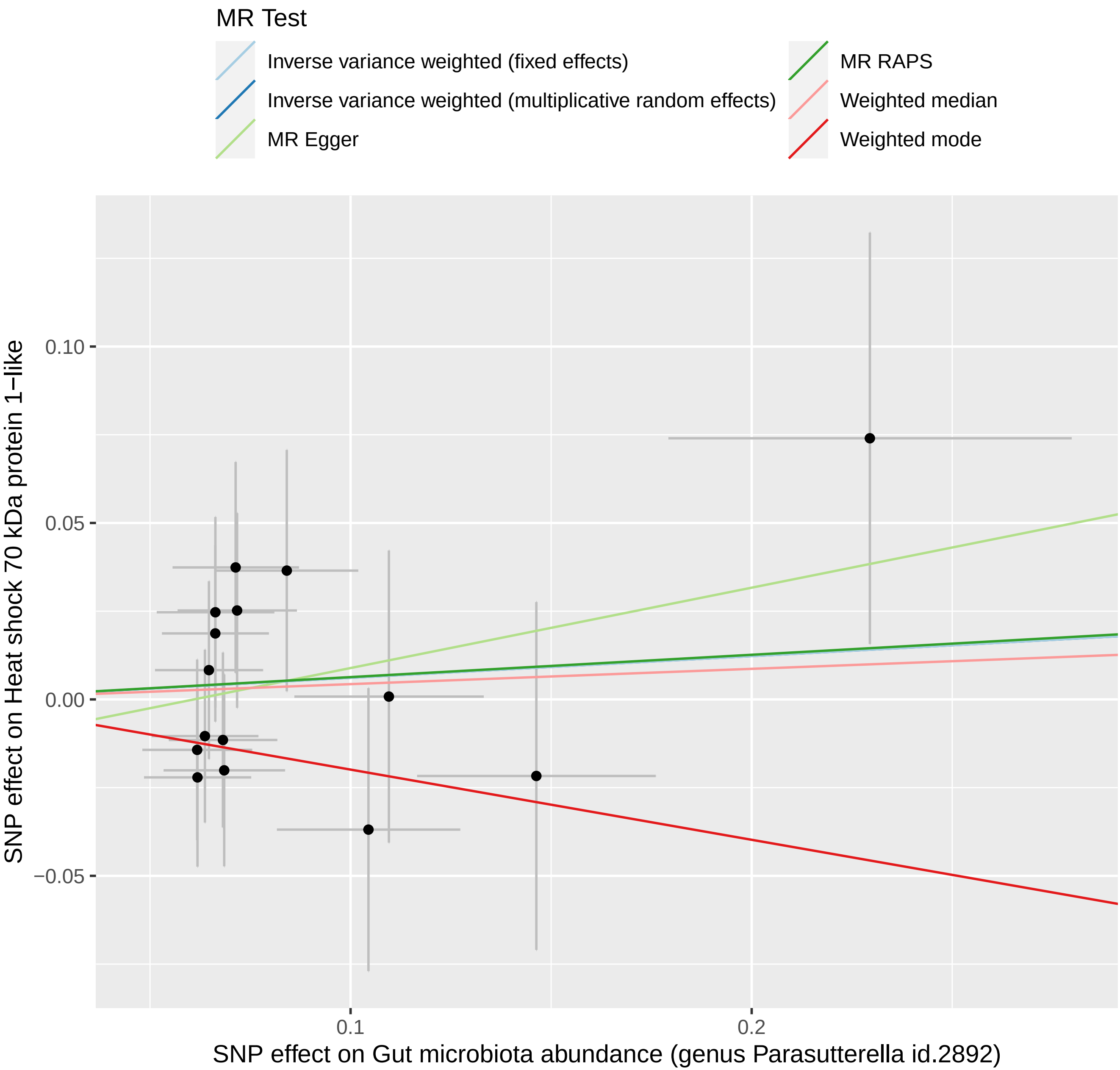

Batch 2−2775 : Gut microbiota abundance (genus Catenibacterium id.2153) on Heat shock 70 kDa protein 6
with a threshold of 1e−05

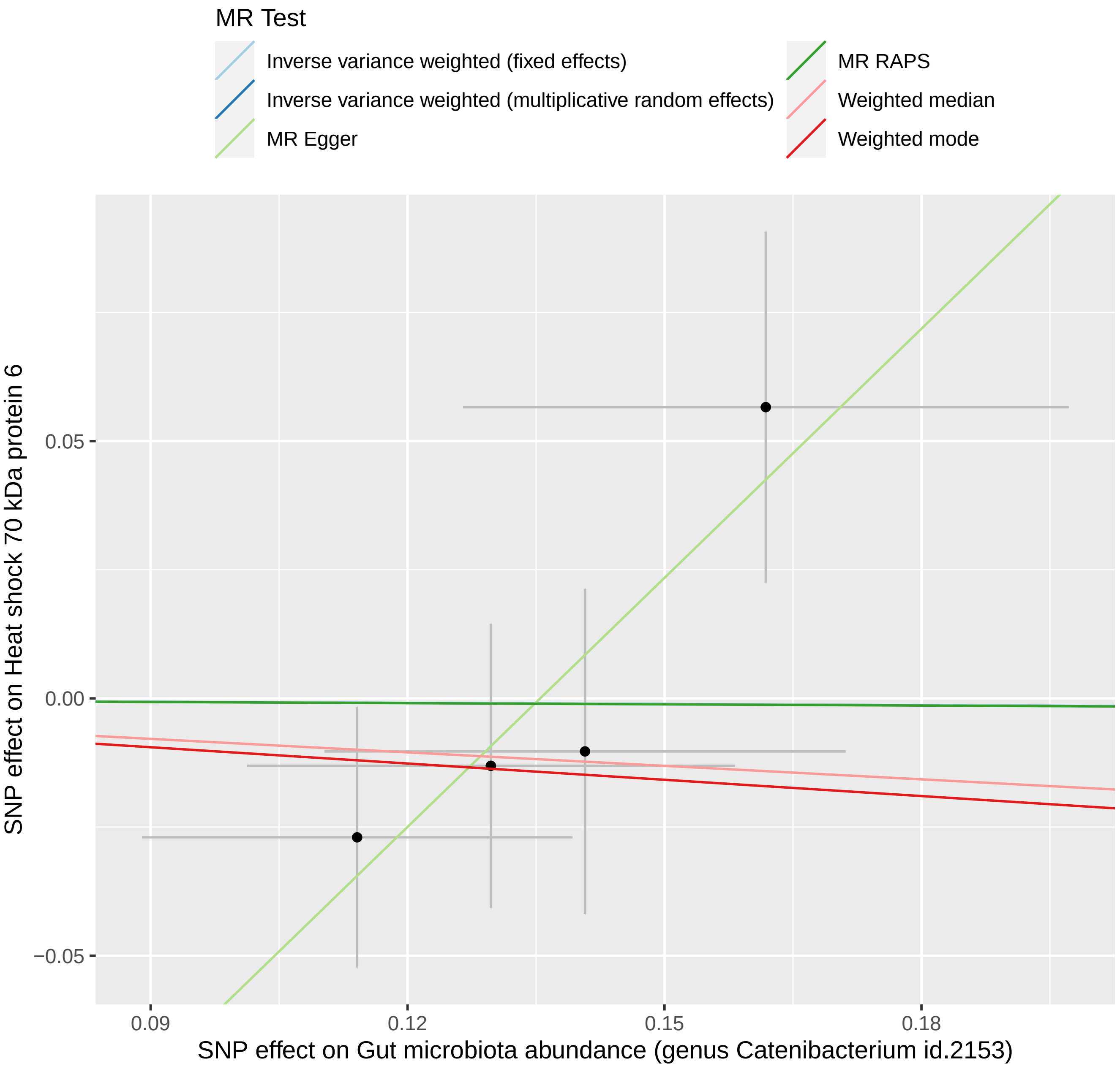

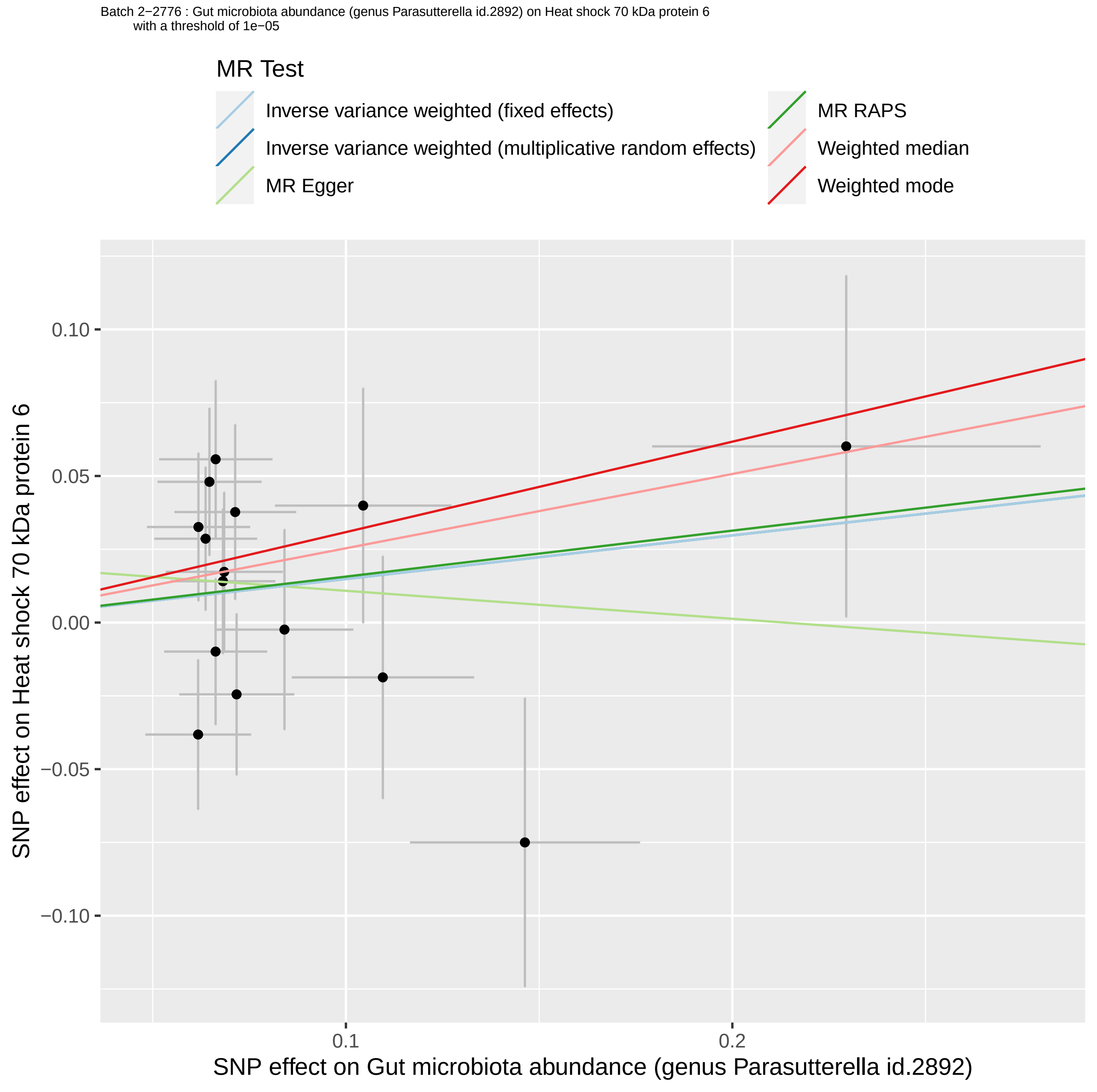

Batch 2−2776 : Gut microbiota abundance (genus Parasutterella id.2892) on Heat shock 70 kDa protein 6
with a threshold of 1e−05
MR Test
Inverse variance weighted (fixed effects)
Inverse variance weighted (multiplicative random effects)
MR Egger
MR RAPS
Weighted median
Weighted mode
0.10
0.05
0.00
−0.05
−0.10
0.1
0.2
SNP effect on Heat shock 70 kDa protein 6
SNP effect on Gut microbiota abundance (genus Parasutterella id.2892)

Batch 2−2777 : Gut microbiota abundance (genus Catenibacterium id.2153) on Oxidoreductase HTATIP2
with a threshold of 1e−05

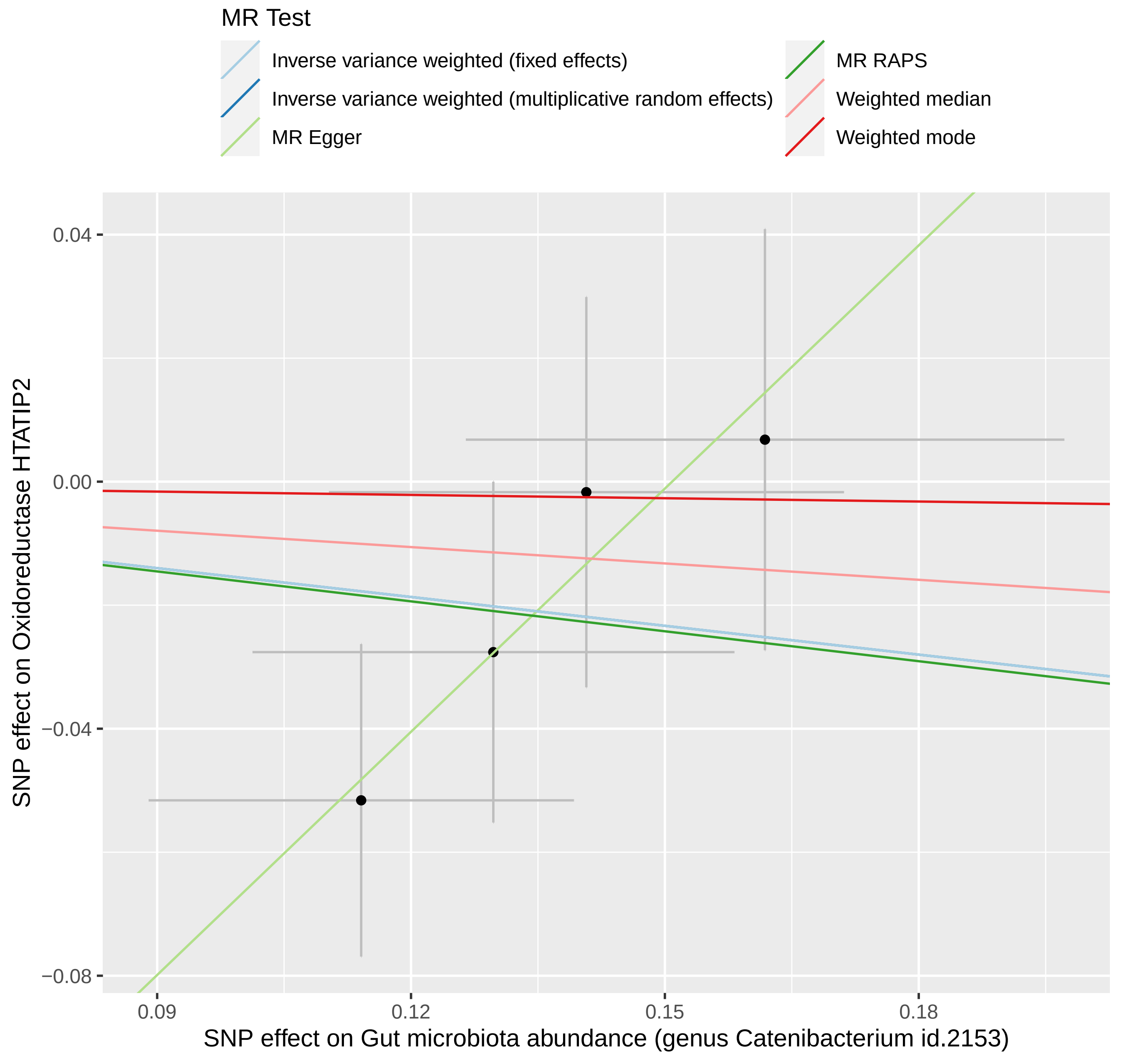

Batch 2−2778 : Gut microbiota abundance (genus Parasutterella id.2892) on Oxidoreductase HTATIP2
with a threshold of 1e−05

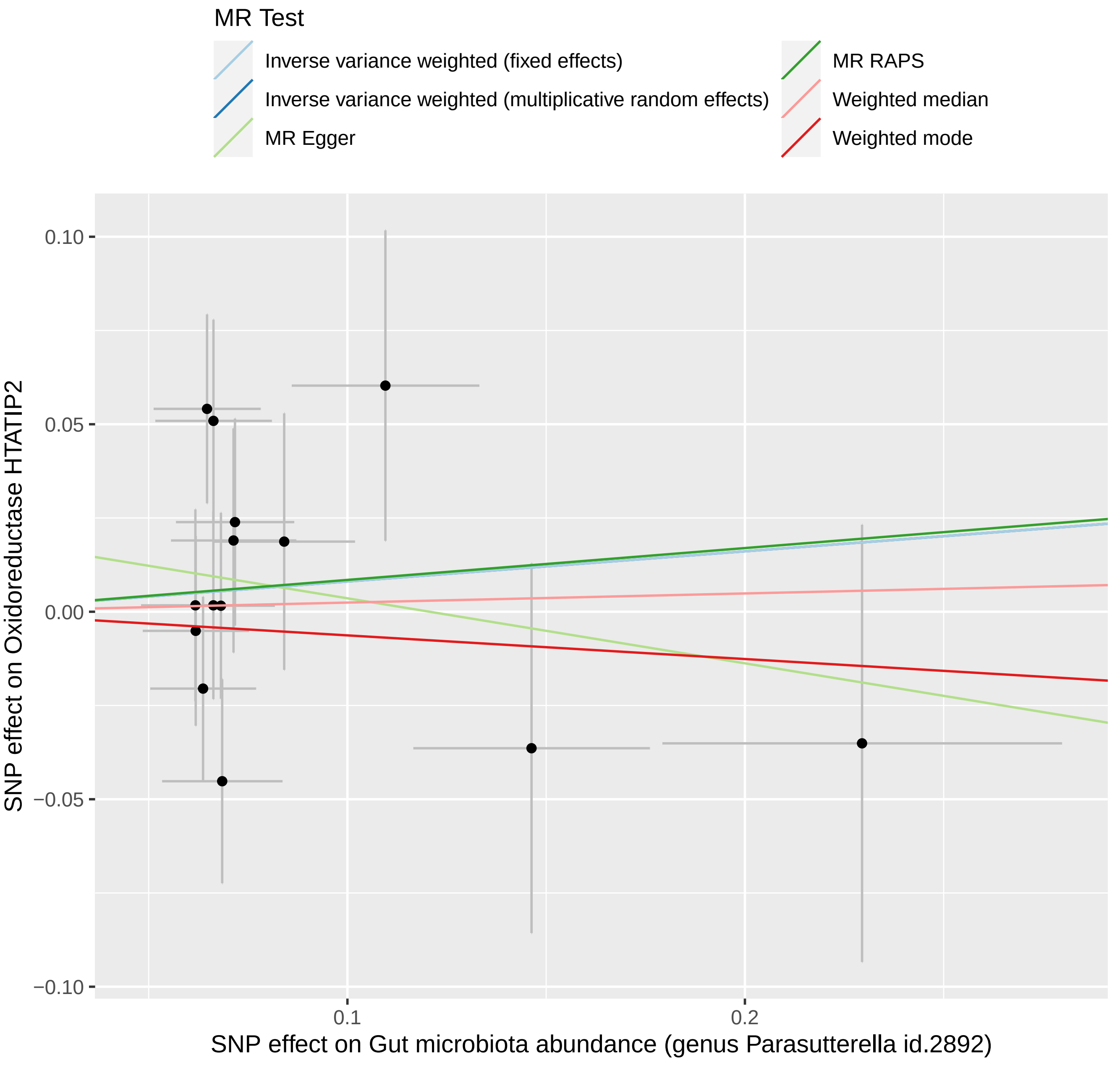

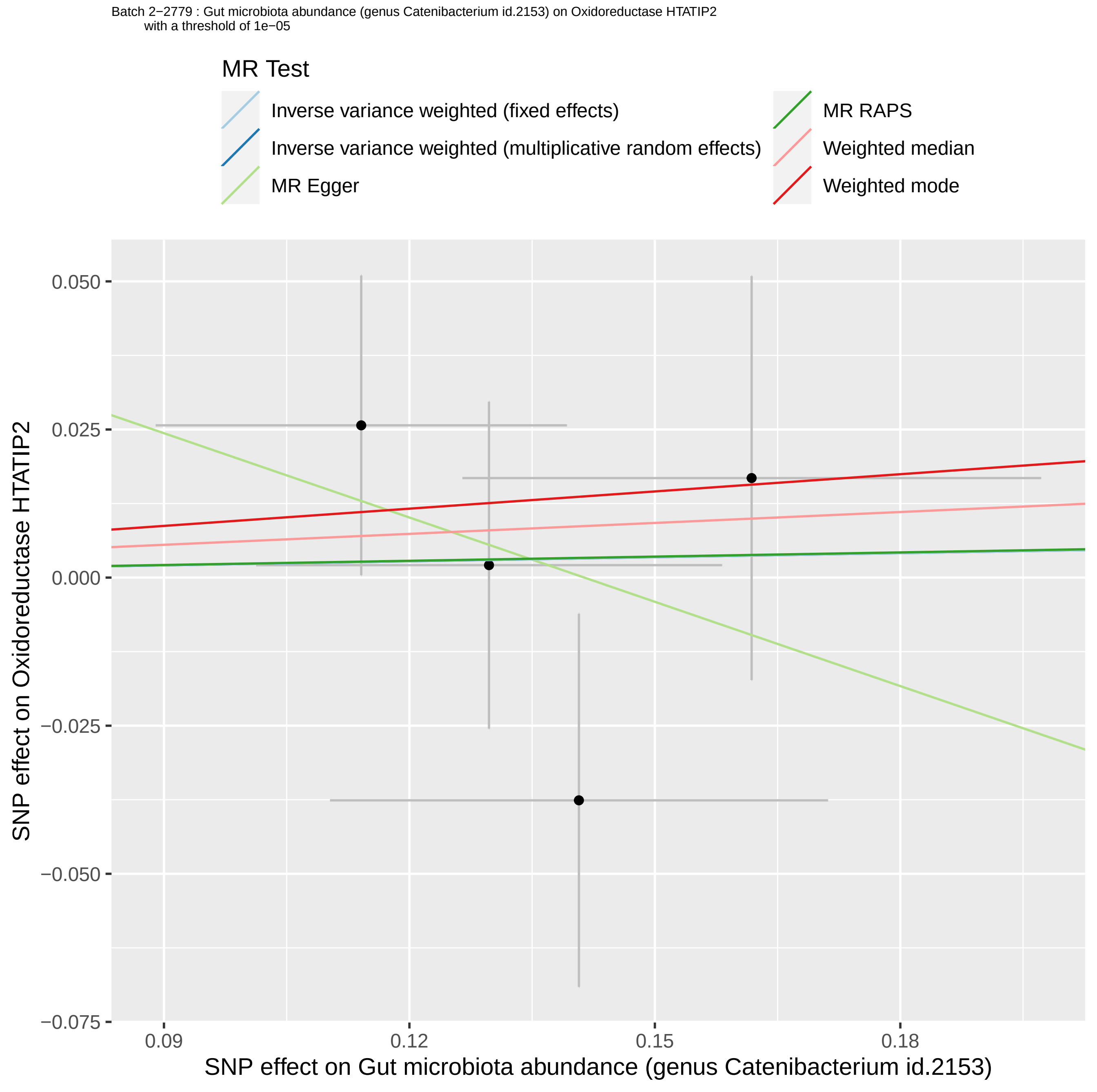

Batch 2−2779 : Gut microbiota abundance (genus Catenibacterium id.2153) on Oxidoreductase HTATIP2
with a threshold of 1e−05
MR Test
Inverse variance weighted (fixed effects)
Inverse variance weighted (multiplicative random effects)
MR Egger
MR RAPS
Weighted median
Weighted mode
0.050
0.025
0.000
−0.025
−0.050
−0.075
0.09
0.12
0.15
0.18
SNP effect on Oxidoreductase HTATIP2
SNP effect on Gut microbiota abundance (genus Catenibacterium id.2153)

Batch 2−2780 : Gut microbiota abundance (genus Parasutterella id.2892) on Oxidoreductase HTATIP2
with a threshold of 1e−05

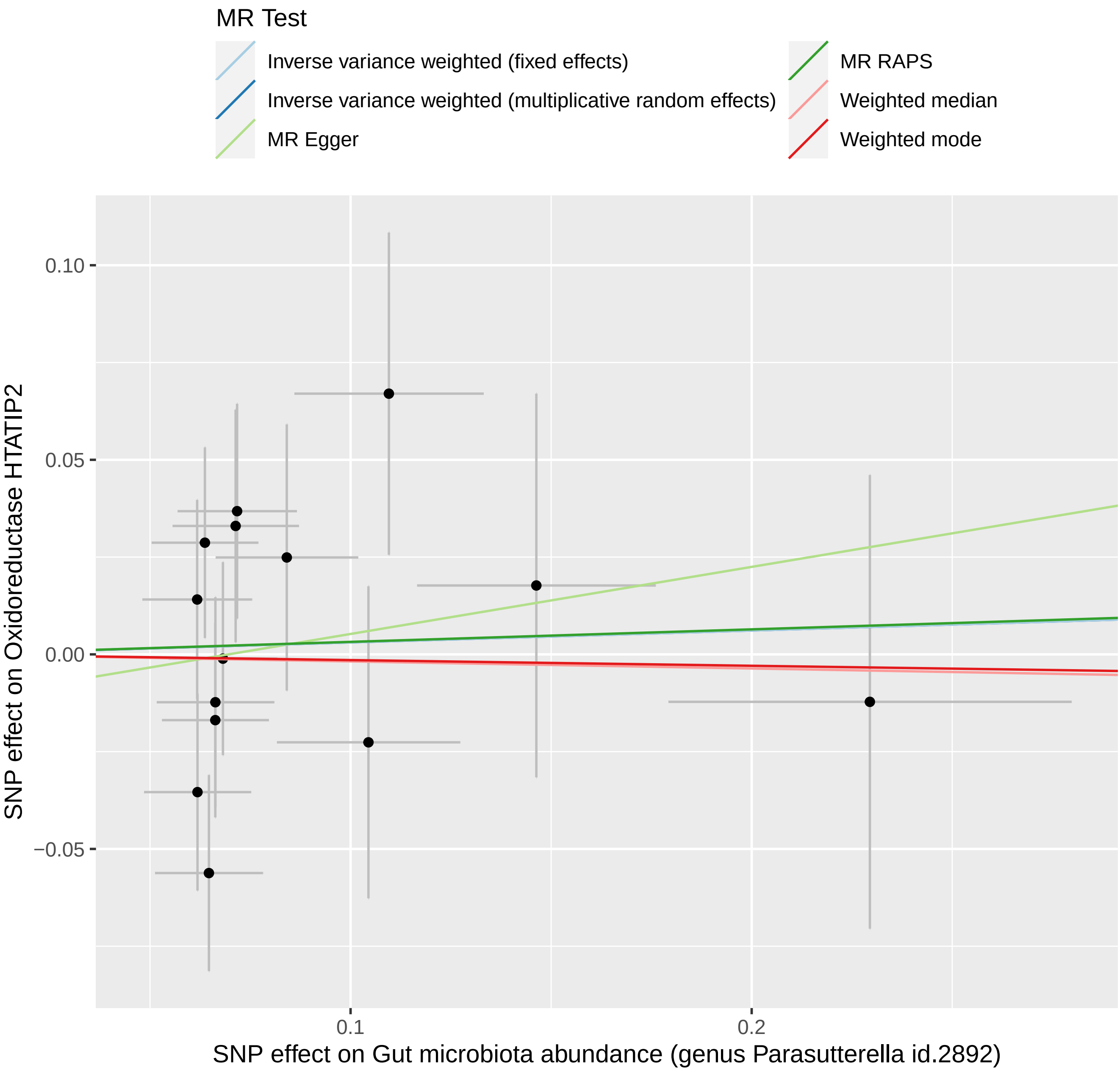

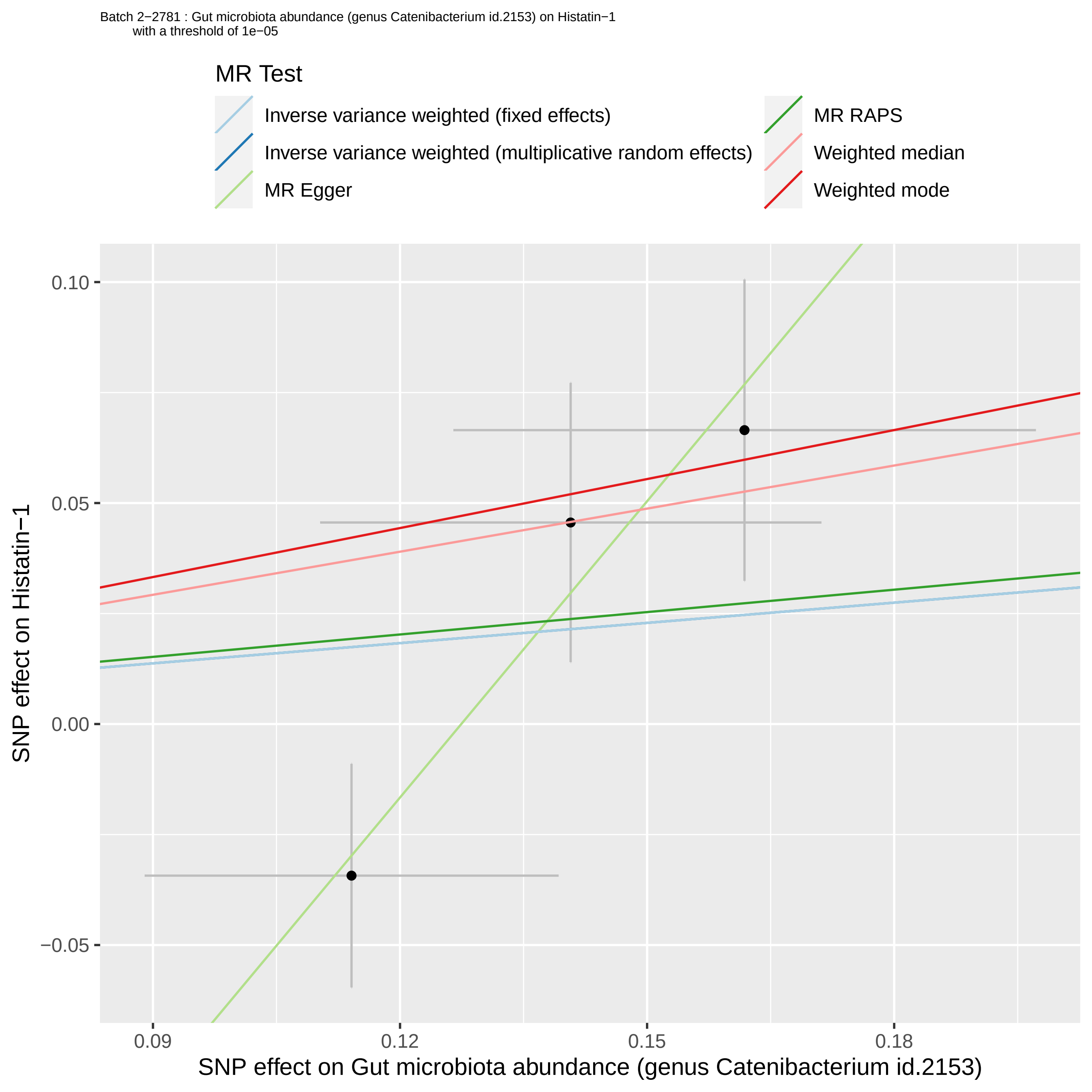

Batch 2−2781 : Gut microbiota abundance (genus Catenibacterium id.2153) on Histatin−1
with a threshold of 1e−05
MR Test
Inverse variance weighted (fixed effects)
Inverse variance weighted (multiplicative random effects)
MR Egger
MR RAPS
Weighted median
Weighted mode
0.10
0.05
0.00
−0.05
0.09
0.12
0.15
0.18
SNP effect on Histatin−1
SNP effect on Gut microbiota abundance (genus Catenibacterium id.2153)

Batch 2−2782 : Gut microbiota abundance (genus Parasutterella id.2892) on Histatin−1
with a threshold of 1e−05

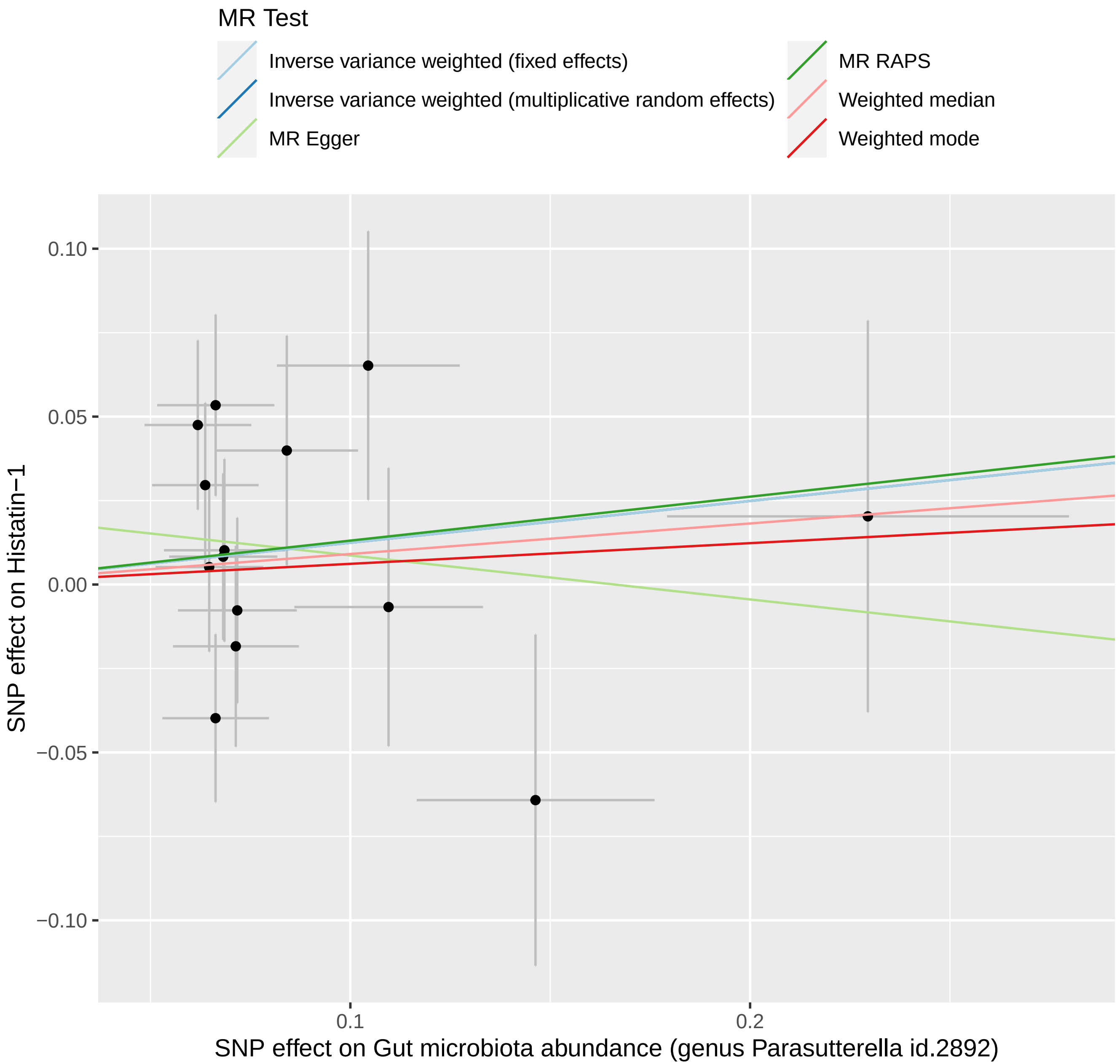

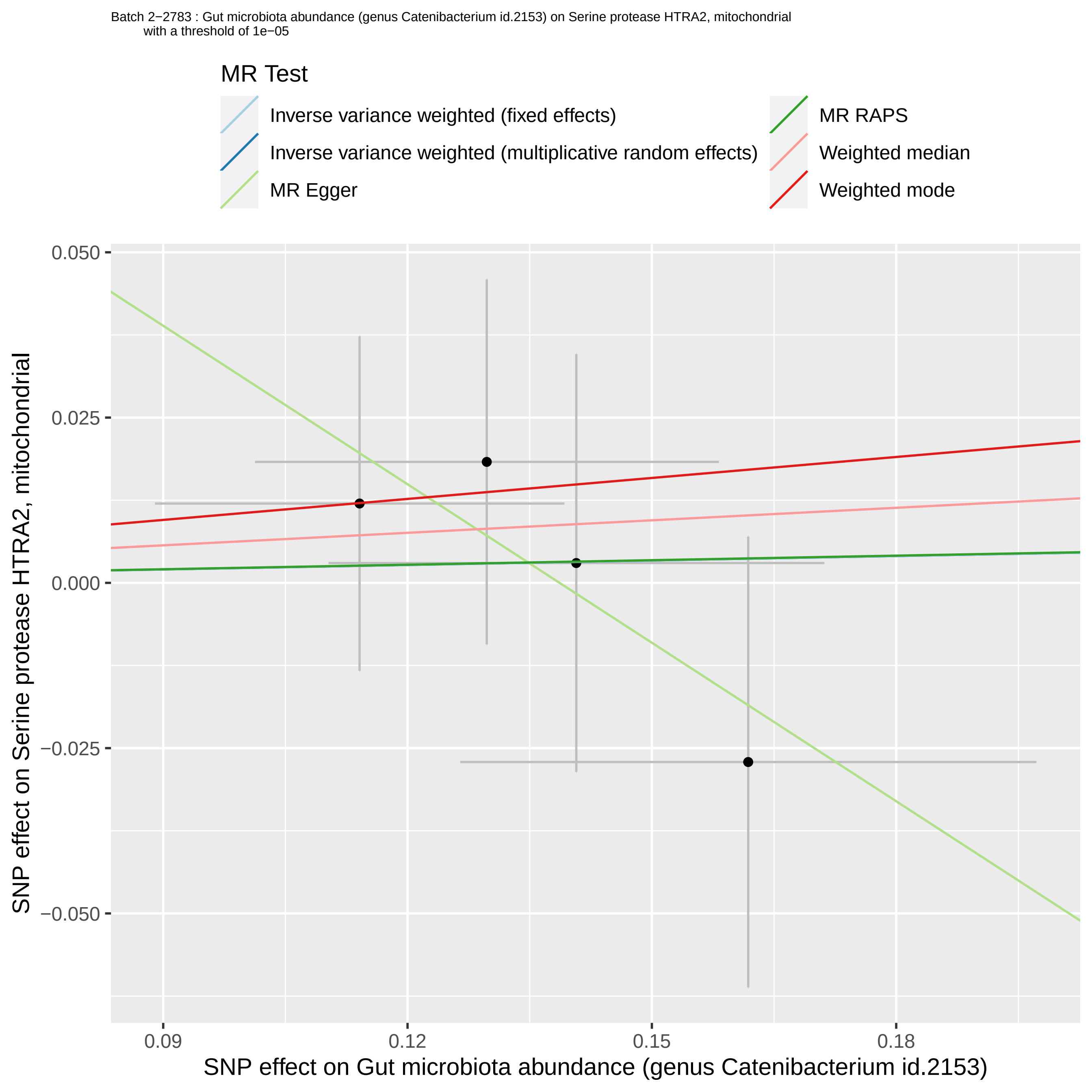

Batch 2−2783 : Gut microbiota abundance (genus Catenibacterium id.2153) on Serine protease HTRA2, mitochondrial
with a threshold of 1e−05
MR Test
Inverse variance weighted (fixed effects)
Inverse variance weighted (multiplicative random effects)
MR Egger
MR RAPS
Weighted median
Weighted mode
0.050
0.025
0.000
−0.025
−0.050
0.09
0.12
0.15
0.18
SNP effect on Serine protease HTRA2, mitochondrial
SNP effect on Gut microbiota abundance (genus Catenibacterium id.2153)

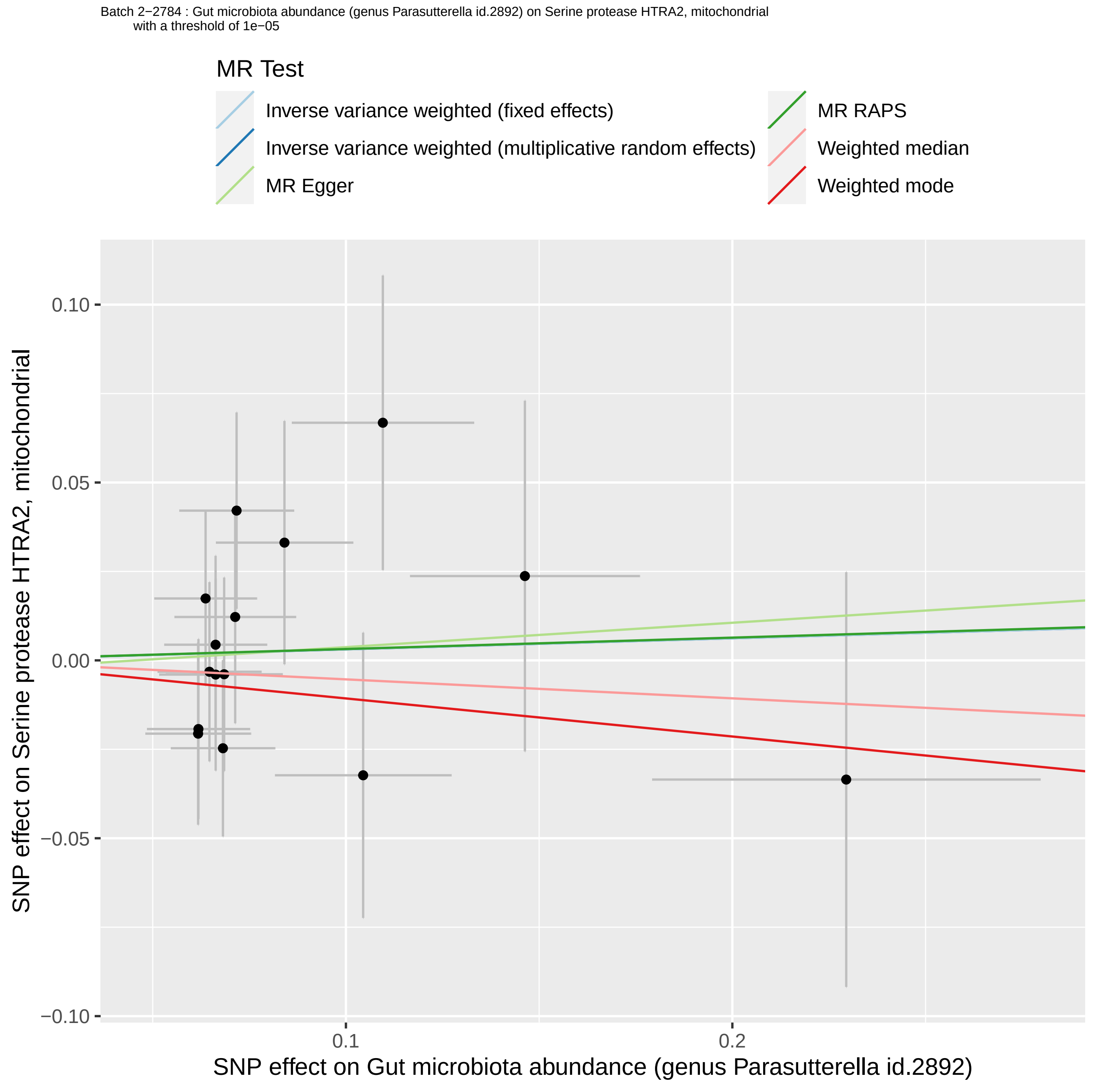
Batch 2−2784 : Gut microbiota abundance (genus Parasutterella id.2892) on Serine protease HTRA2, mitochondrial
with a threshold of 1e−05
MR Test
Inverse variance weighted (fixed effects)
Inverse variance weighted (multiplicative random effects)
MR Egger
MR RAPS
Weighted median
Weighted mode
0.10
0.05
0.00
−0.05
−0.10
0.1
0.2
SNP effect on Serine protease HTRA2, mitochondrial
SNP effect on Gut microbiota abundance (genus Parasutterella id.2892)

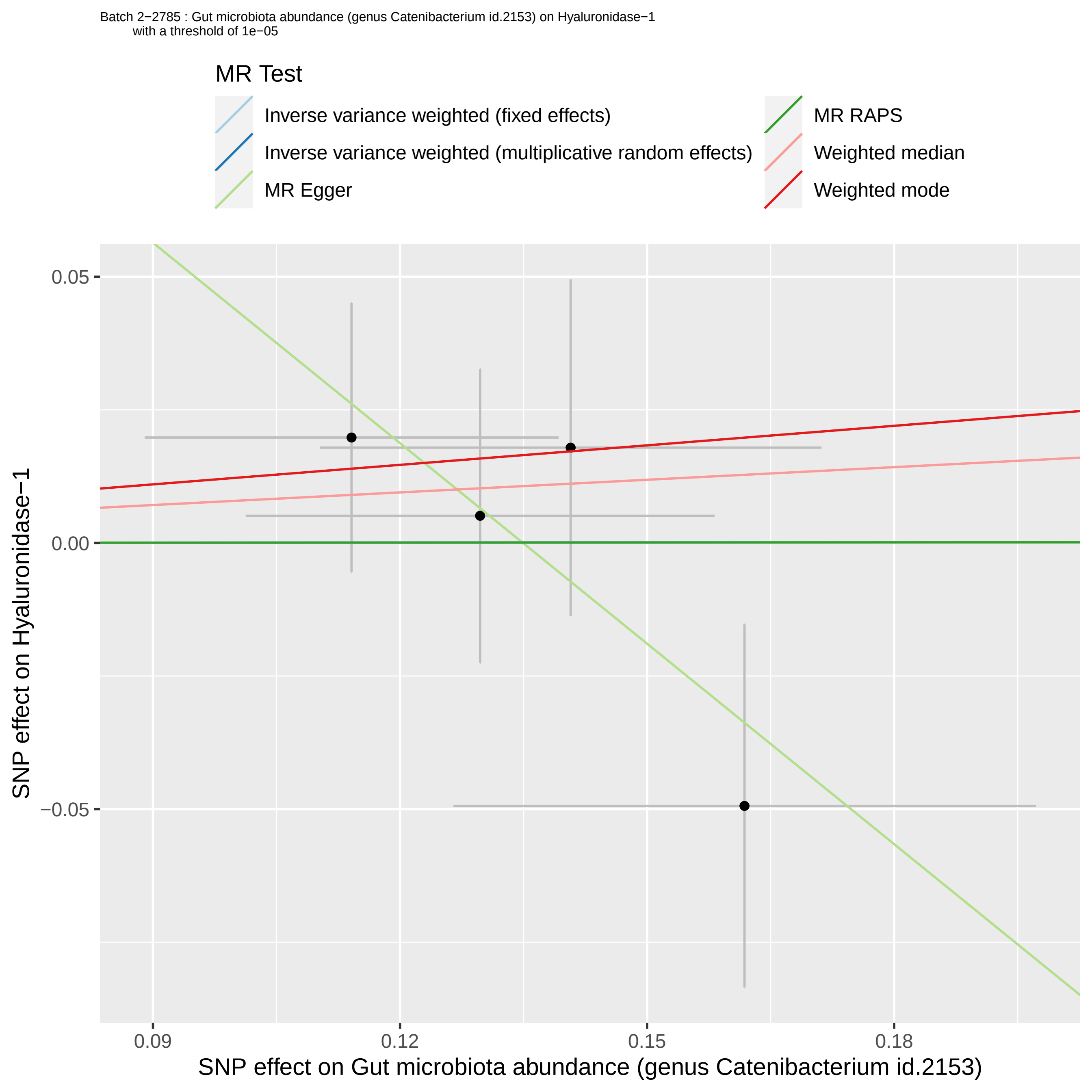

Batch 2−2785 : Gut microbiota abundance (genus Catenibacterium id.2153) on Hyaluronidase−1
with a threshold of 1e−05
MR Test
Inverse variance weighted (fixed effects)
Inverse variance weighted (multiplicative random effects)
MR Egger
MR RAPS
Weighted median
Weighted mode
0.05
0.00
−0.05
0.09
0.12
0.15
0.18
SNP effect on Hyaluronidase−1
SNP effect on Gut microbiota abundance (genus Catenibacterium id.2153)

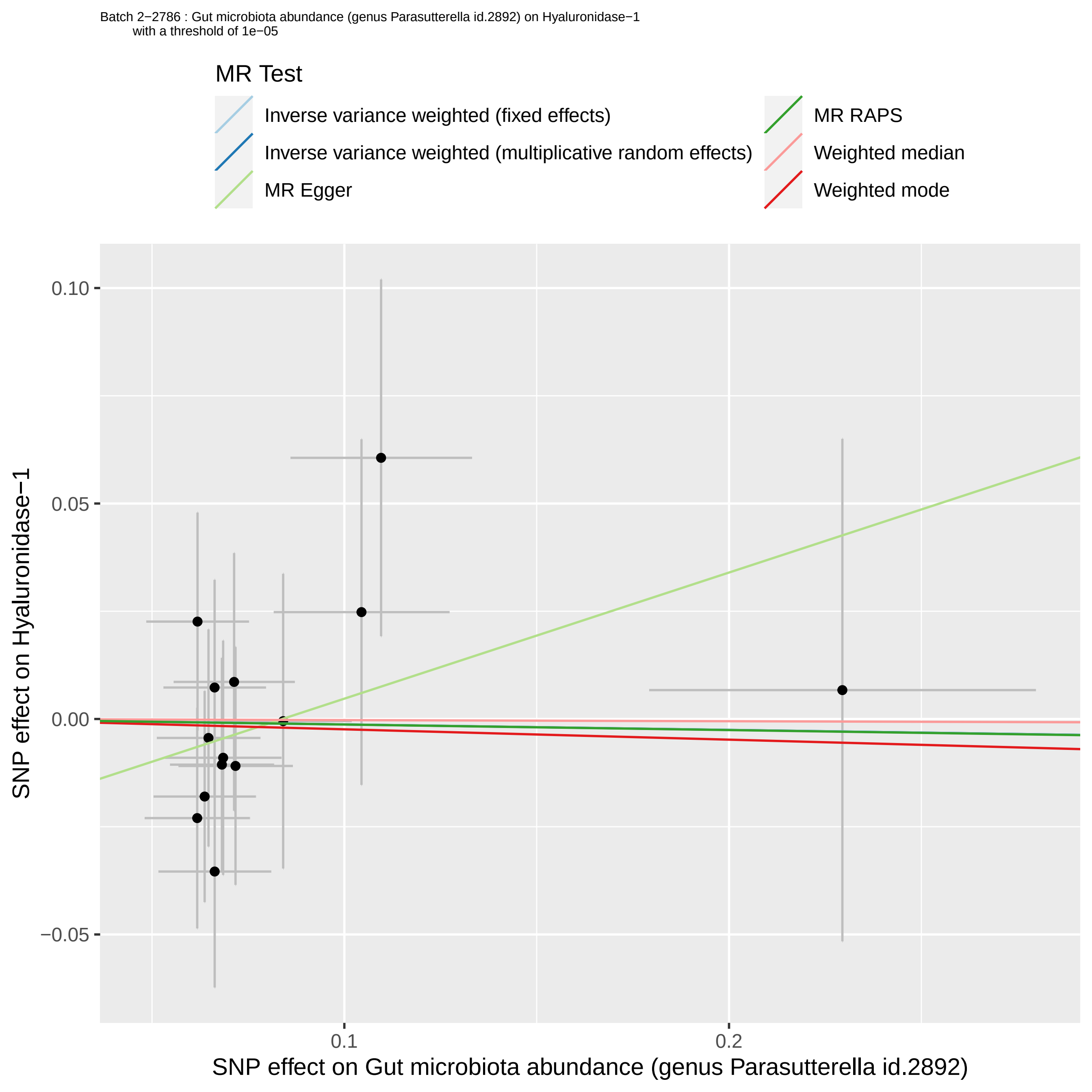

Batch 2−2786 : Gut microbiota abundance (genus Parasutterella id.2892) on Hyaluronidase−1
with a threshold of 1e−05
MR Test
Inverse variance weighted (fixed effects)
Inverse variance weighted (multiplicative random effects)
MR Egger
MR RAPS
Weighted median
Weighted mode
0.10
0.05
0.00
−0.05
0.1
0.2
SNP effect on Hyaluronidase−1
SNP effect on Gut microbiota abundance (genus Parasutterella id.2892)

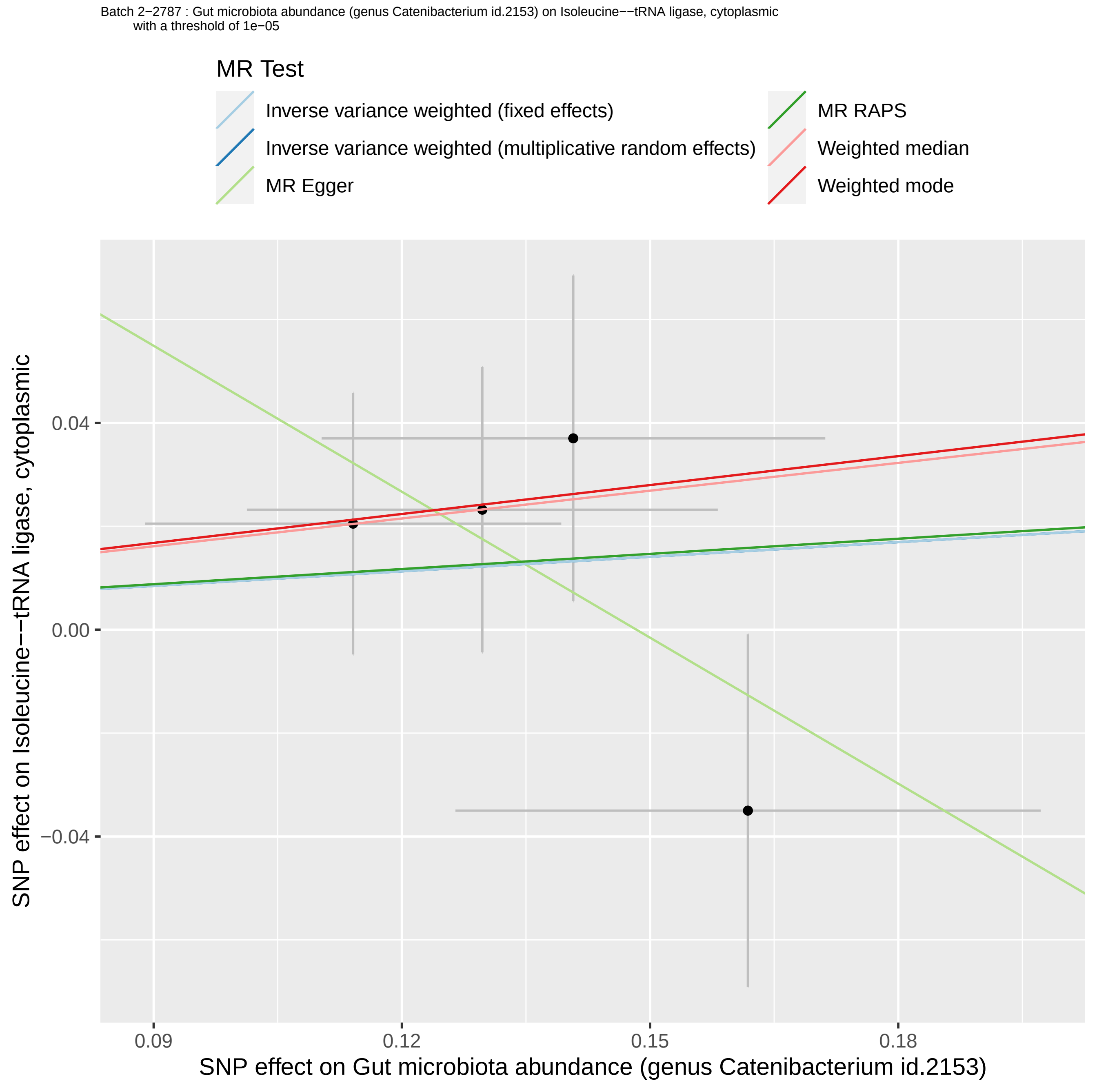

Batch 2−2787 : Gut microbiota abundance (genus Catenibacterium id.2153) on Isoleucine−−tRNA ligase, cytoplasmic
with a threshold of 1e−05
MR Test
Inverse variance weighted (fixed effects)
Inverse variance weighted (multiplicative random effects)
MR Egger
MR RAPS
Weighted median
Weighted mode
0.04
0.00
−0.04
0.09
0.12
0.15
0.18
SNP effect on Isoleucine−−tRNA ligase, cytoplasmic
SNP effect on Gut microbiota abundance (genus Catenibacterium id.2153)

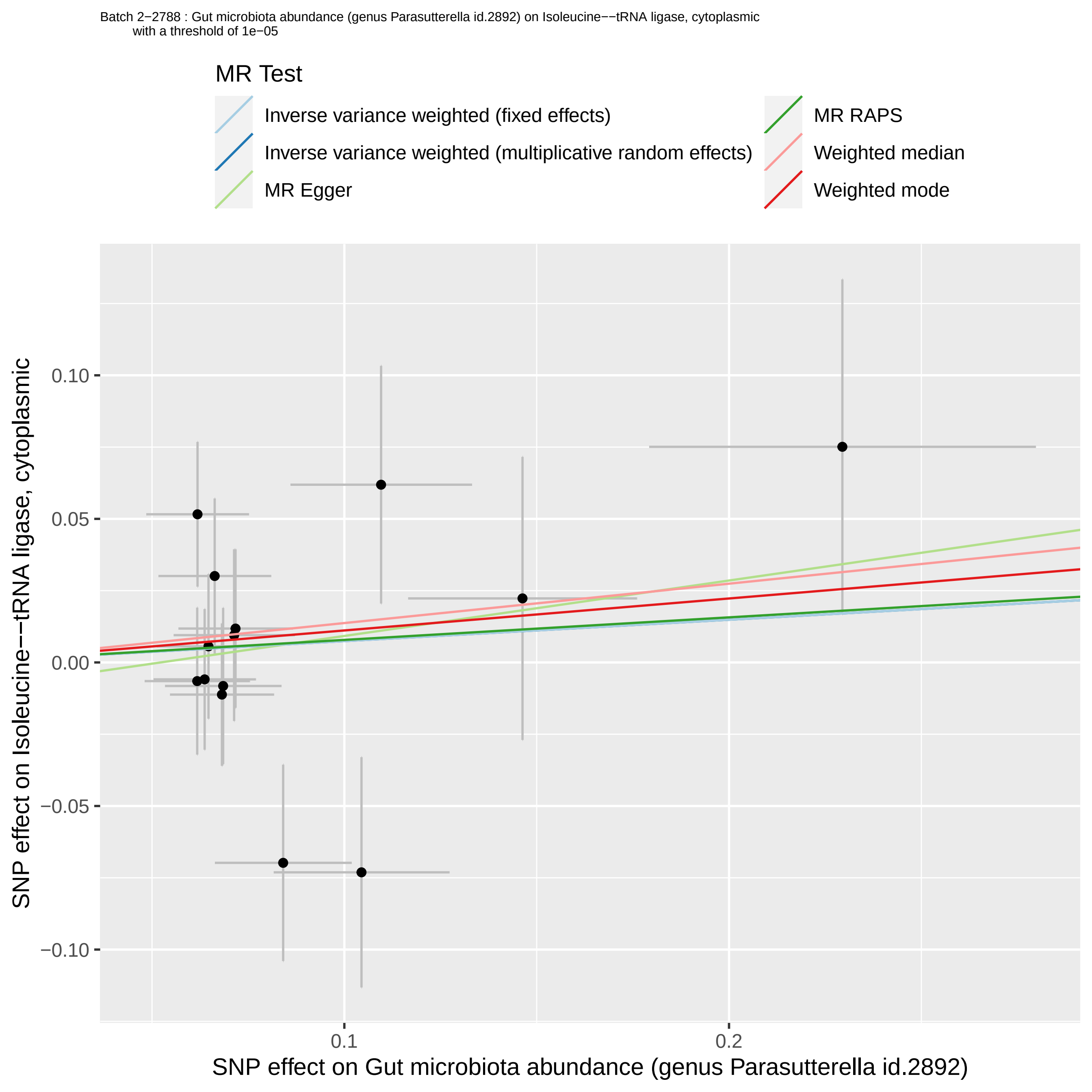
Batch 2−2788 : Gut microbiota abundance (genus Parasutterella id.2892) on Isoleucine−−tRNA ligase, cytoplasmic
with a threshold of 1e−05
MR Test
Inverse variance weighted (fixed effects)
Inverse variance weighted (multiplicative random effects)
MR Egger
MR RAPS
Weighted median
Weighted mode
0.10
0.05
0.00
−0.05
−0.10
0.1
0.2
SNP effect on Isoleucine−−tRNA ligase, cytoplasmic
SNP effect on Gut microbiota abundance (genus Parasutterella id.2892)

Batch 2−2789 : Gut microbiota abundance (genus Catenibacterium id.2153) on Bone sialoprotein 2
with a threshold of 1e−05

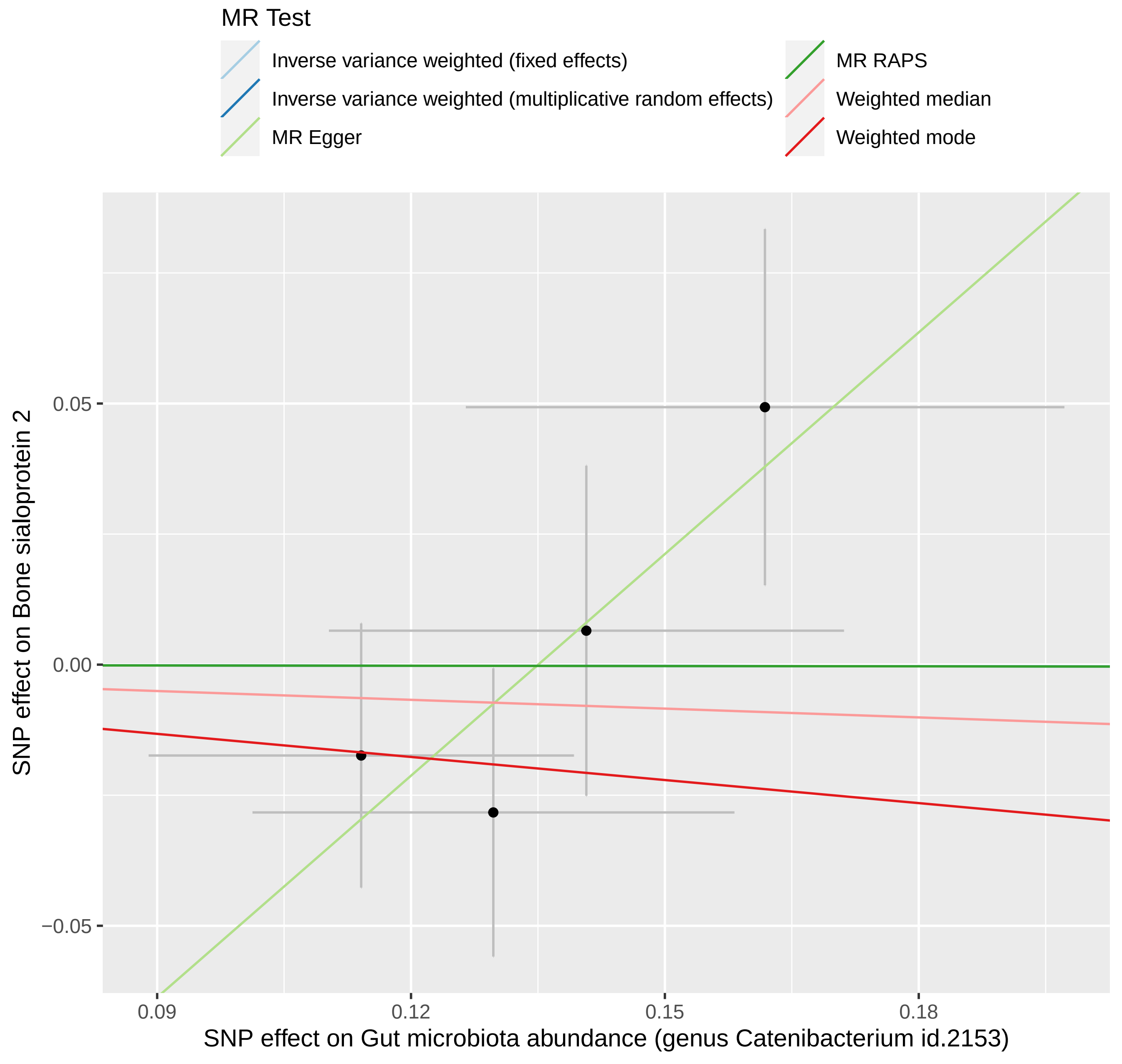

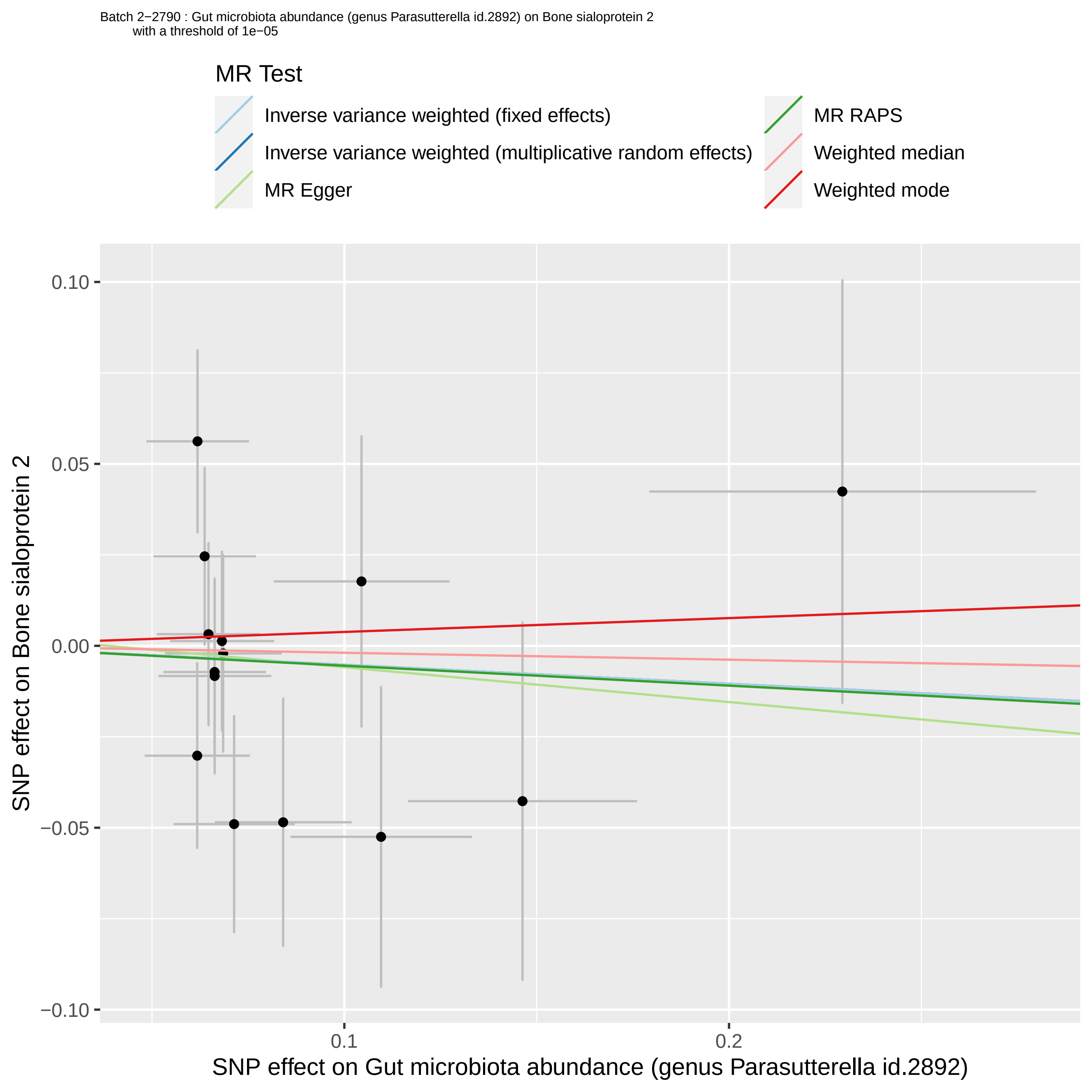
Batch 2−2790 : Gut microbiota abundance (genus Parasutterella id.2892) on Bone sialoprotein 2
with a threshold of 1e−05
MR Test
Inverse variance weighted (fixed effects)
Inverse variance weighted (multiplicative random effects)
MR Egger
MR RAPS
Weighted median
Weighted mode
0.10
0.05
0.00
−0.05
−0.10
0.1
0.2
SNP effect on Bone sialoprotein 2
SNP effect on Gut microbiota abundance (genus Parasutterella id.2892)

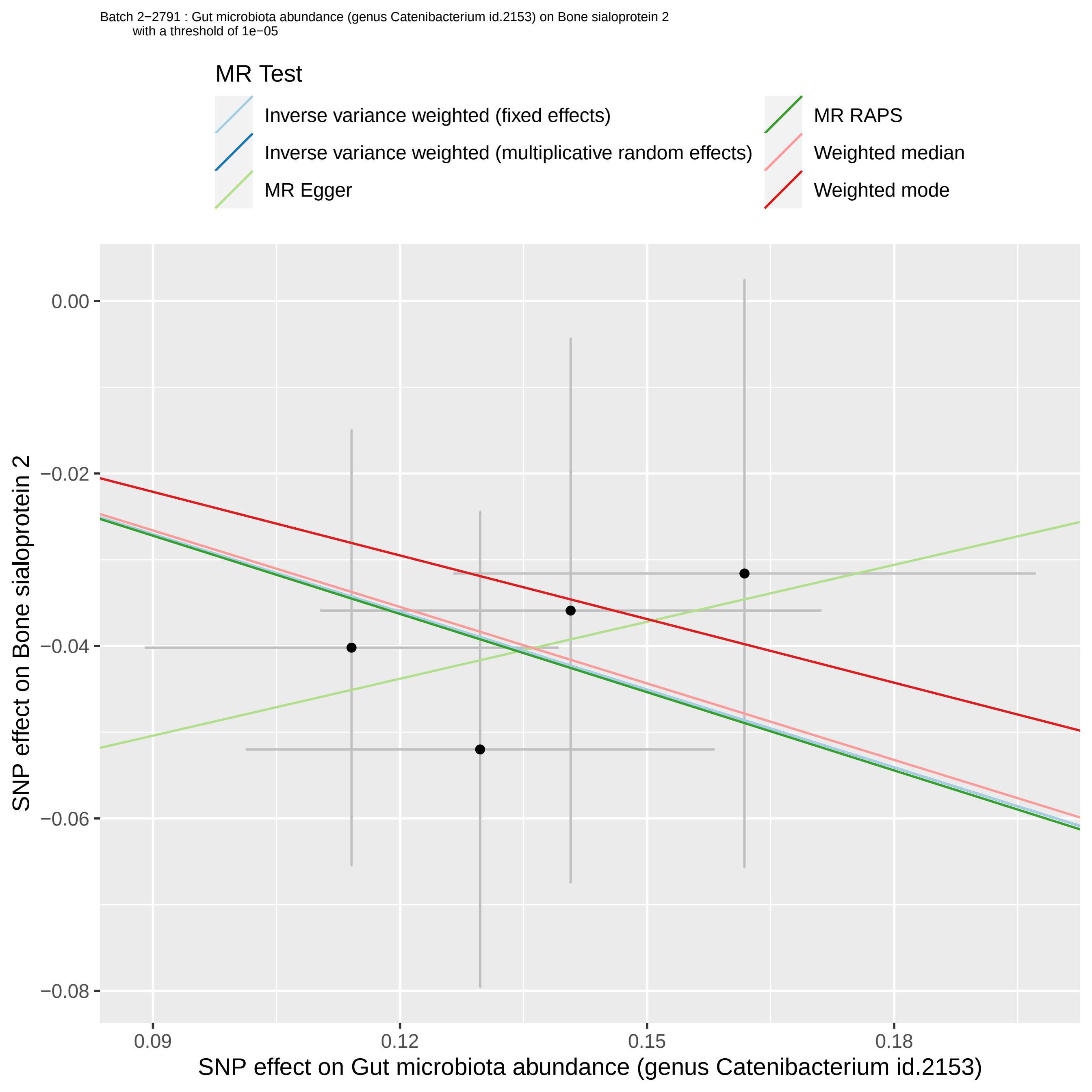

Batch 2−2791 : Gut microbiota abundance (genus Catenibacterium id.2153) on Bone sialoprotein 2
with a threshold of 1e−05
MR Test
Inverse variance weighted (fixed effects)
Inverse variance weighted (multiplicative random effects)
MR Egger
MR RAPS
Weighted median
Weighted mode
0.00
−0.02
−0.04
−0.06
−0.08
0.09
0.12
0.15
0.18
SNP effect on Bone sialoprotein 2
SNP effect on Gut microbiota abundance (genus Catenibacterium id.2153)

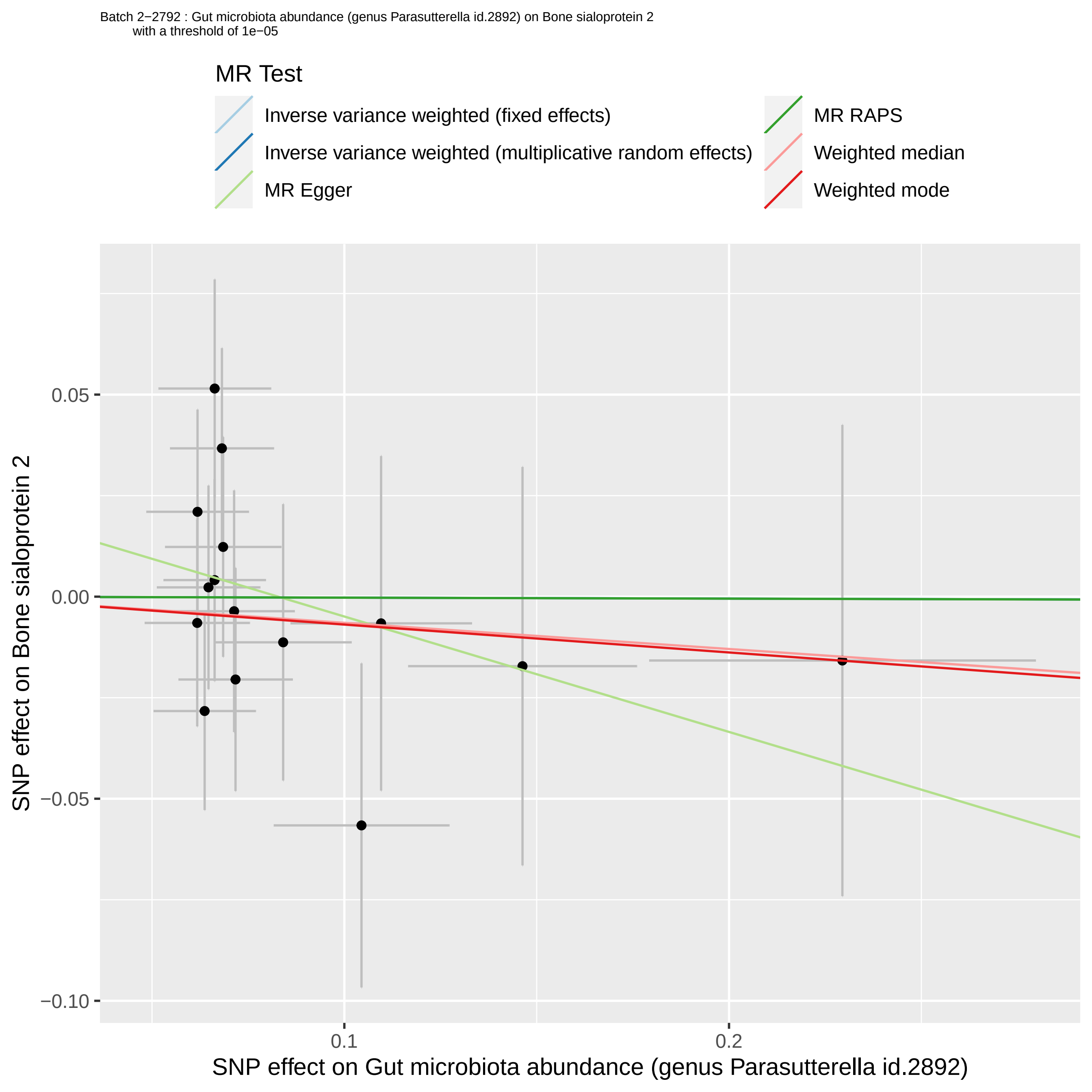
Batch 2−2792 : Gut microbiota abundance (genus Parasutterella id.2892) on Bone sialoprotein 2
with a threshold of 1e−05
MR Test
Inverse variance weighted (fixed effects)
Inverse variance weighted (multiplicative random effects)
MR Egger
MR RAPS
Weighted median
Weighted mode
SNP effect on Bone sialoprotein 2
0.05
0.00
−0.05
−0.10
0.1
0.2
SNP effect on Gut microbiota abundance (genus Parasutterella id.2892)

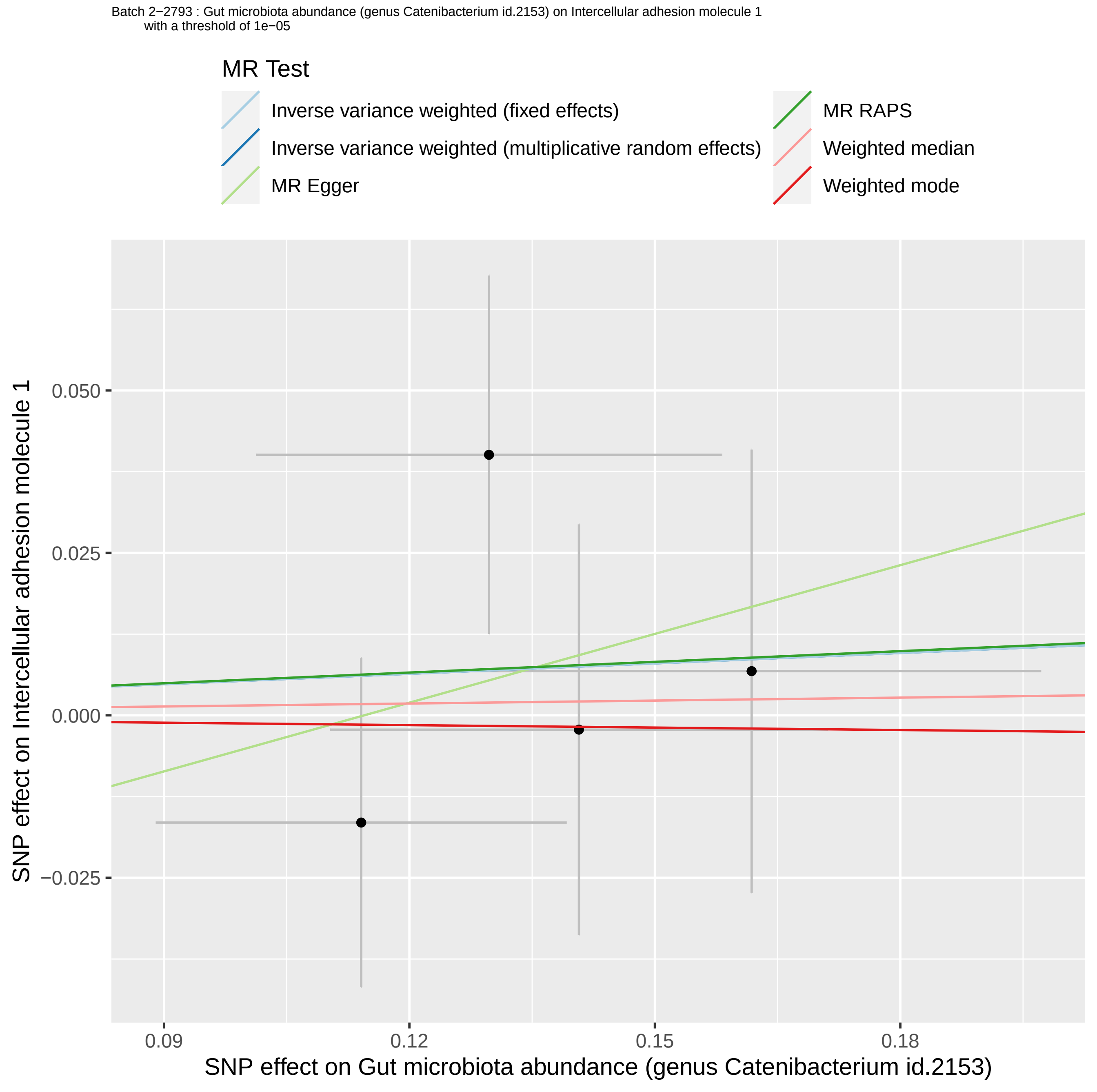

Batch 2−2793 : Gut microbiota abundance (genus Catenibacterium id.2153) on Intercellular adhesion molecule 1
with a threshold of 1e−05
MR Test
Inverse variance weighted (fixed effects)
Inverse variance weighted (multiplicative random effects)
MR Egger
MR RAPS
Weighted median
Weighted mode
SNP effect on Intercellular adhesion molecule 1
0.050
0.025
0.000
−0.025
0.09
0.12
0.15
0.18
SNP effect on Gut microbiota abundance (genus Catenibacterium id.2153)

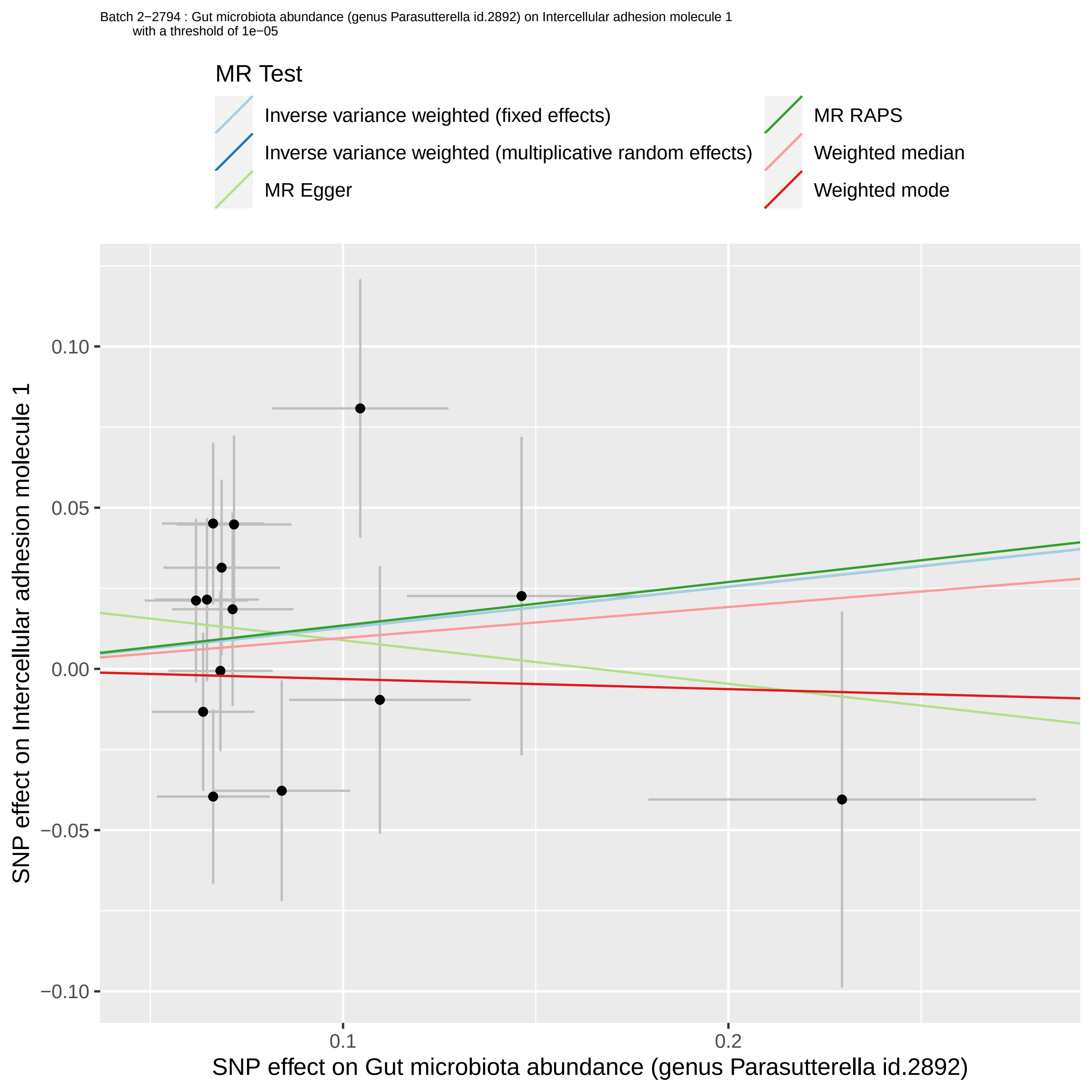

Batch 2−2794 : Gut microbiota abundance (genus Parasutterella id.2892) on Intercellular adhesion molecule 1
with a threshold of 1e−05
MR Test
Inverse variance weighted (fixed effects)
Inverse variance weighted (multiplicative random effects)
MR Egger
MR RAPS
Weighted median
Weighted mode
0.10
0.05
0.00
−0.05
−0.10
0.1
0.2
SNP effect on Intercellular adhesion molecule 1
SNP effect on Gut microbiota abundance (genus Parasutterella id.2892)

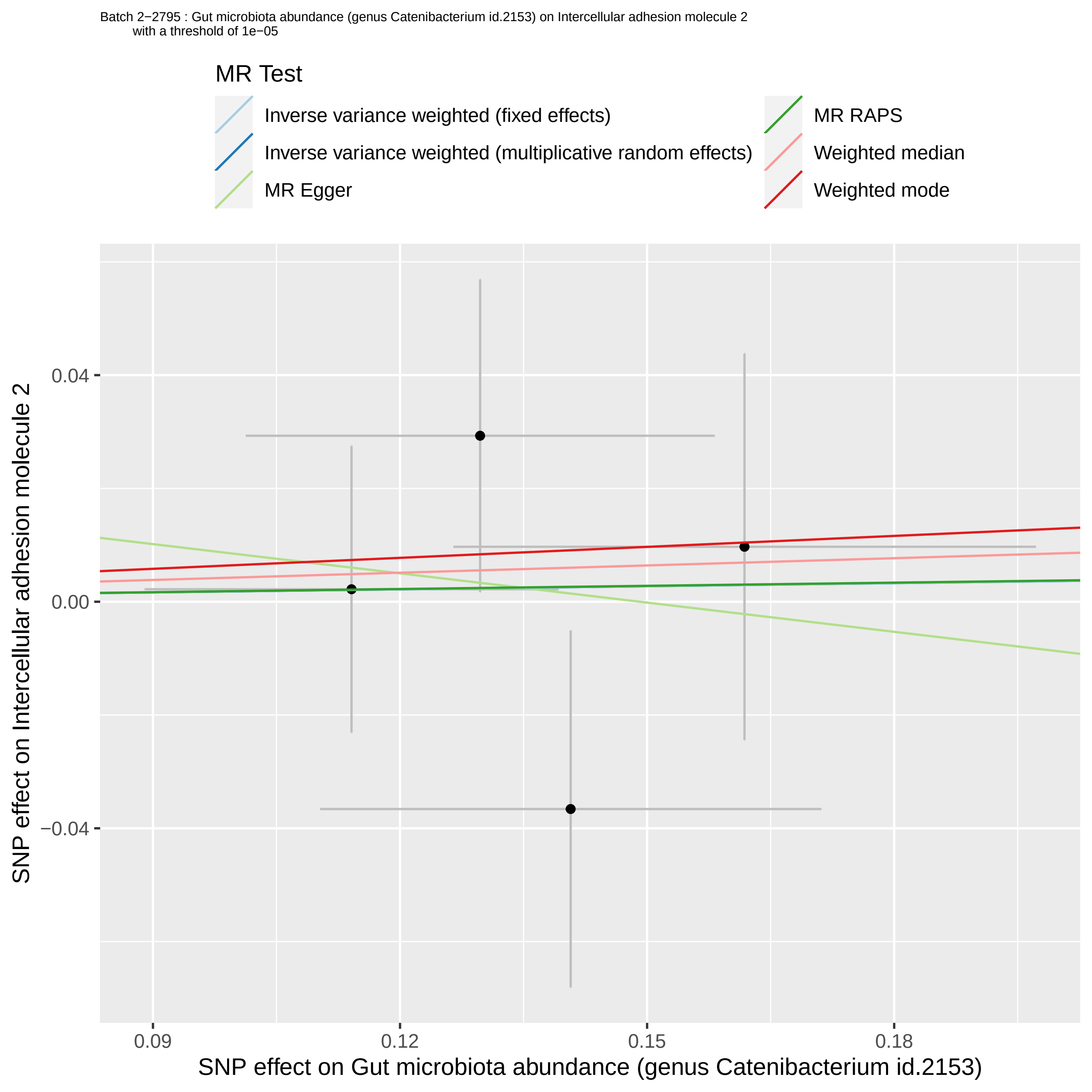

Batch 2−2795 : Gut microbiota abundance (genus Catenibacterium id.2153) on Intercellular adhesion molecule 2
with a threshold of 1e−05
MR Test
Inverse variance weighted (fixed effects)
Inverse variance weighted (multiplicative random effects)
MR Egger
MR RAPS
Weighted median
Weighted mode
0.04
0.00
−0.04
0.09
0.12
0.15
0.18
SNP effect on Intercellular adhesion molecule 2
SNP effect on Gut microbiota abundance (genus Catenibacterium id.2153)

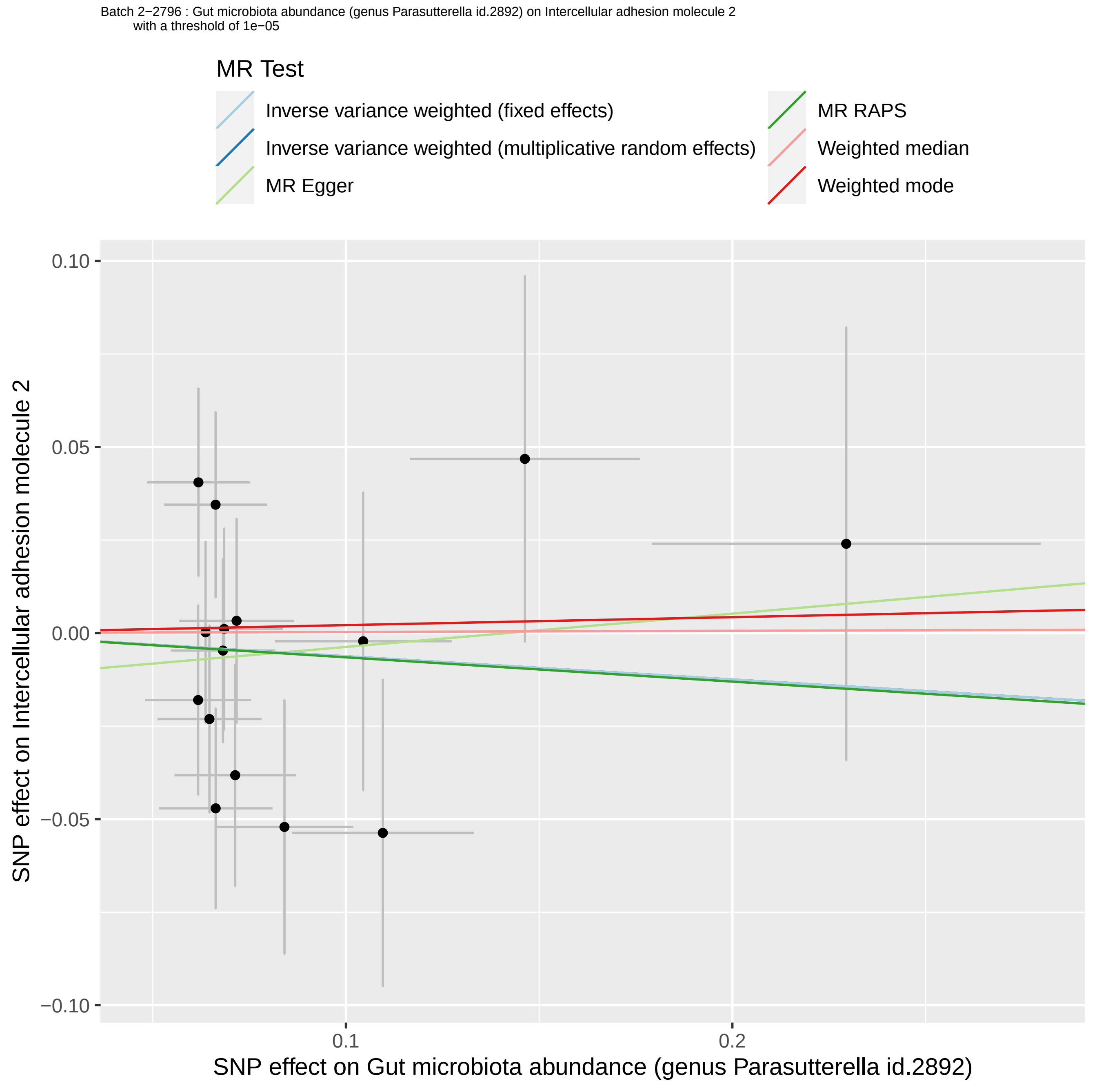

Batch 2−2796 : Gut microbiota abundance (genus Parasutterella id.2892) on Intercellular adhesion molecule 2
with a threshold of 1e−05
MR Test
Inverse variance weighted (fixed effects)
Inverse variance weighted (multiplicative random effects)
MR Egger
MR RAPS
Weighted median
Weighted mode
SNP effect on Intercellular adhesion molecule 2
0.10
0.05
0.00
−0.05
−0.10
0.1
0.2
SNP effect on Gut microbiota abundance (genus Parasutterella id.2892)

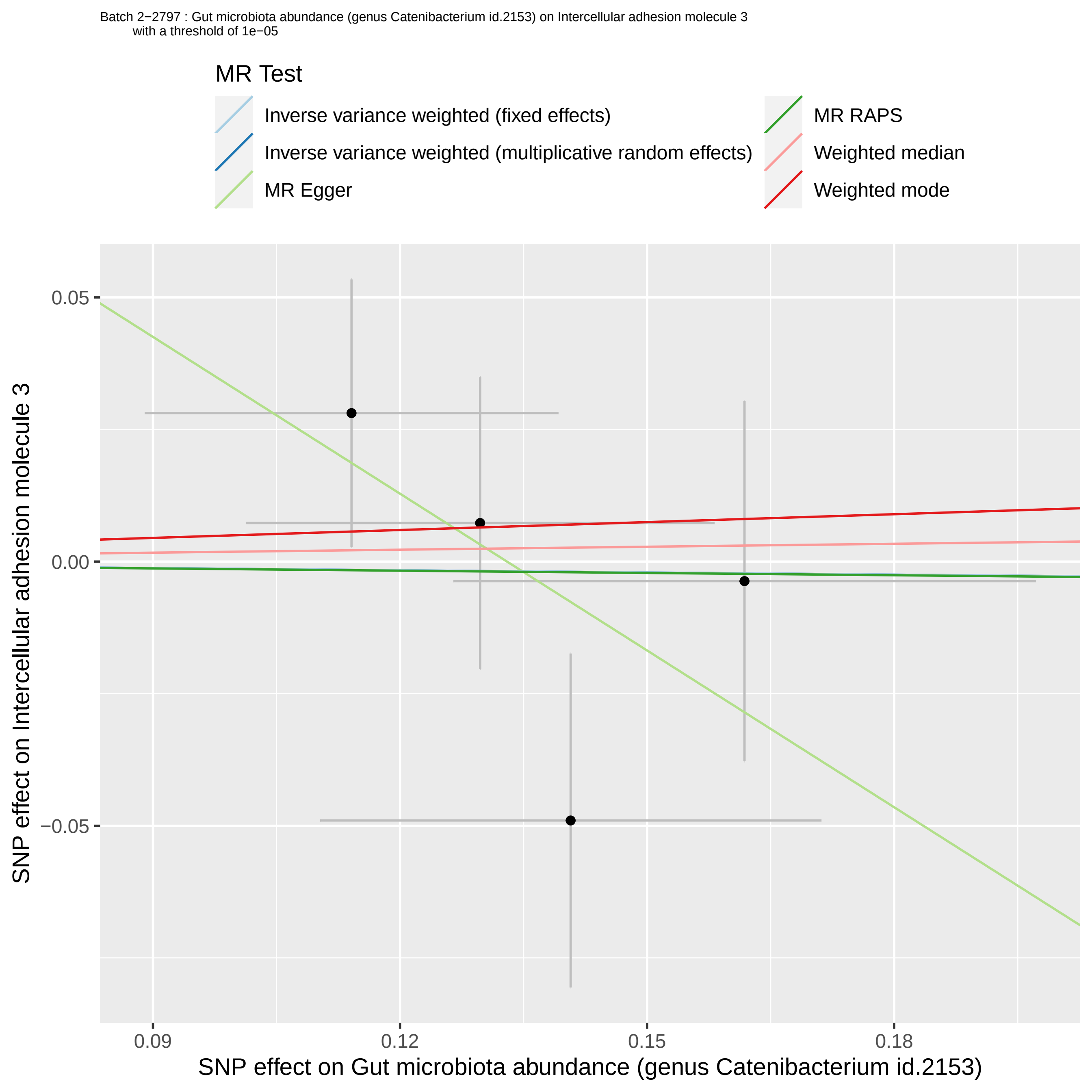
Batch 2−2797 : Gut microbiota abundance (genus Catenibacterium id.2153) on Intercellular adhesion molecule 3
with a threshold of 1e−05
MR Test
Inverse variance weighted (fixed effects)
Inverse variance weighted (multiplicative random effects)
MR Egger
MR RAPS
Weighted median
Weighted mode
0.05
0.00
−0.05
0.09
0.12
0.15
0.18
SNP effect on Intercellular adhesion molecule 3
SNP effect on Gut microbiota abundance (genus Catenibacterium id.2153)

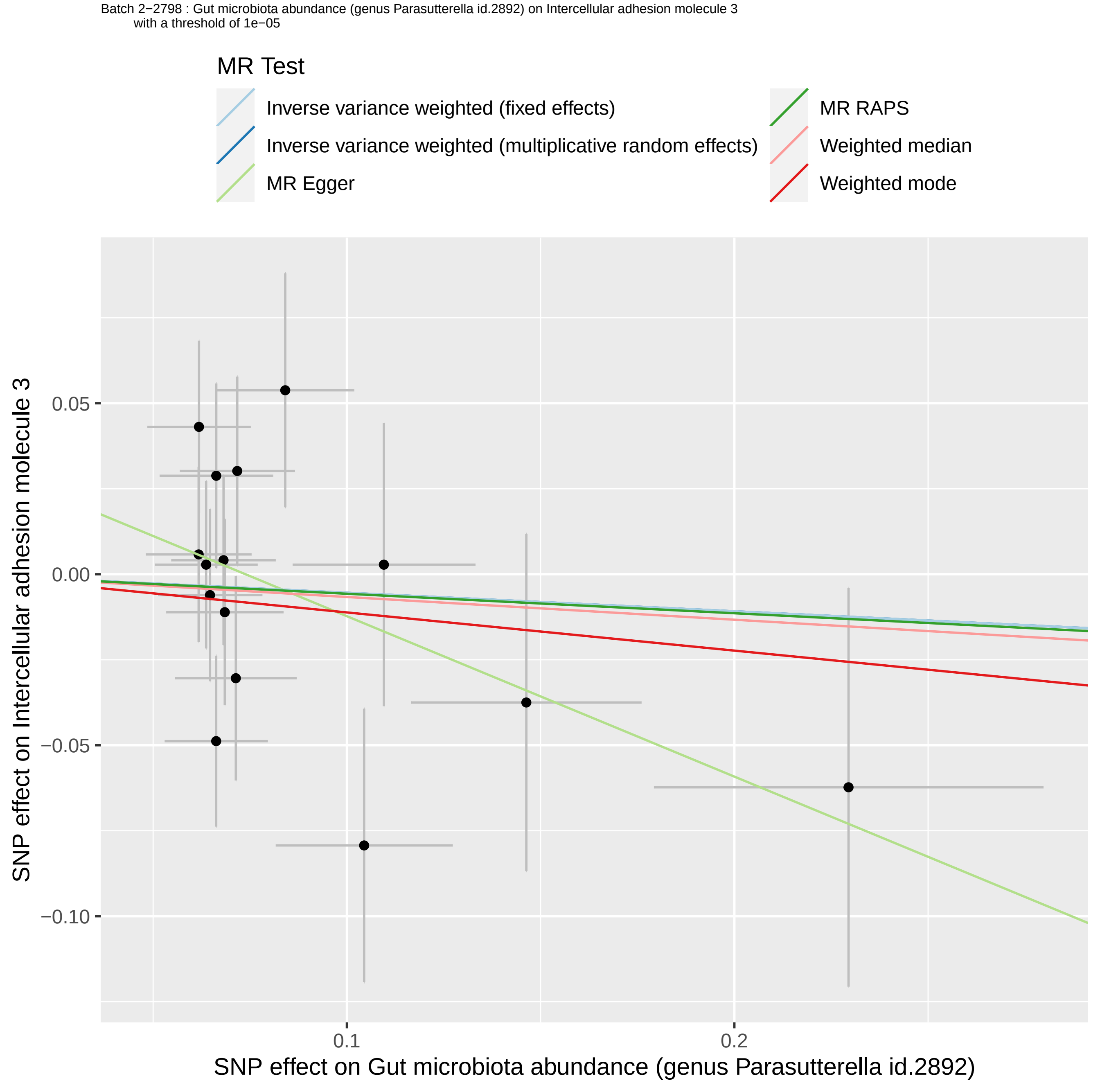

Batch 2−2798 : Gut microbiota abundance (genus Parasutterella id.2892) on Intercellular adhesion molecule 3
with a threshold of 1e−05
MR Test
Inverse variance weighted (fixed effects)
Inverse variance weighted (multiplicative random effects)
MR Egger
MR RAPS
Weighted median
Weighted mode
SNP effect on Intercellular adhesion molecule 3
0.05
0.00
−0.05
−0.10
0.1
0.2
SNP effect on Gut microbiota abundance (genus Parasutterella id.2892)

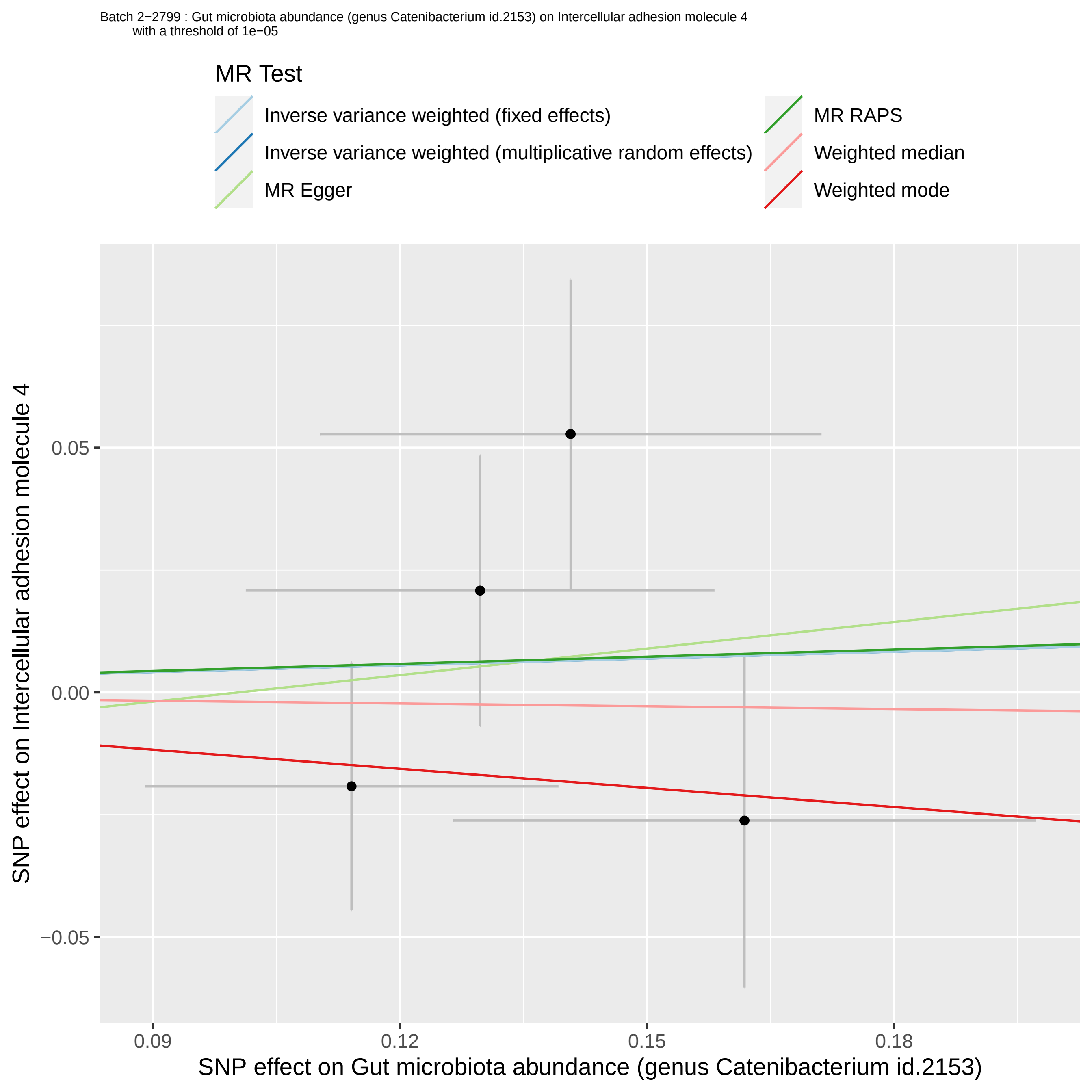
Batch 2−2799 : Gut microbiota abundance (genus Catenibacterium id.2153) on Intercellular adhesion molecule 4
with a threshold of 1e−05
MR Test
Inverse variance weighted (fixed effects)
Inverse variance weighted (multiplicative random effects)
MR Egger
MR RAPS
Weighted median
Weighted mode
SNP effect on Intercellular adhesion molecule 4
0.05
0.00
−0.05
0.09
0.12
0.15
0.18
SNP effect on Gut microbiota abundance (genus Catenibacterium id.2153)

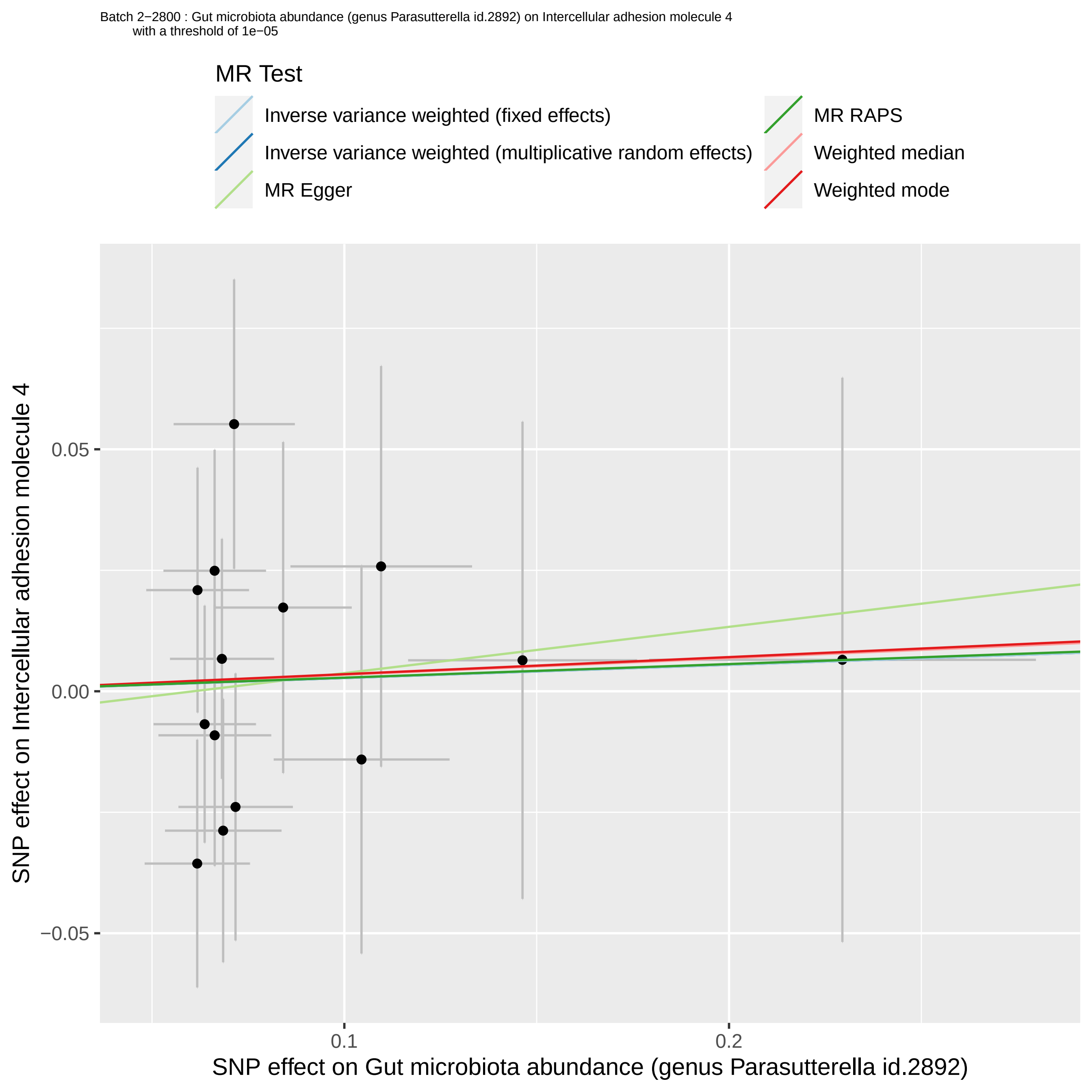

Batch 2−2800 : Gut microbiota abundance (genus Parasutterella id.2892) on Intercellular adhesion molecule 4
with a threshold of 1e−05
MR Test
Inverse variance weighted (fixed effects)
Inverse variance weighted (multiplicative random effects)
MR Egger
MR RAPS
Weighted median
Weighted mode
SNP effect on Intercellular adhesion molecule 4
0.05
0.00
−0.05
0.1
0.2
SNP effect on Gut microbiota abundance (genus Parasutterella id.2892)

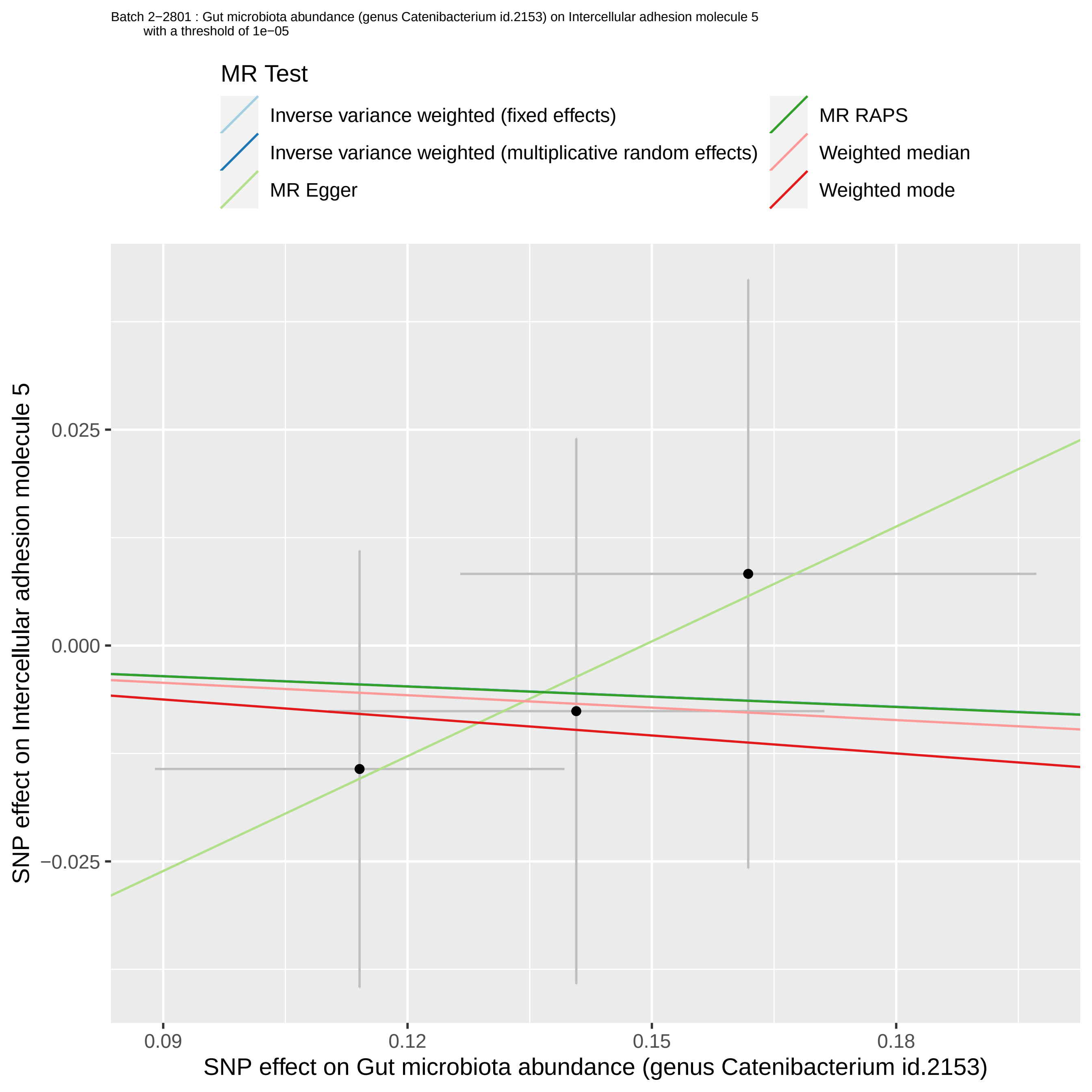
Batch 2−2801 : Gut microbiota abundance (genus Catenibacterium id.2153) on Intercellular adhesion molecule 5
with a threshold of 1e−05
MR Test
Inverse variance weighted (fixed effects)
Inverse variance weighted (multiplicative random effects)
MR Egger
MR RAPS
Weighted median
Weighted mode
0.025
0.000
−0.025
0.09
0.12
0.15
0.18
SNP effect on Intercellular adhesion molecule 5
SNP effect on Gut microbiota abundance (genus Catenibacterium id.2153)

Batch 2−2802 : Gut microbiota abundance (genus Parasutterella id.2892) on Intercellular adhesion molecule 5
with a threshold of 1e−05

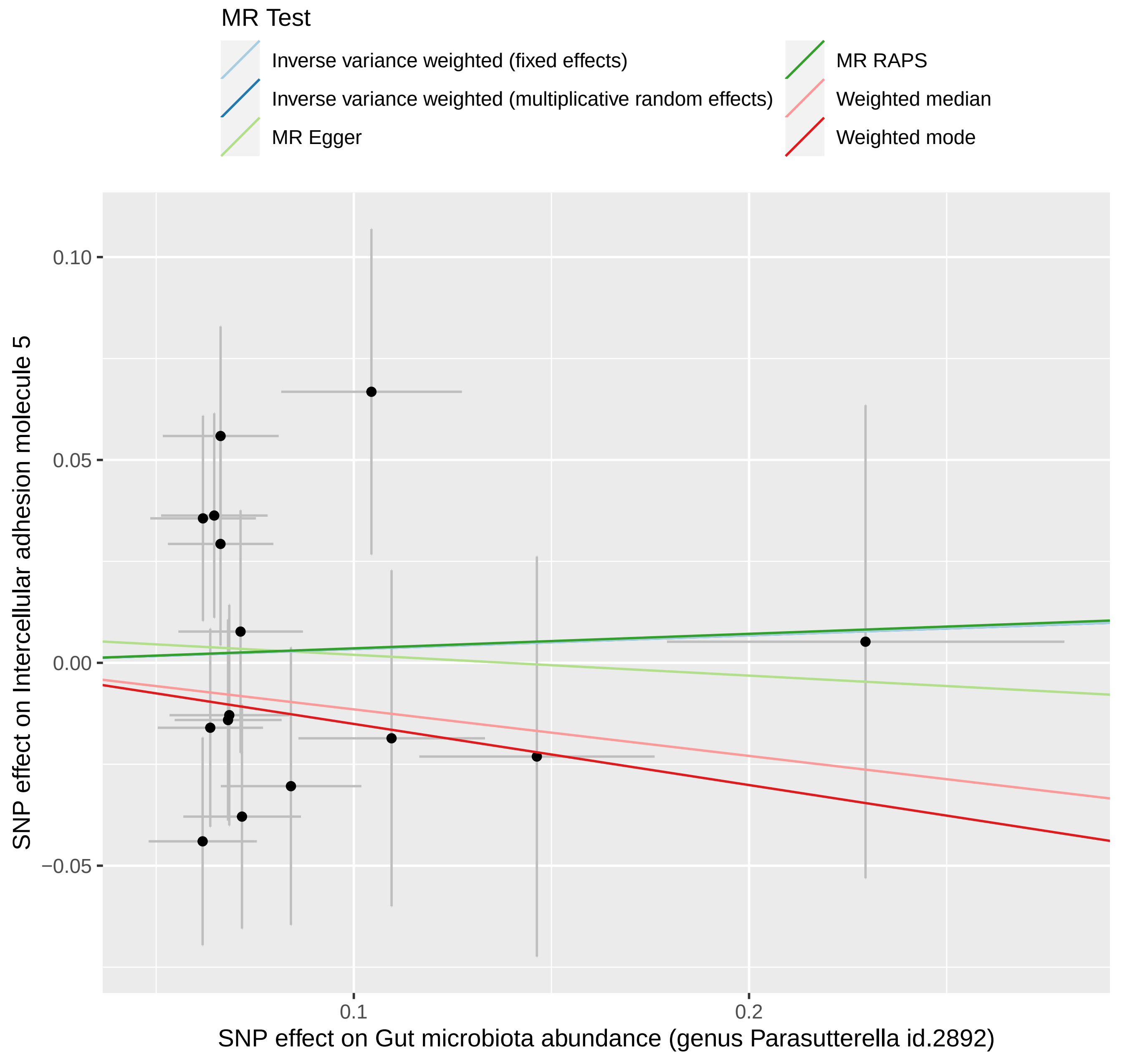

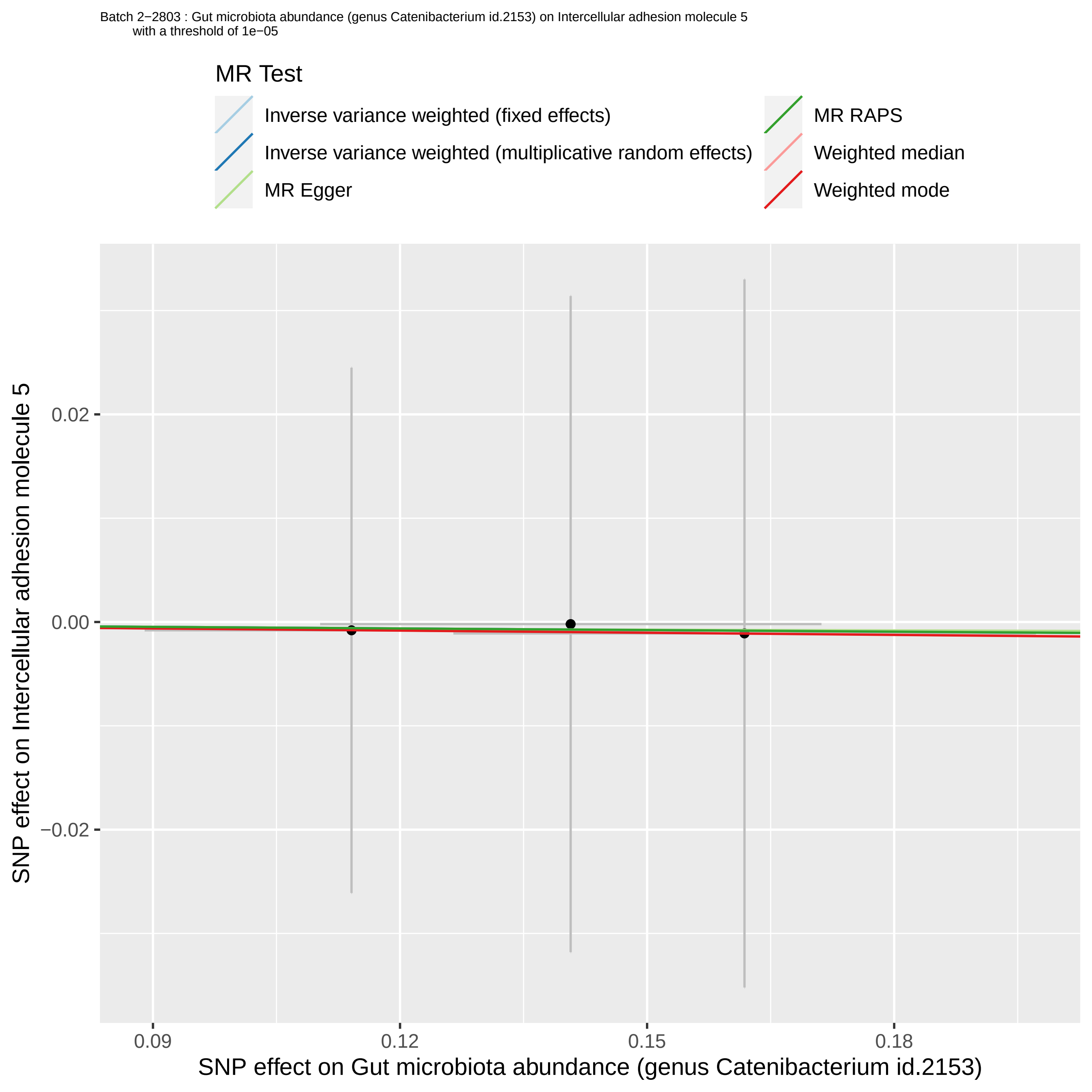
Batch 2−2803 : Gut microbiota abundance (genus Catenibacterium id.2153) on Intercellular adhesion molecule 5
with a threshold of 1e−05
MR Test
Inverse variance weighted (fixed effects)
Inverse variance weighted (multiplicative random effects)
MR Egger
MR RAPS
Weighted median
Weighted mode
0.02
0.00
−0.02
0.09
0.12
0.15
0.18
SNP effect on Intercellular adhesion molecule 5
SNP effect on Gut microbiota abundance (genus Catenibacterium id.2153)

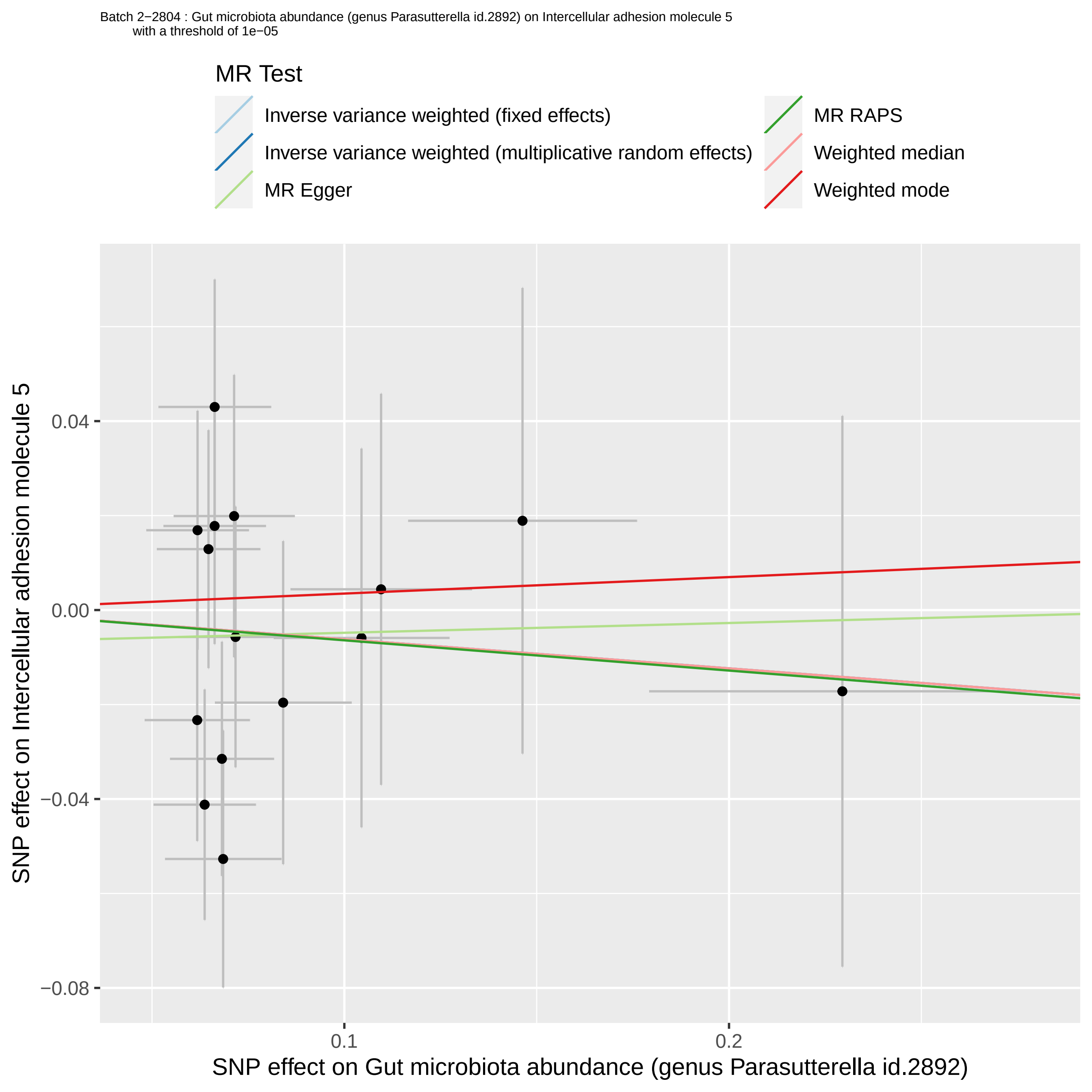

Batch 2−2804 : Gut microbiota abundance (genus Parasutterella id.2892) on Intercellular adhesion molecule 5
with a threshold of 1e−05
MR Test
Inverse variance weighted (fixed effects)
Inverse variance weighted (multiplicative random effects)
MR Egger
MR RAPS
Weighted median
Weighted mode
0.04
0.00
−0.04
−0.08
0.1
0.2
SNP effect on Intercellular adhesion molecule 5
SNP effect on Gut microbiota abundance (genus Parasutterella id.2892)

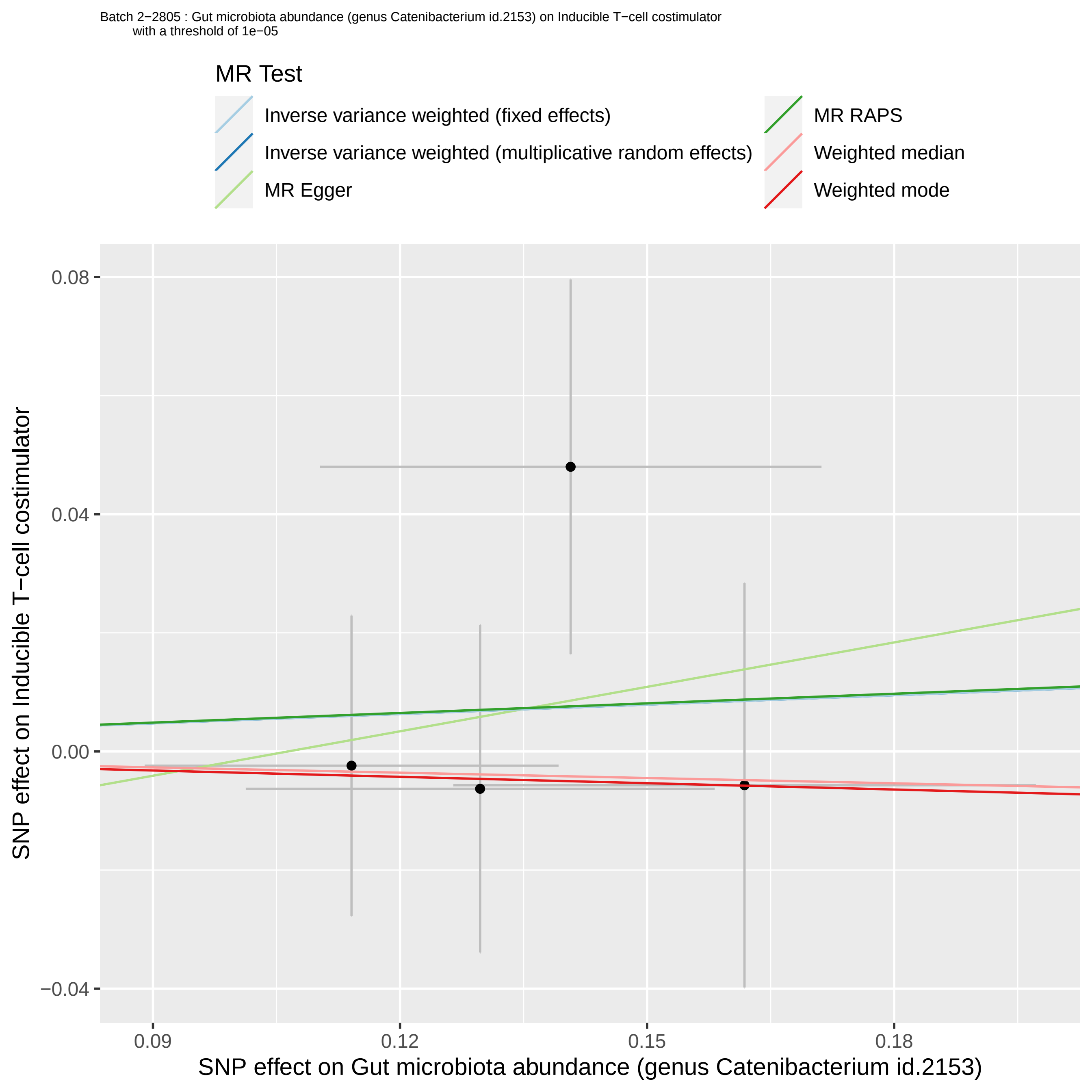
Batch 2−2805 : Gut microbiota abundance (genus Catenibacterium id.2153) on Inducible T−cell costimulator
with a threshold of 1e−05
MR Test
Inverse variance weighted (fixed effects)
Inverse variance weighted (multiplicative random effects)
MR Egger
MR RAPS
Weighted median
Weighted mode
0.08
0.04
0.00
−0.04
0.09
0.12
0.15
0.18
SNP effect on Inducible T−cell costimulator
SNP effect on Gut microbiota abundance (genus Catenibacterium id.2153)

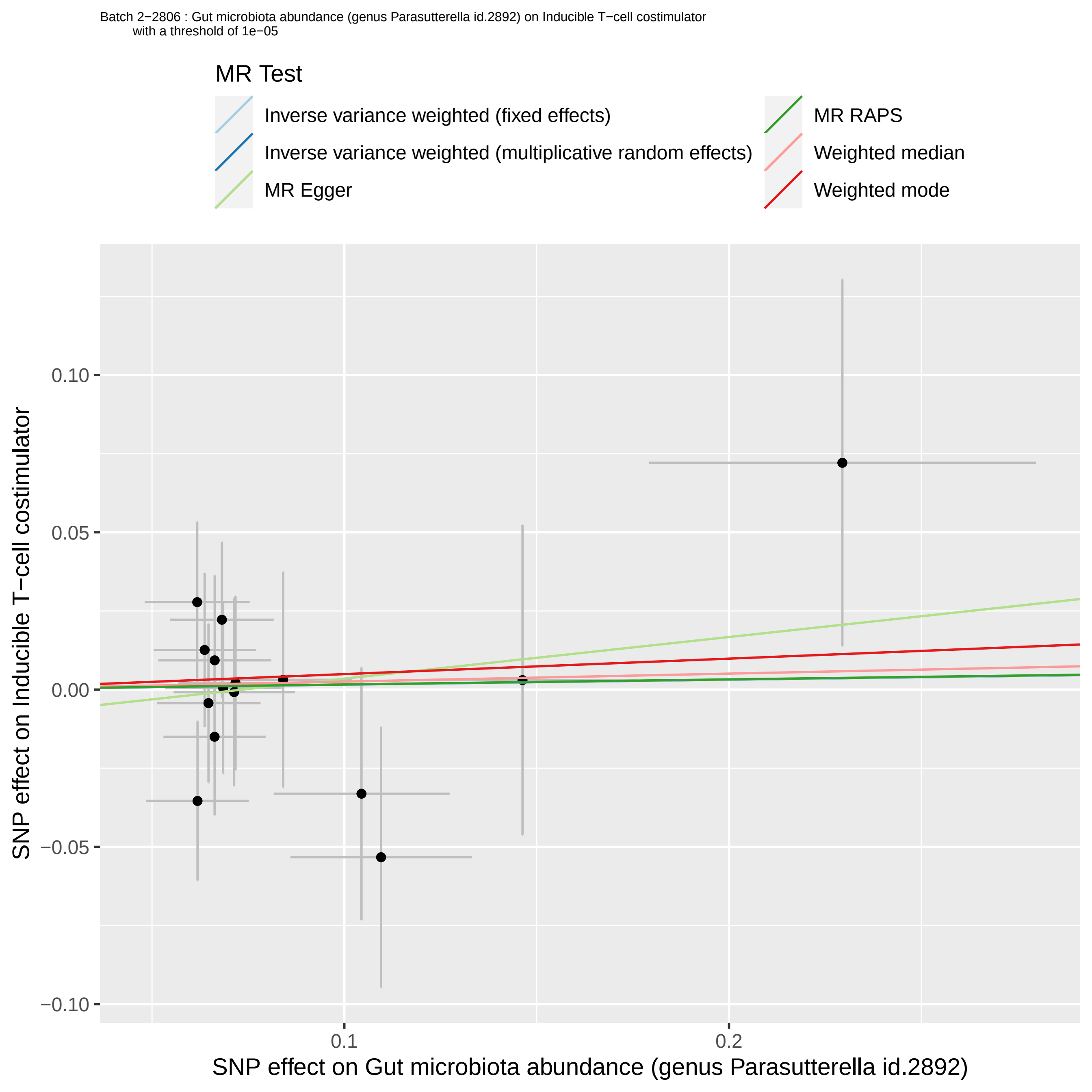
Batch 2−2806 : Gut microbiota abundance (genus Parasutterella id.2892) on Inducible T−cell costimulator
with a threshold of 1e−05
MR Test
Inverse variance weighted (fixed effects)
Inverse variance weighted (multiplicative random effects)
MR Egger
MR RAPS
Weighted median
Weighted mode
0.10
0.05
0.00
−0.05
−0.10
0.1
0.2
SNP effect on Inducible T−cell costimulator
SNP effect on Gut microbiota abundance (genus Parasutterella id.2892)

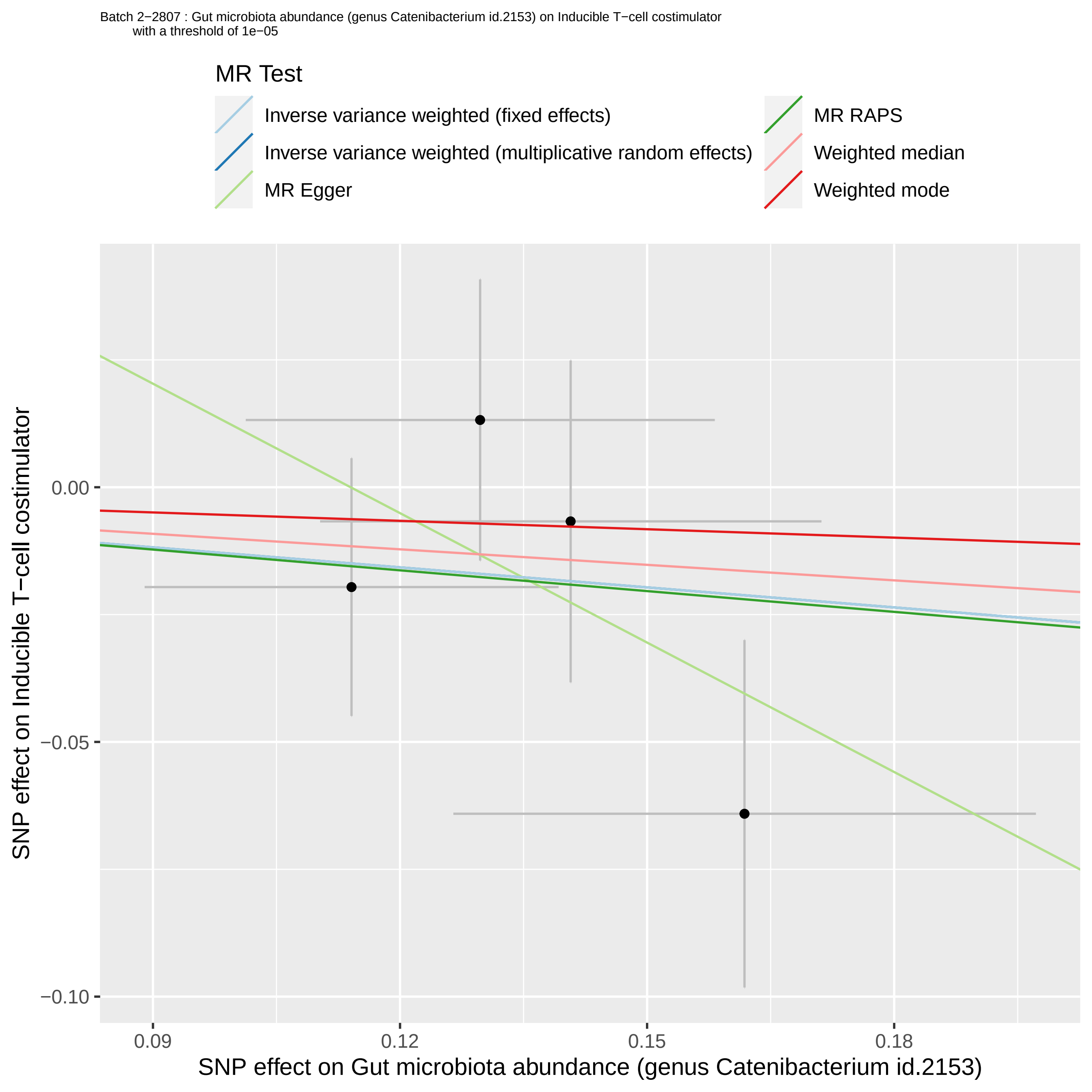
Batch 2−2807 : Gut microbiota abundance (genus Catenibacterium id.2153) on Inducible T−cell costimulator
with a threshold of 1e−05
MR Test
Inverse variance weighted (fixed effects)
Inverse variance weighted (multiplicative random effects)
MR Egger
MR RAPS
Weighted median
Weighted mode
0.00
−0.05
−0.10
0.09
0.12
0.15
0.18
SNP effect on Inducible T−cell costimulator
SNP effect on Gut microbiota abundance (genus Catenibacterium id.2153)

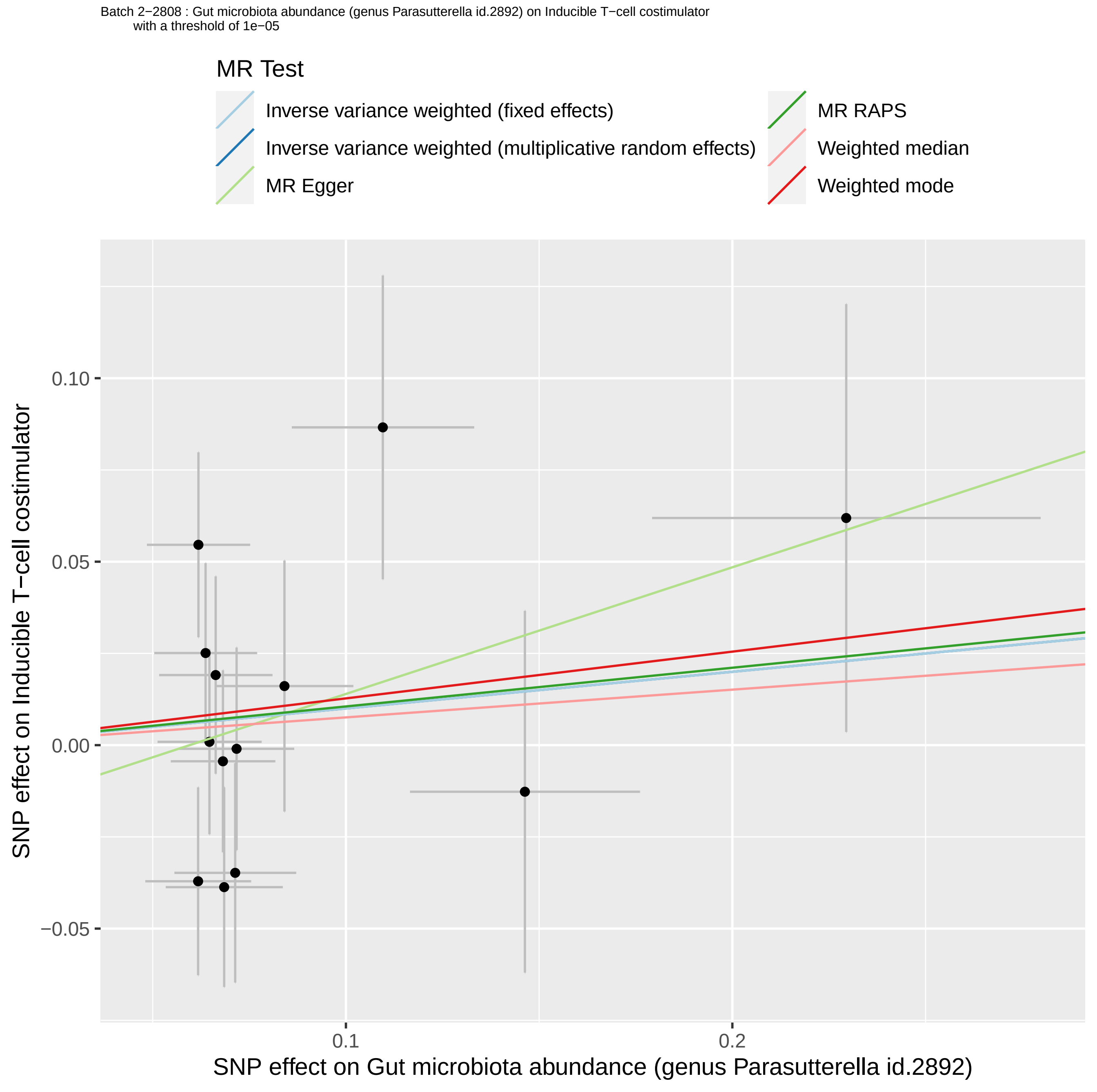

Batch 2−2808 : Gut microbiota abundance (genus Parasutterella id.2892) on Inducible T−cell costimulator
with a threshold of 1e−05
MR Test
Inverse variance weighted (fixed effects)
Inverse variance weighted (multiplicative random effects)
MR Egger
MR RAPS
Weighted median
Weighted mode
0.10
0.05
0.00
−0.05
0.1
0.2
SNP effect on Inducible T−cell costimulator
SNP effect on Gut microbiota abundance (genus Parasutterella id.2892)

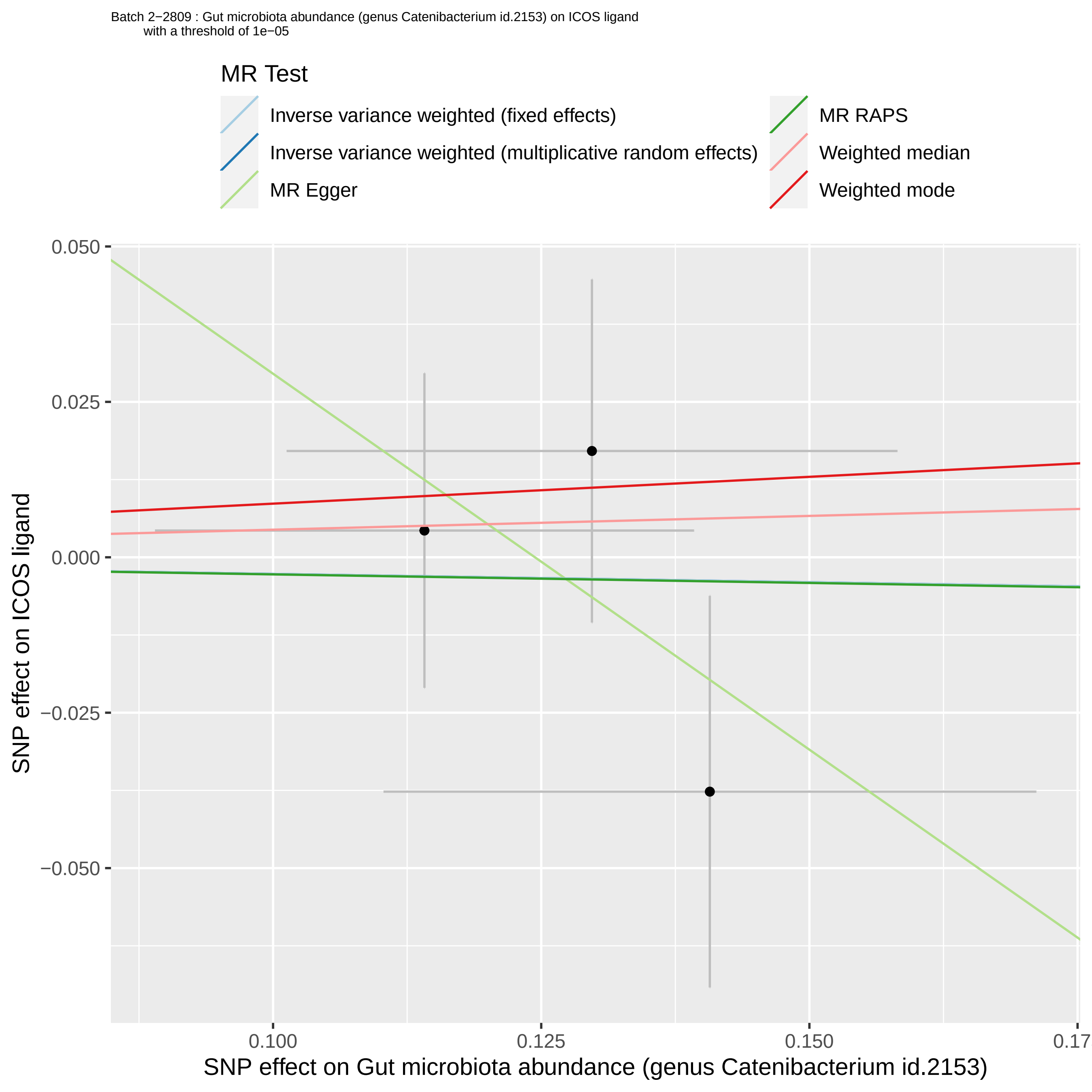
Batch 2−2809 : Gut microbiota abundance (genus Catenibacterium id.2153) on ICOS ligand
with a threshold of 1e−05
MR Test
Inverse variance weighted (fixed effects)
Inverse variance weighted (multiplicative random effects)
MR Egger
MR RAPS
Weighted median
Weighted mode
0.050
0.025
0.000
−0.025
−0.050
SNP effect on ICOS ligand
0.100
0.125
0.150
0.17
SNP effect on Gut microbiota abundance (genus Catenibacterium id.2153)

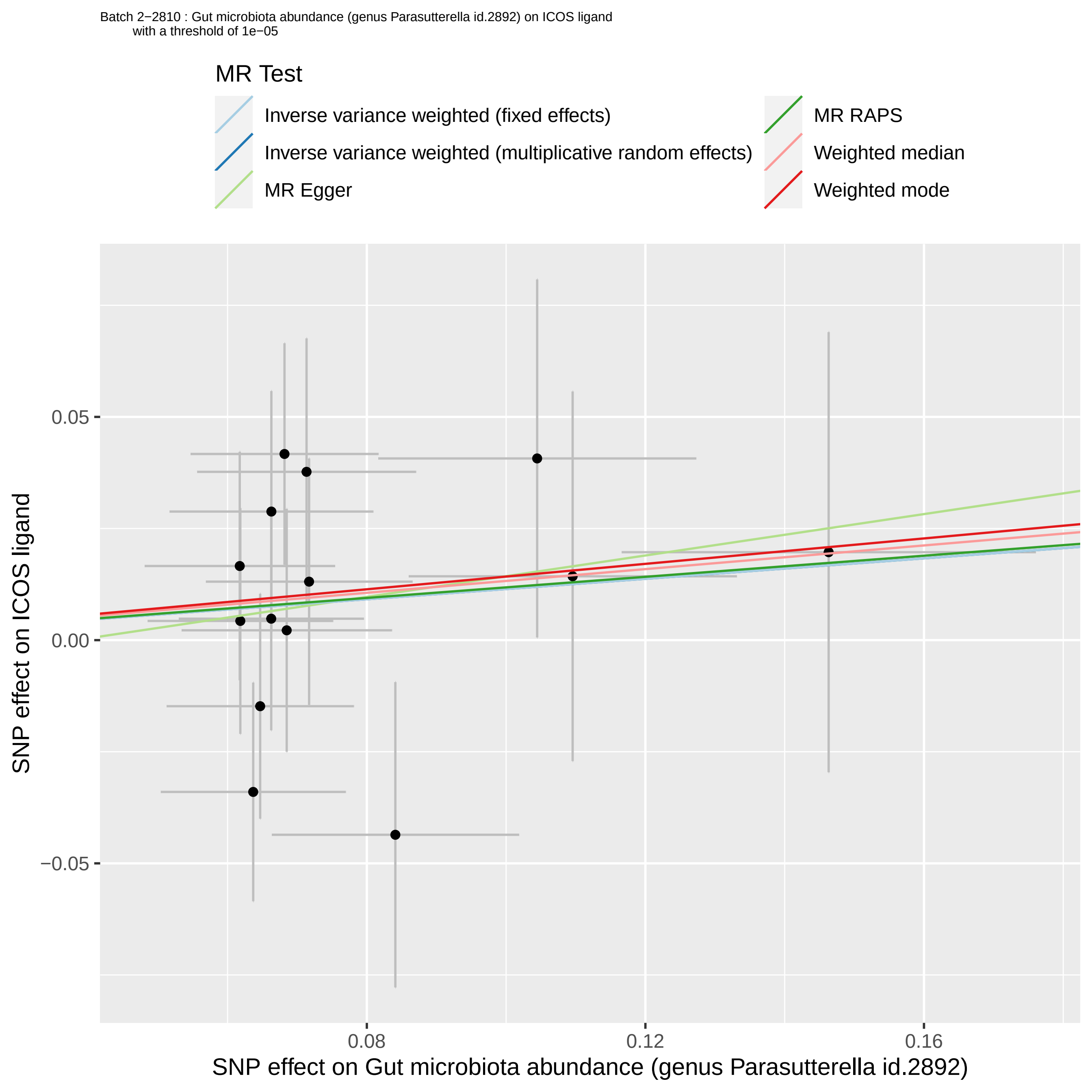
Batch 2−2810 : Gut microbiota abundance (genus Parasutterella id.2892) on ICOS ligand
with a threshold of 1e−05
MR Test
Inverse variance weighted (fixed effects)
Inverse variance weighted (multiplicative random effects)
MR Egger
MR RAPS
Weighted median
Weighted mode
0.05
0.00
−0.05
0.08
0.12
0.16
SNP effect on ICOS ligand
SNP effect on Gut microbiota abundance (genus Parasutterella id.2892)

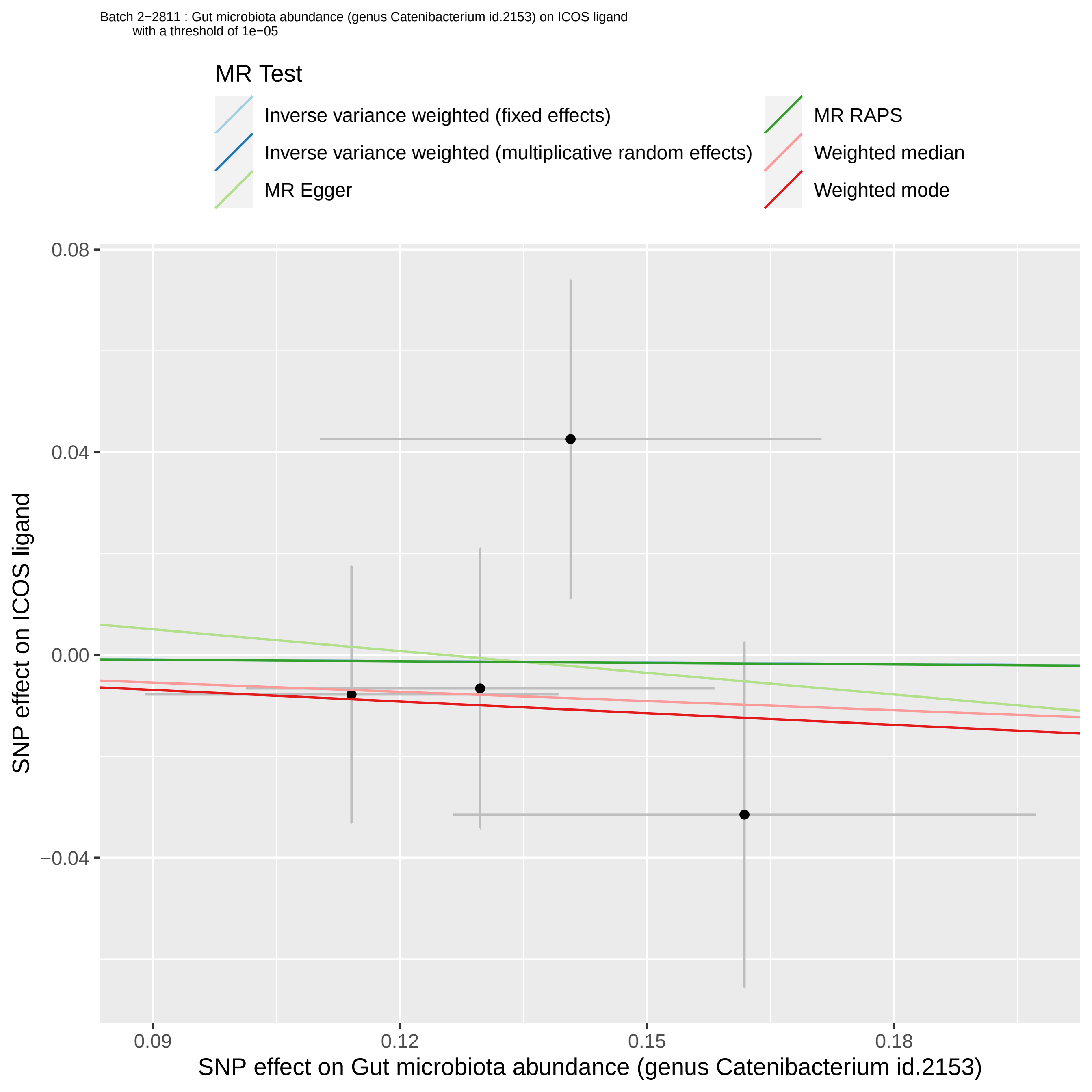
Batch 2−2811 : Gut microbiota abundance (genus Catenibacterium id.2153) on ICOS ligand
with a threshold of 1e−05
MR Test
Inverse variance weighted (fixed effects)
Inverse variance weighted (multiplicative random effects)
MR Egger
MR RAPS
Weighted median
Weighted mode
0.08
0.04
0.00
−0.04
0.09
0.12
0.15
0.18
SNP effect on ICOS ligand
SNP effect on Gut microbiota abundance (genus Catenibacterium id.2153)

Batch 2−2812 : Gut microbiota abundance (genus Parasutterella id.2892) on ICOS ligand
with a threshold of 1e−05

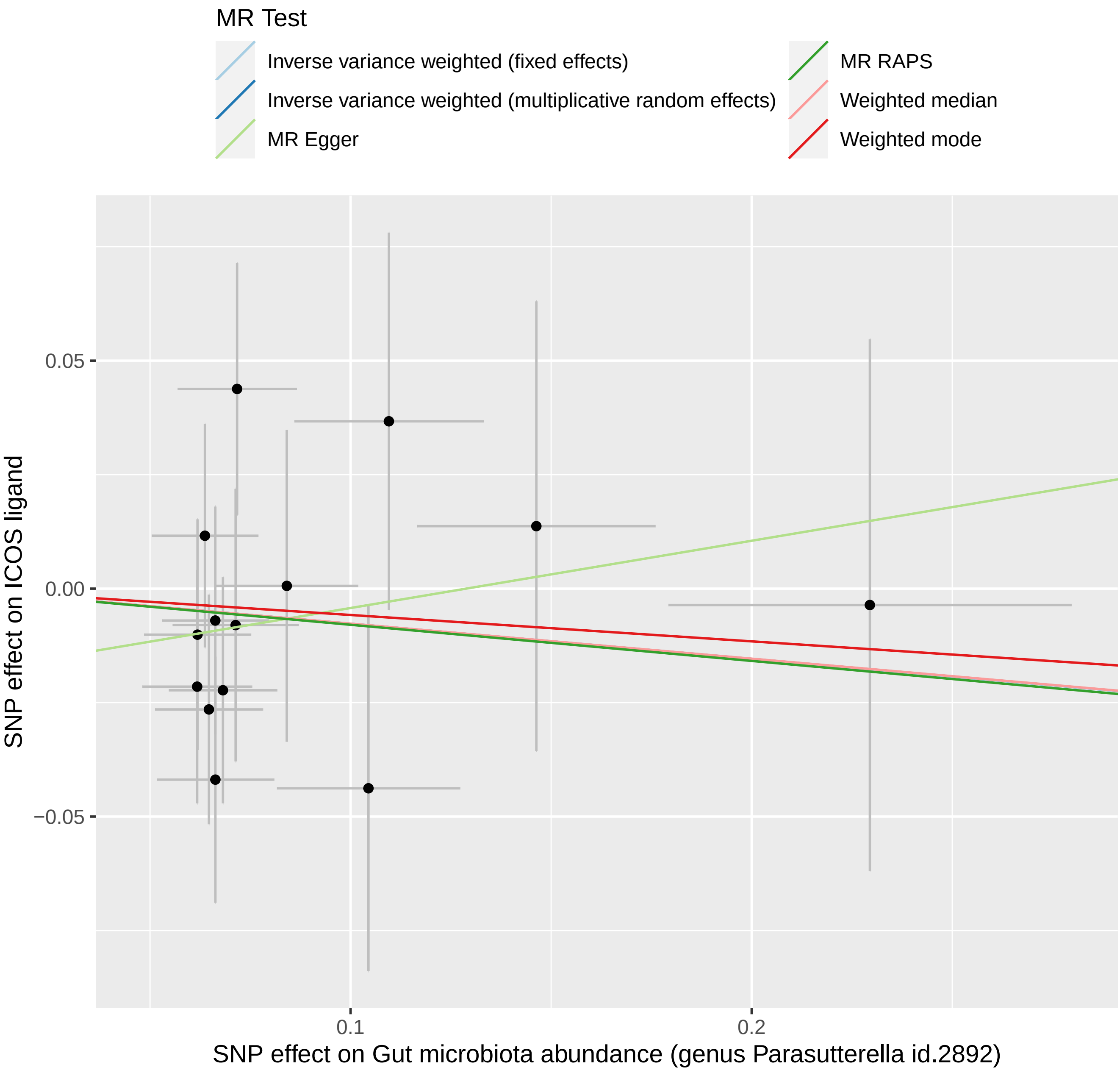

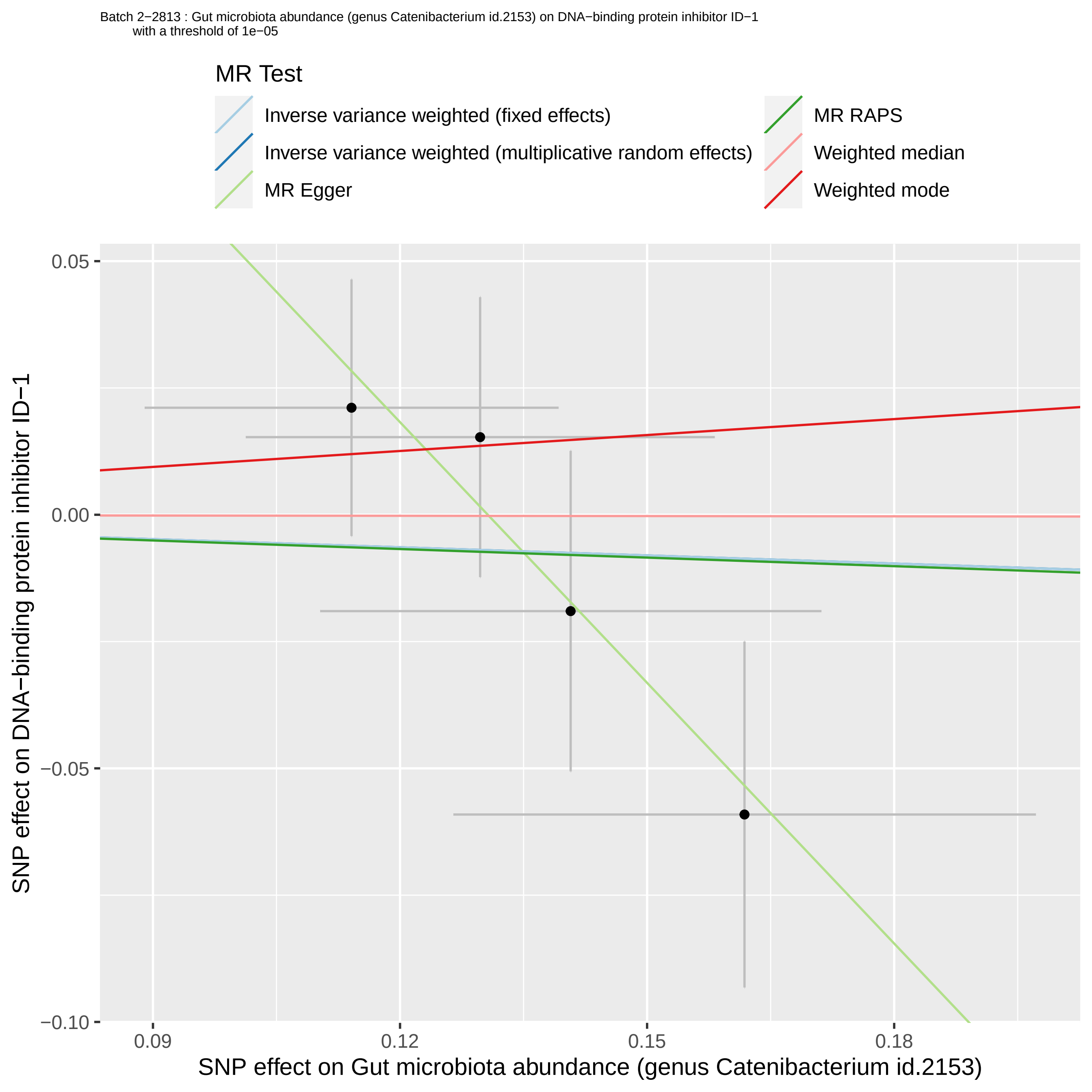
Batch 2−2813 : Gut microbiota abundance (genus Catenibacterium id.2153) on DNA−binding protein inhibitor ID−1
with a threshold of 1e−05
MR Test
Inverse variance weighted (fixed effects)
Inverse variance weighted (multiplicative random effects)
MR Egger
MR RAPS
Weighted median
Weighted mode
0.05
0.00
−0.05
−0.10
0.09
0.12
0.15
0.18
SNP effect on DNA−binding protein inhibitor ID−1
SNP effect on Gut microbiota abundance (genus Catenibacterium id.2153)

Batch 2−2814 : Gut microbiota abundance (genus Parasutterella id.2892) on DNA−binding protein inhibitor ID−1
with a threshold of 1e−05

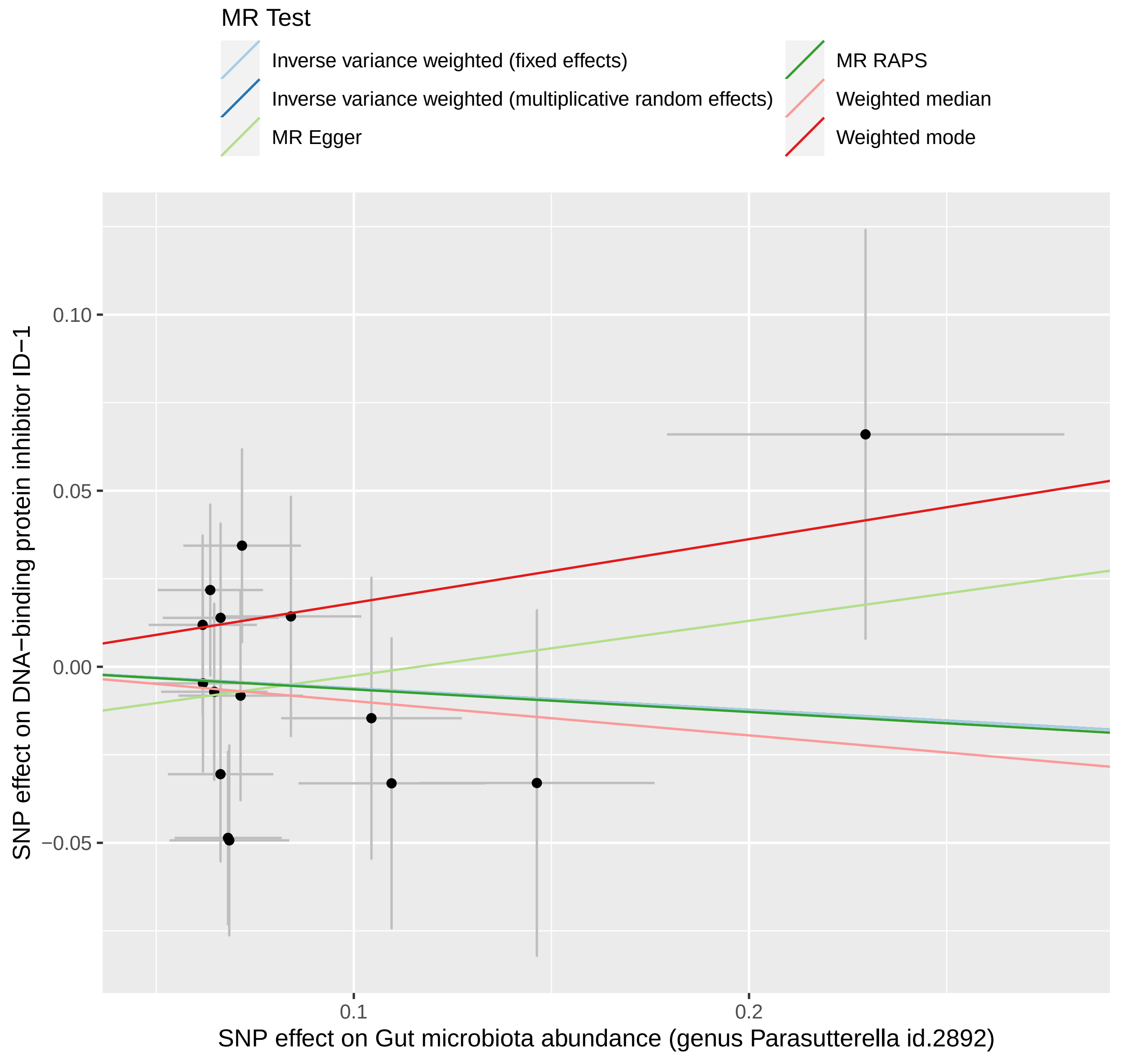

Batch 2−2815 : Gut microbiota abundance (genus Catenibacterium id.2153) on DNA−binding protein inhibitor ID−2
with a threshold of 1e−05

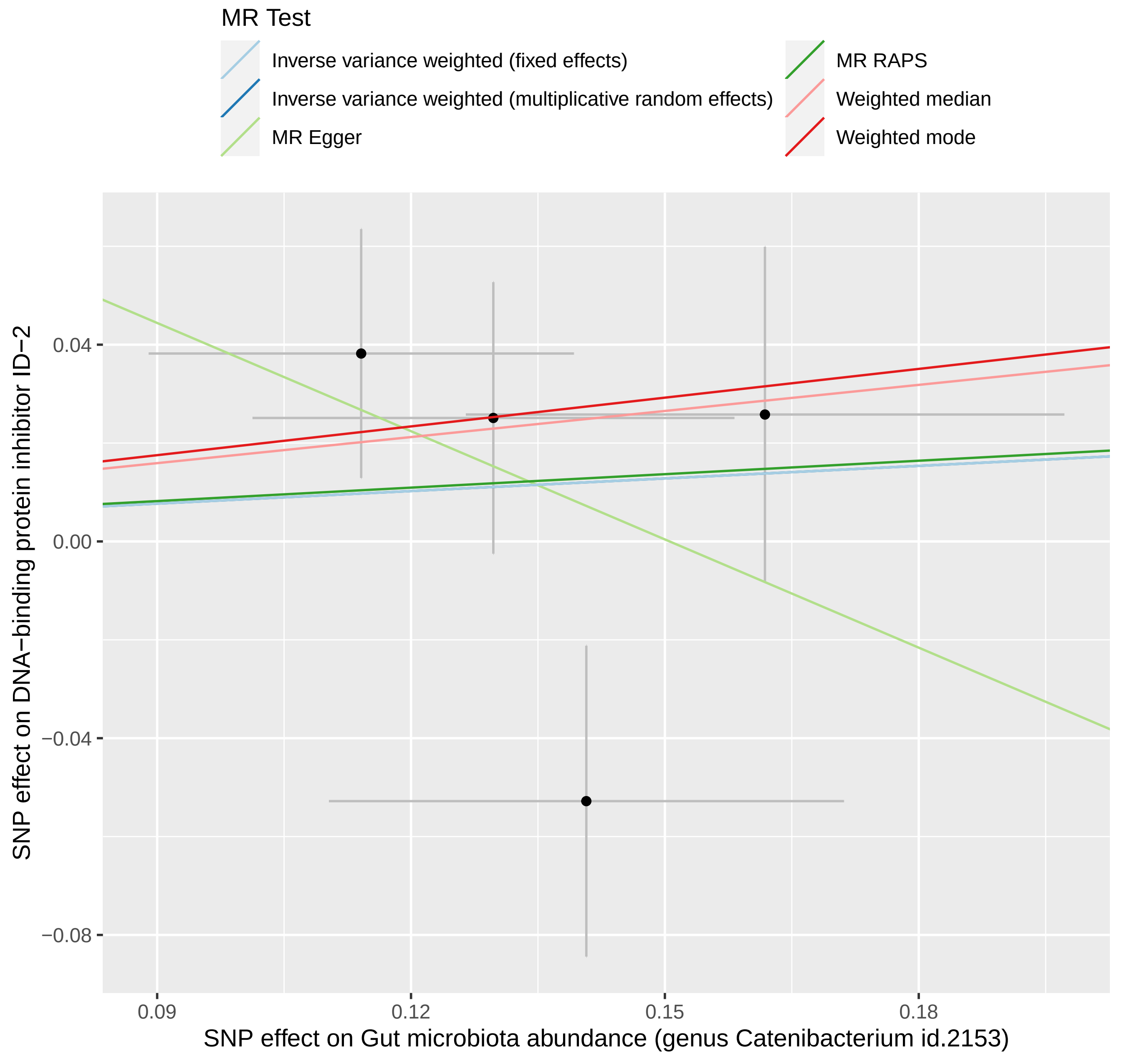

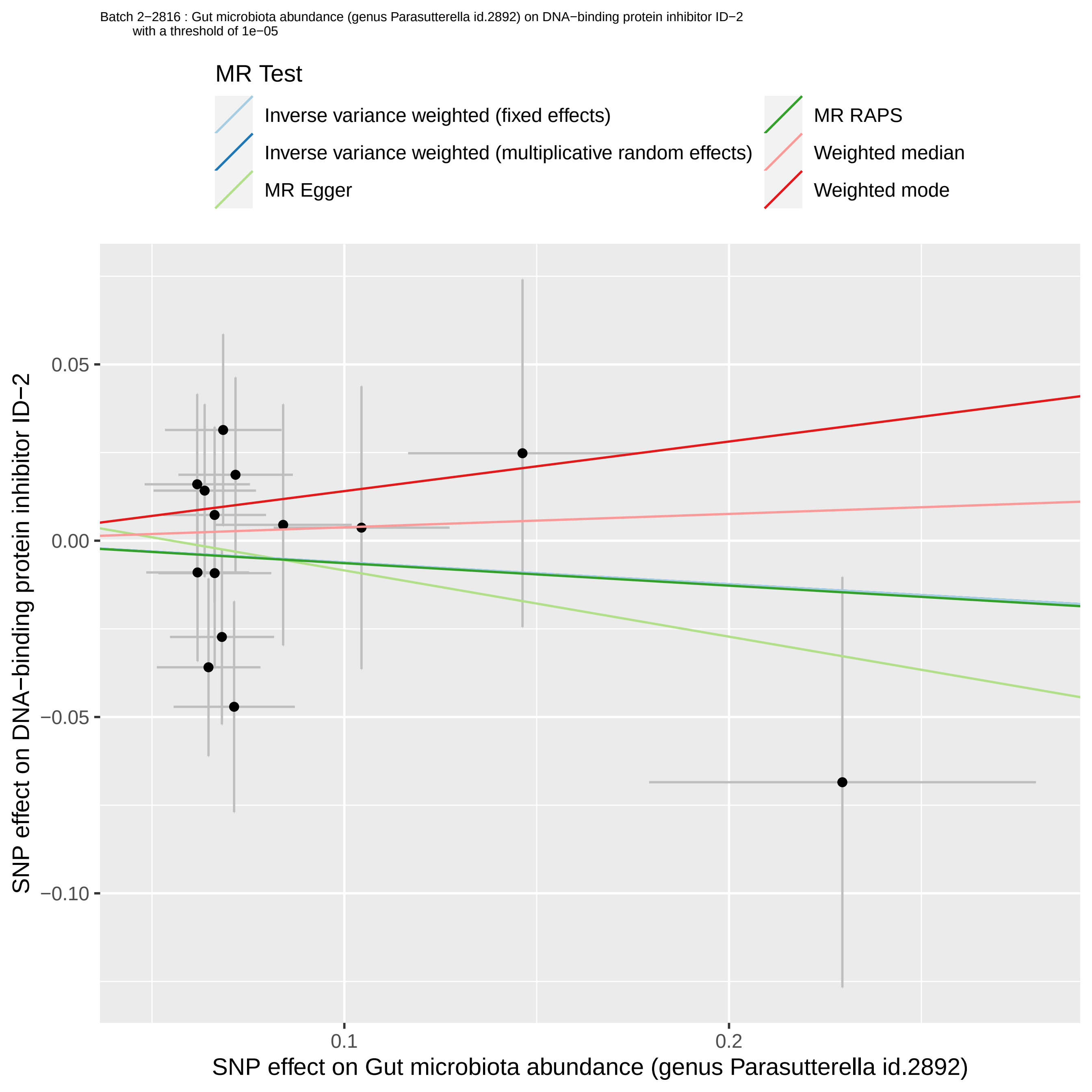
Batch 2−2816 : Gut microbiota abundance (genus Parasutterella id.2892) on DNA−binding protein inhibitor ID−2
with a threshold of 1e−05
MR Test
Inverse variance weighted (fixed effects)
Inverse variance weighted (multiplicative random effects)
MR Egger
MR RAPS
Weighted median
Weighted mode
0.05
0.00
−0.05
−0.10
0.1
0.2
SNP effect on DNA−binding protein inhibitor ID−2
SNP effect on Gut microbiota abundance (genus Parasutterella id.2892)

Batch 2−2817 : Gut microbiota abundance (genus Catenibacterium id.2153) on Insulin−degrading enzyme
with a threshold of 1e−05

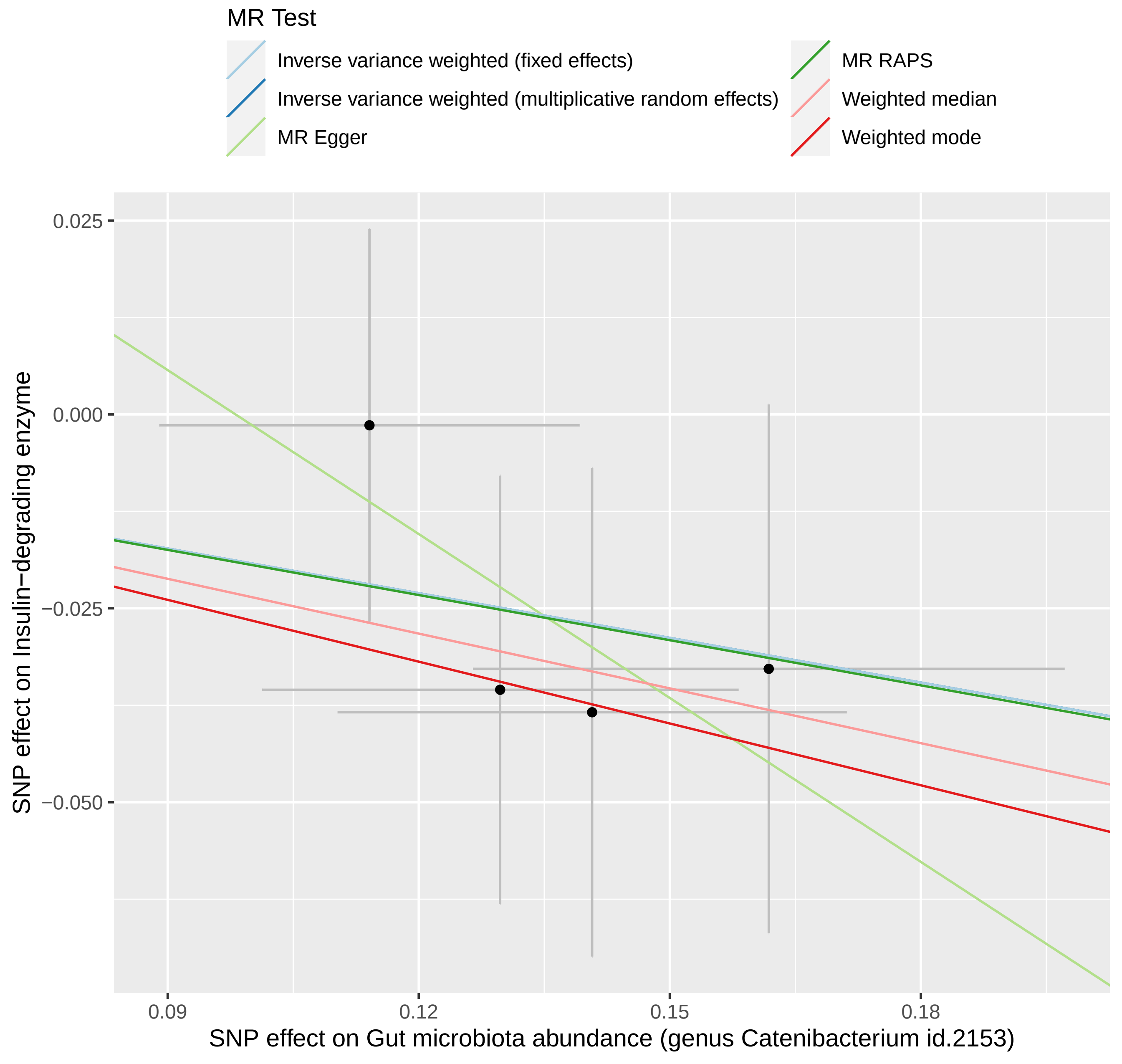

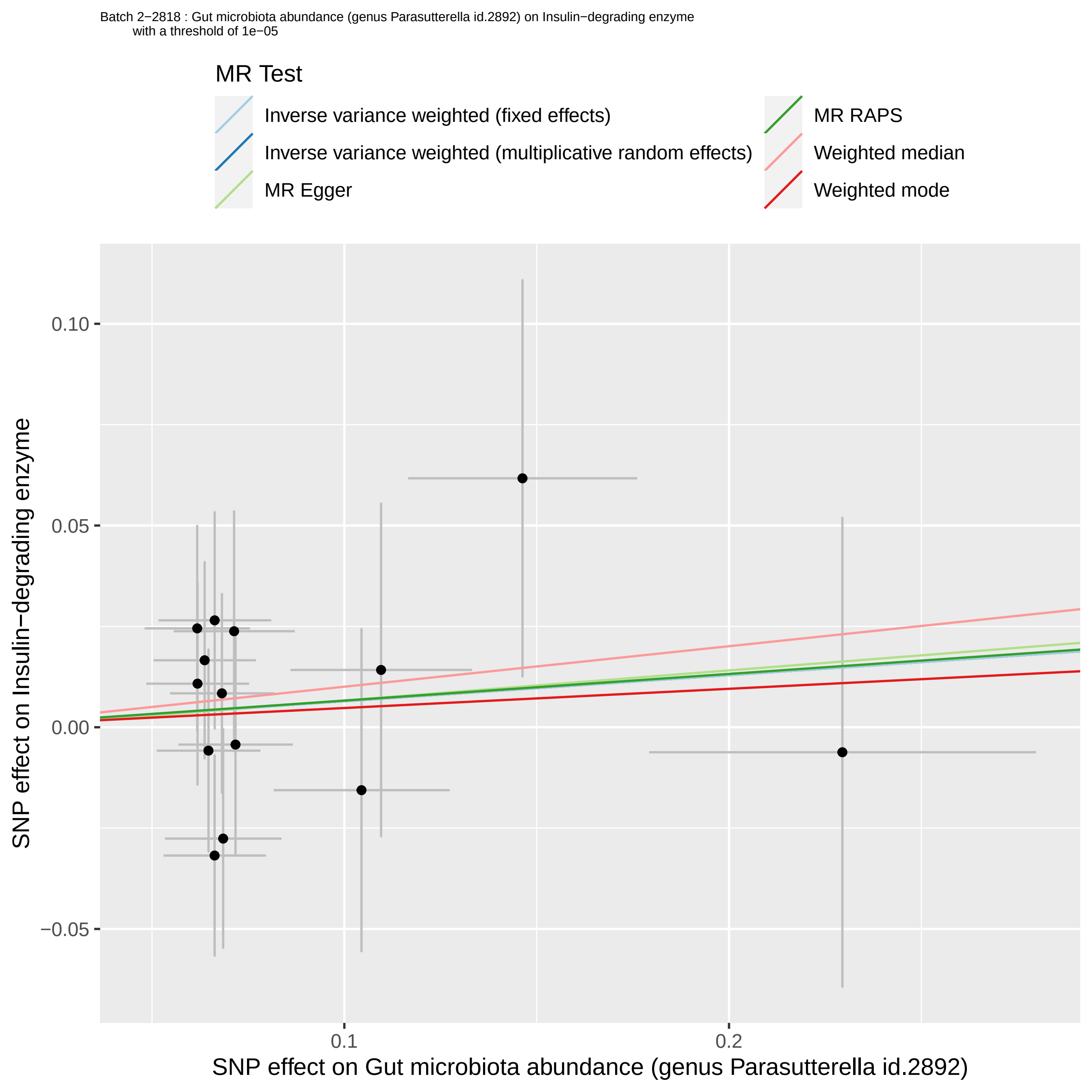
Batch 2−2818 : Gut microbiota abundance (genus Parasutterella id.2892) on Insulin−degrading enzyme
with a threshold of 1e−05
MR Test
Inverse variance weighted (fixed effects)
Inverse variance weighted (multiplicative random effects)
MR Egger
MR RAPS
Weighted median
Weighted mode
0.10
0.05
0.00
−0.05
0.1
0.2
SNP effect on Insulin−degrading enzyme
SNP effect on Gut microbiota abundance (genus Parasutterella id.2892)

Batch 2−2819 : Gut microbiota abundance (genus Catenibacterium id.2153) on Indoleamine 2,3−dioxygenase 1
with a threshold of 1e−05

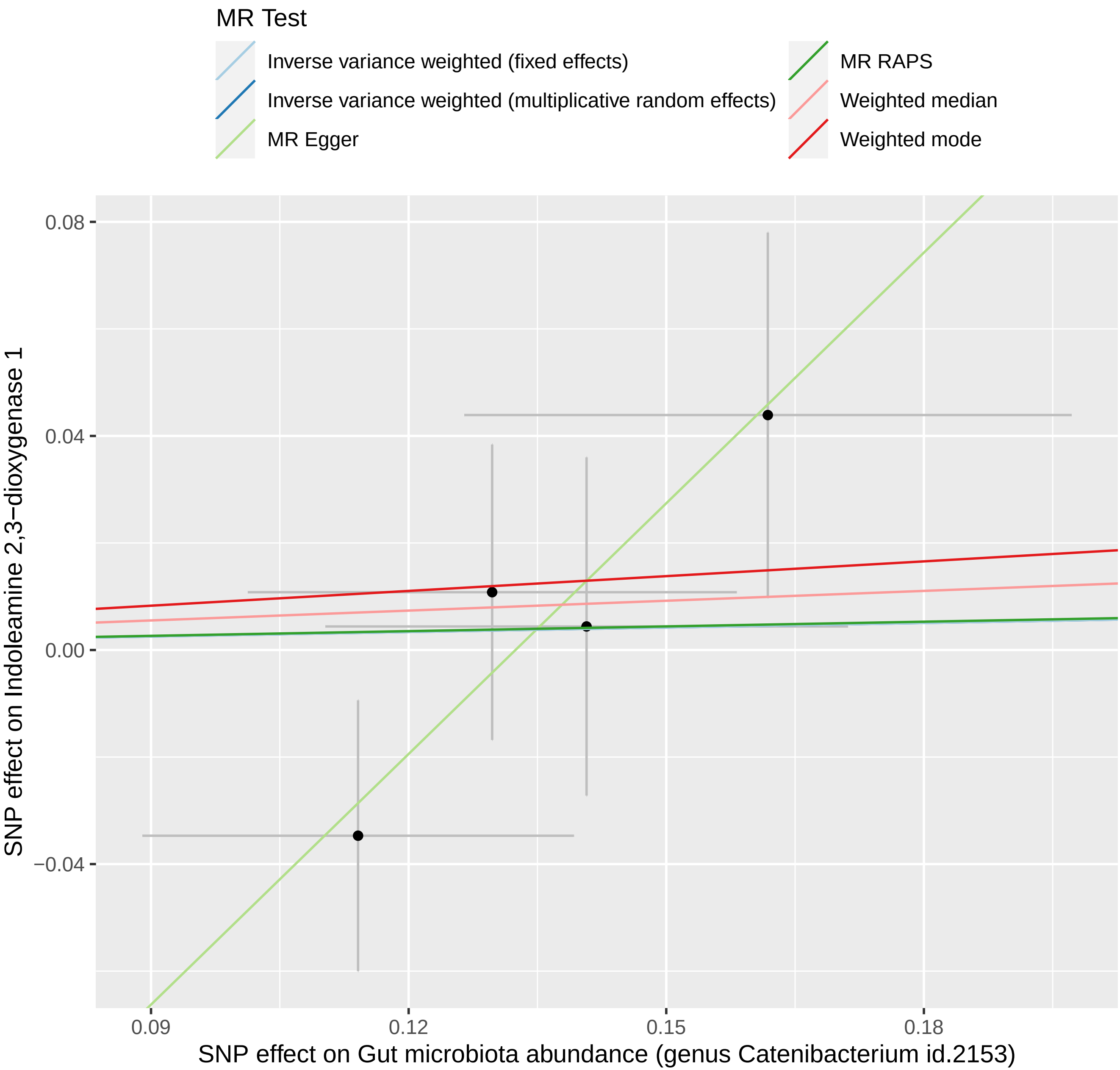

Batch 2−2820 : Gut microbiota abundance (genus Parasutterella id.2892) on Indoleamine 2,3−dioxygenase 1
with a threshold of 1e−05

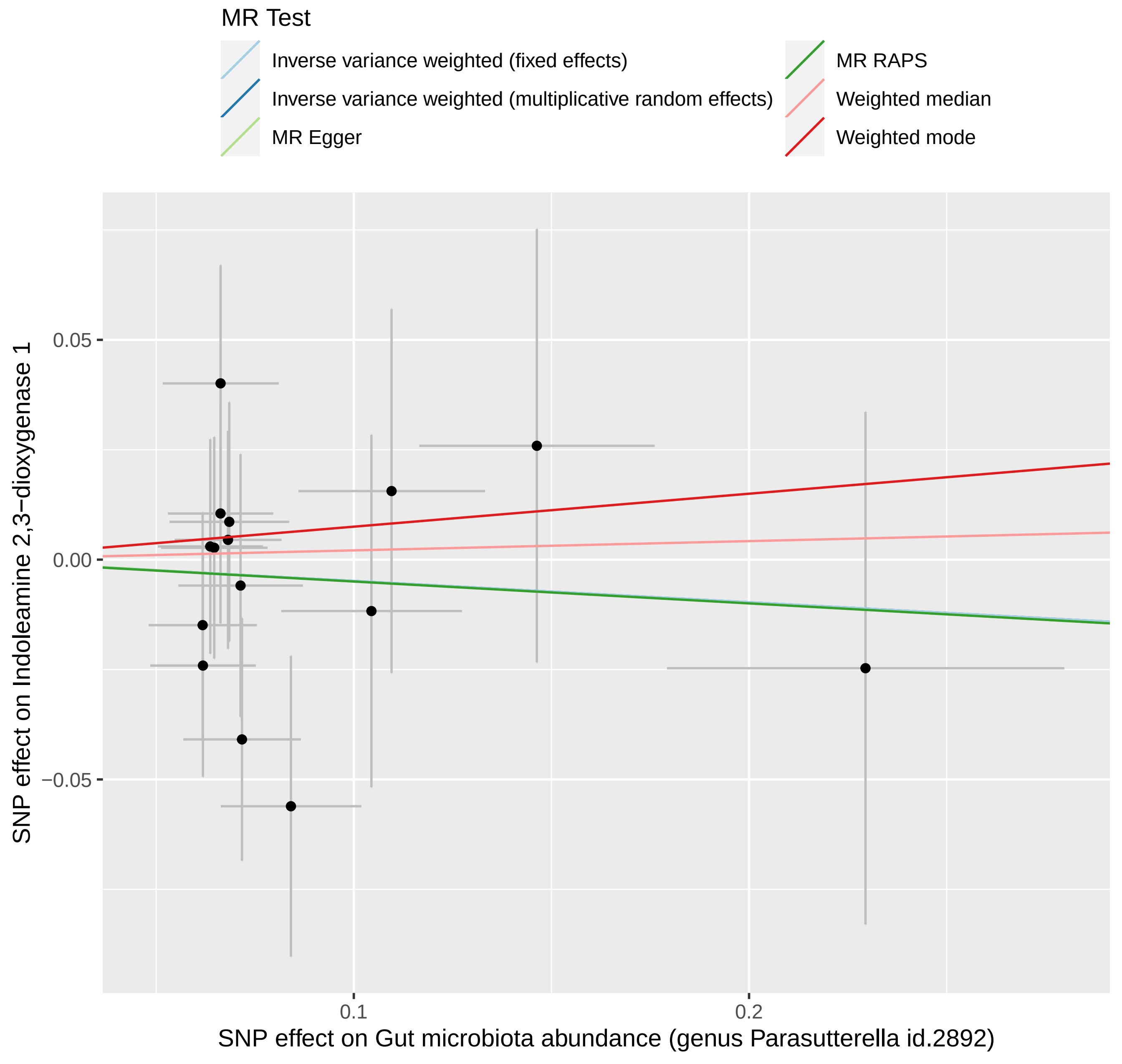

Batch 2−2821 : Gut microbiota abundance (genus Catenibacterium id.2153) on Alpha−L−iduronidase
with a threshold of 1e−05

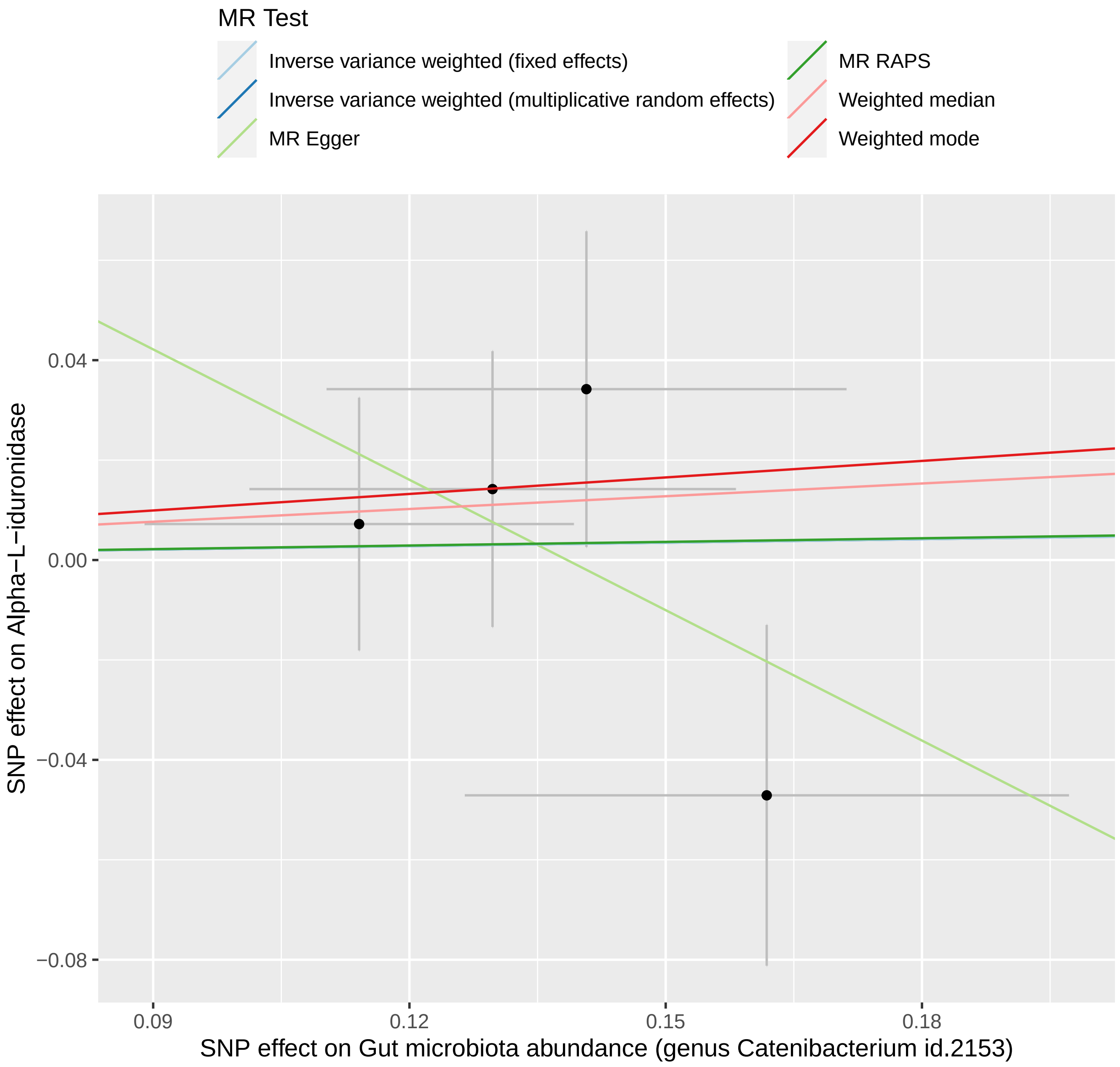

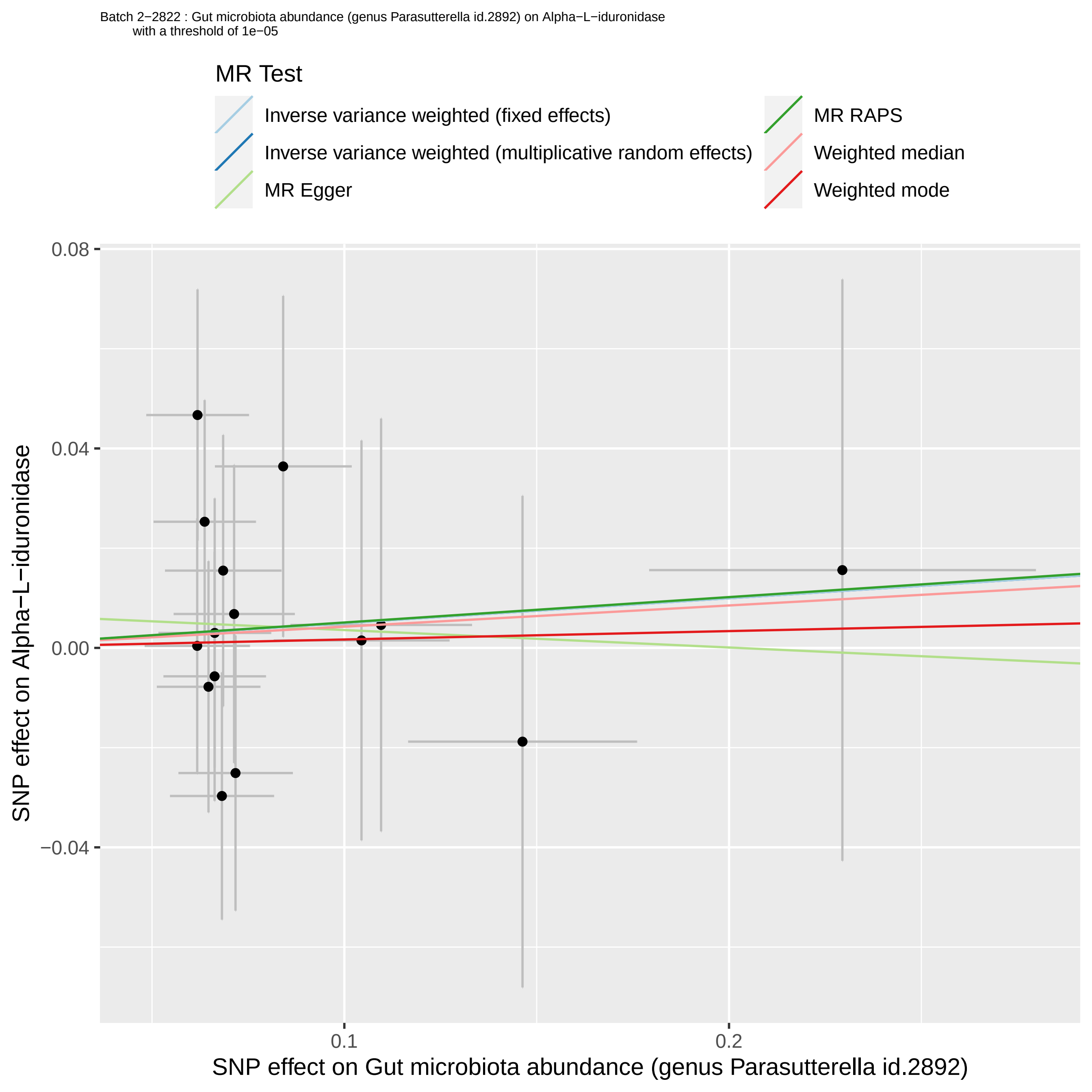
Batch 2−2822 : Gut microbiota abundance (genus Parasutterella id.2892) on Alpha−L−iduronidase
with a threshold of 1e−05
MR Test
Inverse variance weighted (fixed effects)
Inverse variance weighted (multiplicative random effects)
MR Egger
MR RAPS
Weighted median
Weighted mode
SNP effect on Alpha−L−iduronidase
0.08
0.04
0.00
−0.04
0.1
0.2
SNP effect on Gut microbiota abundance (genus Parasutterella id.2892)

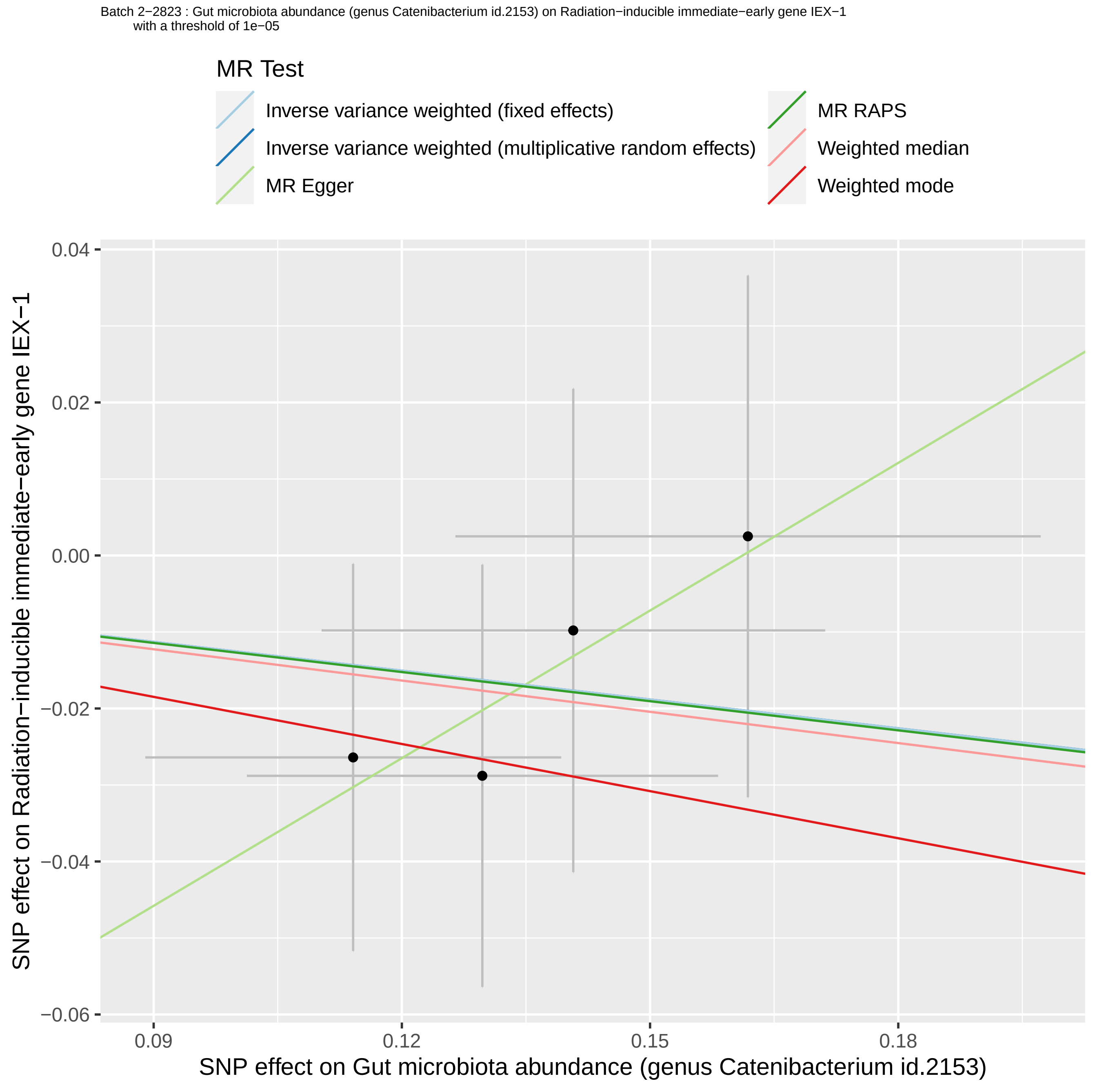
Batch 2−2823 : Gut microbiota abundance (genus Catenibacterium id.2153) on Radiation−inducible immediate−early gene IEX−1
with a threshold of 1e−05
MR Test
Inverse variance weighted (fixed effects)
Inverse variance weighted (multiplicative random effects)
MR Egger
MR RAPS
Weighted median
Weighted mode
0.04
0.02
0.00
−0.02
−0.04
−0.06
0.09
0.12
0.15
0.18
SNP effect on Radiation−inducible immediate−early gene IEX−1
SNP effect on Gut microbiota abundance (genus Catenibacterium id.2153)

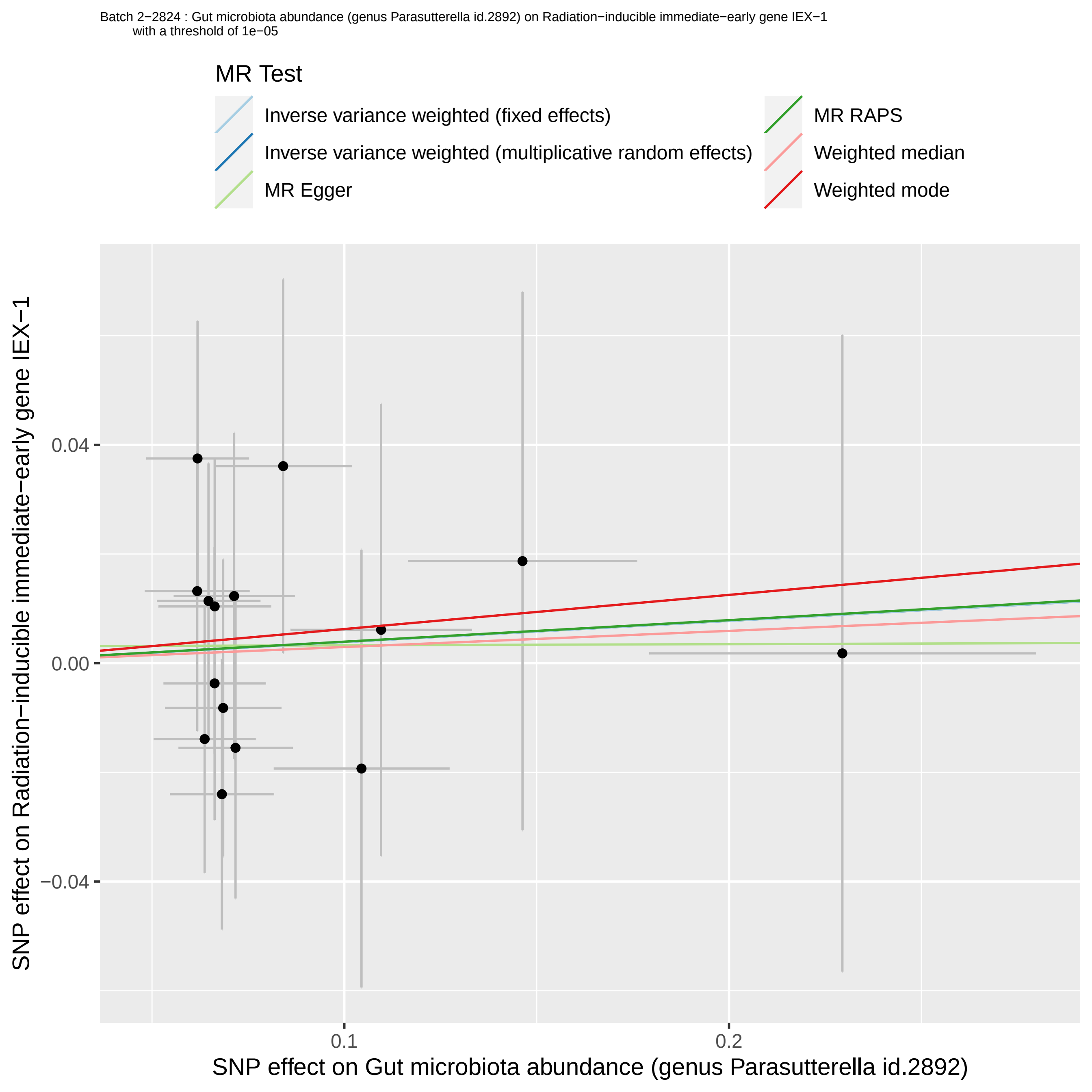
Batch 2−2824 : Gut microbiota abundance (genus Parasutterella id.2892) on Radiation−inducible immediate−early gene IEX−1
with a threshold of 1e−05
MR Test
Inverse variance weighted (fixed effects)
Inverse variance weighted (multiplicative random effects)
MR Egger
MR RAPS
Weighted median
Weighted mode
0.04
0.00
−0.04
0.1
0.2
SNP effect on Radiation−inducible immediate−early gene IEX−1
SNP effect on Gut microbiota abundance (genus Parasutterella id.2892)

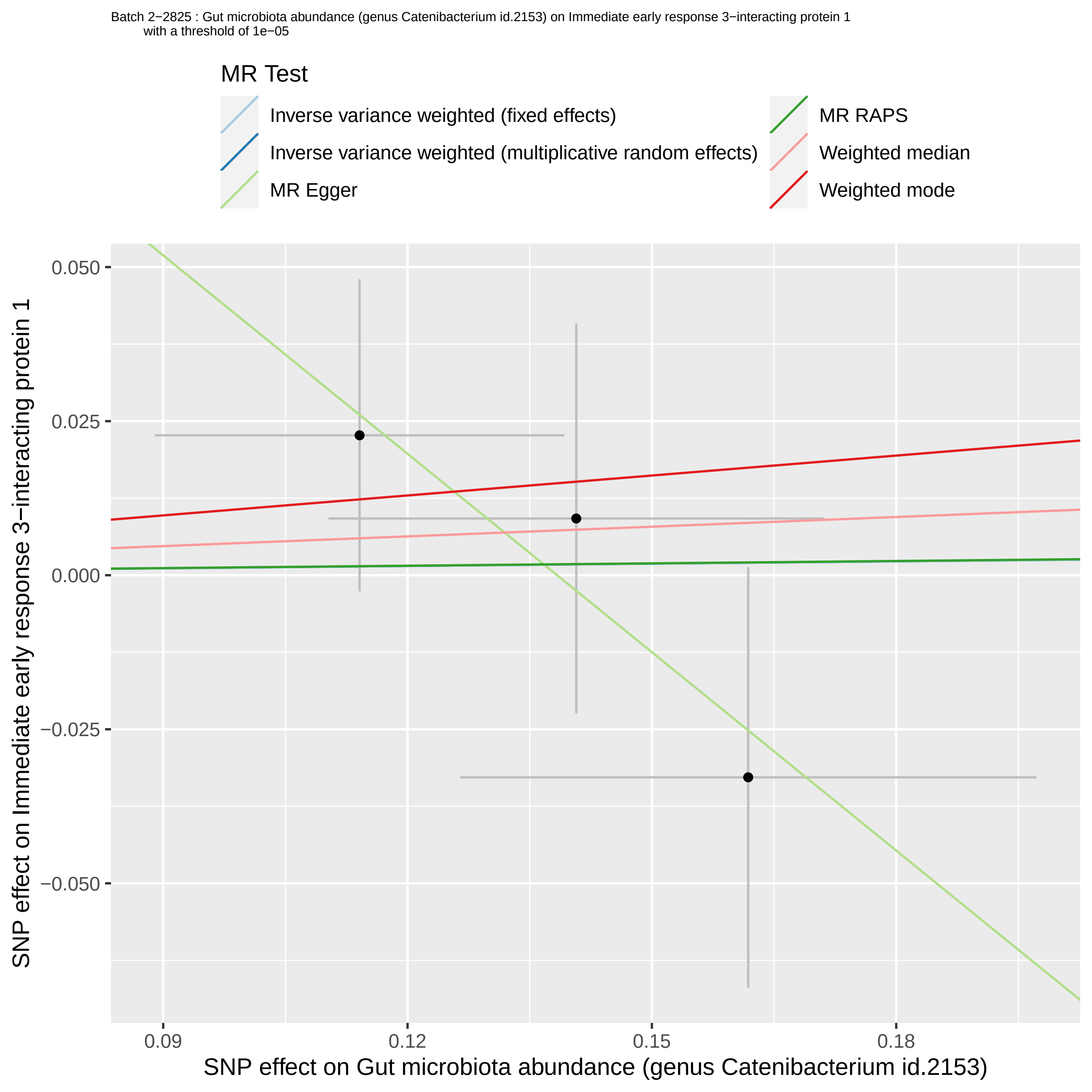

Batch 2−2825 : Gut microbiota abundance (genus Catenibacterium id.2153) on Immediate early response 3−interacting protein 1
with a threshold of 1e−05
MR Test
Inverse variance weighted (fixed effects)
Inverse variance weighted (multiplicative random effects)
MR Egger
MR RAPS
Weighted median
Weighted mode
0.050
0.025
0.000
−0.025
−0.050
0.09
0.12
0.15
0.18
SNP effect on Immediate early response 3−interacting protein 1
SNP effect on Gut microbiota abundance (genus Catenibacterium id.2153)

Batch 2−2826 : Gut microbiota abundance (genus Parasutterella id.2892) on Immediate early response 3−interacting protein 1
with a threshold of 1e−05

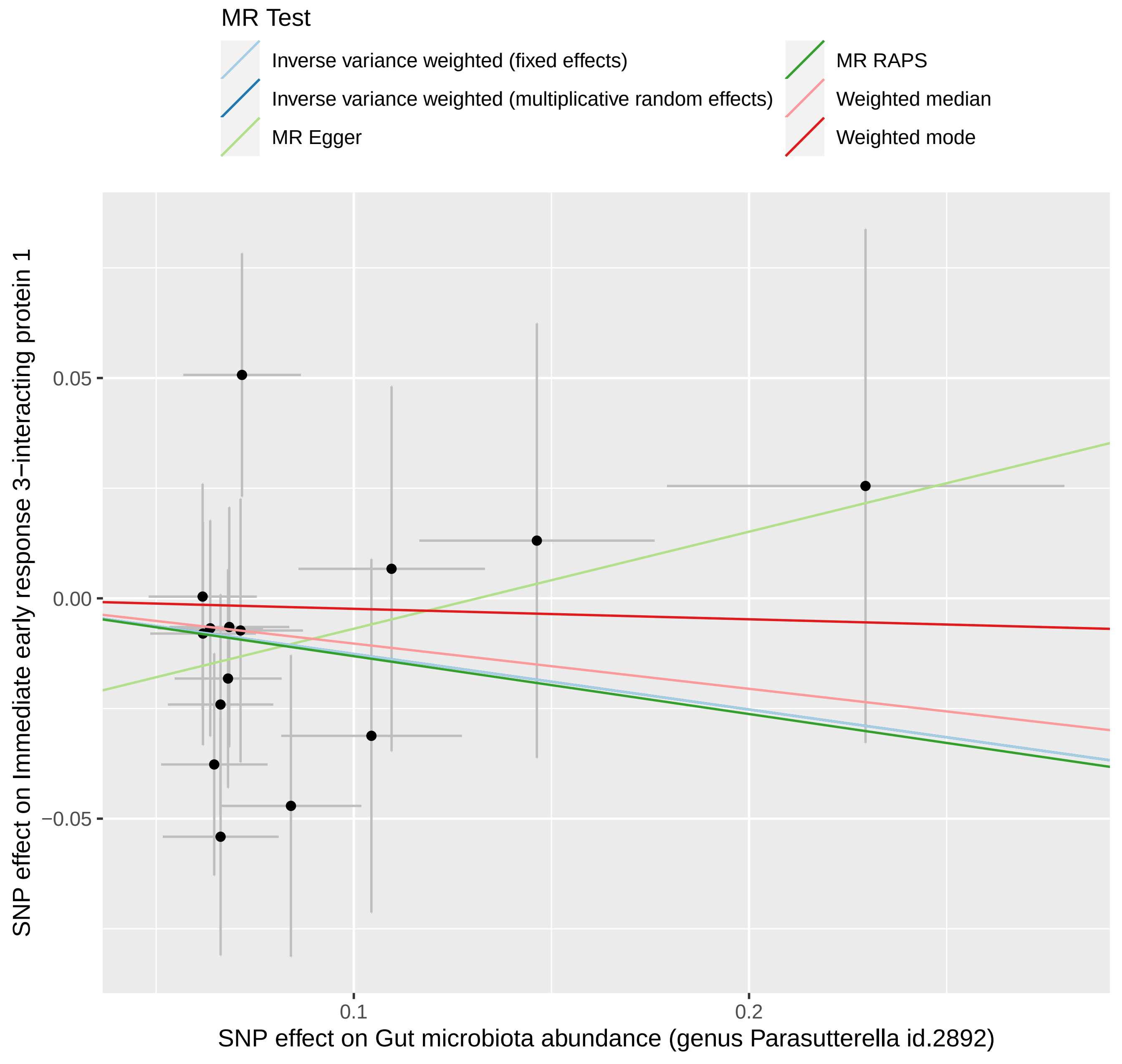

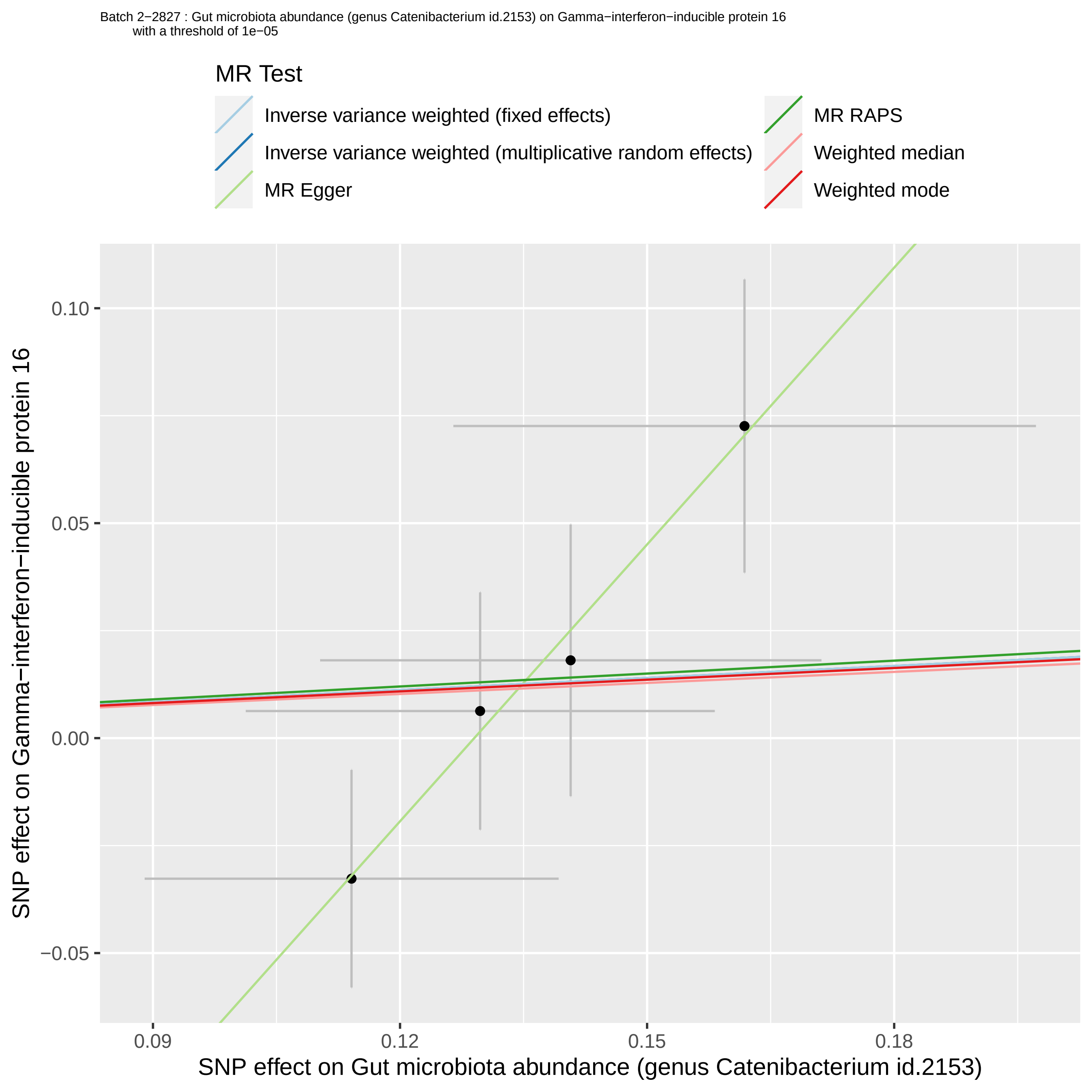
Batch 2−2827 : Gut microbiota abundance (genus Catenibacterium id.2153) on Gamma−interferon−inducible protein 16
with a threshold of 1e−05
MR Test
Inverse variance weighted (fixed effects)
Inverse variance weighted (multiplicative random effects)
MR Egger
MR RAPS
Weighted median
Weighted mode
0.10
0.05
0.00
−0.05
0.09
0.12
0.15
0.18
SNP effect on Gamma−interferon−inducible protein 16
SNP effect on Gut microbiota abundance (genus Catenibacterium id.2153)

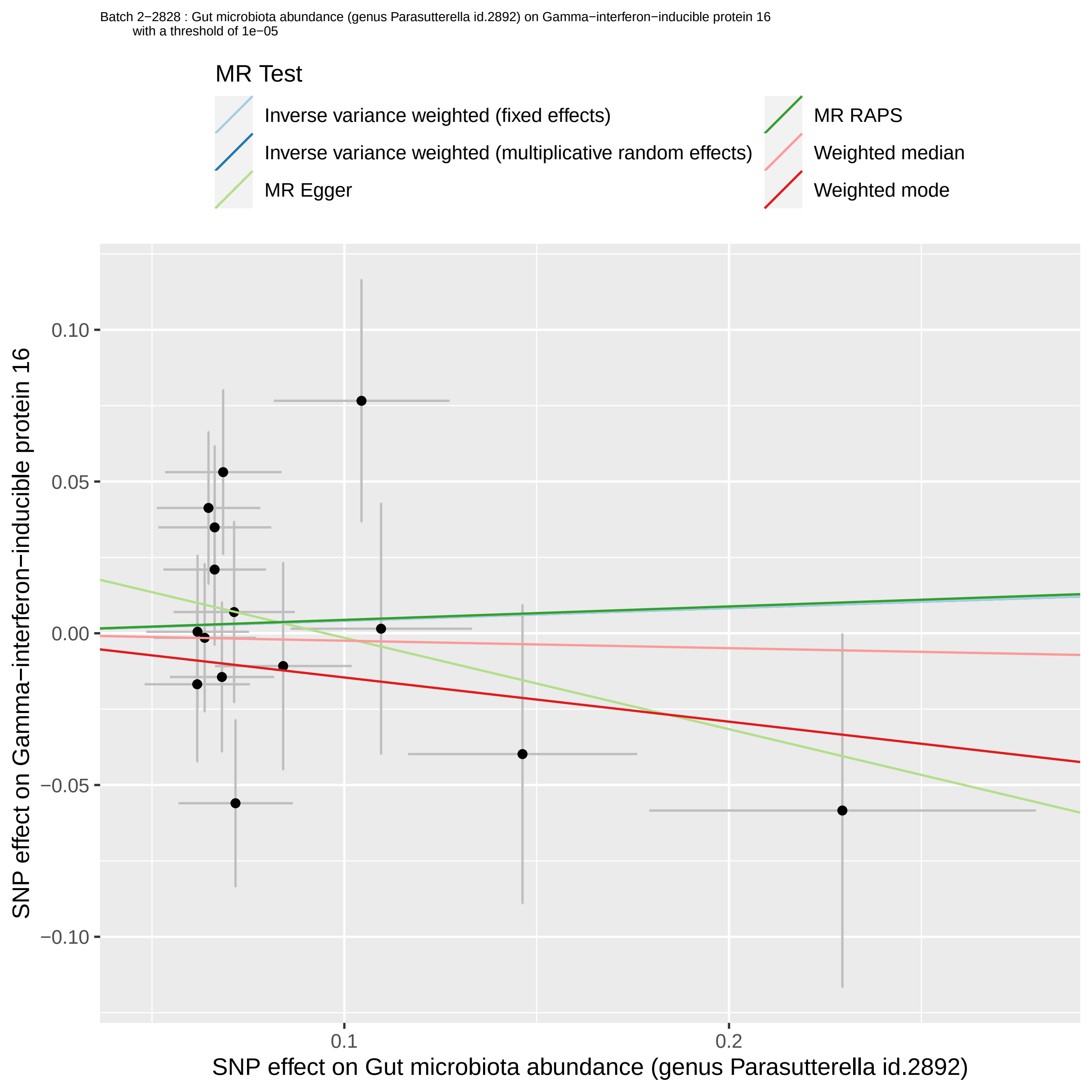

Batch 2−2828 : Gut microbiota abundance (genus Parasutterella id.2892) on Gamma−interferon−inducible protein 16
with a threshold of 1e−05
MR Test
Inverse variance weighted (fixed effects)
Inverse variance weighted (multiplicative random effects)
MR Egger
MR RAPS
Weighted median
Weighted mode
0.10
0.05
0.00
−0.05
−0.10
0.1
0.2
SNP effect on Gamma−interferon−inducible protein 16
SNP effect on Gut microbiota abundance (genus Parasutterella id.2892)

Batch 2−2829 : Gut microbiota abundance (genus Catenibacterium id.2153) on Gamma−interferon−inducible protein 16
with a threshold of 1e−05

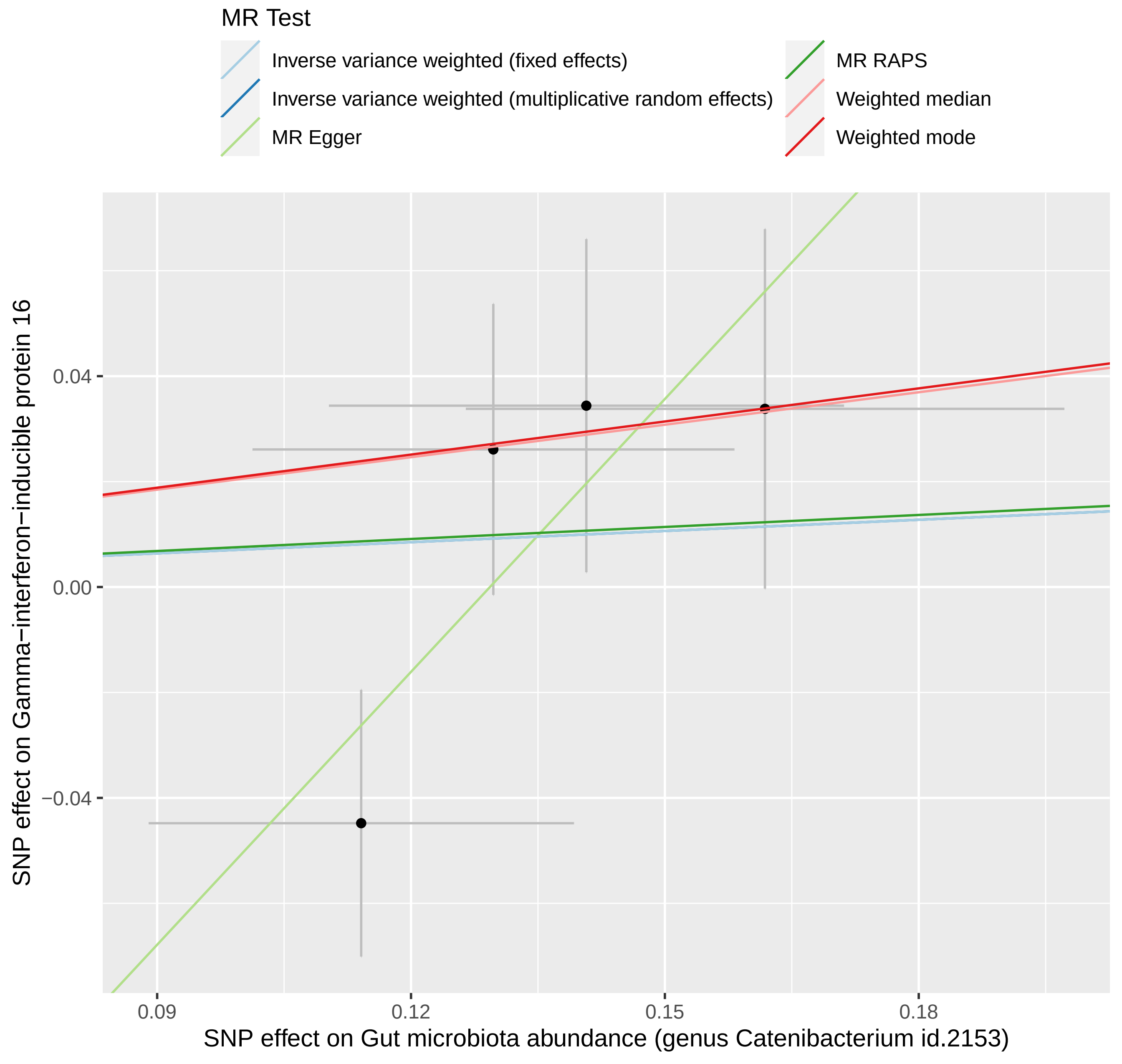

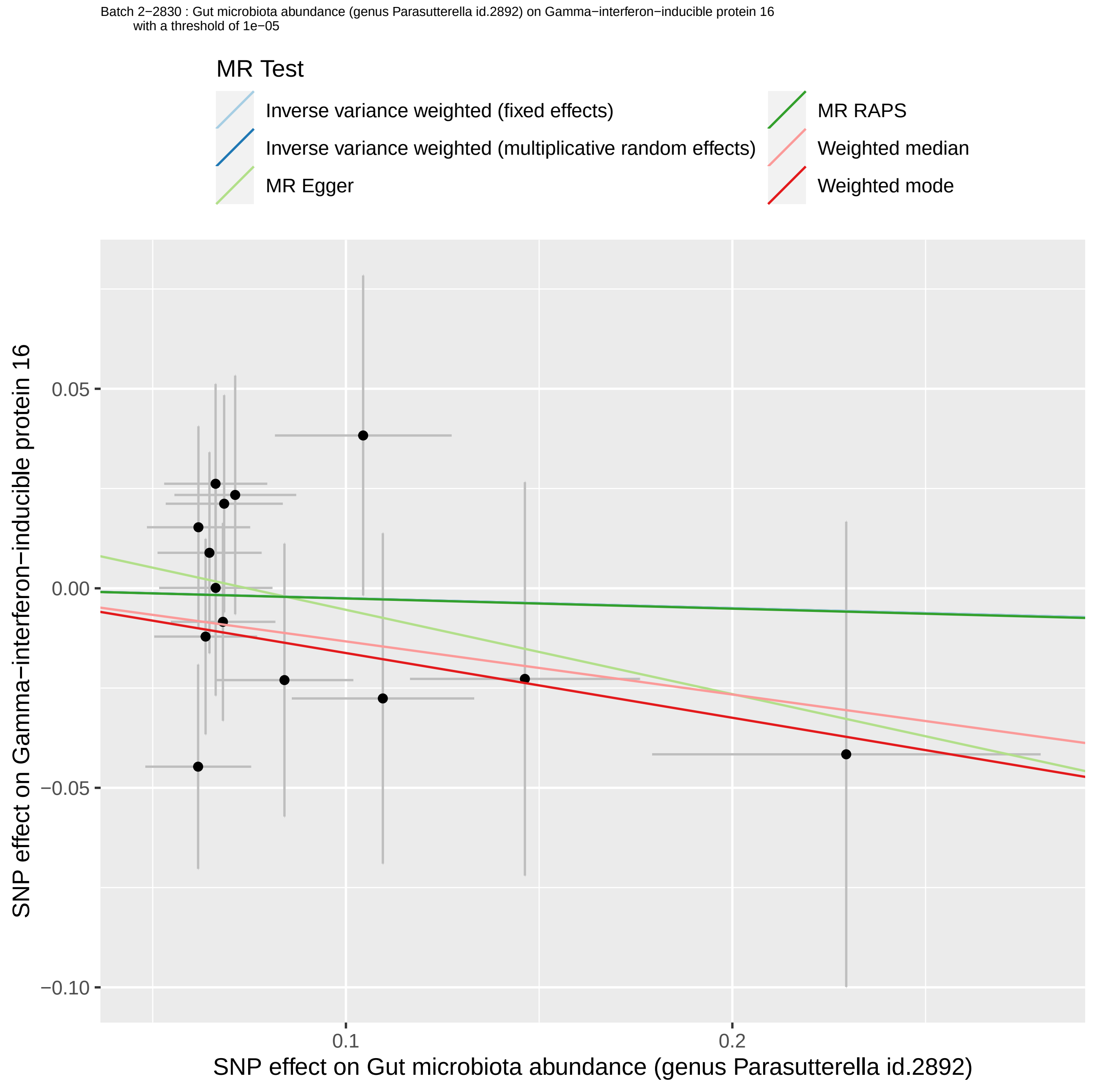

Batch 2−2830 : Gut microbiota abundance (genus Parasutterella id.2892) on Gamma−interferon−inducible protein 16
with a threshold of 1e−05
MR Test
Inverse variance weighted (fixed effects)
Inverse variance weighted (multiplicative random effects)
MR Egger
MR RAPS
Weighted median
Weighted mode
SNP effect on Gamma−interferon−inducible protein 16
0.05
0.00
−0.05
−0.10
0.1
0.2
SNP effect on Gut microbiota abundance (genus Parasutterella id.2892)

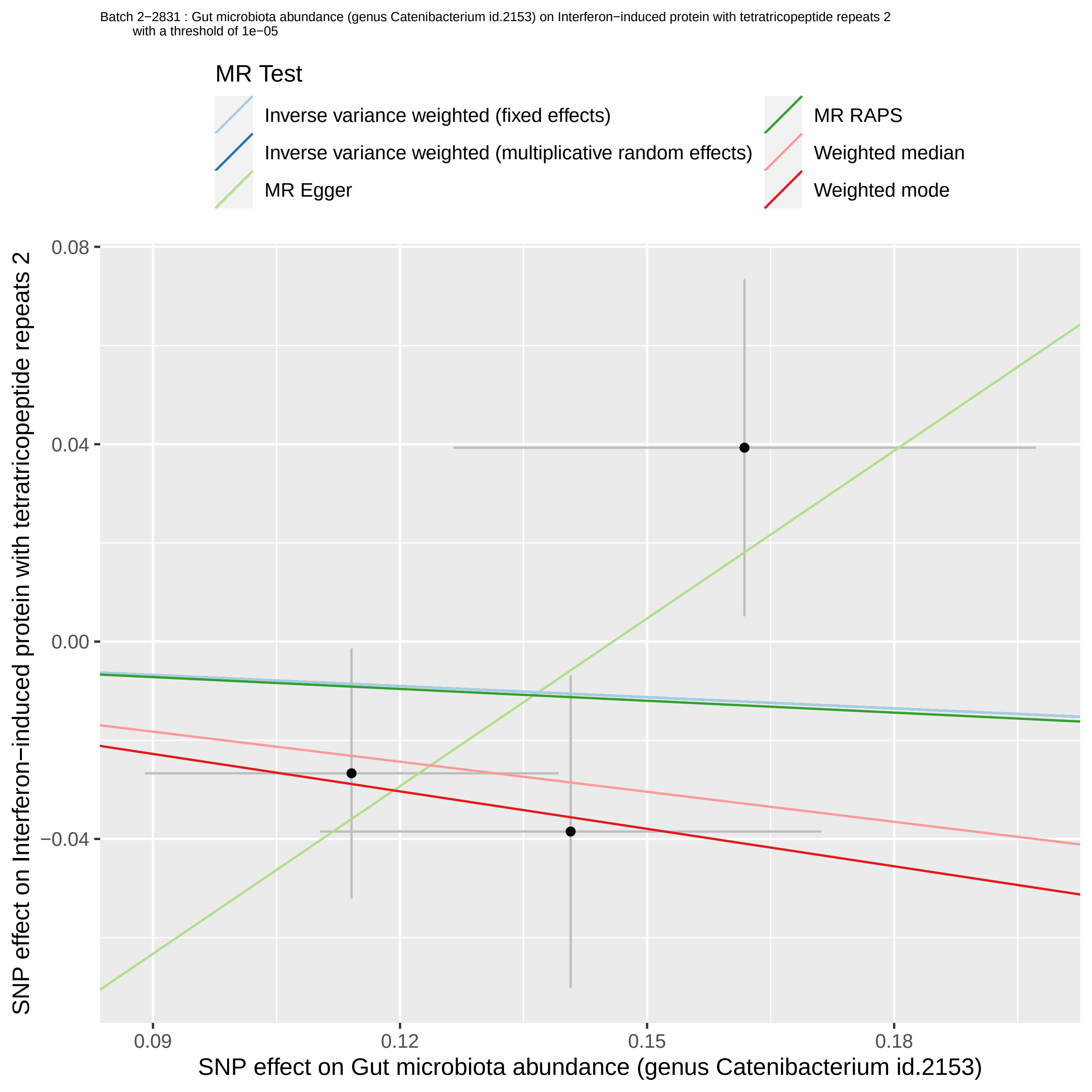
Batch 2−2831 : Gut microbiota abundance (genus Catenibacterium id.2153) on Interferon−induced protein with tetratricopeptide repeats 2
with a threshold of 1e−05
MR Test
Inverse variance weighted (fixed effects)
Inverse variance weighted (multiplicative random effects)
MR Egger
MR RAPS
Weighted median
Weighted mode
0.08
0.04
0.00
−0.04
0.09
0.12
0.15
0.18
SNP effect on Interferon−induced protein with tetratricopeptide repeats 2
SNP effect on Gut microbiota abundance (genus Catenibacterium id.2153)

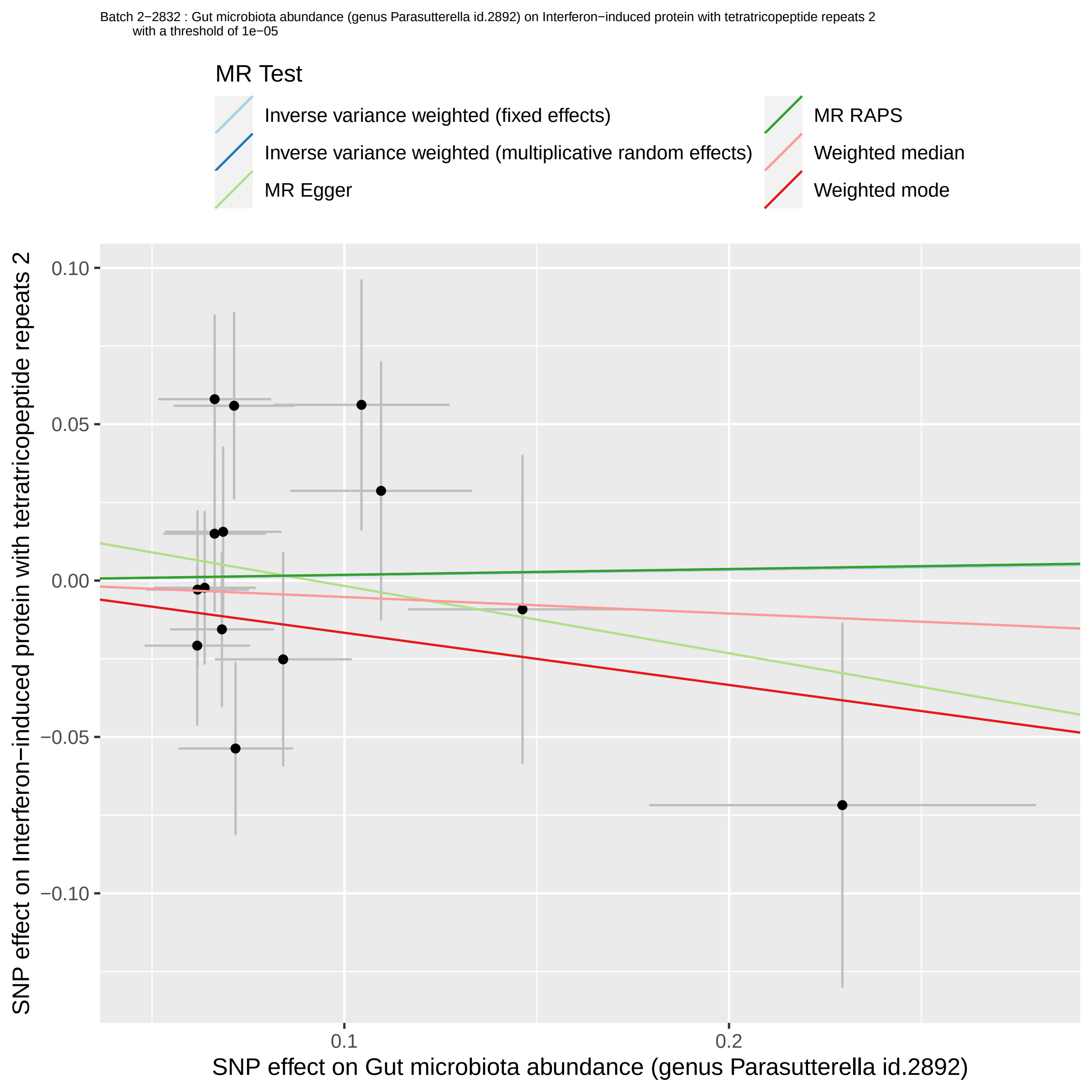
Batch 2−2832 : Gut microbiota abundance (genus Parasutterella id.2892) on Interferon−induced protein with tetratricopeptide repeats 2
with a threshold of 1e−05
MR Test
Inverse variance weighted (fixed effects)
Inverse variance weighted (multiplicative random effects)
MR Egger
MR RAPS
Weighted median
Weighted mode
0.10
0.05
0.00
−0.05
−0.10
0.1
0.2
SNP effect on Interferon−induced protein with tetratricopeptide repeats 2
SNP effect on Gut microbiota abundance (genus Parasutterella id.2892)

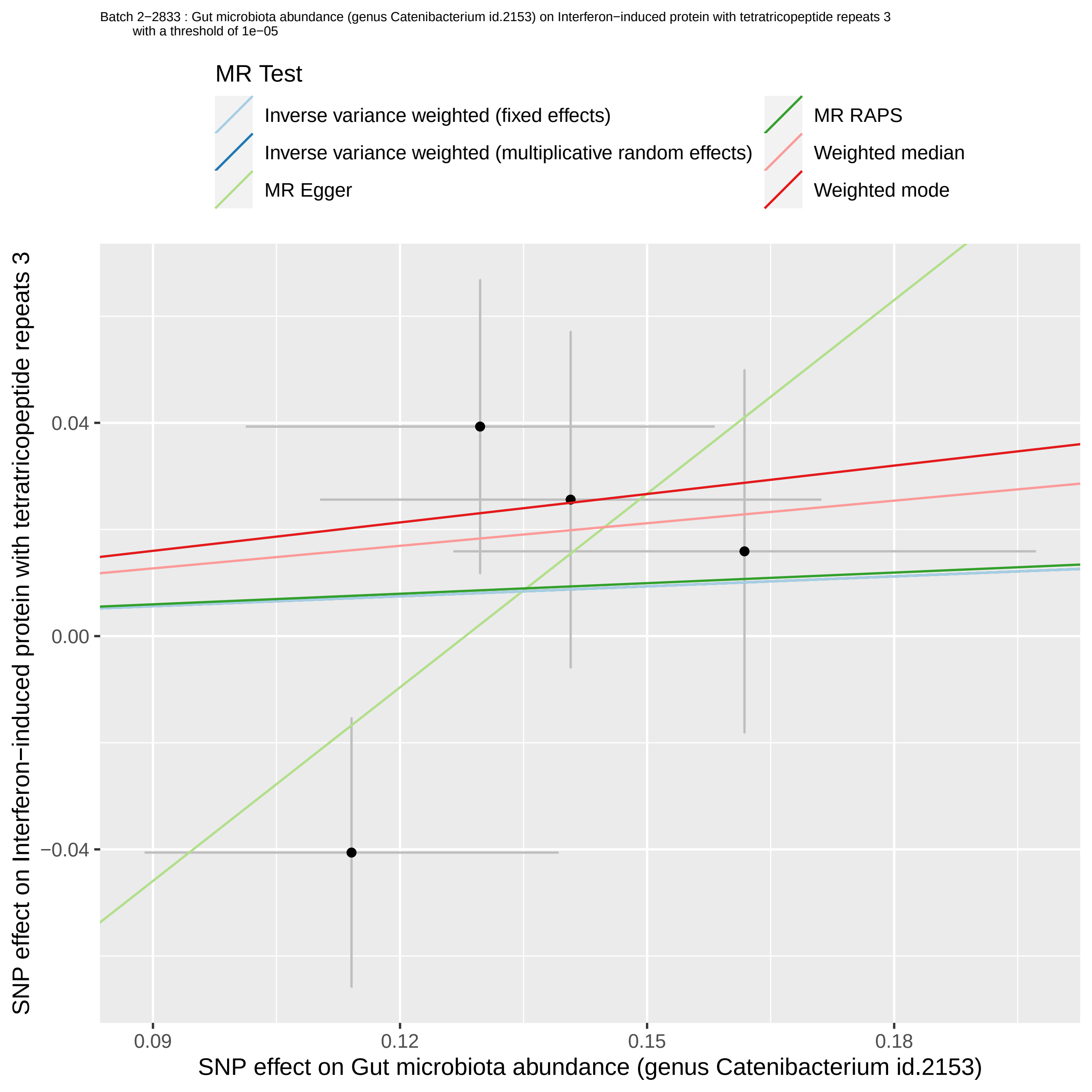

Batch 2−2833 : Gut microbiota abundance (genus Catenibacterium id.2153) on Interferon−induced protein with tetratricopeptide repeats 3
with a threshold of 1e−05
MR Test
Inverse variance weighted (fixed effects)
Inverse variance weighted (multiplicative random effects)
MR Egger
MR RAPS
Weighted median
Weighted mode
0.04
0.00
−0.04
0.09
0.12
0.15
0.18
SNP effect on Interferon−induced protein with tetratricopeptide repeats 3
SNP effect on Gut microbiota abundance (genus Catenibacterium id.2153)

Batch 2−2834 : Gut microbiota abundance (genus Parasutterella id.2892) on Interferon−induced protein with tetratricopeptide repeats 3
with a threshold of 1e−05

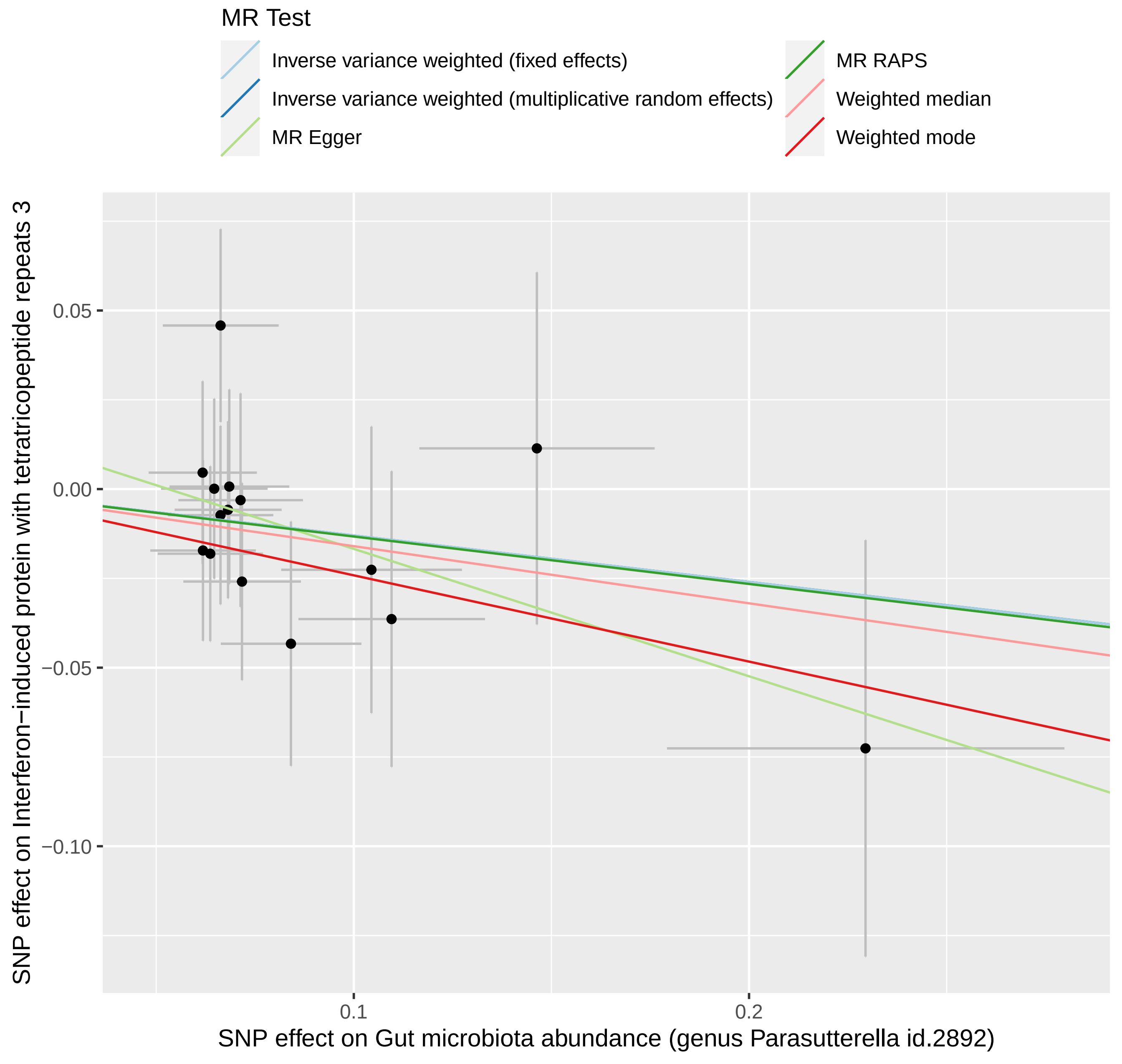

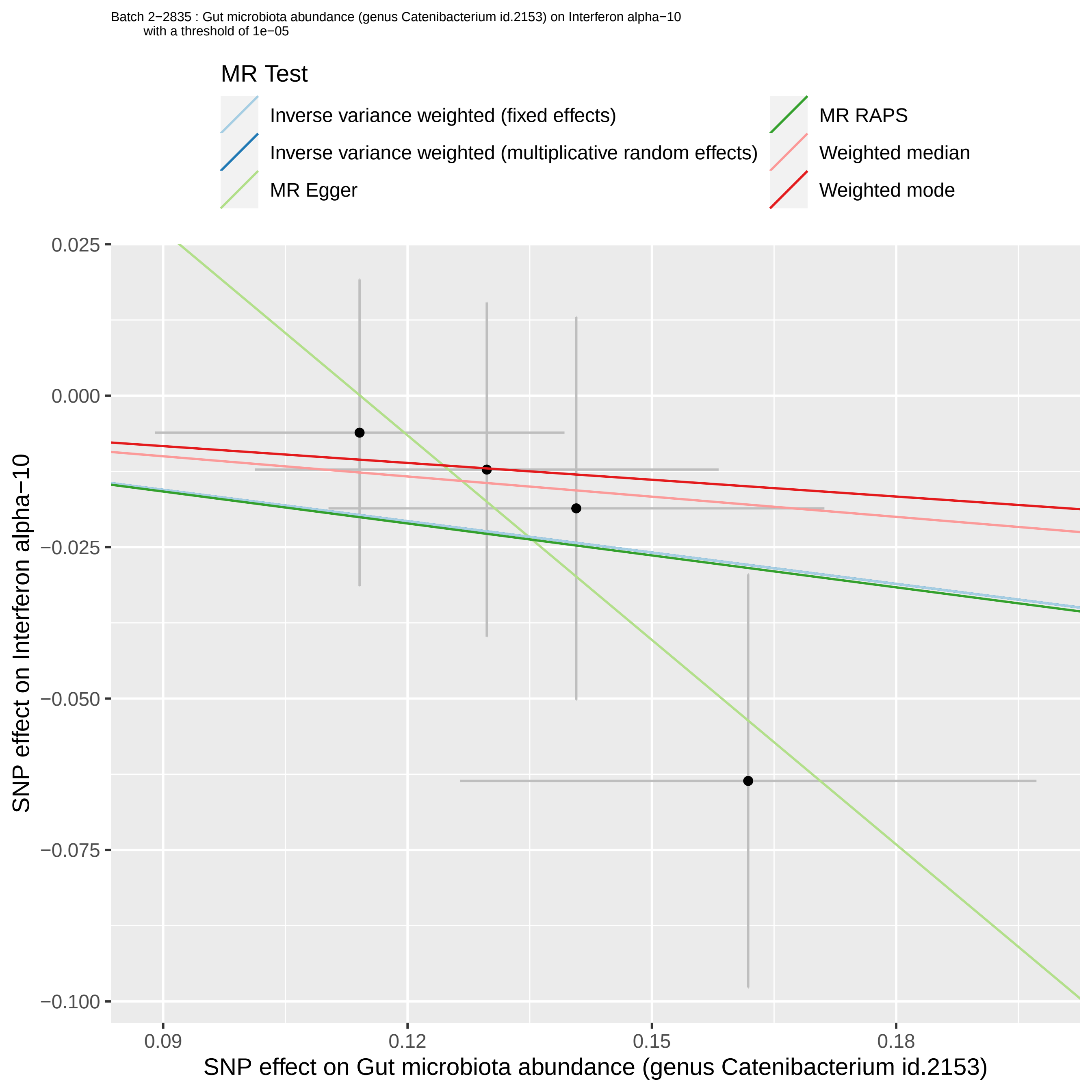
Batch 2−2835 : Gut microbiota abundance (genus Catenibacterium id.2153) on Interferon alpha−10
with a threshold of 1e−05
MR Test
Inverse variance weighted (fixed effects)
Inverse variance weighted (multiplicative random effects)
MR Egger
MR RAPS
Weighted median
Weighted mode
0.025
0.000
−0.025
−0.050
−0.075
−0.100
0.09
0.12
0.15
0.18
SNP effect on Interferon alpha−10
SNP effect on Gut microbiota abundance (genus Catenibacterium id.2153)

Batch 2−2836 : Gut microbiota abundance (genus Parasutterella id.2892) on Interferon alpha−10
with a threshold of 1e−05

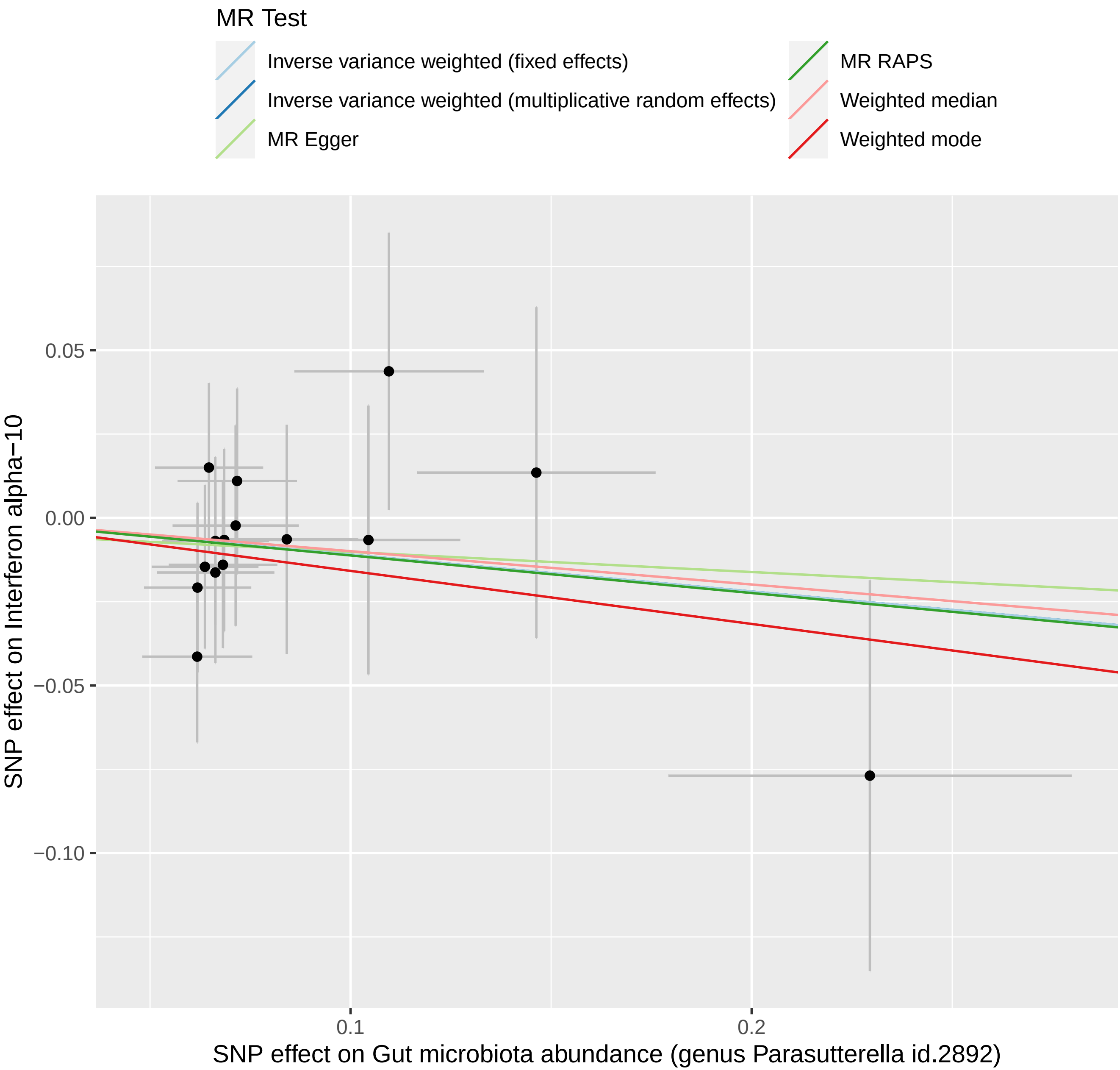

Batch 2−2837 : Gut microbiota abundance (genus Catenibacterium id.2153) on Interferon alpha−10
with a threshold of 1e−05

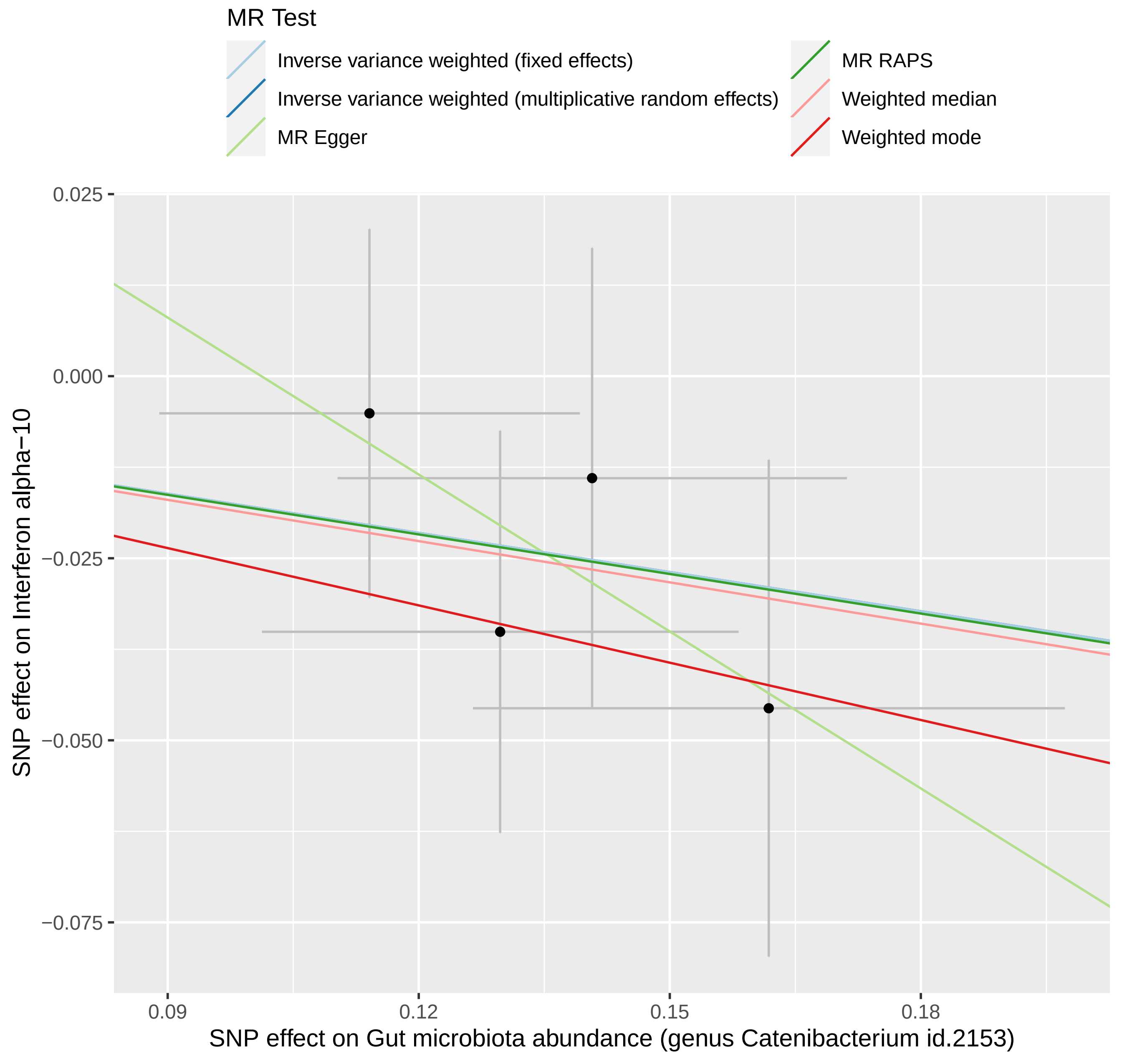

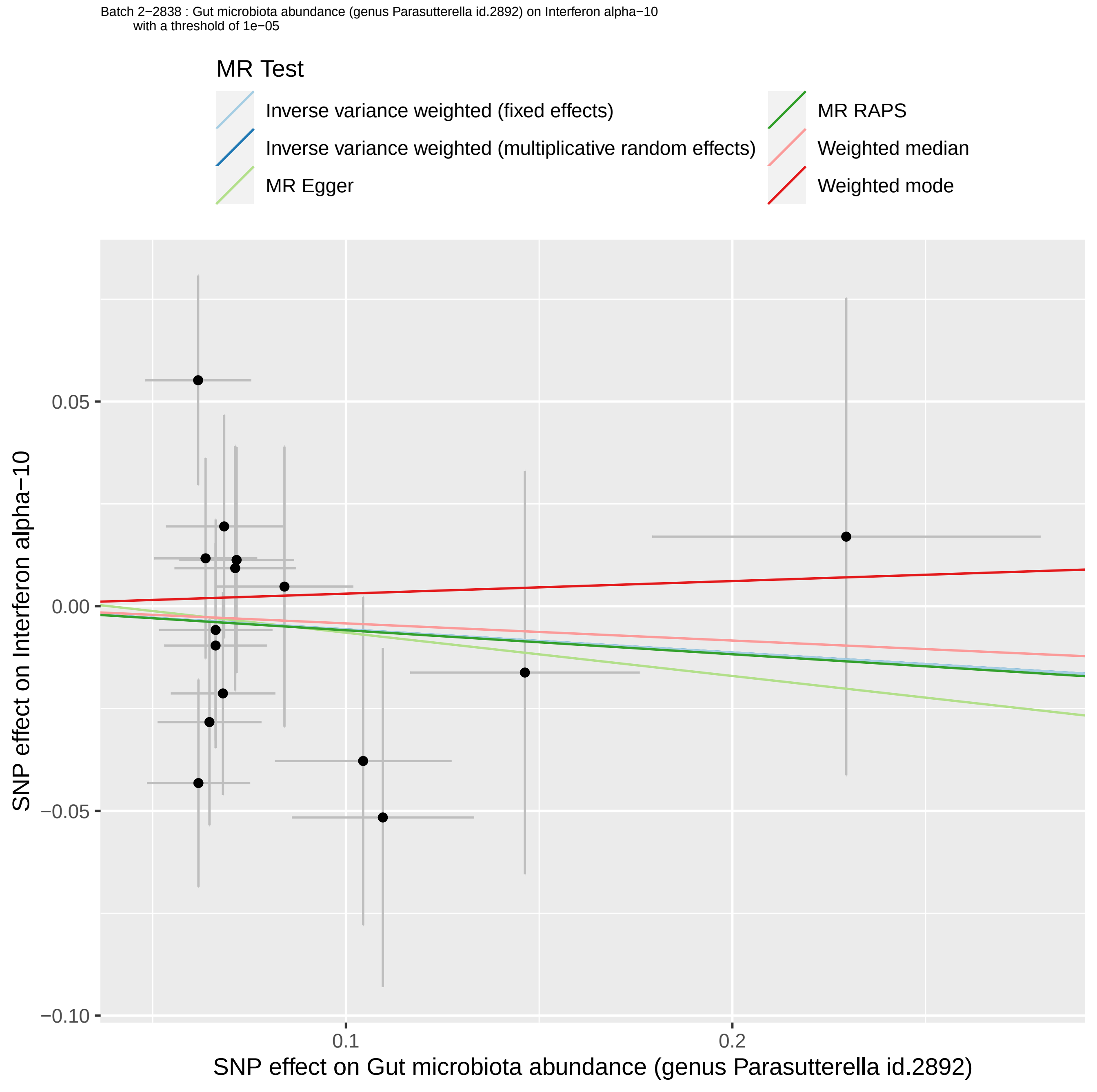

Batch 2−2838 : Gut microbiota abundance (genus Parasutterella id.2892) on Interferon alpha−10
with a threshold of 1e−05
MR Test
Inverse variance weighted (fixed effects)
Inverse variance weighted (multiplicative random effects)
MR Egger
MR RAPS
Weighted median
Weighted mode
SNP effect on Interferon alpha−10
0.05
0.00
−0.05
−0.10
0.1
0.2
SNP effect on Gut microbiota abundance (genus Parasutterella id.2892)

Batch 2−2839 : Gut microbiota abundance (genus Catenibacterium id.2153) on Interferon alpha−14
with a threshold of 1e−05

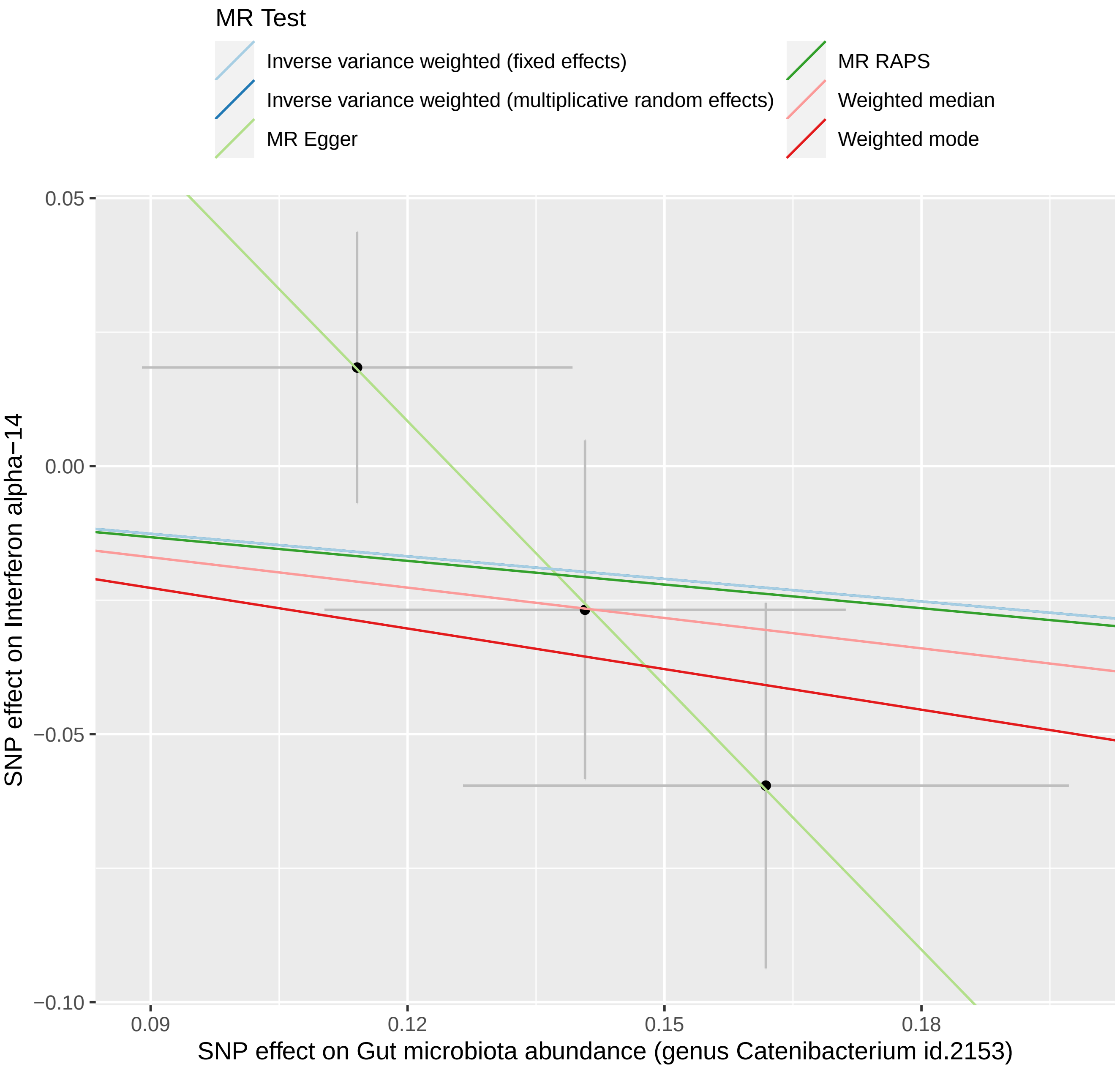

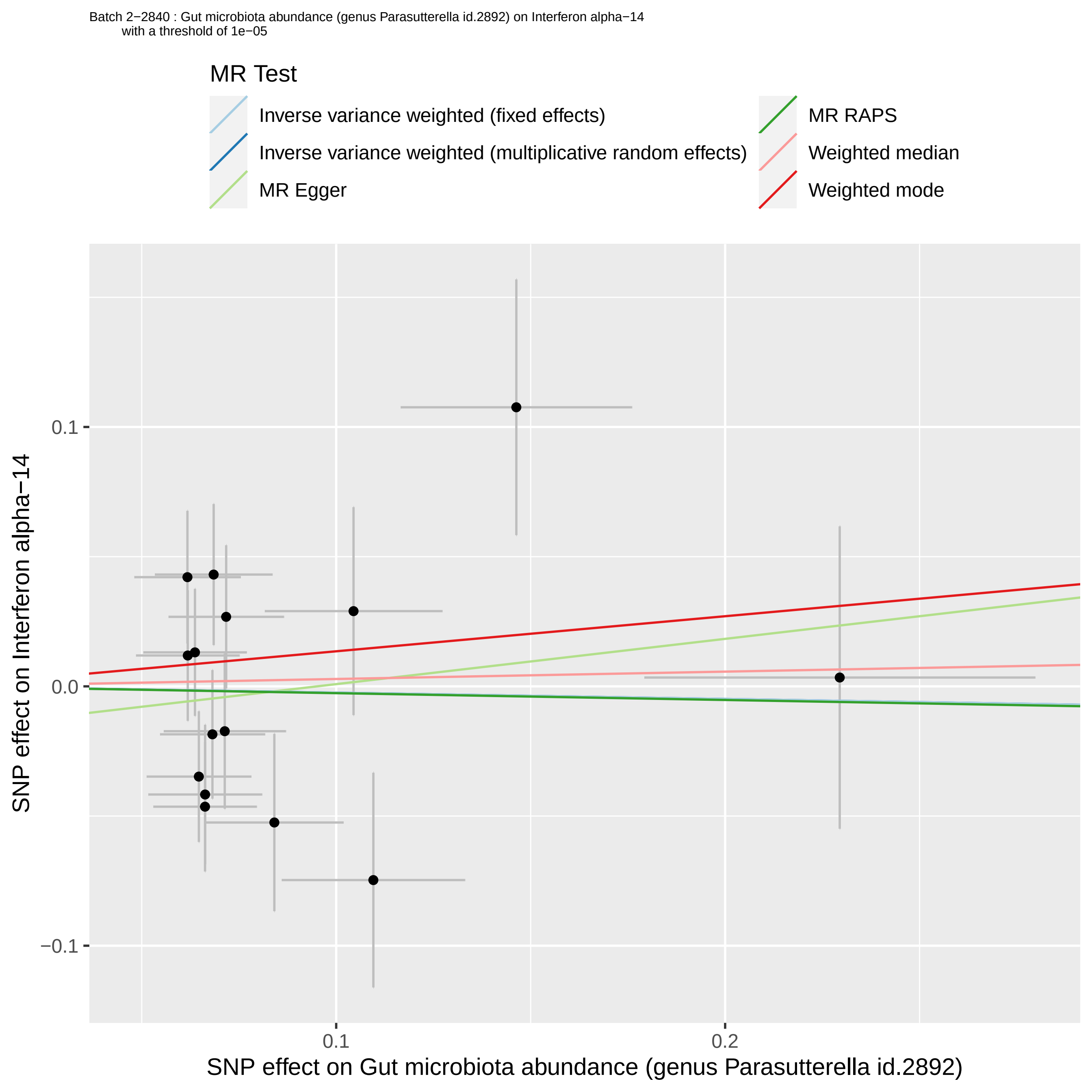

Batch 2−2840 : Gut microbiota abundance (genus Parasutterella id.2892) on Interferon alpha−14
with a threshold of 1e−05
MR Test
Inverse variance weighted (fixed effects)
Inverse variance weighted (multiplicative random effects)
MR Egger
MR RAPS
Weighted median
Weighted mode
0.1
0.0
−0.1
0.1
0.2
SNP effect on Interferon alpha−14
SNP effect on Gut microbiota abundance (genus Parasutterella id.2892)

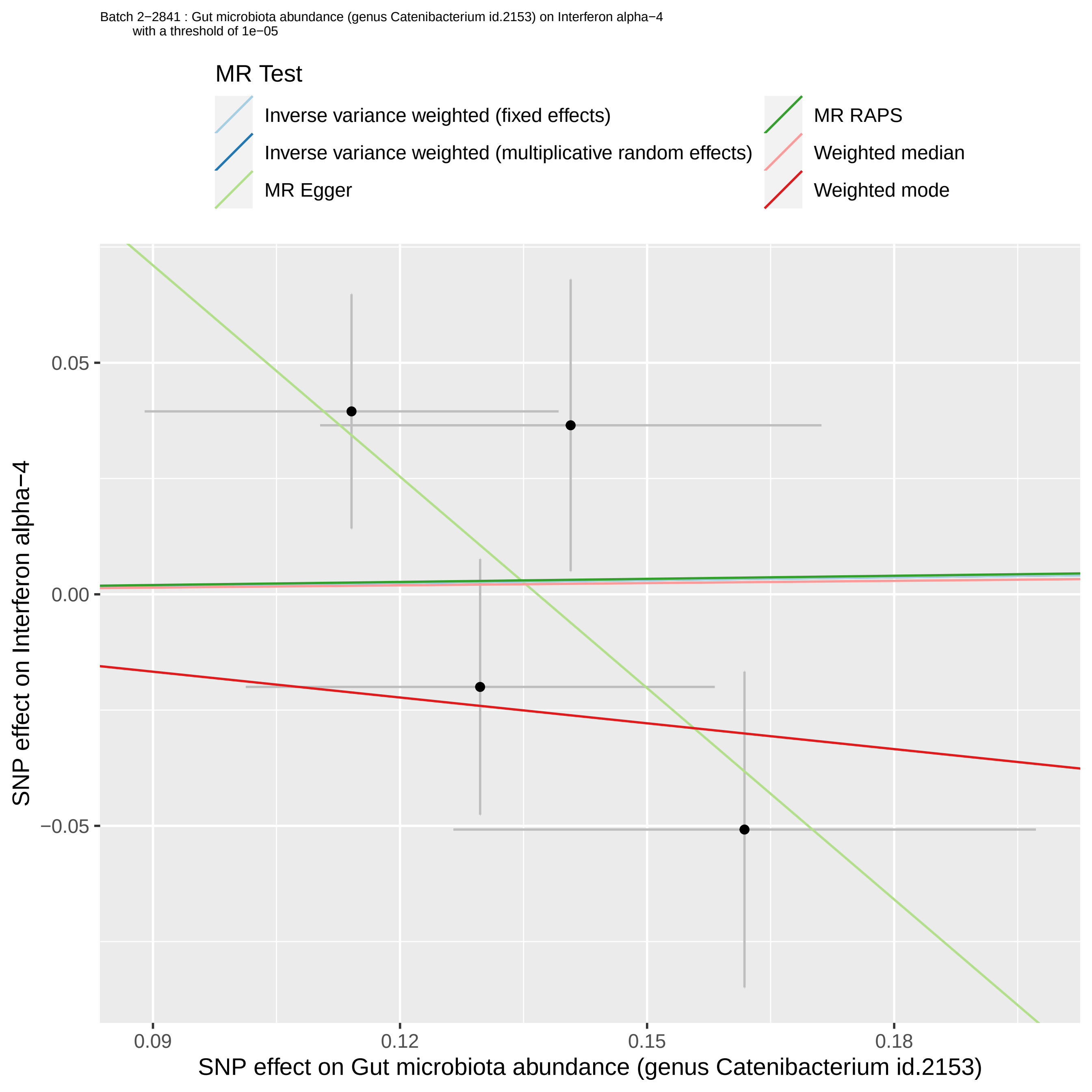
Batch 2−2841 : Gut microbiota abundance (genus Catenibacterium id.2153) on Interferon alpha−4
with a threshold of 1e−05
MR Test
Inverse variance weighted (fixed effects)
Inverse variance weighted (multiplicative random effects)
MR Egger
MR RAPS
Weighted median
Weighted mode
0.05
0.00
−0.05
0.09
0.12
0.15
0.18
SNP effect on Interferon alpha−4
SNP effect on Gut microbiota abundance (genus Catenibacterium id.2153)

Batch 2−2842 : Gut microbiota abundance (genus Parasutterella id.2892) on Interferon alpha−4
with a threshold of 1e−05

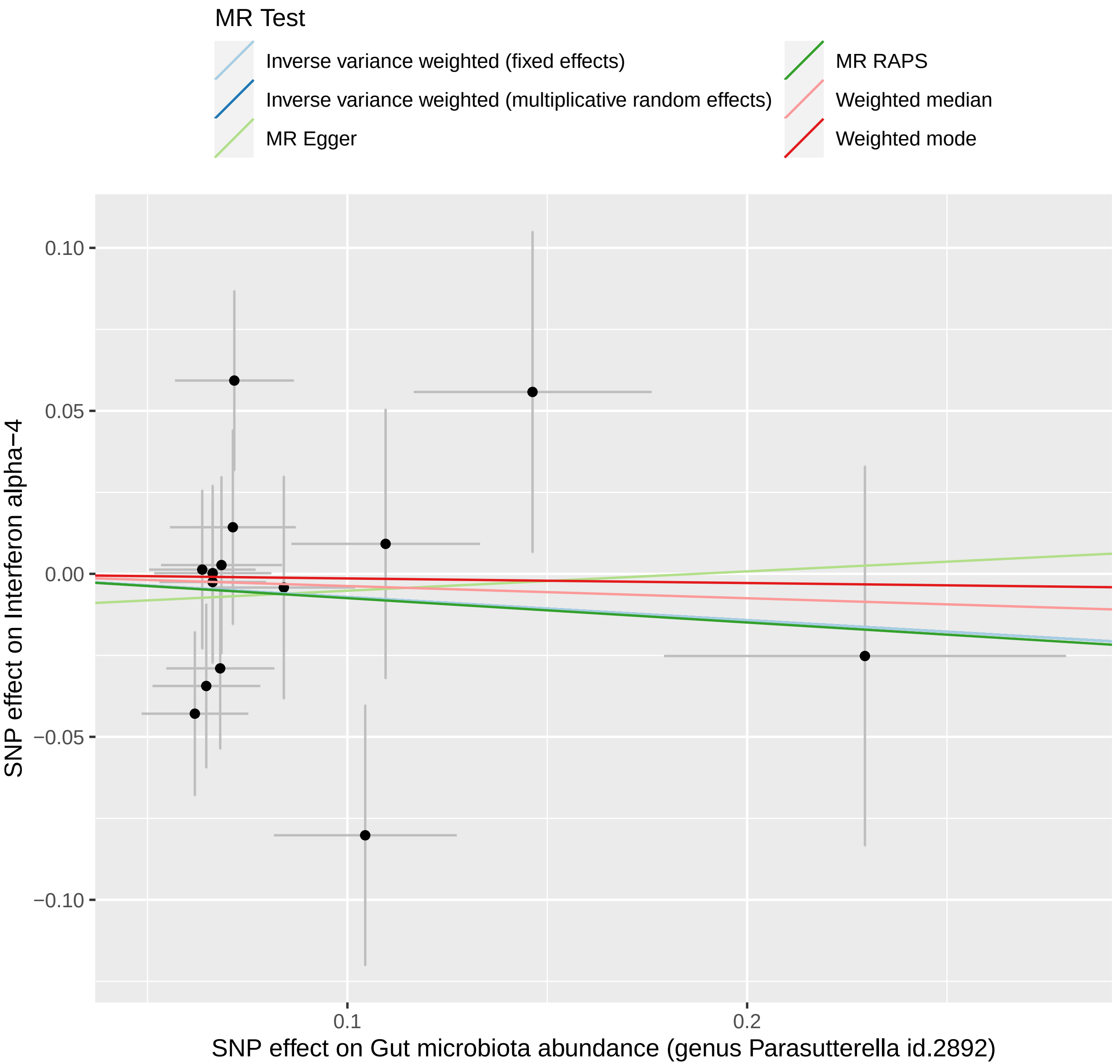

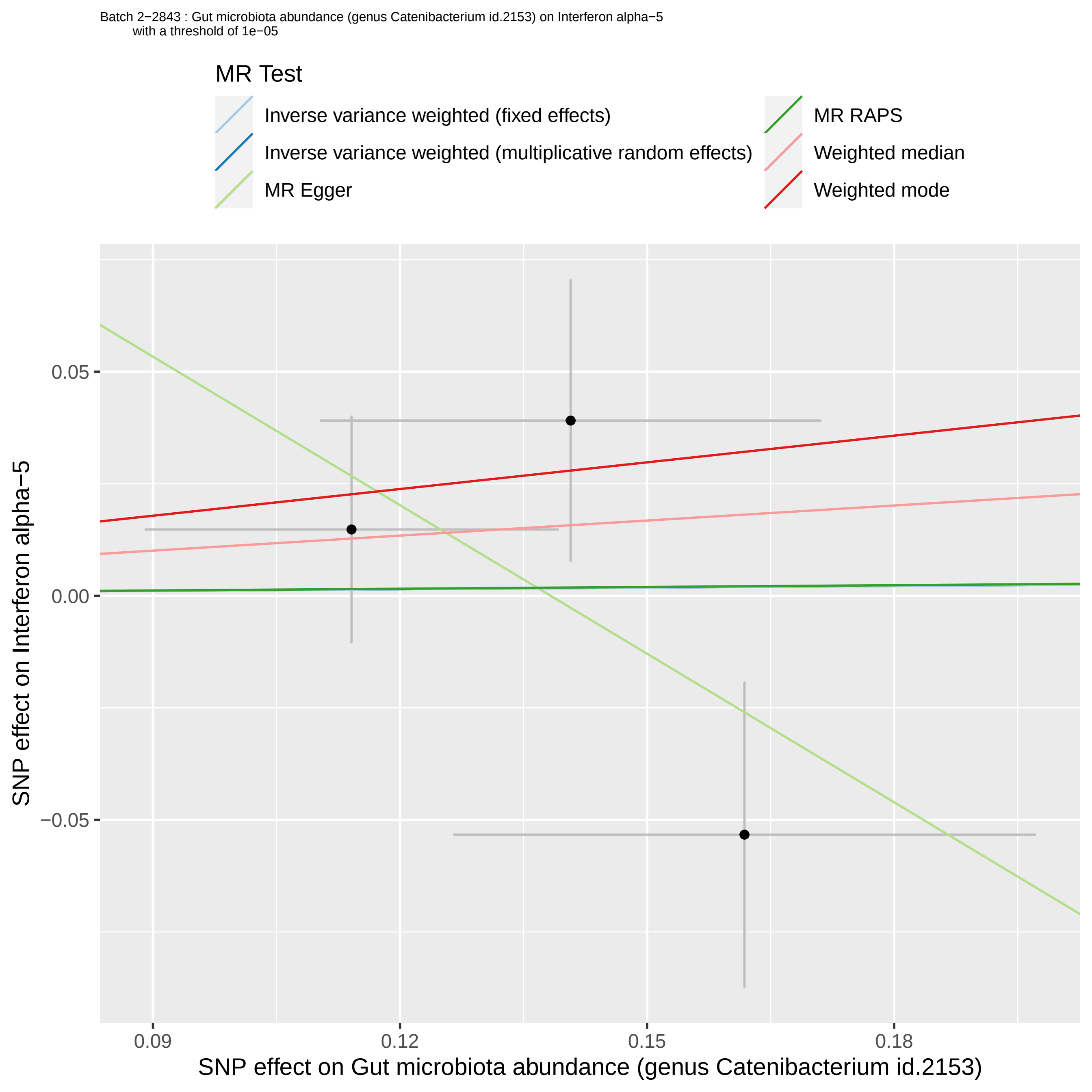

Batch 2−2843 : Gut microbiota abundance (genus Catenibacterium id.2153) on Interferon alpha−5
with a threshold of 1e−05
MR Test
Inverse variance weighted (fixed effects)
Inverse variance weighted (multiplicative random effects)
MR Egger
MR RAPS
Weighted median
Weighted mode
0.05
0.00
−0.05
SNP effect on Interferon alpha−5
0.09
0.12
0.15
0.18
SNP effect on Gut microbiota abundance (genus Catenibacterium id.2153)

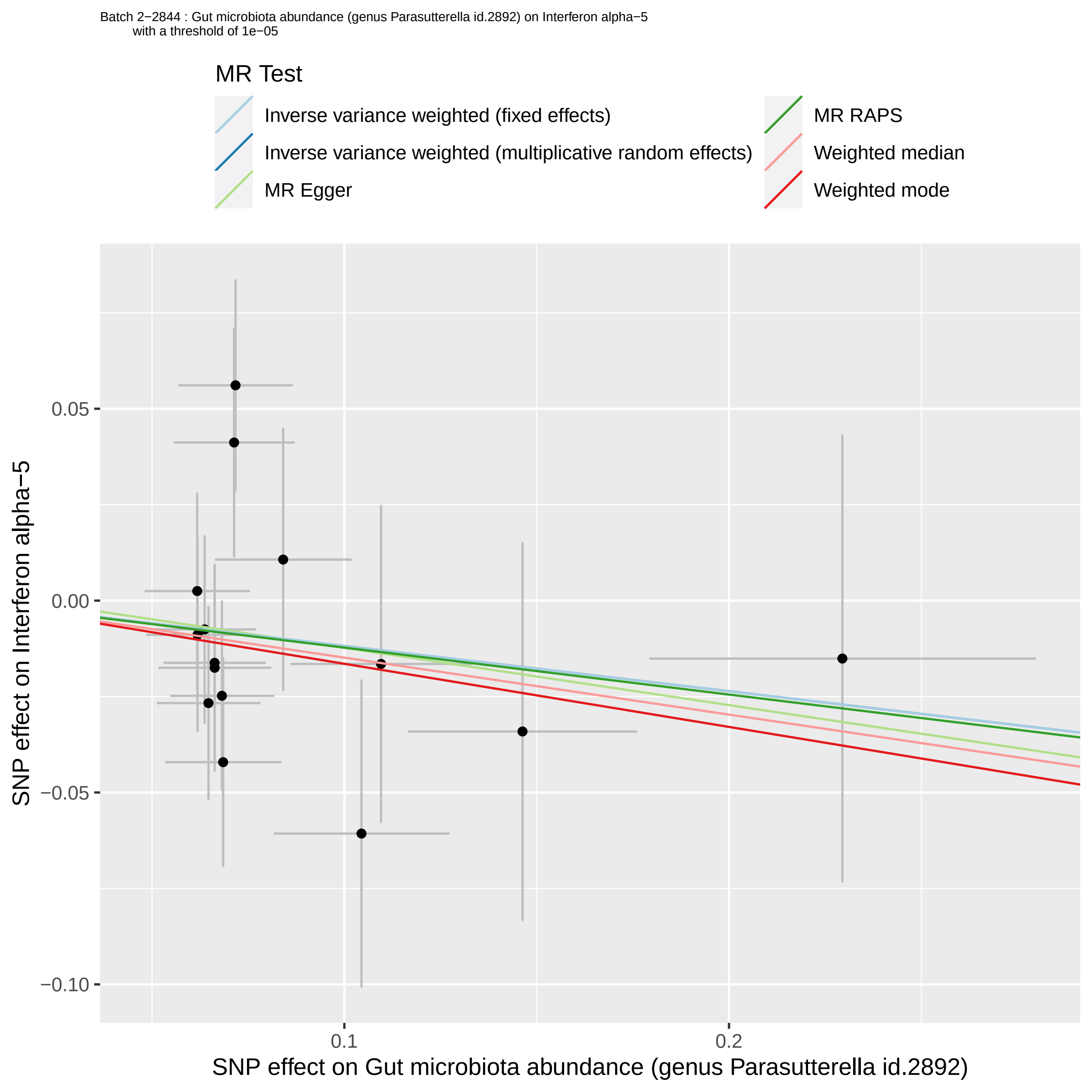

Batch 2−2844 : Gut microbiota abundance (genus Parasutterella id.2892) on Interferon alpha−5
with a threshold of 1e−05
MR Test
Inverse variance weighted (fixed effects)
Inverse variance weighted (multiplicative random effects)
MR Egger
MR RAPS
Weighted median
Weighted mode
0.05
0.00
−0.05
−0.10
0.1
0.2
SNP effect on Interferon alpha−5
SNP effect on Gut microbiota abundance (genus Parasutterella id.2892)

Batch 2−2845 : Gut microbiota abundance (genus Catenibacterium id.2153) on Interferon alpha−6
with a threshold of 1e−05

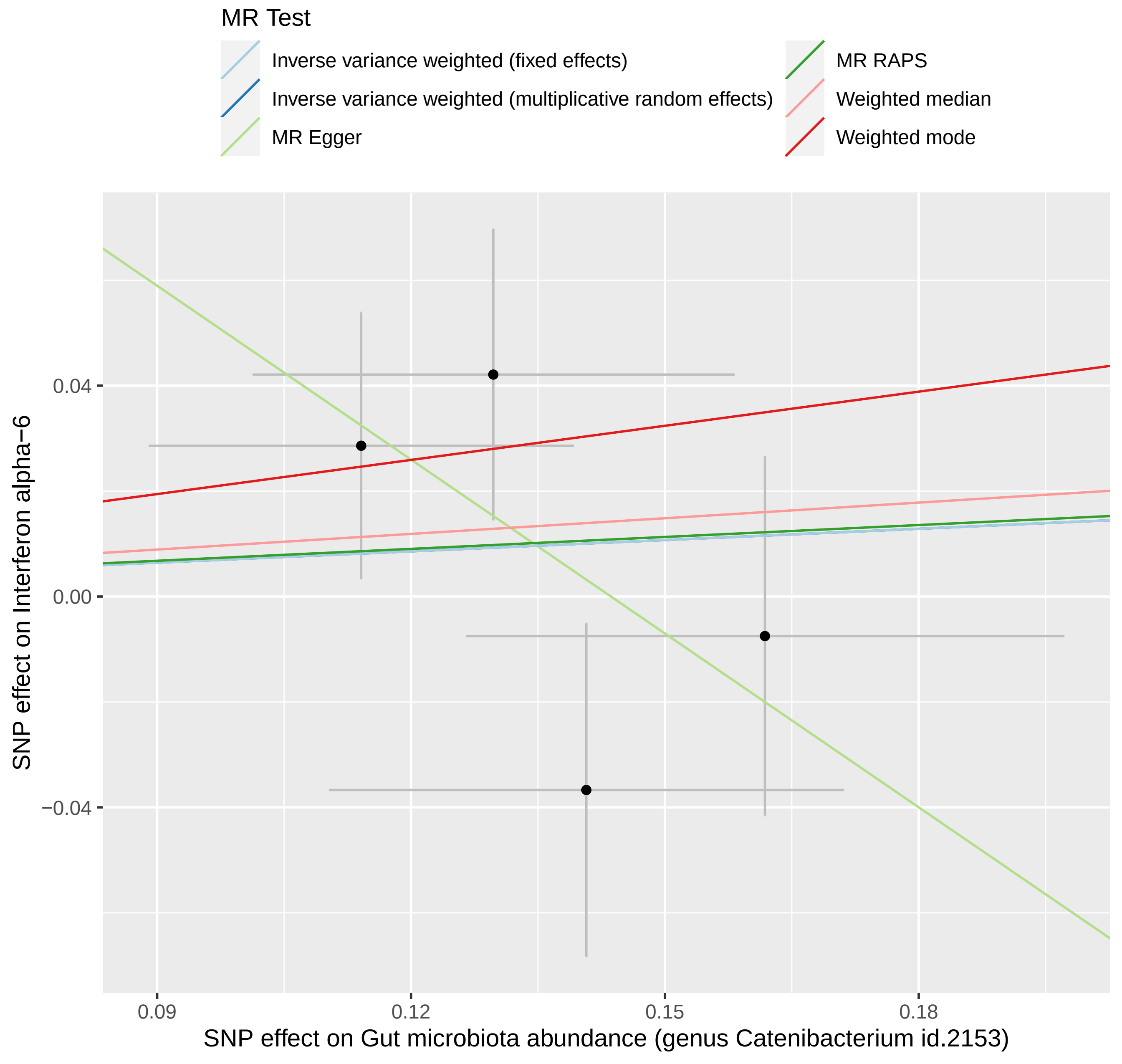

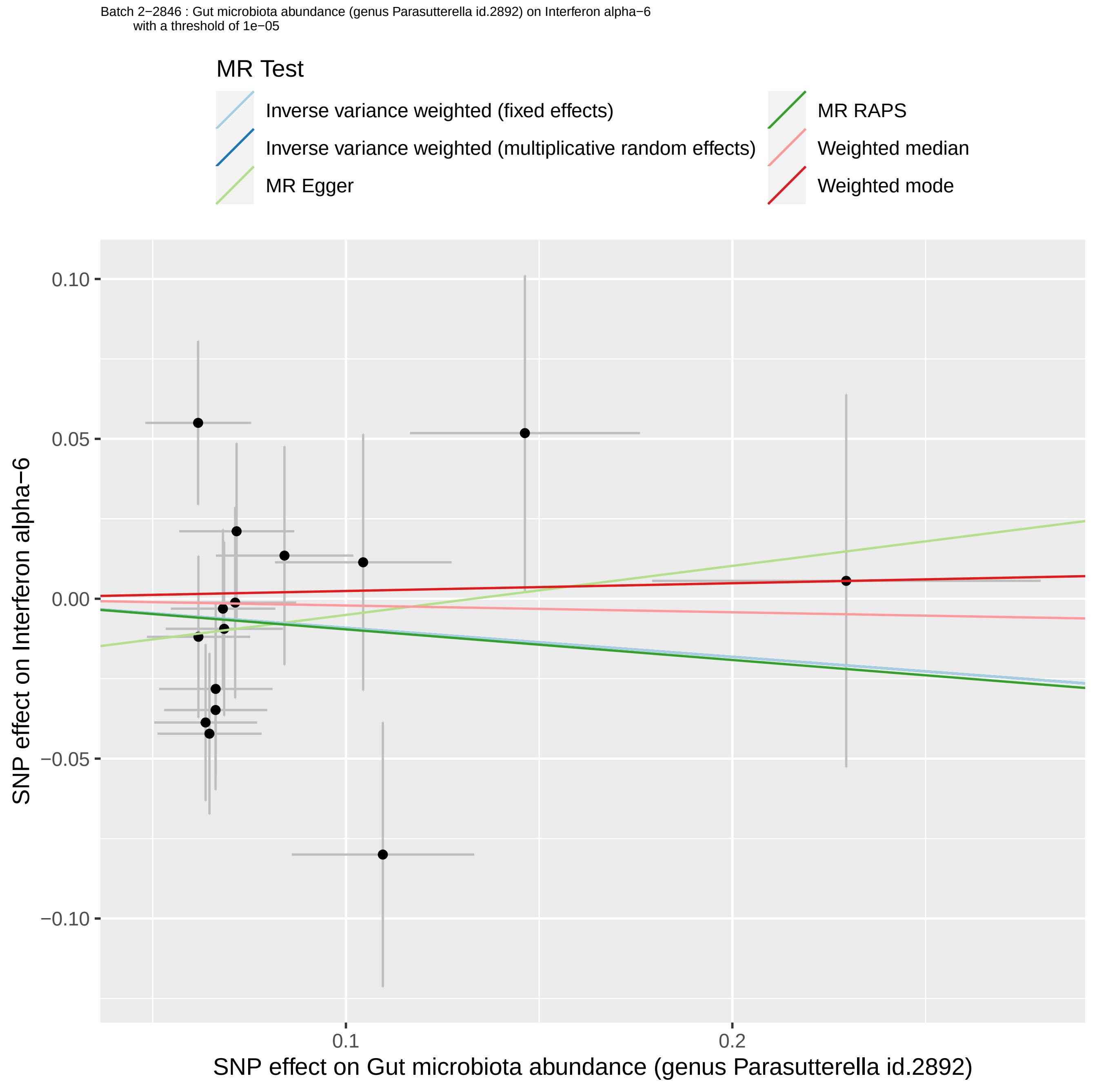
Batch 2−2846 : Gut microbiota abundance (genus Parasutterella id.2892) on Interferon alpha−6
with a threshold of 1e−05
MR Test
Inverse variance weighted (fixed effects)
Inverse variance weighted (multiplicative random effects)
MR Egger
MR RAPS
Weighted median
Weighted mode
0.10
0.05
0.00
−0.05
−0.10
0.1
0.2
SNP effect on Interferon alpha−6
SNP effect on Gut microbiota abundance (genus Parasutterella id.2892)

Batch 2−2847 : Gut microbiota abundance (genus Catenibacterium id.2153) on Interferon alpha−7
with a threshold of 1e−05

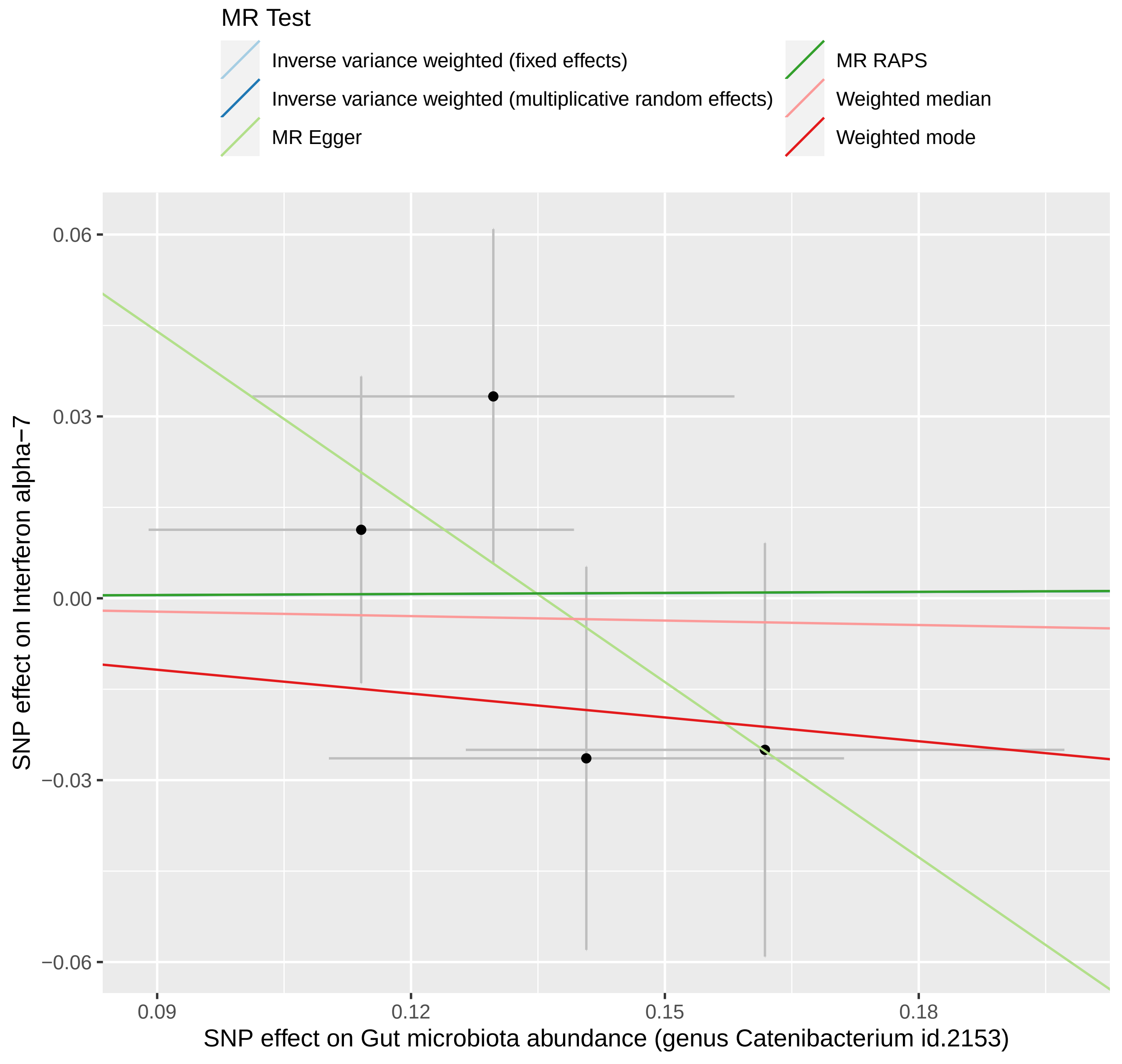

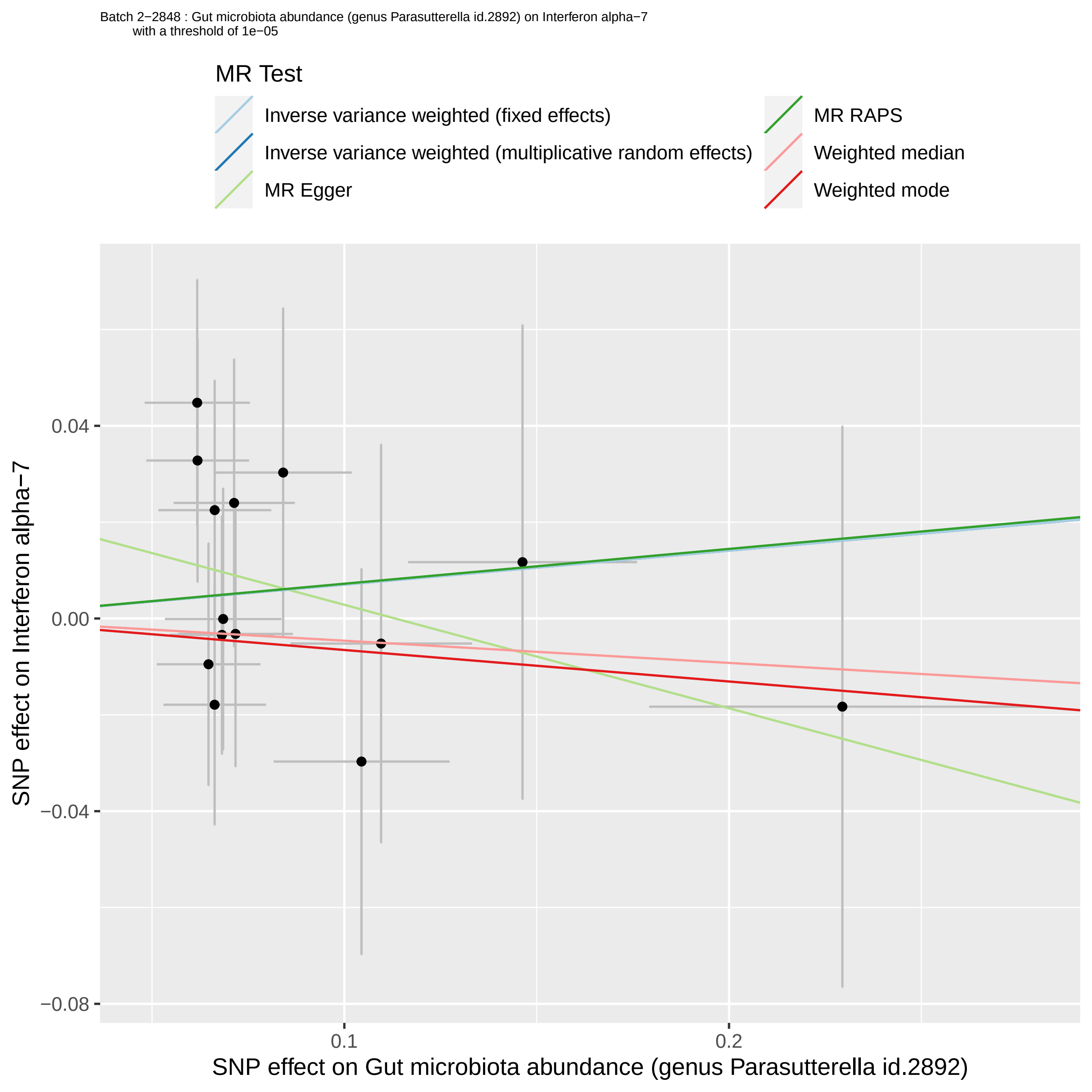

Batch 2−2848 : Gut microbiota abundance (genus Parasutterella id.2892) on Interferon alpha−7
with a threshold of 1e−05
MR Test
Inverse variance weighted (fixed effects)
Inverse variance weighted (multiplicative random effects)
MR Egger
MR RAPS
Weighted median
Weighted mode
0.04
0.00
−0.04
−0.08
0.1
0.2
SNP effect on Interferon alpha−7
SNP effect on Gut microbiota abundance (genus Parasutterella id.2892)

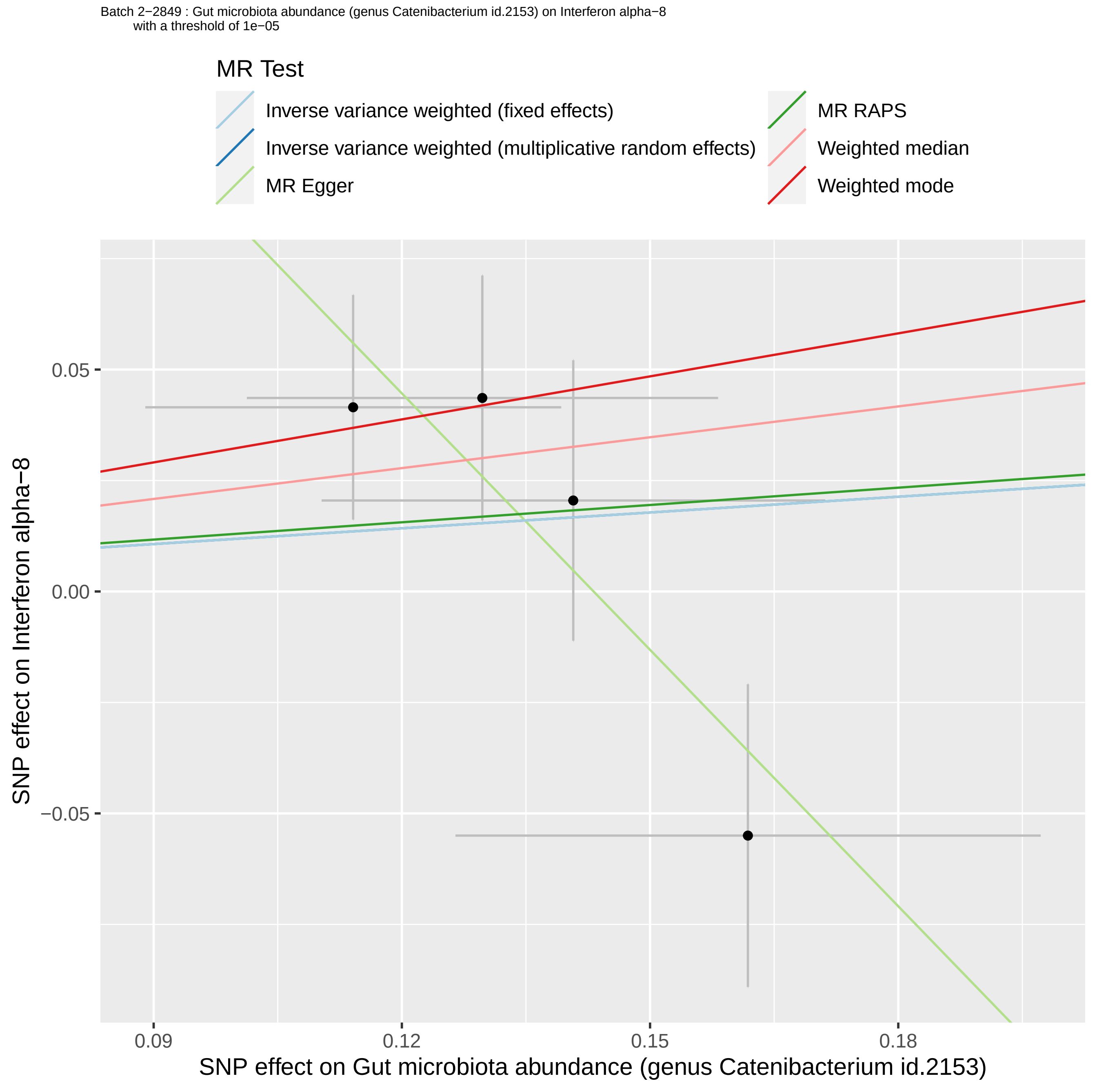

Batch 2−2849 : Gut microbiota abundance (genus Catenibacterium id.2153) on Interferon alpha−8
with a threshold of 1e−05
MR Test
Inverse variance weighted (fixed effects)
Inverse variance weighted (multiplicative random effects)
MR Egger
MR RAPS
Weighted median
Weighted mode
0.05
0.00
−0.05
0.09
0.12
0.15
0.18
SNP effect on Interferon alpha−8
SNP effect on Gut microbiota abundance (genus Catenibacterium id.2153)

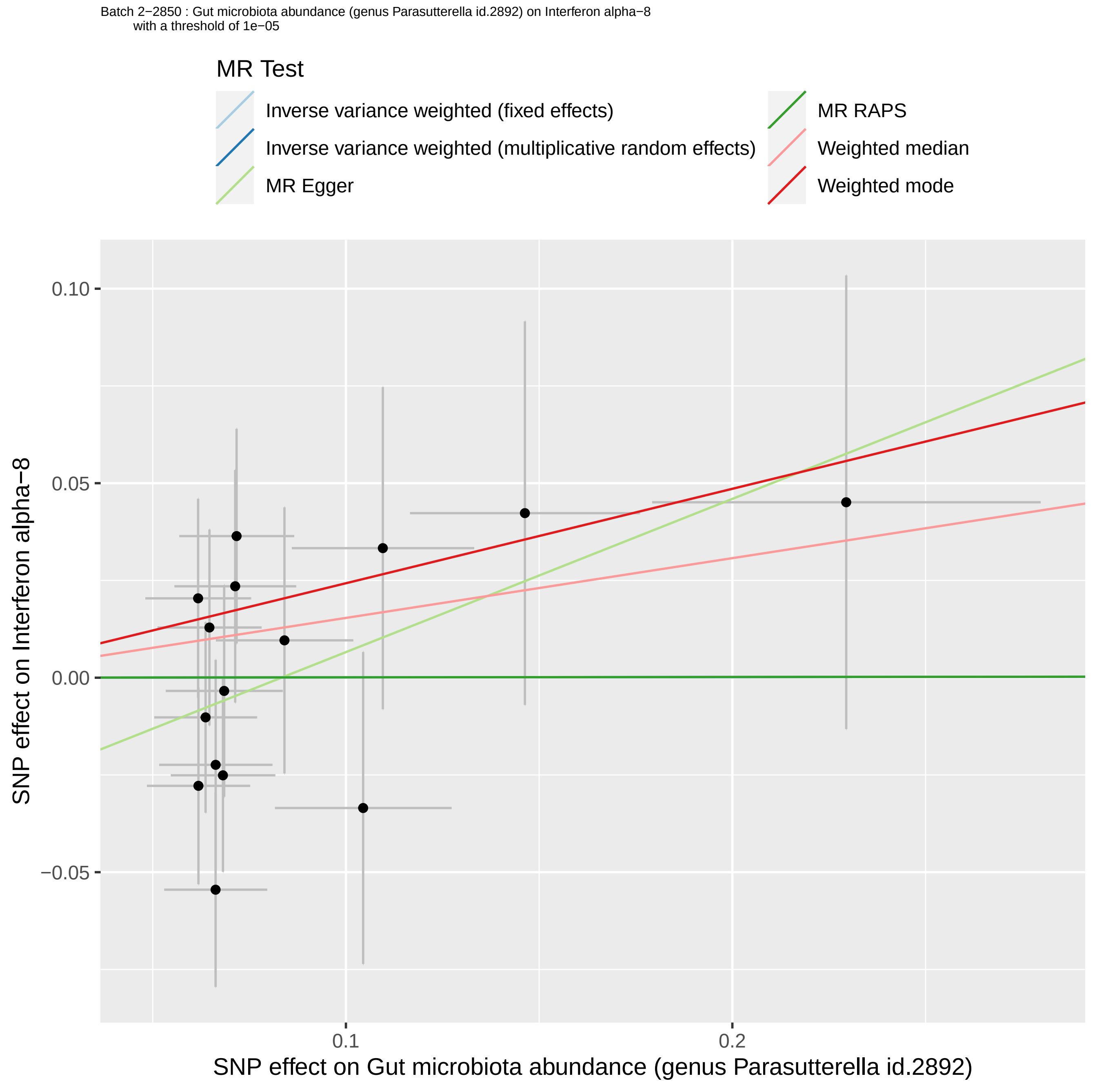
Batch 2−2850 : Gut microbiota abundance (genus Parasutterella id.2892) on Interferon alpha−8
with a threshold of 1e−05
MR Test
Inverse variance weighted (fixed effects)
Inverse variance weighted (multiplicative random effects)
MR Egger
MR RAPS
Weighted median
Weighted mode
0.10
0.05
0.00
−0.05
0.1
0.2
SNP effect on Interferon alpha−8
SNP effect on Gut microbiota abundance (genus Parasutterella id.2892)

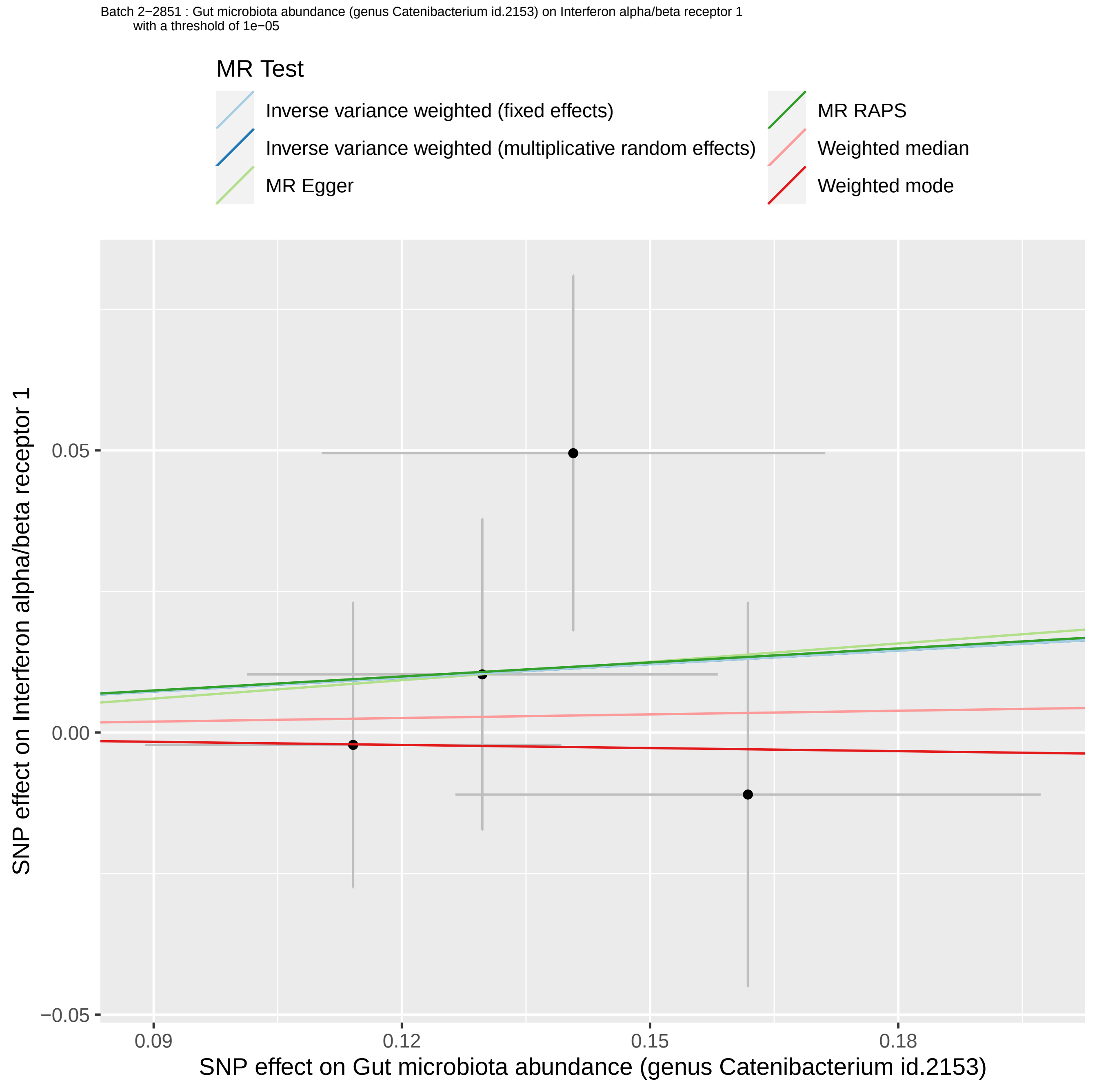

Batch 2−2851 : Gut microbiota abundance (genus Catenibacterium id.2153) on Interferon alpha/beta receptor 1
with a threshold of 1e−05
MR Test
Inverse variance weighted (fixed effects)
Inverse variance weighted (multiplicative random effects)
MR Egger
MR RAPS
Weighted median
Weighted mode
0.05
0.00
−0.05
0.09
0.12
0.15
0.18
SNP effect on Interferon alpha/beta receptor 1
SNP effect on Gut microbiota abundance (genus Catenibacterium id.2153)

Batch 2−2852 : Gut microbiota abundance (genus Parasutterella id.2892) on Interferon alpha/beta receptor 1
with a threshold of 1e−05

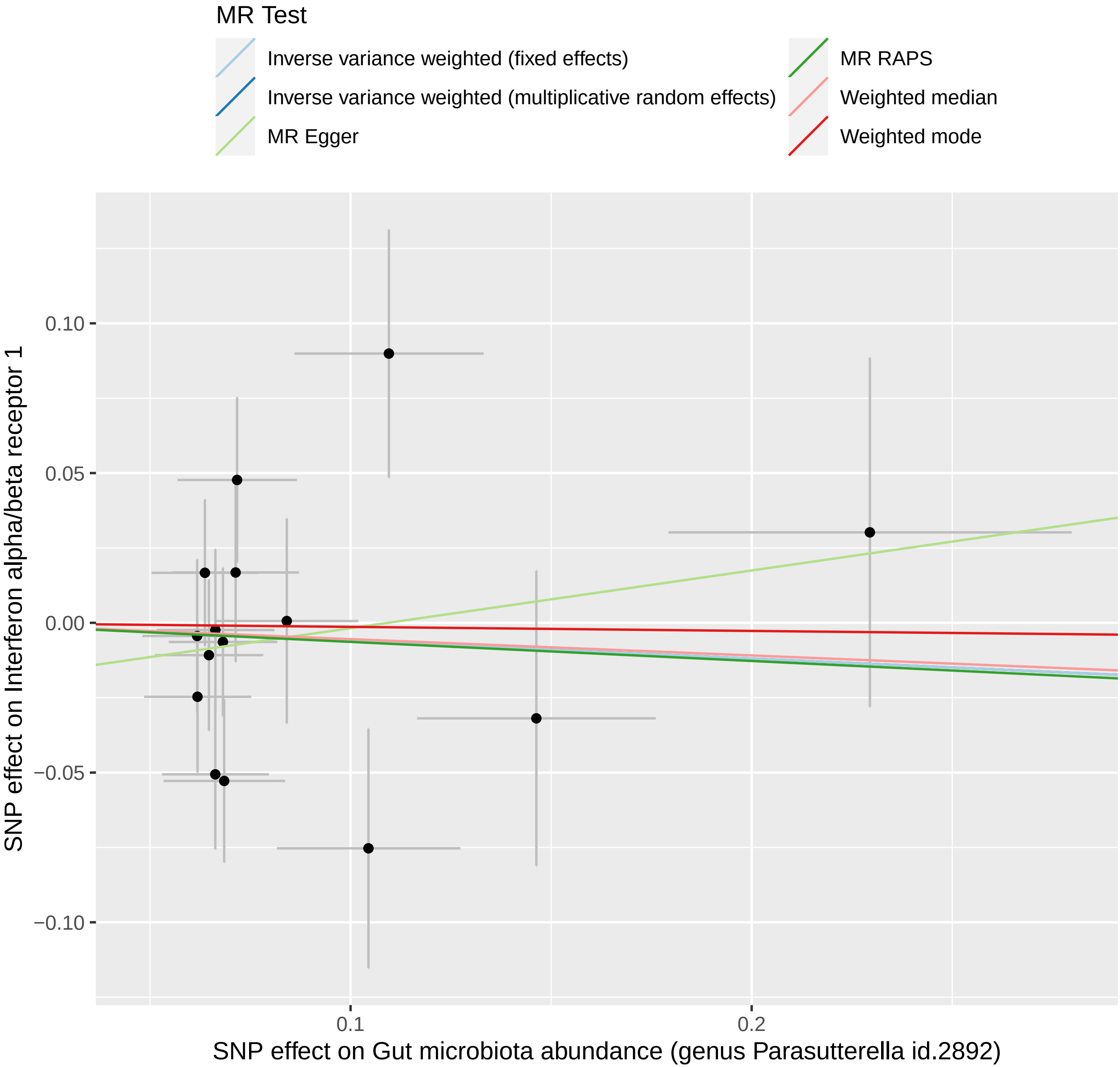

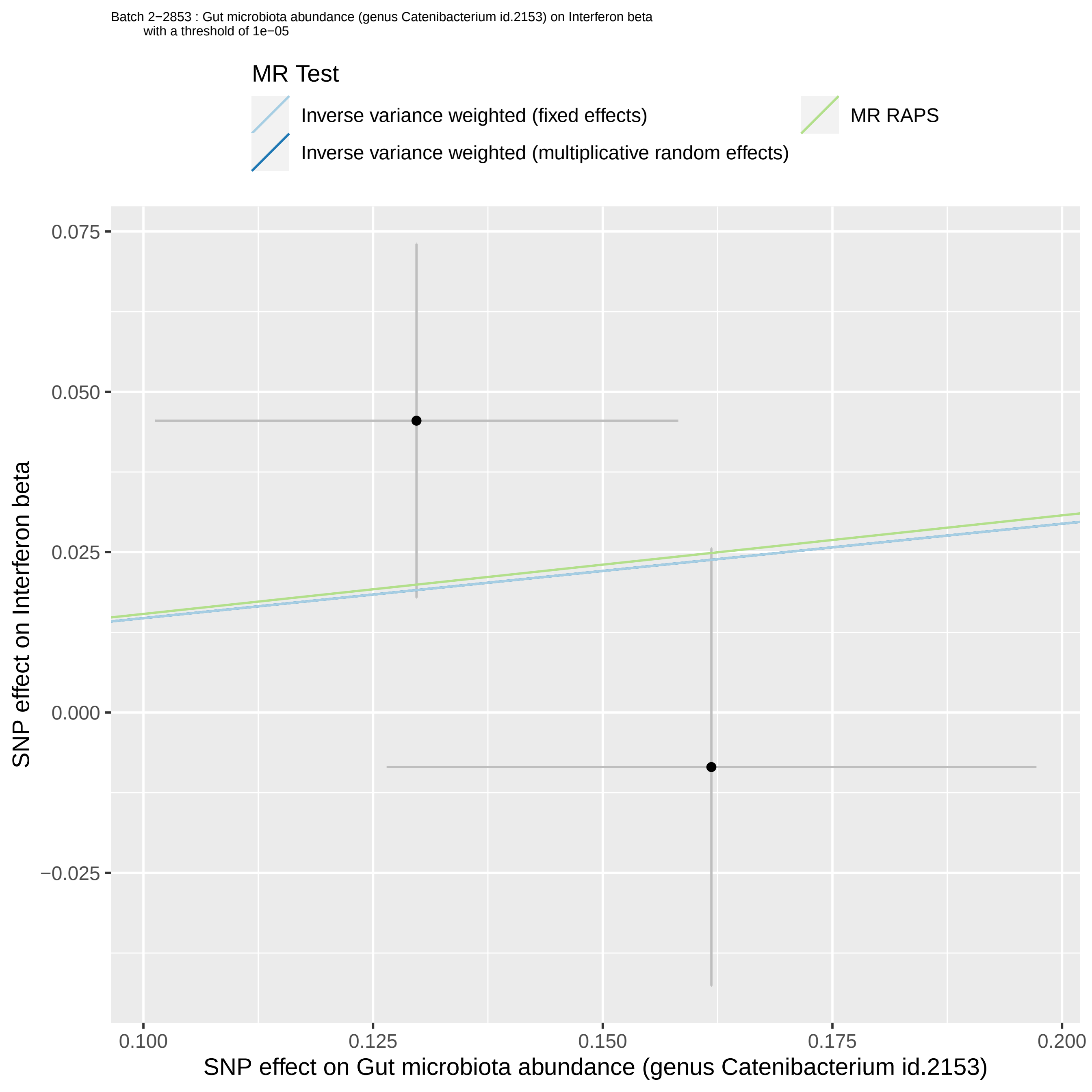

Batch 2−2853 : Gut microbiota abundance (genus Catenibacterium id.2153) on Interferon beta
with a threshold of 1e−05
MR Test
Inverse variance weighted (fixed effects)
Inverse variance weighted (multiplicative random effects)
MR RAPS
0.075
0.050
0.025
0.000
−0.025
0.100
0.125
0.150
0.175
0.200
SNP effect on Interferon beta
SNP effect on Gut microbiota abundance (genus Catenibacterium id.2153)

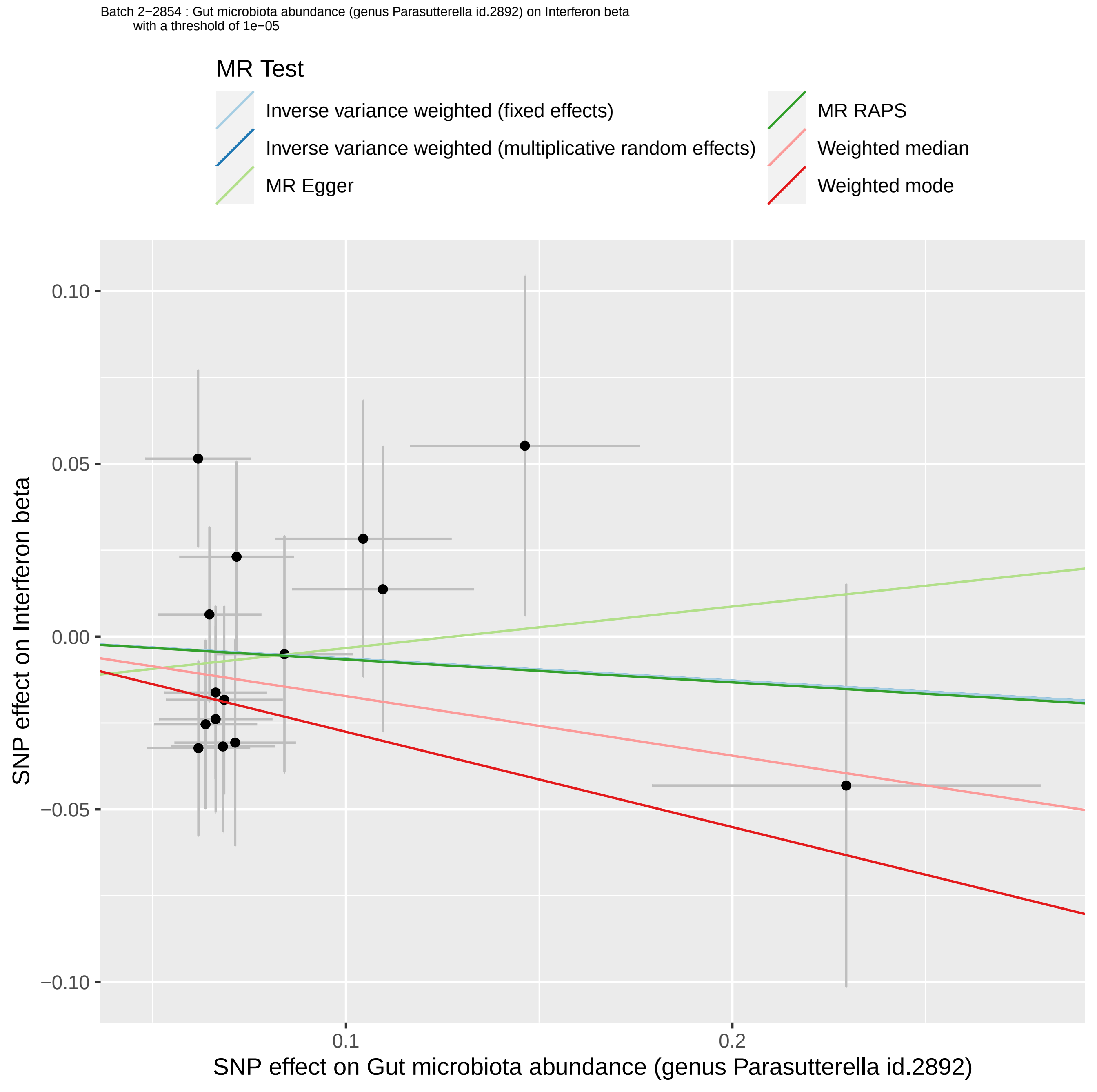
Batch 2−2854 : Gut microbiota abundance (genus Parasutterella id.2892) on Interferon beta
with a threshold of 1e−05
MR Test
Inverse variance weighted (fixed effects)
Inverse variance weighted (multiplicative random effects)
MR Egger
MR RAPS
Weighted median
Weighted mode
0.10
0.05
0.00
−0.05
−0.10
0.1
0.2
SNP effect on Interferon beta
SNP effect on Gut microbiota abundance (genus Parasutterella id.2892)

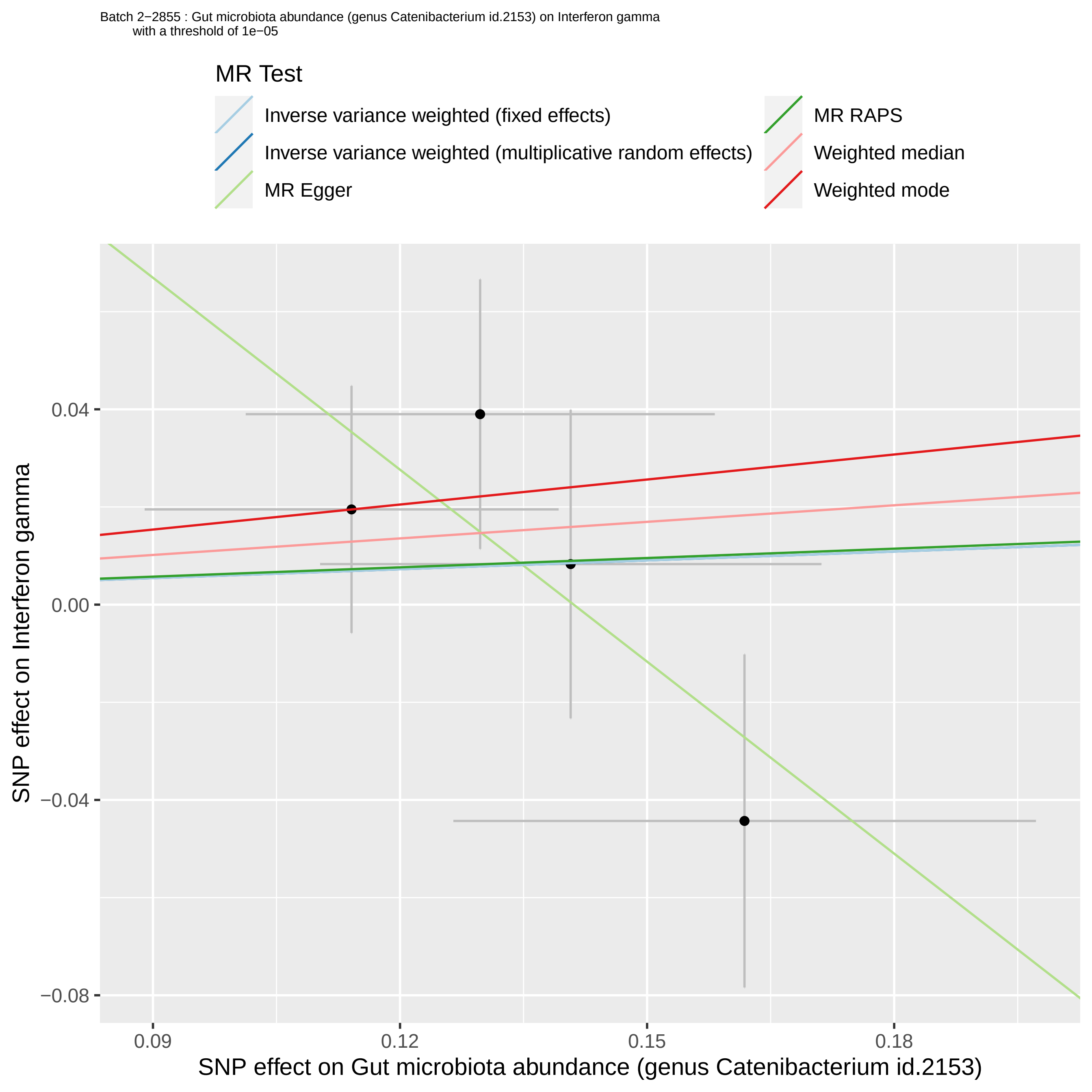
Batch 2−2855 : Gut microbiota abundance (genus Catenibacterium id.2153) on Interferon gamma
with a threshold of 1e−05
MR Test
Inverse variance weighted (fixed effects)
Inverse variance weighted (multiplicative random effects)
MR Egger
MR RAPS
Weighted median
Weighted mode
SNP effect on Interferon gamma
0.04
0.00
−0.04
−0.08
0.09
0.12
0.15
0.18
SNP effect on Gut microbiota abundance (genus Catenibacterium id.2153)

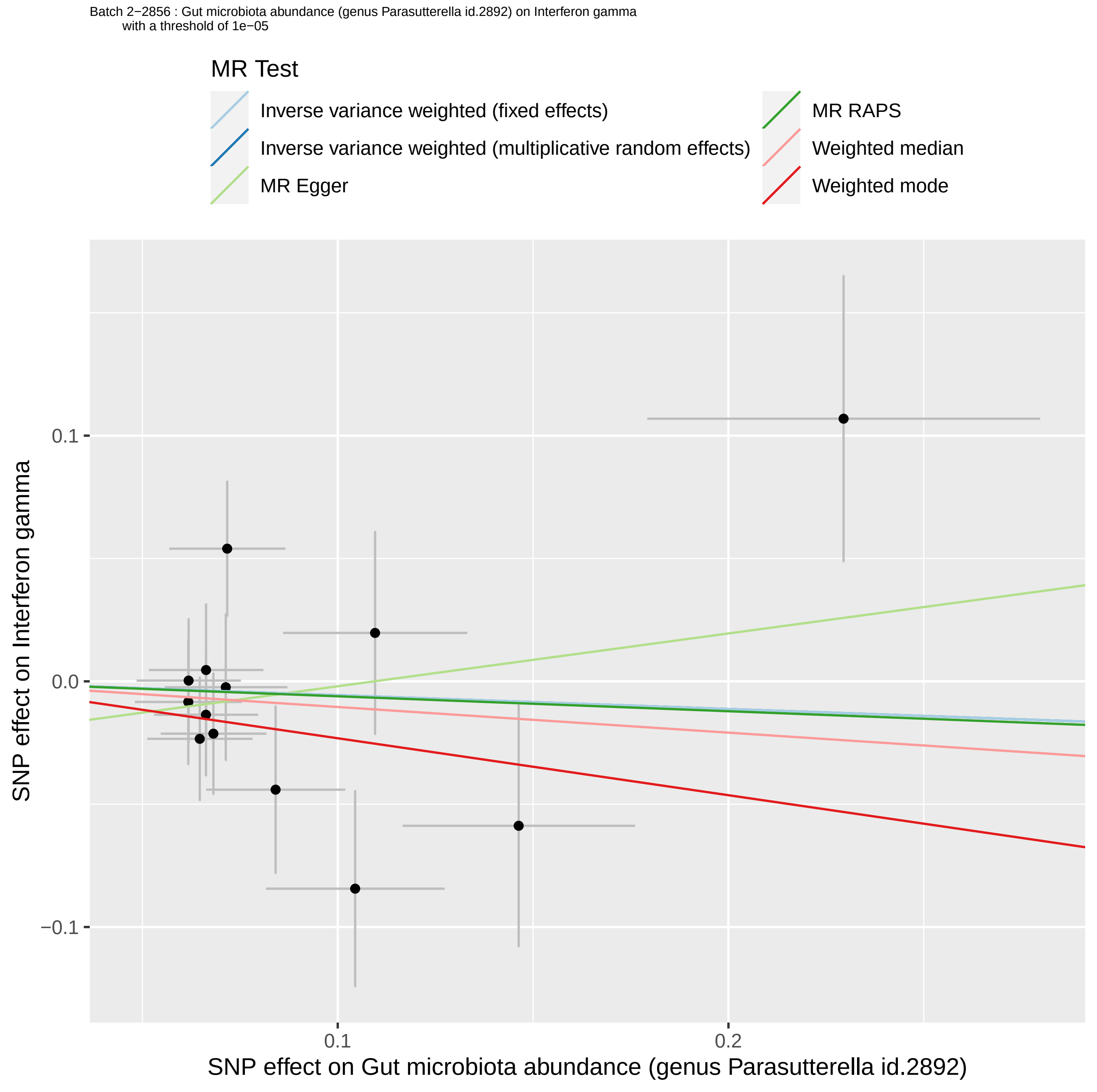

Batch 2−2856 : Gut microbiota abundance (genus Parasutterella id.2892) on Interferon gamma
with a threshold of 1e−05
MR Test
Inverse variance weighted (fixed effects)
Inverse variance weighted (multiplicative random effects)
MR Egger
MR RAPS
Weighted median
Weighted mode
0.1
0.0
−0.1
0.1
0.2
SNP effect on Interferon gamma
SNP effect on Gut microbiota abundance (genus Parasutterella id.2892)

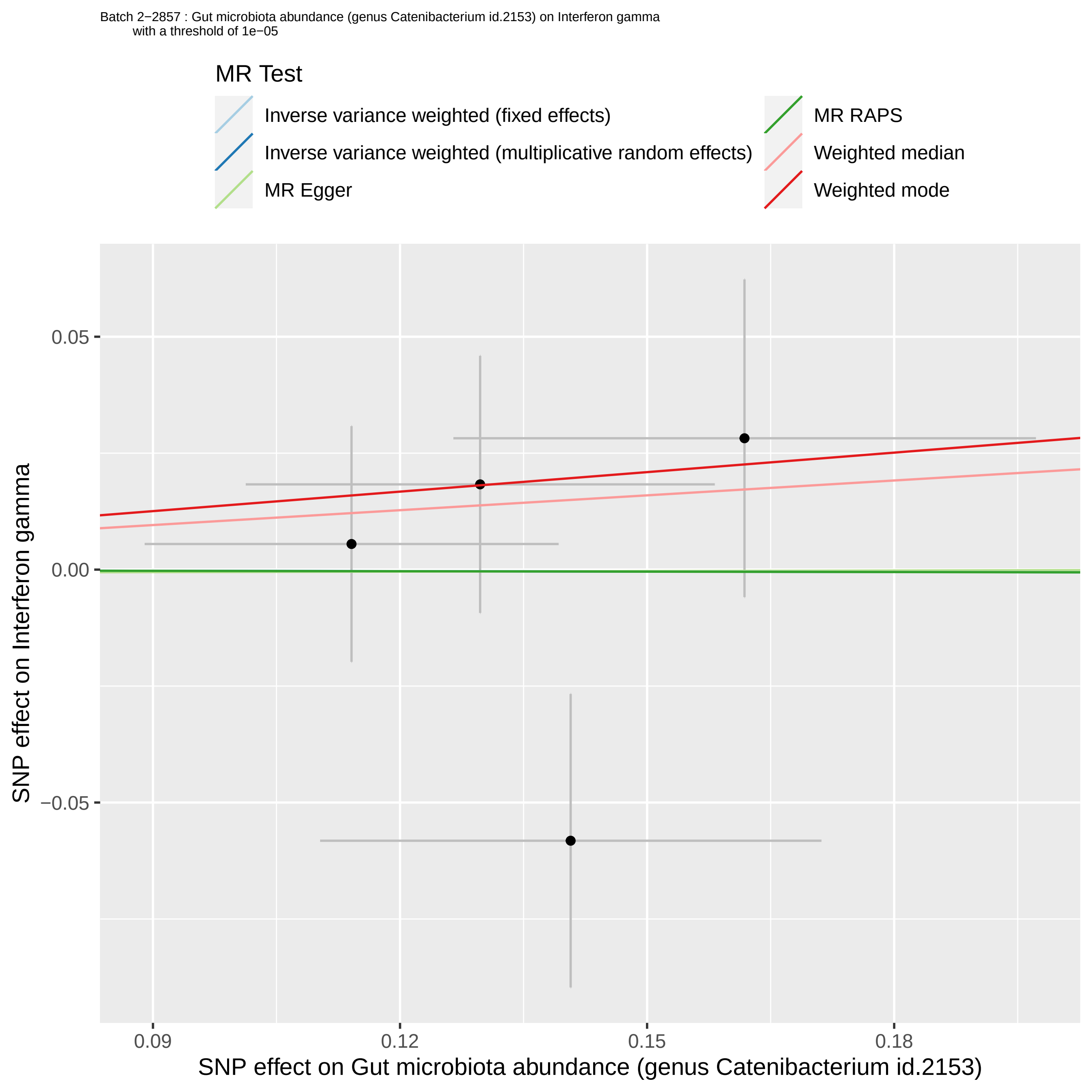
Batch 2−2857 : Gut microbiota abundance (genus Catenibacterium id.2153) on Interferon gamma
with a threshold of 1e−05
MR Test
Inverse variance weighted (fixed effects)
Inverse variance weighted (multiplicative random effects)
MR Egger
MR RAPS
Weighted median
Weighted mode
0.05
0.00
−0.05
0.09
0.12
0.15
0.18
SNP effect on Interferon gamma
SNP effect on Gut microbiota abundance (genus Catenibacterium id.2153)

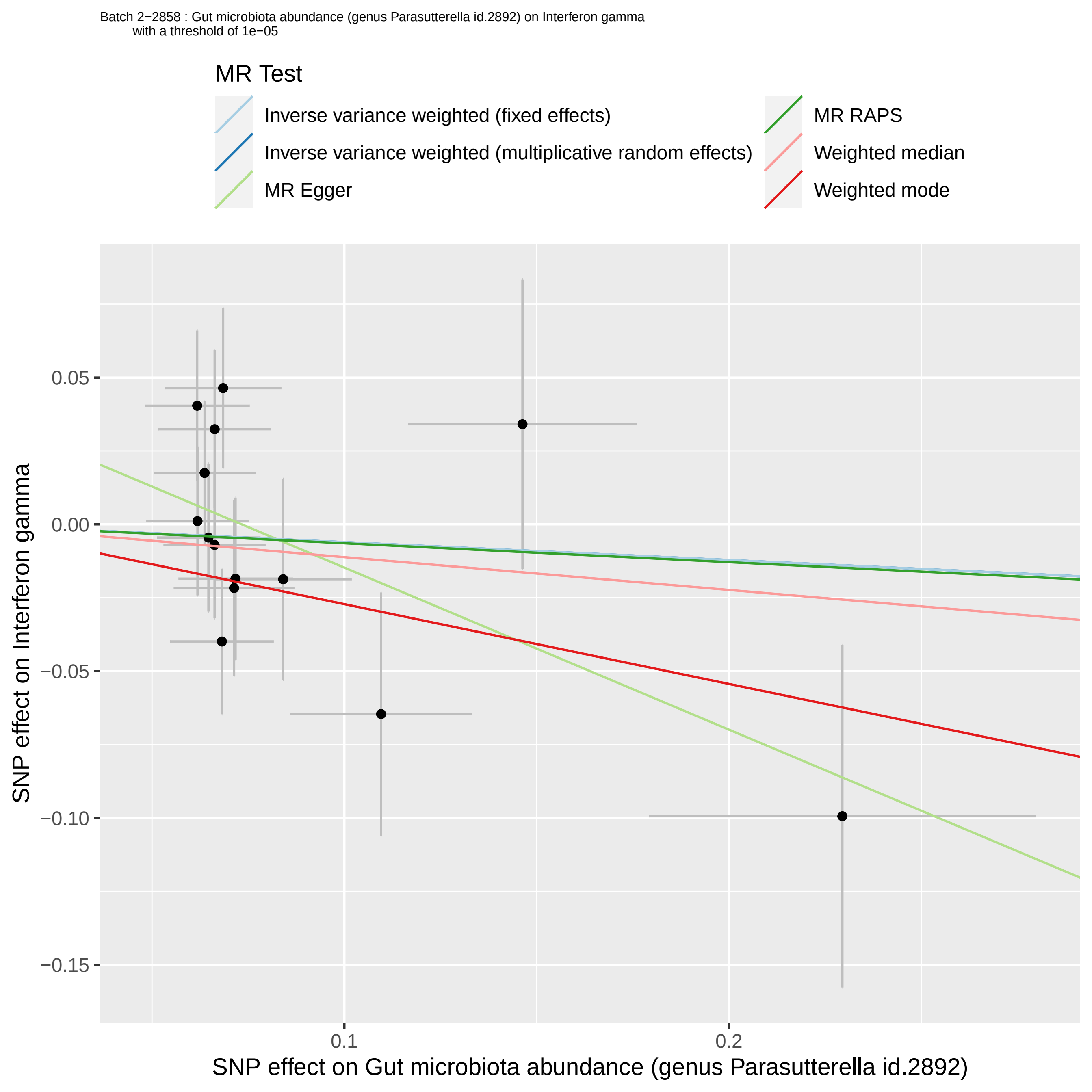

Batch 2−2858 : Gut microbiota abundance (genus Parasutterella id.2892) on Interferon gamma
with a threshold of 1e−05
MR Test
Inverse variance weighted (fixed effects)
Inverse variance weighted (multiplicative random effects)
MR Egger
MR RAPS
Weighted median
Weighted mode
0.05
0.00
−0.05
−0.10
−0.15
0.1
0.2
SNP effect on Interferon gamma
SNP effect on Gut microbiota abundance (genus Parasutterella id.2892)

Batch 2−2859 : Gut microbiota abundance (genus Catenibacterium id.2153) on Interferon gamma receptor 1
with a threshold of 1e−05

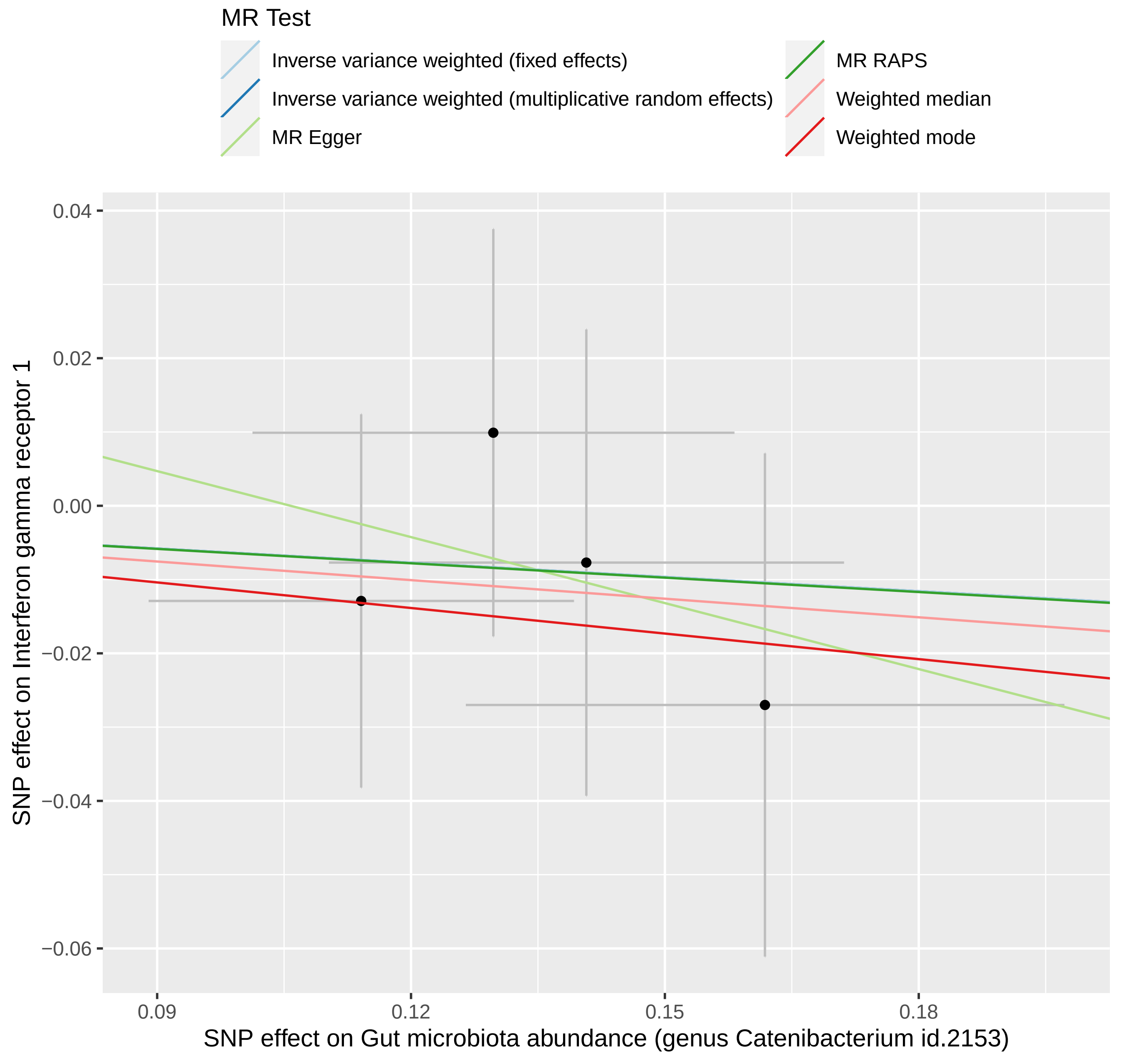

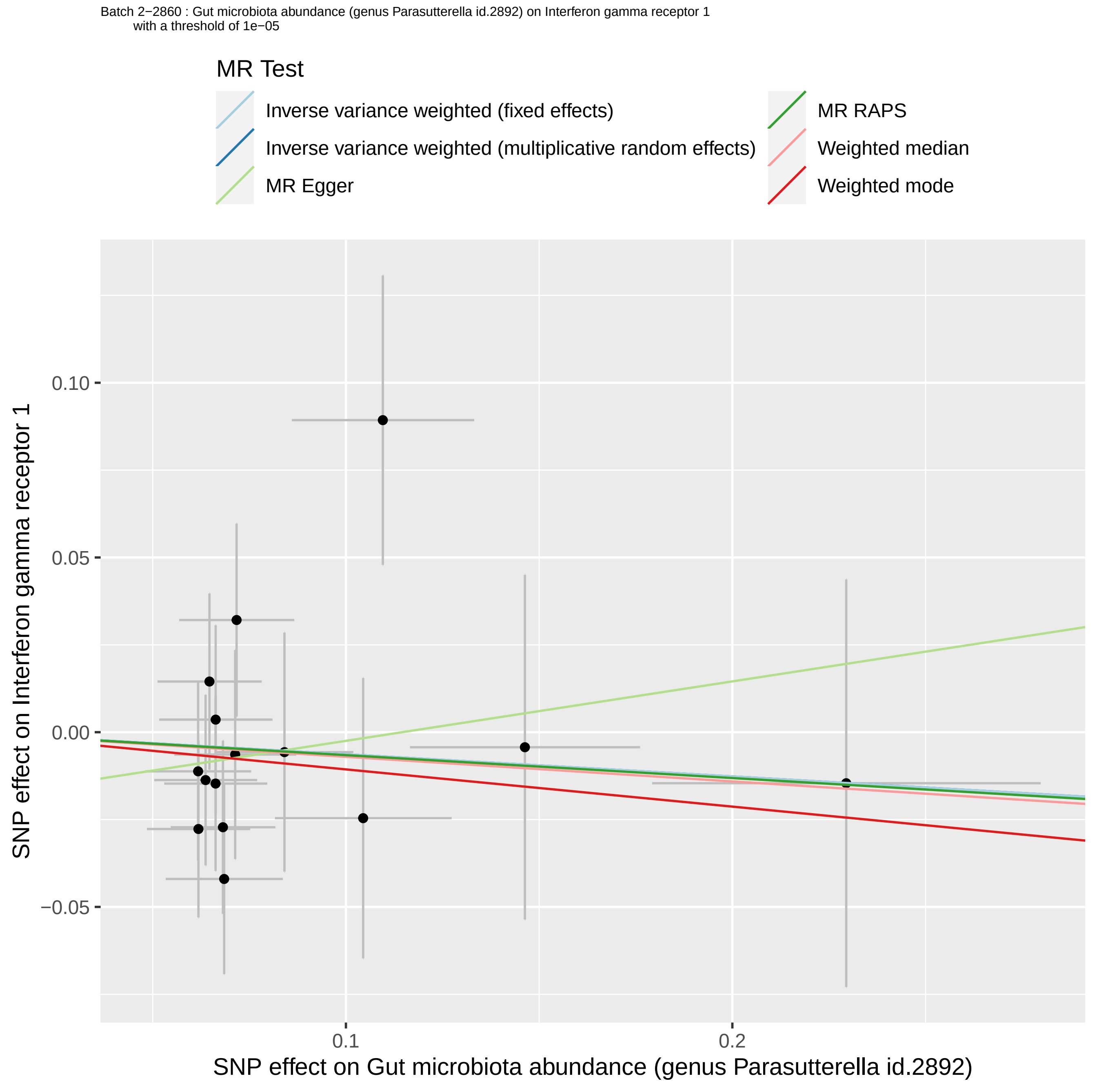

Batch 2−2860 : Gut microbiota abundance (genus Parasutterella id.2892) on Interferon gamma receptor 1
with a threshold of 1e−05
MR Test
Inverse variance weighted (fixed effects)
Inverse variance weighted (multiplicative random effects)
MR Egger
MR RAPS
Weighted median
Weighted mode
0.10
0.05
0.00
−0.05
0.1
0.2
SNP effect on Interferon gamma receptor 1
SNP effect on Gut microbiota abundance (genus Parasutterella id.2892)

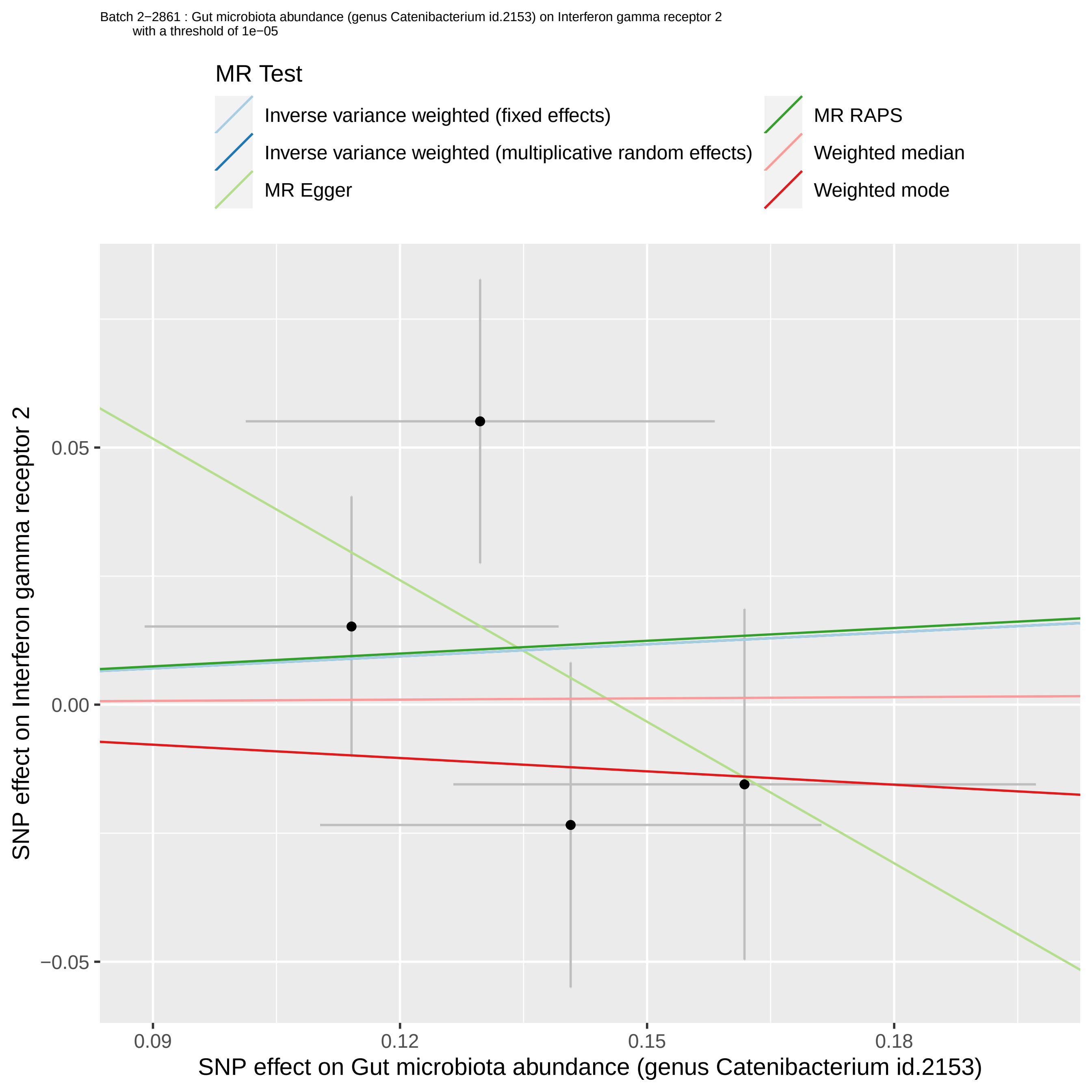

Batch 2−2861 : Gut microbiota abundance (genus Catenibacterium id.2153) on Interferon gamma receptor 2
with a threshold of 1e−05
MR Test
Inverse variance weighted (fixed effects)
Inverse variance weighted (multiplicative random effects)
MR Egger
MR RAPS
Weighted median
Weighted mode
SNP effect on Interferon gamma receptor 2
0.05
0.00
−0.05
0.09
0.12
0.15
0.18
SNP effect on Gut microbiota abundance (genus Catenibacterium id.2153)

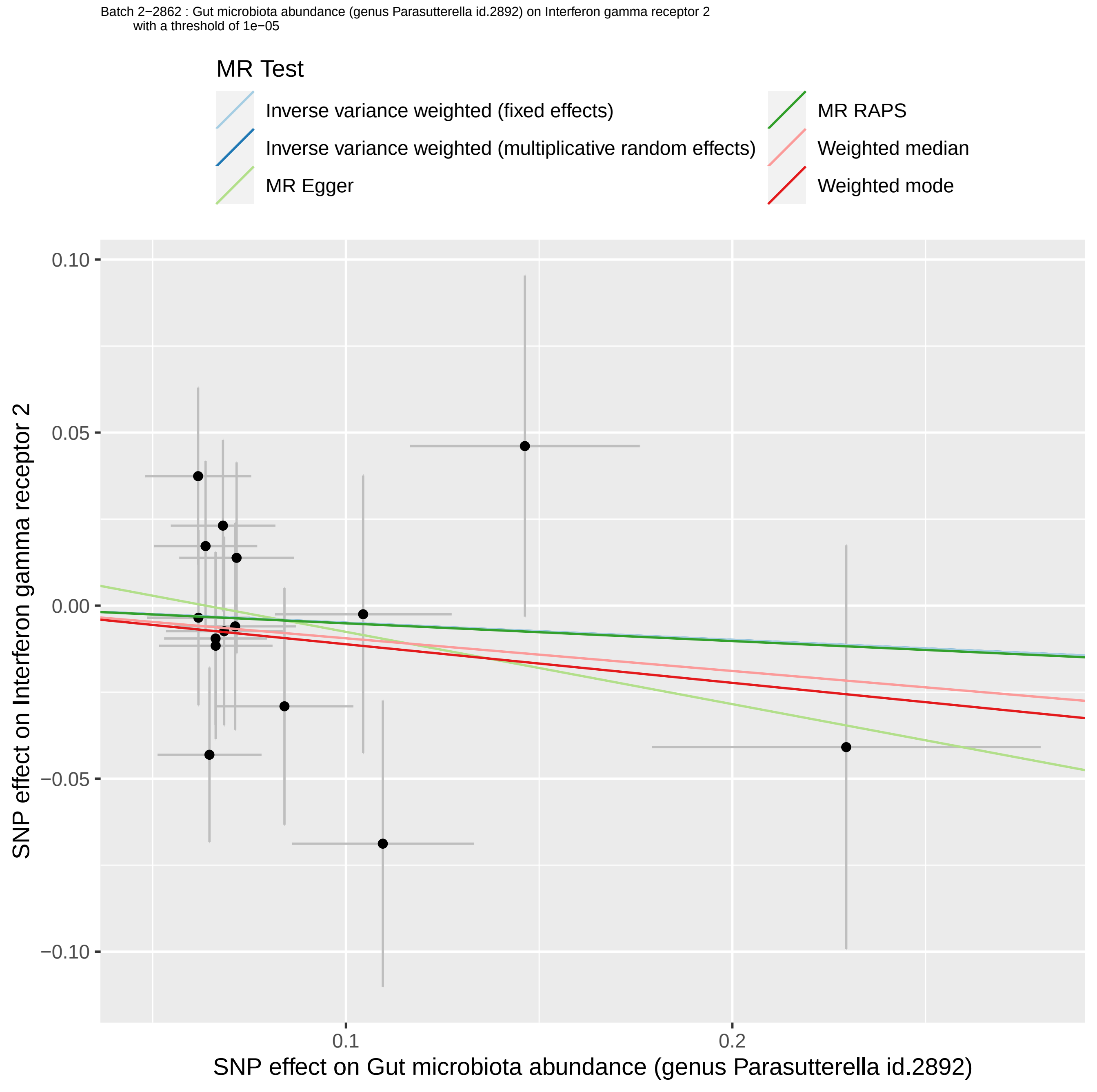
Batch 2−2862 : Gut microbiota abundance (genus Parasutterella id.2892) on Interferon gamma receptor 2
with a threshold of 1e−05
MR Test
Inverse variance weighted (fixed effects)
Inverse variance weighted (multiplicative random effects)
MR Egger
MR RAPS
Weighted median
Weighted mode
0.10
0.05
0.00
−0.05
−0.10
0.1
0.2
SNP effect on Interferon gamma receptor 2
SNP effect on Gut microbiota abundance (genus Parasutterella id.2892)

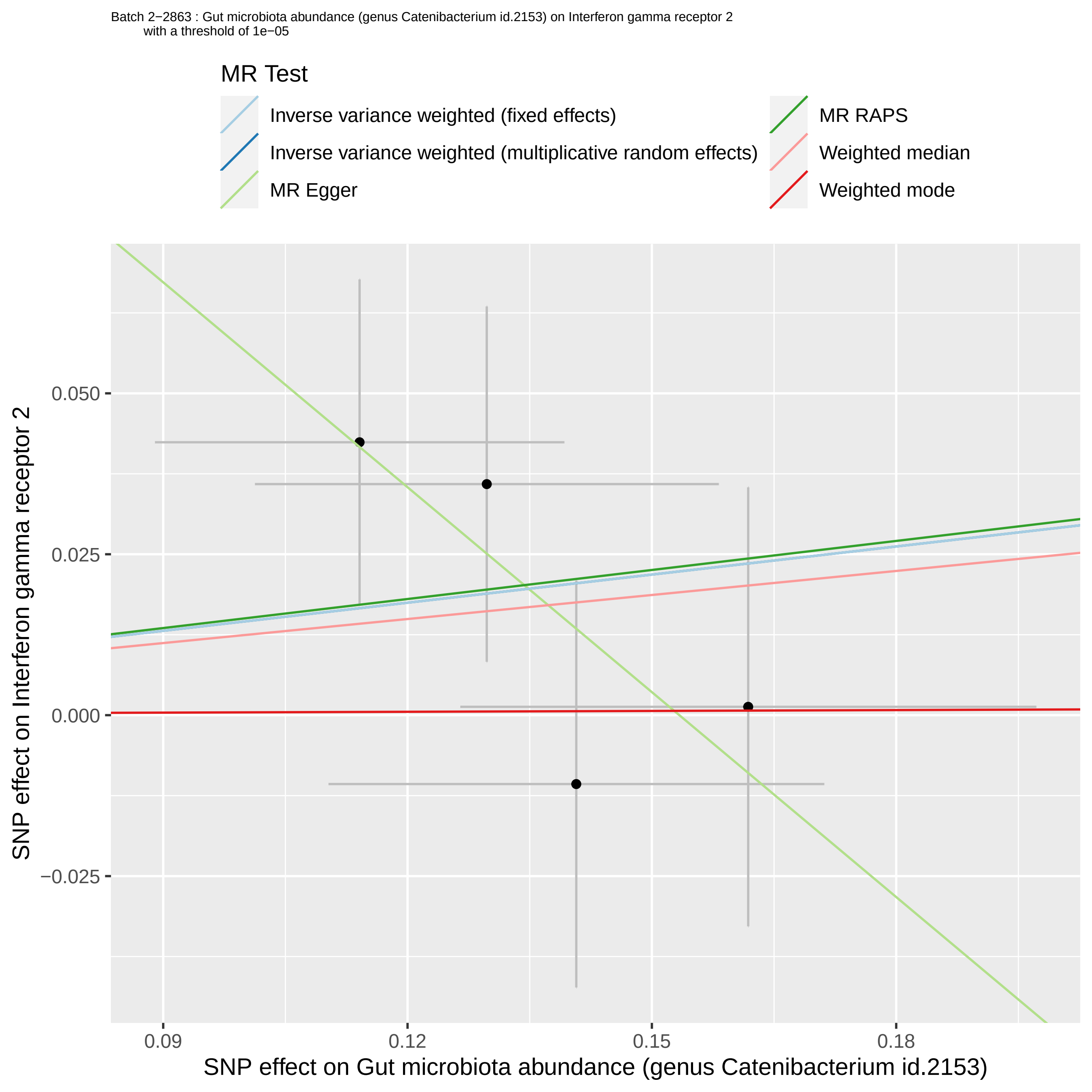
Batch 2−2863 : Gut microbiota abundance (genus Catenibacterium id.2153) on Interferon gamma receptor 2
with a threshold of 1e−05
MR Test
Inverse variance weighted (fixed effects)
Inverse variance weighted (multiplicative random effects)
MR Egger
MR RAPS
Weighted median
Weighted mode
0.050
0.025
0.000
−0.025
0.09
0.12
0.15
0.18
SNP effect on Interferon gamma receptor 2
SNP effect on Gut microbiota abundance (genus Catenibacterium id.2153)

Batch 2−2864 : Gut microbiota abundance (genus Parasutterella id.2892) on Interferon gamma receptor 2
with a threshold of 1e−05

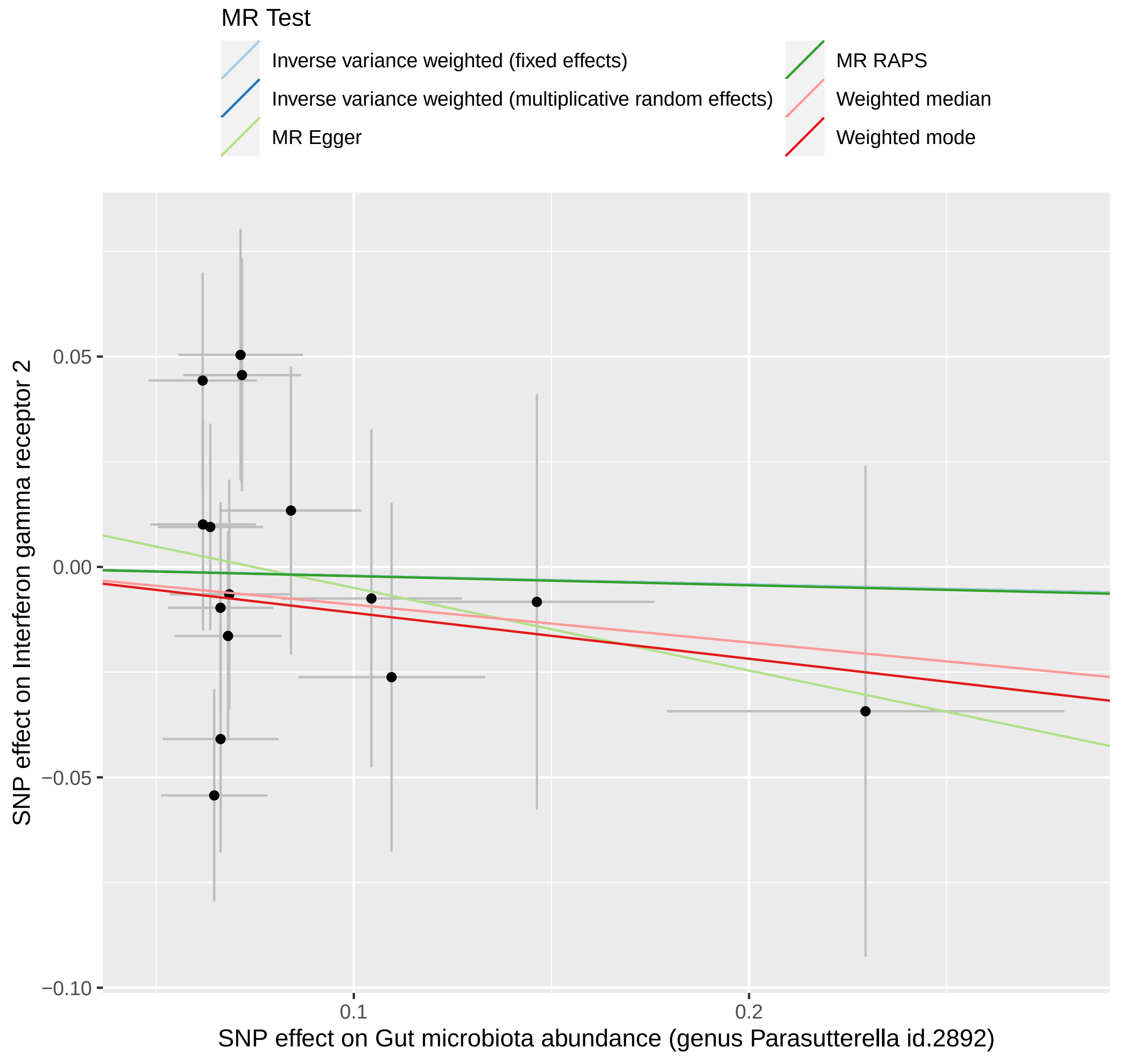

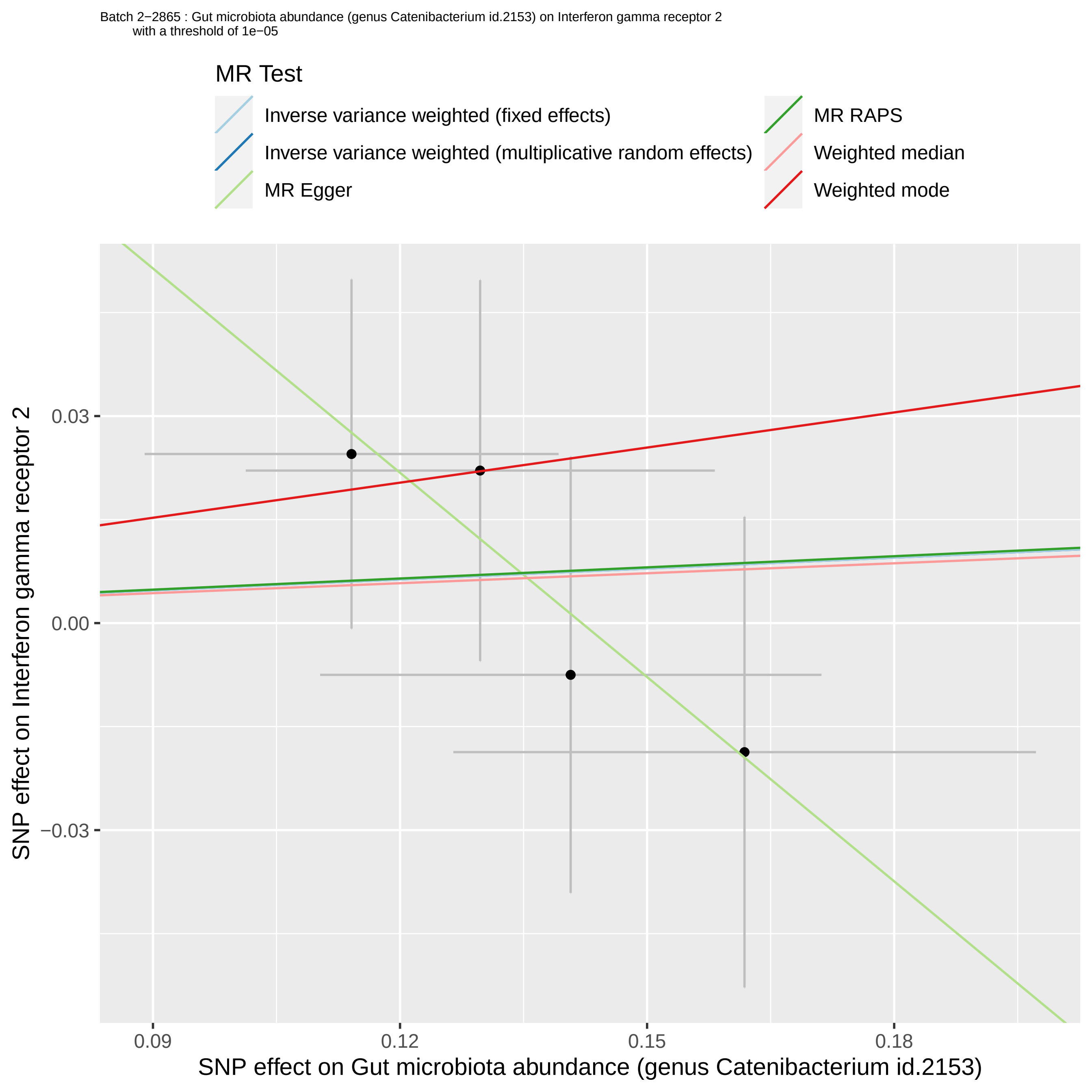
Batch 2−2865 : Gut microbiota abundance (genus Catenibacterium id.2153) on Interferon gamma receptor 2
with a threshold of 1e−05
MR Test
Inverse variance weighted (fixed effects)
Inverse variance weighted (multiplicative random effects)
MR Egger
MR RAPS
Weighted median
Weighted mode
SNP effect on Interferon gamma receptor 2
0.03
0.00
−0.03
0.09
0.12
0.15
0.18
SNP effect on Gut microbiota abundance (genus Catenibacterium id.2153)

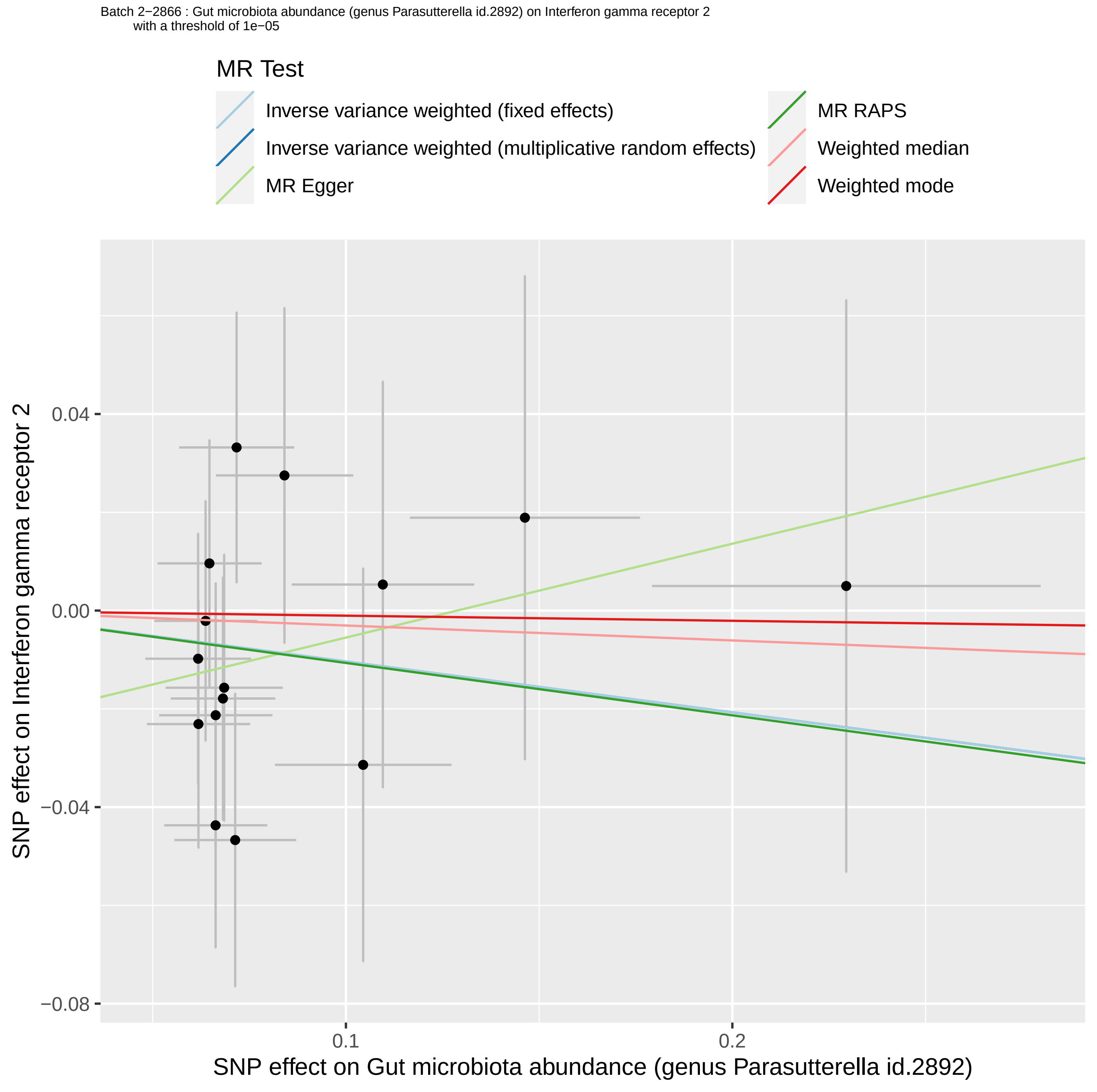
Batch 2−2866 : Gut microbiota abundance (genus Parasutterella id.2892) on Interferon gamma receptor 2
with a threshold of 1e−05
MR Test
Inverse variance weighted (fixed effects)
Inverse variance weighted (multiplicative random effects)
MR Egger
MR RAPS
Weighted median
Weighted mode
0.04
0.00
−0.04
−0.08
0.1
0.2
SNP effect on Interferon gamma receptor 2
SNP effect on Gut microbiota abundance (genus Parasutterella id.2892)

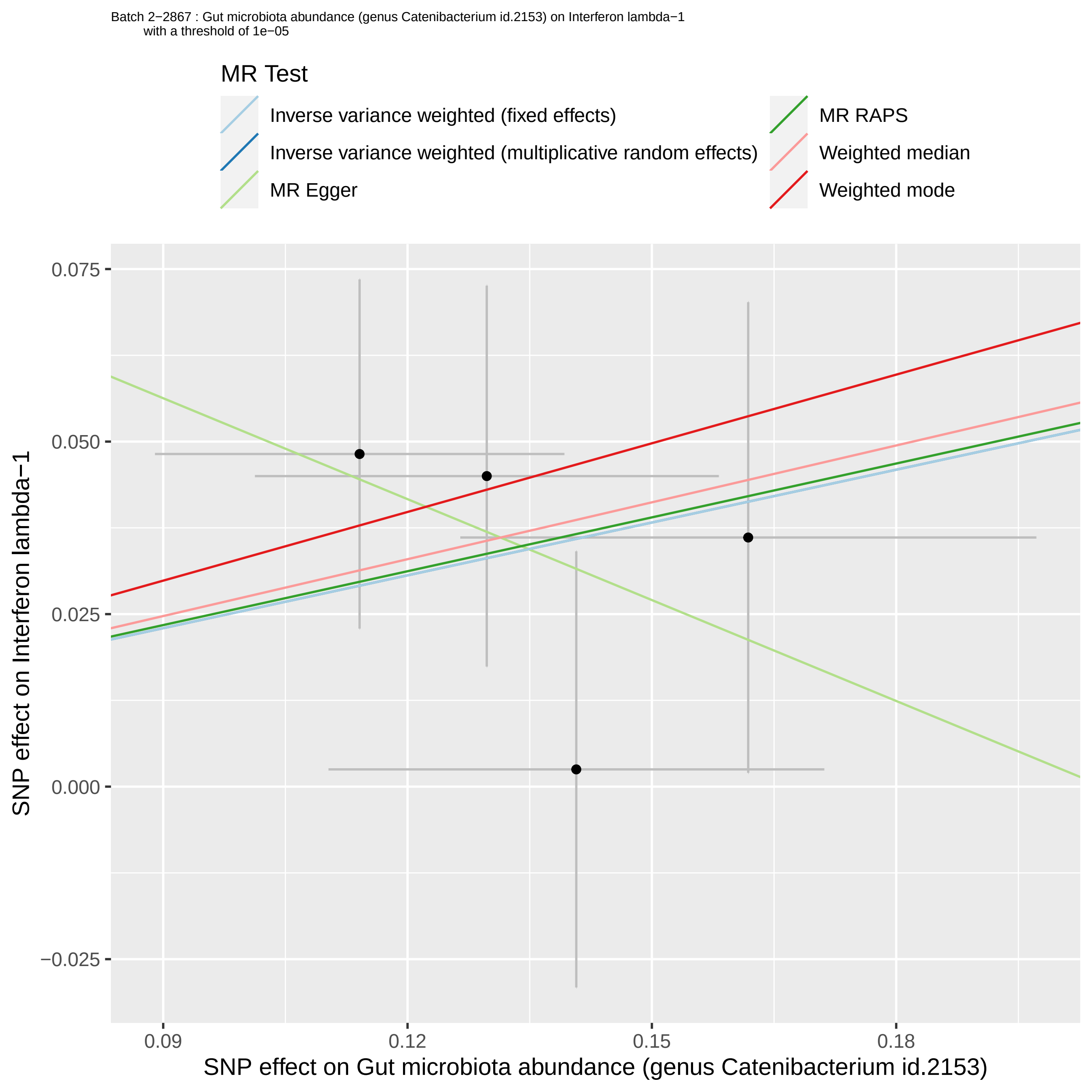

Batch 2−2867 : Gut microbiota abundance (genus Catenibacterium id.2153) on Interferon lambda−1
with a threshold of 1e−05
MR Test
Inverse variance weighted (fixed effects)
Inverse variance weighted (multiplicative random effects)
MR Egger
MR RAPS
Weighted median
Weighted mode
0.075
0.050
0.025
0.000
−0.025
SNP effect on Interferon lambda−1
0.09
0.12
0.15
0.18
SNP effect on Gut microbiota abundance (genus Catenibacterium id.2153)

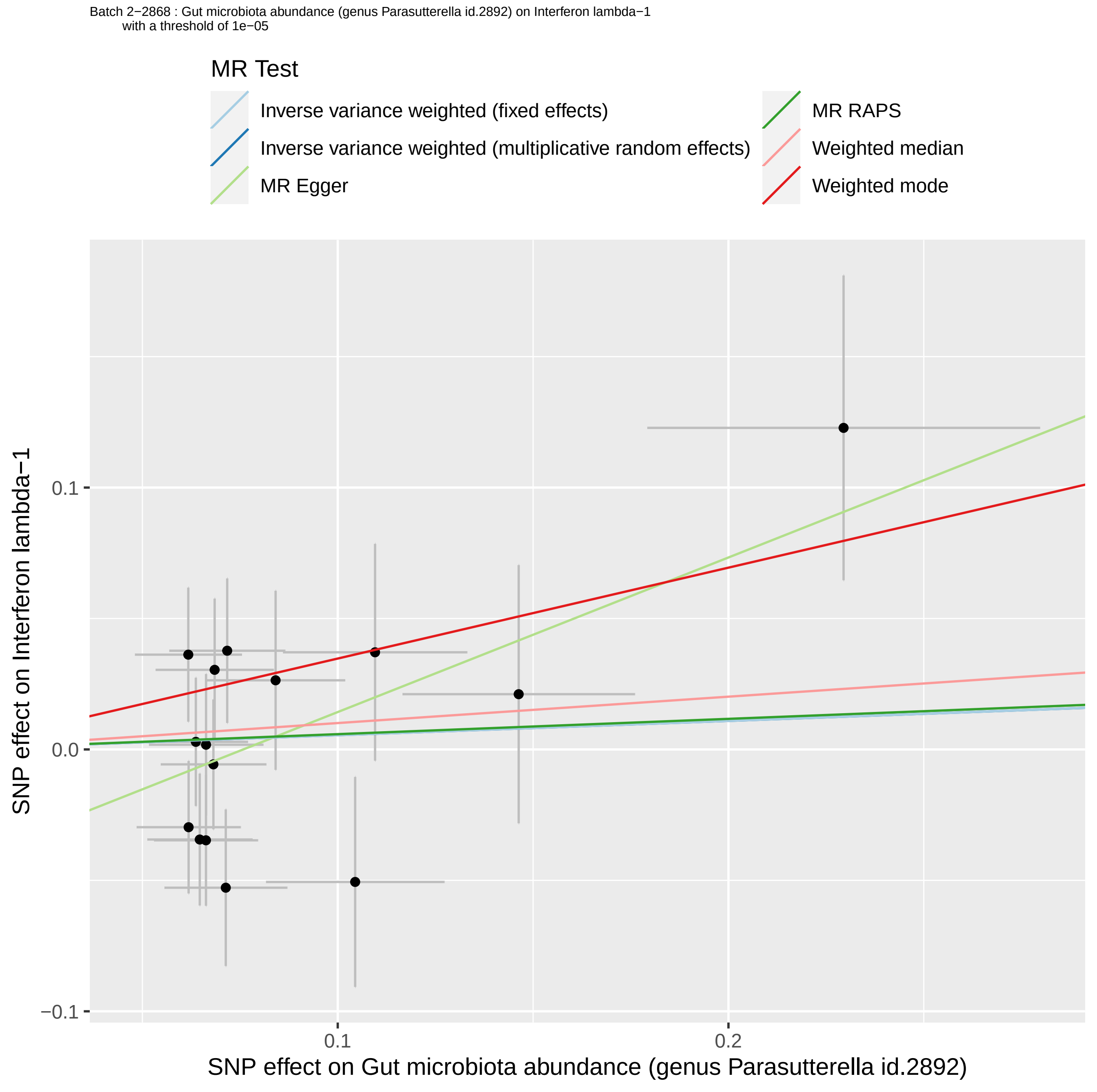
Batch 2−2868 : Gut microbiota abundance (genus Parasutterella id.2892) on Interferon lambda−1
with a threshold of 1e−05
MR Test
Inverse variance weighted (fixed effects)
Inverse variance weighted (multiplicative random effects)
MR Egger
MR RAPS
Weighted median
Weighted mode
SNP effect on Interferon lambda−1
0.1
0.0
−0.1
0.1
0.2
SNP effect on Gut microbiota abundance (genus Parasutterella id.2892)

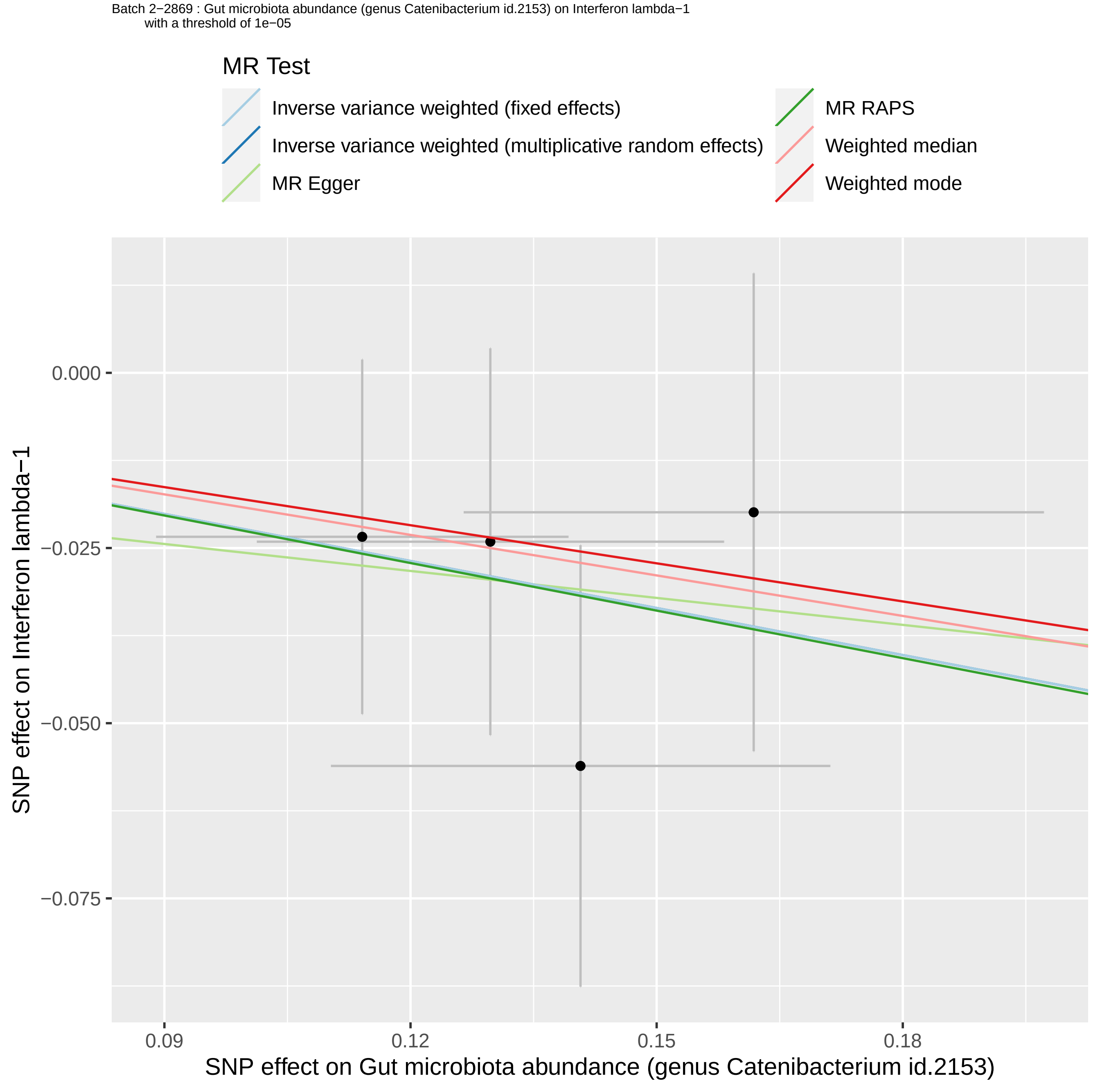

Batch 2−2869 : Gut microbiota abundance (genus Catenibacterium id.2153) on Interferon lambda−1
with a threshold of 1e−05
MR Test
Inverse variance weighted (fixed effects)
Inverse variance weighted (multiplicative random effects)
MR Egger
MR RAPS
Weighted median
Weighted mode
0.000
−0.025
−0.050
−0.075
0.09
0.12
0.15
0.18
SNP effect on Interferon lambda−1
SNP effect on Gut microbiota abundance (genus Catenibacterium id.2153)

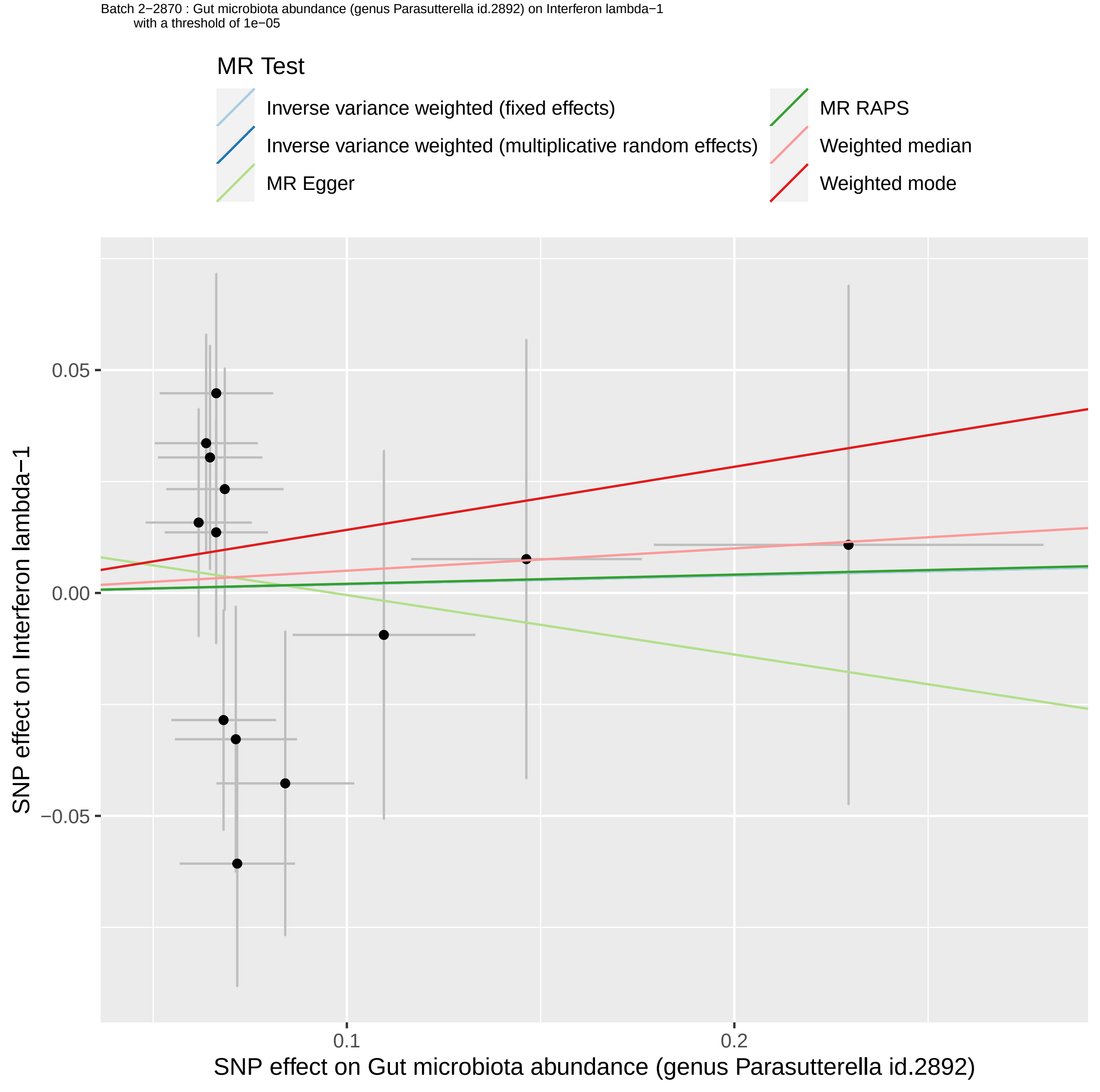
Batch 2−2870 : Gut microbiota abundance (genus Parasutterella id.2892) on Interferon lambda−1
with a threshold of 1e−05
MR Test
Inverse variance weighted (fixed effects)
Inverse variance weighted (multiplicative random effects)
MR Egger
MR RAPS
Weighted median
Weighted mode
0.05
0.00
−0.05
0.1
0.2
SNP effect on Interferon lambda−1
SNP effect on Gut microbiota abundance (genus Parasutterella id.2892)

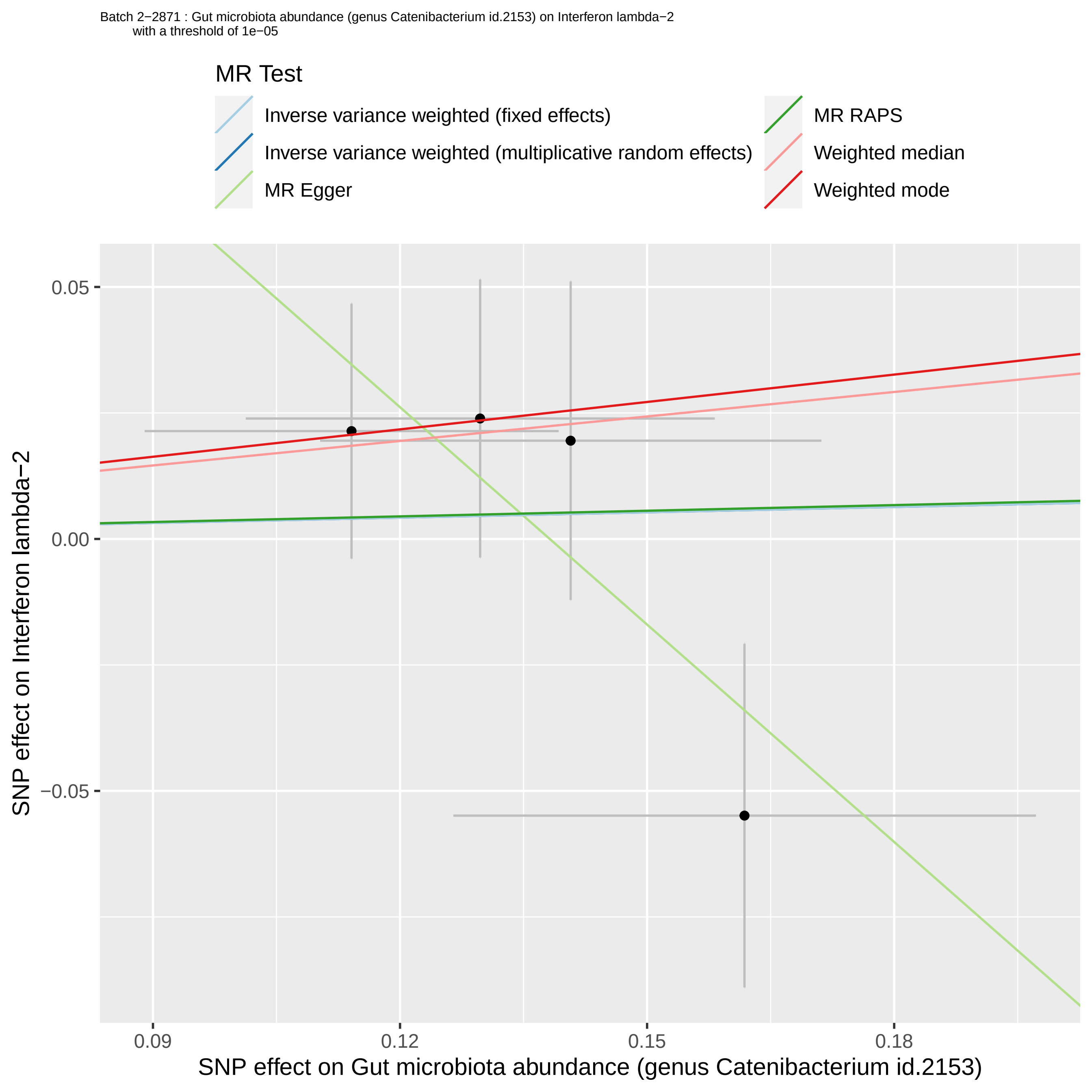

Batch 2−2871 : Gut microbiota abundance (genus Catenibacterium id.2153) on Interferon lambda−2
with a threshold of 1e−05
MR Test
Inverse variance weighted (fixed effects)
Inverse variance weighted (multiplicative random effects)
MR Egger
MR RAPS
Weighted median
Weighted mode
0.05
0.00
−0.05
0.09
0.12
0.15
0.18
SNP effect on Interferon lambda−2
SNP effect on Gut microbiota abundance (genus Catenibacterium id.2153)

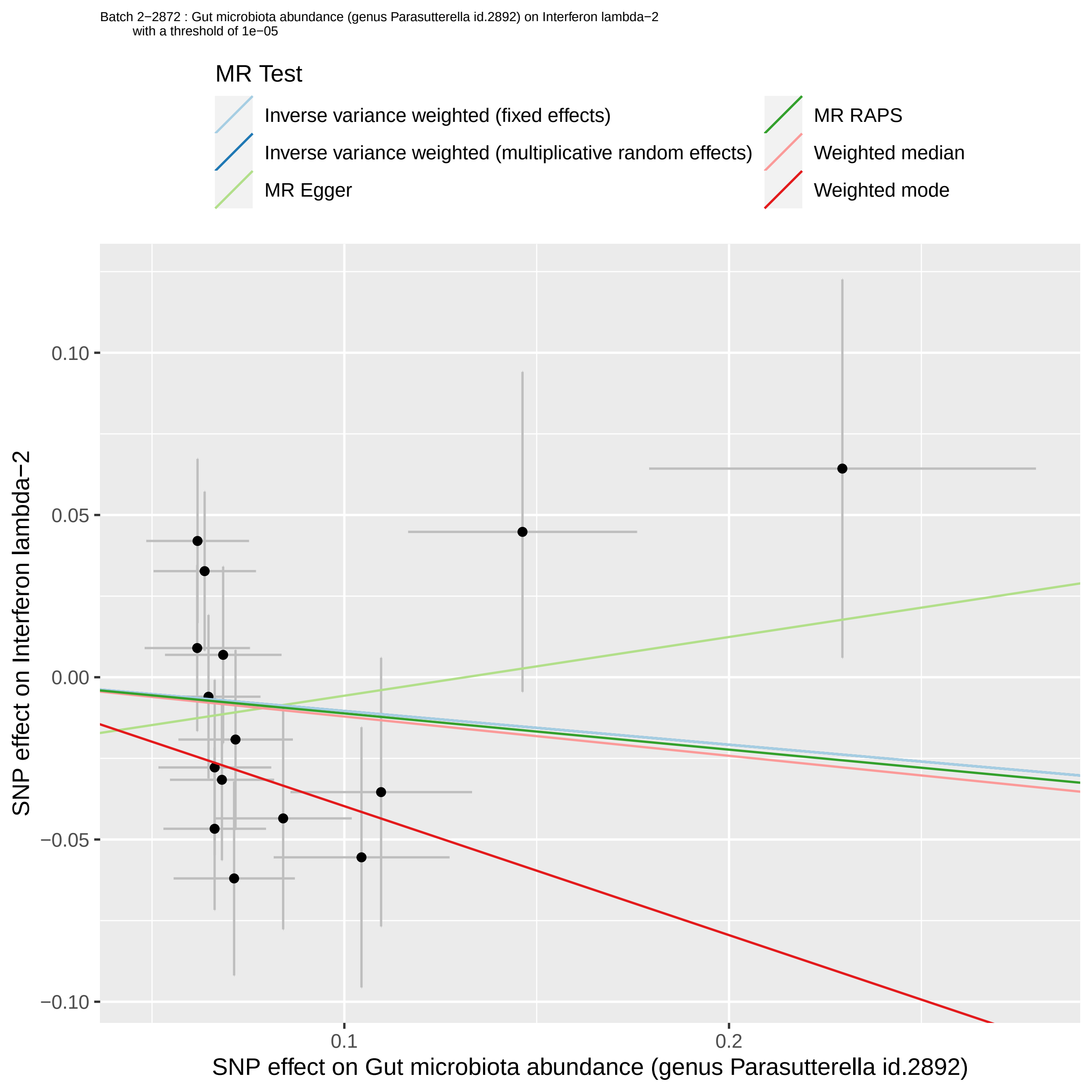
Batch 2−2872 : Gut microbiota abundance (genus Parasutterella id.2892) on Interferon lambda−2
with a threshold of 1e−05
MR Test
Inverse variance weighted (fixed effects)
Inverse variance weighted (multiplicative random effects)
MR Egger
MR RAPS
Weighted median
Weighted mode
0.10
0.05
0.00
−0.05
−0.10
SNP effect on Interferon lambda−2
0.1
0.2
SNP effect on Gut microbiota abundance (genus Parasutterella id.2892)

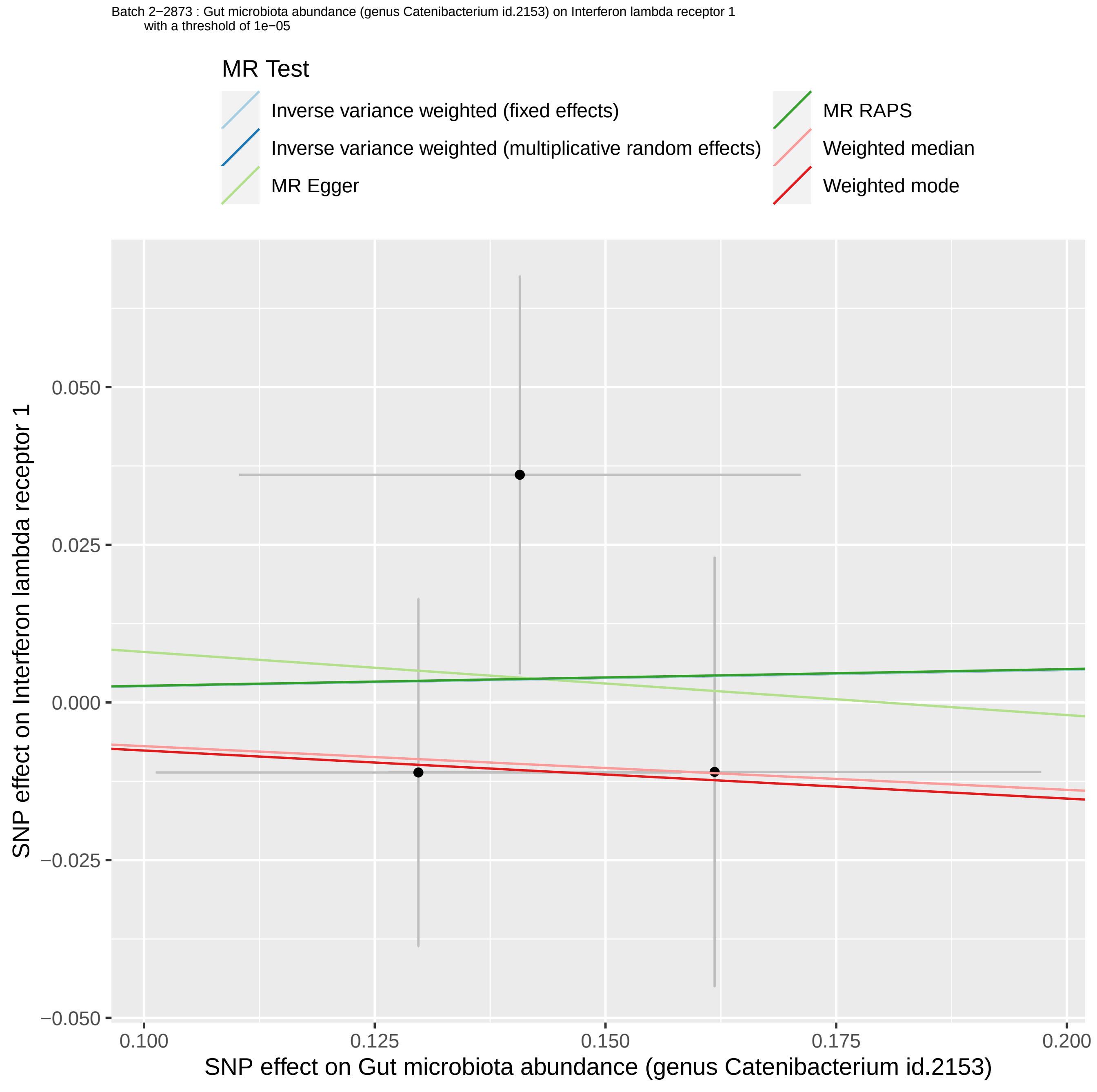
Batch 2−2873 : Gut microbiota abundance (genus Catenibacterium id.2153) on Interferon lambda receptor 1
with a threshold of 1e−05
MR Test
Inverse variance weighted (fixed effects)
Inverse variance weighted (multiplicative random effects)
MR Egger
MR RAPS
Weighted median
Weighted mode
0.050
0.025
0.000
−0.025
−0.050
0.100
0.125
0.150
0.175
0.200
SNP effect on Interferon lambda receptor 1
SNP effect on Gut microbiota abundance (genus Catenibacterium id.2153)

Batch 2−2874 : Gut microbiota abundance (genus Parasutterella id.2892) on Interferon lambda receptor 1
with a threshold of 1e−05

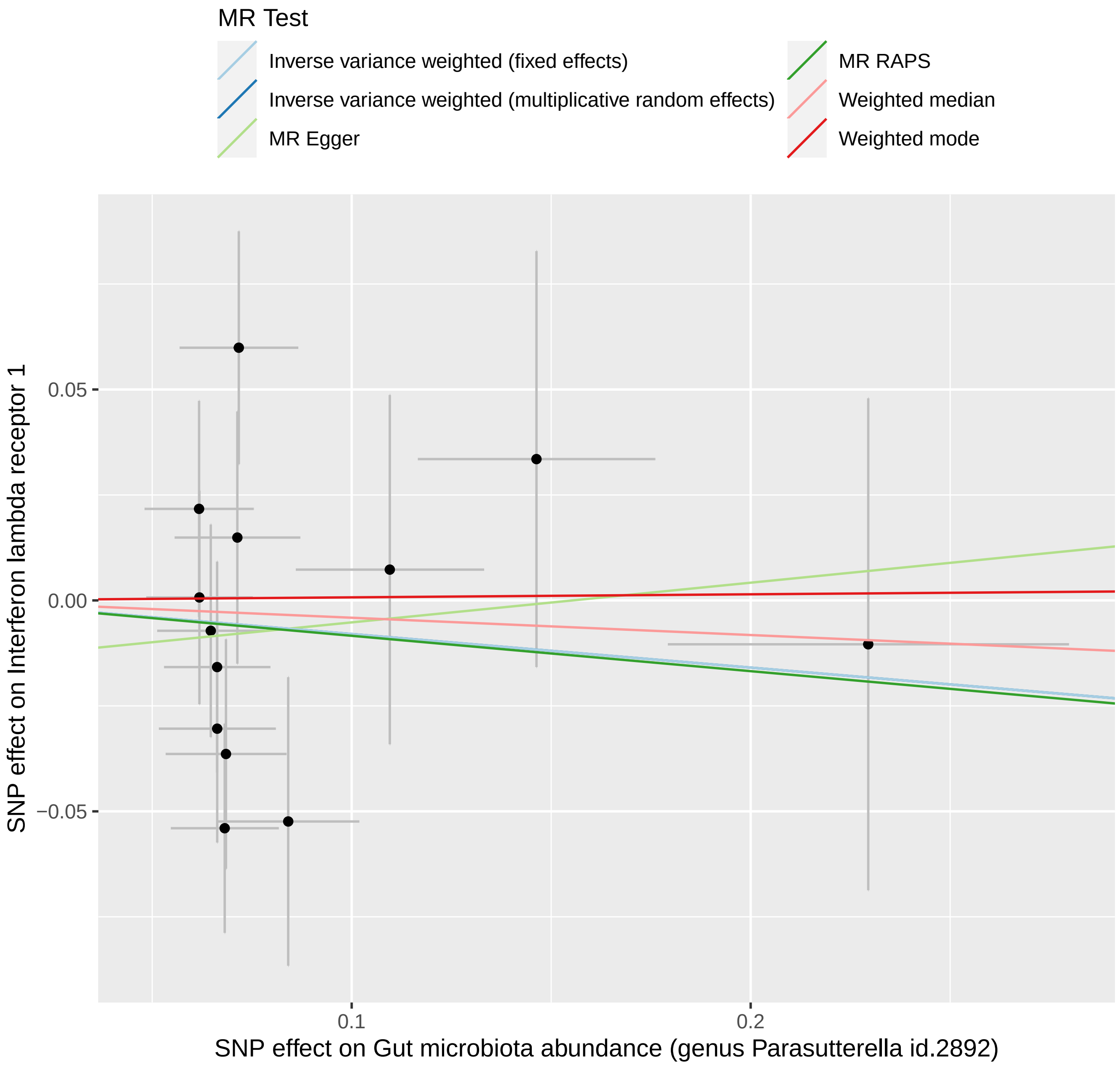

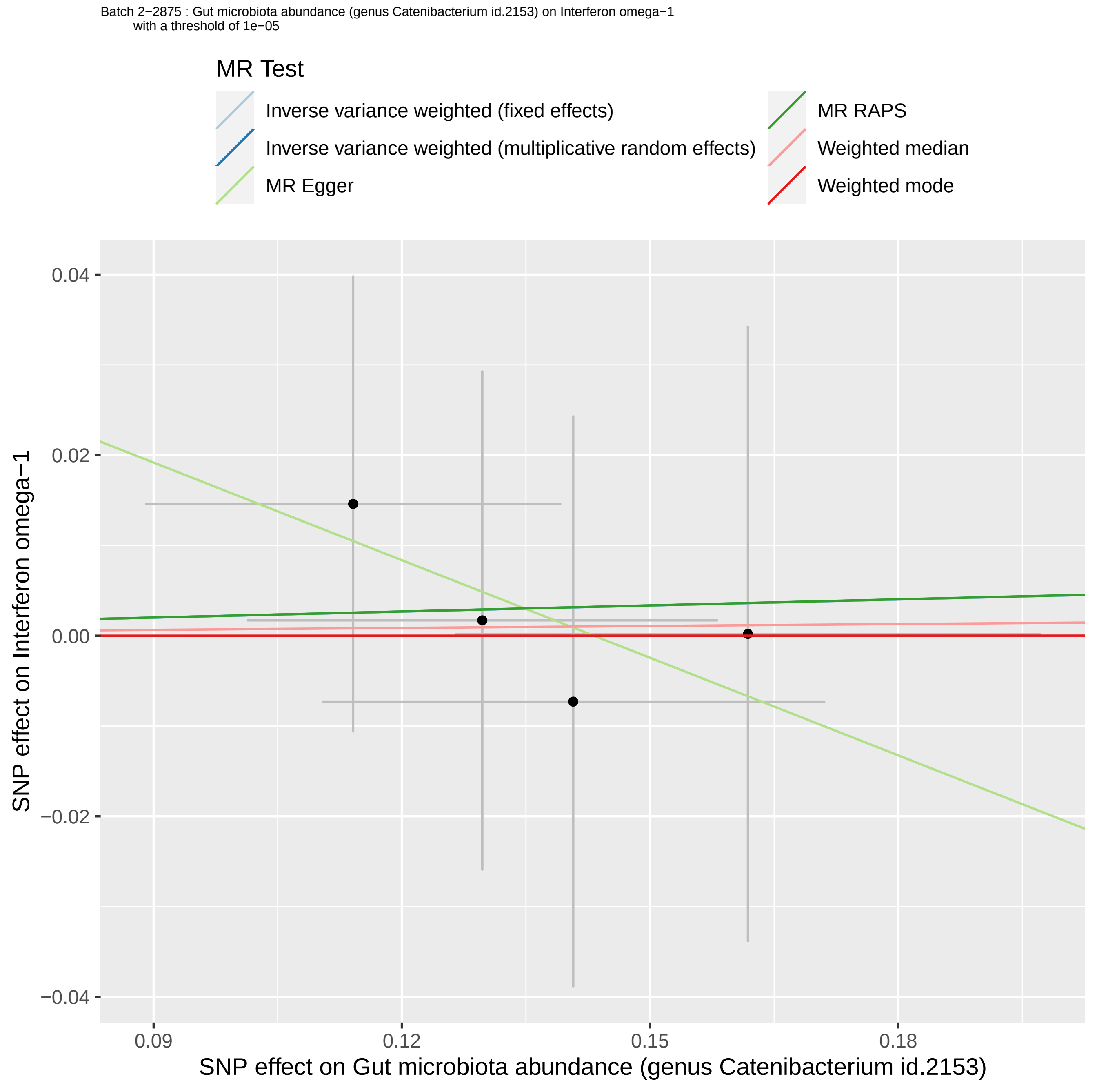
Batch 2−2875 : Gut microbiota abundance (genus Catenibacterium id.2153) on Interferon omega−1
with a threshold of 1e−05
MR Test
Inverse variance weighted (fixed effects)
Inverse variance weighted (multiplicative random effects)
MR Egger
MR RAPS
Weighted median
Weighted mode
0.04
0.02
0.00
−0.02
−0.04
0.09
0.12
0.15
0.18
SNP effect on Interferon omega−1
SNP effect on Gut microbiota abundance (genus Catenibacterium id.2153)

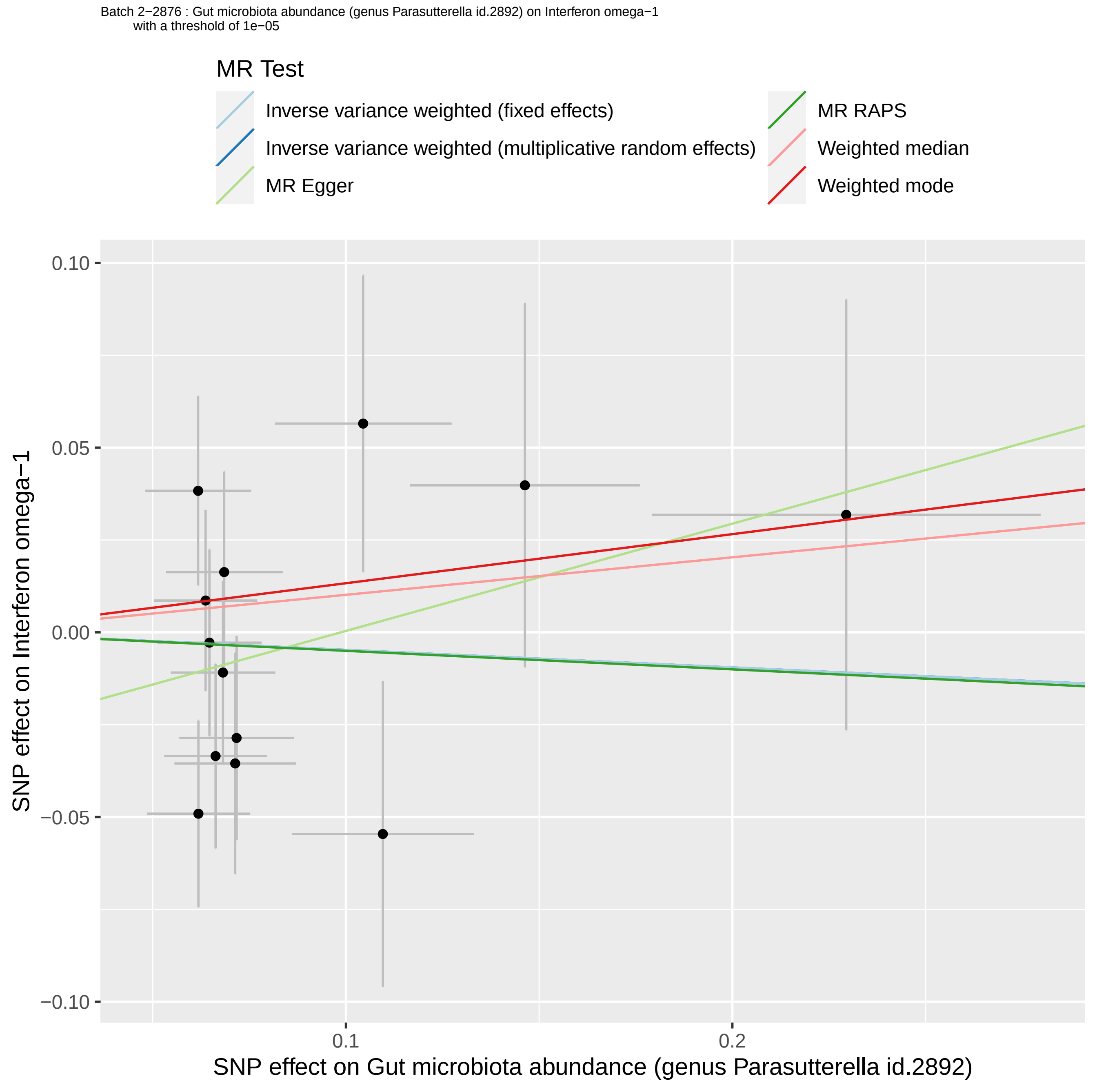

Batch 2−2876 : Gut microbiota abundance (genus Parasutterella id.2892) on Interferon omega−1
with a threshold of 1e−05
MR Test
Inverse variance weighted (fixed effects)
Inverse variance weighted (multiplicative random effects)
MR Egger
MR RAPS
Weighted median
Weighted mode
0.10
0.05
0.00
−0.05
−0.10
0.1
0.2
SNP effect on Interferon omega−1
SNP effect on Gut microbiota abundance (genus Parasutterella id.2892)

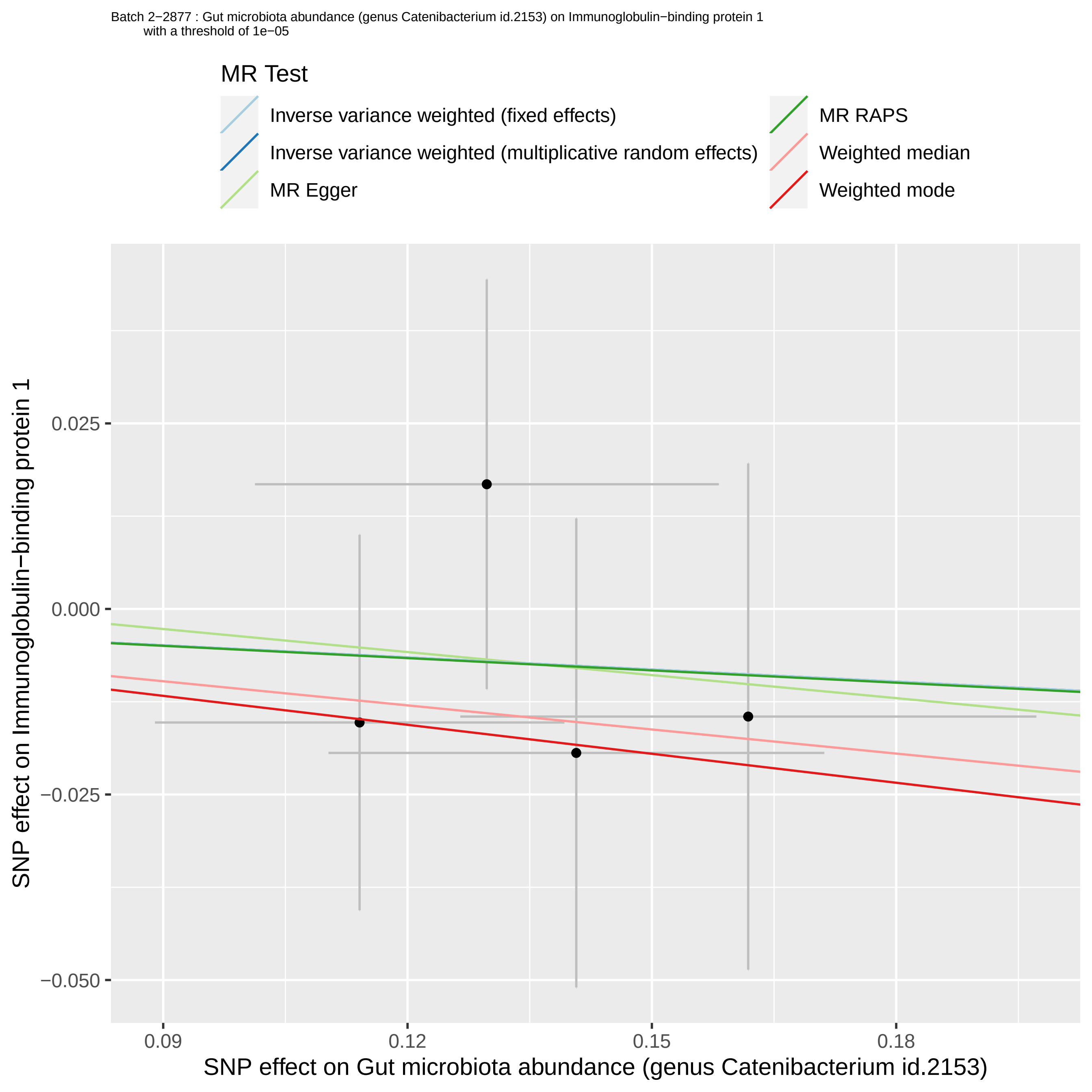
Batch 2−2877 : Gut microbiota abundance (genus Catenibacterium id.2153) on Immunoglobulin−binding protein 1
with a threshold of 1e−05
MR Test
Inverse variance weighted (fixed effects)
Inverse variance weighted (multiplicative random effects)
MR Egger
MR RAPS
Weighted median
Weighted mode
0.025
0.000
−0.025
−0.050
0.09
0.12
0.15
0.18
SNP effect on Immunoglobulin−binding protein 1
SNP effect on Gut microbiota abundance (genus Catenibacterium id.2153)

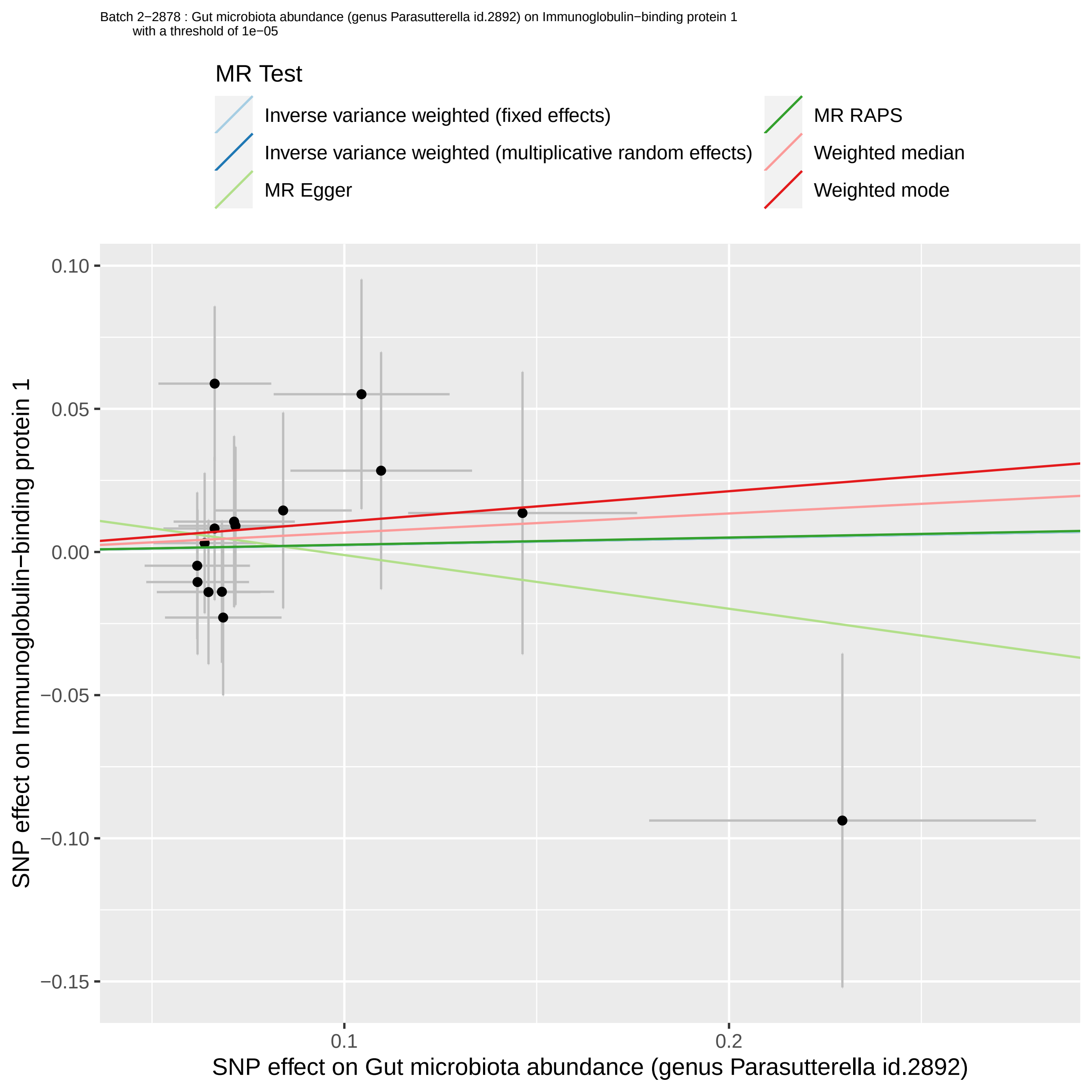
Batch 2−2878 : Gut microbiota abundance (genus Parasutterella id.2892) on Immunoglobulin−binding protein 1
with a threshold of 1e−05
MR Test
Inverse variance weighted (fixed effects)
Inverse variance weighted (multiplicative random effects)
MR Egger
MR RAPS
Weighted median
Weighted mode
0.10
0.05
0.00
−0.05
−0.10
−0.15
0.1
0.2
SNP effect on Immunoglobulin−binding protein 1
SNP effect on Gut microbiota abundance (genus Parasutterella id.2892)

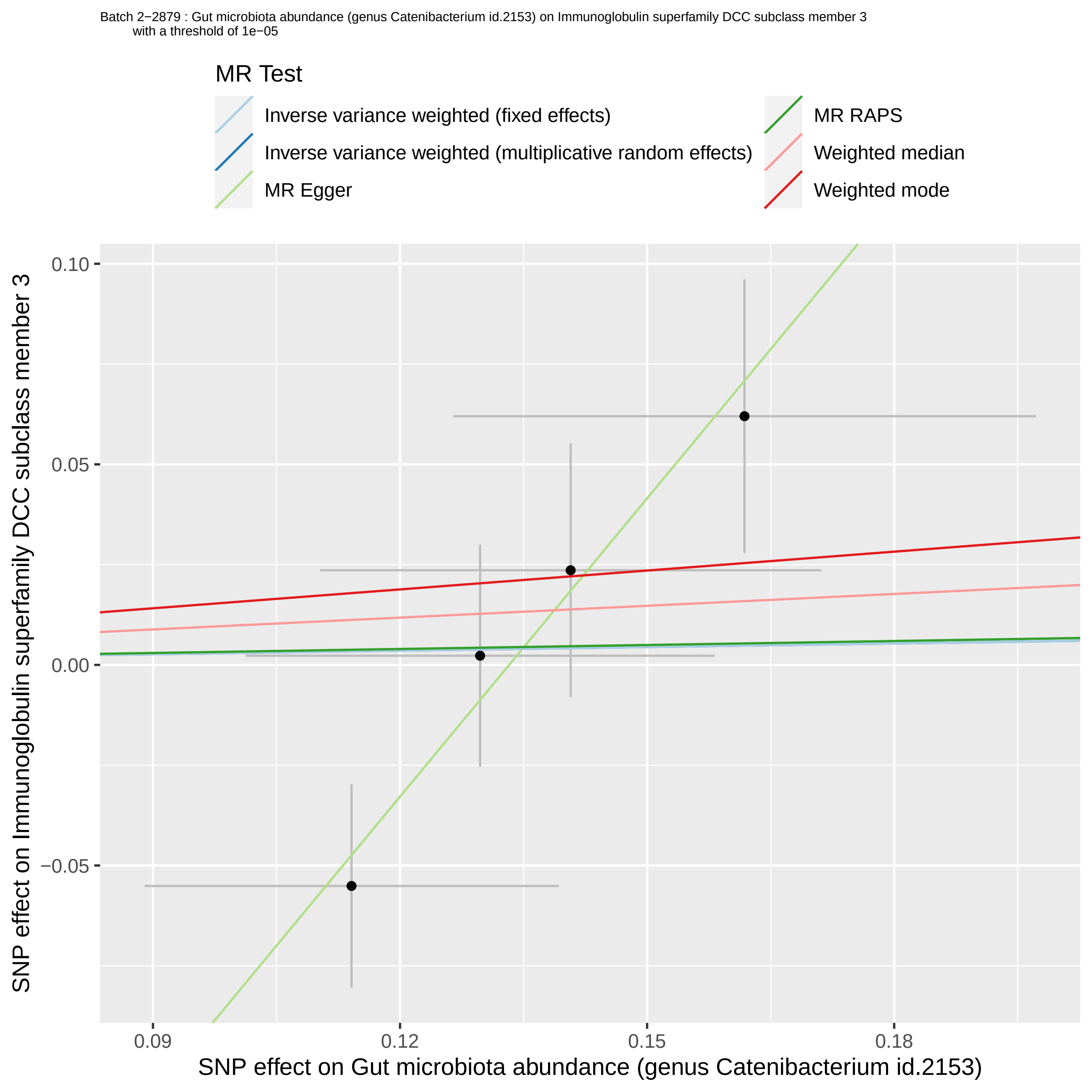
Batch 2−2879 : Gut microbiota abundance (genus Catenibacterium id.2153) on Immunoglobulin superfamily DCC subclass member 3
with a threshold of 1e−05
MR Test
Inverse variance weighted (fixed effects)
Inverse variance weighted (multiplicative random effects)
MR Egger
MR RAPS
Weighted median
Weighted mode
0.10
0.05
0.00
−0.05
0.09
0.12
0.15
0.18
SNP effect on Immunoglobulin superfamily DCC subclass member 3
SNP effect on Gut microbiota abundance (genus Catenibacterium id.2153)

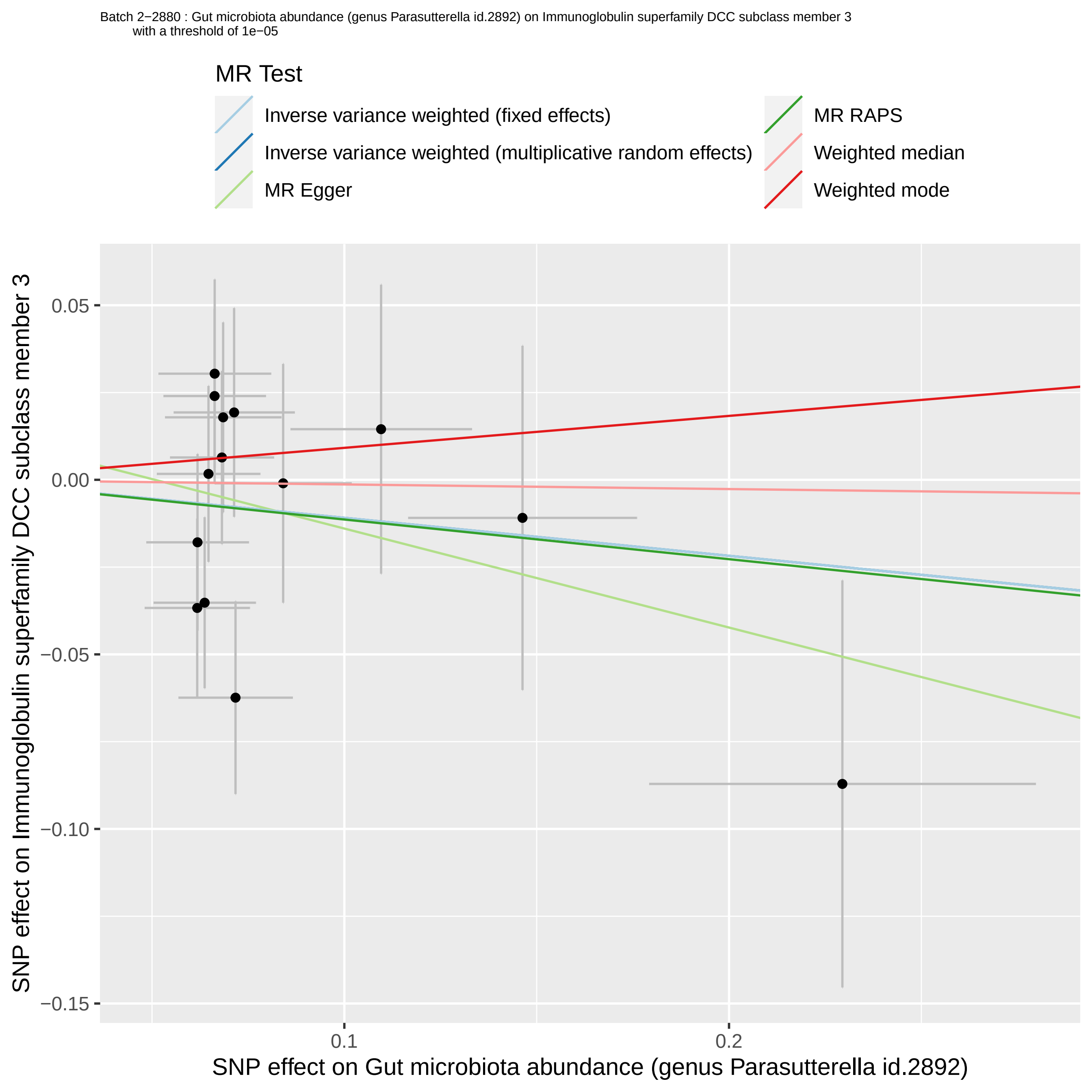
Batch 2−2880 : Gut microbiota abundance (genus Parasutterella id.2892) on Immunoglobulin superfamily DCC subclass member 3
with a threshold of 1e−05
MR Test
Inverse variance weighted (fixed effects)
Inverse variance weighted (multiplicative random effects)
MR Egger
MR RAPS
Weighted median
Weighted mode
SNP effect on Immunoglobulin superfamily DCC subclass member 3
0.05
0.00
−0.05
−0.10
−0.15
0.1
0.2
SNP effect on Gut microbiota abundance (genus Parasutterella id.2892)

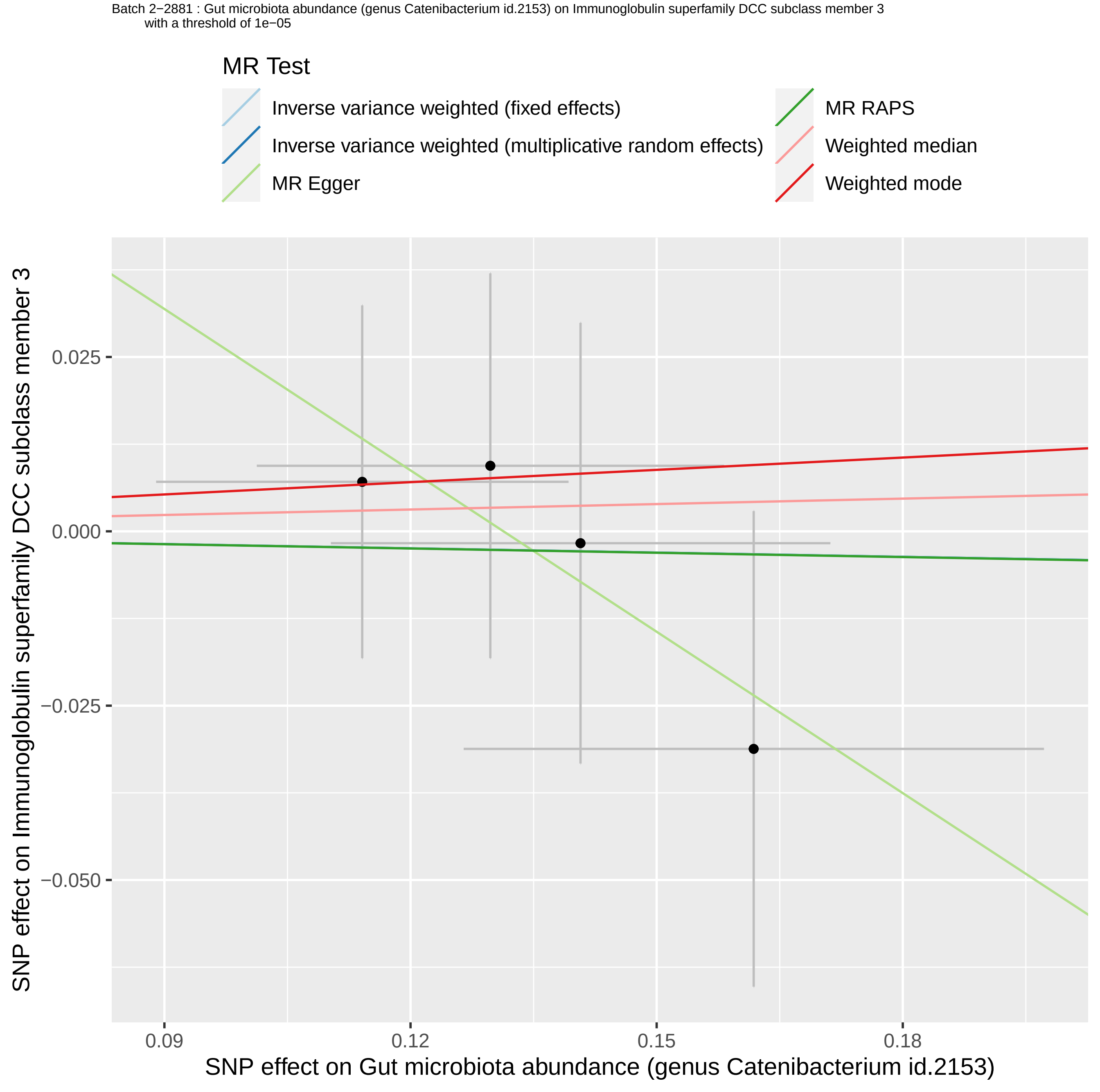
Batch 2−2881 : Gut microbiota abundance (genus Catenibacterium id.2153) on Immunoglobulin superfamily DCC subclass member 3
with a threshold of 1e−05
MR Test
Inverse variance weighted (fixed effects)
Inverse variance weighted (multiplicative random effects)
MR Egger
MR RAPS
Weighted median
Weighted mode
0.025
0.000
−0.025
−0.050
0.09
0.12
0.15
0.18
SNP effect on Immunoglobulin superfamily DCC subclass member 3
SNP effect on Gut microbiota abundance (genus Catenibacterium id.2153)

Batch 2−2882 : Gut microbiota abundance (genus Parasutterella id.2892) on Immunoglobulin superfamily DCC subclass member 3
with a threshold of 1e−05

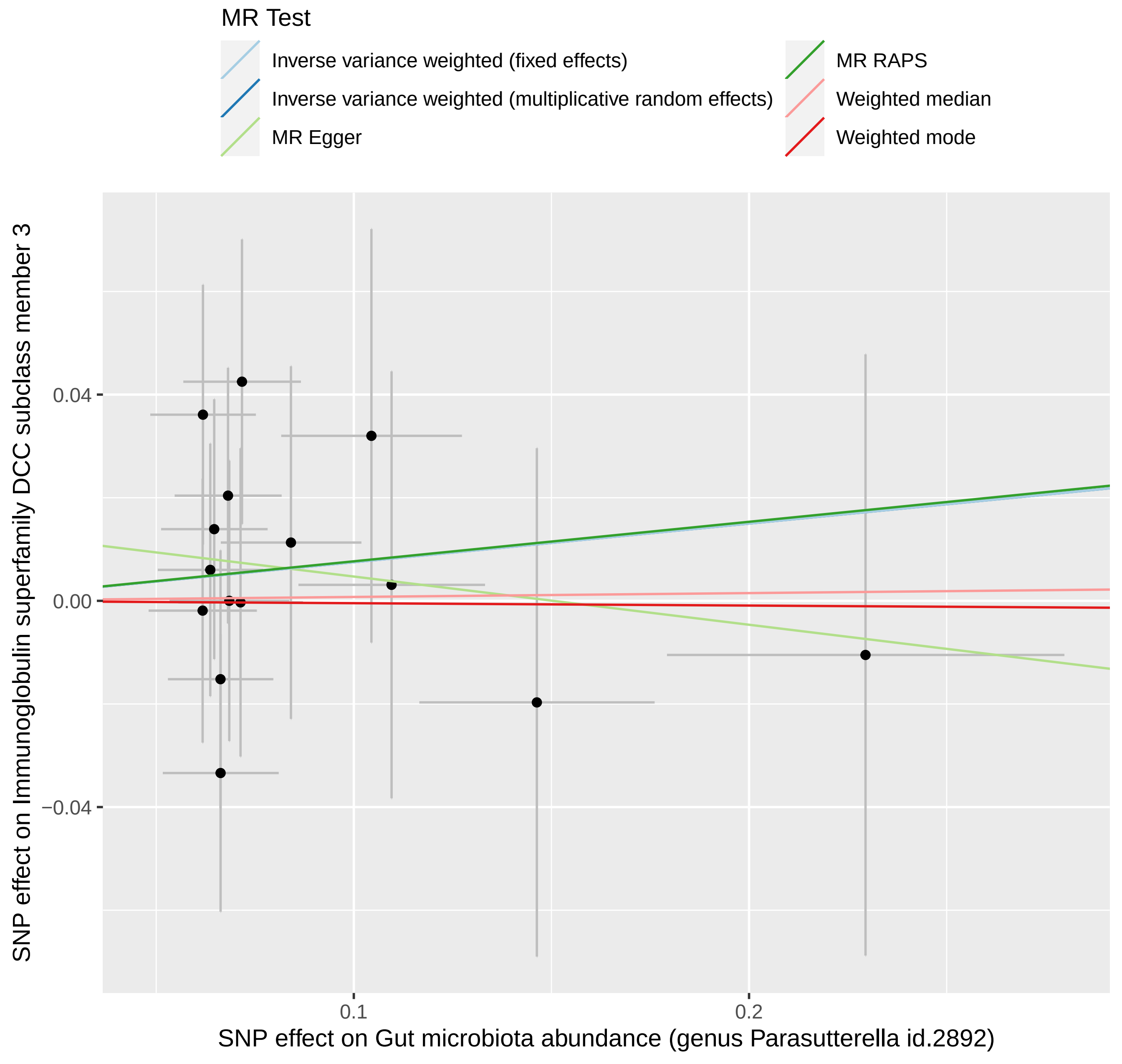

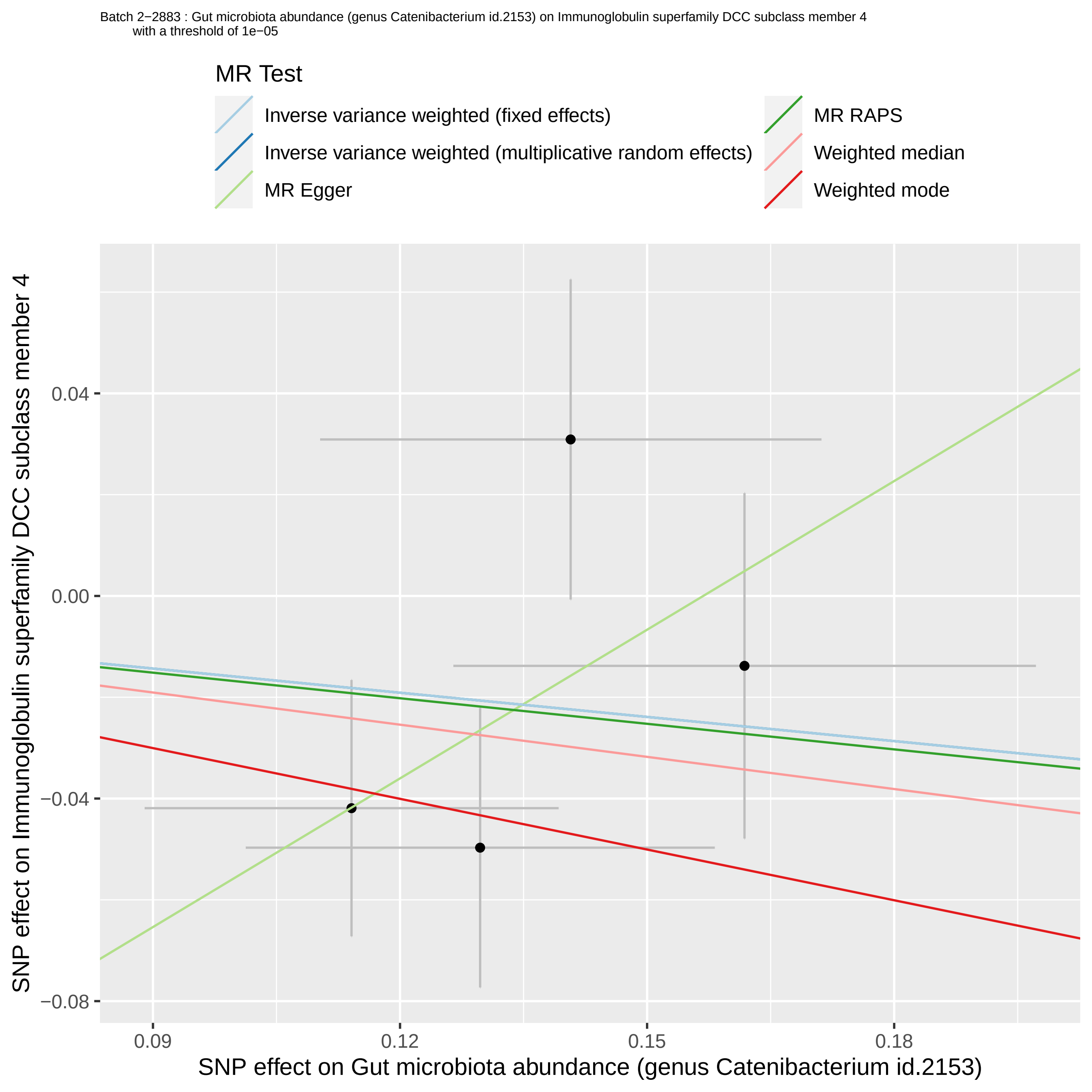
Batch 2−2883 : Gut microbiota abundance (genus Catenibacterium id.2153) on Immunoglobulin superfamily DCC subclass member 4
with a threshold of 1e−05
MR Test
Inverse variance weighted (fixed effects)
Inverse variance weighted (multiplicative random effects)
MR Egger
MR RAPS
Weighted median
Weighted mode
0.04
0.00
−0.04
−0.08
0.09
0.12
0.15
0.18
SNP effect on Immunoglobulin superfamily DCC subclass member 4
SNP effect on Gut microbiota abundance (genus Catenibacterium id.2153)

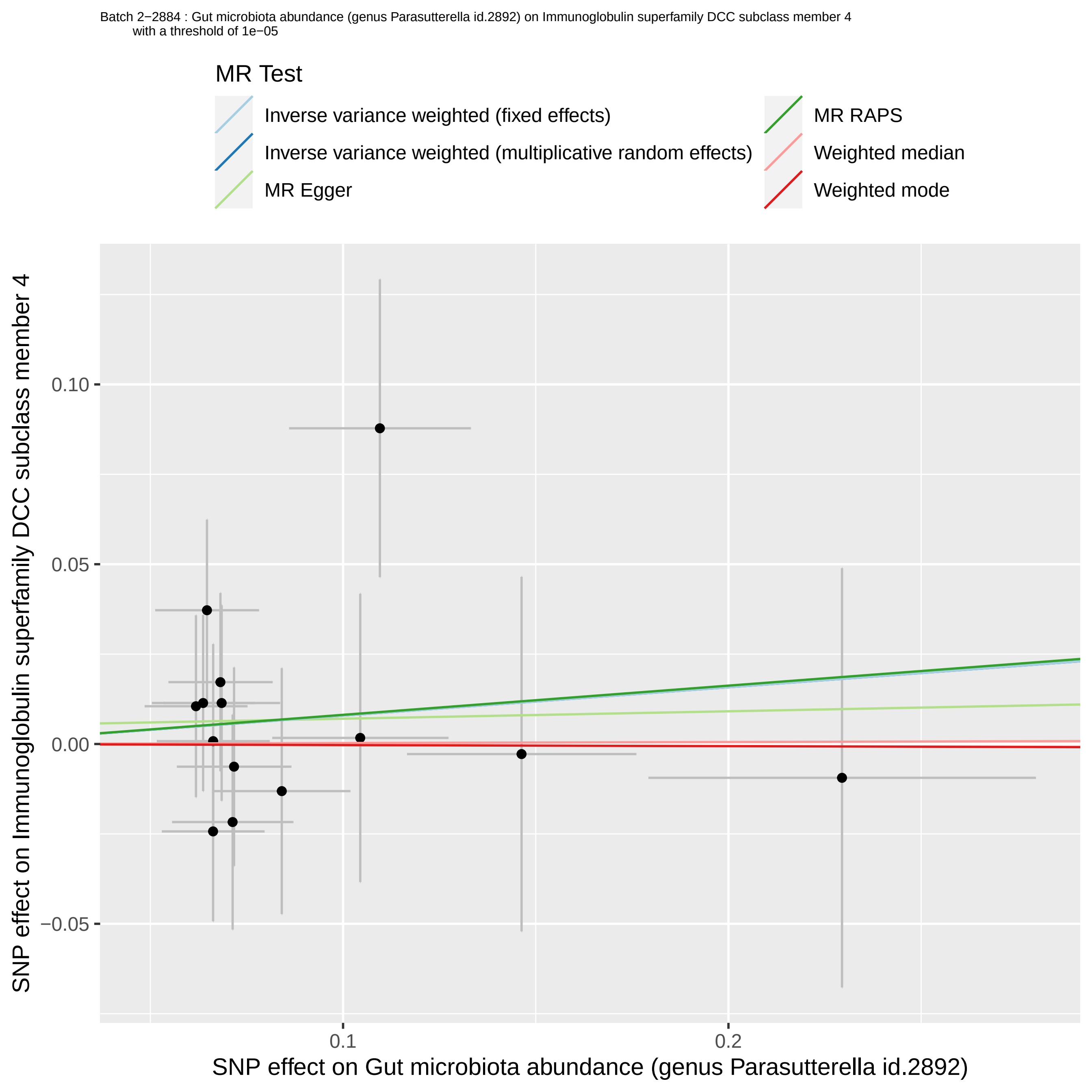
Batch 2−2884 : Gut microbiota abundance (genus Parasutterella id.2892) on Immunoglobulin superfamily DCC subclass member 4
with a threshold of 1e−05
MR Test
Inverse variance weighted (fixed effects)
Inverse variance weighted (multiplicative random effects)
MR Egger
MR RAPS
Weighted median
Weighted mode
0.10
0.05
0.00
−0.05
0.1
0.2
SNP effect on Immunoglobulin superfamily DCC subclass member 4
SNP effect on Gut microbiota abundance (genus Parasutterella id.2892)

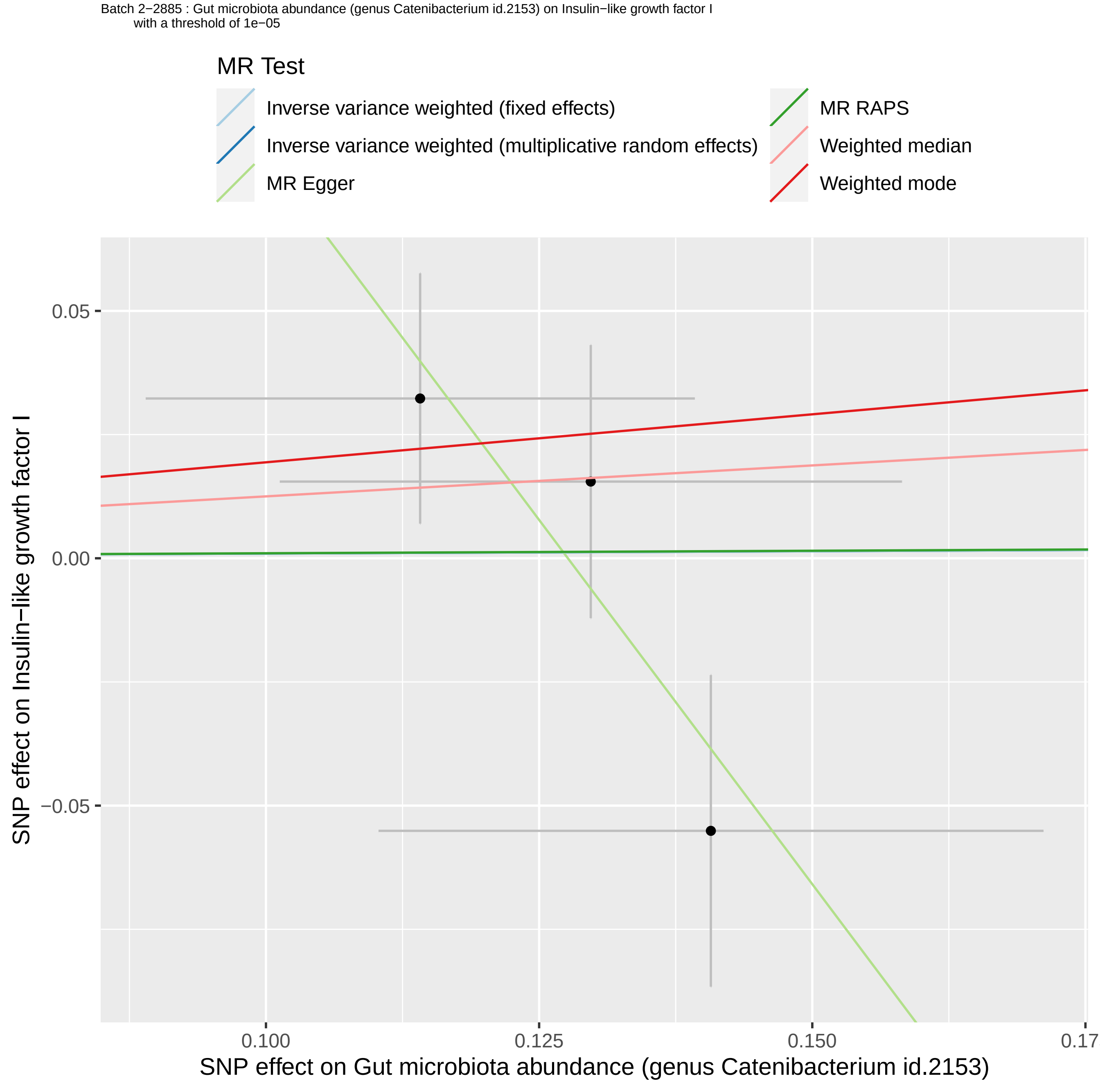
Batch 2−2885 : Gut microbiota abundance (genus Catenibacterium id.2153) on Insulin−like growth factor I
with a threshold of 1e−05
MR Test
Inverse variance weighted (fixed effects)
Inverse variance weighted (multiplicative random effects)
MR Egger
MR RAPS
Weighted median
Weighted mode
0.05
0.00
−0.05
0.100
0.125
0.150
0.17
SNP effect on Insulin−like growth factor I
SNP effect on Gut microbiota abundance (genus Catenibacterium id.2153)

Batch 2−2886 : Gut microbiota abundance (genus Parasutterella id.2892) on Insulin−like growth factor I
with a threshold of 1e−05

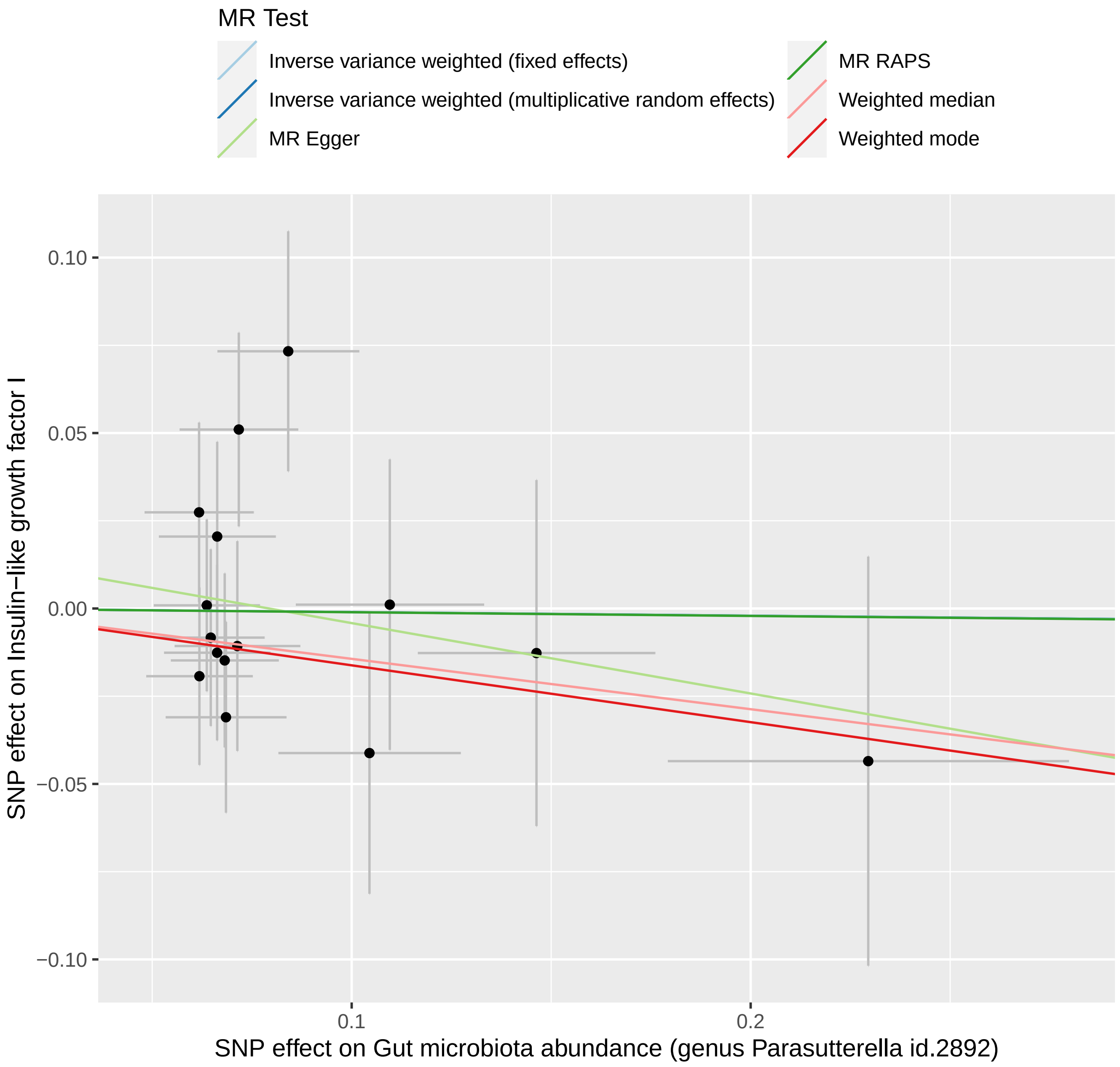

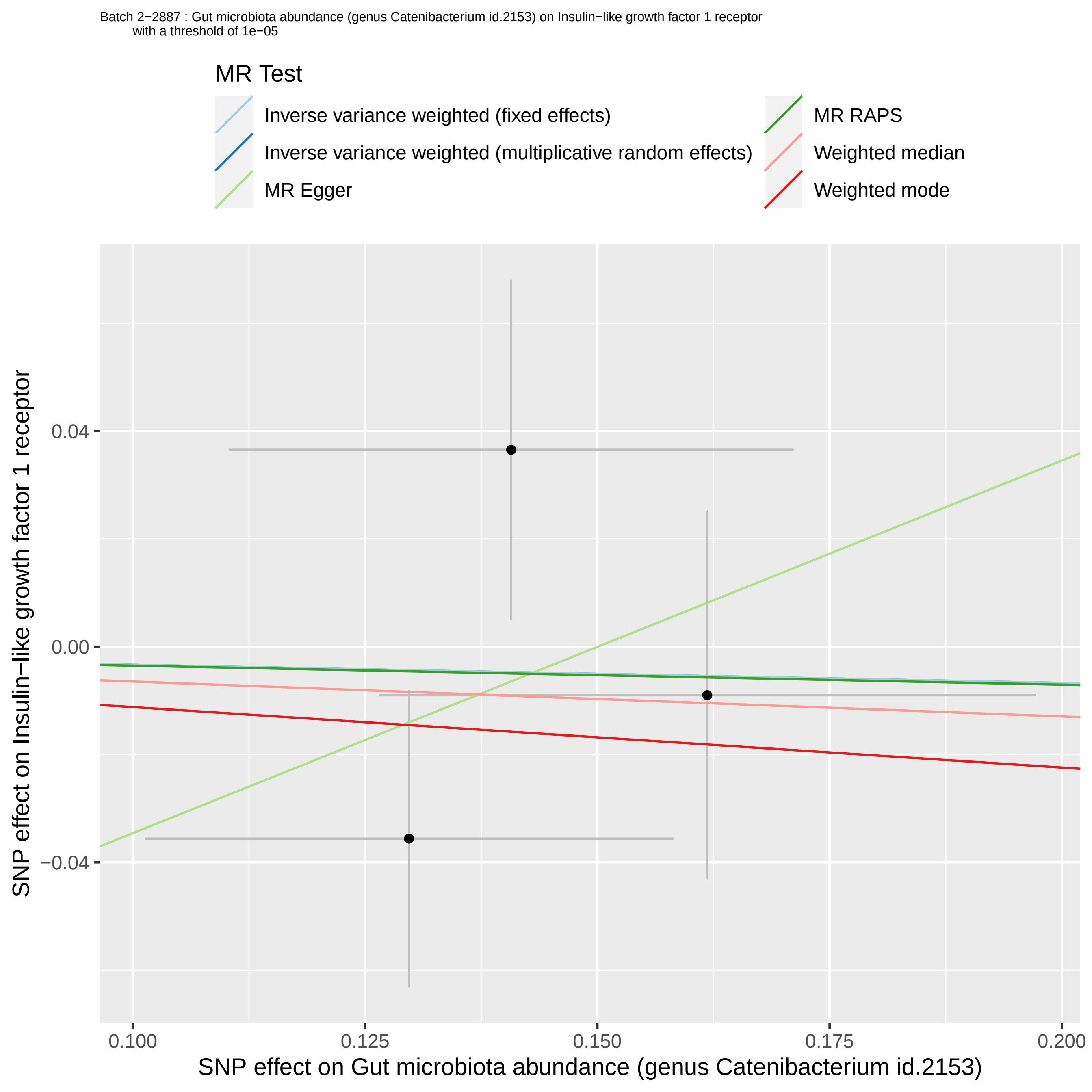
Batch 2−2887 : Gut microbiota abundance (genus Catenibacterium id.2153) on Insulin−like growth factor 1 receptor with a threshold of 1e−05
MR Test
Inverse variance weighted (fixed effects)
Inverse variance weighted (multiplicative random effects)
MR Egger
MR RAPS
Weighted median
Weighted mode
SNP effect on Insulin−like growth factor 1 receptor
0.04
0.00
−0.04
0.100
0.125
0.150
0.175
0.200
SNP effect on Gut microbiota abundance (genus Catenibacterium id.2153)

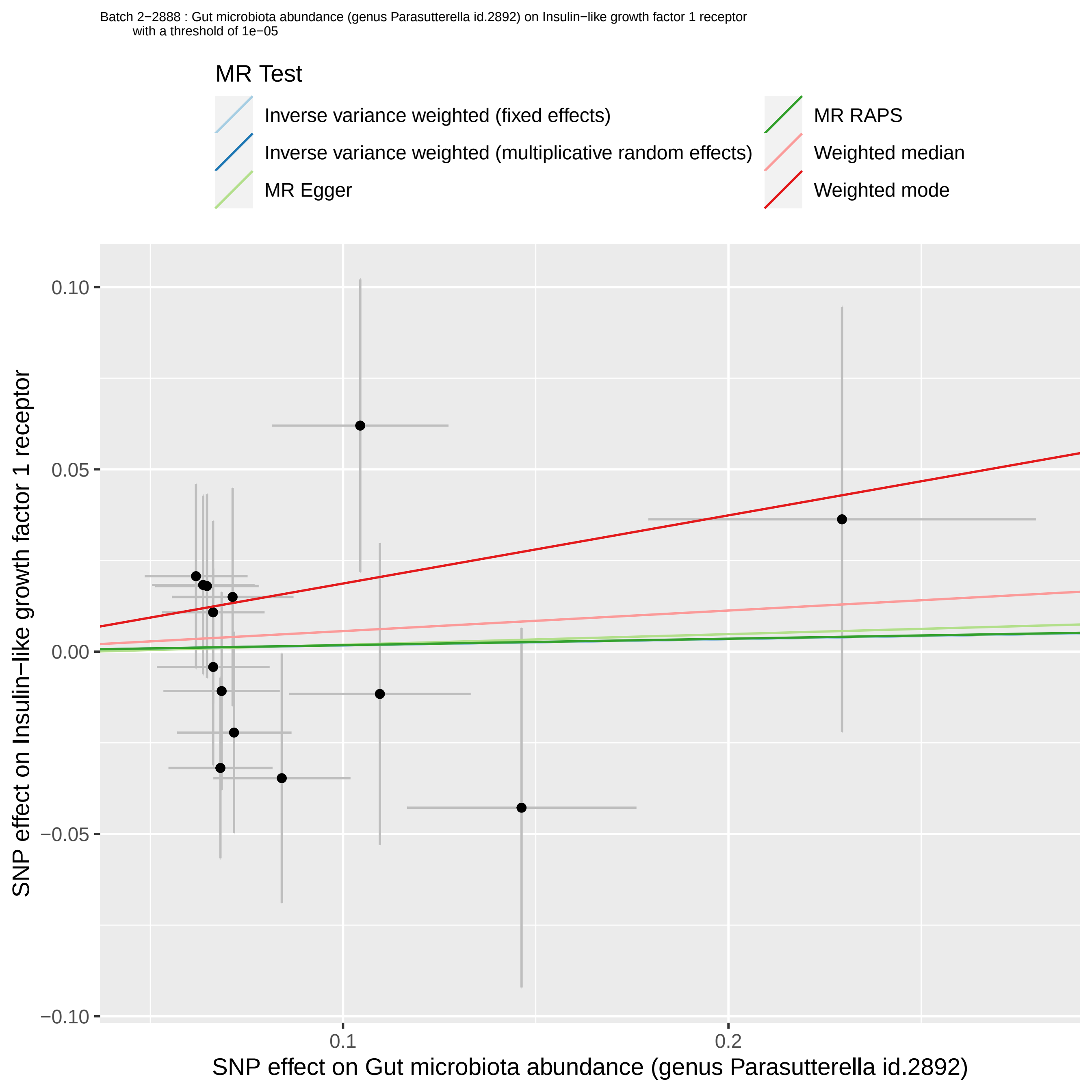
Batch 2−2888 : Gut microbiota abundance (genus Parasutterella id.2892) on Insulin−like growth factor 1 receptor
with a threshold of 1e−05
MR Test
Inverse variance weighted (fixed effects)
Inverse variance weighted (multiplicative random effects)
MR Egger
MR RAPS
Weighted median
Weighted mode
0.10
0.05
0.00
−0.05
−0.10
0.1
0.2
SNP effect on Insulin−like growth factor 1 receptor
SNP effect on Gut microbiota abundance (genus Parasutterella id.2892)

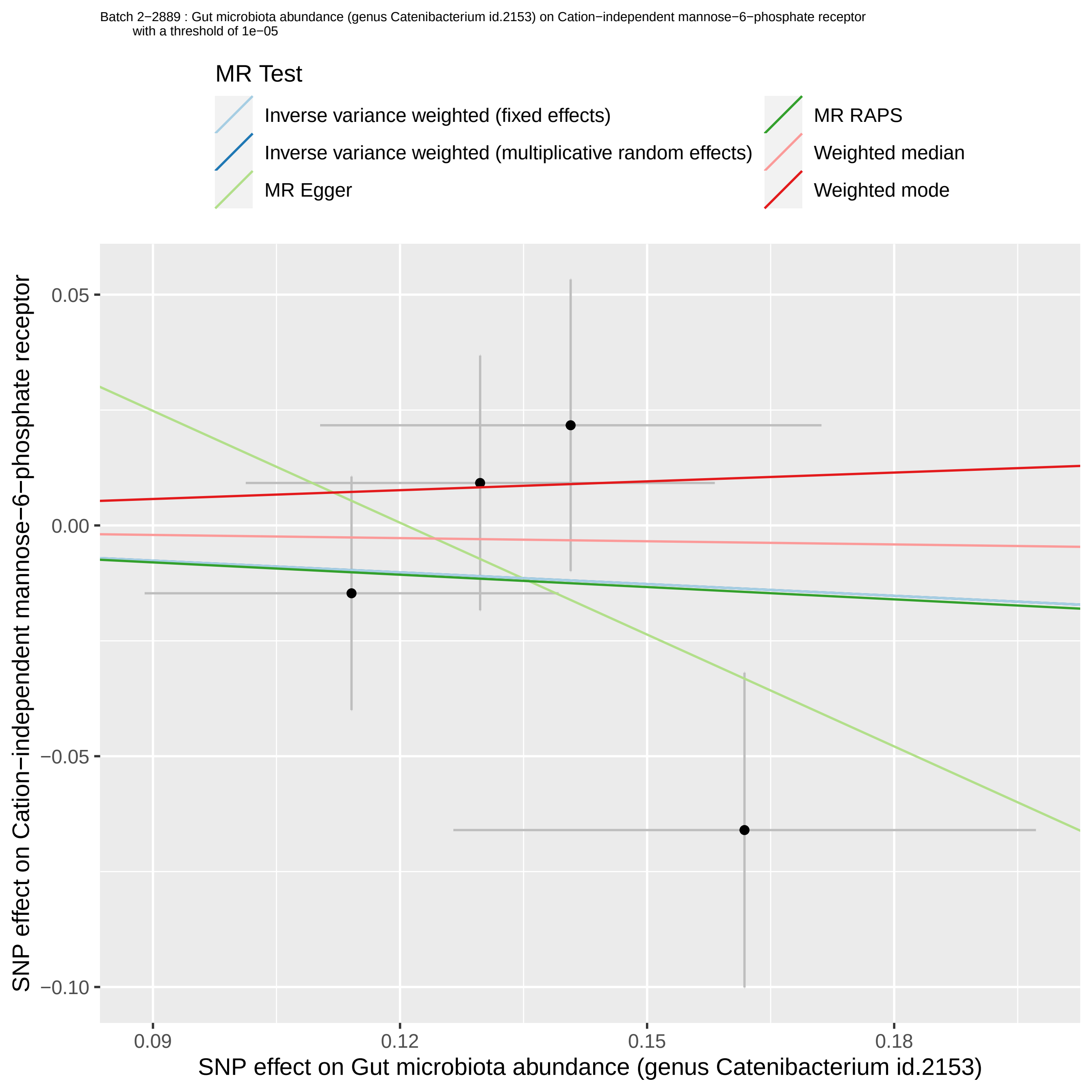

Batch 2−2889 : Gut microbiota abundance (genus Catenibacterium id.2153) on Cation−independent mannose−6−phosphate receptor
with a threshold of 1e−05
MR Test
Inverse variance weighted (fixed effects)
Inverse variance weighted (multiplicative random effects)
MR Egger
MR RAPS
Weighted median
Weighted mode
0.05
0.00
−0.05
−0.10
0.09
0.12
0.15
0.18
SNP effect on Cation−independent mannose−6−phosphate receptor
SNP effect on Gut microbiota abundance (genus Catenibacterium id.2153)

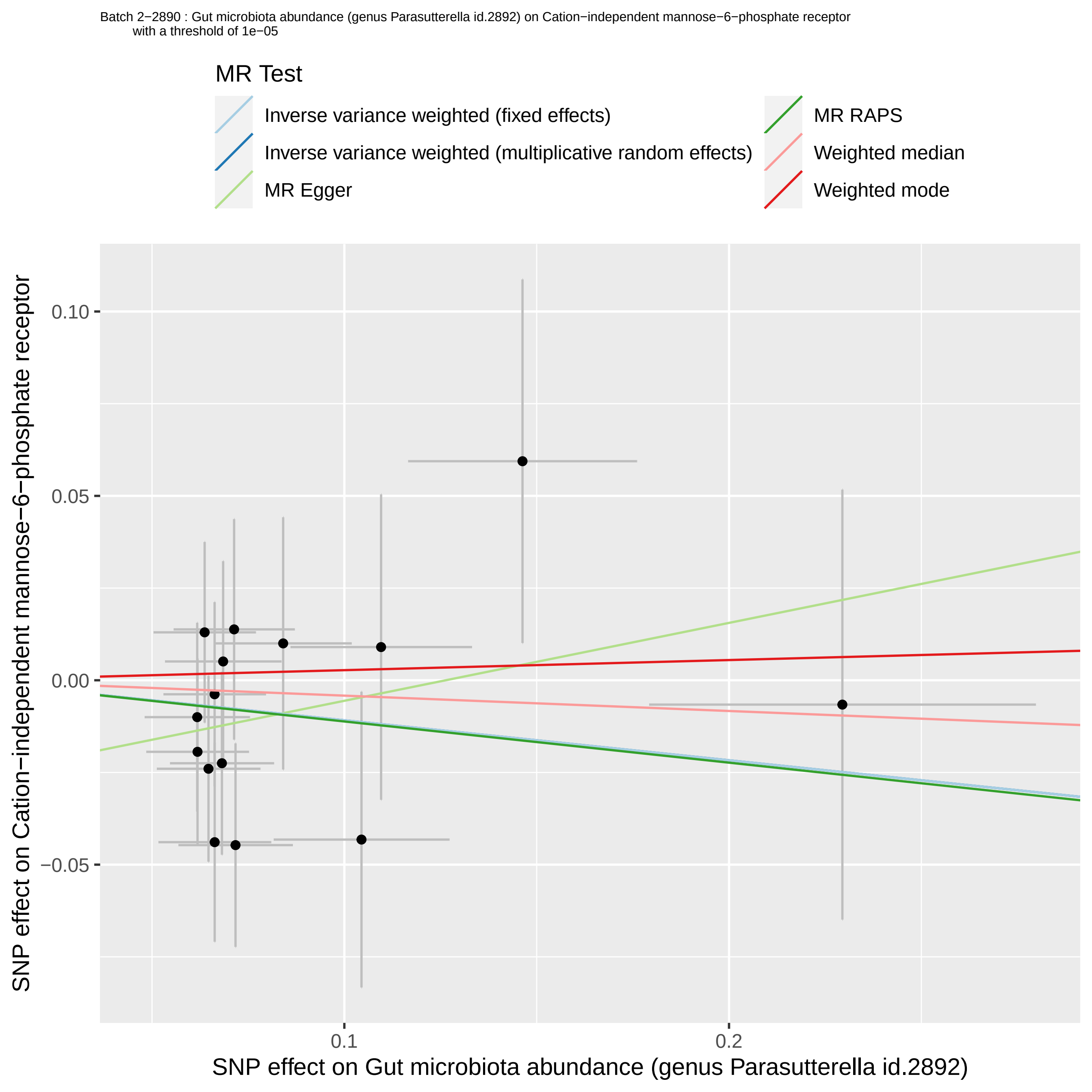
Batch 2−2890 : Gut microbiota abundance (genus Parasutterella id.2892) on Cation−independent mannose−6−phosphate receptor
with a threshold of 1e−05
MR Test
Inverse variance weighted (fixed effects)
Inverse variance weighted (multiplicative random effects)
MR Egger
MR RAPS
Weighted median
Weighted mode
SNP effect on Cation−independent mannose−6−phosphate receptor
0.10
0.05
0.00
−0.05
0.1
0.2
SNP effect on Gut microbiota abundance (genus Parasutterella id.2892)

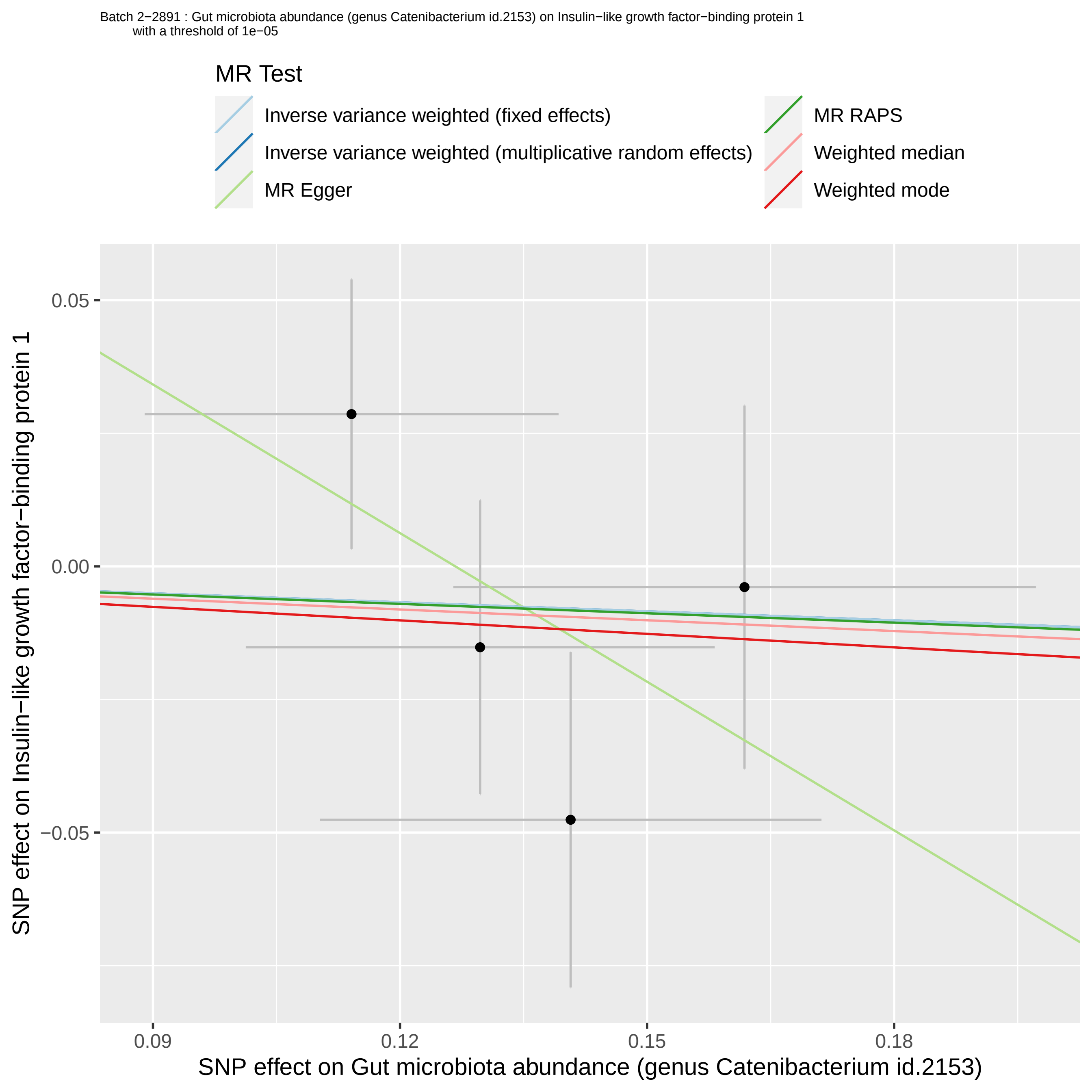

Batch 2−2891 : Gut microbiota abundance (genus Catenibacterium id.2153) on Insulin−like growth factor−binding protein 1
with a threshold of 1e−05
MR Test
Inverse variance weighted (fixed effects)
Inverse variance weighted (multiplicative random effects)
MR Egger
MR RAPS
Weighted median
Weighted mode
0.05
0.00
−0.05
0.09
0.12
0.15
0.18
SNP effect on Insulin−like growth factor−binding protein 1
SNP effect on Gut microbiota abundance (genus Catenibacterium id.2153)

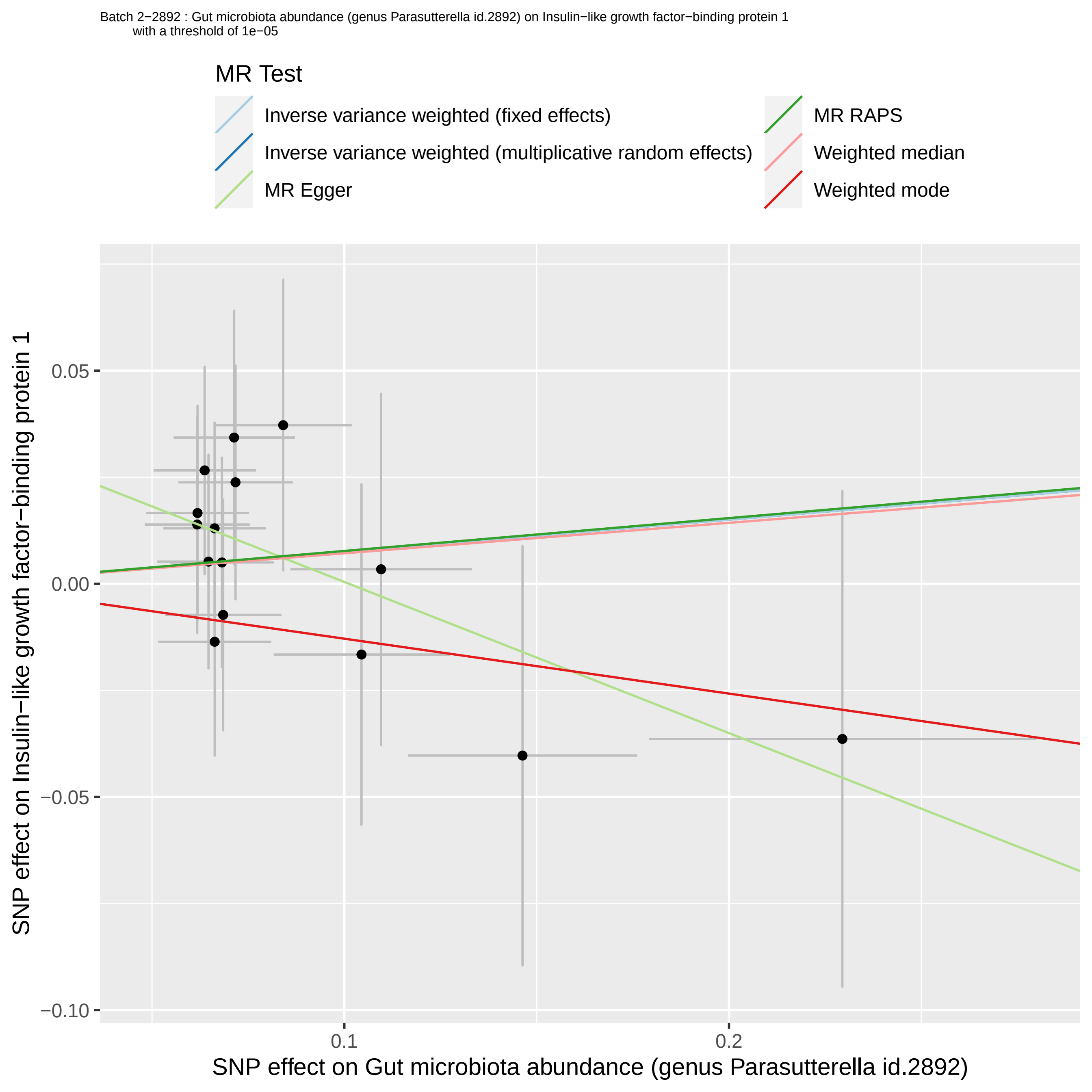
Batch 2−2892 : Gut microbiota abundance (genus Parasutterella id.2892) on Insulin−like growth factor−binding protein 1
with a threshold of 1e−05
MR Test
Inverse variance weighted (fixed effects)
Inverse variance weighted (multiplicative random effects)
MR Egger
MR RAPS
Weighted median
Weighted mode
0.05
0.00
−0.05
−0.10
0.1
0.2
SNP effect on Insulin−like growth factor−binding protein 1
SNP effect on Gut microbiota abundance (genus Parasutterella id.2892)

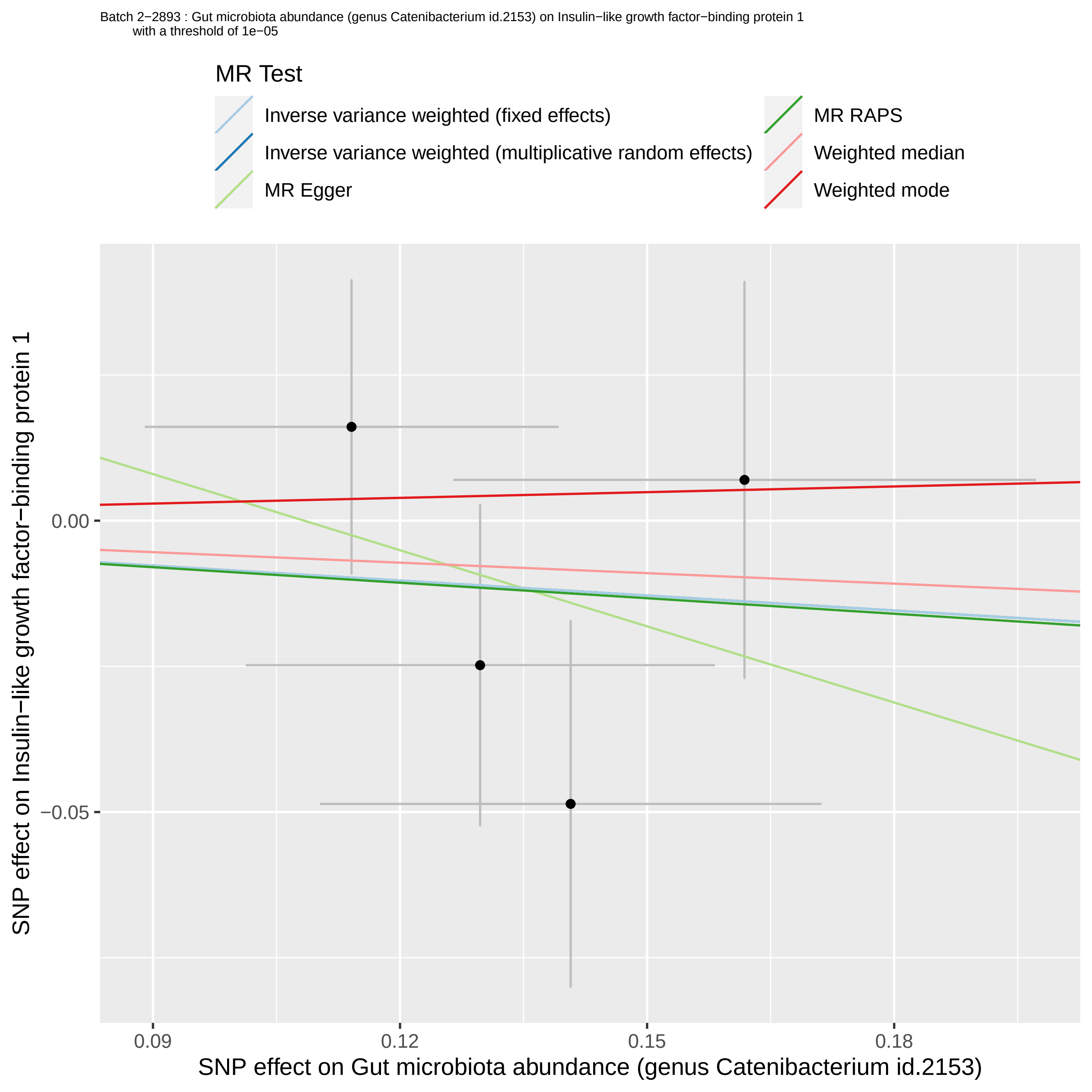
Batch 2−2893 : Gut microbiota abundance (genus Catenibacterium id.2153) on Insulin−like growth factor−binding protein 1
with a threshold of 1e−05
MR Test
Inverse variance weighted (fixed effects)
Inverse variance weighted (multiplicative random effects)
MR Egger
MR RAPS
Weighted median
Weighted mode
0.00
−0.05
0.09
0.12
0.15
0.18
SNP effect on Insulin−like growth factor−binding protein 1
SNP effect on Gut microbiota abundance (genus Catenibacterium id.2153)

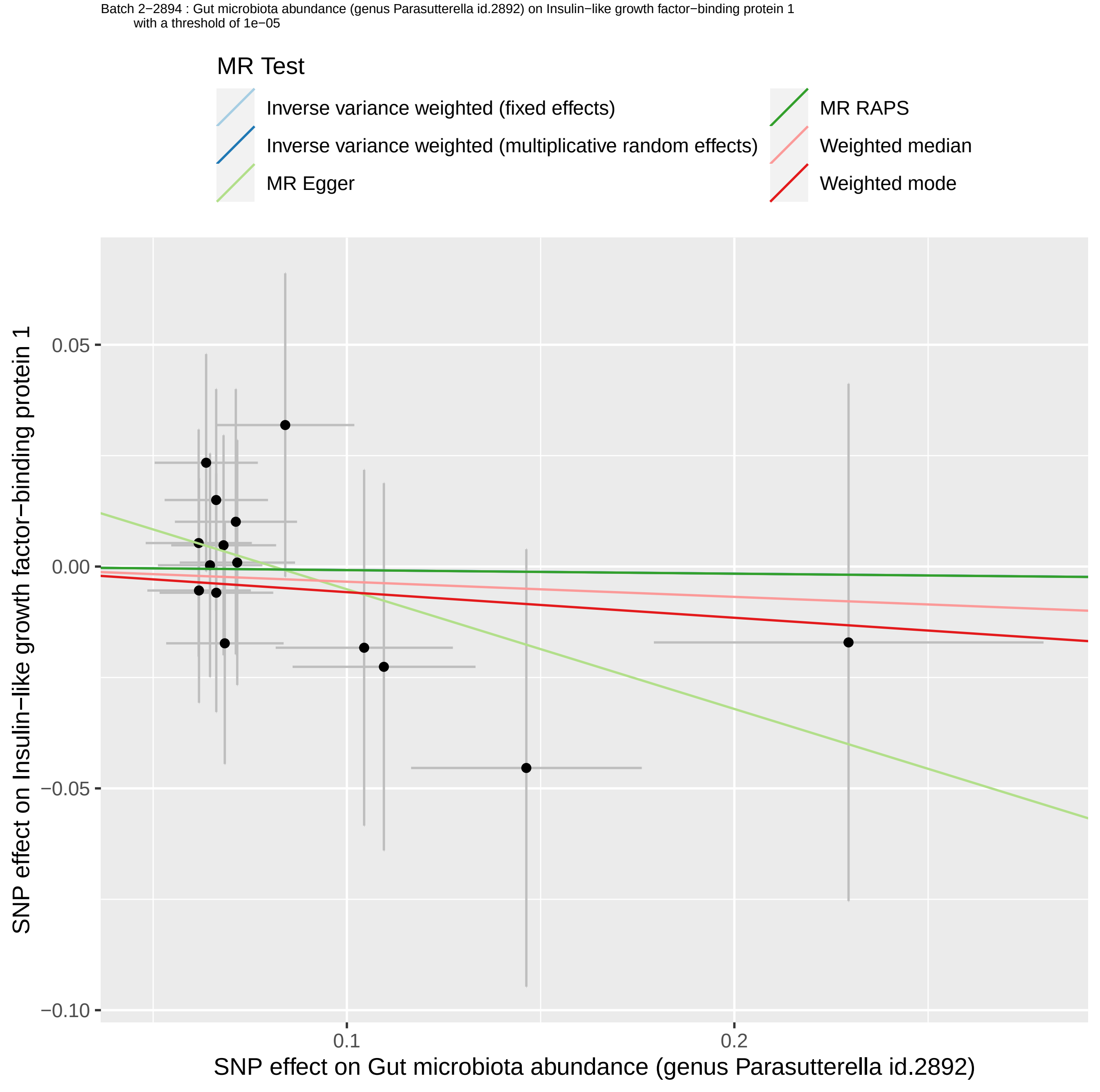

Batch 2−2894 : Gut microbiota abundance (genus Parasutterella id.2892) on Insulin−like growth factor−binding protein 1
with a threshold of 1e−05
MR Test
Inverse variance weighted (fixed effects)
Inverse variance weighted (multiplicative random effects)
MR Egger
MR RAPS
Weighted median
Weighted mode
0.05
0.00
−0.05
−0.10
0.1
0.2
SNP effect on Insulin−like growth factor−binding protein 1
SNP effect on Gut microbiota abundance (genus Parasutterella id.2892)

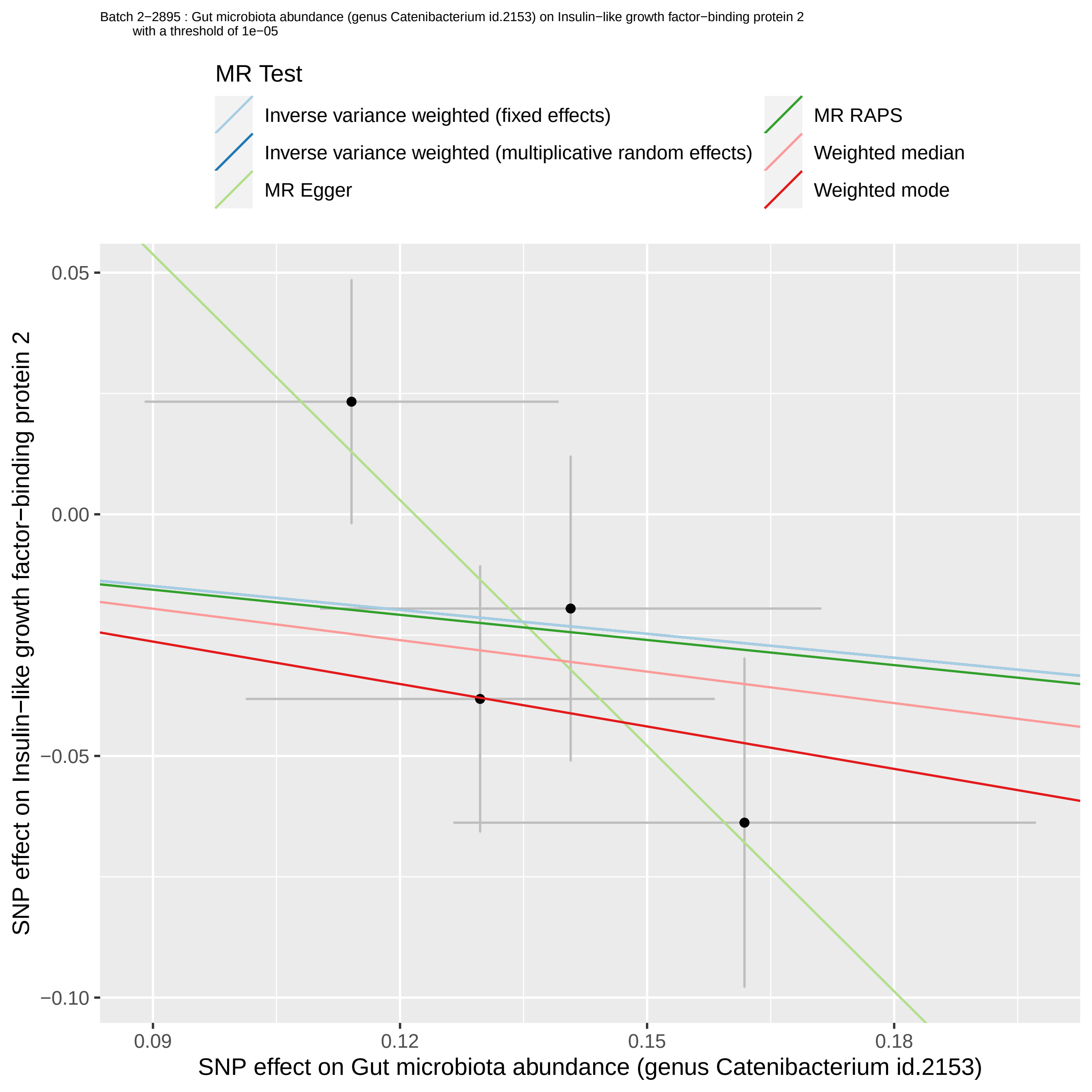
Batch 2−2895 : Gut microbiota abundance (genus Catenibacterium id.2153) on Insulin−like growth factor−binding protein 2
with a threshold of 1e−05
MR Test
Inverse variance weighted (fixed effects)
Inverse variance weighted (multiplicative random effects)
MR Egger
MR RAPS
Weighted median
Weighted mode
SNP effect on Insulin−like growth factor−binding protein 2
SNP effect on Gut microbiota abundance (genus Catenibacterium id.2153)
0.05
0.00
−0.05
−0.10
0.09
0.12
0.15
0.18

Batch 2−2896 : Gut microbiota abundance (genus Parasutterella id.2892) on Insulin−like growth factor−binding protein 2
with a threshold of 1e−05

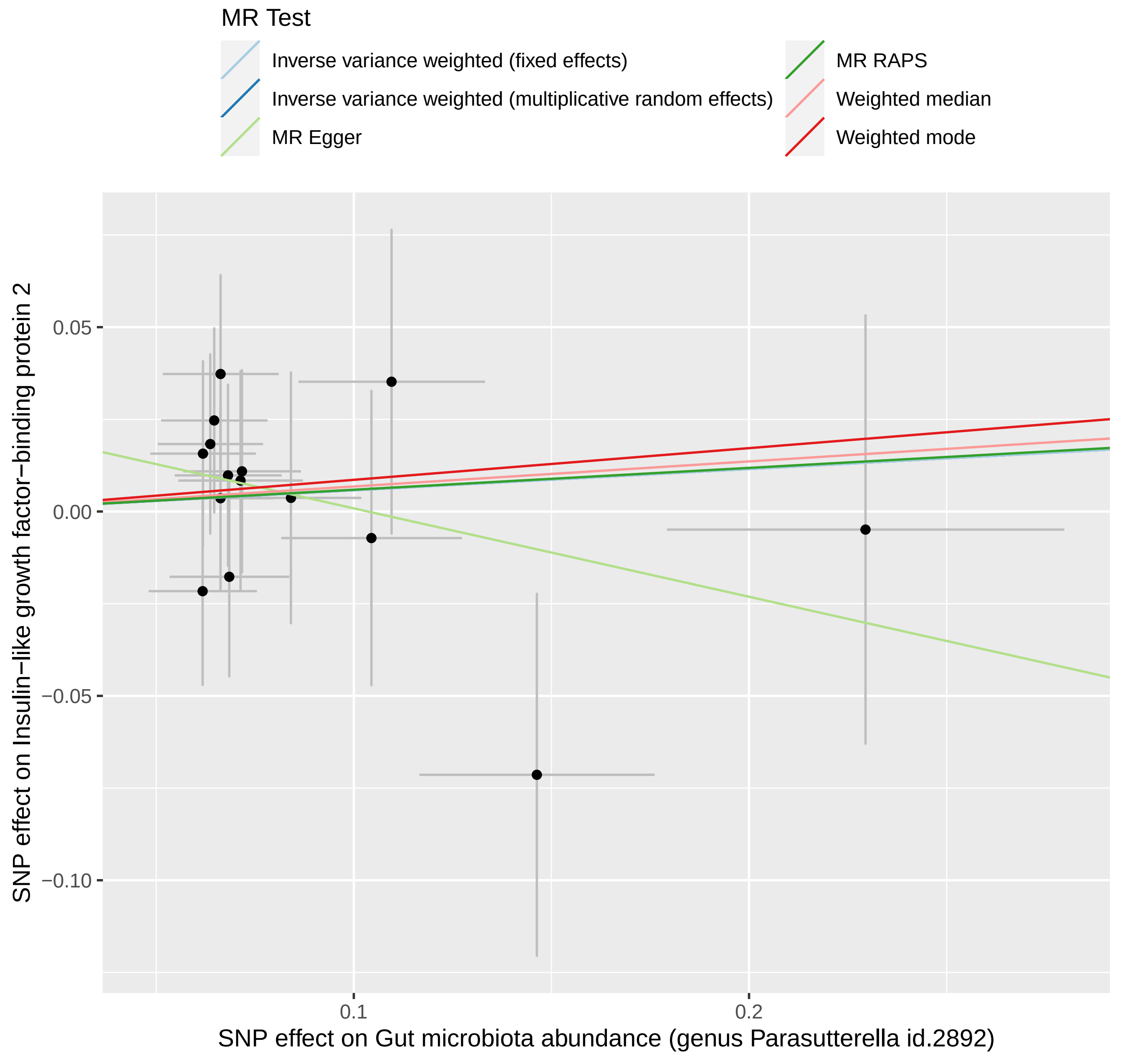

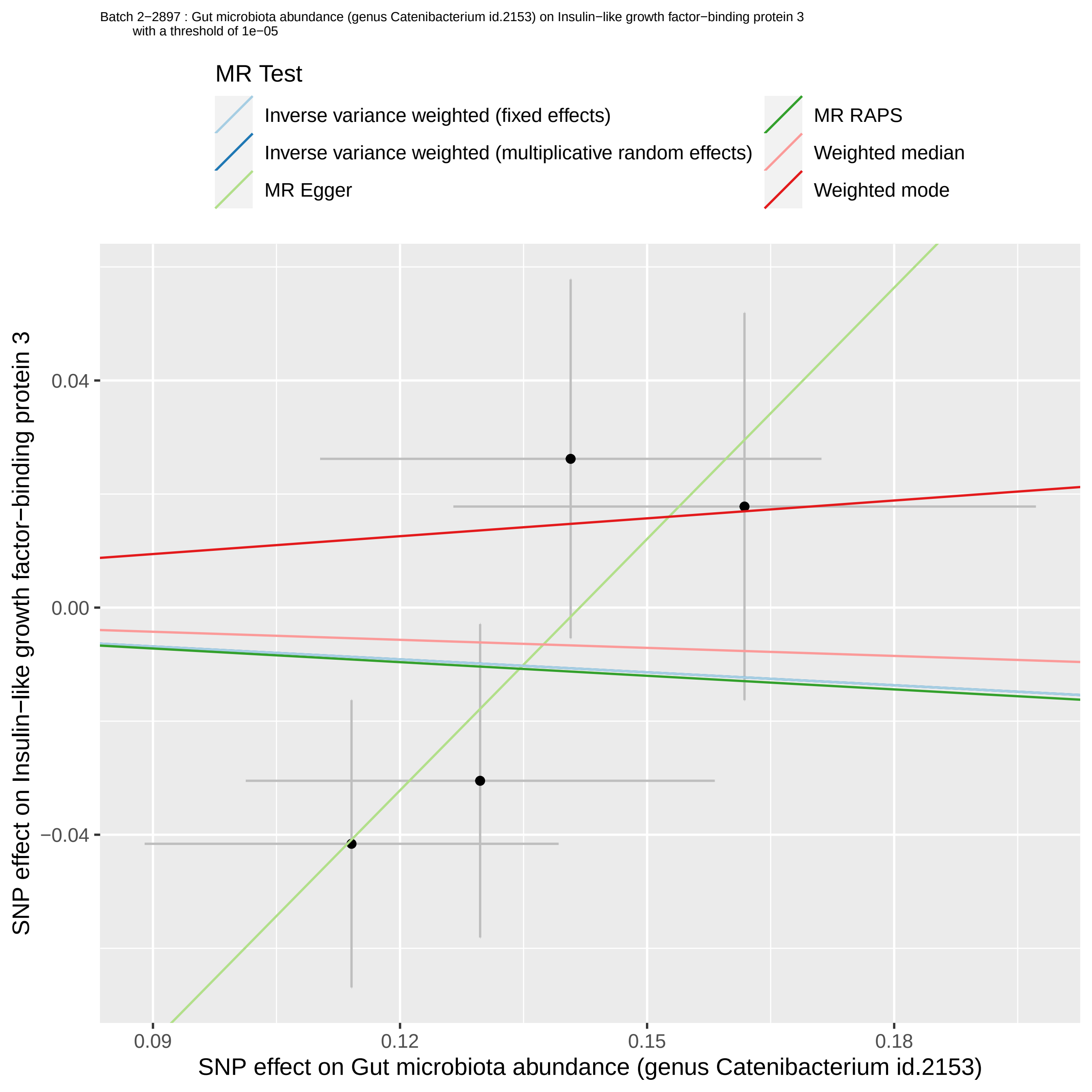
Batch 2−2897 : Gut microbiota abundance (genus Catenibacterium id.2153) on Insulin−like growth factor−binding protein 3
with a threshold of 1e−05
MR Test
Inverse variance weighted (fixed effects)
Inverse variance weighted (multiplicative random effects)
MR Egger
MR RAPS
Weighted median
Weighted mode
0.04
0.00
−0.04
0.09
0.12
0.15
0.18
SNP effect on Insulin−like growth factor−binding protein 3
SNP effect on Gut microbiota abundance (genus Catenibacterium id.2153)

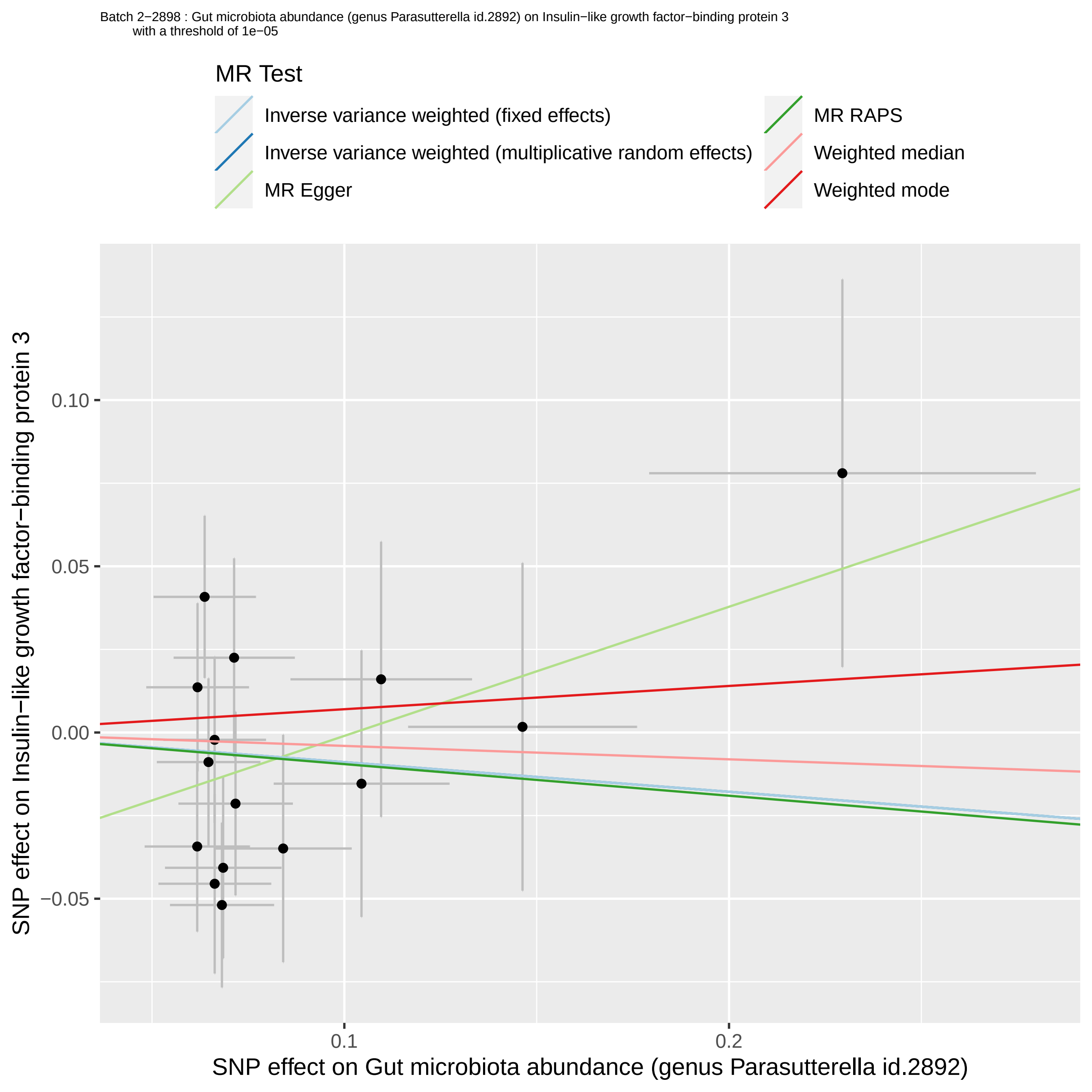

Batch 2−2898 : Gut microbiota abundance (genus Parasutterella id.2892) on Insulin−like growth factor−binding protein 3
with a threshold of 1e−05
MR Test
Inverse variance weighted (fixed effects)
Inverse variance weighted (multiplicative random effects)
MR Egger
MR RAPS
Weighted median
Weighted mode
0.10
0.05
0.00
−0.05
0.1
0.2
SNP effect on Insulin−like growth factor−binding protein 3
SNP effect on Gut microbiota abundance (genus Parasutterella id.2892)

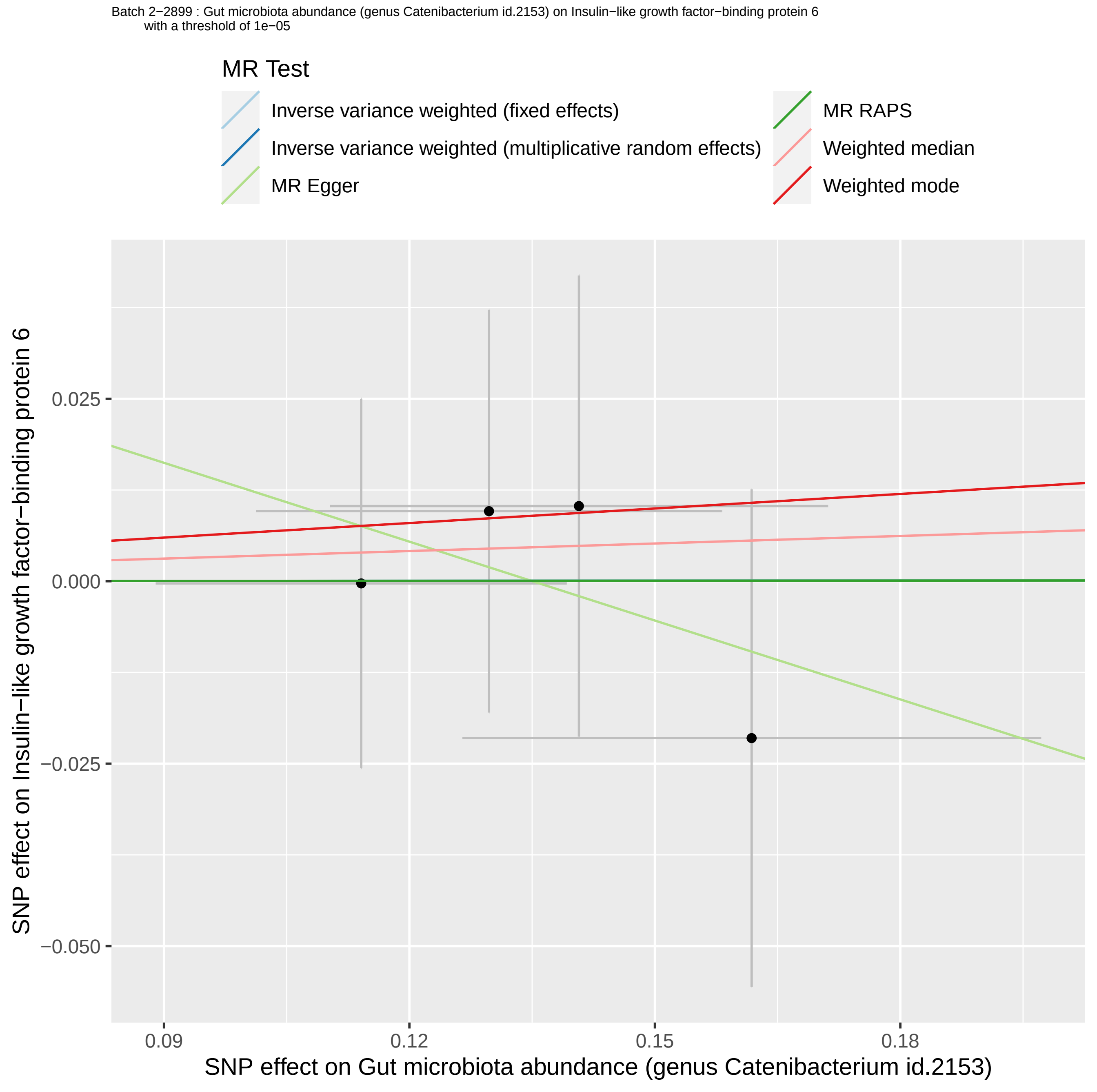
Batch 2−2899 : Gut microbiota abundance (genus Catenibacterium id.2153) on Insulin−like growth factor−binding protein 6
with a threshold of 1e−05
MR Test
Inverse variance weighted (fixed effects)
Inverse variance weighted (multiplicative random effects)
MR Egger
MR RAPS
Weighted median
Weighted mode
SNP effect on Insulin−like growth factor−binding protein 6
0.025
0.000
−0.025
−0.050
0.09
0.12
0.15
0.18
SNP effect on Gut microbiota abundance (genus Catenibacterium id.2153)

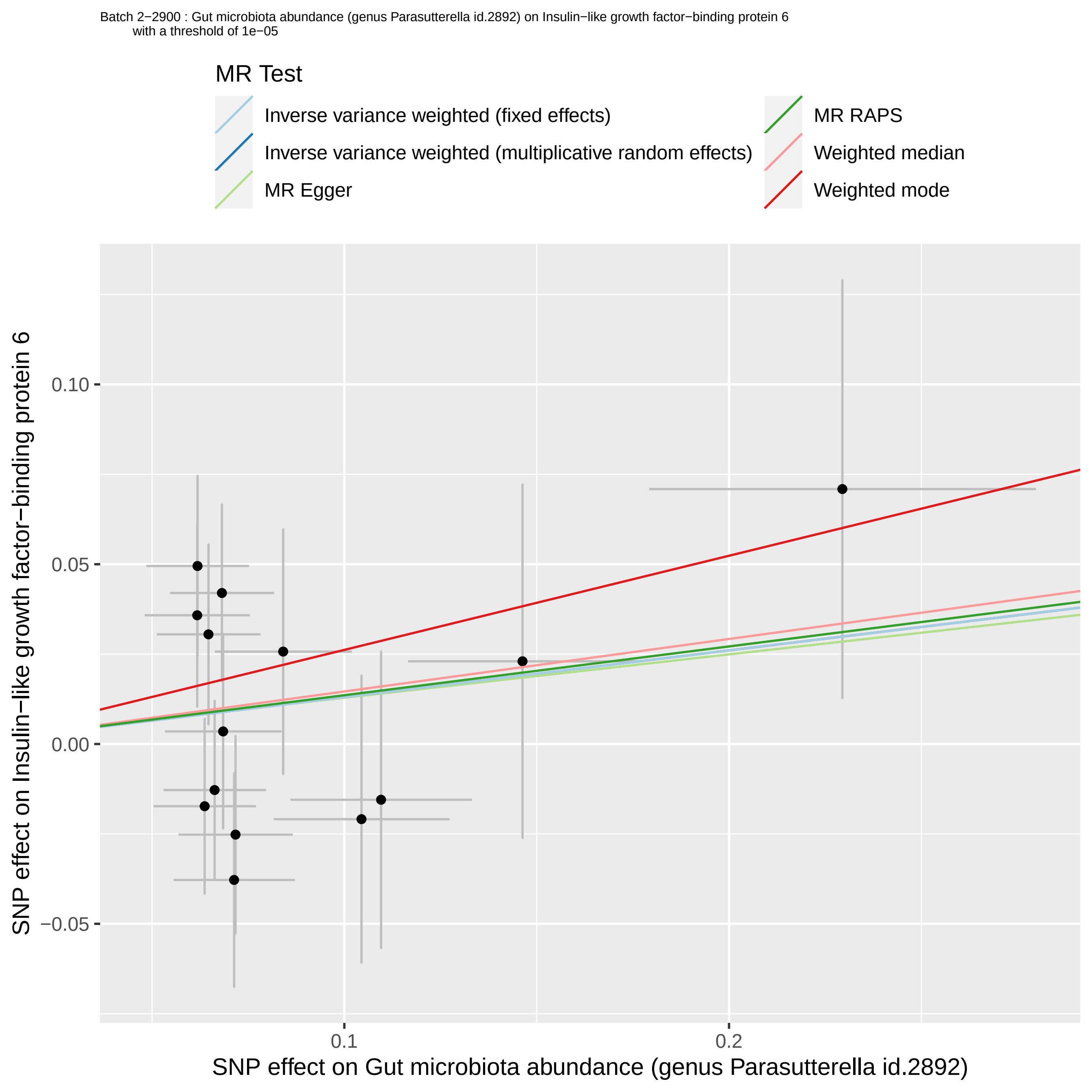
Batch 2−2900 : Gut microbiota abundance (genus Parasutterella id.2892) on Insulin−like growth factor−binding protein 6
with a threshold of 1e−05
MR Test
Inverse variance weighted (fixed effects)
Inverse variance weighted (multiplicative random effects)
MR Egger
MR RAPS
Weighted median
Weighted mode
0.10
0.05
0.00
−0.05
0.1
0.2
SNP effect on Insulin−like growth factor−binding protein 6
SNP effect on Gut microbiota abundance (genus Parasutterella id.2892)

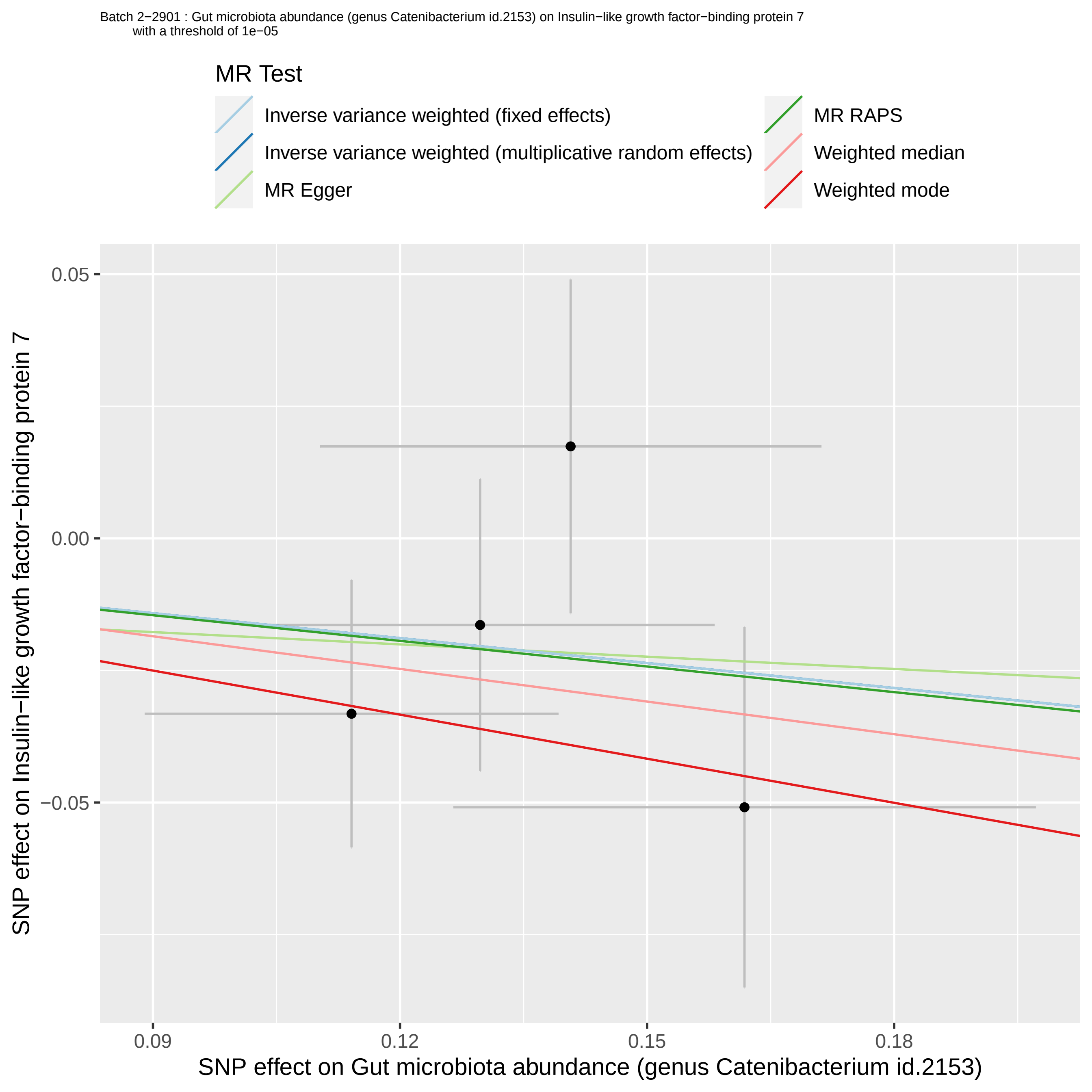
Batch 2−2901 : Gut microbiota abundance (genus Catenibacterium id.2153) on Insulin−like growth factor−binding protein 7
with a threshold of 1e−05
MR Test
Inverse variance weighted (fixed effects)
Inverse variance weighted (multiplicative random effects)
MR Egger
MR RAPS
Weighted median
Weighted mode
0.05
0.00
−0.05
0.09
0.12
0.15
0.18
SNP effect on Insulin−like growth factor−binding protein 7
SNP effect on Gut microbiota abundance (genus Catenibacterium id.2153)

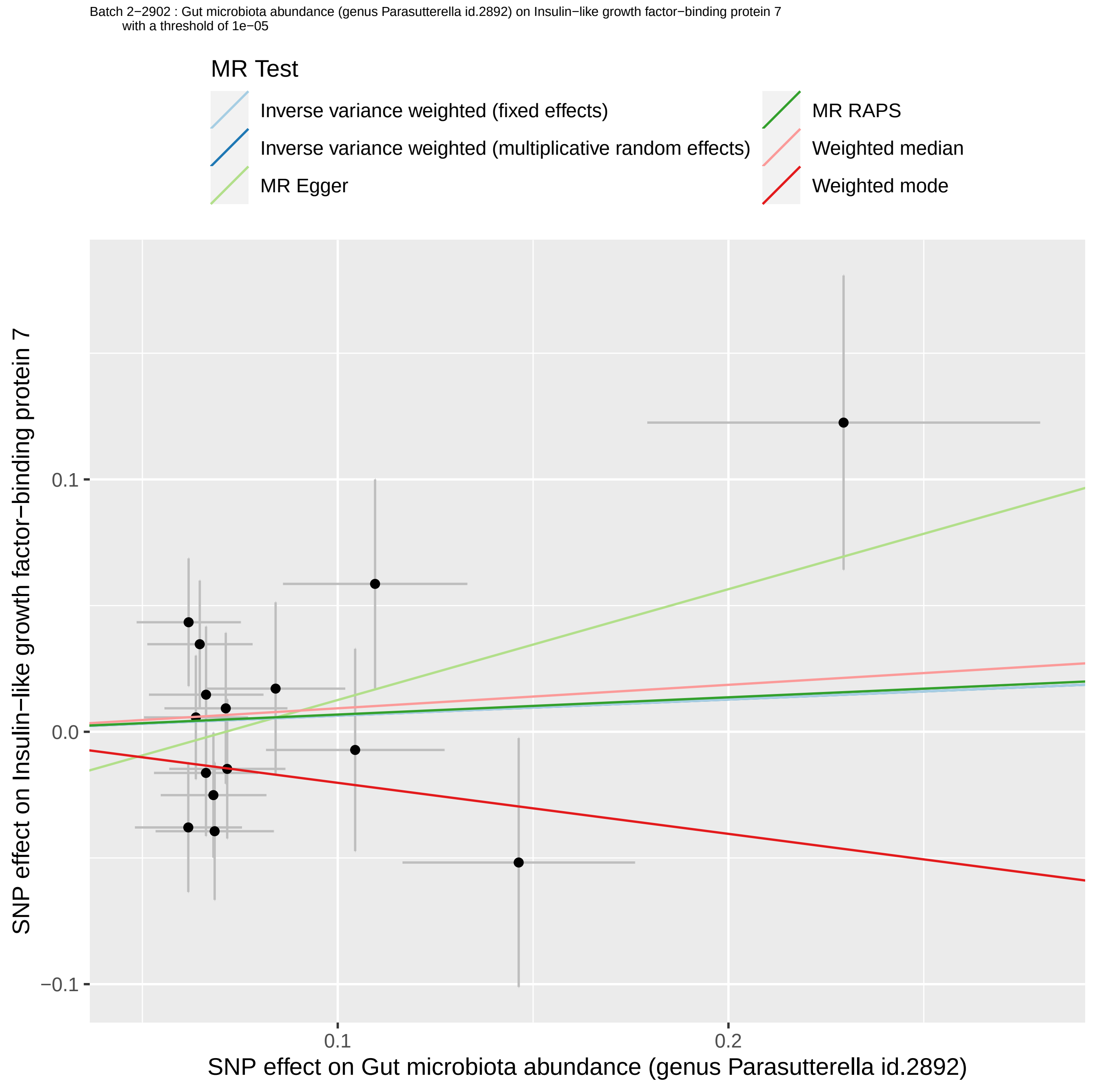

Batch 2−2902 : Gut microbiota abundance (genus Parasutterella id.2892) on Insulin−like growth factor−binding protein 7
with a threshold of 1e−05
MR Test
Inverse variance weighted (fixed effects)
Inverse variance weighted (multiplicative random effects)
MR Egger
MR RAPS
Weighted median
Weighted mode
0.1
0.0
−0.1
0.1
0.2
SNP effect on Insulin−like growth factor−binding protein 7
SNP effect on Gut microbiota abundance (genus Parasutterella id.2892)

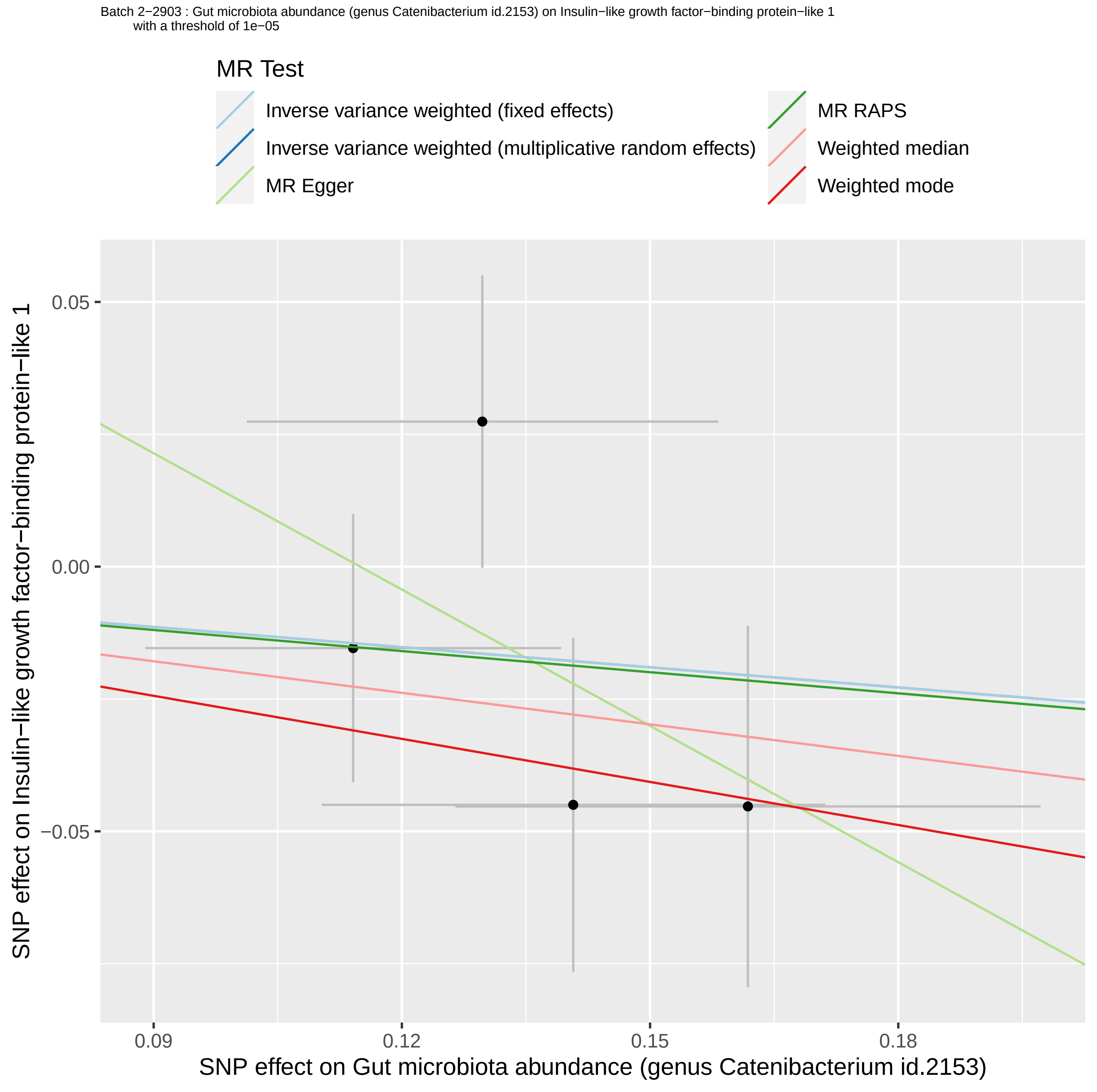

Batch 2−2903 : Gut microbiota abundance (genus Catenibacterium id.2153) on Insulin−like growth factor−binding protein−like 1
with a threshold of 1e−05
MR Test
Inverse variance weighted (fixed effects)
Inverse variance weighted (multiplicative random effects)
MR Egger
MR RAPS
Weighted median
Weighted mode
0.05
0.00
−0.05
0.09
0.12
0.15
0.18
SNP effect on Insulin−like growth factor−binding protein−like 1
SNP effect on Gut microbiota abundance (genus Catenibacterium id.2153)

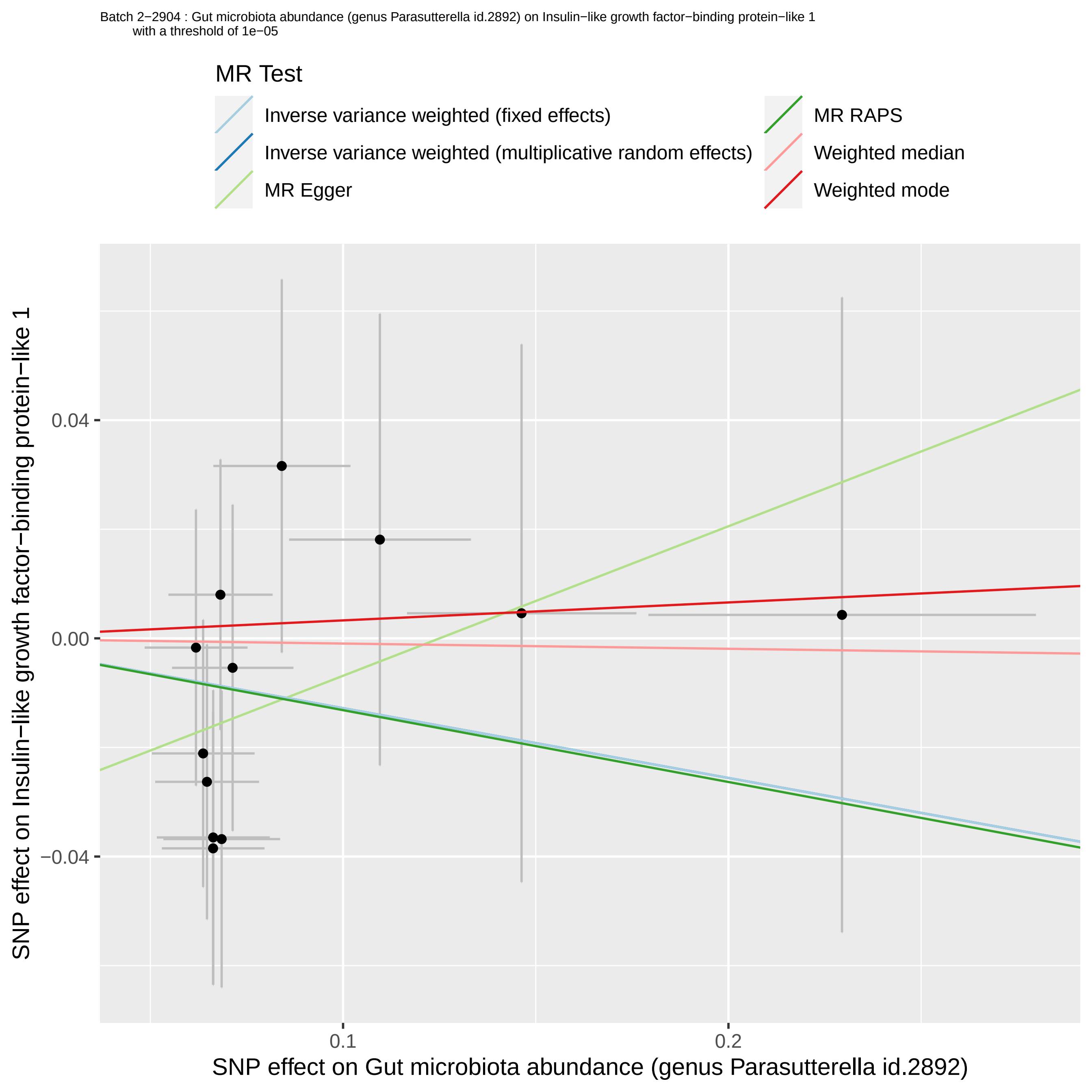

Batch 2−2904 : Gut microbiota abundance (genus Parasutterella id.2892) on Insulin−like growth factor−binding protein−like 1
with a threshold of 1e−05
MR Test
Inverse variance weighted (fixed effects)
Inverse variance weighted (multiplicative random effects)
MR Egger
MR RAPS
Weighted median
Weighted mode
0.04
0.00
−0.04
0.1
0.2
SNP effect on Insulin−like growth factor−binding protein−like 1
SNP effect on Gut microbiota abundance (genus Parasutterella id.2892)

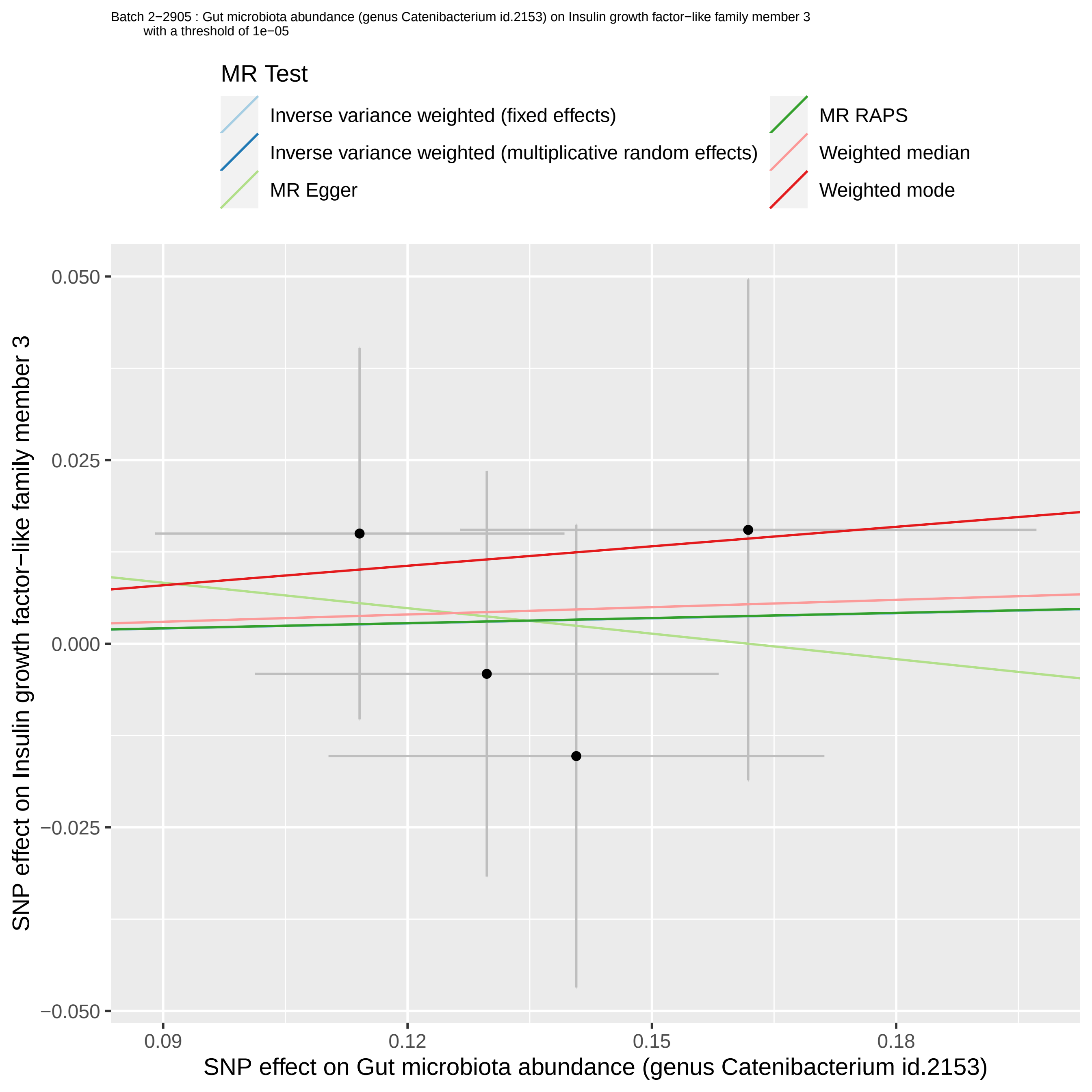
Batch 2−2905 : Gut microbiota abundance (genus Catenibacterium id.2153) on Insulin growth factor−like family member 3
with a threshold of 1e−05
MR Test
Inverse variance weighted (fixed effects)
Inverse variance weighted (multiplicative random effects)
MR Egger
MR RAPS
Weighted median
Weighted mode
0.050
0.025
0.000
−0.025
−0.050
0.09
0.12
0.15
0.18
SNP effect on Insulin growth factor−like family member 3
SNP effect on Gut microbiota abundance (genus Catenibacterium id.2153)

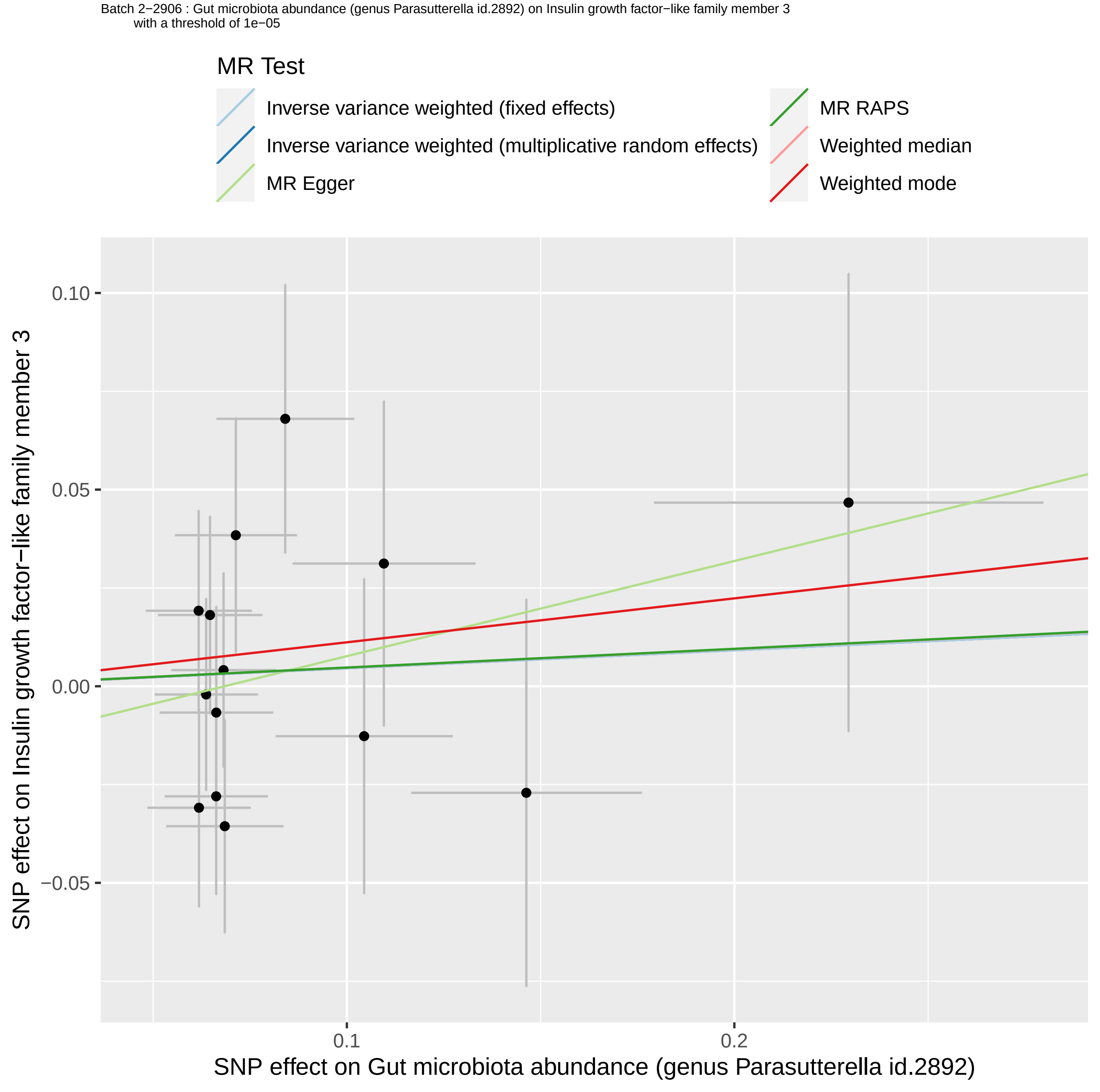

Batch 2−2906 : Gut microbiota abundance (genus Parasutterella id.2892) on Insulin growth factor−like family member 3
with a threshold of 1e−05
MR Test
Inverse variance weighted (fixed effects)
Inverse variance weighted (multiplicative random effects)
MR Egger
MR RAPS
Weighted median
Weighted mode
0.10
0.05
0.00
−0.05
0.1
0.2
SNP effect on Insulin growth factor−like family member 3
SNP effect on Gut microbiota abundance (genus Parasutterella id.2892)

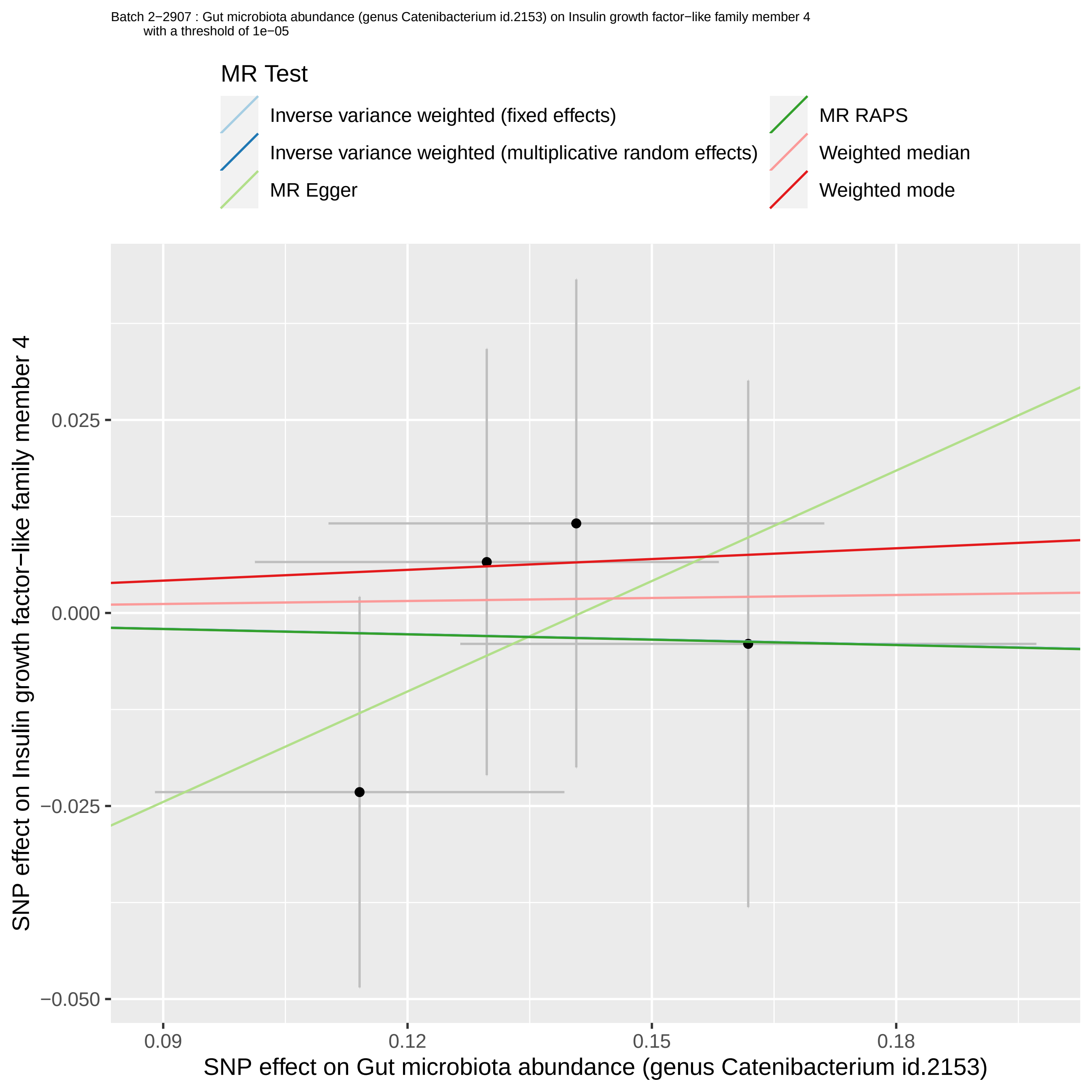
Batch 2−2907 : Gut microbiota abundance (genus Catenibacterium id.2153) on Insulin growth factor−like family member 4
with a threshold of 1e−05
MR Test
Inverse variance weighted (fixed effects)
Inverse variance weighted (multiplicative random effects)
MR Egger
MR RAPS
Weighted median
Weighted mode
0.025
0.000
−0.025
−0.050
0.09
0.12
0.15
0.18
SNP effect on Insulin growth factor−like family member 4
SNP effect on Gut microbiota abundance (genus Catenibacterium id.2153)

Batch 2−2908 : Gut microbiota abundance (genus Parasutterella id.2892) on Insulin growth factor−like family member 4
with a threshold of 1e−05

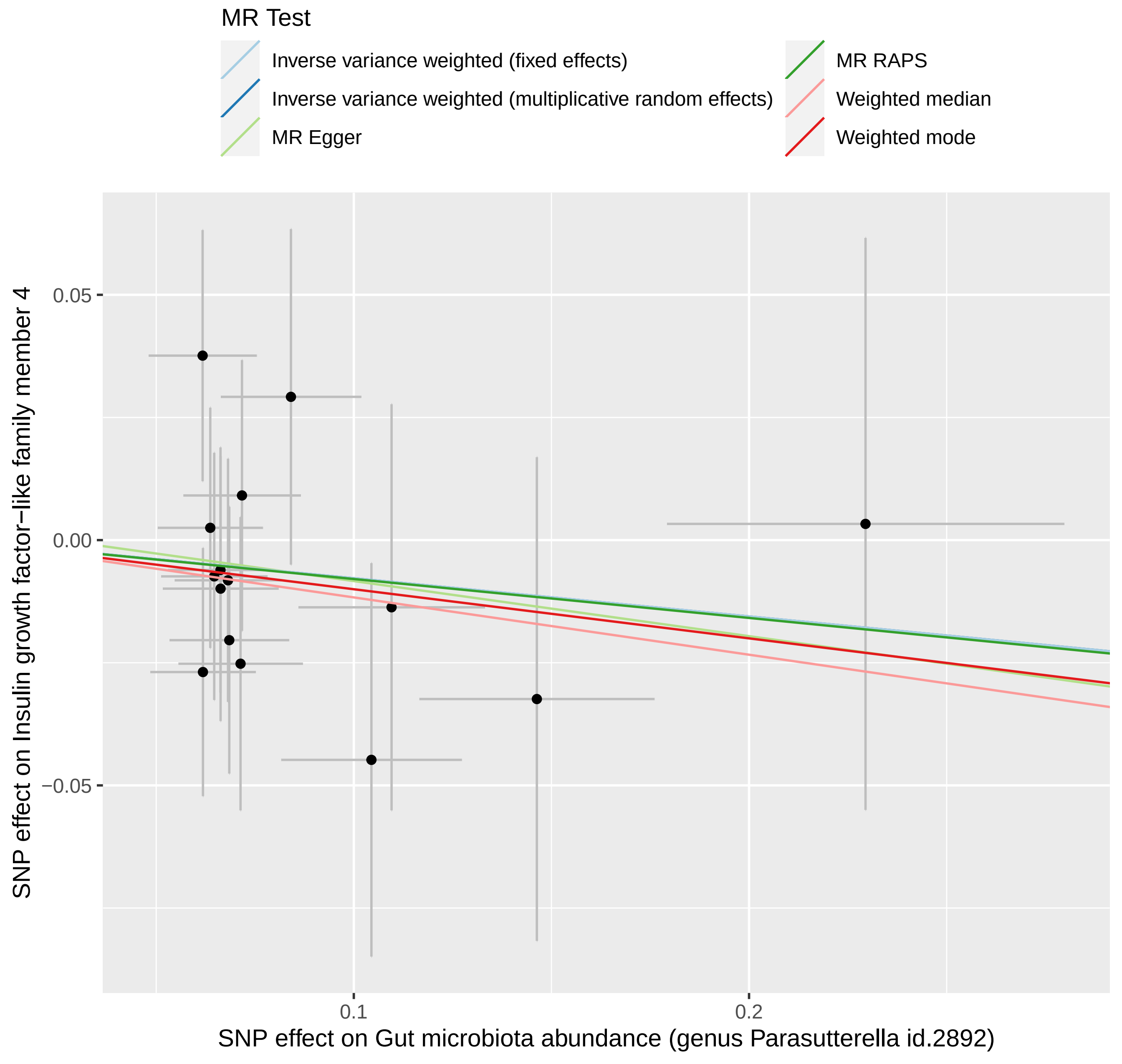

Batch 2−2909 : Gut microbiota abundance (genus Catenibacterium id.2153) on IGF−like family receptor 1
with a threshold of 1e−05

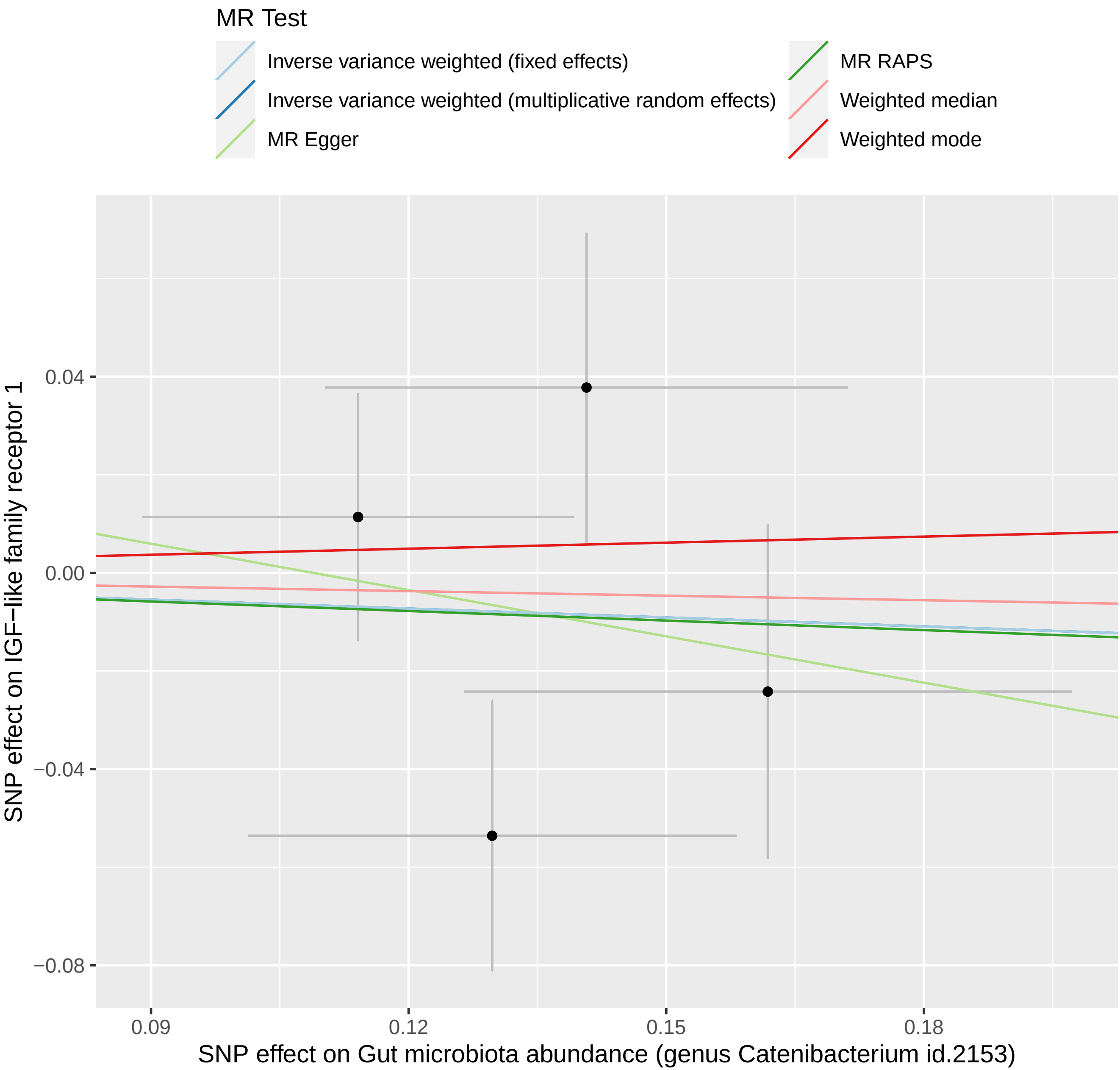

Batch 2−2910 : Gut microbiota abundance (genus Parasutterella id.2892) on IGF−like family receptor 1
with a threshold of 1e−05

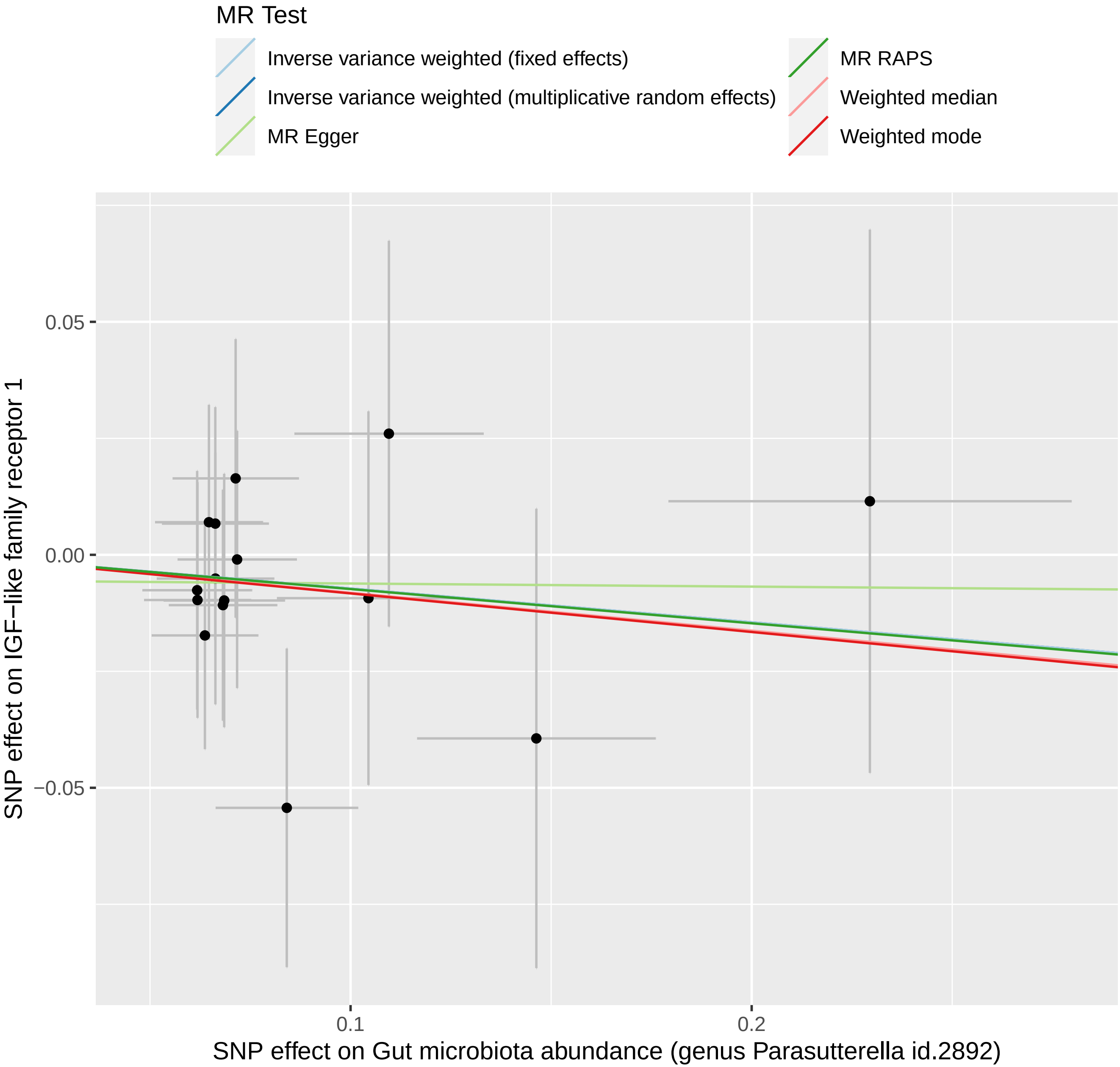

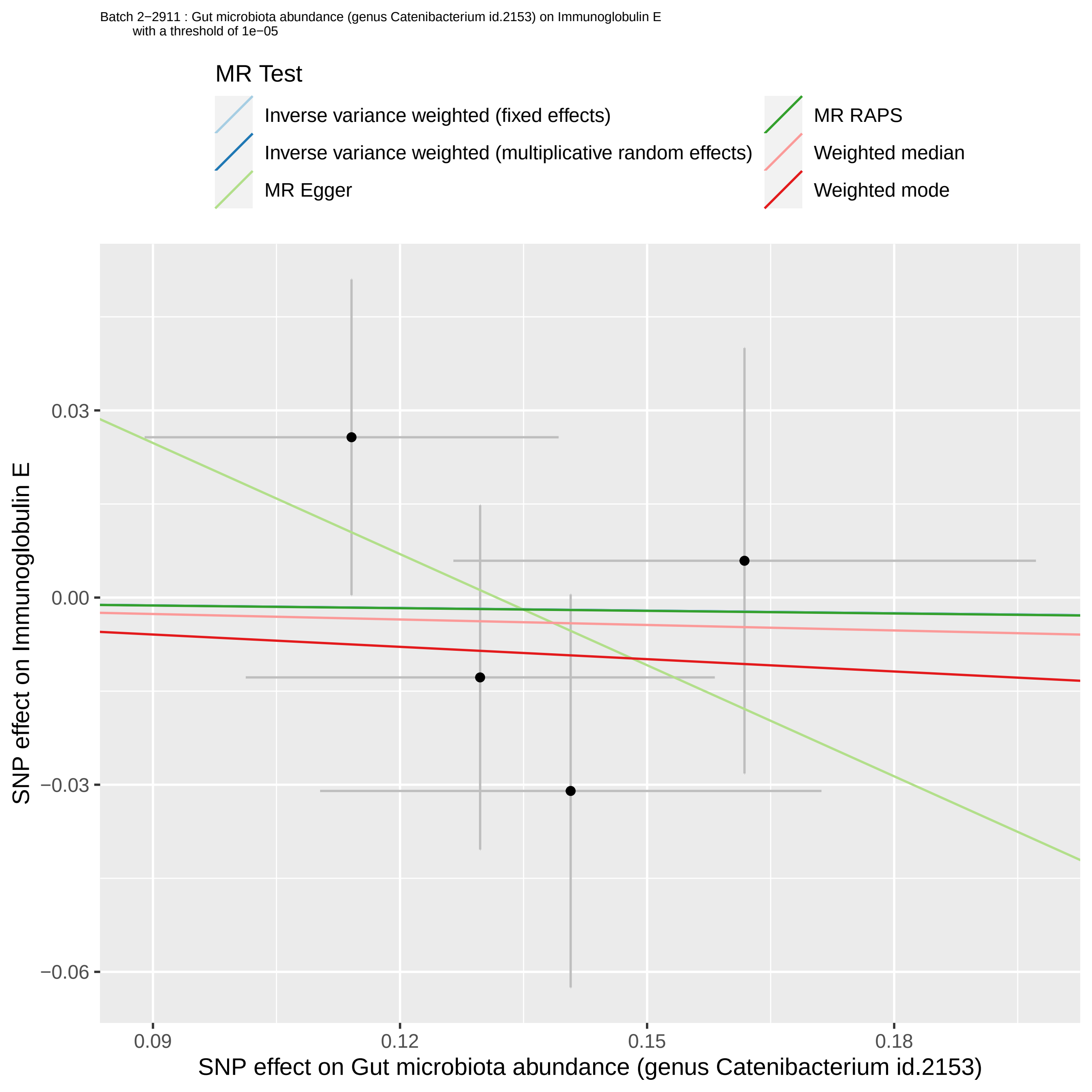
Batch 2−2911 : Gut microbiota abundance (genus Catenibacterium id.2153) on Immunoglobulin E
with a threshold of 1e−05
MR Test
Inverse variance weighted (fixed effects)
Inverse variance weighted (multiplicative random effects)
MR Egger
MR RAPS
Weighted median
Weighted mode
0.03
0.00
−0.03
−0.06
0.09
0.12
0.15
0.18
SNP effect on Immunoglobulin E
SNP effect on Gut microbiota abundance (genus Catenibacterium id.2153)

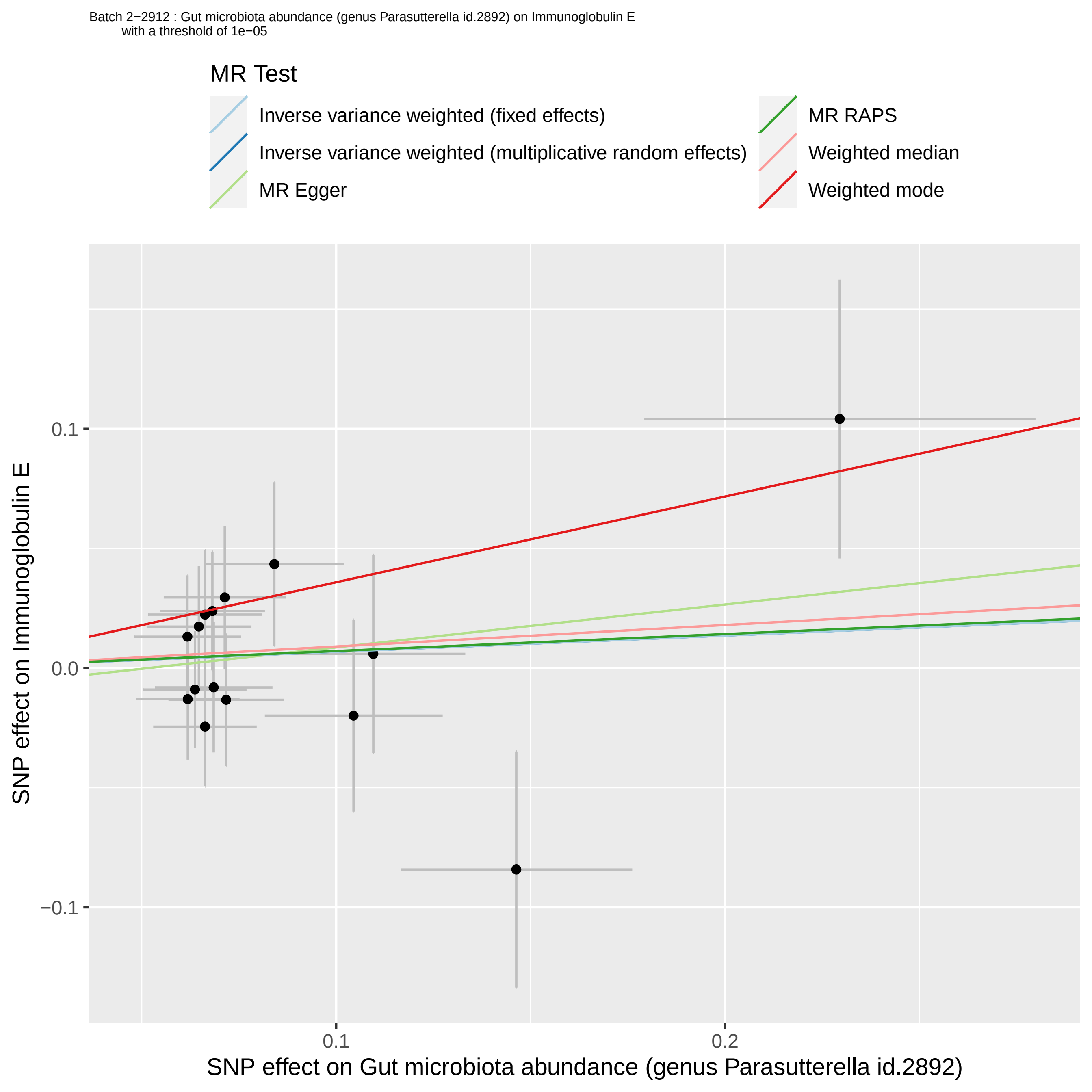

Batch 2−2912 : Gut microbiota abundance (genus Parasutterella id.2892) on Immunoglobulin E
with a threshold of 1e−05
MR Test
Inverse variance weighted (fixed effects)
Inverse variance weighted (multiplicative random effects)
MR Egger
MR RAPS
Weighted median
Weighted mode
0.1
0.0
−0.1
0.1
0.2
SNP effect on Immunoglobulin E
SNP effect on Gut microbiota abundance (genus Parasutterella id.2892)

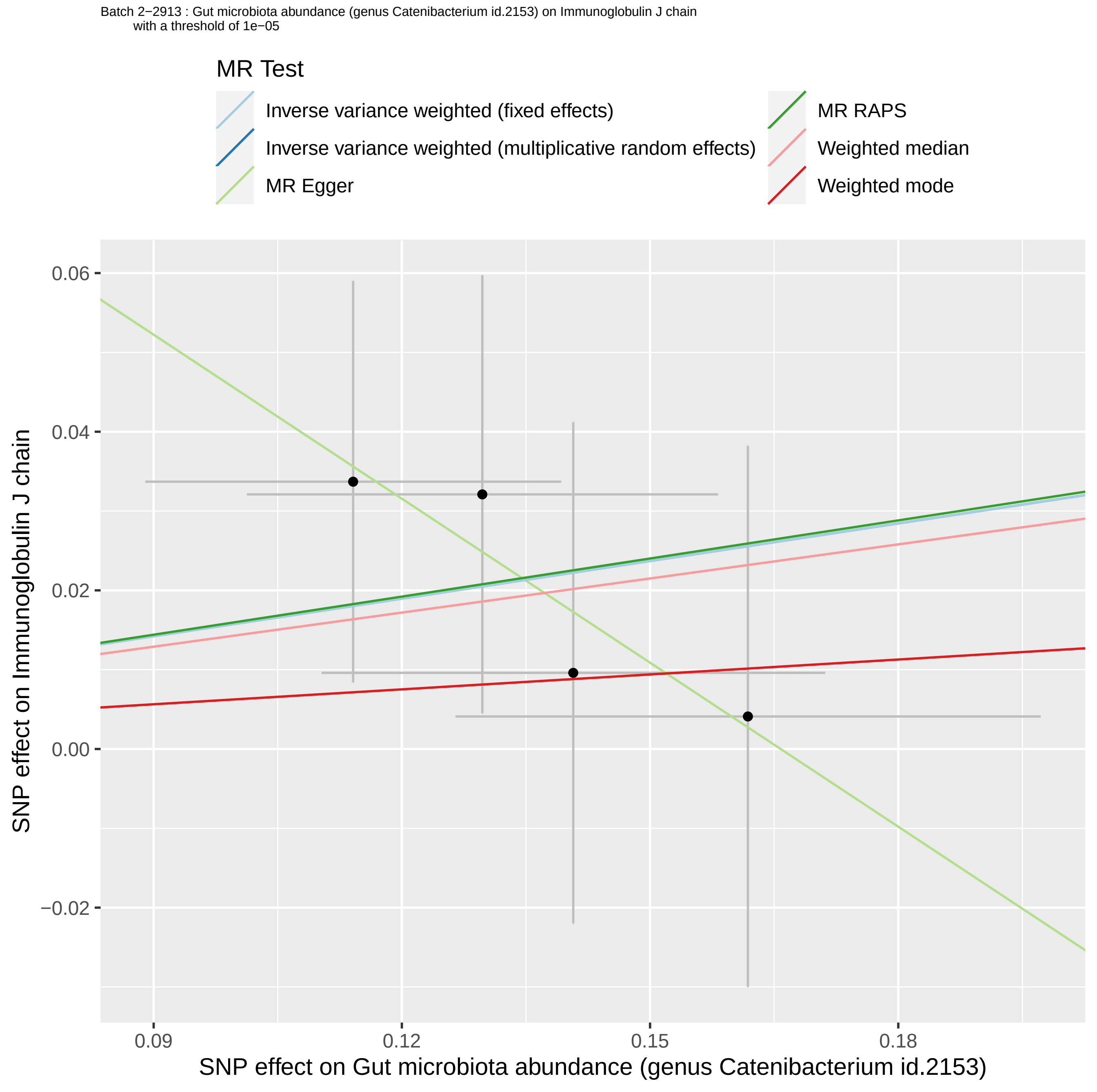
Batch 2−2913 : Gut microbiota abundance (genus Catenibacterium id.2153) on Immunoglobulin J chain
with a threshold of 1e−05
MR Test
Inverse variance weighted (fixed effects)
Inverse variance weighted (multiplicative random effects)
MR Egger
MR RAPS
Weighted median
Weighted mode
0.06
0.04
0.02
0.00
−0.02
0.09
0.12
0.15
0.18
SNP effect on Immunoglobulin J chain
SNP effect on Gut microbiota abundance (genus Catenibacterium id.2153)

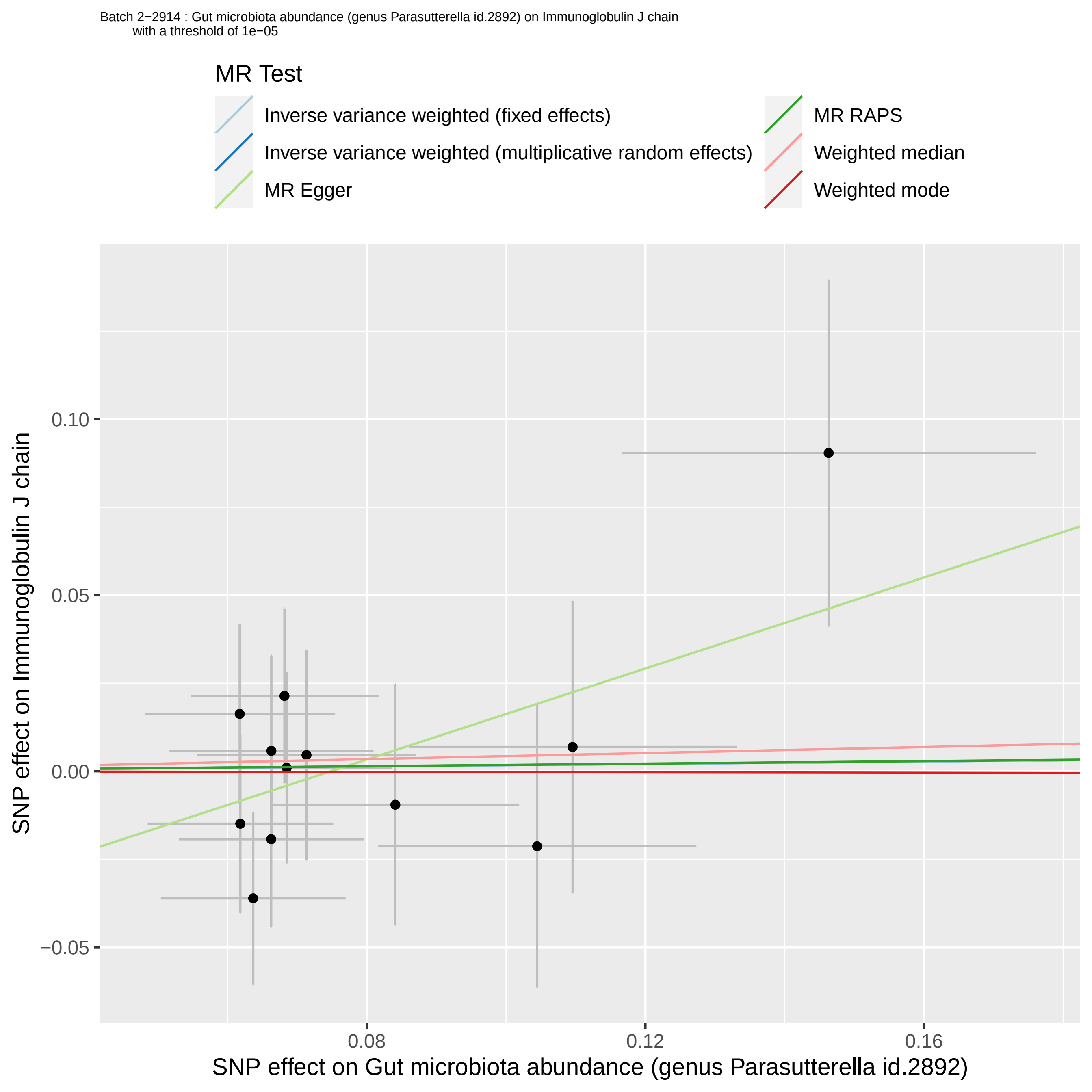

Batch 2−2914 : Gut microbiota abundance (genus Parasutterella id.2892) on Immunoglobulin J chain
with a threshold of 1e−05
MR Test
Inverse variance weighted (fixed effects)
Inverse variance weighted (multiplicative random effects)
MR Egger
MR RAPS
Weighted median
Weighted mode
SNP effect on Immunoglobulin J chain
0.10
0.05
0.00
−0.05
0.08
0.12
0.16
SNP effect on Gut microbiota abundance (genus Parasutterella id.2892)

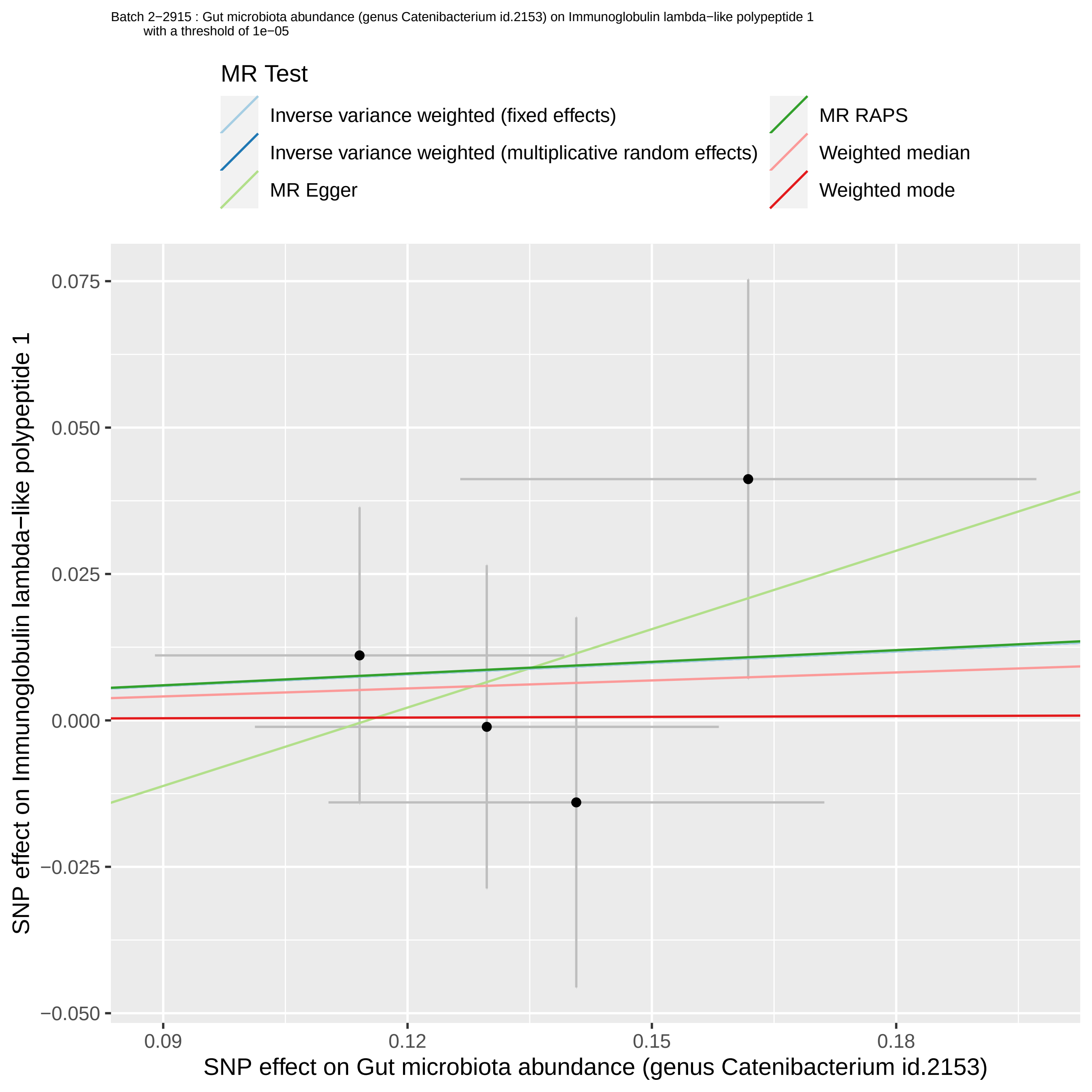

Batch 2−2915 : Gut microbiota abundance (genus Catenibacterium id.2153) on Immunoglobulin lambda−like polypeptide 1
with a threshold of 1e−05
MR Test
Inverse variance weighted (fixed effects)
Inverse variance weighted (multiplicative random effects)
MR Egger
MR RAPS
Weighted median
Weighted mode
0.075
0.050
0.025
0.000
−0.025
−0.050
0.09
0.12
0.15
0.18
SNP effect on Immunoglobulin lambda−like polypeptide 1
SNP effect on Gut microbiota abundance (genus Catenibacterium id.2153)

Batch 2−2916 : Gut microbiota abundance (genus Parasutterella id.2892) on Immunoglobulin lambda−like polypeptide 1
with a threshold of 1e−05

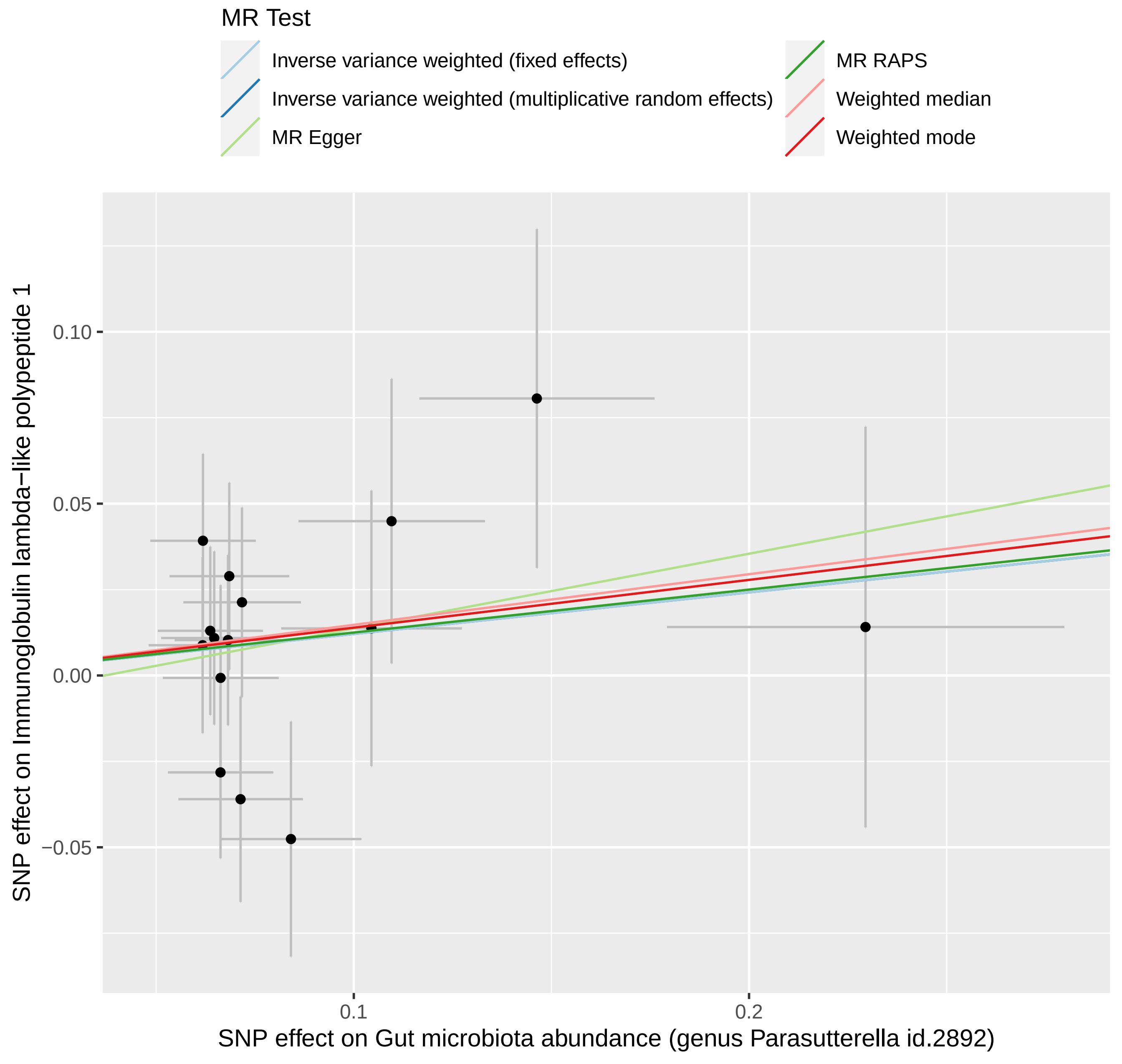

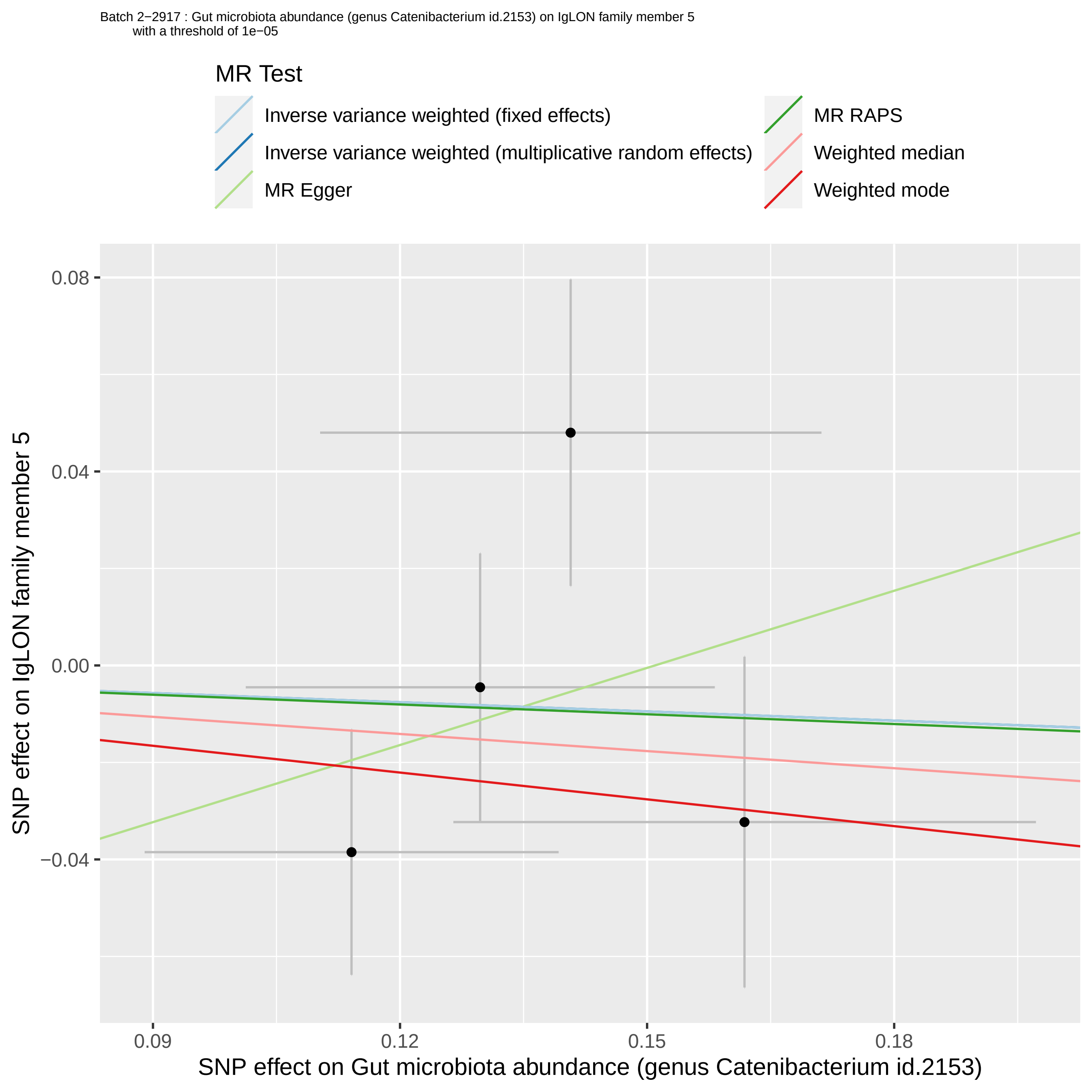
Batch 2−2917 : Gut microbiota abundance (genus Catenibacterium id.2153) on IgLON family member 5
with a threshold of 1e−05
MR Test
Inverse variance weighted (fixed effects)
Inverse variance weighted (multiplicative random effects)
MR Egger
MR RAPS
Weighted median
Weighted mode
0.08
0.04
0.00
−0.04
0.09
0.12
0.15
0.18
SNP effect on IgLON family member 5
SNP effect on Gut microbiota abundance (genus Catenibacterium id.2153)

Batch 2−2918 : Gut microbiota abundance (genus Parasutterella id.2892) on IgLON family member 5
with a threshold of 1e−05

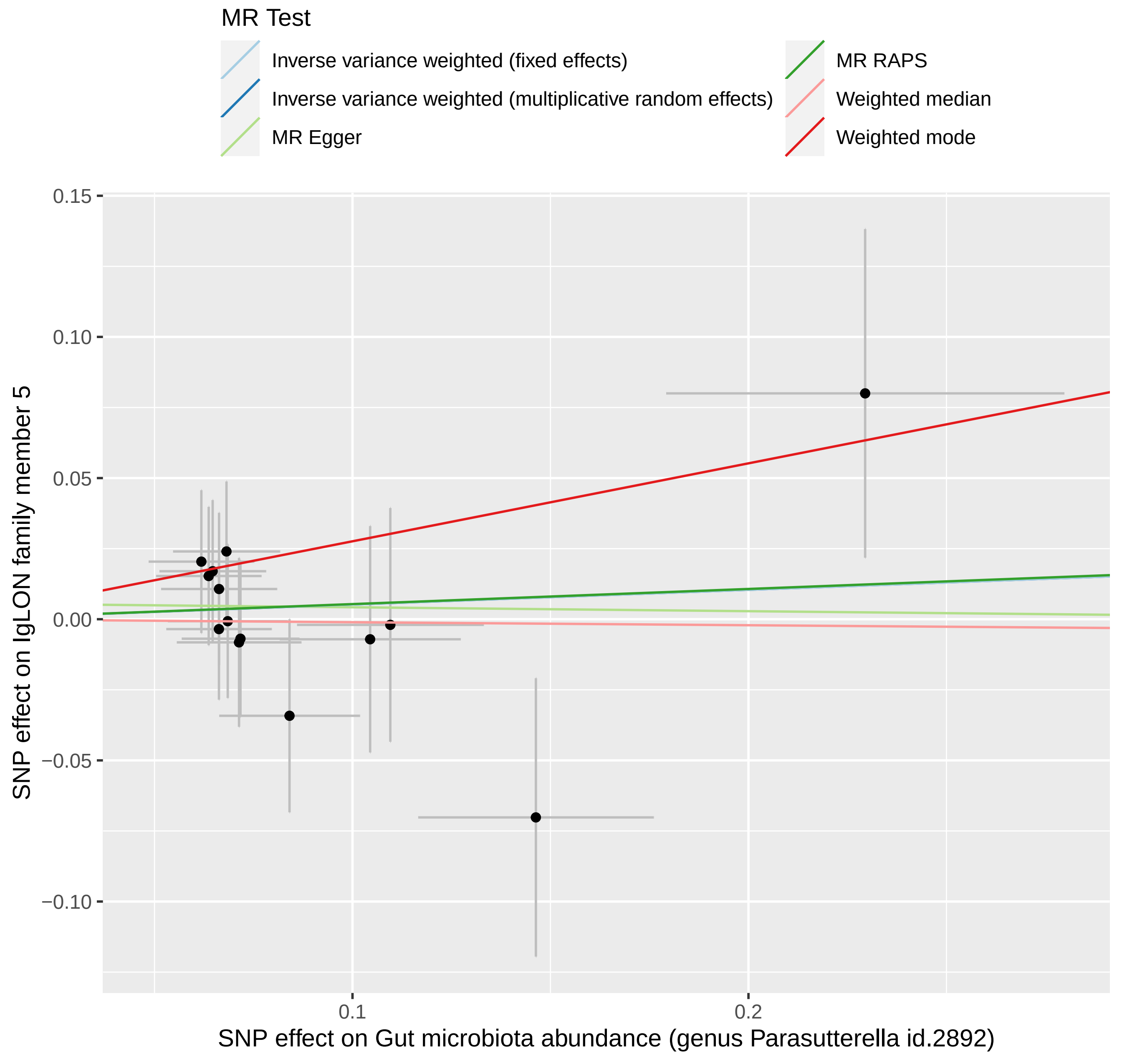

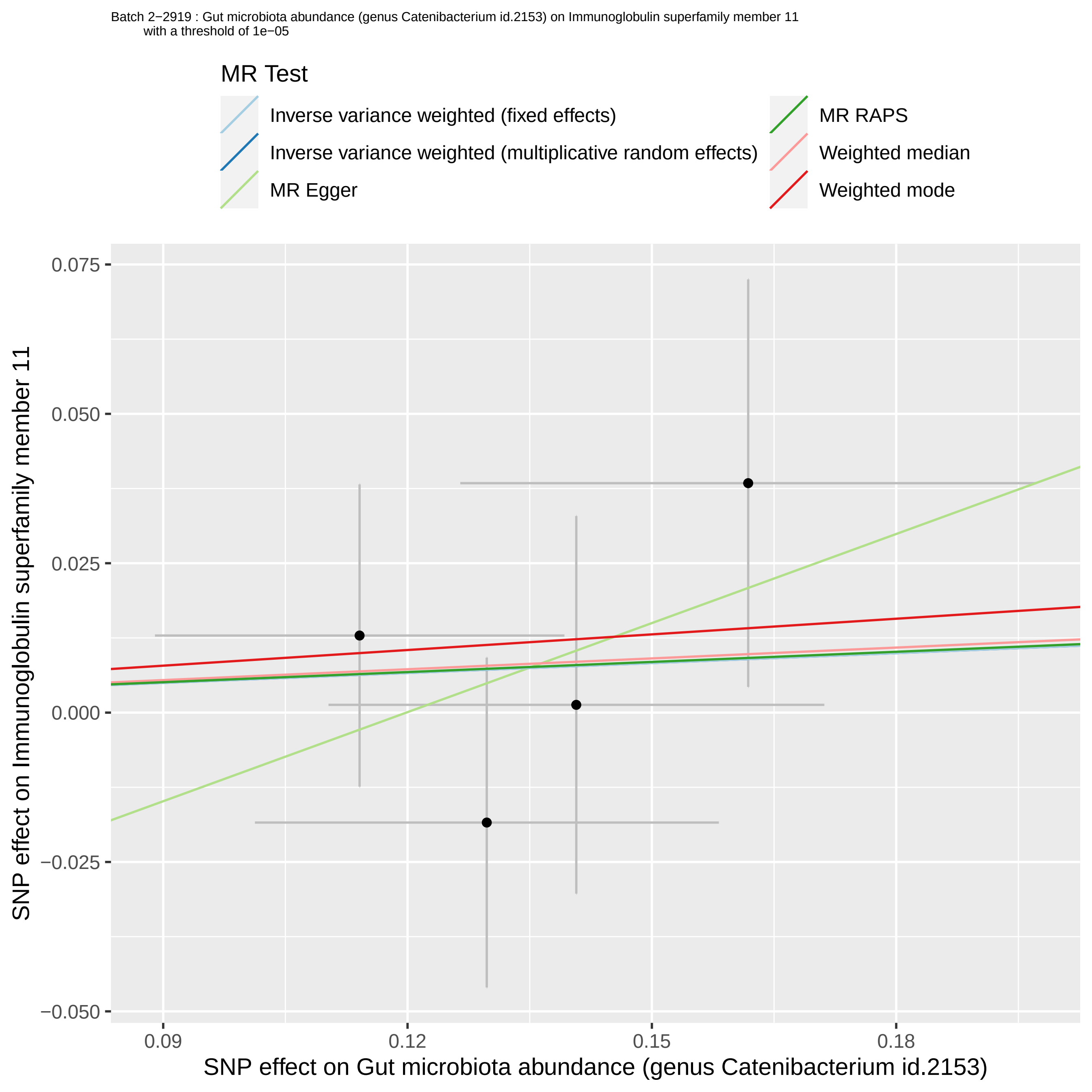
Batch 2−2919 : Gut microbiota abundance (genus Catenibacterium id.2153) on Immunoglobulin superfamily member 11
with a threshold of 1e−05
MR Test
Inverse variance weighted (fixed effects)
Inverse variance weighted (multiplicative random effects)
MR Egger
MR RAPS
Weighted median
Weighted mode
0.075
0.050
0.025
0.000
−0.025
−0.050
SNP effect on Immunoglobulin superfamily member 11
0.09
0.12
0.15
0.18
SNP effect on Gut microbiota abundance (genus Catenibacterium id.2153)

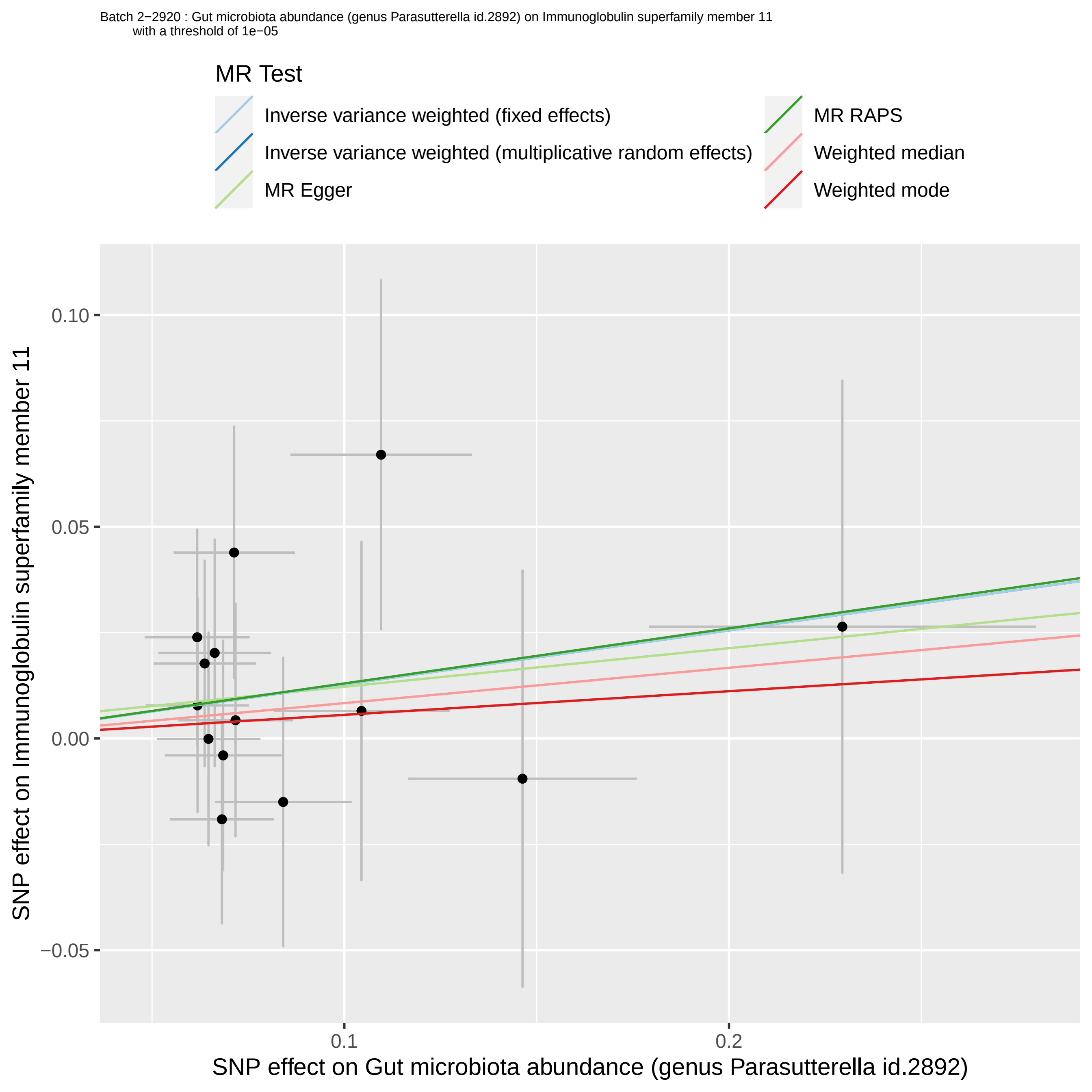
Batch 2−2920 : Gut microbiota abundance (genus Parasutterella id.2892) on Immunoglobulin superfamily member 11
with a threshold of 1e−05
MR Test
Inverse variance weighted (fixed effects)
Inverse variance weighted (multiplicative random effects)
MR Egger
MR RAPS
Weighted median
Weighted mode
0.10
0.05
0.00
−0.05
0.1
0.2
SNP effect on Immunoglobulin superfamily member 11
SNP effect on Gut microbiota abundance (genus Parasutterella id.2892)

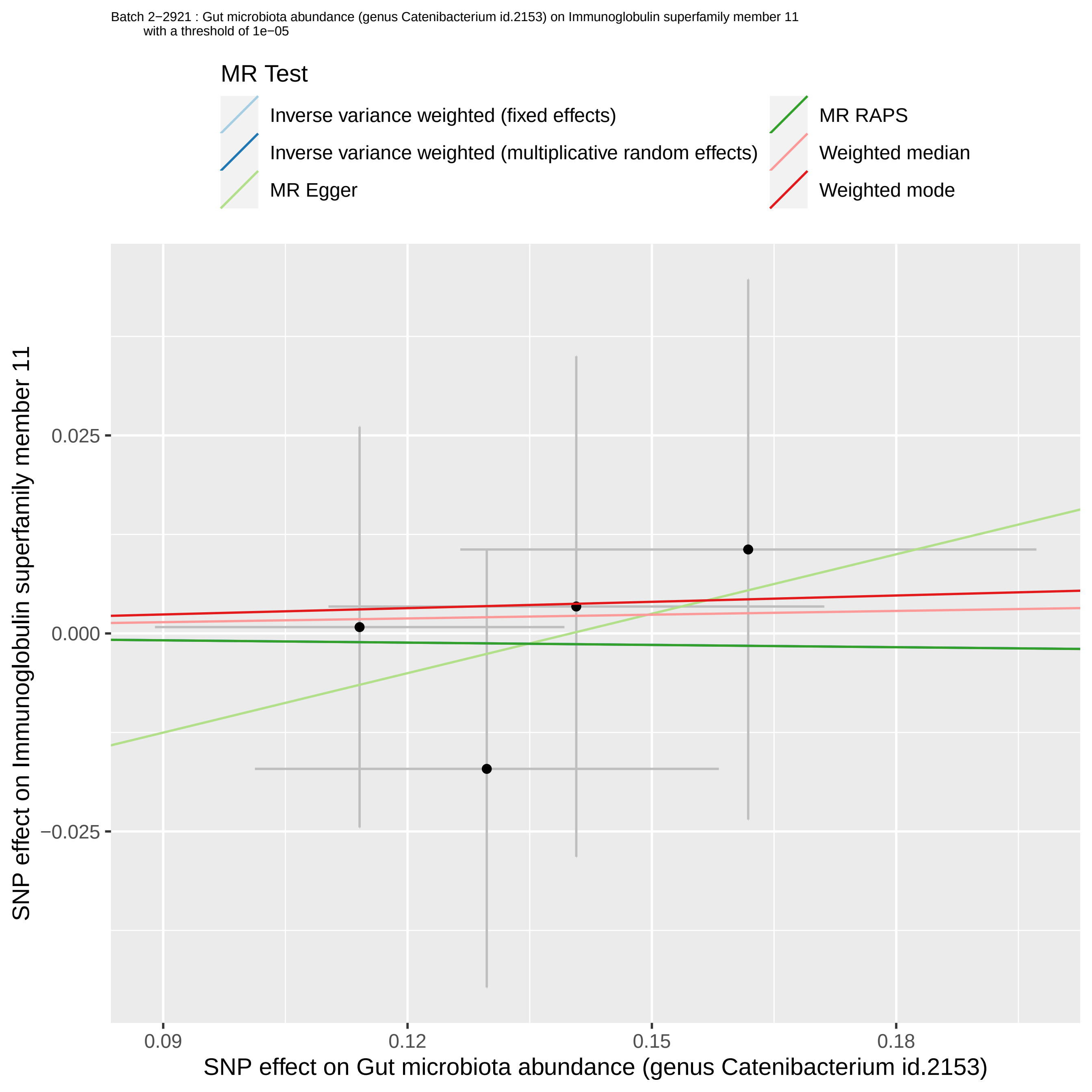

Batch 2−2921 : Gut microbiota abundance (genus Catenibacterium id.2153) on Immunoglobulin superfamily member 11
with a threshold of 1e−05
MR Test
Inverse variance weighted (fixed effects)
Inverse variance weighted (multiplicative random effects)
MR Egger
MR RAPS
Weighted median
Weighted mode
SNP effect on Immunoglobulin superfamily member 11
0.025
0.000
−0.025
0.09
0.12
0.15
0.18
SNP effect on Gut microbiota abundance (genus Catenibacterium id.2153)

Batch 2−2922 : Gut microbiota abundance (genus Parasutterella id.2892) on Immunoglobulin superfamily member 11
with a threshold of 1e−05

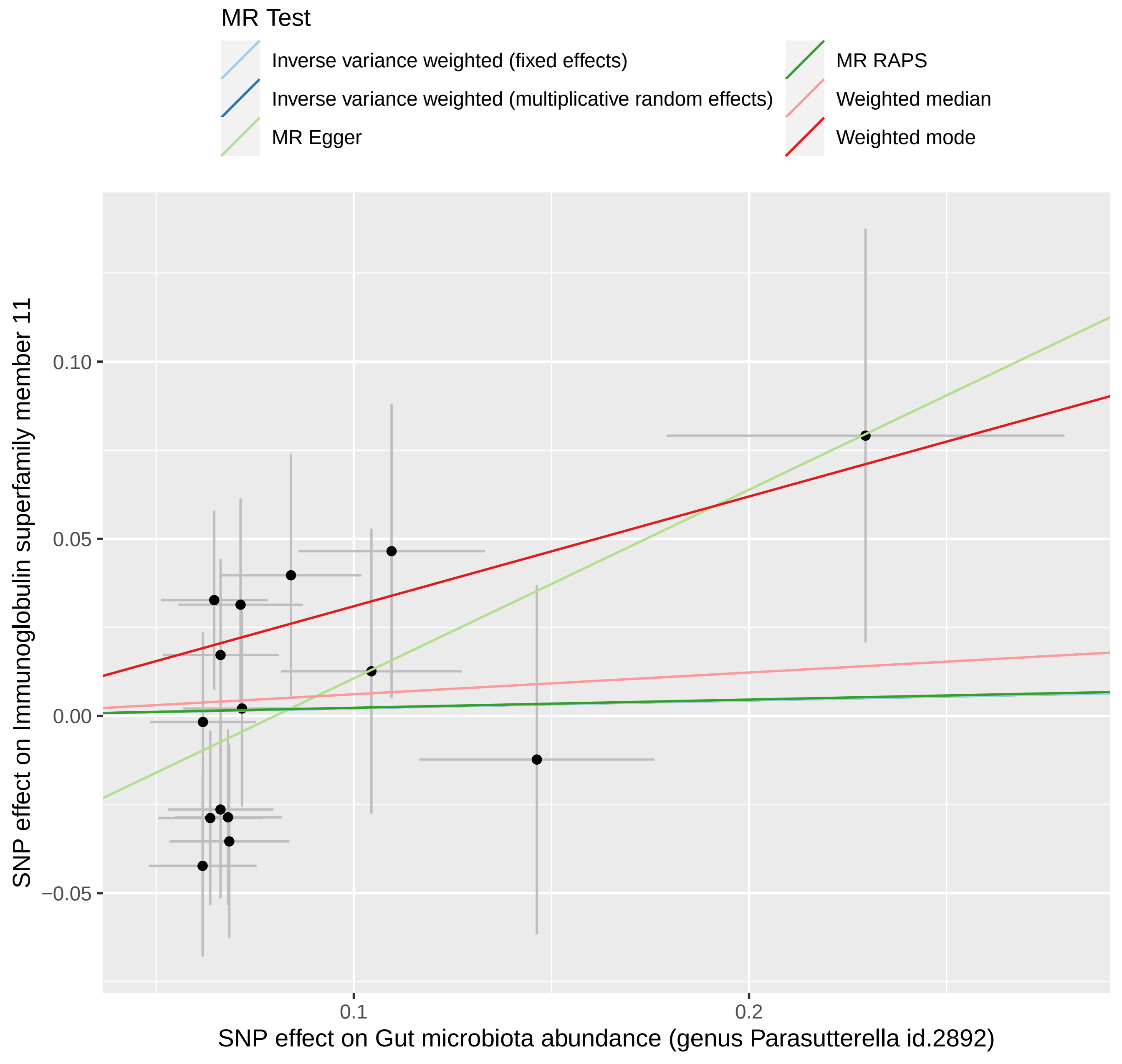

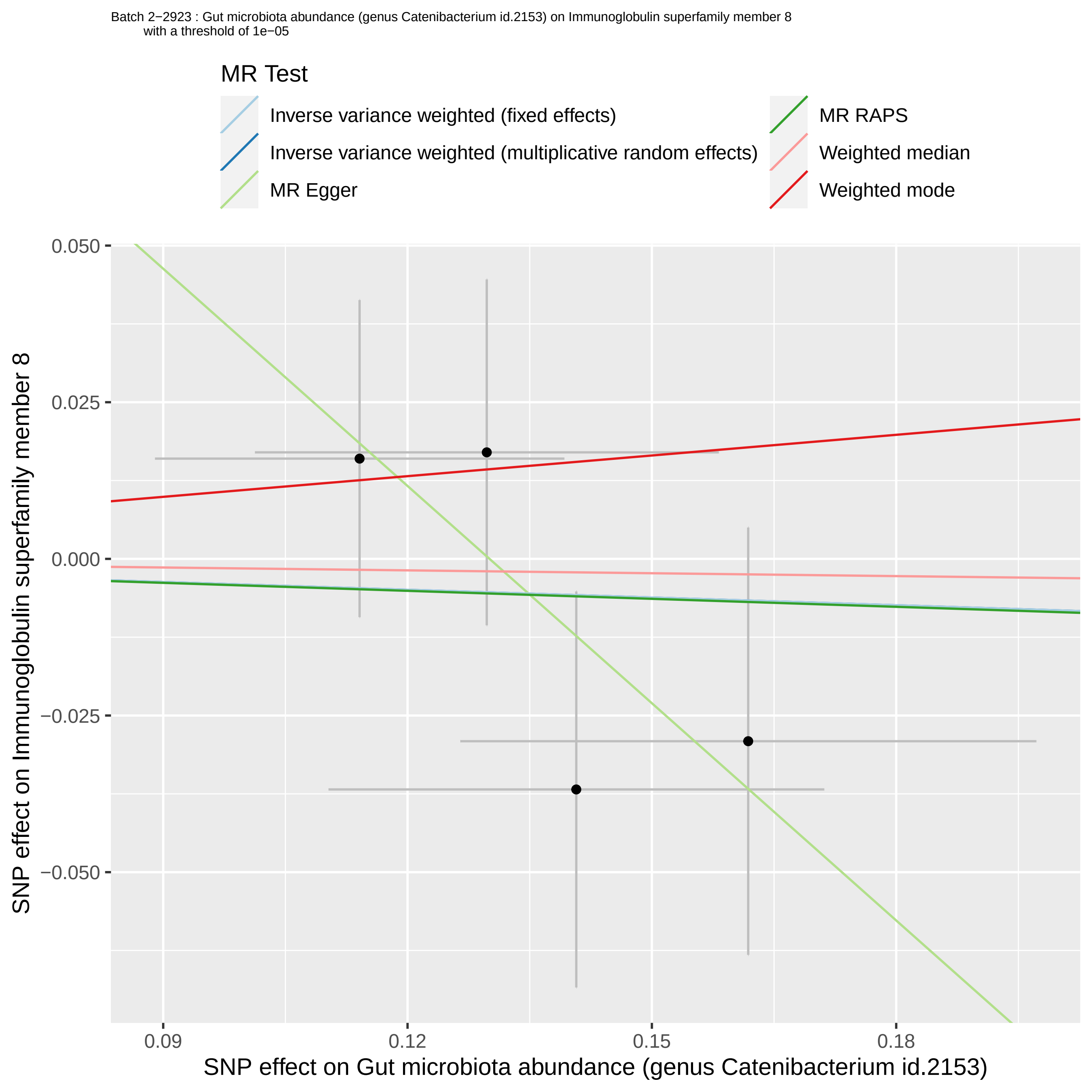

Batch 2−2923 : Gut microbiota abundance (genus Catenibacterium id.2153) on Immunoglobulin superfamily member 8
with a threshold of 1e−05
MR Test
Inverse variance weighted (fixed effects)
Inverse variance weighted (multiplicative random effects)
MR Egger
MR RAPS
Weighted median
Weighted mode
0.050
0.025
0.000
−0.025
−0.050
0.09
0.12
0.15
0.18
SNP effect on Immunoglobulin superfamily member 8
SNP effect on Gut microbiota abundance (genus Catenibacterium id.2153)

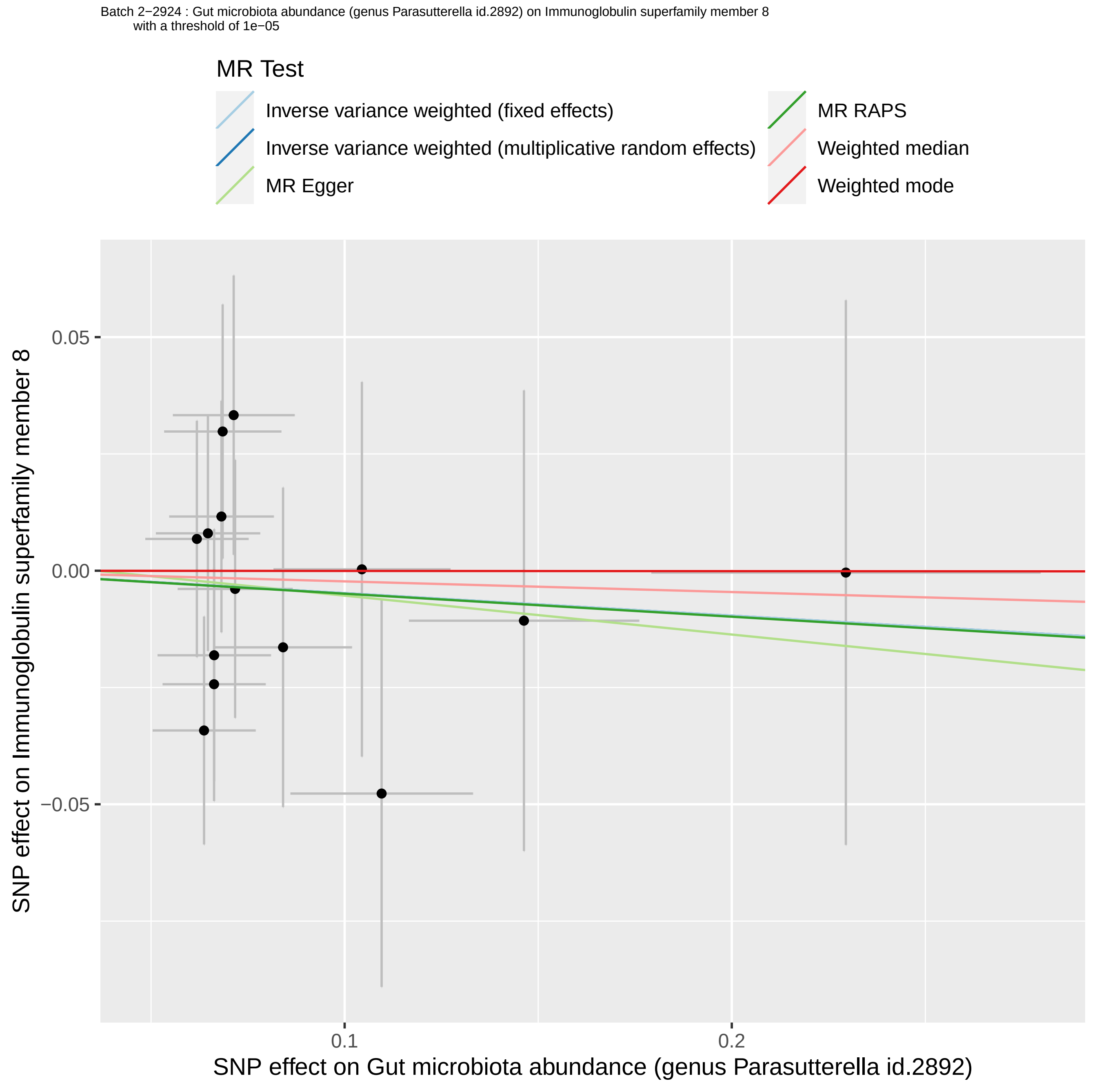
Batch 2−2924 : Gut microbiota abundance (genus Parasutterella id.2892) on Immunoglobulin superfamily member 8
with a threshold of 1e−05
MR Test
Inverse variance weighted (fixed effects)
Inverse variance weighted (multiplicative random effects)
MR Egger
MR RAPS
Weighted median
Weighted mode
0.05
0.00
−0.05
0.1
0.2
SNP effect on Immunoglobulin superfamily member 8
SNP effect on Gut microbiota abundance (genus Parasutterella id.2892)

Batch 2−2925 : Gut microbiota abundance (genus Catenibacterium id.2153) on Protein turtle homolog A
with a threshold of 1e−05

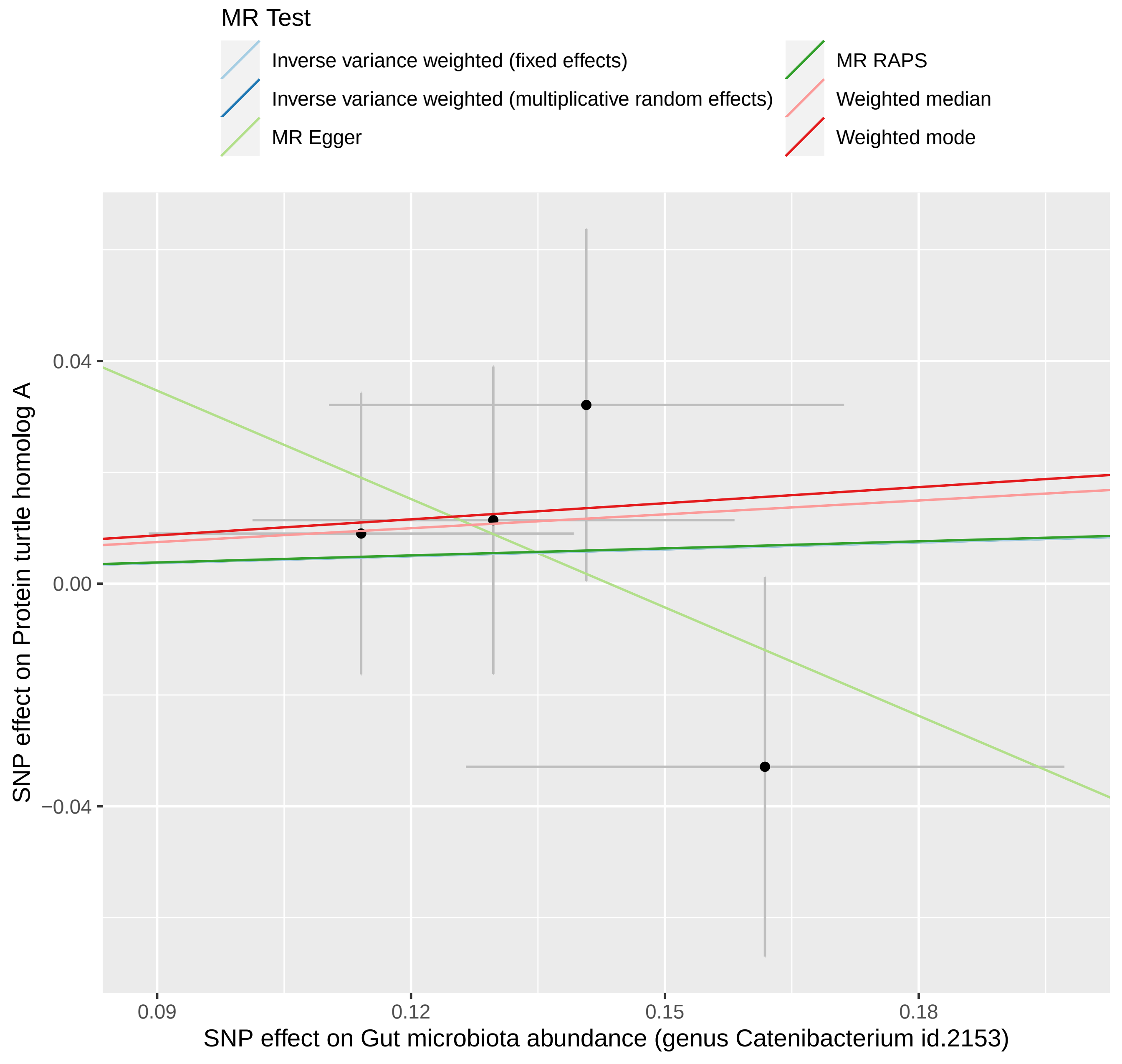

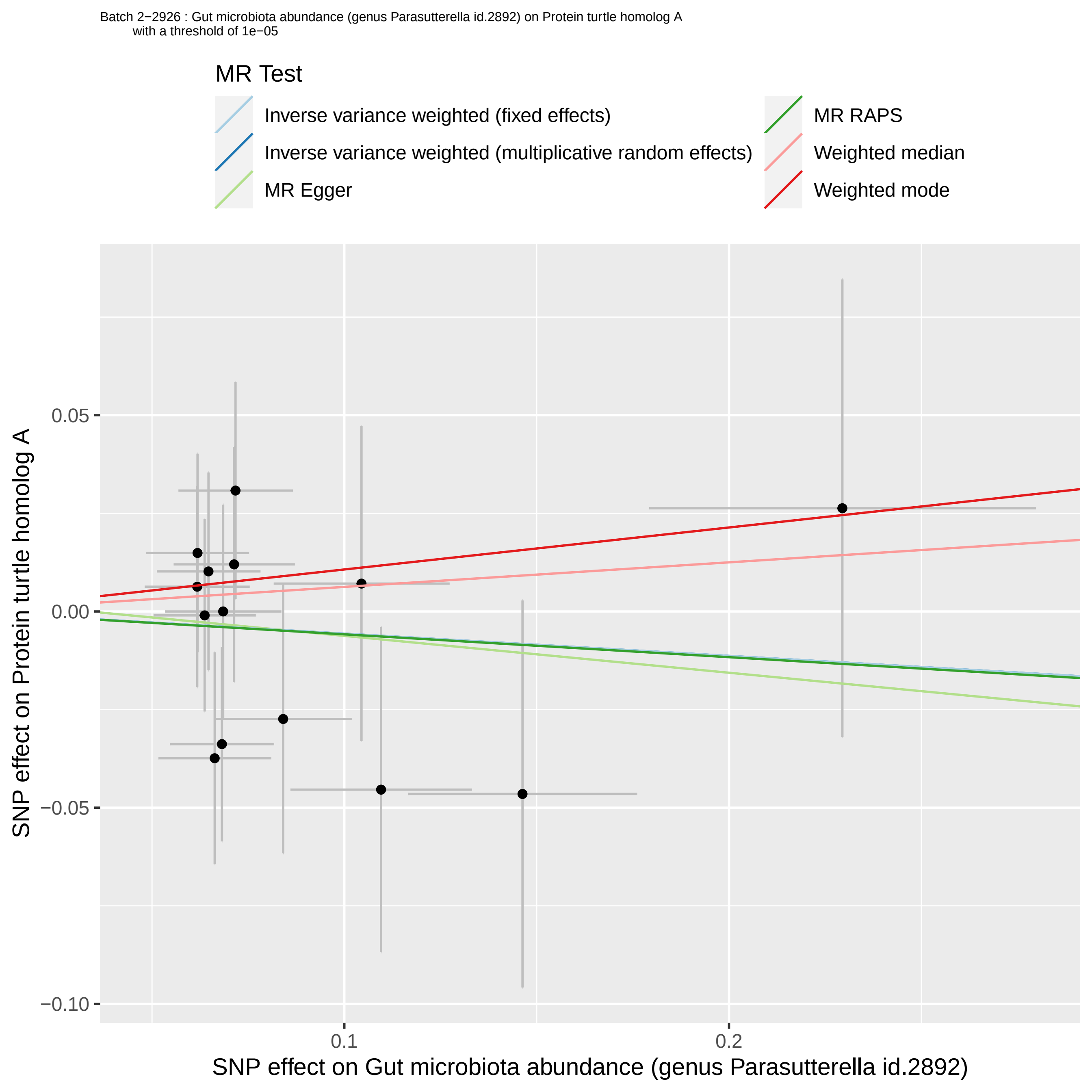
Batch 2−2926 : Gut microbiota abundance (genus Parasutterella id.2892) on Protein turtle homolog A
with a threshold of 1e−05
MR Test
Inverse variance weighted (fixed effects)
Inverse variance weighted (multiplicative random effects)
MR Egger
MR RAPS
Weighted median
Weighted mode
0.05
0.00
−0.05
−0.10
0.1
0.2
SNP effect on Protein turtle homolog A
SNP effect on Gut microbiota abundance (genus Parasutterella id.2892)

Batch 2−2927 : Gut microbiota abundance (genus Catenibacterium id.2153) on Interleukin−10
with a threshold of 1e−05

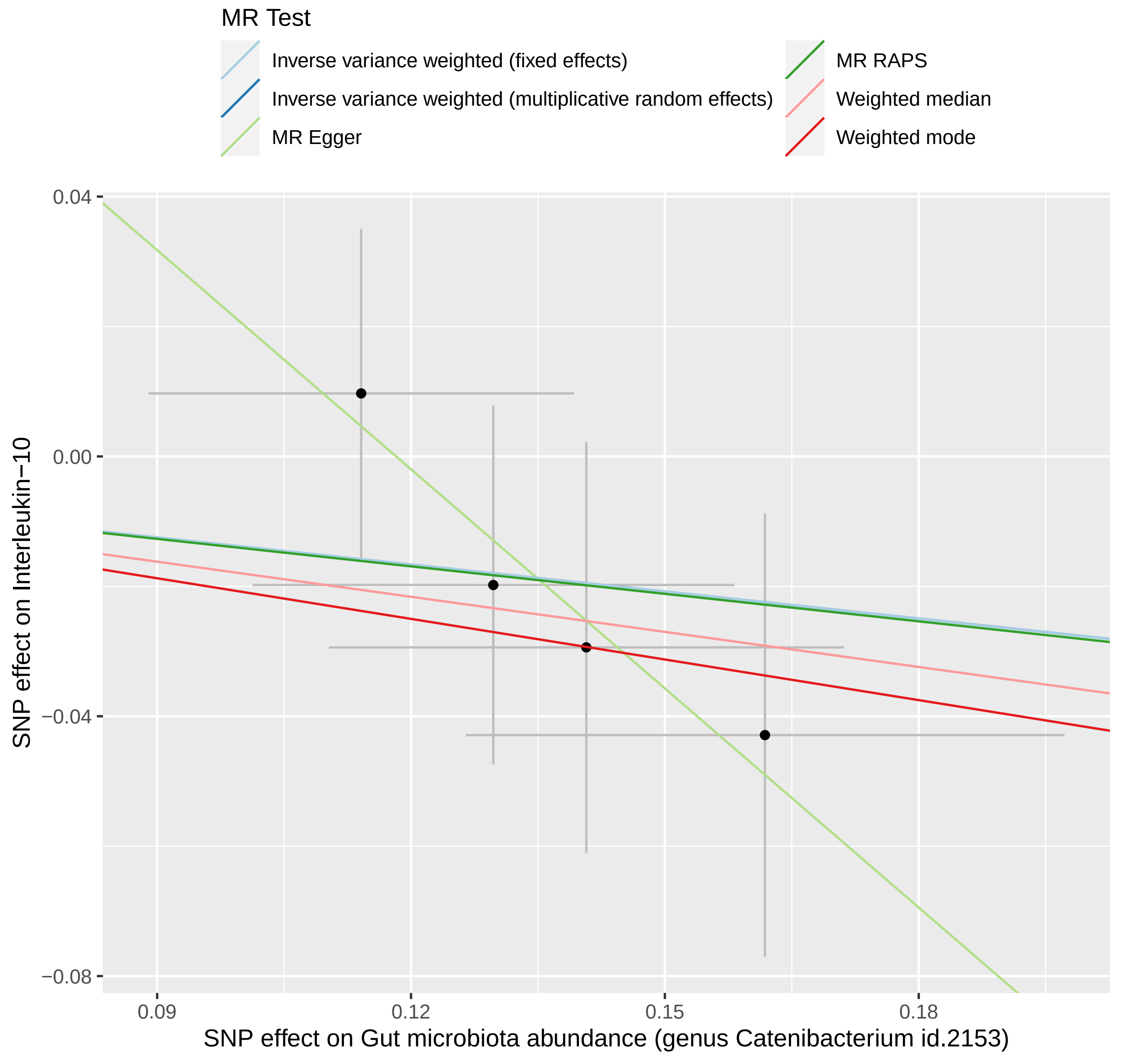

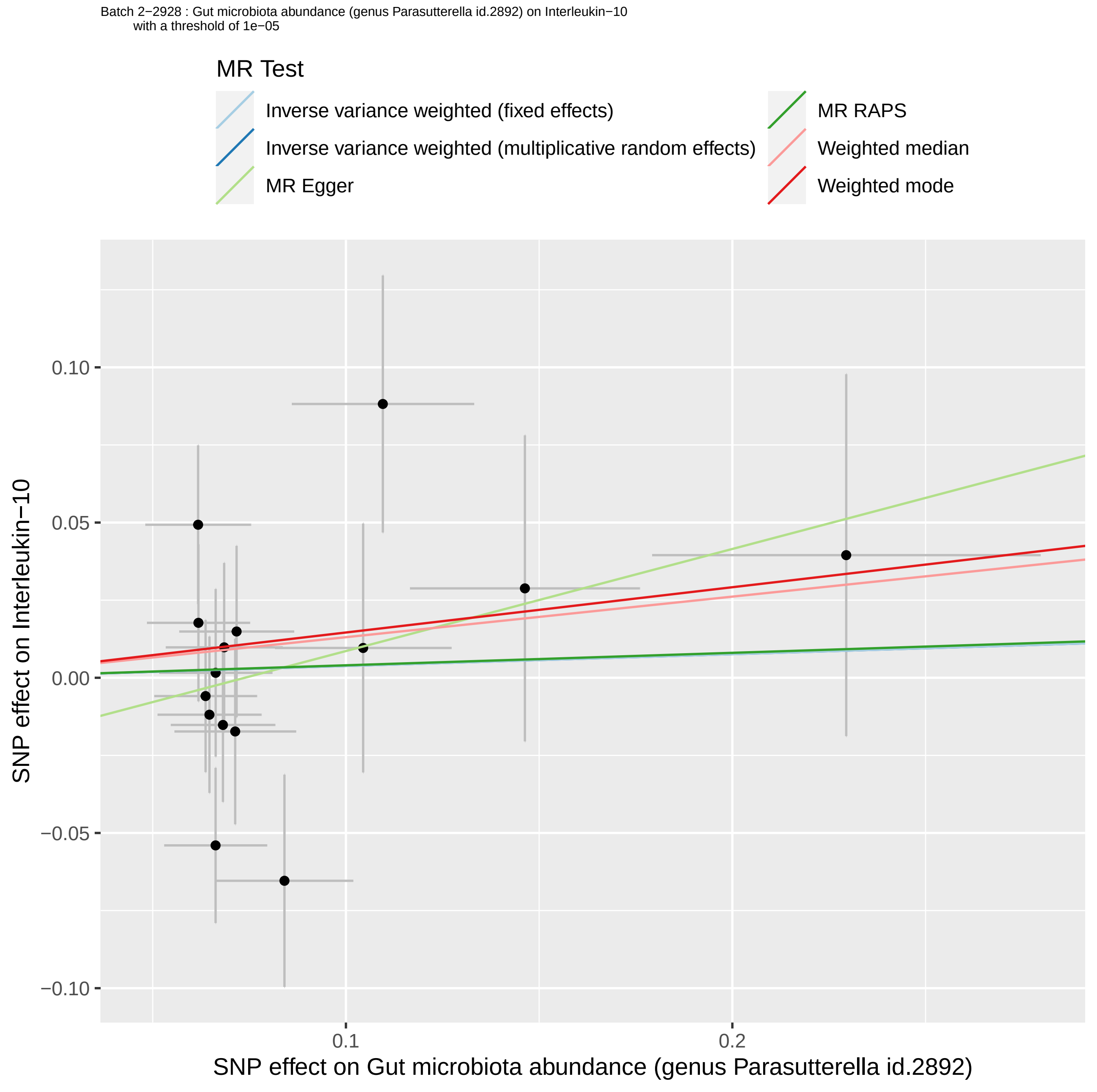

Batch 2−2928 : Gut microbiota abundance (genus Parasutterella id.2892) on Interleukin−10
with a threshold of 1e−05
MR Test
Inverse variance weighted (fixed effects)
Inverse variance weighted (multiplicative random effects)
MR Egger
MR RAPS
Weighted median
Weighted mode
0.10
0.05
0.00
−0.05
−0.10
0.1
0.2
SNP effect on Interleukin−10
SNP effect on Gut microbiota abundance (genus Parasutterella id.2892)

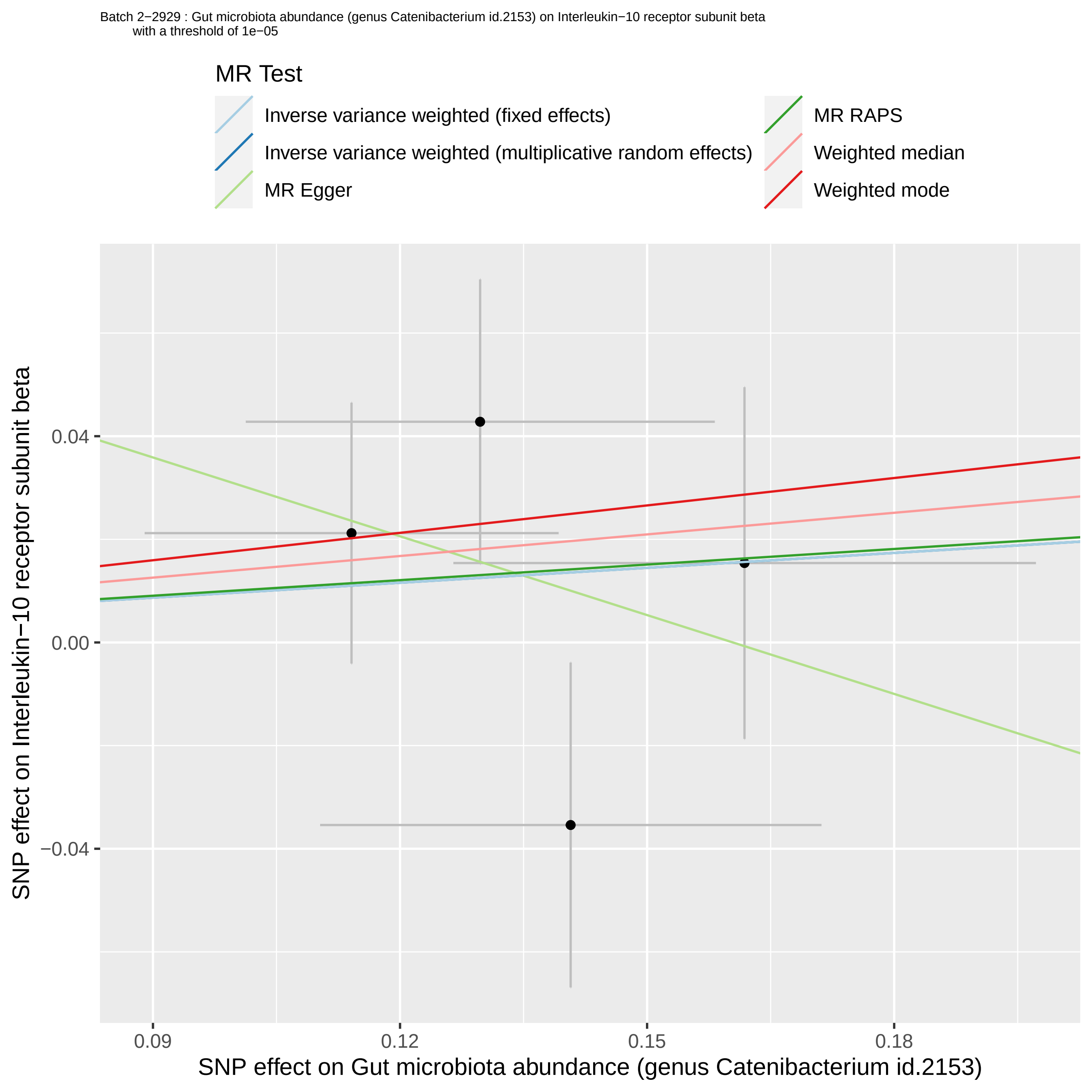
Batch 2−2929 : Gut microbiota abundance (genus Catenibacterium id.2153) on Interleukin−10 receptor subunit beta
with a threshold of 1e−05
MR Test
Inverse variance weighted (fixed effects)
Inverse variance weighted (multiplicative random effects)
MR Egger
MR RAPS
Weighted median
Weighted mode
SNP effect on Interleukin−10 receptor subunit beta
0.04
0.00
−0.04
0.09
0.12
0.15
0.18
SNP effect on Gut microbiota abundance (genus Catenibacterium id.2153)

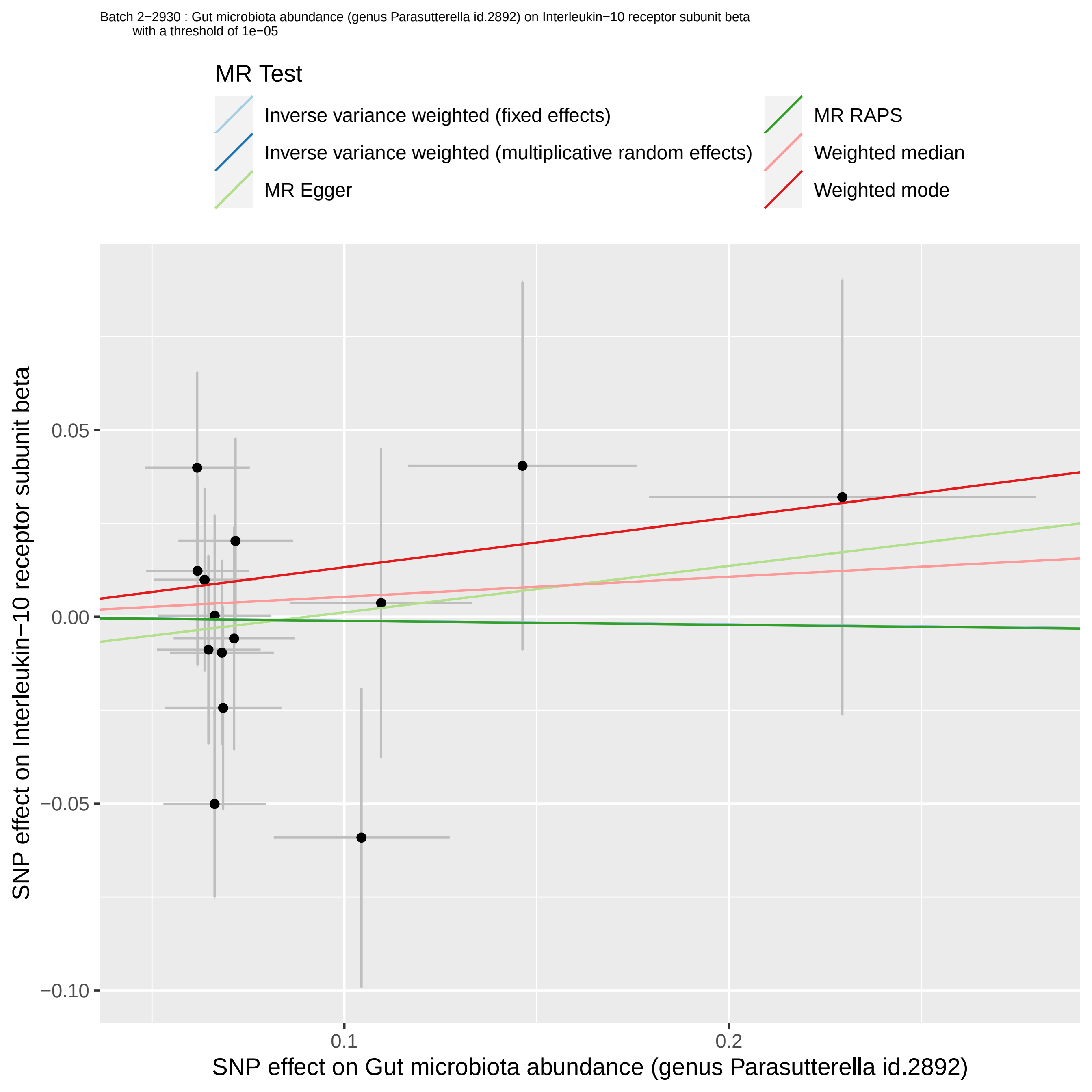
Batch 2−2930 : Gut microbiota abundance (genus Parasutterella id.2892) on Interleukin−10 receptor subunit beta
with a threshold of 1e−05
MR Test
Inverse variance weighted (fixed effects)
Inverse variance weighted (multiplicative random effects)
MR Egger
MR RAPS
Weighted median
Weighted mode
0.05
0.00
−0.05
−0.10
0.1
0.2
SNP effect on Interleukin−10 receptor subunit beta
SNP effect on Gut microbiota abundance (genus Parasutterella id.2892)

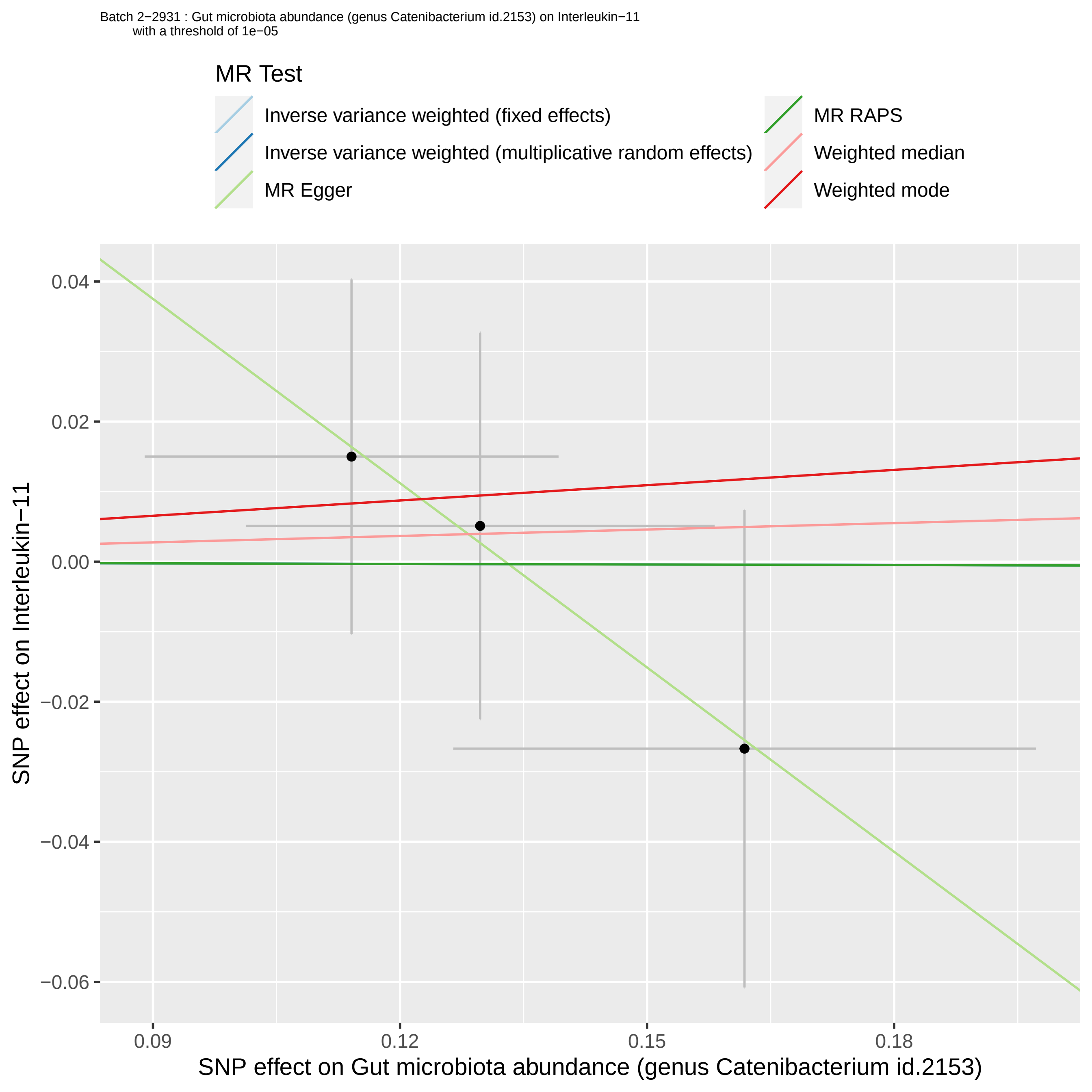

Batch 2−2931 : Gut microbiota abundance (genus Catenibacterium id.2153) on Interleukin−11
with a threshold of 1e−05
MR Test
Inverse variance weighted (fixed effects)
Inverse variance weighted (multiplicative random effects)
MR Egger
MR RAPS
Weighted median
Weighted mode
0.04
0.02
0.00
−0.02
−0.04
−0.06
SNP effect on Interleukin−11
0.09
0.12
0.15
0.18
SNP effect on Gut microbiota abundance (genus Catenibacterium id.2153)

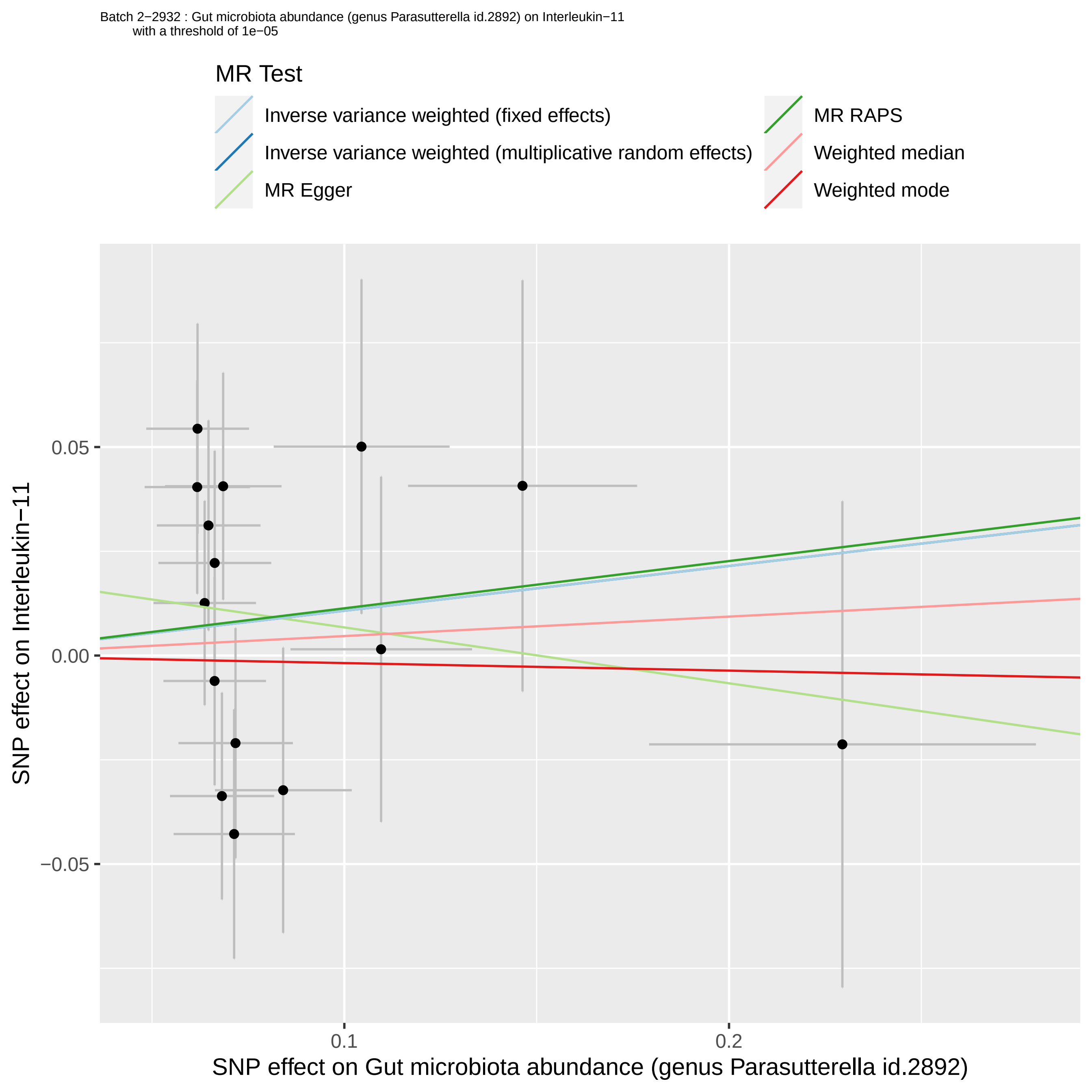

Batch 2−2932 : Gut microbiota abundance (genus Parasutterella id.2892) on Interleukin−11
with a threshold of 1e−05
MR Test
Inverse variance weighted (fixed effects)
Inverse variance weighted (multiplicative random effects)
MR Egger
MR RAPS
Weighted median
Weighted mode
SNP effect on Interleukin−11
0.05
0.00
−0.05
0.1
0.2
SNP effect on Gut microbiota abundance (genus Parasutterella id.2892)

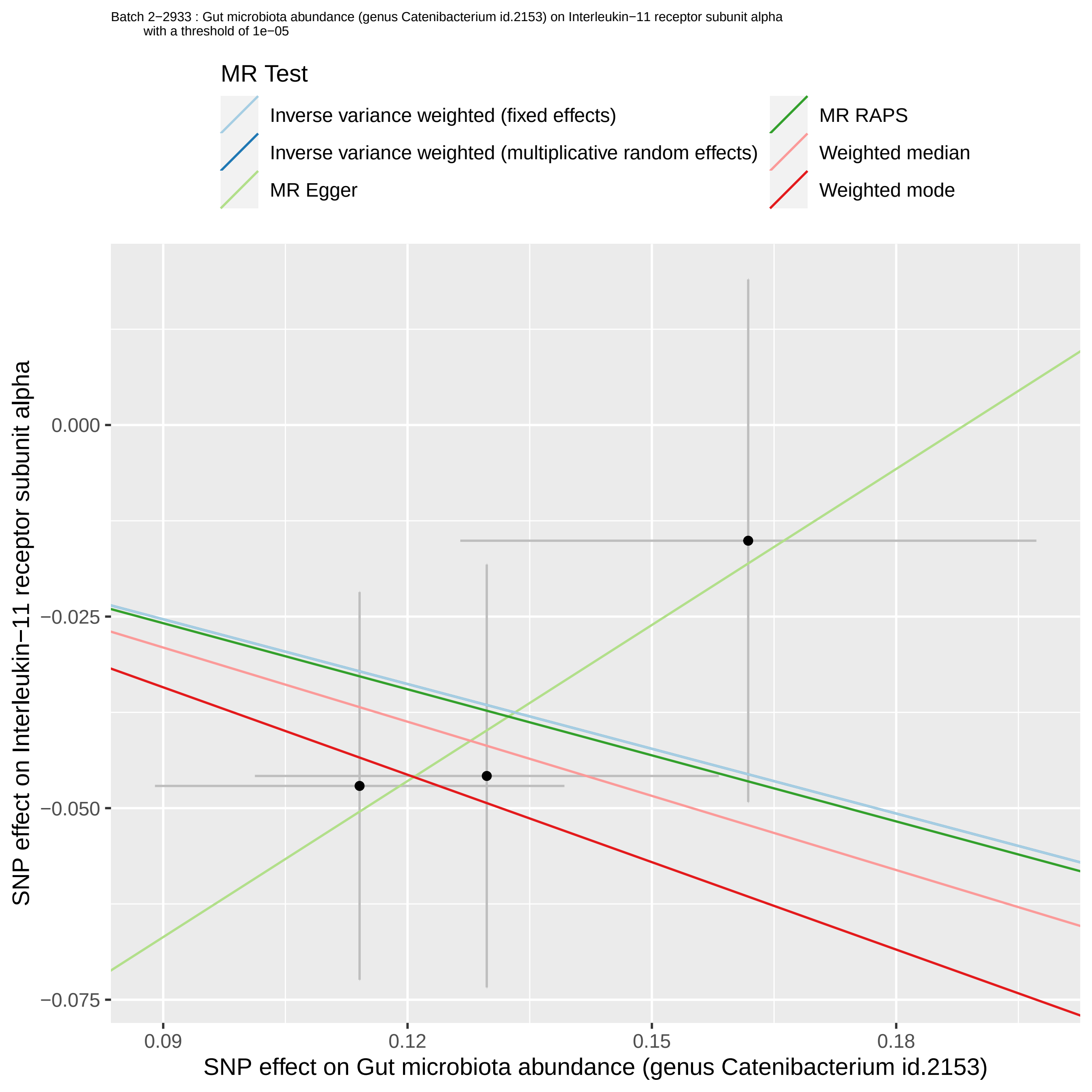
Batch 2−2933 : Gut microbiota abundance (genus Catenibacterium id.2153) on Interleukin−11 receptor subunit alpha
with a threshold of 1e−05
MR Test
Inverse variance weighted (fixed effects)
Inverse variance weighted (multiplicative random effects)
MR Egger
MR RAPS
Weighted median
Weighted mode
SNP effect on Interleukin−11 receptor subunit alpha
0.000
−0.025
−0.050
−0.075
0.09
0.12
0.15
0.18
SNP effect on Gut microbiota abundance (genus Catenibacterium id.2153)

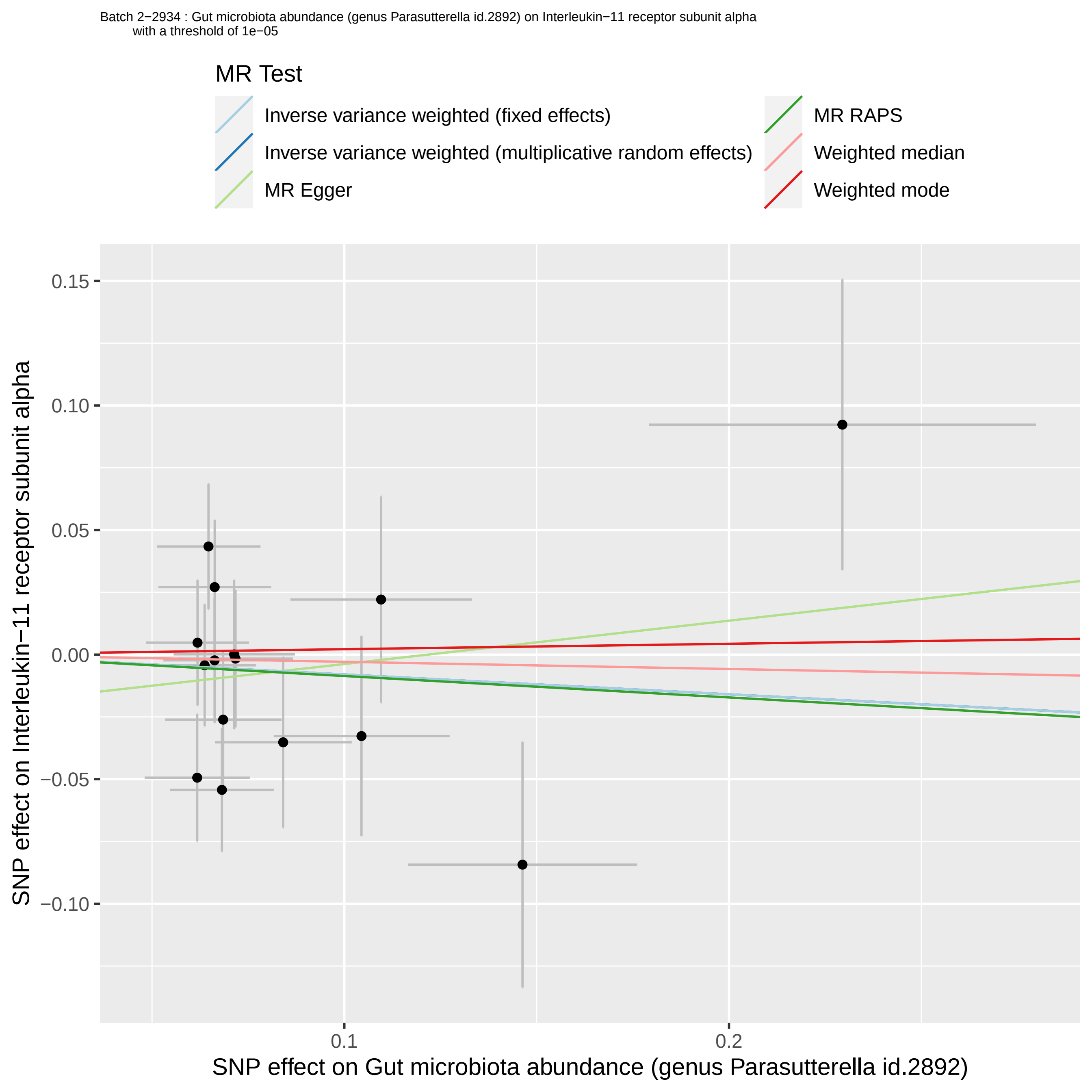

Batch 2−2934 : Gut microbiota abundance (genus Parasutterella id.2892) on Interleukin−11 receptor subunit alpha
with a threshold of 1e−05
MR Test
Inverse variance weighted (fixed effects)
Inverse variance weighted (multiplicative random effects)
MR Egger
MR RAPS
Weighted median
Weighted mode
0.15
0.10
0.05
0.00
−0.05
−0.10
0.1
0.2
SNP effect on Interleukin−11 receptor subunit alpha
SNP effect on Gut microbiota abundance (genus Parasutterella id.2892)

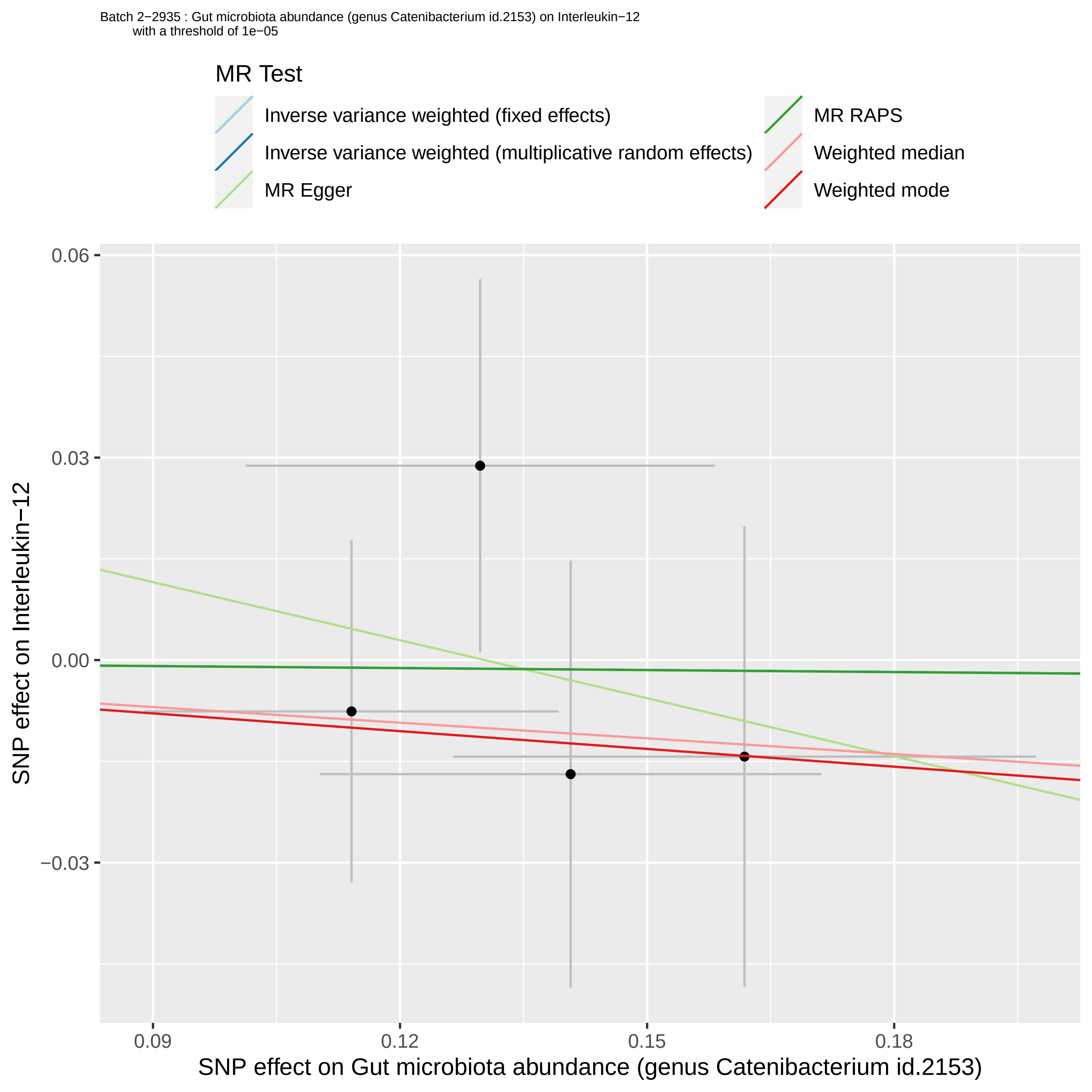

Batch 2−2935 : Gut microbiota abundance (genus Catenibacterium id.2153) on Interleukin−12
with a threshold of 1e−05
MR Test
Inverse variance weighted (fixed effects)
Inverse variance weighted (multiplicative random effects)
MR Egger
MR RAPS
Weighted median
Weighted mode
0.06
0.03
0.00
−0.03
0.09
0.12
0.15
0.18
SNP effect on Interleukin−12
SNP effect on Gut microbiota abundance (genus Catenibacterium id.2153)

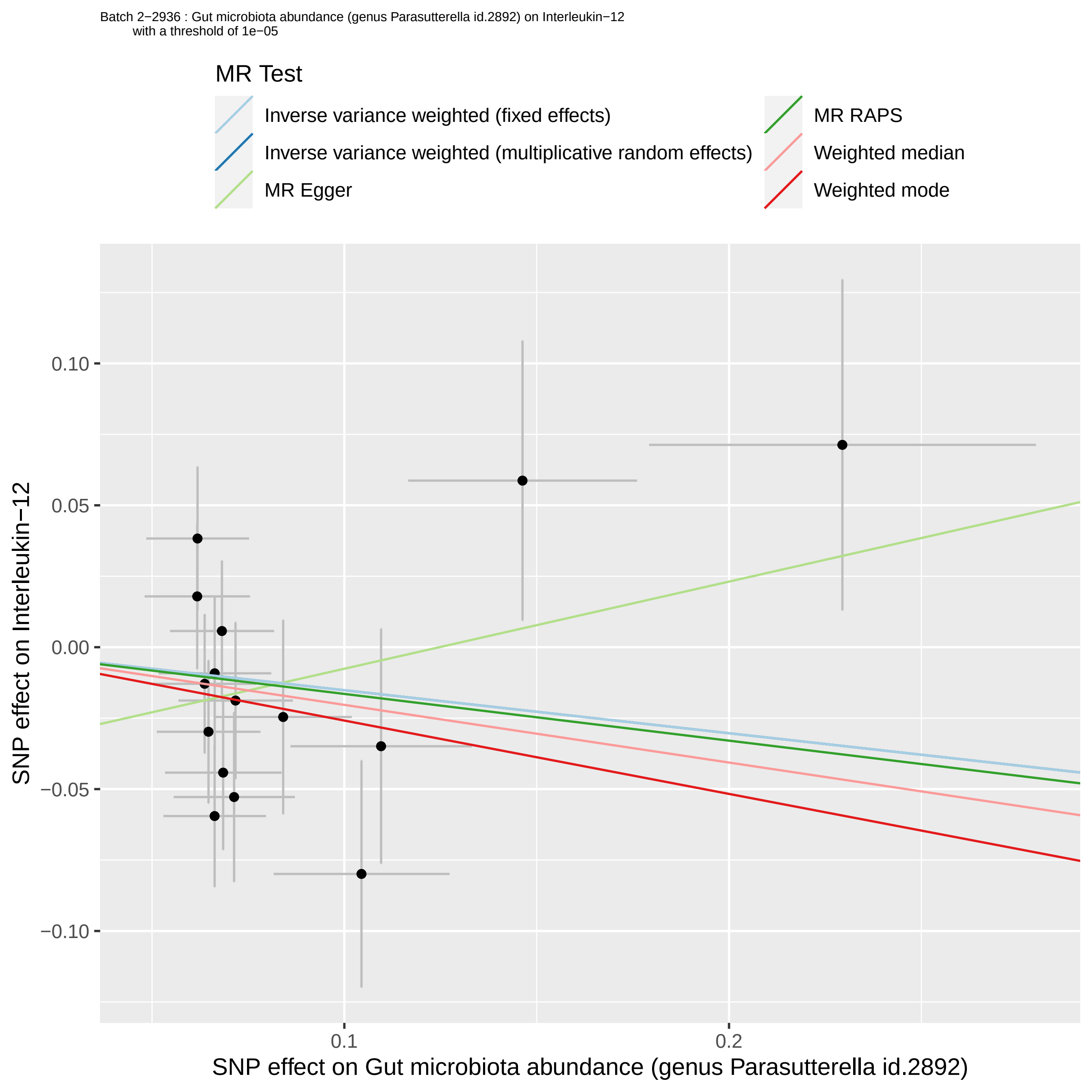

Batch 2−2936 : Gut microbiota abundance (genus Parasutterella id.2892) on Interleukin−12
with a threshold of 1e−05
MR Test
Inverse variance weighted (fixed effects)
Inverse variance weighted (multiplicative random effects)
MR Egger
MR RAPS
Weighted median
Weighted mode
0.10
0.05
0.00
−0.05
−0.10
0.1
0.2
SNP effect on Interleukin−12
SNP effect on Gut microbiota abundance (genus Parasutterella id.2892)

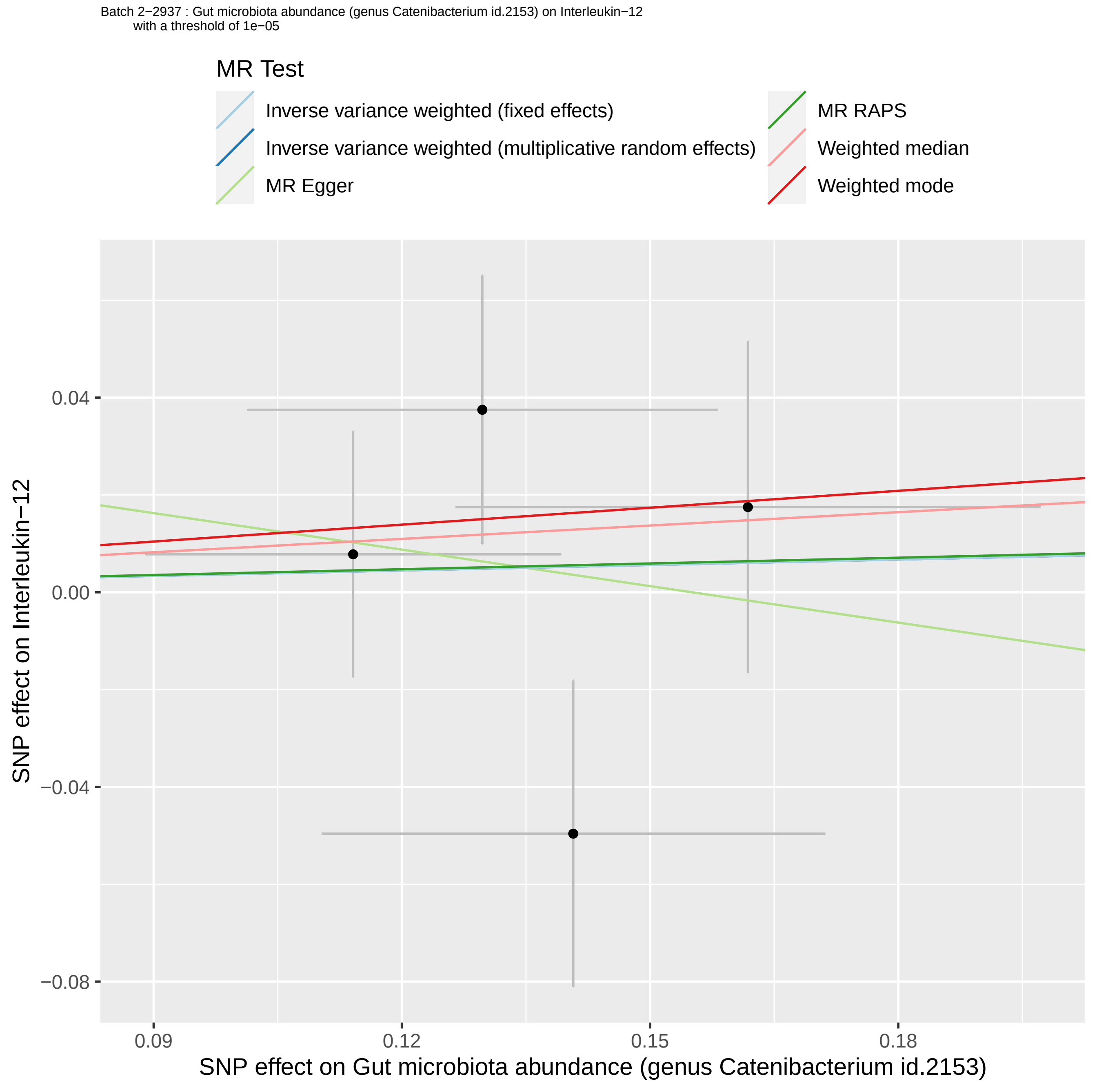

Batch 2−2937 : Gut microbiota abundance (genus Catenibacterium id.2153) on Interleukin−12
with a threshold of 1e−05
MR Test
Inverse variance weighted (fixed effects)
Inverse variance weighted (multiplicative random effects)
MR Egger
MR RAPS
Weighted median
Weighted mode
0.04
0.00
−0.04
−0.08
0.09
0.12
0.15
0.18
SNP effect on Interleukin−12
SNP effect on Gut microbiota abundance (genus Catenibacterium id.2153)

Batch 2−2938 : Gut microbiota abundance (genus Parasutterella id.2892) on Interleukin−12
with a threshold of 1e−05

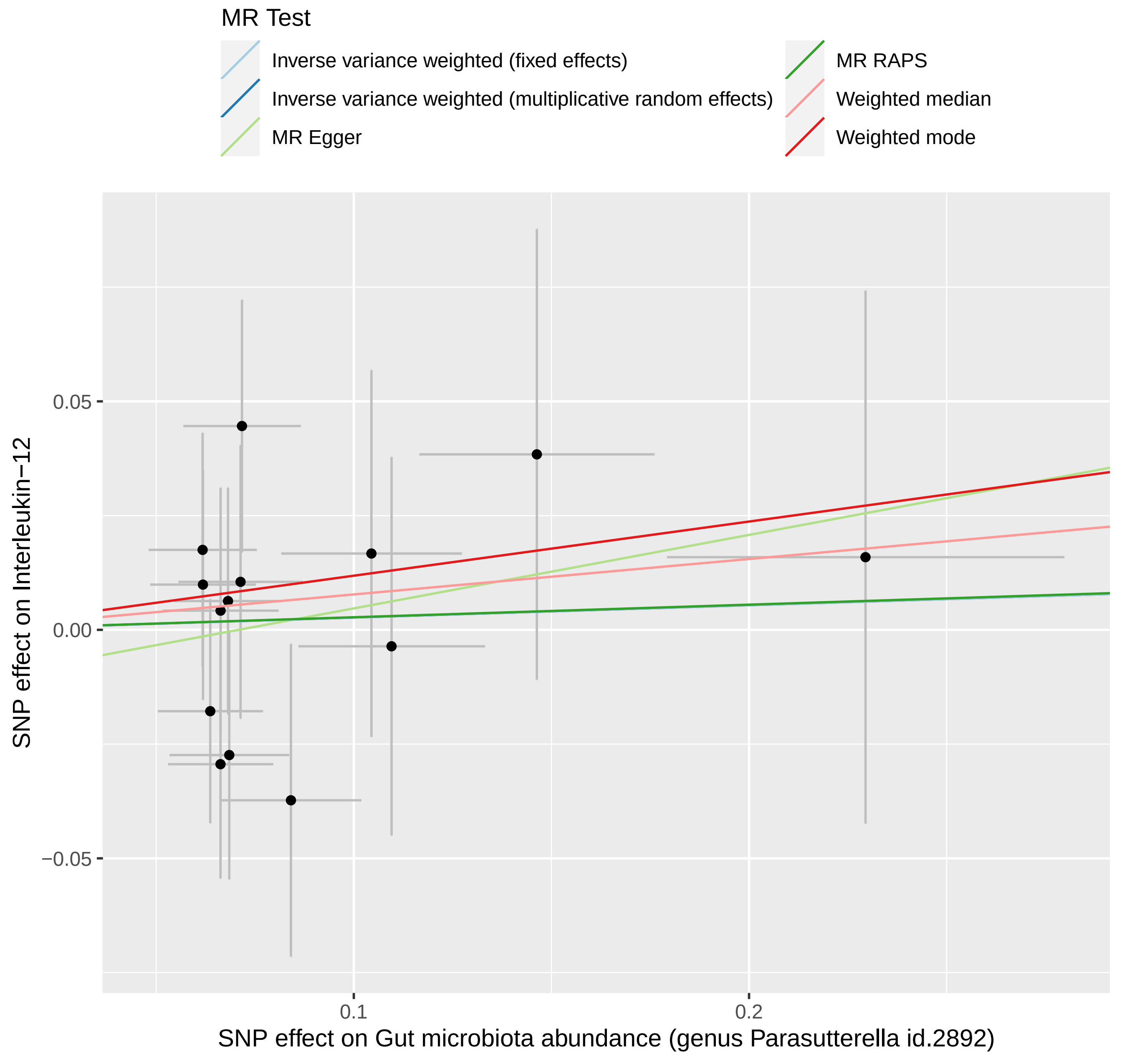

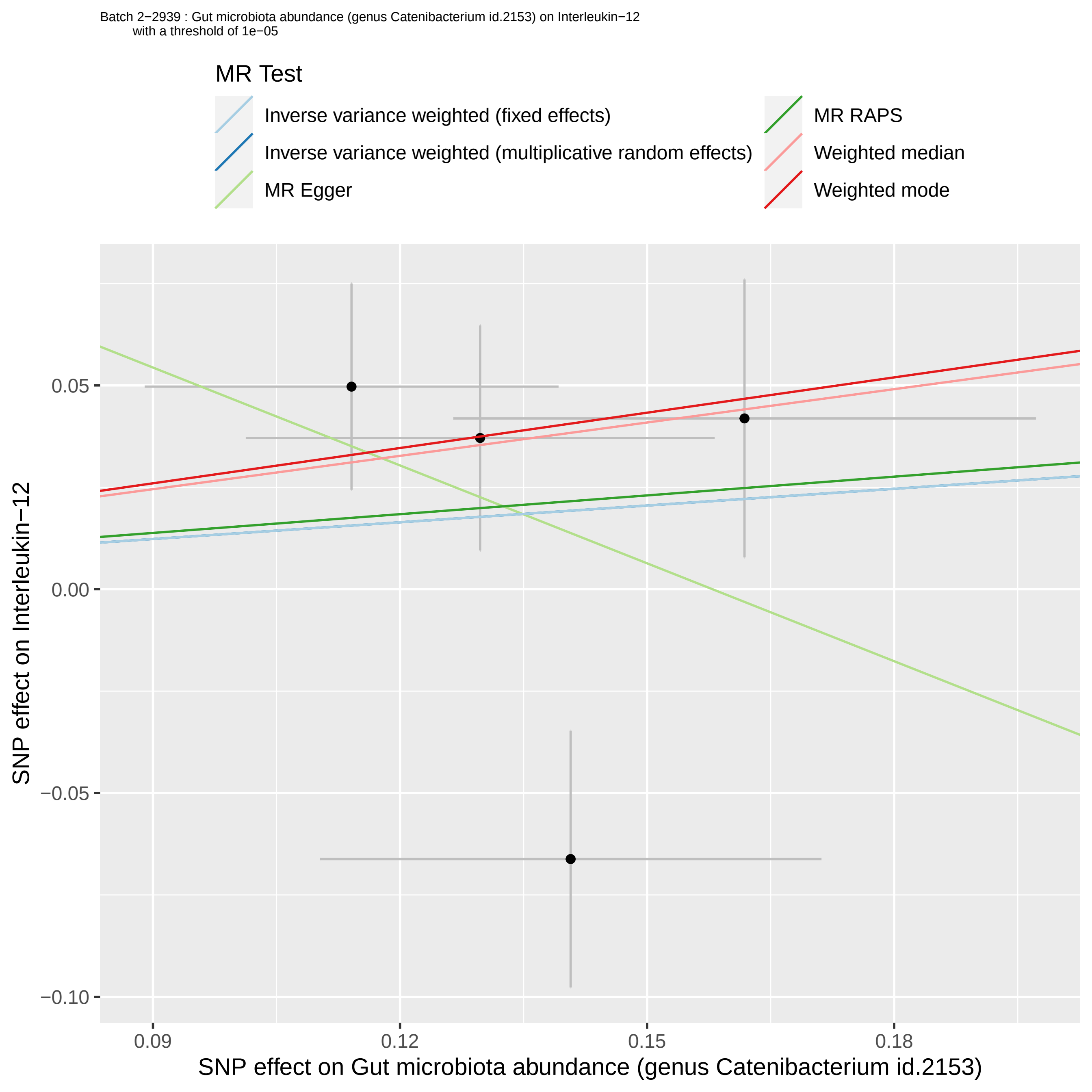

Batch 2−2939 : Gut microbiota abundance (genus Catenibacterium id.2153) on Interleukin−12
with a threshold of 1e−05
MR Test
Inverse variance weighted (fixed effects)
Inverse variance weighted (multiplicative random effects)
MR Egger
MR RAPS
Weighted median
Weighted mode
0.05
0.00
−0.05
−0.10
SNP effect on Interleukin−12
0.09
0.12
0.15
0.18
SNP effect on Gut microbiota abundance (genus Catenibacterium id.2153)

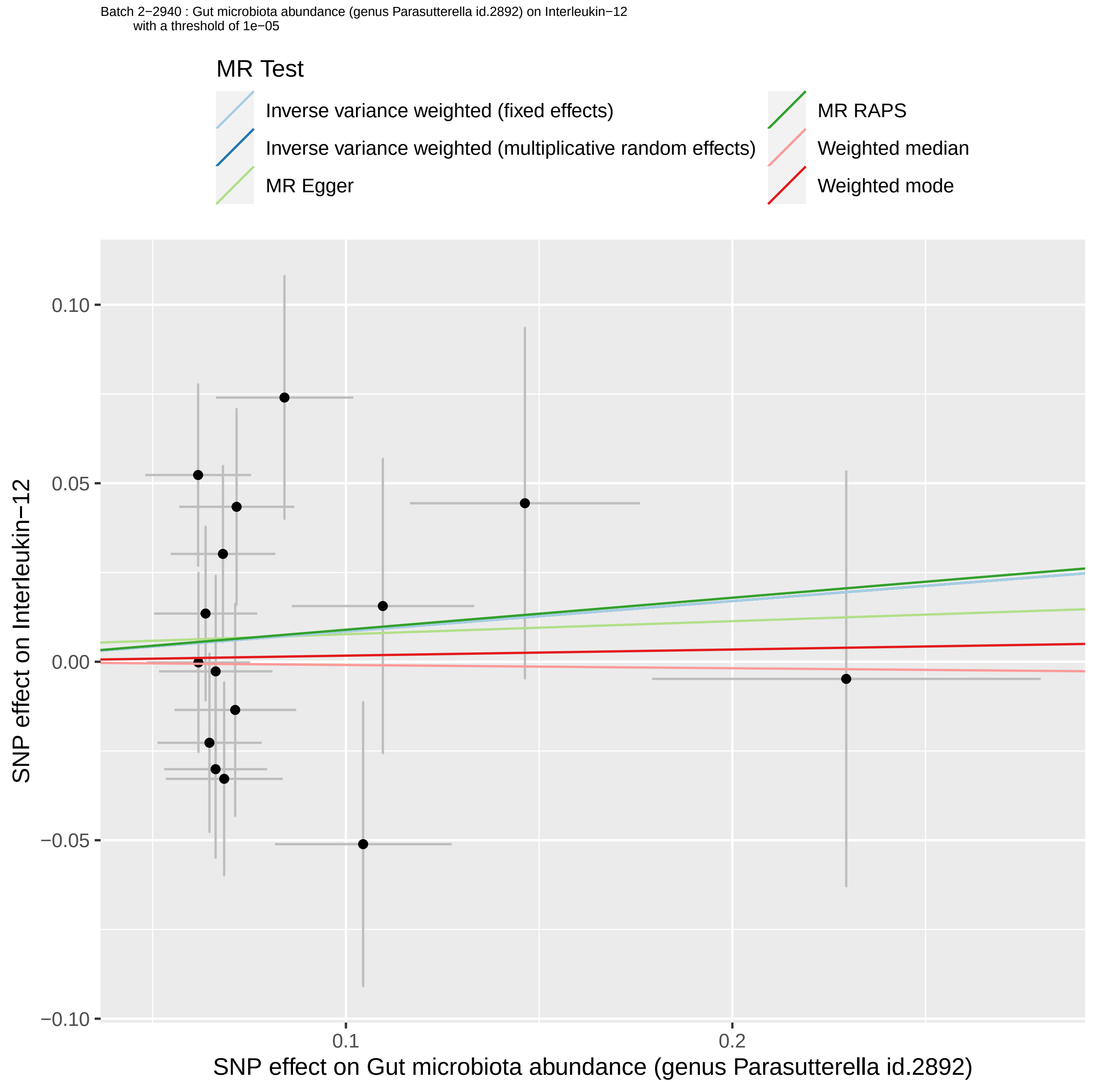

Batch 2−2940 : Gut microbiota abundance (genus Parasutterella id.2892) on Interleukin−12
with a threshold of 1e−05
MR Test
Inverse variance weighted (fixed effects)
Inverse variance weighted (multiplicative random effects)
MR Egger
MR RAPS
Weighted median
Weighted mode
0.10
0.05
0.00
−0.05
−0.10
0.1
0.2
SNP effect on Interleukin−12
SNP effect on Gut microbiota abundance (genus Parasutterella id.2892)

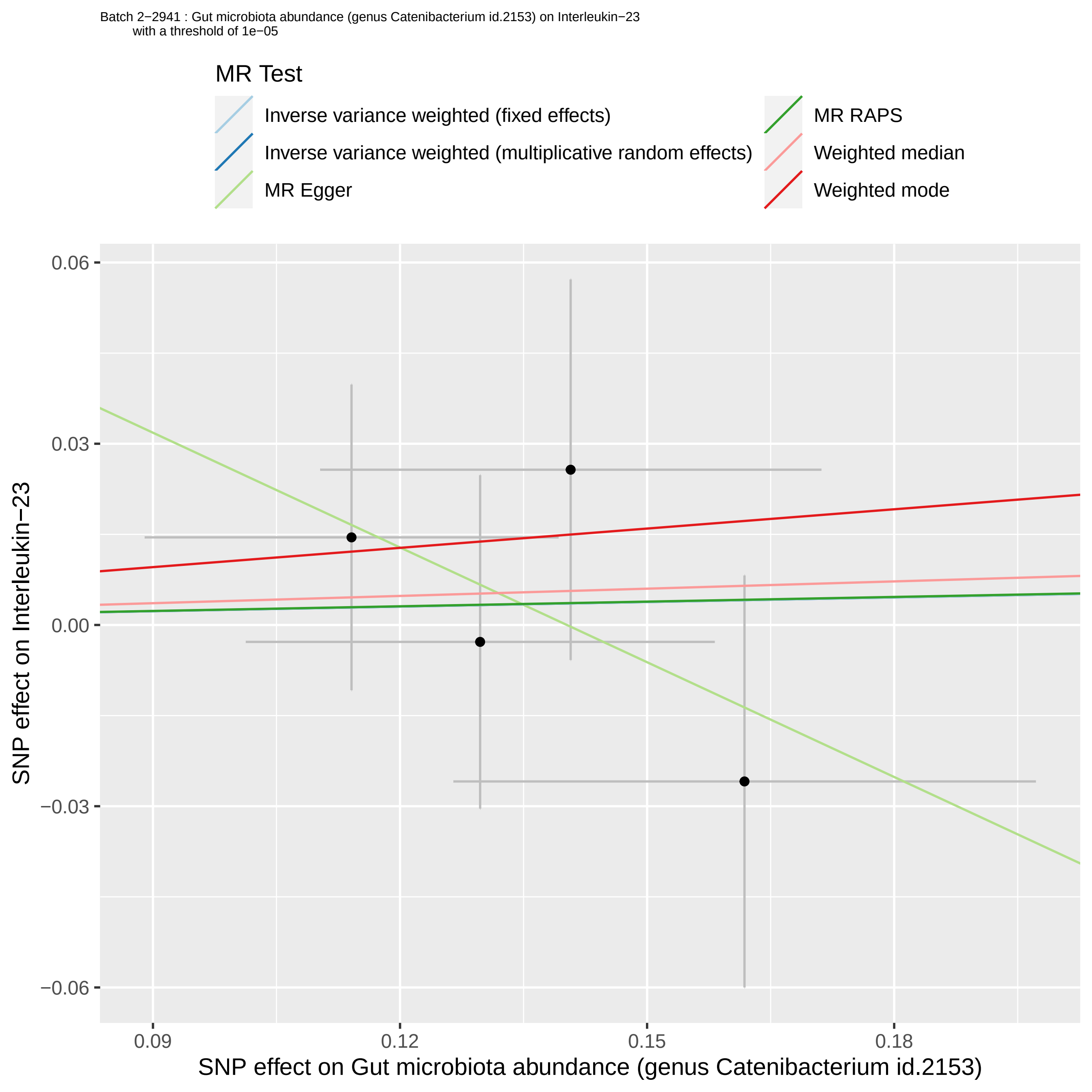
Batch 2−2941 : Gut microbiota abundance (genus Catenibacterium id.2153) on Interleukin−23
with a threshold of 1e−05
MR Test
Inverse variance weighted (fixed effects)
Inverse variance weighted (multiplicative random effects)
MR Egger
MR RAPS
Weighted median
Weighted mode
0.06
0.03
0.00
−0.03
−0.06
0.09
0.12
0.15
0.18
SNP effect on Interleukin−23
SNP effect on Gut microbiota abundance (genus Catenibacterium id.2153)

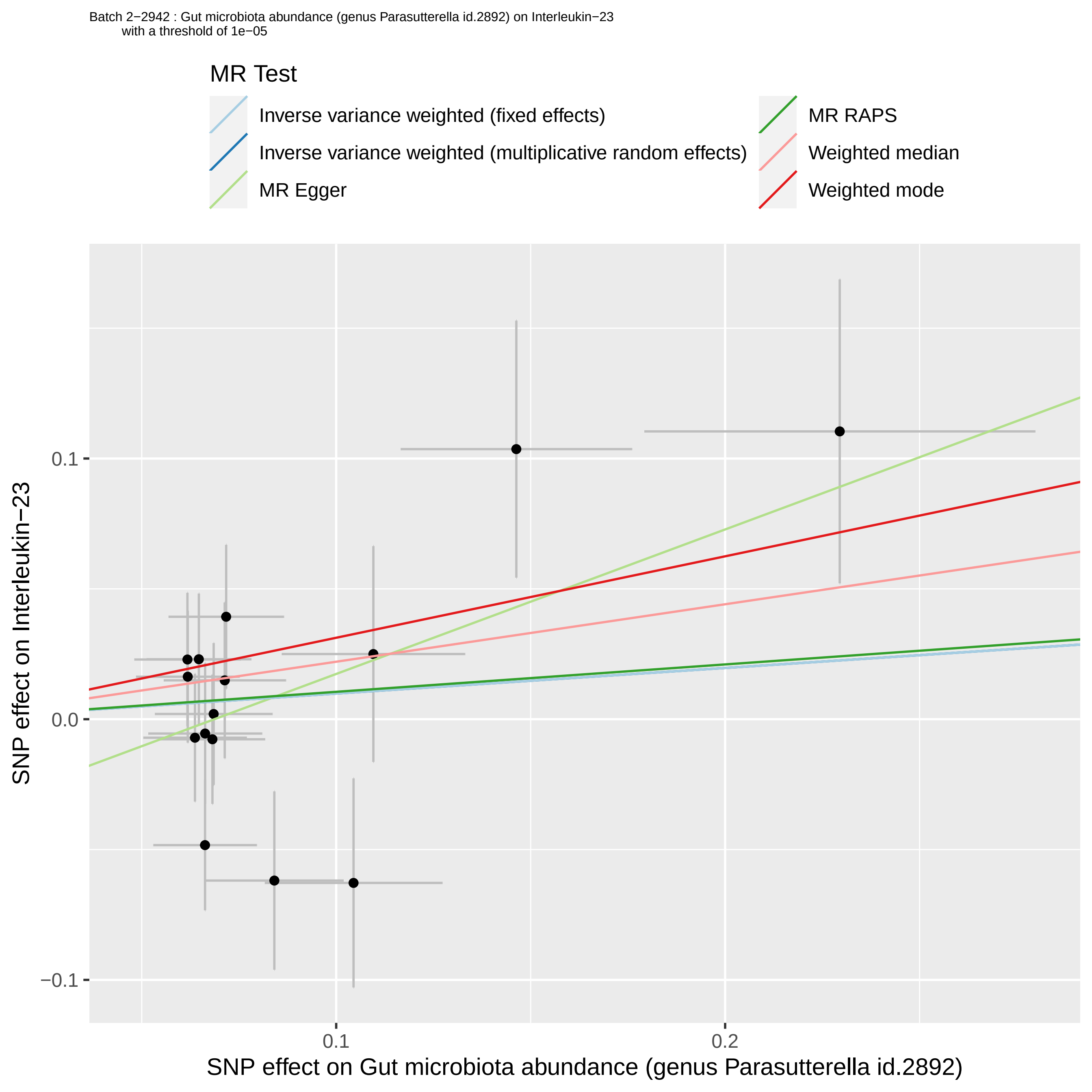

Batch 2−2942 : Gut microbiota abundance (genus Parasutterella id.2892) on Interleukin−23
with a threshold of 1e−05
MR Test
Inverse variance weighted (fixed effects)
Inverse variance weighted (multiplicative random effects)
MR Egger
MR RAPS
Weighted median
Weighted mode
SNP effect on Interleukin−23
0.1
0.0
−0.1
0.1
0.2
SNP effect on Gut microbiota abundance (genus Parasutterella id.2892)

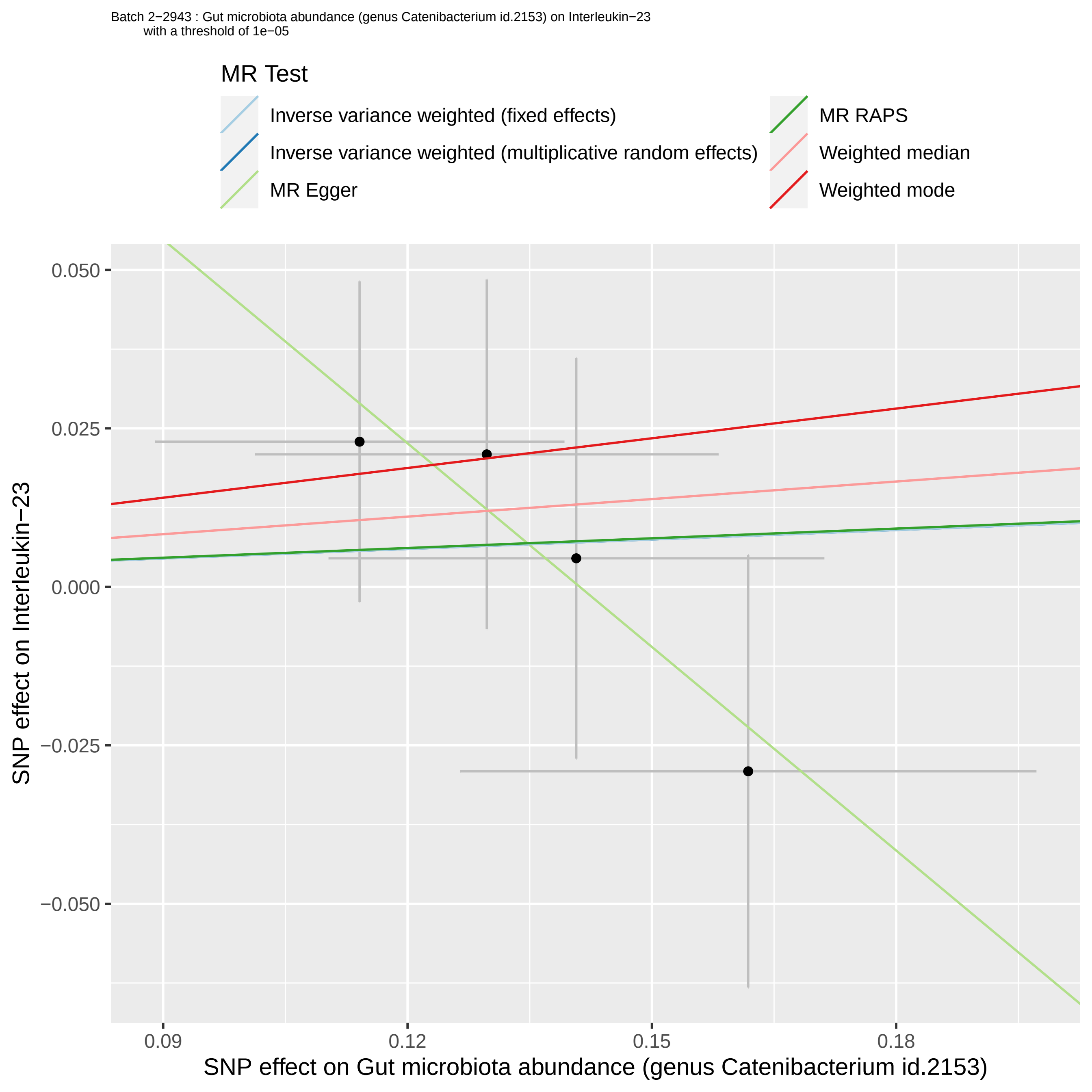
Batch 2−2943 : Gut microbiota abundance (genus Catenibacterium id.2153) on Interleukin−23
with a threshold of 1e−05
MR Test
Inverse variance weighted (fixed effects)
Inverse variance weighted (multiplicative random effects)
MR Egger
MR RAPS
Weighted median
Weighted mode
0.050
0.025
0.000
−0.025
−0.050
0.09
0.12
0.15
0.18
SNP effect on Interleukin−23
SNP effect on Gut microbiota abundance (genus Catenibacterium id.2153)

Batch 2−2944 : Gut microbiota abundance (genus Parasutterella id.2892) on Interleukin−23
with a threshold of 1e−05

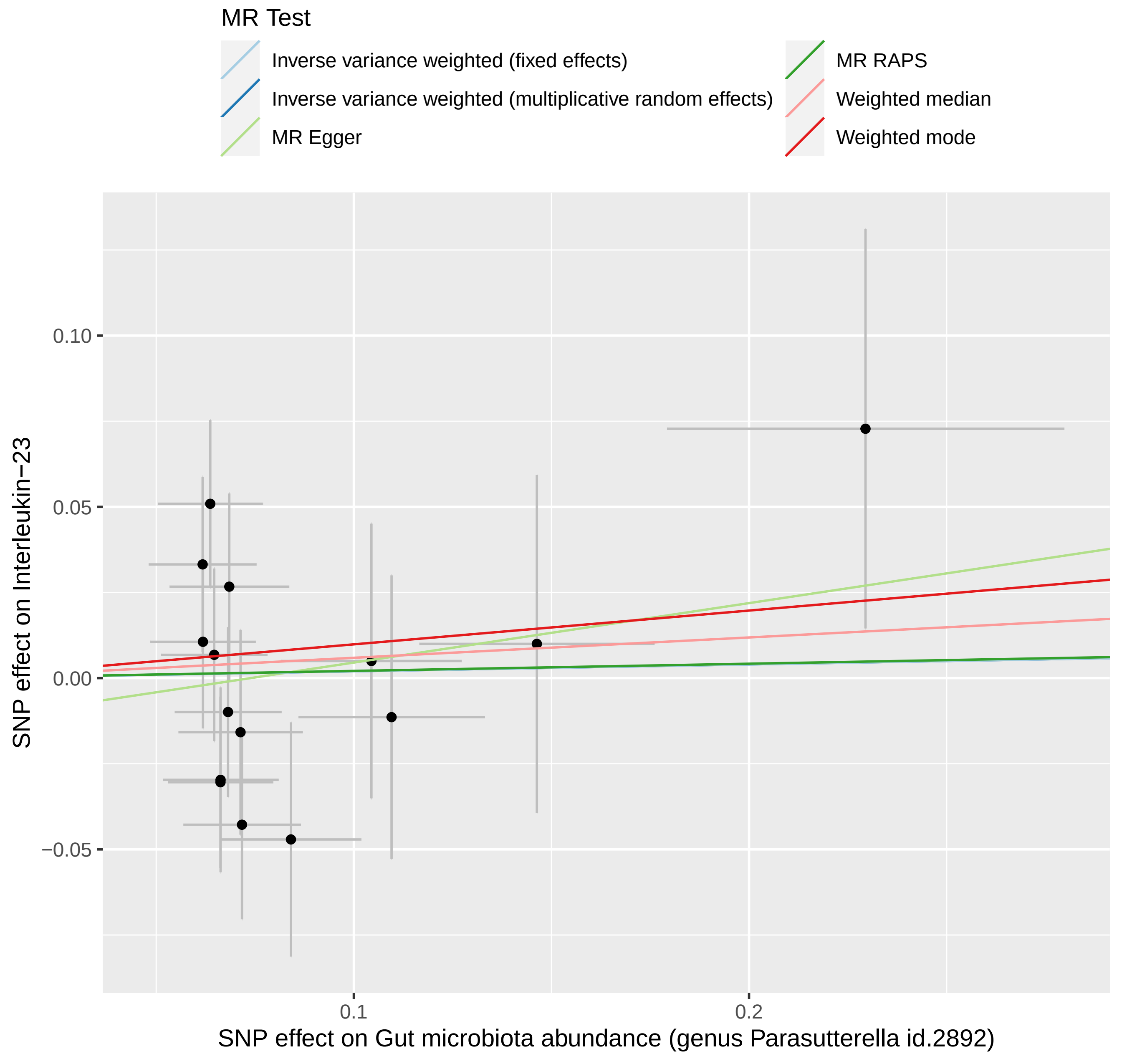

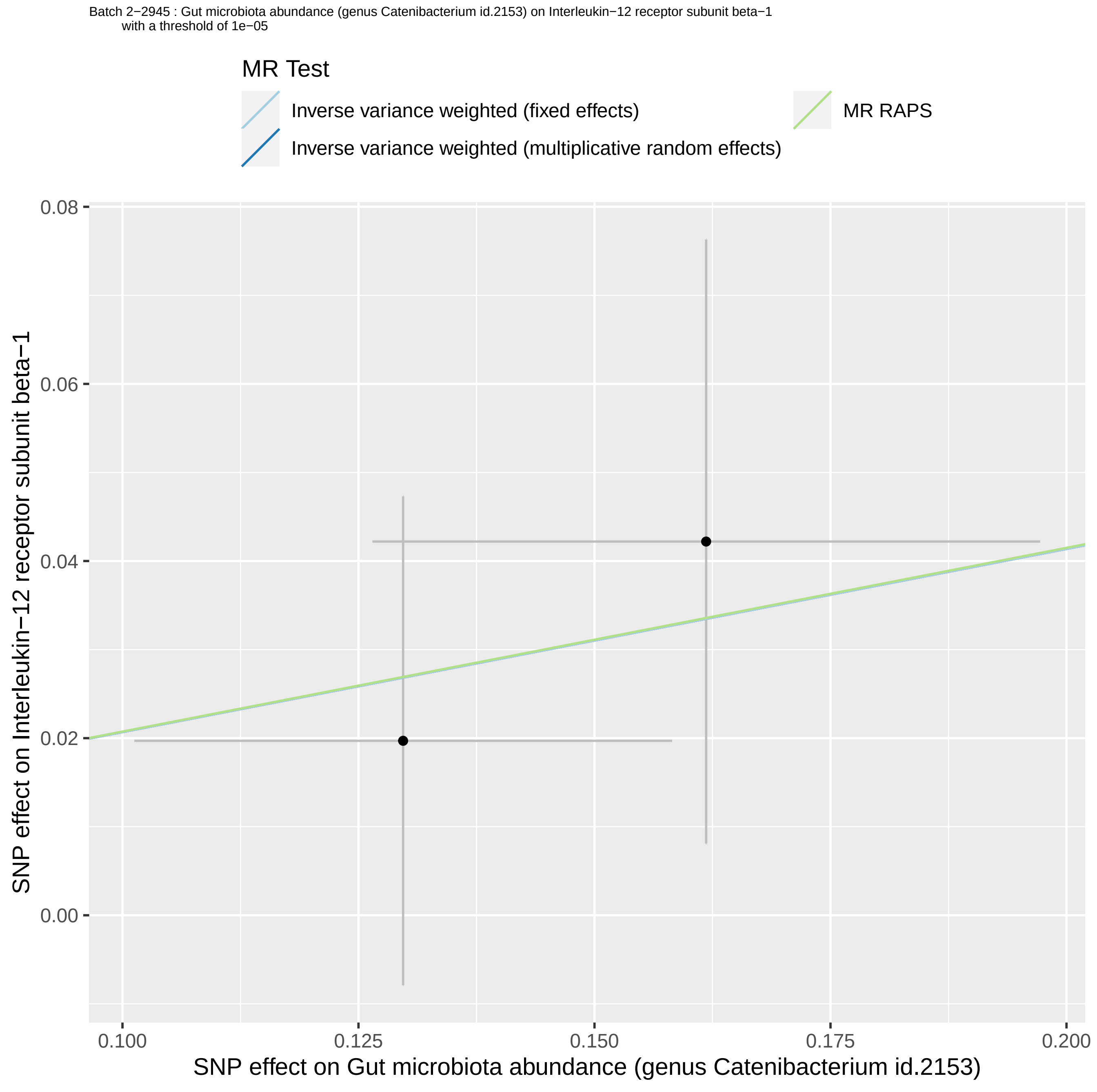
Batch 2−2945 : Gut microbiota abundance (genus Catenibacterium id.2153) on Interleukin−12 receptor subunit beta−1
with a threshold of 1e−05
MR Test
Inverse variance weighted (fixed effects)
Inverse variance weighted (multiplicative random effects)
MR RAPS
0.08
0.06
0.04
0.02
0.00
0.100
0.125
0.150
0.175
0.200
SNP effect on Interleukin−12 receptor subunit beta−1
SNP effect on Gut microbiota abundance (genus Catenibacterium id.2153)

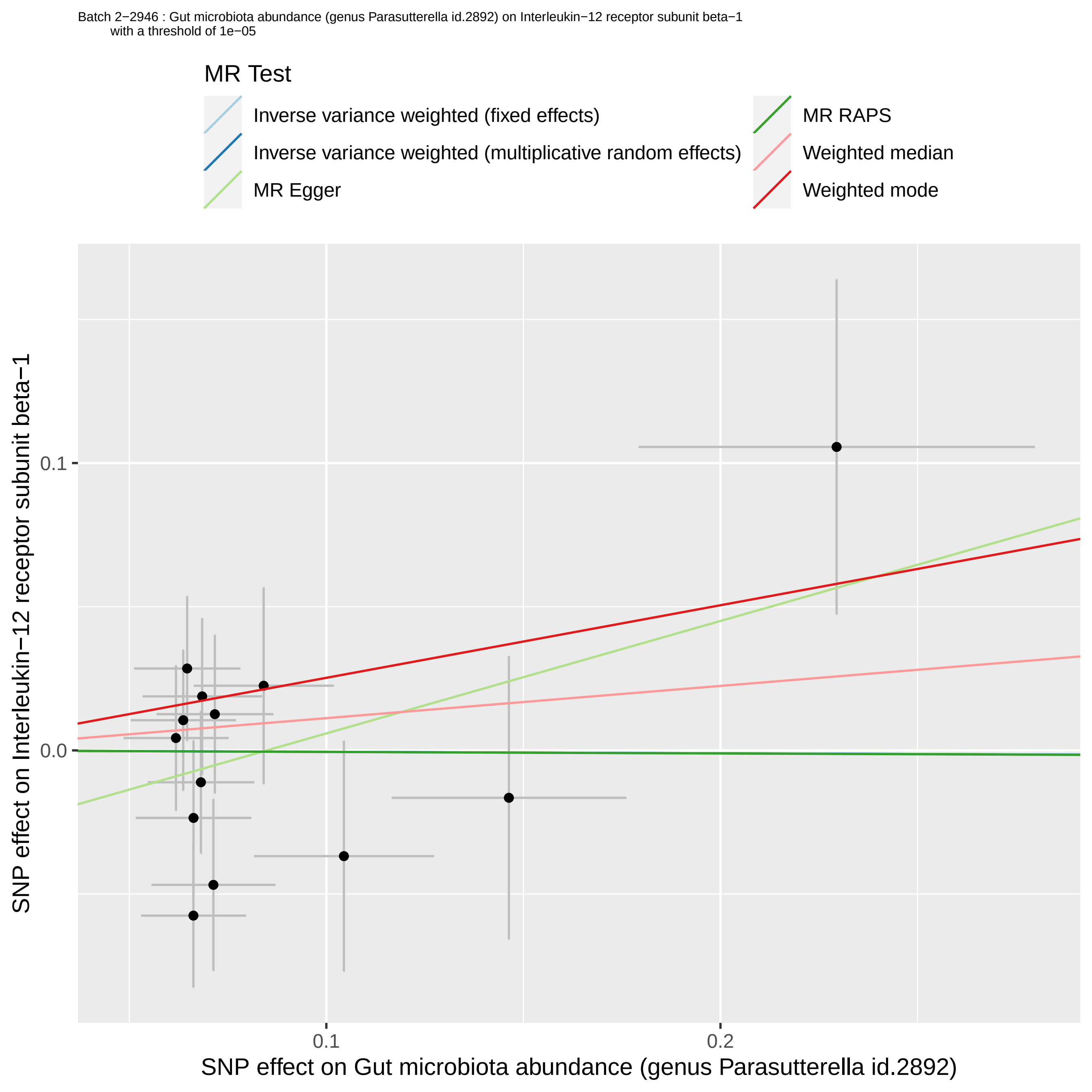
Batch 2−2946 : Gut microbiota abundance (genus Parasutterella id.2892) on Interleukin−12 receptor subunit beta−1
with a threshold of 1e−05
MR Test
Inverse variance weighted (fixed effects)
Inverse variance weighted (multiplicative random effects)
MR Egger
MR RAPS
Weighted median
Weighted mode
0.1
0.0
0.1
0.2
SNP effect on Interleukin−12 receptor subunit beta−1
SNP effect on Gut microbiota abundance (genus Parasutterella id.2892)

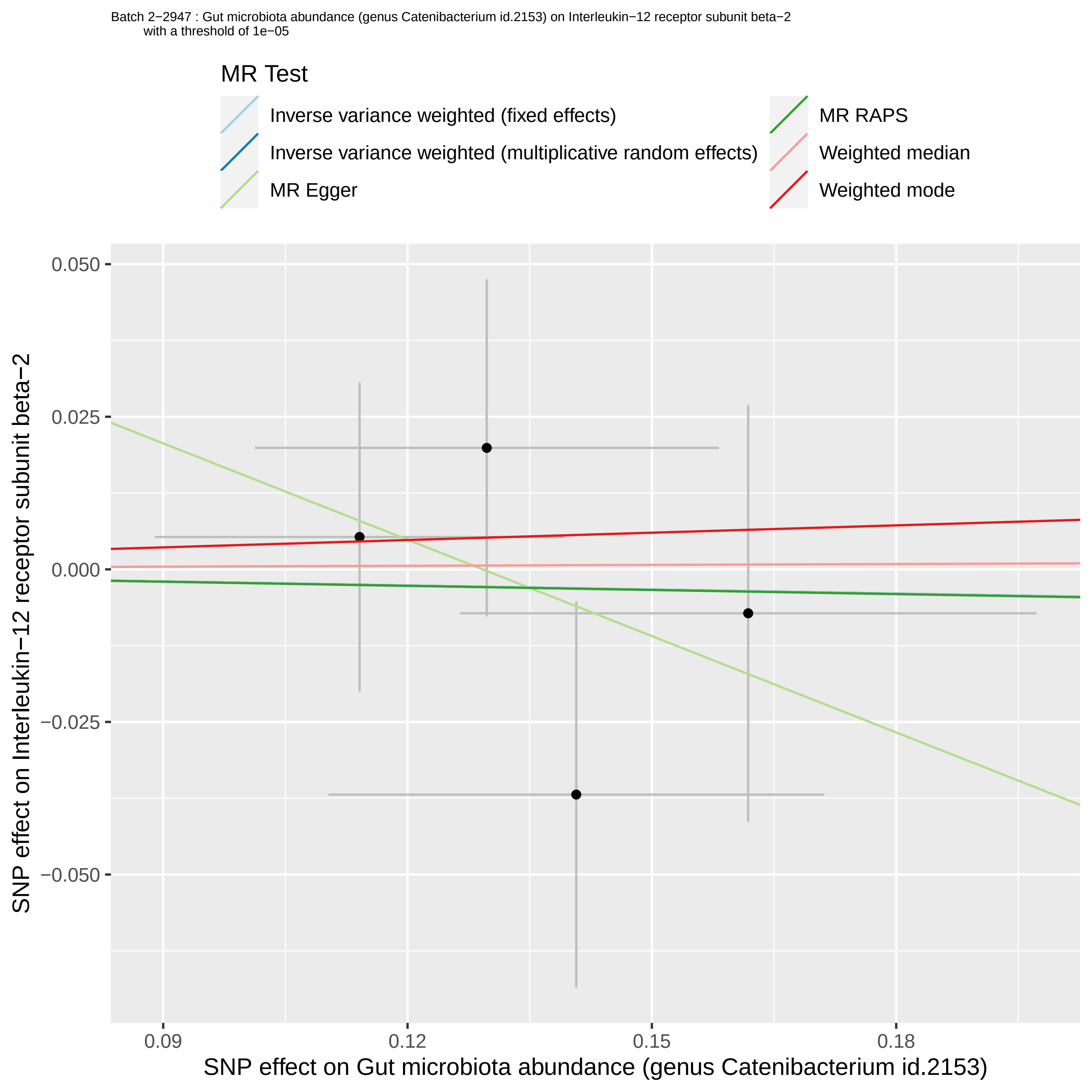

Batch 2−2947 : Gut microbiota abundance (genus Catenibacterium id.2153) on Interleukin−12 receptor subunit beta−2
with a threshold of 1e−05
MR Test
Inverse variance weighted (fixed effects)
Inverse variance weighted (multiplicative random effects)
MR Egger
MR RAPS
Weighted median
Weighted mode
0.050
0.025
0.000
−0.025
−0.050
0.09
0.12
0.15
0.18
SNP effect on Interleukin−12 receptor subunit beta−2
SNP effect on Gut microbiota abundance (genus Catenibacterium id.2153)

Batch 2−2948 : Gut microbiota abundance (genus Parasutterella id.2892) on Interleukin−12 receptor subunit beta−2
with a threshold of 1e−05

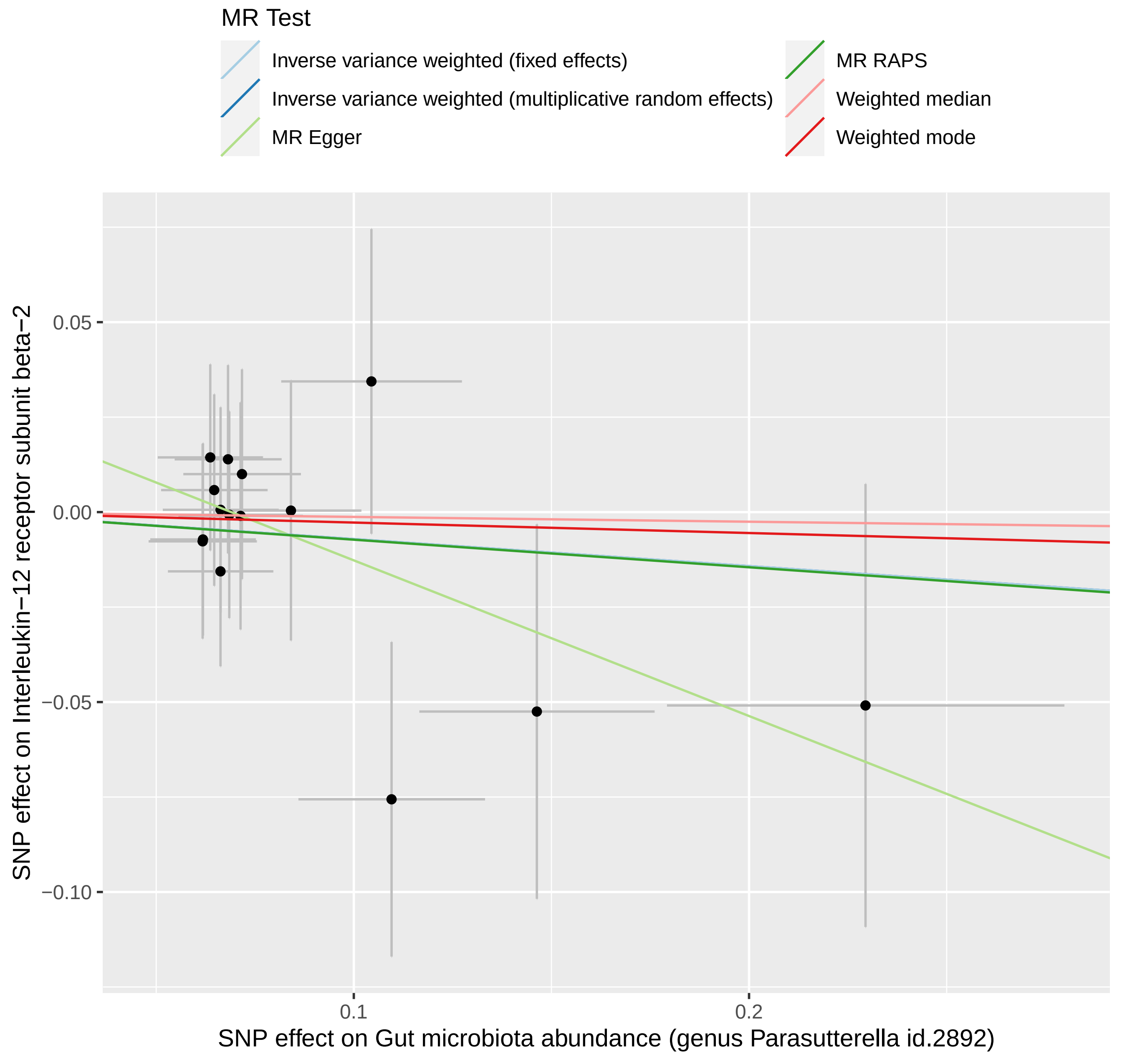

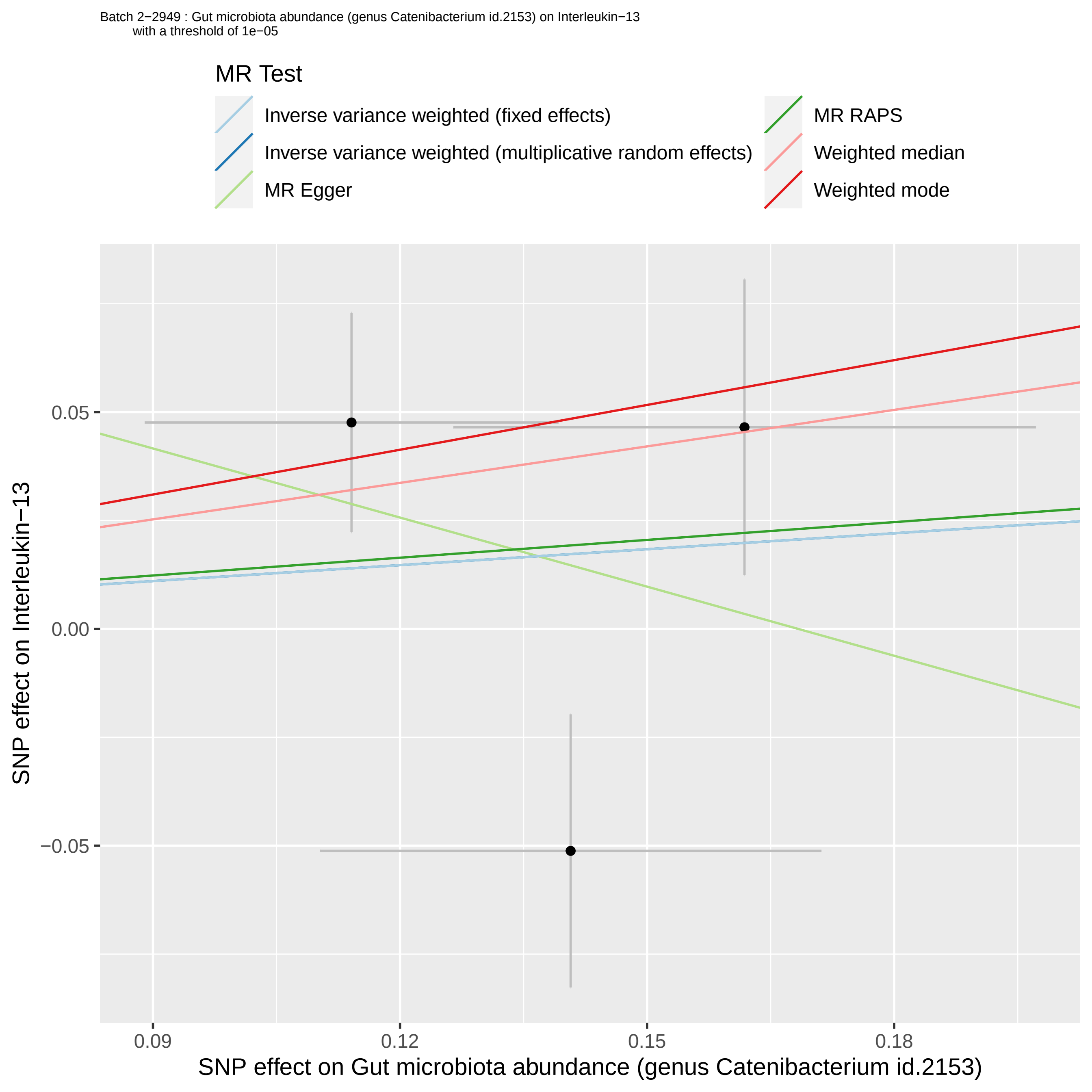

Batch 2−2949 : Gut microbiota abundance (genus Catenibacterium id.2153) on Interleukin−13
with a threshold of 1e−05
MR Test
Inverse variance weighted (fixed effects)
Inverse variance weighted (multiplicative random effects)
MR Egger
MR RAPS
Weighted median
Weighted mode
0.05
0.00
−0.05
SNP effect on Interleukin−13
0.09
0.12
0.15
0.18
SNP effect on Gut microbiota abundance (genus Catenibacterium id.2153)

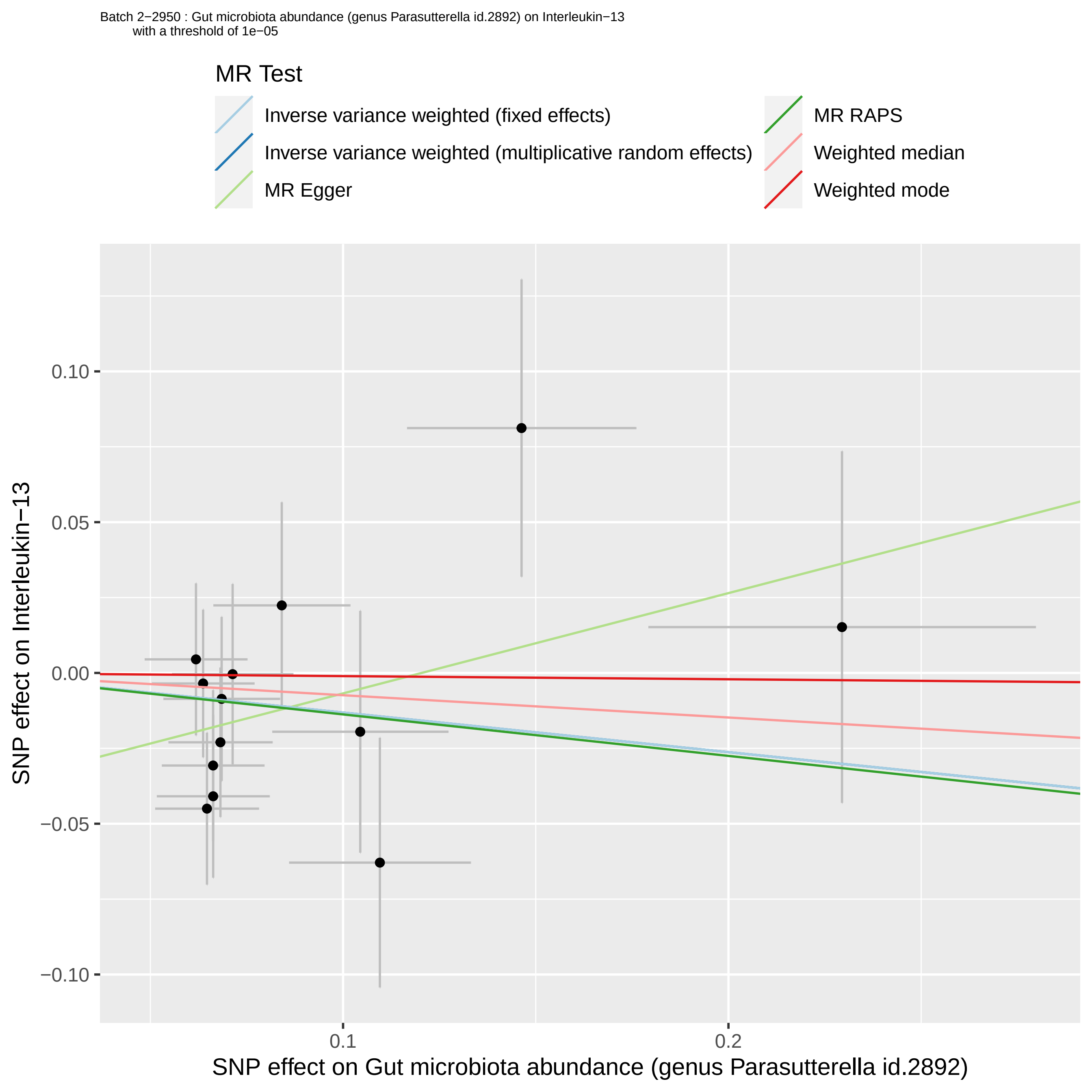

Batch 2−2950 : Gut microbiota abundance (genus Parasutterella id.2892) on Interleukin−13
with a threshold of 1e−05
MR Test
Inverse variance weighted (fixed effects)
Inverse variance weighted (multiplicative random effects)
MR Egger
MR RAPS
Weighted median
Weighted mode
0.10
0.05
0.00
−0.05
−0.10
0.1
0.2
SNP effect on Interleukin−13
SNP effect on Gut microbiota abundance (genus Parasutterella id.2892)

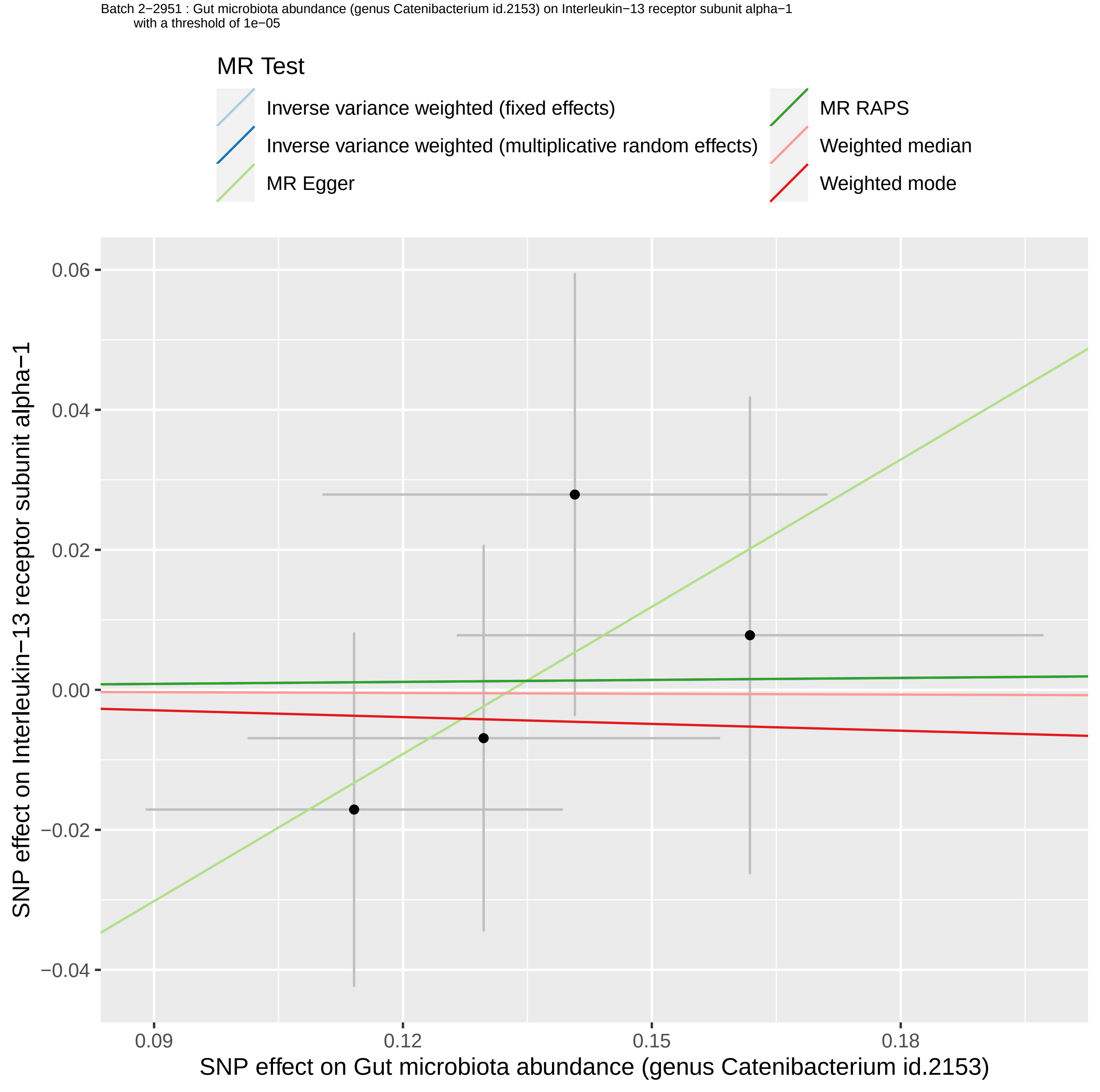

Batch 2−2951 : Gut microbiota abundance (genus Catenibacterium id.2153) on Interleukin−13 receptor subunit alpha−1
with a threshold of 1e−05
MR Test
Inverse variance weighted (fixed effects)
Inverse variance weighted (multiplicative random effects)
MR Egger
MR RAPS
Weighted median
Weighted mode
0.06
0.04
0.02
0.00
−0.02
−0.04
0.09
0.12
0.15
0.18
SNP effect on Interleukin−13 receptor subunit alpha−1
SNP effect on Gut microbiota abundance (genus Catenibacterium id.2153)

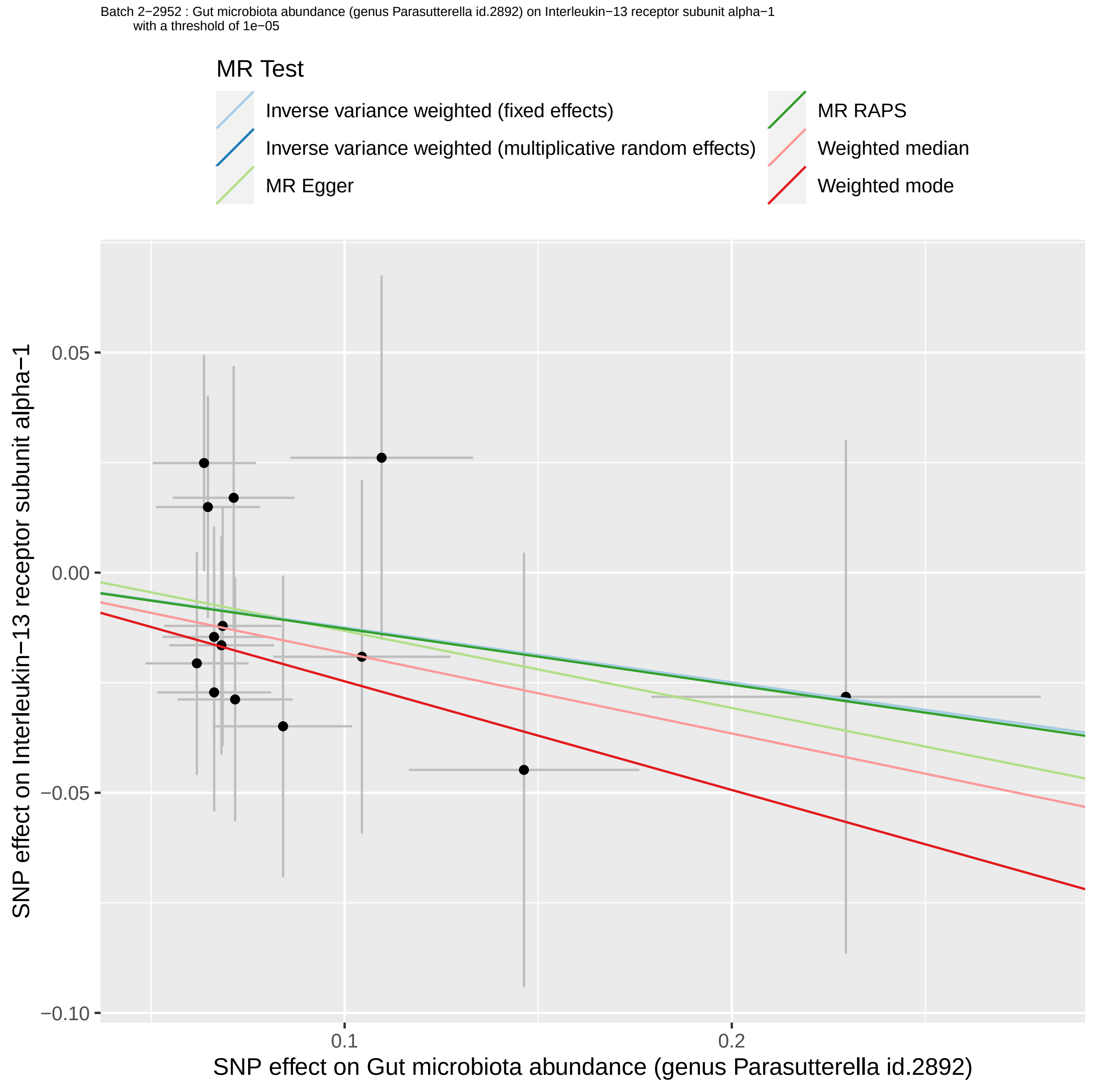
Batch 2−2952 : Gut microbiota abundance (genus Parasutterella id.2892) on Interleukin−13 receptor subunit alpha−1
with a threshold of 1e−05
MR Test
Inverse variance weighted (fixed effects)
Inverse variance weighted (multiplicative random effects)
MR Egger
MR RAPS
Weighted median
Weighted mode
0.05
0.00
−0.05
−0.10
0.1
0.2
SNP effect on Interleukin−13 receptor subunit alpha−1
SNP effect on Gut microbiota abundance (genus Parasutterella id.2892)

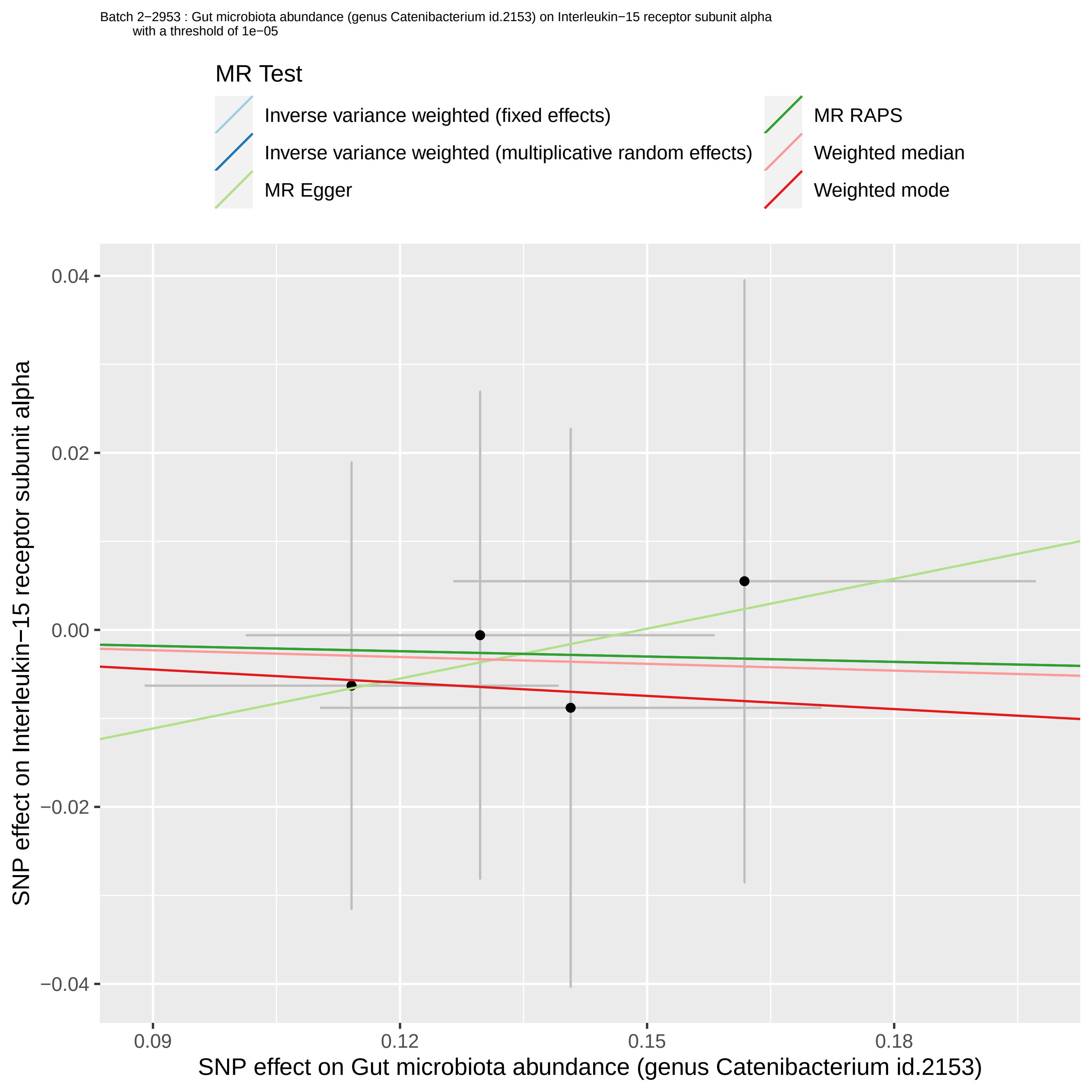
Batch 2−2953 : Gut microbiota abundance (genus Catenibacterium id.2153) on Interleukin−15 receptor subunit alpha
with a threshold of 1e−05
MR Test
Inverse variance weighted (fixed effects)
Inverse variance weighted (multiplicative random effects)
MR Egger
MR RAPS
Weighted median
Weighted mode
0.04
0.02
0.00
−0.02
−0.04
0.09
0.12
0.15
0.18
SNP effect on Interleukin−15 receptor subunit alpha
SNP effect on Gut microbiota abundance (genus Catenibacterium id.2153)

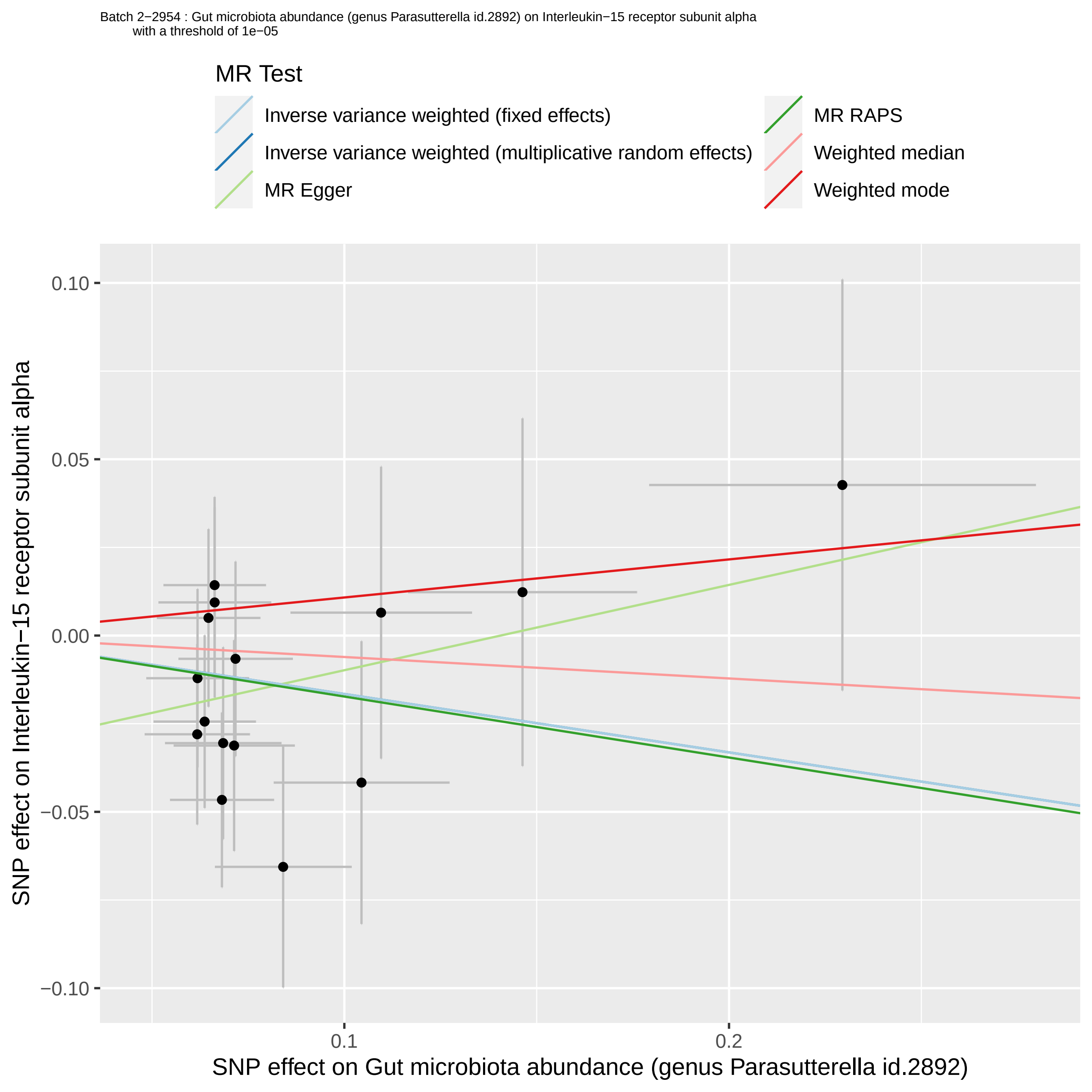
Batch 2−2954 : Gut microbiota abundance (genus Parasutterella id.2892) on Interleukin−15 receptor subunit alpha
with a threshold of 1e−05
MR Test
Inverse variance weighted (fixed effects)
Inverse variance weighted (multiplicative random effects)
MR Egger
MR RAPS
Weighted median
Weighted mode
0.10
0.05
0.00
−0.05
−0.10
0.1
0.2
SNP effect on Interleukin−15 receptor subunit alpha
SNP effect on Gut microbiota abundance (genus Parasutterella id.2892)

Batch 2−2955 : Gut microbiota abundance (genus Catenibacterium id.2153) on Interleukin−15 receptor subunit alpha
with a threshold of 1e−05

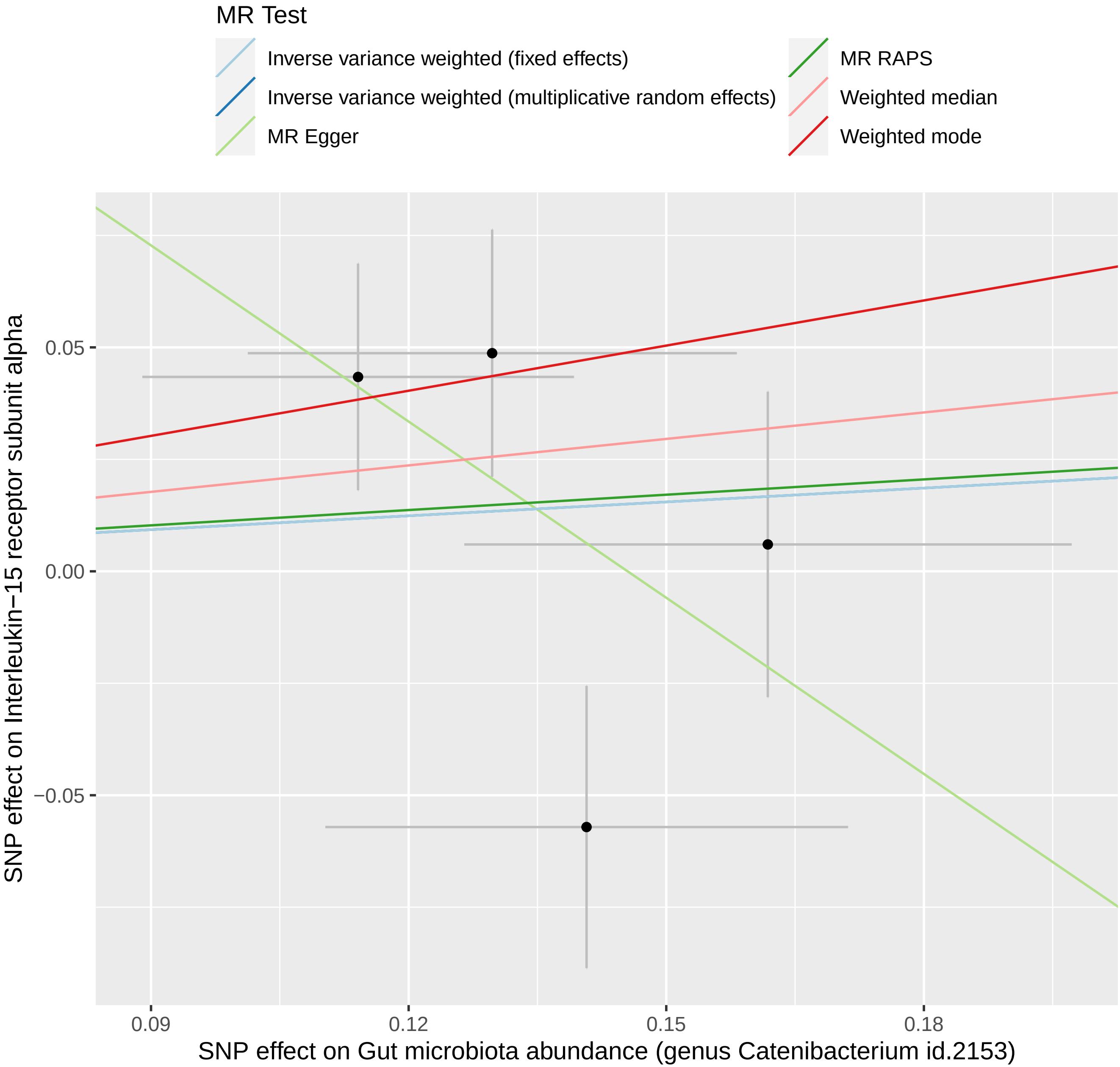

Batch 2−2956 : Gut microbiota abundance (genus Parasutterella id.2892) on Interleukin−15 receptor subunit alpha
with a threshold of 1e−05

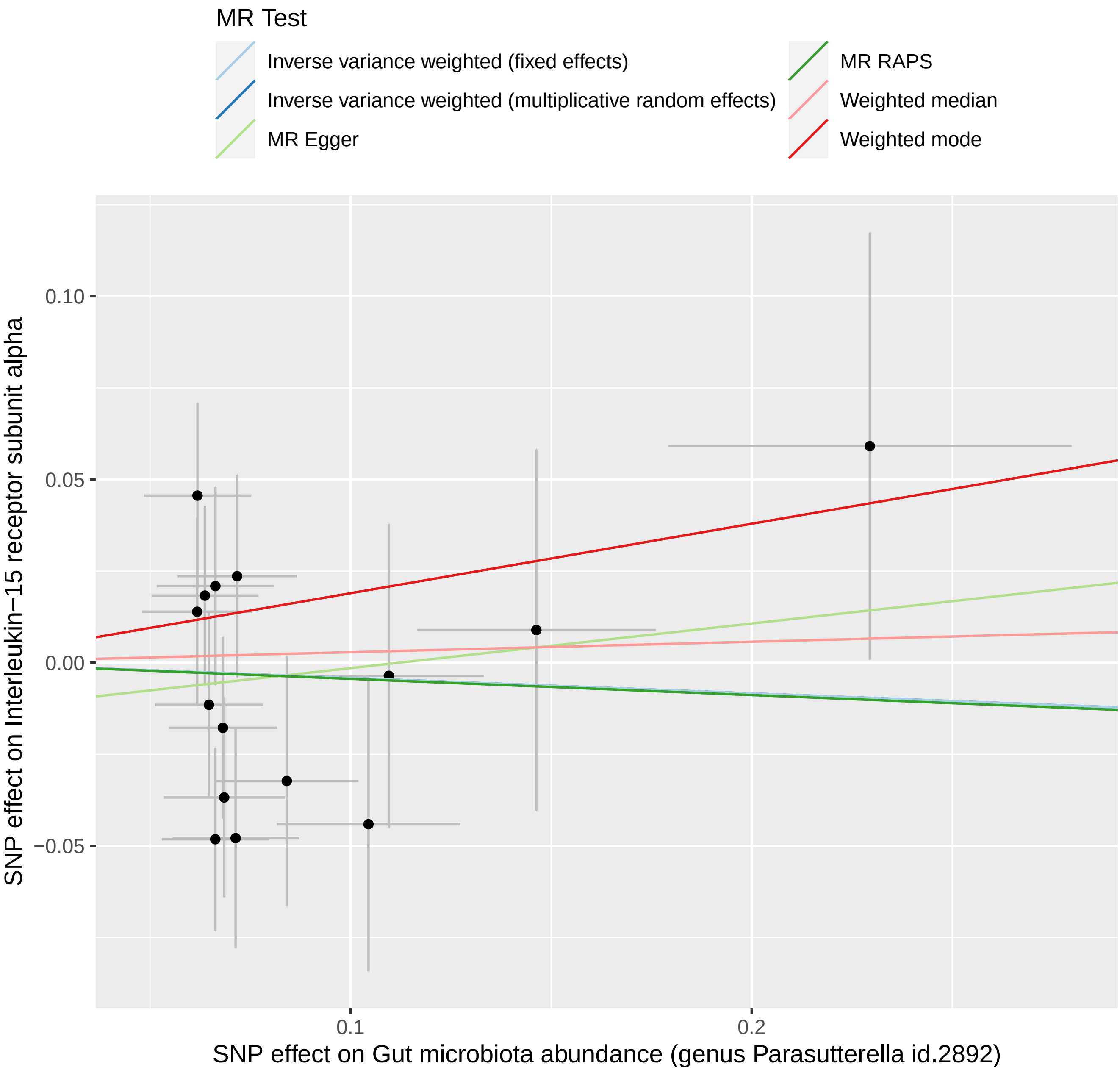

Batch 2−2957 : Gut microbiota abundance (genus Catenibacterium id.2153) on Interleukin−16
with a threshold of 1e−05

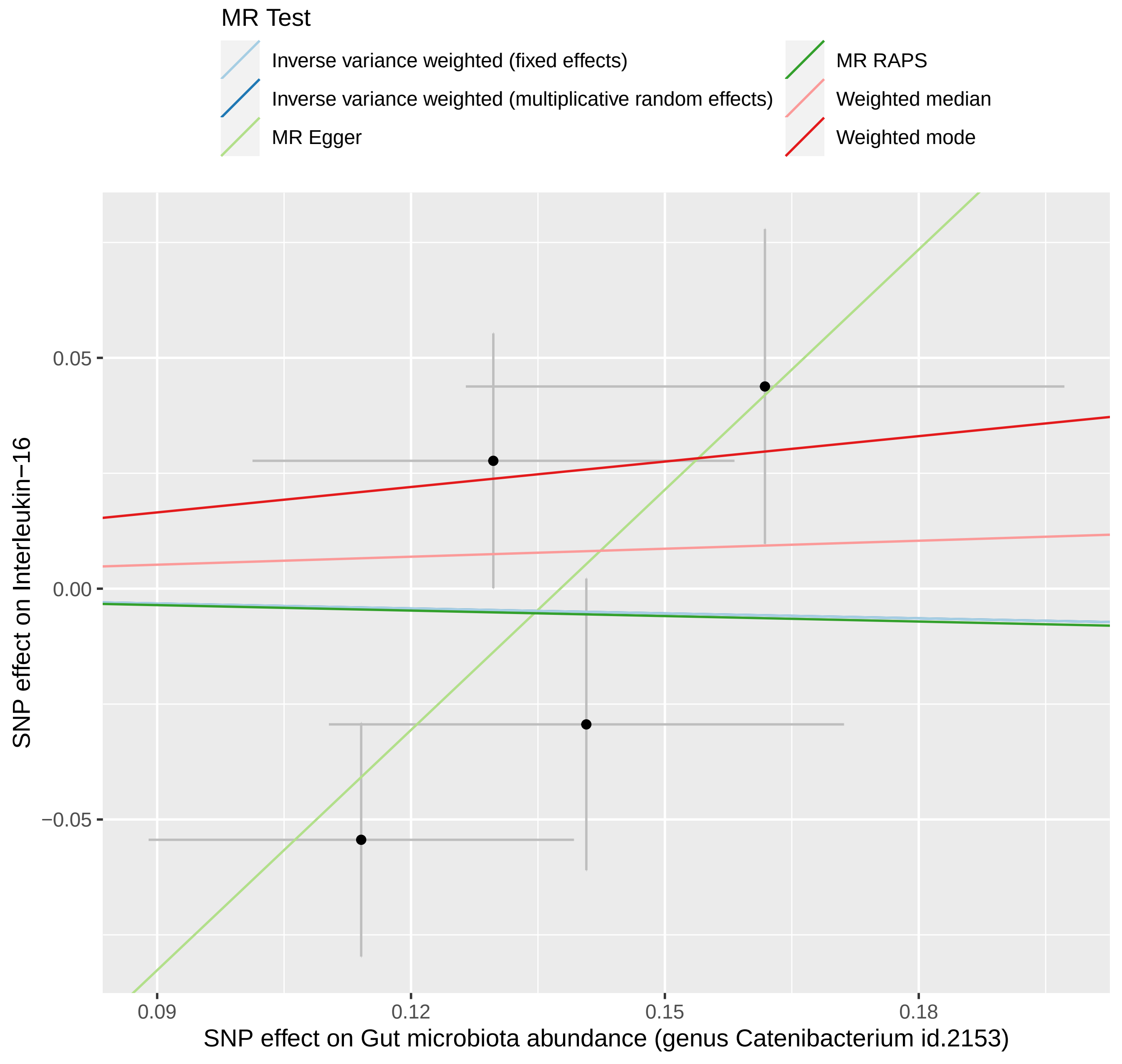

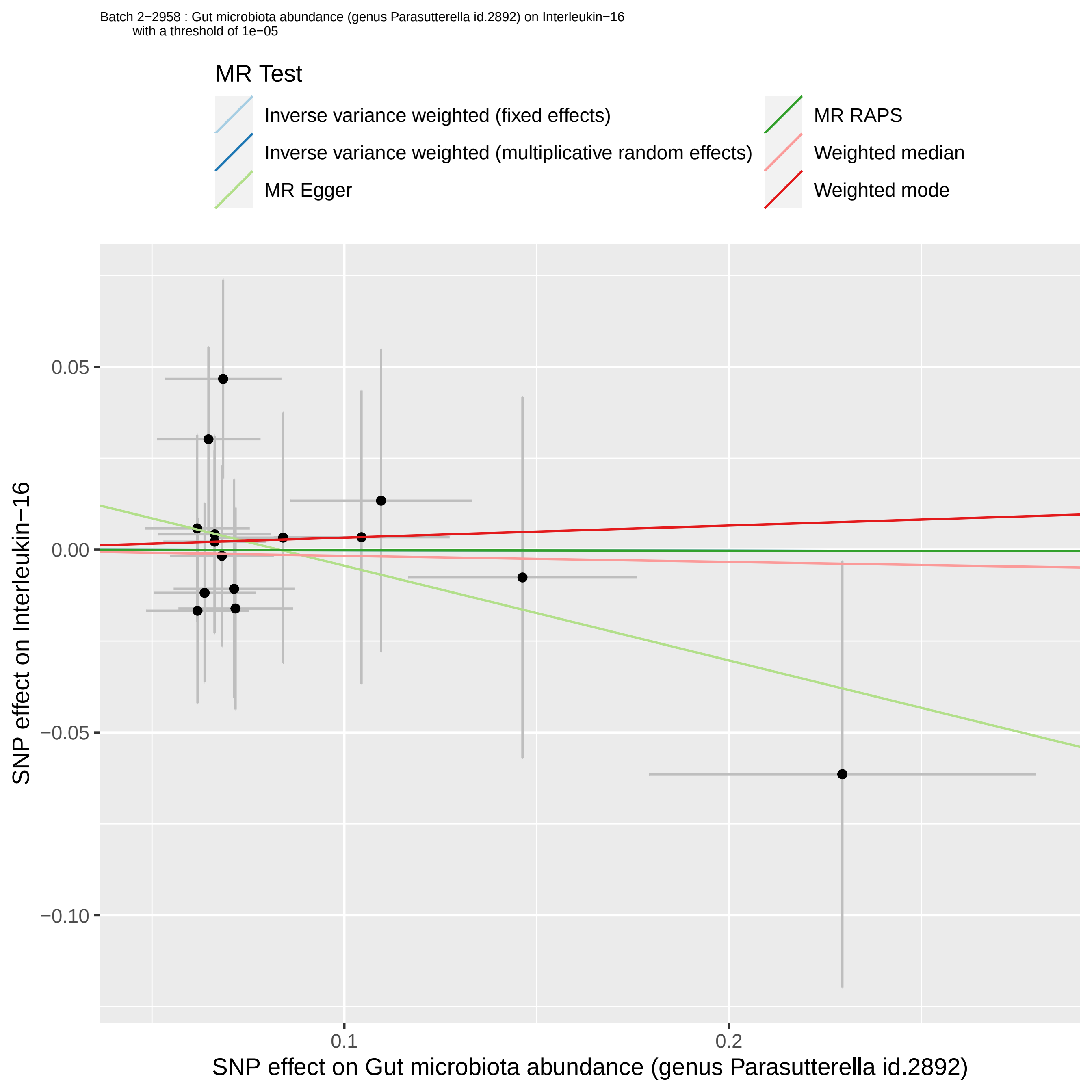

Batch 2−2958 : Gut microbiota abundance (genus Parasutterella id.2892) on Interleukin−16
with a threshold of 1e−05
MR Test
Inverse variance weighted (fixed effects)
Inverse variance weighted (multiplicative random effects)
MR Egger
MR RAPS
Weighted median
Weighted mode
0.05
0.00
−0.05
−0.10
0.1
0.2
SNP effect on Interleukin−16
SNP effect on Gut microbiota abundance (genus Parasutterella id.2892)

Batch 2−2959 : Gut microbiota abundance (genus Catenibacterium id.2153) on Interleukin−17A
with a threshold of 1e−05

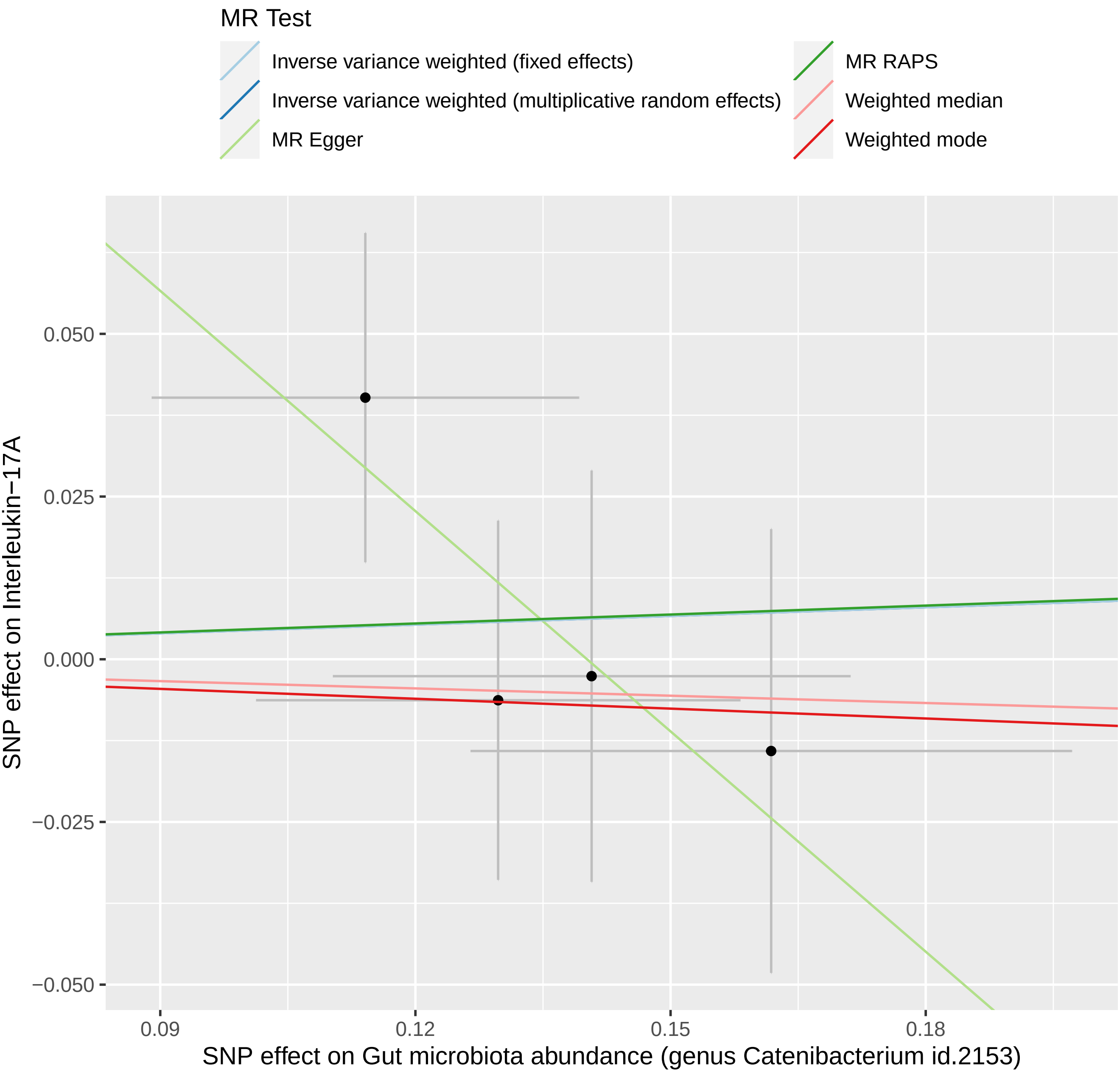

Batch 2−2960 : Gut microbiota abundance (genus Parasutterella id.2892) on Interleukin−17A
with a threshold of 1e−05

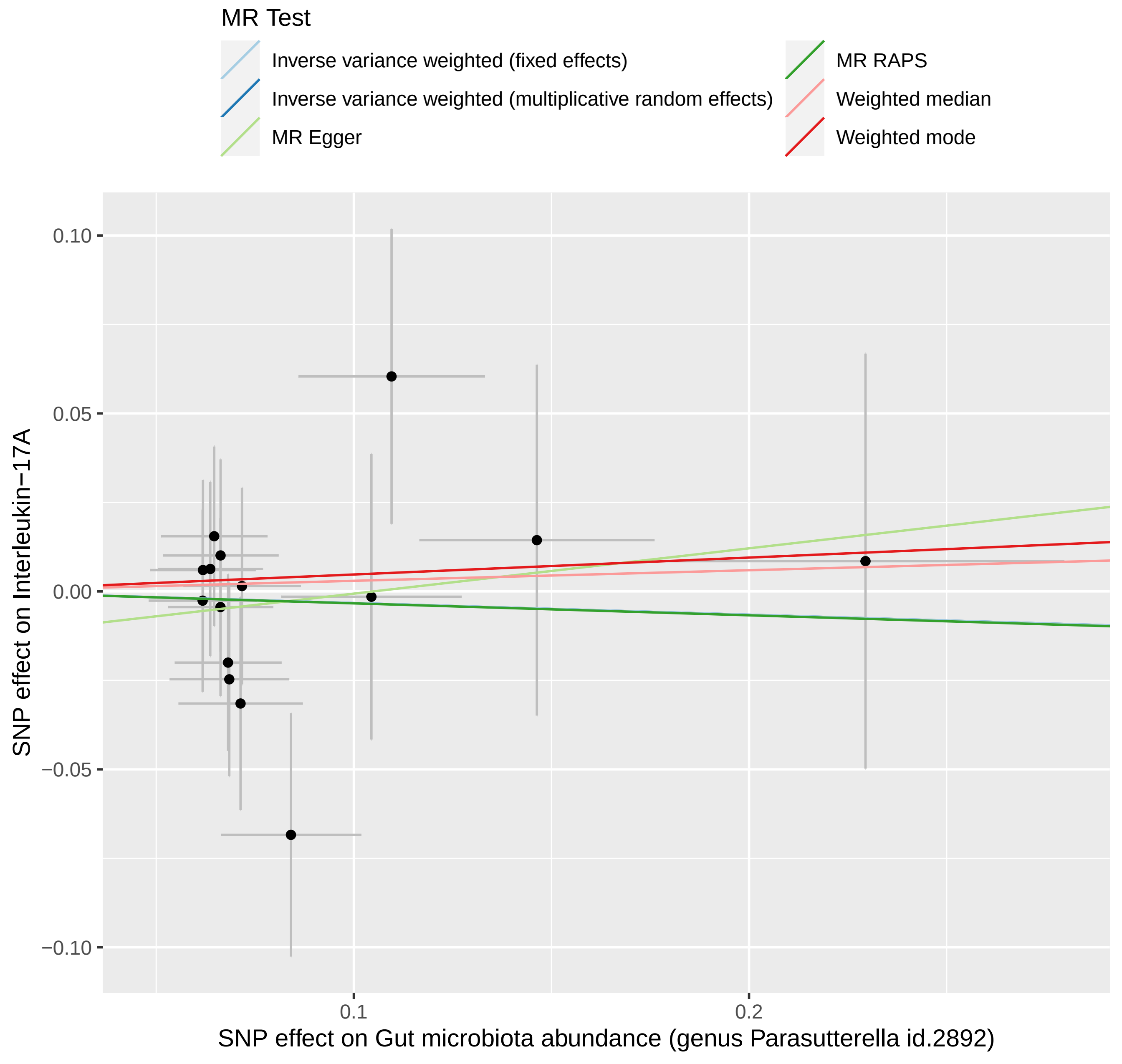

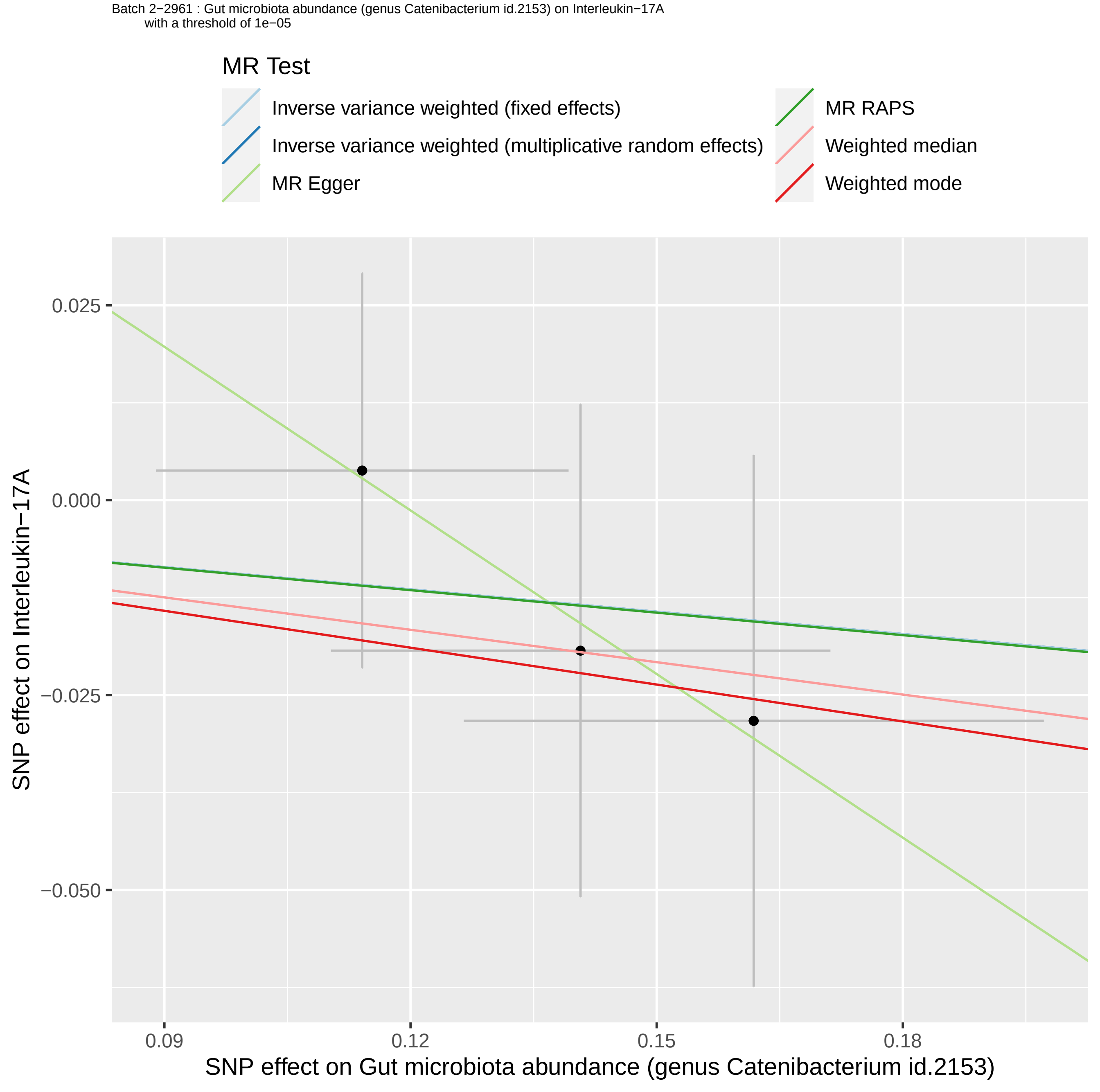

Batch 2−2961 : Gut microbiota abundance (genus Catenibacterium id.2153) on Interleukin−17A
with a threshold of 1e−05
MR Test
Inverse variance weighted (fixed effects)
Inverse variance weighted (multiplicative random effects)
MR Egger
MR RAPS
Weighted median
Weighted mode
0.025
0.000
−0.025
−0.050
SNP effect on Interleukin−17A
0.09
0.12
0.15
0.18
SNP effect on Gut microbiota abundance (genus Catenibacterium id.2153)

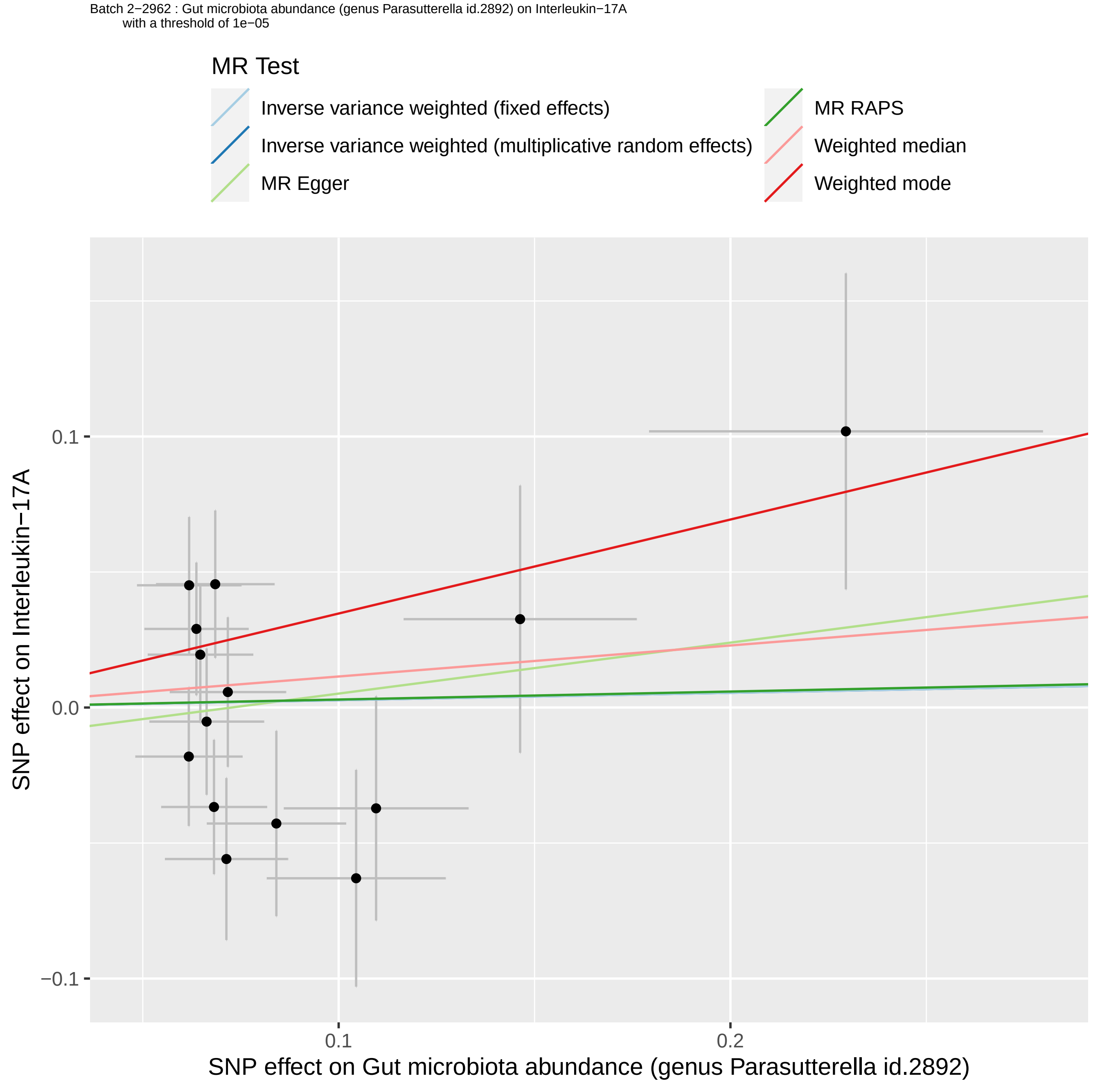
Batch 2−2962 : Gut microbiota abundance (genus Parasutterella id.2892) on Interleukin−17A
with a threshold of 1e−05
MR Test
Inverse variance weighted (fixed effects)
Inverse variance weighted (multiplicative random effects)
MR Egger
MR RAPS
Weighted median
Weighted mode
0.1
0.0
−0.1
0.1
0.2
SNP effect on Interleukin−17A
SNP effect on Gut microbiota abundance (genus Parasutterella id.2892)

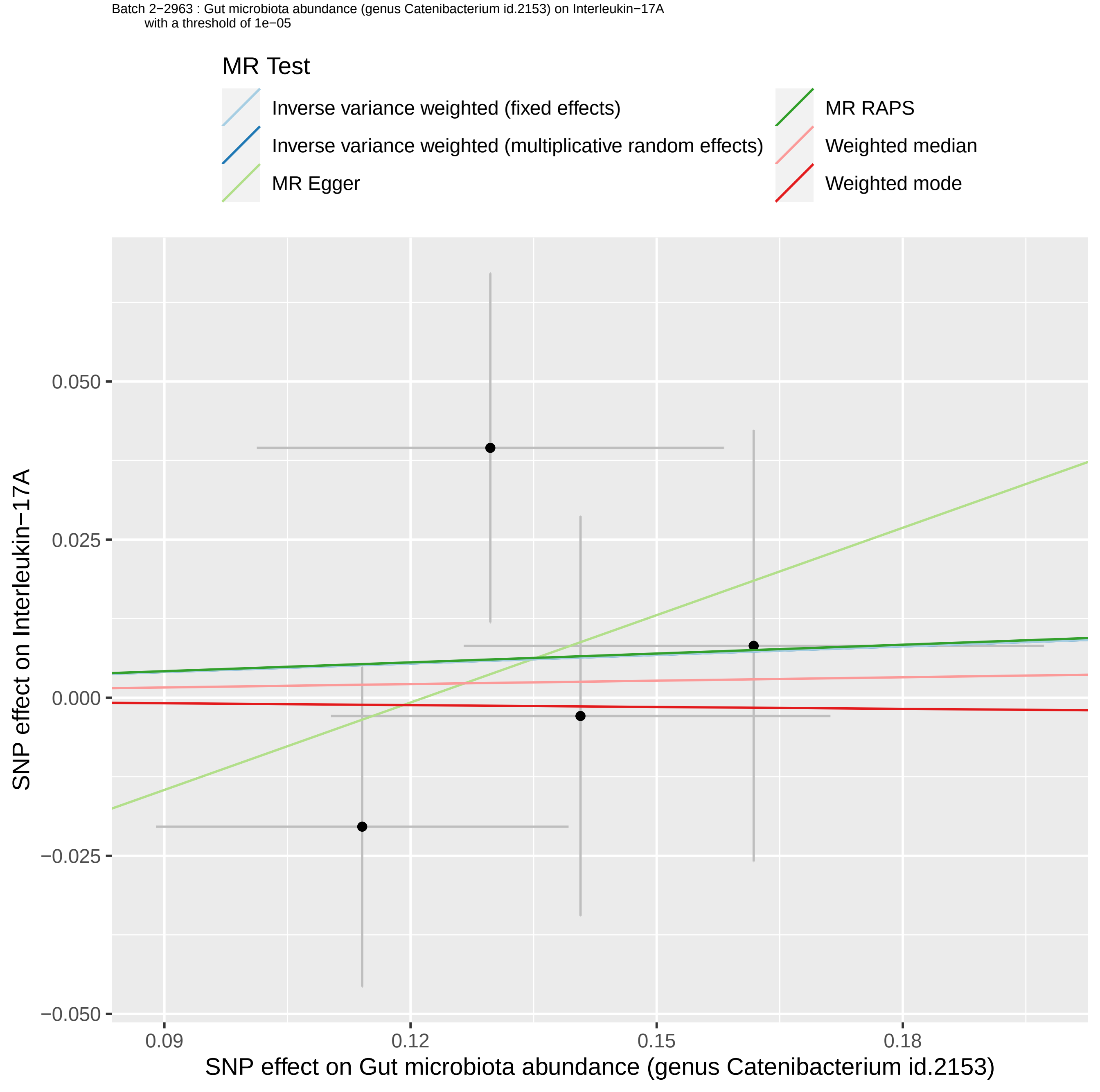
Batch 2−2963 : Gut microbiota abundance (genus Catenibacterium id.2153) on Interleukin−17A
with a threshold of 1e−05
MR Test
Inverse variance weighted (fixed effects)
Inverse variance weighted (multiplicative random effects)
MR Egger
MR RAPS
Weighted median
Weighted mode
SNP effect on Interleukin−17A
0.050
0.025
0.000
−0.025
−0.050
0.09
0.12
0.15
0.18
SNP effect on Gut microbiota abundance (genus Catenibacterium id.2153)

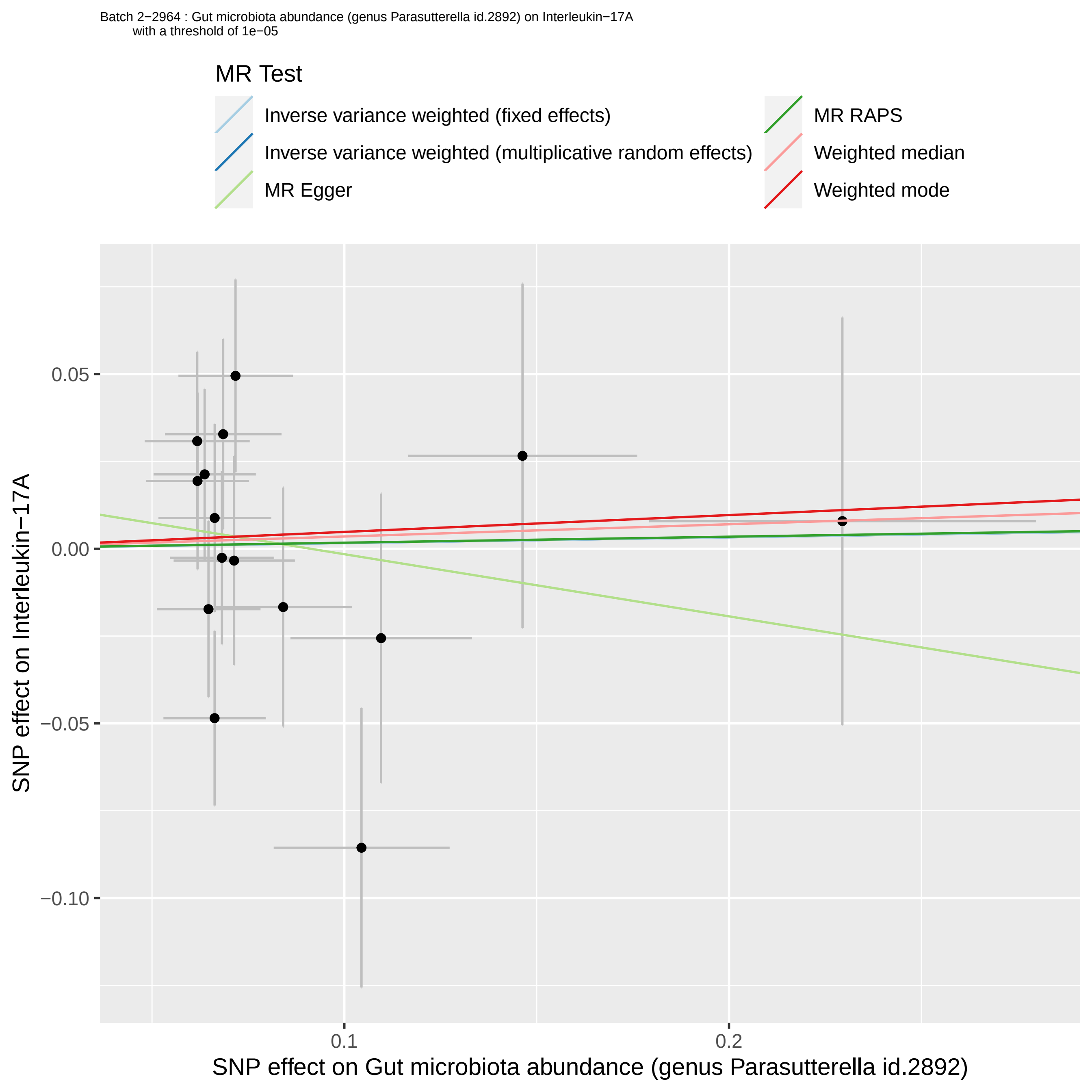

Batch 2−2964 : Gut microbiota abundance (genus Parasutterella id.2892) on Interleukin−17A
with a threshold of 1e−05
MR Test
Inverse variance weighted (fixed effects)
Inverse variance weighted (multiplicative random effects)
MR Egger
MR RAPS
Weighted median
Weighted mode
0.05
0.00
−0.05
−0.10
0.1
0.2
SNP effect on Interleukin−17A
SNP effect on Gut microbiota abundance (genus Parasutterella id.2892)

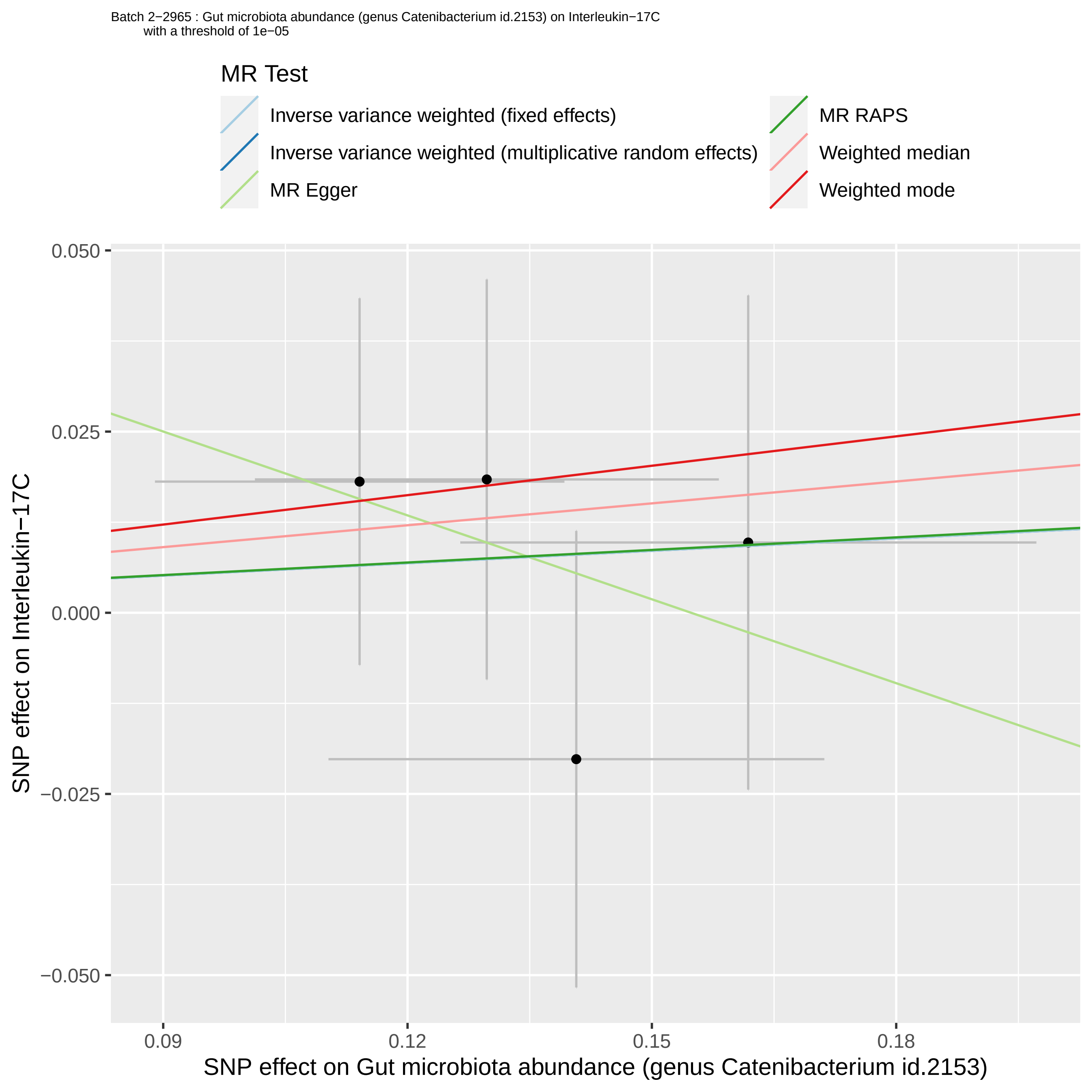
Batch 2−2965 : Gut microbiota abundance (genus Catenibacterium id.2153) on Interleukin−17C
with a threshold of 1e−05
MR Test
Inverse variance weighted (fixed effects)
Inverse variance weighted (multiplicative random effects)
MR Egger
MR RAPS
Weighted median
Weighted mode
0.050
0.025
0.000
−0.025
−0.050
0.09
0.12
0.15
0.18
SNP effect on Interleukin−17C
SNP effect on Gut microbiota abundance (genus Catenibacterium id.2153)

Batch 2−2966 : Gut microbiota abundance (genus Parasutterella id.2892) on Interleukin−17C
with a threshold of 1e−05

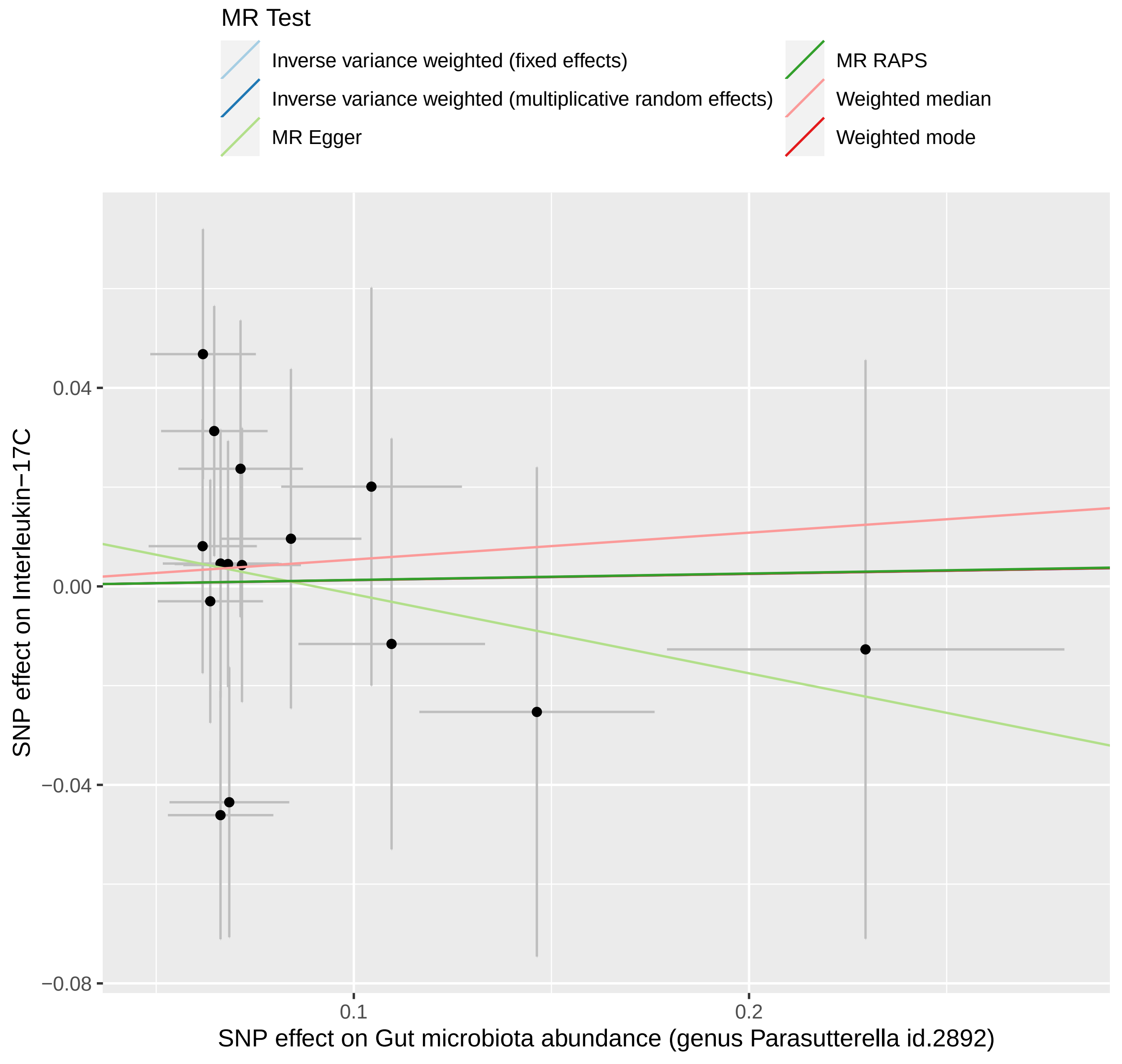

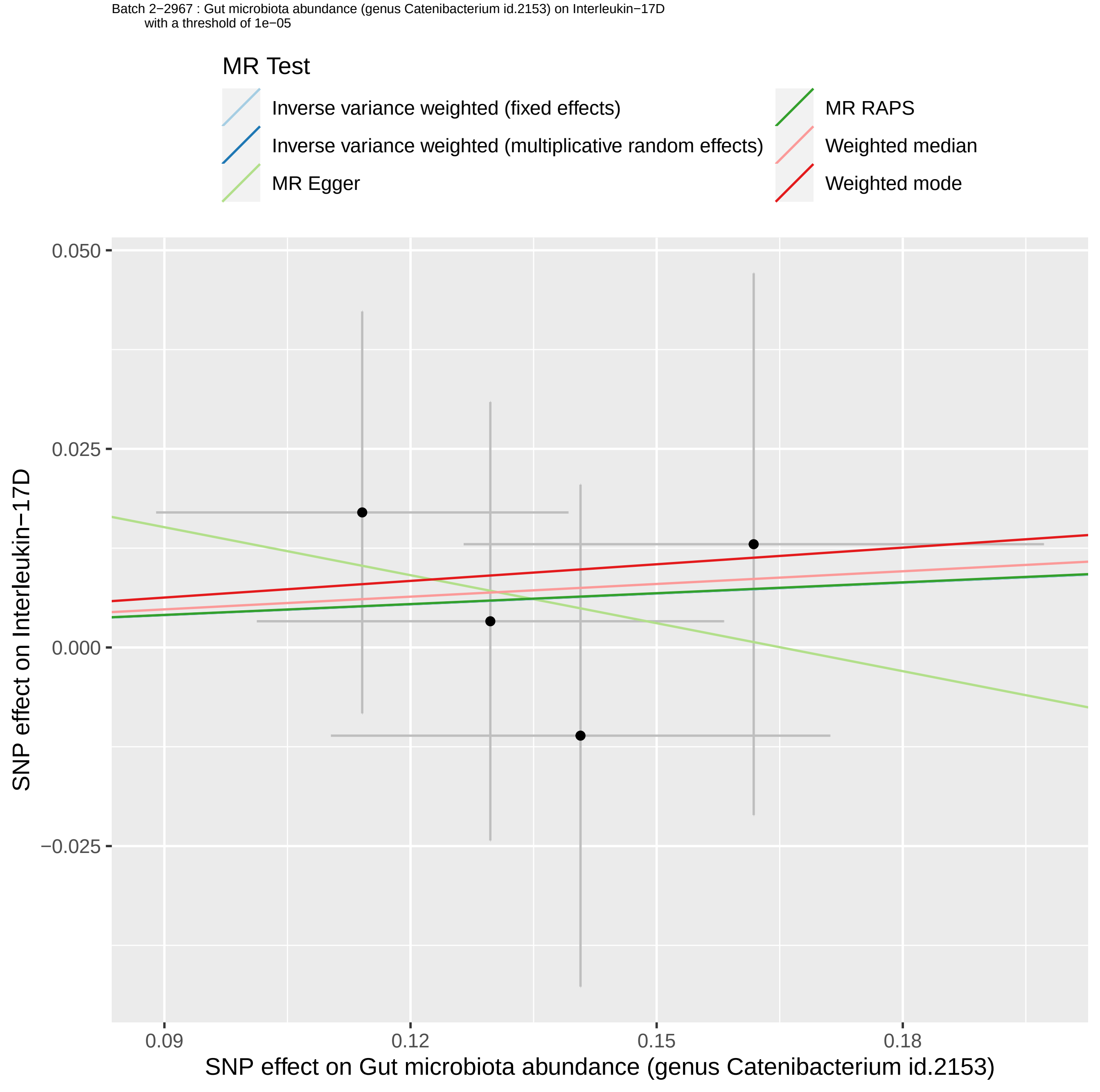
Batch 2−2967 : Gut microbiota abundance (genus Catenibacterium id.2153) on Interleukin−17D
with a threshold of 1e−05
MR Test
Inverse variance weighted (fixed effects)
Inverse variance weighted (multiplicative random effects)
MR Egger
MR RAPS
Weighted median
Weighted mode
0.050
0.025
0.000
−0.025
SNP effect on Interleukin−17D
0.09
0.12
0.15
0.18
SNP effect on Gut microbiota abundance (genus Catenibacterium id.2153)

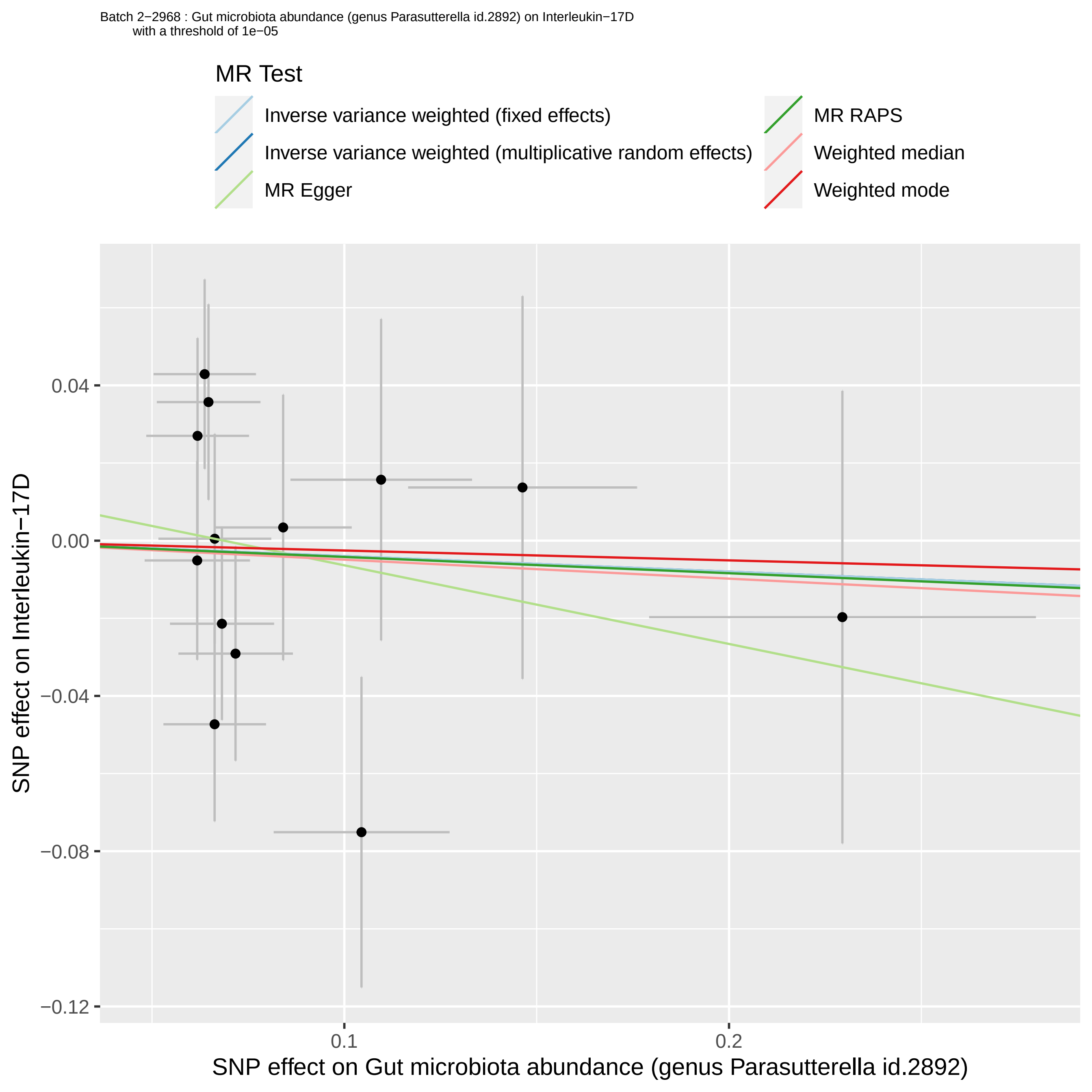
Batch 2−2968 : Gut microbiota abundance (genus Parasutterella id.2892) on Interleukin−17D
with a threshold of 1e−05
MR Test
Inverse variance weighted (fixed effects)
Inverse variance weighted (multiplicative random effects)
MR Egger
MR RAPS
Weighted median
Weighted mode
0.04
0.00
−0.04
−0.08
−0.12
0.1
0.2
SNP effect on Interleukin−17D
SNP effect on Gut microbiota abundance (genus Parasutterella id.2892)

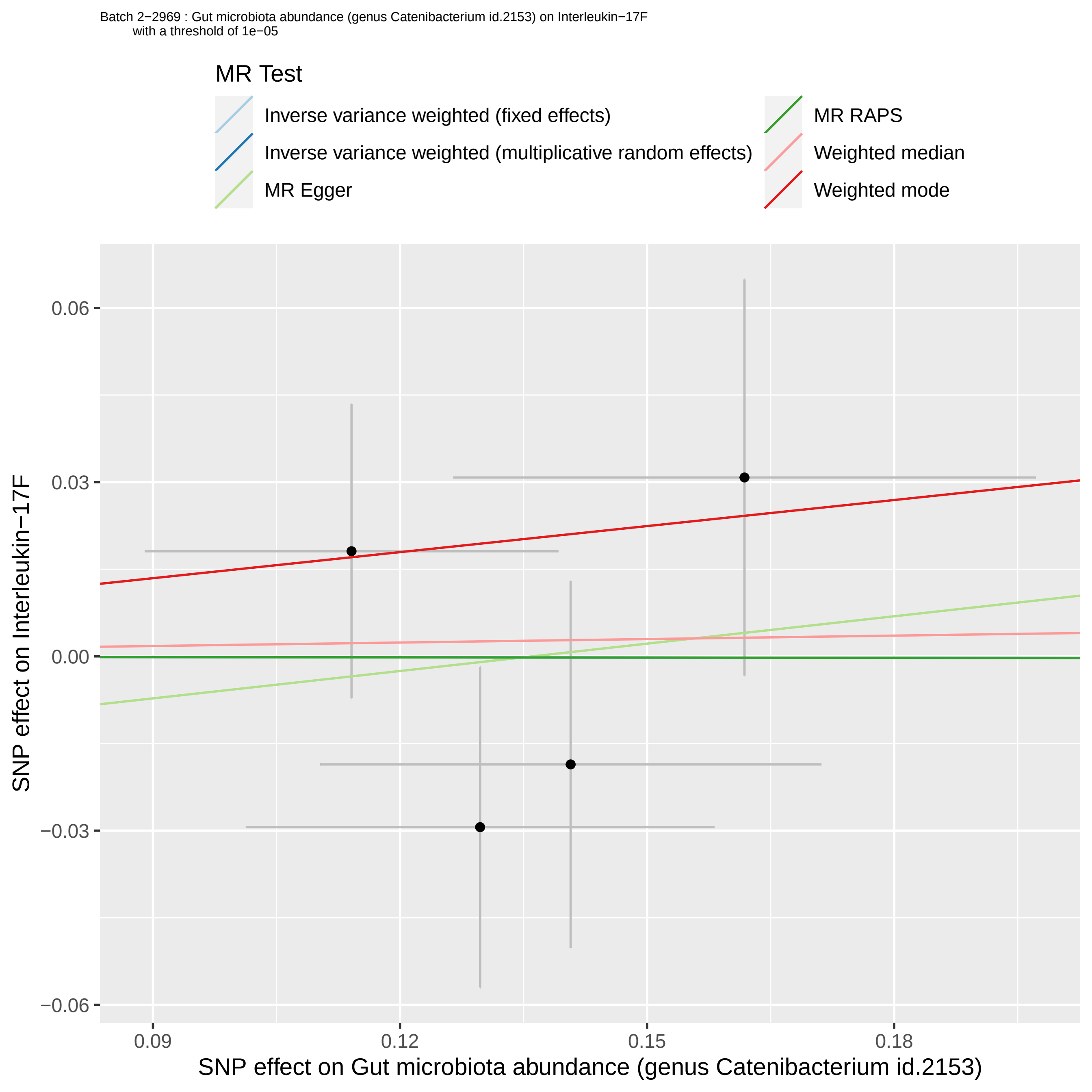

Batch 2−2969 : Gut microbiota abundance (genus Catenibacterium id.2153) on Interleukin−17F
with a threshold of 1e−05
MR Test
Inverse variance weighted (fixed effects)
Inverse variance weighted (multiplicative random effects)
MR Egger
MR RAPS
Weighted median
Weighted mode
0.06
0.03
0.00
−0.03
−0.06
SNP effect on Interleukin−17F
0.09
0.12
0.15
0.18
SNP effect on Gut microbiota abundance (genus Catenibacterium id.2153)

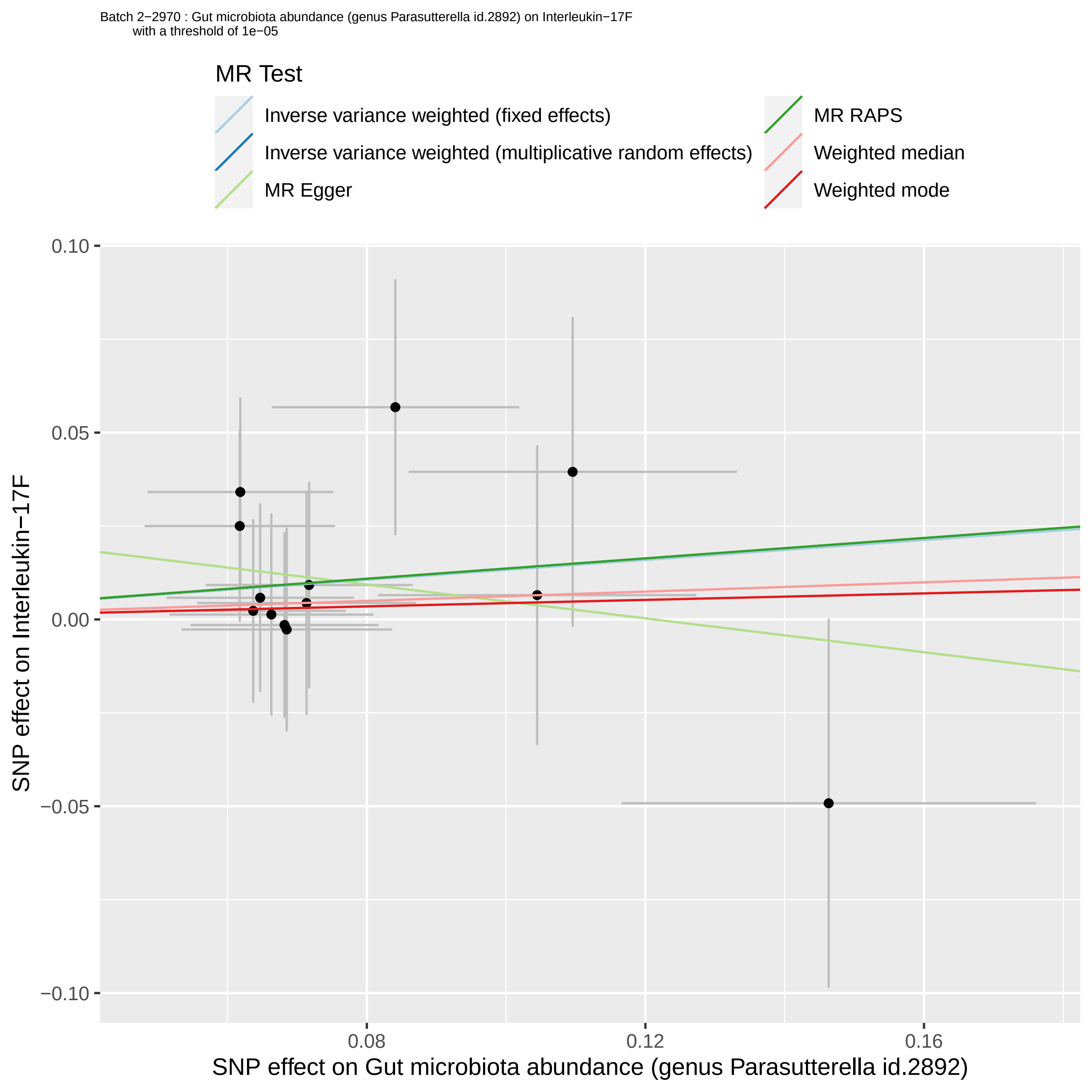

Batch 2−2970 : Gut microbiota abundance (genus Parasutterella id.2892) on Interleukin−17F
with a threshold of 1e−05
MR Test
Inverse variance weighted (fixed effects)
Inverse variance weighted (multiplicative random effects)
MR Egger
MR RAPS
Weighted median
Weighted mode
0.10
0.05
0.00
−0.05
−0.10
0.08
0.12
0.16
SNP effect on Interleukin−17F
SNP effect on Gut microbiota abundance (genus Parasutterella id.2892)

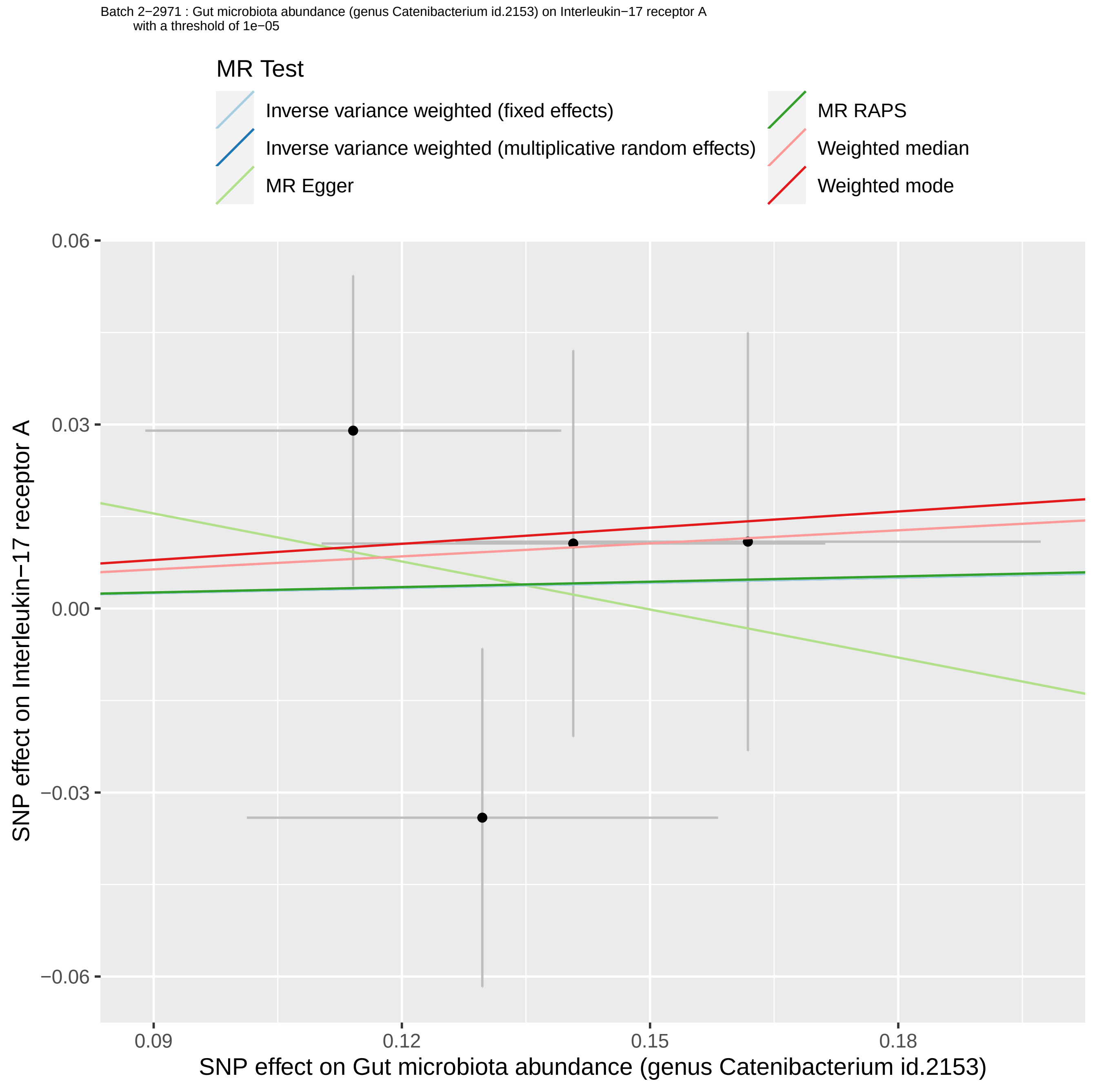

Batch 2−2971 : Gut microbiota abundance (genus Catenibacterium id.2153) on Interleukin−17 receptor A
with a threshold of 1e−05
MR Test
Inverse variance weighted (fixed effects)
Inverse variance weighted (multiplicative random effects)
MR Egger
MR RAPS
Weighted median
Weighted mode
0.06
0.03
0.00
−0.03
−0.06
0.09
0.12
0.15
0.18
SNP effect on Interleukin−17 receptor A
SNP effect on Gut microbiota abundance (genus Catenibacterium id.2153)

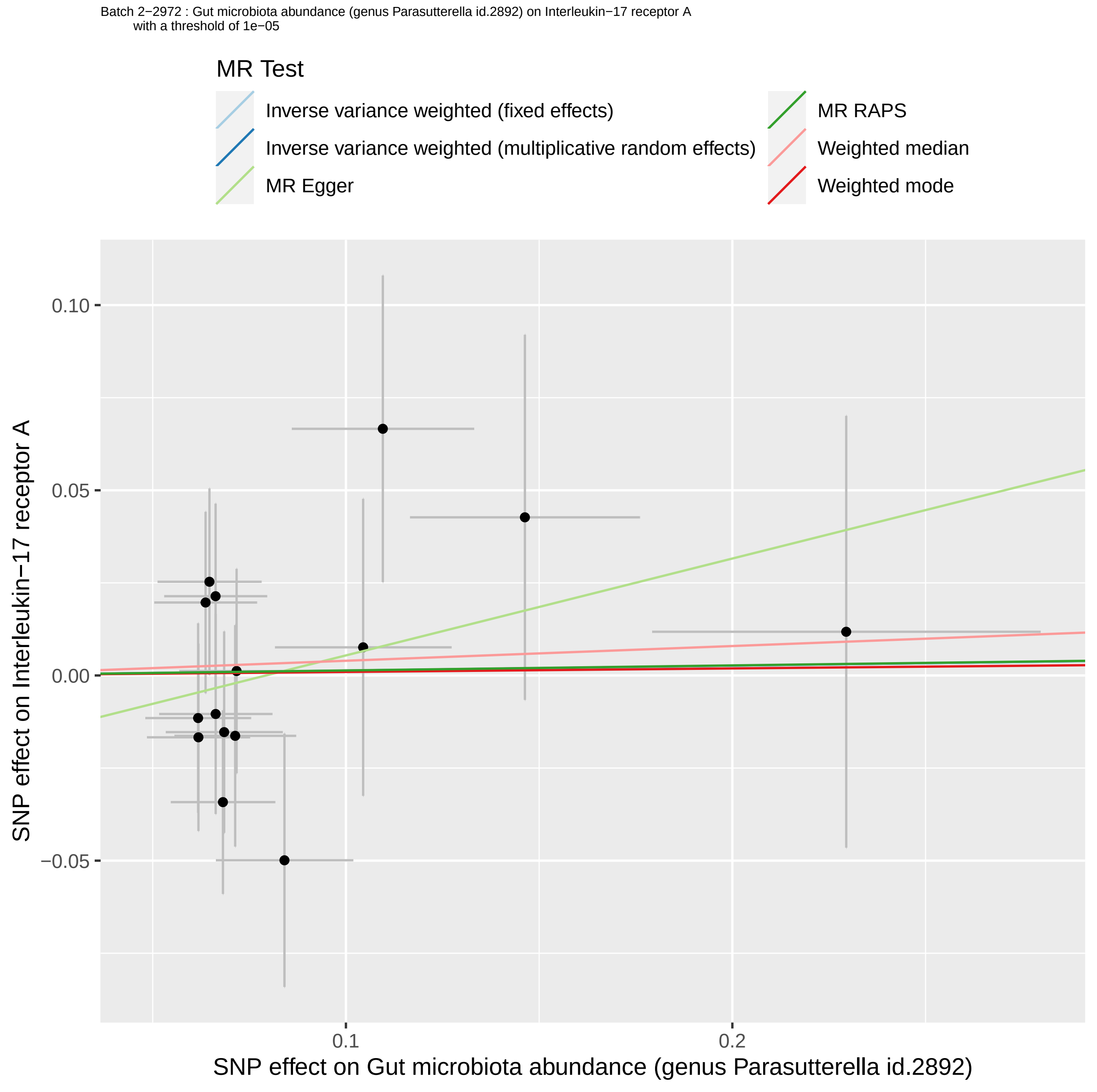

Batch 2−2972 : Gut microbiota abundance (genus Parasutterella id.2892) on Interleukin−17 receptor A
with a threshold of 1e−05
MR Test
Inverse variance weighted (fixed effects)
Inverse variance weighted (multiplicative random effects)
MR Egger
MR RAPS
Weighted median
Weighted mode
0.10
0.05
0.00
−0.05
0.1
0.2
SNP effect on Interleukin−17 receptor A
SNP effect on Gut microbiota abundance (genus Parasutterella id.2892)

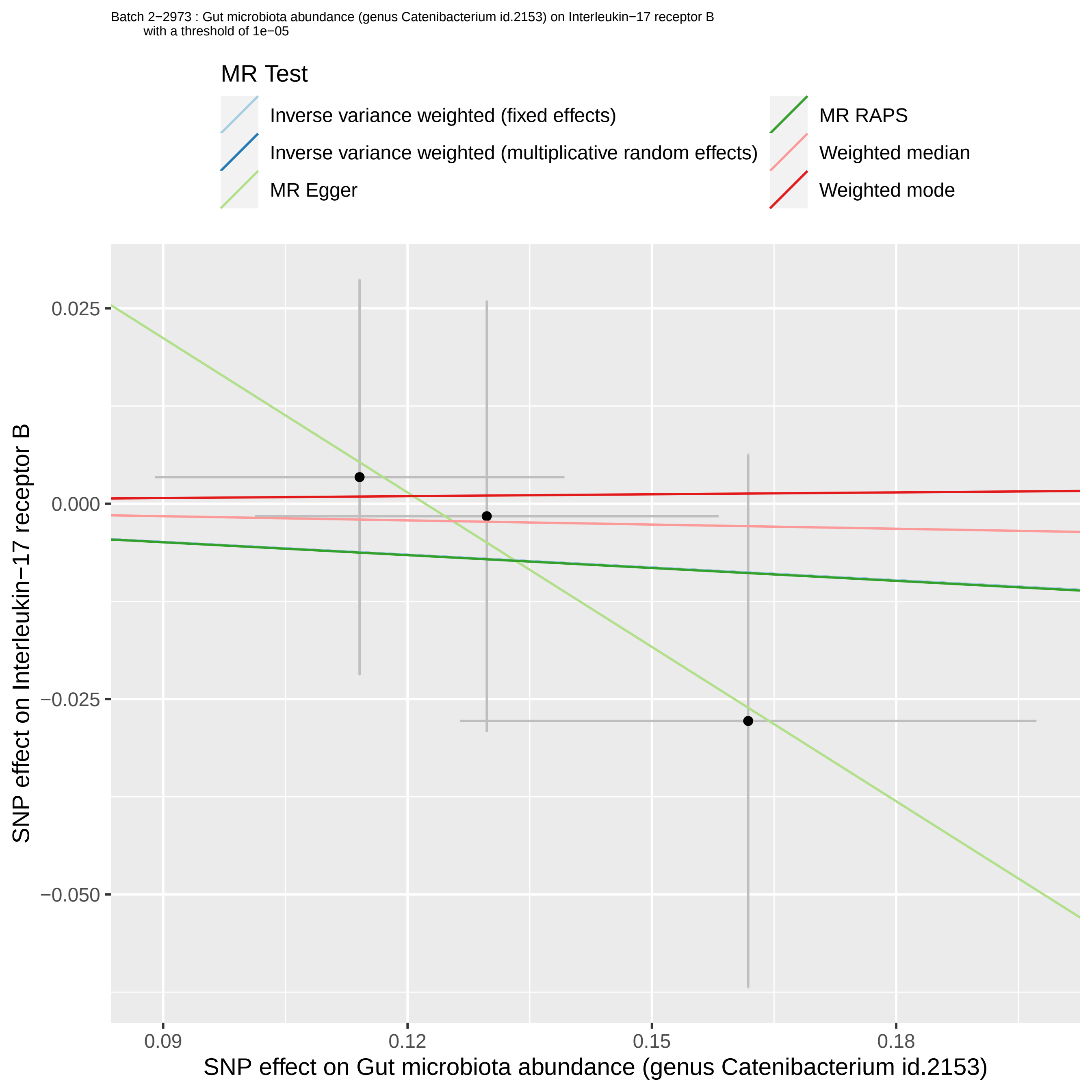
Batch 2−2973 : Gut microbiota abundance (genus Catenibacterium id.2153) on Interleukin−17 receptor B
with a threshold of 1e−05
MR Test
Inverse variance weighted (fixed effects)
Inverse variance weighted (multiplicative random effects)
MR Egger
MR RAPS
Weighted median
Weighted mode
0.025
0.000
−0.025
−0.050
0.09
0.12
0.15
0.18
SNP effect on Interleukin−17 receptor B
SNP effect on Gut microbiota abundance (genus Catenibacterium id.2153)

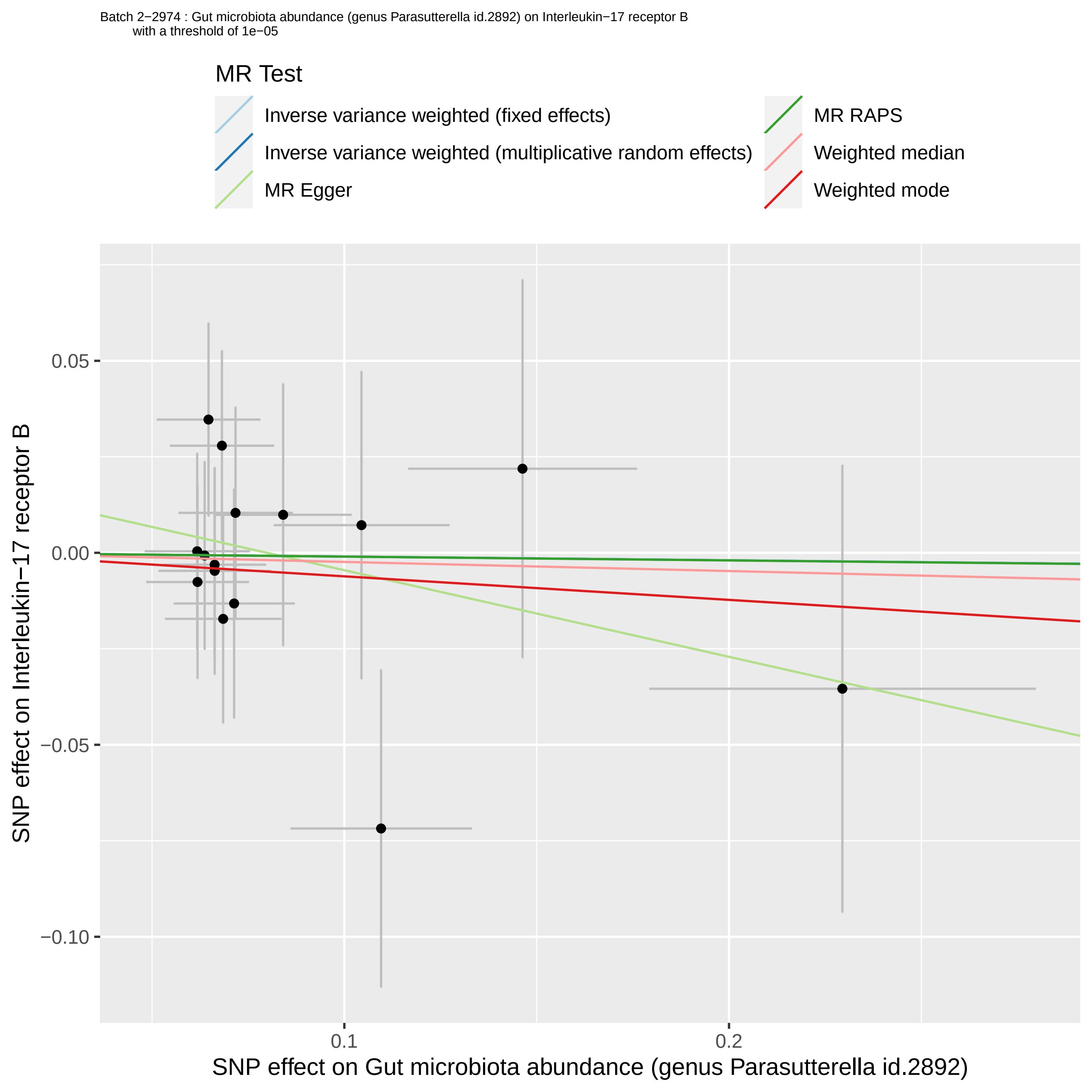
Batch 2−2974 : Gut microbiota abundance (genus Parasutterella id.2892) on Interleukin−17 receptor B
with a threshold of 1e−05
MR Test
Inverse variance weighted (fixed effects)
Inverse variance weighted (multiplicative random effects)
MR Egger
MR RAPS
Weighted median
Weighted mode
0.05
0.00
−0.05
−0.10
0.1
0.2
SNP effect on Interleukin−17 receptor B
SNP effect on Gut microbiota abundance (genus Parasutterella id.2892)

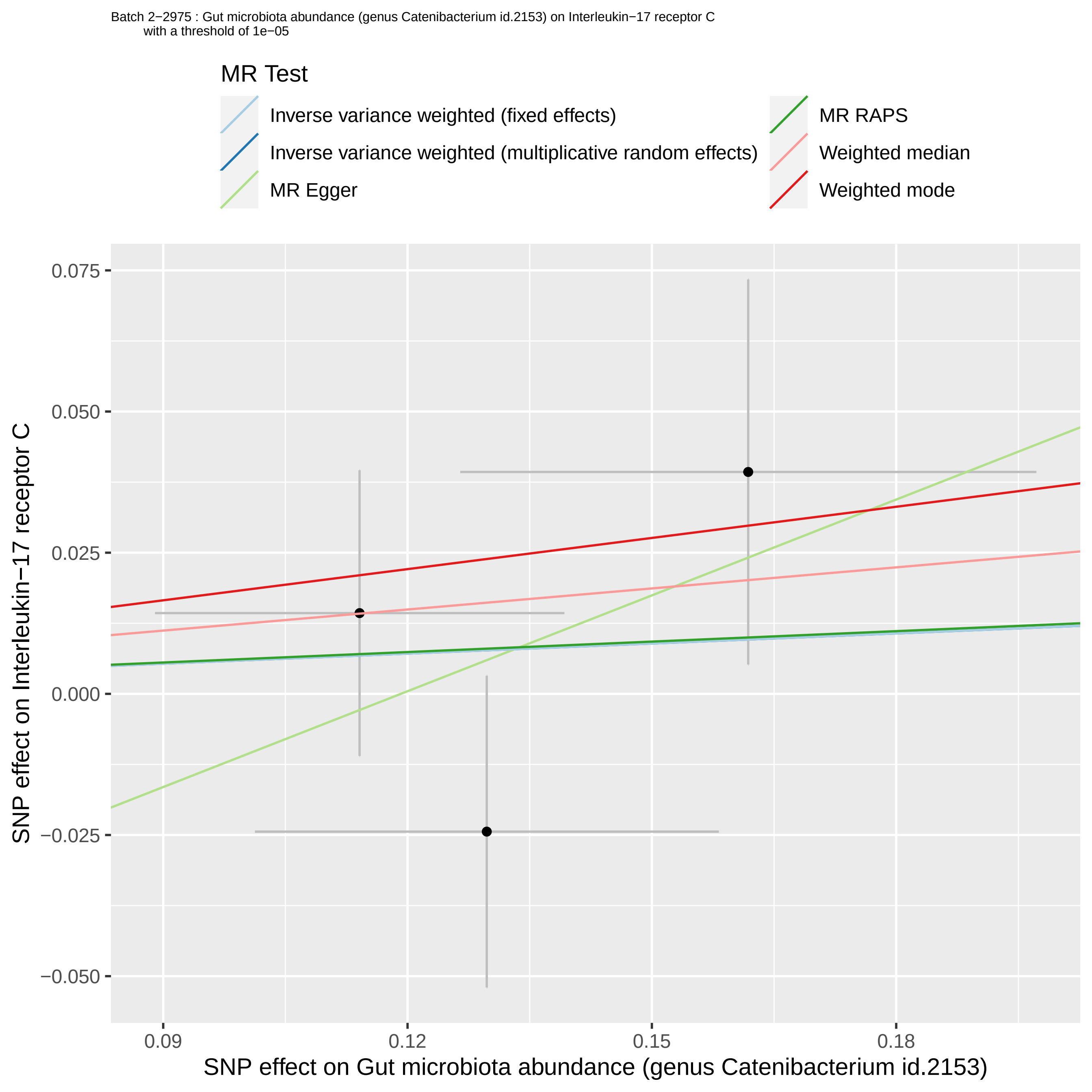

Batch 2−2975 : Gut microbiota abundance (genus Catenibacterium id.2153) on Interleukin−17 receptor C
with a threshold of 1e−05
MR Test
Inverse variance weighted (fixed effects)
Inverse variance weighted (multiplicative random effects)
MR Egger
MR RAPS
Weighted median
Weighted mode
0.075
0.050
0.025
0.000
−0.025
−0.050
SNP effect on Interleukin−17 receptor C
0.09
0.12
0.15
0.18
SNP effect on Gut microbiota abundance (genus Catenibacterium id.2153)

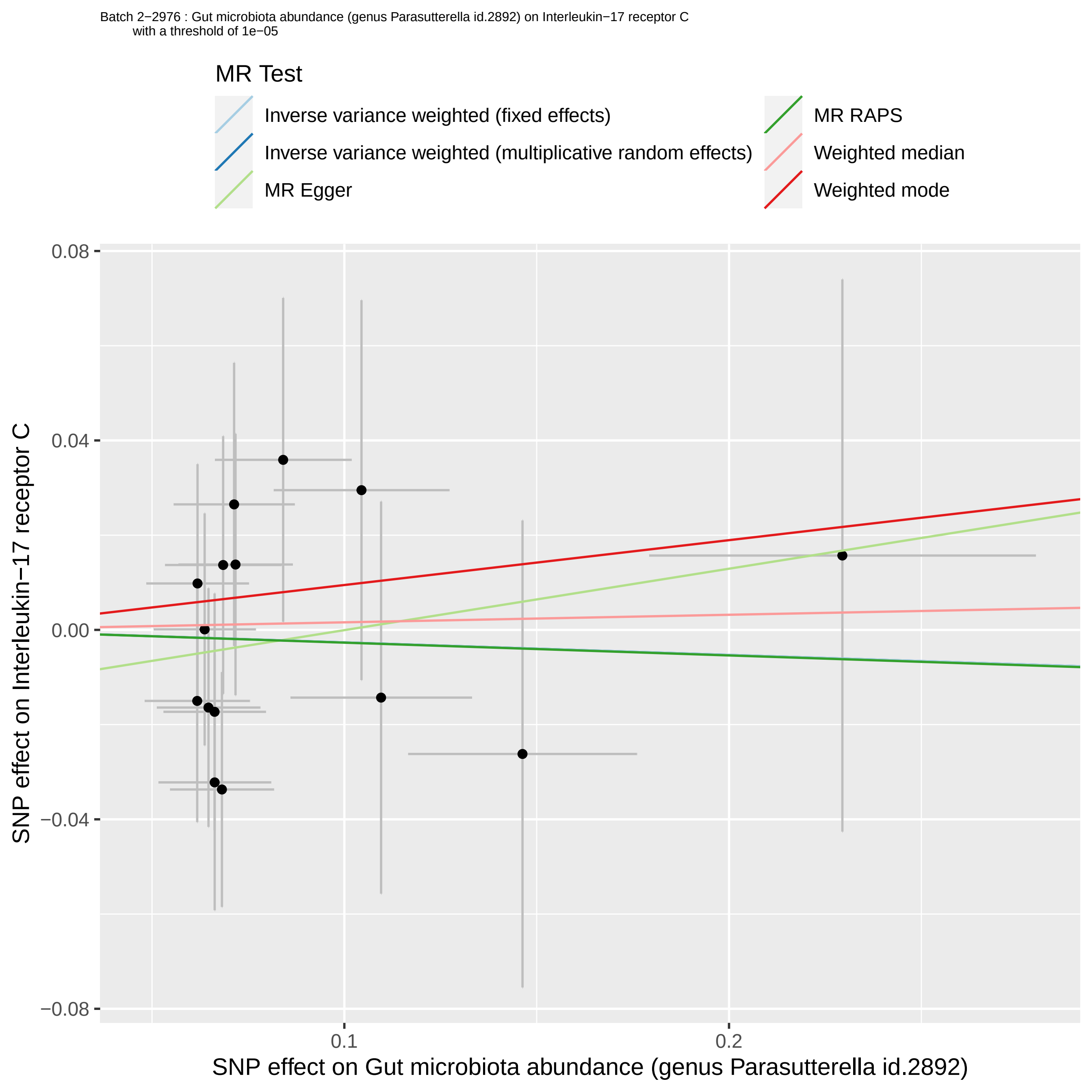

Batch 2−2976 : Gut microbiota abundance (genus Parasutterella id.2892) on Interleukin−17 receptor C
with a threshold of 1e−05
MR Test
Inverse variance weighted (fixed effects)
Inverse variance weighted (multiplicative random effects)
MR Egger
MR RAPS
Weighted median
Weighted mode
0.08
0.04
0.00
−0.04
−0.08
0.1
0.2
SNP effect on Interleukin−17 receptor C
SNP effect on Gut microbiota abundance (genus Parasutterella id.2892)

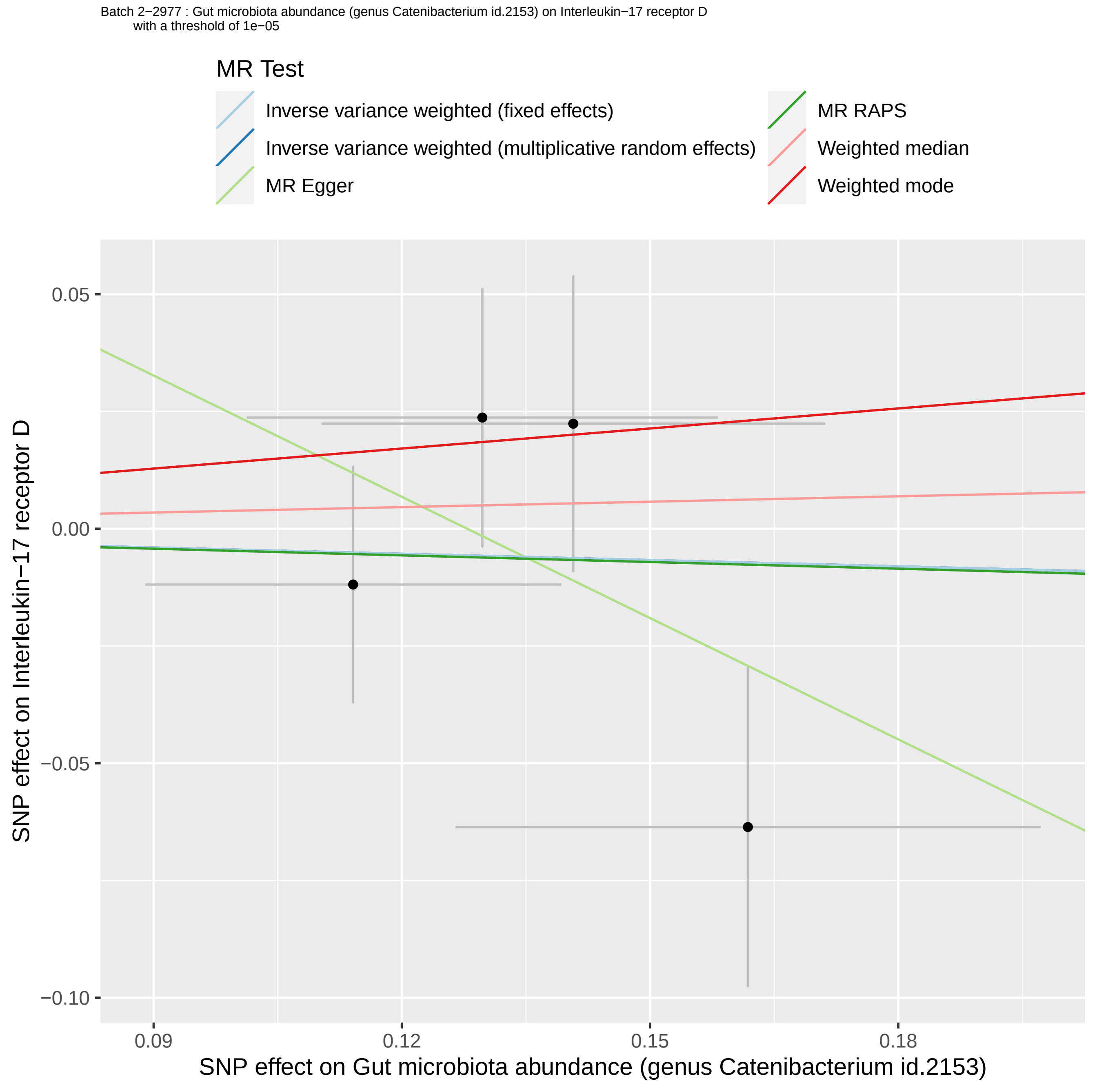

Batch 2−2977 : Gut microbiota abundance (genus Catenibacterium id.2153) on Interleukin−17 receptor D
with a threshold of 1e−05
MR Test
Inverse variance weighted (fixed effects)
Inverse variance weighted (multiplicative random effects)
MR Egger
MR RAPS
Weighted median
Weighted mode
0.05
0.00
−0.05
−0.10
0.09
0.12
0.15
0.18
SNP effect on Interleukin−17 receptor D
SNP effect on Gut microbiota abundance (genus Catenibacterium id.2153)

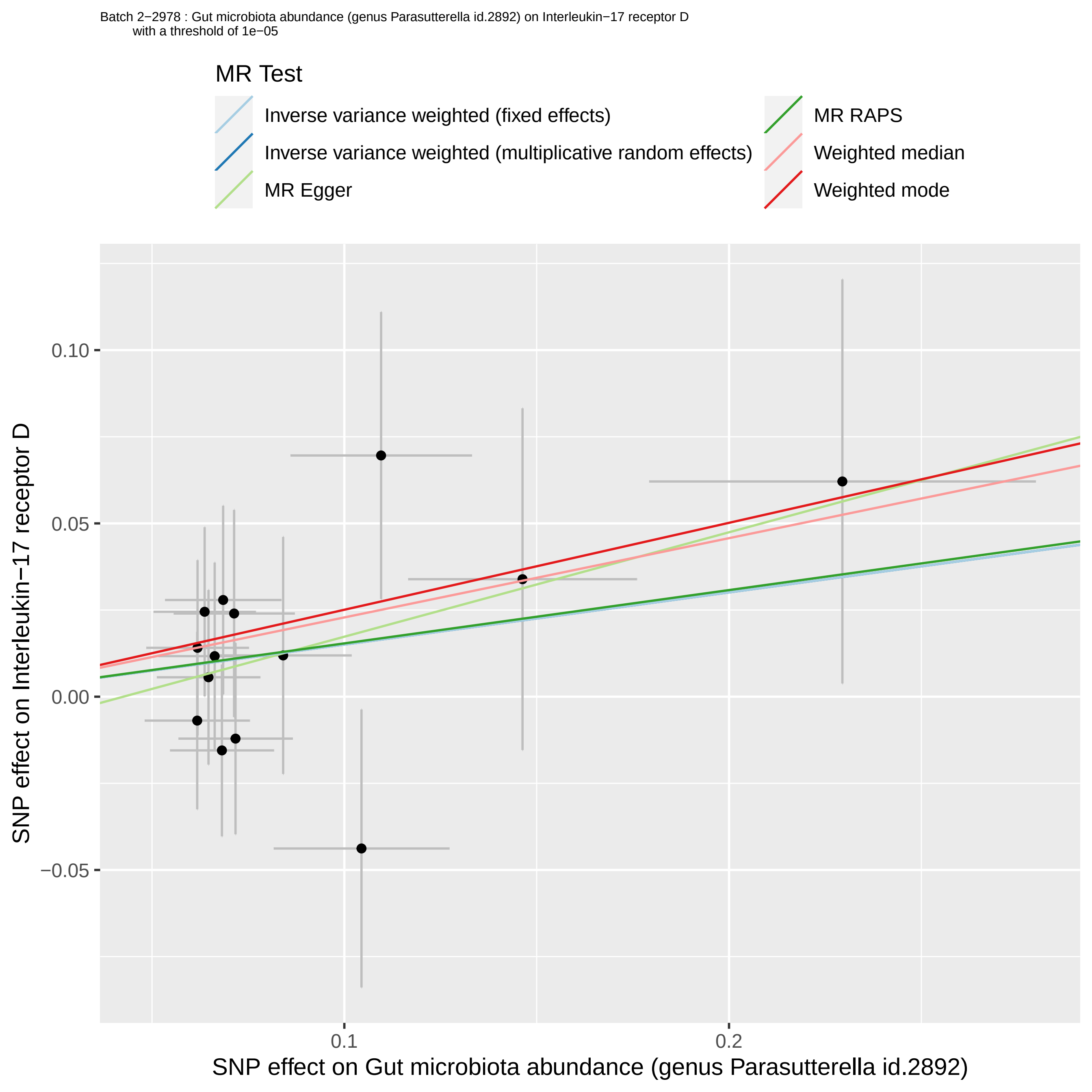
Batch 2−2978 : Gut microbiota abundance (genus Parasutterella id.2892) on Interleukin−17 receptor D
with a threshold of 1e−05
MR Test
Inverse variance weighted (fixed effects)
Inverse variance weighted (multiplicative random effects)
MR Egger
MR RAPS
Weighted median
Weighted mode
0.10
0.05
0.00
−0.05
0.1
0.2
SNP effect on Interleukin−17 receptor D
SNP effect on Gut microbiota abundance (genus Parasutterella id.2892)

Batch 2−2979 : Gut microbiota abundance (genus Catenibacterium id.2153) on Interleukin−18−binding protein
with a threshold of 1e−05

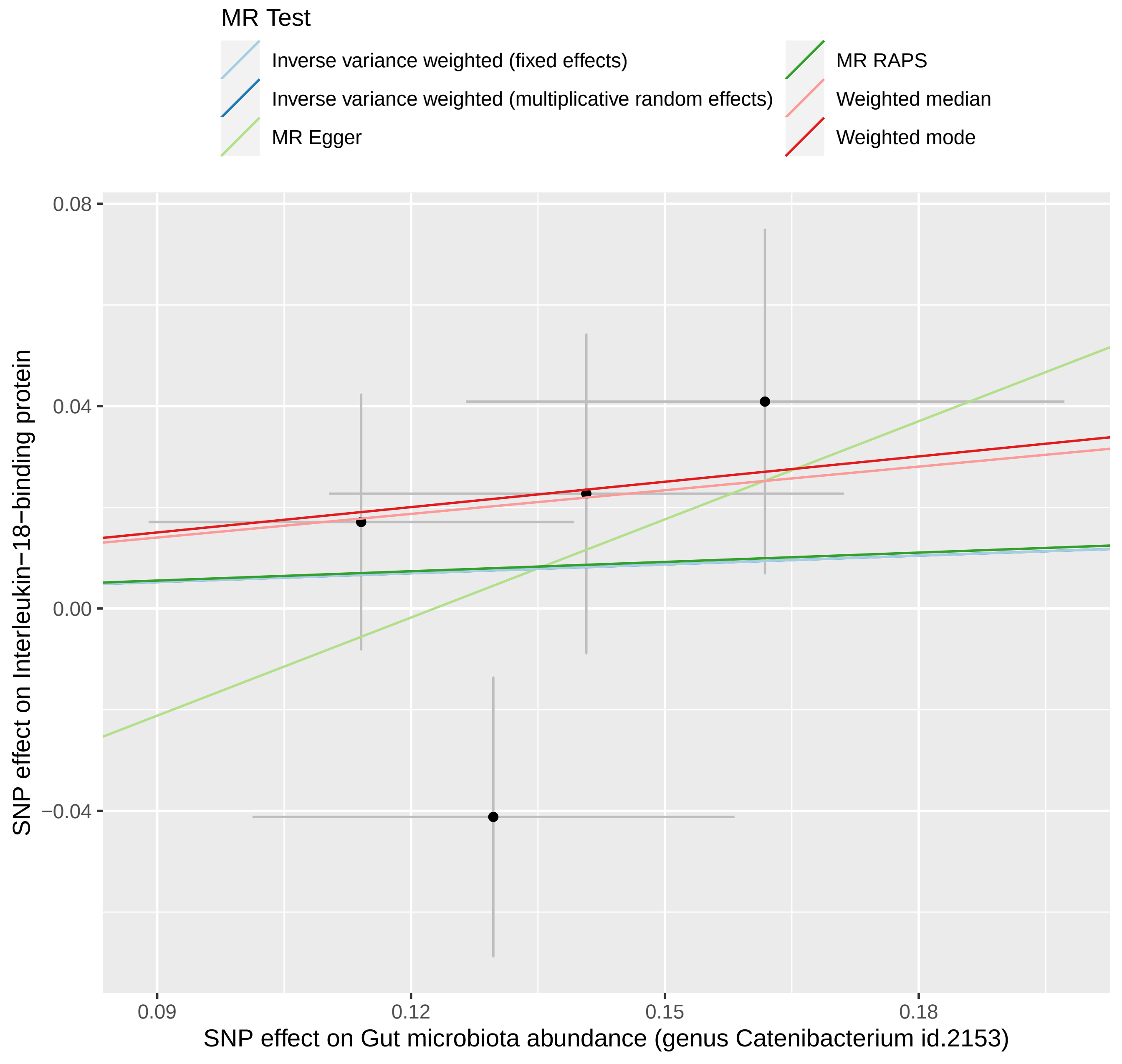

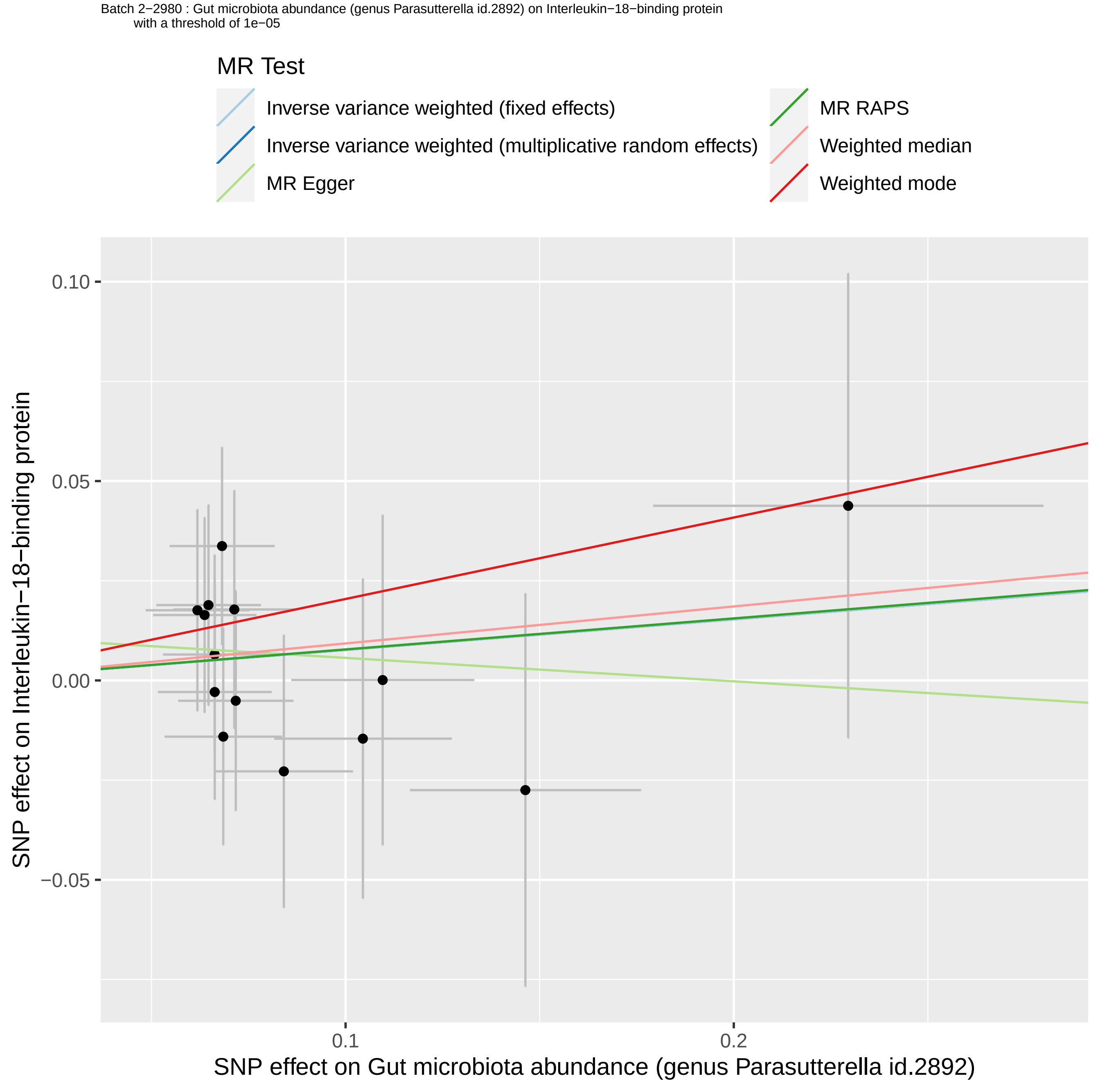
Batch 2−2980 : Gut microbiota abundance (genus Parasutterella id.2892) on Interleukin−18−binding protein
with a threshold of 1e−05
MR Test
Inverse variance weighted (fixed effects)
Inverse variance weighted (multiplicative random effects)
MR Egger
MR RAPS
Weighted median
Weighted mode
0.10
0.05
0.00
−0.05
0.1
0.2
SNP effect on Interleukin−18−binding protein
SNP effect on Gut microbiota abundance (genus Parasutterella id.2892)

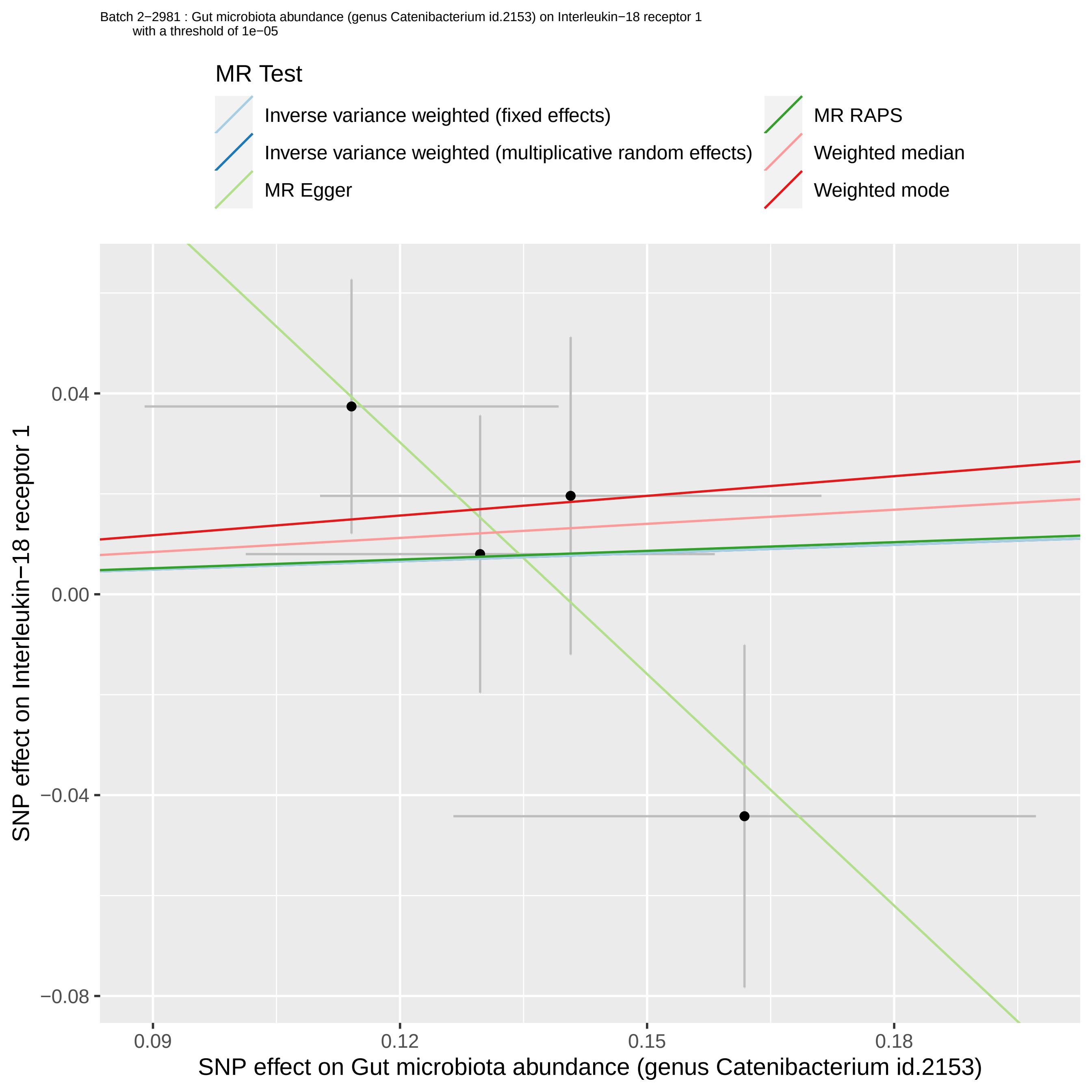
Batch 2−2981 : Gut microbiota abundance (genus Catenibacterium id.2153) on Interleukin−18 receptor 1
with a threshold of 1e−05
MR Test
Inverse variance weighted (fixed effects)
Inverse variance weighted (multiplicative random effects)
MR Egger
MR RAPS
Weighted median
Weighted mode
0.04
0.00
−0.04
−0.08
0.09
0.12
0.15
0.18
SNP effect on Interleukin−18 receptor 1
SNP effect on Gut microbiota abundance (genus Catenibacterium id.2153)

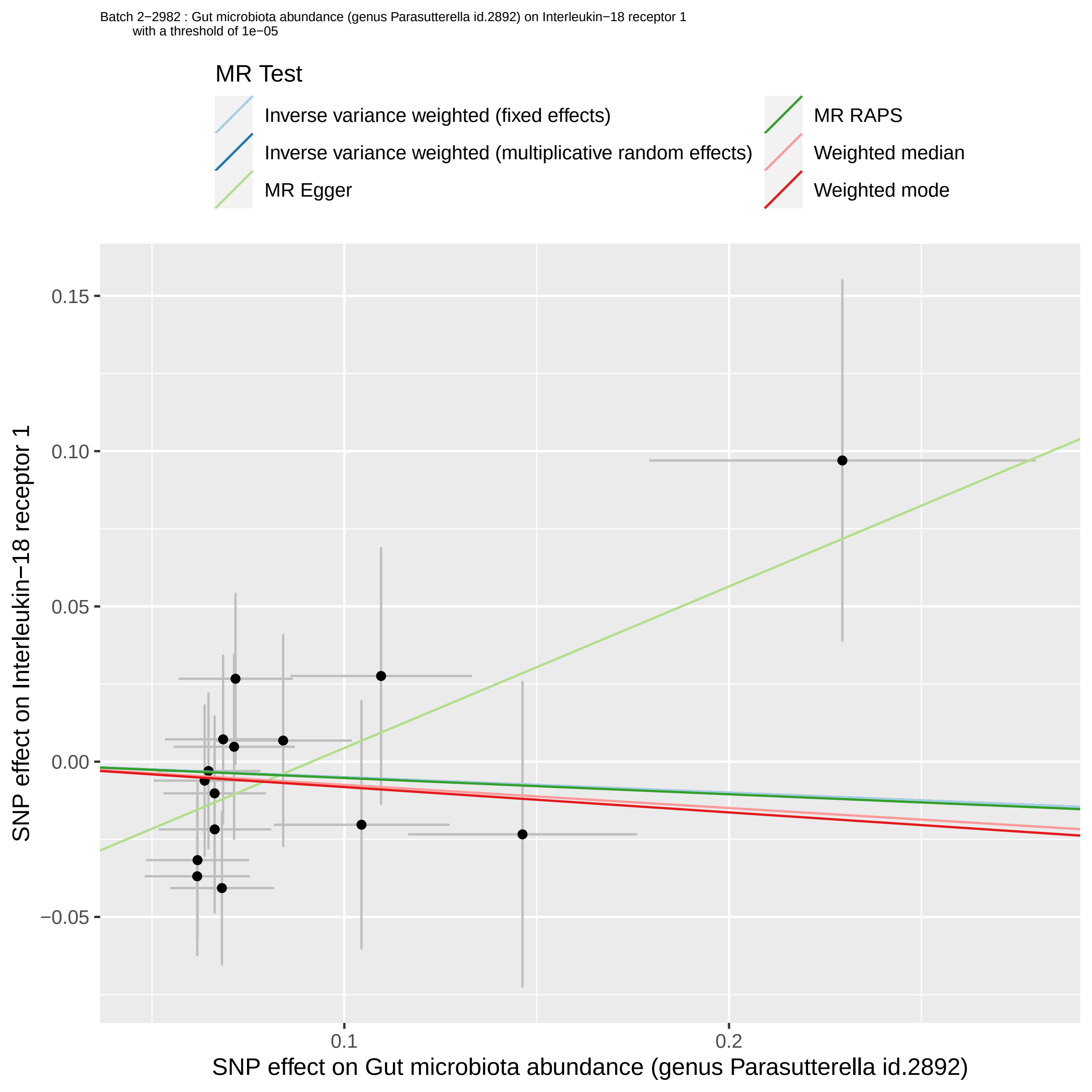

Batch 2−2982 : Gut microbiota abundance (genus Parasutterella id.2892) on Interleukin−18 receptor 1
with a threshold of 1e−05
MR Test
Inverse variance weighted (fixed effects)
Inverse variance weighted (multiplicative random effects)
MR Egger
MR RAPS
Weighted median
Weighted mode
0.15
0.10
0.05
0.00
−0.05
0.1
0.2
SNP effect on Interleukin−18 receptor 1
SNP effect on Gut microbiota abundance (genus Parasutterella id.2892)

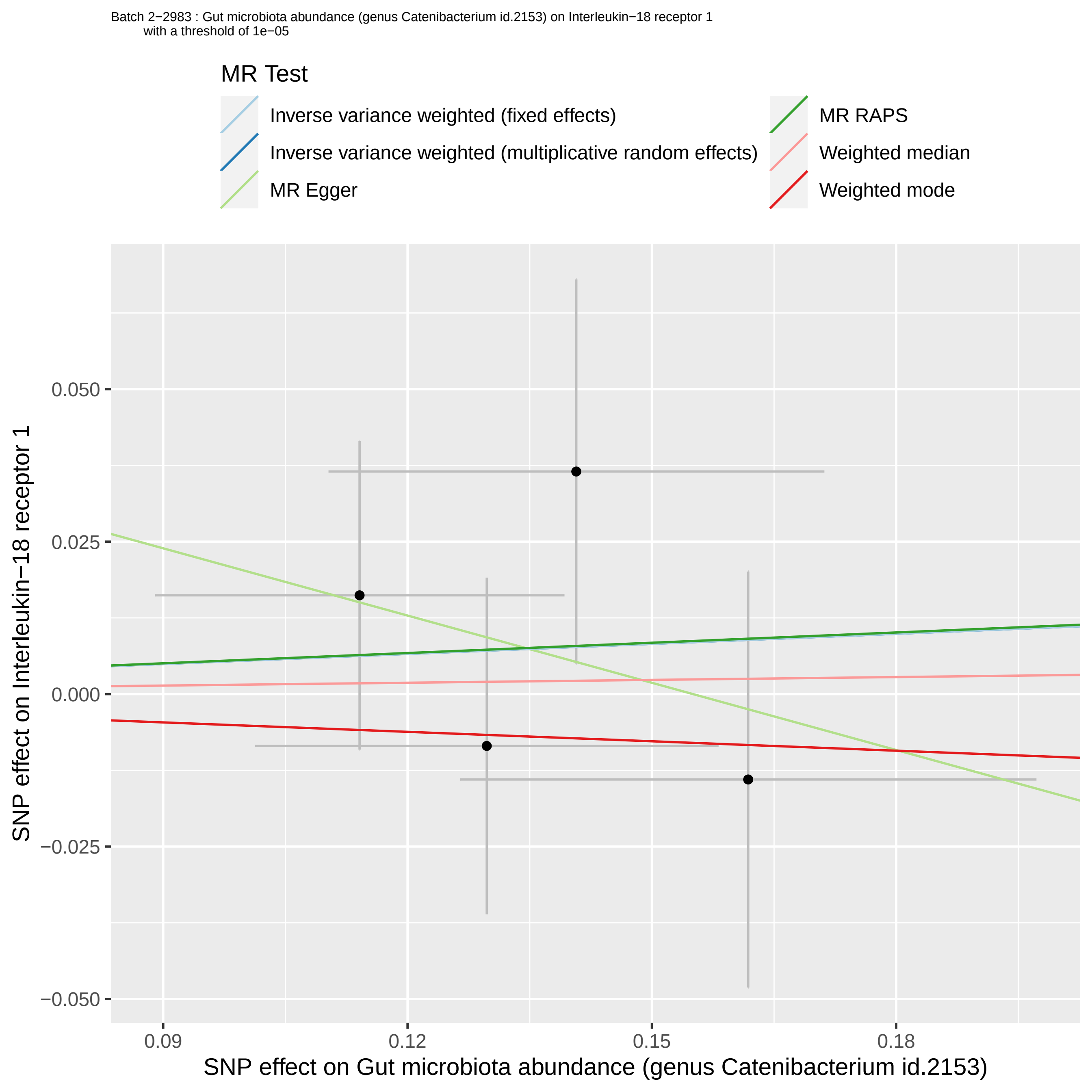
Batch 2−2983 : Gut microbiota abundance (genus Catenibacterium id.2153) on Interleukin−18 receptor 1
with a threshold of 1e−05
MR Test
Inverse variance weighted (fixed effects)
Inverse variance weighted (multiplicative random effects)
MR Egger
MR RAPS
Weighted median
Weighted mode
0.050
0.025
0.000
−0.025
−0.050
0.09
0.12
0.15
0.18
SNP effect on Interleukin−18 receptor 1
SNP effect on Gut microbiota abundance (genus Catenibacterium id.2153)

Batch 2−2984 : Gut microbiota abundance (genus Parasutterella id.2892) on Interleukin−18 receptor 1
with a threshold of 1e−05

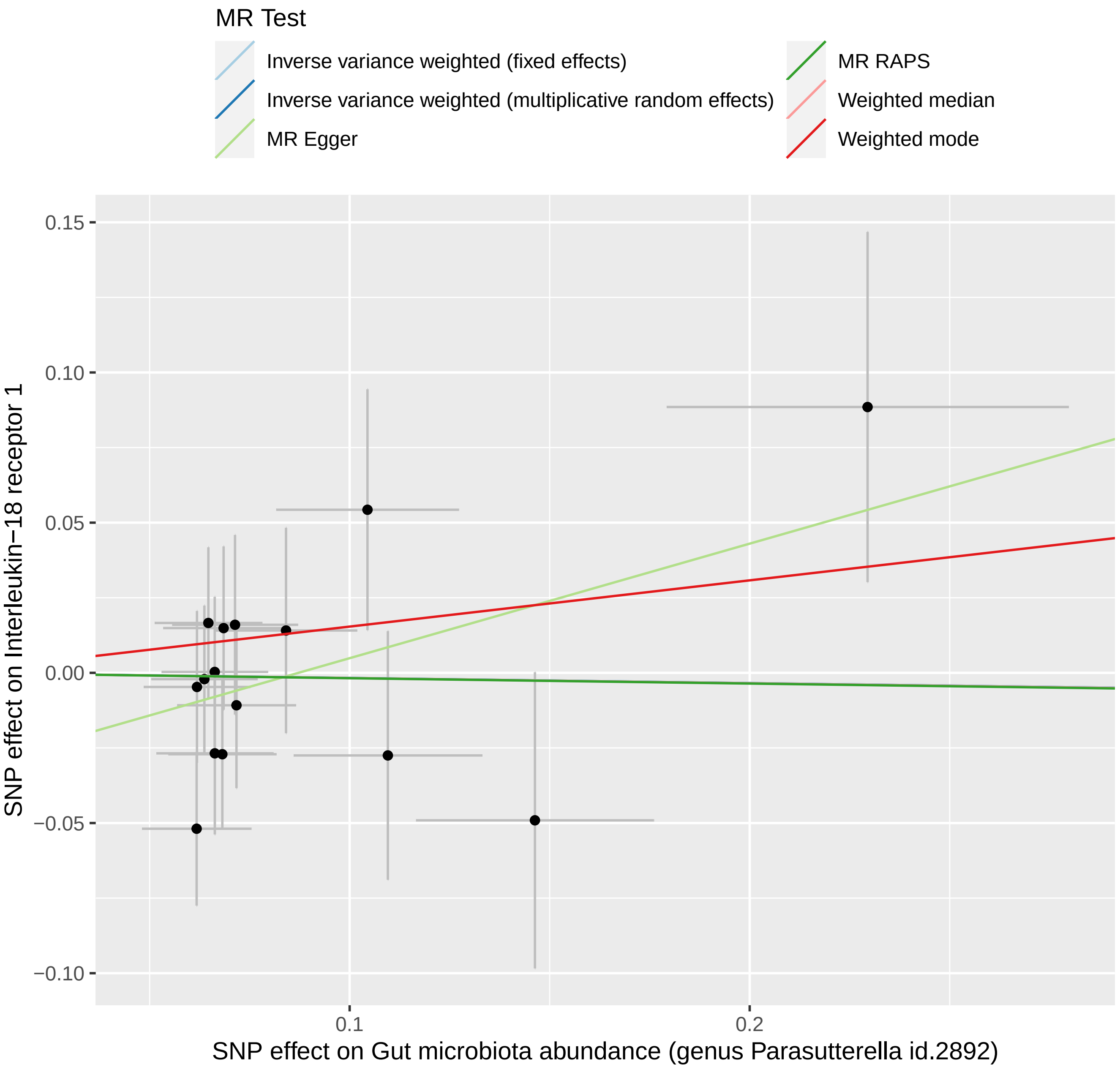

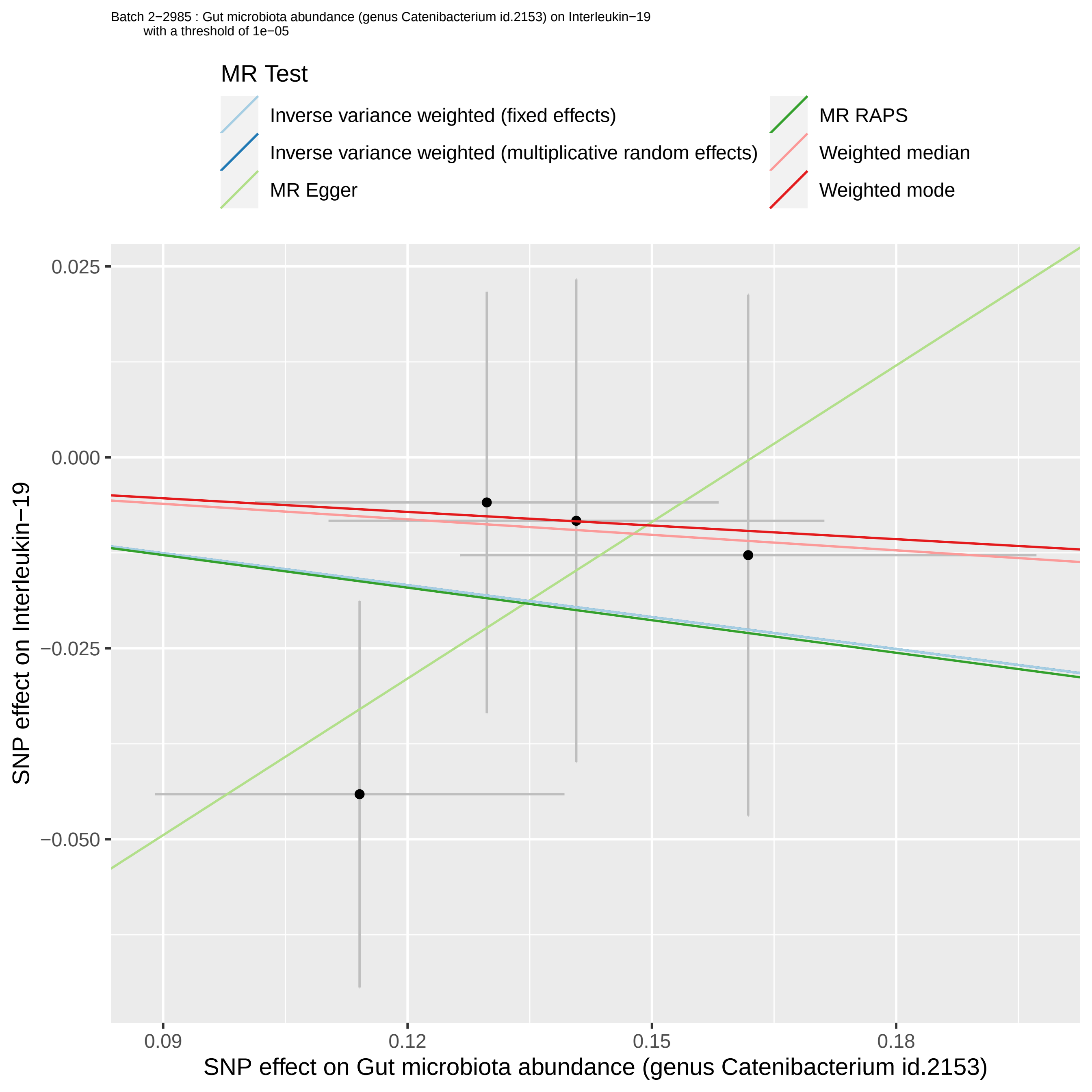

Batch 2−2985 : Gut microbiota abundance (genus Catenibacterium id.2153) on Interleukin−19
with a threshold of 1e−05
MR Test
Inverse variance weighted (fixed effects)
Inverse variance weighted (multiplicative random effects)
MR Egger
MR RAPS
Weighted median
Weighted mode
0.025
0.000
−0.025
−0.050
0.09
0.12
0.15
0.18
SNP effect on Interleukin−19
SNP effect on Gut microbiota abundance (genus Catenibacterium id.2153)

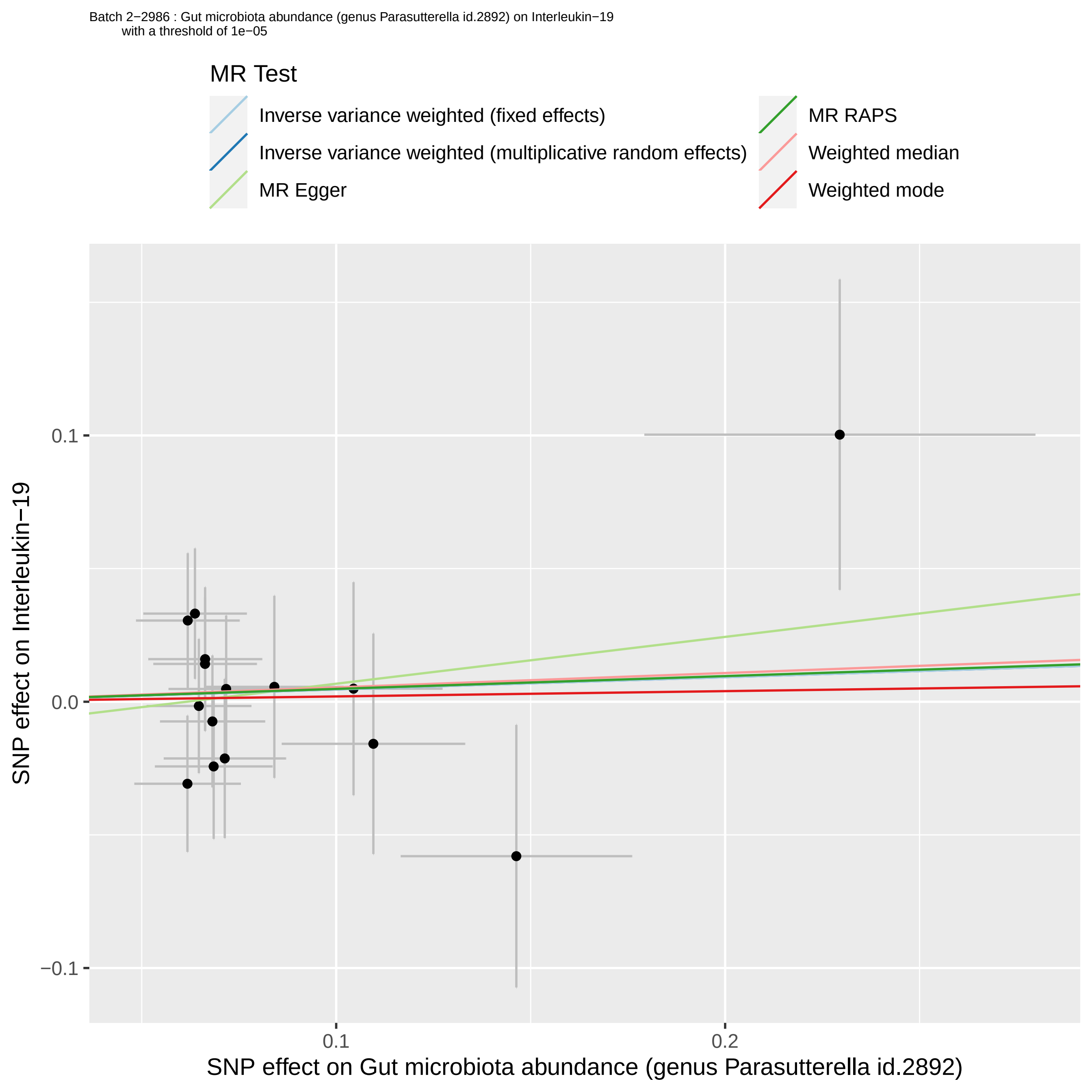

Batch 2−2986 : Gut microbiota abundance (genus Parasutterella id.2892) on Interleukin−19
with a threshold of 1e−05
MR Test
Inverse variance weighted (fixed effects)
Inverse variance weighted (multiplicative random effects)
MR Egger
MR RAPS
Weighted median
Weighted mode
0.1
0.0
−0.1
0.1
0.2
SNP effect on Interleukin−19
SNP effect on Gut microbiota abundance (genus Parasutterella id.2892)

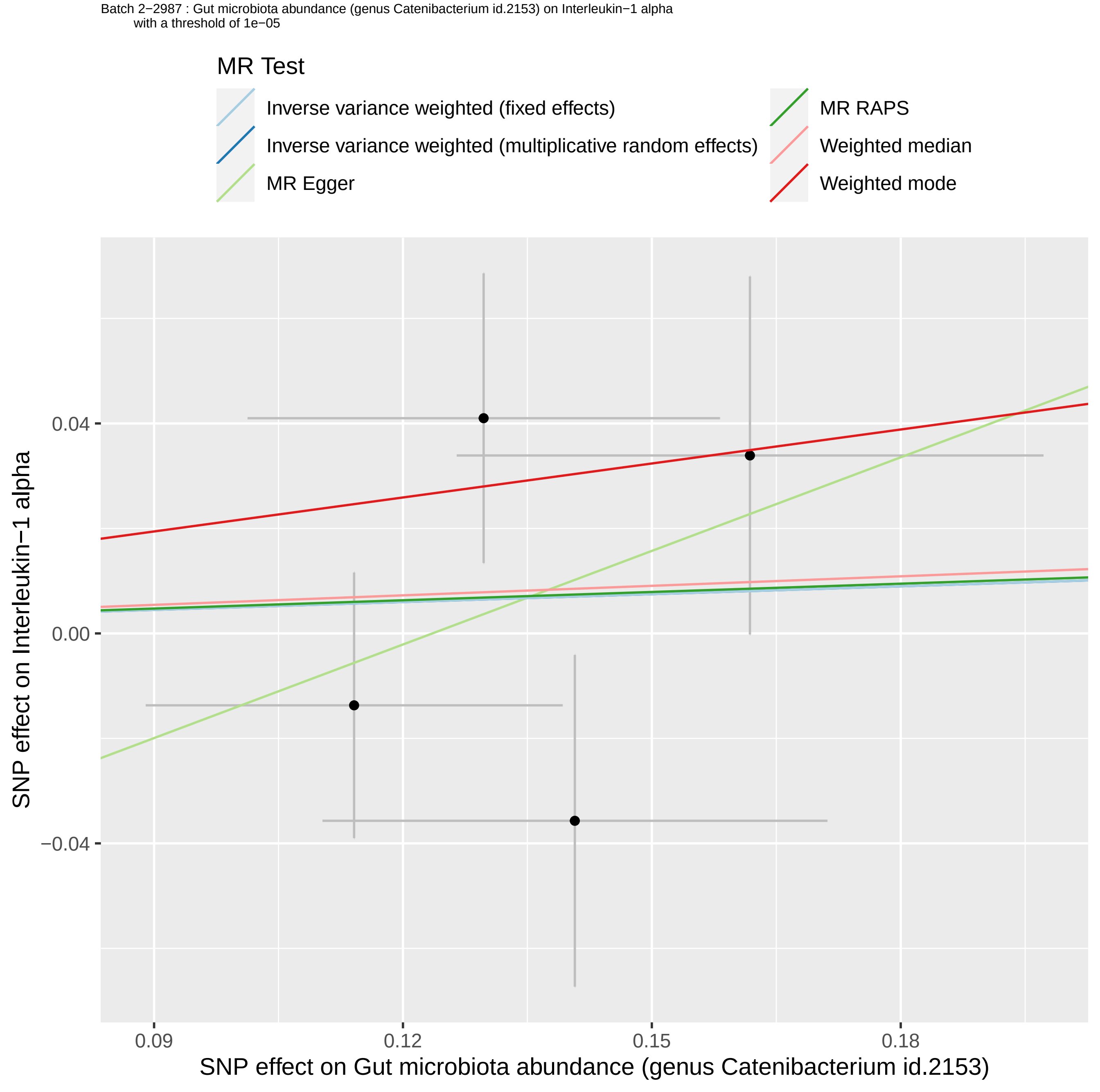

Batch 2−2987 : Gut microbiota abundance (genus Catenibacterium id.2153) on Interleukin−1 alpha
with a threshold of 1e−05
MR Test
Inverse variance weighted (fixed effects)
Inverse variance weighted (multiplicative random effects)
MR Egger
MR RAPS
Weighted median
Weighted mode
0.04
0.00
−0.04
0.09
0.12
0.15
0.18
SNP effect on Interleukin−1 alpha
SNP effect on Gut microbiota abundance (genus Catenibacterium id.2153)

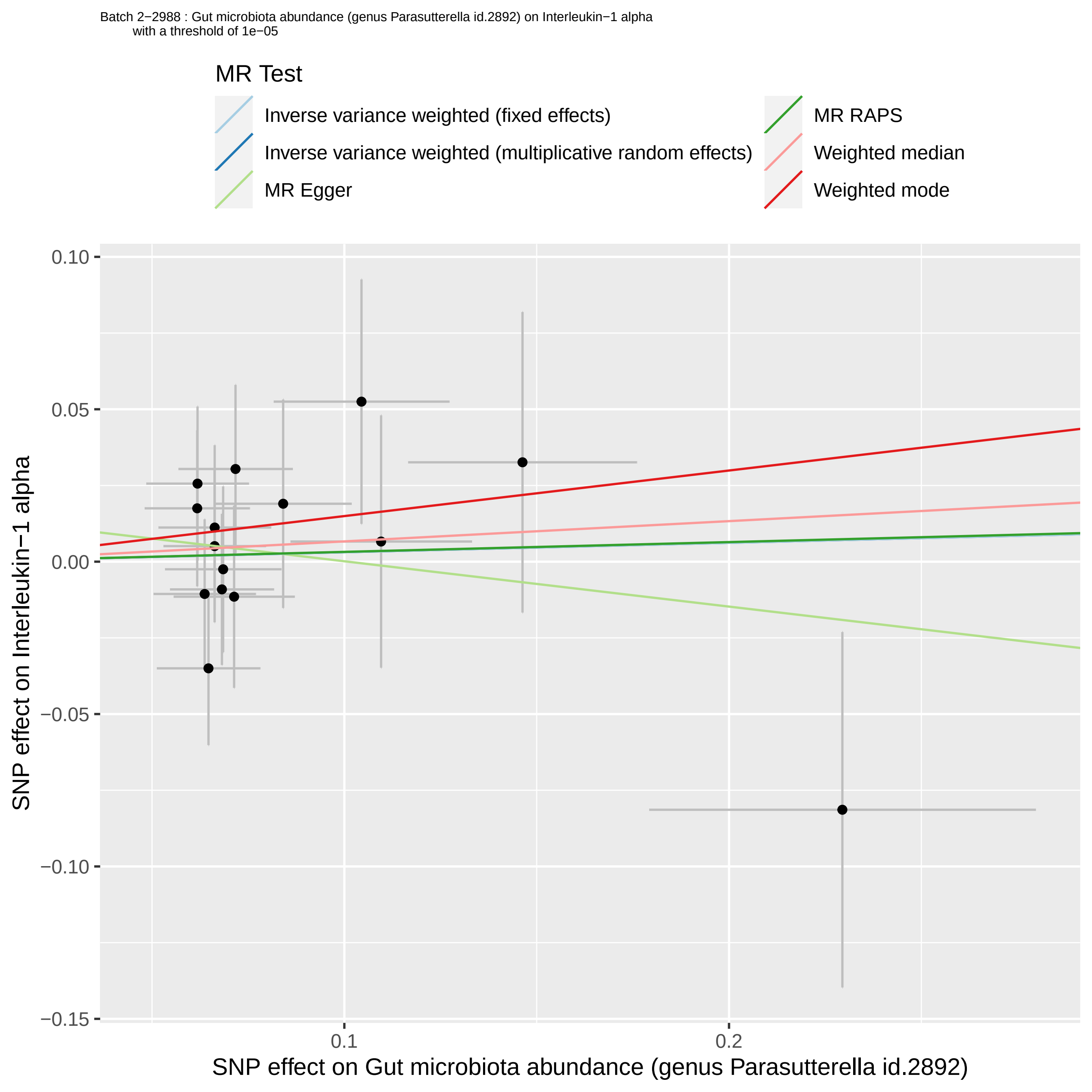

Batch 2−2988 : Gut microbiota abundance (genus Parasutterella id.2892) on Interleukin−1 alpha
with a threshold of 1e−05
MR Test
Inverse variance weighted (fixed effects)
Inverse variance weighted (multiplicative random effects)
MR Egger
MR RAPS
Weighted median
Weighted mode
0.10
0.05
0.00
−0.05
−0.10
−0.15
0.1
0.2
SNP effect on Interleukin−1 alpha
SNP effect on Gut microbiota abundance (genus Parasutterella id.2892)

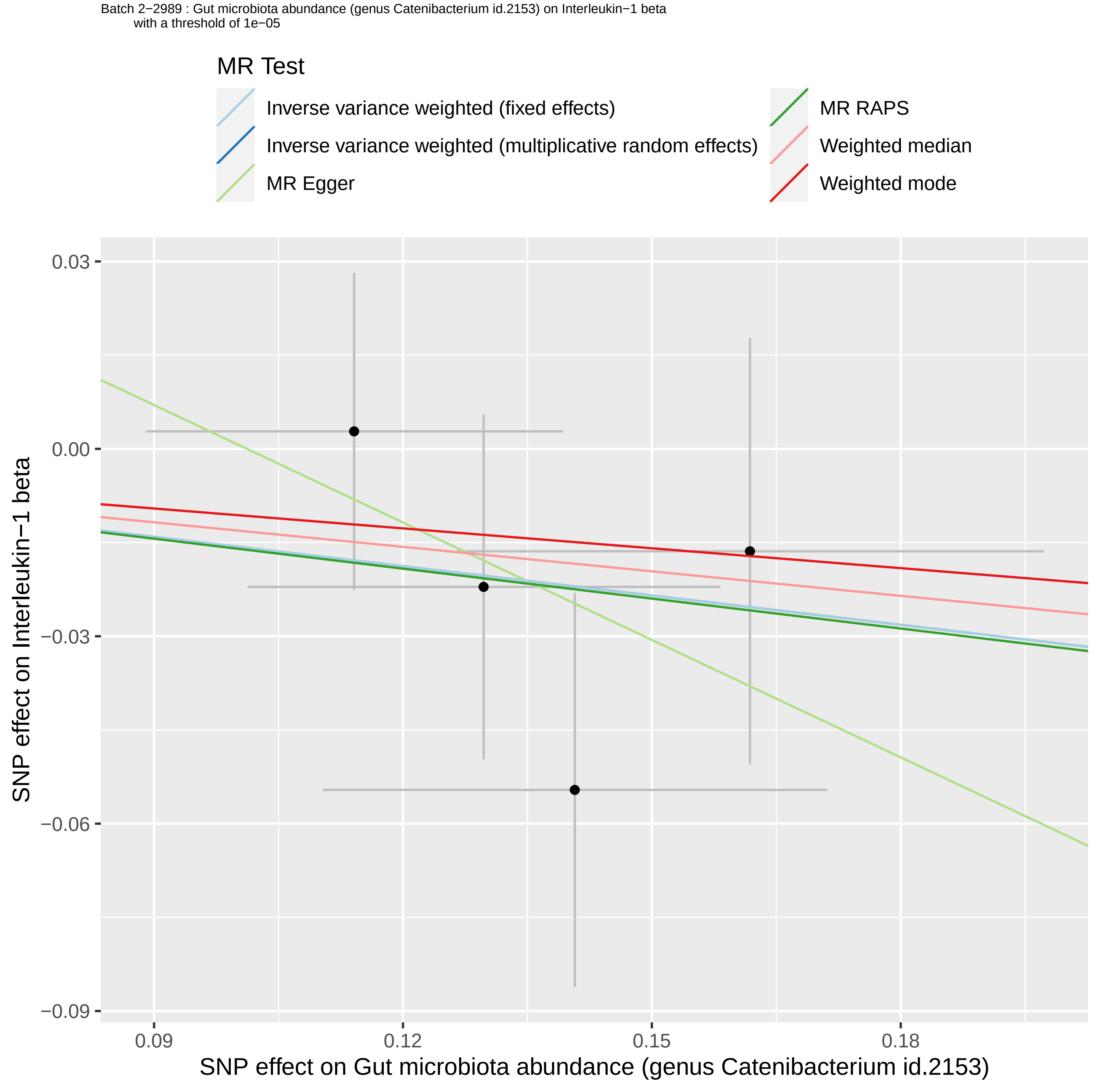
Batch 2−2989 : Gut microbiota abundance (genus Catenibacterium id.2153) on Interleukin−1 beta
with a threshold of 1e−05
MR Test
Inverse variance weighted (fixed effects)
Inverse variance weighted (multiplicative random effects)
MR Egger
MR RAPS
Weighted median
Weighted mode
0.03
0.00
−0.03
−0.06
−0.09
0.09
0.12
0.15
0.18
SNP effect on Interleukin−1 beta
SNP effect on Gut microbiota abundance (genus Catenibacterium id.2153)

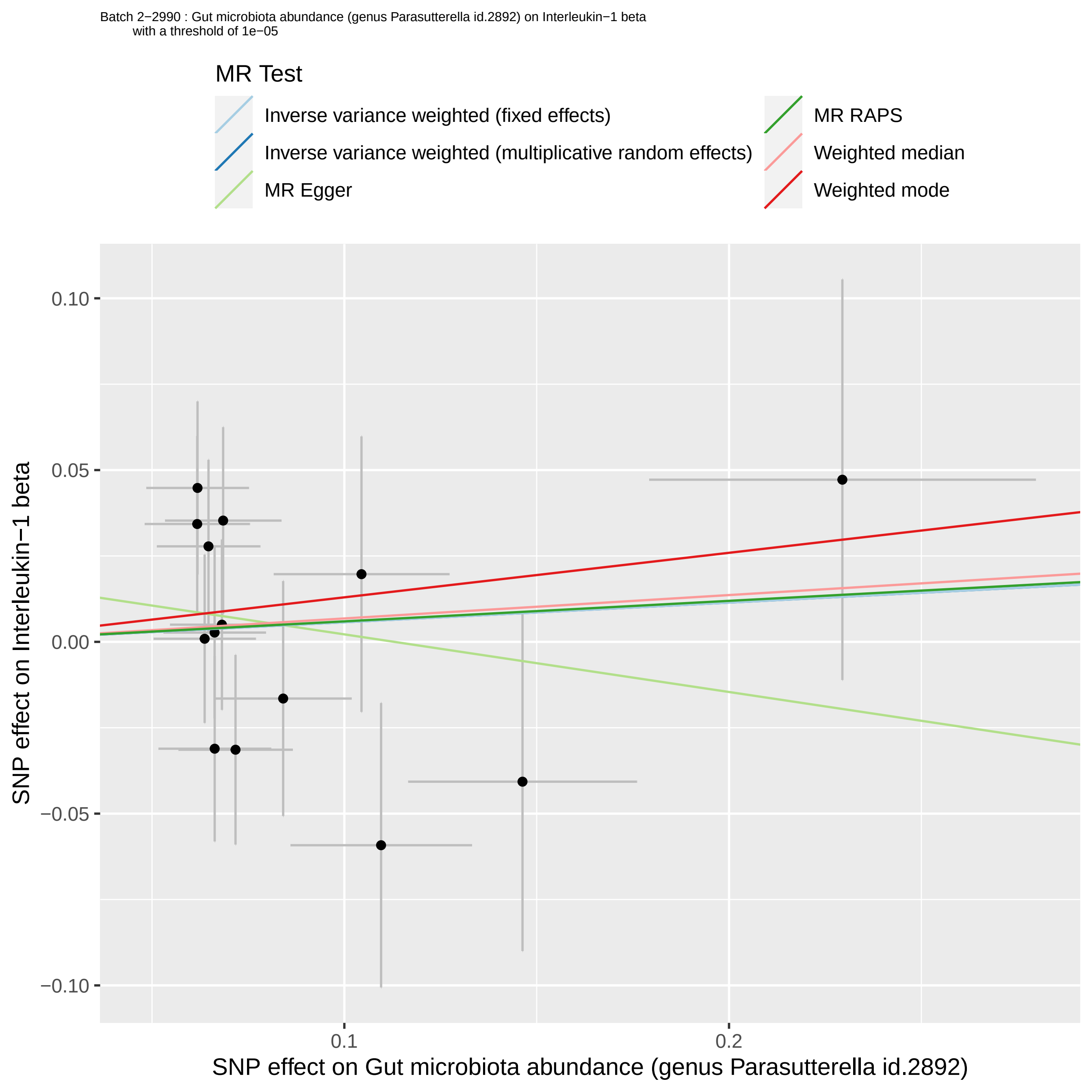
Batch 2−2990 : Gut microbiota abundance (genus Parasutterella id.2892) on Interleukin−1 beta
with a threshold of 1e−05
MR Test
Inverse variance weighted (fixed effects)
Inverse variance weighted (multiplicative random effects)
MR Egger
MR RAPS
Weighted median
Weighted mode
0.10
0.05
0.00
−0.05
−0.10
0.1
0.2
SNP effect on Interleukin−1 beta
SNP effect on Gut microbiota abundance (genus Parasutterella id.2892)

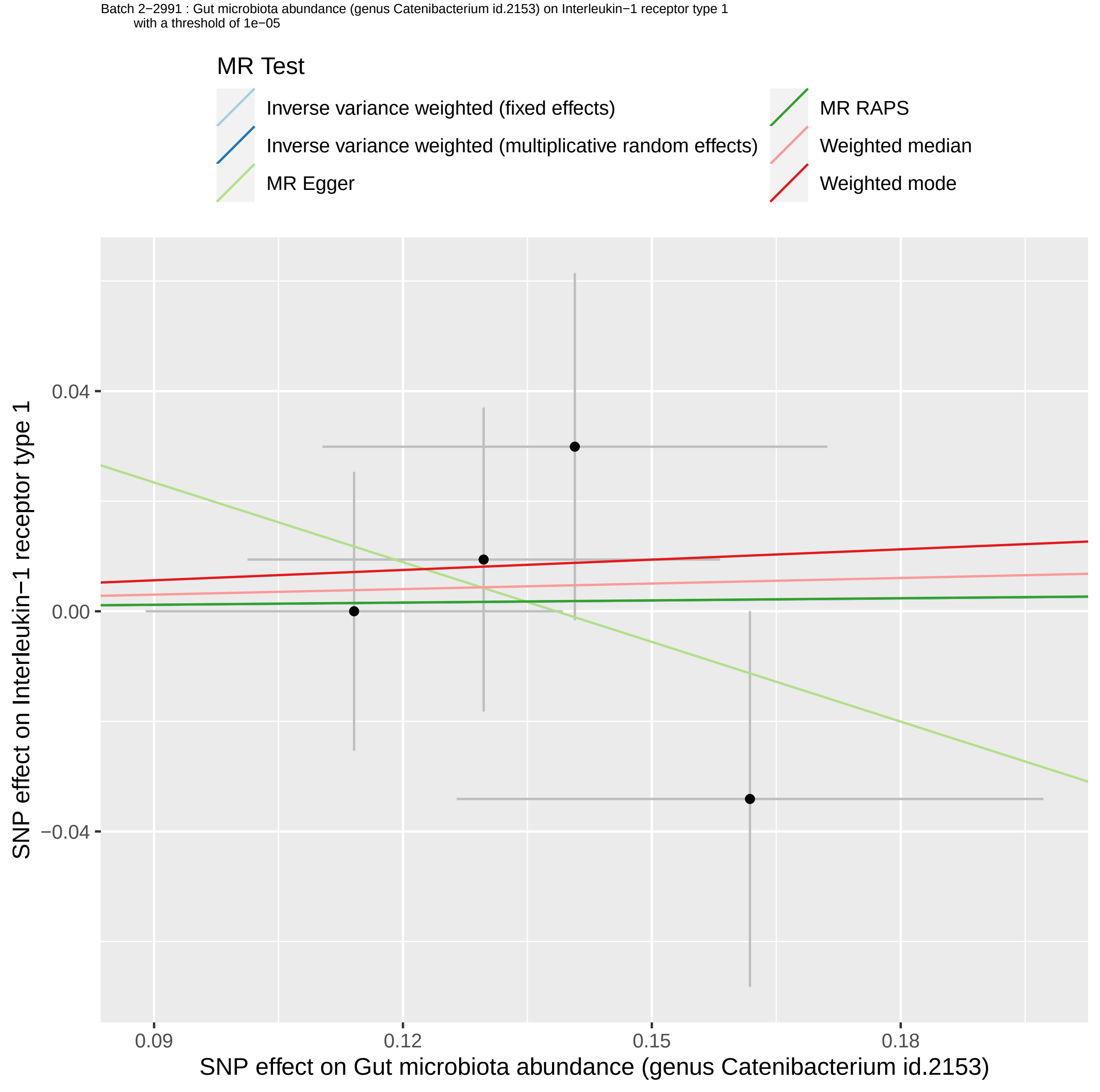
Batch 2−2991 : Gut microbiota abundance (genus Catenibacterium id.2153) on Interleukin−1 receptor type 1
with a threshold of 1e−05
MR Test
Inverse variance weighted (fixed effects)
Inverse variance weighted (multiplicative random effects)
MR Egger
MR RAPS
Weighted median
Weighted mode
0.04
0.00
−0.04
0.09
0.12
0.15
0.18
SNP effect on Interleukin−1 receptor type 1
SNP effect on Gut microbiota abundance (genus Catenibacterium id.2153)

Batch 2−2992 : Gut microbiota abundance (genus Parasutterella id.2892) on Interleukin−1 receptor type 1
with a threshold of 1e−05

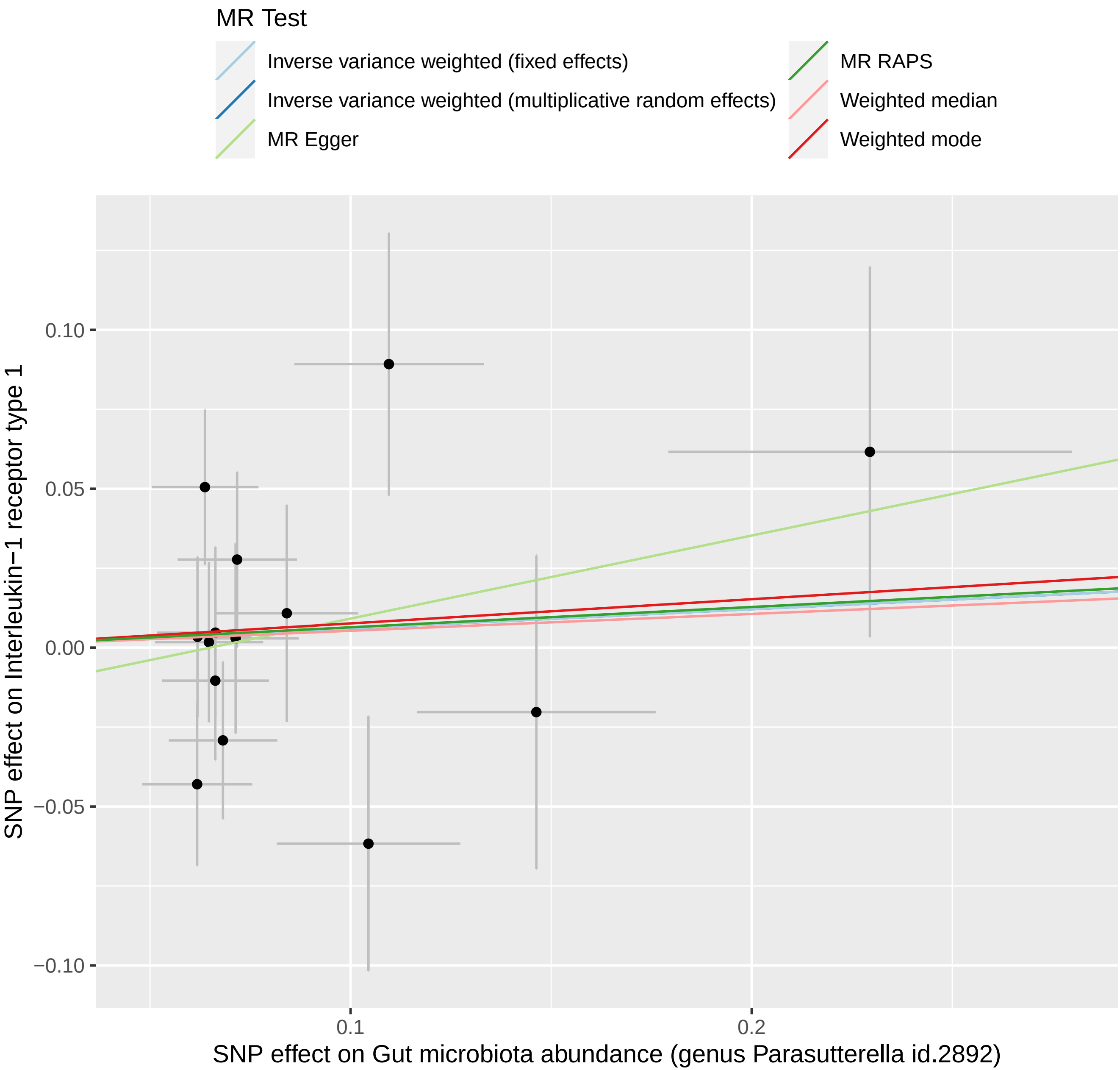

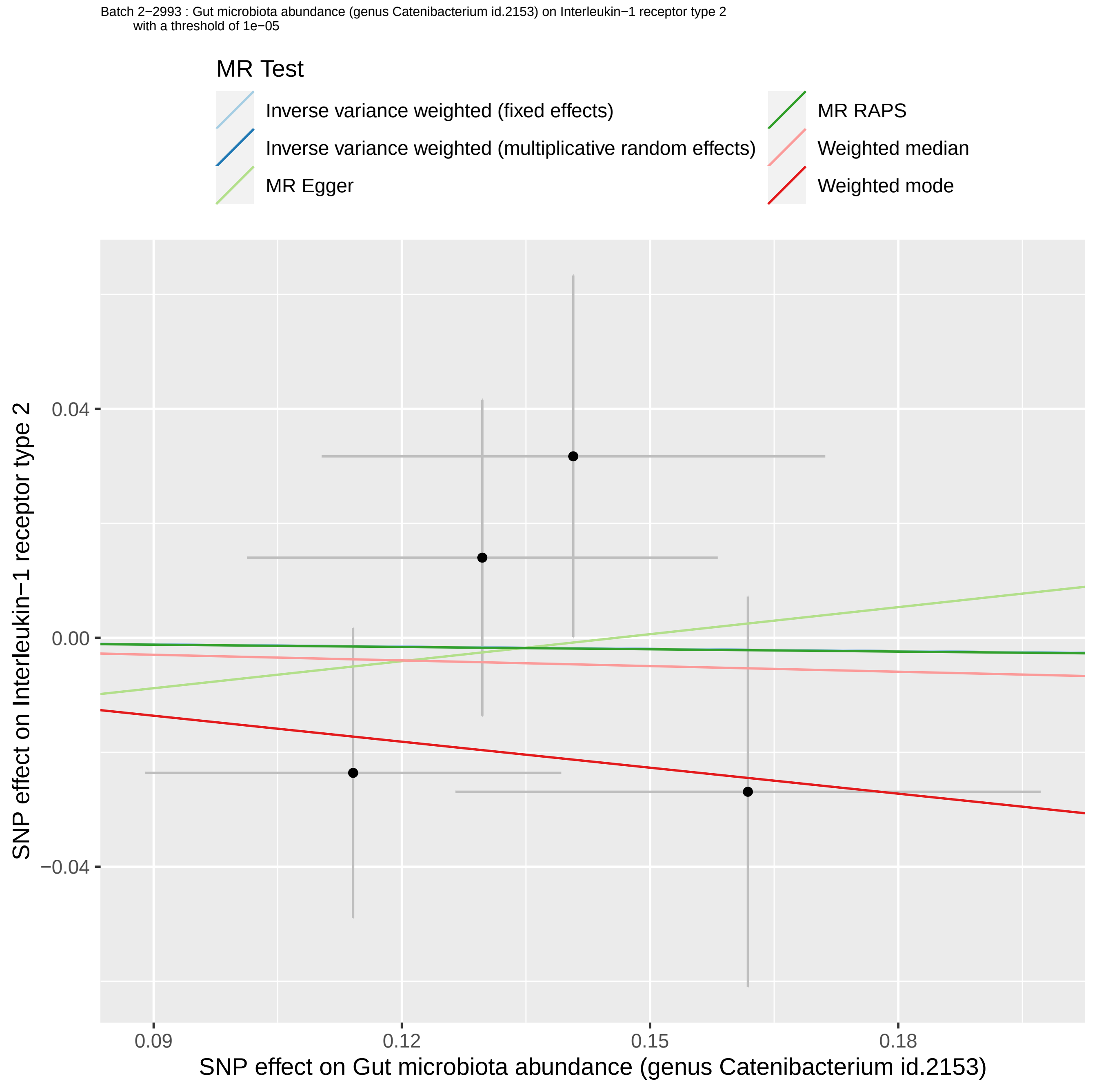
Batch 2−2993 : Gut microbiota abundance (genus Catenibacterium id.2153) on Interleukin−1 receptor type 2
with a threshold of 1e−05
MR Test
Inverse variance weighted (fixed effects)
Inverse variance weighted (multiplicative random effects)
MR Egger
MR RAPS
Weighted median
Weighted mode
0.04
0.00
−0.04
0.09
0.12
0.15
0.18
SNP effect on Interleukin−1 receptor type 2
SNP effect on Gut microbiota abundance (genus Catenibacterium id.2153)

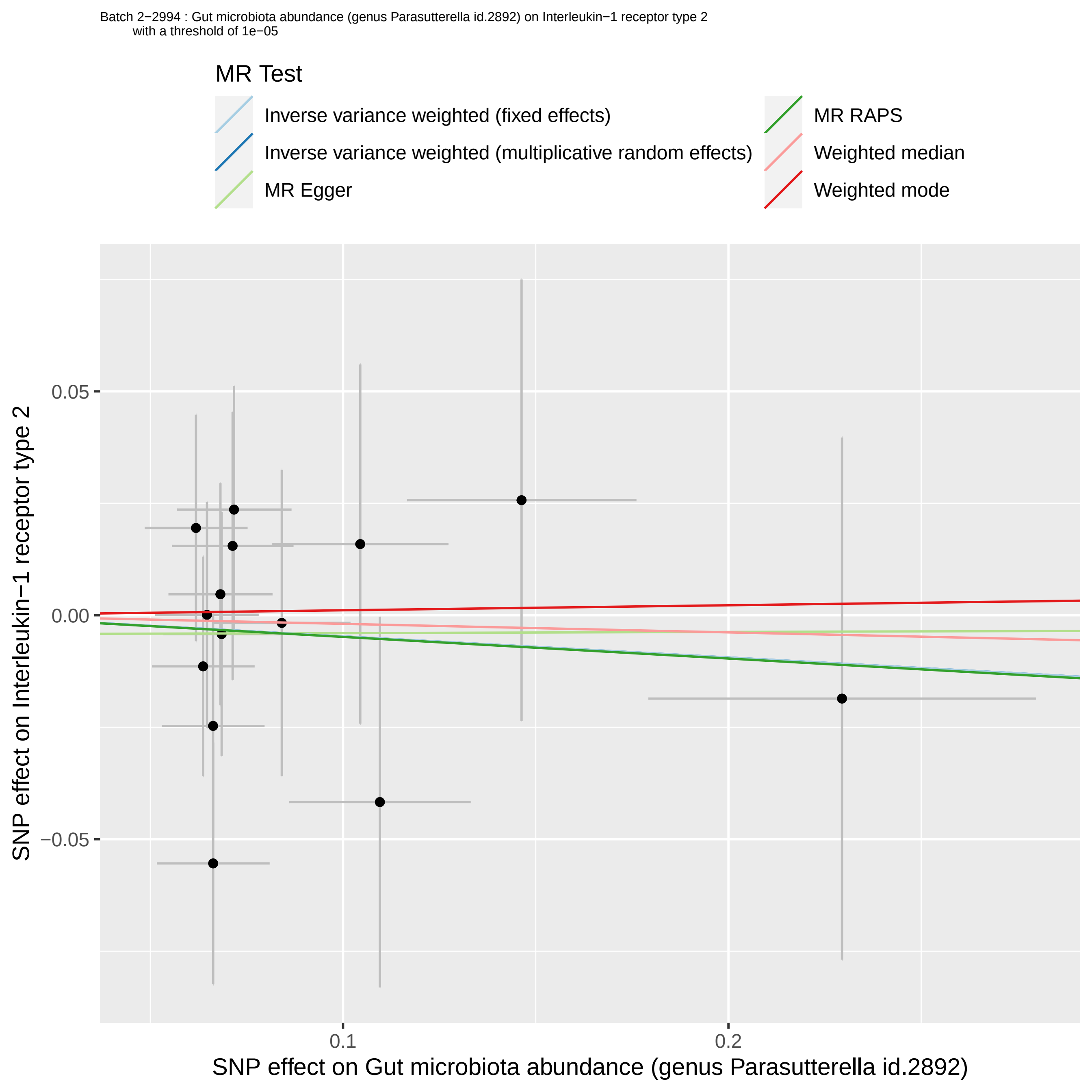
Batch 2−2994 : Gut microbiota abundance (genus Parasutterella id.2892) on Interleukin−1 receptor type 2
with a threshold of 1e−05
MR Test
Inverse variance weighted (fixed effects)
Inverse variance weighted (multiplicative random effects)
MR Egger
MR RAPS
Weighted median
Weighted mode
0.05
0.00
−0.05
0.1
0.2
SNP effect on Interleukin−1 receptor type 2
SNP effect on Gut microbiota abundance (genus Parasutterella id.2892)

Batch 2−2995 : Gut microbiota abundance (genus Catenibacterium id.2153) on Interleukin−1 receptor type 2
with a threshold of 1e−05

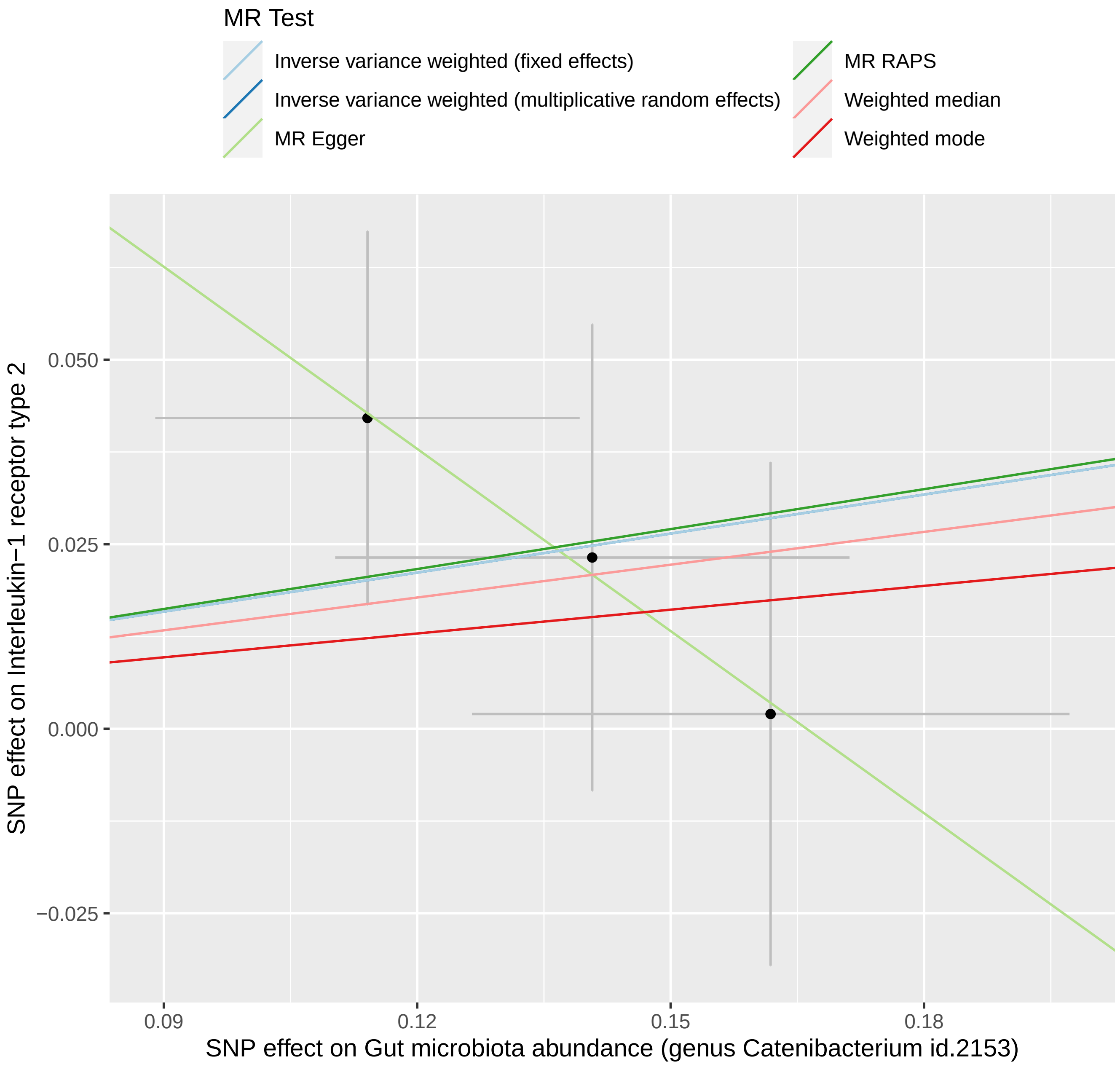

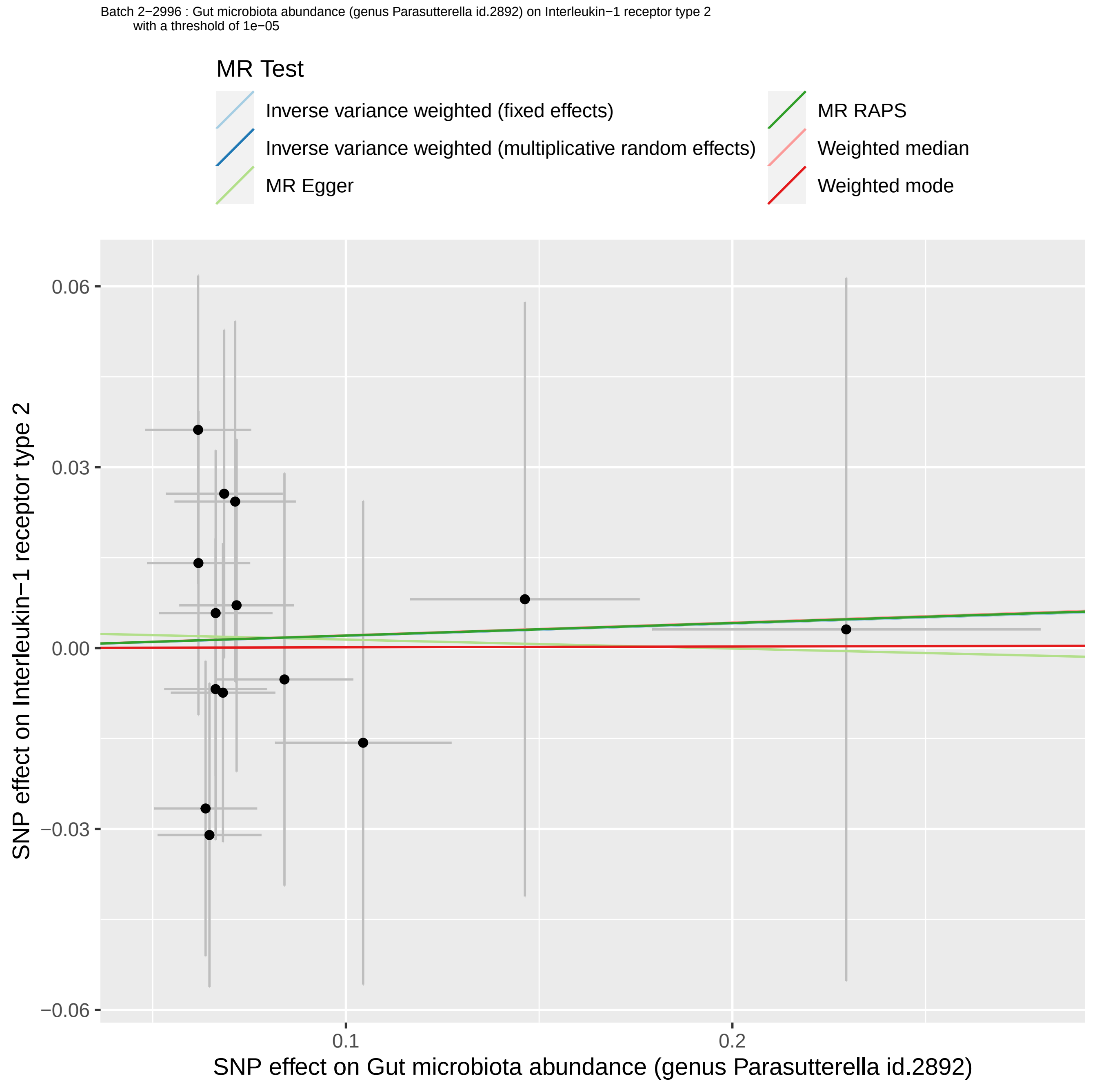

Batch 2−2996 : Gut microbiota abundance (genus Parasutterella id.2892) on Interleukin−1 receptor type 2
with a threshold of 1e−05
MR Test
Inverse variance weighted (fixed effects)
Inverse variance weighted (multiplicative random effects)
MR Egger
MR RAPS
Weighted median
Weighted mode
0.06
0.03
0.00
−0.03
−0.06
0.1
0.2
SNP effect on Interleukin−1 receptor type 2
SNP effect on Gut microbiota abundance (genus Parasutterella id.2892)

Batch 2−2997 : Gut microbiota abundance (genus Catenibacterium id.2153) on Interleukin−1 Receptor accessory protein
with a threshold of 1e−05

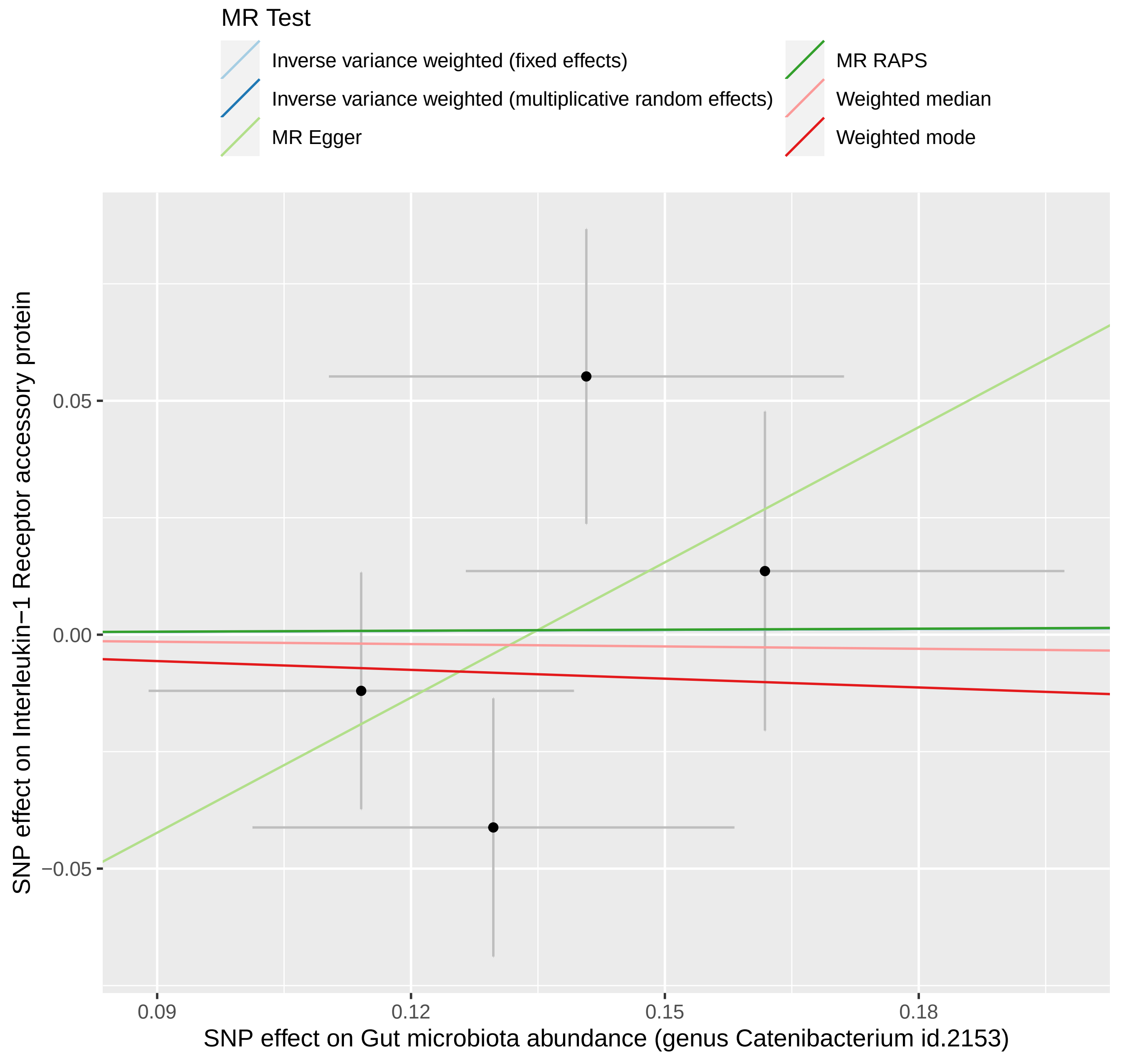

Batch 2−2998 : Gut microbiota abundance (genus Parasutterella id.2892) on Interleukin−1 Receptor accessory protein
with a threshold of 1e−05

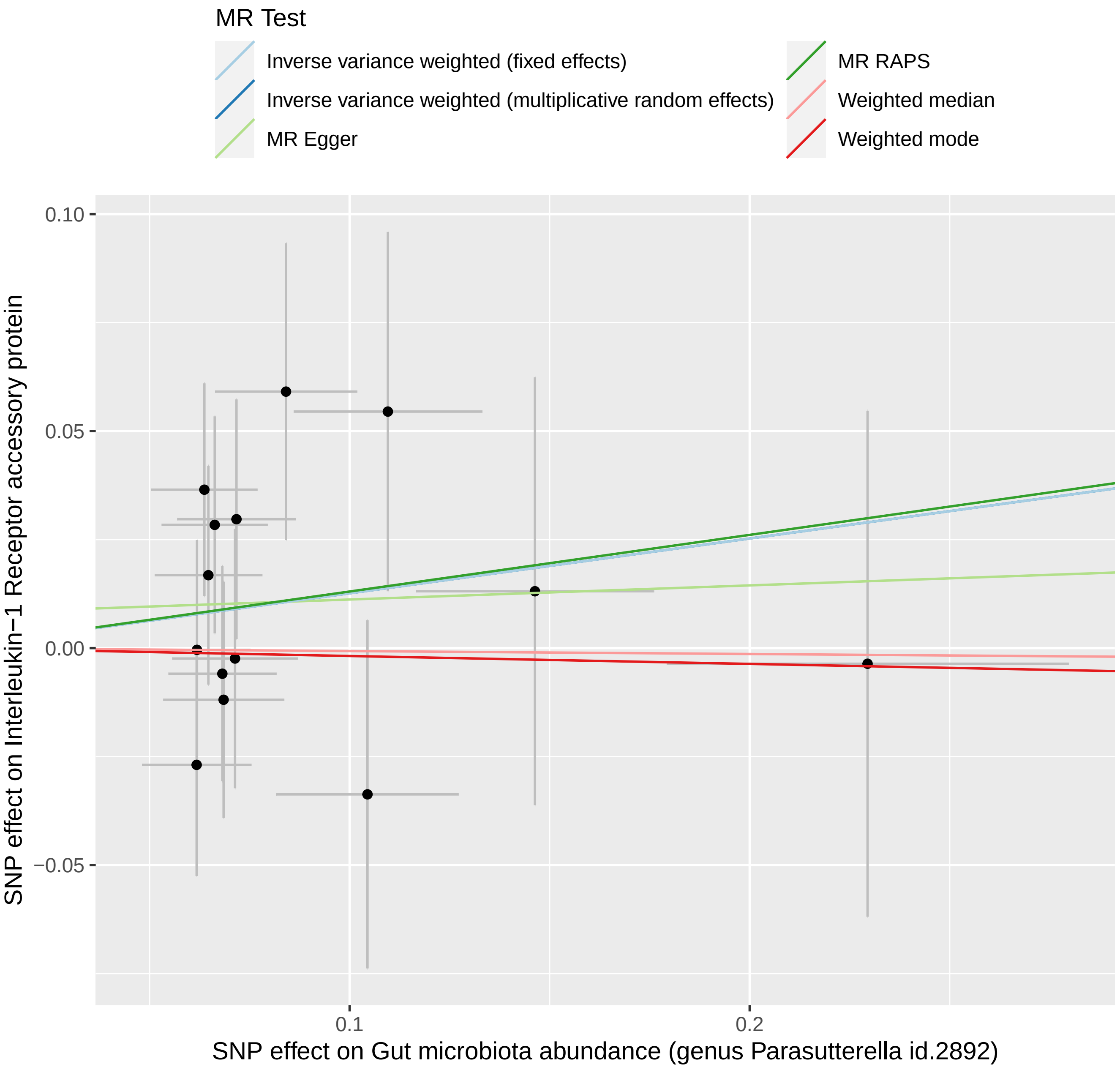

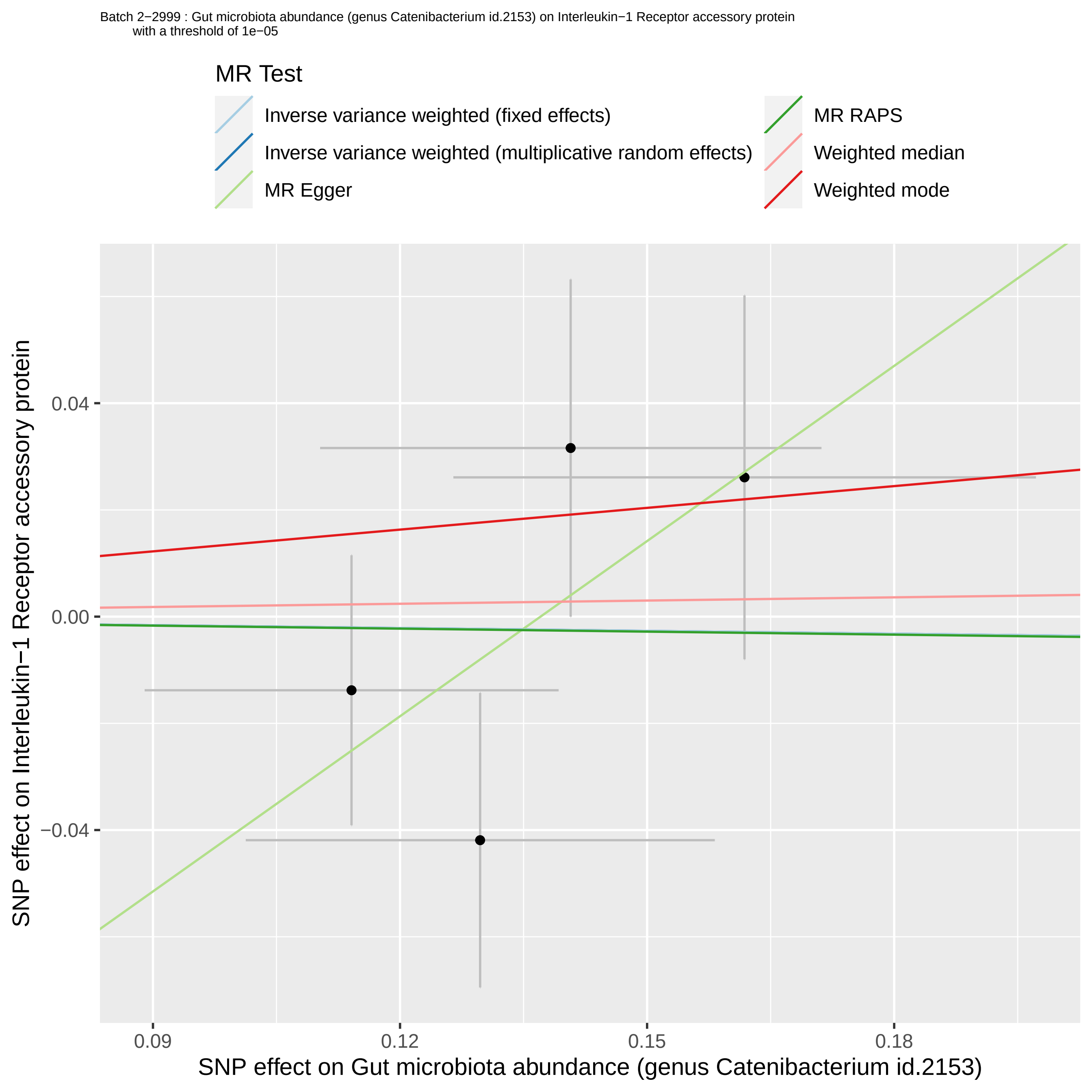

Batch 2−2999 : Gut microbiota abundance (genus Catenibacterium id.2153) on Interleukin−1 Receptor accessory protein
with a threshold of 1e−05
MR Test
Inverse variance weighted (fixed effects)
Inverse variance weighted (multiplicative random effects)
MR Egger
MR RAPS
Weighted median
Weighted mode
0.04
0.00
−0.04
0.09
0.12
0.15
0.18
SNP effect on Interleukin−1 Receptor accessory protein
SNP effect on Gut microbiota abundance (genus Catenibacterium id.2153)

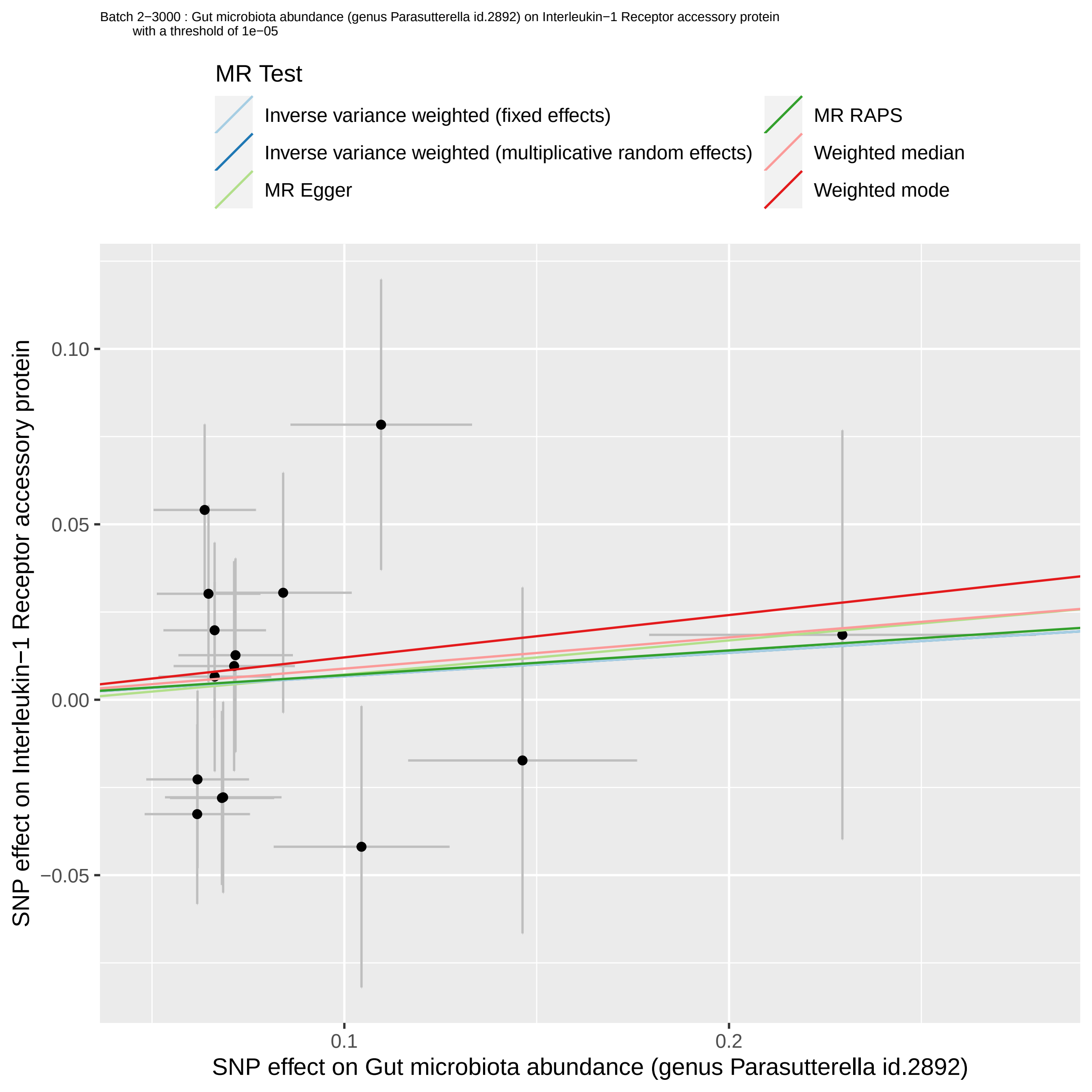
Batch 2−3000 : Gut microbiota abundance (genus Parasutterella id.2892) on Interleukin−1 Receptor accessory protein
with a threshold of 1e−05
MR Test
Inverse variance weighted (fixed effects)
Inverse variance weighted (multiplicative random effects)
MR Egger
MR RAPS
Weighted median
Weighted mode
0.10
0.05
0.00
−0.05
0.1
0.2
SNP effect on Interleukin−1 Receptor accessory protein
SNP effect on Gut microbiota abundance (genus Parasutterella id.2892)

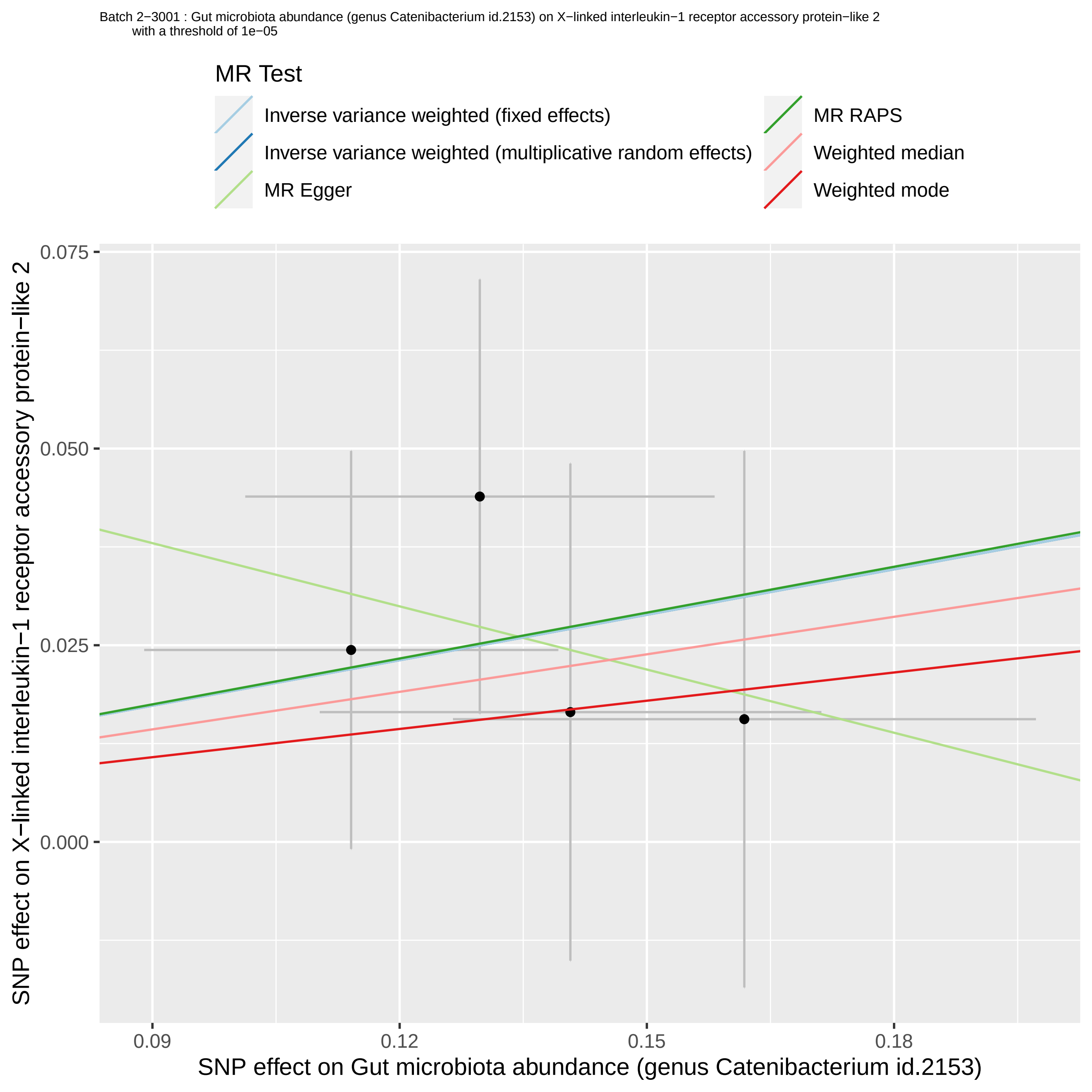

Batch 2−3001 : Gut microbiota abundance (genus Catenibacterium id.2153) on X−linked interleukin−1 receptor accessory protein−like 2
with a threshold of 1e−05
MR Test
Inverse variance weighted (fixed effects)
Inverse variance weighted (multiplicative random effects)
MR Egger
MR RAPS
Weighted median
Weighted mode
0.075
0.050
0.025
0.000
0.09
0.12
0.15
0.18
SNP effect on X−linked interleukin−1 receptor accessory protein−like 2
SNP effect on Gut microbiota abundance (genus Catenibacterium id.2153)

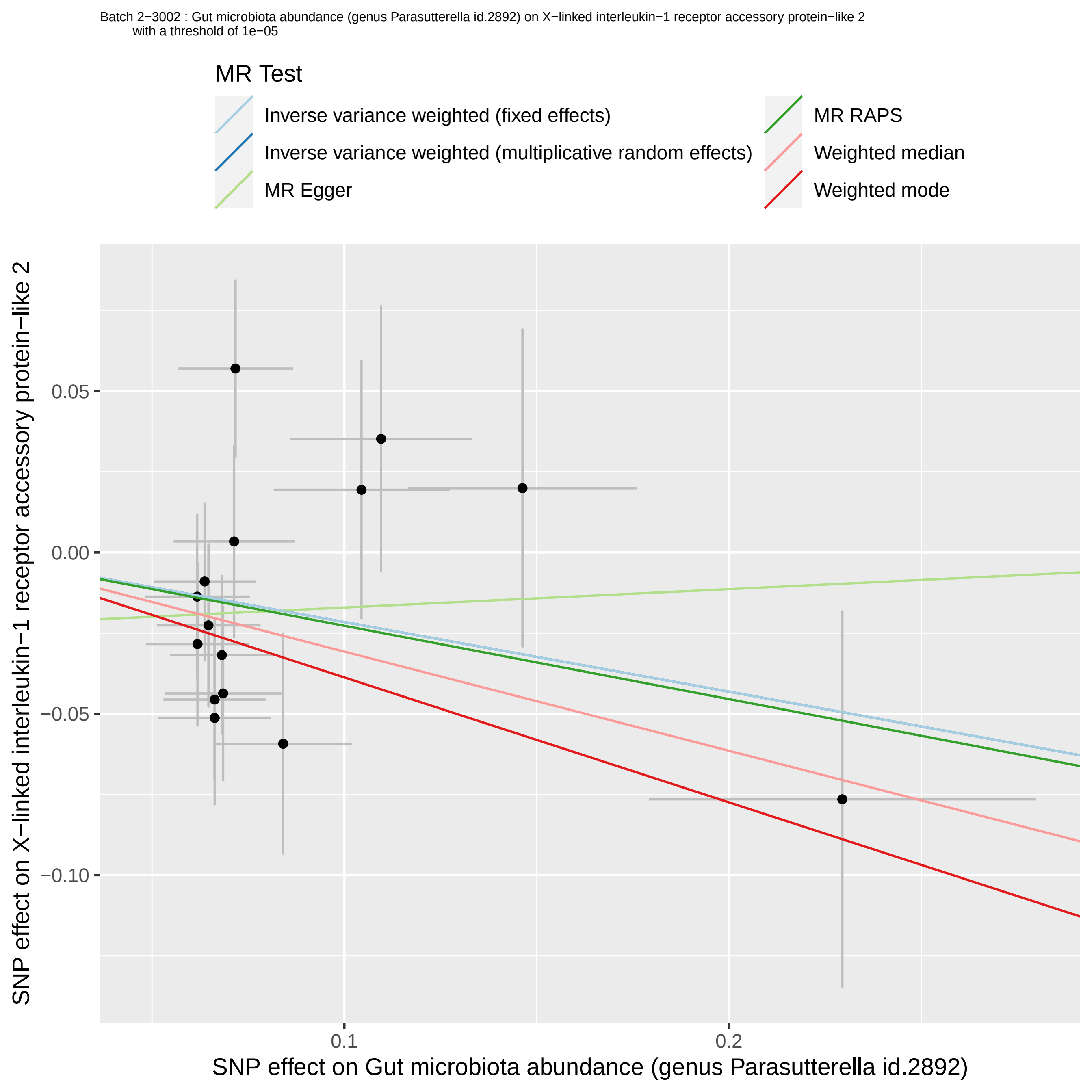

Batch 2−3002 : Gut microbiota abundance (genus Parasutterella id.2892) on X−linked interleukin−1 receptor accessory protein−like 2
with a threshold of 1e−05
MR Test
Inverse variance weighted (fixed effects)
Inverse variance weighted (multiplicative random effects)
MR Egger
MR RAPS
Weighted median
Weighted mode
0.05
0.00
−0.05
−0.10
0.1
0.2
SNP effect on X−linked interleukin−1 receptor accessory protein−like 2
SNP effect on Gut microbiota abundance (genus Parasutterella id.2892)

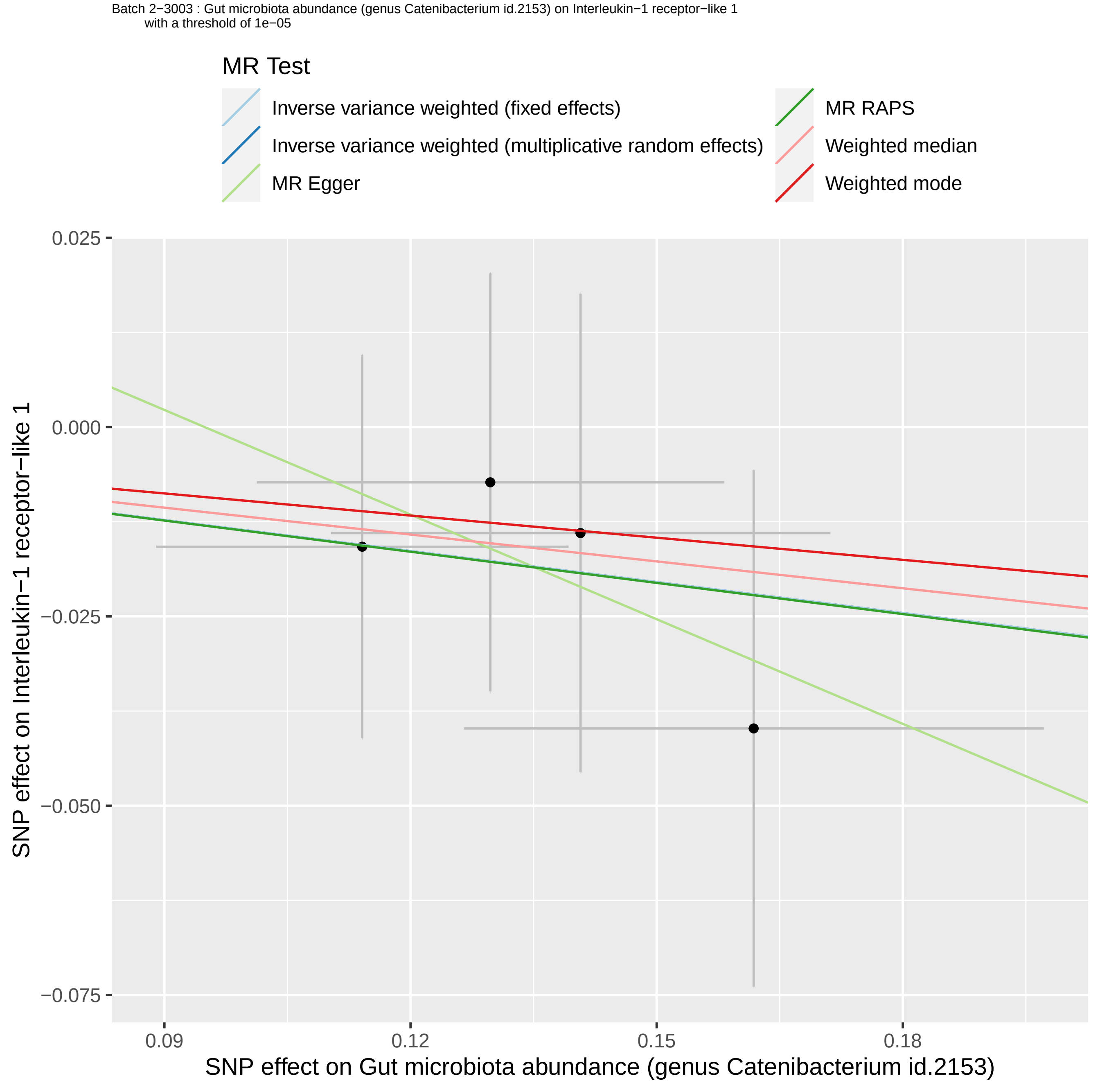
Batch 2−3003 : Gut microbiota abundance (genus Catenibacterium id.2153) on Interleukin−1 receptor−like 1
with a threshold of 1e−05
MR Test
Inverse variance weighted (fixed effects)
Inverse variance weighted (multiplicative random effects)
MR Egger
MR RAPS
Weighted median
Weighted mode
0.025
0.000
−0.025
−0.050
−0.075
0.09
0.12
0.15
0.18
SNP effect on Interleukin−1 receptor−like 1
SNP effect on Gut microbiota abundance (genus Catenibacterium id.2153)

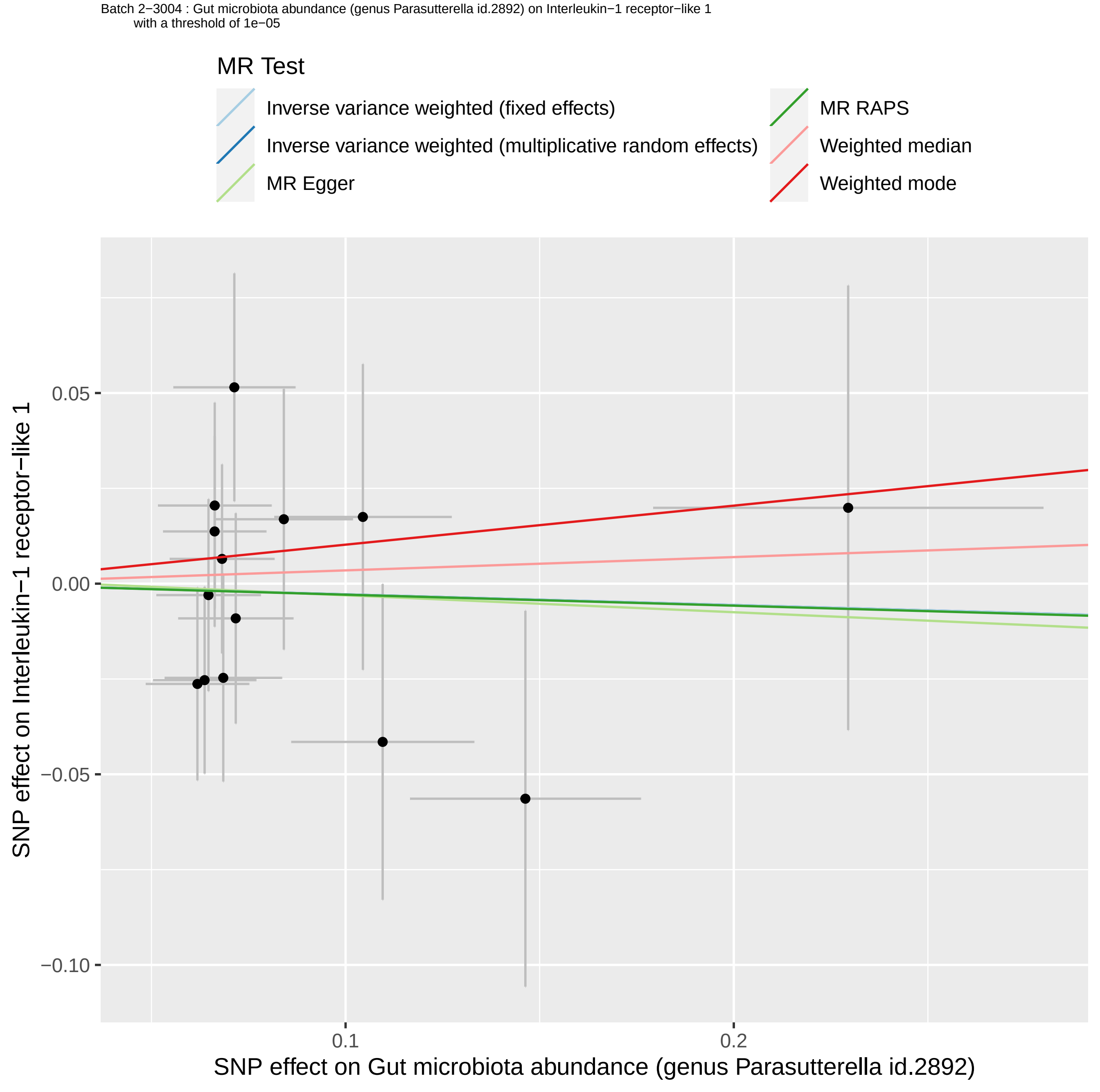
Batch 2−3004 : Gut microbiota abundance (genus Parasutterella id.2892) on Interleukin−1 receptor−like 1
with a threshold of 1e−05
MR Test
Inverse variance weighted (fixed effects)
Inverse variance weighted (multiplicative random effects)
MR Egger
MR RAPS
Weighted median
Weighted mode
0.05
0.00
−0.05
−0.10
0.1
0.2
SNP effect on Interleukin−1 receptor−like 1
SNP effect on Gut microbiota abundance (genus Parasutterella id.2892)

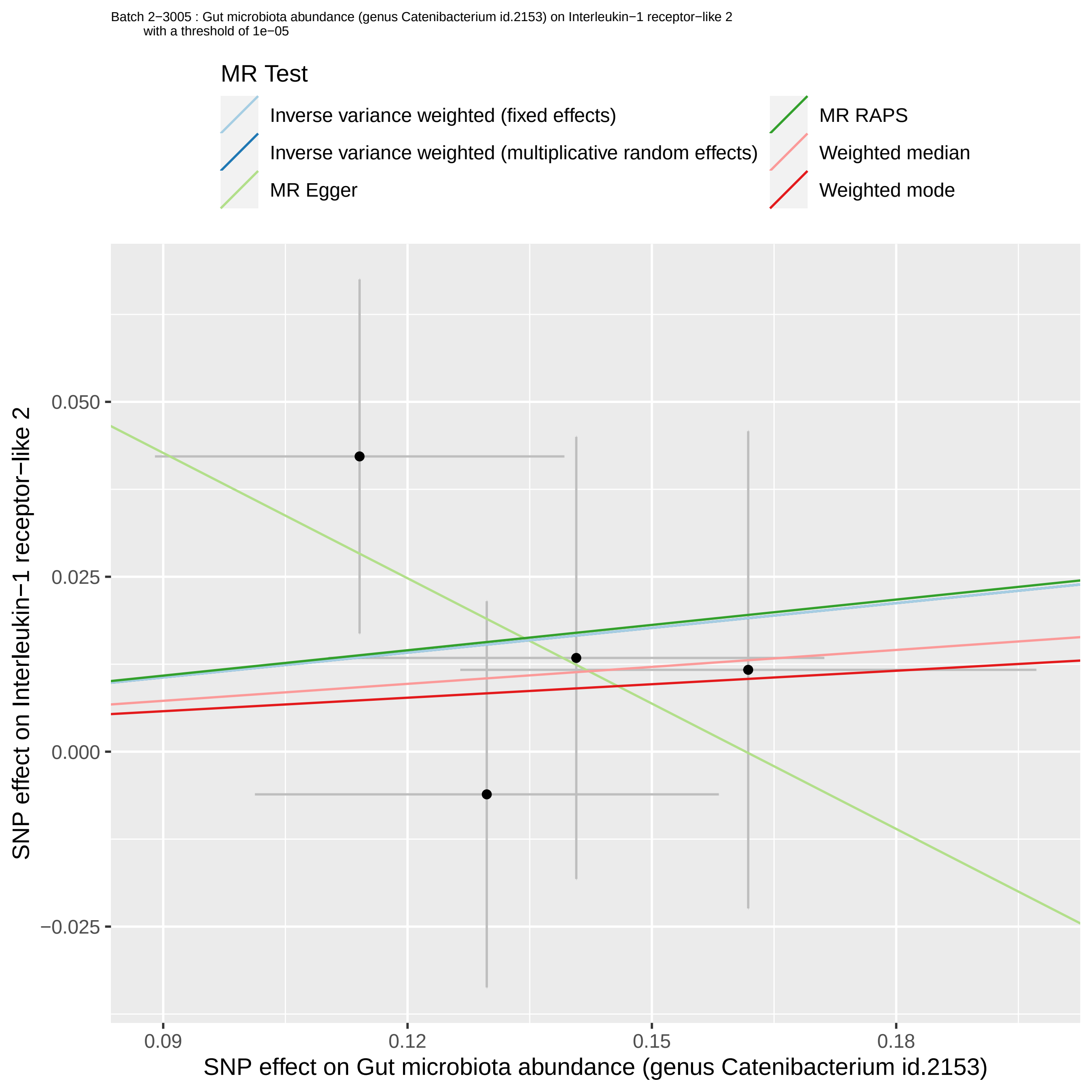
Batch 2−3005 : Gut microbiota abundance (genus Catenibacterium id.2153) on Interleukin−1 receptor−like 2
with a threshold of 1e−05
MR Test
Inverse variance weighted (fixed effects)
Inverse variance weighted (multiplicative random effects)
MR Egger
MR RAPS
Weighted median
Weighted mode
0.050
0.025
0.000
−0.025
0.09
0.12
0.15
0.18
SNP effect on Interleukin−1 receptor−like 2
SNP effect on Gut microbiota abundance (genus Catenibacterium id.2153)

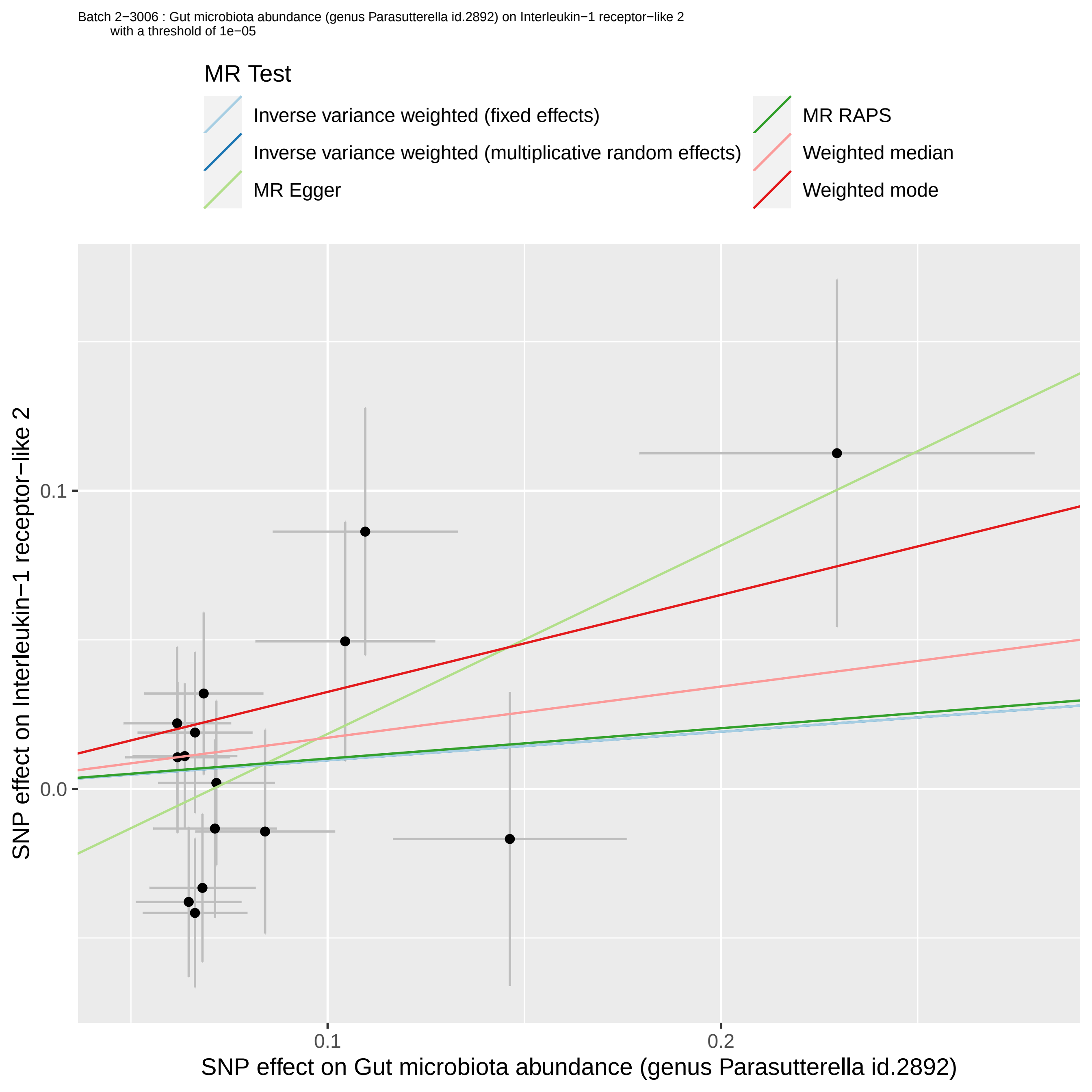

Batch 2−3006 : Gut microbiota abundance (genus Parasutterella id.2892) on Interleukin−1 receptor−like 2
with a threshold of 1e−05
MR Test
Inverse variance weighted (fixed effects)
Inverse variance weighted (multiplicative random effects)
MR Egger
MR RAPS
Weighted median
Weighted mode
SNP effect on Interleukin−1 receptor−like 2
0.1
0.0
0.1
0.2
SNP effect on Gut microbiota abundance (genus Parasutterella id.2892)

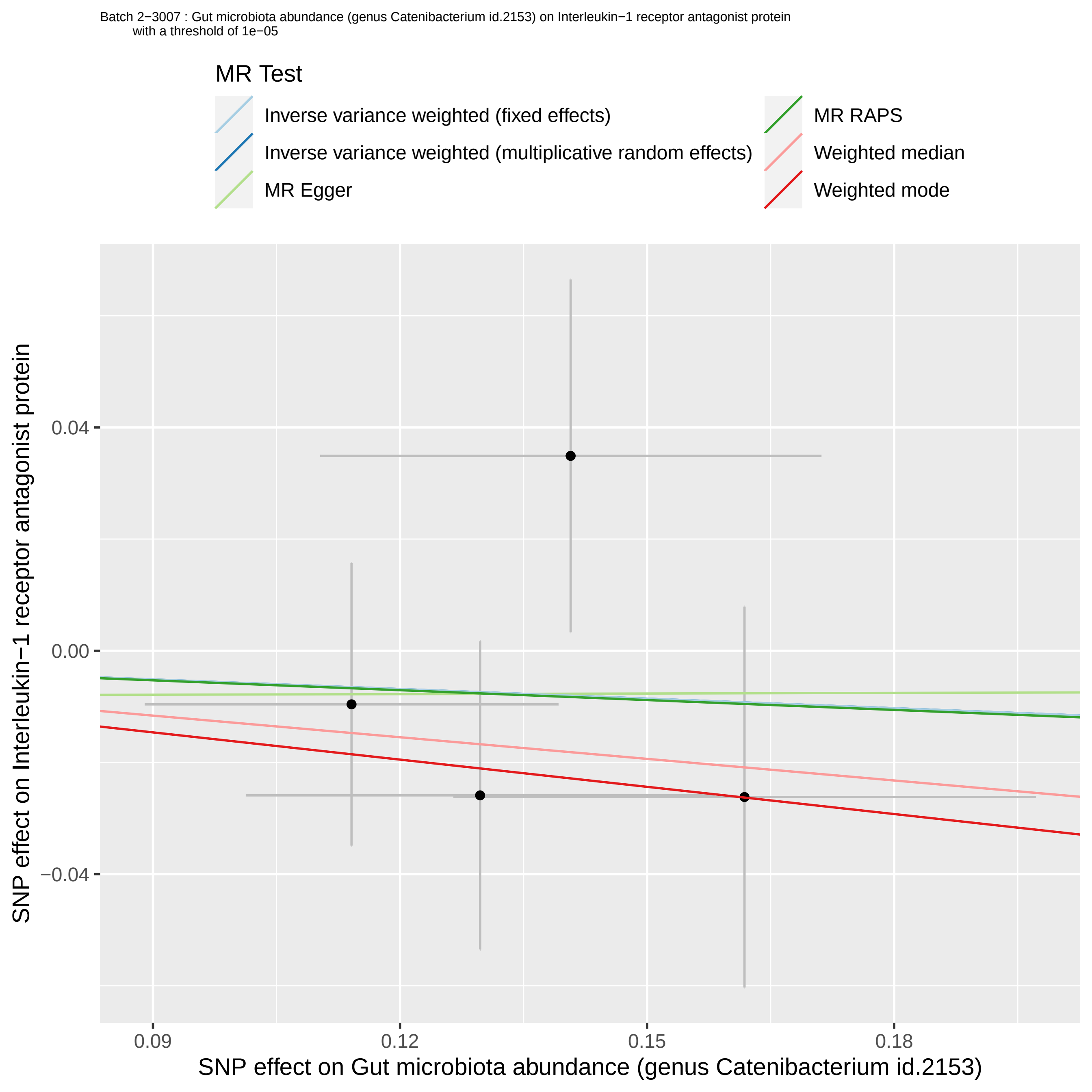

Batch 2−3007 : Gut microbiota abundance (genus Catenibacterium id.2153) on Interleukin−1 receptor antagonist protein
with a threshold of 1e−05
MR Test
Inverse variance weighted (fixed effects)
Inverse variance weighted (multiplicative random effects)
MR Egger
MR RAPS
Weighted median
Weighted mode
0.04
0.00
−0.04
0.09
0.12
0.15
0.18
SNP effect on Interleukin−1 receptor antagonist protein
SNP effect on Gut microbiota abundance (genus Catenibacterium id.2153)

Batch 2−3008 : Gut microbiota abundance (genus Parasutterella id.2892) on Interleukin−1 receptor antagonist protein
with a threshold of 1e−05

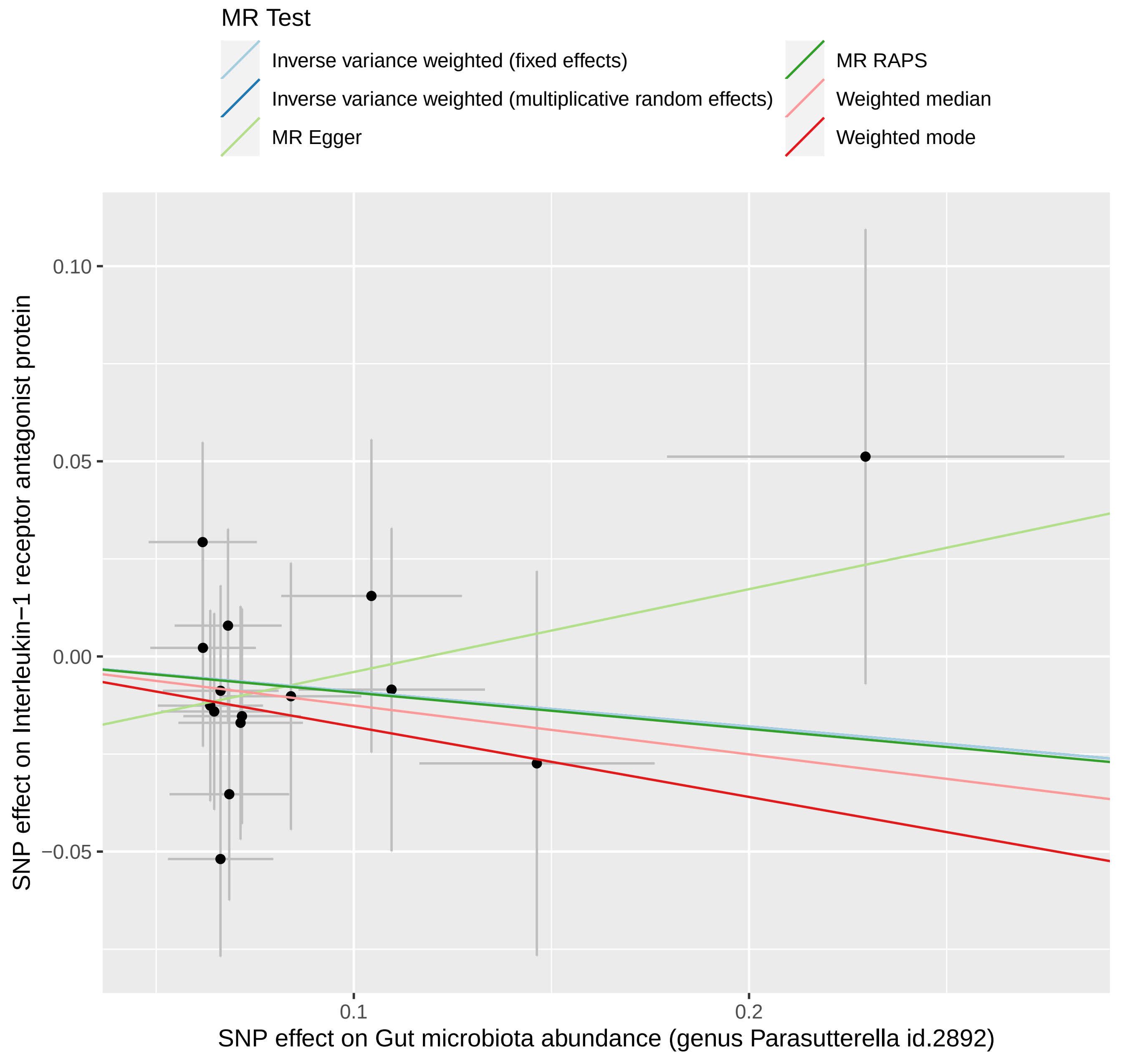

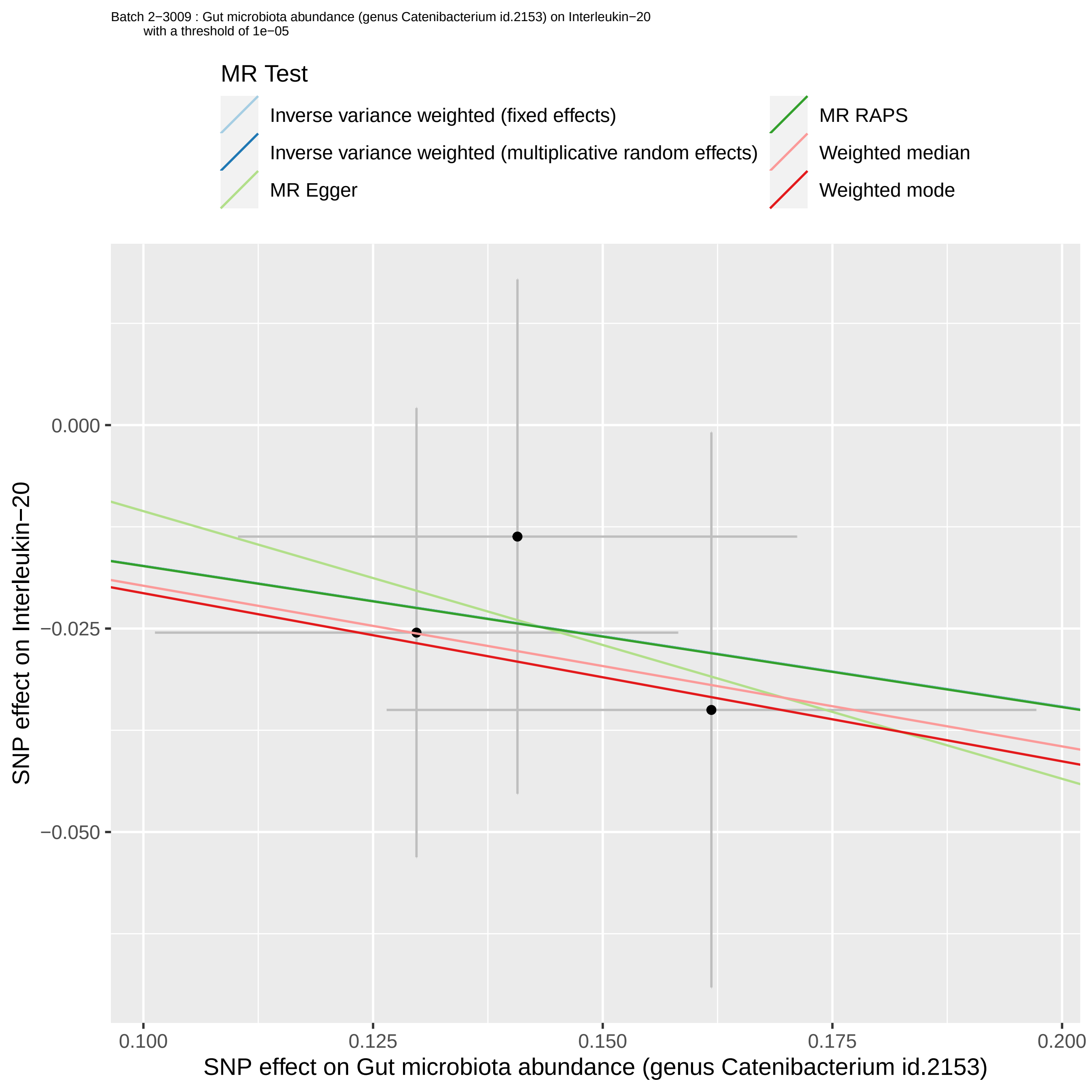
Batch 2−3009 : Gut microbiota abundance (genus Catenibacterium id.2153) on Interleukin−20
with a threshold of 1e−05
MR Test
Inverse variance weighted (fixed effects)
Inverse variance weighted (multiplicative random effects)
MR Egger
MR RAPS
Weighted median
Weighted mode
0.000
−0.025
−0.050
0.100
0.125
0.150
0.175
0.200
SNP effect on Interleukin−20
SNP effect on Gut microbiota abundance (genus Catenibacterium id.2153)

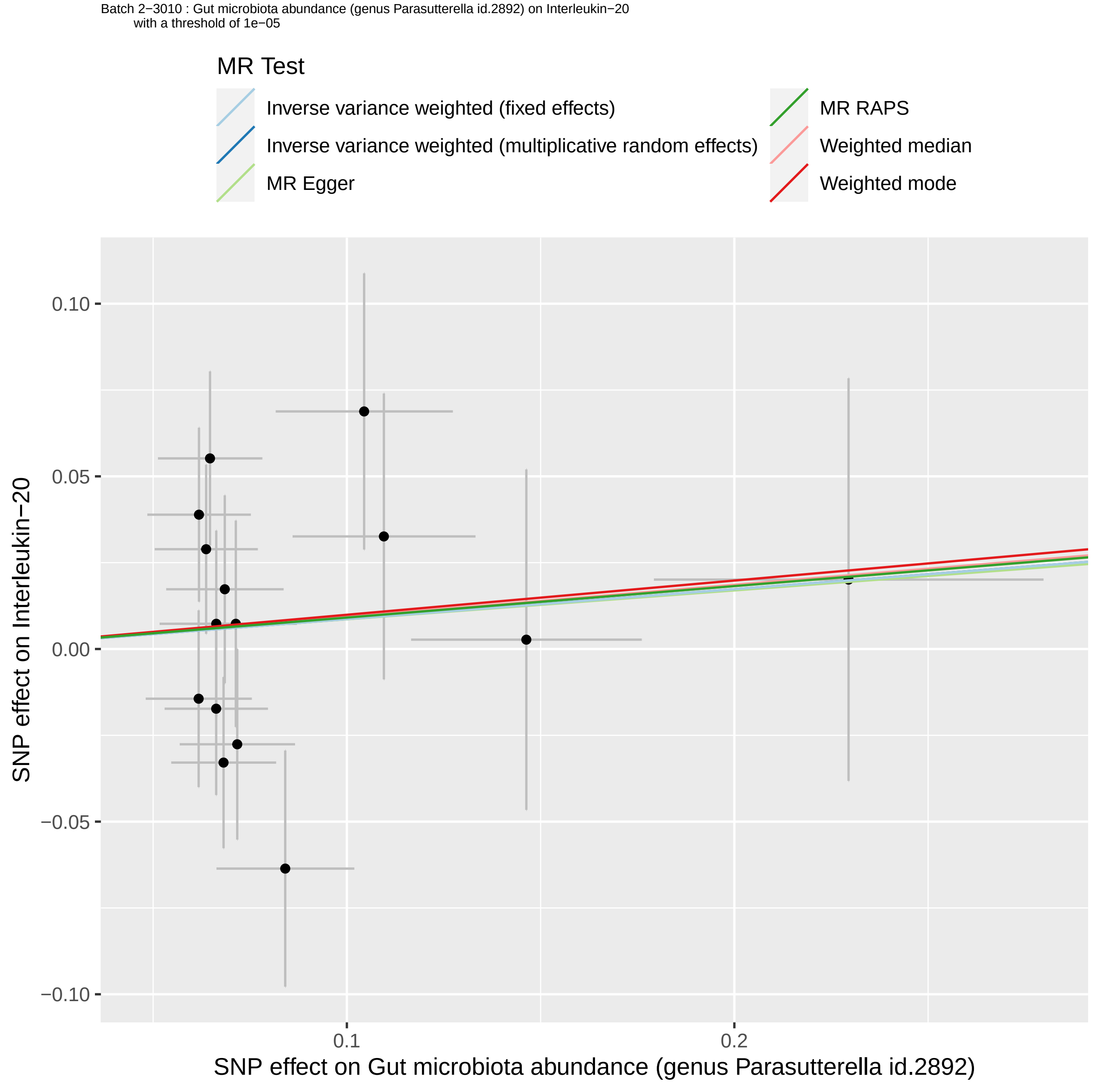

Batch 2−3010 : Gut microbiota abundance (genus Parasutterella id.2892) on Interleukin−20
with a threshold of 1e−05
MR Test
Inverse variance weighted (fixed effects)
Inverse variance weighted (multiplicative random effects)
MR Egger
MR RAPS
Weighted median
Weighted mode
0.10
0.05
0.00
−0.05
−0.10
0.1
0.2
SNP effect on Interleukin−20
SNP effect on Gut microbiota abundance (genus Parasutterella id.2892)

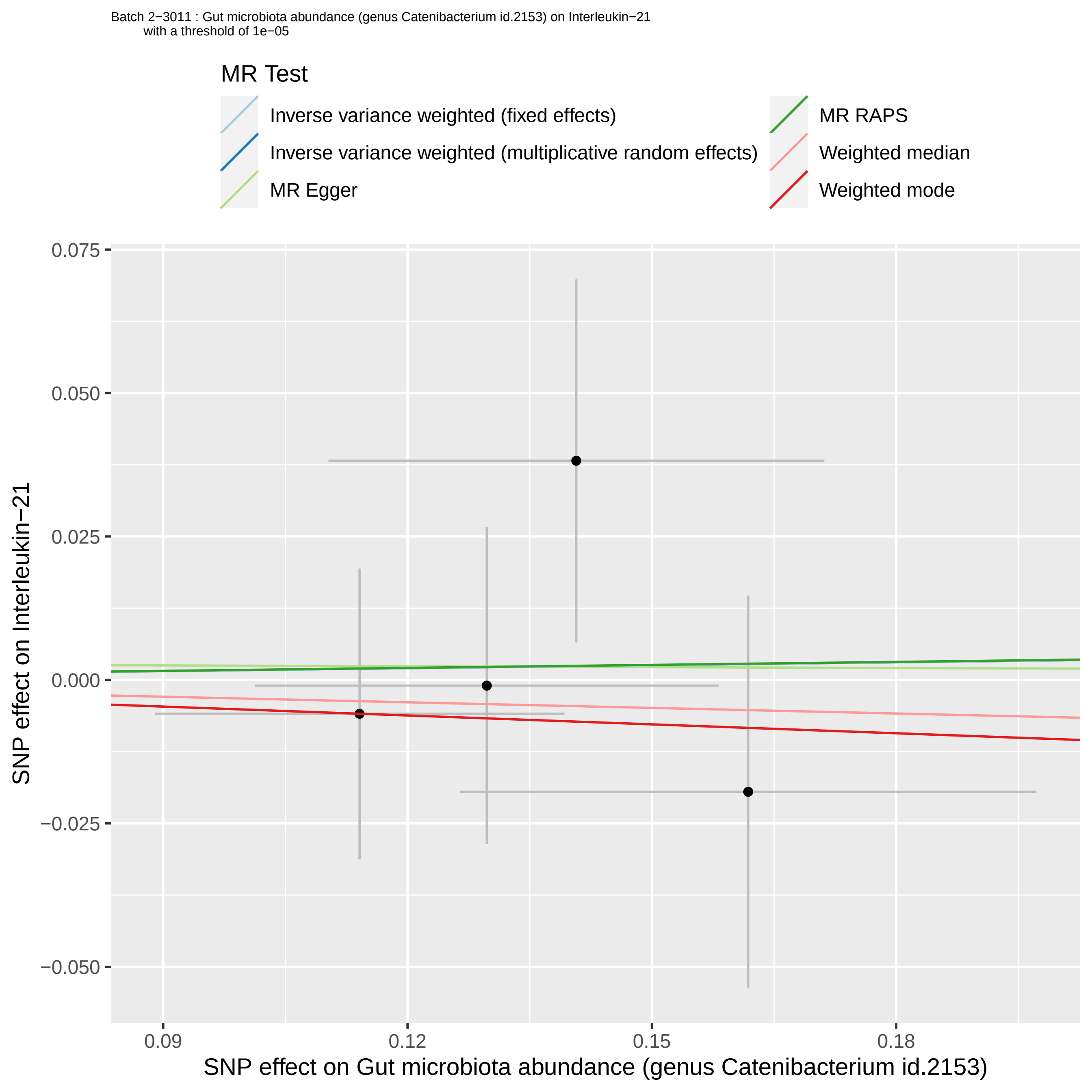

Batch 2−3011 : Gut microbiota abundance (genus Catenibacterium id.2153) on Interleukin−21
with a threshold of 1e−05
MR Test
Inverse variance weighted (fixed effects)
Inverse variance weighted (multiplicative random effects)
MR Egger
MR RAPS
Weighted median
Weighted mode
0.075
0.050
0.025
0.000
−0.025
−0.050
0.09
0.12
0.15
0.18
SNP effect on Interleukin−21
SNP effect on Gut microbiota abundance (genus Catenibacterium id.2153)

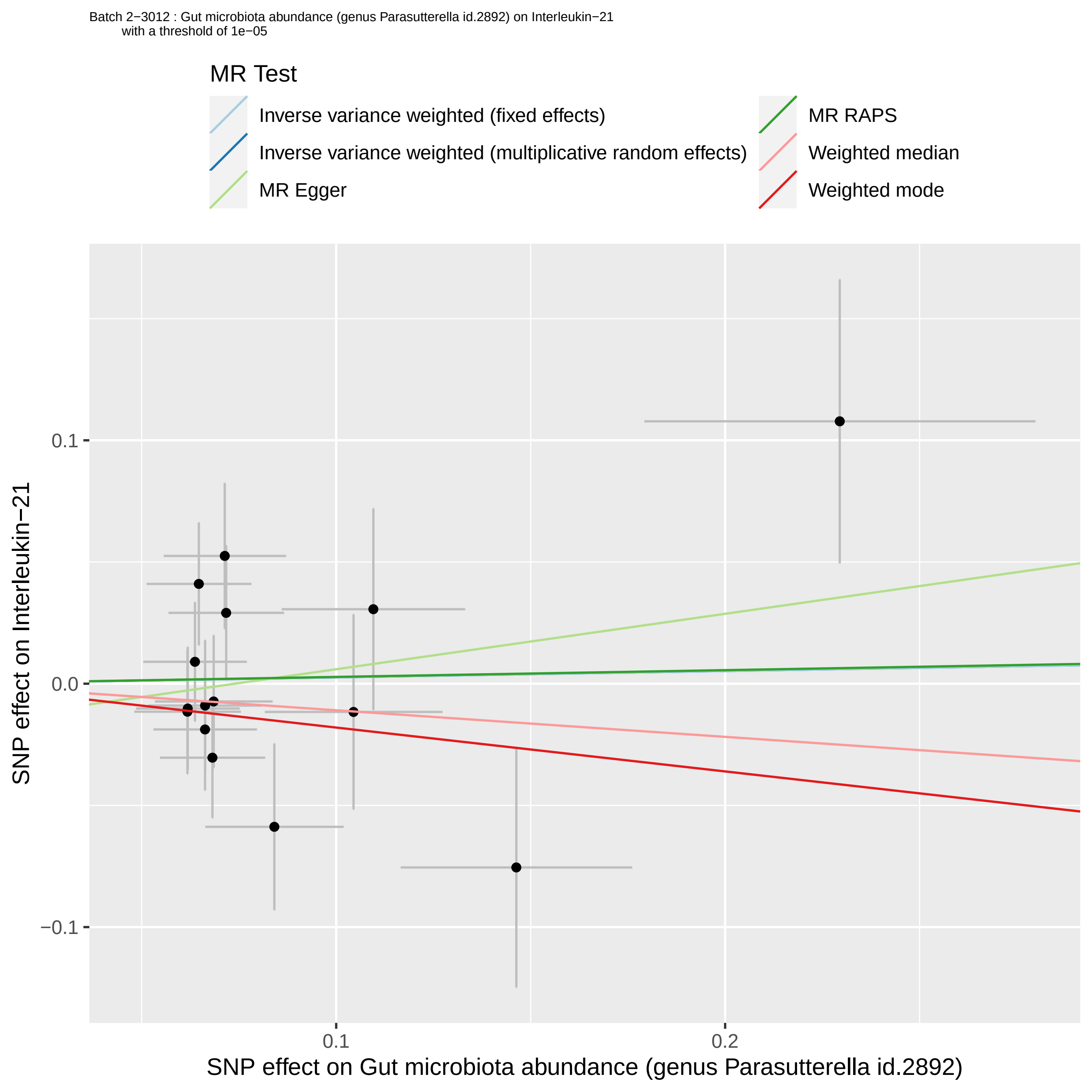

Batch 2−3012 : Gut microbiota abundance (genus Parasutterella id.2892) on Interleukin−21
with a threshold of 1e−05
MR Test
Inverse variance weighted (fixed effects)
Inverse variance weighted (multiplicative random effects)
MR Egger
MR RAPS
Weighted median
Weighted mode
0.1
0.0
−0.1
0.1
0.2
SNP effect on Interleukin−21
SNP effect on Gut microbiota abundance (genus Parasutterella id.2892)

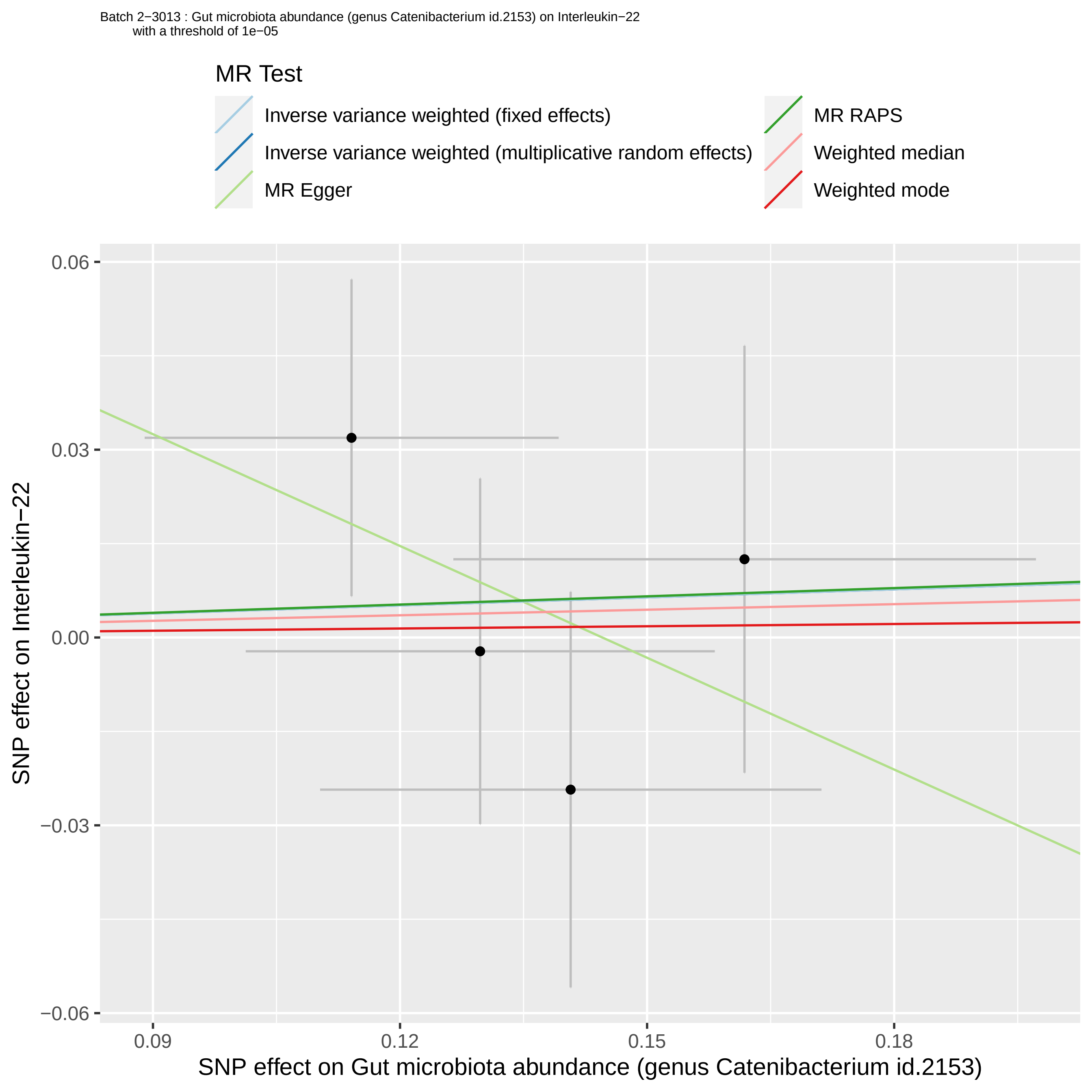
Batch 2−3013 : Gut microbiota abundance (genus Catenibacterium id.2153) on Interleukin−22
with a threshold of 1e−05
MR Test
Inverse variance weighted (fixed effects)
Inverse variance weighted (multiplicative random effects)
MR Egger
MR RAPS
Weighted median
Weighted mode
0.06
0.03
0.00
−0.03
−0.06
0.09
0.12
0.15
0.18
SNP effect on Interleukin−22
SNP effect on Gut microbiota abundance (genus Catenibacterium id.2153)

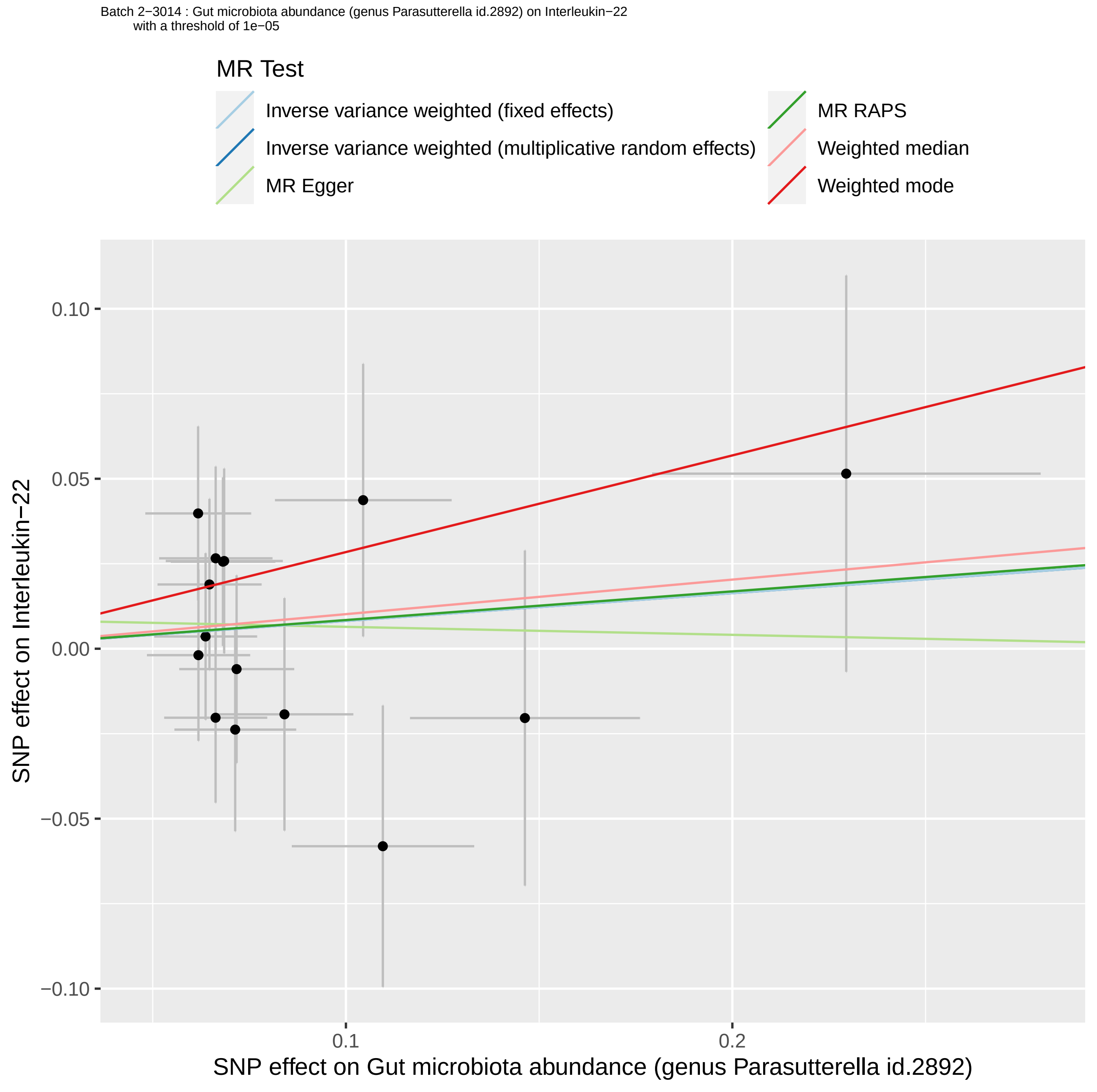

Batch 2−3014 : Gut microbiota abundance (genus Parasutterella id.2892) on Interleukin−22
with a threshold of 1e−05
MR Test
Inverse variance weighted (fixed effects)
Inverse variance weighted (multiplicative random effects)
MR Egger
MR RAPS
Weighted median
Weighted mode
0.10
0.05
0.00
−0.05
−0.10
0.1
0.2
SNP effect on Interleukin−22
SNP effect on Gut microbiota abundance (genus Parasutterella id.2892)

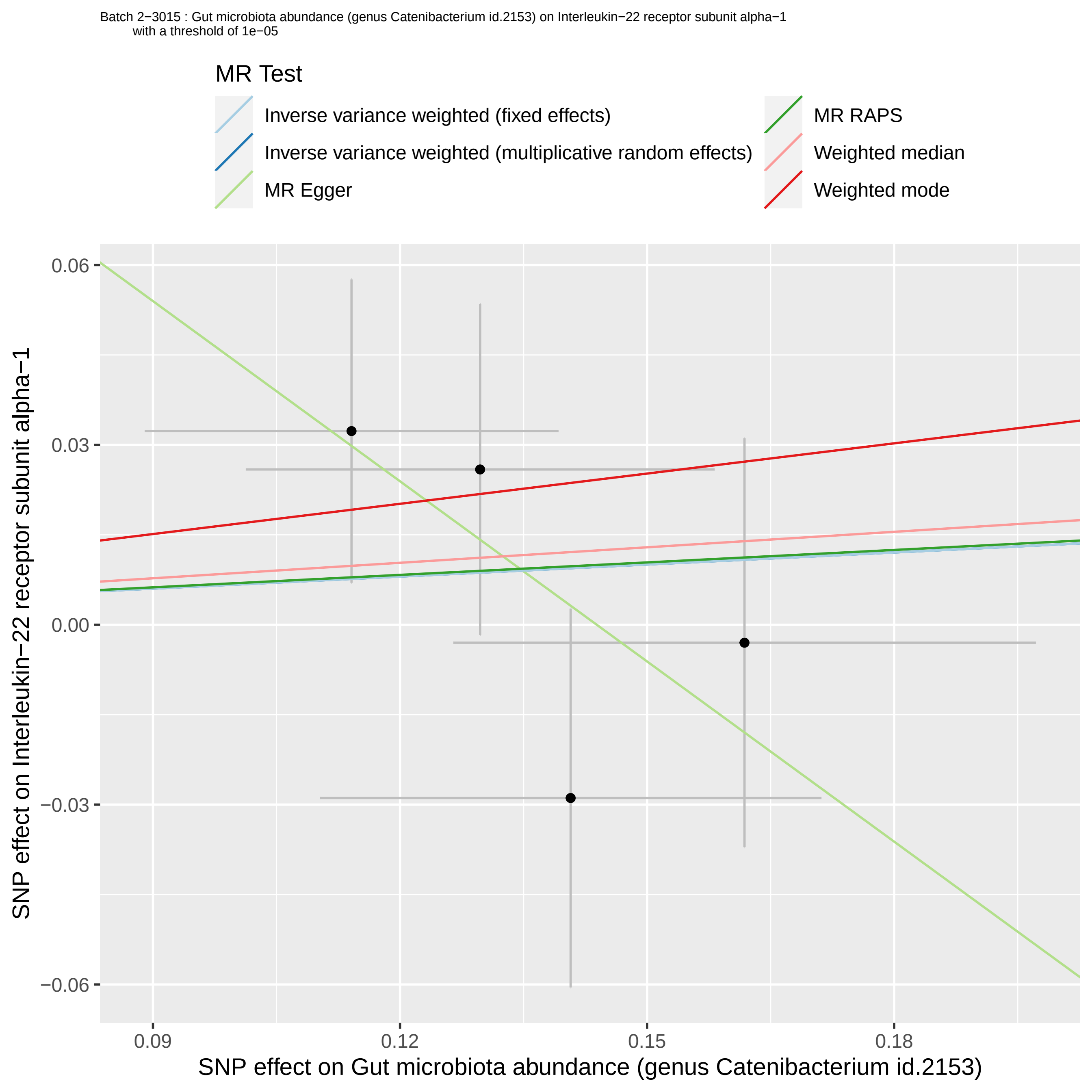
Batch 2−3015 : Gut microbiota abundance (genus Catenibacterium id.2153) on Interleukin−22 receptor subunit alpha−1
with a threshold of 1e−05
MR Test
Inverse variance weighted (fixed effects)
Inverse variance weighted (multiplicative random effects)
MR Egger
MR RAPS
Weighted median
Weighted mode
0.06
0.03
0.00
−0.03
−0.06
0.09
0.12
0.15
0.18
SNP effect on Interleukin−22 receptor subunit alpha−1
SNP effect on Gut microbiota abundance (genus Catenibacterium id.2153)

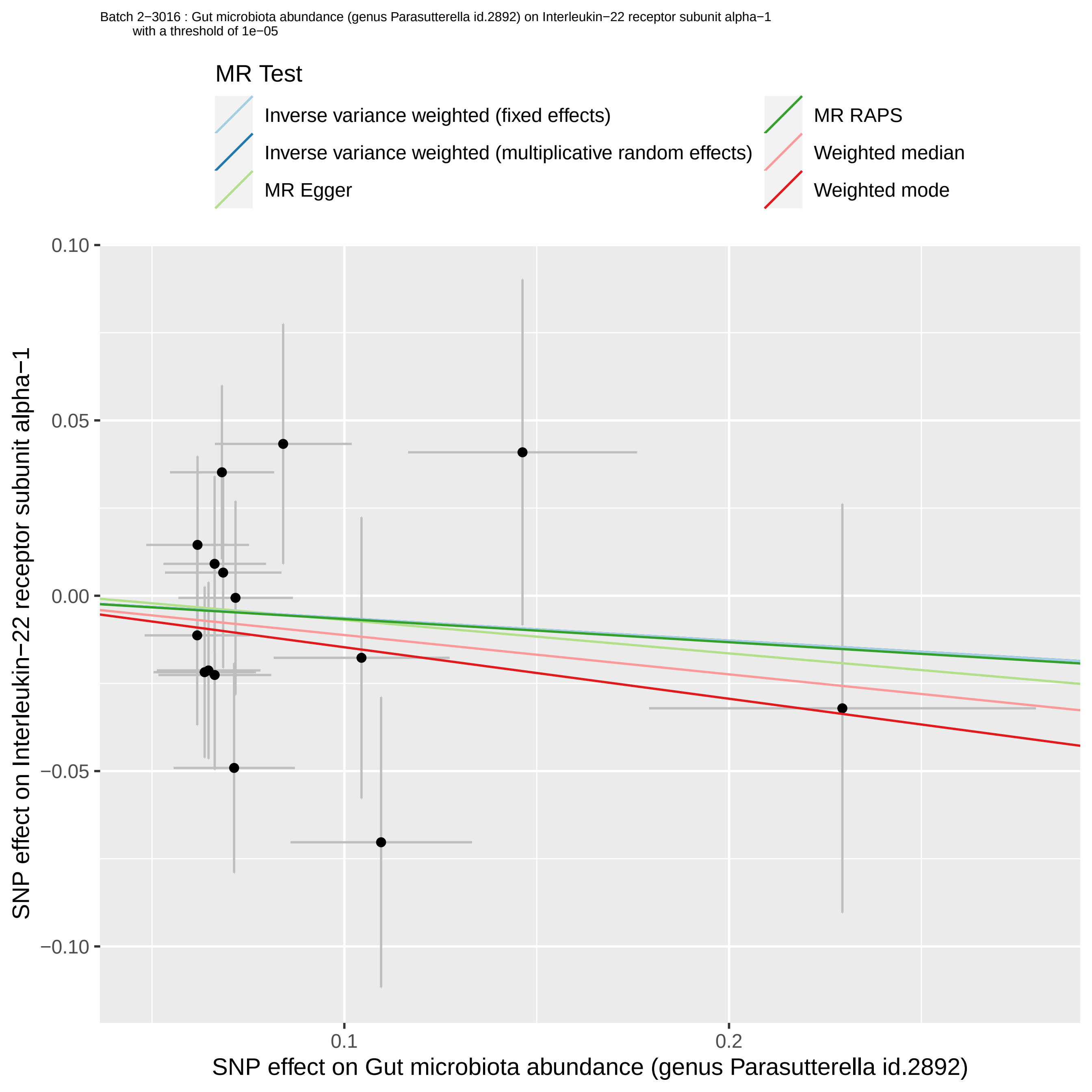

Batch 2−3016 : Gut microbiota abundance (genus Parasutterella id.2892) on Interleukin−22 receptor subunit alpha−1
with a threshold of 1e−05
MR Test
Inverse variance weighted (fixed effects)
Inverse variance weighted (multiplicative random effects)
MR Egger
MR RAPS
Weighted median
Weighted mode
0.10
0.05
0.00
−0.05
−0.10
0.1
0.2
SNP effect on Interleukin−22 receptor subunit alpha−1
SNP effect on Gut microbiota abundance (genus Parasutterella id.2892)

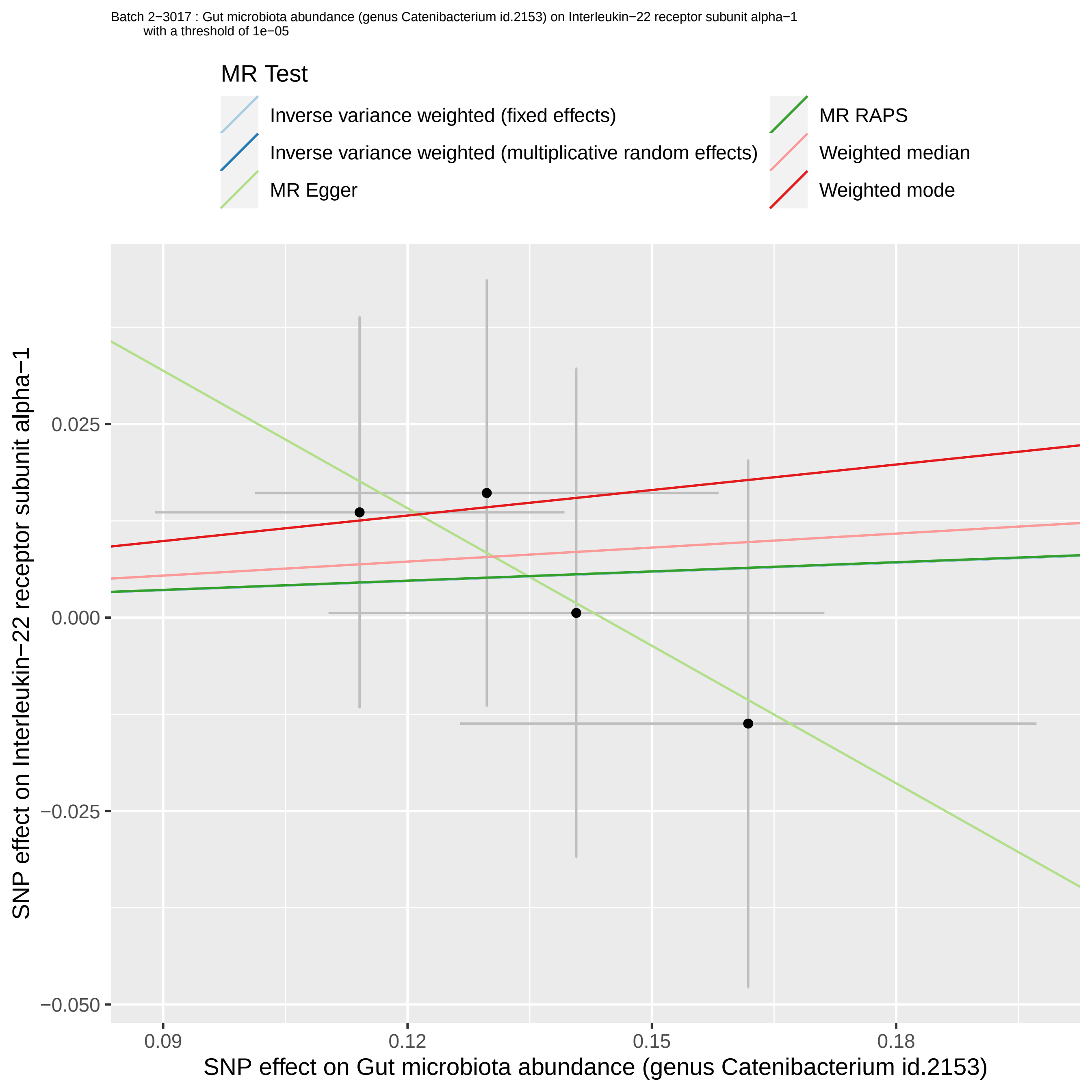
Batch 2−3017 : Gut microbiota abundance (genus Catenibacterium id.2153) on Interleukin−22 receptor subunit alpha−1
with a threshold of 1e−05
MR Test
Inverse variance weighted (fixed effects)
Inverse variance weighted (multiplicative random effects)
MR Egger
MR RAPS
Weighted median
Weighted mode
SNP effect on Interleukin−22 receptor subunit alpha−1
0.025
0.000
−0.025
−0.050
0.09
0.12
0.15
0.18
SNP effect on Gut microbiota abundance (genus Catenibacterium id.2153)

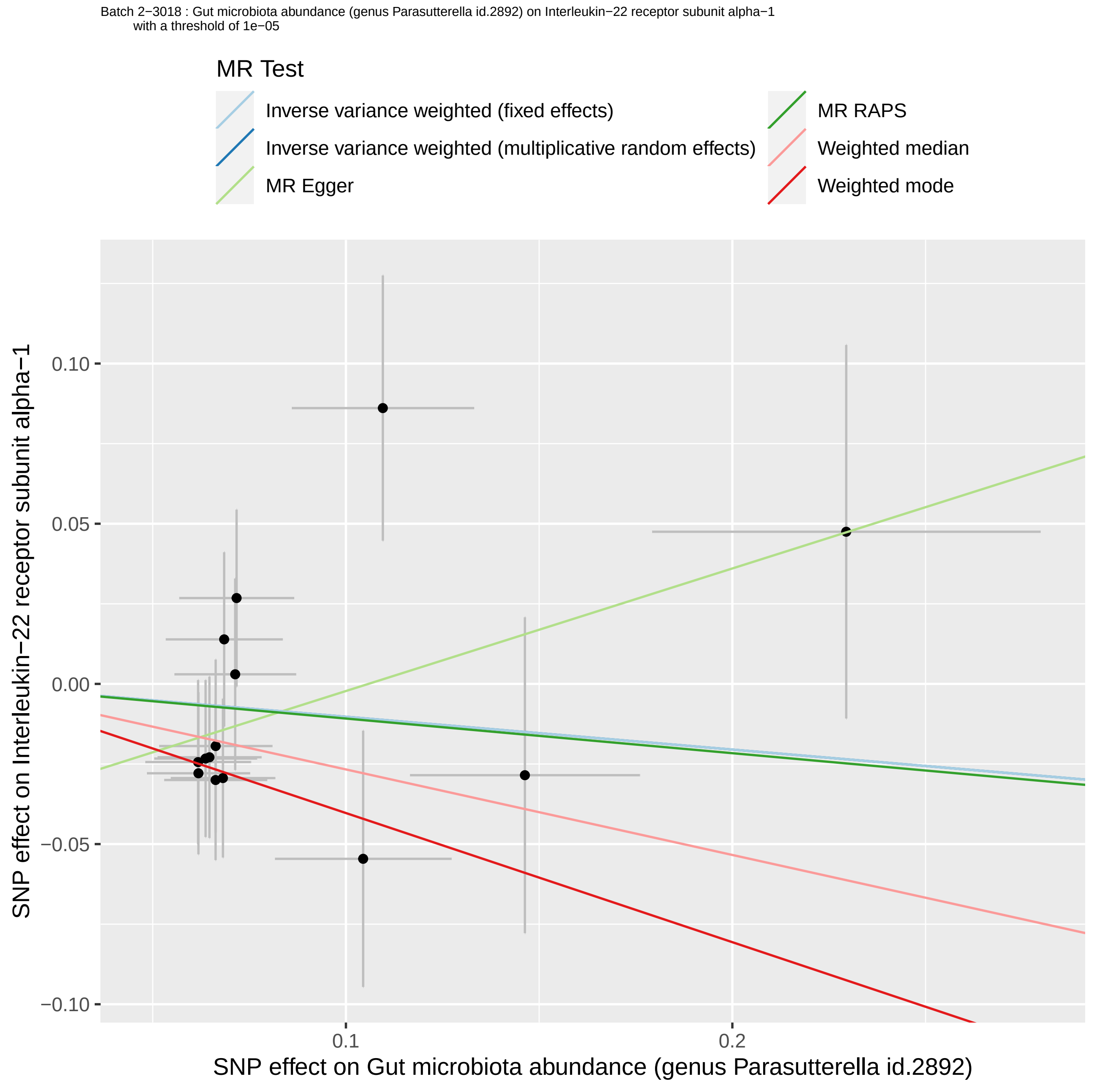
Batch 2−3018 : Gut microbiota abundance (genus Parasutterella id.2892) on Interleukin−22 receptor subunit alpha−1
with a threshold of 1e−05
MR Test
Inverse variance weighted (fixed effects)
Inverse variance weighted (multiplicative random effects)
MR Egger
MR RAPS
Weighted median
Weighted mode
0.10
0.05
0.00
−0.05
−0.10
0.1
0.2
SNP effect on Interleukin−22 receptor subunit alpha−1
SNP effect on Gut microbiota abundance (genus Parasutterella id.2892)

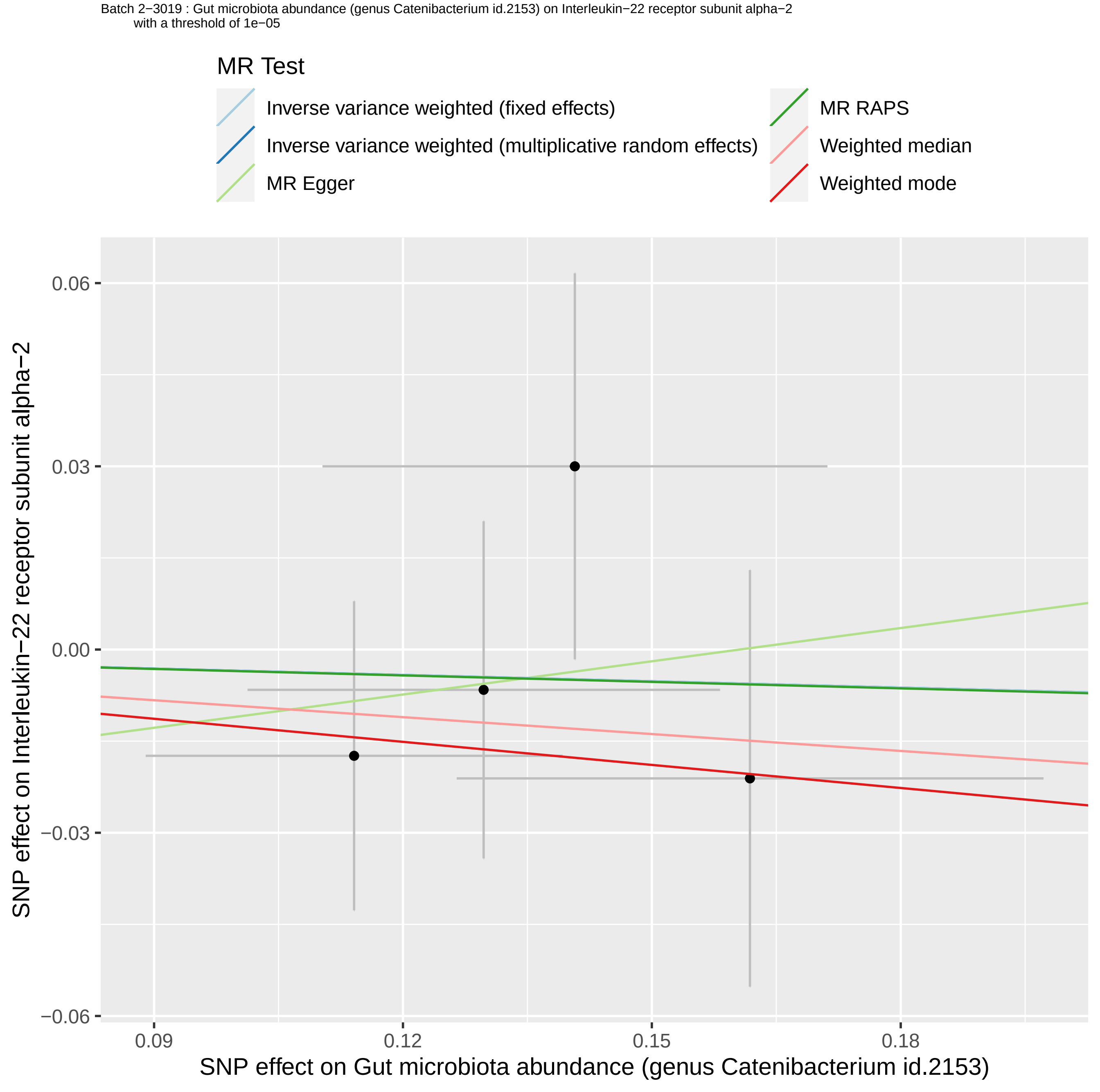
Batch 2−3019 : Gut microbiota abundance (genus Catenibacterium id.2153) on Interleukin−22 receptor subunit alpha−2
with a threshold of 1e−05
MR Test
Inverse variance weighted (fixed effects)
Inverse variance weighted (multiplicative random effects)
MR Egger
MR RAPS
Weighted median
Weighted mode
0.06
0.03
0.00
−0.03
−0.06
0.09
0.12
0.15
0.18
SNP effect on Interleukin−22 receptor subunit alpha−2
SNP effect on Gut microbiota abundance (genus Catenibacterium id.2153)

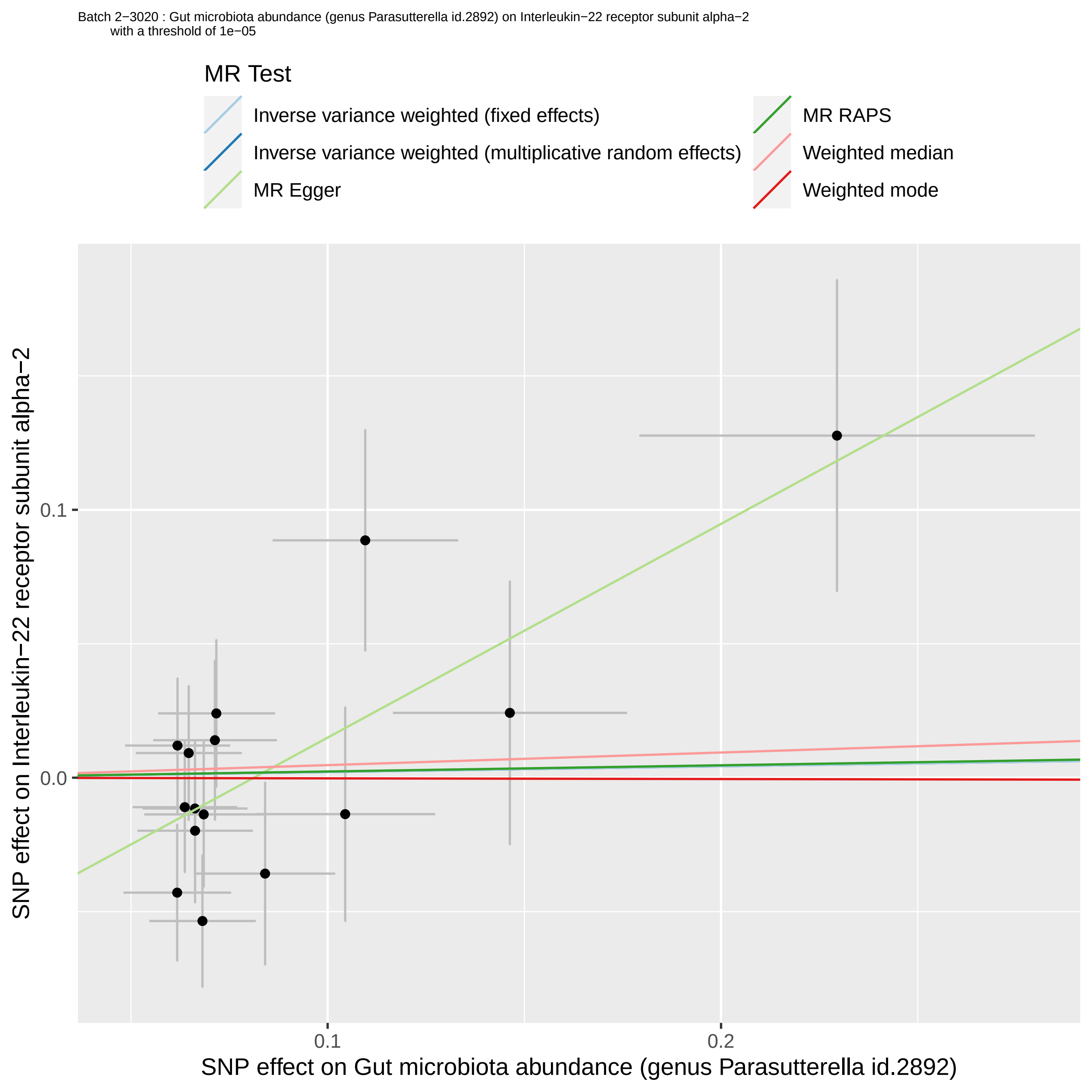

Batch 2−3020 : Gut microbiota abundance (genus Parasutterella id.2892) on Interleukin−22 receptor subunit alpha−2
with a threshold of 1e−05
MR Test
Inverse variance weighted (fixed effects)
Inverse variance weighted (multiplicative random effects)
MR Egger
MR RAPS
Weighted median
Weighted mode
SNP effect on Interleukin−22 receptor subunit alpha−2
0.1
0.0
0.1
0.2
SNP effect on Gut microbiota abundance (genus Parasutterella id.2892)

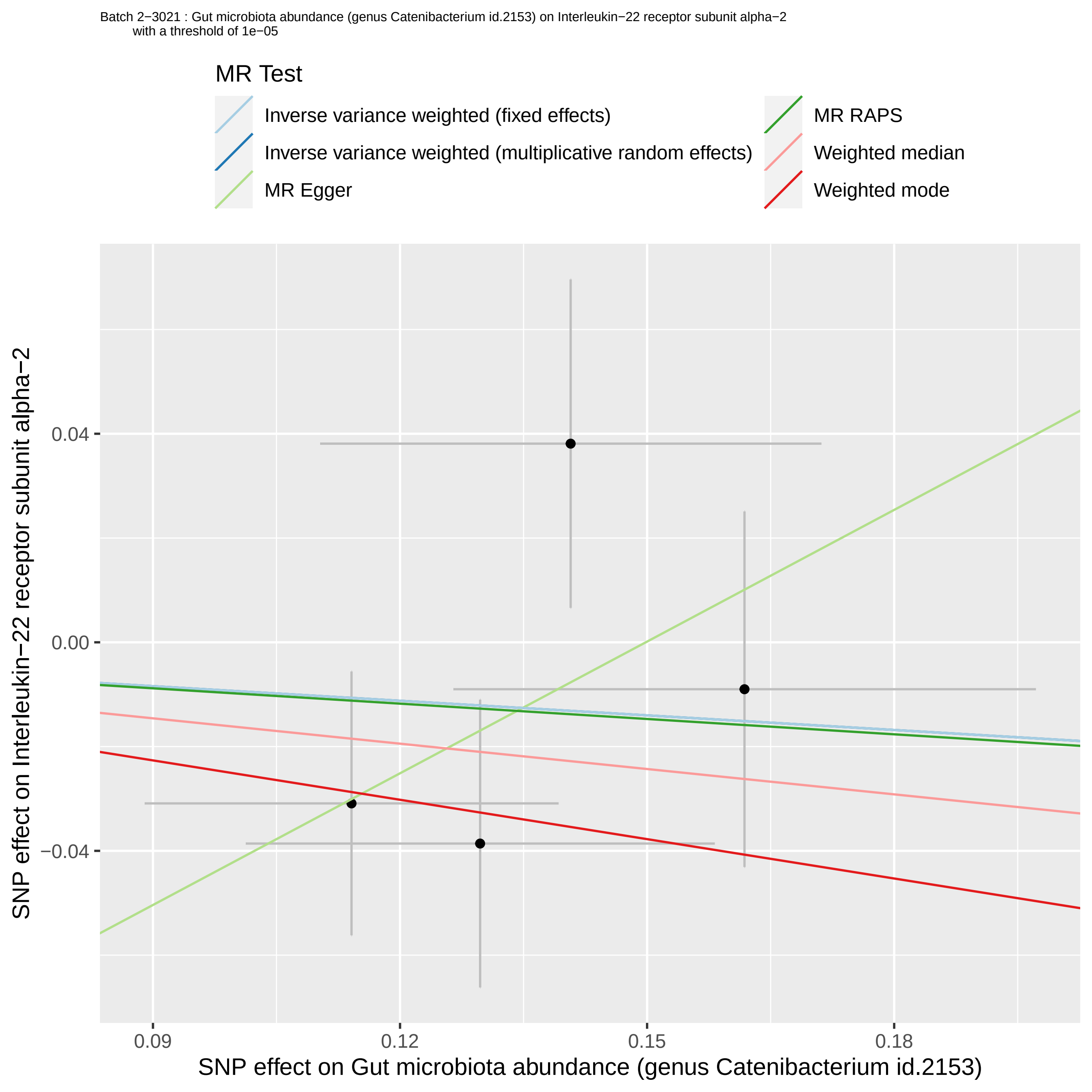

Batch 2−3021 : Gut microbiota abundance (genus Catenibacterium id.2153) on Interleukin−22 receptor subunit alpha−2
with a threshold of 1e−05
MR Test
Inverse variance weighted (fixed effects)
Inverse variance weighted (multiplicative random effects)
MR Egger
MR RAPS
Weighted median
Weighted mode
0.04
0.00
−0.04
0.09
0.12
0.15
0.18
SNP effect on Interleukin−22 receptor subunit alpha−2
SNP effect on Gut microbiota abundance (genus Catenibacterium id.2153)

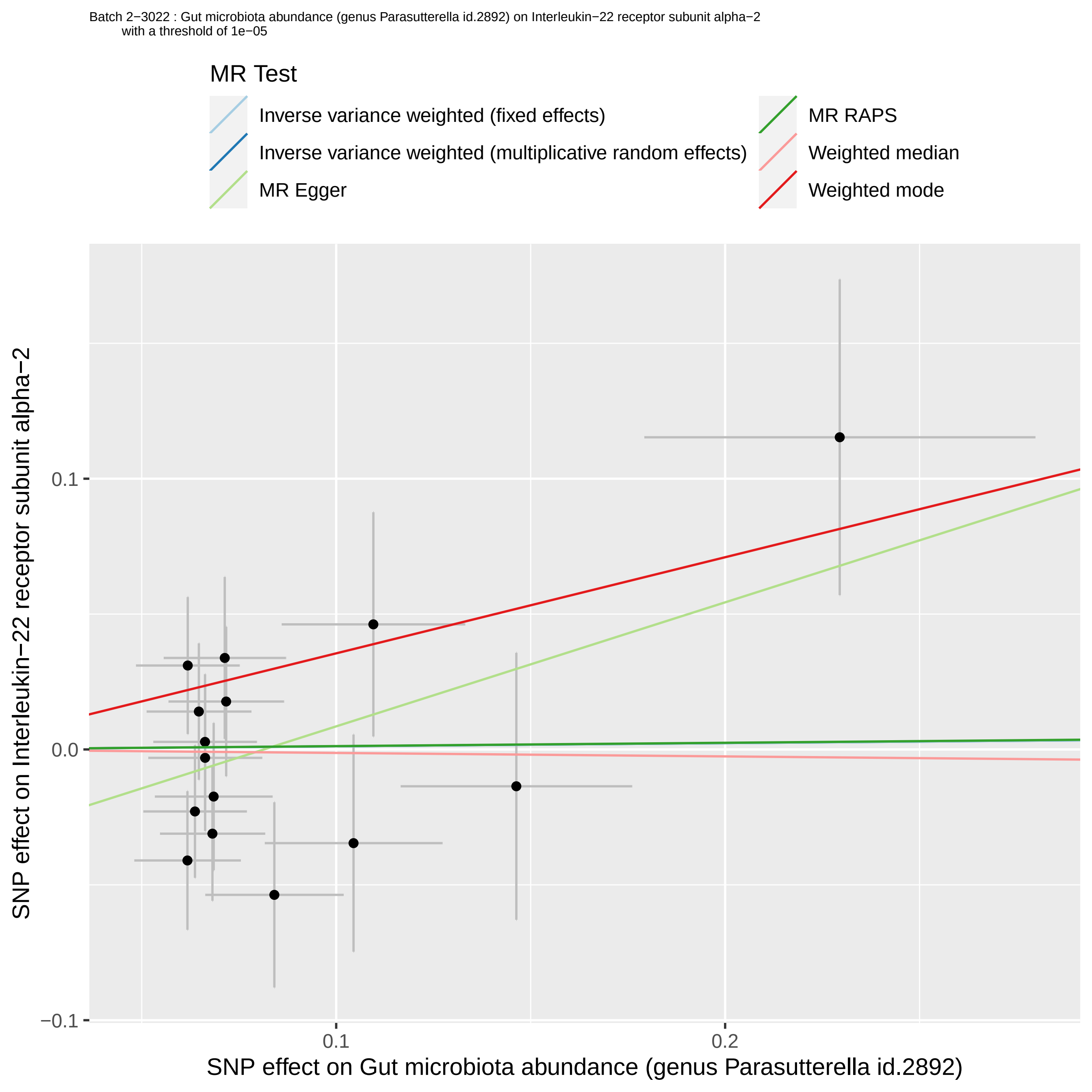

Batch 2−3022 : Gut microbiota abundance (genus Parasutterella id.2892) on Interleukin−22 receptor subunit alpha−2
with a threshold of 1e−05
MR Test
Inverse variance weighted (fixed effects)
Inverse variance weighted (multiplicative random effects)
MR Egger
MR RAPS
Weighted median
Weighted mode
0.1
0.0
−0.1
0.1
0.2
SNP effect on Interleukin−22 receptor subunit alpha−2
SNP effect on Gut microbiota abundance (genus Parasutterella id.2892)

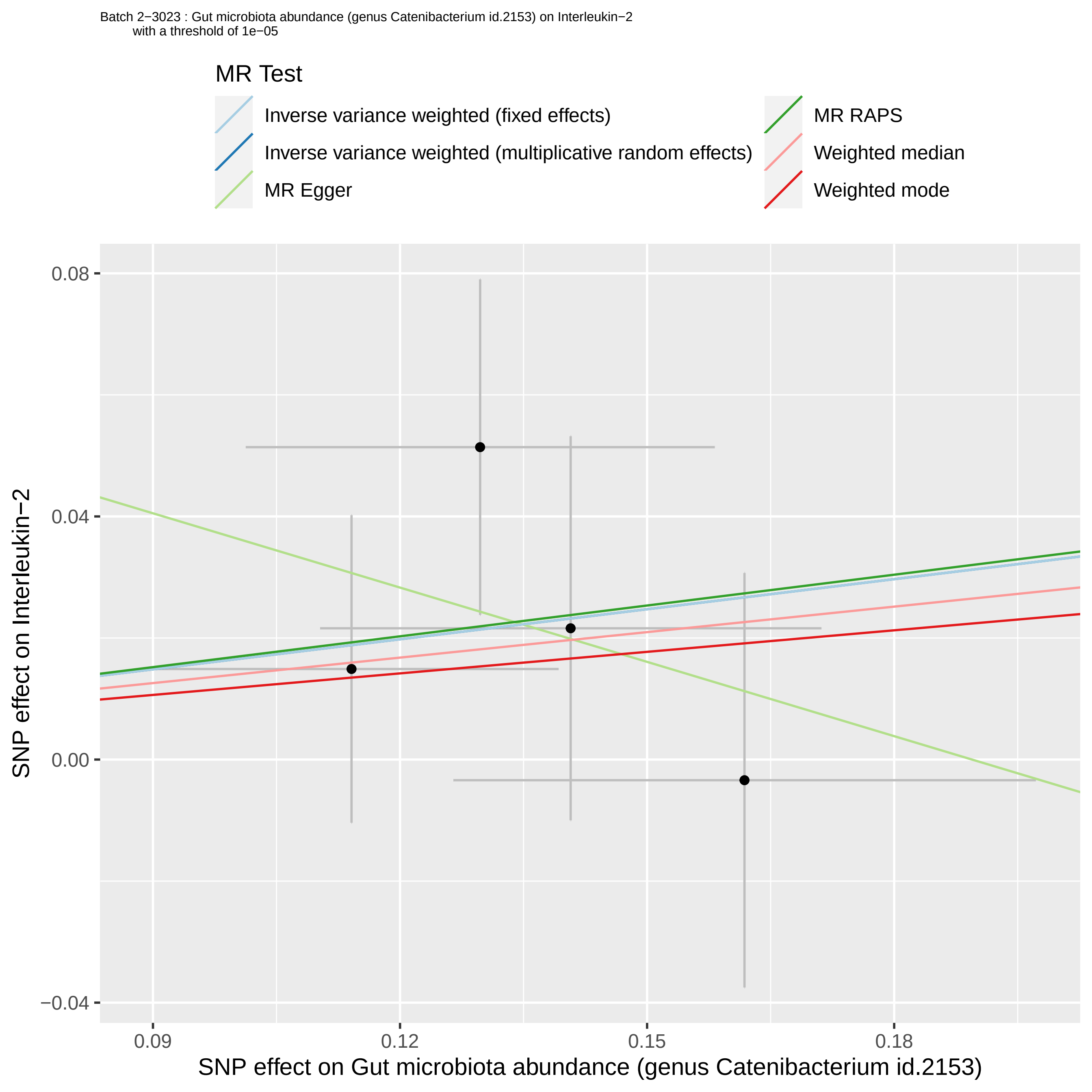

Batch 2−3023 : Gut microbiota abundance (genus Catenibacterium id.2153) on Interleukin−2
with a threshold of 1e−05
MR Test
Inverse variance weighted (fixed effects)
Inverse variance weighted (multiplicative random effects)
MR Egger
MR RAPS
Weighted median
Weighted mode
0.08
0.04
0.00
−0.04
0.09
0.12
0.15
0.18
SNP effect on Interleukin−2
SNP effect on Gut microbiota abundance (genus Catenibacterium id.2153)

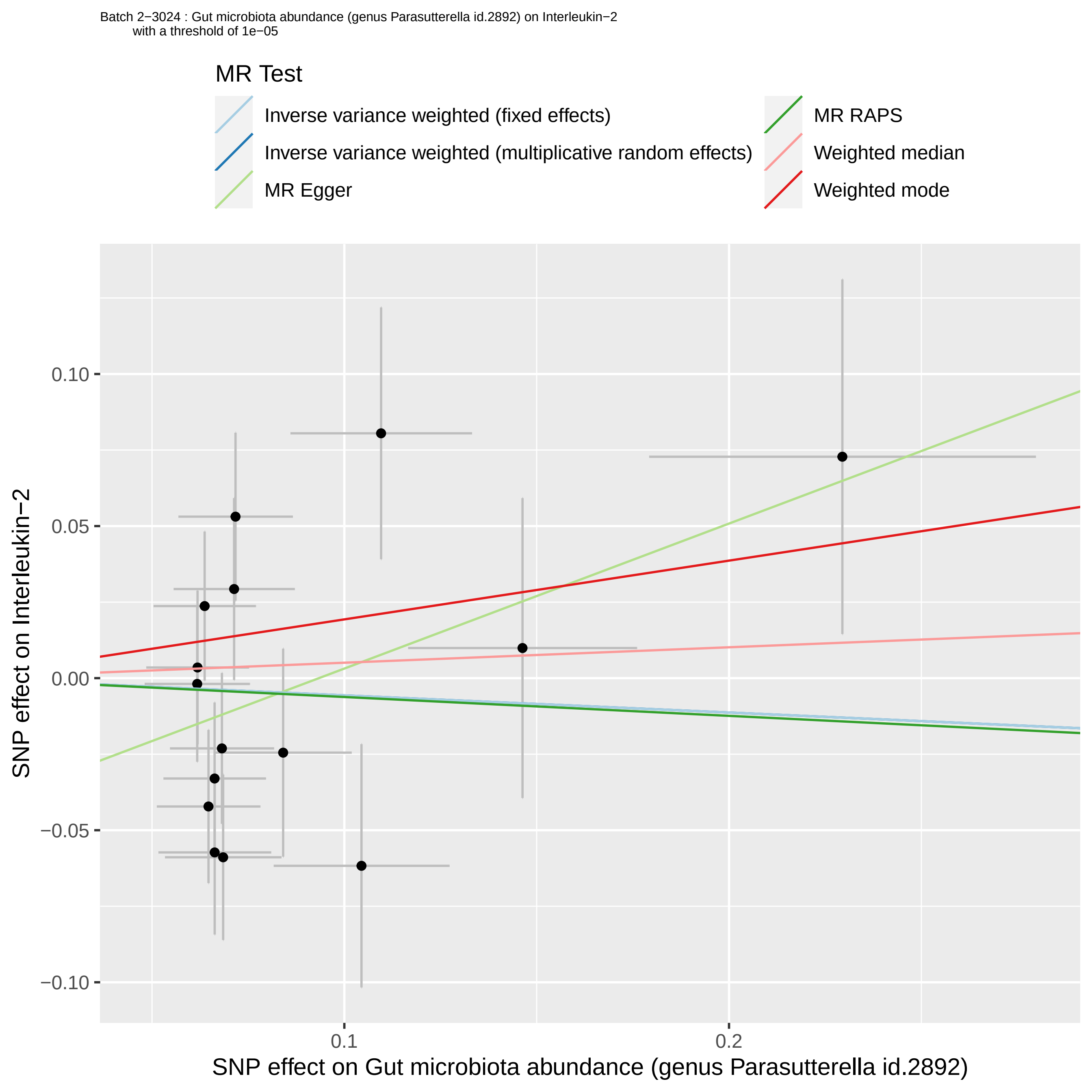

Batch 2−3024 : Gut microbiota abundance (genus Parasutterella id.2892) on Interleukin−2
with a threshold of 1e−05
MR Test
Inverse variance weighted (fixed effects)
Inverse variance weighted (multiplicative random effects)
MR Egger
MR RAPS
Weighted median
Weighted mode
SNP effect on Interleukin−2
0.10
0.05
0.00
−0.05
−0.10
0.1
0.2
SNP effect on Gut microbiota abundance (genus Parasutterella id.2892)

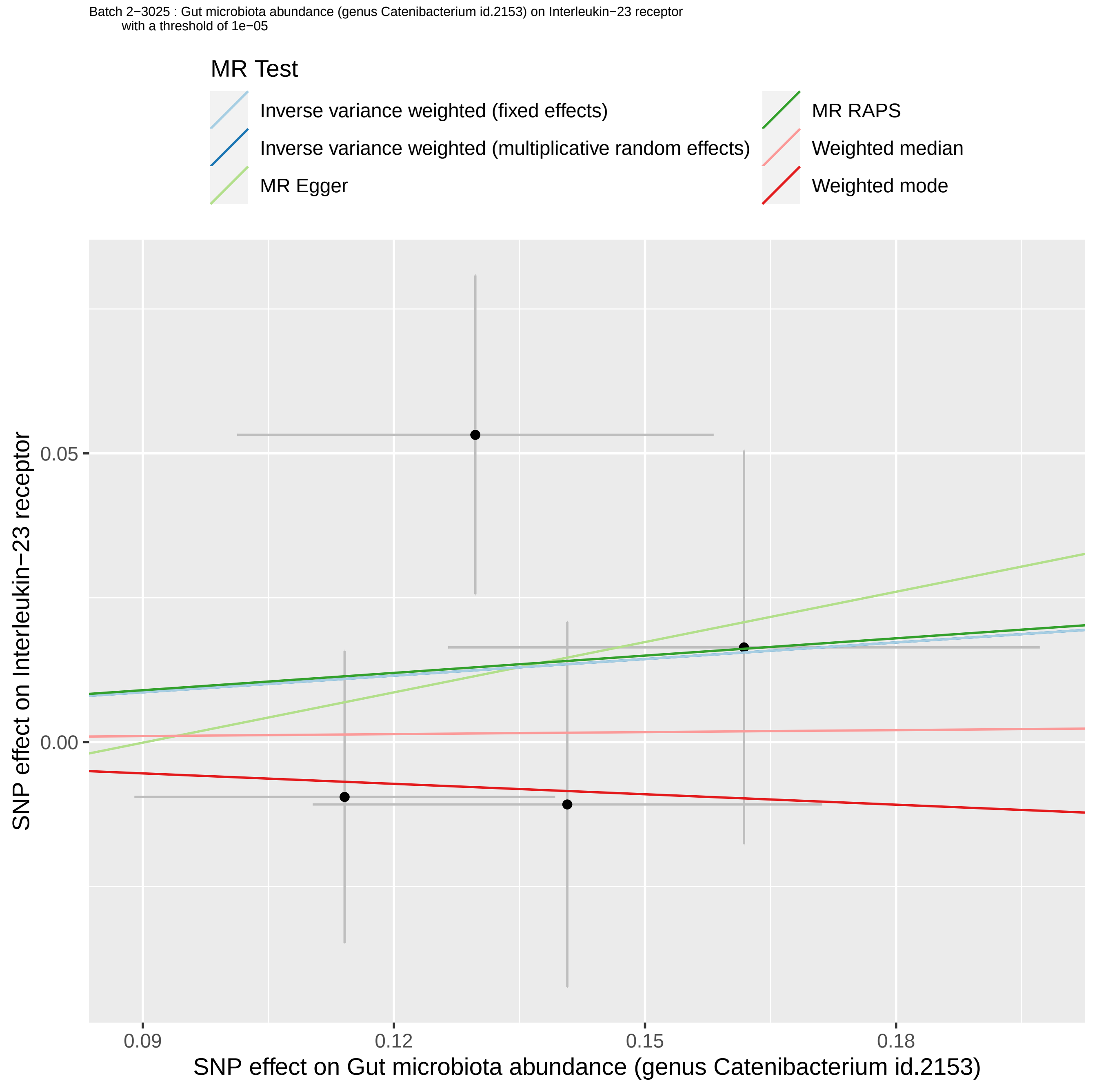
Batch 2−3025 : Gut microbiota abundance (genus Catenibacterium id.2153) on Interleukin−23 receptor
with a threshold of 1e−05
MR Test
Inverse variance weighted (fixed effects)
Inverse variance weighted (multiplicative random effects)
MR Egger
MR RAPS
Weighted median
Weighted mode
SNP effect on Interleukin−23 receptor
0.05
0.00
0.09
0.12
0.15
0.18
SNP effect on Gut microbiota abundance (genus Catenibacterium id.2153)

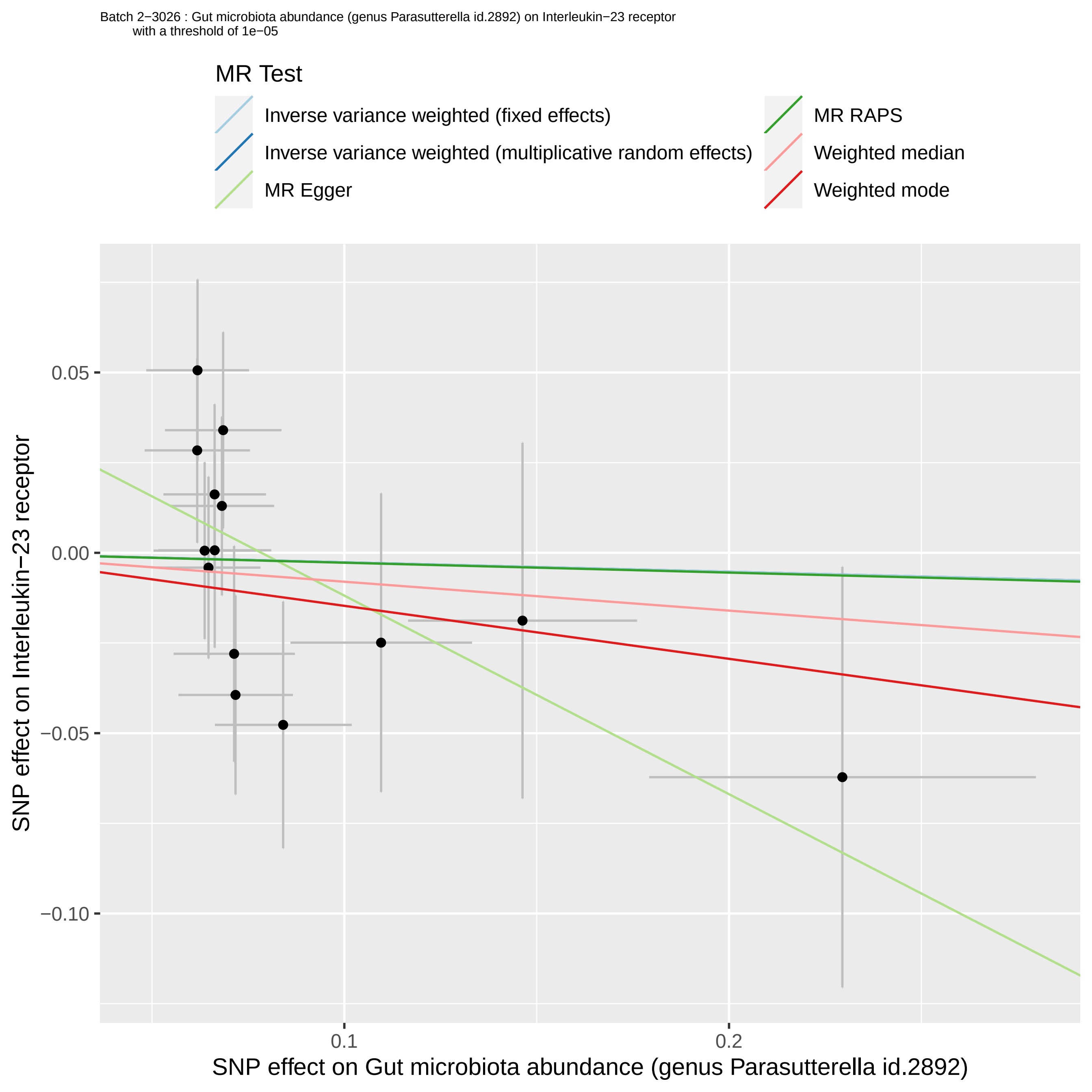
Batch 2−3026 : Gut microbiota abundance (genus Parasutterella id.2892) on Interleukin−23 receptor
with a threshold of 1e−05
MR Test
Inverse variance weighted (fixed effects)
Inverse variance weighted (multiplicative random effects)
MR Egger
MR RAPS
Weighted median
Weighted mode
0.05
0.00
−0.05
−0.10
0.1
0.2
SNP effect on Interleukin−23 receptor
SNP effect on Gut microbiota abundance (genus Parasutterella id.2892)

Batch 2−3027 : Gut microbiota abundance (genus Catenibacterium id.2153) on Interleukin−24
with a threshold of 1e−05

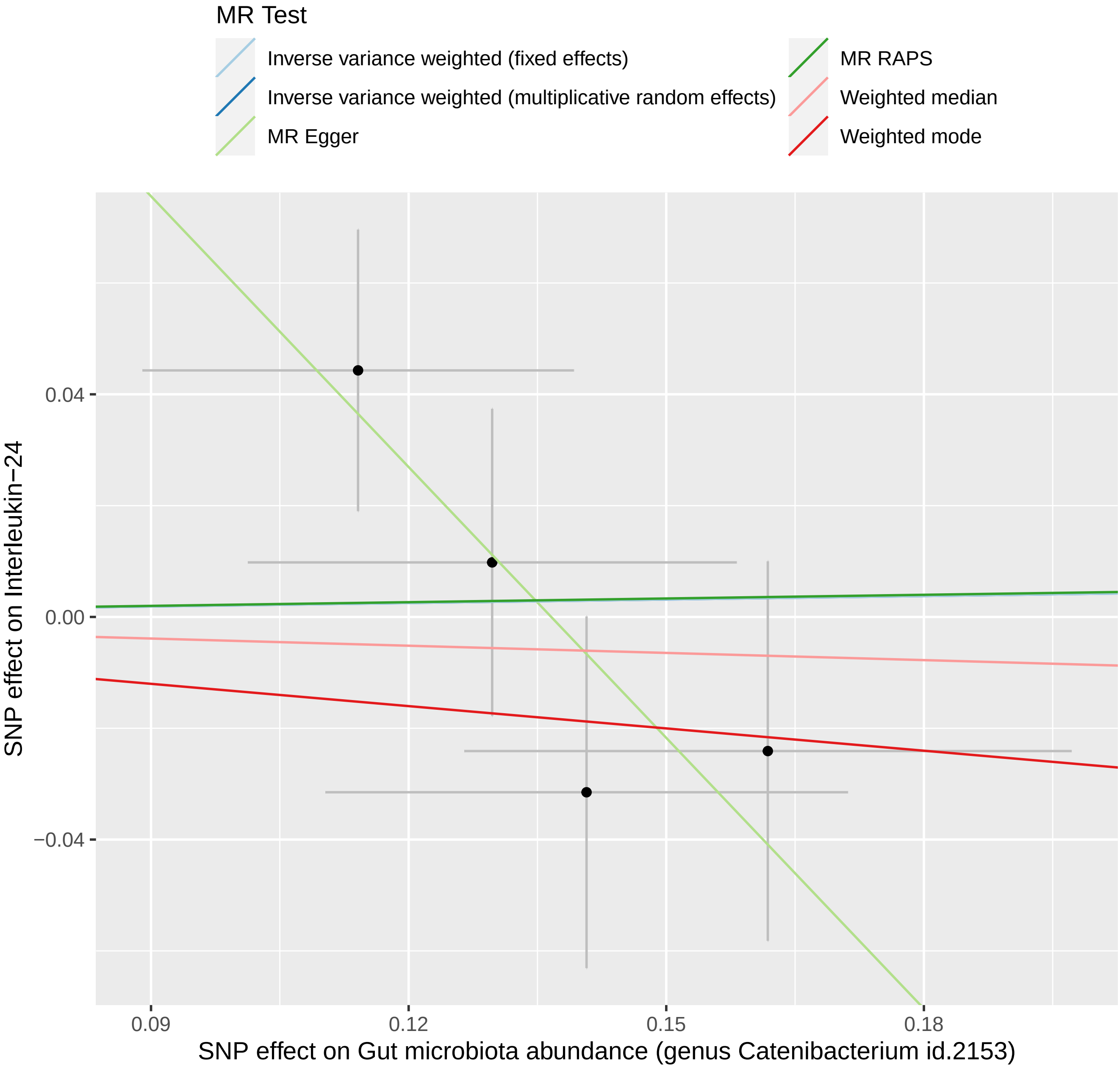

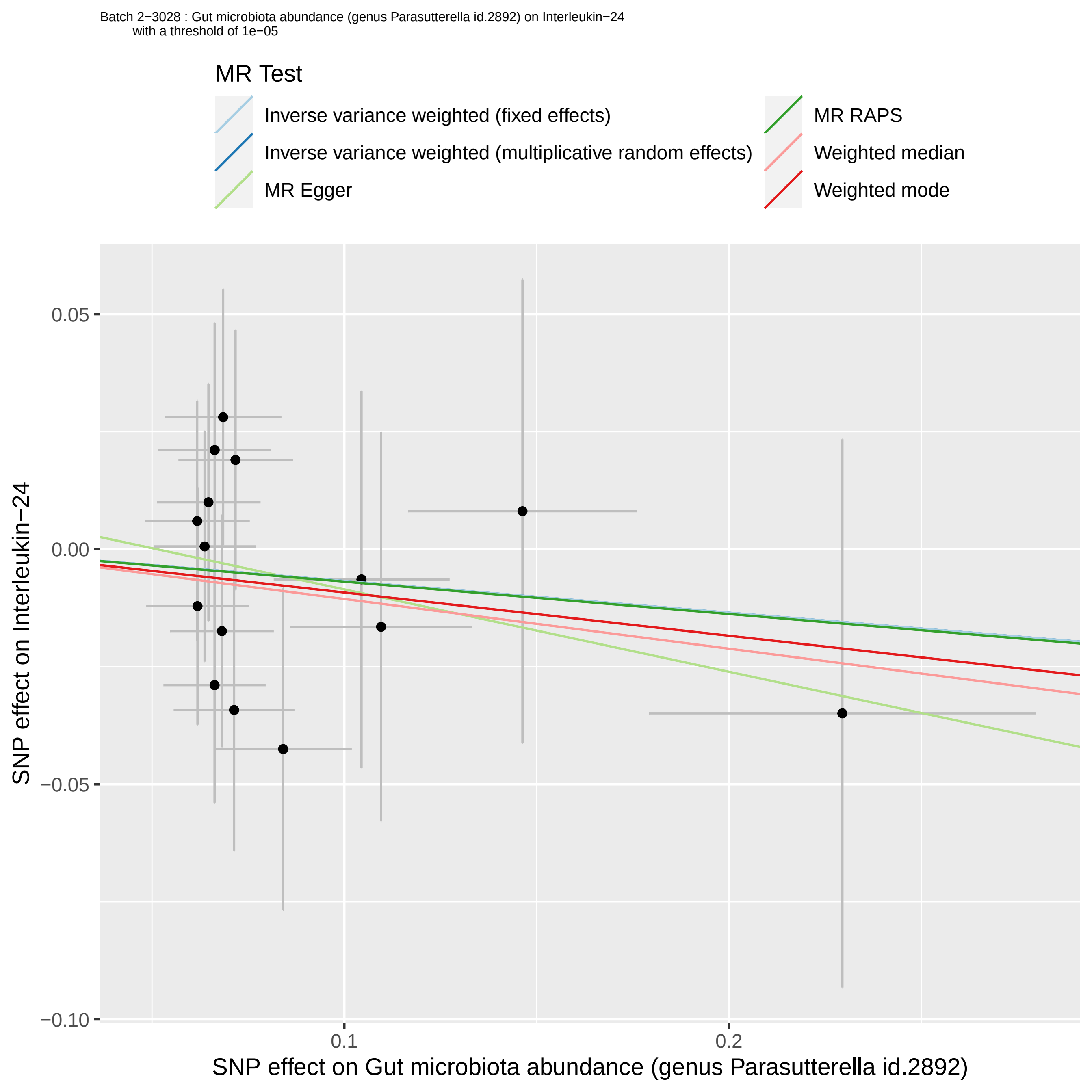

Batch 2−3028 : Gut microbiota abundance (genus Parasutterella id.2892) on Interleukin−24
with a threshold of 1e−05
MR Test
Inverse variance weighted (fixed effects)
Inverse variance weighted (multiplicative random effects)
MR Egger
MR RAPS
Weighted median
Weighted mode
0.05
0.00
−0.05
−0.10
0.1
0.2
SNP effect on Interleukin−24
SNP effect on Gut microbiota abundance (genus Parasutterella id.2892)

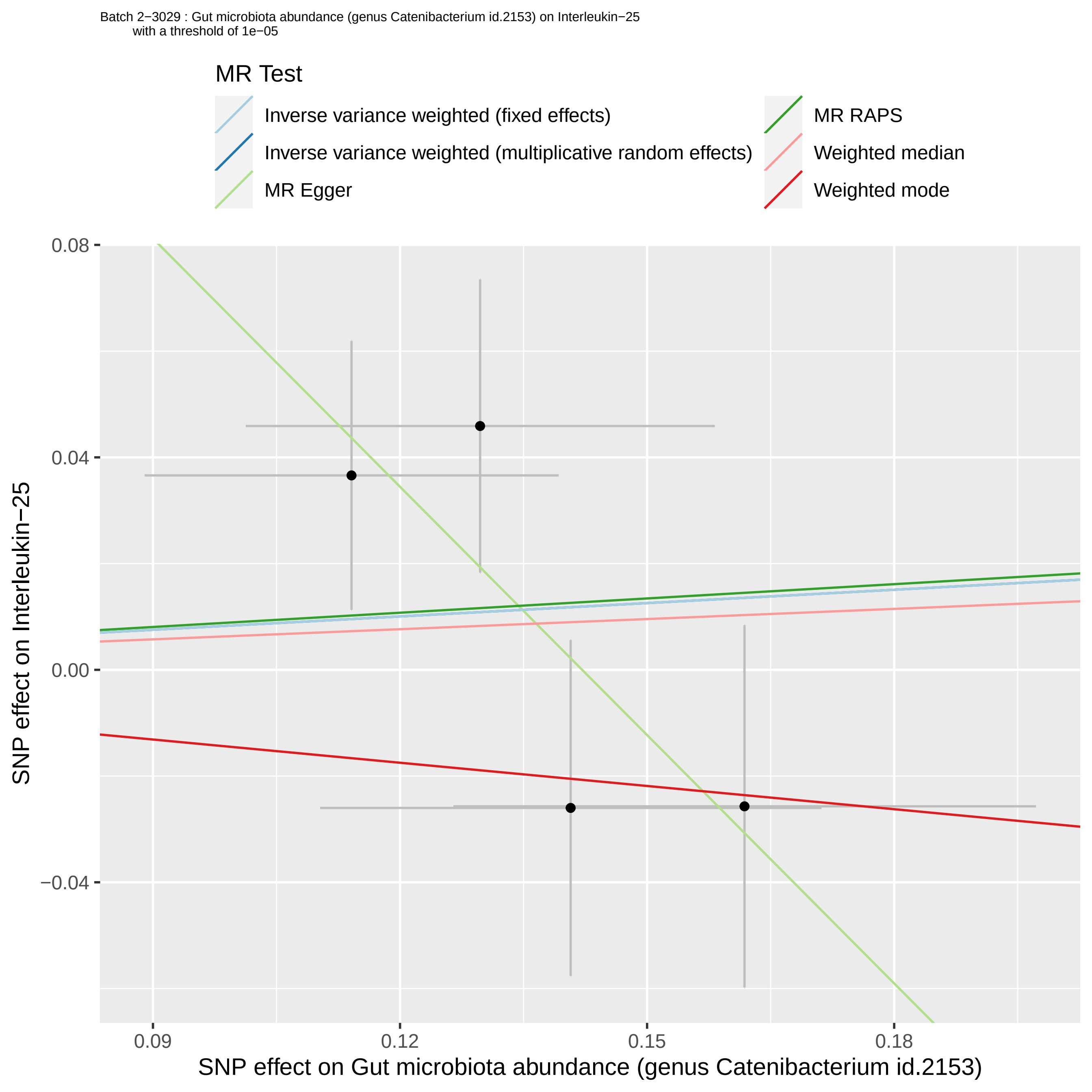

Batch 2−3029 : Gut microbiota abundance (genus Catenibacterium id.2153) on Interleukin−25
with a threshold of 1e−05
MR Test
Inverse variance weighted (fixed effects)
Inverse variance weighted (multiplicative random effects)
MR Egger
MR RAPS
Weighted median
Weighted mode
0.08
0.04
0.00
−0.04
0.09
0.12
0.15
0.18
SNP effect on Interleukin−25
SNP effect on Gut microbiota abundance (genus Catenibacterium id.2153)

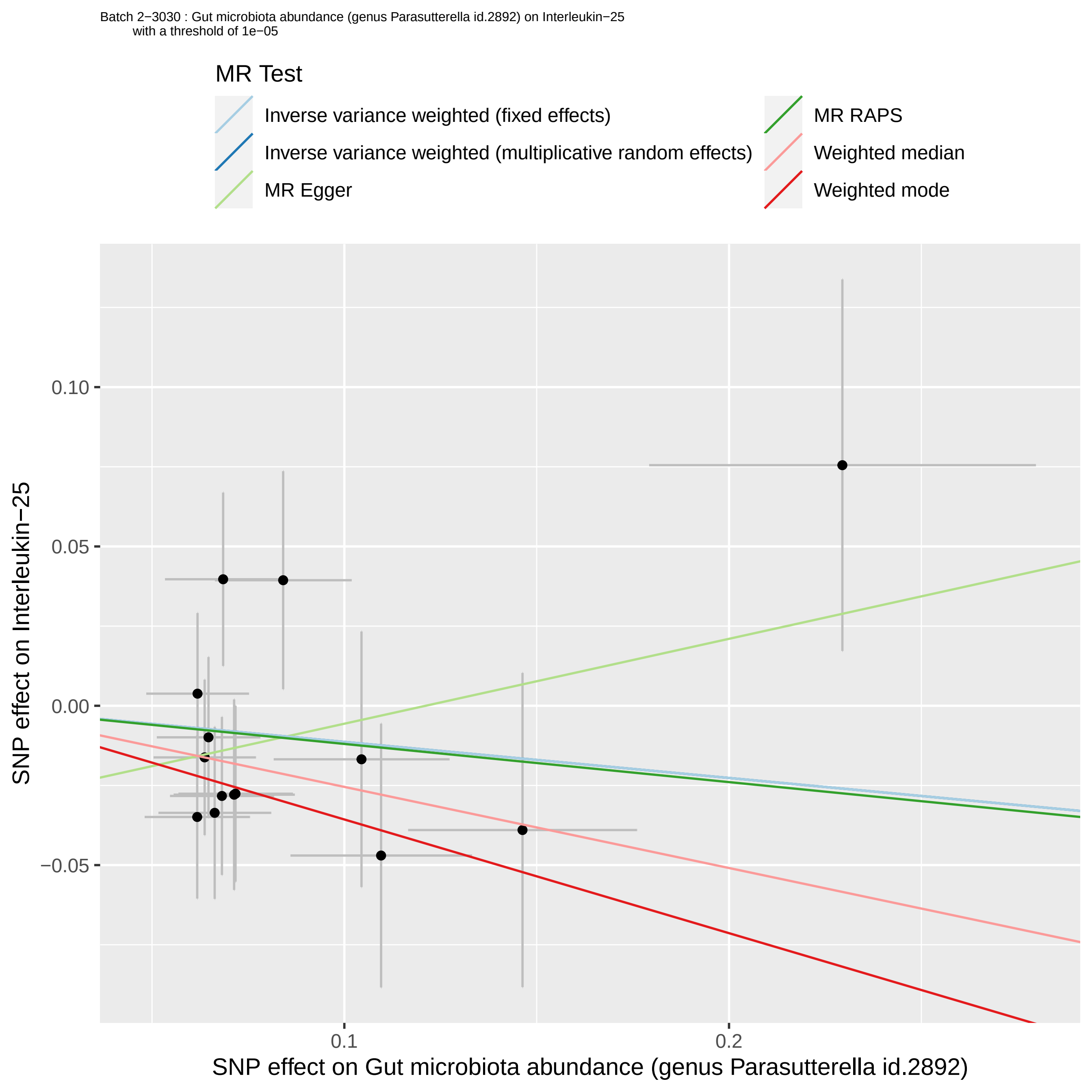
Batch 2−3030 : Gut microbiota abundance (genus Parasutterella id.2892) on Interleukin−25
with a threshold of 1e−05
MR Test
Inverse variance weighted (fixed effects)
Inverse variance weighted (multiplicative random effects)
MR Egger
MR RAPS
Weighted median
Weighted mode
0.10
0.05
0.00
−0.05
SNP effect on Interleukin−25
0.1
0.2
SNP effect on Gut microbiota abundance (genus Parasutterella id.2892)

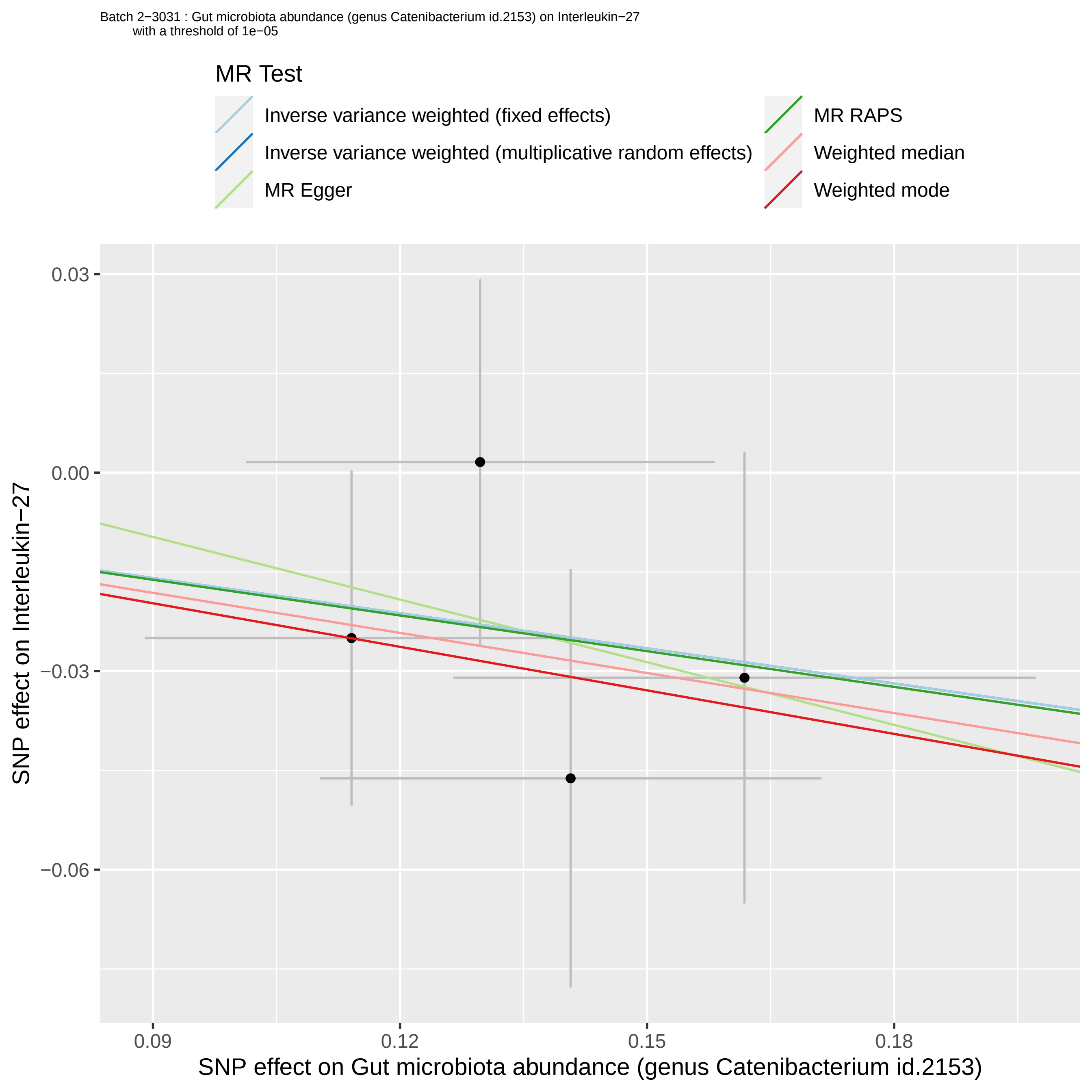

Batch 2−3031 : Gut microbiota abundance (genus Catenibacterium id.2153) on Interleukin−27
with a threshold of 1e−05
MR Test
Inverse variance weighted (fixed effects)
Inverse variance weighted (multiplicative random effects)
MR Egger
MR RAPS
Weighted median
Weighted mode
0.03
0.00
−0.03
−0.06
0.09
0.12
0.15
0.18
SNP effect on Interleukin−27
SNP effect on Gut microbiota abundance (genus Catenibacterium id.2153)

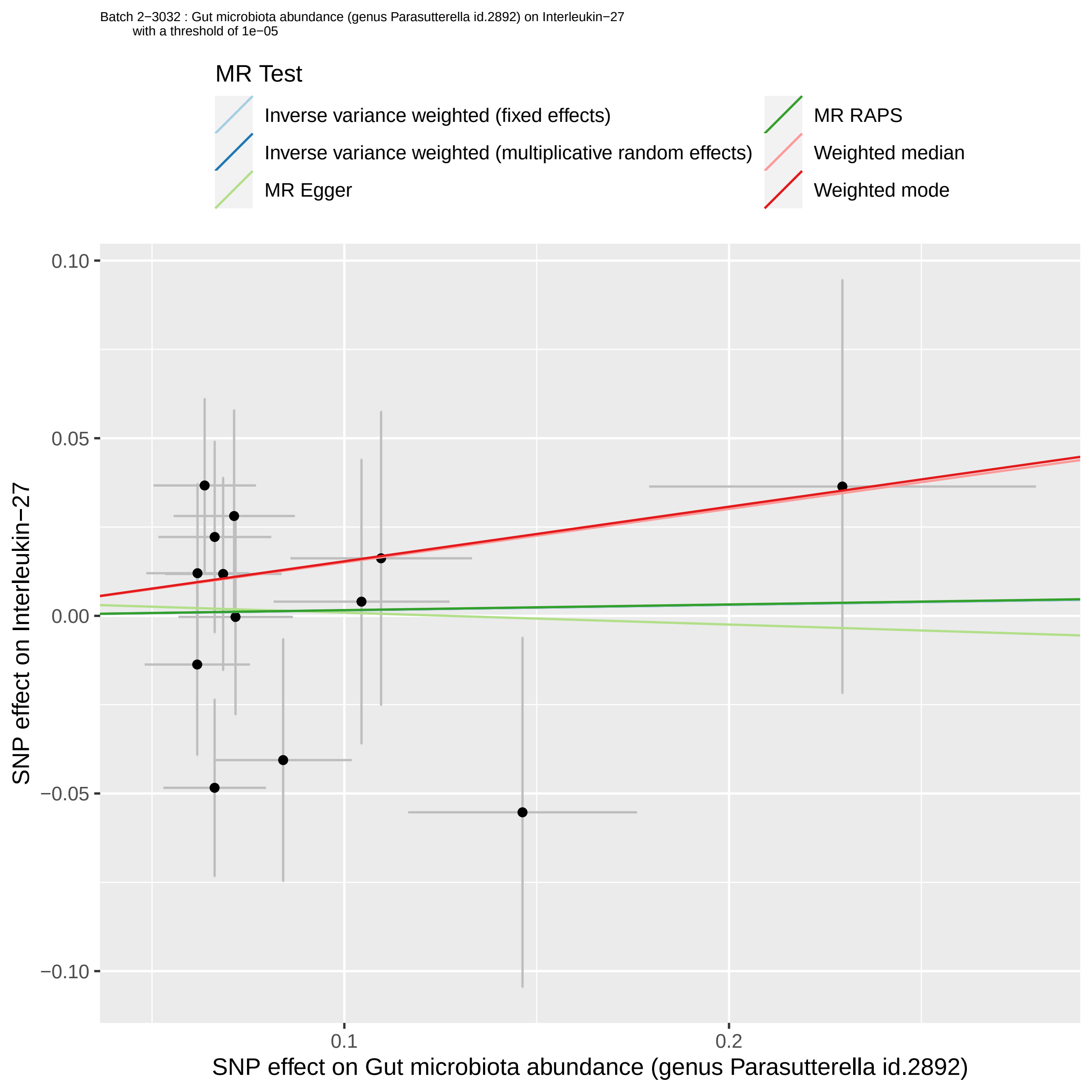

Batch 2−3032 : Gut microbiota abundance (genus Parasutterella id.2892) on Interleukin−27
with a threshold of 1e−05
MR Test
Inverse variance weighted (fixed effects)
Inverse variance weighted (multiplicative random effects)
MR Egger
MR RAPS
Weighted median
Weighted mode
0.10
0.05
0.00
−0.05
−0.10
0.1
0.2
SNP effect on Interleukin−27
SNP effect on Gut microbiota abundance (genus Parasutterella id.2892)

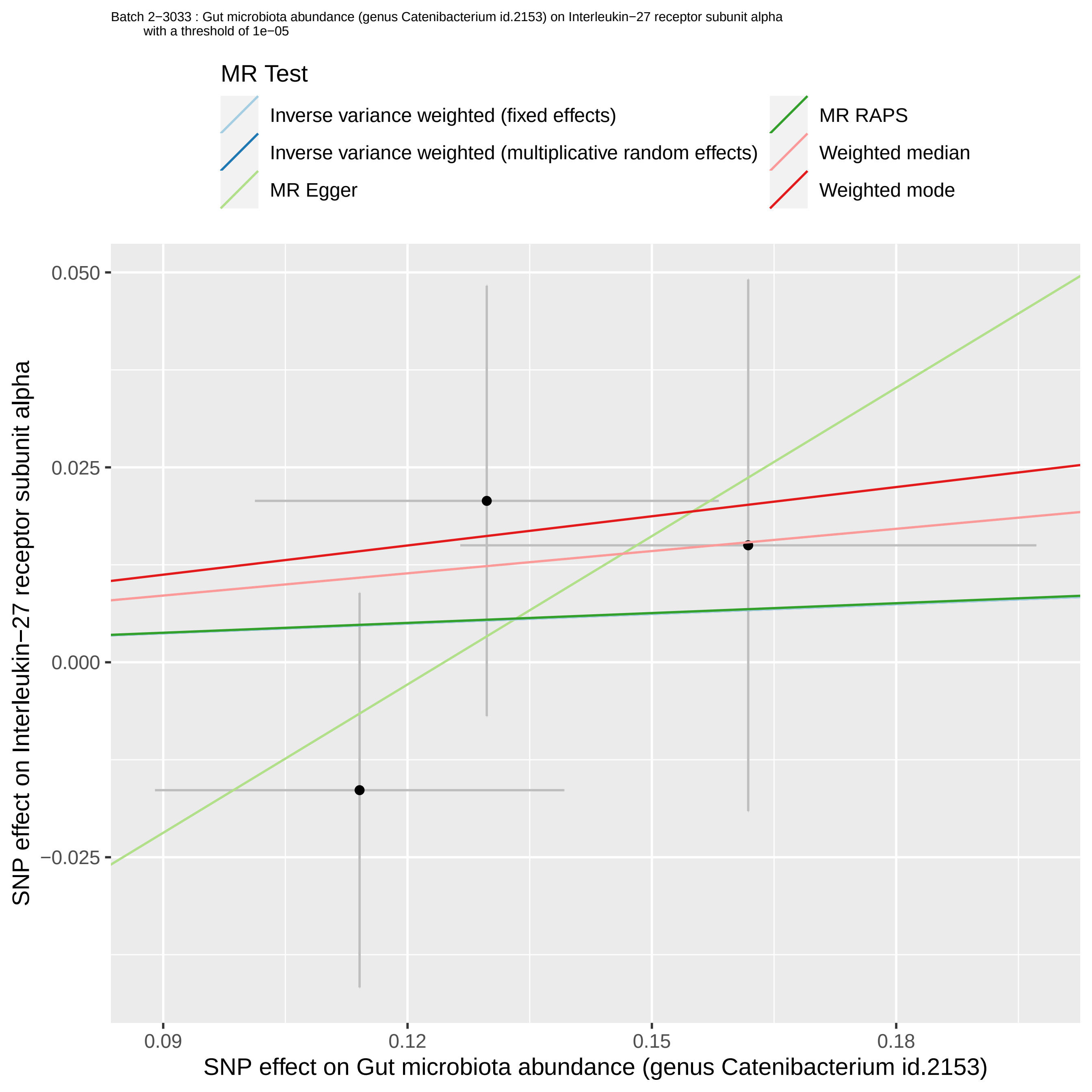

Batch 2−3033 : Gut microbiota abundance (genus Catenibacterium id.2153) on Interleukin−27 receptor subunit alpha
with a threshold of 1e−05
MR Test
Inverse variance weighted (fixed effects)
Inverse variance weighted (multiplicative random effects)
MR Egger
MR RAPS
Weighted median
Weighted mode
0.050
0.025
0.000
−0.025
0.09
0.12
0.15
0.18
SNP effect on Interleukin−27 receptor subunit alpha
SNP effect on Gut microbiota abundance (genus Catenibacterium id.2153)

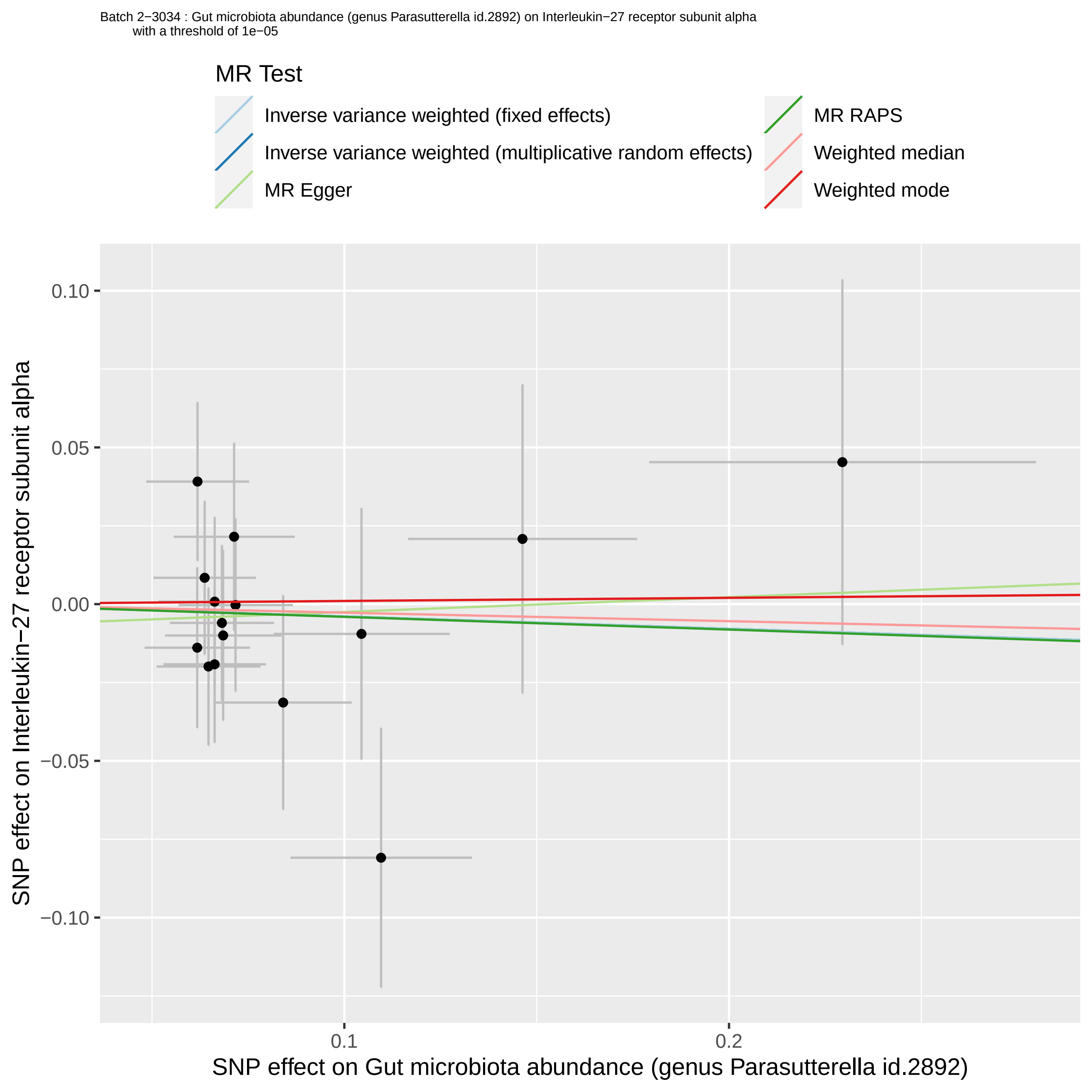
Batch 2−3034 : Gut microbiota abundance (genus Parasutterella id.2892) on Interleukin−27 receptor subunit alpha
with a threshold of 1e−05
MR Test
Inverse variance weighted (fixed effects)
Inverse variance weighted (multiplicative random effects)
MR Egger
MR RAPS
Weighted median
Weighted mode
0.10
0.05
0.00
−0.05
−0.10
0.1
0.2
SNP effect on Interleukin−27 receptor subunit alpha
SNP effect on Gut microbiota abundance (genus Parasutterella id.2892)

Batch 2−3035 : Gut microbiota abundance (genus Catenibacterium id.2153) on Interleukin−2 receptor subunit alpha
with a threshold of 1e−05

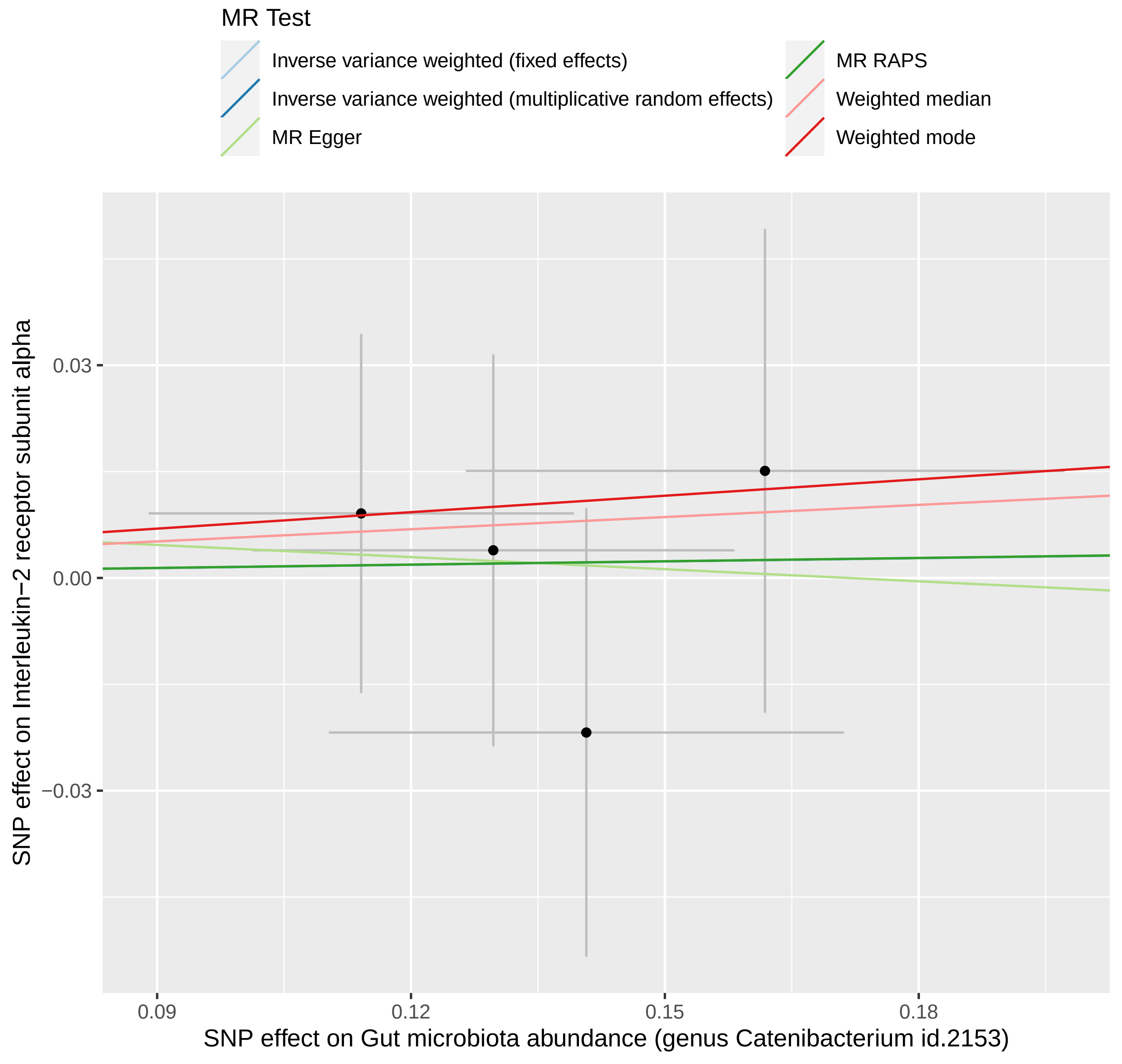

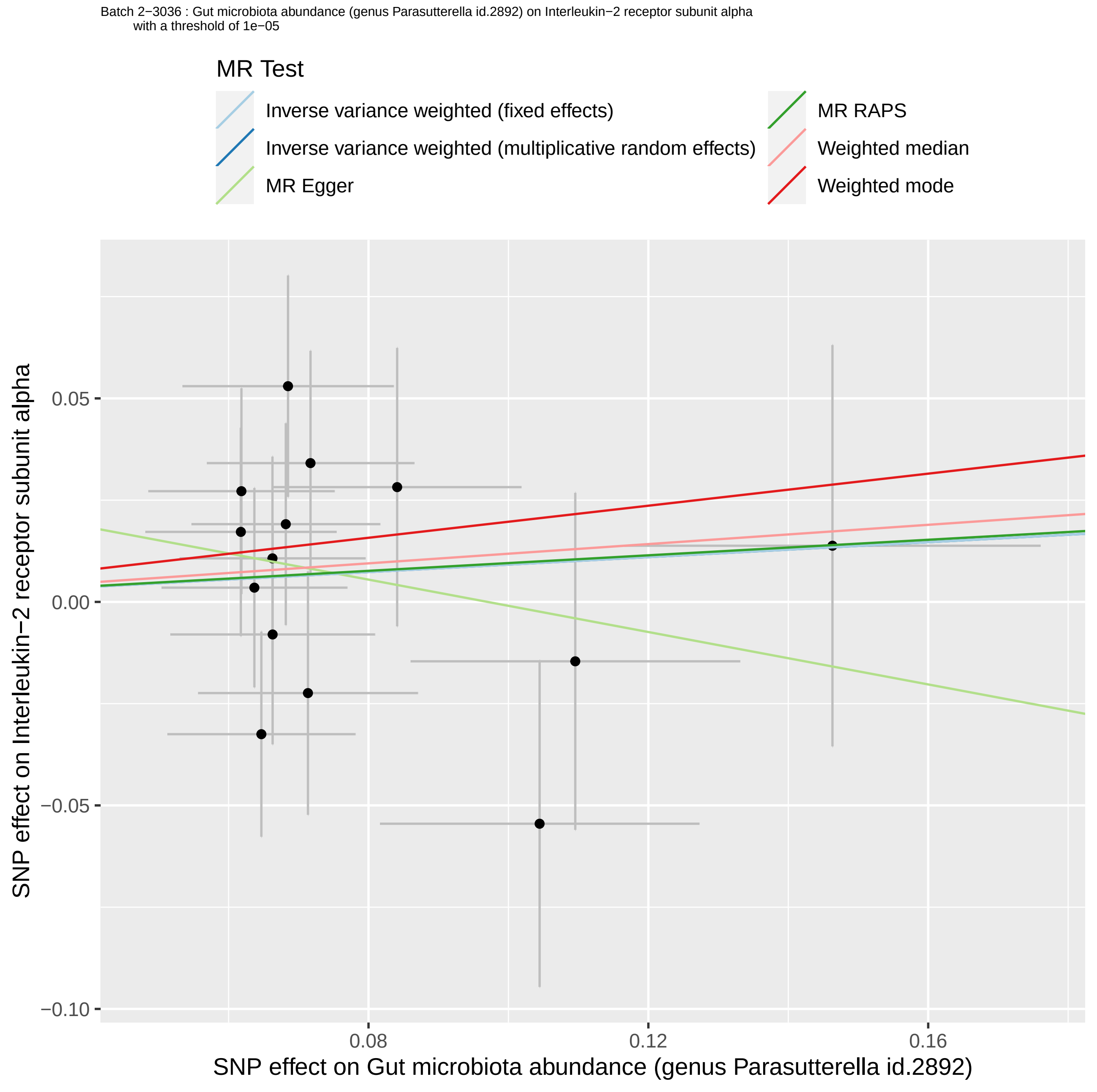

Batch 2−3036 : Gut microbiota abundance (genus Parasutterella id.2892) on Interleukin−2 receptor subunit alpha
with a threshold of 1e−05
MR Test
Inverse variance weighted (fixed effects)
Inverse variance weighted (multiplicative random effects)
MR Egger
MR RAPS
Weighted median
Weighted mode
0.05
0.00
−0.05
−0.10
0.08
0.12
0.16
SNP effect on Interleukin−2 receptor subunit alpha
SNP effect on Gut microbiota abundance (genus Parasutterella id.2892)

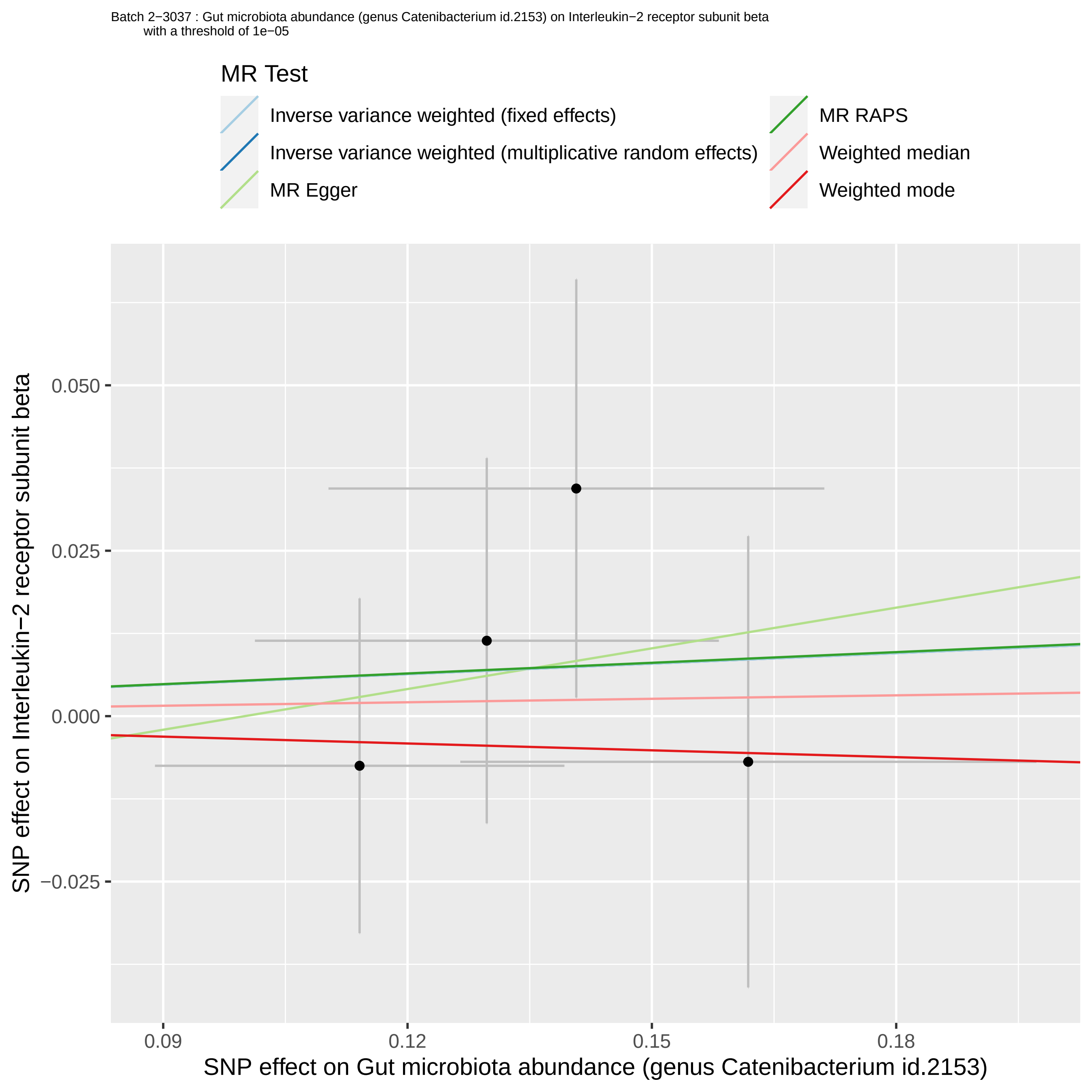
Batch 2−3037 : Gut microbiota abundance (genus Catenibacterium id.2153) on Interleukin−2 receptor subunit beta
with a threshold of 1e−05
MR Test
Inverse variance weighted (fixed effects)
Inverse variance weighted (multiplicative random effects)
MR Egger
MR RAPS
Weighted median
Weighted mode
0.050
0.025
0.000
−0.025
0.09
0.12
0.15
0.18
SNP effect on Interleukin−2 receptor subunit beta
SNP effect on Gut microbiota abundance (genus Catenibacterium id.2153)

Batch 2−3038 : Gut microbiota abundance (genus Parasutterella id.2892) on Interleukin−2 receptor subunit beta
with a threshold of 1e−05

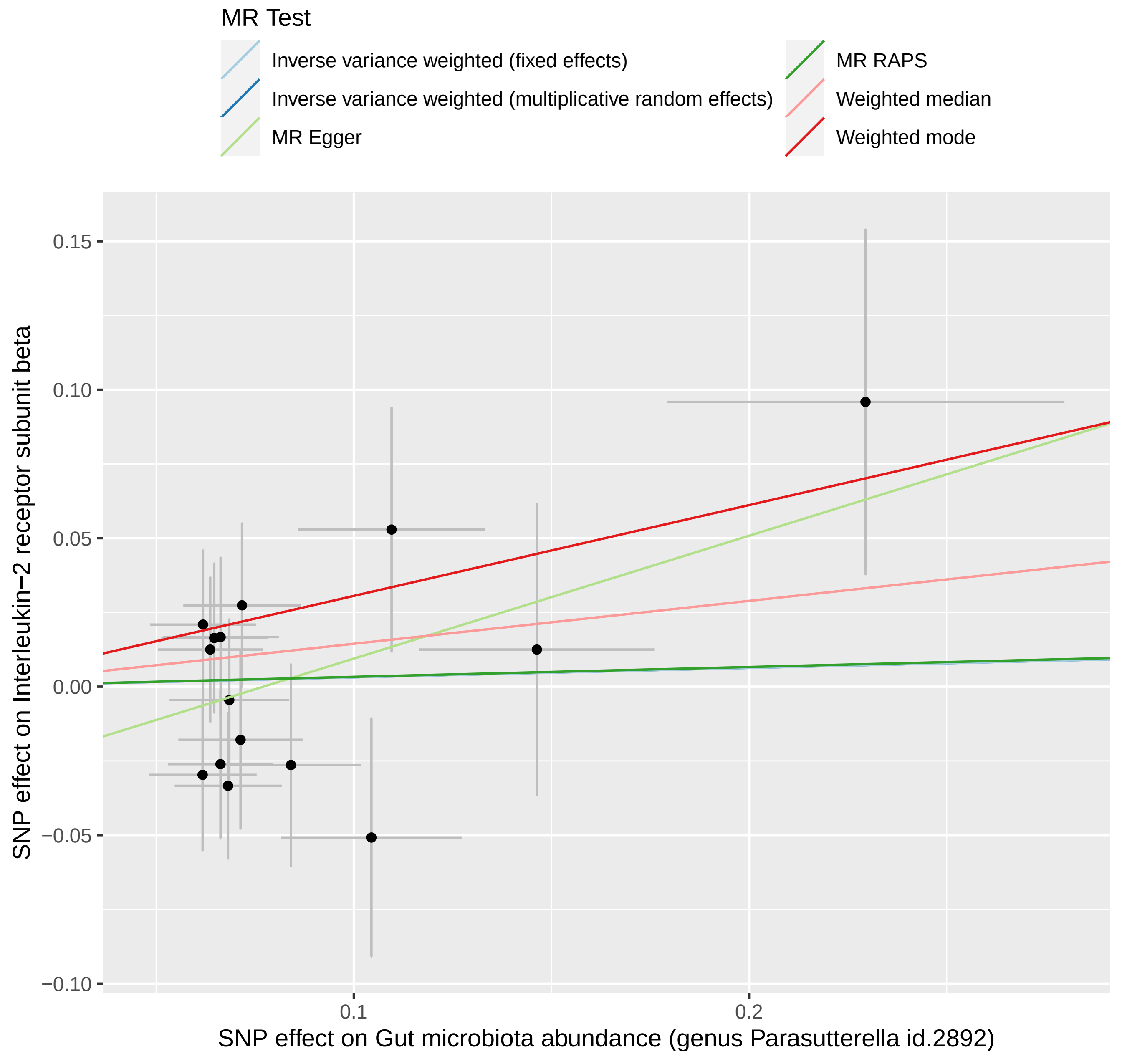

Batch 2−3039 : Gut microbiota abundance (genus Catenibacterium id.2153) on Cytokine receptor common subunit gamma
with a threshold of 1e−05

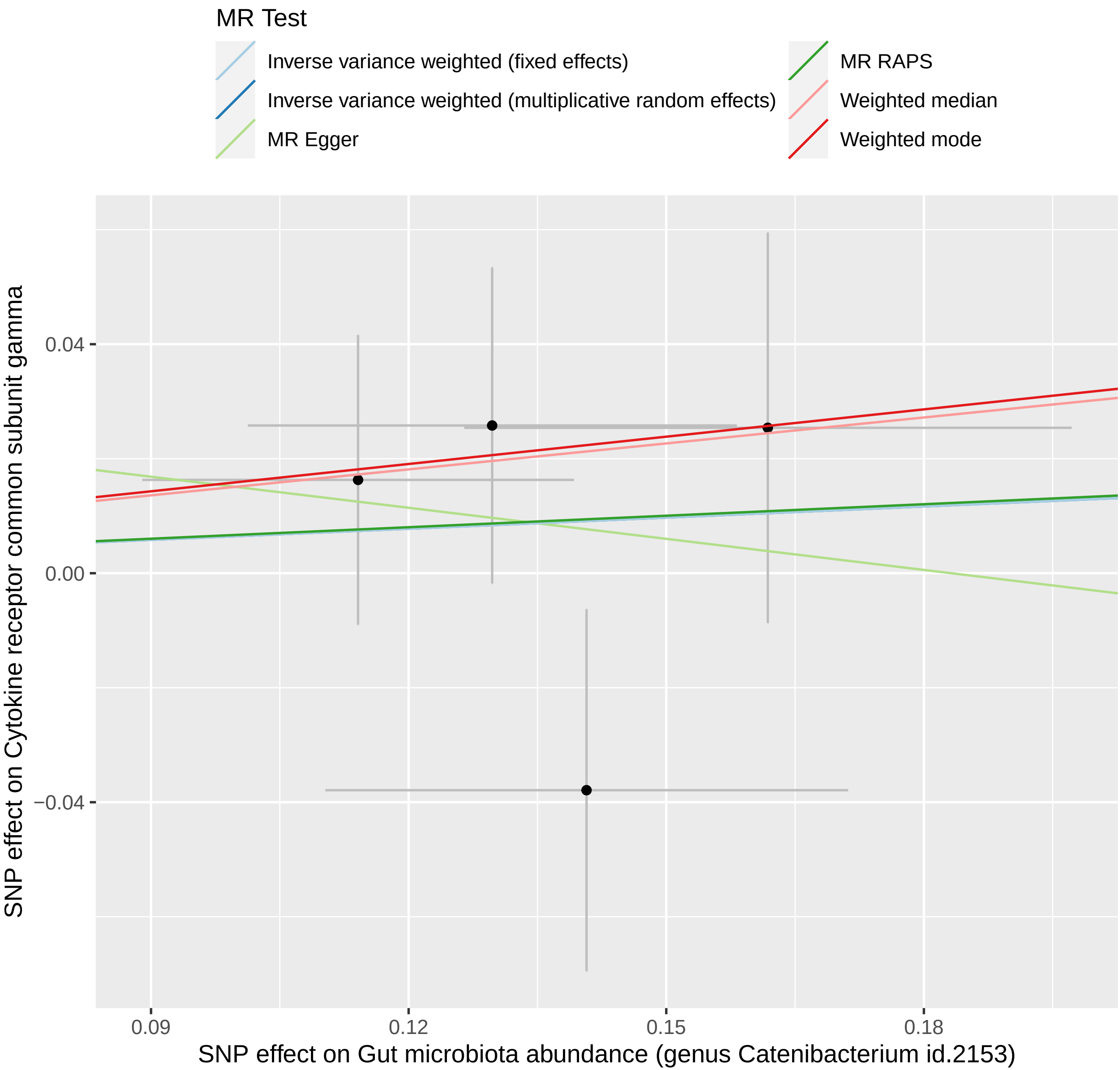

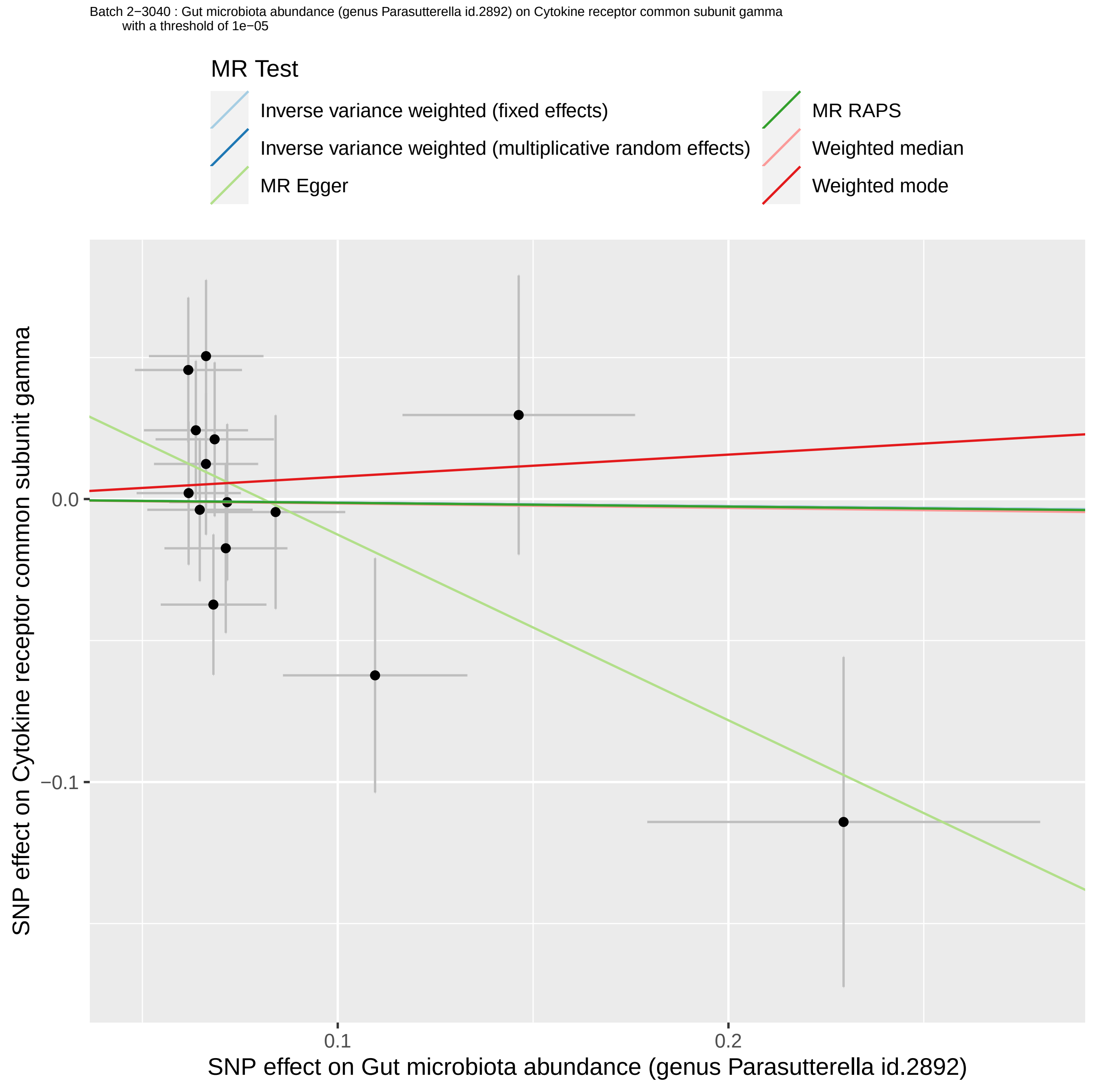
Batch 2−3040 : Gut microbiota abundance (genus Parasutterella id.2892) on Cytokine receptor common subunit gamma
with a threshold of 1e−05
MR Test
Inverse variance weighted (fixed effects)
Inverse variance weighted (multiplicative random effects)
MR Egger
MR RAPS
Weighted median
Weighted mode
0.0
−0.1
0.1
0.2
SNP effect on Cytokine receptor common subunit gamma
SNP effect on Gut microbiota abundance (genus Parasutterella id.2892)

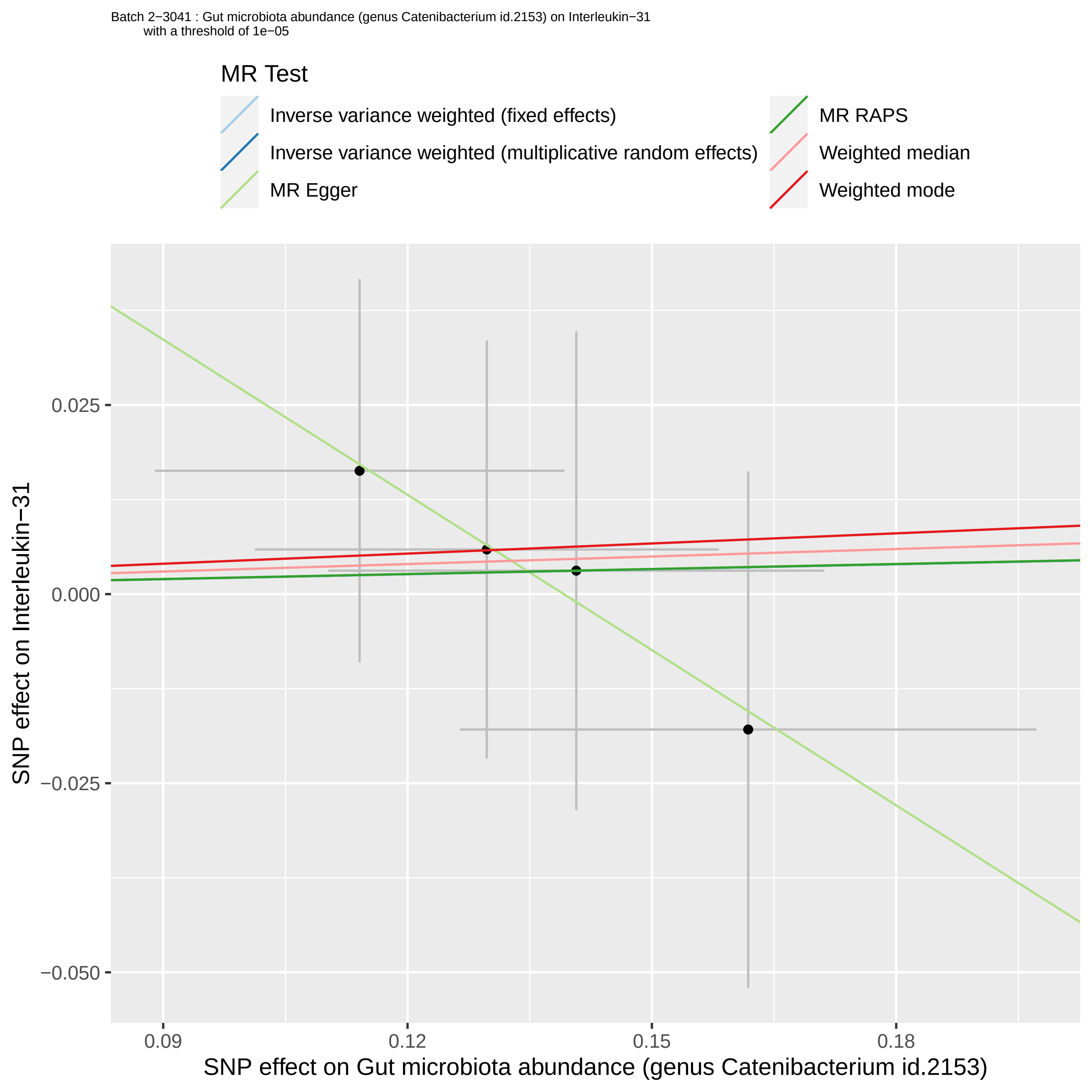
Batch 2−3041 : Gut microbiota abundance (genus Catenibacterium id.2153) on Interleukin−31
with a threshold of 1e−05
MR Test
Inverse variance weighted (fixed effects)
Inverse variance weighted (multiplicative random effects)
MR Egger
MR RAPS
Weighted median
Weighted mode
0.025
0.000
−0.025
−0.050
0.09
0.12
0.15
0.18
SNP effect on Interleukin−31
SNP effect on Gut microbiota abundance (genus Catenibacterium id.2153)

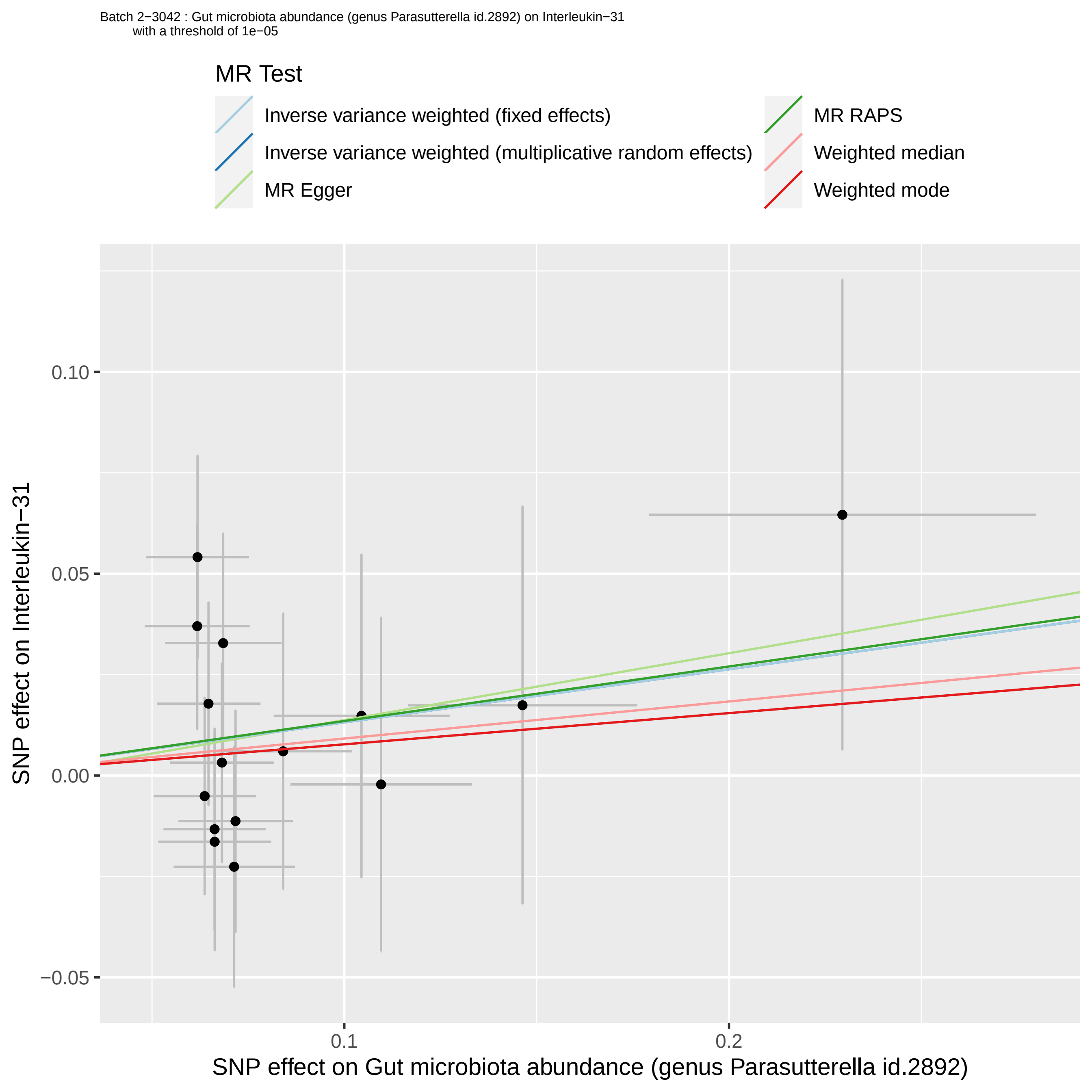
Batch 2−3042 : Gut microbiota abundance (genus Parasutterella id.2892) on Interleukin−31
with a threshold of 1e−05
MR Test
Inverse variance weighted (fixed effects)
Inverse variance weighted (multiplicative random effects)
MR Egger
MR RAPS
Weighted median
Weighted mode
0.10
0.05
0.00
−0.05
0.1
0.2
SNP effect on Interleukin−31
SNP effect on Gut microbiota abundance (genus Parasutterella id.2892)

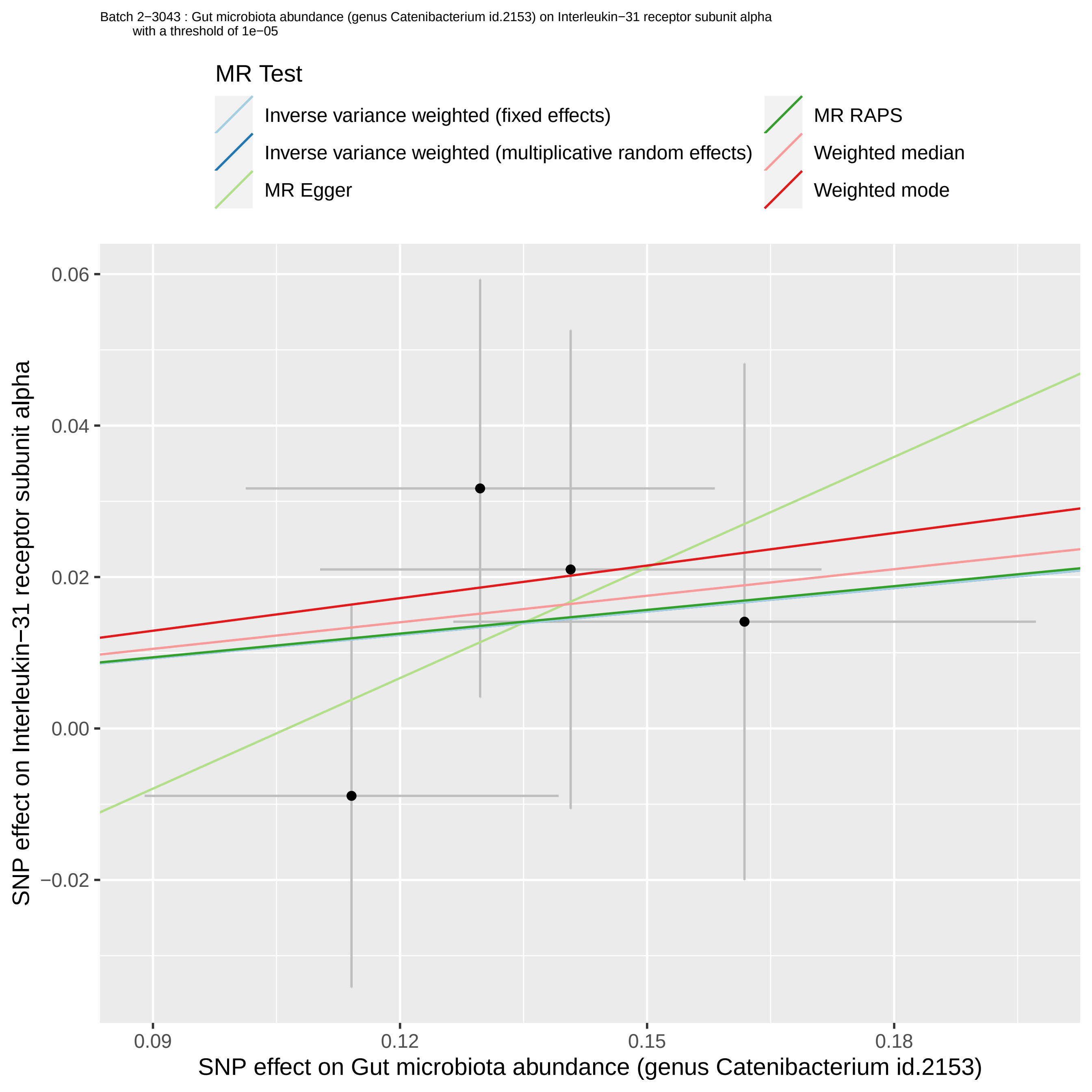

Batch 2−3043 : Gut microbiota abundance (genus Catenibacterium id.2153) on Interleukin−31 receptor subunit alpha
with a threshold of 1e−05
MR Test
Inverse variance weighted (fixed effects)
Inverse variance weighted (multiplicative random effects)
MR Egger
MR RAPS
Weighted median
Weighted mode
0.06
0.04
0.02
0.00
−0.02
0.09
0.12
0.15
0.18
SNP effect on Interleukin−31 receptor subunit alpha
SNP effect on Gut microbiota abundance (genus Catenibacterium id.2153)

Batch 2−3044 : Gut microbiota abundance (genus Parasutterella id.2892) on Interleukin−31 receptor subunit alpha
with a threshold of 1e−05

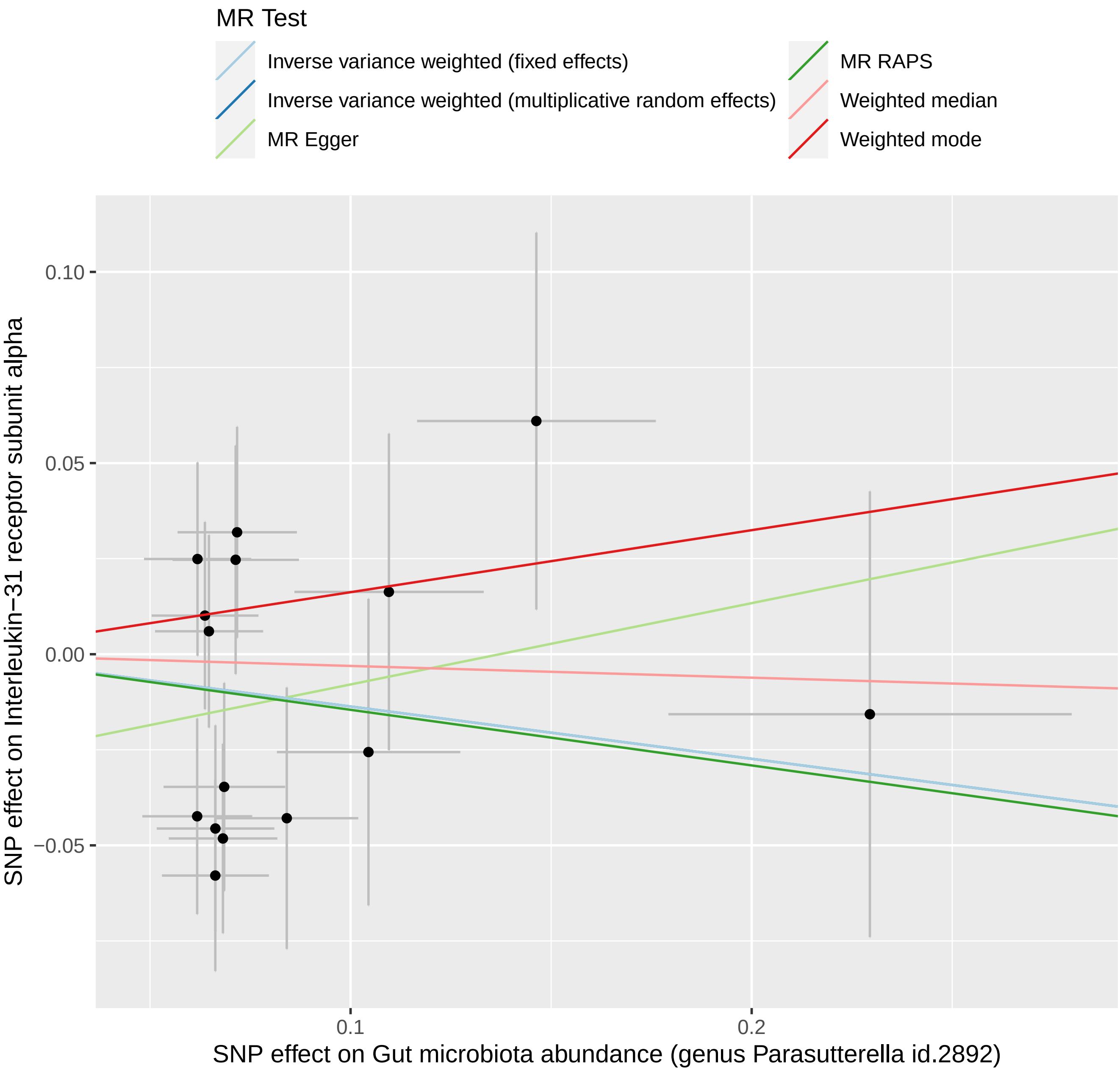

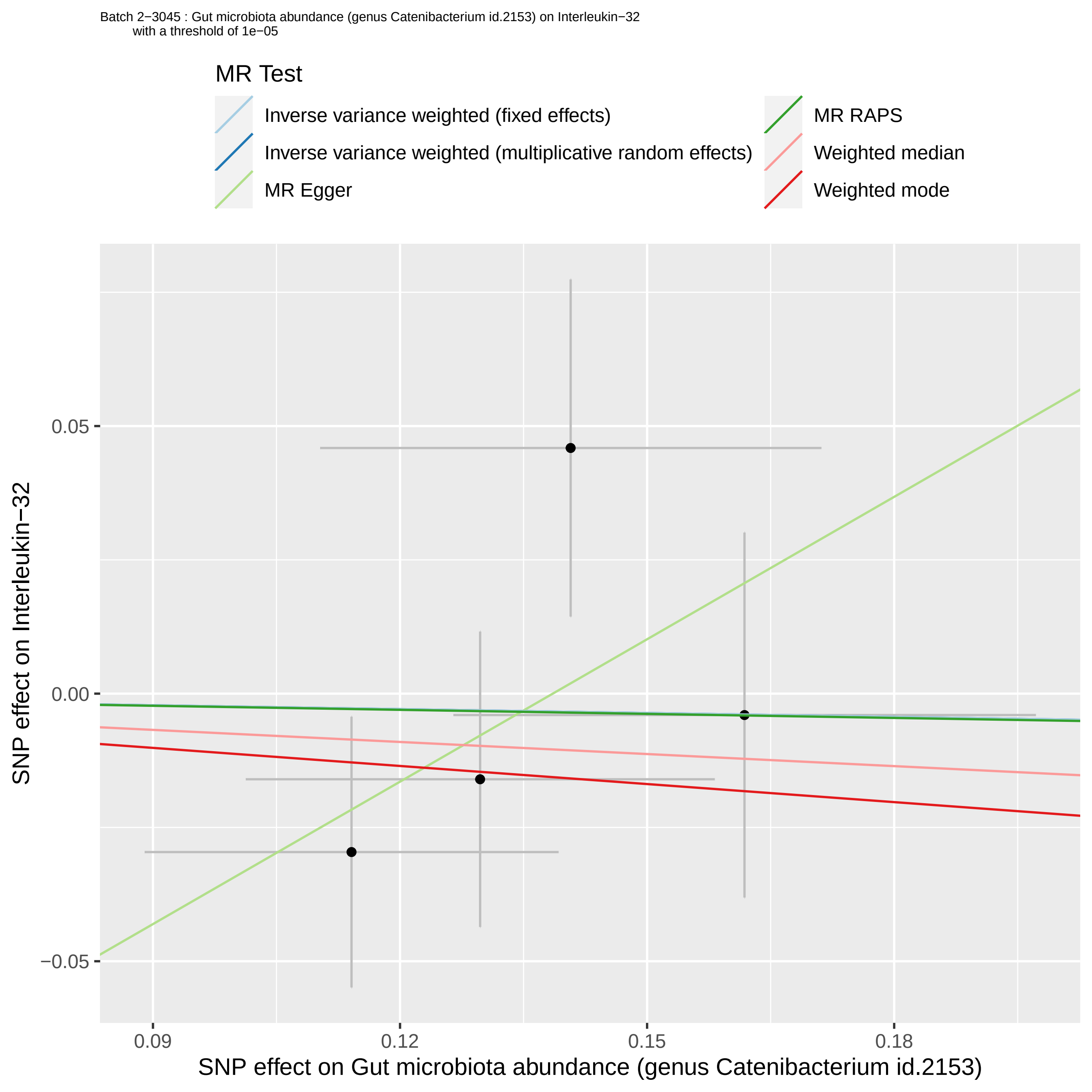
Batch 2−3045 : Gut microbiota abundance (genus Catenibacterium id.2153) on Interleukin−32
with a threshold of 1e−05
MR Test
Inverse variance weighted (fixed effects)
Inverse variance weighted (multiplicative random effects)
MR Egger
MR RAPS
Weighted median
Weighted mode
0.05
0.00
−0.05
0.09
0.12
0.15
0.18
SNP effect on Interleukin−32
SNP effect on Gut microbiota abundance (genus Catenibacterium id.2153)

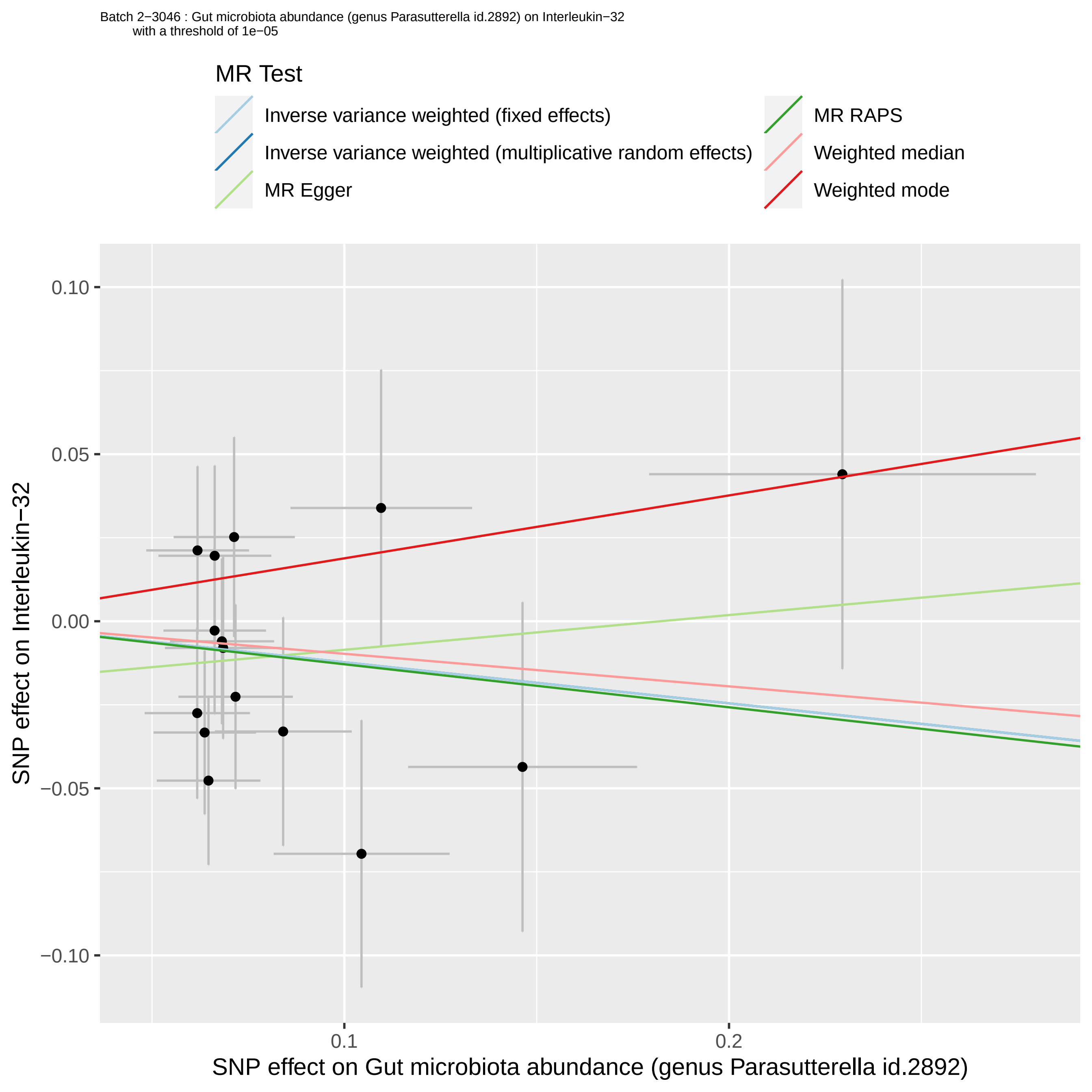
Batch 2−3046 : Gut microbiota abundance (genus Parasutterella id.2892) on Interleukin−32
with a threshold of 1e−05
MR Test
Inverse variance weighted (fixed effects)
Inverse variance weighted (multiplicative random effects)
MR Egger
MR RAPS
Weighted median
Weighted mode
0.10
0.05
0.00
−0.05
−0.10
0.1
0.2
SNP effect on Interleukin−32
SNP effect on Gut microbiota abundance (genus Parasutterella id.2892)

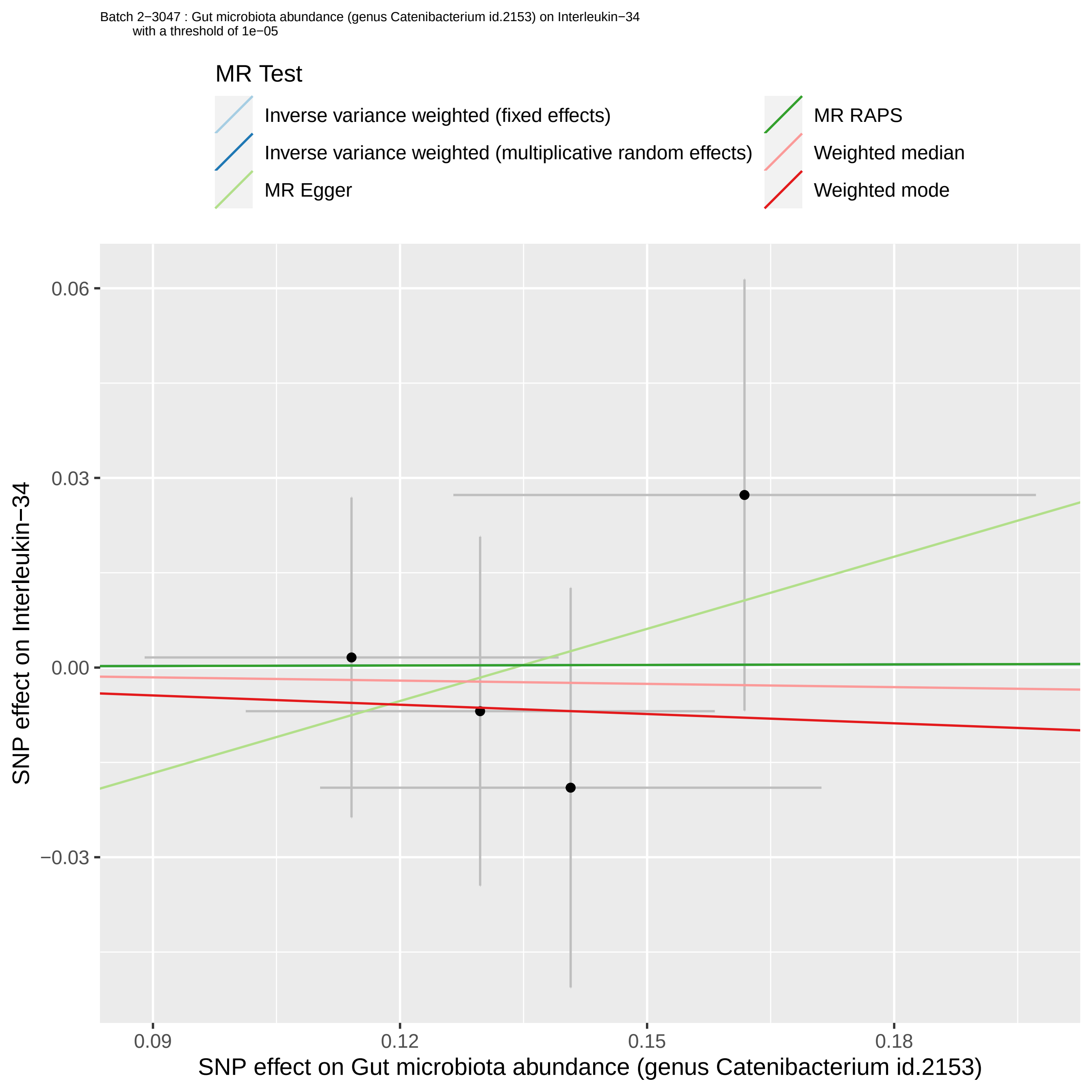

Batch 2−3047 : Gut microbiota abundance (genus Catenibacterium id.2153) on Interleukin−34
with a threshold of 1e−05
MR Test
Inverse variance weighted (fixed effects)
Inverse variance weighted (multiplicative random effects)
MR Egger
MR RAPS
Weighted median
Weighted mode
0.06
0.03
0.00
−0.03
0.09
0.12
0.15
0.18
SNP effect on Interleukin−34
SNP effect on Gut microbiota abundance (genus Catenibacterium id.2153)

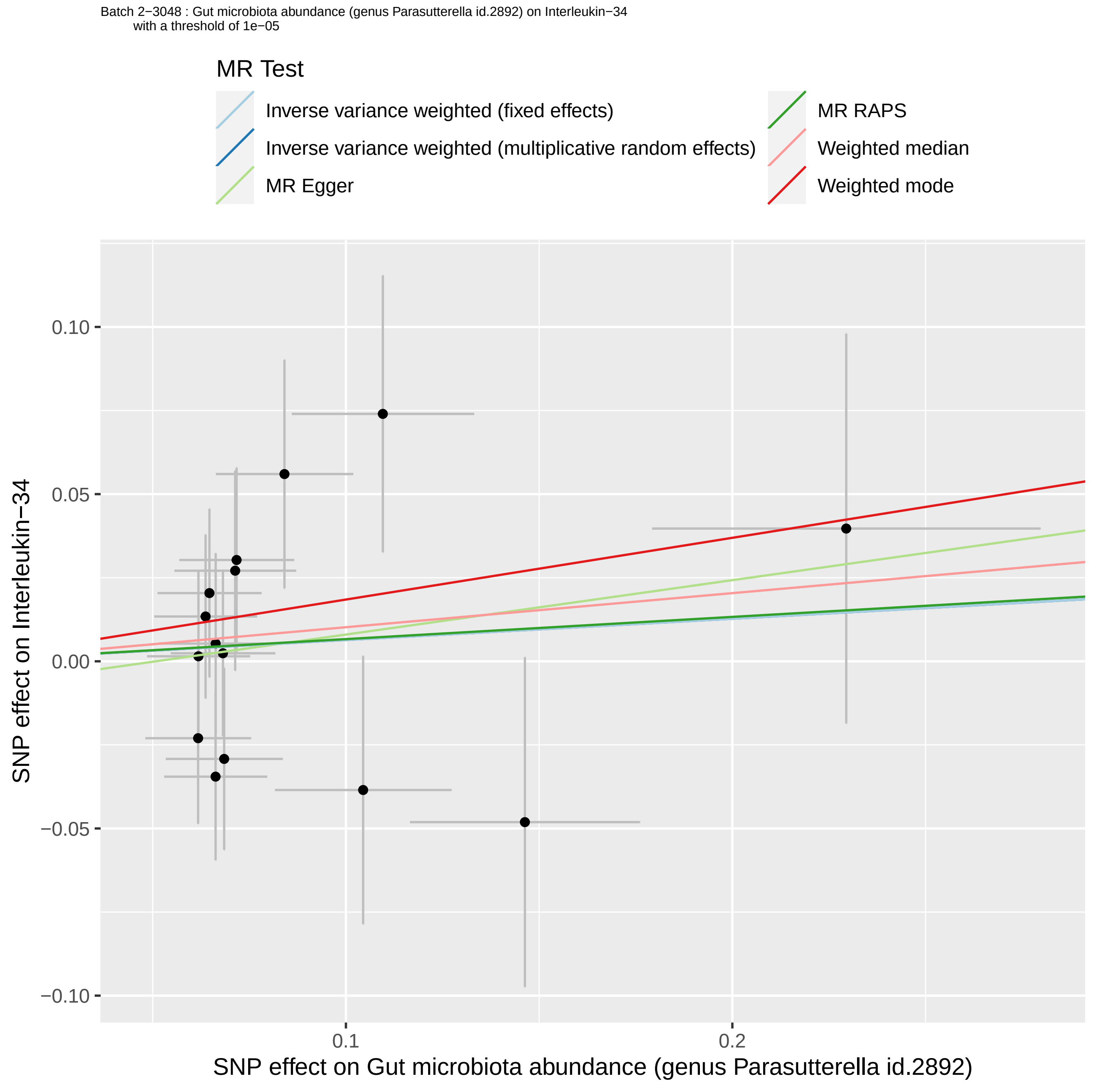
Batch 2−3048 : Gut microbiota abundance (genus Parasutterella id.2892) on Interleukin−34
with a threshold of 1e−05
MR Test
Inverse variance weighted (fixed effects)
Inverse variance weighted (multiplicative random effects)
MR Egger
MR RAPS
Weighted median
Weighted mode
0.10
0.05
0.00
−0.05
−0.10
0.1
0.2
SNP effect on Interleukin−34
SNP effect on Gut microbiota abundance (genus Parasutterella id.2892)

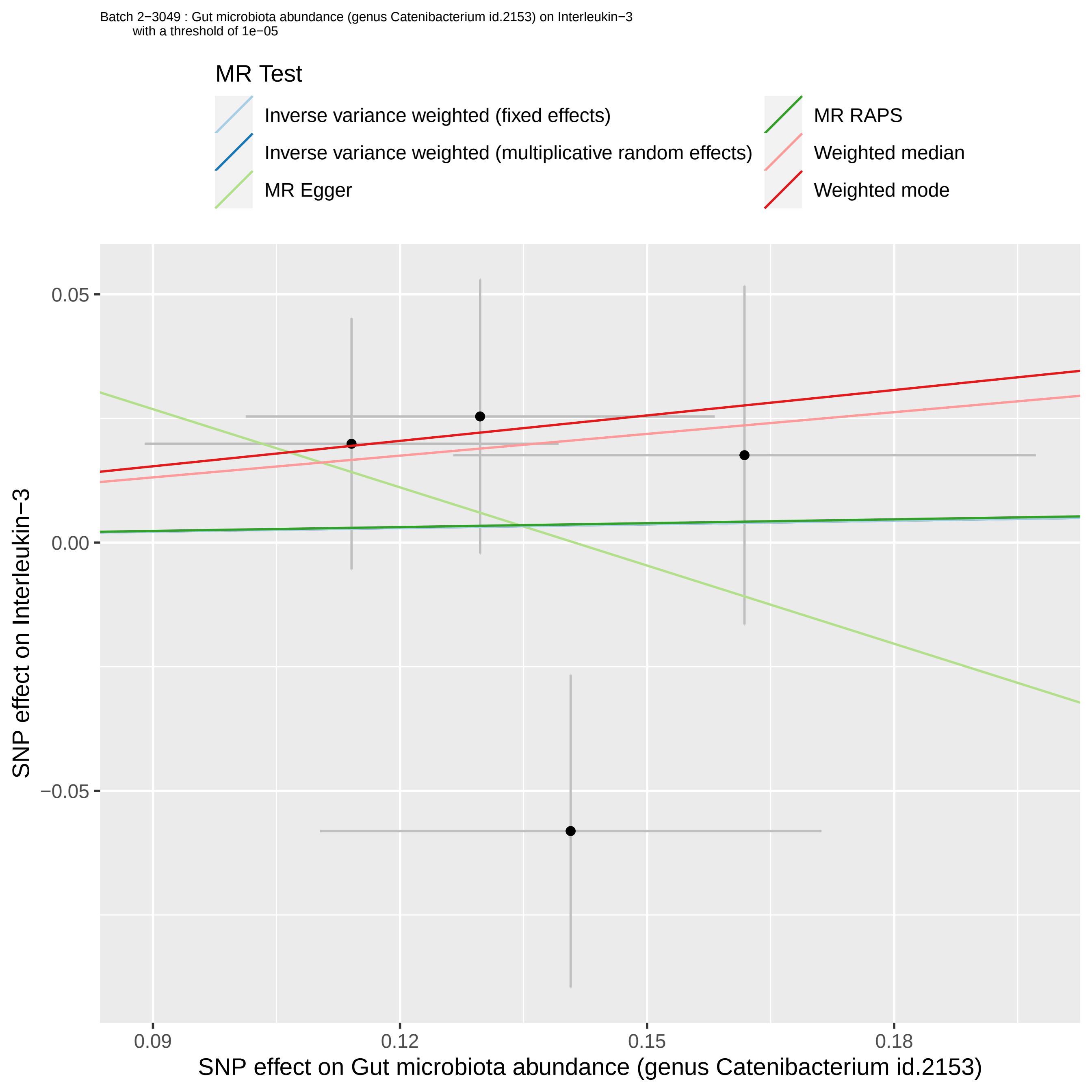

Batch 2−3049 : Gut microbiota abundance (genus Catenibacterium id.2153) on Interleukin−3
with a threshold of 1e−05
MR Test
Inverse variance weighted (fixed effects)
Inverse variance weighted (multiplicative random effects)
MR Egger
MR RAPS
Weighted median
Weighted mode
0.05
0.00
−0.05
SNP effect on Interleukin−3
0.09
0.12
0.15
0.18
SNP effect on Gut microbiota abundance (genus Catenibacterium id.2153)

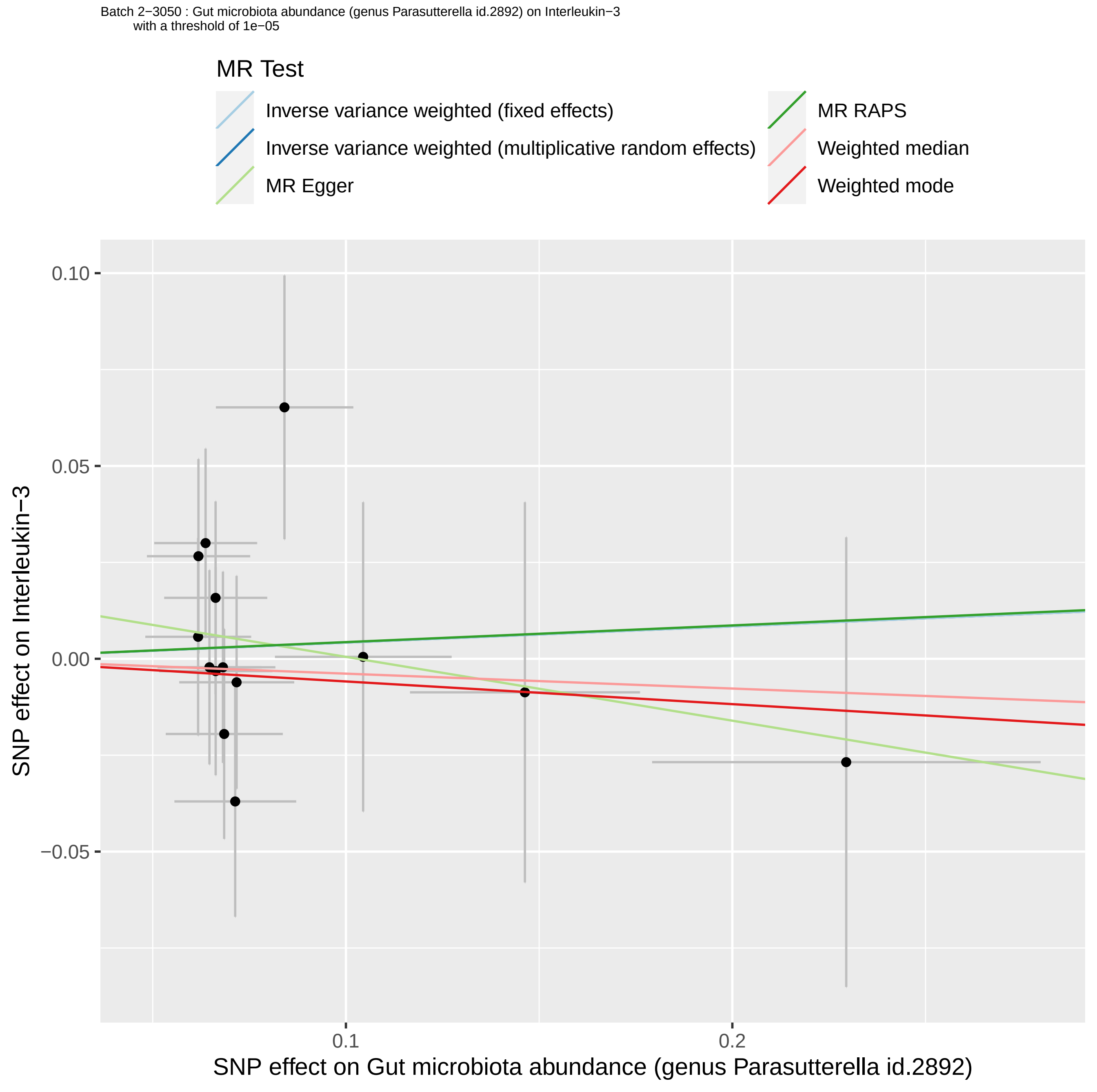

Batch 2−3050 : Gut microbiota abundance (genus Parasutterella id.2892) on Interleukin−3
with a threshold of 1e−05
MR Test
Inverse variance weighted (fixed effects)
Inverse variance weighted (multiplicative random effects)
MR Egger
MR RAPS
Weighted median
Weighted mode
0.10
0.05
0.00
−0.05
SNP effect on Interleukin−3
0.1
0.2
SNP effect on Gut microbiota abundance (genus Parasutterella id.2892)

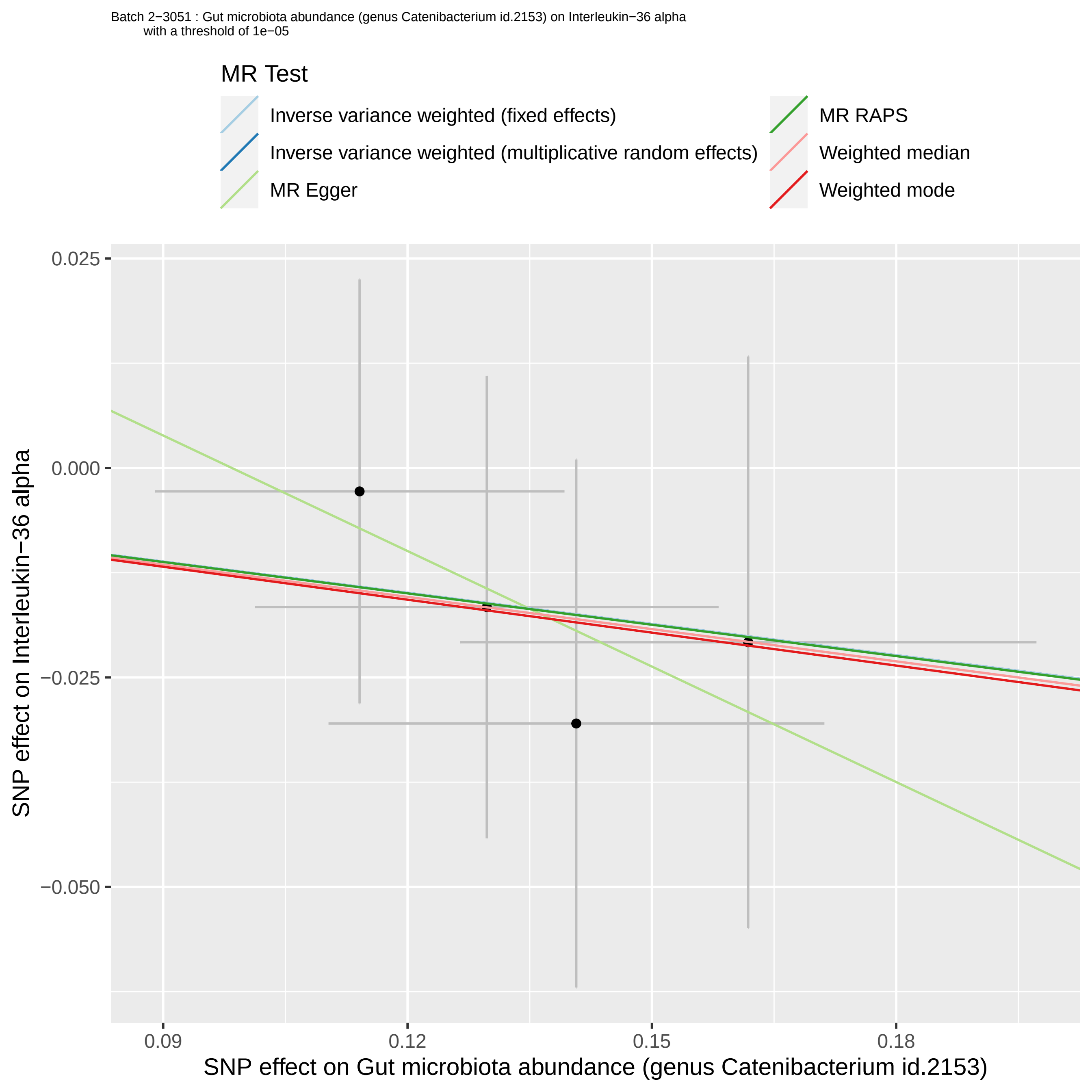
Batch 2−3051 : Gut microbiota abundance (genus Catenibacterium id.2153) on Interleukin−36 alpha
with a threshold of 1e−05
MR Test
Inverse variance weighted (fixed effects)
Inverse variance weighted (multiplicative random effects)
MR Egger
MR RAPS
Weighted median
Weighted mode
0.025
0.000
−0.025
−0.050
0.09
0.12
0.15
0.18
SNP effect on Interleukin−36 alpha
SNP effect on Gut microbiota abundance (genus Catenibacterium id.2153)

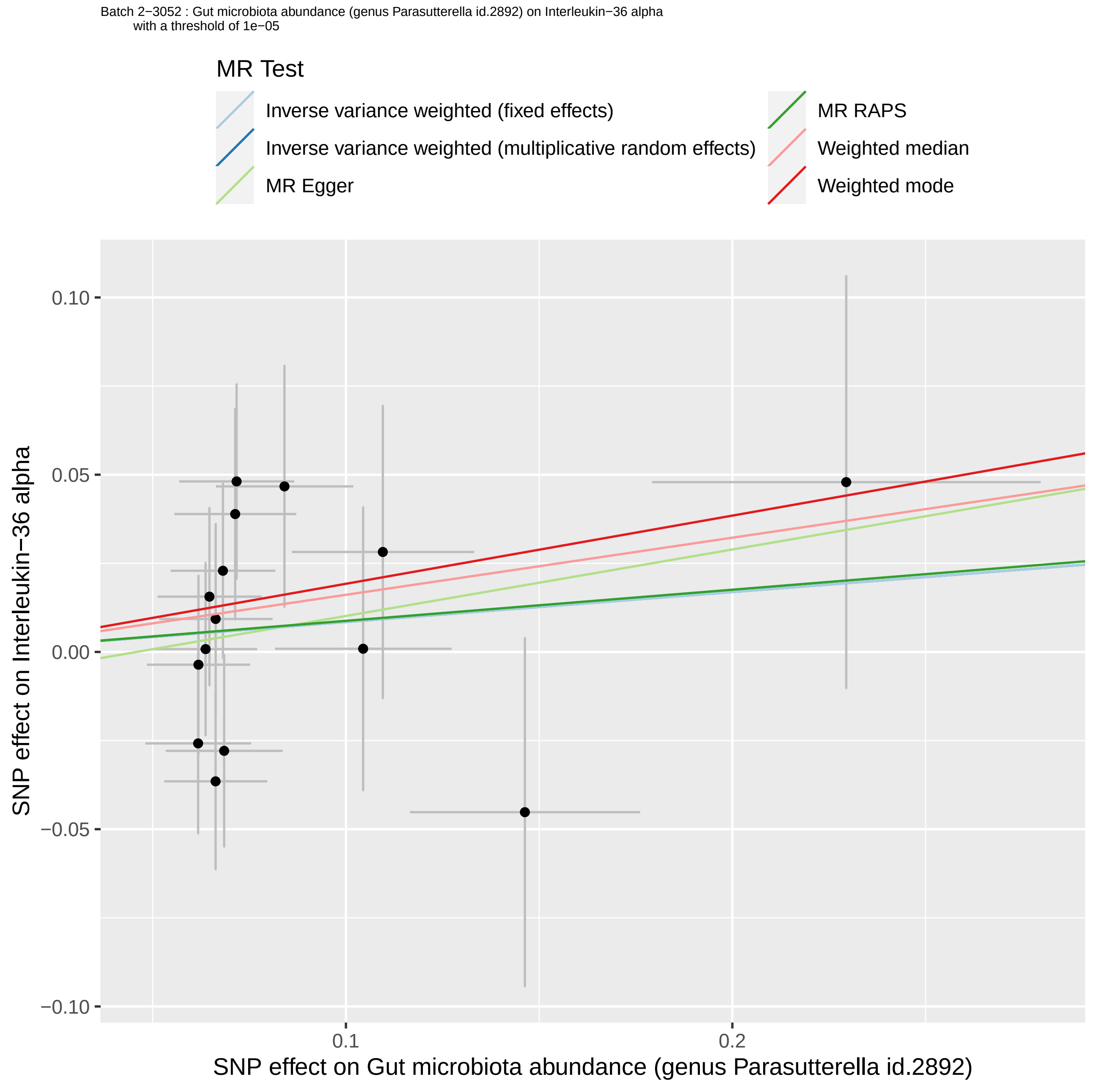

Batch 2−3052 : Gut microbiota abundance (genus Parasutterella id.2892) on Interleukin−36 alpha
with a threshold of 1e−05
MR Test
Inverse variance weighted (fixed effects)
Inverse variance weighted (multiplicative random effects)
MR Egger
MR RAPS
Weighted median
Weighted mode
0.10
0.05
0.00
−0.05
−0.10
0.1
0.2
SNP effect on Interleukin−36 alpha
SNP effect on Gut microbiota abundance (genus Parasutterella id.2892)

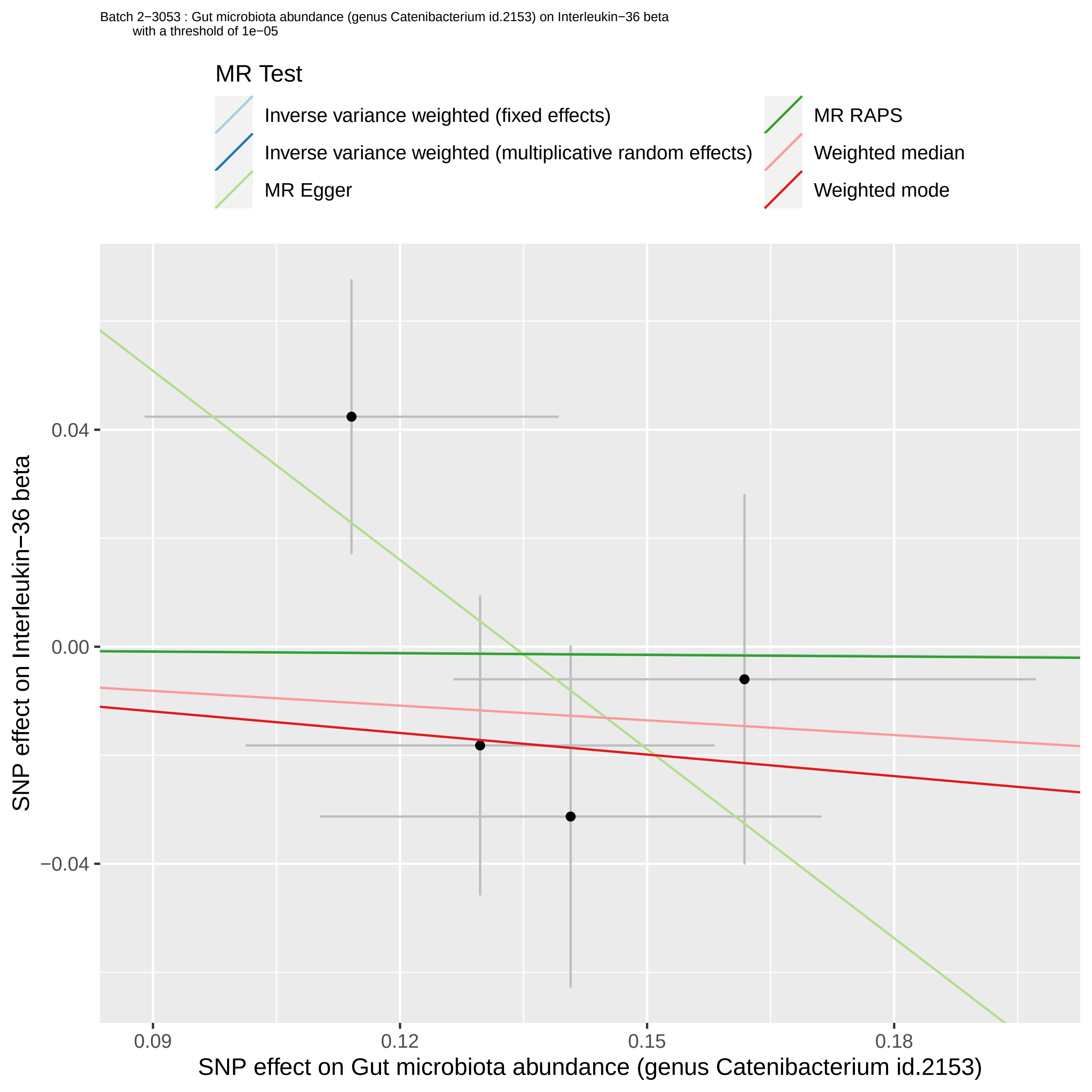
Batch 2−3053 : Gut microbiota abundance (genus Catenibacterium id.2153) on Interleukin−36 beta
with a threshold of 1e−05
MR Test
Inverse variance weighted (fixed effects)
Inverse variance weighted (multiplicative random effects)
MR Egger
MR RAPS
Weighted median
Weighted mode
0.04
0.00
−0.04
0.09
0.12
0.15
0.18
SNP effect on Interleukin−36 beta
SNP effect on Gut microbiota abundance (genus Catenibacterium id.2153)

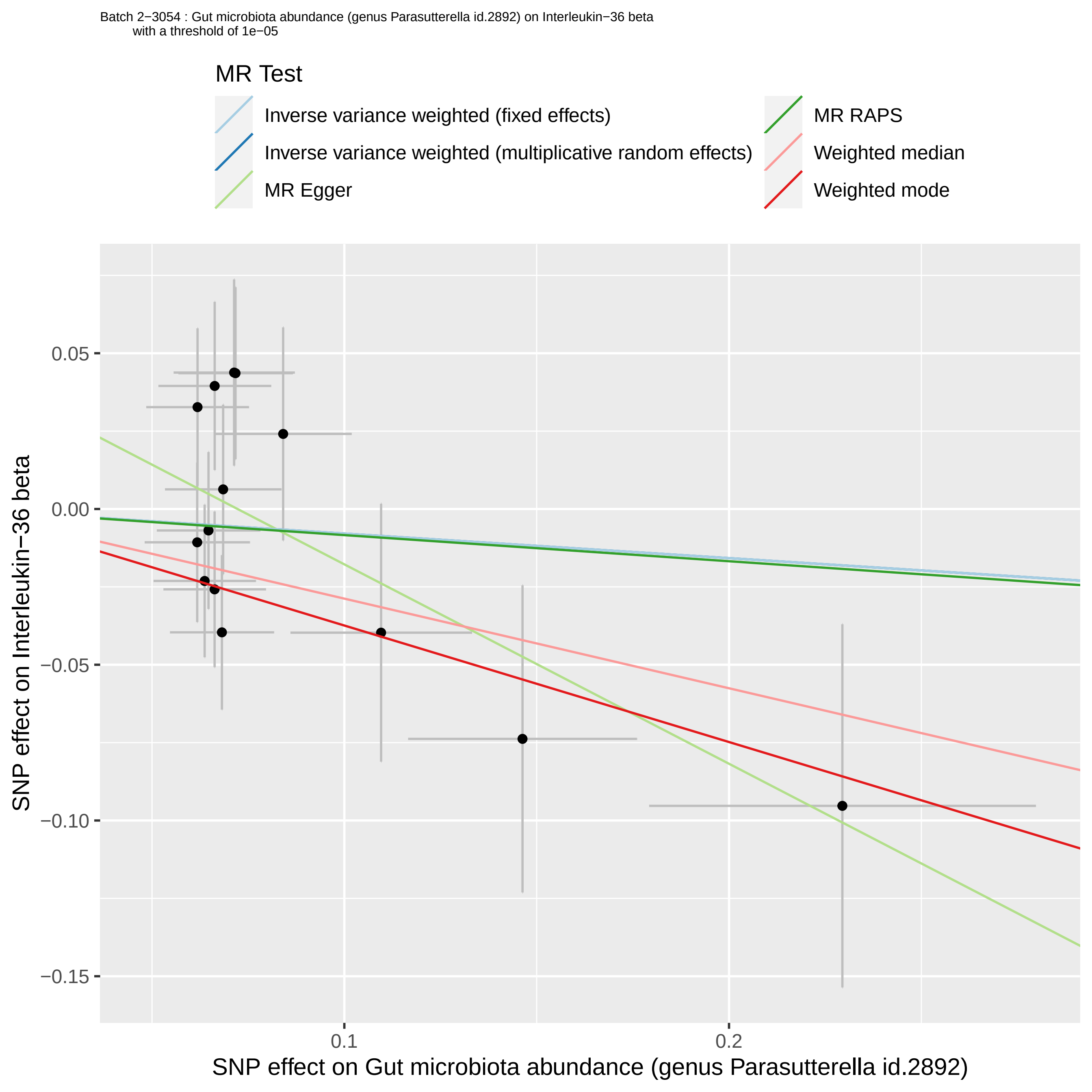
Batch 2−3054 : Gut microbiota abundance (genus Parasutterella id.2892) on Interleukin−36 beta
with a threshold of 1e−05
MR Test
Inverse variance weighted (fixed effects)
Inverse variance weighted (multiplicative random effects)
MR Egger
MR RAPS
Weighted median
Weighted mode
0.05
0.00
−0.05
−0.10
−0.15
0.1
0.2
SNP effect on Interleukin−36 beta
SNP effect on Gut microbiota abundance (genus Parasutterella id.2892)

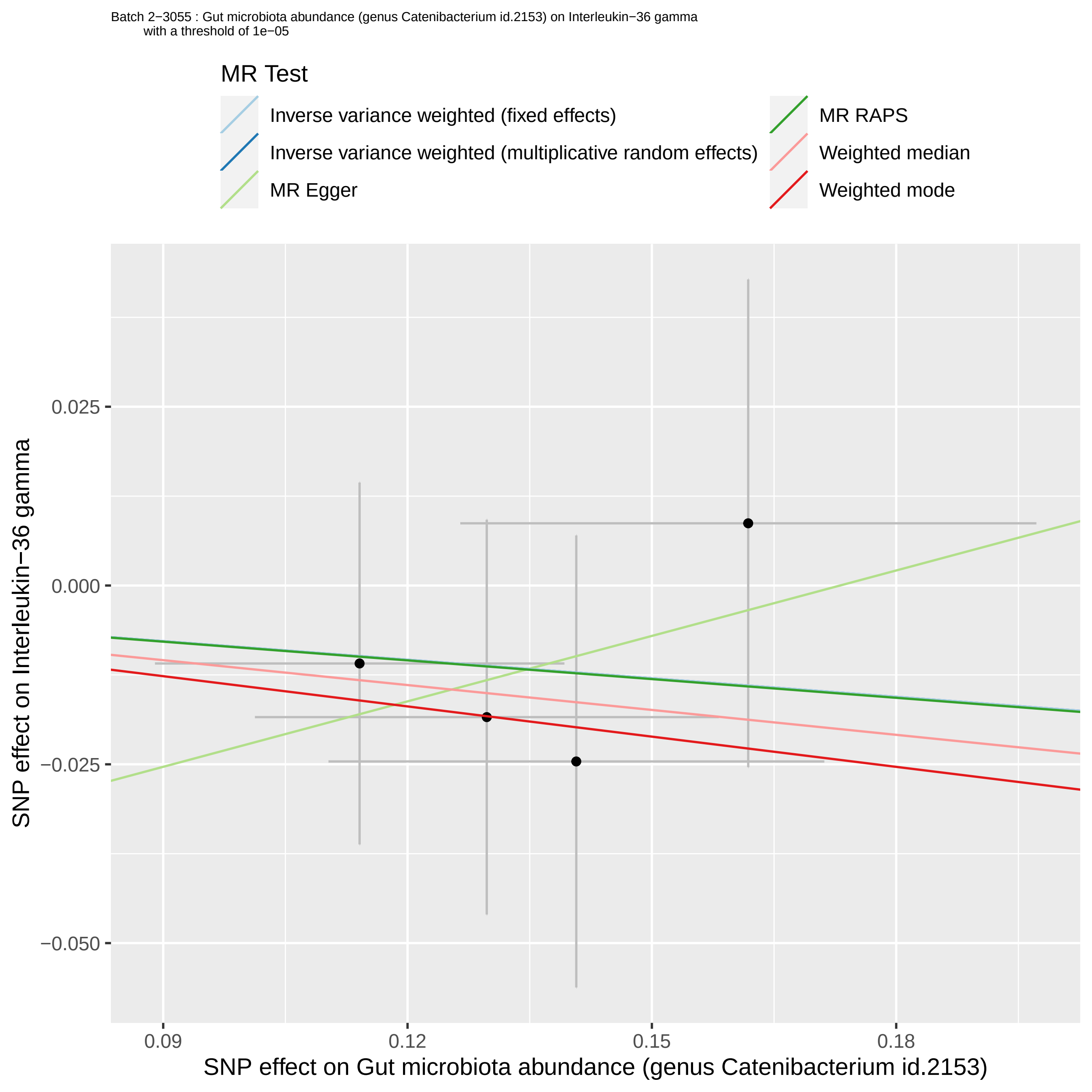
Batch 2−3055 : Gut microbiota abundance (genus Catenibacterium id.2153) on Interleukin−36 gamma
with a threshold of 1e−05
MR Test
Inverse variance weighted (fixed effects)
Inverse variance weighted (multiplicative random effects)
MR Egger
MR RAPS
Weighted median
Weighted mode
0.025
0.000
−0.025
−0.050
0.09
0.12
0.15
0.18
SNP effect on Interleukin−36 gamma
SNP effect on Gut microbiota abundance (genus Catenibacterium id.2153)

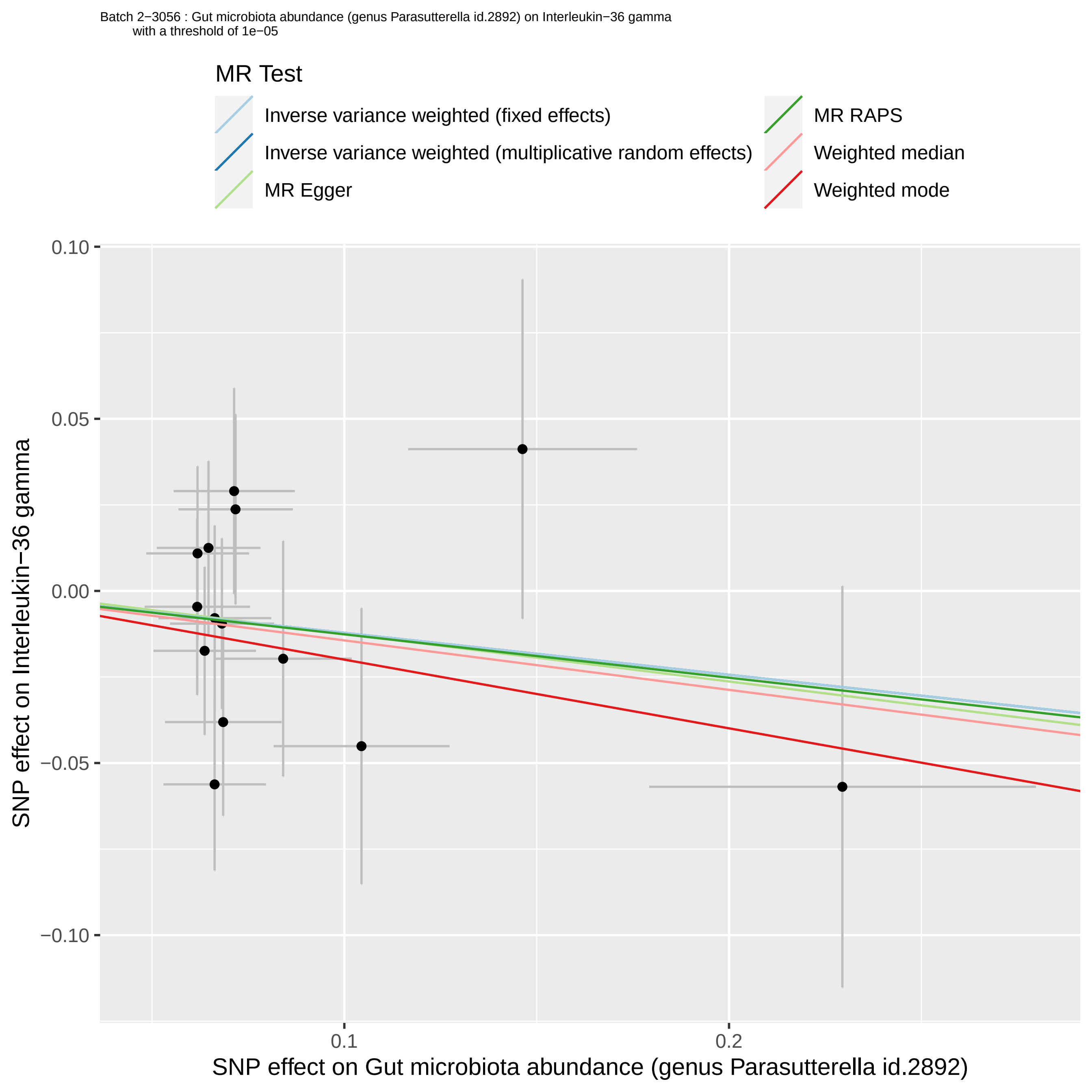

Batch 2−3056 : Gut microbiota abundance (genus Parasutterella id.2892) on Interleukin−36 gamma
with a threshold of 1e−05
MR Test
Inverse variance weighted (fixed effects)
Inverse variance weighted (multiplicative random effects)
MR Egger
MR RAPS
Weighted median
Weighted mode
0.10
0.05
0.00
−0.05
−0.10
SNP effect on Interleukin−36 gamma
0.1
0.2
SNP effect on Gut microbiota abundance (genus Parasutterella id.2892)

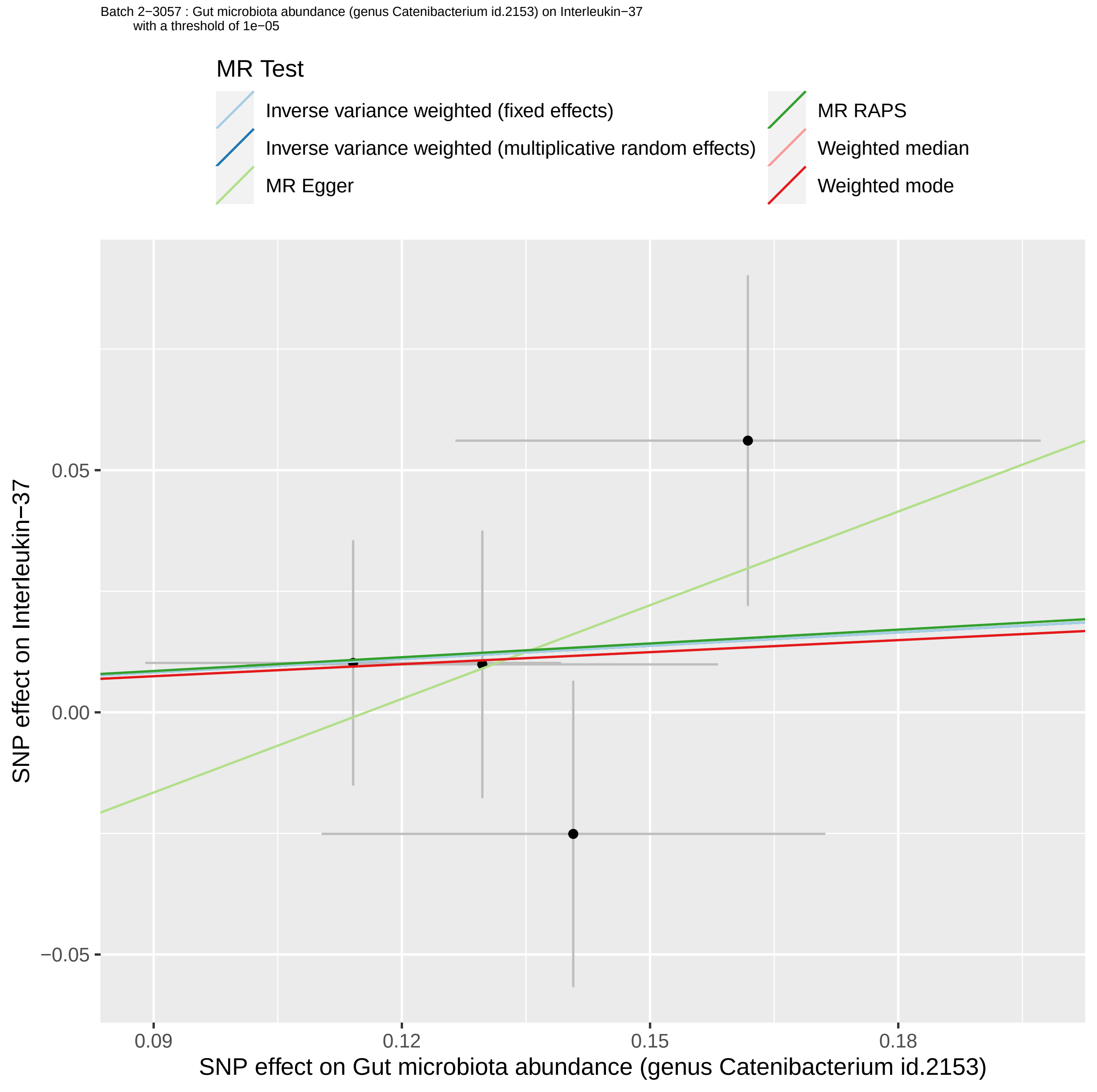
Batch 2−3057 : Gut microbiota abundance (genus Catenibacterium id.2153) on Interleukin−37
with a threshold of 1e−05
MR Test
Inverse variance weighted (fixed effects)
Inverse variance weighted (multiplicative random effects)
MR Egger
MR RAPS
Weighted median
Weighted mode
SNP effect on Interleukin−37
0.05
0.00
−0.05
0.09
0.12
0.15
0.18
SNP effect on Gut microbiota abundance (genus Catenibacterium id.2153)

Batch 2−3058 : Gut microbiota abundance (genus Parasutterella id.2892) on Interleukin−37
with a threshold of 1e−05

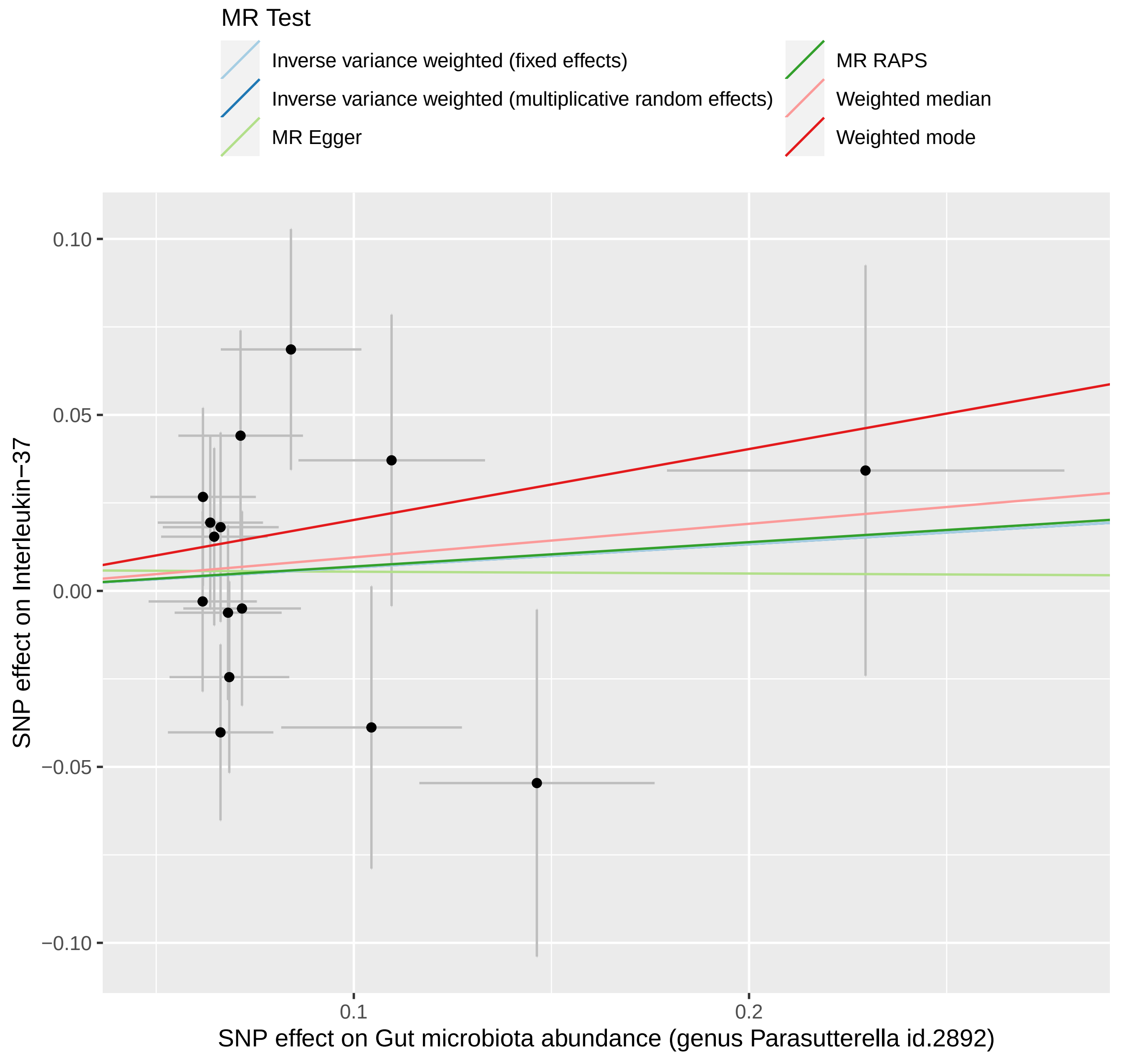

Batch 2−3059 : Gut microbiota abundance (genus Catenibacterium id.2153) on Interleukin−3 receptor subunit alpha
with a threshold of 1e−05

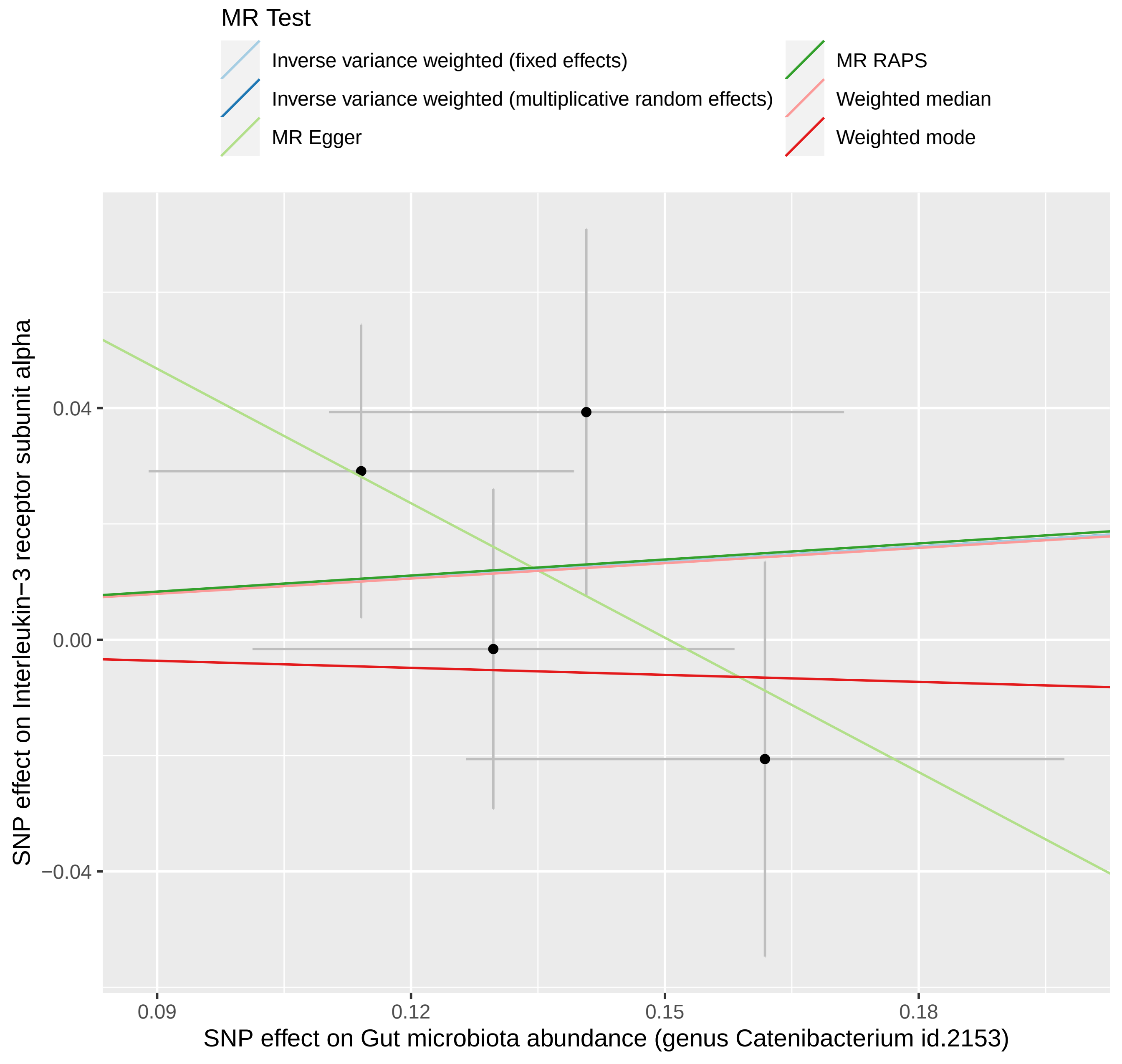

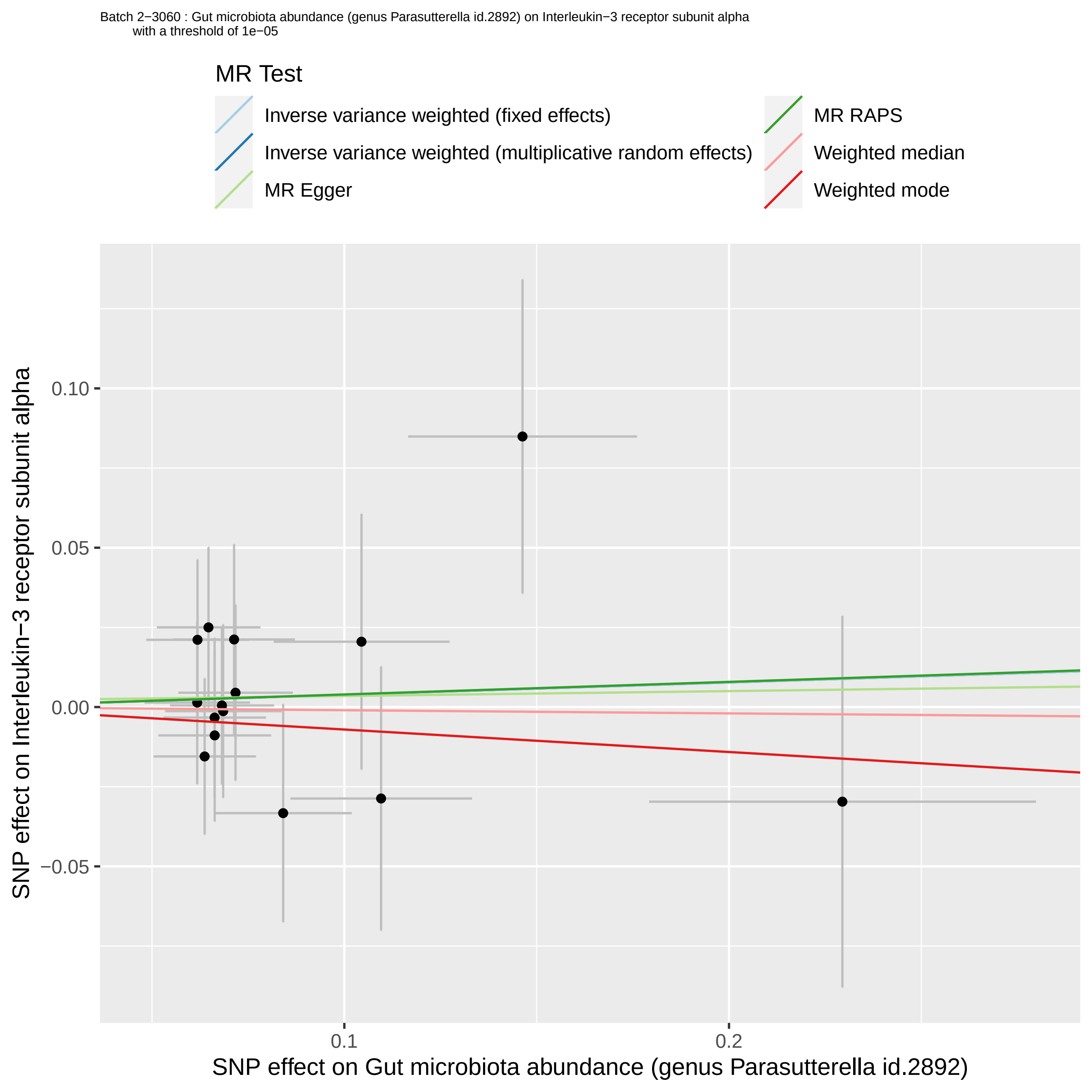

Batch 2−3060 : Gut microbiota abundance (genus Parasutterella id.2892) on Interleukin−3 receptor subunit alpha
with a threshold of 1e−05
MR Test
Inverse variance weighted (fixed effects)
Inverse variance weighted (multiplicative random effects)
MR Egger
MR RAPS
Weighted median
Weighted mode
0.10
0.05
0.00
−0.05
0.1
0.2
SNP effect on Interleukin−3 receptor subunit alpha
SNP effect on Gut microbiota abundance (genus Parasutterella id.2892)

Batch 2−3061 : Gut microbiota abundance (genus Catenibacterium id.2153) on Interleukin−3 receptor subunit alpha
with a threshold of 1e−05

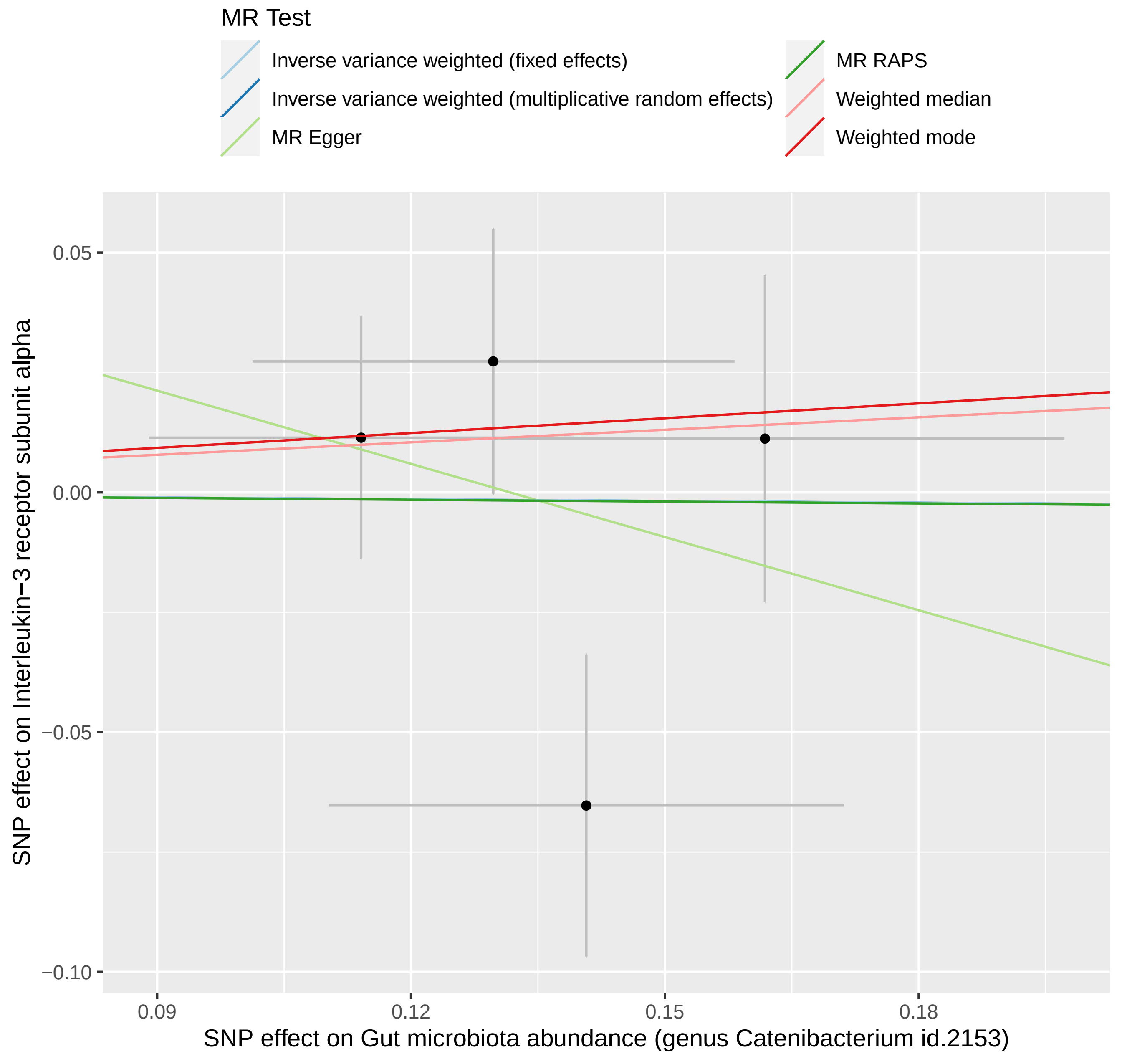

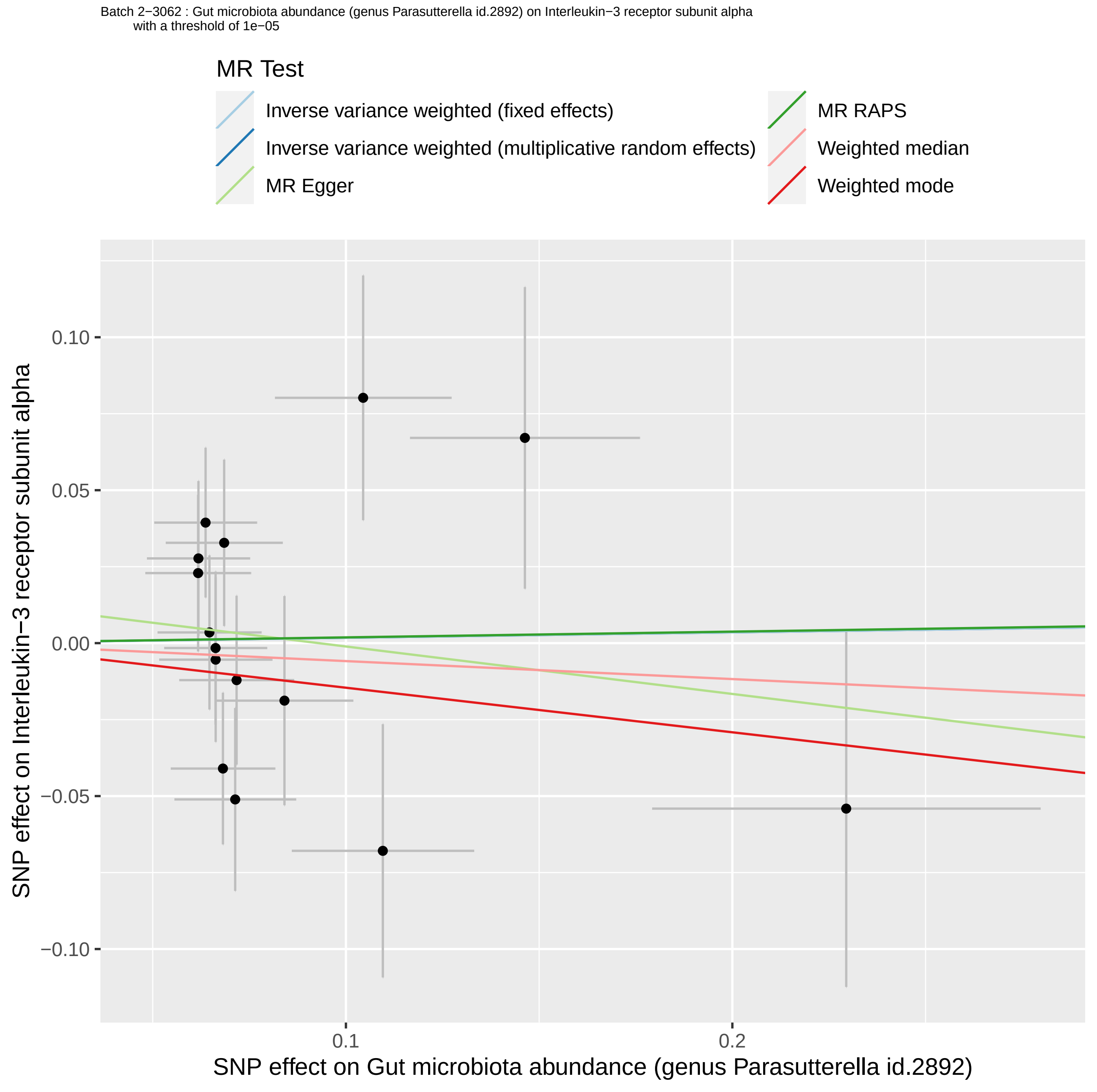
Batch 2−3062 : Gut microbiota abundance (genus Parasutterella id.2892) on Interleukin−3 receptor subunit alpha
with a threshold of 1e−05
MR Test
Inverse variance weighted (fixed effects)
Inverse variance weighted (multiplicative random effects)
MR Egger
MR RAPS
Weighted median
Weighted mode
0.10
0.05
0.00
−0.05
−0.10
0.1
0.2
SNP effect on Interleukin−3 receptor subunit alpha
SNP effect on Gut microbiota abundance (genus Parasutterella id.2892)

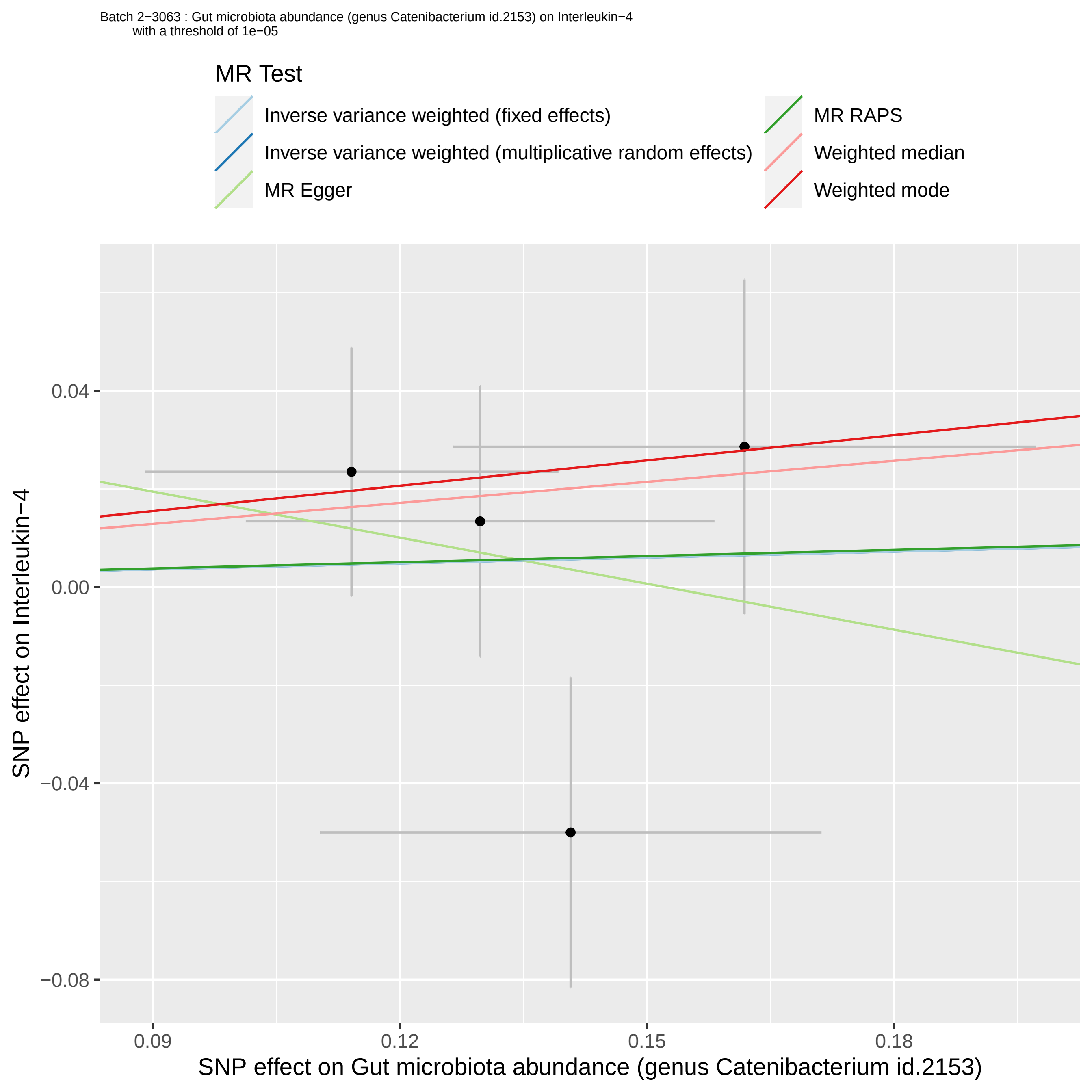
Batch 2−3063 : Gut microbiota abundance (genus Catenibacterium id.2153) on Interleukin−4
with a threshold of 1e−05
MR Test
Inverse variance weighted (fixed effects)
Inverse variance weighted (multiplicative random effects)
MR Egger
MR RAPS
Weighted median
Weighted mode
SNP effect on Interleukin−4
0.04
0.00
−0.04
−0.08
0.09
0.12
0.15
0.18
SNP effect on Gut microbiota abundance (genus Catenibacterium id.2153)

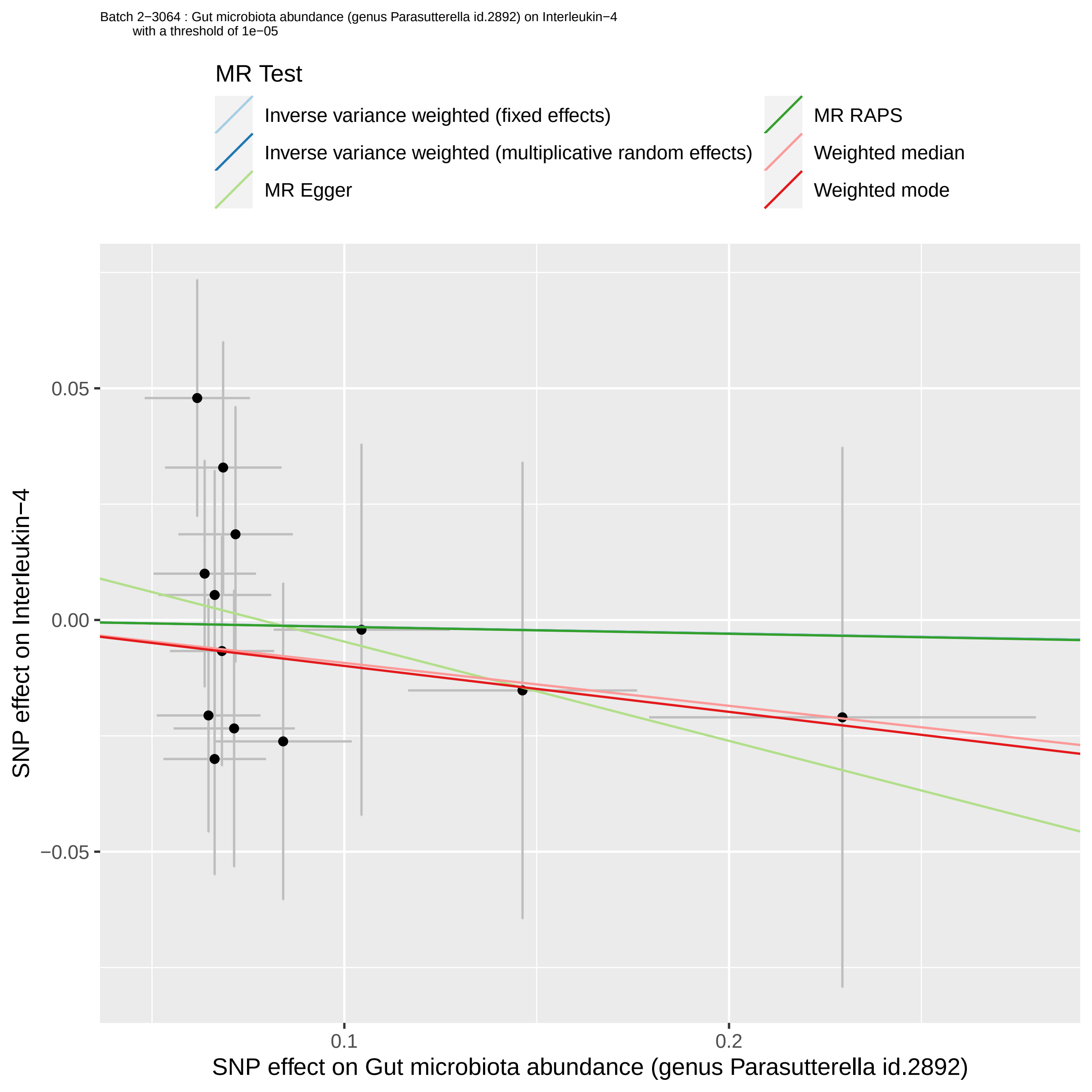
Batch 2−3064 : Gut microbiota abundance (genus Parasutterella id.2892) on Interleukin−4
with a threshold of 1e−05
MR Test
Inverse variance weighted (fixed effects)
Inverse variance weighted (multiplicative random effects)
MR Egger
MR RAPS
Weighted median
Weighted mode
0.05
0.00
−0.05
0.1
0.2
SNP effect on Interleukin−4
SNP effect on Gut microbiota abundance (genus Parasutterella id.2892)

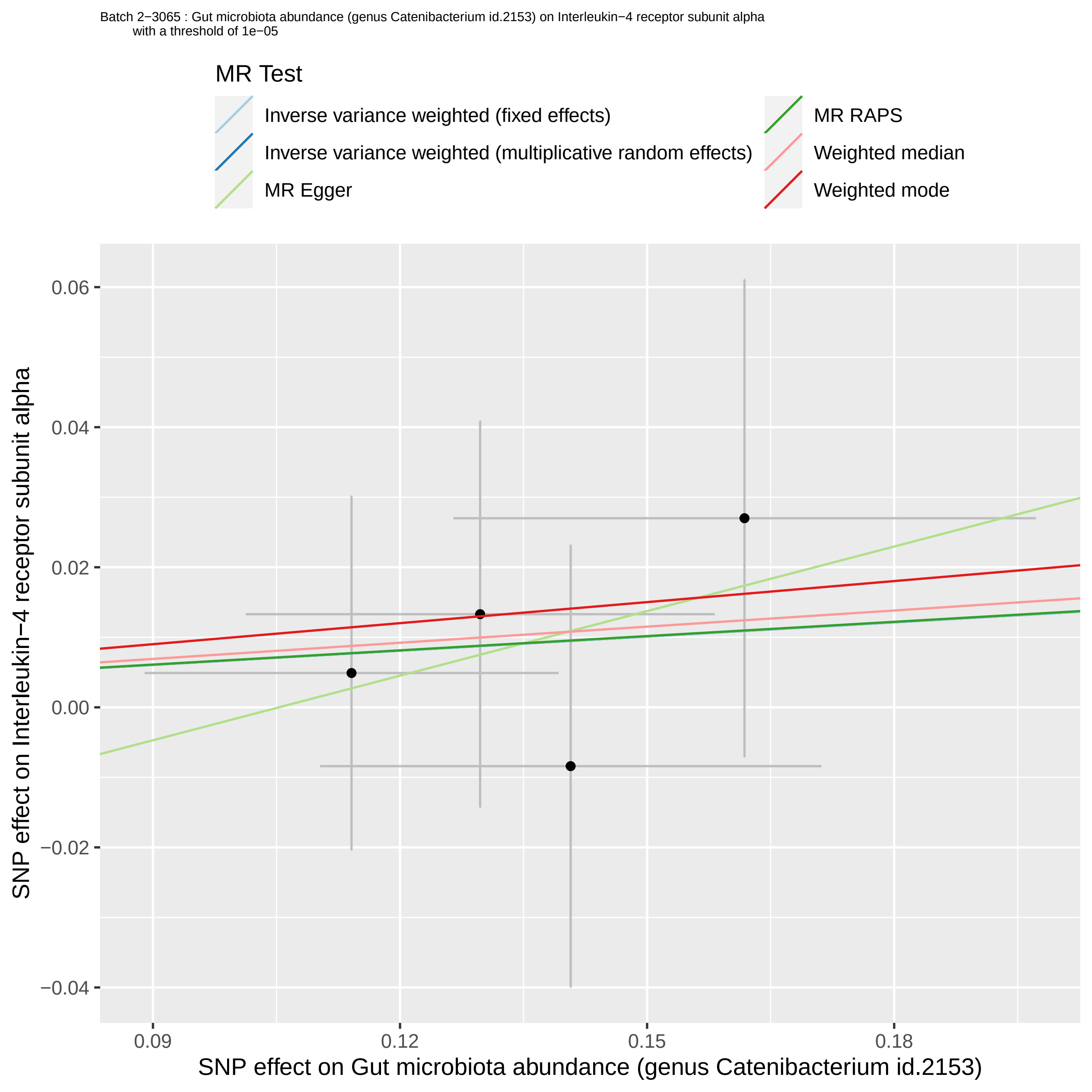
Batch 2−3065 : Gut microbiota abundance (genus Catenibacterium id.2153) on Interleukin−4 receptor subunit alpha
with a threshold of 1e−05
MR Test
Inverse variance weighted (fixed effects)
Inverse variance weighted (multiplicative random effects)
MR Egger
MR RAPS
Weighted median
Weighted mode
0.06
0.04
0.02
0.00
−0.02
−0.04
0.09
0.12
0.15
0.18
SNP effect on Interleukin−4 receptor subunit alpha
SNP effect on Gut microbiota abundance (genus Catenibacterium id.2153)

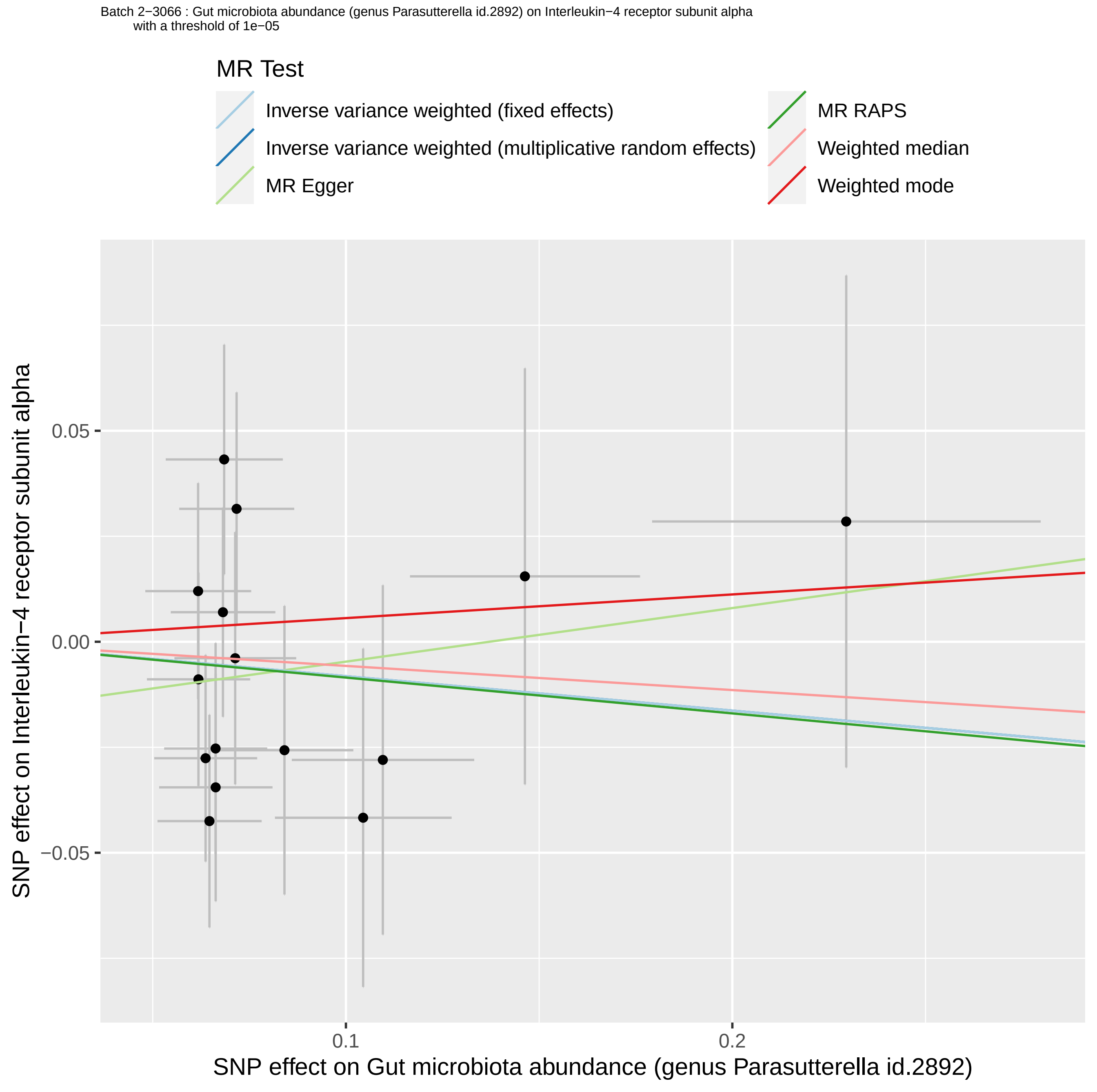

Batch 2−3066 : Gut microbiota abundance (genus Parasutterella id.2892) on Interleukin−4 receptor subunit alpha
with a threshold of 1e−05
MR Test
Inverse variance weighted (fixed effects)
Inverse variance weighted (multiplicative random effects)
MR Egger
MR RAPS
Weighted median
Weighted mode
SNP effect on Interleukin−4 receptor subunit alpha
0.05
0.00
−0.05
0.1
0.2
SNP effect on Gut microbiota abundance (genus Parasutterella id.2892)

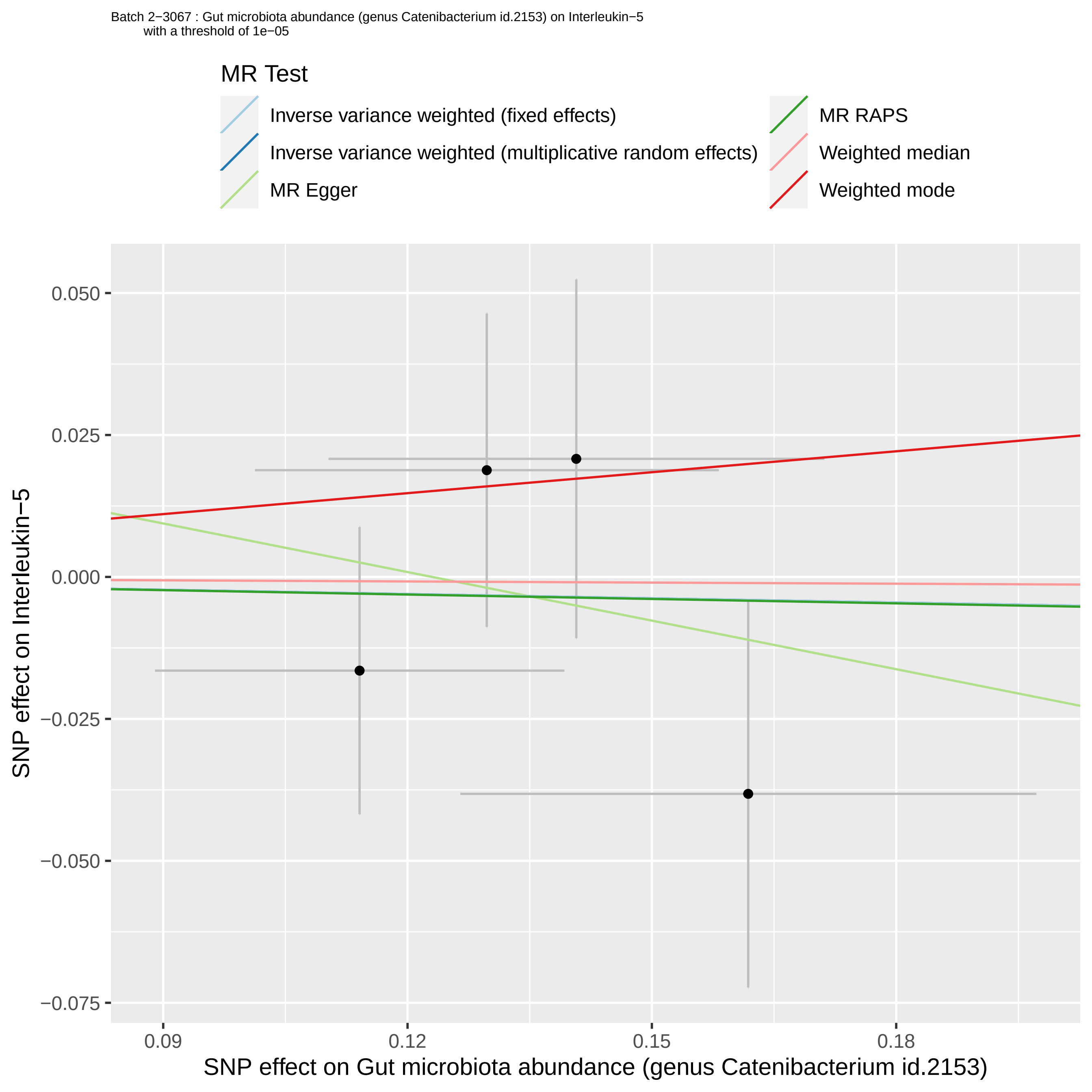
Batch 2−3067 : Gut microbiota abundance (genus Catenibacterium id.2153) on Interleukin−5
with a threshold of 1e−05
MR Test
Inverse variance weighted (fixed effects)
Inverse variance weighted (multiplicative random effects)
MR Egger
MR RAPS
Weighted median
Weighted mode
0.050
0.025
0.000
−0.025
−0.050
−0.075
0.09
0.12
0.15
0.18
SNP effect on Interleukin−5
SNP effect on Gut microbiota abundance (genus Catenibacterium id.2153)

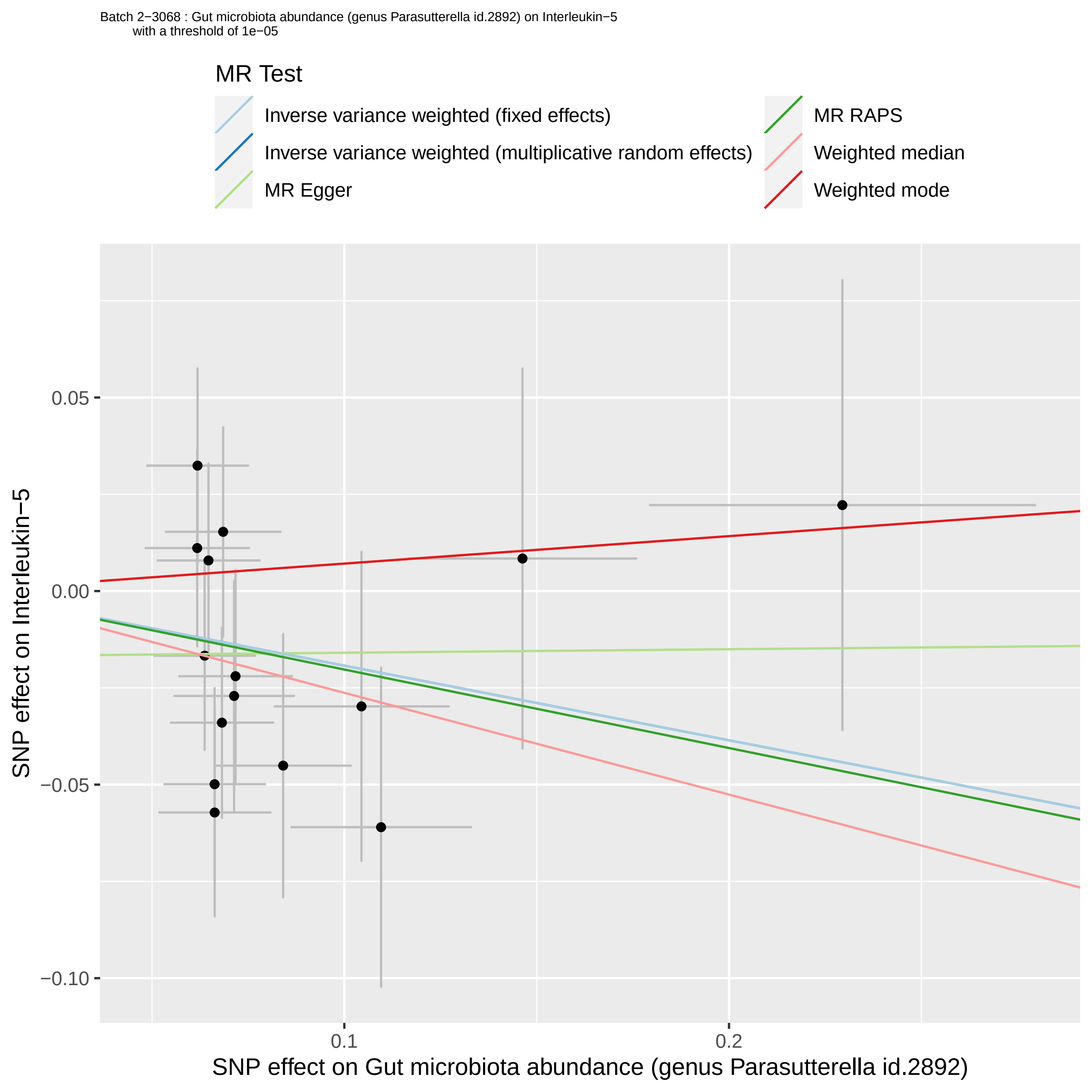
Batch 2−3068 : Gut microbiota abundance (genus Parasutterella id.2892) on Interleukin−5
with a threshold of 1e−05
MR Test
Inverse variance weighted (fixed effects)
Inverse variance weighted (multiplicative random effects)
MR Egger
MR RAPS
Weighted median
Weighted mode
0.05
0.00
−0.05
−0.10
0.1
0.2
SNP effect on Interleukin−5
SNP effect on Gut microbiota abundance (genus Parasutterella id.2892)

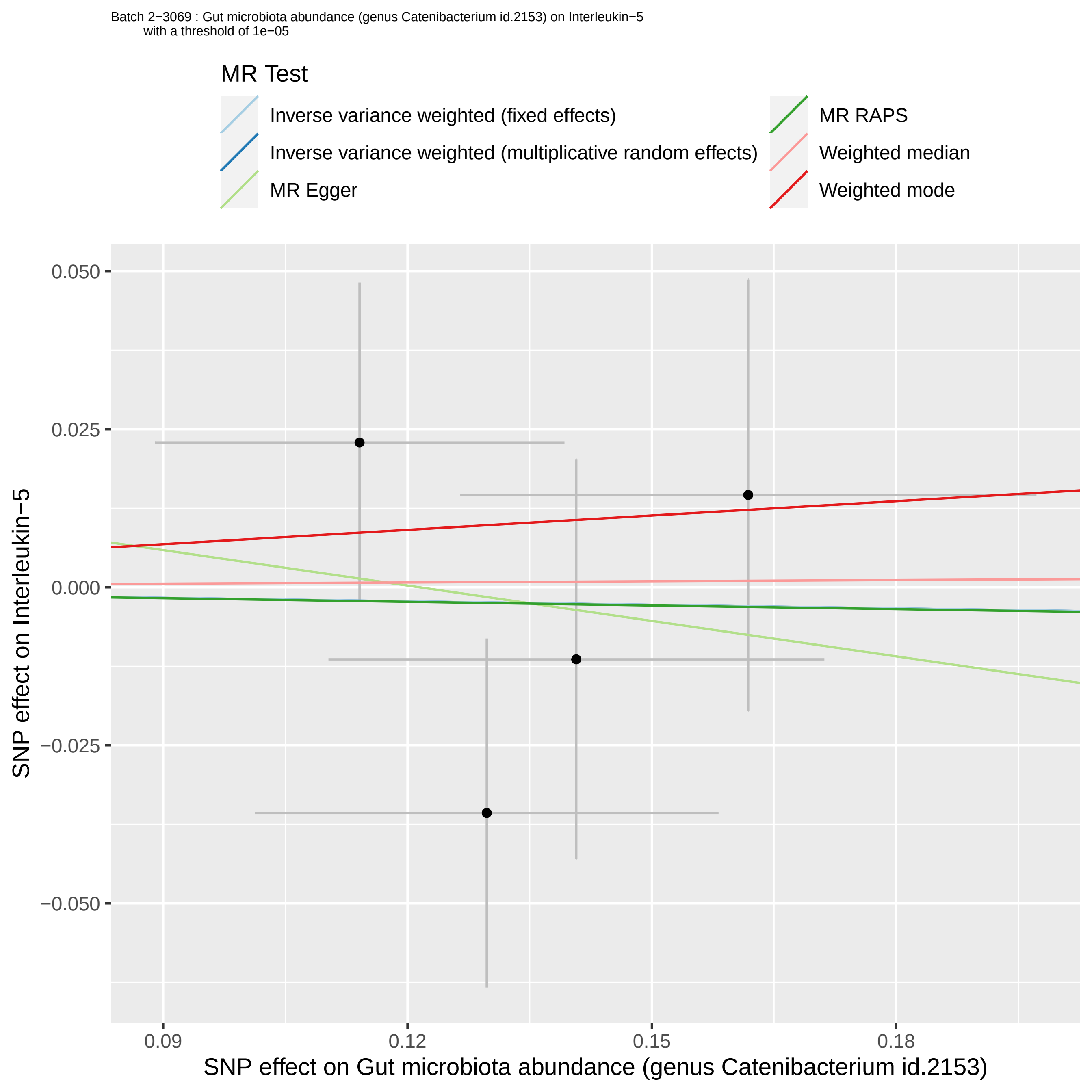
Batch 2−3069 : Gut microbiota abundance (genus Catenibacterium id.2153) on Interleukin−5
with a threshold of 1e−05
MR Test
Inverse variance weighted (fixed effects)
Inverse variance weighted (multiplicative random effects)
MR Egger
MR RAPS
Weighted median
Weighted mode
0.050
0.025
0.000
−0.025
−0.050
0.09
0.12
0.15
0.18
SNP effect on Interleukin−5
SNP effect on Gut microbiota abundance (genus Catenibacterium id.2153)

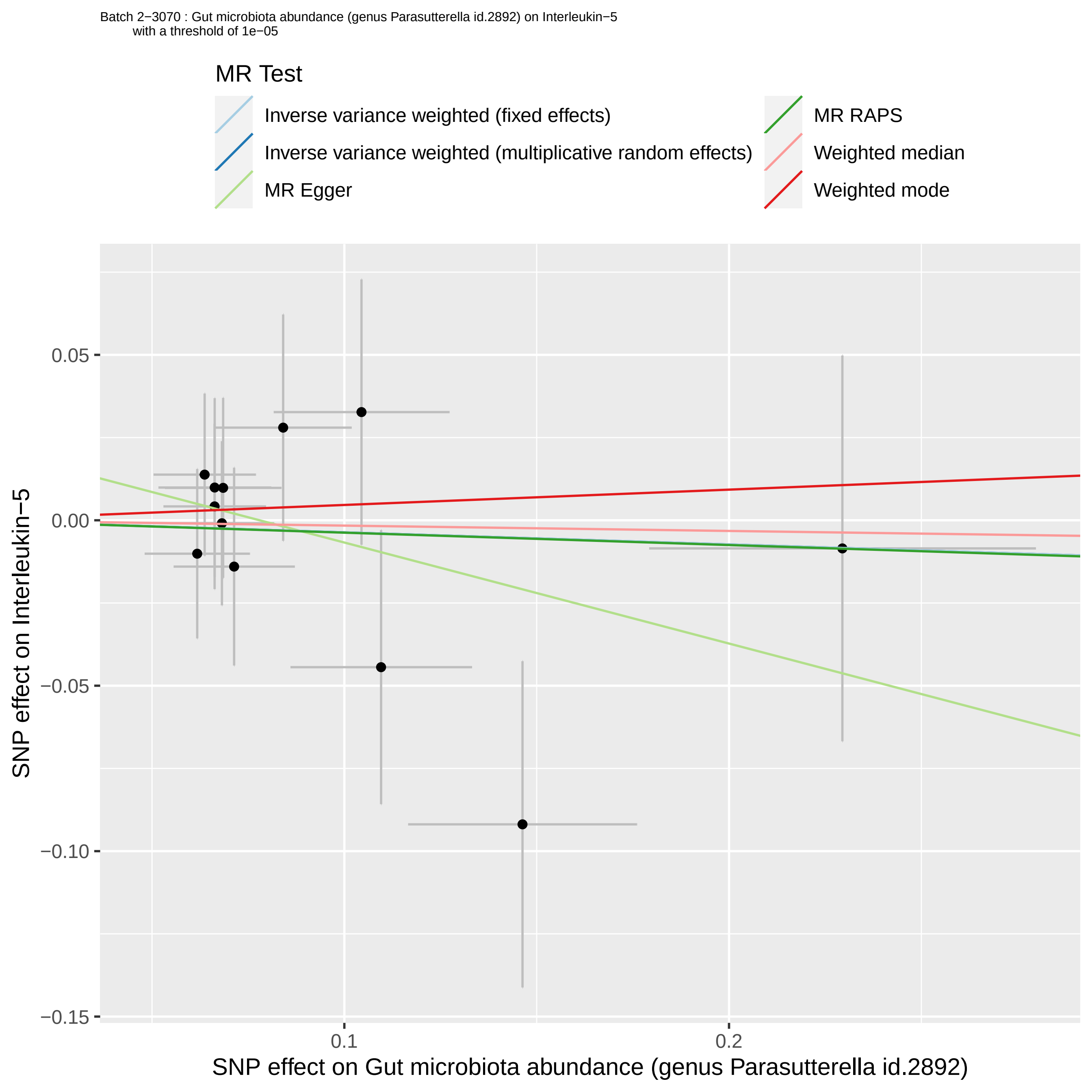
Batch 2−3070 : Gut microbiota abundance (genus Parasutterella id.2892) on Interleukin−5
with a threshold of 1e−05
MR Test
Inverse variance weighted (fixed effects)
Inverse variance weighted (multiplicative random effects)
MR Egger
MR RAPS
Weighted median
Weighted mode
SNP effect on Interleukin−5
0.05
0.00
−0.05
−0.10
−0.15
0.1
0.2
SNP effect on Gut microbiota abundance (genus Parasutterella id.2892)

Batch 2−3071 : Gut microbiota abundance (genus Catenibacterium id.2153) on Interleukin−5 receptor subunit alpha
with a threshold of 1e−05

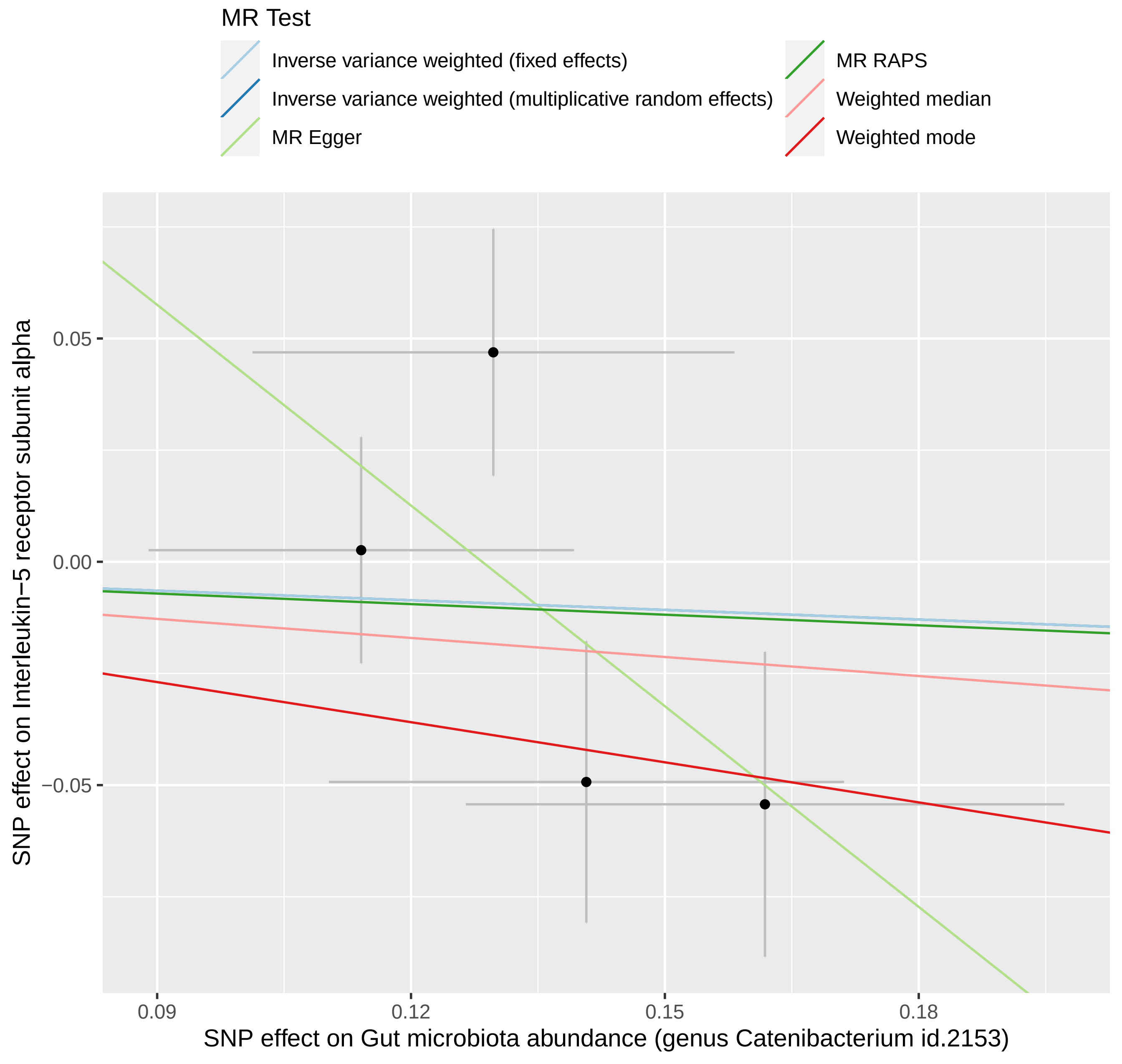

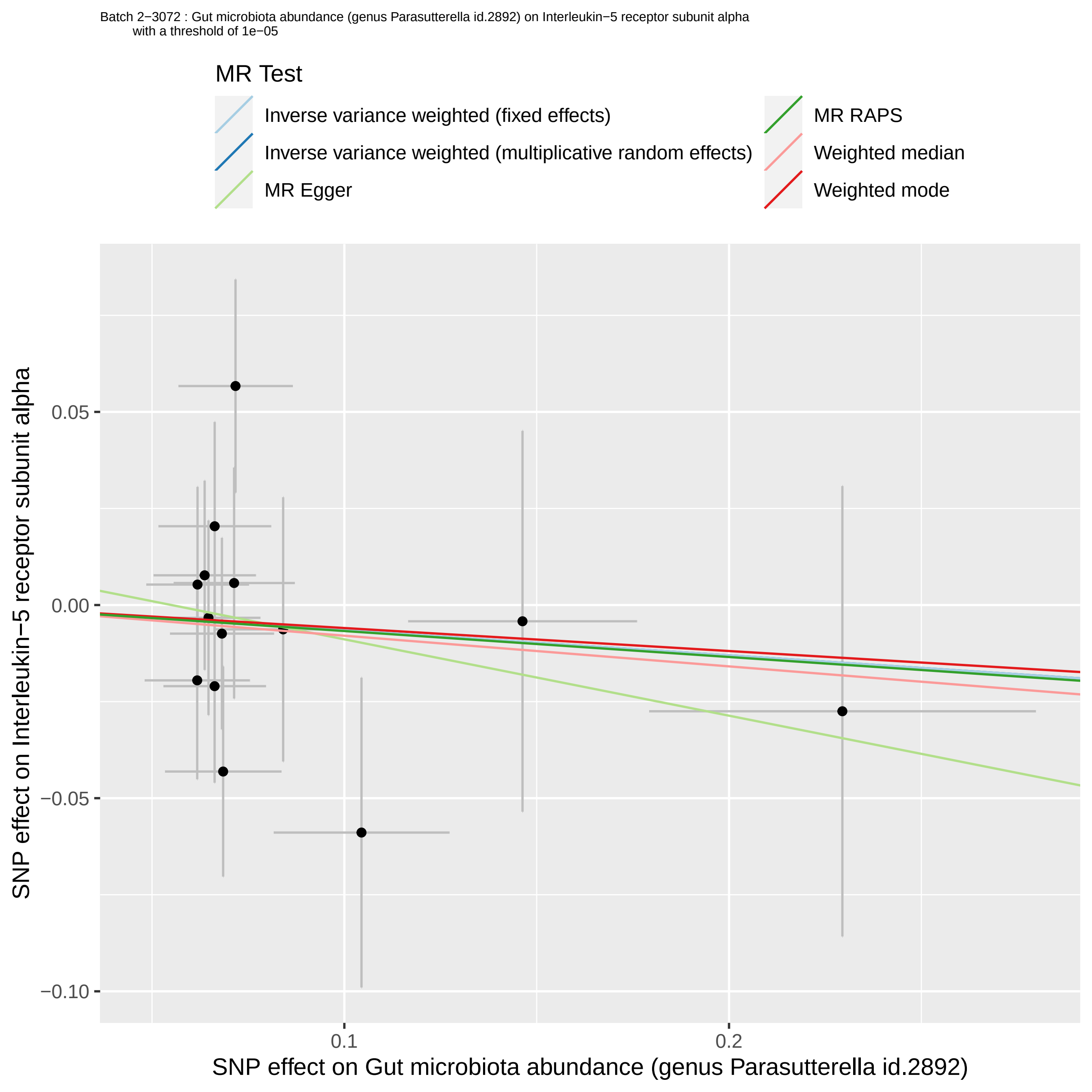

Batch 2−3072 : Gut microbiota abundance (genus Parasutterella id.2892) on Interleukin−5 receptor subunit alpha
with a threshold of 1e−05
MR Test
Inverse variance weighted (fixed effects)
Inverse variance weighted (multiplicative random effects)
MR Egger
MR RAPS
Weighted median
Weighted mode
SNP effect on Interleukin−5 receptor subunit alpha
0.05
0.00
−0.05
−0.10
0.1
0.2
SNP effect on Gut microbiota abundance (genus Parasutterella id.2892)

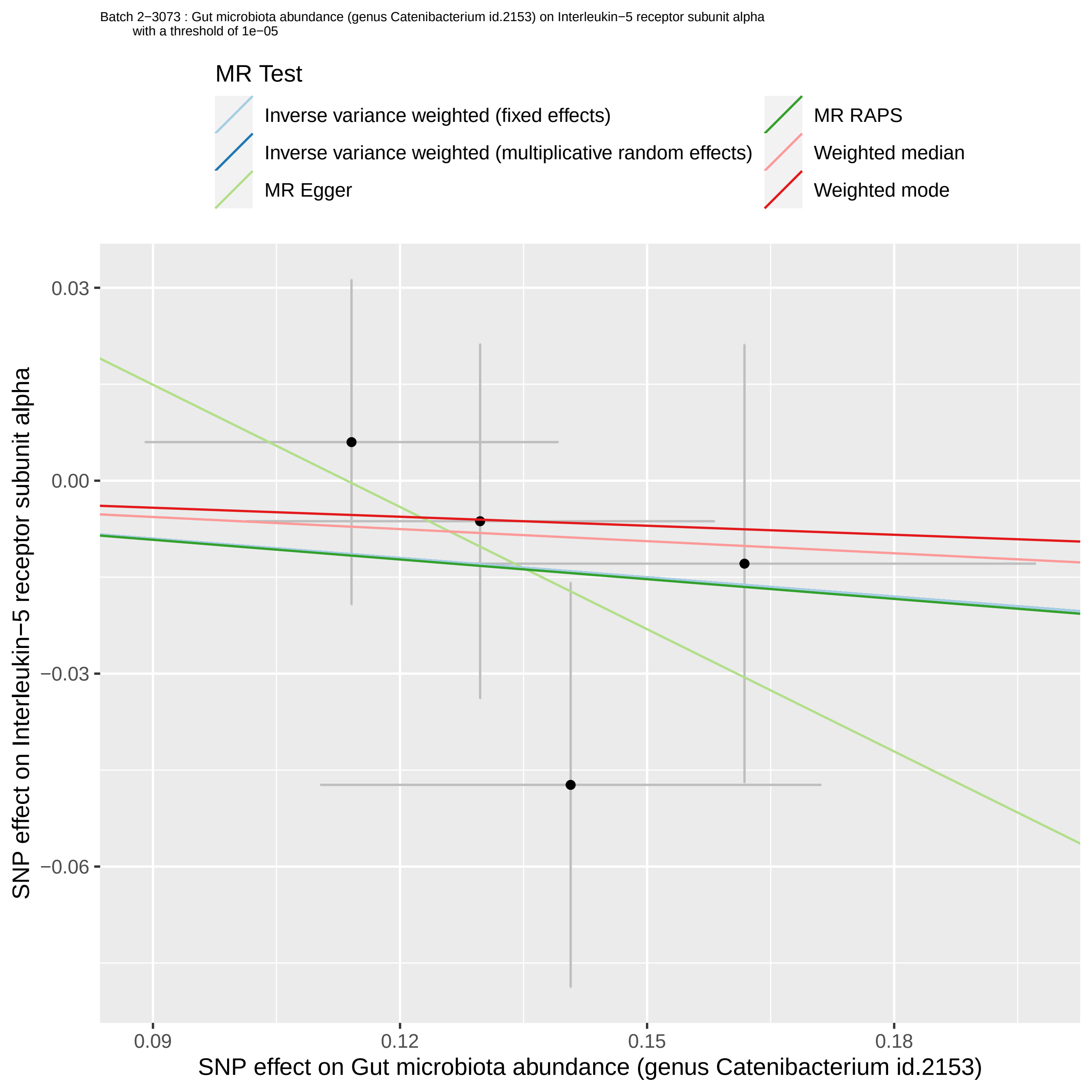

Batch 2−3073 : Gut microbiota abundance (genus Catenibacterium id.2153) on Interleukin−5 receptor subunit alpha
with a threshold of 1e−05
MR Test
Inverse variance weighted (fixed effects)
Inverse variance weighted (multiplicative random effects)
MR Egger
MR RAPS
Weighted median
Weighted mode
0.03
0.00
−0.03
−0.06
0.09
0.12
0.15
0.18
SNP effect on Interleukin−5 receptor subunit alpha
SNP effect on Gut microbiota abundance (genus Catenibacterium id.2153)

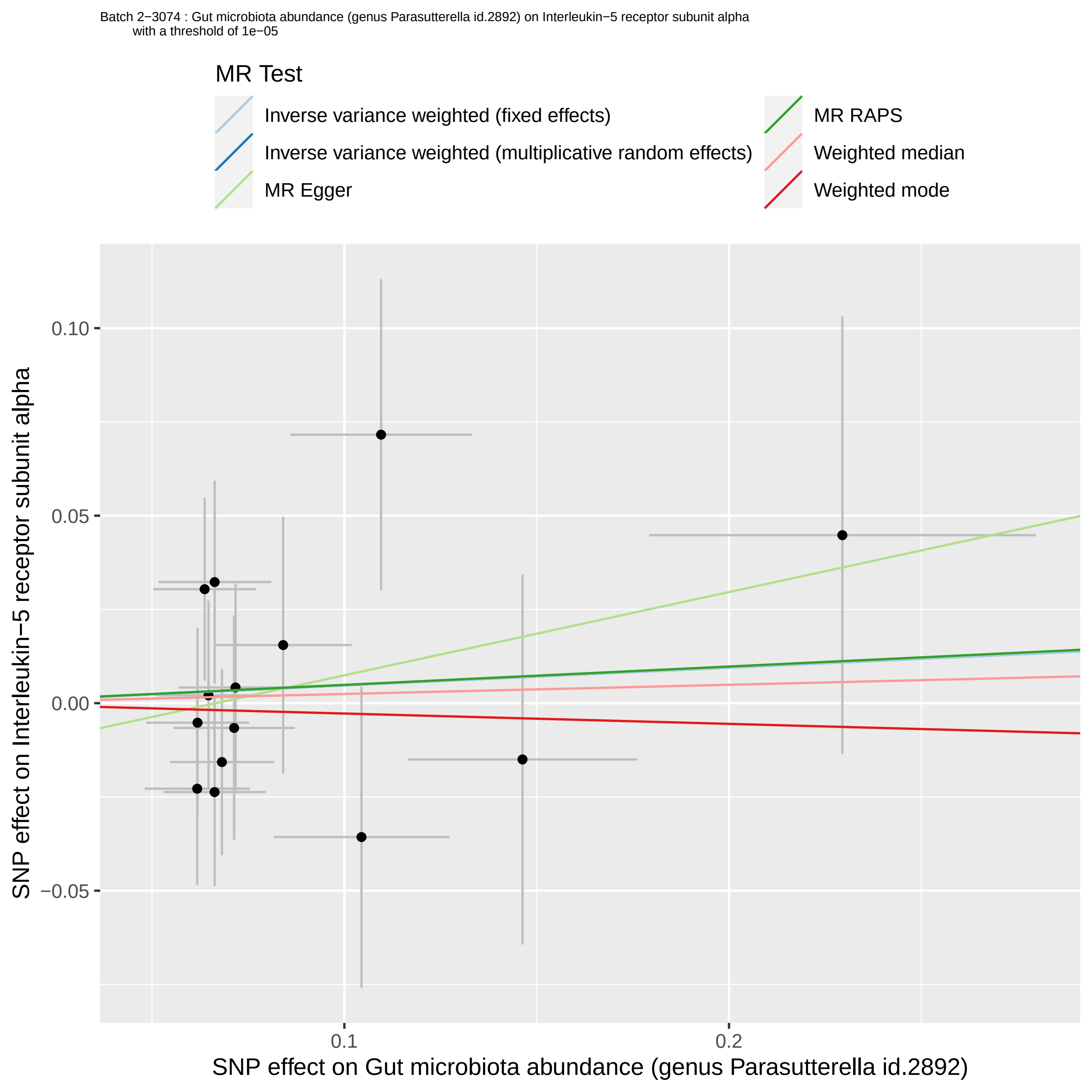
Batch 2−3074 : Gut microbiota abundance (genus Parasutterella id.2892) on Interleukin−5 receptor subunit alpha
with a threshold of 1e−05
MR Test
Inverse variance weighted (fixed effects)
Inverse variance weighted (multiplicative random effects)
MR Egger
MR RAPS
Weighted median
Weighted mode
0.10
0.05
0.00
−0.05
0.1
0.2
SNP effect on Interleukin−5 receptor subunit alpha
SNP effect on Gut microbiota abundance (genus Parasutterella id.2892)

Batch 2−3075 : Gut microbiota abundance (genus Catenibacterium id.2153) on Interleukin−6
with a threshold of 1e−05

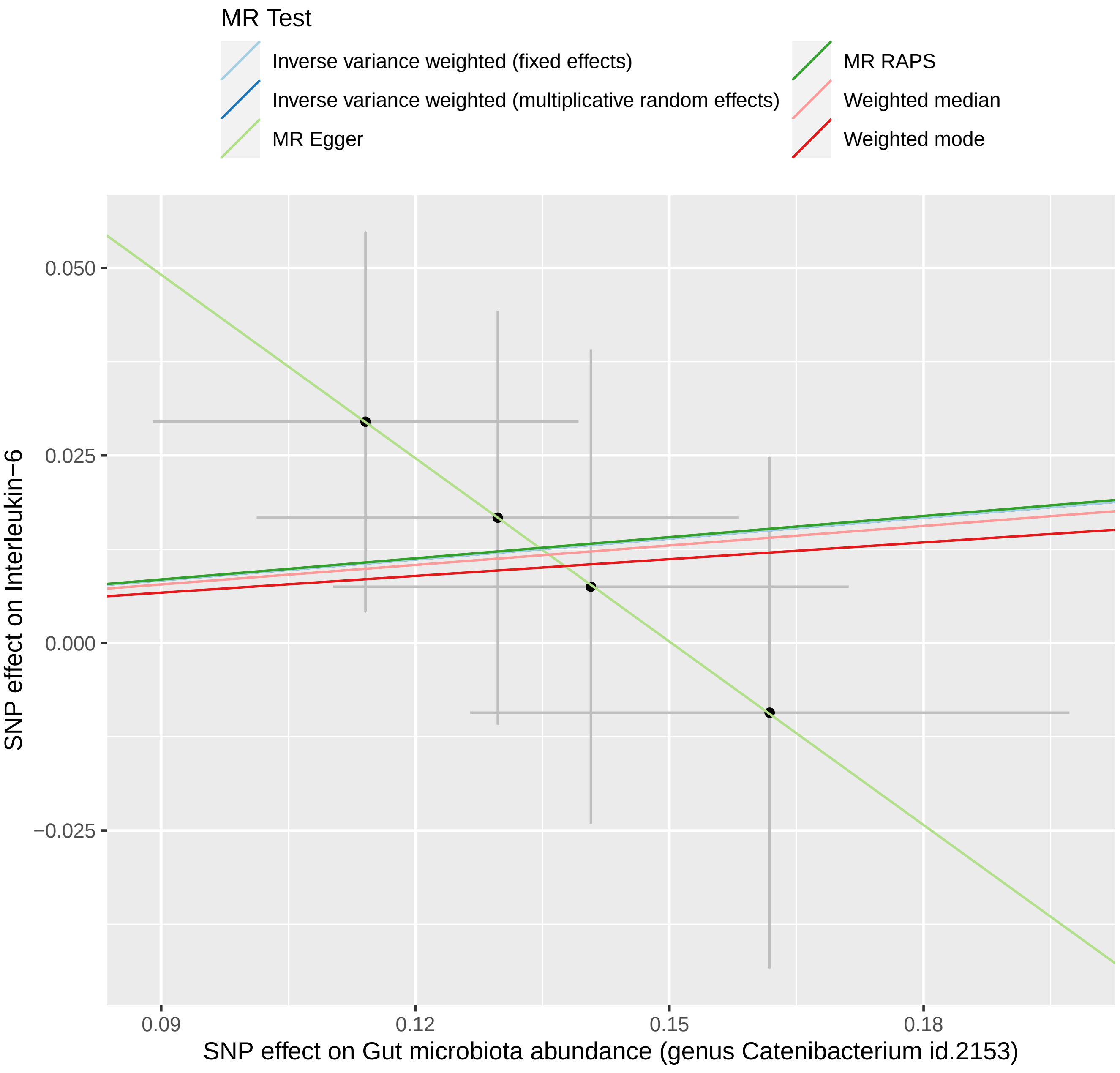

Batch 2−3076 : Gut microbiota abundance (genus Parasutterella id.2892) on Interleukin−6
with a threshold of 1e−05

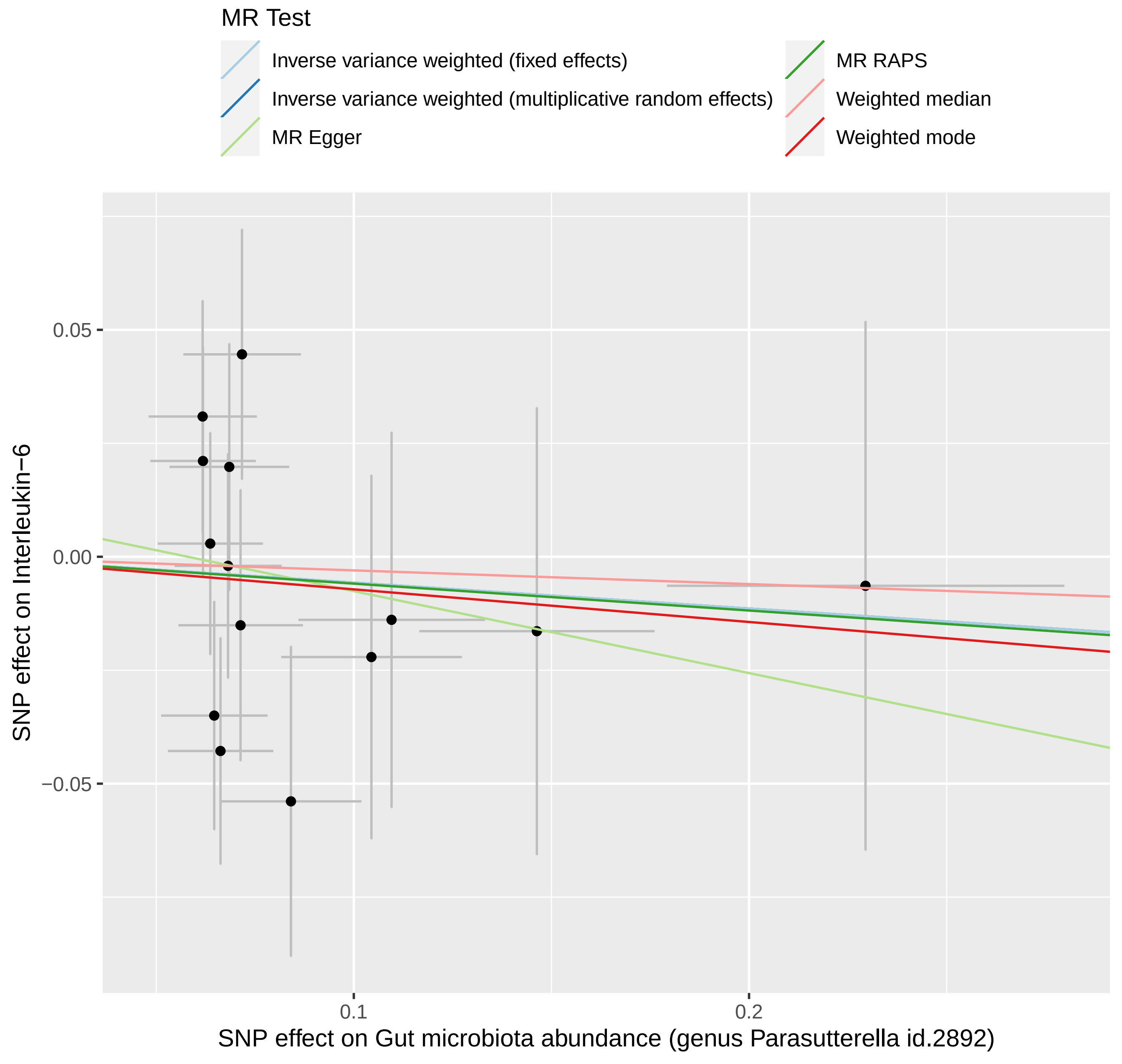

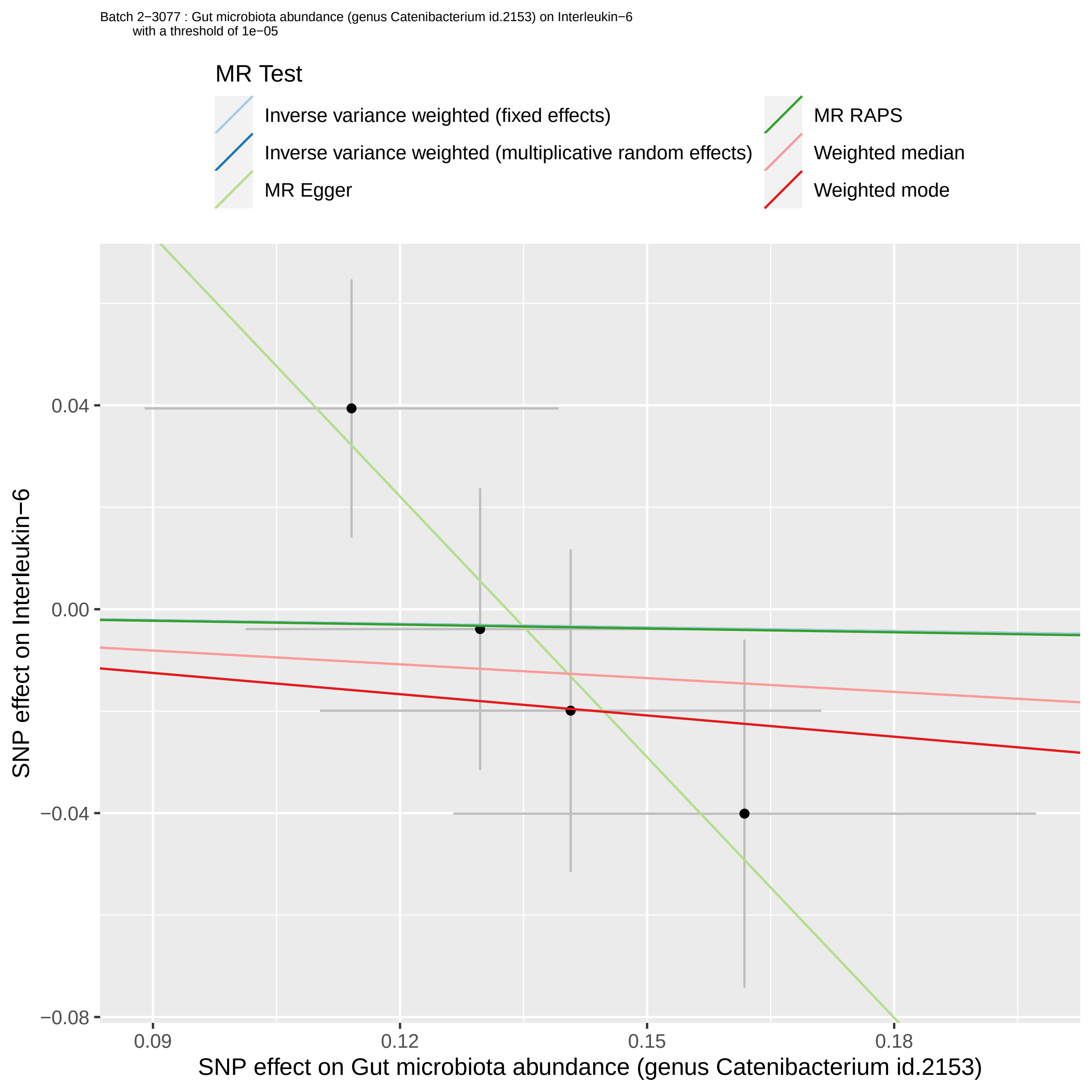
Batch 2−3077 : Gut microbiota abundance (genus Catenibacterium id.2153) on Interleukin−6
with a threshold of 1e−05
MR Test
Inverse variance weighted (fixed effects)
Inverse variance weighted (multiplicative random effects)
MR Egger
MR RAPS
Weighted median
Weighted mode
0.04
0.00
−0.04
−0.08
0.09
0.12
0.15
0.18
SNP effect on Interleukin−6
SNP effect on Gut microbiota abundance (genus Catenibacterium id.2153)

Batch 2−3078 : Gut microbiota abundance (genus Parasutterella id.2892) on Interleukin−6
with a threshold of 1e−05

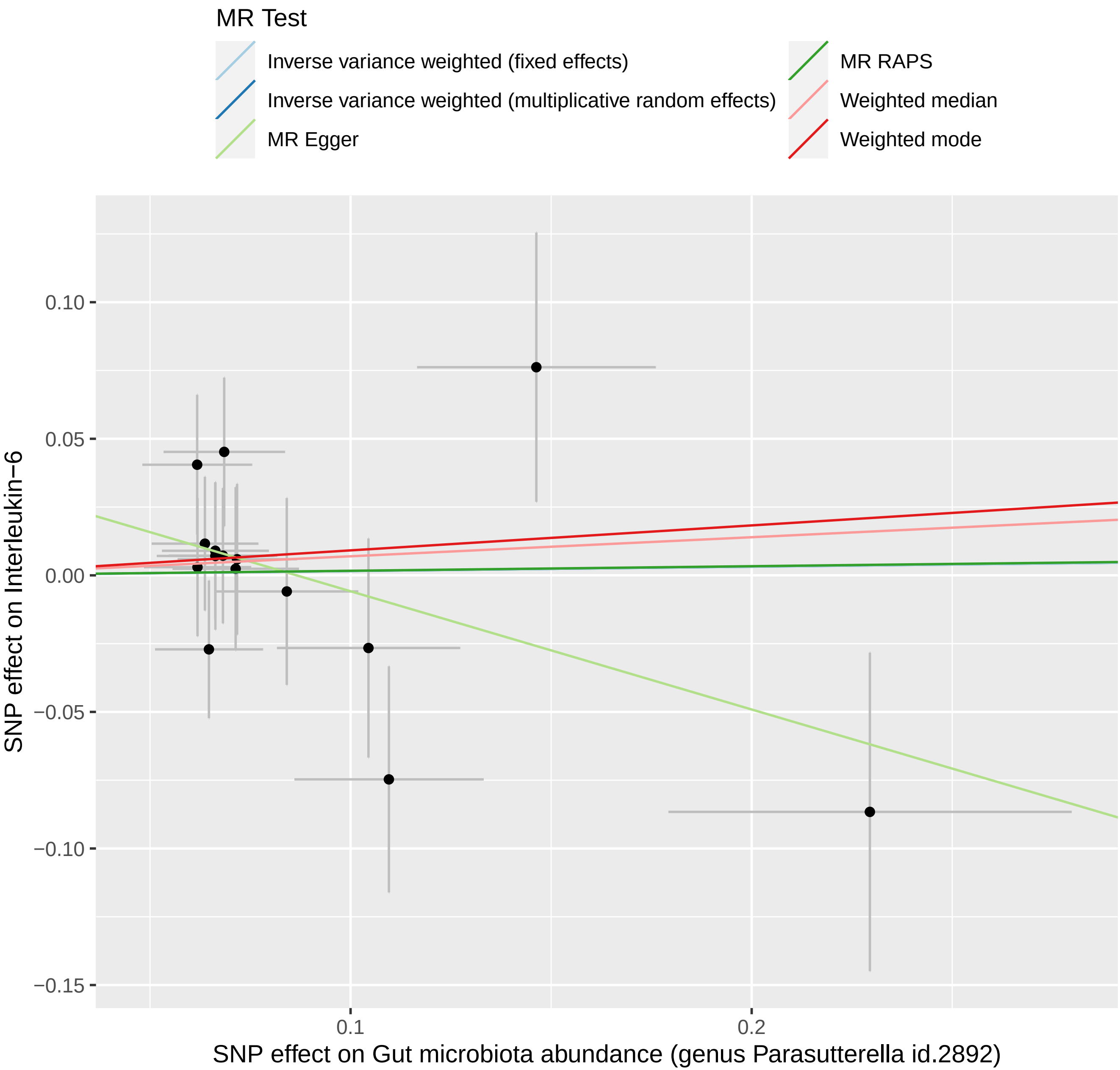

Batch 2−3079 : Gut microbiota abundance (genus Catenibacterium id.2153) on Interleukin−6 receptor subunit alpha
with a threshold of 1e−05

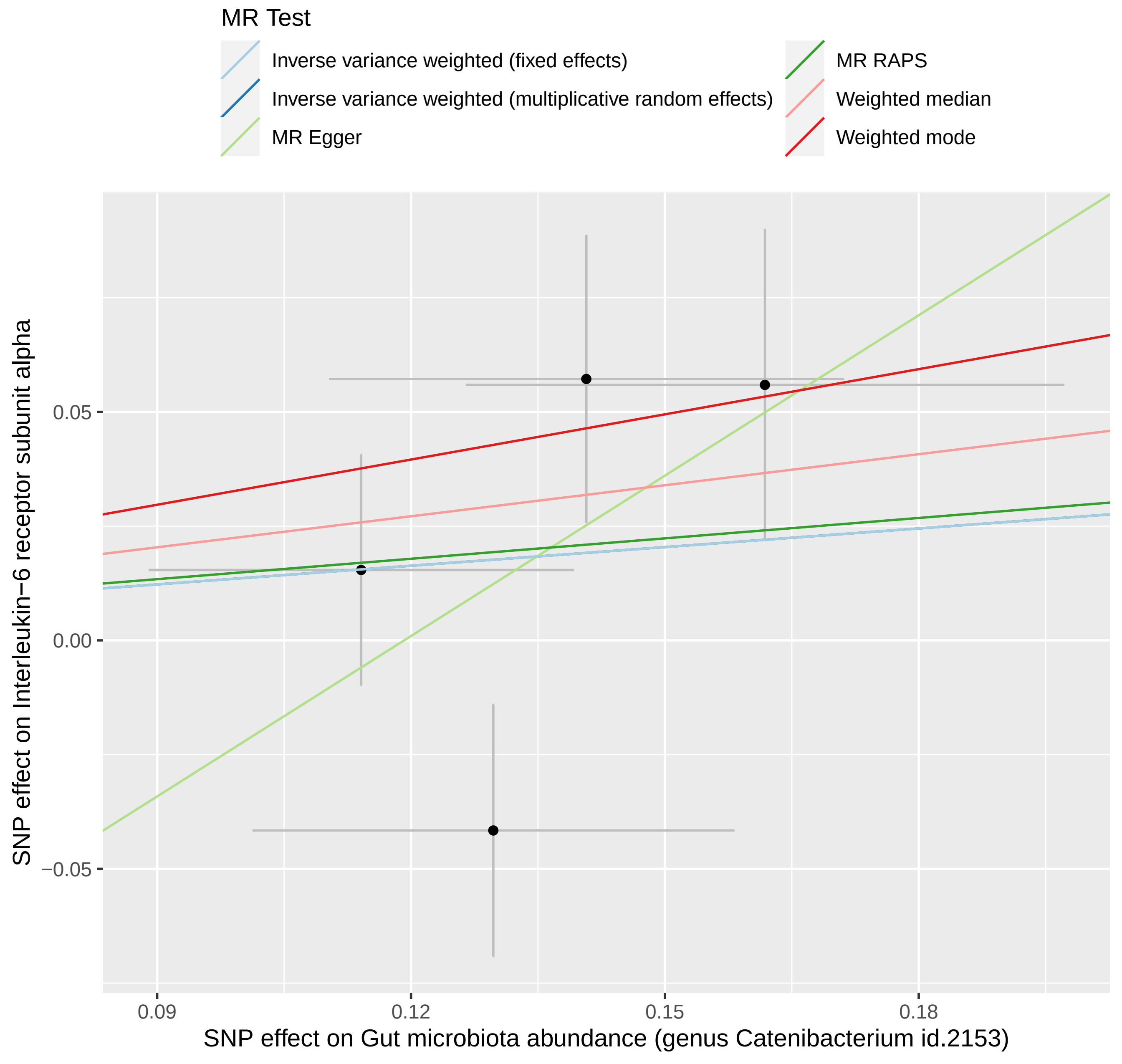

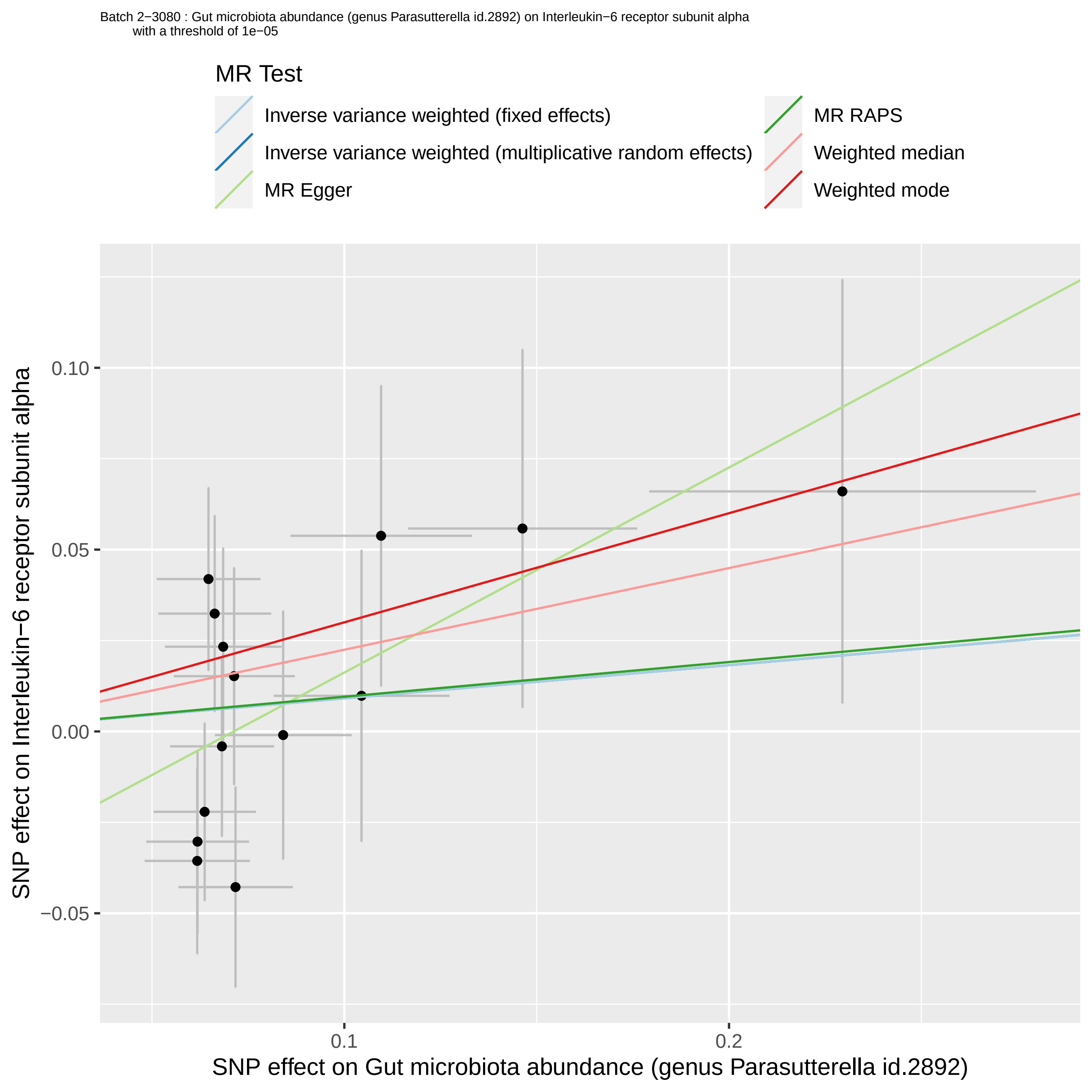

Batch 2−3080 : Gut microbiota abundance (genus Parasutterella id.2892) on Interleukin−6 receptor subunit alpha
with a threshold of 1e−05
MR Test
Inverse variance weighted (fixed effects)
Inverse variance weighted (multiplicative random effects)
MR Egger
MR RAPS
Weighted median
Weighted mode
SNP effect on Interleukin−6 receptor subunit alpha
0.10
0.05
0.00
−0.05
0.1
0.2
SNP effect on Gut microbiota abundance (genus Parasutterella id.2892)

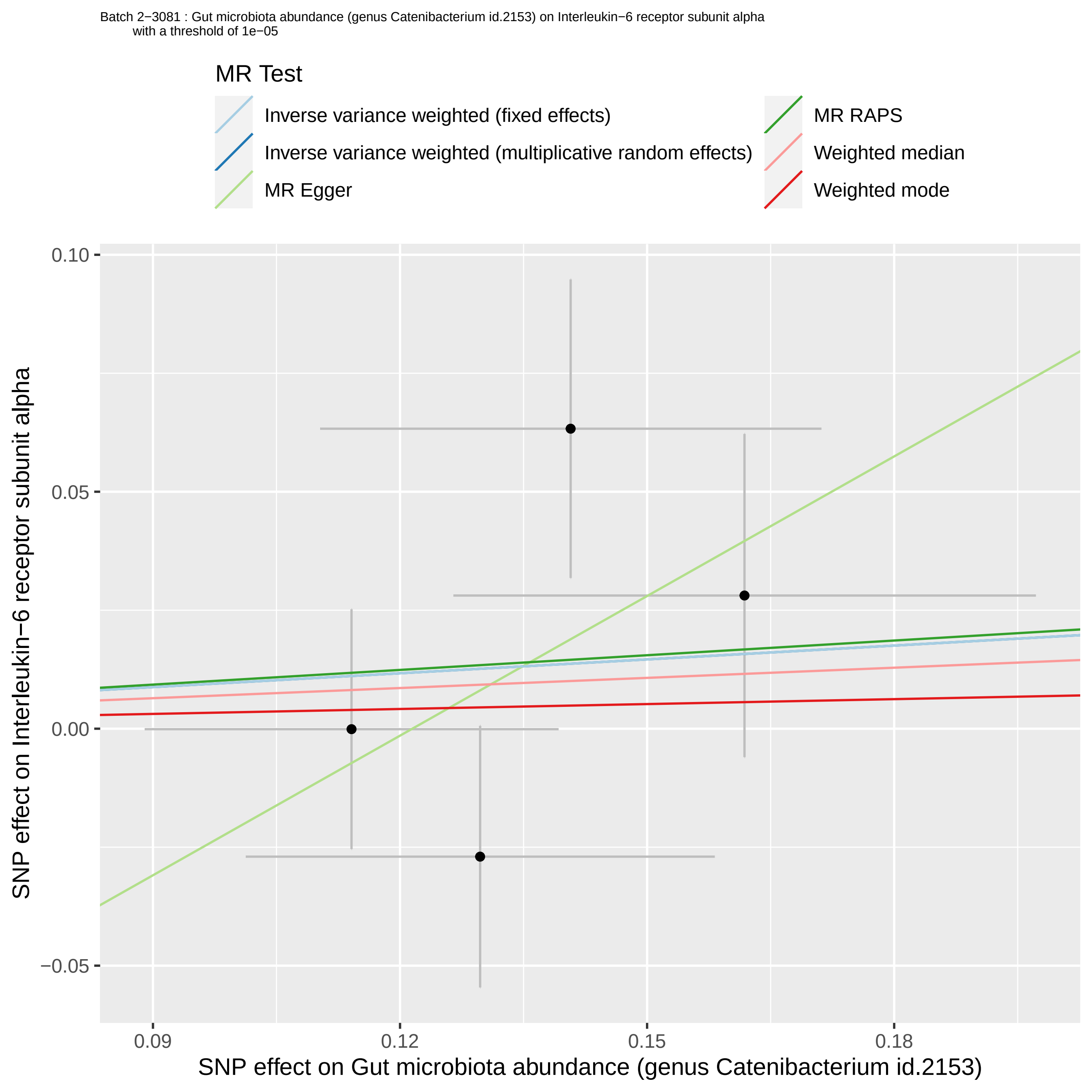
Batch 2−3081 : Gut microbiota abundance (genus Catenibacterium id.2153) on Interleukin−6 receptor subunit alpha
with a threshold of 1e−05
MR Test
Inverse variance weighted (fixed effects)
Inverse variance weighted (multiplicative random effects)
MR Egger
MR RAPS
Weighted median
Weighted mode
0.10
0.05
0.00
−0.05
0.09
0.12
0.15
0.18
SNP effect on Interleukin−6 receptor subunit alpha
SNP effect on Gut microbiota abundance (genus Catenibacterium id.2153)

Batch 2−3082 : Gut microbiota abundance (genus Parasutterella id.2892) on Interleukin−6 receptor subunit alpha
with a threshold of 1e−05

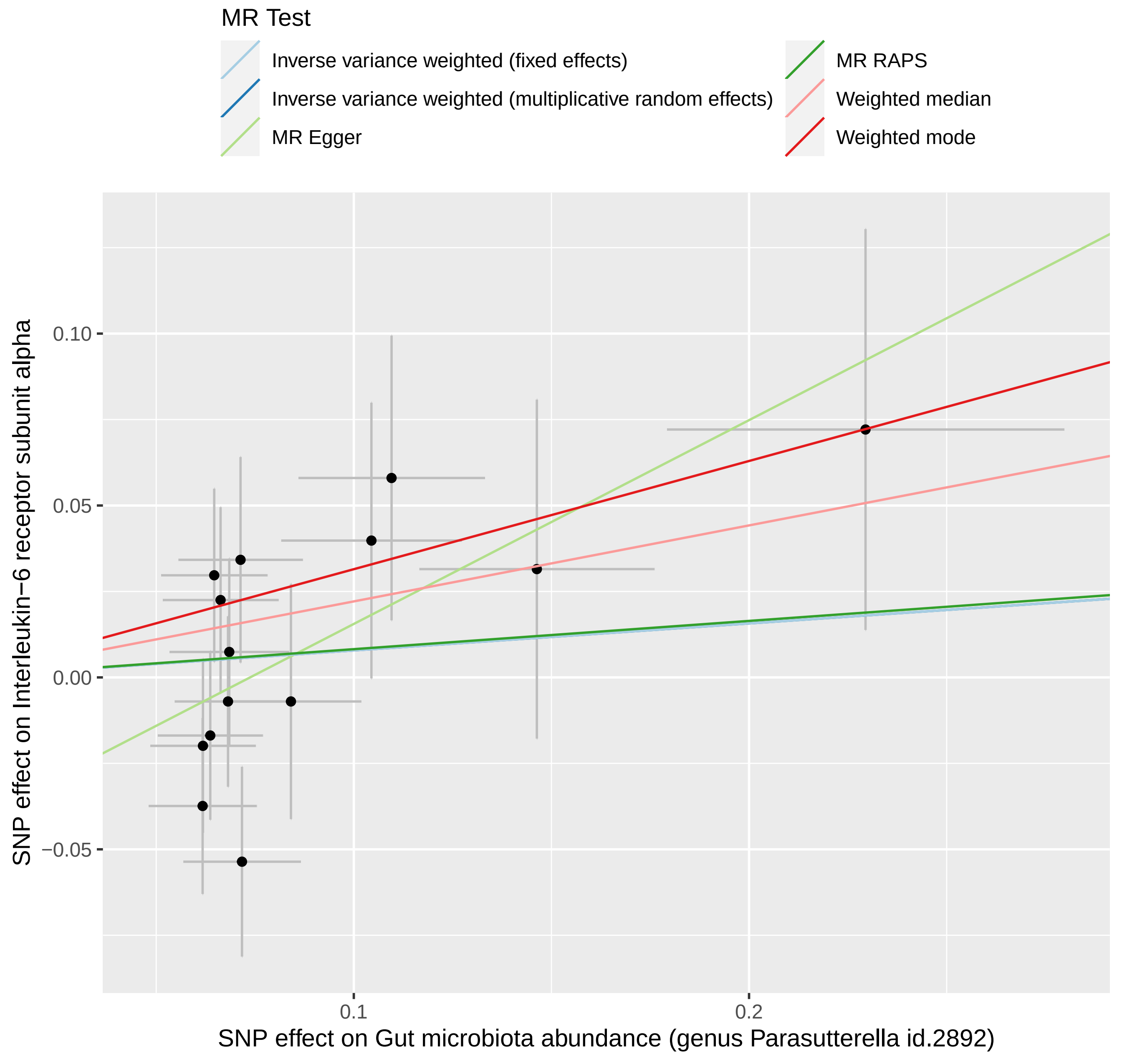

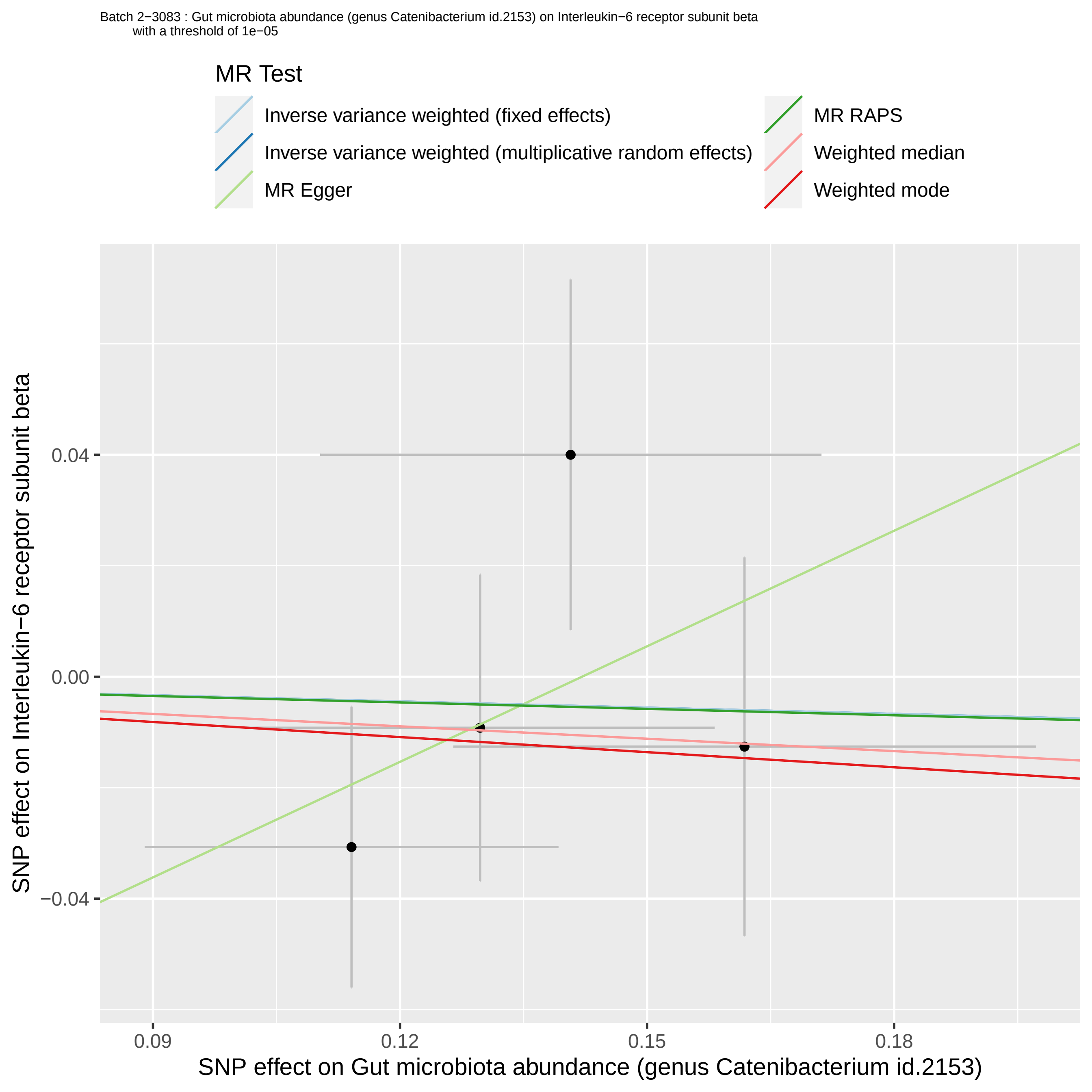

Batch 2−3083 : Gut microbiota abundance (genus Catenibacterium id.2153) on Interleukin−6 receptor subunit beta
with a threshold of 1e−05
MR Test
Inverse variance weighted (fixed effects)
Inverse variance weighted (multiplicative random effects)
MR Egger
MR RAPS
Weighted median
Weighted mode
0.04
0.00
−0.04
0.09
0.12
0.15
0.18
SNP effect on Interleukin−6 receptor subunit beta
SNP effect on Gut microbiota abundance (genus Catenibacterium id.2153)

Batch 2−3084 : Gut microbiota abundance (genus Parasutterella id.2892) on Interleukin−6 receptor subunit beta
with a threshold of 1e−05

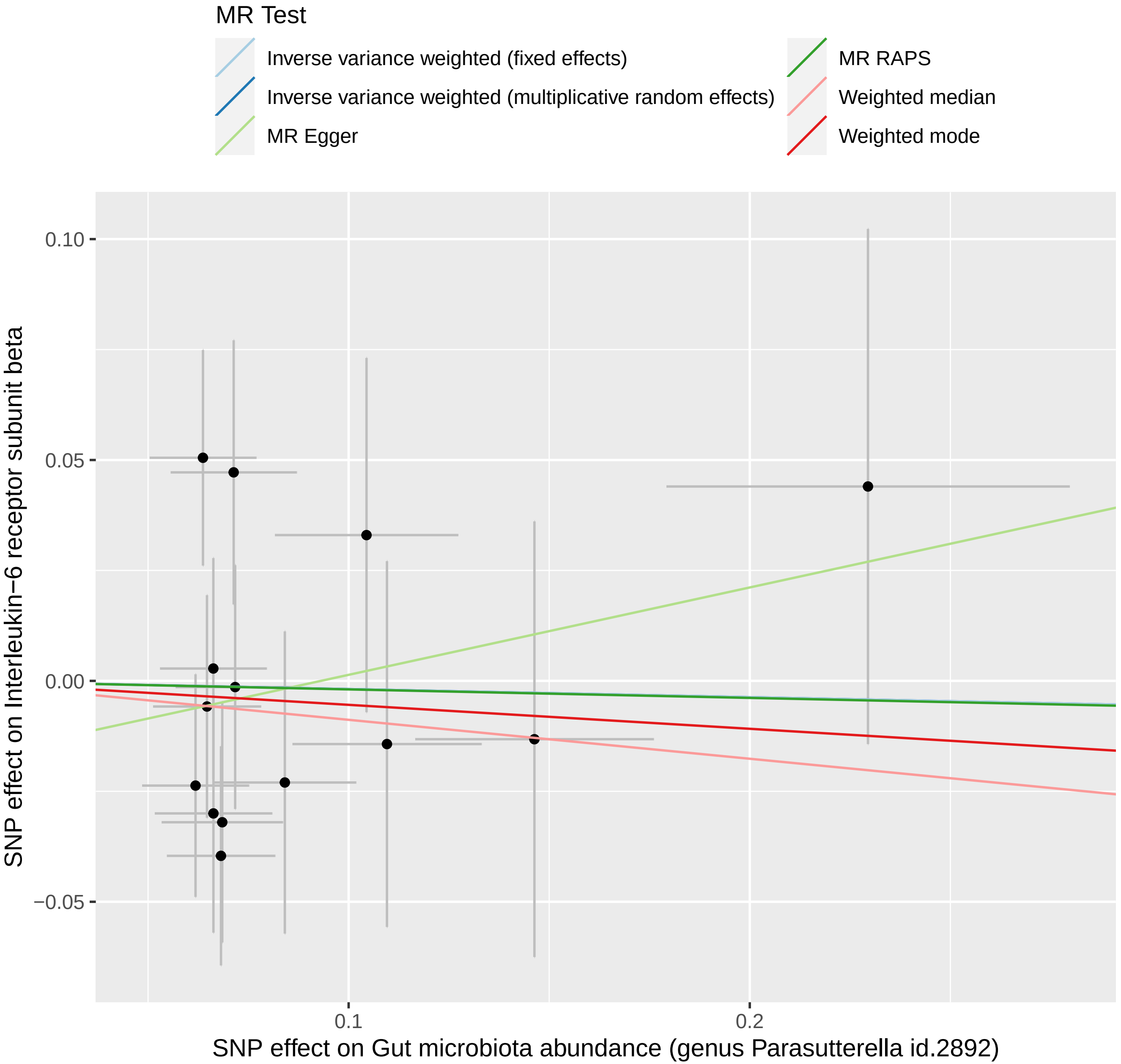

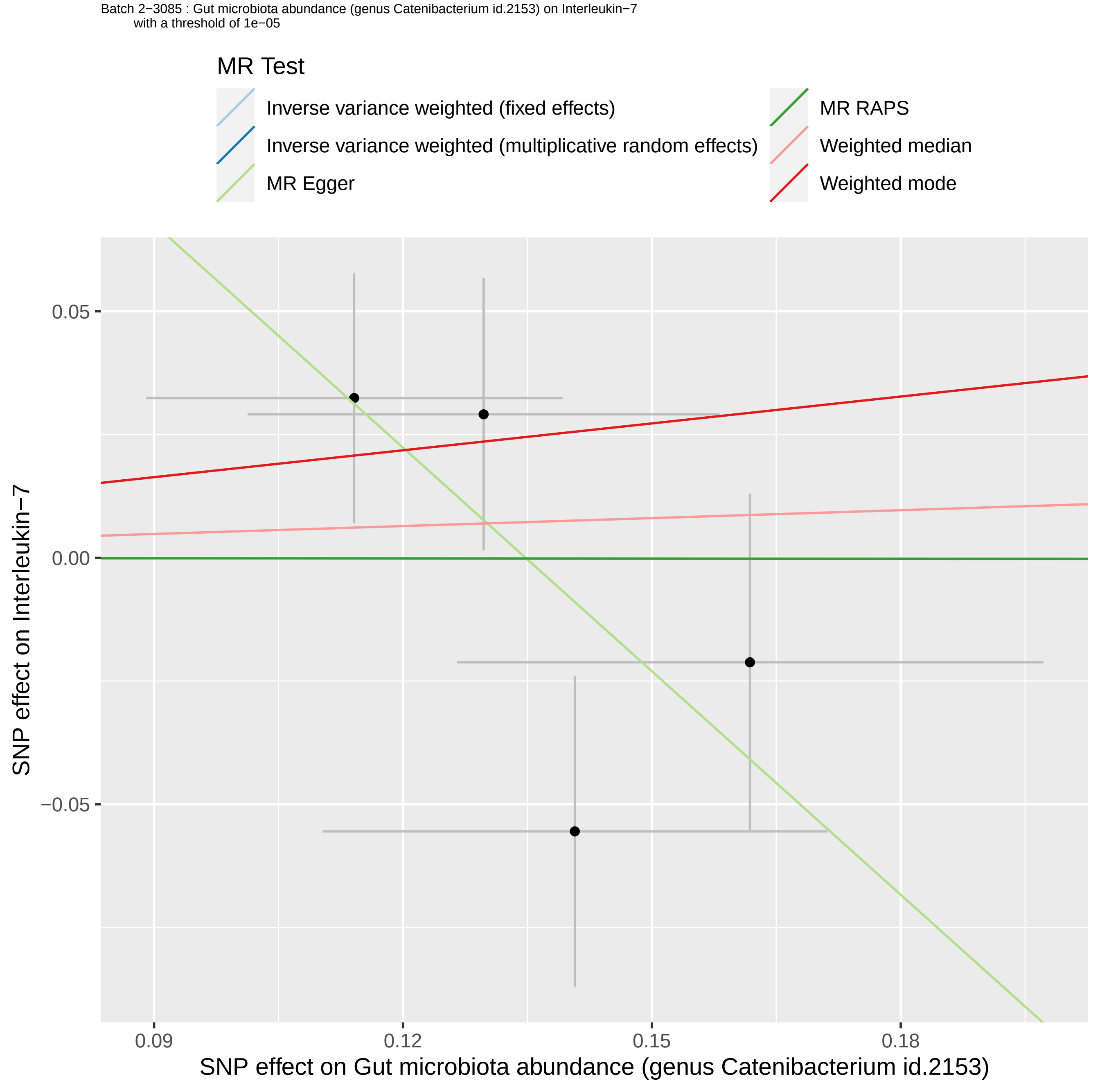

Batch 2−3085 : Gut microbiota abundance (genus Catenibacterium id.2153) on Interleukin−7
with a threshold of 1e−05
MR Test
Inverse variance weighted (fixed effects)
Inverse variance weighted (multiplicative random effects)
MR Egger
MR RAPS
Weighted median
Weighted mode
0.05
0.00
−0.05
0.09
0.12
0.15
0.18
SNP effect on Interleukin−7
SNP effect on Gut microbiota abundance (genus Catenibacterium id.2153)

Batch 2−3086 : Gut microbiota abundance (genus Parasutterella id.2892) on Interleukin−7
with a threshold of 1e−05

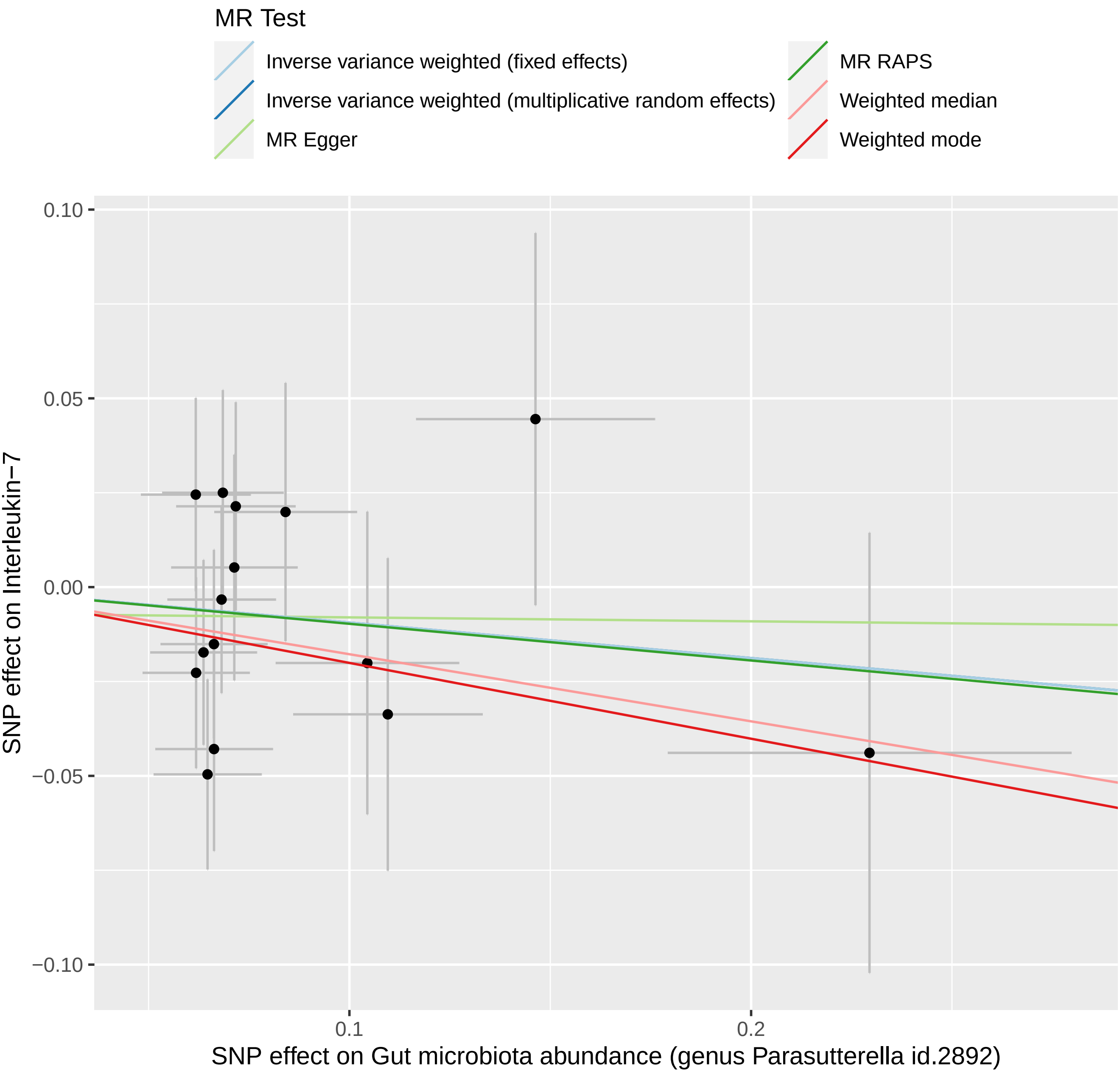

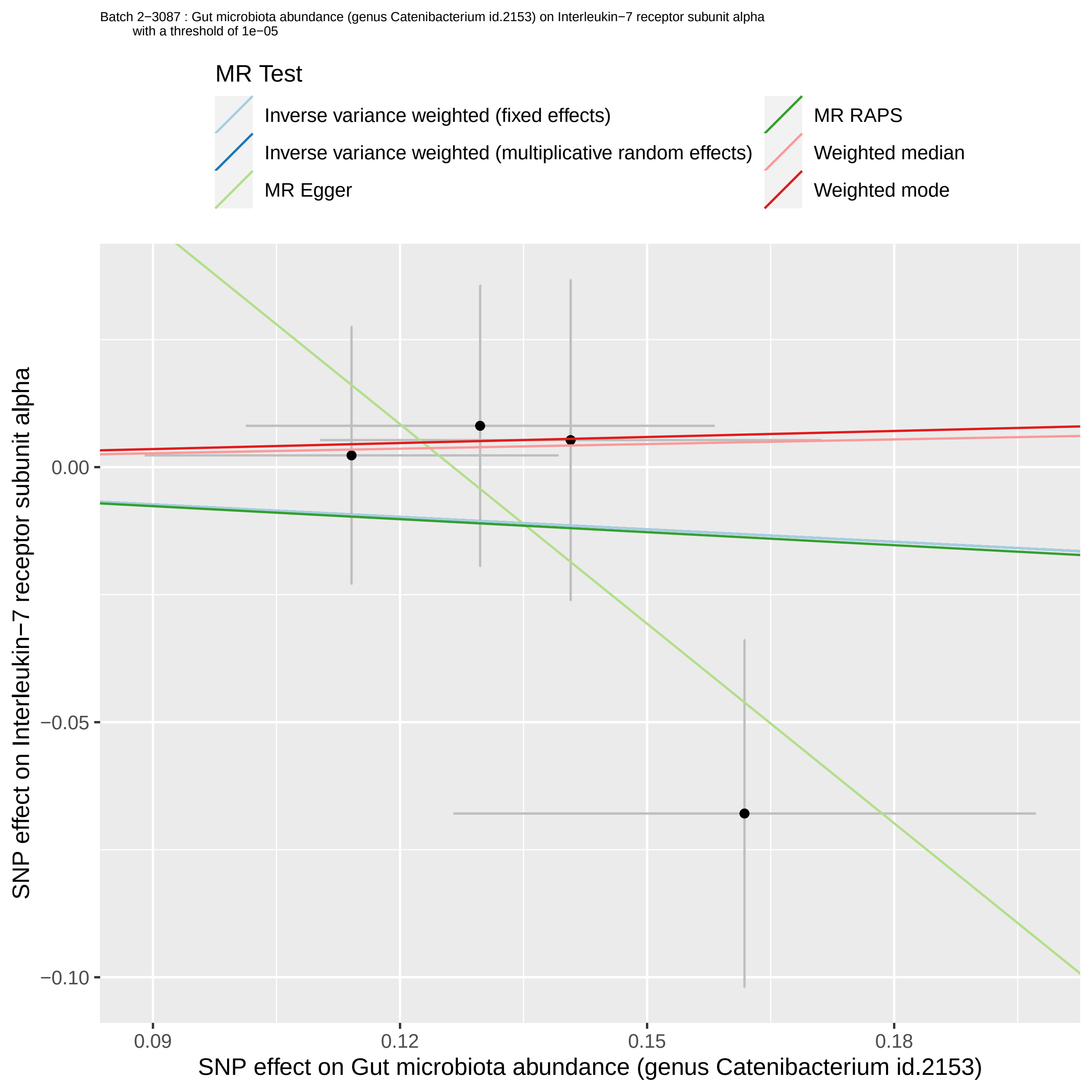
Batch 2−3087 : Gut microbiota abundance (genus Catenibacterium id.2153) on Interleukin−7 receptor subunit alpha
with a threshold of 1e−05
MR Test
Inverse variance weighted (fixed effects)
Inverse variance weighted (multiplicative random effects)
MR Egger
MR RAPS
Weighted median
Weighted mode
0.00
−0.05
−0.10
0.09
0.12
0.15
0.18
SNP effect on Interleukin−7 receptor subunit alpha
SNP effect on Gut microbiota abundance (genus Catenibacterium id.2153)

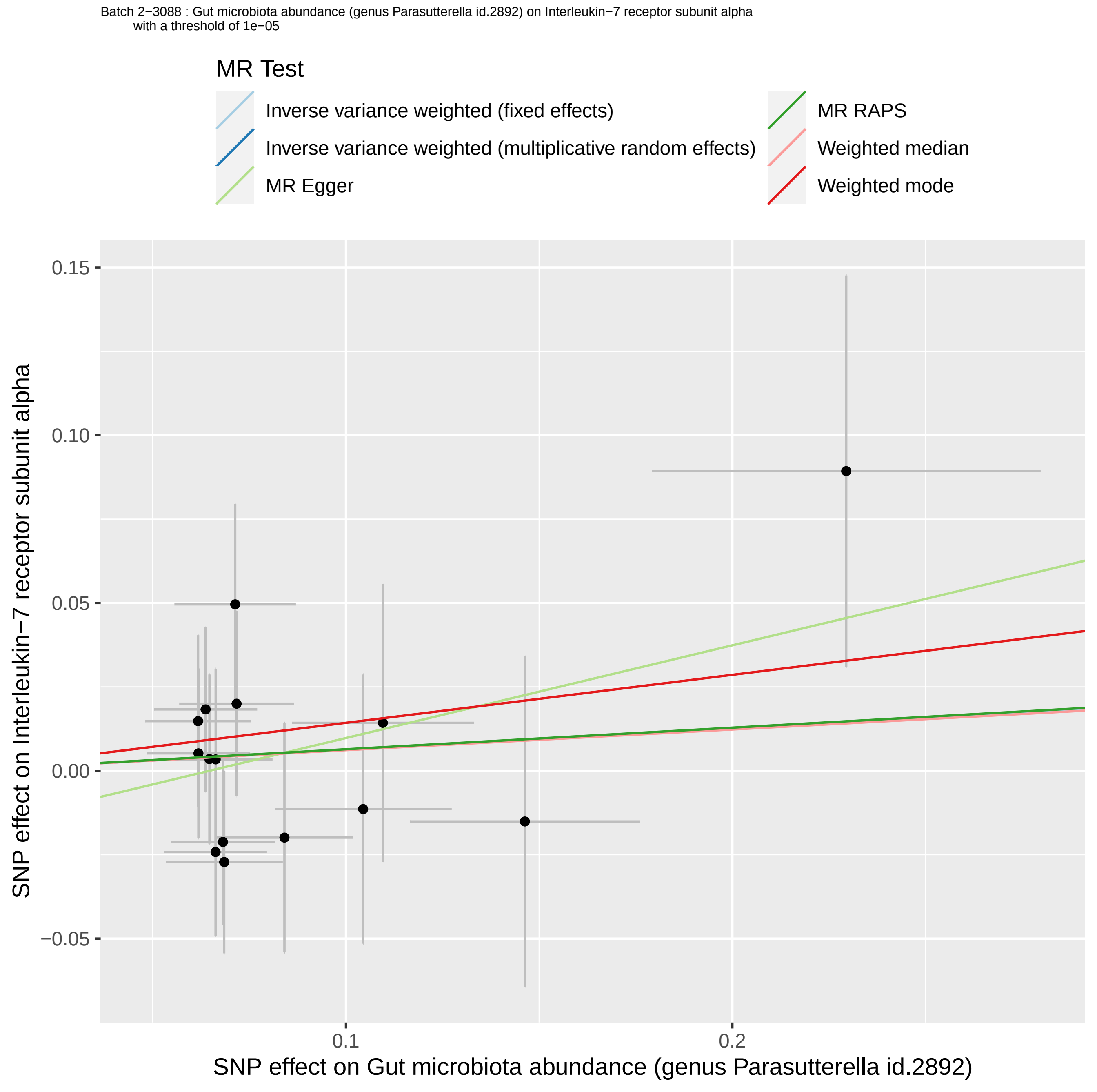
Batch 2−3088 : Gut microbiota abundance (genus Parasutterella id.2892) on Interleukin−7 receptor subunit alpha
with a threshold of 1e−05
MR Test
Inverse variance weighted (fixed effects)
Inverse variance weighted (multiplicative random effects)
MR Egger
MR RAPS
Weighted median
Weighted mode
SNP effect on Interleukin−7 receptor subunit alpha
0.15
0.10
0.05
0.00
−0.05
0.1
0.2
SNP effect on Gut microbiota abundance (genus Parasutterella id.2892)

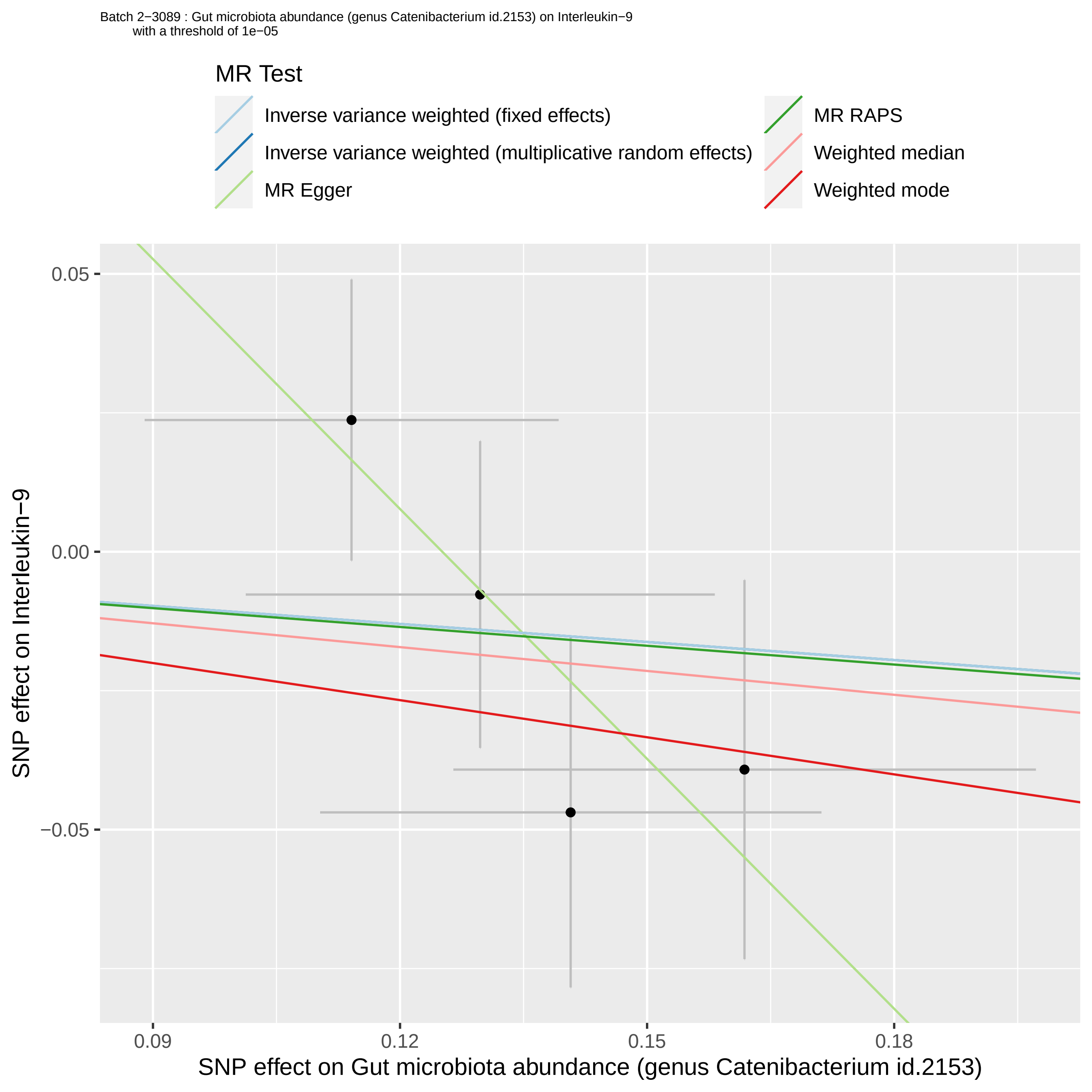
Batch 2−3089 : Gut microbiota abundance (genus Catenibacterium id.2153) on Interleukin−9
with a threshold of 1e−05
MR Test
Inverse variance weighted (fixed effects)
Inverse variance weighted (multiplicative random effects)
MR Egger
MR RAPS
Weighted median
Weighted mode
0.05
0.00
−0.05
0.09
0.12
0.15
0.18
SNP effect on Interleukin−9
SNP effect on Gut microbiota abundance (genus Catenibacterium id.2153)

Batch 2−3090 : Gut microbiota abundance (genus Parasutterella id.2892) on Interleukin−9
with a threshold of 1e−05

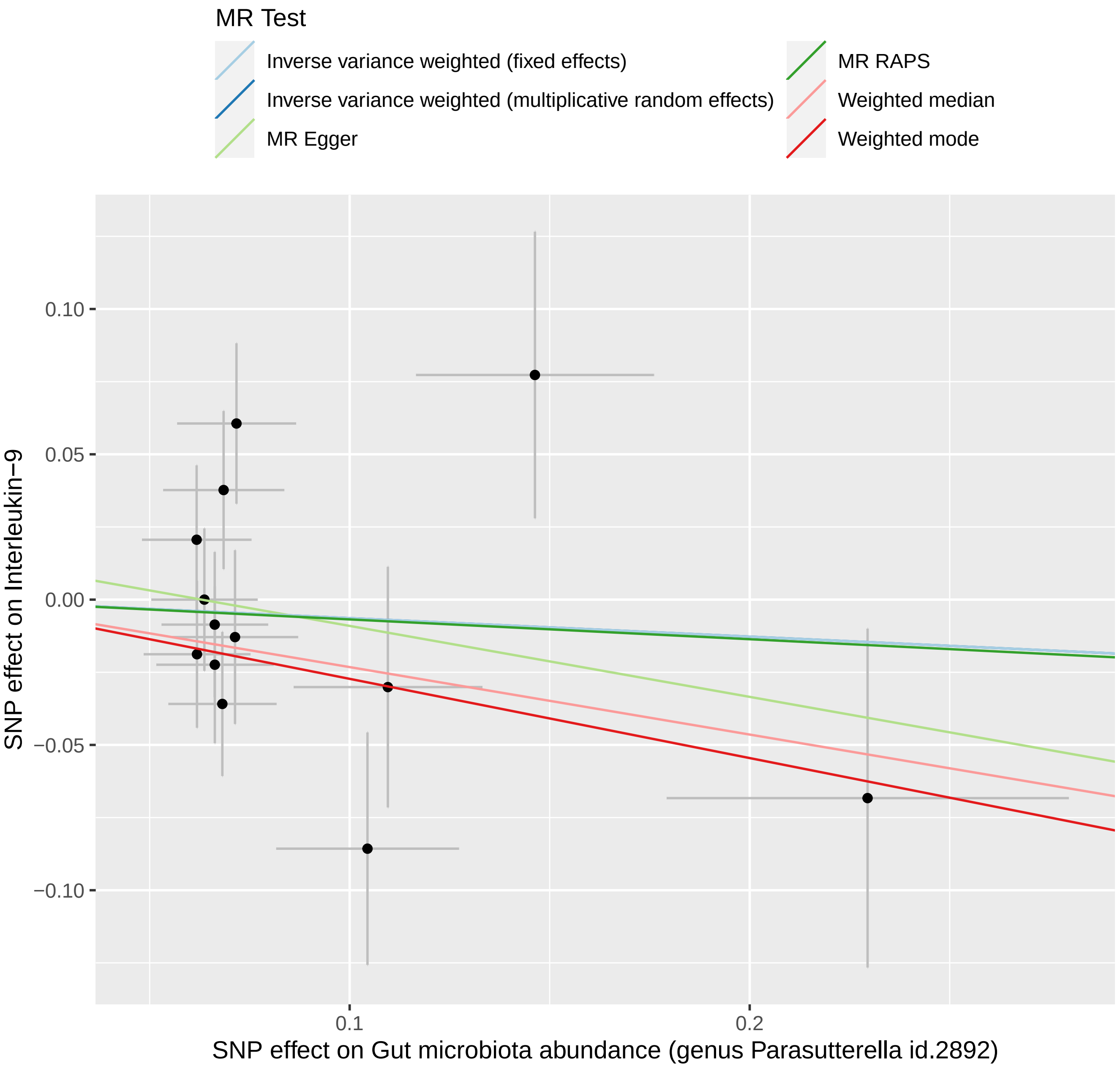

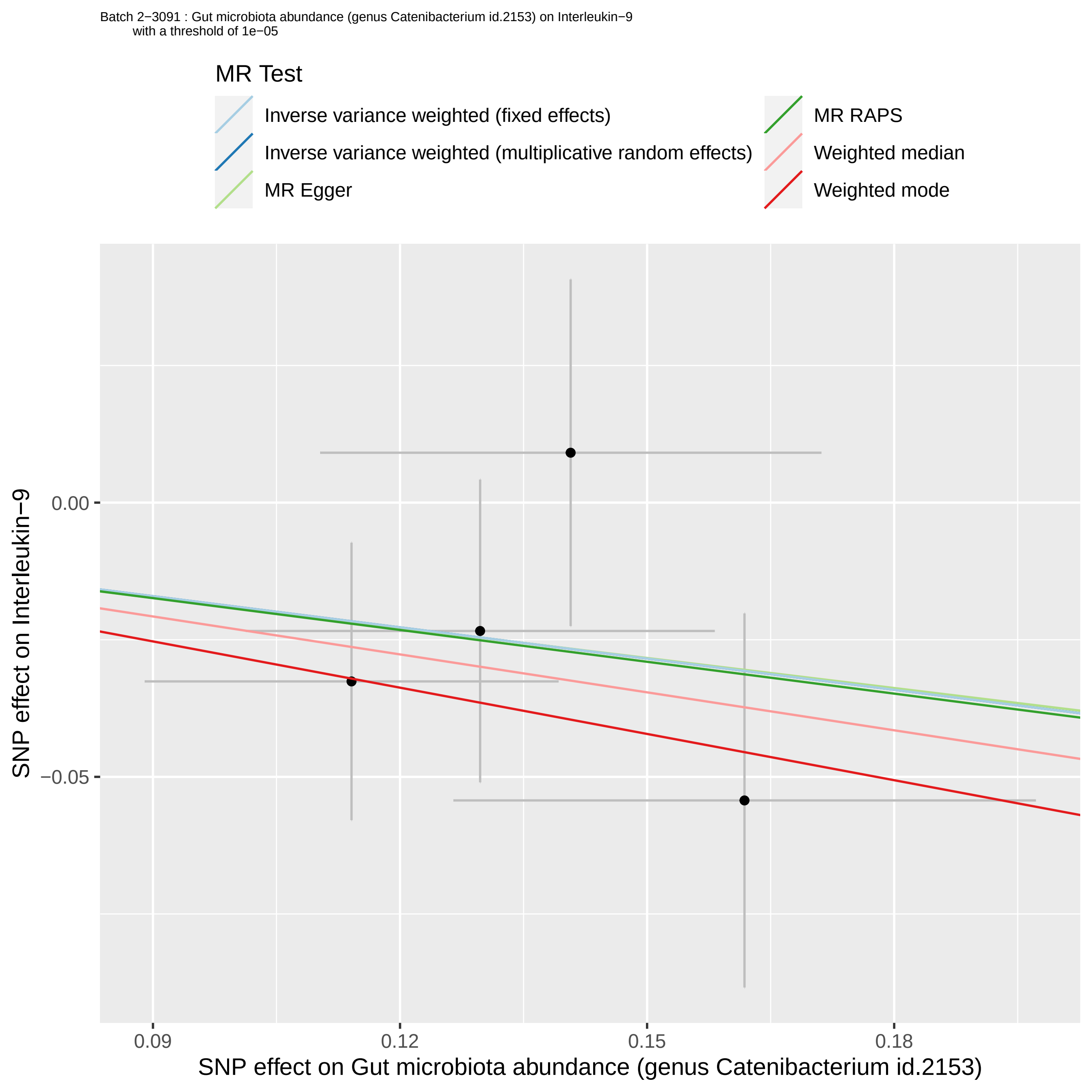

Batch 2−3091 : Gut microbiota abundance (genus Catenibacterium id.2153) on Interleukin−9
with a threshold of 1e−05
MR Test
Inverse variance weighted (fixed effects)
Inverse variance weighted (multiplicative random effects)
MR Egger
MR RAPS
Weighted median
Weighted mode
SNP effect on Interleukin−9
0.00
−0.05
0.09
0.12
0.15
0.18
SNP effect on Gut microbiota abundance (genus Catenibacterium id.2153)

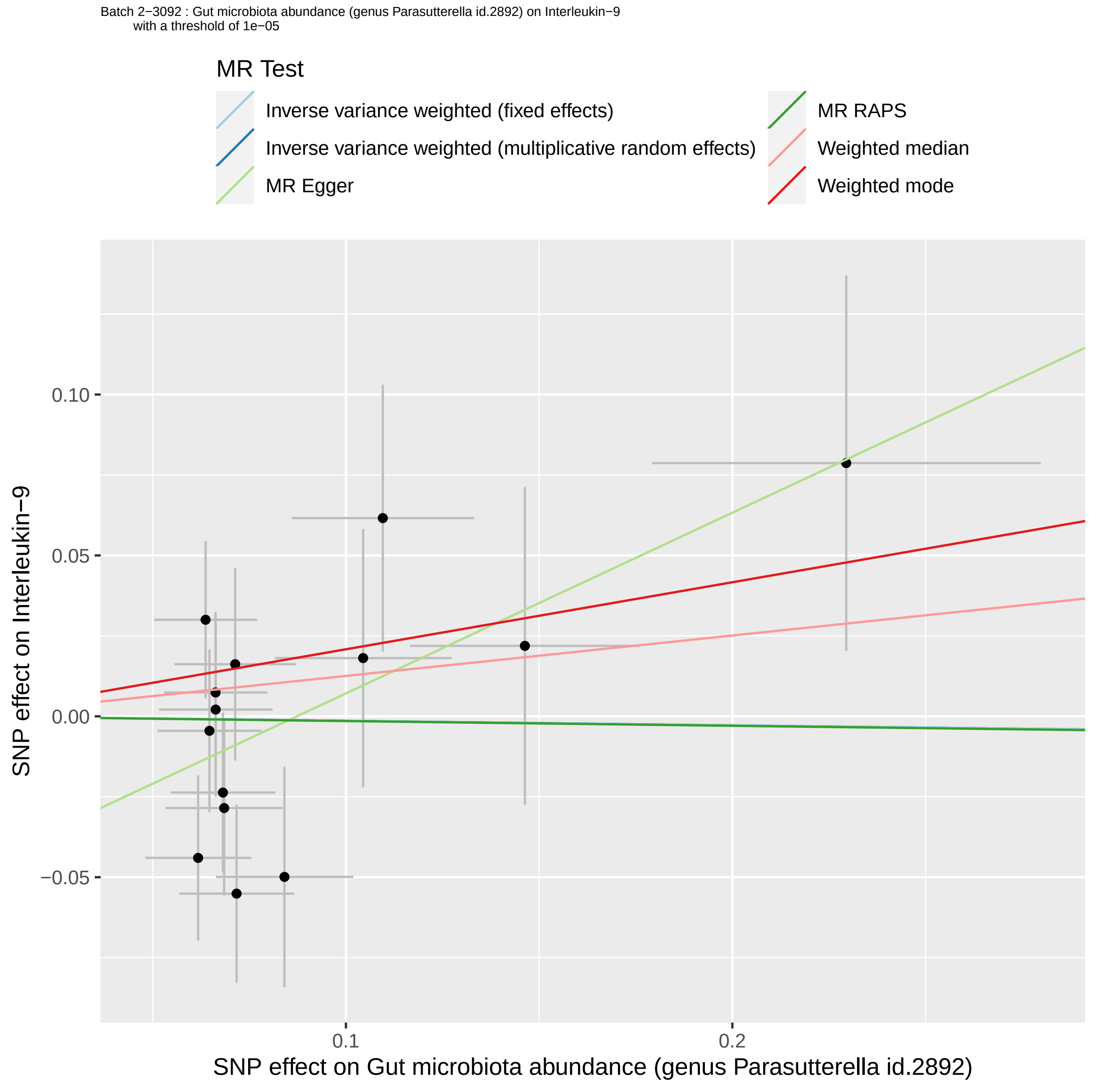

Batch 2−3092 : Gut microbiota abundance (genus Parasutterella id.2892) on Interleukin−9
with a threshold of 1e−05
MR Test
Inverse variance weighted (fixed effects)
Inverse variance weighted (multiplicative random effects)
MR Egger
MR RAPS
Weighted median
Weighted mode
0.10
0.05
0.00
−0.05
0.1
0.2
SNP effect on Interleukin−9
SNP effect on Gut microbiota abundance (genus Parasutterella id.2892)

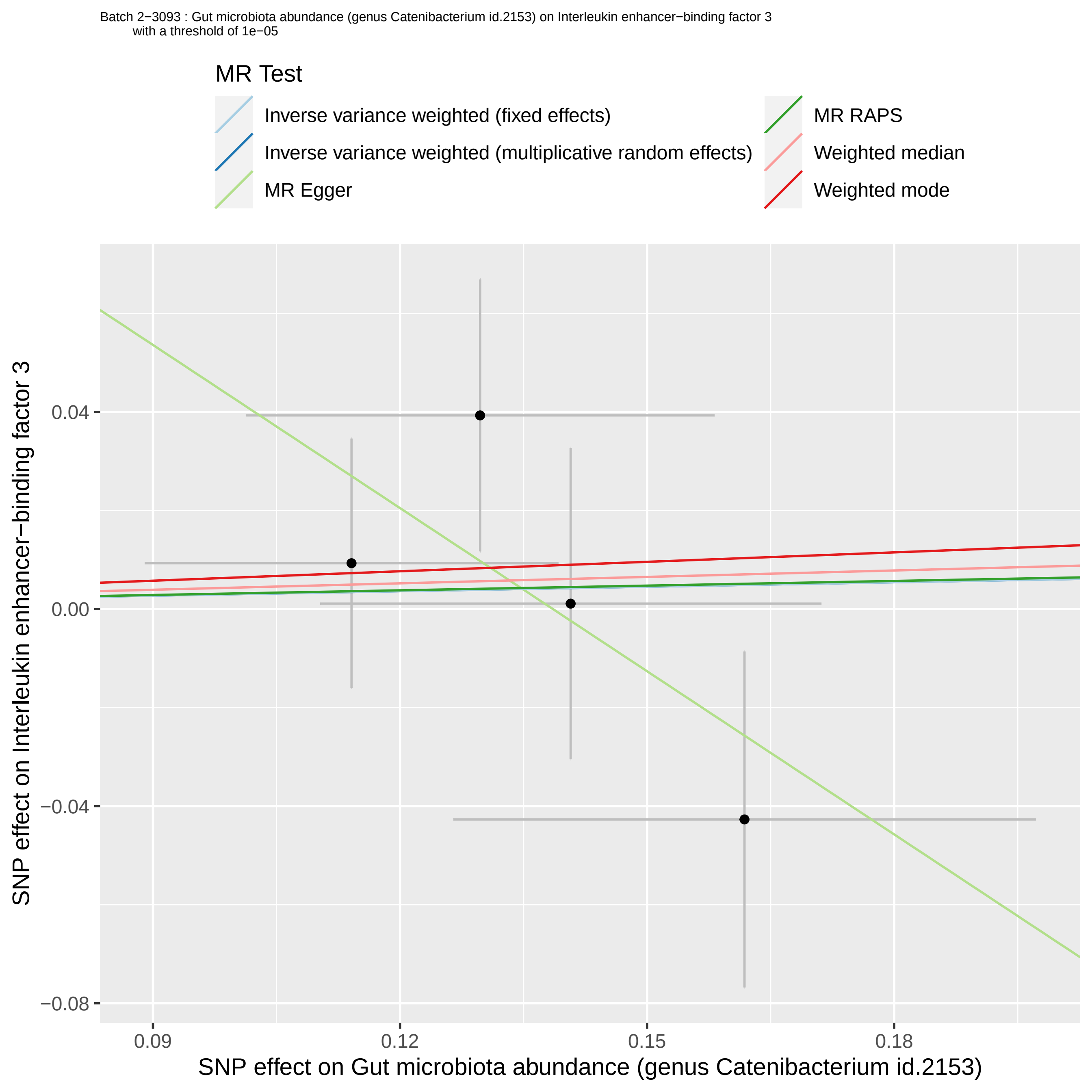
Batch 2−3093 : Gut microbiota abundance (genus Catenibacterium id.2153) on Interleukin enhancer−binding factor 3
with a threshold of 1e−05
MR Test
Inverse variance weighted (fixed effects)
Inverse variance weighted (multiplicative random effects)
MR Egger
MR RAPS
Weighted median
Weighted mode
SNP effect on Interleukin enhancer−binding factor 3
0.04
0.00
−0.04
−0.08
0.09
0.12
0.15
0.18
SNP effect on Gut microbiota abundance (genus Catenibacterium id.2153)

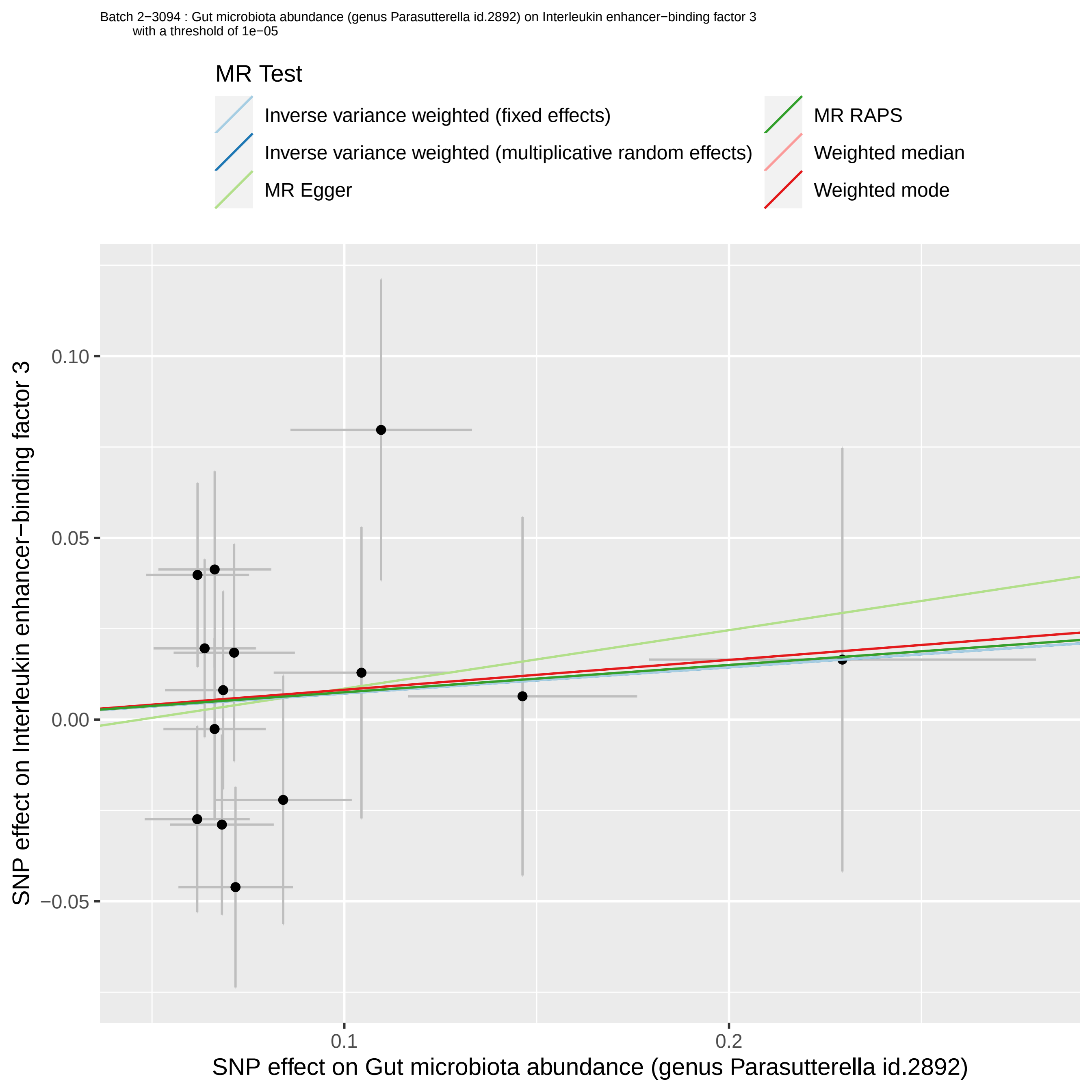

Batch 2−3094 : Gut microbiota abundance (genus Parasutterella id.2892) on Interleukin enhancer−binding factor 3
with a threshold of 1e−05
MR Test
Inverse variance weighted (fixed effects)
Inverse variance weighted (multiplicative random effects)
MR Egger
MR RAPS
Weighted median
Weighted mode
0.10
0.05
0.00
−0.05
0.1
0.2
SNP effect on Interleukin enhancer−binding factor 3
SNP effect on Gut microbiota abundance (genus Parasutterella id.2892)

Batch 2−3095 : Gut microbiota abundance (genus Catenibacterium id.2153) on Inositol monophosphatase 3
with a threshold of 1e−05

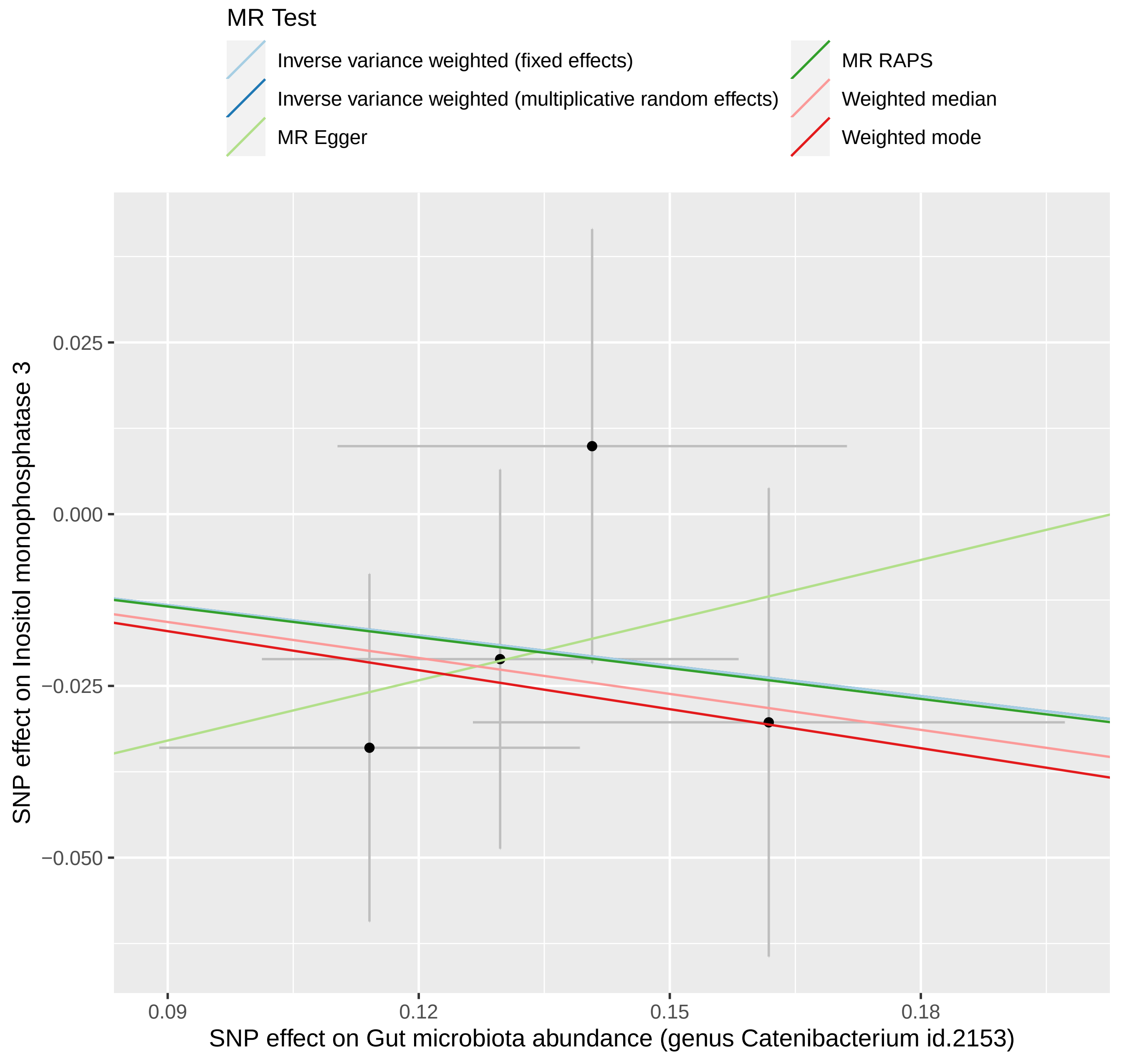

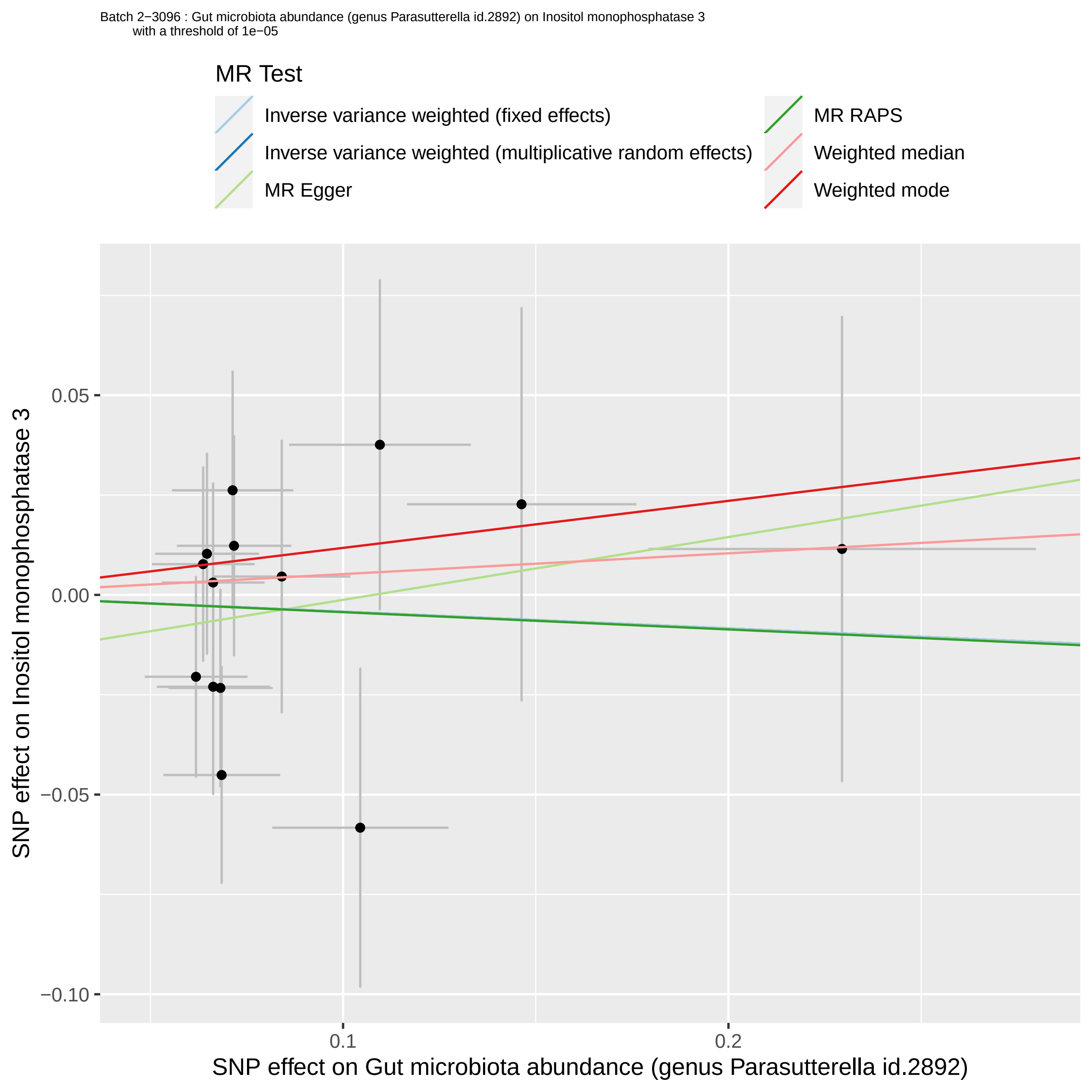
Batch 2−3096 : Gut microbiota abundance (genus Parasutterella id.2892) on Inositol monophosphatase 3
with a threshold of 1e−05
MR Test
Inverse variance weighted (fixed effects)
Inverse variance weighted (multiplicative random effects)
MR Egger
MR RAPS
Weighted median
Weighted mode
0.05
0.00
−0.05
−0.10
0.1
0.2
SNP effect on Inositol monophosphatase 3
SNP effect on Gut microbiota abundance (genus Parasutterella id.2892)

Batch 2−3097 : Gut microbiota abundance (genus Catenibacterium id.2153) on Inosine−5'−monophosphate dehydrogenase 1
with a threshold of 1e−05

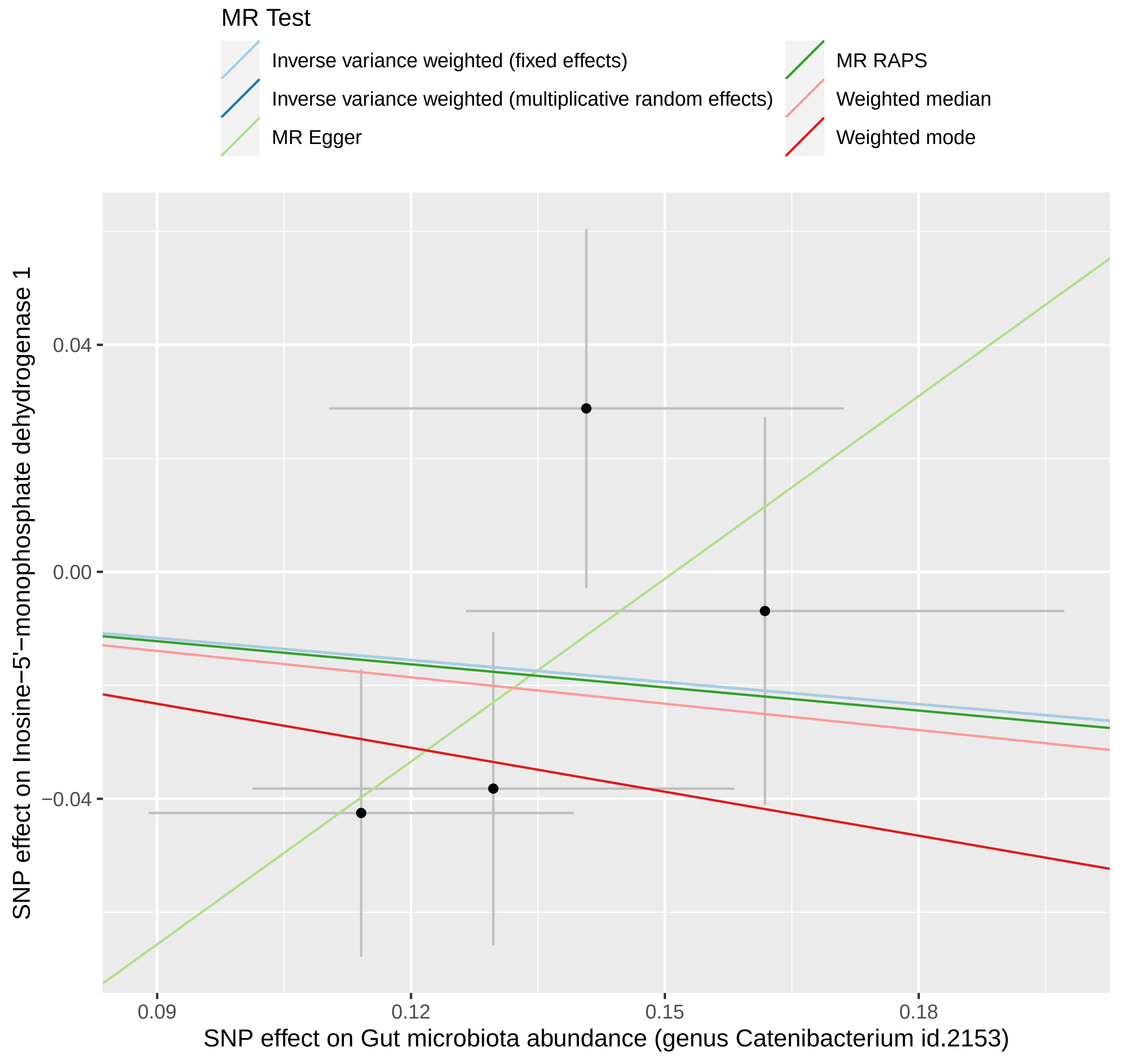

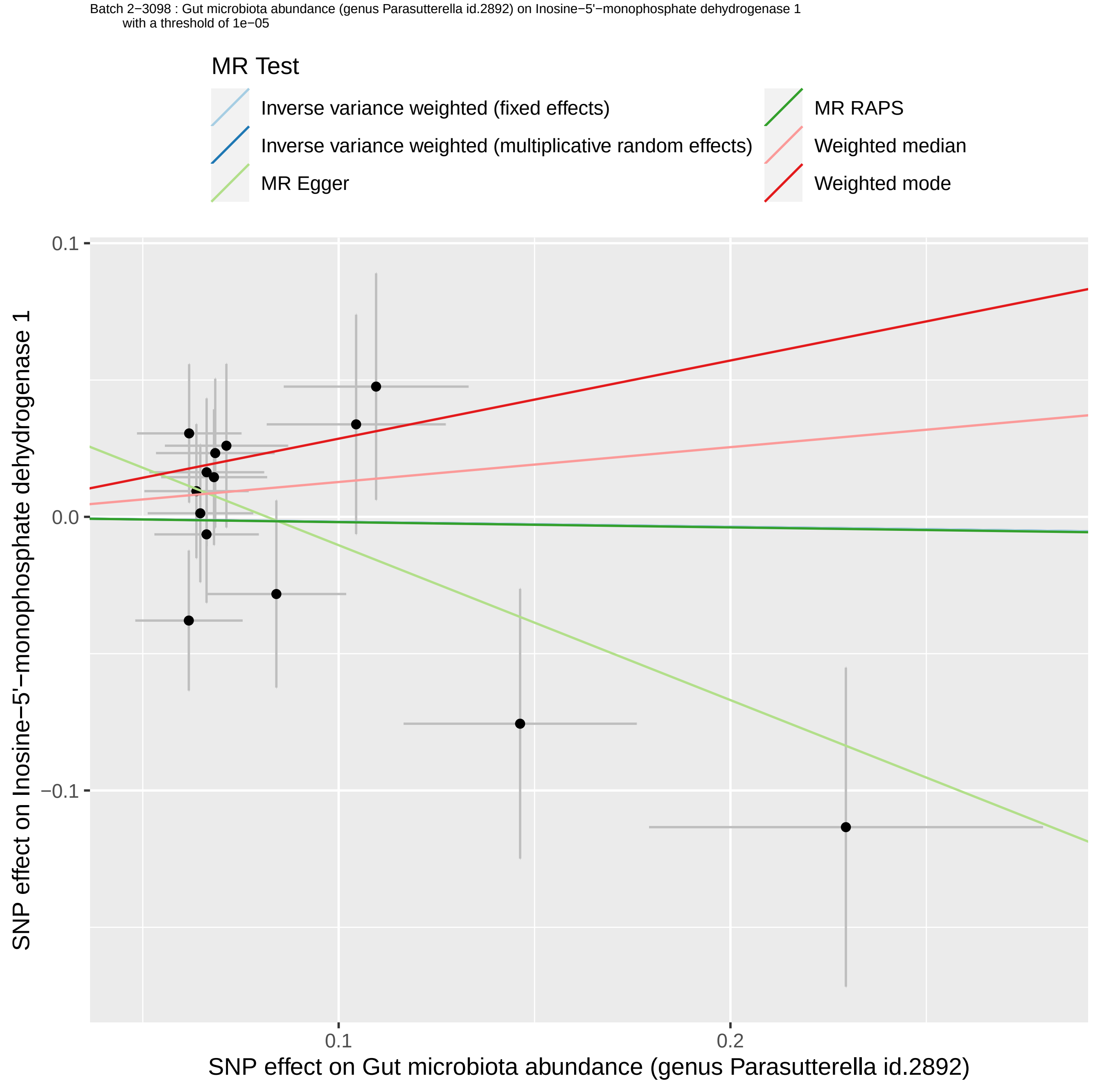
Batch 2−3098 : Gut microbiota abundance (genus Parasutterella id.2892) on Inosine−5'−monophosphate dehydrogenase 1
with a threshold of 1e−05
MR Test
Inverse variance weighted (fixed effects)
Inverse variance weighted (multiplicative random effects)
MR Egger
MR RAPS
Weighted median
Weighted mode
0.1
0.0
−0.1
0.1
0.2
SNP effect on Inosine−5'−monophosphate dehydrogenase 1
SNP effect on Gut microbiota abundance (genus Parasutterella id.2892)

Batch 2−3099 : Gut microbiota abundance (genus Catenibacterium id.2153) on Inosine−5'−monophosphate dehydrogenase 2
with a threshold of 1e−05

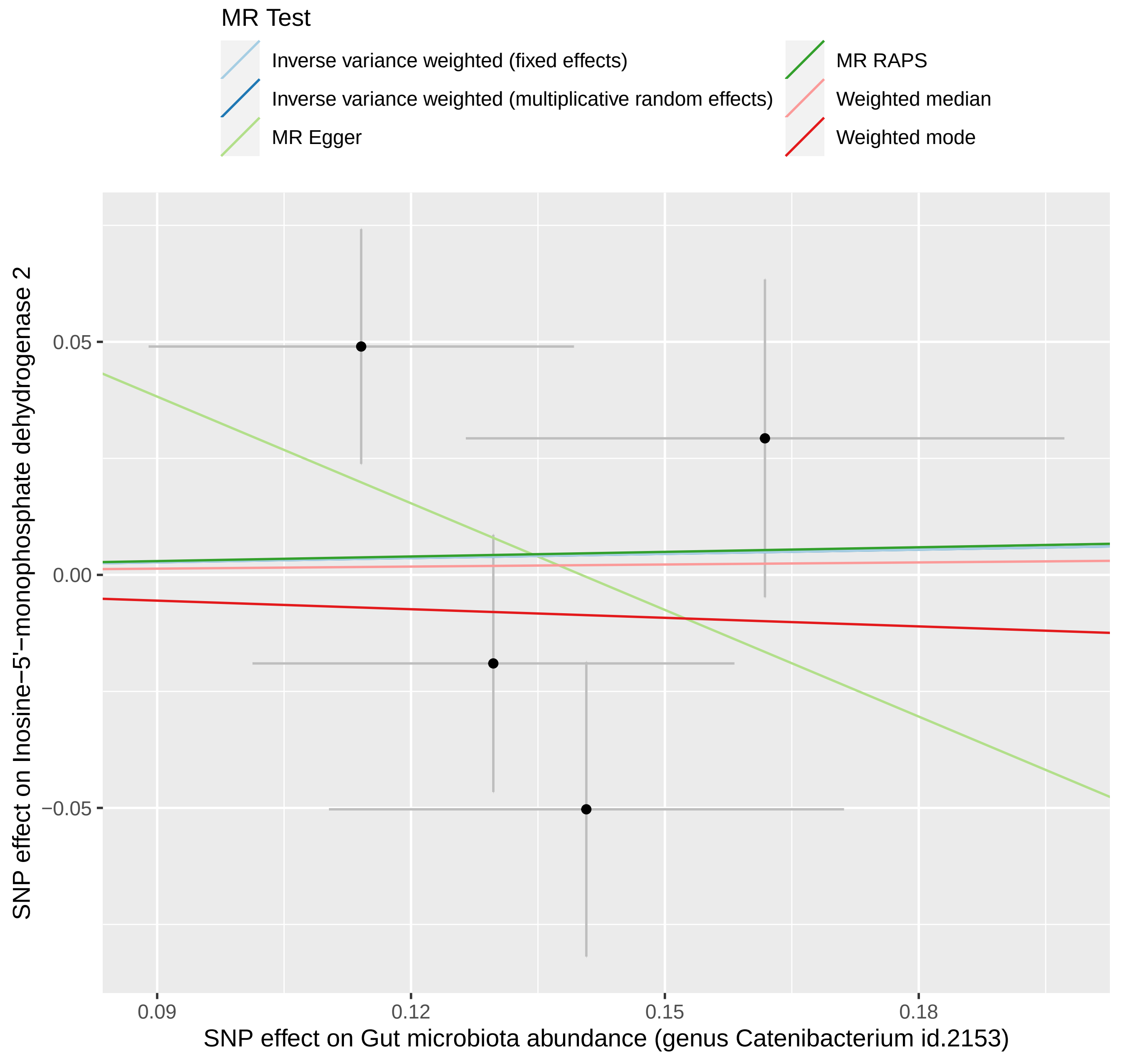

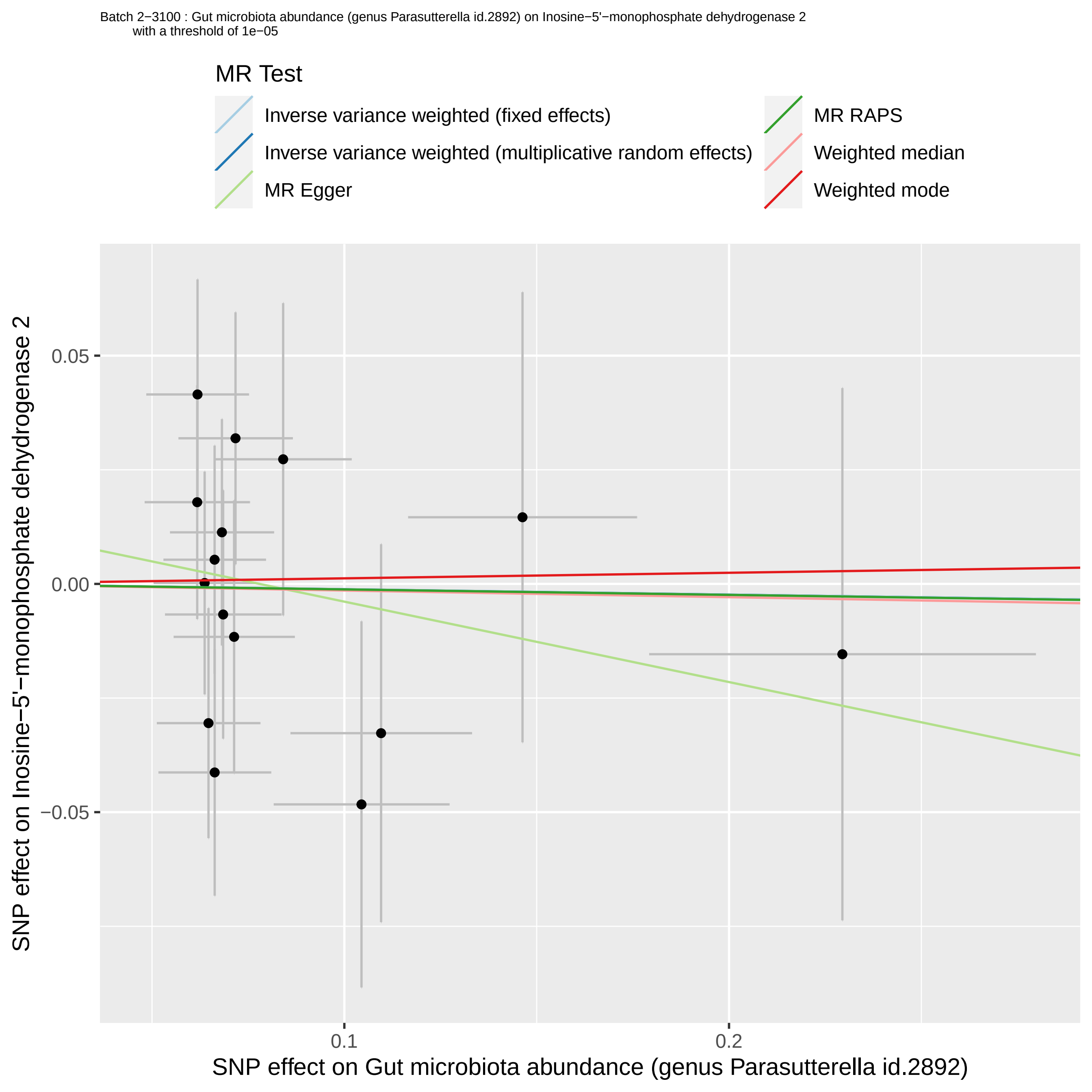
Batch 2−3100 : Gut microbiota abundance (genus Parasutterella id.2892) on Inosine−5'−monophosphate dehydrogenase 2
with a threshold of 1e−05
MR Test
Inverse variance weighted (fixed effects)
Inverse variance weighted (multiplicative random effects)
MR Egger
MR RAPS
Weighted median
Weighted mode
0.05
0.00
−0.05
0.1
0.2
SNP effect on Inosine−5'−monophosphate dehydrogenase 2
SNP effect on Gut microbiota abundance (genus Parasutterella id.2892)

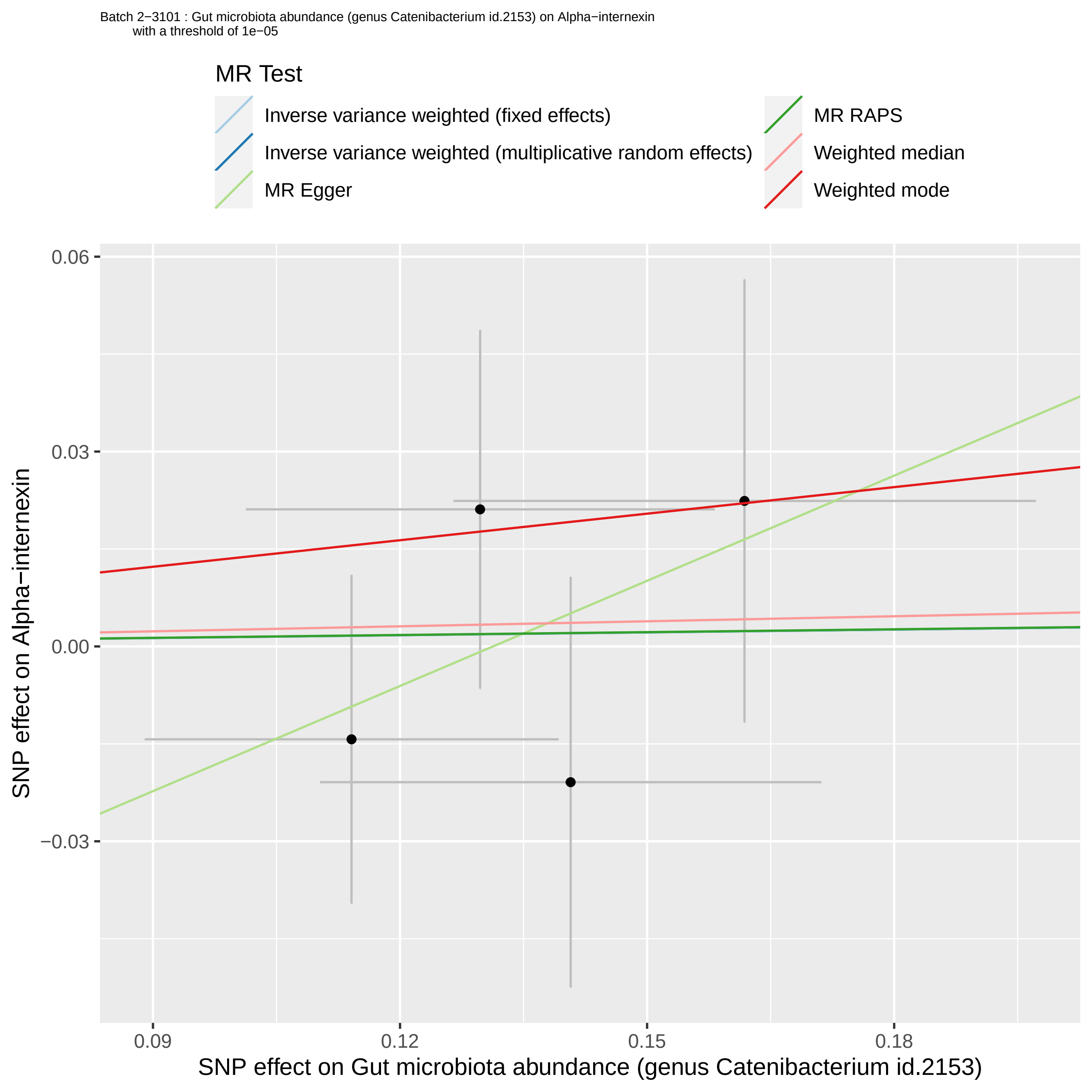
Batch 2−3101 : Gut microbiota abundance (genus Catenibacterium id.2153) on Alpha−internexin
with a threshold of 1e−05
MR Test
Inverse variance weighted (fixed effects)
Inverse variance weighted (multiplicative random effects)
MR Egger
MR RAPS
Weighted median
Weighted mode
0.06
0.03
0.00
−0.03
0.09
0.12
0.15
0.18
SNP effect on Alpha−internexin
SNP effect on Gut microbiota abundance (genus Catenibacterium id.2153)

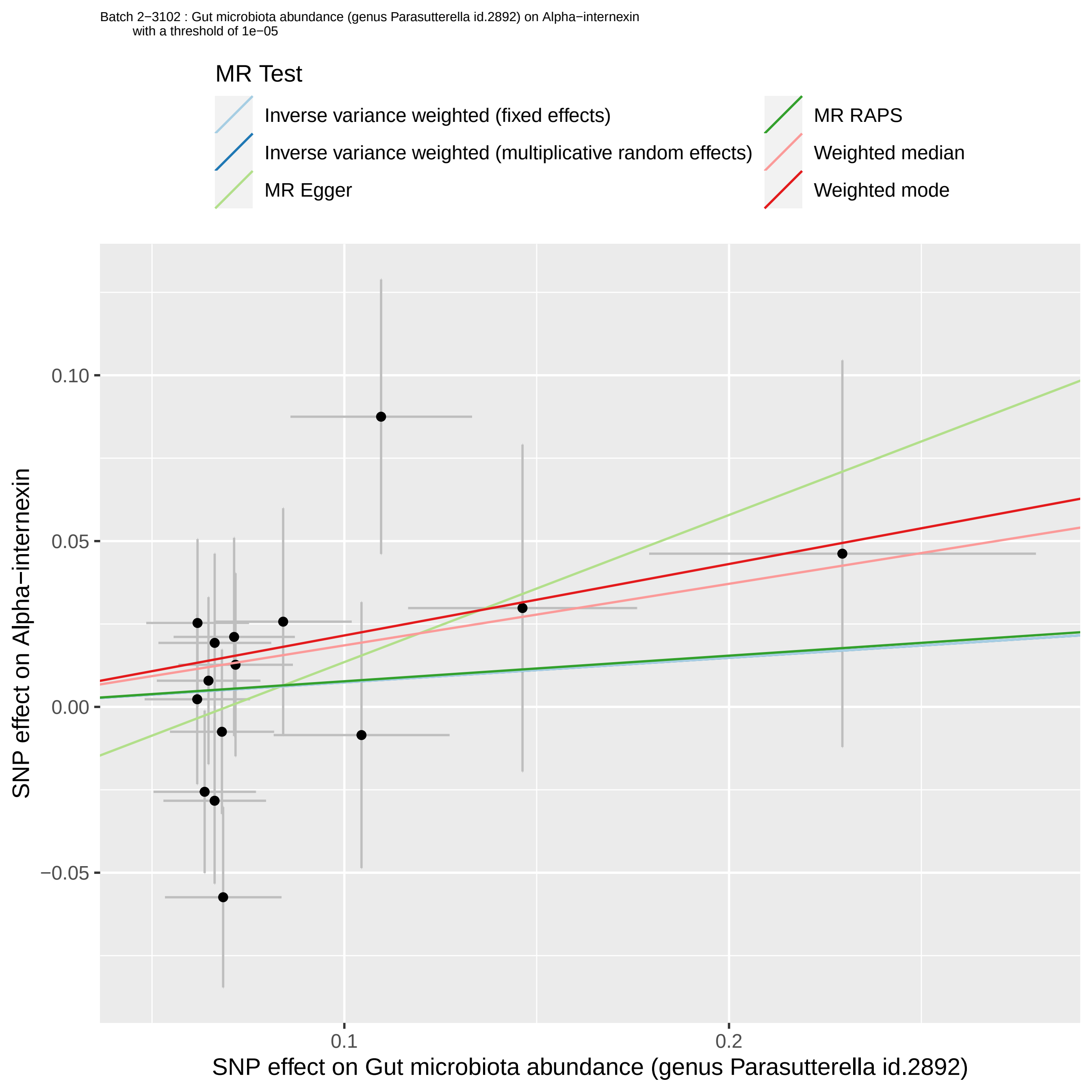
Batch 2−3102 : Gut microbiota abundance (genus Parasutterella id.2892) on Alpha−internexin
with a threshold of 1e−05
MR Test
Inverse variance weighted (fixed effects)
Inverse variance weighted (multiplicative random effects)
MR Egger
MR RAPS
Weighted median
Weighted mode
0.10
0.05
0.00
−0.05
0.1
0.2
SNP effect on Alpha−internexin
SNP effect on Gut microbiota abundance (genus Parasutterella id.2892)

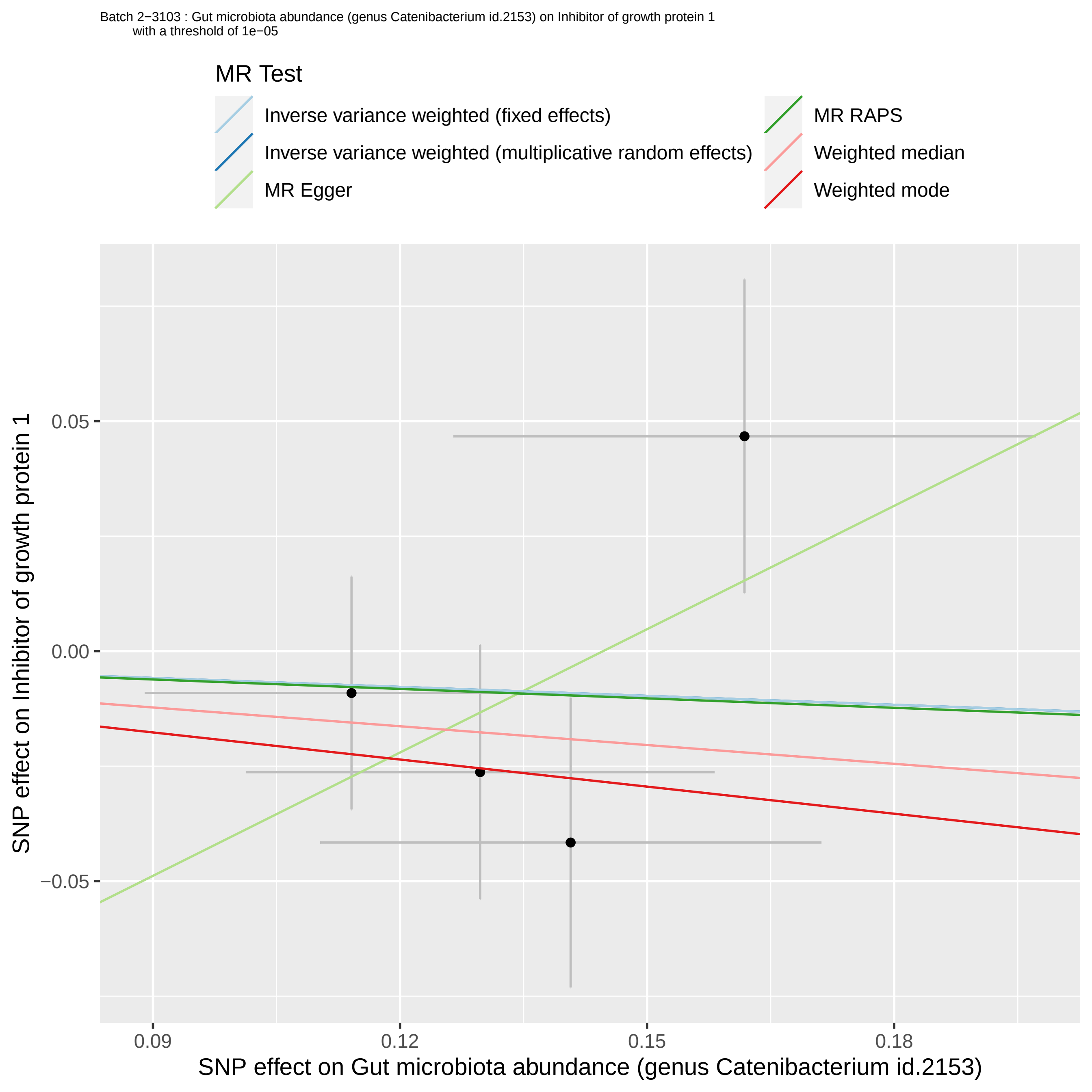

Batch 2−3103 : Gut microbiota abundance (genus Catenibacterium id.2153) on Inhibitor of growth protein 1
with a threshold of 1e−05
MR Test
Inverse variance weighted (fixed effects)
Inverse variance weighted (multiplicative random effects)
MR Egger
MR RAPS
Weighted median
Weighted mode
0.05
0.00
−0.05
0.09
0.12
0.15
0.18
SNP effect on Inhibitor of growth protein 1
SNP effect on Gut microbiota abundance (genus Catenibacterium id.2153)

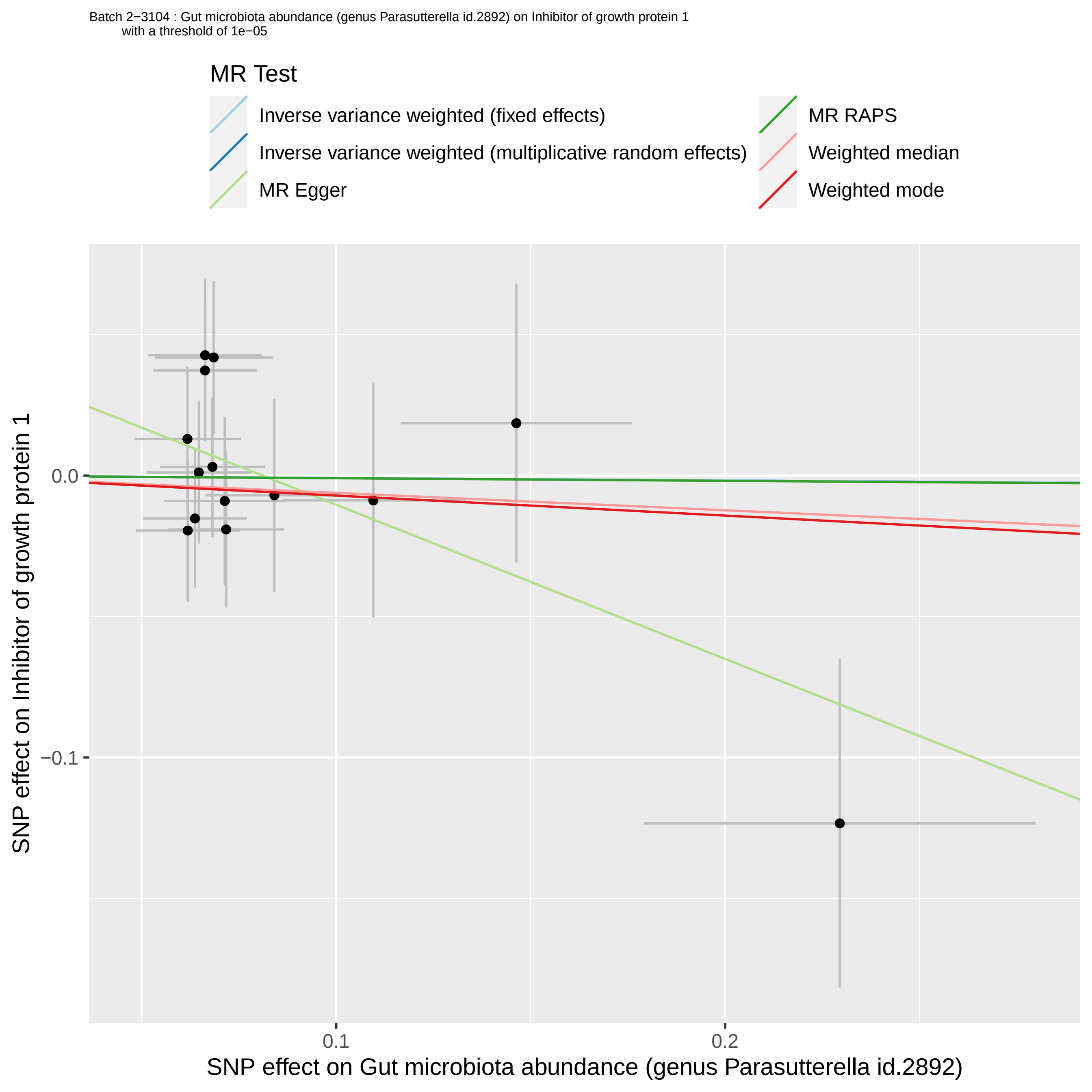

Batch 2−3104 : Gut microbiota abundance (genus Parasutterella id.2892) on Inhibitor of growth protein 1
with a threshold of 1e−05
MR Test
Inverse variance weighted (fixed effects)
Inverse variance weighted (multiplicative random effects)
MR Egger
MR RAPS
Weighted median
Weighted mode
0.0
−0.1
0.1
0.2
SNP effect on Inhibitor of growth protein 1
SNP effect on Gut microbiota abundance (genus Parasutterella id.2892)

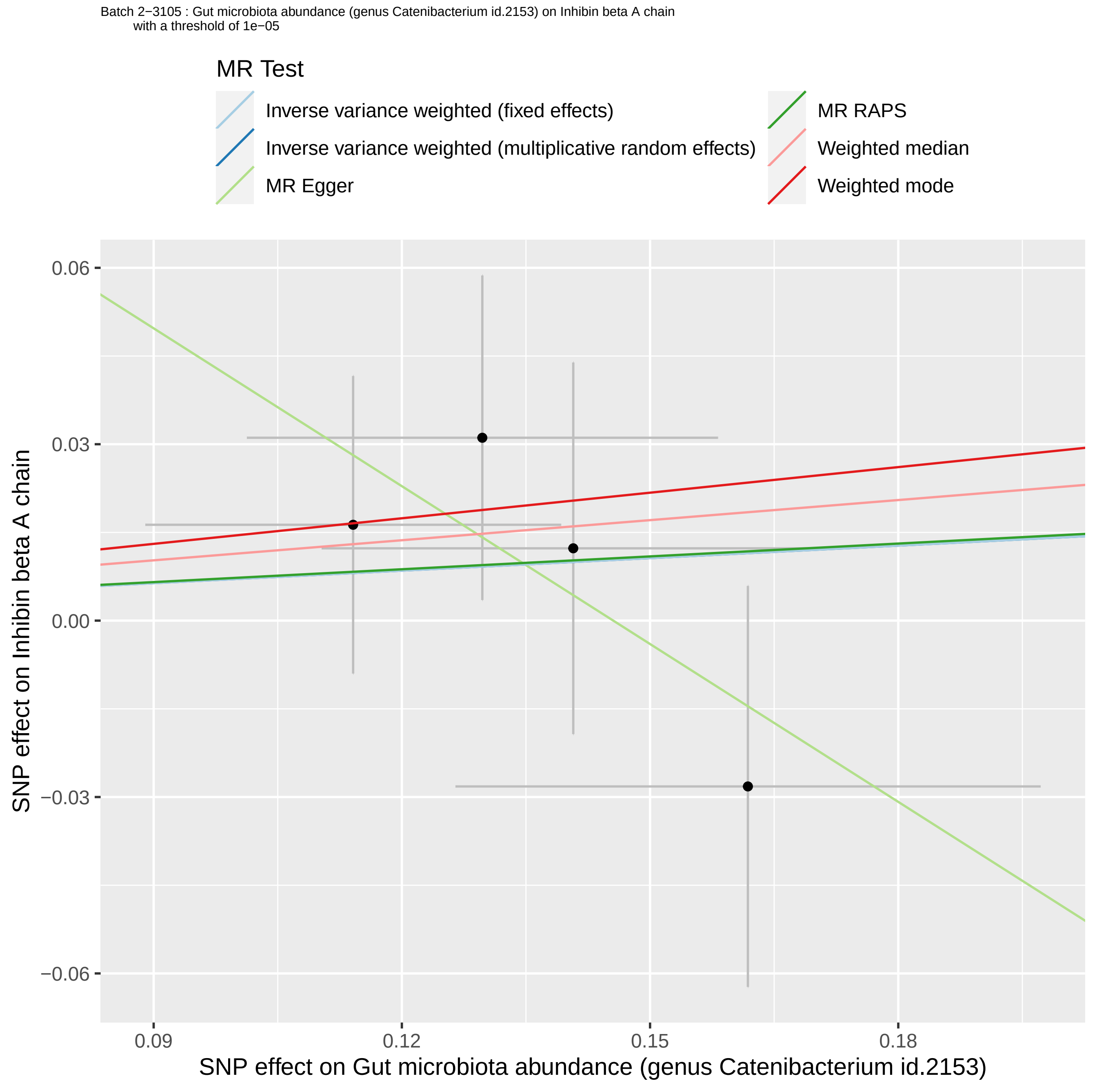

Batch 2−3105 : Gut microbiota abundance (genus Catenibacterium id.2153) on Inhibin beta A chain
with a threshold of 1e−05
MR Test
Inverse variance weighted (fixed effects)
Inverse variance weighted (multiplicative random effects)
MR Egger
MR RAPS
Weighted median
Weighted mode
0.06
0.03
0.00
−0.03
−0.06
0.09
0.12
0.15
0.18
SNP effect on Inhibin beta A chain
SNP effect on Gut microbiota abundance (genus Catenibacterium id.2153)

Batch 2−3106 : Gut microbiota abundance (genus Parasutterella id.2892) on Inhibin beta A chain
with a threshold of 1e−05

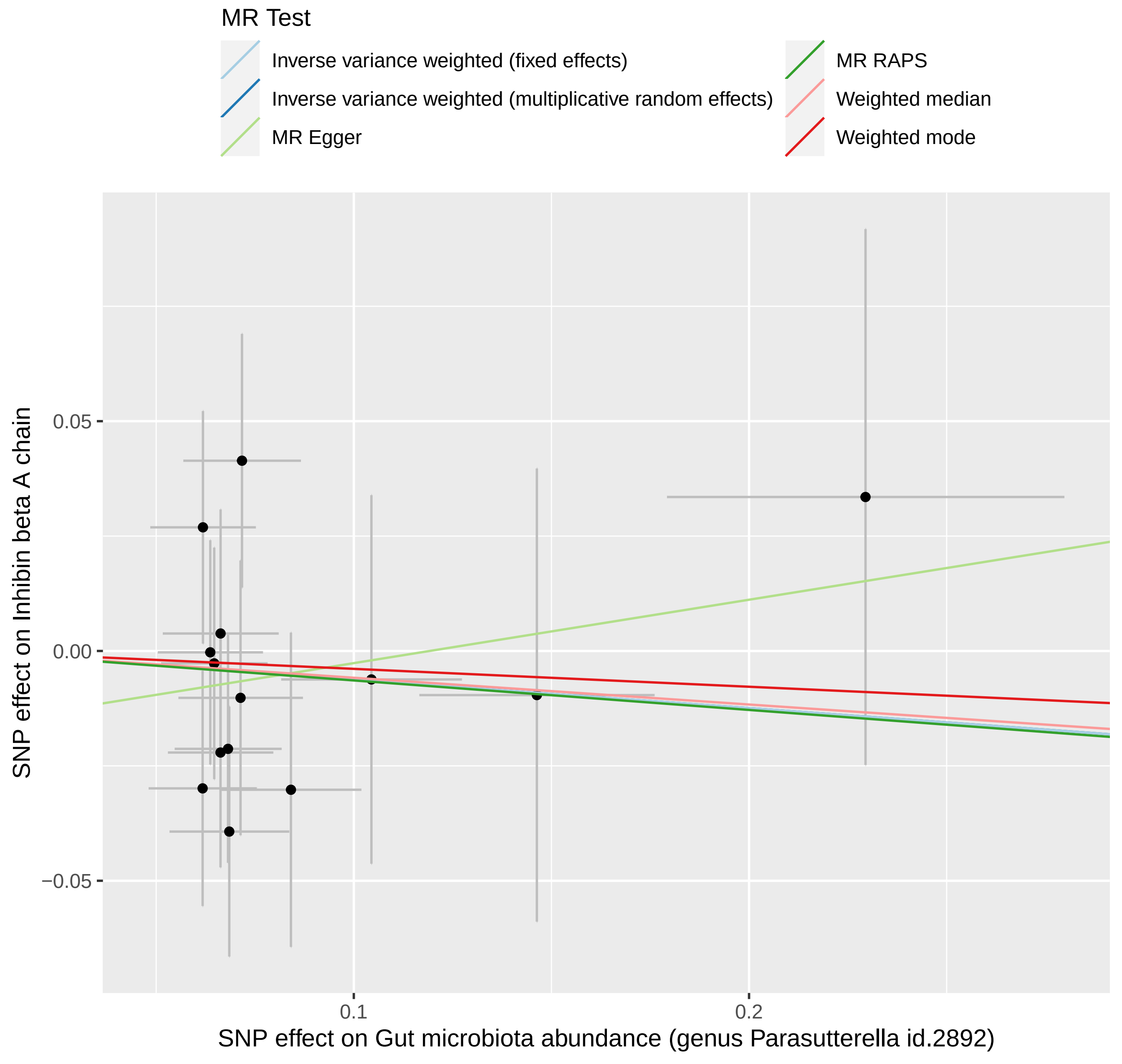

Batch 2−3107 : Gut microbiota abundance (genus Catenibacterium id.2153) on Inhibin beta A chain:Inhibin beta B chain heterodimer
with a threshold of 1e−05

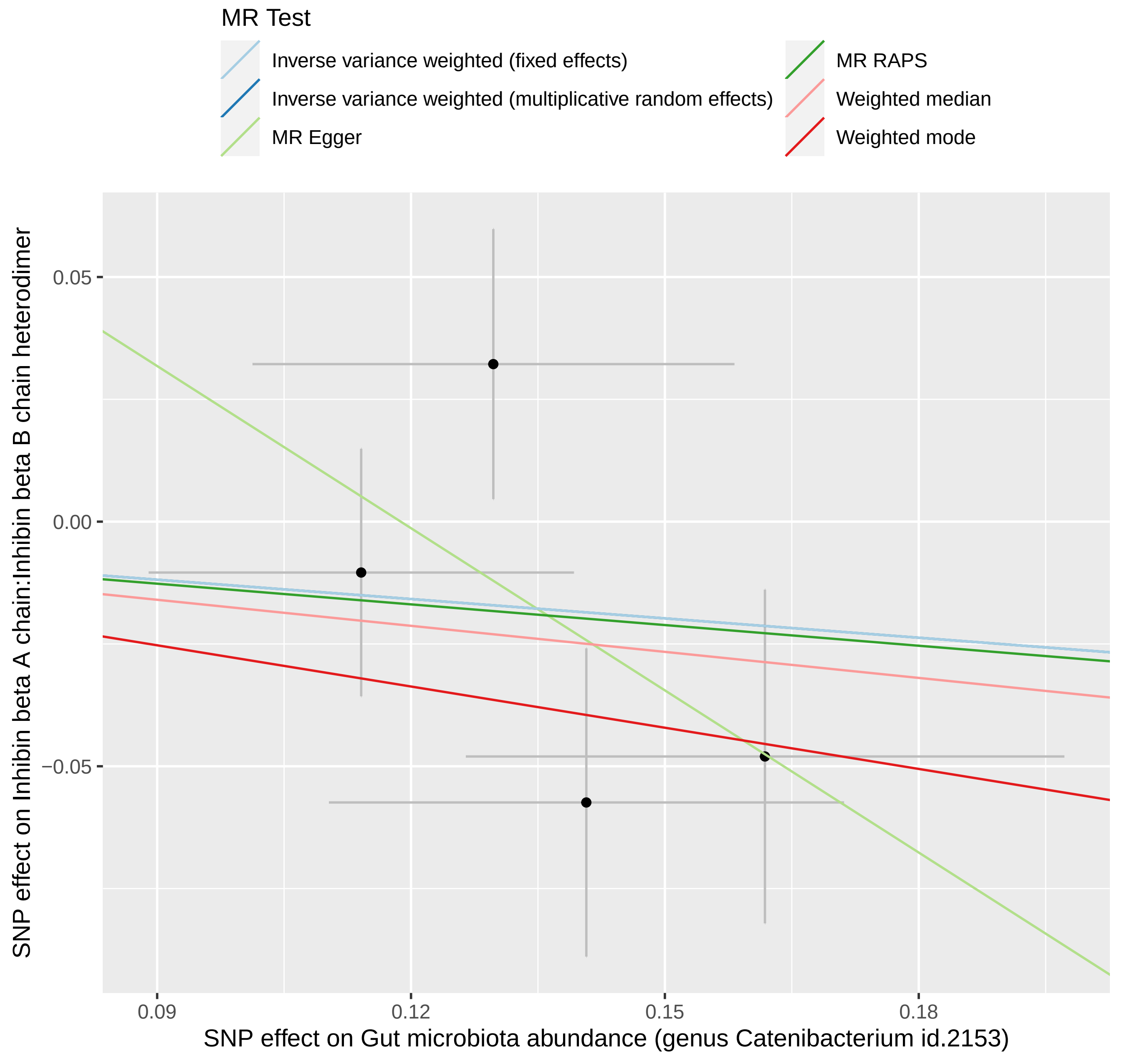

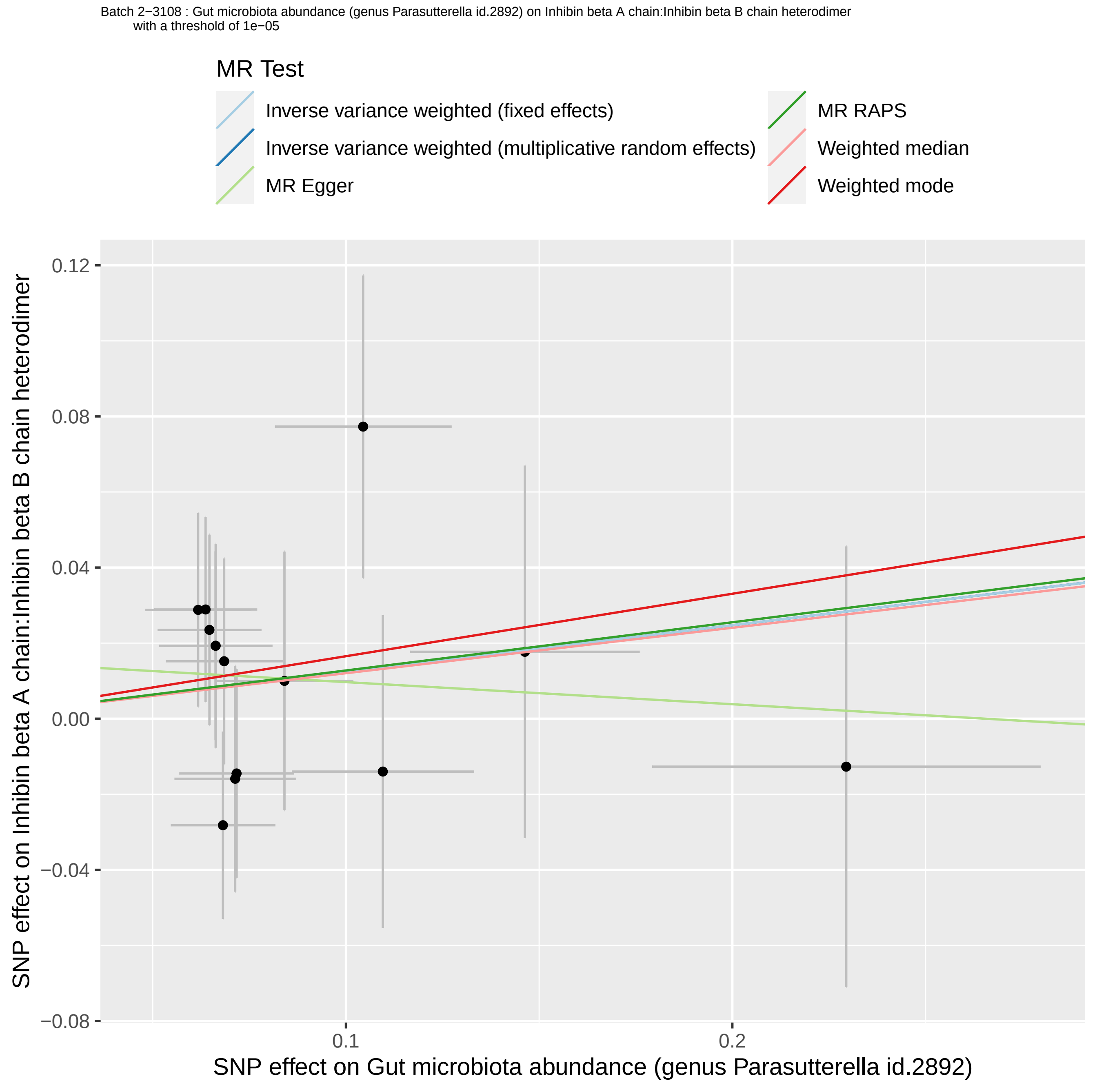

Batch 2−3108 : Gut microbiota abundance (genus Parasutterella id.2892) on Inhibin beta A chain:Inhibin beta B chain heterodimer
with a threshold of 1e−05
MR Test
Inverse variance weighted (fixed effects)
Inverse variance weighted (multiplicative random effects)
MR Egger
MR RAPS
Weighted median
Weighted mode
0.12
0.08
0.04
0.00
−0.04
−0.08
0.1
0.2
SNP effect on Inhibin beta A chain:Inhibin beta B chain heterodimer
SNP effect on Gut microbiota abundance (genus Parasutterella id.2892)

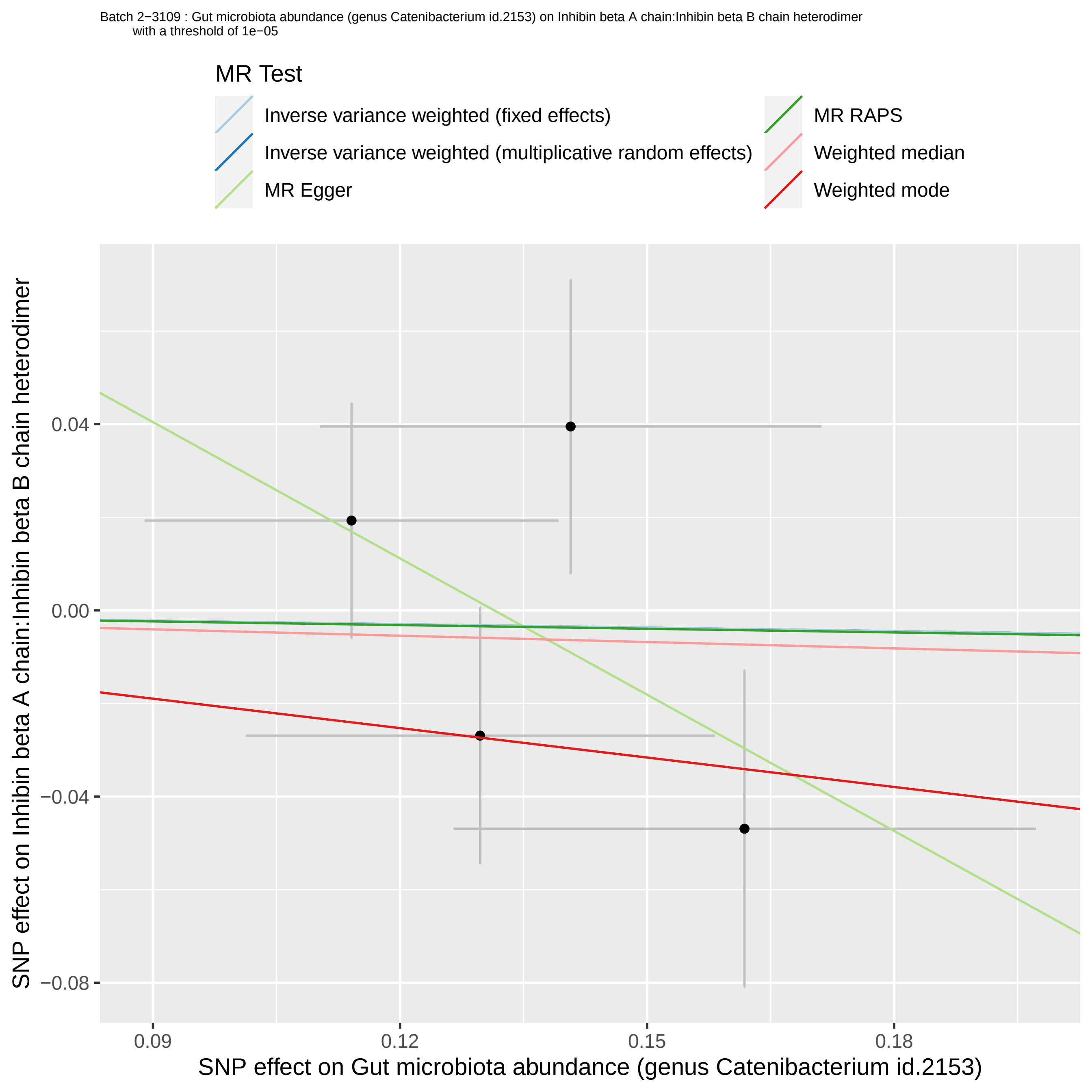

Batch 2−3109 : Gut microbiota abundance (genus Catenibacterium id.2153) on Inhibin beta A chain:Inhibin beta B chain heterodimer
with a threshold of 1e−05
MR Test
Inverse variance weighted (fixed effects)
Inverse variance weighted (multiplicative random effects)
MR Egger
MR RAPS
Weighted median
Weighted mode
0.04
0.00
−0.04
−0.08
0.09
0.12
0.15
0.18
SNP effect on Inhibin beta A chain:Inhibin beta B chain heterodimer
SNP effect on Gut microbiota abundance (genus Catenibacterium id.2153)

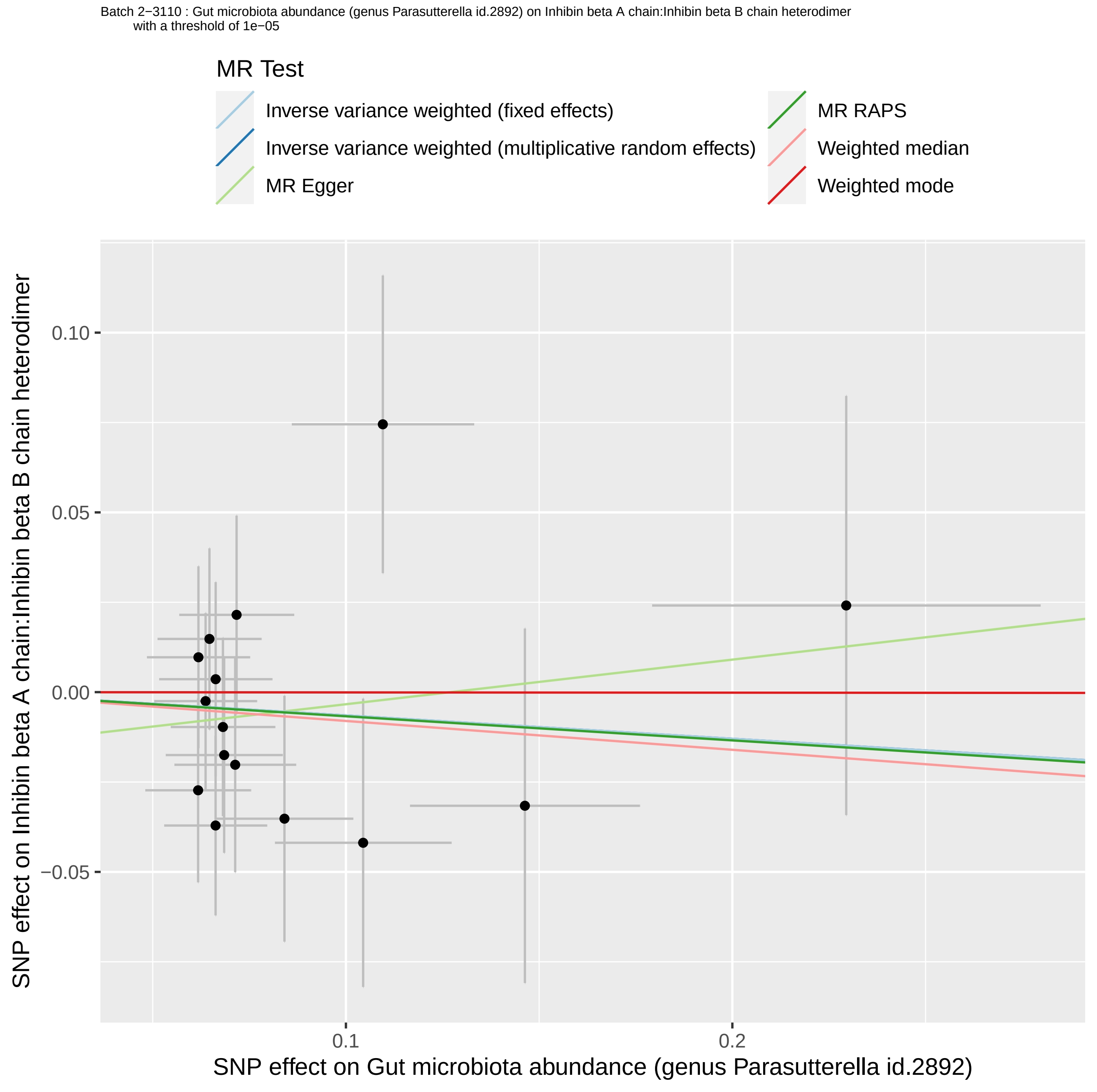
Batch 2−3110 : Gut microbiota abundance (genus Parasutterella id.2892) on Inhibin beta A chain:Inhibin beta B chain heterodimer
with a threshold of 1e−05
MR Test
Inverse variance weighted (fixed effects)
Inverse variance weighted (multiplicative random effects)
MR Egger
MR RAPS
Weighted median
Weighted mode
0.10
0.05
0.00
−0.05
0.1
0.2
SNP effect on Inhibin beta A chain:Inhibin beta B chain heterodimer
SNP effect on Gut microbiota abundance (genus Parasutterella id.2892)

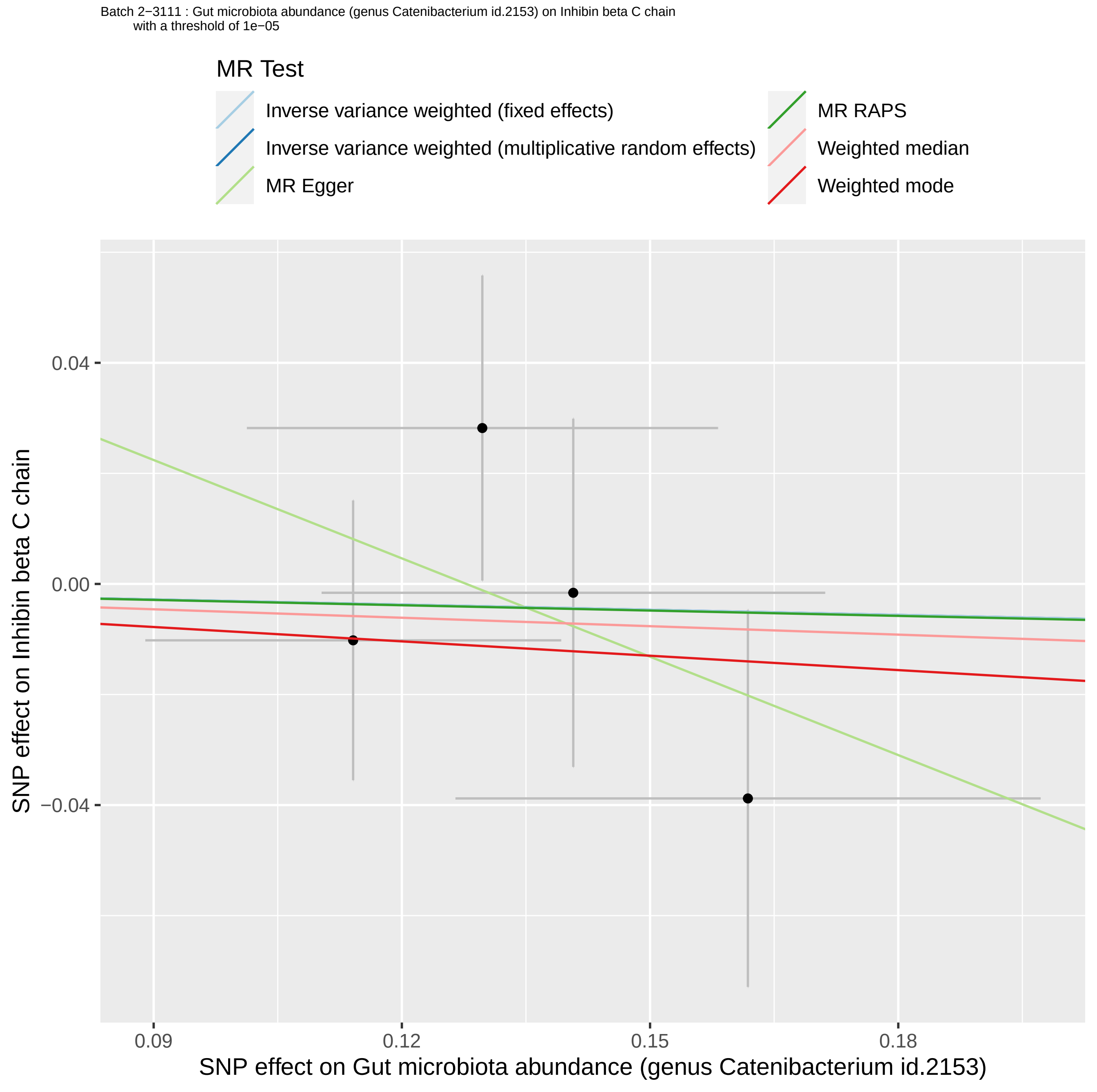
Batch 2−3111 : Gut microbiota abundance (genus Catenibacterium id.2153) on Inhibin beta C chain
with a threshold of 1e−05
MR Test
Inverse variance weighted (fixed effects)
Inverse variance weighted (multiplicative random effects)
MR Egger
MR RAPS
Weighted median
Weighted mode
0.04
0.00
−0.04
0.09
0.12
0.15
0.18
SNP effect on Inhibin beta C chain
SNP effect on Gut microbiota abundance (genus Catenibacterium id.2153)

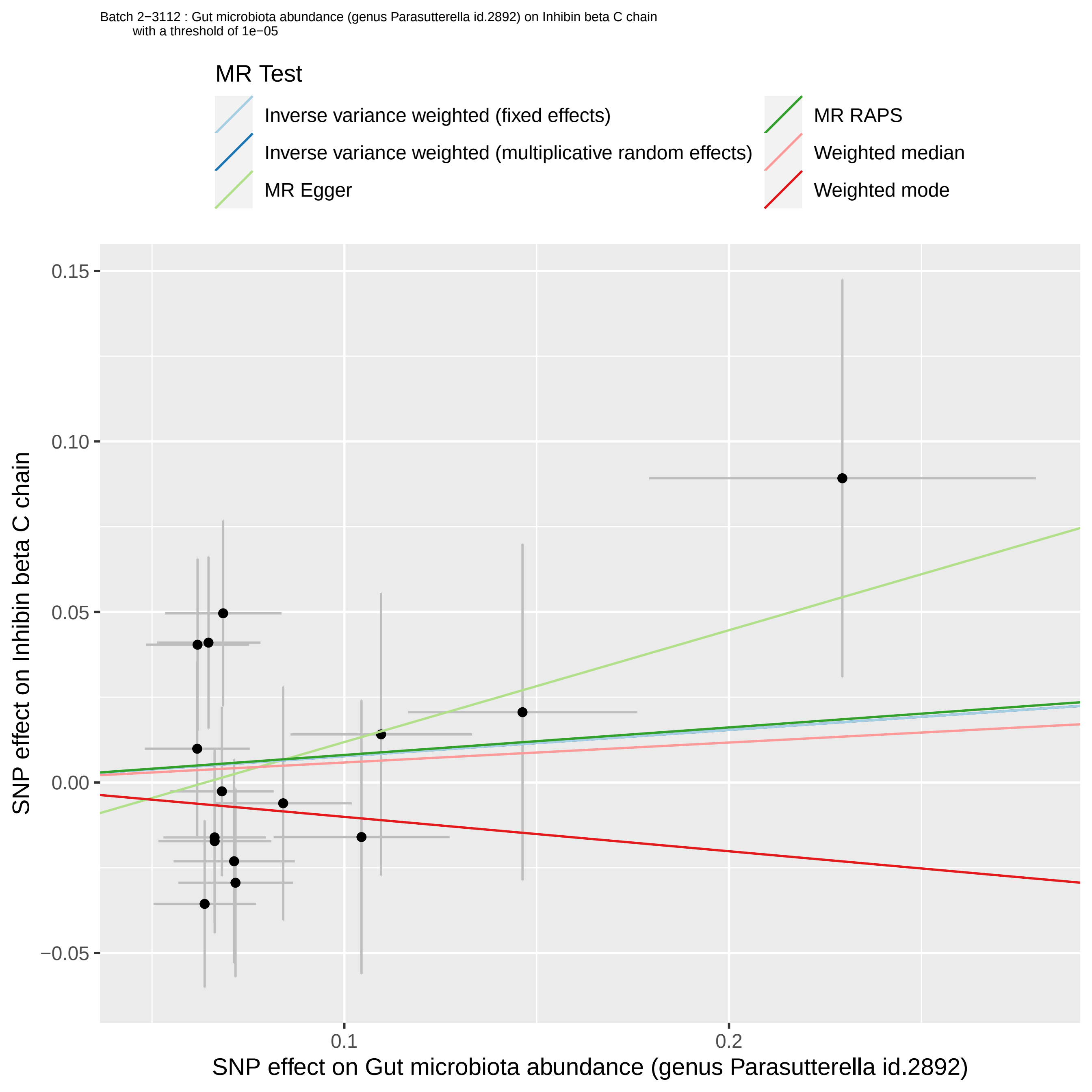
Batch 2−3112 : Gut microbiota abundance (genus Parasutterella id.2892) on Inhibin beta C chain
with a threshold of 1e−05
MR Test
Inverse variance weighted (fixed effects)
Inverse variance weighted (multiplicative random effects)
MR Egger
MR RAPS
Weighted median
Weighted mode
0.15
0.10
0.05
0.00
−0.05
0.1
0.2
SNP effect on Inhibin beta C chain
SNP effect on Gut microbiota abundance (genus Parasutterella id.2892)

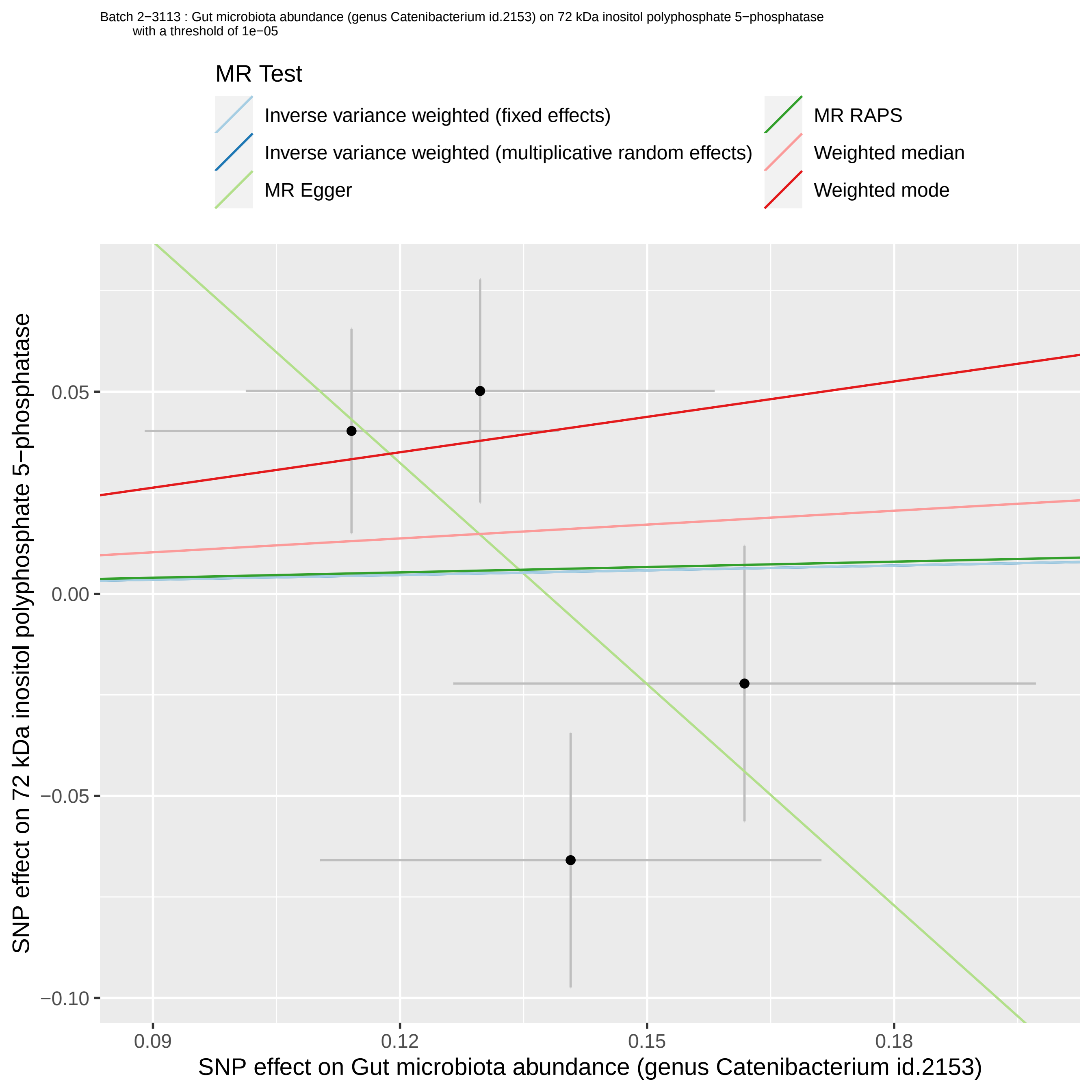
Batch 2−3113 : Gut microbiota abundance (genus Catenibacterium id.2153) on 72 kDa inositol polyphosphate 5−phosphatase
with a threshold of 1e−05
MR Test
Inverse variance weighted (fixed effects)
Inverse variance weighted (multiplicative random effects)
MR Egger
MR RAPS
Weighted median
Weighted mode
0.05
0.00
−0.05
−0.10
0.09
0.12
0.15
0.18
SNP effect on 72 kDa inositol polyphosphate 5−phosphatase
SNP effect on Gut microbiota abundance (genus Catenibacterium id.2153)

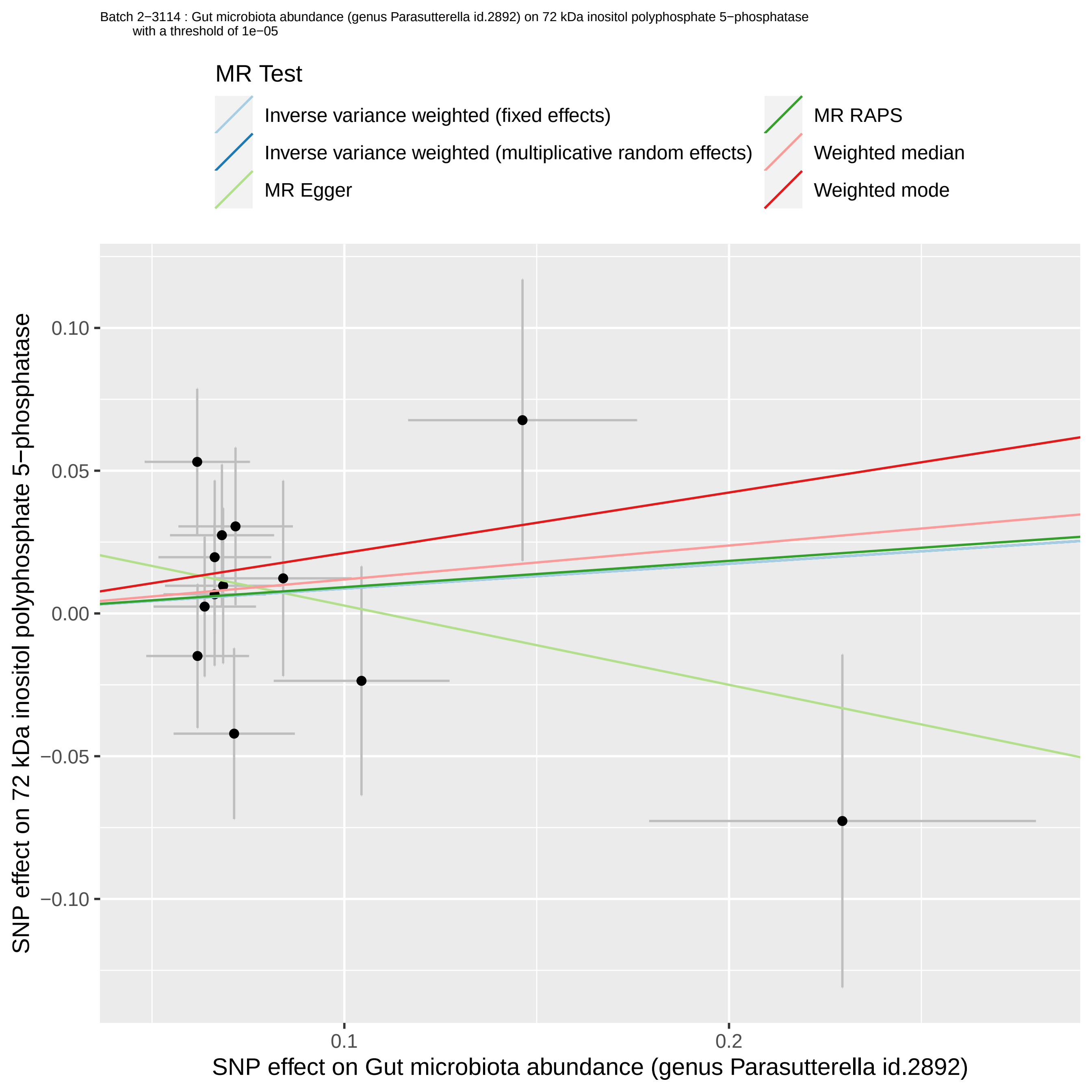

Batch 2−3114 : Gut microbiota abundance (genus Parasutterella id.2892) on 72 kDa inositol polyphosphate 5−phosphatase
with a threshold of 1e−05
MR Test
Inverse variance weighted (fixed effects)
Inverse variance weighted (multiplicative random effects)
MR Egger
MR RAPS
Weighted median
Weighted mode
0.10
0.05
0.00
−0.05
−0.10
0.1
0.2
SNP effect on 72 kDa inositol polyphosphate 5−phosphatase
SNP effect on Gut microbiota abundance (genus Parasutterella id.2892)

Batch 2−3115 : Gut microbiota abundance (genus Catenibacterium id.2153) on Insulin
with a threshold of 1e−05

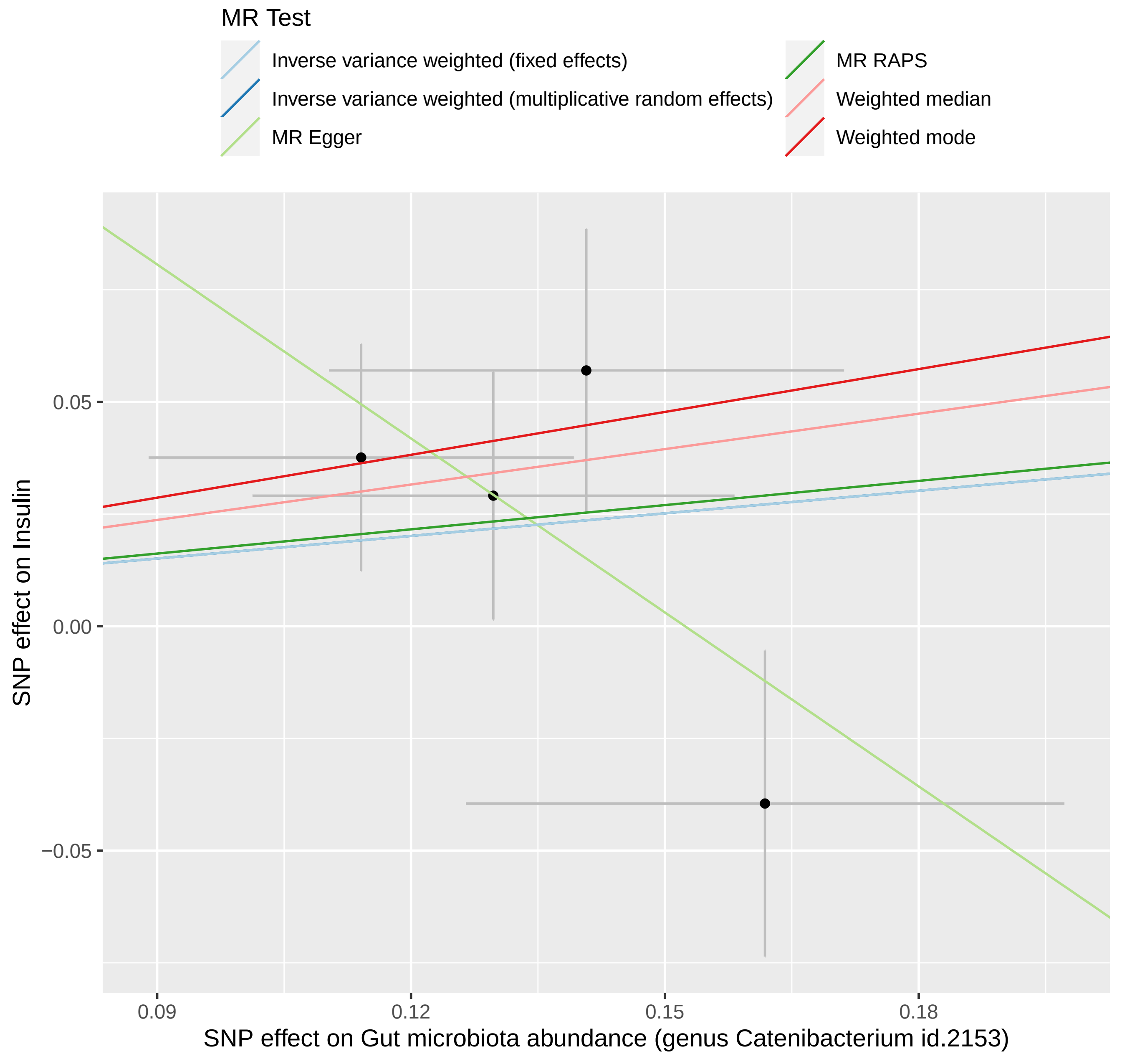

Batch 2−3116 : Gut microbiota abundance (genus Parasutterella id.2892) on Insulin
with a threshold of 1e−05

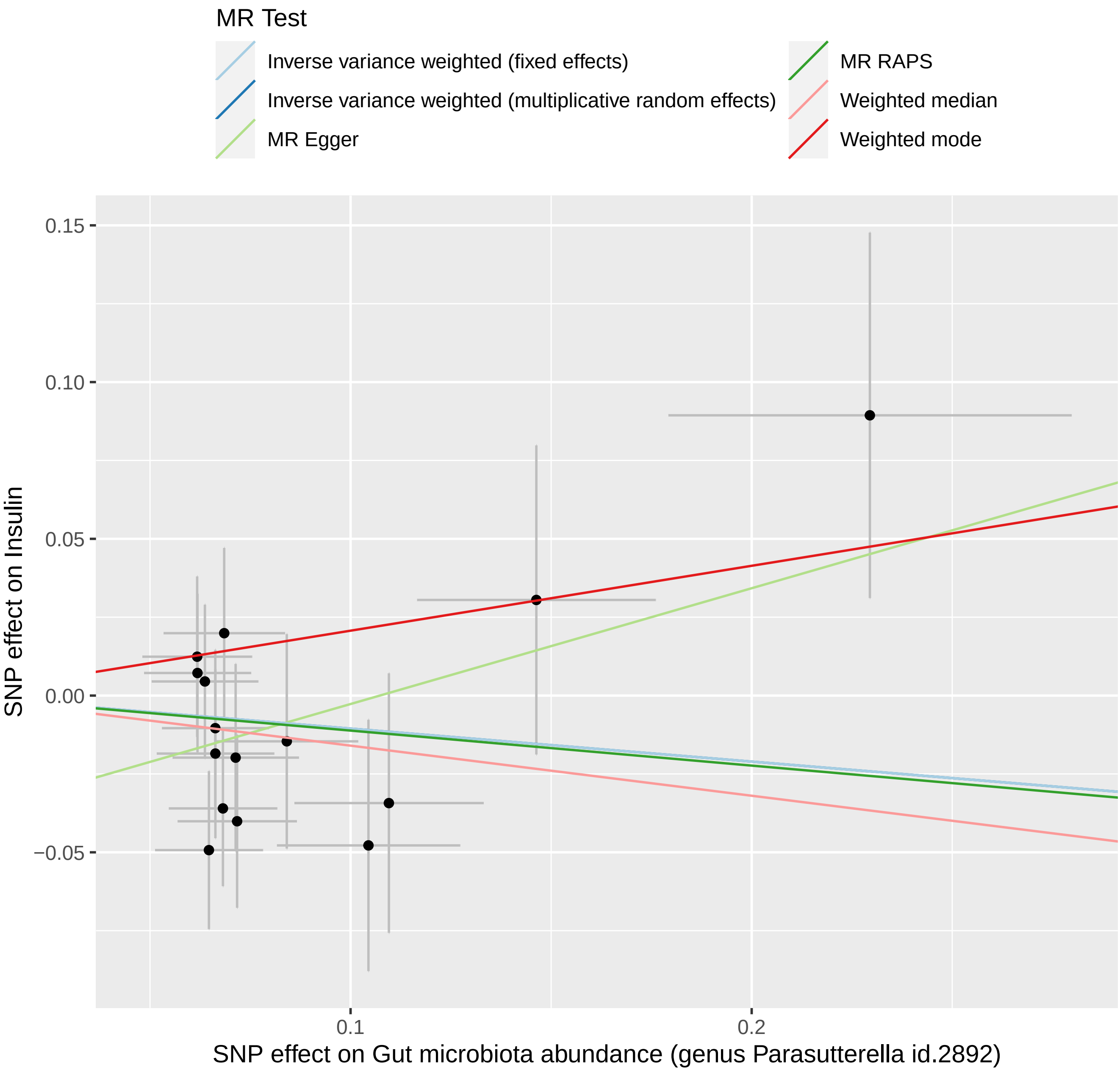

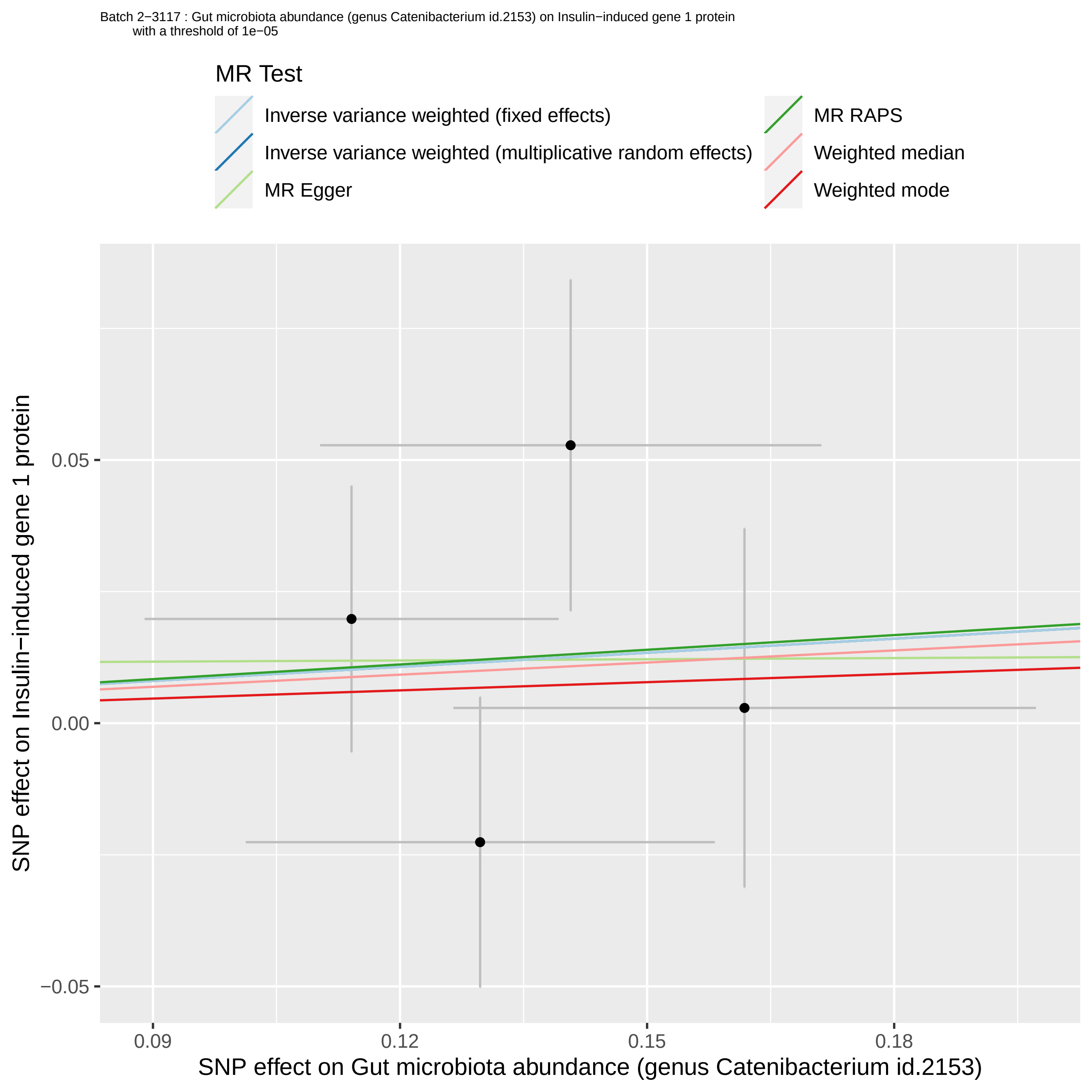
Batch 2−3117 : Gut microbiota abundance (genus Catenibacterium id.2153) on Insulin−induced gene 1 protein
with a threshold of 1e−05
MR Test
Inverse variance weighted (fixed effects)
Inverse variance weighted (multiplicative random effects)
MR Egger
MR RAPS
Weighted median
Weighted mode
SNP effect on Insulin−induced gene 1 protein
0.05
0.00
−0.05
0.09
0.12
0.15
0.18
SNP effect on Gut microbiota abundance (genus Catenibacterium id.2153)

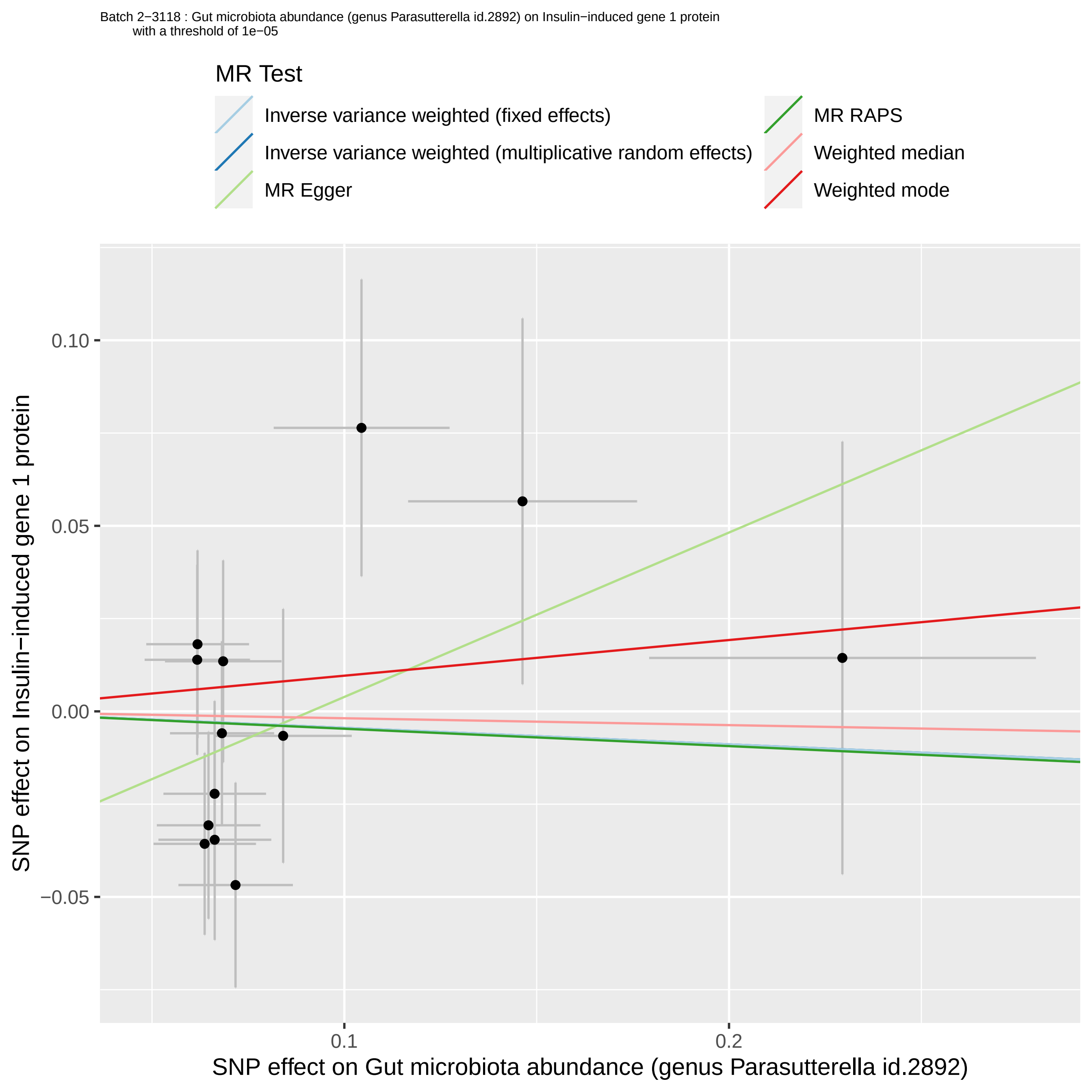

Batch 2−3118 : Gut microbiota abundance (genus Parasutterella id.2892) on Insulin−induced gene 1 protein
with a threshold of 1e−05
MR Test
Inverse variance weighted (fixed effects)
Inverse variance weighted (multiplicative random effects)
MR Egger
MR RAPS
Weighted median
Weighted mode
SNP effect on Insulin−induced gene 1 protein
0.10
0.05
0.00
−0.05
0.1
0.2
SNP effect on Gut microbiota abundance (genus Parasutterella id.2892)

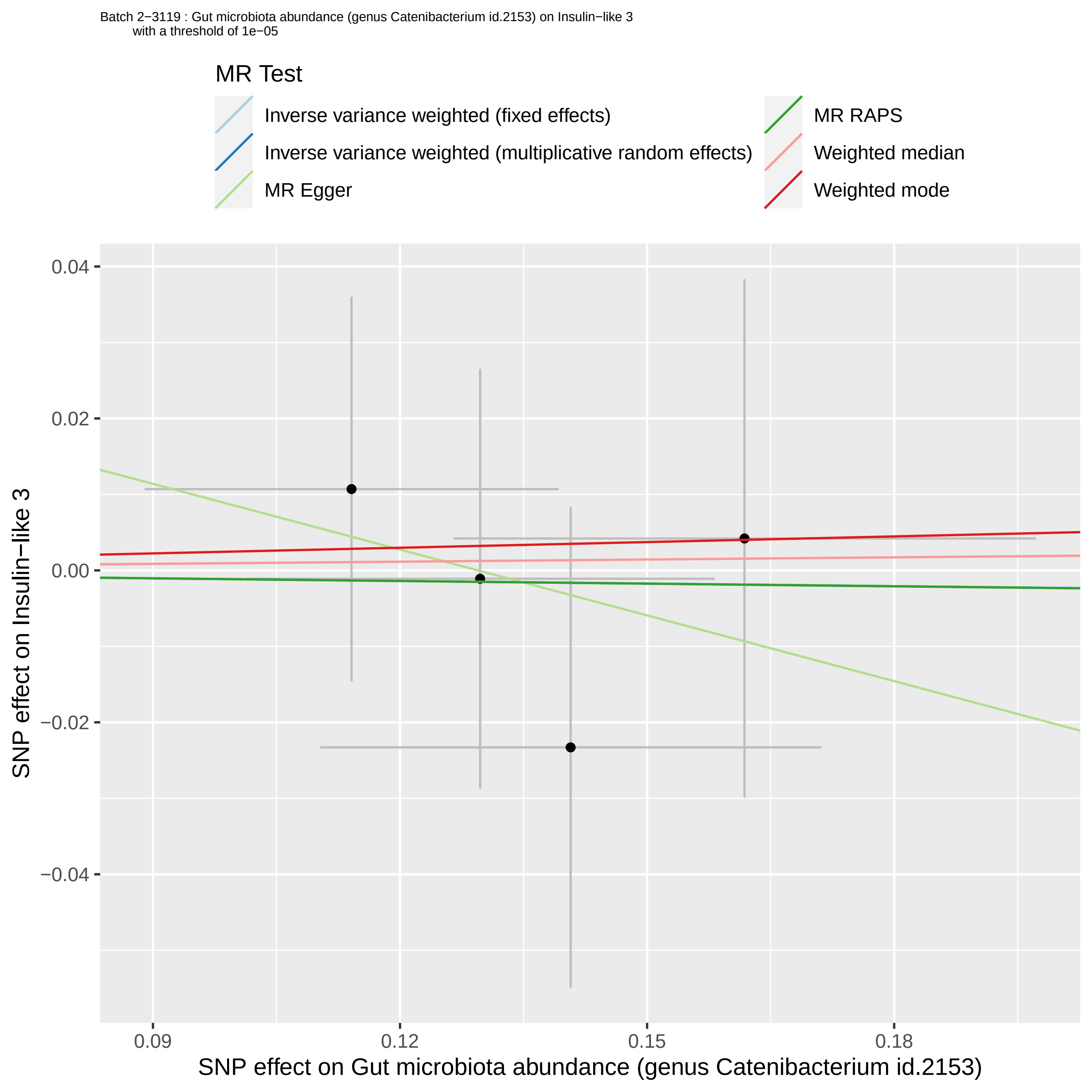
Batch 2−3119 : Gut microbiota abundance (genus Catenibacterium id.2153) on Insulin−like 3
with a threshold of 1e−05
MR Test
Inverse variance weighted (fixed effects)
Inverse variance weighted (multiplicative random effects)
MR Egger
MR RAPS
Weighted median
Weighted mode
0.04
0.02
0.00
−0.02
−0.04
SNP effect on Insulin−like 3
0.09
0.12
0.15
0.18
SNP effect on Gut microbiota abundance (genus Catenibacterium id.2153)

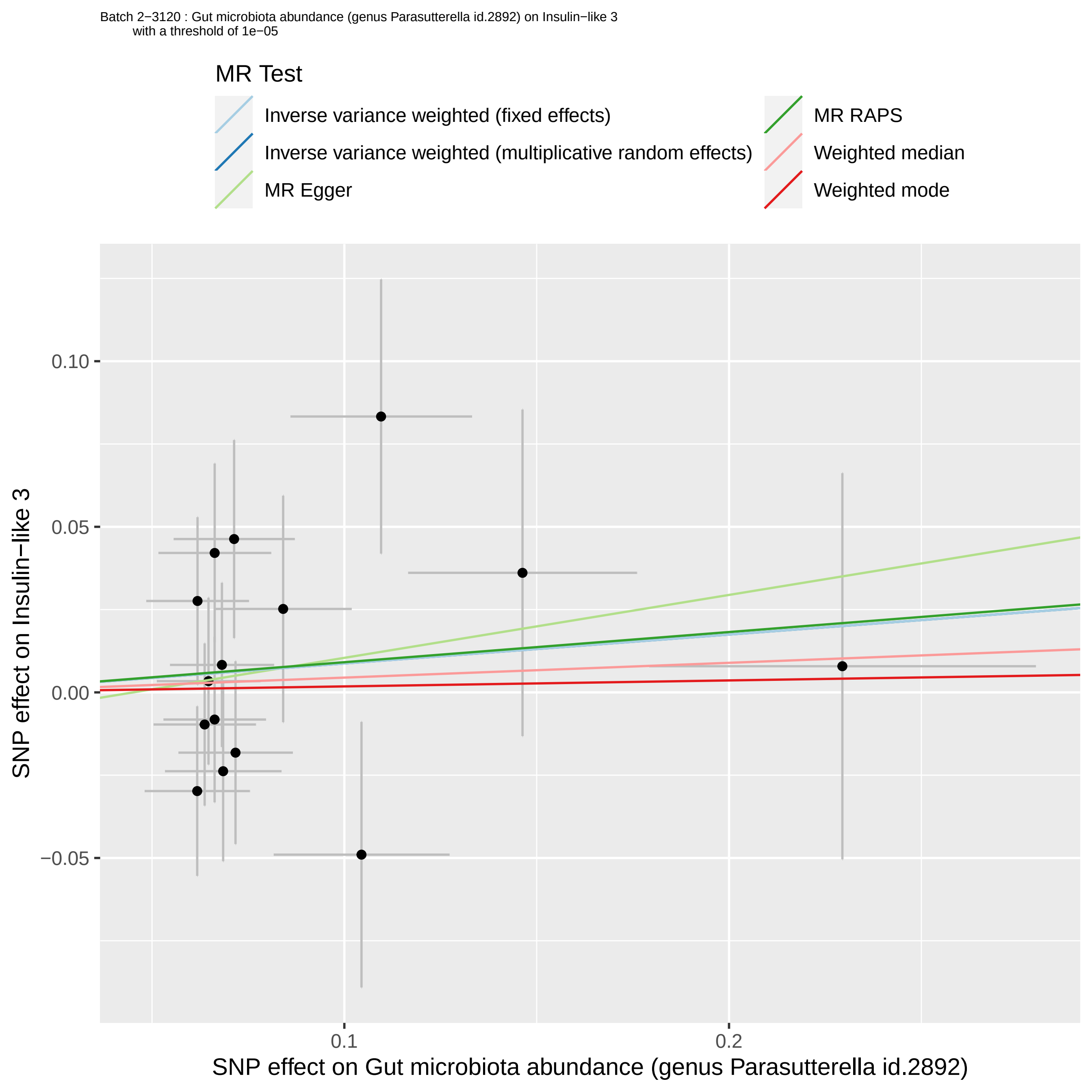

Batch 2−3120 : Gut microbiota abundance (genus Parasutterella id.2892) on Insulin−like 3
with a threshold of 1e−05
MR Test
Inverse variance weighted (fixed effects)
Inverse variance weighted (multiplicative random effects)
MR Egger
MR RAPS
Weighted median
Weighted mode
0.10
0.05
0.00
−0.05
0.1
0.2
SNP effect on Insulin−like 3
SNP effect on Gut microbiota abundance (genus Parasutterella id.2892)

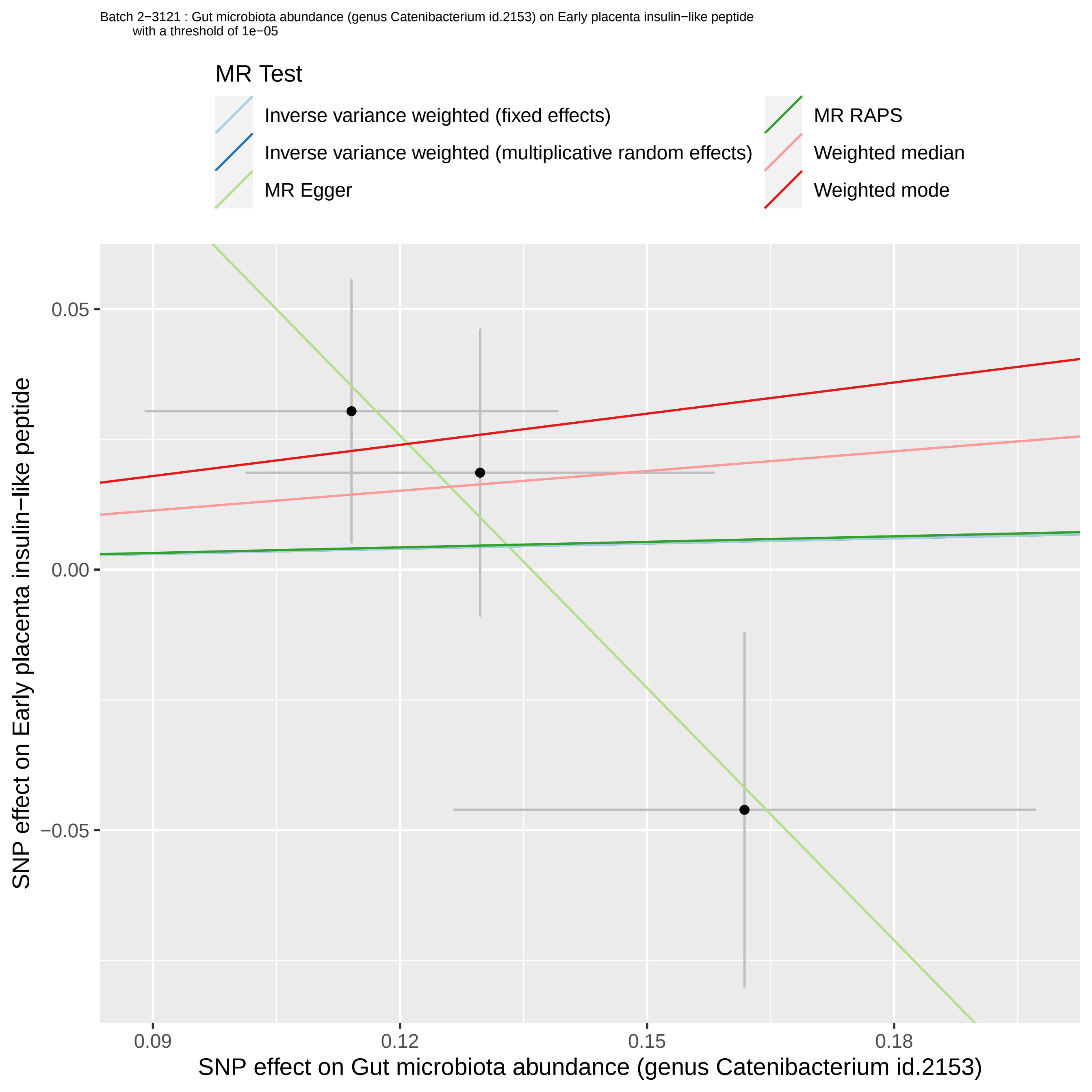
Batch 2−3121 : Gut microbiota abundance (genus Catenibacterium id.2153) on Early placenta insulin−like peptide
with a threshold of 1e−05
MR Test
Inverse variance weighted (fixed effects)
Inverse variance weighted (multiplicative random effects)
MR Egger
MR RAPS
Weighted median
Weighted mode
0.05
0.00
−0.05
0.09
0.12
0.15
0.18
SNP effect on Early placenta insulin−like peptide
SNP effect on Gut microbiota abundance (genus Catenibacterium id.2153)

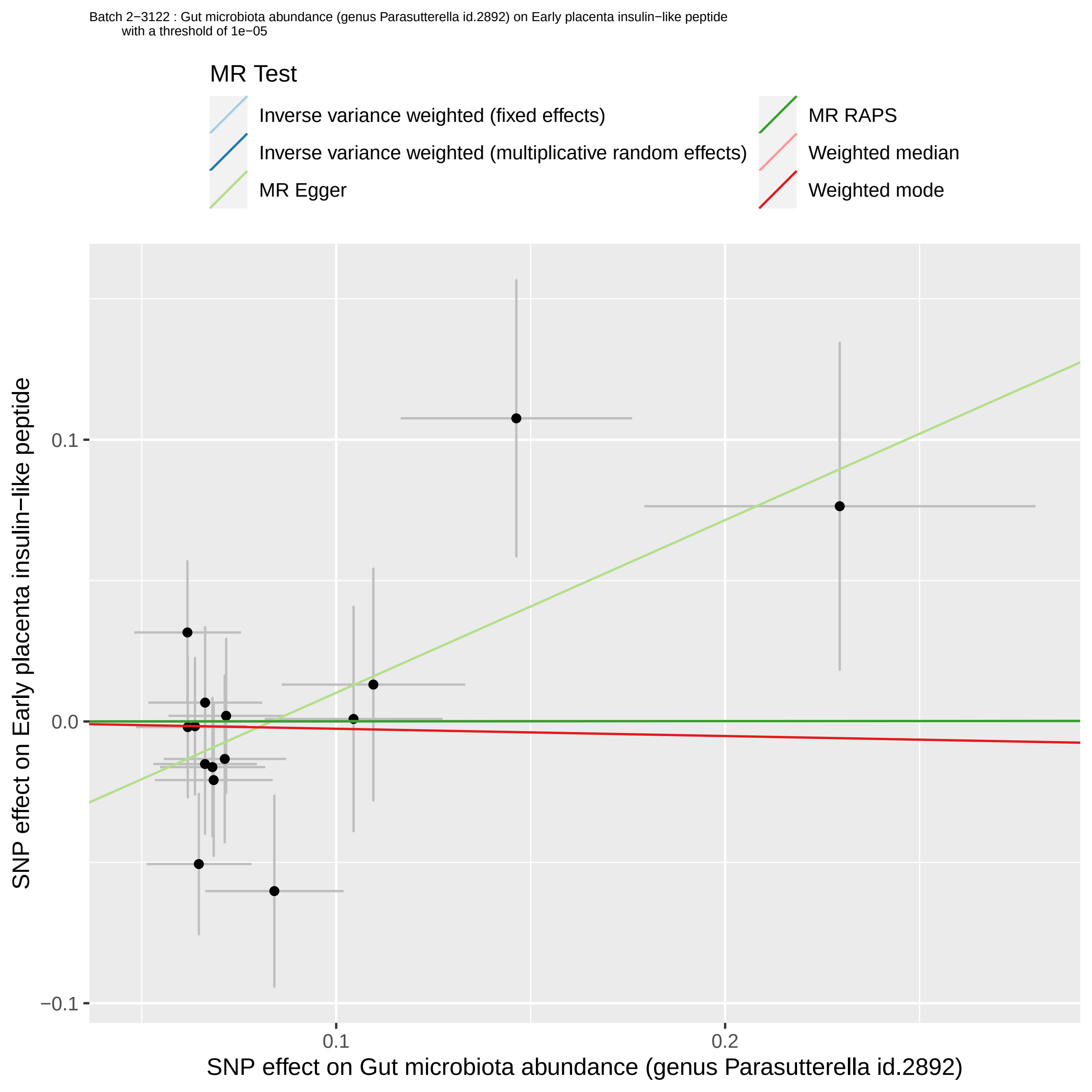
Batch 2−3122 : Gut microbiota abundance (genus Parasutterella id.2892) on Early placenta insulin−like peptide
with a threshold of 1e−05
MR Test
Inverse variance weighted (fixed effects)
Inverse variance weighted (multiplicative random effects)
MR Egger
MR RAPS
Weighted median
Weighted mode
SNP effect on Early placenta insulin−like peptide
0.1
0.0
−0.1
0.1
0.2
SNP effect on Gut microbiota abundance (genus Parasutterella id.2892)

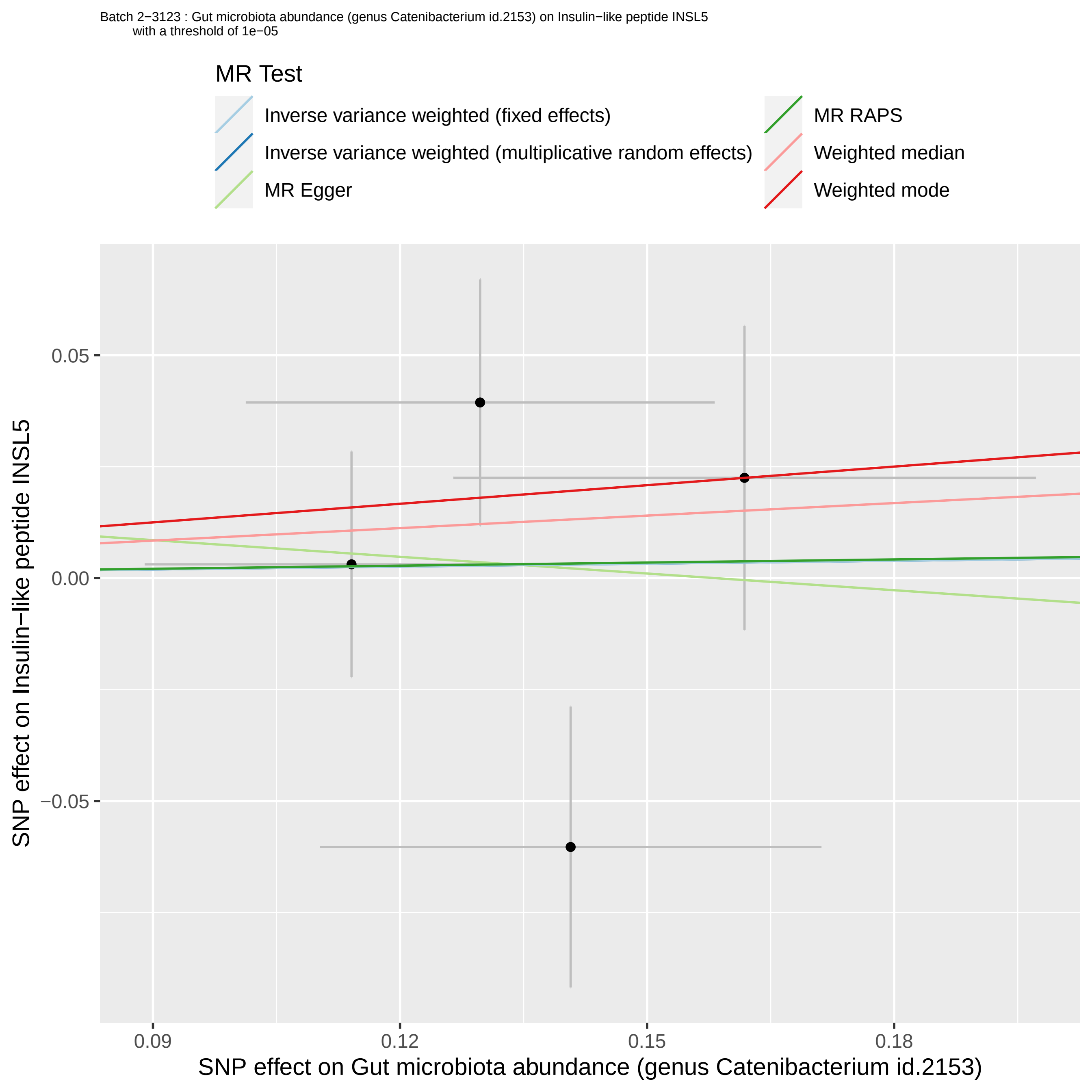
Batch 2−3123 : Gut microbiota abundance (genus Catenibacterium id.2153) on Insulin−like peptide INSL5
with a threshold of 1e−05
MR Test
Inverse variance weighted (fixed effects)
Inverse variance weighted (multiplicative random effects)
MR Egger
MR RAPS
Weighted median
Weighted mode
0.05
0.00
−0.05
0.09
0.12
0.15
0.18
SNP effect on Insulin−like peptide INSL5
SNP effect on Gut microbiota abundance (genus Catenibacterium id.2153)

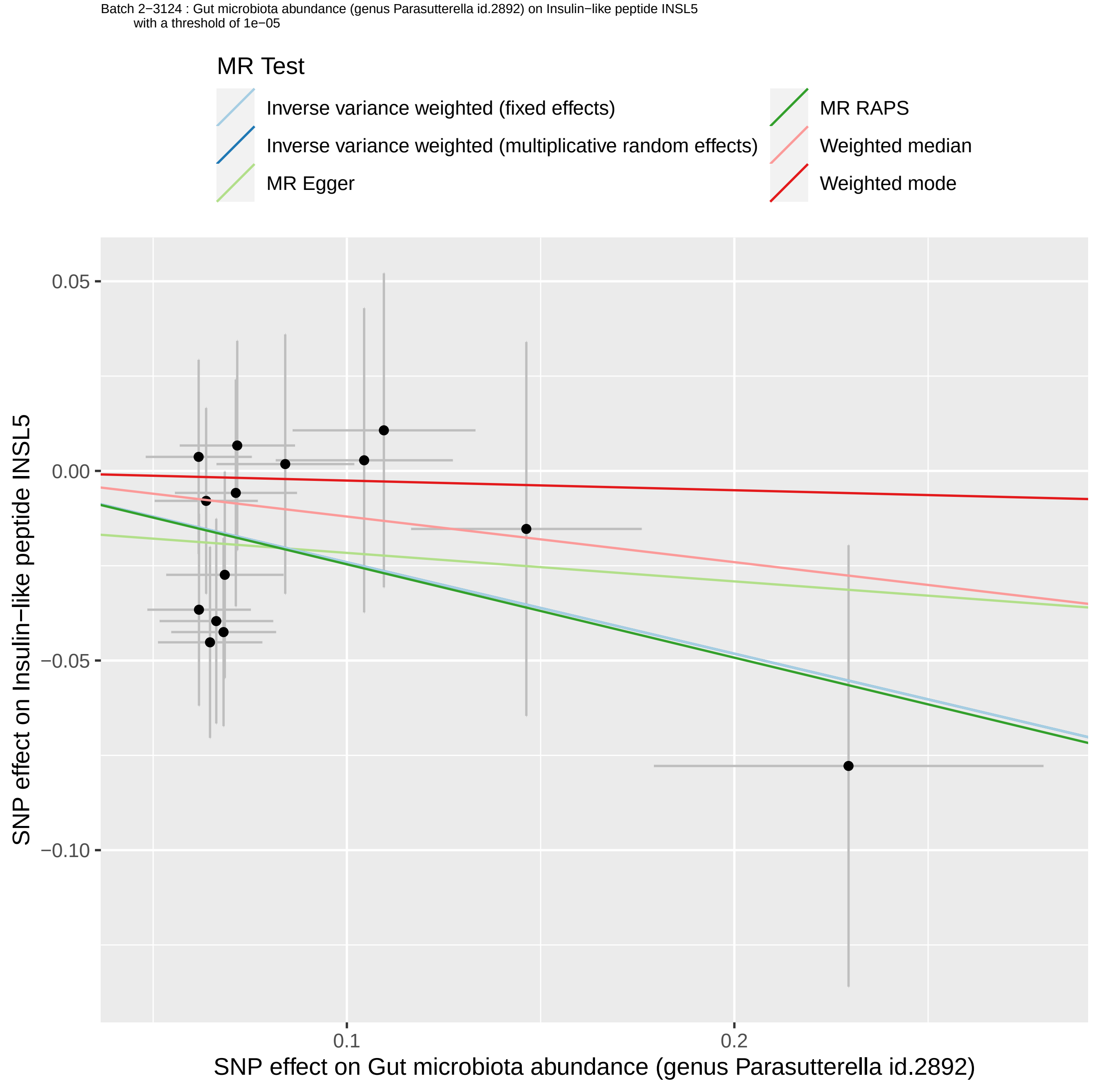

Batch 2−3124 : Gut microbiota abundance (genus Parasutterella id.2892) on Insulin−like peptide INSL5
with a threshold of 1e−05
MR Test
Inverse variance weighted (fixed effects)
Inverse variance weighted (multiplicative random effects)
MR Egger
MR RAPS
Weighted median
Weighted mode
0.05
0.00
−0.05
−0.10
0.1
0.2
SNP effect on Insulin−like peptide INSL5
SNP effect on Gut microbiota abundance (genus Parasutterella id.2892)

Batch 2−3125 : Gut microbiota abundance (genus Catenibacterium id.2153) on Insulin−like peptide INSL6
with a threshold of 1e−05

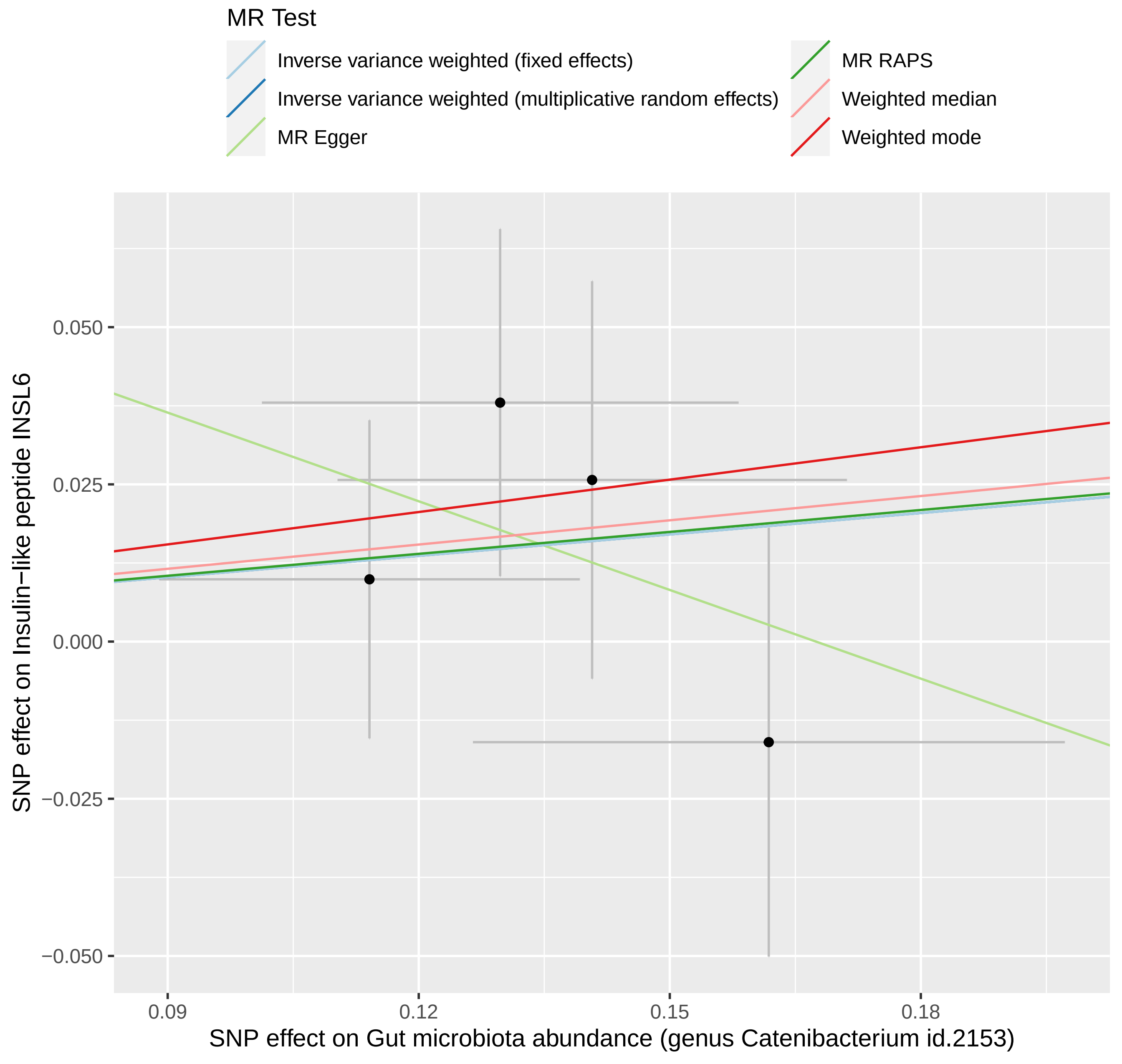

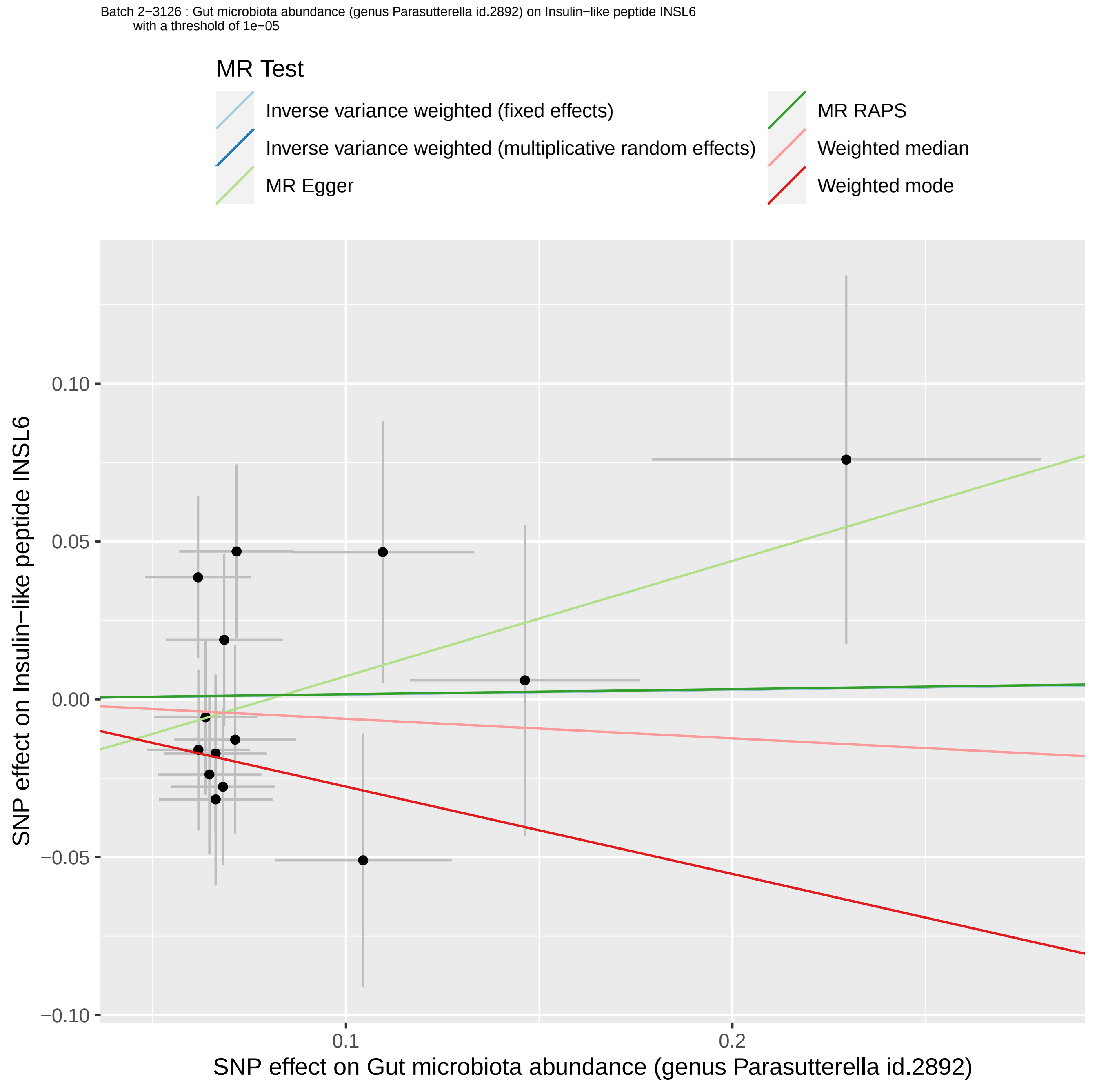
Batch 2−3126 : Gut microbiota abundance (genus Parasutterella id.2892) on Insulin−like peptide INSL6
with a threshold of 1e−05
MR Test
Inverse variance weighted (fixed effects)
Inverse variance weighted (multiplicative random effects)
MR Egger
MR RAPS
Weighted median
Weighted mode
0.10
0.05
0.00
−0.05
−0.10
0.1
0.2
SNP effect on Insulin−like peptide INSL6
SNP effect on Gut microbiota abundance (genus Parasutterella id.2892)

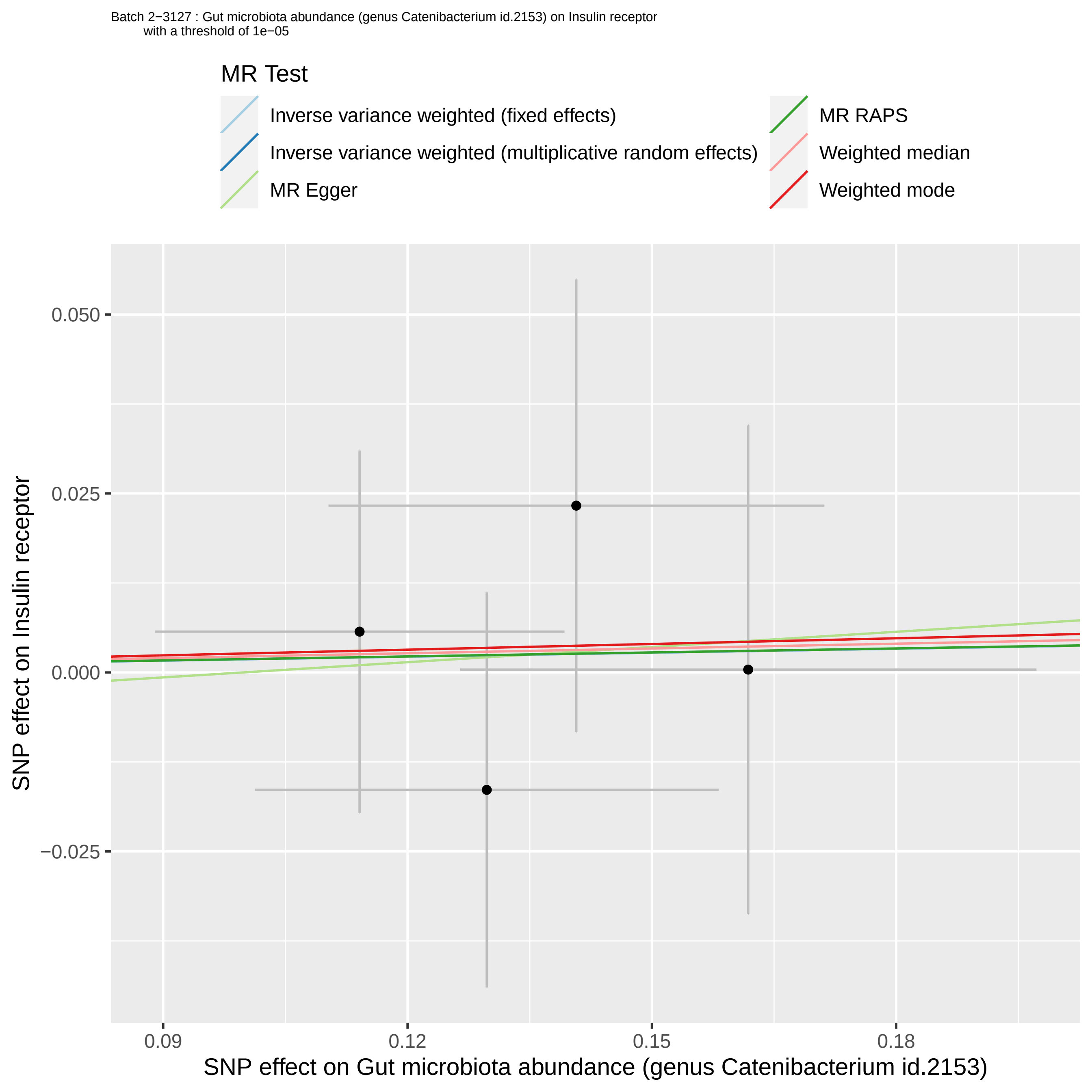
Batch 2−3127 : Gut microbiota abundance (genus Catenibacterium id.2153) on Insulin receptor
with a threshold of 1e−05
MR Test
Inverse variance weighted (fixed effects)
Inverse variance weighted (multiplicative random effects)
MR Egger
MR RAPS
Weighted median
Weighted mode
0.050
0.025
0.000
−0.025
0.09
0.12
0.15
0.18
SNP effect on Insulin receptor
SNP effect on Gut microbiota abundance (genus Catenibacterium id.2153)

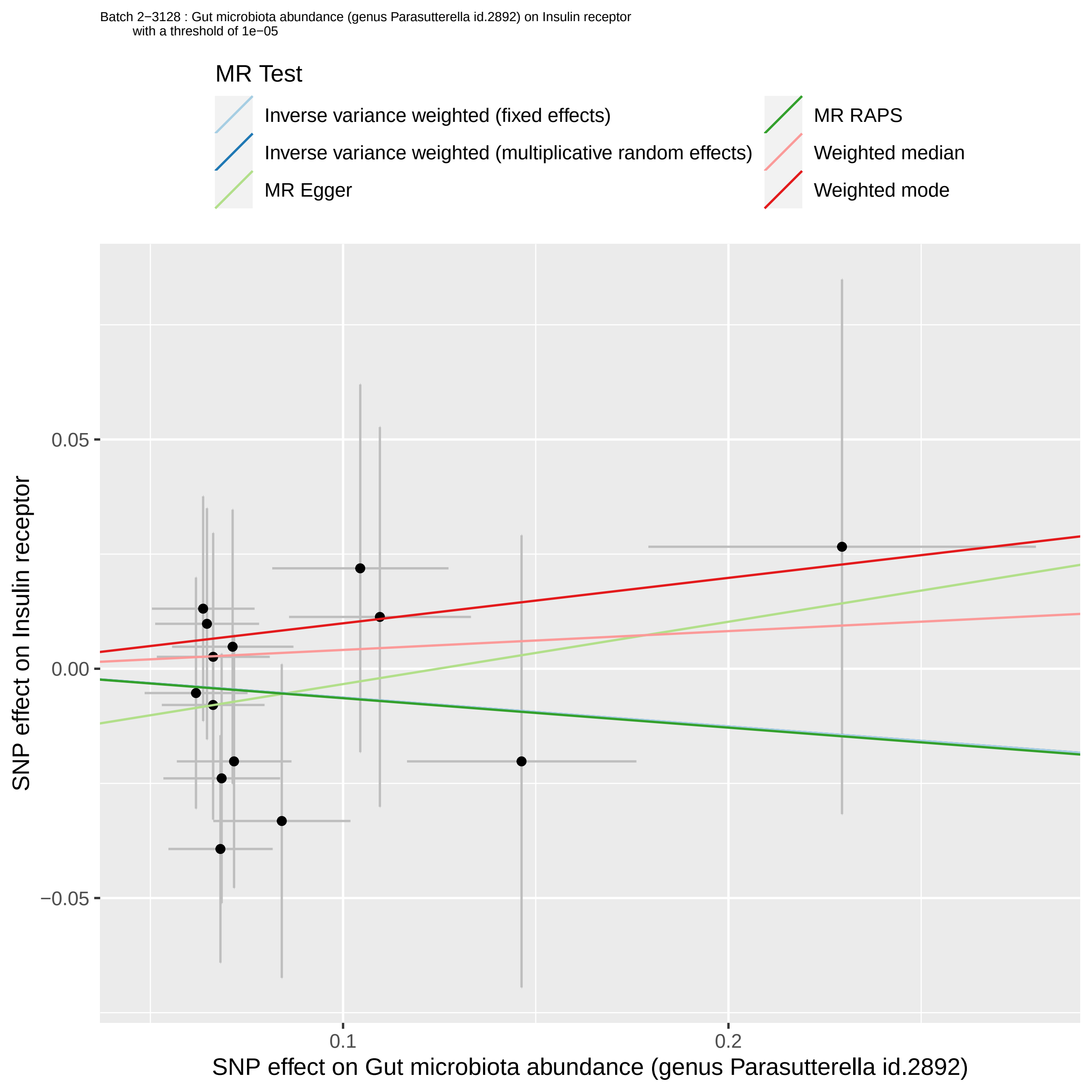

Batch 2−3128 : Gut microbiota abundance (genus Parasutterella id.2892) on Insulin receptor
with a threshold of 1e−05
MR Test
Inverse variance weighted (fixed effects)
Inverse variance weighted (multiplicative random effects)
MR Egger
MR RAPS
Weighted median
Weighted mode
0.05
0.00
−0.05
0.1
0.2
SNP effect on Insulin receptor
SNP effect on Gut microbiota abundance (genus Parasutterella id.2892)

Batch 2−3129 : Gut microbiota abundance (genus Catenibacterium id.2153) on IQ domain−containing protein F1
with a threshold of 1e−05

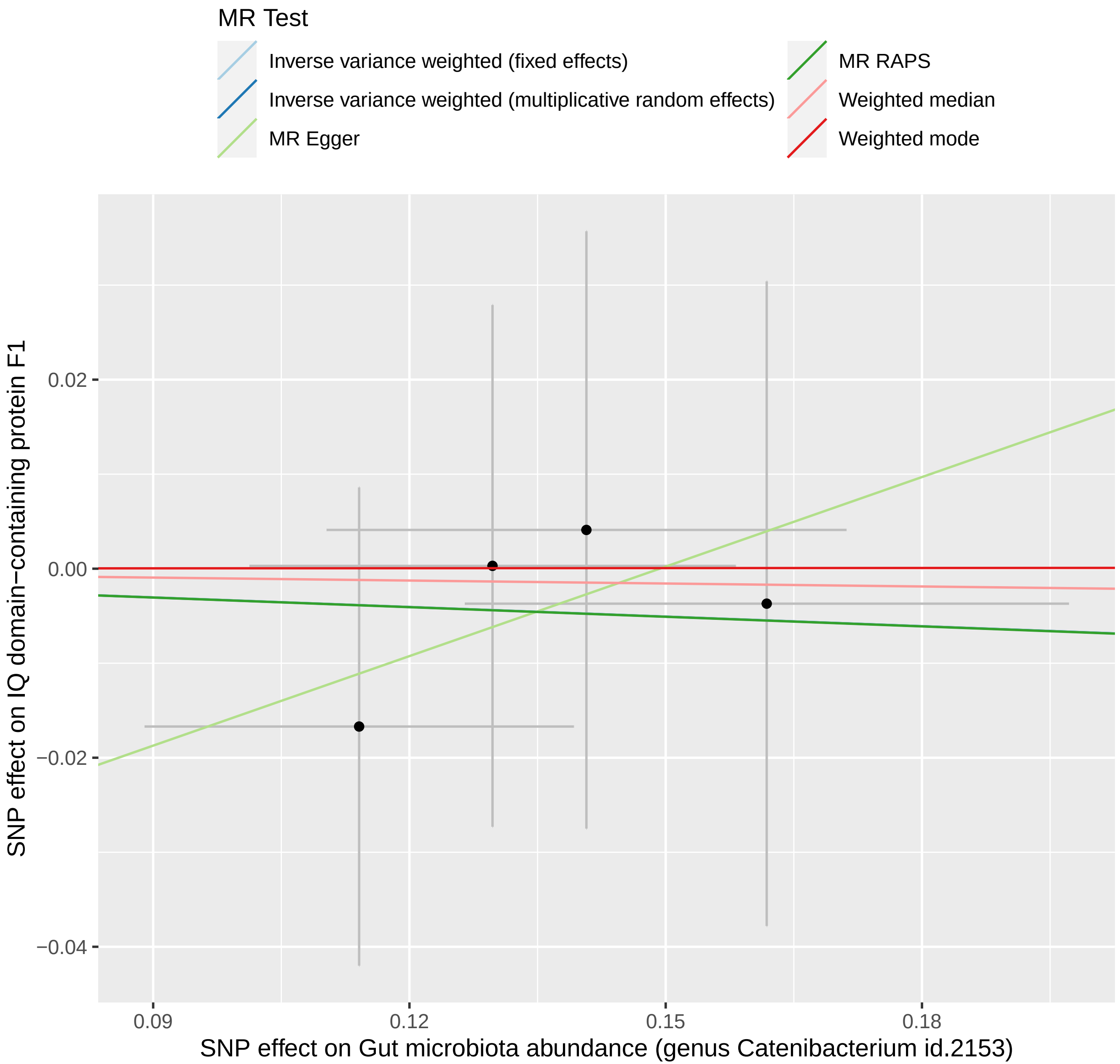

Batch 2−3130 : Gut microbiota abundance (genus Parasutterella id.2892) on IQ domain−containing protein F1
with a threshold of 1e−05

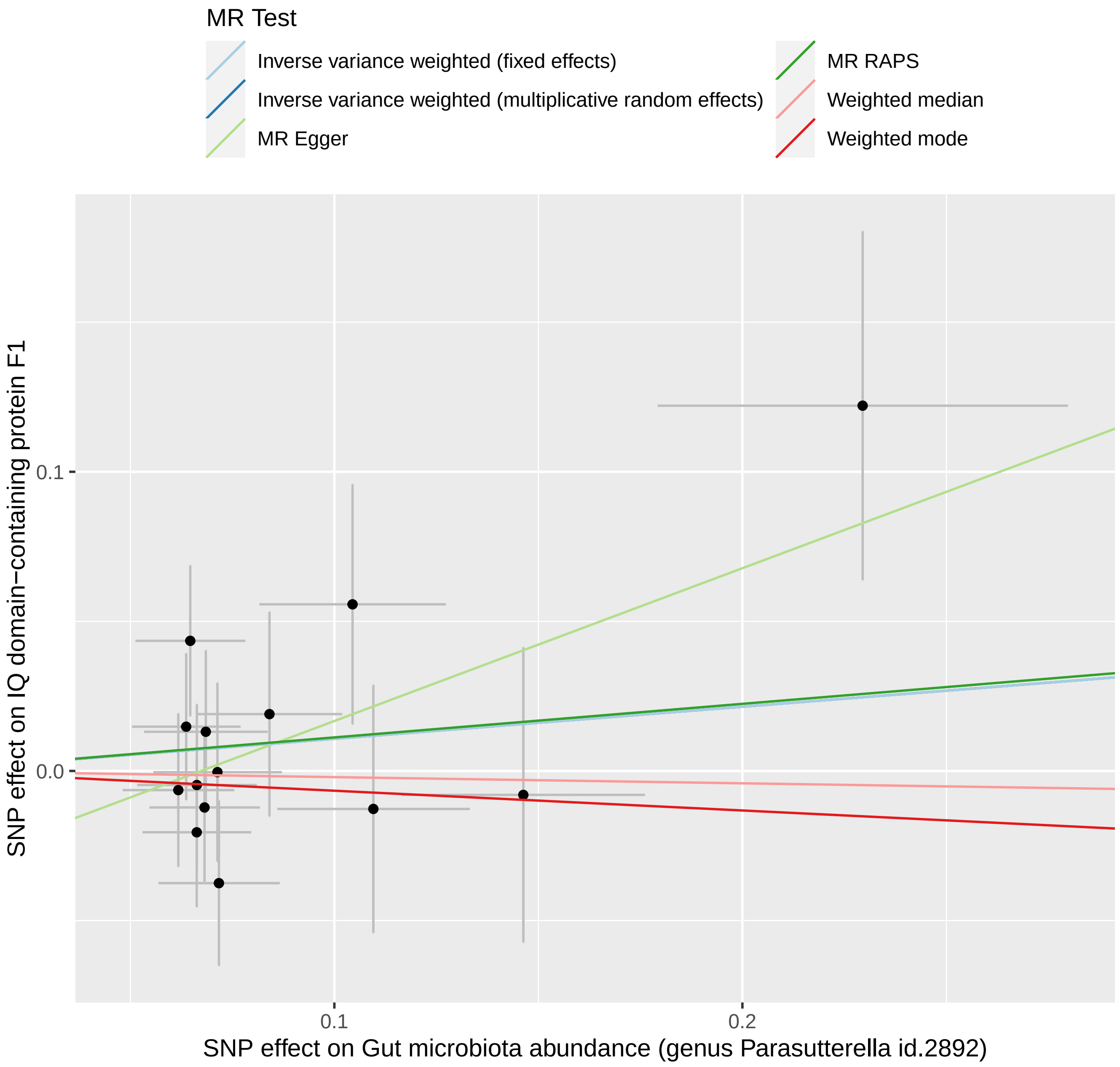

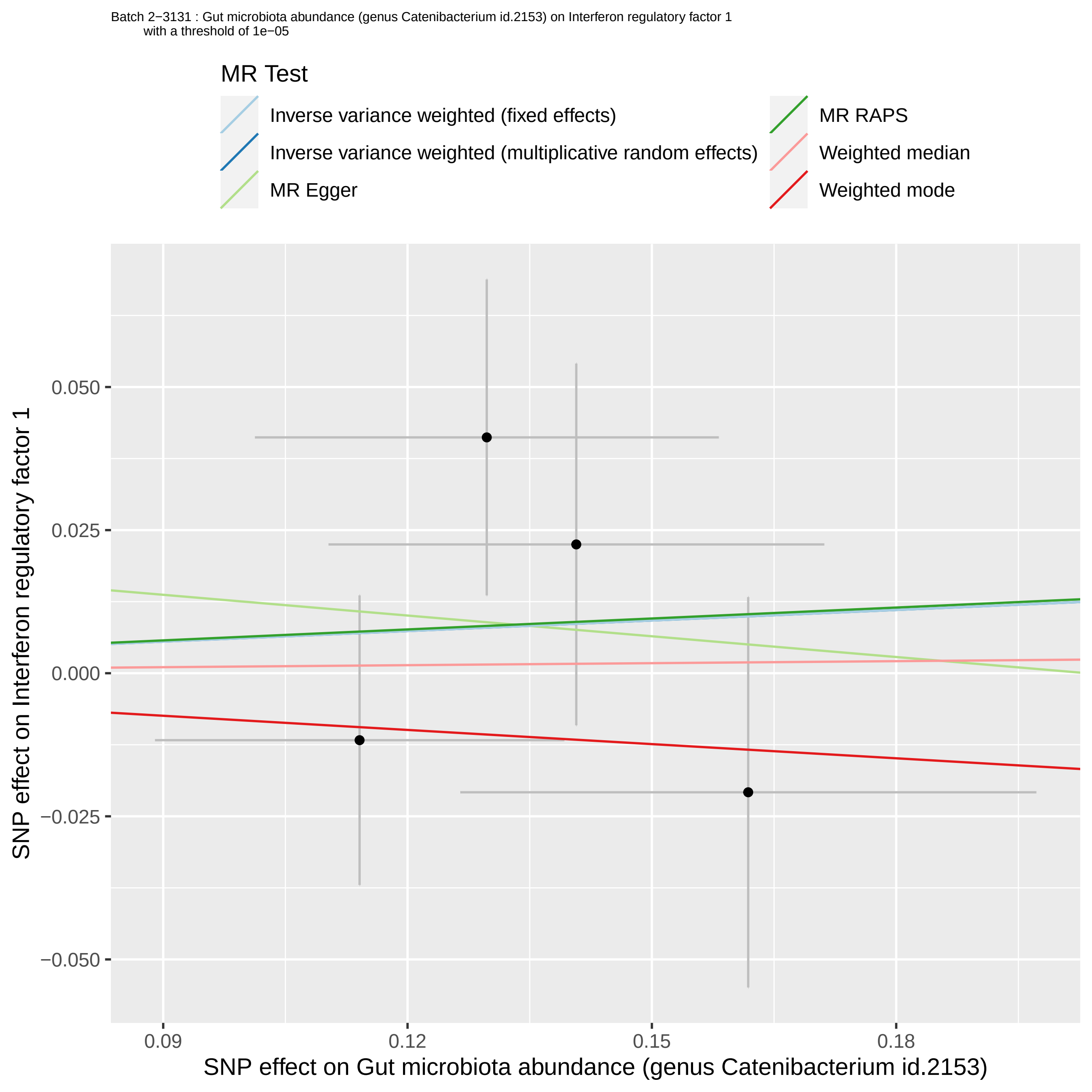
Batch 2−3131 : Gut microbiota abundance (genus Catenibacterium id.2153) on Interferon regulatory factor 1
with a threshold of 1e−05
MR Test
Inverse variance weighted (fixed effects)
Inverse variance weighted (multiplicative random effects)
MR Egger
MR RAPS
Weighted median
Weighted mode
0.050
0.025
0.000
−0.025
−0.050
0.09
0.12
0.15
0.18
SNP effect on Interferon regulatory factor 1
SNP effect on Gut microbiota abundance (genus Catenibacterium id.2153)

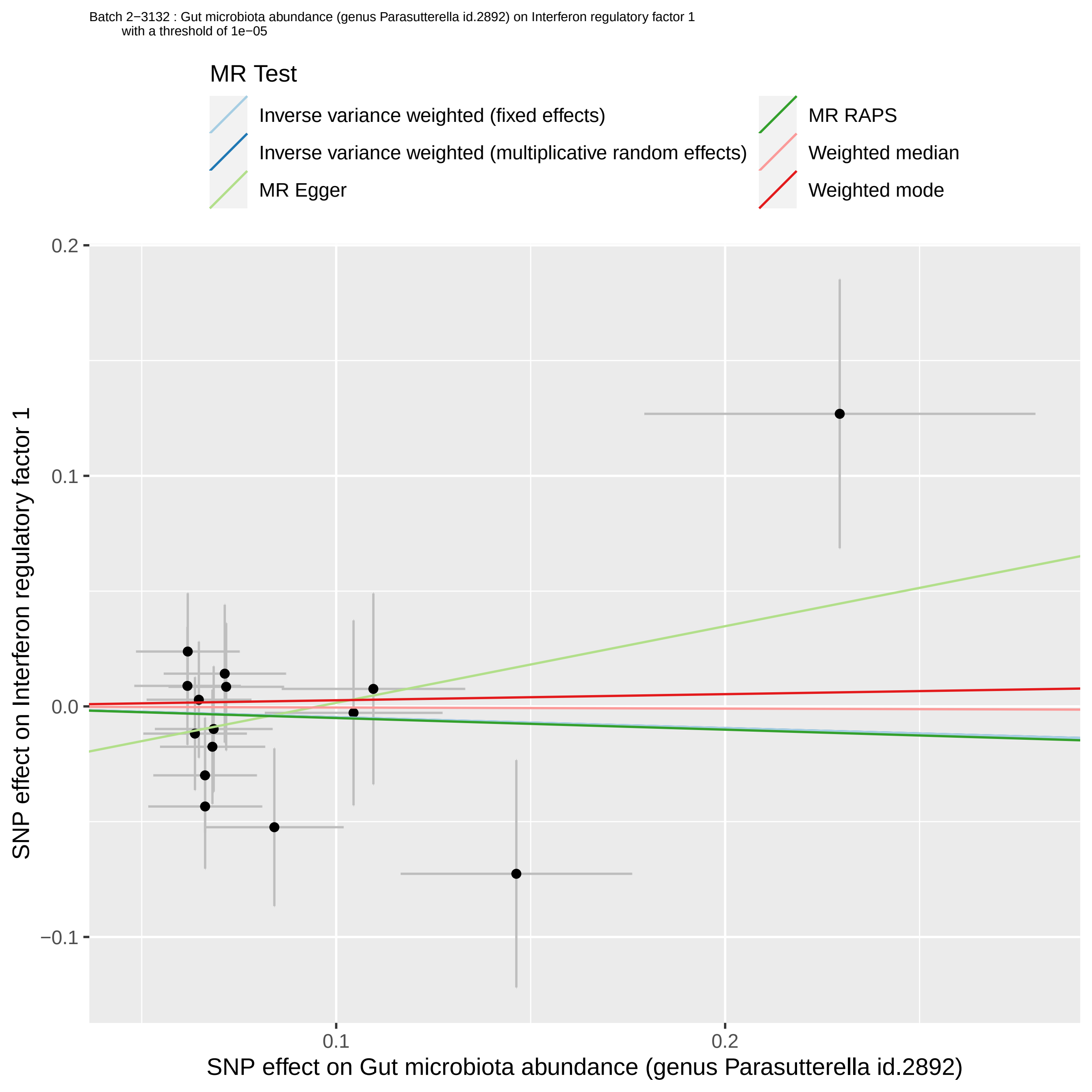

Batch 2−3132 : Gut microbiota abundance (genus Parasutterella id.2892) on Interferon regulatory factor 1
with a threshold of 1e−05
MR Test
Inverse variance weighted (fixed effects)
Inverse variance weighted (multiplicative random effects)
MR Egger
MR RAPS
Weighted median
Weighted mode
0.2
0.1
0.0
−0.1
SNP effect on Interferon regulatory factor 1
0.1
0.2
SNP effect on Gut microbiota abundance (genus Parasutterella id.2892)

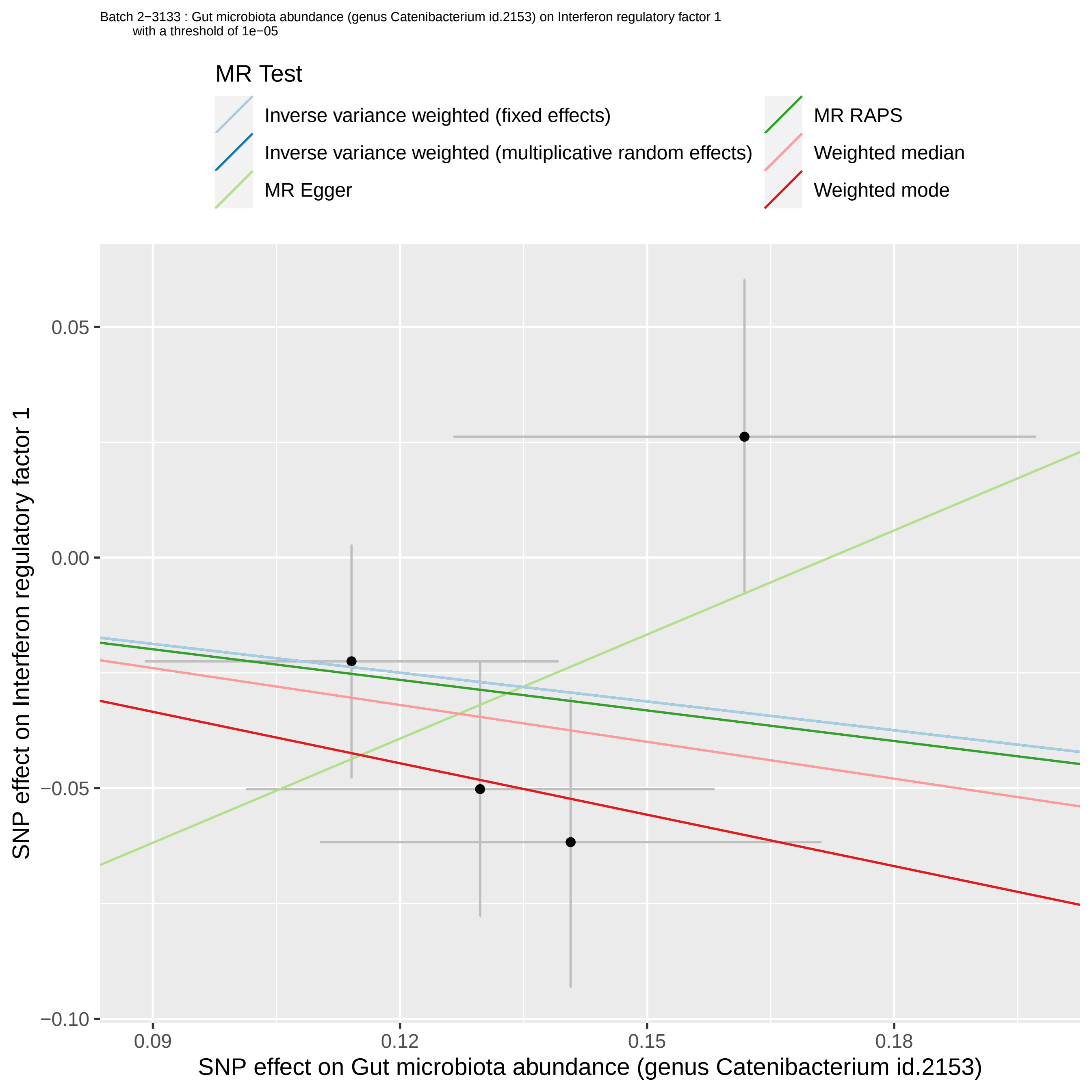

Batch 2−3133 : Gut microbiota abundance (genus Catenibacterium id.2153) on Interferon regulatory factor 1
with a threshold of 1e−05
MR Test
Inverse variance weighted (fixed effects)
Inverse variance weighted (multiplicative random effects)
MR Egger
MR RAPS
Weighted median
Weighted mode
0.05
0.00
−0.05
−0.10
0.09
0.12
0.15
0.18
SNP effect on Interferon regulatory factor 1
SNP effect on Gut microbiota abundance (genus Catenibacterium id.2153)

Batch 2−3134 : Gut microbiota abundance (genus Parasutterella id.2892) on Interferon regulatory factor 1
with a threshold of 1e−05

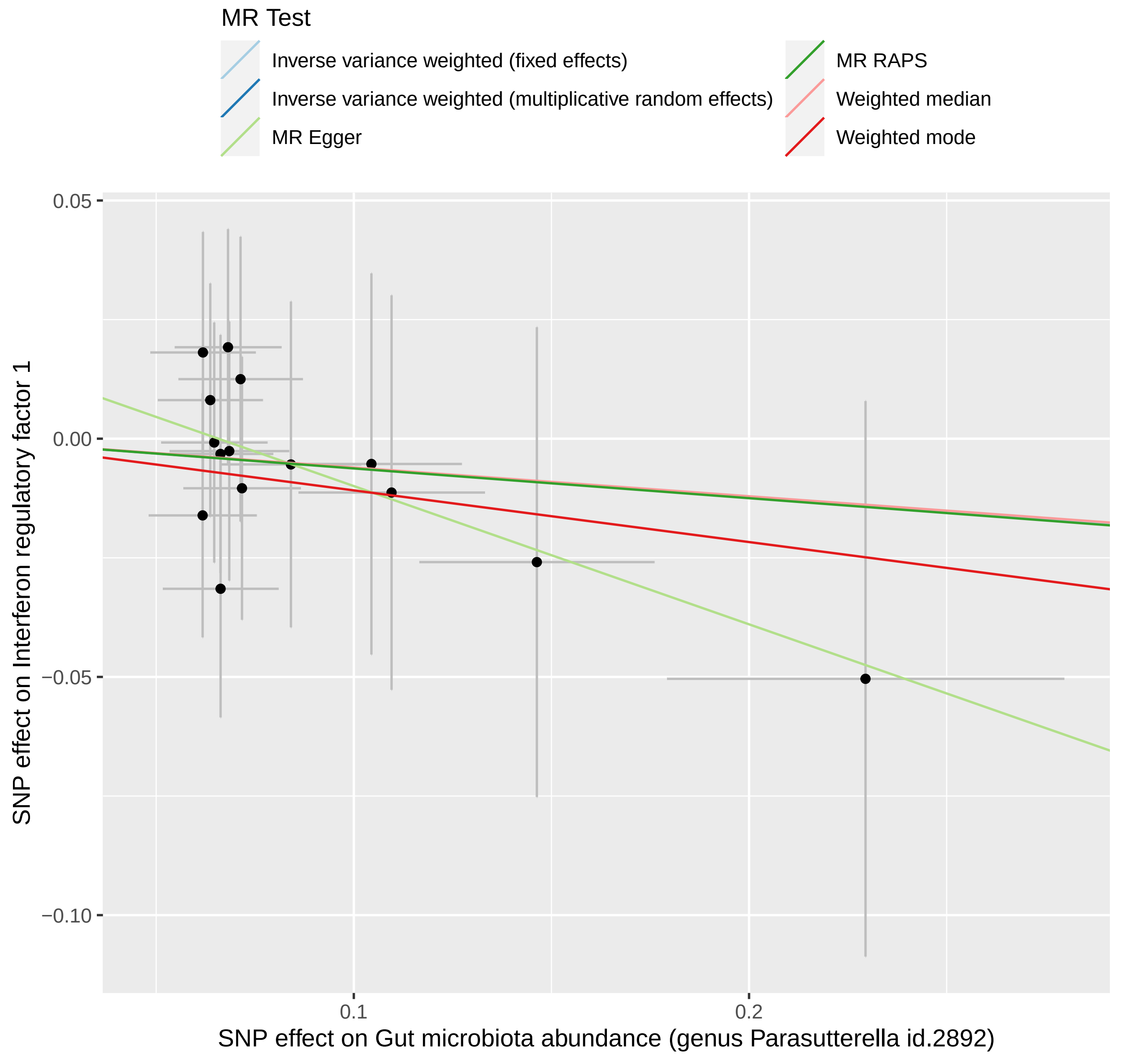

Batch 2−3135 : Gut microbiota abundance (genus Catenibacterium id.2153) on Interferon regulatory factor 2
with a threshold of 1e−05

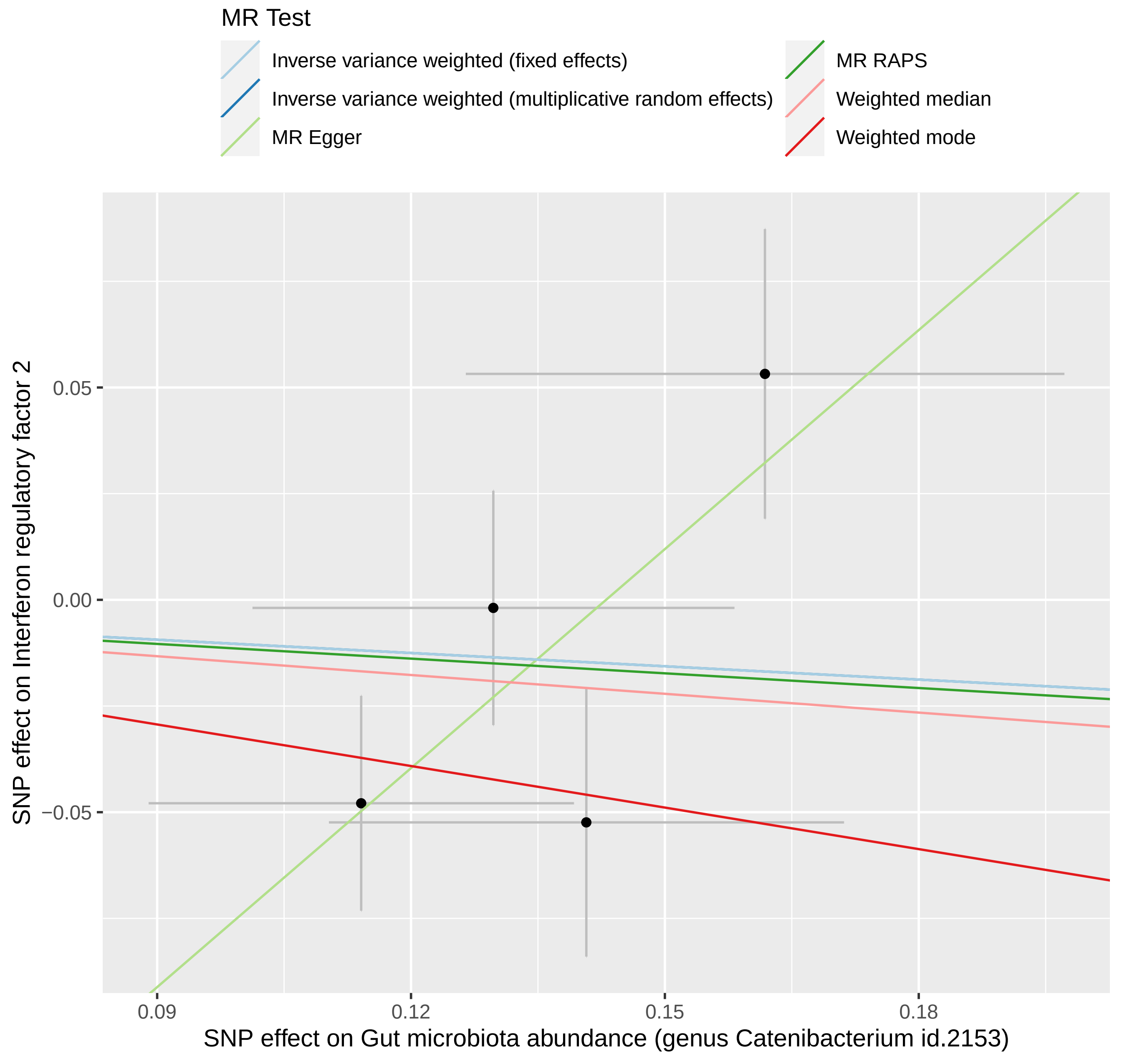

Batch 2−3136 : Gut microbiota abundance (genus Parasutterella id.2892) on Interferon regulatory factor 2
with a threshold of 1e−05

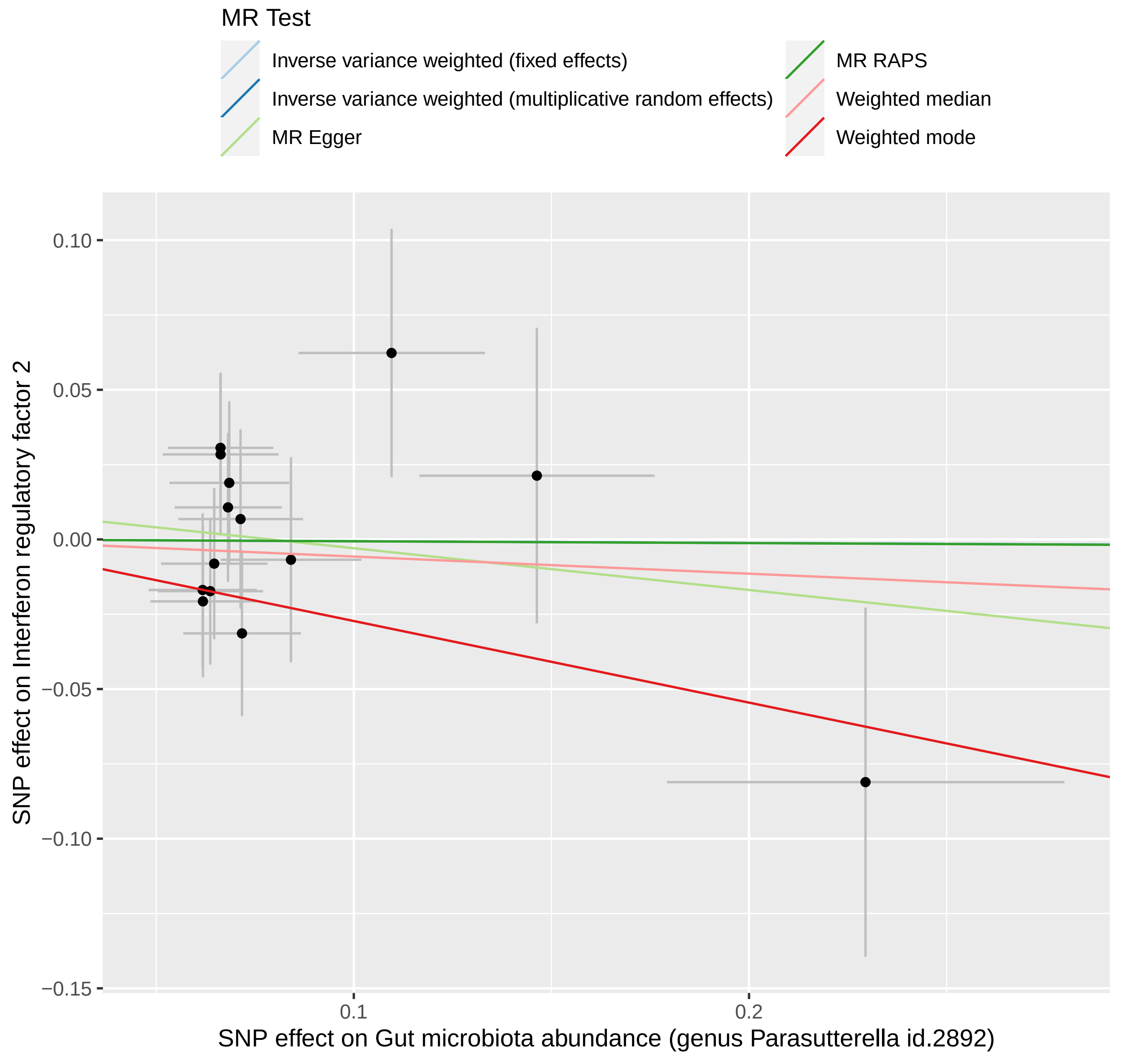

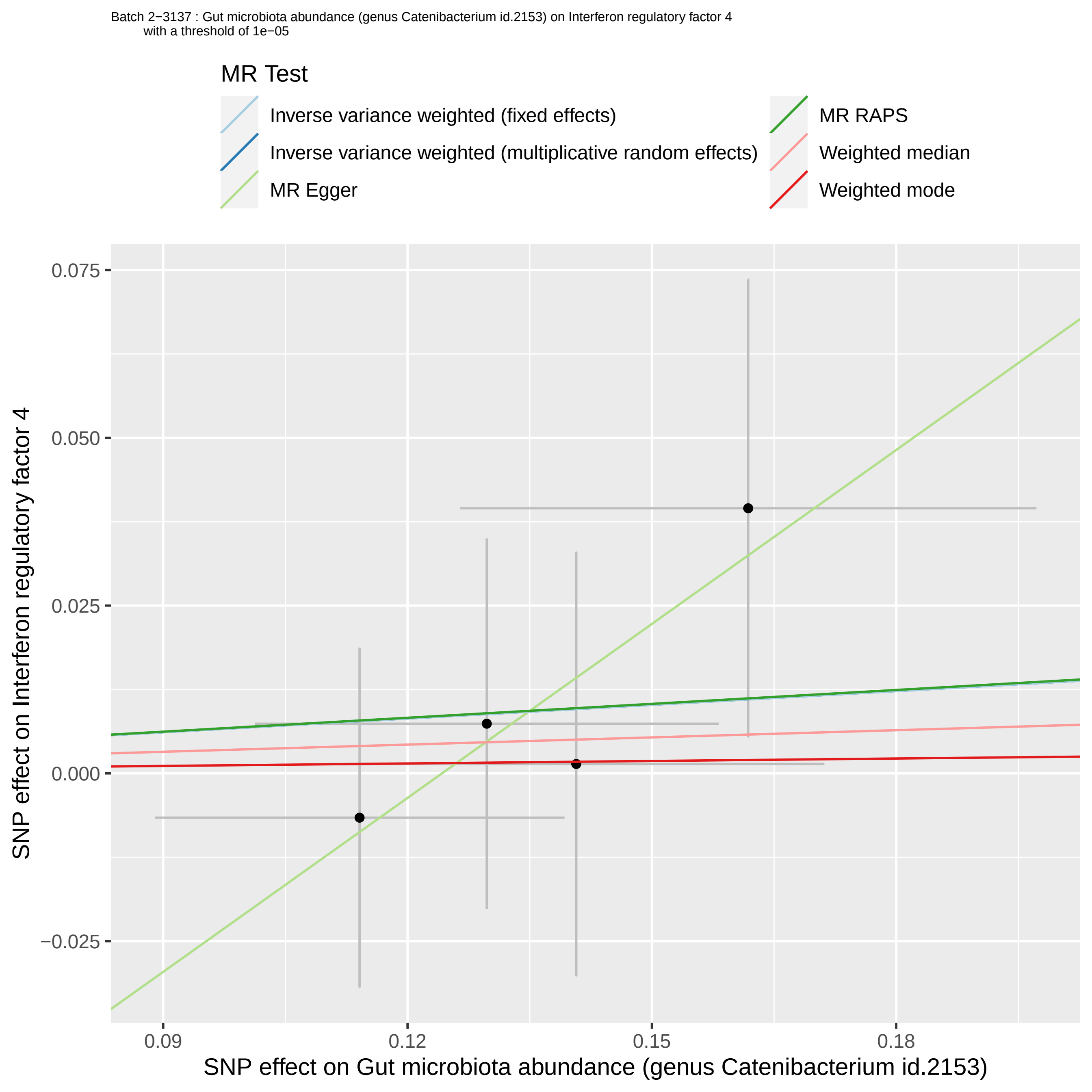

Batch 2−3137 : Gut microbiota abundance (genus Catenibacterium id.2153) on Interferon regulatory factor 4
with a threshold of 1e−05
MR Test
Inverse variance weighted (fixed effects)
Inverse variance weighted (multiplicative random effects)
MR Egger
MR RAPS
Weighted median
Weighted mode
0.075
0.050
0.025
0.000
−0.025
0.09
0.12
0.15
0.18
SNP effect on Interferon regulatory factor 4
SNP effect on Gut microbiota abundance (genus Catenibacterium id.2153)

Batch 2−3138 : Gut microbiota abundance (genus Parasutterella id.2892) on Interferon regulatory factor 4
with a threshold of 1e−05

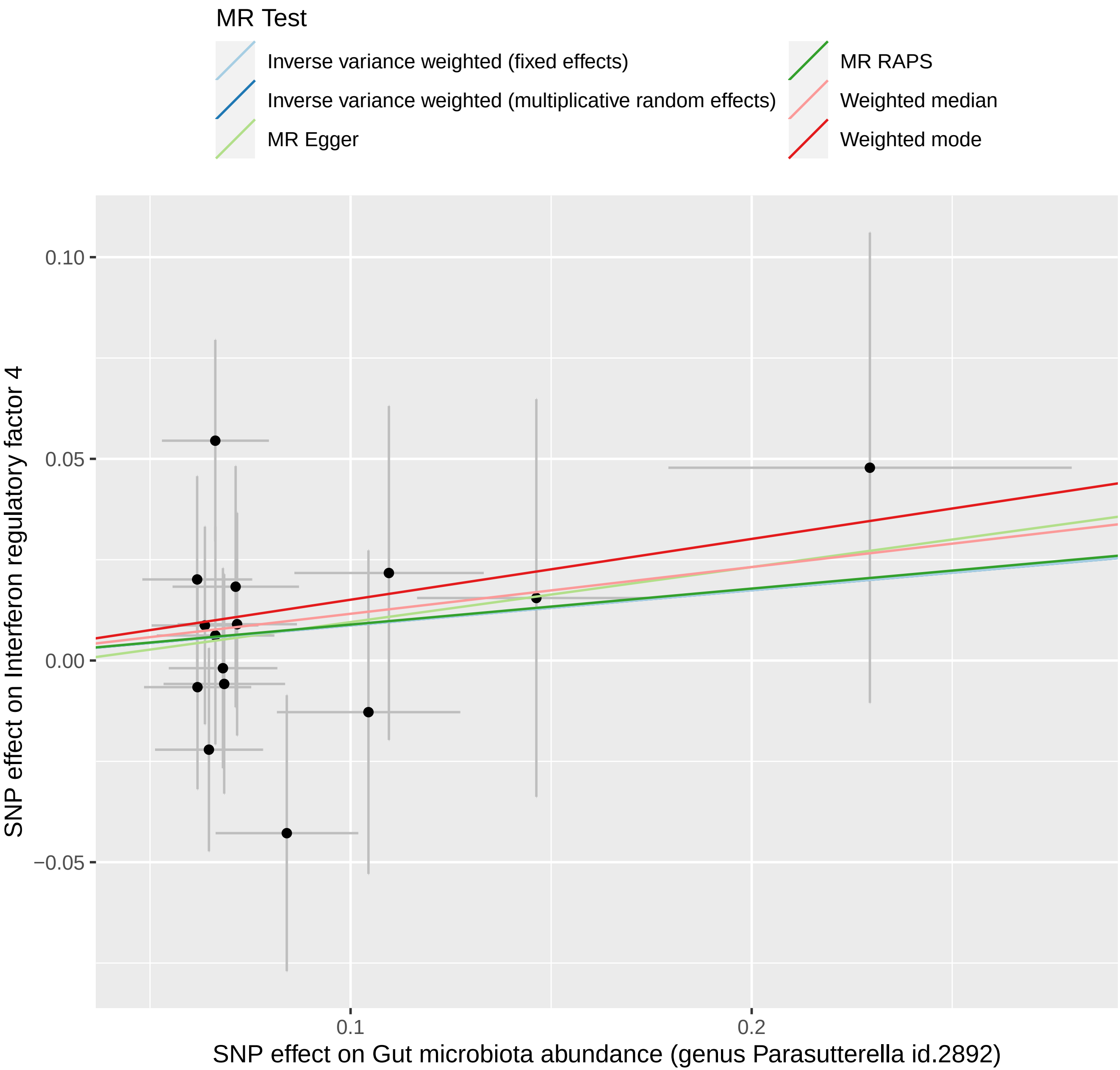

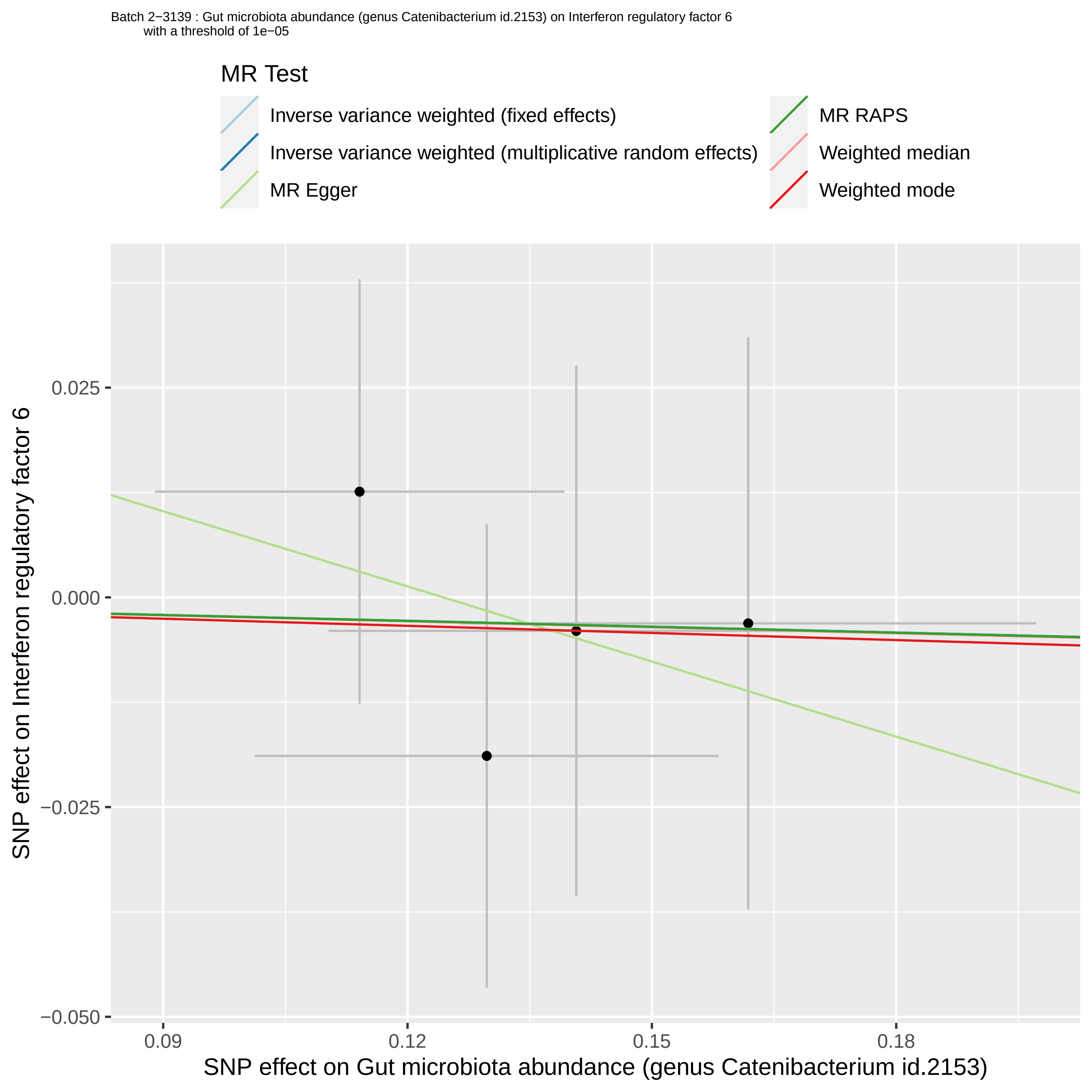
Batch 2−3139 : Gut microbiota abundance (genus Catenibacterium id.2153) on Interferon regulatory factor 6
with a threshold of 1e−05
MR Test
Inverse variance weighted (fixed effects)
Inverse variance weighted (multiplicative random effects)
MR Egger
MR RAPS
Weighted median
Weighted mode
SNP effect on Interferon regulatory factor 6
0.025
0.000
−0.025
−0.050
0.09
0.12
0.15
0.18
SNP effect on Gut microbiota abundance (genus Catenibacterium id.2153)

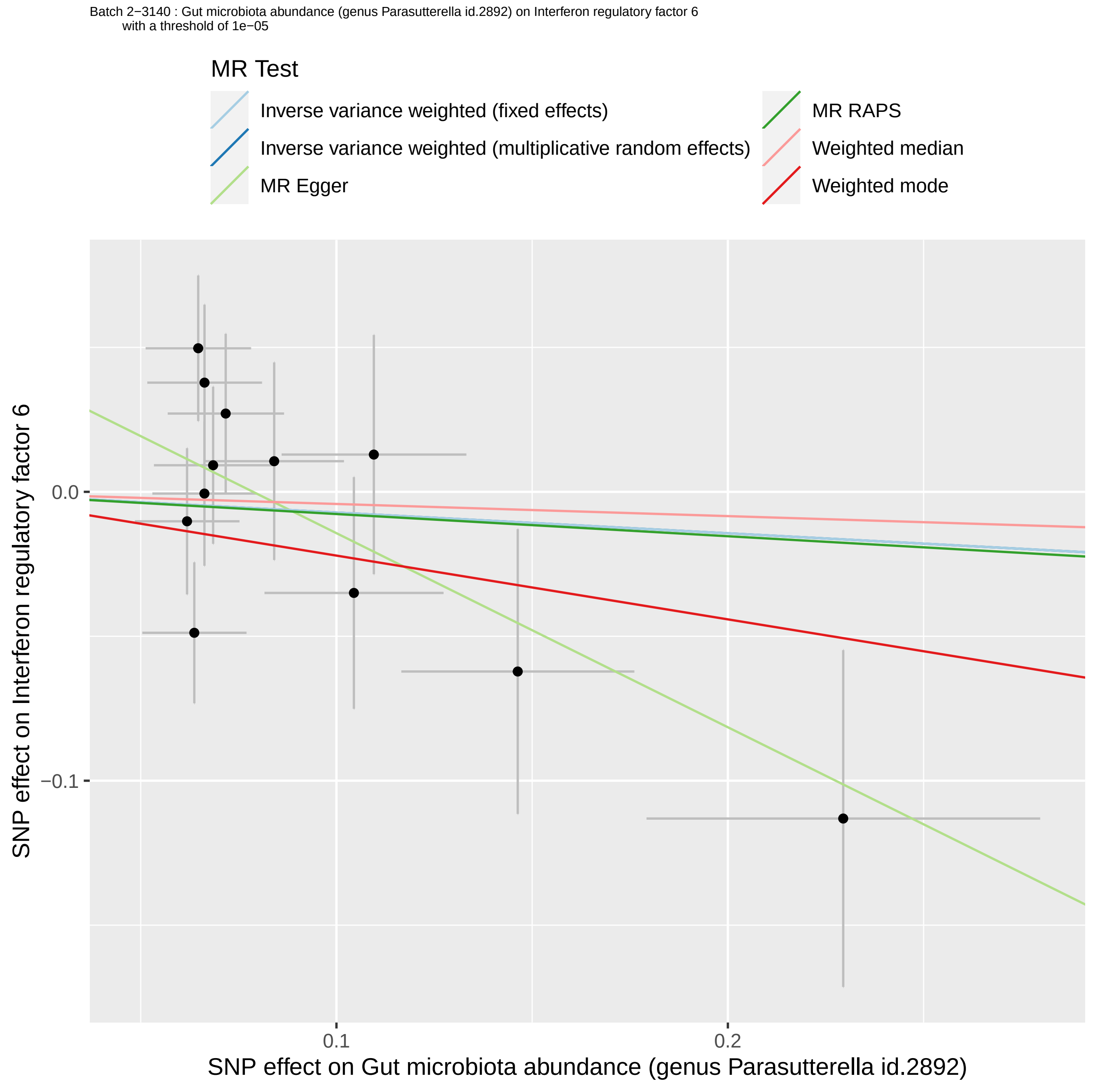
Batch 2−3140 : Gut microbiota abundance (genus Parasutterella id.2892) on Interferon regulatory factor 6
with a threshold of 1e−05
MR Test
Inverse variance weighted (fixed effects)
Inverse variance weighted (multiplicative random effects)
MR Egger
MR RAPS
Weighted median
Weighted mode
SNP effect on Interferon regulatory factor 6
0.0
−0.1
0.1
0.2
SNP effect on Gut microbiota abundance (genus Parasutterella id.2892)

Batch 2−3141 : Gut microbiota abundance (genus Catenibacterium id.2153) on Interferon regulatory factor 9
with a threshold of 1e−05

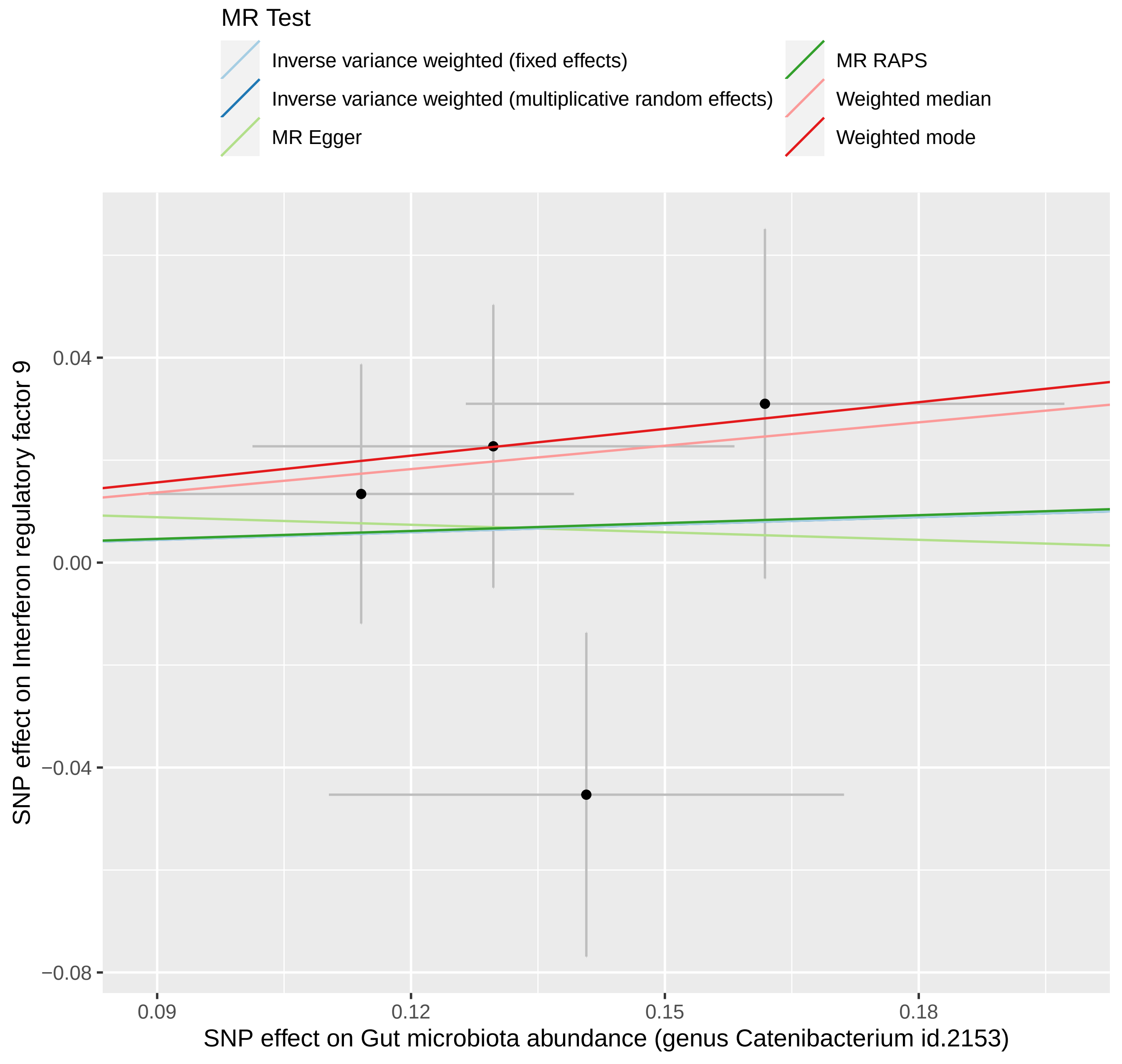

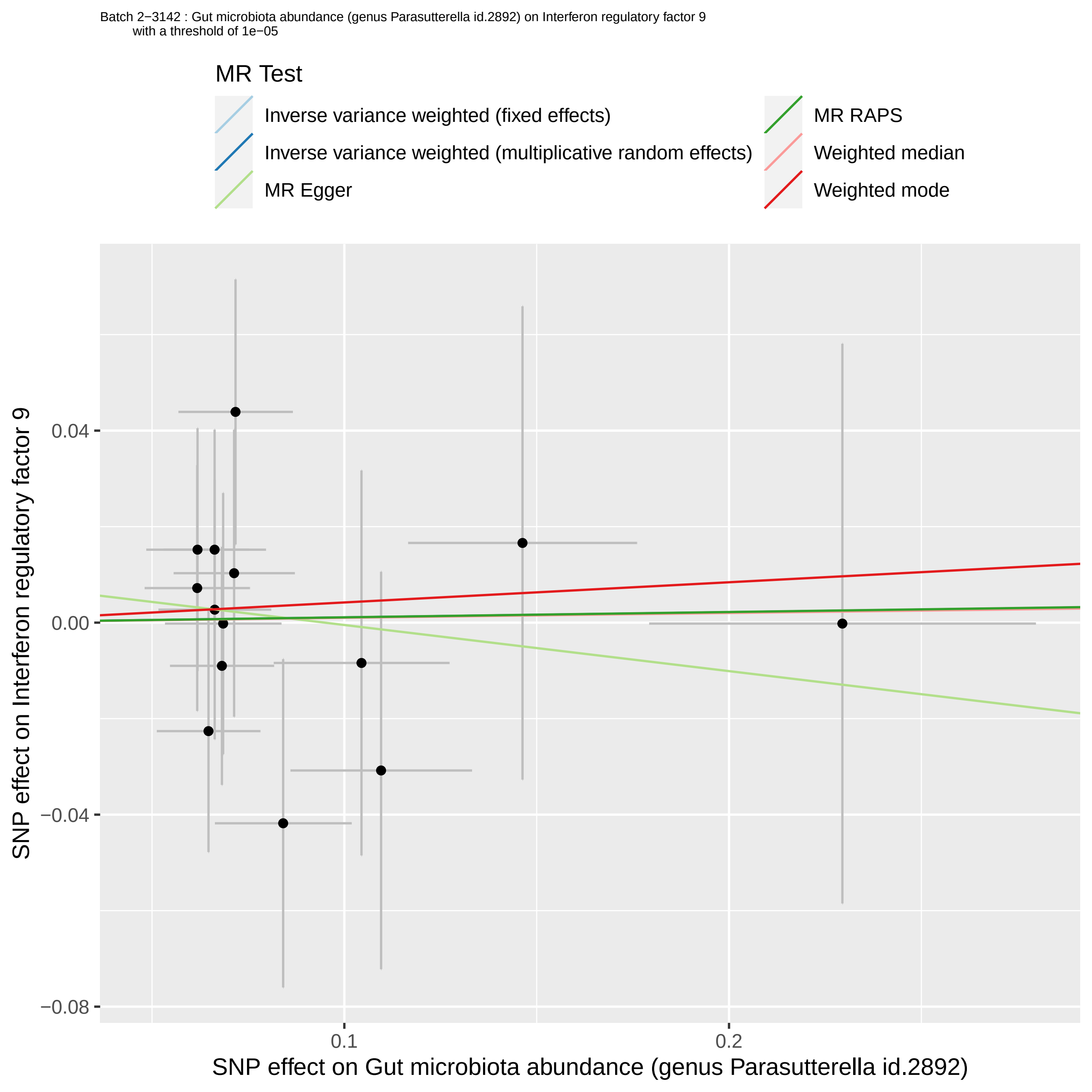
Batch 2−3142 : Gut microbiota abundance (genus Parasutterella id.2892) on Interferon regulatory factor 9
with a threshold of 1e−05
MR Test
Inverse variance weighted (fixed effects)
Inverse variance weighted (multiplicative random effects)
MR Egger
MR RAPS
Weighted median
Weighted mode
0.04
0.00
−0.04
−0.08
0.1
0.2
SNP effect on Interferon regulatory factor 9
SNP effect on Gut microbiota abundance (genus Parasutterella id.2892)

Batch 2−3143 : Gut microbiota abundance (genus Catenibacterium id.2153) on Iron−sulfur cluster assembly enzyme ISCU, mitochondrial
with a threshold of 1e−05

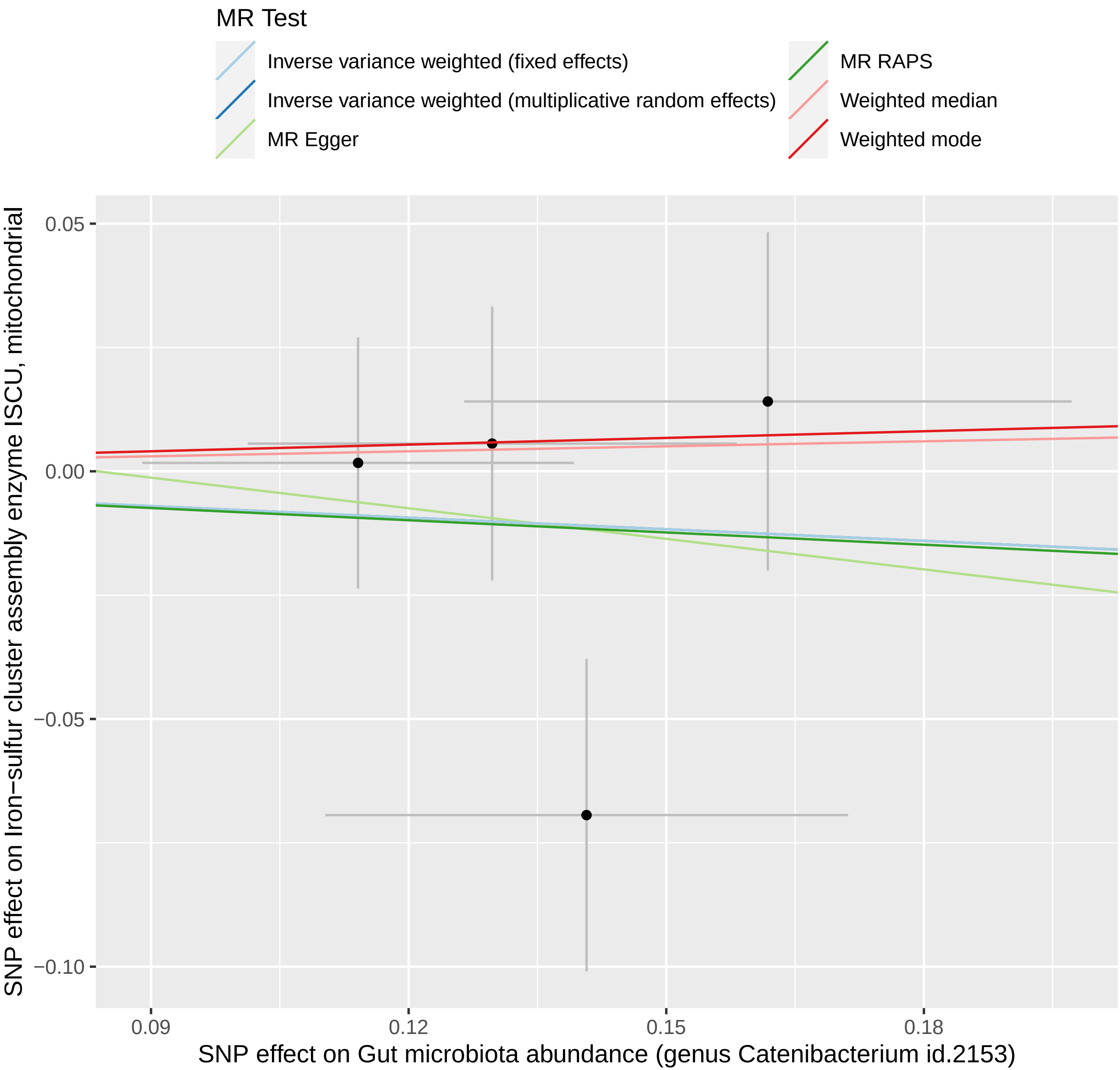

Batch 2−3144 : Gut microbiota abundance (genus Parasutterella id.2892) on Iron−sulfur cluster assembly enzyme ISCU, mitochondrial
with a threshold of 1e−05

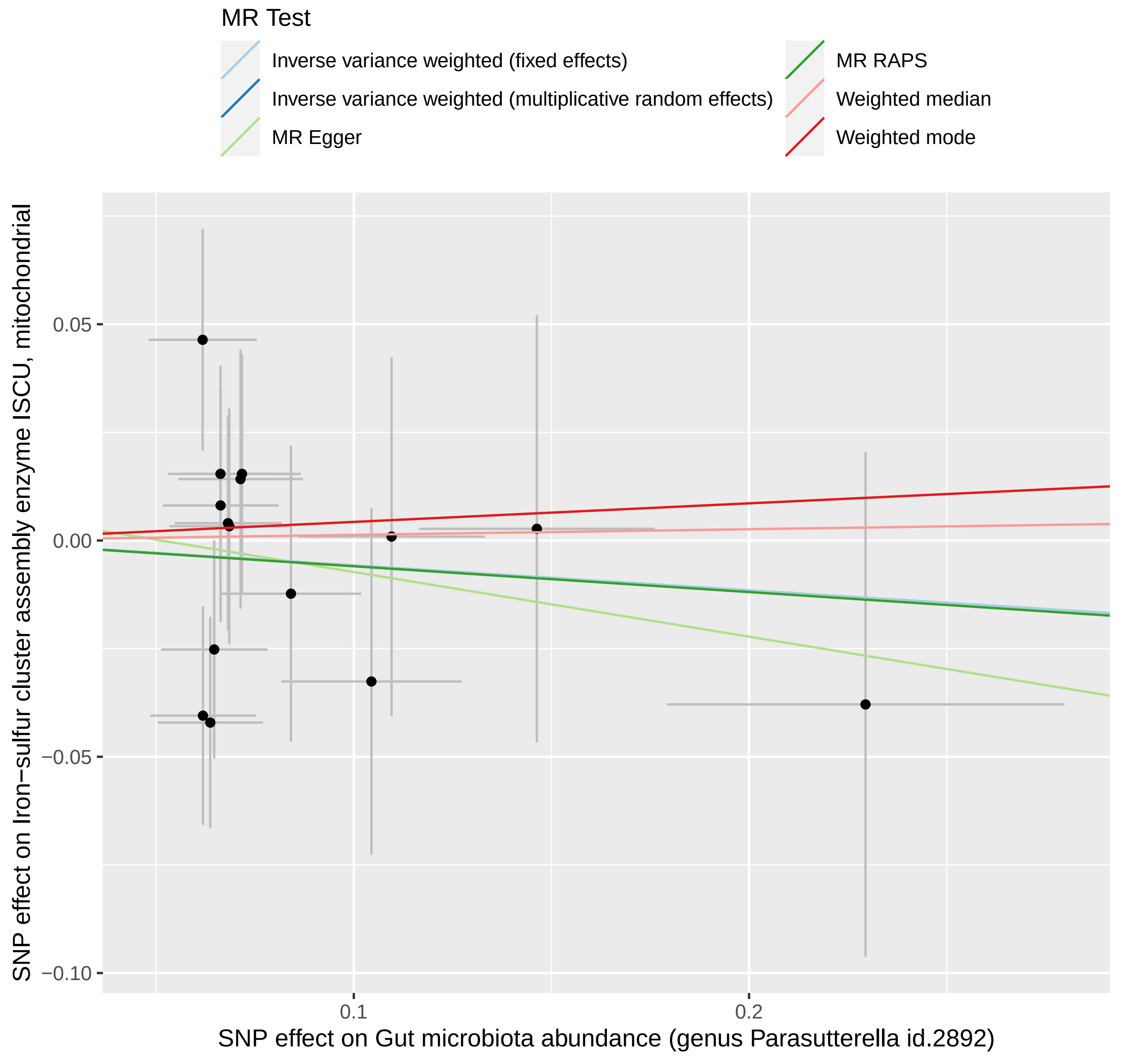

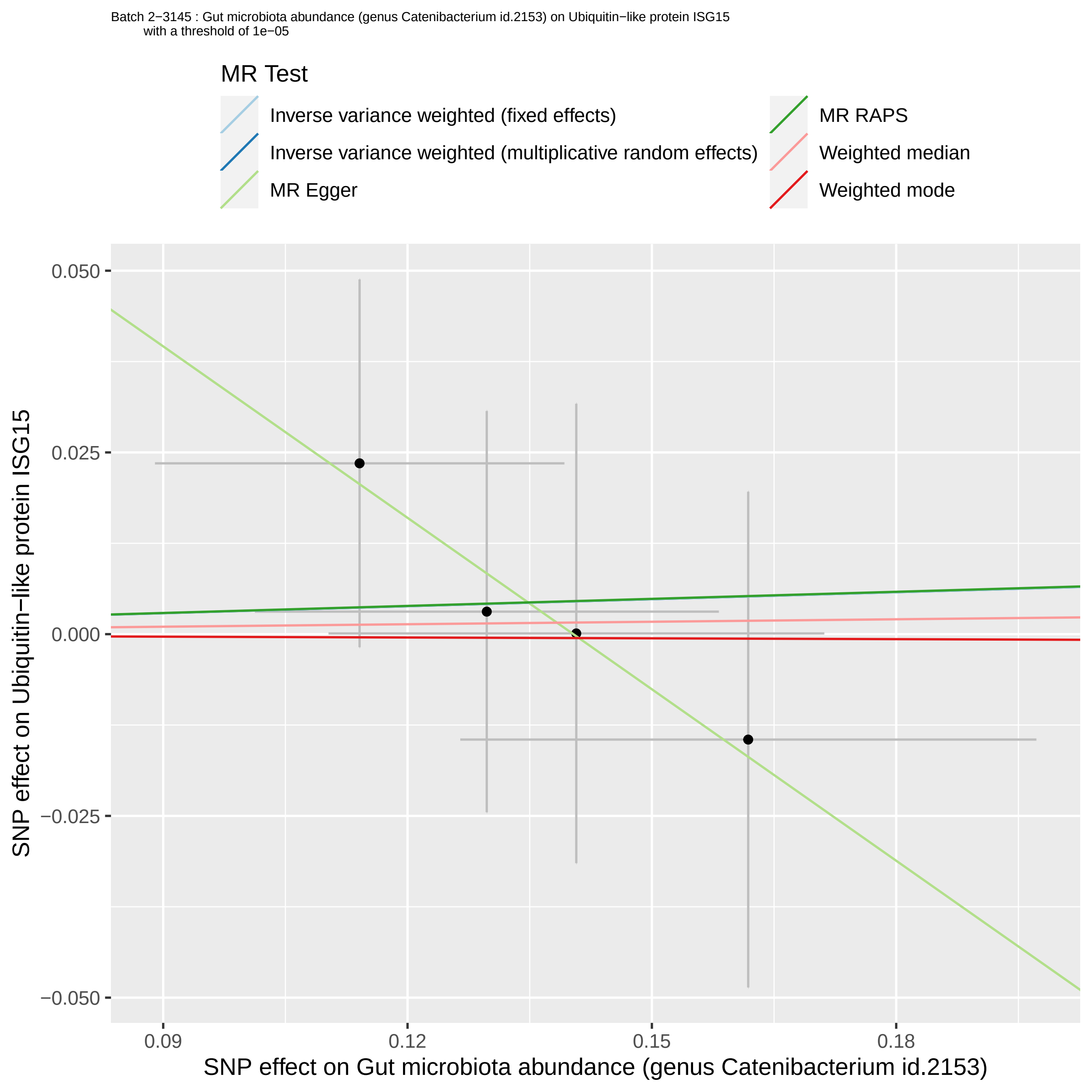
Batch 2−3145 : Gut microbiota abundance (genus Catenibacterium id.2153) on Ubiquitin−like protein ISG15
with a threshold of 1e−05
MR Test
Inverse variance weighted (fixed effects)
Inverse variance weighted (multiplicative random effects)
MR Egger
MR RAPS
Weighted median
Weighted mode
0.050
0.025
0.000
−0.025
−0.050
0.09
0.12
0.15
0.18
SNP effect on Ubiquitin−like protein ISG15
SNP effect on Gut microbiota abundance (genus Catenibacterium id.2153)

Batch 2−3146 : Gut microbiota abundance (genus Parasutterella id.2892) on Ubiquitin−like protein ISG15
with a threshold of 1e−05

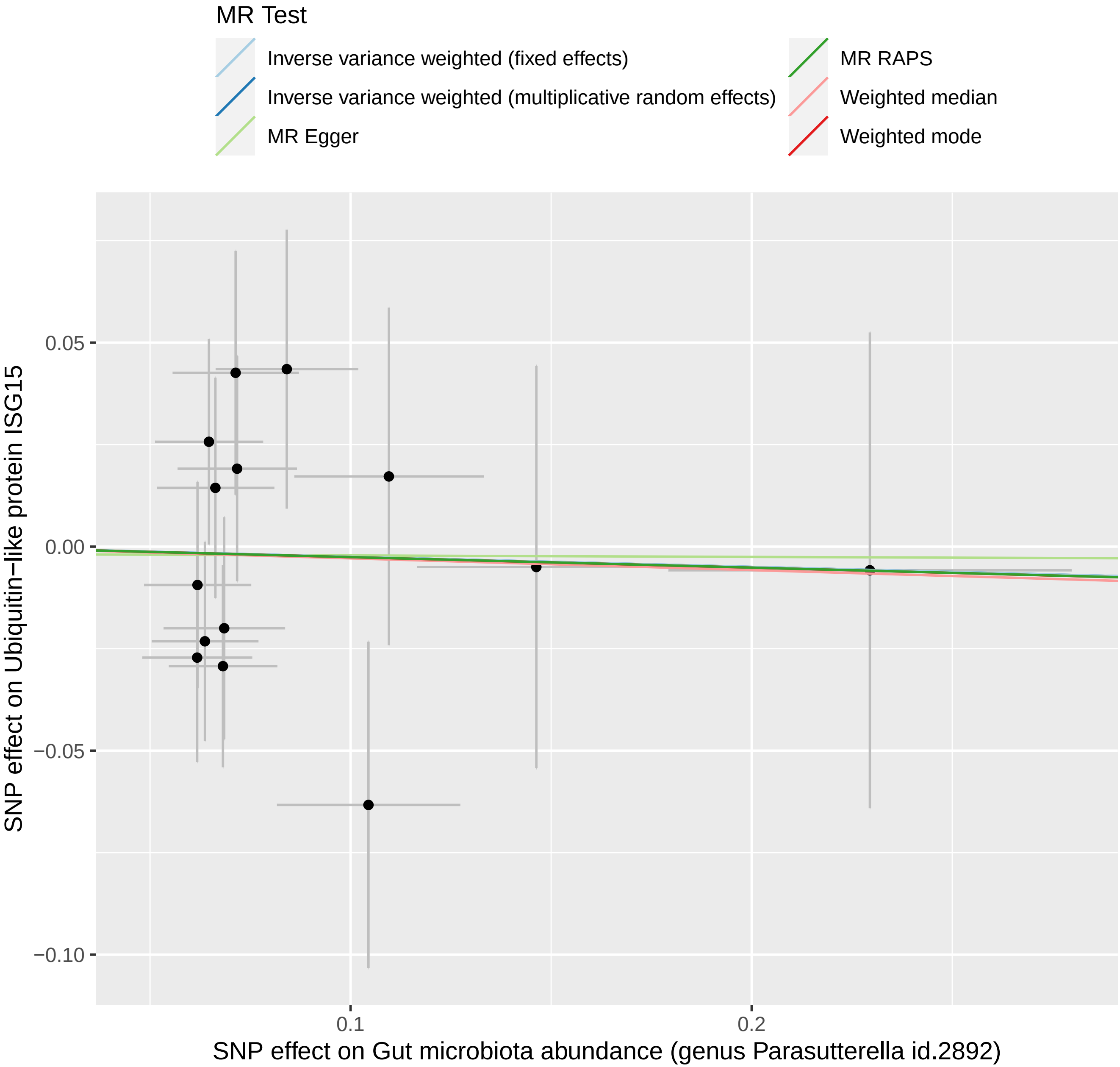

Batch 2−3147 : Gut microbiota abundance (genus Catenibacterium id.2153) on Ubiquitin−like protein ISG15
with a threshold of 1e−05

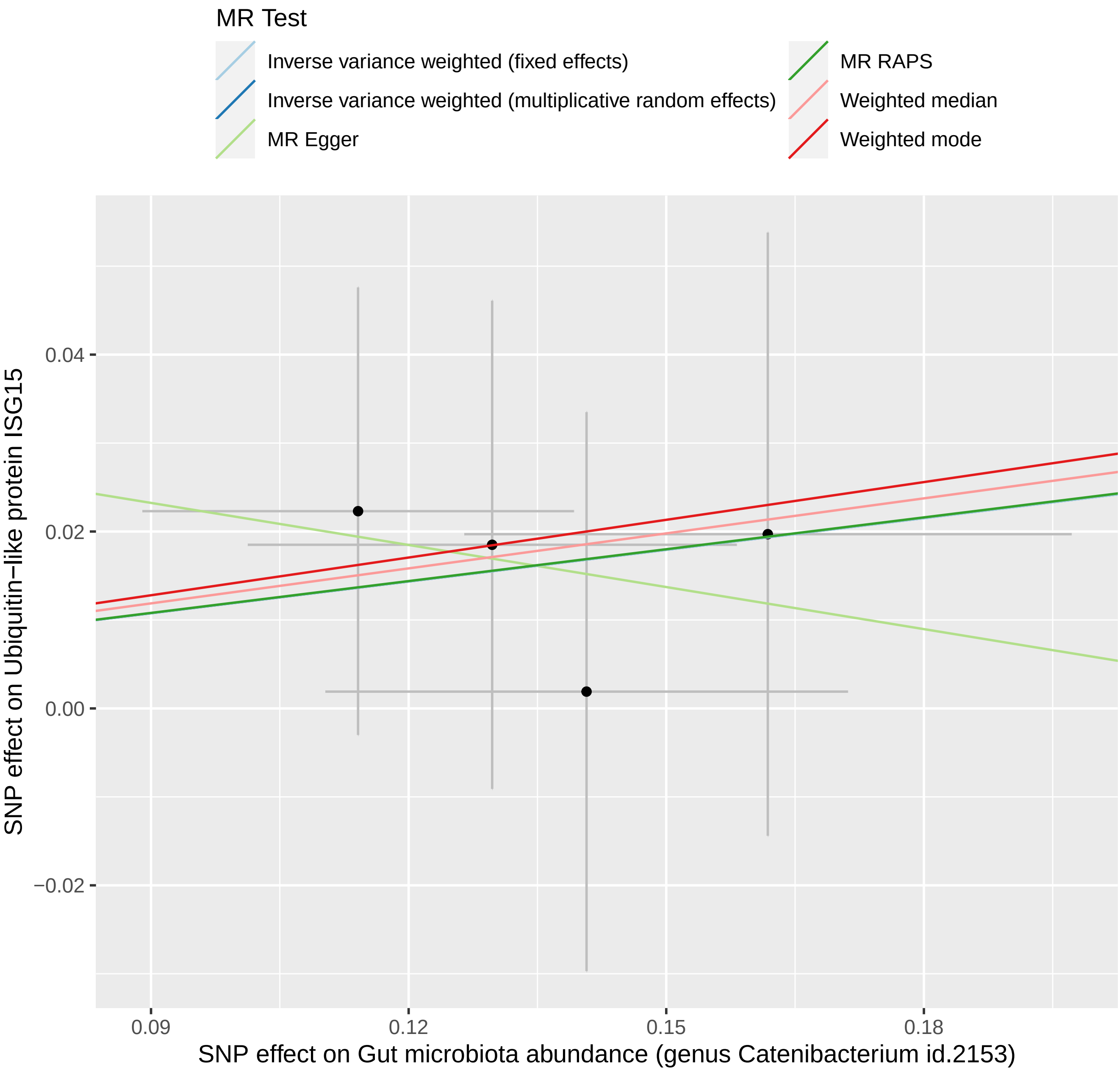

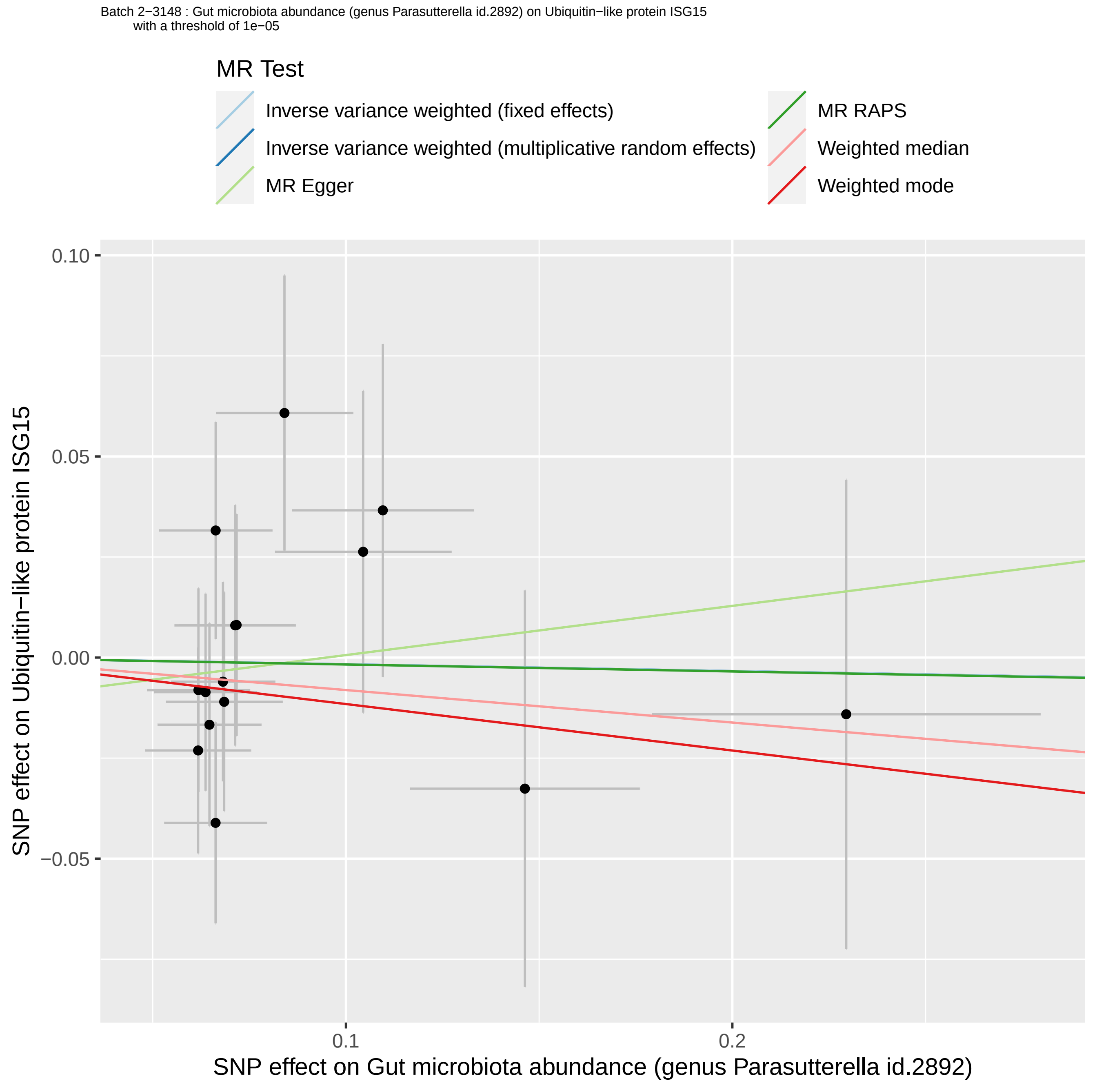
Batch 2−3148 : Gut microbiota abundance (genus Parasutterella id.2892) on Ubiquitin−like protein ISG15
with a threshold of 1e−05
MR Test
Inverse variance weighted (fixed effects)
Inverse variance weighted (multiplicative random effects)
MR Egger
MR RAPS
Weighted median
Weighted mode
0.10
0.05
0.00
−0.05
0.1
0.2
SNP effect on Ubiquitin−like protein ISG15
SNP effect on Gut microbiota abundance (genus Parasutterella id.2892)

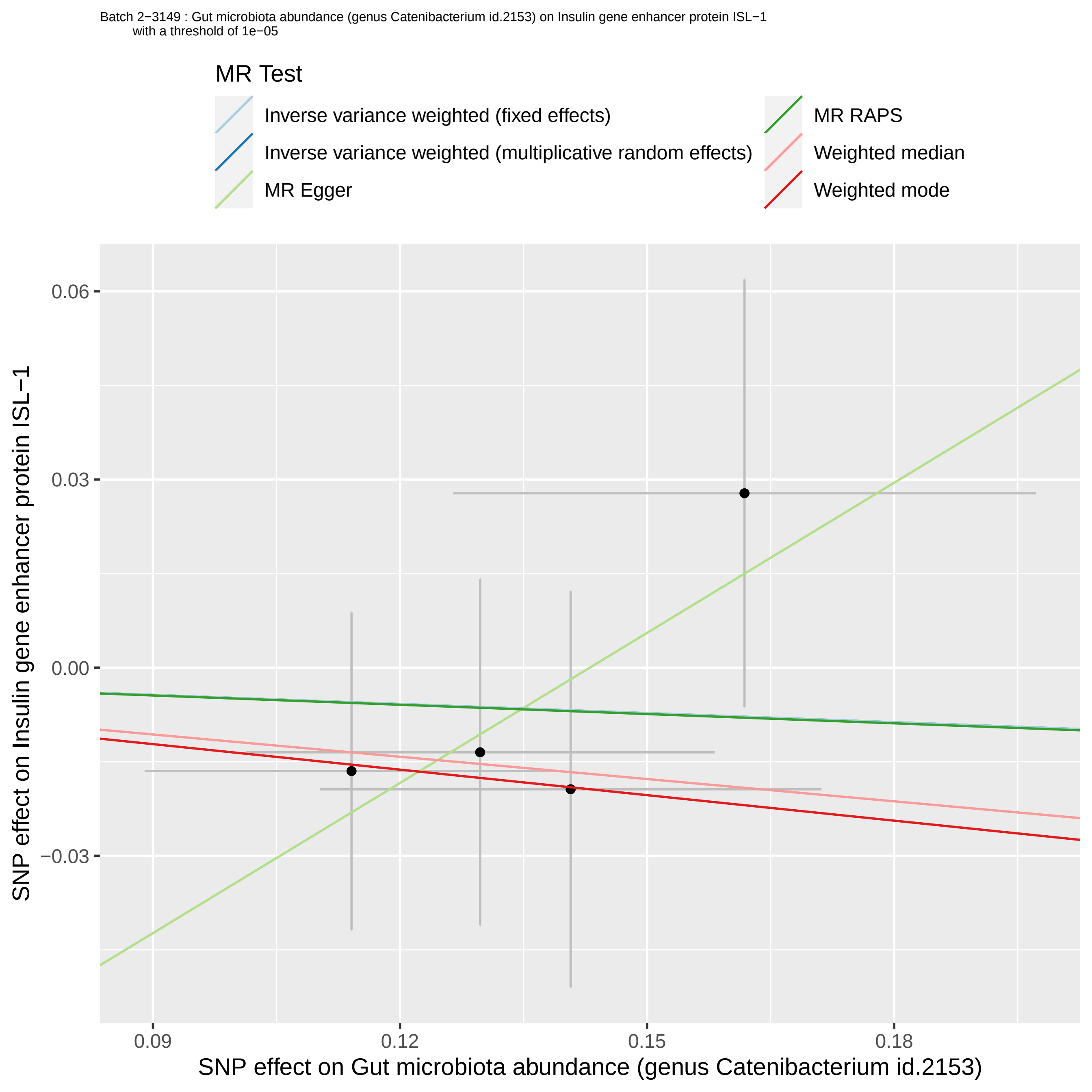

Batch 2−3149 : Gut microbiota abundance (genus Catenibacterium id.2153) on Insulin gene enhancer protein ISL−1
with a threshold of 1e−05
MR Test
Inverse variance weighted (fixed effects)
Inverse variance weighted (multiplicative random effects)
MR Egger
MR RAPS
Weighted median
Weighted mode
0.06
0.03
0.00
−0.03
0.09
0.12
0.15
0.18
SNP effect on Insulin gene enhancer protein ISL−1
SNP effect on Gut microbiota abundance (genus Catenibacterium id.2153)

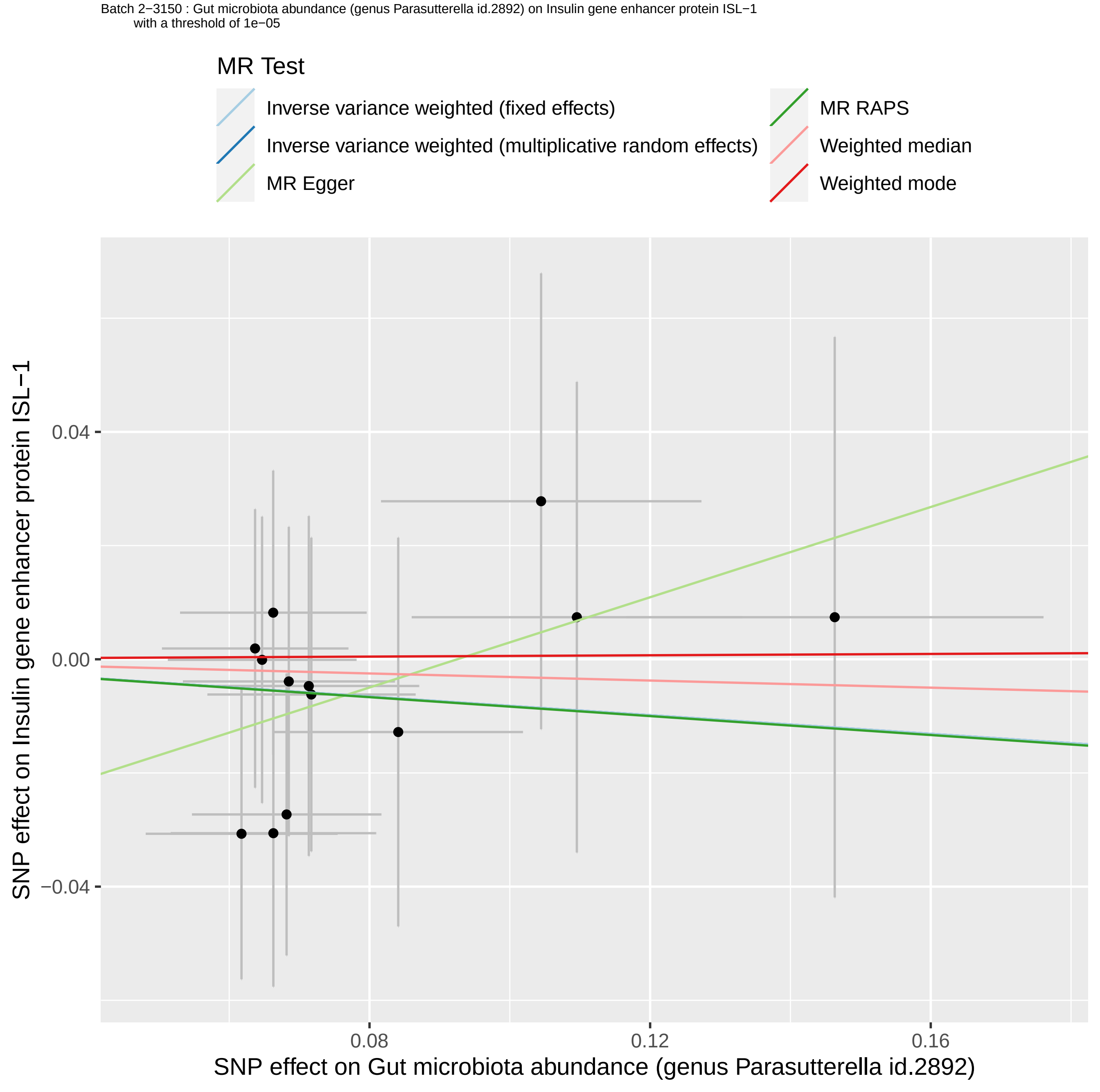

Batch 2−3150 : Gut microbiota abundance (genus Parasutterella id.2892) on Insulin gene enhancer protein ISL−1
with a threshold of 1e−05
MR Test
Inverse variance weighted (fixed effects)
Inverse variance weighted (multiplicative random effects)
MR Egger
MR RAPS
Weighted median
Weighted mode
SNP effect on Insulin gene enhancer protein ISL−1
0.04
0.00
−0.04
0.08
0.12
0.16
SNP effect on Gut microbiota abundance (genus Parasutterella id.2892)

Batch 2−3151 : Gut microbiota abundance (genus Catenibacterium id.2153) on Immunoglobulin superfamily containing leucine−rich repeat protein 2
with a threshold of 1e−05

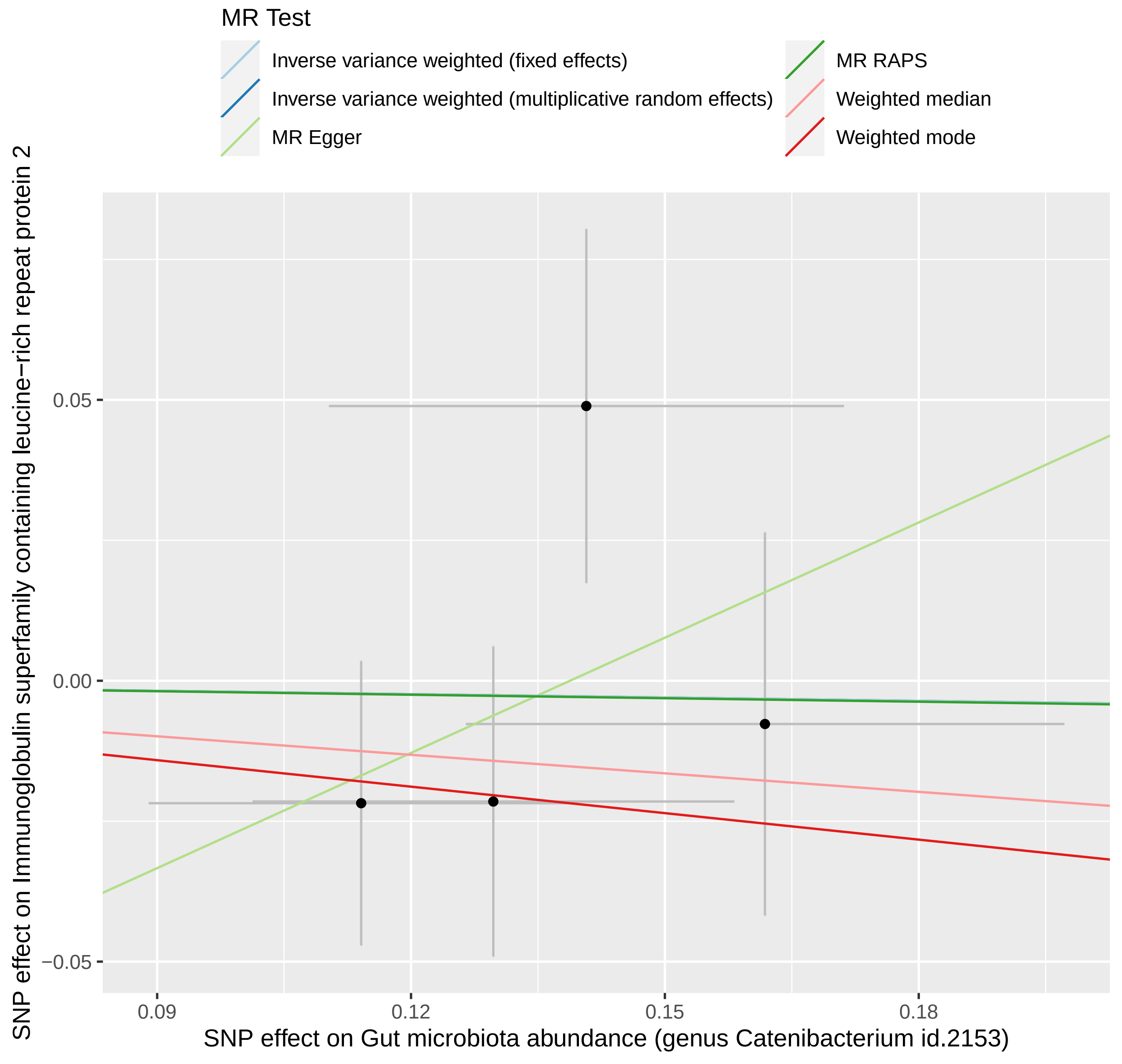

Batch 2−3152 : Gut microbiota abundance (genus Parasutterella id.2892) on Immunoglobulin superfamily containing leucine−rich repeat protein 2
with a threshold of 1e−05

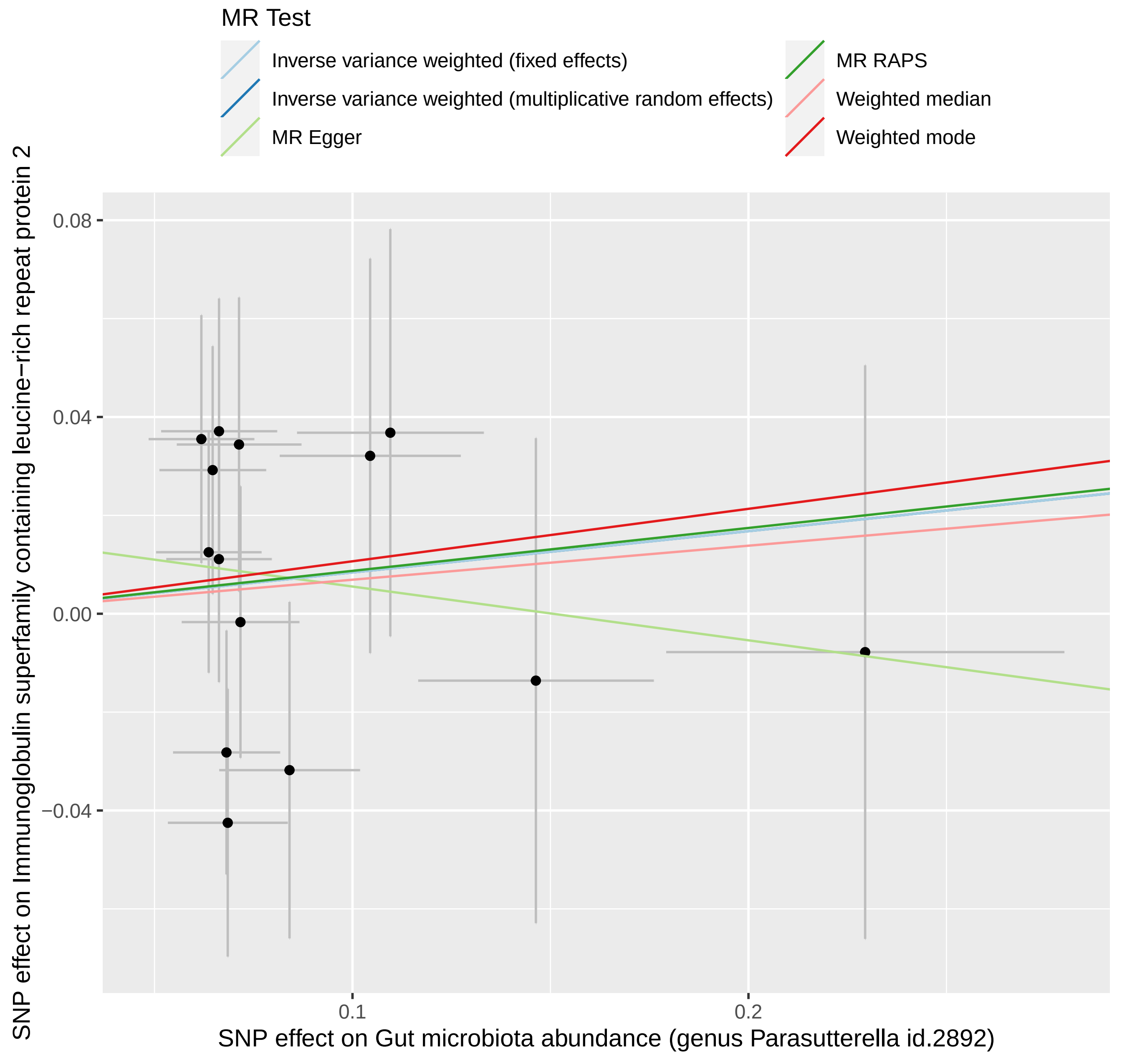

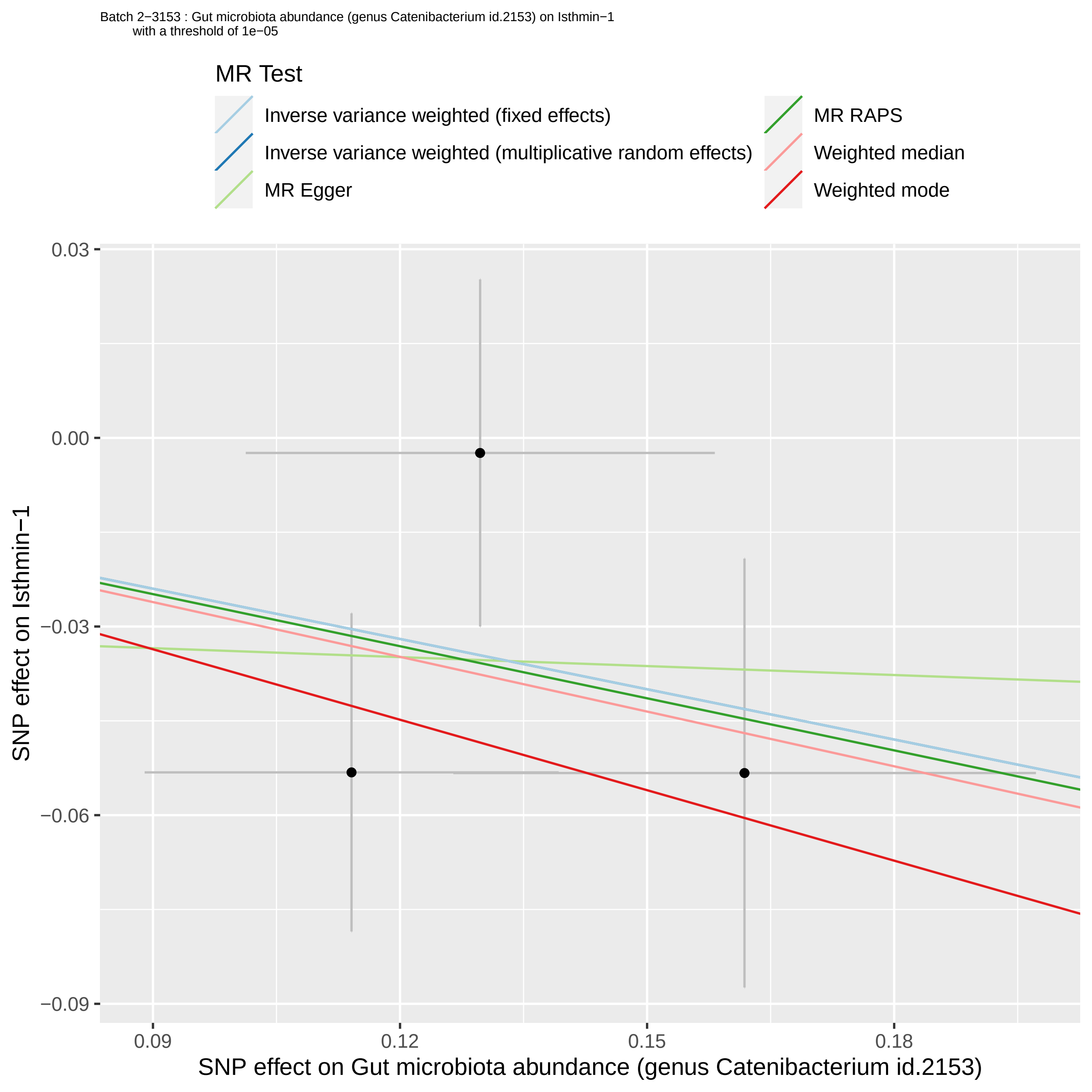
Batch 2−3153 : Gut microbiota abundance (genus Catenibacterium id.2153) on Isthmin−1
with a threshold of 1e−05
MR Test
Inverse variance weighted (fixed effects)
Inverse variance weighted (multiplicative random effects)
MR Egger
MR RAPS
Weighted median
Weighted mode
0.03
0.00
−0.03
−0.06
−0.09
0.09
0.12
0.15
0.18
SNP effect on Isthmin−1
SNP effect on Gut microbiota abundance (genus Catenibacterium id.2153)

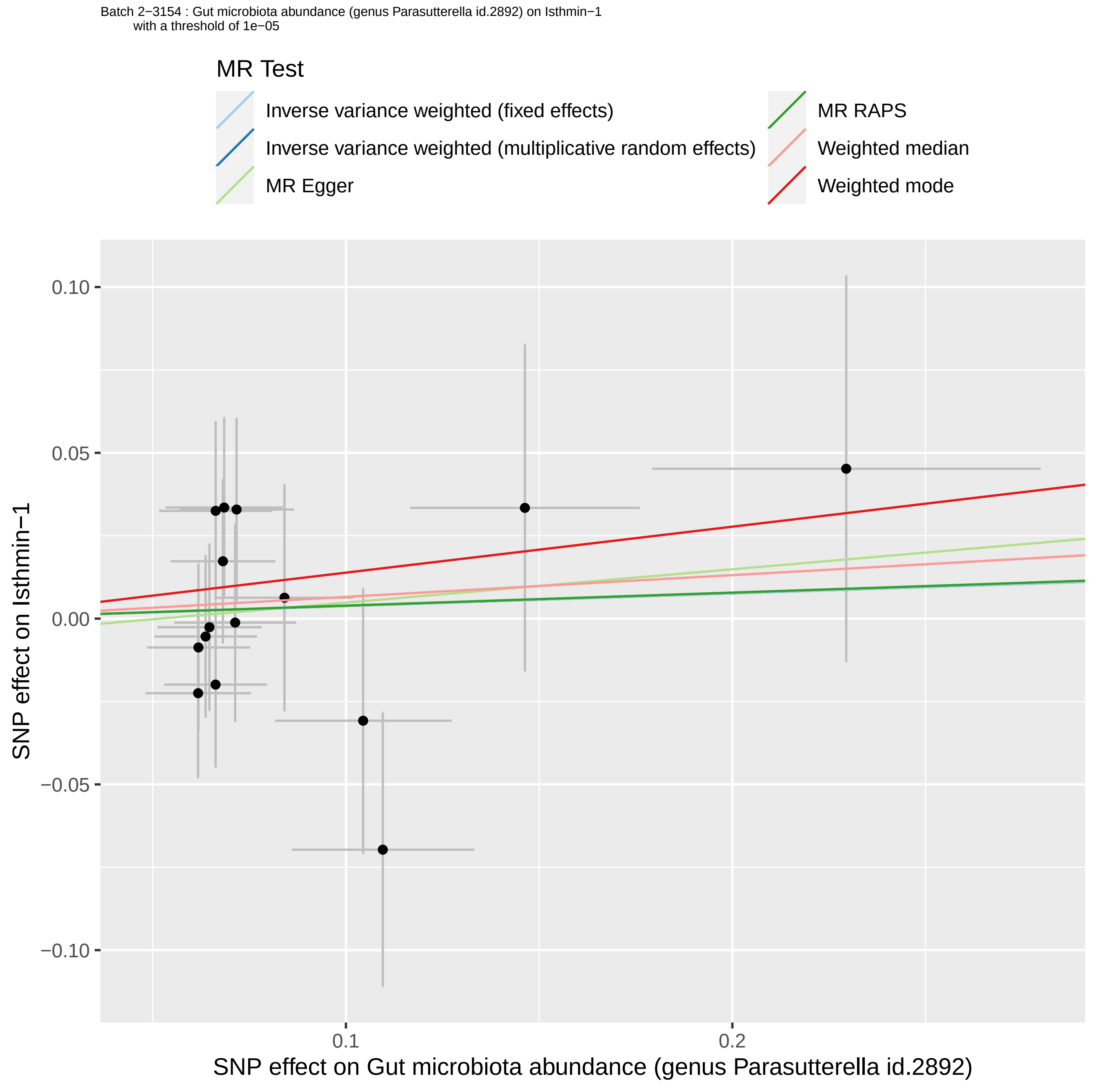
Batch 2−3154 : Gut microbiota abundance (genus Parasutterella id.2892) on Isthmin−1
with a threshold of 1e−05
MR Test
Inverse variance weighted (fixed effects)
Inverse variance weighted (multiplicative random effects)
MR Egger
MR RAPS
Weighted median
Weighted mode
0.10
0.05
0.00
−0.05
−0.10
0.1
0.2
SNP effect on Isthmin−1
SNP effect on Gut microbiota abundance (genus Parasutterella id.2892)

Batch 2−3155 : Gut microbiota abundance (genus Catenibacterium id.2153) on Isochorismatase domain−containing protein 1 with a threshold of 1e−05

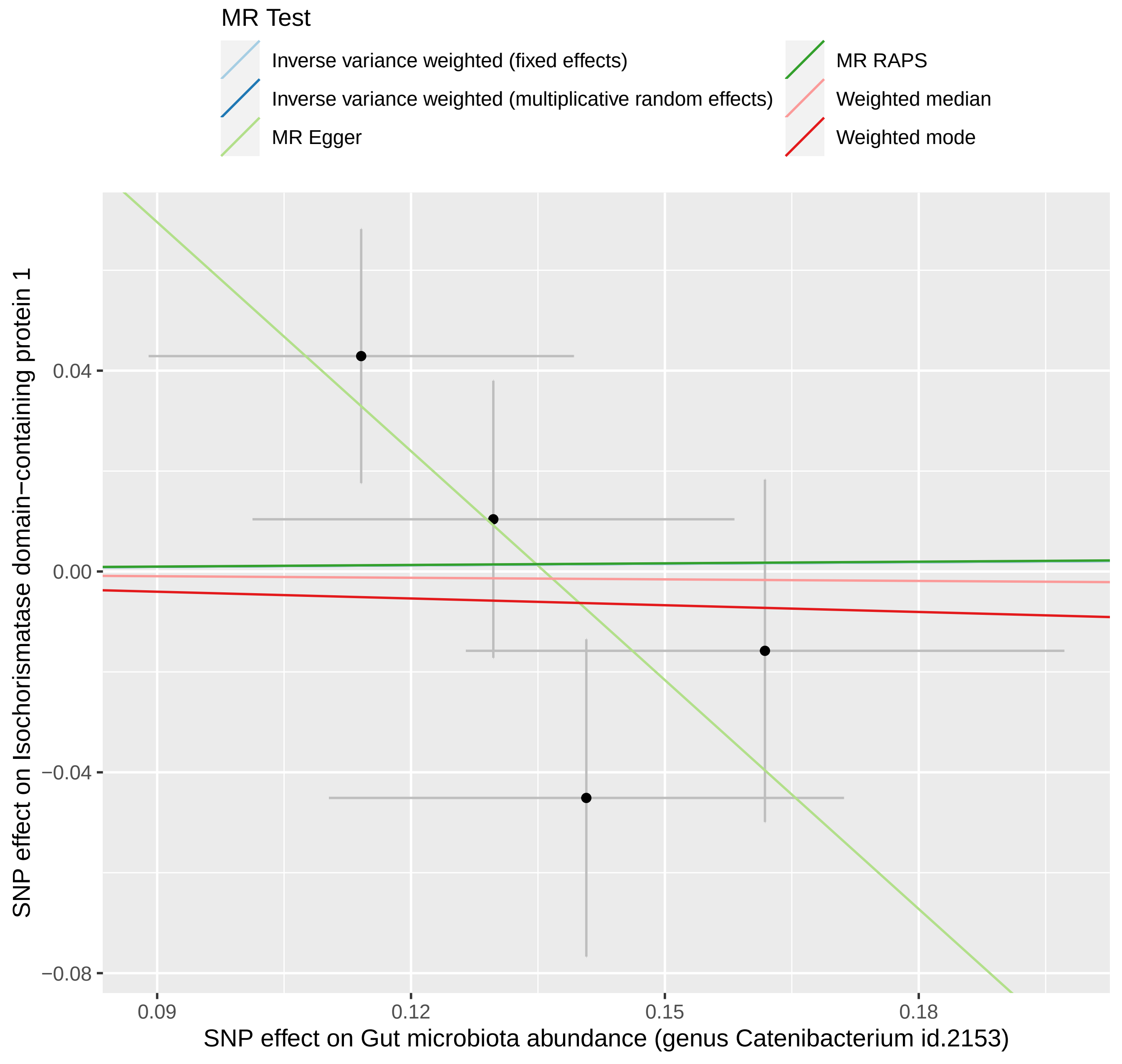

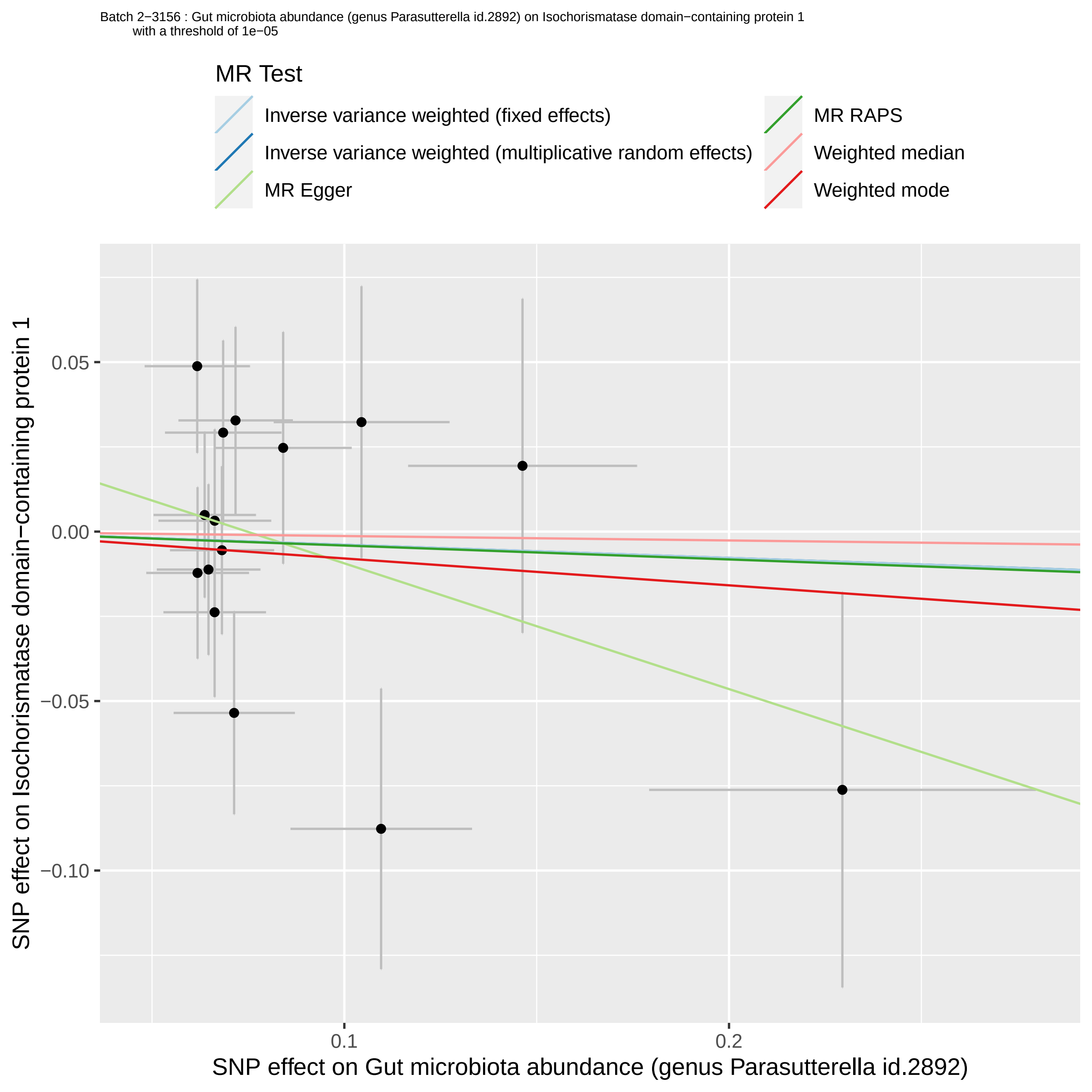
Batch 2−3156 : Gut microbiota abundance (genus Parasutterella id.2892) on Isochorismatase domain−containing protein 1
with a threshold of 1e−05
MR Test
Inverse variance weighted (fixed effects)
Inverse variance weighted (multiplicative random effects)
MR Egger
MR RAPS
Weighted median
Weighted mode
0.05
0.00
−0.05
−0.10
0.1
0.2
SNP effect on Isochorismatase domain−containing protein 1
SNP effect on Gut microbiota abundance (genus Parasutterella id.2892)

Batch 2−3157 : Gut microbiota abundance (genus Catenibacterium id.2153) on IST1 homolog
with a threshold of 1e−05

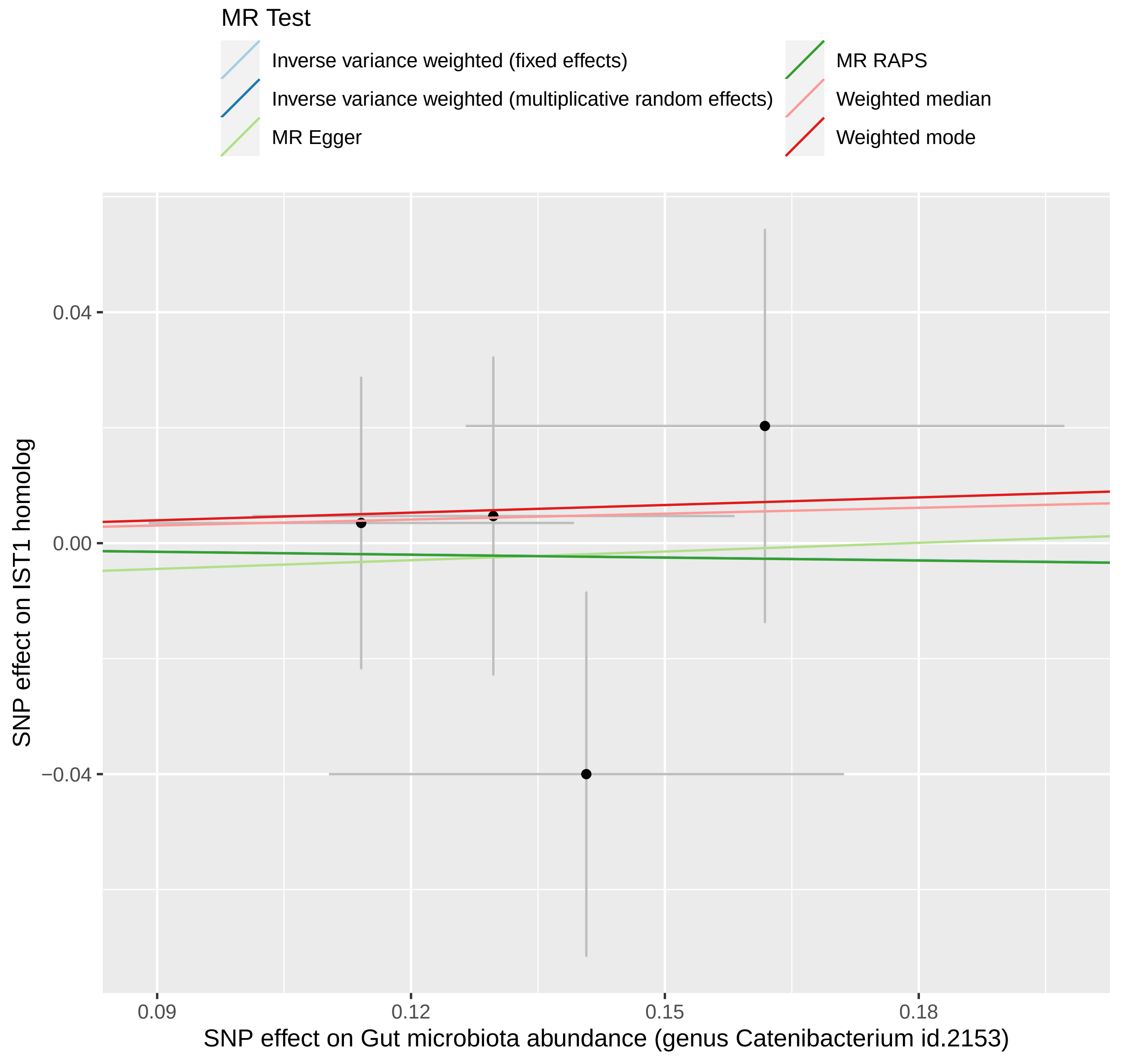

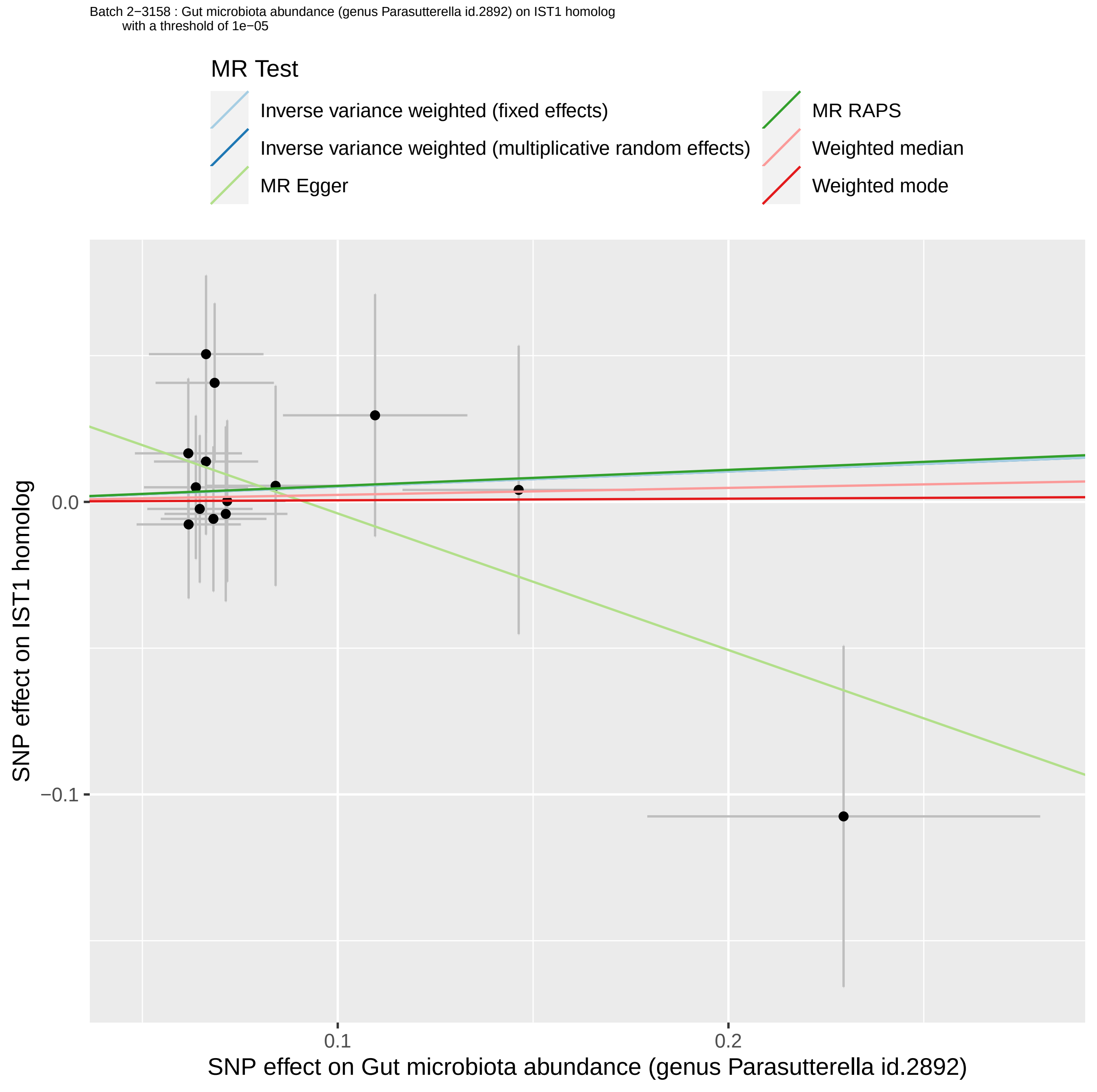
Batch 2−3158 : Gut microbiota abundance (genus Parasutterella id.2892) on IST1 homolog
with a threshold of 1e−05
MR Test
Inverse variance weighted (fixed effects)
Inverse variance weighted (multiplicative random effects)
MR Egger
MR RAPS
Weighted median
Weighted mode
0.0
−0.1
0.1
0.2
SNP effect on IST1 homolog
SNP effect on Gut microbiota abundance (genus Parasutterella id.2892)

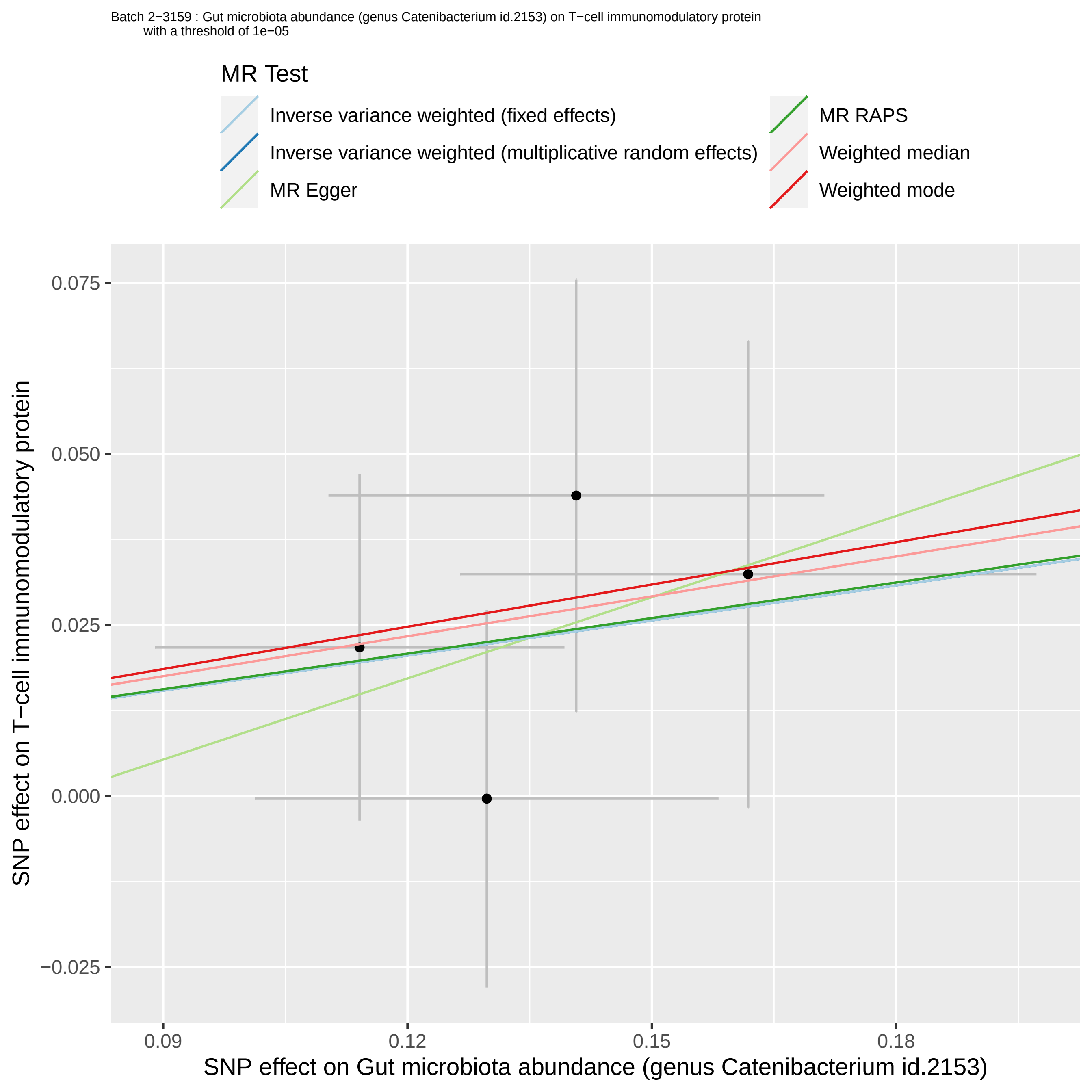
Batch 2−3159 : Gut microbiota abundance (genus Catenibacterium id.2153) on T−cell immunomodulatory protein
with a threshold of 1e−05
MR Test
Inverse variance weighted (fixed effects)
Inverse variance weighted (multiplicative random effects)
MR Egger
MR RAPS
Weighted median
Weighted mode
0.075
0.050
0.025
0.000
−0.025
0.09
0.12
0.15
0.18
SNP effect on T−cell immunomodulatory protein
SNP effect on Gut microbiota abundance (genus Catenibacterium id.2153)

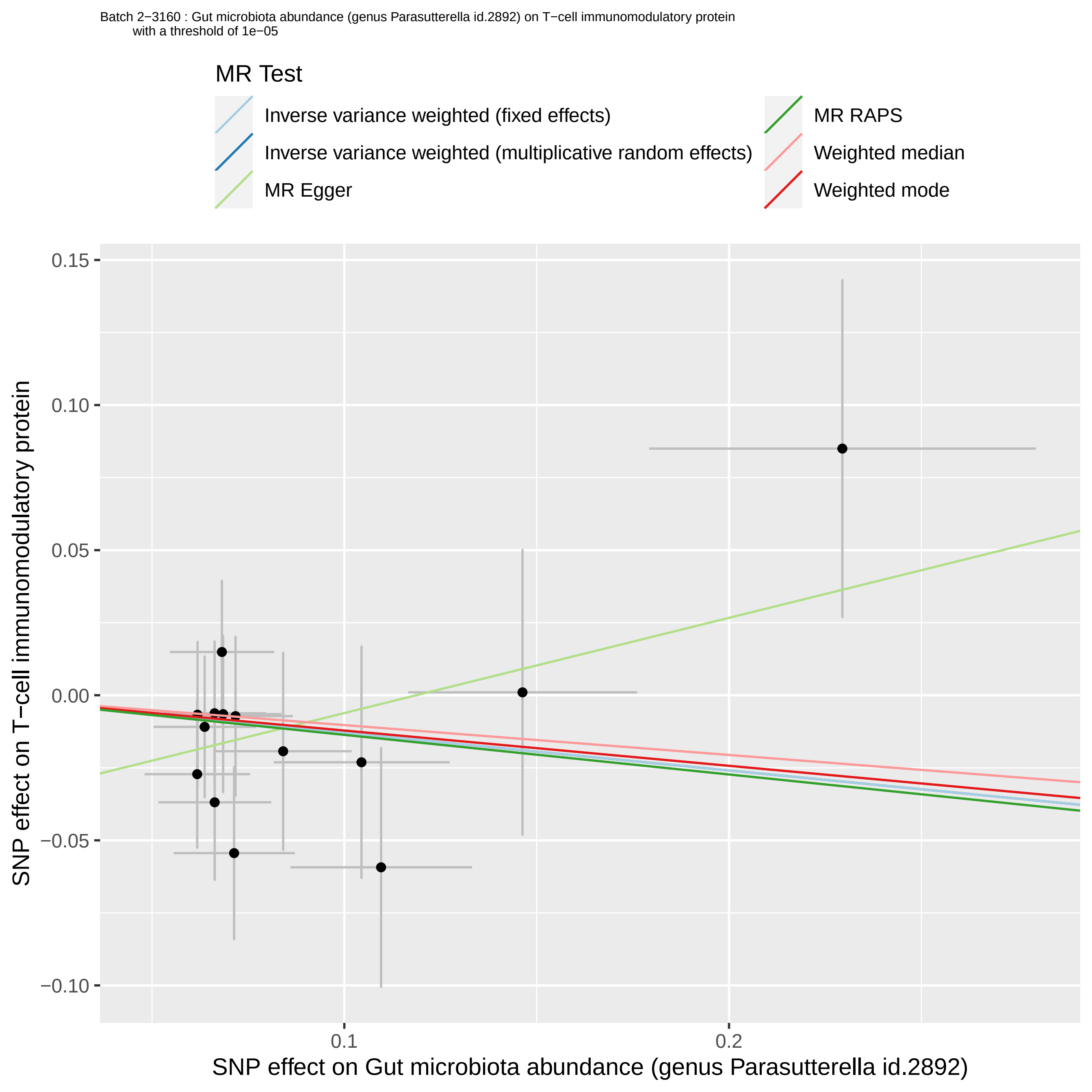

Batch 2−3160 : Gut microbiota abundance (genus Parasutterella id.2892) on T−cell immunomodulatory protein
with a threshold of 1e−05
MR Test
Inverse variance weighted (fixed effects)
Inverse variance weighted (multiplicative random effects)
MR Egger
MR RAPS
Weighted median
Weighted mode
0.15
0.10
0.05
0.00
−0.05
−0.10
0.1
0.2
SNP effect on T−cell immunomodulatory protein
SNP effect on Gut microbiota abundance (genus Parasutterella id.2892)

Batch 2−3161 : Gut microbiota abundance (genus Catenibacterium id.2153) on Integrin alpha−I: beta−1 complex
with a threshold of 1e−05

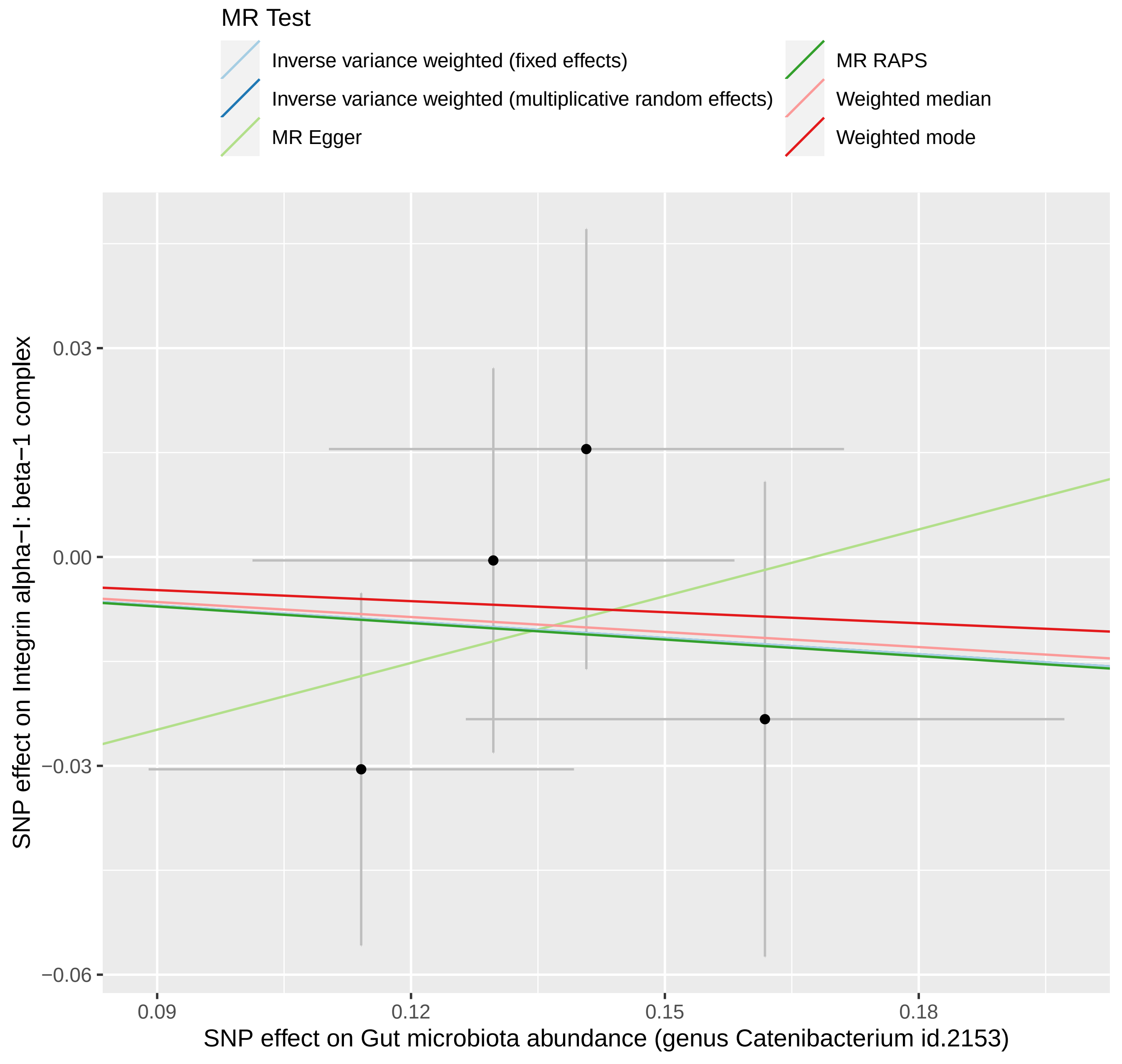

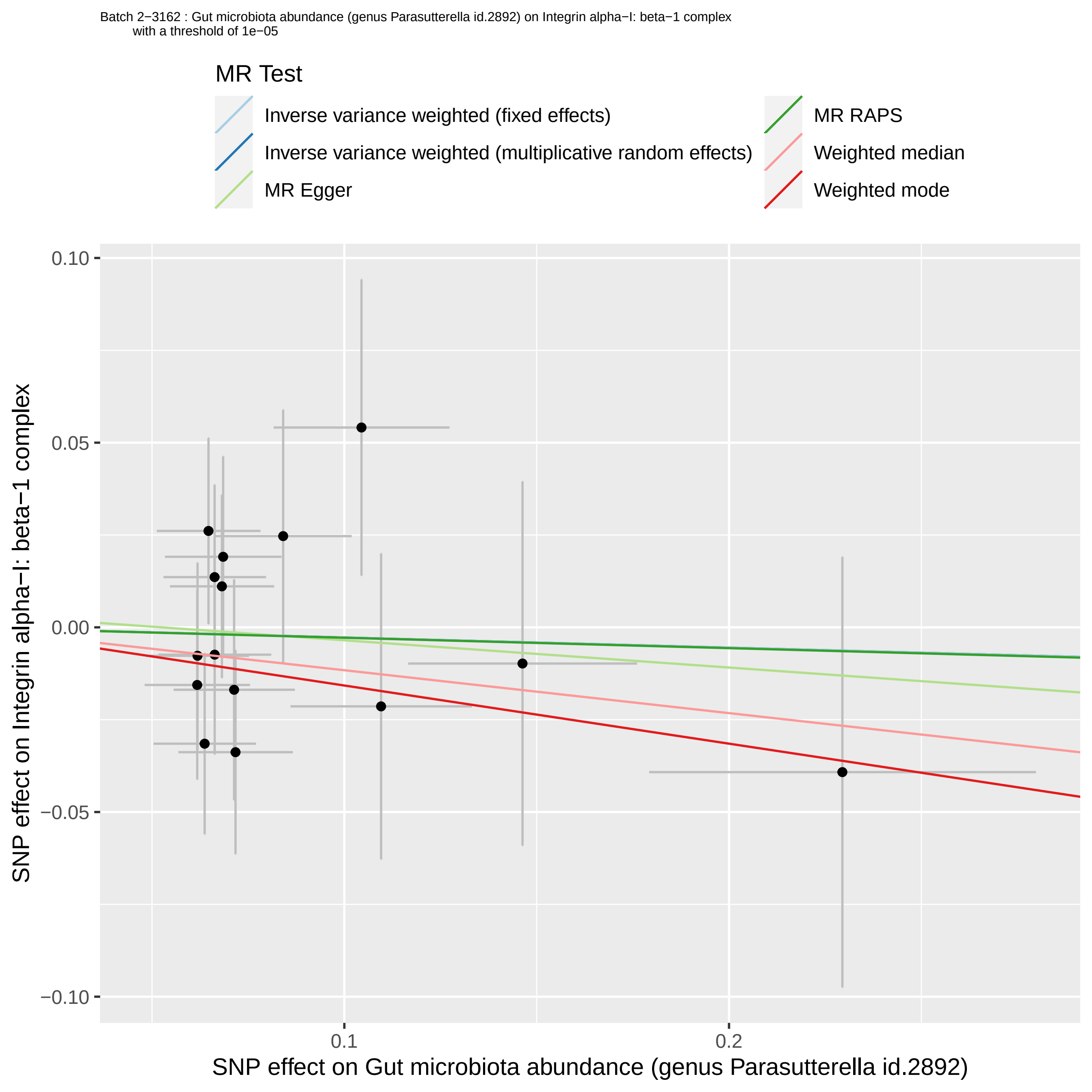

Batch 2−3162 : Gut microbiota abundance (genus Parasutterella id.2892) on Integrin alpha−I: beta−1 complex
with a threshold of 1e−05
MR Test
Inverse variance weighted (fixed effects)
Inverse variance weighted (multiplicative random effects)
MR Egger
MR RAPS
Weighted median
Weighted mode
0.10
0.05
0.00
−0.05
−0.10
0.1
0.2
SNP effect on Integrin alpha−I: beta−1 complex
SNP effect on Gut microbiota abundance (genus Parasutterella id.2892)

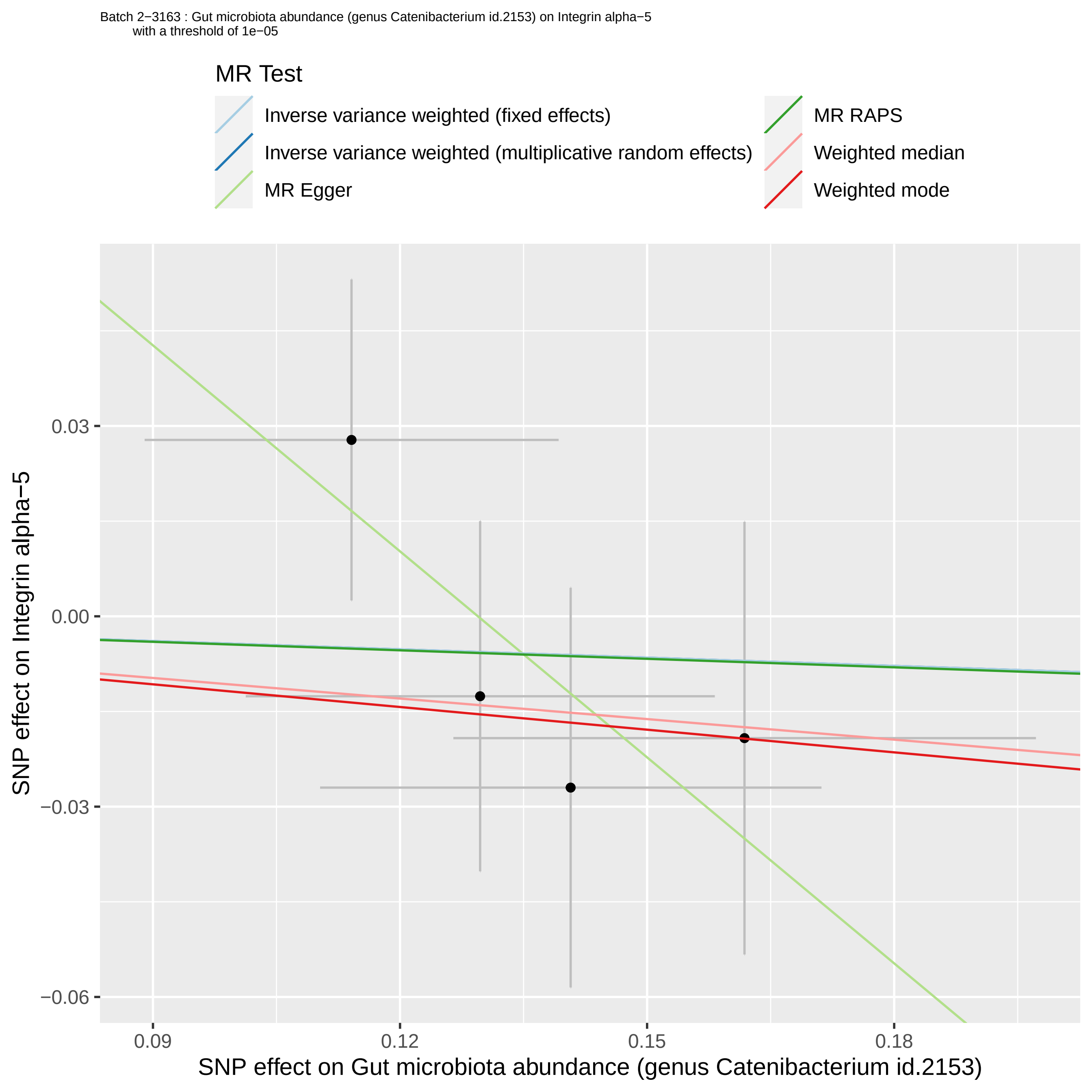
Batch 2−3163 : Gut microbiota abundance (genus Catenibacterium id.2153) on Integrin alpha−5
with a threshold of 1e−05
MR Test
Inverse variance weighted (fixed effects)
Inverse variance weighted (multiplicative random effects)
MR Egger
MR RAPS
Weighted median
Weighted mode
0.03
0.00
−0.03
−0.06
0.09
0.12
0.15
0.18
SNP effect on Integrin alpha−5
SNP effect on Gut microbiota abundance (genus Catenibacterium id.2153)

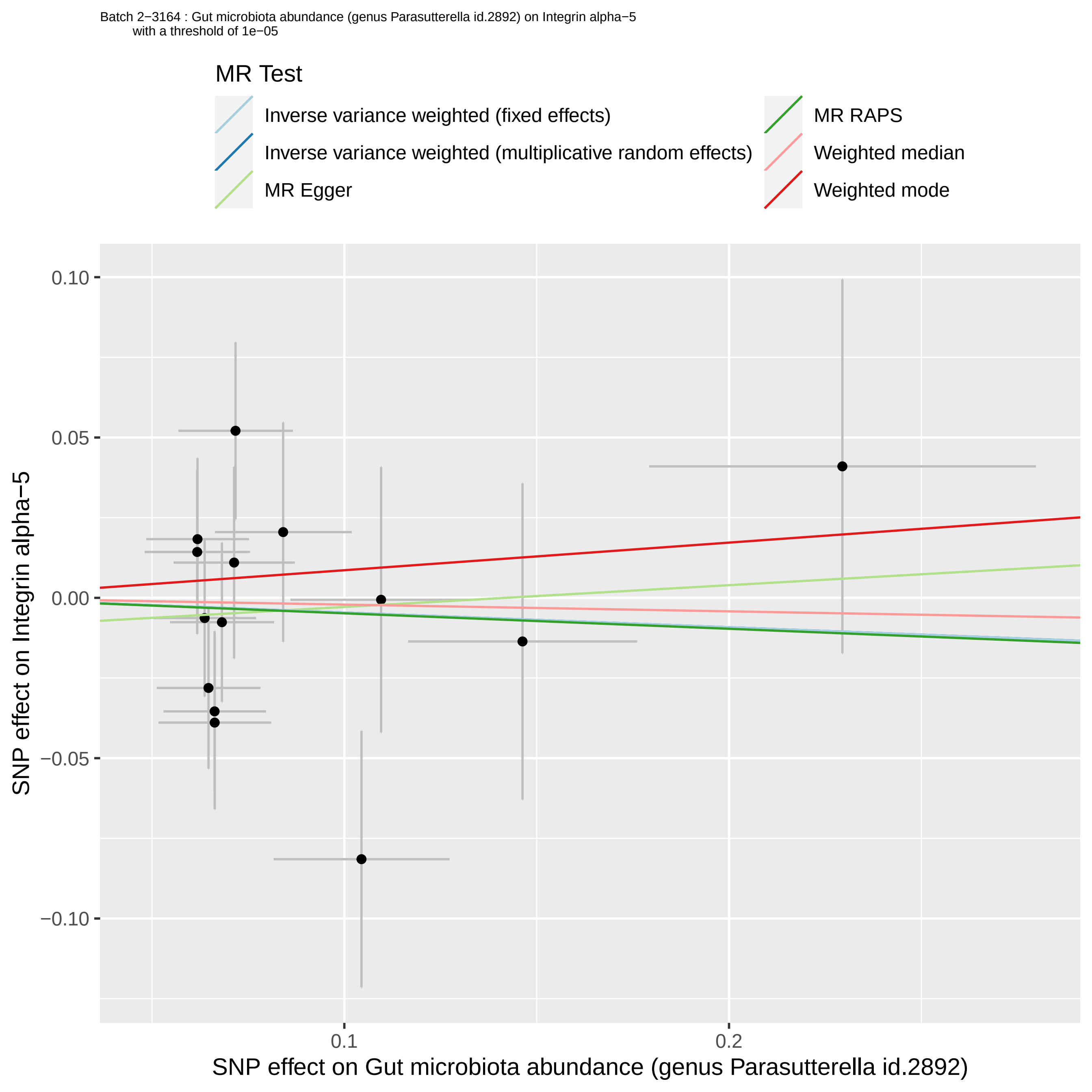

Batch 2−3164 : Gut microbiota abundance (genus Parasutterella id.2892) on Integrin alpha−5
with a threshold of 1e−05
MR Test
Inverse variance weighted (fixed effects)
Inverse variance weighted (multiplicative random effects)
MR Egger
MR RAPS
Weighted median
Weighted mode
0.10
0.05
0.00
−0.05
−0.10
0.1
0.2
SNP effect on Integrin alpha−5
SNP effect on Gut microbiota abundance (genus Parasutterella id.2892)

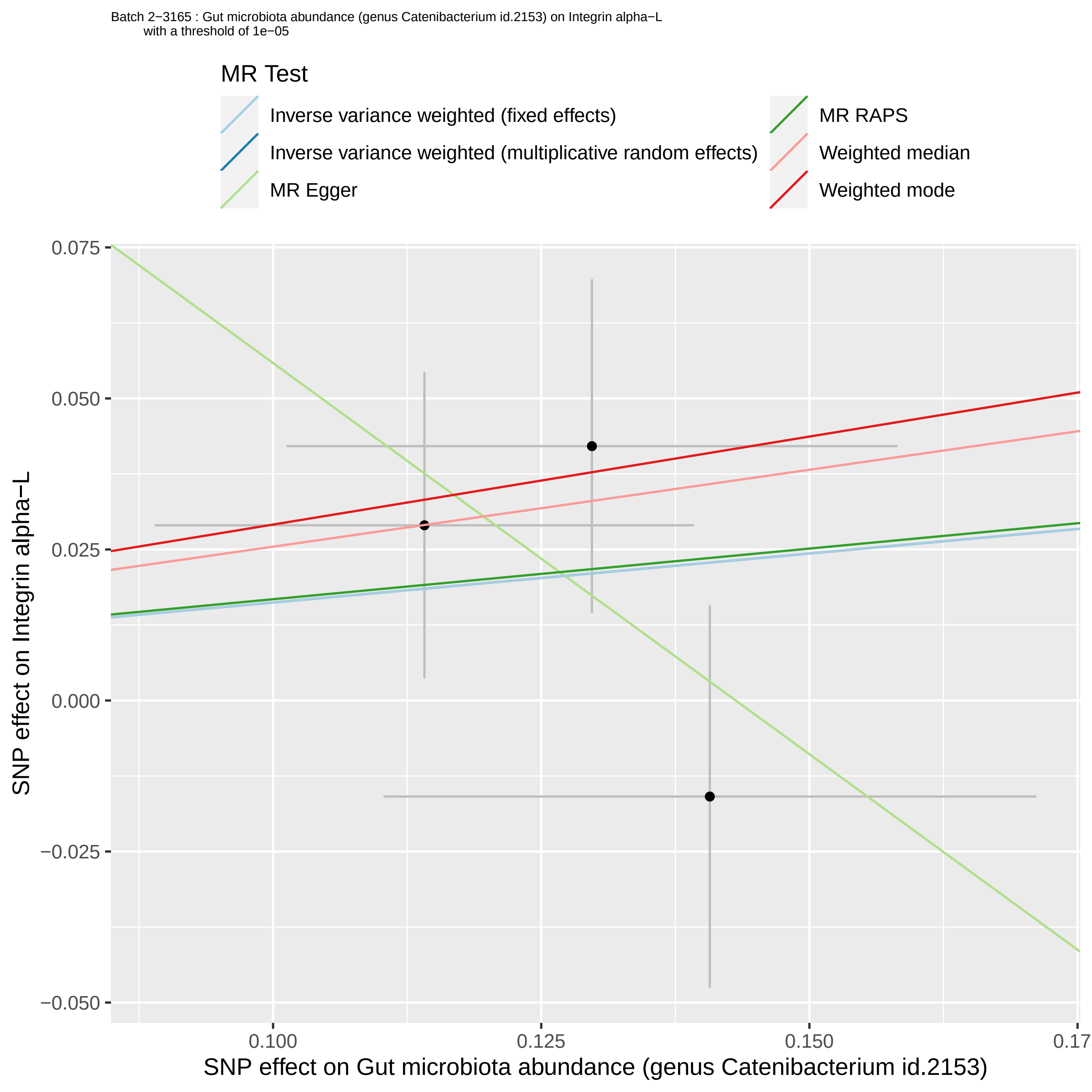
Batch 2−3165 : Gut microbiota abundance (genus Catenibacterium id.2153) on Integrin alpha−L
with a threshold of 1e−05
MR Test
Inverse variance weighted (fixed effects)
Inverse variance weighted (multiplicative random effects)
MR Egger
MR RAPS
Weighted median
Weighted mode
0.075
0.050
0.025
0.000
−0.025
−0.050
0.100
0.125
0.150
0.17
SNP effect on Integrin alpha−L
SNP effect on Gut microbiota abundance (genus Catenibacterium id.2153)

Batch 2−3166 : Gut microbiota abundance (genus Parasutterella id.2892) on Integrin alpha−L
with a threshold of 1e−05

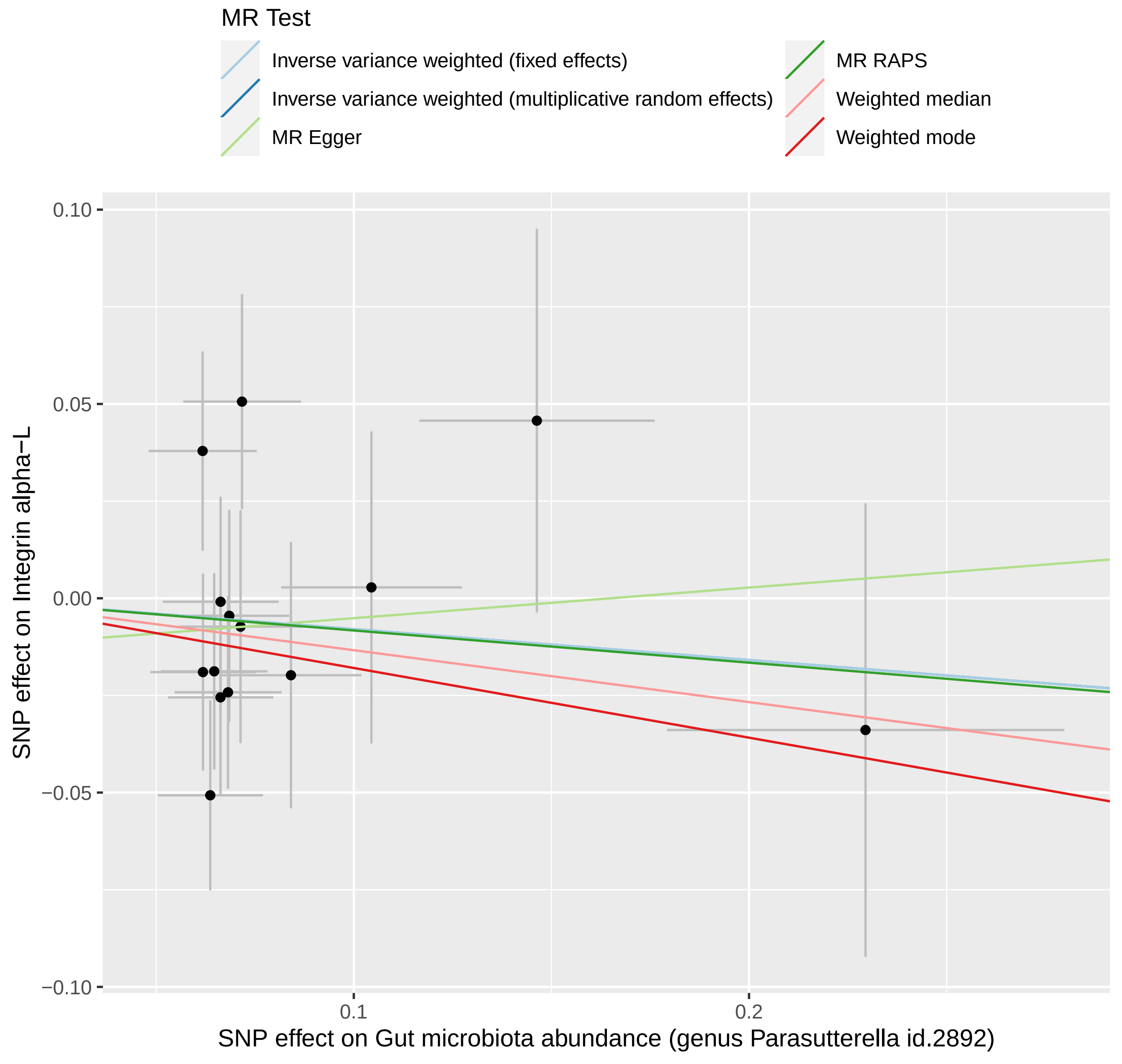

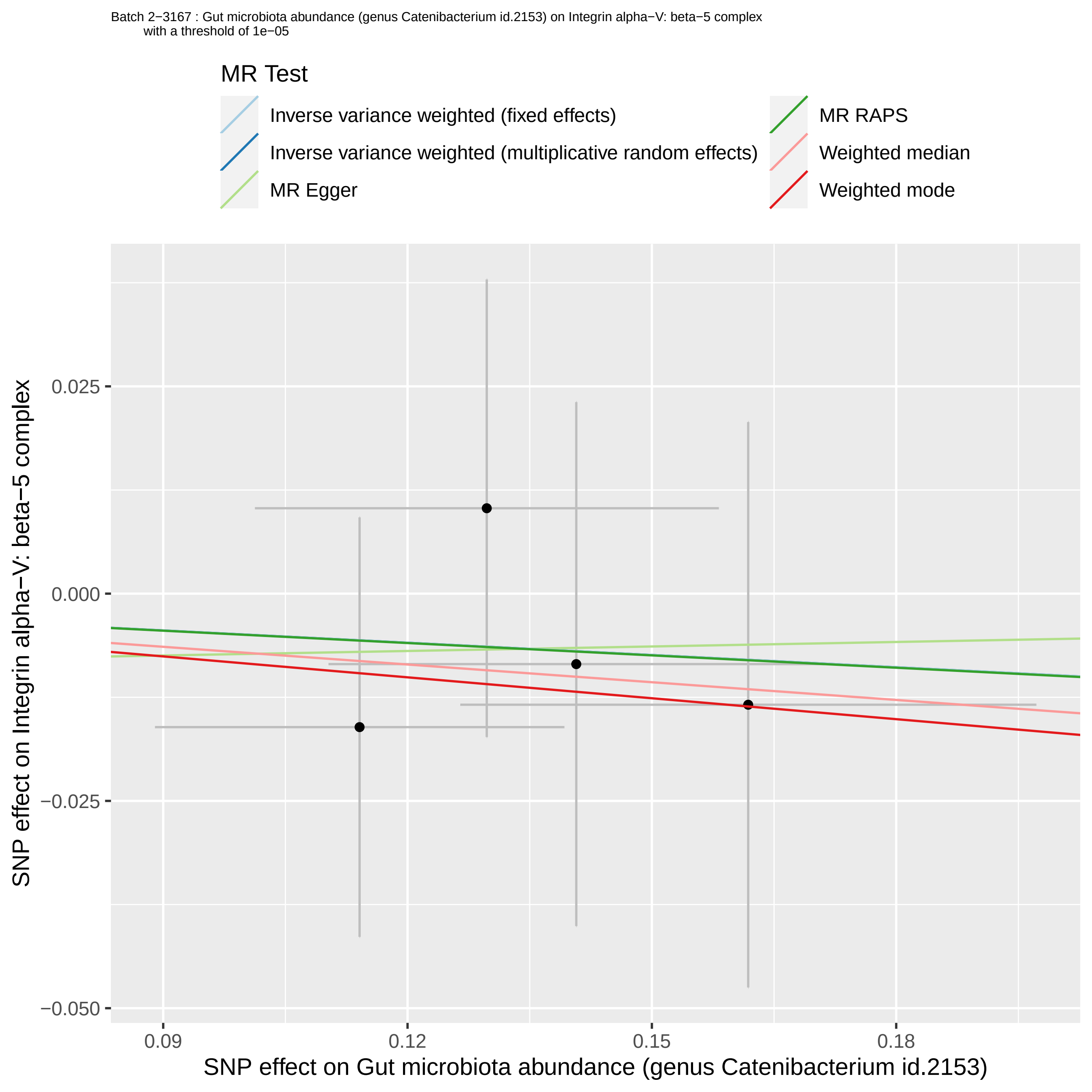
Batch 2−3167 : Gut microbiota abundance (genus Catenibacterium id.2153) on Integrin alpha−V: beta−5 complex
with a threshold of 1e−05
MR Test
Inverse variance weighted (fixed effects)
Inverse variance weighted (multiplicative random effects)
MR Egger
MR RAPS
Weighted median
Weighted mode
0.025
0.000
−0.025
−0.050
0.09
0.12
0.15
0.18
SNP effect on Integrin alpha−V: beta−5 complex
SNP effect on Gut microbiota abundance (genus Catenibacterium id.2153)

Batch 2−3168 : Gut microbiota abundance (genus Parasutterella id.2892) on Integrin alpha−V: beta−5 complex
with a threshold of 1e−05

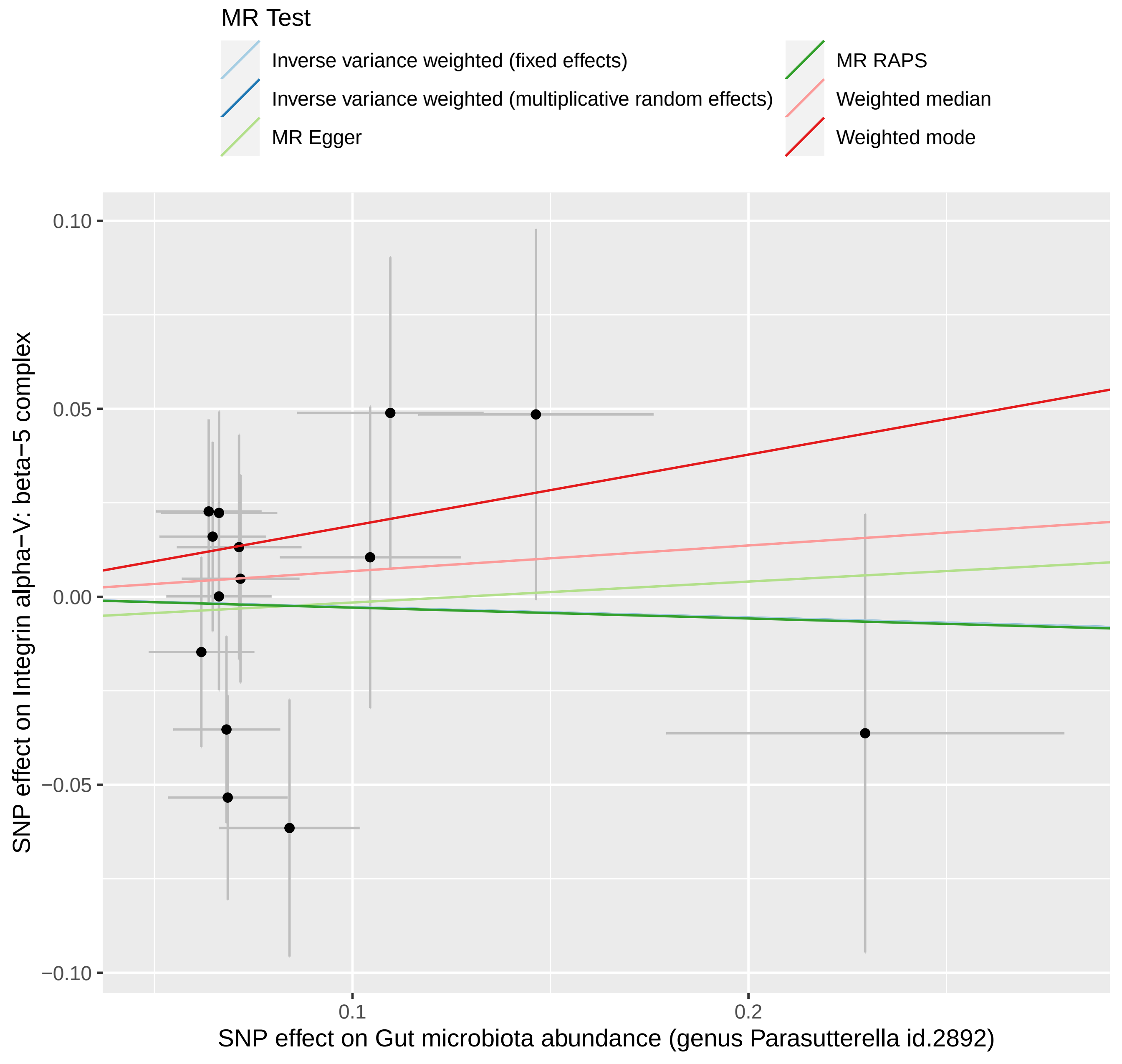

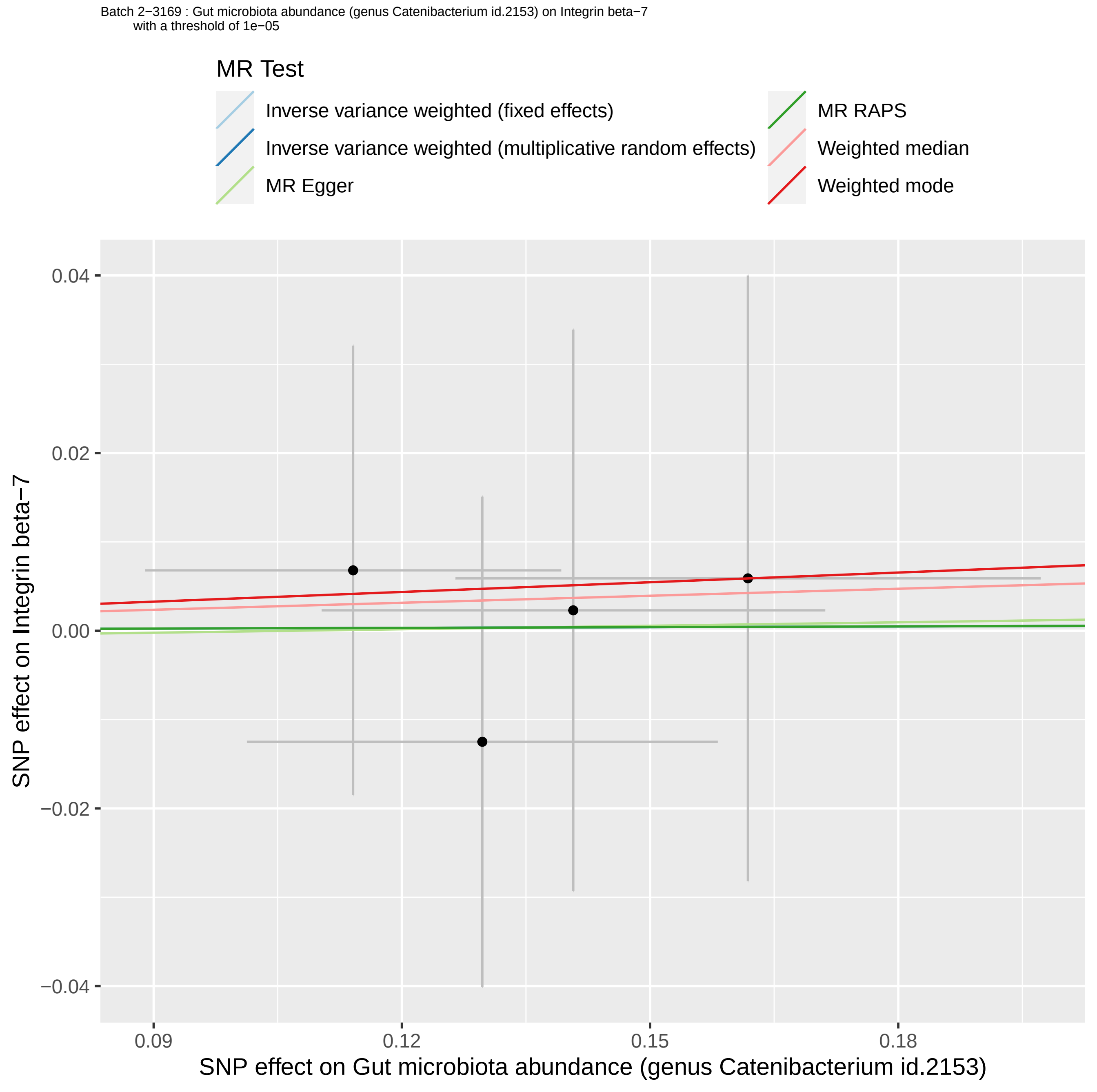
Batch 2−3169 : Gut microbiota abundance (genus Catenibacterium id.2153) on Integrin beta−7
with a threshold of 1e−05
MR Test
Inverse variance weighted (fixed effects)
Inverse variance weighted (multiplicative random effects)
MR Egger
MR RAPS
Weighted median
Weighted mode
0.04
0.02
0.00
−0.02
−0.04
SNP effect on Integrin beta−7
0.09
0.12
0.15
0.18
SNP effect on Gut microbiota abundance (genus Catenibacterium id.2153)

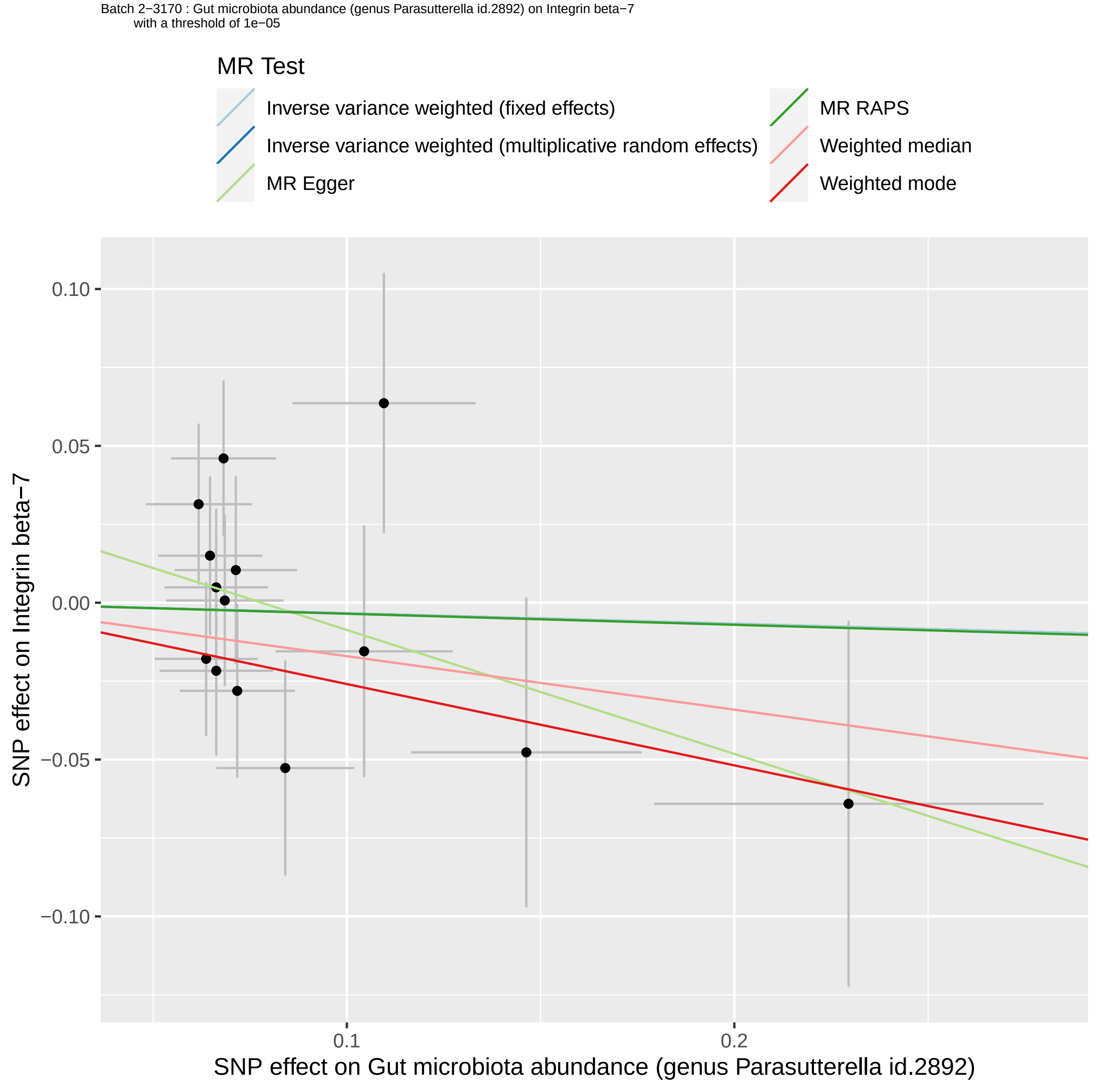
Batch 2−3170 : Gut microbiota abundance (genus Parasutterella id.2892) on Integrin beta−7
with a threshold of 1e−05
MR Test
Inverse variance weighted (fixed effects)
Inverse variance weighted (multiplicative random effects)
MR Egger
MR RAPS
Weighted median
Weighted mode
0.10
0.05
0.00
−0.05
−0.10
SNP effect on Integrin beta−7
0.1
0.2
SNP effect on Gut microbiota abundance (genus Parasutterella id.2892)

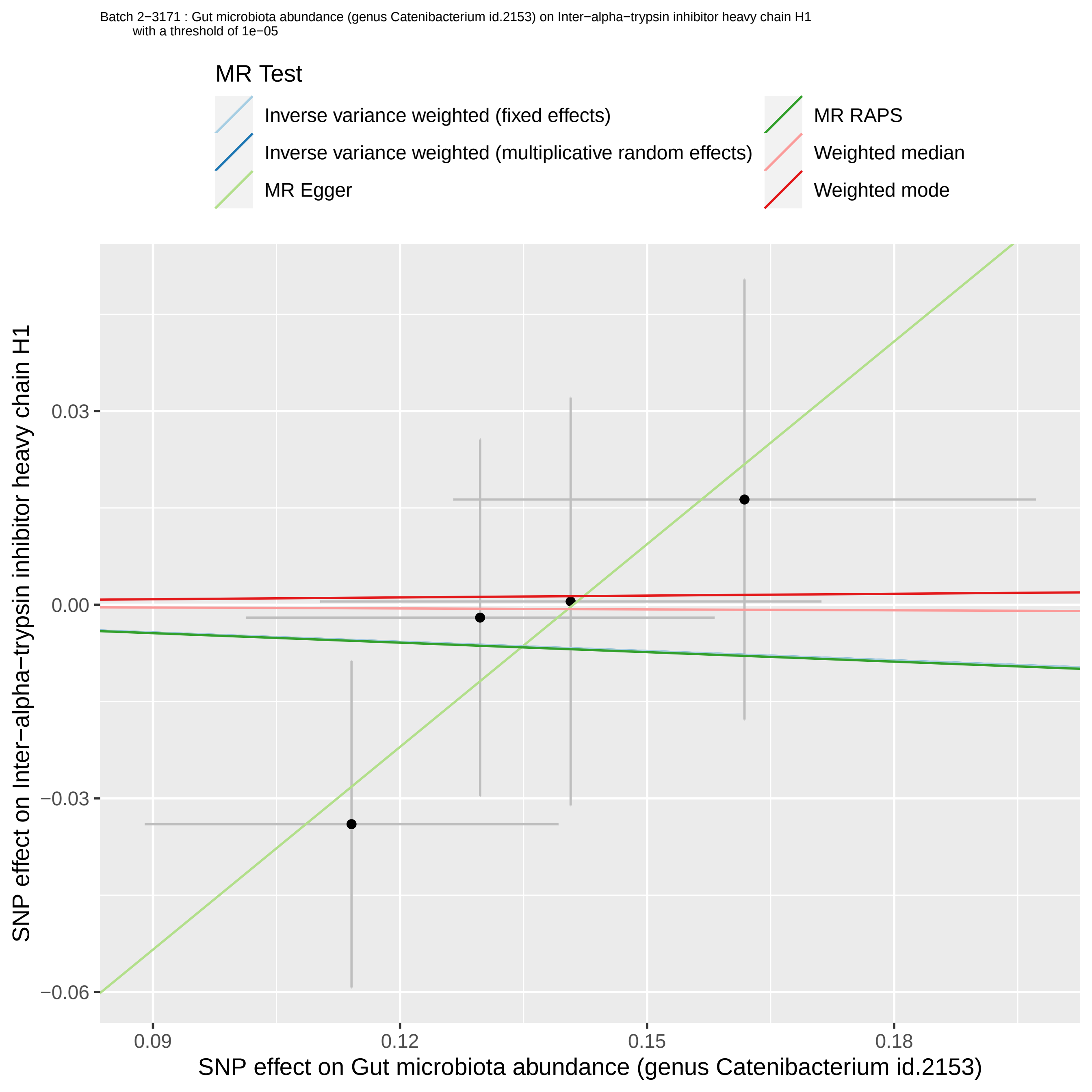

Batch 2−3171 : Gut microbiota abundance (genus Catenibacterium id.2153) on Inter−alpha−trypsin inhibitor heavy chain H1
with a threshold of 1e−05
MR Test
Inverse variance weighted (fixed effects)
Inverse variance weighted (multiplicative random effects)
MR Egger
MR RAPS
Weighted median
Weighted mode
SNP effect on Inter−alpha−trypsin inhibitor heavy chain H1
0.03
0.00
−0.03
−0.06
0.09
0.12
0.15
0.18
SNP effect on Gut microbiota abundance (genus Catenibacterium id.2153)

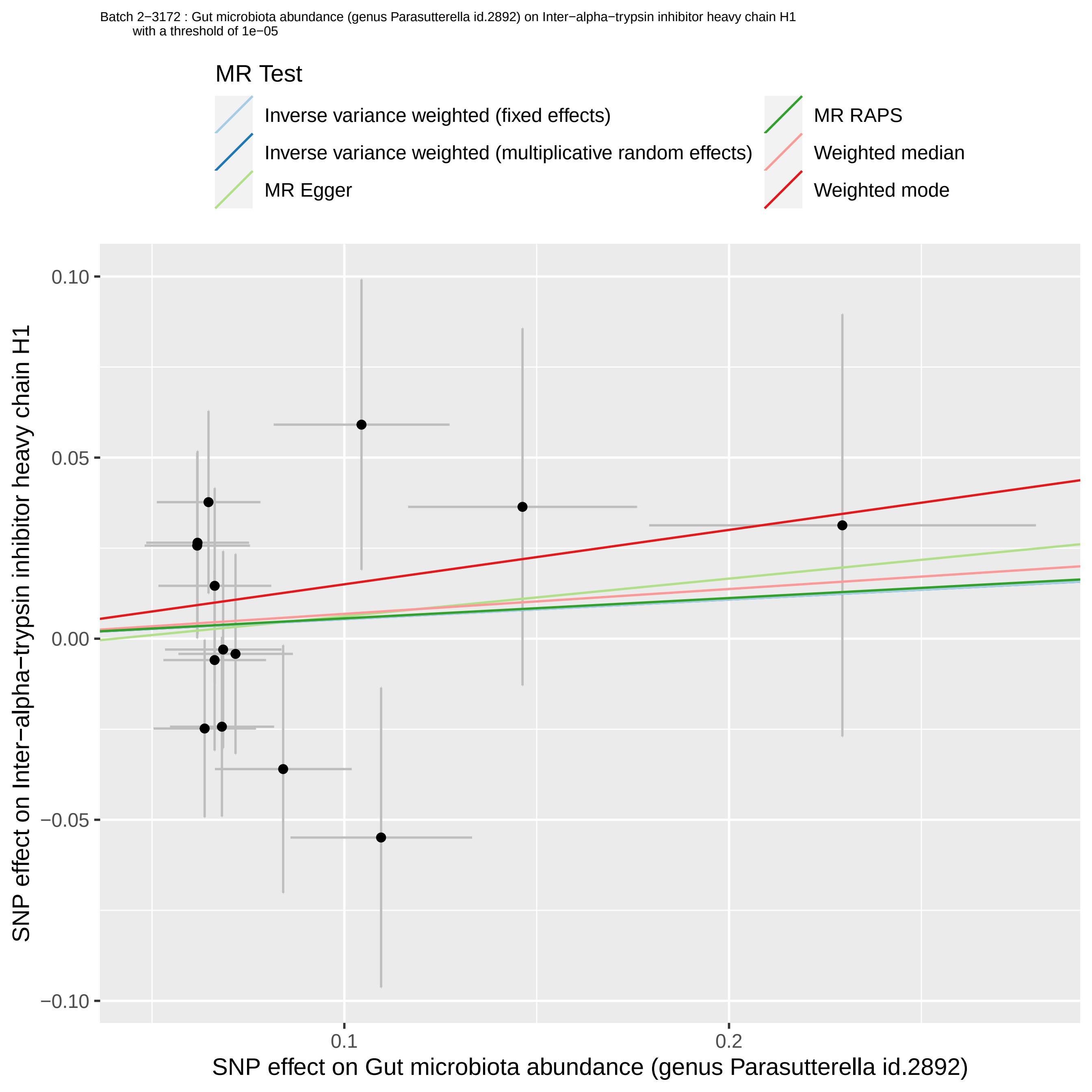
Batch 2−3172 : Gut microbiota abundance (genus Parasutterella id.2892) on Inter−alpha−trypsin inhibitor heavy chain H1
with a threshold of 1e−05
MR Test
Inverse variance weighted (fixed effects)
Inverse variance weighted (multiplicative random effects)
MR Egger
MR RAPS
Weighted median
Weighted mode
0.10
0.05
0.00
−0.05
−0.10
0.1
0.2
SNP effect on Inter−alpha−trypsin inhibitor heavy chain H1
SNP effect on Gut microbiota abundance (genus Parasutterella id.2892)

Batch 2−3173 : Gut microbiota abundance (genus Catenibacterium id.2153) on Inter−alpha−trypsin inhibitor heavy chain H5
with a threshold of 1e−05

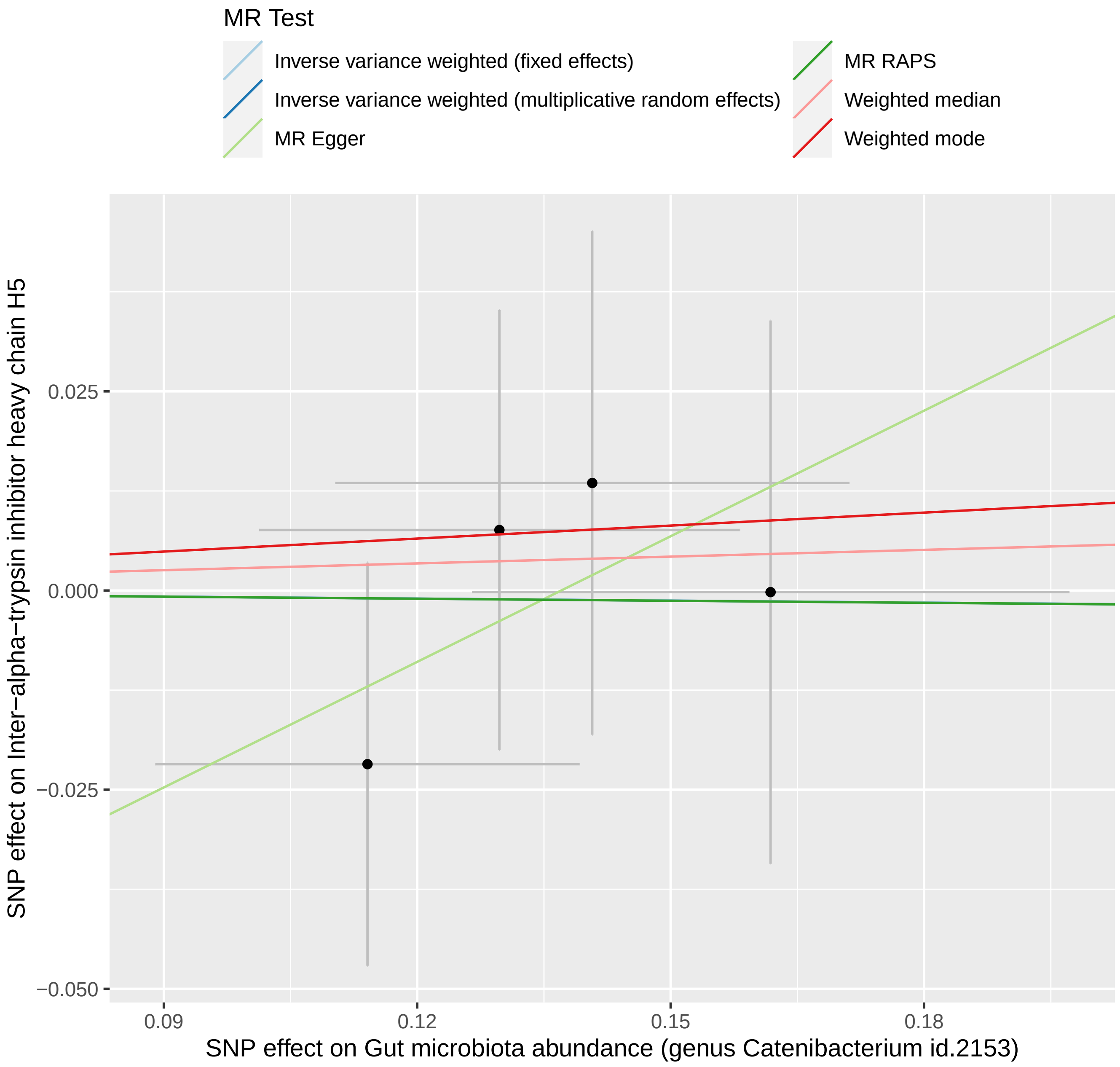

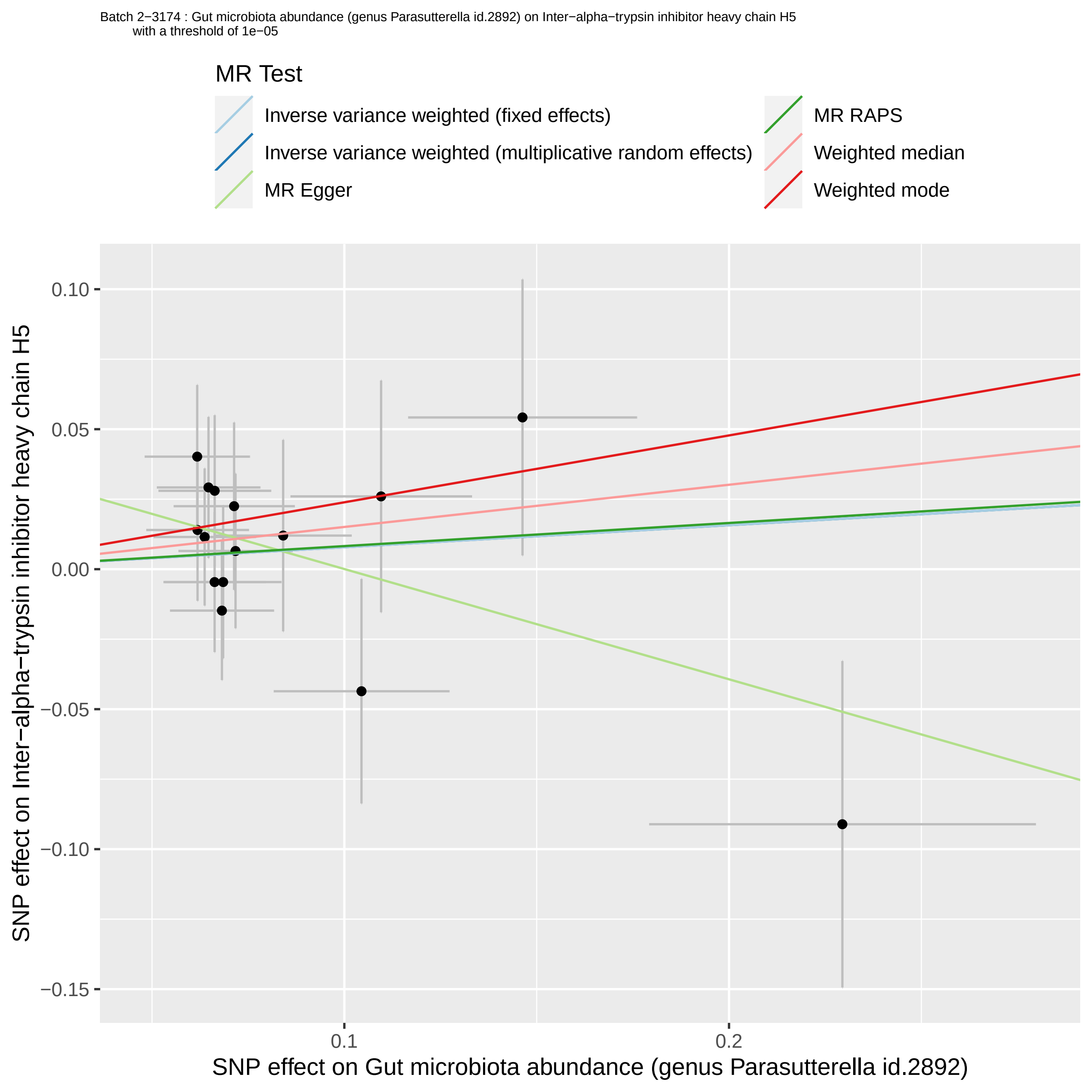
Batch 2−3174 : Gut microbiota abundance (genus Parasutterella id.2892) on Inter−alpha−trypsin inhibitor heavy chain H5
with a threshold of 1e−05
MR Test
Inverse variance weighted (fixed effects)
Inverse variance weighted (multiplicative random effects)
MR Egger
MR RAPS
Weighted median
Weighted mode
0.10
0.05
0.00
−0.05
−0.10
−0.15
0.1
0.2
SNP effect on Inter−alpha−trypsin inhibitor heavy chain H5
SNP effect on Gut microbiota abundance (genus Parasutterella id.2892)

Batch 2−3175 : Gut microbiota abundance (genus Catenibacterium id.2153) on Integral membrane protein 2A
with a threshold of 1e−05

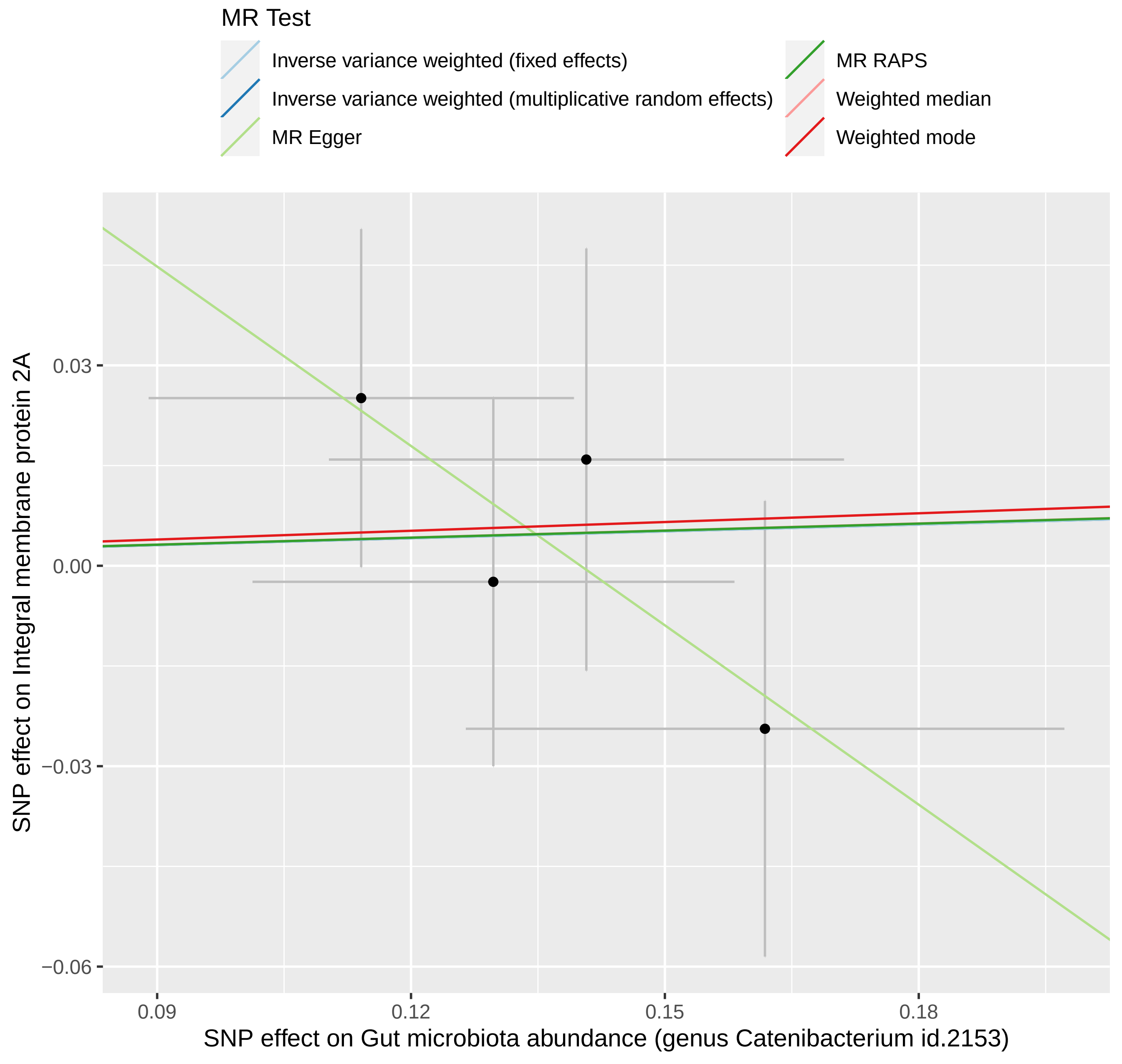

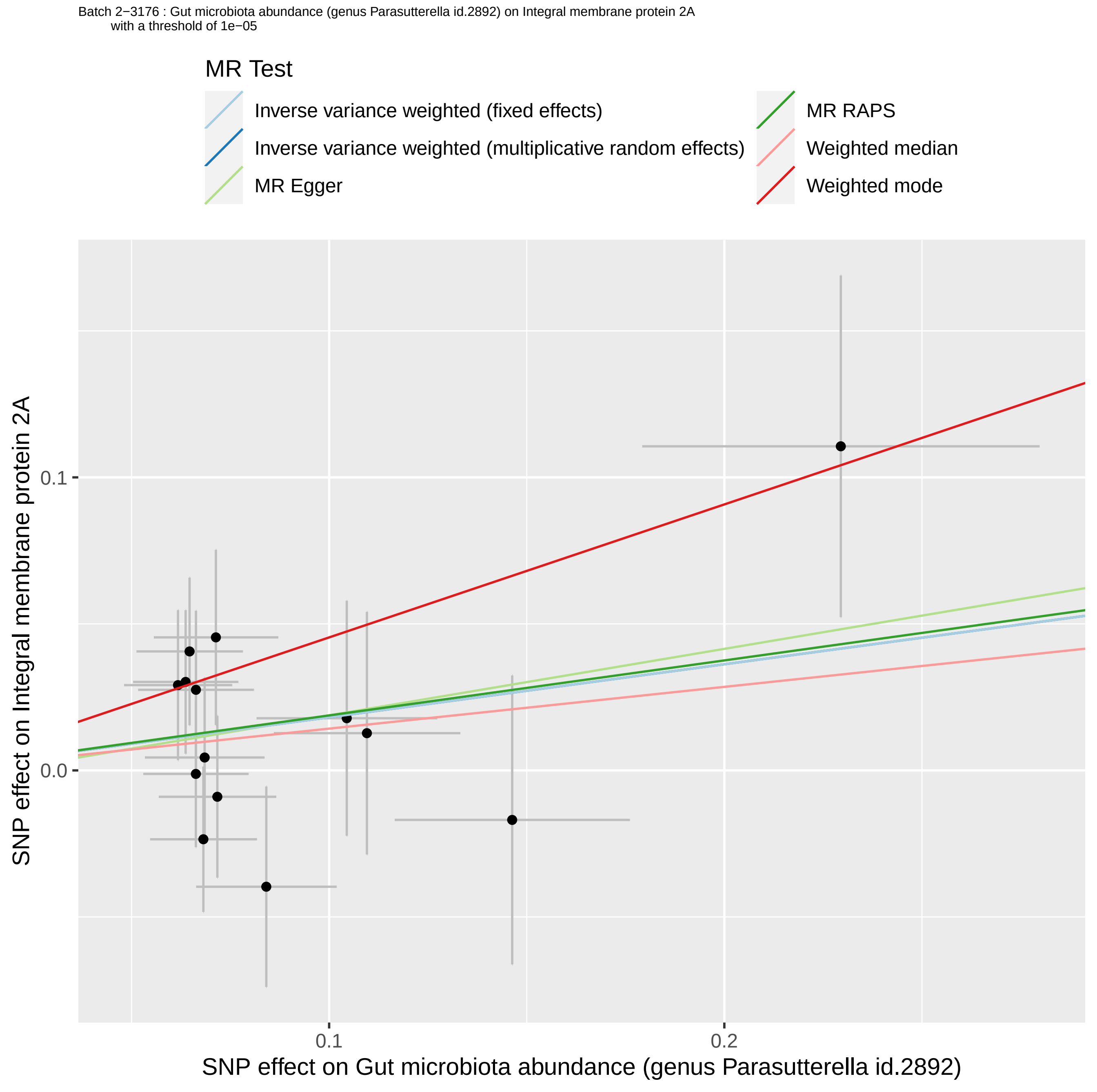

Batch 2−3176 : Gut microbiota abundance (genus Parasutterella id.2892) on Integral membrane protein 2A
with a threshold of 1e−05
MR Test
Inverse variance weighted (fixed effects)
Inverse variance weighted (multiplicative random effects)
MR Egger
MR RAPS
Weighted median
Weighted mode
0.1
0.0
0.1
0.2
SNP effect on Integral membrane protein 2A
SNP effect on Gut microbiota abundance (genus Parasutterella id.2892)

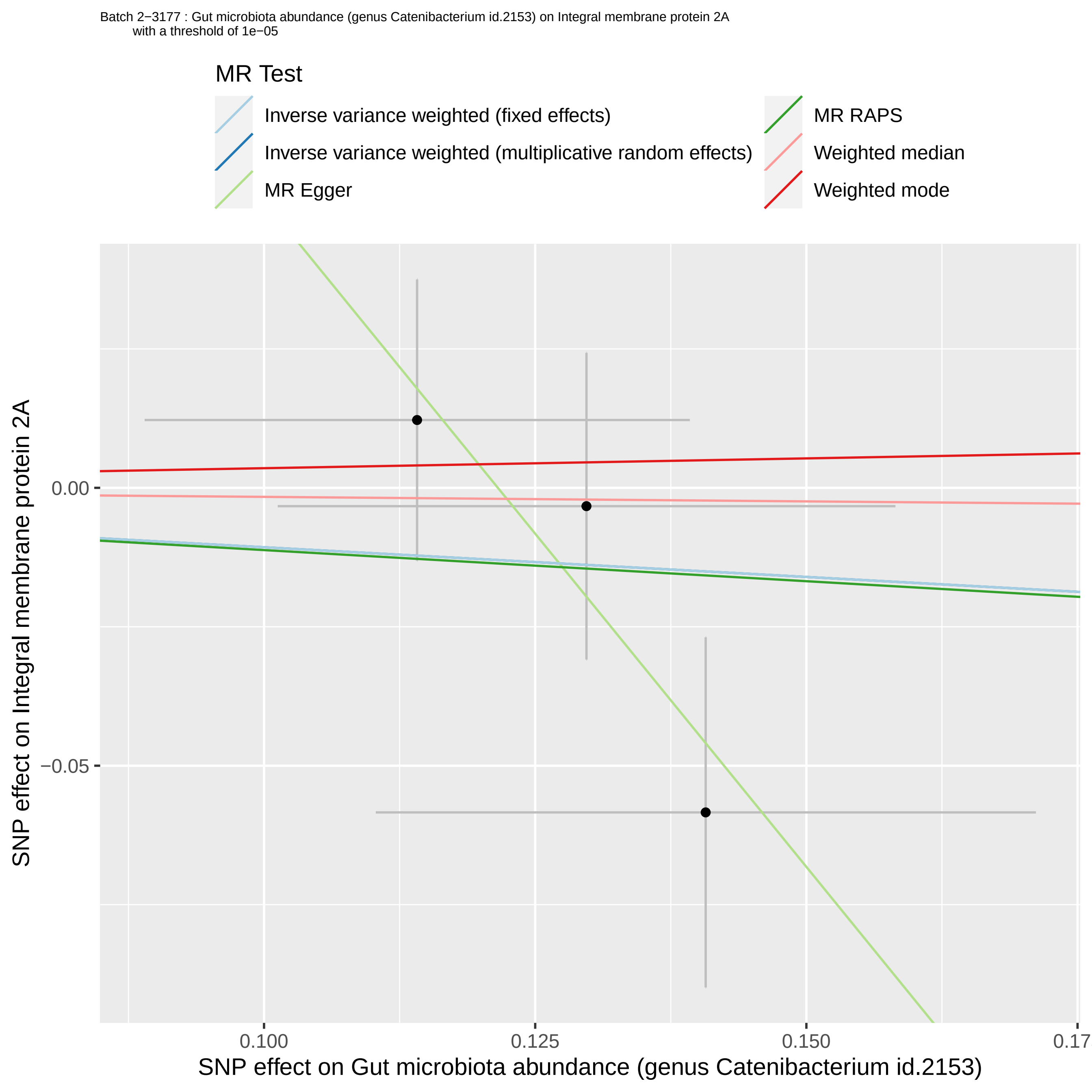

Batch 2−3177 : Gut microbiota abundance (genus Catenibacterium id.2153) on Integral membrane protein 2A
with a threshold of 1e−05
MR Test
Inverse variance weighted (fixed effects)
Inverse variance weighted (multiplicative random effects)
MR Egger
MR RAPS
Weighted median
Weighted mode
0.00
−0.05
0.100
0.125
0.150
0.17
SNP effect on Gut microbiota abundance (genus Catenibacterium id.2153)
SNP effect on Integral membrane protein 2A

Batch 2−3178 : Gut microbiota abundance (genus Parasutterella id.2892) on Integral membrane protein 2A
with a threshold of 1e−05

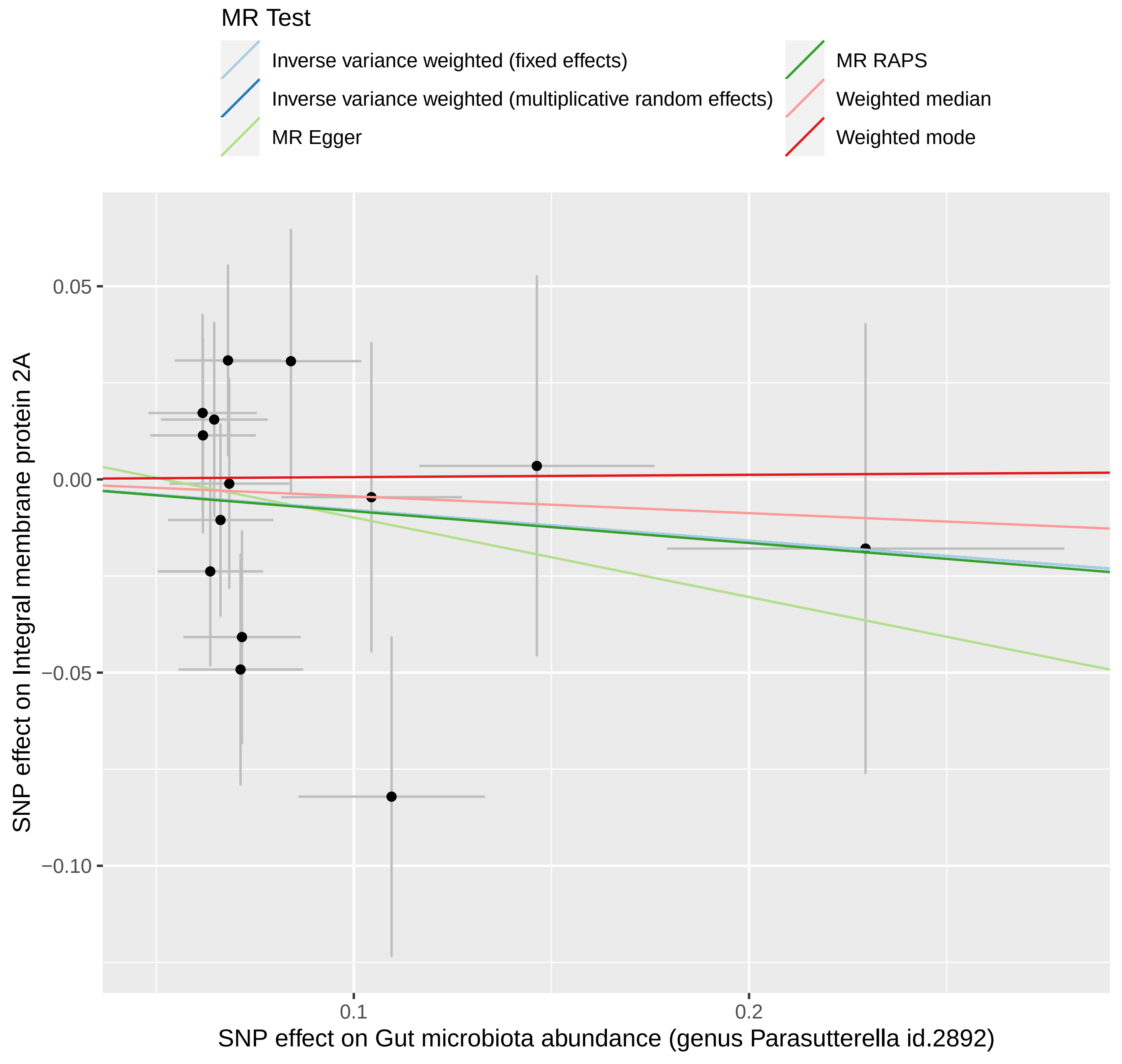

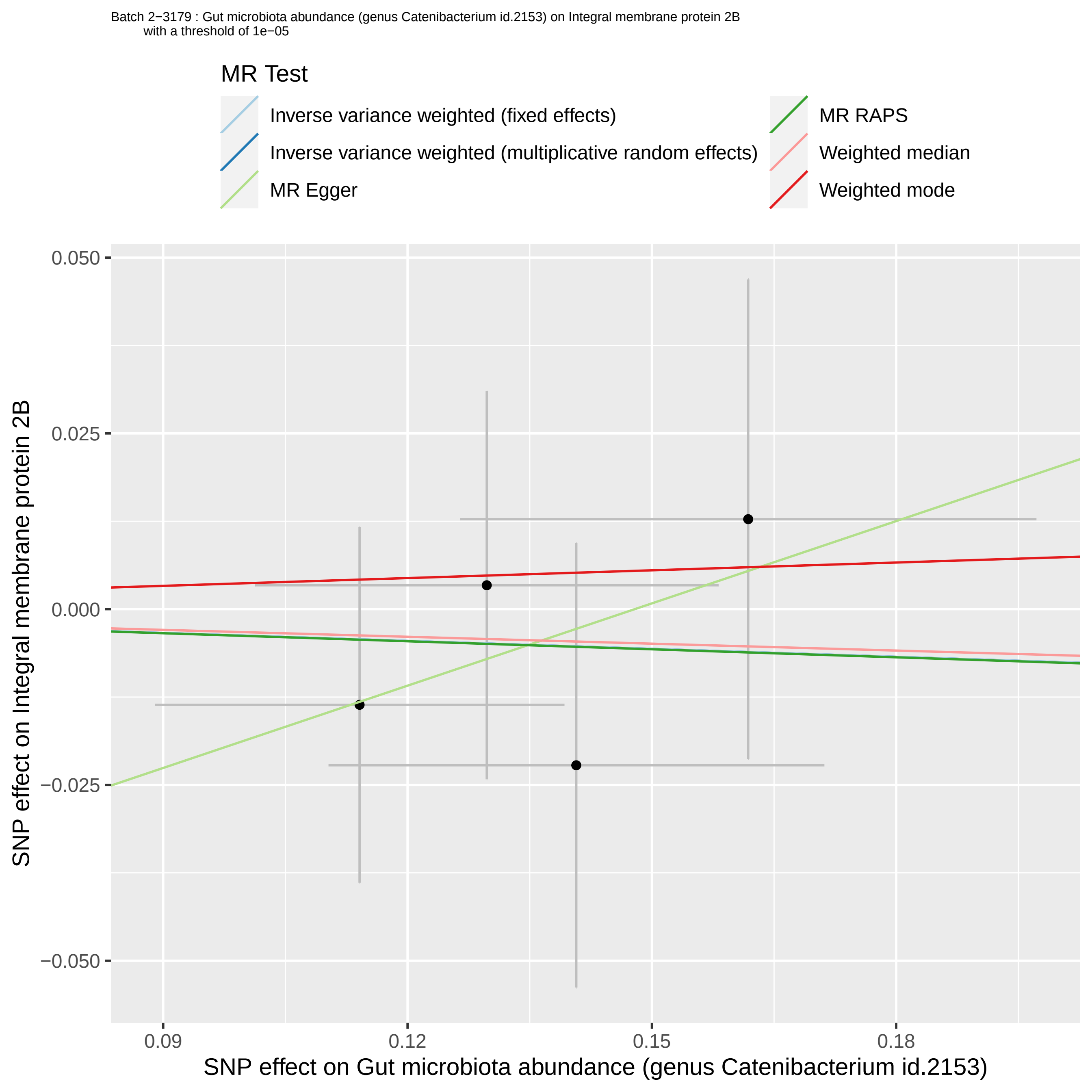
Batch 2−3179 : Gut microbiota abundance (genus Catenibacterium id.2153) on Integral membrane protein 2B
with a threshold of 1e−05
MR Test
Inverse variance weighted (fixed effects)
Inverse variance weighted (multiplicative random effects)
MR Egger
MR RAPS
Weighted median
Weighted mode
0.050
0.025
0.000
−0.025
−0.050
SNP effect on Integral membrane protein 2B
0.09
0.12
0.15
0.18
SNP effect on Gut microbiota abundance (genus Catenibacterium id.2153)

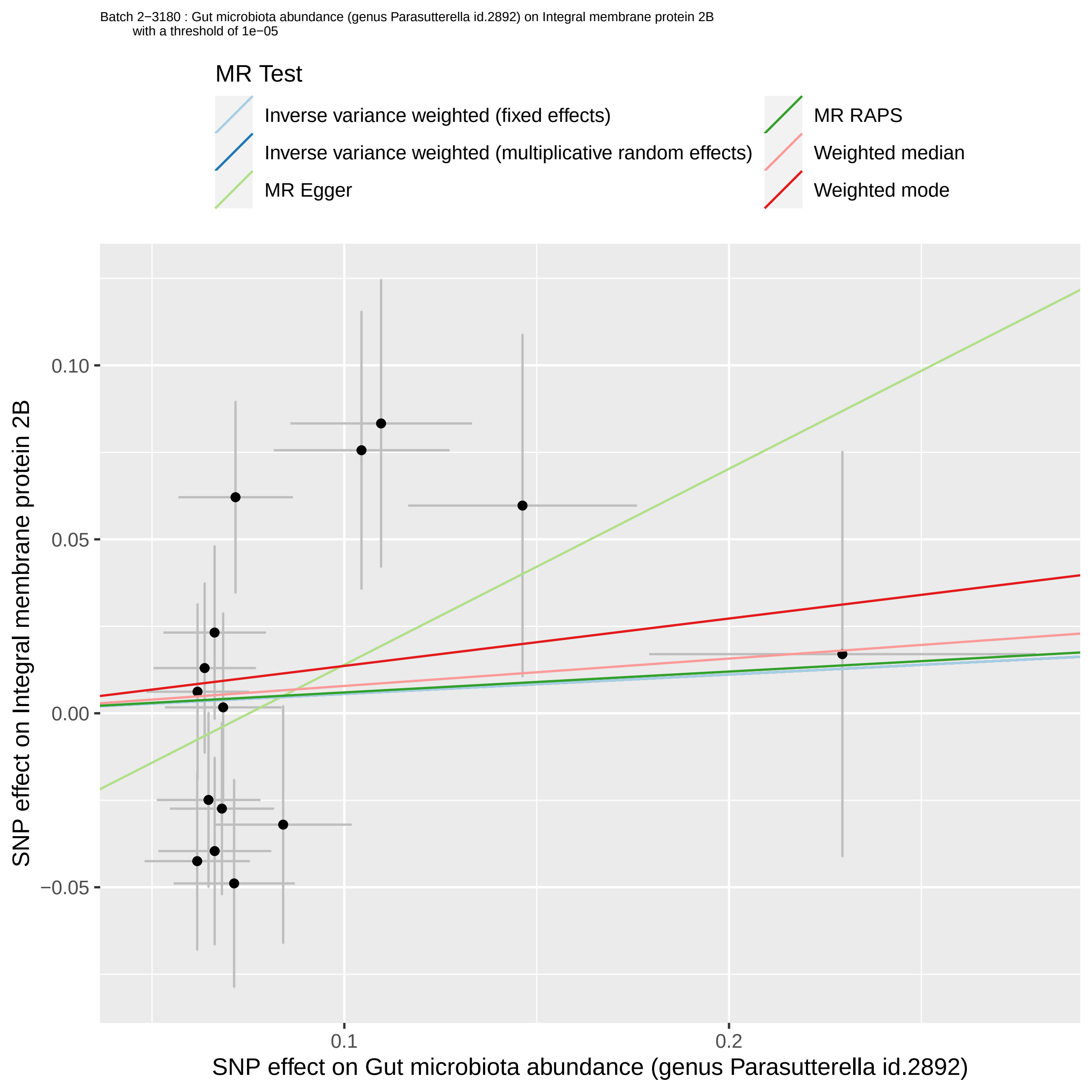
Batch 2−3180 : Gut microbiota abundance (genus Parasutterella id.2892) on Integral membrane protein 2B
with a threshold of 1e−05
MR Test
Inverse variance weighted (fixed effects)
Inverse variance weighted (multiplicative random effects)
MR Egger
MR RAPS
Weighted median
Weighted mode
0.10
0.05
0.00
−0.05
SNP effect on Integral membrane protein 2B
0.1
0.2
SNP effect on Gut microbiota abundance (genus Parasutterella id.2892)

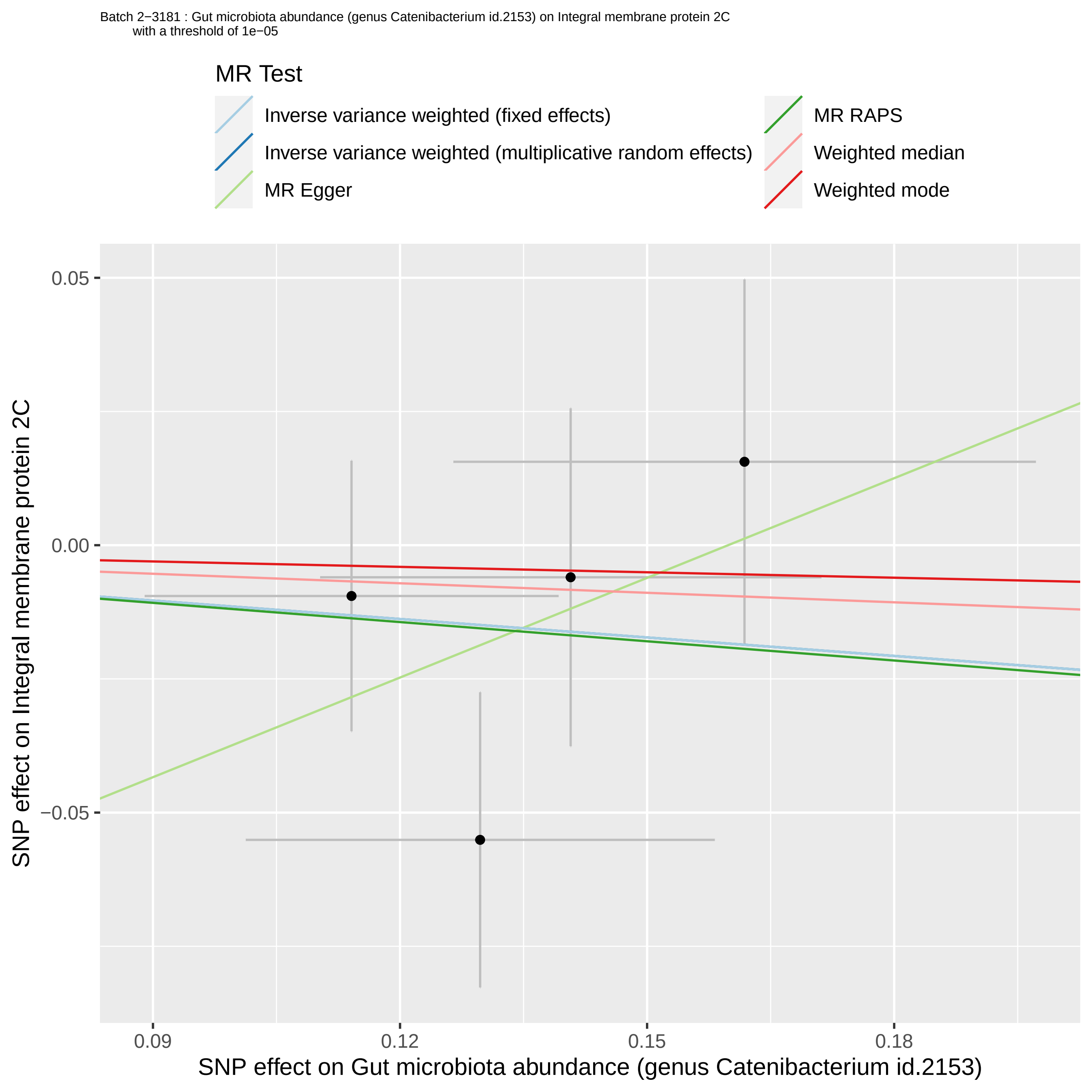

Batch 2−3181 : Gut microbiota abundance (genus Catenibacterium id.2153) on Integral membrane protein 2C
with a threshold of 1e−05
MR Test
Inverse variance weighted (fixed effects)
Inverse variance weighted (multiplicative random effects)
MR Egger
MR RAPS
Weighted median
Weighted mode
0.05
0.00
−0.05
0.09
0.12
0.15
0.18
SNP effect on Integral membrane protein 2C
SNP effect on Gut microbiota abundance (genus Catenibacterium id.2153)

Batch 2−3182 : Gut microbiota abundance (genus Parasutterella id.2892) on Integral membrane protein 2C
with a threshold of 1e−05

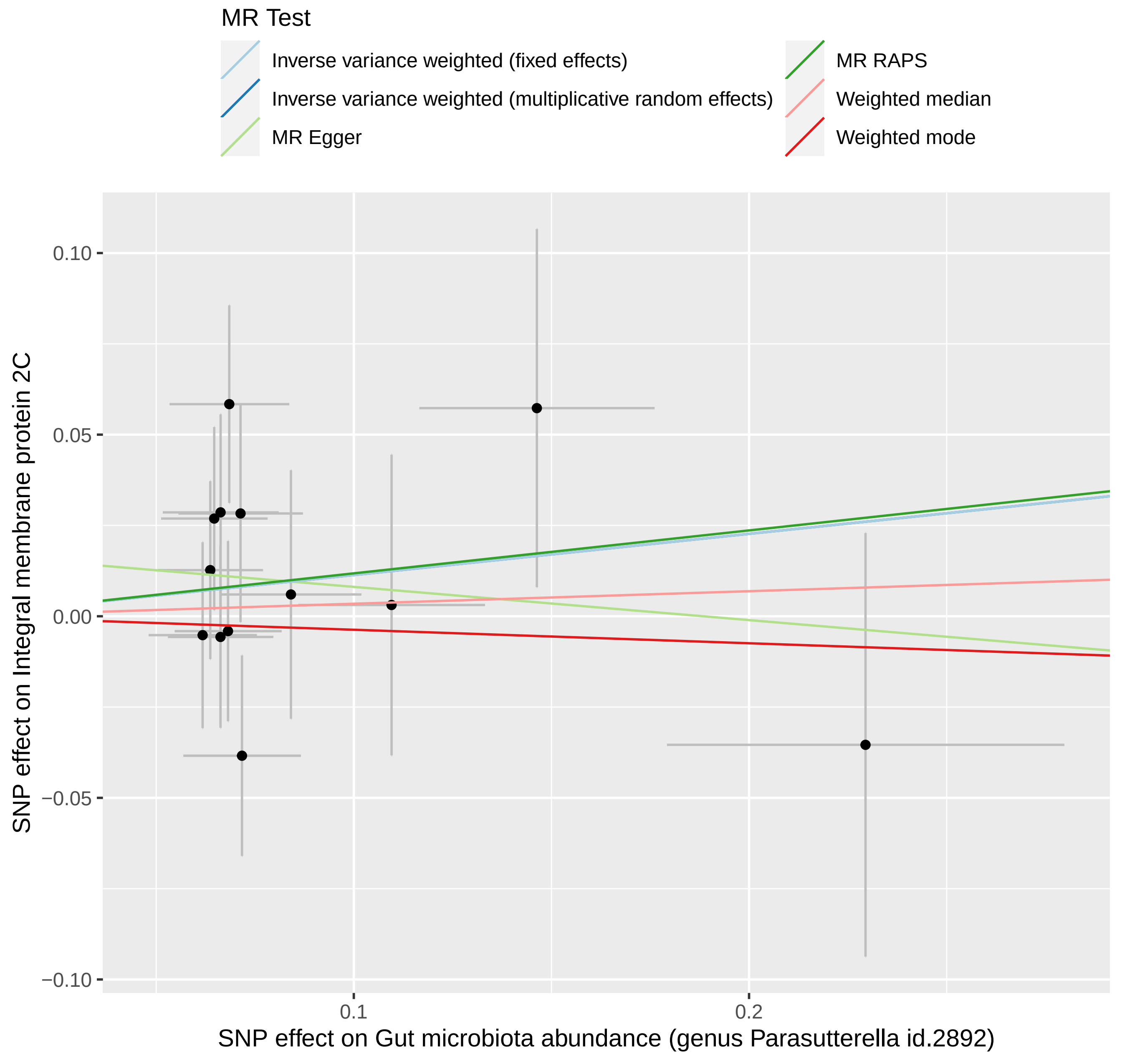

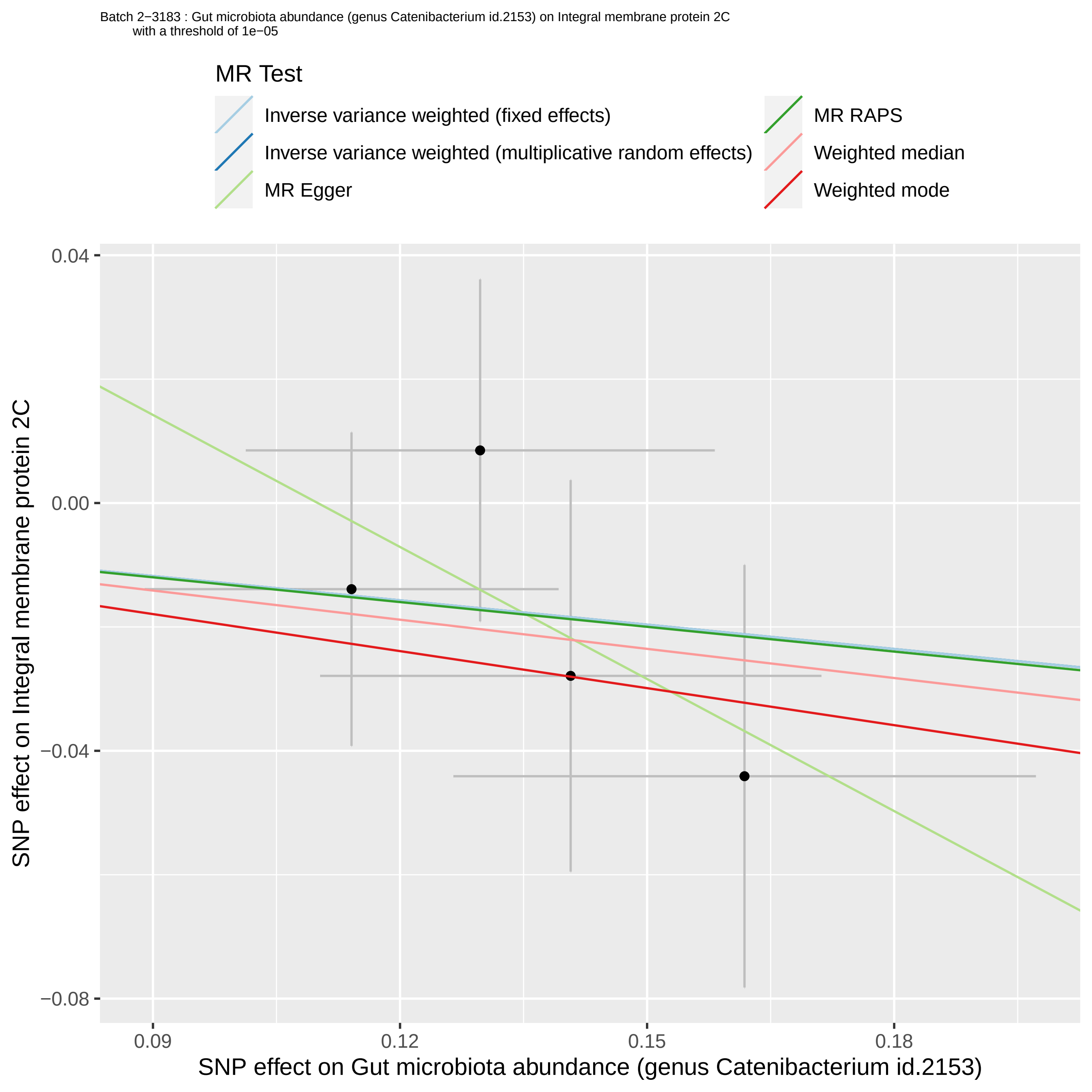
Batch 2−3183 : Gut microbiota abundance (genus Catenibacterium id.2153) on Integral membrane protein 2C
with a threshold of 1e−05
MR Test
Inverse variance weighted (fixed effects)
Inverse variance weighted (multiplicative random effects)
MR Egger
MR RAPS
Weighted median
Weighted mode
0.04
0.00
−0.04
−0.08
0.09
0.12
0.15
0.18
SNP effect on Integral membrane protein 2C
SNP effect on Gut microbiota abundance (genus Catenibacterium id.2153)

Batch 2−3184 : Gut microbiota abundance (genus Parasutterella id.2892) on Integral membrane protein 2C
with a threshold of 1e−05

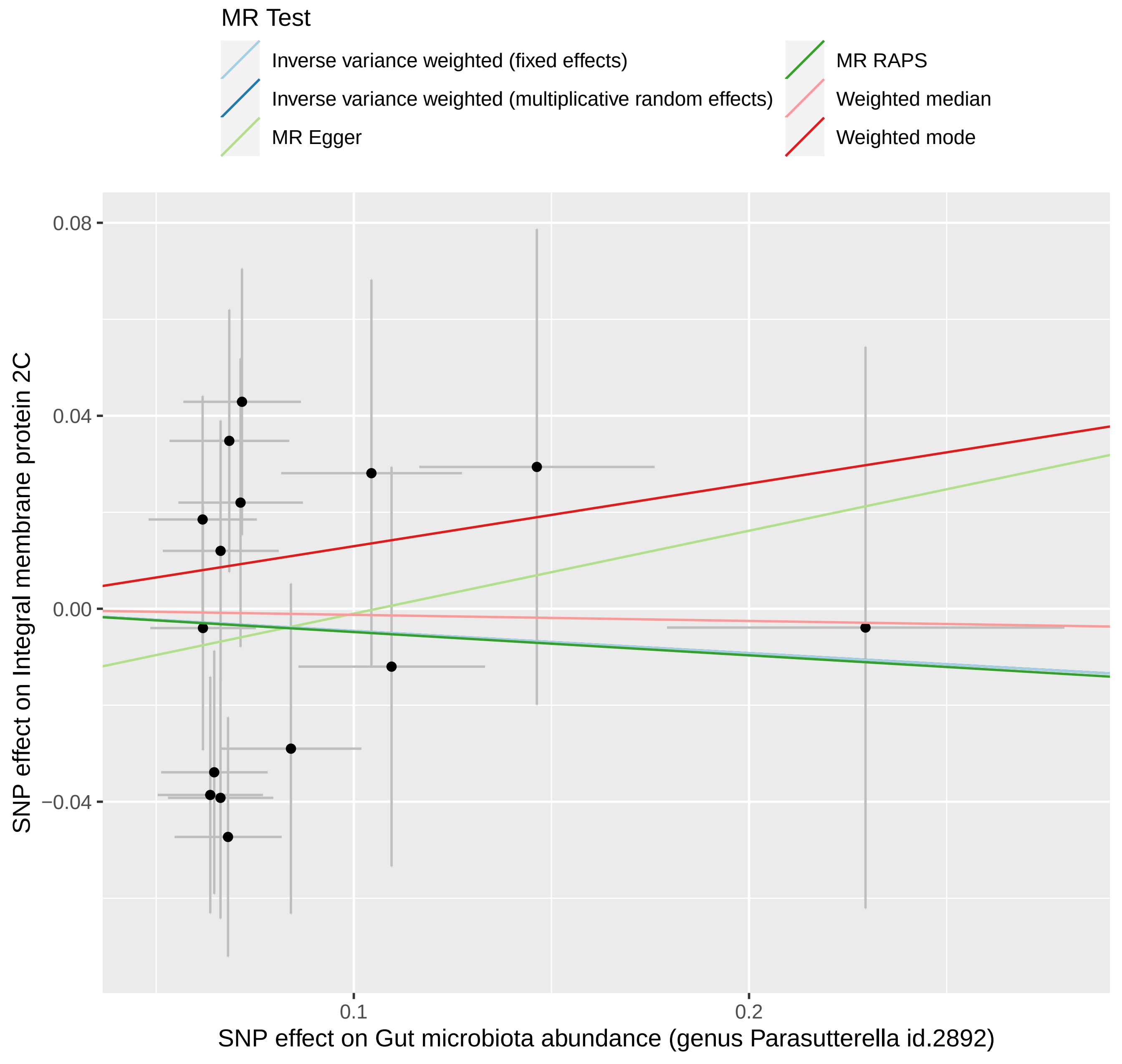

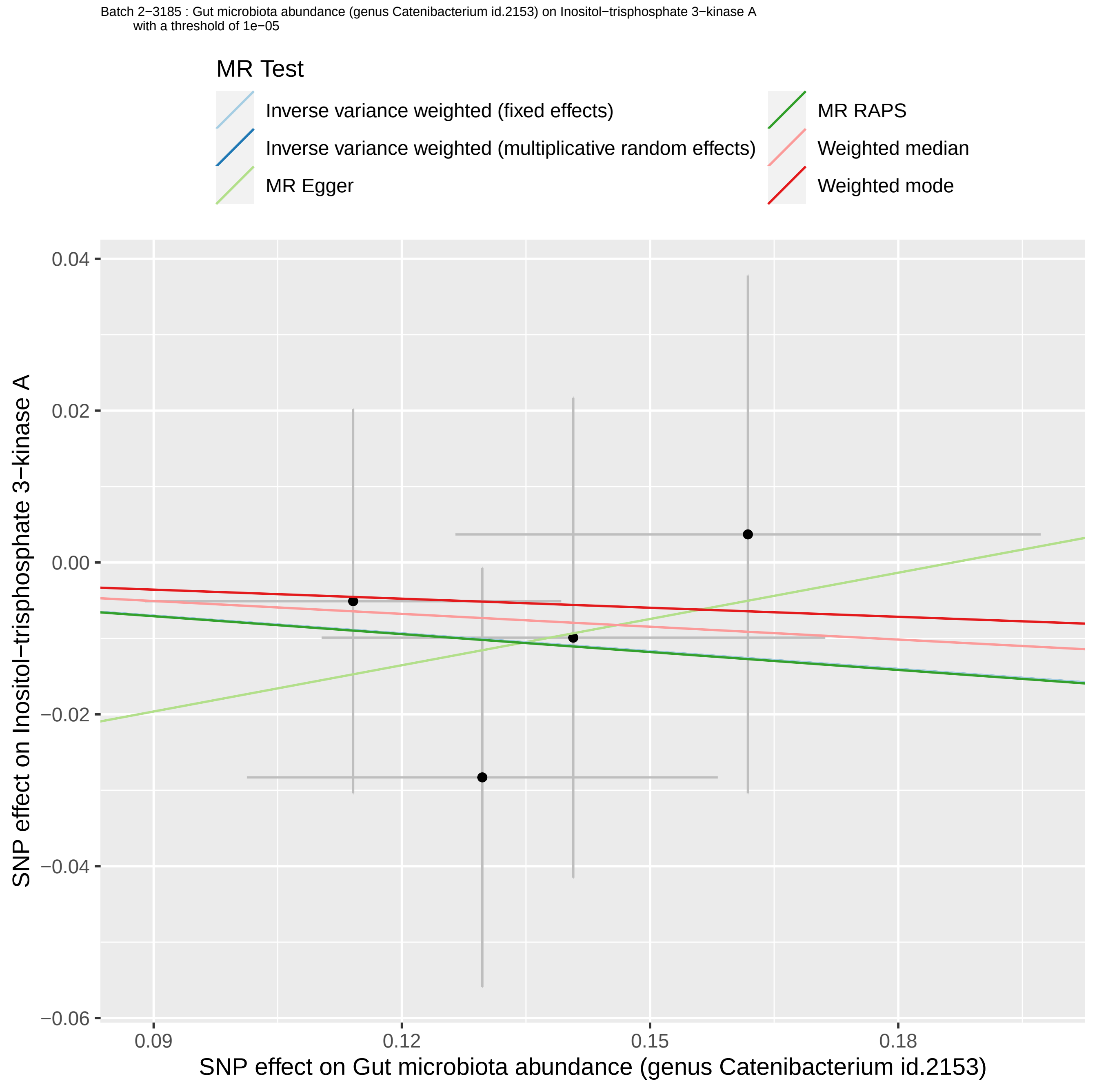

Batch 2−3185 : Gut microbiota abundance (genus Catenibacterium id.2153) on Inositol−trisphosphate 3−kinase A
with a threshold of 1e−05
MR Test
Inverse variance weighted (fixed effects)
Inverse variance weighted (multiplicative random effects)
MR Egger
MR RAPS
Weighted median
Weighted mode
0.04
0.02
0.00
−0.02
−0.04
−0.06
0.09
0.12
0.15
0.18
SNP effect on Inositol−trisphosphate 3−kinase A
SNP effect on Gut microbiota abundance (genus Catenibacterium id.2153)

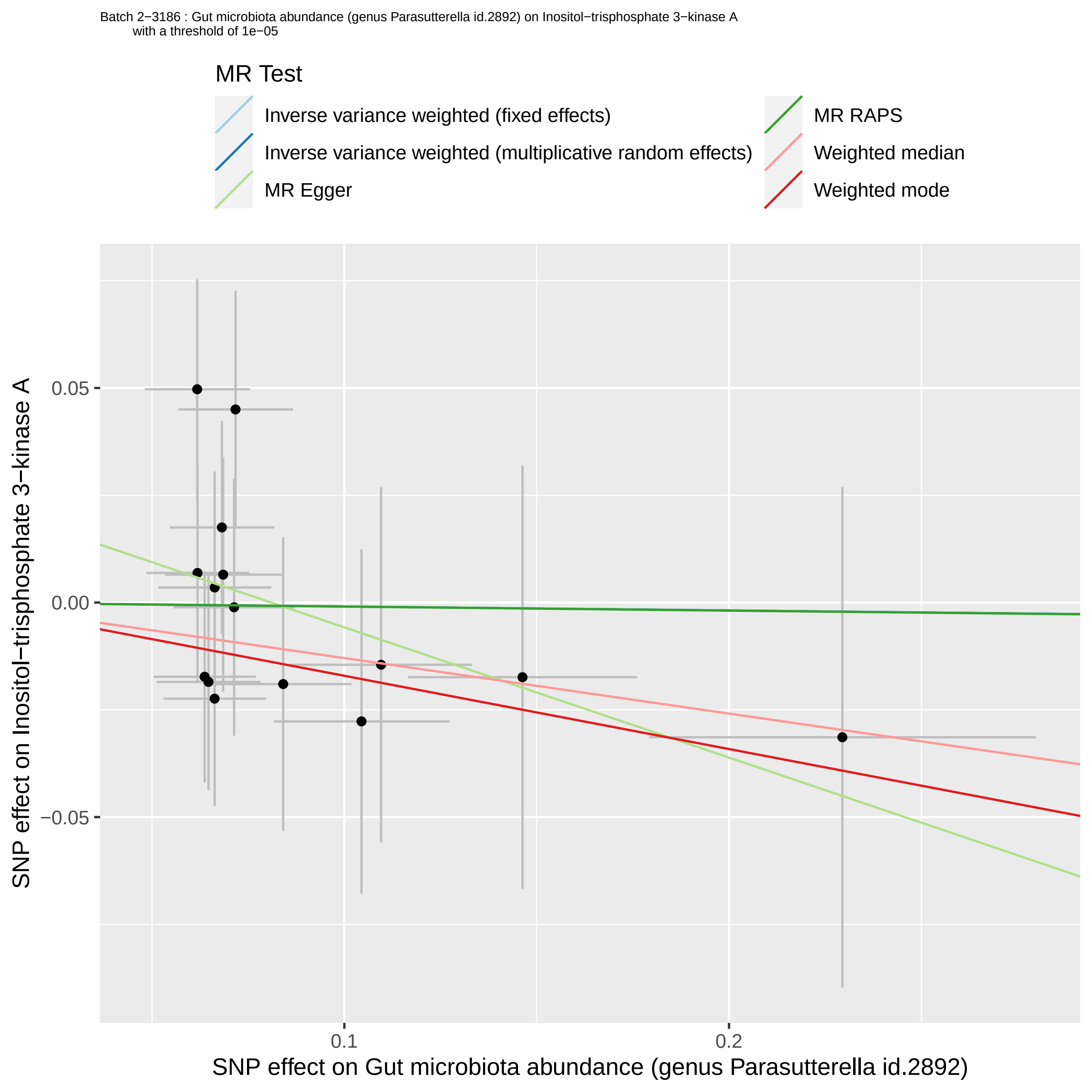

Batch 2−3186 : Gut microbiota abundance (genus Parasutterella id.2892) on Inositol−trisphosphate 3−kinase A
with a threshold of 1e−05
MR Test
Inverse variance weighted (fixed effects)
Inverse variance weighted (multiplicative random effects)
MR Egger
MR RAPS
Weighted median
Weighted mode
0.05
0.00
−0.05
0.1
0.2
SNP effect on Inositol−trisphosphate 3−kinase A
SNP effect on Gut microbiota abundance (genus Parasutterella id.2892)

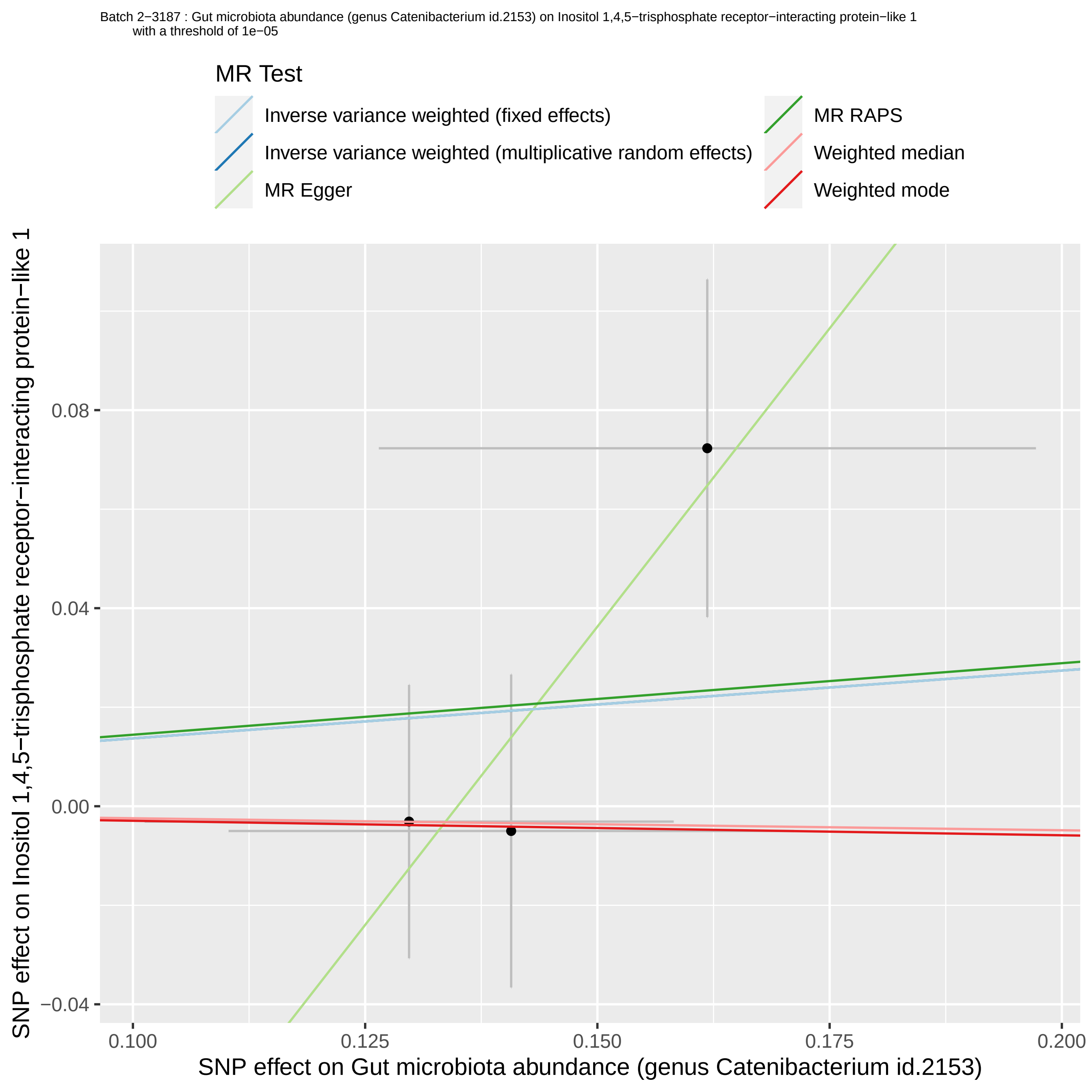

Batch 2−3187 : Gut microbiota abundance (genus Catenibacterium id.2153) on Inositol 1,4,5−trisphosphate receptor−interacting protein−like 1
with a threshold of 1e−05
MR Test
Inverse variance weighted (fixed effects)
Inverse variance weighted (multiplicative random effects)
MR Egger
MR RAPS
Weighted median
Weighted mode
0.08
0.04
0.00
−0.04
0.100
0.125
0.150
0.175
0.200
SNP effect on Inositol 1,4,5−trisphosphate receptor−interacting protein−like 1
SNP effect on Gut microbiota abundance (genus Catenibacterium id.2153)

Batch 2−3188 : Gut microbiota abundance (genus Parasutterella id.2892) on Inositol 1,4,5−trisphosphate receptor−interacting protein−like 1
with a threshold of 1e−05

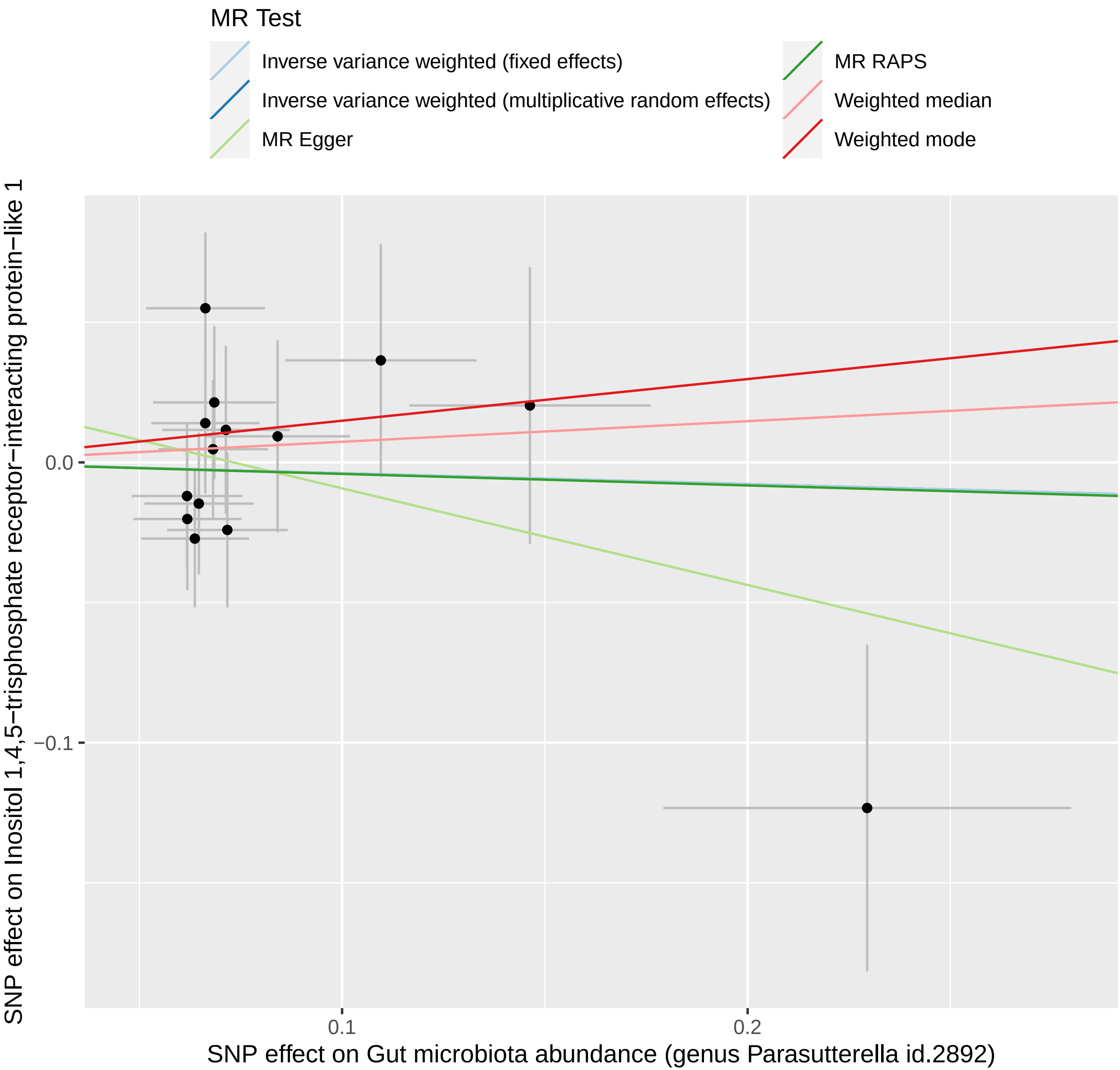

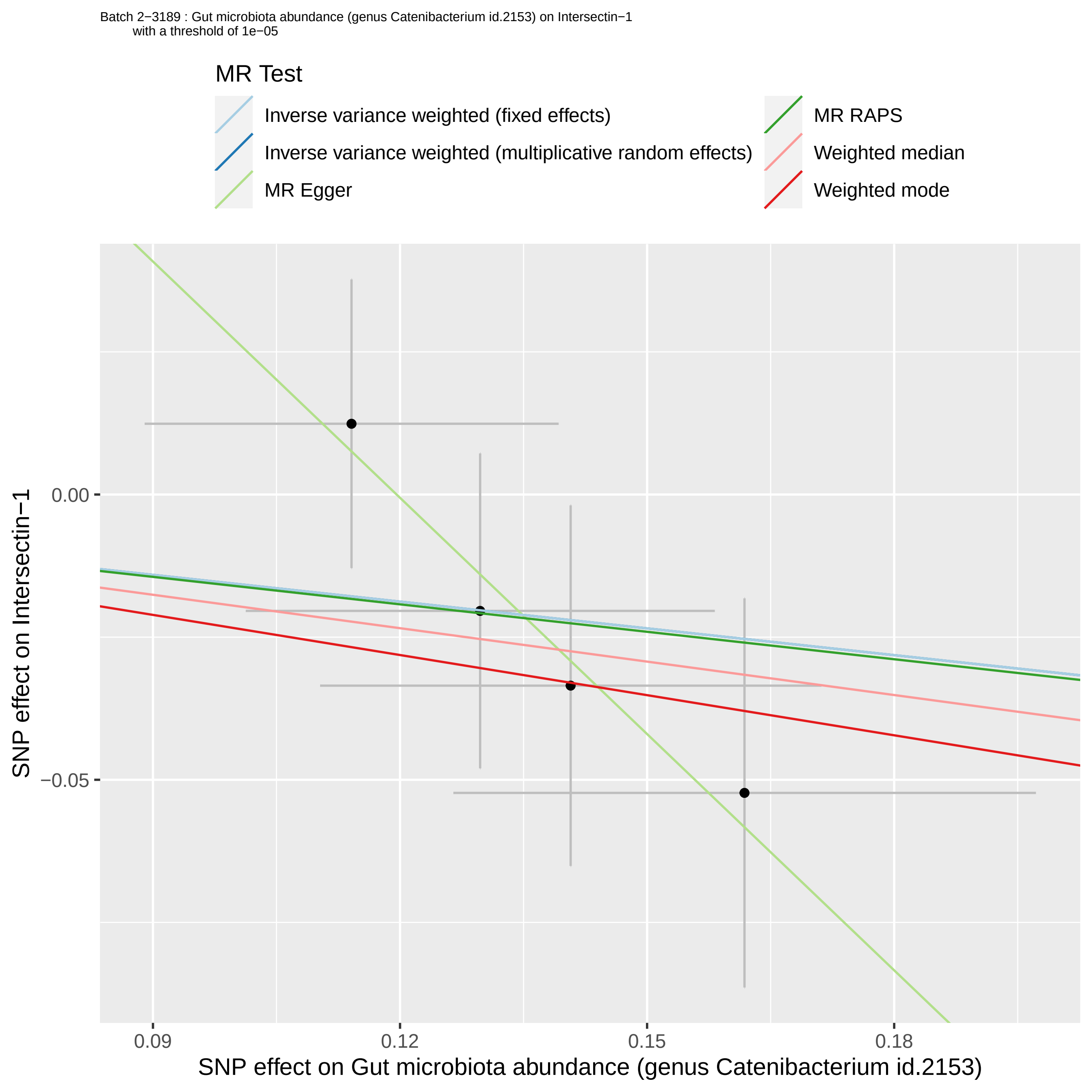

Batch 2−3189 : Gut microbiota abundance (genus Catenibacterium id.2153) on Intersectin−1
with a threshold of 1e−05
MR Test
Inverse variance weighted (fixed effects)
Inverse variance weighted (multiplicative random effects)
MR Egger
MR RAPS
Weighted median
Weighted mode
0.00
−0.05
0.09
0.12
0.15
0.18
SNP effect on Intersectin−1
SNP effect on Gut microbiota abundance (genus Catenibacterium id.2153)

Batch 2−3190 : Gut microbiota abundance (genus Parasutterella id.2892) on Intersectin−1
with a threshold of 1e−05

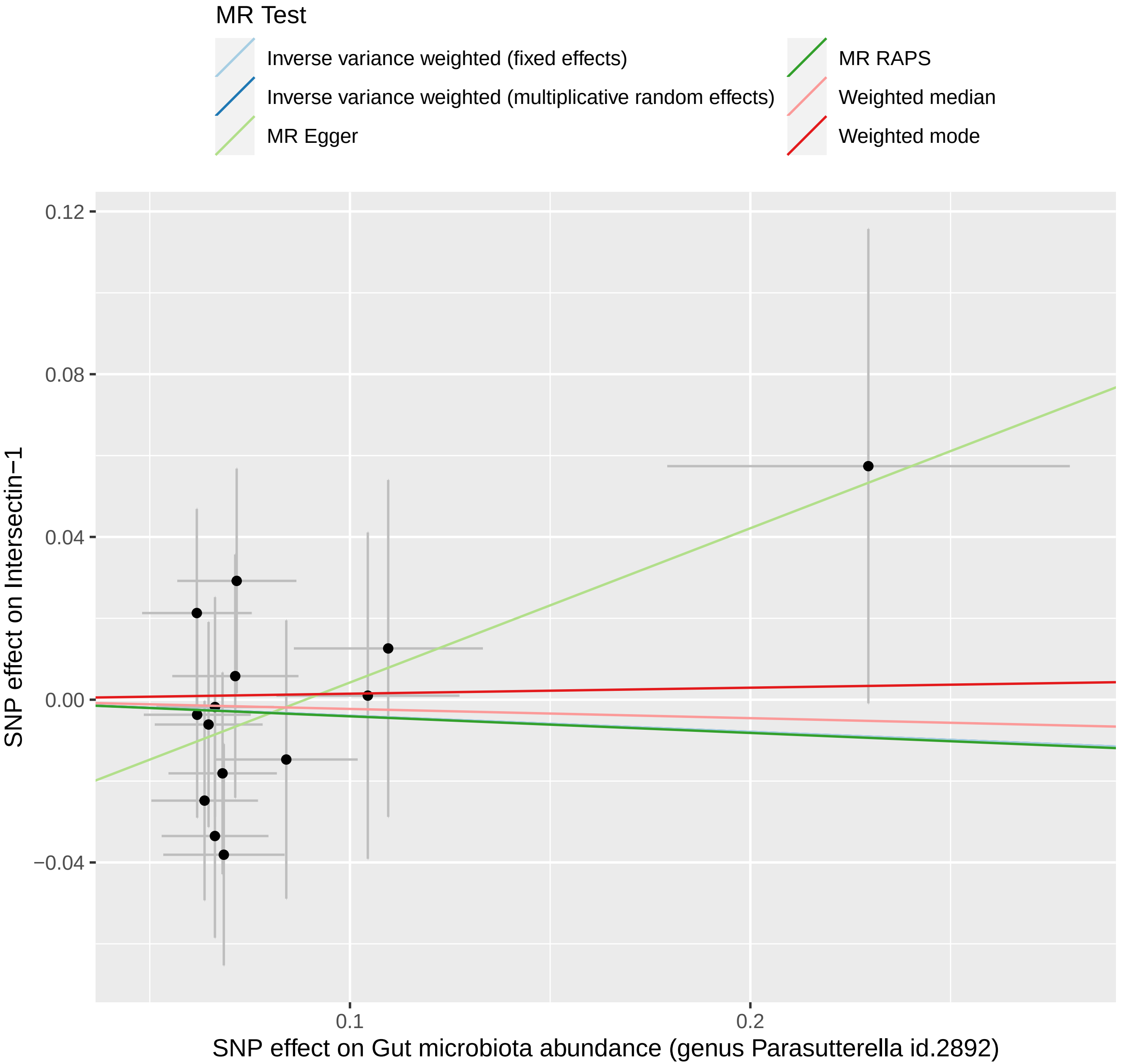

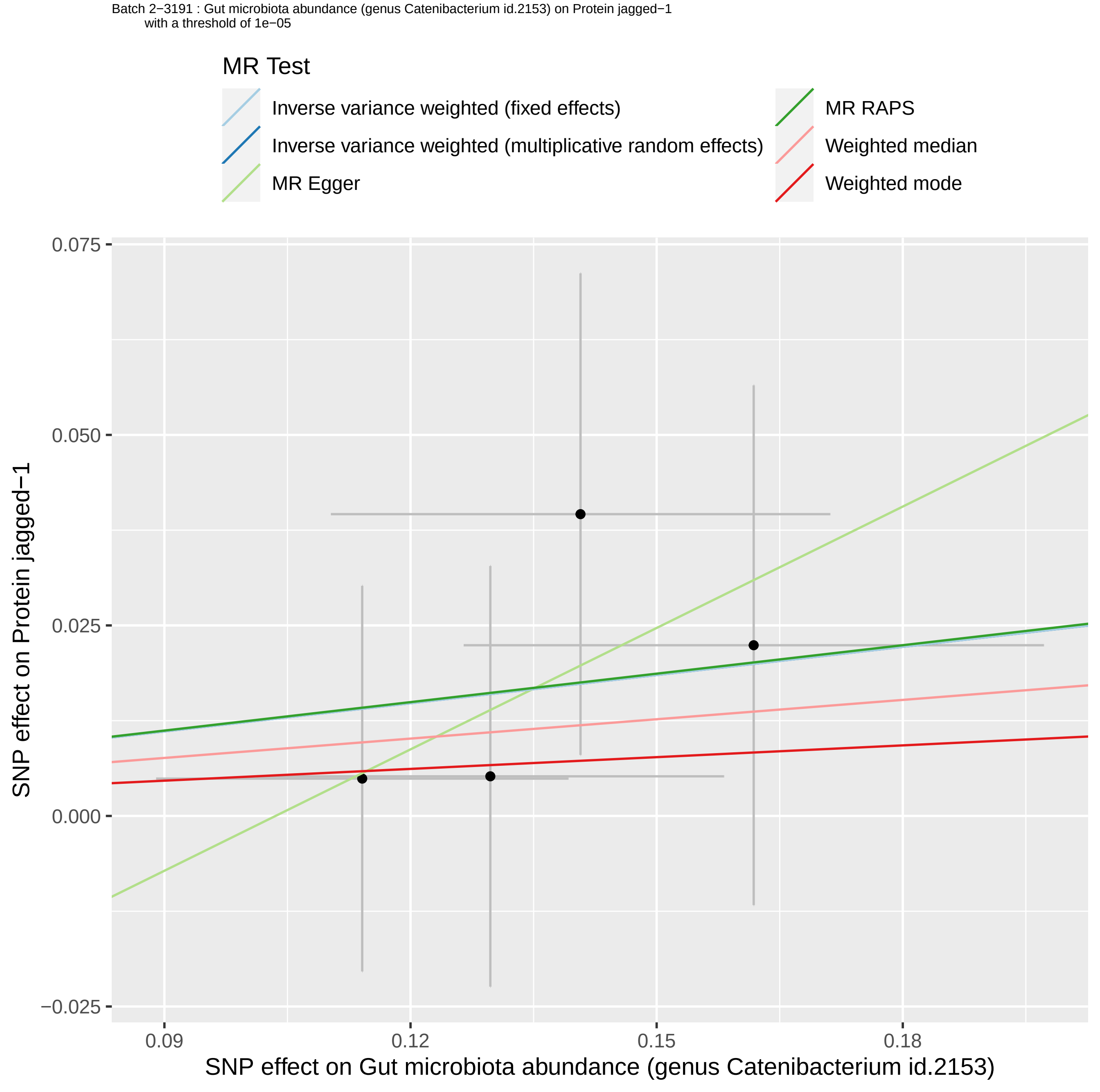
Batch 2−3191 : Gut microbiota abundance (genus Catenibacterium id.2153) on Protein jagged−1
with a threshold of 1e−05
MR Test
Inverse variance weighted (fixed effects)
Inverse variance weighted (multiplicative random effects)
MR Egger
MR RAPS
Weighted median
Weighted mode
0.075
0.050
0.025
0.000
−0.025
SNP effect on Protein jagged−1
0.09
0.12
0.15
0.18
SNP effect on Gut microbiota abundance (genus Catenibacterium id.2153)

Batch 2−3192 : Gut microbiota abundance (genus Parasutterella id.2892) on Protein jagged−1
with a threshold of 1e−05

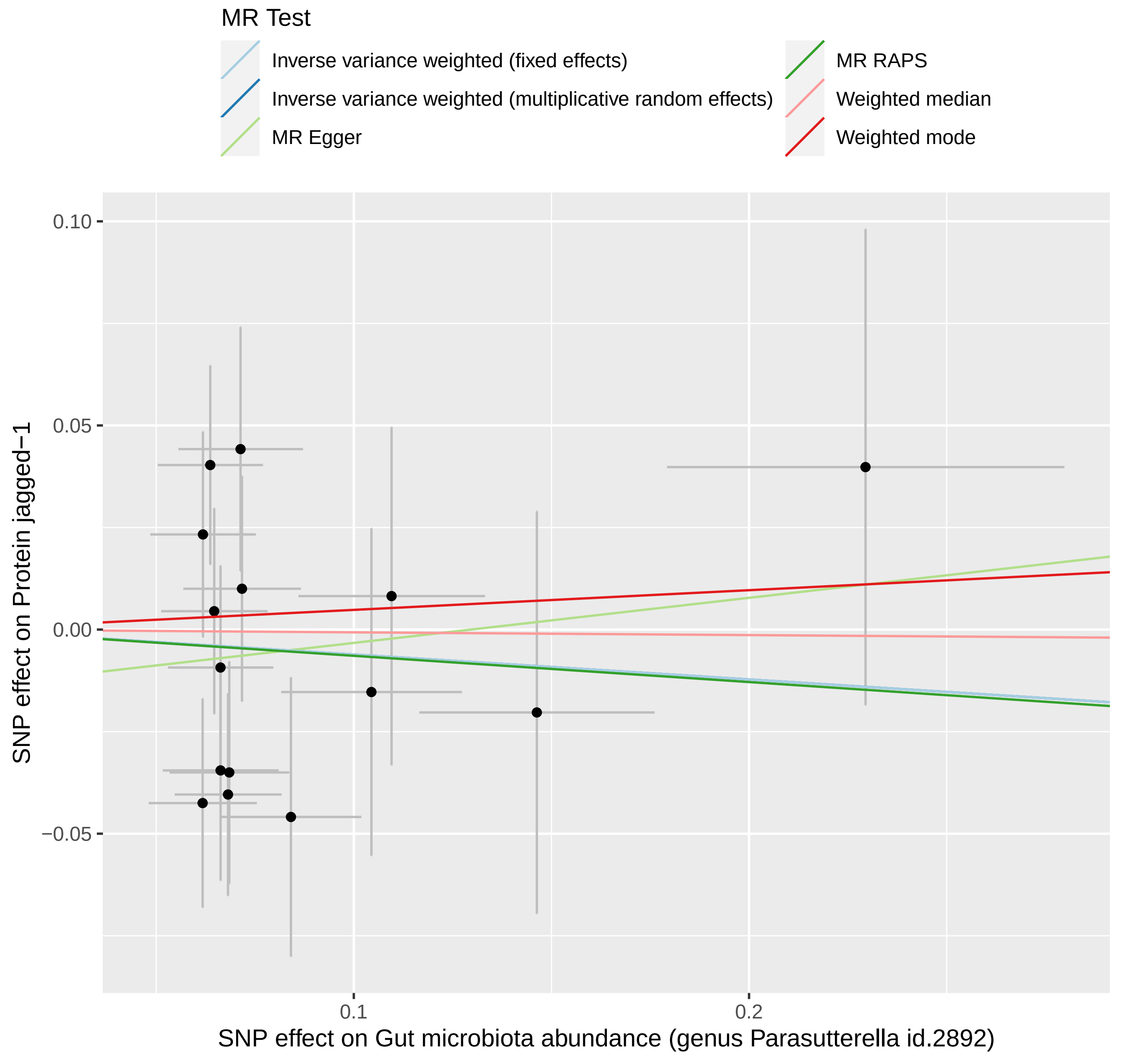

Batch 2−3193 : Gut microbiota abundance (genus Catenibacterium id.2153) on Protein jagged−2
with a threshold of 1e−05

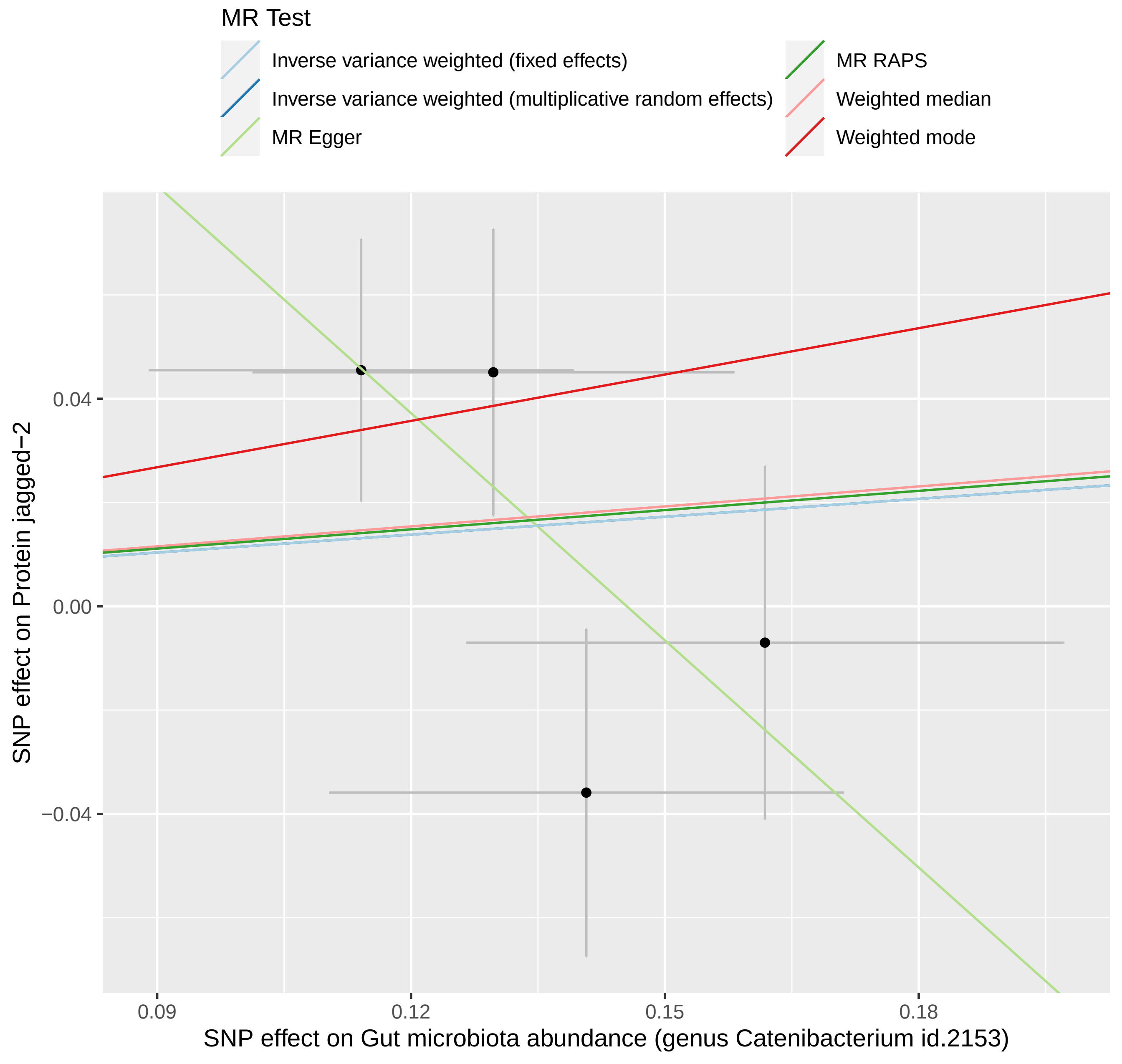

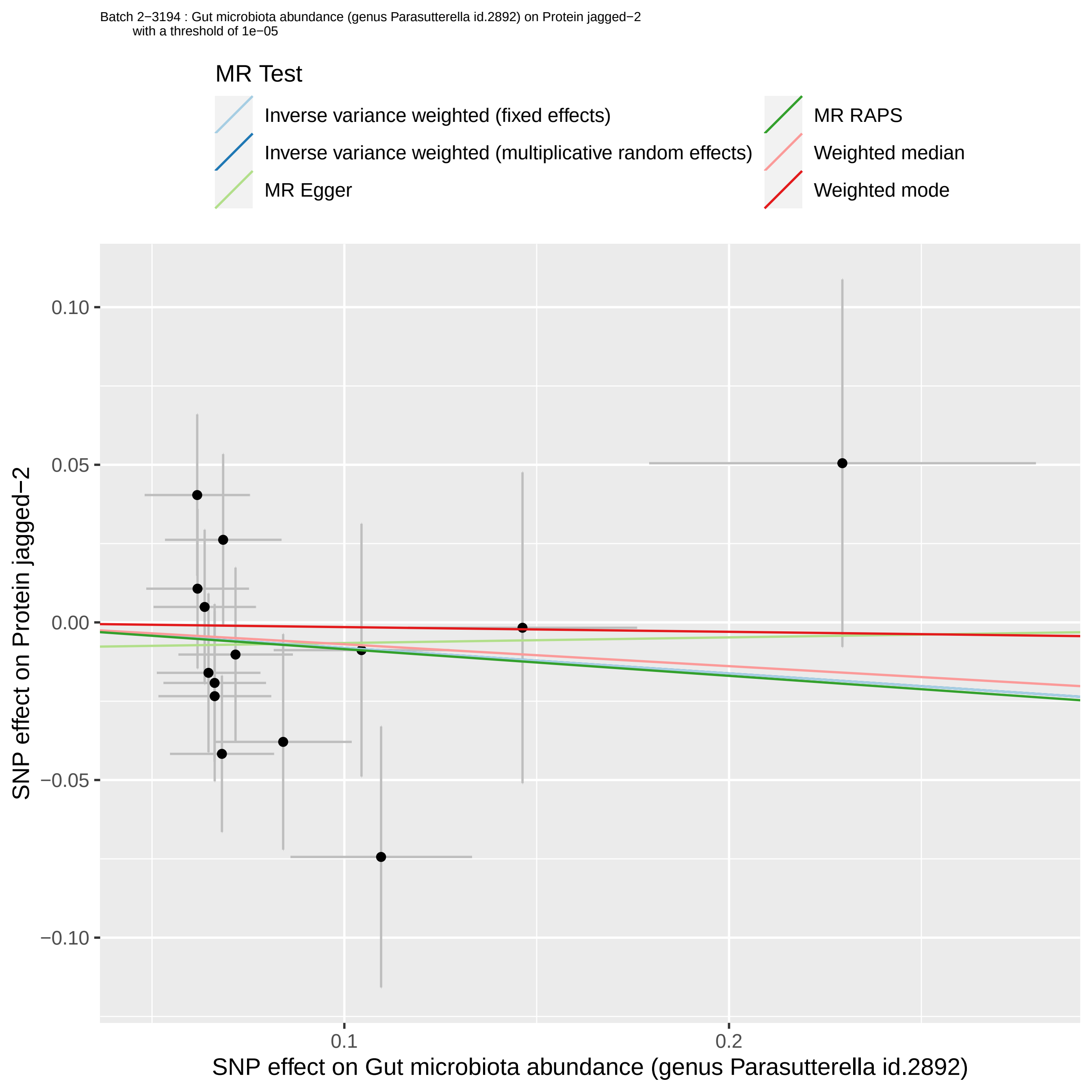

Batch 2−3194 : Gut microbiota abundance (genus Parasutterella id.2892) on Protein jagged−2
with a threshold of 1e−05
MR Test
Inverse variance weighted (fixed effects)
Inverse variance weighted (multiplicative random effects)
MR Egger
MR RAPS
Weighted median
Weighted mode
SNP effect on Protein jagged−2
0.10
0.05
0.00
−0.05
−0.10
0.1
0.2
SNP effect on Gut microbiota abundance (genus Parasutterella id.2892)

Batch 2−3195 : Gut microbiota abundance (genus Catenibacterium id.2153) on Tyrosine−protein kinase JAK2
with a threshold of 1e−05

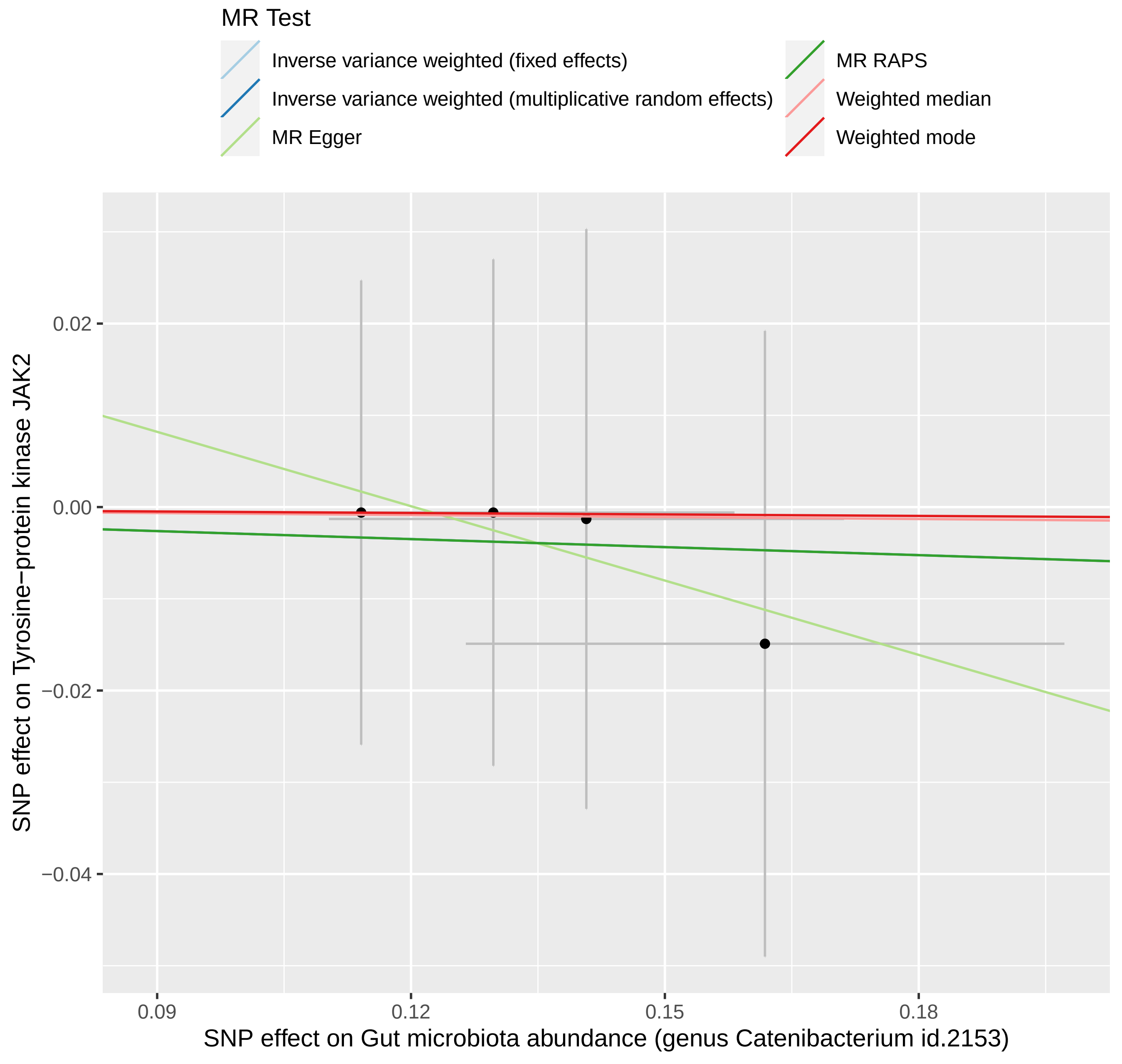

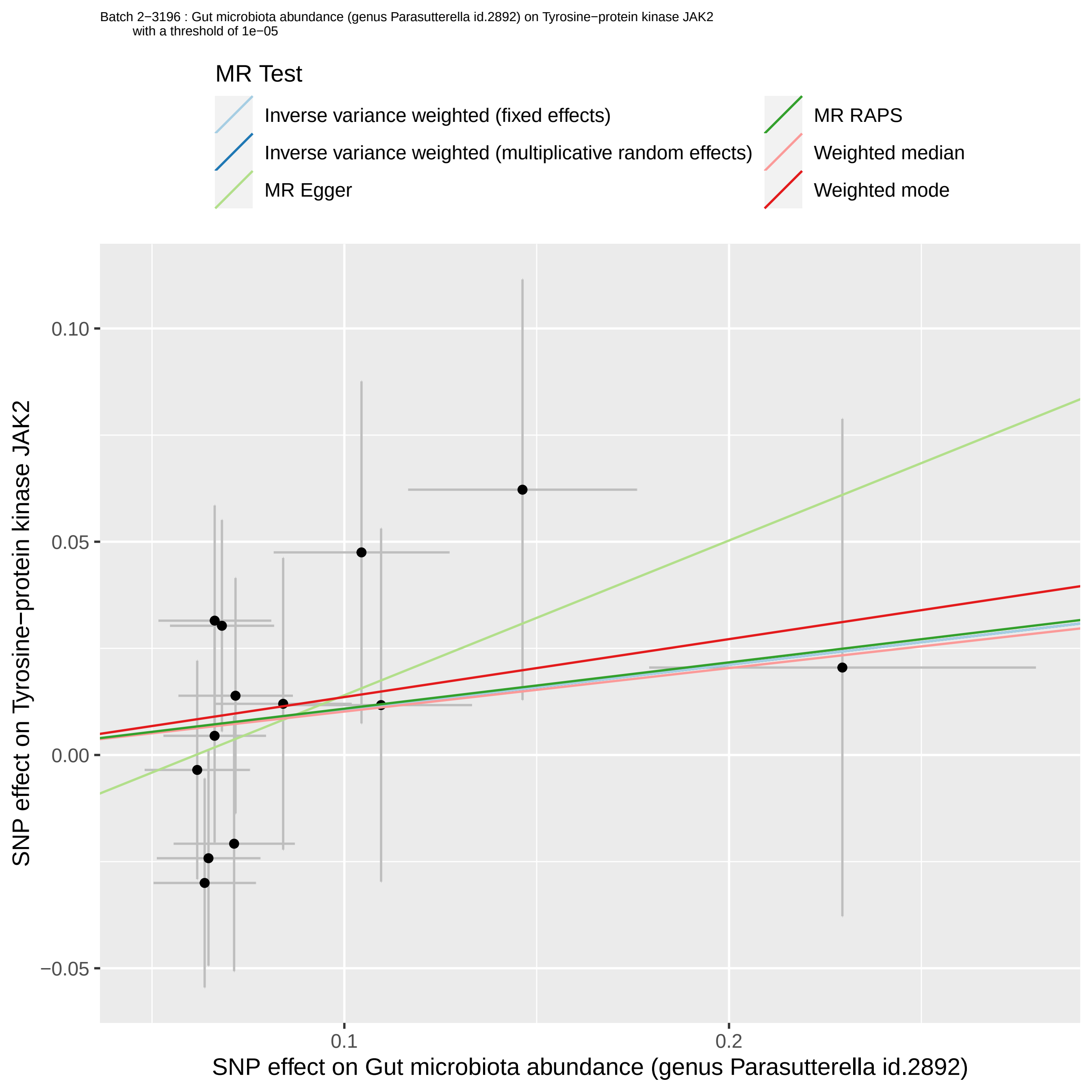

Batch 2−3196 : Gut microbiota abundance (genus Parasutterella id.2892) on Tyrosine−protein kinase JAK2
with a threshold of 1e−05
MR Test
Inverse variance weighted (fixed effects)
Inverse variance weighted (multiplicative random effects)
MR Egger
MR RAPS
Weighted median
Weighted mode
0.10
0.05
0.00
−0.05
0.1
0.2
SNP effect on Tyrosine−protein kinase JAK2
SNP effect on Gut microbiota abundance (genus Parasutterella id.2892)

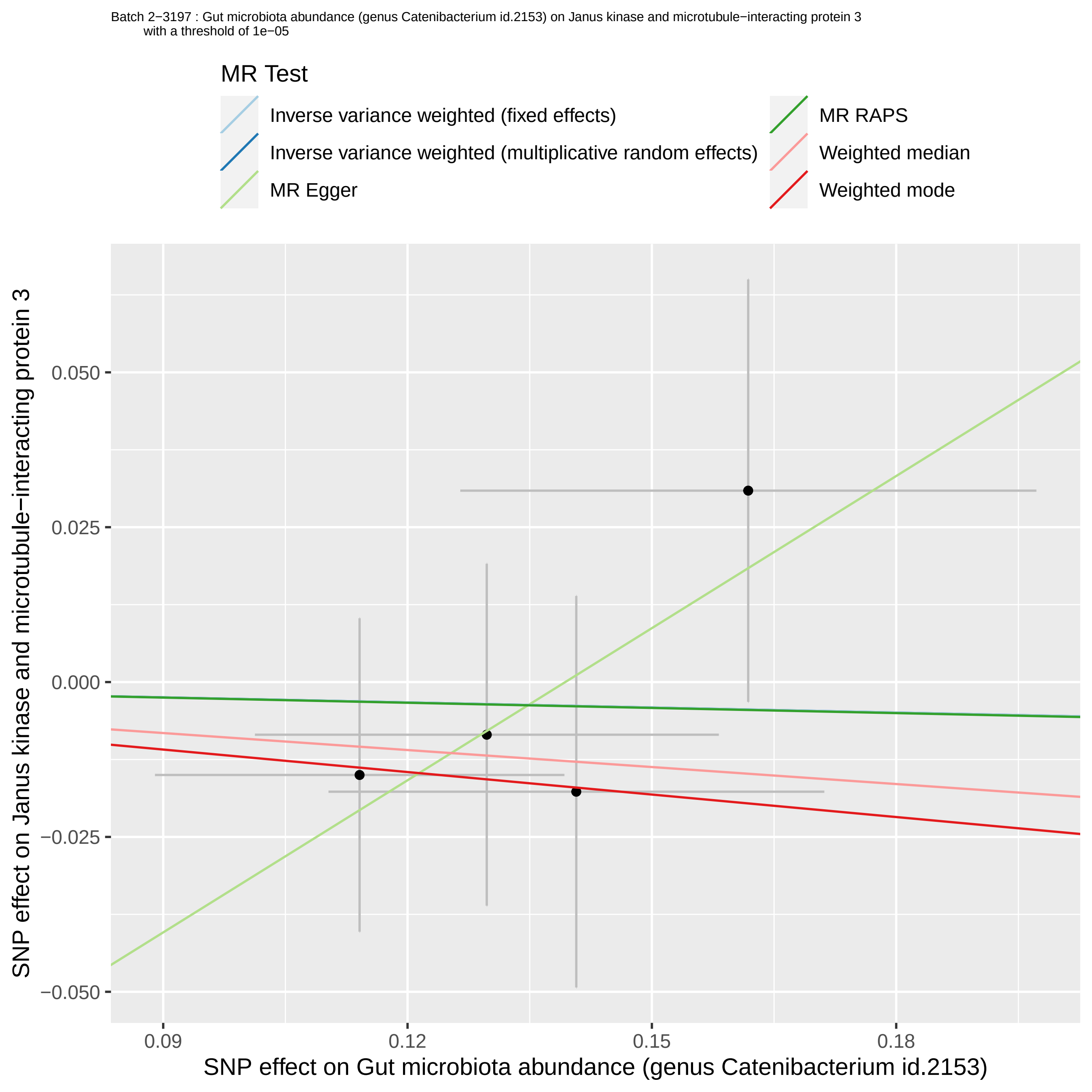
Batch 2−3197 : Gut microbiota abundance (genus Catenibacterium id.2153) on Janus kinase and microtubule−interacting protein 3
with a threshold of 1e−05
MR Test
Inverse variance weighted (fixed effects)
Inverse variance weighted (multiplicative random effects)
MR Egger
MR RAPS
Weighted median
Weighted mode
0.050
0.025
0.000
−0.025
−0.050
0.09
0.12
0.15
0.18
SNP effect on Janus kinase and microtubule−interacting protein 3
SNP effect on Gut microbiota abundance (genus Catenibacterium id.2153)

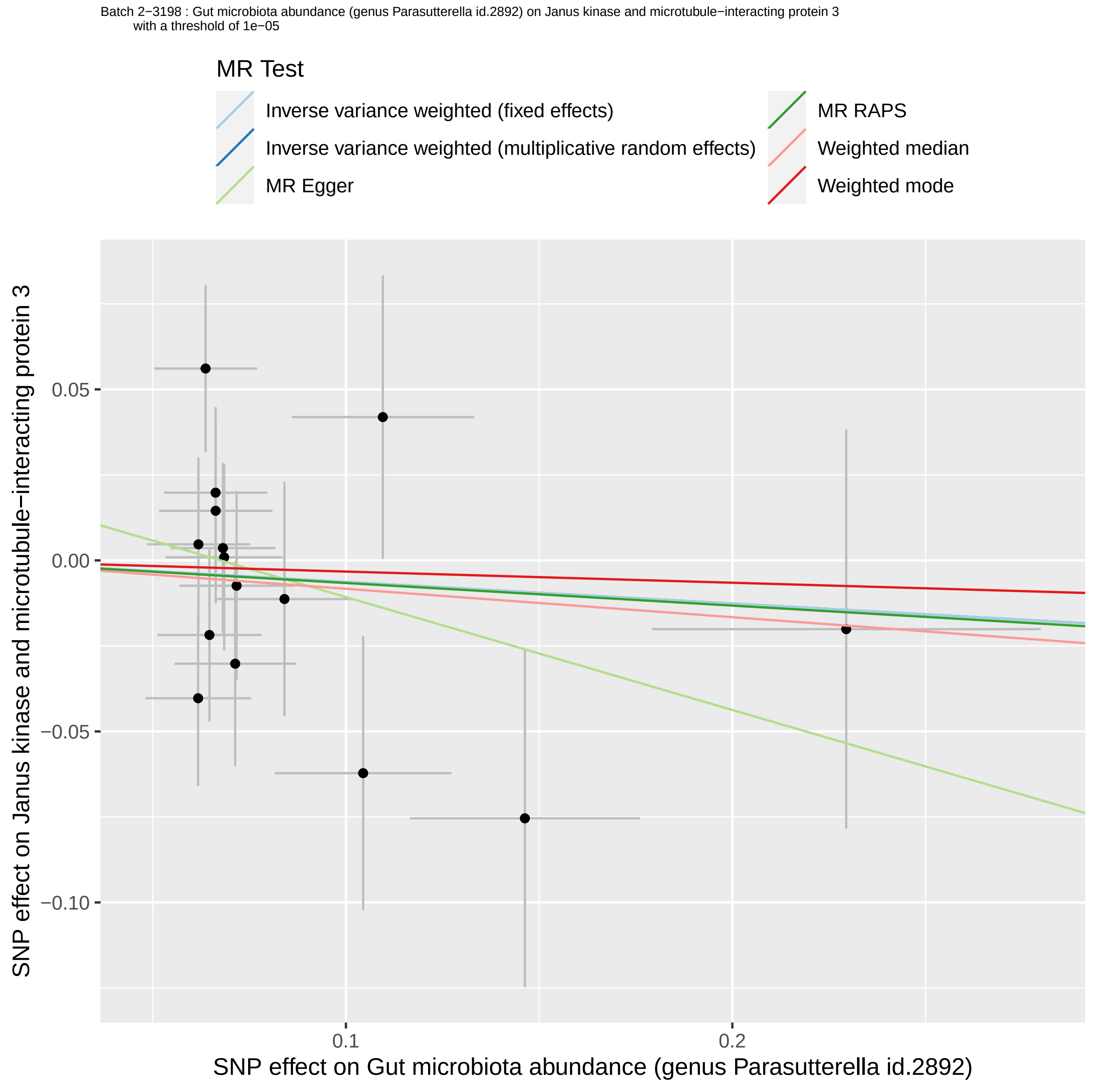

Batch 2−3198 : Gut microbiota abundance (genus Parasutterella id.2892) on Janus kinase and microtubule−interacting protein 3
with a threshold of 1e−05
MR Test
Inverse variance weighted (fixed effects)
Inverse variance weighted (multiplicative random effects)
MR Egger
MR RAPS
Weighted median
Weighted mode
0.05
0.00
−0.05
−0.10
0.1
0.2
SNP effect on Gut microbiota abundance (genus Parasutterella id.2892)
SNP effect on Janus kinase and microtubule−interacting protein 3

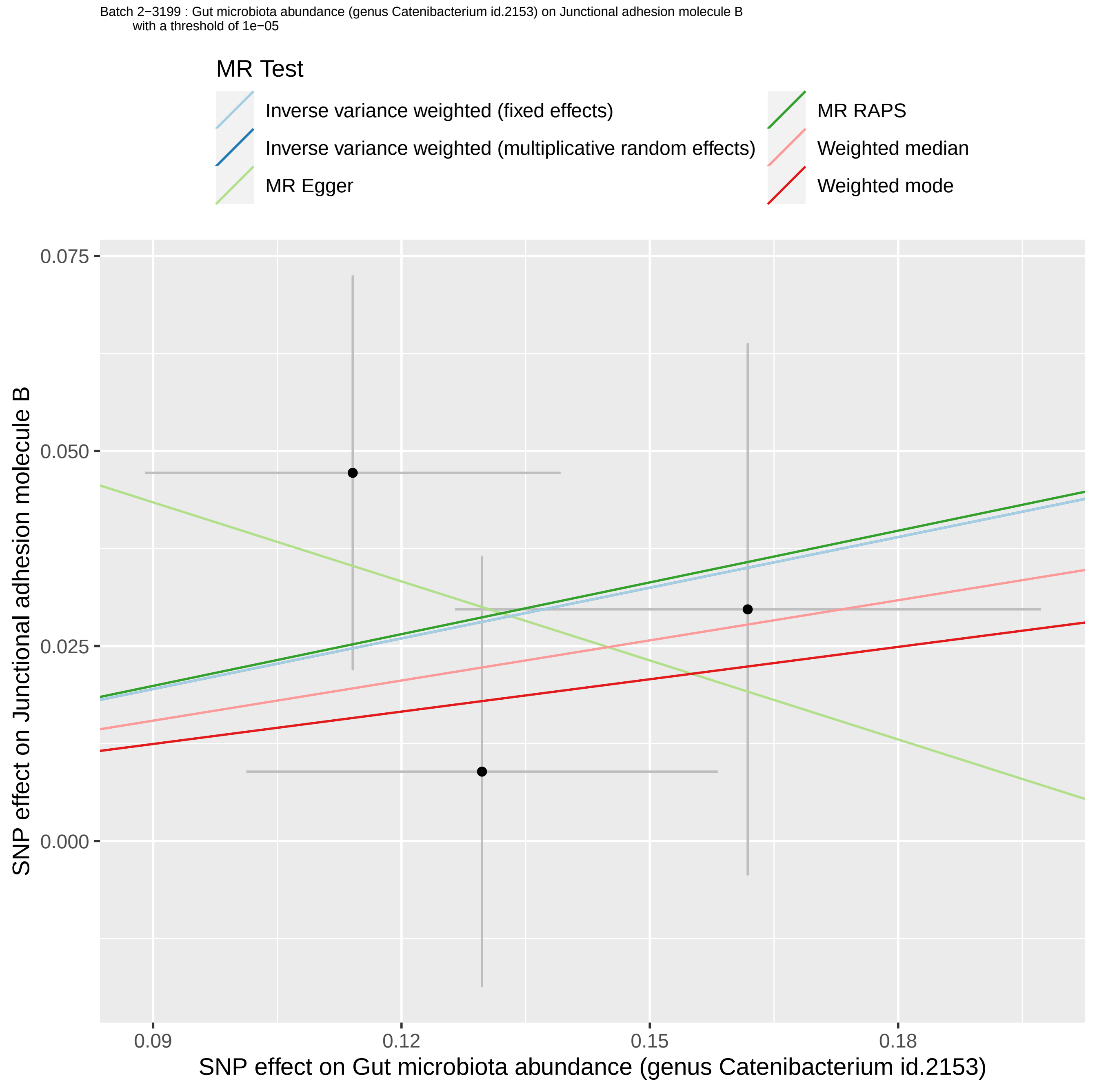
Batch 2−3199 : Gut microbiota abundance (genus Catenibacterium id.2153) on Junctional adhesion molecule B
with a threshold of 1e−05
MR Test
Inverse variance weighted (fixed effects)
Inverse variance weighted (multiplicative random effects)
MR Egger
MR RAPS
Weighted median
Weighted mode
0.075
0.050
0.025
0.000
0.09
0.12
0.15
0.18
SNP effect on Junctional adhesion molecule B
SNP effect on Gut microbiota abundance (genus Catenibacterium id.2153)

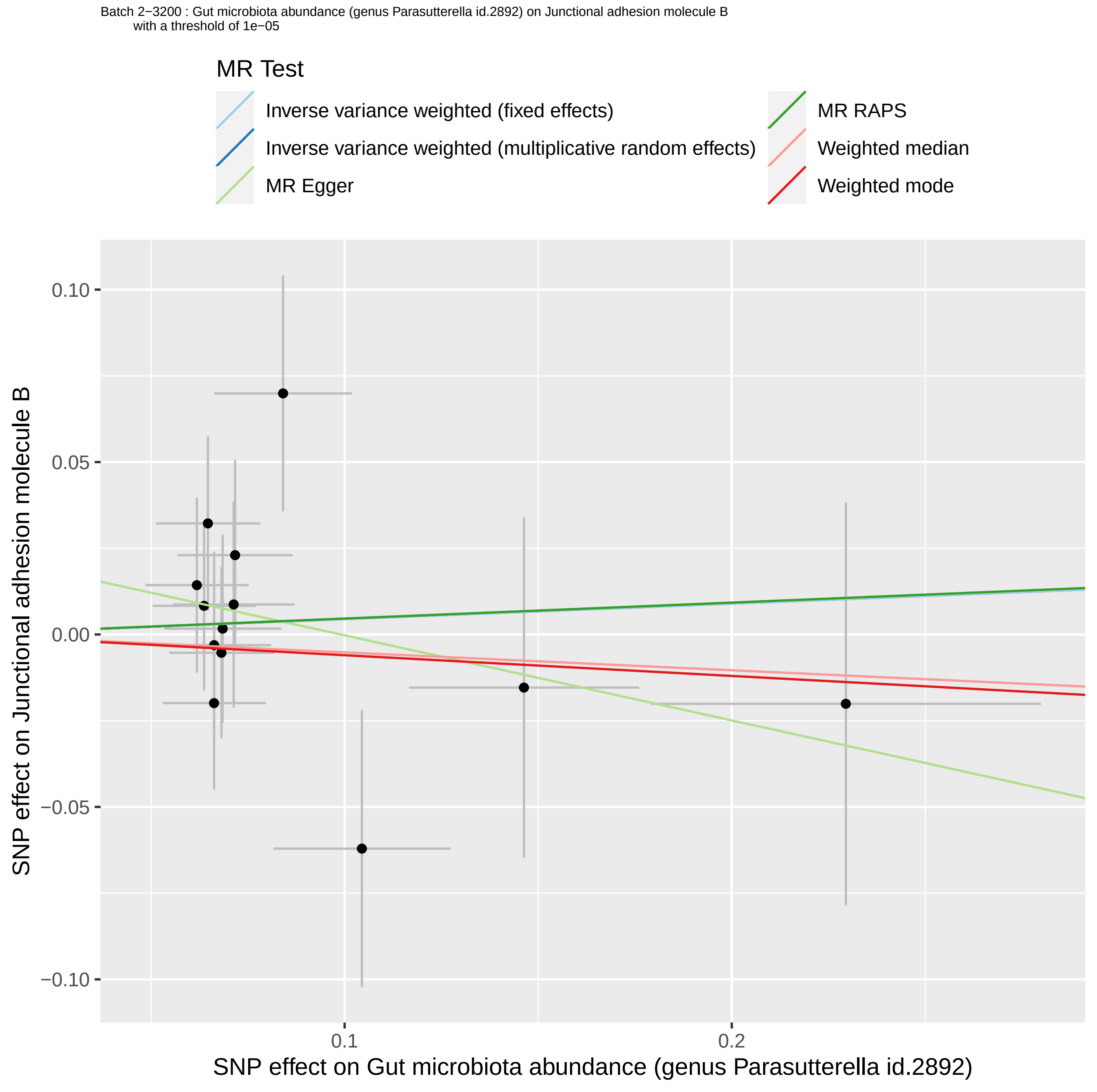
Batch 2−3200 : Gut microbiota abundance (genus Parasutterella id.2892) on Junctional adhesion molecule B
with a threshold of 1e−05
MR Test
Inverse variance weighted (fixed effects)
Inverse variance weighted (multiplicative random effects)
MR Egger
MR RAPS
Weighted median
Weighted mode
0.10
0.05
0.00
−0.05
−0.10
0.1
0.2
SNP effect on Junctional adhesion molecule B
SNP effect on Gut microbiota abundance (genus Parasutterella id.2892)

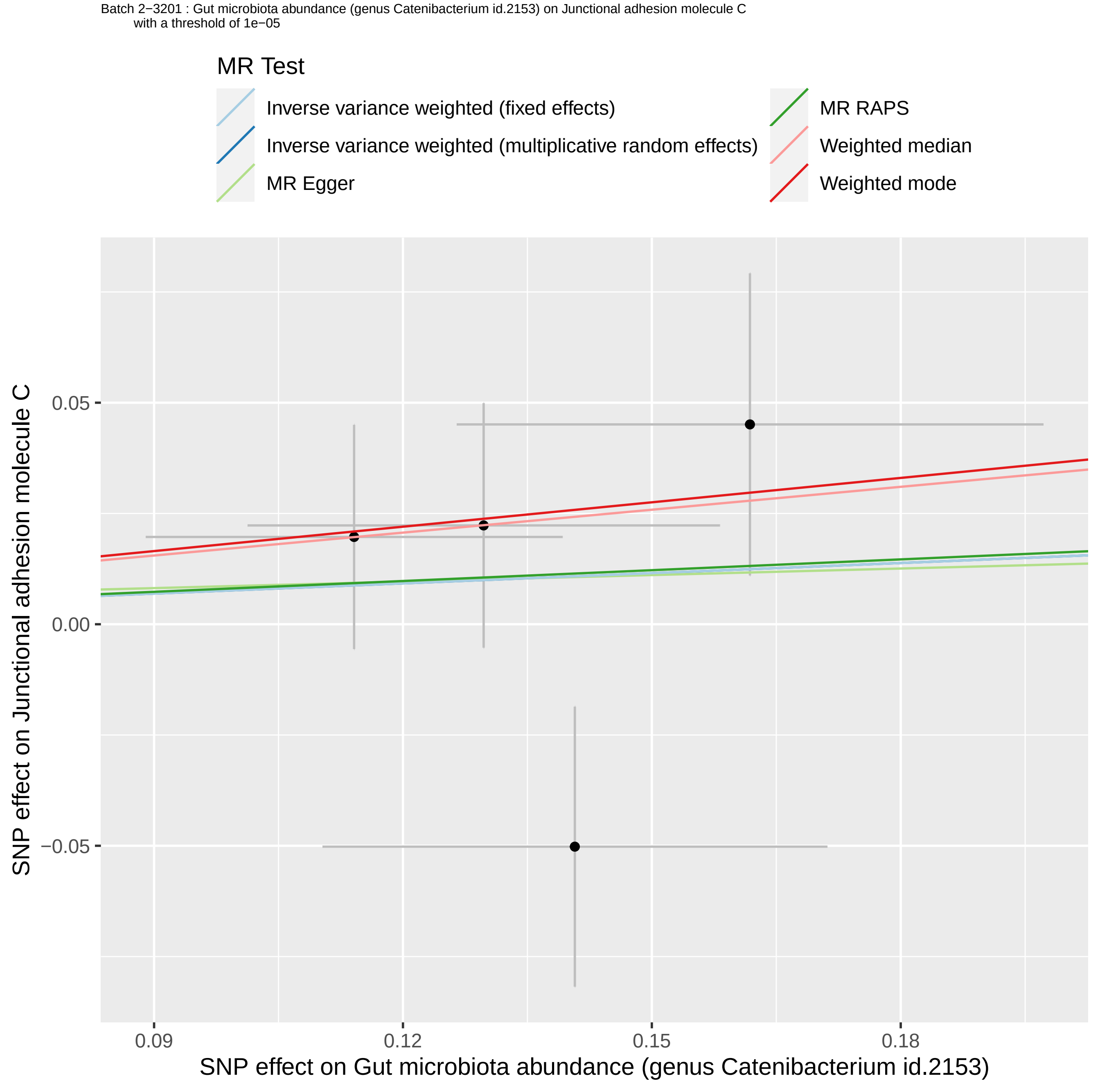

Batch 2−3201 : Gut microbiota abundance (genus Catenibacterium id.2153) on Junctional adhesion molecule C
with a threshold of 1e−05
MR Test
Inverse variance weighted (fixed effects)
Inverse variance weighted (multiplicative random effects)
MR Egger
MR RAPS
Weighted median
Weighted mode
SNP effect on Junctional adhesion molecule C
0.05
0.00
−0.05
0.09
0.12
0.15
0.18
SNP effect on Gut microbiota abundance (genus Catenibacterium id.2153)

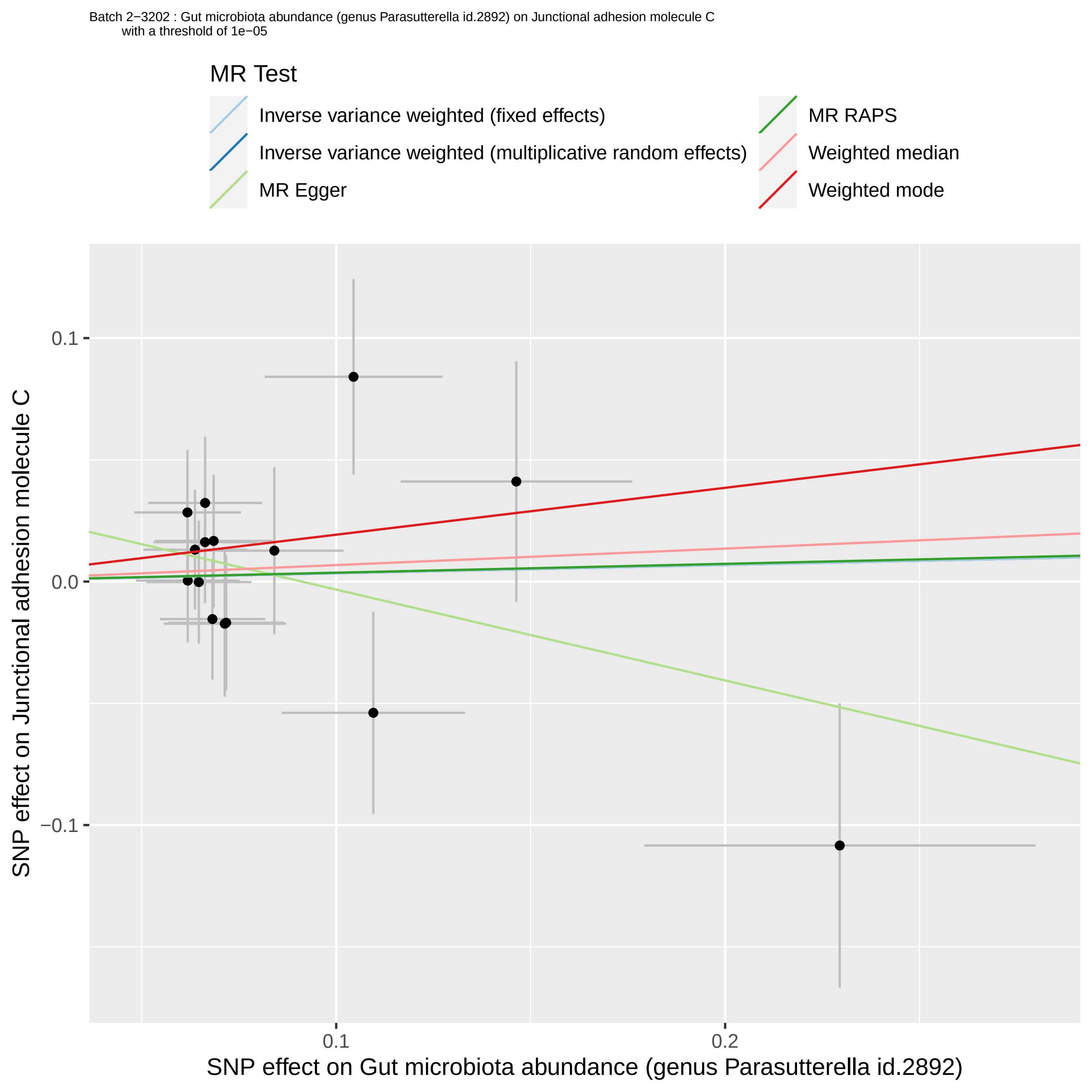
Batch 2−3202 : Gut microbiota abundance (genus Parasutterella id.2892) on Junctional adhesion molecule C
with a threshold of 1e−05
MR Test
Inverse variance weighted (fixed effects)
Inverse variance weighted (multiplicative random effects)
MR Egger
MR RAPS
Weighted median
Weighted mode
0.1
0.0
−0.1
0.1
0.2
SNP effect on Junctional adhesion molecule C
SNP effect on Gut microbiota abundance (genus Parasutterella id.2892)

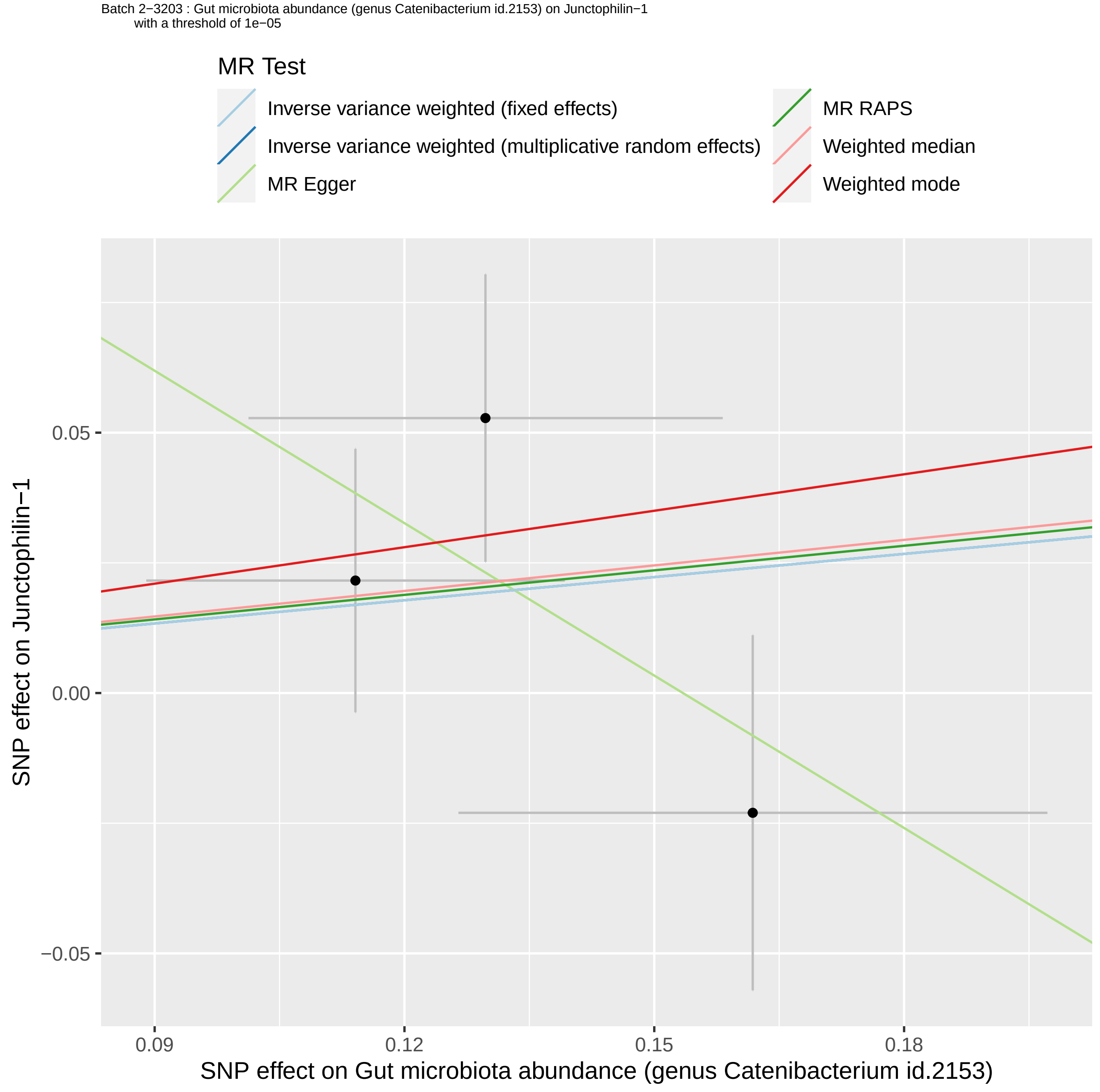

Batch 2−3203 : Gut microbiota abundance (genus Catenibacterium id.2153) on Junctophilin−1
with a threshold of 1e−05
MR Test
Inverse variance weighted (fixed effects)
Inverse variance weighted (multiplicative random effects)
MR Egger
MR RAPS
Weighted median
Weighted mode
SNP effect on Junctophilin−1
0.05
0.00
−0.05
0.09
0.12
0.15
0.18
SNP effect on Gut microbiota abundance (genus Catenibacterium id.2153)

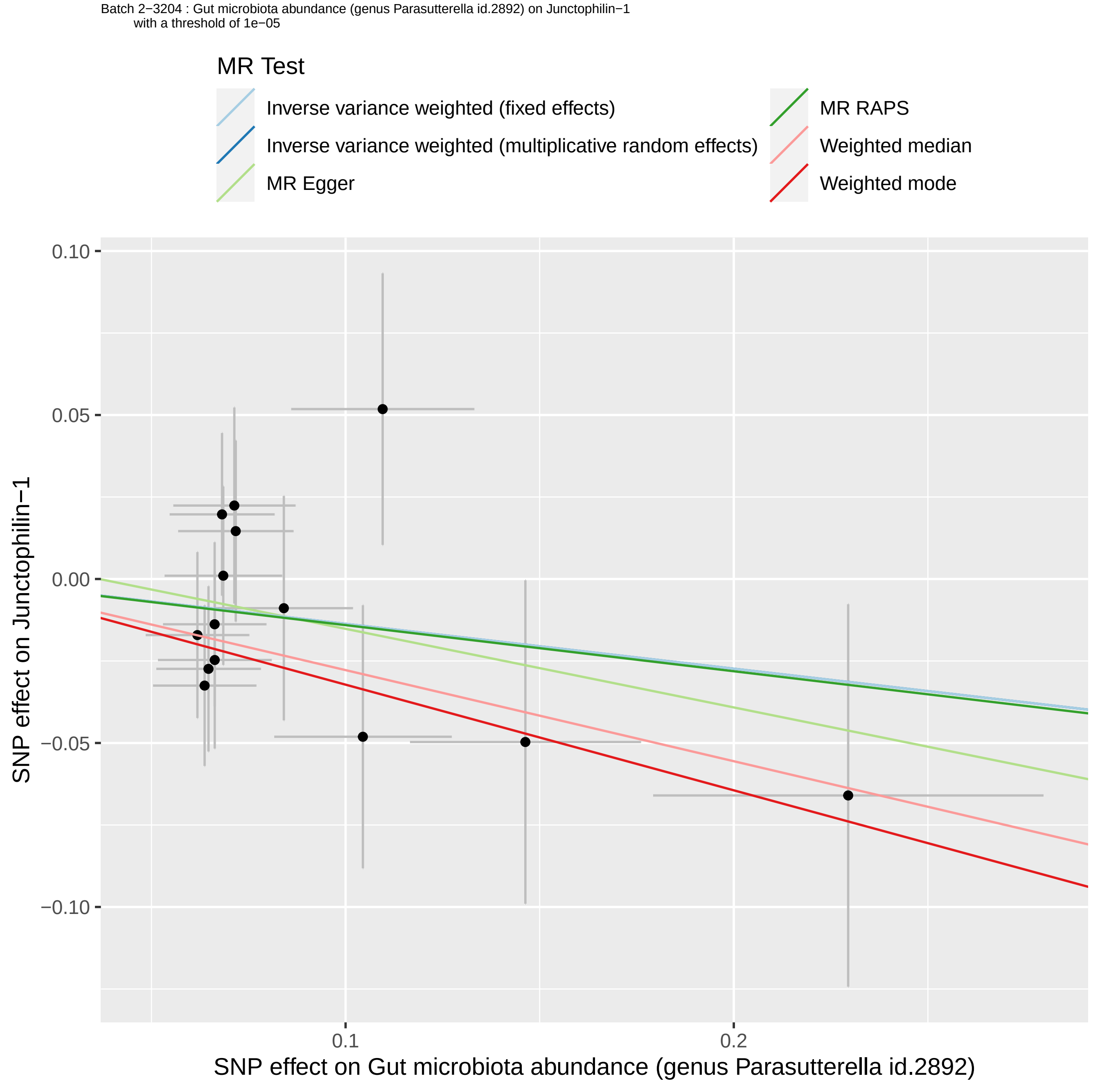
Batch 2−3204 : Gut microbiota abundance (genus Parasutterella id.2892) on Junctophilin−1
with a threshold of 1e−05
MR Test
Inverse variance weighted (fixed effects)
Inverse variance weighted (multiplicative random effects)
MR Egger
MR RAPS
Weighted median
Weighted mode
0.10
0.05
0.00
−0.05
−0.10
0.1
0.2
SNP effect on Junctophilin−1
SNP effect on Gut microbiota abundance (genus Parasutterella id.2892)

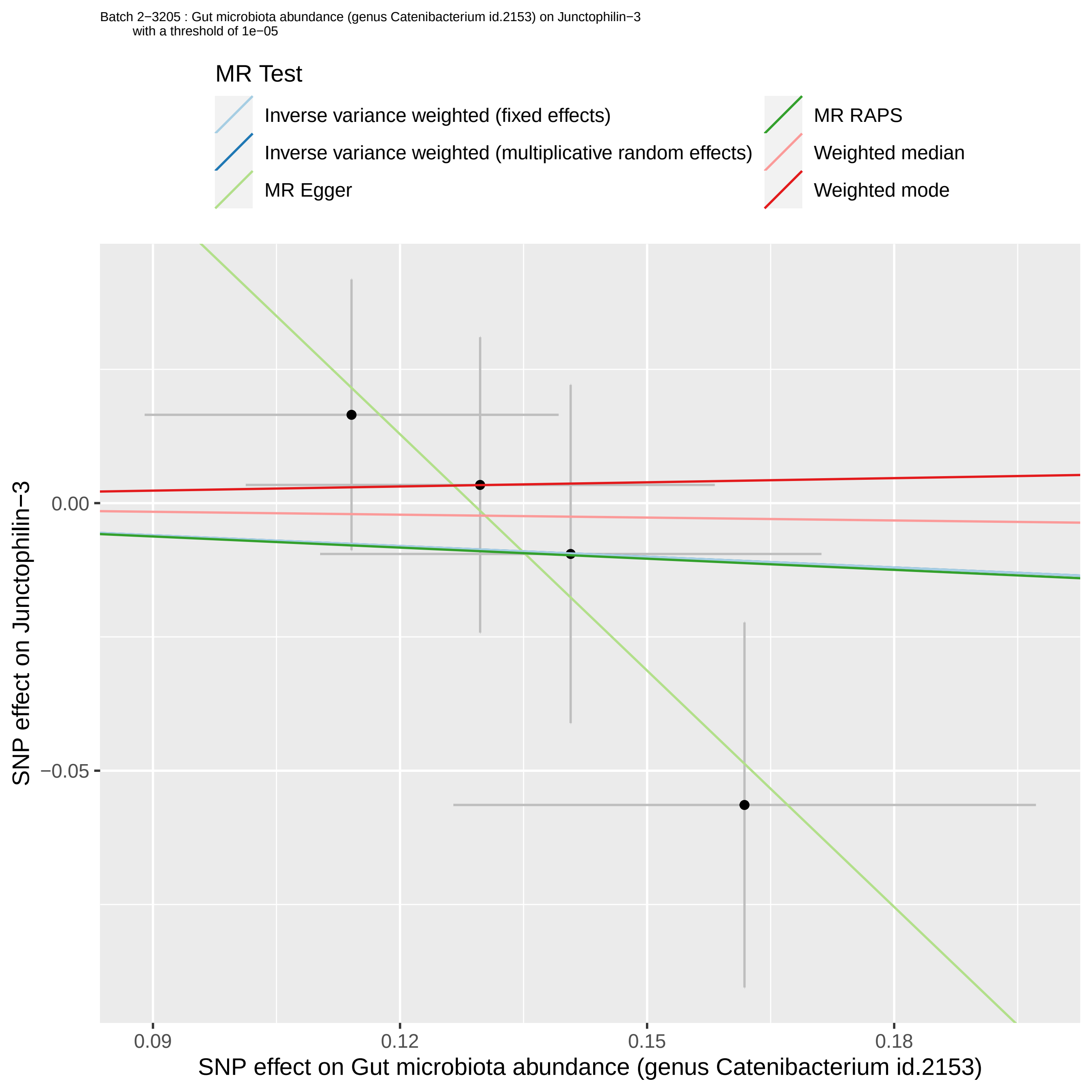
Batch 2−3205 : Gut microbiota abundance (genus Catenibacterium id.2153) on Junctophilin−3
with a threshold of 1e−05
MR Test
Inverse variance weighted (fixed effects)
Inverse variance weighted (multiplicative random effects)
MR Egger
MR RAPS
Weighted median
Weighted mode
0.00
−0.05
0.09
0.12
0.15
0.18
SNP effect on Junctophilin−3
SNP effect on Gut microbiota abundance (genus Catenibacterium id.2153)

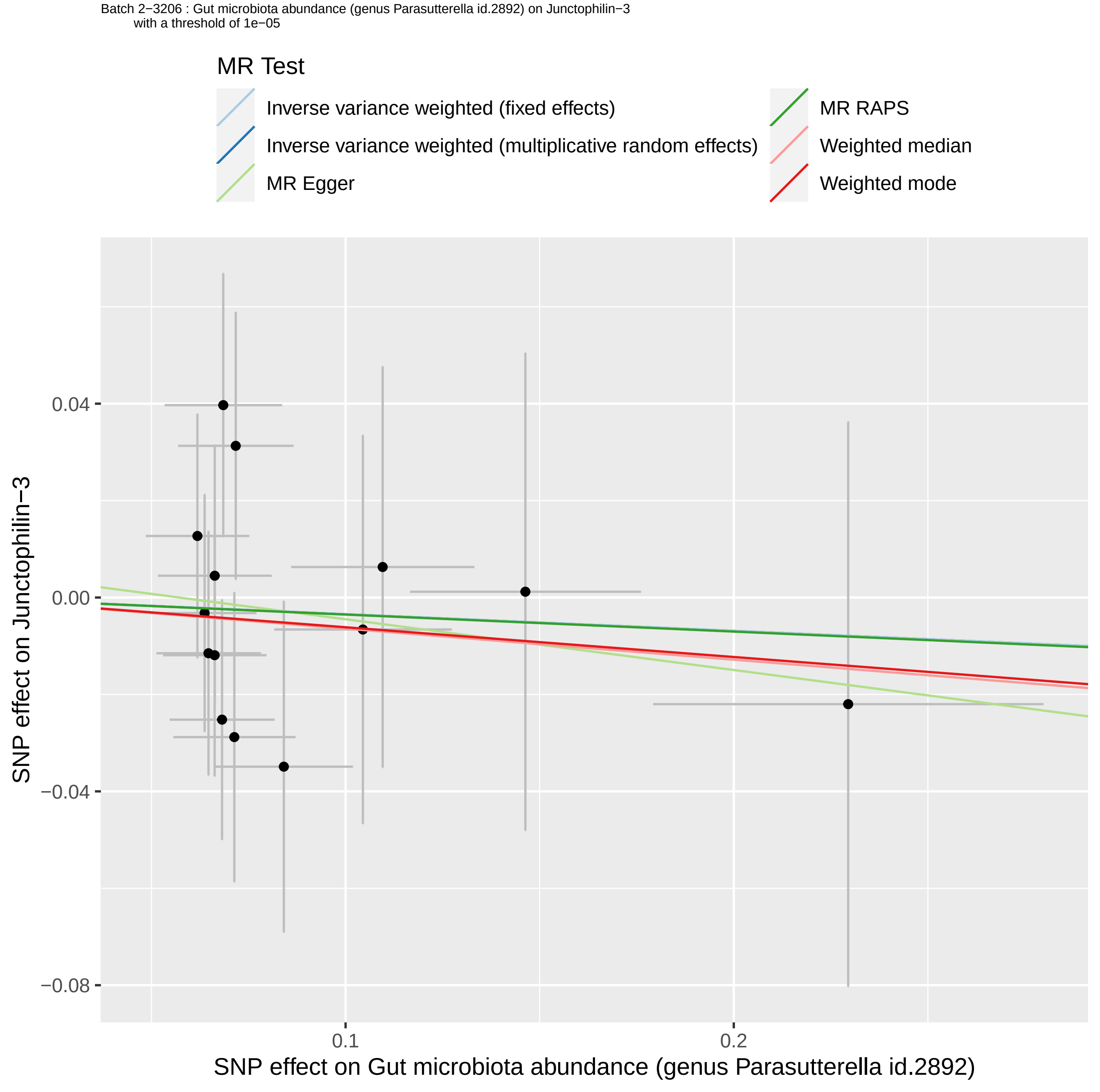

Batch 2−3206 : Gut microbiota abundance (genus Parasutterella id.2892) on Junctophilin−3
with a threshold of 1e−05
MR Test
Inverse variance weighted (fixed effects)
Inverse variance weighted (multiplicative random effects)
MR Egger
MR RAPS
Weighted median
Weighted mode
0.04
0.00
−0.04
−0.08
0.1
0.2
SNP effect on Junctophilin−3
SNP effect on Gut microbiota abundance (genus Parasutterella id.2892)

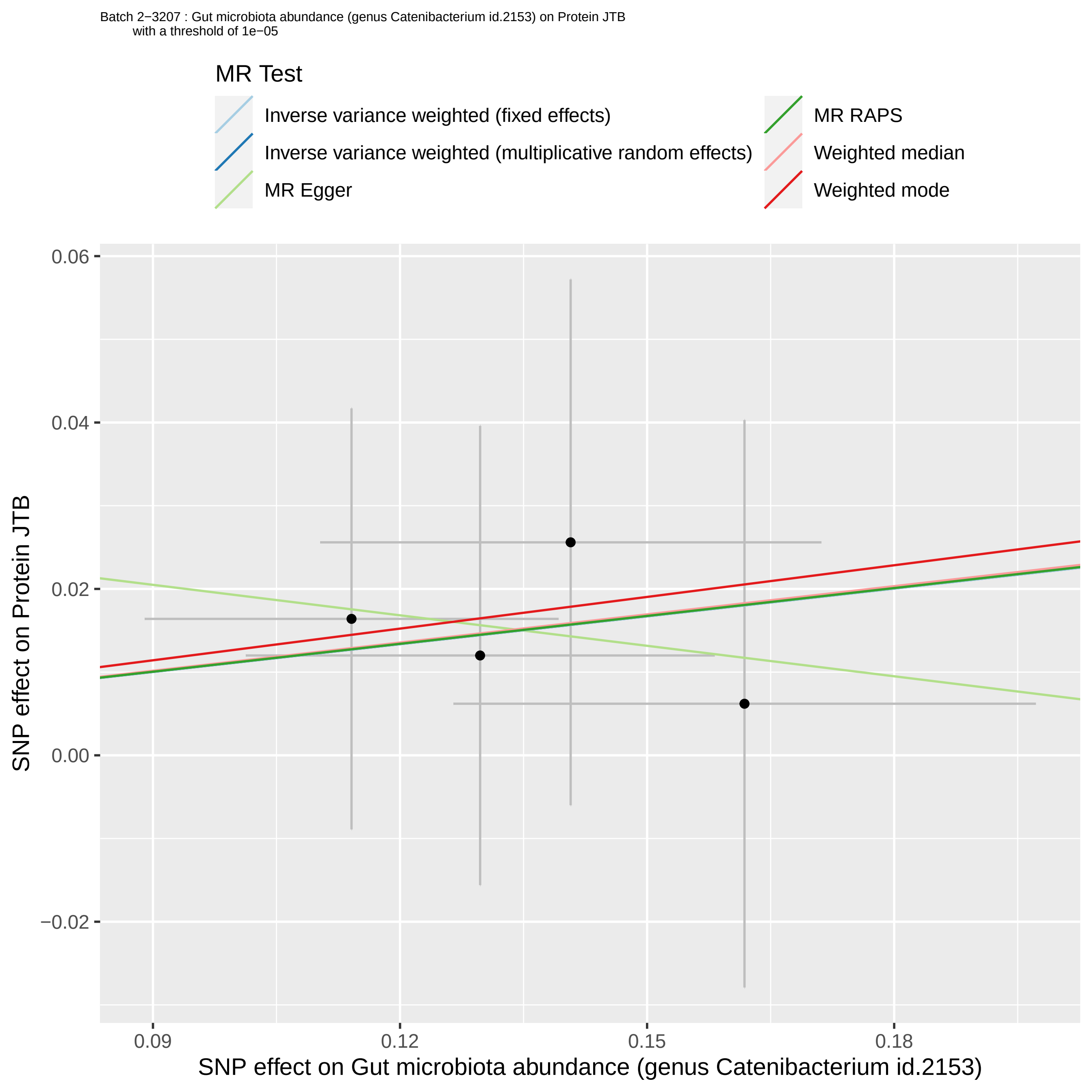
Batch 2−3207 : Gut microbiota abundance (genus Catenibacterium id.2153) on Protein JTB
with a threshold of 1e−05
MR Test
Inverse variance weighted (fixed effects)
Inverse variance weighted (multiplicative random effects)
MR Egger
MR RAPS
Weighted median
Weighted mode
0.06
0.04
0.02
0.00
−0.02
0.09
0.12
0.15
0.18
SNP effect on Protein JTB
SNP effect on Gut microbiota abundance (genus Catenibacterium id.2153)

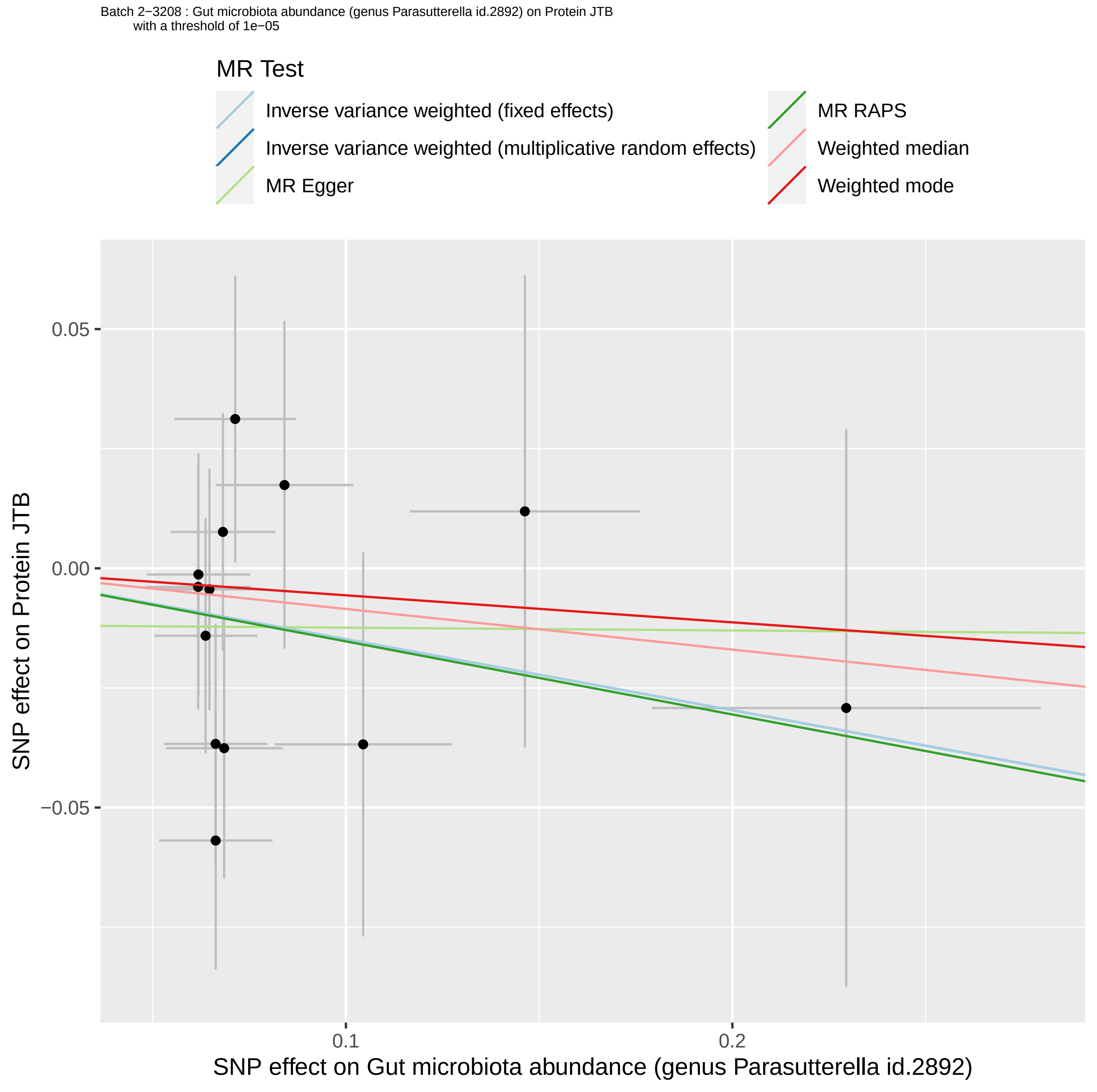

Batch 2−3208 : Gut microbiota abundance (genus Parasutterella id.2892) on Protein JTB
with a threshold of 1e−05
MR Test
Inverse variance weighted (fixed effects)
Inverse variance weighted (multiplicative random effects)
MR Egger
MR RAPS
Weighted median
Weighted mode
0.05
0.00
−0.05
0.1
0.2
SNP effect on Protein JTB
SNP effect on Gut microbiota abundance (genus Parasutterella id.2892)

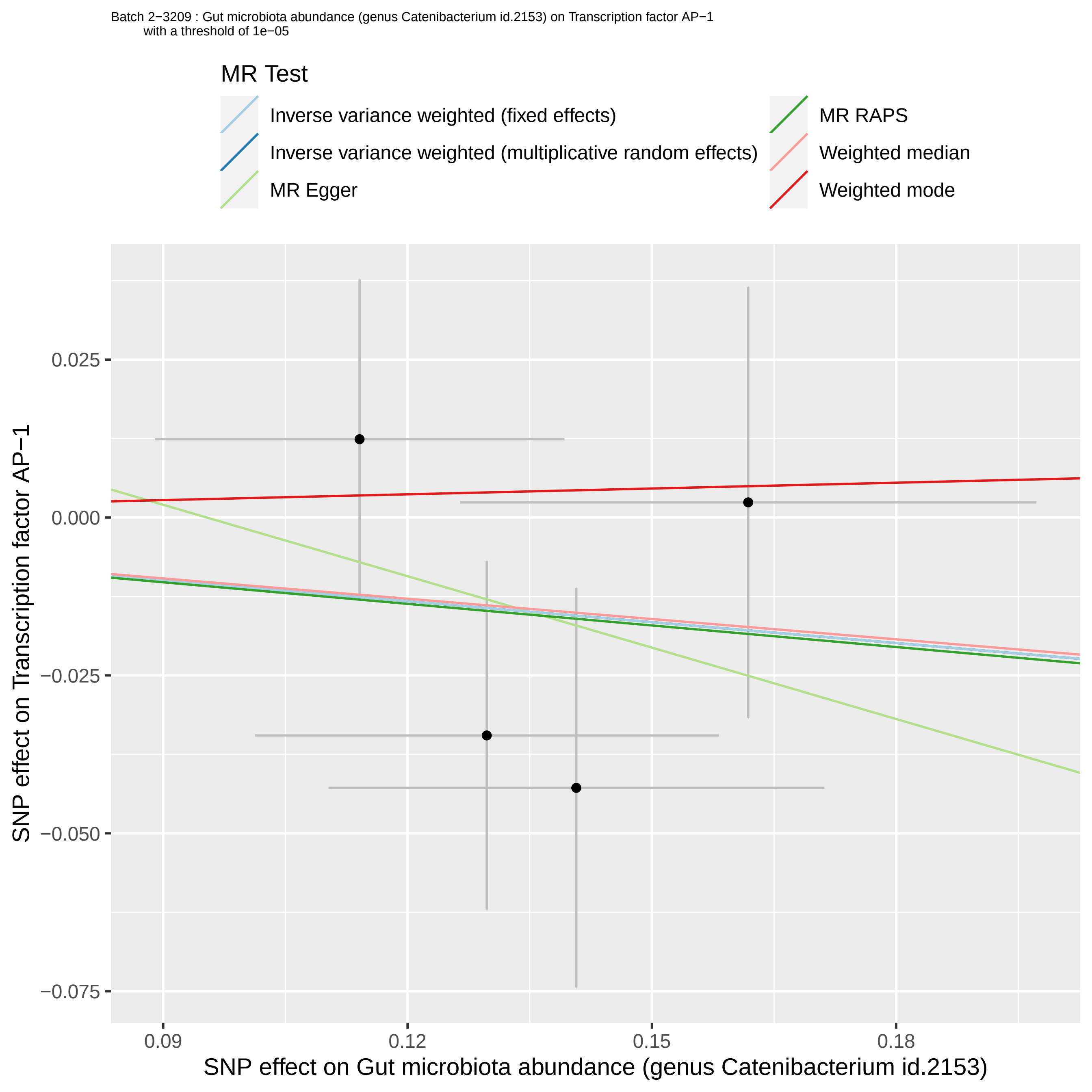
Batch 2−3209 : Gut microbiota abundance (genus Catenibacterium id.2153) on Transcription factor AP−1
with a threshold of 1e−05
MR Test
Inverse variance weighted (fixed effects)
Inverse variance weighted (multiplicative random effects)
MR Egger
MR RAPS
Weighted median
Weighted mode
SNP effect on Transcription factor AP−1
0.025
0.000
−0.025
−0.050
−0.075
0.09
0.12
0.15
0.18
SNP effect on Gut microbiota abundance (genus Catenibacterium id.2153)

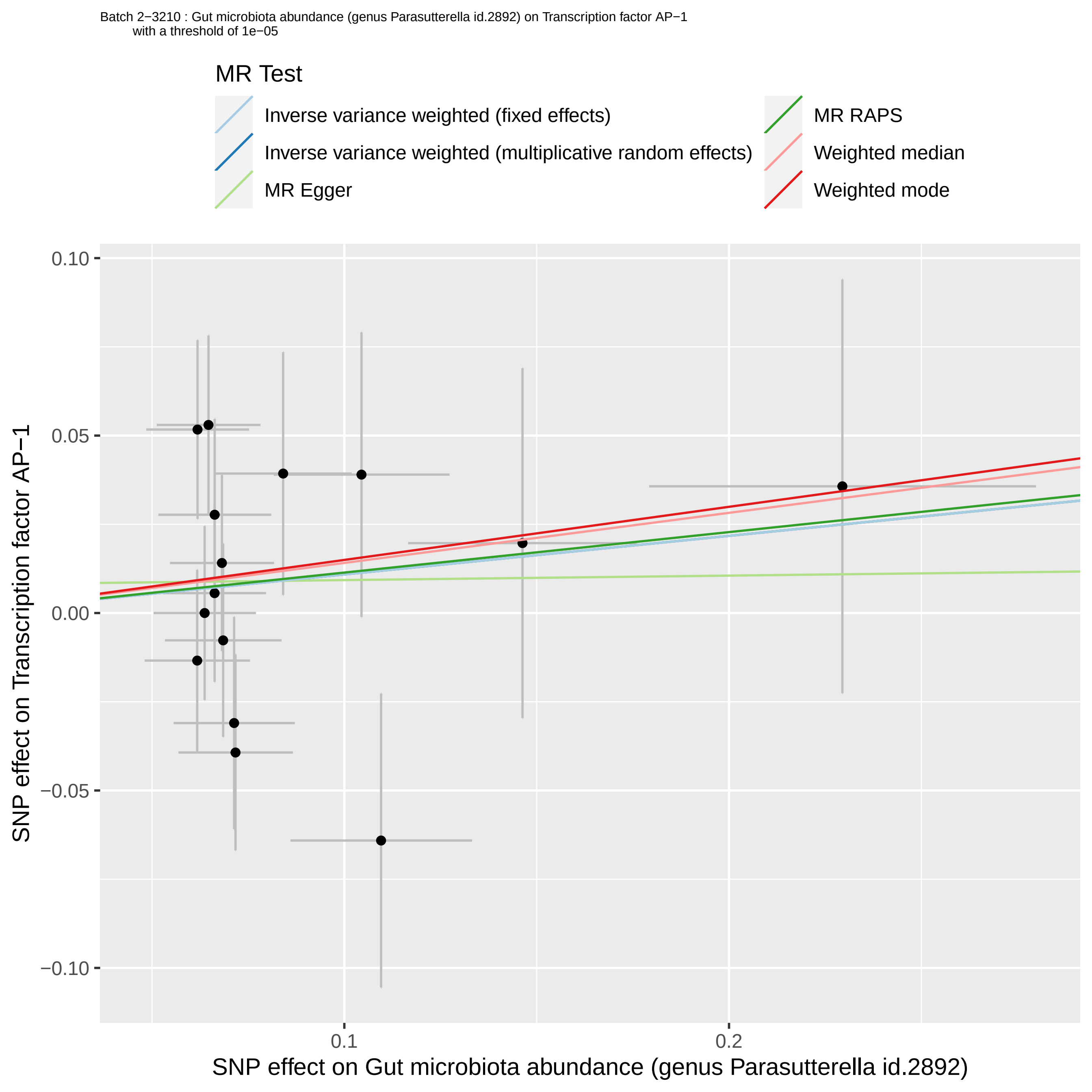

Batch 2−3210 : Gut microbiota abundance (genus Parasutterella id.2892) on Transcription factor AP−1
with a threshold of 1e−05
MR Test
Inverse variance weighted (fixed effects)
Inverse variance weighted (multiplicative random effects)
MR Egger
MR RAPS
Weighted median
Weighted mode
0.10
0.05
0.00
−0.05
−0.10
SNP effect on Transcription factor AP−1
0.1
0.2
SNP effect on Gut microbiota abundance (genus Parasutterella id.2892)

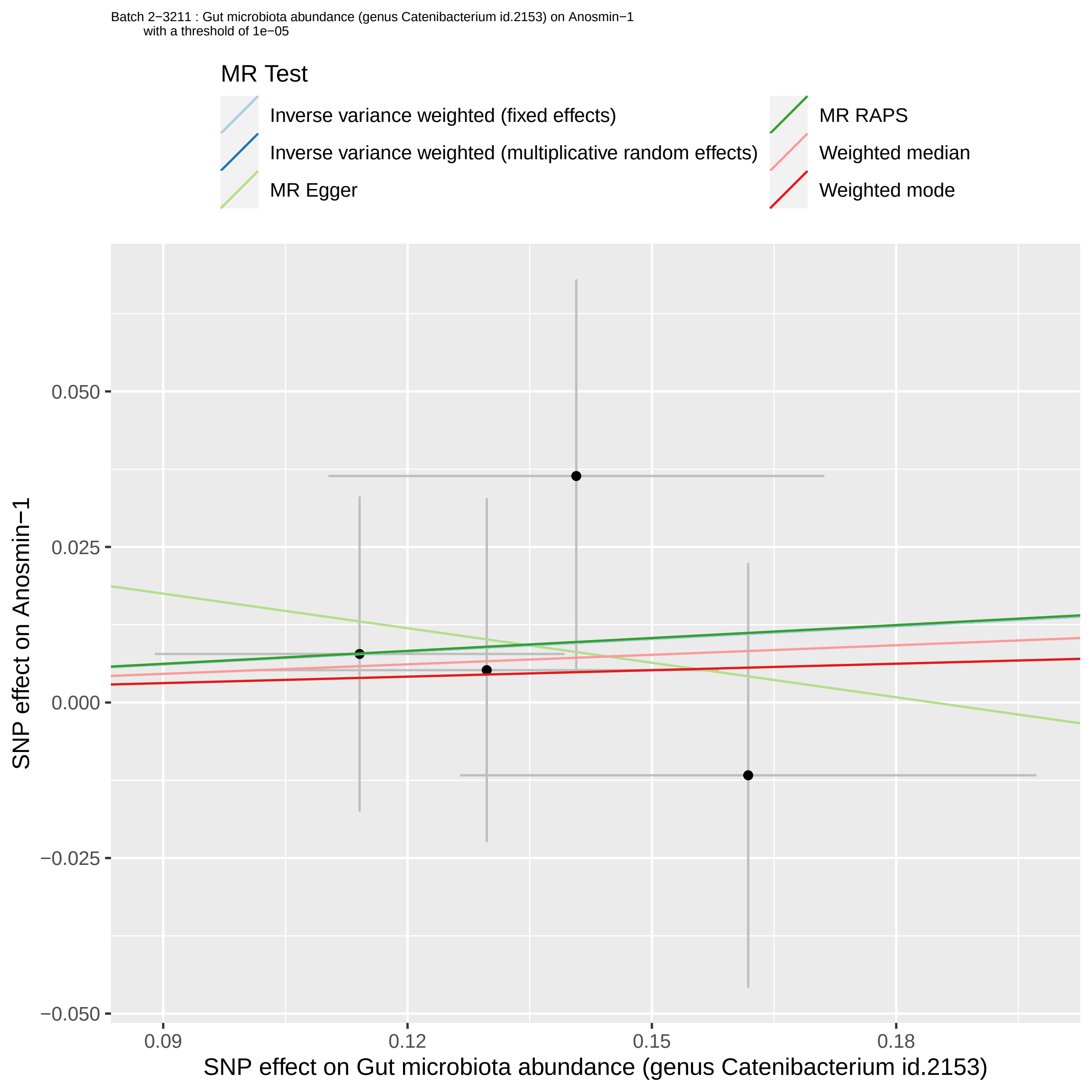
Batch 2−3211 : Gut microbiota abundance (genus Catenibacterium id.2153) on Anosmin−1
with a threshold of 1e−05
MR Test
Inverse variance weighted (fixed effects)
Inverse variance weighted (multiplicative random effects)
MR Egger
MR RAPS
Weighted median
Weighted mode
0.050
0.025
0.000
−0.025
−0.050
0.09
0.12
0.15
0.18
SNP effect on Anosmin−1
SNP effect on Gut microbiota abundance (genus Catenibacterium id.2153)

Batch 2−3212 : Gut microbiota abundance (genus Parasutterella id.2892) on Anosmin−1
with a threshold of 1e−05

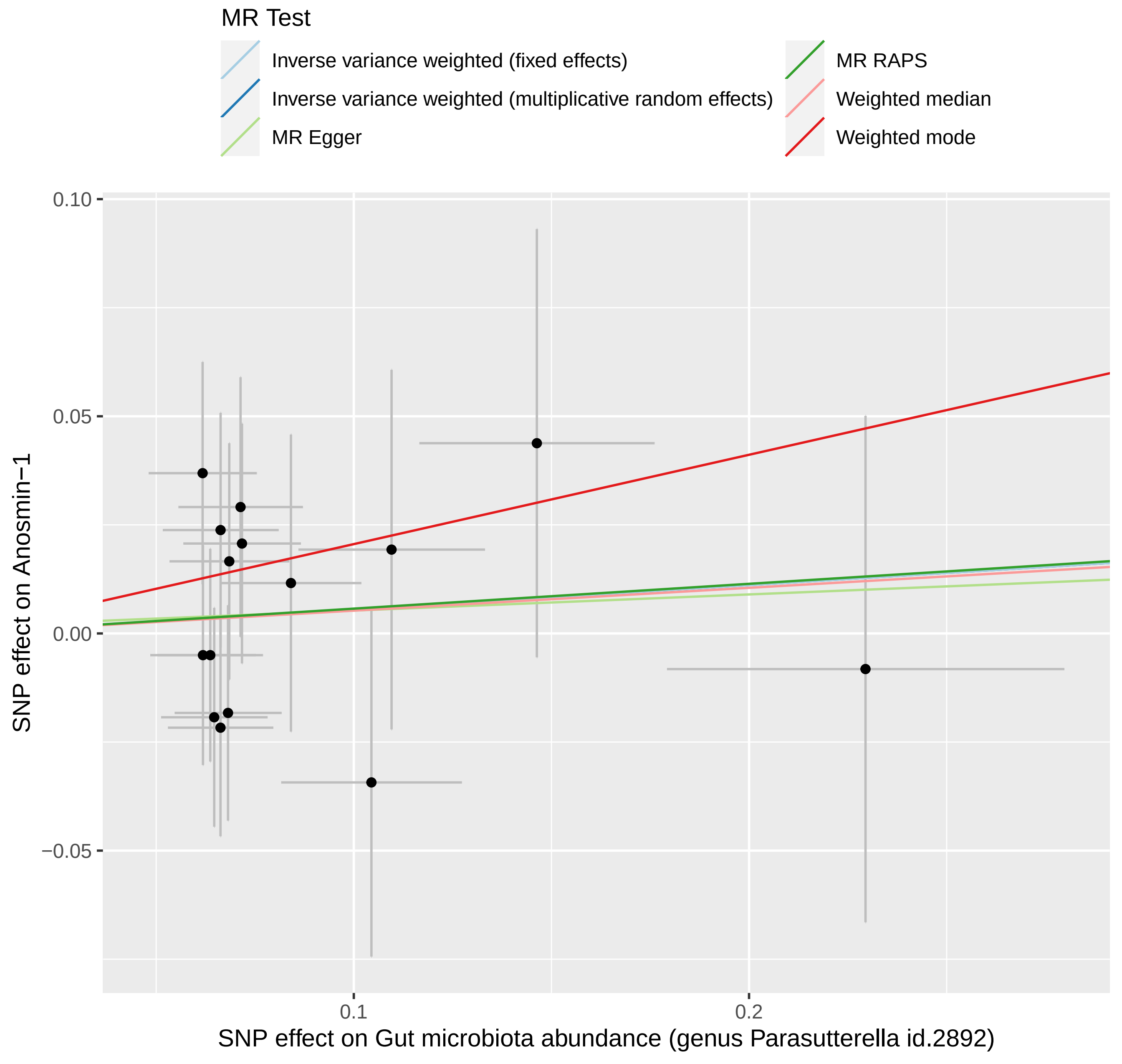

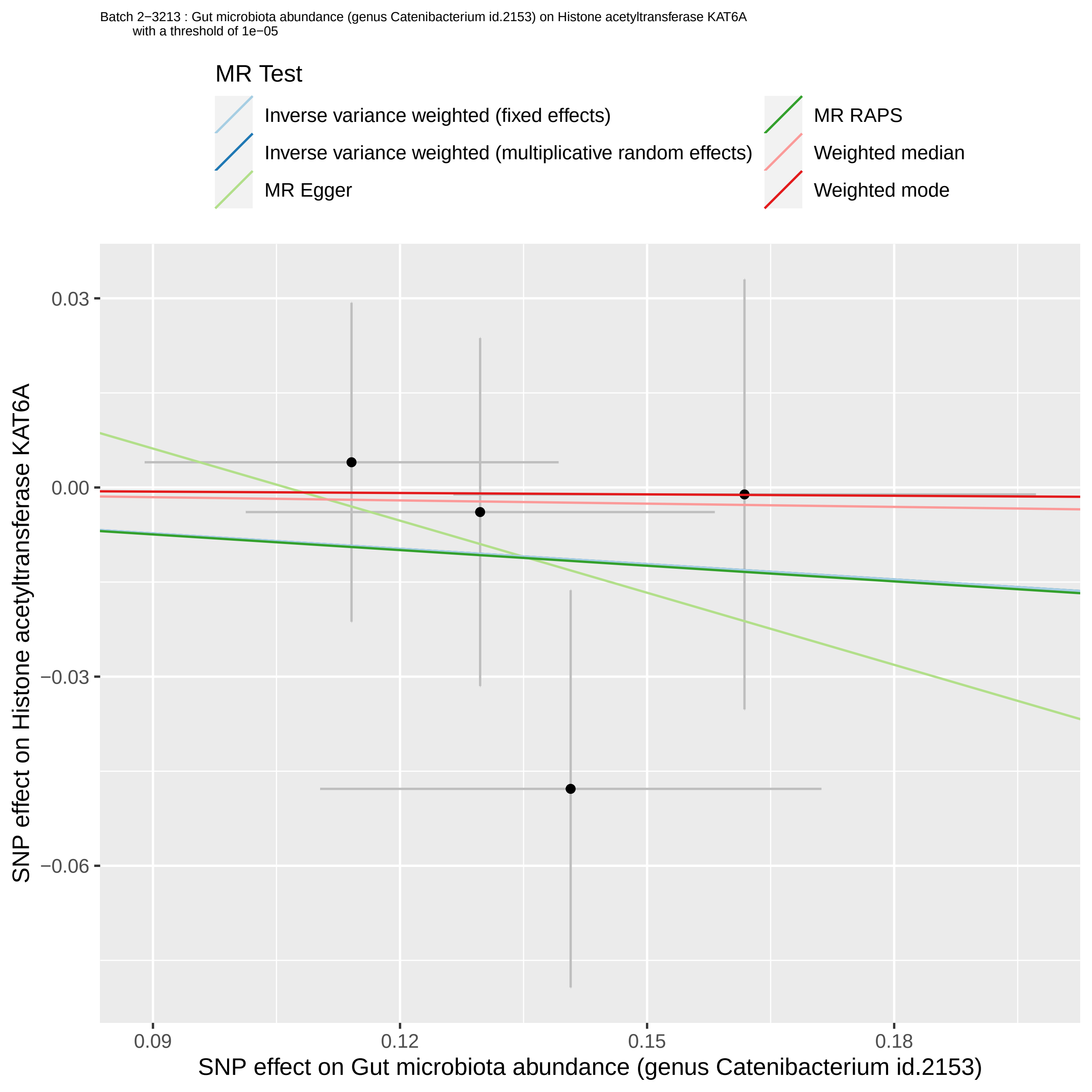
Batch 2−3213 : Gut microbiota abundance (genus Catenibacterium id.2153) on Histone acetyltransferase KAT6A
with a threshold of 1e−05
MR Test
Inverse variance weighted (fixed effects)
Inverse variance weighted (multiplicative random effects)
MR Egger
MR RAPS
Weighted median
Weighted mode
0.03
0.00
−0.03
−0.06
0.09
0.12
0.15
0.18
SNP effect on Histone acetyltransferase KAT6A
SNP effect on Gut microbiota abundance (genus Catenibacterium id.2153)

Batch 2−3214 : Gut microbiota abundance (genus Parasutterella id.2892) on Histone acetyltransferase KAT6A
with a threshold of 1e−05

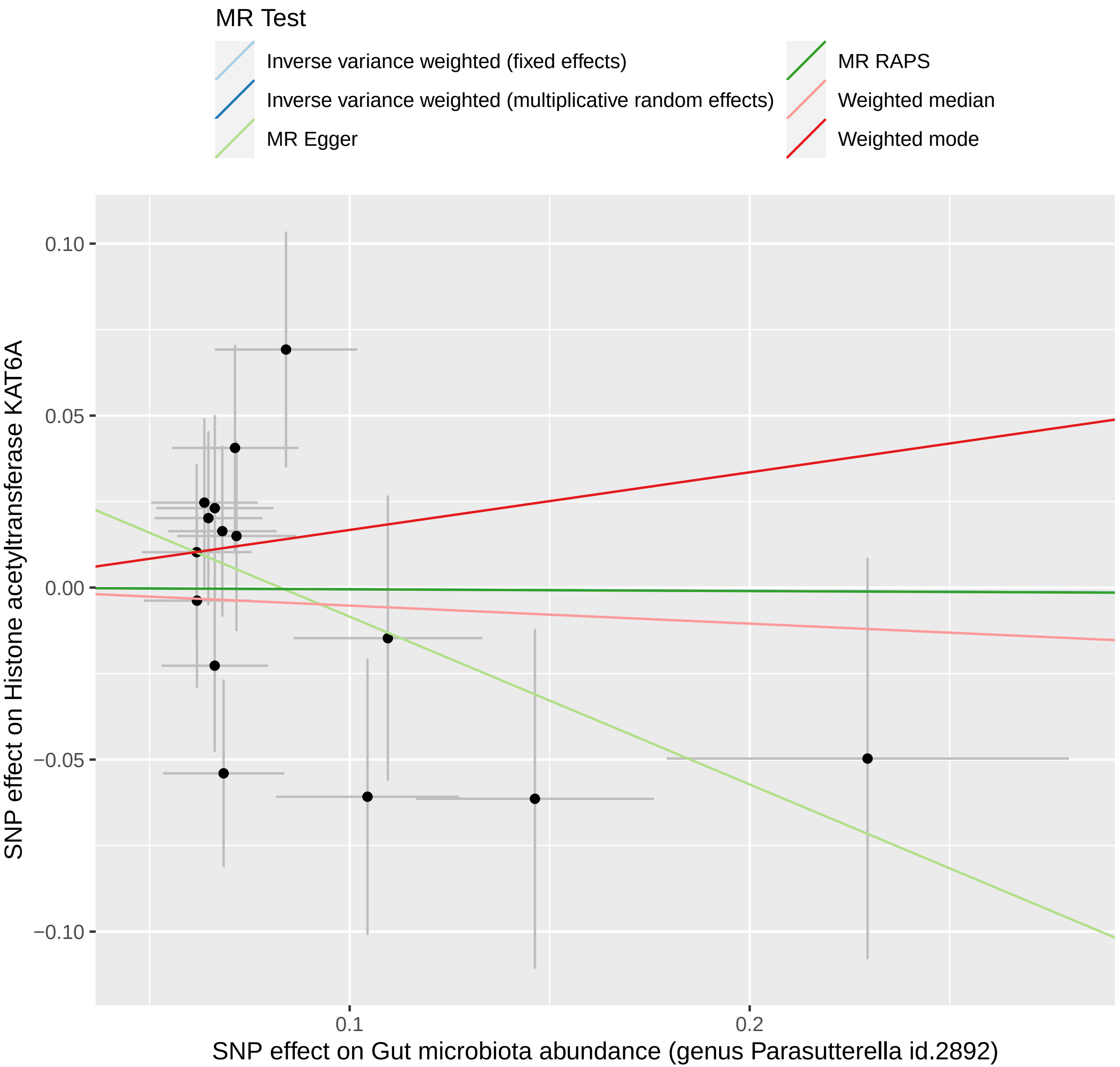

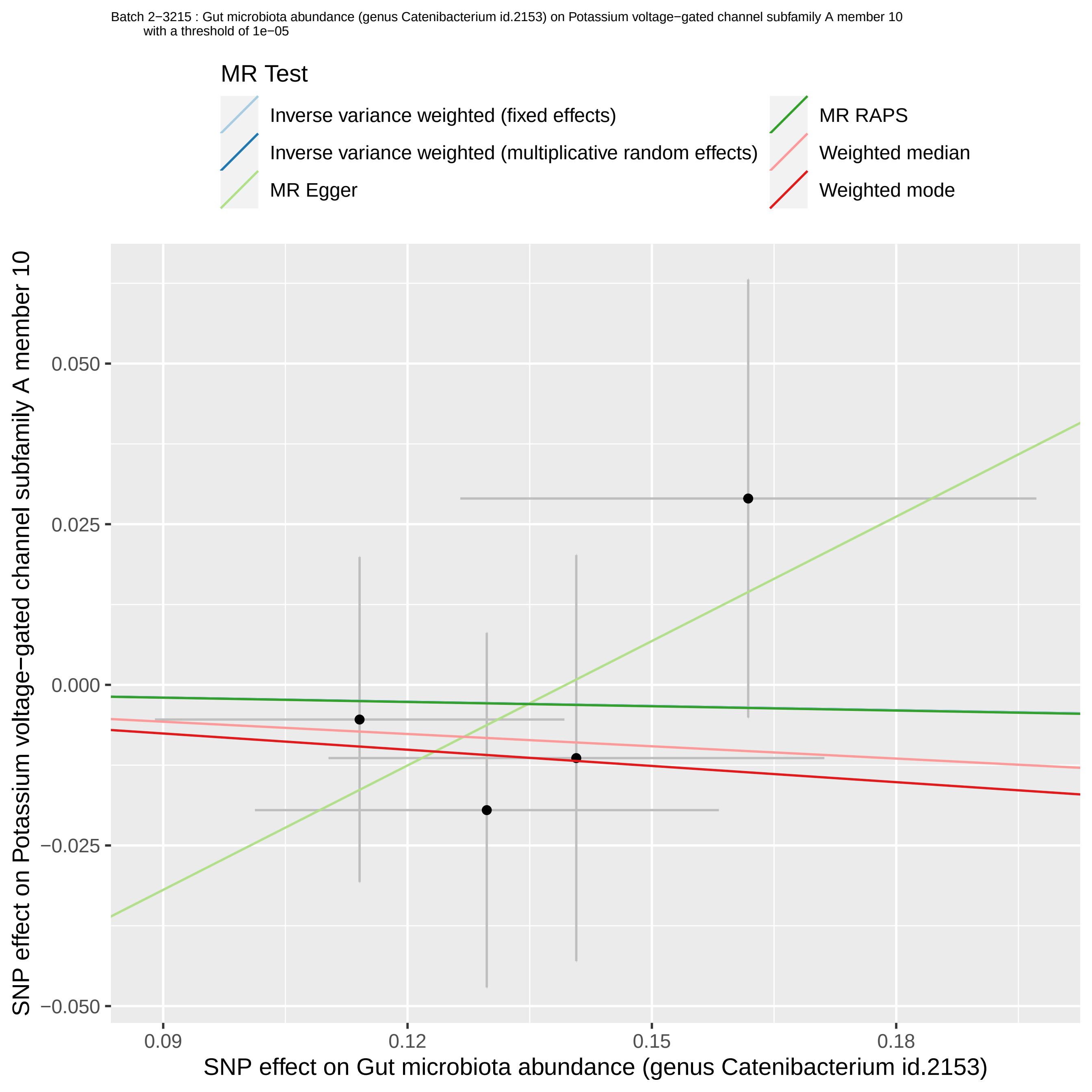
Batch 2−3215 : Gut microbiota abundance (genus Catenibacterium id.2153) on Potassium voltage−gated channel subfamily A member 10
with a threshold of 1e−05
MR Test
Inverse variance weighted (fixed effects)
Inverse variance weighted (multiplicative random effects)
MR Egger
MR RAPS
Weighted median
Weighted mode
SNP effect on Potassium voltage−gated channel subfamily A member 10
0.050
0.025
0.000
−0.025
−0.050
0.09
0.12
0.15
0.18
SNP effect on Gut microbiota abundance (genus Catenibacterium id.2153)

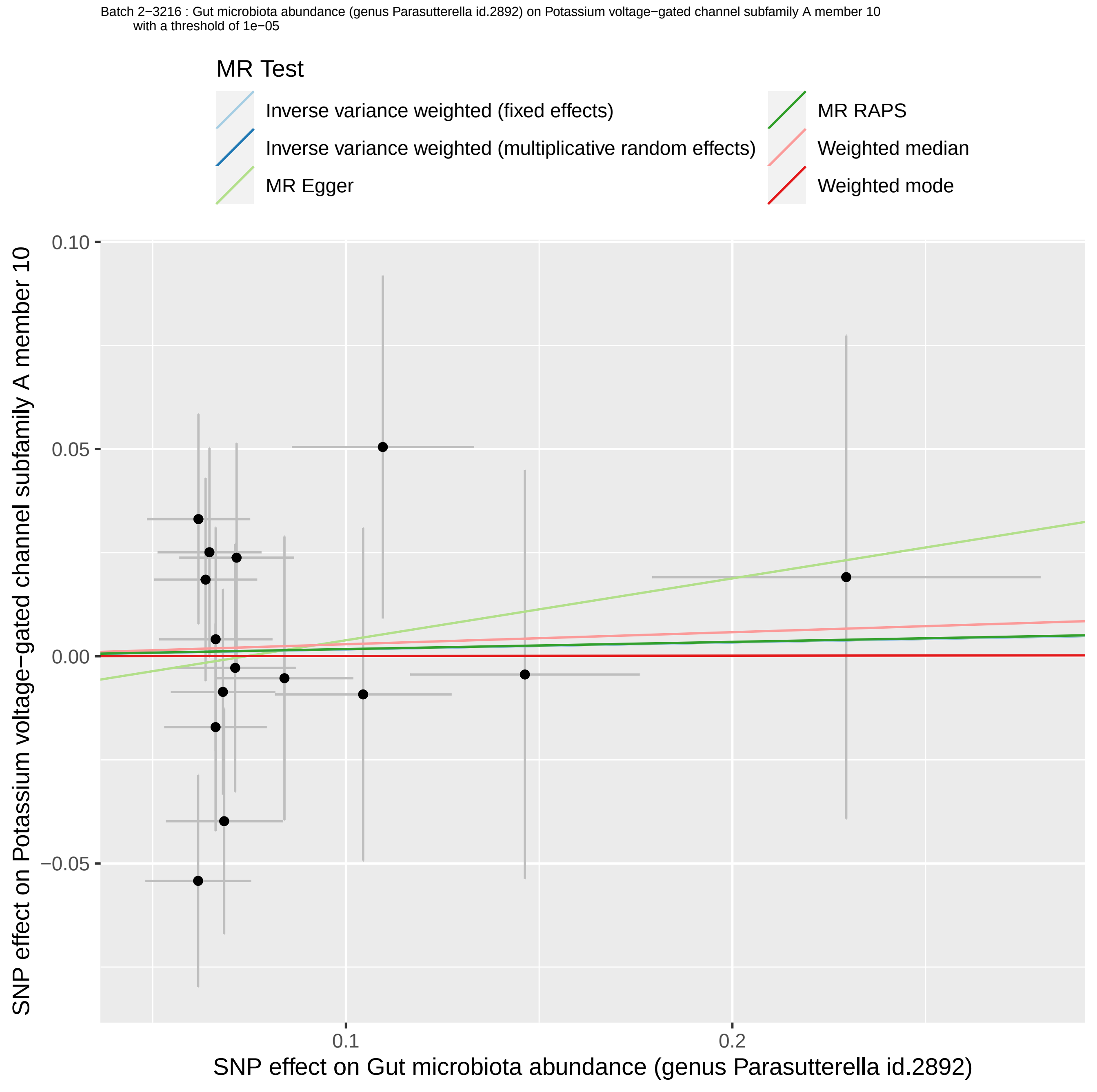
Batch 2−3216 : Gut microbiota abundance (genus Parasutterella id.2892) on Potassium voltage−gated channel subfamily A member 10
with a threshold of 1e−05
MR Test
Inverse variance weighted (fixed effects)
Inverse variance weighted (multiplicative random effects)
MR Egger
MR RAPS
Weighted median
Weighted mode
0.10
0.05
0.00
−0.05
0.1
0.2
SNP effect on Potassium voltage−gated channel subfamily A member 10
SNP effect on Gut microbiota abundance (genus Parasutterella id.2892)

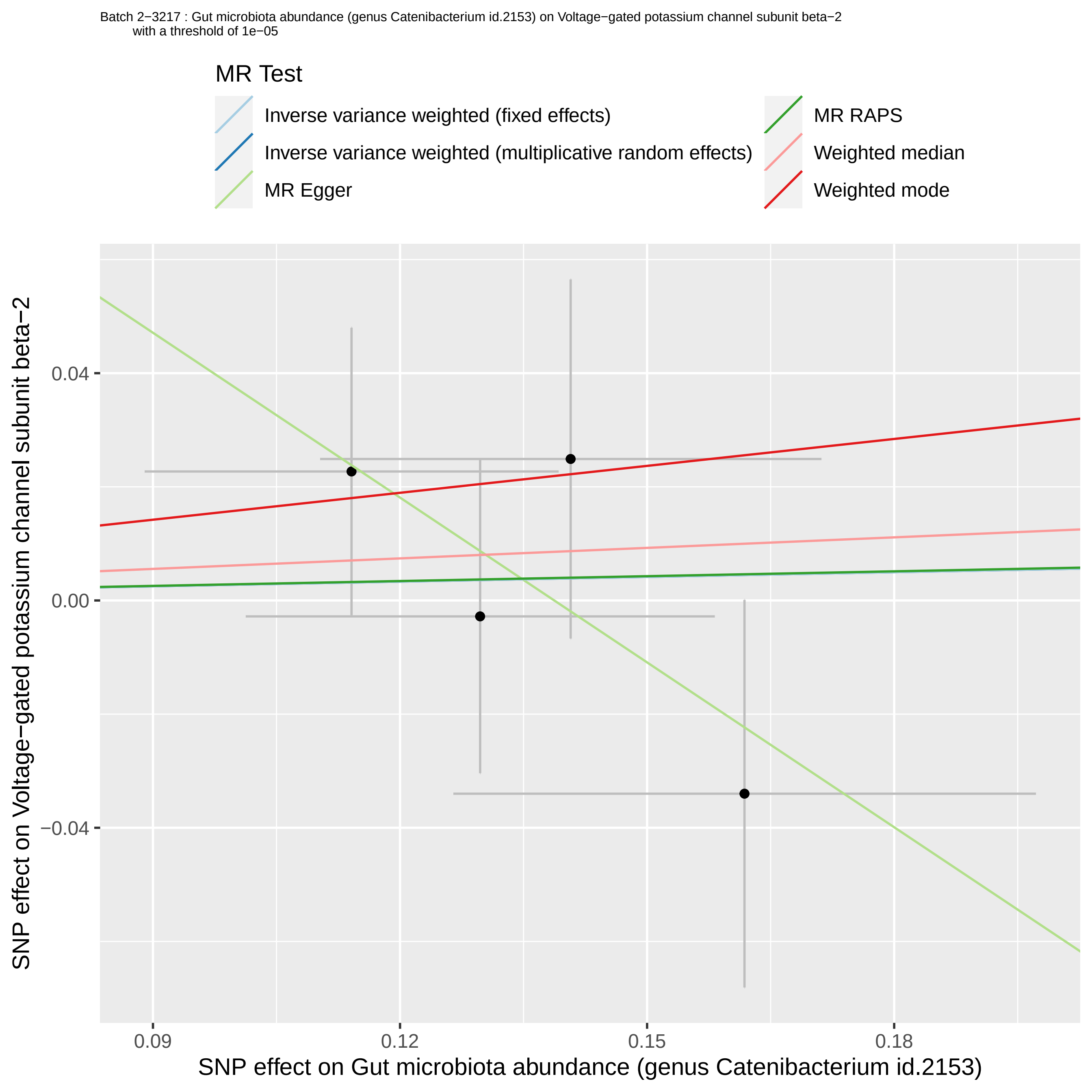
Batch 2−3217 : Gut microbiota abundance (genus Catenibacterium id.2153) on Voltage−gated potassium channel subunit beta−2
with a threshold of 1e−05
MR Test
Inverse variance weighted (fixed effects)
Inverse variance weighted (multiplicative random effects)
MR Egger
MR RAPS
Weighted median
Weighted mode
0.04
0.00
−0.04
0.09
0.12
0.15
0.18
SNP effect on Voltage−gated potassium channel subunit beta−2
SNP effect on Gut microbiota abundance (genus Catenibacterium id.2153)

Batch 2−3218 : Gut microbiota abundance (genus Parasutterella id.2892) on Voltage−gated potassium channel subunit beta−2
with a threshold of 1e−05

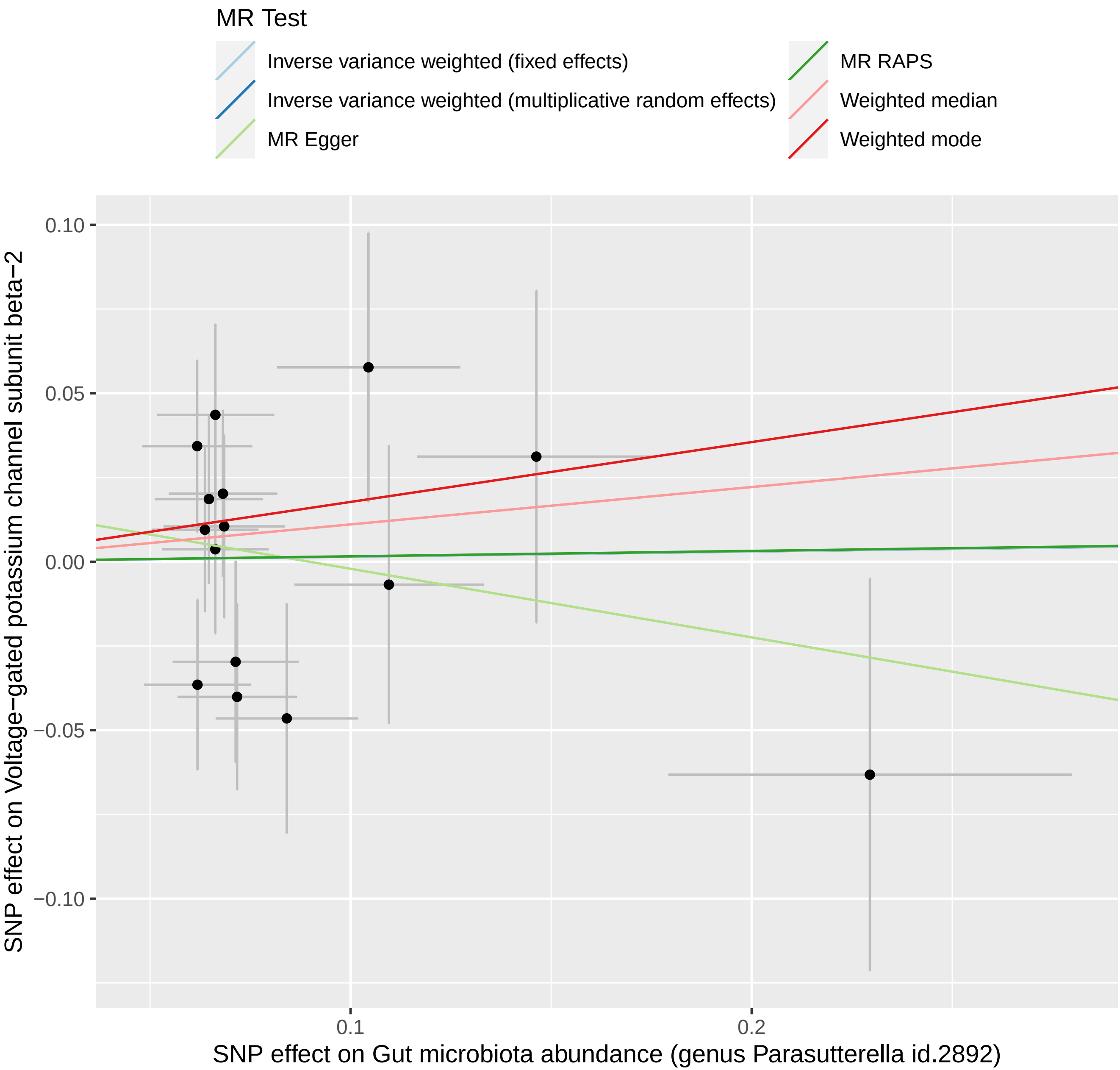

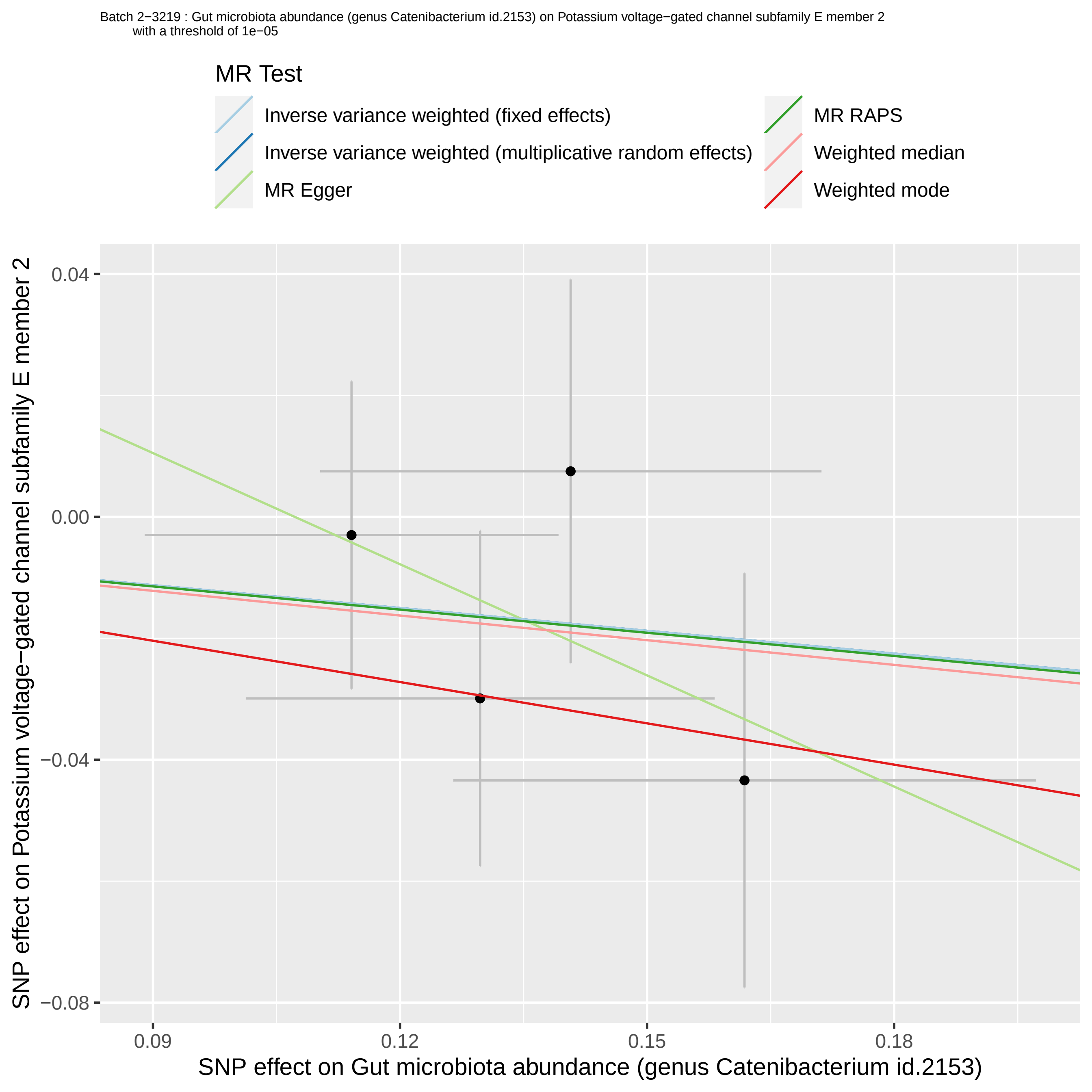

Batch 2−3219 : Gut microbiota abundance (genus Catenibacterium id.2153) on Potassium voltage−gated channel subfamily E member 2
with a threshold of 1e−05
MR Test
Inverse variance weighted (fixed effects)
Inverse variance weighted (multiplicative random effects)
MR Egger
MR RAPS
Weighted median
Weighted mode
0.04
0.00
−0.04
−0.08
0.09
0.12
0.15
0.18
SNP effect on Potassium voltage−gated channel subfamily E member 2
SNP effect on Gut microbiota abundance (genus Catenibacterium id.2153)

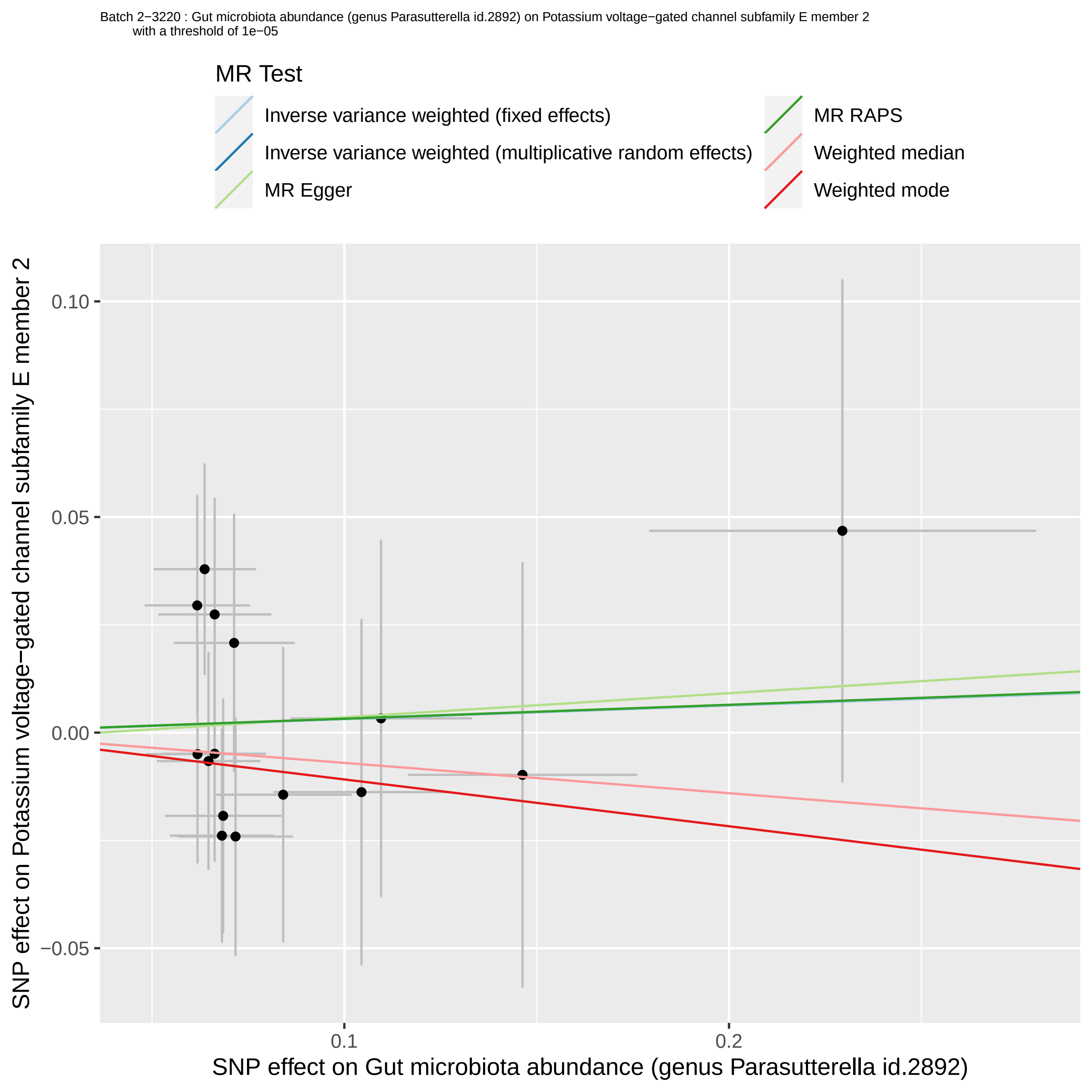

Batch 2−3220 : Gut microbiota abundance (genus Parasutterella id.2892) on Potassium voltage−gated channel subfamily E member 2
with a threshold of 1e−05
MR Test
Inverse variance weighted (fixed effects)
Inverse variance weighted (multiplicative random effects)
MR Egger
MR RAPS
Weighted median
Weighted mode
SNP effect on Potassium voltage−gated channel subfamily E member 2
0.10
0.05
0.00
−0.05
0.1
0.2
SNP effect on Gut microbiota abundance (genus Parasutterella id.2892)

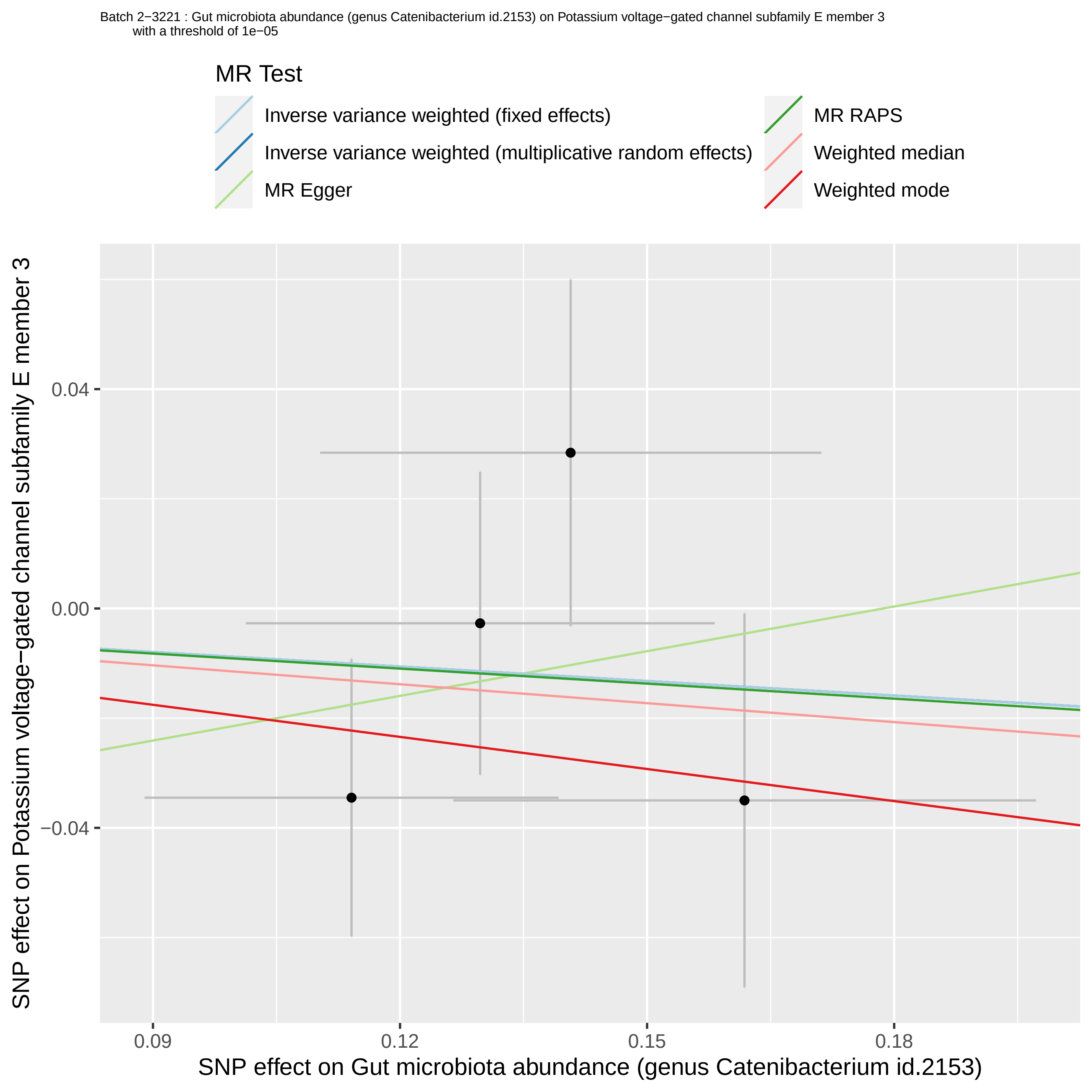

Batch 2−3221 : Gut microbiota abundance (genus Catenibacterium id.2153) on Potassium voltage−gated channel subfamily E member 3
with a threshold of 1e−05
MR Test
Inverse variance weighted (fixed effects)
Inverse variance weighted (multiplicative random effects)
MR Egger
MR RAPS
Weighted median
Weighted mode
0.04
0.00
−0.04
0.09
0.12
0.15
0.18
SNP effect on Potassium voltage−gated channel subfamily E member 3
SNP effect on Gut microbiota abundance (genus Catenibacterium id.2153)

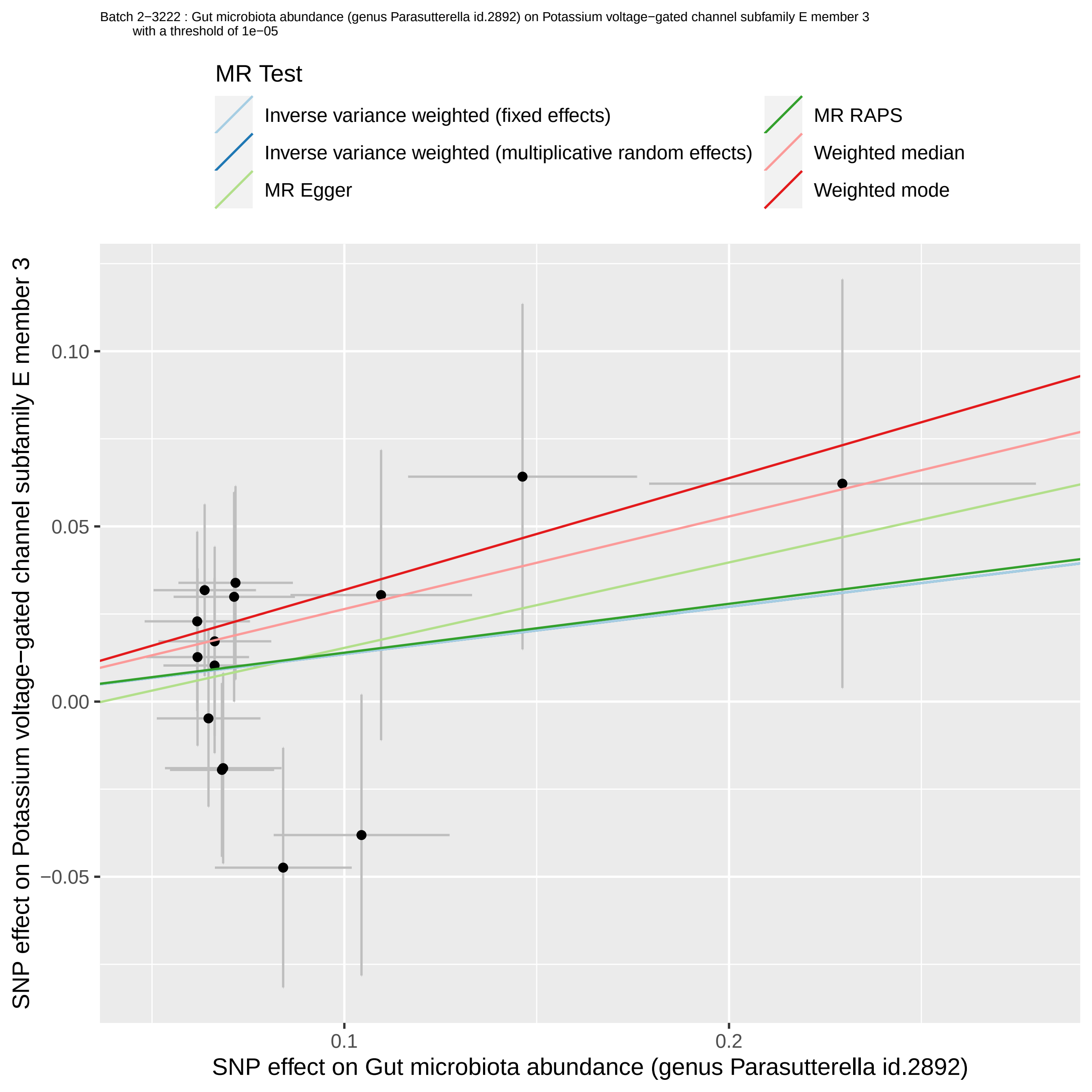

Batch 2−3222 : Gut microbiota abundance (genus Parasutterella id.2892) on Potassium voltage−gated channel subfamily E member 3
with a threshold of 1e−05
MR Test
Inverse variance weighted (fixed effects)
Inverse variance weighted (multiplicative random effects)
MR Egger
MR RAPS
Weighted median
Weighted mode
SNP effect on Potassium voltage−gated channel subfamily E member 3
0.10
0.05
0.00
−0.05
0.1
0.2
SNP effect on Gut microbiota abundance (genus Parasutterella id.2892)

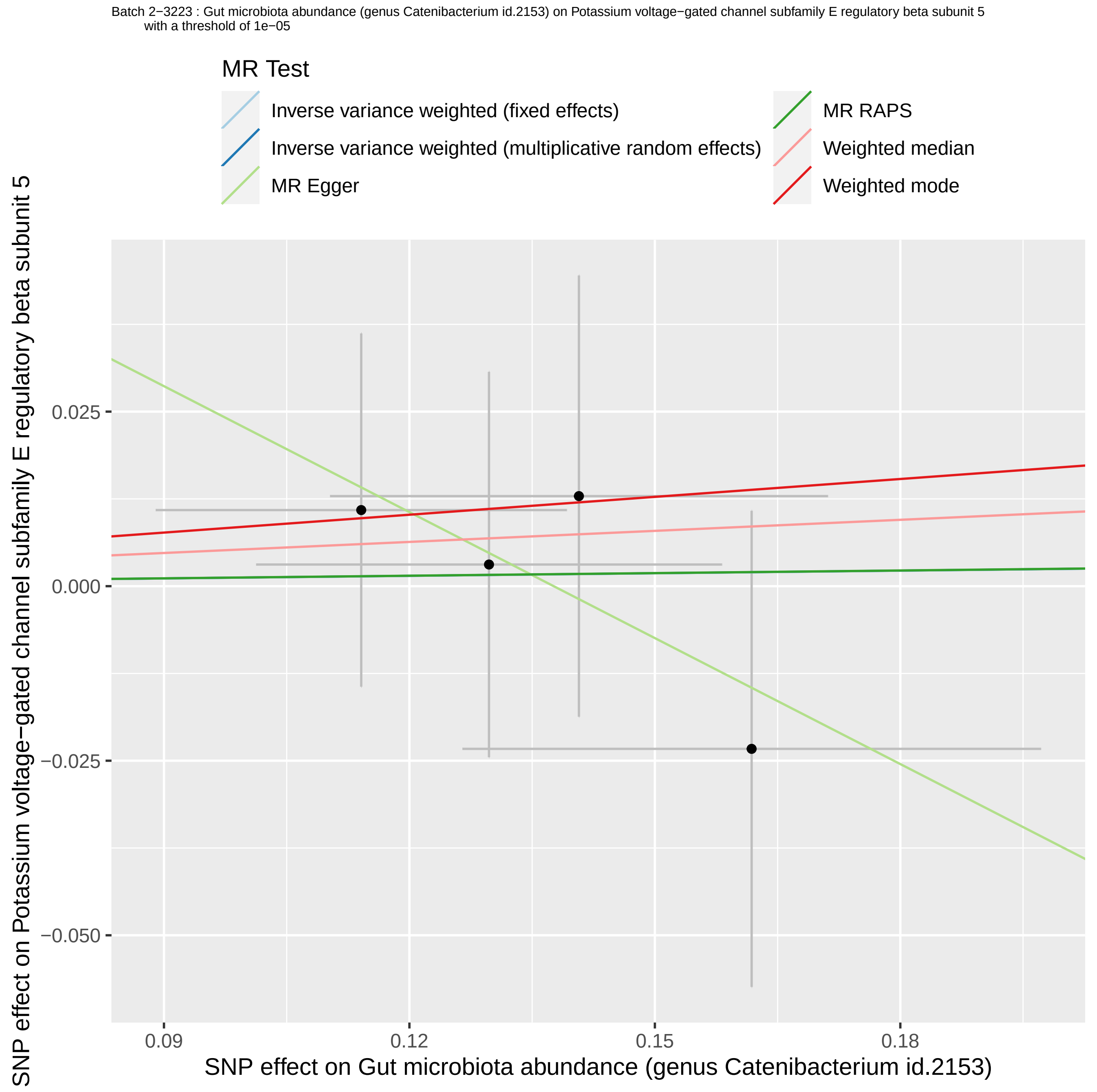

Batch 2−3223 : Gut microbiota abundance (genus Catenibacterium id.2153) on Potassium voltage−gated channel subfamily E regulatory beta subunit 5
with a threshold of 1e−05
MR Test
Inverse variance weighted (fixed effects)
Inverse variance weighted (multiplicative random effects)
MR Egger
MR RAPS
Weighted median
Weighted mode
0.025
0.000
−0.025
−0.050
0.09
0.12
0.15
0.18
SNP effect on Potassium voltage−gated channel subfamily E regulatory beta subunit 5
SNP effect on Gut microbiota abundance (genus Catenibacterium id.2153)

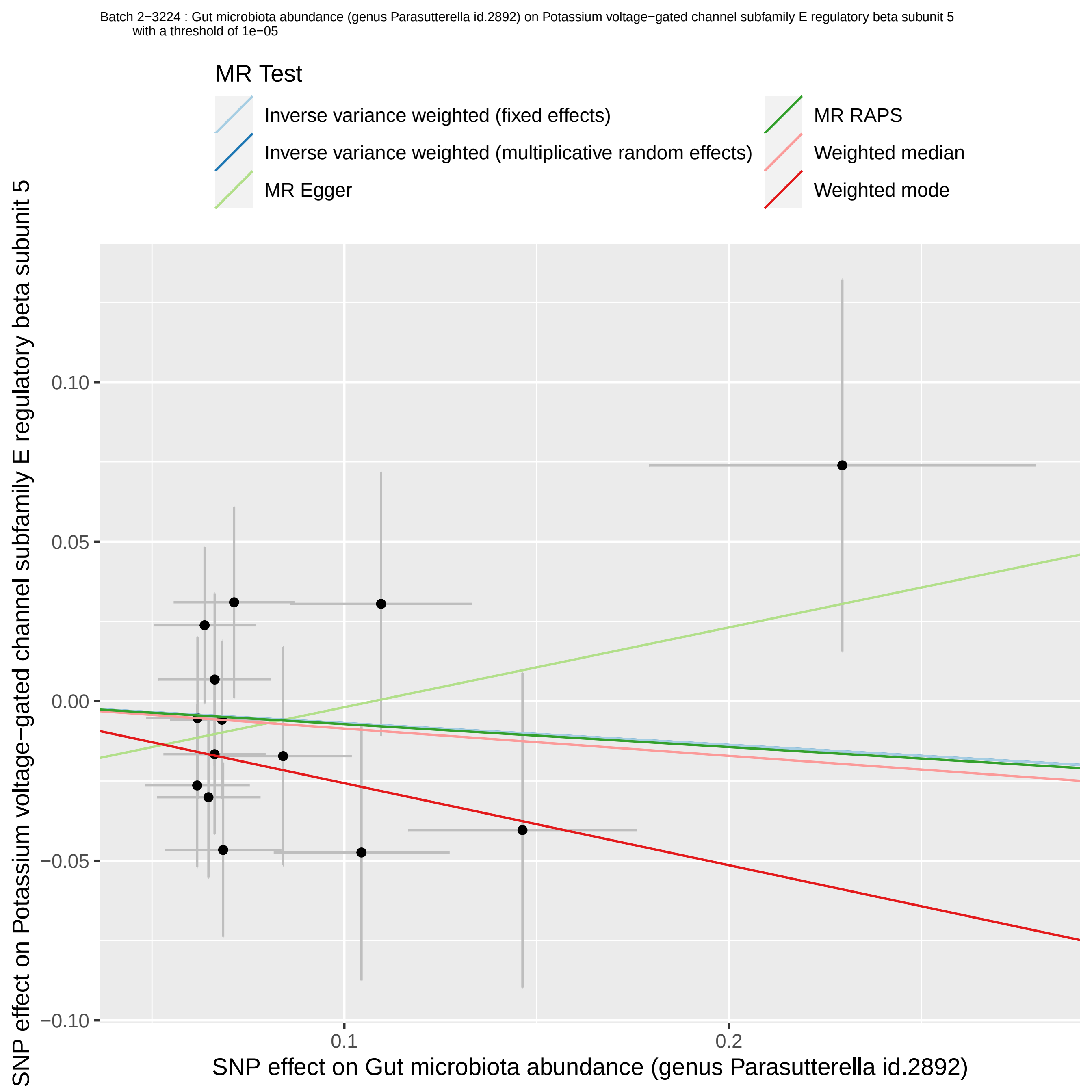

Batch 2−3224 : Gut microbiota abundance (genus Parasutterella id.2892) on Potassium voltage−gated channel subfamily E regulatory beta subunit 5
with a threshold of 1e−05
MR Test
Inverse variance weighted (fixed effects)
Inverse variance weighted (multiplicative random effects)
MR Egger
MR RAPS
Weighted median
Weighted mode
0.10
0.05
0.00
−0.05
−0.10
0.1
0.2
SNP effect on Potassium voltage−gated channel subfamily E regulatory beta subunit 5
SNP effect on Gut microbiota abundance (genus Parasutterella id.2892)

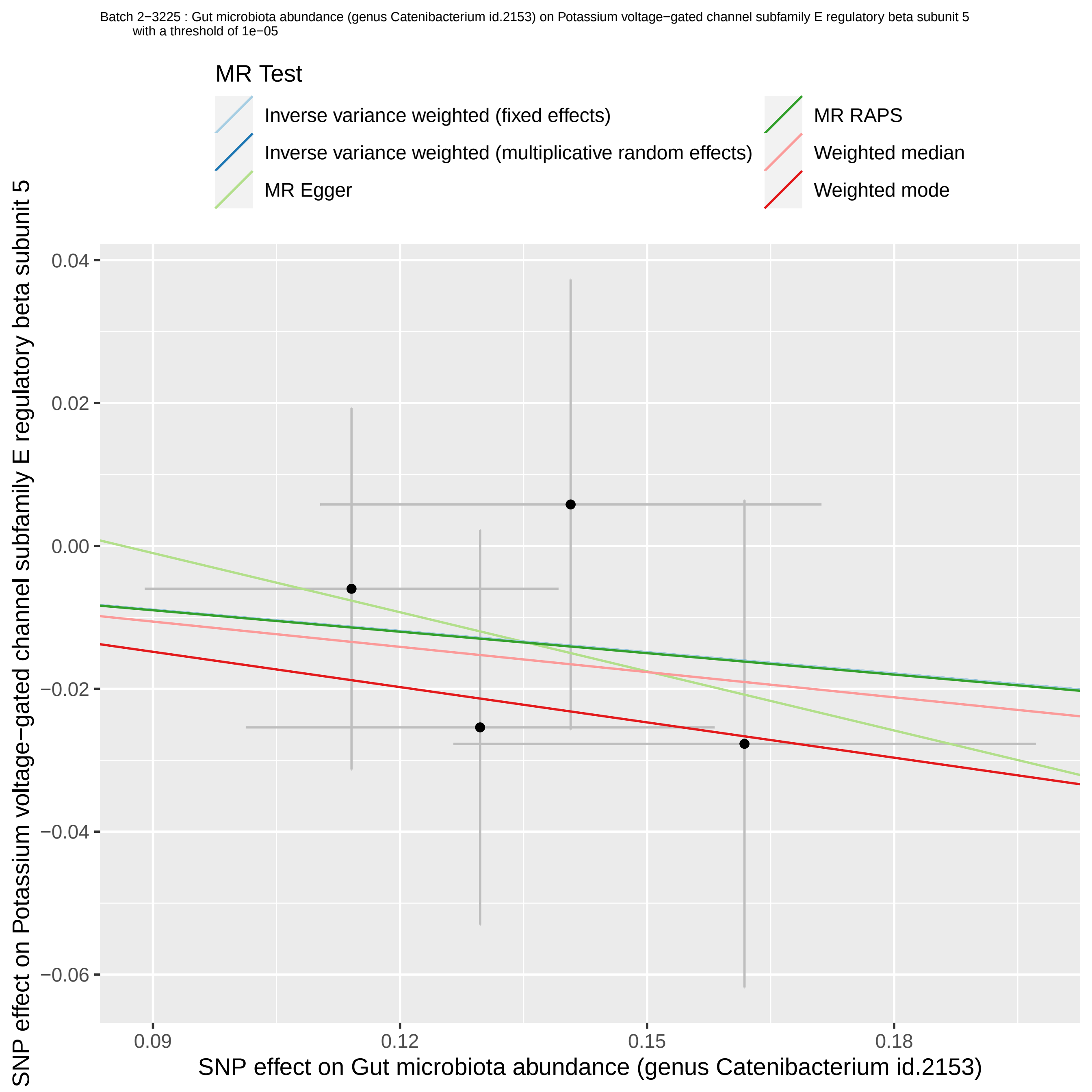
Batch 2−3225 : Gut microbiota abundance (genus Catenibacterium id.2153) on Potassium voltage−gated channel subfamily E regulatory beta subunit 5 with a threshold of 1e−05
MR Test
Inverse variance weighted (fixed effects)
Inverse variance weighted (multiplicative random effects)
MR Egger
MR RAPS
Weighted median
Weighted mode
0.04
0.02
0.00
−0.02
−0.04
−0.06
0.09
0.12
0.15
0.18
SNP effect on Potassium voltage−gated channel subfamily E regulatory beta subunit 5
SNP effect on Gut microbiota abundance (genus Catenibacterium id.2153)

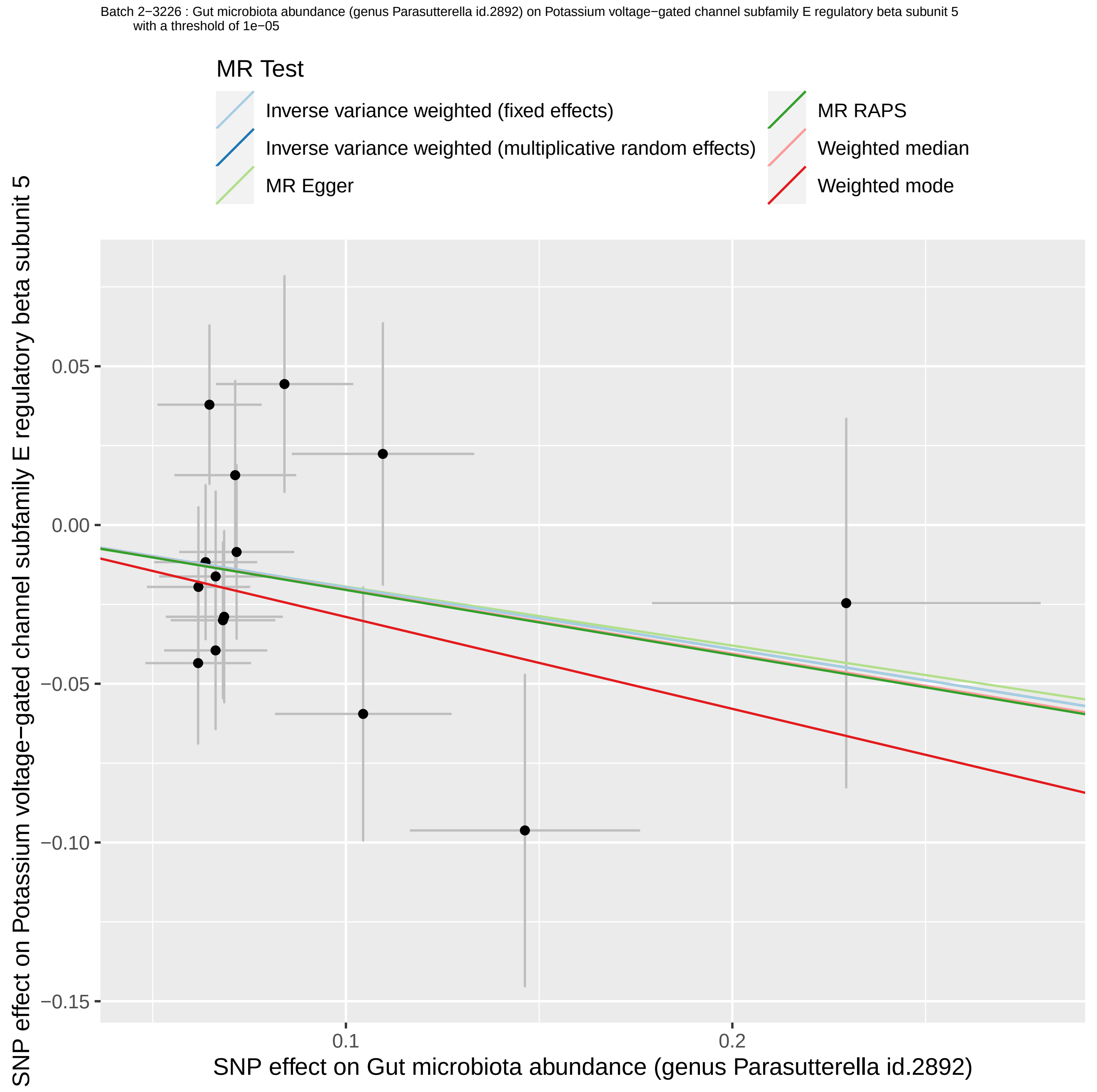
Batch 2−3226 : Gut microbiota abundance (genus Parasutterella id.2892) on Potassium voltage−gated channel subfamily E regulatory beta subunit 5
with a threshold of 1e−05
MR Test
Inverse variance weighted (fixed effects)
Inverse variance weighted (multiplicative random effects)
MR Egger
MR RAPS
Weighted median
Weighted mode
0.05
0.00
−0.05
−0.10
−0.15
0.1
0.2
SNP effect on Potassium voltage−gated channel subfamily E regulatory beta subunit 5
SNP effect on Gut microbiota abundance (genus Parasutterella id.2892)

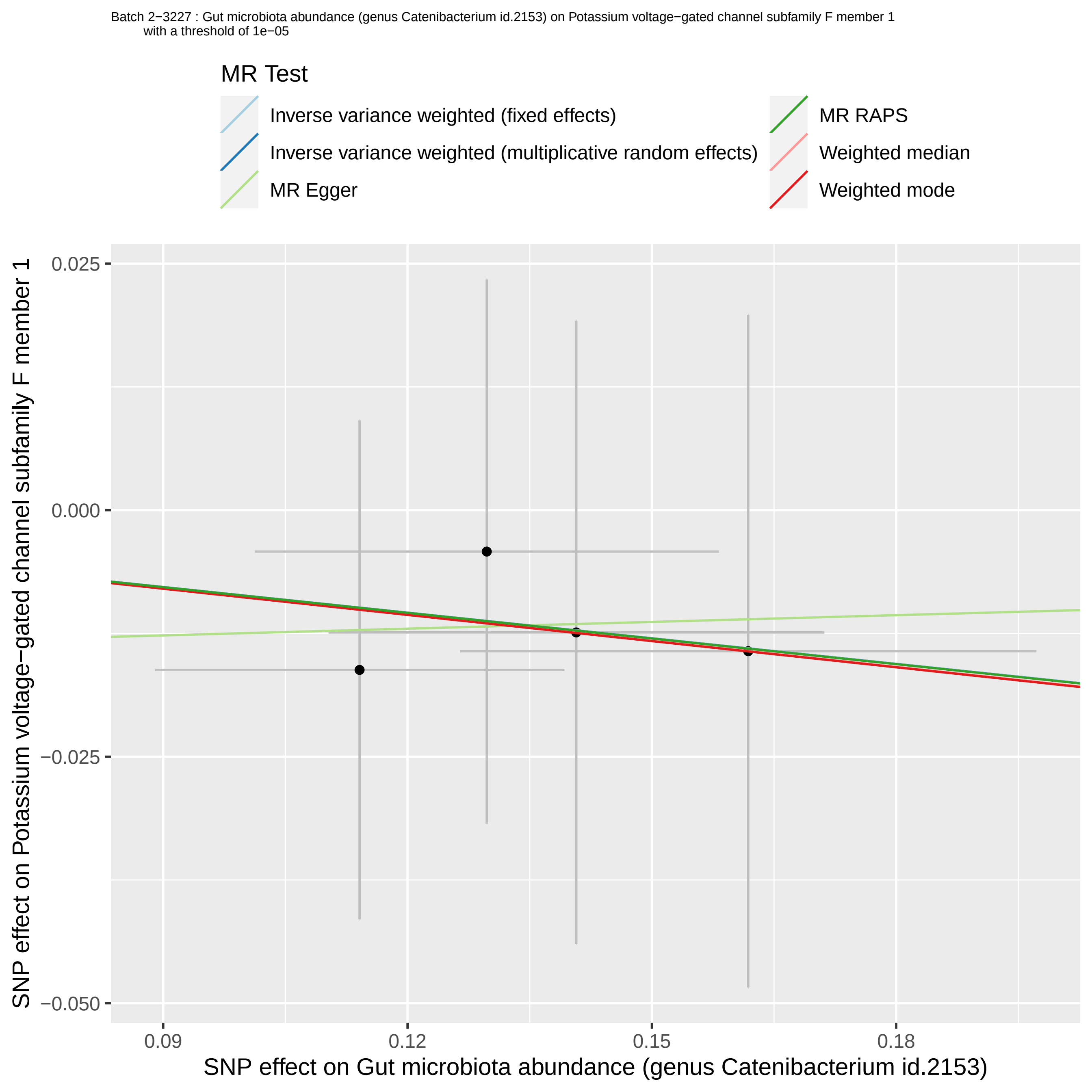
Batch 2−3227 : Gut microbiota abundance (genus Catenibacterium id.2153) on Potassium voltage−gated channel subfamily F member 1 with a threshold of 1e−05
MR Test
Inverse variance weighted (fixed effects)
Inverse variance weighted (multiplicative random effects)
MR Egger
MR RAPS
Weighted median
Weighted mode
0.025
0.000
−0.025
−0.050
0.09
0.12
0.15
0.18
SNP effect on Potassium voltage−gated channel subfamily F member 1
SNP effect on Gut microbiota abundance (genus Catenibacterium id.2153)

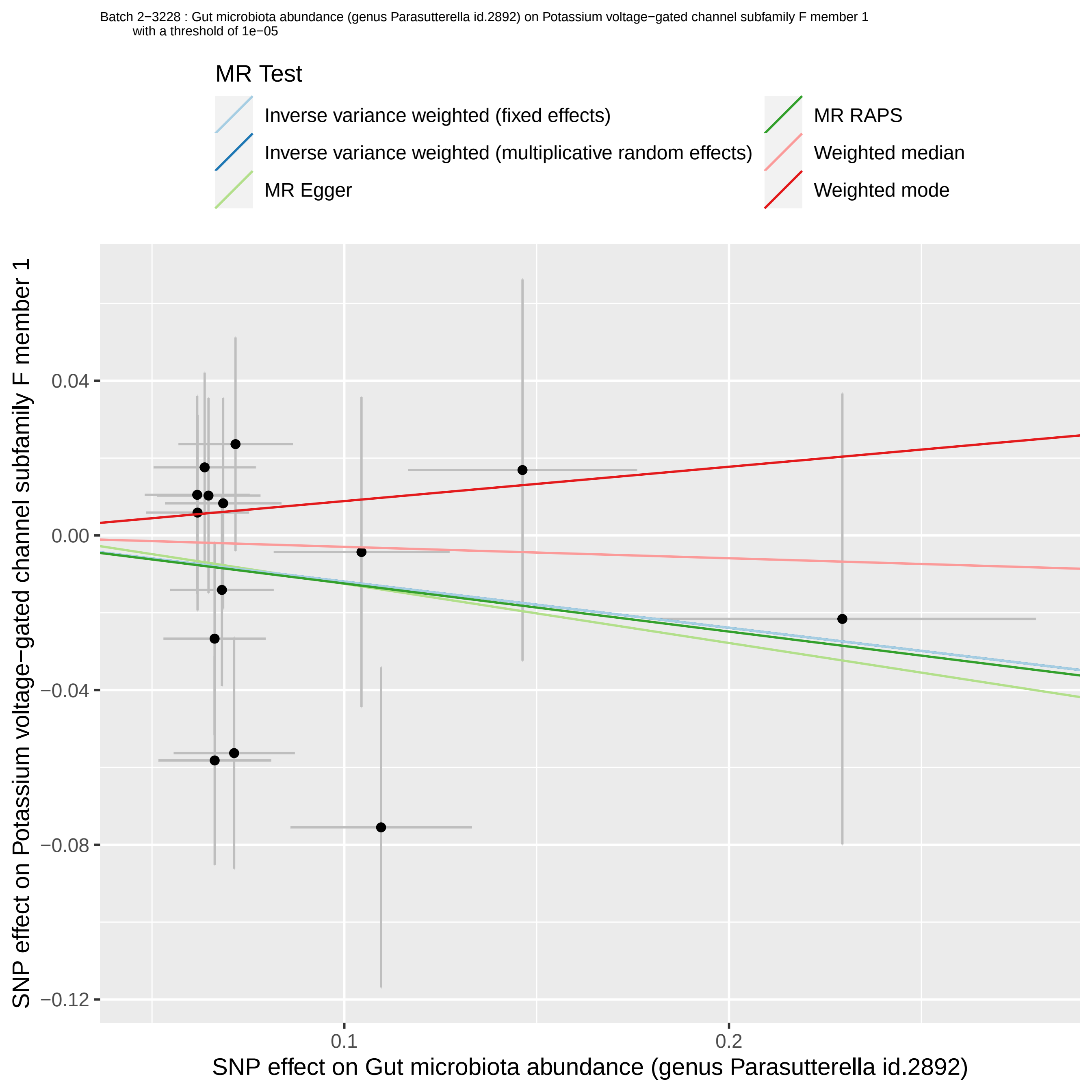

Batch 2−3228 : Gut microbiota abundance (genus Parasutterella id.2892) on Potassium voltage−gated channel subfamily F member 1
with a threshold of 1e−05
MR Test
Inverse variance weighted (fixed effects)
Inverse variance weighted (multiplicative random effects)
MR Egger
MR RAPS
Weighted median
Weighted mode
0.04
0.00
−0.04
−0.08
−0.12
0.1
0.2
SNP effect on Potassium voltage−gated channel subfamily F member 1
SNP effect on Gut microbiota abundance (genus Parasutterella id.2892)

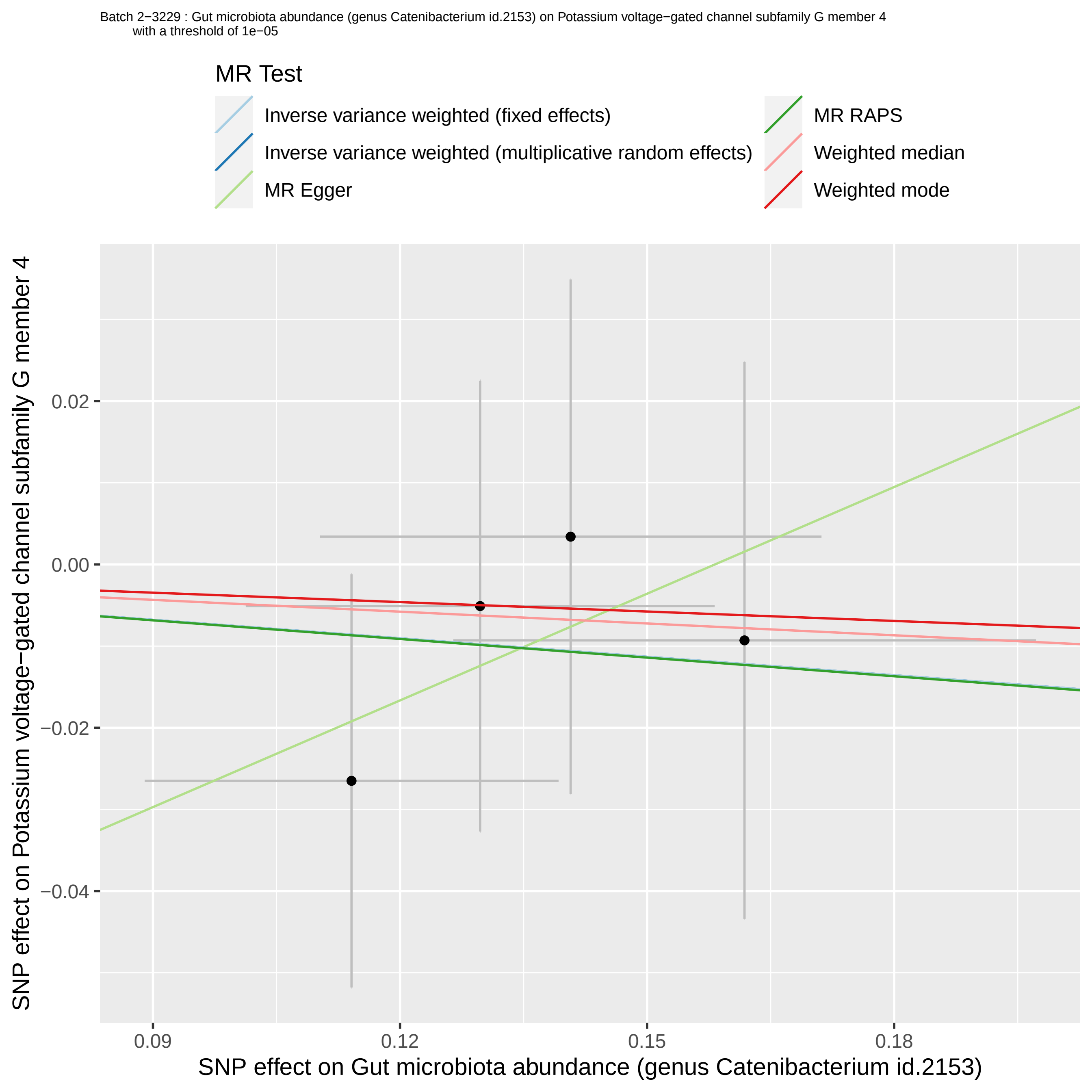
Batch 2−3229 : Gut microbiota abundance (genus Catenibacterium id.2153) on Potassium voltage−gated channel subfamily G member 4
with a threshold of 1e−05
MR Test
Inverse variance weighted (fixed effects)
Inverse variance weighted (multiplicative random effects)
MR Egger
MR RAPS
Weighted median
Weighted mode
SNP effect on Potassium voltage−gated channel subfamily G member 4
0.02
0.00
−0.02
−0.04
0.09
0.12
0.15
0.18
SNP effect on Gut microbiota abundance (genus Catenibacterium id.2153)

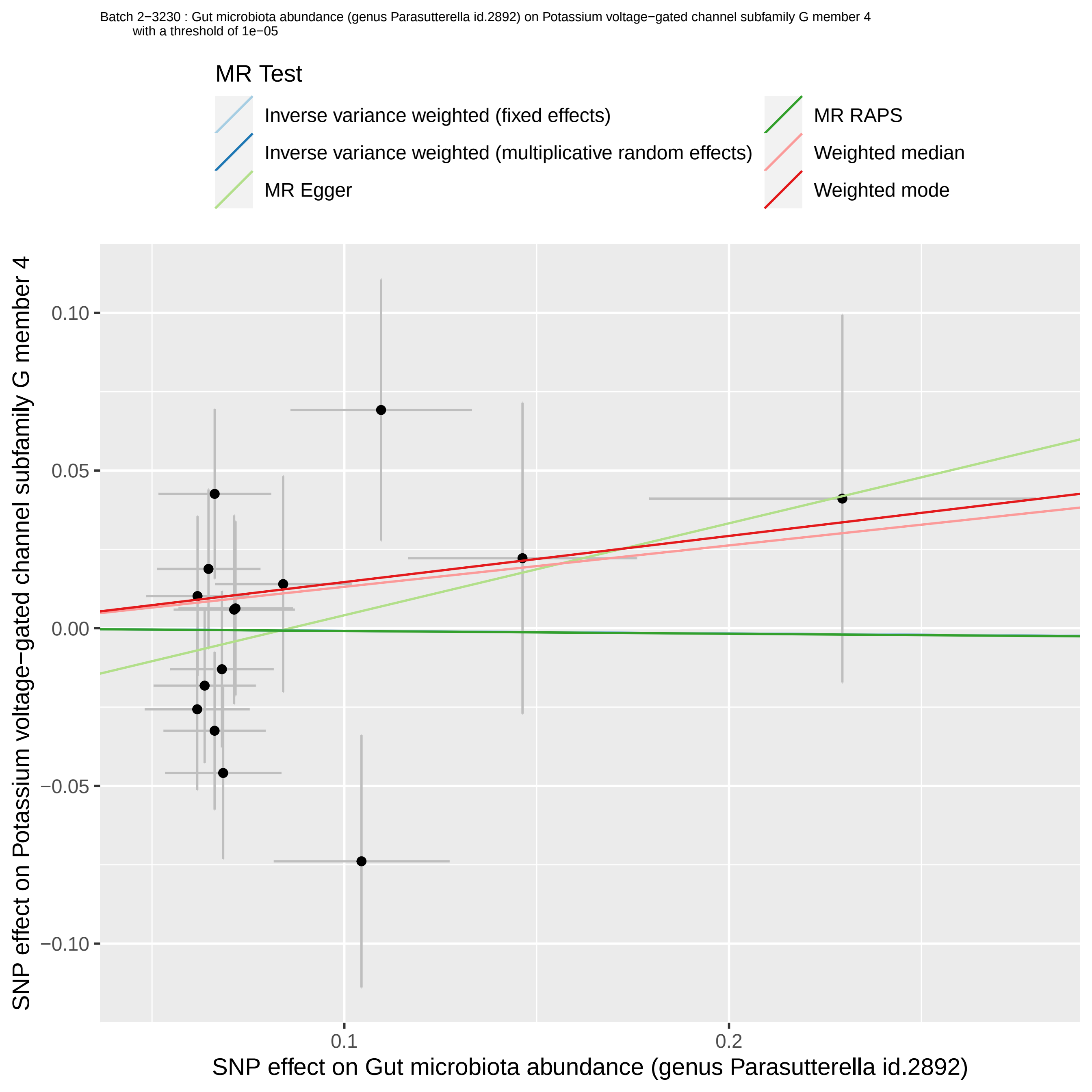

Batch 2−3230 : Gut microbiota abundance (genus Parasutterella id.2892) on Potassium voltage−gated channel subfamily G member 4
with a threshold of 1e−05
MR Test
Inverse variance weighted (fixed effects)
Inverse variance weighted (multiplicative random effects)
MR Egger
MR RAPS
Weighted median
Weighted mode
0.10
0.05
0.00
−0.05
−0.10
0.1
0.2
SNP effect on Potassium voltage−gated channel subfamily G member 4
SNP effect on Gut microbiota abundance (genus Parasutterella id.2892)

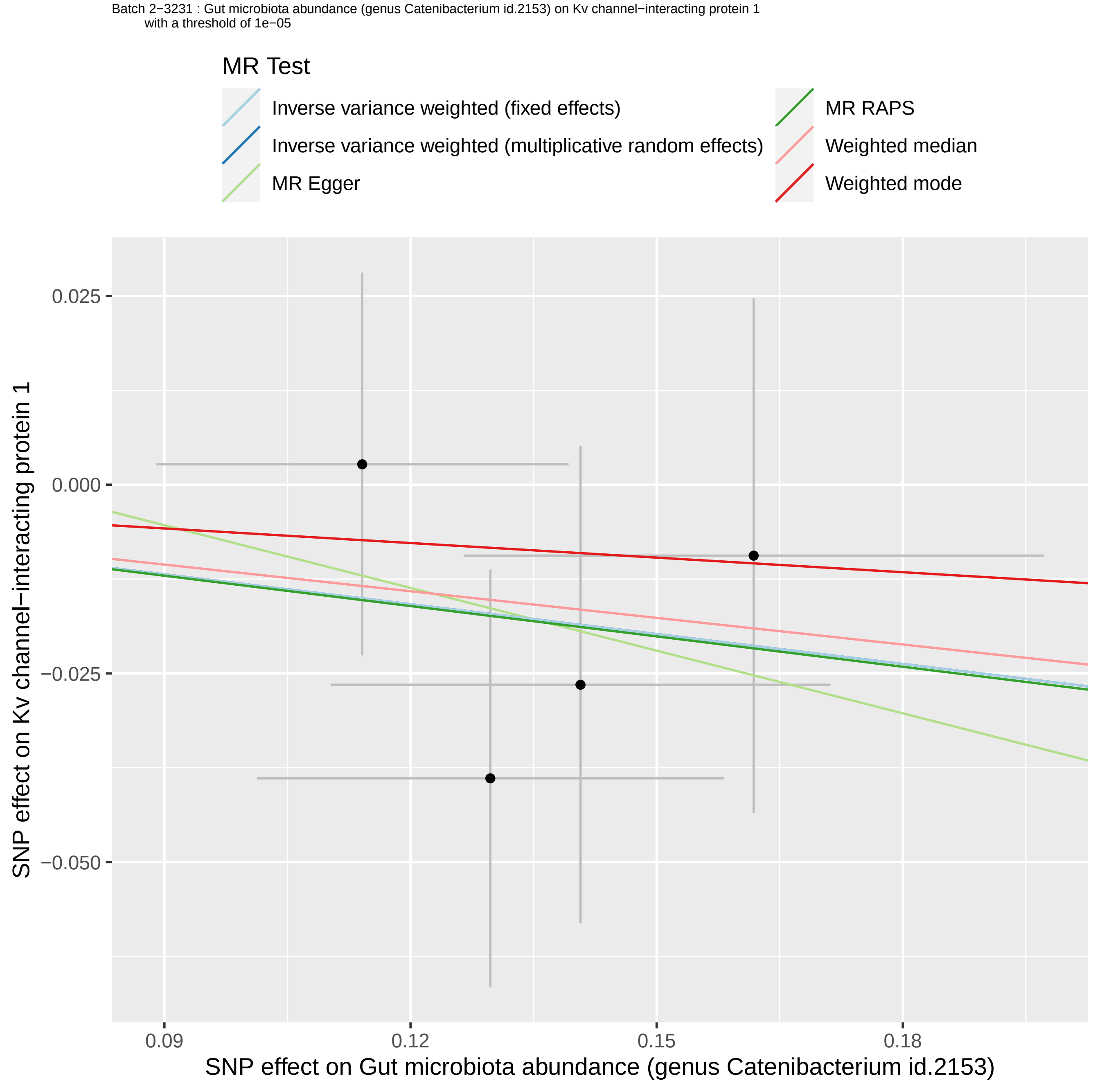
Batch 2−3231 : Gut microbiota abundance (genus Catenibacterium id.2153) on Kv channel−interacting protein 1
with a threshold of 1e−05
MR Test
Inverse variance weighted (fixed effects)
Inverse variance weighted (multiplicative random effects)
MR Egger
MR RAPS
Weighted median
Weighted mode
SNP effect on Kv channel−interacting protein 1
0.025
0.000
−0.025
−0.050
0.09
0.12
0.15
0.18
SNP effect on Gut microbiota abundance (genus Catenibacterium id.2153)

Batch 2−3232 : Gut microbiota abundance (genus Parasutterella id.2892) on Kv channel−interacting protein 1
with a threshold of 1e−05

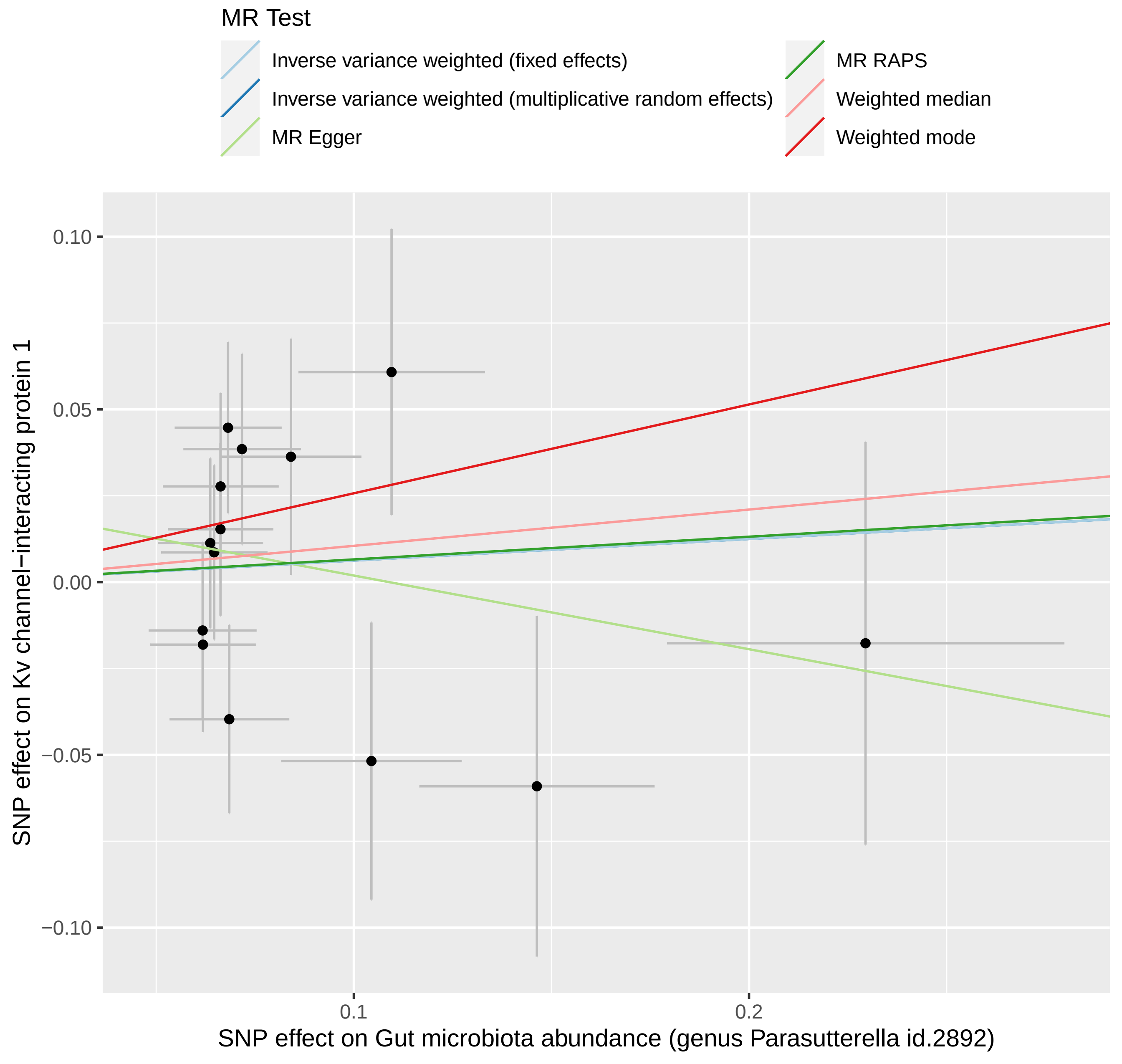

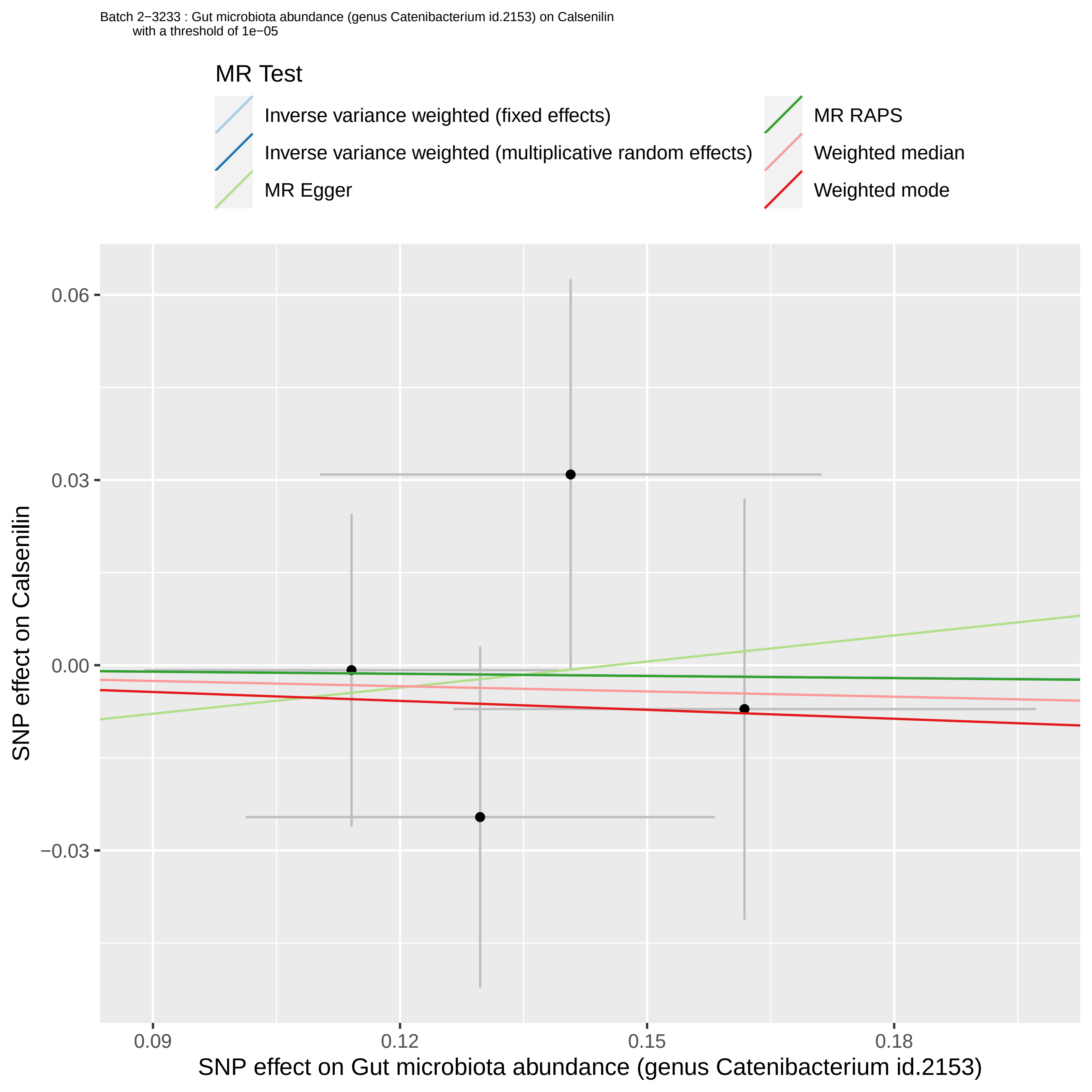
Batch 2−3233 : Gut microbiota abundance (genus Catenibacterium id.2153) on Calsenilin
with a threshold of 1e−05
MR Test
Inverse variance weighted (fixed effects)
Inverse variance weighted (multiplicative random effects)
MR Egger
MR RAPS
Weighted median
Weighted mode
0.06
0.03
0.00
−0.03
0.09
0.12
0.15
0.18
SNP effect on Calsenilin
SNP effect on Gut microbiota abundance (genus Catenibacterium id.2153)

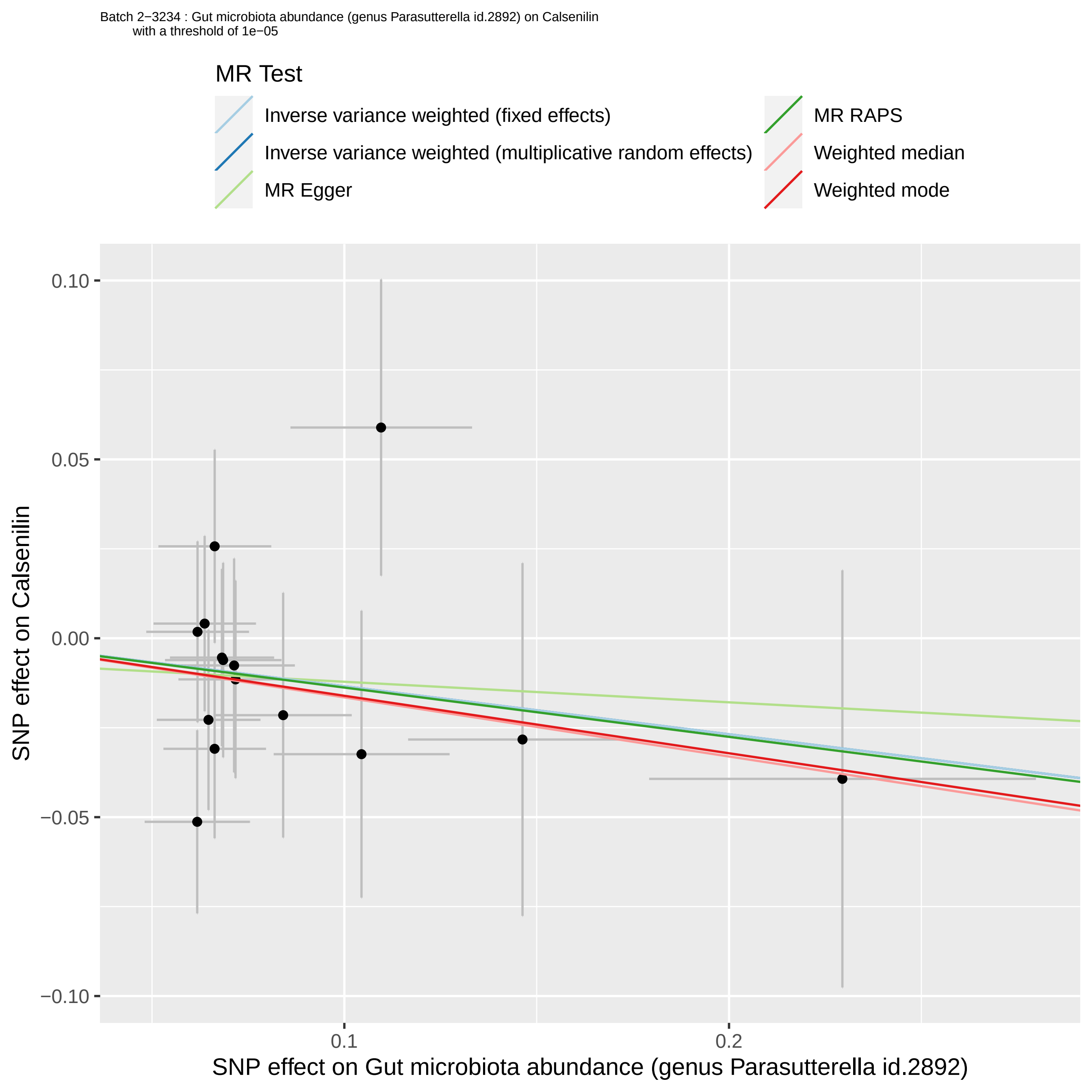
Batch 2−3234 : Gut microbiota abundance (genus Parasutterella id.2892) on Calsenilin
with a threshold of 1e−05
MR Test
Inverse variance weighted (fixed effects)
Inverse variance weighted (multiplicative random effects)
MR Egger
MR RAPS
Weighted median
Weighted mode
0.10
0.05
0.00
−0.05
−0.10
0.1
0.2
SNP effect on Calsenilin
SNP effect on Gut microbiota abundance (genus Parasutterella id.2892)

Batch 2−3235 : Gut microbiota abundance (genus Catenibacterium id.2153) on Small conductance calcium−activated potassium channel protein 1 with a threshold of 1e−05

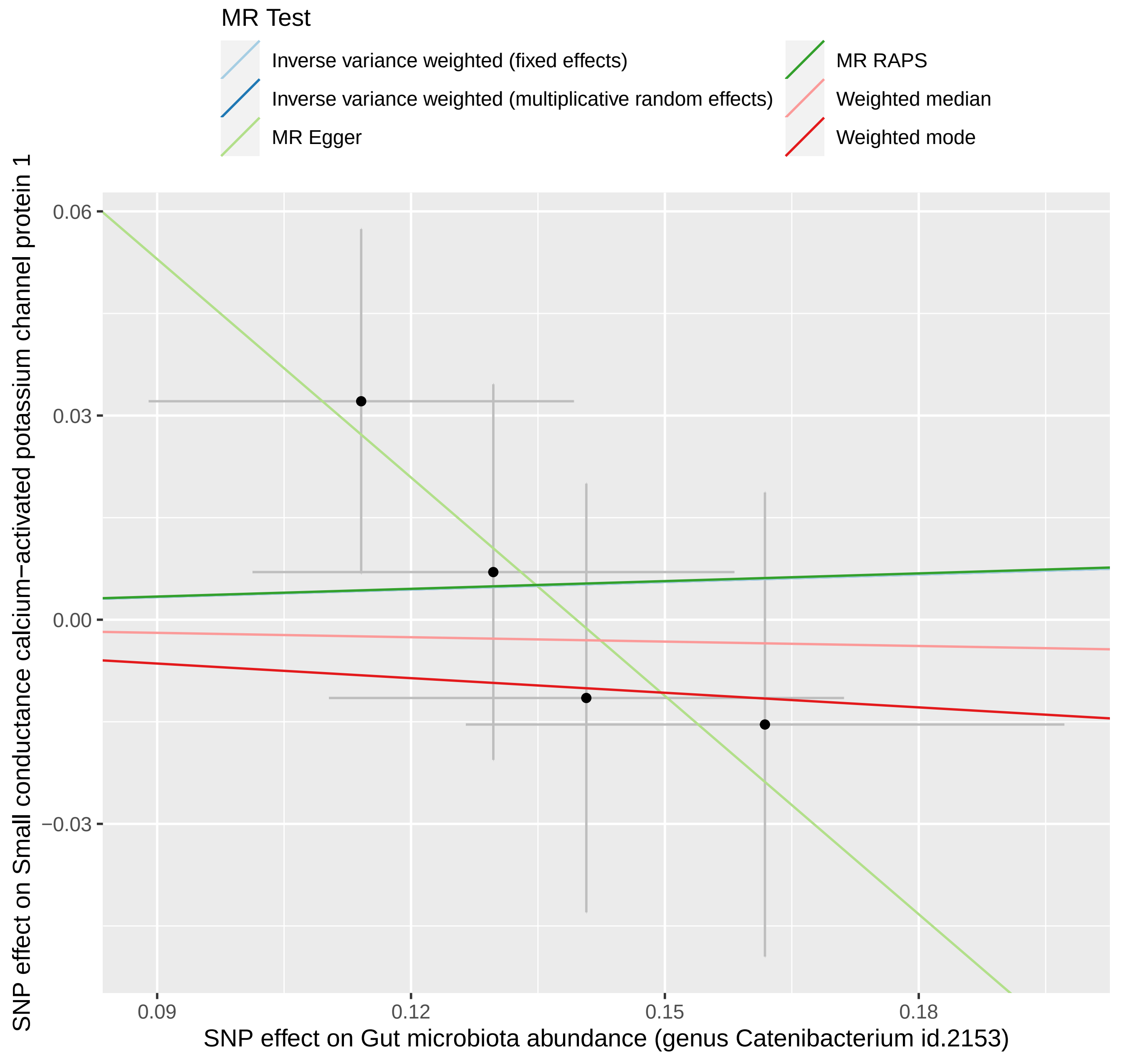

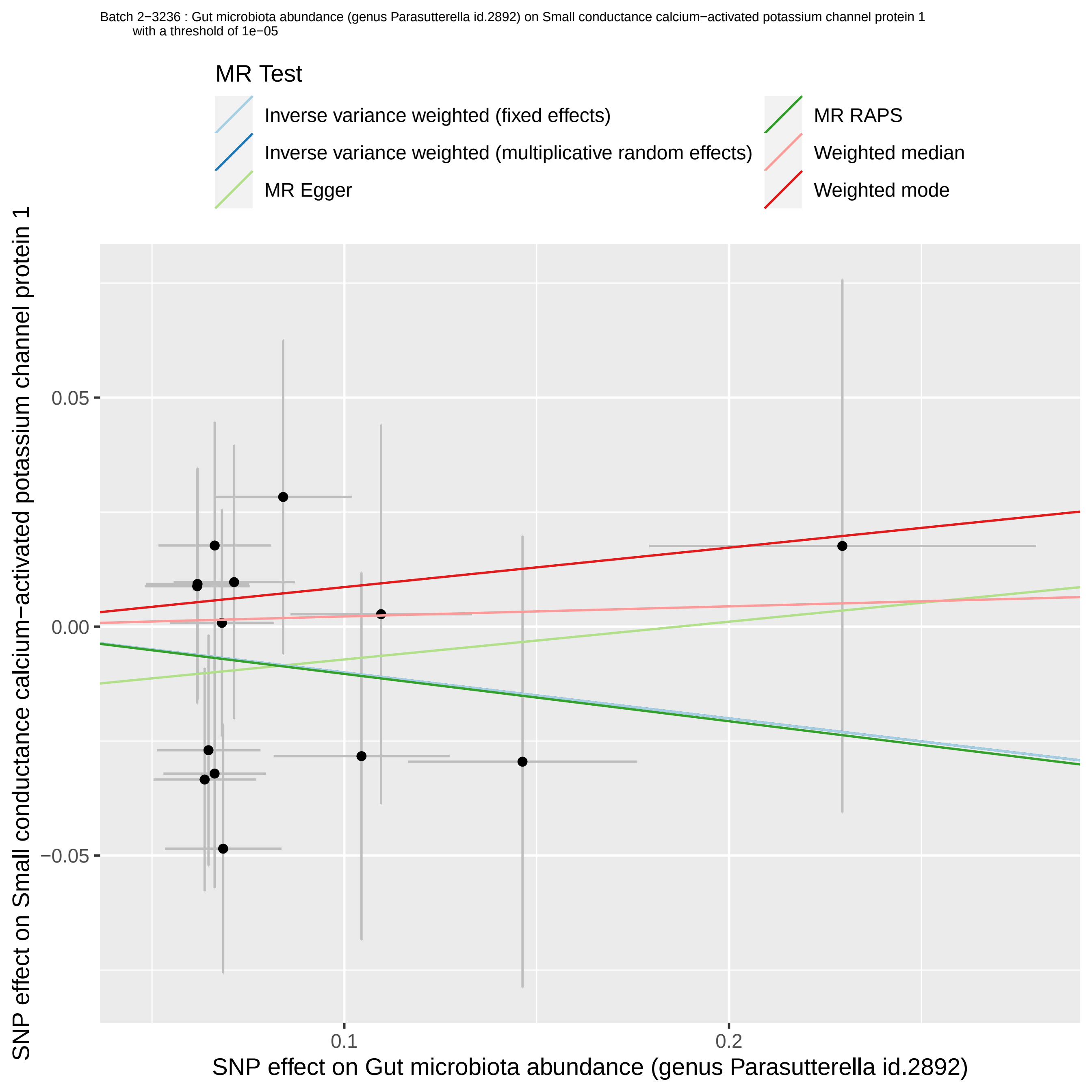
Batch 2−3236 : Gut microbiota abundance (genus Parasutterella id.2892) on Small conductance calcium−activated potassium channel protein 1
with a threshold of 1e−05
MR Test
Inverse variance weighted (fixed effects)
Inverse variance weighted (multiplicative random effects)
MR Egger
MR RAPS
Weighted median
Weighted mode
SNP effect on Small conductance calcium−activated potassium channel protein 1
0.05
0.00
−0.05
0.1
0.2
SNP effect on Gut microbiota abundance (genus Parasutterella id.2892)

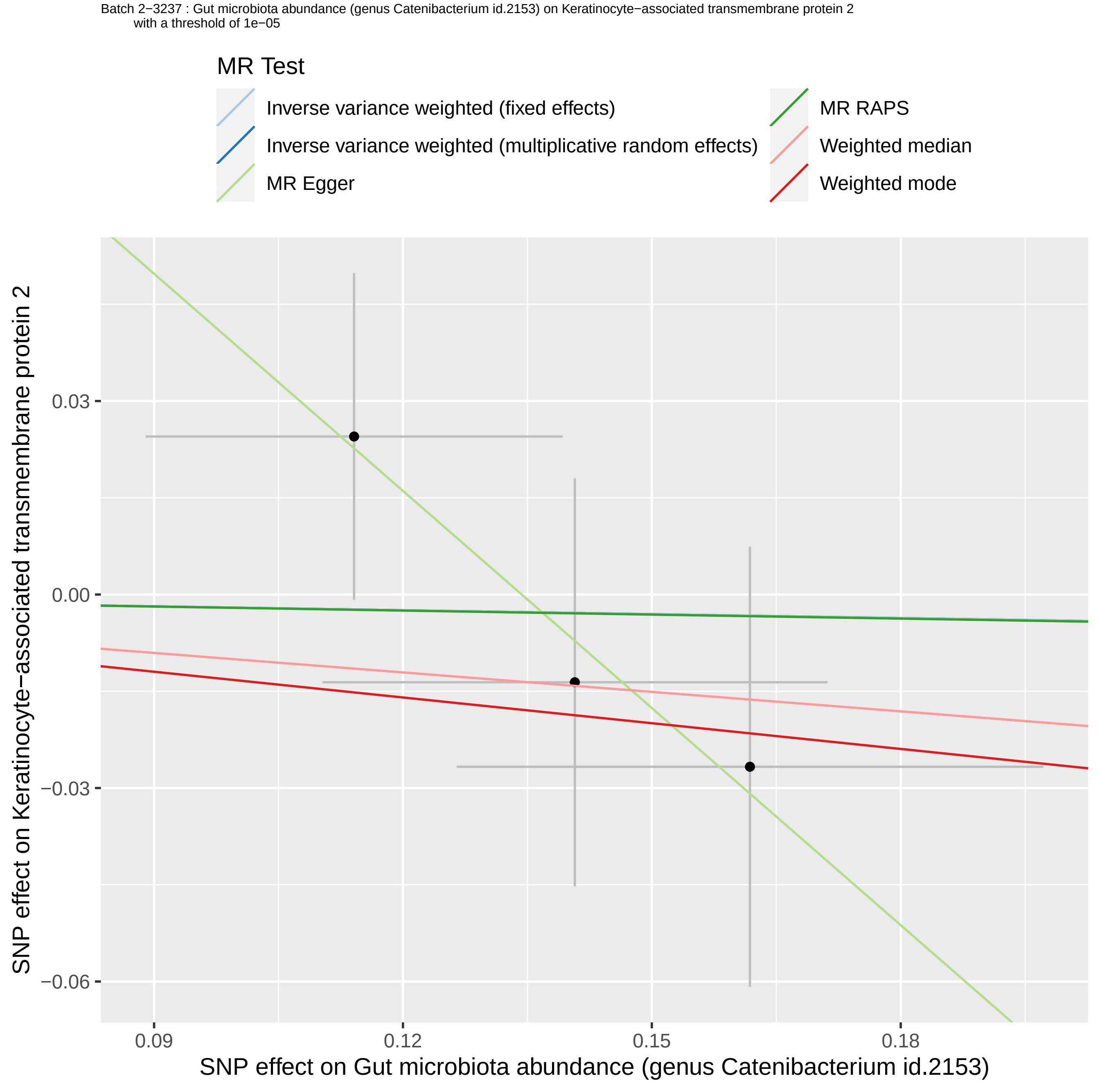
Batch 2−3237 : Gut microbiota abundance (genus Catenibacterium id.2153) on Keratinocyte−associated transmembrane protein 2
with a threshold of 1e−05
MR Test
Inverse variance weighted (fixed effects)
Inverse variance weighted (multiplicative random effects)
MR Egger
MR RAPS
Weighted median
Weighted mode
SNP effect on Keratinocyte−associated transmembrane protein 2
0.03
0.00
−0.03
−0.06
0.09
0.12
0.15
0.18
SNP effect on Gut microbiota abundance (genus Catenibacterium id.2153)

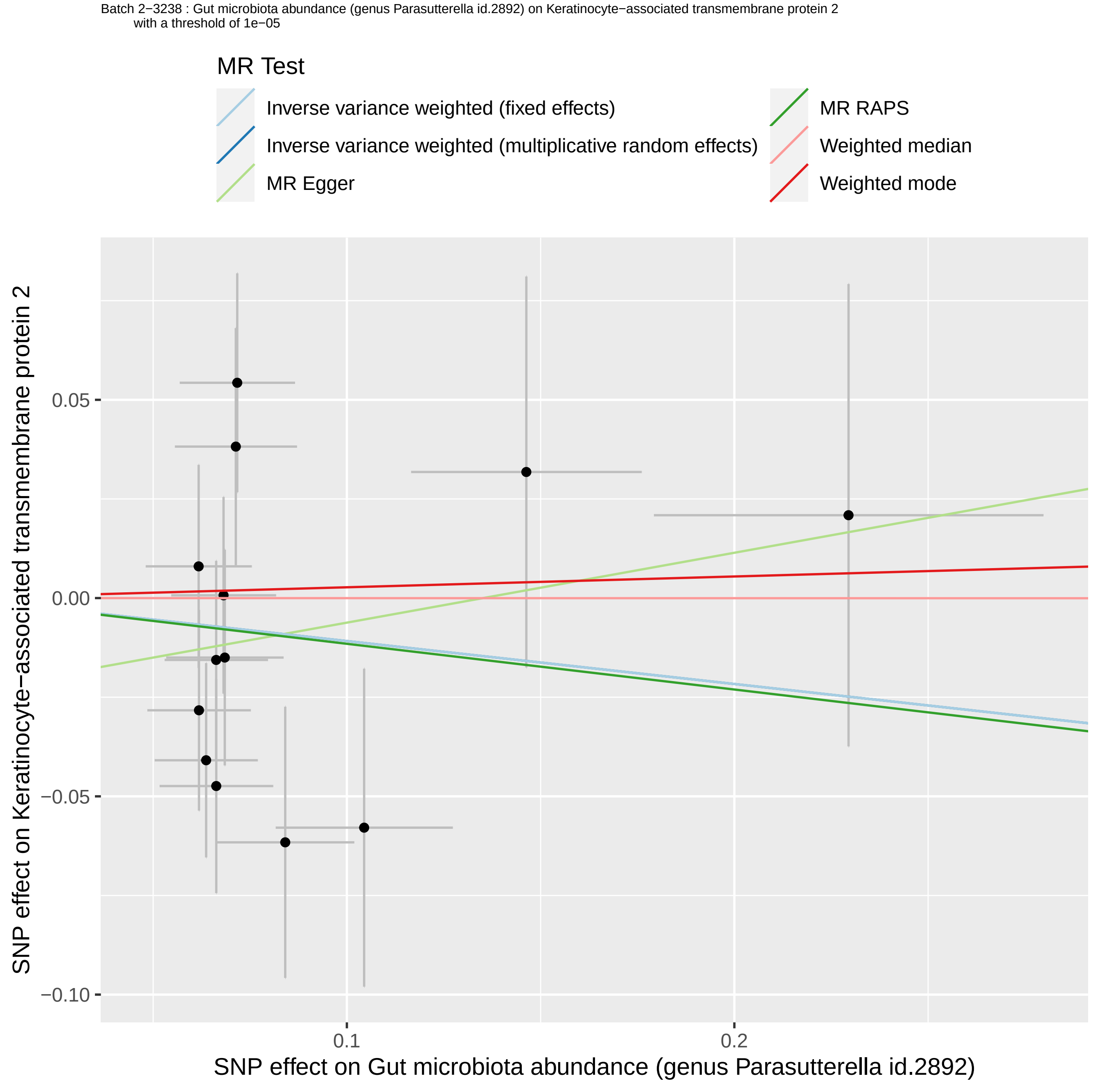

Batch 2−3238 : Gut microbiota abundance (genus Parasutterella id.2892) on Keratinocyte−associated transmembrane protein 2
with a threshold of 1e−05
MR Test
Inverse variance weighted (fixed effects)
Inverse variance weighted (multiplicative random effects)
MR Egger
MR RAPS
Weighted median
Weighted mode
0.05
0.00
−0.05
−0.10
0.1
0.2
SNP effect on Keratinocyte−associated transmembrane protein 2
SNP effect on Gut microbiota abundance (genus Parasutterella id.2892)

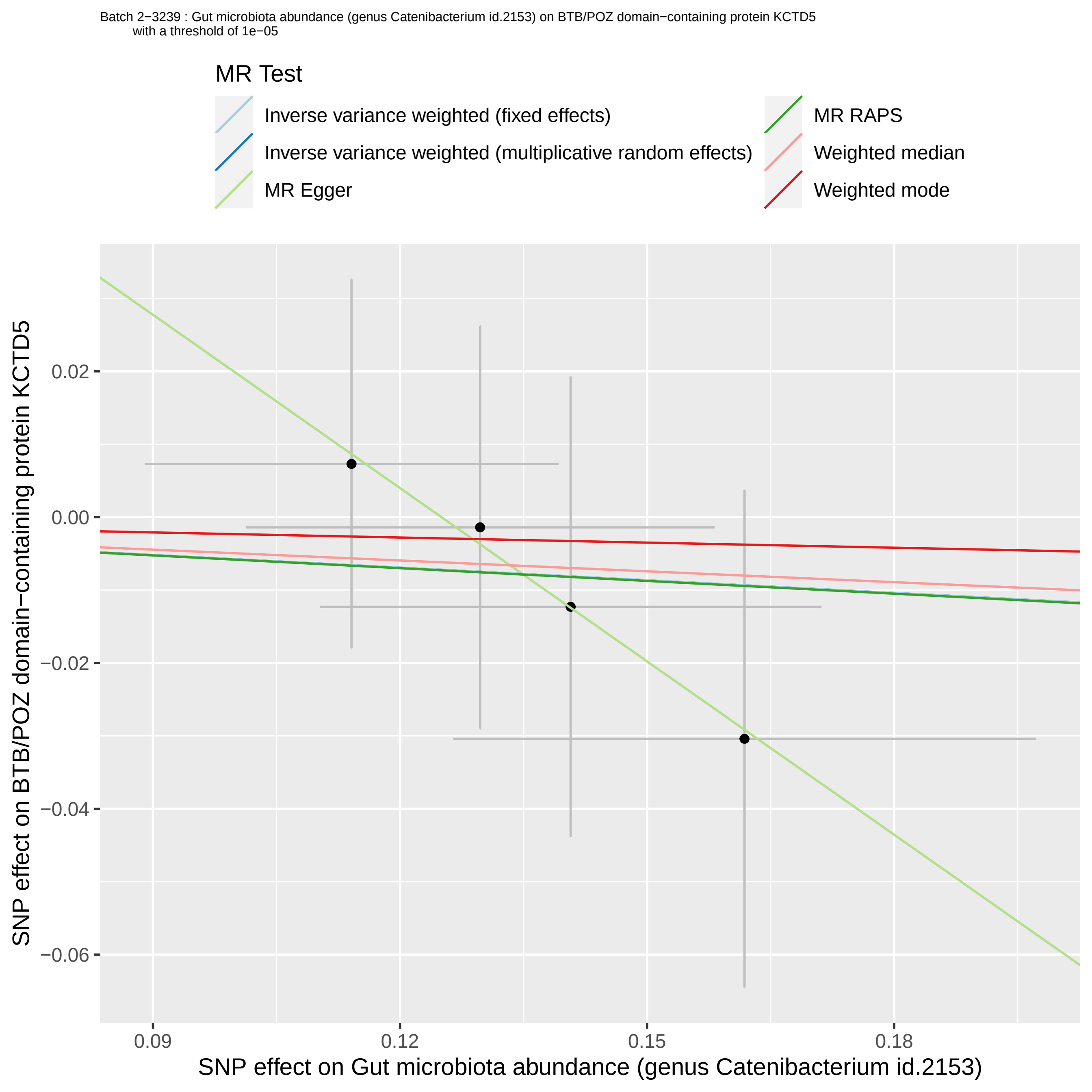
Batch 2−3239 : Gut microbiota abundance (genus Catenibacterium id.2153) on BTB/POZ domain−containing protein KCTD5 with a threshold of 1e−05
MR Test
Inverse variance weighted (fixed effects)
Inverse variance weighted (multiplicative random effects)
MR Egger
MR RAPS
Weighted median
Weighted mode
SNP effect on BTB/POZ domain−containing protein KCTD5
0.02
0.00
−0.02
−0.04
−0.06
0.09
0.12
0.15
0.18
SNP effect on Gut microbiota abundance (genus Catenibacterium id.2153)

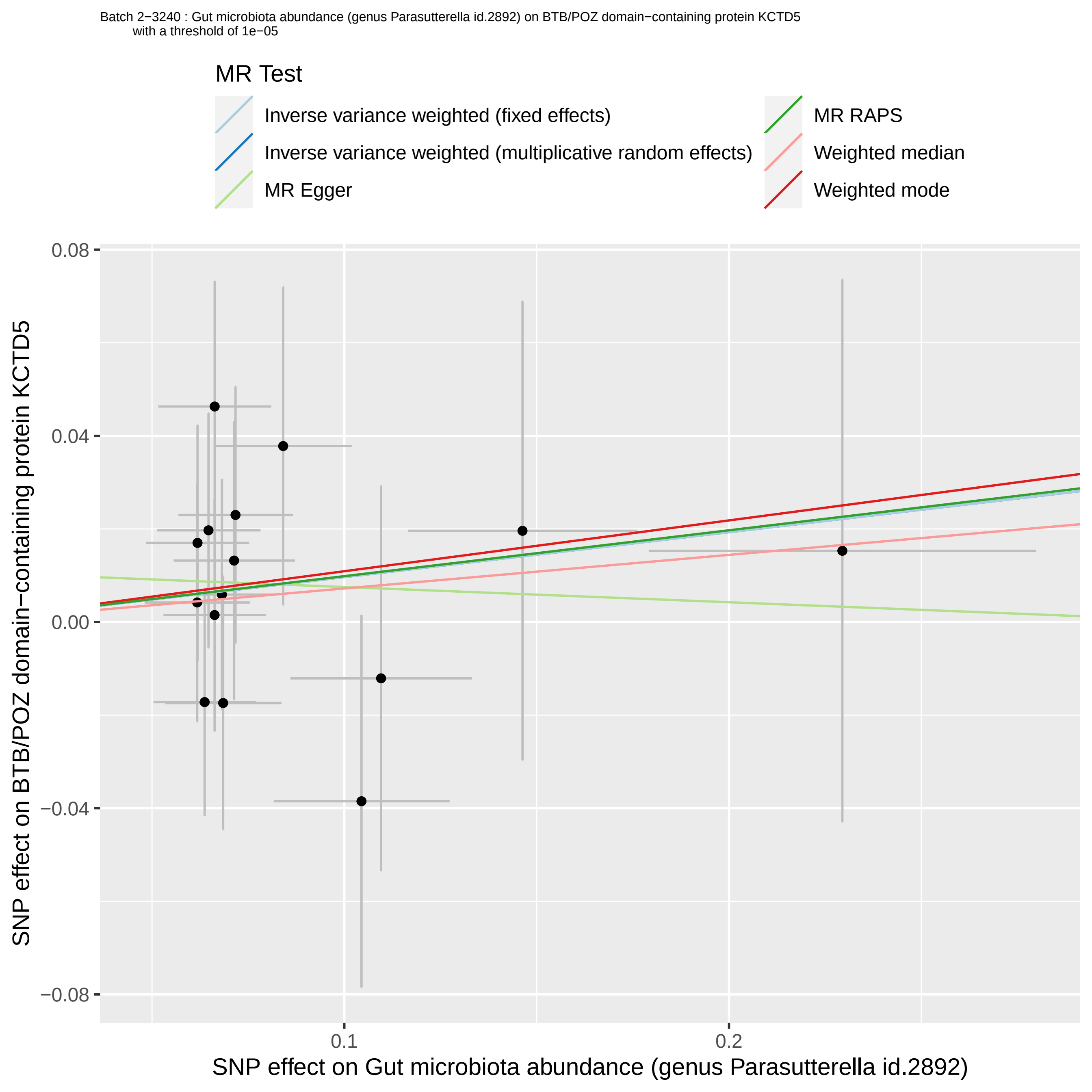
Batch 2−3240 : Gut microbiota abundance (genus Parasutterella id.2892) on BTB/POZ domain−containing protein KCTD5
with a threshold of 1e−05
MR Test
Inverse variance weighted (fixed effects)
Inverse variance weighted (multiplicative random effects)
MR Egger
MR RAPS
Weighted median
Weighted mode
0.08
0.04
0.00
−0.04
−0.08
0.1
0.2
SNP effect on BTB/POZ domain−containing protein KCTD5
SNP effect on Gut microbiota abundance (genus Parasutterella id.2892)

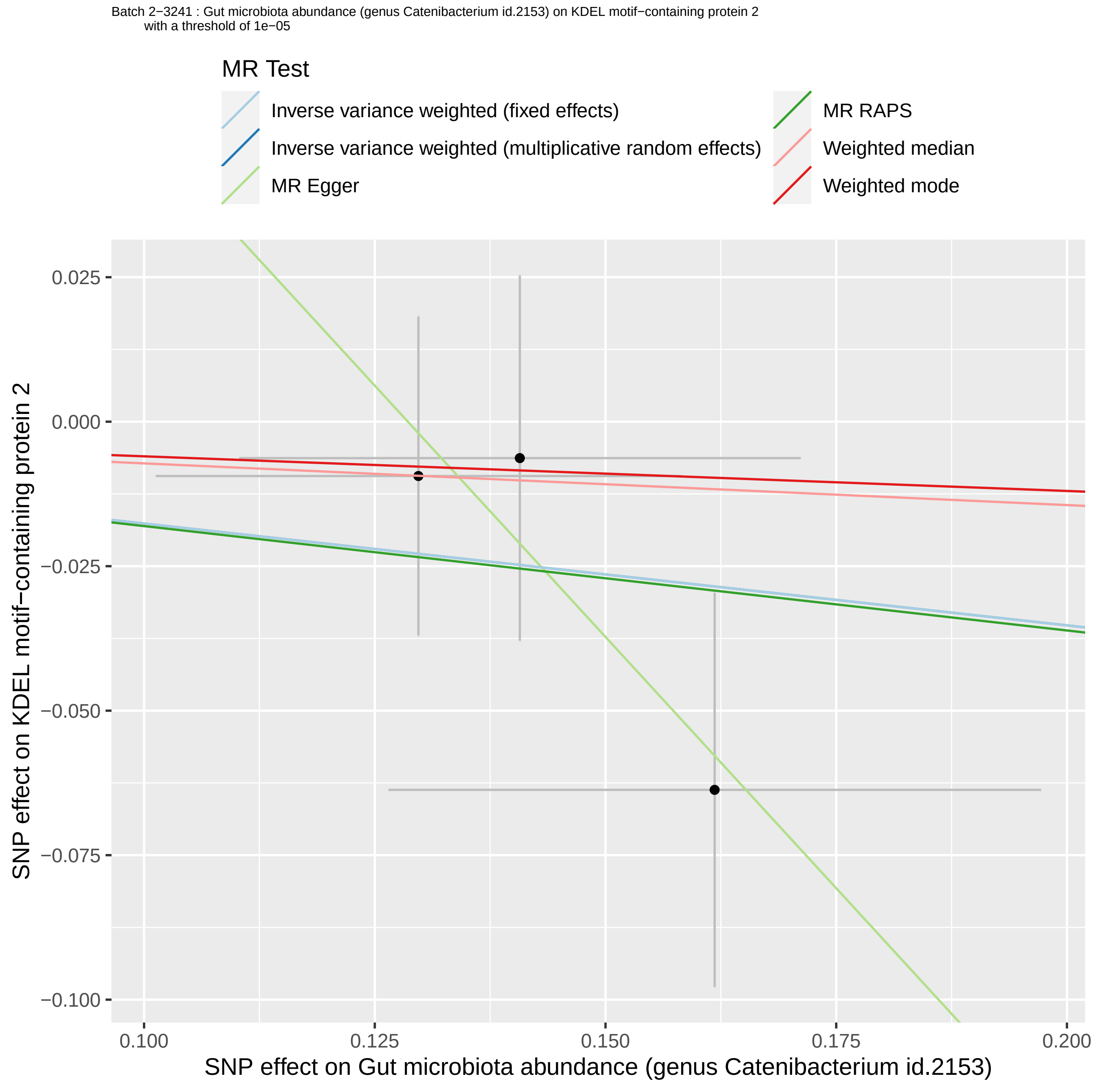
Batch 2−3241 : Gut microbiota abundance (genus Catenibacterium id.2153) on KDEL motif−containing protein 2
with a threshold of 1e−05
MR Test
Inverse variance weighted (fixed effects)
Inverse variance weighted (multiplicative random effects)
MR Egger
MR RAPS
Weighted median
Weighted mode
0.025
0.000
−0.025
−0.050
−0.075
−0.100
0.100
0.125
0.150
0.175
0.200
SNP effect on KDEL motif−containing protein 2
SNP effect on Gut microbiota abundance (genus Catenibacterium id.2153)

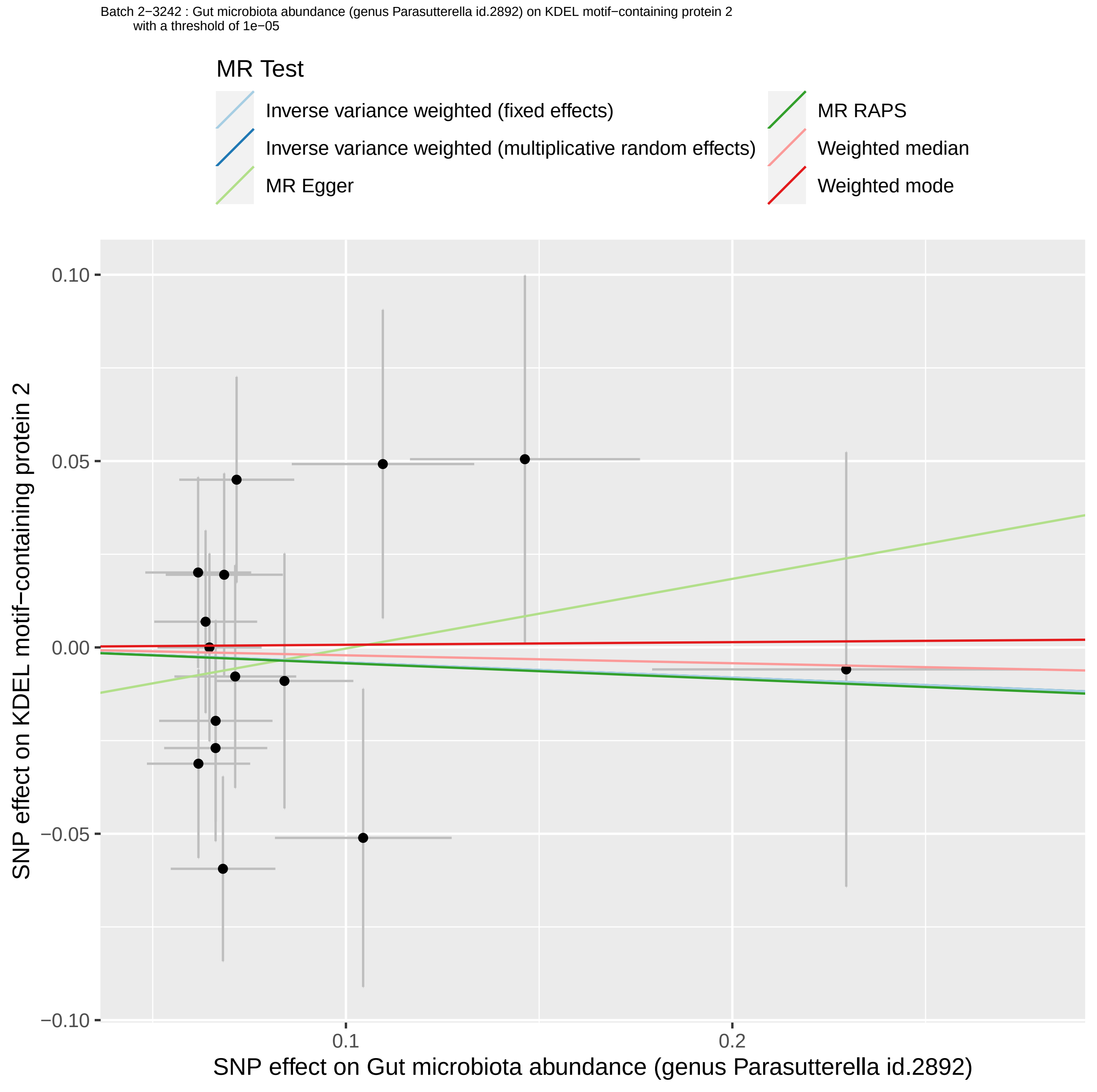
Batch 2−3242 : Gut microbiota abundance (genus Parasutterella id.2892) on KDEL motif−containing protein 2
with a threshold of 1e−05
MR Test
Inverse variance weighted (fixed effects)
Inverse variance weighted (multiplicative random effects)
MR Egger
MR RAPS
Weighted median
Weighted mode
SNP effect on KDEL motif−containing protein 2
0.10
0.05
0.00
−0.05
−0.10
0.1
0.2
SNP effect on Gut microbiota abundance (genus Parasutterella id.2892)

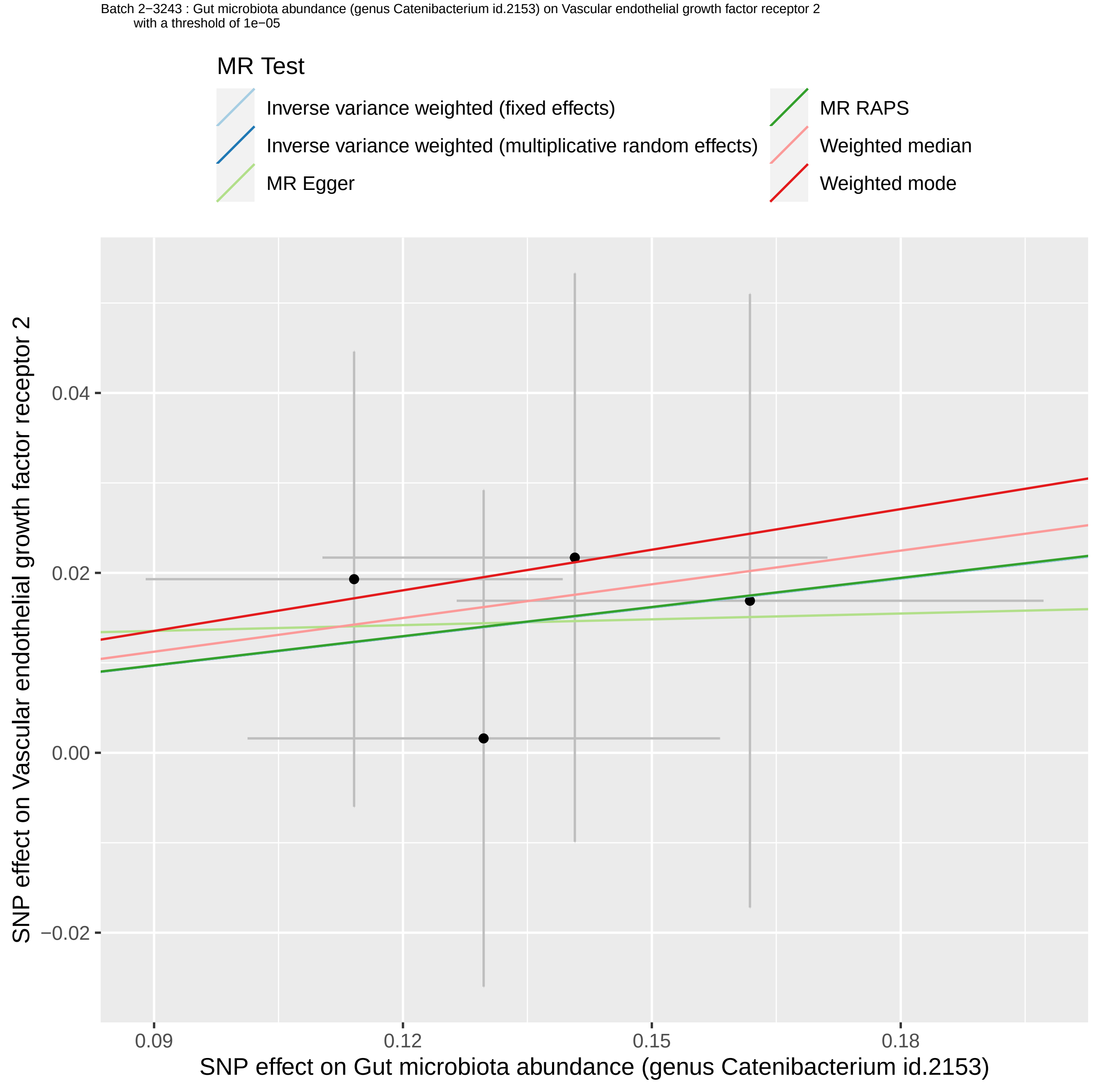
Batch 2−3243 : Gut microbiota abundance (genus Catenibacterium id.2153) on Vascular endothelial growth factor receptor 2
with a threshold of 1e−05
MR Test
Inverse variance weighted (fixed effects)
Inverse variance weighted (multiplicative random effects)
MR Egger
MR RAPS
Weighted median
Weighted mode
0.04
0.02
0.00
−0.02
0.09
0.12
0.15
0.18
SNP effect on Vascular endothelial growth factor receptor 2
SNP effect on Gut microbiota abundance (genus Catenibacterium id.2153)

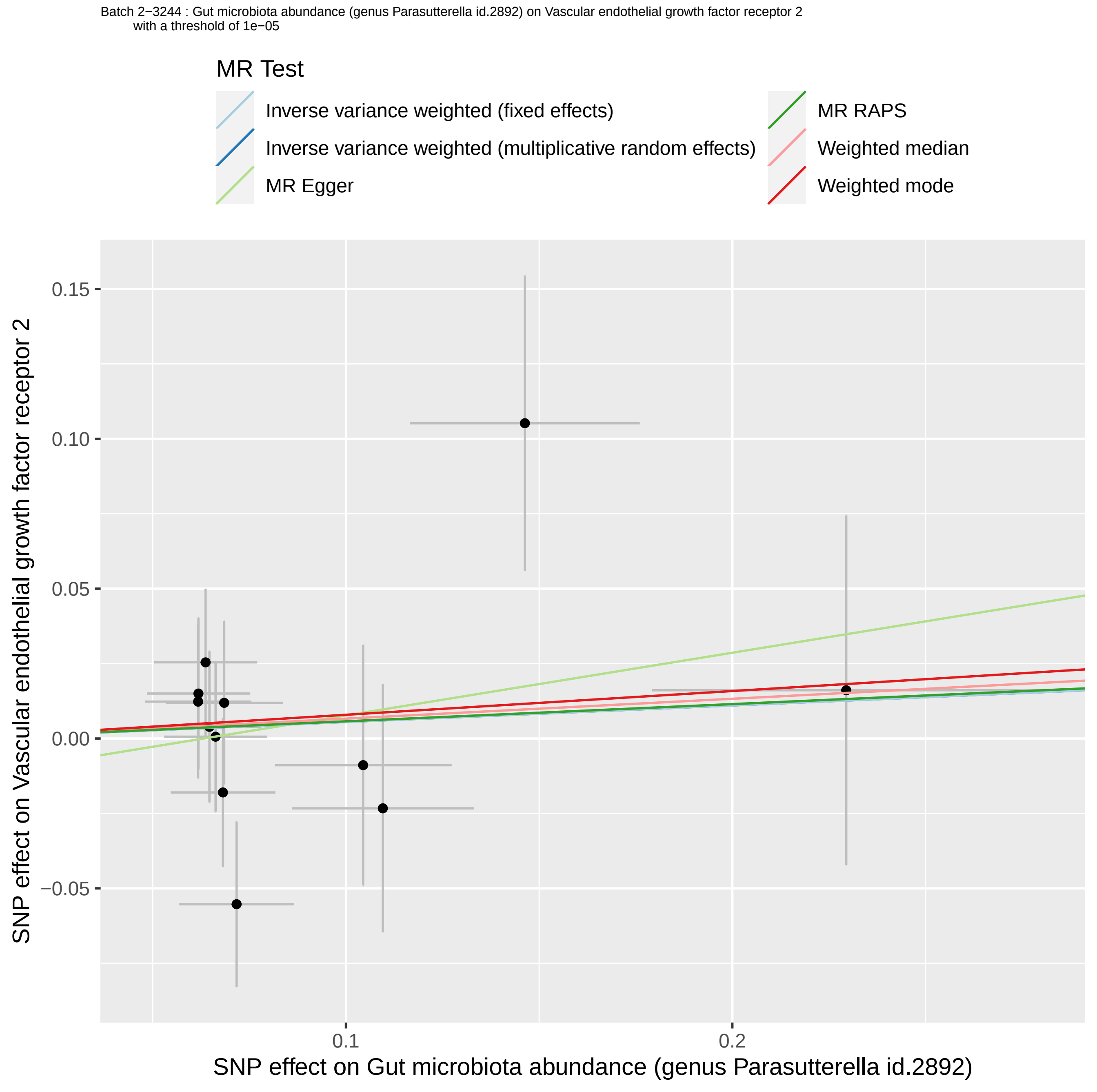
Batch 2−3244 : Gut microbiota abundance (genus Parasutterella id.2892) on Vascular endothelial growth factor receptor 2
with a threshold of 1e−05
MR Test
Inverse variance weighted (fixed effects)
Inverse variance weighted (multiplicative random effects)
MR Egger
MR RAPS
Weighted median
Weighted mode
0.15
0.10
0.05
0.00
−0.05
0.1
0.2
SNP effect on Vascular endothelial growth factor receptor 2
SNP effect on Gut microbiota abundance (genus Parasutterella id.2892)

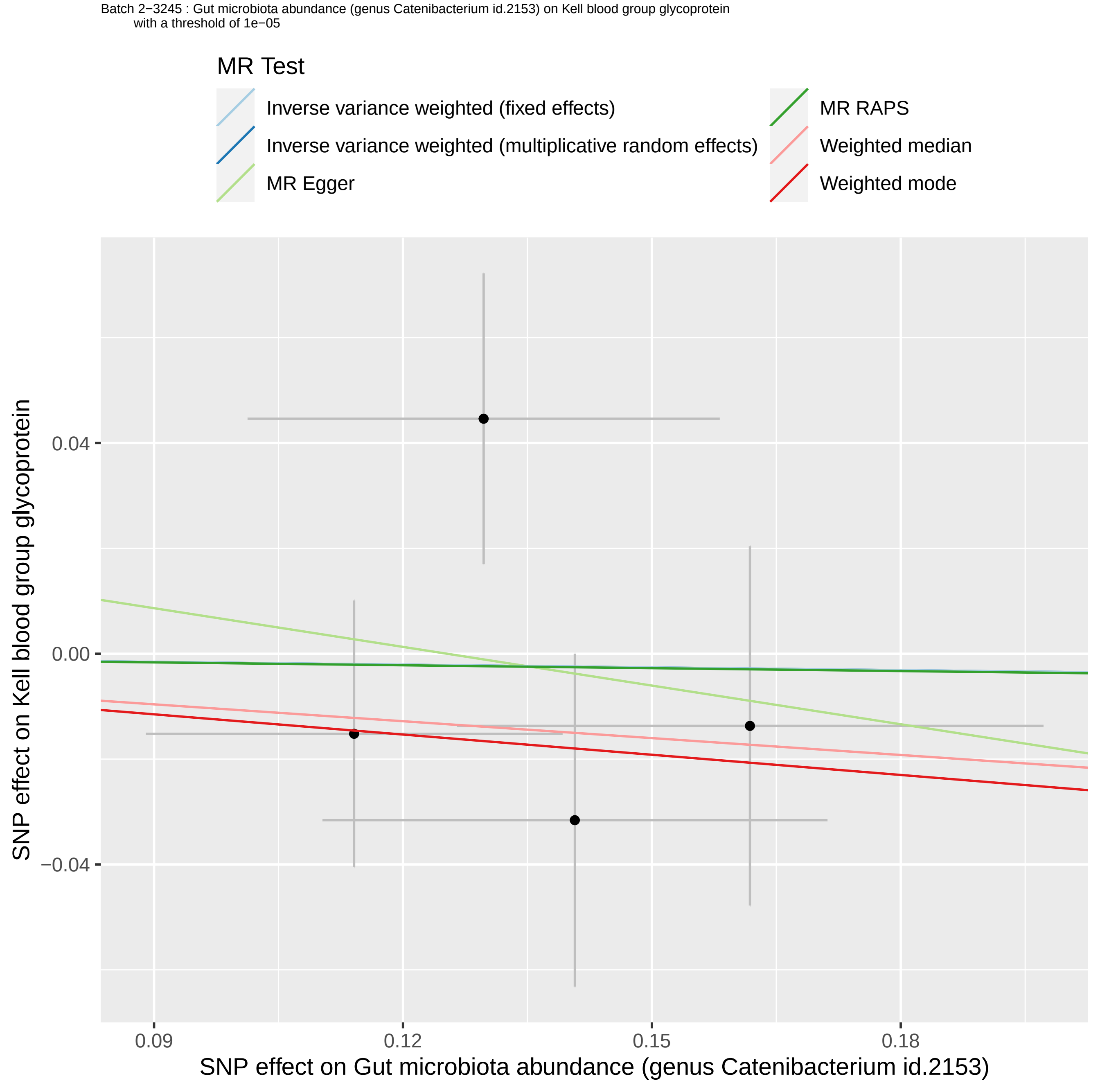

Batch 2−3245 : Gut microbiota abundance (genus Catenibacterium id.2153) on Kell blood group glycoprotein
with a threshold of 1e−05
MR Test
Inverse variance weighted (fixed effects)
Inverse variance weighted (multiplicative random effects)
MR Egger
MR RAPS
Weighted median
Weighted mode
0.04
0.00
−0.04
0.09
0.12
0.15
0.18
SNP effect on Kell blood group glycoprotein
SNP effect on Gut microbiota abundance (genus Catenibacterium id.2153)

Batch 2−3246 : Gut microbiota abundance (genus Parasutterella id.2892) on Kell blood group glycoprotein
with a threshold of 1e−05

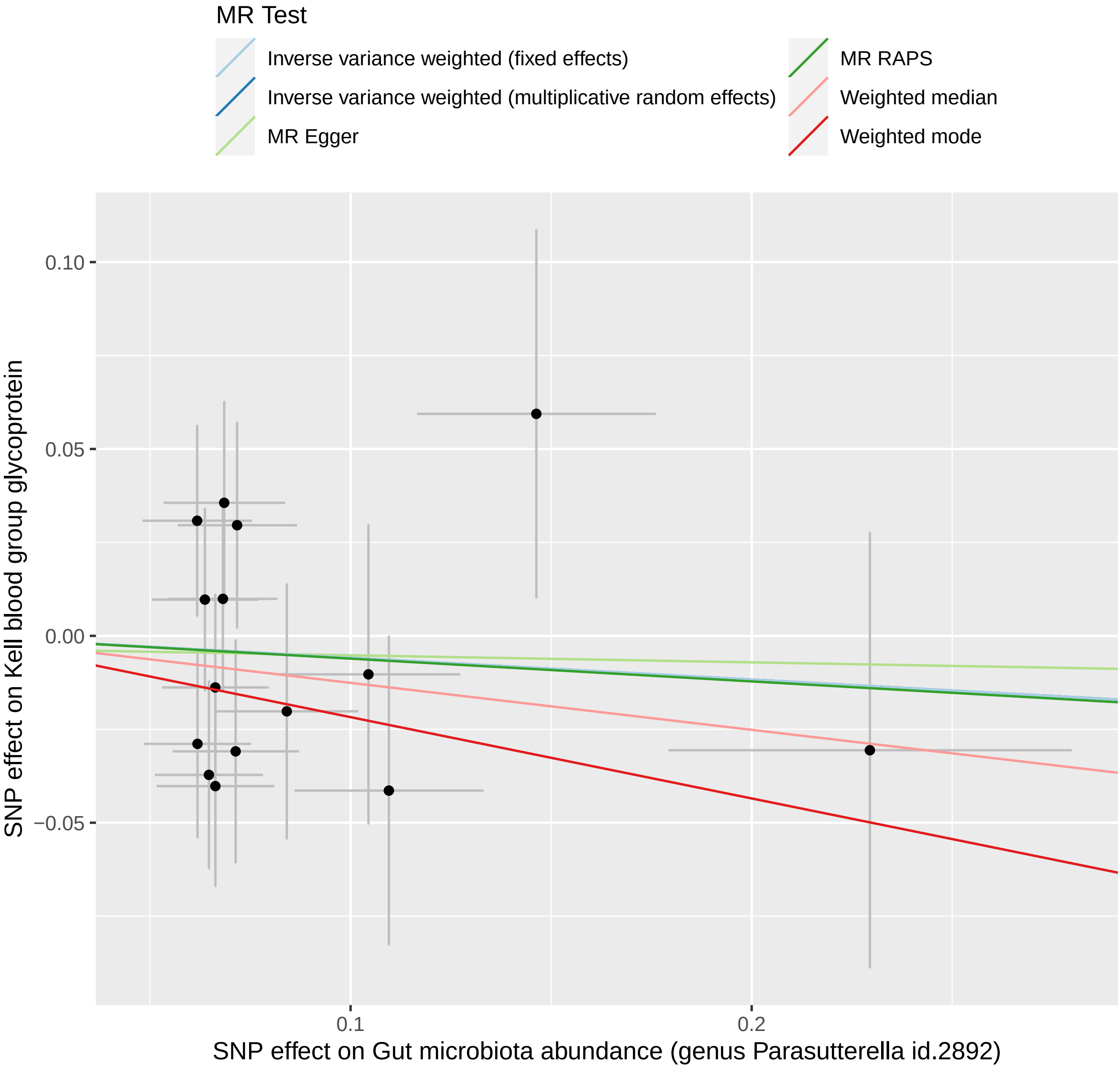

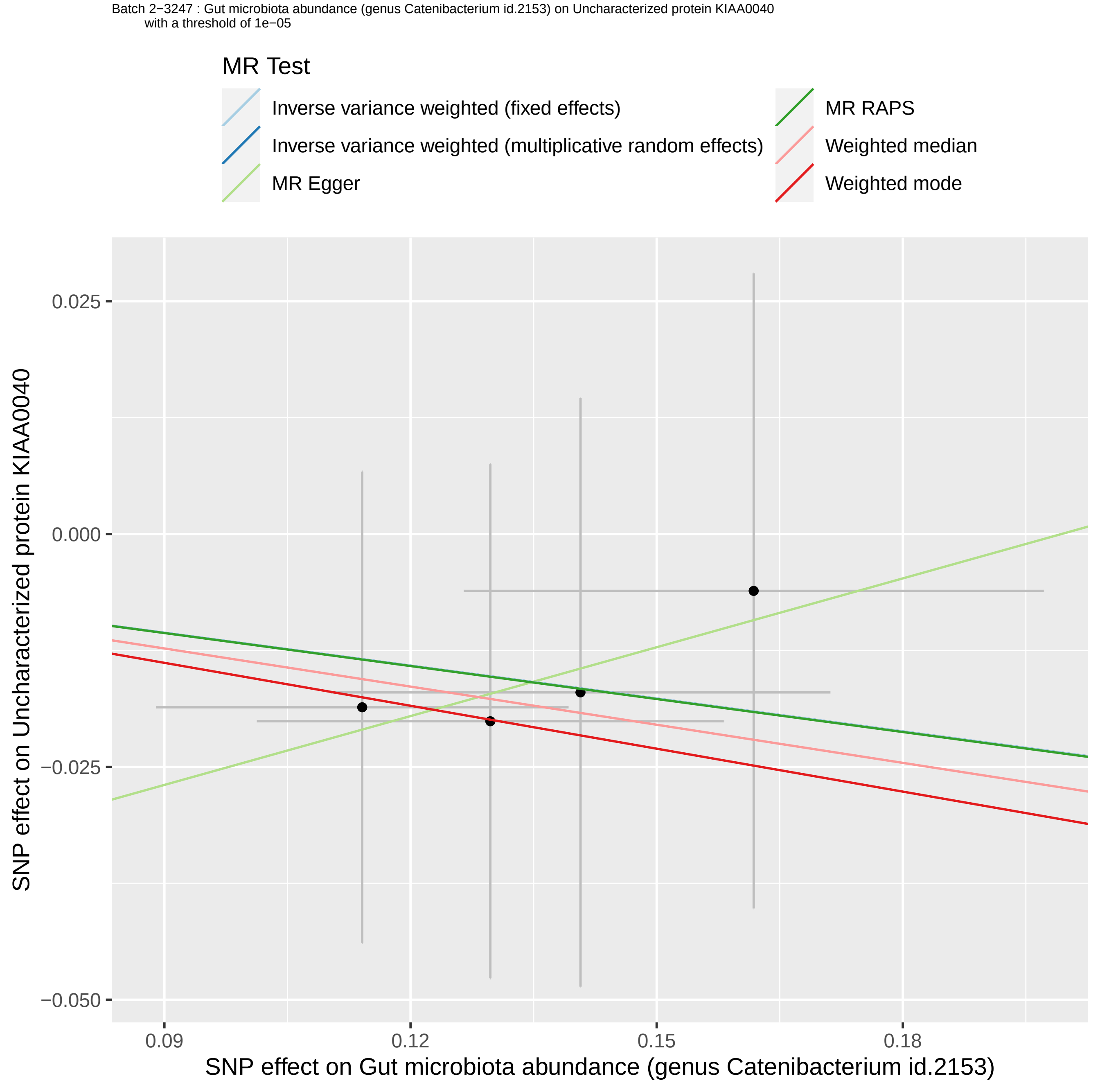

Batch 2−3247 : Gut microbiota abundance (genus Catenibacterium id.2153) on Uncharacterized protein KIAA0040
with a threshold of 1e−05
MR Test
Inverse variance weighted (fixed effects)
Inverse variance weighted (multiplicative random effects)
MR Egger
MR RAPS
Weighted median
Weighted mode
0.025
0.000
−0.025
−0.050
0.09
0.12
0.15
0.18
SNP effect on Uncharacterized protein KIAA0040
SNP effect on Gut microbiota abundance (genus Catenibacterium id.2153)

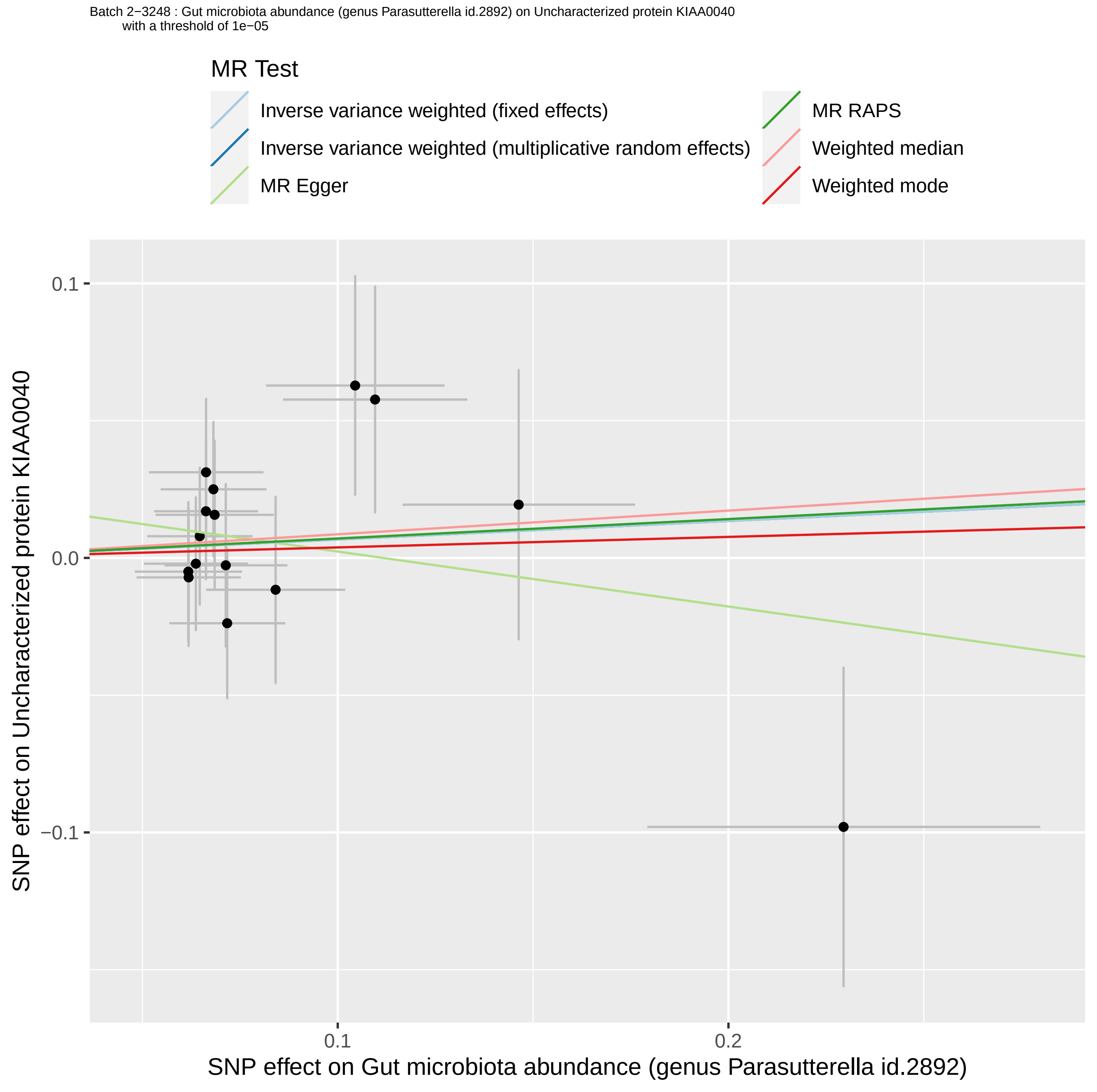

Batch 2−3248 : Gut microbiota abundance (genus Parasutterella id.2892) on Uncharacterized protein KIAA0040
with a threshold of 1e−05
MR Test
Inverse variance weighted (fixed effects)
Inverse variance weighted (multiplicative random effects)
MR Egger
MR RAPS
Weighted median
Weighted mode
0.1
0.0
−0.1
0.1
0.2
SNP effect on Uncharacterized protein KIAA0040
SNP effect on Gut microbiota abundance (genus Parasutterella id.2892)

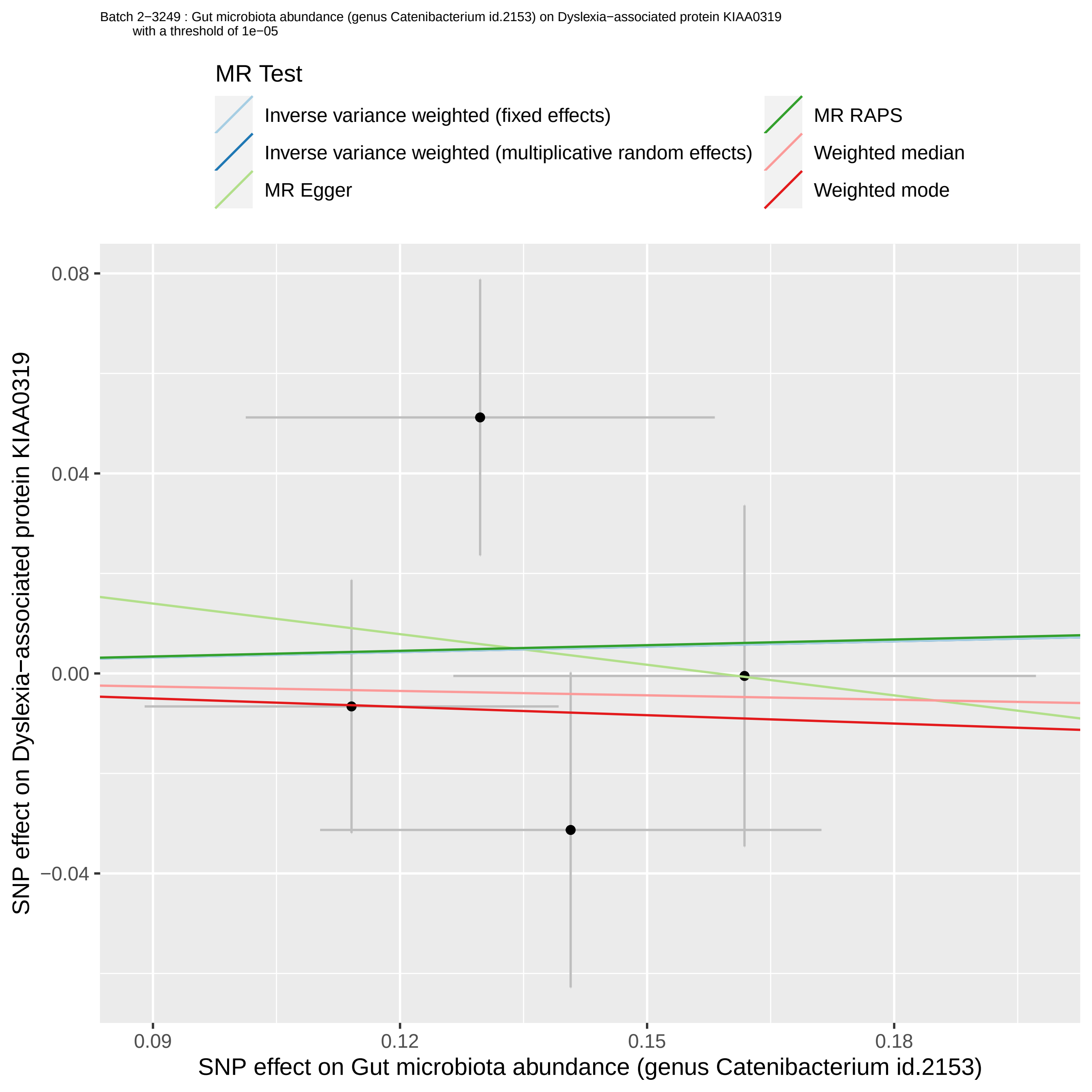

Batch 2−3249 : Gut microbiota abundance (genus Catenibacterium id.2153) on Dyslexia−associated protein KIAA0319
with a threshold of 1e−05
MR Test
Inverse variance weighted (fixed effects)
Inverse variance weighted (multiplicative random effects)
MR Egger
MR RAPS
Weighted median
Weighted mode
0.08
0.04
0.00
−0.04
0.09
0.12
0.15
0.18
SNP effect on Dyslexia−associated protein KIAA0319
SNP effect on Gut microbiota abundance (genus Catenibacterium id.2153)

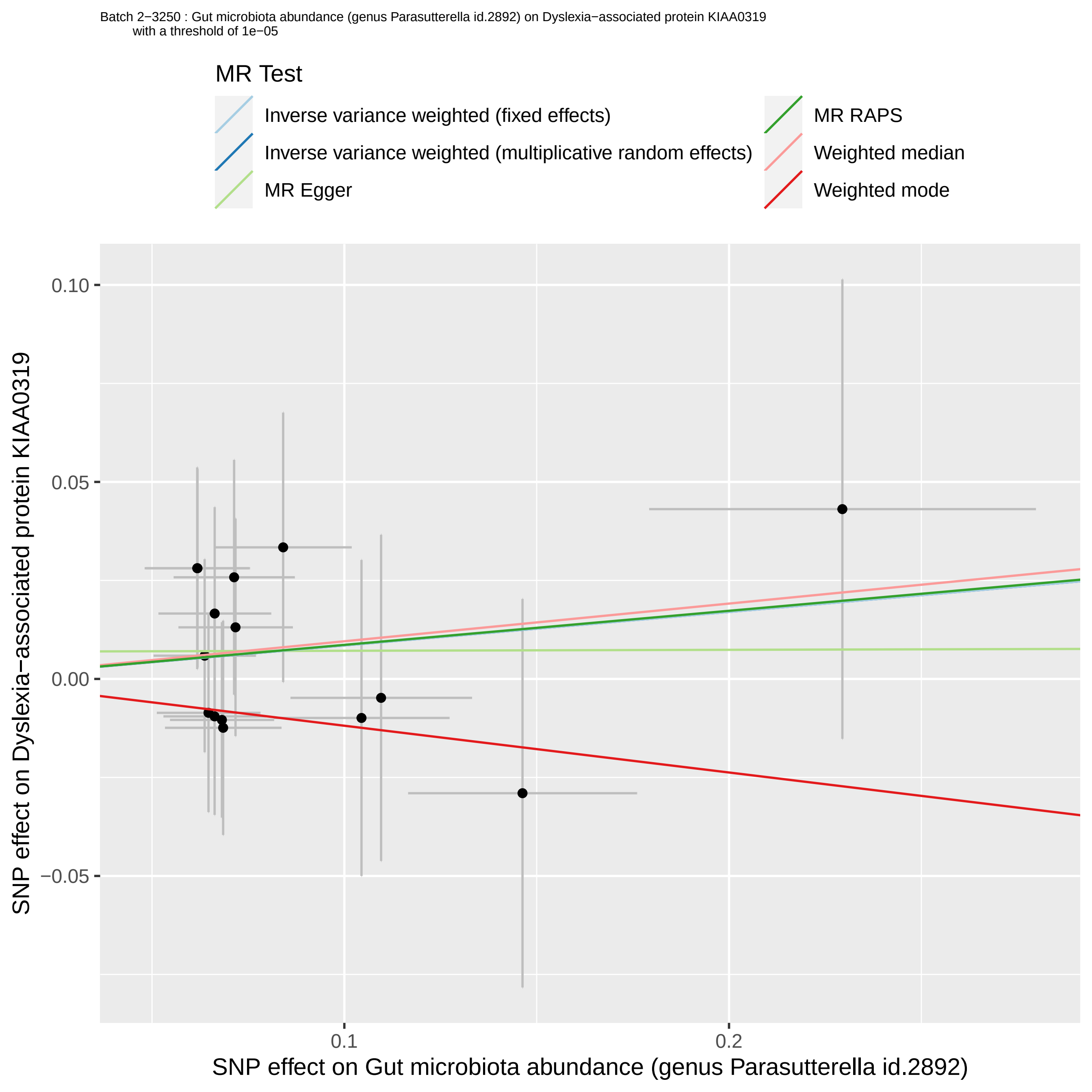
Batch 2−3250 : Gut microbiota abundance (genus Parasutterella id.2892) on Dyslexia−associated protein KIAA0319
with a threshold of 1e−05
MR Test
Inverse variance weighted (fixed effects)
Inverse variance weighted (multiplicative random effects)
MR Egger
MR RAPS
Weighted median
Weighted mode
0.10
0.05
0.00
−0.05
0.1
0.2
SNP effect on Dyslexia−associated protein KIAA0319
SNP effect on Gut microbiota abundance (genus Parasutterella id.2892)

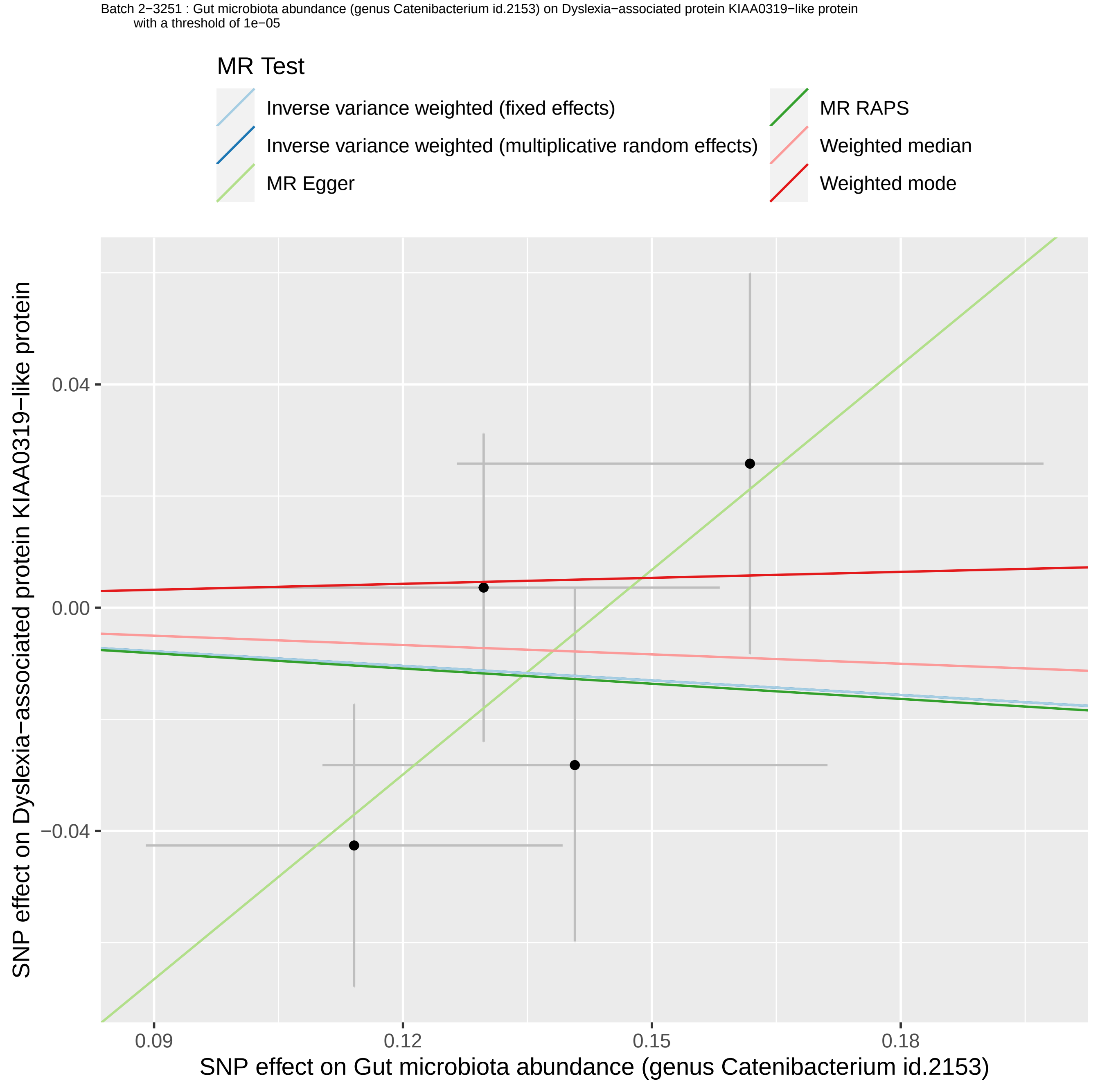
Batch 2−3251 : Gut microbiota abundance (genus Catenibacterium id.2153) on Dyslexia−associated protein KIAA0319−like protein with a threshold of 1e−05
MR Test
Inverse variance weighted (fixed effects)
Inverse variance weighted (multiplicative random effects)
MR Egger
MR RAPS
Weighted median
Weighted mode
SNP effect on Dyslexia−associated protein KIAA0319−like protein
0.04
0.00
−0.04
0.09
0.12
0.15
0.18
SNP effect on Gut microbiota abundance (genus Catenibacterium id.2153)

Batch 2−3252 : Gut microbiota abundance (genus Parasutterella id.2892) on Dyslexia−associated protein KIAA0319−like protein
with a threshold of 1e−05

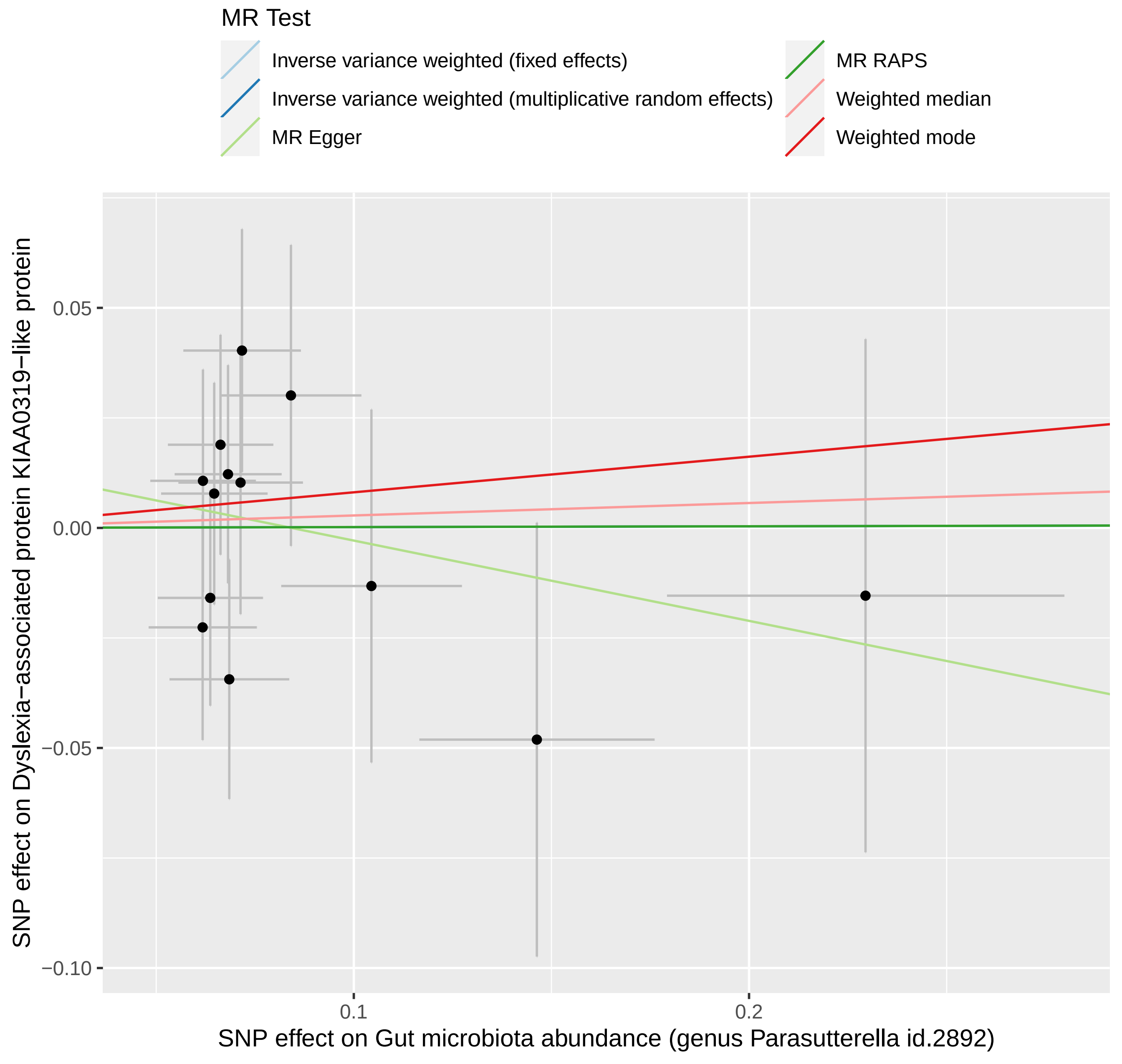

Batch 2−3253 : Gut microbiota abundance (genus Catenibacterium id.2153) on UPF0258 protein KIAA1024
with a threshold of 1e−05

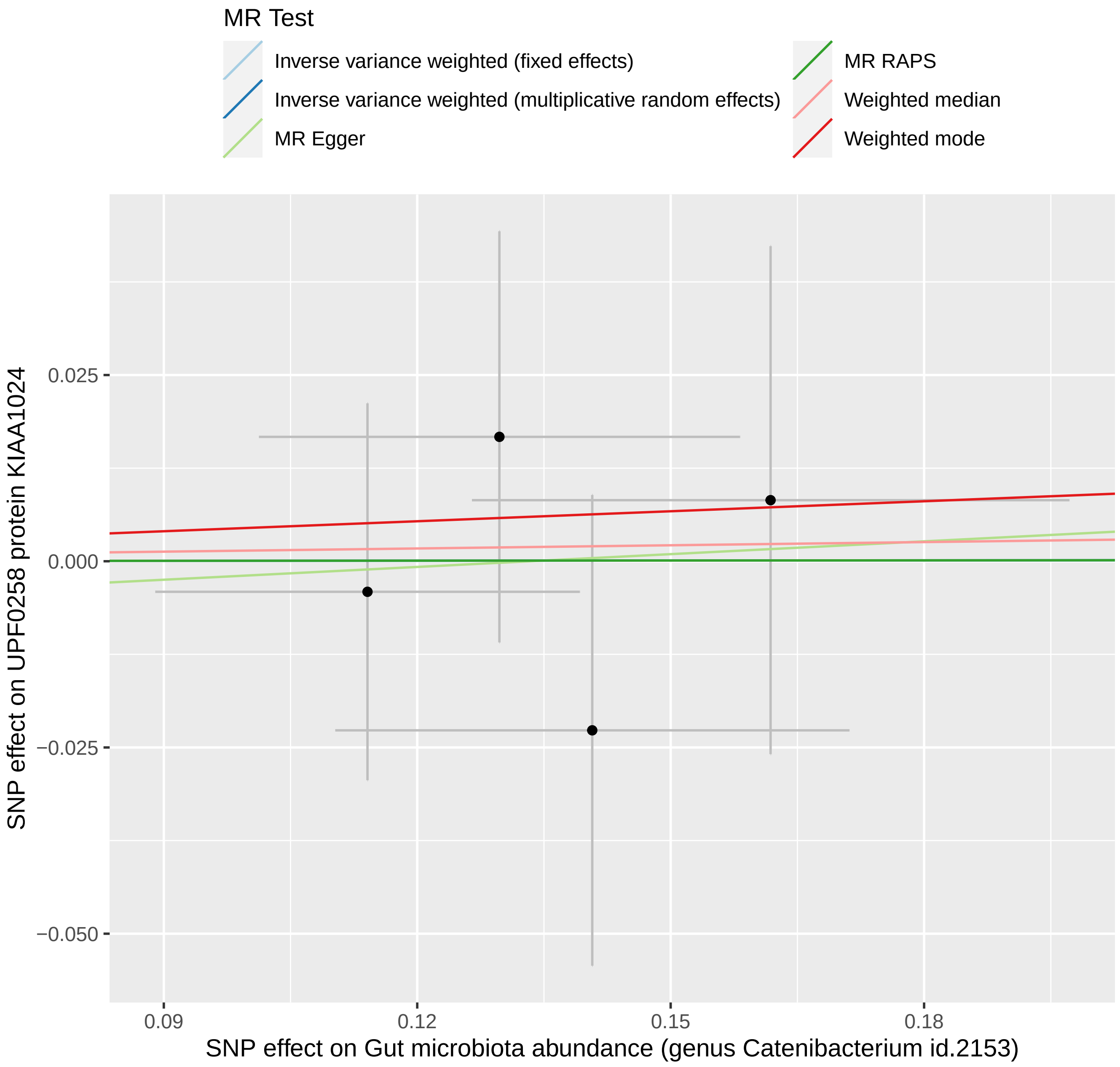

Batch 2−3254 : Gut microbiota abundance (genus Parasutterella id.2892) on UPF0258 protein KIAA1024
with a threshold of 1e−05

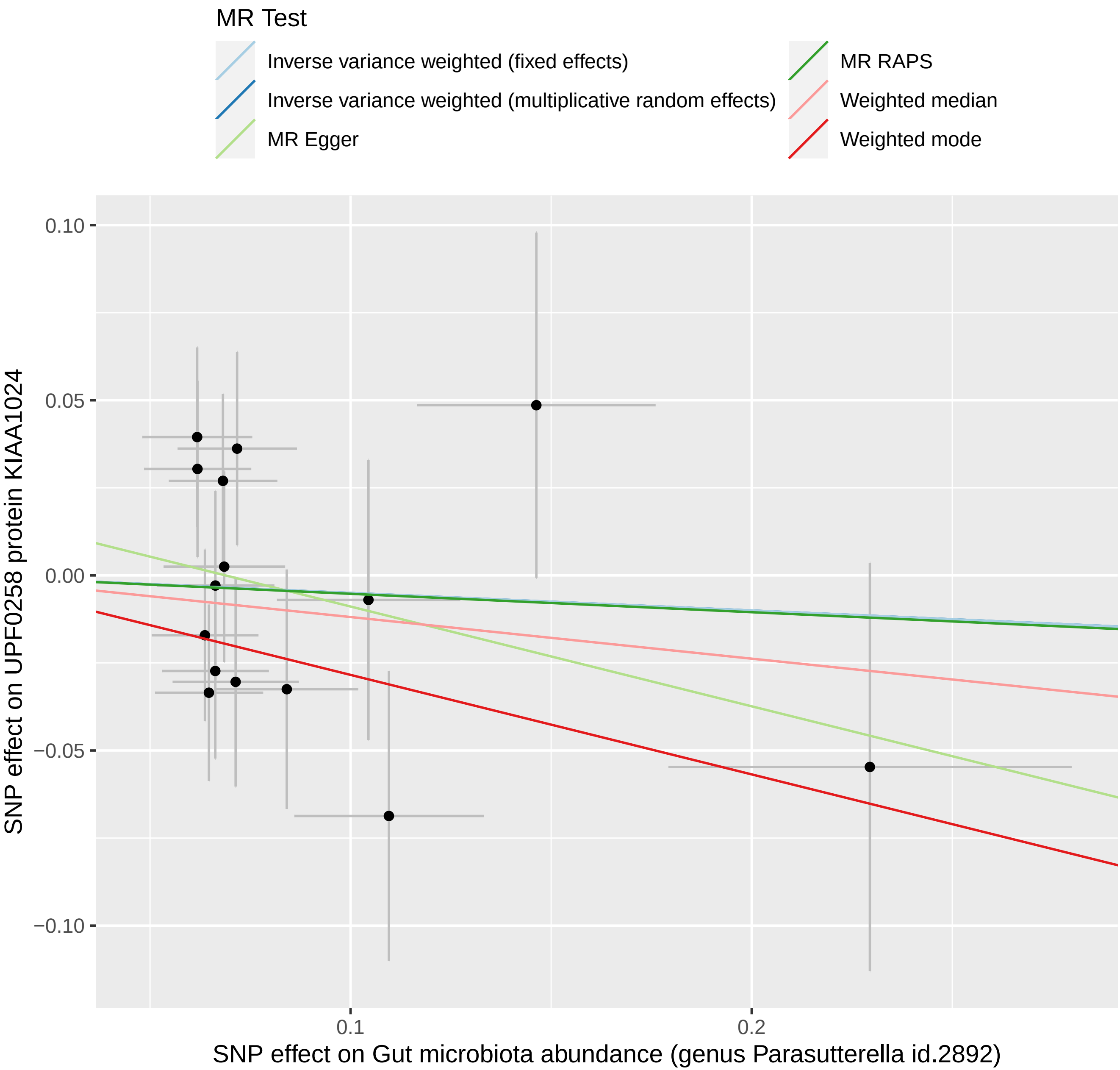

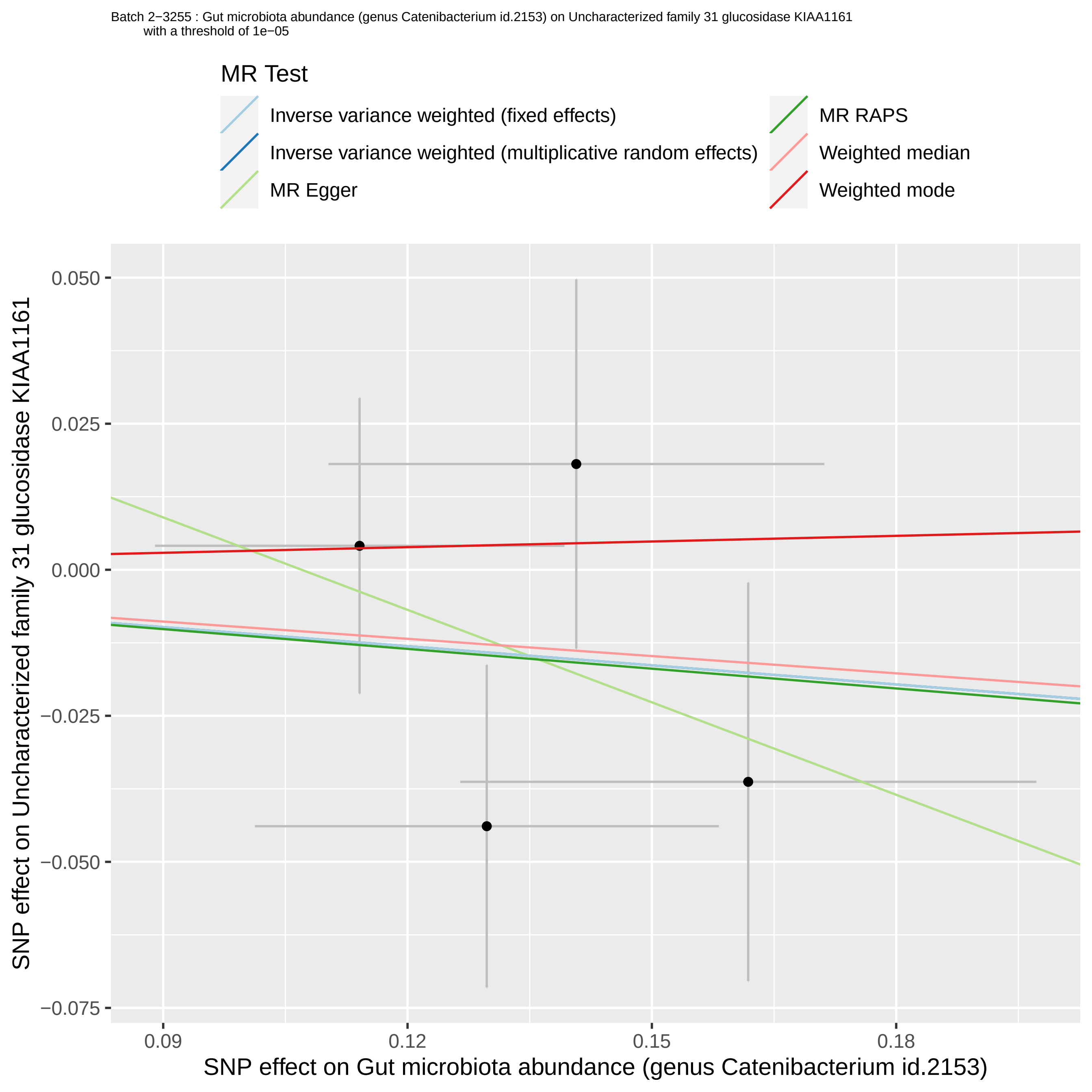
Batch 2−3255 : Gut microbiota abundance (genus Catenibacterium id.2153) on Uncharacterized family 31 glucosidase KIAA1161
with a threshold of 1e−05
MR Test
Inverse variance weighted (fixed effects)
Inverse variance weighted (multiplicative random effects)
MR Egger
MR RAPS
Weighted median
Weighted mode
0.050
0.025
0.000
−0.025
−0.050
−0.075
0.09
0.12
0.15
0.18
SNP effect on Uncharacterized family 31 glucosidase KIAA1161
SNP effect on Gut microbiota abundance (genus Catenibacterium id.2153)

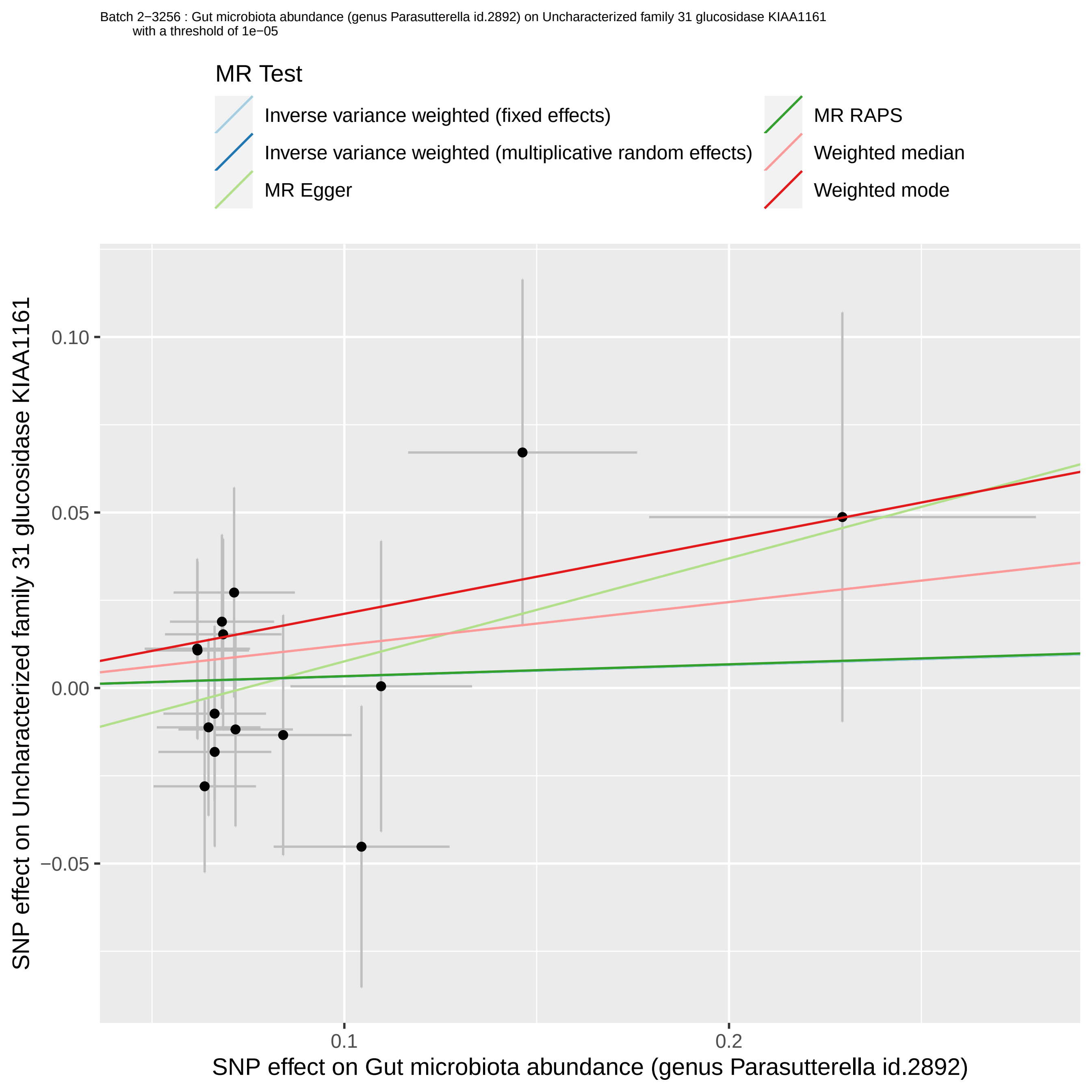
Batch 2−3256 : Gut microbiota abundance (genus Parasutterella id.2892) on Uncharacterized family 31 glucosidase KIAA1161
with a threshold of 1e−05
MR Test
Inverse variance weighted (fixed effects)
Inverse variance weighted (multiplicative random effects)
MR Egger
MR RAPS
Weighted median
Weighted mode
0.10
0.05
0.00
−0.05
0.1
0.2
SNP effect on Uncharacterized family 31 glucosidase KIAA1161
SNP effect on Gut microbiota abundance (genus Parasutterella id.2892)

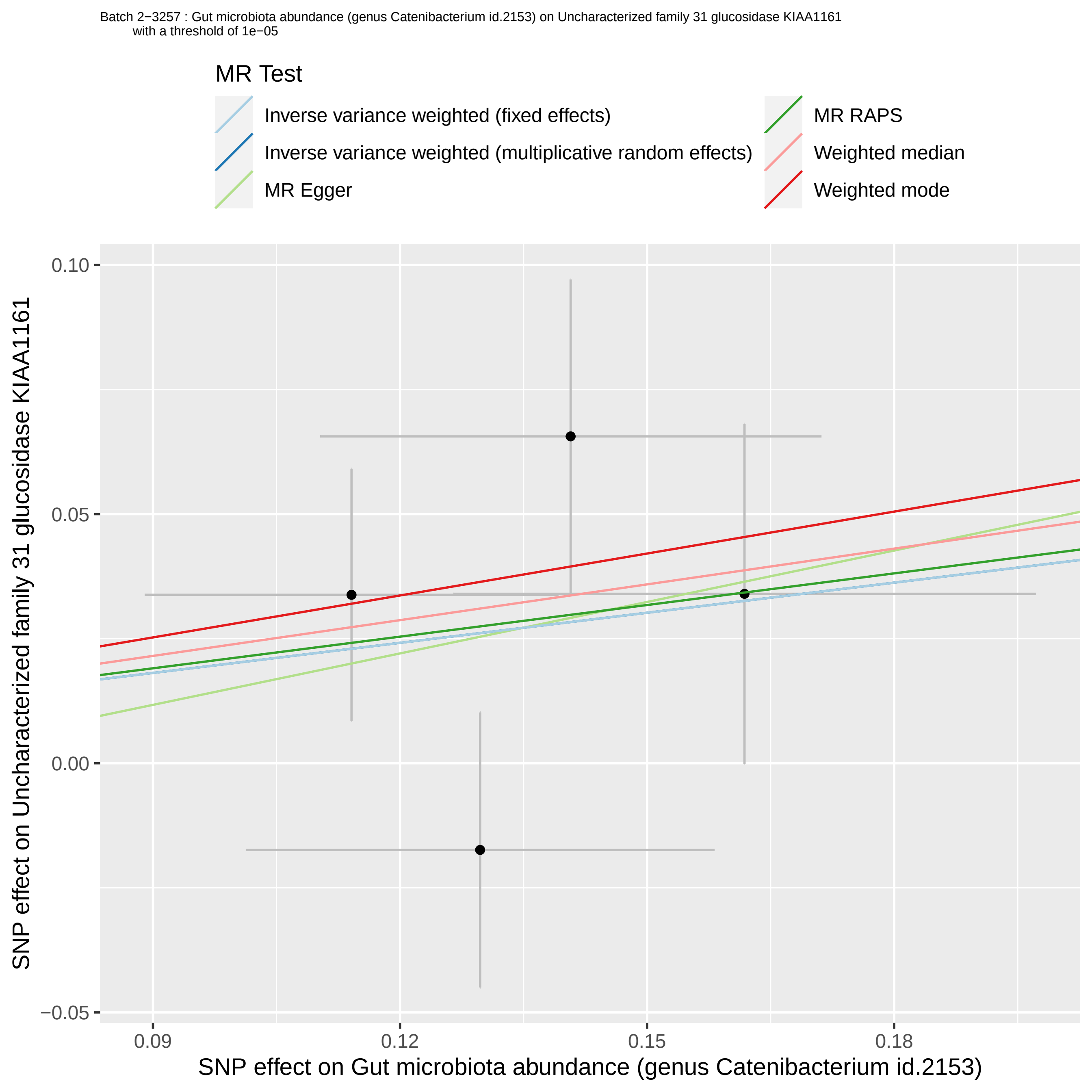

Batch 2−3257 : Gut microbiota abundance (genus Catenibacterium id.2153) on Uncharacterized family 31 glucosidase KIAA1161
with a threshold of 1e−05
MR Test
Inverse variance weighted (fixed effects)
Inverse variance weighted (multiplicative random effects)
MR Egger
MR RAPS
Weighted median
Weighted mode
0.10
0.05
0.00
−0.05
0.09
0.12
0.15
0.18
SNP effect on Uncharacterized family 31 glucosidase KIAA1161
SNP effect on Gut microbiota abundance (genus Catenibacterium id.2153)

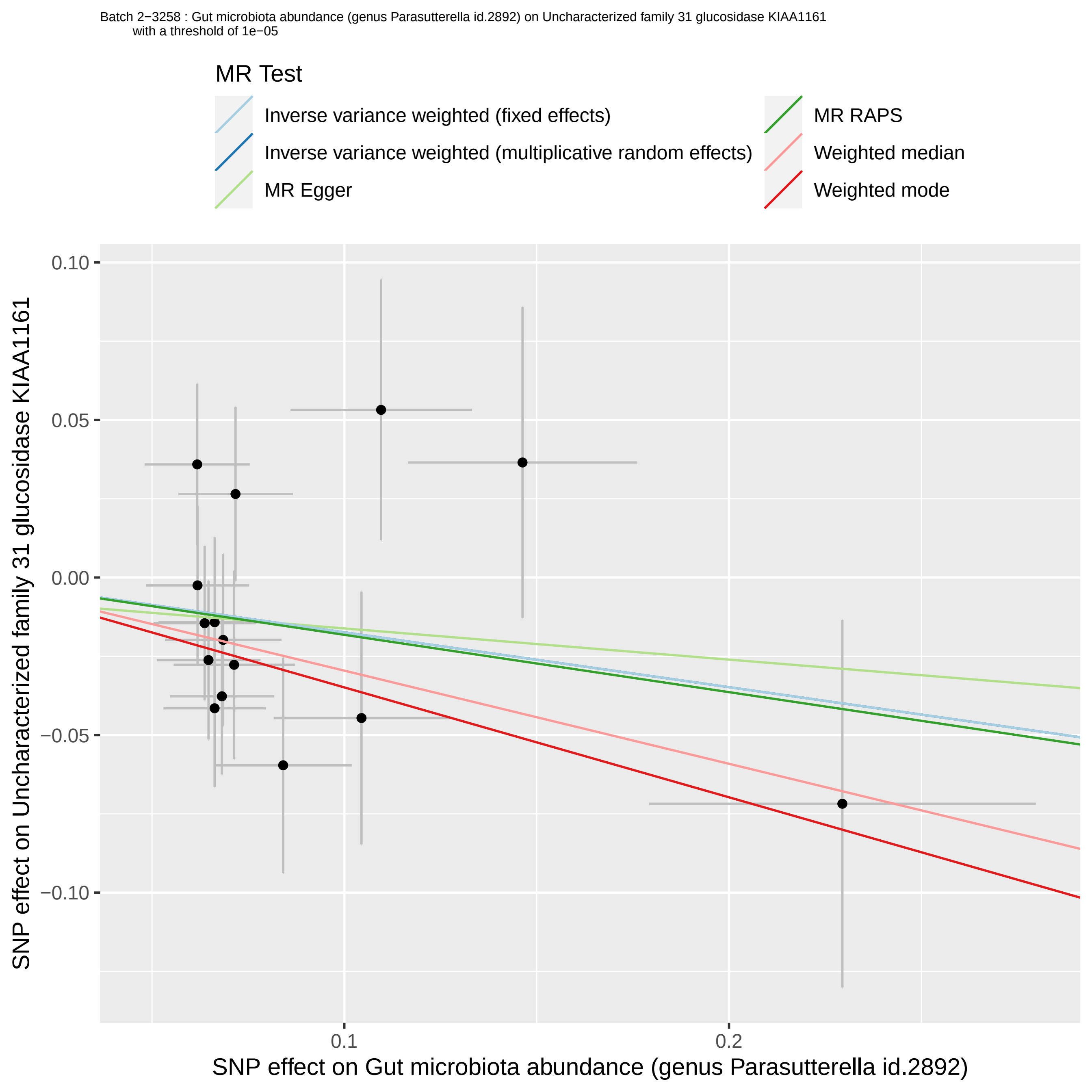

Batch 2−3258 : Gut microbiota abundance (genus Parasutterella id.2892) on Uncharacterized family 31 glucosidase KIAA1161
with a threshold of 1e−05
MR Test
Inverse variance weighted (fixed effects)
Inverse variance weighted (multiplicative random effects)
MR Egger
MR RAPS
Weighted median
Weighted mode
0.10
0.05
0.00
−0.05
−0.10
0.1
0.2
SNP effect on Uncharacterized family 31 glucosidase KIAA1161
SNP effect on Gut microbiota abundance (genus Parasutterella id.2892)

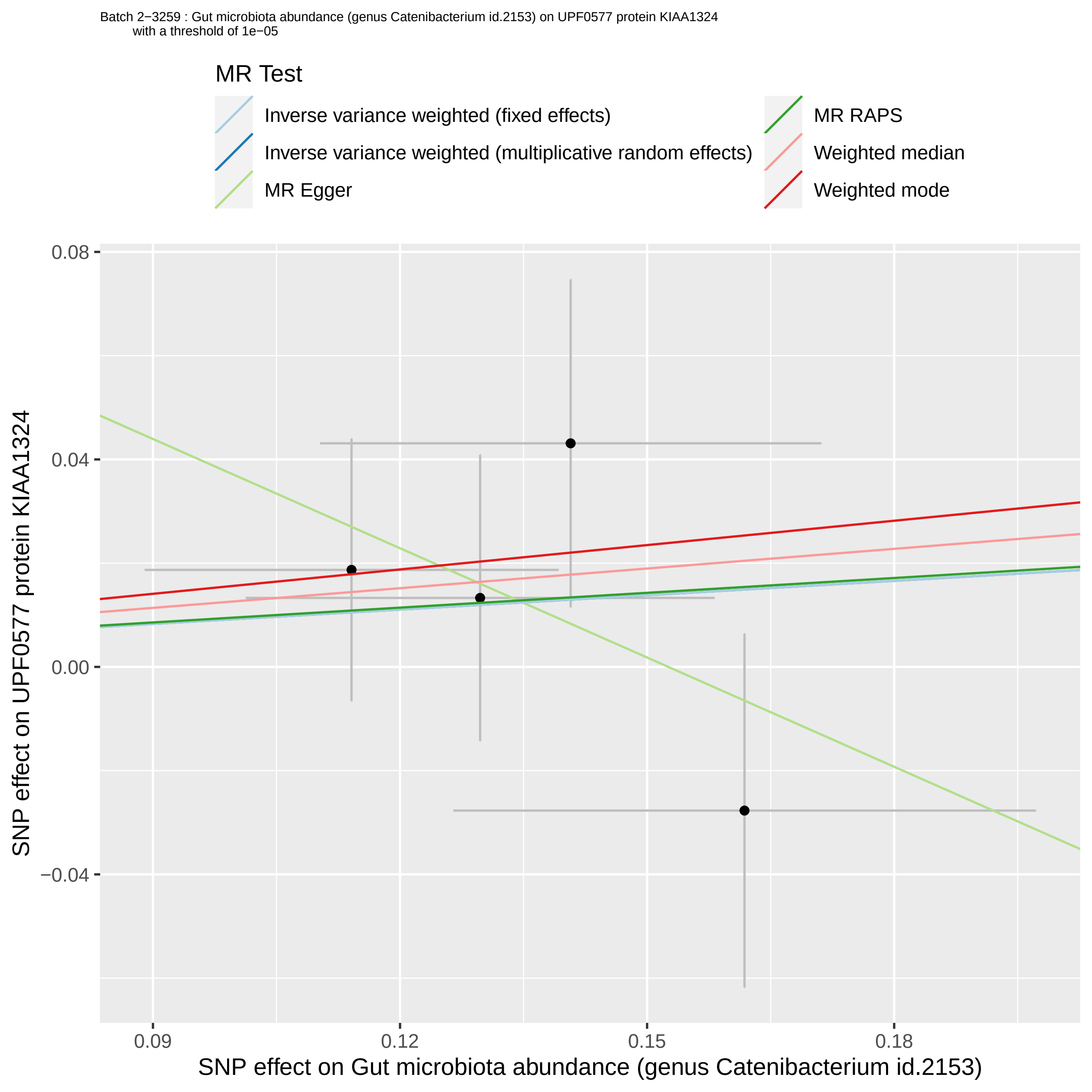

Batch 2−3259 : Gut microbiota abundance (genus Catenibacterium id.2153) on UPF0577 protein KIAA1324
with a threshold of 1e−05
MR Test
Inverse variance weighted (fixed effects)
Inverse variance weighted (multiplicative random effects)
MR Egger
MR RAPS
Weighted median
Weighted mode
0.08
0.04
0.00
−0.04
0.09
0.12
0.15
0.18
SNP effect on UPF0577 protein KIAA1324
SNP effect on Gut microbiota abundance (genus Catenibacterium id.2153)

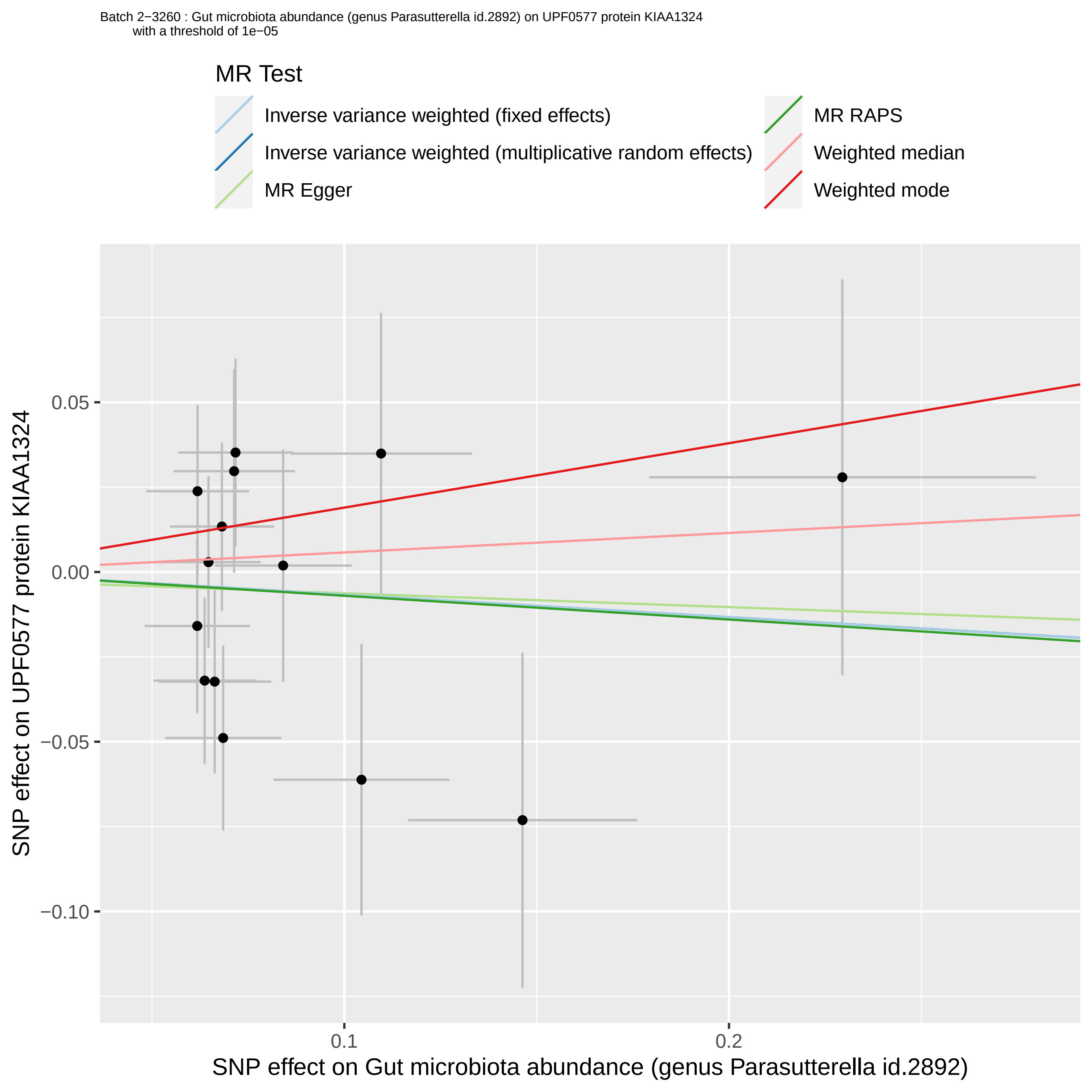
Batch 2−3260 : Gut microbiota abundance (genus Parasutterella id.2892) on UPF0577 protein KIAA1324
with a threshold of 1e−05
MR Test
Inverse variance weighted (fixed effects)
Inverse variance weighted (multiplicative random effects)
MR Egger
MR RAPS
Weighted median
Weighted mode
0.05
0.00
−0.05
−0.10
0.1
0.2
SNP effect on UPF0577 protein KIAA1324
SNP effect on Gut microbiota abundance (genus Parasutterella id.2892)

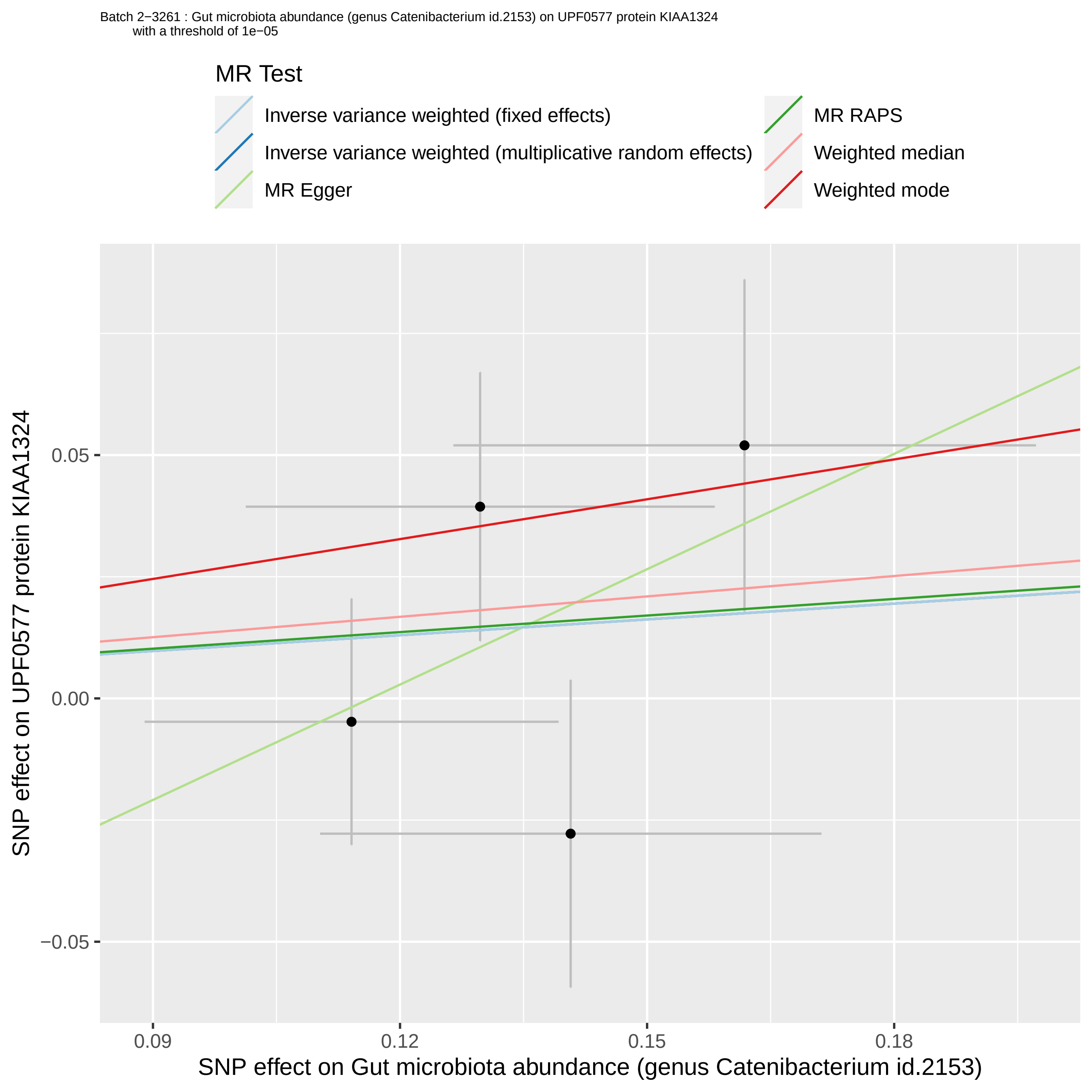

Batch 2−3261 : Gut microbiota abundance (genus Catenibacterium id.2153) on UPF0577 protein KIAA1324
with a threshold of 1e−05
MR Test
Inverse variance weighted (fixed effects)
Inverse variance weighted (multiplicative random effects)
MR Egger
MR RAPS
Weighted median
Weighted mode
SNP effect on UPF0577 protein KIAA1324
0.05
0.00
−0.05
0.09
0.12
0.15
0.18
SNP effect on Gut microbiota abundance (genus Catenibacterium id.2153)

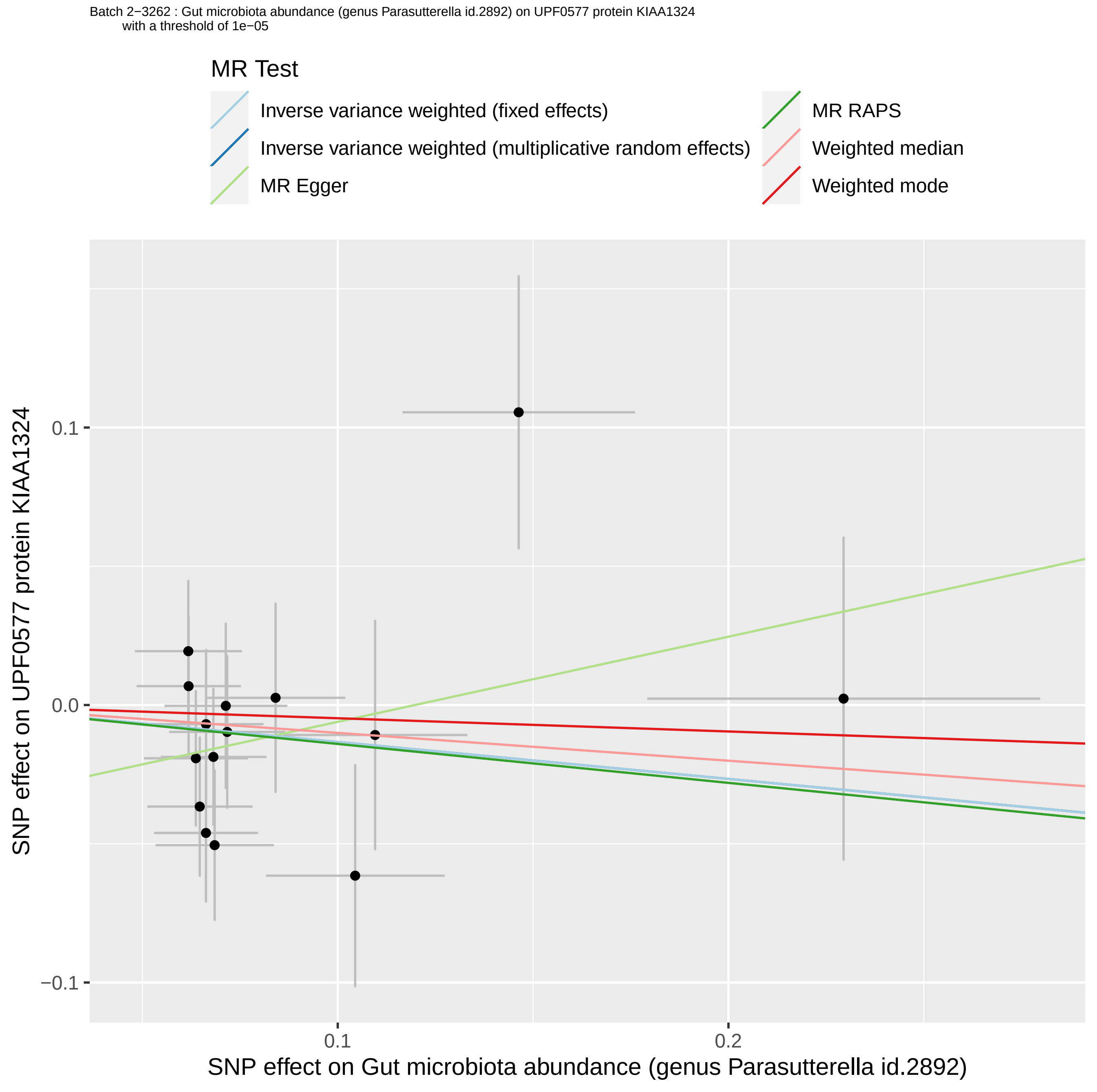
Batch 2−3262 : Gut microbiota abundance (genus Parasutterella id.2892) on UPF0577 protein KIAA1324
with a threshold of 1e−05
MR Test
Inverse variance weighted (fixed effects)
Inverse variance weighted (multiplicative random effects)
MR Egger
MR RAPS
Weighted median
Weighted mode
SNP effect on UPF0577 protein KIAA1324
0.1
0.0
−0.1
0.1
0.2
SNP effect on Gut microbiota abundance (genus Parasutterella id.2892)

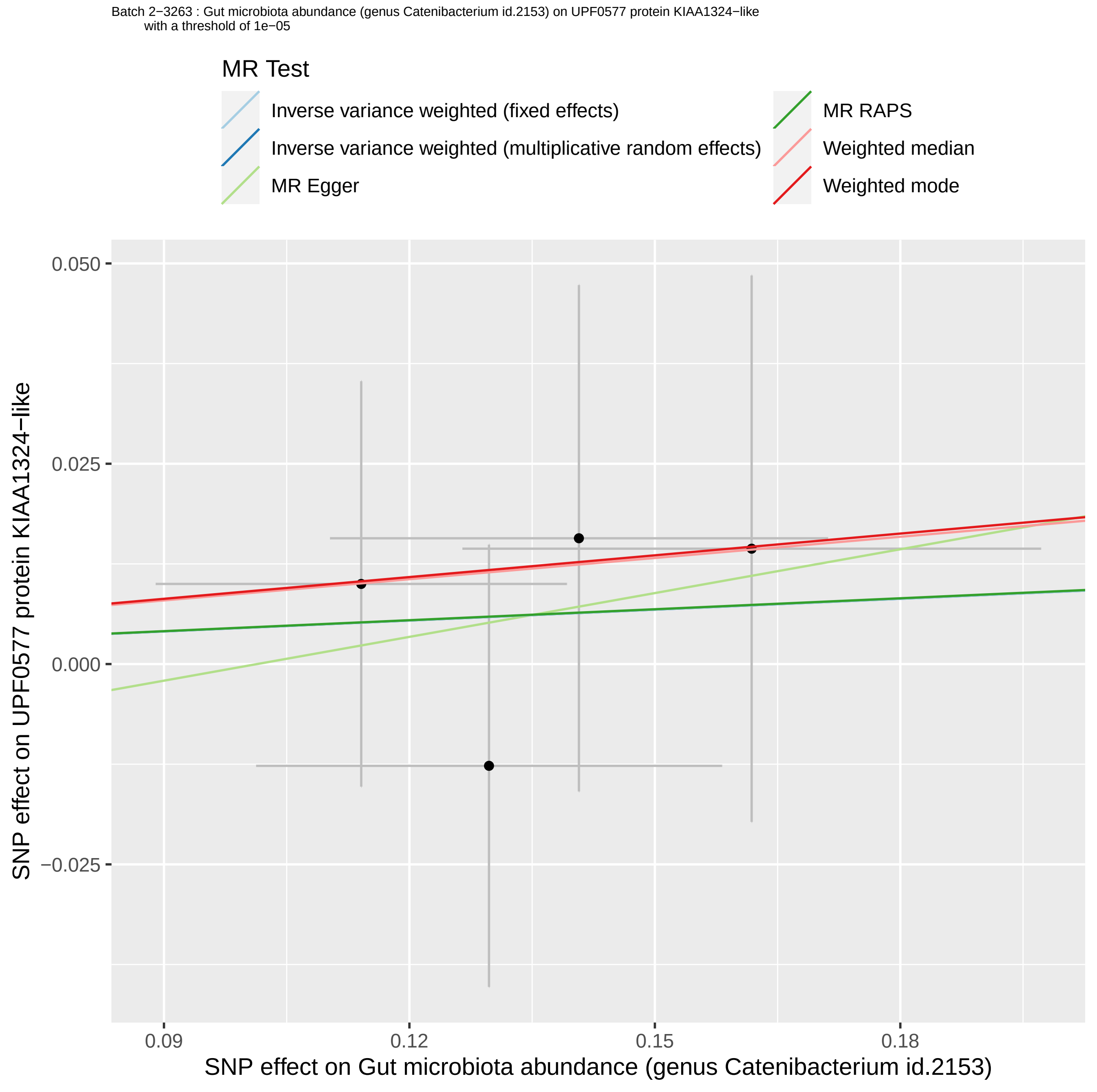

Batch 2−3263 : Gut microbiota abundance (genus Catenibacterium id.2153) on UPF0577 protein KIAA1324−like
with a threshold of 1e−05
MR Test
Inverse variance weighted (fixed effects)
Inverse variance weighted (multiplicative random effects)
MR Egger
MR RAPS
Weighted median
Weighted mode
0.050
0.025
0.000
−0.025
0.09
0.12
0.15
0.18
SNP effect on UPF0577 protein KIAA1324−like
SNP effect on Gut microbiota abundance (genus Catenibacterium id.2153)

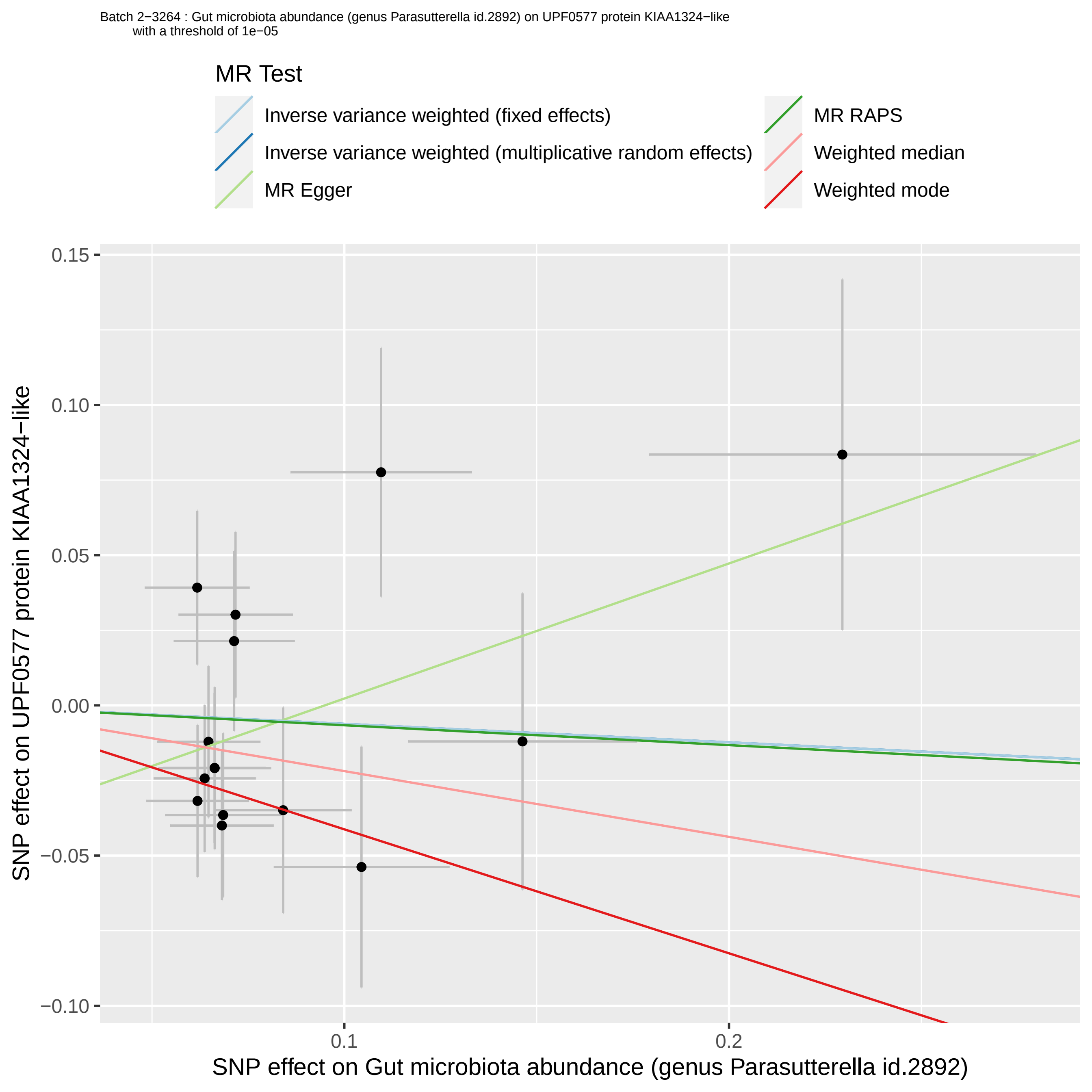

Batch 2−3264 : Gut microbiota abundance (genus Parasutterella id.2892) on UPF0577 protein KIAA1324−like
with a threshold of 1e−05
MR Test
Inverse variance weighted (fixed effects)
Inverse variance weighted (multiplicative random effects)
MR Egger
MR RAPS
Weighted median
Weighted mode
0.15
0.10
0.05
0.00
−0.05
−0.10
0.1
0.2
SNP effect on UPF0577 protein KIAA1324−like
SNP effect on Gut microbiota abundance (genus Parasutterella id.2892)

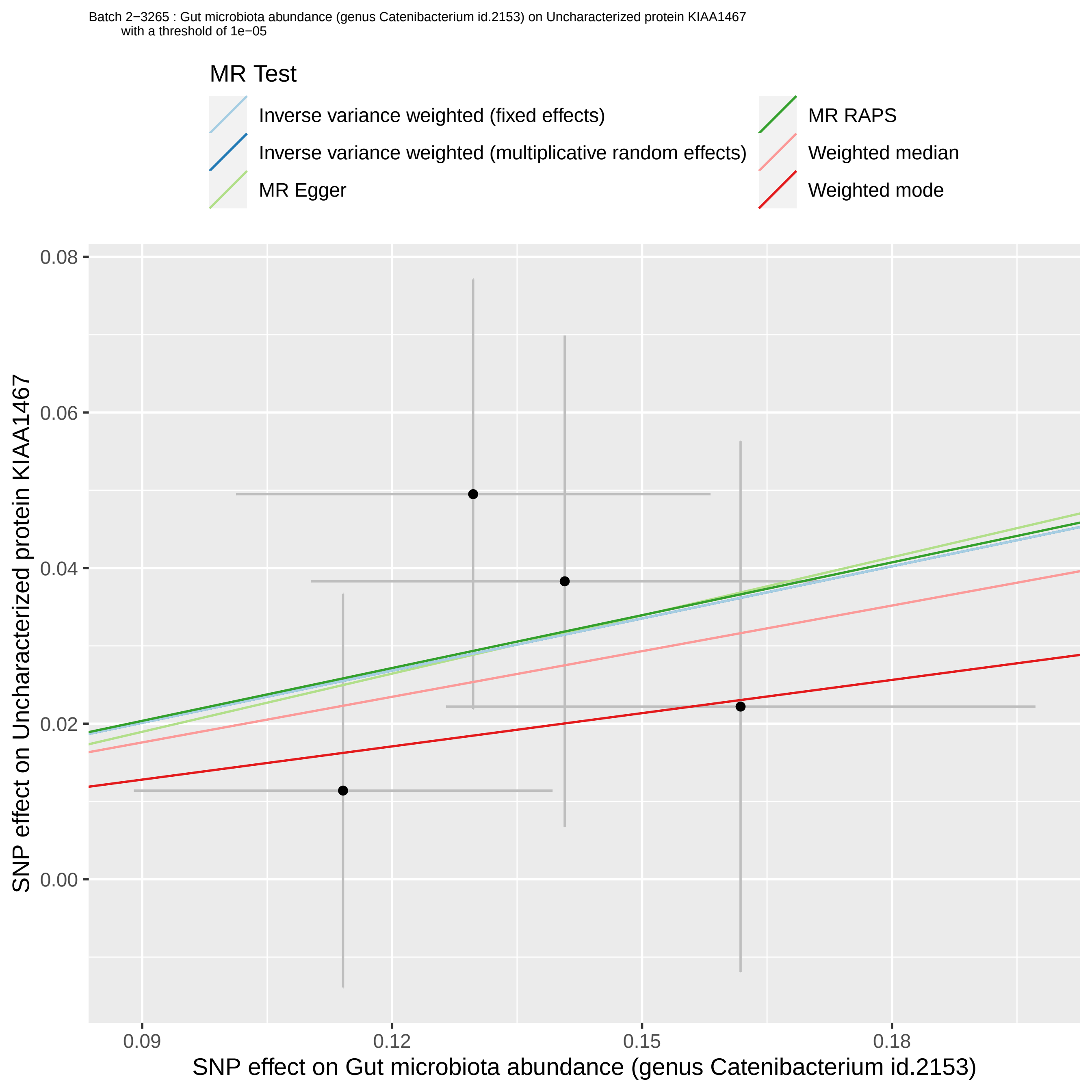
Batch 2−3265 : Gut microbiota abundance (genus Catenibacterium id.2153) on Uncharacterized protein KIAA1467
with a threshold of 1e−05
MR Test
Inverse variance weighted (fixed effects)
Inverse variance weighted (multiplicative random effects)
MR Egger
MR RAPS
Weighted median
Weighted mode
SNP effect on Uncharacterized protein KIAA1467
0.08
0.06
0.04
0.02
0.00
0.09
0.12
0.15
0.18
SNP effect on Gut microbiota abundance (genus Catenibacterium id.2153)

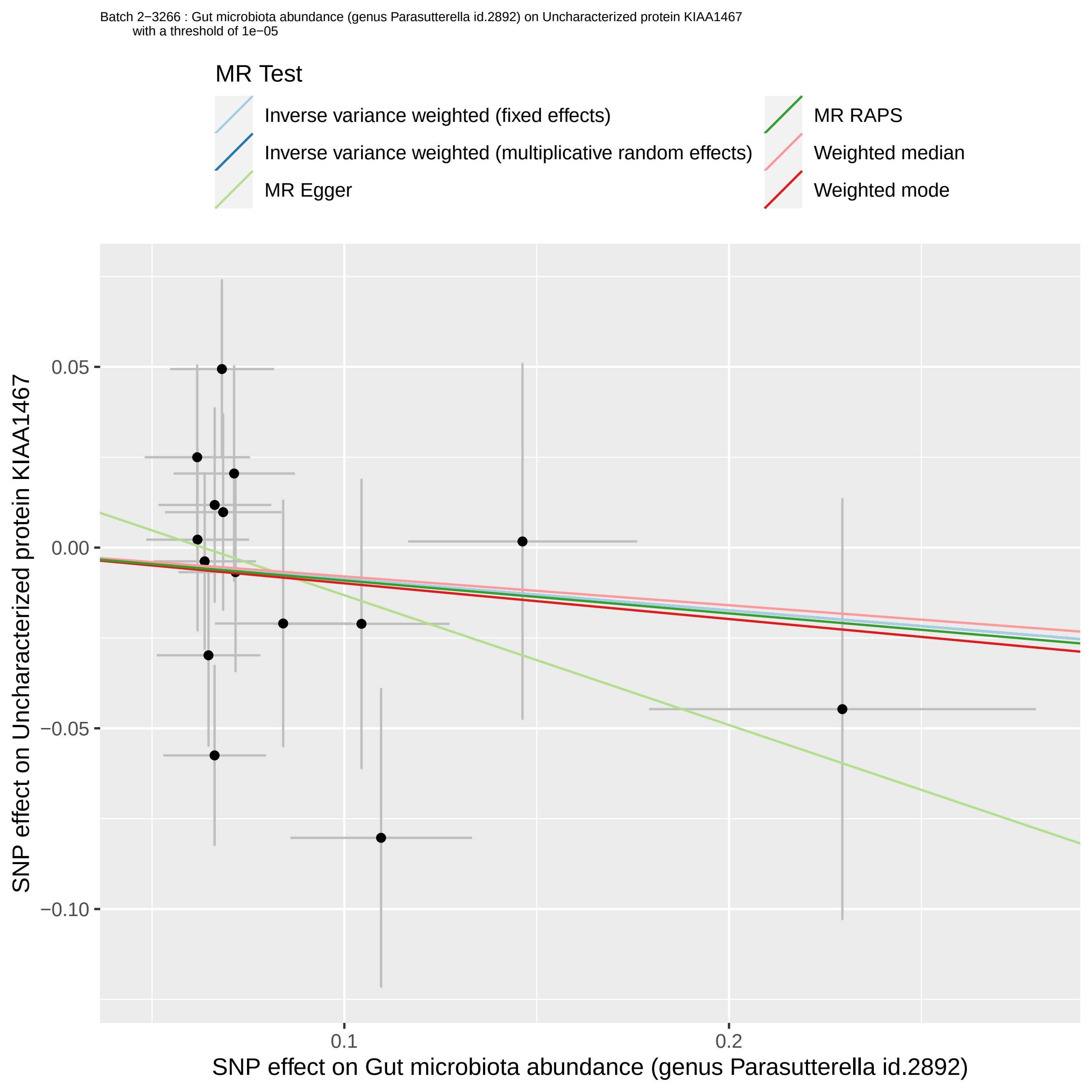
Batch 2−3266 : Gut microbiota abundance (genus Parasutterella id.2892) on Uncharacterized protein KIAA1467
with a threshold of 1e−05
MR Test
Inverse variance weighted (fixed effects)
Inverse variance weighted (multiplicative random effects)
MR Egger
MR RAPS
Weighted median
Weighted mode
0.05
0.00
−0.05
−0.10
0.1
0.2
SNP effect on Uncharacterized protein KIAA1467
SNP effect on Gut microbiota abundance (genus Parasutterella id.2892)

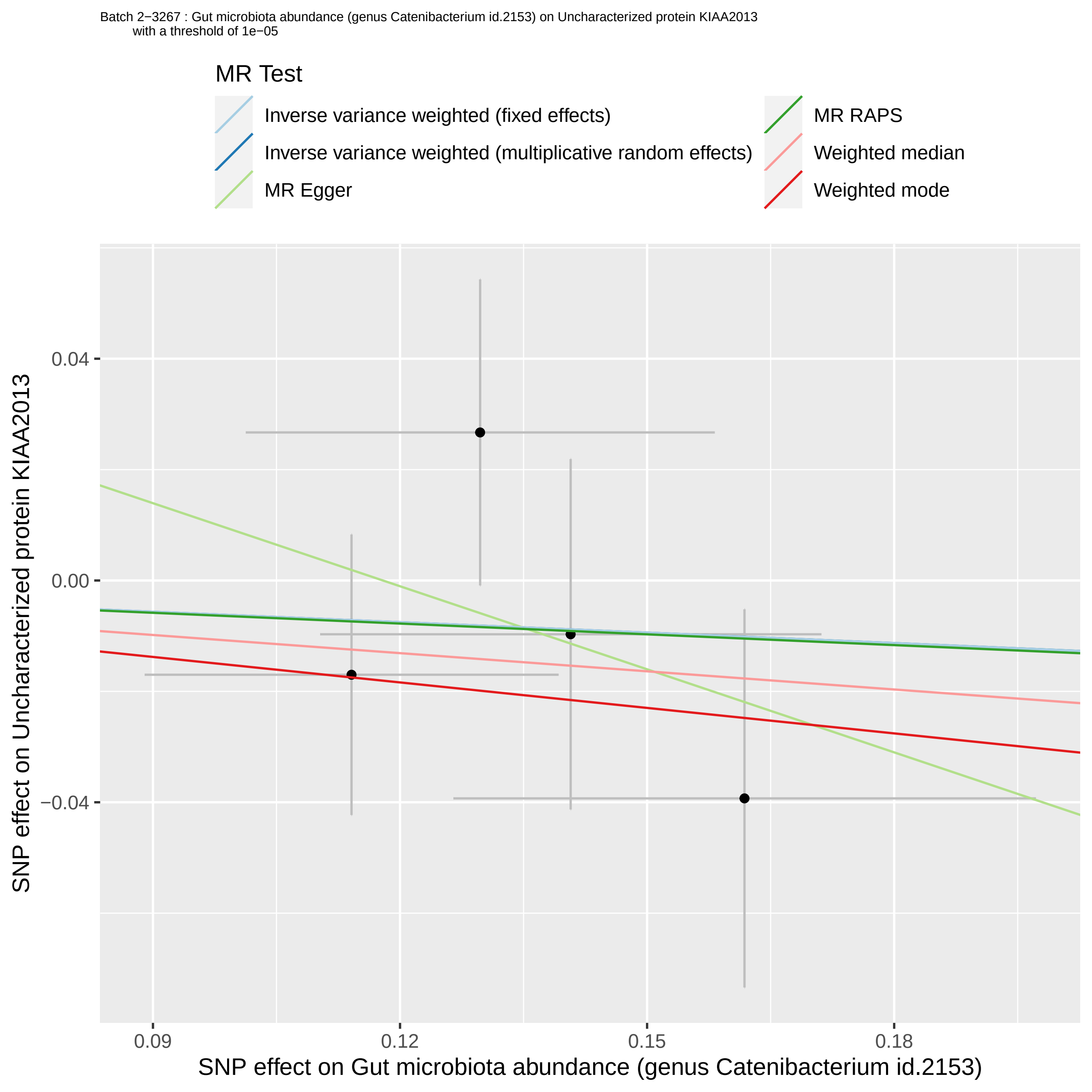

Batch 2−3267 : Gut microbiota abundance (genus Catenibacterium id.2153) on Uncharacterized protein KIAA2013
with a threshold of 1e−05
MR Test
Inverse variance weighted (fixed effects)
Inverse variance weighted (multiplicative random effects)
MR Egger
MR RAPS
Weighted median
Weighted mode
0.04
0.00
−0.04
0.09
0.12
0.15
0.18
SNP effect on Uncharacterized protein KIAA2013
SNP effect on Gut microbiota abundance (genus Catenibacterium id.2153)

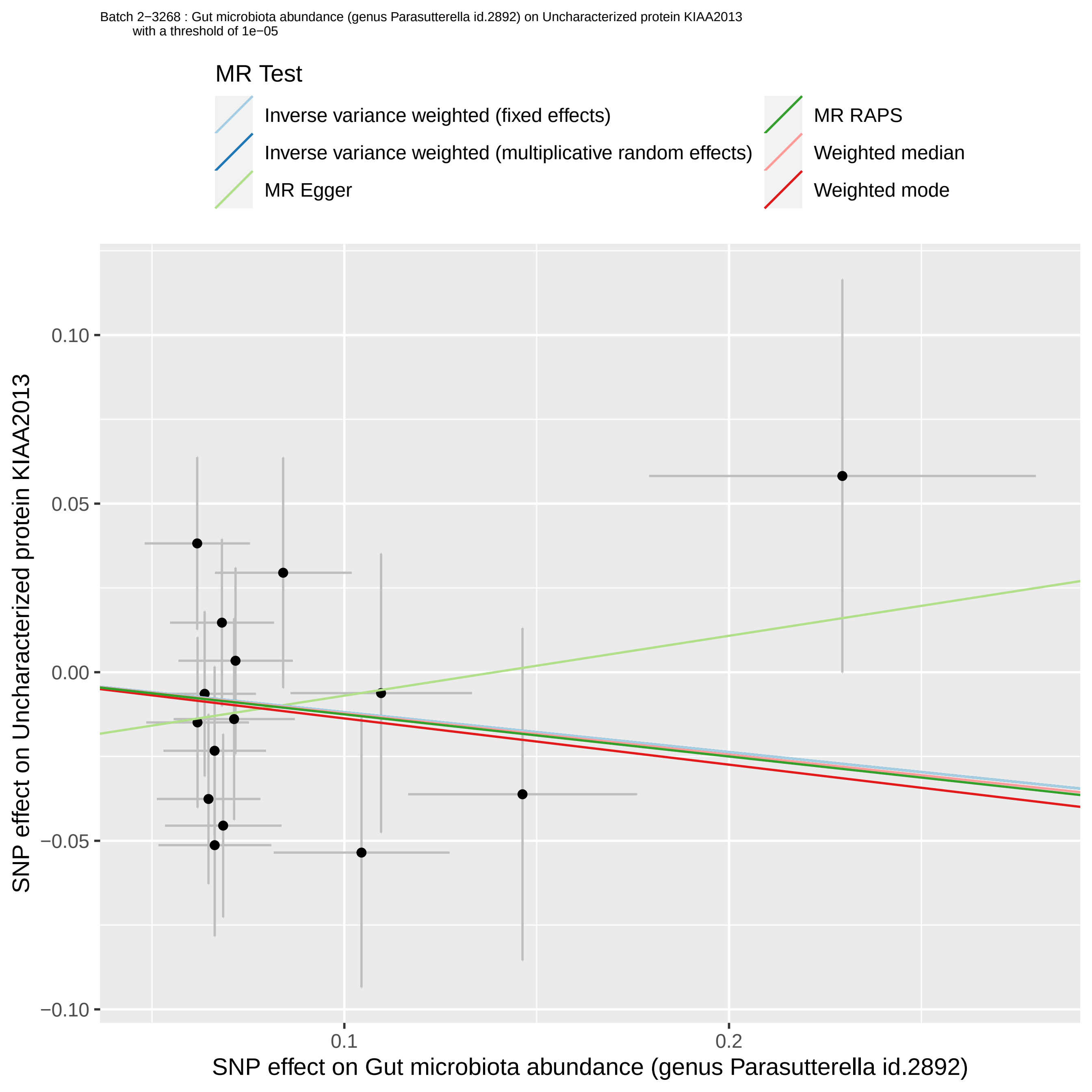

Batch 2−3268 : Gut microbiota abundance (genus Parasutterella id.2892) on Uncharacterized protein KIAA2013
with a threshold of 1e−05
MR Test
Inverse variance weighted (fixed effects)
Inverse variance weighted (multiplicative random effects)
MR Egger
MR RAPS
Weighted median
Weighted mode
0.10
0.05
0.00
−0.05
−0.10
0.1
0.2
SNP effect on Uncharacterized protein KIAA2013
SNP effect on Gut microbiota abundance (genus Parasutterella id.2892)

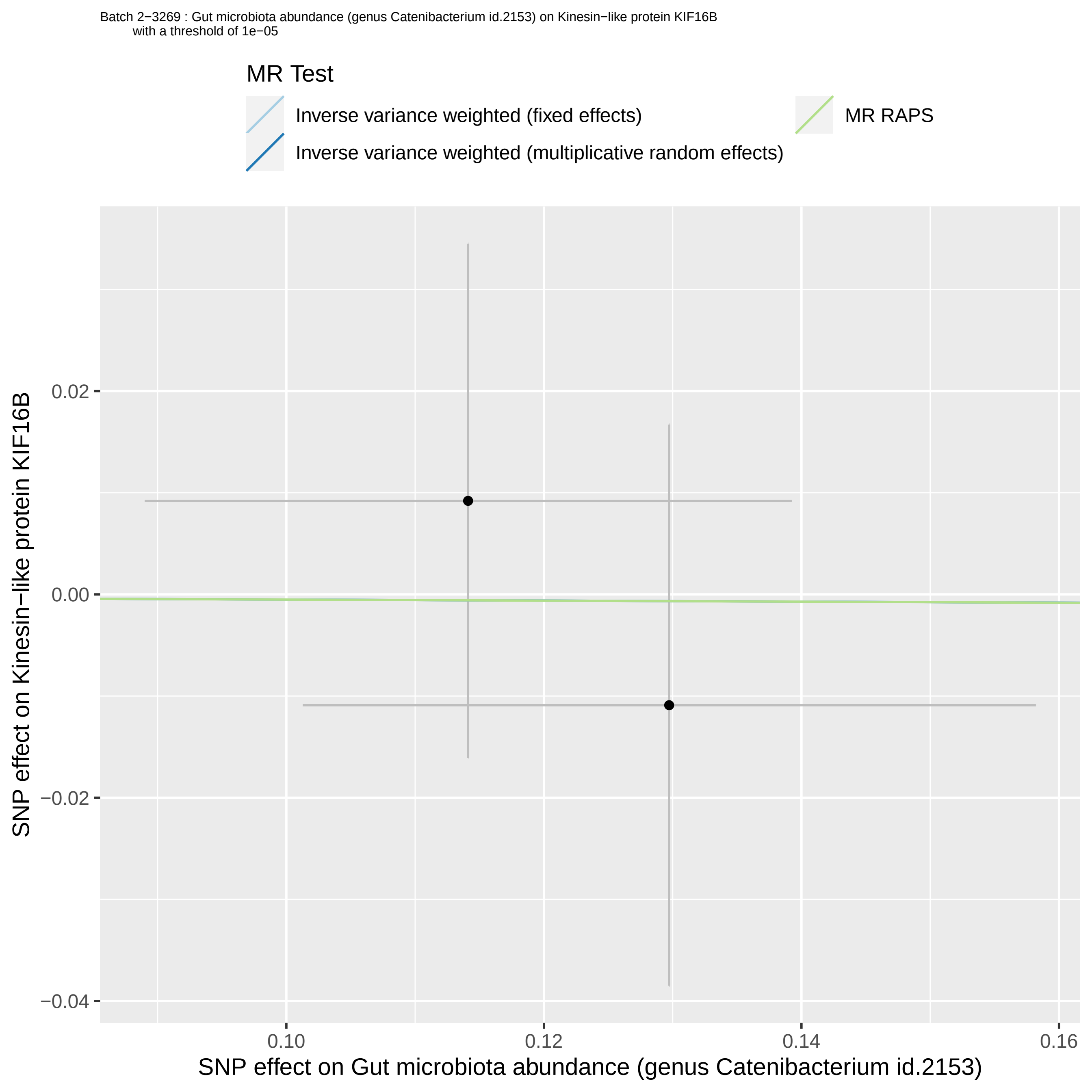

Batch 2−3269 : Gut microbiota abundance (genus Catenibacterium id.2153) on Kinesin−like protein KIF16B
with a threshold of 1e−05
MR Test
Inverse variance weighted (fixed effects)
Inverse variance weighted (multiplicative random effects)
MR RAPS
0.02
0.00
−0.02
−0.04
0.10
0.12
0.14
0.16
SNP effect on Kinesin−like protein KIF16B
SNP effect on Gut microbiota abundance (genus Catenibacterium id.2153)

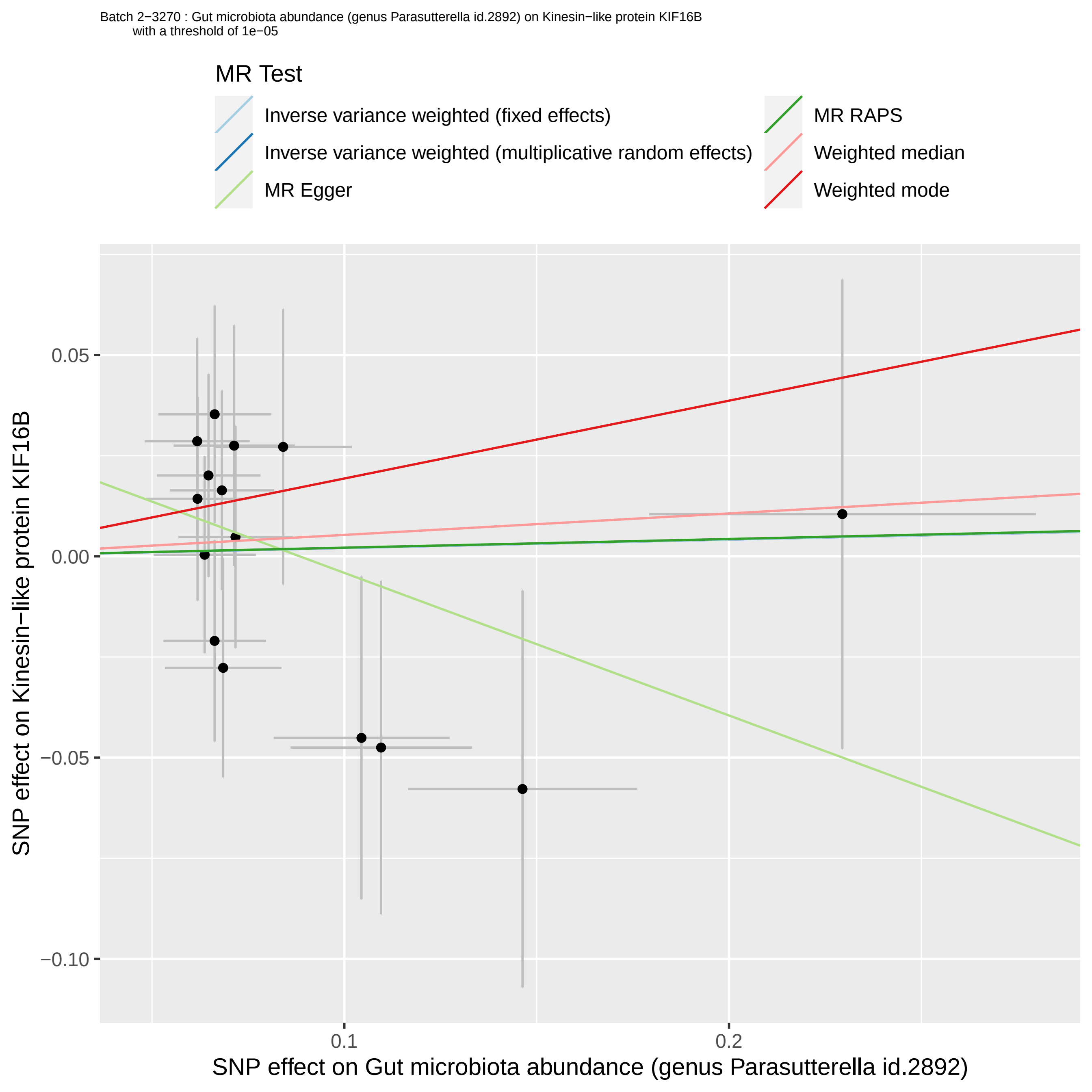

Batch 2−3270 : Gut microbiota abundance (genus Parasutterella id.2892) on Kinesin−like protein KIF16B
with a threshold of 1e−05
MR Test
Inverse variance weighted (fixed effects)
Inverse variance weighted (multiplicative random effects)
MR Egger
MR RAPS
Weighted median
Weighted mode
0.05
0.00
−0.05
−0.10
0.1
0.2
SNP effect on Kinesin−like protein KIF16B
SNP effect on Gut microbiota abundance (genus Parasutterella id.2892)

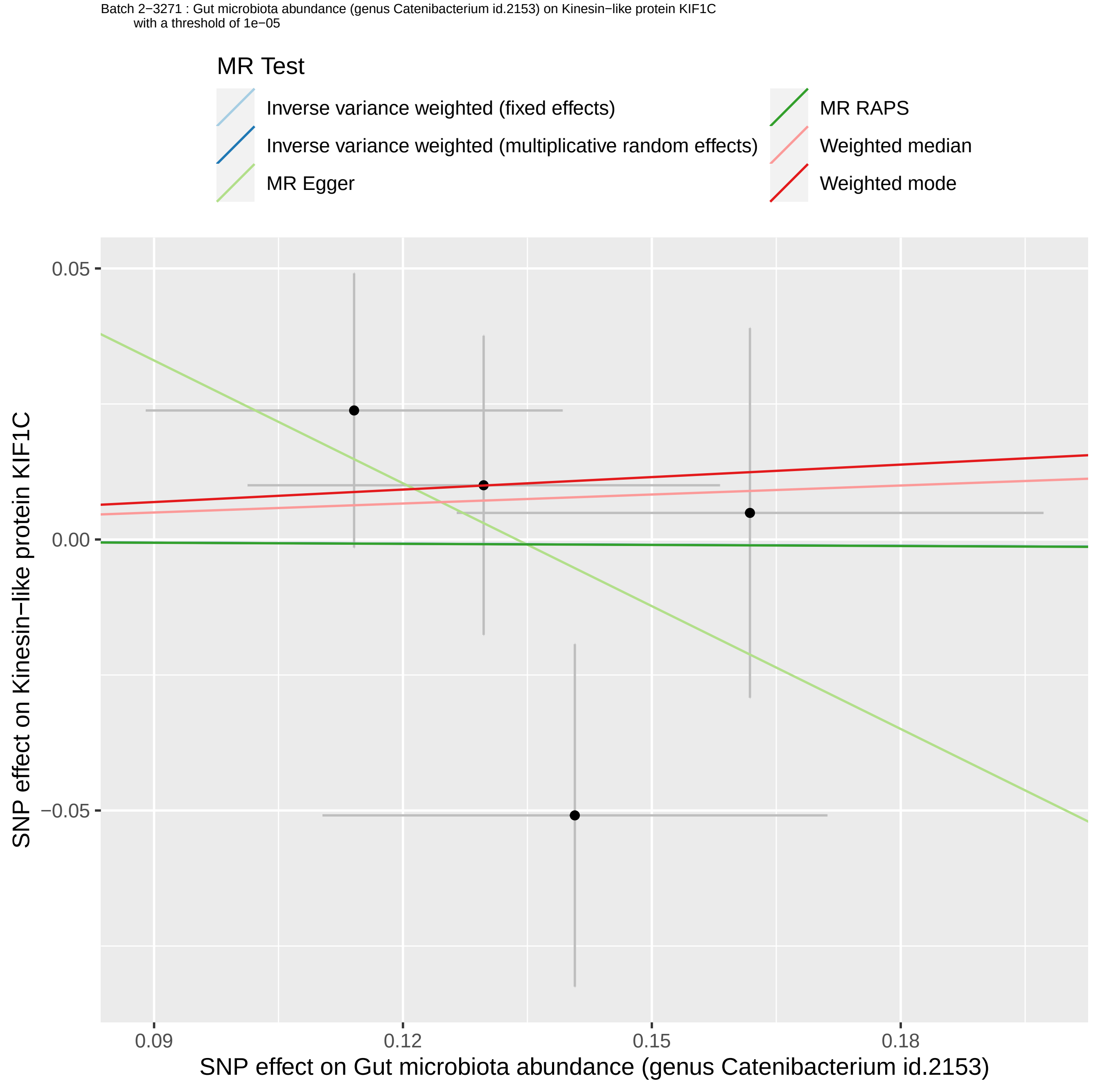
Batch 2−3271 : Gut microbiota abundance (genus Catenibacterium id.2153) on Kinesin−like protein KIF1C
with a threshold of 1e−05
MR Test
Inverse variance weighted (fixed effects)
Inverse variance weighted (multiplicative random effects)
MR Egger
MR RAPS
Weighted median
Weighted mode
0.05
0.00
−0.05
0.09
0.12
0.15
0.18
SNP effect on Kinesin−like protein KIF1C
SNP effect on Gut microbiota abundance (genus Catenibacterium id.2153)

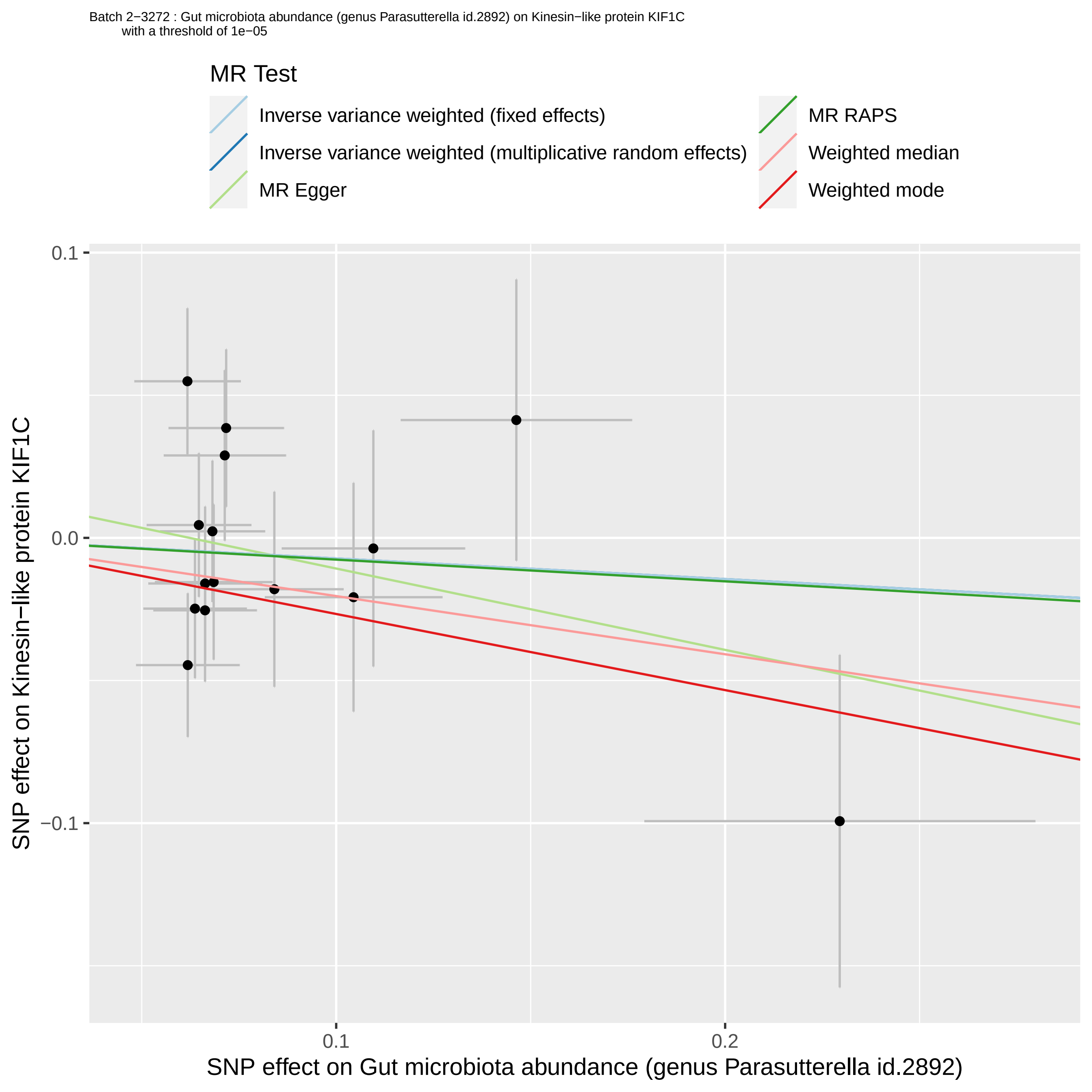

Batch 2−3272 : Gut microbiota abundance (genus Parasutterella id.2892) on Kinesin−like protein KIF1C
with a threshold of 1e−05
MR Test
Inverse variance weighted (fixed effects)
Inverse variance weighted (multiplicative random effects)
MR Egger
MR RAPS
Weighted median
Weighted mode
0.1
0.0
−0.1
0.1
0.2
SNP effect on Kinesin−like protein KIF1C
SNP effect on Gut microbiota abundance (genus Parasutterella id.2892)

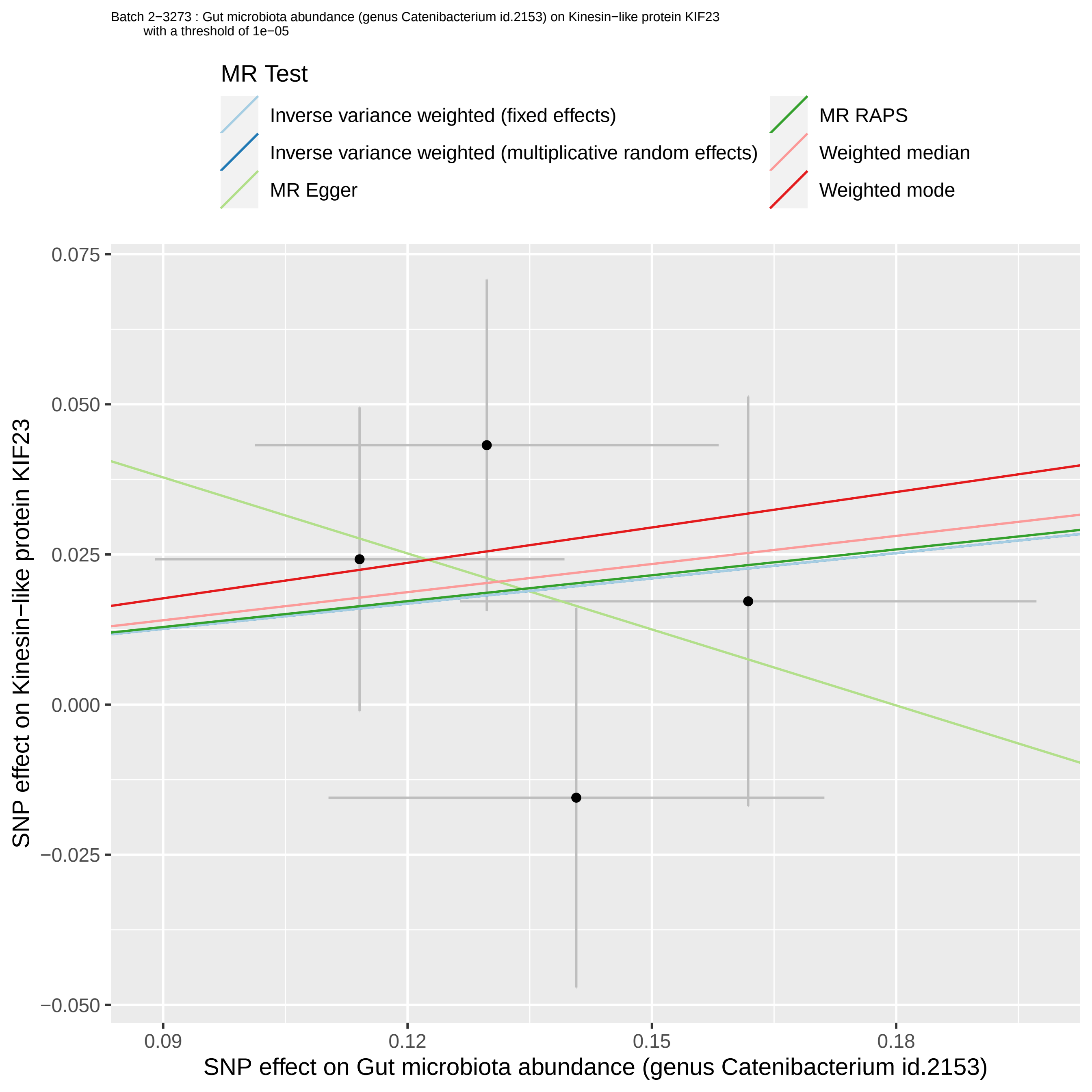
Batch 2−3273 : Gut microbiota abundance (genus Catenibacterium id.2153) on Kinesin−like protein KIF23
with a threshold of 1e−05
MR Test
Inverse variance weighted (fixed effects)
Inverse variance weighted (multiplicative random effects)
MR Egger
MR RAPS
Weighted median
Weighted mode
0.075
0.050
0.025
0.000
−0.025
−0.050
0.09
0.12
0.15
0.18
SNP effect on Kinesin−like protein KIF23
SNP effect on Gut microbiota abundance (genus Catenibacterium id.2153)

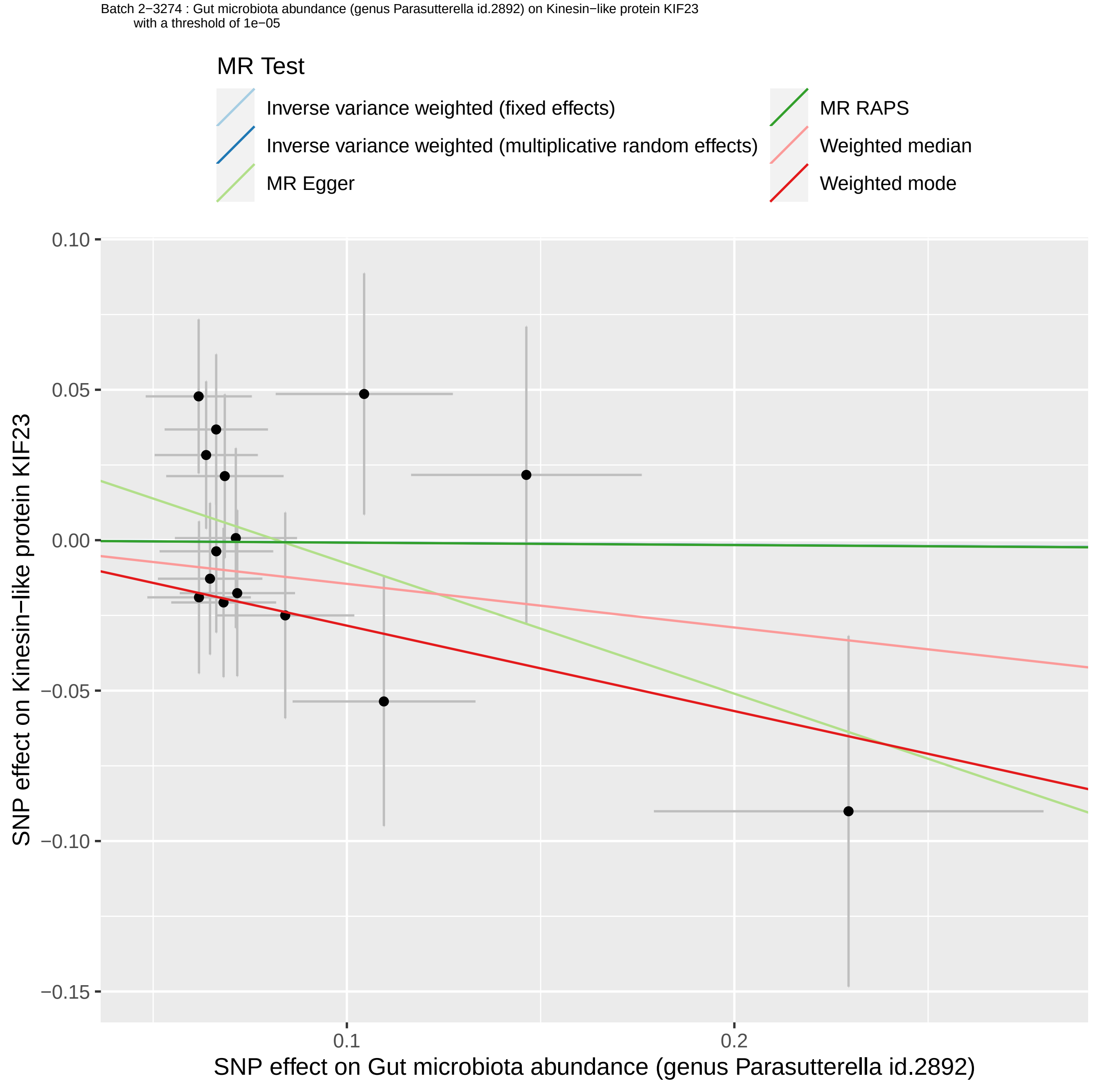
Batch 2−3274 : Gut microbiota abundance (genus Parasutterella id.2892) on Kinesin−like protein KIF23
with a threshold of 1e−05
MR Test
Inverse variance weighted (fixed effects)
Inverse variance weighted (multiplicative random effects)
MR Egger
MR RAPS
Weighted median
Weighted mode
0.10
0.05
0.00
−0.05
−0.10
−0.15
0.1
0.2
SNP effect on Kinesin−like protein KIF23
SNP effect on Gut microbiota abundance (genus Parasutterella id.2892)

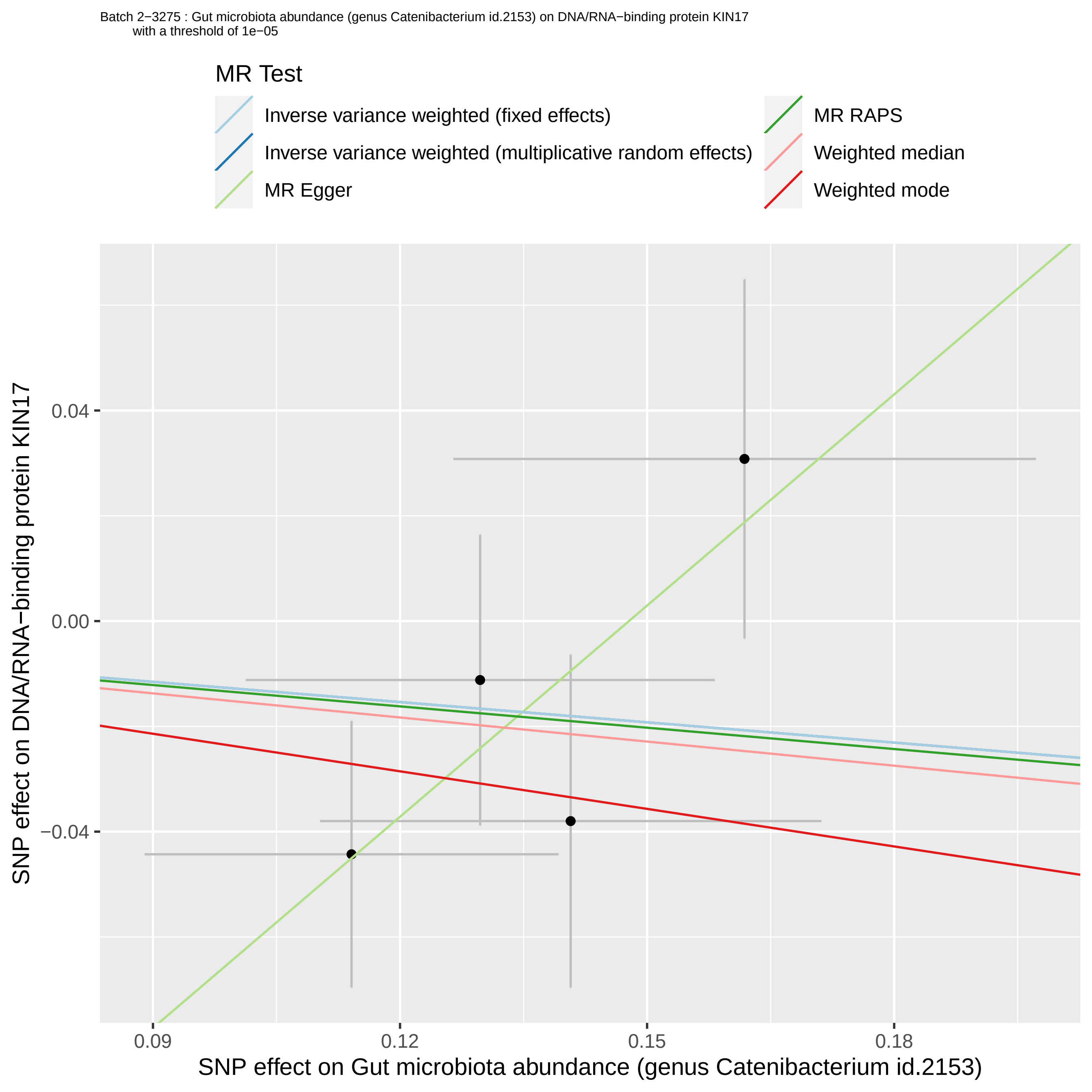

Batch 2−3275 : Gut microbiota abundance (genus Catenibacterium id.2153) on DNA/RNA−binding protein KIN17
with a threshold of 1e−05
MR Test
Inverse variance weighted (fixed effects)
Inverse variance weighted (multiplicative random effects)
MR Egger
MR RAPS
Weighted median
Weighted mode
0.04
0.00
−0.04
0.09
0.12
0.15
0.18
SNP effect on DNA/RNA−binding protein KIN17
SNP effect on Gut microbiota abundance (genus Catenibacterium id.2153)

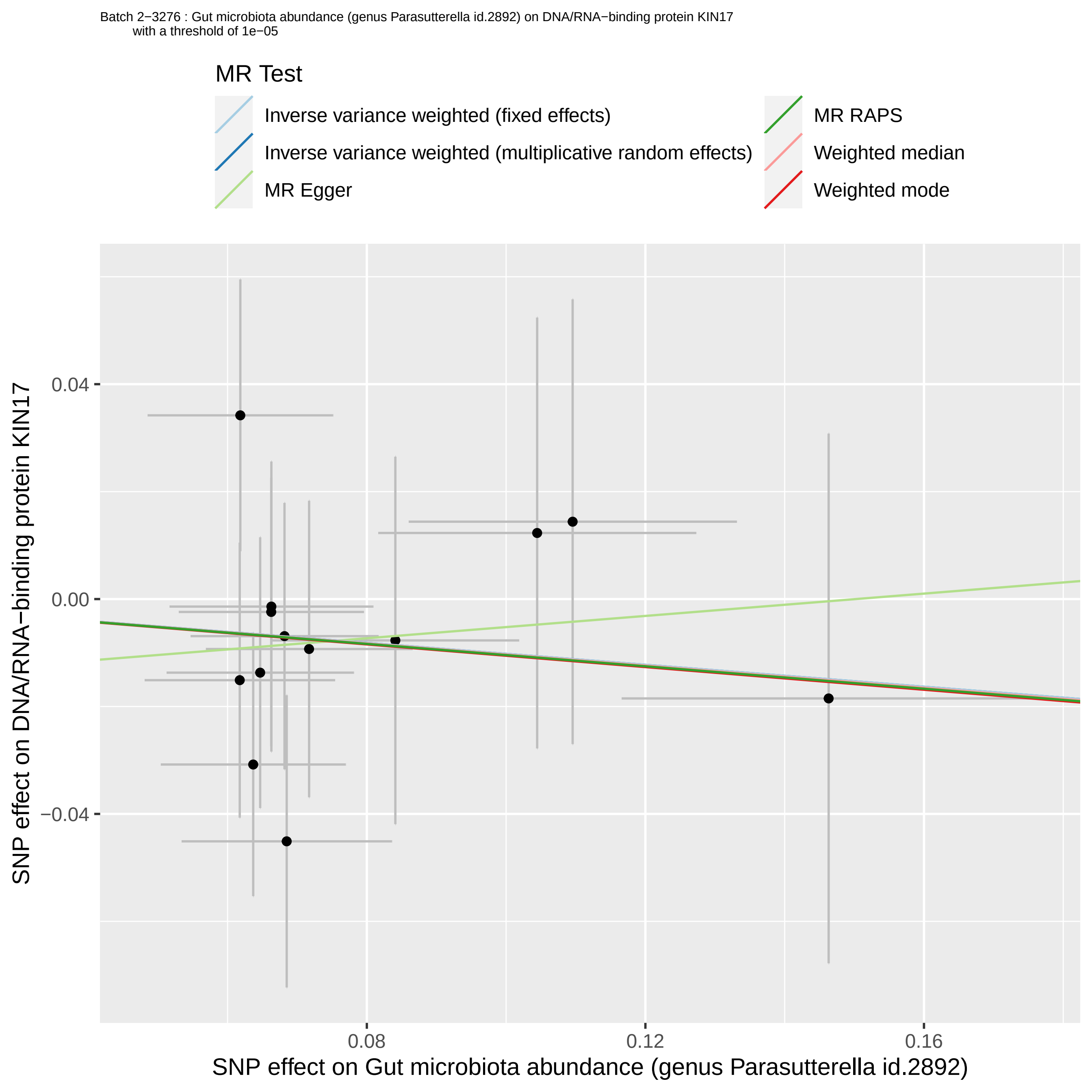

Batch 2−3276 : Gut microbiota abundance (genus Parasutterella id.2892) on DNA/RNA−binding protein KIN17
with a threshold of 1e−05
MR Test
Inverse variance weighted (fixed effects)
Inverse variance weighted (multiplicative random effects)
MR Egger
MR RAPS
Weighted median
Weighted mode
0.04
0.00
−0.04
0.08
0.12
0.16
SNP effect on DNA/RNA−binding protein KIN17
SNP effect on Gut microbiota abundance (genus Parasutterella id.2892)

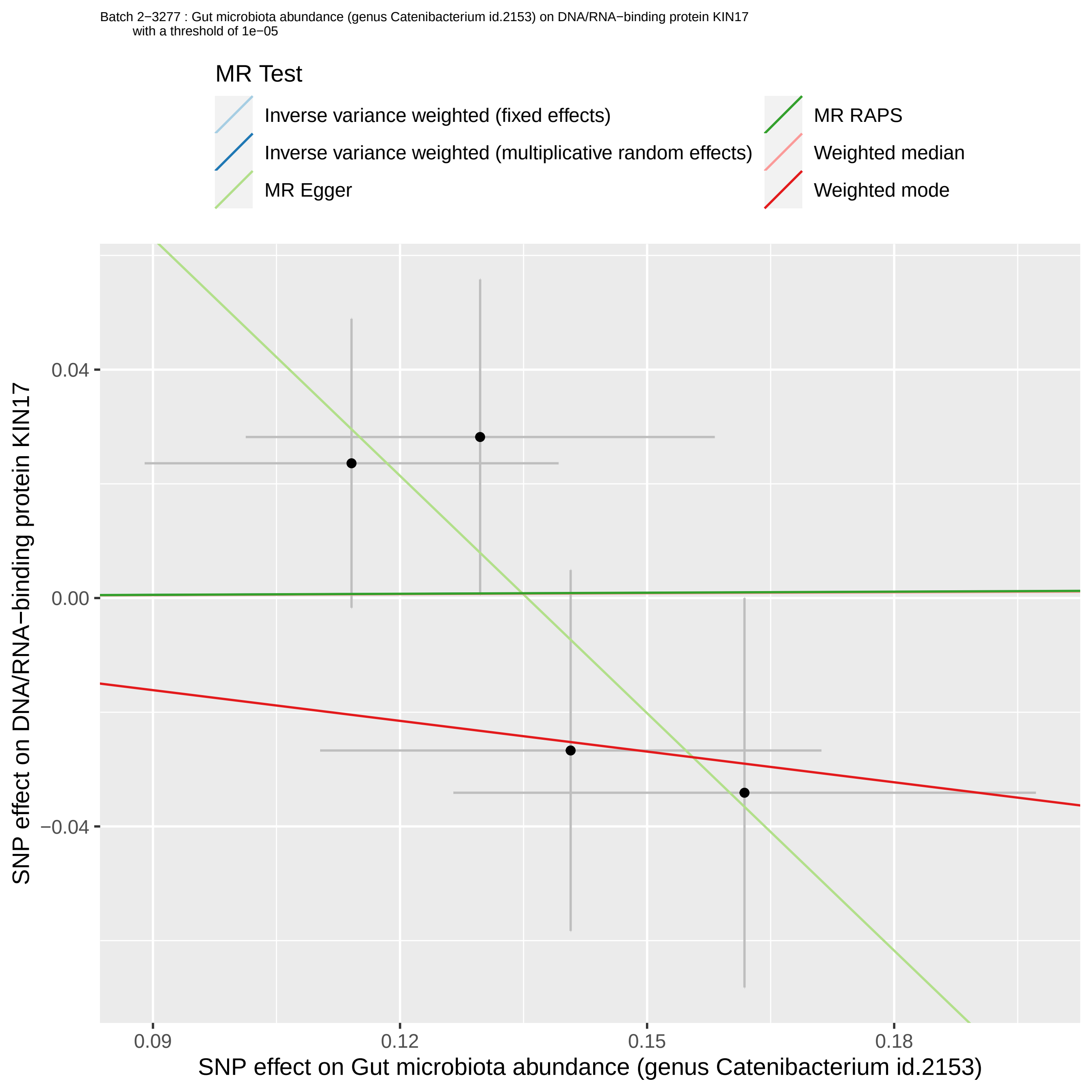
Batch 2−3277 : Gut microbiota abundance (genus Catenibacterium id.2153) on DNA/RNA−binding protein KIN17
with a threshold of 1e−05
MR Test
Inverse variance weighted (fixed effects)
Inverse variance weighted (multiplicative random effects)
MR Egger
MR RAPS
Weighted median
Weighted mode
0.04
0.00
−0.04
0.09
0.12
0.15
0.18
SNP effect on DNA/RNA−binding protein KIN17
SNP effect on Gut microbiota abundance (genus Catenibacterium id.2153)

Batch 2−3278 : Gut microbiota abundance (genus Parasutterella id.2892) on DNA/RNA−binding protein KIN17
with a threshold of 1e−05

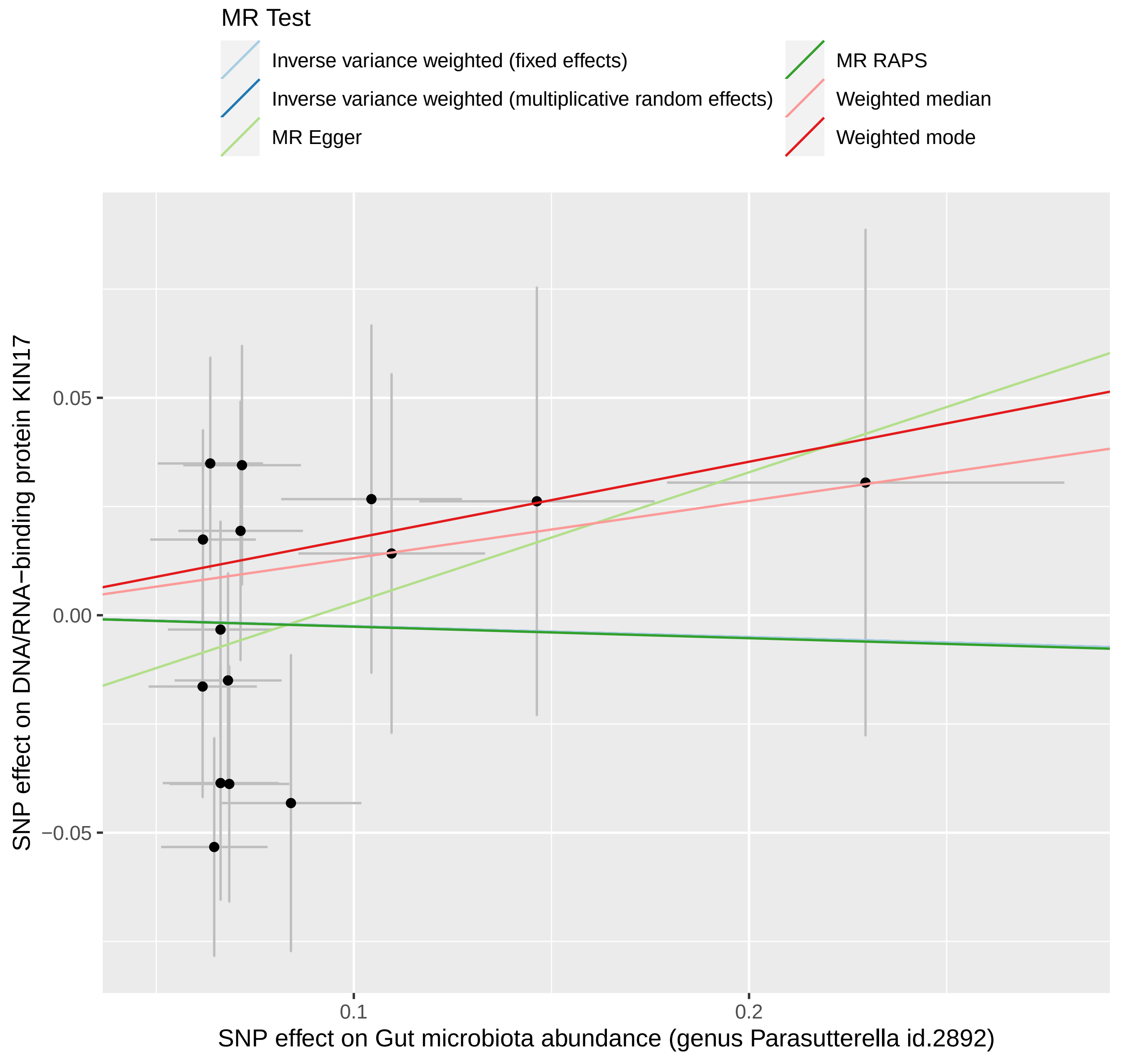

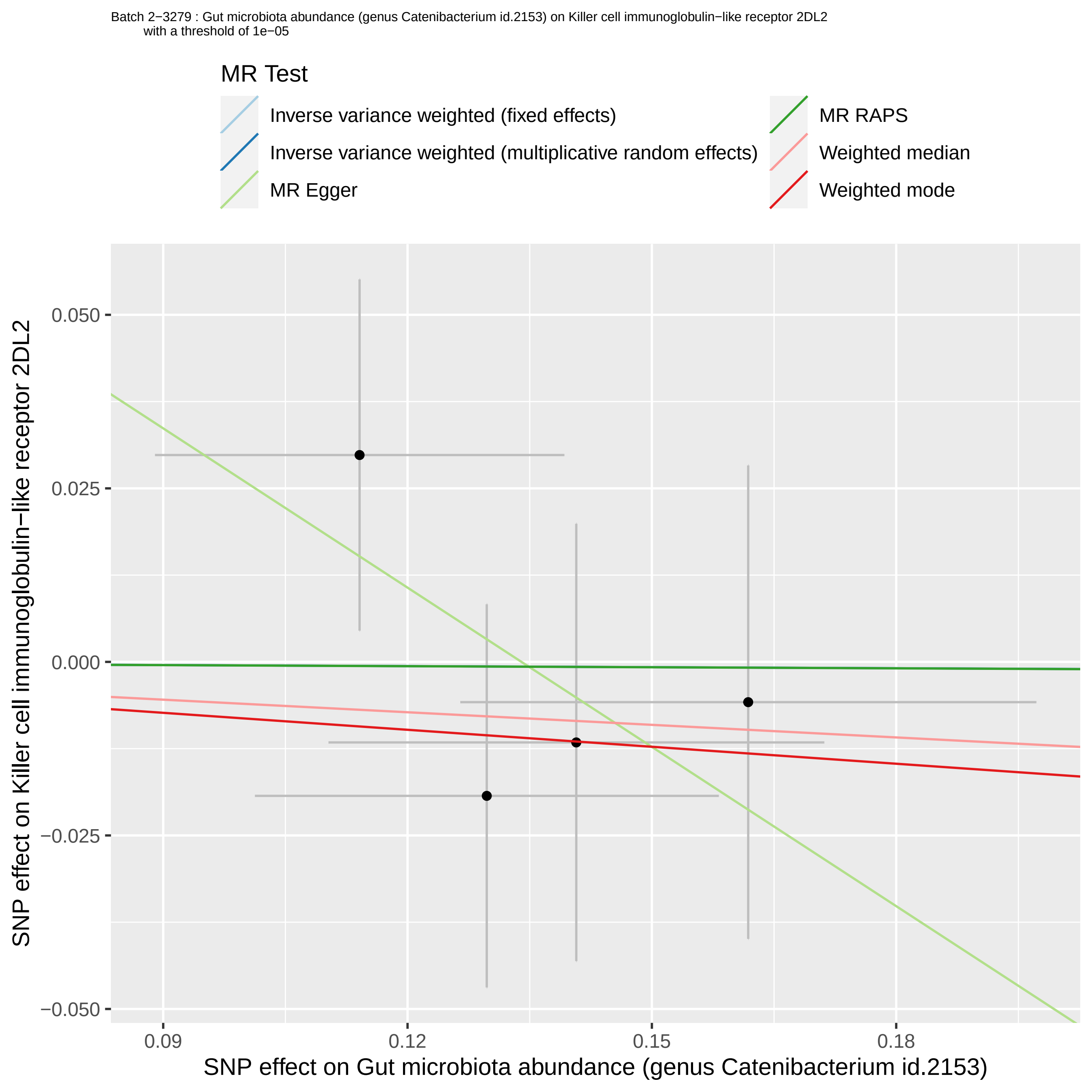
Batch 2−3279 : Gut microbiota abundance (genus Catenibacterium id.2153) on Killer cell immunoglobulin−like receptor 2DL2
with a threshold of 1e−05
MR Test
Inverse variance weighted (fixed effects)
Inverse variance weighted (multiplicative random effects)
MR Egger
MR RAPS
Weighted median
Weighted mode
0.050
0.025
0.000
−0.025
−0.050
0.09
0.12
0.15
0.18
SNP effect on Killer cell immunoglobulin−like receptor 2DL2
SNP effect on Gut microbiota abundance (genus Catenibacterium id.2153)

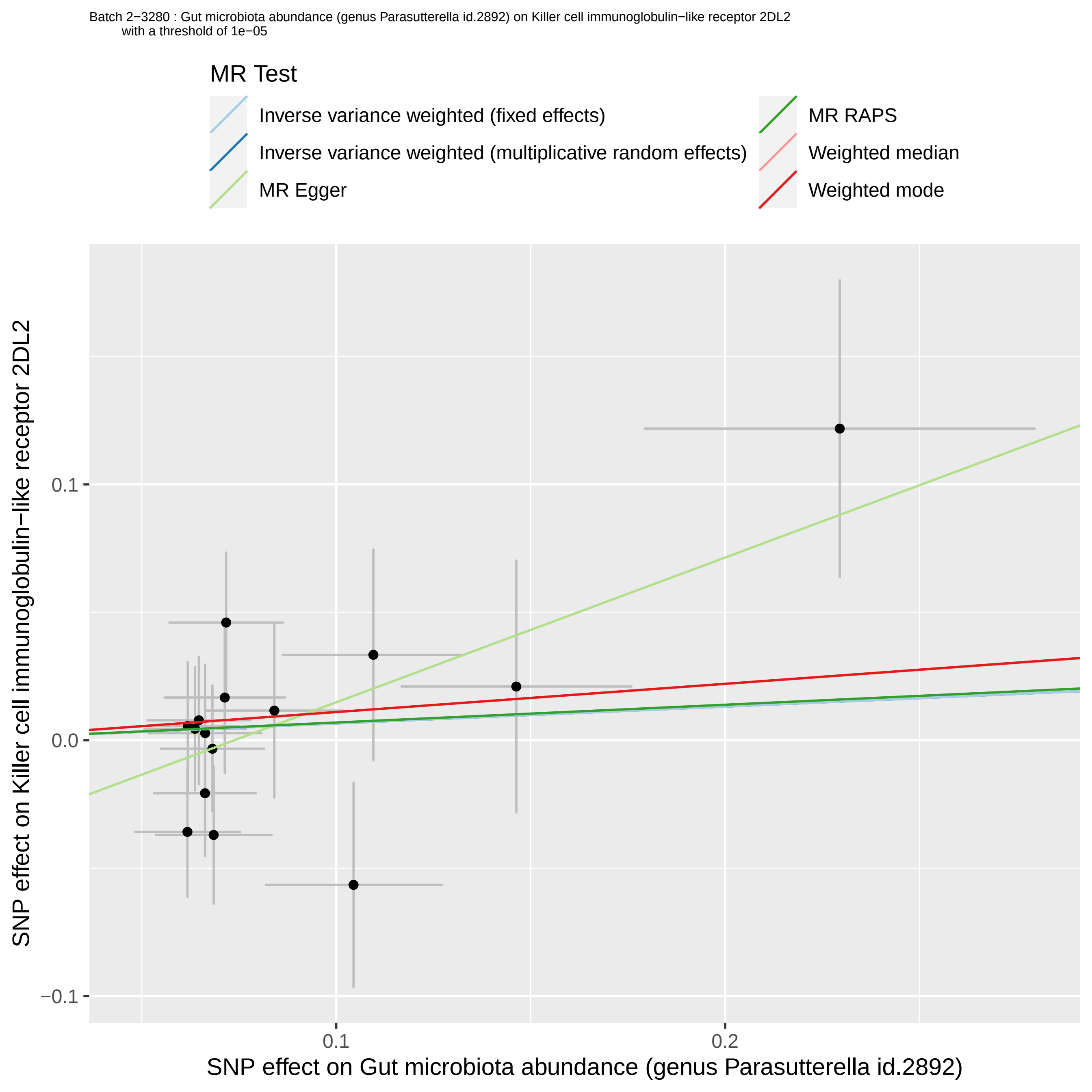
Batch 2−3280 : Gut microbiota abundance (genus Parasutterella id.2892) on Killer cell immunoglobulin−like receptor 2DL2
with a threshold of 1e−05
MR Test
Inverse variance weighted (fixed effects)
Inverse variance weighted (multiplicative random effects)
MR Egger
MR RAPS
Weighted median
Weighted mode
0.1
0.0
−0.1
0.1
0.2
SNP effect on Killer cell immunoglobulin−like receptor 2DL2
SNP effect on Gut microbiota abundance (genus Parasutterella id.2892)

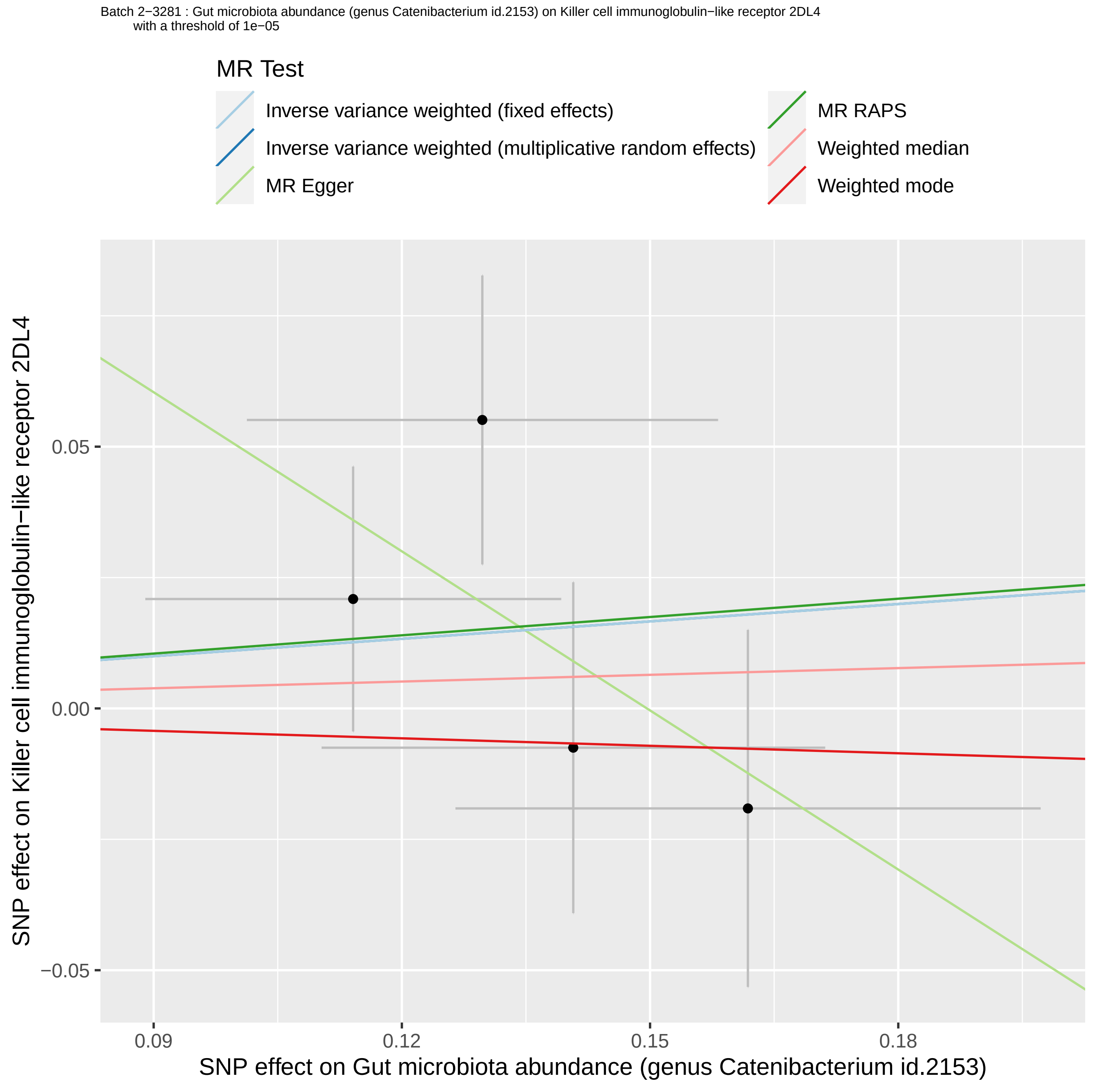
Batch 2−3281 : Gut microbiota abundance (genus Catenibacterium id.2153) on Killer cell immunoglobulin−like receptor 2DL4
with a threshold of 1e−05
MR Test
Inverse variance weighted (fixed effects)
Inverse variance weighted (multiplicative random effects)
MR Egger
MR RAPS
Weighted median
Weighted mode
0.05
0.00
−0.05
0.09
0.12
0.15
0.18
SNP effect on Killer cell immunoglobulin−like receptor 2DL4
SNP effect on Gut microbiota abundance (genus Catenibacterium id.2153)

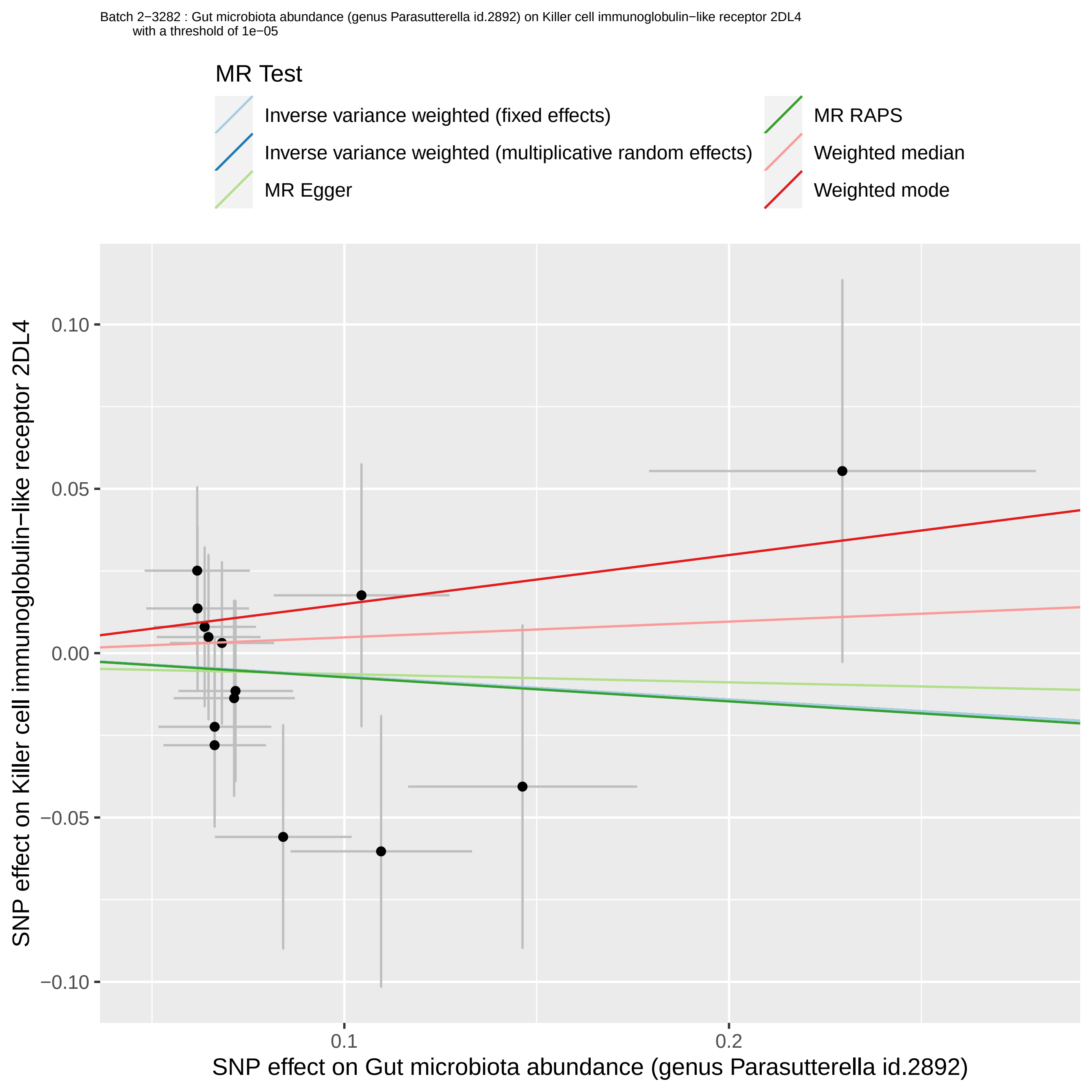
Batch 2−3282 : Gut microbiota abundance (genus Parasutterella id.2892) on Killer cell immunoglobulin−like receptor 2DL4
with a threshold of 1e−05
MR Test
Inverse variance weighted (fixed effects)
Inverse variance weighted (multiplicative random effects)
MR Egger
MR RAPS
Weighted median
Weighted mode
SNP effect on Killer cell immunoglobulin−like receptor 2DL4
0.10
0.05
0.00
−0.05
−0.10
0.1
0.2
SNP effect on Gut microbiota abundance (genus Parasutterella id.2892)

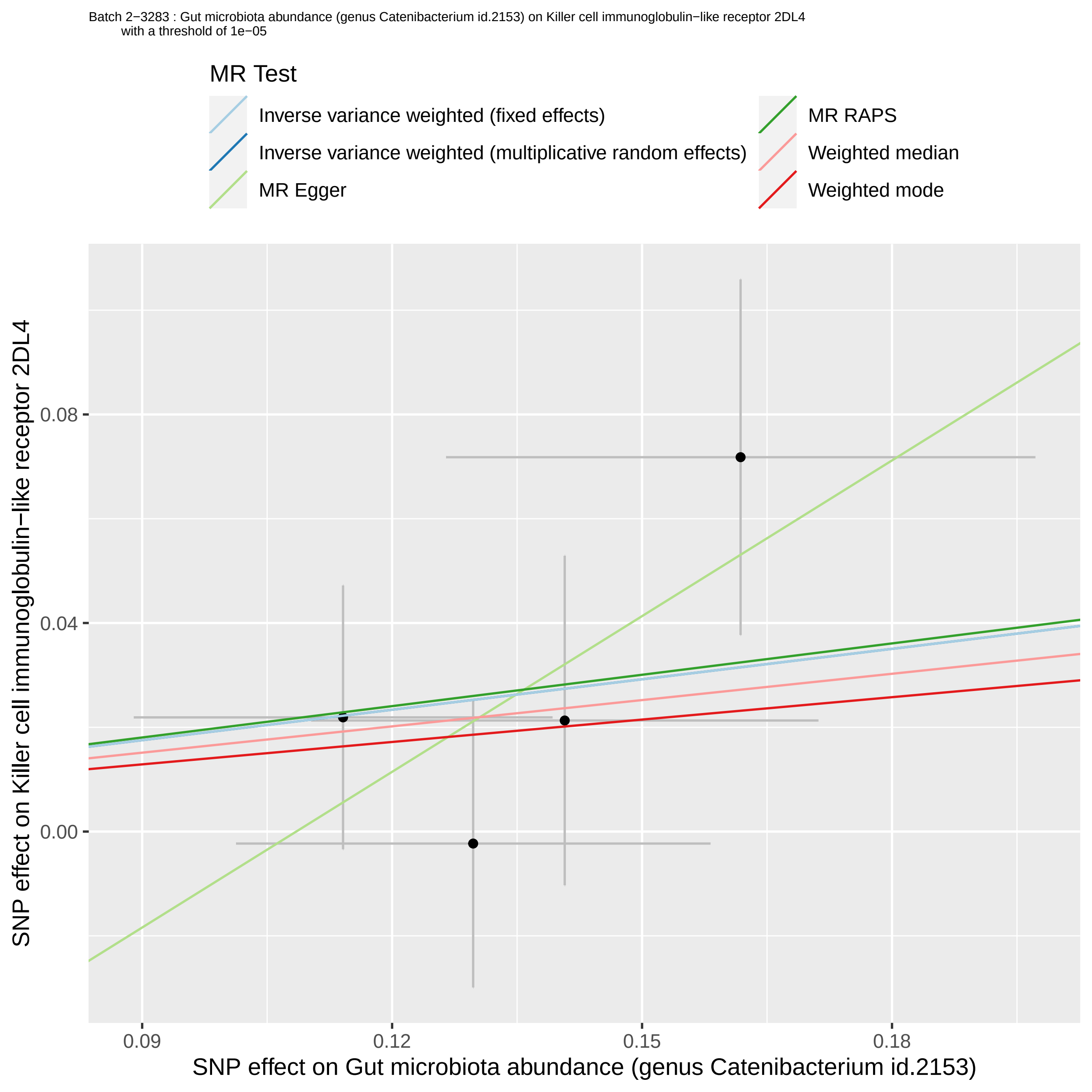
Batch 2−3283 : Gut microbiota abundance (genus Catenibacterium id.2153) on Killer cell immunoglobulin−like receptor 2DL4
with a threshold of 1e−05
MR Test
Inverse variance weighted (fixed effects)
Inverse variance weighted (multiplicative random effects)
MR Egger
MR RAPS
Weighted median
Weighted mode
SNP effect on Killer cell immunoglobulin−like receptor 2DL4
0.08
0.04
0.00
0.09
0.12
0.15
0.18
SNP effect on Gut microbiota abundance (genus Catenibacterium id.2153)

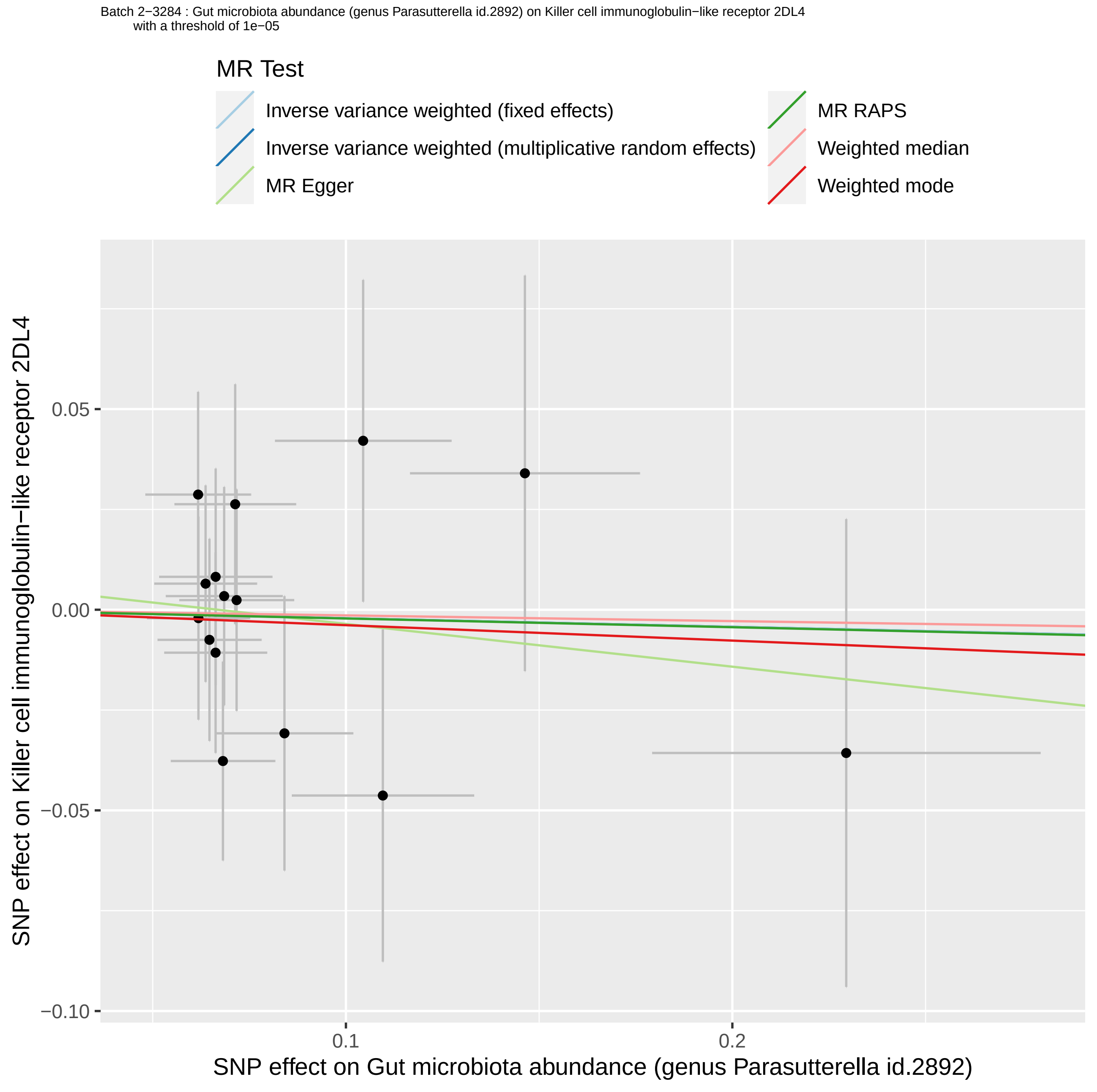
Batch 2−3284 : Gut microbiota abundance (genus Parasutterella id.2892) on Killer cell immunoglobulin−like receptor 2DL4
with a threshold of 1e−05
MR Test
Inverse variance weighted (fixed effects)
Inverse variance weighted (multiplicative random effects)
MR Egger
MR RAPS
Weighted median
Weighted mode
0.05
0.00
−0.05
−0.10
0.1
0.2
SNP effect on Killer cell immunoglobulin−like receptor 2DL4
SNP effect on Gut microbiota abundance (genus Parasutterella id.2892)

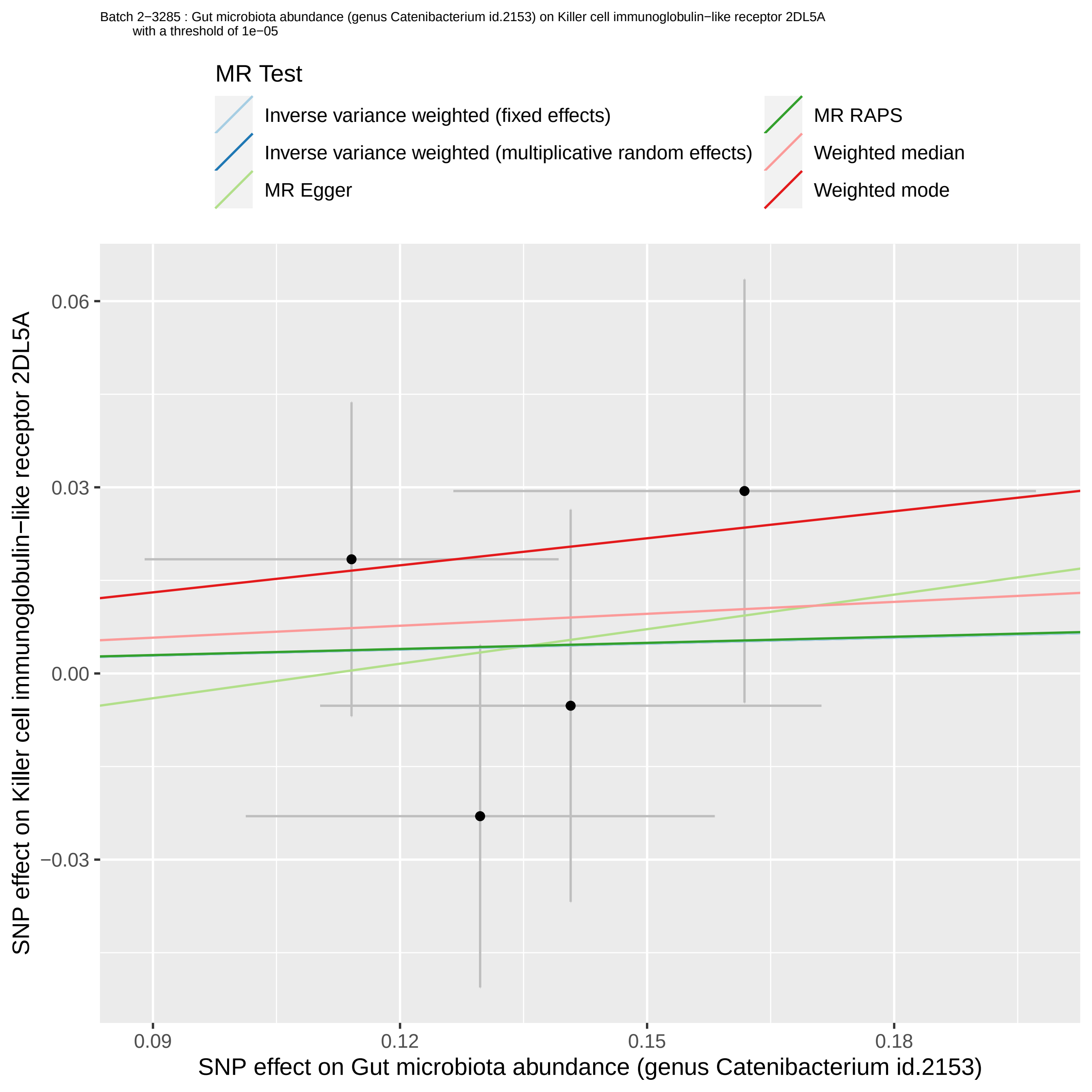
Batch 2−3285 : Gut microbiota abundance (genus Catenibacterium id.2153) on Killer cell immunoglobulin−like receptor 2DL5A
with a threshold of 1e−05
MR Test
Inverse variance weighted (fixed effects)
Inverse variance weighted (multiplicative random effects)
MR Egger
MR RAPS
Weighted median
Weighted mode
0.06
0.03
0.00
−0.03
0.09
0.12
0.15
0.18
SNP effect on Killer cell immunoglobulin−like receptor 2DL5A
SNP effect on Gut microbiota abundance (genus Catenibacterium id.2153)

Batch 2−3286 : Gut microbiota abundance (genus Parasutterella id.2892) on Killer cell immunoglobulin−like receptor 2DL5A
with a threshold of 1e−05

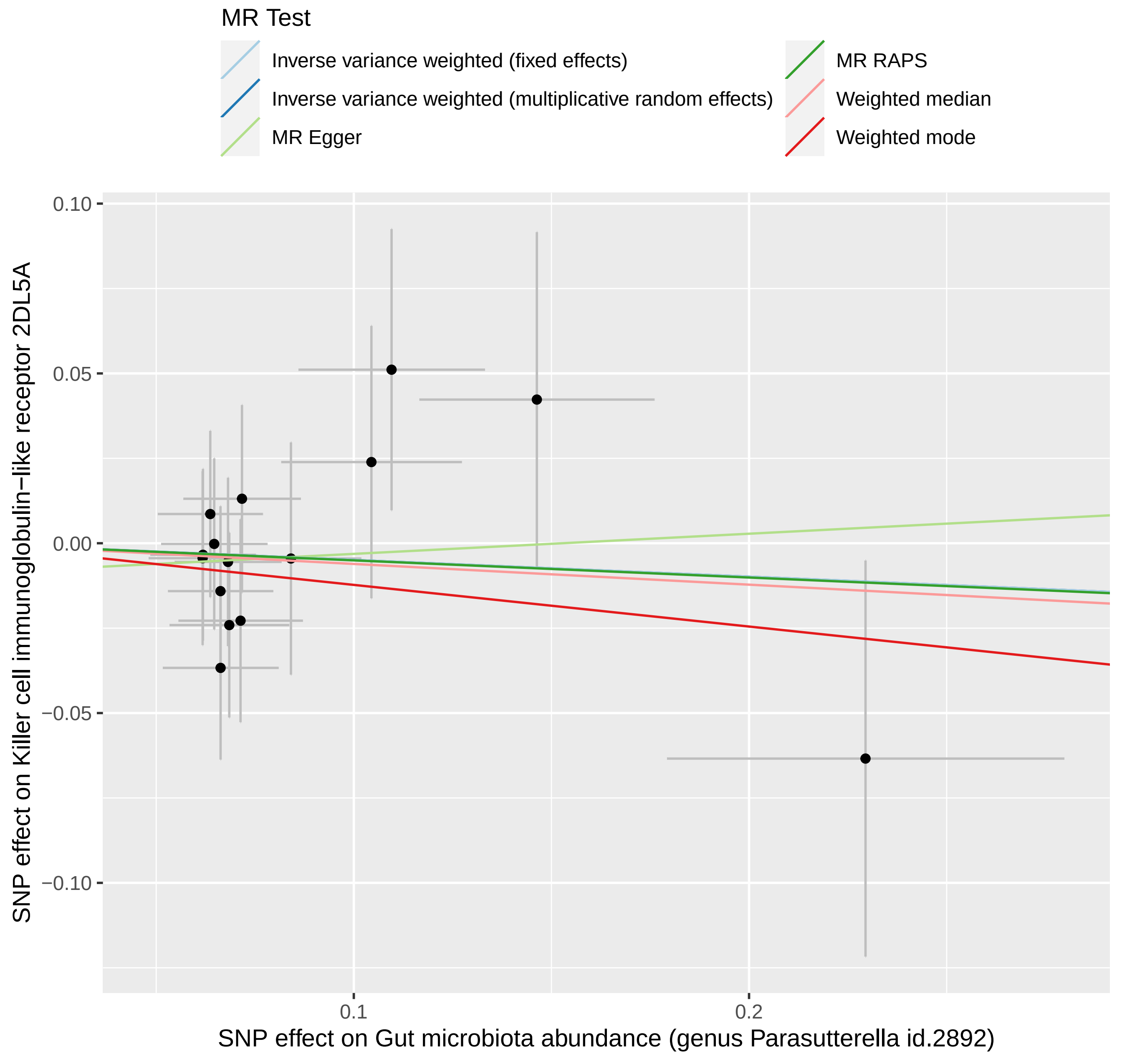

Batch 2−3287 : Gut microbiota abundance (genus Catenibacterium id.2153) on Killer cell immunoglobulin−like receptor 2DL5A
with a threshold of 1e−05

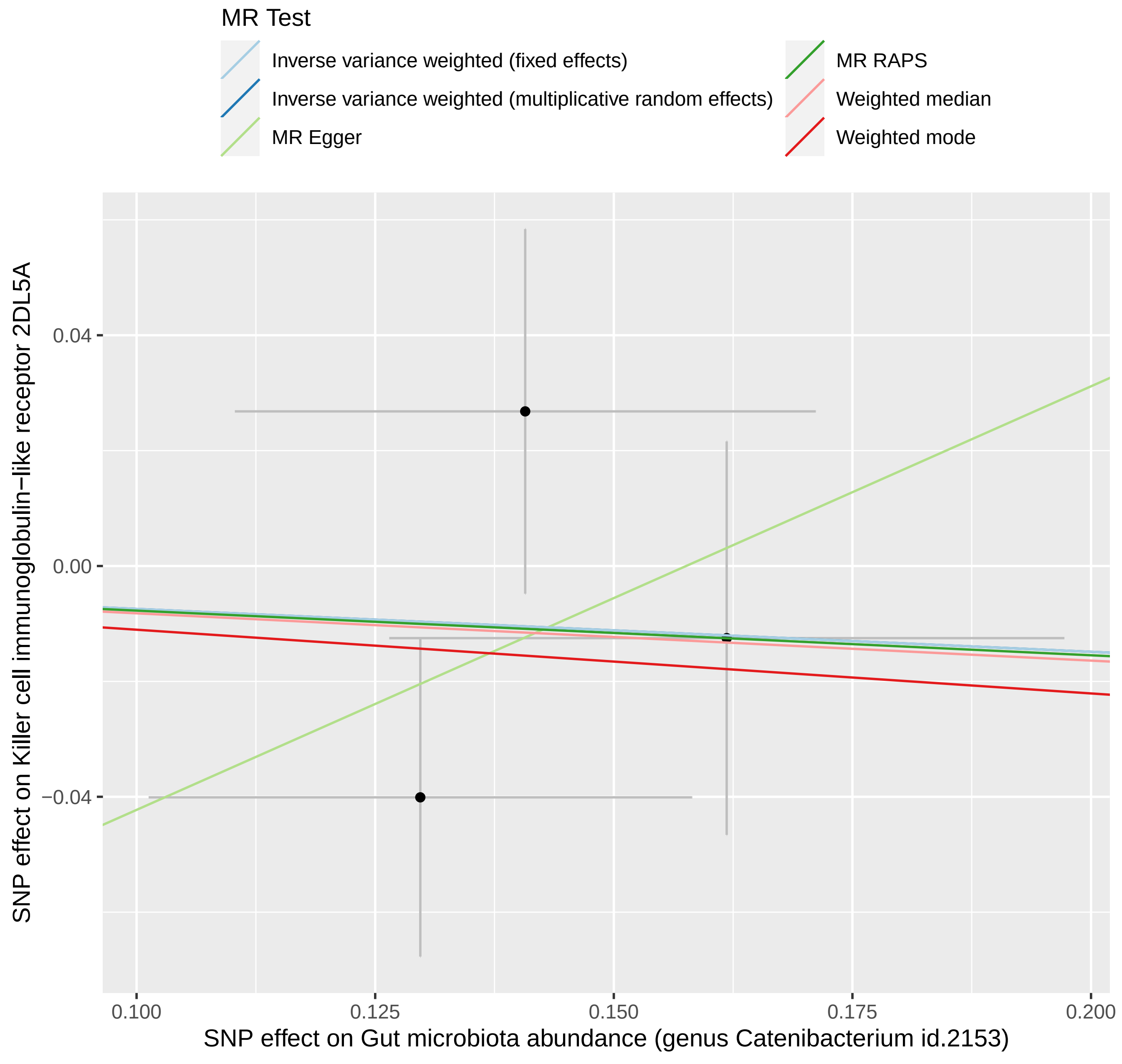

Batch 2−3288 : Gut microbiota abundance (genus Parasutterella id.2892) on Killer cell immunoglobulin−like receptor 2DL5A
with a threshold of 1e−05

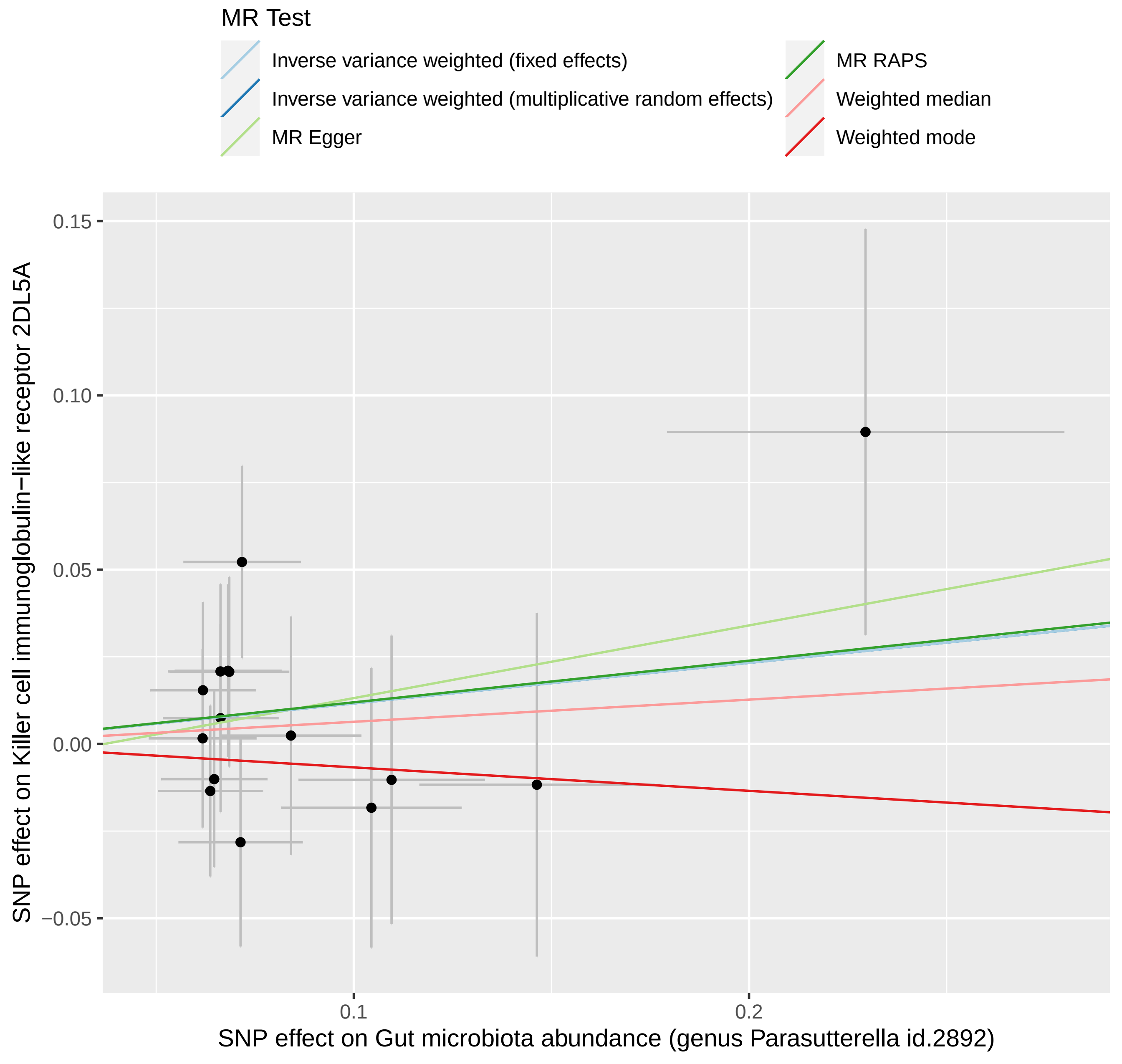

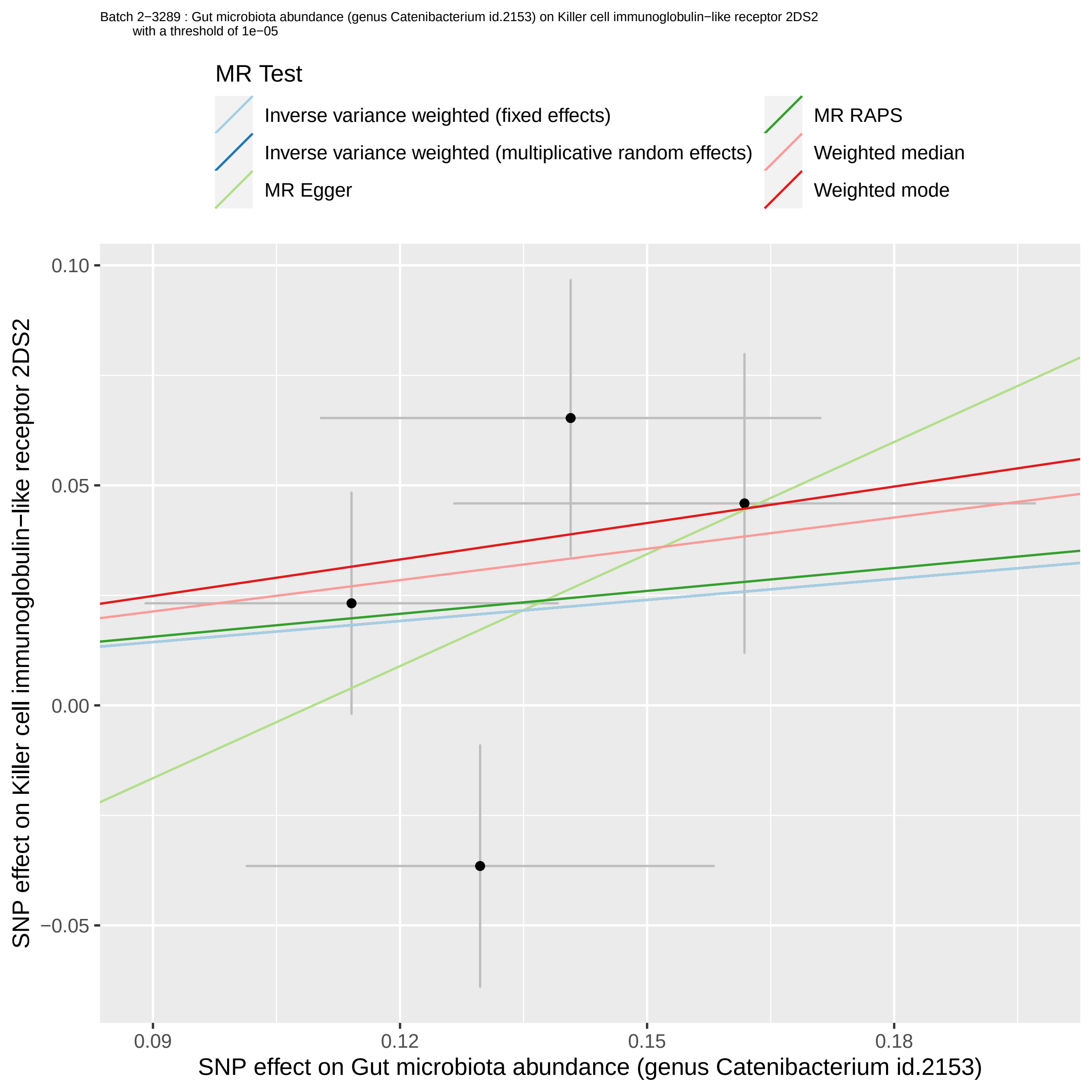

Batch 2−3289 : Gut microbiota abundance (genus Catenibacterium id.2153) on Killer cell immunoglobulin−like receptor 2DS2
with a threshold of 1e−05
MR Test
Inverse variance weighted (fixed effects)
Inverse variance weighted (multiplicative random effects)
MR Egger
MR RAPS
Weighted median
Weighted mode
0.10
0.05
0.00
−0.05
0.09
0.12
0.15
0.18
SNP effect on Killer cell immunoglobulin−like receptor 2DS2
SNP effect on Gut microbiota abundance (genus Catenibacterium id.2153)

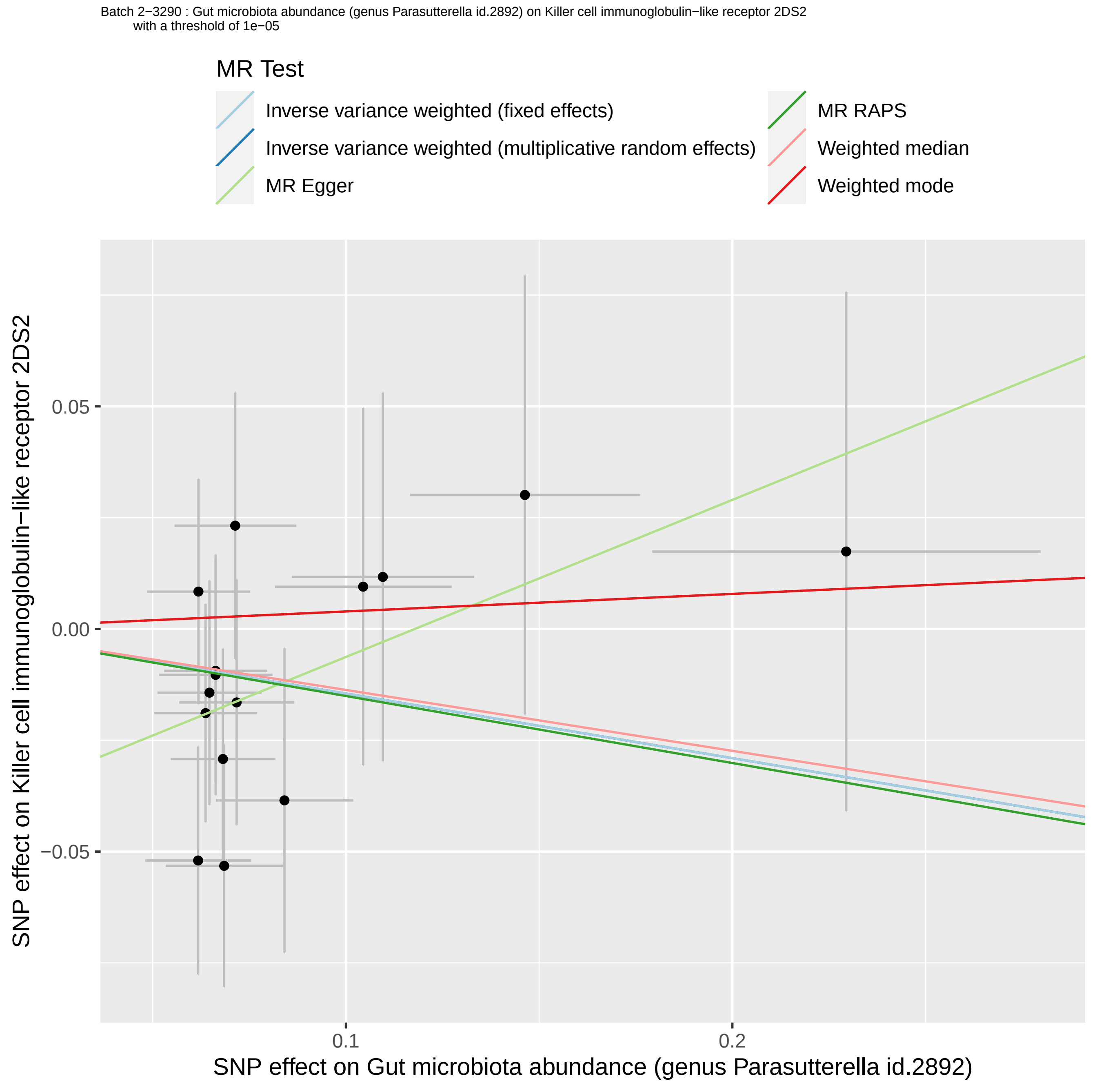

Batch 2−3290 : Gut microbiota abundance (genus Parasutterella id.2892) on Killer cell immunoglobulin−like receptor 2DS2
with a threshold of 1e−05
MR Test
Inverse variance weighted (fixed effects)
Inverse variance weighted (multiplicative random effects)
MR Egger
MR RAPS
Weighted median
Weighted mode
0.05
0.00
−0.05
0.1
0.2
SNP effect on Killer cell immunoglobulin−like receptor 2DS2
SNP effect on Gut microbiota abundance (genus Parasutterella id.2892)

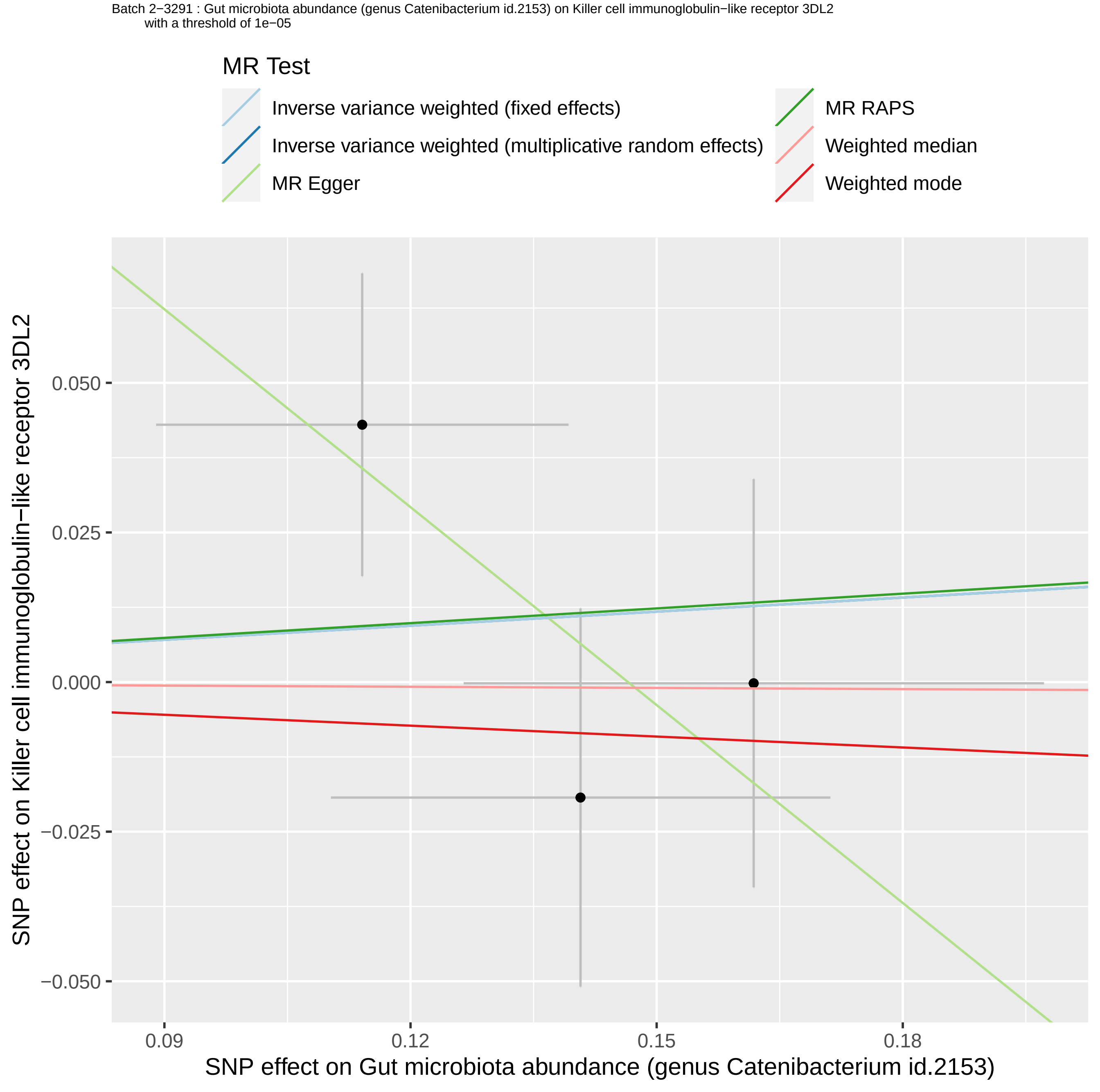

Batch 2−3291 : Gut microbiota abundance (genus Catenibacterium id.2153) on Killer cell immunoglobulin−like receptor 3DL2
with a threshold of 1e−05
MR Test
Inverse variance weighted (fixed effects)
Inverse variance weighted (multiplicative random effects)
MR Egger
MR RAPS
Weighted median
Weighted mode
0.050
0.025
0.000
−0.025
−0.050
0.09
0.12
0.15
0.18
SNP effect on Killer cell immunoglobulin−like receptor 3DL2
SNP effect on Gut microbiota abundance (genus Catenibacterium id.2153)

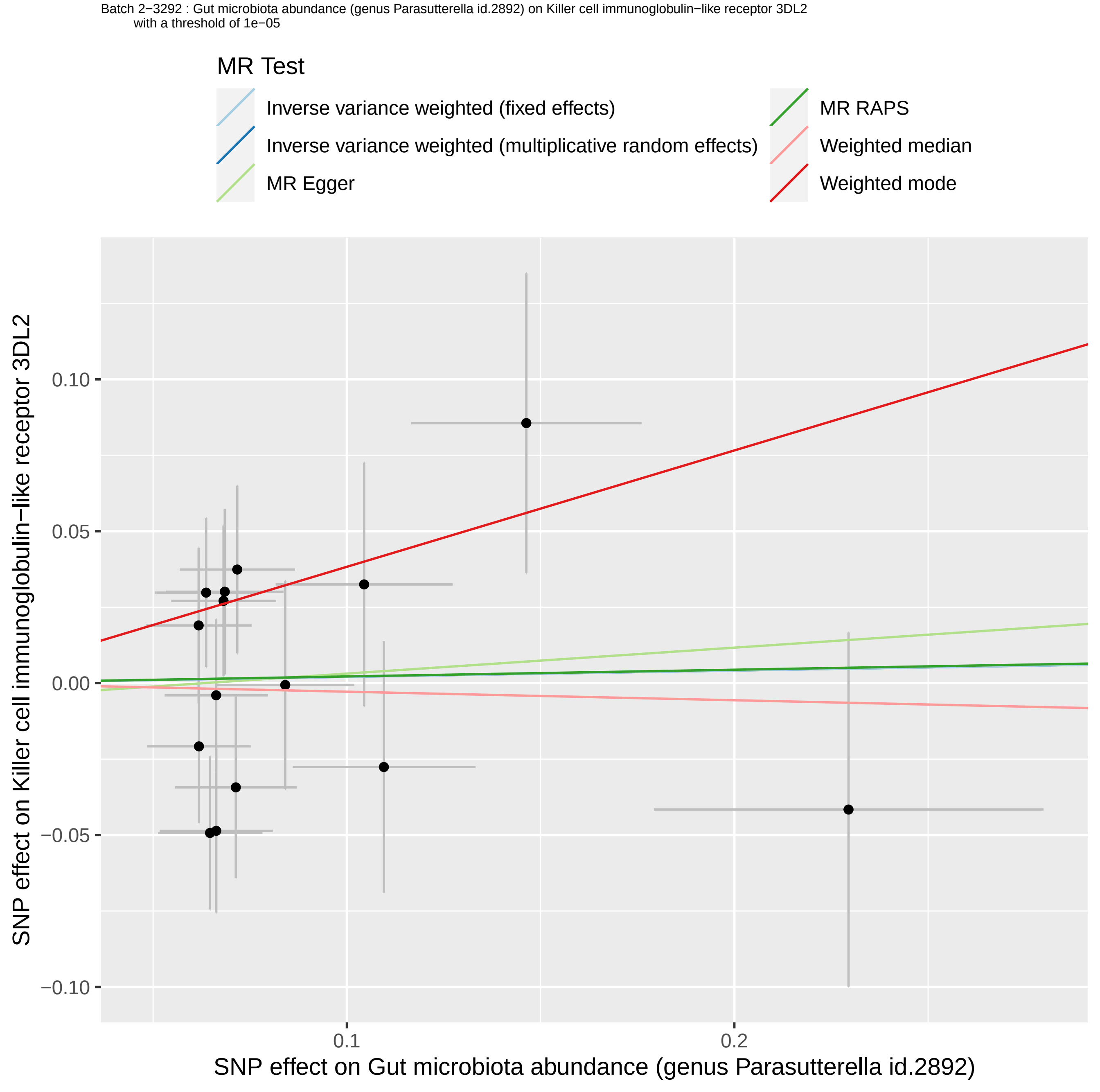
Batch 2−3292 : Gut microbiota abundance (genus Parasutterella id.2892) on Killer cell immunoglobulin−like receptor 3DL2
with a threshold of 1e−05
MR Test
Inverse variance weighted (fixed effects)
Inverse variance weighted (multiplicative random effects)
MR Egger
MR RAPS
Weighted median
Weighted mode
SNP effect on Killer cell immunoglobulin−like receptor 3DL2
0.10
0.05
0.00
−0.05
−0.10
0.1
0.2
SNP effect on Gut microbiota abundance (genus Parasutterella id.2892)

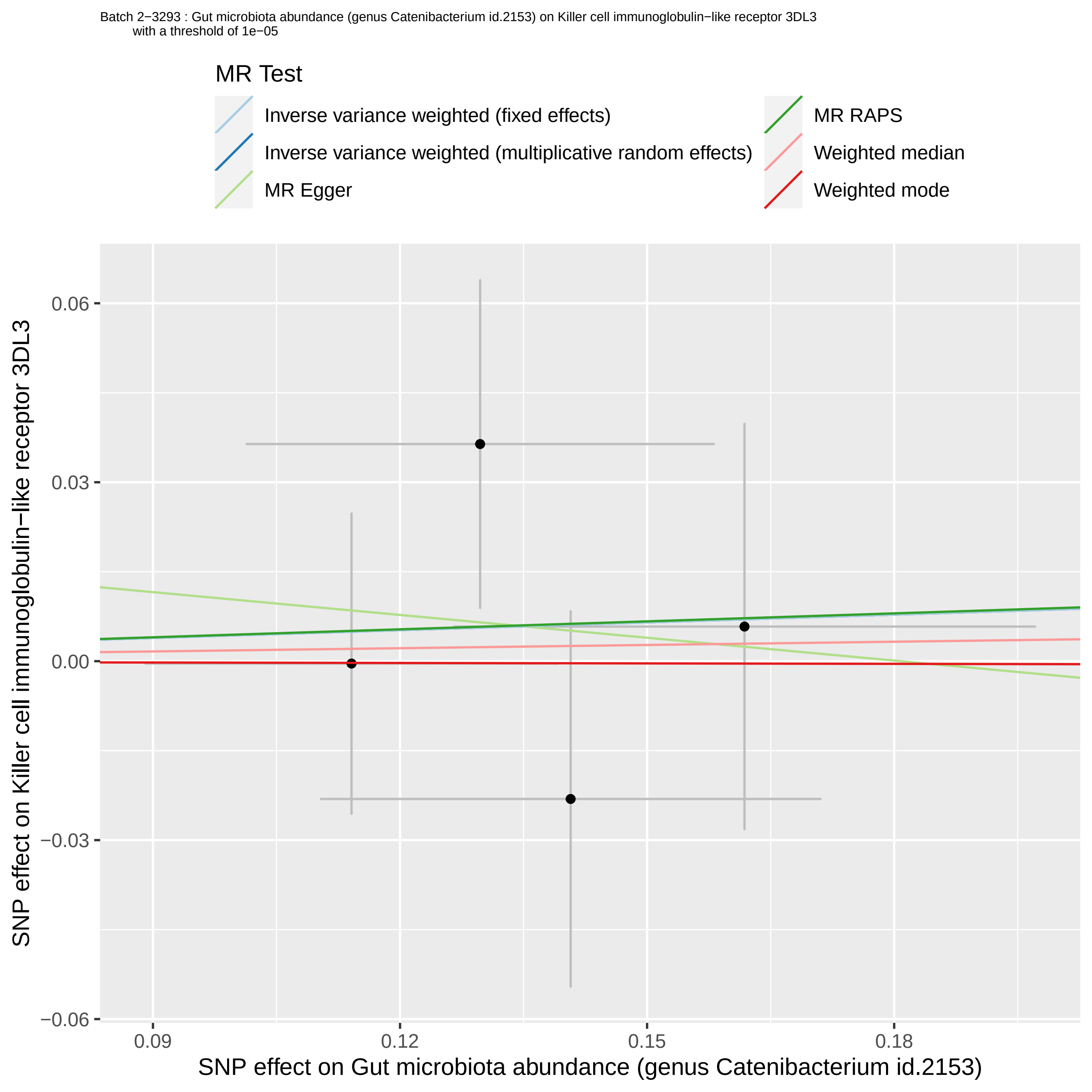

Batch 2−3293 : Gut microbiota abundance (genus Catenibacterium id.2153) on Killer cell immunoglobulin−like receptor 3DL3
with a threshold of 1e−05
MR Test
Inverse variance weighted (fixed effects)
Inverse variance weighted (multiplicative random effects)
MR Egger
MR RAPS
Weighted median
Weighted mode
0.06
0.03
0.00
−0.03
−0.06
0.09
0.12
0.15
0.18
SNP effect on Killer cell immunoglobulin−like receptor 3DL3
SNP effect on Gut microbiota abundance (genus Catenibacterium id.2153)

Batch 2−3294 : Gut microbiota abundance (genus Parasutterella id.2892) on Killer cell immunoglobulin−like receptor 3DL3
with a threshold of 1e−05

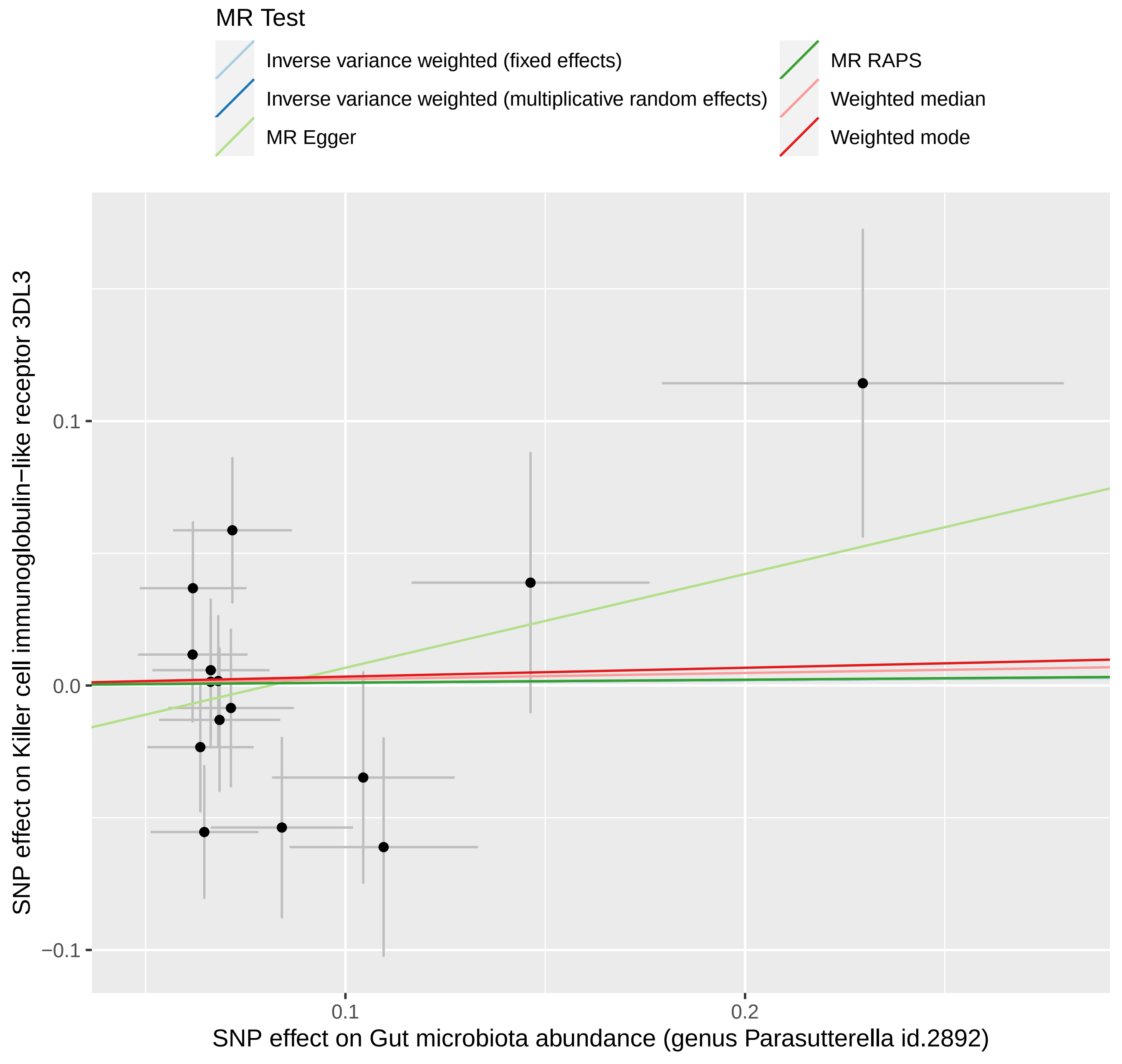

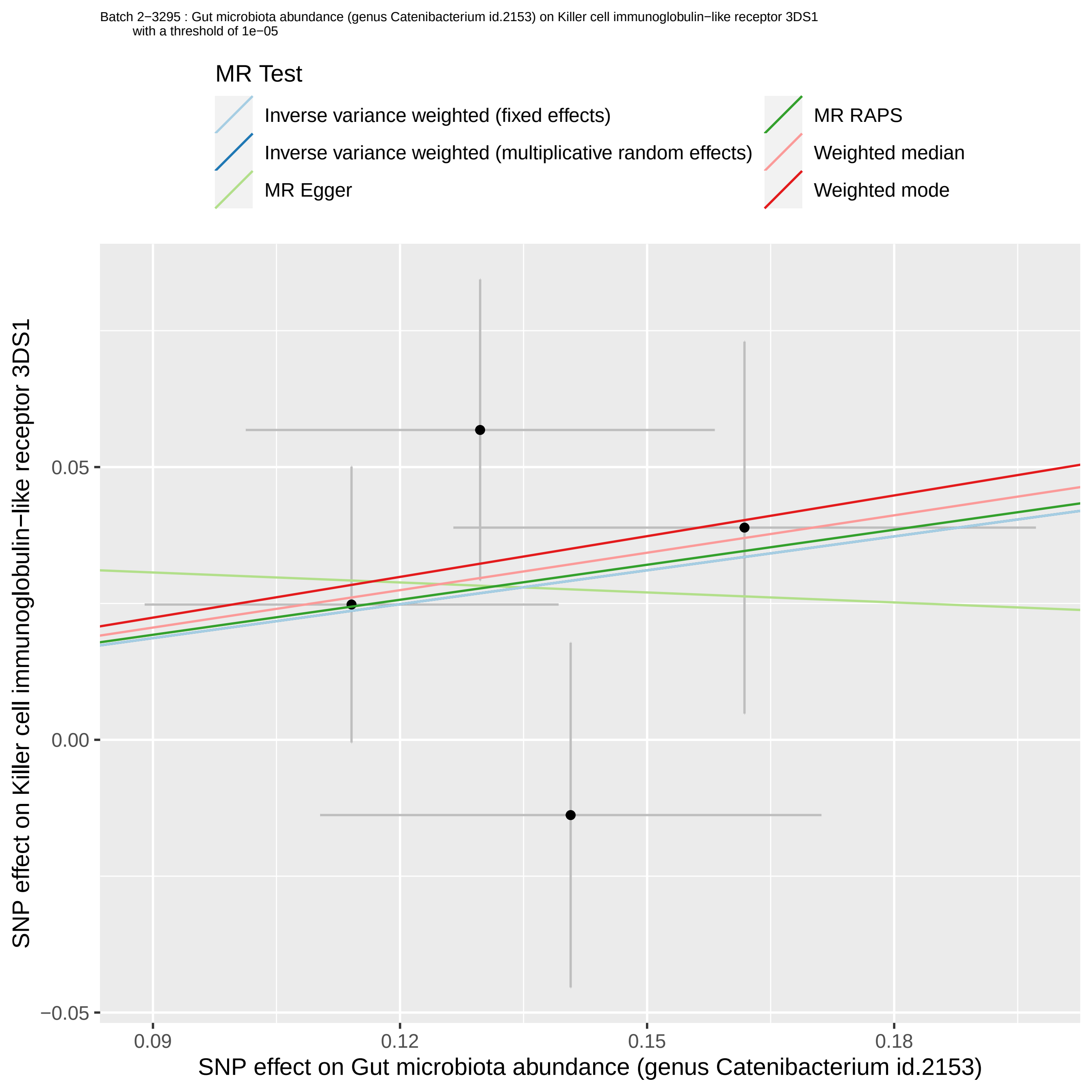
Batch 2−3295 : Gut microbiota abundance (genus Catenibacterium id.2153) on Killer cell immunoglobulin−like receptor 3DS1
with a threshold of 1e−05
MR Test
Inverse variance weighted (fixed effects)
Inverse variance weighted (multiplicative random effects)
MR Egger
MR RAPS
Weighted median
Weighted mode
0.05
0.00
−0.05
0.09
0.12
0.15
0.18
SNP effect on Killer cell immunoglobulin−like receptor 3DS1
SNP effect on Gut microbiota abundance (genus Catenibacterium id.2153)

Batch 2−3296 : Gut microbiota abundance (genus Parasutterella id.2892) on Killer cell immunoglobulin−like receptor 3DS1
with a threshold of 1e−05

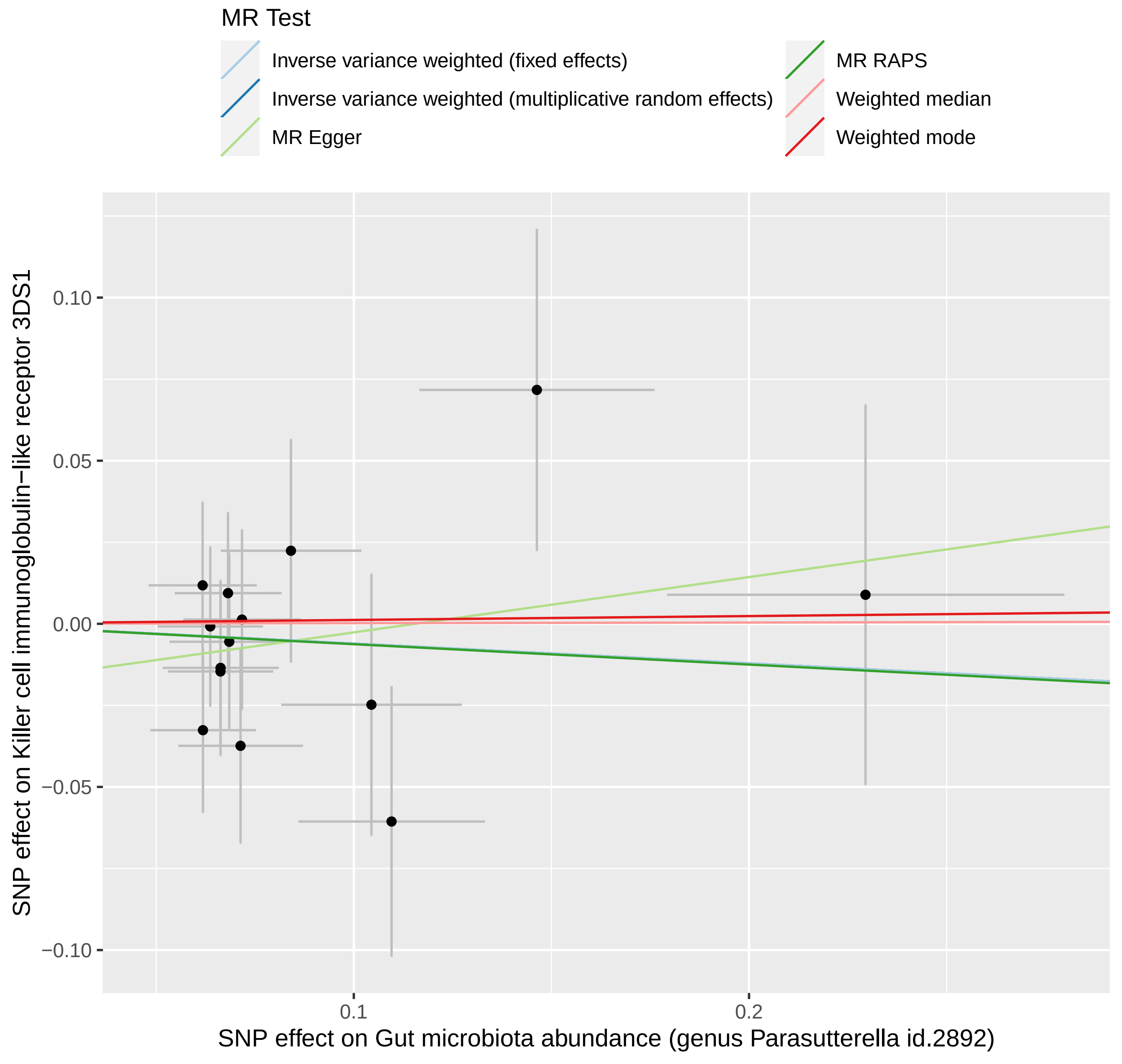

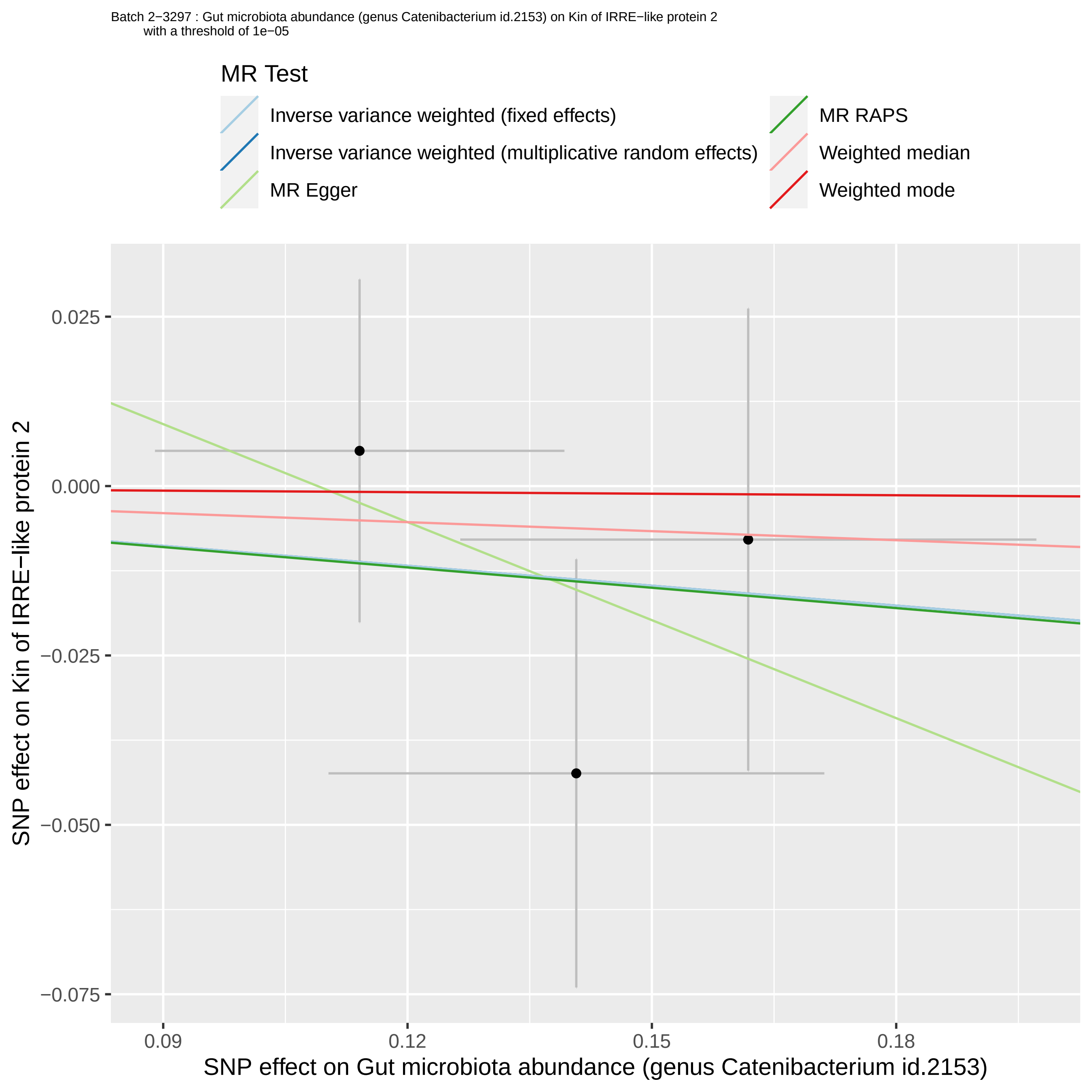
Batch 2−3297 : Gut microbiota abundance (genus Catenibacterium id.2153) on Kin of IRRE−like protein 2
with a threshold of 1e−05
MR Test
Inverse variance weighted (fixed effects)
Inverse variance weighted (multiplicative random effects)
MR Egger
MR RAPS
Weighted median
Weighted mode
SNP effect on Kin of IRRE−like protein 2
0.025
0.000
−0.025
−0.050
−0.075
0.09
0.12
0.15
0.18
SNP effect on Gut microbiota abundance (genus Catenibacterium id.2153)

Batch 2−3298 : Gut microbiota abundance (genus Parasutterella id.2892) on Kin of IRRE−like protein 2
with a threshold of 1e−05

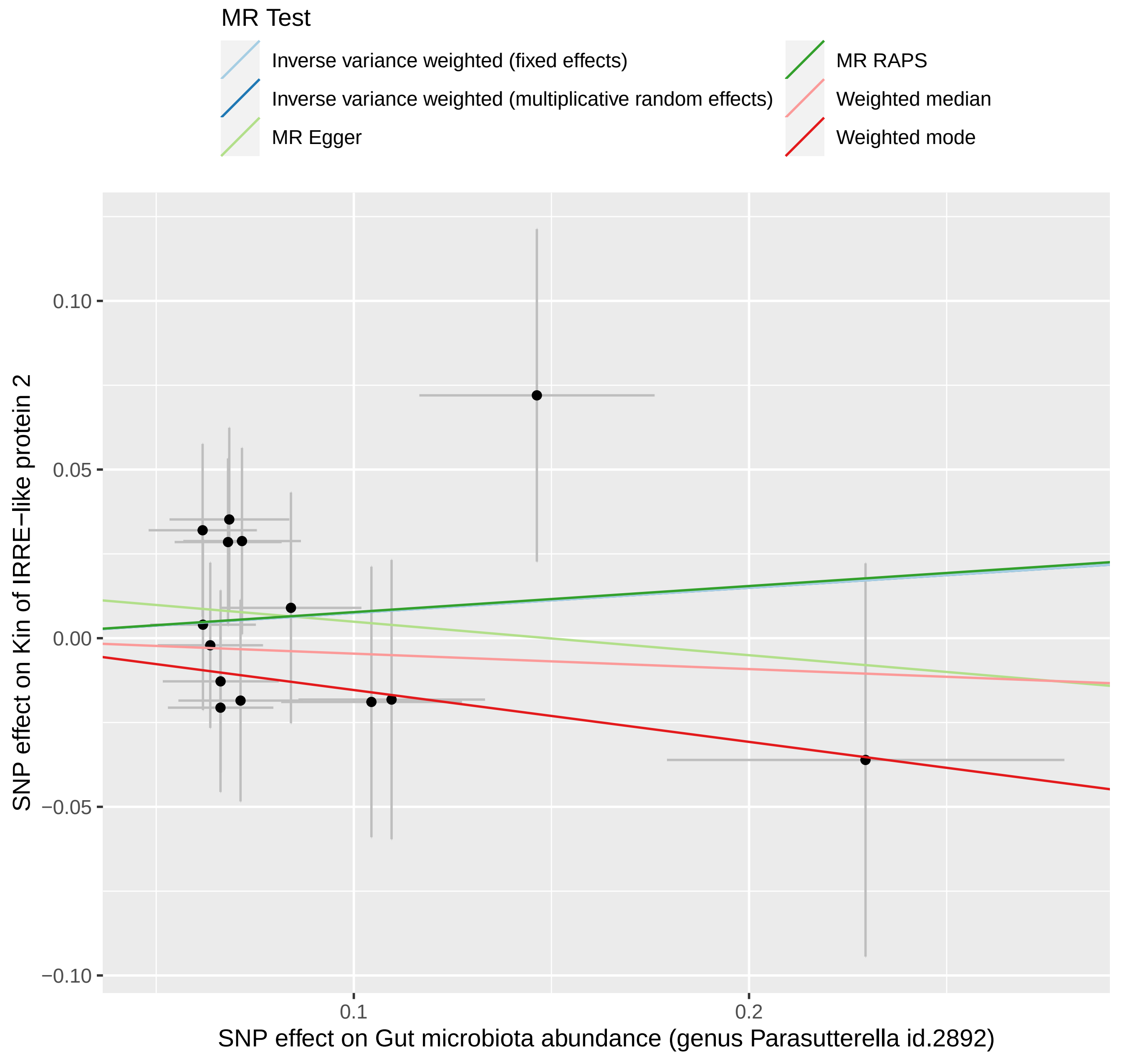

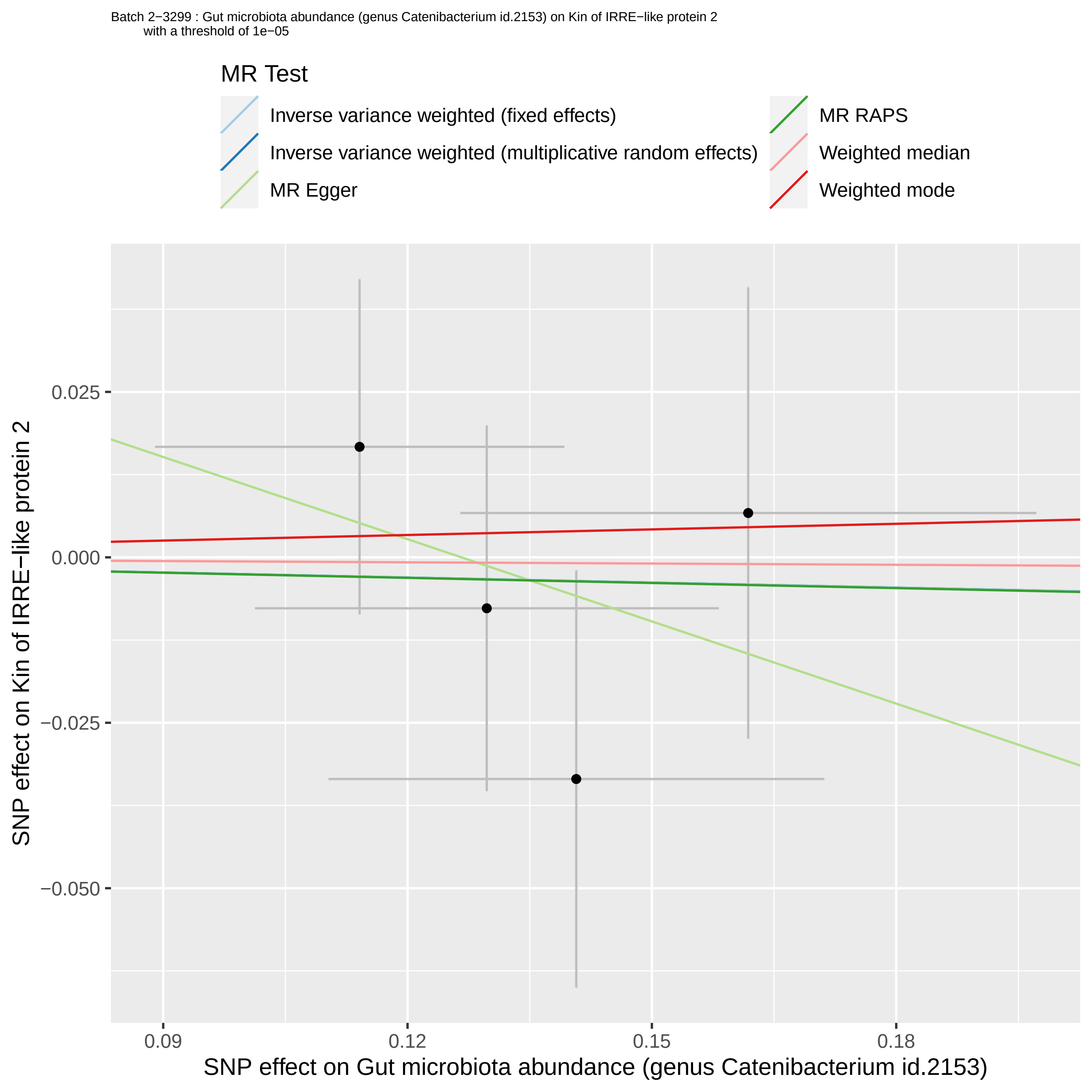

Batch 2−3299 : Gut microbiota abundance (genus Catenibacterium id.2153) on Kin of IRRE−like protein 2
with a threshold of 1e−05
MR Test
Inverse variance weighted (fixed effects)
Inverse variance weighted (multiplicative random effects)
MR Egger
MR RAPS
Weighted median
Weighted mode
0.025
0.000
−0.025
−0.050
0.09
0.12
0.15
0.18
SNP effect on Kin of IRRE−like protein 2
SNP effect on Gut microbiota abundance (genus Catenibacterium id.2153)

Batch 2−3300 : Gut microbiota abundance (genus Parasutterella id.2892) on Kin of IRRE−like protein 2
with a threshold of 1e−05

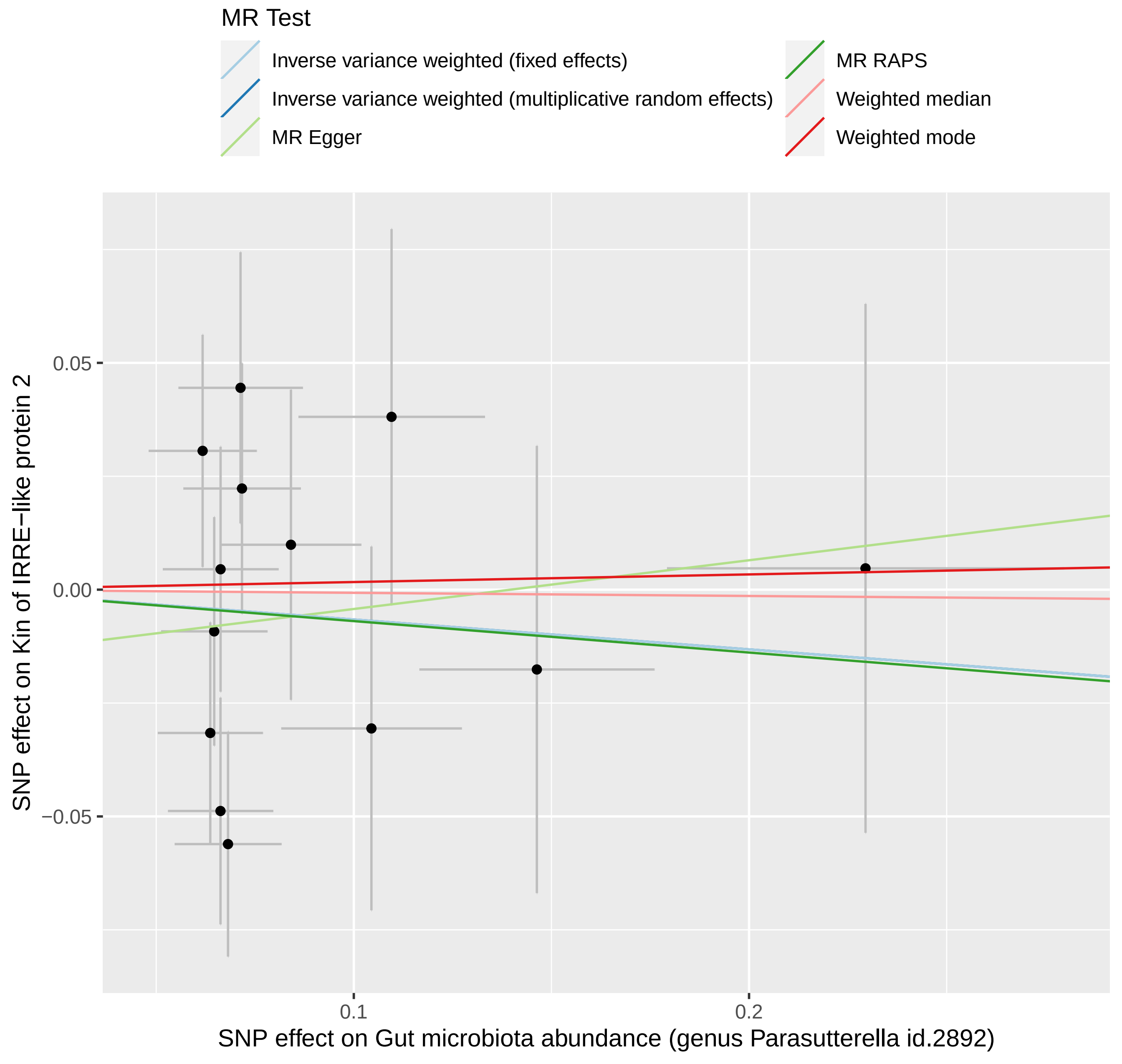

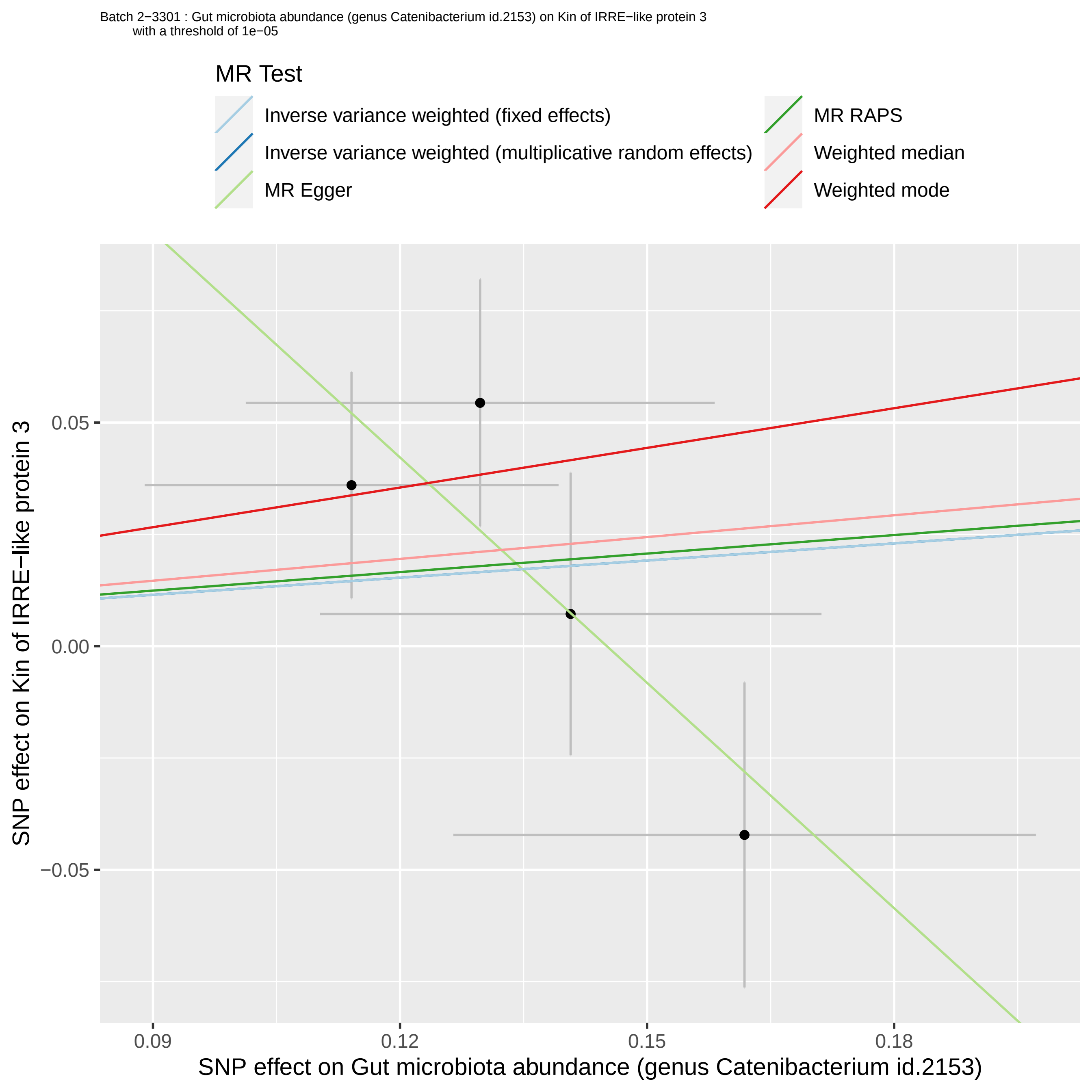
Batch 2−3301 : Gut microbiota abundance (genus Catenibacterium id.2153) on Kin of IRRE−like protein 3
with a threshold of 1e−05
MR Test
Inverse variance weighted (fixed effects)
Inverse variance weighted (multiplicative random effects)
MR Egger
MR RAPS
Weighted median
Weighted mode
SNP effect on Kin of IRRE−like protein 3
0.05
0.00
−0.05
0.09
0.12
0.15
0.18
SNP effect on Gut microbiota abundance (genus Catenibacterium id.2153)

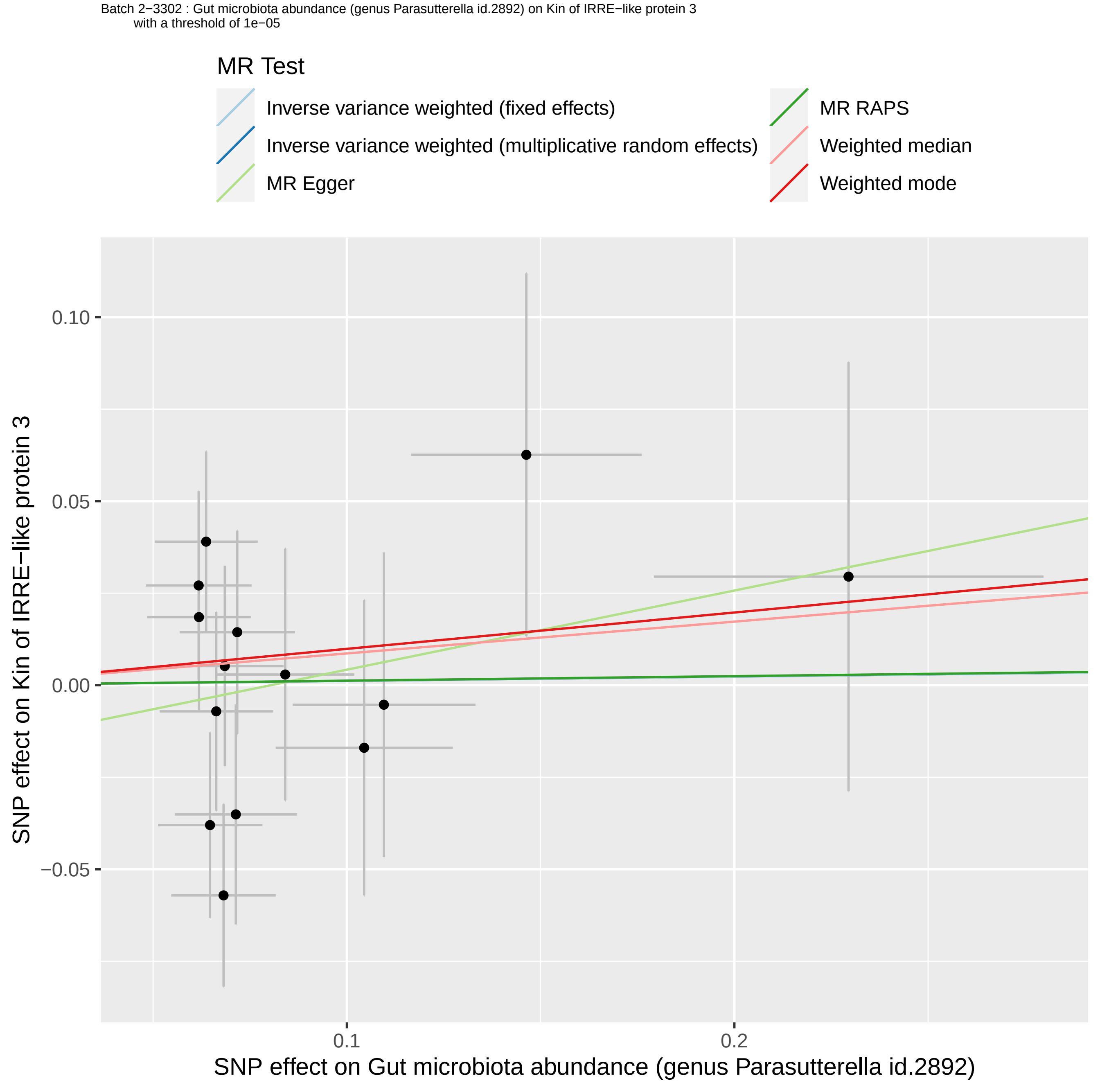
Batch 2−3302 : Gut microbiota abundance (genus Parasutterella id.2892) on Kin of IRRE−like protein 3
with a threshold of 1e−05
MR Test
Inverse variance weighted (fixed effects)
Inverse variance weighted (multiplicative random effects)
MR Egger
MR RAPS
Weighted median
Weighted mode
0.10
0.05
0.00
−0.05
0.1
0.2
SNP effect on Kin of IRRE−like protein 3
SNP effect on Gut microbiota abundance (genus Parasutterella id.2892)

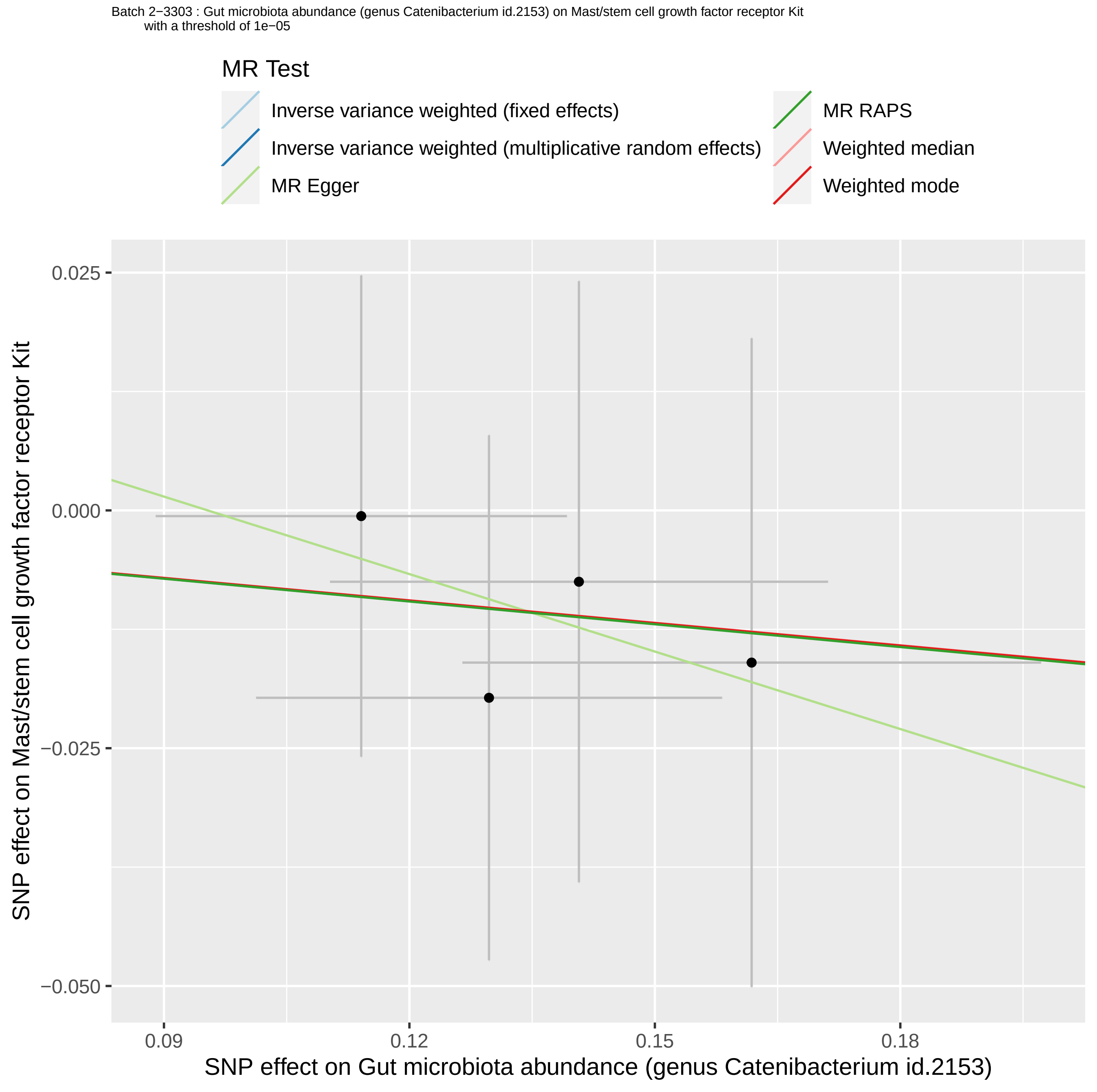
Batch 2−3303 : Gut microbiota abundance (genus Catenibacterium id.2153) on Mast/stem cell growth factor receptor Kit
with a threshold of 1e−05
MR Test
Inverse variance weighted (fixed effects)
Inverse variance weighted (multiplicative random effects)
MR Egger
MR RAPS
Weighted median
Weighted mode
0.025
0.000
−0.025
−0.050
0.09
0.12
0.15
0.18
SNP effect on Mast/stem cell growth factor receptor Kit
SNP effect on Gut microbiota abundance (genus Catenibacterium id.2153)

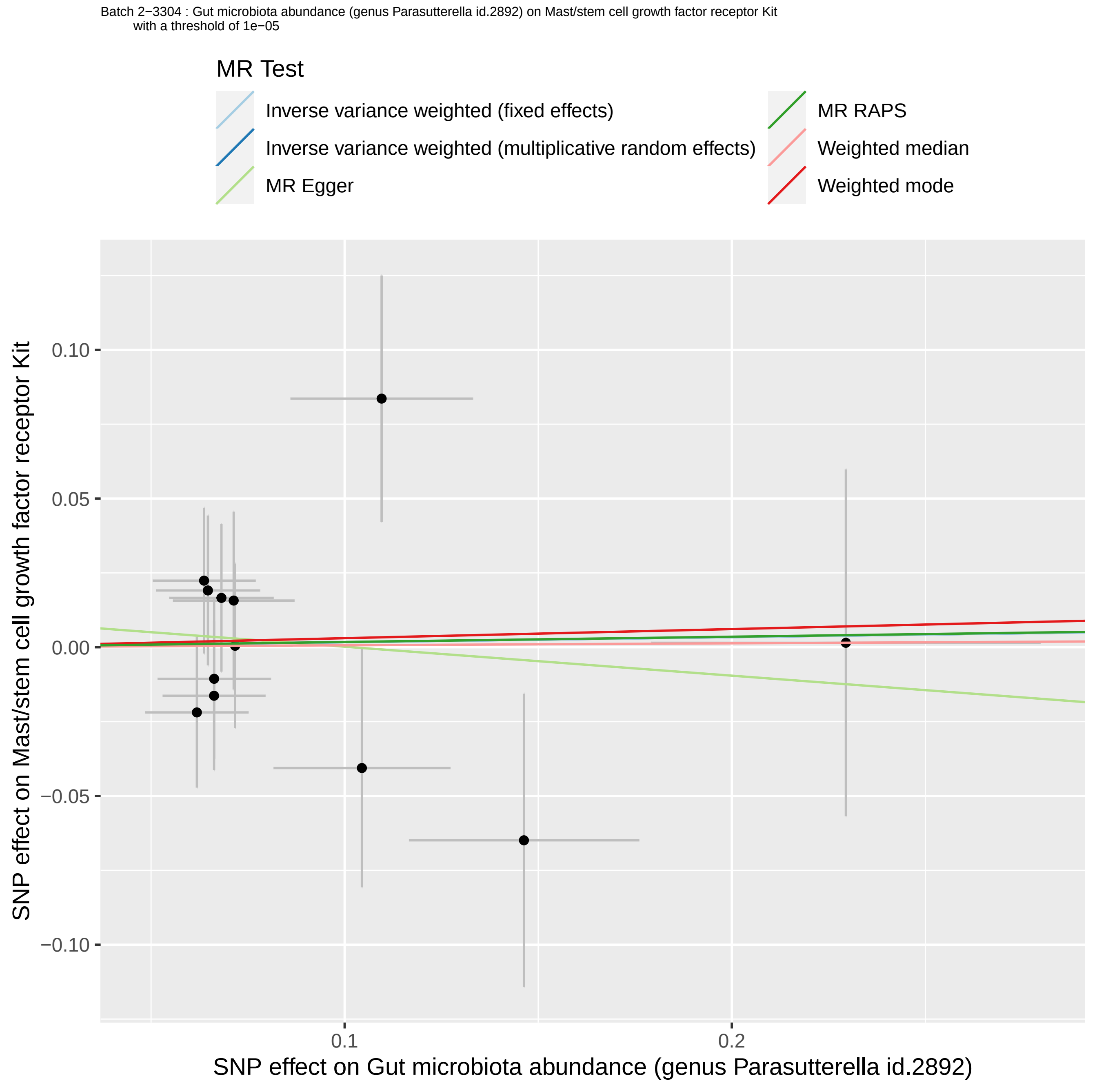
Batch 2−3304 : Gut microbiota abundance (genus Parasutterella id.2892) on Mast/stem cell growth factor receptor Kit
with a threshold of 1e−05
MR Test
Inverse variance weighted (fixed effects)
Inverse variance weighted (multiplicative random effects)
MR Egger
MR RAPS
Weighted median
Weighted mode
0.10
0.05
0.00
−0.05
−0.10
0.1
0.2
SNP effect on Mast/stem cell growth factor receptor Kit
SNP effect on Gut microbiota abundance (genus Parasutterella id.2892)

Batch 2−3305 : Gut microbiota abundance (genus Catenibacterium id.2153) on Kit ligand
with a threshold of 1e−05

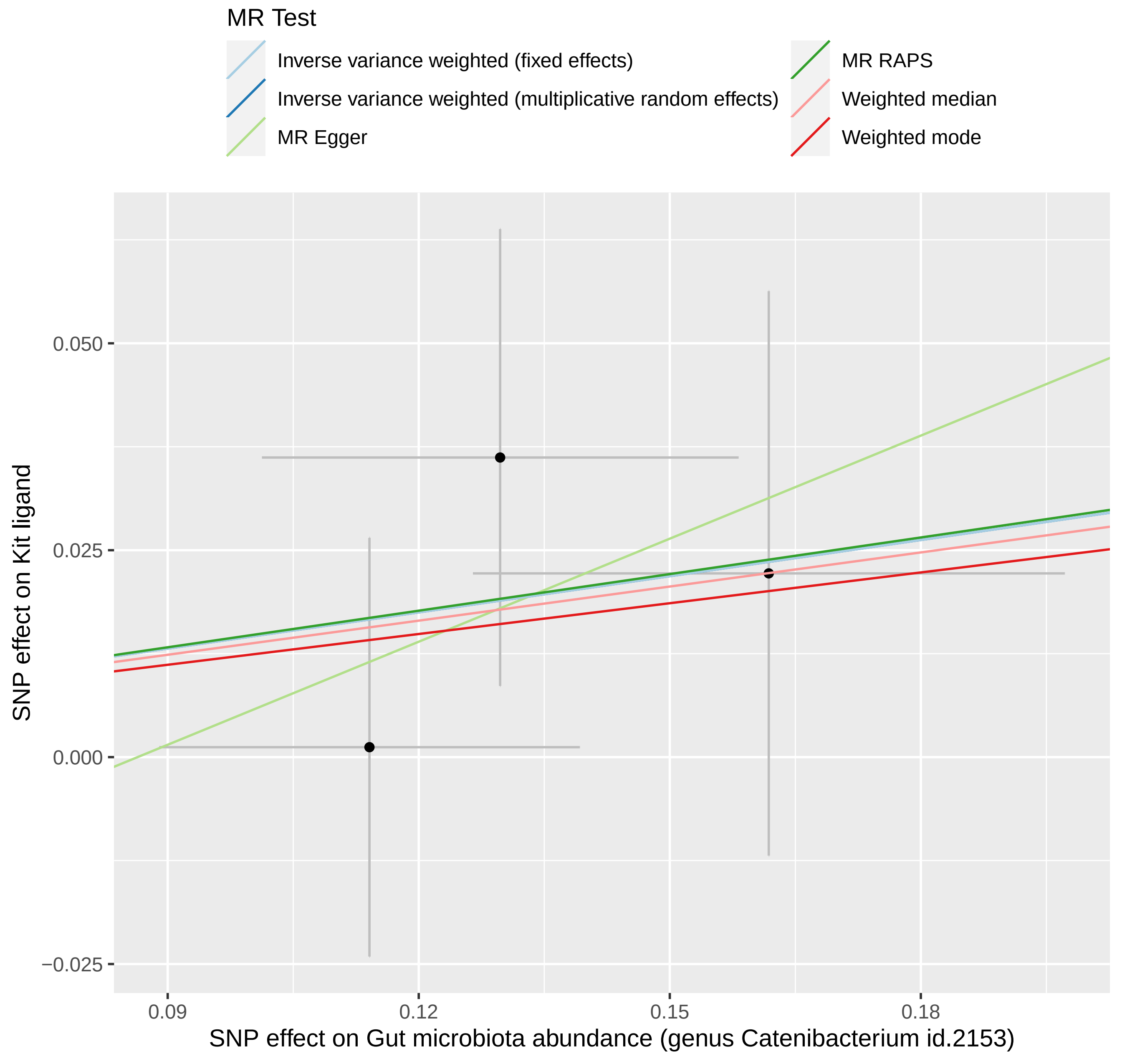

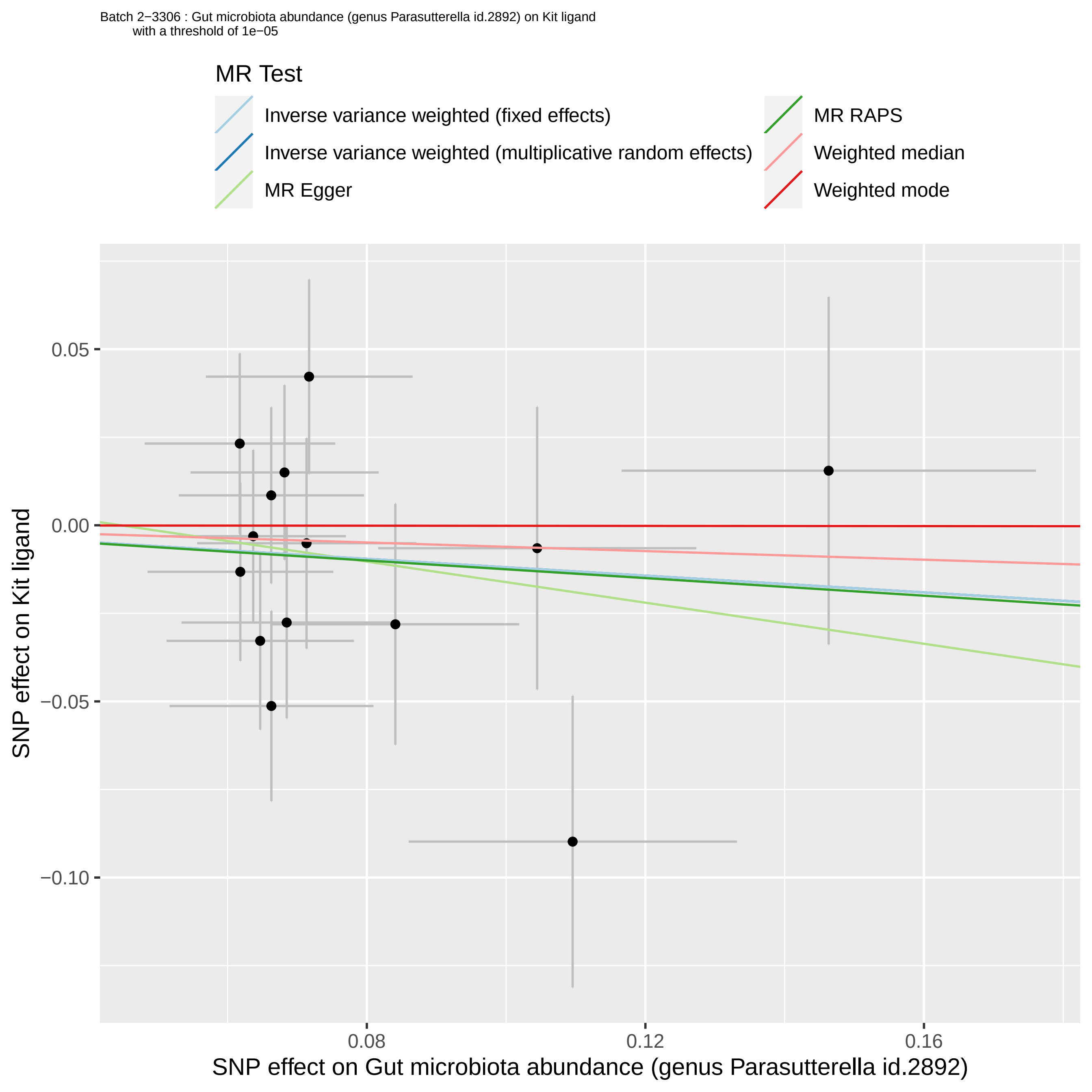
Batch 2−3306 : Gut microbiota abundance (genus Parasutterella id.2892) on Kit ligand
with a threshold of 1e−05
MR Test
Inverse variance weighted (fixed effects)
Inverse variance weighted (multiplicative random effects)
MR Egger
MR RAPS
Weighted median
Weighted mode
0.05
0.00
−0.05
−0.10
0.08
0.12
0.16
SNP effect on Kit ligand
SNP effect on Gut microbiota abundance (genus Parasutterella id.2892)

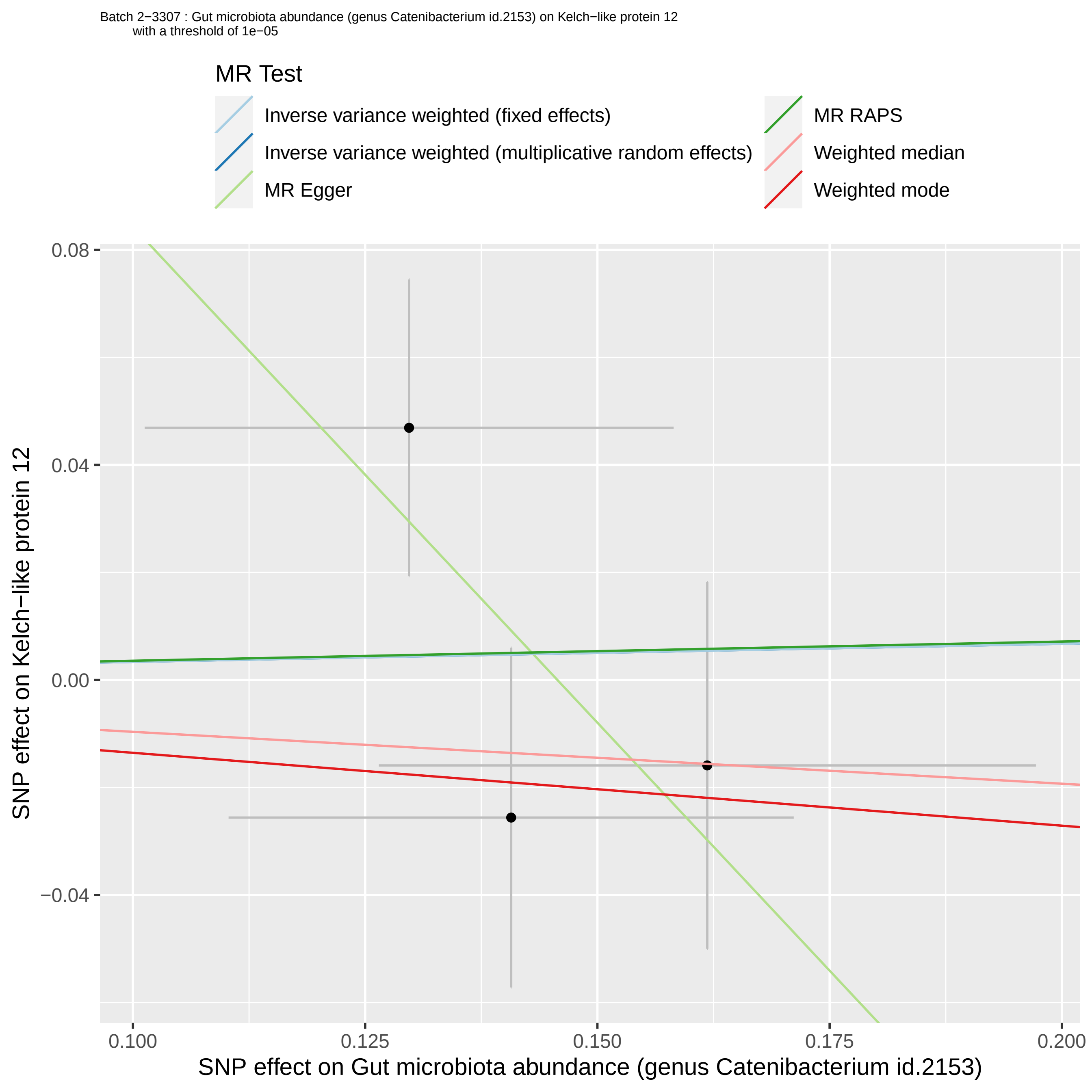
Batch 2−3307 : Gut microbiota abundance (genus Catenibacterium id.2153) on Kelch−like protein 12
with a threshold of 1e−05
MR Test
Inverse variance weighted (fixed effects)
Inverse variance weighted (multiplicative random effects)
MR Egger
MR RAPS
Weighted median
Weighted mode
0.08
0.04
0.00
−0.04
SNP effect on Kelch−like protein 12
0.100
0.125
0.150
0.175
0.200
SNP effect on Gut microbiota abundance (genus Catenibacterium id.2153)

Batch 2−3308 : Gut microbiota abundance (genus Parasutterella id.2892) on Kelch−like protein 12
with a threshold of 1e−05

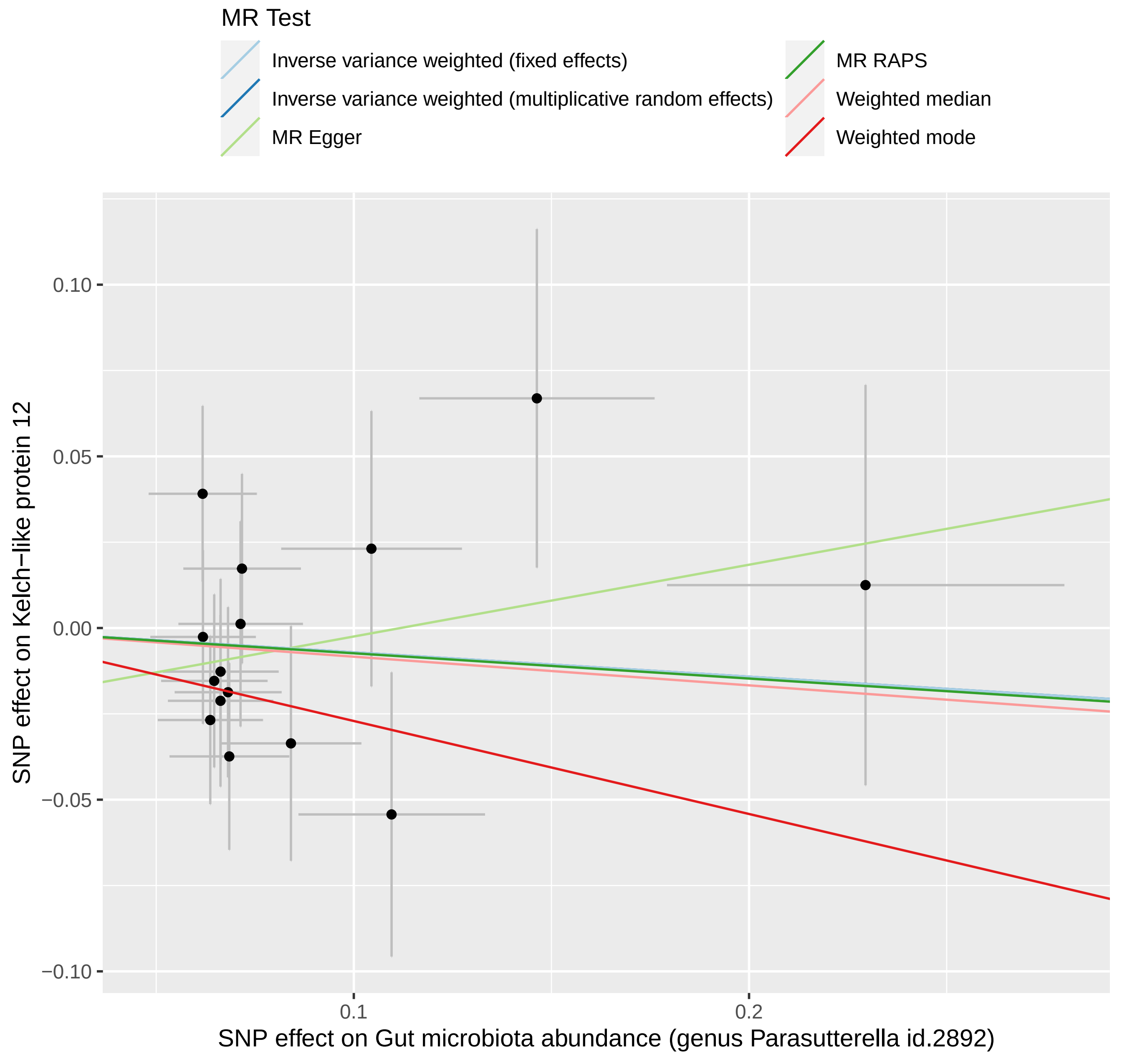

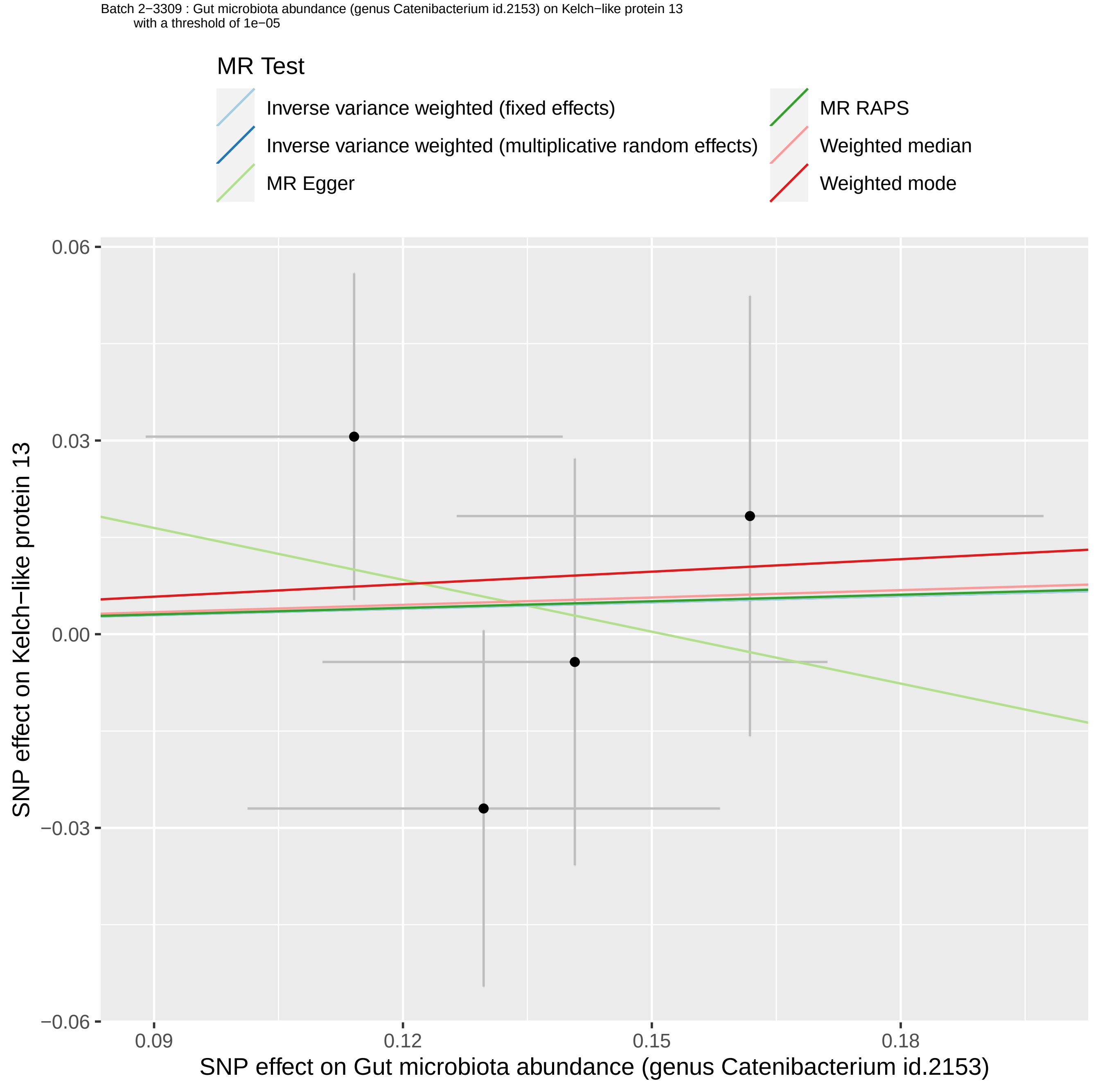
Batch 2−3309 : Gut microbiota abundance (genus Catenibacterium id.2153) on Kelch−like protein 13
with a threshold of 1e−05
MR Test
Inverse variance weighted (fixed effects)
Inverse variance weighted (multiplicative random effects)
MR Egger
MR RAPS
Weighted median
Weighted mode
0.06
0.03
0.00
−0.03
−0.06
0.09
0.12
0.15
0.18
SNP effect on Kelch−like protein 13
SNP effect on Gut microbiota abundance (genus Catenibacterium id.2153)

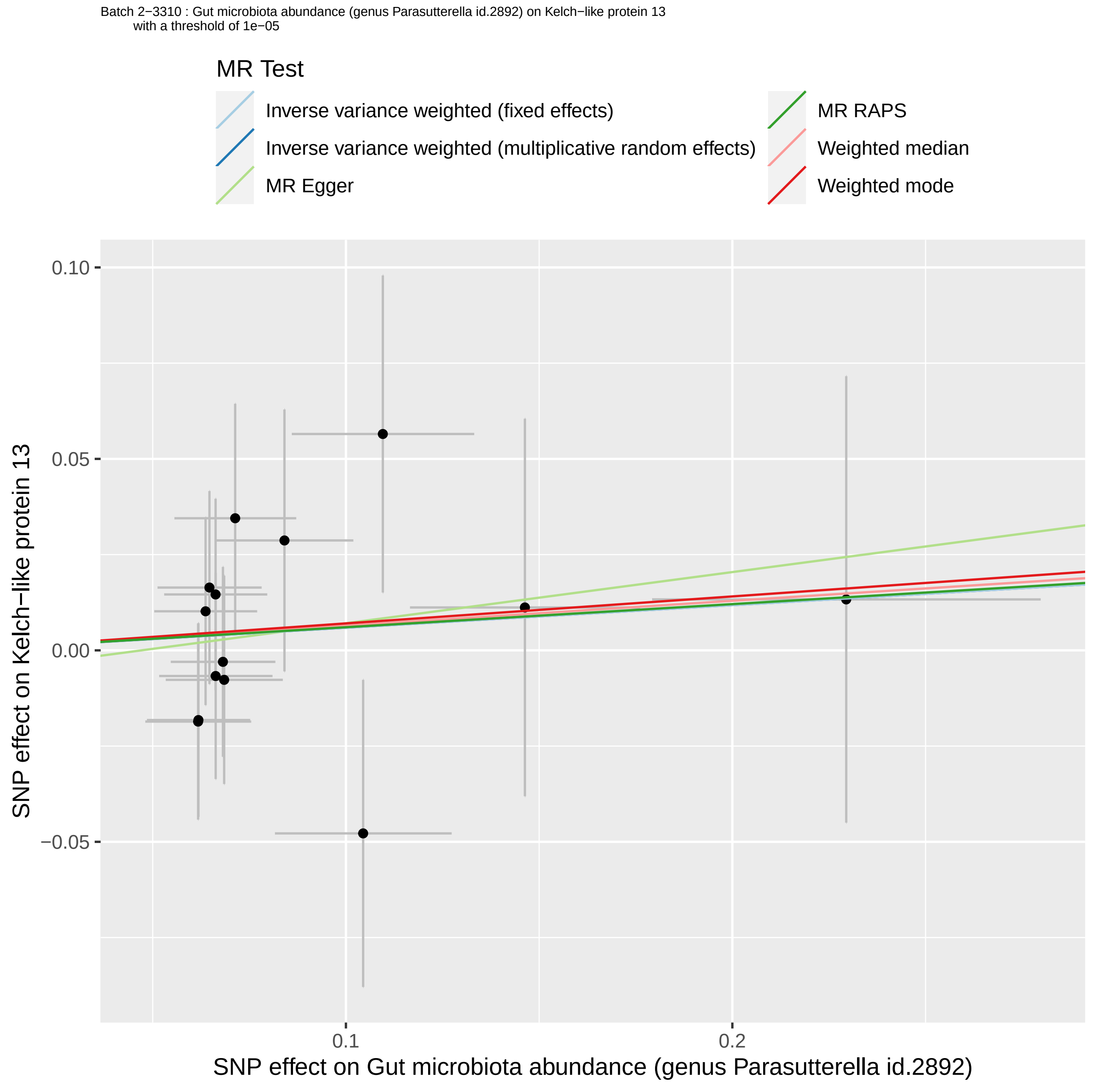

Batch 2−3310 : Gut microbiota abundance (genus Parasutterella id.2892) on Kelch−like protein 13
with a threshold of 1e−05
MR Test
Inverse variance weighted (fixed effects)
Inverse variance weighted (multiplicative random effects)
MR Egger
MR RAPS
Weighted median
Weighted mode
0.10
0.05
0.00
−0.05
0.1
0.2
SNP effect on Kelch−like protein 13
SNP effect on Gut microbiota abundance (genus Parasutterella id.2892)

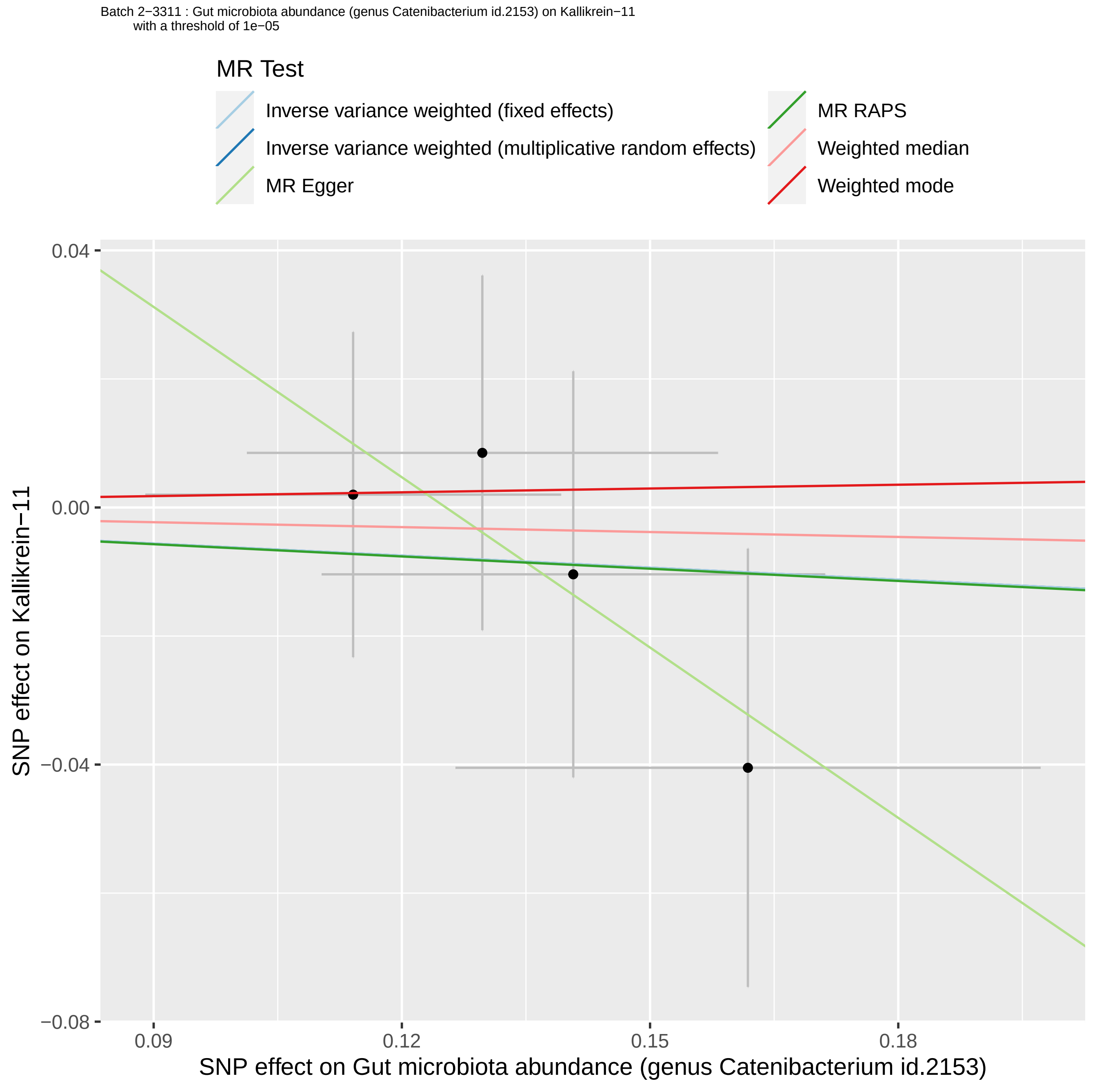

Batch 2−3311 : Gut microbiota abundance (genus Catenibacterium id.2153) on Kallikrein−11
with a threshold of 1e−05
MR Test
Inverse variance weighted (fixed effects)
Inverse variance weighted (multiplicative random effects)
MR Egger
MR RAPS
Weighted median
Weighted mode
0.04
0.00
−0.04
−0.08
0.09
0.12
0.15
0.18
SNP effect on Kallikrein−11
SNP effect on Gut microbiota abundance (genus Catenibacterium id.2153)

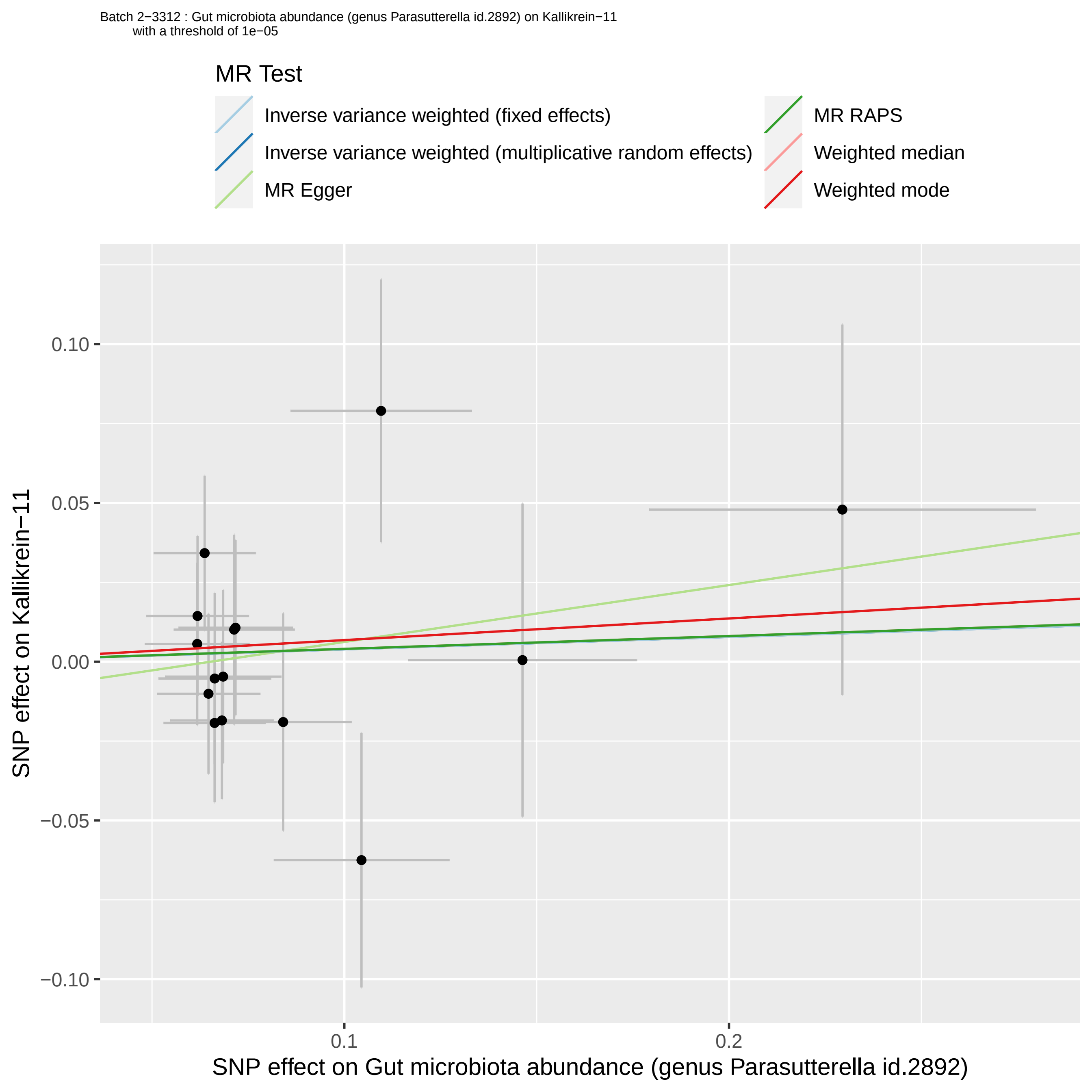

Batch 2−3312 : Gut microbiota abundance (genus Parasutterella id.2892) on Kallikrein−11
with a threshold of 1e−05
MR Test
Inverse variance weighted (fixed effects)
Inverse variance weighted (multiplicative random effects)
MR Egger
MR RAPS
Weighted median
Weighted mode
0.10
0.05
0.00
−0.05
−0.10
0.1
0.2
SNP effect on Kallikrein−11
SNP effect on Gut microbiota abundance (genus Parasutterella id.2892)

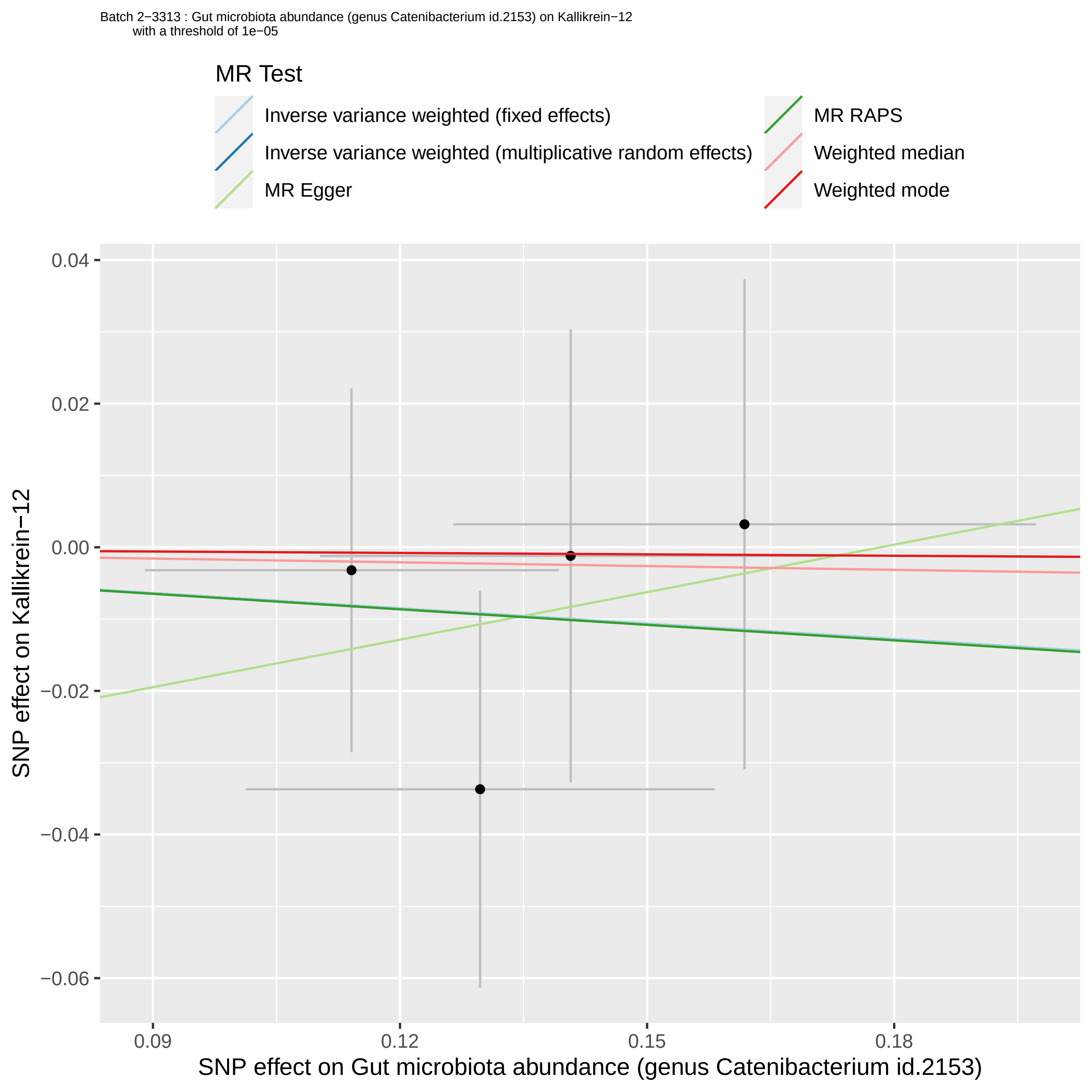

Batch 2−3313 : Gut microbiota abundance (genus Catenibacterium id.2153) on Kallikrein−12
with a threshold of 1e−05
MR Test
Inverse variance weighted (fixed effects)
Inverse variance weighted (multiplicative random effects)
MR Egger
MR RAPS
Weighted median
Weighted mode
0.04
0.02
0.00
−0.02
−0.04
−0.06
SNP effect on Kallikrein−12
0.09
0.12
0.15
0.18
SNP effect on Gut microbiota abundance (genus Catenibacterium id.2153)

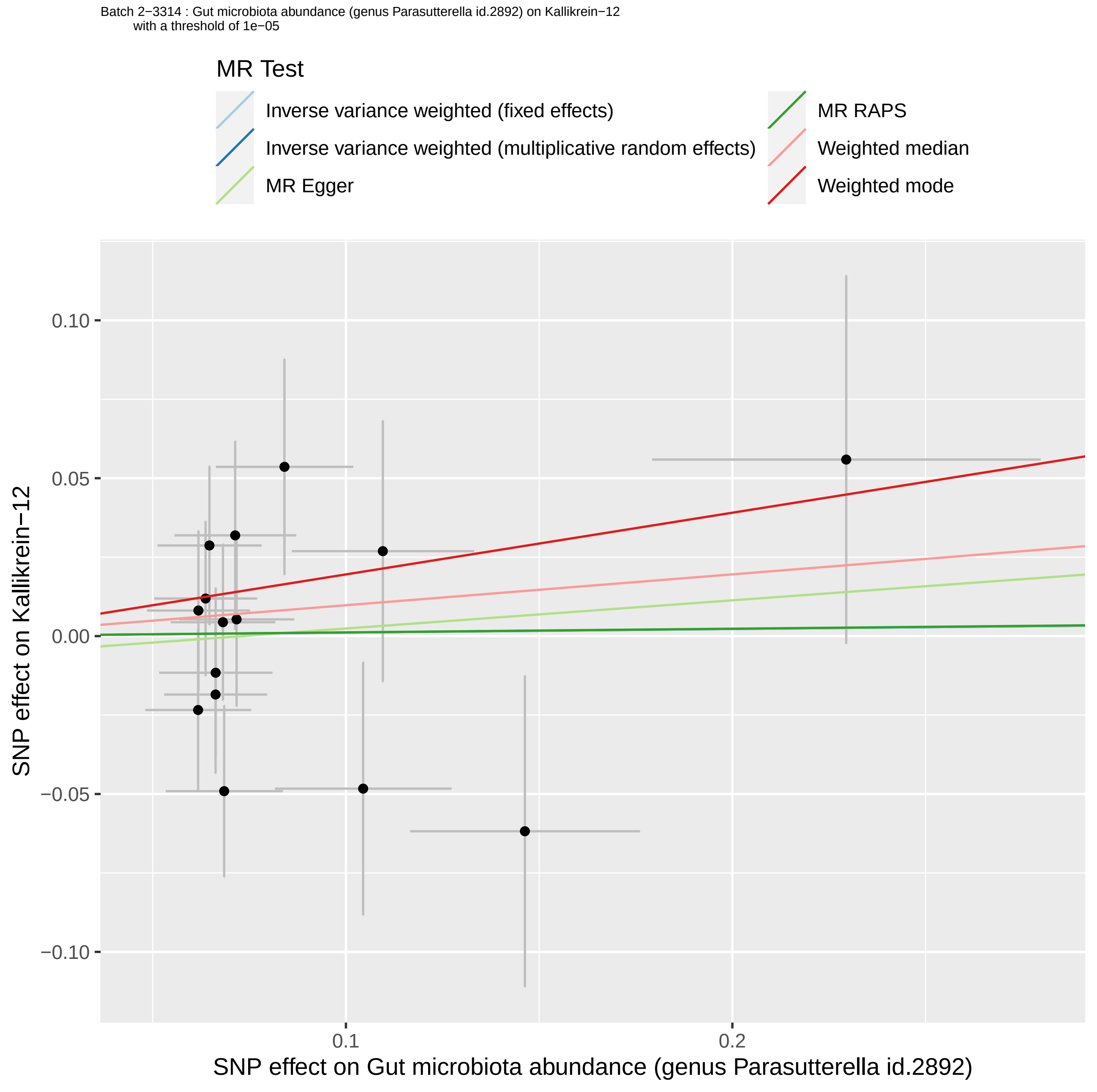
Batch 2−3314 : Gut microbiota abundance (genus Parasutterella id.2892) on Kallikrein−12
with a threshold of 1e−05
MR Test
Inverse variance weighted (fixed effects)
Inverse variance weighted (multiplicative random effects)
MR Egger
MR RAPS
Weighted median
Weighted mode
0.10
0.05
0.00
−0.05
−0.10
0.1
0.2
SNP effect on Kallikrein−12
SNP effect on Gut microbiota abundance (genus Parasutterella id.2892)

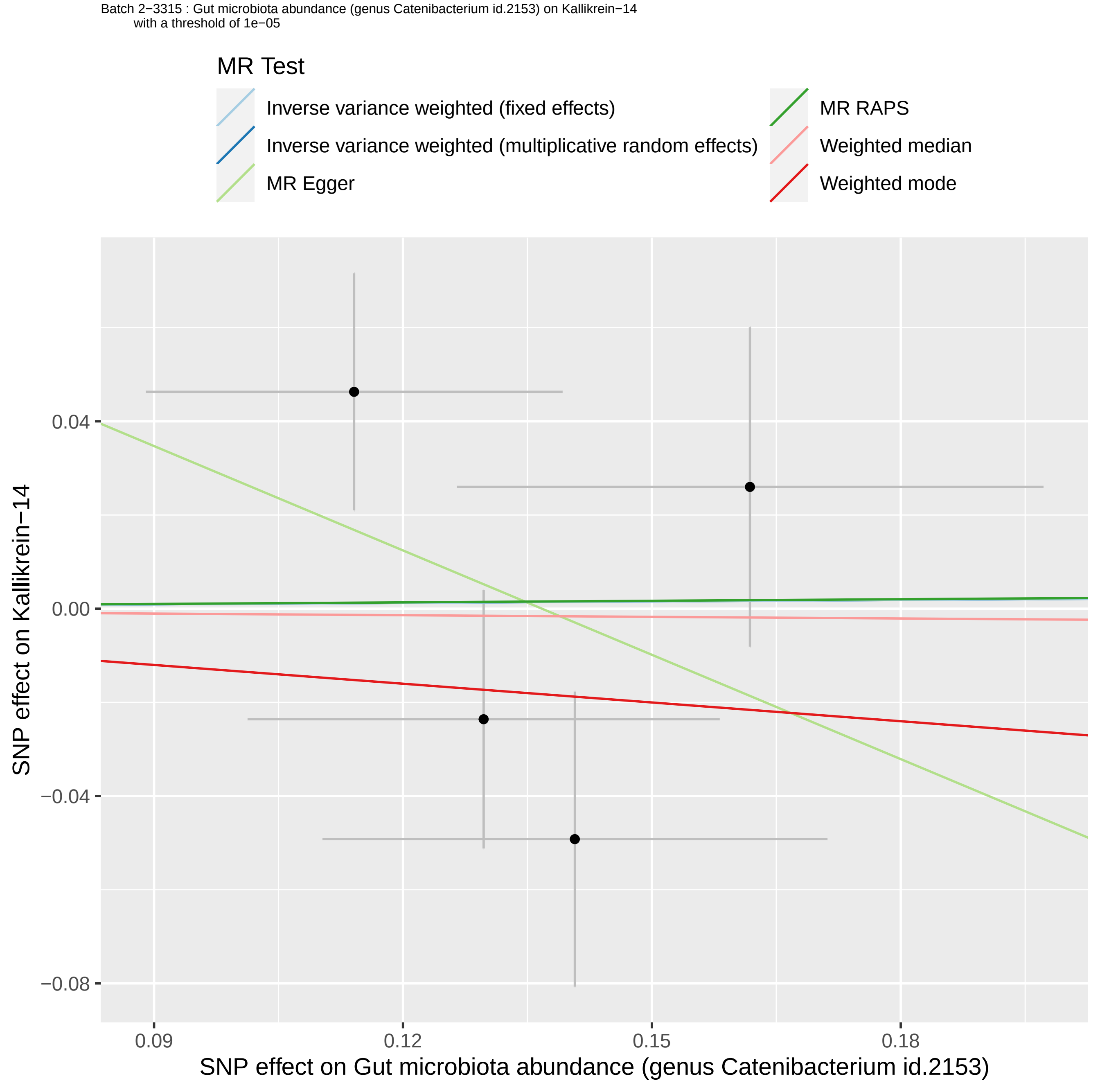

Batch 2−3315 : Gut microbiota abundance (genus Catenibacterium id.2153) on Kallikrein−14
with a threshold of 1e−05
MR Test
Inverse variance weighted (fixed effects)
Inverse variance weighted (multiplicative random effects)
MR Egger
MR RAPS
Weighted median
Weighted mode
0.04
0.00
−0.04
−0.08
0.09
0.12
0.15
0.18
SNP effect on Kallikrein−14
SNP effect on Gut microbiota abundance (genus Catenibacterium id.2153)

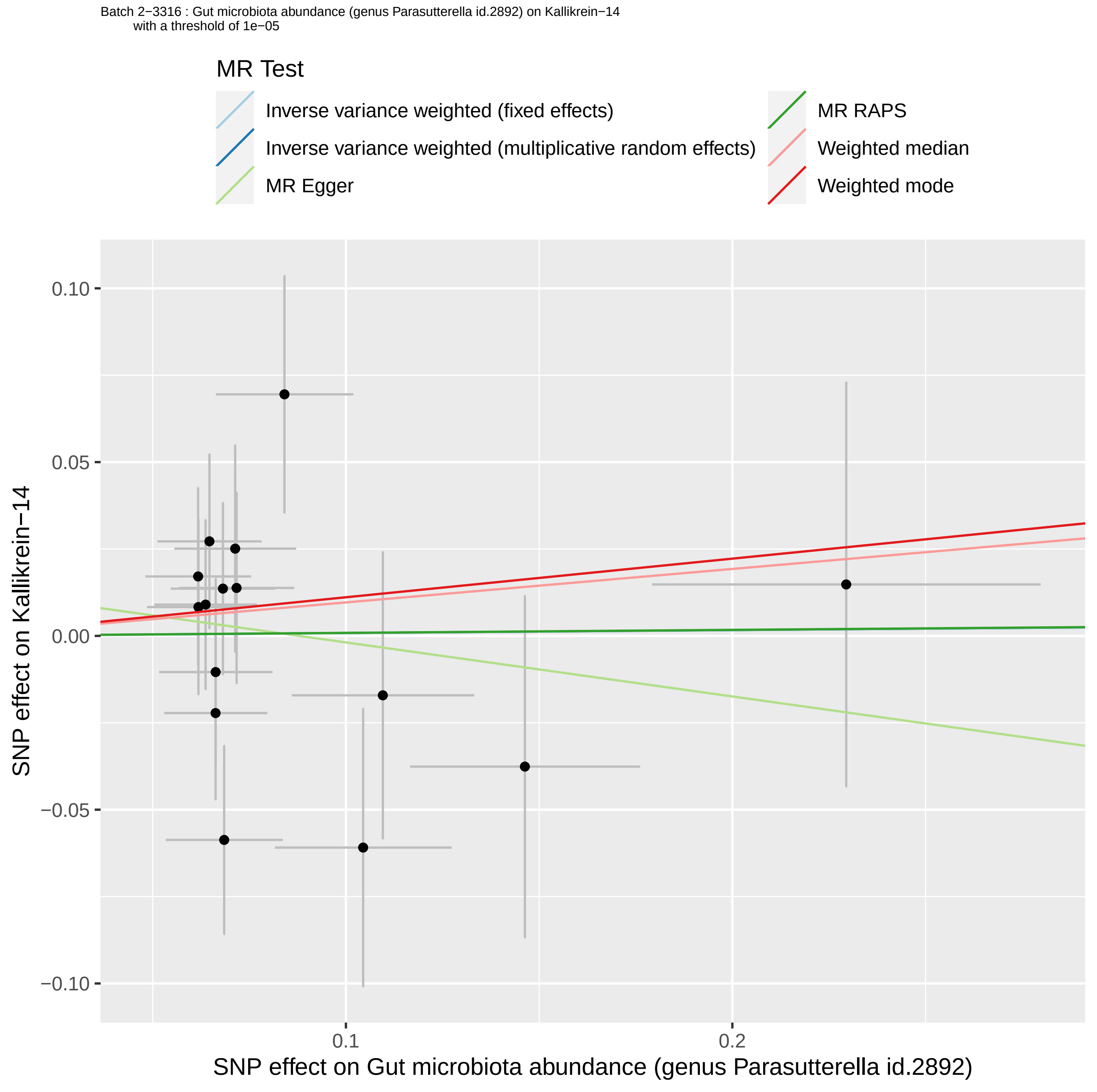
Batch 2−3316 : Gut microbiota abundance (genus Parasutterella id.2892) on Kallikrein−14
with a threshold of 1e−05
MR Test
Inverse variance weighted (fixed effects)
Inverse variance weighted (multiplicative random effects)
MR Egger
MR RAPS
Weighted median
Weighted mode
0.10
0.05
0.00
−0.05
−0.10
0.1
0.2
SNP effect on Kallikrein−14
SNP effect on Gut microbiota abundance (genus Parasutterella id.2892)

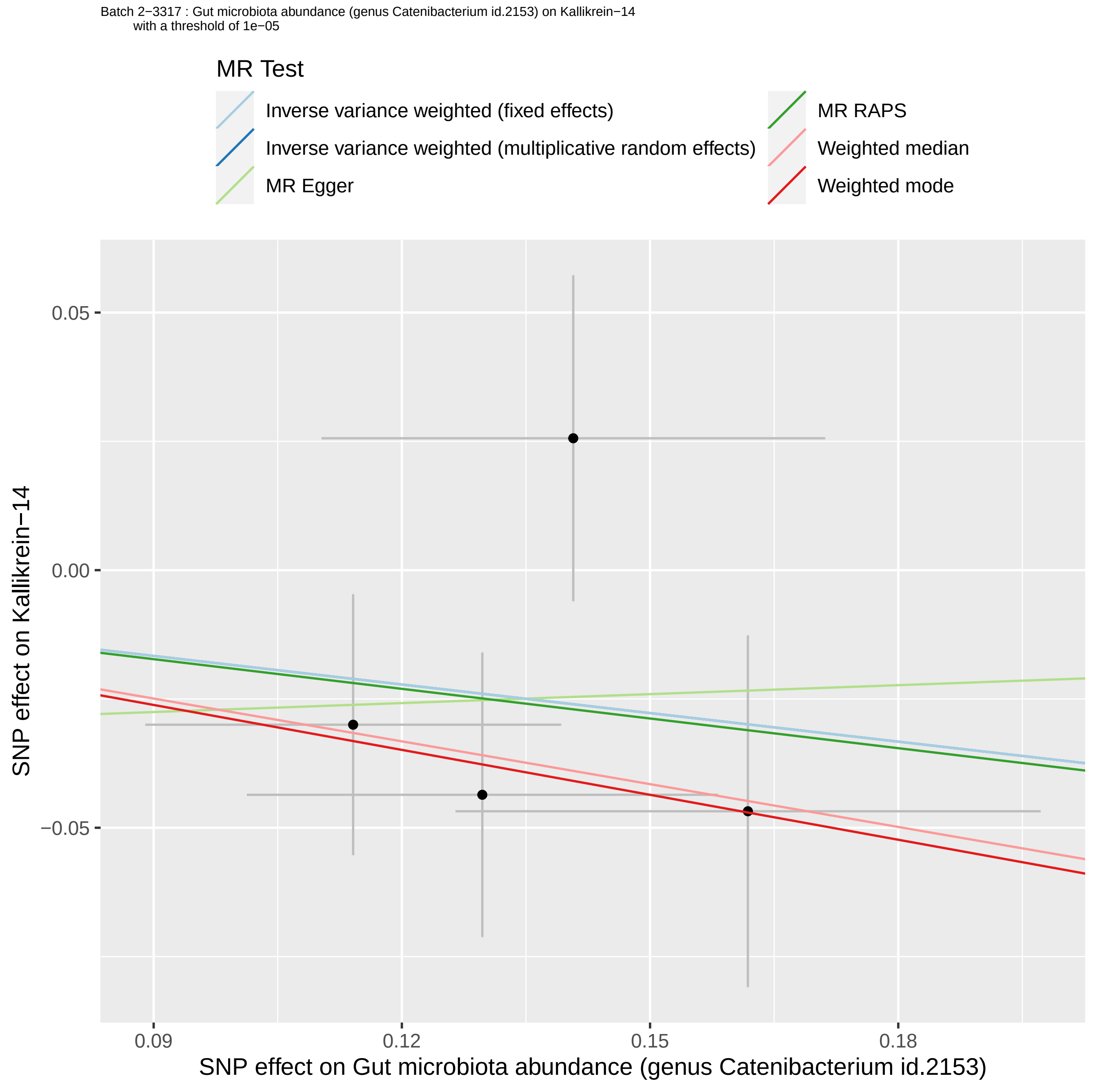
Batch 2−3317 : Gut microbiota abundance (genus Catenibacterium id.2153) on Kallikrein−14
with a threshold of 1e−05
MR Test
Inverse variance weighted (fixed effects)
Inverse variance weighted (multiplicative random effects)
MR Egger
MR RAPS
Weighted median
Weighted mode
0.05
0.00
−0.05
0.09
0.12
0.15
0.18
SNP effect on Kallikrein−14
SNP effect on Gut microbiota abundance (genus Catenibacterium id.2153)

Batch 2−3318 : Gut microbiota abundance (genus Parasutterella id.2892) on Kallikrein−14
with a threshold of 1e−05

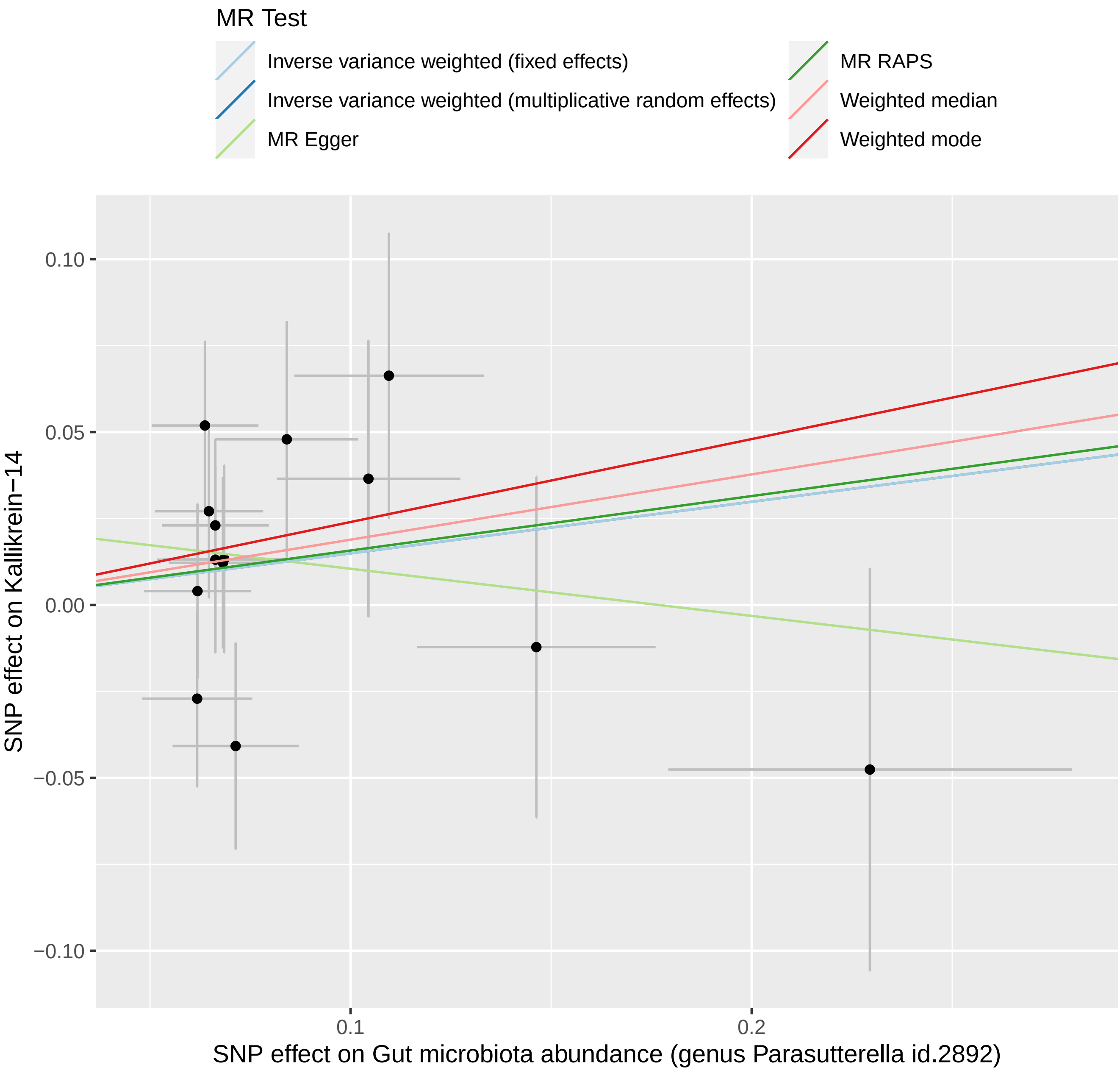

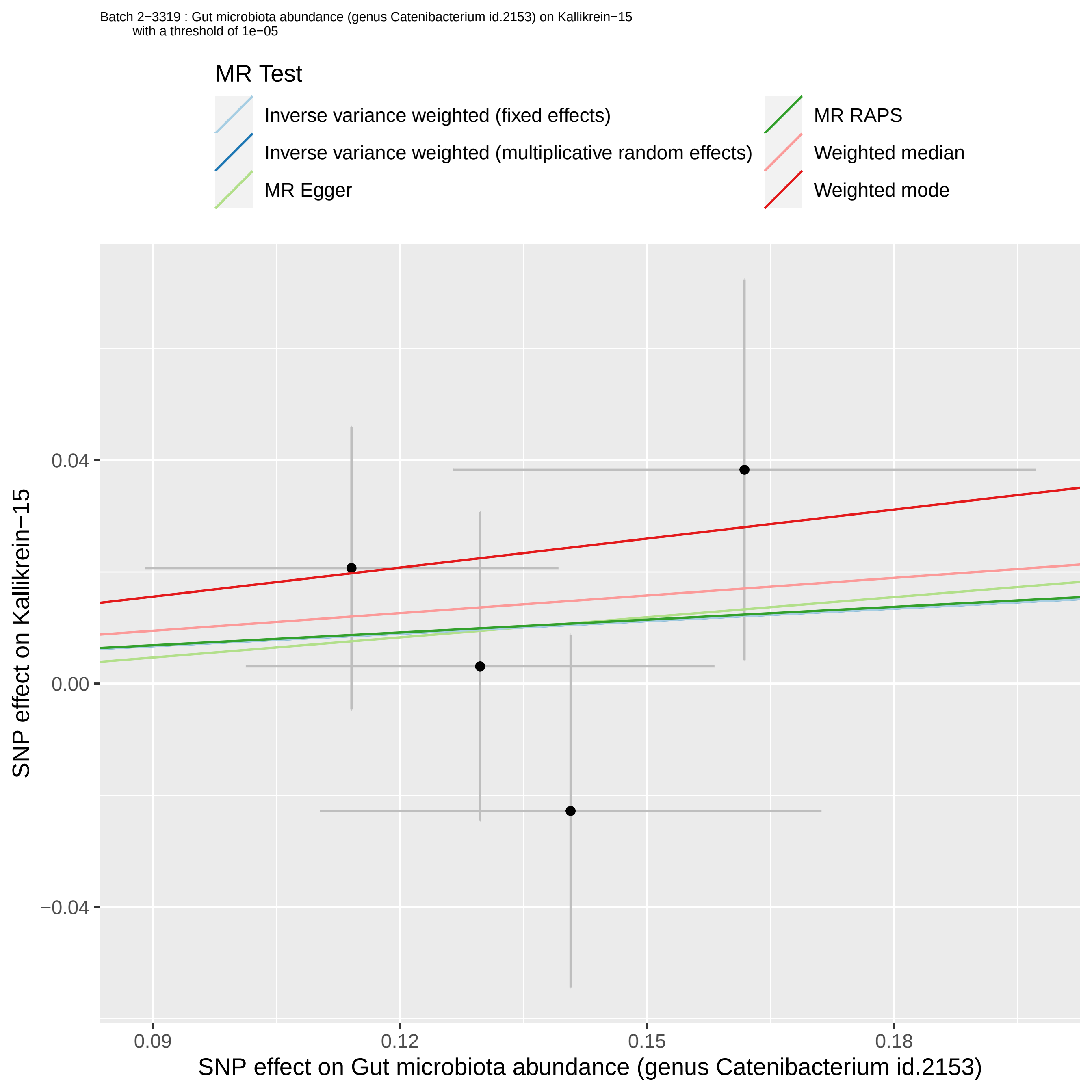
Batch 2−3319 : Gut microbiota abundance (genus Catenibacterium id.2153) on Kallikrein−15
with a threshold of 1e−05
MR Test
Inverse variance weighted (fixed effects)
Inverse variance weighted (multiplicative random effects)
MR Egger
MR RAPS
Weighted median
Weighted mode
0.04
0.00
−0.04
0.09
0.12
0.15
0.18
SNP effect on Kallikrein−15
SNP effect on Gut microbiota abundance (genus Catenibacterium id.2153)

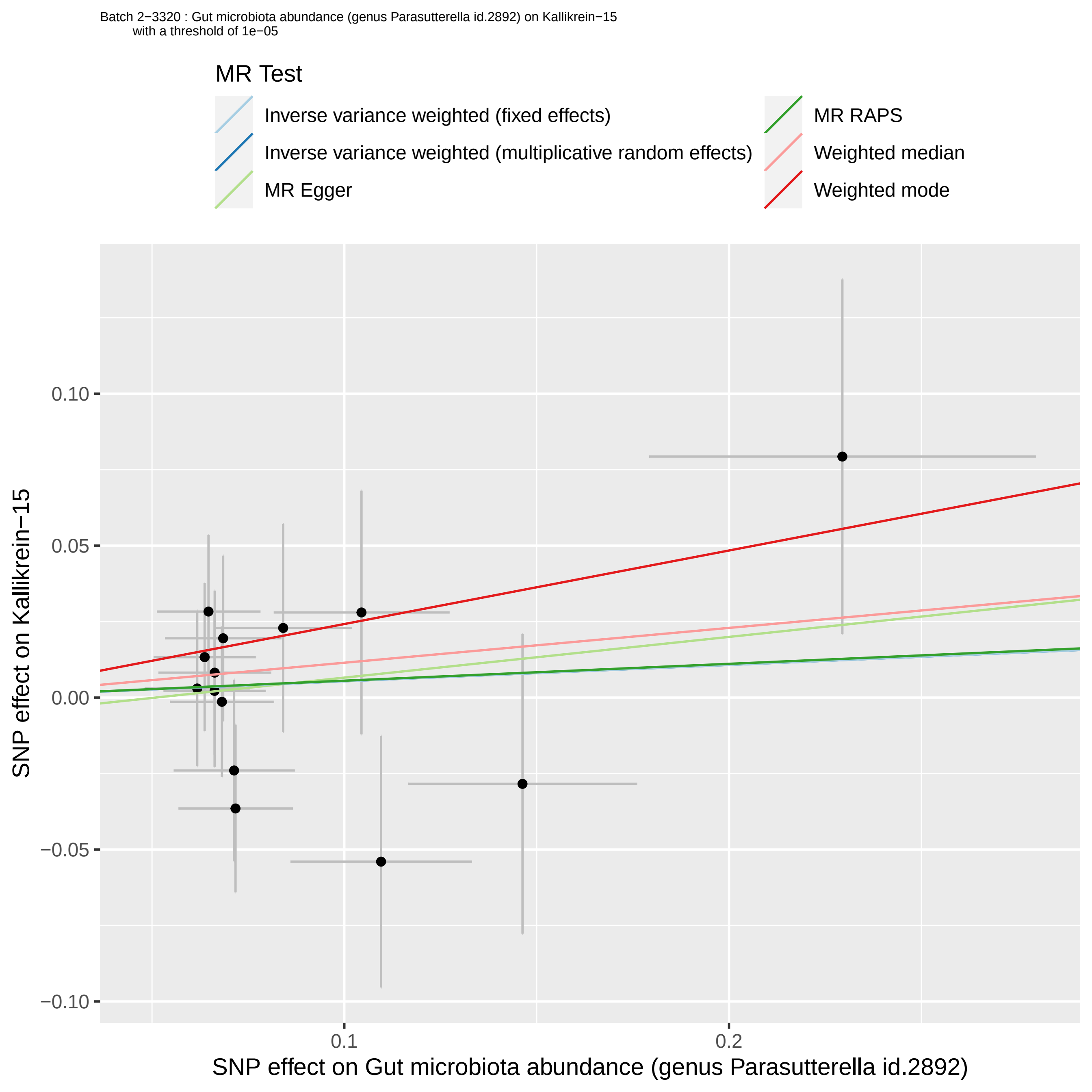
Batch 2−3320 : Gut microbiota abundance (genus Parasutterella id.2892) on Kallikrein−15
with a threshold of 1e−05
MR Test
Inverse variance weighted (fixed effects)
Inverse variance weighted (multiplicative random effects)
MR Egger
MR RAPS
Weighted median
Weighted mode
0.10
0.05
0.00
−0.05
−0.10
0.1
0.2
SNP effect on Kallikrein−15
SNP effect on Gut microbiota abundance (genus Parasutterella id.2892)

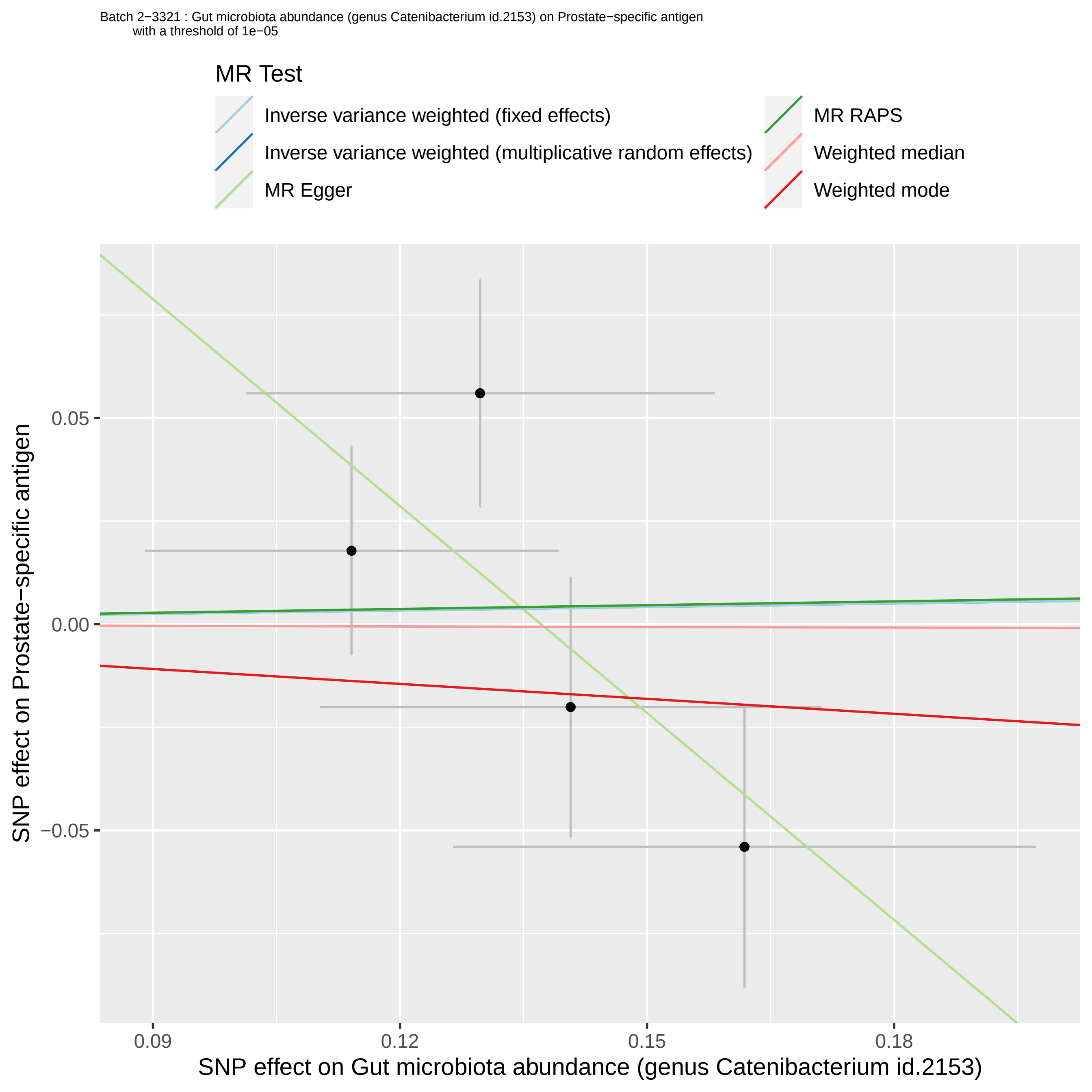

Batch 2−3321 : Gut microbiota abundance (genus Catenibacterium id.2153) on Prostate−specific antigen
with a threshold of 1e−05
MR Test
Inverse variance weighted (fixed effects)
Inverse variance weighted (multiplicative random effects)
MR Egger
MR RAPS
Weighted median
Weighted mode
0.05
0.00
−0.05
0.09
0.12
0.15
0.18
SNP effect on Prostate−specific antigen
SNP effect on Gut microbiota abundance (genus Catenibacterium id.2153)

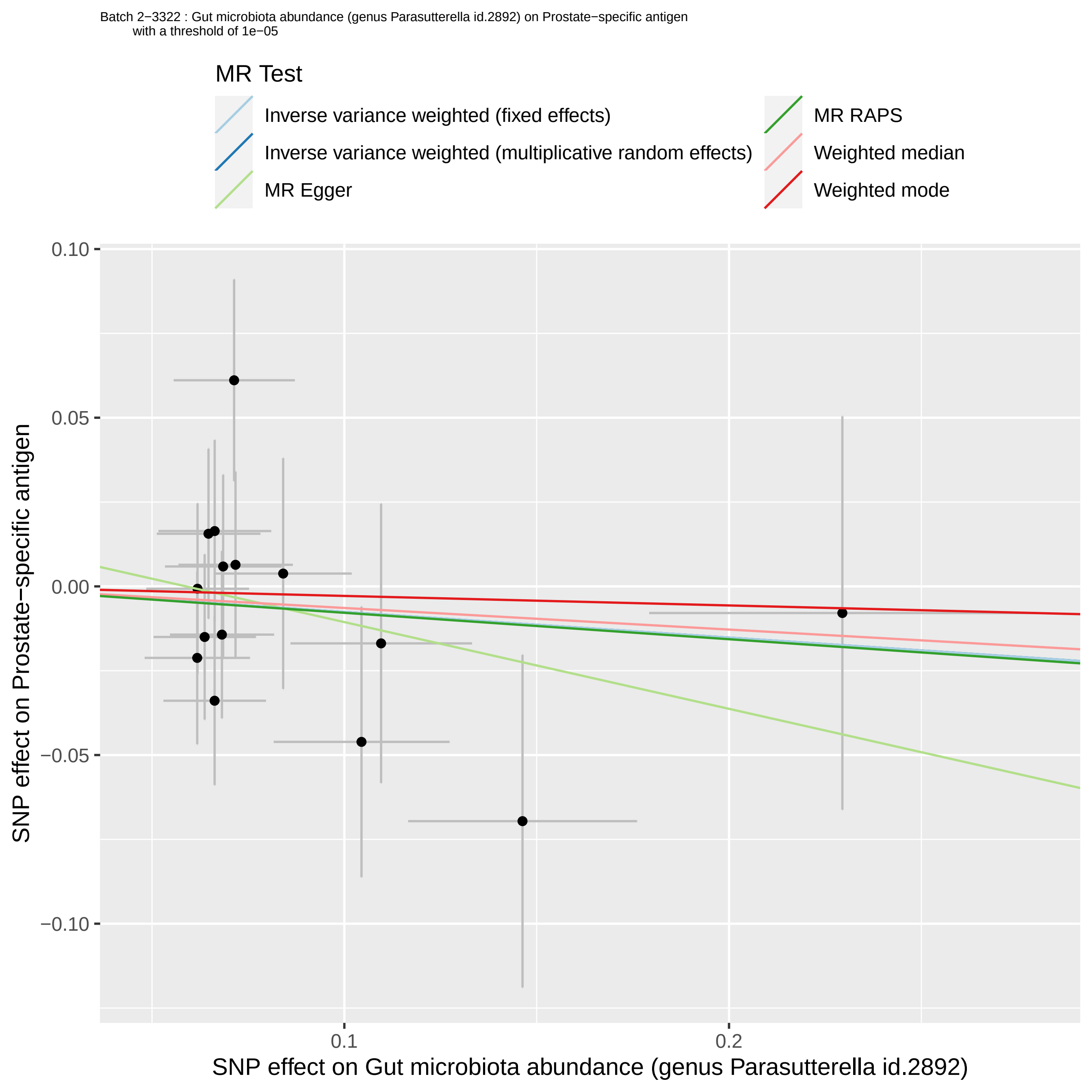

Batch 2−3322 : Gut microbiota abundance (genus Parasutterella id.2892) on Prostate−specific antigen
with a threshold of 1e−05
MR Test
Inverse variance weighted (fixed effects)
Inverse variance weighted (multiplicative random effects)
MR Egger
MR RAPS
Weighted median
Weighted mode
0.10
0.05
0.00
−0.05
−0.10
0.1
0.2
SNP effect on Prostate−specific antigen
SNP effect on Gut microbiota abundance (genus Parasutterella id.2892)

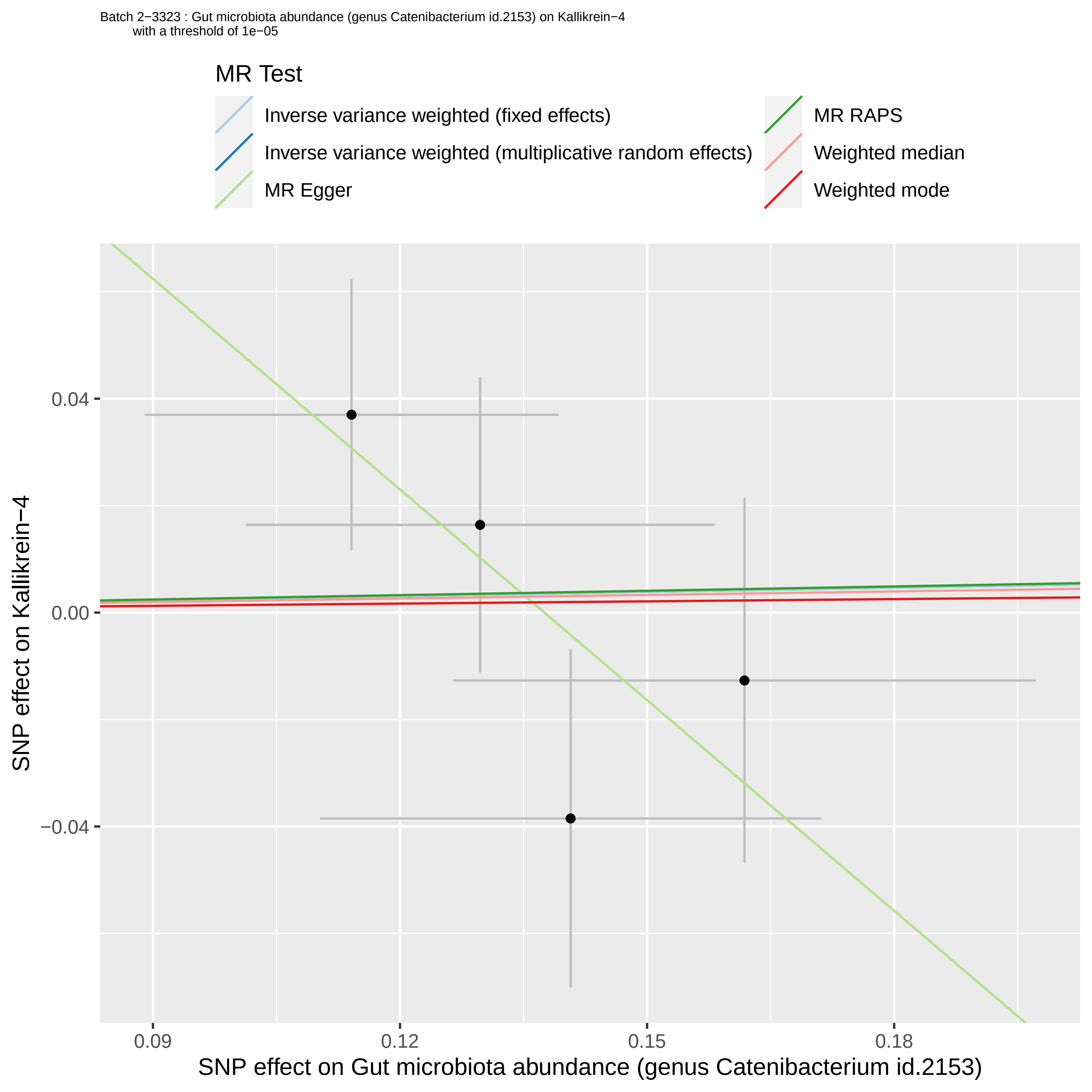
Batch 2−3323 : Gut microbiota abundance (genus Catenibacterium id.2153) on Kallikrein−4
with a threshold of 1e−05
MR Test
Inverse variance weighted (fixed effects)
Inverse variance weighted (multiplicative random effects)
MR Egger
MR RAPS
Weighted median
Weighted mode
0.04
0.00
−0.04
0.09
0.12
0.15
0.18
SNP effect on Kallikrein−4
SNP effect on Gut microbiota abundance (genus Catenibacterium id.2153)

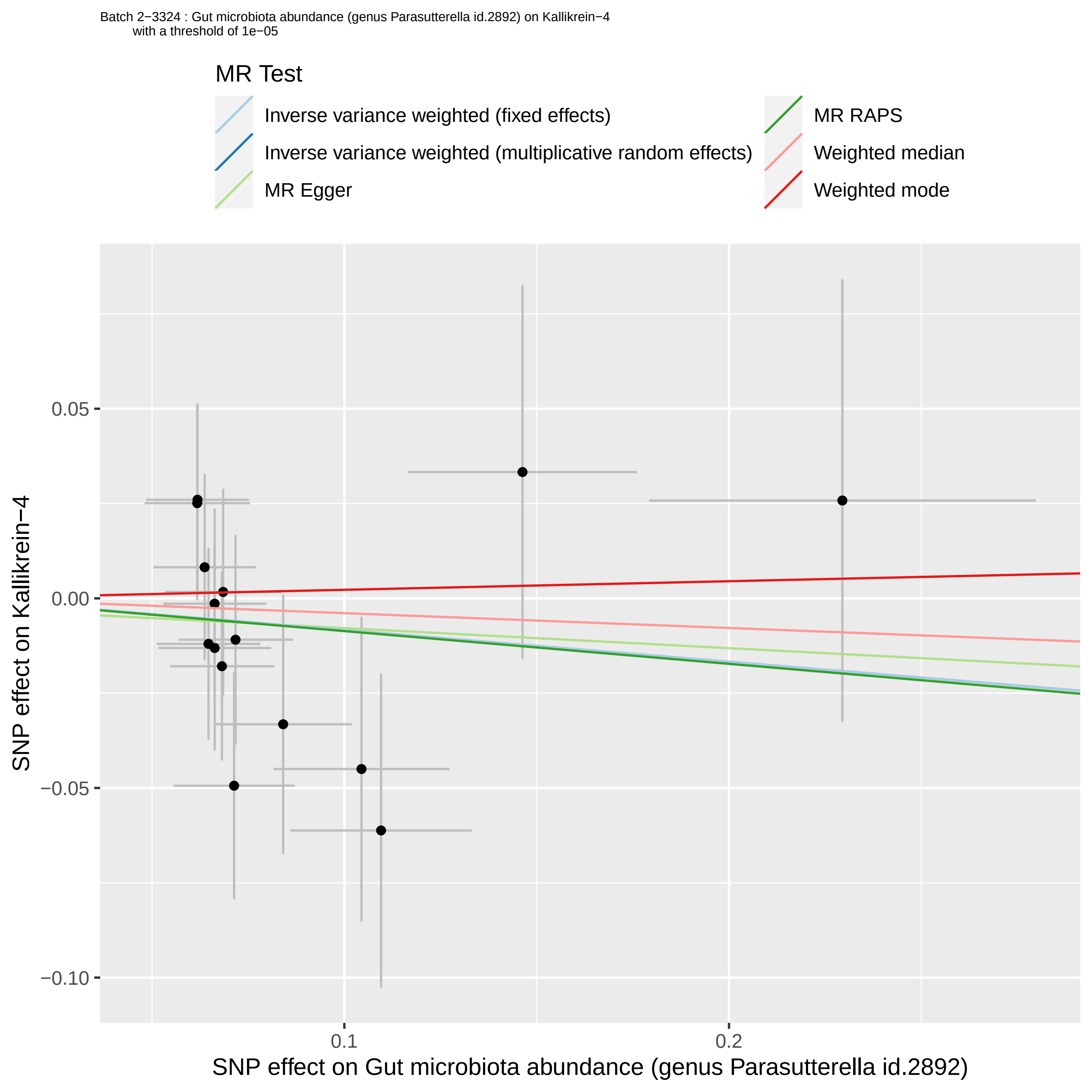
Batch 2−3324 : Gut microbiota abundance (genus Parasutterella id.2892) on Kallikrein−4
with a threshold of 1e−05
MR Test
Inverse variance weighted (fixed effects)
Inverse variance weighted (multiplicative random effects)
MR Egger
MR RAPS
Weighted median
Weighted mode
SNP effect on Kallikrein−4
0.05
0.00
−0.05
−0.10
0.1
0.2
SNP effect on Gut microbiota abundance (genus Parasutterella id.2892)

Batch 2−3325 : Gut microbiota abundance (genus Catenibacterium id.2153) on Kallikrein−5
with a threshold of 1e−05

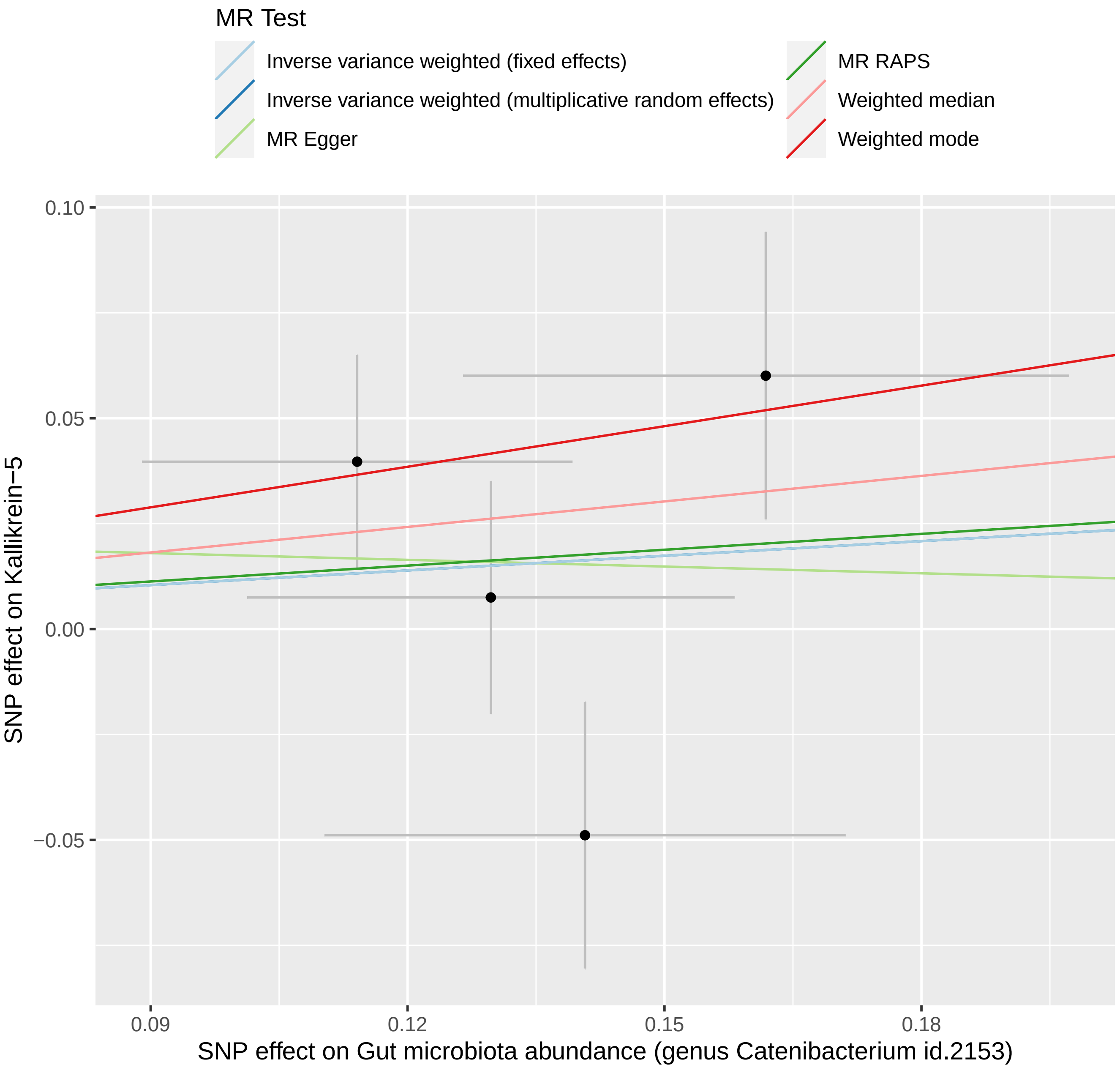

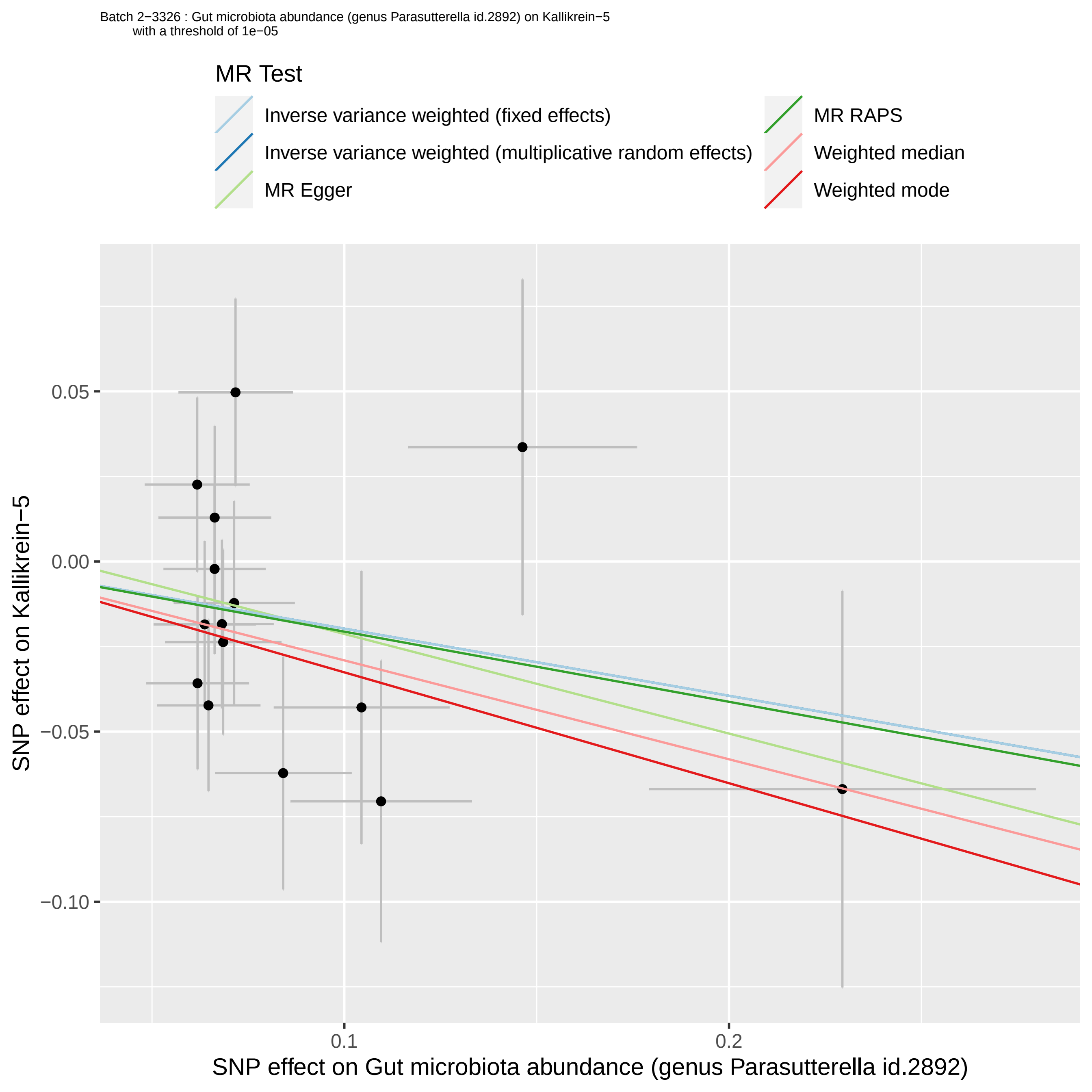

Batch 2−3326 : Gut microbiota abundance (genus Parasutterella id.2892) on Kallikrein−5
with a threshold of 1e−05
MR Test
Inverse variance weighted (fixed effects)
Inverse variance weighted (multiplicative random effects)
MR Egger
MR RAPS
Weighted median
Weighted mode
SNP effect on Kallikrein−5
0.05
0.00
−0.05
−0.10
0.1
0.2
SNP effect on Gut microbiota abundance (genus Parasutterella id.2892)

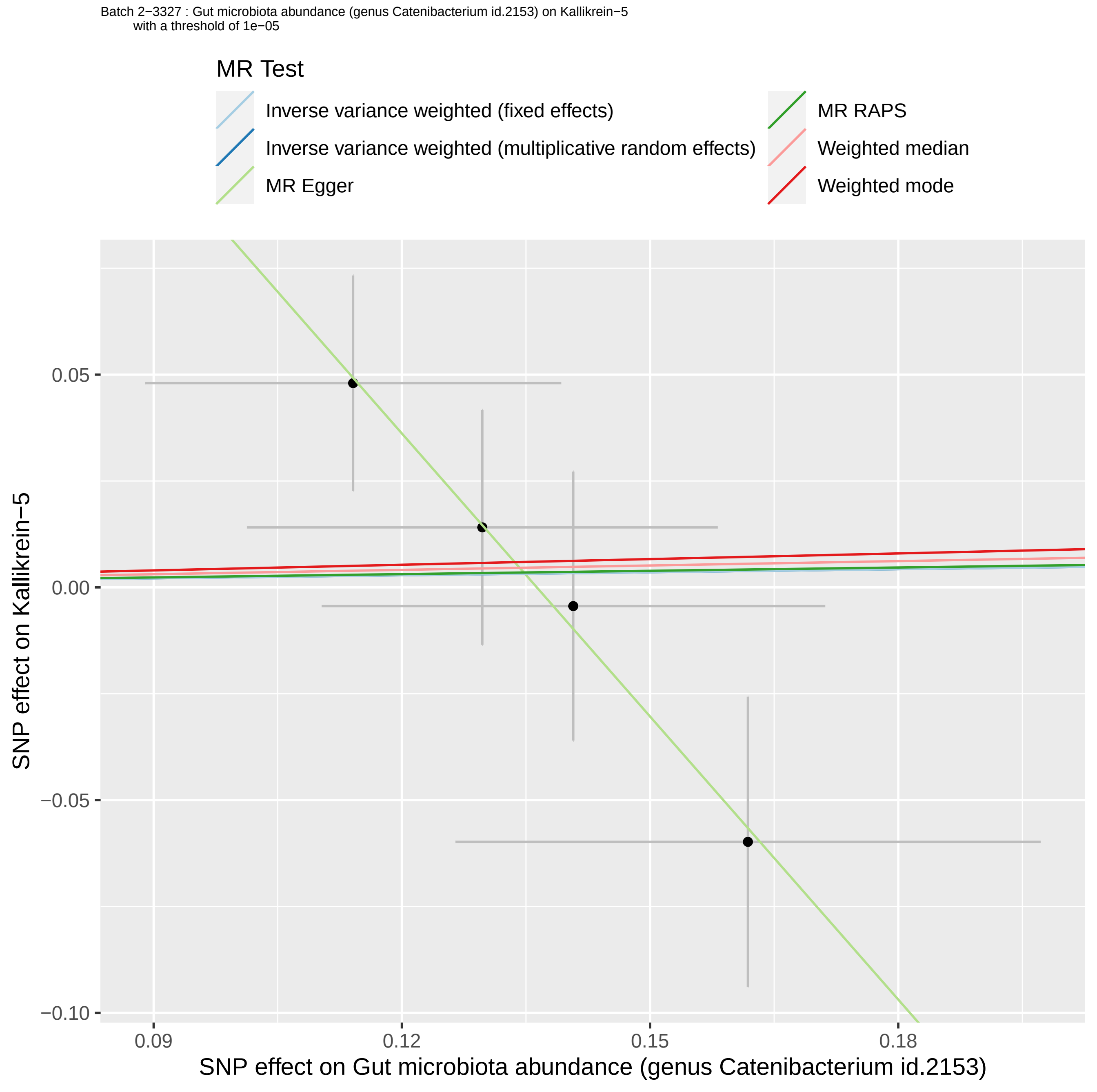
Batch 2−3327 : Gut microbiota abundance (genus Catenibacterium id.2153) on Kallikrein−5
with a threshold of 1e−05
MR Test
Inverse variance weighted (fixed effects)
Inverse variance weighted (multiplicative random effects)
MR Egger
MR RAPS
Weighted median
Weighted mode
0.05
0.00
−0.05
−0.10
0.09
0.12
0.15
0.18
SNP effect on Kallikrein−5
SNP effect on Gut microbiota abundance (genus Catenibacterium id.2153)

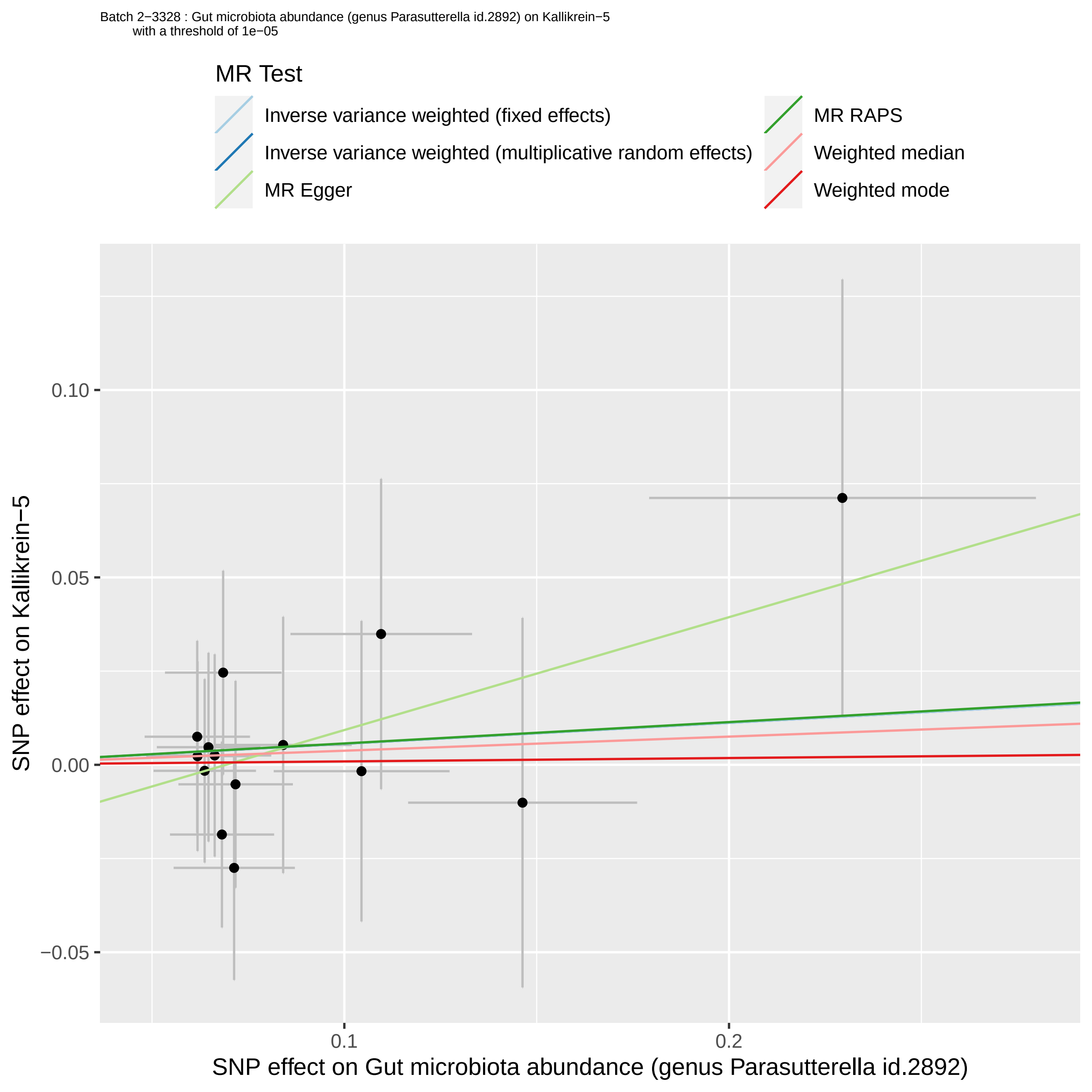
Batch 2−3328 : Gut microbiota abundance (genus Parasutterella id.2892) on Kallikrein−5
with a threshold of 1e−05
MR Test
Inverse variance weighted (fixed effects)
Inverse variance weighted (multiplicative random effects)
MR Egger
MR RAPS
Weighted median
Weighted mode
0.10
0.05
0.00
−0.05
0.1
0.2
SNP effect on Kallikrein−5
SNP effect on Gut microbiota abundance (genus Parasutterella id.2892)

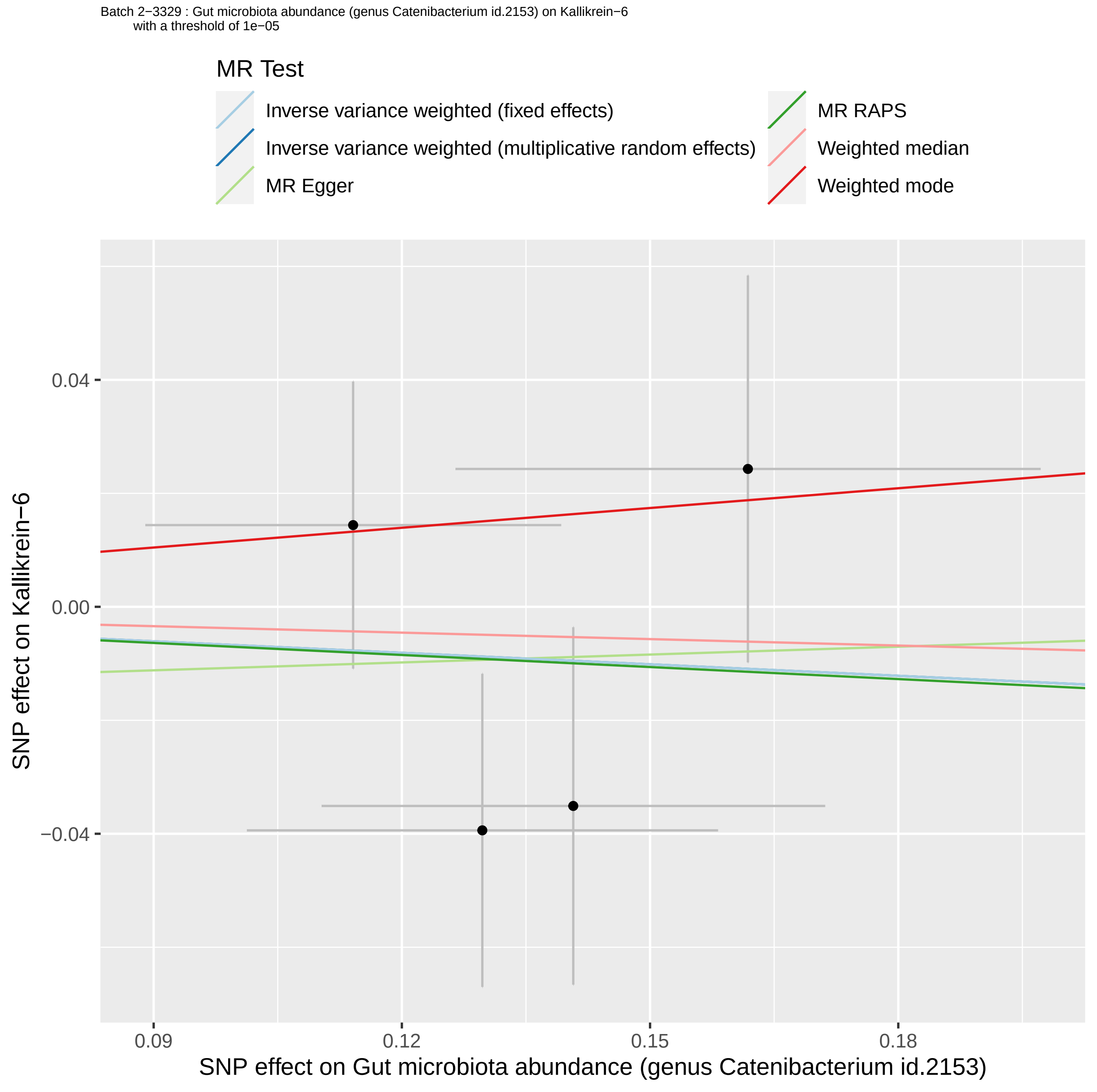

Batch 2−3329 : Gut microbiota abundance (genus Catenibacterium id.2153) on Kallikrein−6
with a threshold of 1e−05
MR Test
Inverse variance weighted (fixed effects)
Inverse variance weighted (multiplicative random effects)
MR Egger
MR RAPS
Weighted median
Weighted mode
0.04
0.00
−0.04
0.09
0.12
0.15
0.18
SNP effect on Kallikrein−6
SNP effect on Gut microbiota abundance (genus Catenibacterium id.2153)

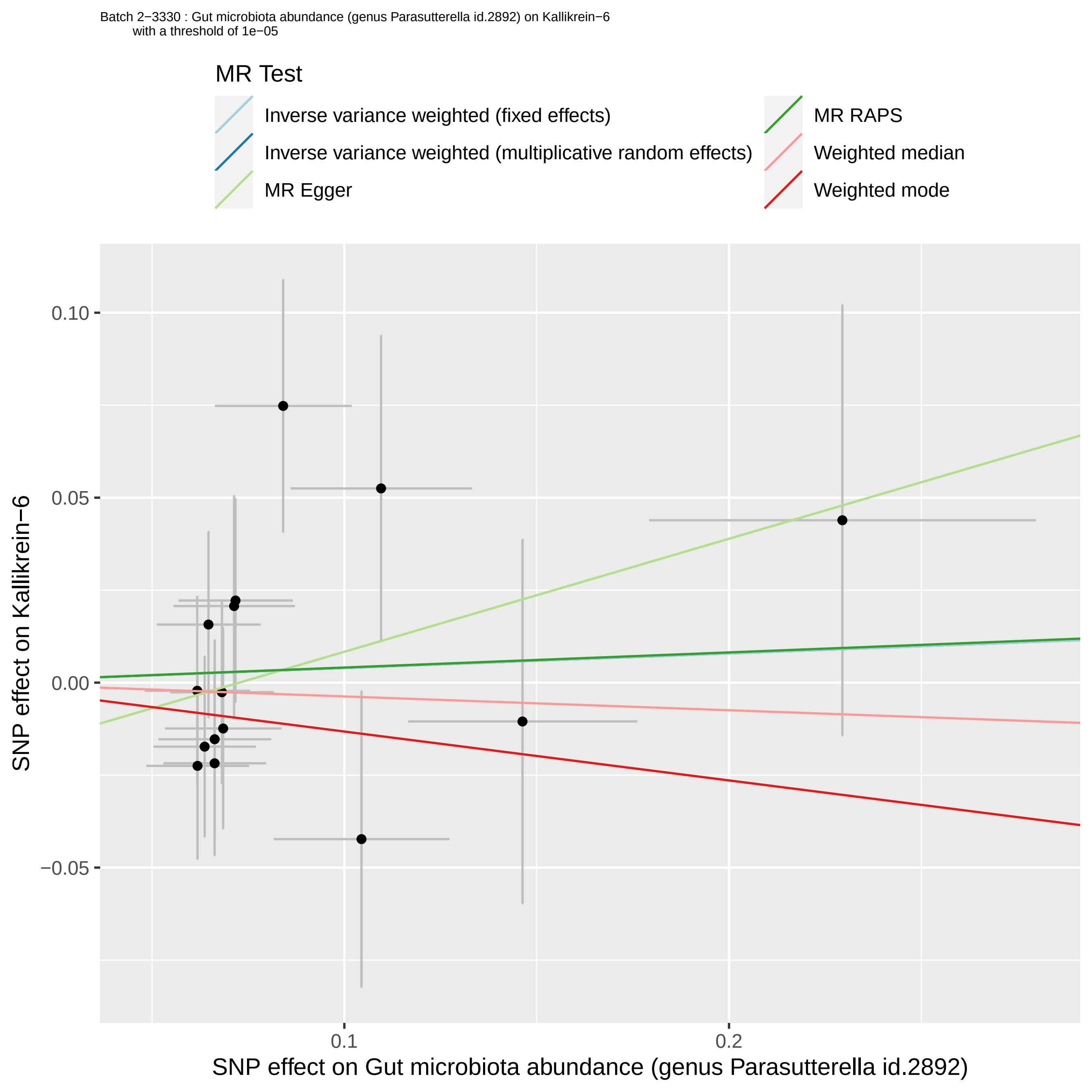

Batch 2−3330 : Gut microbiota abundance (genus Parasutterella id.2892) on Kallikrein−6
with a threshold of 1e−05
MR Test
Inverse variance weighted (fixed effects)
Inverse variance weighted (multiplicative random effects)
MR Egger
MR RAPS
Weighted median
Weighted mode
0.10
0.05
0.00
−0.05
0.1
0.2
SNP effect on Kallikrein−6
SNP effect on Gut microbiota abundance (genus Parasutterella id.2892)

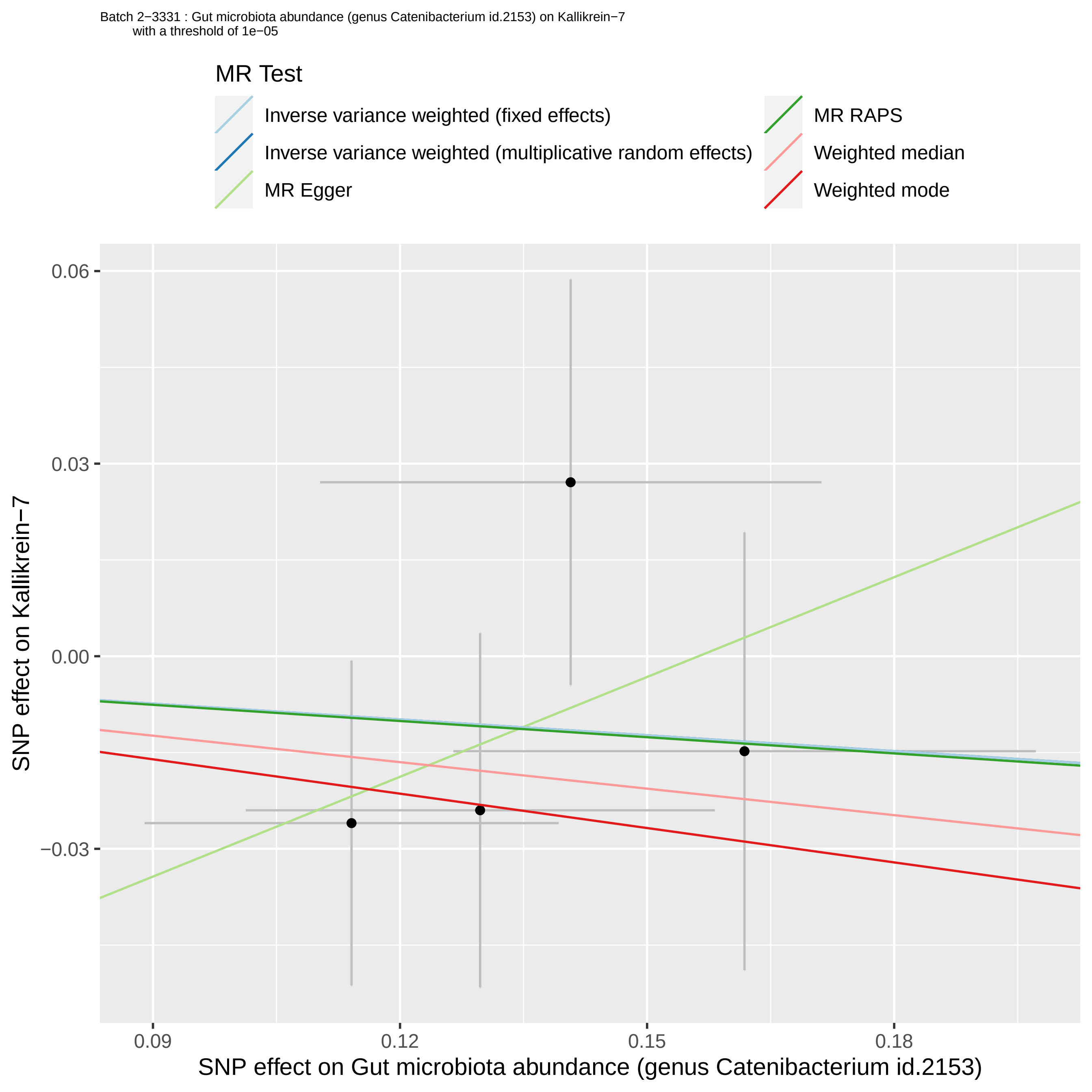
Batch 2−3331 : Gut microbiota abundance (genus Catenibacterium id.2153) on Kallikrein−7
with a threshold of 1e−05
MR Test
Inverse variance weighted (fixed effects)
Inverse variance weighted (multiplicative random effects)
MR Egger
MR RAPS
Weighted median
Weighted mode
0.06
0.03
0.00
−0.03
0.09
0.12
0.15
0.18
SNP effect on Kallikrein−7
SNP effect on Gut microbiota abundance (genus Catenibacterium id.2153)

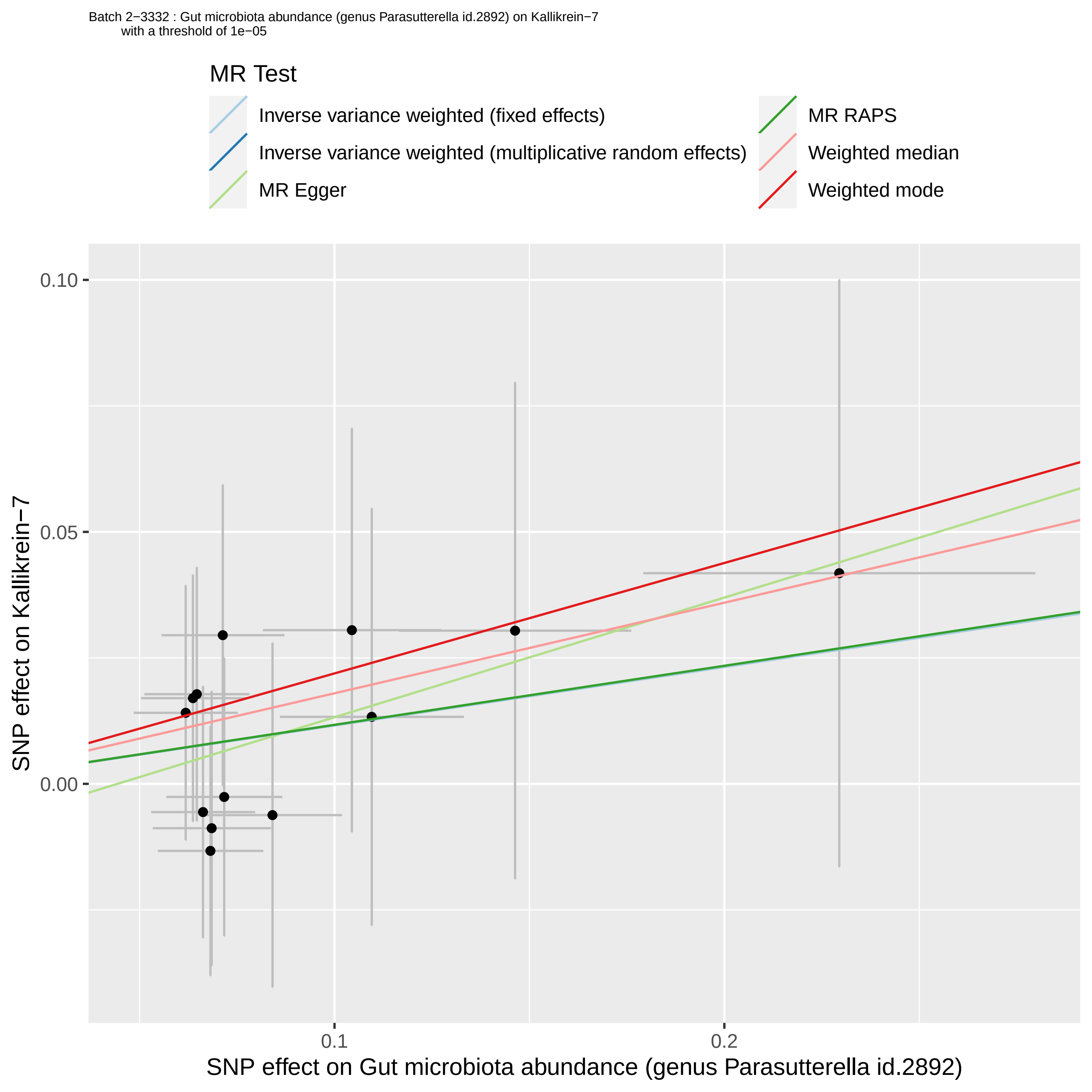

Batch 2−3332 : Gut microbiota abundance (genus Parasutterella id.2892) on Kallikrein−7
with a threshold of 1e−05
MR Test
Inverse variance weighted (fixed effects)
Inverse variance weighted (multiplicative random effects)
MR Egger
MR RAPS
Weighted median
Weighted mode
0.10
0.05
0.00
0.1
0.2
SNP effect on Kallikrein−7
SNP effect on Gut microbiota abundance (genus Parasutterella id.2892)

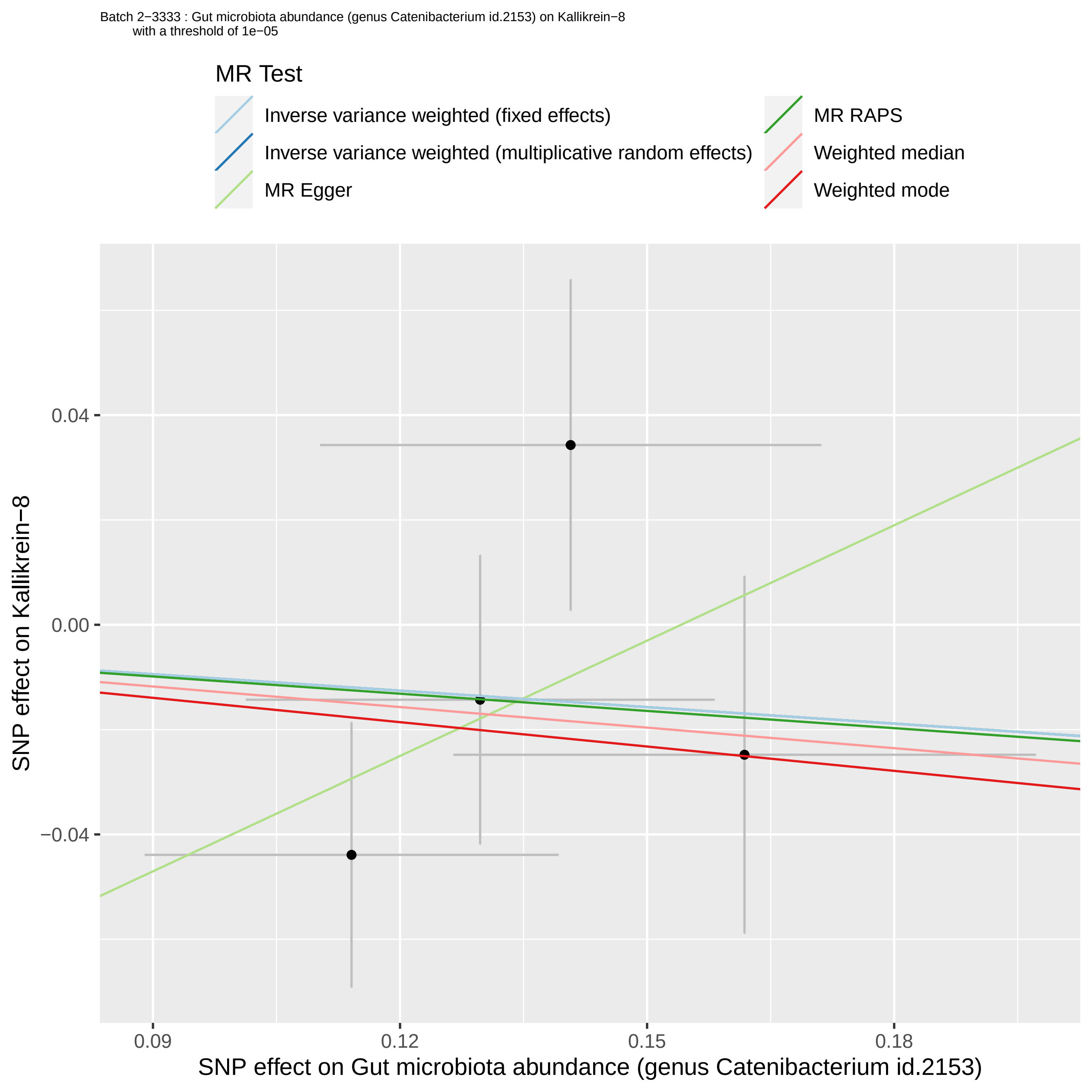
Batch 2−3333 : Gut microbiota abundance (genus Catenibacterium id.2153) on Kallikrein−8
with a threshold of 1e−05
MR Test
Inverse variance weighted (fixed effects)
Inverse variance weighted (multiplicative random effects)
MR Egger
MR RAPS
Weighted median
Weighted mode
0.04
0.00
−0.04
0.09
0.12
0.15
0.18
SNP effect on Kallikrein−8
SNP effect on Gut microbiota abundance (genus Catenibacterium id.2153)

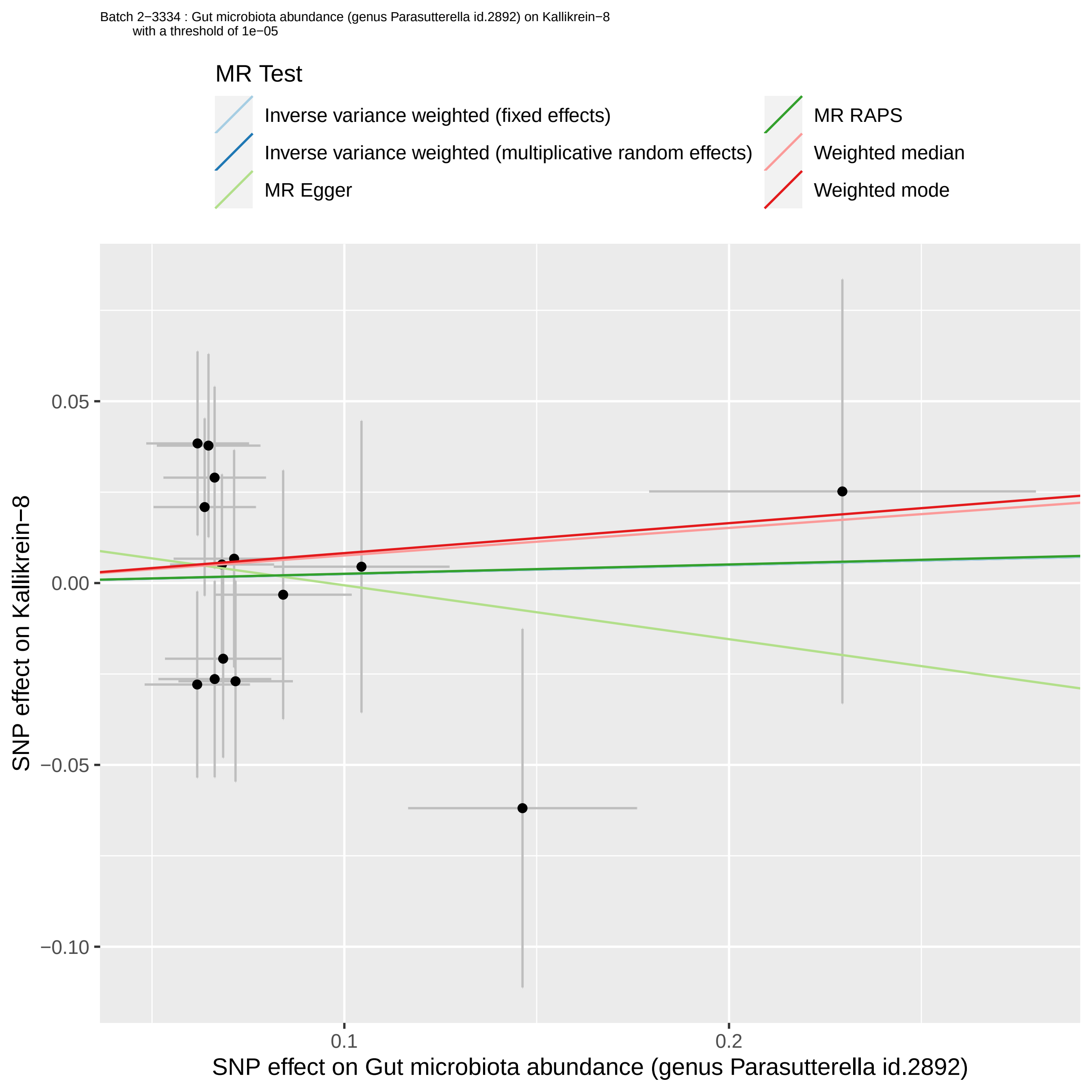
Batch 2−3334 : Gut microbiota abundance (genus Parasutterella id.2892) on Kallikrein−8
with a threshold of 1e−05
MR Test
Inverse variance weighted (fixed effects)
Inverse variance weighted (multiplicative random effects)
MR Egger
MR RAPS
Weighted median
Weighted mode
0.05
0.00
−0.05
−0.10
0.1
0.2
SNP effect on Kallikrein−8
SNP effect on Gut microbiota abundance (genus Parasutterella id.2892)

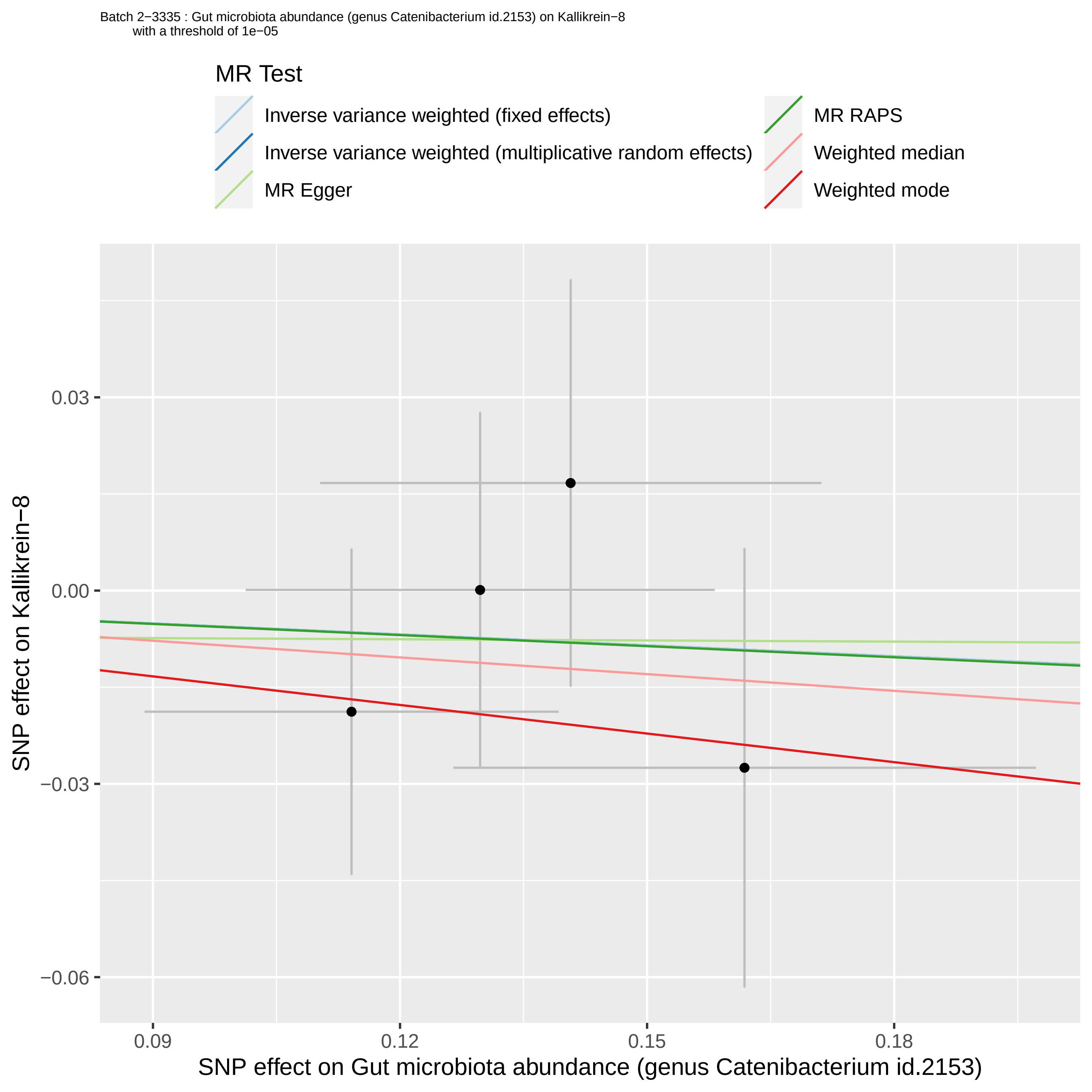

Batch 2−3335 : Gut microbiota abundance (genus Catenibacterium id.2153) on Kallikrein−8
with a threshold of 1e−05
MR Test
Inverse variance weighted (fixed effects)
Inverse variance weighted (multiplicative random effects)
MR Egger
MR RAPS
Weighted median
Weighted mode
0.03
0.00
−0.03
−0.06
0.09
0.12
0.15
0.18
SNP effect on Kallikrein−8
SNP effect on Gut microbiota abundance (genus Catenibacterium id.2153)

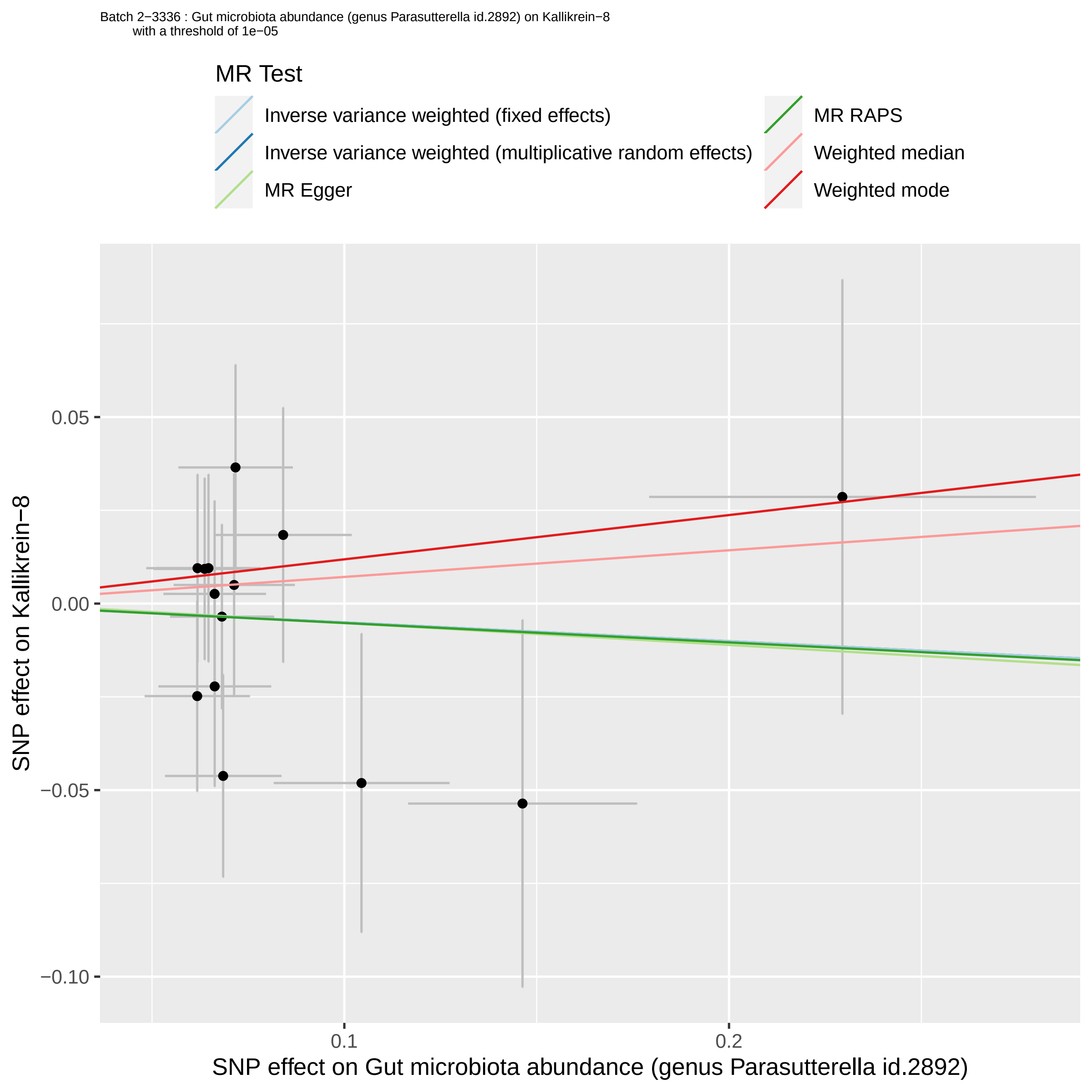

Batch 2−3336 : Gut microbiota abundance (genus Parasutterella id.2892) on Kallikrein−8
with a threshold of 1e−05
MR Test
Inverse variance weighted (fixed effects)
Inverse variance weighted (multiplicative random effects)
MR Egger
MR RAPS
Weighted median
Weighted mode
0.05
0.00
−0.05
−0.10
0.1
0.2
SNP effect on Kallikrein−8
SNP effect on Gut microbiota abundance (genus Parasutterella id.2892)

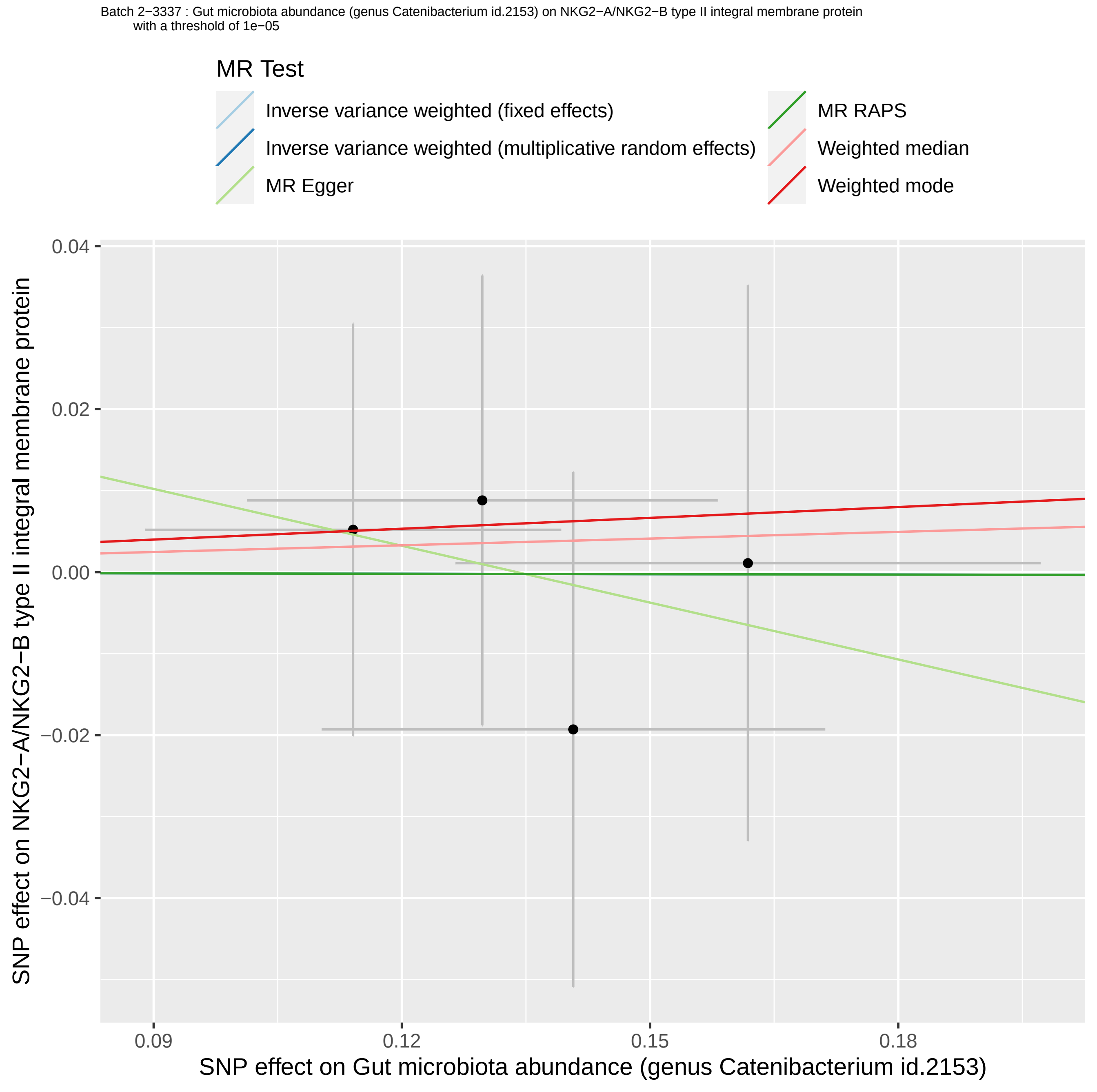
Batch 2−3337 : Gut microbiota abundance (genus Catenibacterium id.2153) on NKG2−A/NKG2−B type II integral membrane protein
with a threshold of 1e−05
MR Test
Inverse variance weighted (fixed effects)
Inverse variance weighted (multiplicative random effects)
MR Egger
MR RAPS
Weighted median
Weighted mode
0.04
0.02
0.00
−0.02
−0.04
0.09
0.12
0.15
0.18
SNP effect on NKG2−A/NKG2−B type II integral membrane protein
SNP effect on Gut microbiota abundance (genus Catenibacterium id.2153)

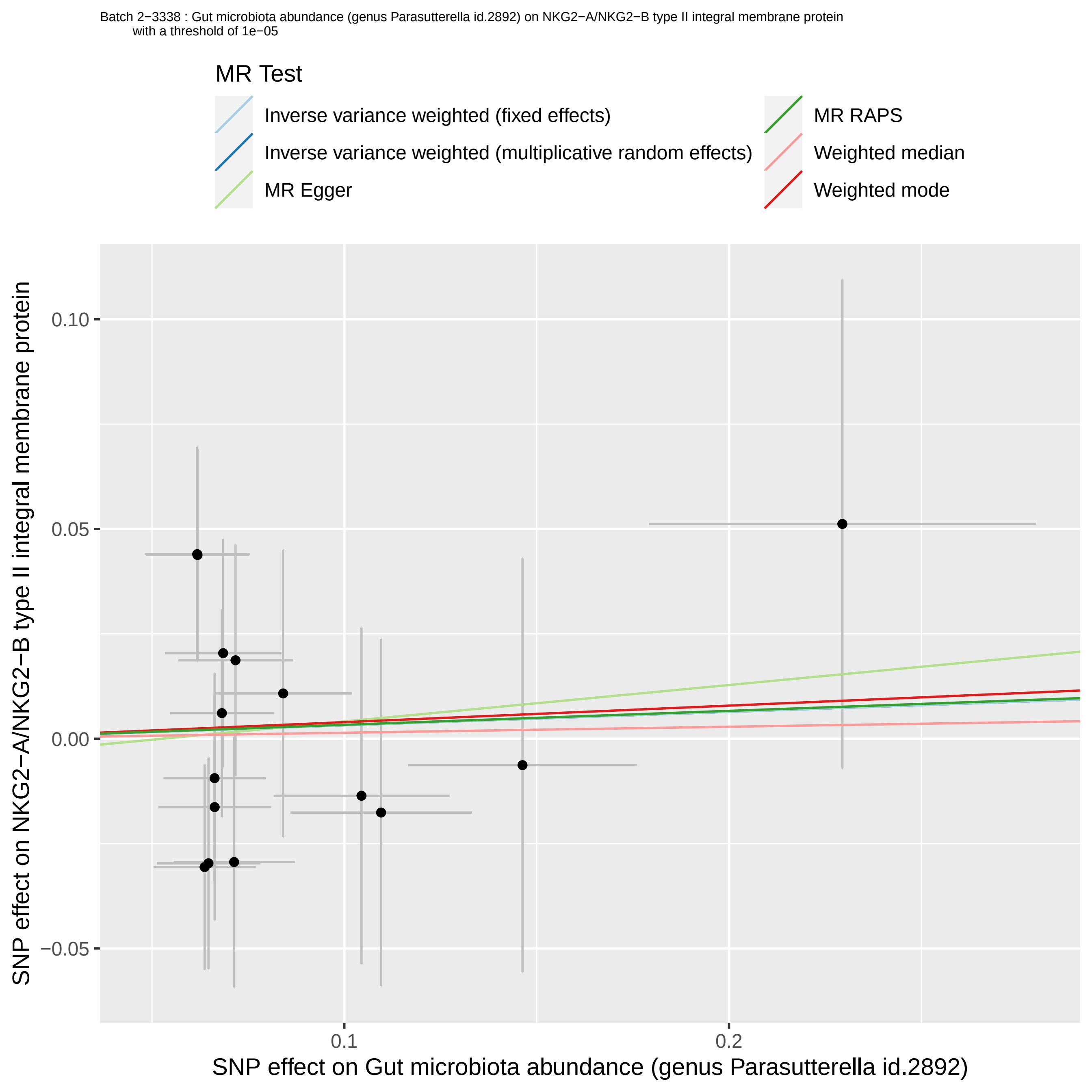

Batch 2−3338 : Gut microbiota abundance (genus Parasutterella id.2892) on NKG2−A/NKG2−B type II integral membrane protein
with a threshold of 1e−05
MR Test
Inverse variance weighted (fixed effects)
Inverse variance weighted (multiplicative random effects)
MR Egger
MR RAPS
Weighted median
Weighted mode
SNP effect on NKG2−A/NKG2−B type II integral membrane protein
0.10
0.05
0.00
−0.05
0.1
0.2
SNP effect on Gut microbiota abundance (genus Parasutterella id.2892)

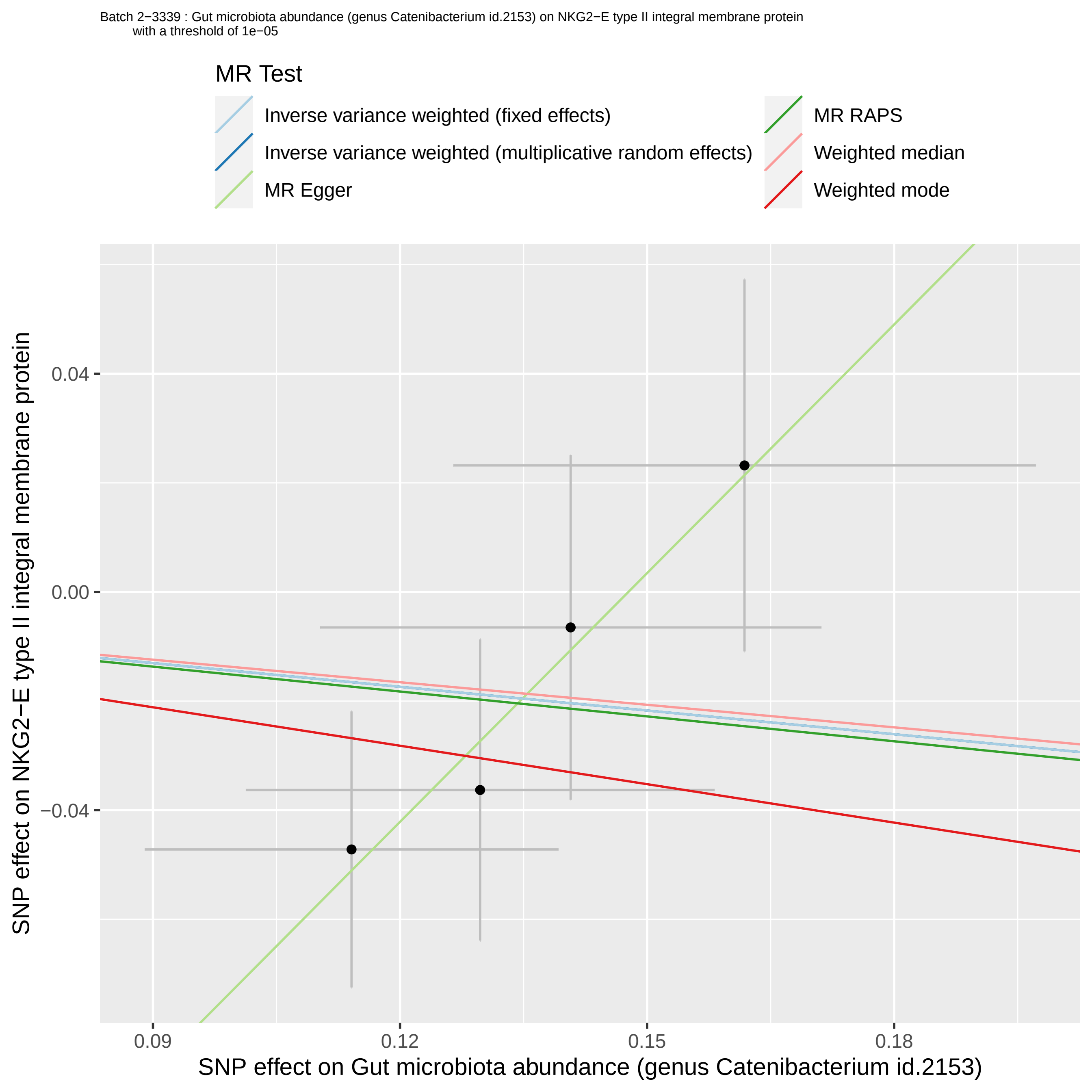

Batch 2−3339 : Gut microbiota abundance (genus Catenibacterium id.2153) on NKG2−E type II integral membrane protein
with a threshold of 1e−05
MR Test
Inverse variance weighted (fixed effects)
Inverse variance weighted (multiplicative random effects)
MR Egger
MR RAPS
Weighted median
Weighted mode
0.04
0.00
−0.04
0.09
0.12
0.15
0.18
SNP effect on NKG2−E type II integral membrane protein
SNP effect on Gut microbiota abundance (genus Catenibacterium id.2153)

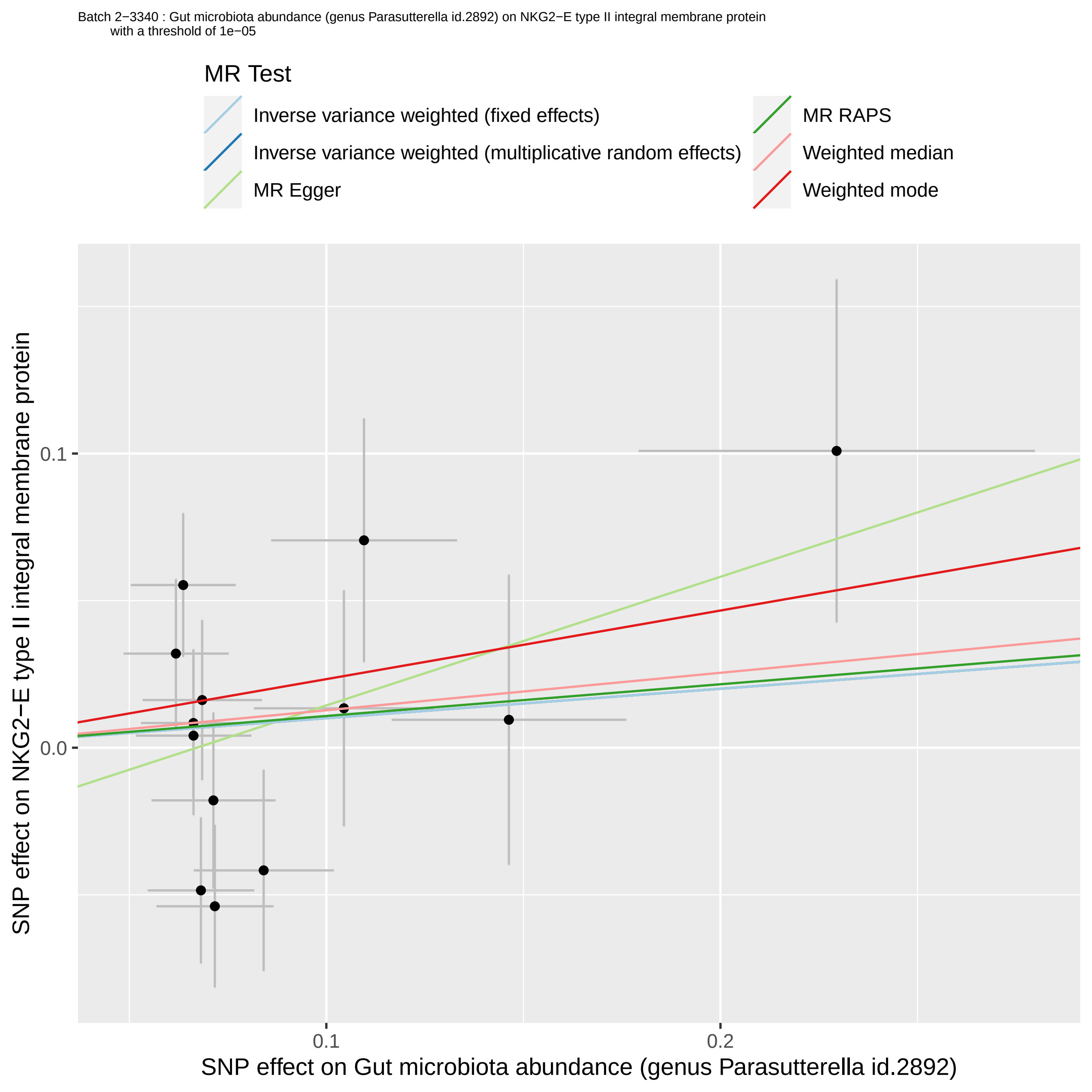

Batch 2−3340 : Gut microbiota abundance (genus Parasutterella id.2892) on NKG2−E type II integral membrane protein
with a threshold of 1e−05
MR Test
Inverse variance weighted (fixed effects)
Inverse variance weighted (multiplicative random effects)
MR Egger
MR RAPS
Weighted median
Weighted mode
0.1
0.0
0.1
0.2
SNP effect on NKG2−E type II integral membrane protein
SNP effect on Gut microbiota abundance (genus Parasutterella id.2892)

Batch 2−3341 : Gut microbiota abundance (genus Catenibacterium id.2153) on NKG2−E type II integral membrane protein
with a threshold of 1e−05

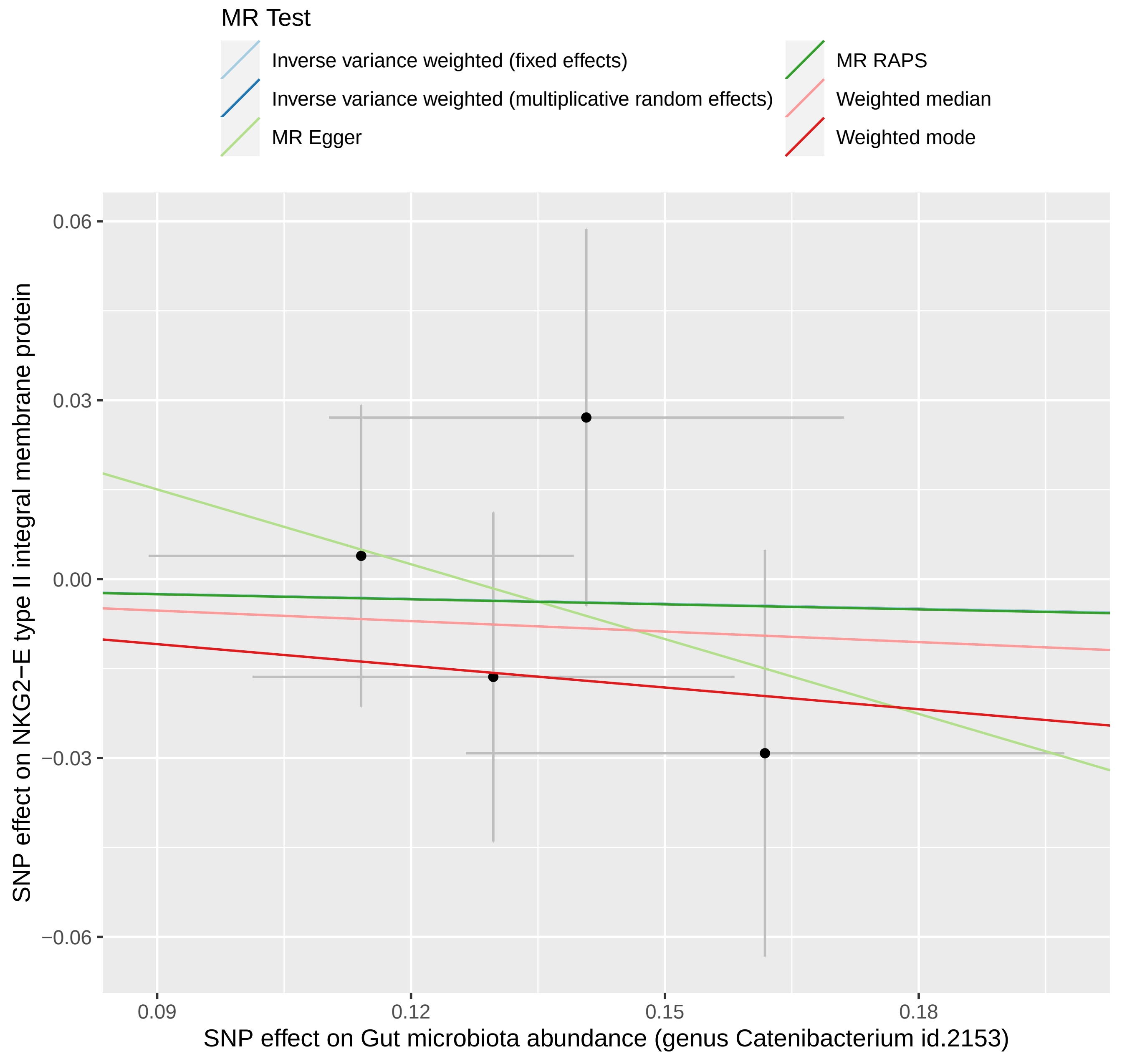

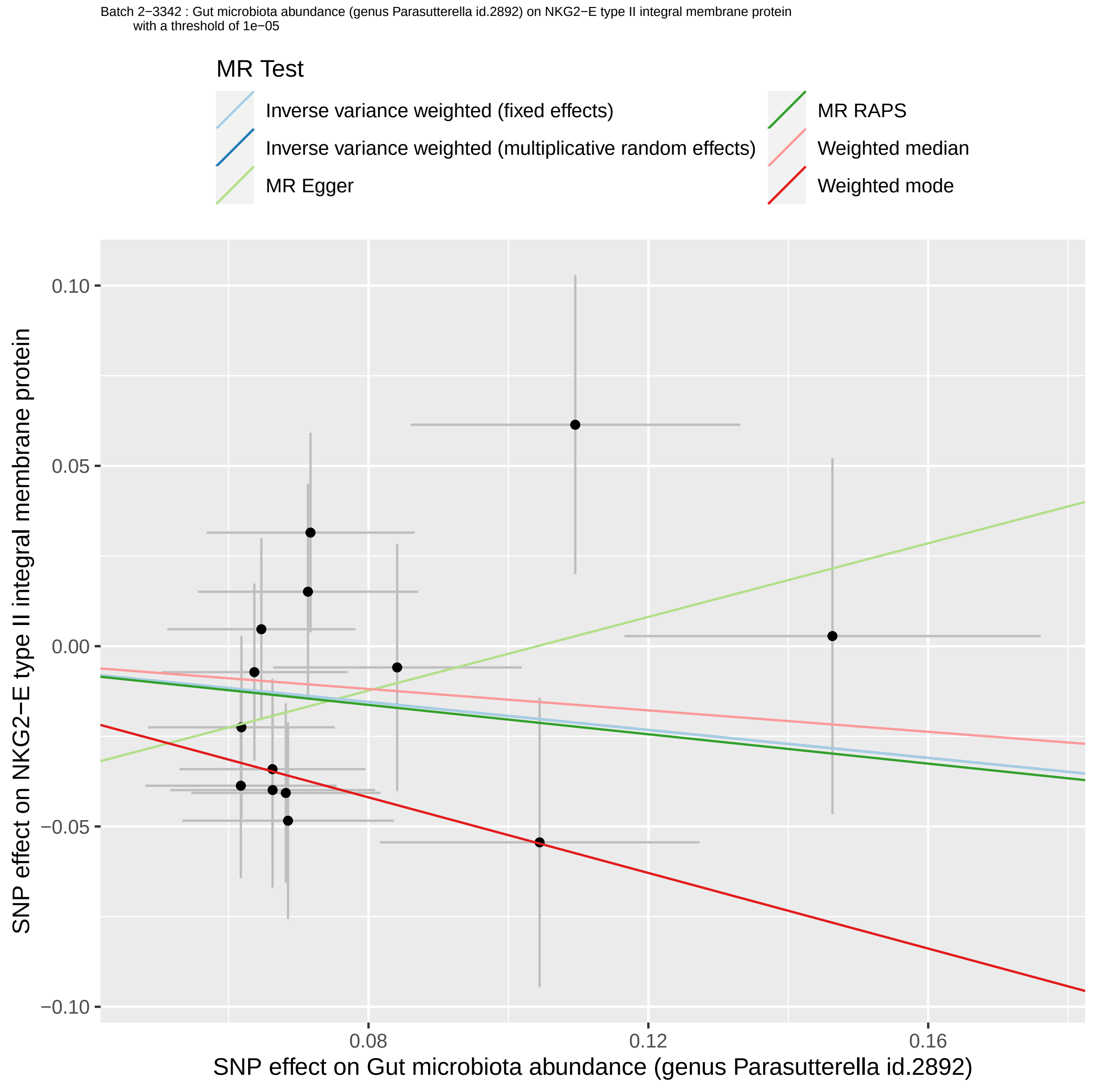

Batch 2−3342 : Gut microbiota abundance (genus Parasutterella id.2892) on NKG2−E type II integral membrane protein
with a threshold of 1e−05
MR Test
Inverse variance weighted (fixed effects)
Inverse variance weighted (multiplicative random effects)
MR Egger
MR RAPS
Weighted median
Weighted mode
0.10
0.05
0.00
−0.05
−0.10
0.08
0.12
0.16
SNP effect on NKG2−E type II integral membrane protein
SNP effect on Gut microbiota abundance (genus Parasutterella id.2892)

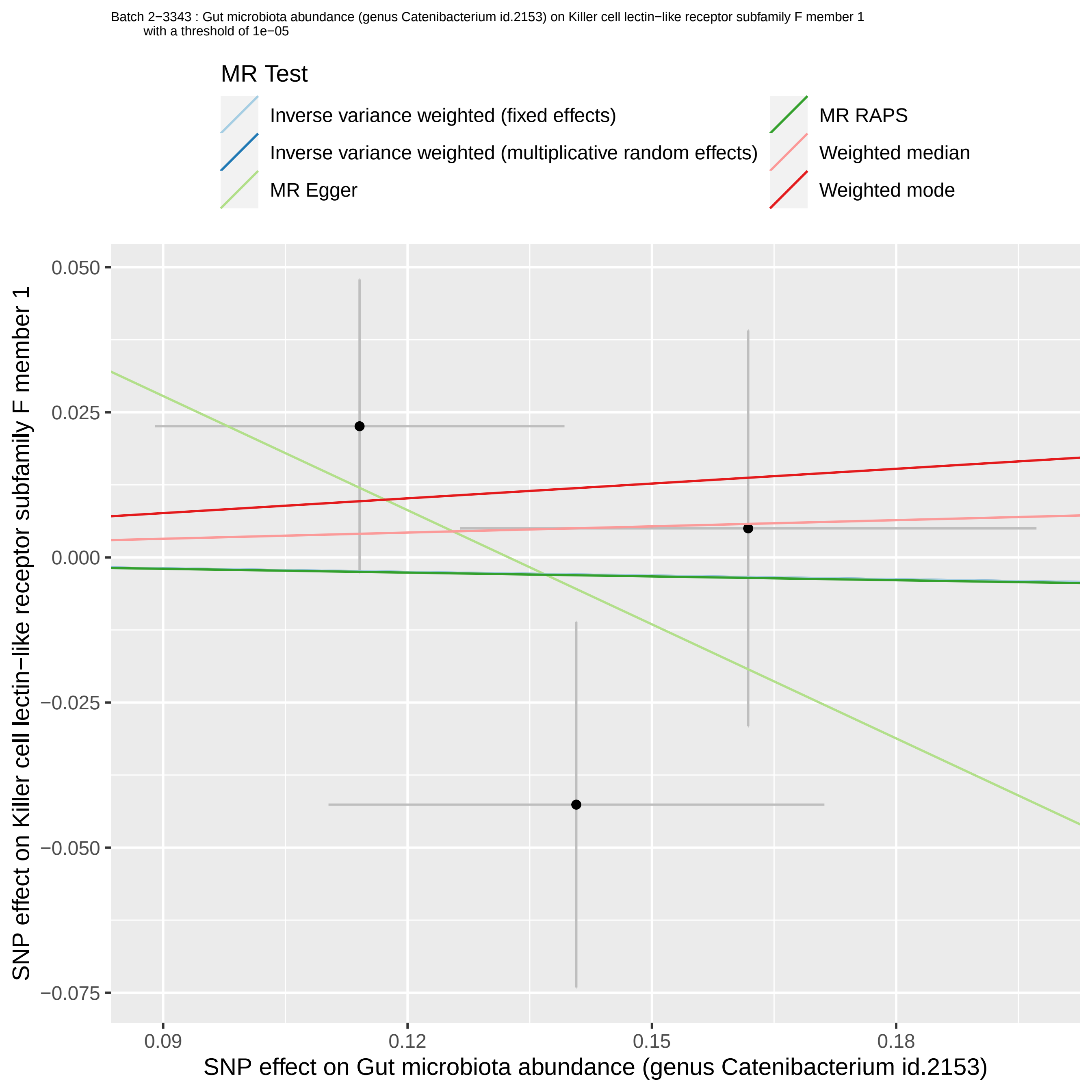

Batch 2−3343 : Gut microbiota abundance (genus Catenibacterium id.2153) on Killer cell lectin−like receptor subfamily F member 1
with a threshold of 1e−05
MR Test
Inverse variance weighted (fixed effects)
Inverse variance weighted (multiplicative random effects)
MR Egger
MR RAPS
Weighted median
Weighted mode
0.050
0.025
0.000
−0.025
−0.050
−0.075
0.09
0.12
0.15
0.18
SNP effect on Killer cell lectin−like receptor subfamily F member 1
SNP effect on Gut microbiota abundance (genus Catenibacterium id.2153)

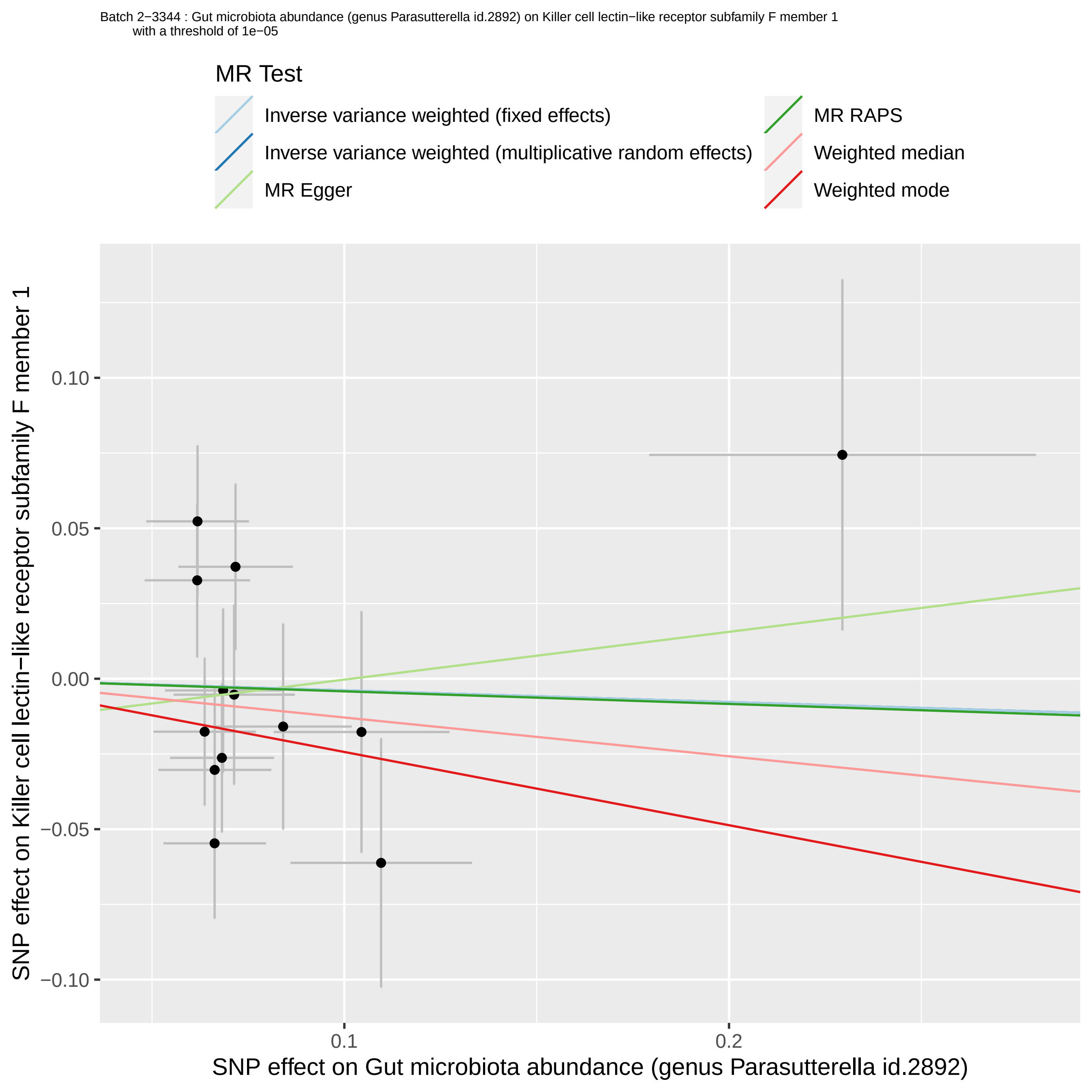
Batch 2−3344 : Gut microbiota abundance (genus Parasutterella id.2892) on Killer cell lectin−like receptor subfamily F member 1
with a threshold of 1e−05
MR Test
Inverse variance weighted (fixed effects)
Inverse variance weighted (multiplicative random effects)
MR Egger
MR RAPS
Weighted median
Weighted mode
0.10
0.05
0.00
−0.05
−0.10
0.1
0.2
SNP effect on Killer cell lectin−like receptor subfamily F member 1
SNP effect on Gut microbiota abundance (genus Parasutterella id.2892)

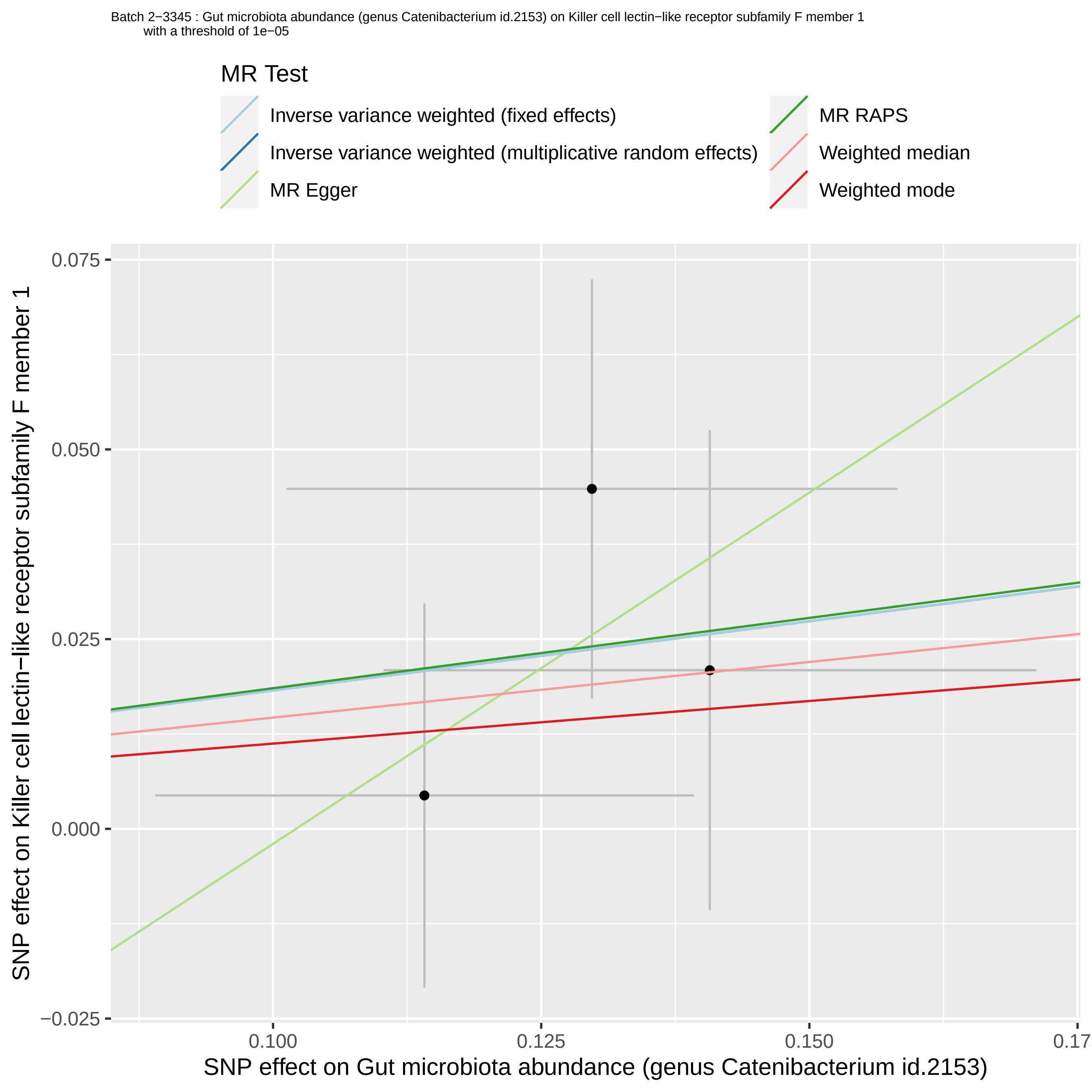
Batch 2−3345 : Gut microbiota abundance (genus Catenibacterium id.2153) on Killer cell lectin−like receptor subfamily F member 1
with a threshold of 1e−05
MR Test
Inverse variance weighted (fixed effects)
Inverse variance weighted (multiplicative random effects)
MR Egger
MR RAPS
Weighted median
Weighted mode
0.075
0.050
0.025
0.000
−0.025
0.100
0.125
0.150
0.17
SNP effect on Killer cell lectin−like receptor subfamily F member 1
SNP effect on Gut microbiota abundance (genus Catenibacterium id.2153)

Batch 2−3346 : Gut microbiota abundance (genus Parasutterella id.2892) on Killer cell lectin−like receptor subfamily F member 1
with a threshold of 1e−05

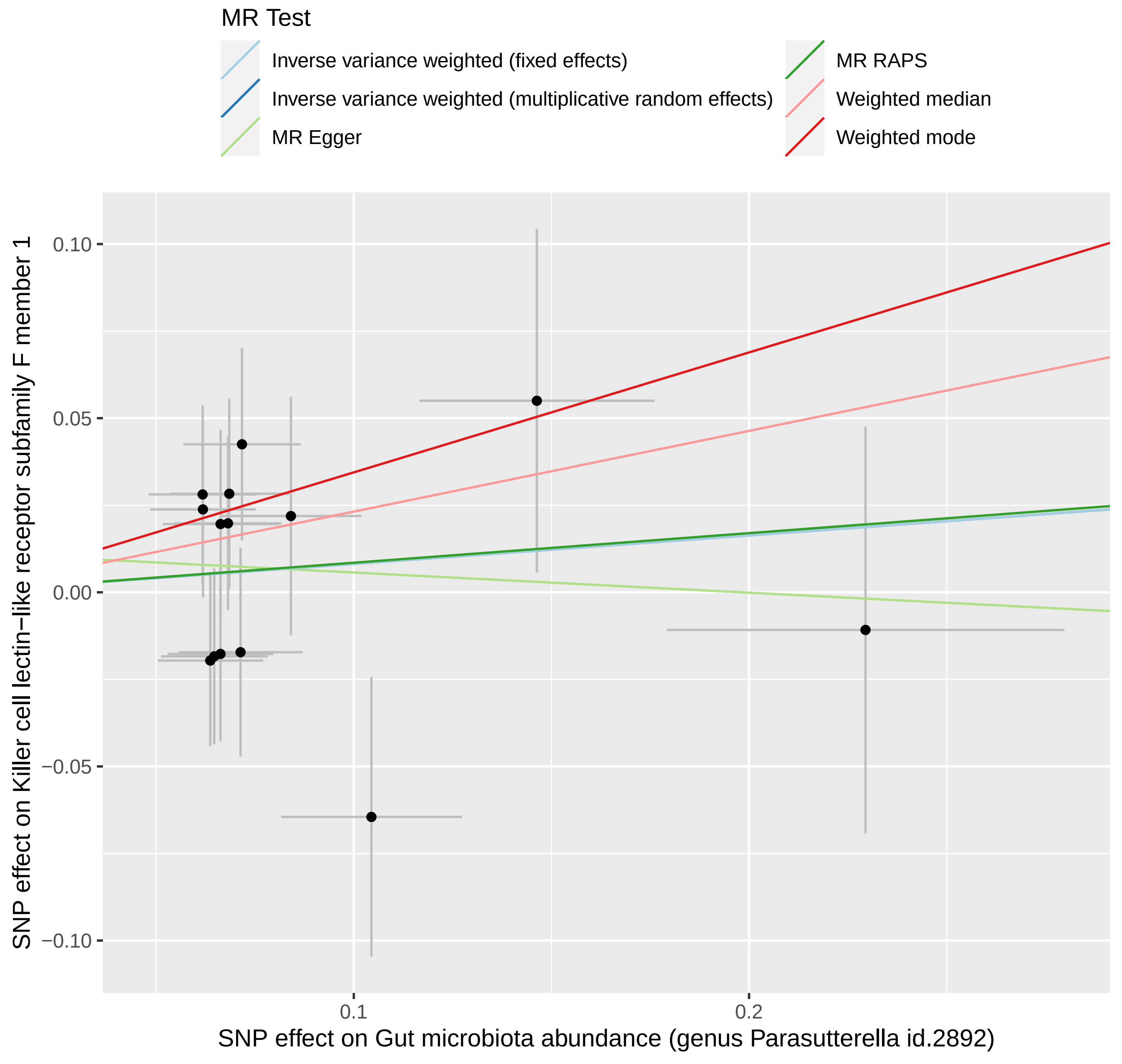

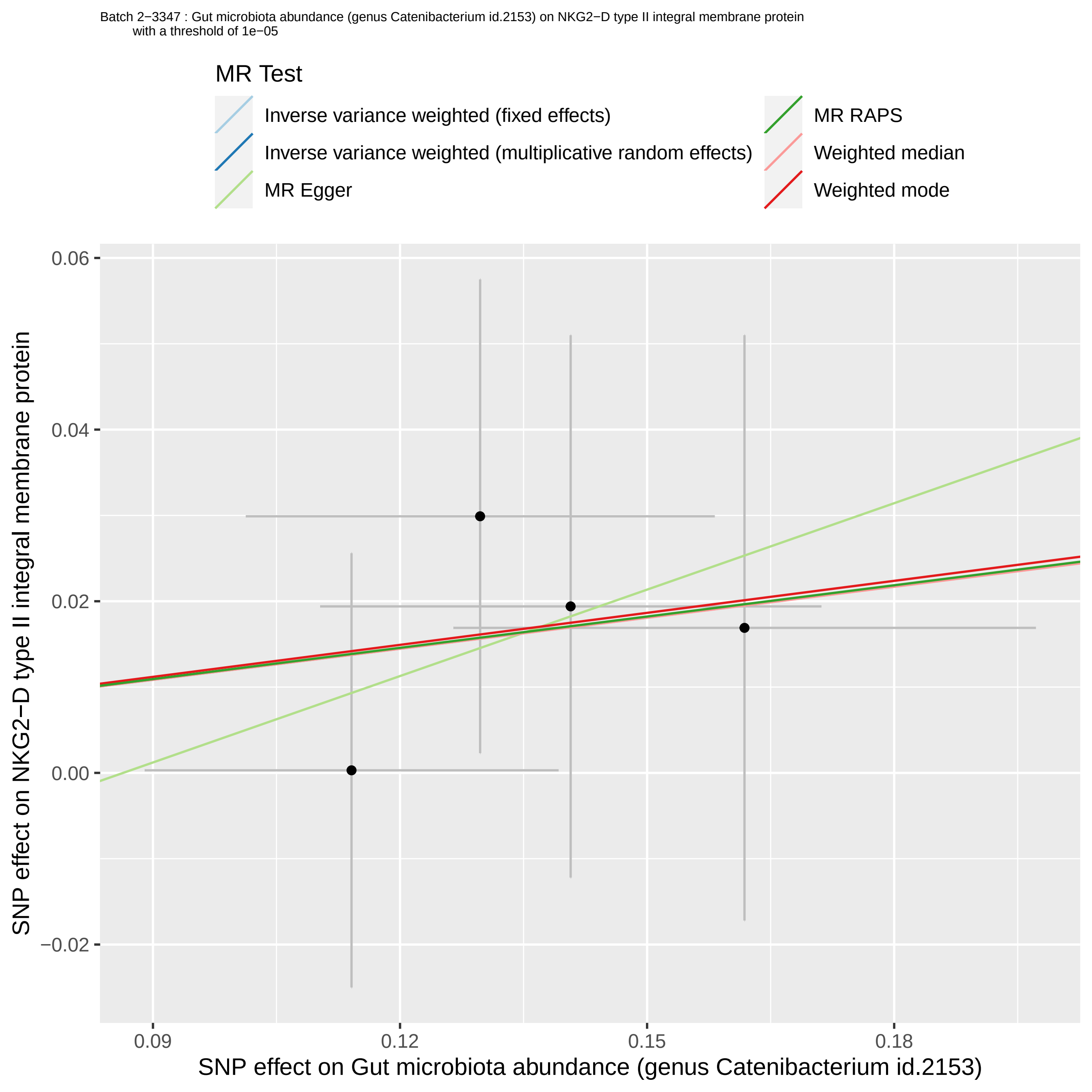
Batch 2−3347 : Gut microbiota abundance (genus Catenibacterium id.2153) on NKG2−D type II integral membrane protein
with a threshold of 1e−05
MR Test
Inverse variance weighted (fixed effects)
Inverse variance weighted (multiplicative random effects)
MR Egger
MR RAPS
Weighted median
Weighted mode
0.06
0.04
0.02
0.00
−0.02
0.09
0.12
0.15
0.18
SNP effect on NKG2−D type II integral membrane protein
SNP effect on Gut microbiota abundance (genus Catenibacterium id.2153)

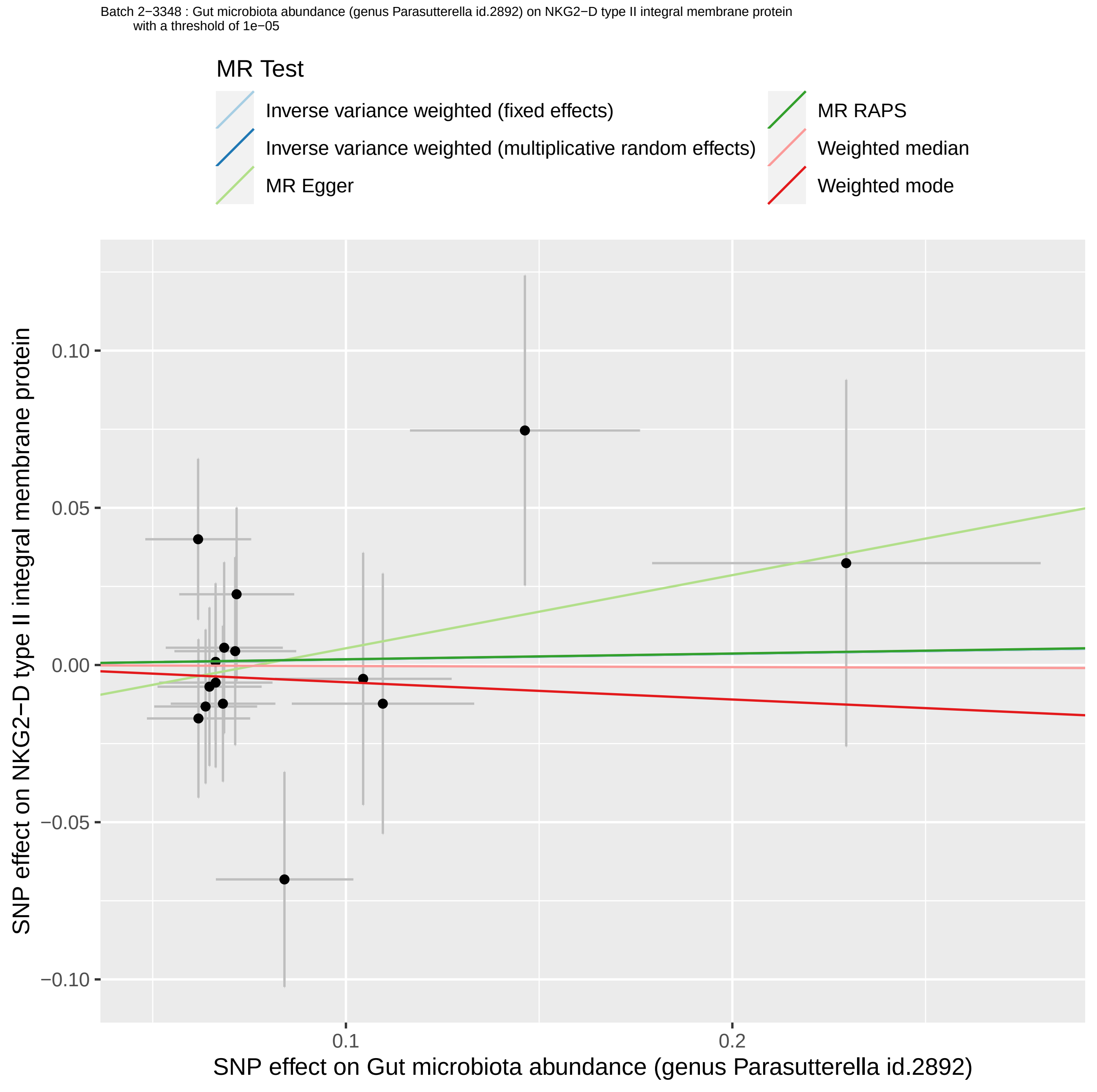

Batch 2−3348 : Gut microbiota abundance (genus Parasutterella id.2892) on NKG2−D type II integral membrane protein
with a threshold of 1e−05
MR Test
Inverse variance weighted (fixed effects)
Inverse variance weighted (multiplicative random effects)
MR Egger
MR RAPS
Weighted median
Weighted mode
0.10
0.05
0.00
−0.05
−0.10
0.1
0.2
SNP effect on NKG2−D type II integral membrane protein
SNP effect on Gut microbiota abundance (genus Parasutterella id.2892)

Batch 2−3349 : Gut microbiota abundance (genus Catenibacterium id.2153) on NKG2−D type II integral membrane protein
with a threshold of 1e−05

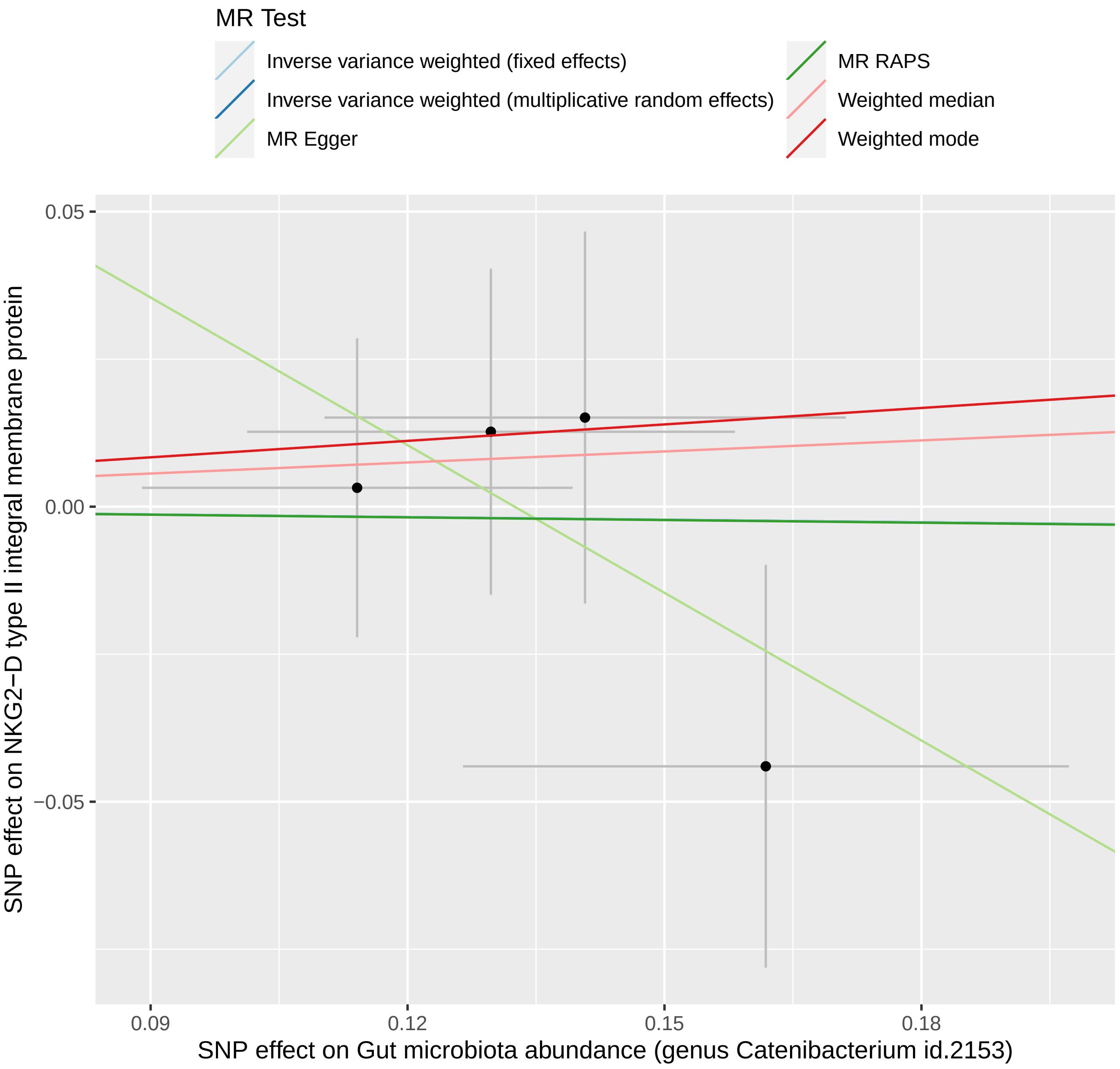

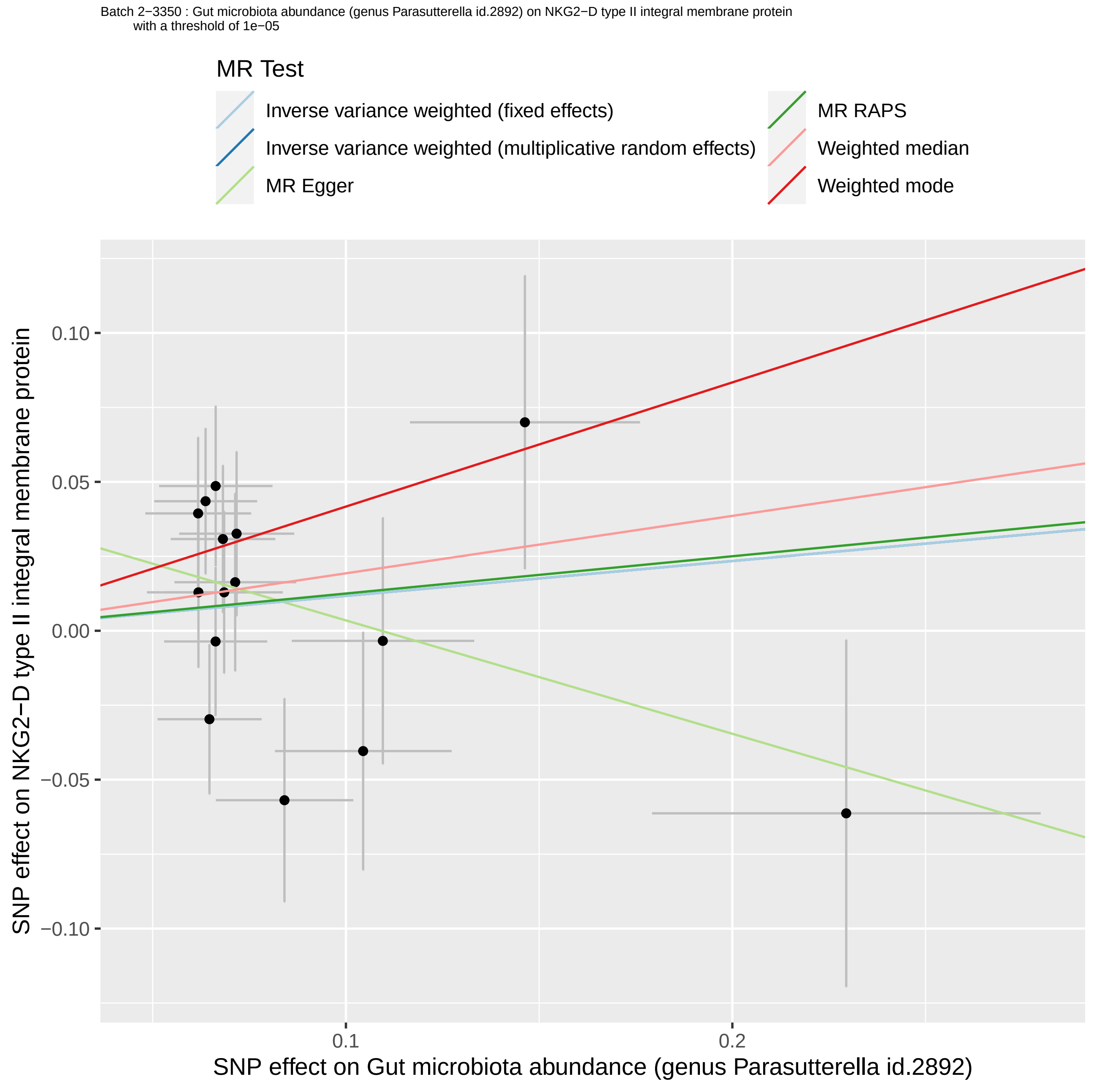
Batch 2−3350 : Gut microbiota abundance (genus Parasutterella id.2892) on NKG2−D type II integral membrane protein
with a threshold of 1e−05
MR Test
Inverse variance weighted (fixed effects)
Inverse variance weighted (multiplicative random effects)
MR Egger
MR RAPS
Weighted median
Weighted mode
0.10
0.05
0.00
−0.05
−0.10
0.1
0.2
SNP effect on NKG2−D type II integral membrane protein
SNP effect on Gut microbiota abundance (genus Parasutterella id.2892)

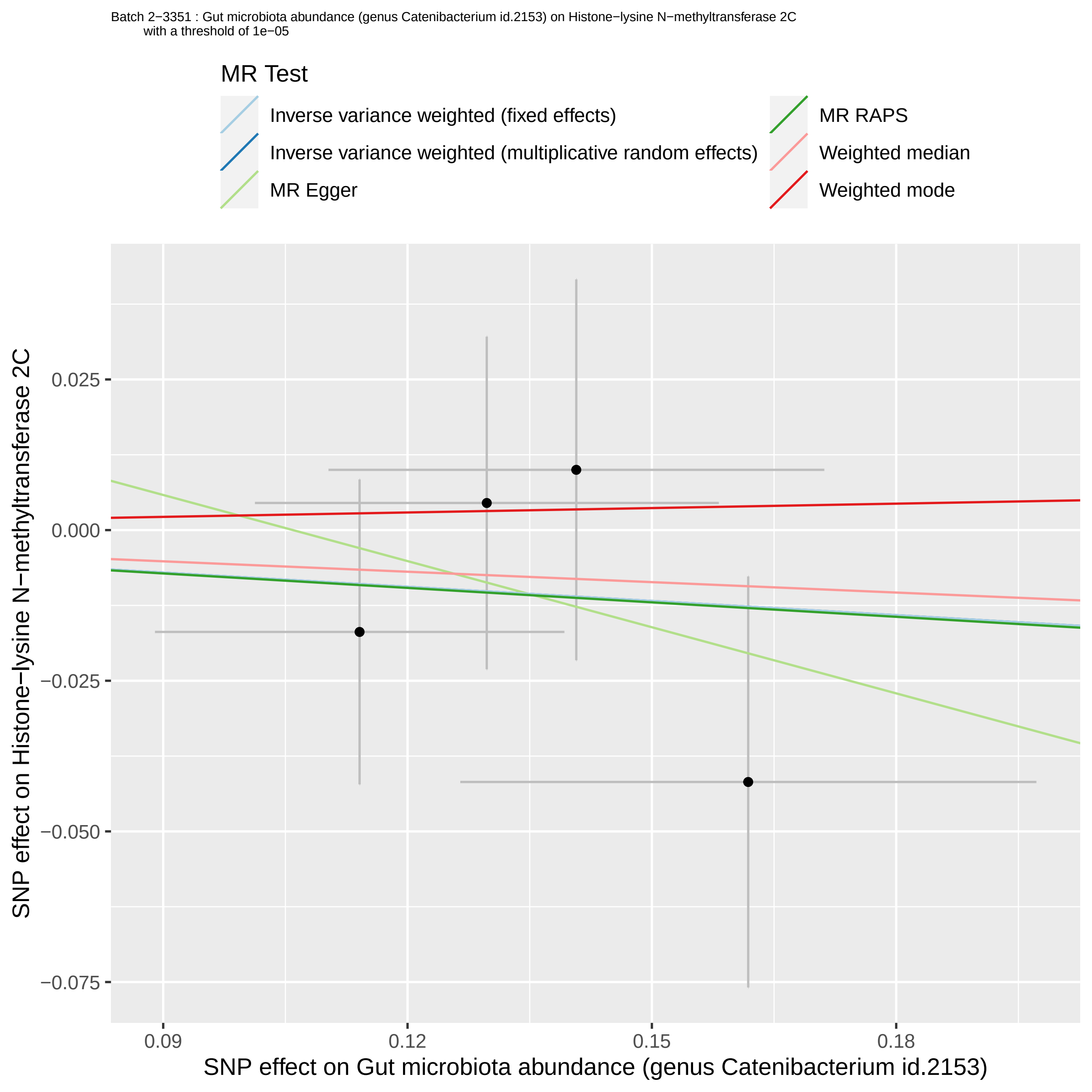

Batch 2−3351 : Gut microbiota abundance (genus Catenibacterium id.2153) on Histone−lysine N−methyltransferase 2C
with a threshold of 1e−05
MR Test
Inverse variance weighted (fixed effects)
Inverse variance weighted (multiplicative random effects)
MR Egger
MR RAPS
Weighted median
Weighted mode
SNP effect on Histone−lysine N−methyltransferase 2C
0.025
0.000
−0.025
−0.050
−0.075
0.09
0.12
0.15
0.18
SNP effect on Gut microbiota abundance (genus Catenibacterium id.2153)

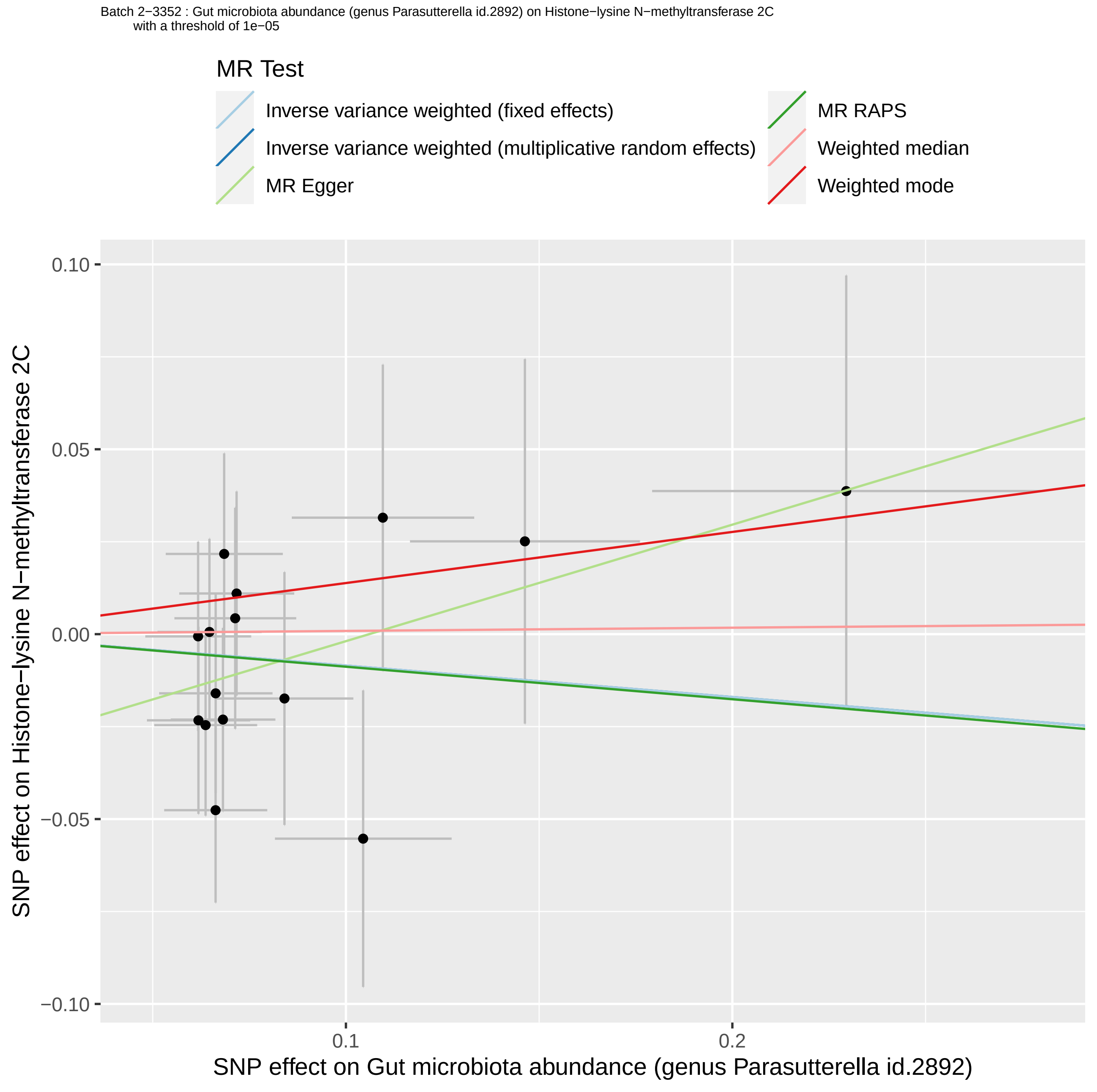

Batch 2−3352 : Gut microbiota abundance (genus Parasutterella id.2892) on Histone−lysine N−methyltransferase 2C
with a threshold of 1e−05
MR Test
Inverse variance weighted (fixed effects)
Inverse variance weighted (multiplicative random effects)
MR Egger
MR RAPS
Weighted median
Weighted mode
0.10
0.05
0.00
−0.05
−0.10
0.1
0.2
SNP effect on Histone−lysine N−methyltransferase 2C
SNP effect on Gut microbiota abundance (genus Parasutterella id.2892)

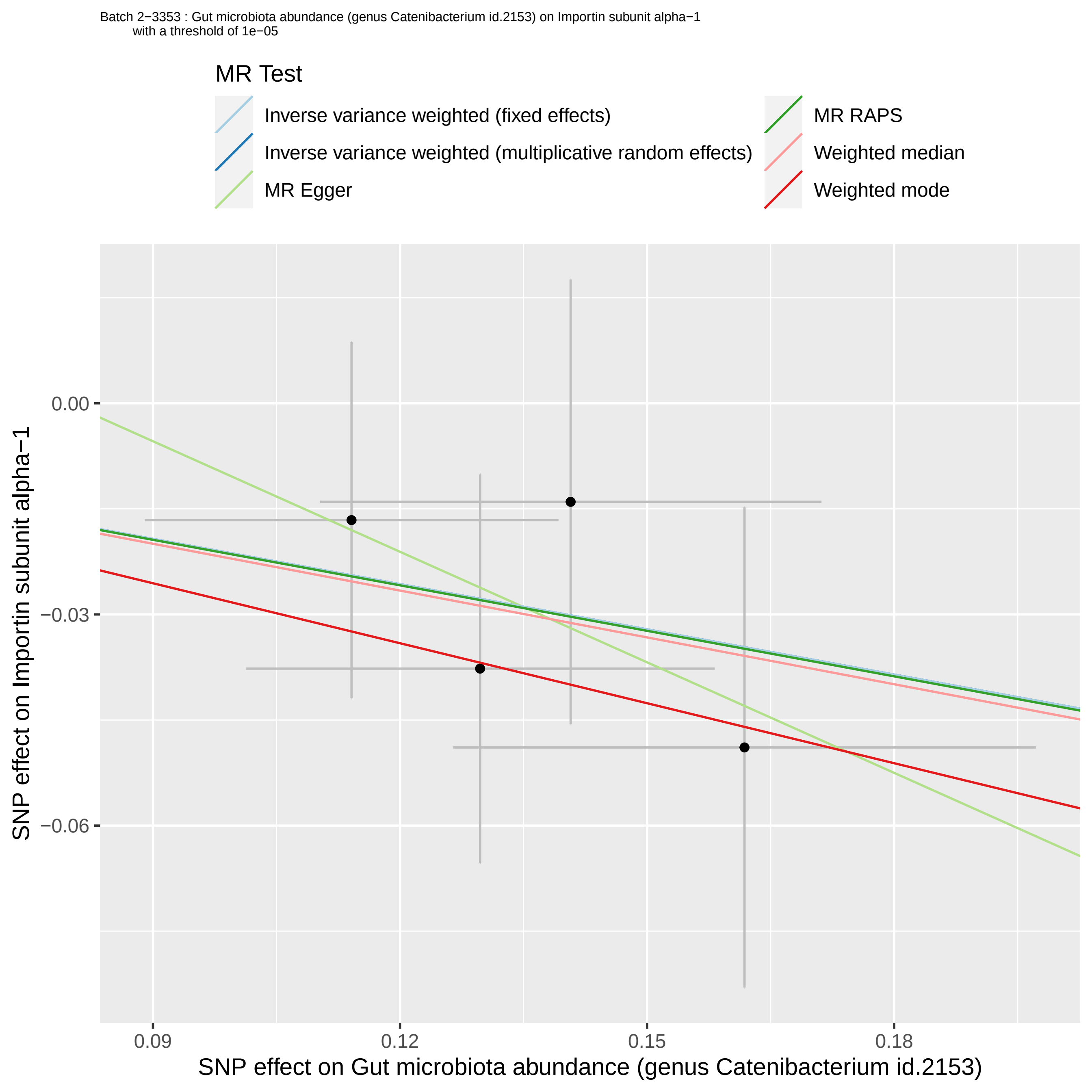
Batch 2−3353 : Gut microbiota abundance (genus Catenibacterium id.2153) on Importin subunit alpha−1
with a threshold of 1e−05
MR Test
Inverse variance weighted (fixed effects)
Inverse variance weighted (multiplicative random effects)
MR Egger
MR RAPS
Weighted median
Weighted mode
0.00
−0.03
−0.06
0.09
0.12
0.15
0.18
SNP effect on Importin subunit alpha−1
SNP effect on Gut microbiota abundance (genus Catenibacterium id.2153)

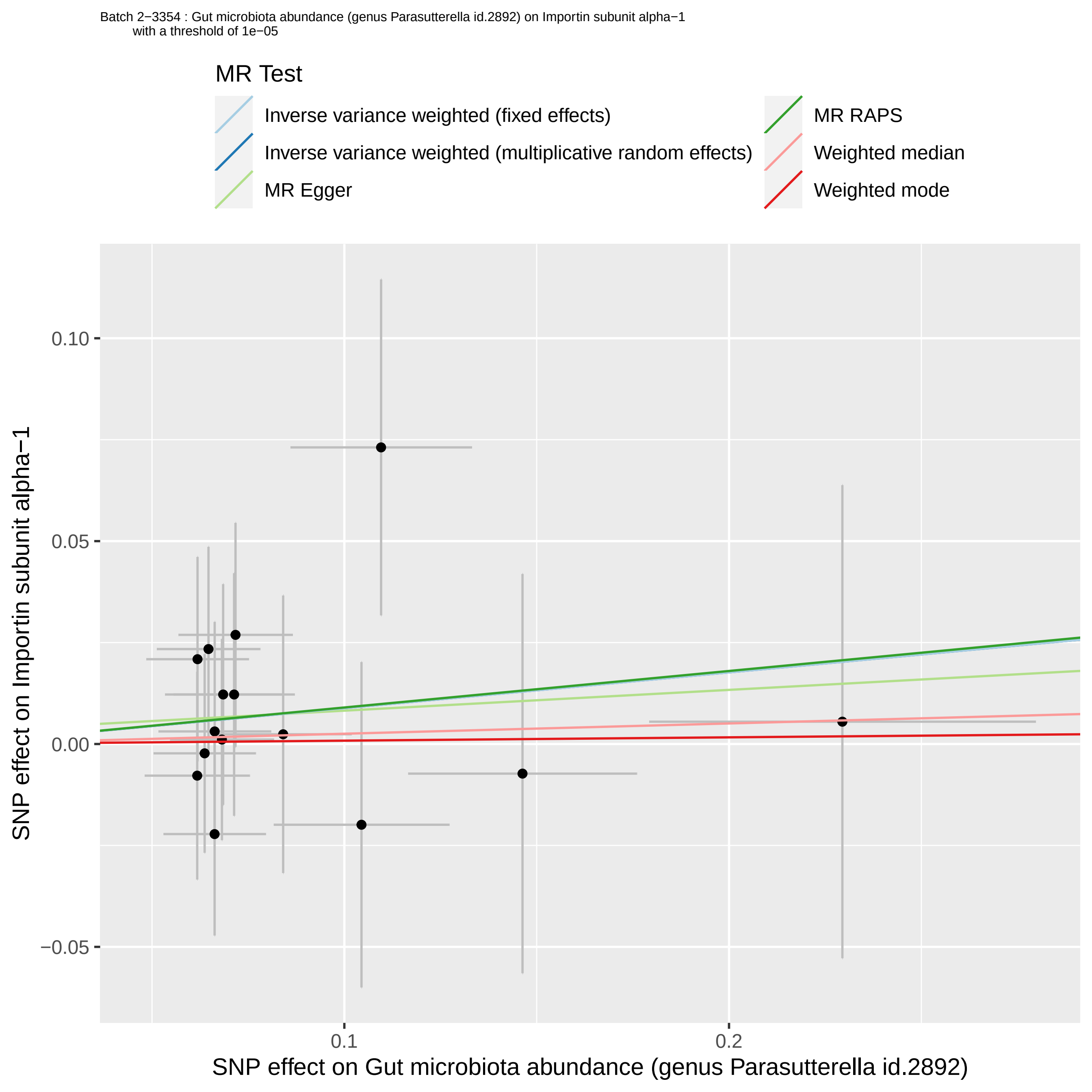
Batch 2−3354 : Gut microbiota abundance (genus Parasutterella id.2892) on Importin subunit alpha−1
with a threshold of 1e−05
MR Test
Inverse variance weighted (fixed effects)
Inverse variance weighted (multiplicative random effects)
MR Egger
MR RAPS
Weighted median
Weighted mode
0.10
0.05
0.00
−0.05
0.1
0.2
SNP effect on Importin subunit alpha−1
SNP effect on Gut microbiota abundance (genus Parasutterella id.2892)

Batch 2−3355 : Gut microbiota abundance (genus Catenibacterium id.2153) on Importin subunit alpha−3
with a threshold of 1e−05

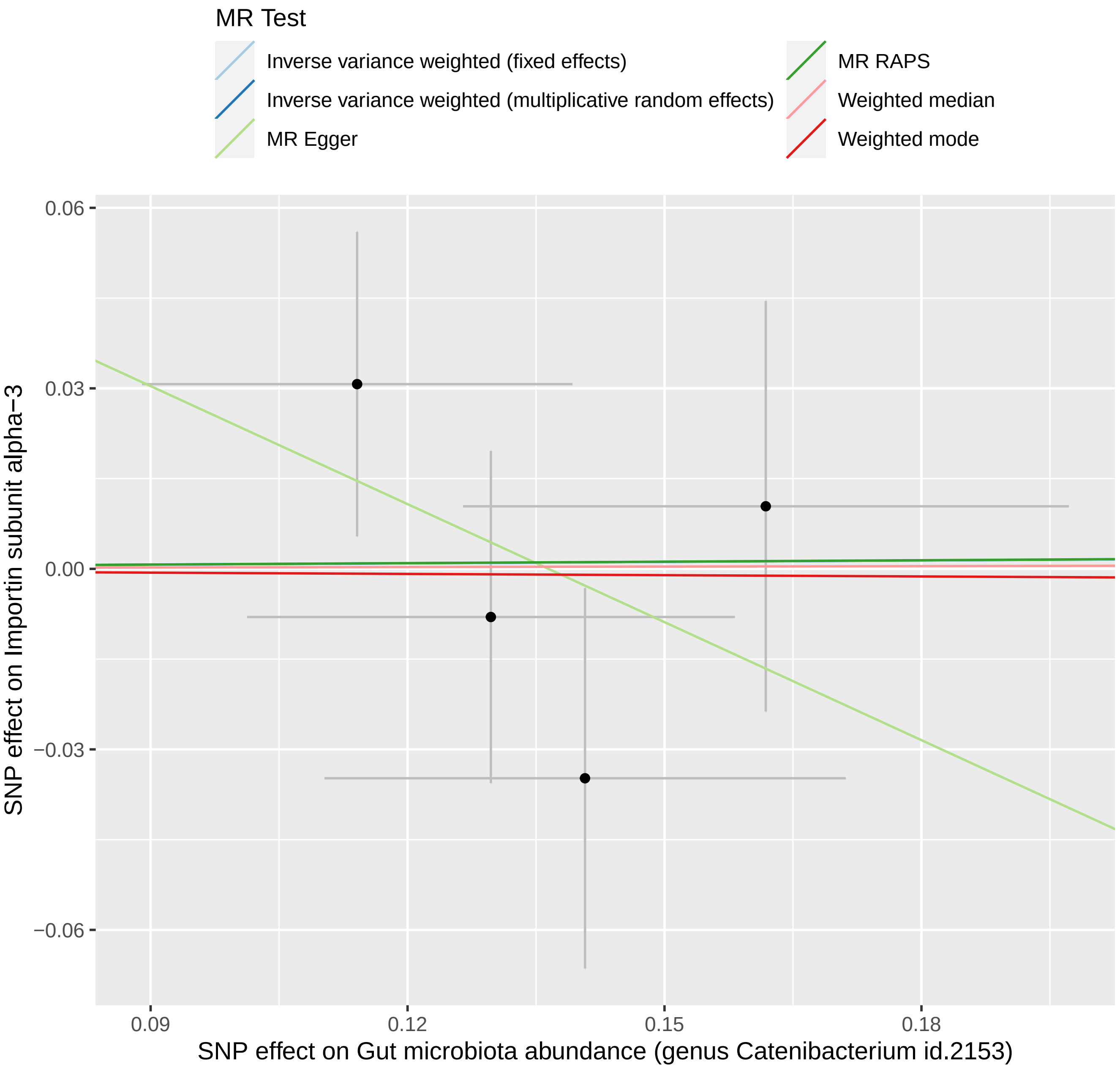

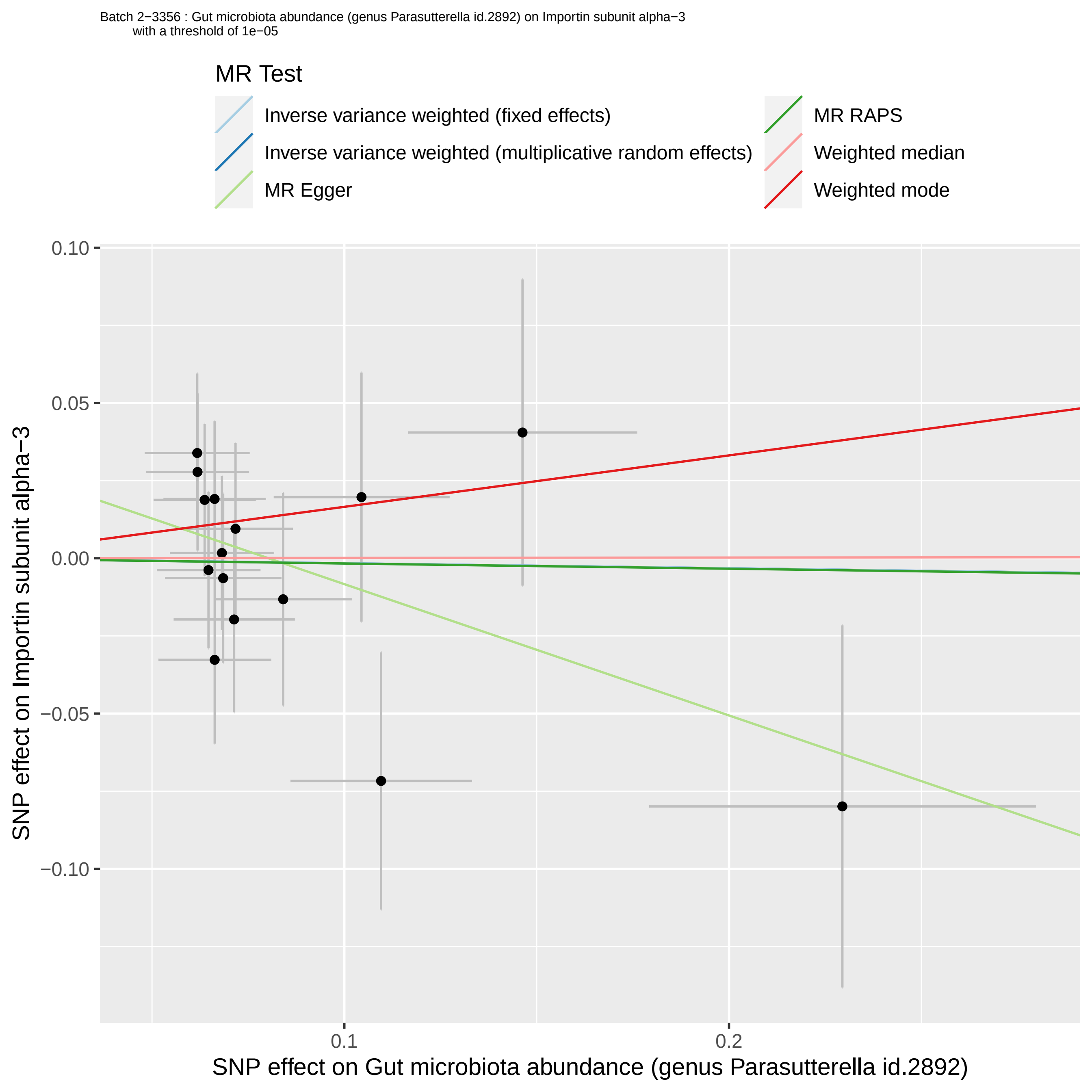

Batch 2−3356 : Gut microbiota abundance (genus Parasutterella id.2892) on Importin subunit alpha−3
with a threshold of 1e−05
MR Test
Inverse variance weighted (fixed effects)
Inverse variance weighted (multiplicative random effects)
MR Egger
MR RAPS
Weighted median
Weighted mode
0.10
0.05
0.00
−0.05
−0.10
0.1
0.2
SNP effect on Importin subunit alpha−3
SNP effect on Gut microbiota abundance (genus Parasutterella id.2892)

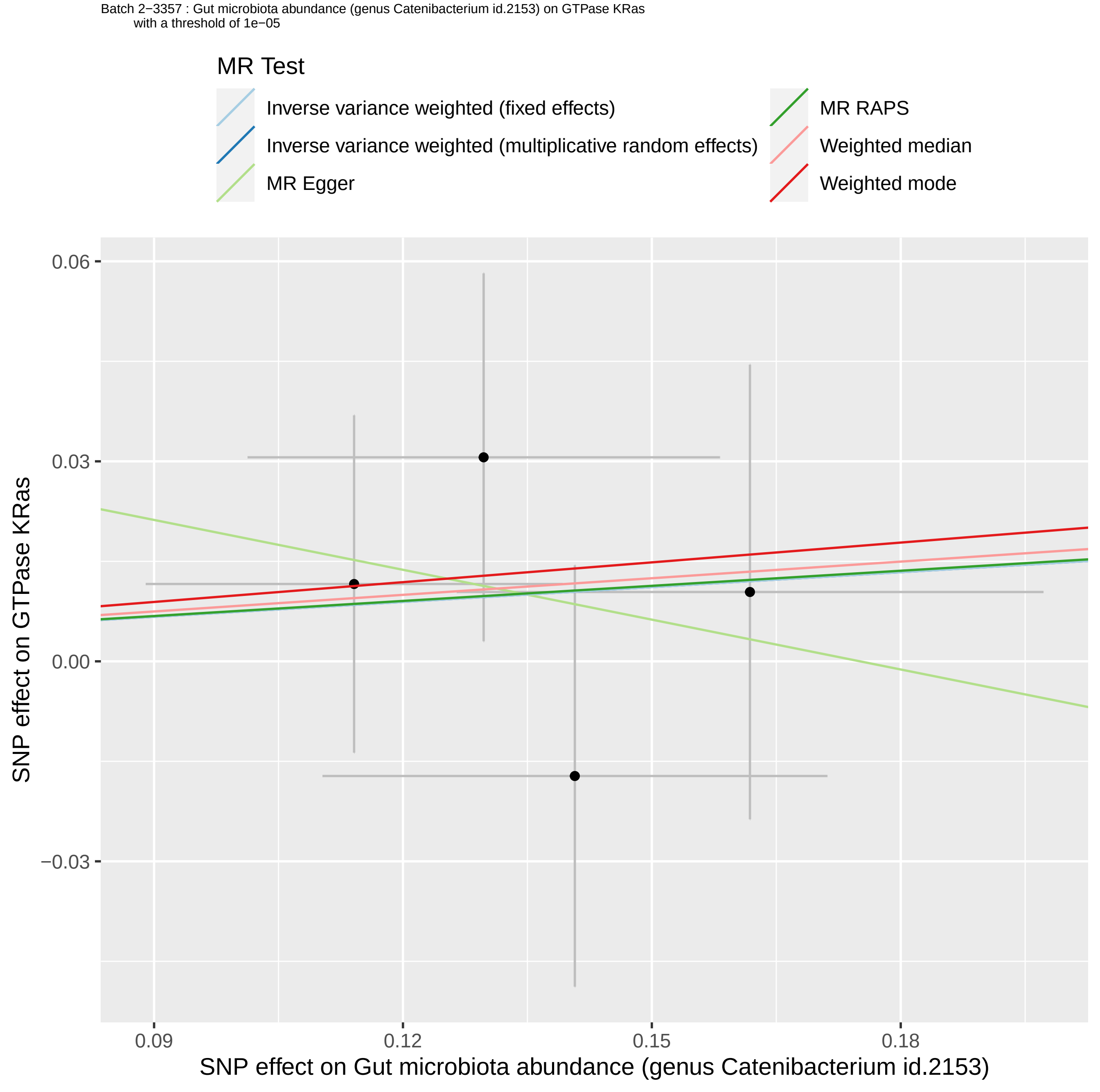

Batch 2−3357 : Gut microbiota abundance (genus Catenibacterium id.2153) on GTPase KRas
with a threshold of 1e−05
MR Test
Inverse variance weighted (fixed effects)
Inverse variance weighted (multiplicative random effects)
MR Egger
MR RAPS
Weighted median
Weighted mode
0.06
0.03
0.00
−0.03
0.09
0.12
0.15
0.18
SNP effect on GTPase KRas
SNP effect on Gut microbiota abundance (genus Catenibacterium id.2153)

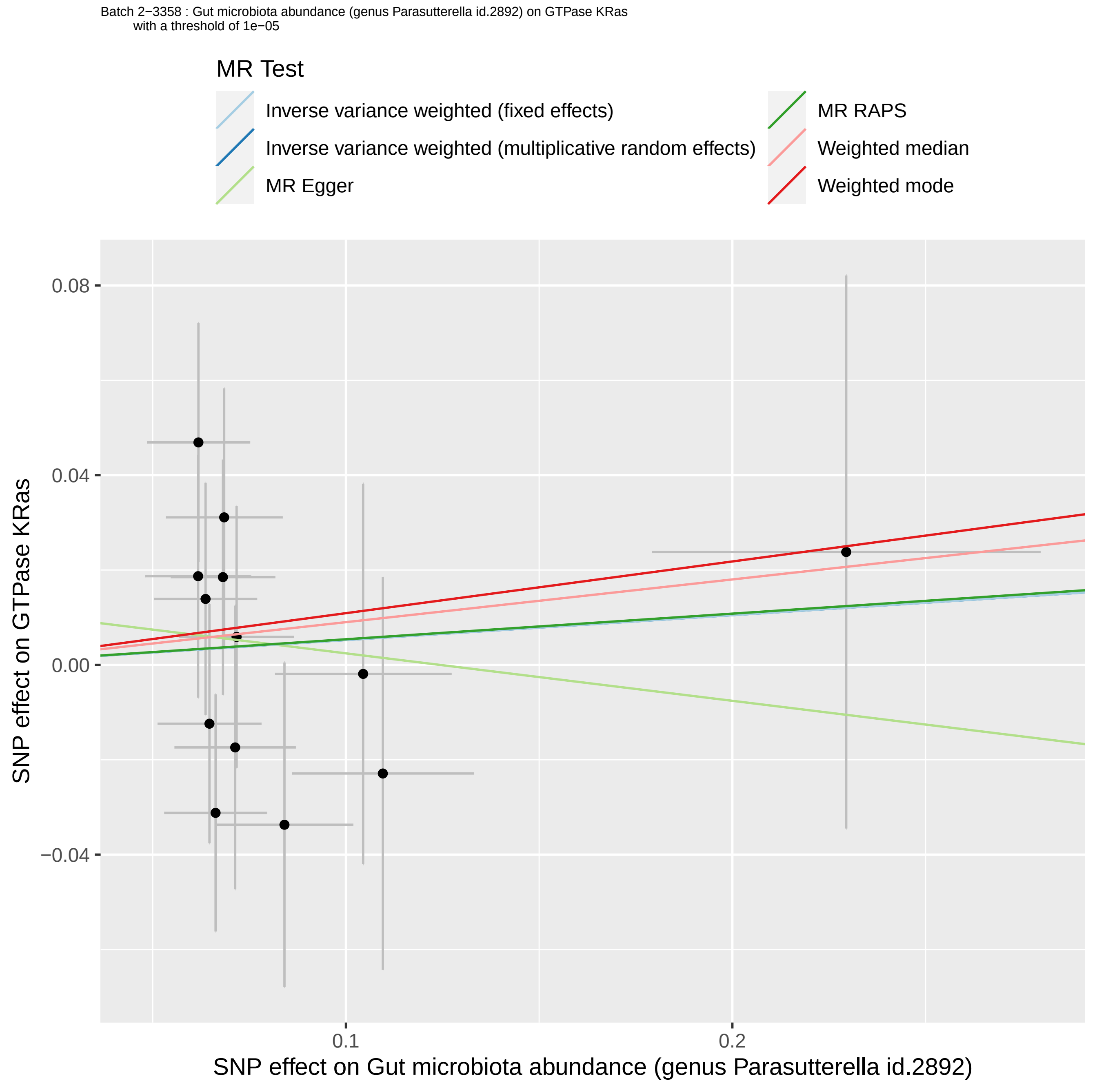

Batch 2−3358 : Gut microbiota abundance (genus Parasutterella id.2892) on GTPase KRas
with a threshold of 1e−05
MR Test
Inverse variance weighted (fixed effects)
Inverse variance weighted (multiplicative random effects)
MR Egger
MR RAPS
Weighted median
Weighted mode
0.08
0.04
0.00
−0.04
0.1
0.2
SNP effect on GTPase KRas
SNP effect on Gut microbiota abundance (genus Parasutterella id.2892)

Batch 2−3359 : Gut microbiota abundance (genus Catenibacterium id.2153) on Kremen protein 1
with a threshold of 1e−05

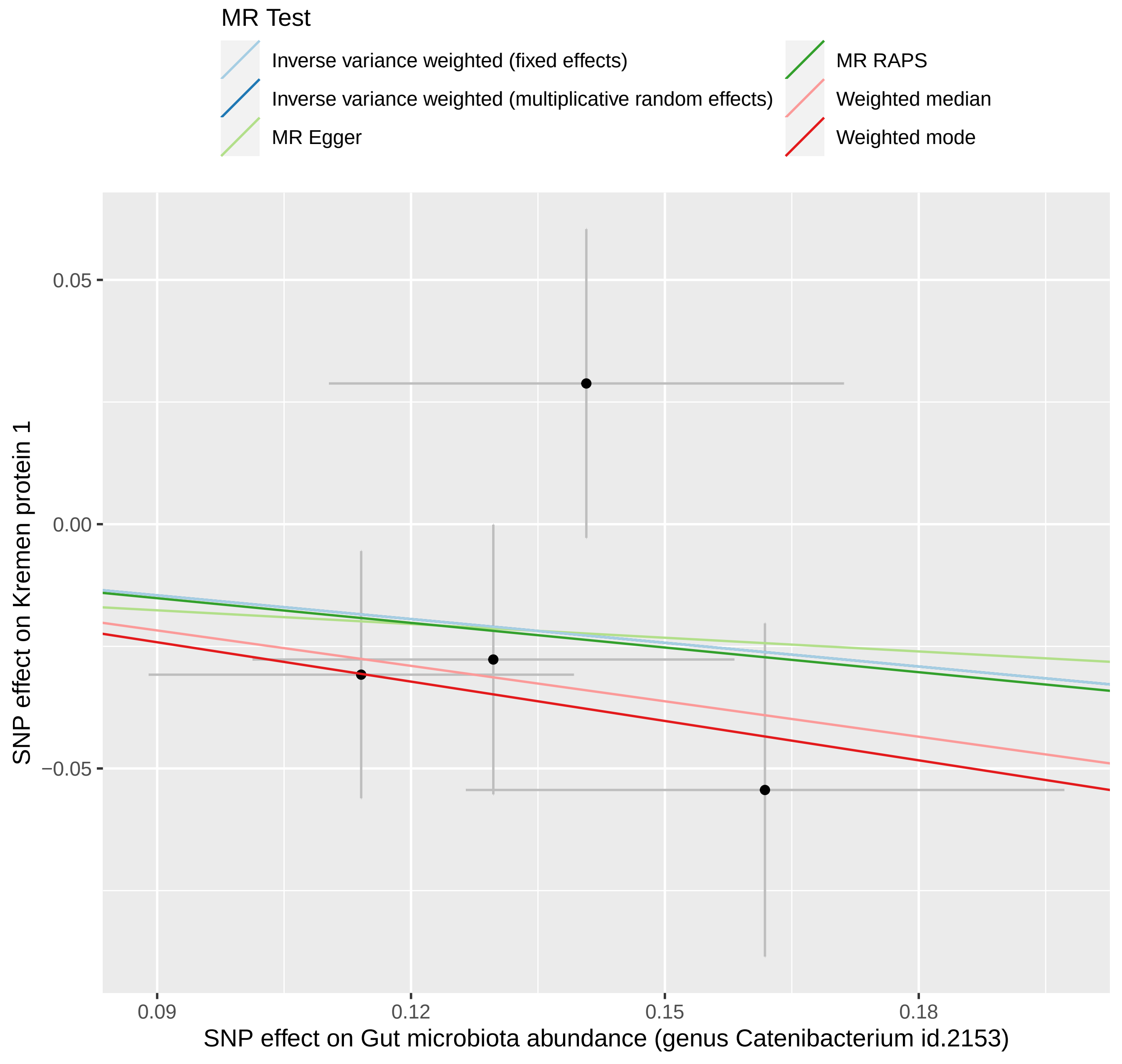

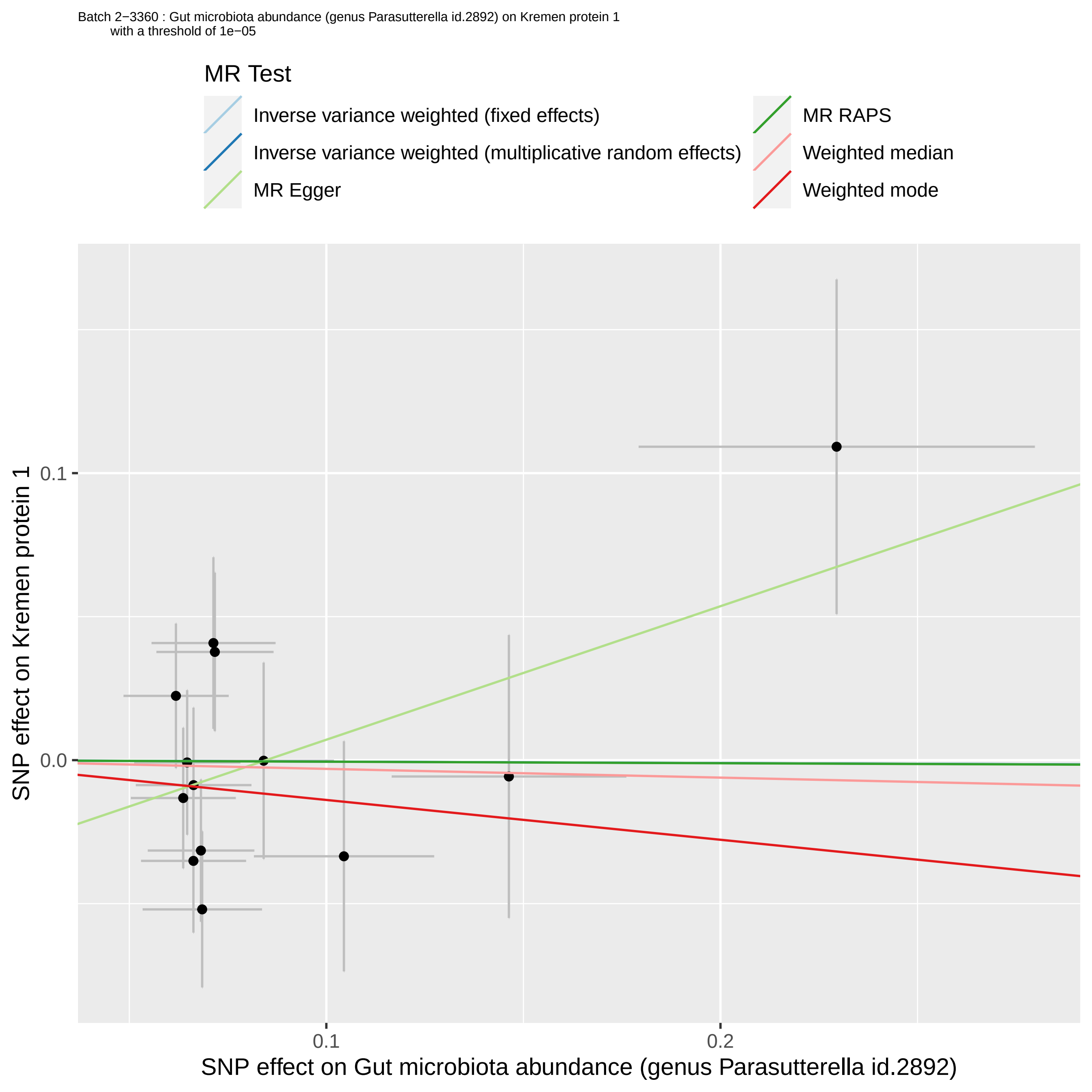

Batch 2−3360 : Gut microbiota abundance (genus Parasutterella id.2892) on Kremen protein 1
with a threshold of 1e−05
MR Test
Inverse variance weighted (fixed effects)
Inverse variance weighted (multiplicative random effects)
MR Egger
MR RAPS
Weighted median
Weighted mode
0.1
0.0
0.1
0.2
SNP effect on Kremen protein 1
SNP effect on Gut microbiota abundance (genus Parasutterella id.2892)

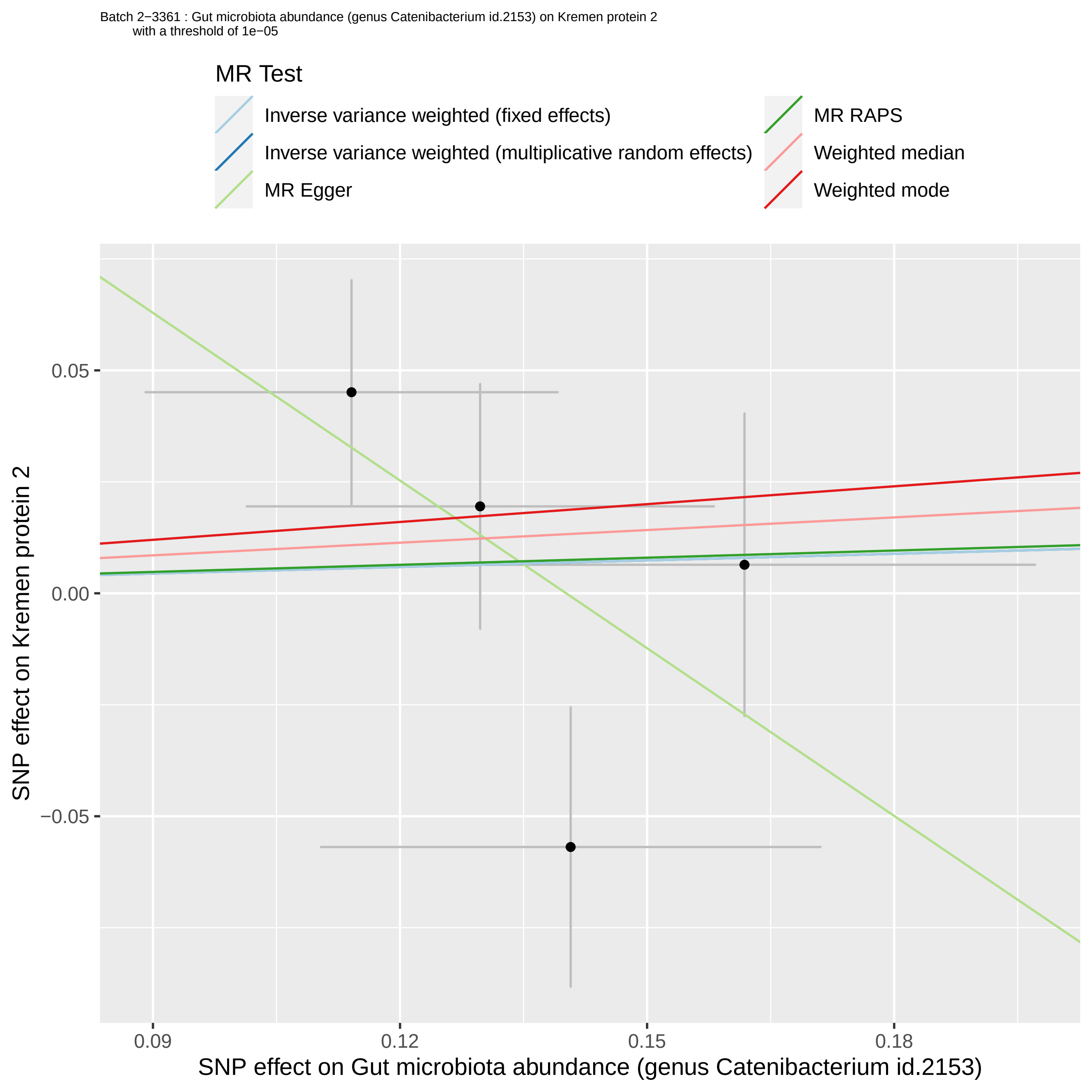
Batch 2−3361 : Gut microbiota abundance (genus Catenibacterium id.2153) on Kremen protein 2
with a threshold of 1e−05
MR Test
Inverse variance weighted (fixed effects)
Inverse variance weighted (multiplicative random effects)
MR Egger
MR RAPS
Weighted median
Weighted mode
0.05
0.00
−0.05
0.09
0.12
0.15
0.18
SNP effect on Kremen protein 2
SNP effect on Gut microbiota abundance (genus Catenibacterium id.2153)

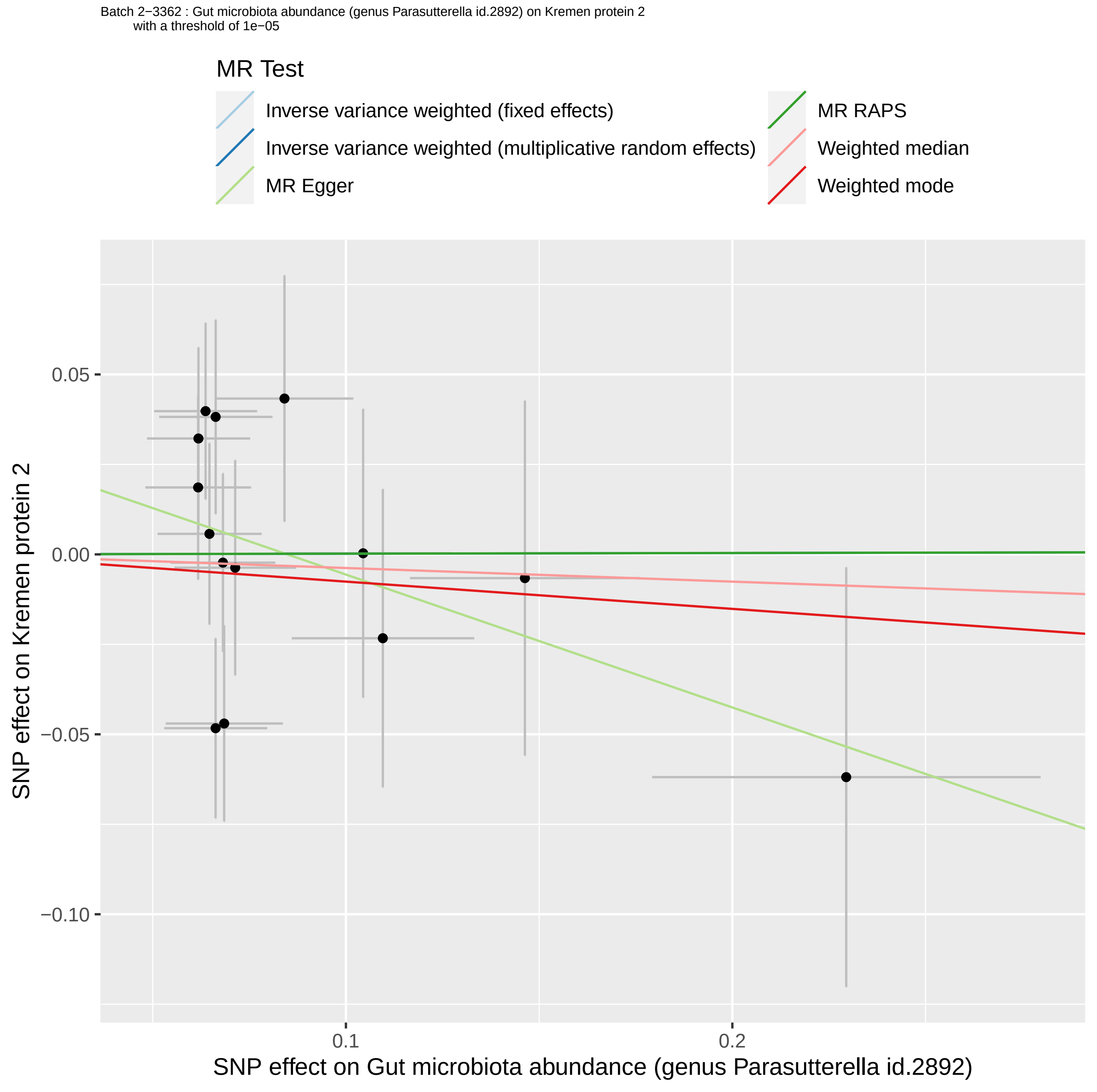

Batch 2−3362 : Gut microbiota abundance (genus Parasutterella id.2892) on Kremen protein 2
with a threshold of 1e−05
MR Test
Inverse variance weighted (fixed effects)
Inverse variance weighted (multiplicative random effects)
MR Egger
MR RAPS
Weighted median
Weighted mode
0.05
0.00
−0.05
−0.10
0.1
0.2
SNP effect on Kremen protein 2
SNP effect on Gut microbiota abundance (genus Parasutterella id.2892)

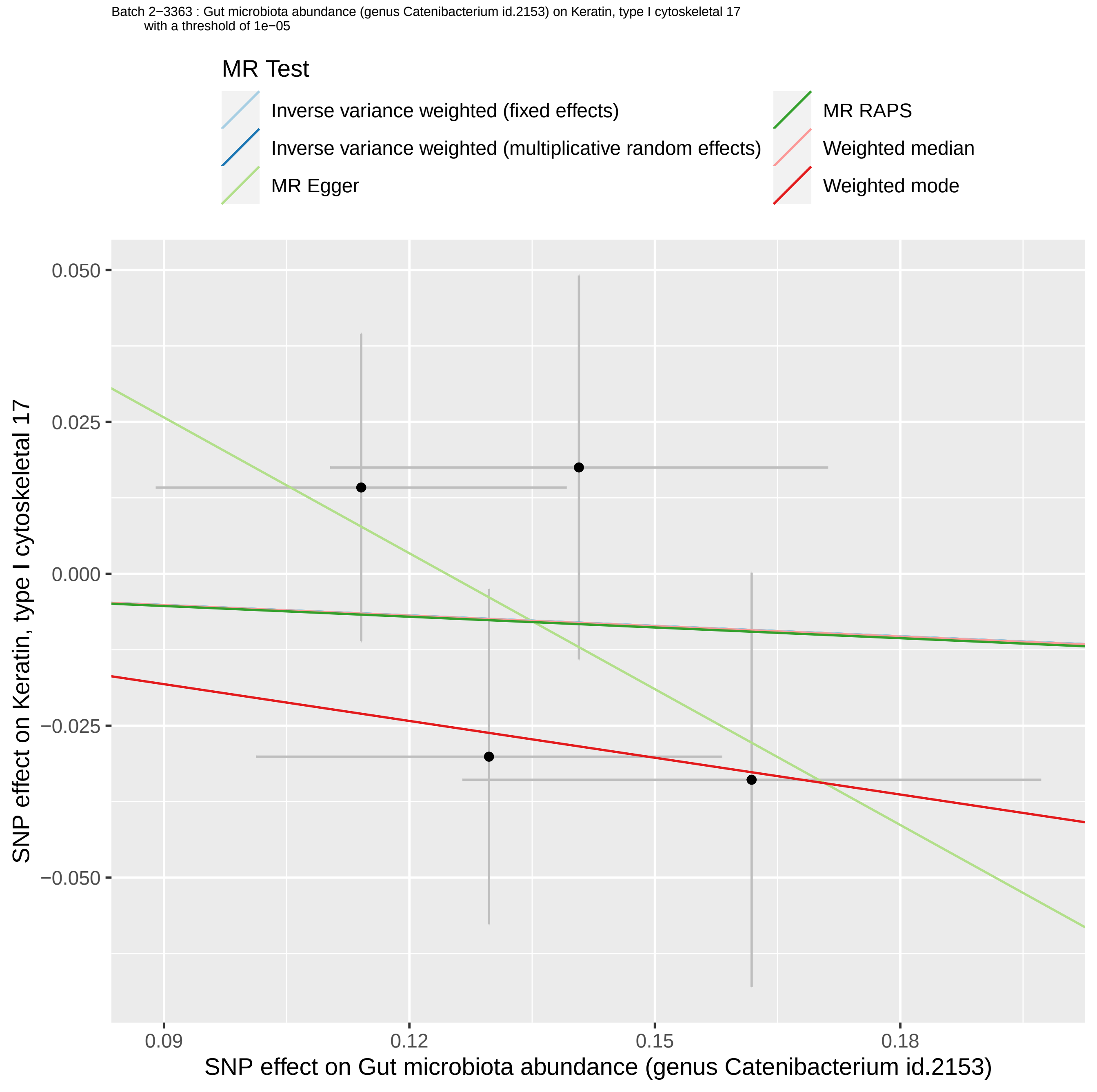

Batch 2−3363 : Gut microbiota abundance (genus Catenibacterium id.2153) on Keratin, type I cytoskeletal 17
with a threshold of 1e−05
MR Test
Inverse variance weighted (fixed effects)
Inverse variance weighted (multiplicative random effects)
MR Egger
MR RAPS
Weighted median
Weighted mode
0.050
0.025
0.000
−0.025
−0.050
0.09
0.12
0.15
0.18
SNP effect on Keratin, type I cytoskeletal 17
SNP effect on Gut microbiota abundance (genus Catenibacterium id.2153)

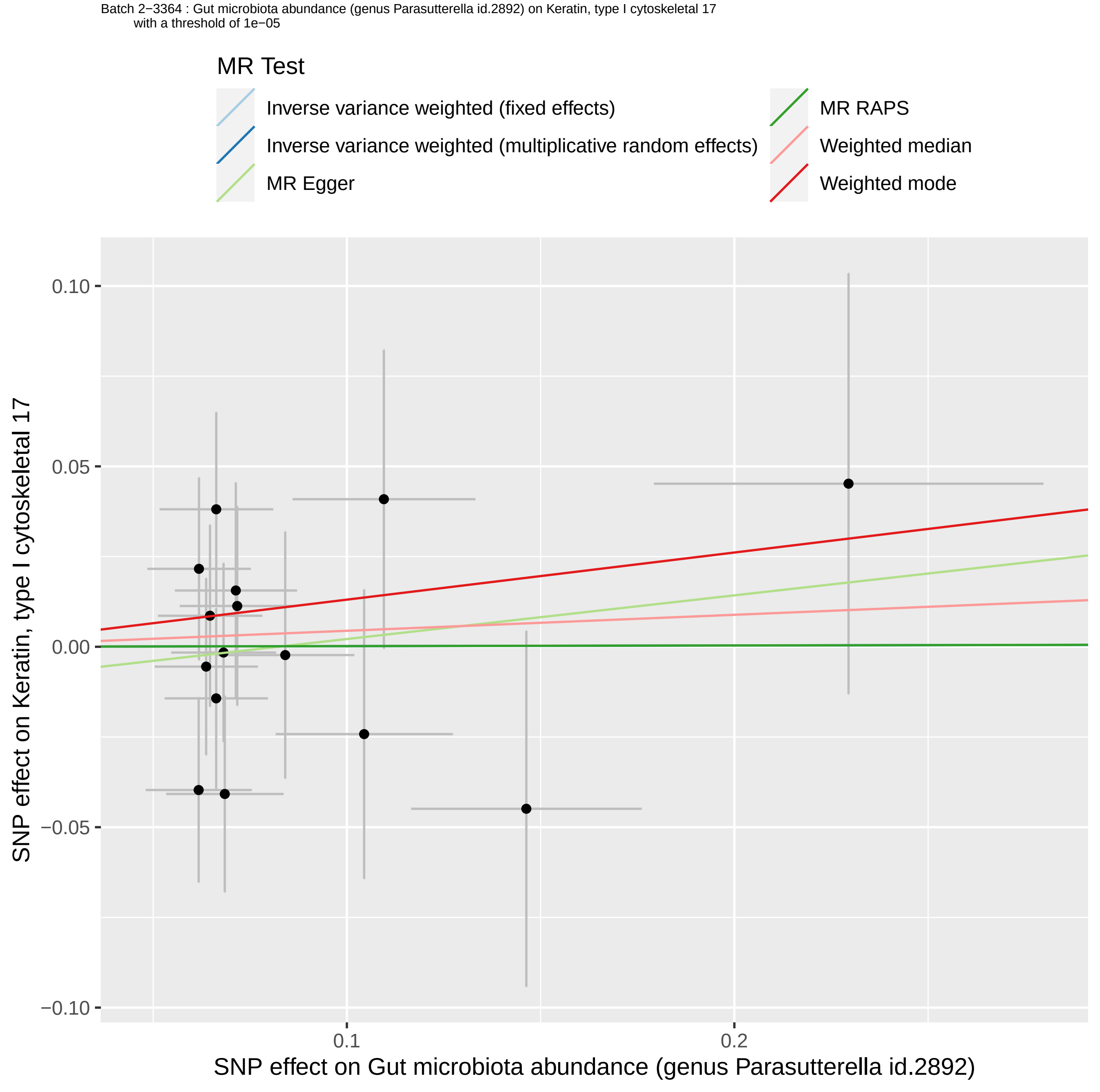
Batch 2−3364 : Gut microbiota abundance (genus Parasutterella id.2892) on Keratin, type I cytoskeletal 17
with a threshold of 1e−05
MR Test
Inverse variance weighted (fixed effects)
Inverse variance weighted (multiplicative random effects)
MR Egger
MR RAPS
Weighted median
Weighted mode
0.10
0.05
0.00
−0.05
−0.10
0.1
0.2
SNP effect on Keratin, type I cytoskeletal 17
SNP effect on Gut microbiota abundance (genus Parasutterella id.2892)

Batch 2−3365 : Gut microbiota abundance (genus Catenibacterium id.2153) on Keratin, type I cytoskeletal 18
with a threshold of 1e−05

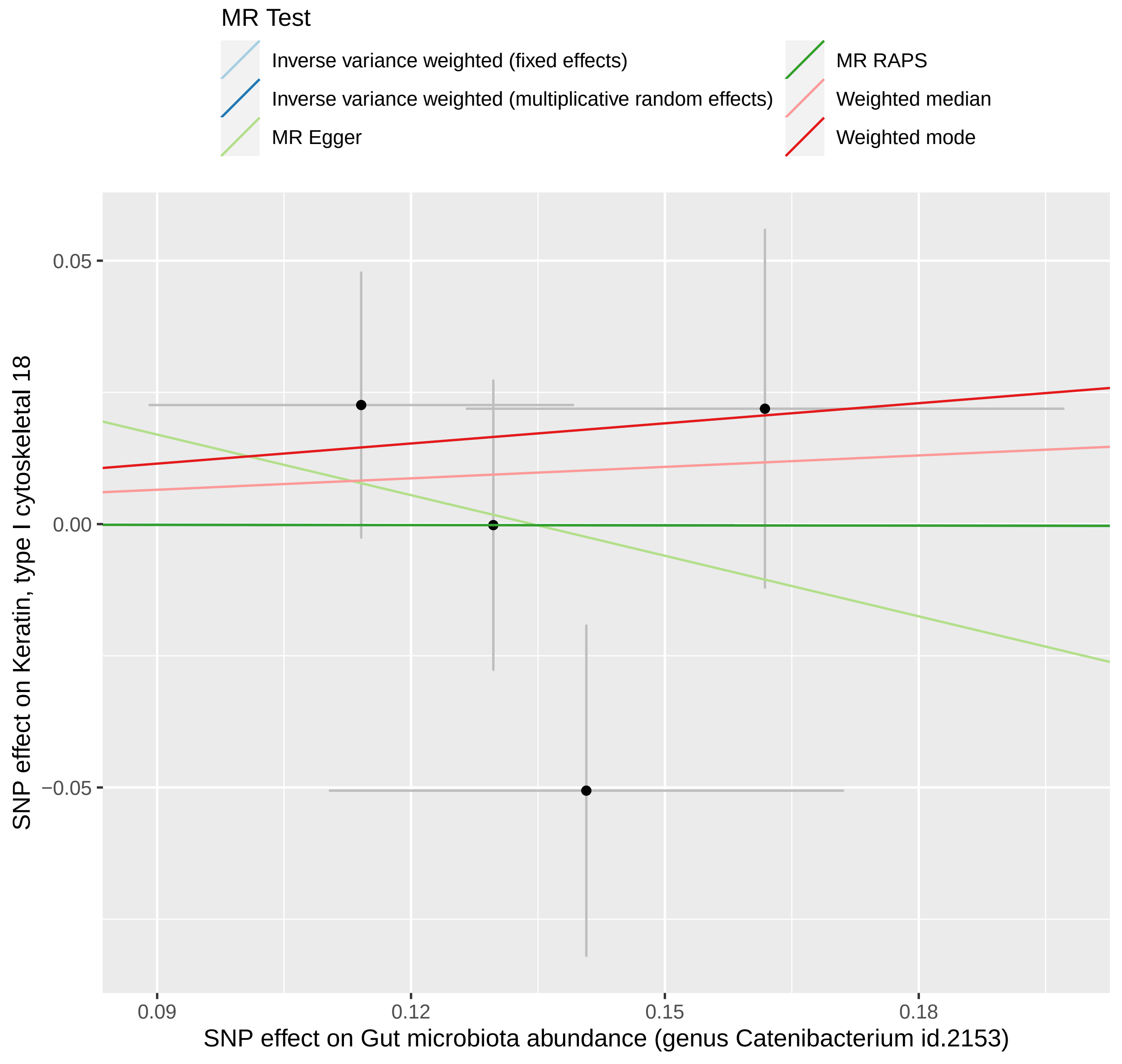

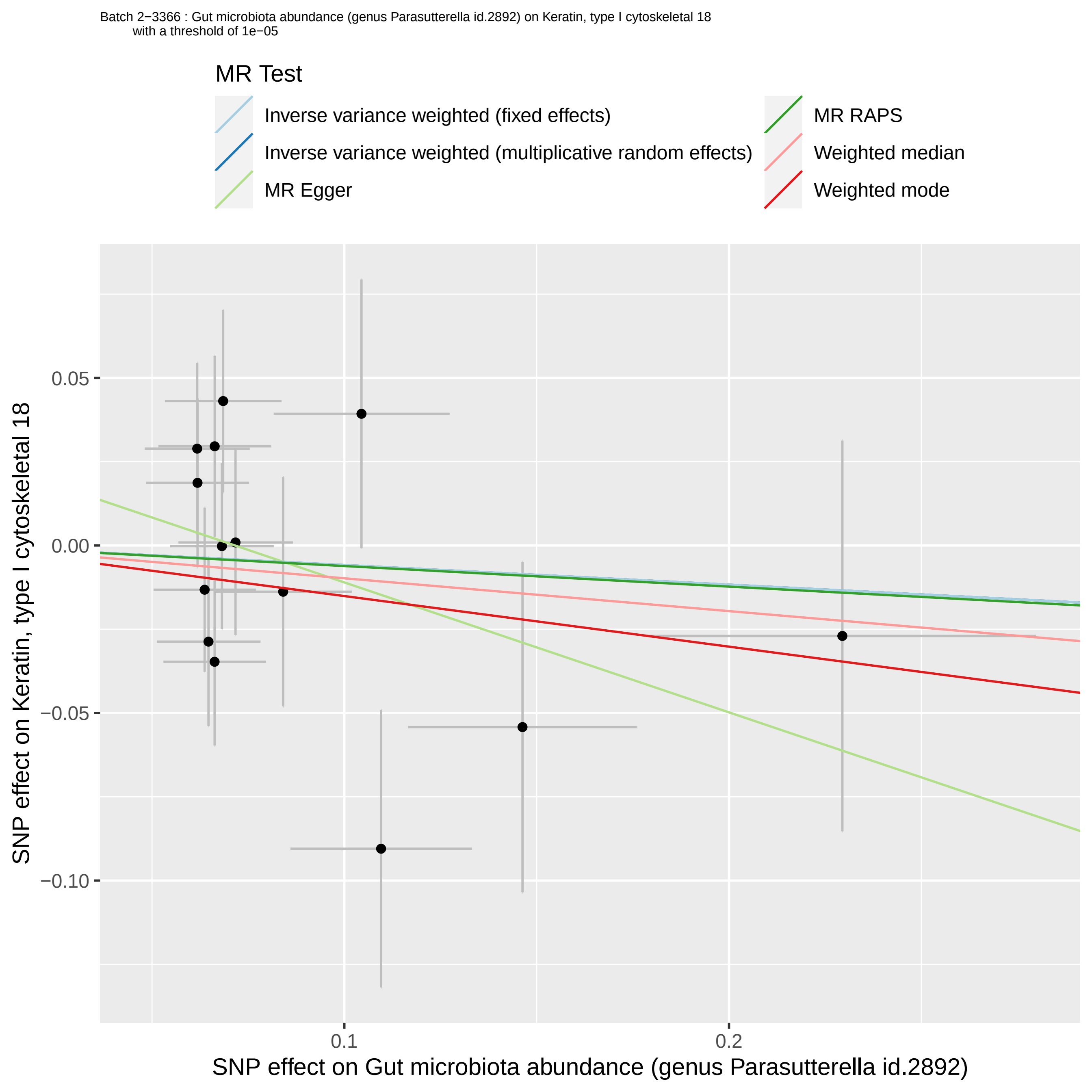

Batch 2−3366 : Gut microbiota abundance (genus Parasutterella id.2892) on Keratin, type I cytoskeletal 18
with a threshold of 1e−05
MR Test
Inverse variance weighted (fixed effects)
Inverse variance weighted (multiplicative random effects)
MR Egger
MR RAPS
Weighted median
Weighted mode
0.05
0.00
−0.05
−0.10
0.1
0.2
SNP effect on Keratin, type I cytoskeletal 18
SNP effect on Gut microbiota abundance (genus Parasutterella id.2892)

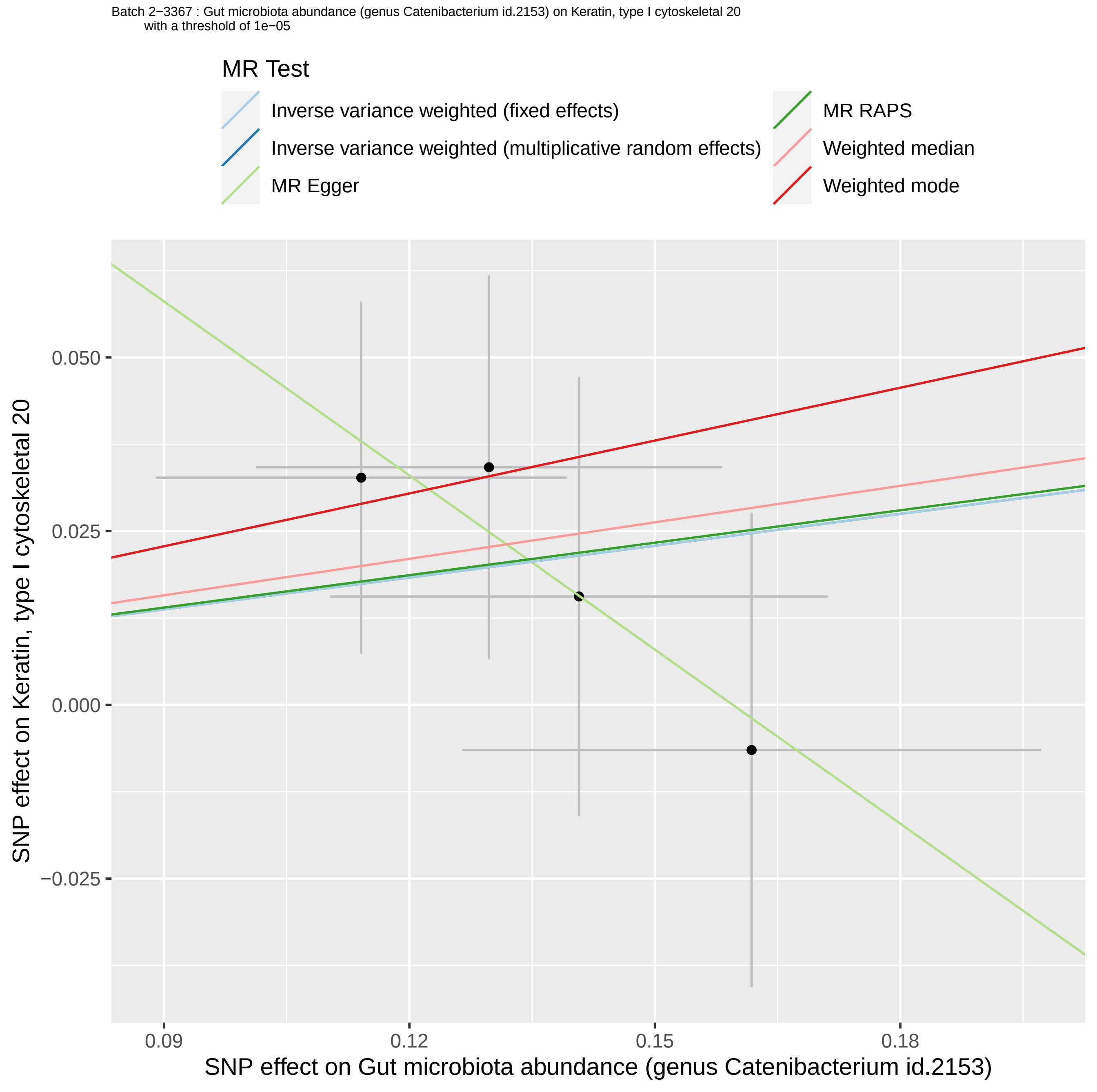
Batch 2−3367 : Gut microbiota abundance (genus Catenibacterium id.2153) on Keratin, type I cytoskeletal 20
with a threshold of 1e−05
MR Test
Inverse variance weighted (fixed effects)
Inverse variance weighted (multiplicative random effects)
MR Egger
MR RAPS
Weighted median
Weighted mode
0.050
0.025
0.000
−0.025
0.09
0.12
0.15
0.18
SNP effect on Keratin, type I cytoskeletal 20
SNP effect on Gut microbiota abundance (genus Catenibacterium id.2153)

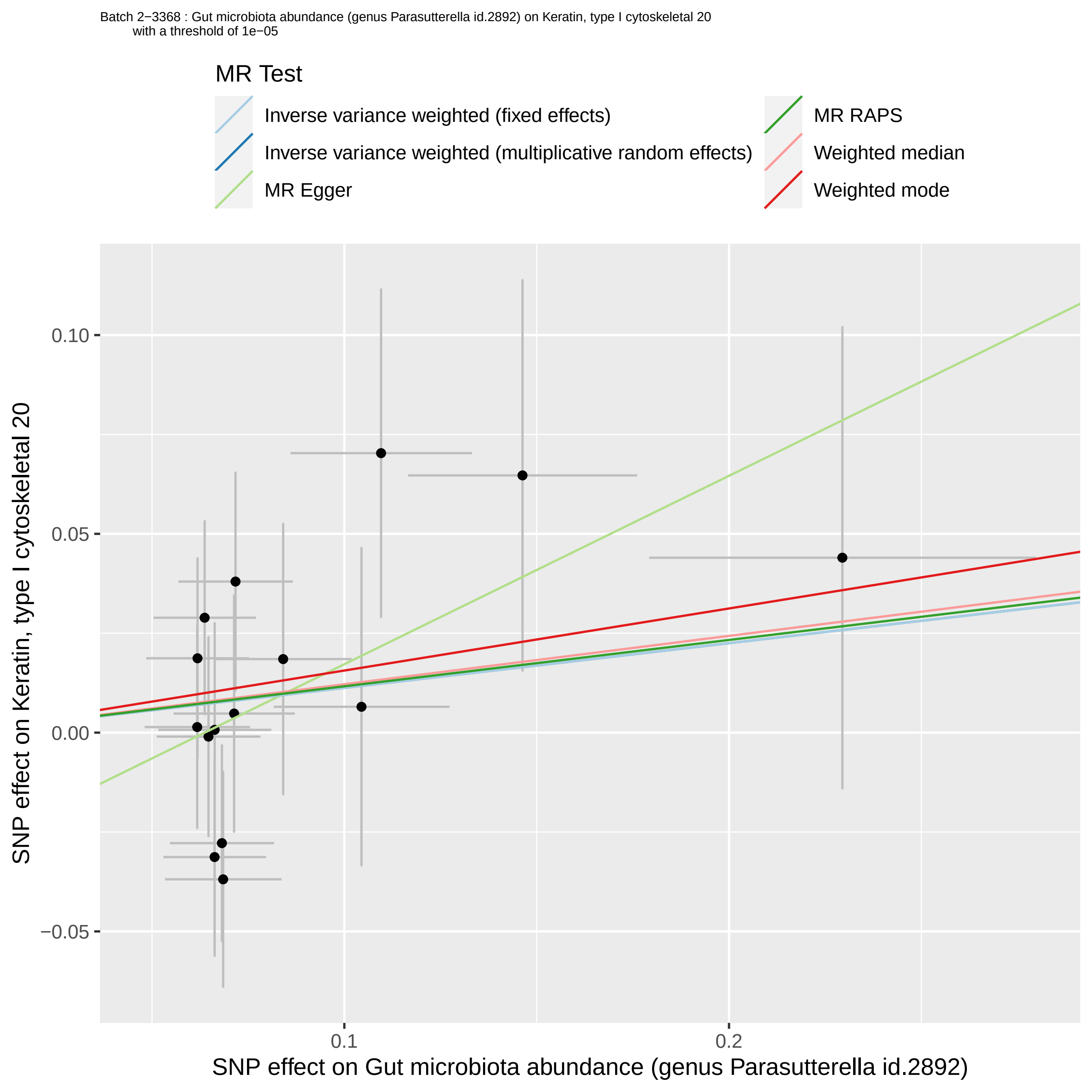

Batch 2−3368 : Gut microbiota abundance (genus Parasutterella id.2892) on Keratin, type I cytoskeletal 20
with a threshold of 1e−05
MR Test
Inverse variance weighted (fixed effects)
Inverse variance weighted (multiplicative random effects)
MR Egger
MR RAPS
Weighted median
Weighted mode
0.10
0.05
0.00
−0.05
0.1
0.2
SNP effect on Keratin, type I cytoskeletal 20
SNP effect on Gut microbiota abundance (genus Parasutterella id.2892)

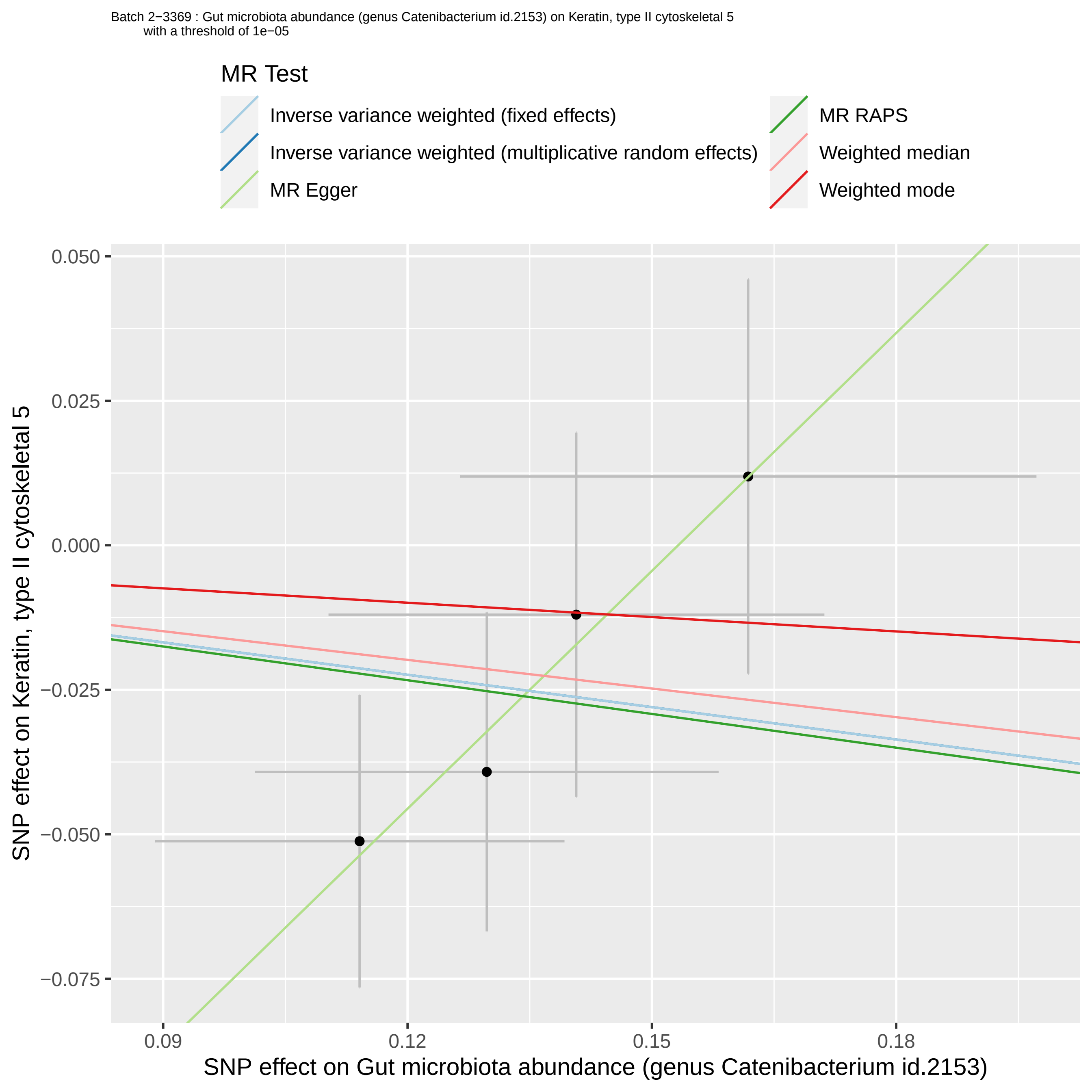
Batch 2−3369 : Gut microbiota abundance (genus Catenibacterium id.2153) on Keratin, type II cytoskeletal 5
with a threshold of 1e−05
MR Test
Inverse variance weighted (fixed effects)
Inverse variance weighted (multiplicative random effects)
MR Egger
MR RAPS
Weighted median
Weighted mode
0.050
0.025
0.000
−0.025
−0.050
−0.075
0.09
0.12
0.15
0.18
SNP effect on Keratin, type II cytoskeletal 5
SNP effect on Gut microbiota abundance (genus Catenibacterium id.2153)

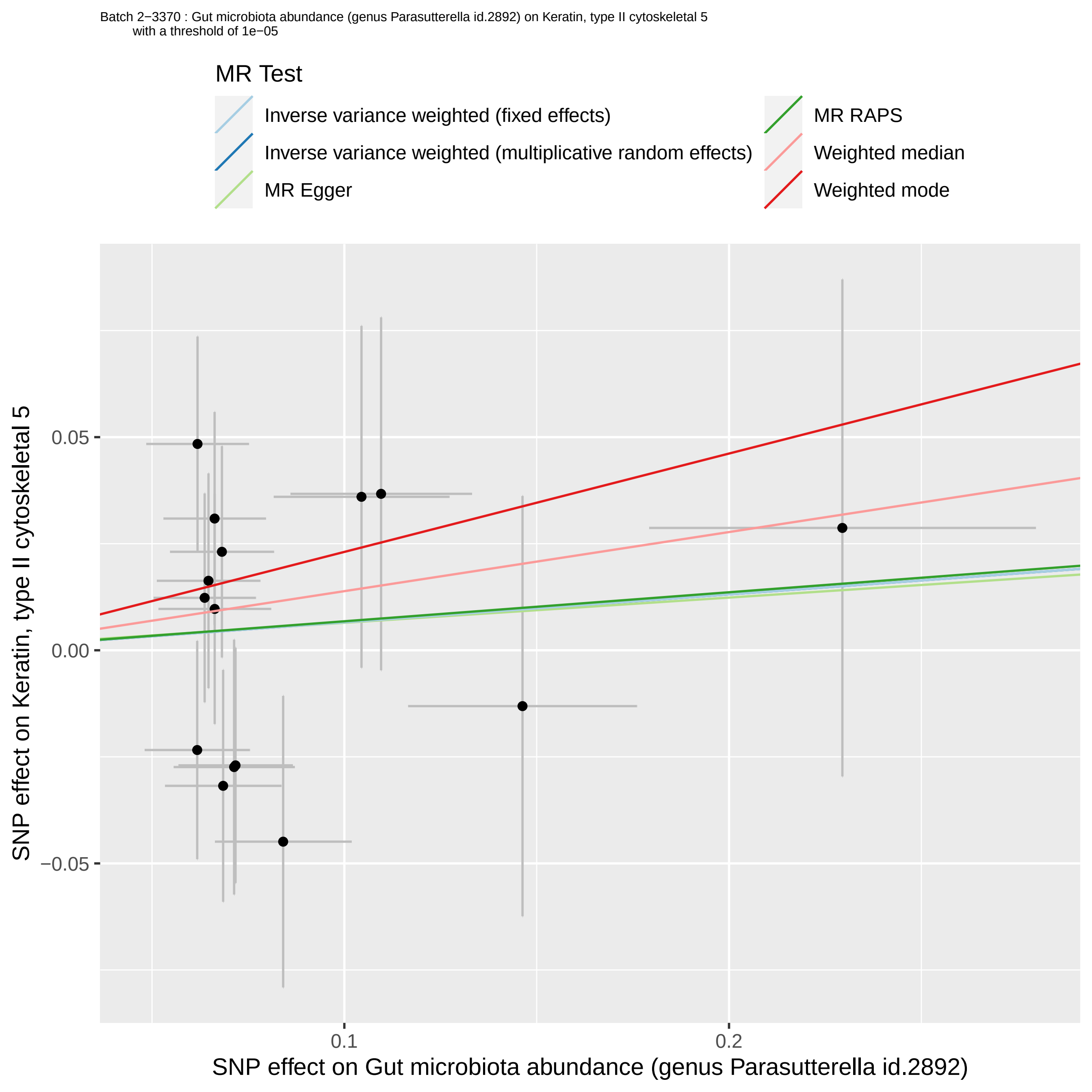
Batch 2−3370 : Gut microbiota abundance (genus Parasutterella id.2892) on Keratin, type II cytoskeletal 5
with a threshold of 1e−05
MR Test
Inverse variance weighted (fixed effects)
Inverse variance weighted (multiplicative random effects)
MR Egger
MR RAPS
Weighted median
Weighted mode
0.05
0.00
−0.05
0.1
0.2
SNP effect on Keratin, type II cytoskeletal 5
SNP effect on Gut microbiota abundance (genus Parasutterella id.2892)

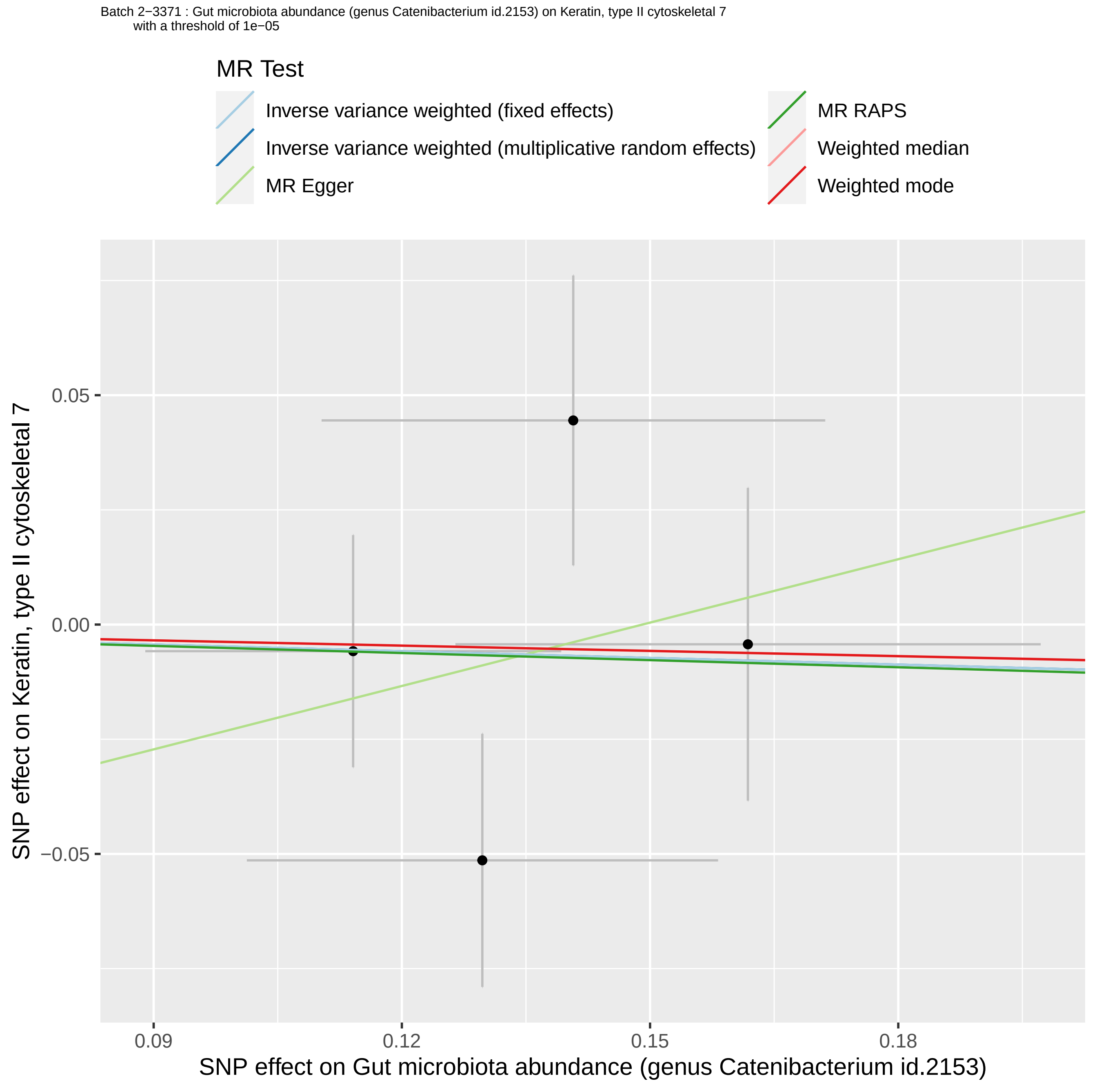
Batch 2−3371 : Gut microbiota abundance (genus Catenibacterium id.2153) on Keratin, type II cytoskeletal 7
with a threshold of 1e−05
MR Test
Inverse variance weighted (fixed effects)
Inverse variance weighted (multiplicative random effects)
MR Egger
MR RAPS
Weighted median
Weighted mode
SNP effect on Keratin, type II cytoskeletal 7
0.05
0.00
−0.05
0.09
0.12
0.15
0.18
SNP effect on Gut microbiota abundance (genus Catenibacterium id.2153)

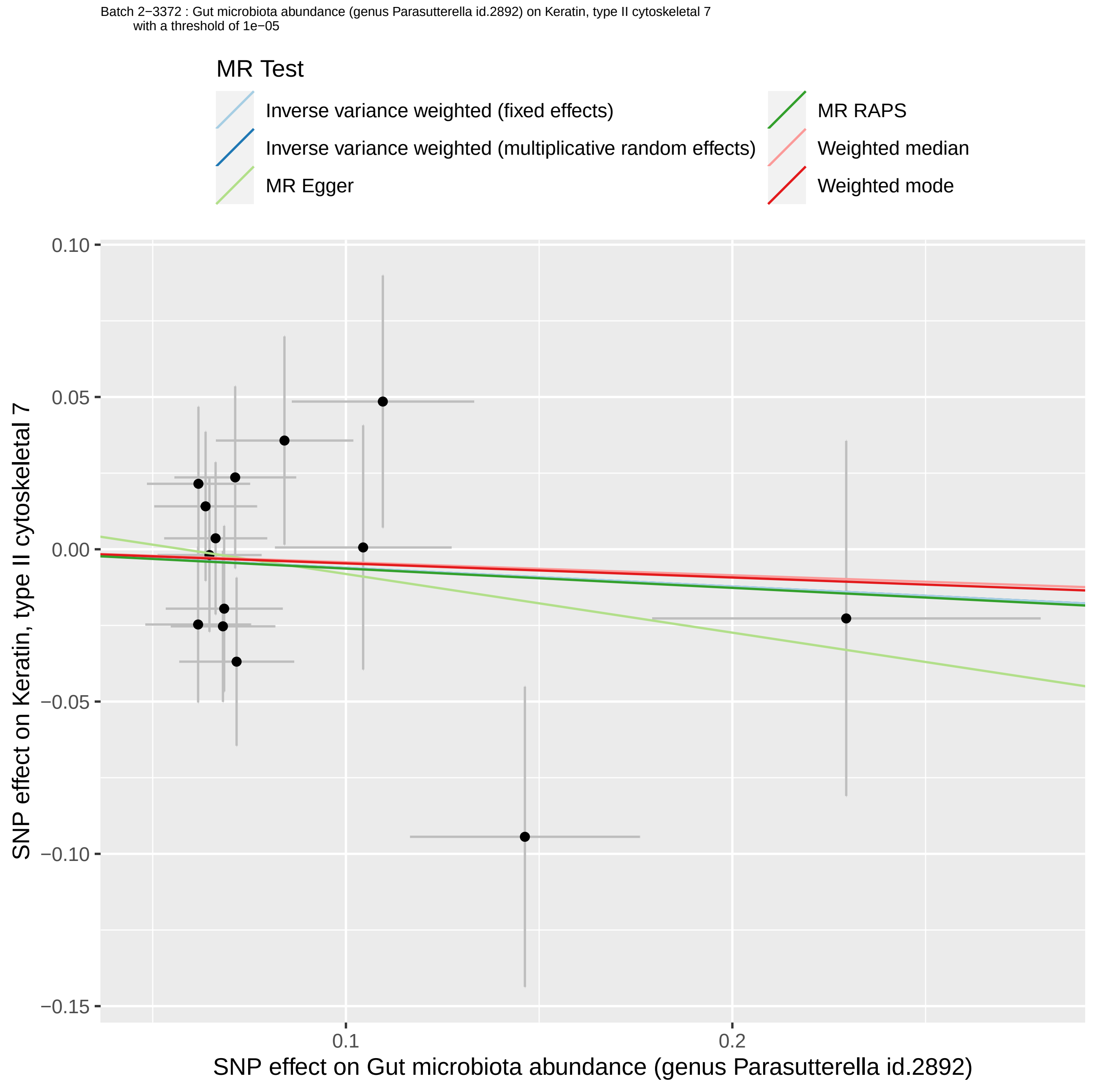

Batch 2−3372 : Gut microbiota abundance (genus Parasutterella id.2892) on Keratin, type II cytoskeletal 7
with a threshold of 1e−05
MR Test
Inverse variance weighted (fixed effects)
Inverse variance weighted (multiplicative random effects)
MR Egger
MR RAPS
Weighted median
Weighted mode
0.10
0.05
0.00
−0.05
−0.10
−0.15
0.1
0.2
SNP effect on Keratin, type II cytoskeletal 7
SNP effect on Gut microbiota abundance (genus Parasutterella id.2892)

Batch 2−3373 : Gut microbiota abundance (genus Catenibacterium id.2153) on Keratin−associated protein 2−4
with a threshold of 1e−05

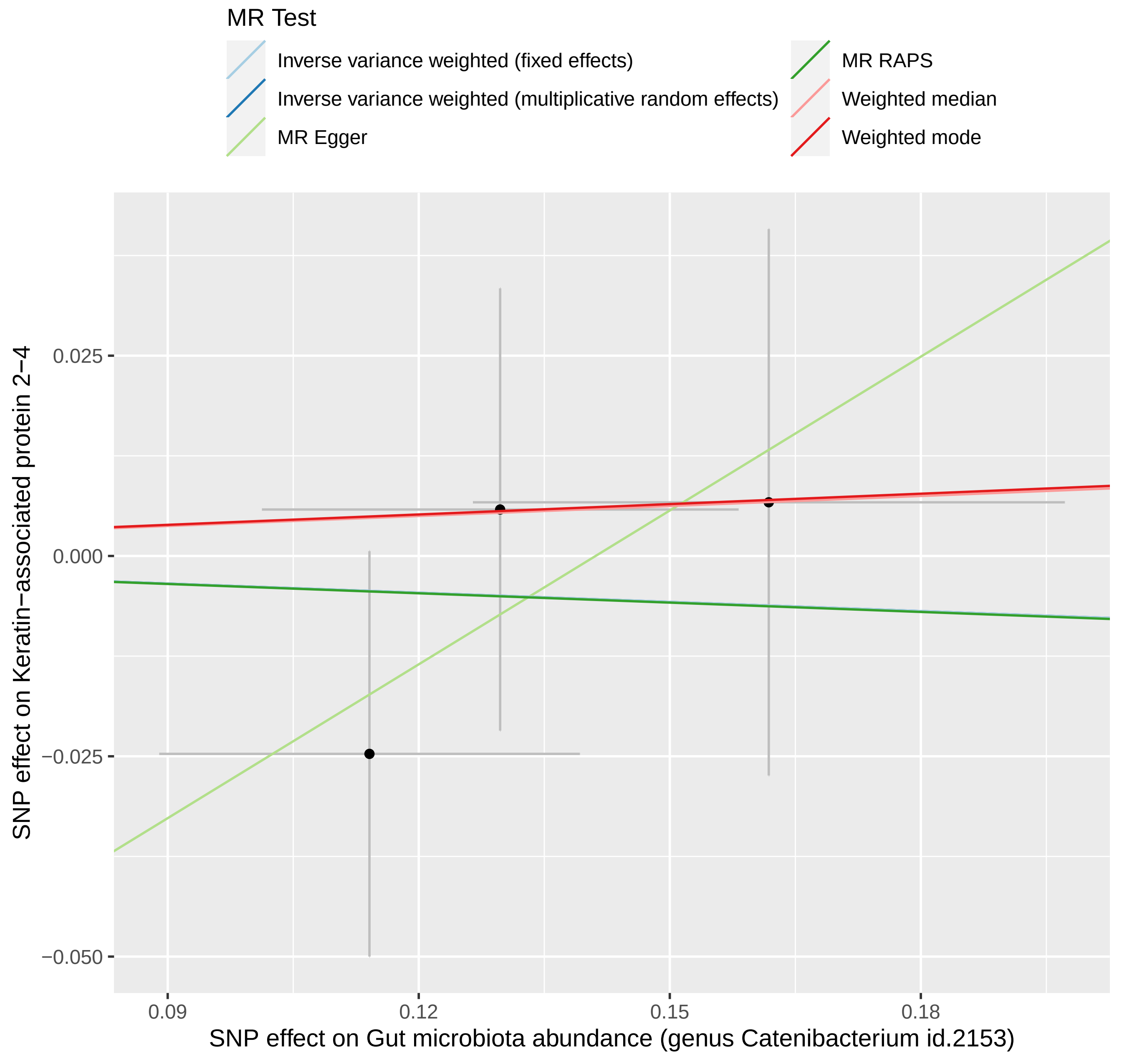

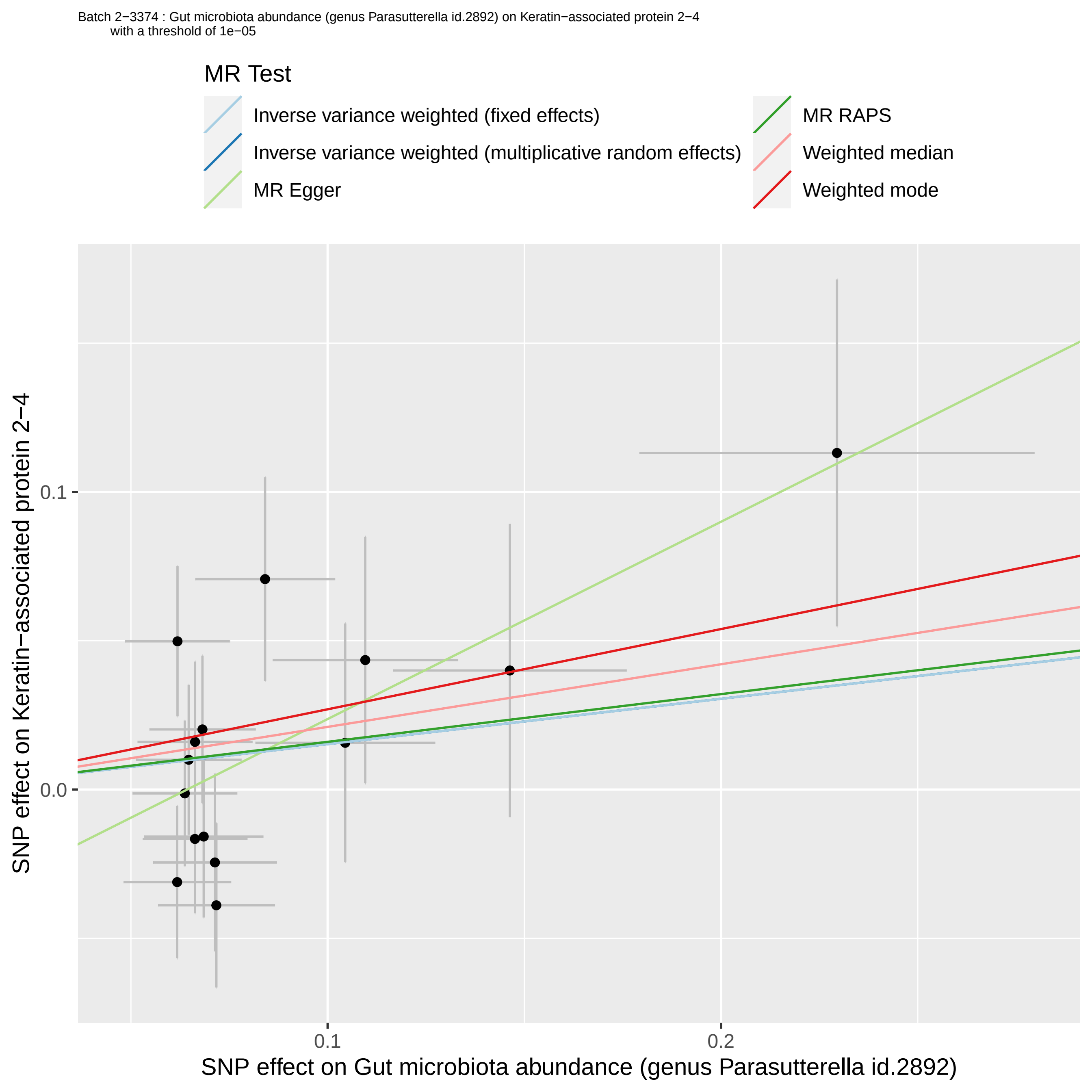

Batch 2−3374 : Gut microbiota abundance (genus Parasutterella id.2892) on Keratin−associated protein 2−4
with a threshold of 1e−05
MR Test
Inverse variance weighted (fixed effects)
Inverse variance weighted (multiplicative random effects)
MR Egger
MR RAPS
Weighted median
Weighted mode
0.1
0.0
0.1
0.2
SNP effect on Keratin−associated protein 2−4
SNP effect on Gut microbiota abundance (genus Parasutterella id.2892)

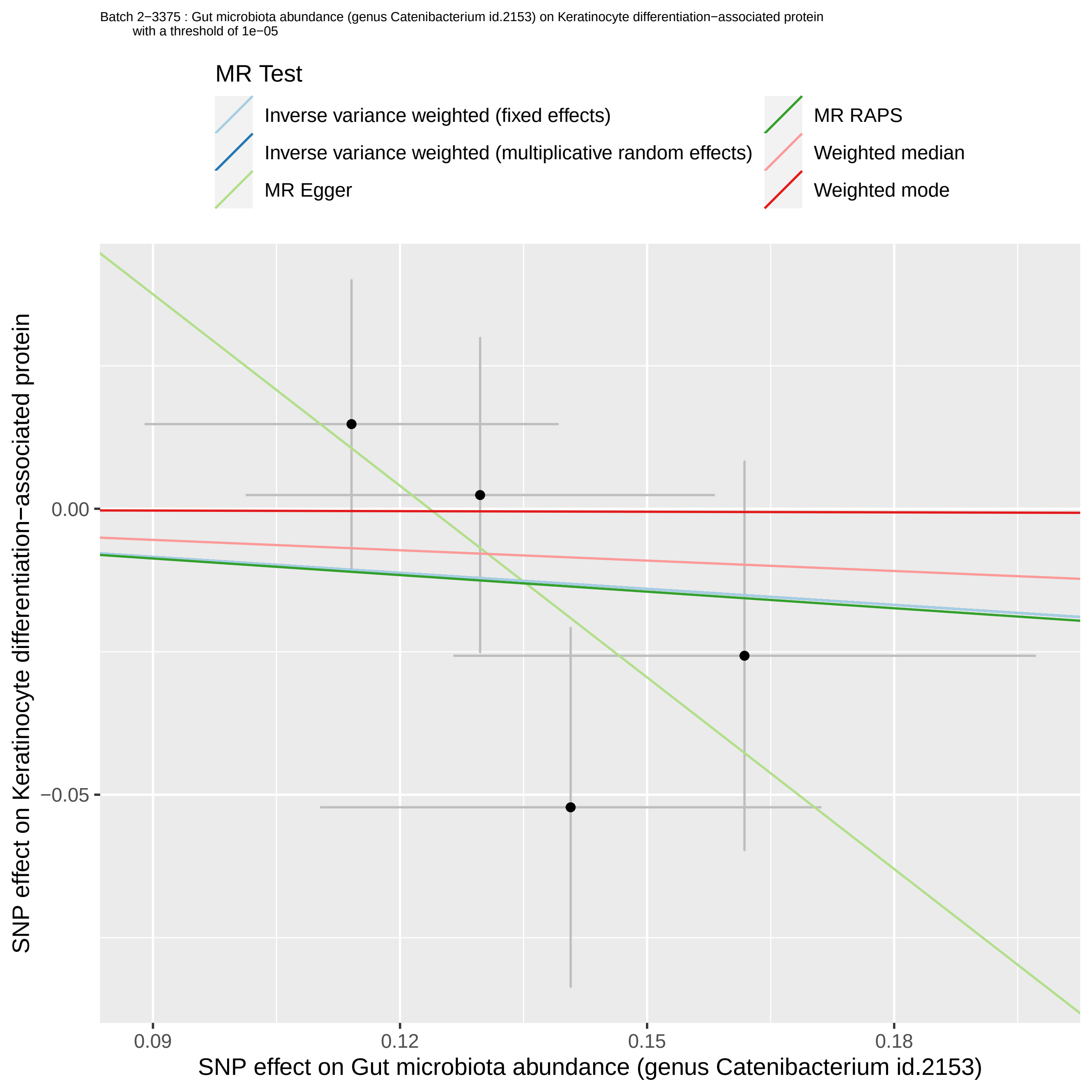
Batch 2−3375 : Gut microbiota abundance (genus Catenibacterium id.2153) on Keratinocyte differentiation−associated protein with a threshold of 1e−05
MR Test
Inverse variance weighted (fixed effects)
Inverse variance weighted (multiplicative random effects)
MR Egger
MR RAPS
Weighted median
Weighted mode
0.00
−0.05
0.09
0.12
0.15
0.18
SNP effect on Gut microbiota abundance (genus Catenibacterium id.2153)
SNP effect on Keratinocyte differentiation−associated protein

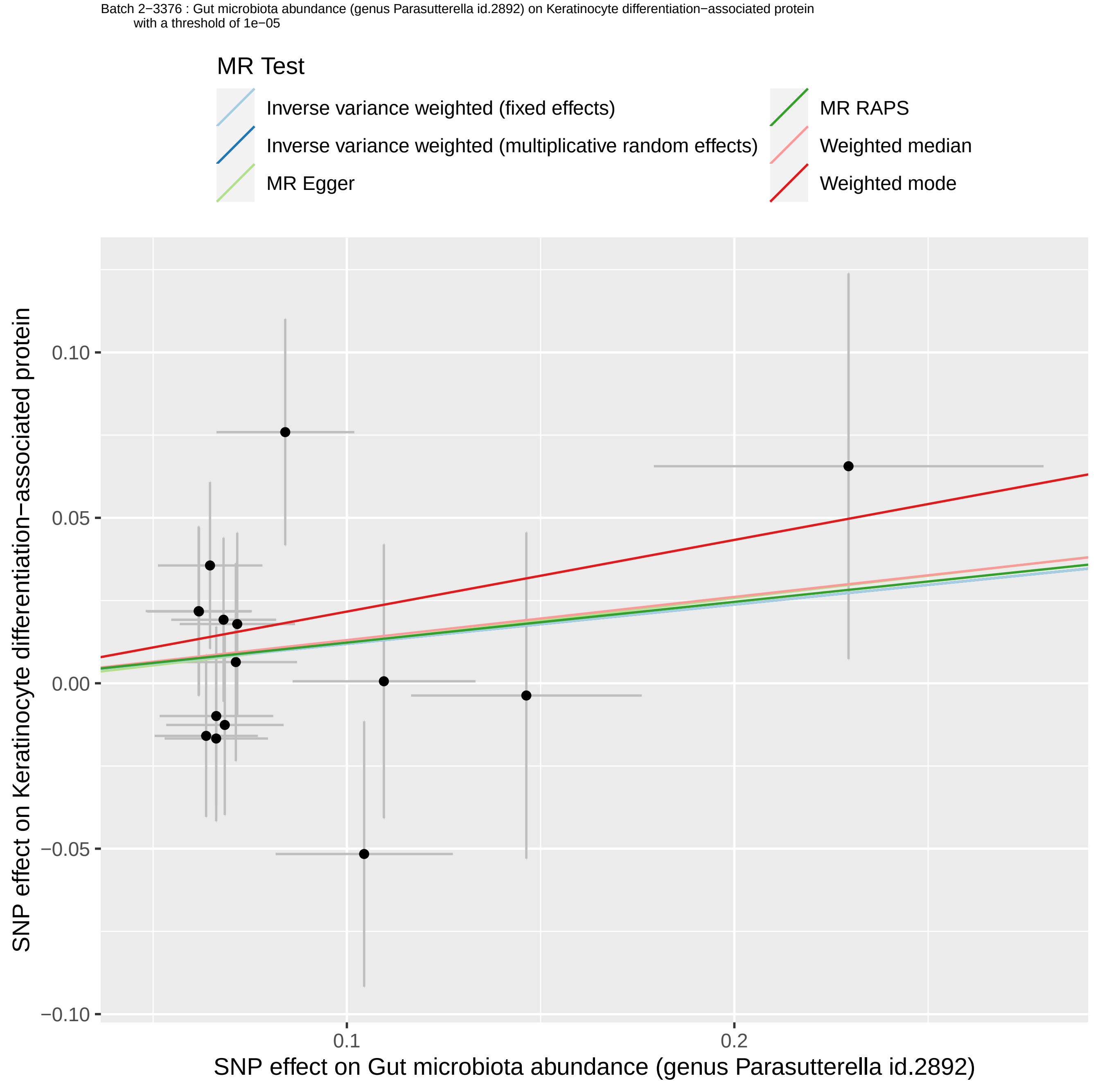

Batch 2−3376 : Gut microbiota abundance (genus Parasutterella id.2892) on Keratinocyte differentiation−associated protein
with a threshold of 1e−05
MR Test
Inverse variance weighted (fixed effects)
Inverse variance weighted (multiplicative random effects)
MR Egger
MR RAPS
Weighted median
Weighted mode
0.10
0.05
0.00
−0.05
−0.10
0.1
0.2
SNP effect on Keratinocyte differentiation−associated protein
SNP effect on Gut microbiota abundance (genus Parasutterella id.2892)

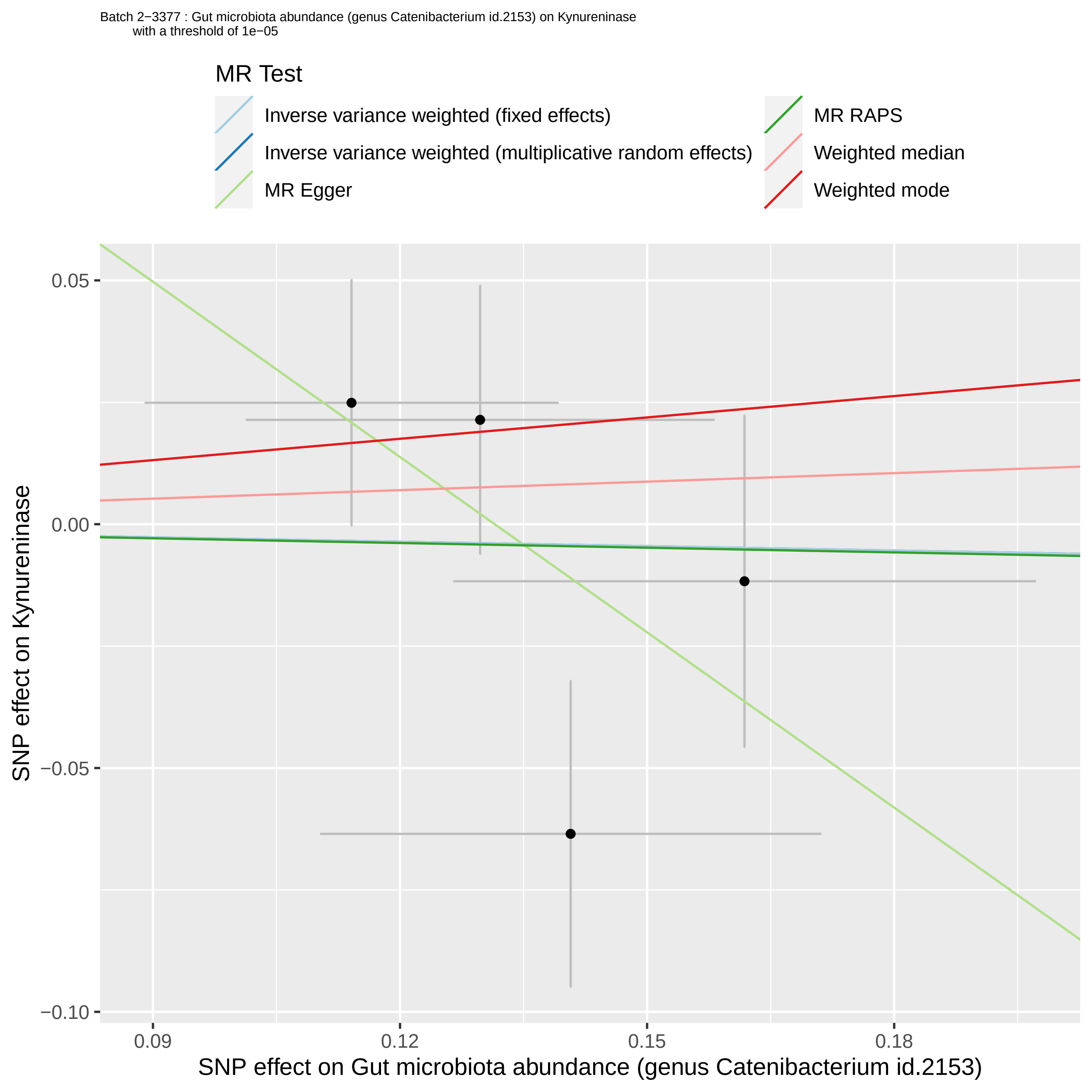

Batch 2−3377 : Gut microbiota abundance (genus Catenibacterium id.2153) on Kynureninase
with a threshold of 1e−05
MR Test
Inverse variance weighted (fixed effects)
Inverse variance weighted (multiplicative random effects)
MR Egger
MR RAPS
Weighted median
Weighted mode
0.05
0.00
−0.05
−0.10
0.09
0.12
0.15
0.18
SNP effect on Kynureninase
SNP effect on Gut microbiota abundance (genus Catenibacterium id.2153)

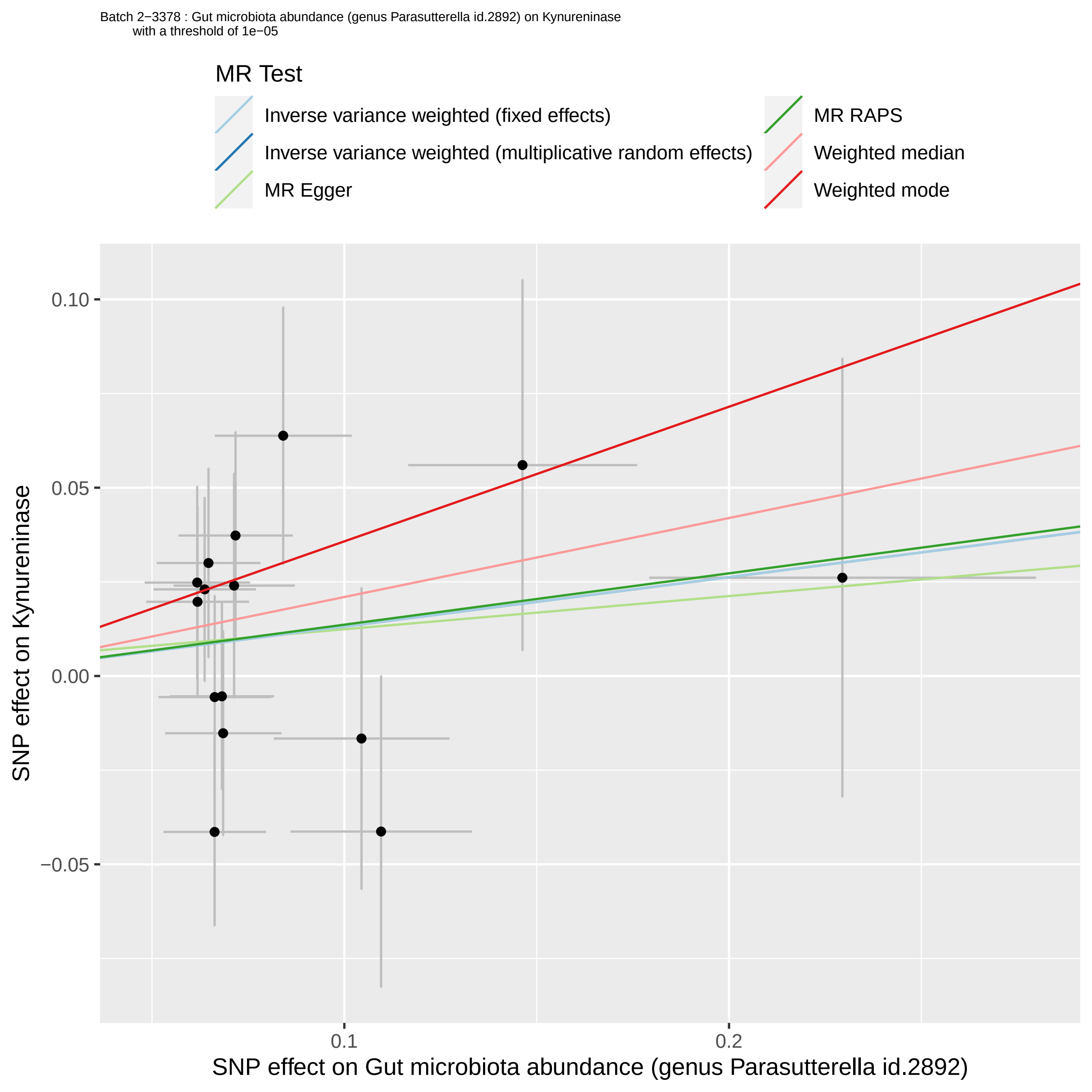

Batch 2−3378 : Gut microbiota abundance (genus Parasutterella id.2892) on Kynureninase
with a threshold of 1e−05
MR Test
Inverse variance weighted (fixed effects)
Inverse variance weighted (multiplicative random effects)
MR Egger
MR RAPS
Weighted median
Weighted mode
0.10
0.05
0.00
−0.05
0.1
0.2
SNP effect on Kynureninase
SNP effect on Gut microbiota abundance (genus Parasutterella id.2892)

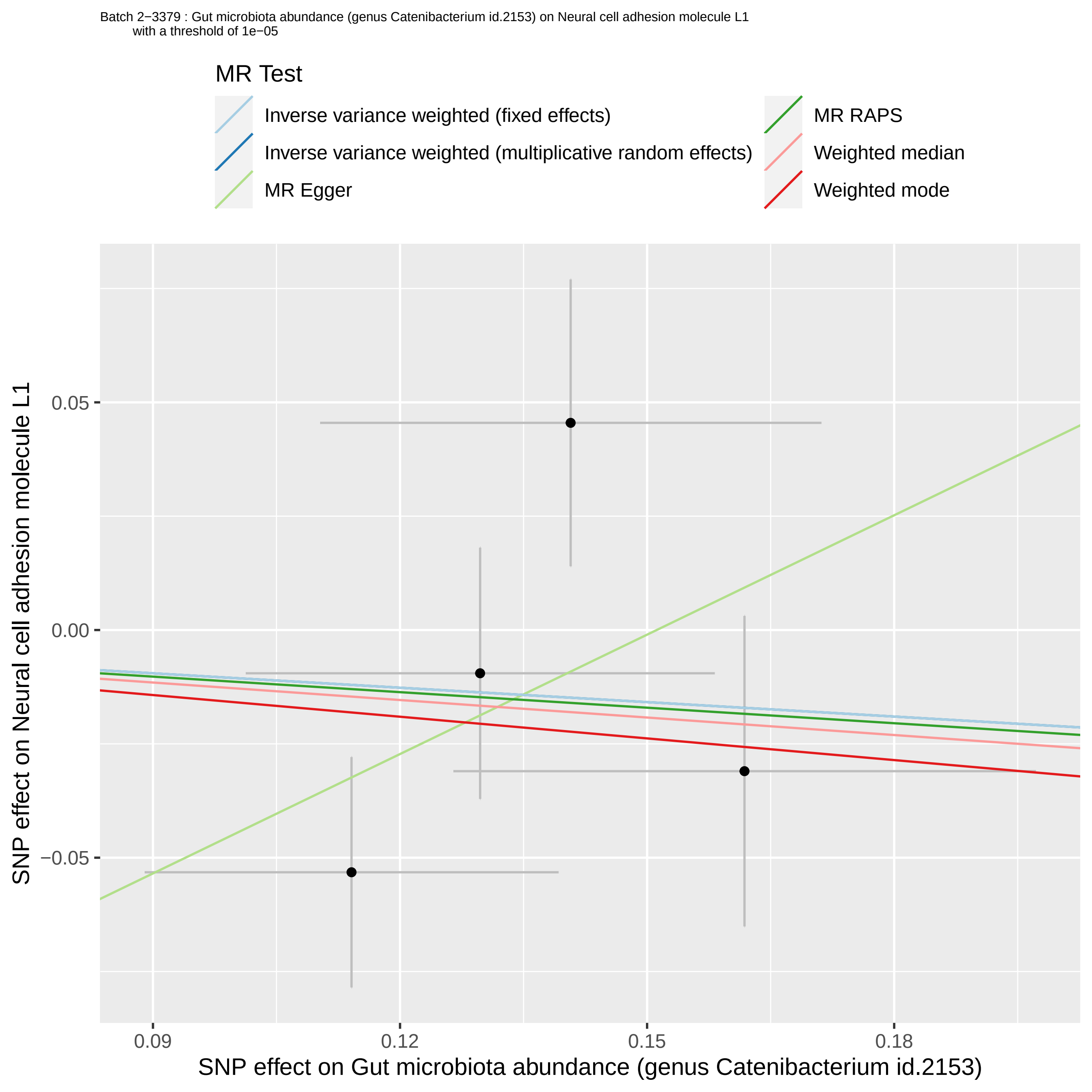

Batch 2−3379 : Gut microbiota abundance (genus Catenibacterium id.2153) on Neural cell adhesion molecule L1
with a threshold of 1e−05
MR Test
Inverse variance weighted (fixed effects)
Inverse variance weighted (multiplicative random effects)
MR Egger
MR RAPS
Weighted median
Weighted mode
0.05
0.00
−0.05
0.09
0.12
0.15
0.18
SNP effect on Neural cell adhesion molecule L1
SNP effect on Gut microbiota abundance (genus Catenibacterium id.2153)

Batch 2−3380 : Gut microbiota abundance (genus Parasutterella id.2892) on Neural cell adhesion molecule L1
with a threshold of 1e−05

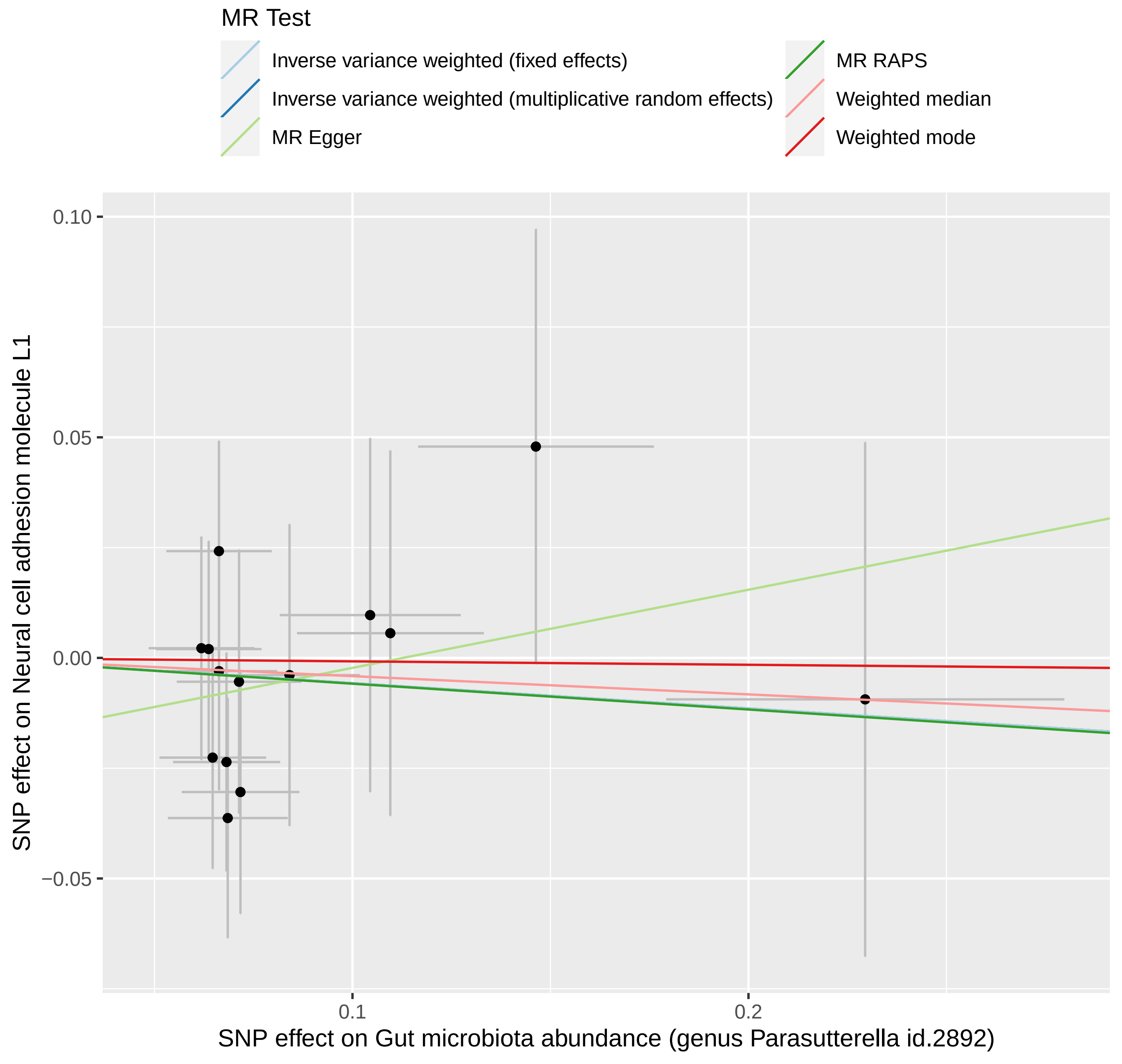

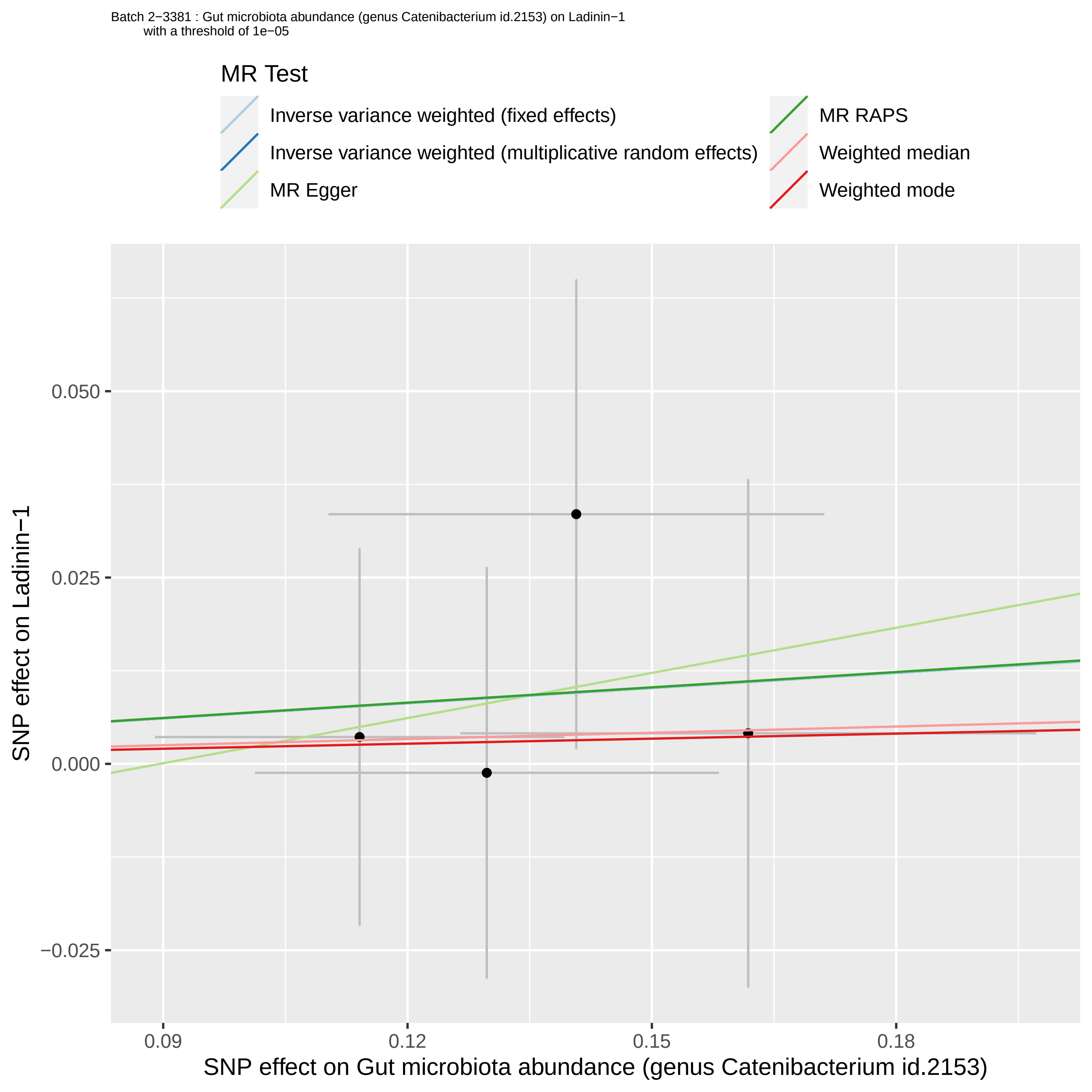

Batch 2−3381 : Gut microbiota abundance (genus Catenibacterium id.2153) on Ladinin−1
with a threshold of 1e−05
MR Test
Inverse variance weighted (fixed effects)
Inverse variance weighted (multiplicative random effects)
MR Egger
MR RAPS
Weighted median
Weighted mode
0.050
0.025
0.000
−0.025
0.09
0.12
0.15
0.18
SNP effect on Ladinin−1
SNP effect on Gut microbiota abundance (genus Catenibacterium id.2153)

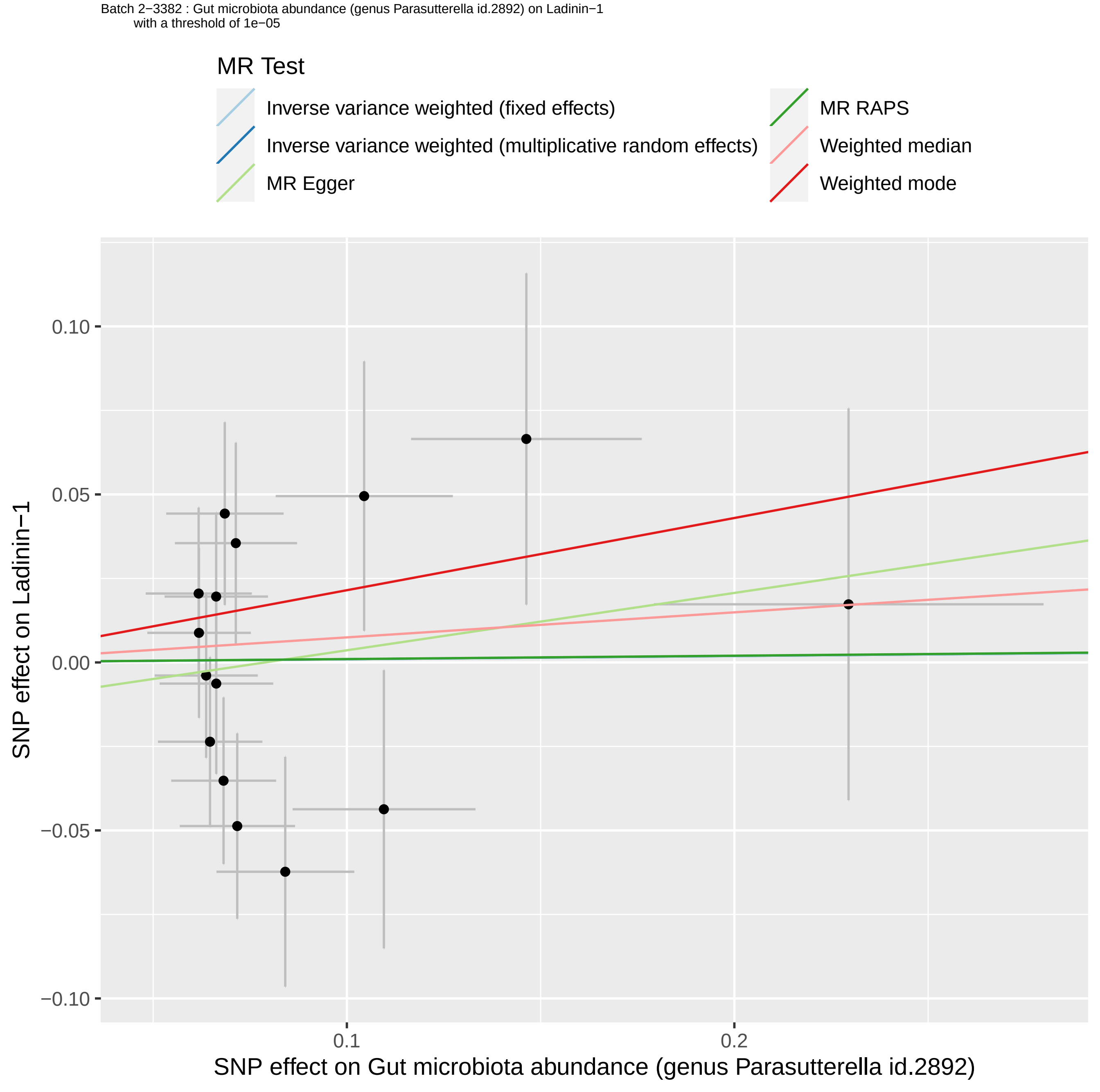
Batch 2−3382 : Gut microbiota abundance (genus Parasutterella id.2892) on Ladinin−1
with a threshold of 1e−05
MR Test
Inverse variance weighted (fixed effects)
Inverse variance weighted (multiplicative random effects)
MR Egger
MR RAPS
Weighted median
Weighted mode
0.10
0.05
0.00
−0.05
−0.10
0.1
0.2
SNP effect on Ladinin−1
SNP effect on Gut microbiota abundance (genus Parasutterella id.2892)

Batch 2−3383 : Gut microbiota abundance (genus Catenibacterium id.2153) on Lymphocyte activation gene 3 protein
with a threshold of 1e−05

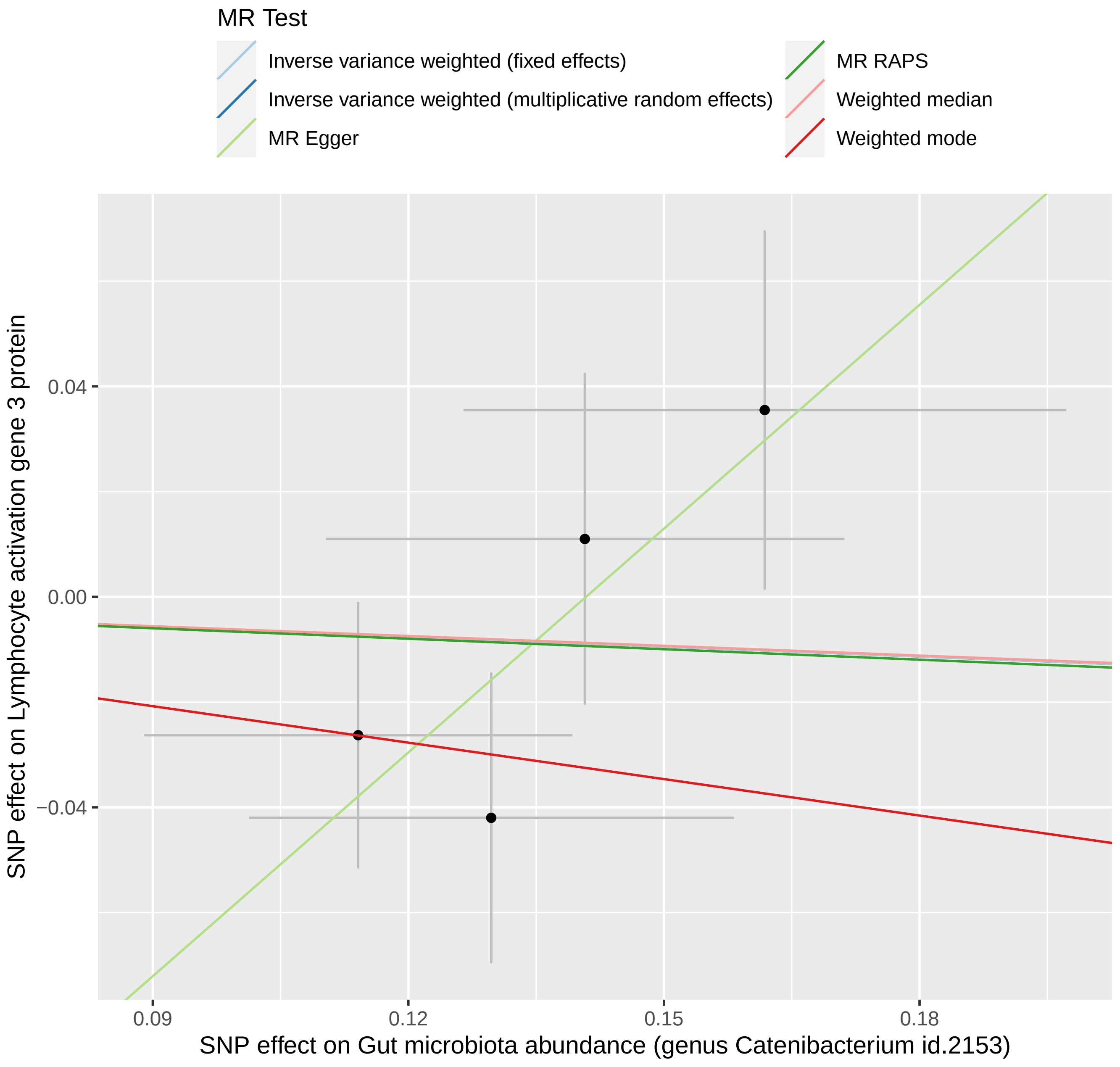

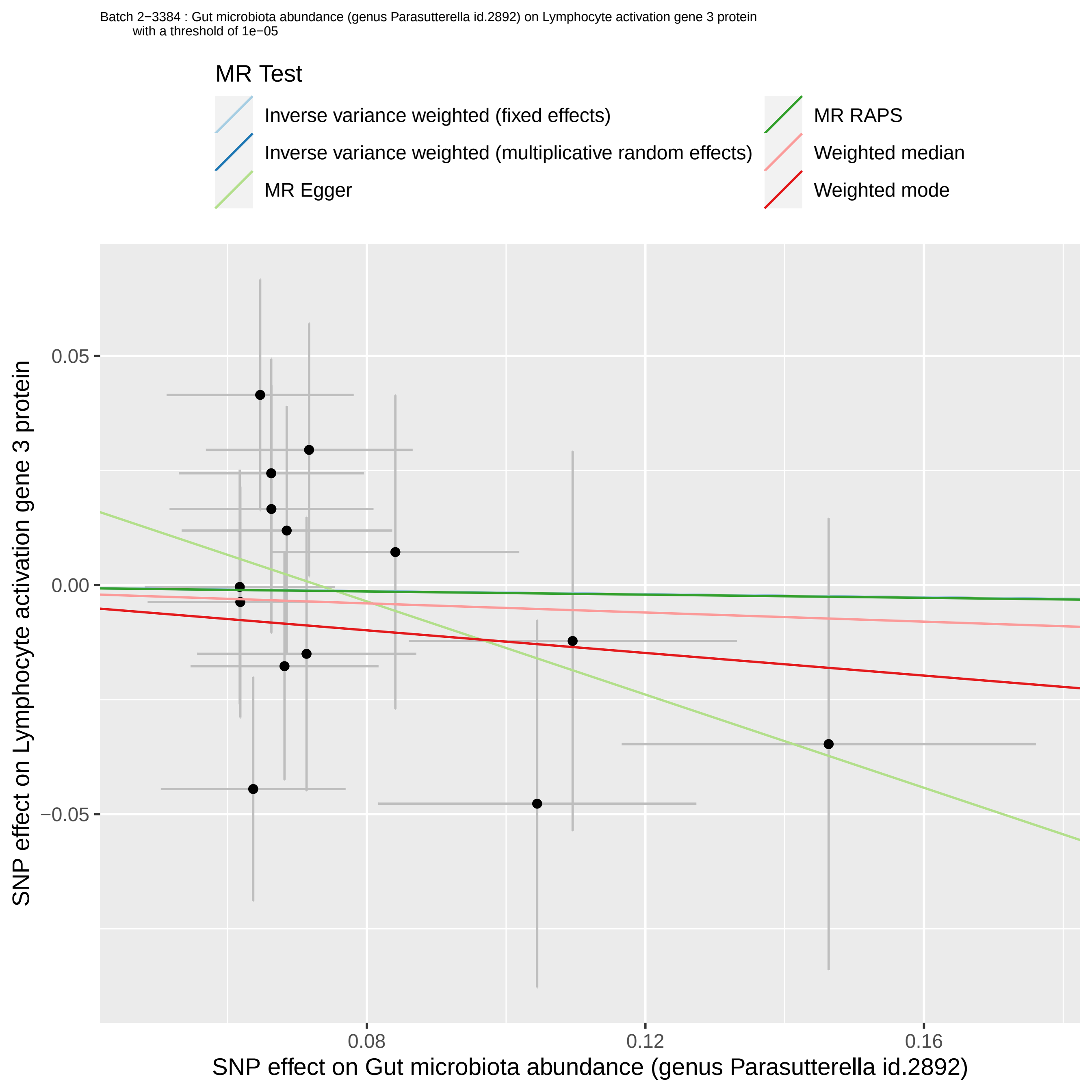

Batch 2−3384 : Gut microbiota abundance (genus Parasutterella id.2892) on Lymphocyte activation gene 3 protein
with a threshold of 1e−05
MR Test
Inverse variance weighted (fixed effects)
Inverse variance weighted (multiplicative random effects)
MR Egger
MR RAPS
Weighted median
Weighted mode
0.05
0.00
−0.05
0.08
0.12
0.16
SNP effect on Lymphocyte activation gene 3 protein
SNP effect on Gut microbiota abundance (genus Parasutterella id.2892)

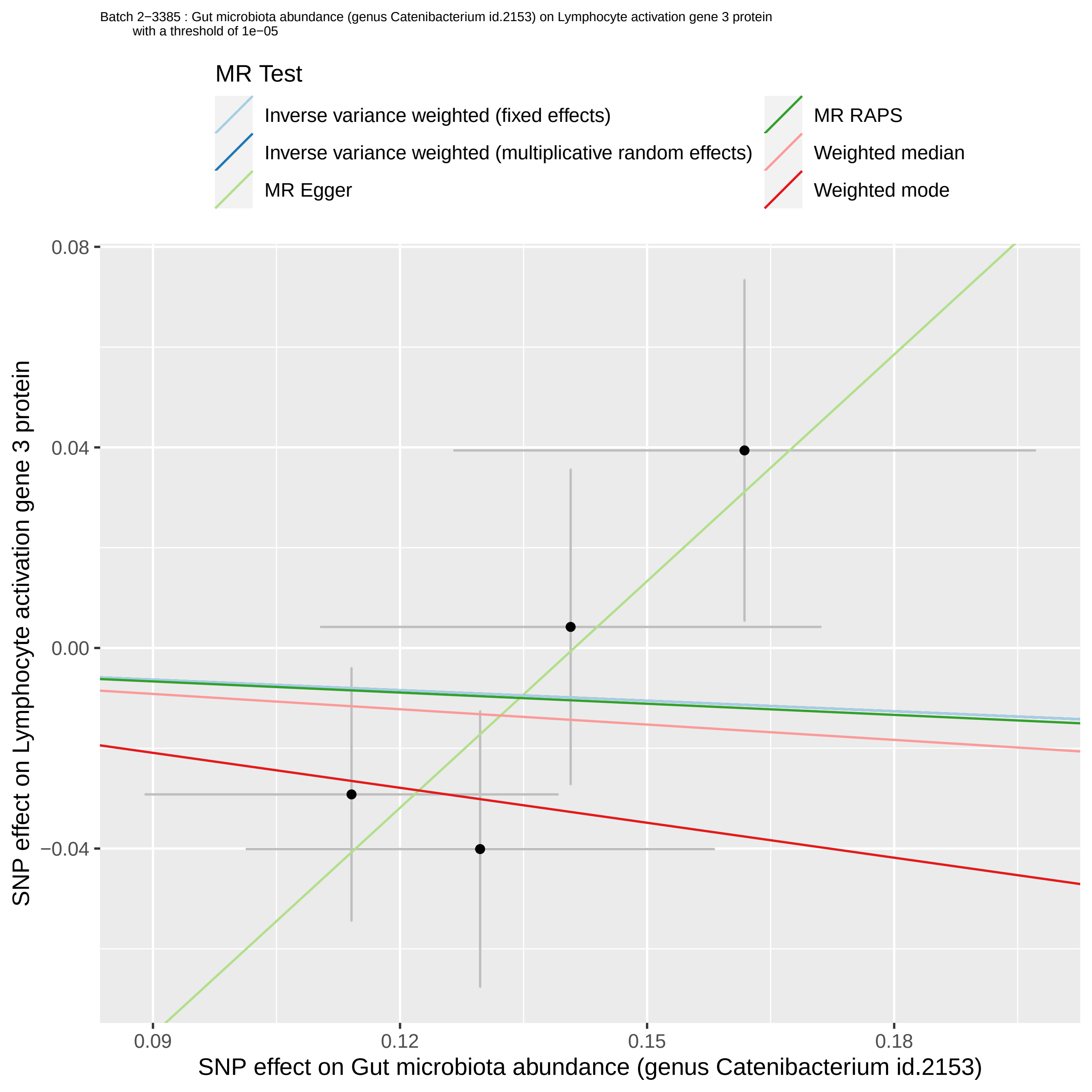

Batch 2−3385 : Gut microbiota abundance (genus Catenibacterium id.2153) on Lymphocyte activation gene 3 protein
with a threshold of 1e−05
MR Test
Inverse variance weighted (fixed effects)
Inverse variance weighted (multiplicative random effects)
MR Egger
MR RAPS
Weighted median
Weighted mode
0.08
0.04
0.00
−0.04
0.09
0.12
0.15
0.18
SNP effect on Lymphocyte activation gene 3 protein
SNP effect on Gut microbiota abundance (genus Catenibacterium id.2153)

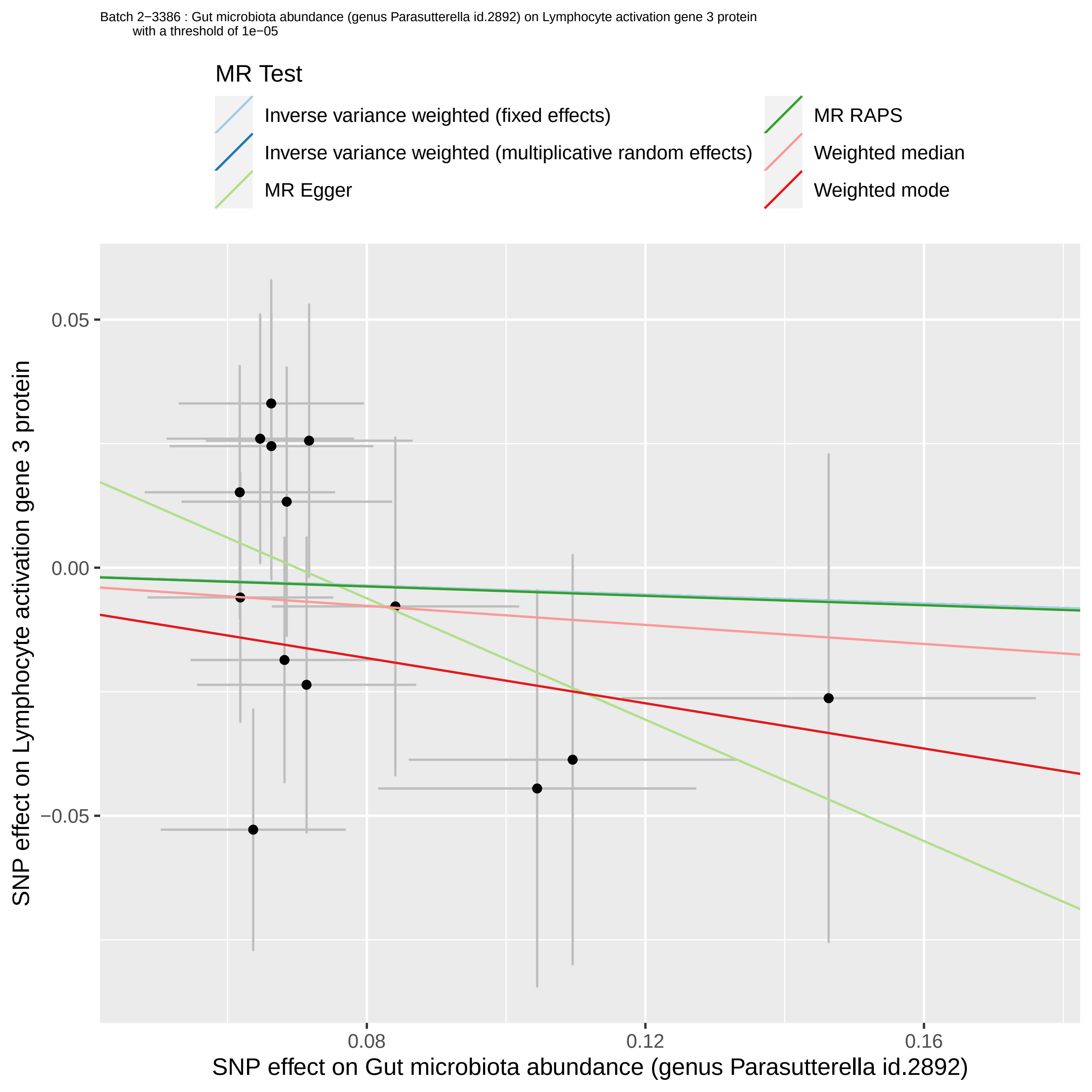

Batch 2−3386 : Gut microbiota abundance (genus Parasutterella id.2892) on Lymphocyte activation gene 3 protein
with a threshold of 1e−05
MR Test
Inverse variance weighted (fixed effects)
Inverse variance weighted (multiplicative random effects)
MR Egger
MR RAPS
Weighted median
Weighted mode
0.05
0.00
−0.05
0.08
0.12
0.16
SNP effect on Lymphocyte activation gene 3 protein
SNP effect on Gut microbiota abundance (genus Parasutterella id.2892)

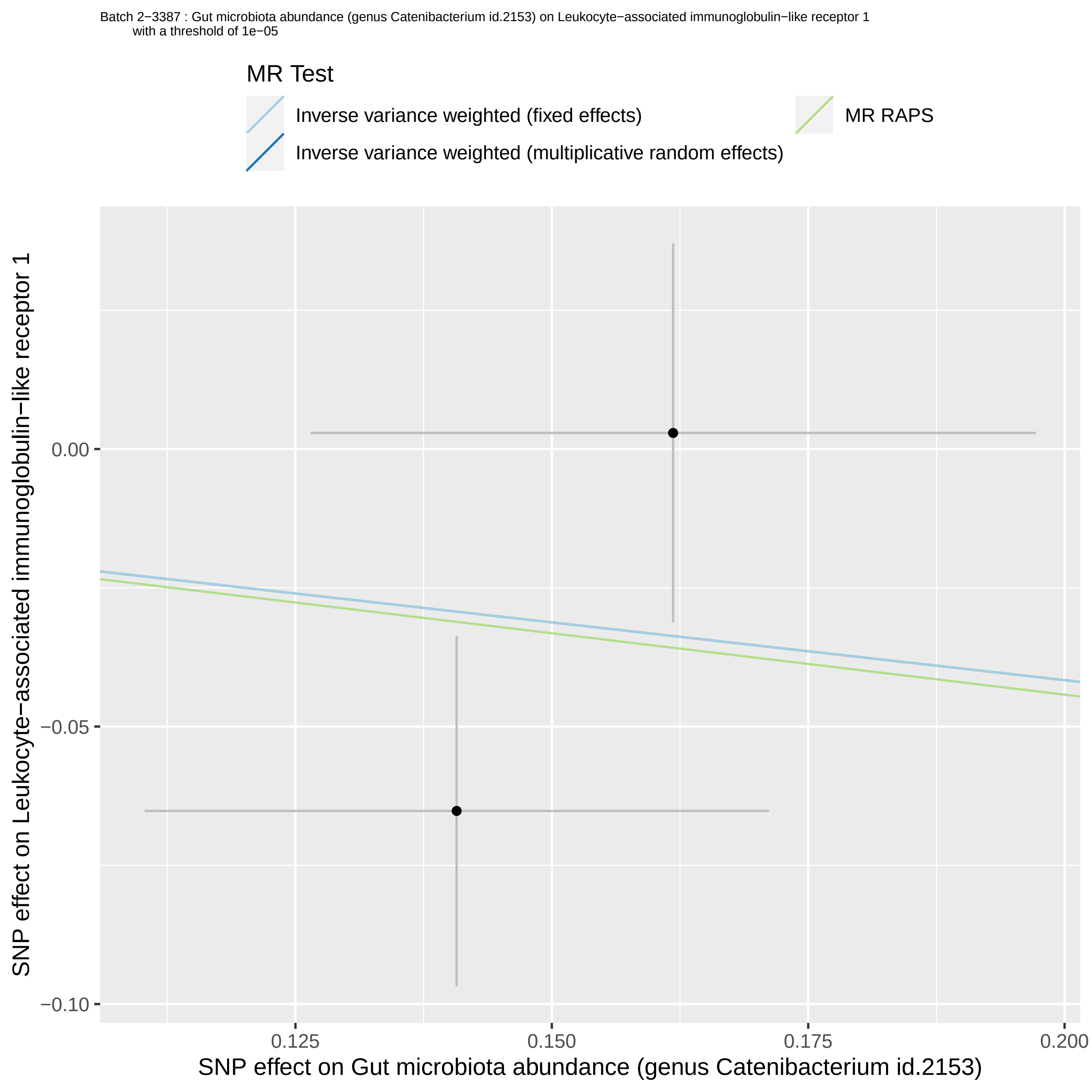
Batch 2−3387 : Gut microbiota abundance (genus Catenibacterium id.2153) on Leukocyte−associated immunoglobulin−like receptor 1
with a threshold of 1e−05
MR Test
Inverse variance weighted (fixed effects)
Inverse variance weighted (multiplicative random effects)
MR RAPS
0.00
−0.05
−0.10
0.125
0.150
0.175
0.200
SNP effect on Gut microbiota abundance (genus Catenibacterium id.2153)
SNP effect on Leukocyte−associated immunoglobulin−like receptor 1

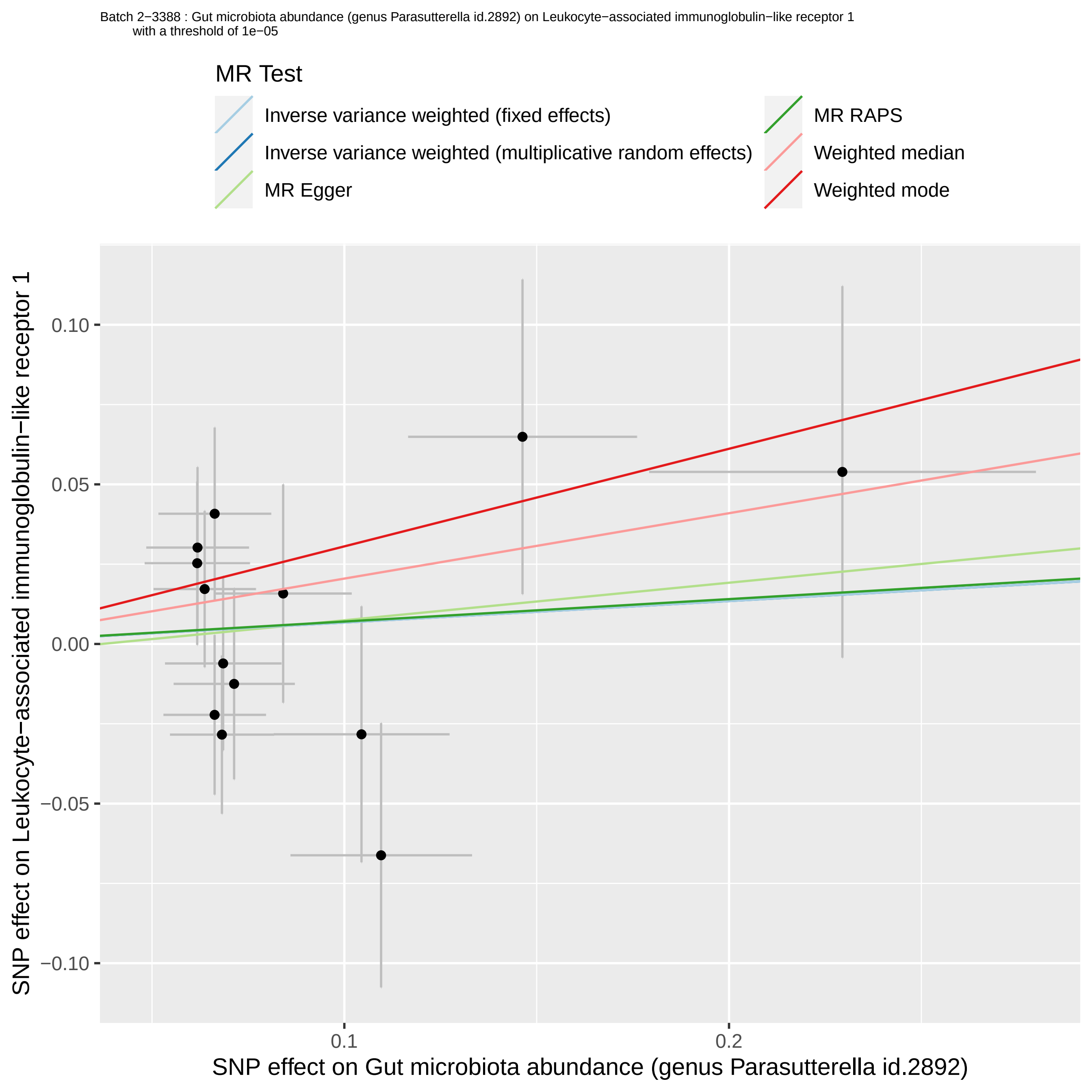
Batch 2−3388 : Gut microbiota abundance (genus Parasutterella id.2892) on Leukocyte−associated immunoglobulin−like receptor 1
with a threshold of 1e−05
MR Test
Inverse variance weighted (fixed effects)
Inverse variance weighted (multiplicative random effects)
MR Egger
MR RAPS
Weighted median
Weighted mode
SNP effect on Leukocyte−associated immunoglobulin−like receptor 1
0.10
0.05
0.00
−0.05
−0.10
0.1
0.2
SNP effect on Gut microbiota abundance (genus Parasutterella id.2892)

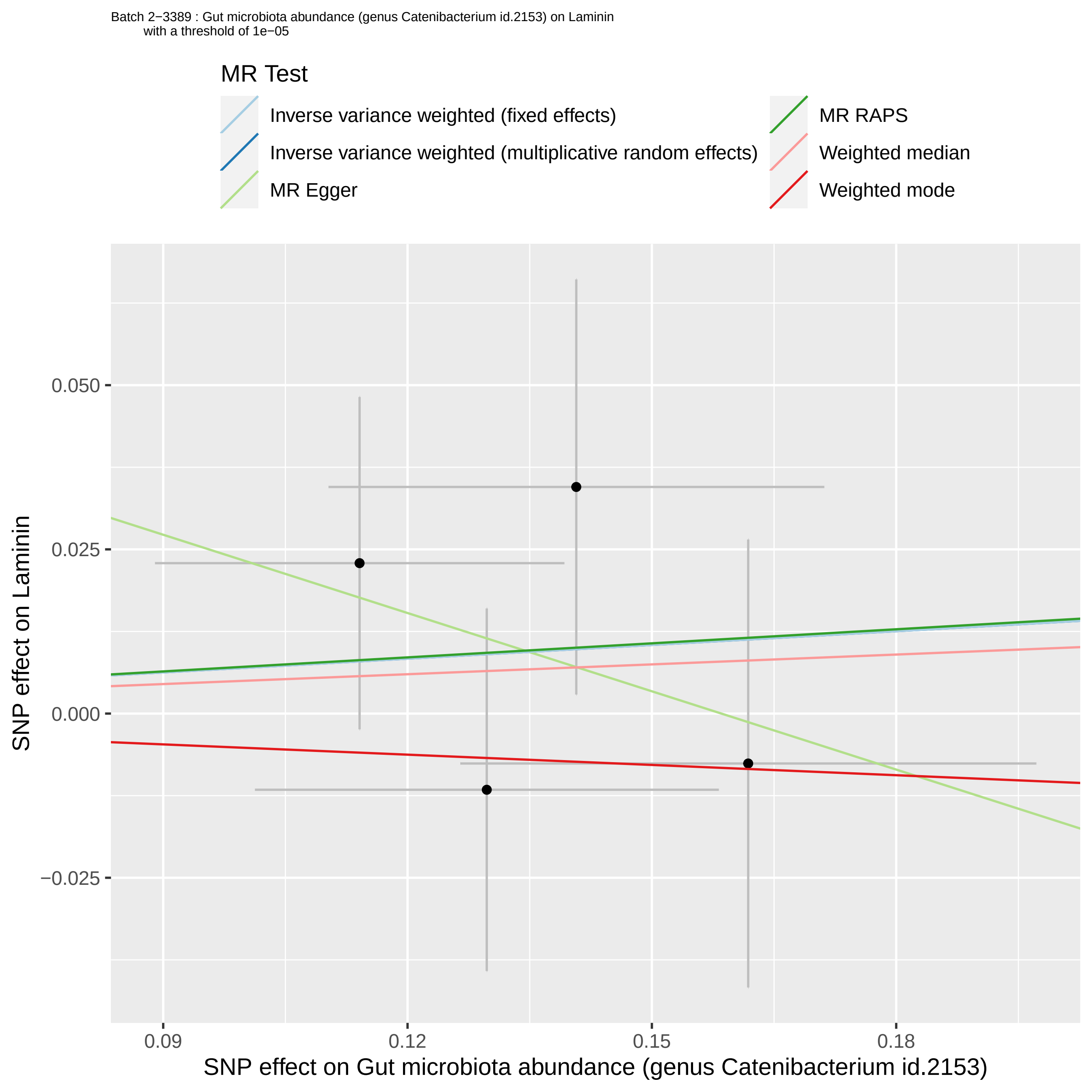
Batch 2−3389 : Gut microbiota abundance (genus Catenibacterium id.2153) on Laminin
with a threshold of 1e−05
MR Test
Inverse variance weighted (fixed effects)
Inverse variance weighted (multiplicative random effects)
MR Egger
MR RAPS
Weighted median
Weighted mode
0.050
0.025
0.000
−0.025
0.09
0.12
0.15
0.18
SNP effect on Laminin
SNP effect on Gut microbiota abundance (genus Catenibacterium id.2153)

Batch 2−3390 : Gut microbiota abundance (genus Parasutterella id.2892) on Laminin
with a threshold of 1e−05

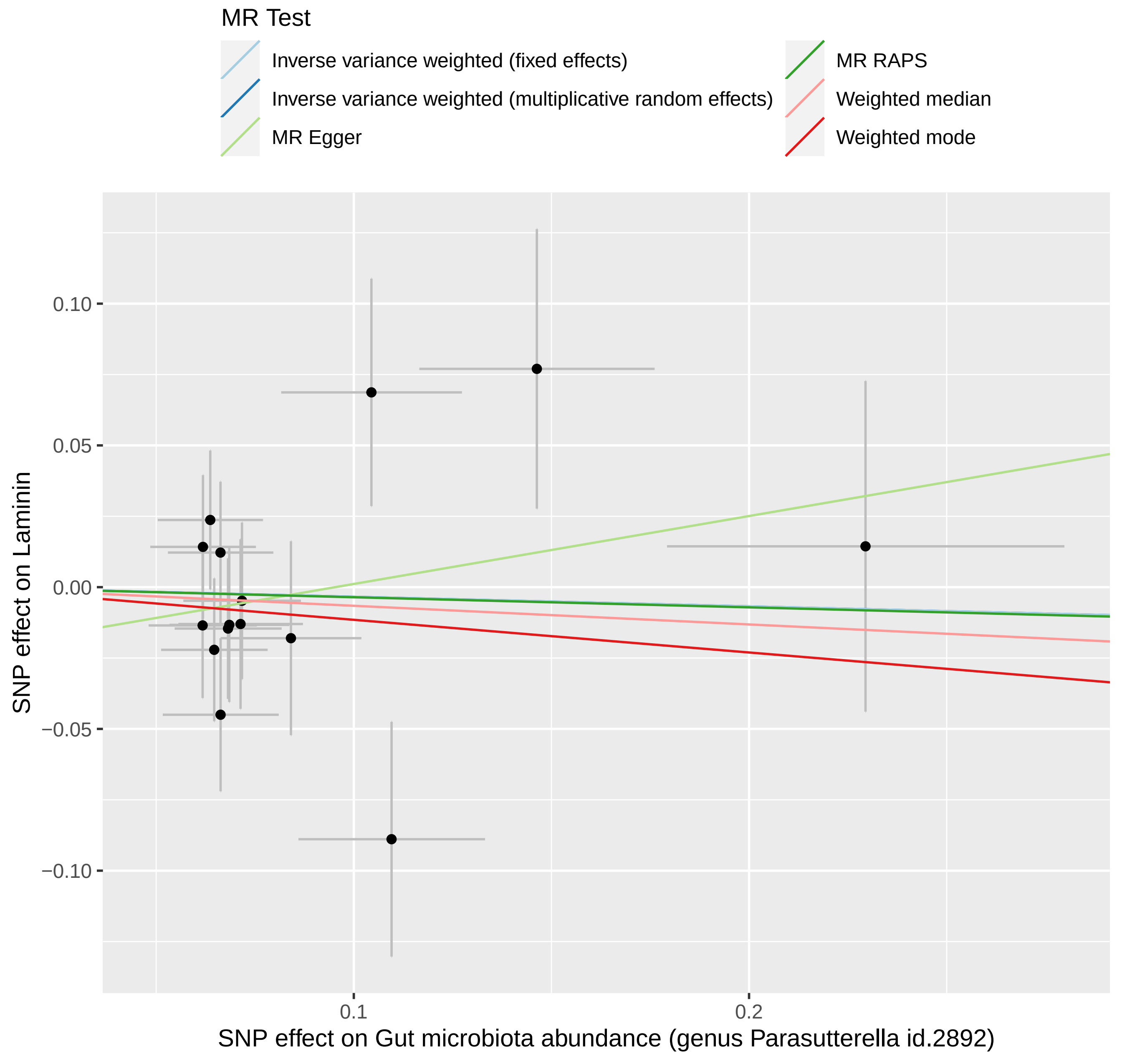

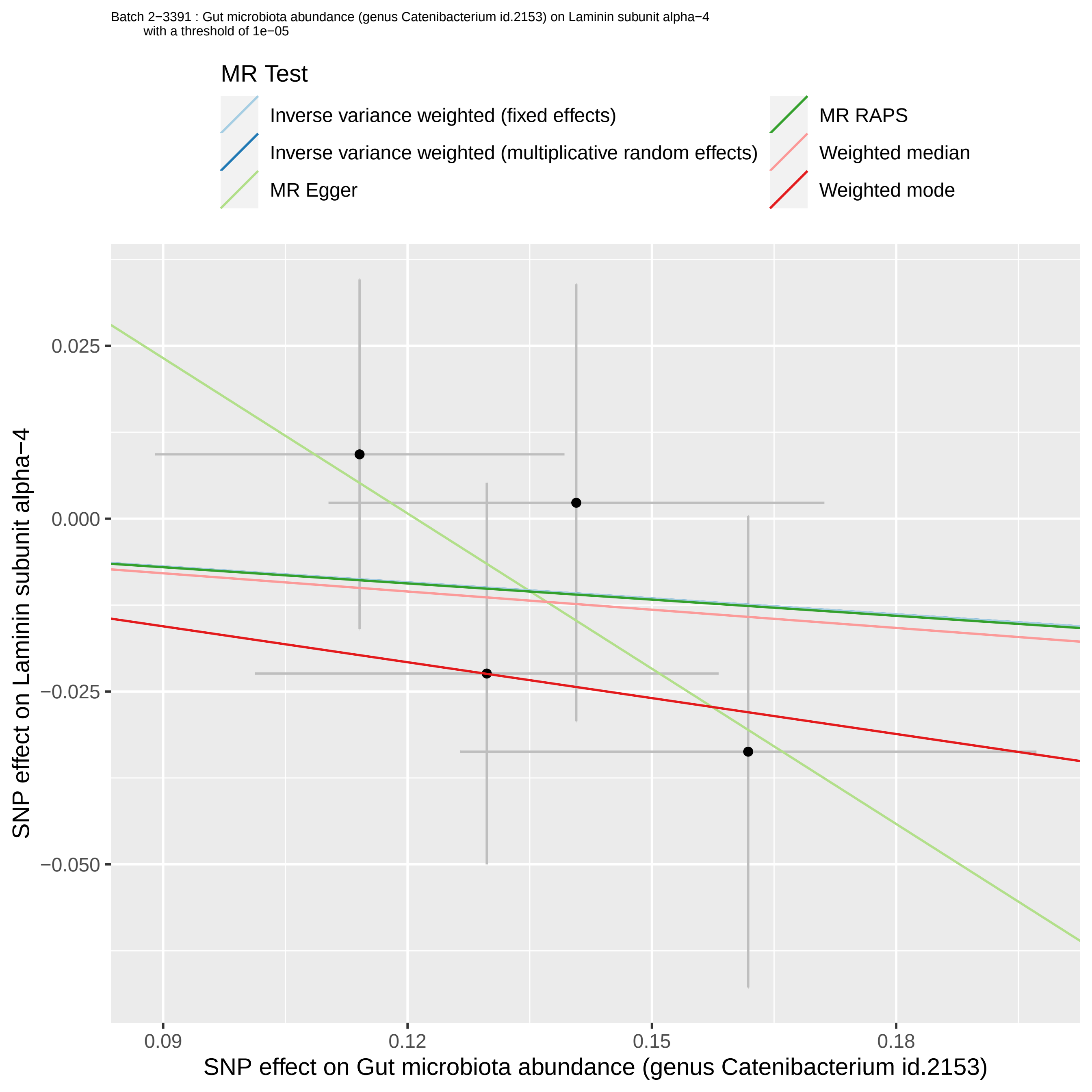
Batch 2−3391 : Gut microbiota abundance (genus Catenibacterium id.2153) on Laminin subunit alpha−4
with a threshold of 1e−05
MR Test
Inverse variance weighted (fixed effects)
Inverse variance weighted (multiplicative random effects)
MR Egger
MR RAPS
Weighted median
Weighted mode
0.025
0.000
−0.025
−0.050
0.09
0.12
0.15
0.18
SNP effect on Laminin subunit alpha−4
SNP effect on Gut microbiota abundance (genus Catenibacterium id.2153)

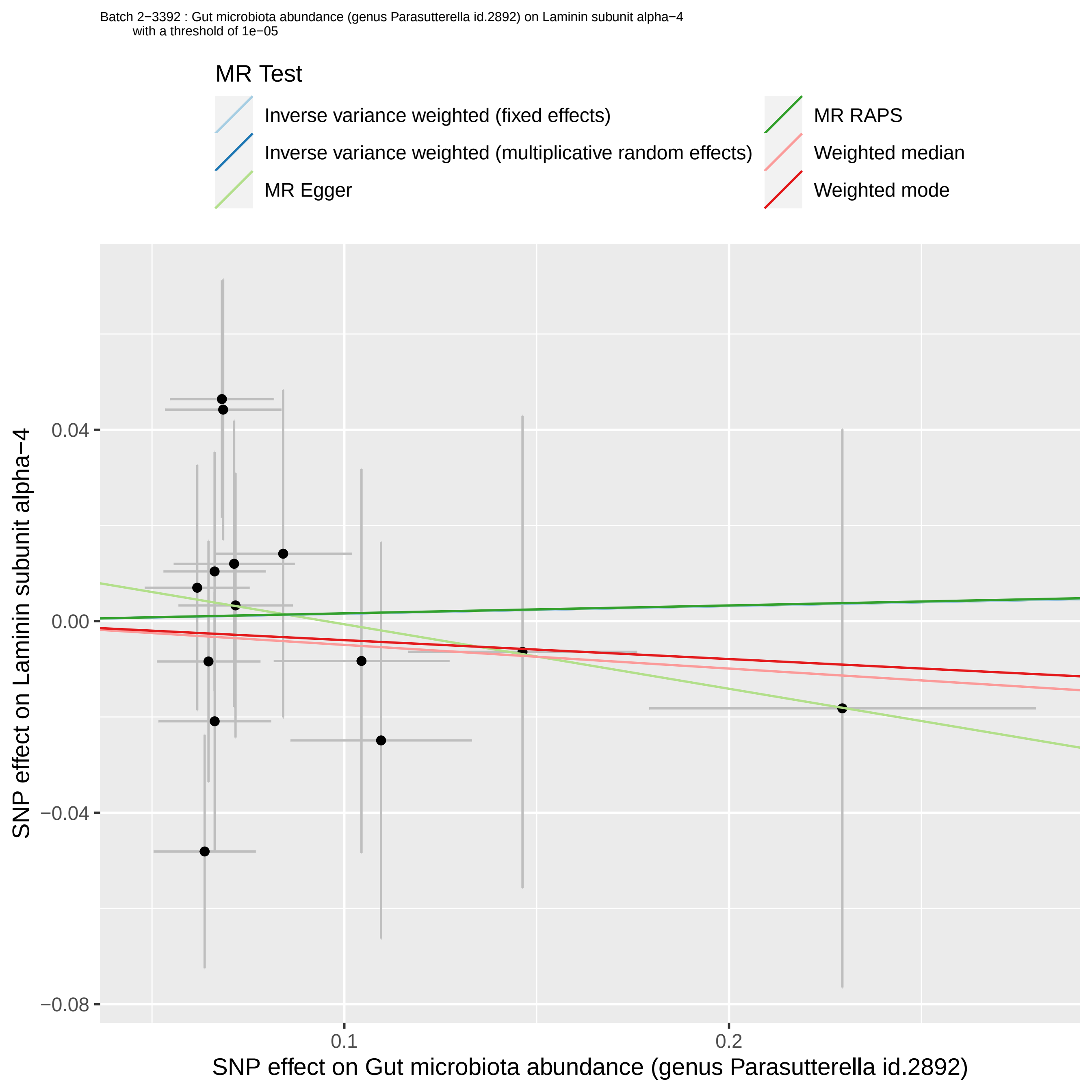
Batch 2−3392 : Gut microbiota abundance (genus Parasutterella id.2892) on Laminin subunit alpha−4
with a threshold of 1e−05
MR Test
Inverse variance weighted (fixed effects)
Inverse variance weighted (multiplicative random effects)
MR Egger
MR RAPS
Weighted median
Weighted mode
0.04
0.00
−0.04
−0.08
0.1
0.2
SNP effect on Laminin subunit alpha−4
SNP effect on Gut microbiota abundance (genus Parasutterella id.2892)

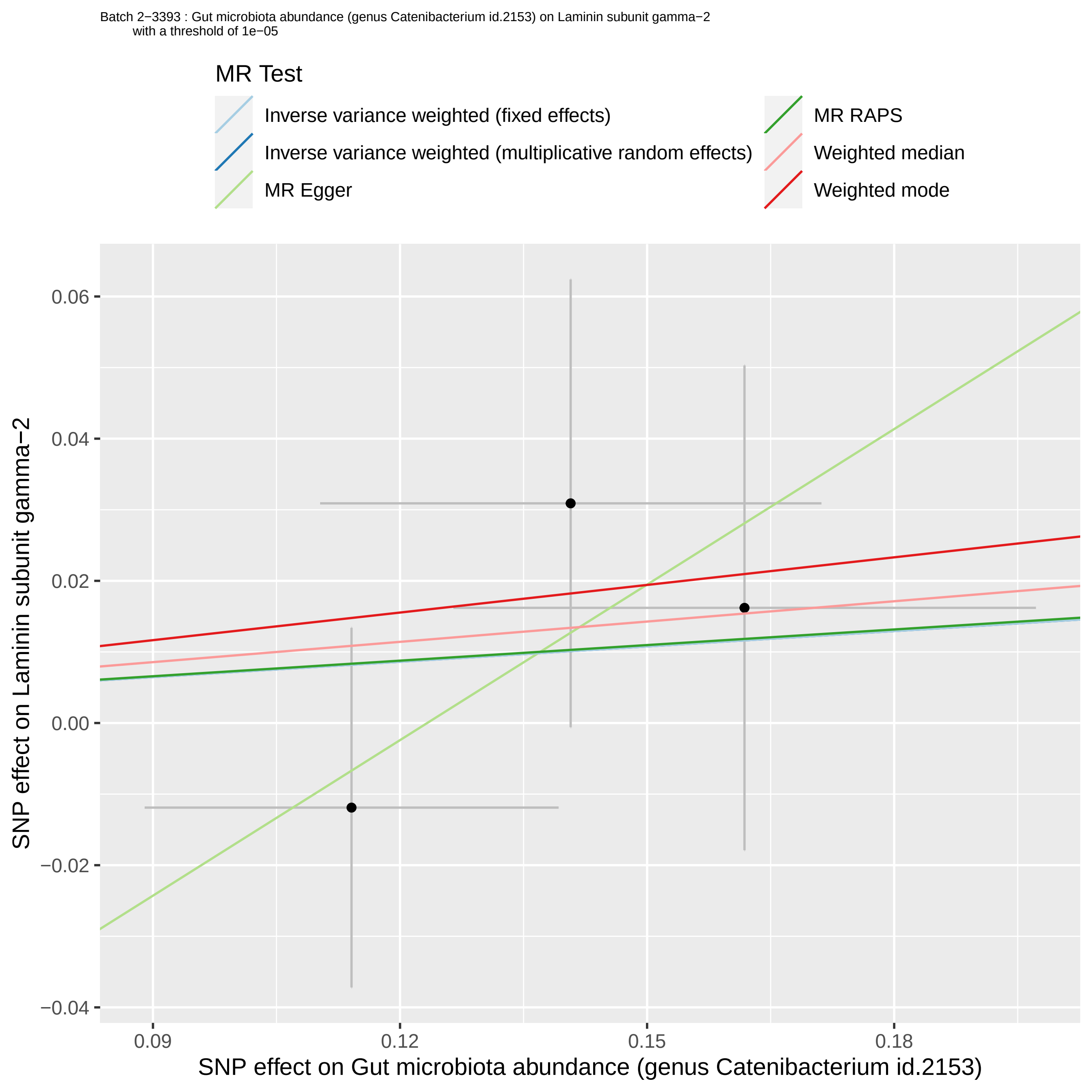
Batch 2−3393 : Gut microbiota abundance (genus Catenibacterium id.2153) on Laminin subunit gamma−2
with a threshold of 1e−05
MR Test
Inverse variance weighted (fixed effects)
Inverse variance weighted (multiplicative random effects)
MR Egger
MR RAPS
Weighted median
Weighted mode
0.06
0.04
0.02
0.00
−0.02
−0.04
0.09
0.12
0.15
0.18
SNP effect on Laminin subunit gamma−2
SNP effect on Gut microbiota abundance (genus Catenibacterium id.2153)

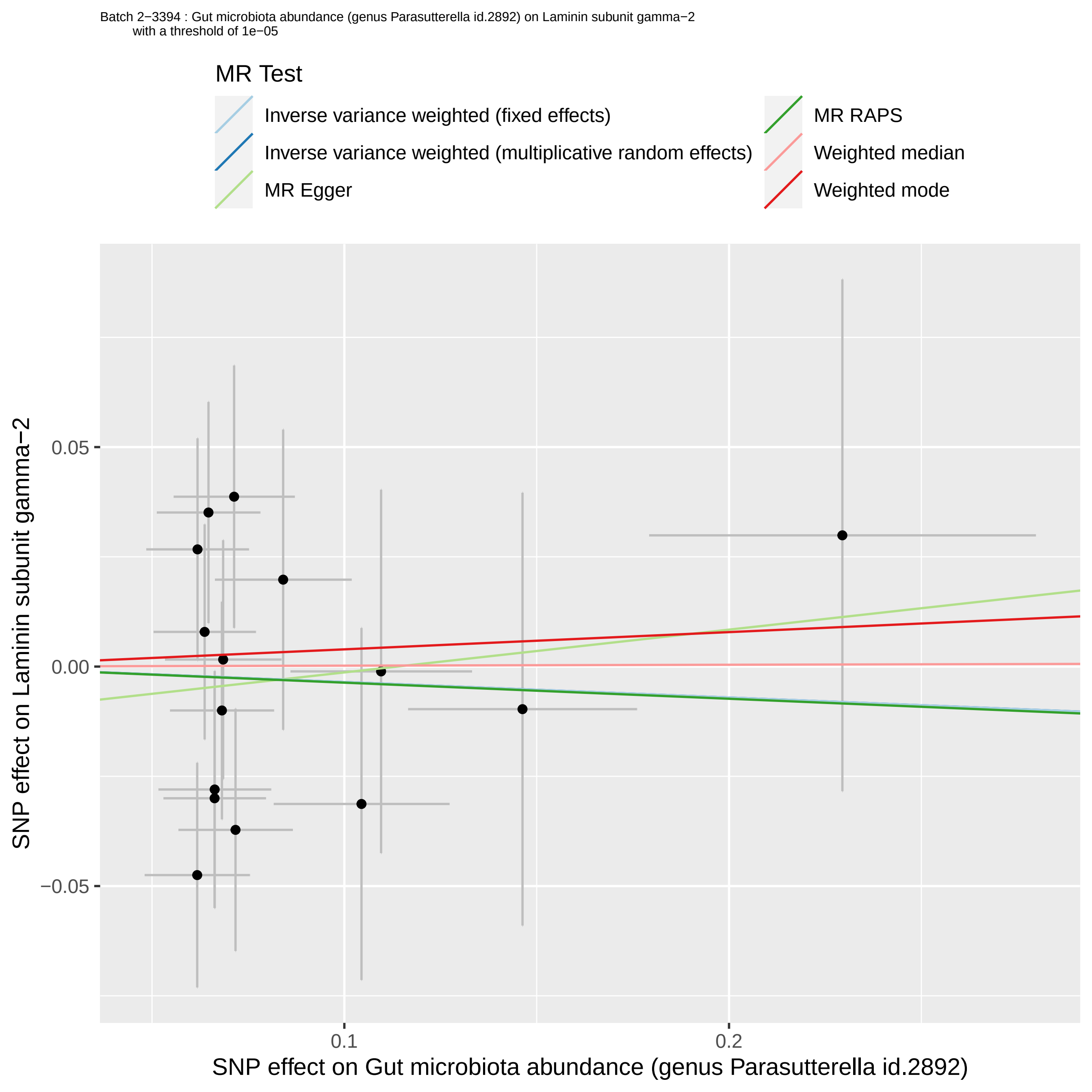

Batch 2−3394 : Gut microbiota abundance (genus Parasutterella id.2892) on Laminin subunit gamma−2
with a threshold of 1e−05
MR Test
Inverse variance weighted (fixed effects)
Inverse variance weighted (multiplicative random effects)
MR Egger
MR RAPS
Weighted median
Weighted mode
0.05
0.00
−0.05
0.1
0.2
SNP effect on Laminin subunit gamma−2
SNP effect on Gut microbiota abundance (genus Parasutterella id.2892)

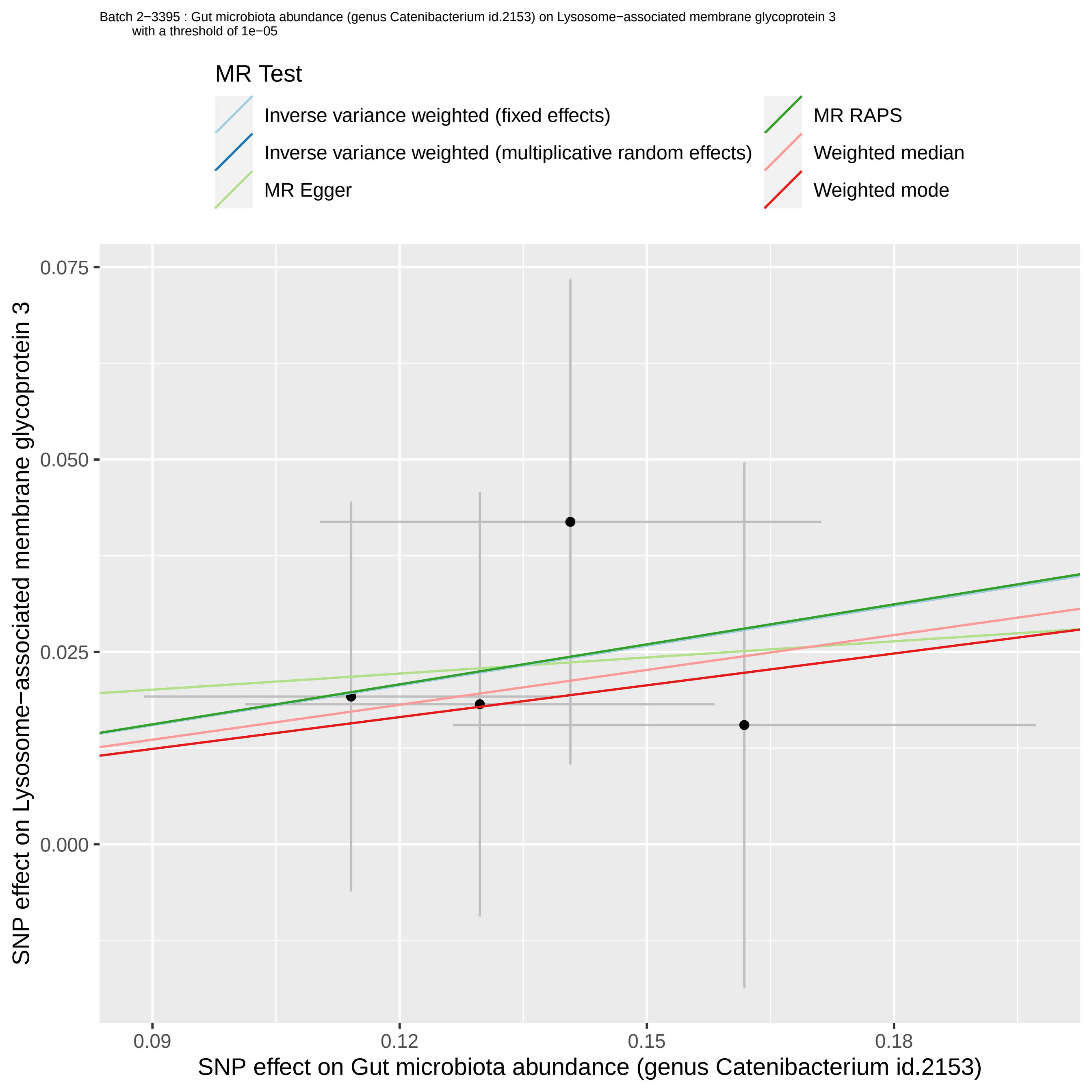

Batch 2−3395 : Gut microbiota abundance (genus Catenibacterium id.2153) on Lysosome−associated membrane glycoprotein 3
with a threshold of 1e−05
MR Test
Inverse variance weighted (fixed effects)
Inverse variance weighted (multiplicative random effects)
MR Egger
MR RAPS
Weighted median
Weighted mode
0.075
0.050
0.025
0.000
0.09
0.12
0.15
0.18
SNP effect on Lysosome−associated membrane glycoprotein 3
SNP effect on Gut microbiota abundance (genus Catenibacterium id.2153)

Batch 2−3396 : Gut microbiota abundance (genus Parasutterella id.2892) on Lysosome−associated membrane glycoprotein 3
with a threshold of 1e−05

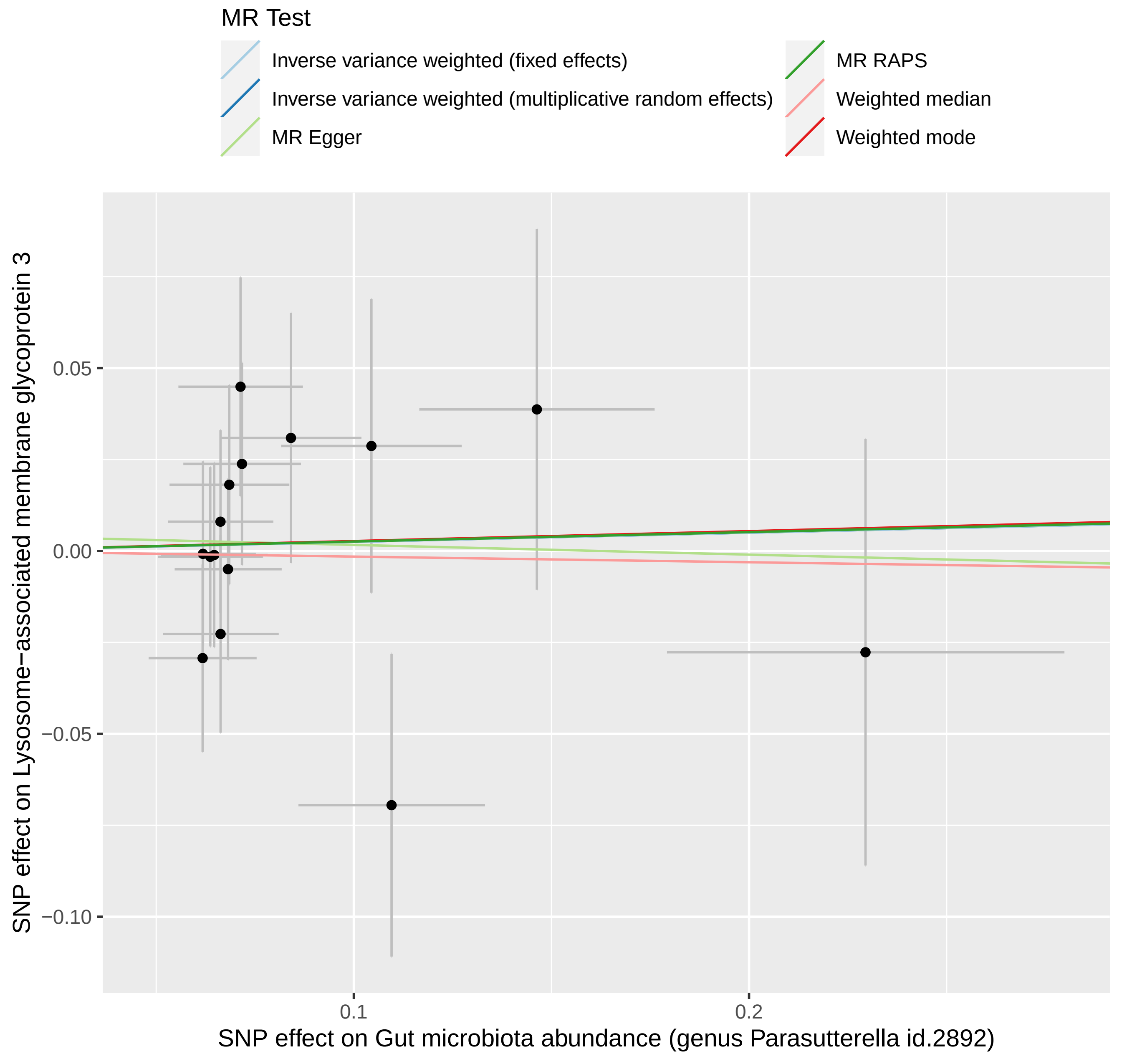

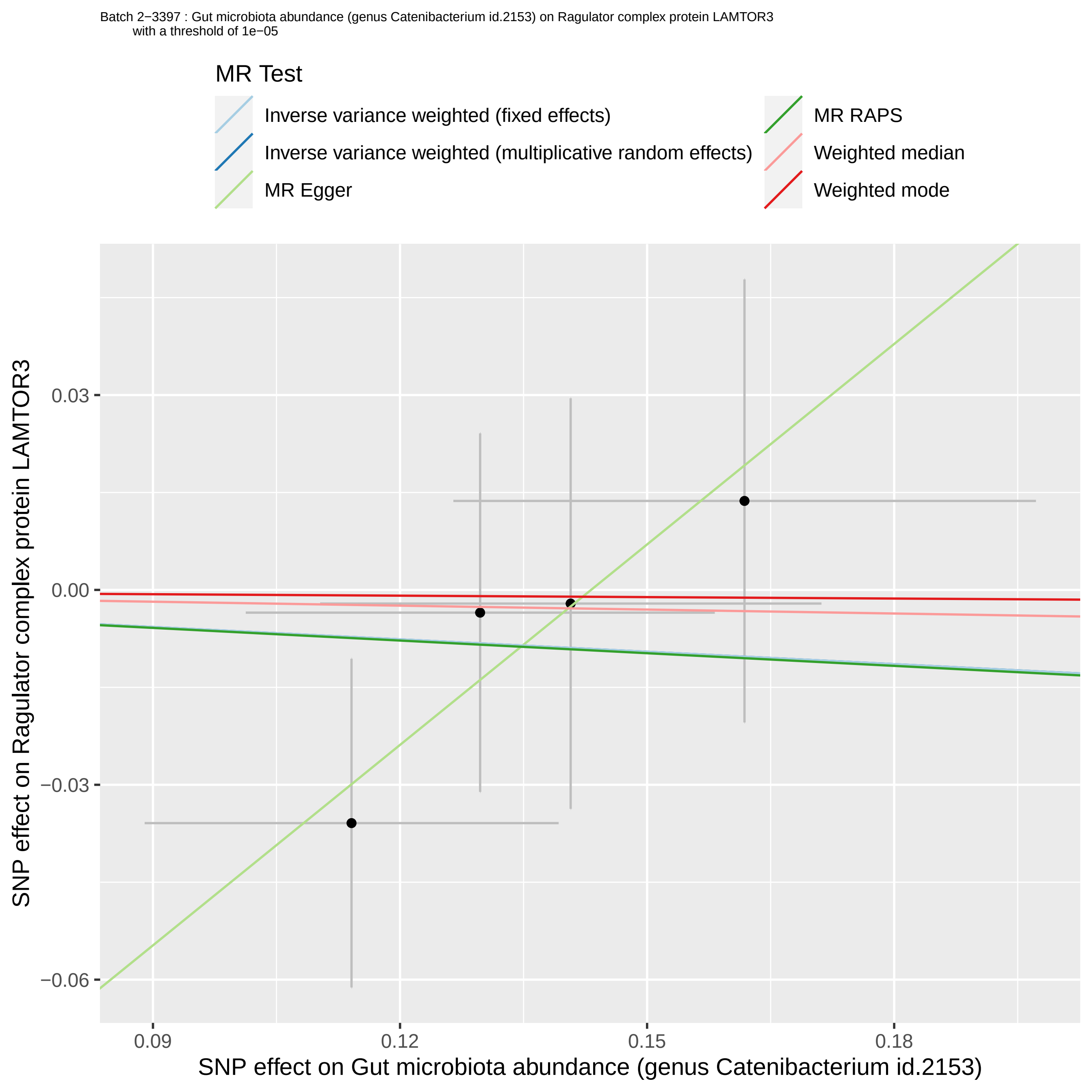
Batch 2−3397 : Gut microbiota abundance (genus Catenibacterium id.2153) on Ragulator complex protein LAMTOR3
with a threshold of 1e−05
MR Test
Inverse variance weighted (fixed effects)
Inverse variance weighted (multiplicative random effects)
MR Egger
MR RAPS
Weighted median
Weighted mode
SNP effect on Ragulator complex protein LAMTOR3
0.03
0.00
−0.03
−0.06
0.09
0.12
0.15
0.18
SNP effect on Gut microbiota abundance (genus Catenibacterium id.2153)

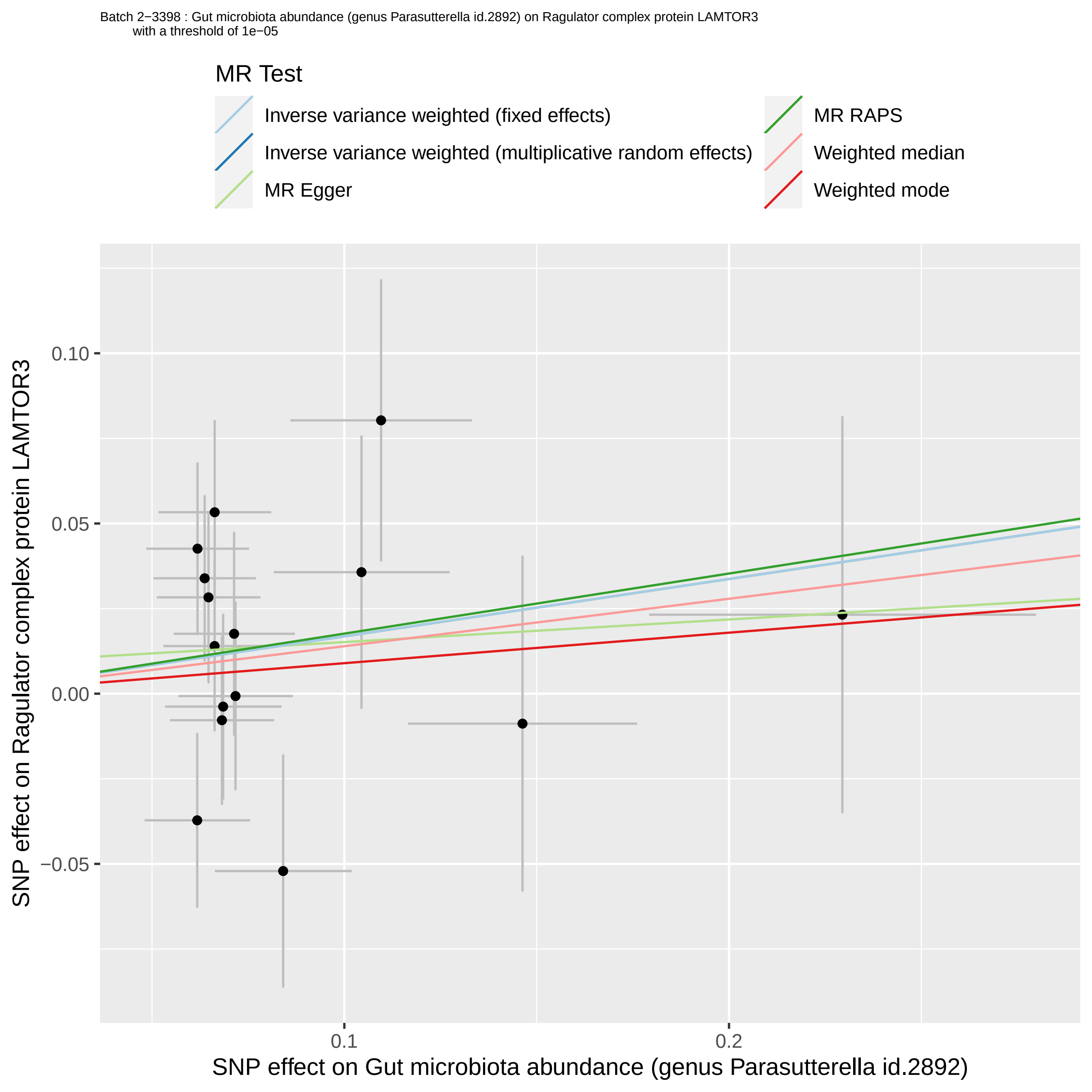

Batch 2−3398 : Gut microbiota abundance (genus Parasutterella id.2892) on Ragulator complex protein LAMTOR3
with a threshold of 1e−05
MR Test
Inverse variance weighted (fixed effects)
Inverse variance weighted (multiplicative random effects)
MR Egger
MR RAPS
Weighted median
Weighted mode
0.10
0.05
0.00
−0.05
0.1
0.2
SNP effect on Ragulator complex protein LAMTOR3
SNP effect on Gut microbiota abundance (genus Parasutterella id.2892)

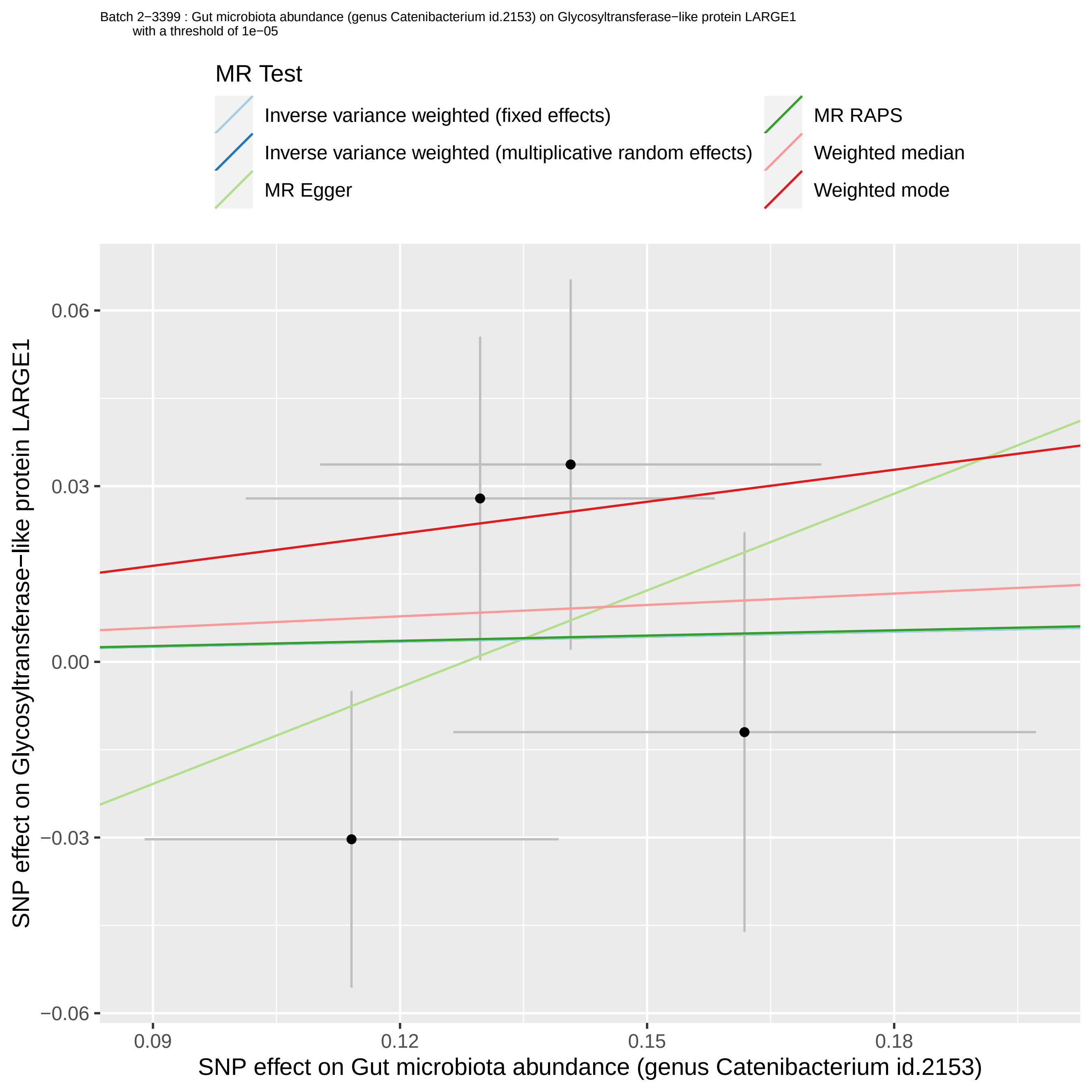
Batch 2−3399 : Gut microbiota abundance (genus Catenibacterium id.2153) on Glycosyltransferase−like protein LARGE1
with a threshold of 1e−05
MR Test
Inverse variance weighted (fixed effects)
Inverse variance weighted (multiplicative random effects)
MR Egger
MR RAPS
Weighted median
Weighted mode
0.06
0.03
0.00
−0.03
−0.06
0.09
0.12
0.15
0.18
SNP effect on Glycosyltransferase−like protein LARGE1
SNP effect on Gut microbiota abundance (genus Catenibacterium id.2153)

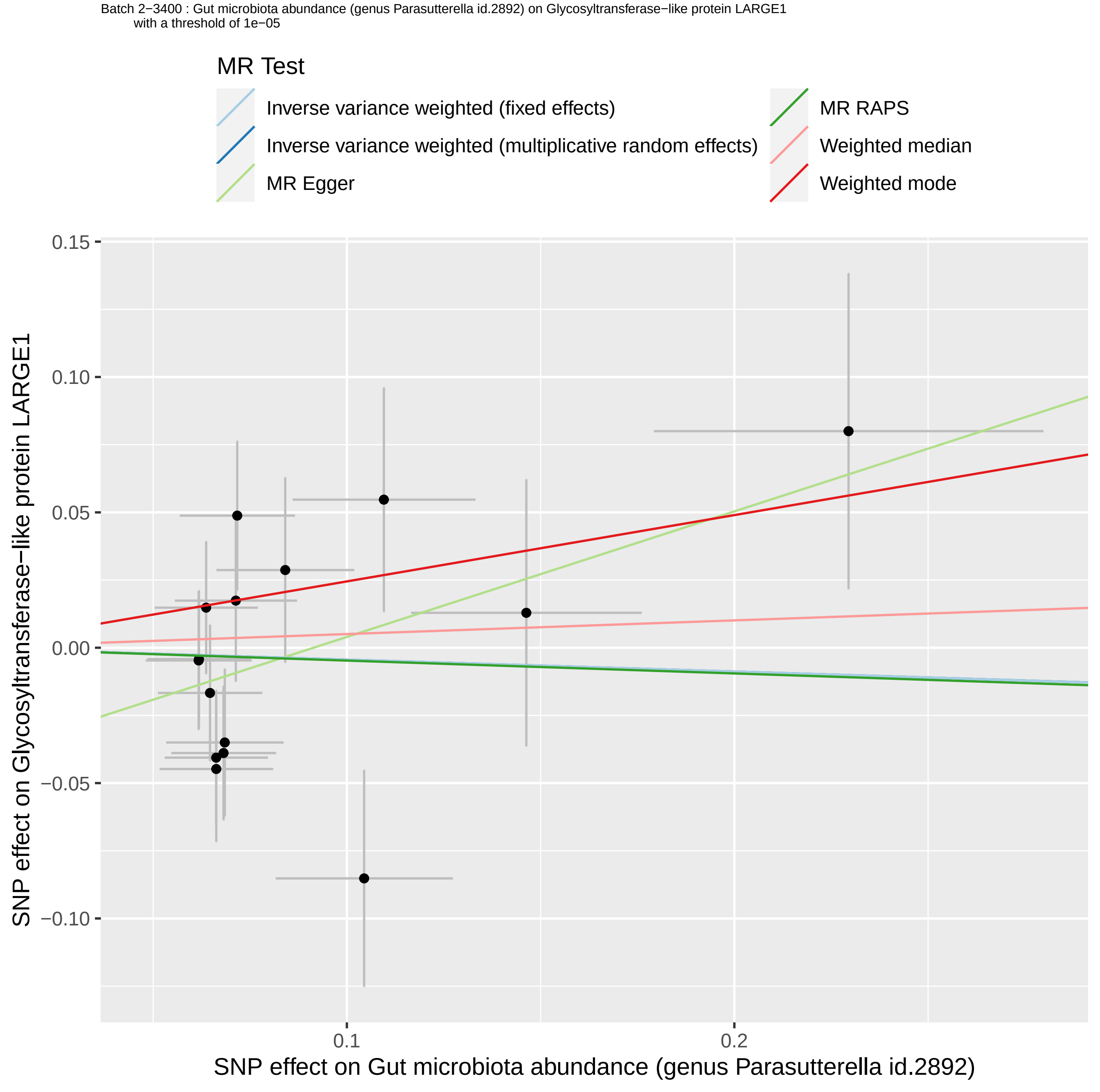
Batch 2−3400 : Gut microbiota abundance (genus Parasutterella id.2892) on Glycosyltransferase−like protein LARGE1
with a threshold of 1e−05
MR Test
Inverse variance weighted (fixed effects)
Inverse variance weighted (multiplicative random effects)
MR Egger
MR RAPS
Weighted median
Weighted mode
0.15
0.10
0.05
0.00
−0.05
−0.10
0.1
0.2
SNP effect on Glycosyltransferase−like protein LARGE1
SNP effect on Gut microbiota abundance (genus Parasutterella id.2892)

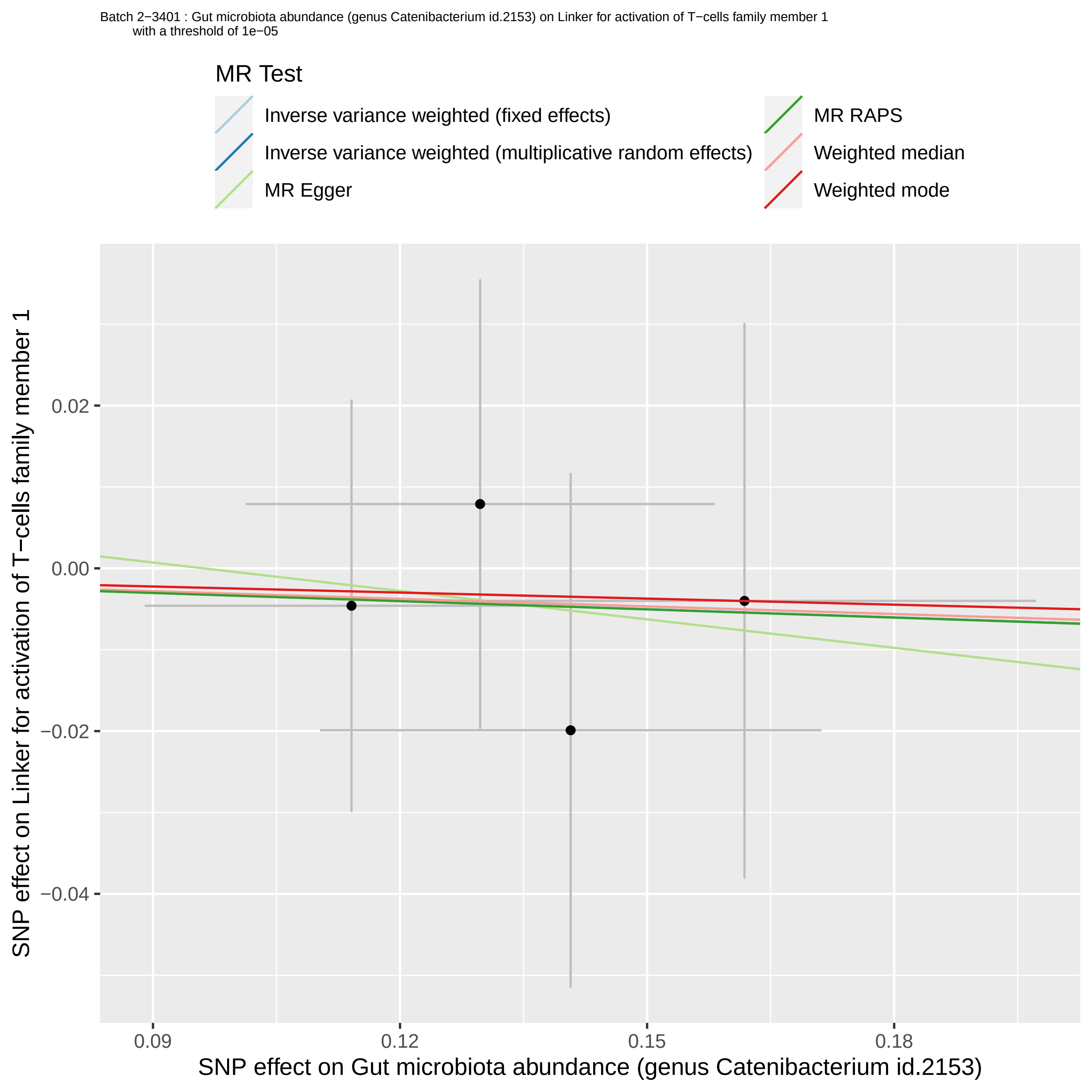
Batch 2−3401 : Gut microbiota abundance (genus Catenibacterium id.2153) on Linker for activation of T−cells family member 1
with a threshold of 1e−05
MR Test
Inverse variance weighted (fixed effects)
Inverse variance weighted (multiplicative random effects)
MR Egger
MR RAPS
Weighted median
Weighted mode
SNP effect on Linker for activation of T−cells family member 1
0.02
0.00
−0.02
−0.04
0.09
0.12
0.15
0.18
SNP effect on Gut microbiota abundance (genus Catenibacterium id.2153)

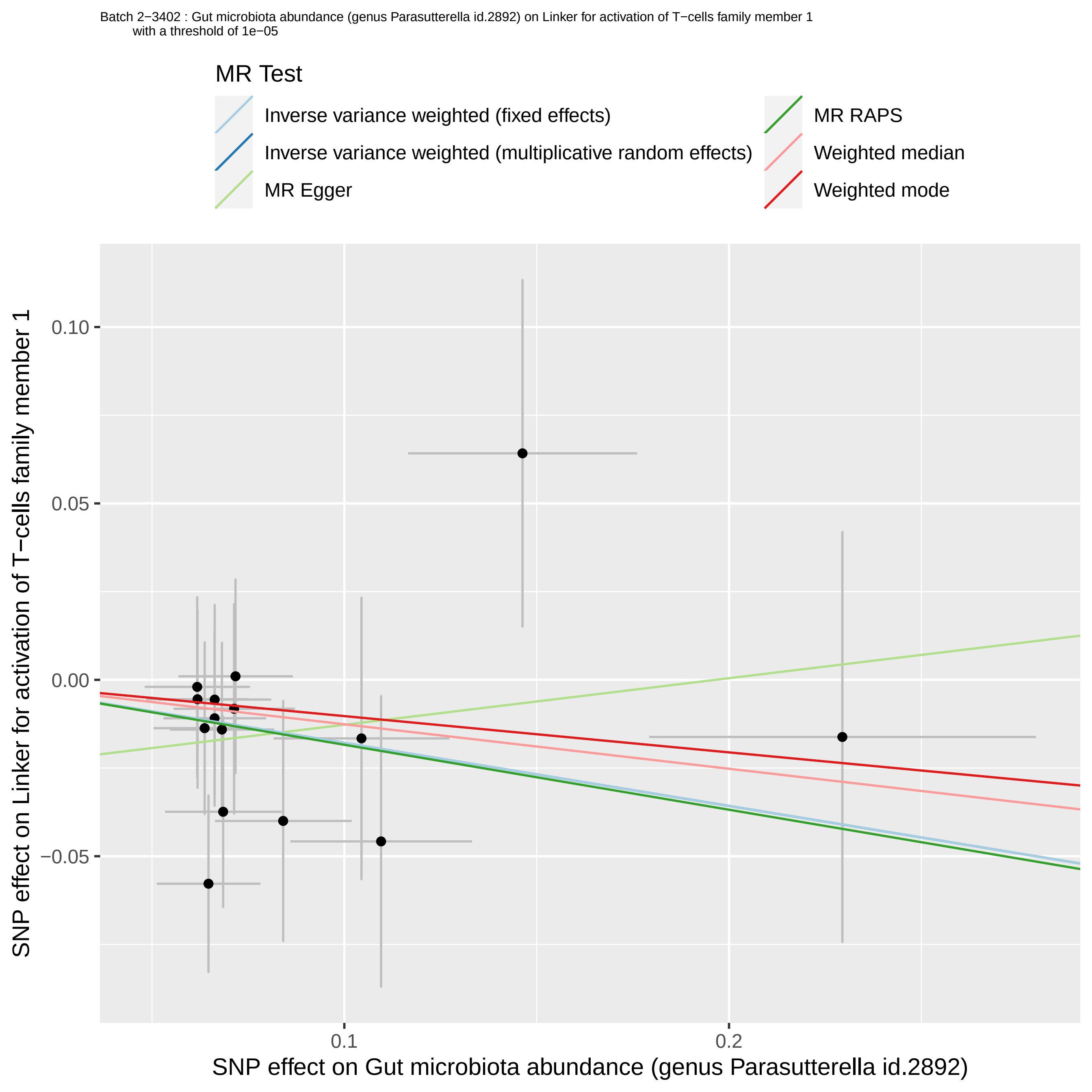

Batch 2−3402 : Gut microbiota abundance (genus Parasutterella id.2892) on Linker for activation of T−cells family member 1
with a threshold of 1e−05
MR Test
Inverse variance weighted (fixed effects)
Inverse variance weighted (multiplicative random effects)
MR Egger
MR RAPS
Weighted median
Weighted mode
0.10
0.05
0.00
−0.05
0.1
0.2
SNP effect on Linker for activation of T−cells family member 1
SNP effect on Gut microbiota abundance (genus Parasutterella id.2892)

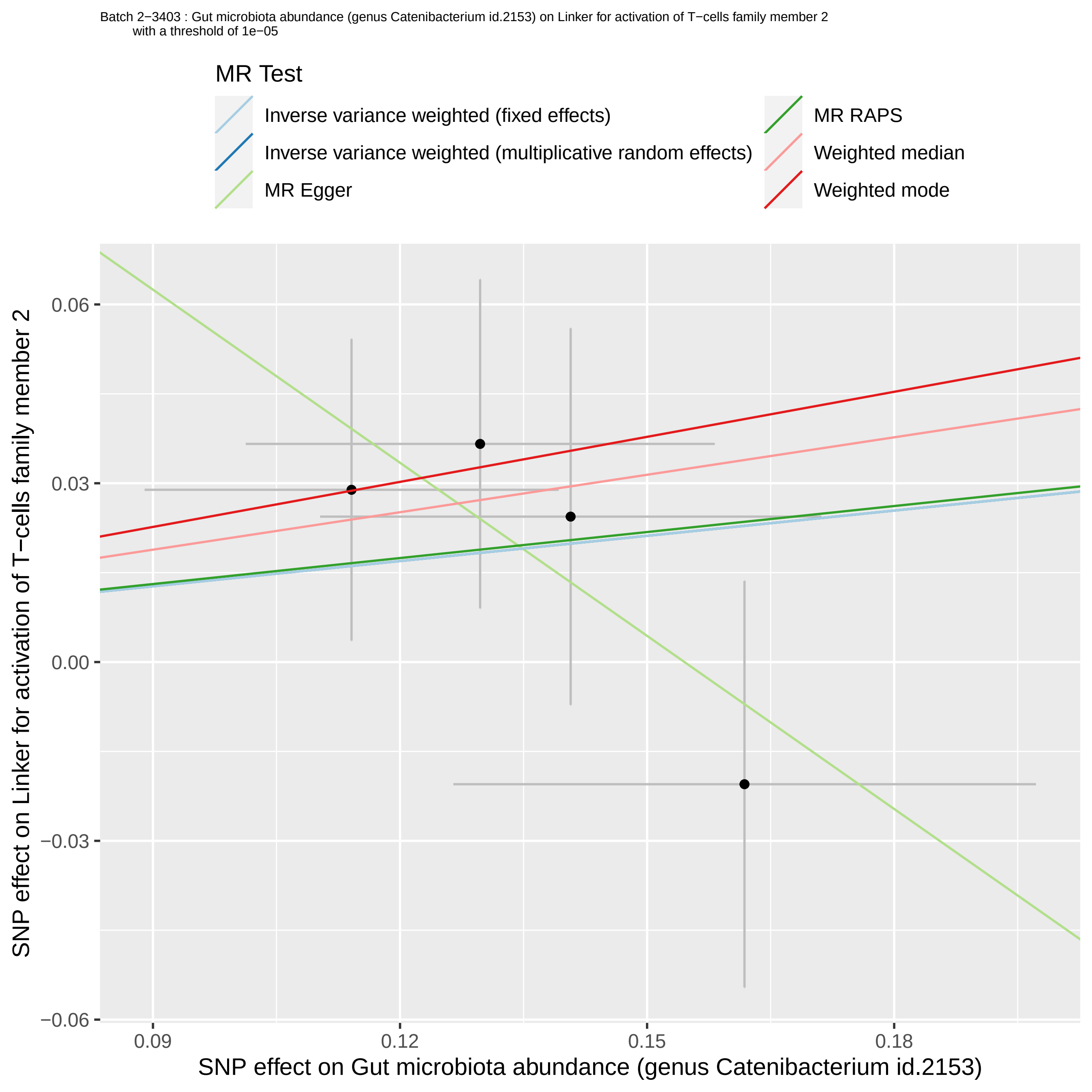

Batch 2−3403 : Gut microbiota abundance (genus Catenibacterium id.2153) on Linker for activation of T−cells family member 2
with a threshold of 1e−05
MR Test
Inverse variance weighted (fixed effects)
Inverse variance weighted (multiplicative random effects)
MR Egger
MR RAPS
Weighted median
Weighted mode
SNP effect on Linker for activation of T−cells family member 2
0.06
0.03
0.00
−0.03
−0.06
0.09
0.12
0.15
0.18
SNP effect on Gut microbiota abundance (genus Catenibacterium id.2153)

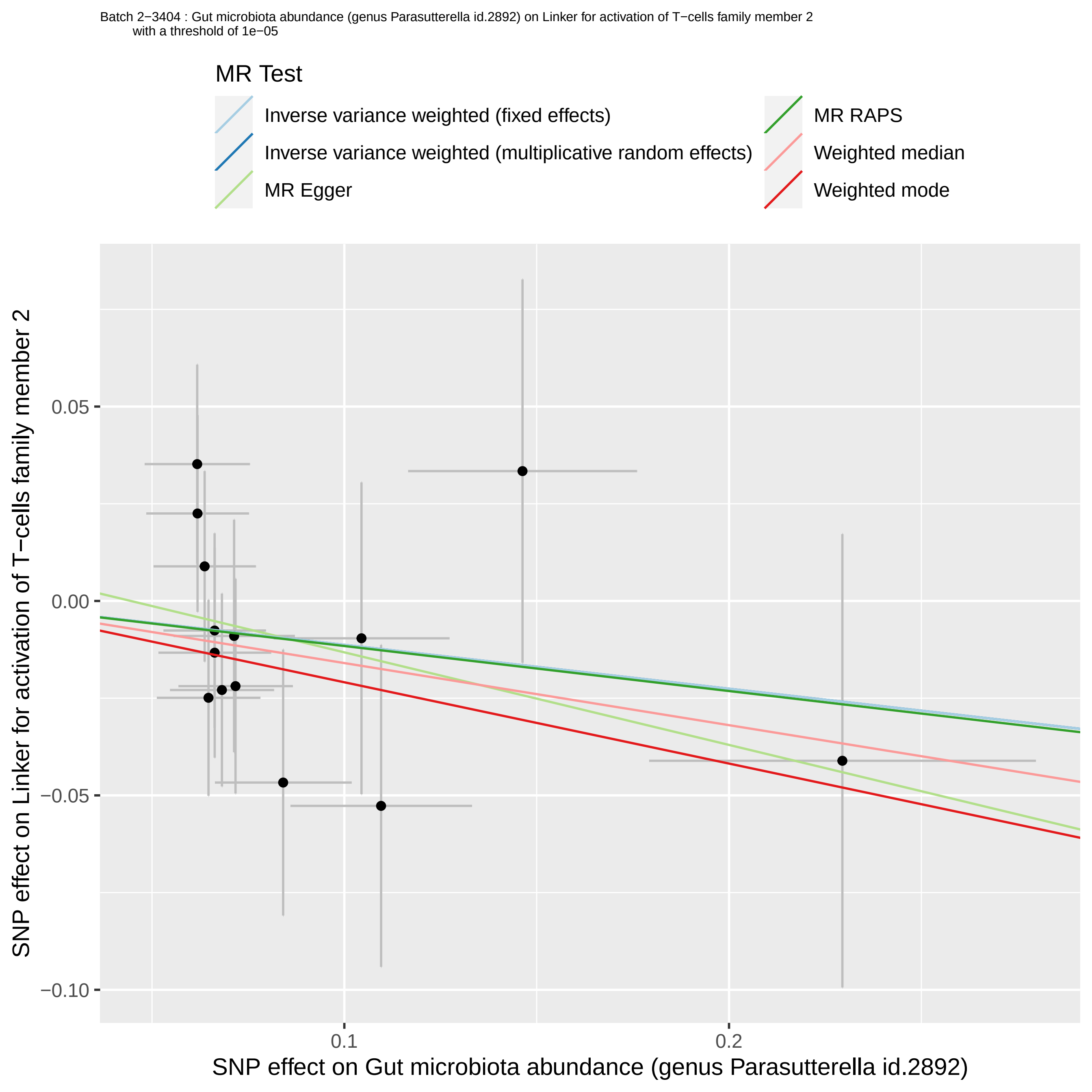

Batch 2−3404 : Gut microbiota abundance (genus Parasutterella id.2892) on Linker for activation of T−cells family member 2
with a threshold of 1e−05
MR Test
Inverse variance weighted (fixed effects)
Inverse variance weighted (multiplicative random effects)
MR Egger
MR RAPS
Weighted median
Weighted mode
0.05
0.00
−0.05
−0.10
0.1
0.2
SNP effect on Linker for activation of T−cells family member 2
SNP effect on Gut microbiota abundance (genus Parasutterella id.2892)

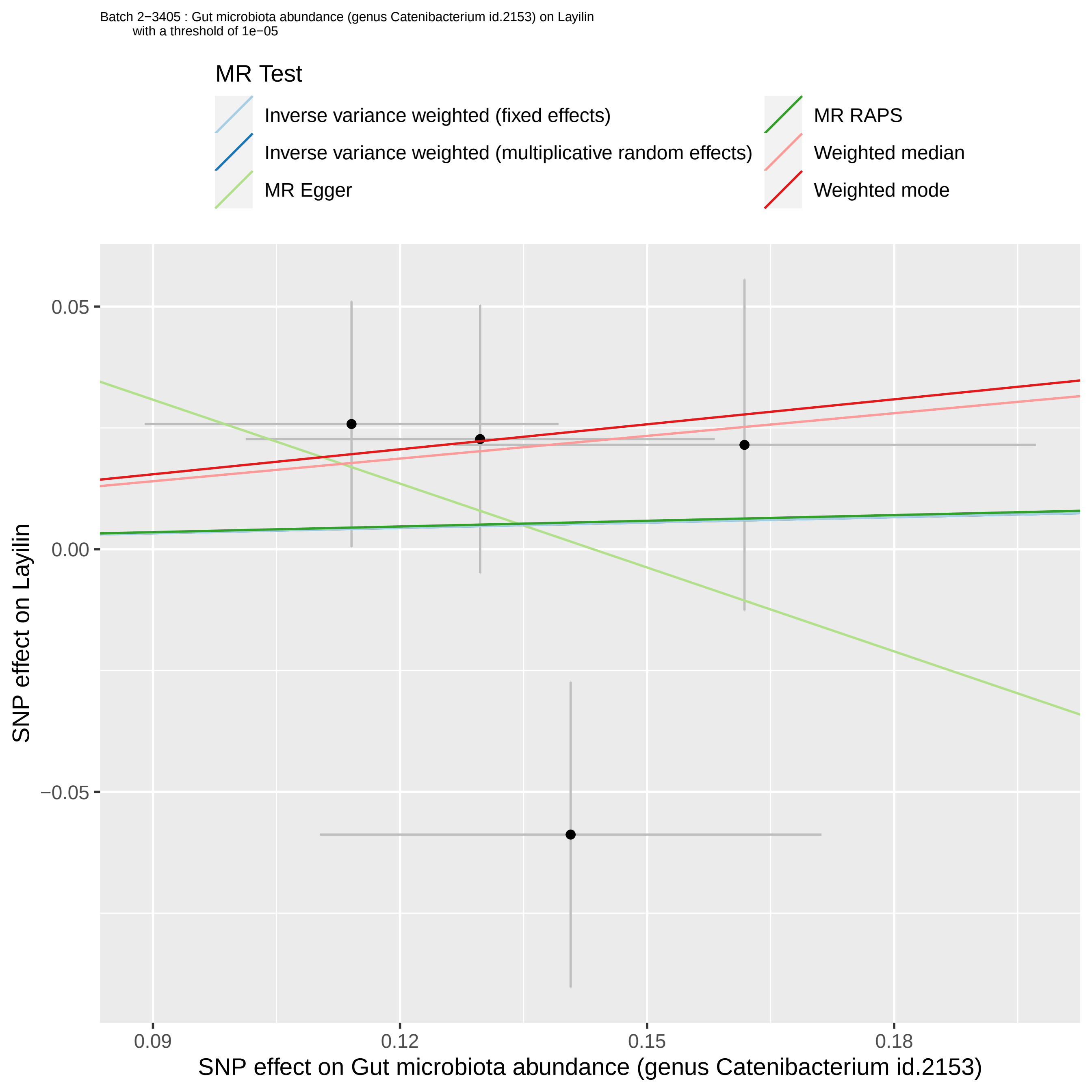

Batch 2−3405 : Gut microbiota abundance (genus Catenibacterium id.2153) on Layilin
with a threshold of 1e−05
MR Test
Inverse variance weighted (fixed effects)
Inverse variance weighted (multiplicative random effects)
MR Egger
MR RAPS
Weighted median
Weighted mode
0.05
0.00
−0.05
0.09
0.12
0.15
0.18
SNP effect on Layilin
SNP effect on Gut microbiota abundance (genus Catenibacterium id.2153)

Batch 2−3406 : Gut microbiota abundance (genus Parasutterella id.2892) on Layilin
with a threshold of 1e−05

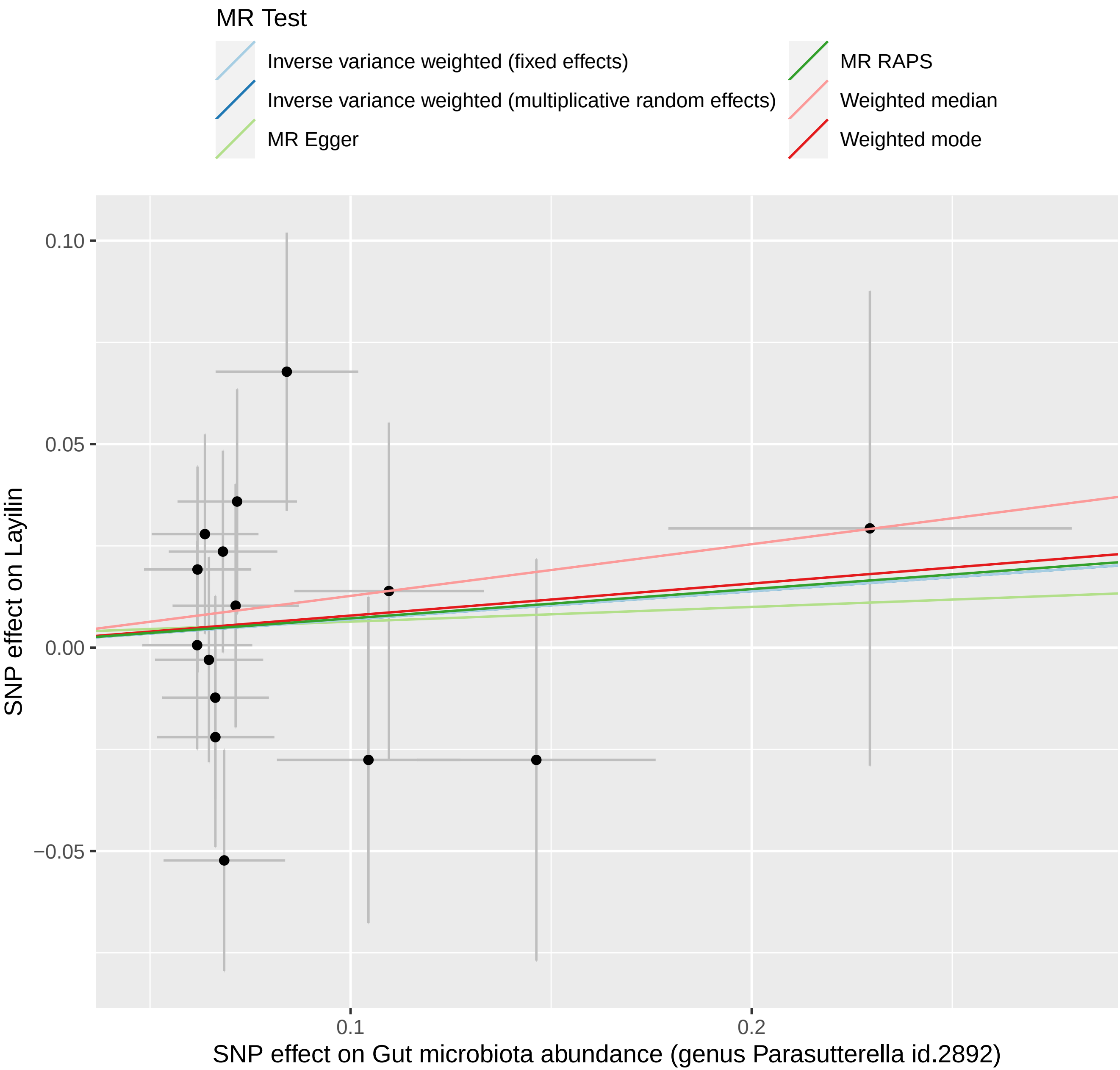

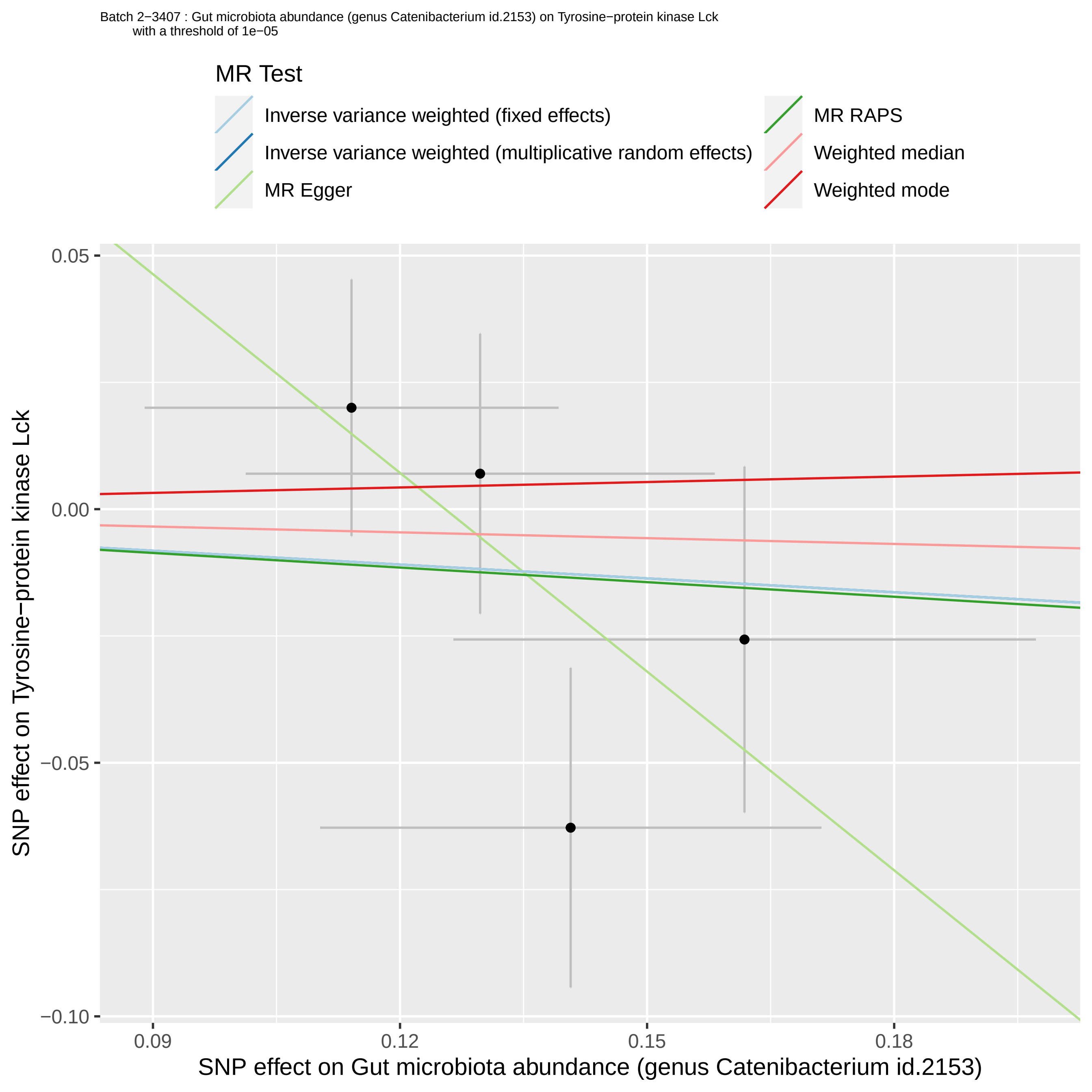
Batch 2−3407 : Gut microbiota abundance (genus Catenibacterium id.2153) on Tyrosine−protein kinase Lck
with a threshold of 1e−05
MR Test
Inverse variance weighted (fixed effects)
Inverse variance weighted (multiplicative random effects)
MR Egger
MR RAPS
Weighted median
Weighted mode
0.05
0.00
−0.05
−0.10
0.09
0.12
0.15
0.18
SNP effect on Tyrosine−protein kinase Lck
SNP effect on Gut microbiota abundance (genus Catenibacterium id.2153)

Batch 2−3408 : Gut microbiota abundance (genus Parasutterella id.2892) on Tyrosine−protein kinase Lck
with a threshold of 1e−05

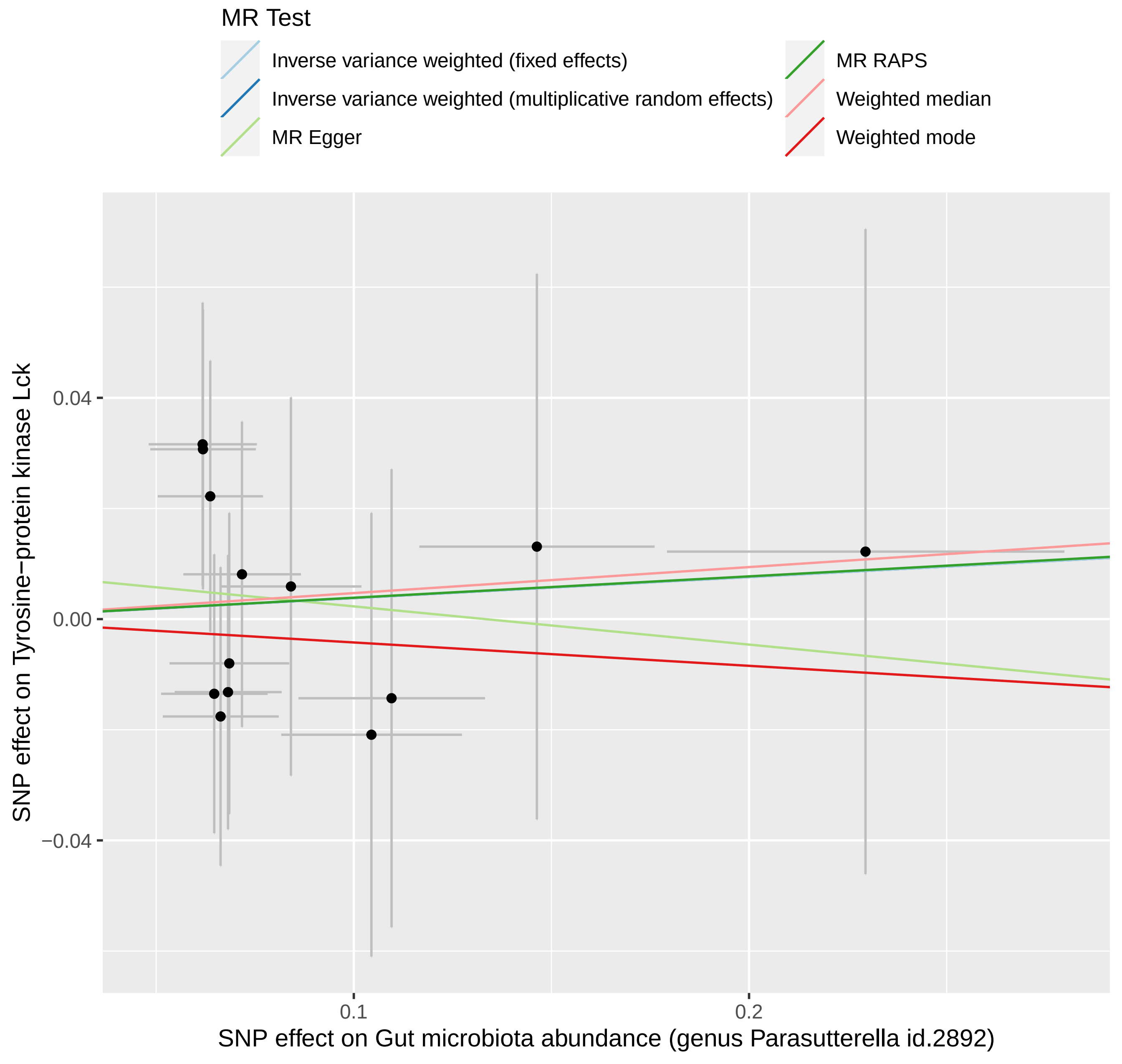

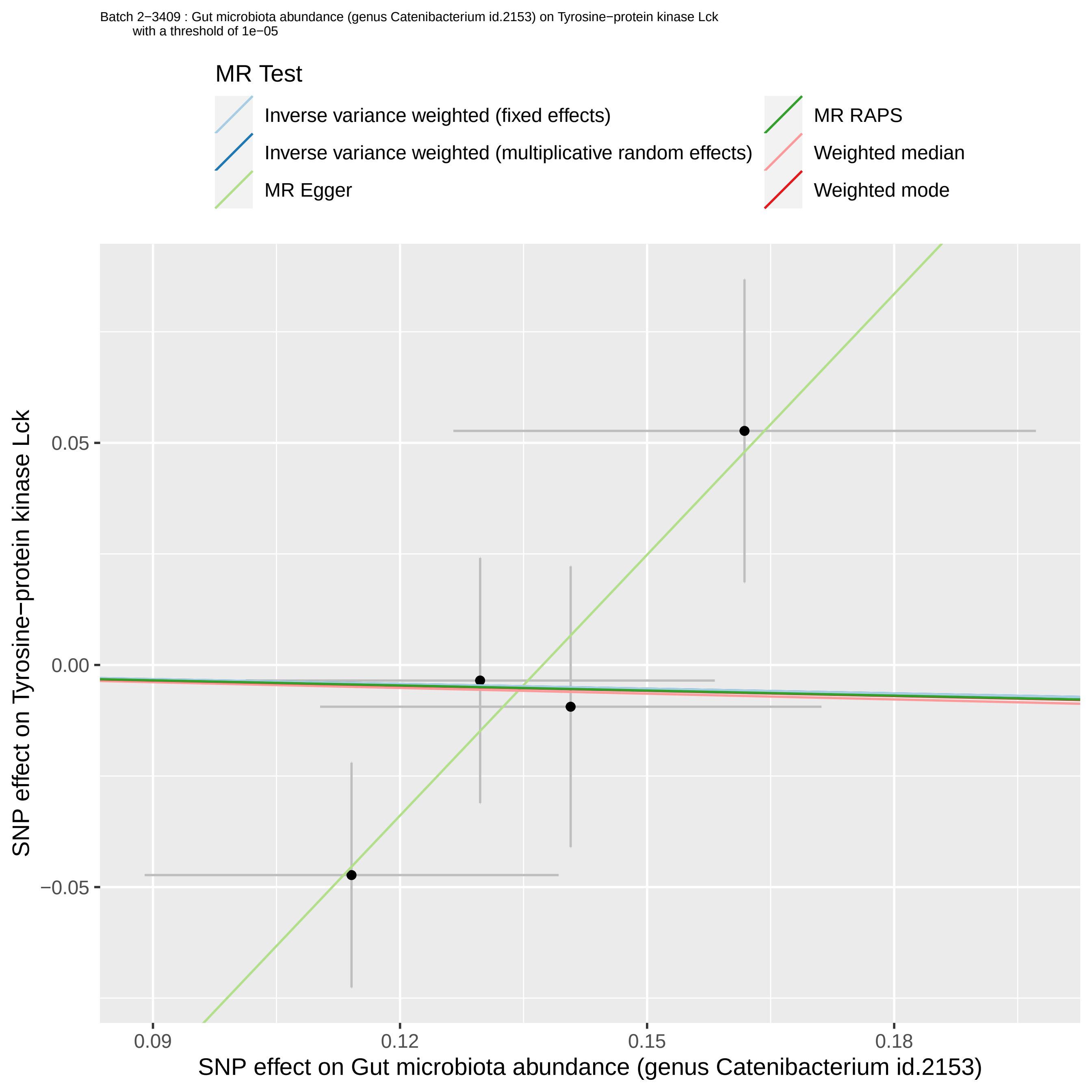
Batch 2−3409 : Gut microbiota abundance (genus Catenibacterium id.2153) on Tyrosine−protein kinase Lck
with a threshold of 1e−05
MR Test
Inverse variance weighted (fixed effects)
Inverse variance weighted (multiplicative random effects)
MR Egger
MR RAPS
Weighted median
Weighted mode
0.05
0.00
−0.05
0.09
0.12
0.15
0.18
SNP effect on Tyrosine−protein kinase Lck
SNP effect on Gut microbiota abundance (genus Catenibacterium id.2153)

Batch 2−3410 : Gut microbiota abundance (genus Parasutterella id.2892) on Tyrosine−protein kinase Lck
with a threshold of 1e−05

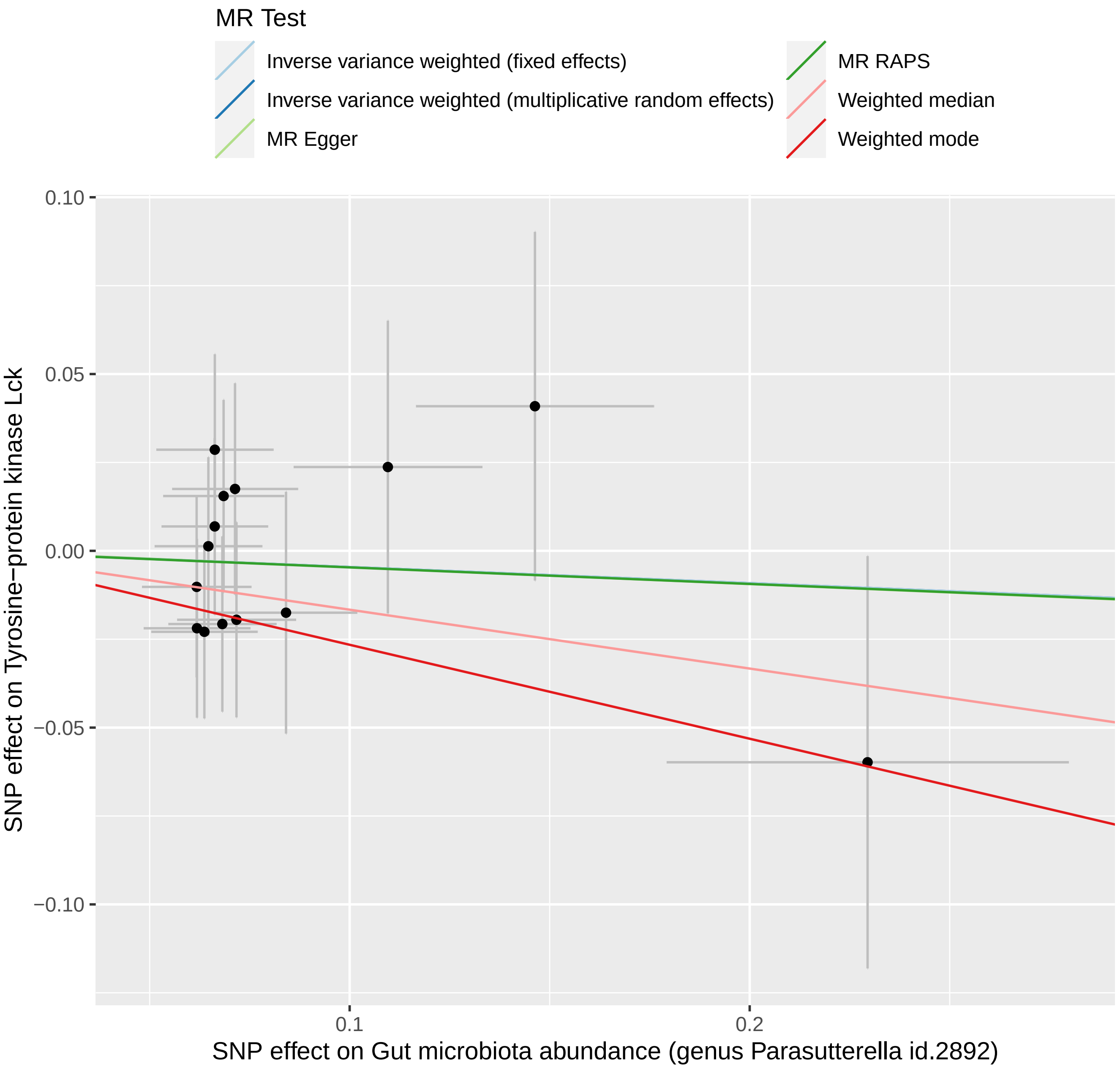

Batch 2−3411 : Gut microbiota abundance (genus Catenibacterium id.2153) on Leucine carboxyl methyltransferase 1
with a threshold of 1e−05

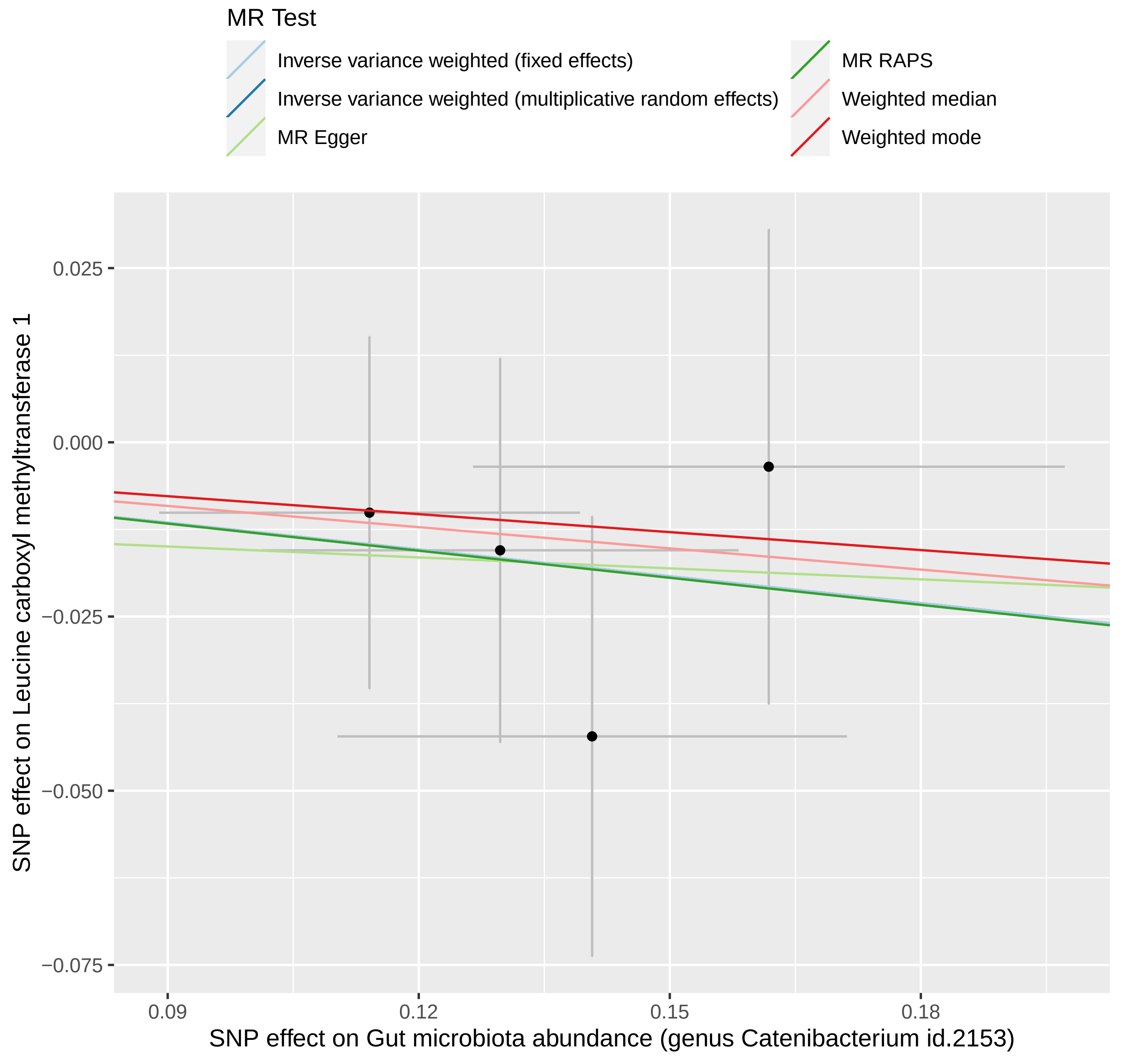

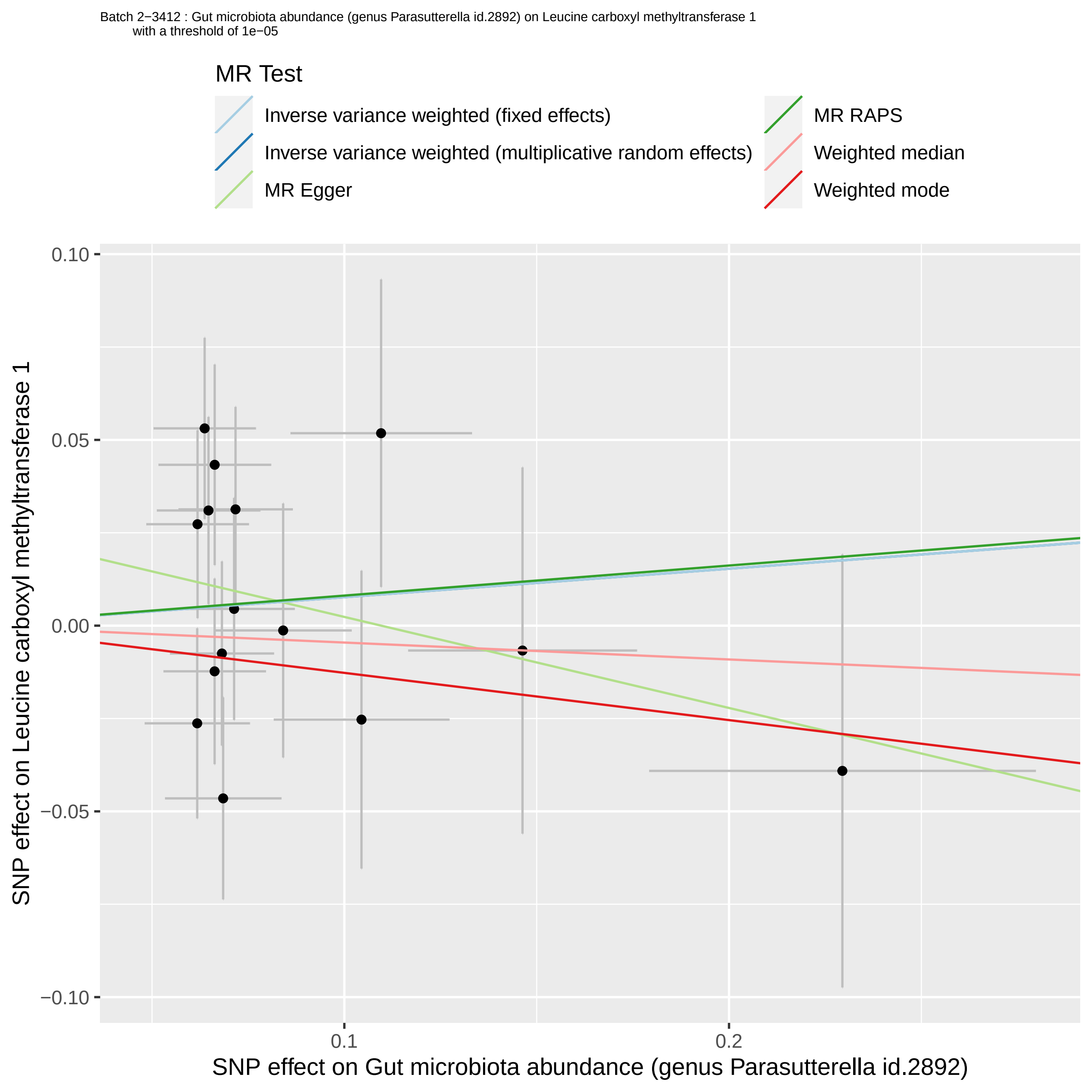

Batch 2−3412 : Gut microbiota abundance (genus Parasutterella id.2892) on Leucine carboxyl methyltransferase 1
with a threshold of 1e−05
MR Test
Inverse variance weighted (fixed effects)
Inverse variance weighted (multiplicative random effects)
MR Egger
MR RAPS
Weighted median
Weighted mode
0.10
0.05
0.00
−0.05
−0.10
0.1
0.2
SNP effect on Leucine carboxyl methyltransferase 1
SNP effect on Gut microbiota abundance (genus Parasutterella id.2892)

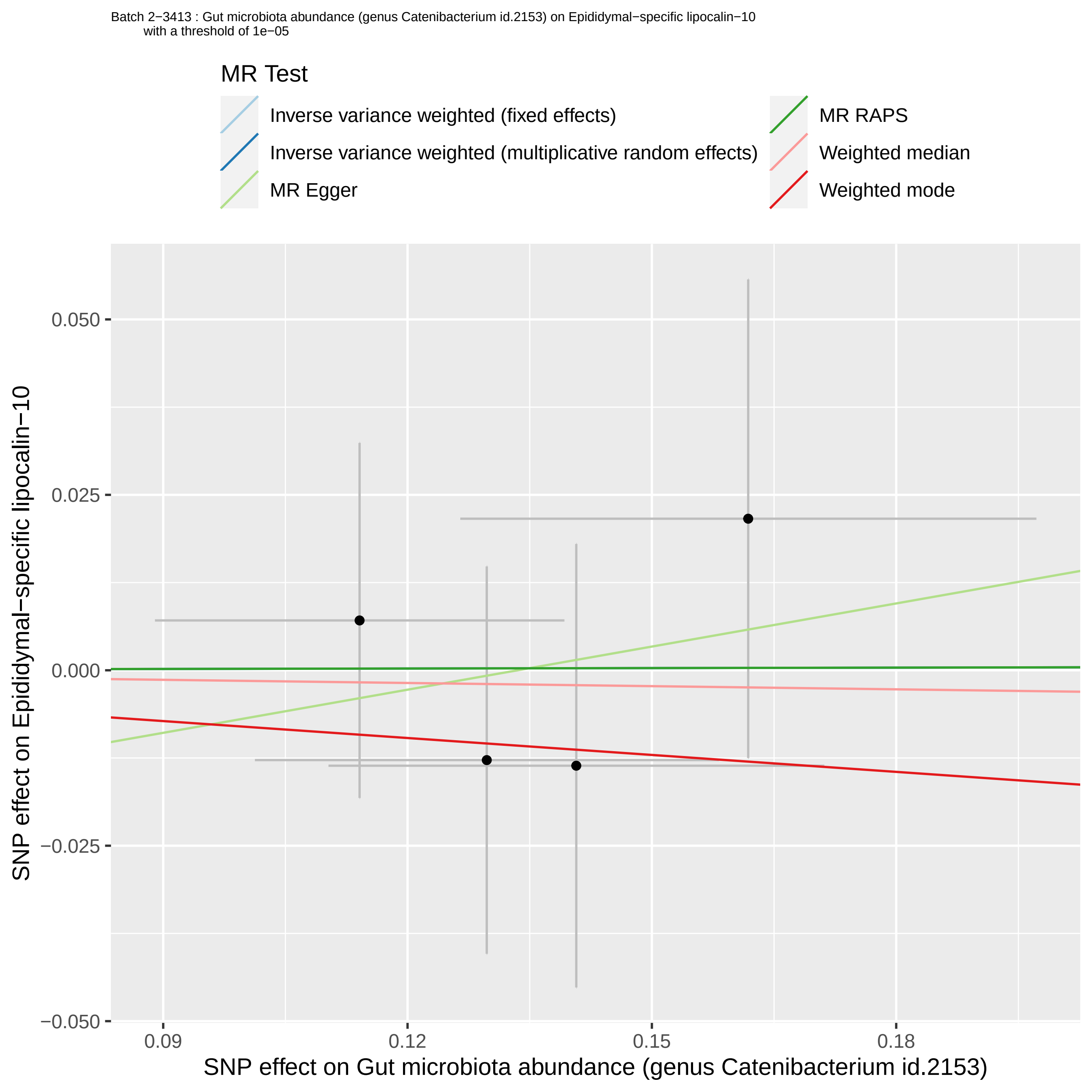
Batch 2−3413 : Gut microbiota abundance (genus Catenibacterium id.2153) on Epididymal−specific lipocalin−10
with a threshold of 1e−05
MR Test
Inverse variance weighted (fixed effects)
Inverse variance weighted (multiplicative random effects)
MR Egger
MR RAPS
Weighted median
Weighted mode
0.050
0.025
0.000
−0.025
−0.050
0.09
0.12
0.15
0.18
SNP effect on Epididymal−specific lipocalin−10
SNP effect on Gut microbiota abundance (genus Catenibacterium id.2153)

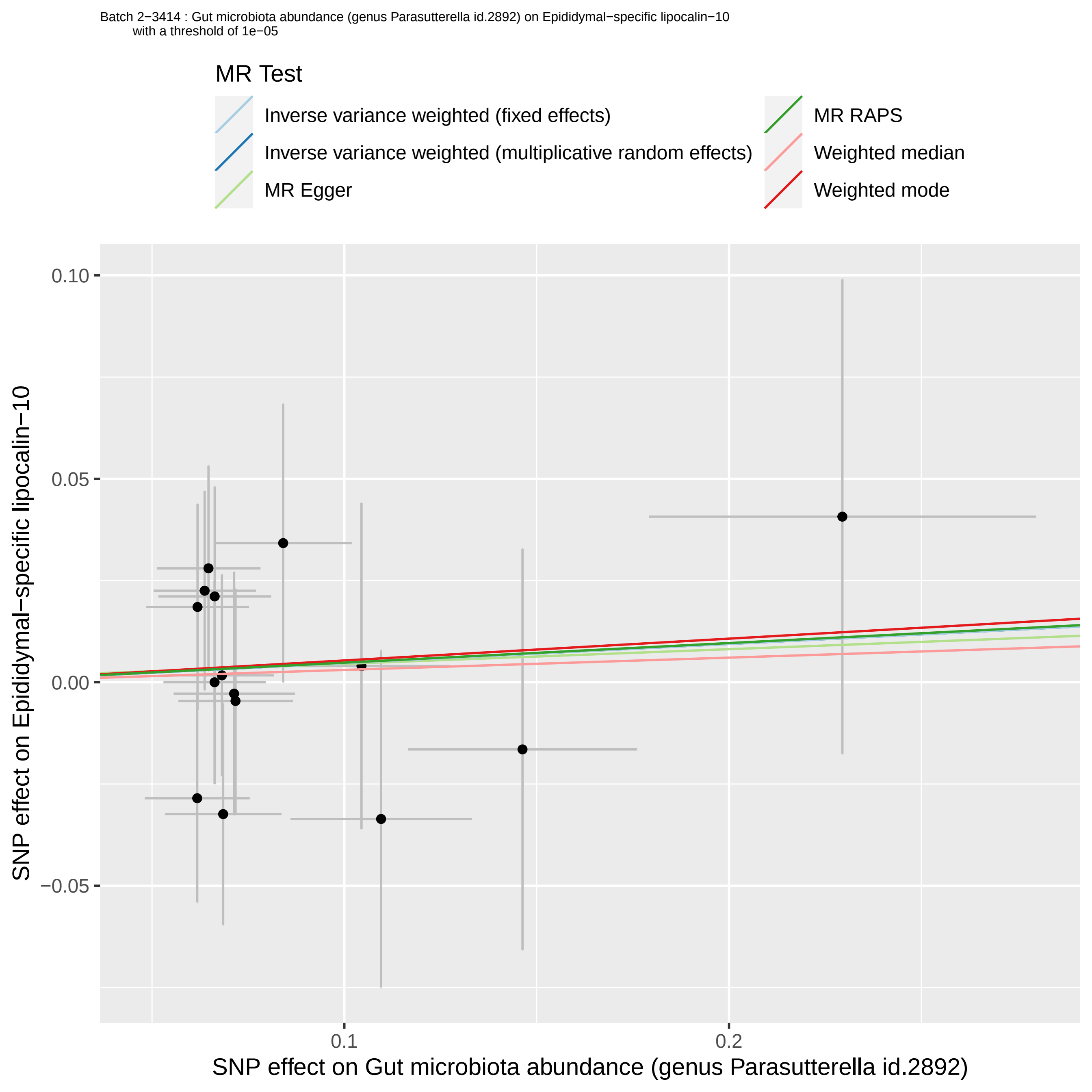
Batch 2−3414 : Gut microbiota abundance (genus Parasutterella id.2892) on Epididymal−specific lipocalin−10
with a threshold of 1e−05
MR Test
Inverse variance weighted (fixed effects)
Inverse variance weighted (multiplicative random effects)
MR Egger
MR RAPS
Weighted median
Weighted mode
0.10
0.05
0.00
−0.05
0.1
0.2
SNP effect on Epididymal−specific lipocalin−10
SNP effect on Gut microbiota abundance (genus Parasutterella id.2892)

Batch 2−3415 : Gut microbiota abundance (genus Catenibacterium id.2153) on Lipocalin−1
with a threshold of 1e−05

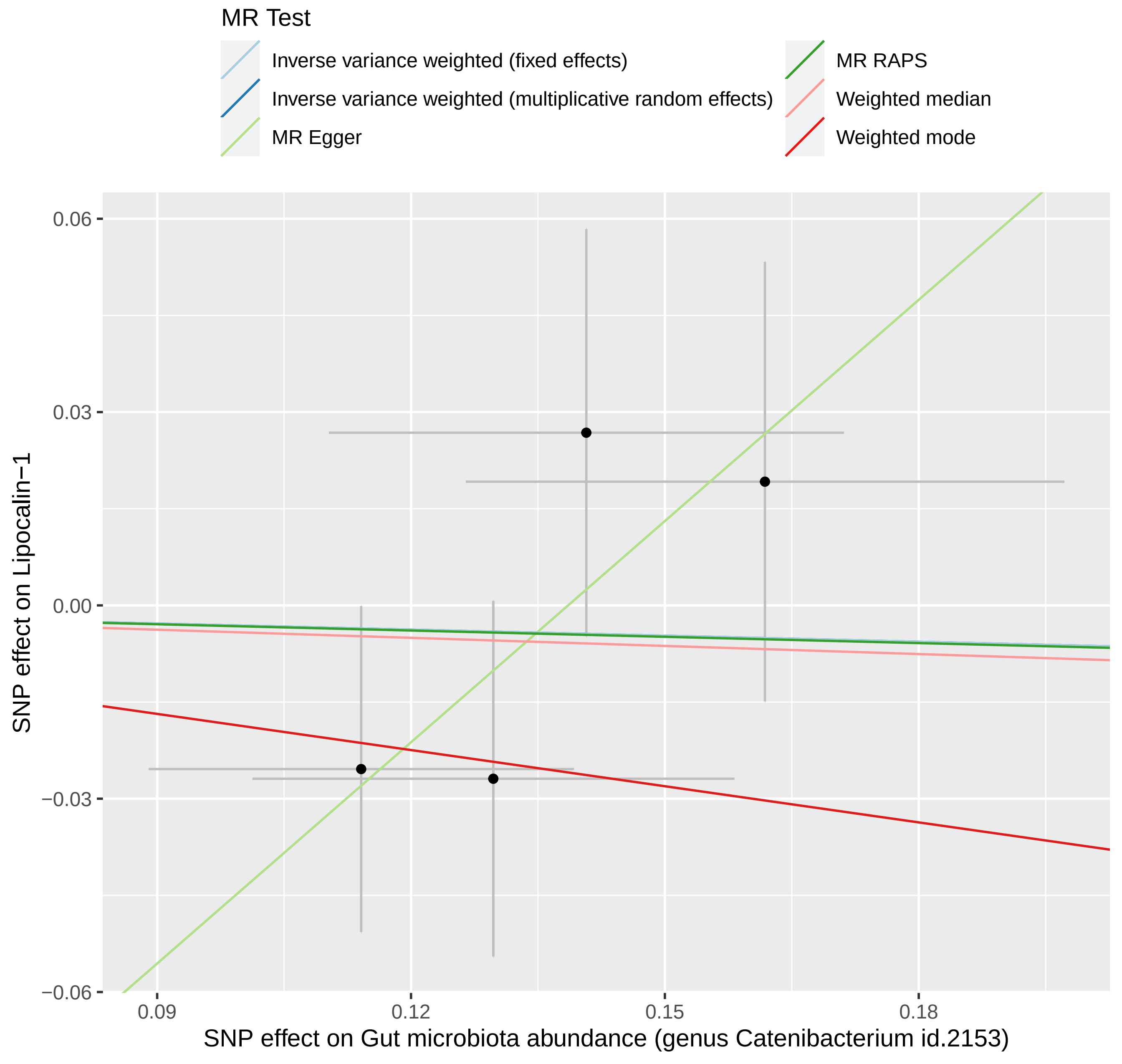

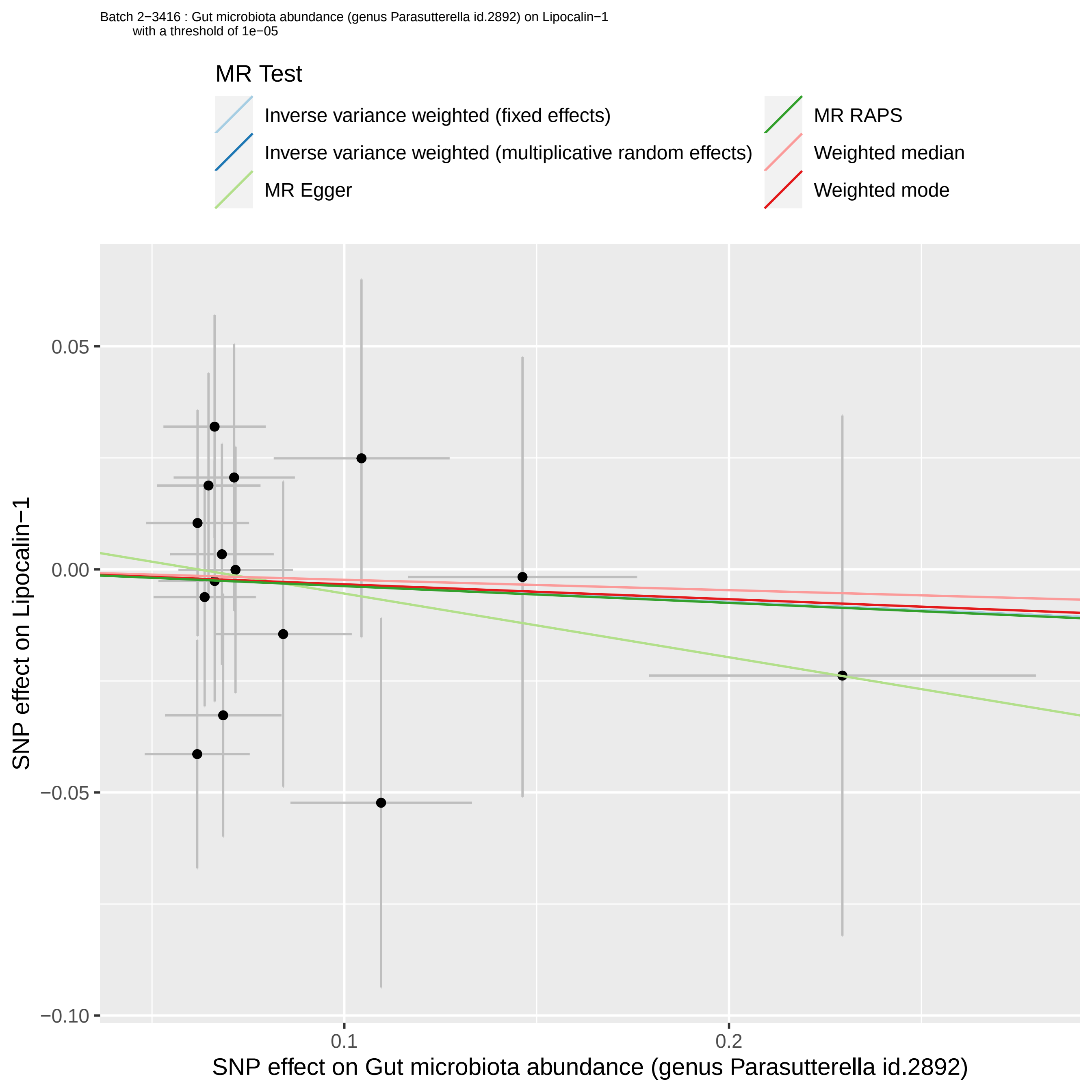

Batch 2−3416 : Gut microbiota abundance (genus Parasutterella id.2892) on Lipocalin−1
with a threshold of 1e−05
MR Test
Inverse variance weighted (fixed effects)
Inverse variance weighted (multiplicative random effects)
MR Egger
MR RAPS
Weighted median
Weighted mode
0.05
0.00
−0.05
−0.10
0.1
0.2
SNP effect on Lipocalin−1
SNP effect on Gut microbiota abundance (genus Parasutterella id.2892)

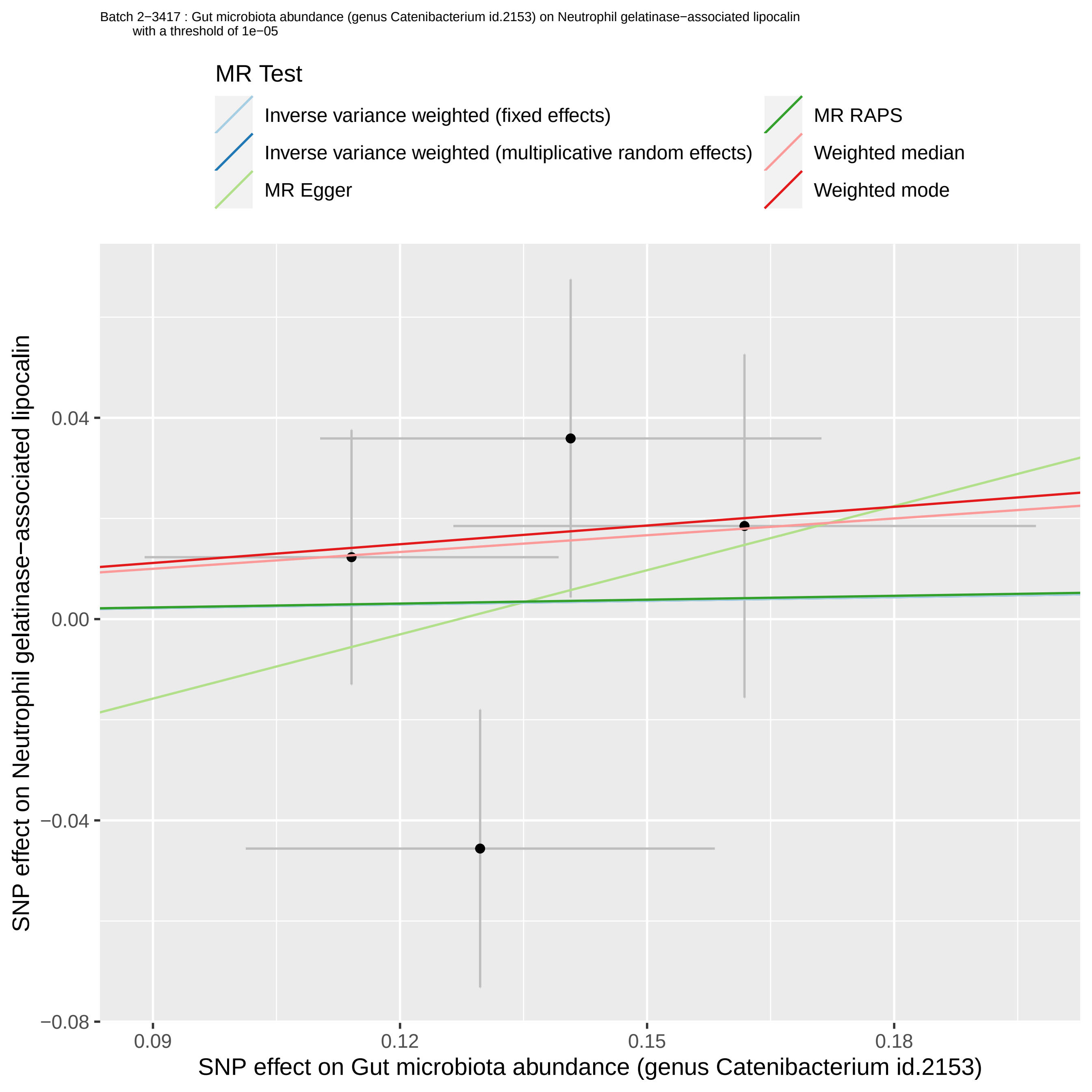

Batch 2−3417 : Gut microbiota abundance (genus Catenibacterium id.2153) on Neutrophil gelatinase−associated lipocalin
with a threshold of 1e−05
MR Test
Inverse variance weighted (fixed effects)
Inverse variance weighted (multiplicative random effects)
MR Egger
MR RAPS
Weighted median
Weighted mode
0.04
0.00
−0.04
−0.08
0.09
0.12
0.15
0.18
SNP effect on Neutrophil gelatinase−associated lipocalin
SNP effect on Gut microbiota abundance (genus Catenibacterium id.2153)

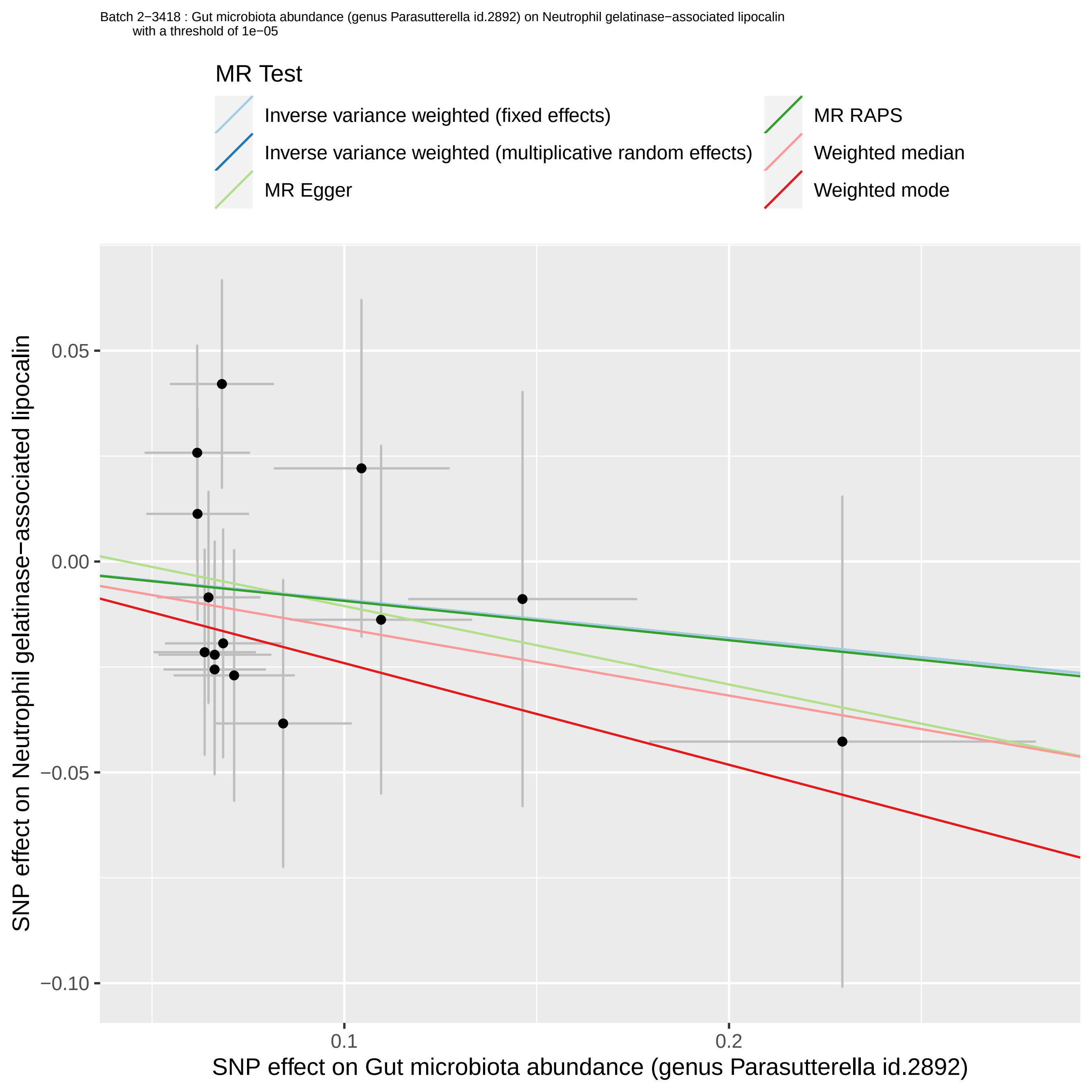

Batch 2−3418 : Gut microbiota abundance (genus Parasutterella id.2892) on Neutrophil gelatinase−associated lipocalin
with a threshold of 1e−05
MR Test
Inverse variance weighted (fixed effects)
Inverse variance weighted (multiplicative random effects)
MR Egger
MR RAPS
Weighted median
Weighted mode
0.05
0.00
−0.05
−0.10
0.1
0.2
SNP effect on Neutrophil gelatinase−associated lipocalin
SNP effect on Gut microbiota abundance (genus Parasutterella id.2892)

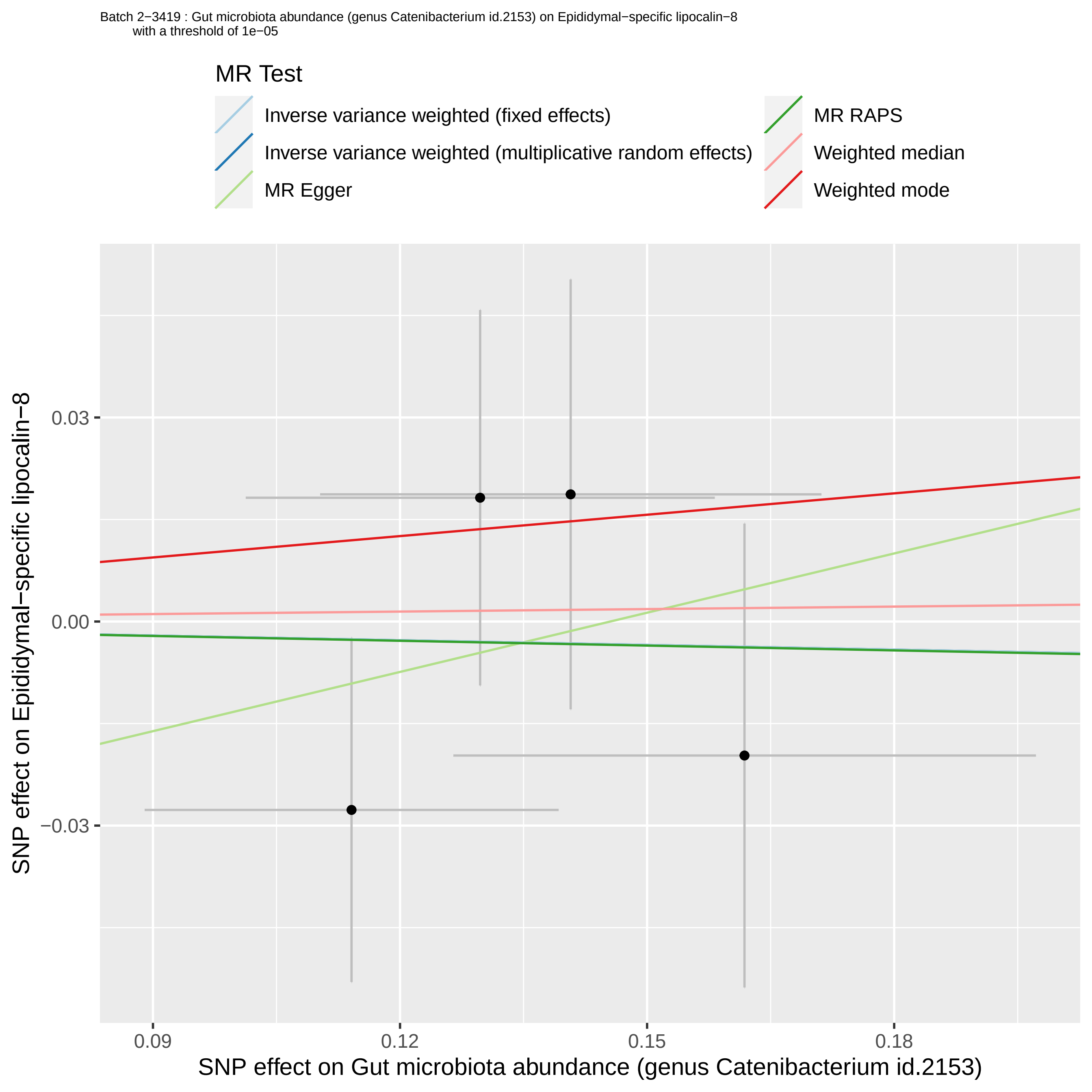

Batch 2−3419 : Gut microbiota abundance (genus Catenibacterium id.2153) on Epididymal−specific lipocalin−8
with a threshold of 1e−05
MR Test
Inverse variance weighted (fixed effects)
Inverse variance weighted (multiplicative random effects)
MR Egger
MR RAPS
Weighted median
Weighted mode
0.03
0.00
−0.03
0.09
0.12
0.15
0.18
SNP effect on Epididymal−specific lipocalin−8
SNP effect on Gut microbiota abundance (genus Catenibacterium id.2153)

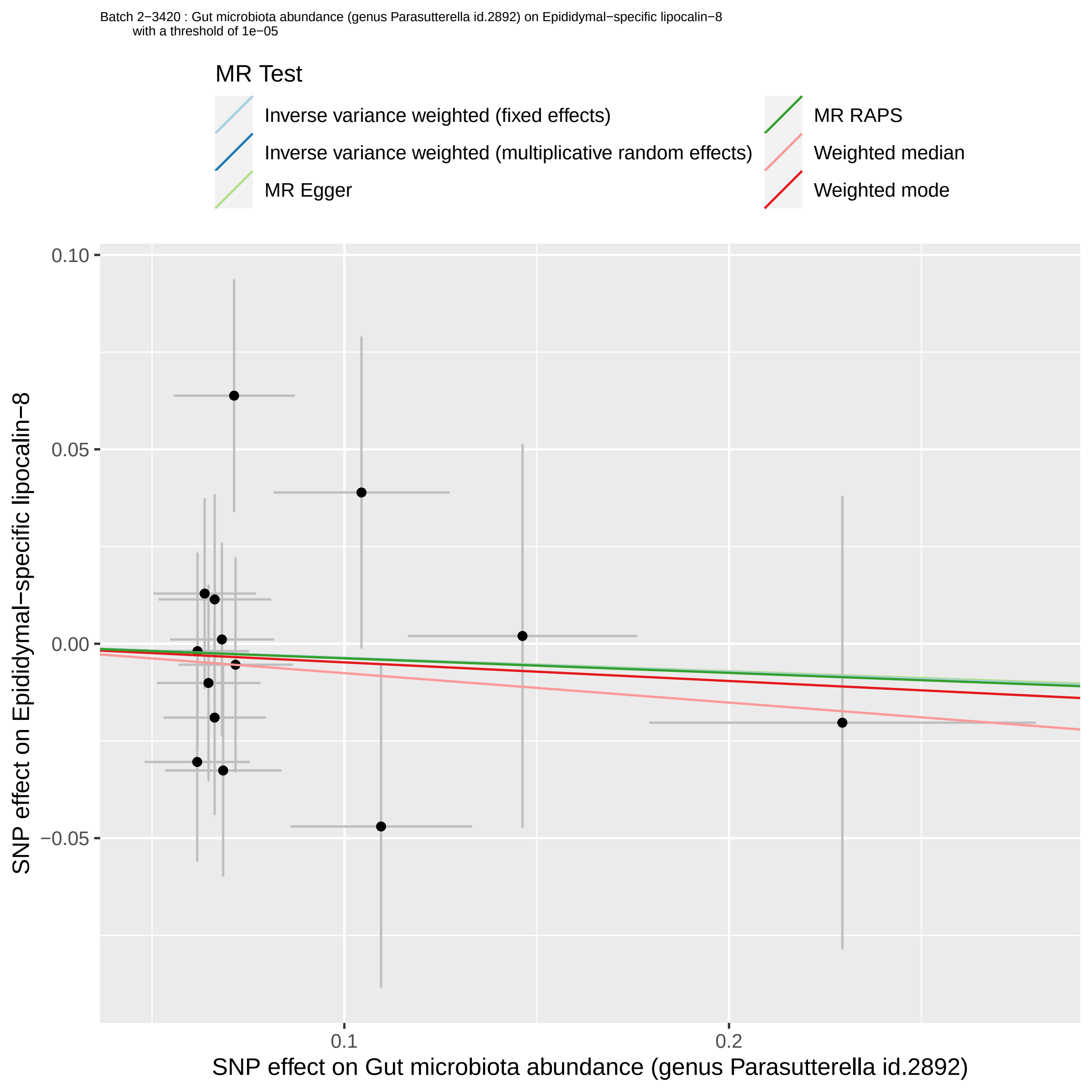

Batch 2−3420 : Gut microbiota abundance (genus Parasutterella id.2892) on Epididymal−specific lipocalin−8
with a threshold of 1e−05
MR Test
Inverse variance weighted (fixed effects)
Inverse variance weighted (multiplicative random effects)
MR Egger
MR RAPS
Weighted median
Weighted mode
0.10
0.05
0.00
−0.05
0.1
0.2
SNP effect on Epididymal−specific lipocalin−8
SNP effect on Gut microbiota abundance (genus Parasutterella id.2892)

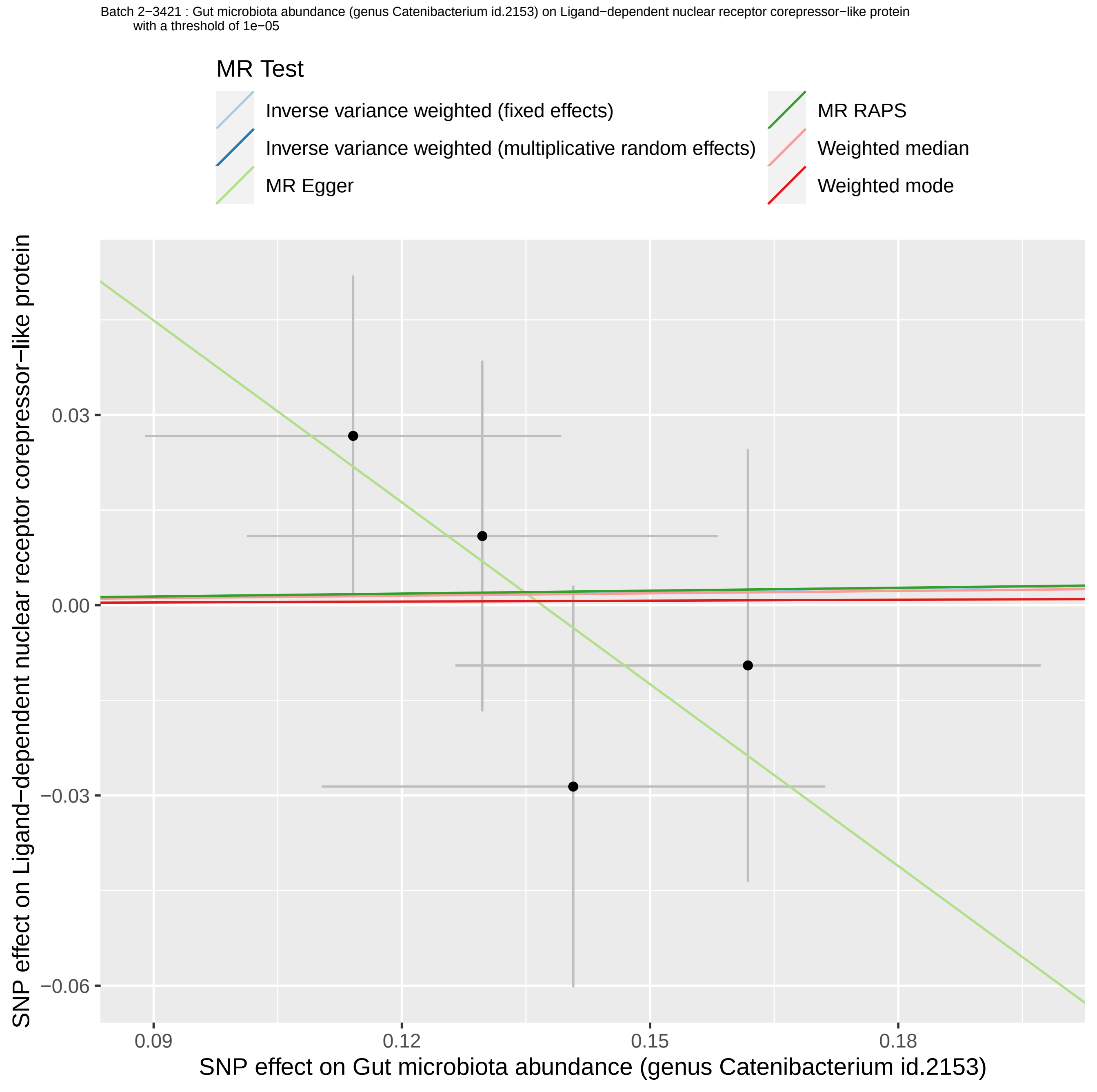
Batch 2−3421 : Gut microbiota abundance (genus Catenibacterium id.2153) on Ligand−dependent nuclear receptor corepressor−like protein
with a threshold of 1e−05
MR Test
Inverse variance weighted (fixed effects)
Inverse variance weighted (multiplicative random effects)
MR Egger
MR RAPS
Weighted median
Weighted mode
0.03
0.00
−0.03
−0.06
0.09
0.12
0.15
0.18
SNP effect on Ligand−dependent nuclear receptor corepressor−like protein
SNP effect on Gut microbiota abundance (genus Catenibacterium id.2153)

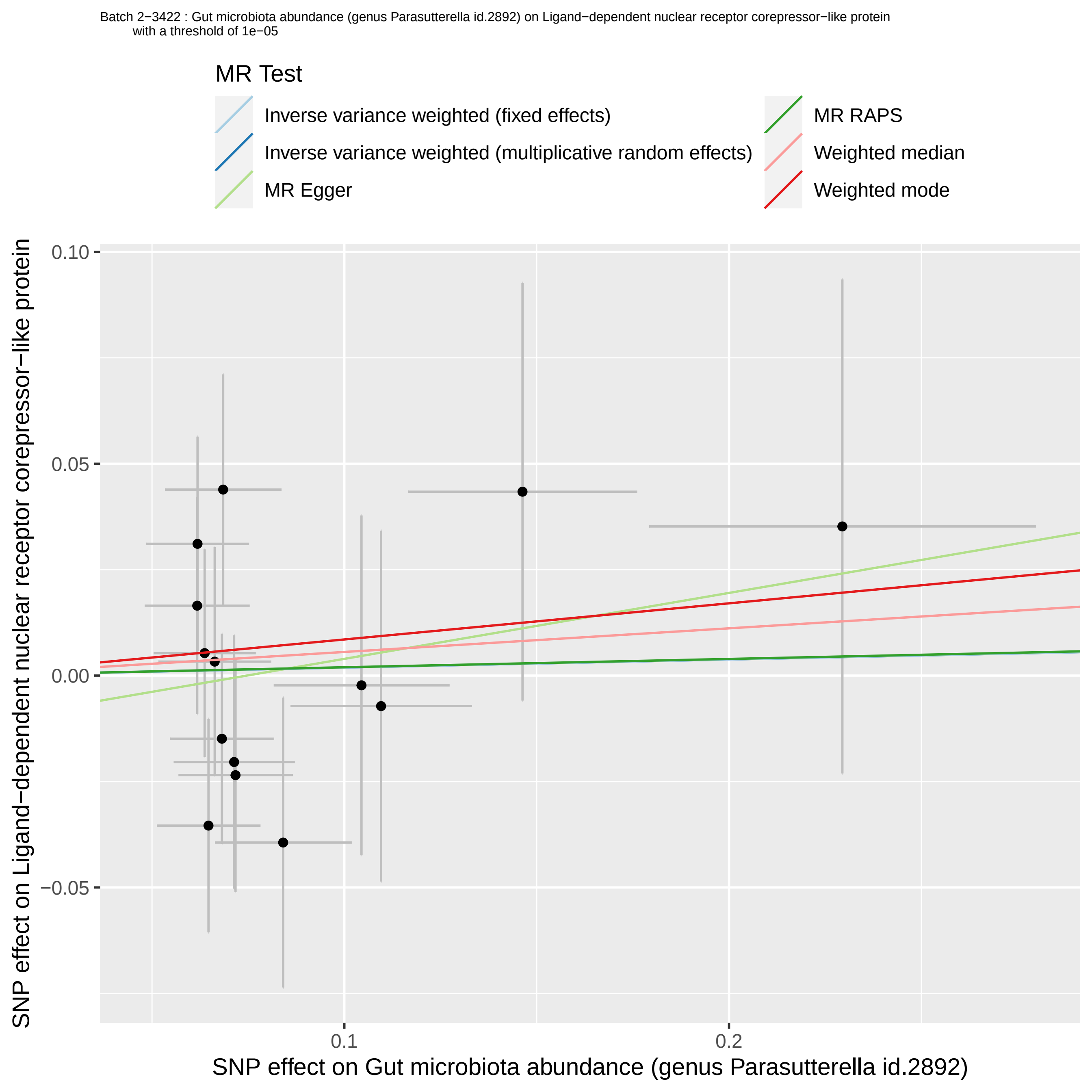

Batch 2−3422 : Gut microbiota abundance (genus Parasutterella id.2892) on Ligand−dependent nuclear receptor corepressor−like protein
with a threshold of 1e−05
MR Test
Inverse variance weighted (fixed effects)
Inverse variance weighted (multiplicative random effects)
MR Egger
MR RAPS
Weighted median
Weighted mode
0.10
0.05
0.00
−0.05
0.1
0.2
SNP effect on Gut microbiota abundance (genus Parasutterella id.2892)
SNP effect on Ligand−dependent nuclear receptor corepressor−like protein

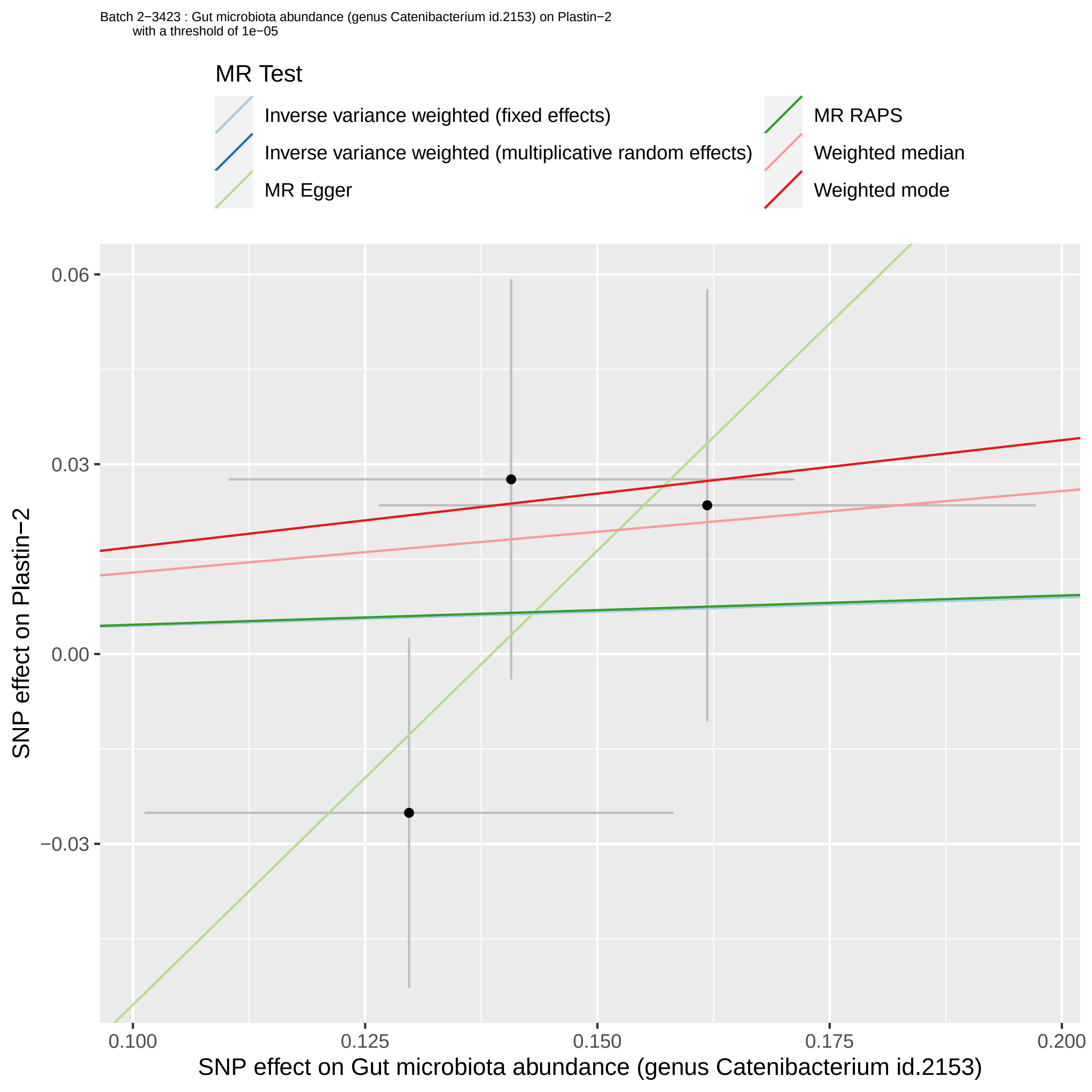
Batch 2−3423 : Gut microbiota abundance (genus Catenibacterium id.2153) on Plastin−2
with a threshold of 1e−05
MR Test
Inverse variance weighted (fixed effects)
Inverse variance weighted (multiplicative random effects)
MR Egger
MR RAPS
Weighted median
Weighted mode
0.06
0.03
0.00
−0.03
0.100
0.125
0.150
0.175
0.200
SNP effect on Plastin−2
SNP effect on Gut microbiota abundance (genus Catenibacterium id.2153)

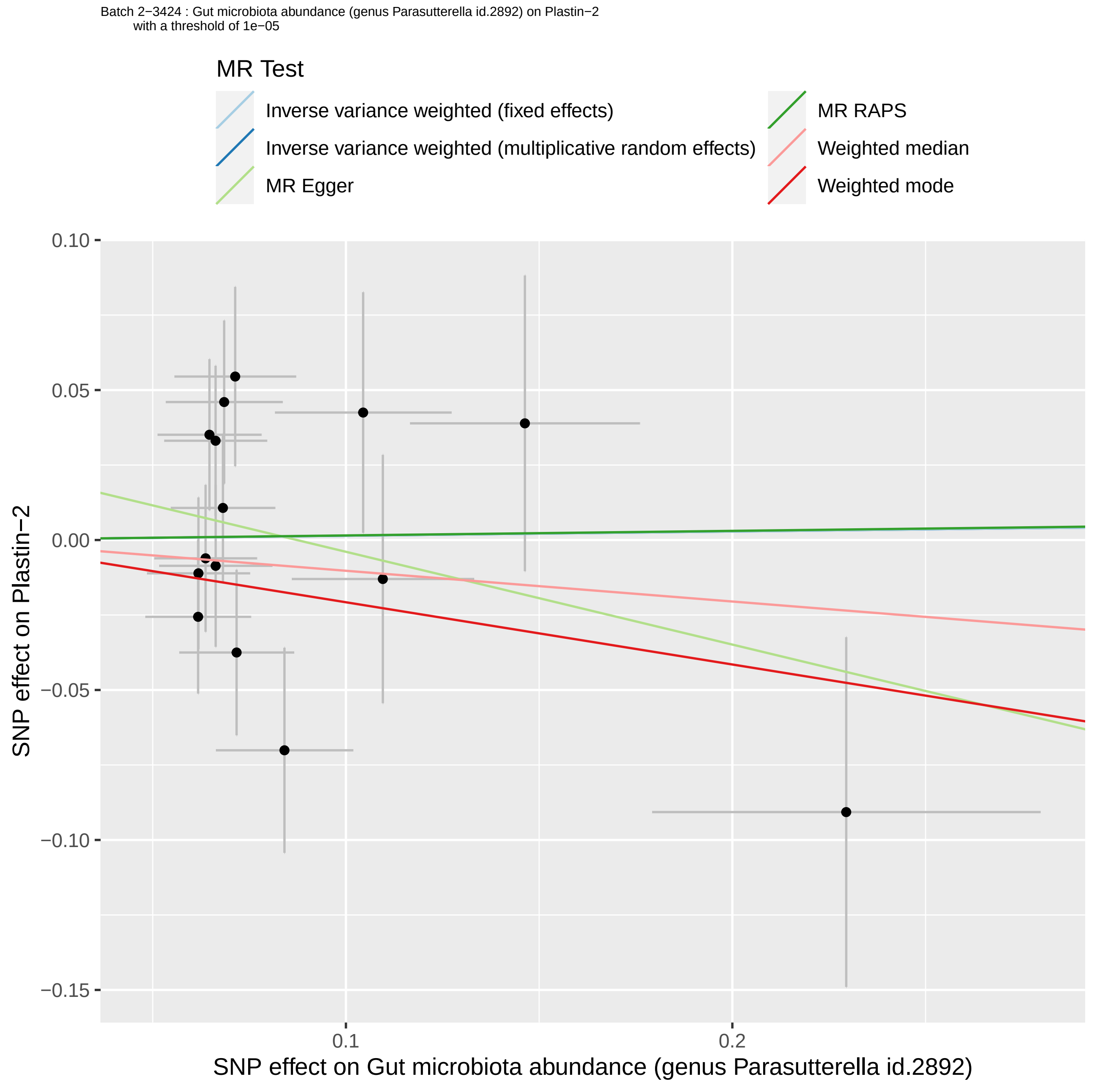

Batch 2−3424 : Gut microbiota abundance (genus Parasutterella id.2892) on Plastin−2
with a threshold of 1e−05
MR Test
Inverse variance weighted (fixed effects)
Inverse variance weighted (multiplicative random effects)
MR Egger
MR RAPS
Weighted median
Weighted mode
0.10
0.05
0.00
−0.05
−0.10
−0.15
0.1
0.2
SNP effect on Plastin−2
SNP effect on Gut microbiota abundance (genus Parasutterella id.2892)

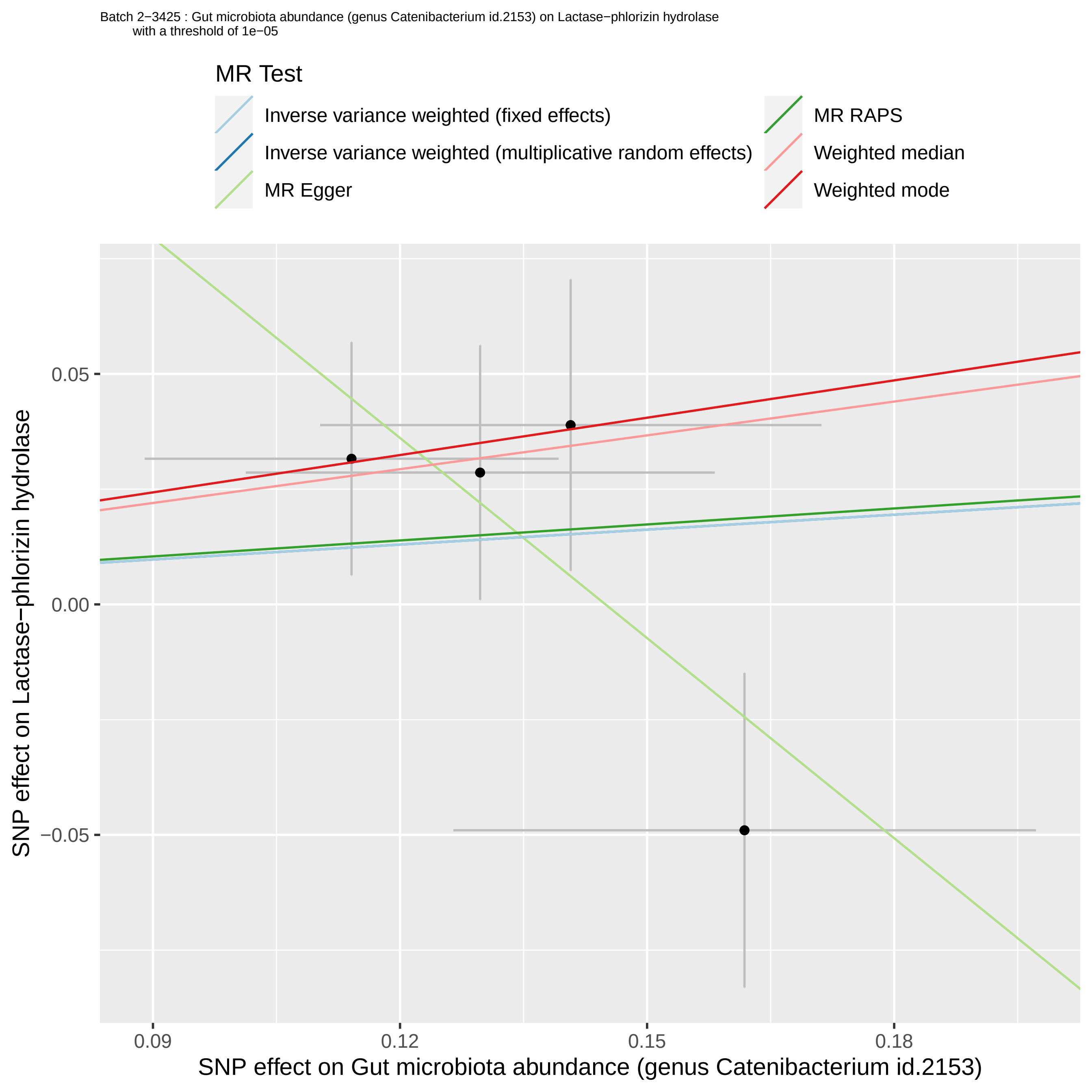
Batch 2−3425 : Gut microbiota abundance (genus Catenibacterium id.2153) on Lactase−phlorizin hydrolase
with a threshold of 1e−05
MR Test
Inverse variance weighted (fixed effects)
Inverse variance weighted (multiplicative random effects)
MR Egger
MR RAPS
Weighted median
Weighted mode
SNP effect on Lactase−phlorizin hydrolase
0.05
0.00
−0.05
0.09
0.12
0.15
0.18
SNP effect on Gut microbiota abundance (genus Catenibacterium id.2153)

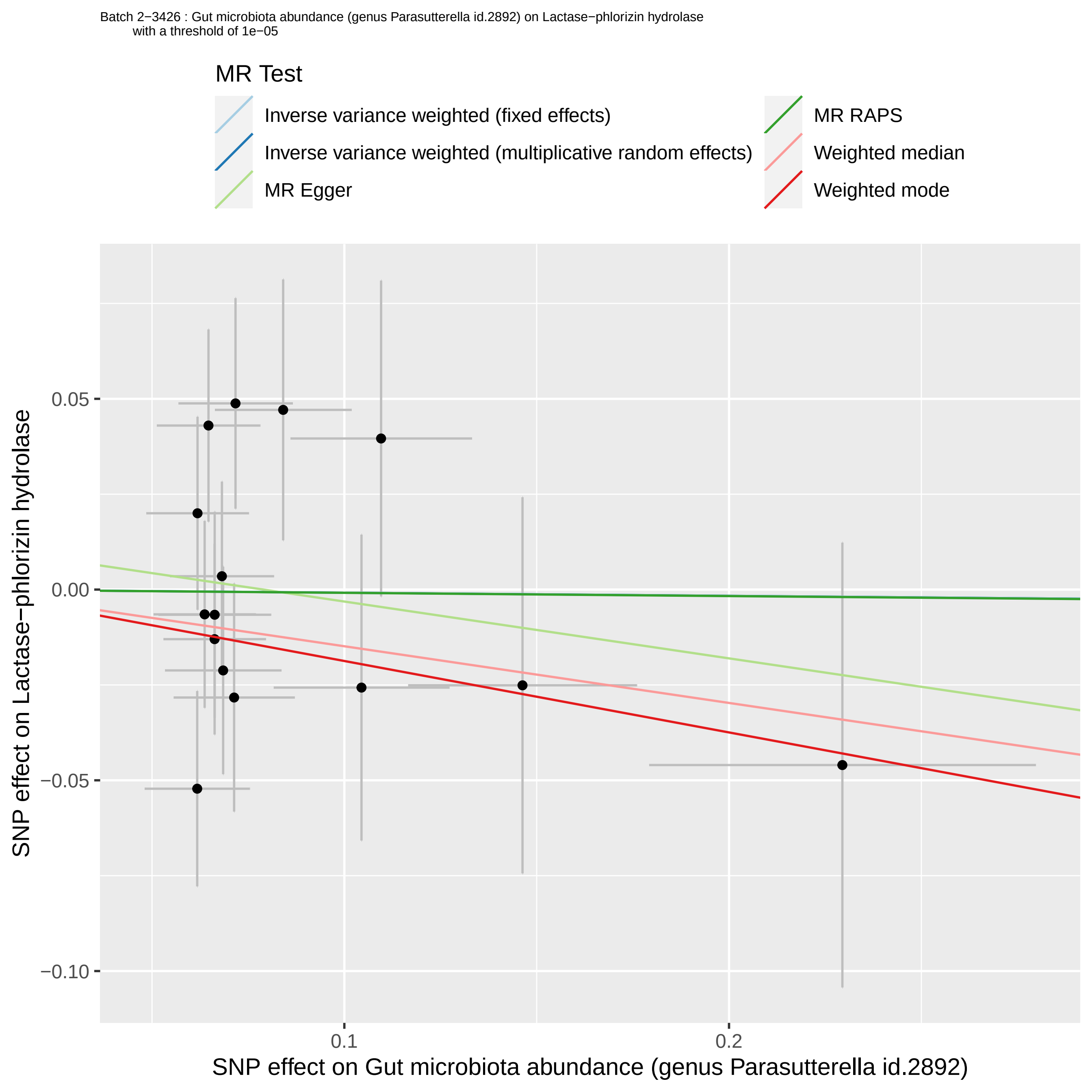
Batch 2−3426 : Gut microbiota abundance (genus Parasutterella id.2892) on Lactase−phlorizin hydrolase
with a threshold of 1e−05
MR Test
Inverse variance weighted (fixed effects)
Inverse variance weighted (multiplicative random effects)
MR Egger
MR RAPS
Weighted median
Weighted mode
SNP effect on Lactase−phlorizin hydrolase
0.05
0.00
−0.05
−0.10
0.1
0.2
SNP effect on Gut microbiota abundance (genus Parasutterella id.2892)

Batch 2−3427 : Gut microbiota abundance (genus Catenibacterium id.2153) on Lactase−like protein
with a threshold of 1e−05

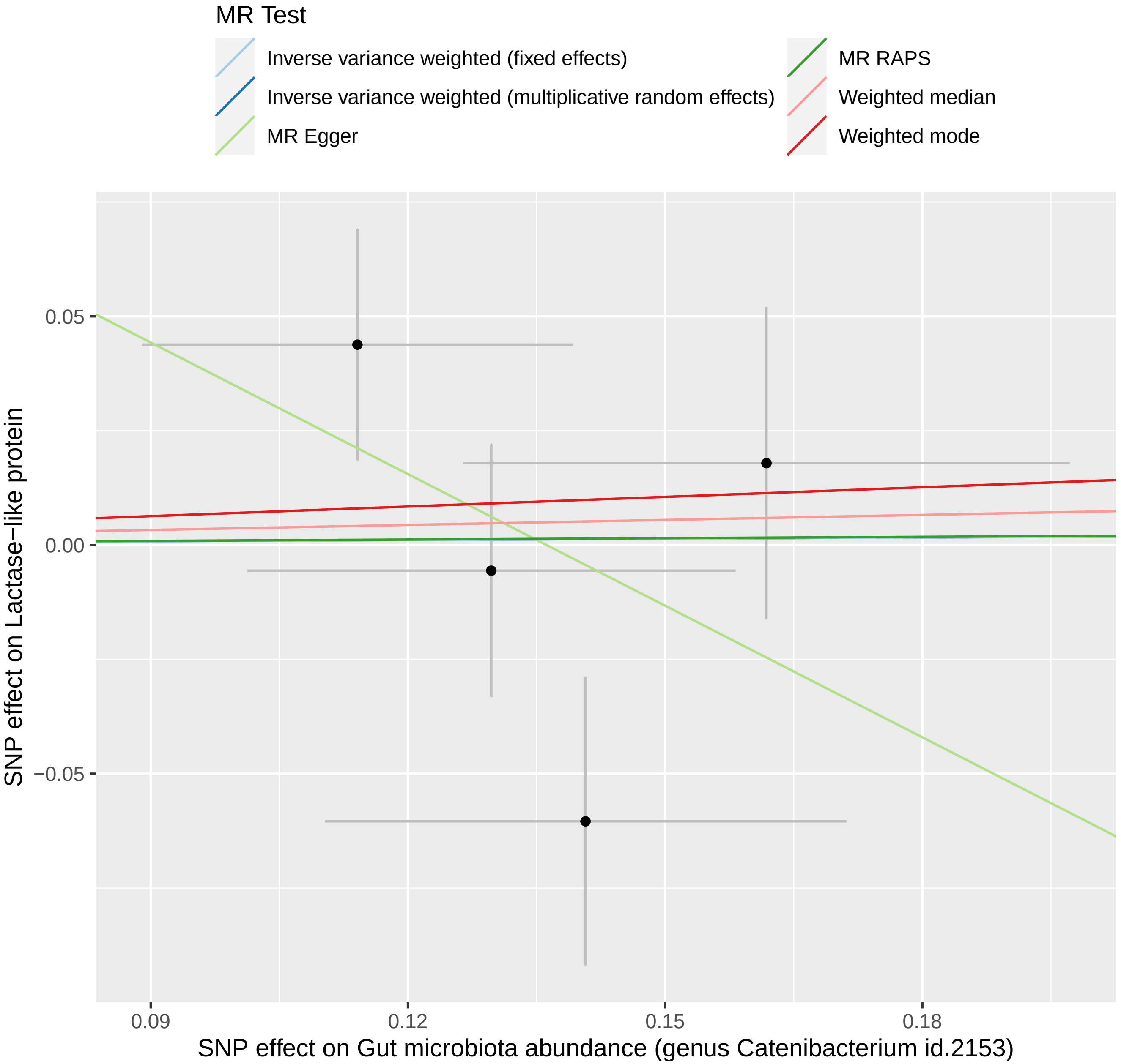

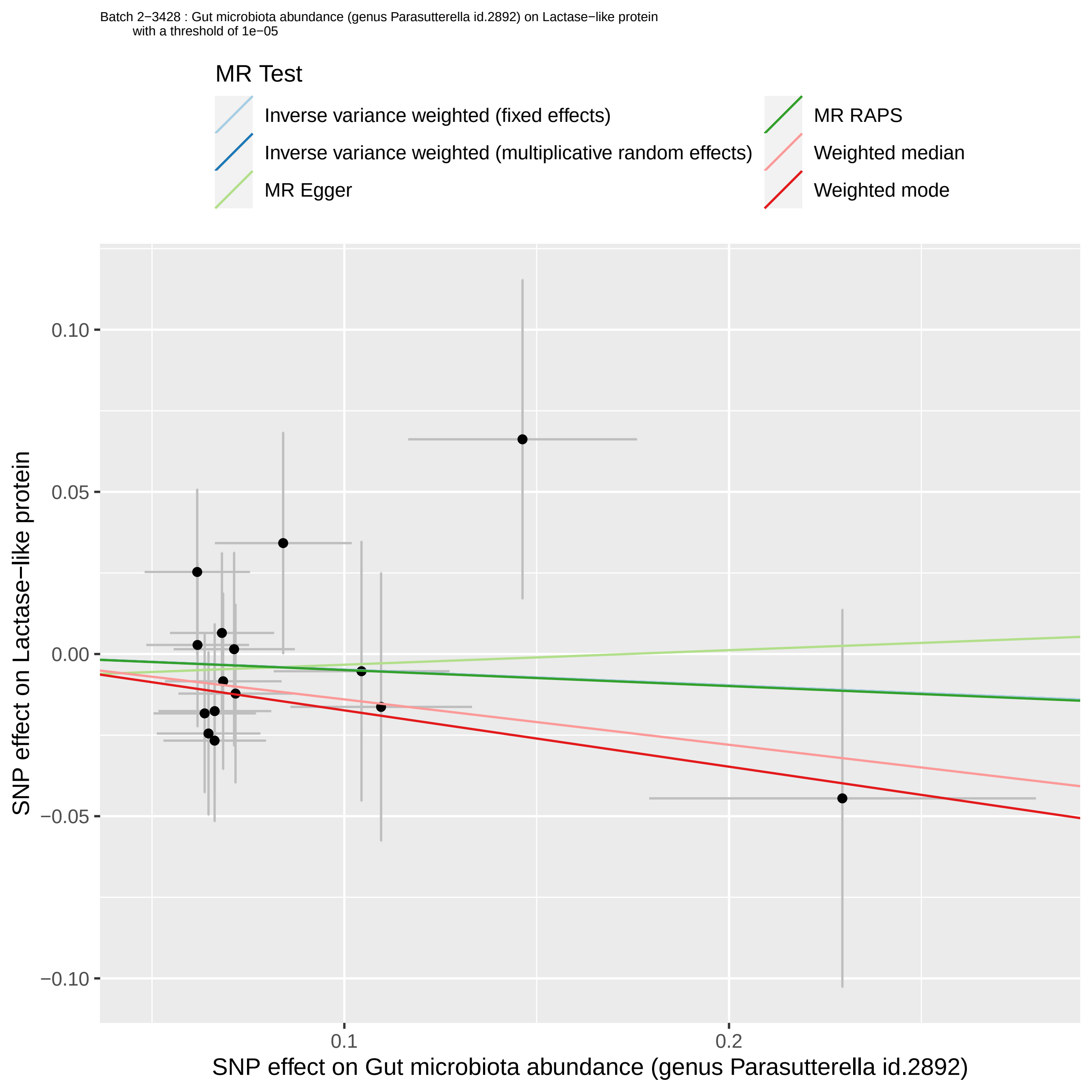
Batch 2−3428 : Gut microbiota abundance (genus Parasutterella id.2892) on Lactase−like protein
with a threshold of 1e−05
MR Test
Inverse variance weighted (fixed effects)
Inverse variance weighted (multiplicative random effects)
MR Egger
MR RAPS
Weighted median
Weighted mode
0.10
0.05
0.00
−0.05
−0.10
0.1
0.2
SNP effect on Lactase−like protein
SNP effect on Gut microbiota abundance (genus Parasutterella id.2892)

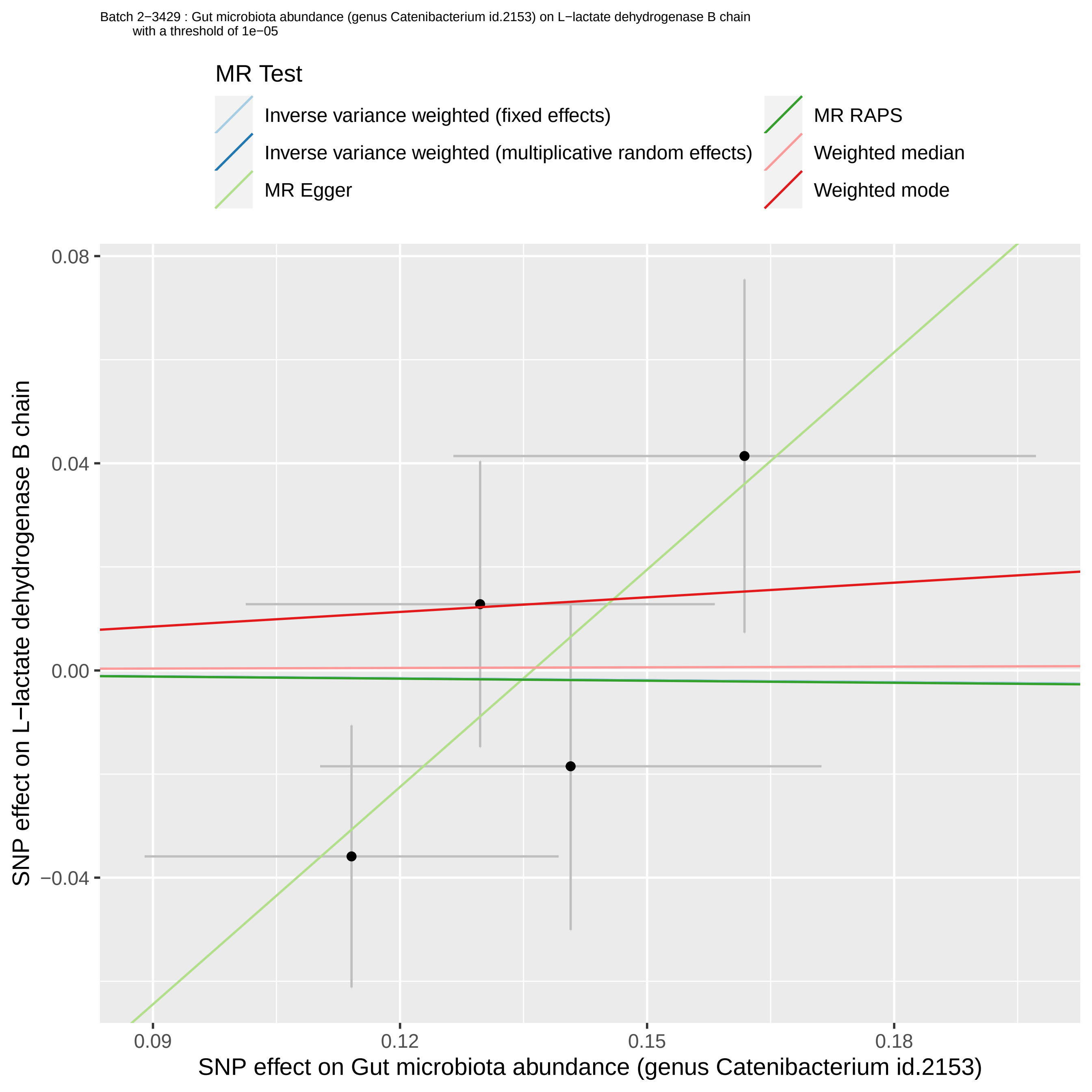
Batch 2−3429 : Gut microbiota abundance (genus Catenibacterium id.2153) on L−lactate dehydrogenase B chain with a threshold of 1e−05
MR Test
Inverse variance weighted (fixed effects)
Inverse variance weighted (multiplicative random effects)
MR Egger
MR RAPS
Weighted median
Weighted mode
0.08
0.04
0.00
−0.04
0.09
0.12
0.15
0.18
SNP effect on L−lactate dehydrogenase B chain
SNP effect on Gut microbiota abundance (genus Catenibacterium id.2153)

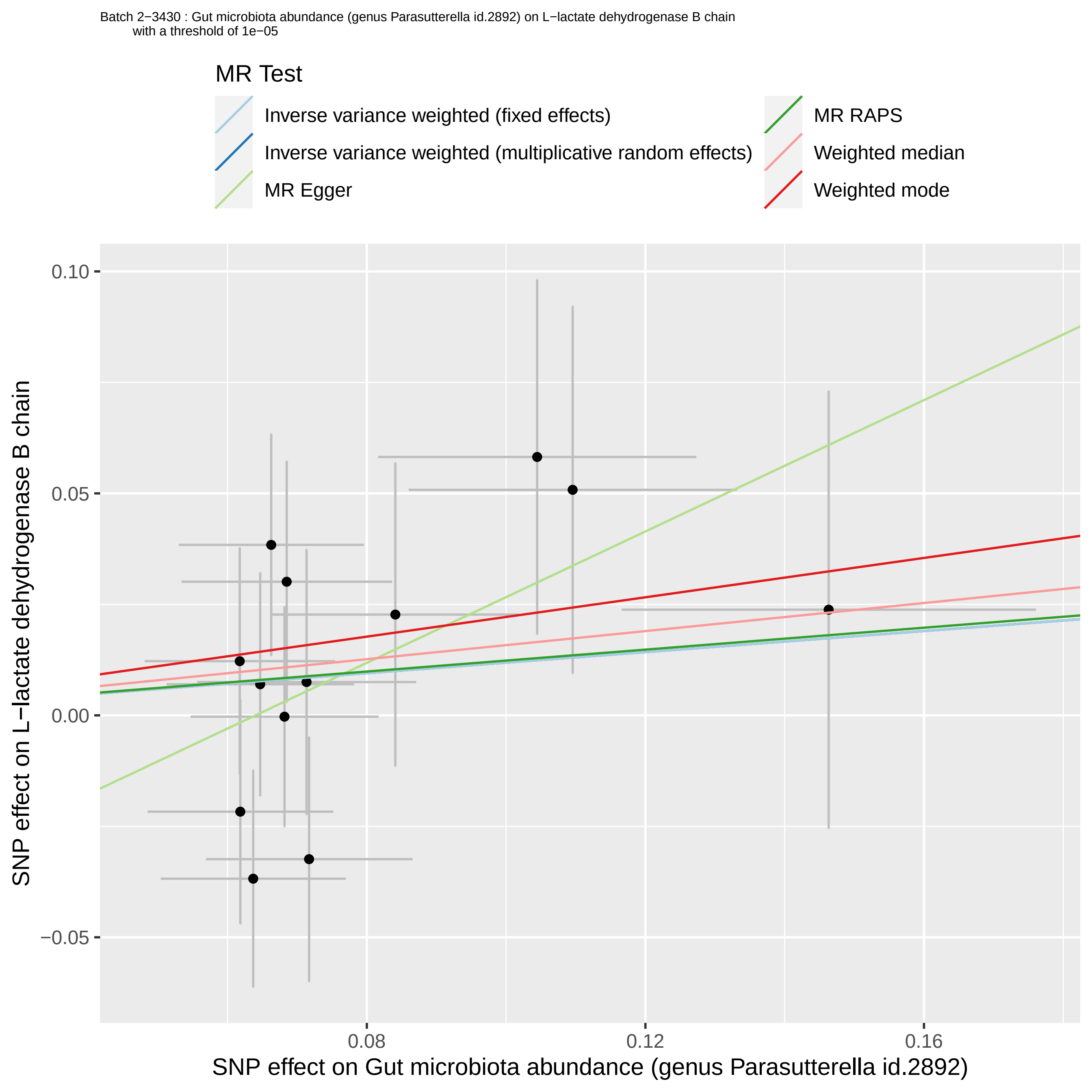

Batch 2−3430 : Gut microbiota abundance (genus Parasutterella id.2892) on L−lactate dehydrogenase B chain
with a threshold of 1e−05
MR Test
Inverse variance weighted (fixed effects)
Inverse variance weighted (multiplicative random effects)
MR Egger
MR RAPS
Weighted median
Weighted mode
0.10
0.05
0.00
−0.05
0.08
0.12
0.16
SNP effect on L−lactate dehydrogenase B chain
SNP effect on Gut microbiota abundance (genus Parasutterella id.2892)

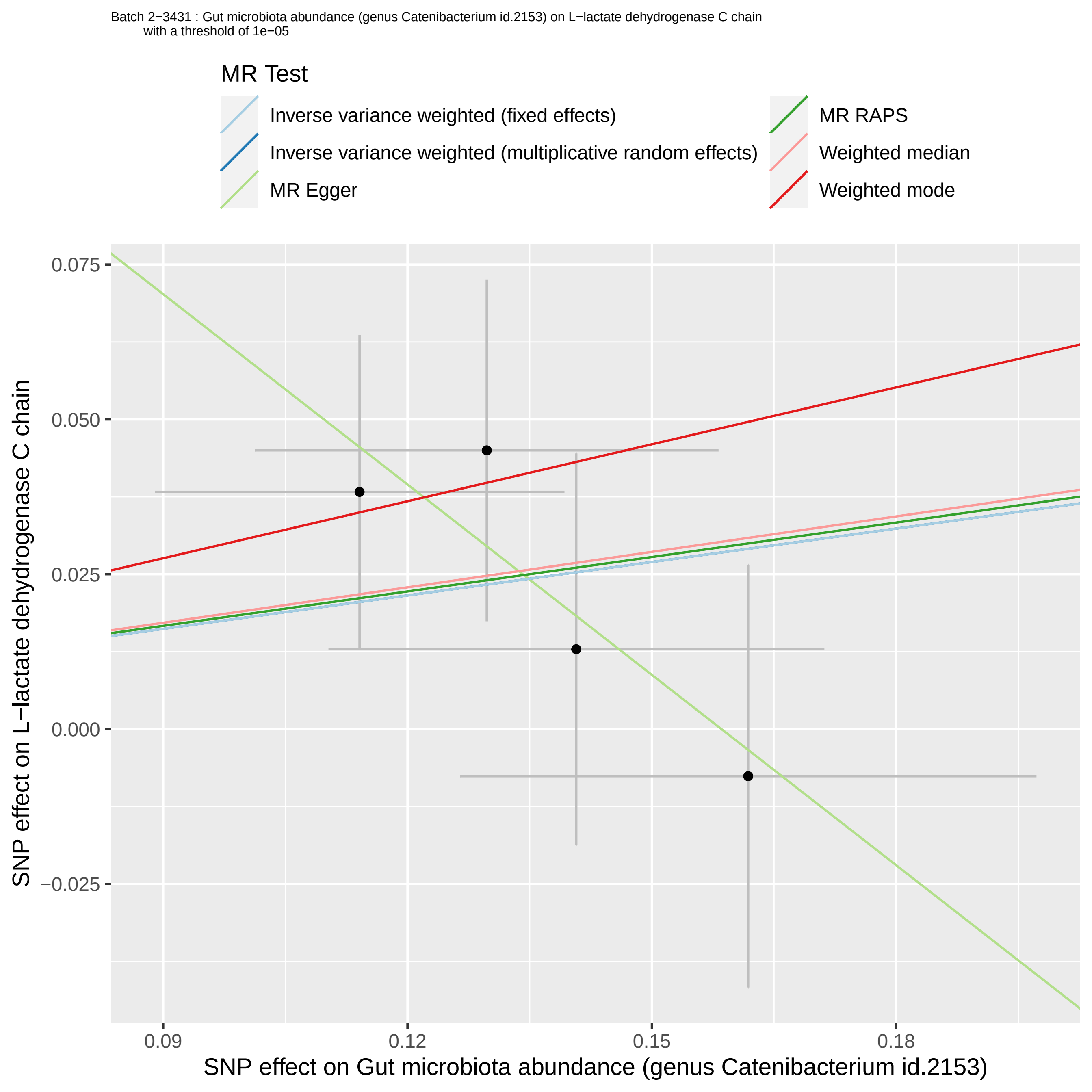
Batch 2−3431 : Gut microbiota abundance (genus Catenibacterium id.2153) on L−lactate dehydrogenase C chain
with a threshold of 1e−05
MR Test
Inverse variance weighted (fixed effects)
Inverse variance weighted (multiplicative random effects)
MR Egger
MR RAPS
Weighted median
Weighted mode
0.075
0.050
0.025
0.000
−0.025
0.09
0.12
0.15
0.18
SNP effect on L−lactate dehydrogenase C chain
SNP effect on Gut microbiota abundance (genus Catenibacterium id.2153)

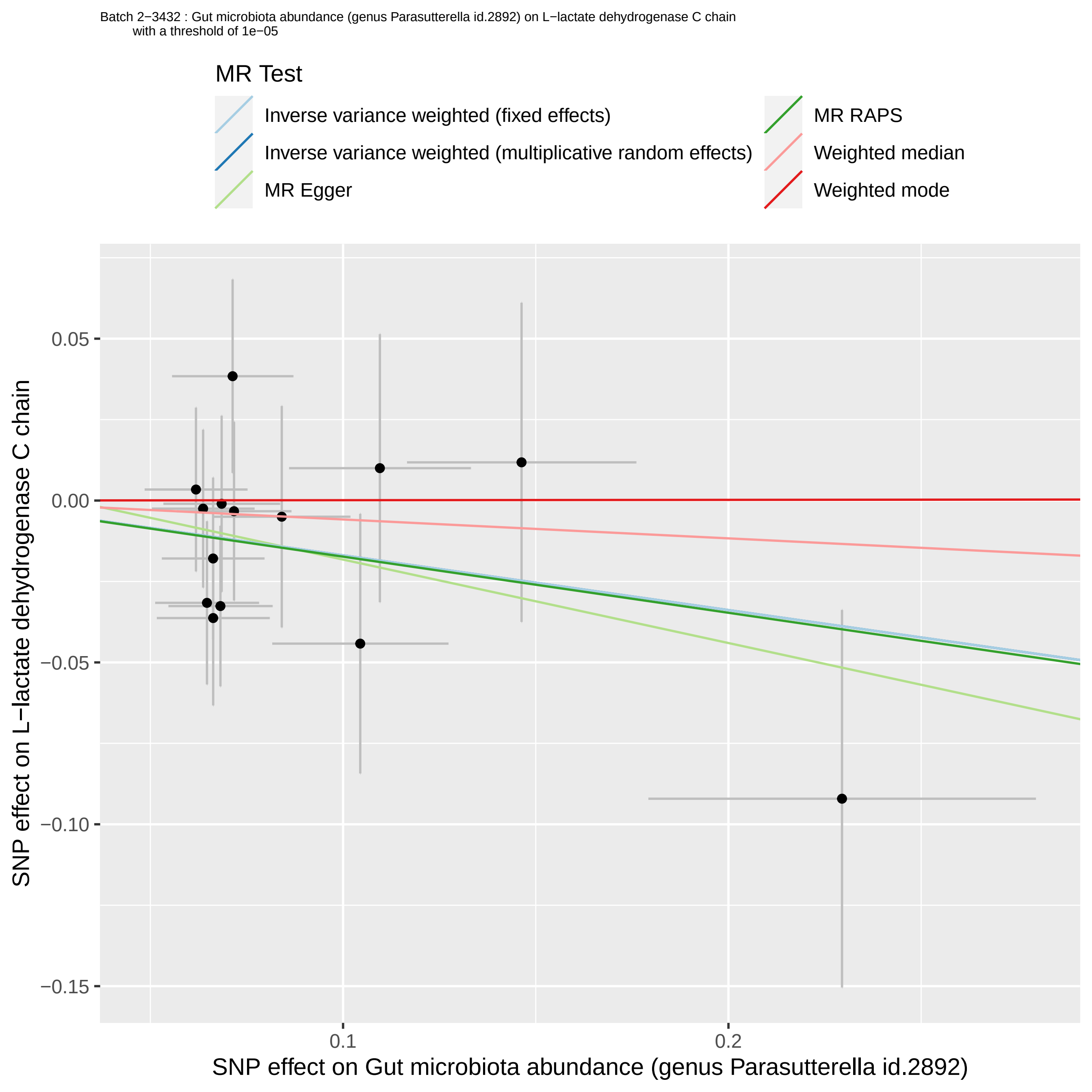
Batch 2−3432 : Gut microbiota abundance (genus Parasutterella id.2892) on L−lactate dehydrogenase C chain
with a threshold of 1e−05
MR Test
Inverse variance weighted (fixed effects)
Inverse variance weighted (multiplicative random effects)
MR Egger
MR RAPS
Weighted median
Weighted mode
SNP effect on L−lactate dehydrogenase C chain
0.05
0.00
−0.05
−0.10
−0.15
0.1
0.2
SNP effect on Gut microbiota abundance (genus Parasutterella id.2892)

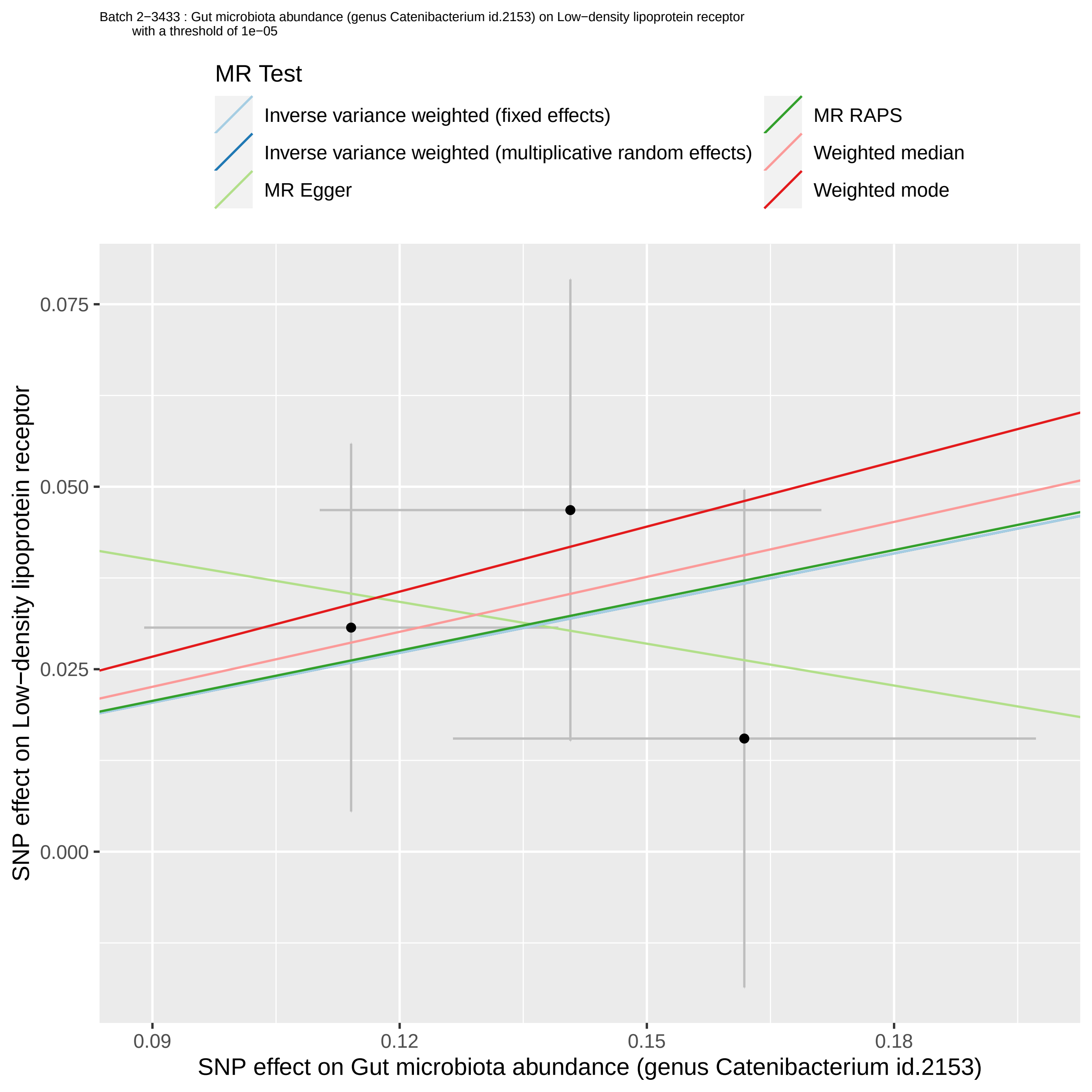

Batch 2−3433 : Gut microbiota abundance (genus Catenibacterium id.2153) on Low−density lipoprotein receptor
with a threshold of 1e−05
MR Test
Inverse variance weighted (fixed effects)
Inverse variance weighted (multiplicative random effects)
MR Egger
MR RAPS
Weighted median
Weighted mode
0.075
0.050
0.025
0.000
0.09
0.12
0.15
0.18
SNP effect on Low−density lipoprotein receptor
SNP effect on Gut microbiota abundance (genus Catenibacterium id.2153)

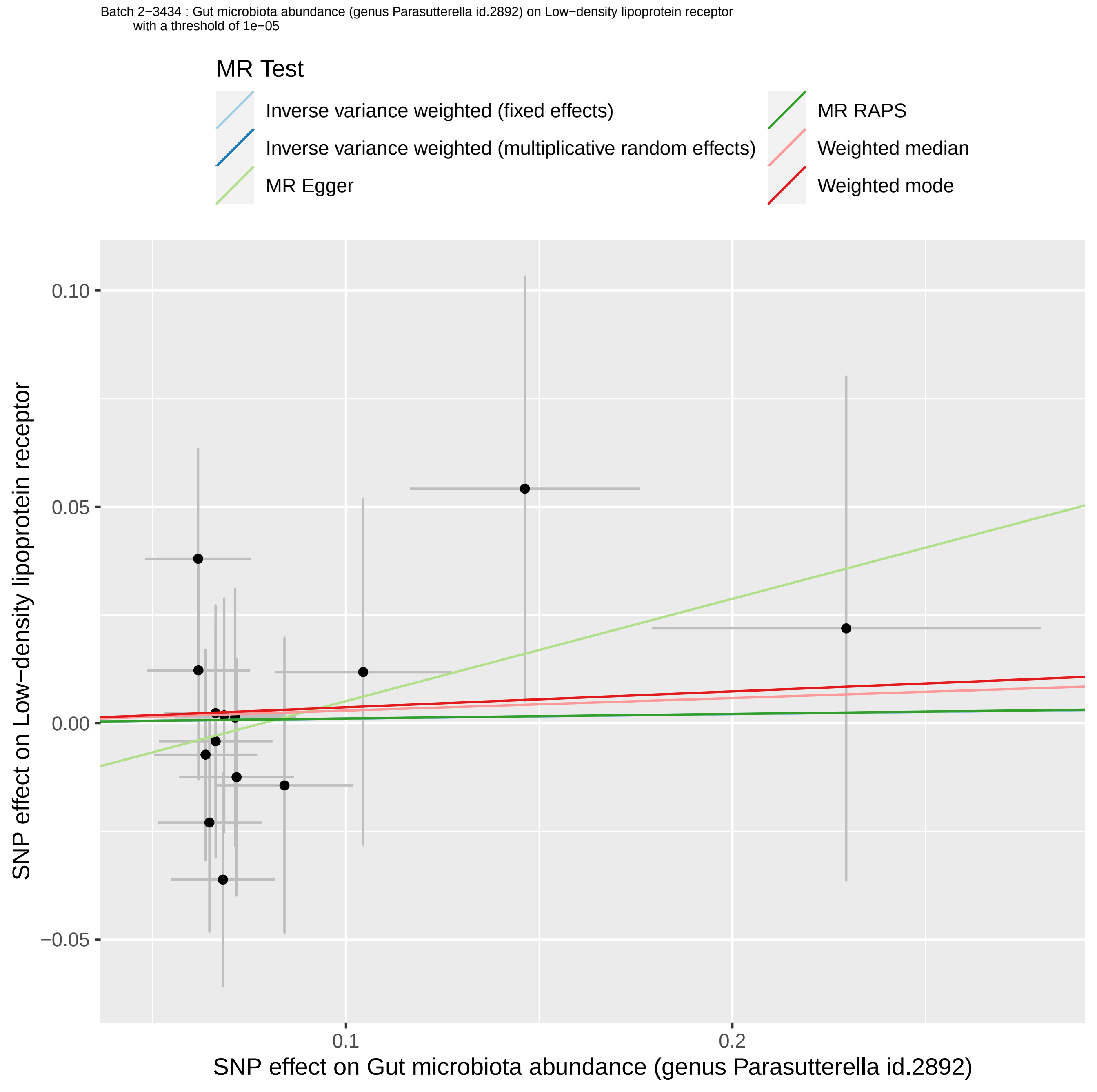
Batch 2−3434 : Gut microbiota abundance (genus Parasutterella id.2892) on Low−density lipoprotein receptor
with a threshold of 1e−05
MR Test
Inverse variance weighted (fixed effects)
Inverse variance weighted (multiplicative random effects)
MR Egger
MR RAPS
Weighted median
Weighted mode
0.10
0.05
0.00
−0.05
0.1
0.2
SNP effect on Low−density lipoprotein receptor
SNP effect on Gut microbiota abundance (genus Parasutterella id.2892)

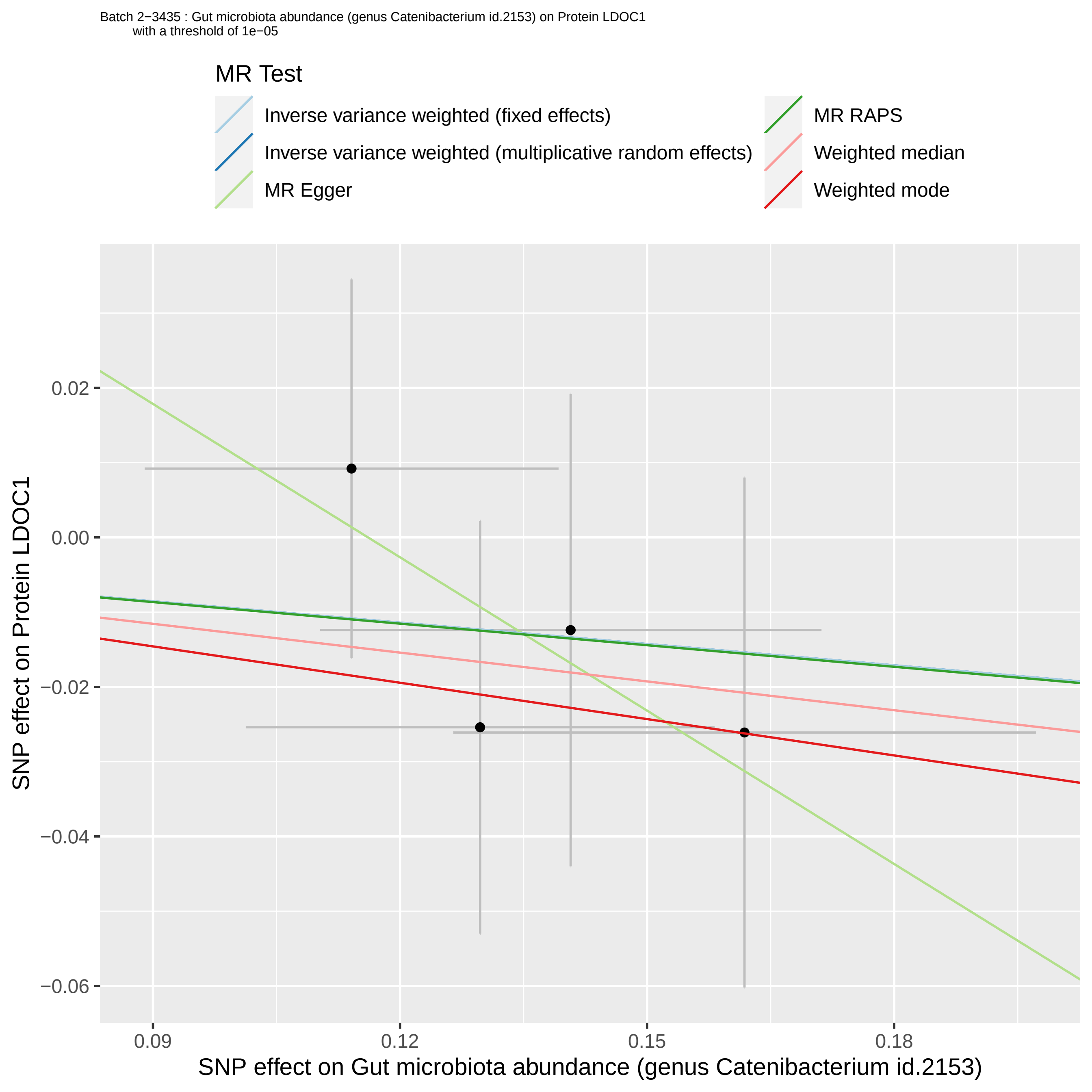
Batch 2−3435 : Gut microbiota abundance (genus Catenibacterium id.2153) on Protein LDOC1
with a threshold of 1e−05
MR Test
Inverse variance weighted (fixed effects)
Inverse variance weighted (multiplicative random effects)
MR Egger
MR RAPS
Weighted median
Weighted mode
0.02
0.00
−0.02
−0.04
−0.06
0.09
0.12
0.15
0.18
SNP effect on Protein LDOC1
SNP effect on Gut microbiota abundance (genus Catenibacterium id.2153)

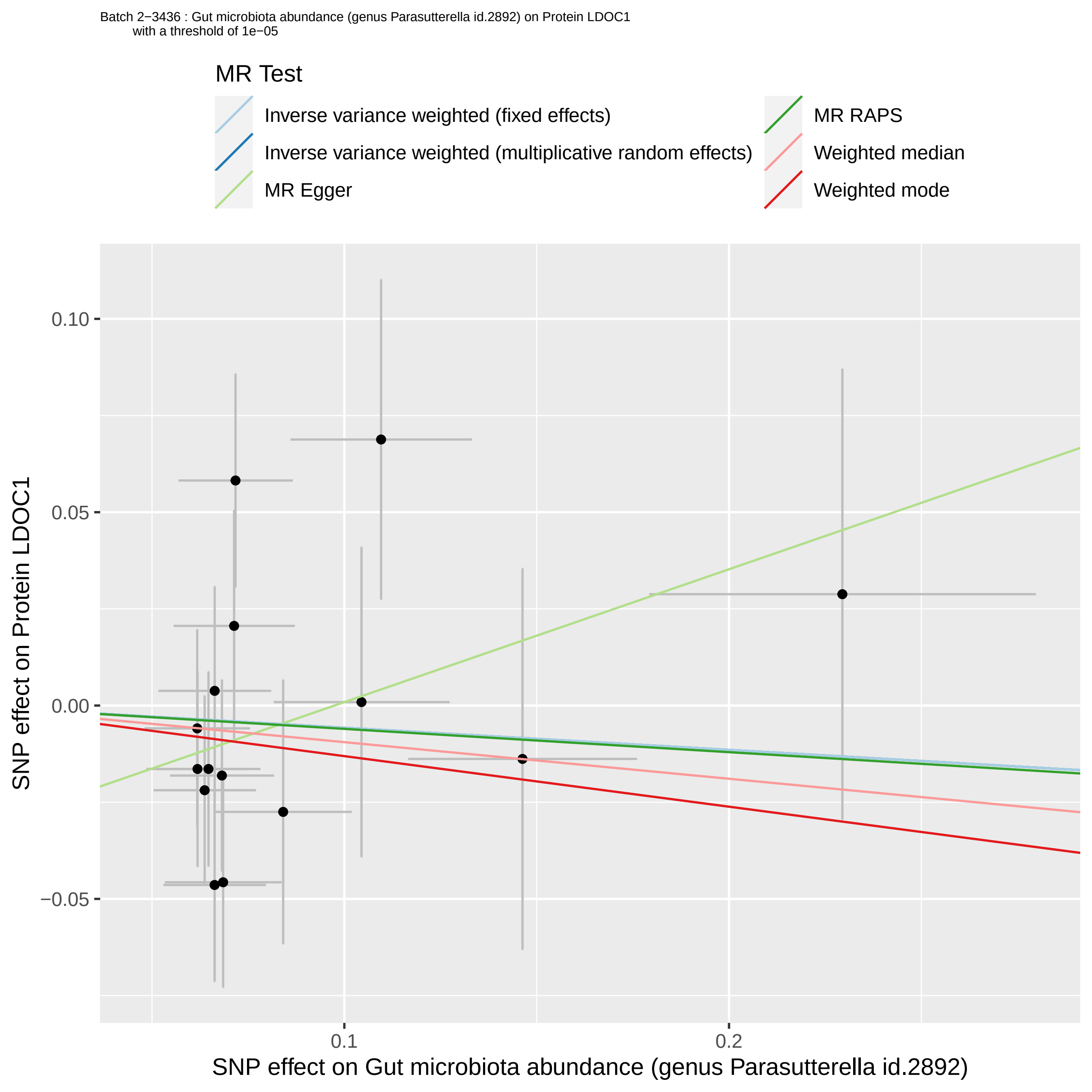
Batch 2−3436 : Gut microbiota abundance (genus Parasutterella id.2892) on Protein LDOC1
with a threshold of 1e−05
MR Test
Inverse variance weighted (fixed effects)
Inverse variance weighted (multiplicative random effects)
MR Egger
MR RAPS
Weighted median
Weighted mode
0.10
0.05
0.00
−0.05
0.1
0.2
SNP effect on Protein LDOC1
SNP effect on Gut microbiota abundance (genus Parasutterella id.2892)

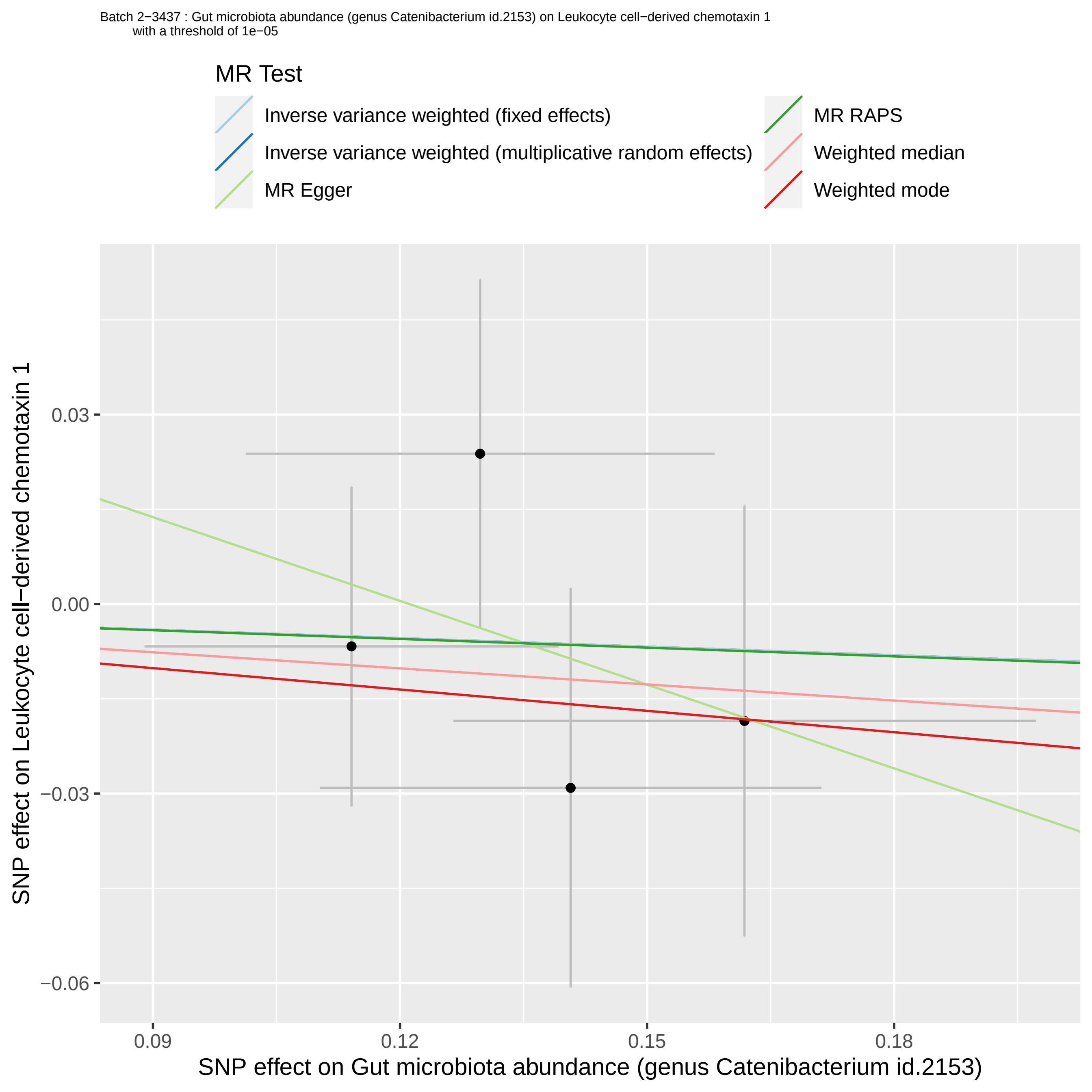
Batch 2−3437 : Gut microbiota abundance (genus Catenibacterium id.2153) on Leukocyte cell−derived chemotaxin 1 with a threshold of 1e−05
MR Test
Inverse variance weighted (fixed effects)
Inverse variance weighted (multiplicative random effects)
MR Egger
MR RAPS
Weighted median
Weighted mode
SNP effect on Leukocyte cell−derived chemotaxin 1
0.03
0.00
−0.03
−0.06
0.09
0.12
0.15
0.18
SNP effect on Gut microbiota abundance (genus Catenibacterium id.2153)

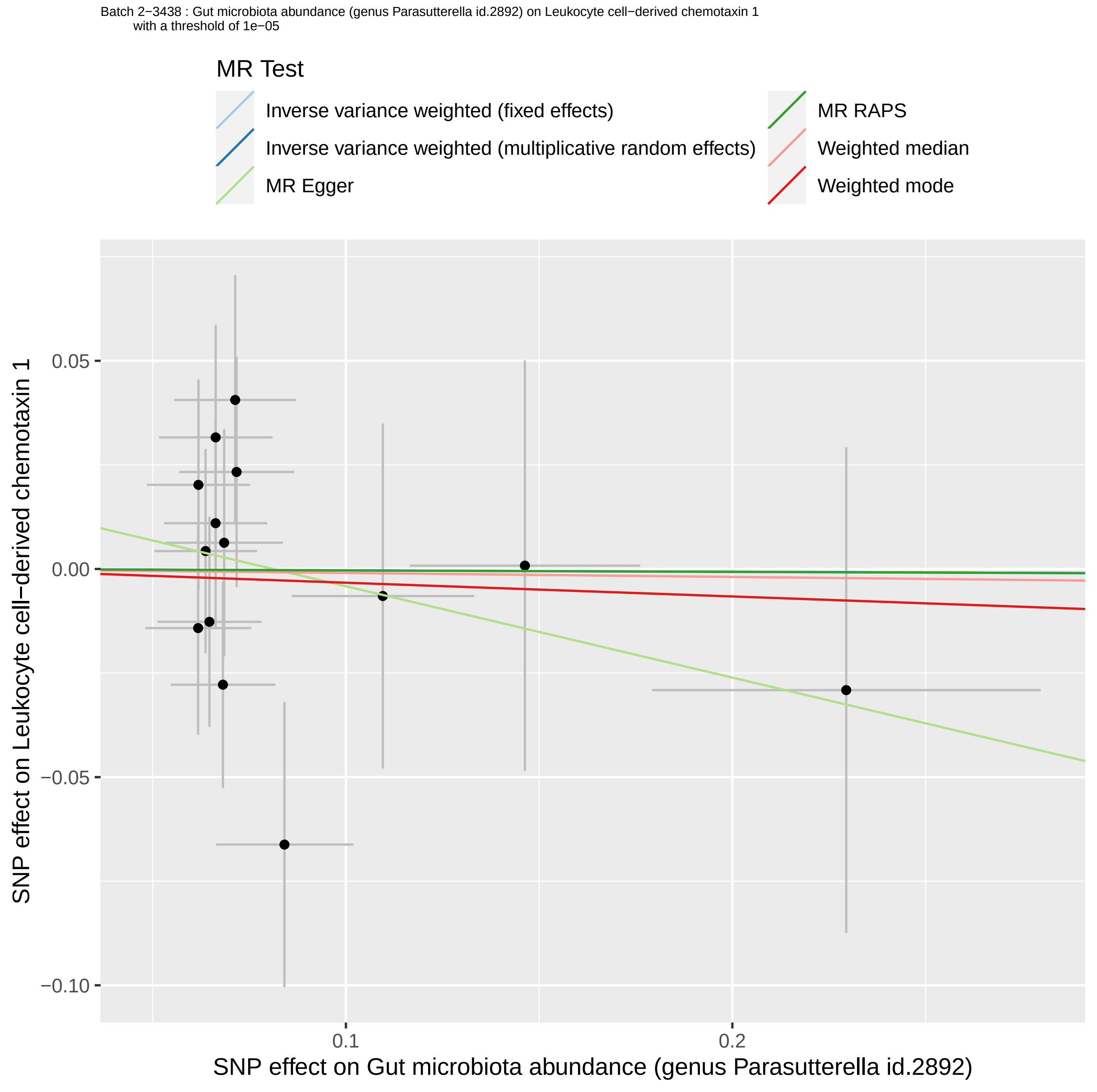
Batch 2−3438 : Gut microbiota abundance (genus Parasutterella id.2892) on Leukocyte cell−derived chemotaxin 1
with a threshold of 1e−05
MR Test
Inverse variance weighted (fixed effects)
Inverse variance weighted (multiplicative random effects)
MR Egger
MR RAPS
Weighted median
Weighted mode
0.05
0.00
−0.05
−0.10
0.1
0.2
SNP effect on Leukocyte cell−derived chemotaxin 1
SNP effect on Gut microbiota abundance (genus Parasutterella id.2892)

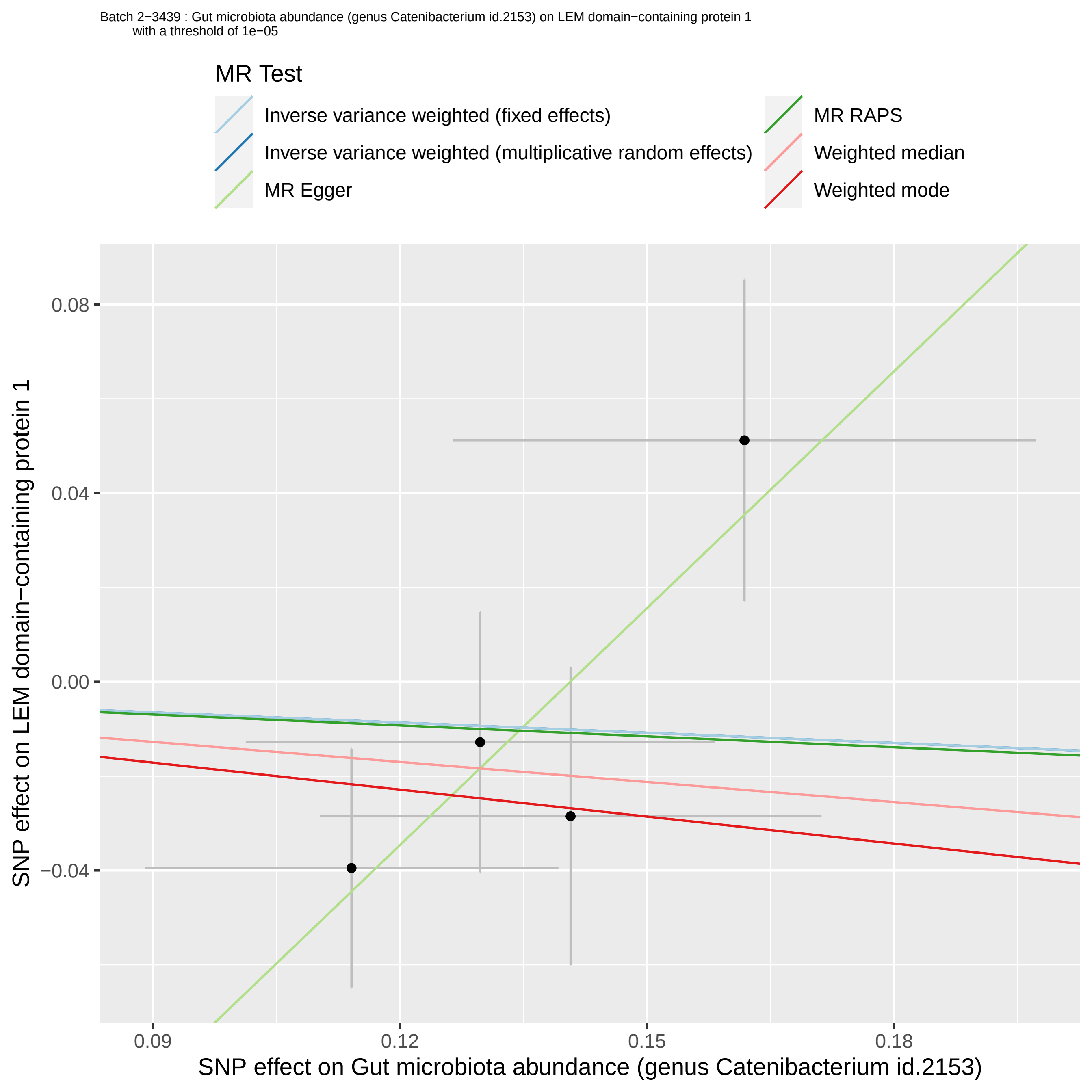
Batch 2−3439 : Gut microbiota abundance (genus Catenibacterium id.2153) on LEM domain−containing protein 1
with a threshold of 1e−05
MR Test
Inverse variance weighted (fixed effects)
Inverse variance weighted (multiplicative random effects)
MR Egger
MR RAPS
Weighted median
Weighted mode
0.08
0.04
0.00
−0.04
0.09
0.12
0.15
0.18
SNP effect on LEM domain−containing protein 1
SNP effect on Gut microbiota abundance (genus Catenibacterium id.2153)

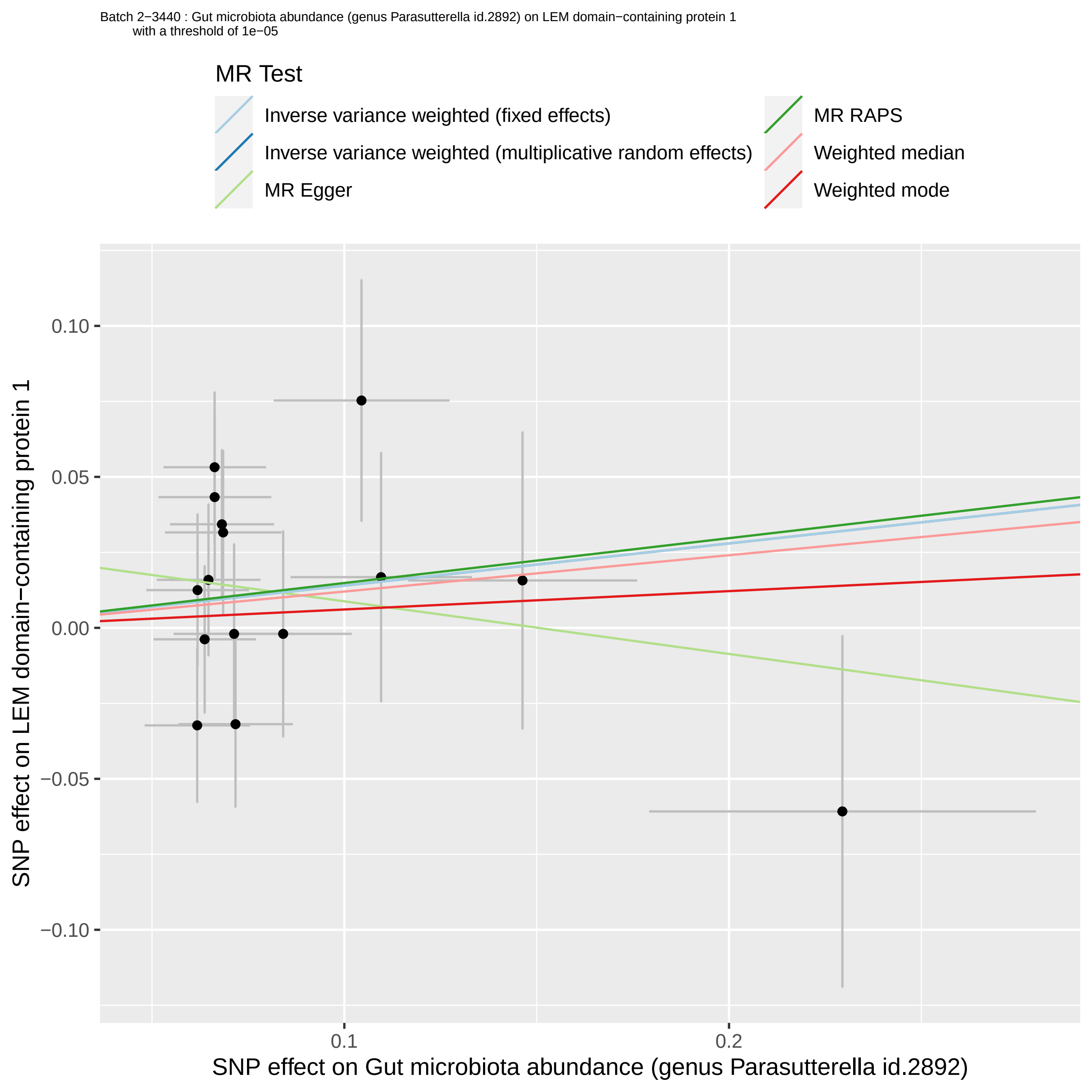

Batch 2−3440 : Gut microbiota abundance (genus Parasutterella id.2892) on LEM domain−containing protein 1
with a threshold of 1e−05
MR Test
Inverse variance weighted (fixed effects)
Inverse variance weighted (multiplicative random effects)
MR Egger
MR RAPS
Weighted median
Weighted mode
0.10
0.05
0.00
−0.05
−0.10
0.1
0.2
SNP effect on LEM domain−containing protein 1
SNP effect on Gut microbiota abundance (genus Parasutterella id.2892)

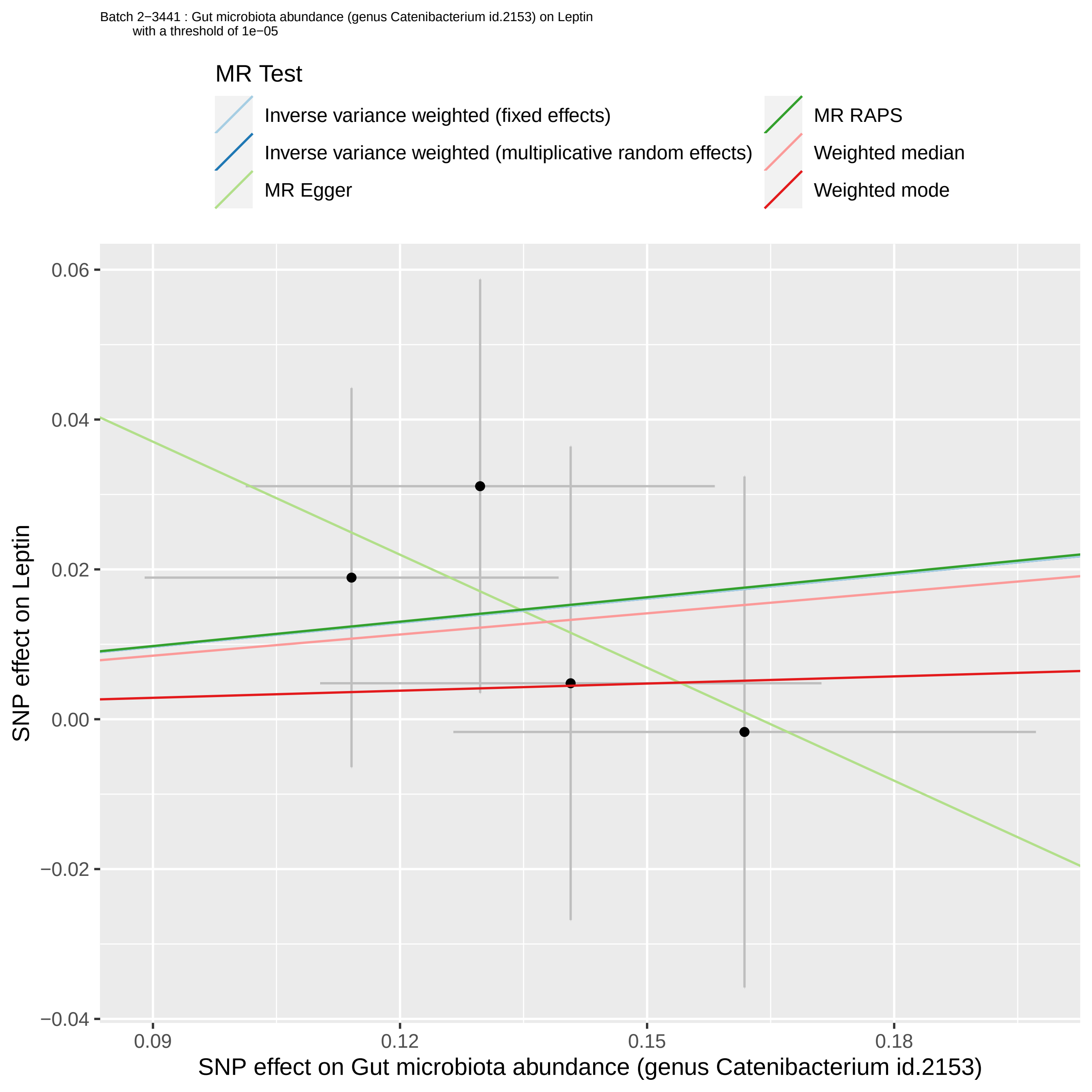
Batch 2−3441 : Gut microbiota abundance (genus Catenibacterium id.2153) on Leptin
with a threshold of 1e−05
MR Test
Inverse variance weighted (fixed effects)
Inverse variance weighted (multiplicative random effects)
MR Egger
MR RAPS
Weighted median
Weighted mode
SNP effect on Leptin
0.06
0.04
0.02
0.00
−0.02
−0.04
0.09
0.12
0.15
0.18
SNP effect on Gut microbiota abundance (genus Catenibacterium id.2153)

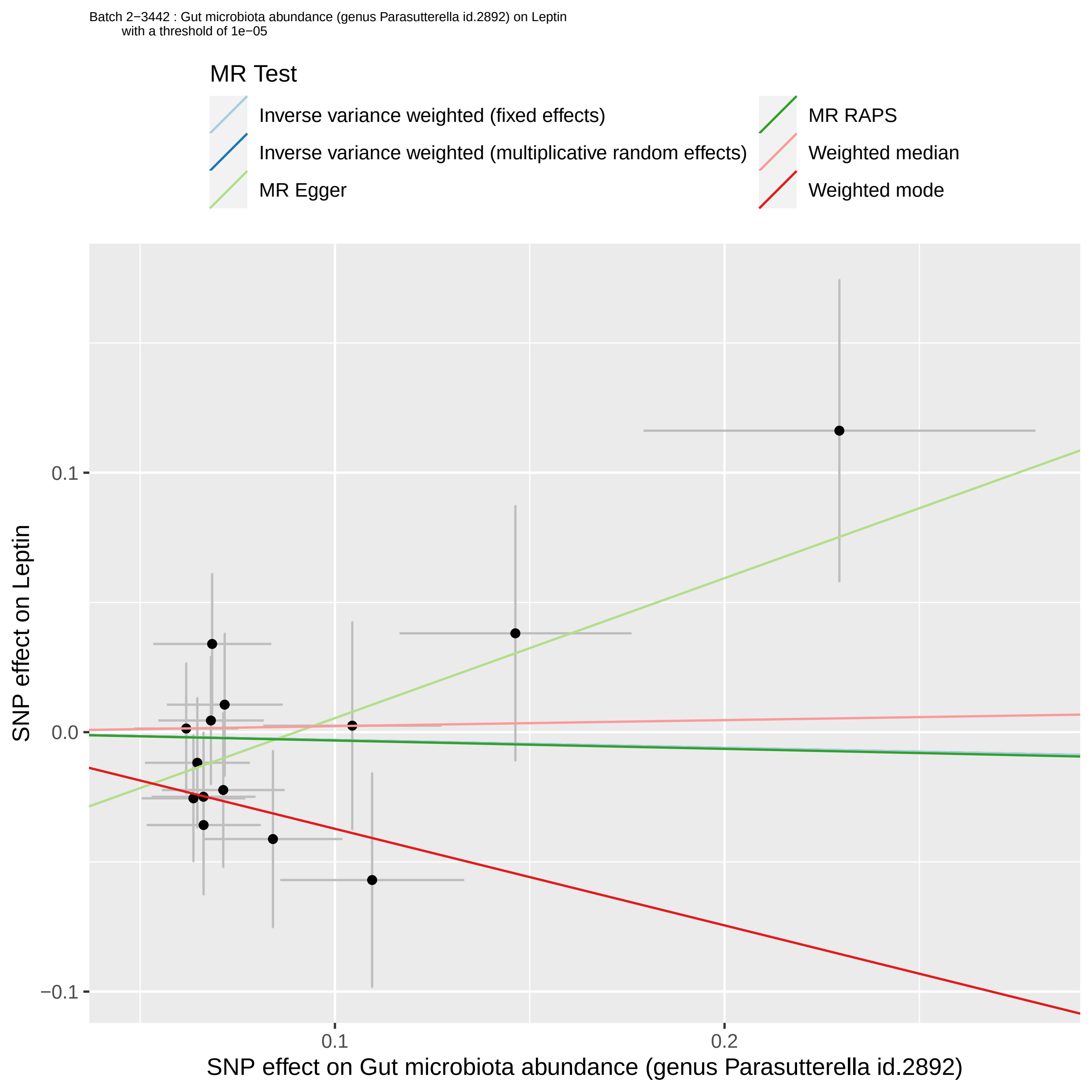

Batch 2−3442 : Gut microbiota abundance (genus Parasutterella id.2892) on Leptin
with a threshold of 1e−05
MR Test
Inverse variance weighted (fixed effects)
Inverse variance weighted (multiplicative random effects)
MR Egger
MR RAPS
Weighted median
Weighted mode
0.1
0.0
−0.1
SNP effect on Leptin
0.1
0.2
SNP effect on Gut microbiota abundance (genus Parasutterella id.2892)

Batch 2−3443 : Gut microbiota abundance (genus Catenibacterium id.2153) on Leptin
with a threshold of 1e−05

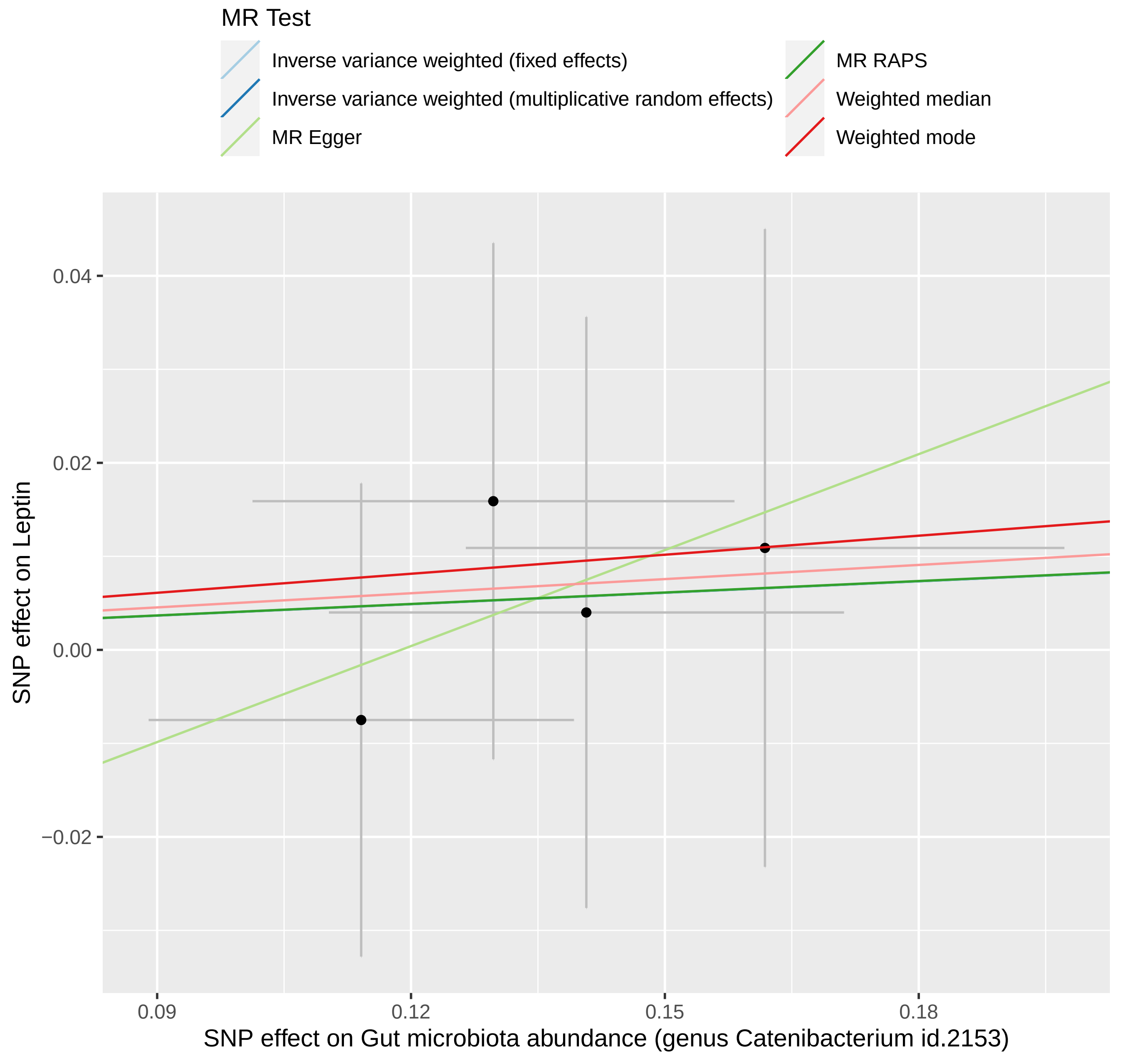

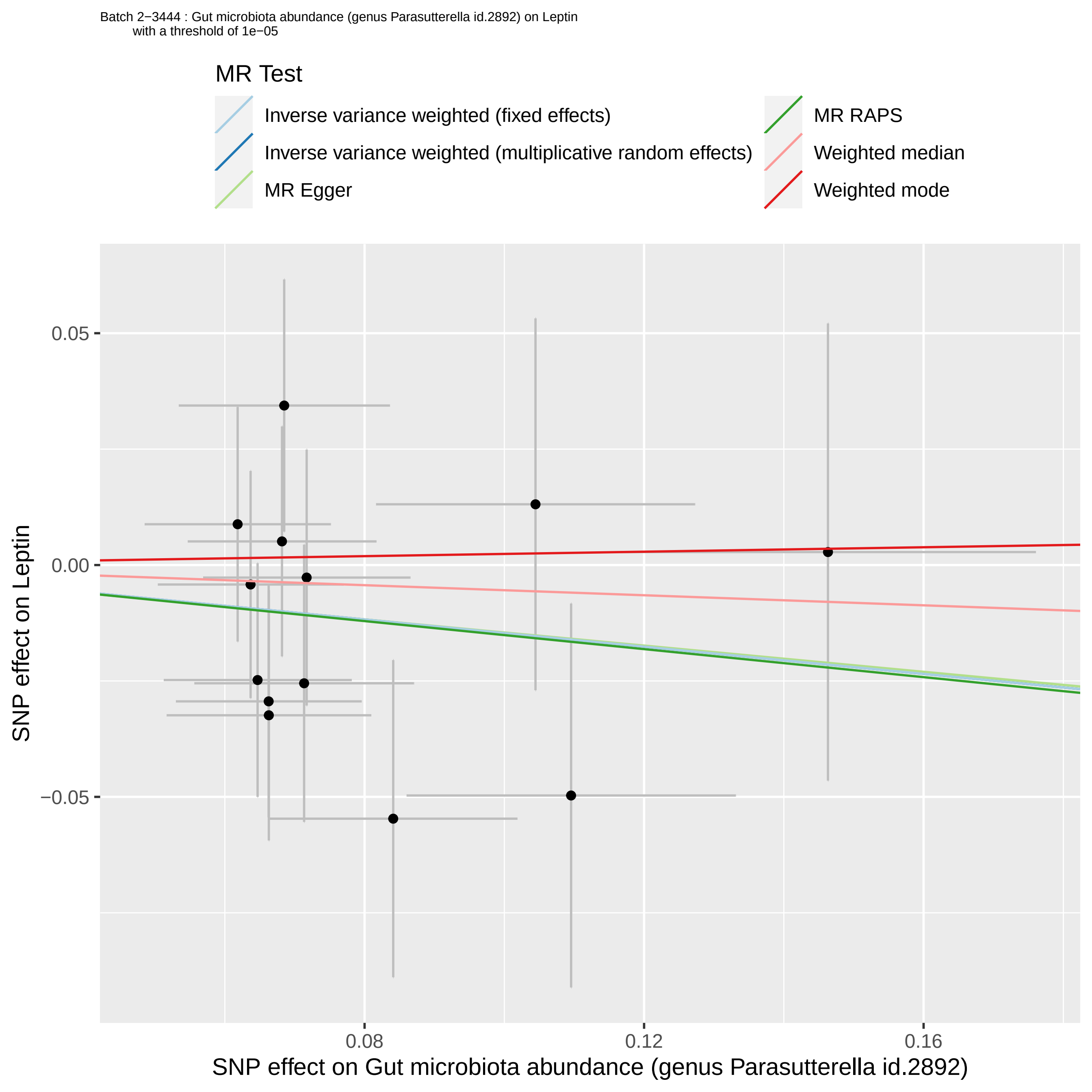
Batch 2−3444 : Gut microbiota abundance (genus Parasutterella id.2892) on Leptin
with a threshold of 1e−05
MR Test
Inverse variance weighted (fixed effects)
Inverse variance weighted (multiplicative random effects)
MR Egger
MR RAPS
Weighted median
Weighted mode
0.05
0.00
−0.05
0.08
0.12
0.16
SNP effect on Leptin
SNP effect on Gut microbiota abundance (genus Parasutterella id.2892)

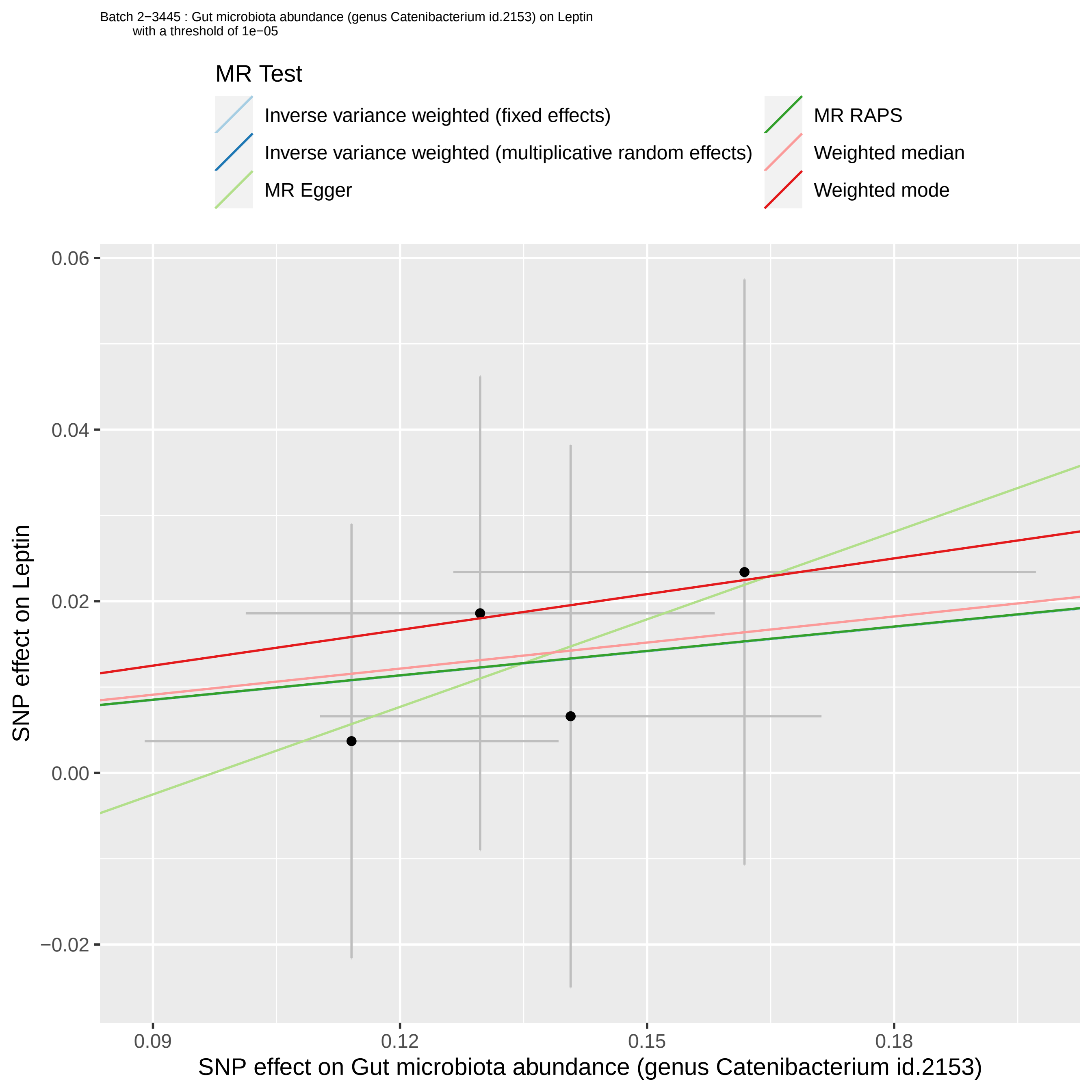
Batch 2−3445 : Gut microbiota abundance (genus Catenibacterium id.2153) on Leptin
with a threshold of 1e−05
MR Test
Inverse variance weighted (fixed effects)
Inverse variance weighted (multiplicative random effects)
MR Egger
MR RAPS
Weighted median
Weighted mode
SNP effect on Leptin
0.06
0.04
0.02
0.00
−0.02
0.09
0.12
0.15
0.18
SNP effect on Gut microbiota abundance (genus Catenibacterium id.2153)

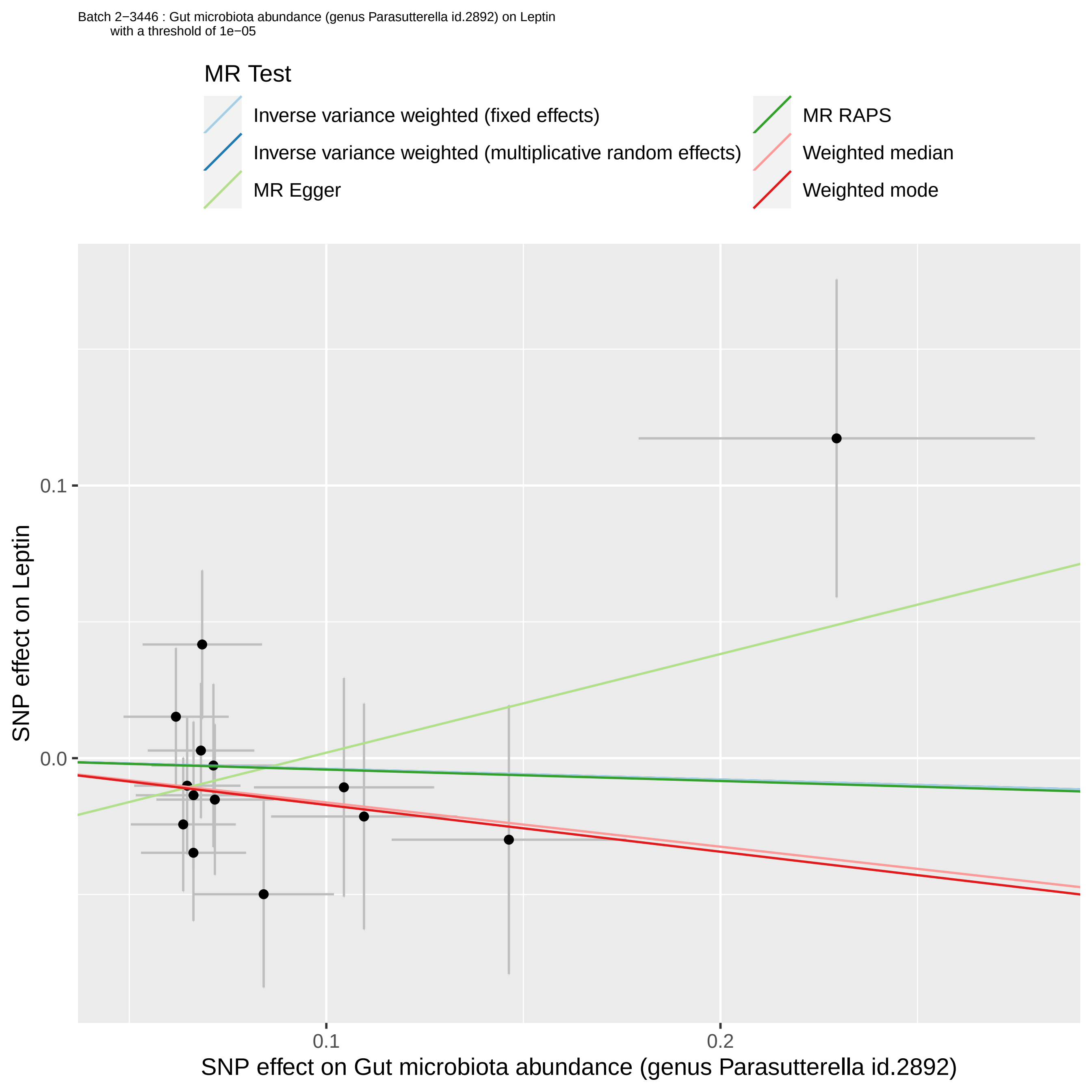
Batch 2−3446 : Gut microbiota abundance (genus Parasutterella id.2892) on Leptin
with a threshold of 1e−05
MR Test
Inverse variance weighted (fixed effects)
Inverse variance weighted (multiplicative random effects)
MR Egger
MR RAPS
Weighted median
Weighted mode
SNP effect on Leptin
0.1
0.0
0.1
0.2
SNP effect on Gut microbiota abundance (genus Parasutterella id.2892)

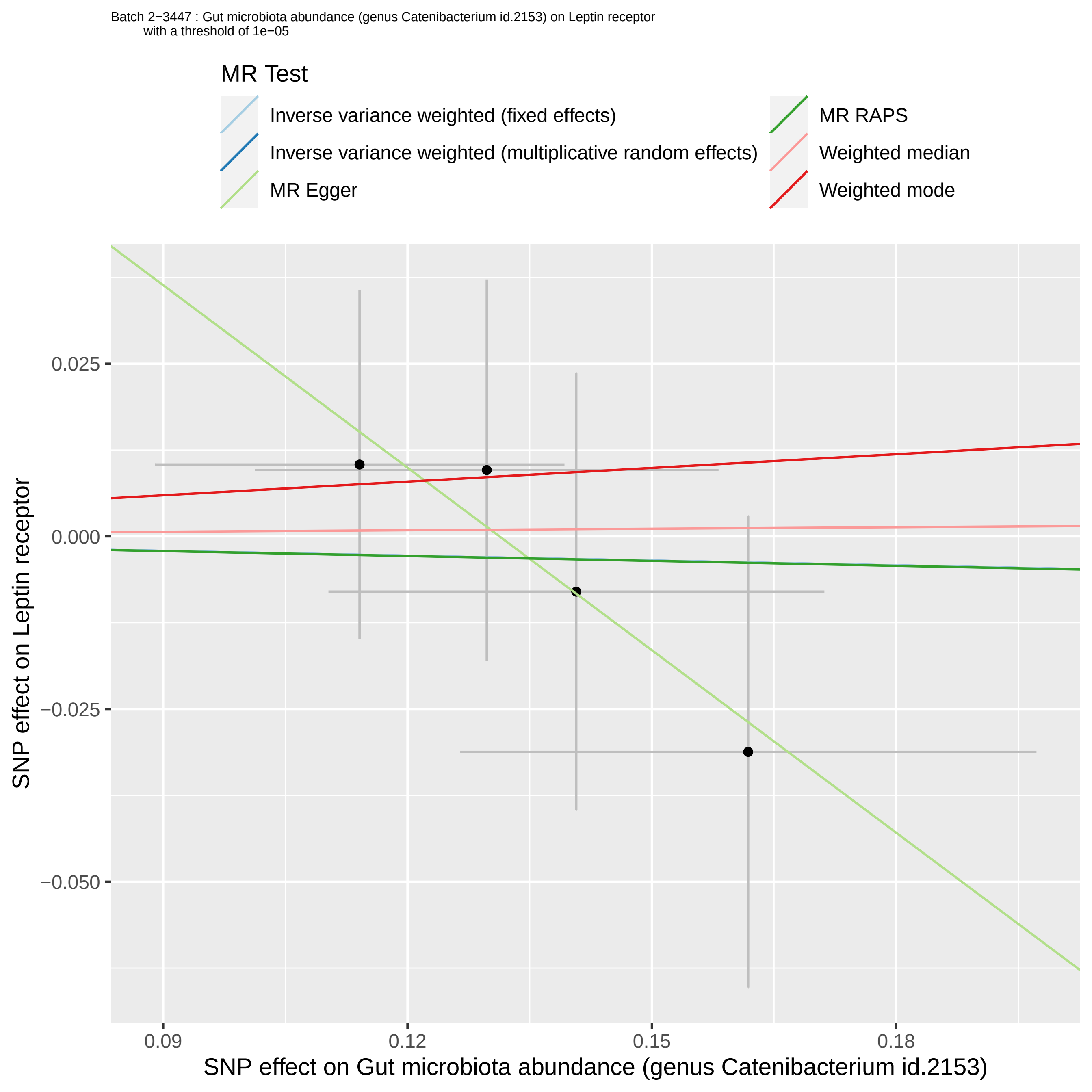

Batch 2−3447 : Gut microbiota abundance (genus Catenibacterium id.2153) on Leptin receptor
with a threshold of 1e−05
MR Test
Inverse variance weighted (fixed effects)
Inverse variance weighted (multiplicative random effects)
MR Egger
MR RAPS
Weighted median
Weighted mode
0.025
0.000
−0.025
−0.050
0.09
0.12
0.15
0.18
SNP effect on Leptin receptor
SNP effect on Gut microbiota abundance (genus Catenibacterium id.2153)

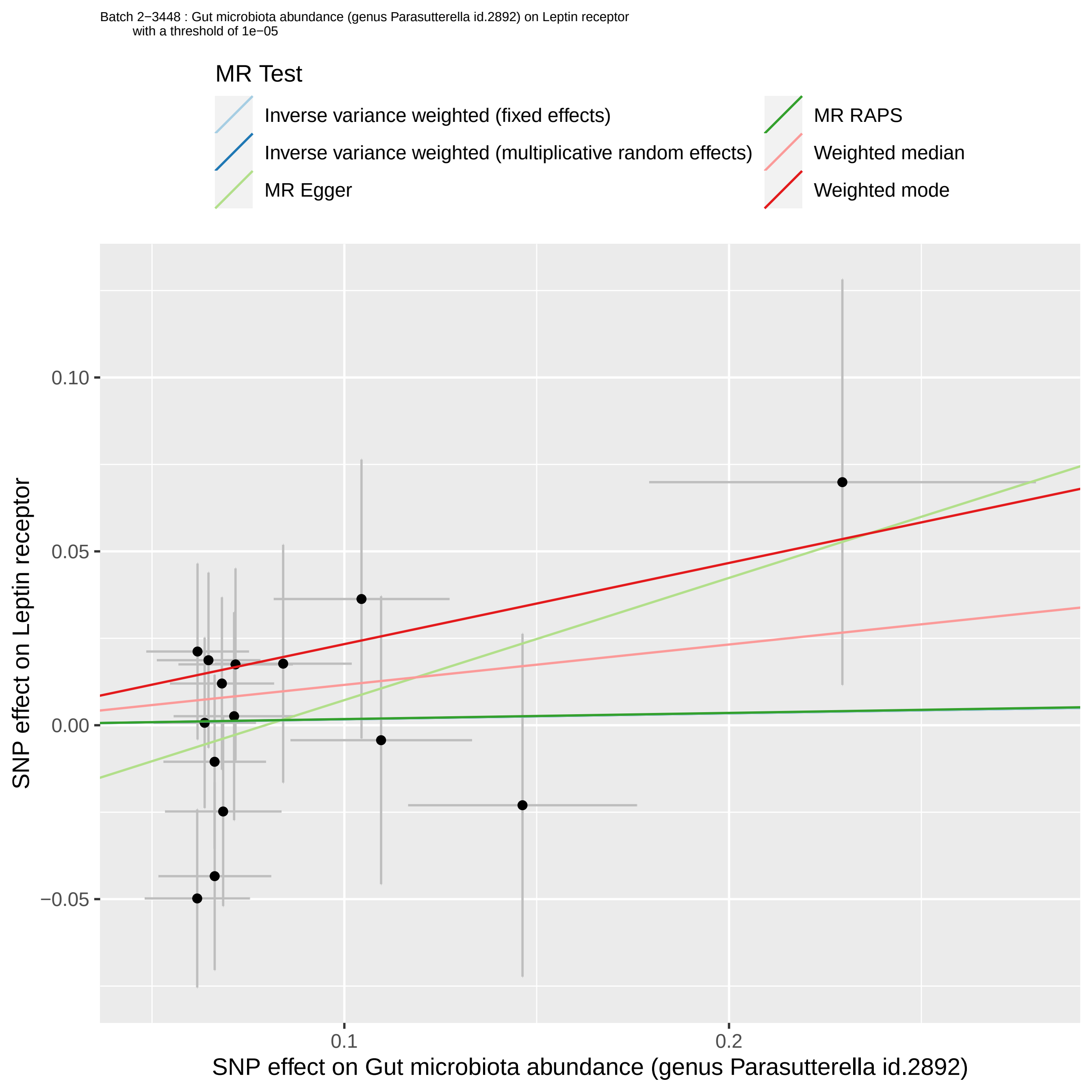
Batch 2−3448 : Gut microbiota abundance (genus Parasutterella id.2892) on Leptin receptor
with a threshold of 1e−05
MR Test
Inverse variance weighted (fixed effects)
Inverse variance weighted (multiplicative random effects)
MR Egger
MR RAPS
Weighted median
Weighted mode
SNP effect on Leptin receptor
0.10
0.05
0.00
−0.05
0.1
0.2
SNP effect on Gut microbiota abundance (genus Parasutterella id.2892)

Batch 2−3449 : Gut microbiota abundance (genus Catenibacterium id.2153) on Leptin receptor
with a threshold of 1e−05

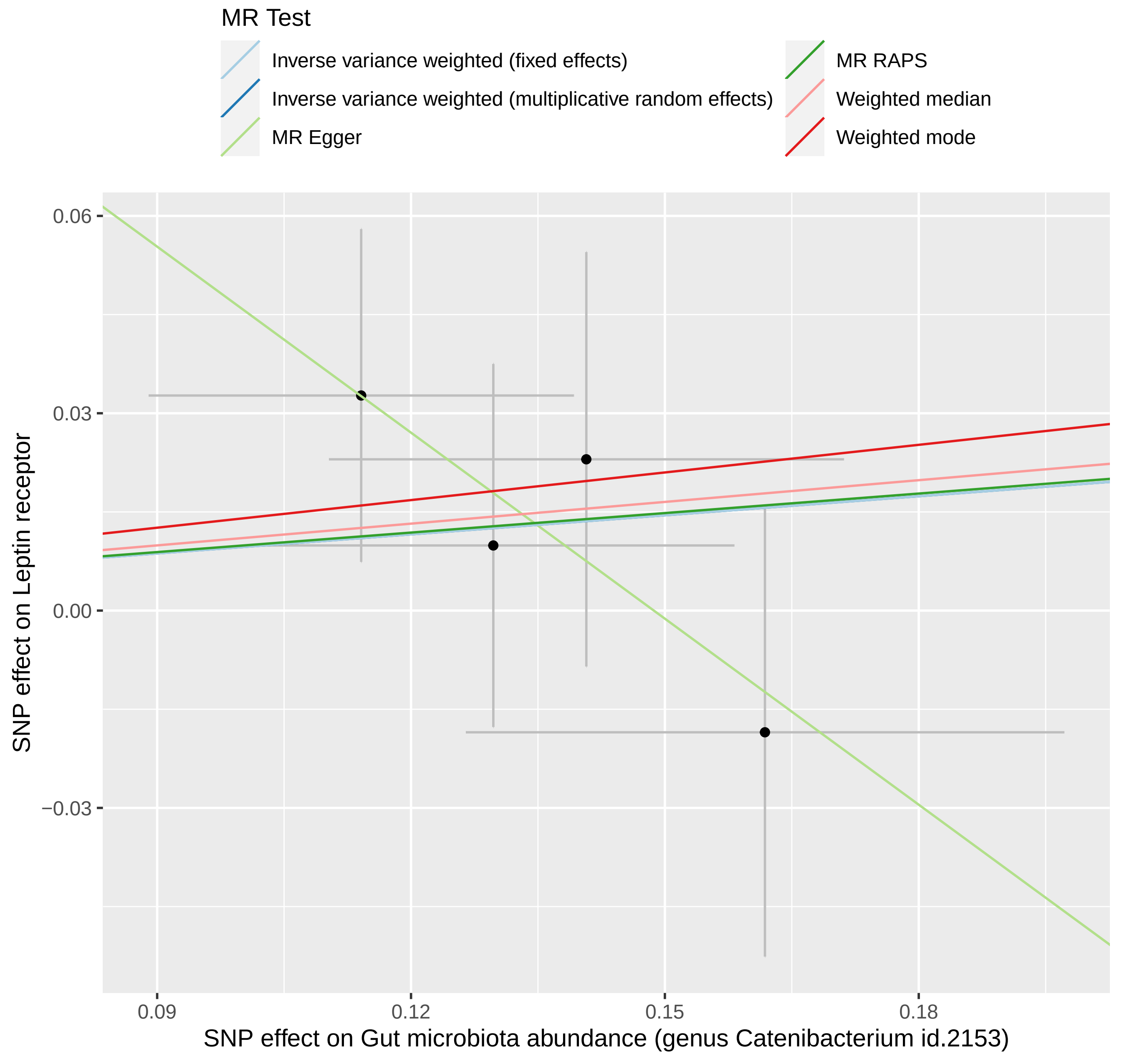

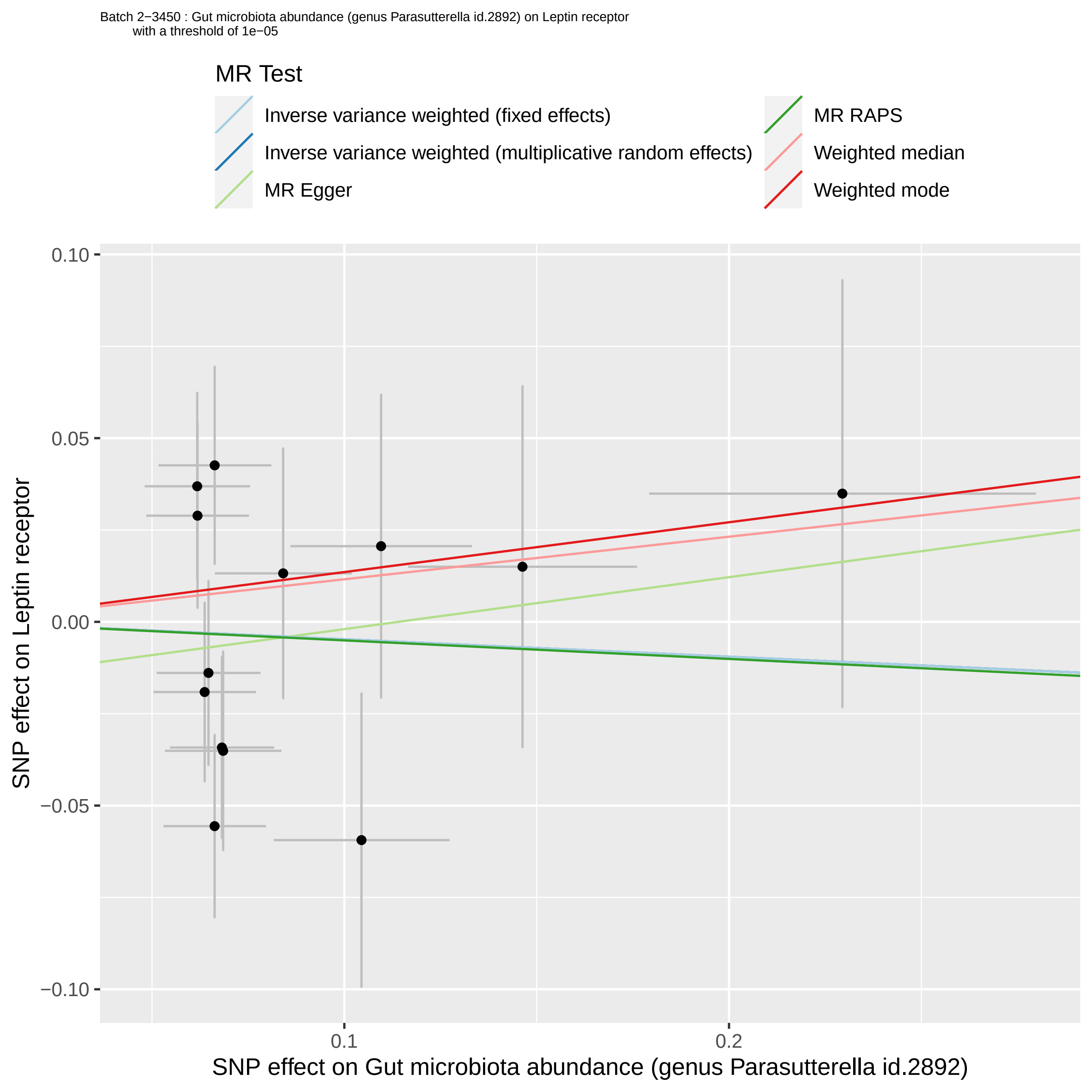
Batch 2−3450 : Gut microbiota abundance (genus Parasutterella id.2892) on Leptin receptor
with a threshold of 1e−05
MR Test
Inverse variance weighted (fixed effects)
Inverse variance weighted (multiplicative random effects)
MR Egger
MR RAPS
Weighted median
Weighted mode
0.10
0.05
0.00
−0.05
−0.10
0.1
0.2
SNP effect on Leptin receptor
SNP effect on Gut microbiota abundance (genus Parasutterella id.2892)

Batch 2−3451 : Gut microbiota abundance (genus Catenibacterium id.2153) on Galectin−1
with a threshold of 1e−05

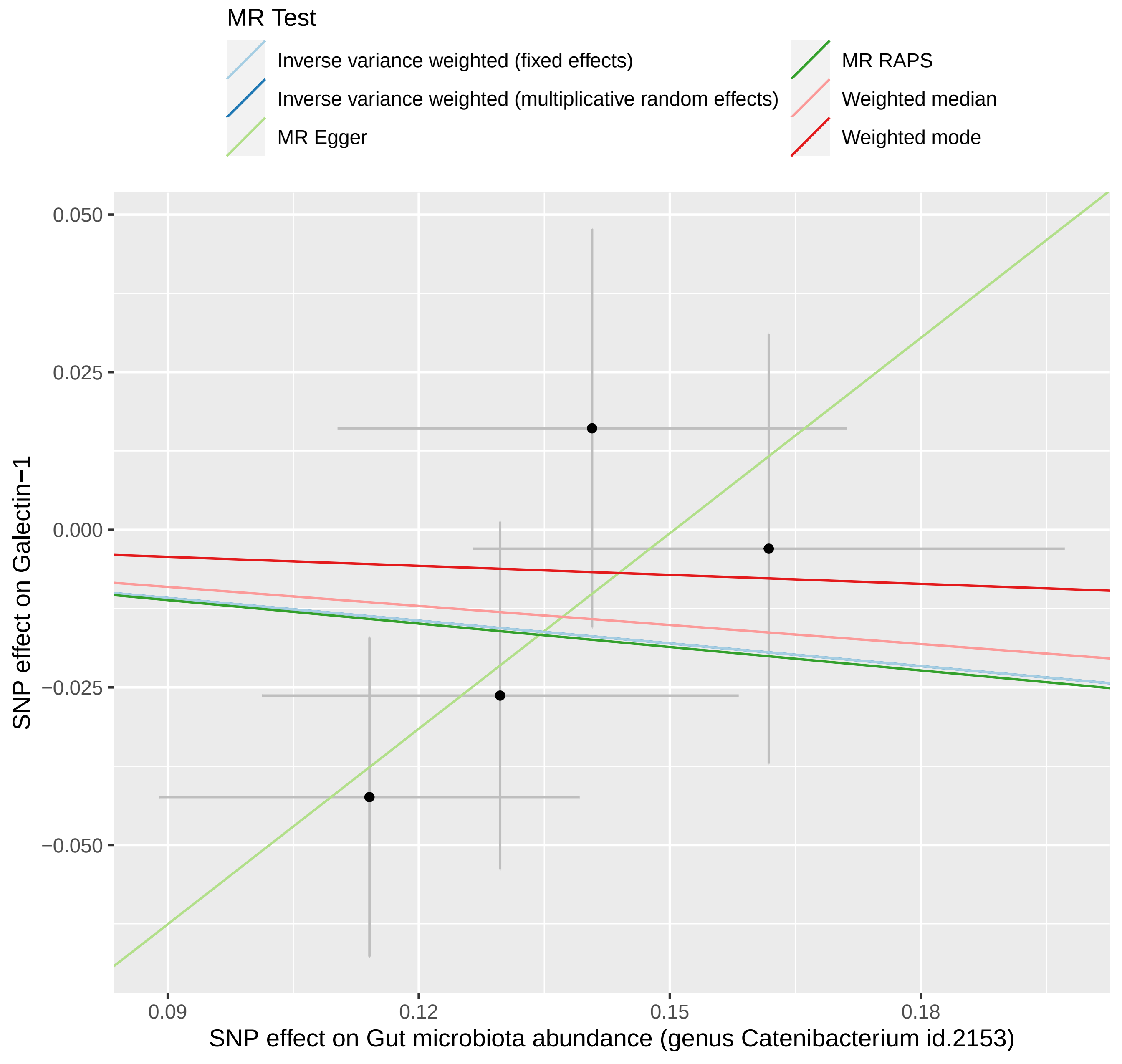

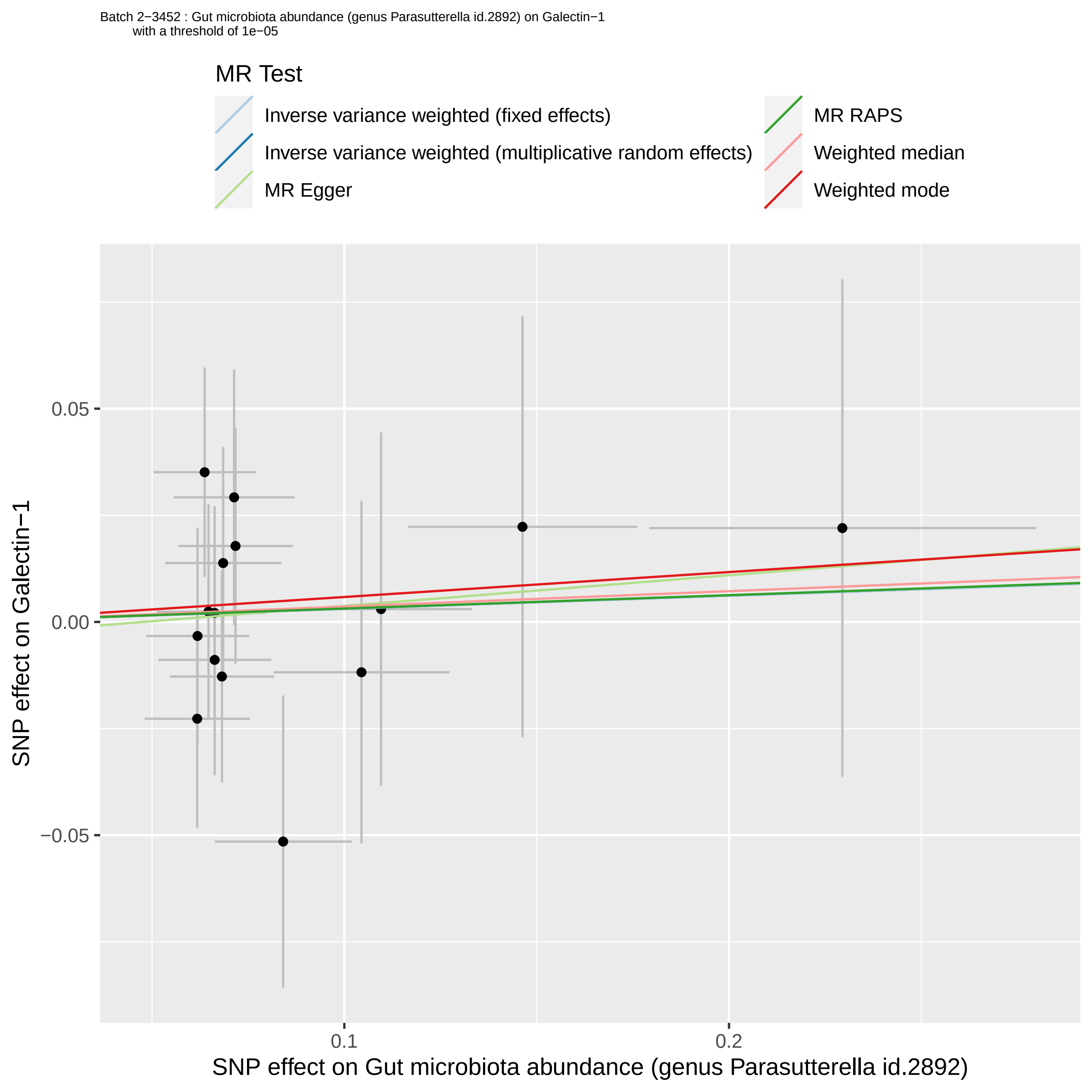

Batch 2−3452 : Gut microbiota abundance (genus Parasutterella id.2892) on Galectin−1
with a threshold of 1e−05
MR Test
Inverse variance weighted (fixed effects)
Inverse variance weighted (multiplicative random effects)
MR Egger
MR RAPS
Weighted median
Weighted mode
0.05
0.00
−0.05
0.1
0.2
SNP effect on Galectin−1
SNP effect on Gut microbiota abundance (genus Parasutterella id.2892)

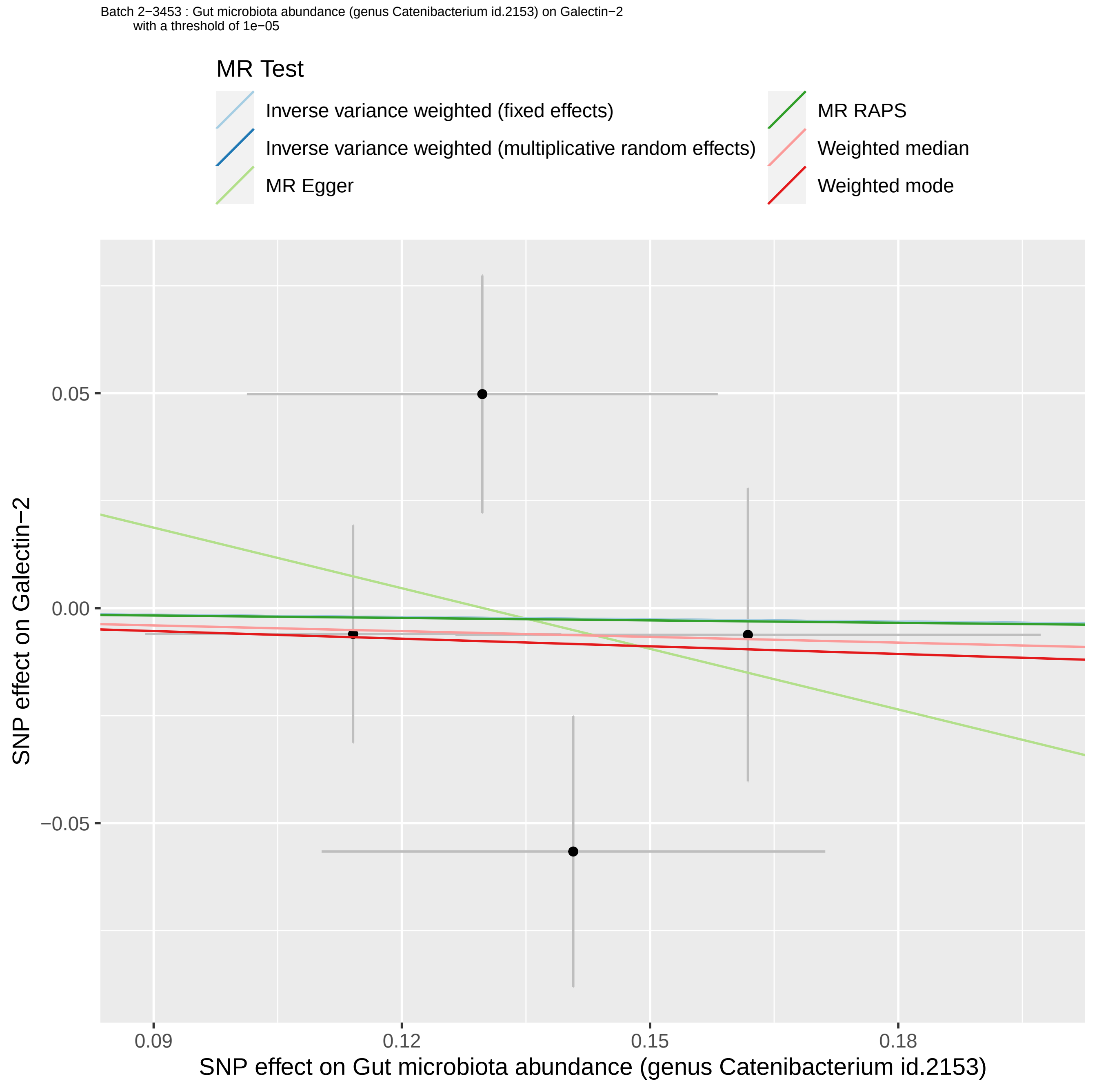
Batch 2−3453 : Gut microbiota abundance (genus Catenibacterium id.2153) on Galectin−2
with a threshold of 1e−05
MR Test
Inverse variance weighted (fixed effects)
Inverse variance weighted (multiplicative random effects)
MR Egger
MR RAPS
Weighted median
Weighted mode
0.05
0.00
−0.05
SNP effect on Galectin−2
0.09
0.12
0.15
0.18
SNP effect on Gut microbiota abundance (genus Catenibacterium id.2153)

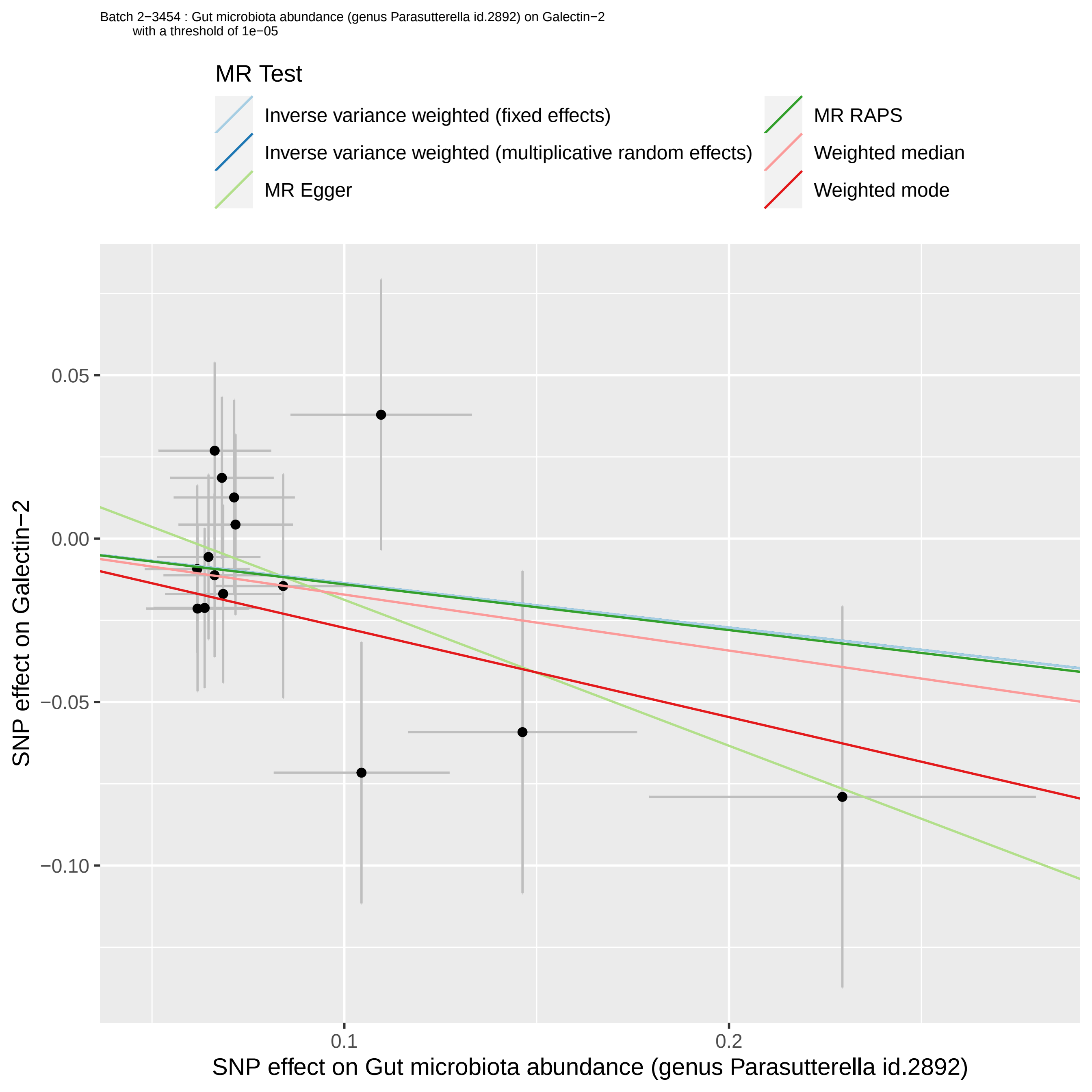
Batch 2−3454 : Gut microbiota abundance (genus Parasutterella id.2892) on Galectin−2
with a threshold of 1e−05
MR Test
Inverse variance weighted (fixed effects)
Inverse variance weighted (multiplicative random effects)
MR Egger
MR RAPS
Weighted median
Weighted mode
0.05
0.00
−0.05
−0.10
0.1
0.2
SNP effect on Galectin−2
SNP effect on Gut microbiota abundance (genus Parasutterella id.2892)

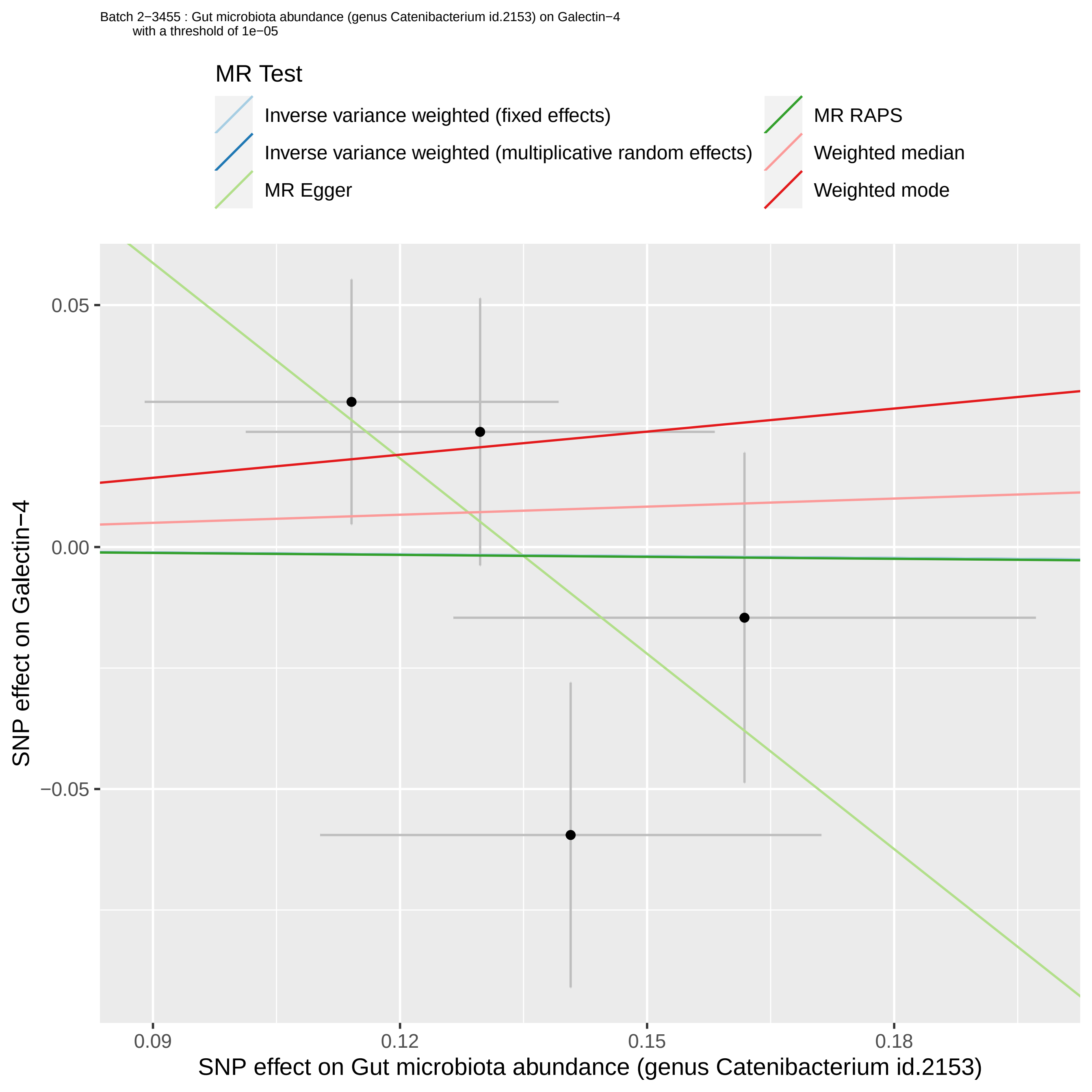

Batch 2−3455 : Gut microbiota abundance (genus Catenibacterium id.2153) on Galectin−4
with a threshold of 1e−05
MR Test
Inverse variance weighted (fixed effects)
Inverse variance weighted (multiplicative random effects)
MR Egger
MR RAPS
Weighted median
Weighted mode
0.05
0.00
−0.05
SNP effect on Galectin−4
0.09
0.12
0.15
0.18
SNP effect on Gut microbiota abundance (genus Catenibacterium id.2153)

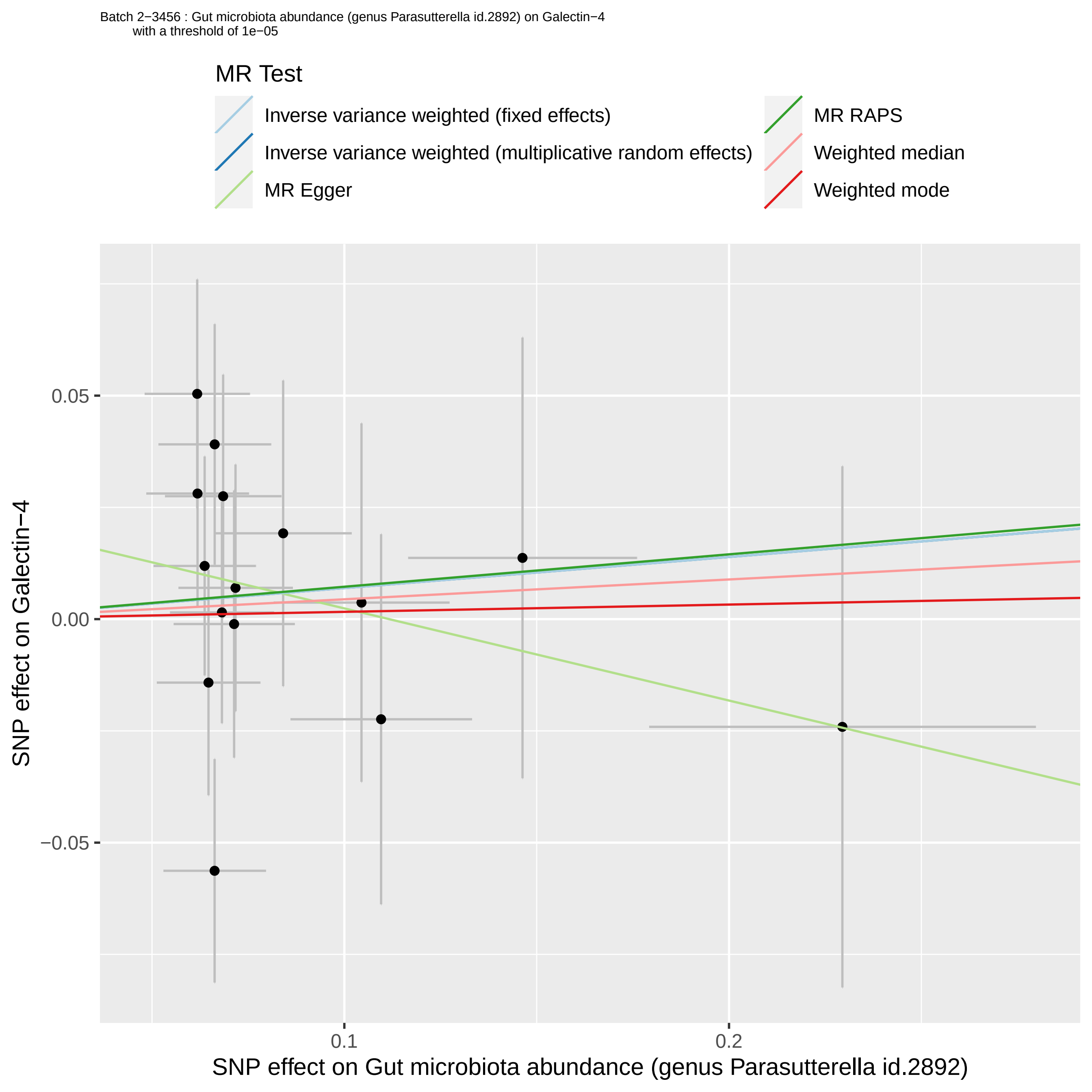

Batch 2−3456 : Gut microbiota abundance (genus Parasutterella id.2892) on Galectin−4
with a threshold of 1e−05
MR Test
Inverse variance weighted (fixed effects)
Inverse variance weighted (multiplicative random effects)
MR Egger
MR RAPS
Weighted median
Weighted mode
0.05
0.00
−0.05
0.1
0.2
SNP effect on Galectin−4
SNP effect on Gut microbiota abundance (genus Parasutterella id.2892)

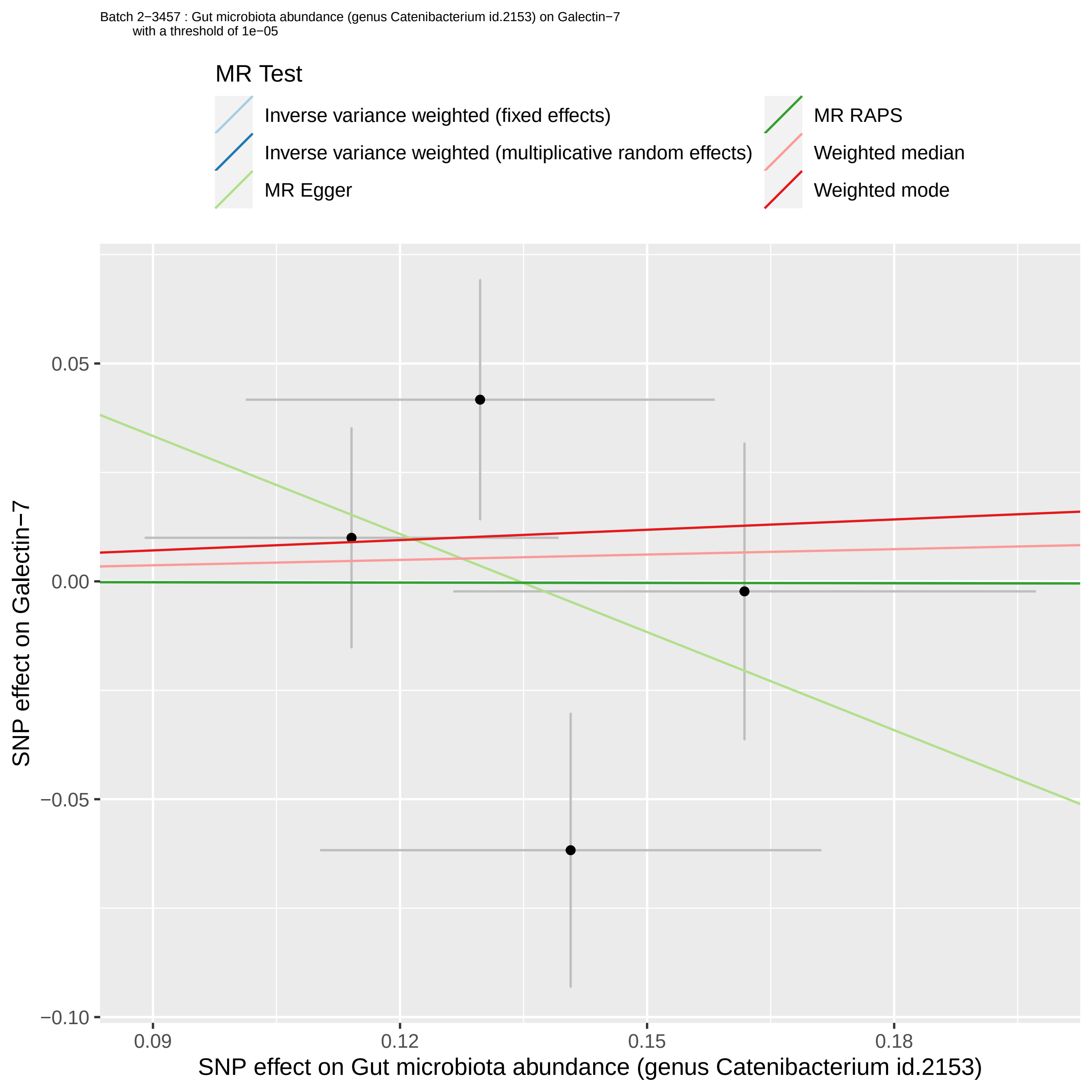
Batch 2−3457 : Gut microbiota abundance (genus Catenibacterium id.2153) on Galectin−7
with a threshold of 1e−05
MR Test
Inverse variance weighted (fixed effects)
Inverse variance weighted (multiplicative random effects)
MR Egger
MR RAPS
Weighted median
Weighted mode
0.05
0.00
−0.05
−0.10
0.09
0.12
0.15
0.18
SNP effect on Galectin−7
SNP effect on Gut microbiota abundance (genus Catenibacterium id.2153)

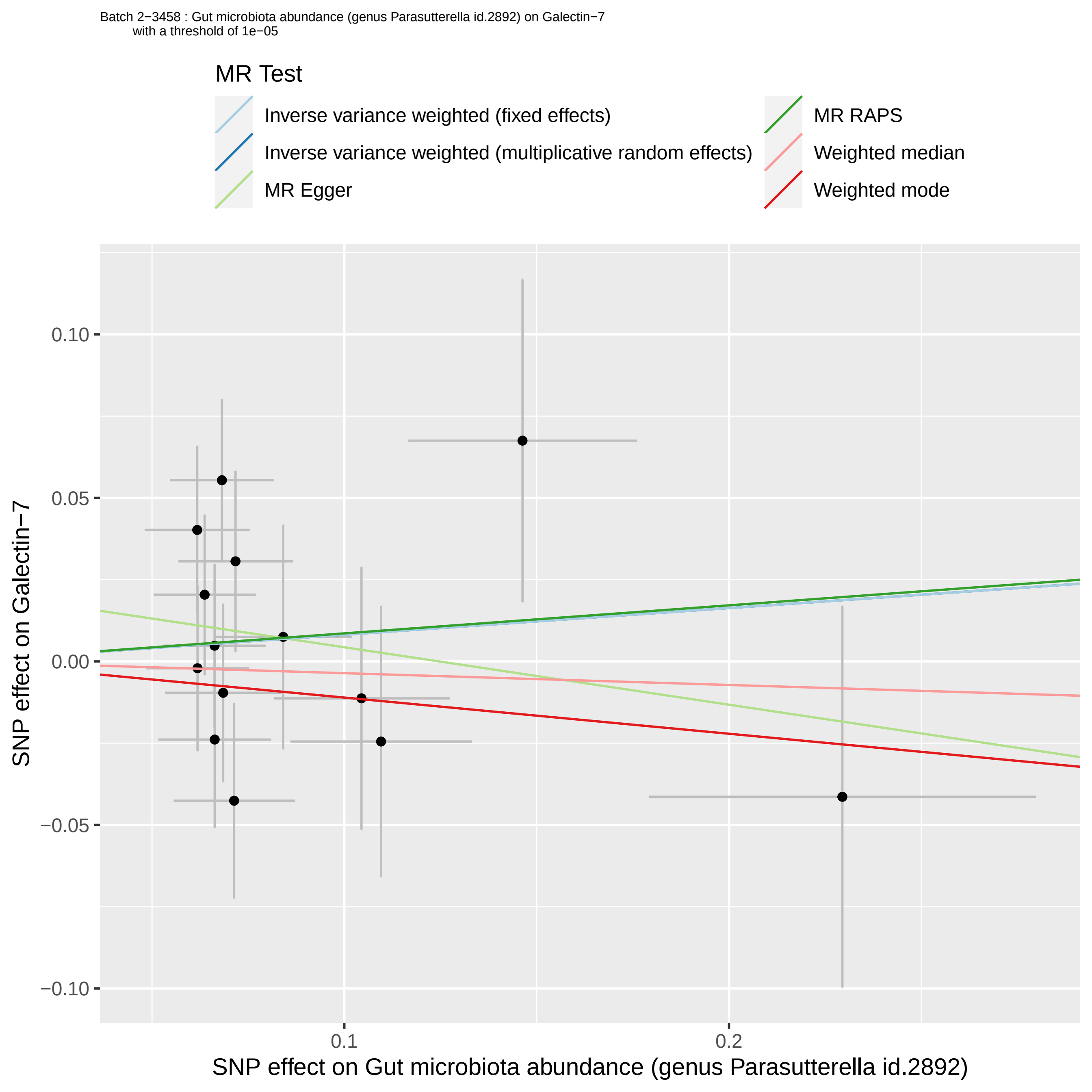

Batch 2−3458 : Gut microbiota abundance (genus Parasutterella id.2892) on Galectin−7
with a threshold of 1e−05
MR Test
Inverse variance weighted (fixed effects)
Inverse variance weighted (multiplicative random effects)
MR Egger
MR RAPS
Weighted median
Weighted mode
0.10
0.05
0.00
−0.05
−0.10
0.1
0.2
SNP effect on Galectin−7
SNP effect on Gut microbiota abundance (genus Parasutterella id.2892)

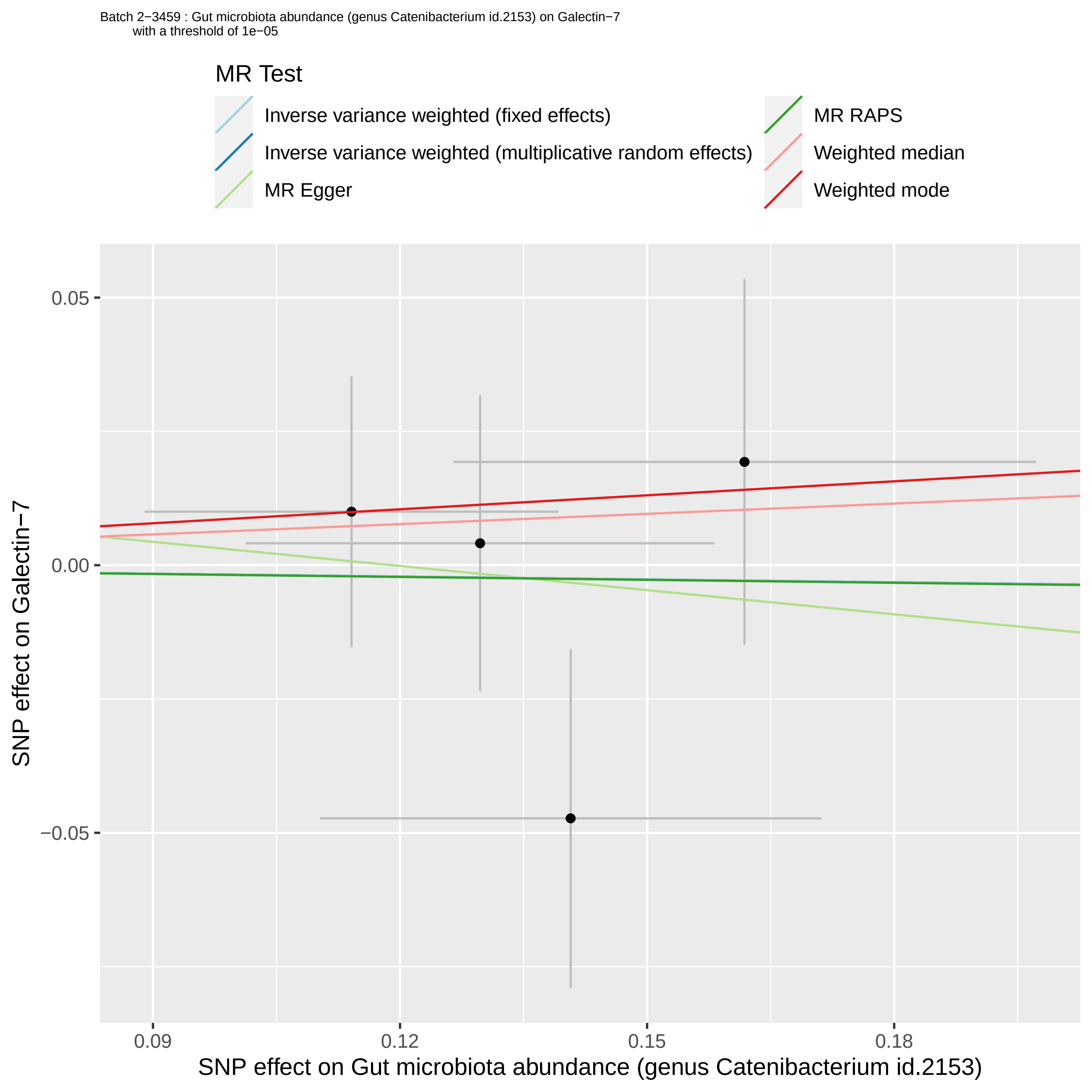

Batch 2−3459 : Gut microbiota abundance (genus Catenibacterium id.2153) on Galectin−7
with a threshold of 1e−05
MR Test
Inverse variance weighted (fixed effects)
Inverse variance weighted (multiplicative random effects)
MR Egger
MR RAPS
Weighted median
Weighted mode
0.05
0.00
−0.05
0.09
0.12
0.15
0.18
SNP effect on Galectin−7
SNP effect on Gut microbiota abundance (genus Catenibacterium id.2153)

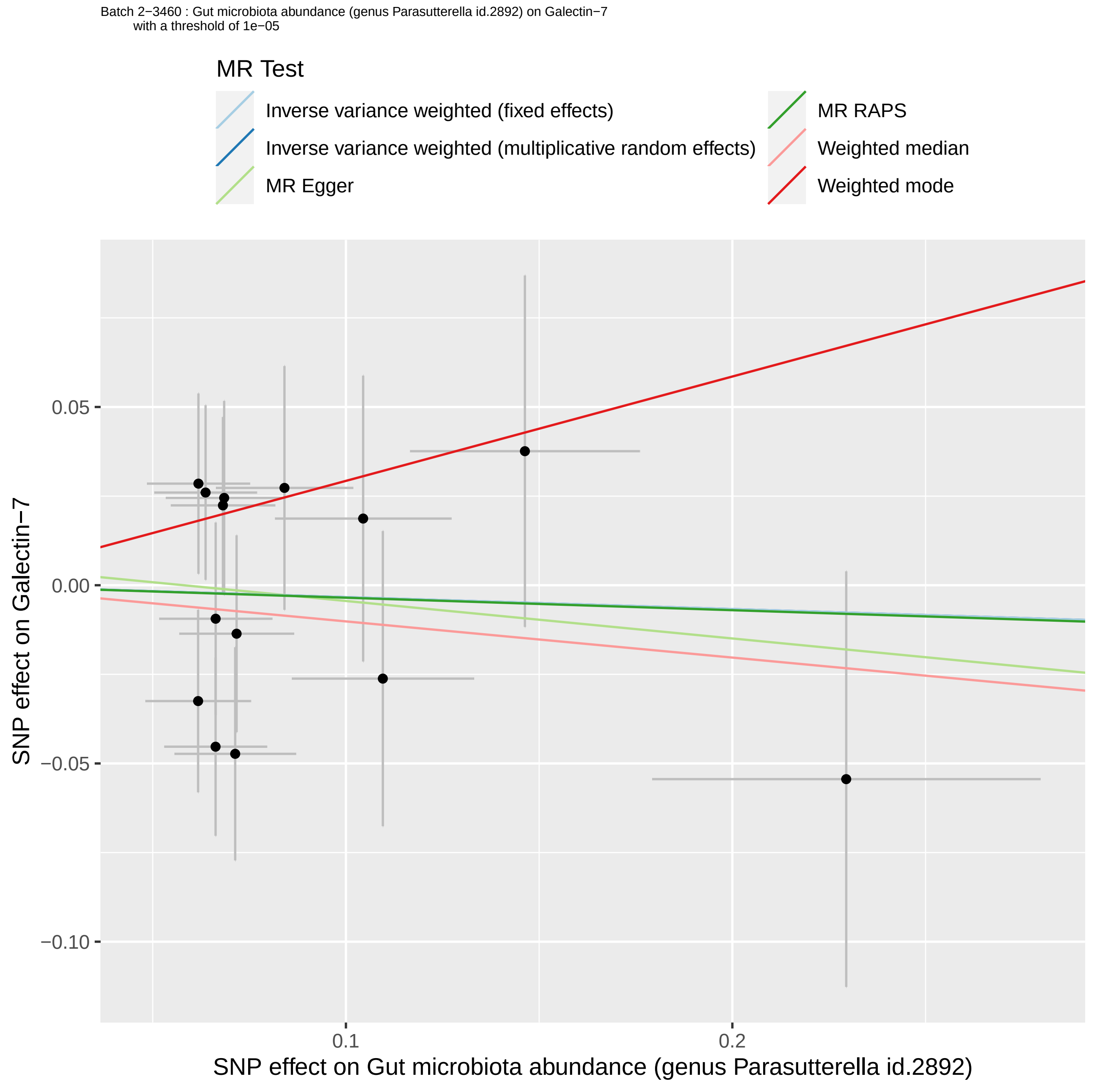

Batch 2−3460 : Gut microbiota abundance (genus Parasutterella id.2892) on Galectin−7
with a threshold of 1e−05
MR Test
Inverse variance weighted (fixed effects)
Inverse variance weighted (multiplicative random effects)
MR Egger
MR RAPS
Weighted median
Weighted mode
0.05
0.00
−0.05
−0.10
SNP effect on Galectin−7
0.1
0.2
SNP effect on Gut microbiota abundance (genus Parasutterella id.2892)

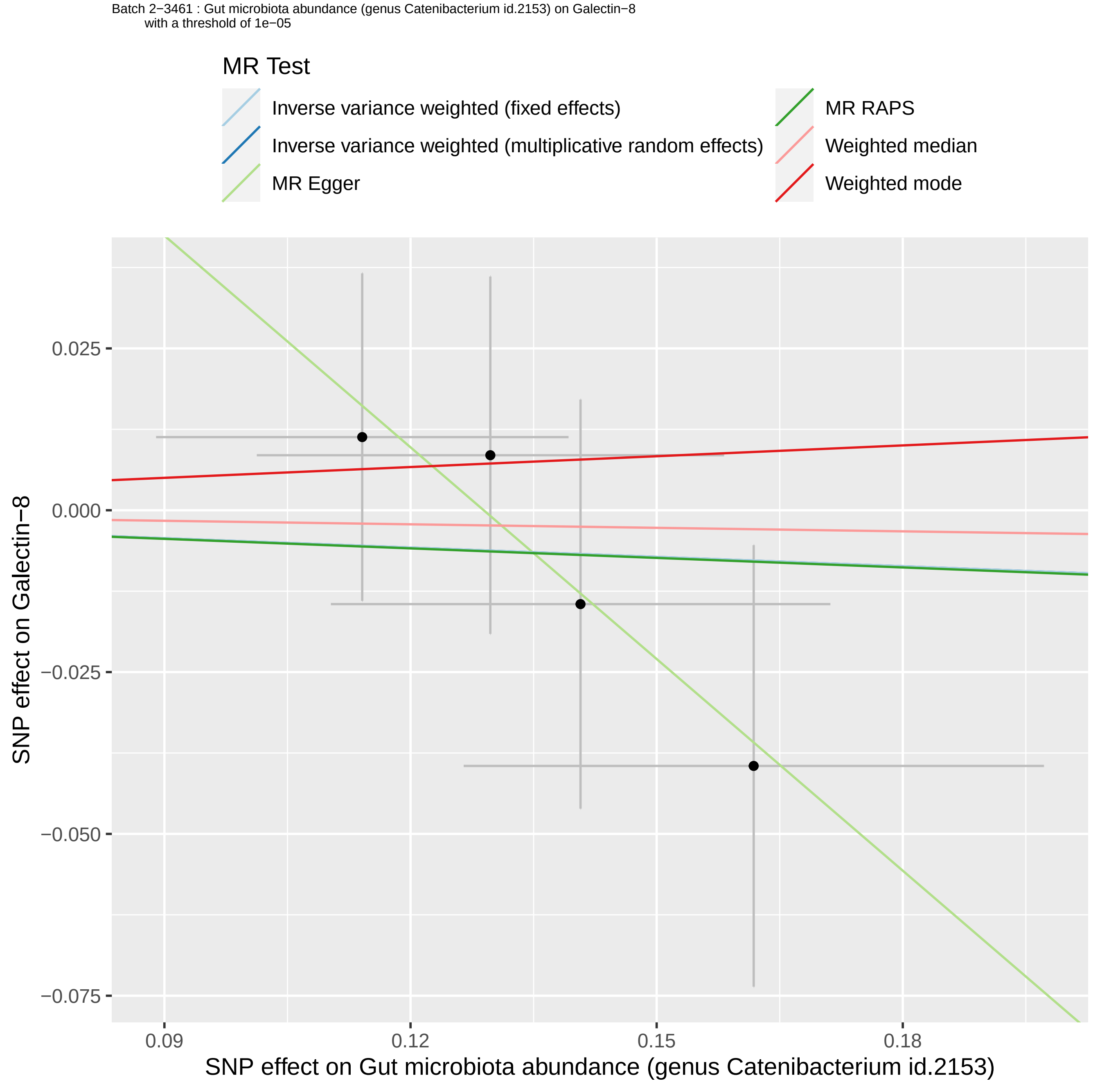

Batch 2−3461 : Gut microbiota abundance (genus Catenibacterium id.2153) on Galectin−8
with a threshold of 1e−05
MR Test
Inverse variance weighted (fixed effects)
Inverse variance weighted (multiplicative random effects)
MR Egger
MR RAPS
Weighted median
Weighted mode
0.025
0.000
−0.025
−0.050
−0.075
0.09
0.12
0.15
0.18
SNP effect on Galectin−8
SNP effect on Gut microbiota abundance (genus Catenibacterium id.2153)

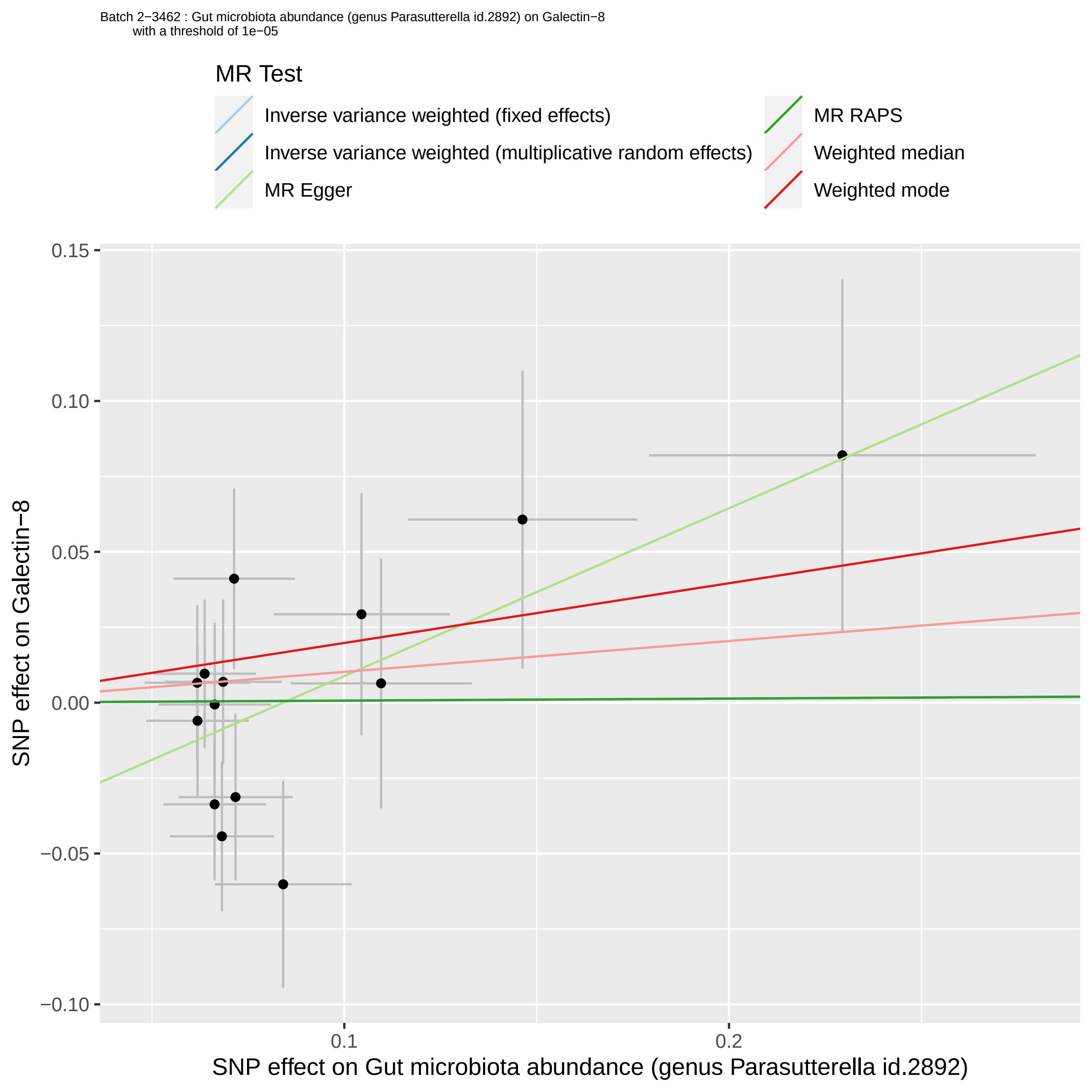

Batch 2−3462 : Gut microbiota abundance (genus Parasutterella id.2892) on Galectin−8
with a threshold of 1e−05
MR Test
Inverse variance weighted (fixed effects)
Inverse variance weighted (multiplicative random effects)
MR Egger
MR RAPS
Weighted median
Weighted mode
0.15
0.10
0.05
0.00
−0.05
−0.10
0.1
0.2
SNP effect on Galectin−8
SNP effect on Gut microbiota abundance (genus Parasutterella id.2892)

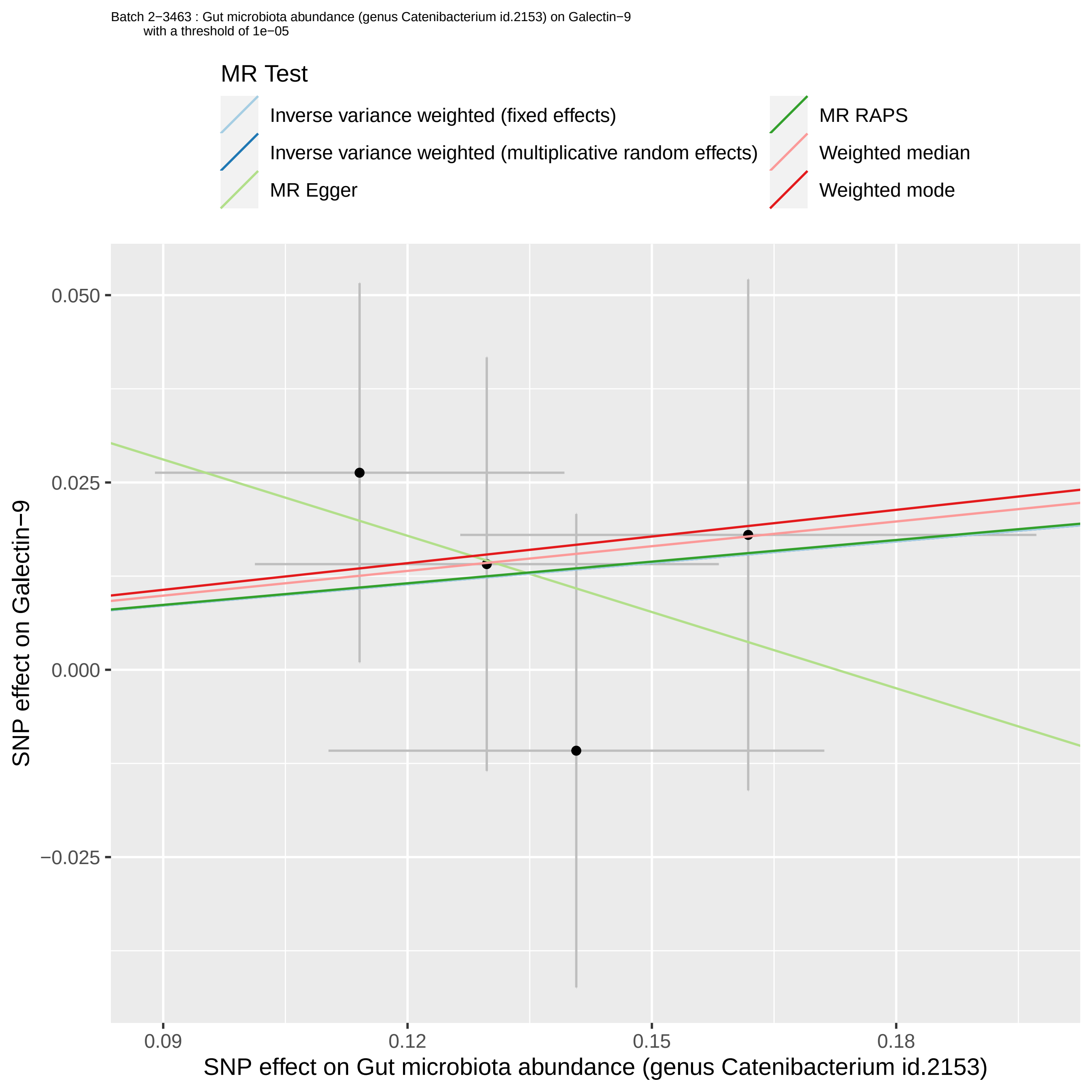
Batch 2−3463 : Gut microbiota abundance (genus Catenibacterium id.2153) on Galectin−9
with a threshold of 1e−05
MR Test
Inverse variance weighted (fixed effects)
Inverse variance weighted (multiplicative random effects)
MR Egger
MR RAPS
Weighted median
Weighted mode
0.050
0.025
0.000
−0.025
0.09
0.12
0.15
0.18
SNP effect on Galectin−9
SNP effect on Gut microbiota abundance (genus Catenibacterium id.2153)

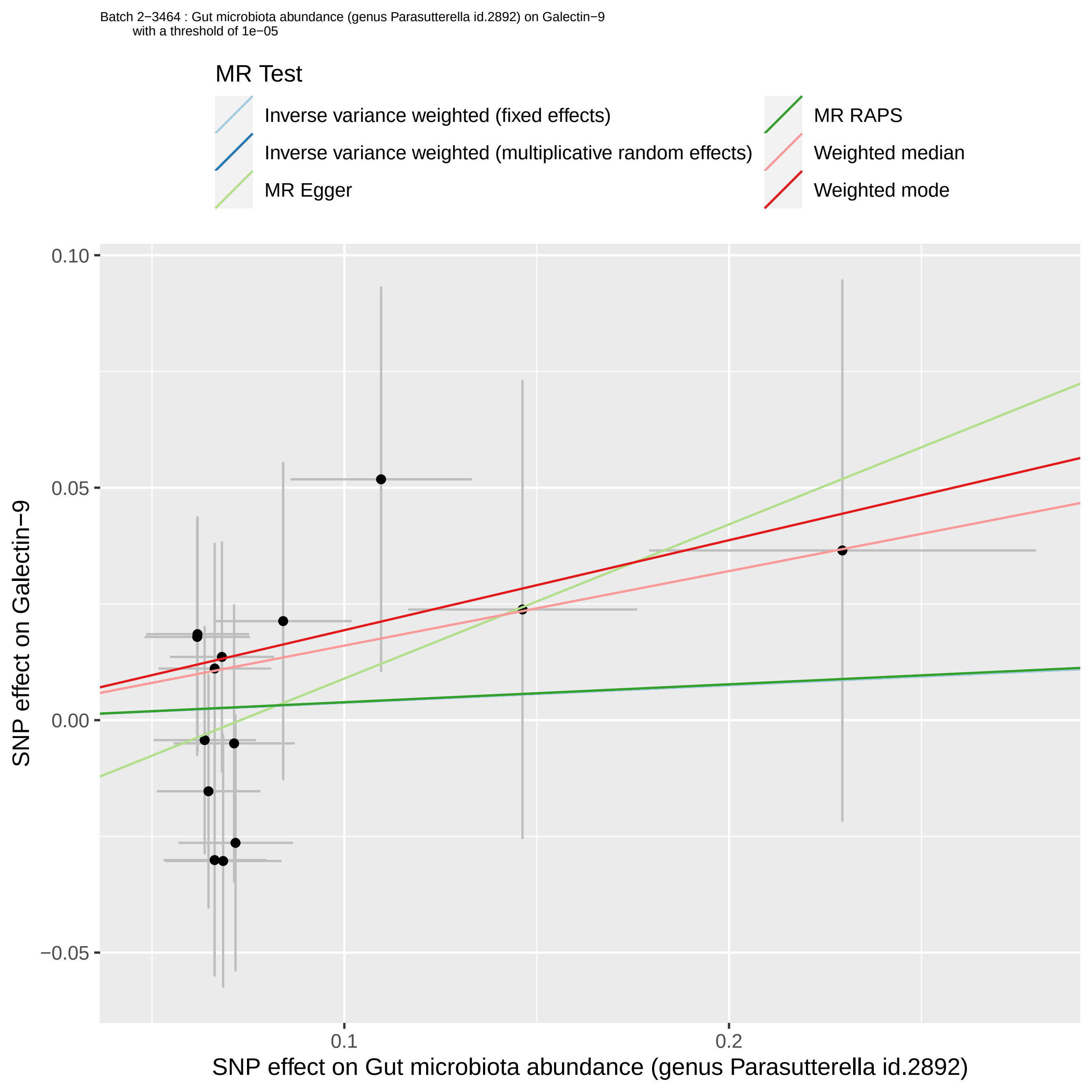
Batch 2−3464 : Gut microbiota abundance (genus Parasutterella id.2892) on Galectin−9
with a threshold of 1e−05
MR Test
Inverse variance weighted (fixed effects)
Inverse variance weighted (multiplicative random effects)
MR Egger
MR RAPS
Weighted median
Weighted mode
SNP effect on Galectin−9
0.10
0.05
0.00
−0.05
0.1
0.2
SNP effect on Gut microbiota abundance (genus Parasutterella id.2892)

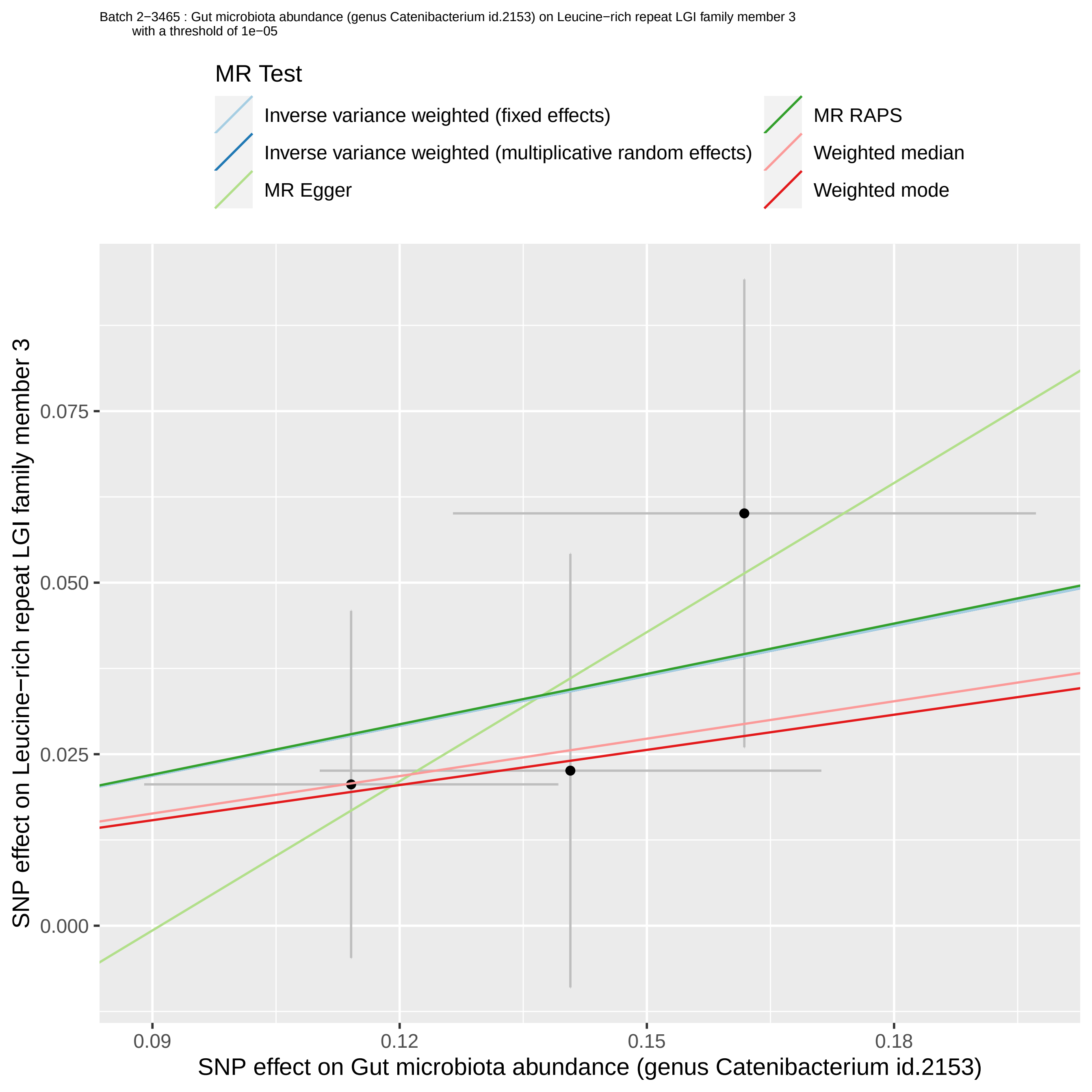
Batch 2−3465 : Gut microbiota abundance (genus Catenibacterium id.2153) on Leucine−rich repeat LGI family member 3
with a threshold of 1e−05
MR Test
Inverse variance weighted (fixed effects)
Inverse variance weighted (multiplicative random effects)
MR Egger
MR RAPS
Weighted median
Weighted mode
SNP effect on Leucine−rich repeat LGI family member 3
0.075
0.050
0.025
0.000
0.09
0.12
0.15
0.18
SNP effect on Gut microbiota abundance (genus Catenibacterium id.2153)

Batch 2−3466 : Gut microbiota abundance (genus Parasutterella id.2892) on Leucine−rich repeat LGI family member 3
with a threshold of 1e−05

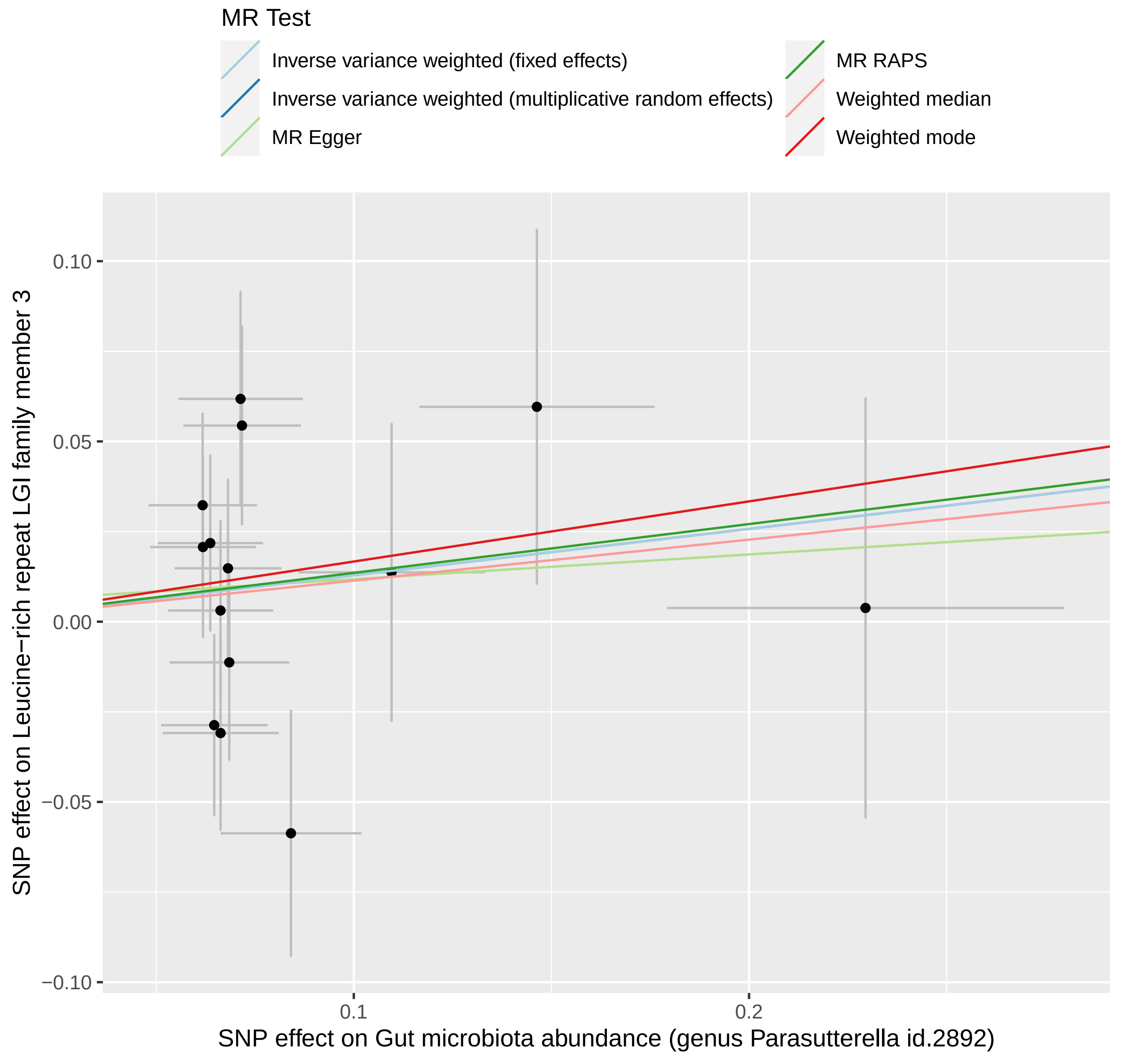

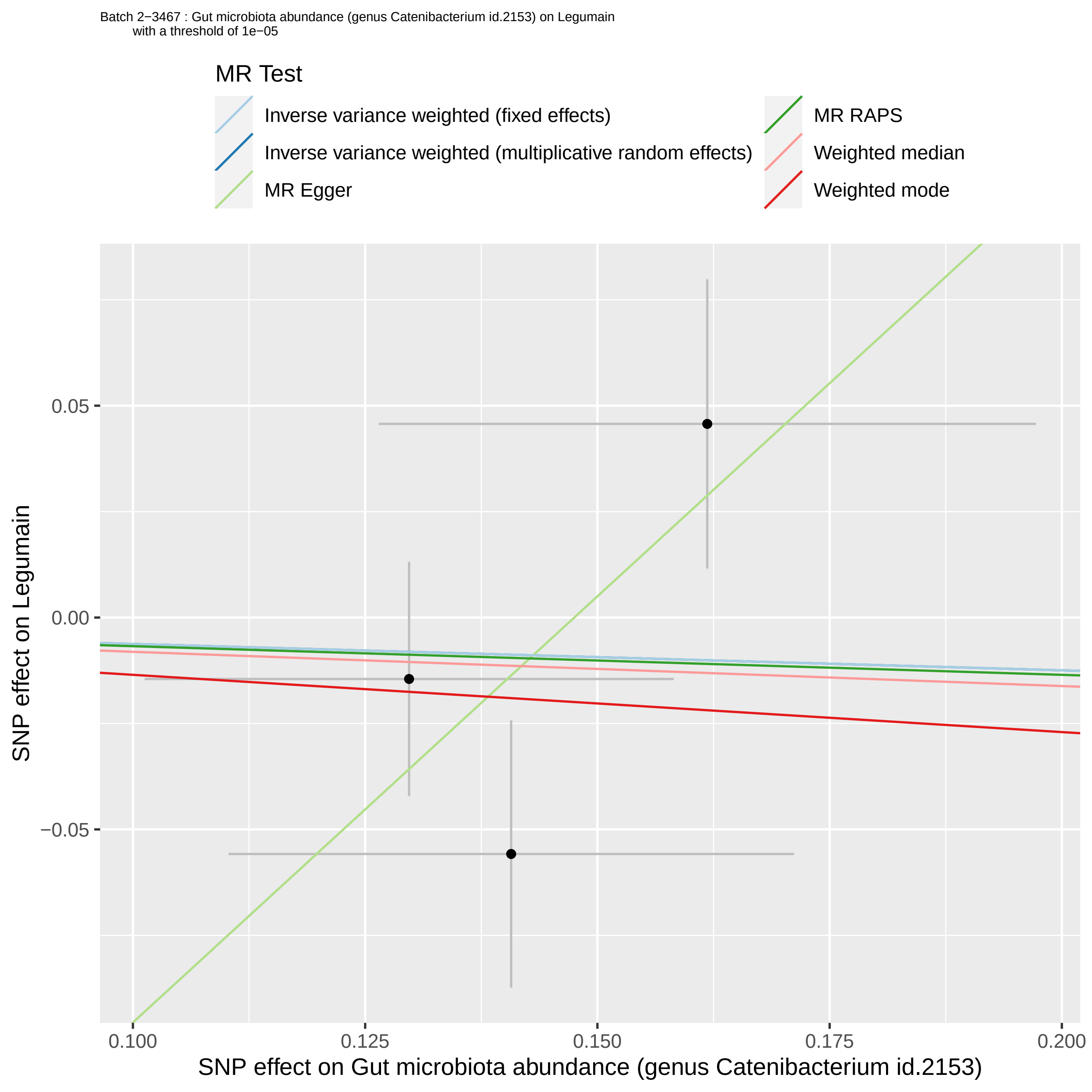

Batch 2−3467 : Gut microbiota abundance (genus Catenibacterium id.2153) on Legumain
with a threshold of 1e−05
MR Test
Inverse variance weighted (fixed effects)
Inverse variance weighted (multiplicative random effects)
MR Egger
MR RAPS
Weighted median
Weighted mode
0.05
0.00
−0.05
0.100
0.125
0.150
0.175
0.200
SNP effect on Legumain
SNP effect on Gut microbiota abundance (genus Catenibacterium id.2153)

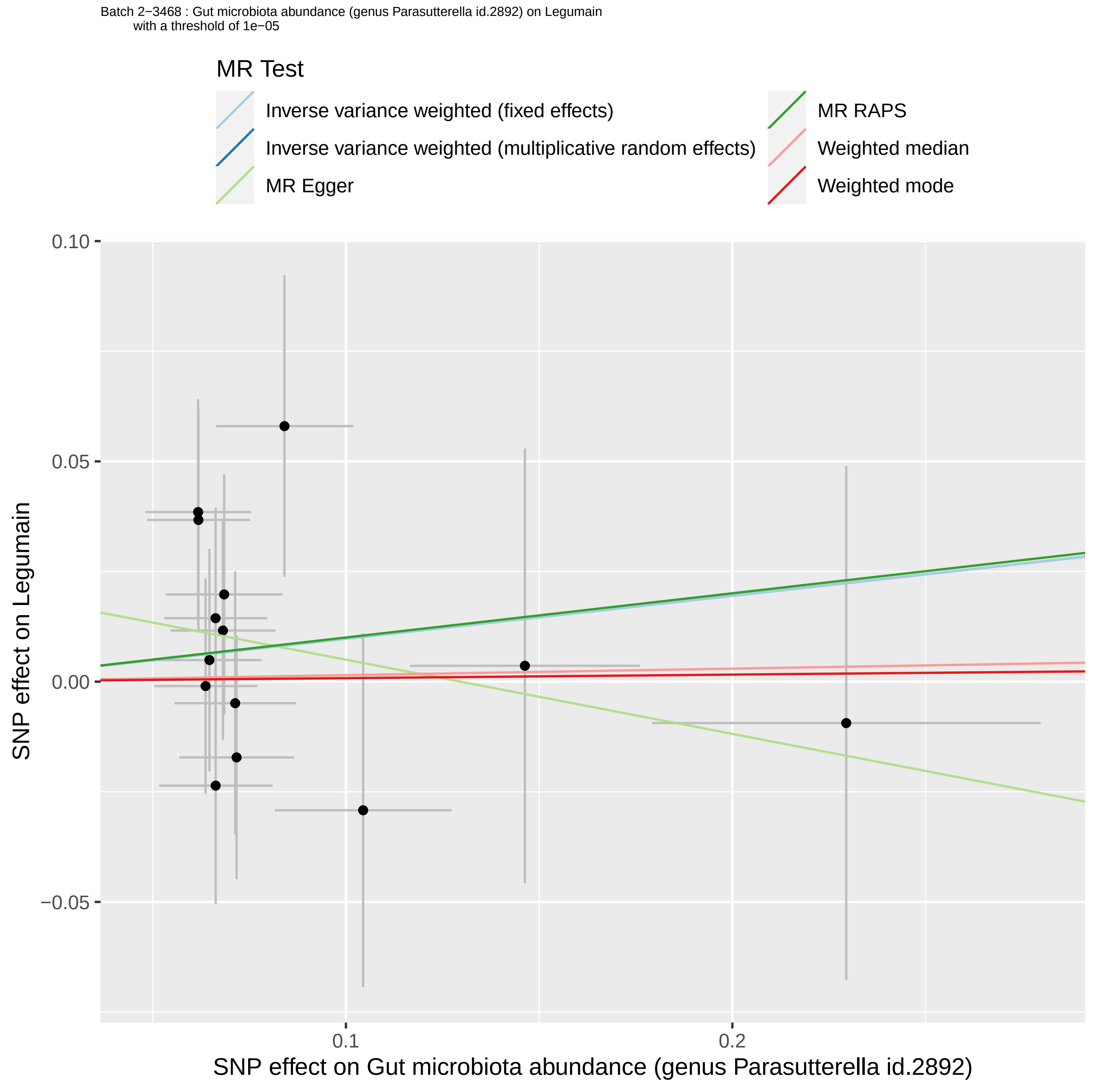

Batch 2−3468 : Gut microbiota abundance (genus Parasutterella id.2892) on Legumain
with a threshold of 1e−05
MR Test
Inverse variance weighted (fixed effects)
Inverse variance weighted (multiplicative random effects)
MR Egger
MR RAPS
Weighted median
Weighted mode
0.10
0.05
0.00
−0.05
0.1
0.2
SNP effect on Legumain
SNP effect on Gut microbiota abundance (genus Parasutterella id.2892)

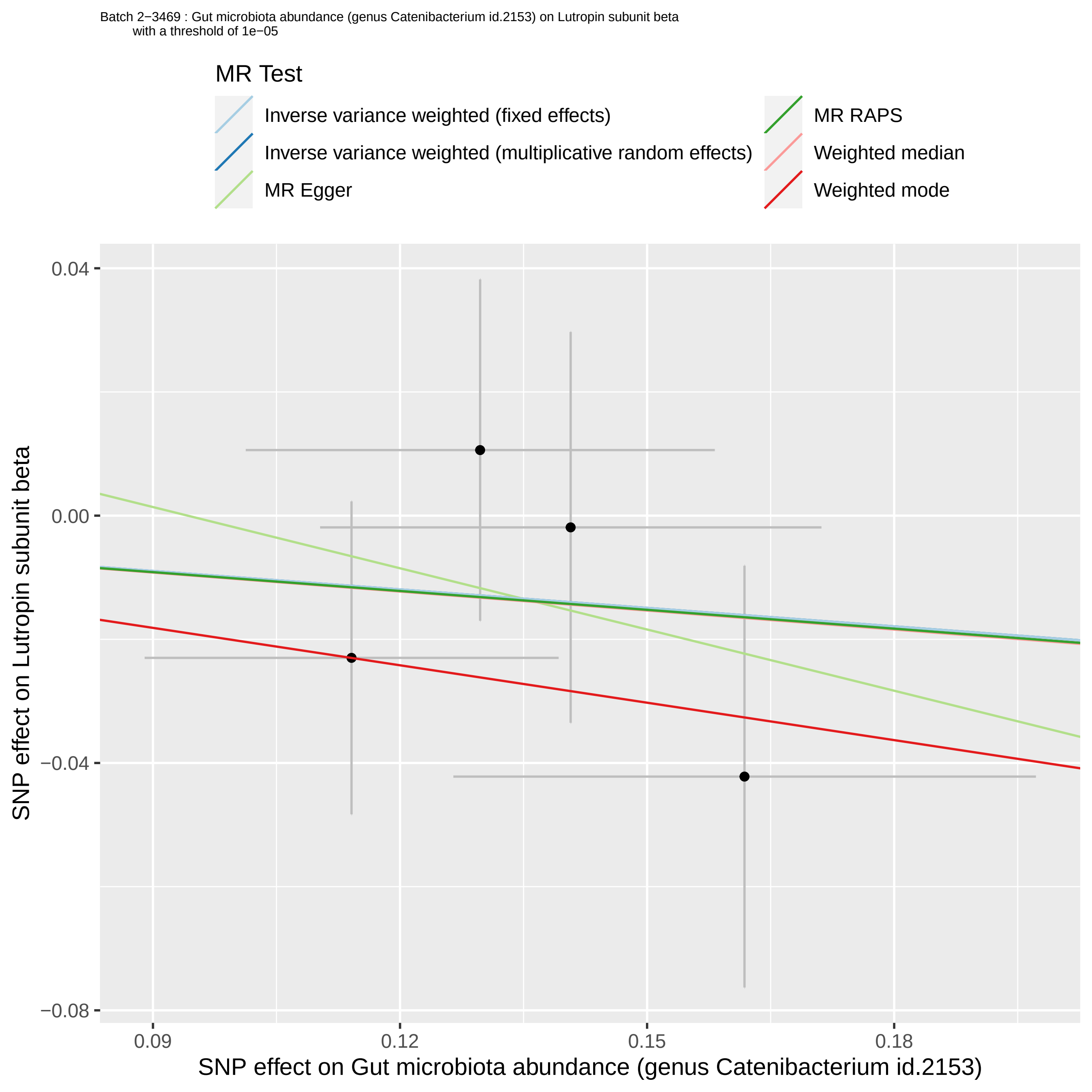

Batch 2−3469 : Gut microbiota abundance (genus Catenibacterium id.2153) on Lutropin subunit beta
with a threshold of 1e−05
MR Test
Inverse variance weighted (fixed effects)
Inverse variance weighted (multiplicative random effects)
MR Egger
MR RAPS
Weighted median
Weighted mode
0.04
0.00
−0.04
−0.08
0.09
0.12
0.15
0.18
SNP effect on Lutropin subunit beta
SNP effect on Gut microbiota abundance (genus Catenibacterium id.2153)

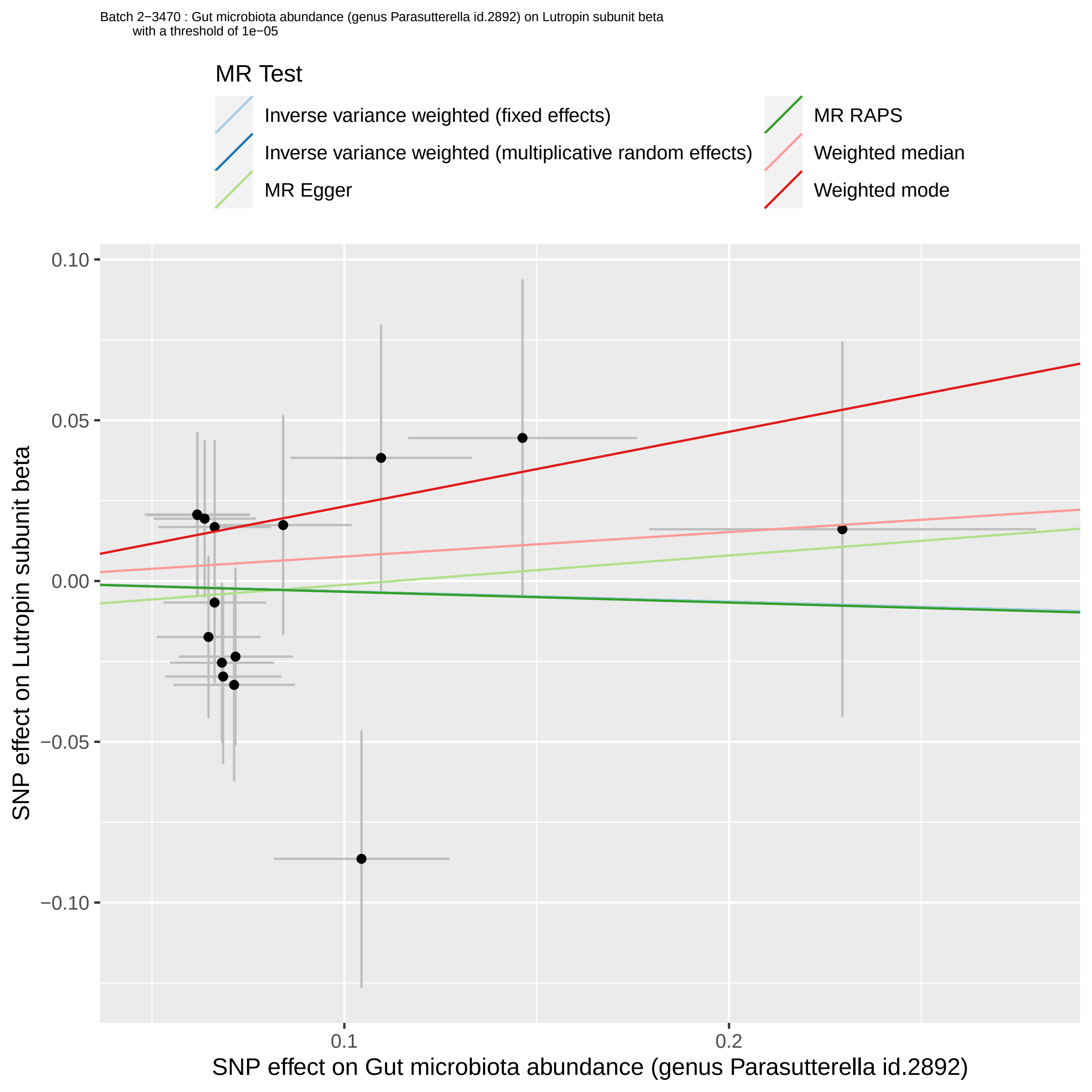

Batch 2−3470 : Gut microbiota abundance (genus Parasutterella id.2892) on Lutropin subunit beta
with a threshold of 1e−05
MR Test
Inverse variance weighted (fixed effects)
Inverse variance weighted (multiplicative random effects)
MR Egger
MR RAPS
Weighted median
Weighted mode
0.10
0.05
0.00
−0.05
−0.10
0.1
0.2
SNP effect on Lutropin subunit beta
SNP effect on Gut microbiota abundance (genus Parasutterella id.2892)

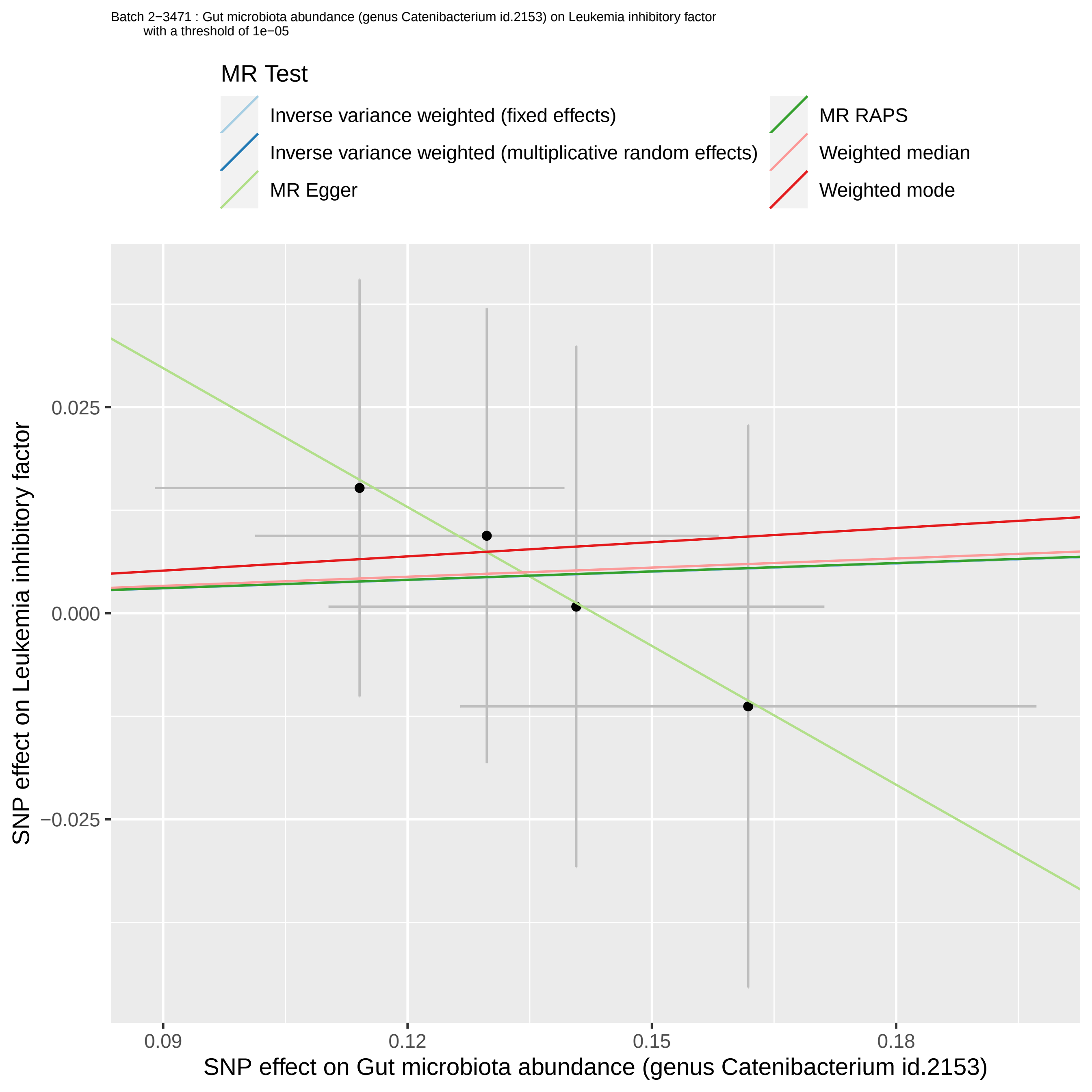

Batch 2−3471 : Gut microbiota abundance (genus Catenibacterium id.2153) on Leukemia inhibitory factor
with a threshold of 1e−05
MR Test
Inverse variance weighted (fixed effects)
Inverse variance weighted (multiplicative random effects)
MR Egger
MR RAPS
Weighted median
Weighted mode
0.025
0.000
−0.025
0.09
0.12
0.15
0.18
SNP effect on Leukemia inhibitory factor
SNP effect on Gut microbiota abundance (genus Catenibacterium id.2153)

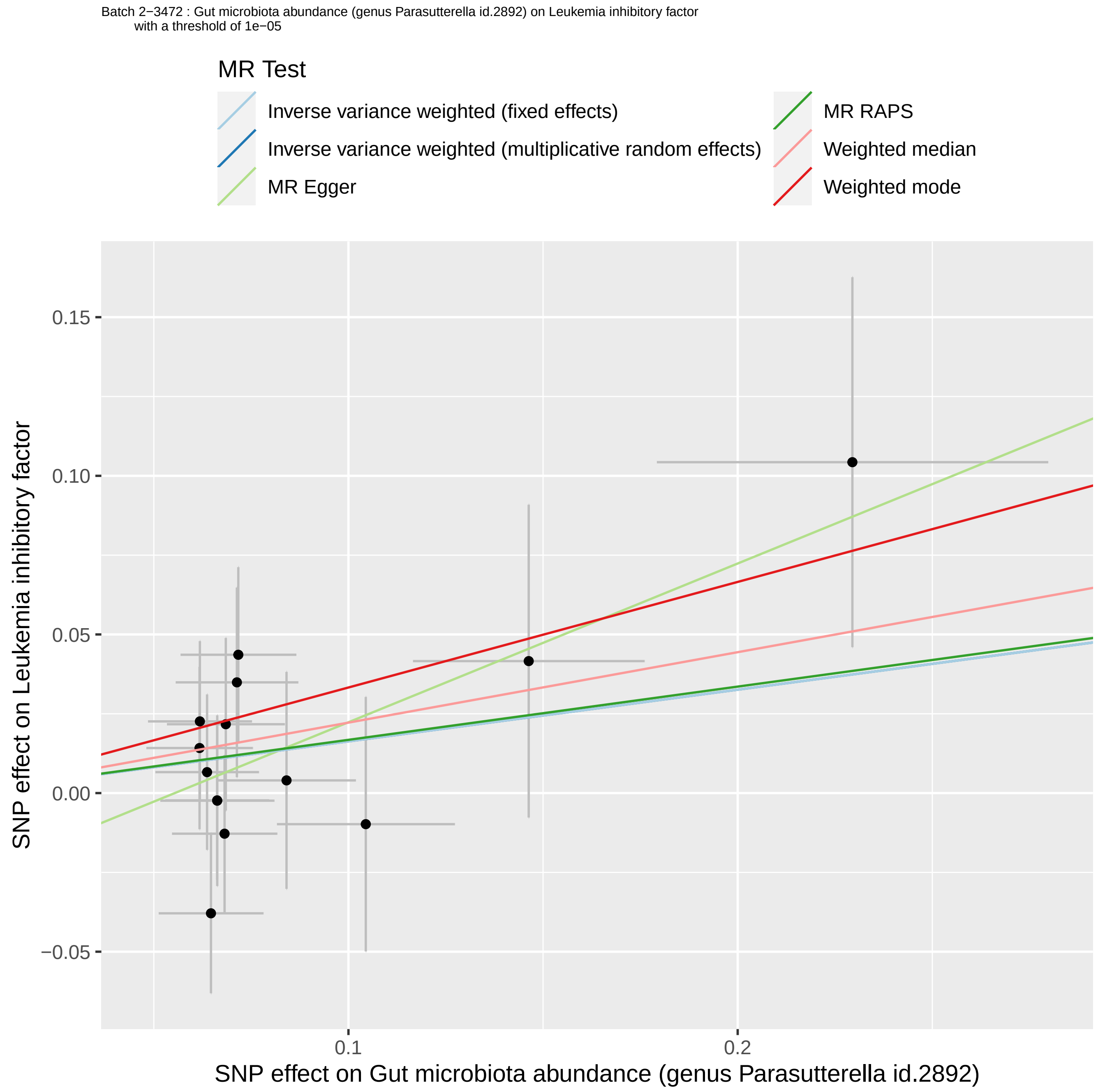

Batch 2−3472 : Gut microbiota abundance (genus Parasutterella id.2892) on Leukemia inhibitory factor
with a threshold of 1e−05
MR Test
Inverse variance weighted (fixed effects)
Inverse variance weighted (multiplicative random effects)
MR Egger
MR RAPS
Weighted median
Weighted mode
0.15
0.10
0.05
0.00
−0.05
0.1
0.2
SNP effect on Leukemia inhibitory factor
SNP effect on Gut microbiota abundance (genus Parasutterella id.2892)

Batch 2−3473 : Gut microbiota abundance (genus Catenibacterium id.2153) on Leukemia inhibitory factor receptor
with a threshold of 1e−05

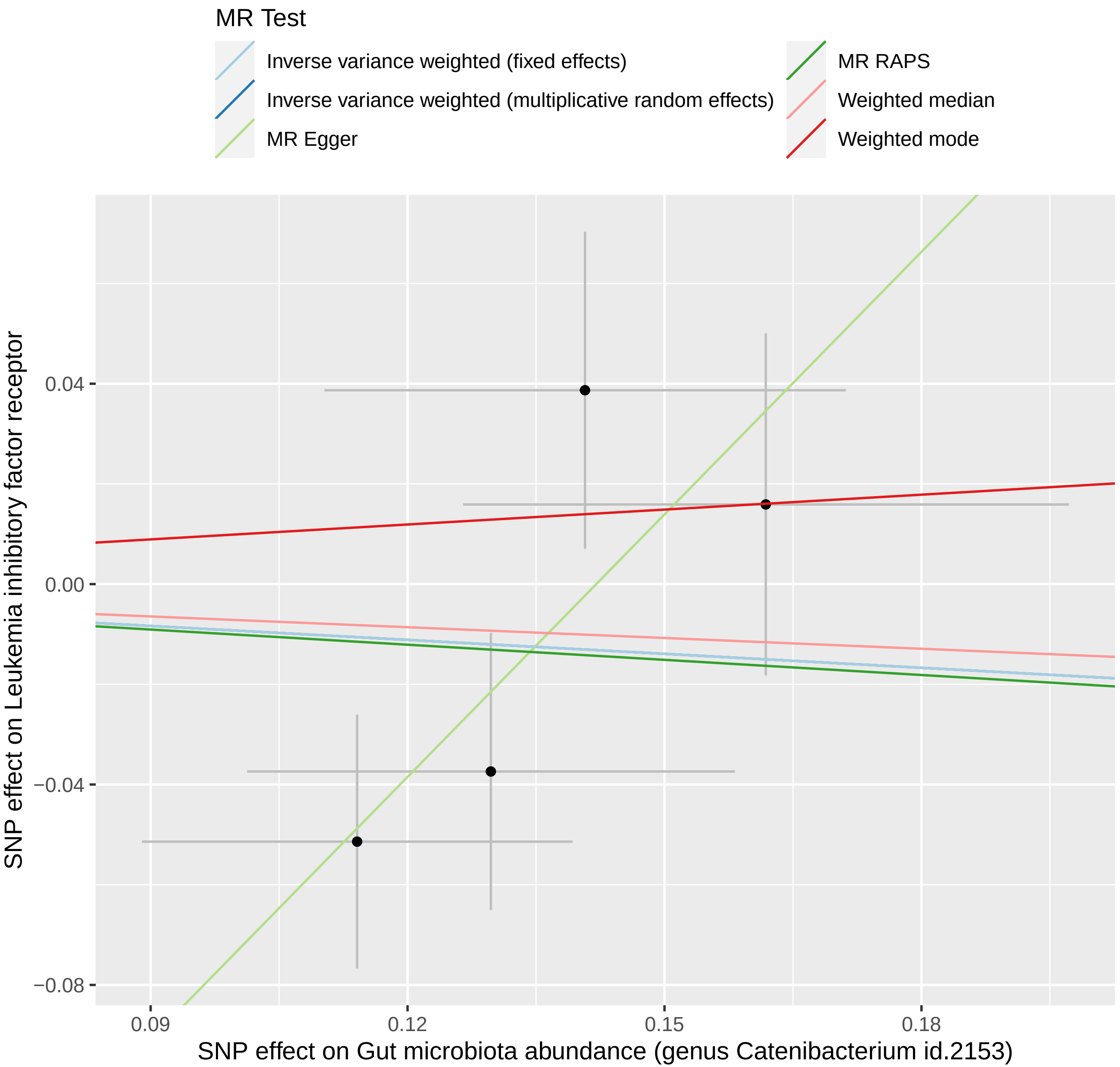

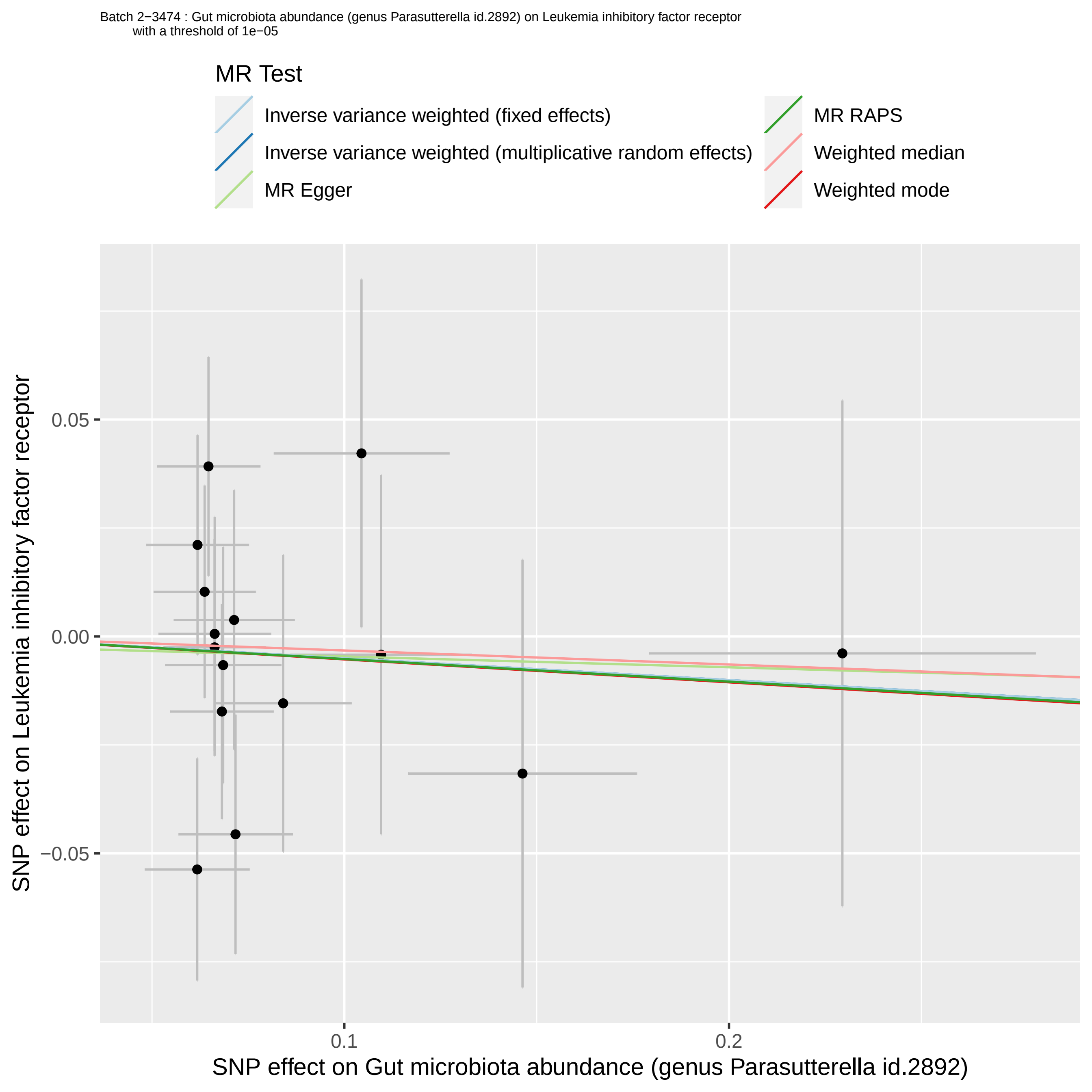

Batch 2−3474 : Gut microbiota abundance (genus Parasutterella id.2892) on Leukemia inhibitory factor receptor
with a threshold of 1e−05
MR Test
Inverse variance weighted (fixed effects)
Inverse variance weighted (multiplicative random effects)
MR Egger
MR RAPS
Weighted median
Weighted mode
SNP effect on Leukemia inhibitory factor receptor
0.05
0.00
−0.05
0.1
0.2
SNP effect on Gut microbiota abundance (genus Parasutterella id.2892)

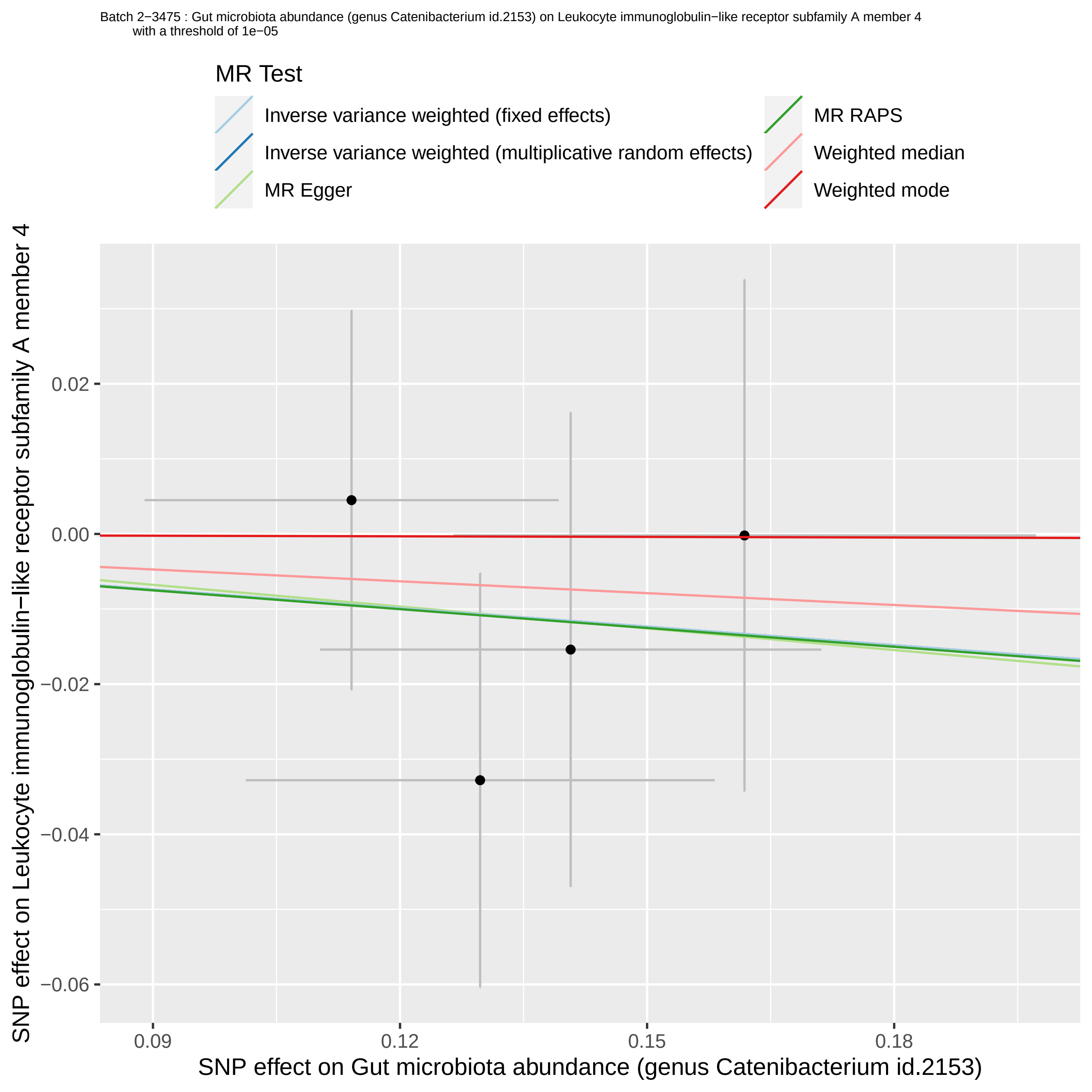
Batch 2−3475 : Gut microbiota abundance (genus Catenibacterium id.2153) on Leukocyte immunoglobulin−like receptor subfamily A member 4
with a threshold of 1e−05
MR Test
Inverse variance weighted (fixed effects)
Inverse variance weighted (multiplicative random effects)
MR Egger
MR RAPS
Weighted median
Weighted mode
SNP effect on Leukocyte immunoglobulin−like receptor subfamily A member 4
0.02
0.00
−0.02
−0.04
−0.06
0.09
0.12
0.15
0.18
SNP effect on Gut microbiota abundance (genus Catenibacterium id.2153)

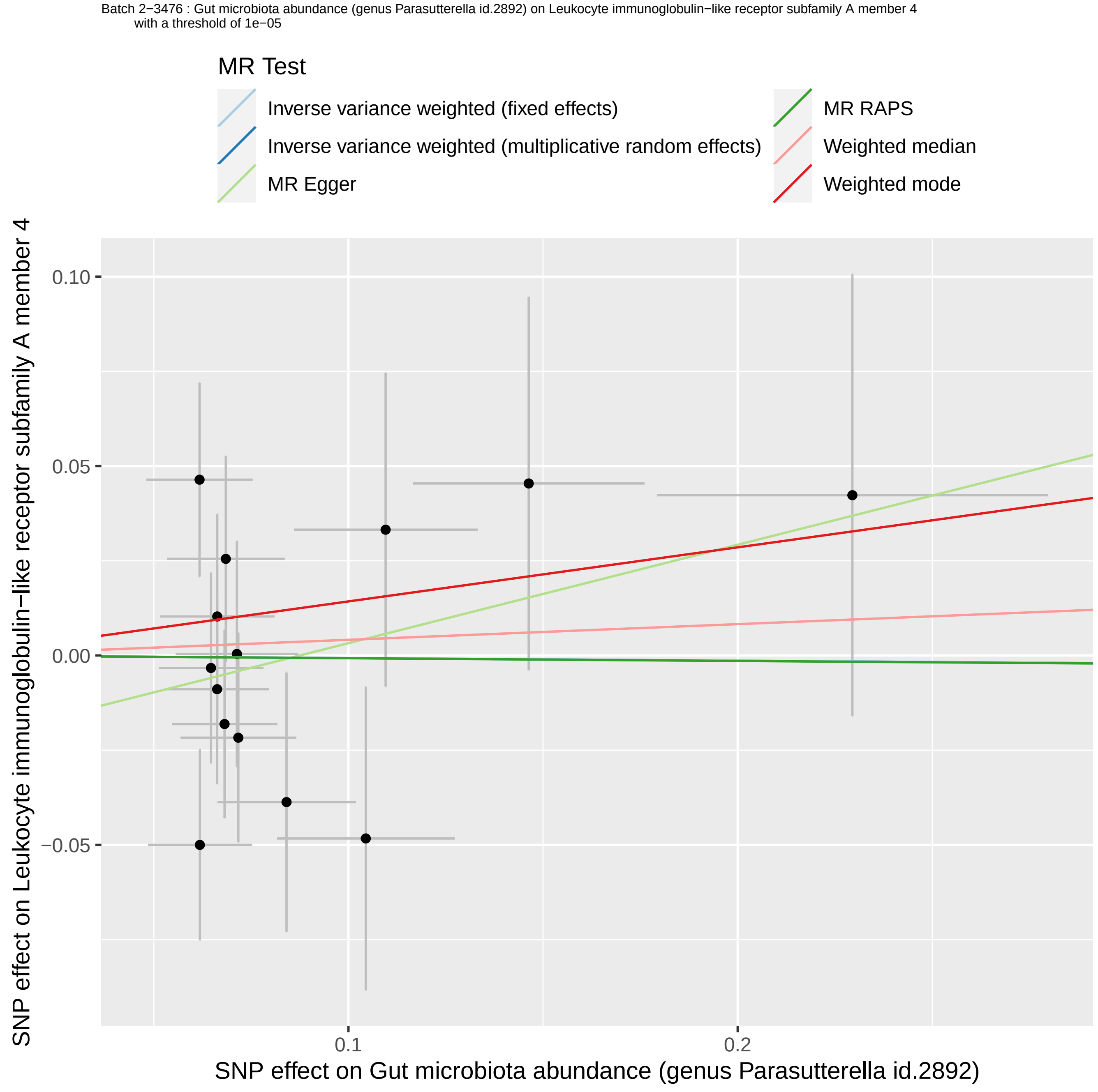

Batch 2−3476 : Gut microbiota abundance (genus Parasutterella id.2892) on Leukocyte immunoglobulin−like receptor subfamily A member 4
with a threshold of 1e−05
MR Test
Inverse variance weighted (fixed effects)
Inverse variance weighted (multiplicative random effects)
MR Egger
MR RAPS
Weighted median
Weighted mode
0.10
0.05
0.00
−0.05
0.1
0.2
SNP effect on Leukocyte immunoglobulin−like receptor subfamily A member 4
SNP effect on Gut microbiota abundance (genus Parasutterella id.2892)

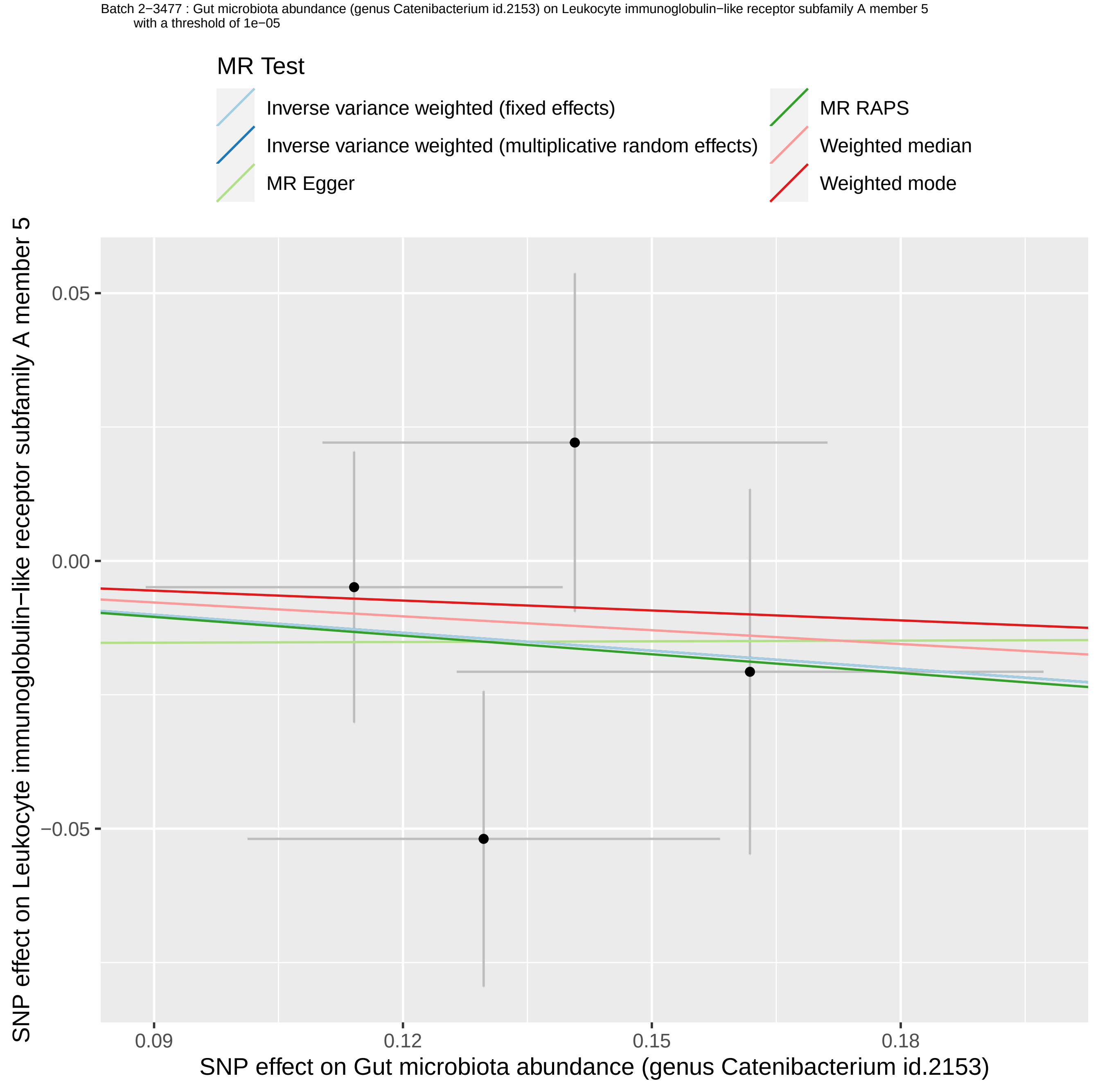

Batch 2−3477 : Gut microbiota abundance (genus Catenibacterium id.2153) on Leukocyte immunoglobulin−like receptor subfamily A member 5
with a threshold of 1e−05
MR Test
Inverse variance weighted (fixed effects)
Inverse variance weighted (multiplicative random effects)
MR Egger
MR RAPS
Weighted median
Weighted mode
0.05
0.00
−0.05
0.09
0.12
0.15
0.18
SNP effect on Leukocyte immunoglobulin−like receptor subfamily A member 5
SNP effect on Gut microbiota abundance (genus Catenibacterium id.2153)

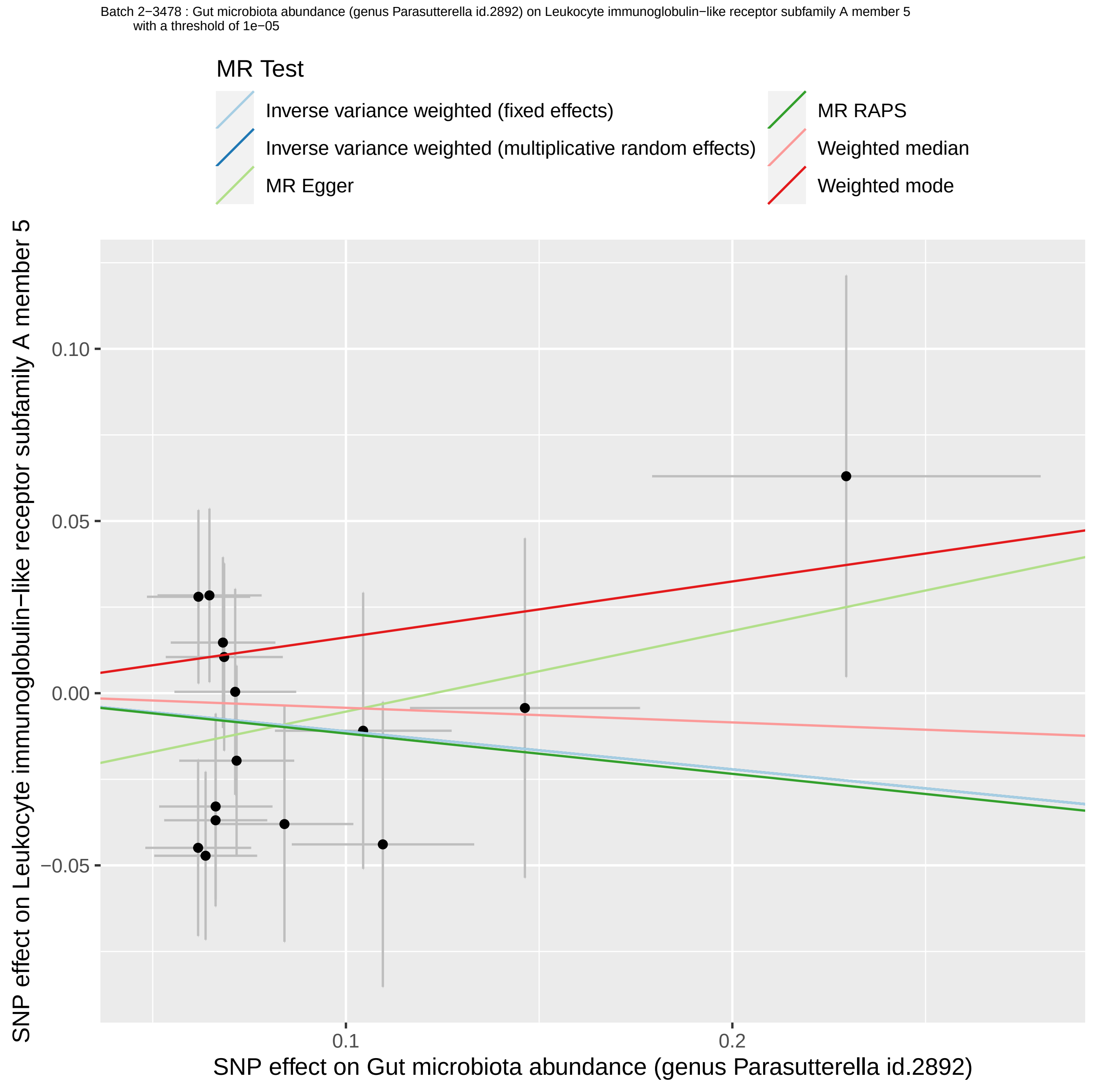

Batch 2−3478 : Gut microbiota abundance (genus Parasutterella id.2892) on Leukocyte immunoglobulin−like receptor subfamily A member 5
with a threshold of 1e−05
MR Test
Inverse variance weighted (fixed effects)
Inverse variance weighted (multiplicative random effects)
MR Egger
MR RAPS
Weighted median
Weighted mode
0.10
0.05
0.00
−0.05
0.1
0.2
SNP effect on Gut microbiota abundance (genus Parasutterella id.2892)
SNP effect on Leukocyte immunoglobulin−like receptor subfamily A member 5

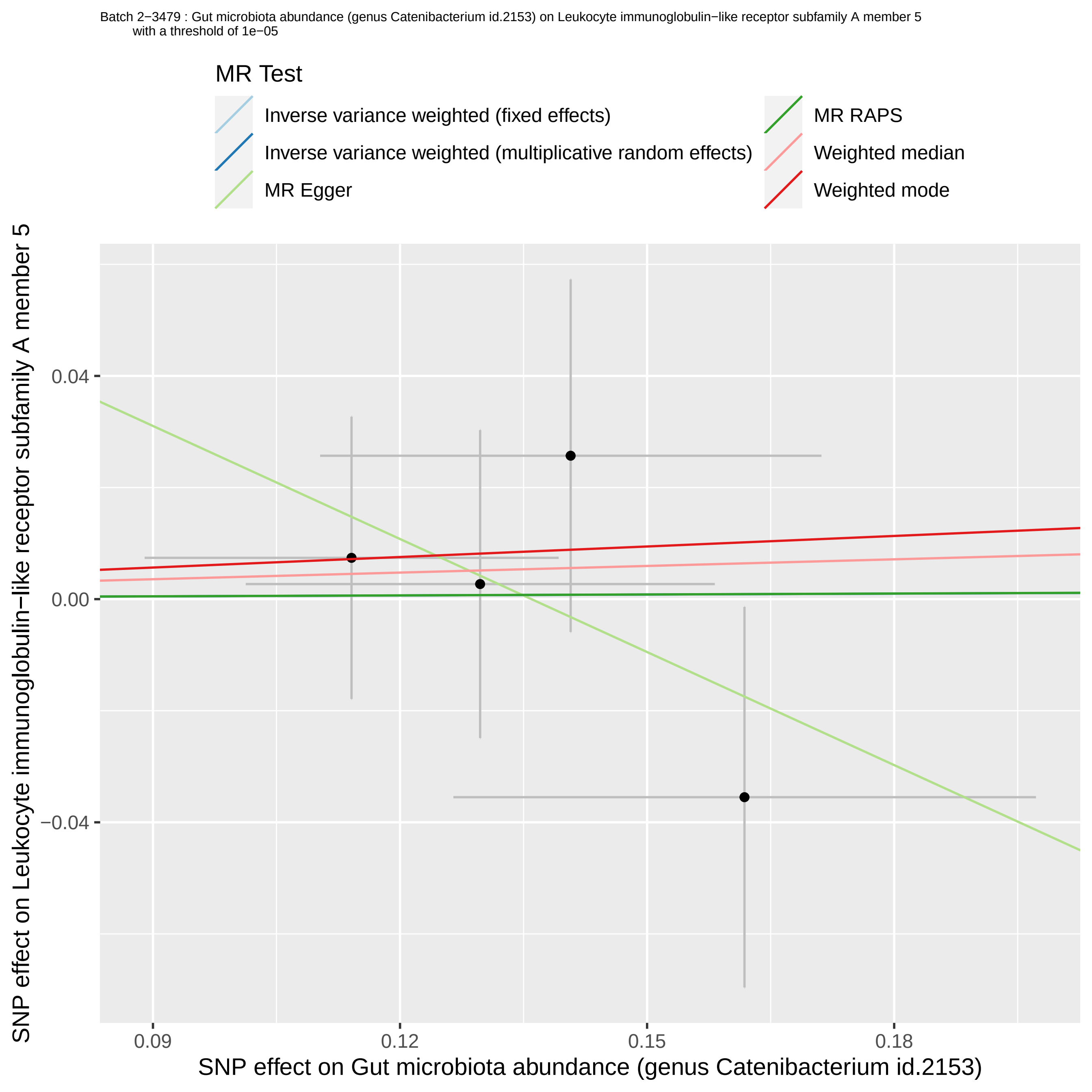
Batch 2−3479 : Gut microbiota abundance (genus Catenibacterium id.2153) on Leukocyte immunoglobulin−like receptor subfamily A member 5
with a threshold of 1e−05
MR Test
Inverse variance weighted (fixed effects)
Inverse variance weighted (multiplicative random effects)
MR Egger
MR RAPS
Weighted median
Weighted mode
SNP effect on Leukocyte immunoglobulin−like receptor subfamily A member 5
0.04
0.00
−0.04
0.09
0.12
0.15
0.18
SNP effect on Gut microbiota abundance (genus Catenibacterium id.2153)

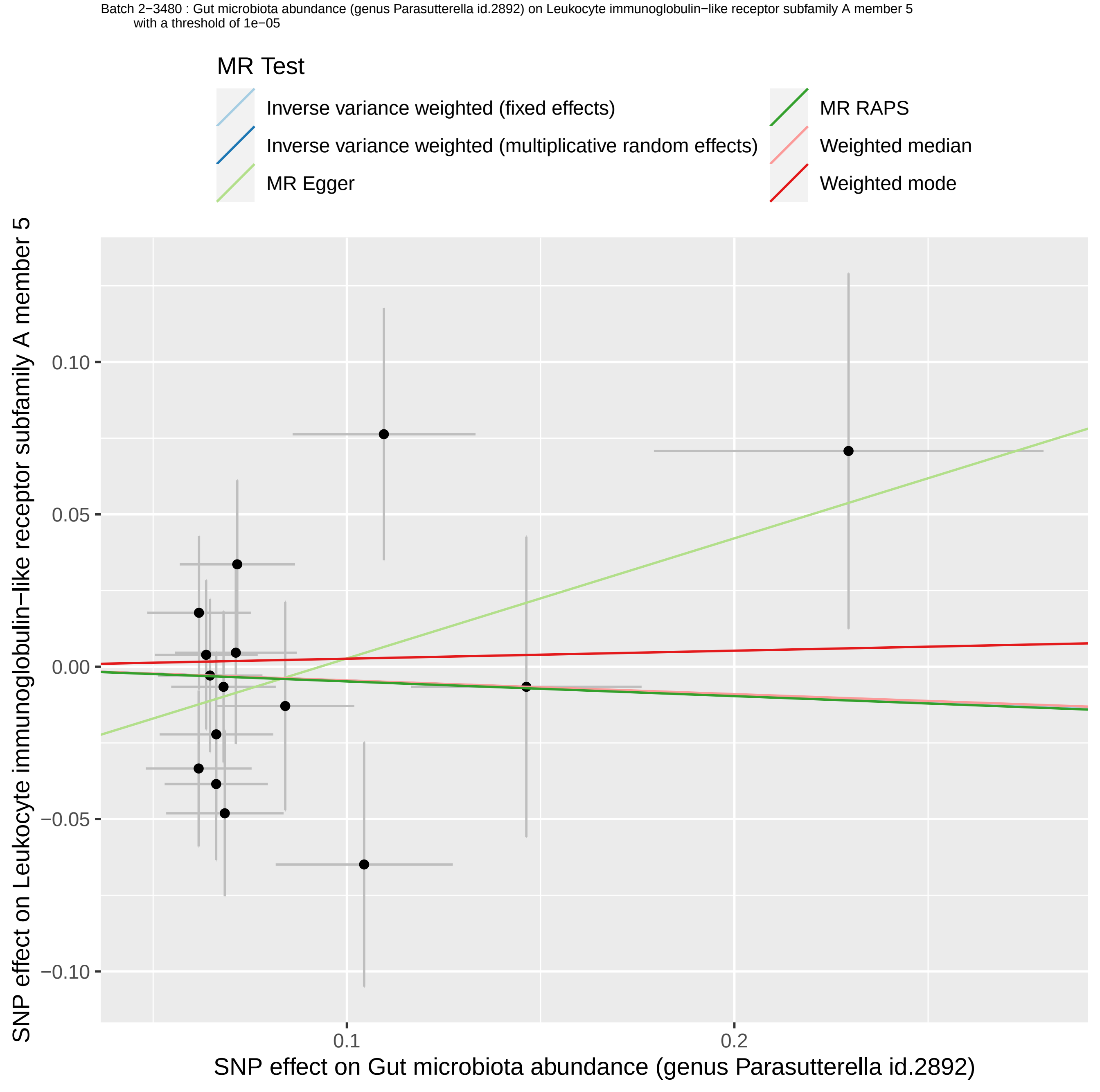
Batch 2−3480 : Gut microbiota abundance (genus Parasutterella id.2892) on Leukocyte immunoglobulin−like receptor subfamily A member 5
with a threshold of 1e−05
MR Test
Inverse variance weighted (fixed effects)
Inverse variance weighted (multiplicative random effects)
MR Egger
MR RAPS
Weighted median
Weighted mode
0.10
0.05
0.00
−0.05
−0.10
0.1
0.2
SNP effect on Leukocyte immunoglobulin−like receptor subfamily A member 5
SNP effect on Gut microbiota abundance (genus Parasutterella id.2892)

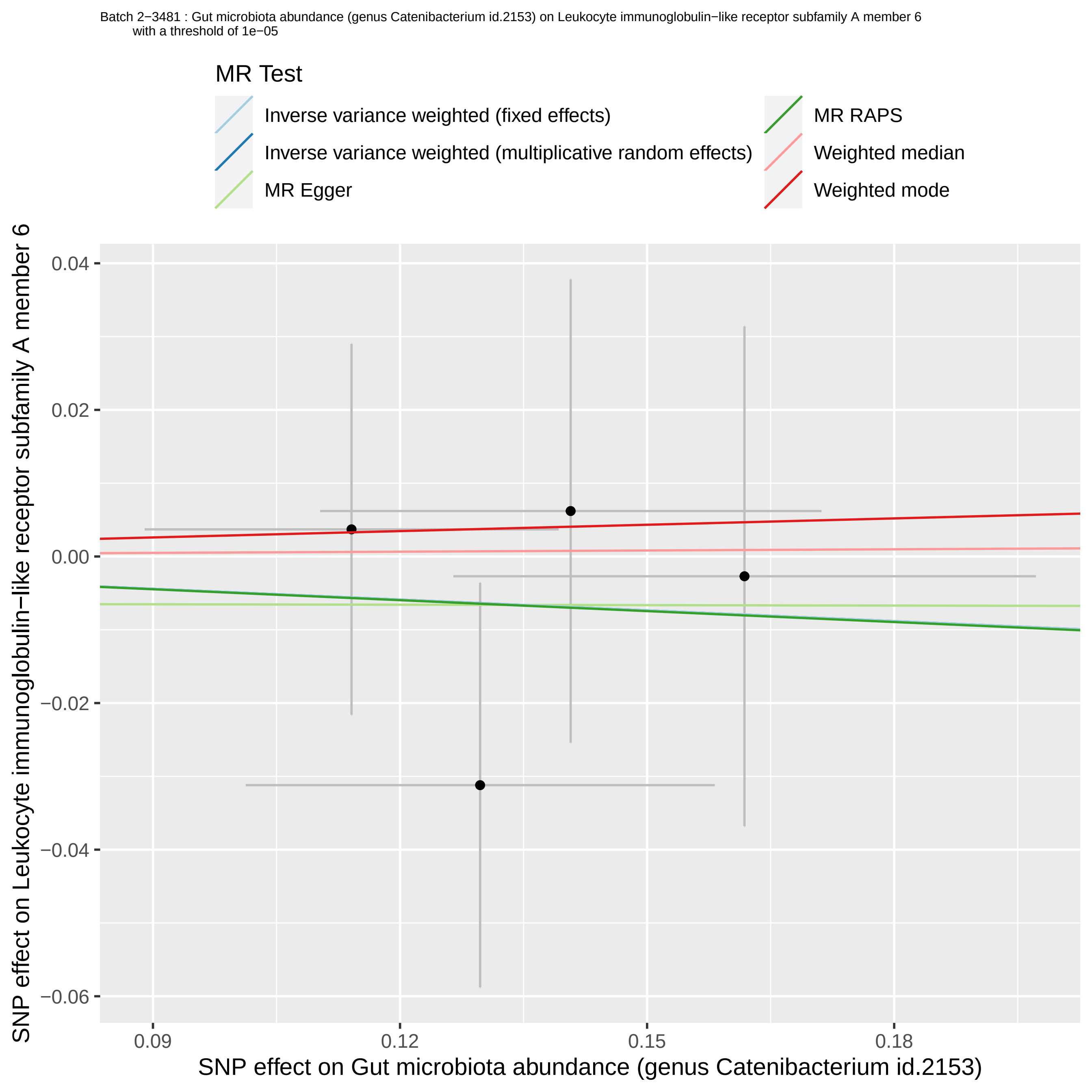
Batch 2−3481 : Gut microbiota abundance (genus Catenibacterium id.2153) on Leukocyte immunoglobulin−like receptor subfamily A member 6 with a threshold of 1e−05
MR Test
Inverse variance weighted (fixed effects)
Inverse variance weighted (multiplicative random effects)
MR Egger
MR RAPS
Weighted median
Weighted mode
SNP effect on Leukocyte immunoglobulin−like receptor subfamily A member 6
0.04
0.02
0.00
−0.02
−0.04
−0.06
0.09
0.12
0.15
0.18
SNP effect on Gut microbiota abundance (genus Catenibacterium id.2153)

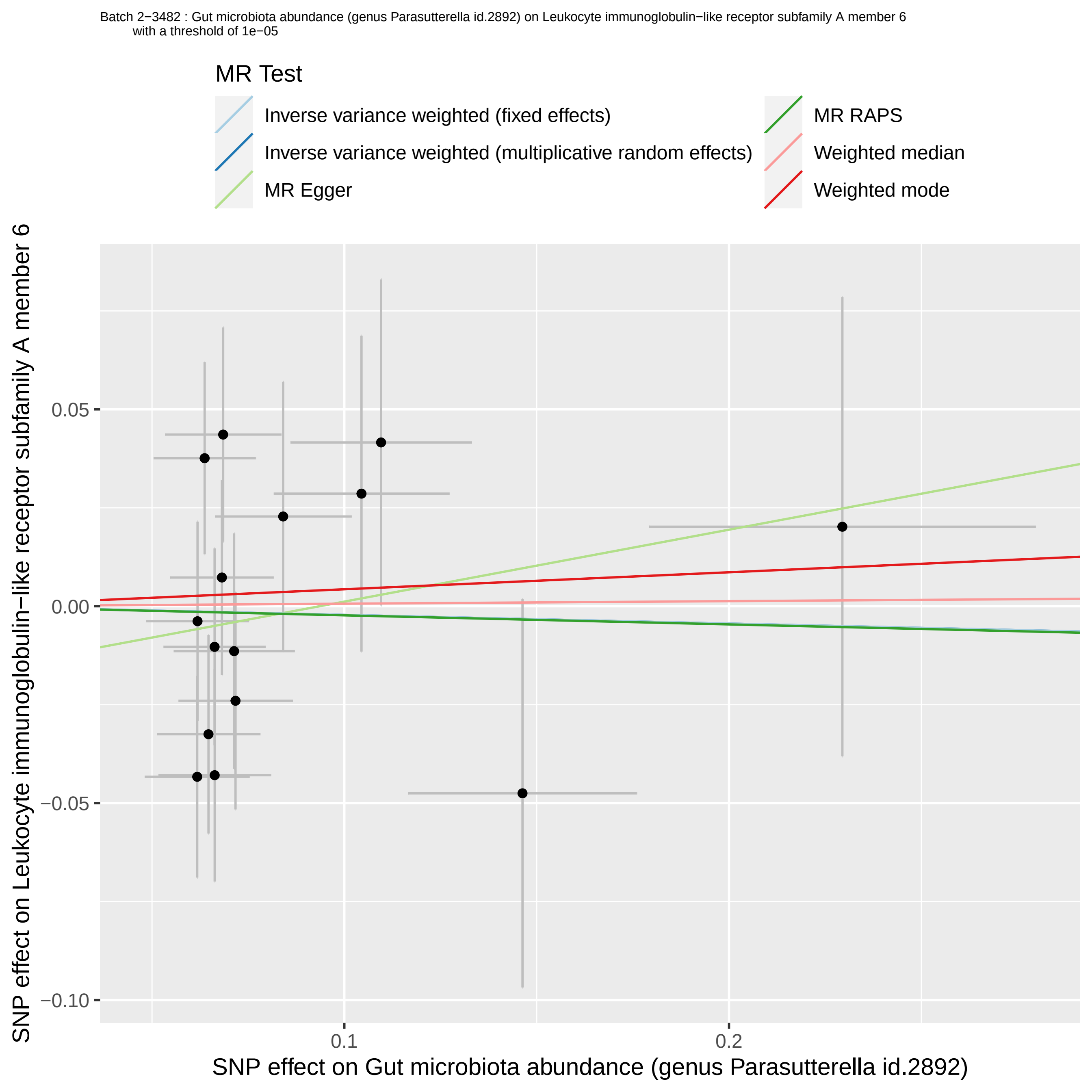
Batch 2−3482 : Gut microbiota abundance (genus Parasutterella id.2892) on Leukocyte immunoglobulin−like receptor subfamily A member 6
with a threshold of 1e−05
MR Test
Inverse variance weighted (fixed effects)
Inverse variance weighted (multiplicative random effects)
MR Egger
MR RAPS
Weighted median
Weighted mode
0.05
0.00
−0.05
−0.10
0.1
0.2
SNP effect on Leukocyte immunoglobulin−like receptor subfamily A member 6
SNP effect on Gut microbiota abundance (genus Parasutterella id.2892)

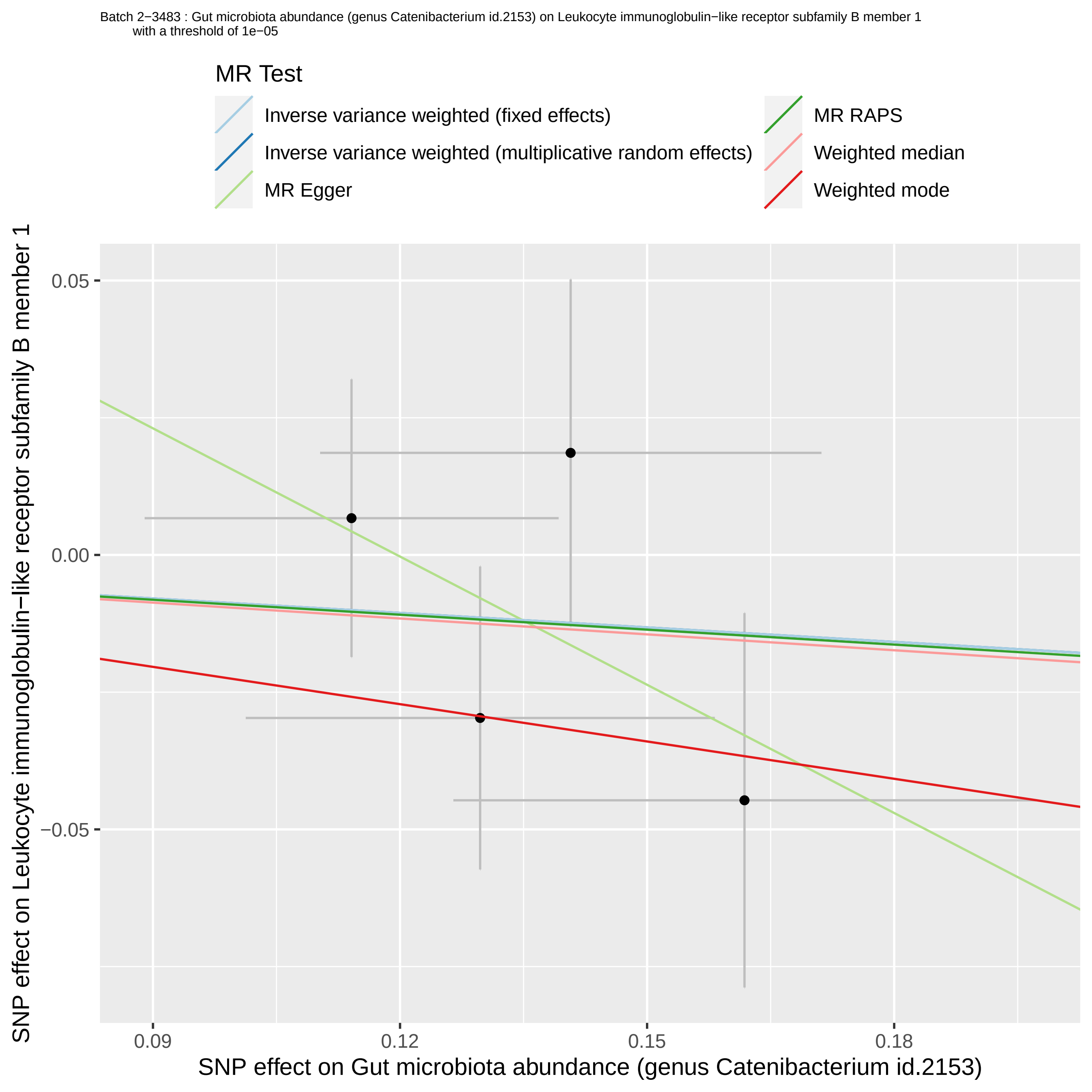

Batch 2−3483 : Gut microbiota abundance (genus Catenibacterium id.2153) on Leukocyte immunoglobulin−like receptor subfamily B member 1
with a threshold of 1e−05
MR Test
Inverse variance weighted (fixed effects)
Inverse variance weighted (multiplicative random effects)
MR Egger
MR RAPS
Weighted median
Weighted mode
SNP effect on Leukocyte immunoglobulin−like receptor subfamily B member 1
0.05
0.00
−0.05
0.09
0.12
0.15
0.18
SNP effect on Gut microbiota abundance (genus Catenibacterium id.2153)

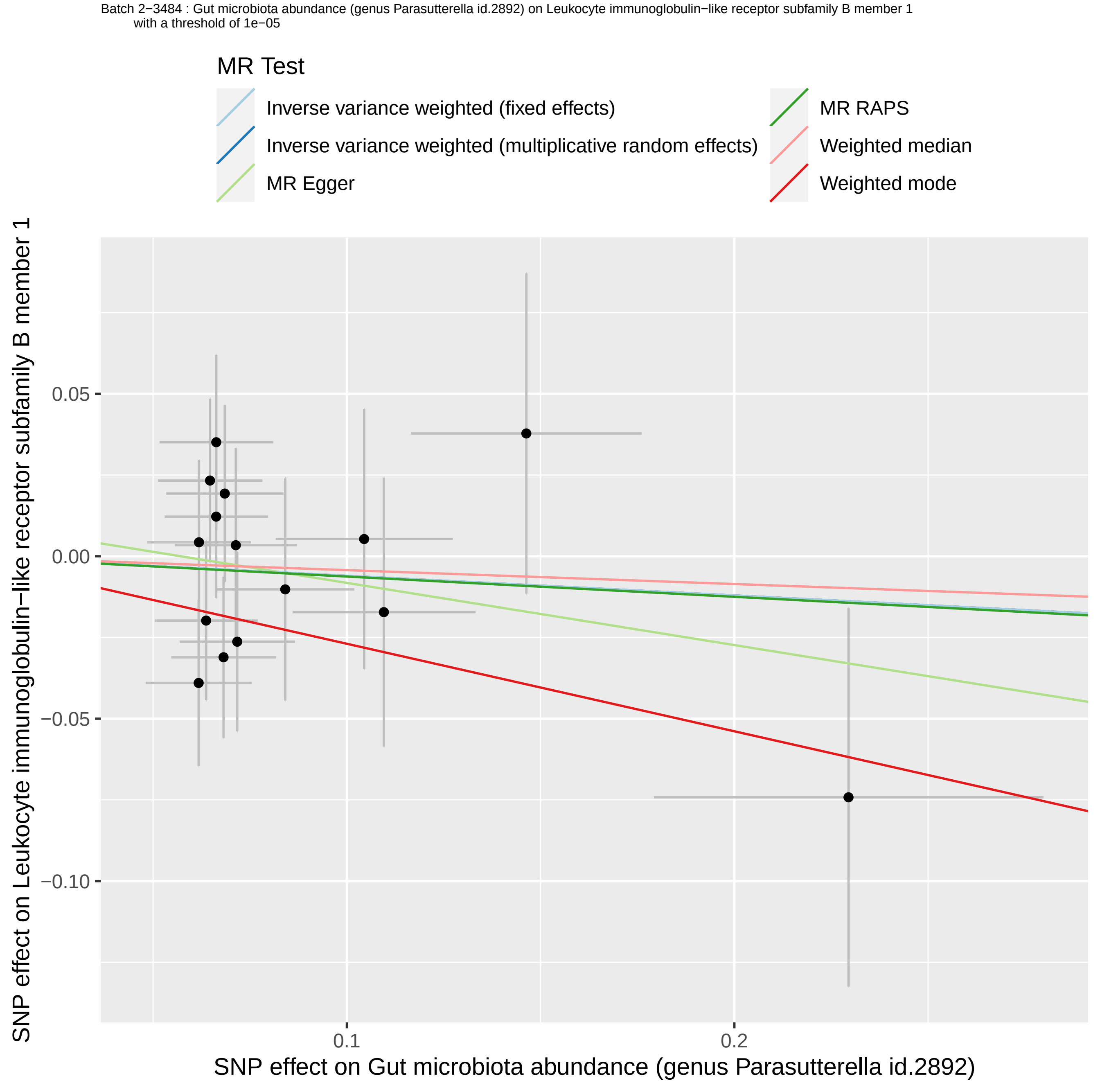

Batch 2−3484 : Gut microbiota abundance (genus Parasutterella id.2892) on Leukocyte immunoglobulin−like receptor subfamily B member 1
with a threshold of 1e−05
MR Test
Inverse variance weighted (fixed effects)
Inverse variance weighted (multiplicative random effects)
MR Egger
MR RAPS
Weighted median
Weighted mode
0.05
0.00
−0.05
−0.10
0.1
0.2
SNP effect on Leukocyte immunoglobulin−like receptor subfamily B member 1
SNP effect on Gut microbiota abundance (genus Parasutterella id.2892)

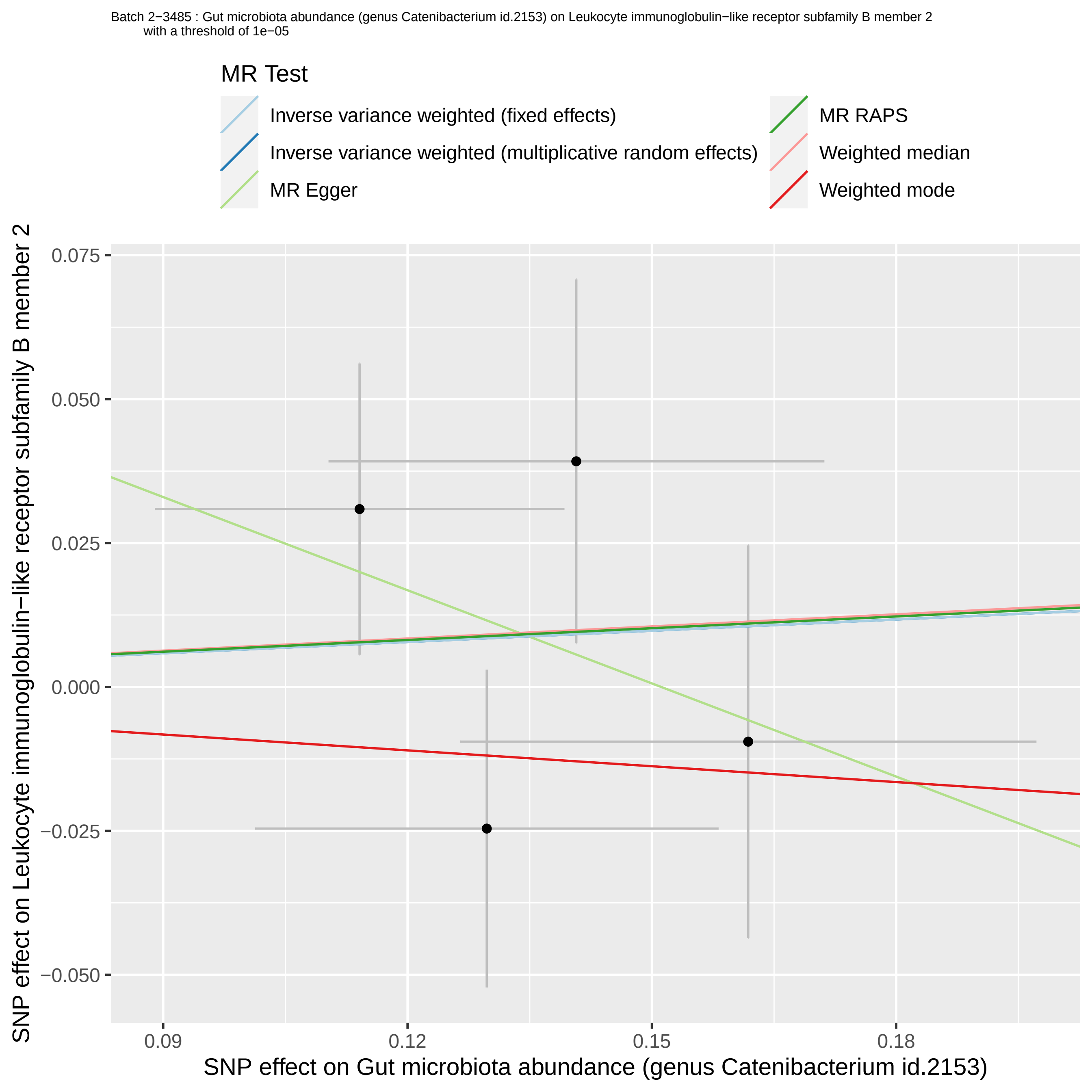
Batch 2−3485 : Gut microbiota abundance (genus Catenibacterium id.2153) on Leukocyte immunoglobulin−like receptor subfamily B member 2
with a threshold of 1e−05
MR Test
Inverse variance weighted (fixed effects)
Inverse variance weighted (multiplicative random effects)
MR Egger
MR RAPS
Weighted median
Weighted mode
SNP effect on Leukocyte immunoglobulin−like receptor subfamily B member 2
0.075
0.050
0.025
0.000
−0.025
−0.050
0.09
0.12
0.15
0.18
SNP effect on Gut microbiota abundance (genus Catenibacterium id.2153)

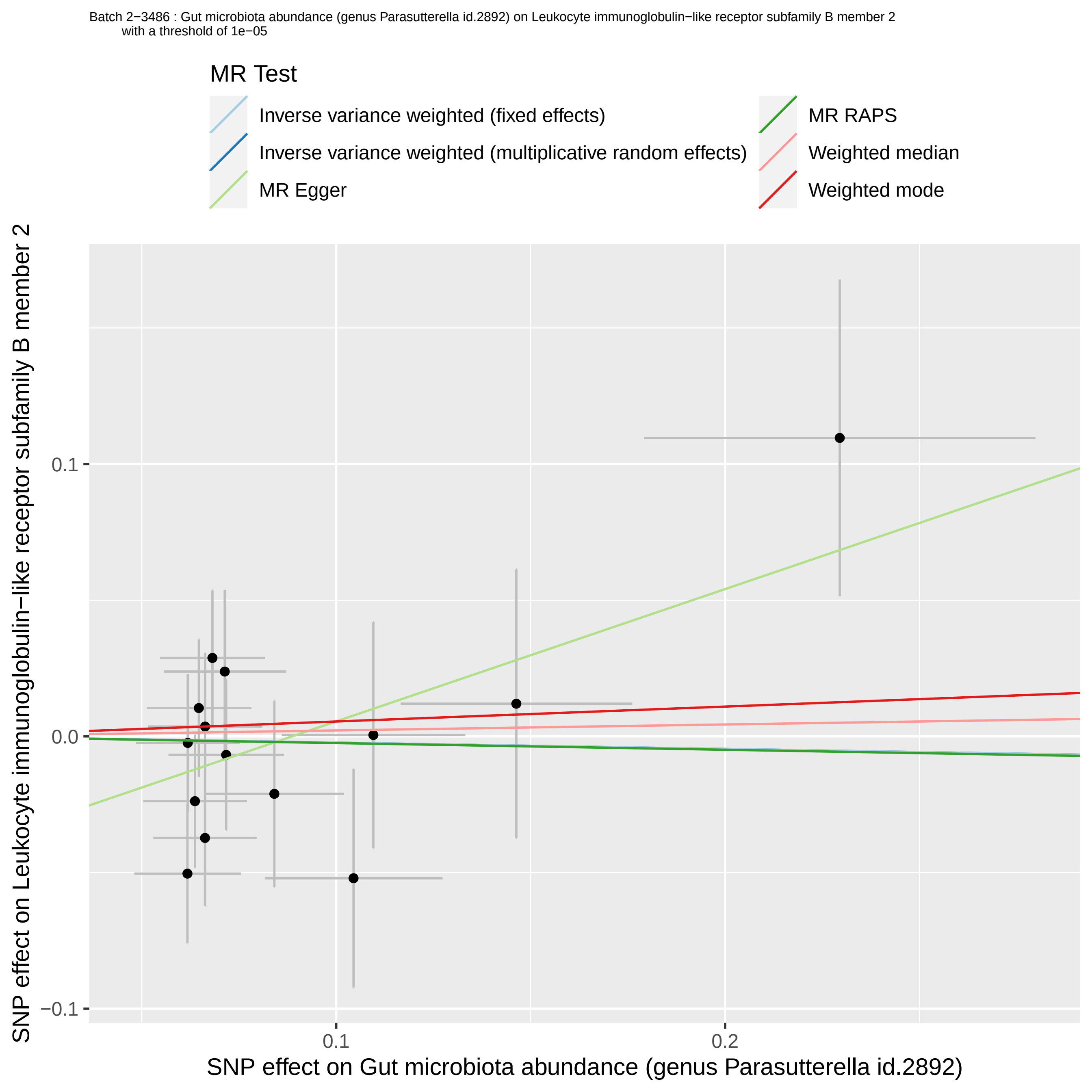
Batch 2−3486 : Gut microbiota abundance (genus Parasutterella id.2892) on Leukocyte immunoglobulin−like receptor subfamily B member 2
with a threshold of 1e−05
MR Test
Inverse variance weighted (fixed effects)
Inverse variance weighted (multiplicative random effects)
MR Egger
MR RAPS
Weighted median
Weighted mode
SNP effect on Leukocyte immunoglobulin−like receptor subfamily B member 2
0.1
0.0
−0.1
0.1
0.2
SNP effect on Gut microbiota abundance (genus Parasutterella id.2892)

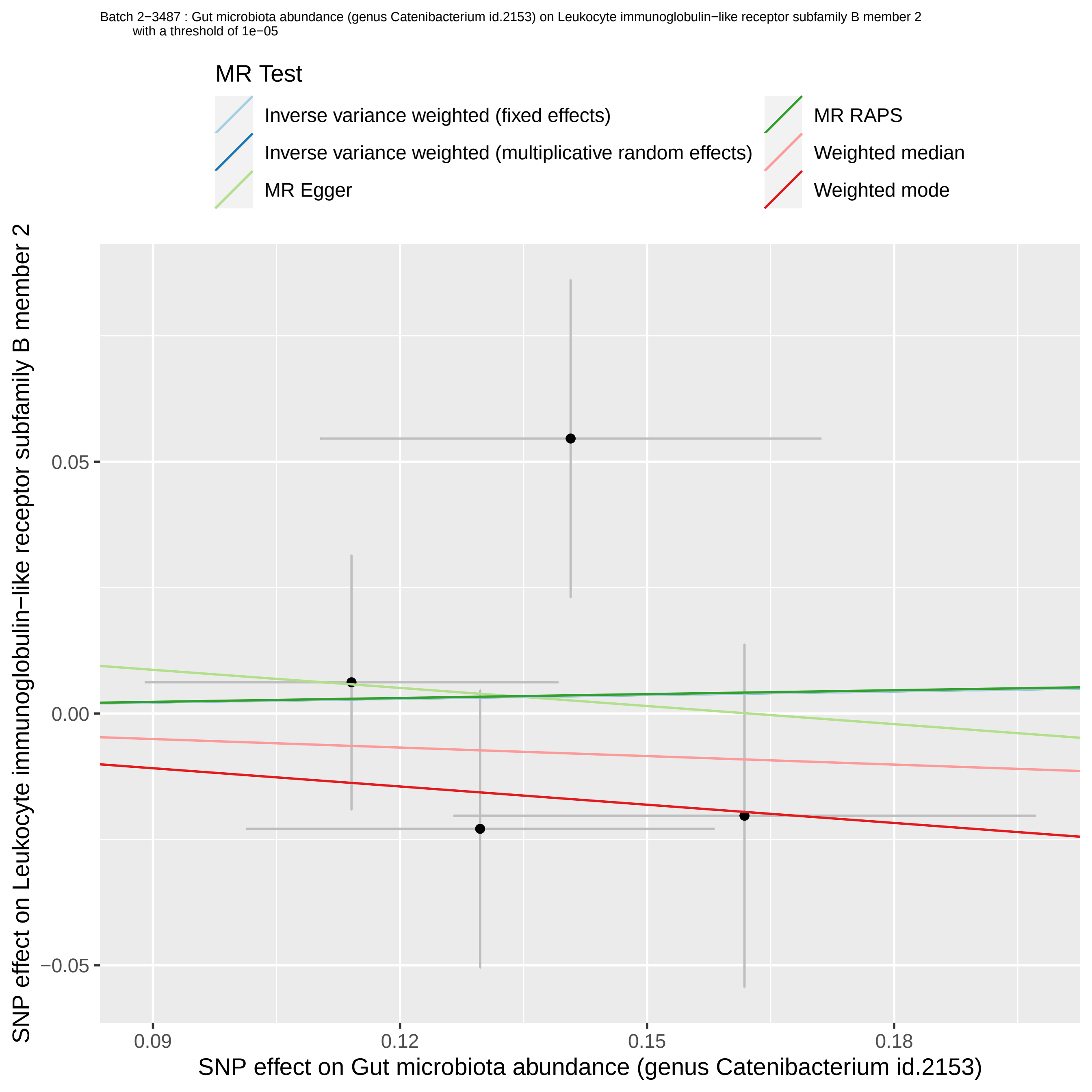
Batch 2−3487 : Gut microbiota abundance (genus Catenibacterium id.2153) on Leukocyte immunoglobulin−like receptor subfamily B member 2
with a threshold of 1e−05
MR Test
Inverse variance weighted (fixed effects)
Inverse variance weighted (multiplicative random effects)
MR Egger
MR RAPS
Weighted median
Weighted mode
SNP effect on Leukocyte immunoglobulin−like receptor subfamily B member 2
0.05
0.00
−0.05
0.09
0.12
0.15
0.18
SNP effect on Gut microbiota abundance (genus Catenibacterium id.2153)

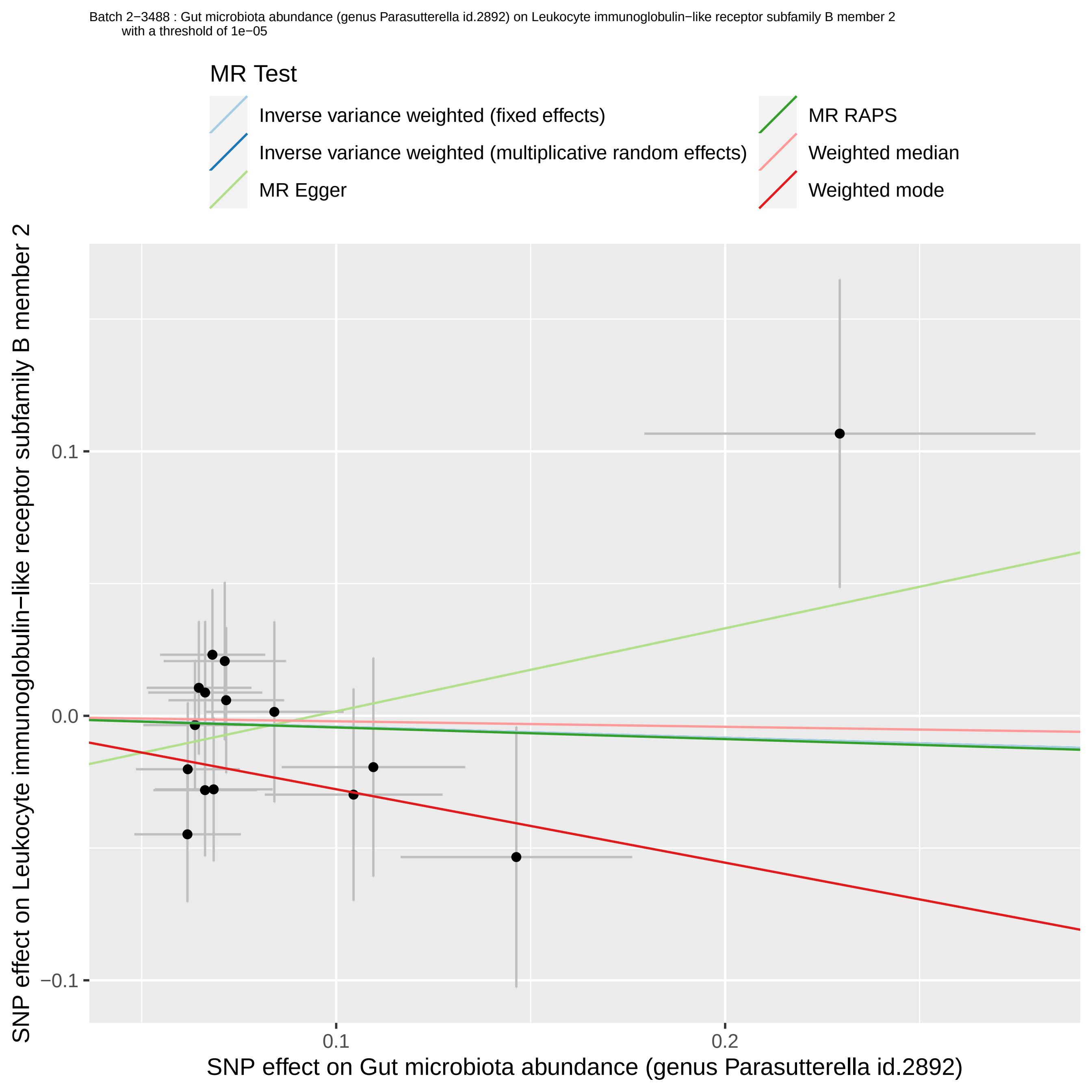
Batch 2−3488 : Gut microbiota abundance (genus Parasutterella id.2892) on Leukocyte immunoglobulin−like receptor subfamily B member 2
with a threshold of 1e−05
MR Test
Inverse variance weighted (fixed effects)
Inverse variance weighted (multiplicative random effects)
MR Egger
MR RAPS
Weighted median
Weighted mode
0.1
0.0
−0.1
0.1
0.2
SNP effect on Leukocyte immunoglobulin−like receptor subfamily B member 2
SNP effect on Gut microbiota abundance (genus Parasutterella id.2892)

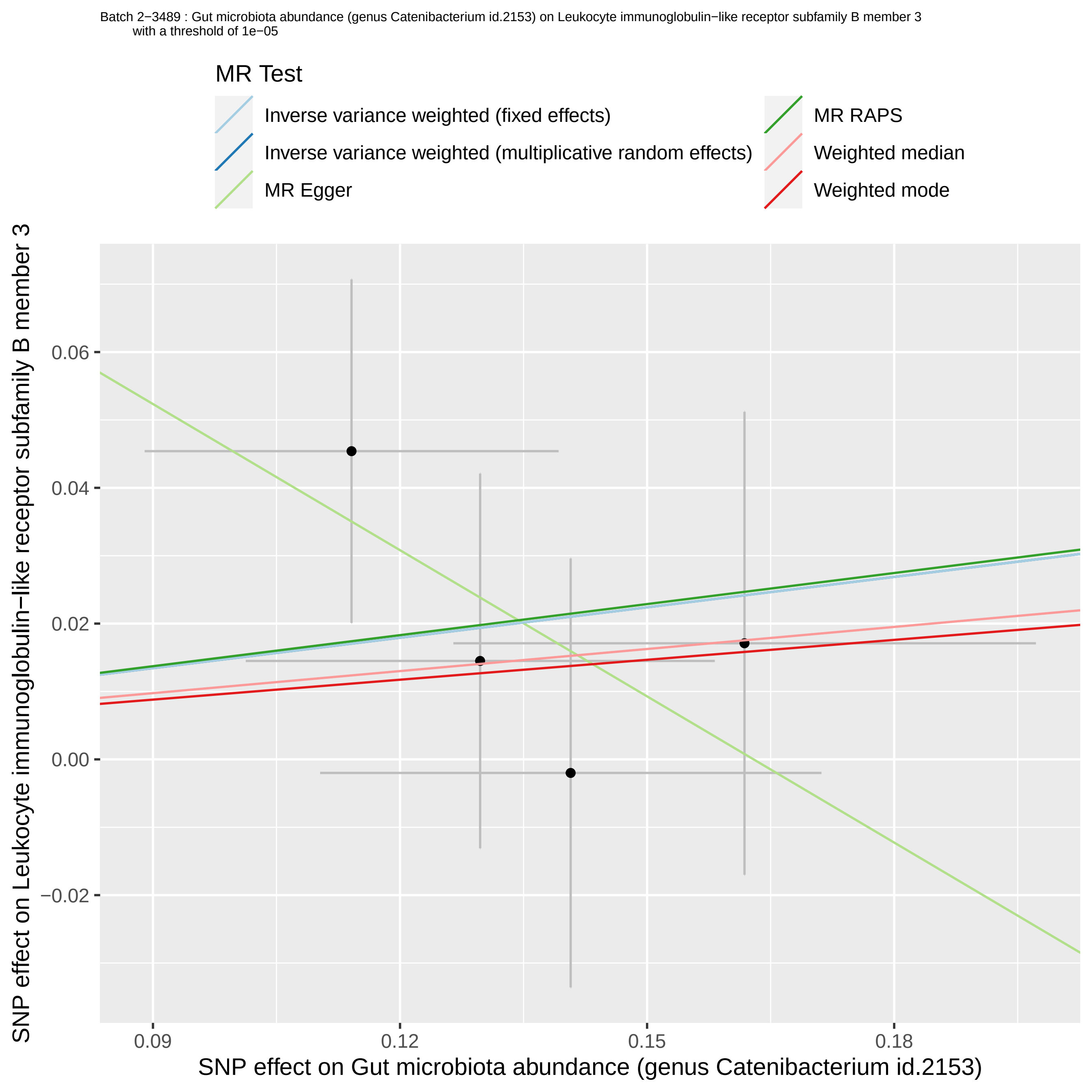

Batch 2−3489 : Gut microbiota abundance (genus Catenibacterium id.2153) on Leukocyte immunoglobulin−like receptor subfamily B member 3
with a threshold of 1e−05
MR Test
Inverse variance weighted (fixed effects)
Inverse variance weighted (multiplicative random effects)
MR Egger
MR RAPS
Weighted median
Weighted mode
SNP effect on Leukocyte immunoglobulin−like receptor subfamily B member 3
0.06
0.04
0.02
0.00
−0.02
0.09
0.12
0.15
0.18
SNP effect on Gut microbiota abundance (genus Catenibacterium id.2153)

Batch 2−3490 : Gut microbiota abundance (genus Parasutterella id.2892) on Leukocyte immunoglobulin−like receptor subfamily B member 3
with a threshold of 1e−05

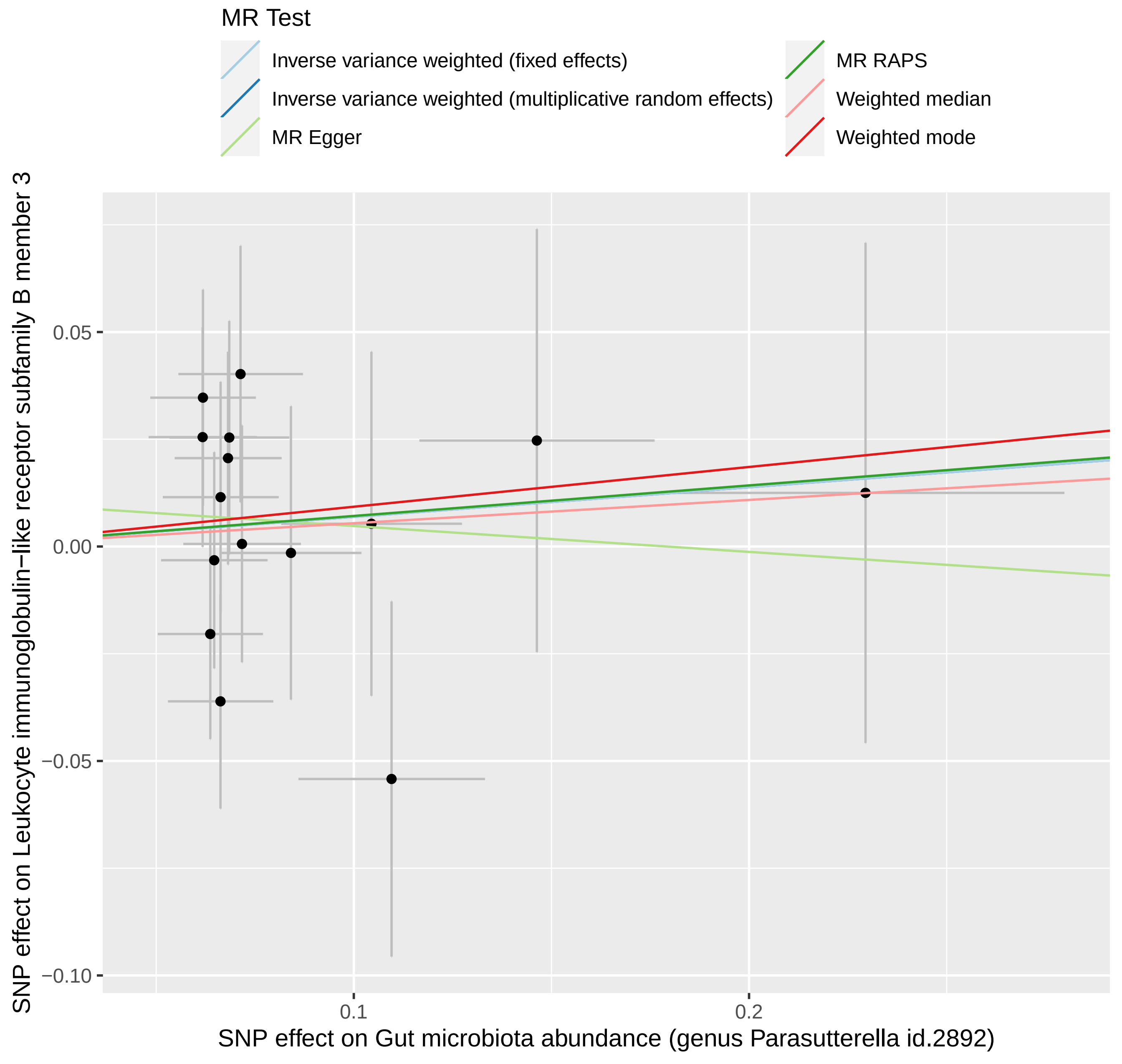

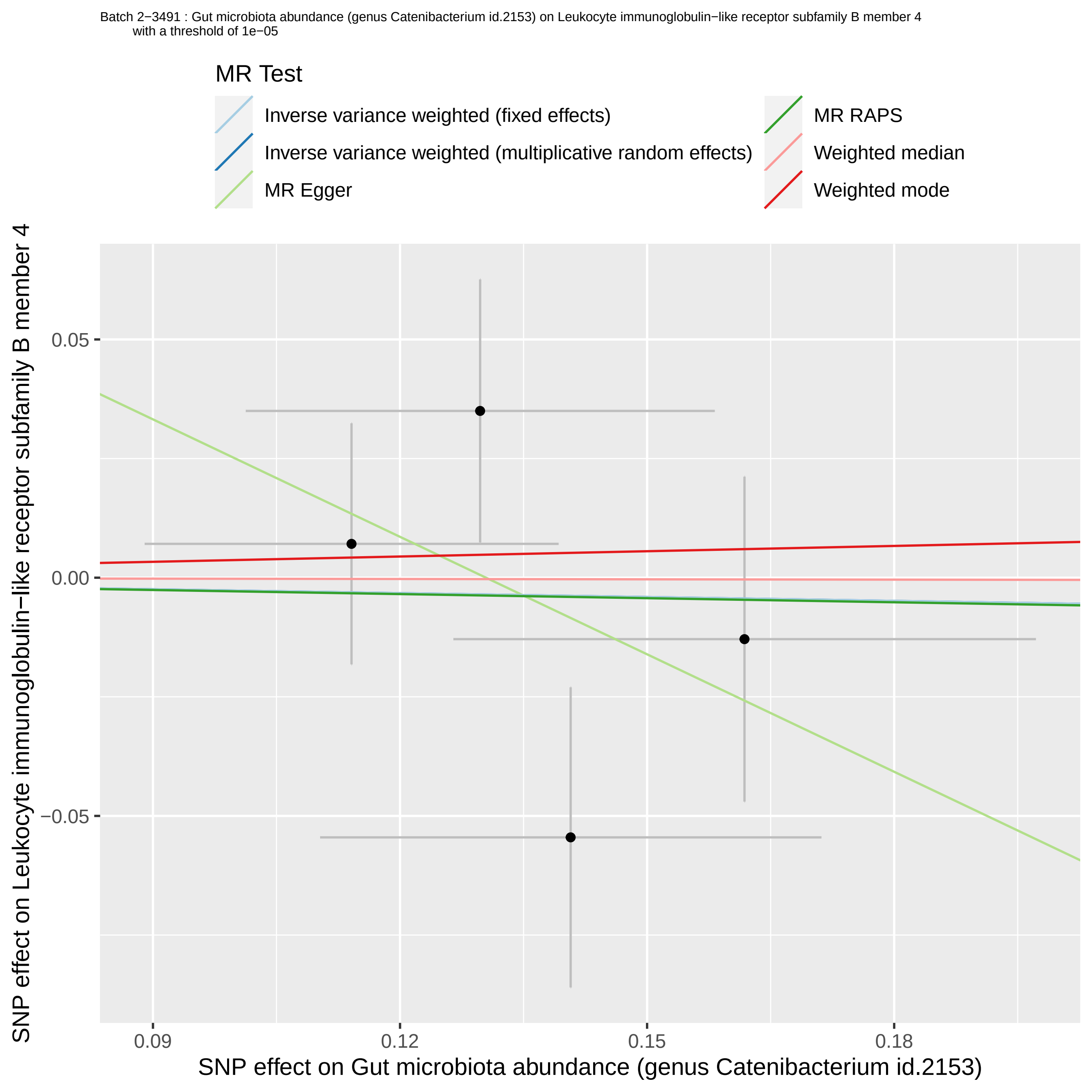

Batch 2−3491 : Gut microbiota abundance (genus Catenibacterium id.2153) on Leukocyte immunoglobulin−like receptor subfamily B member 4
with a threshold of 1e−05
MR Test
Inverse variance weighted (fixed effects)
Inverse variance weighted (multiplicative random effects)
MR Egger
MR RAPS
Weighted median
Weighted mode
SNP effect on Leukocyte immunoglobulin−like receptor subfamily B member 4
0.05
0.00
−0.05
0.09
0.12
0.15
0.18
SNP effect on Gut microbiota abundance (genus Catenibacterium id.2153)

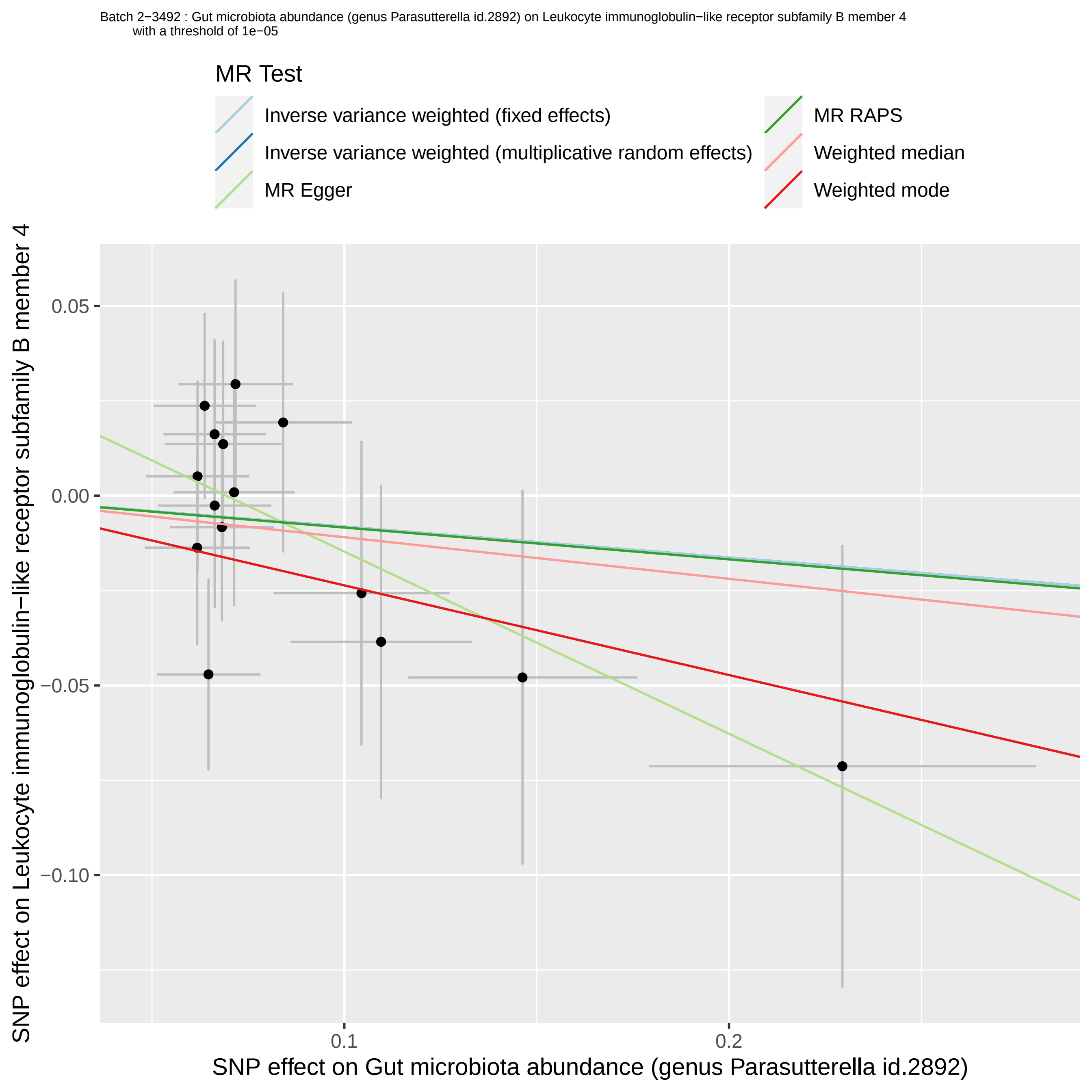

Batch 2−3492 : Gut microbiota abundance (genus Parasutterella id.2892) on Leukocyte immunoglobulin−like receptor subfamily B member 4
with a threshold of 1e−05
MR Test
Inverse variance weighted (fixed effects)
Inverse variance weighted (multiplicative random effects)
MR Egger
MR RAPS
Weighted median
Weighted mode
0.05
0.00
−0.05
−0.10
0.1
0.2
SNP effect on Leukocyte immunoglobulin−like receptor subfamily B member 4
SNP effect on Gut microbiota abundance (genus Parasutterella id.2892)

Batch 2−3493 : Gut microbiota abundance (genus Catenibacterium id.2153) on Leukocyte immunoglobulin−like receptor subfamily B member 5 with a threshold of 1e−05

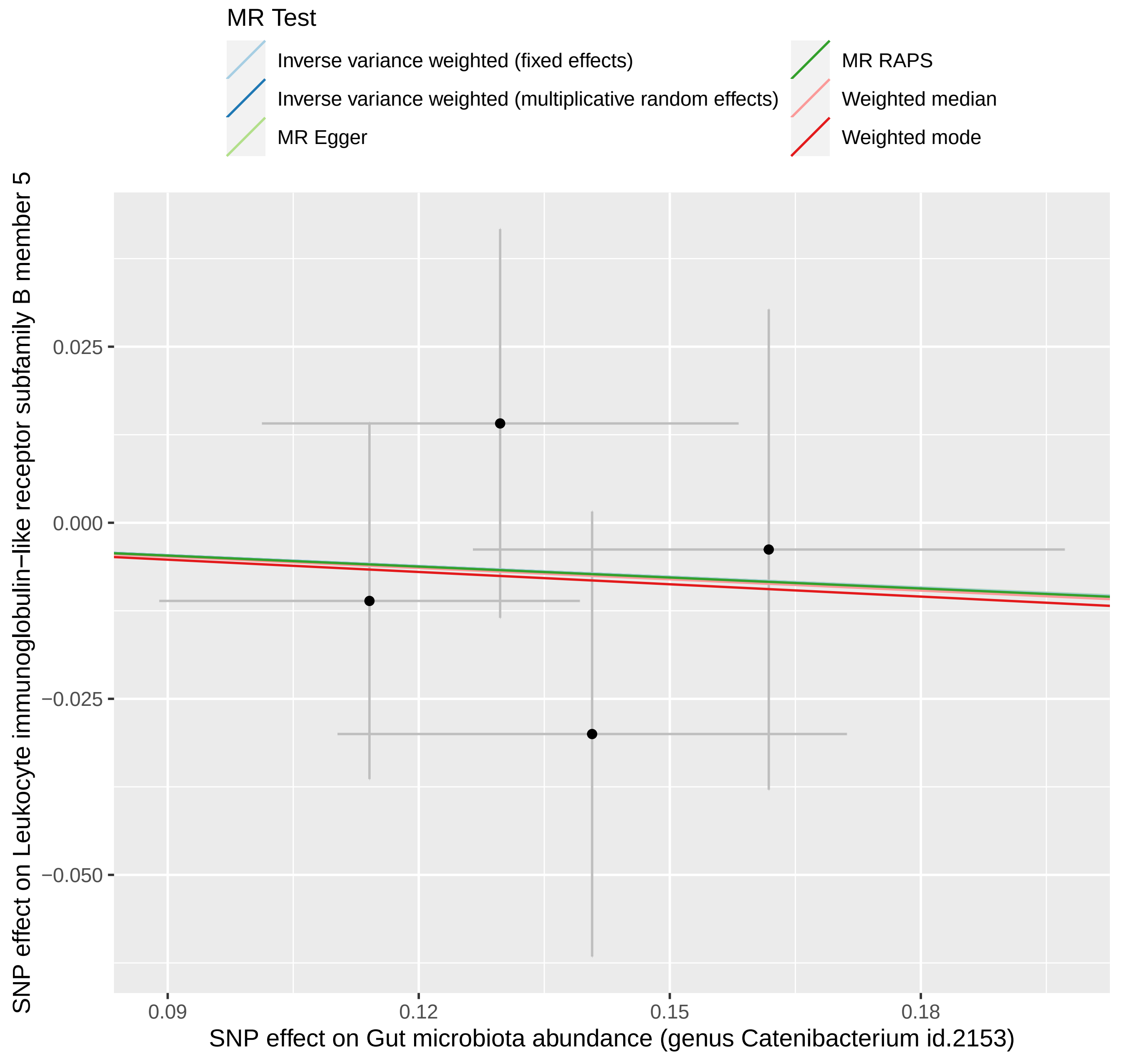

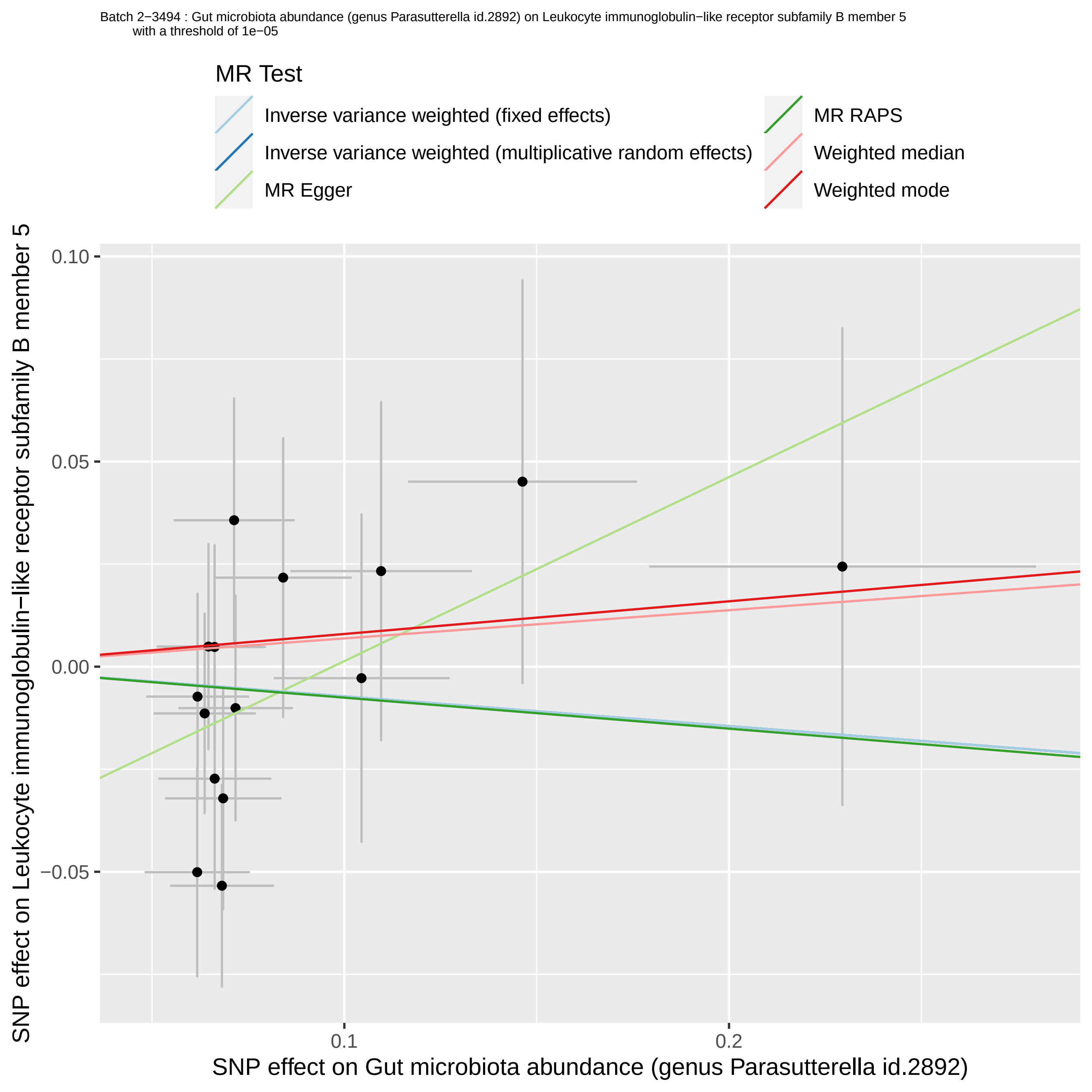
Batch 2−3494 : Gut microbiota abundance (genus Parasutterella id.2892) on Leukocyte immunoglobulin−like receptor subfamily B member 5
with a threshold of 1e−05
MR Test
Inverse variance weighted (fixed effects)
Inverse variance weighted (multiplicative random effects)
MR Egger
MR RAPS
Weighted median
Weighted mode
0.10
0.05
0.00
−0.05
0.1
0.2
SNP effect on Leukocyte immunoglobulin−like receptor subfamily B member 5
SNP effect on Gut microbiota abundance (genus Parasutterella id.2892)

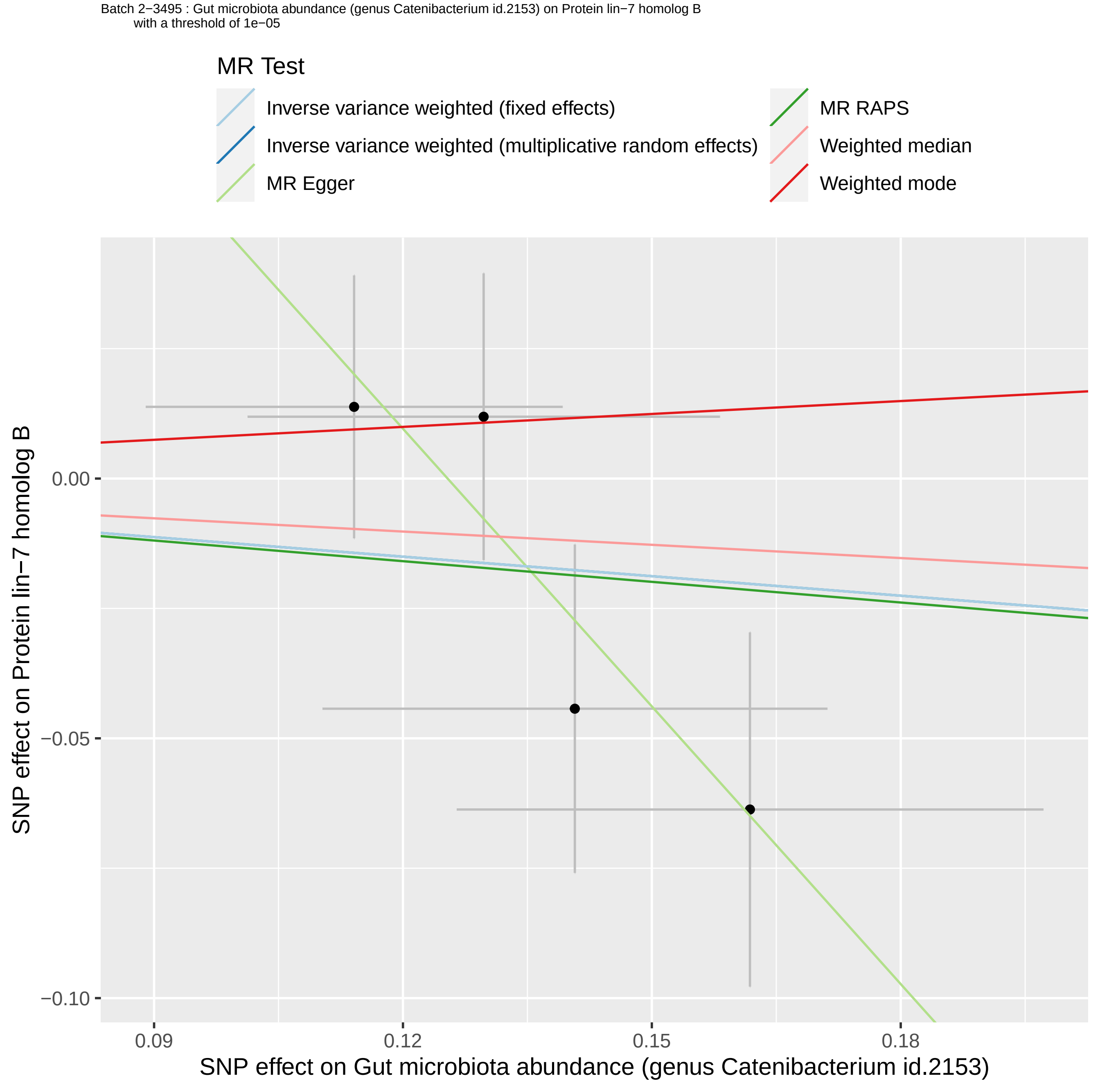

Batch 2−3495 : Gut microbiota abundance (genus Catenibacterium id.2153) on Protein lin−7 homolog B
with a threshold of 1e−05
MR Test
Inverse variance weighted (fixed effects)
Inverse variance weighted (multiplicative random effects)
MR Egger
MR RAPS
Weighted median
Weighted mode
0.00
−0.05
−0.10
0.09
0.12
0.15
0.18
SNP effect on Protein lin−7 homolog B
SNP effect on Gut microbiota abundance (genus Catenibacterium id.2153)

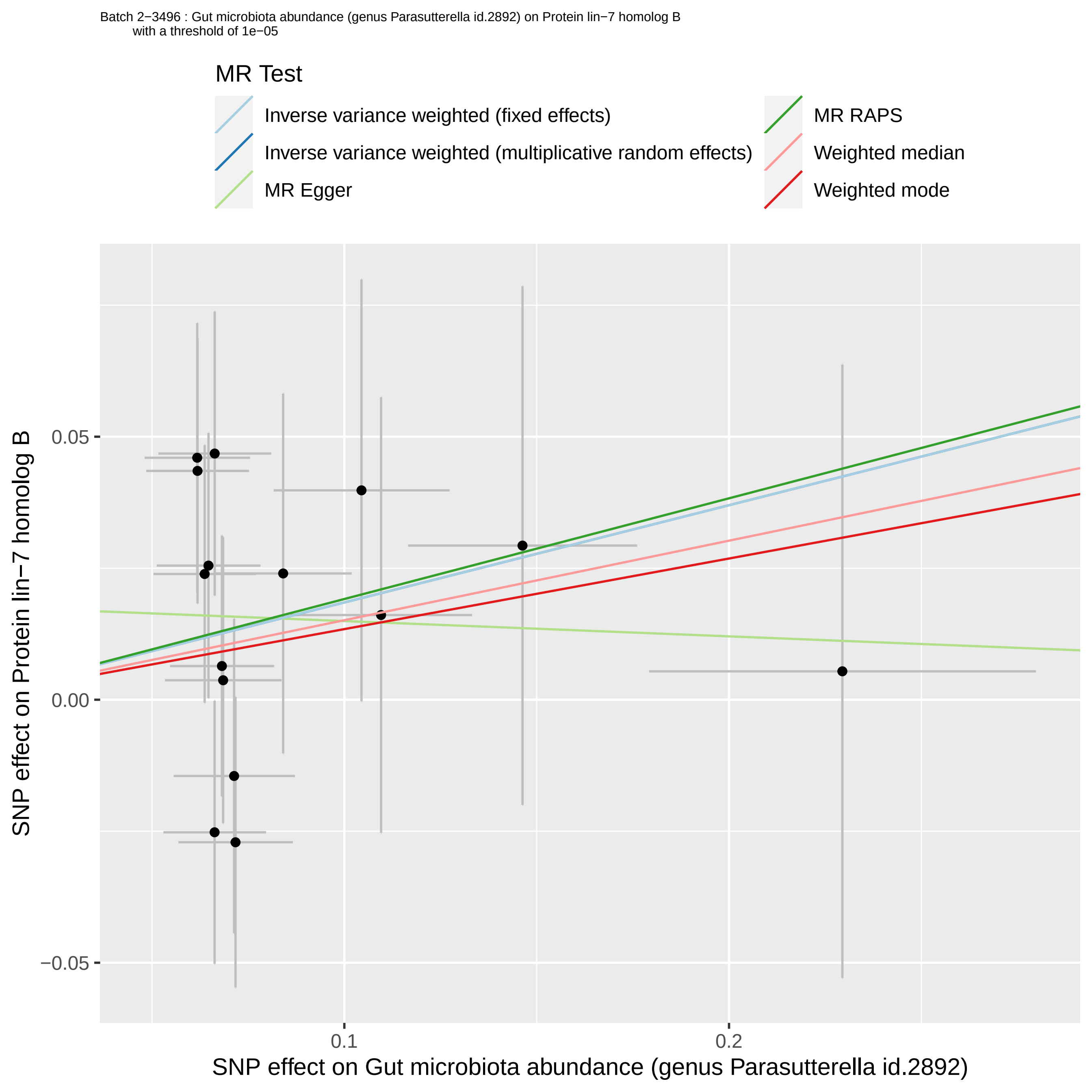
Batch 2−3496 : Gut microbiota abundance (genus Parasutterella id.2892) on Protein lin−7 homolog B
with a threshold of 1e−05
MR Test
Inverse variance weighted (fixed effects)
Inverse variance weighted (multiplicative random effects)
MR Egger
MR RAPS
Weighted median
Weighted mode
0.05
0.00
−0.05
0.1
0.2
SNP effect on Protein lin−7 homolog B
SNP effect on Gut microbiota abundance (genus Parasutterella id.2892)

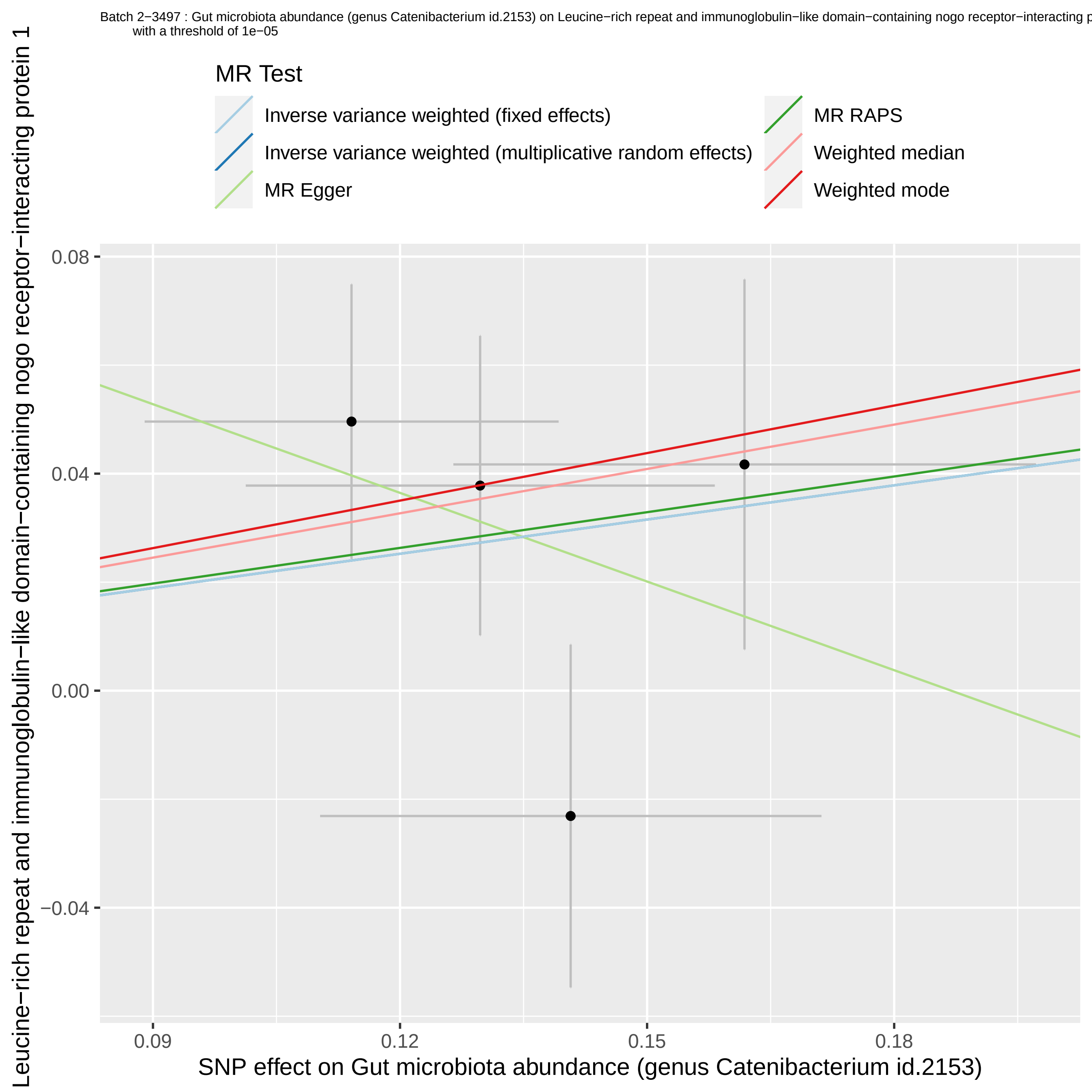
Batch 2−3497 : Gut microbiota abundance (genus Catenibacterium id.2153) on Leucine−rich repeat and immunoglobulin−like domain−containing nogo receptor−interacting p
with a threshold of 1e−05
MR Test
Inverse variance weighted (fixed effects)
Inverse variance weighted (multiplicative random effects)
MR Egger
MR RAPS
Weighted median
Weighted mode
0.08
0.04
0.00
−0.04
0.09
0.12
0.15
0.18
SNP effect on Gut microbiota abundance (genus Catenibacterium id.2153)
Leucine−rich repeat and immunoglobulin−like domain−containing nogo receptor−interacting protein 1

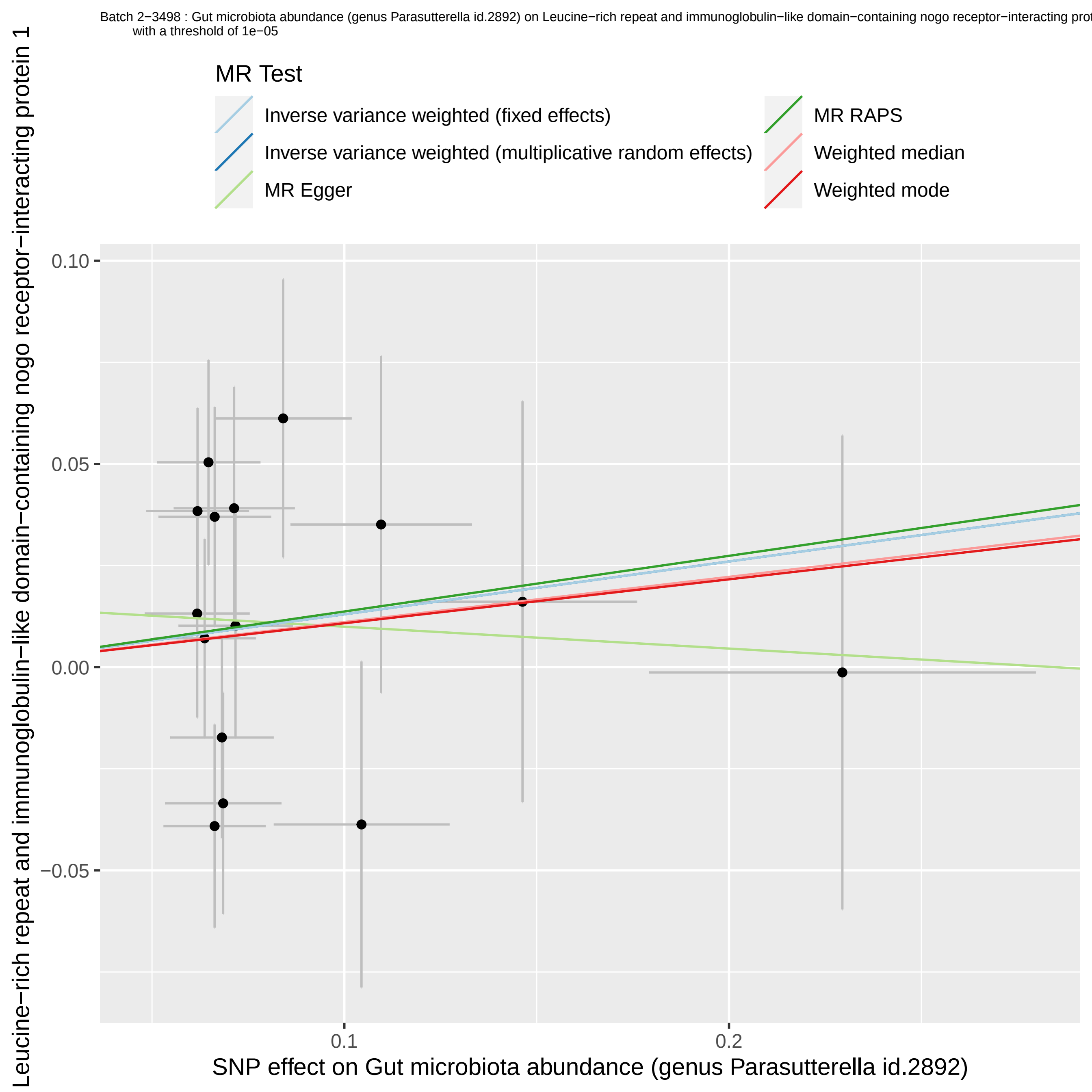

Batch 2−3498 : Gut microbiota abundance (genus Parasutterella id.2892) on Leucine−rich repeat and immunoglobulin−like domain−containing nogo receptor−interacting prot
with a threshold of 1e−05
MR Test
Inverse variance weighted (fixed effects)
Inverse variance weighted (multiplicative random effects)
MR Egger
MR RAPS
Weighted median
Weighted mode
0.10
0.05
0.00
−0.05
0.1
0.2
SNP effect on Gut microbiota abundance (genus Parasutterella id.2892)
Leucine−rich repeat and immunoglobulin−like domain−containing nogo receptor−interacting protein 1

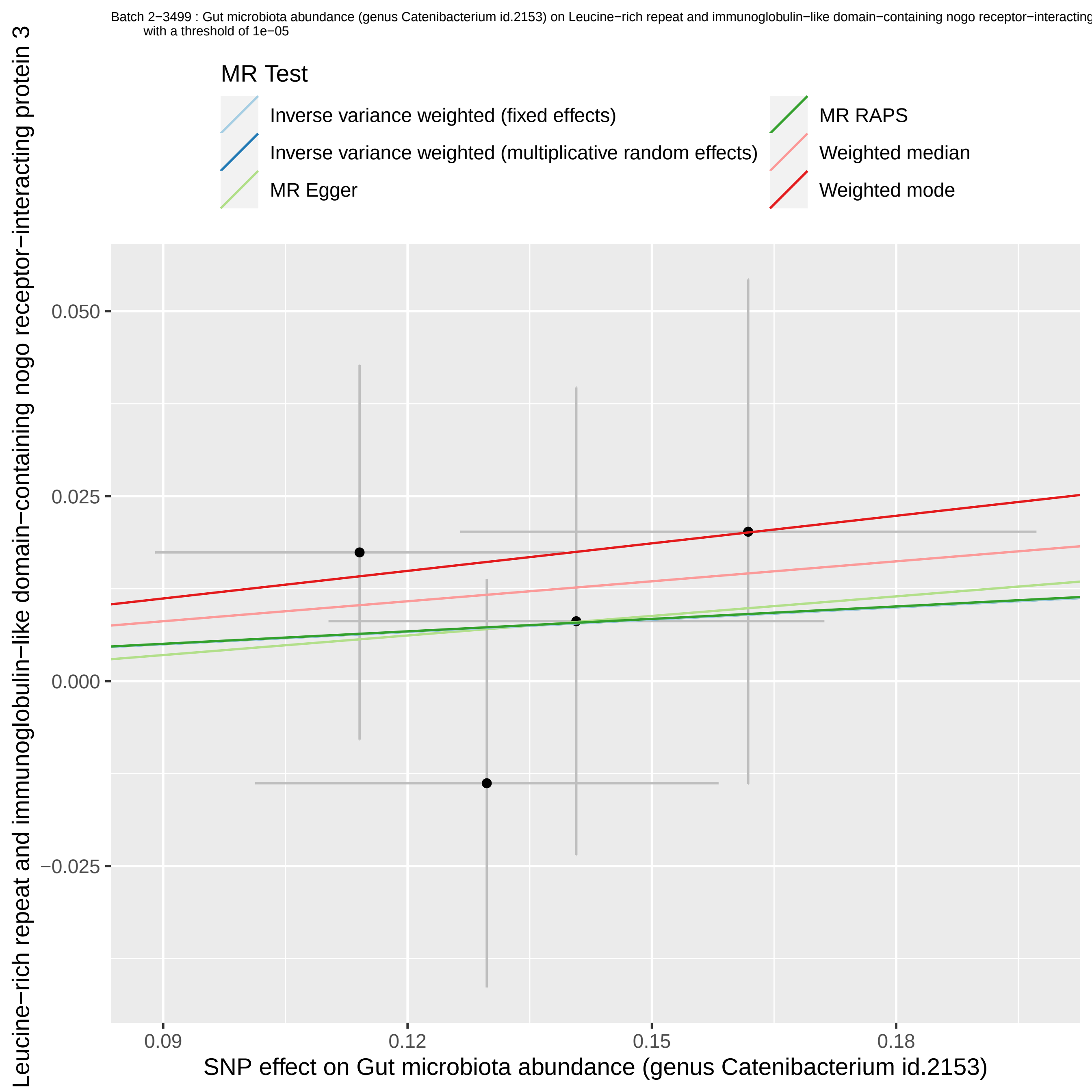
Batch 2−3499 : Gut microbiota abundance (genus Catenibacterium id.2153) on Leucine−rich repeat and immunoglobulin−like domain−containing nogo receptor−interacting
with a threshold of 1e−05
MR Test
Inverse variance weighted (fixed effects)
Inverse variance weighted (multiplicative random effects)
MR Egger
MR RAPS
Weighted median
Weighted mode
0.050
0.025
0.000
−0.025
0.09
0.12
0.15
0.18
SNP effect on Gut microbiota abundance (genus Catenibacterium id.2153)
Leucine−rich repeat and immunoglobulin−like domain−containing nogo receptor−interacting protein 3

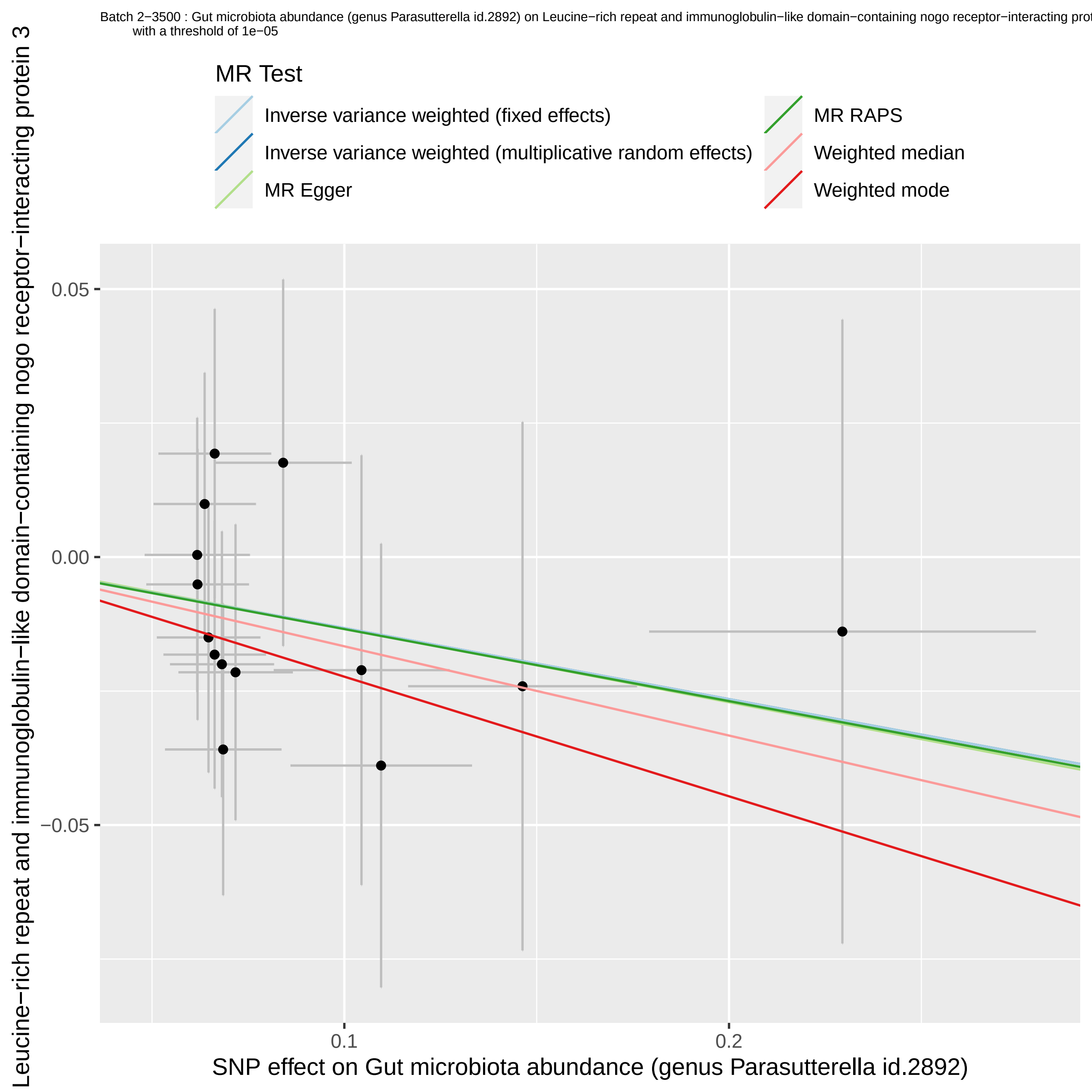

Batch 2−3500 : Gut microbiota abundance (genus Parasutterella id.2892) on Leucine−rich repeat and immunoglobulin−like domain−containing nogo receptor−interacting prot
with a threshold of 1e−05
MR Test
Inverse variance weighted (fixed effects)
Inverse variance weighted (multiplicative random effects)
MR Egger
MR RAPS
Weighted median
Weighted mode
0.05
0.00
−0.05
0.1
0.2
SNP effect on Gut microbiota abundance (genus Parasutterella id.2892)
Leucine−rich repeat and immunoglobulin−like domain−containing nogo receptor−interacting protein 3

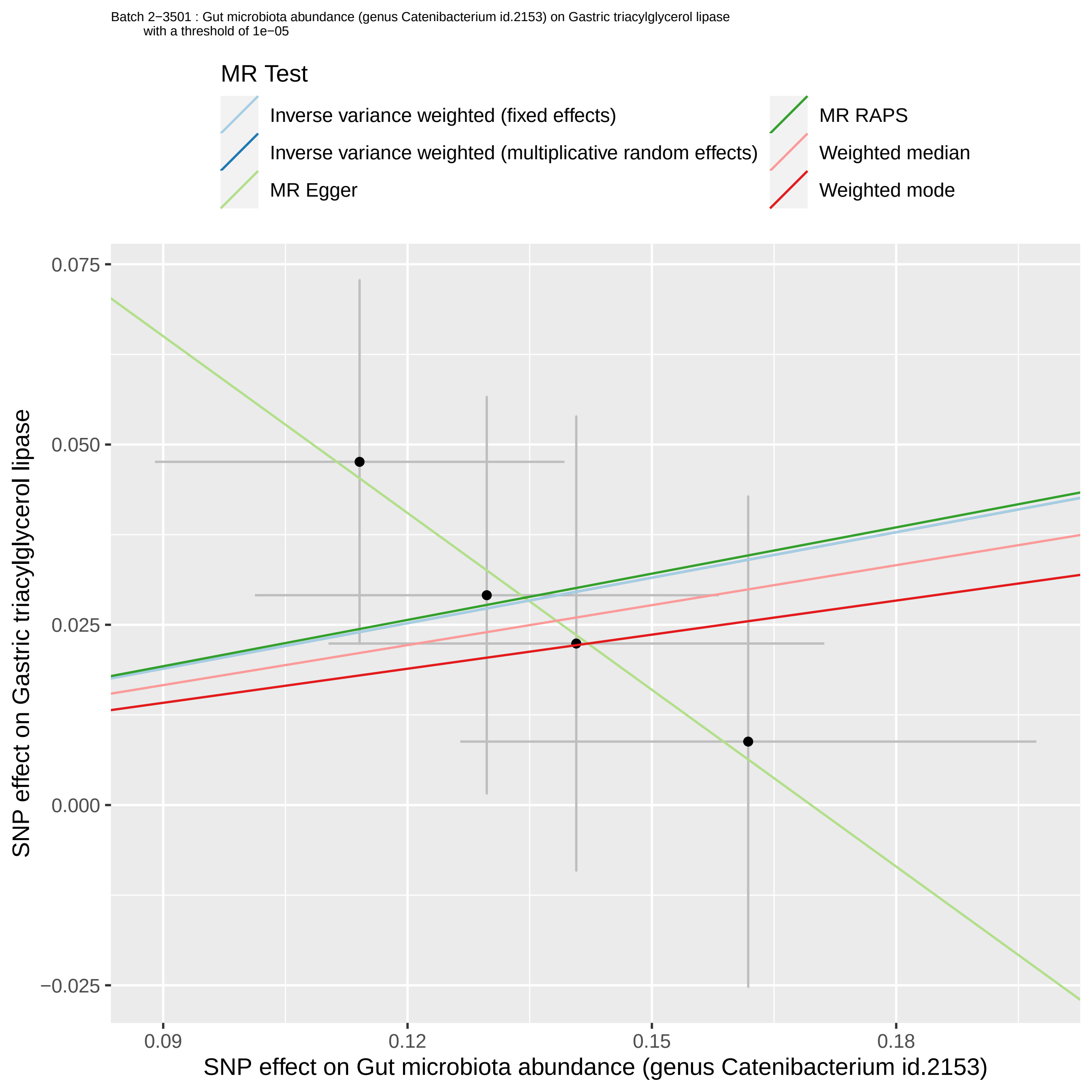

Batch 2−3501 : Gut microbiota abundance (genus Catenibacterium id.2153) on Gastric triacylglycerol lipase
with a threshold of 1e−05
MR Test
Inverse variance weighted (fixed effects)
Inverse variance weighted (multiplicative random effects)
MR Egger
MR RAPS
Weighted median
Weighted mode
0.075
0.050
0.025
0.000
−0.025
0.09
0.12
0.15
0.18
SNP effect on Gastric triacylglycerol lipase
SNP effect on Gut microbiota abundance (genus Catenibacterium id.2153)

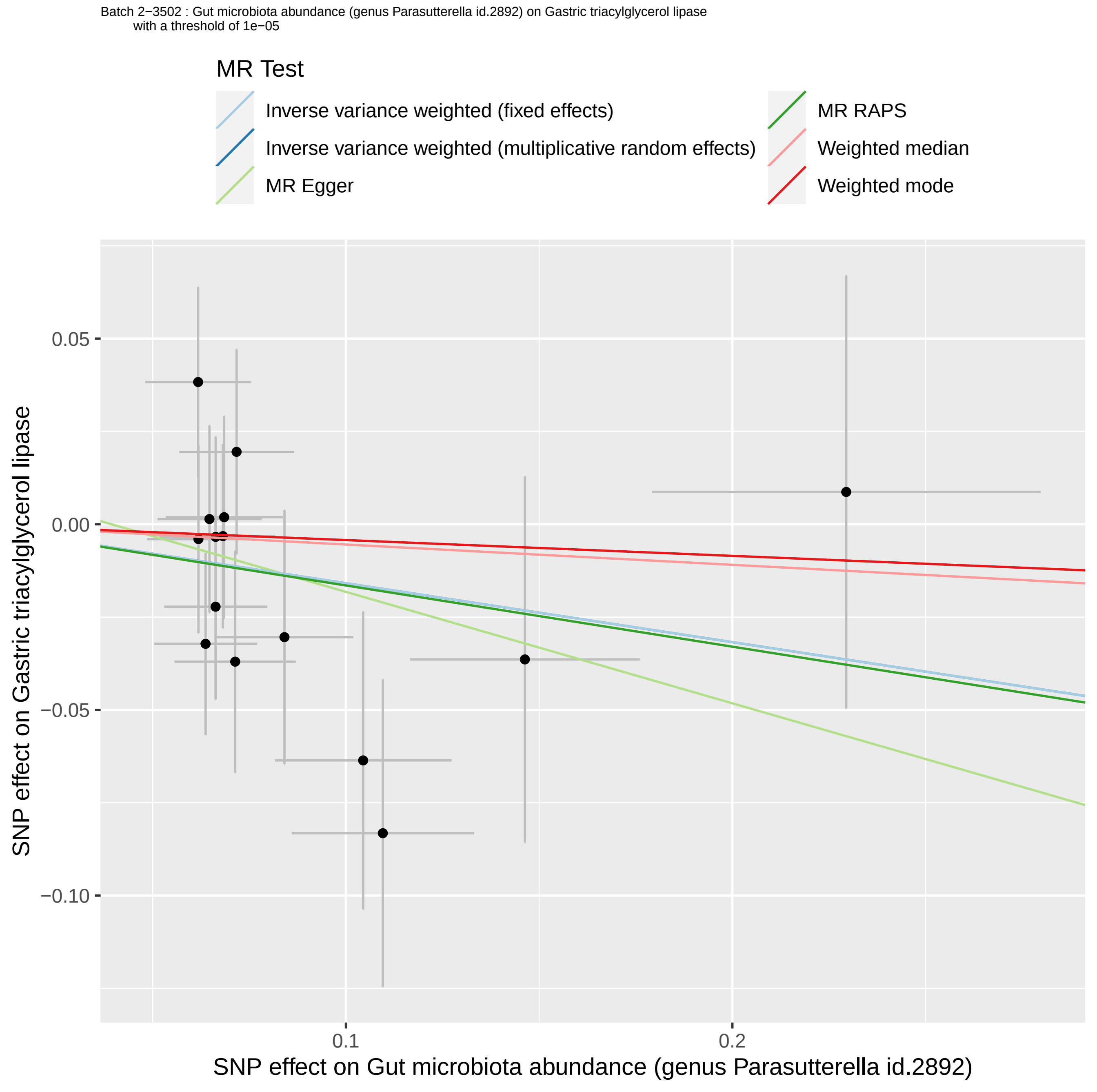
Batch 2−3502 : Gut microbiota abundance (genus Parasutterella id.2892) on Gastric triacylglycerol lipase
with a threshold of 1e−05
MR Test
Inverse variance weighted (fixed effects)
Inverse variance weighted (multiplicative random effects)
MR Egger
MR RAPS
Weighted median
Weighted mode
0.05
0.00
−0.05
−0.10
0.1
0.2
SNP effect on Gastric triacylglycerol lipase
SNP effect on Gut microbiota abundance (genus Parasutterella id.2892)

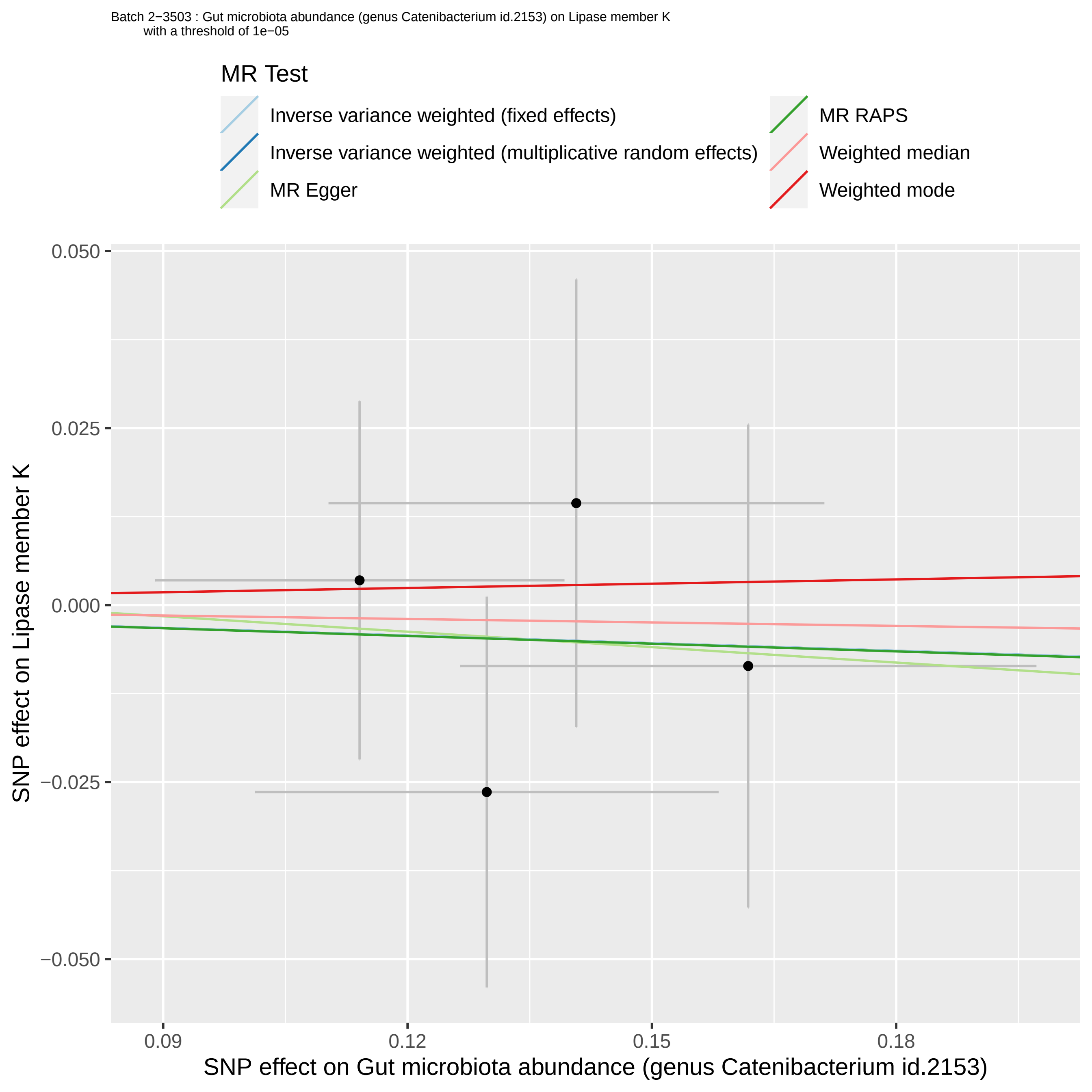

Batch 2−3503 : Gut microbiota abundance (genus Catenibacterium id.2153) on Lipase member K
with a threshold of 1e−05
MR Test
Inverse variance weighted (fixed effects)
Inverse variance weighted (multiplicative random effects)
MR Egger
MR RAPS
Weighted median
Weighted mode
0.050
0.025
0.000
−0.025
−0.050
SNP effect on Lipase member K
0.09
0.12
0.15
0.18
SNP effect on Gut microbiota abundance (genus Catenibacterium id.2153)

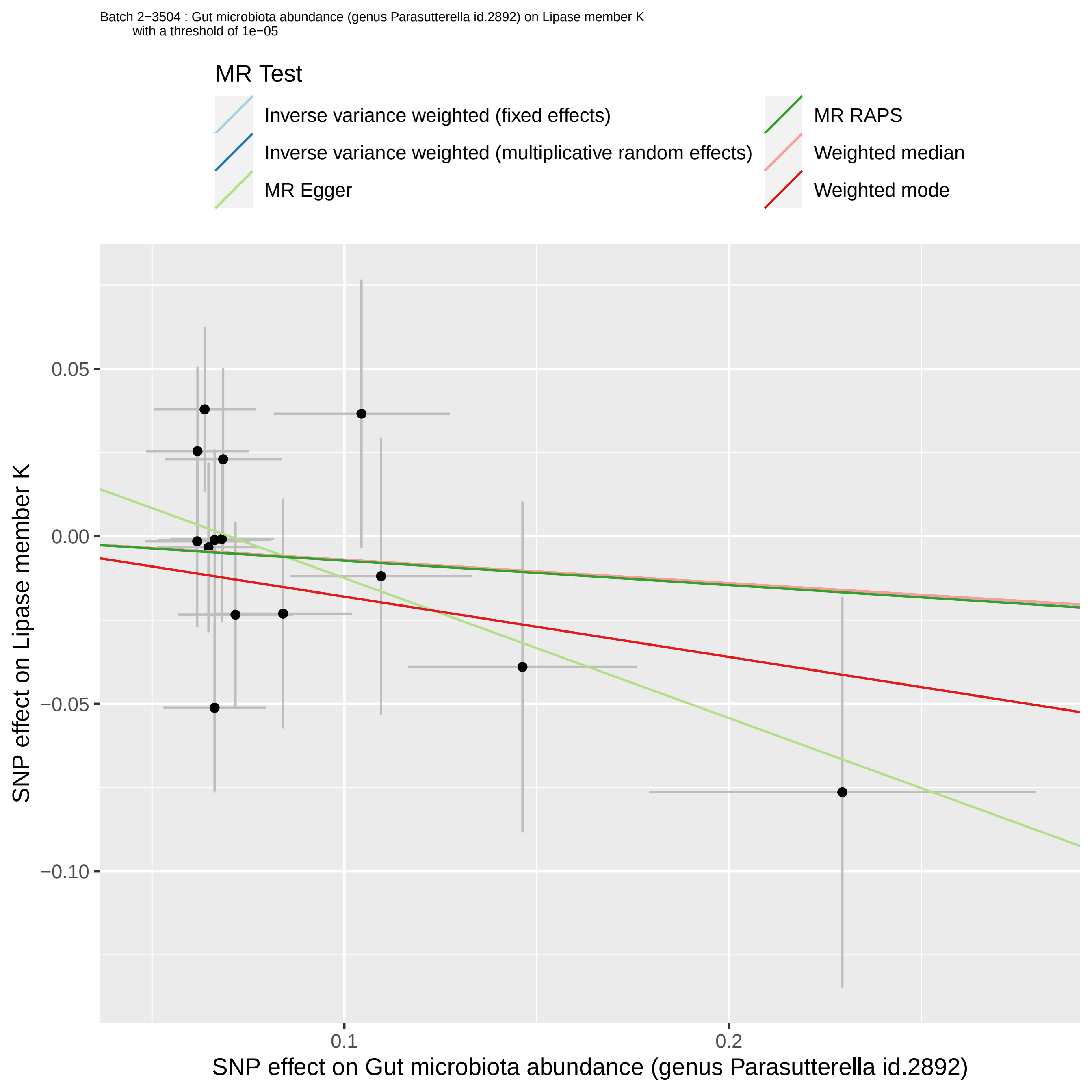

Batch 2−3504 : Gut microbiota abundance (genus Parasutterella id.2892) on Lipase member K
with a threshold of 1e−05
MR Test
Inverse variance weighted (fixed effects)
Inverse variance weighted (multiplicative random effects)
MR Egger
MR RAPS
Weighted median
Weighted mode
0.05
0.00
−0.05
−0.10
0.1
0.2
SNP effect on Lipase member K
SNP effect on Gut microbiota abundance (genus Parasutterella id.2892)

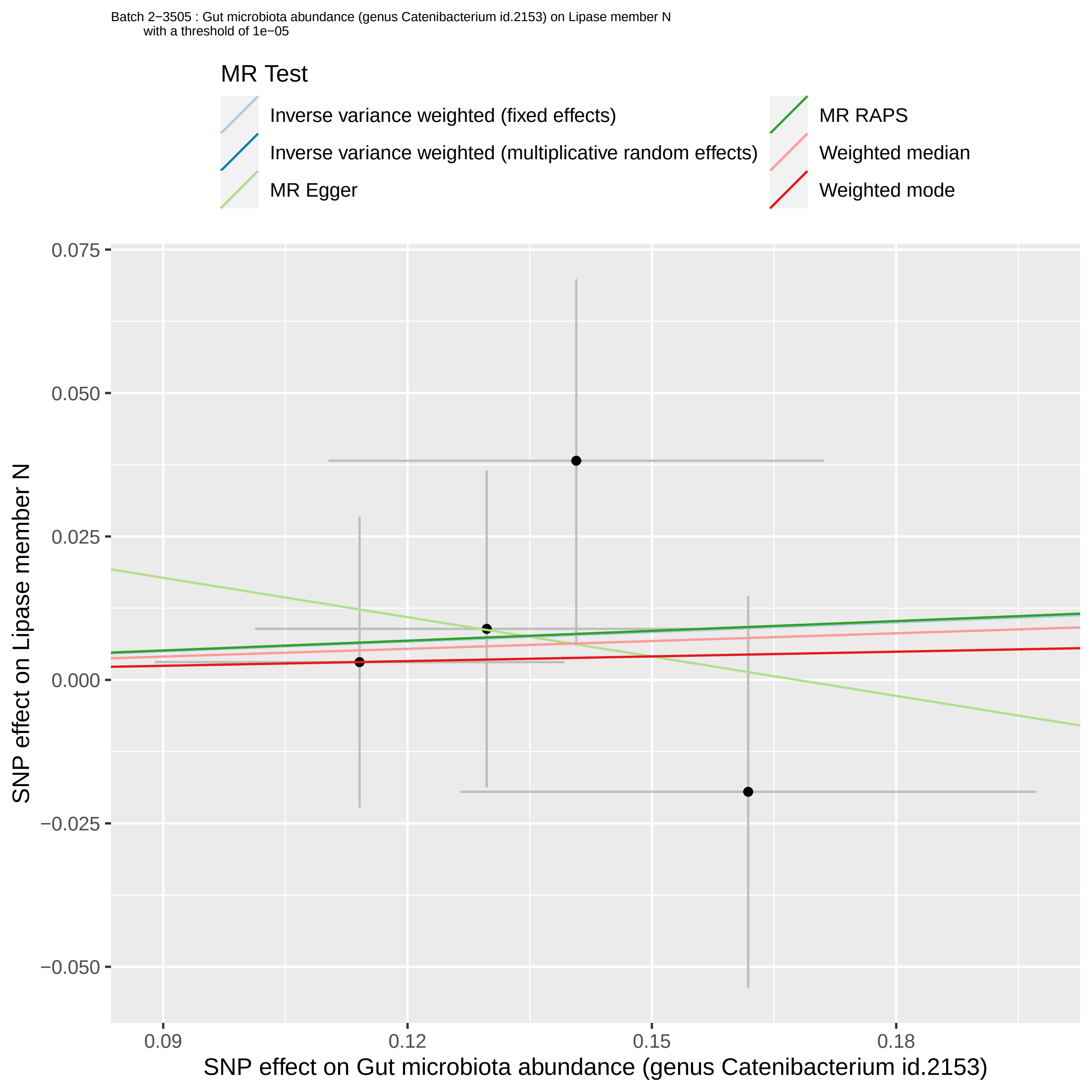
Batch 2−3505 : Gut microbiota abundance (genus Catenibacterium id.2153) on Lipase member N
with a threshold of 1e−05
MR Test
Inverse variance weighted (fixed effects)
Inverse variance weighted (multiplicative random effects)
MR Egger
MR RAPS
Weighted median
Weighted mode
0.075
0.050
0.025
0.000
−0.025
−0.050
0.09
0.12
0.15
0.18
SNP effect on Lipase member N
SNP effect on Gut microbiota abundance (genus Catenibacterium id.2153)

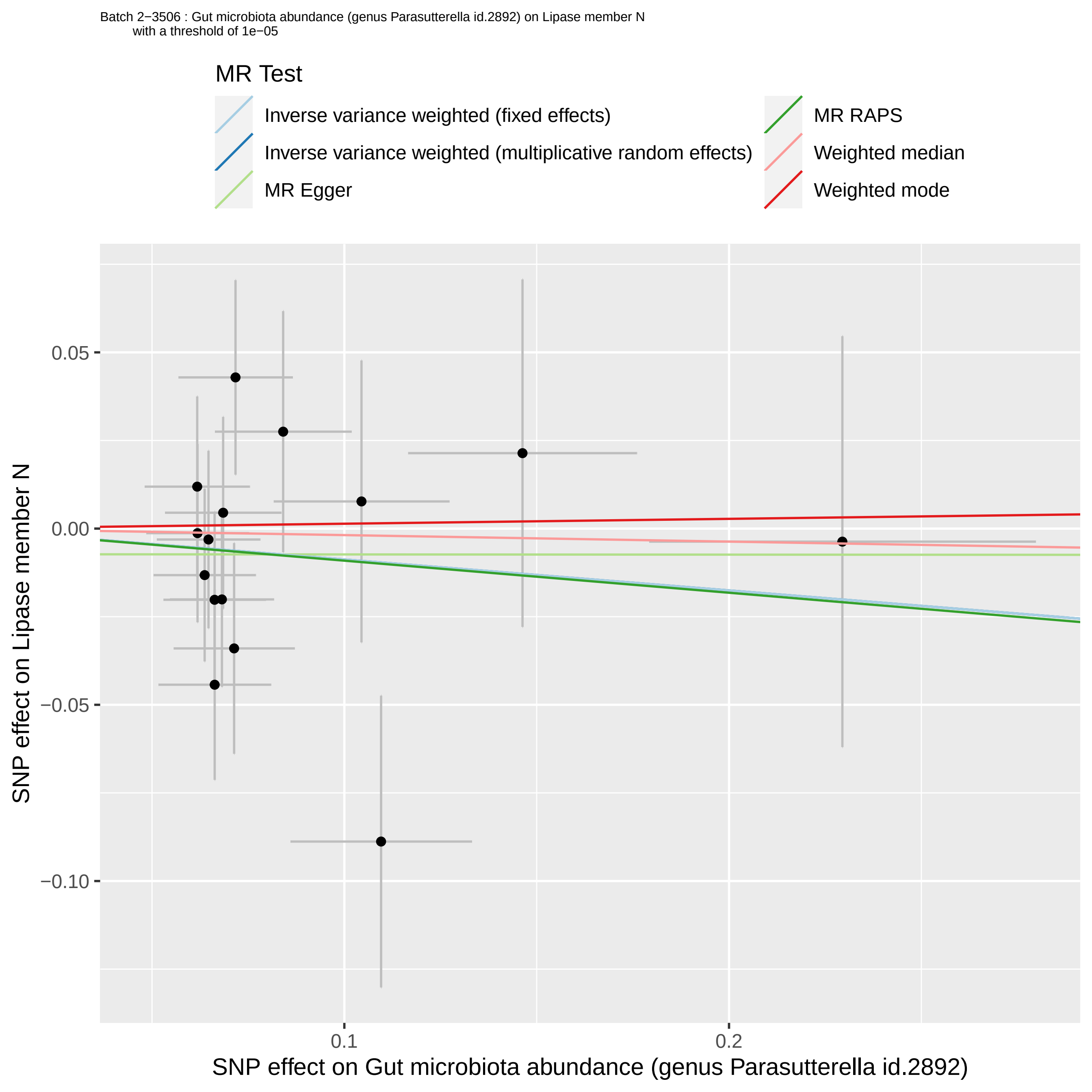
Batch 2−3506 : Gut microbiota abundance (genus Parasutterella id.2892) on Lipase member N
with a threshold of 1e−05
MR Test
Inverse variance weighted (fixed effects)
Inverse variance weighted (multiplicative random effects)
MR Egger
MR RAPS
Weighted median
Weighted mode
0.05
0.00
−0.05
−0.10
0.1
0.2
SNP effect on Lipase member N
SNP effect on Gut microbiota abundance (genus Parasutterella id.2892)

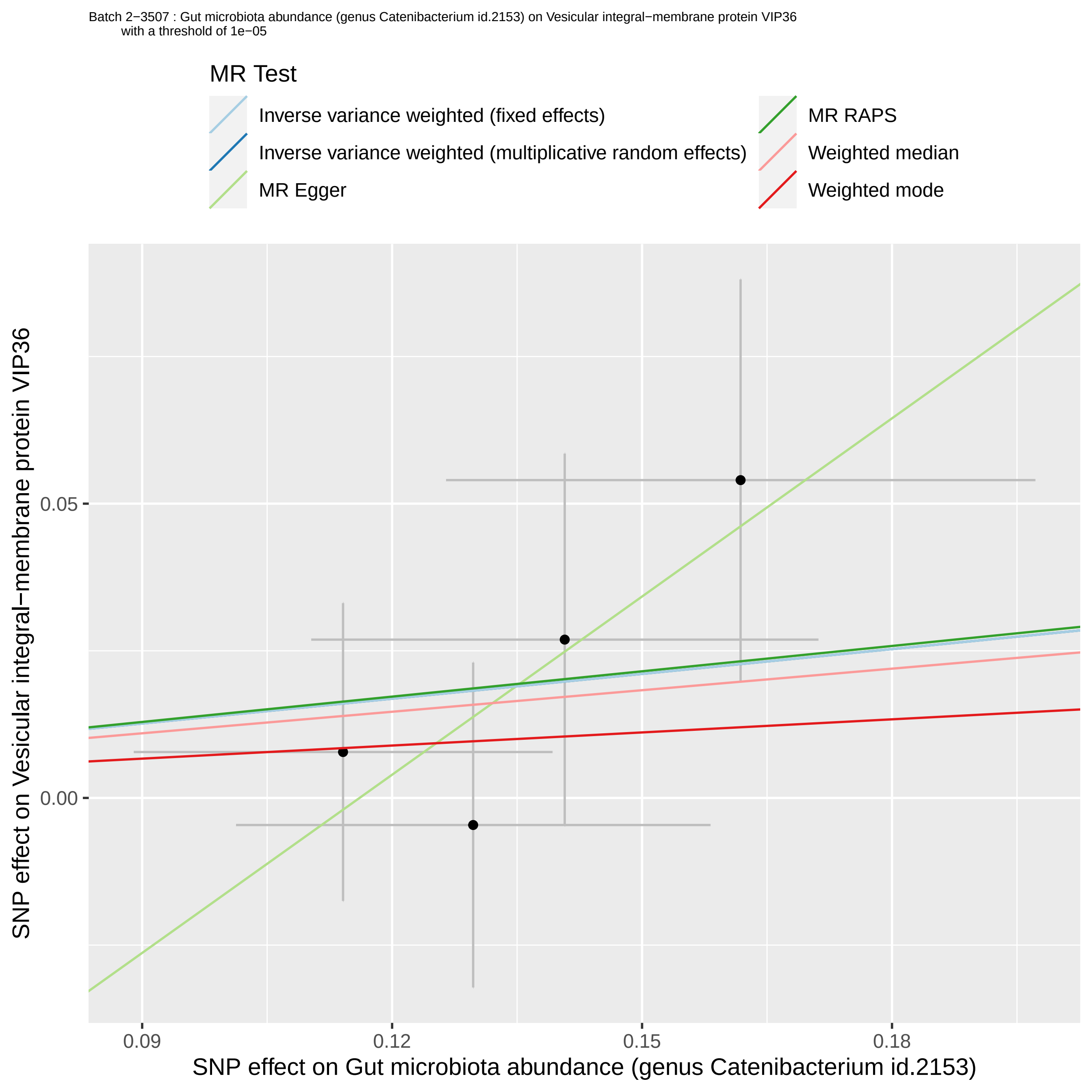
Batch 2−3507 : Gut microbiota abundance (genus Catenibacterium id.2153) on Vesicular integral−membrane protein VIP36
with a threshold of 1e−05
MR Test
Inverse variance weighted (fixed effects)
Inverse variance weighted (multiplicative random effects)
MR Egger
MR RAPS
Weighted median
Weighted mode
0.05
0.00
0.09
0.12
0.15
0.18
SNP effect on Gut microbiota abundance (genus Catenibacterium id.2153)
SNP effect on Vesicular integral−membrane protein VIP36

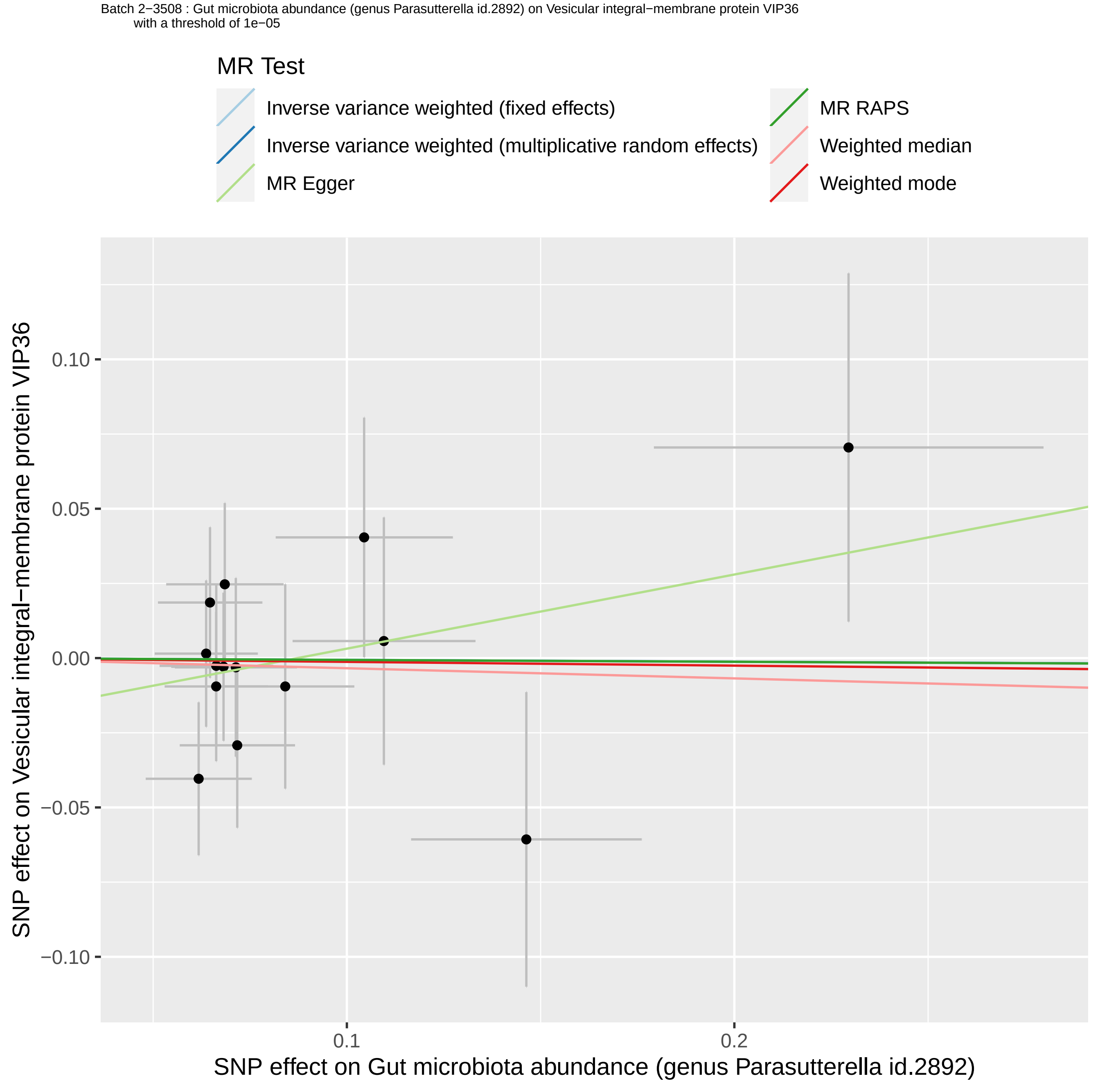
Batch 2−3508 : Gut microbiota abundance (genus Parasutterella id.2892) on Vesicular integral−membrane protein VIP36
with a threshold of 1e−05
MR Test
Inverse variance weighted (fixed effects)
Inverse variance weighted (multiplicative random effects)
MR Egger
MR RAPS
Weighted median
Weighted mode
0.10
0.05
0.00
−0.05
−0.10
0.1
0.2
SNP effect on Vesicular integral−membrane protein VIP36
SNP effect on Gut microbiota abundance (genus Parasutterella id.2892)

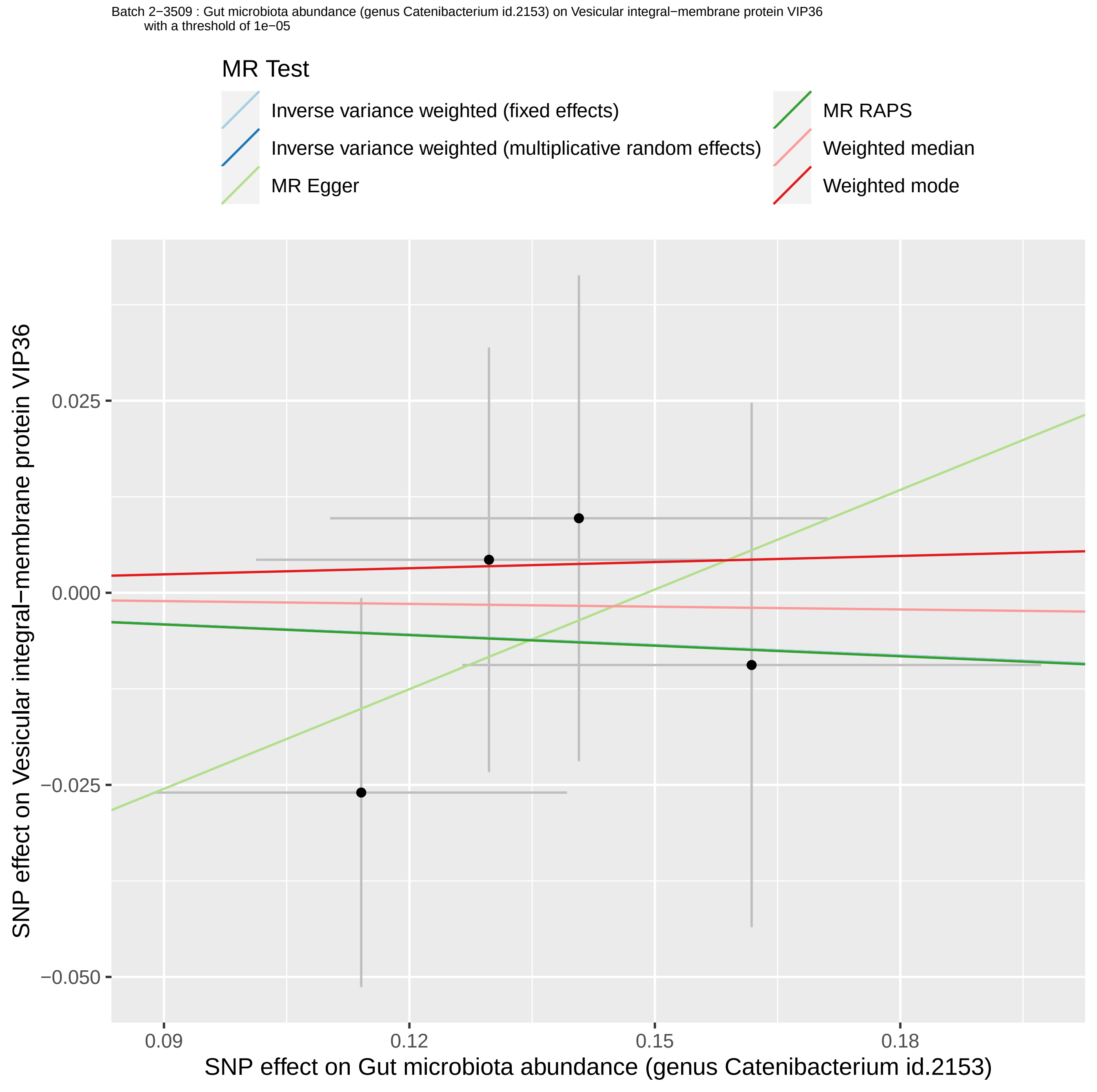

Batch 2−3509 : Gut microbiota abundance (genus Catenibacterium id.2153) on Vesicular integral−membrane protein VIP36
with a threshold of 1e−05
MR Test
Inverse variance weighted (fixed effects)
Inverse variance weighted (multiplicative random effects)
MR Egger
MR RAPS
Weighted median
Weighted mode
0.025
0.000
−0.025
−0.050
0.09
0.12
0.15
0.18
SNP effect on Vesicular integral−membrane protein VIP36
SNP effect on Gut microbiota abundance (genus Catenibacterium id.2153)

Batch 2−3510 : Gut microbiota abundance (genus Parasutterella id.2892) on Vesicular integral−membrane protein VIP36
with a threshold of 1e−05

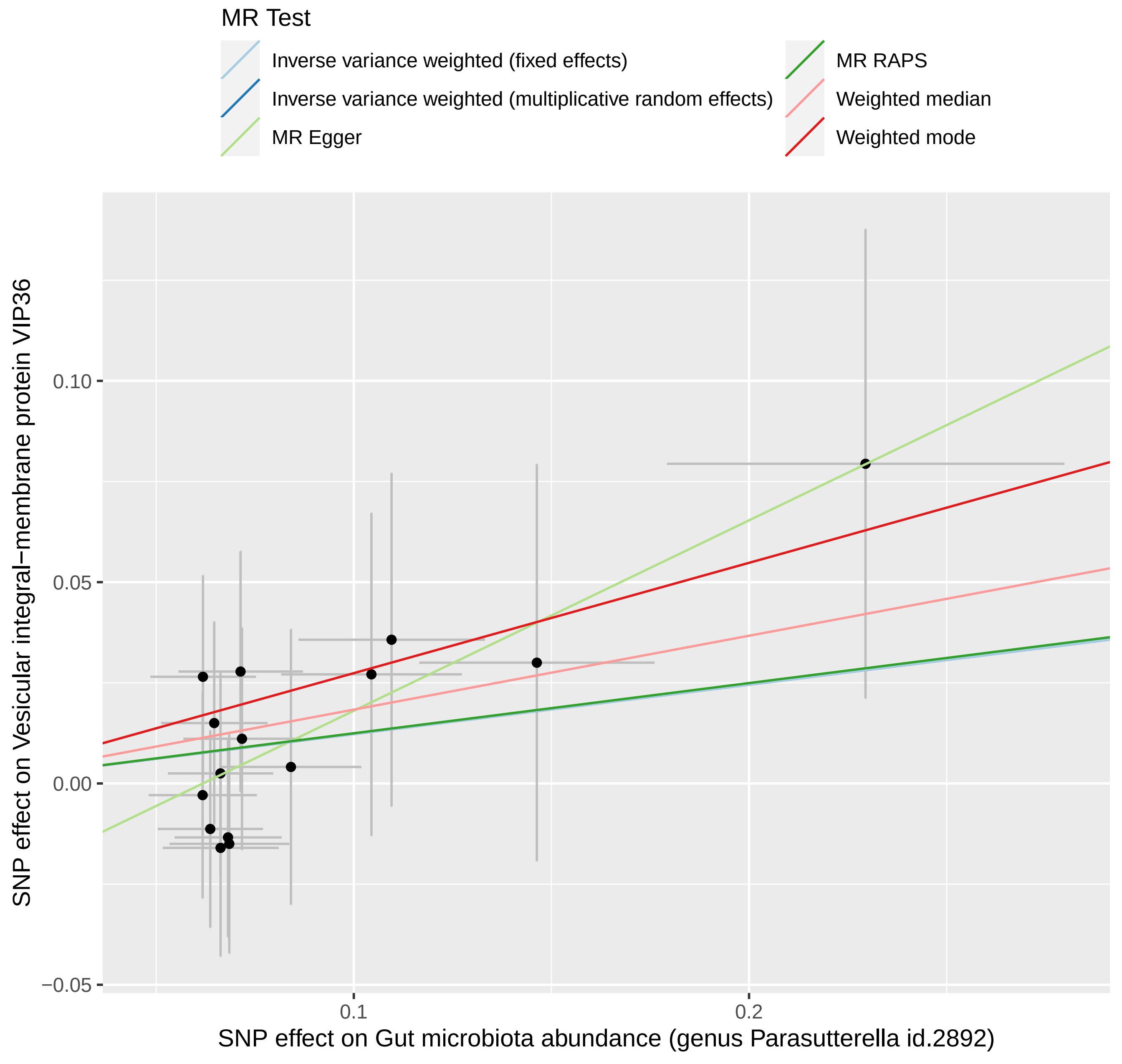

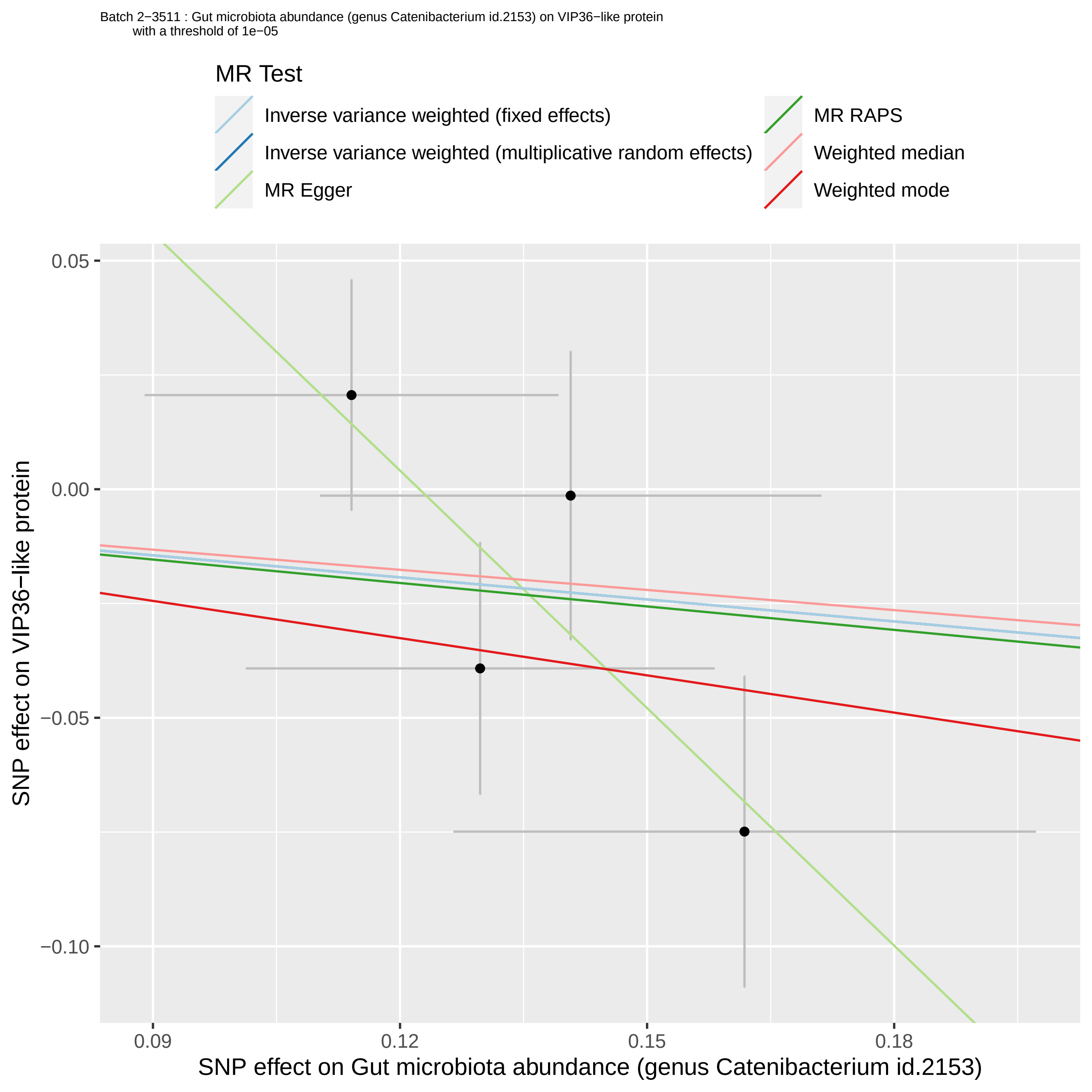
Batch 2−3511 : Gut microbiota abundance (genus Catenibacterium id.2153) on VIP36−like protein
with a threshold of 1e−05
MR Test
Inverse variance weighted (fixed effects)
Inverse variance weighted (multiplicative random effects)
MR Egger
MR RAPS
Weighted median
Weighted mode
0.05
0.00
−0.05
−0.10
0.09
0.12
0.15
0.18
SNP effect on VIP36−like protein
SNP effect on Gut microbiota abundance (genus Catenibacterium id.2153)

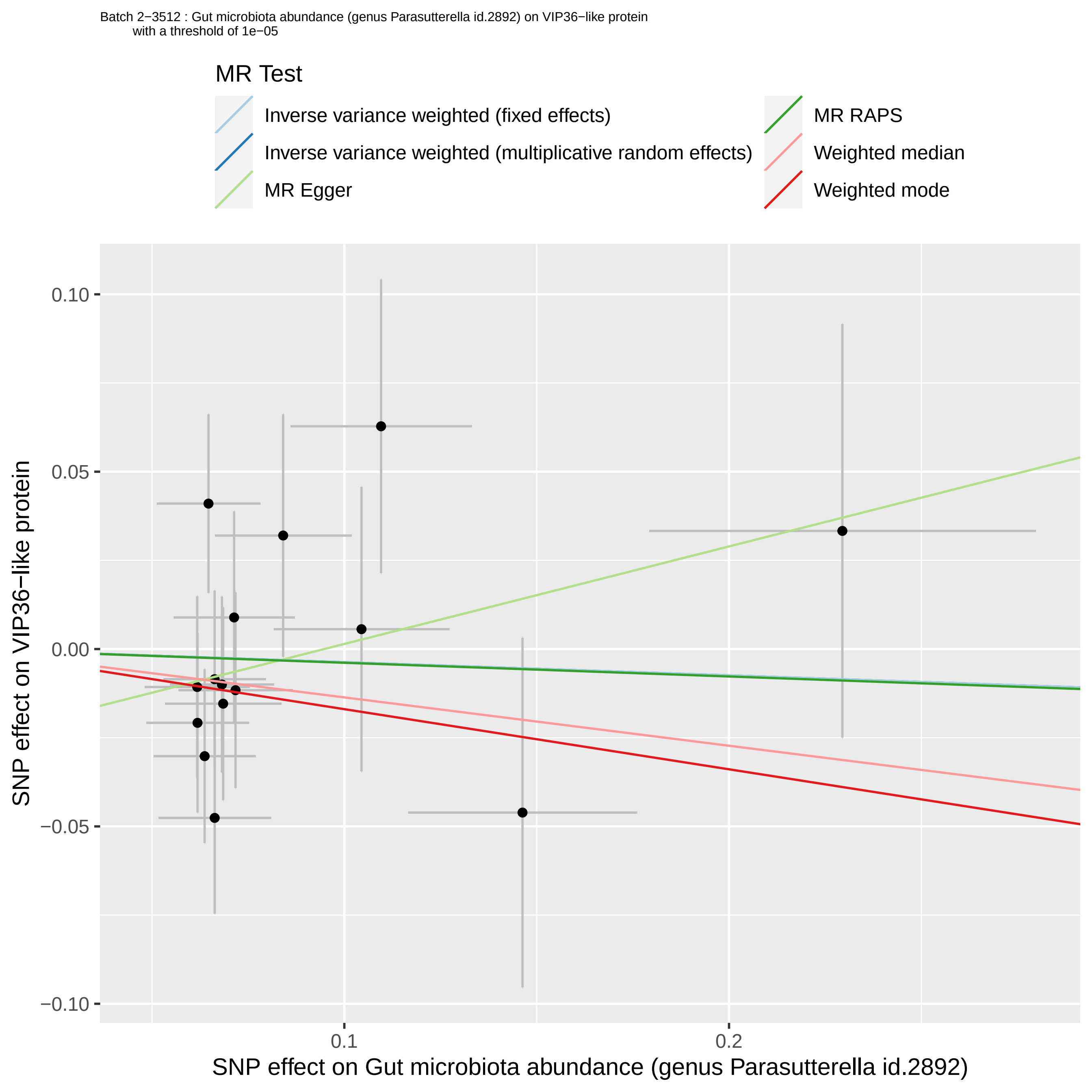

Batch 2−3512 : Gut microbiota abundance (genus Parasutterella id.2892) on VIP36−like protein
with a threshold of 1e−05
MR Test
Inverse variance weighted (fixed effects)
Inverse variance weighted (multiplicative random effects)
MR Egger
MR RAPS
Weighted median
Weighted mode
SNP effect on VIP36−like protein
0.10
0.05
0.00
−0.05
−0.10
0.1
0.2
SNP effect on Gut microbiota abundance (genus Parasutterella id.2892)

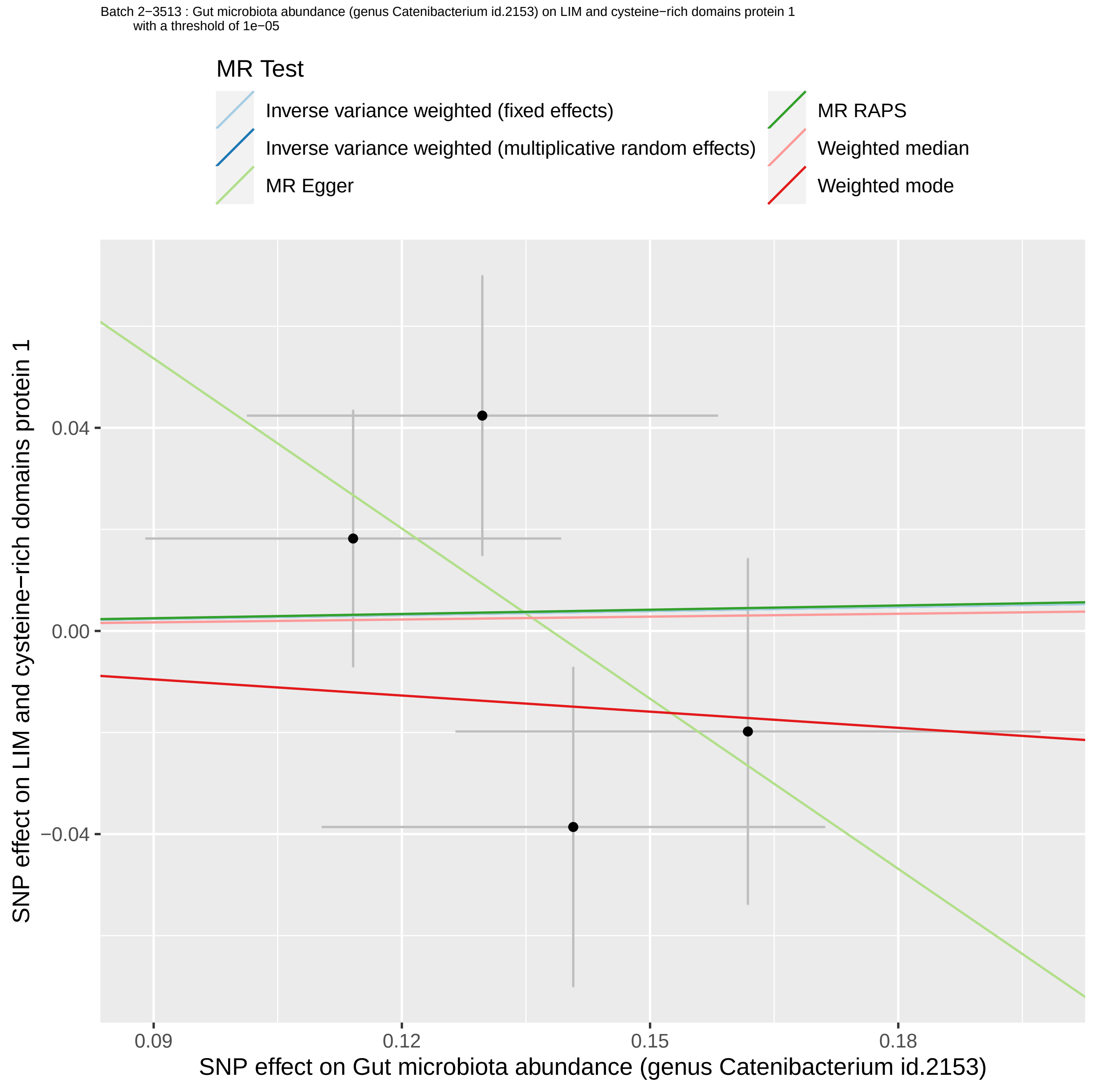
Batch 2−3513 : Gut microbiota abundance (genus Catenibacterium id.2153) on LIM and cysteine−rich domains protein 1
with a threshold of 1e−05
MR Test
Inverse variance weighted (fixed effects)
Inverse variance weighted (multiplicative random effects)
MR Egger
MR RAPS
Weighted median
Weighted mode
0.04
0.00
−0.04
0.09
0.12
0.15
0.18
SNP effect on LIM and cysteine−rich domains protein 1
SNP effect on Gut microbiota abundance (genus Catenibacterium id.2153)

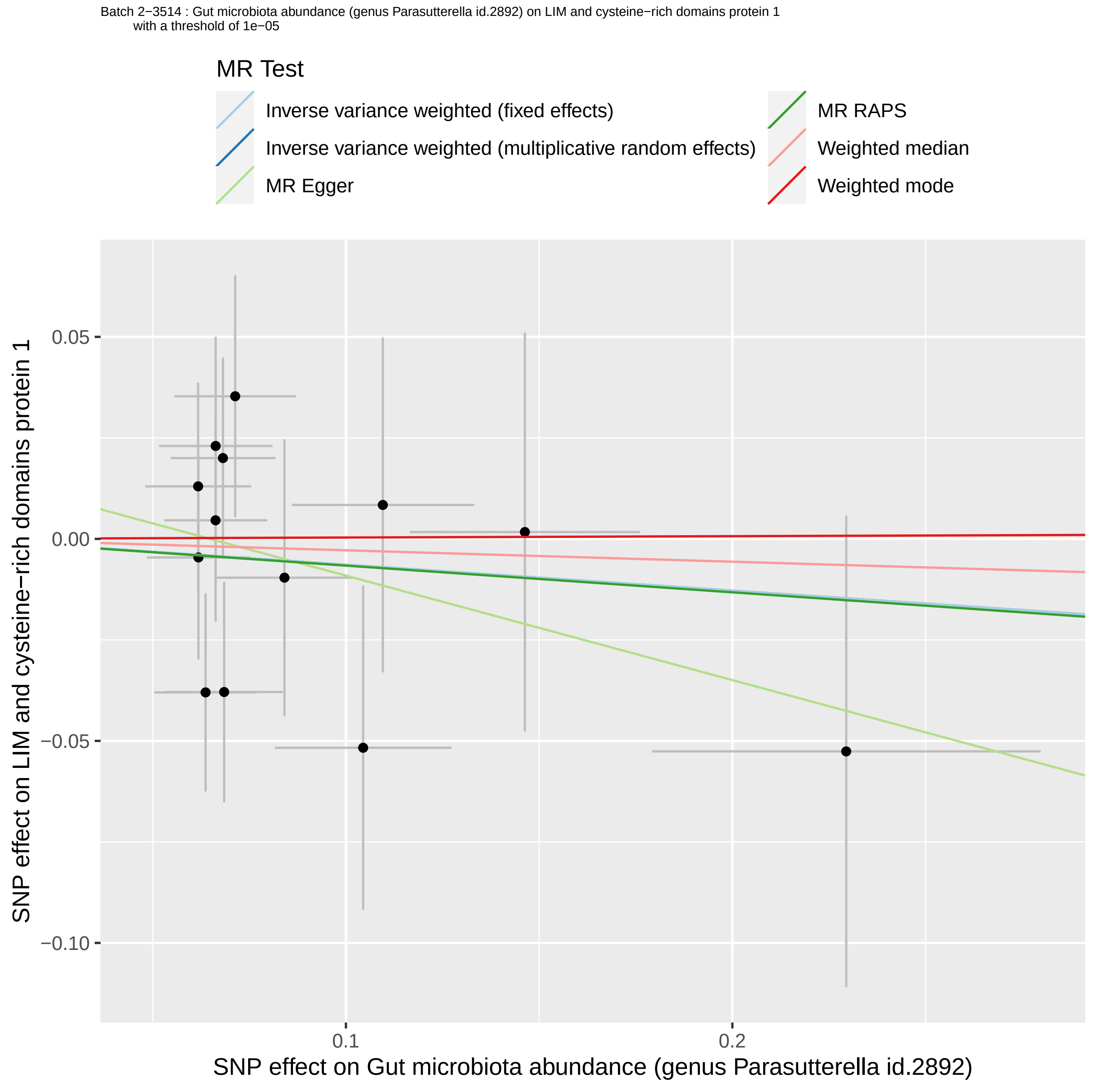
Batch 2−3514 : Gut microbiota abundance (genus Parasutterella id.2892) on LIM and cysteine−rich domains protein 1
with a threshold of 1e−05
MR Test
Inverse variance weighted (fixed effects)
Inverse variance weighted (multiplicative random effects)
MR Egger
MR RAPS
Weighted median
Weighted mode
0.05
0.00
−0.05
−0.10
0.1
0.2
SNP effect on LIM and cysteine−rich domains protein 1
SNP effect on Gut microbiota abundance (genus Parasutterella id.2892)

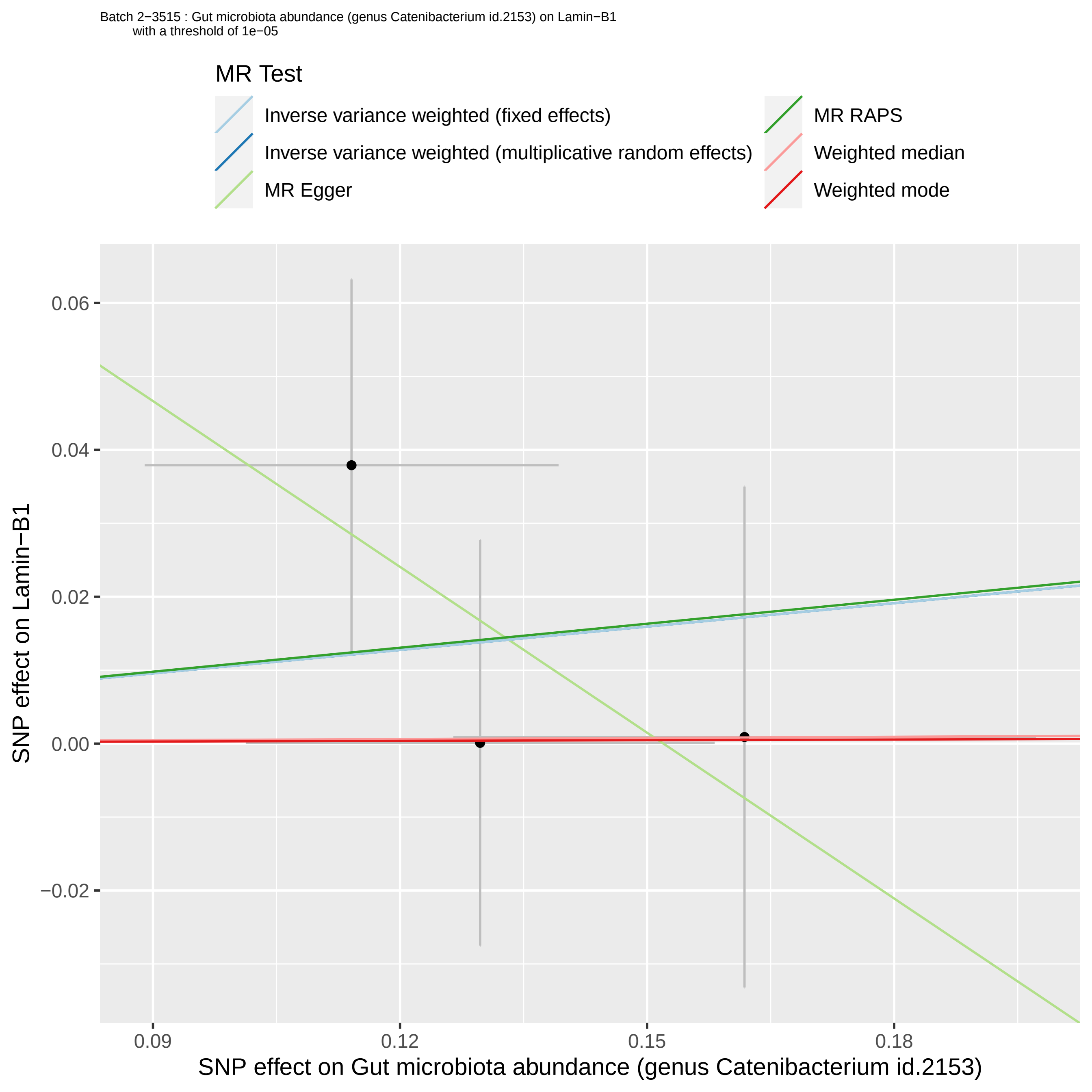
Batch 2−3515 : Gut microbiota abundance (genus Catenibacterium id.2153) on Lamin−B1
with a threshold of 1e−05
MR Test
Inverse variance weighted (fixed effects)
Inverse variance weighted (multiplicative random effects)
MR Egger
MR RAPS
Weighted median
Weighted mode
0.06
0.04
0.02
0.00
−0.02
0.09
0.12
0.15
0.18
SNP effect on Lamin−B1
SNP effect on Gut microbiota abundance (genus Catenibacterium id.2153)

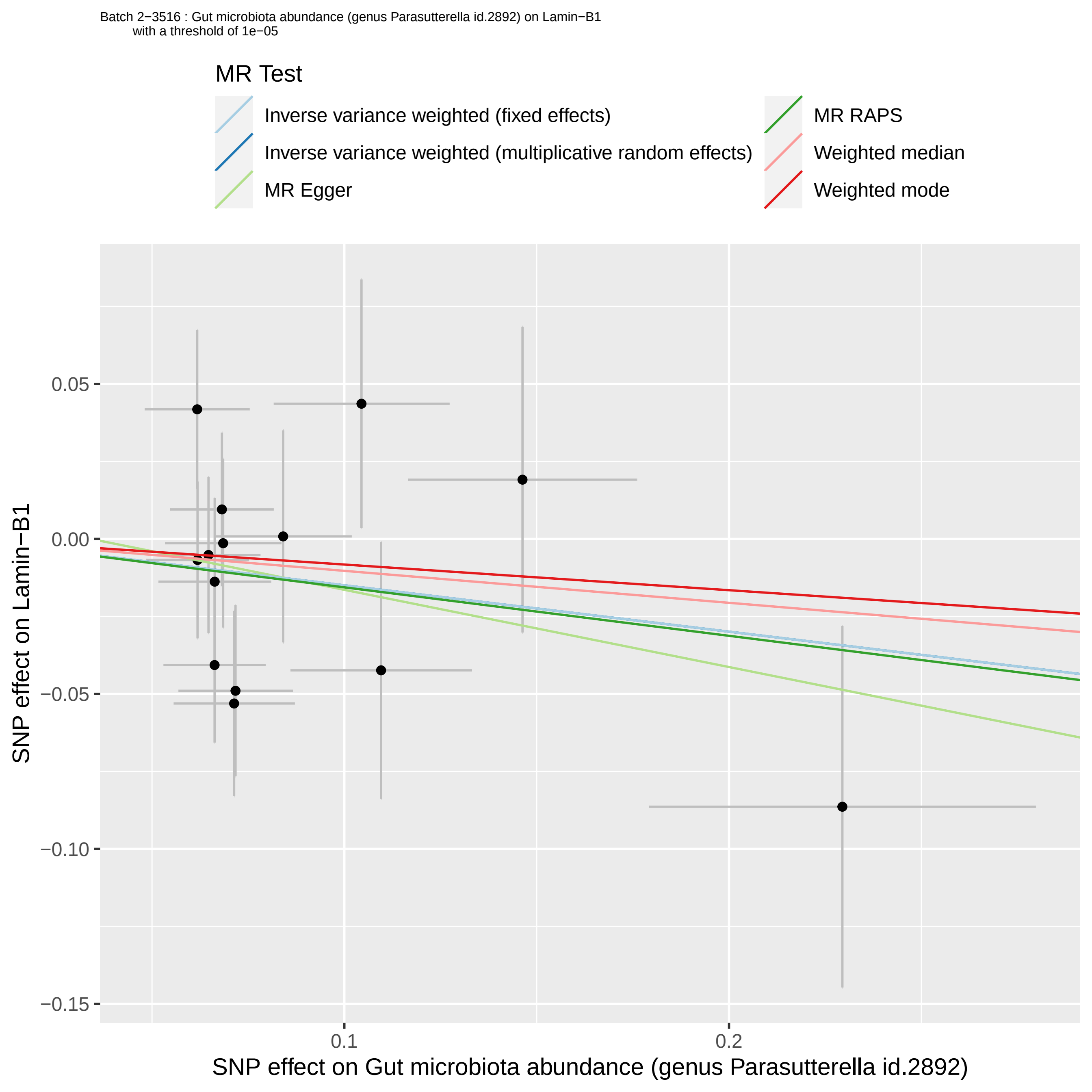

Batch 2−3516 : Gut microbiota abundance (genus Parasutterella id.2892) on Lamin−B1
with a threshold of 1e−05
MR Test
Inverse variance weighted (fixed effects)
Inverse variance weighted (multiplicative random effects)
MR Egger
MR RAPS
Weighted median
Weighted mode
0.05
0.00
−0.05
−0.10
−0.15
0.1
0.2
SNP effect on Lamin−B1
SNP effect on Gut microbiota abundance (genus Parasutterella id.2892)

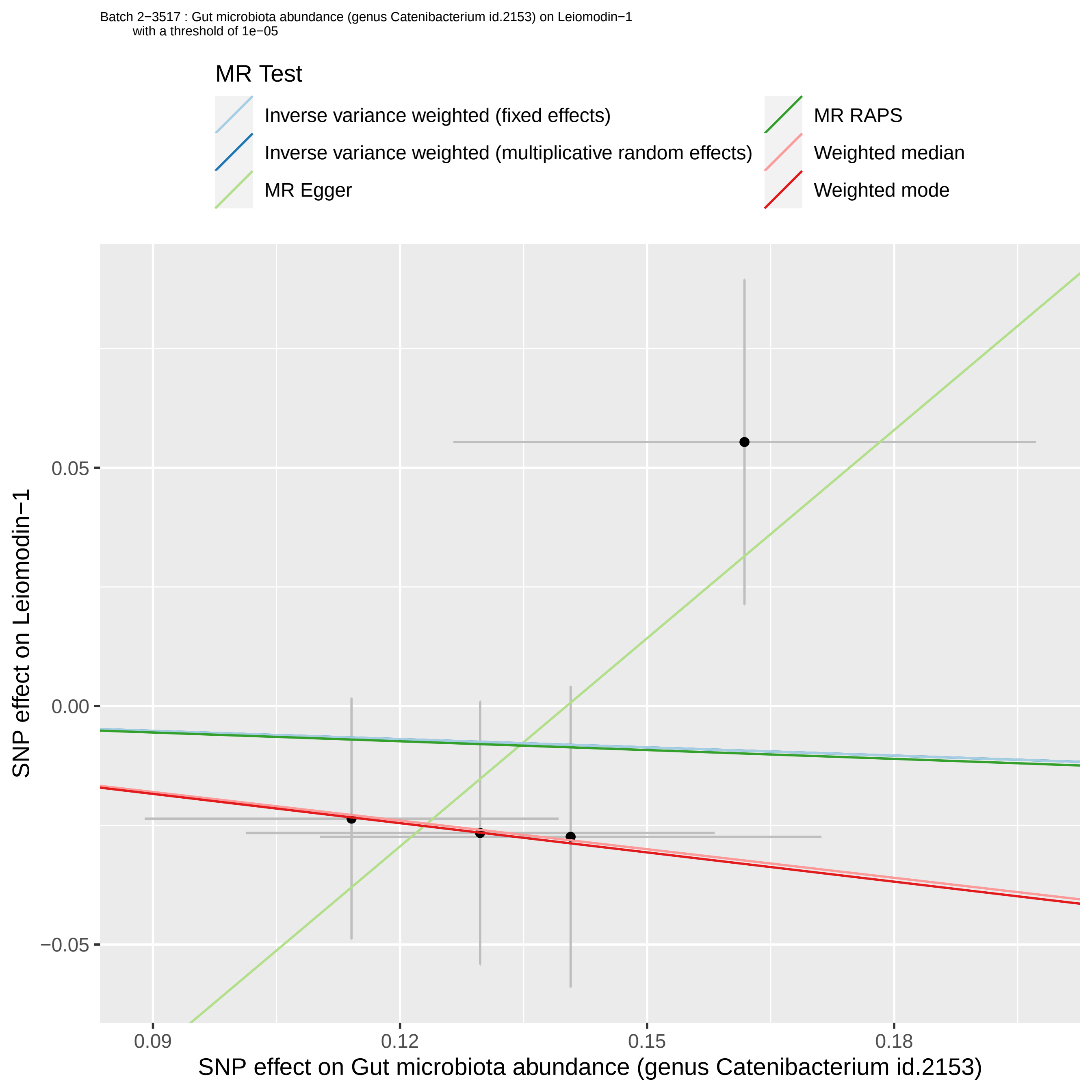

Batch 2−3517 : Gut microbiota abundance (genus Catenibacterium id.2153) on Leiomodin−1
with a threshold of 1e−05
MR Test
Inverse variance weighted (fixed effects)
Inverse variance weighted (multiplicative random effects)
MR Egger
MR RAPS
Weighted median
Weighted mode
0.05
0.00
−0.05
0.09
0.12
0.15
0.18
SNP effect on Leiomodin−1
SNP effect on Gut microbiota abundance (genus Catenibacterium id.2153)

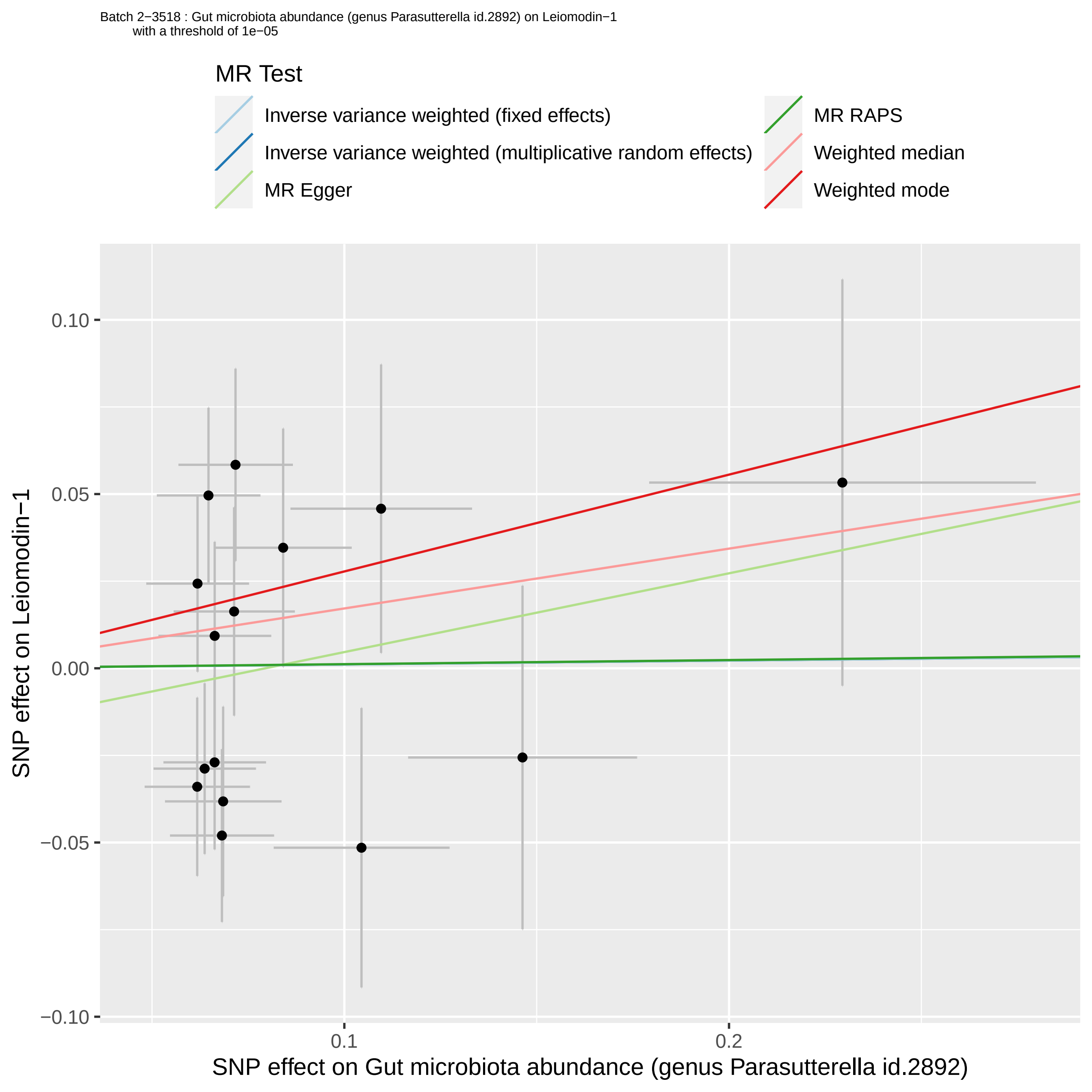
Batch 2−3518 : Gut microbiota abundance (genus Parasutterella id.2892) on Leiomodin−1
with a threshold of 1e−05
MR Test
Inverse variance weighted (fixed effects)
Inverse variance weighted (multiplicative random effects)
MR Egger
MR RAPS
Weighted median
Weighted mode
SNP effect on Leiomodin−1
0.10
0.05
0.00
−0.05
−0.10
0.1
0.2
SNP effect on Gut microbiota abundance (genus Parasutterella id.2892)

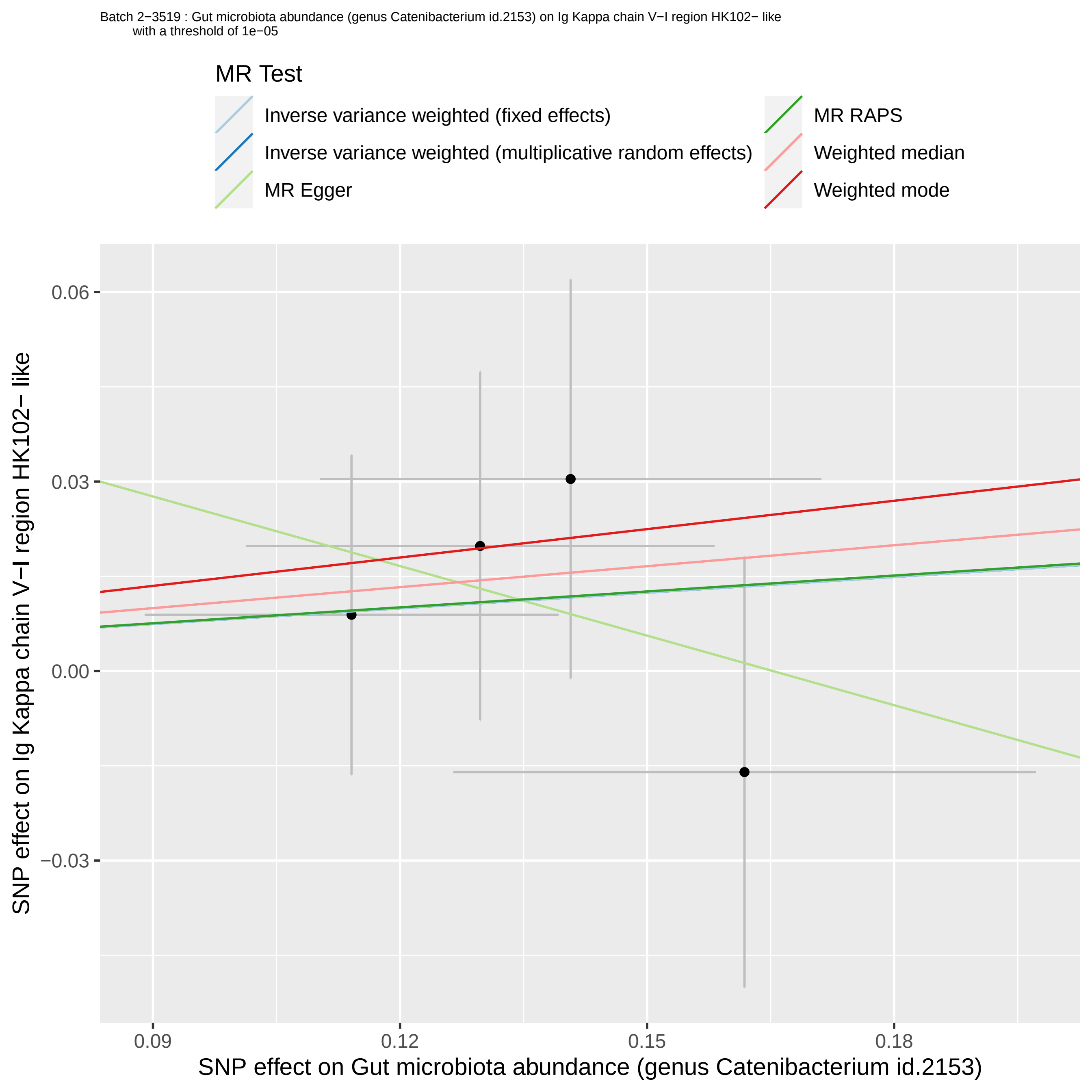
Batch 2−3519 : Gut microbiota abundance (genus Catenibacterium id.2153) on Ig Kappa chain V−I region HK102− like
with a threshold of 1e−05
MR Test
Inverse variance weighted (fixed effects)
Inverse variance weighted (multiplicative random effects)
MR Egger
MR RAPS
Weighted median
Weighted mode
0.06
0.03
0.00
−0.03
0.09
0.12
0.15
0.18
SNP effect on Ig Kappa chain V−I region HK102− like
SNP effect on Gut microbiota abundance (genus Catenibacterium id.2153)

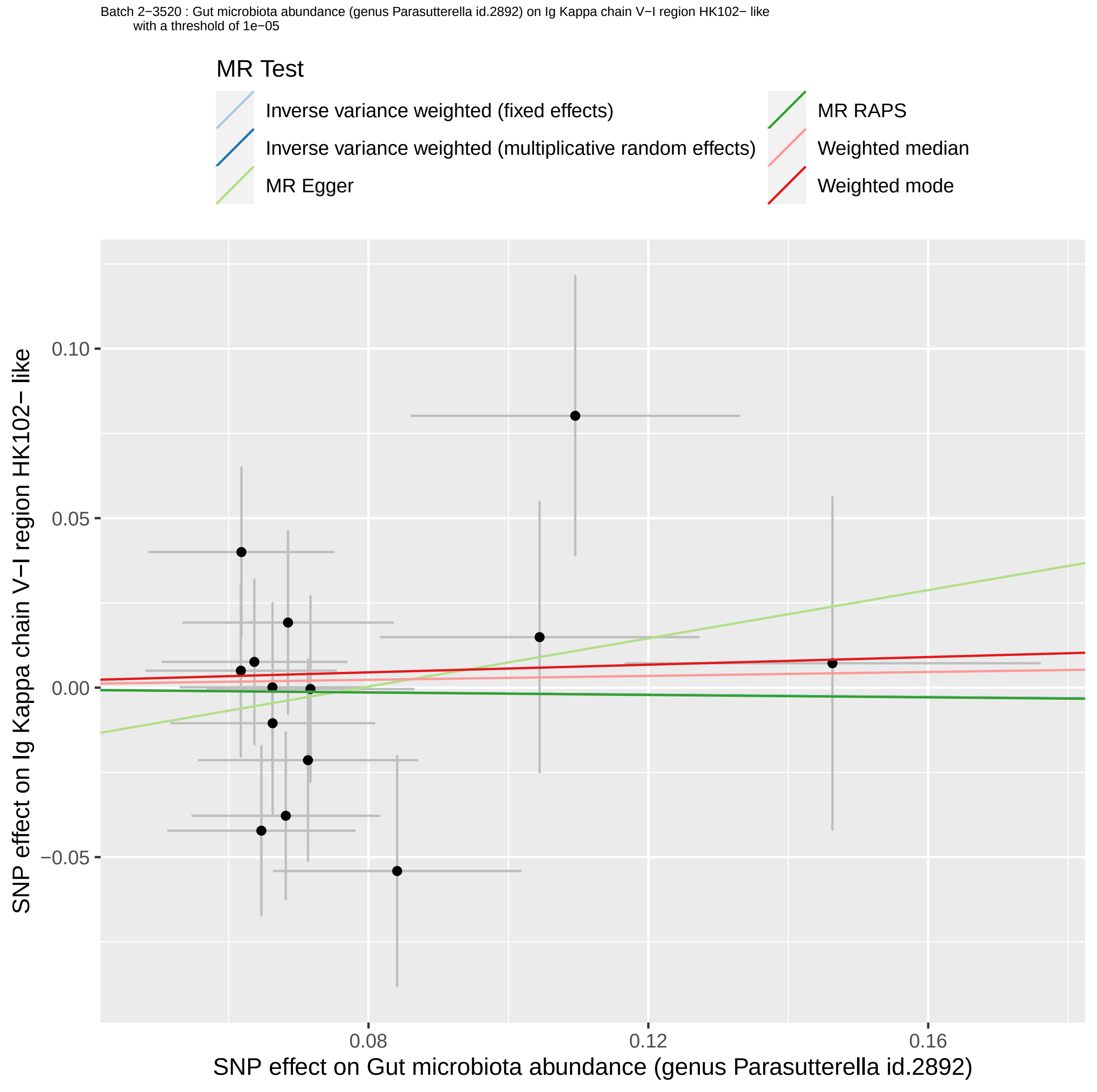

Batch 2−3520 : Gut microbiota abundance (genus Parasutterella id.2892) on Ig Kappa chain V−I region HK102− like
with a threshold of 1e−05
MR Test
Inverse variance weighted (fixed effects)
Inverse variance weighted (multiplicative random effects)
MR Egger
MR RAPS
Weighted median
Weighted mode
0.10
0.05
0.00
−0.05
0.08
0.12
0.16
SNP effect on Ig Kappa chain V−I region HK102− like
SNP effect on Gut microbiota abundance (genus Parasutterella id.2892)

Batch 2−3521 : Gut microbiota abundance (genus Catenibacterium id.2153) on Lon protease homolog, mitochondrial
with a threshold of 1e−05

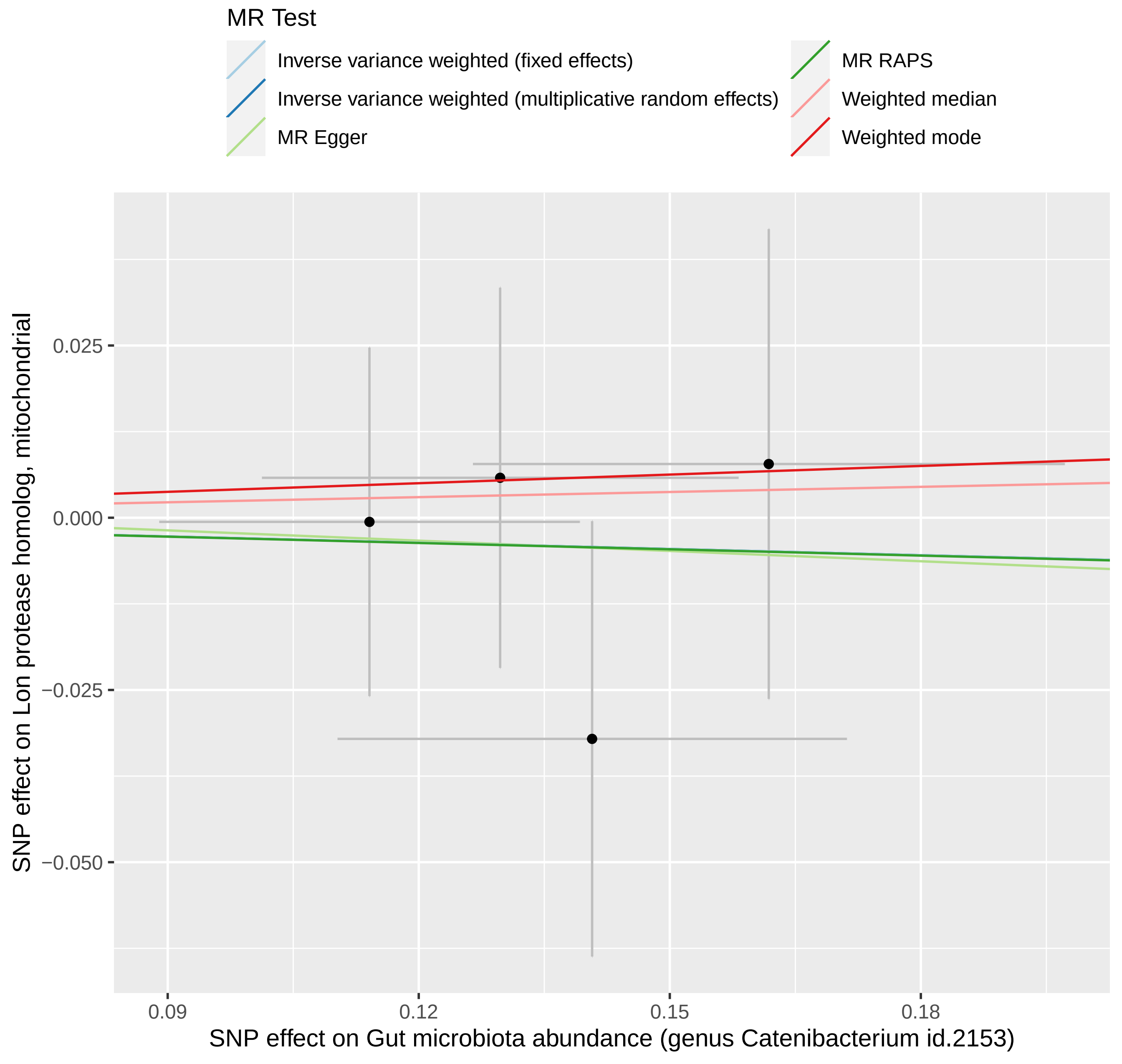

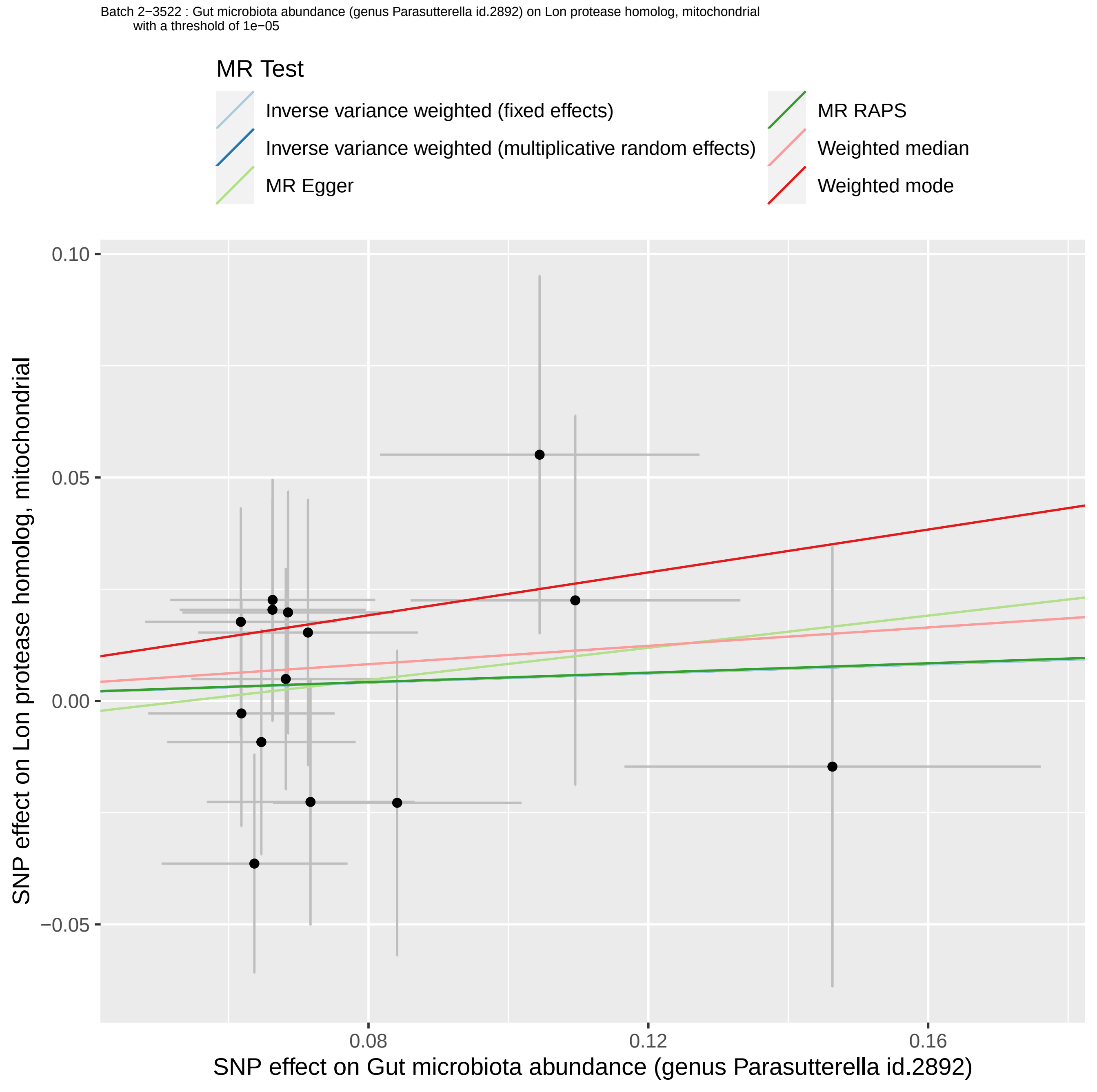
Batch 2−3522 : Gut microbiota abundance (genus Parasutterella id.2892) on Lon protease homolog, mitochondrial
with a threshold of 1e−05
MR Test
Inverse variance weighted (fixed effects)
Inverse variance weighted (multiplicative random effects)
MR Egger
MR RAPS
Weighted median
Weighted mode
0.10
0.05
0.00
−0.05
0.08
0.12
0.16
SNP effect on Lon protease homolog, mitochondrial
SNP effect on Gut microbiota abundance (genus Parasutterella id.2892)

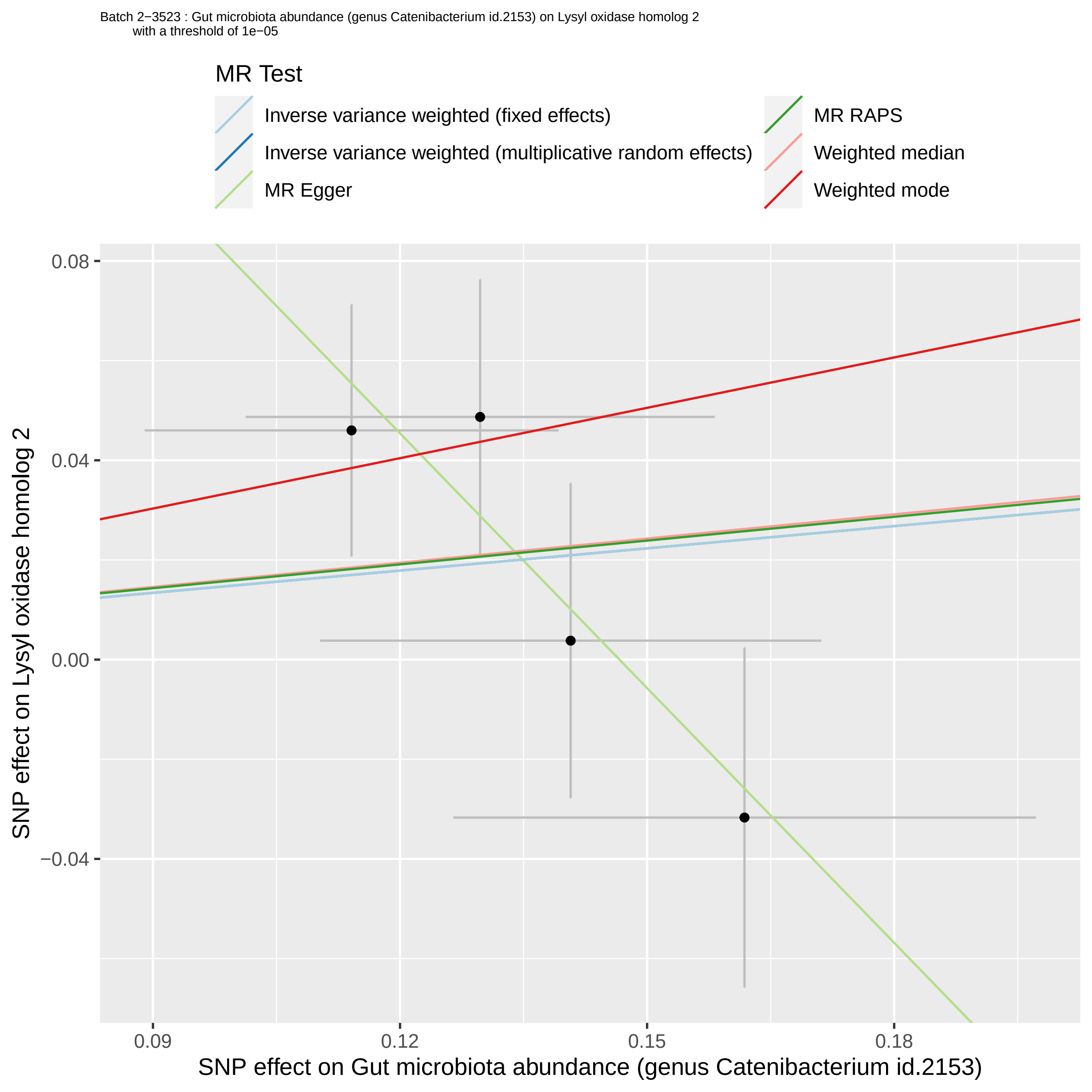
Batch 2−3523 : Gut microbiota abundance (genus Catenibacterium id.2153) on Lysyl oxidase homolog 2
with a threshold of 1e−05
MR Test
Inverse variance weighted (fixed effects)
Inverse variance weighted (multiplicative random effects)
MR Egger
MR RAPS
Weighted median
Weighted mode
0.08
0.04
0.00
−0.04
0.09
0.12
0.15
0.18
SNP effect on Lysyl oxidase homolog 2
SNP effect on Gut microbiota abundance (genus Catenibacterium id.2153)

Batch 2−3524 : Gut microbiota abundance (genus Parasutterella id.2892) on Lysyl oxidase homolog 2
with a threshold of 1e−05

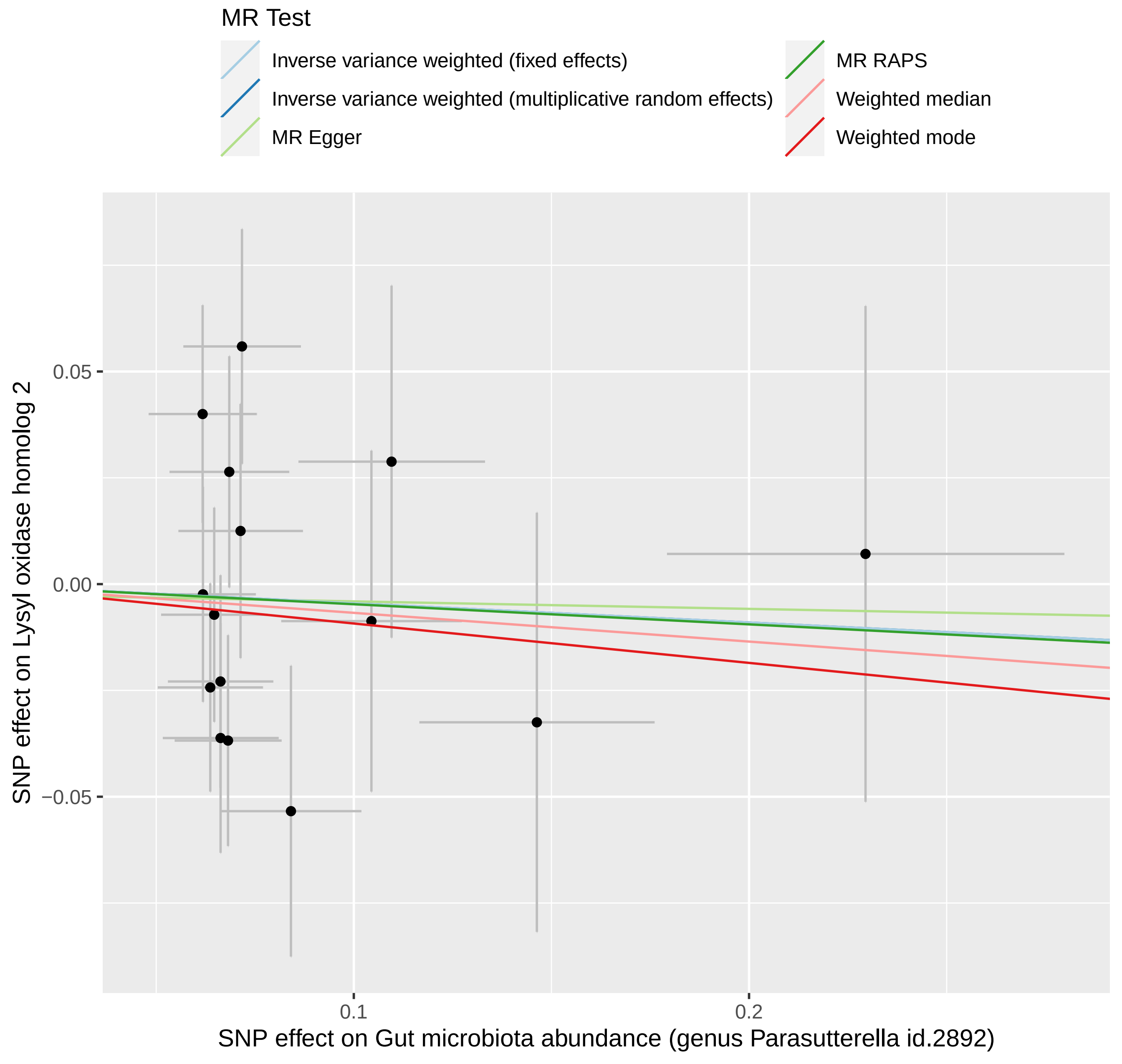

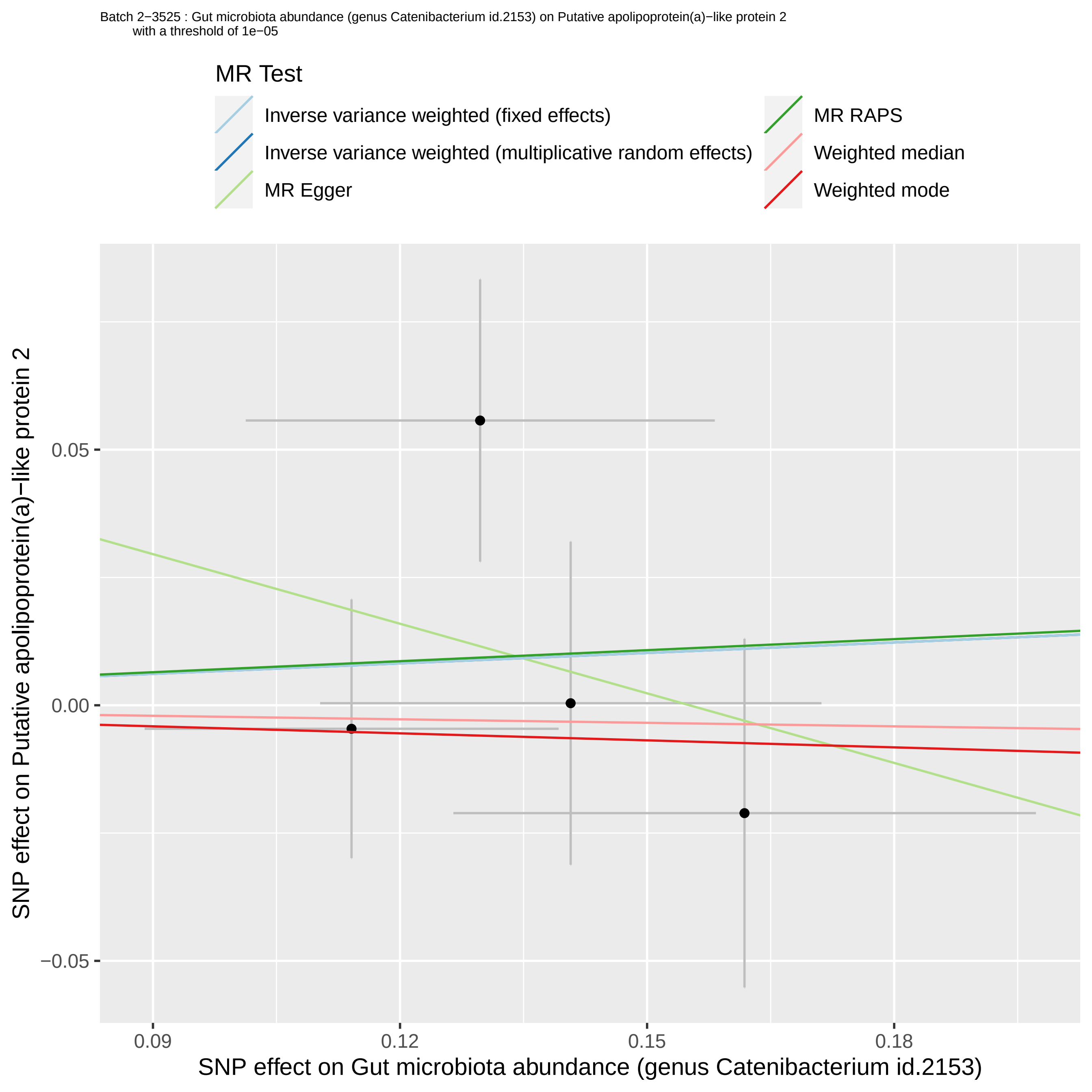

Batch 2−3525 : Gut microbiota abundance (genus Catenibacterium id.2153) on Putative apolipoprotein(a)−like protein 2
with a threshold of 1e−05
MR Test
Inverse variance weighted (fixed effects)
Inverse variance weighted (multiplicative random effects)
MR Egger
MR RAPS
Weighted median
Weighted mode
SNP effect on Putative apolipoprotein(a)−like protein 2
0.05
0.00
−0.05
0.09
0.12
0.15
0.18
SNP effect on Gut microbiota abundance (genus Catenibacterium id.2153)

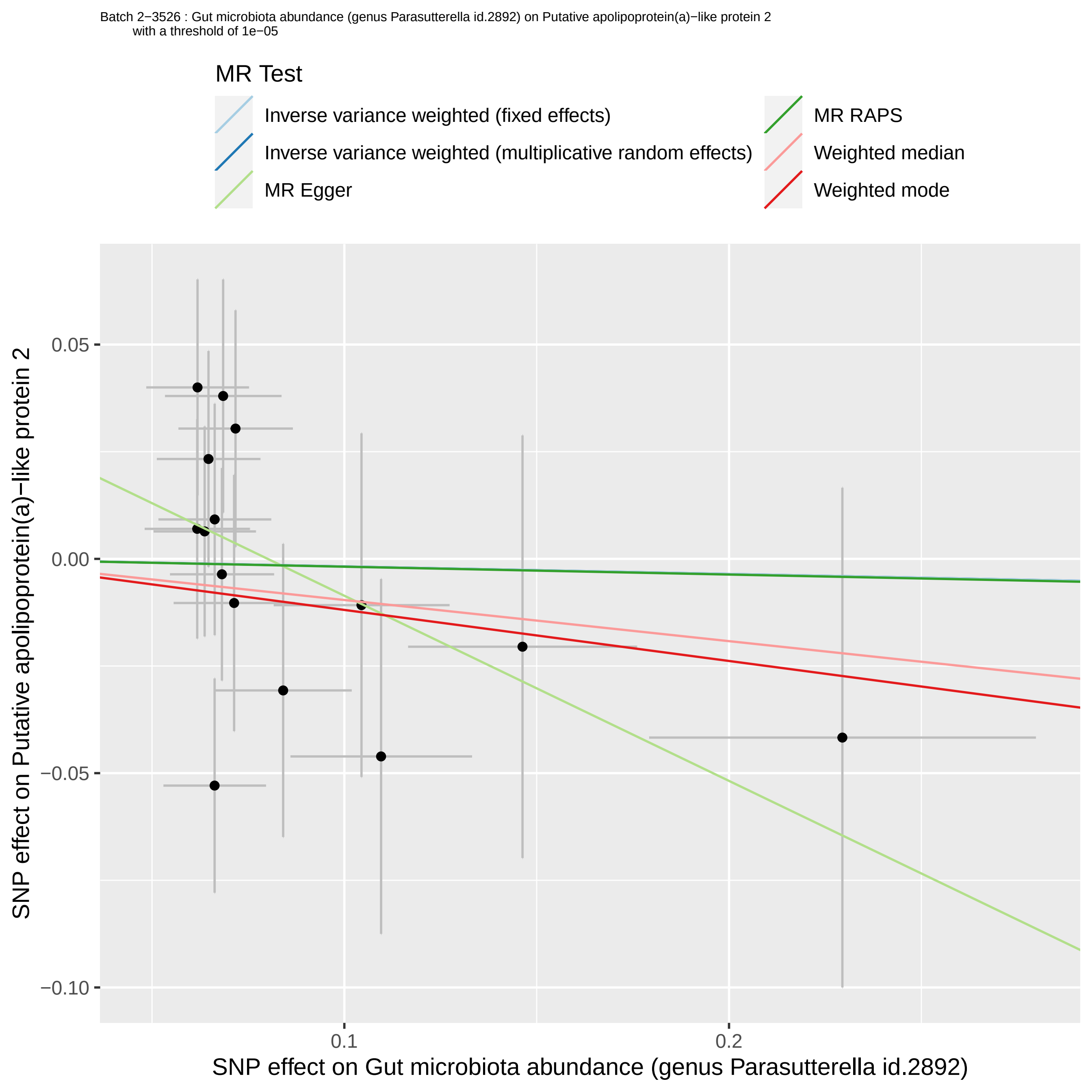
Batch 2−3526 : Gut microbiota abundance (genus Parasutterella id.2892) on Putative apolipoprotein(a)−like protein 2
with a threshold of 1e−05
MR Test
Inverse variance weighted (fixed effects)
Inverse variance weighted (multiplicative random effects)
MR Egger
MR RAPS
Weighted median
Weighted mode
0.05
0.00
−0.05
−0.10
0.1
0.2
SNP effect on Putative apolipoprotein(a)−like protein 2
SNP effect on Gut microbiota abundance (genus Parasutterella id.2892)

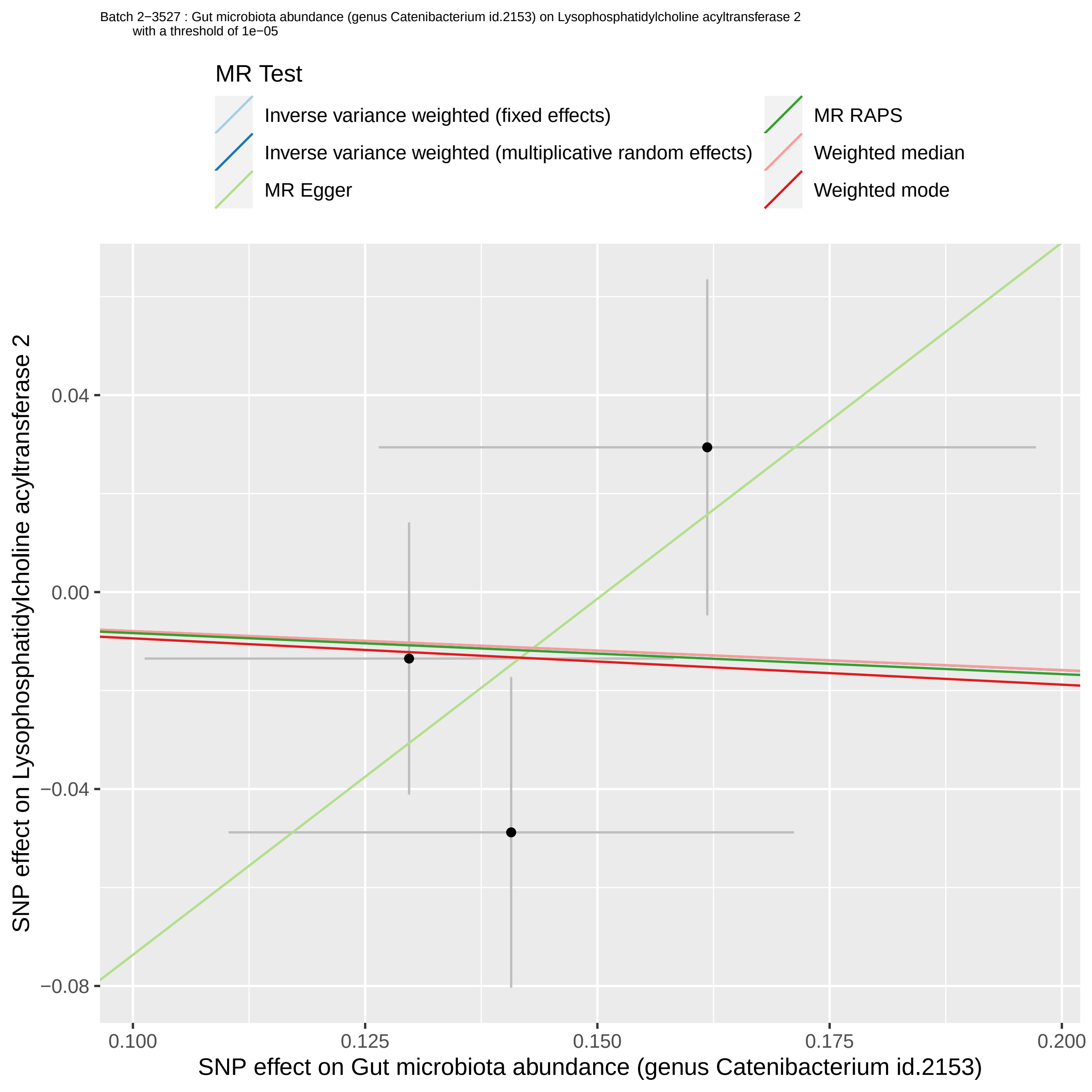
Batch 2−3527 : Gut microbiota abundance (genus Catenibacterium id.2153) on Lysophosphatidylcholine acyltransferase 2
with a threshold of 1e−05
MR Test
Inverse variance weighted (fixed effects)
Inverse variance weighted (multiplicative random effects)
MR Egger
MR RAPS
Weighted median
Weighted mode
0.04
0.00
−0.04
−0.08
0.100
0.125
0.150
0.175
0.200
SNP effect on Lysophosphatidylcholine acyltransferase 2
SNP effect on Gut microbiota abundance (genus Catenibacterium id.2153)

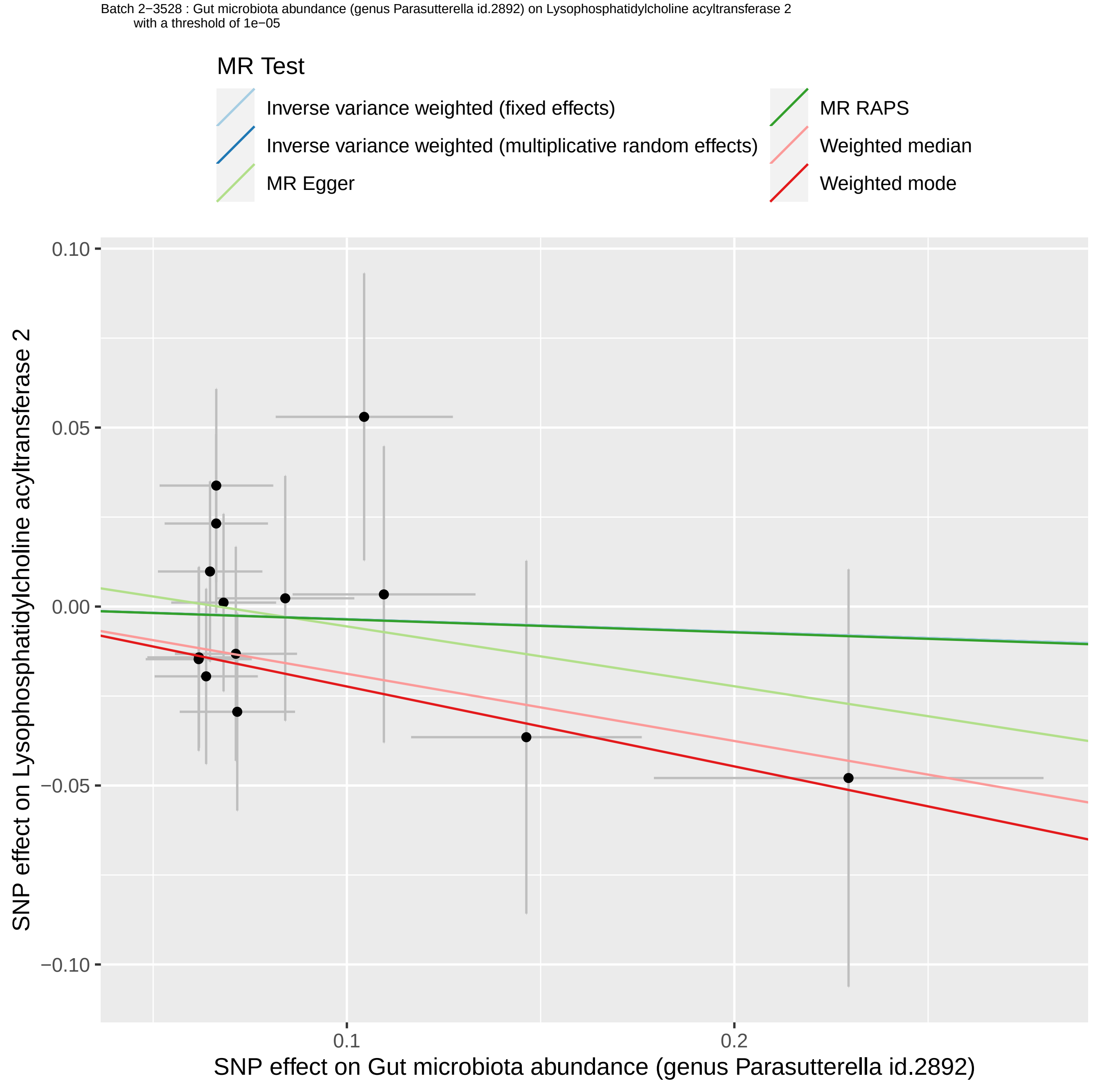
Batch 2−3528 : Gut microbiota abundance (genus Parasutterella id.2892) on Lysophosphatidylcholine acyltransferase 2
with a threshold of 1e−05
MR Test
Inverse variance weighted (fixed effects)
Inverse variance weighted (multiplicative random effects)
MR Egger
MR RAPS
Weighted median
Weighted mode
0.10
0.05
0.00
−0.05
−0.10
0.1
0.2
SNP effect on Lysophosphatidylcholine acyltransferase 2
SNP effect on Gut microbiota abundance (genus Parasutterella id.2892)

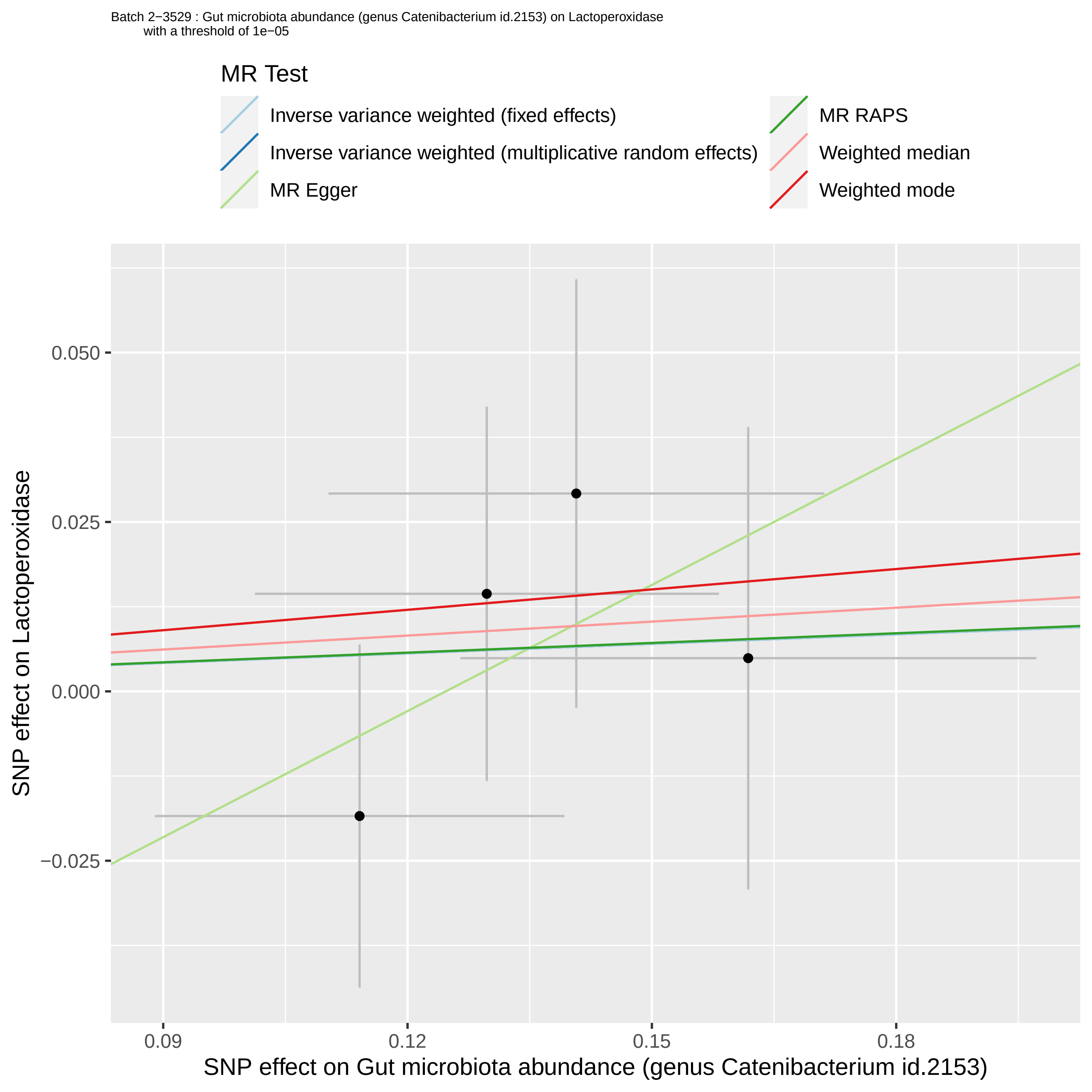
Batch 2−3529 : Gut microbiota abundance (genus Catenibacterium id.2153) on Lactoperoxidase
with a threshold of 1e−05
MR Test
Inverse variance weighted (fixed effects)
Inverse variance weighted (multiplicative random effects)
MR Egger
MR RAPS
Weighted median
Weighted mode
0.050
0.025
0.000
−0.025
0.09
0.12
0.15
0.18
SNP effect on Lactoperoxidase
SNP effect on Gut microbiota abundance (genus Catenibacterium id.2153)

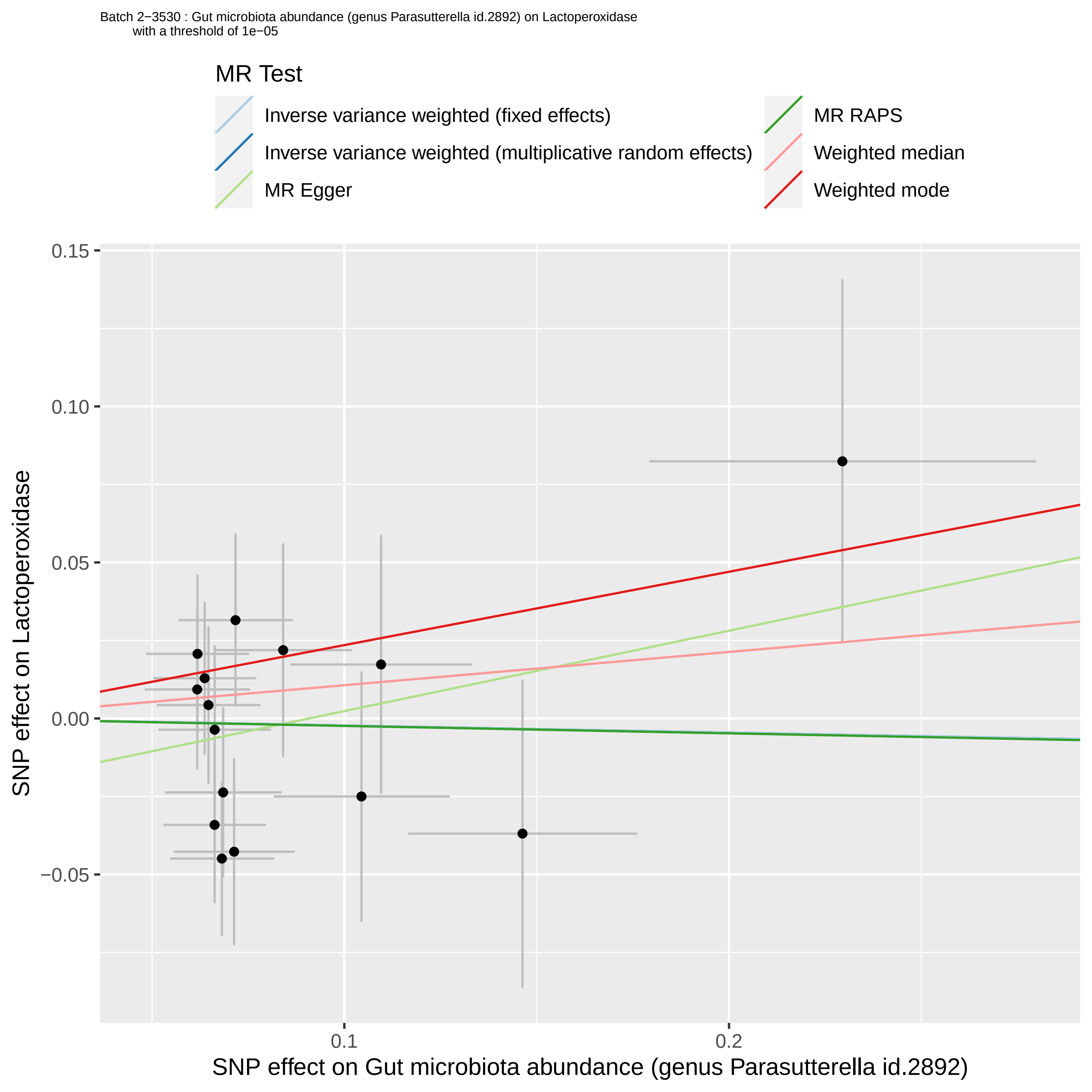

Batch 2−3530 : Gut microbiota abundance (genus Parasutterella id.2892) on Lactoperoxidase
with a threshold of 1e−05
MR Test
Inverse variance weighted (fixed effects)
Inverse variance weighted (multiplicative random effects)
MR Egger
MR RAPS
Weighted median
Weighted mode
0.15
0.10
0.05
0.00
−0.05
0.1
0.2
SNP effect on Lactoperoxidase
SNP effect on Gut microbiota abundance (genus Parasutterella id.2892)

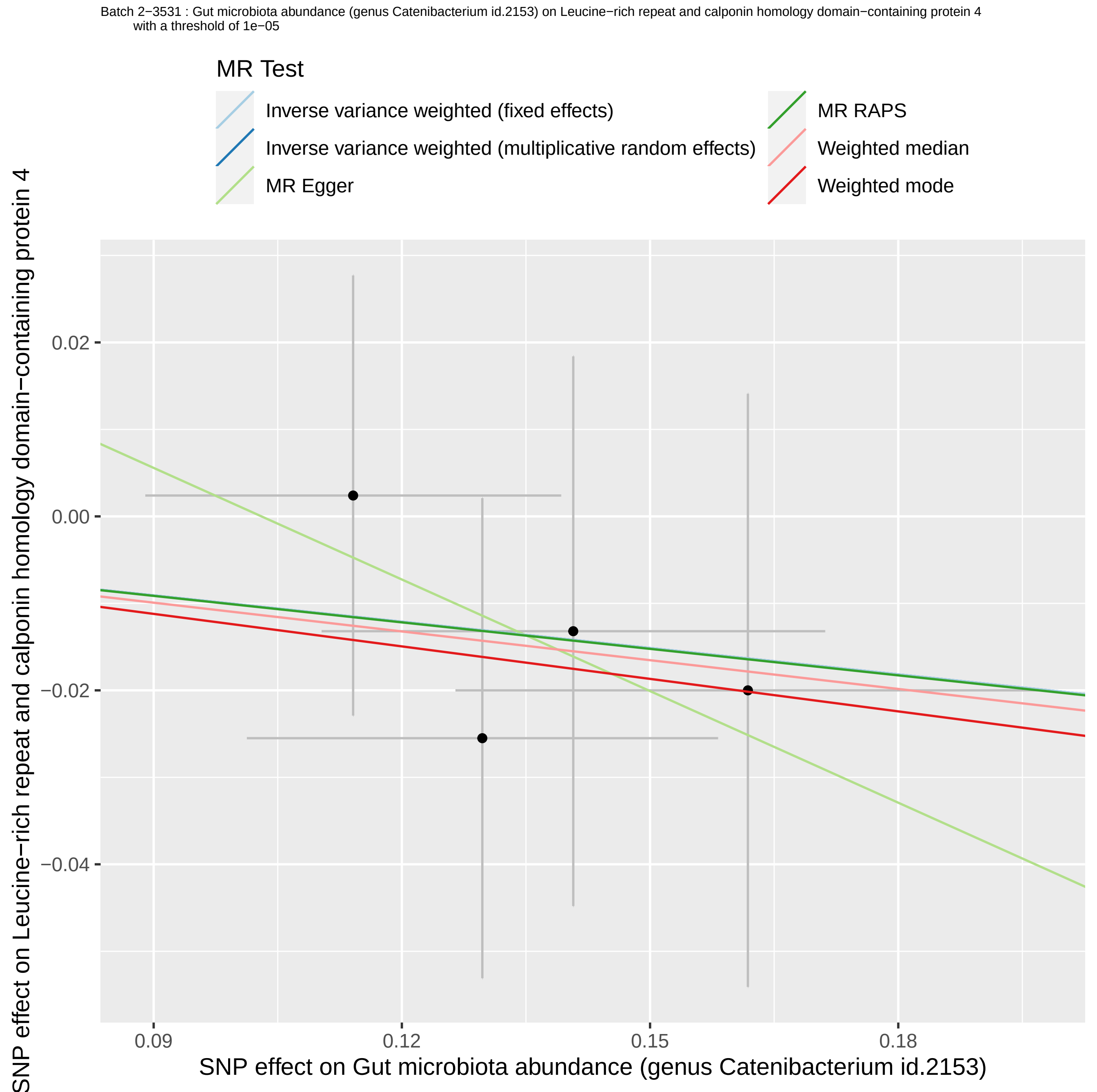
Batch 2−3531 : Gut microbiota abundance (genus Catenibacterium id.2153) on Leucine−rich repeat and calponin homology domain−containing protein 4
with a threshold of 1e−05
MR Test
Inverse variance weighted (fixed effects)
Inverse variance weighted (multiplicative random effects)
MR Egger
MR RAPS
Weighted median
Weighted mode
0.02
0.00
−0.02
−0.04
0.09
0.12
0.15
0.18
SNP effect on Gut microbiota abundance (genus Catenibacterium id.2153)
SNP effect on Leucine−rich repeat and calponin homology domain−containing protein 4

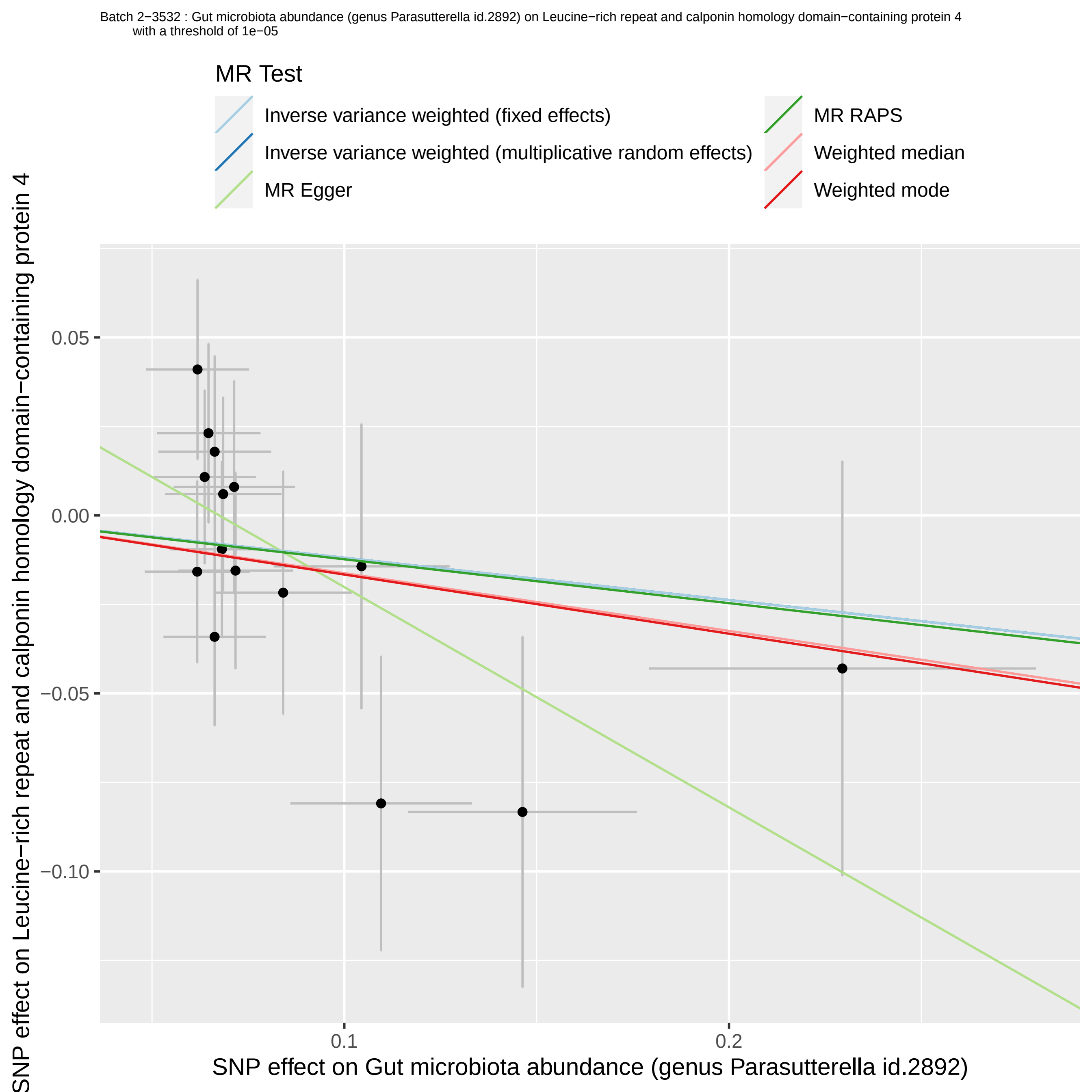

Batch 2−3532 : Gut microbiota abundance (genus Parasutterella id.2892) on Leucine−rich repeat and calponin homology domain−containing protein 4
with a threshold of 1e−05
MR Test
Inverse variance weighted (fixed effects)
Inverse variance weighted (multiplicative random effects)
MR Egger
MR RAPS
Weighted median
Weighted mode
0.05
0.00
−0.05
−0.10
0.1
0.2
SNP effect on Leucine−rich repeat and calponin homology domain−containing protein 4
SNP effect on Gut microbiota abundance (genus Parasutterella id.2892)

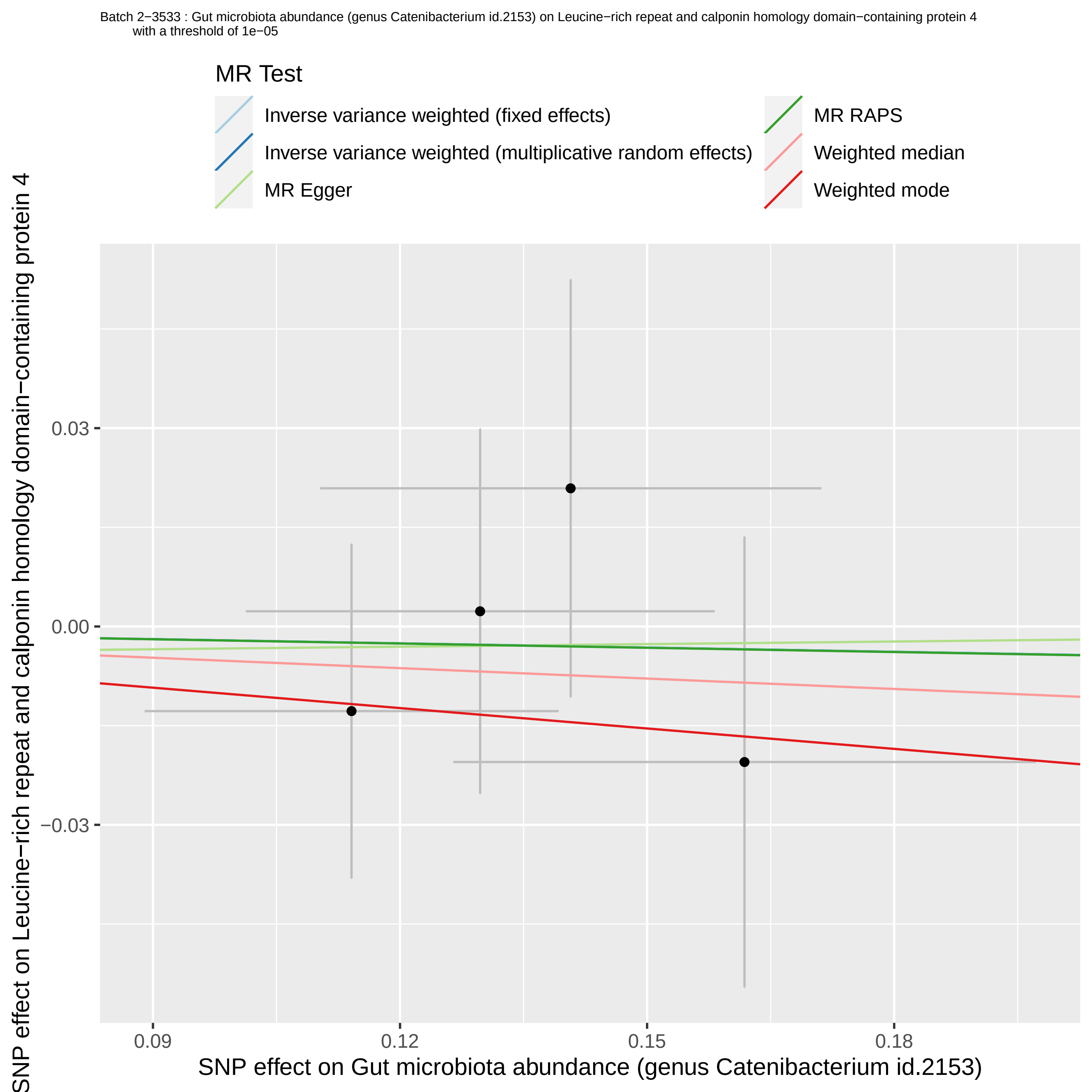
Batch 2−3533 : Gut microbiota abundance (genus Catenibacterium id.2153) on Leucine−rich repeat and calponin homology domain−containing protein 4 with a threshold of 1e−05
MR Test
Inverse variance weighted (fixed effects)
Inverse variance weighted (multiplicative random effects)
MR Egger
MR RAPS
Weighted median
Weighted mode
0.03
0.00
−0.03
0.09
0.12
0.15
0.18
SNP effect on Gut microbiota abundance (genus Catenibacterium id.2153)
SNP effect on Leucine−rich repeat and calponin homology domain−containing protein 4

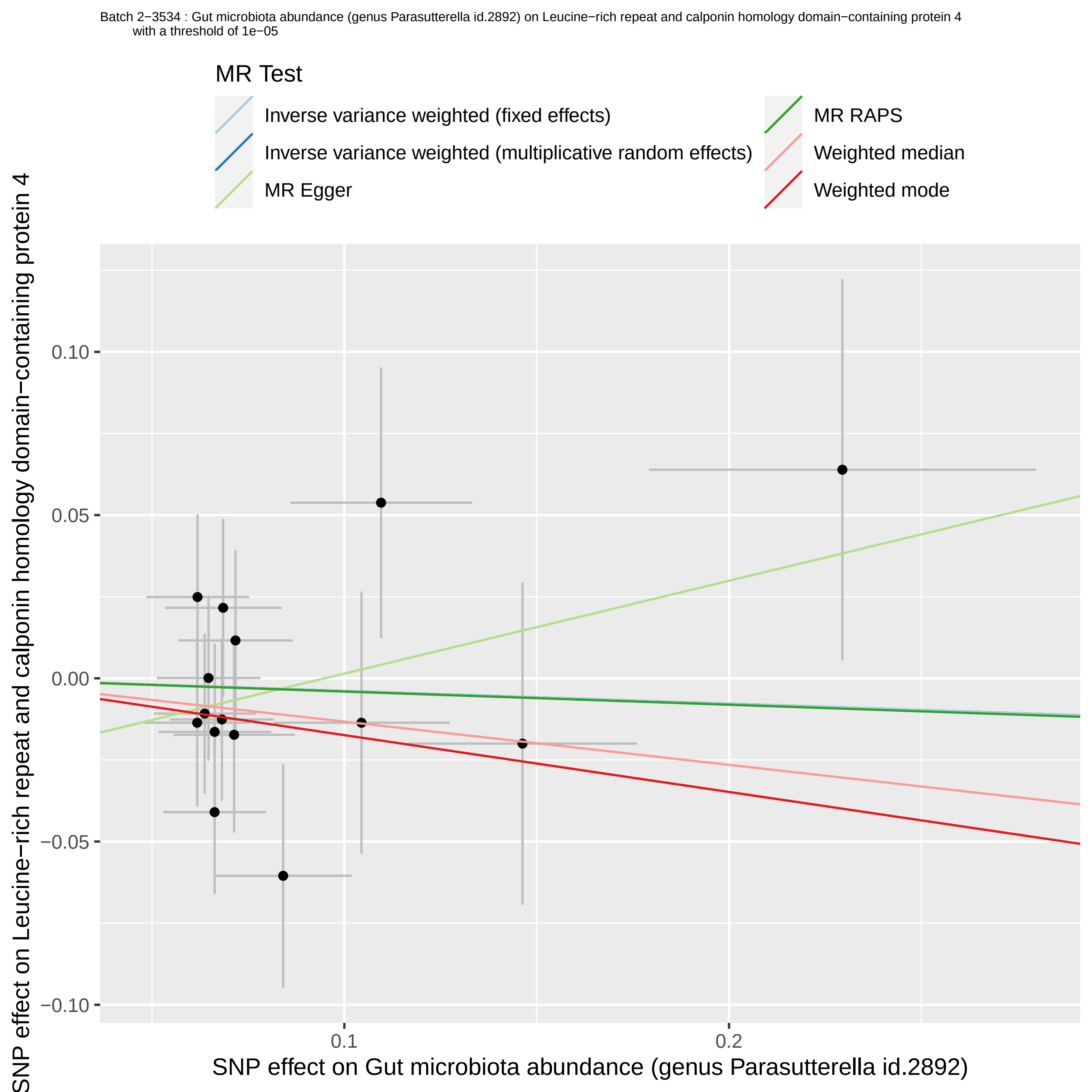
Batch 2−3534 : Gut microbiota abundance (genus Parasutterella id.2892) on Leucine−rich repeat and calponin homology domain−containing protein 4
with a threshold of 1e−05
MR Test
Inverse variance weighted (fixed effects)
Inverse variance weighted (multiplicative random effects)
MR Egger
MR RAPS
Weighted median
Weighted mode
SNP effect on Leucine−rich repeat and calponin homology domain−containing protein 4
0.10
0.05
0.00
−0.05
−0.10
0.1
0.2
SNP effect on Gut microbiota abundance (genus Parasutterella id.2892)

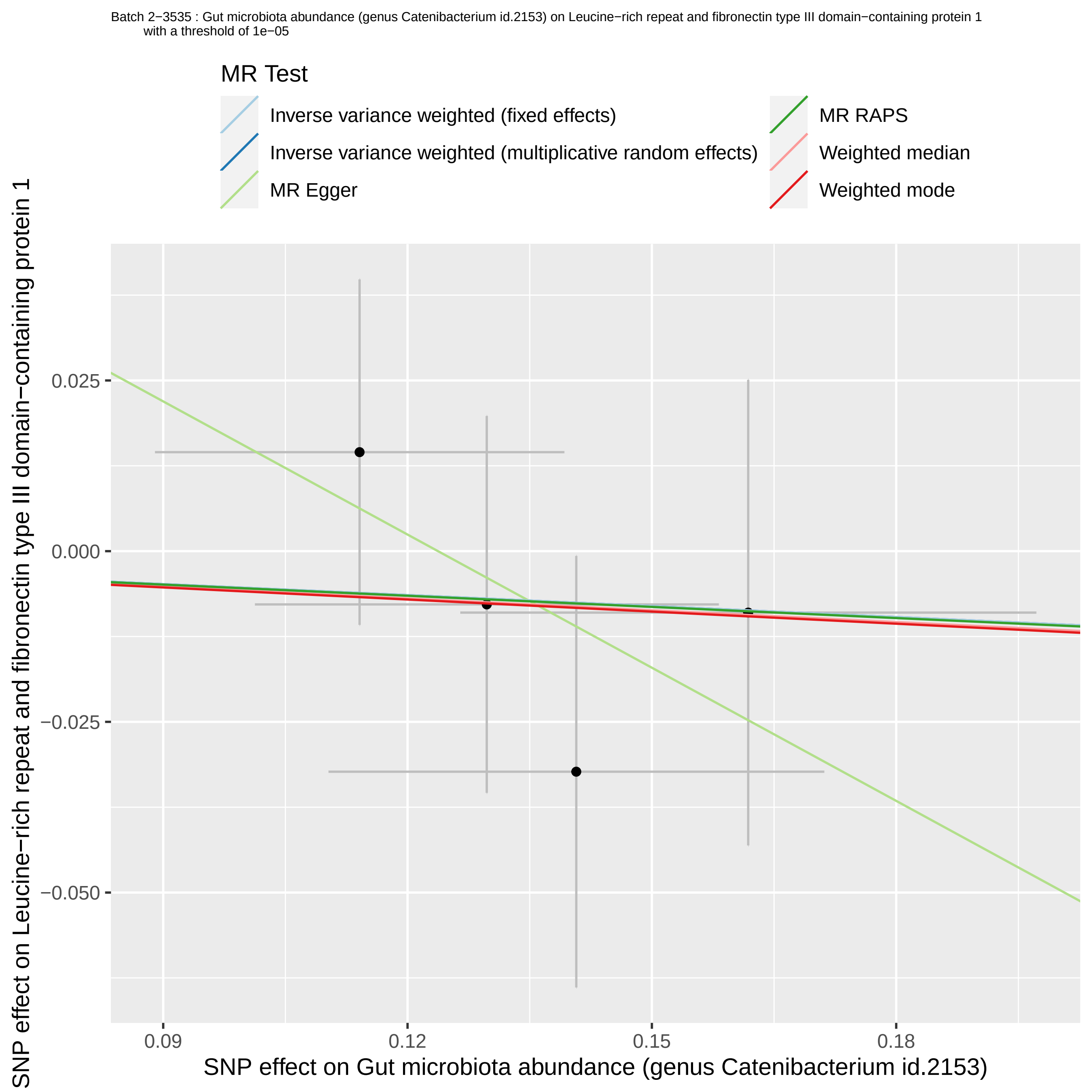

Batch 2−3535 : Gut microbiota abundance (genus Catenibacterium id.2153) on Leucine−rich repeat and fibronectin type III domain−containing protein 1 with a threshold of 1e−05
MR Test
Inverse variance weighted (fixed effects)
Inverse variance weighted (multiplicative random effects)
MR Egger
MR RAPS
Weighted median
Weighted mode
0.025
0.000
−0.025
−0.050
0.09
0.12
0.15
0.18
SNP effect on Gut microbiota abundance (genus Catenibacterium id.2153)
SNP effect on Leucine−rich repeat and fibronectin type III domain−containing protein 1

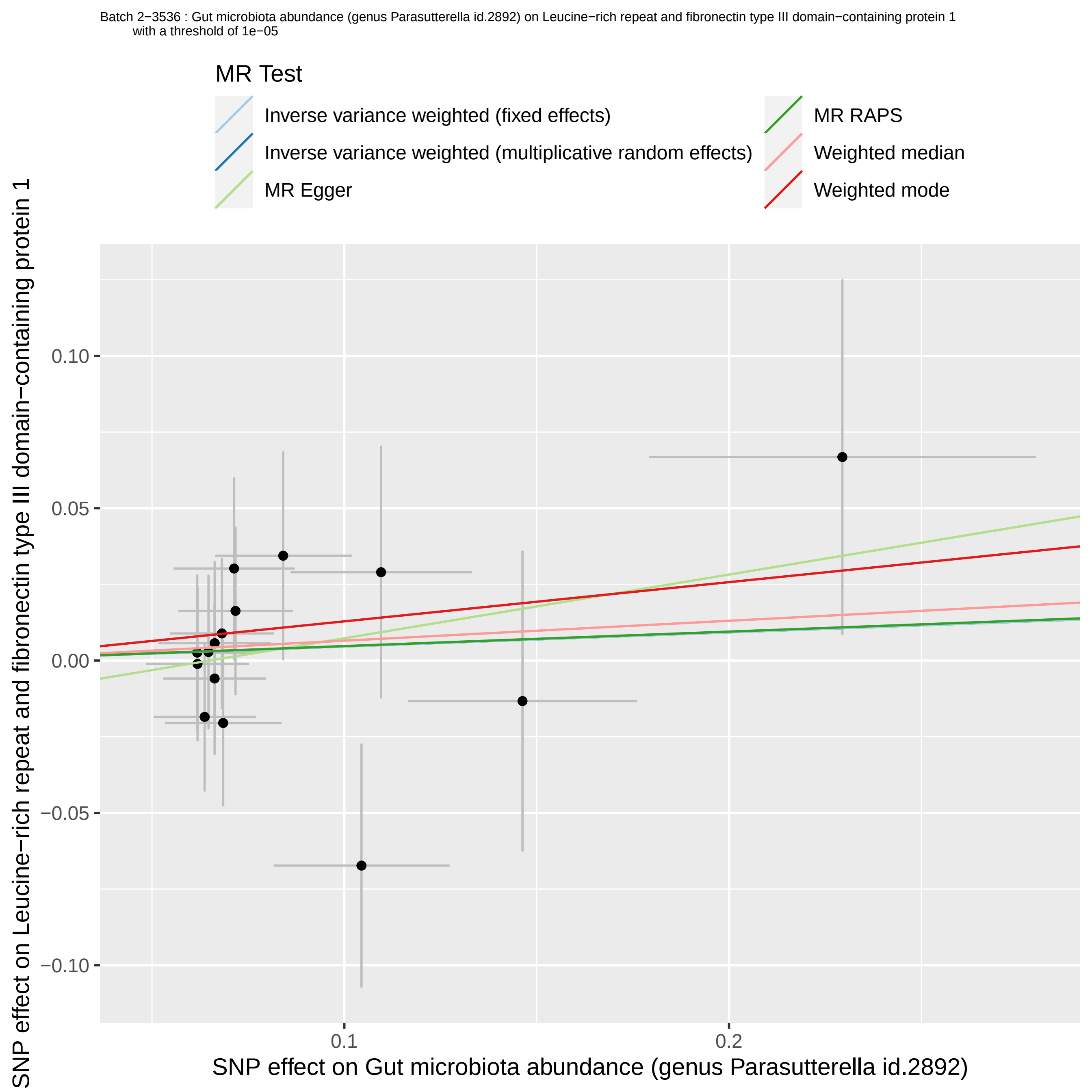

Batch 2−3536 : Gut microbiota abundance (genus Parasutterella id.2892) on Leucine−rich repeat and fibronectin type III domain−containing protein 1
with a threshold of 1e−05
MR Test
Inverse variance weighted (fixed effects)
Inverse variance weighted (multiplicative random effects)
MR Egger
MR RAPS
Weighted median
Weighted mode
0.10
0.05
0.00
−0.05
−0.10
0.1
0.2
SNP effect on Gut microbiota abundance (genus Parasutterella id.2892)
SNP effect on Leucine−rich repeat and fibronectin type III domain−containing protein 1

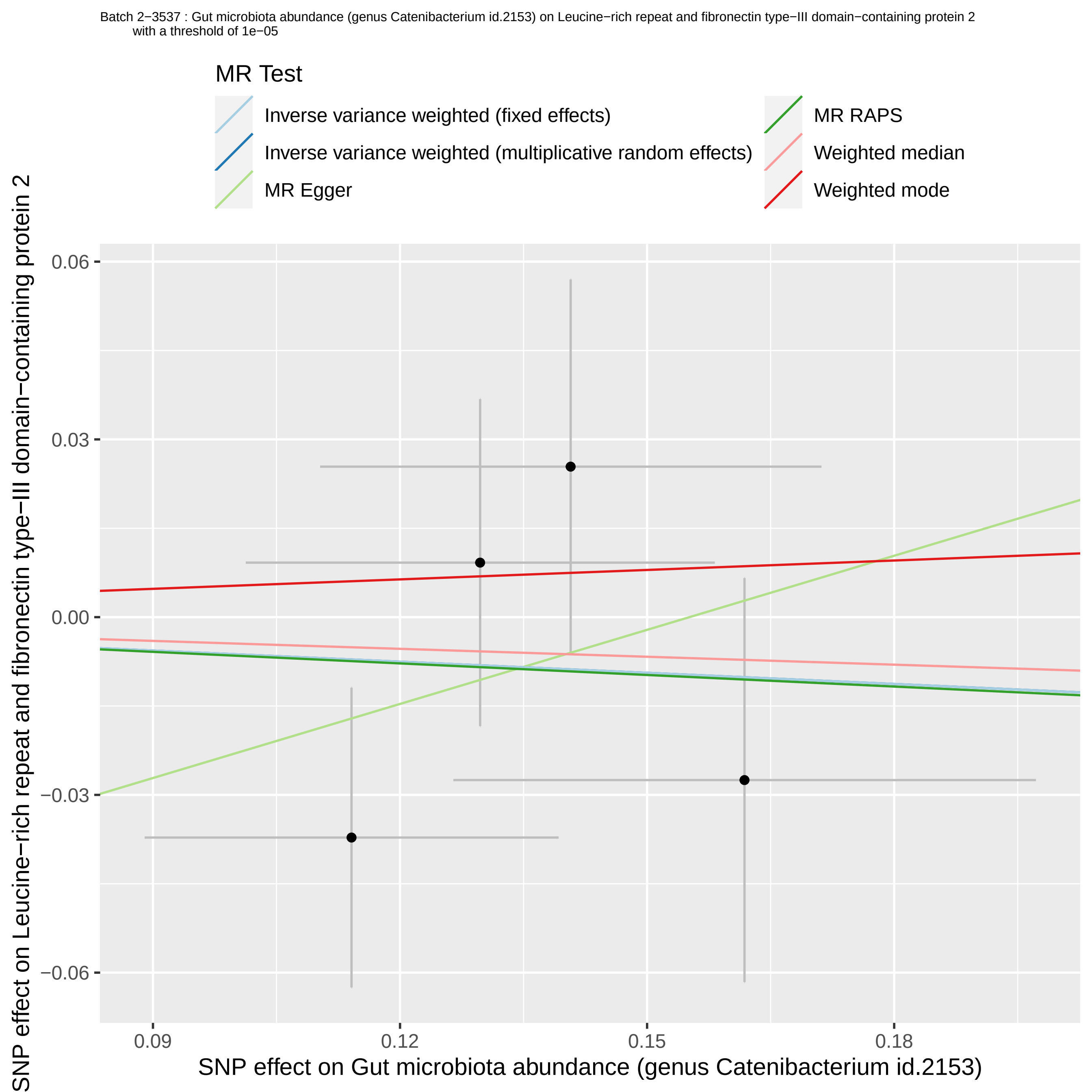
Batch 2−3537 : Gut microbiota abundance (genus Catenibacterium id.2153) on Leucine−rich repeat and fibronectin type−III domain−containing protein 2 with a threshold of 1e−05
MR Test
Inverse variance weighted (fixed effects)
Inverse variance weighted (multiplicative random effects)
MR Egger
MR RAPS
Weighted median
Weighted mode
0.06
0.03
0.00
−0.03
−0.06
0.09
0.12
0.15
0.18
SNP effect on Leucine−rich repeat and fibronectin type−III domain−containing protein 2
SNP effect on Gut microbiota abundance (genus Catenibacterium id.2153)

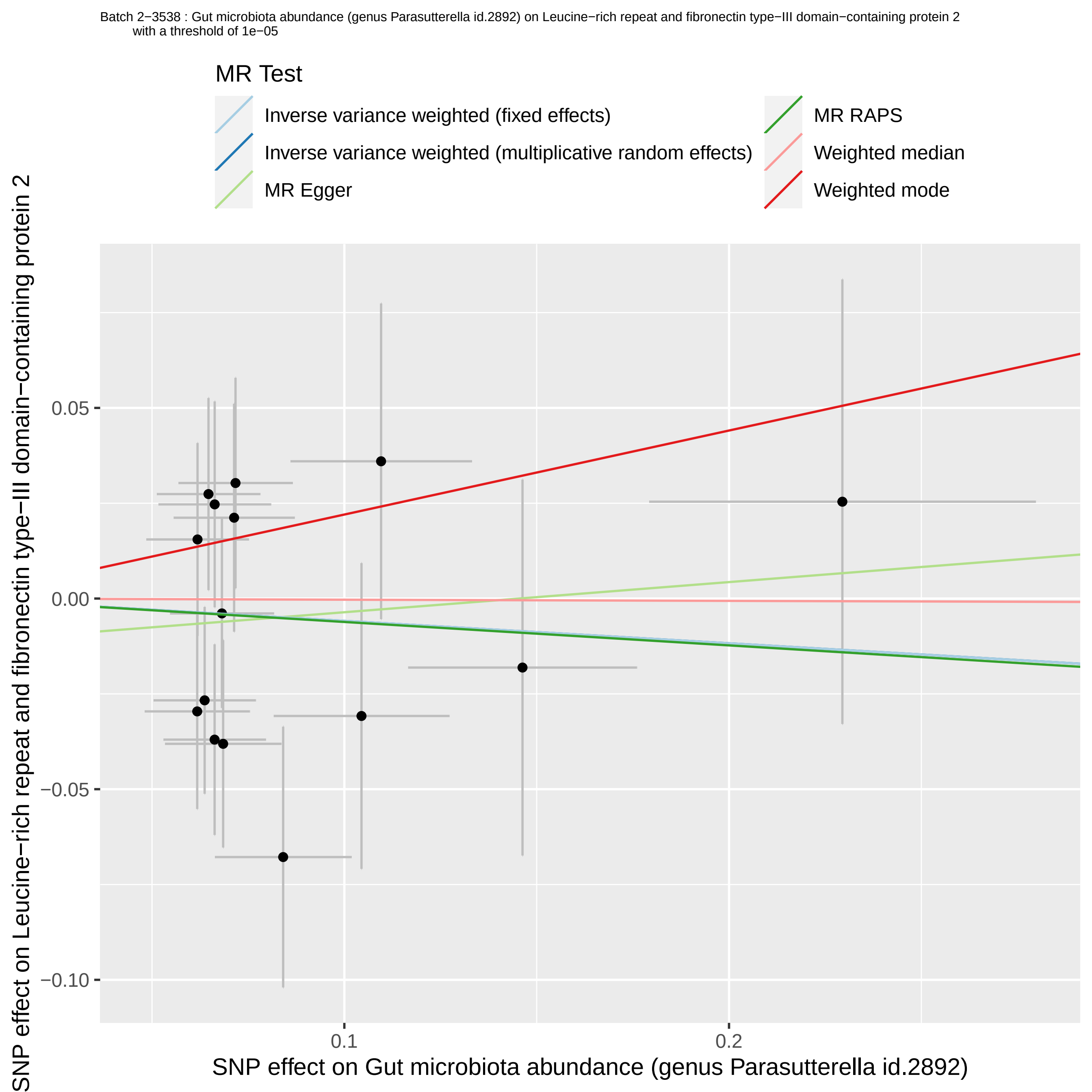
Batch 2−3538 : Gut microbiota abundance (genus Parasutterella id.2892) on Leucine−rich repeat and fibronectin type−III domain−containing protein 2
with a threshold of 1e−05
MR Test
Inverse variance weighted (fixed effects)
Inverse variance weighted (multiplicative random effects)
MR Egger
MR RAPS
Weighted median
Weighted mode
0.05
0.00
−0.05
−0.10
0.1
0.2
SNP effect on Gut microbiota abundance (genus Parasutterella id.2892)
SNP effect on Leucine−rich repeat and fibronectin type−III domain−containing protein 2

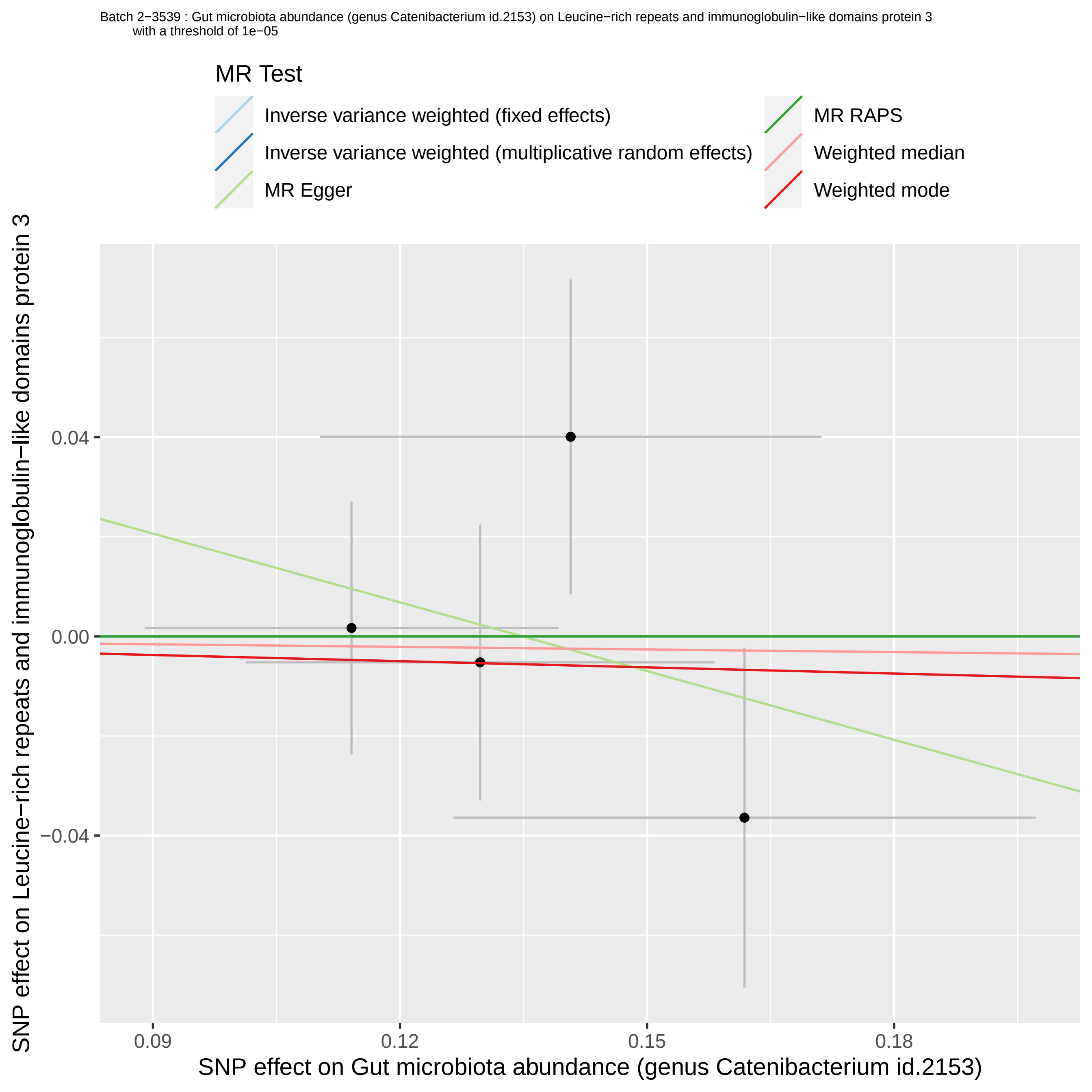

Batch 2−3539 : Gut microbiota abundance (genus Catenibacterium id.2153) on Leucine−rich repeats and immunoglobulin−like domains protein 3
with a threshold of 1e−05
MR Test
Inverse variance weighted (fixed effects)
Inverse variance weighted (multiplicative random effects)
MR Egger
MR RAPS
Weighted median
Weighted mode
0.04
0.00
−0.04
0.09
0.12
0.15
0.18
SNP effect on Leucine−rich repeats and immunoglobulin−like domains protein 3
SNP effect on Gut microbiota abundance (genus Catenibacterium id.2153)

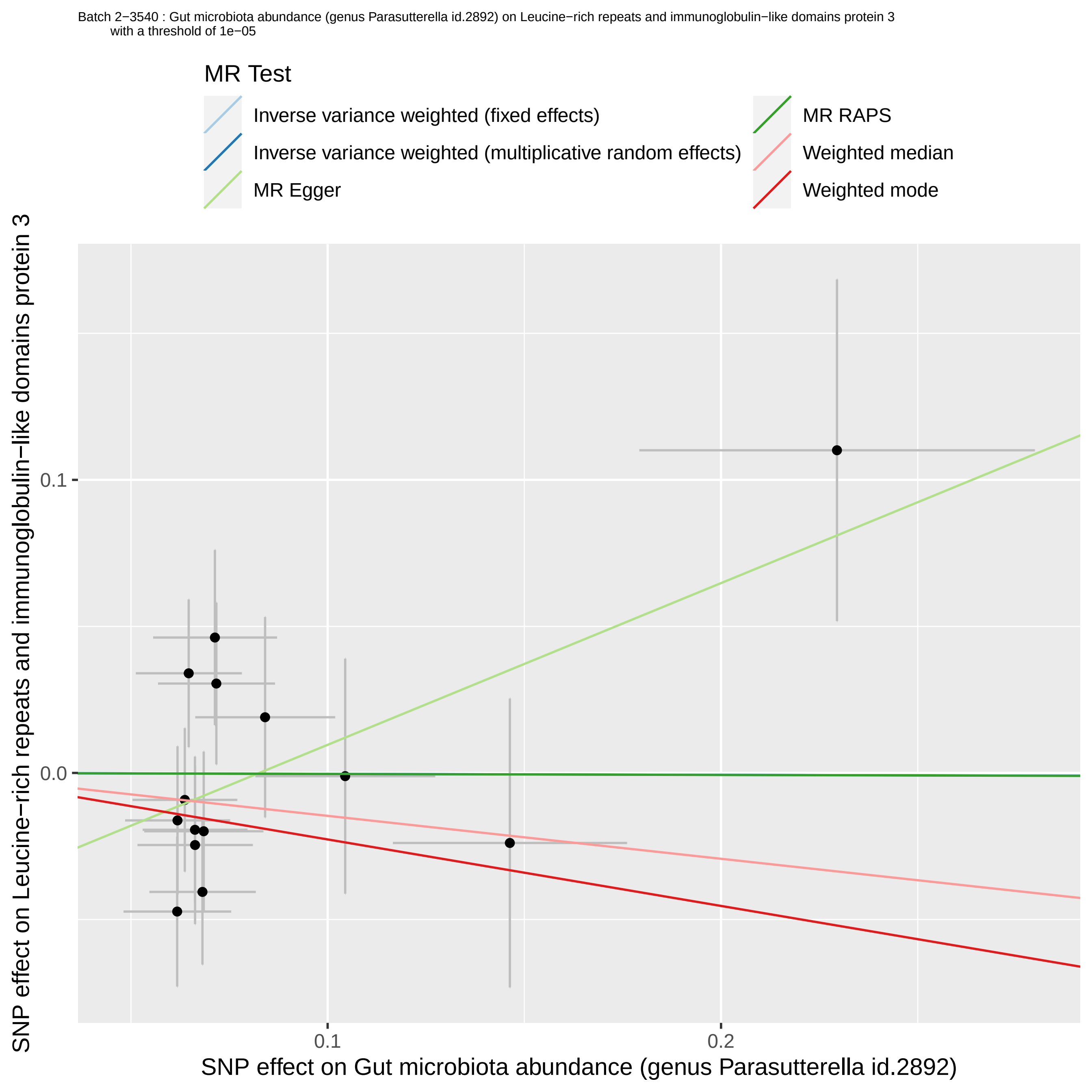

Batch 2−3540 : Gut microbiota abundance (genus Parasutterella id.2892) on Leucine−rich repeats and immunoglobulin−like domains protein 3
with a threshold of 1e−05
MR Test
Inverse variance weighted (fixed effects)
Inverse variance weighted (multiplicative random effects)
MR Egger
MR RAPS
Weighted median
Weighted mode
SNP effect on Leucine−rich repeats and immunoglobulin−like domains protein 3
0.1
0.0
0.1
0.2
SNP effect on Gut microbiota abundance (genus Parasutterella id.2892)

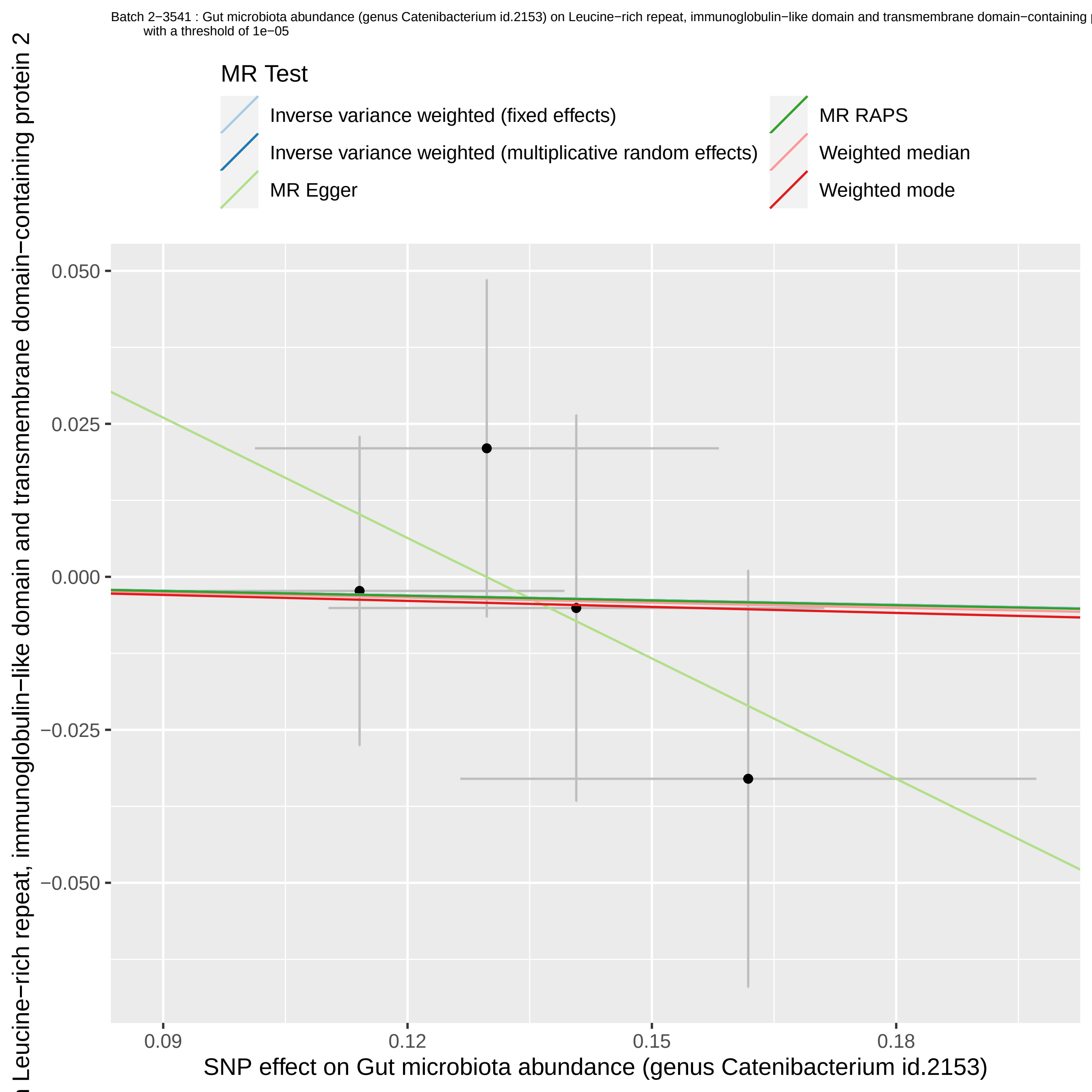
Batch 2−3541 : Gut microbiota abundance (genus Catenibacterium id.2153) on Leucine−rich repeat, immunoglobulin−like domain and transmembrane domain−containing p
with a threshold of 1e−05
MR Test
Inverse variance weighted (fixed effects)
Inverse variance weighted (multiplicative random effects)
MR Egger
MR RAPS
Weighted median
Weighted mode
0.050
0.025
0.000
−0.025
−0.050
0.09
0.12
0.15
0.18
SNP effect on Gut microbiota abundance (genus Catenibacterium id.2153)
n Leucine−rich repeat, immunoglobulin−like domain and transmembrane domain−containing protein 2

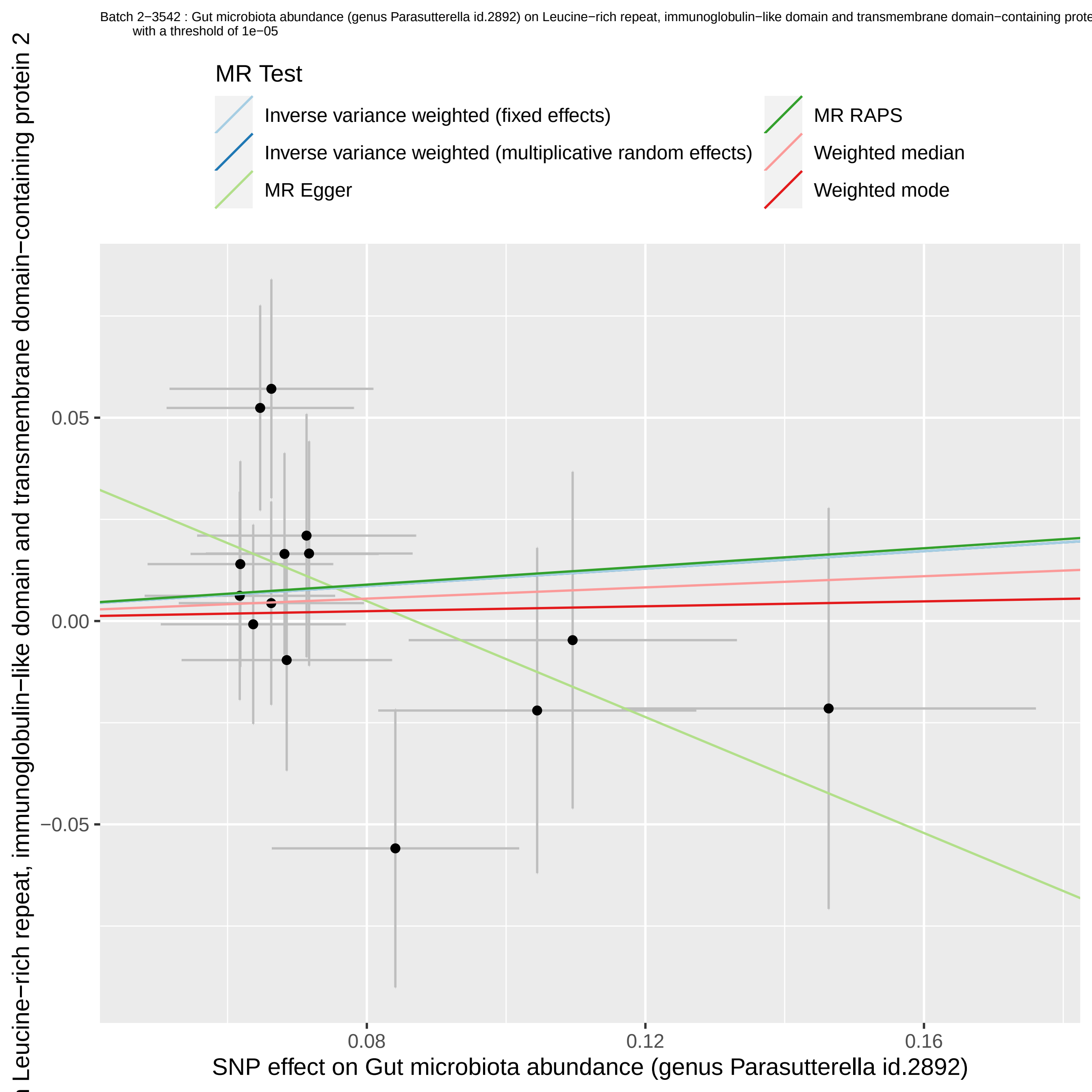

Batch 2−3542 : Gut microbiota abundance (genus Parasutterella id.2892) on Leucine−rich repeat, immunoglobulin−like domain and transmembrane domain−containing prote
with a threshold of 1e−05
MR Test
Inverse variance weighted (fixed effects)
Inverse variance weighted (multiplicative random effects)
MR Egger
MR RAPS
Weighted median
Weighted mode
0.05
0.00
−0.05
0.08
0.12
0.16
SNP effect on Gut microbiota abundance (genus Parasutterella id.2892)
n Leucine−rich repeat, immunoglobulin−like domain and transmembrane domain−containing protein 2

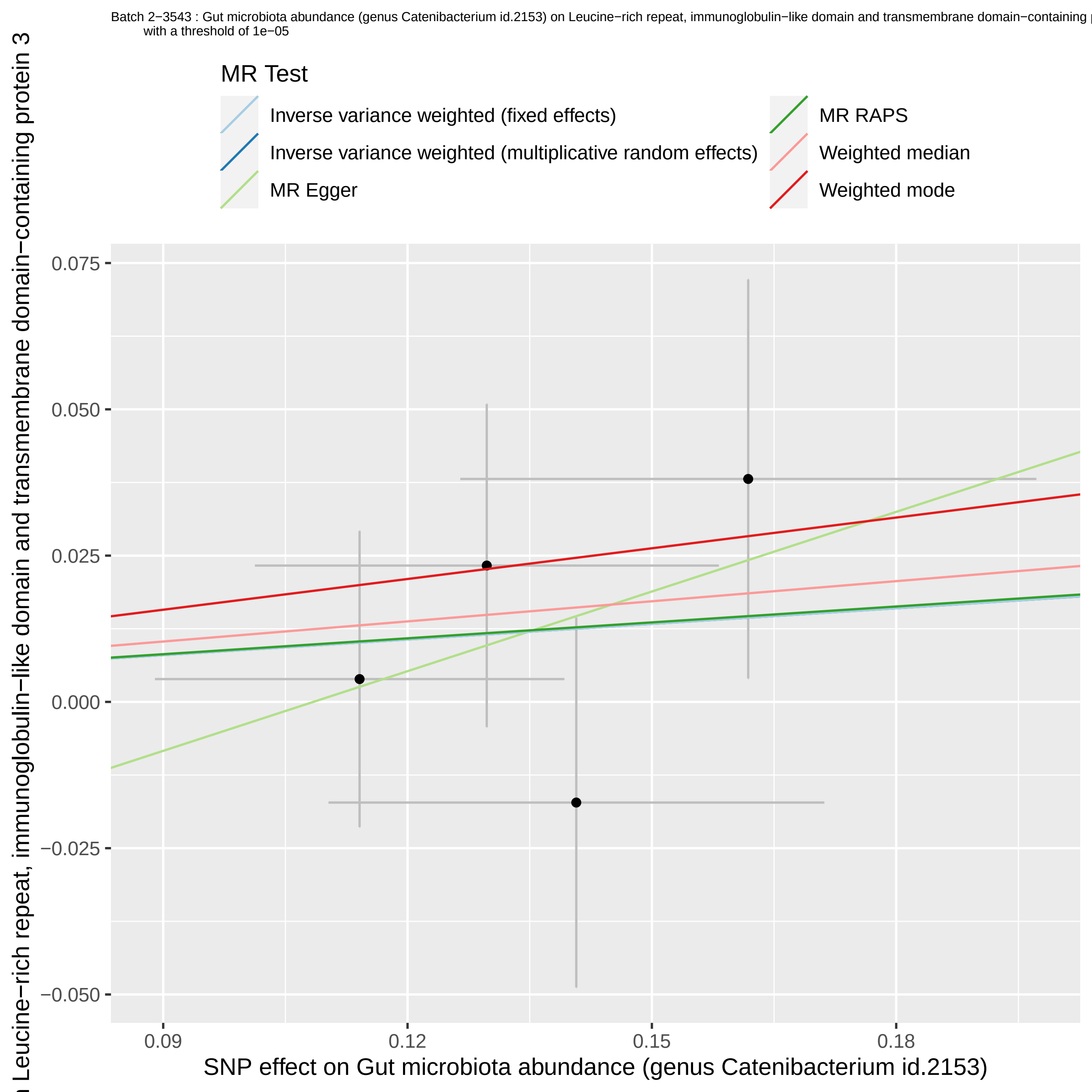

Batch 2−3543 : Gut microbiota abundance (genus Catenibacterium id.2153) on Leucine−rich repeat, immunoglobulin−like domain and transmembrane domain−containing p
with a threshold of 1e−05
MR Test
Inverse variance weighted (fixed effects)
Inverse variance weighted (multiplicative random effects)
MR Egger
MR RAPS
Weighted median
Weighted mode
0.075
0.050
0.025
0.000
−0.025
−0.050
0.09
0.12
0.15
0.18
SNP effect on Gut microbiota abundance (genus Catenibacterium id.2153)
n Leucine−rich repeat, immunoglobulin−like domain and transmembrane domain−containing protein 3

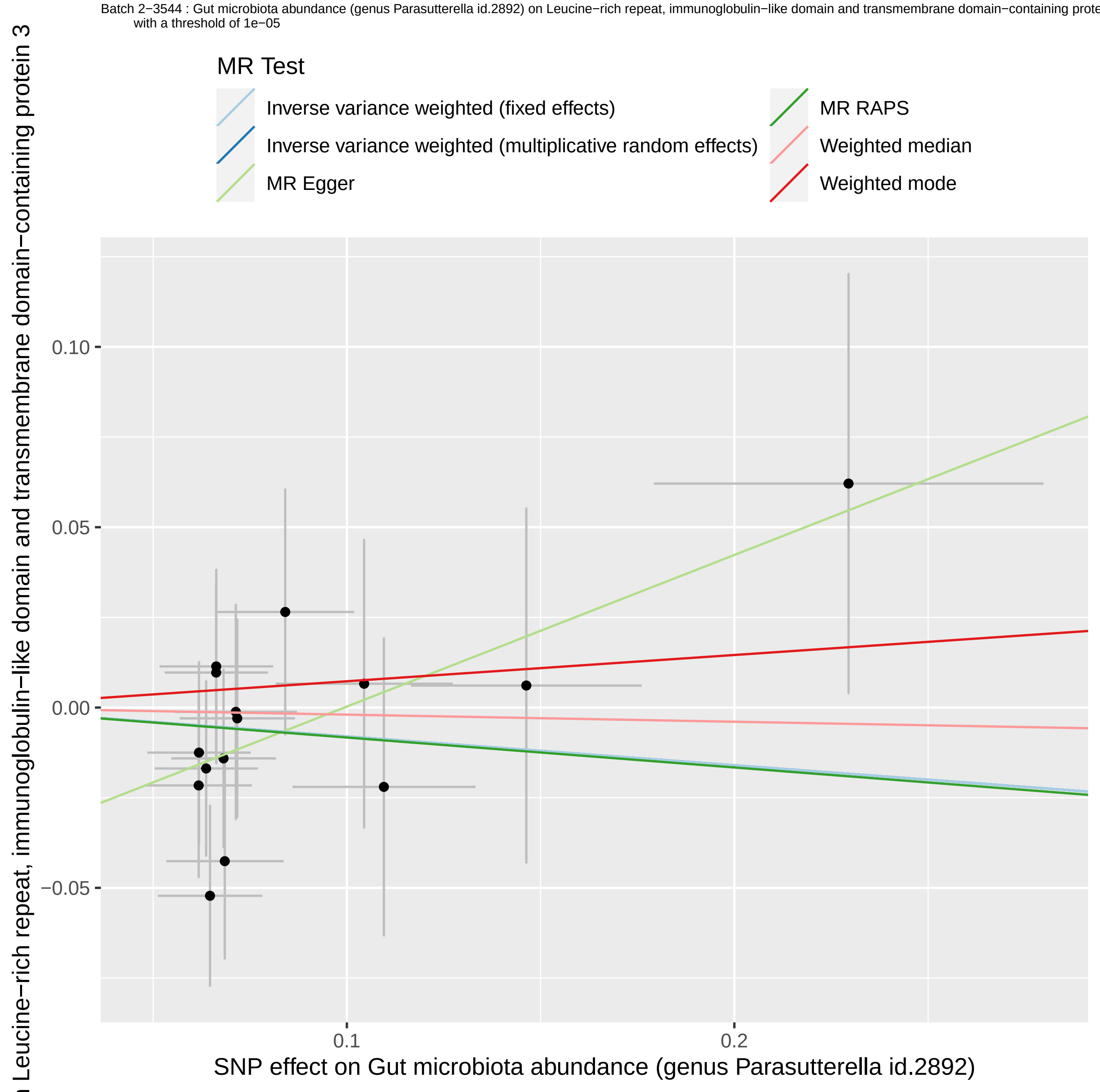
Batch 2−3544 : Gut microbiota abundance (genus Parasutterella id.2892) on Leucine−rich repeat, immunoglobulin−like domain and transmembrane domain−containing prote
with a threshold of 1e−05
MR Test
Inverse variance weighted (fixed effects)
Inverse variance weighted (multiplicative random effects)
MR Egger
MR RAPS
Weighted median
Weighted mode
0.10
0.05
0.00
−0.05
0.1
0.2
SNP effect on Gut microbiota abundance (genus Parasutterella id.2892)
n Leucine−rich repeat, immunoglobulin−like domain and transmembrane domain−containing protein 3

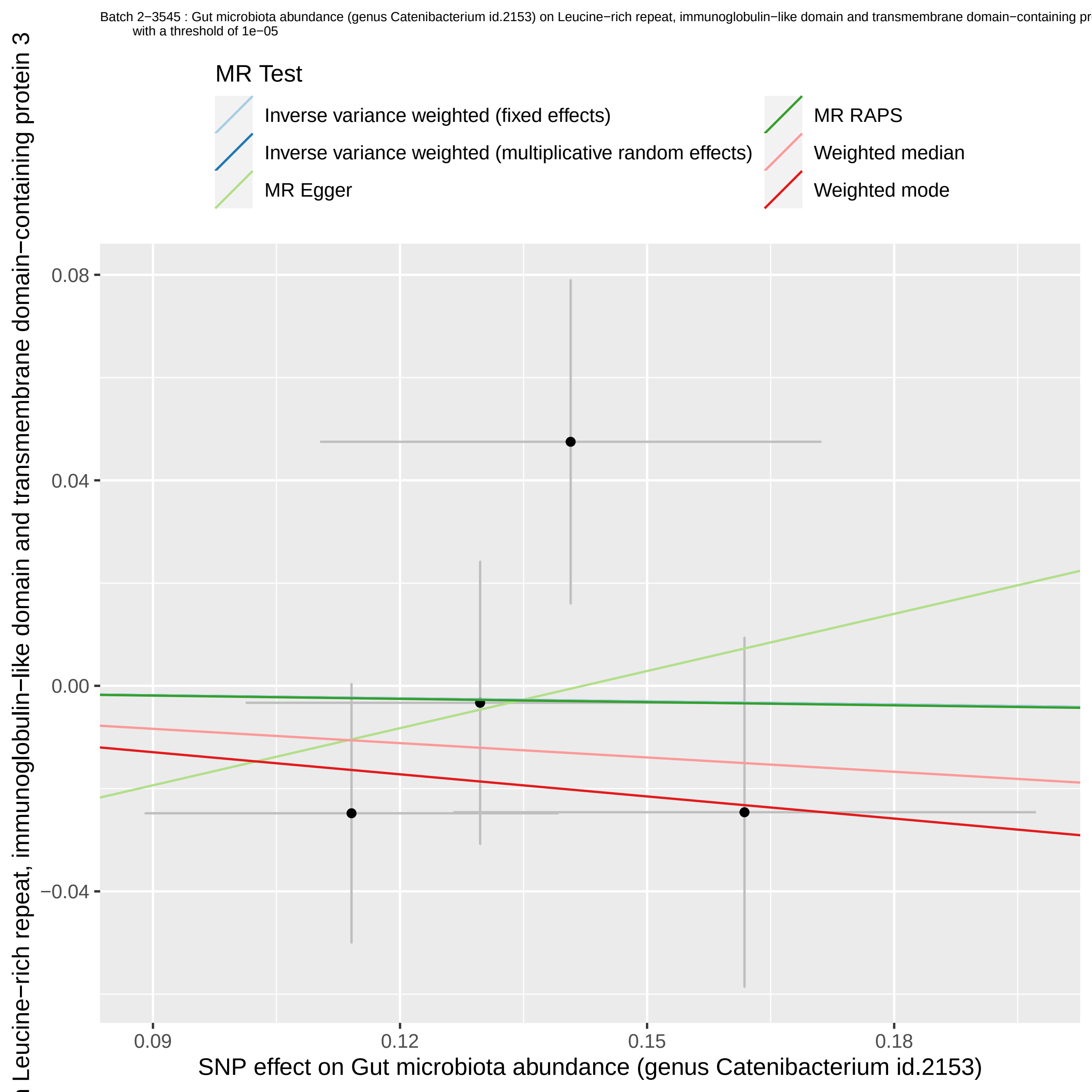
Batch 2−3545 : Gut microbiota abundance (genus Catenibacterium id.2153) on Leucine−rich repeat, immunoglobulin−like domain and transmembrane domain−containing pr
with a threshold of 1e−05
MR Test
Inverse variance weighted (fixed effects)
Inverse variance weighted (multiplicative random effects)
MR Egger
MR RAPS
Weighted median
Weighted mode
0.08
0.04
0.00
−0.04
0.09
0.12
0.15
0.18
SNP effect on Gut microbiota abundance (genus Catenibacterium id.2153)
Leucine−rich repeat, immunoglobulin−like domain and transmembrane domain−containing protein 3

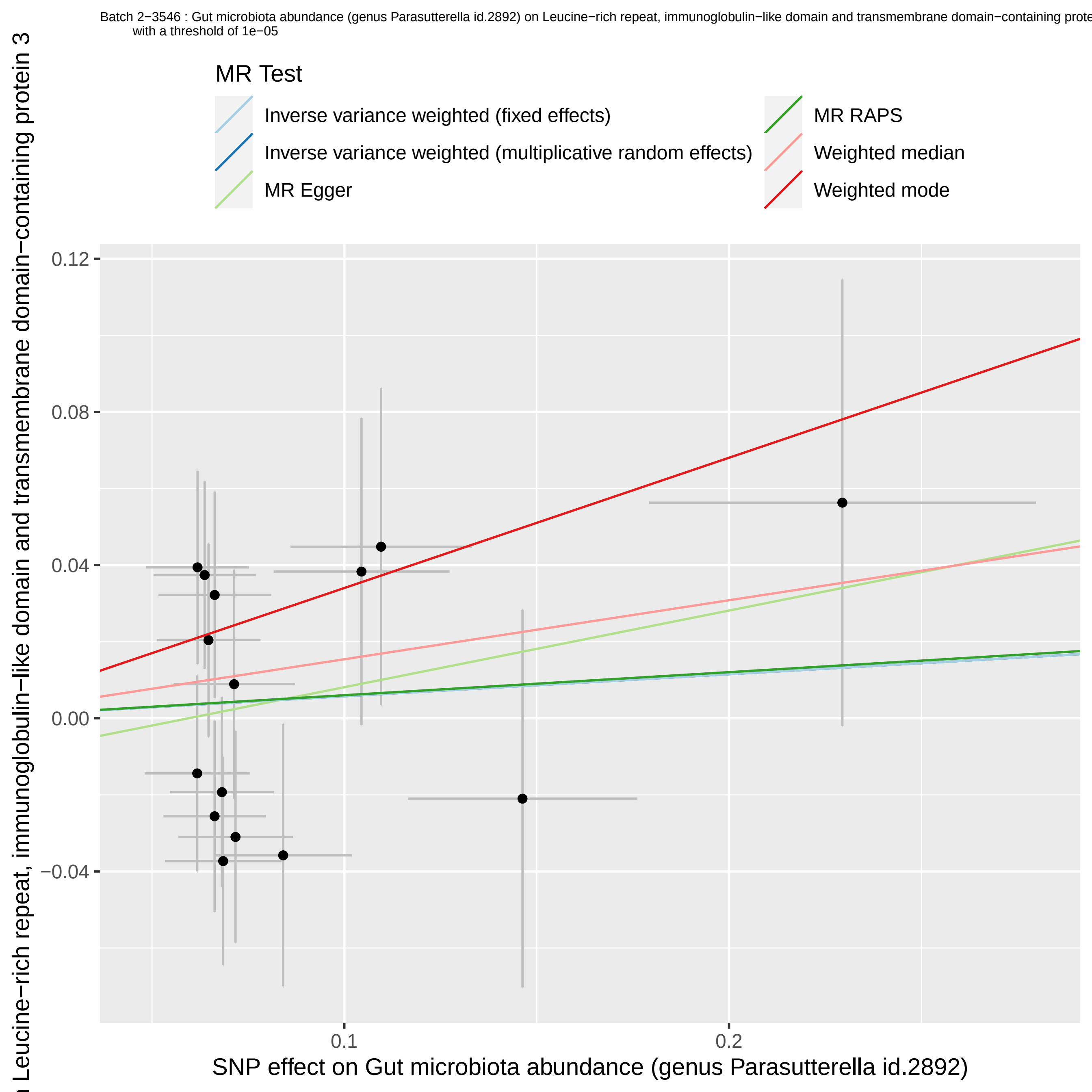

Batch 2−3546 : Gut microbiota abundance (genus Parasutterella id.2892) on Leucine−rich repeat, immunoglobulin−like domain and transmembrane domain−containing prote
with a threshold of 1e−05
MR Test
Inverse variance weighted (fixed effects)
Inverse variance weighted (multiplicative random effects)
MR Egger
MR RAPS
Weighted median
Weighted mode
0.12
0.08
0.04
0.00
−0.04
0.1
0.2
SNP effect on Gut microbiota abundance (genus Parasutterella id.2892)
n Leucine−rich repeat, immunoglobulin−like domain and transmembrane domain−containing protein 3

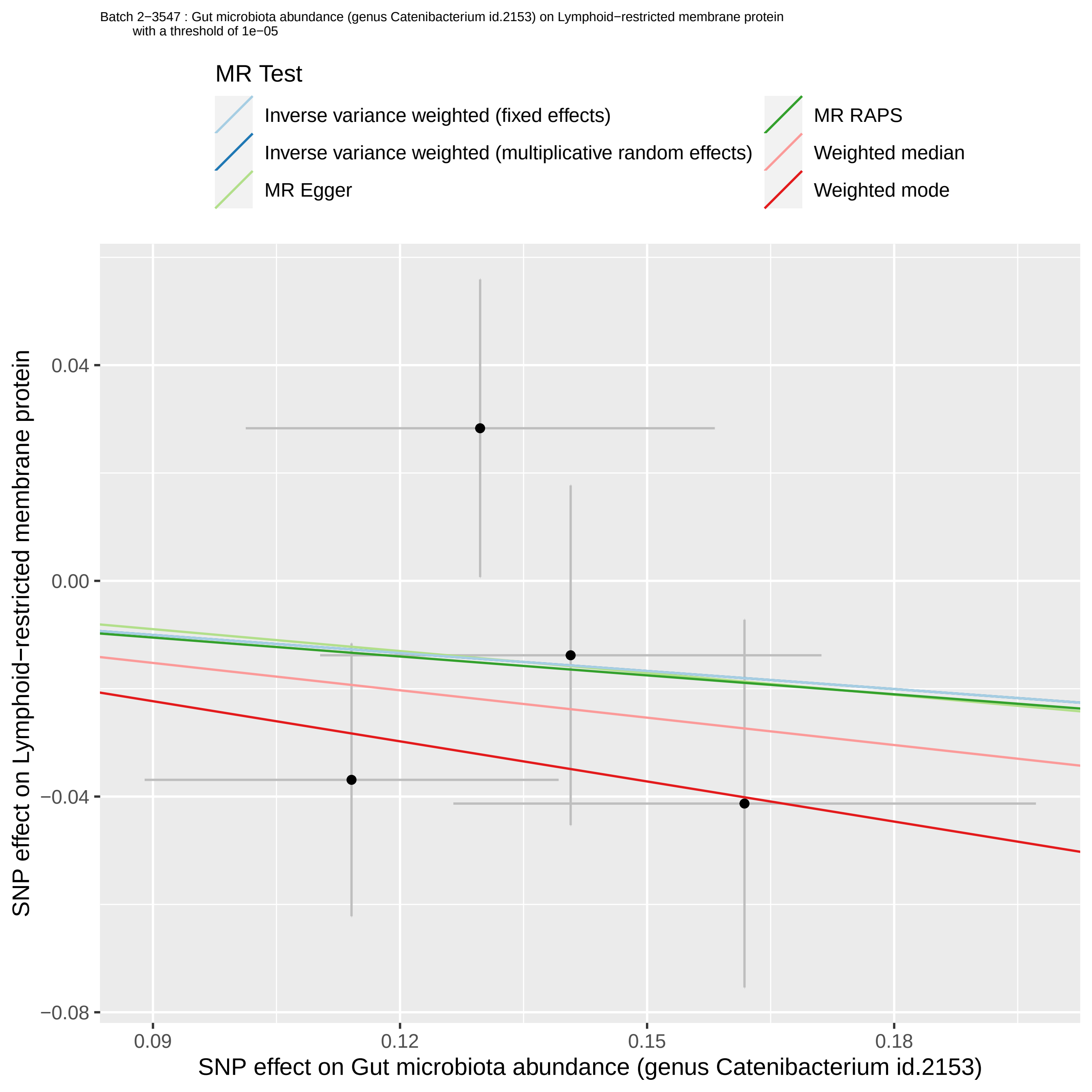

Batch 2−3547 : Gut microbiota abundance (genus Catenibacterium id.2153) on Lymphoid−restricted membrane protein
with a threshold of 1e−05
MR Test
Inverse variance weighted (fixed effects)
Inverse variance weighted (multiplicative random effects)
MR Egger
MR RAPS
Weighted median
Weighted mode
0.04
0.00
−0.04
−0.08
0.09
0.12
0.15
0.18
SNP effect on Lymphoid−restricted membrane protein
SNP effect on Gut microbiota abundance (genus Catenibacterium id.2153)

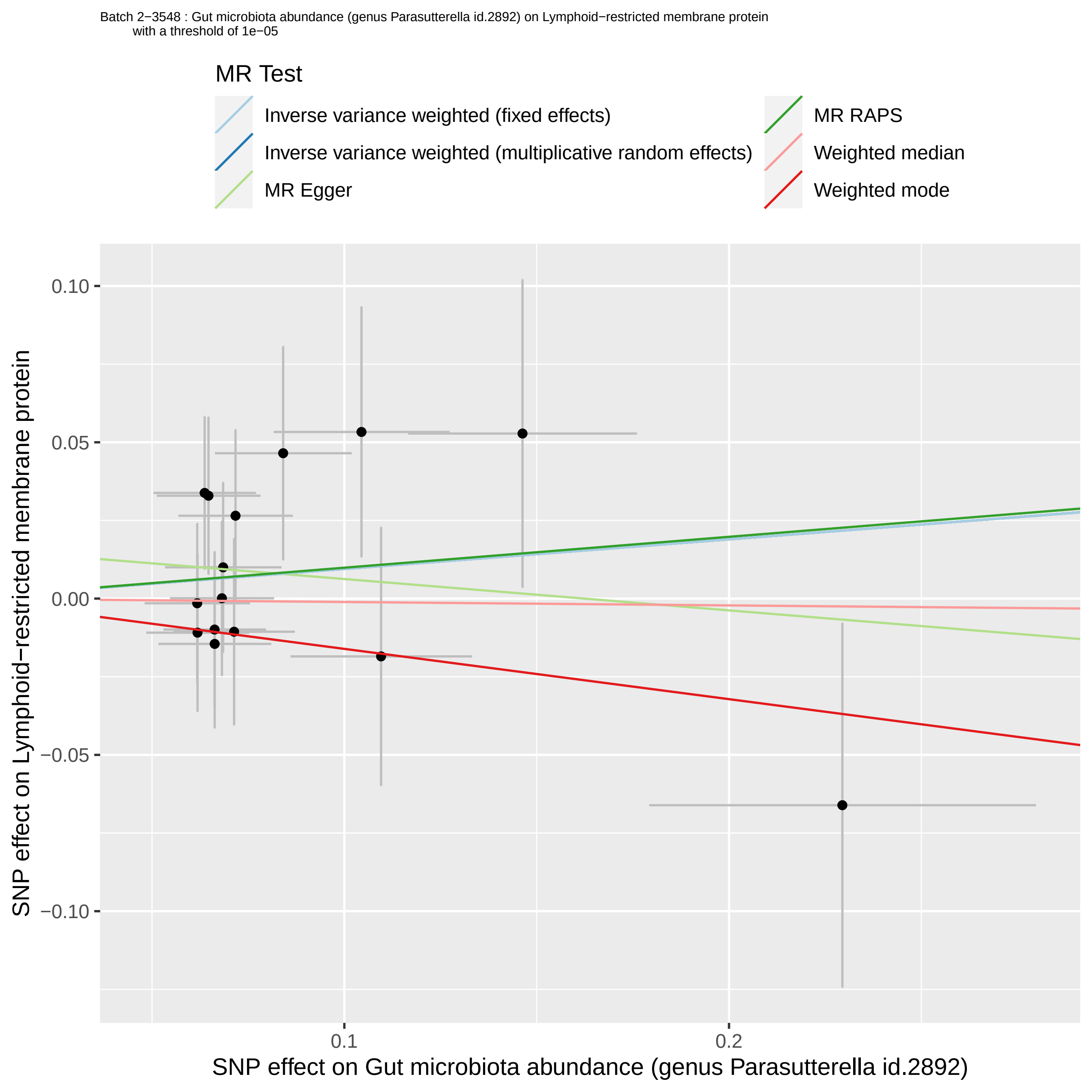

Batch 2−3548 : Gut microbiota abundance (genus Parasutterella id.2892) on Lymphoid−restricted membrane protein
with a threshold of 1e−05
MR Test
Inverse variance weighted (fixed effects)
Inverse variance weighted (multiplicative random effects)
MR Egger
MR RAPS
Weighted median
Weighted mode
SNP effect on Lymphoid−restricted membrane protein
0.10
0.05
0.00
−0.05
−0.10
0.1
0.2
SNP effect on Gut microbiota abundance (genus Parasutterella id.2892)

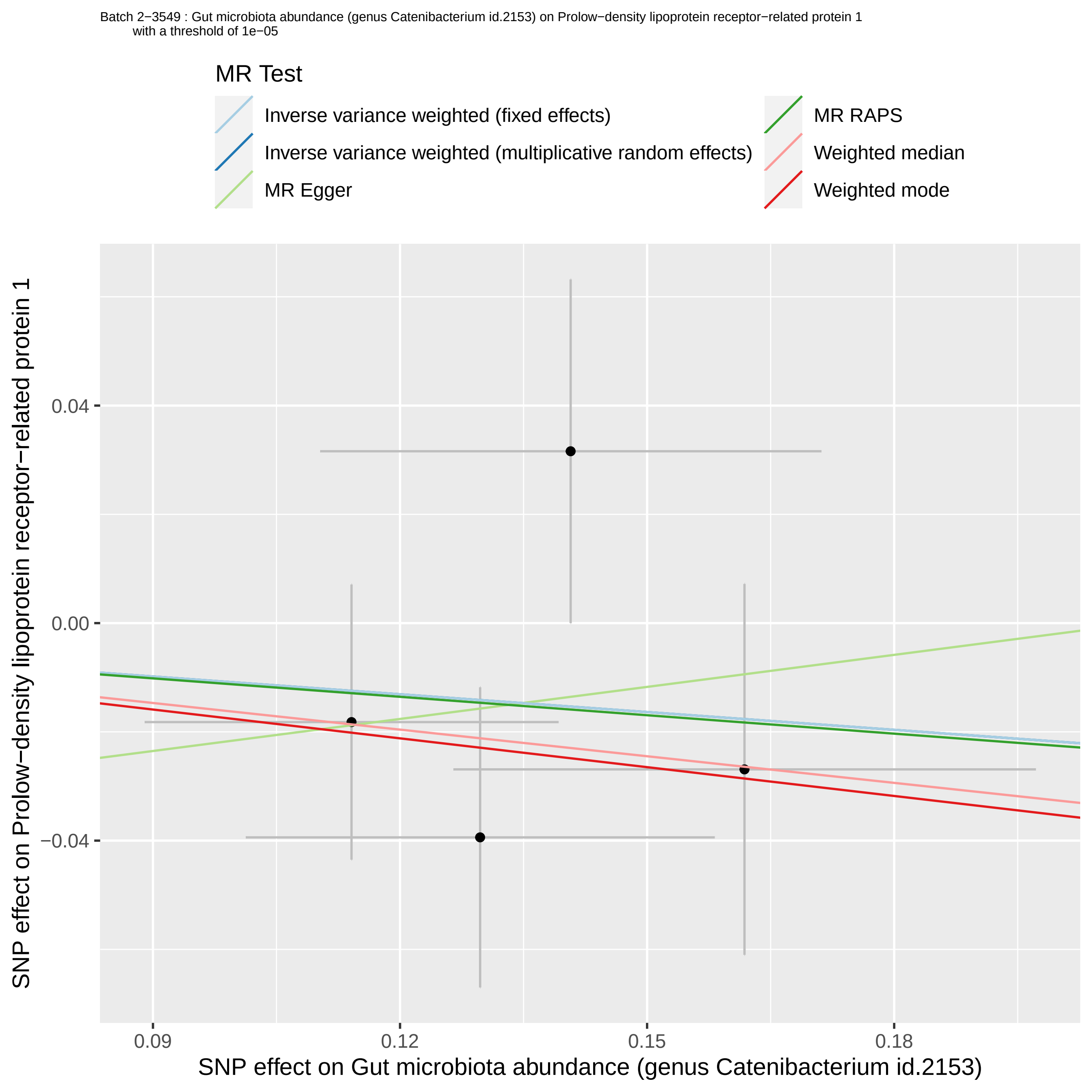
Batch 2−3549 : Gut microbiota abundance (genus Catenibacterium id.2153) on Prolow−density lipoprotein receptor−related protein 1
with a threshold of 1e−05
MR Test
Inverse variance weighted (fixed effects)
Inverse variance weighted (multiplicative random effects)
MR Egger
MR RAPS
Weighted median
Weighted mode
0.04
0.00
−0.04
0.09
0.12
0.15
0.18
SNP effect on Prolow−density lipoprotein receptor−related protein 1
SNP effect on Gut microbiota abundance (genus Catenibacterium id.2153)

Batch 2−3550 : Gut microbiota abundance (genus Parasutterella id.2892) on Prolow−density lipoprotein receptor−related protein 1 with a threshold of 1e−05

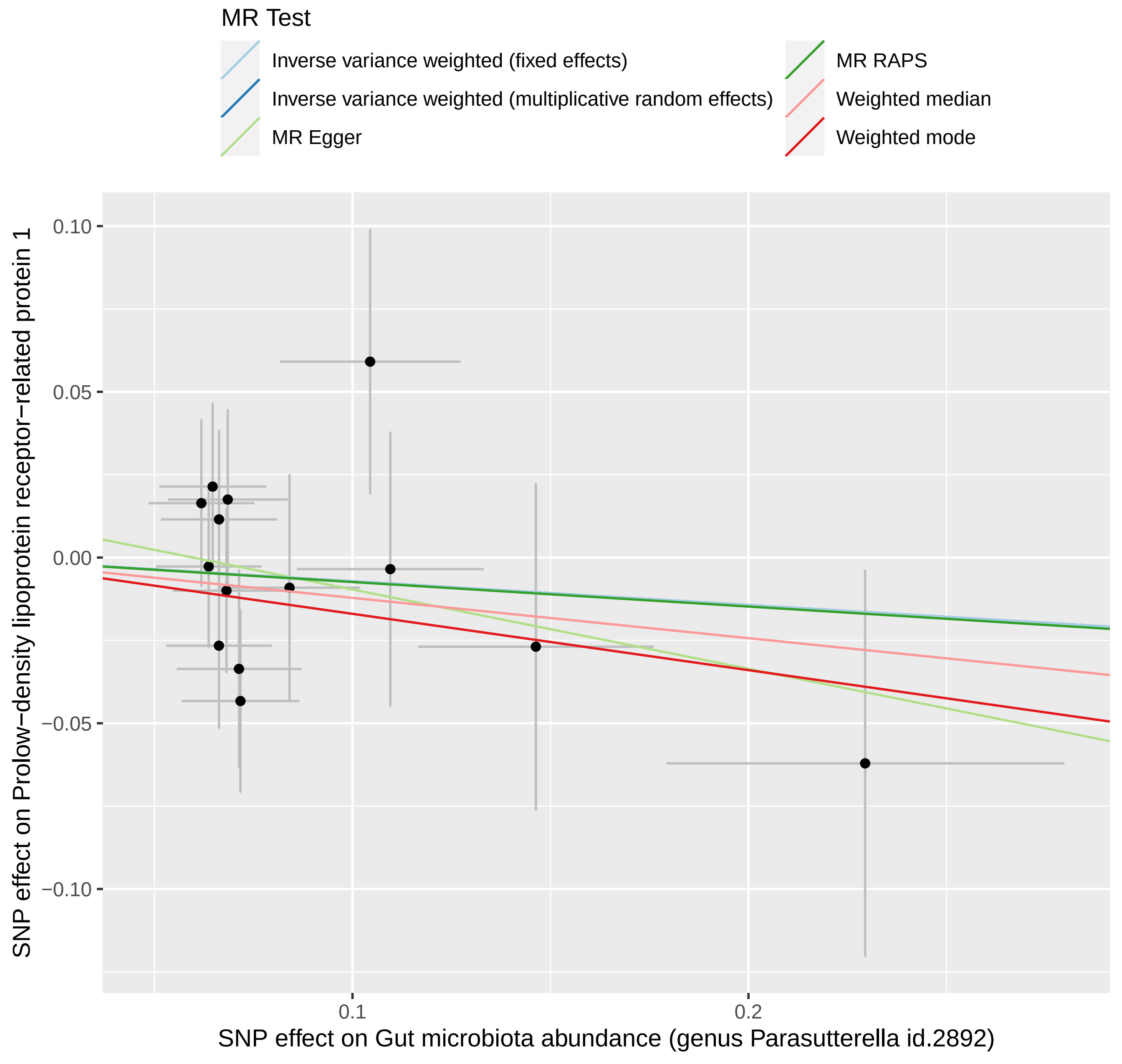

Batch 2−3551 : Gut microbiota abundance (genus Catenibacterium id.2153) on Low−density lipoprotein receptor−related protein 1, soluble
with a threshold of 1e−05

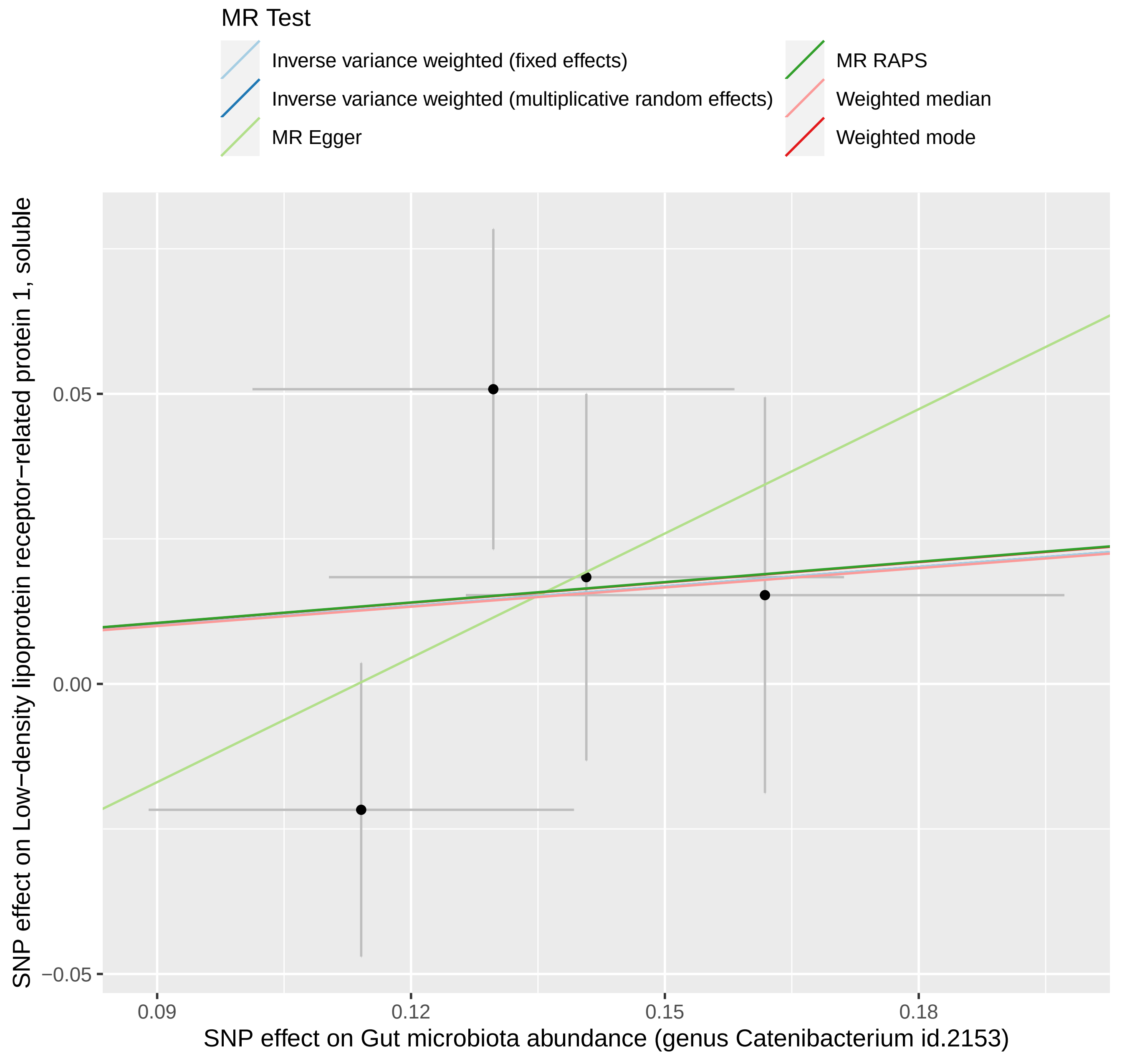

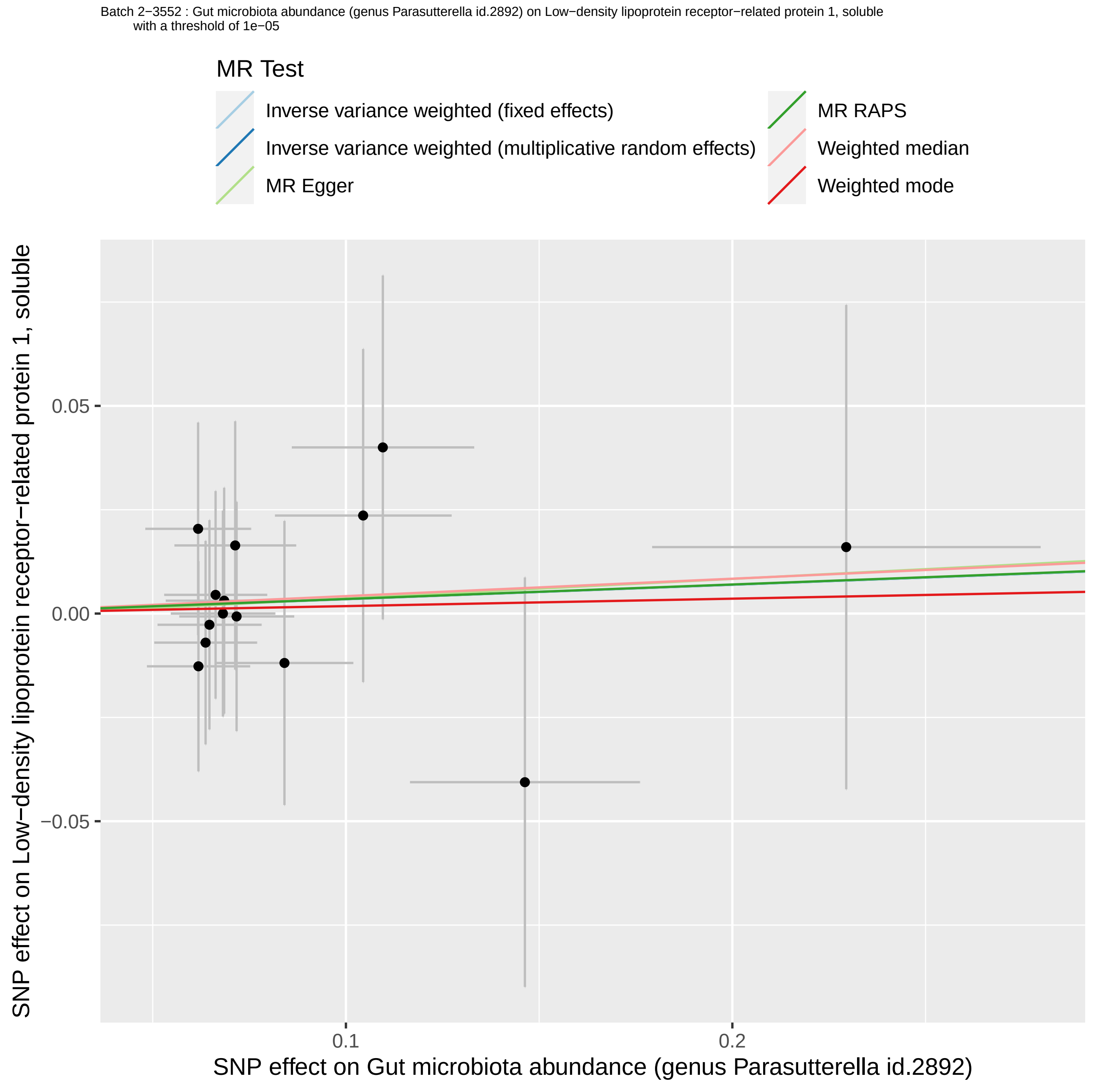
Batch 2−3552 : Gut microbiota abundance (genus Parasutterella id.2892) on Low−density lipoprotein receptor−related protein 1, soluble
with a threshold of 1e−05
MR Test
Inverse variance weighted (fixed effects)
Inverse variance weighted (multiplicative random effects)
MR Egger
MR RAPS
Weighted median
Weighted mode
SNP effect on Low−density lipoprotein receptor−related protein 1, soluble
0.05
0.00
−0.05
0.1
0.2
SNP effect on Gut microbiota abundance (genus Parasutterella id.2892)

Batch 2−3553 : Gut microbiota abundance (genus Catenibacterium id.2153) on Low−density lipoprotein receptor−related protein 1, soluble
with a threshold of 1e−05

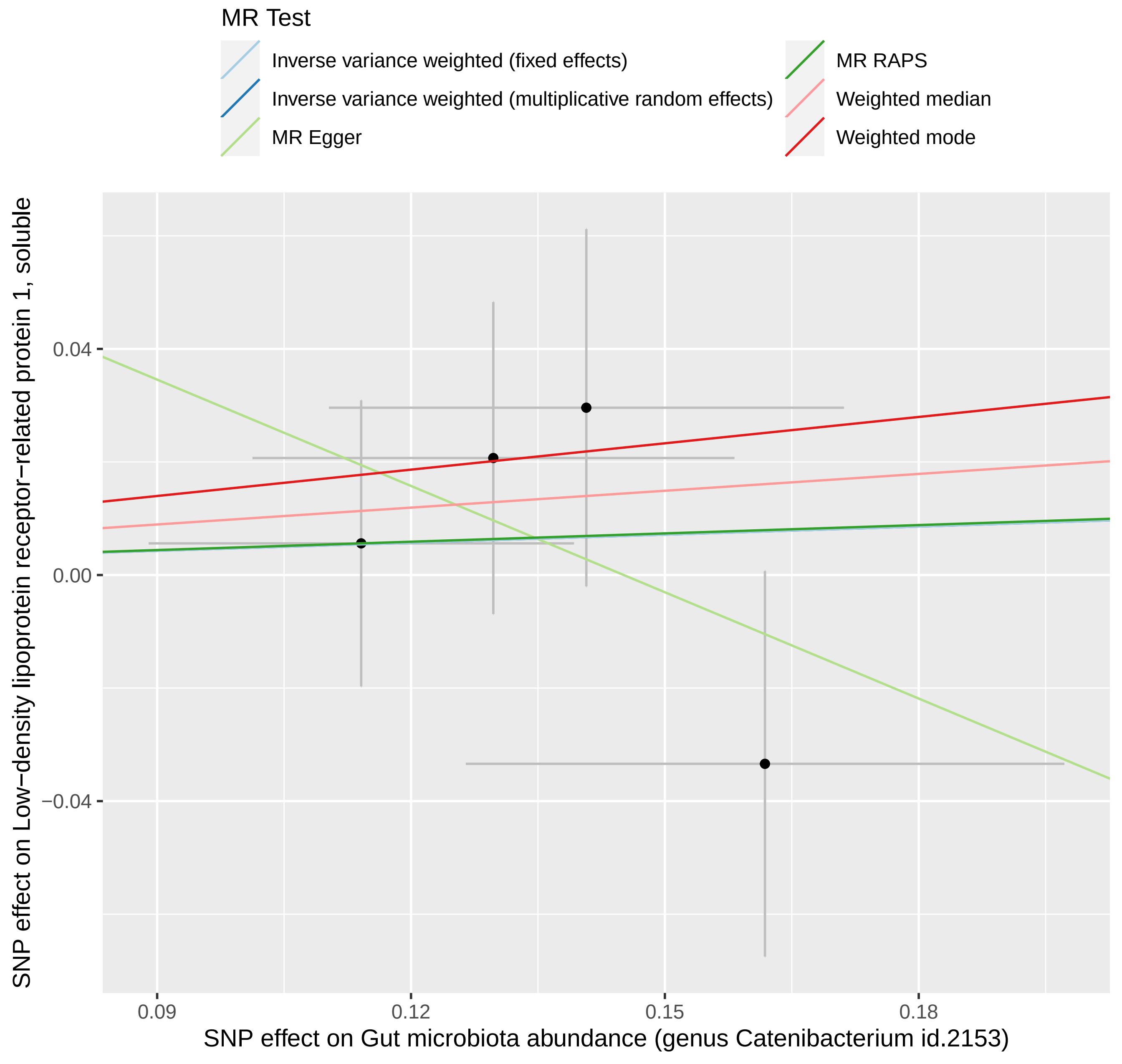

Batch 2−3554 : Gut microbiota abundance (genus Parasutterella id.2892) on Low−density lipoprotein receptor−related protein 1, soluble with a threshold of 1e−05

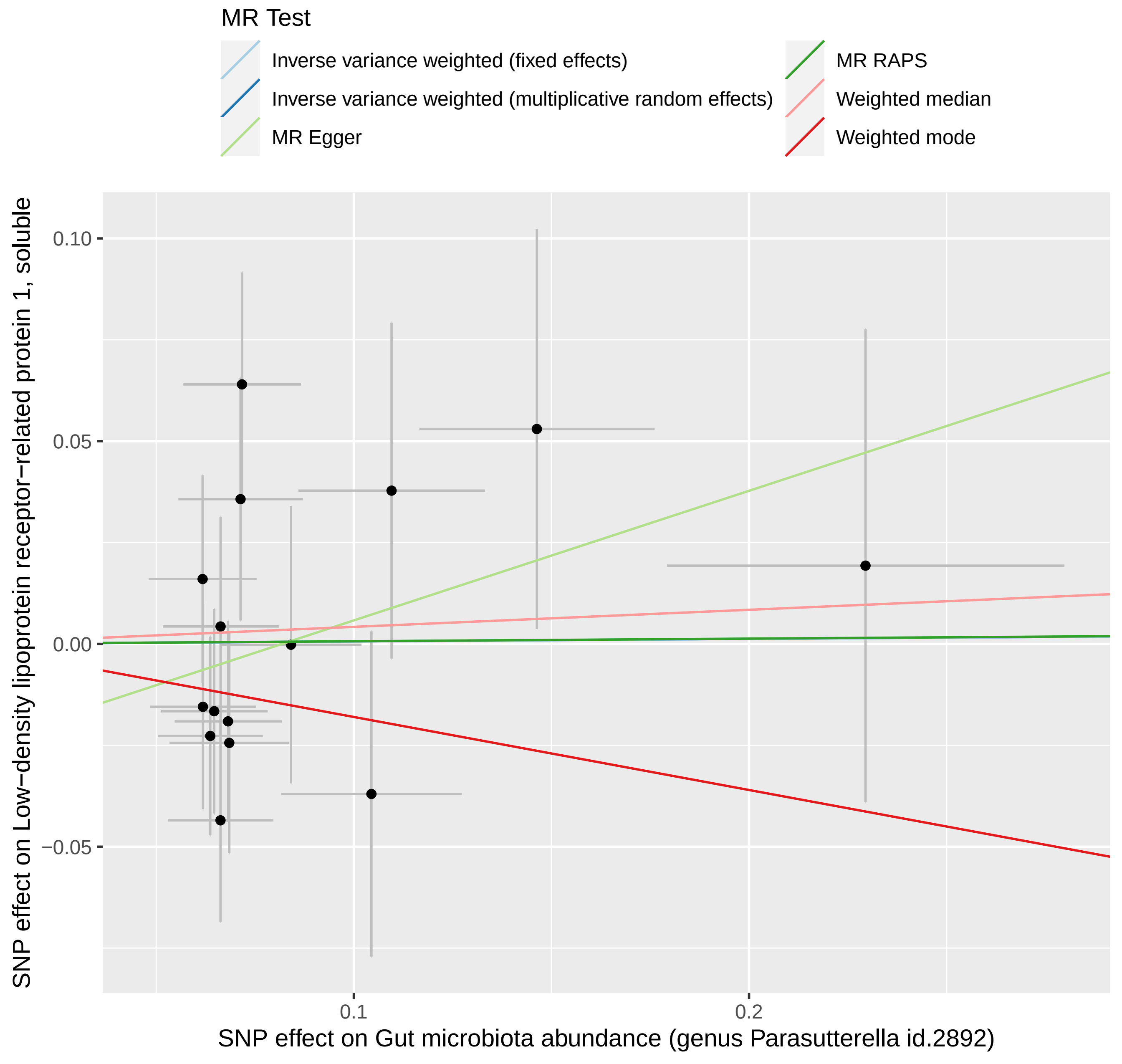

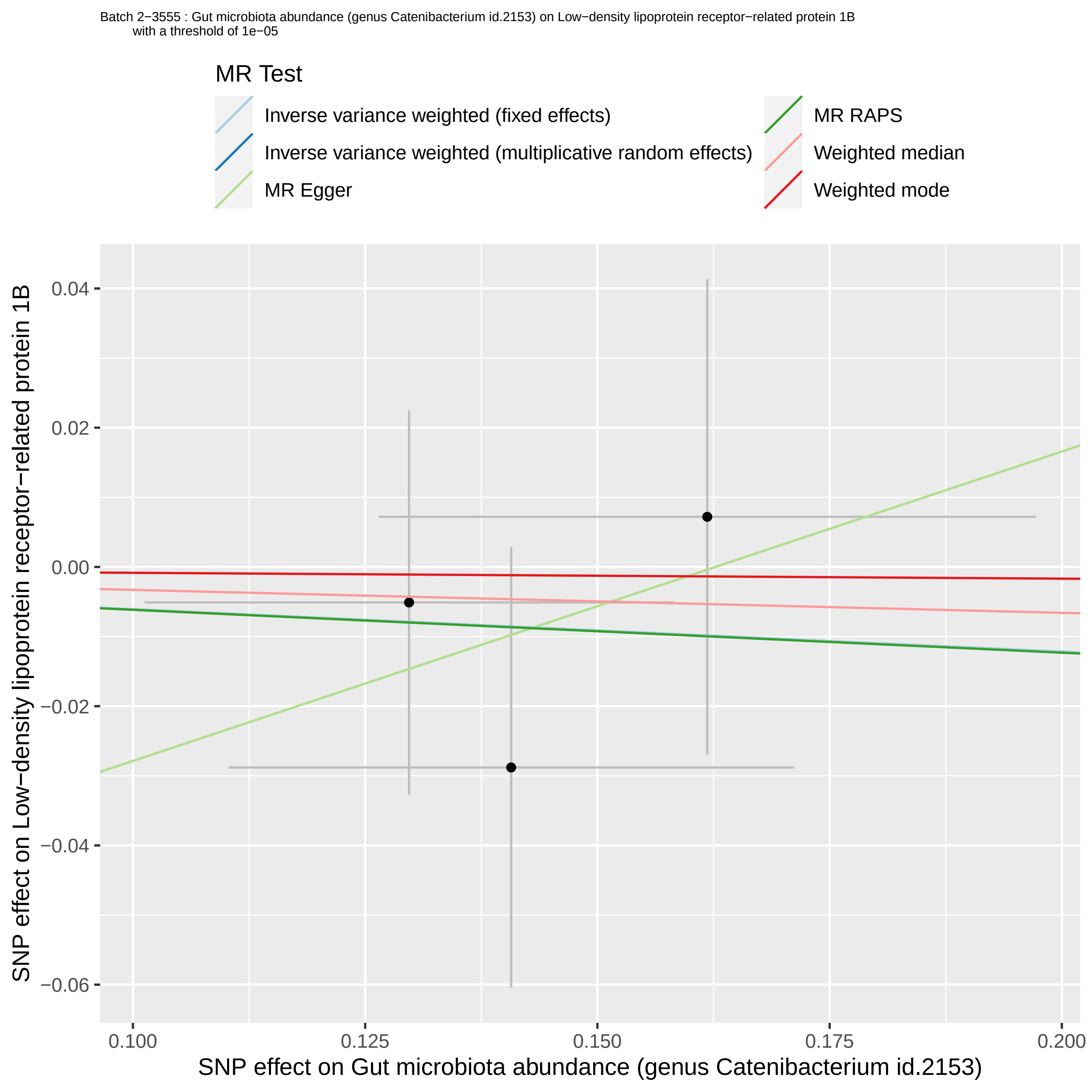

Batch 2−3555 : Gut microbiota abundance (genus Catenibacterium id.2153) on Low−density lipoprotein receptor−related protein 1B
with a threshold of 1e−05
MR Test
Inverse variance weighted (fixed effects)
Inverse variance weighted (multiplicative random effects)
MR Egger
MR RAPS
Weighted median
Weighted mode
0.04
0.02
0.00
−0.02
−0.04
−0.06
0.100
0.125
0.150
0.175
0.200
SNP effect on Low−density lipoprotein receptor−related protein 1B
SNP effect on Gut microbiota abundance (genus Catenibacterium id.2153)

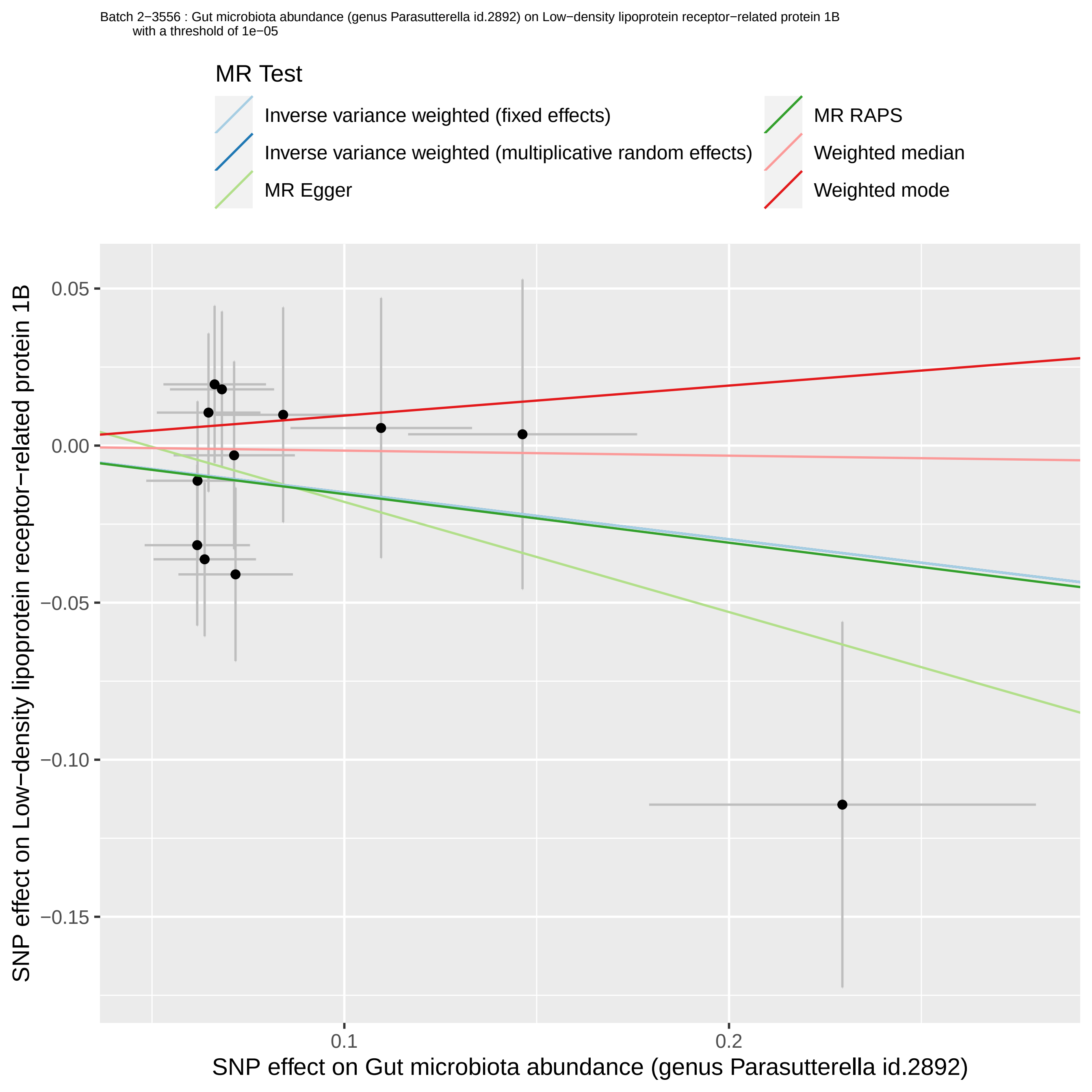
Batch 2−3556 : Gut microbiota abundance (genus Parasutterella id.2892) on Low−density lipoprotein receptor−related protein 1B
with a threshold of 1e−05
MR Test
Inverse variance weighted (fixed effects)
Inverse variance weighted (multiplicative random effects)
MR Egger
MR RAPS
Weighted median
Weighted mode
0.05
0.00
−0.05
−0.10
−0.15
0.1
0.2
SNP effect on Low−density lipoprotein receptor−related protein 1B
SNP effect on Gut microbiota abundance (genus Parasutterella id.2892)

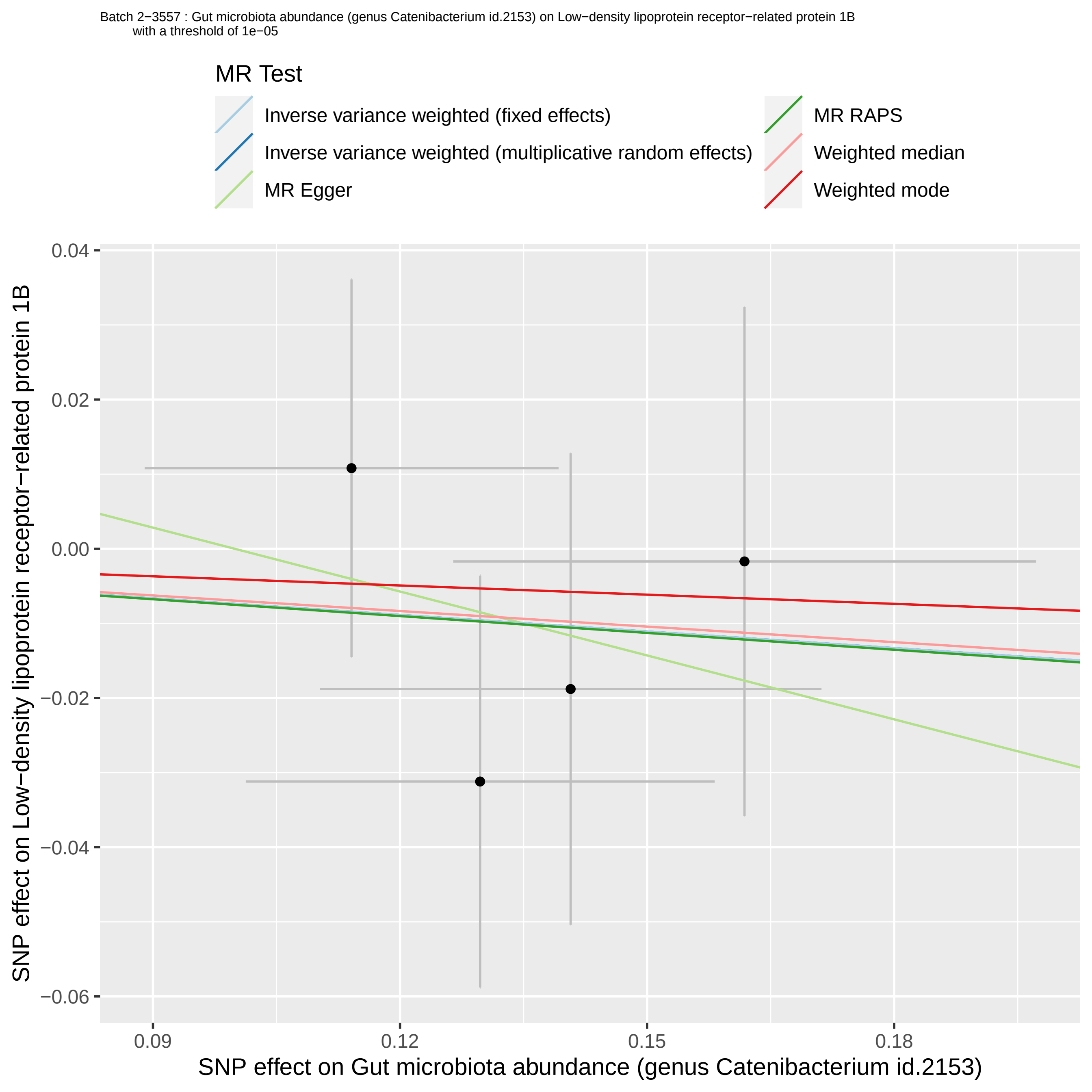
Batch 2−3557 : Gut microbiota abundance (genus Catenibacterium id.2153) on Low−density lipoprotein receptor−related protein 1B
with a threshold of 1e−05
MR Test
Inverse variance weighted (fixed effects)
Inverse variance weighted (multiplicative random effects)
MR Egger
MR RAPS
Weighted median
Weighted mode
0.04
0.02
0.00
−0.02
−0.04
−0.06
0.09
0.12
0.15
0.18
SNP effect on Low−density lipoprotein receptor−related protein 1B
SNP effect on Gut microbiota abundance (genus Catenibacterium id.2153)

Batch 2−3558 : Gut microbiota abundance (genus Parasutterella id.2892) on Low−density lipoprotein receptor−related protein 1B with a threshold of 1e−05

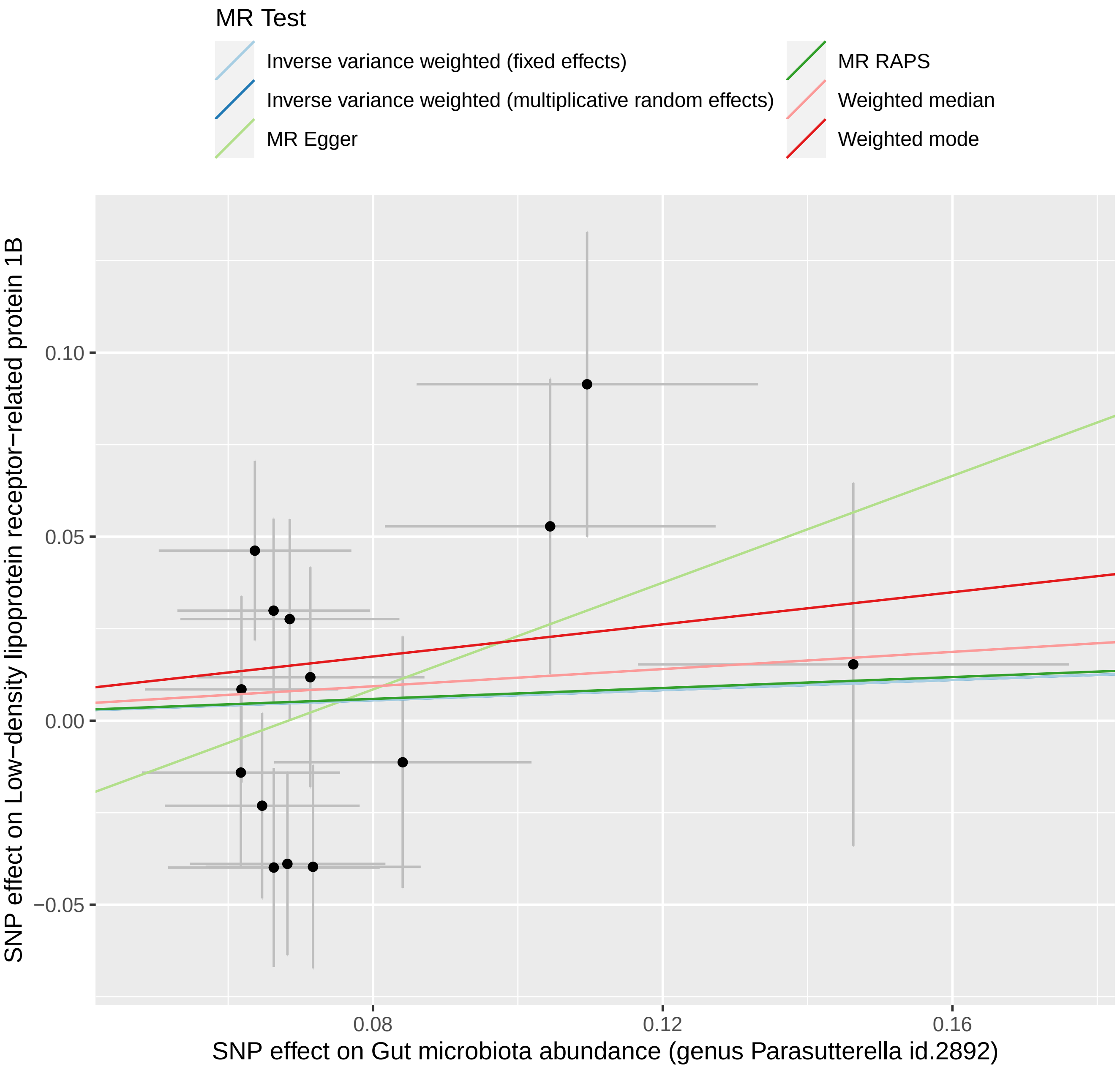

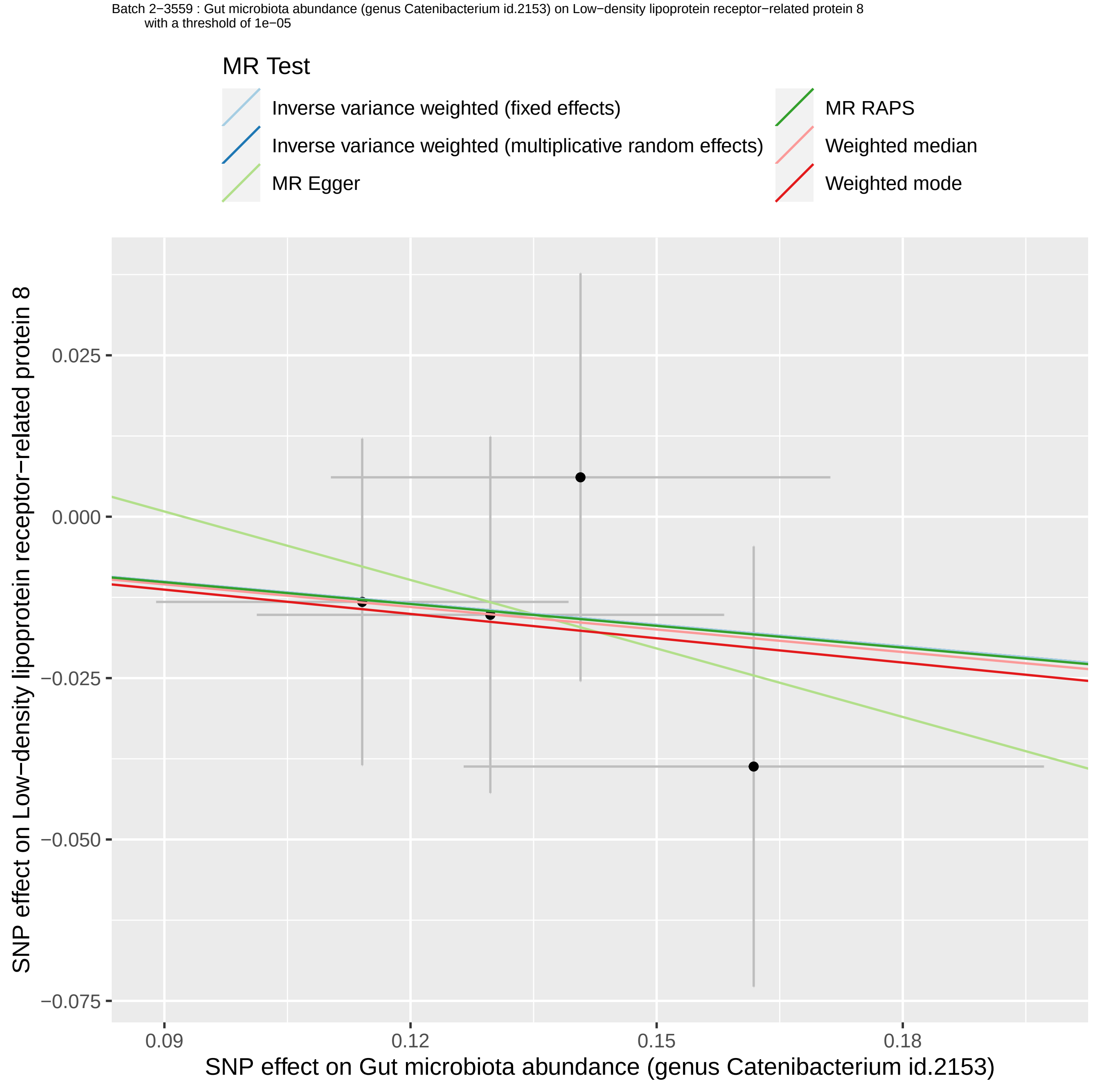

Batch 2−3559 : Gut microbiota abundance (genus Catenibacterium id.2153) on Low−density lipoprotein receptor−related protein 8
with a threshold of 1e−05
MR Test
Inverse variance weighted (fixed effects)
Inverse variance weighted (multiplicative random effects)
MR Egger
MR RAPS
Weighted median
Weighted mode
SNP effect on Low−density lipoprotein receptor−related protein 8
0.025
0.000
−0.025
−0.050
−0.075
0.09
0.12
0.15
0.18
SNP effect on Gut microbiota abundance (genus Catenibacterium id.2153)

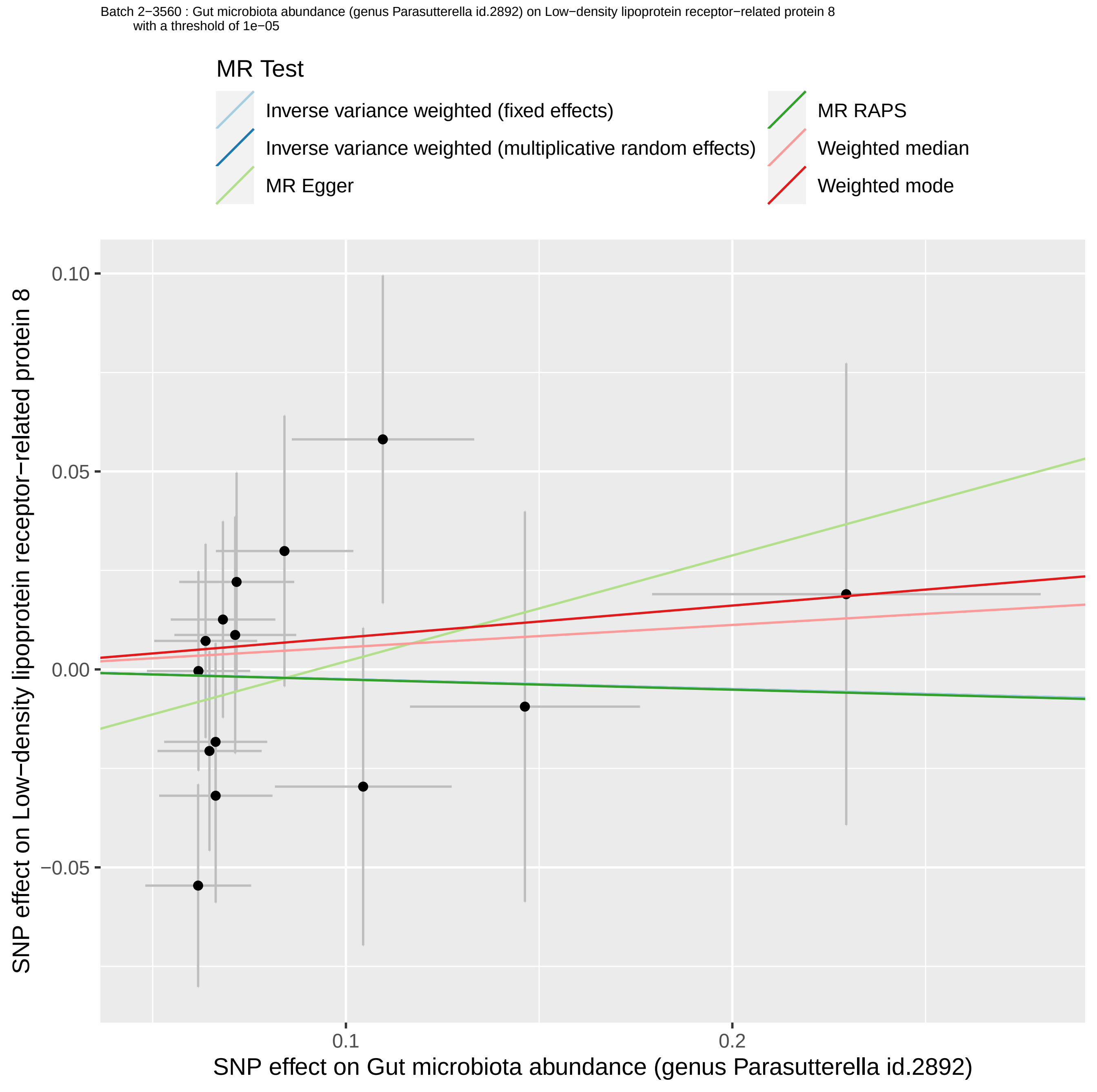
Batch 2−3560 : Gut microbiota abundance (genus Parasutterella id.2892) on Low−density lipoprotein receptor−related protein 8
with a threshold of 1e−05
MR Test
Inverse variance weighted (fixed effects)
Inverse variance weighted (multiplicative random effects)
MR Egger
MR RAPS
Weighted median
Weighted mode
0.10
0.05
0.00
−0.05
0.1
0.2
SNP effect on Gut microbiota abundance (genus Parasutterella id.2892)
SNP effect on Low−density lipoprotein receptor−related protein 8

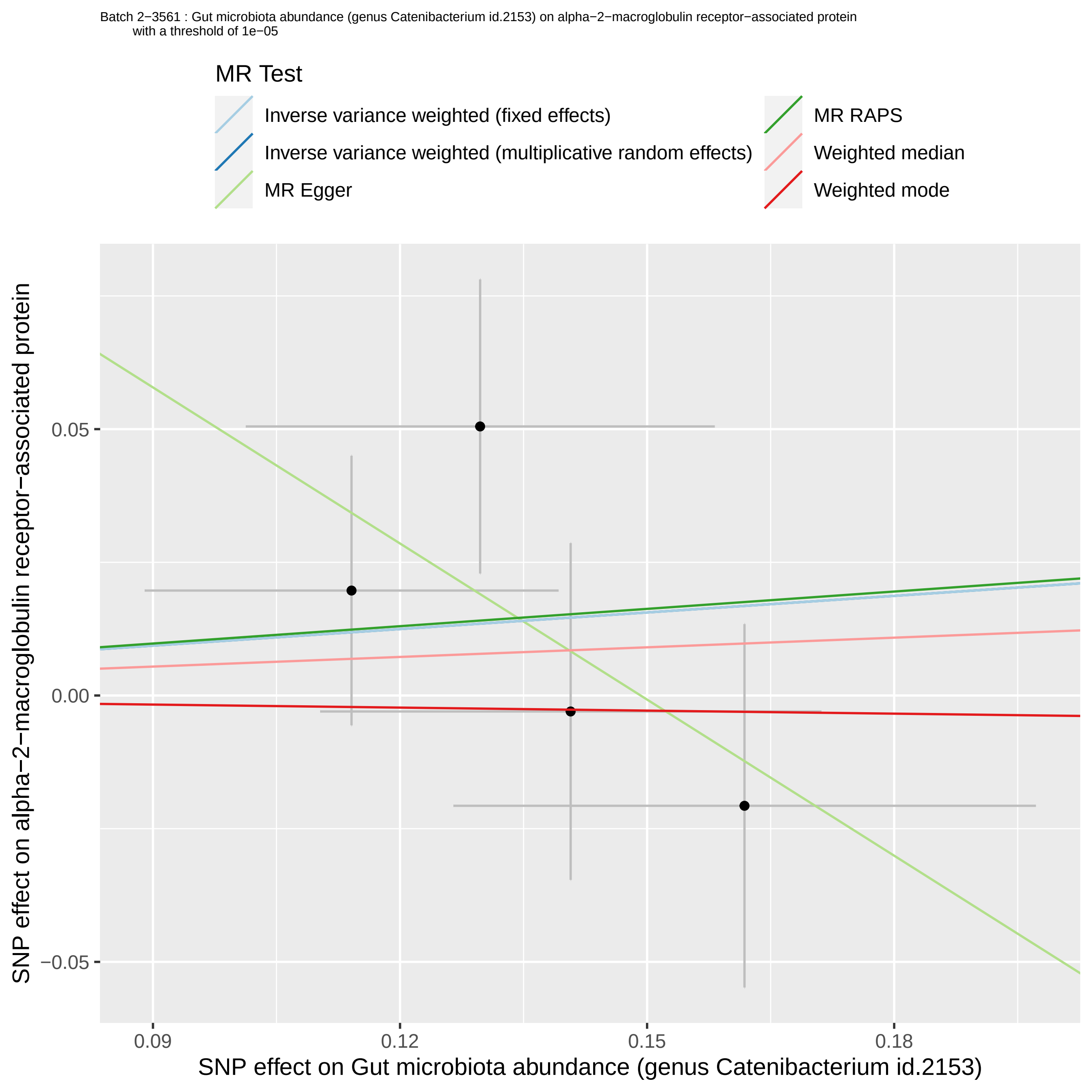
Batch 2−3561 : Gut microbiota abundance (genus Catenibacterium id.2153) on alpha−2−macroglobulin receptor−associated protein
with a threshold of 1e−05
MR Test
Inverse variance weighted (fixed effects)
Inverse variance weighted (multiplicative random effects)
MR Egger
MR RAPS
Weighted median
Weighted mode
SNP effect on alpha−2−macroglobulin receptor−associated protein
0.05
0.00
−0.05
0.09
0.12
0.15
0.18
SNP effect on Gut microbiota abundance (genus Catenibacterium id.2153)

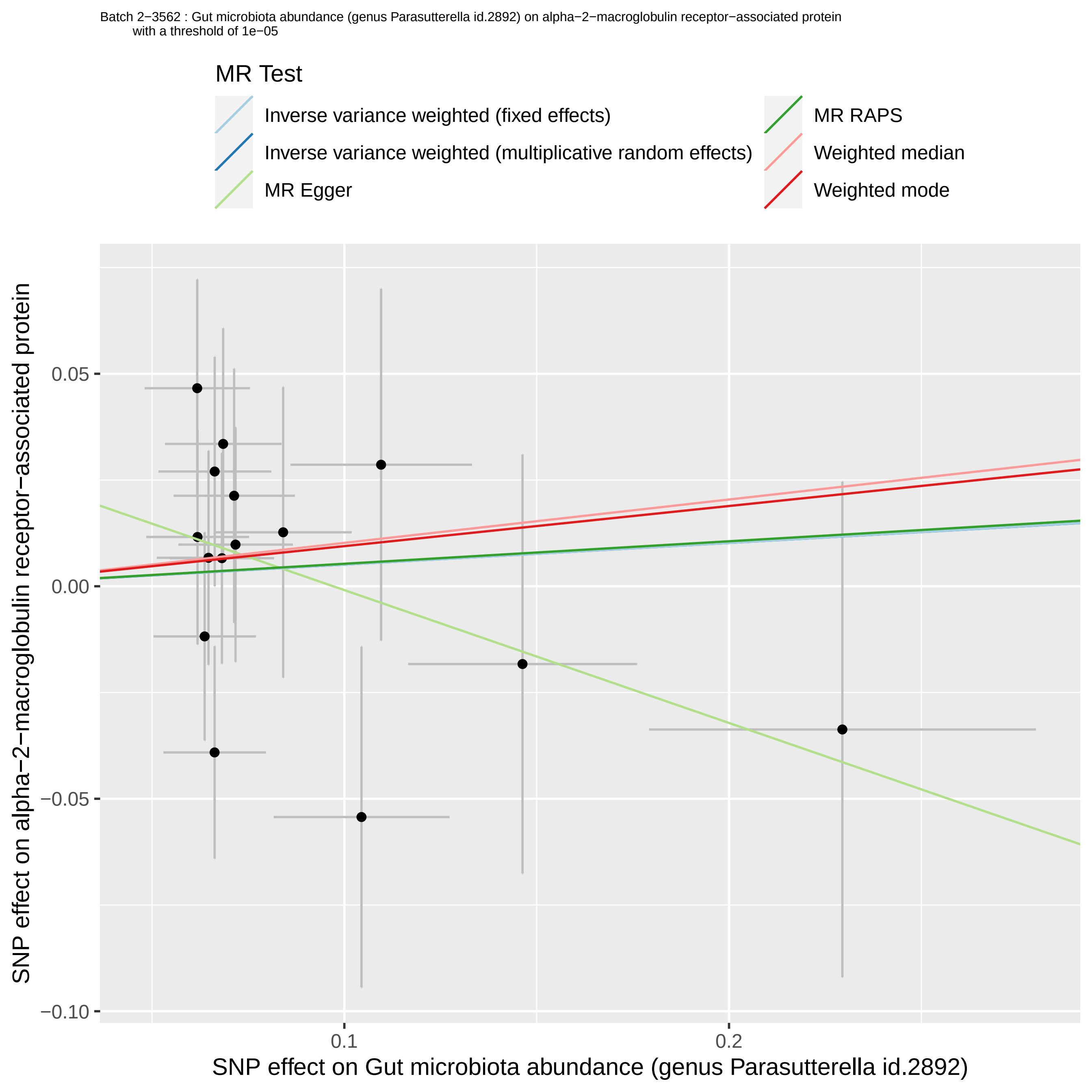
Batch 2−3562 : Gut microbiota abundance (genus Parasutterella id.2892) on alpha−2−macroglobulin receptor−associated protein
with a threshold of 1e−05
MR Test
Inverse variance weighted (fixed effects)
Inverse variance weighted (multiplicative random effects)
MR Egger
MR RAPS
Weighted median
Weighted mode
0.05
0.00
−0.05
−0.10
0.1
0.2
SNP effect on alpha−2−macroglobulin receptor−associated protein
SNP effect on Gut microbiota abundance (genus Parasutterella id.2892)

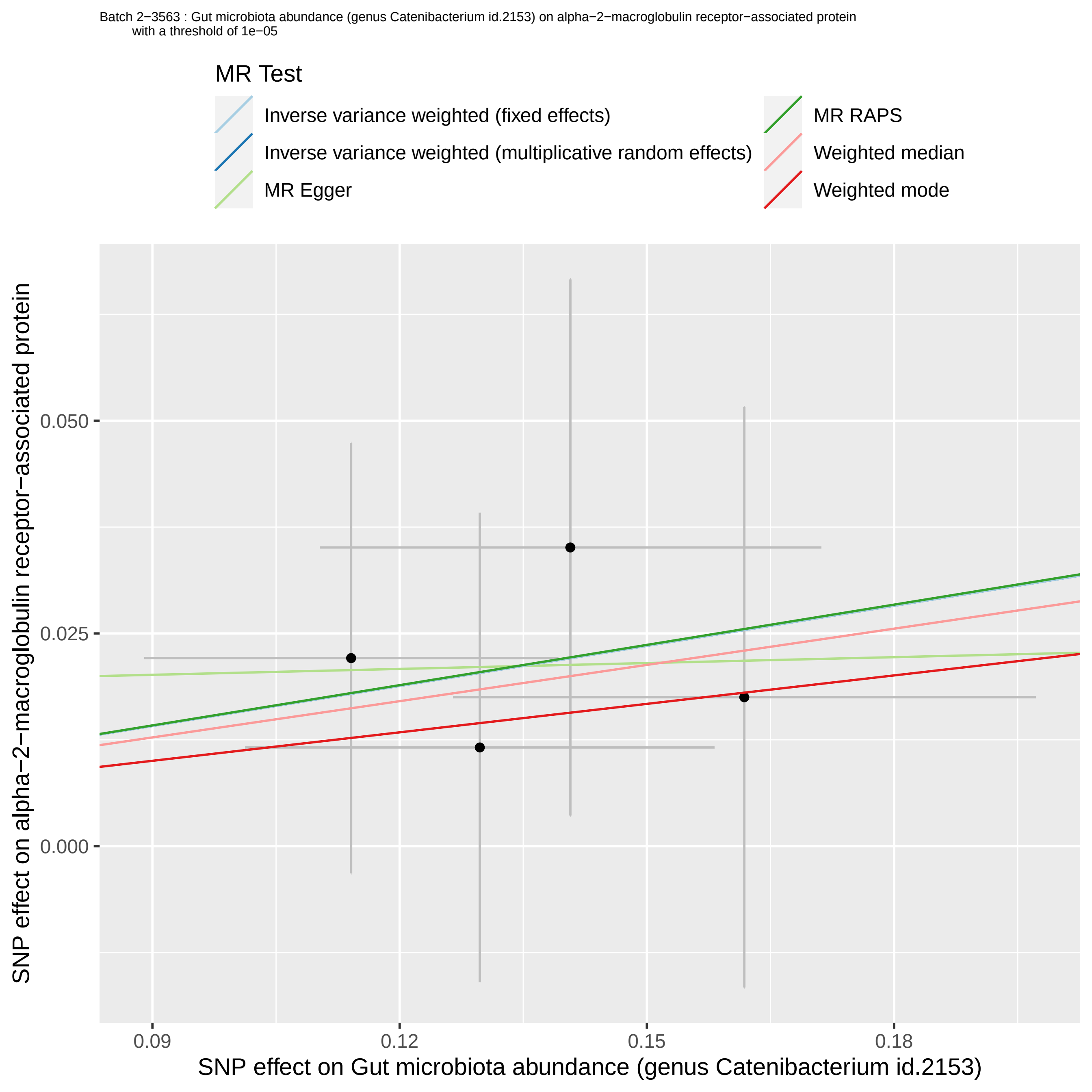
Batch 2−3563 : Gut microbiota abundance (genus Catenibacterium id.2153) on alpha−2−macroglobulin receptor−associated protein
with a threshold of 1e−05
MR Test
Inverse variance weighted (fixed effects)
Inverse variance weighted (multiplicative random effects)
MR Egger
MR RAPS
Weighted median
Weighted mode
SNP effect on alpha−2−macroglobulin receptor−associated protein
0.050
0.025
0.000
0.09
0.12
0.15
0.18
SNP effect on Gut microbiota abundance (genus Catenibacterium id.2153)

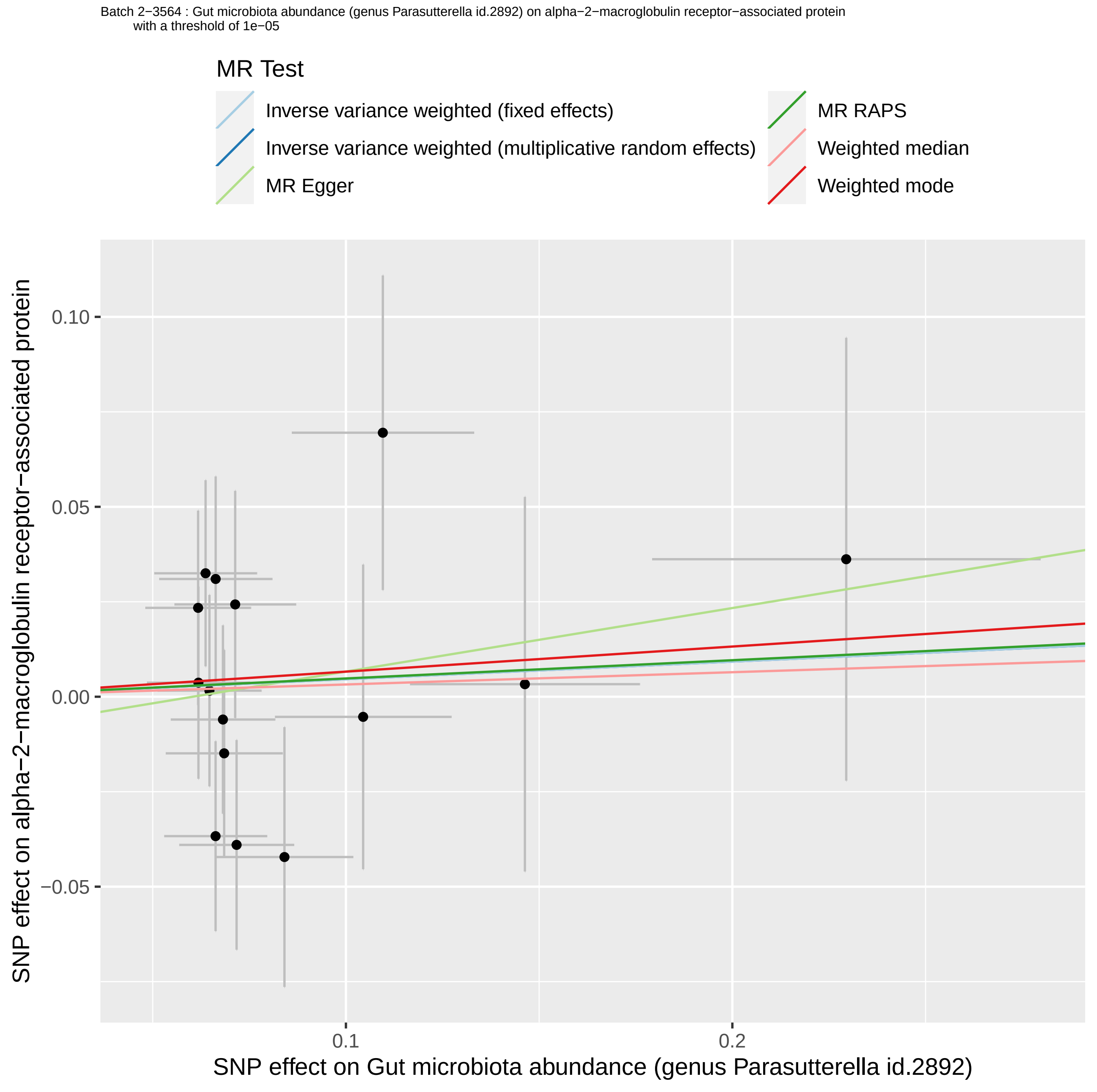
Batch 2−3564 : Gut microbiota abundance (genus Parasutterella id.2892) on alpha−2−macroglobulin receptor−associated protein
with a threshold of 1e−05
MR Test
Inverse variance weighted (fixed effects)
Inverse variance weighted (multiplicative random effects)
MR Egger
MR RAPS
Weighted median
Weighted mode
0.10
0.05
0.00
−0.05
0.1
0.2
SNP effect on alpha−2−macroglobulin receptor−associated protein
SNP effect on Gut microbiota abundance (genus Parasutterella id.2892)

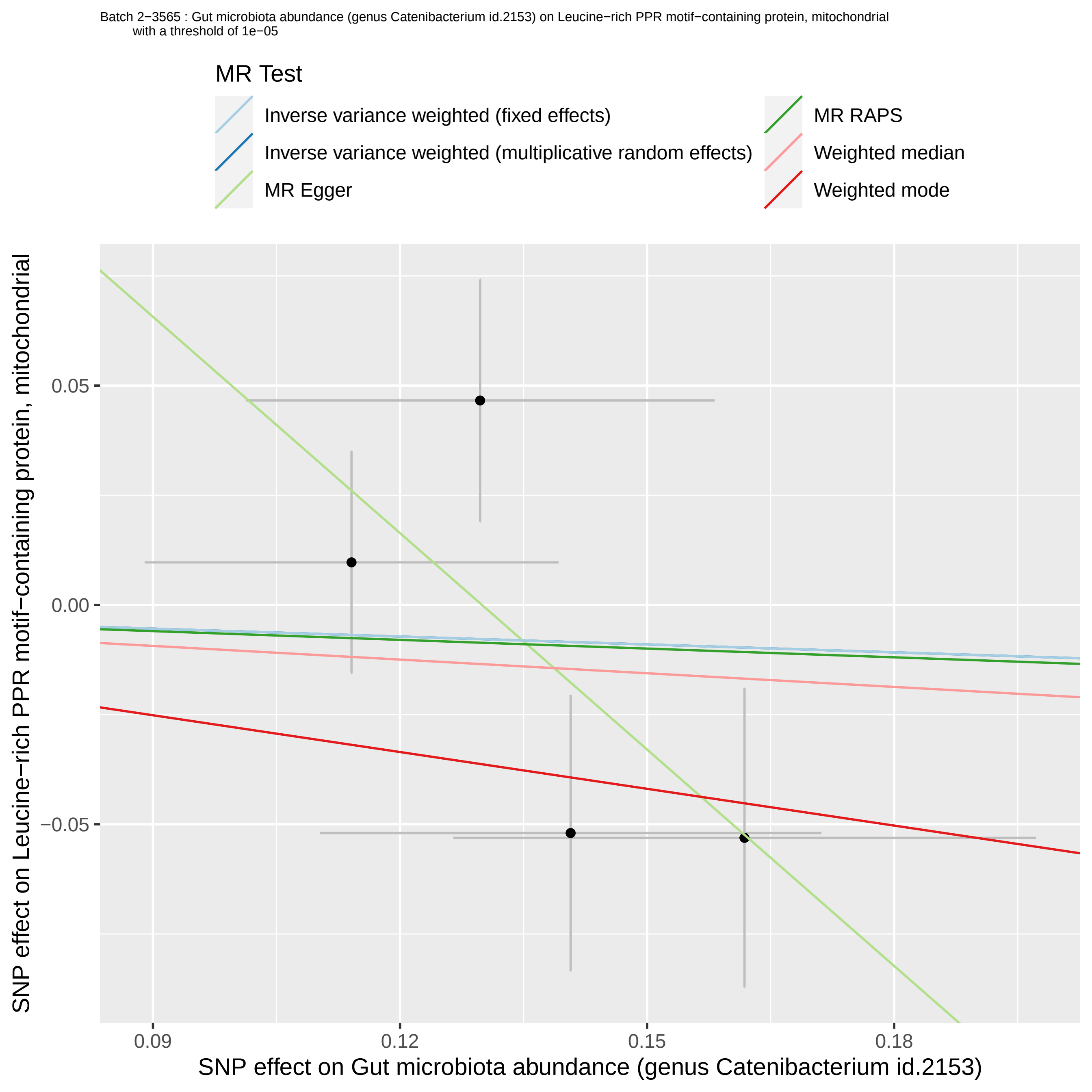

Batch 2−3565 : Gut microbiota abundance (genus Catenibacterium id.2153) on Leucine−rich PPR motif−containing protein, mitochondrial with a threshold of 1e−05
MR Test
Inverse variance weighted (fixed effects)
Inverse variance weighted (multiplicative random effects)
MR Egger
MR RAPS
Weighted median
Weighted mode
0.05
0.00
−0.05
0.09
0.12
0.15
0.18
SNP effect on Leucine−rich PPR motif−containing protein, mitochondrial
SNP effect on Gut microbiota abundance (genus Catenibacterium id.2153)

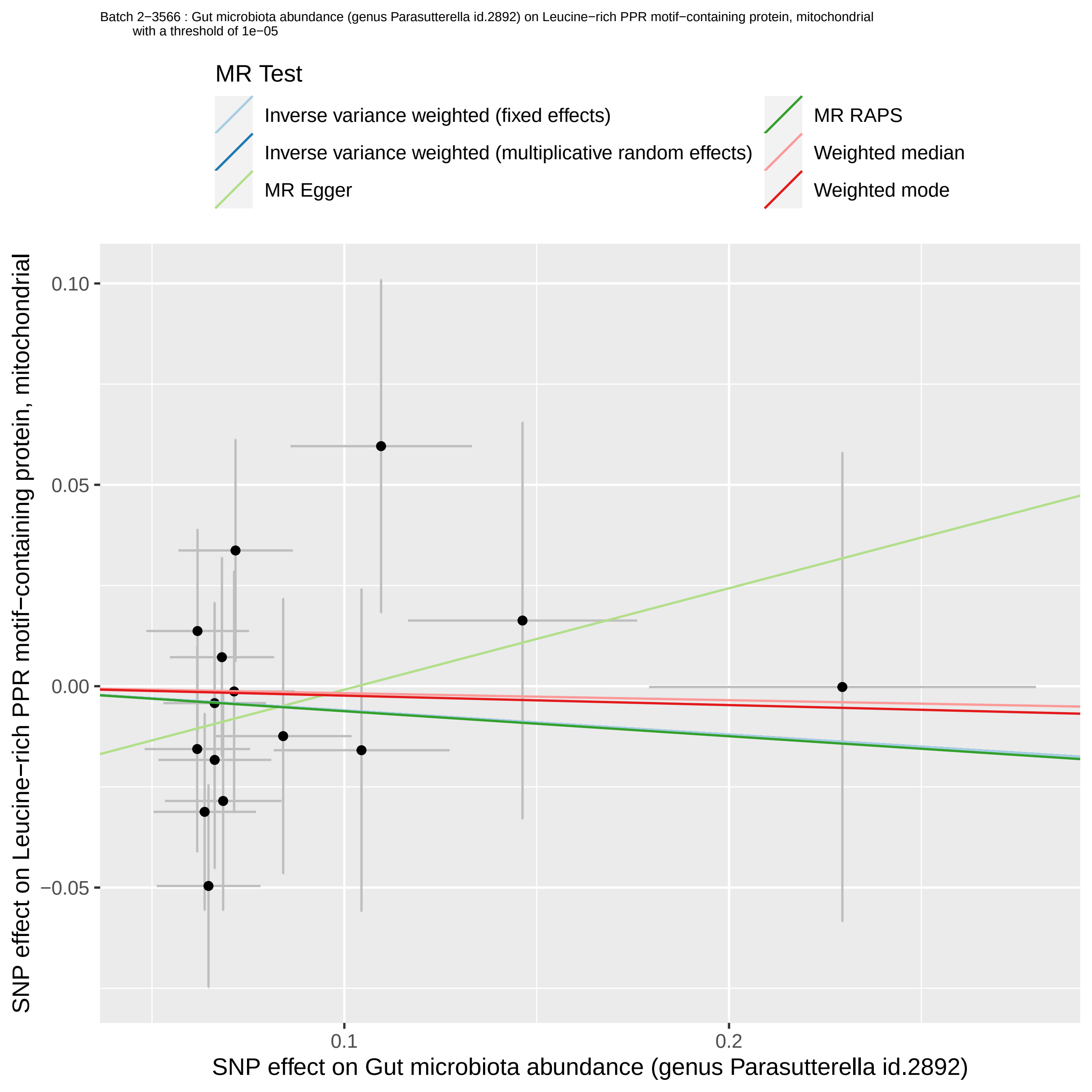
Batch 2−3566 : Gut microbiota abundance (genus Parasutterella id.2892) on Leucine−rich PPR motif−containing protein, mitochondrial
with a threshold of 1e−05
MR Test
Inverse variance weighted (fixed effects)
Inverse variance weighted (multiplicative random effects)
MR Egger
MR RAPS
Weighted median
Weighted mode
0.10
0.05
0.00
−0.05
0.1
0.2
SNP effect on Leucine−rich PPR motif−containing protein, mitochondrial
SNP effect on Gut microbiota abundance (genus Parasutterella id.2892)

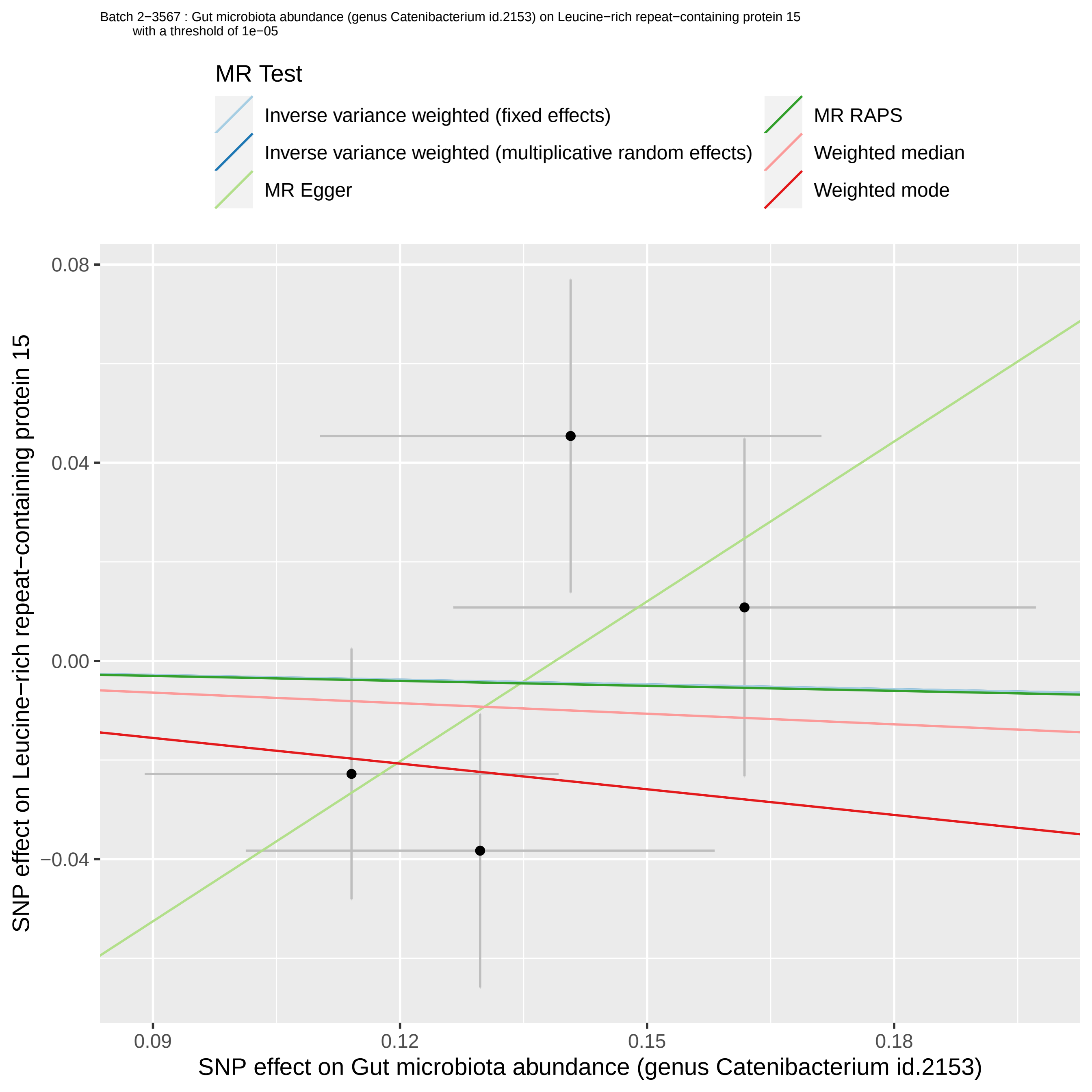
Batch 2−3567 : Gut microbiota abundance (genus Catenibacterium id.2153) on Leucine−rich repeat−containing protein 15
with a threshold of 1e−05
MR Test
Inverse variance weighted (fixed effects)
Inverse variance weighted (multiplicative random effects)
MR Egger
MR RAPS
Weighted median
Weighted mode
0.08
0.04
0.00
−0.04
0.09
0.12
0.15
0.18
SNP effect on Leucine−rich repeat−containing protein 15
SNP effect on Gut microbiota abundance (genus Catenibacterium id.2153)

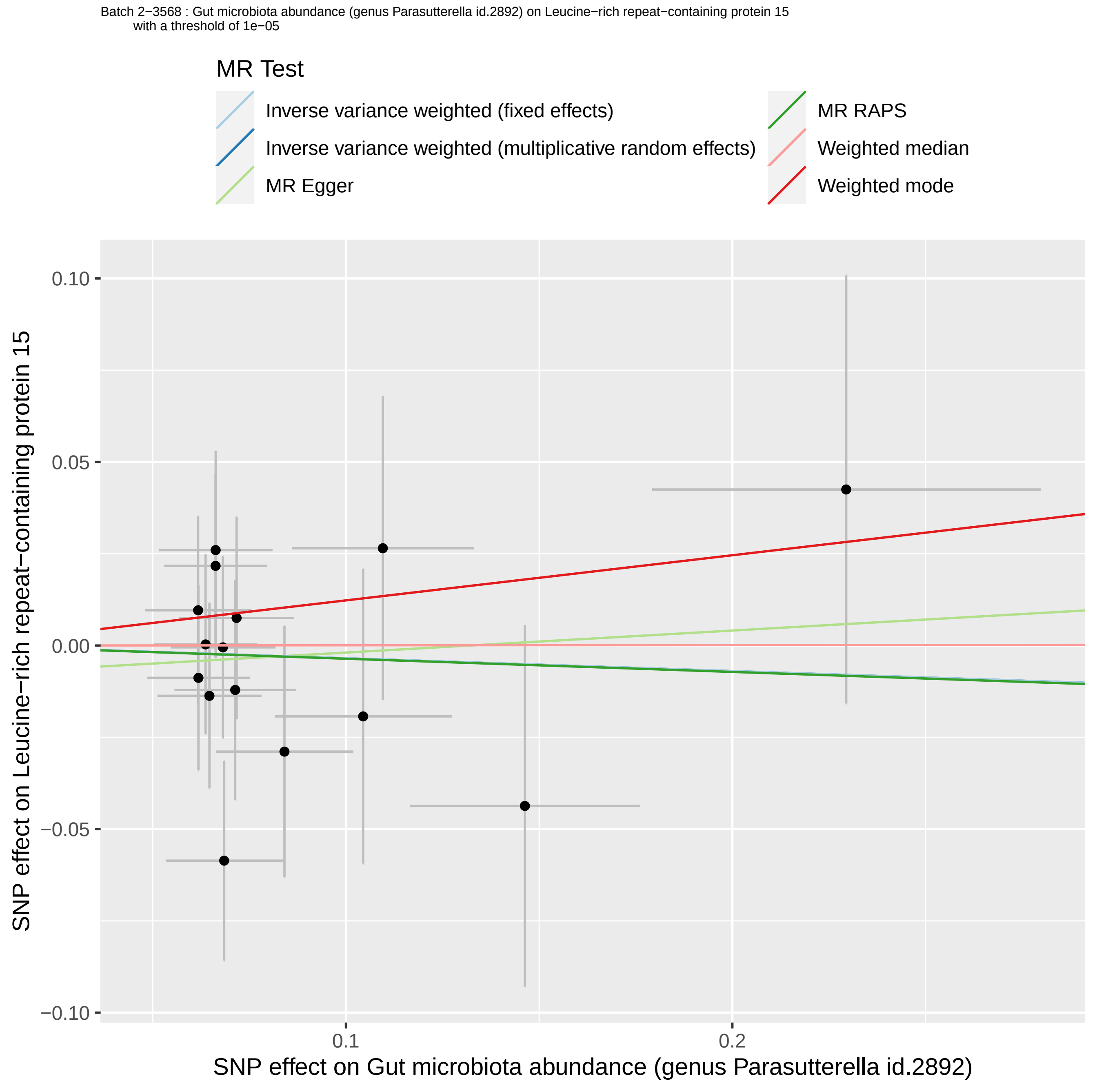
Batch 2−3568 : Gut microbiota abundance (genus Parasutterella id.2892) on Leucine−rich repeat−containing protein 15
with a threshold of 1e−05
MR Test
Inverse variance weighted (fixed effects)
Inverse variance weighted (multiplicative random effects)
MR Egger
MR RAPS
Weighted median
Weighted mode
0.10
0.05
0.00
−0.05
−0.10
0.1
0.2
SNP effect on Leucine−rich repeat−containing protein 15
SNP effect on Gut microbiota abundance (genus Parasutterella id.2892)

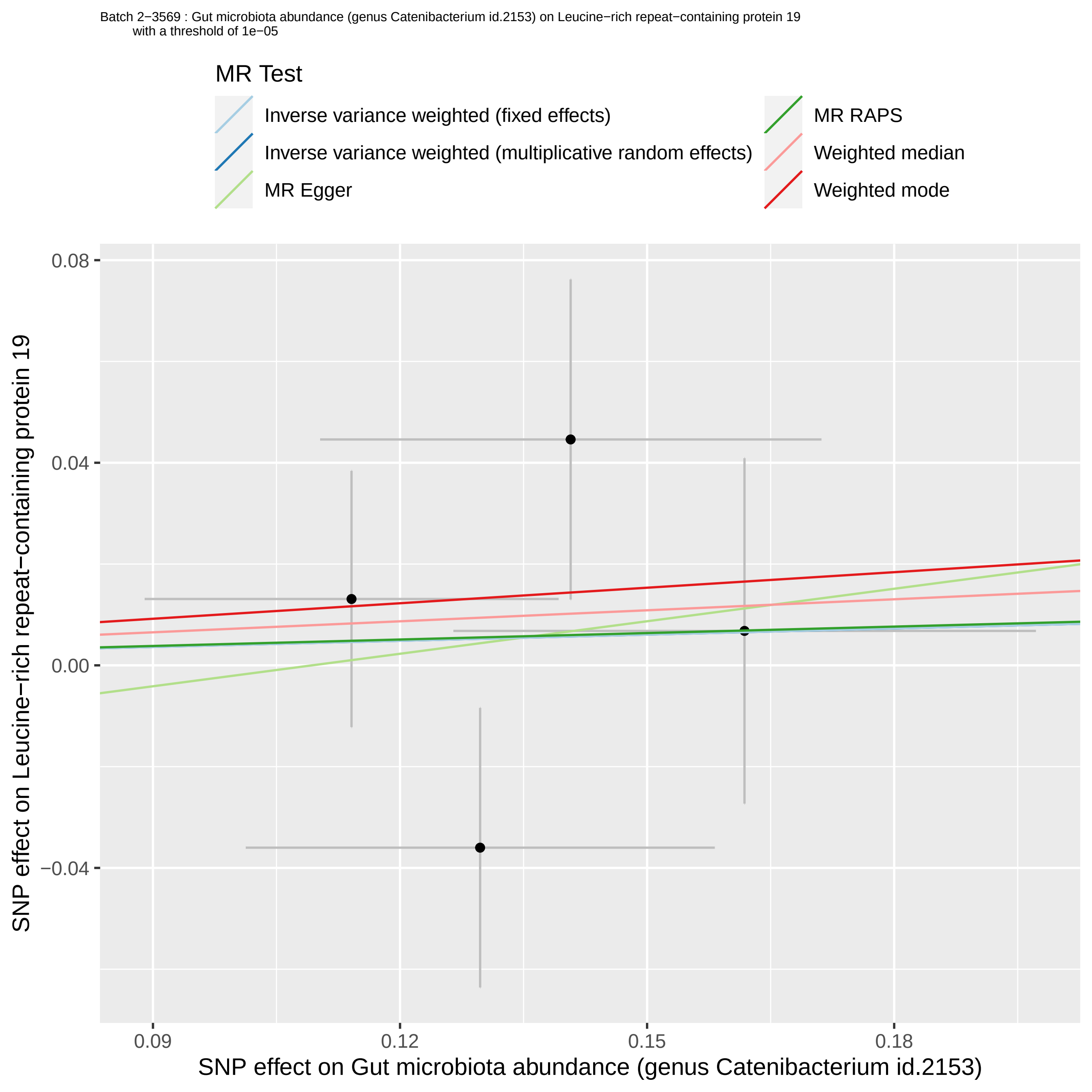
Batch 2−3569 : Gut microbiota abundance (genus Catenibacterium id.2153) on Leucine−rich repeat−containing protein 19
with a threshold of 1e−05
MR Test
Inverse variance weighted (fixed effects)
Inverse variance weighted (multiplicative random effects)
MR Egger
MR RAPS
Weighted median
Weighted mode
0.08
0.04
0.00
−0.04
0.09
0.12
0.15
0.18
SNP effect on Leucine−rich repeat−containing protein 19
SNP effect on Gut microbiota abundance (genus Catenibacterium id.2153)

Batch 2−3570 : Gut microbiota abundance (genus Parasutterella id.2892) on Leucine−rich repeat−containing protein 19
with a threshold of 1e−05

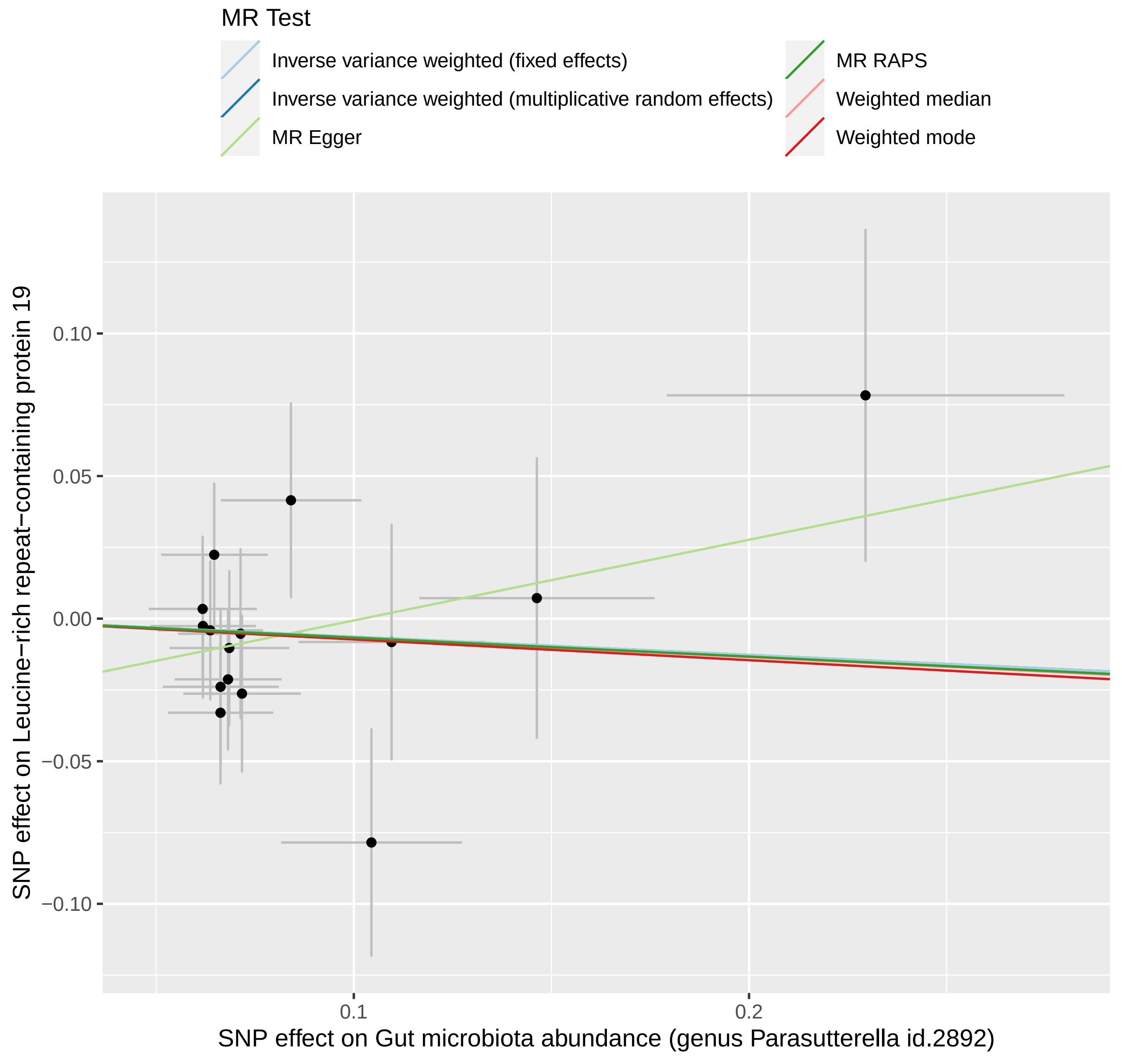

Batch 2−3571 : Gut microbiota abundance (genus Catenibacterium id.2153) on Leucine−rich repeat−containing protein 3
with a threshold of 1e−05

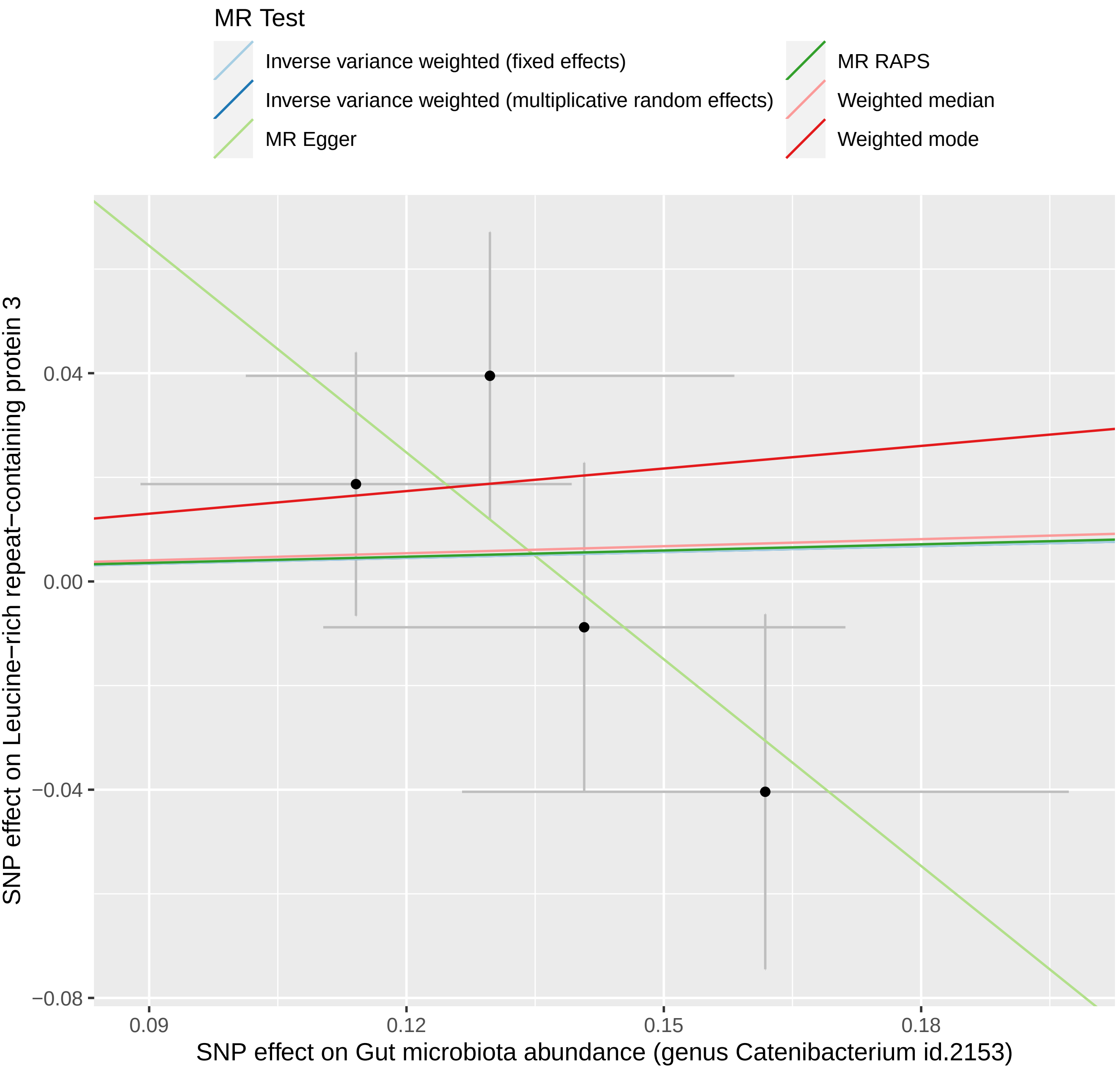

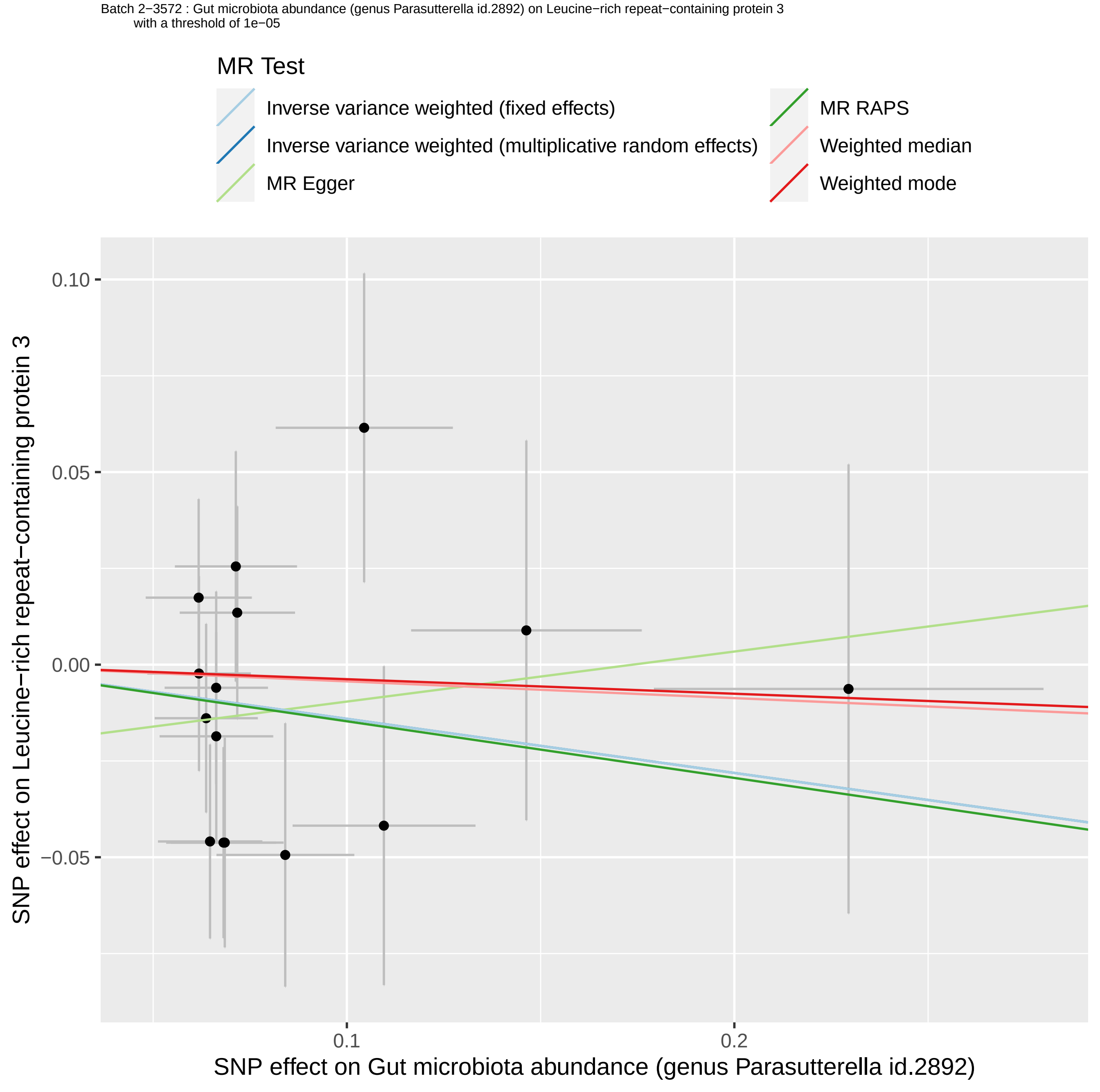

Batch 2−3572 : Gut microbiota abundance (genus Parasutterella id.2892) on Leucine−rich repeat−containing protein 3
with a threshold of 1e−05
MR Test
Inverse variance weighted (fixed effects)
Inverse variance weighted (multiplicative random effects)
MR Egger
MR RAPS
Weighted median
Weighted mode
0.10
0.05
0.00
−0.05
0.1
0.2
SNP effect on Leucine−rich repeat−containing protein 3
SNP effect on Gut microbiota abundance (genus Parasutterella id.2892)

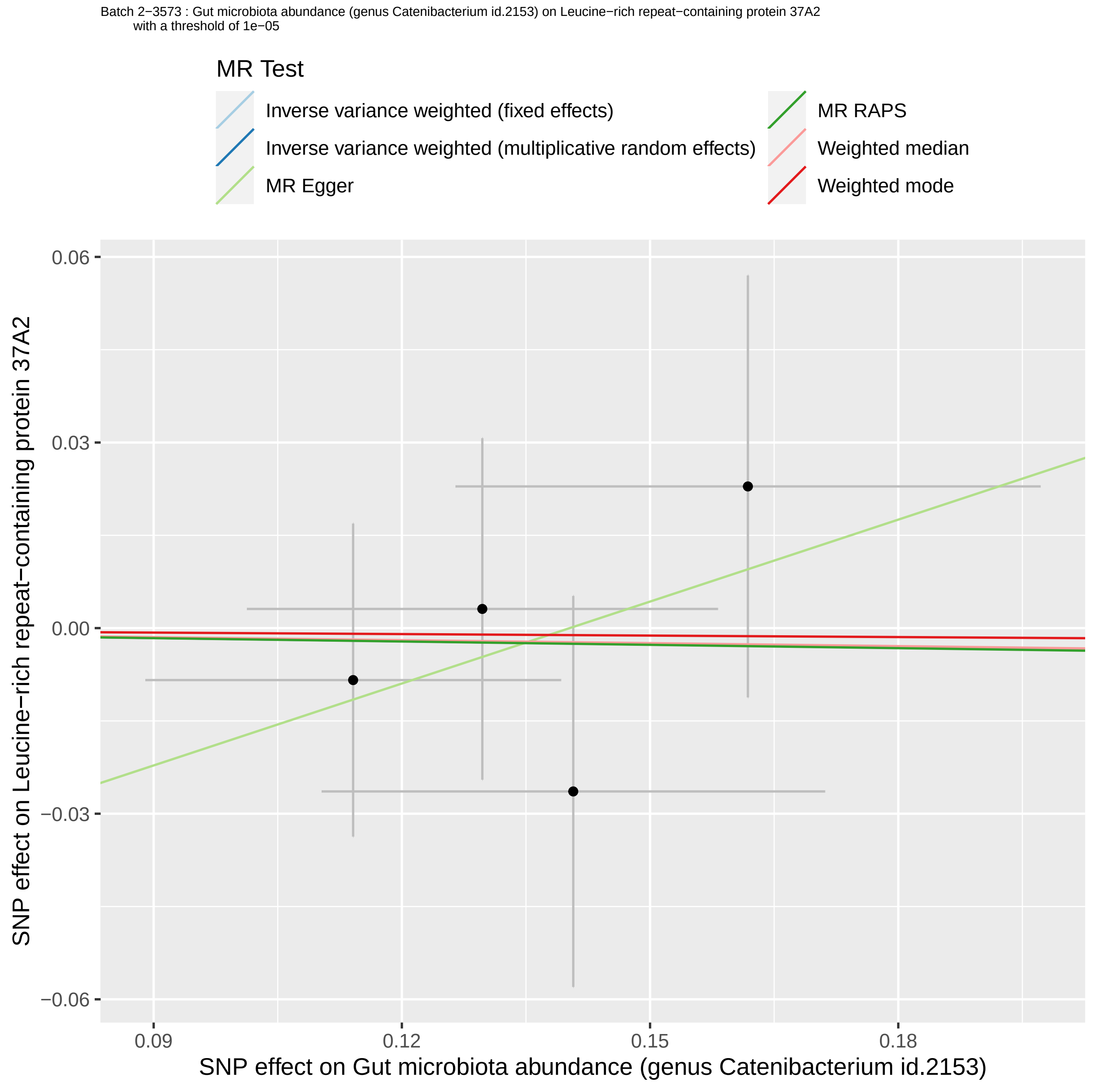
Batch 2−3573 : Gut microbiota abundance (genus Catenibacterium id.2153) on Leucine−rich repeat−containing protein 37A2
with a threshold of 1e−05
MR Test
Inverse variance weighted (fixed effects)
Inverse variance weighted (multiplicative random effects)
MR Egger
MR RAPS
Weighted median
Weighted mode
0.06
0.03
0.00
−0.03
−0.06
0.09
0.12
0.15
0.18
SNP effect on Leucine−rich repeat−containing protein 37A2
SNP effect on Gut microbiota abundance (genus Catenibacterium id.2153)

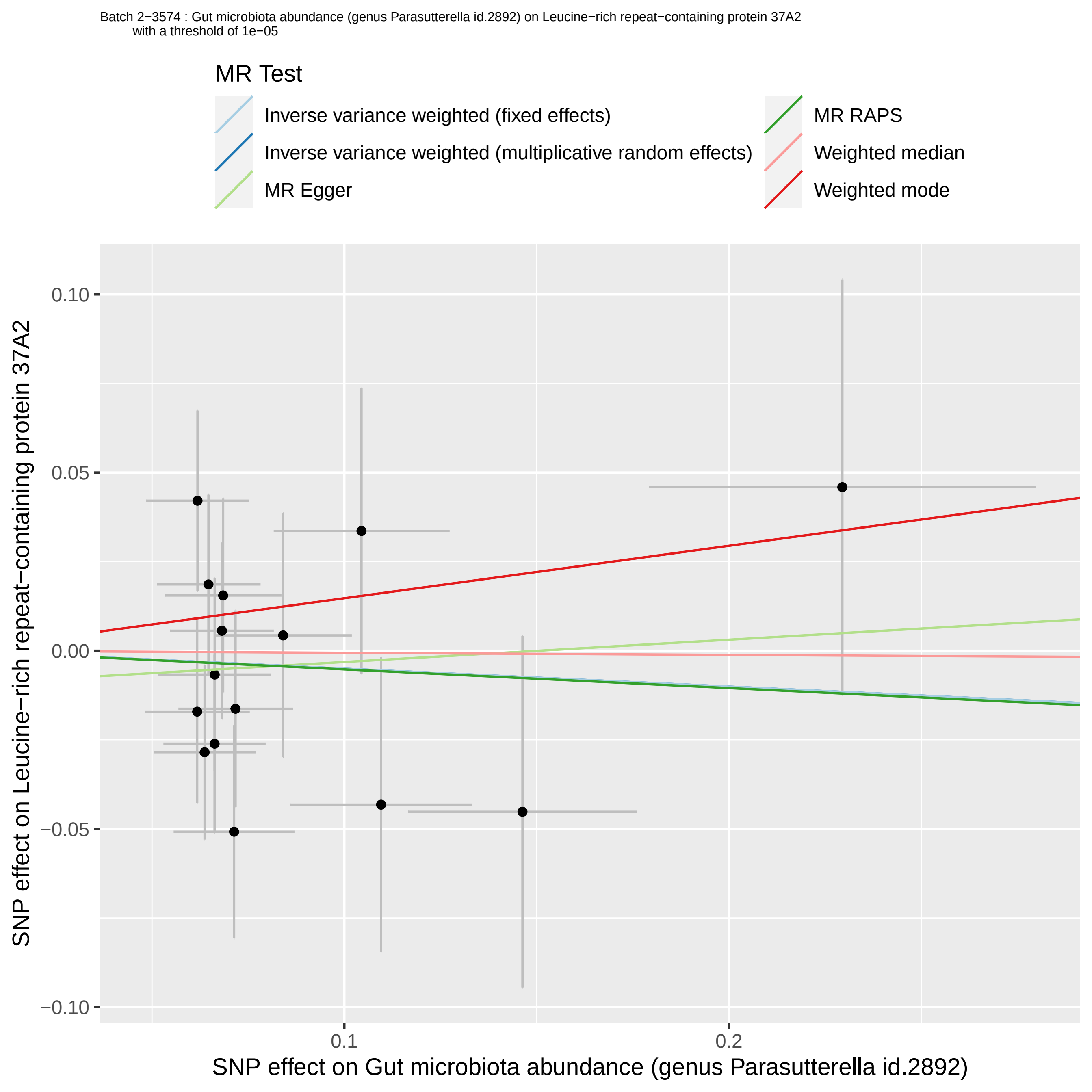
Batch 2−3574 : Gut microbiota abundance (genus Parasutterella id.2892) on Leucine−rich repeat−containing protein 37A2
with a threshold of 1e−05
MR Test
Inverse variance weighted (fixed effects)
Inverse variance weighted (multiplicative random effects)
MR Egger
MR RAPS
Weighted median
Weighted mode
0.10
0.05
0.00
−0.05
−0.10
0.1
0.2
SNP effect on Leucine−rich repeat−containing protein 37A2
SNP effect on Gut microbiota abundance (genus Parasutterella id.2892)

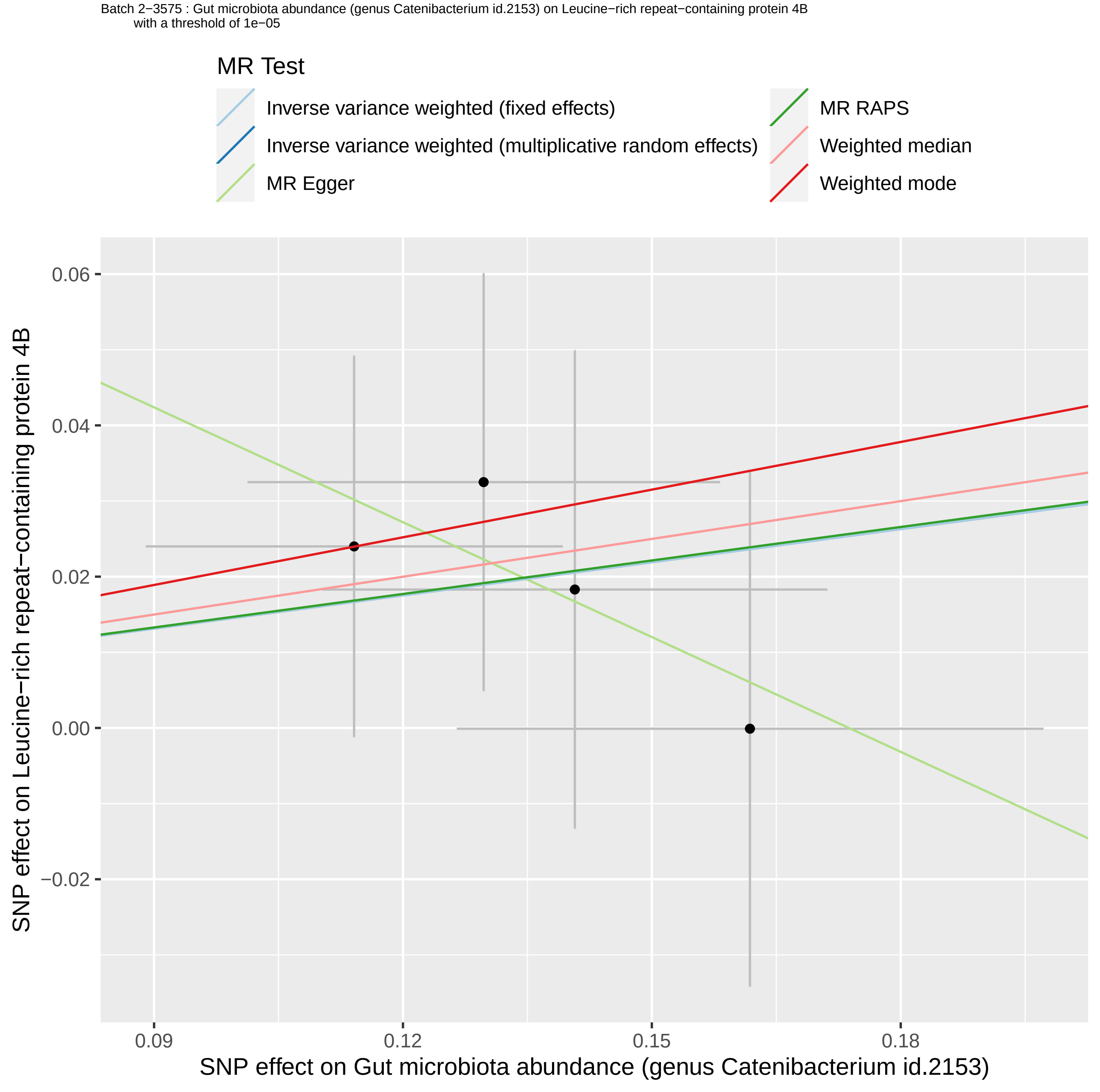
Batch 2−3575 : Gut microbiota abundance (genus Catenibacterium id.2153) on Leucine−rich repeat−containing protein 4B
with a threshold of 1e−05
MR Test
Inverse variance weighted (fixed effects)
Inverse variance weighted (multiplicative random effects)
MR Egger
MR RAPS
Weighted median
Weighted mode
0.06
0.04
0.02
0.00
−0.02
0.09
0.12
0.15
0.18
SNP effect on Leucine−rich repeat−containing protein 4B
SNP effect on Gut microbiota abundance (genus Catenibacterium id.2153)

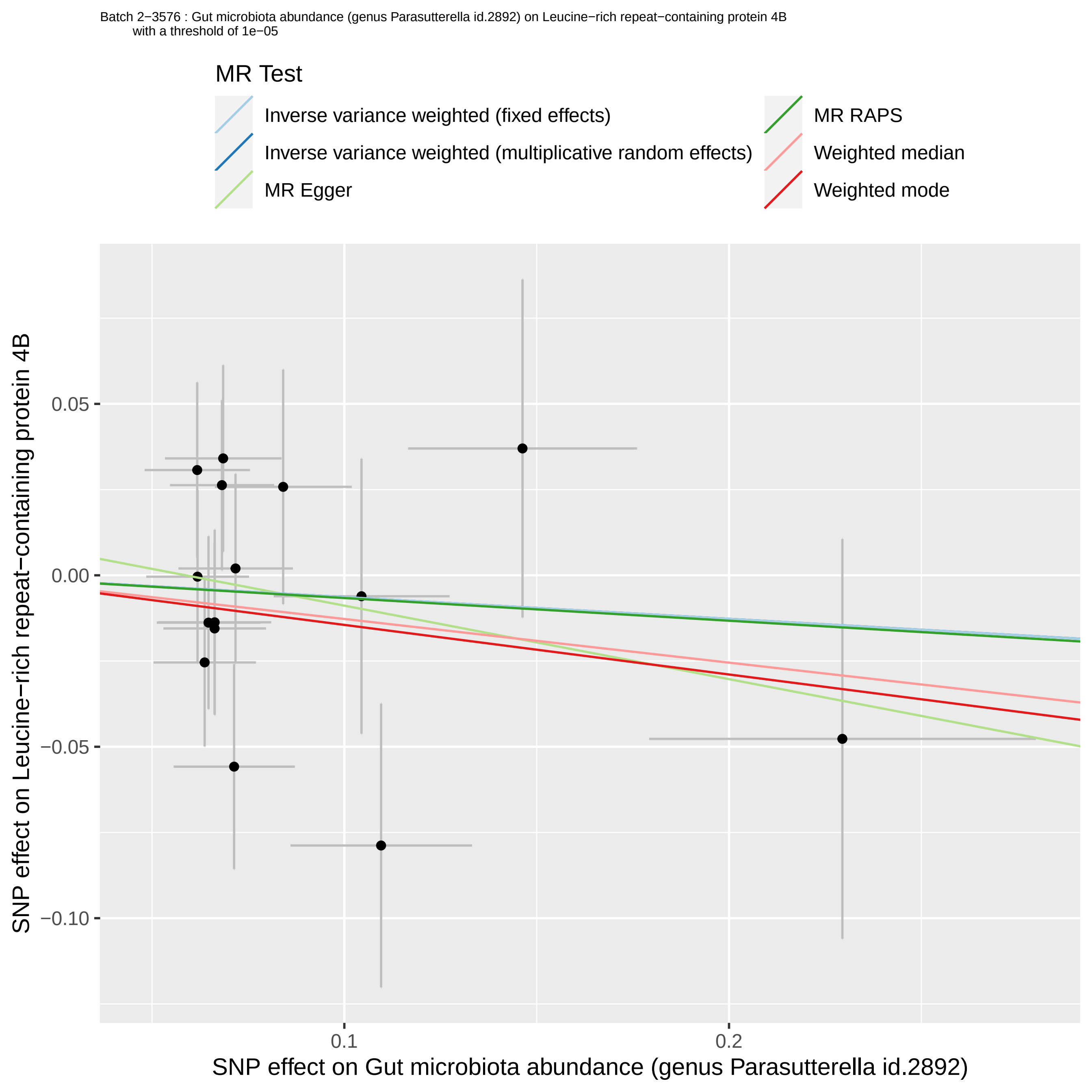
Batch 2−3576 : Gut microbiota abundance (genus Parasutterella id.2892) on Leucine−rich repeat−containing protein 4B
with a threshold of 1e−05
MR Test
Inverse variance weighted (fixed effects)
Inverse variance weighted (multiplicative random effects)
MR Egger
MR RAPS
Weighted median
Weighted mode
0.05
0.00
−0.05
−0.10
0.1
0.2
SNP effect on Leucine−rich repeat−containing protein 4B
SNP effect on Gut microbiota abundance (genus Parasutterella id.2892)

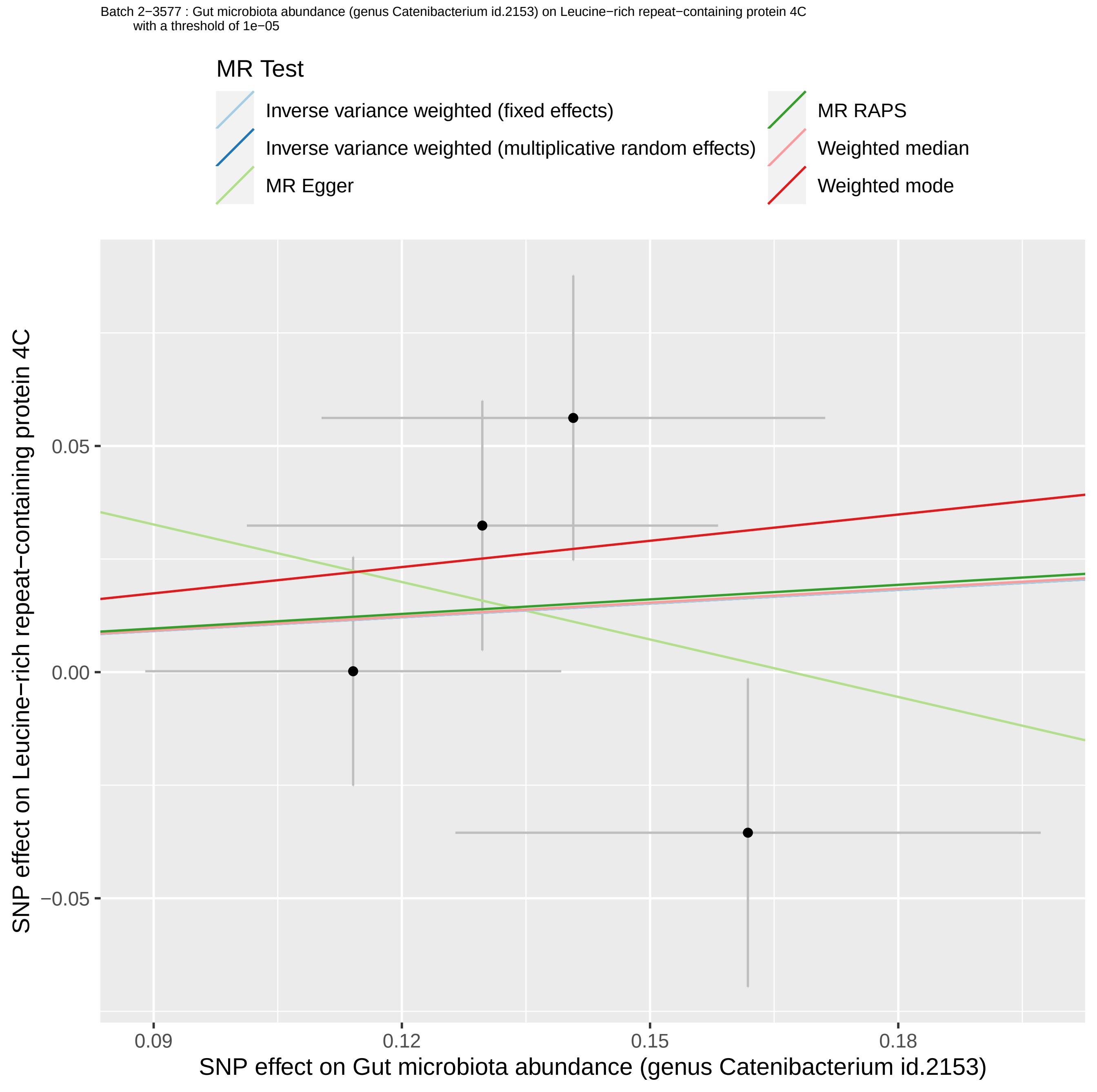
Batch 2−3577 : Gut microbiota abundance (genus Catenibacterium id.2153) on Leucine−rich repeat−containing protein 4C
with a threshold of 1e−05
MR Test
Inverse variance weighted (fixed effects)
Inverse variance weighted (multiplicative random effects)
MR Egger
MR RAPS
Weighted median
Weighted mode
0.05
0.00
−0.05
0.09
0.12
0.15
0.18
SNP effect on Leucine−rich repeat−containing protein 4C
SNP effect on Gut microbiota abundance (genus Catenibacterium id.2153)

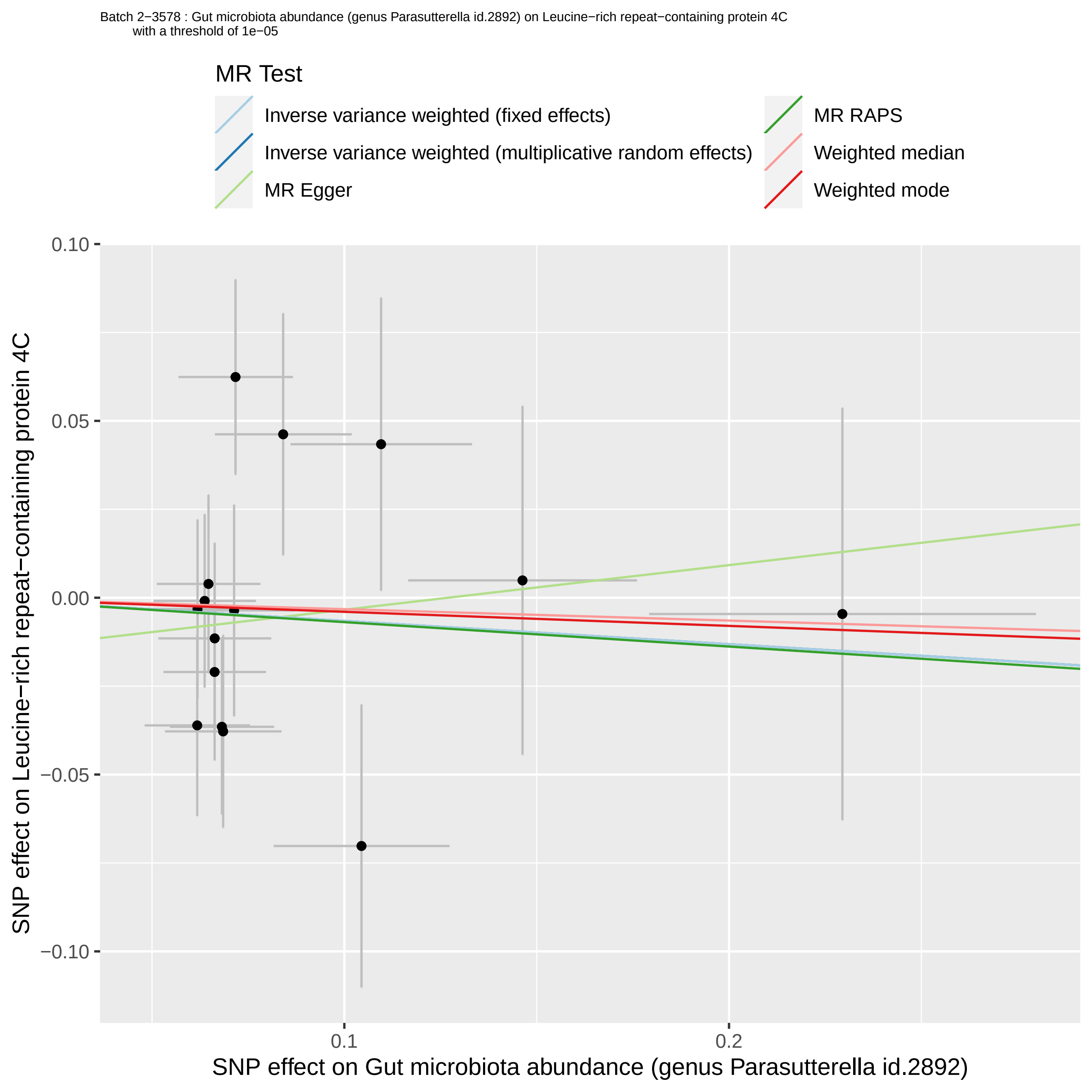

Batch 2−3578 : Gut microbiota abundance (genus Parasutterella id.2892) on Leucine−rich repeat−containing protein 4C
with a threshold of 1e−05
MR Test
Inverse variance weighted (fixed effects)
Inverse variance weighted (multiplicative random effects)
MR Egger
MR RAPS
Weighted median
Weighted mode
0.10
0.05
0.00
−0.05
−0.10
0.1
0.2
SNP effect on Leucine−rich repeat−containing protein 4C
SNP effect on Gut microbiota abundance (genus Parasutterella id.2892)

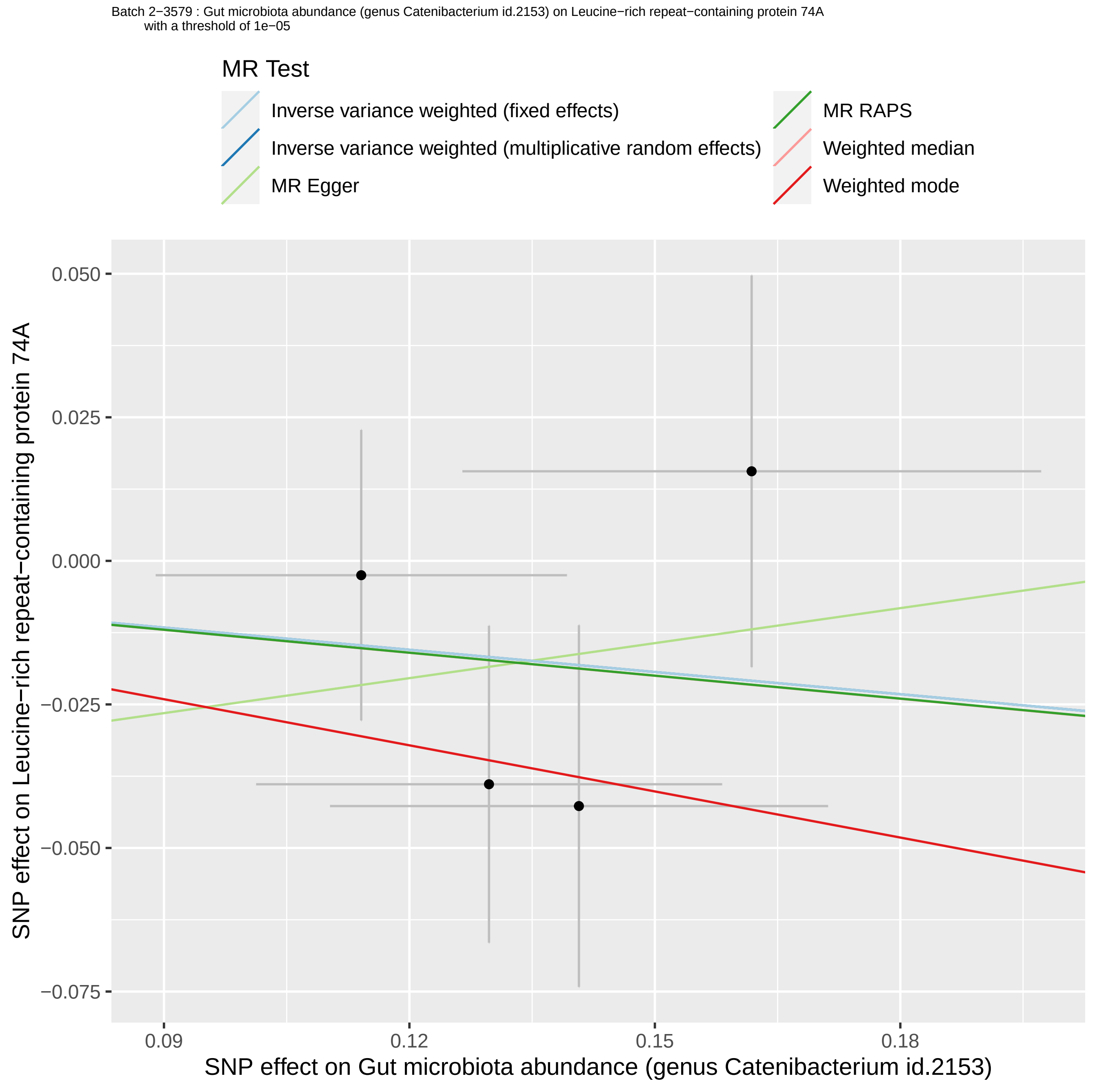

Batch 2−3579 : Gut microbiota abundance (genus Catenibacterium id.2153) on Leucine−rich repeat−containing protein 74A
with a threshold of 1e−05
MR Test
Inverse variance weighted (fixed effects)
Inverse variance weighted (multiplicative random effects)
MR Egger
MR RAPS
Weighted median
Weighted mode
0.050
0.025
0.000
−0.025
−0.050
−0.075
0.09
0.12
0.15
0.18
SNP effect on Leucine−rich repeat−containing protein 74A
SNP effect on Gut microbiota abundance (genus Catenibacterium id.2153)

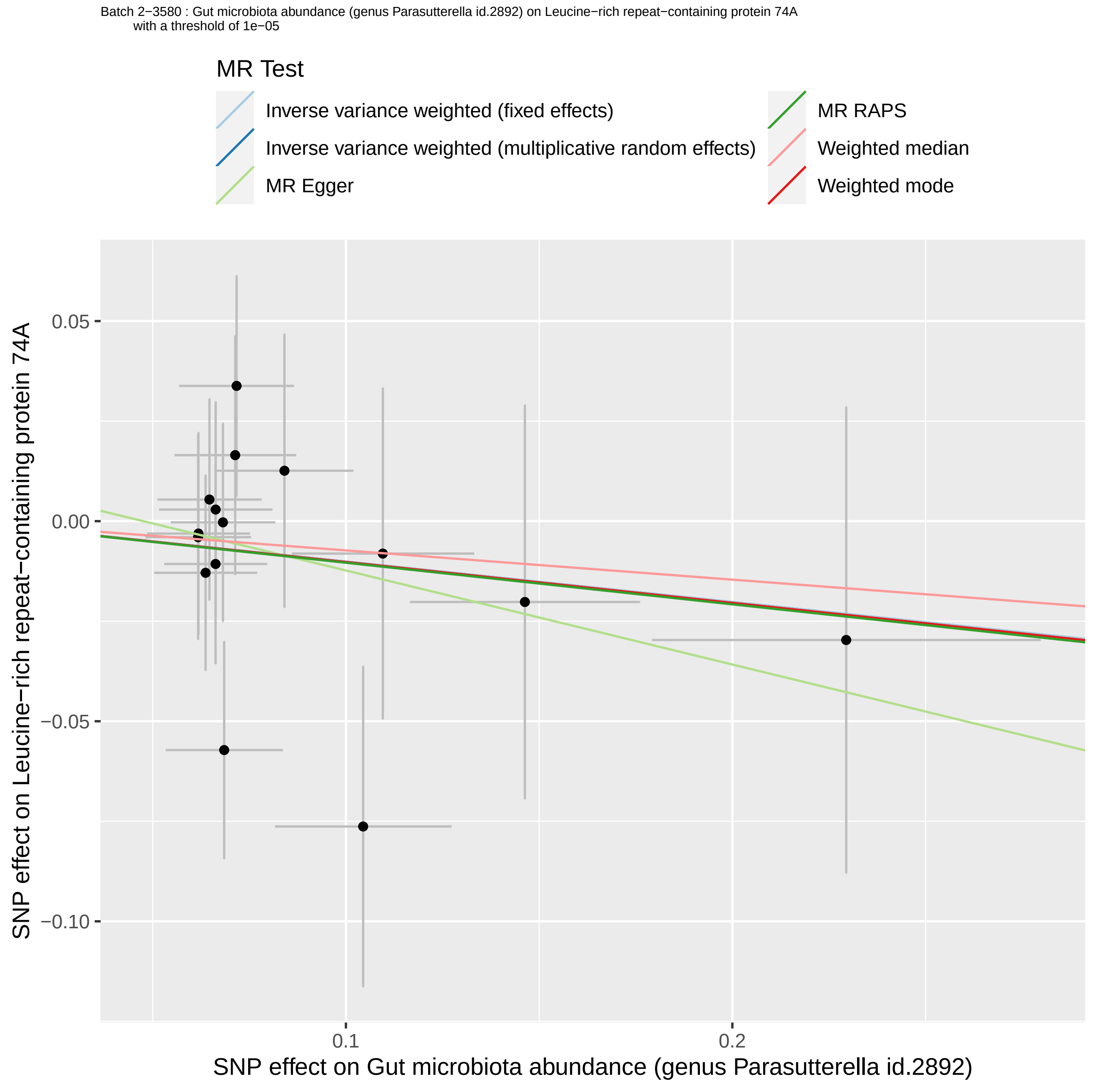

Batch 2−3580 : Gut microbiota abundance (genus Parasutterella id.2892) on Leucine−rich repeat−containing protein 74A
with a threshold of 1e−05
MR Test
Inverse variance weighted (fixed effects)
Inverse variance weighted (multiplicative random effects)
MR Egger
MR RAPS
Weighted median
Weighted mode
0.05
0.00
−0.05
−0.10
0.1
0.2
SNP effect on Leucine−rich repeat−containing protein 74A
SNP effect on Gut microbiota abundance (genus Parasutterella id.2892)

Batch 2−3581 : Gut microbiota abundance (genus Catenibacterium id.2153) on Leucine−rich repeat serine/threonine−protein kinase 2
with a threshold of 1e−05

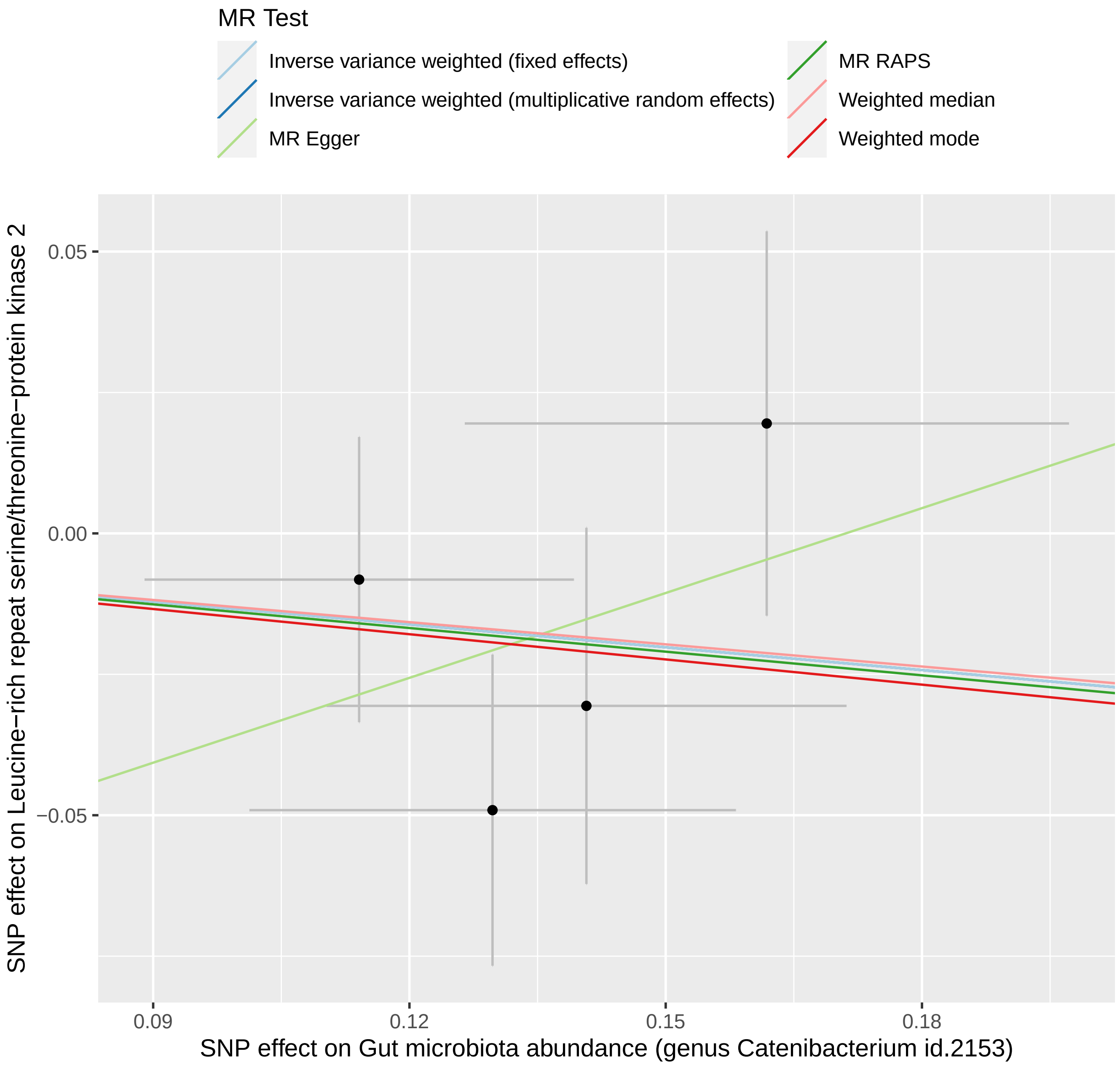

Batch 2−3582 : Gut microbiota abundance (genus Parasutterella id.2892) on Leucine−rich repeat serine/threonine−protein kinase 2
with a threshold of 1e−05

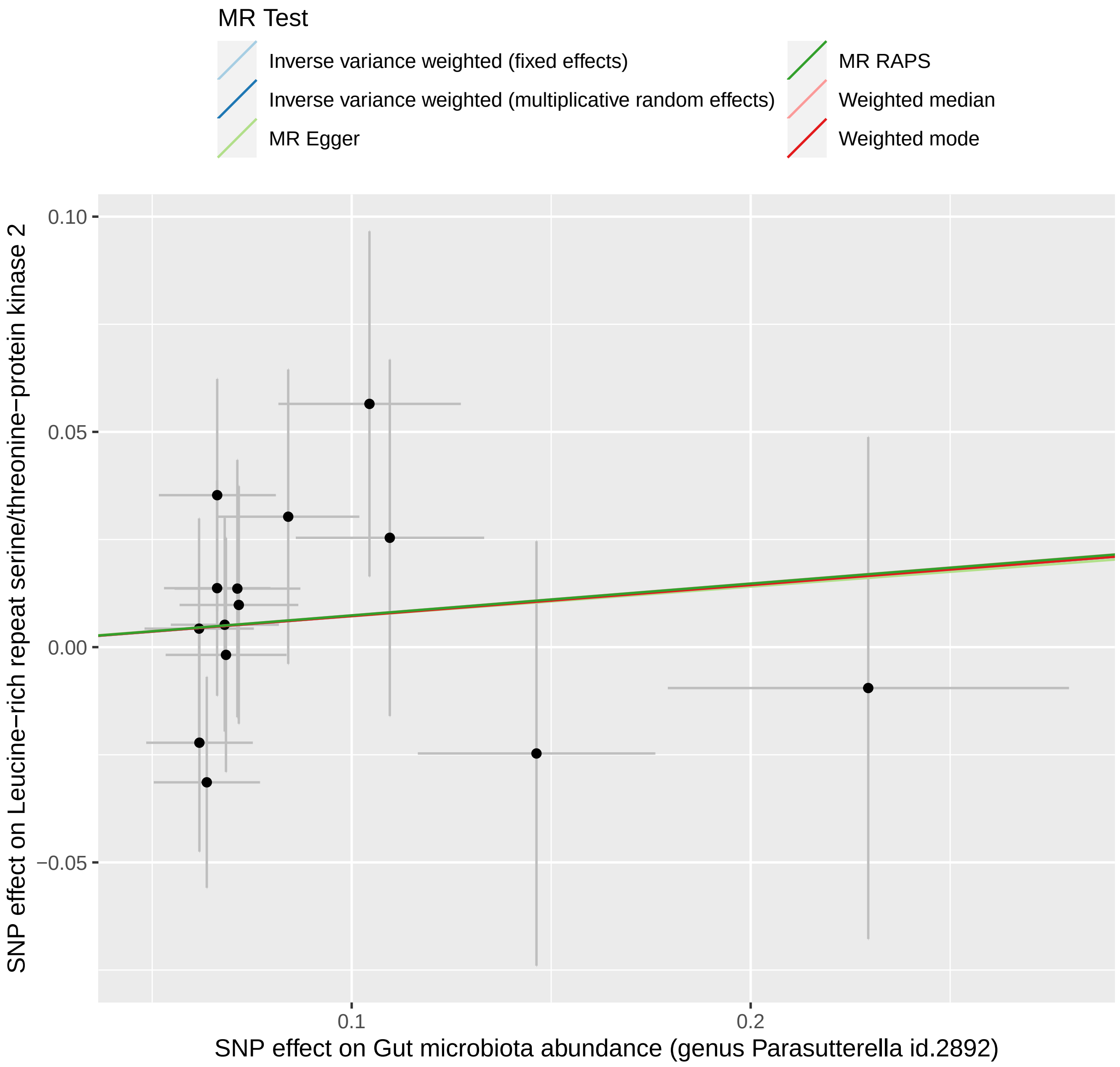

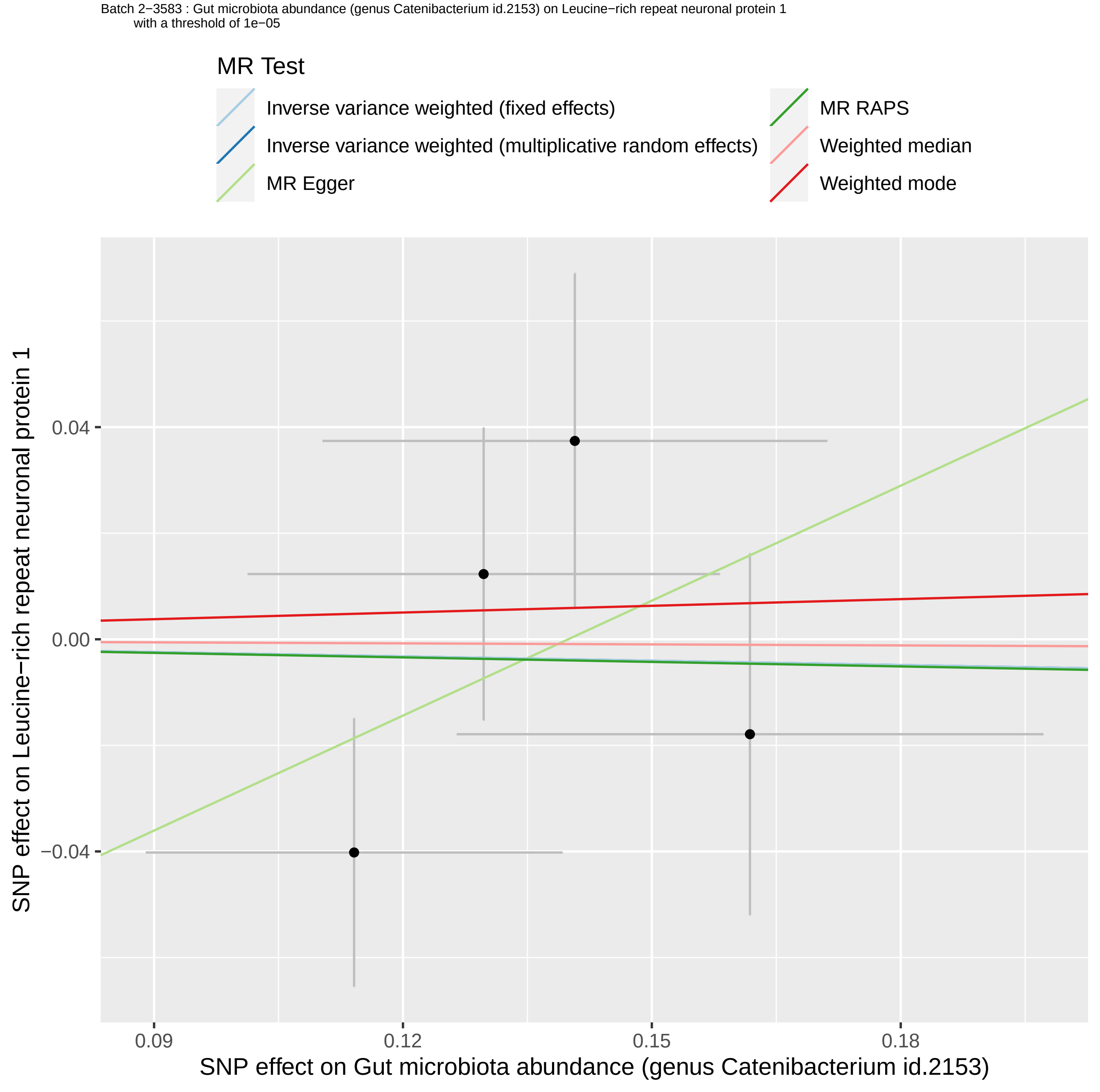
Batch 2−3583 : Gut microbiota abundance (genus Catenibacterium id.2153) on Leucine−rich repeat neuronal protein 1
with a threshold of 1e−05
MR Test
Inverse variance weighted (fixed effects)
Inverse variance weighted (multiplicative random effects)
MR Egger
MR RAPS
Weighted median
Weighted mode
0.04
0.00
−0.04
0.09
0.12
0.15
0.18
SNP effect on Leucine−rich repeat neuronal protein 1
SNP effect on Gut microbiota abundance (genus Catenibacterium id.2153)

Batch 2−3584 : Gut microbiota abundance (genus Parasutterella id.2892) on Leucine−rich repeat neuronal protein 1
with a threshold of 1e−05

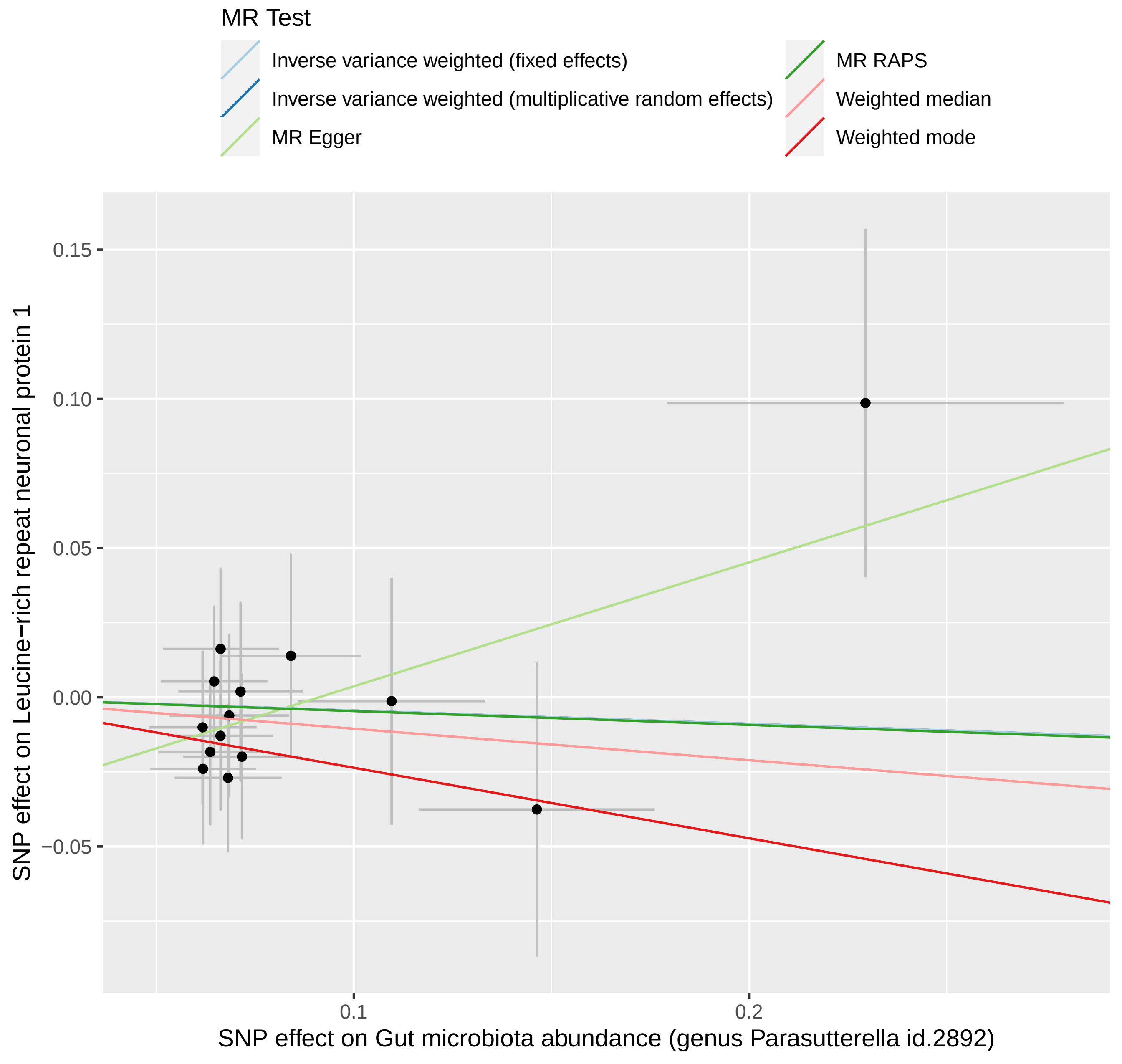

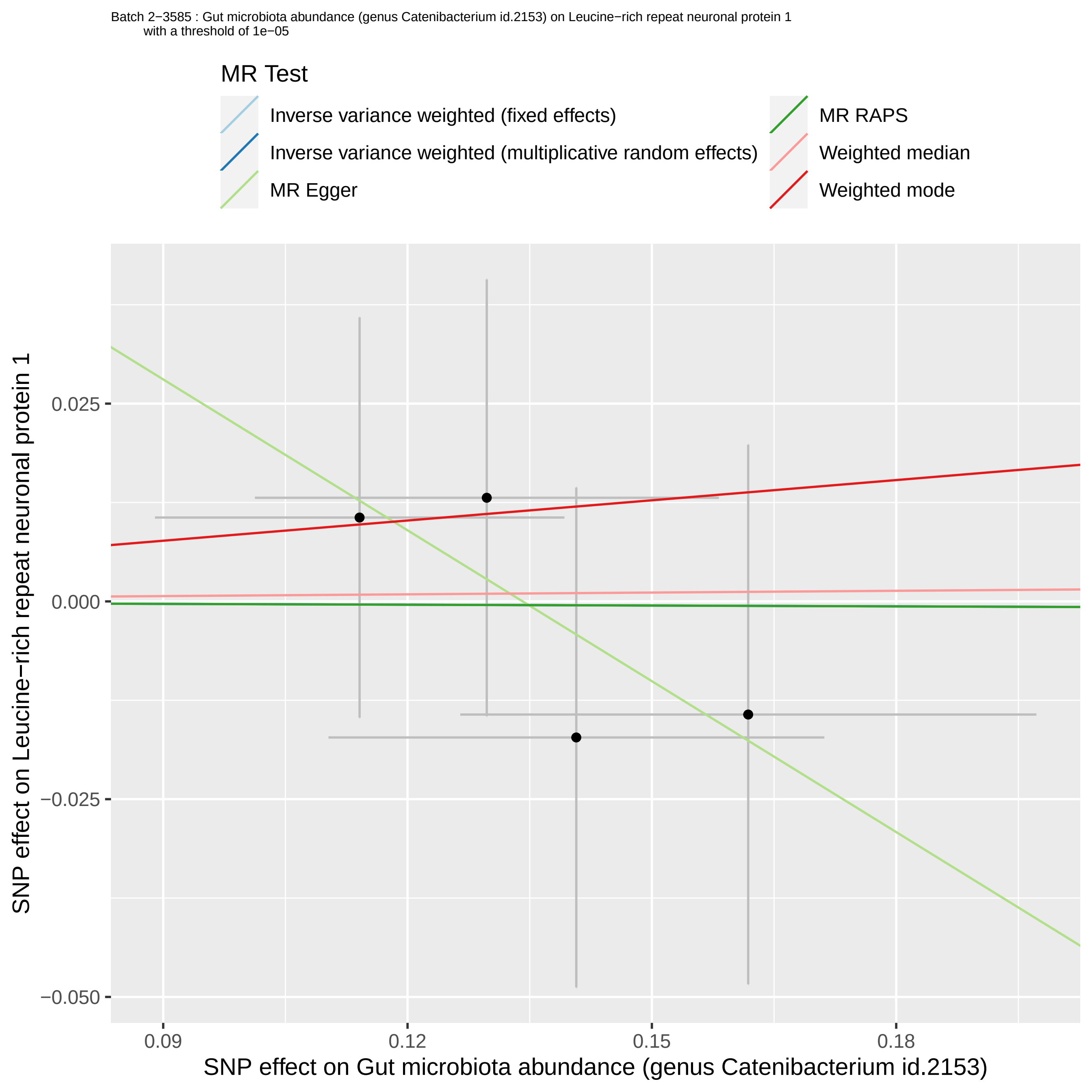
Batch 2−3585 : Gut microbiota abundance (genus Catenibacterium id.2153) on Leucine−rich repeat neuronal protein 1
with a threshold of 1e−05
MR Test
Inverse variance weighted (fixed effects)
Inverse variance weighted (multiplicative random effects)
MR Egger
MR RAPS
Weighted median
Weighted mode
SNP effect on Leucine−rich repeat neuronal protein 1
0.025
0.000
−0.025
−0.050
0.09
0.12
0.15
0.18
SNP effect on Gut microbiota abundance (genus Catenibacterium id.2153)

Batch 2−3586 : Gut microbiota abundance (genus Parasutterella id.2892) on Leucine−rich repeat neuronal protein 1 with a threshold of 1e−05

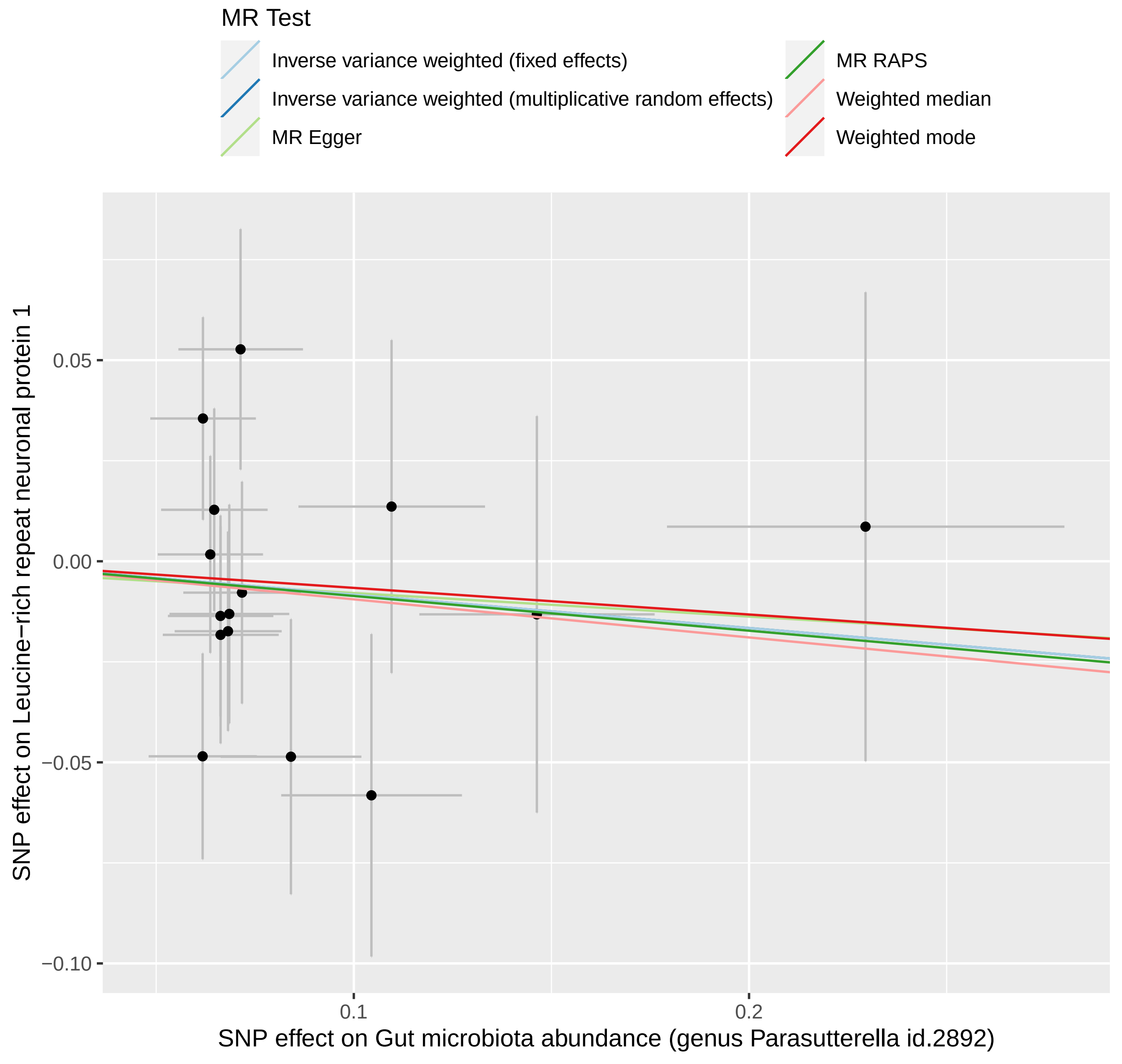

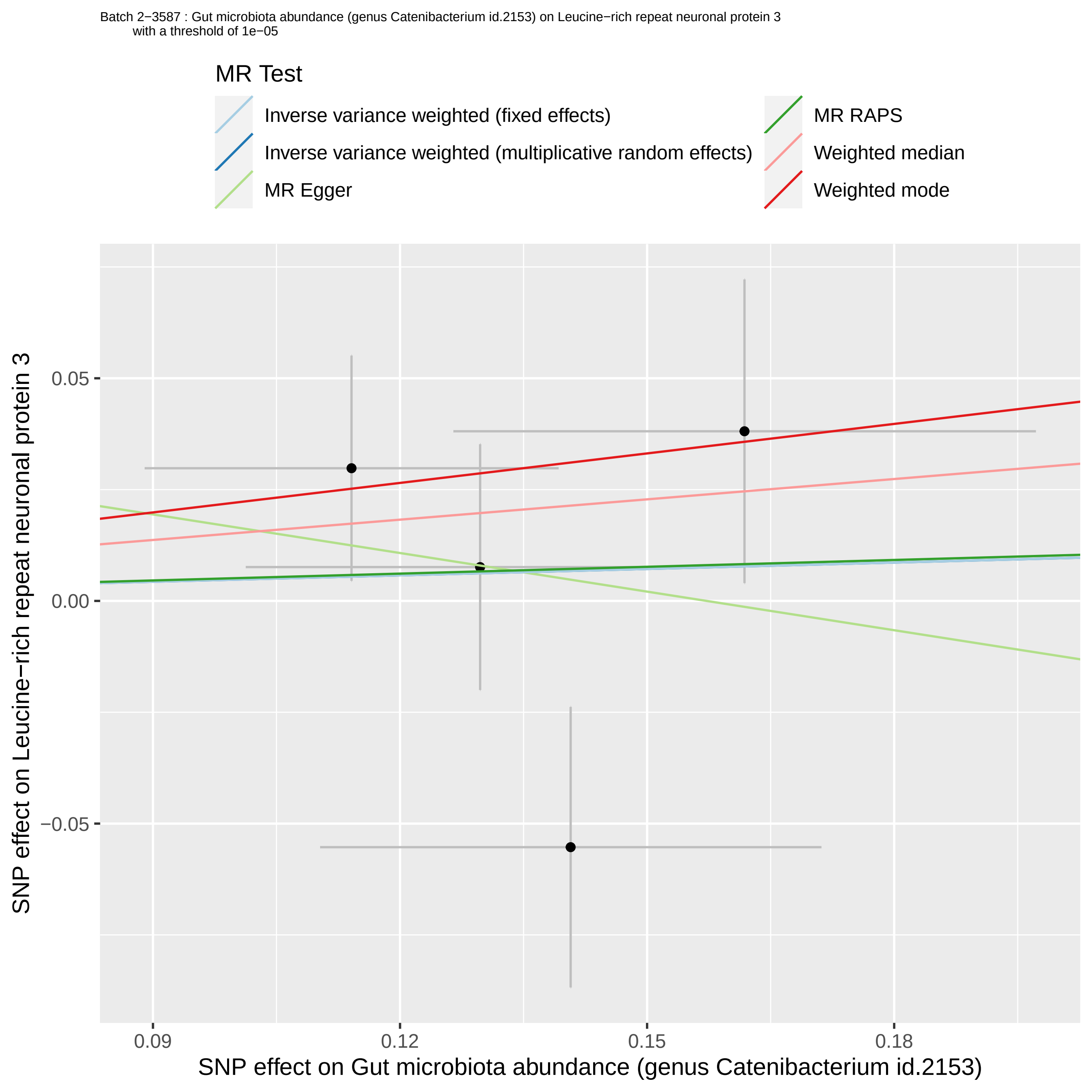
Batch 2−3587 : Gut microbiota abundance (genus Catenibacterium id.2153) on Leucine−rich repeat neuronal protein 3
with a threshold of 1e−05
MR Test
Inverse variance weighted (fixed effects)
Inverse variance weighted (multiplicative random effects)
MR Egger
MR RAPS
Weighted median
Weighted mode
0.05
0.00
−0.05
0.09
0.12
0.15
0.18
SNP effect on Leucine−rich repeat neuronal protein 3
SNP effect on Gut microbiota abundance (genus Catenibacterium id.2153)

Batch 2−3588 : Gut microbiota abundance (genus Parasutterella id.2892) on Leucine−rich repeat neuronal protein 3
with a threshold of 1e−05

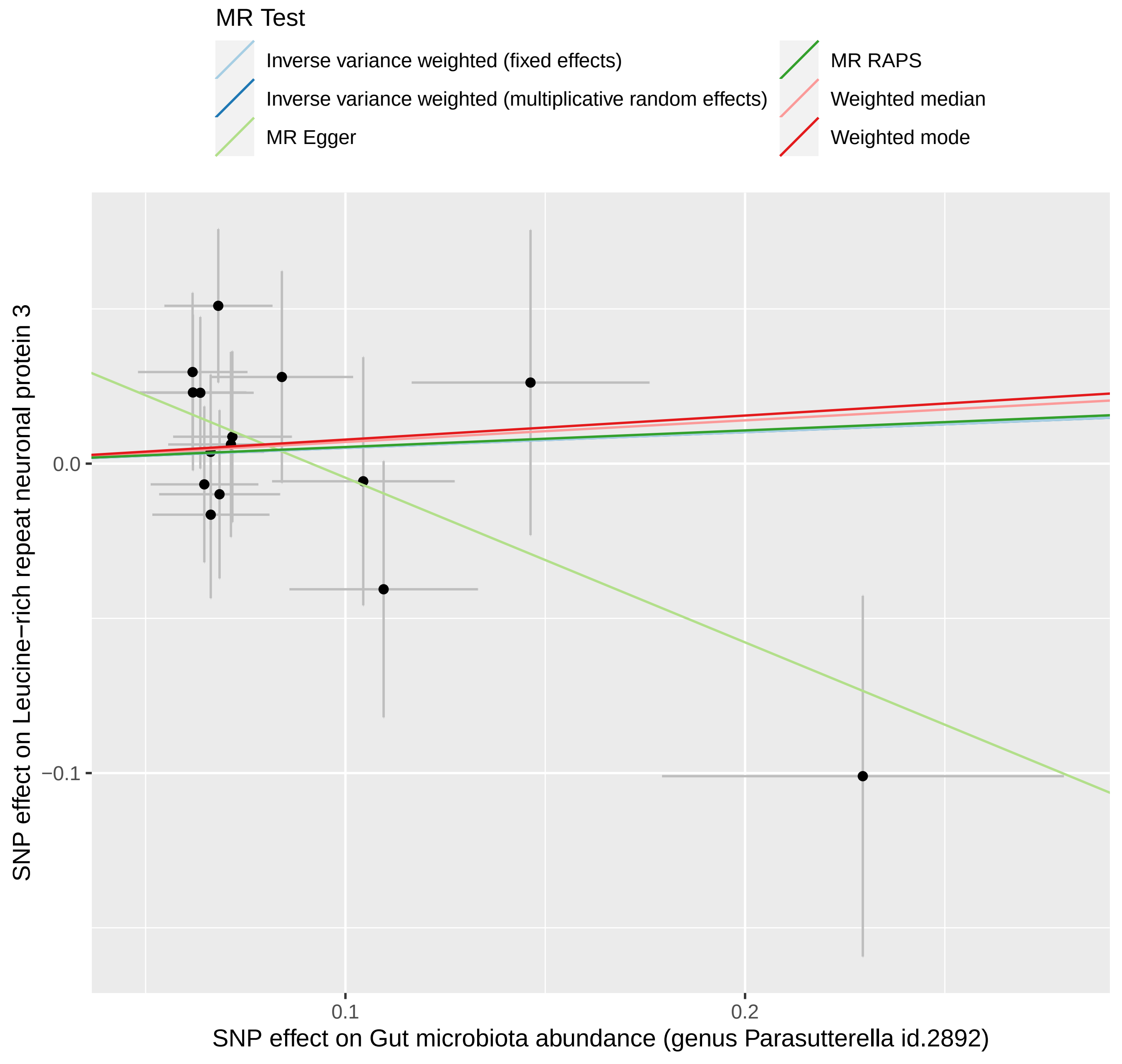

Batch 2−3589 : Gut microbiota abundance (genus Catenibacterium id.2153) on Leucine−rich repeat transmembrane neuronal protein 1 with a threshold of 1e−05

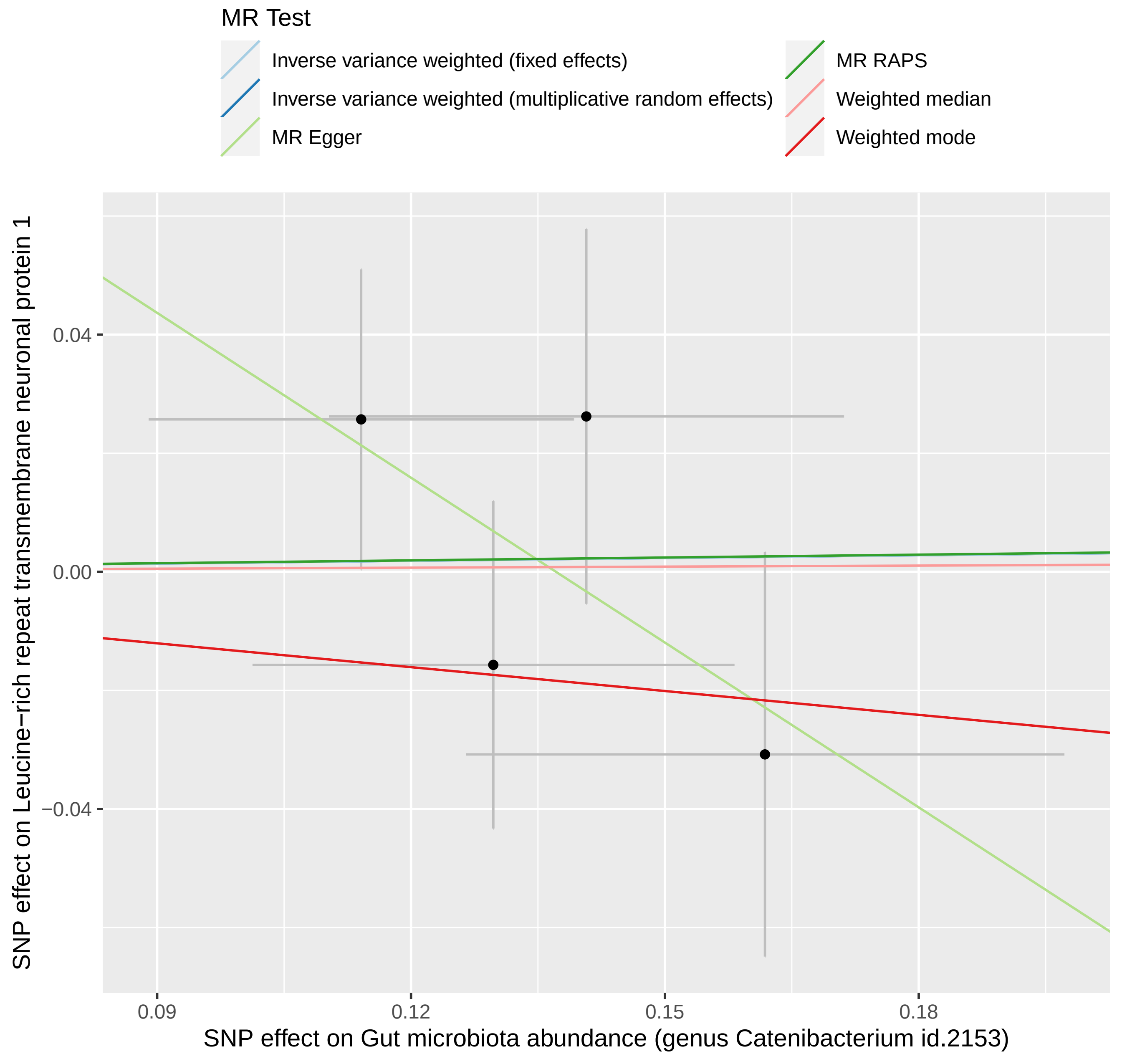

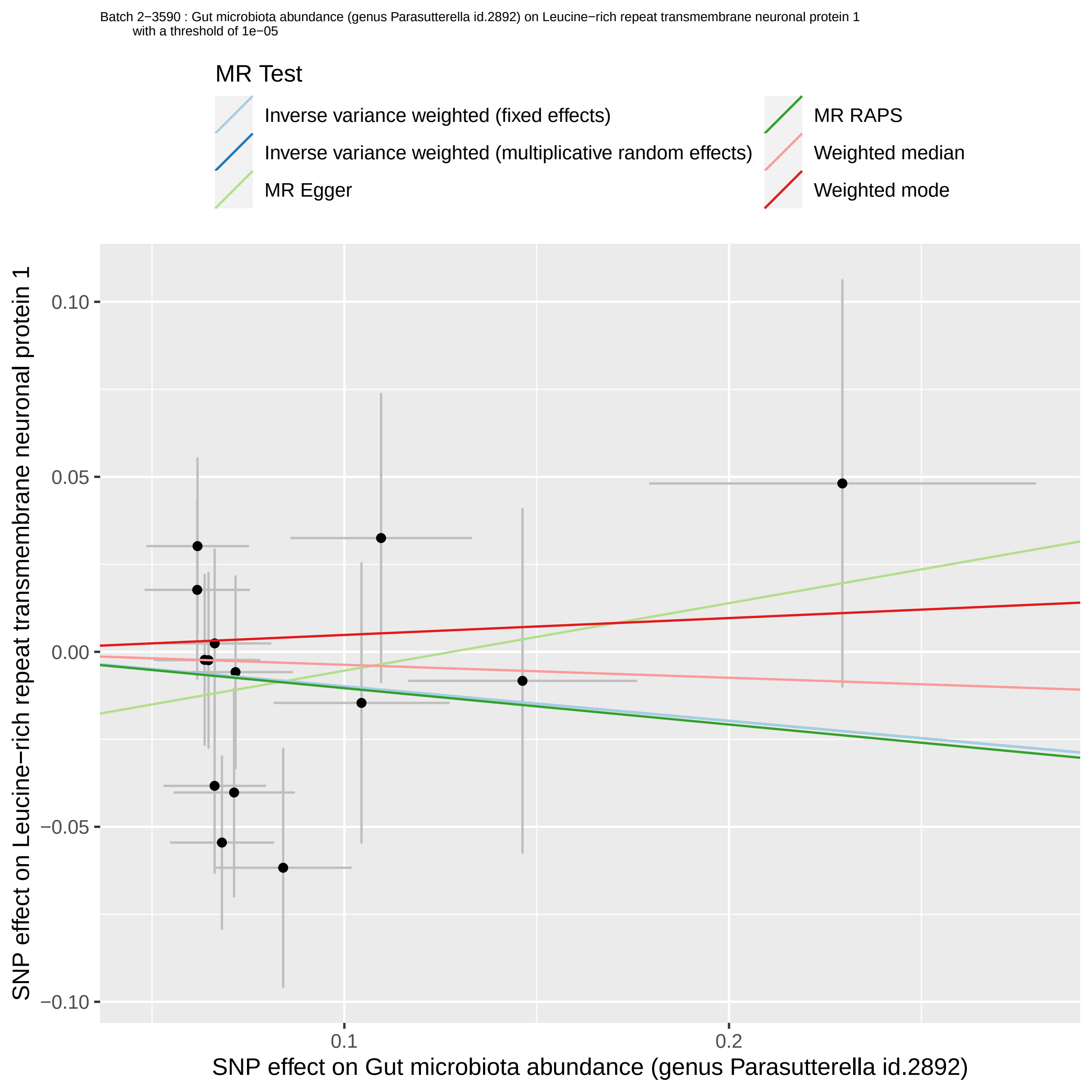
Batch 2−3590 : Gut microbiota abundance (genus Parasutterella id.2892) on Leucine−rich repeat transmembrane neuronal protein 1
with a threshold of 1e−05
MR Test
Inverse variance weighted (fixed effects)
Inverse variance weighted (multiplicative random effects)
MR Egger
MR RAPS
Weighted median
Weighted mode
0.10
0.05
0.00
−0.05
−0.10
0.1
0.2
SNP effect on Leucine−rich repeat transmembrane neuronal protein 1
SNP effect on Gut microbiota abundance (genus Parasutterella id.2892)

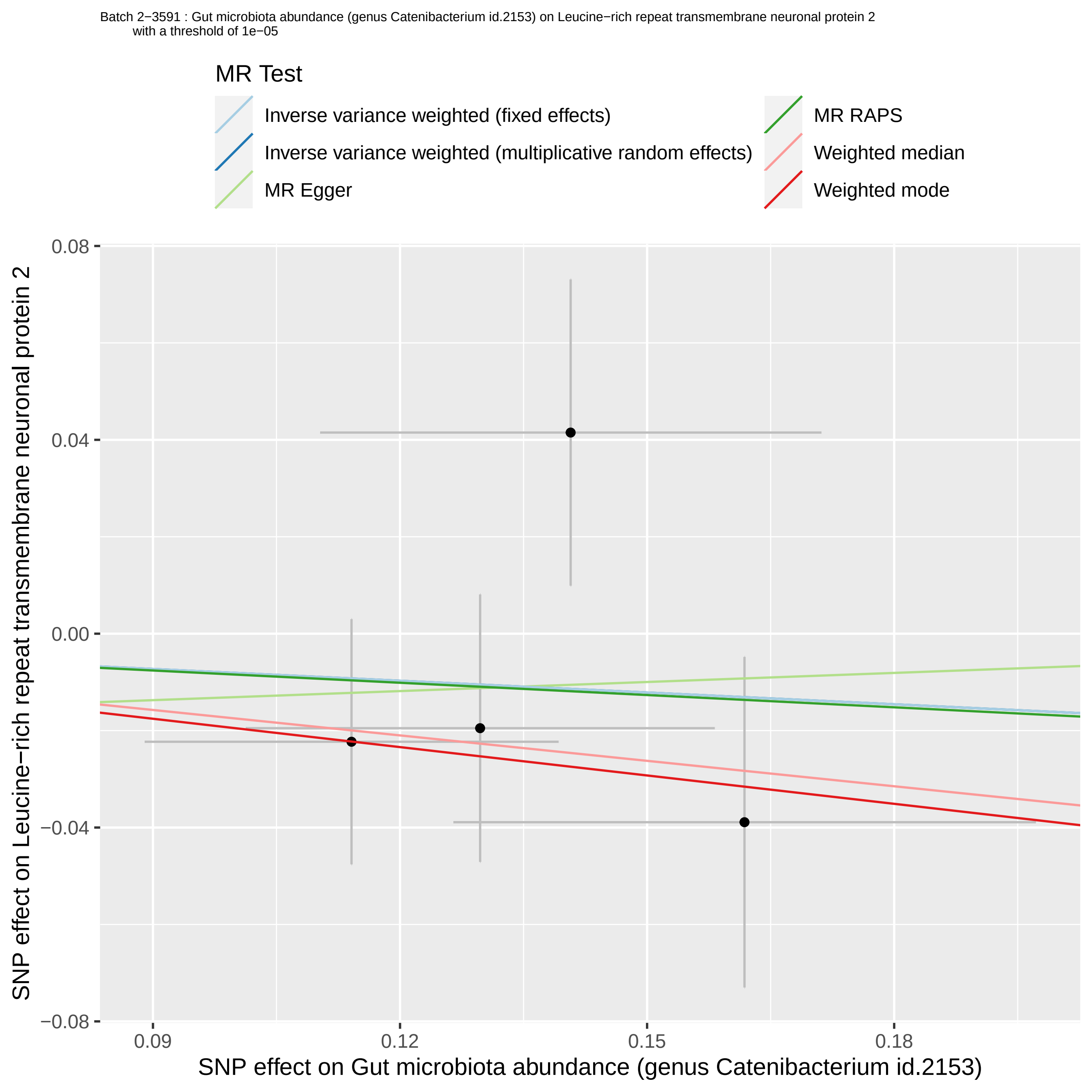
Batch 2−3591 : Gut microbiota abundance (genus Catenibacterium id.2153) on Leucine−rich repeat transmembrane neuronal protein 2
with a threshold of 1e−05
MR Test
Inverse variance weighted (fixed effects)
Inverse variance weighted (multiplicative random effects)
MR Egger
MR RAPS
Weighted median
Weighted mode
0.08
0.04
0.00
−0.04
−0.08
0.09
0.12
0.15
0.18
SNP effect on Leucine−rich repeat transmembrane neuronal protein 2
SNP effect on Gut microbiota abundance (genus Catenibacterium id.2153)

Batch 2−3592 : Gut microbiota abundance (genus Parasutterella id.2892) on Leucine−rich repeat transmembrane neuronal protein 2
with a threshold of 1e−05

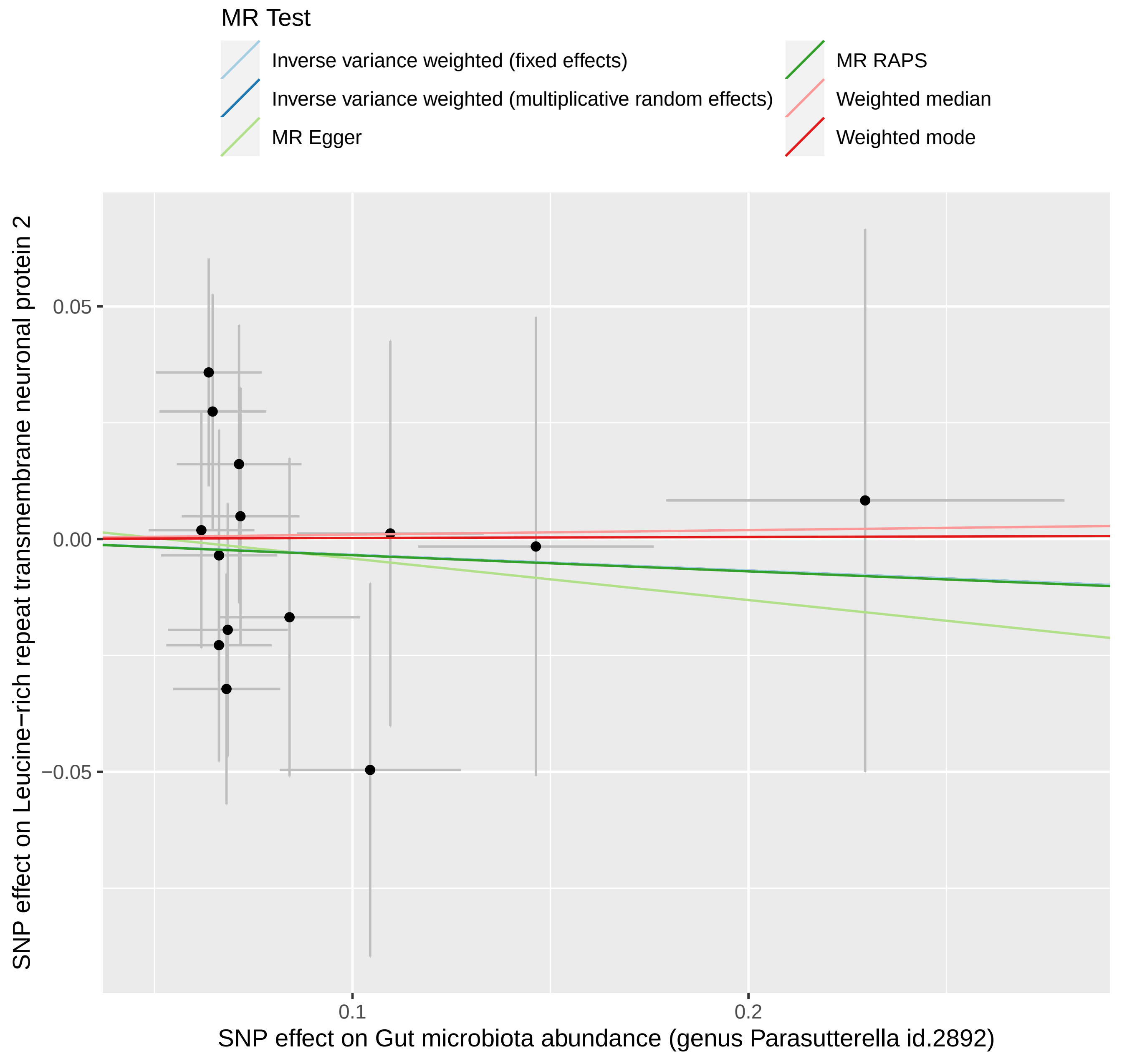

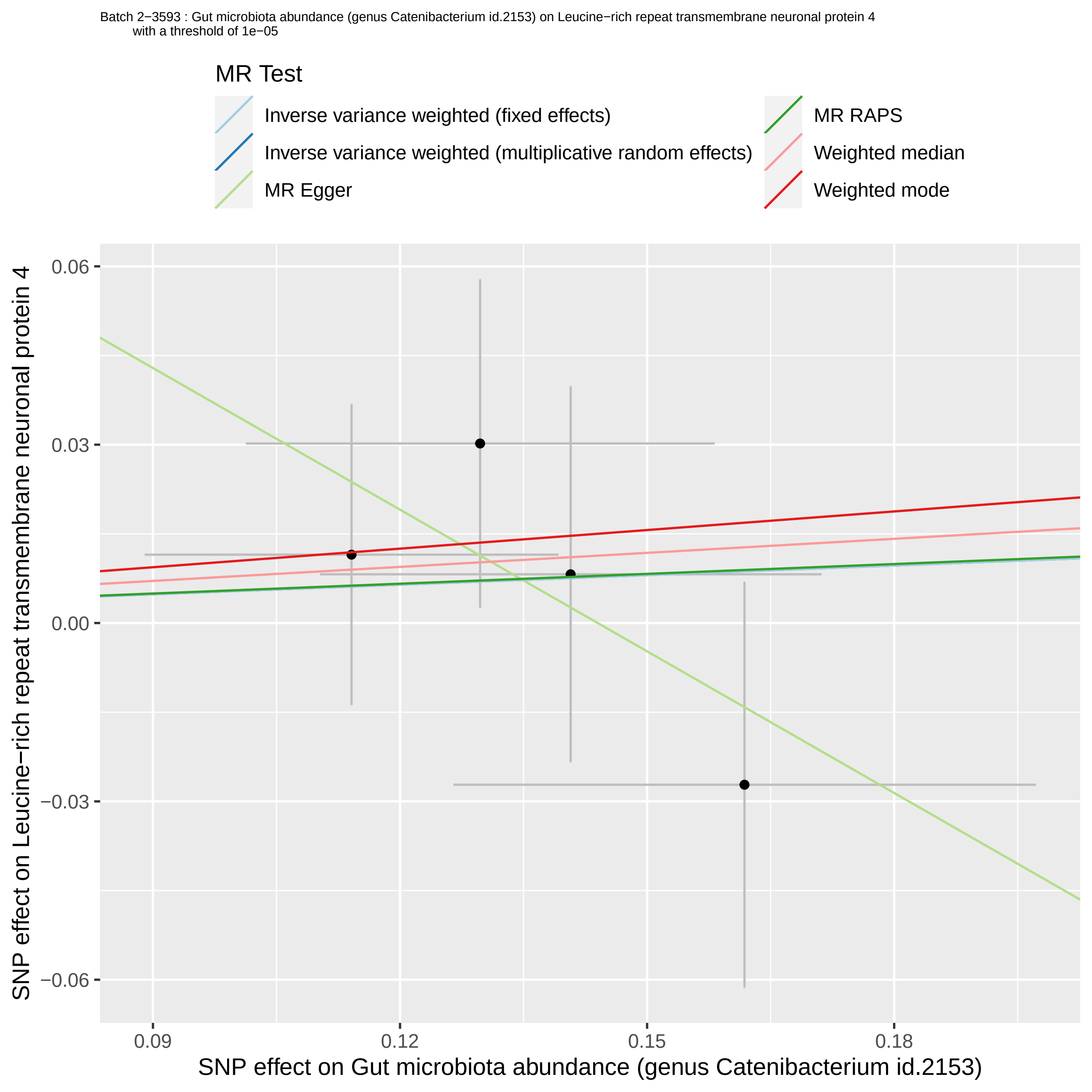
Batch 2−3593 : Gut microbiota abundance (genus Catenibacterium id.2153) on Leucine−rich repeat transmembrane neuronal protein 4
with a threshold of 1e−05
MR Test
Inverse variance weighted (fixed effects)
Inverse variance weighted (multiplicative random effects)
MR Egger
MR RAPS
Weighted median
Weighted mode
0.06
0.03
0.00
−0.03
−0.06
0.09
0.12
0.15
0.18
SNP effect on Leucine−rich repeat transmembrane neuronal protein 4
SNP effect on Gut microbiota abundance (genus Catenibacterium id.2153)

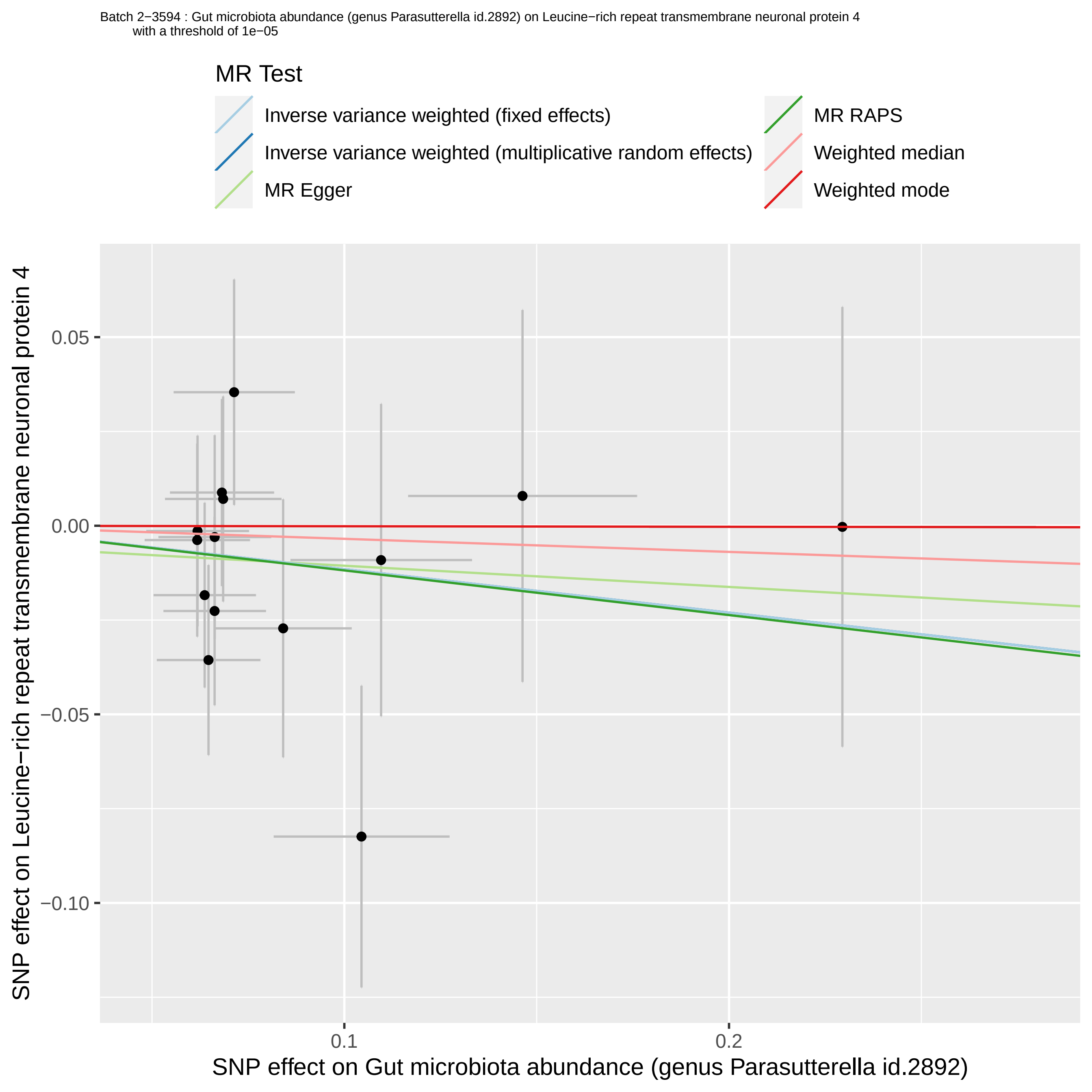
Batch 2−3594 : Gut microbiota abundance (genus Parasutterella id.2892) on Leucine−rich repeat transmembrane neuronal protein 4
with a threshold of 1e−05
MR Test
Inverse variance weighted (fixed effects)
Inverse variance weighted (multiplicative random effects)
MR Egger
MR RAPS
Weighted median
Weighted mode
SNP effect on Leucine−rich repeat transmembrane neuronal protein 4
0.05
0.00
−0.05
−0.10
0.1
0.2
SNP effect on Gut microbiota abundance (genus Parasutterella id.2892)

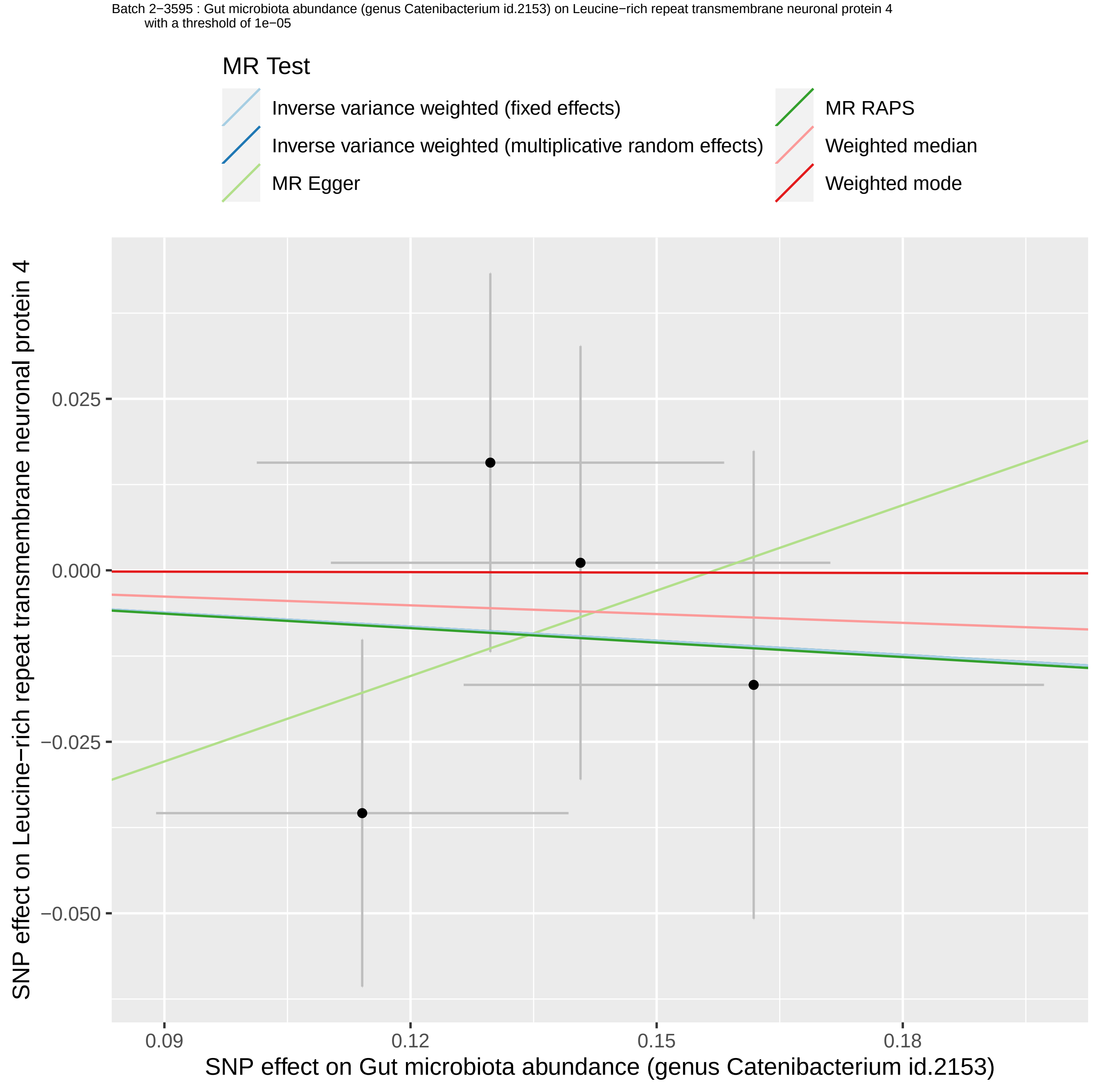
Batch 2−3595 : Gut microbiota abundance (genus Catenibacterium id.2153) on Leucine−rich repeat transmembrane neuronal protein 4
with a threshold of 1e−05
MR Test
Inverse variance weighted (fixed effects)
Inverse variance weighted (multiplicative random effects)
MR Egger
MR RAPS
Weighted median
Weighted mode
0.025
0.000
−0.025
−0.050
0.09
0.12
0.15
0.18
SNP effect on Leucine−rich repeat transmembrane neuronal protein 4
SNP effect on Gut microbiota abundance (genus Catenibacterium id.2153)

Batch 2−3596 : Gut microbiota abundance (genus Parasutterella id.2892) on Leucine−rich repeat transmembrane neuronal protein 4
with a threshold of 1e−05

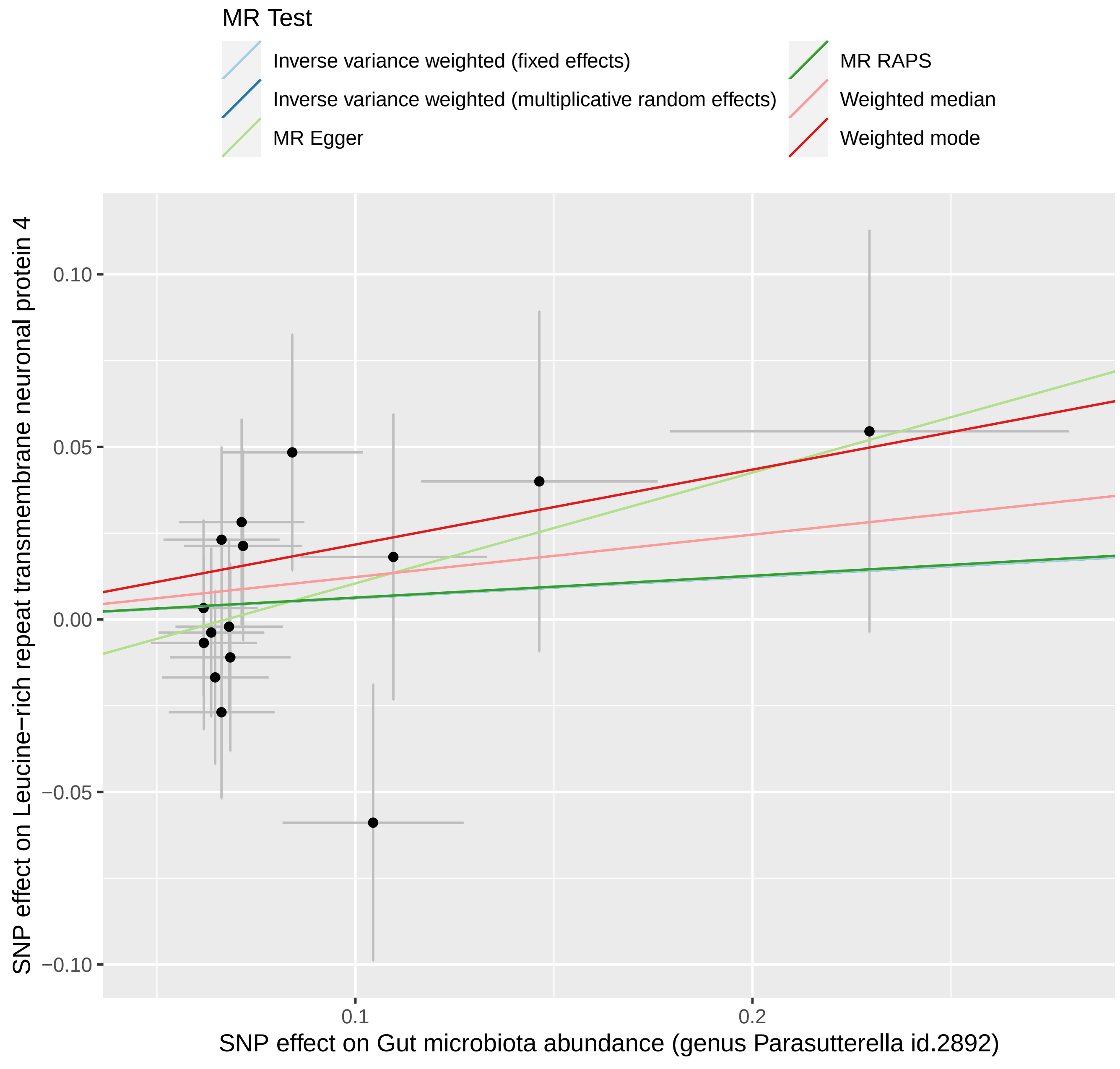

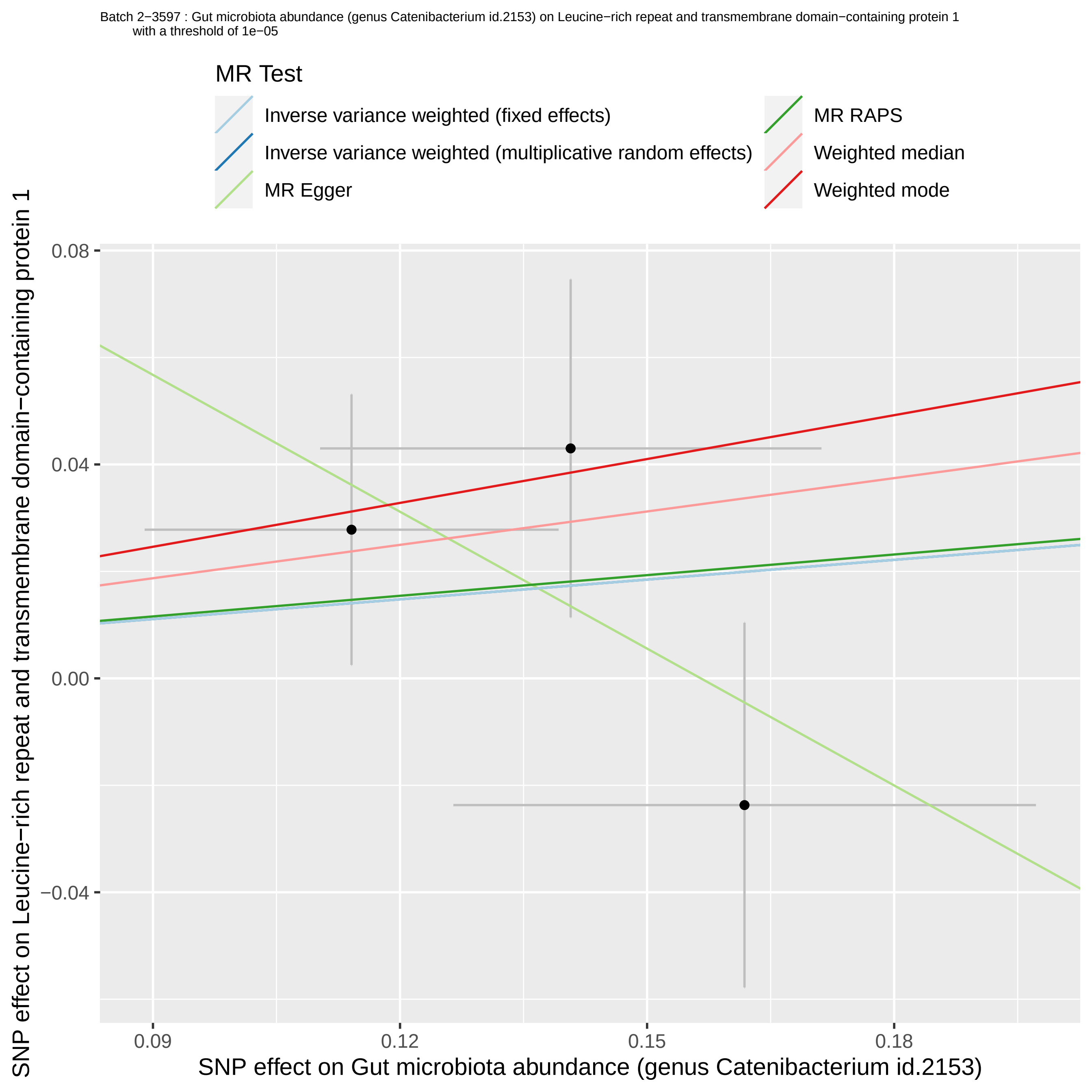

Batch 2−3597 : Gut microbiota abundance (genus Catenibacterium id.2153) on Leucine−rich repeat and transmembrane domain−containing protein 1 with a threshold of 1e−05
MR Test
Inverse variance weighted (fixed effects)
Inverse variance weighted (multiplicative random effects)
MR Egger
MR RAPS
Weighted median
Weighted mode
SNP effect on Leucine−rich repeat and transmembrane domain−containing protein 1
0.08
0.04
0.00
−0.04
0.09
0.12
0.15
0.18
SNP effect on Gut microbiota abundance (genus Catenibacterium id.2153)

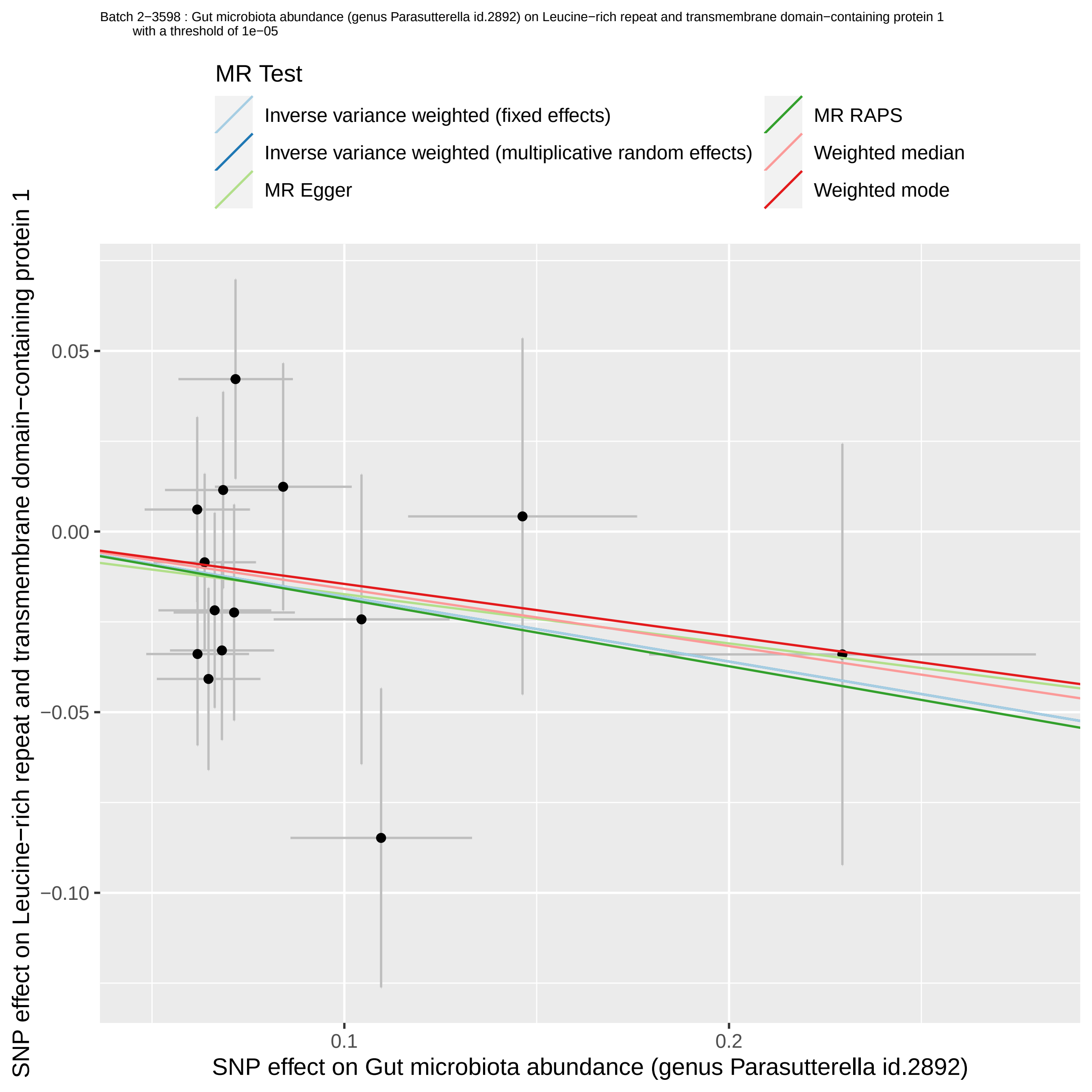
Batch 2−3598 : Gut microbiota abundance (genus Parasutterella id.2892) on Leucine−rich repeat and transmembrane domain−containing protein 1
with a threshold of 1e−05
MR Test
Inverse variance weighted (fixed effects)
Inverse variance weighted (multiplicative random effects)
MR Egger
MR RAPS
Weighted median
Weighted mode
0.05
0.00
−0.05
−0.10
0.1
0.2
SNP effect on Leucine−rich repeat and transmembrane domain−containing protein 1
SNP effect on Gut microbiota abundance (genus Parasutterella id.2892)

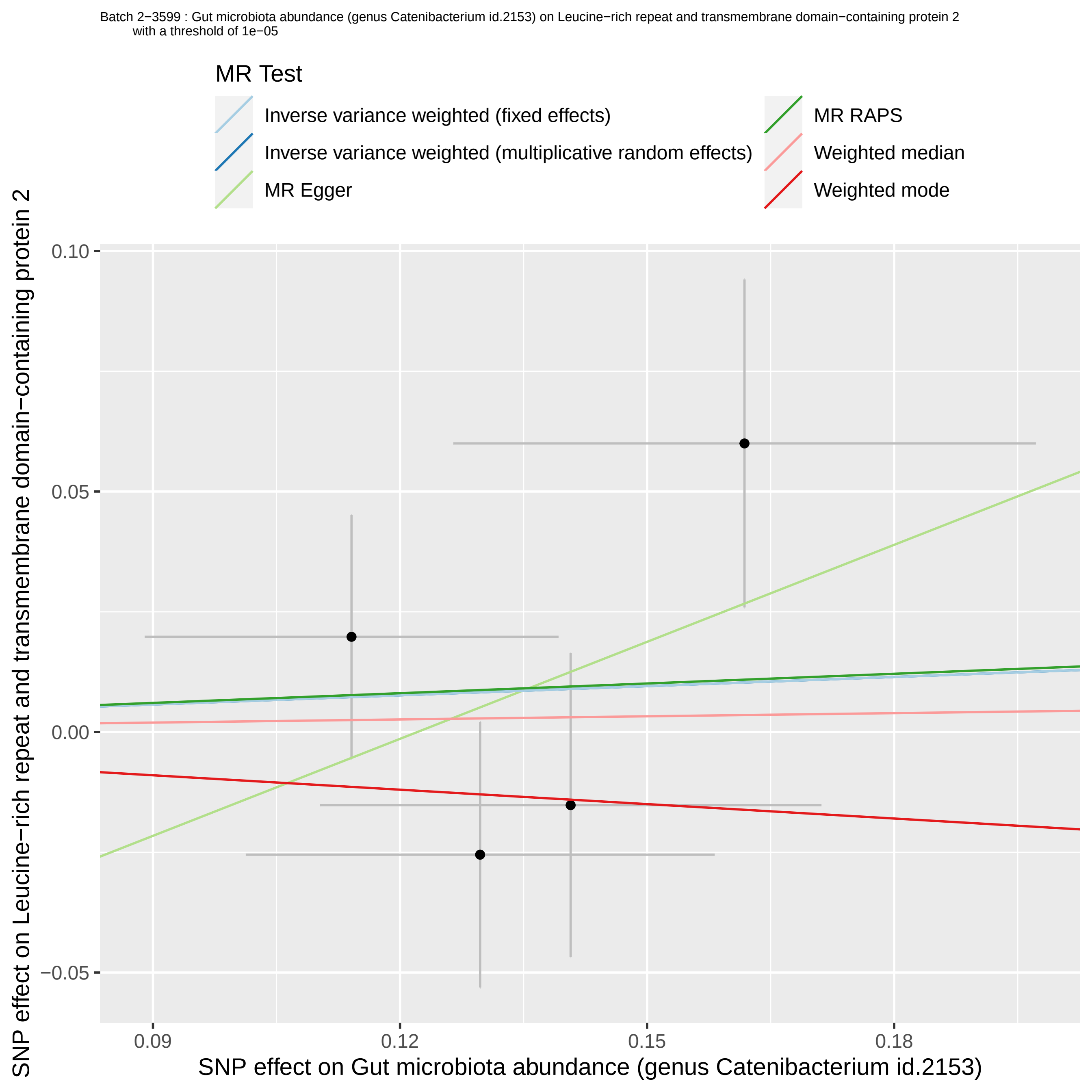

Batch 2−3599 : Gut microbiota abundance (genus Catenibacterium id.2153) on Leucine−rich repeat and transmembrane domain−containing protein 2 with a threshold of 1e−05
MR Test
Inverse variance weighted (fixed effects)
Inverse variance weighted (multiplicative random effects)
MR Egger
MR RAPS
Weighted median
Weighted mode
0.10
0.05
0.00
−0.05
0.09
0.12
0.15
0.18
SNP effect on Leucine−rich repeat and transmembrane domain−containing protein 2
SNP effect on Gut microbiota abundance (genus Catenibacterium id.2153)

Batch 2−3600 : Gut microbiota abundance (genus Parasutterella id.2892) on Leucine−rich repeat and transmembrane domain−containing protein 2
with a threshold of 1e−05

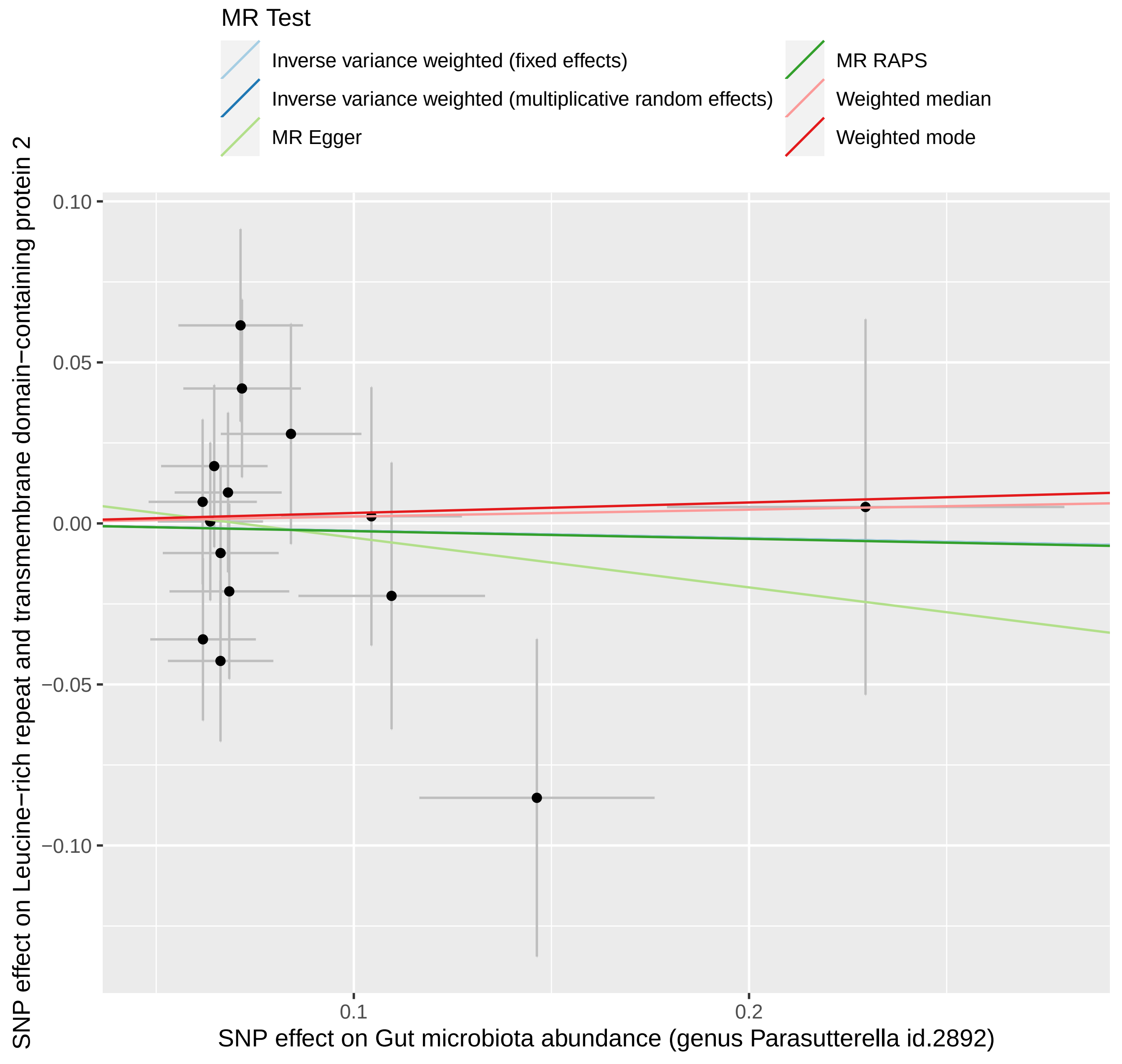

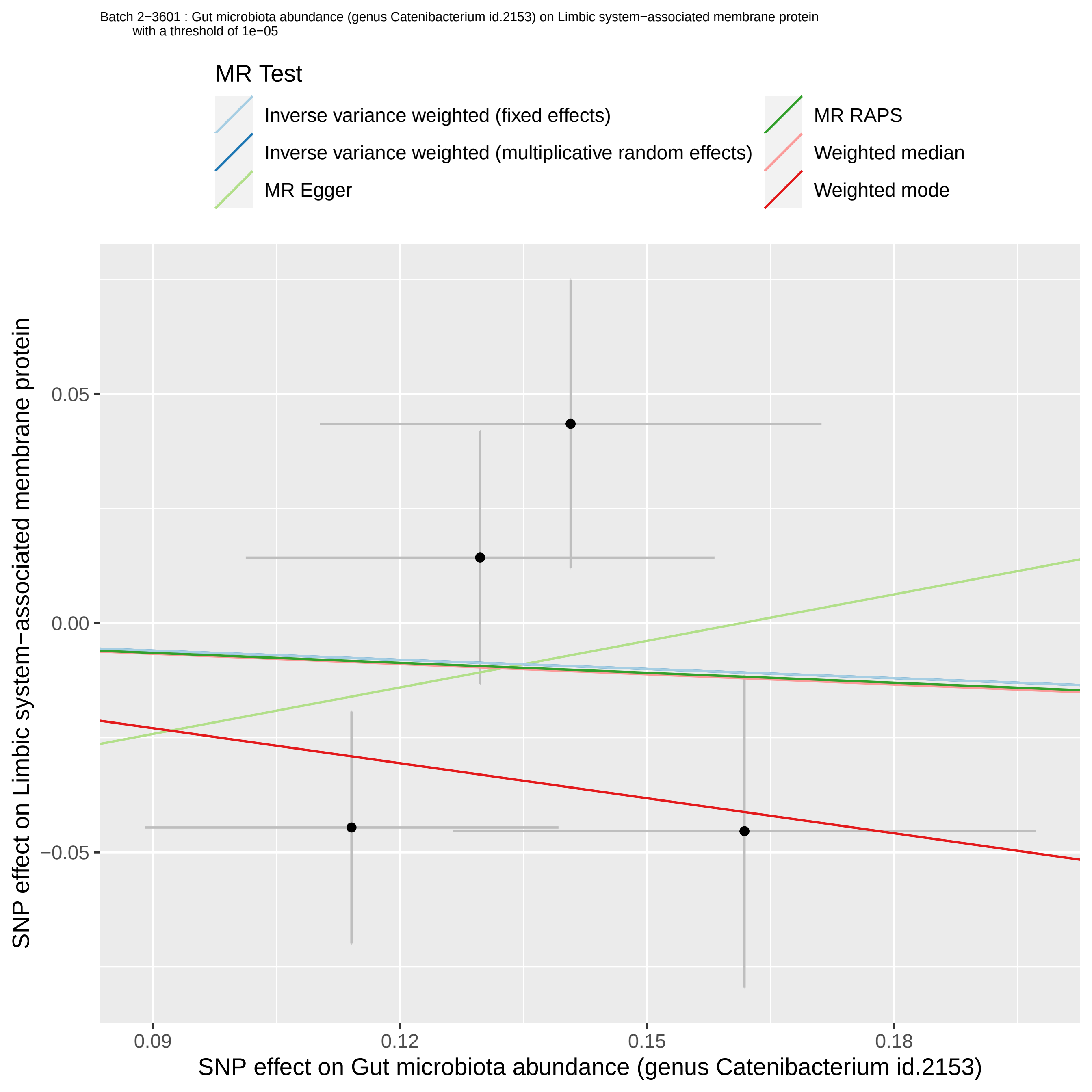

Batch 2−3601 : Gut microbiota abundance (genus Catenibacterium id.2153) on Limbic system−associated membrane protein
with a threshold of 1e−05
MR Test
Inverse variance weighted (fixed effects)
Inverse variance weighted (multiplicative random effects)
MR Egger
MR RAPS
Weighted median
Weighted mode
0.05
0.00
−0.05
0.09
0.12
0.15
0.18
SNP effect on Limbic system−associated membrane protein
SNP effect on Gut microbiota abundance (genus Catenibacterium id.2153)

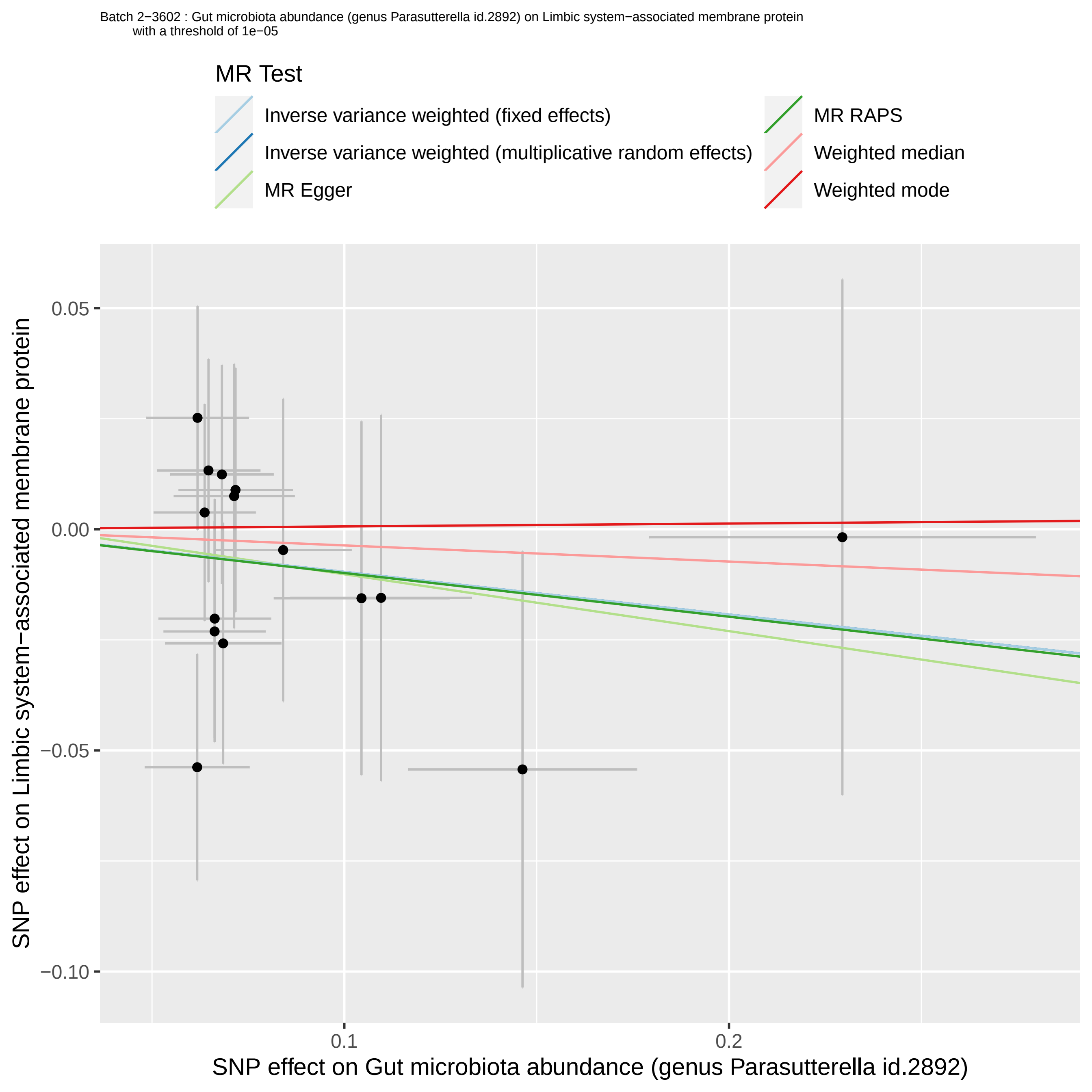

Batch 2−3602 : Gut microbiota abundance (genus Parasutterella id.2892) on Limbic system−associated membrane protein
with a threshold of 1e−05
MR Test
Inverse variance weighted (fixed effects)
Inverse variance weighted (multiplicative random effects)
MR Egger
MR RAPS
Weighted median
Weighted mode
0.05
0.00
−0.05
−0.10
0.1
0.2
SNP effect on Limbic system−associated membrane protein
SNP effect on Gut microbiota abundance (genus Parasutterella id.2892)

Batch 2−3603 : Gut microbiota abundance (genus Catenibacterium id.2153) on Leukocyte−specific transcript 1 protein with a threshold of 1e−05

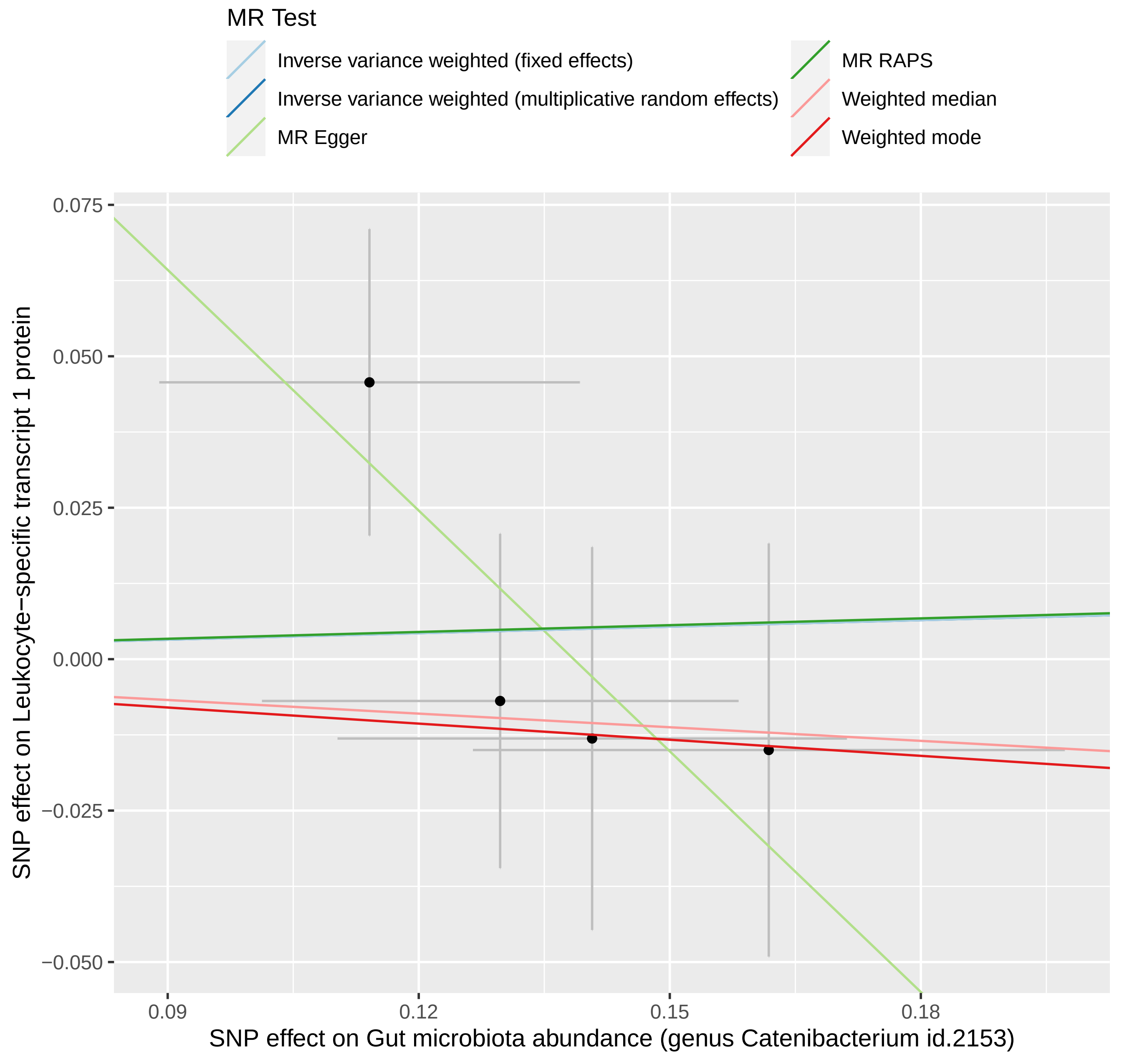

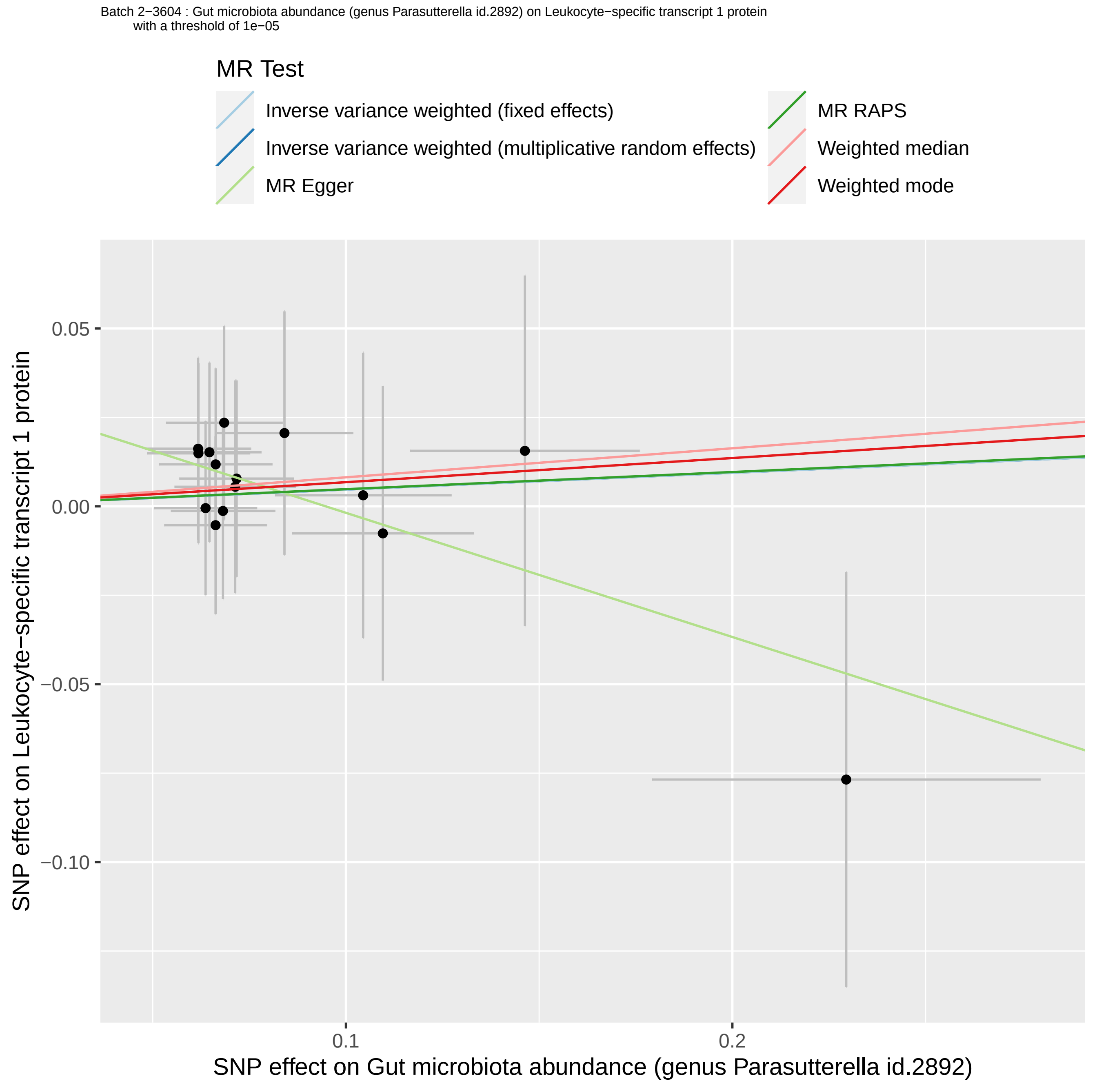

Batch 2−3604 : Gut microbiota abundance (genus Parasutterella id.2892) on Leukocyte−specific transcript 1 protein
with a threshold of 1e−05
MR Test
Inverse variance weighted (fixed effects)
Inverse variance weighted (multiplicative random effects)
MR Egger
MR RAPS
Weighted median
Weighted mode
0.05
0.00
−0.05
−0.10
0.1
0.2
SNP effect on Leukocyte−specific transcript 1 protein
SNP effect on Gut microbiota abundance (genus Parasutterella id.2892)

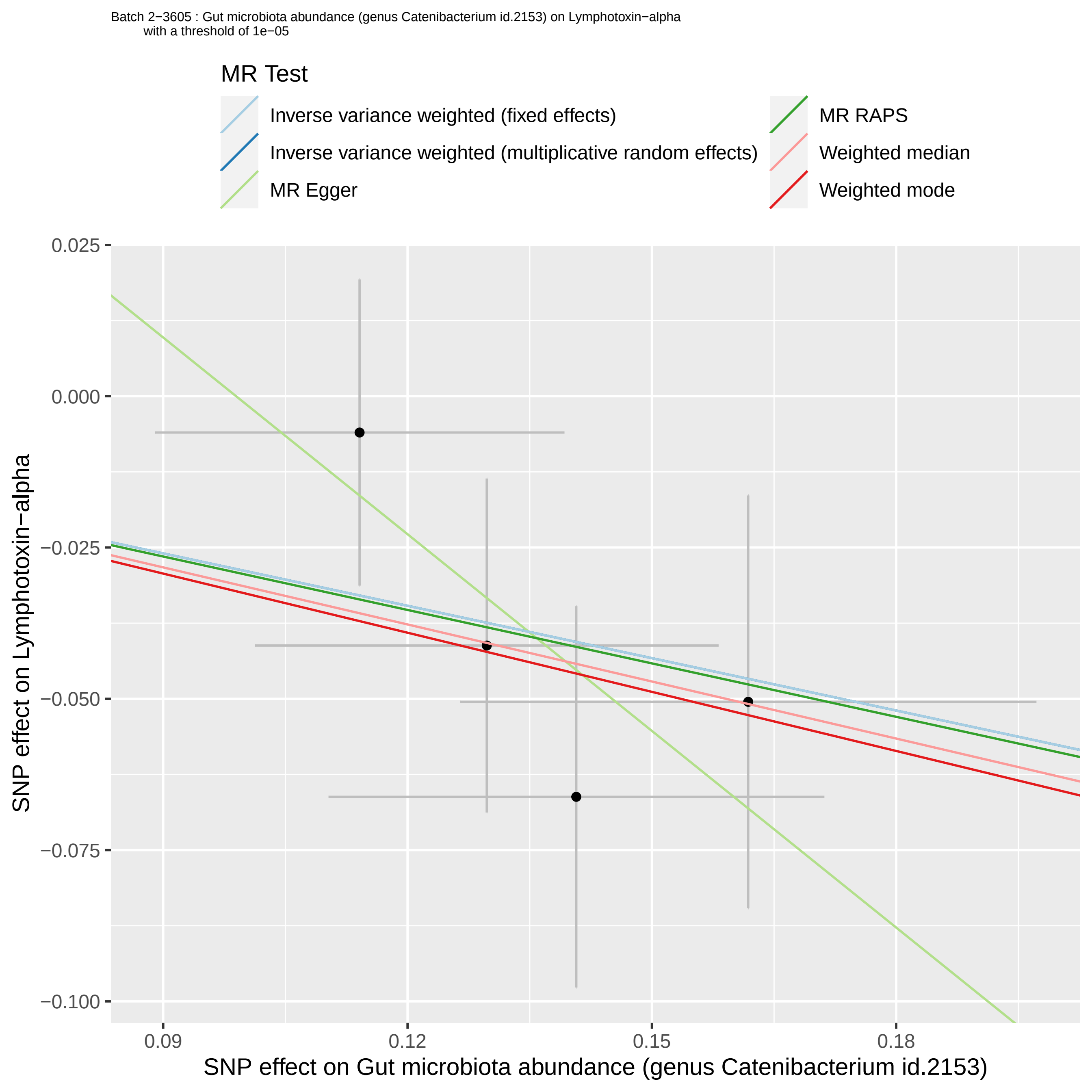
Batch 2−3605 : Gut microbiota abundance (genus Catenibacterium id.2153) on Lymphotoxin−alpha
with a threshold of 1e−05
MR Test
Inverse variance weighted (fixed effects)
Inverse variance weighted (multiplicative random effects)
MR Egger
MR RAPS
Weighted median
Weighted mode
0.025
0.000
−0.025
−0.050
−0.075
−0.100
0.09
0.12
0.15
0.18
SNP effect on Gut microbiota abundance (genus Catenibacterium id.2153)
SNP effect on Lymphotoxin−alpha

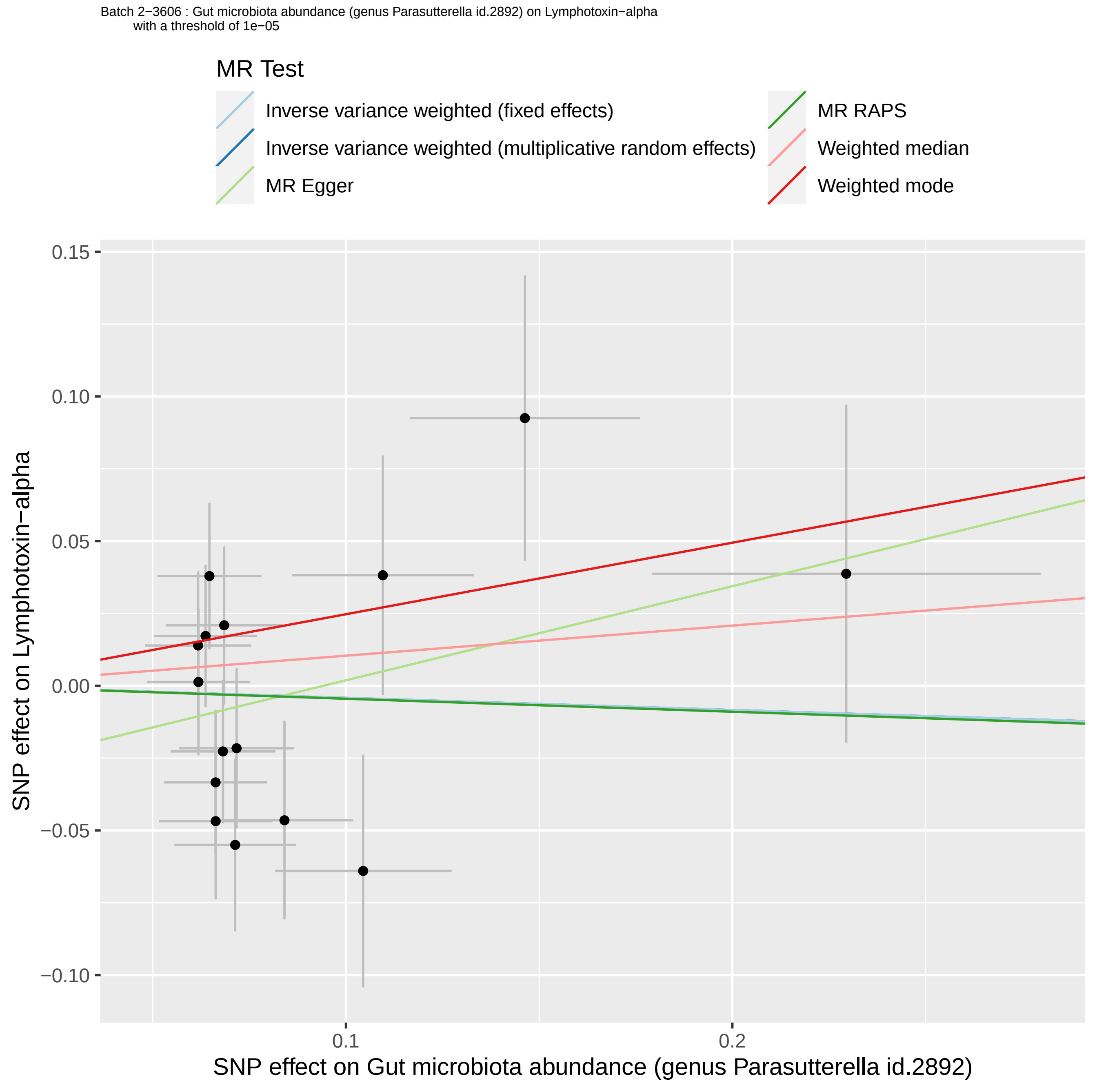
Batch 2−3606 : Gut microbiota abundance (genus Parasutterella id.2892) on Lymphotoxin−alpha
with a threshold of 1e−05
MR Test
Inverse variance weighted (fixed effects)
Inverse variance weighted (multiplicative random effects)
MR Egger
MR RAPS
Weighted median
Weighted mode
0.15
0.10
0.05
0.00
−0.05
−0.10
0.1
0.2
SNP effect on Lymphotoxin−alpha
SNP effect on Gut microbiota abundance (genus Parasutterella id.2892)

Batch 2−3607 : Gut microbiota abundance (genus Catenibacterium id.2153) on Leukotriene A−4 hydrolase
with a threshold of 1e−05

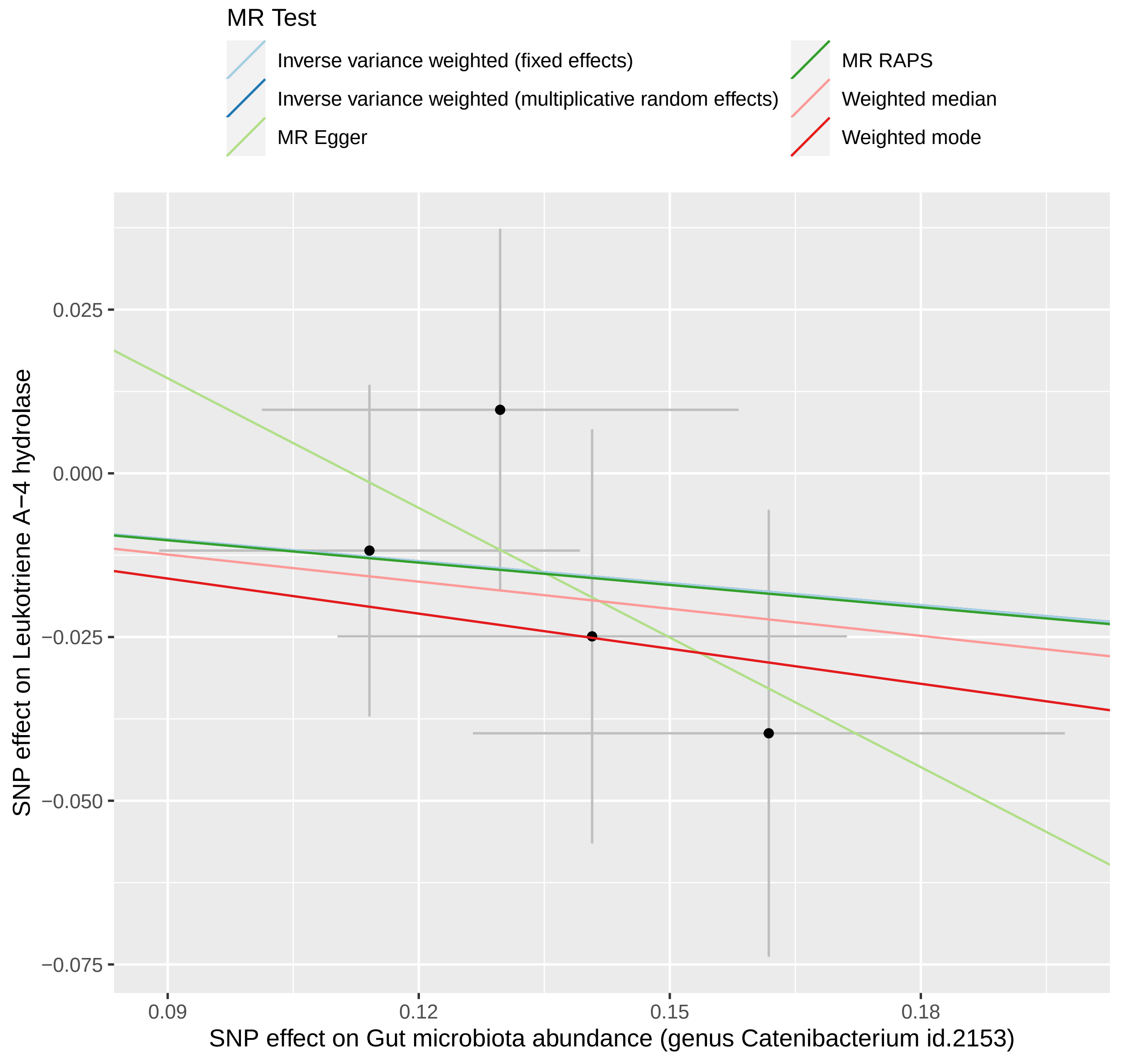

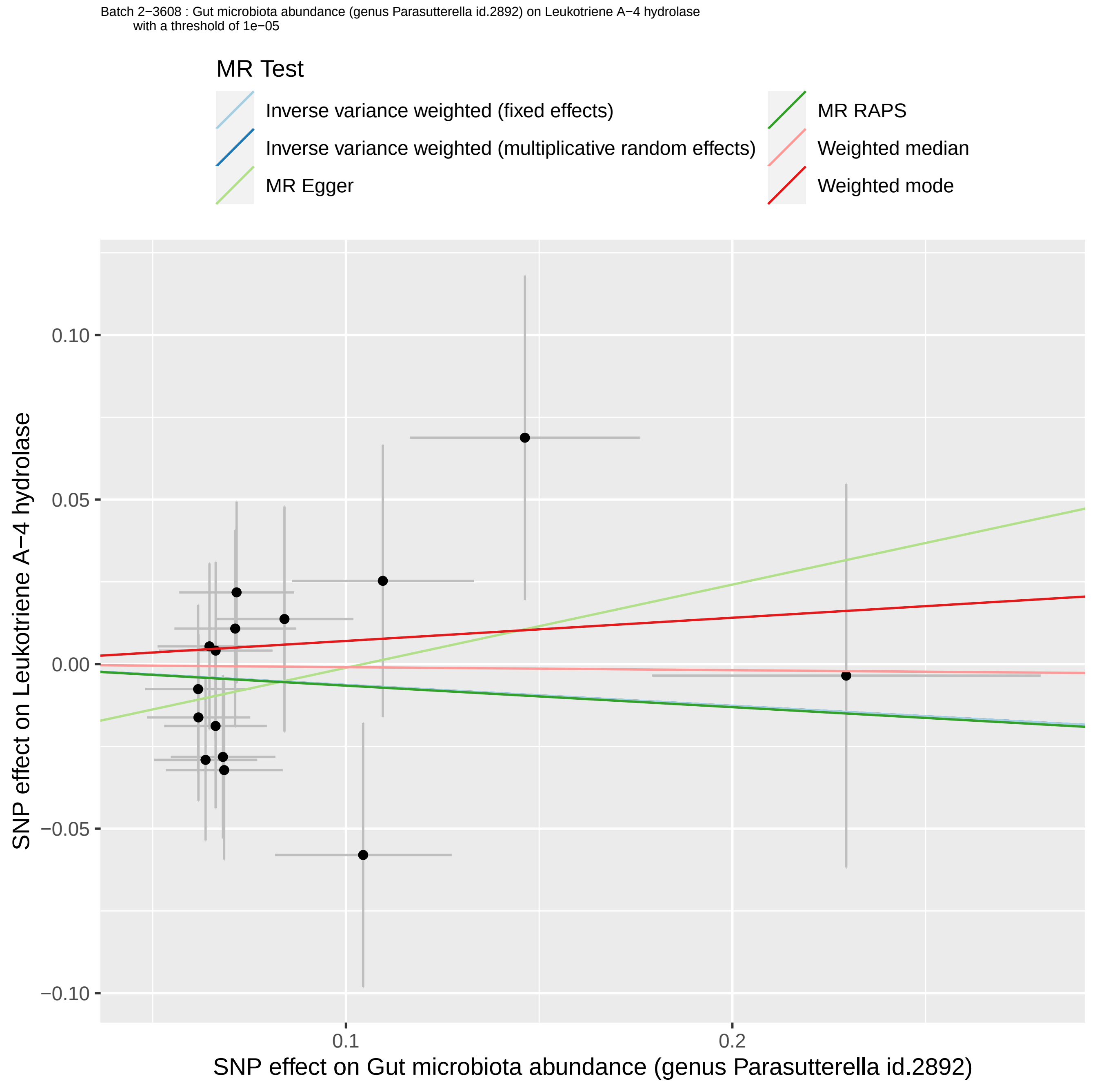

Batch 2−3608 : Gut microbiota abundance (genus Parasutterella id.2892) on Leukotriene A−4 hydrolase
with a threshold of 1e−05
MR Test
Inverse variance weighted (fixed effects)
Inverse variance weighted (multiplicative random effects)
MR Egger
MR RAPS
Weighted median
Weighted mode
0.10
0.05
0.00
−0.05
−0.10
0.1
0.2
SNP effect on Leukotriene A−4 hydrolase
SNP effect on Gut microbiota abundance (genus Parasutterella id.2892)

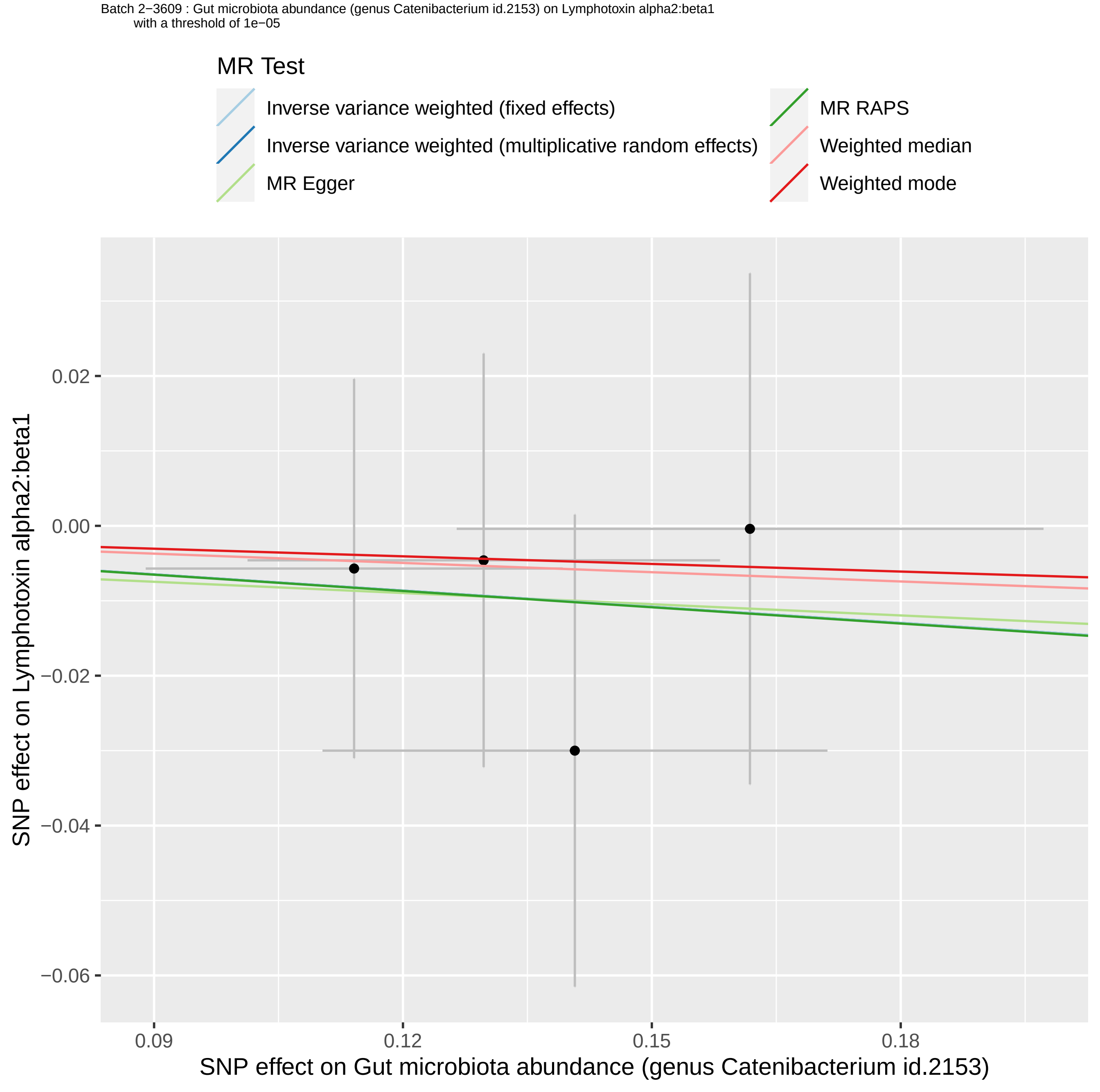

Batch 2−3609 : Gut microbiota abundance (genus Catenibacterium id.2153) on Lymphotoxin alpha2:beta1
with a threshold of 1e−05
MR Test
Inverse variance weighted (fixed effects)
Inverse variance weighted (multiplicative random effects)
MR Egger
MR RAPS
Weighted median
Weighted mode
0.02
0.00
−0.02
−0.04
−0.06
SNP effect on Lymphotoxin alpha2:beta1
0.09
0.12
0.15
0.18
SNP effect on Gut microbiota abundance (genus Catenibacterium id.2153)

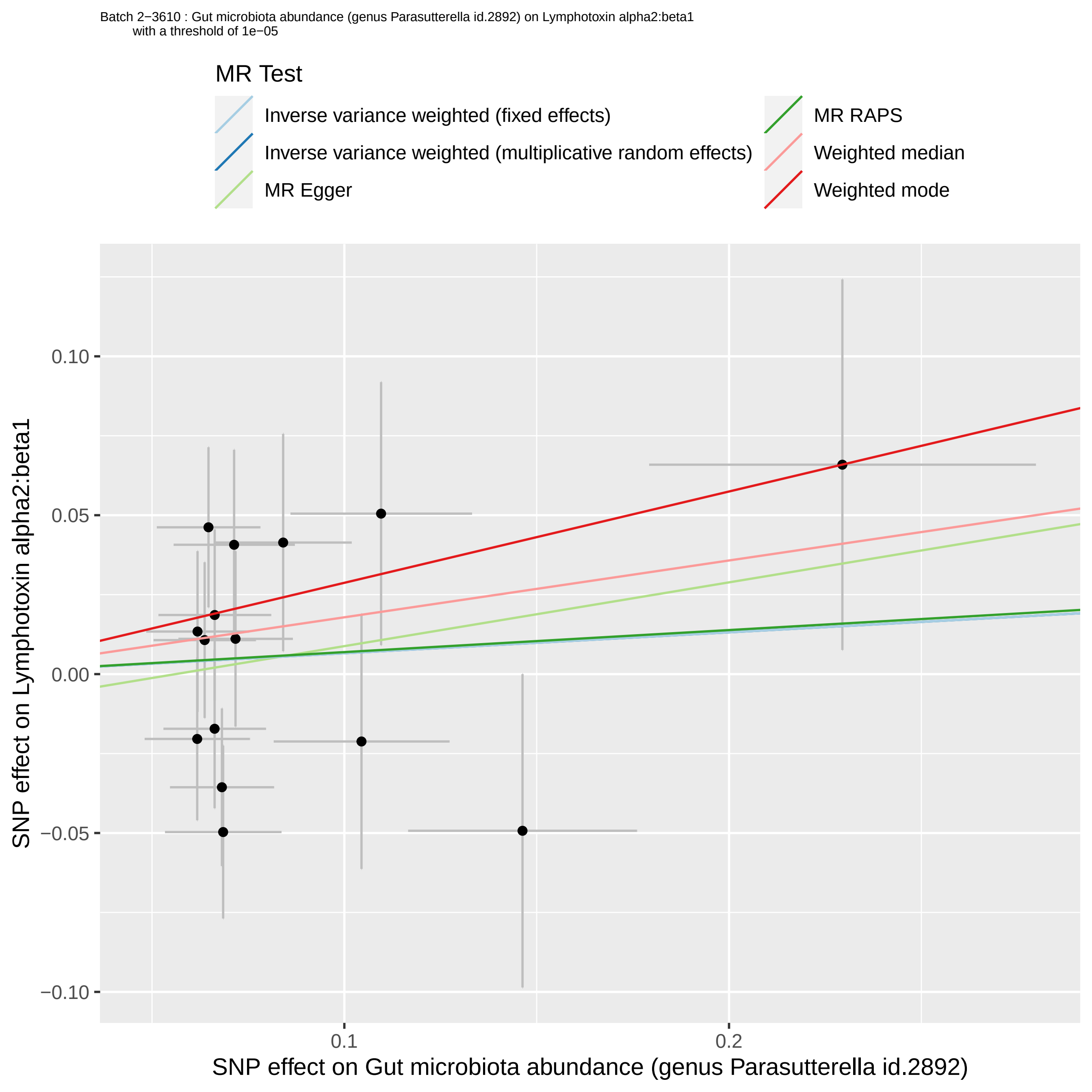
Batch 2−3610 : Gut microbiota abundance (genus Parasutterella id.2892) on Lymphotoxin alpha2:beta1
with a threshold of 1e−05
MR Test
Inverse variance weighted (fixed effects)
Inverse variance weighted (multiplicative random effects)
MR Egger
MR RAPS
Weighted median
Weighted mode
0.10
0.05
0.00
−0.05
−0.10
0.1
0.2
SNP effect on Lymphotoxin alpha2:beta1
SNP effect on Gut microbiota abundance (genus Parasutterella id.2892)

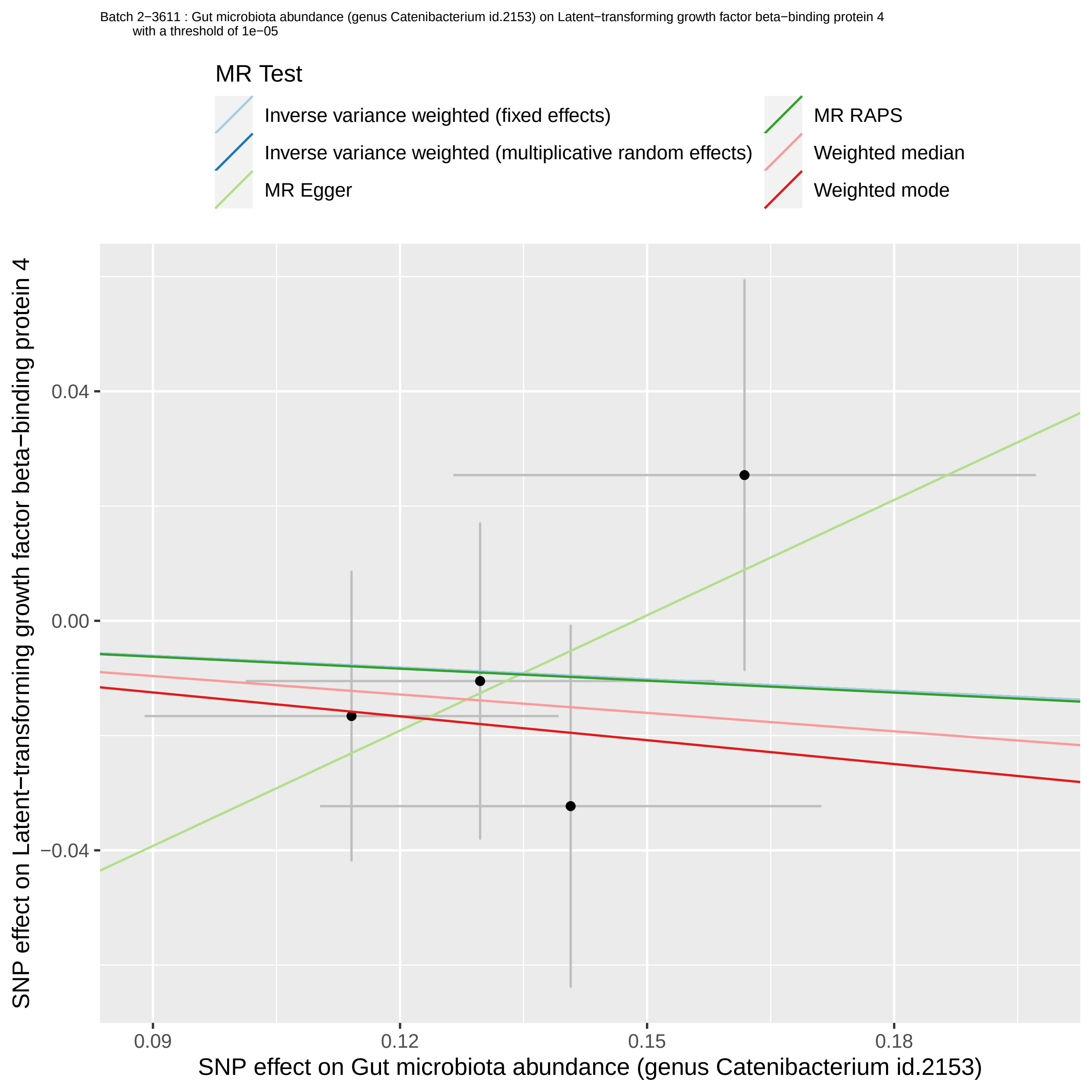

Batch 2−3611 : Gut microbiota abundance (genus Catenibacterium id.2153) on Latent−transforming growth factor beta−binding protein 4
with a threshold of 1e−05
MR Test
Inverse variance weighted (fixed effects)
Inverse variance weighted (multiplicative random effects)
MR Egger
MR RAPS
Weighted median
Weighted mode
0.04
0.00
−0.04
0.09
0.12
0.15
0.18
SNP effect on Gut microbiota abundance (genus Catenibacterium id.2153)
SNP effect on Latent−transforming growth factor beta−binding protein 4

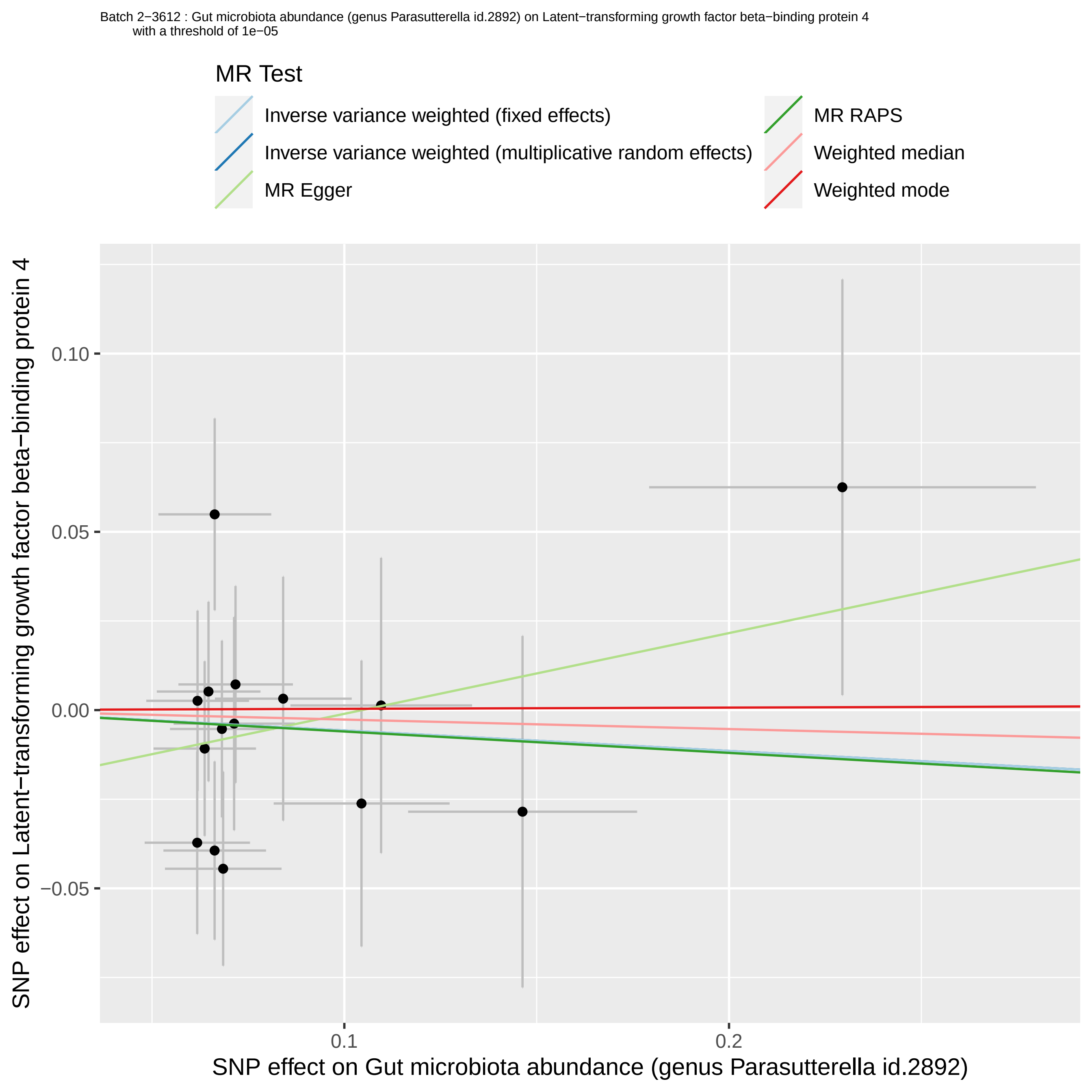

Batch 2−3612 : Gut microbiota abundance (genus Parasutterella id.2892) on Latent−transforming growth factor beta−binding protein 4
with a threshold of 1e−05
MR Test
Inverse variance weighted (fixed effects)
Inverse variance weighted (multiplicative random effects)
MR Egger
MR RAPS
Weighted median
Weighted mode
SNP effect on Latent−transforming growth factor beta−binding protein 4
0.10
0.05
0.00
−0.05
0.1
0.2
SNP effect on Gut microbiota abundance (genus Parasutterella id.2892)

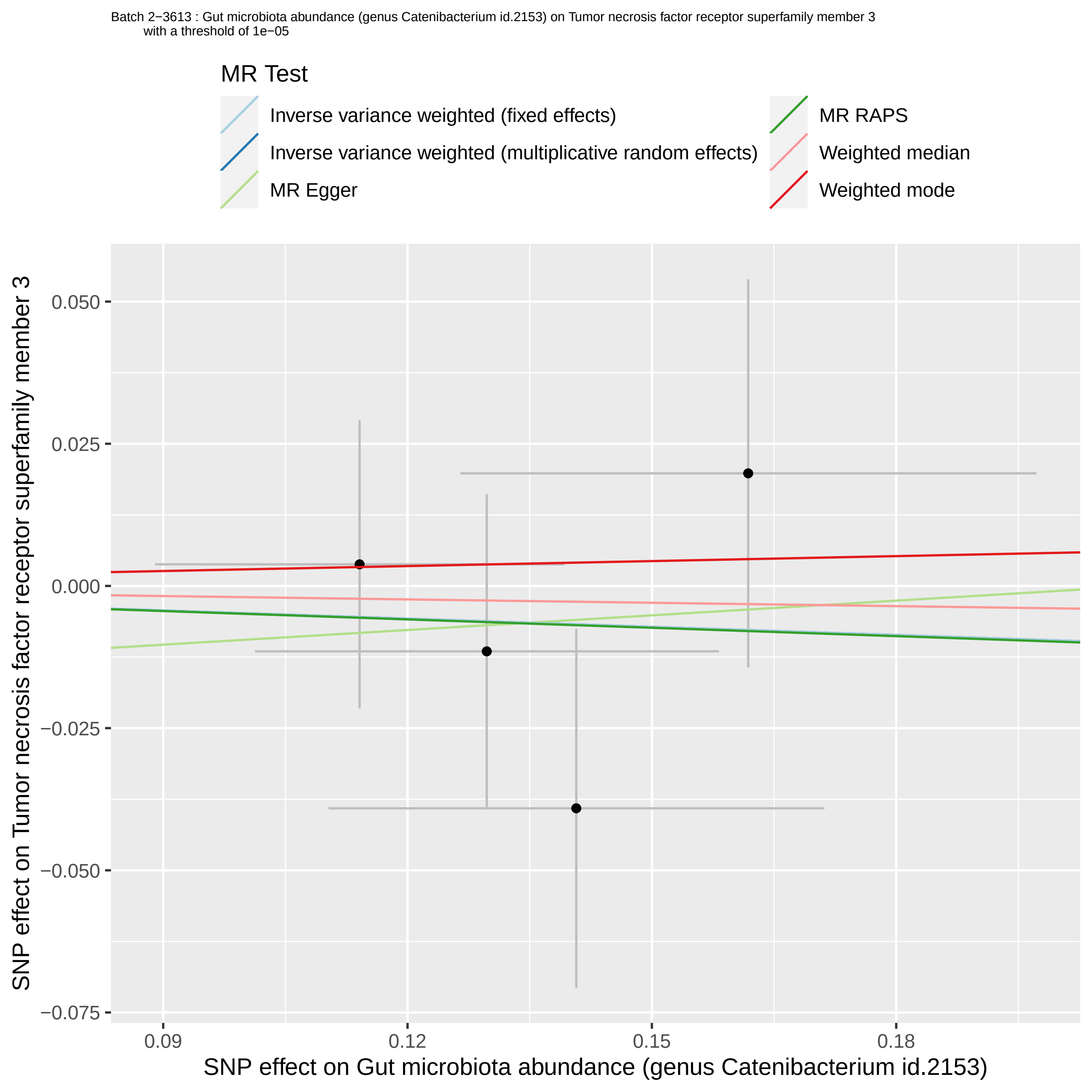
Batch 2−3613 : Gut microbiota abundance (genus Catenibacterium id.2153) on Tumor necrosis factor receptor superfamily member 3
with a threshold of 1e−05
MR Test
Inverse variance weighted (fixed effects)
Inverse variance weighted (multiplicative random effects)
MR Egger
MR RAPS
Weighted median
Weighted mode
SNP effect on Tumor necrosis factor receptor superfamily member 3
0.050
0.025
0.000
−0.025
−0.050
−0.075
0.09
0.12
0.15
0.18
SNP effect on Gut microbiota abundance (genus Catenibacterium id.2153)

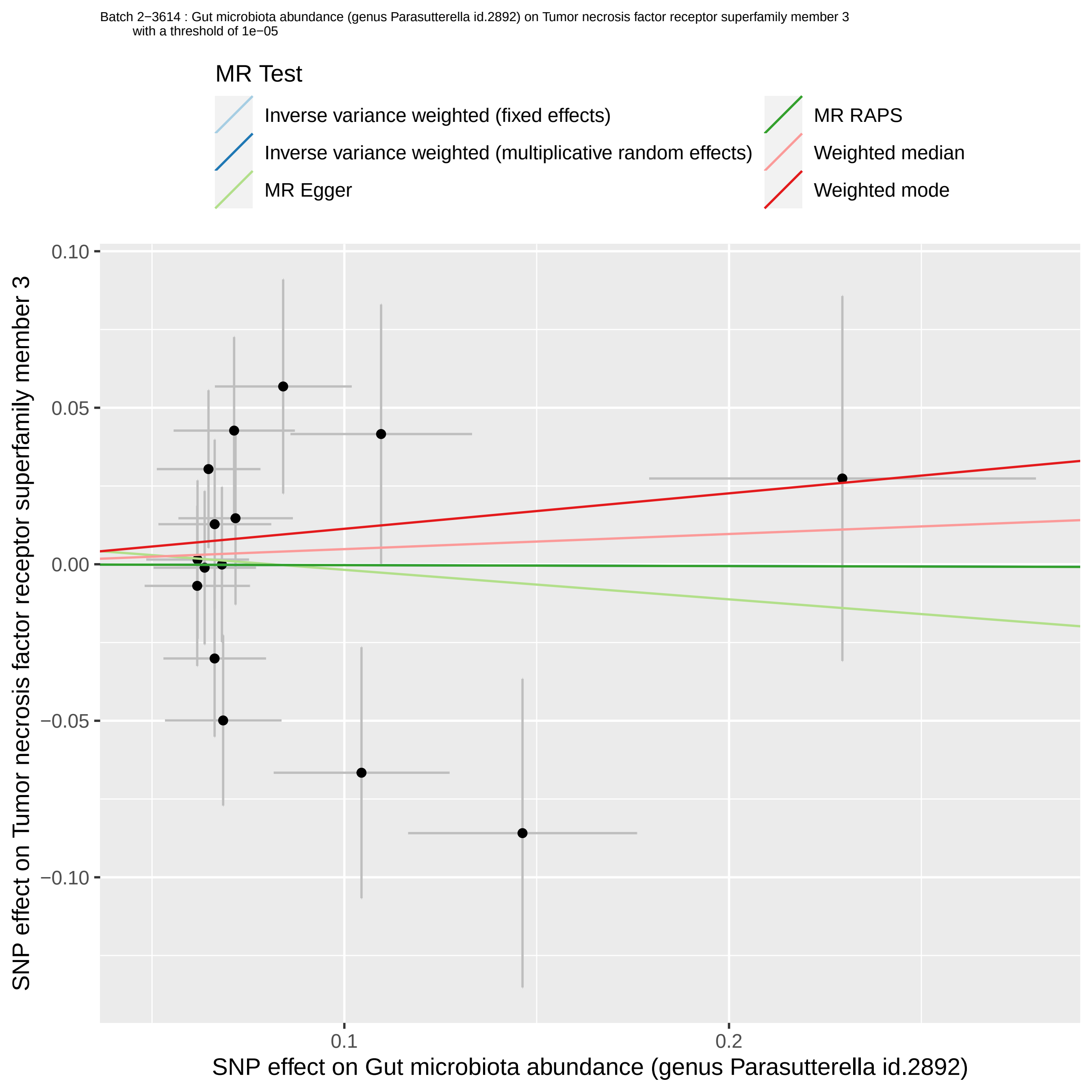
Batch 2−3614 : Gut microbiota abundance (genus Parasutterella id.2892) on Tumor necrosis factor receptor superfamily member 3
with a threshold of 1e−05
MR Test
Inverse variance weighted (fixed effects)
Inverse variance weighted (multiplicative random effects)
MR Egger
MR RAPS
Weighted median
Weighted mode
0.10
0.05
0.00
−0.05
−0.10
0.1
0.2
SNP effect on Tumor necrosis factor receptor superfamily member 3
SNP effect on Gut microbiota abundance (genus Parasutterella id.2892)

Batch 2−3615 : Gut microbiota abundance (genus Catenibacterium id.2153) on Lactotransferrin
with a threshold of 1e−05

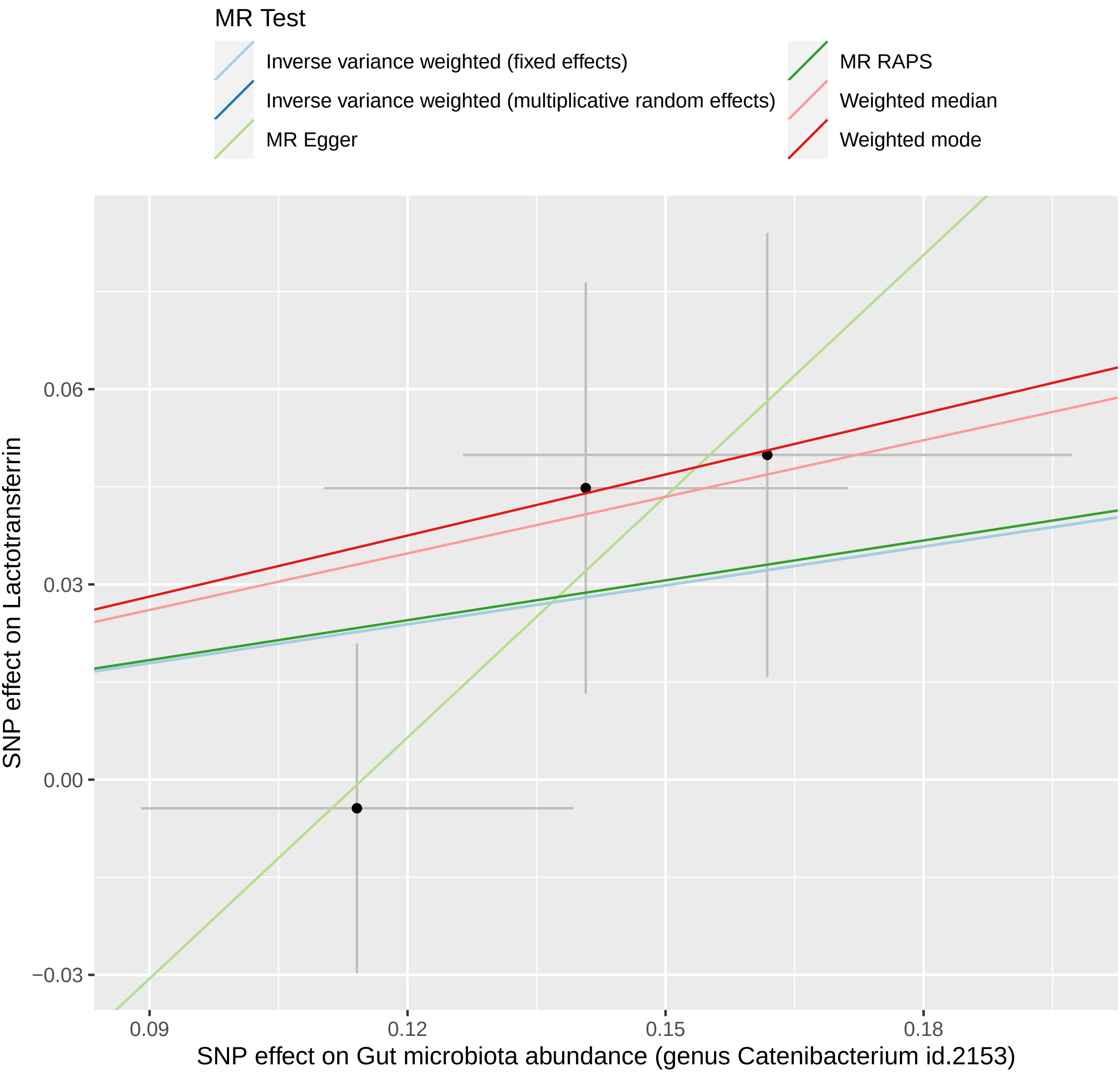

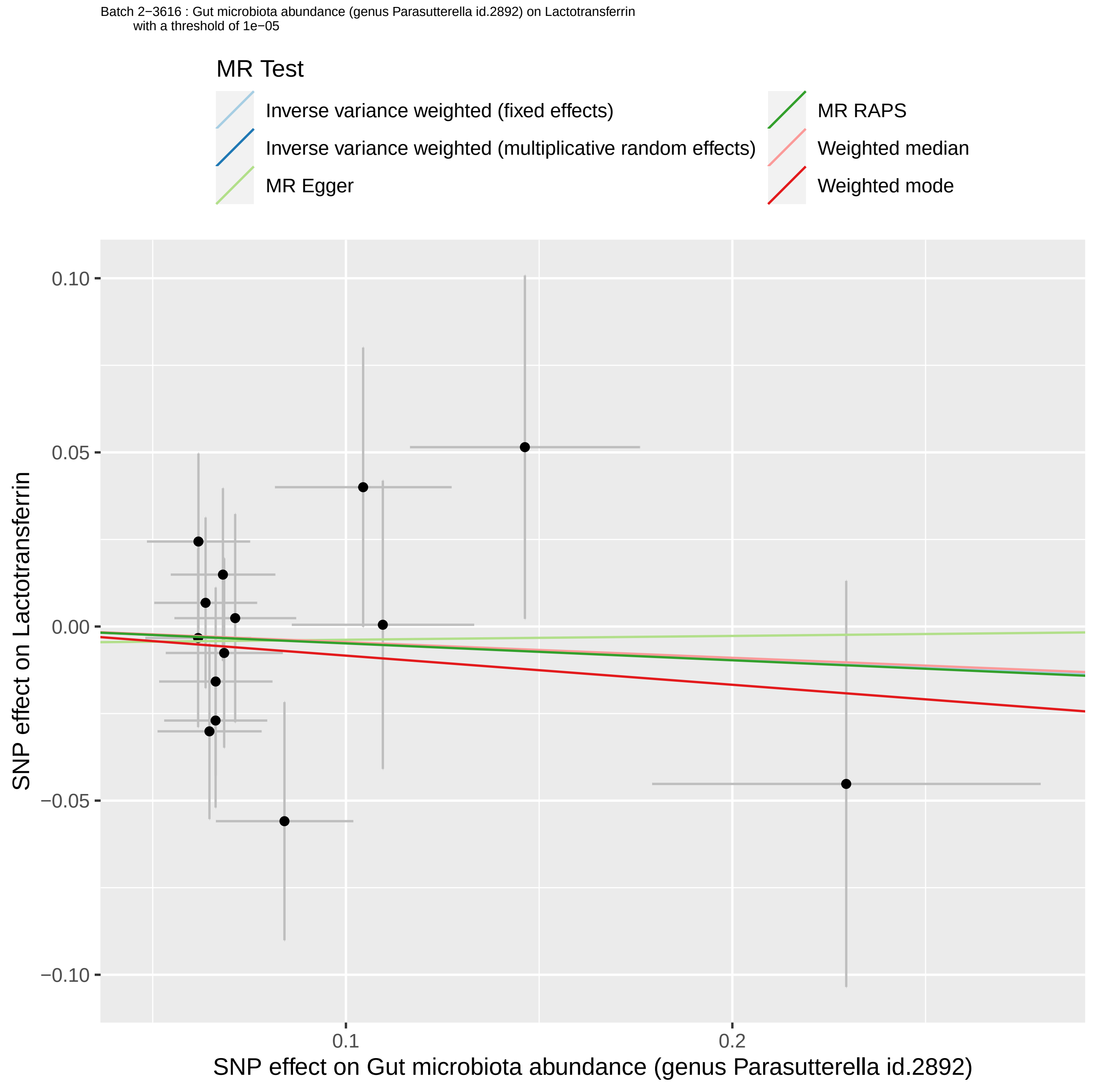
Batch 2−3616 : Gut microbiota abundance (genus Parasutterella id.2892) on Lactotransferrin
with a threshold of 1e−05
MR Test
Inverse variance weighted (fixed effects)
Inverse variance weighted (multiplicative random effects)
MR Egger
MR RAPS
Weighted median
Weighted mode
0.10
0.05
0.00
−0.05
−0.10
0.1
0.2
SNP effect on Lactotransferrin
SNP effect on Gut microbiota abundance (genus Parasutterella id.2892)

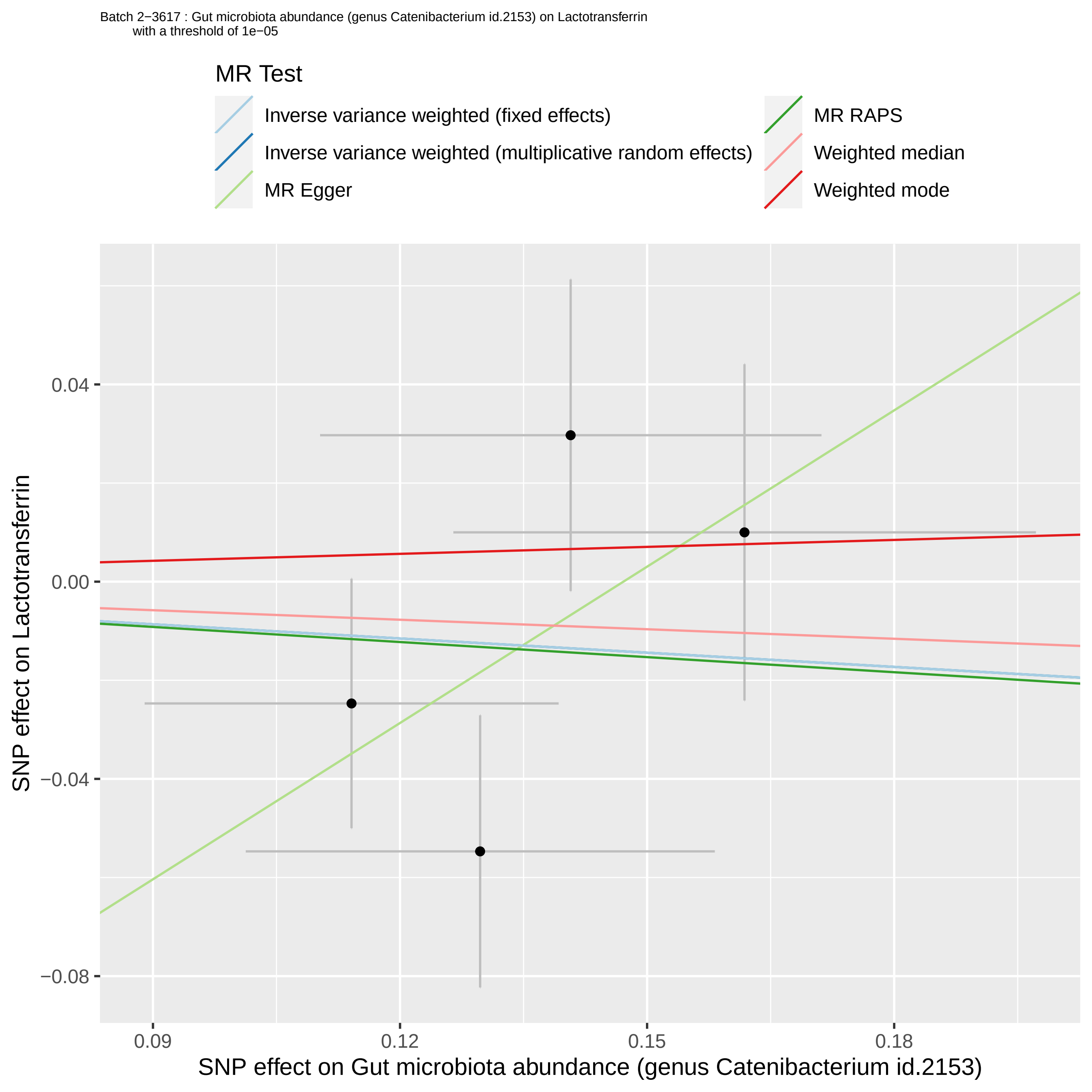
Batch 2−3617 : Gut microbiota abundance (genus Catenibacterium id.2153) on Lactotransferrin
with a threshold of 1e−05
MR Test
Inverse variance weighted (fixed effects)
Inverse variance weighted (multiplicative random effects)
MR Egger
MR RAPS
Weighted median
Weighted mode
SNP effect on Lactotransferrin
0.04
0.00
−0.04
−0.08
0.09
0.12
0.15
0.18
SNP effect on Gut microbiota abundance (genus Catenibacterium id.2153)

Batch 2−3618 : Gut microbiota abundance (genus Parasutterella id.2892) on Lactotransferrin
with a threshold of 1e−05

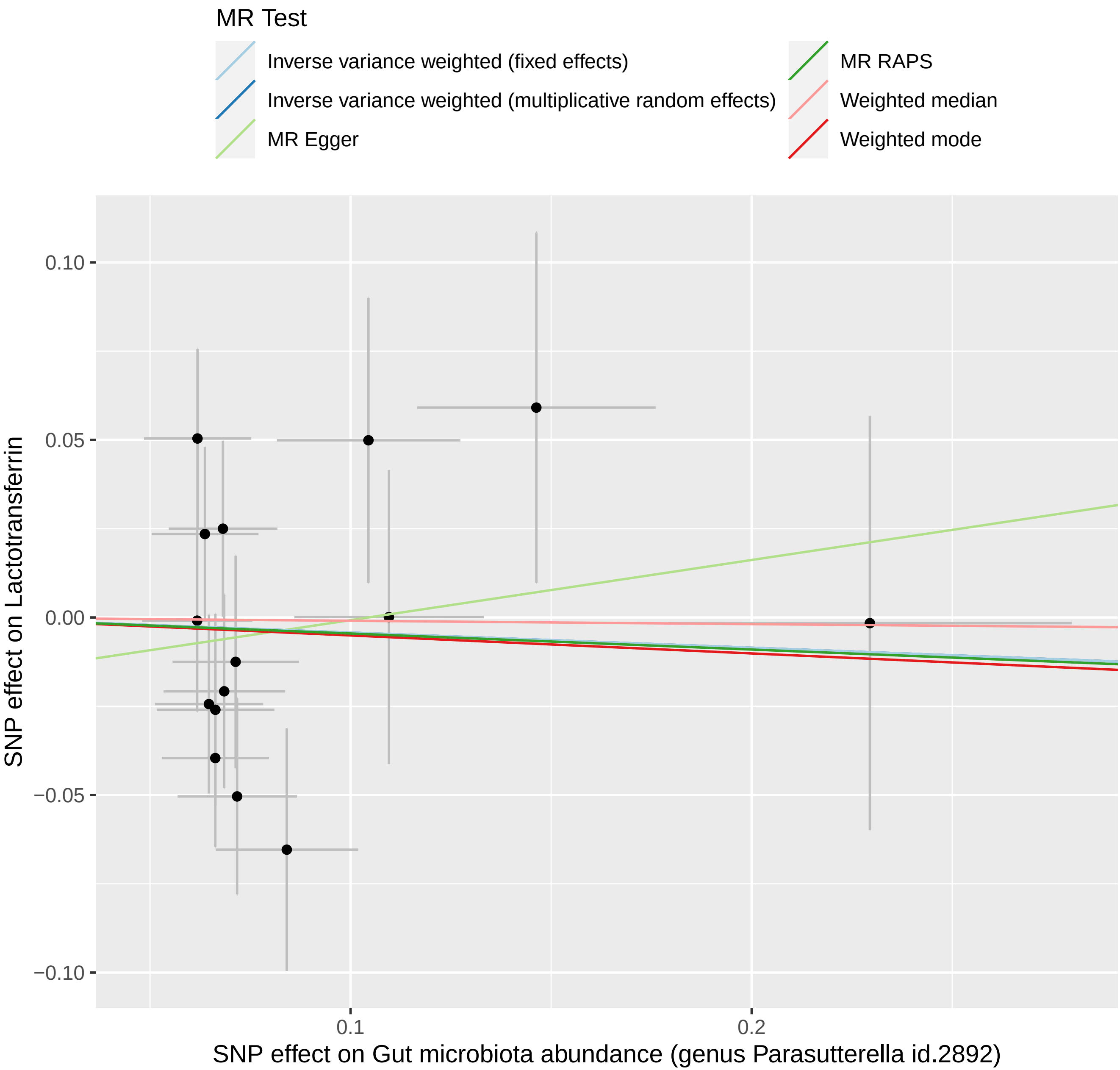

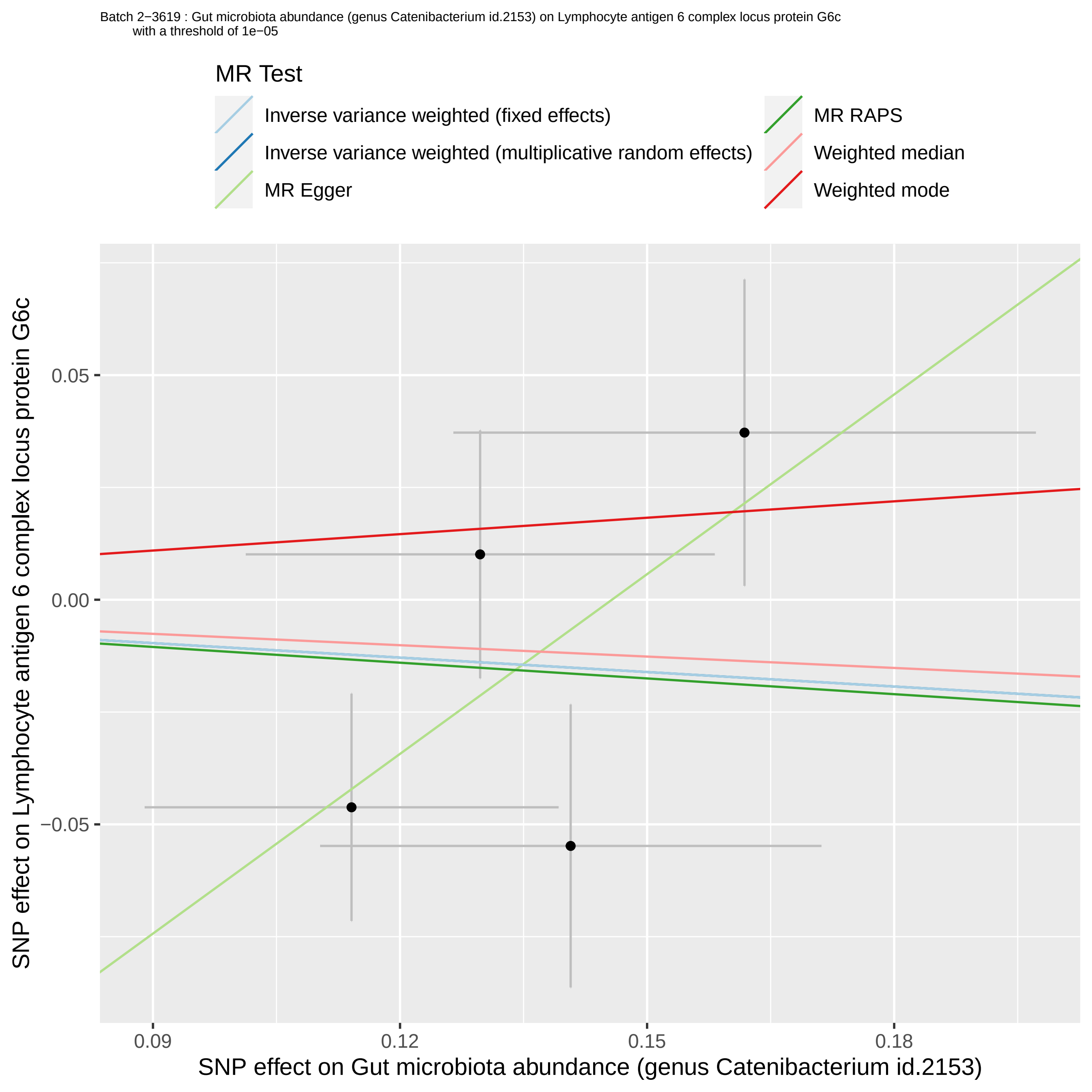
Batch 2−3619 : Gut microbiota abundance (genus Catenibacterium id.2153) on Lymphocyte antigen 6 complex locus protein G6c
with a threshold of 1e−05
MR Test
Inverse variance weighted (fixed effects)
Inverse variance weighted (multiplicative random effects)
MR Egger
MR RAPS
Weighted median
Weighted mode
SNP effect on Lymphocyte antigen 6 complex locus protein G6c
0.05
0.00
−0.05
0.09
0.12
0.15
0.18
SNP effect on Gut microbiota abundance (genus Catenibacterium id.2153)

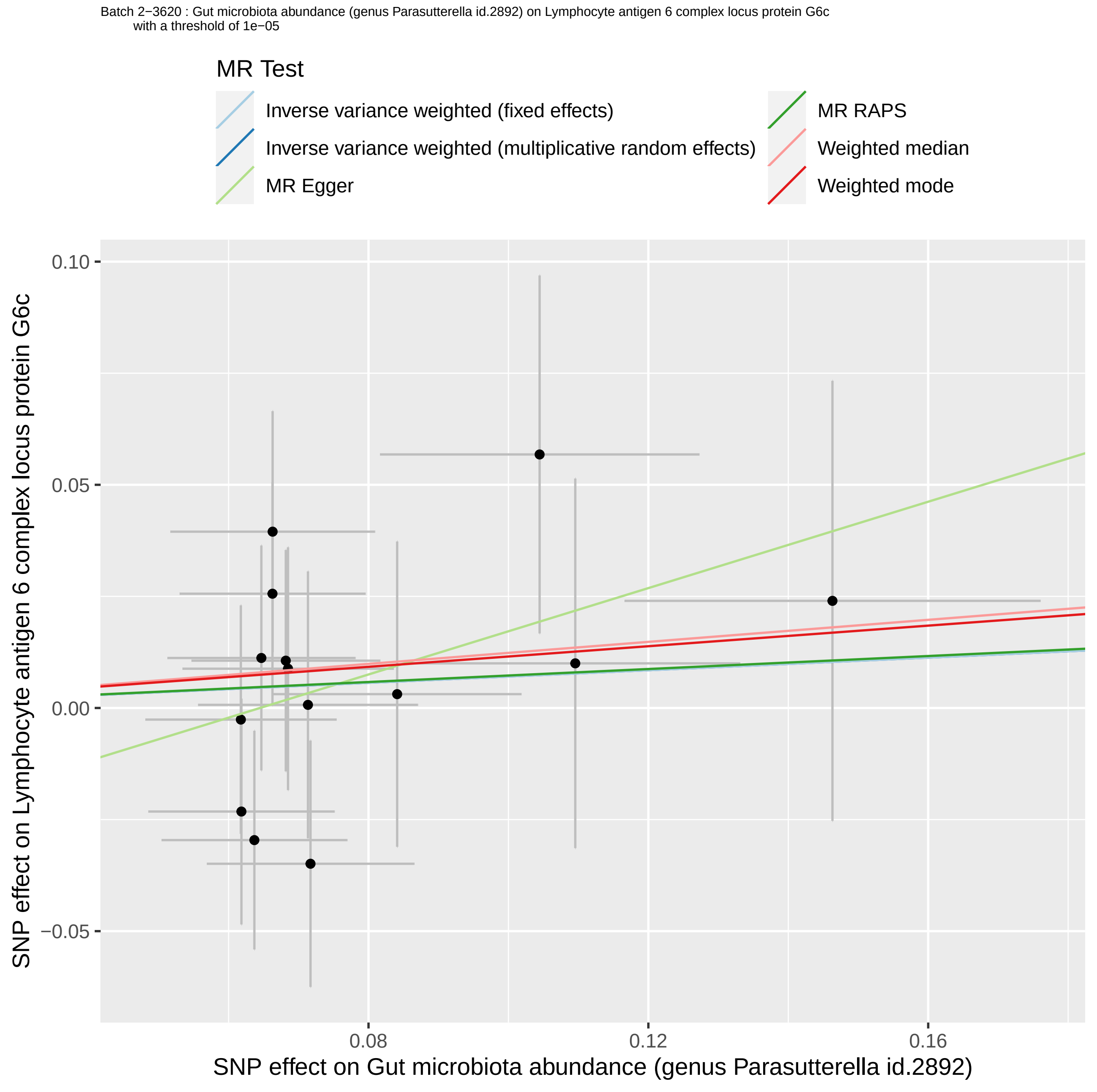

Batch 2−3620 : Gut microbiota abundance (genus Parasutterella id.2892) on Lymphocyte antigen 6 complex locus protein G6c
with a threshold of 1e−05
MR Test
Inverse variance weighted (fixed effects)
Inverse variance weighted (multiplicative random effects)
MR Egger
MR RAPS
Weighted median
Weighted mode
0.10
0.05
0.00
−0.05
0.08
0.12
0.16
SNP effect on Lymphocyte antigen 6 complex locus protein G6c
SNP effect on Gut microbiota abundance (genus Parasutterella id.2892)

Batch 2−3621 : Gut microbiota abundance (genus Catenibacterium id.2153) on Lymphocyte antigen 6 complex locus protein G6d
with a threshold of 1e−05

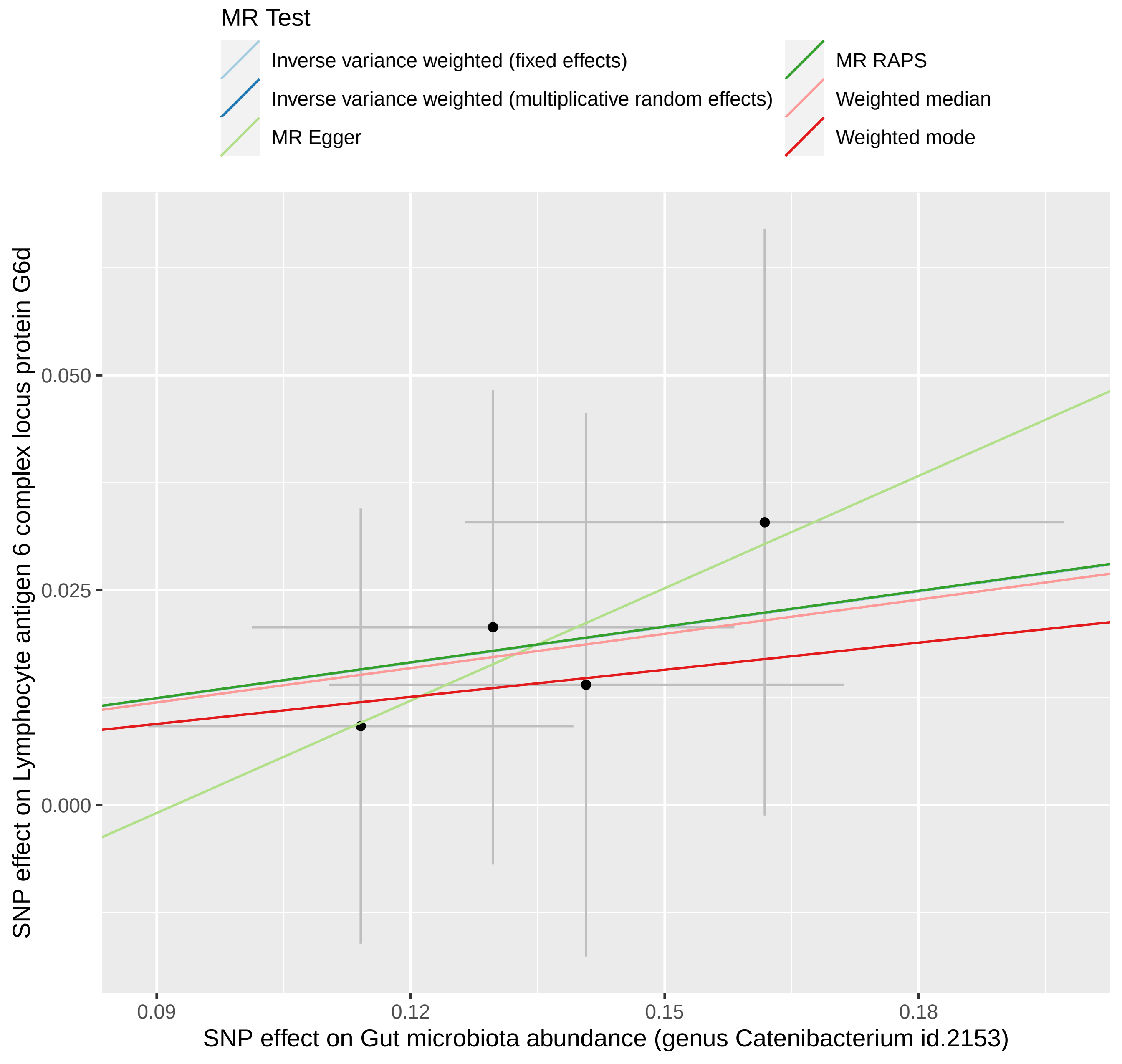

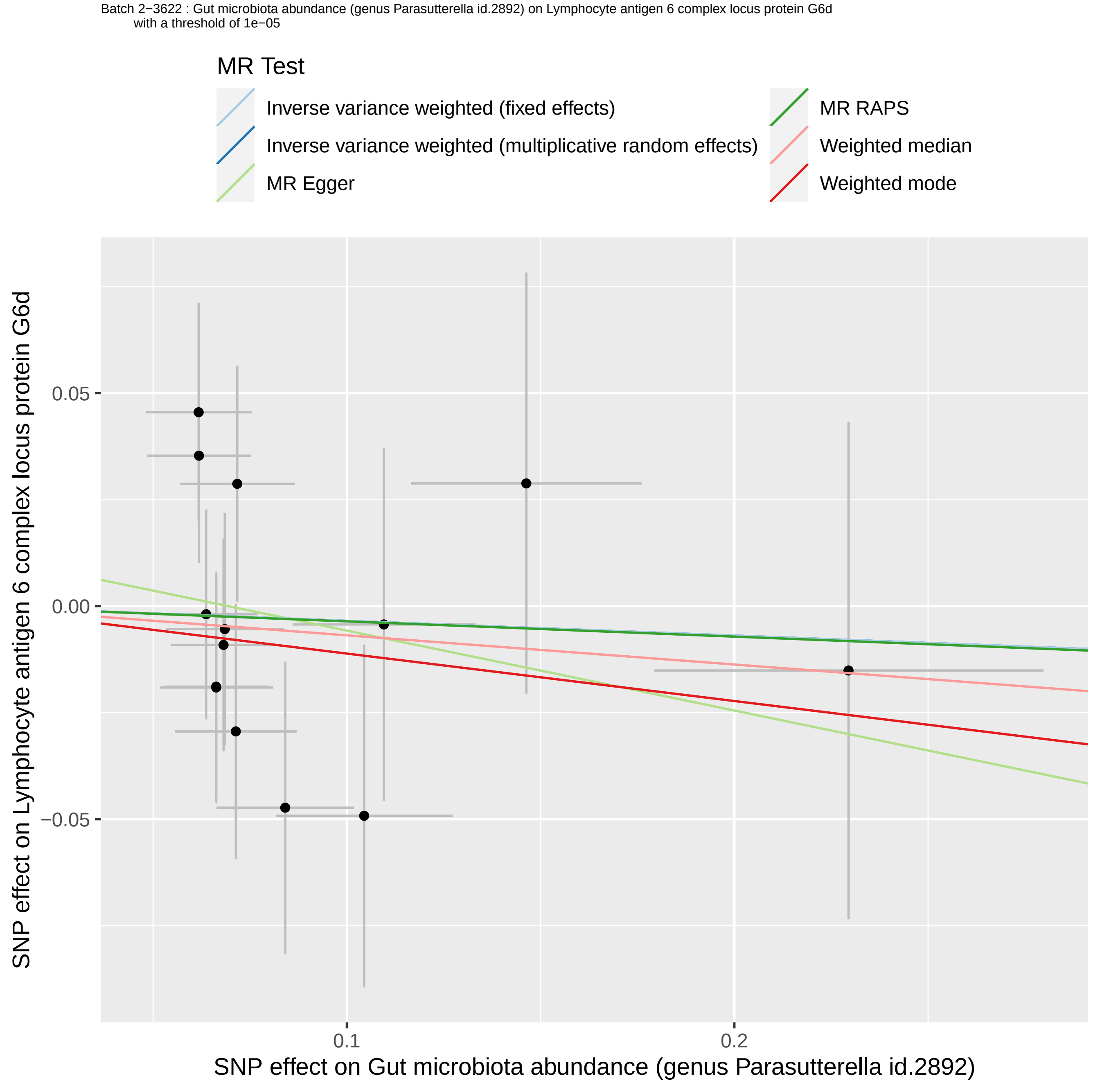
Batch 2−3622 : Gut microbiota abundance (genus Parasutterella id.2892) on Lymphocyte antigen 6 complex locus protein G6d
with a threshold of 1e−05
MR Test
Inverse variance weighted (fixed effects)
Inverse variance weighted (multiplicative random effects)
MR Egger
MR RAPS
Weighted median
Weighted mode
SNP effect on Lymphocyte antigen 6 complex locus protein G6d
0.05
0.00
−0.05
0.1
0.2
SNP effect on Gut microbiota abundance (genus Parasutterella id.2892)

Batch 2−3623 : Gut microbiota abundance (genus Catenibacterium id.2153) on Lymphocyte antigen 86
with a threshold of 1e−05

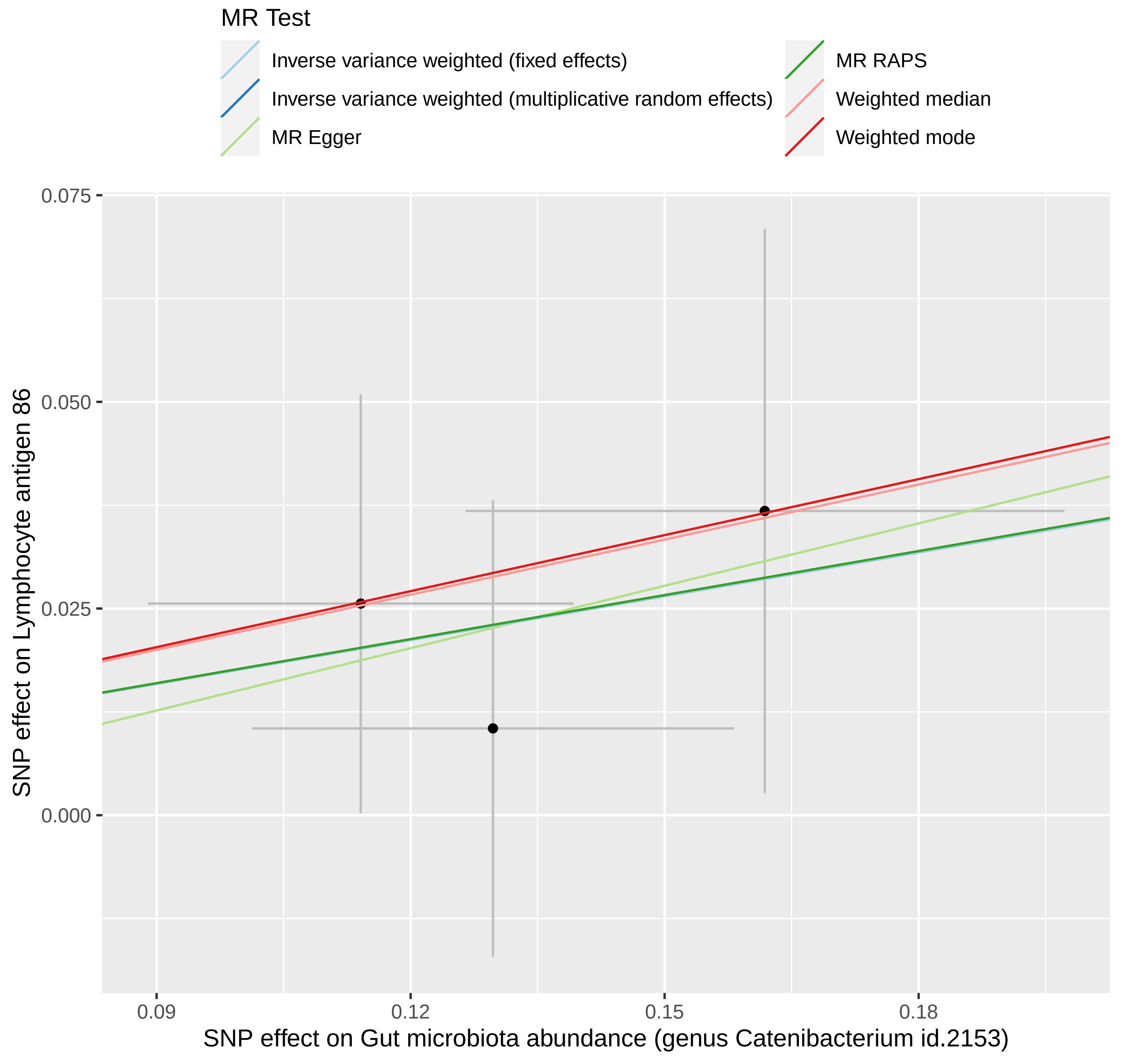

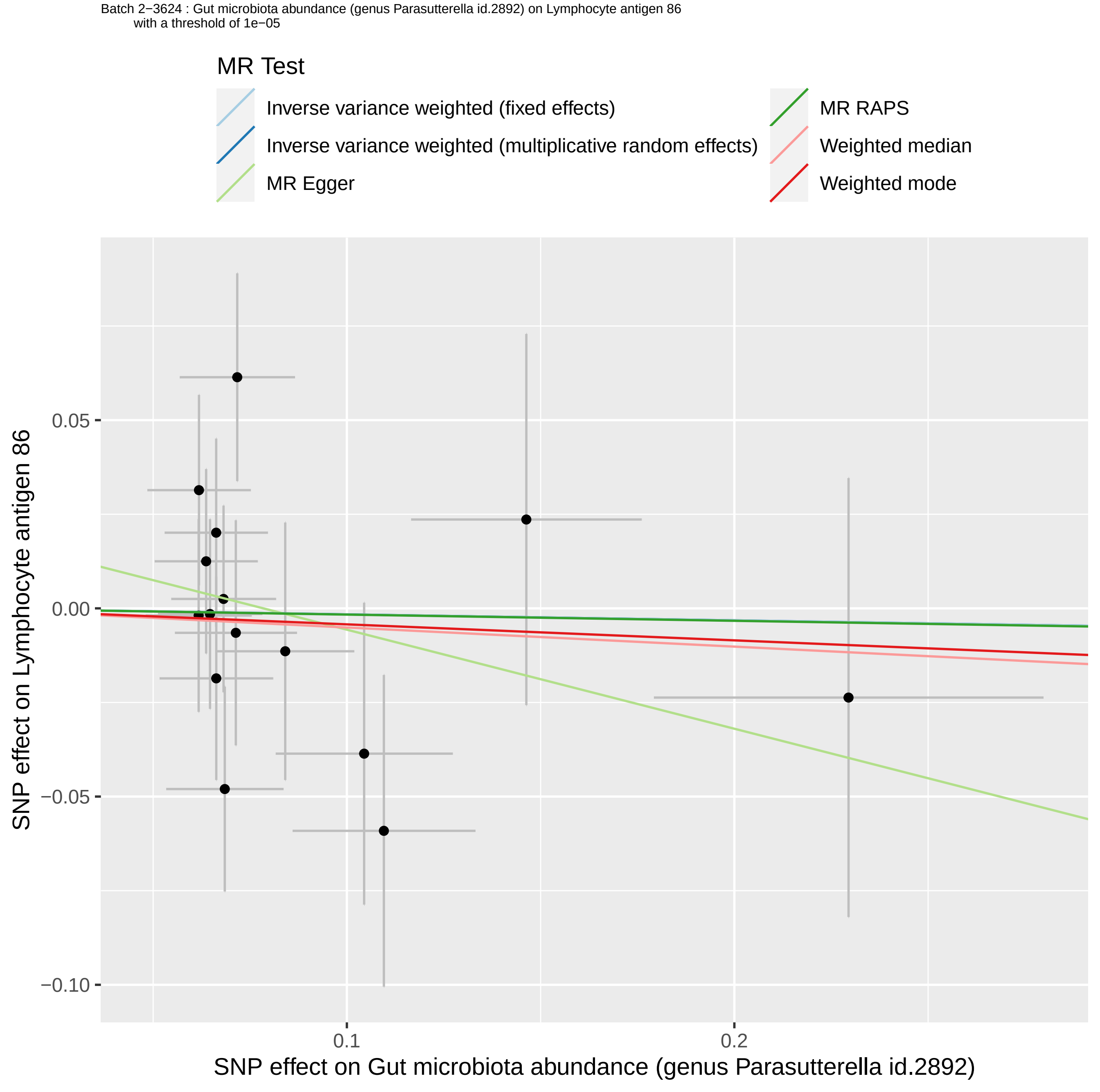
Batch 2−3624 : Gut microbiota abundance (genus Parasutterella id.2892) on Lymphocyte antigen 86
with a threshold of 1e−05
MR Test
Inverse variance weighted (fixed effects)
Inverse variance weighted (multiplicative random effects)
MR Egger
MR RAPS
Weighted median
Weighted mode
SNP effect on Lymphocyte antigen 86
0.05
0.00
−0.05
−0.10
0.1
0.2
SNP effect on Gut microbiota abundance (genus Parasutterella id.2892)

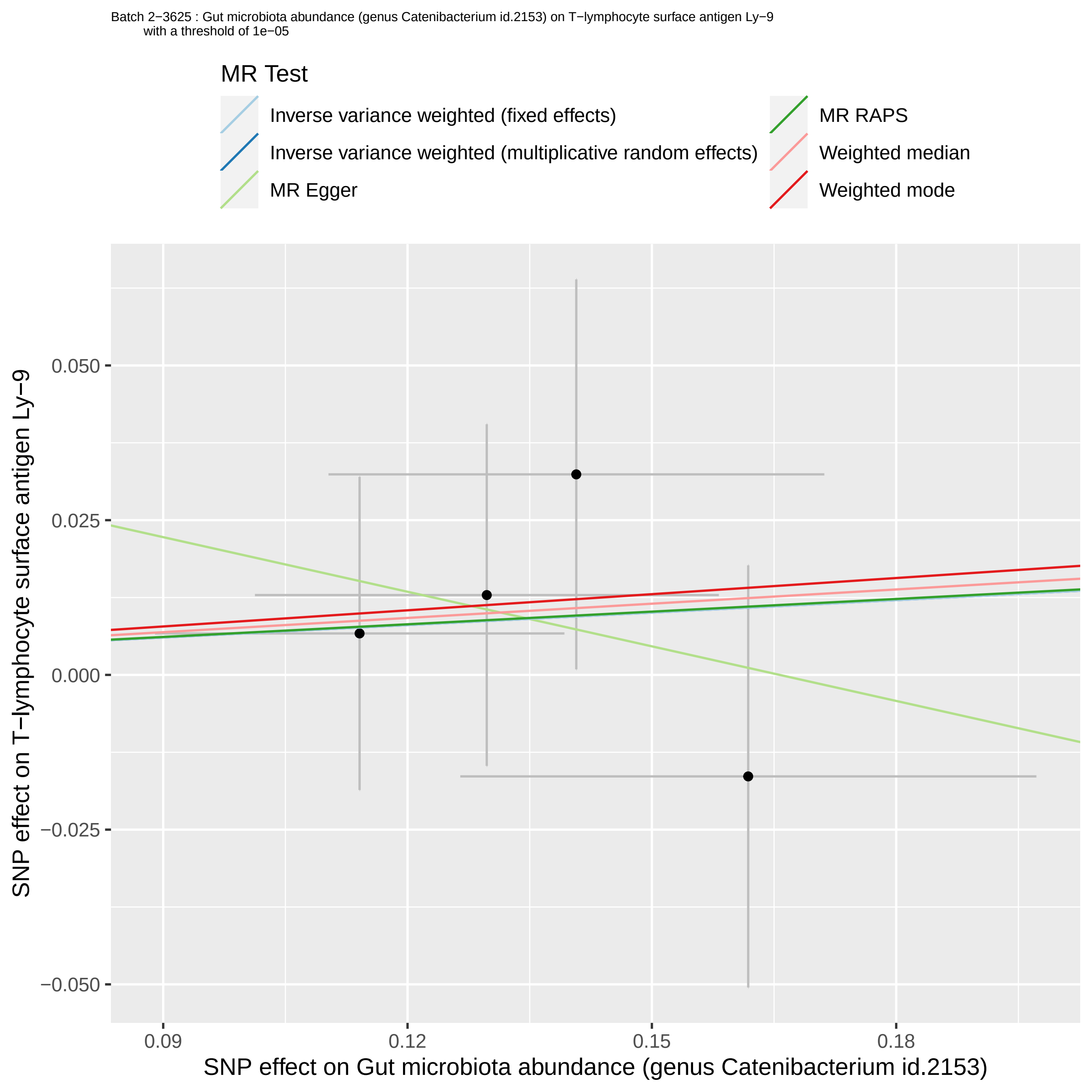
Batch 2−3625 : Gut microbiota abundance (genus Catenibacterium id.2153) on T−lymphocyte surface antigen Ly−9
with a threshold of 1e−05
MR Test
Inverse variance weighted (fixed effects)
Inverse variance weighted (multiplicative random effects)
MR Egger
MR RAPS
Weighted median
Weighted mode
SNP effect on T−lymphocyte surface antigen Ly−9
0.050
0.025
0.000
−0.025
−0.050
0.09
0.12
0.15
0.18
SNP effect on Gut microbiota abundance (genus Catenibacterium id.2153)

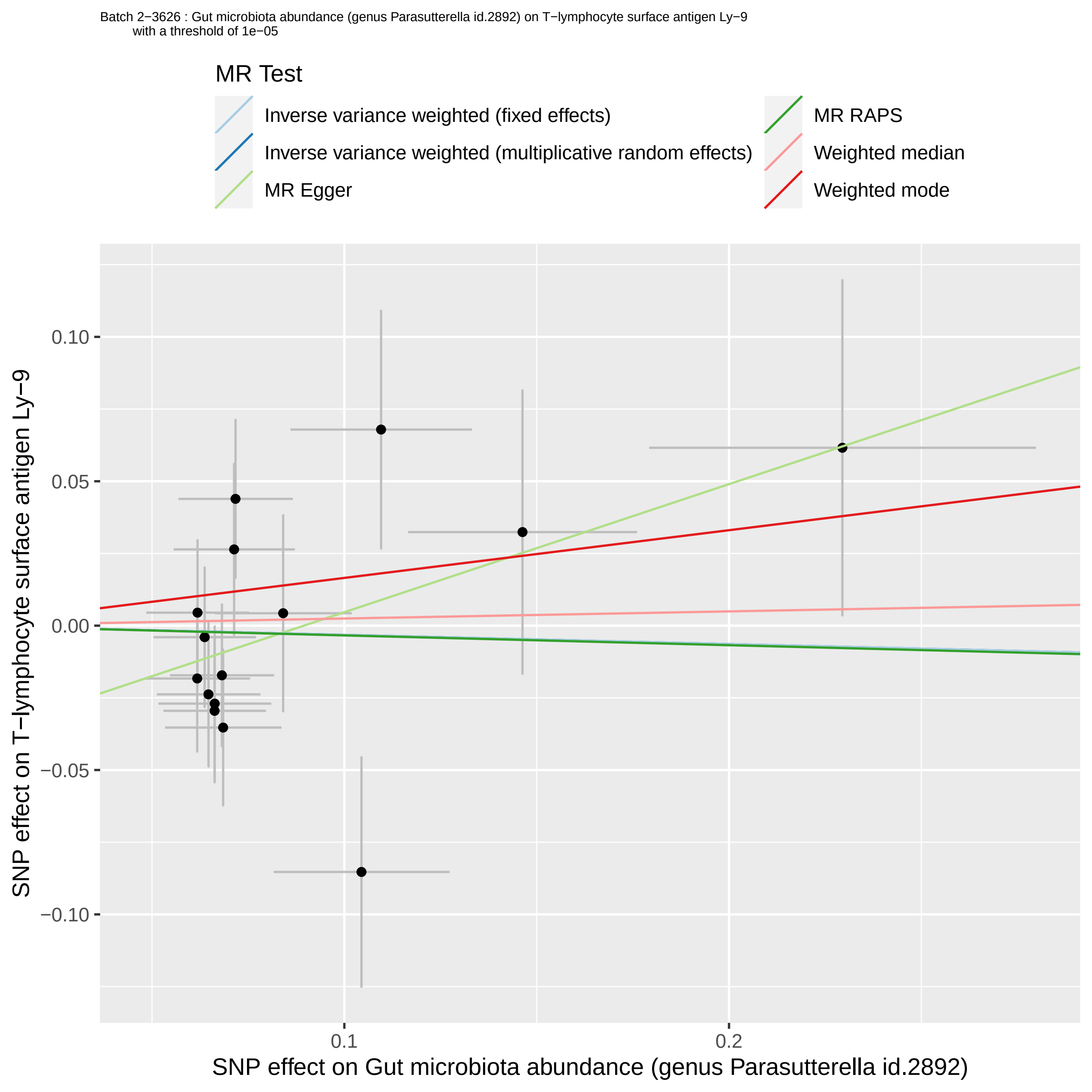

Batch 2−3626 : Gut microbiota abundance (genus Parasutterella id.2892) on T−lymphocyte surface antigen Ly−9
with a threshold of 1e−05
MR Test
Inverse variance weighted (fixed effects)
Inverse variance weighted (multiplicative random effects)
MR Egger
MR RAPS
Weighted median
Weighted mode
0.10
0.05
0.00
−0.05
−0.10
0.1
0.2
SNP effect on T−lymphocyte surface antigen Ly−9
SNP effect on Gut microbiota abundance (genus Parasutterella id.2892)

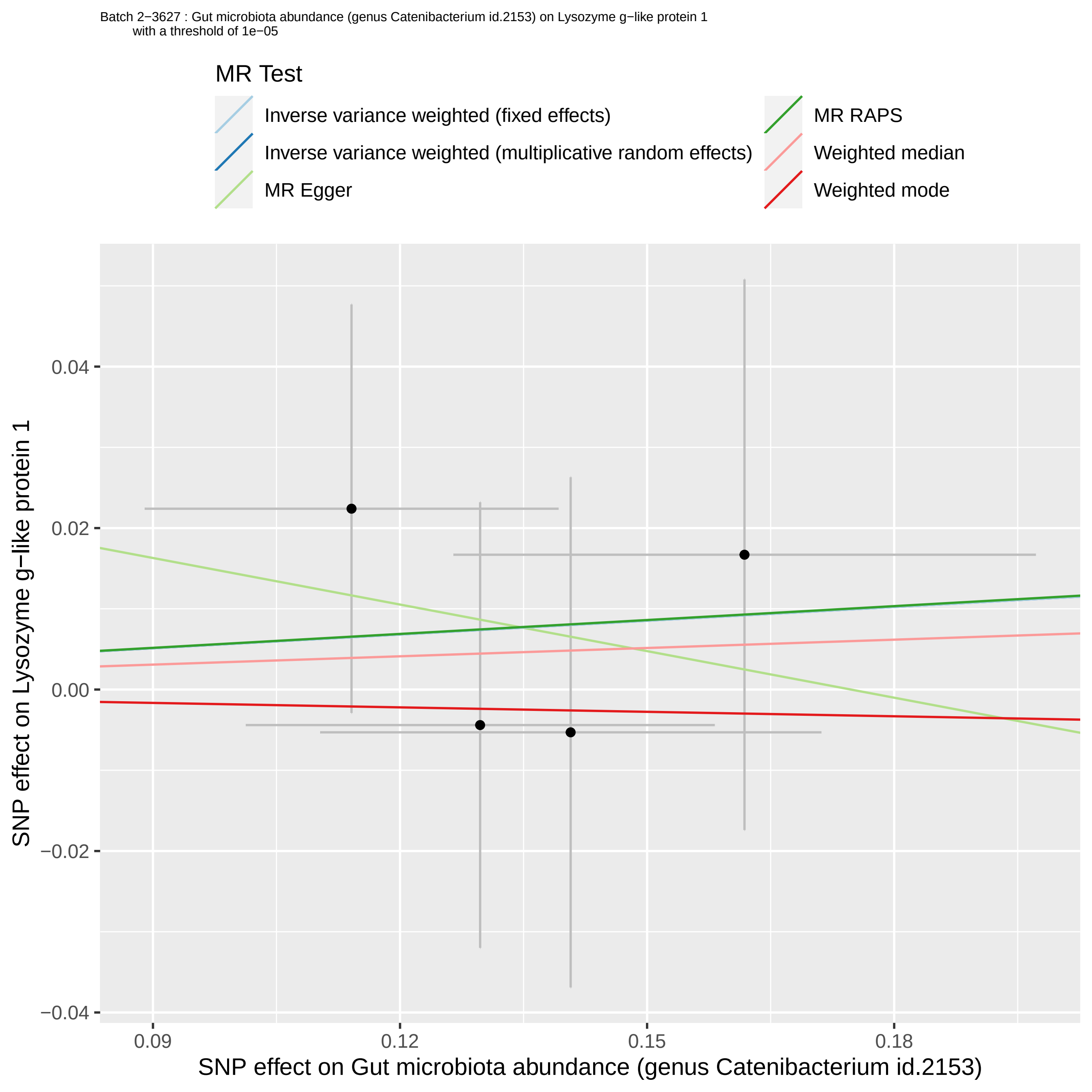

Batch 2−3627 : Gut microbiota abundance (genus Catenibacterium id.2153) on Lysozyme g−like protein 1
with a threshold of 1e−05
MR Test
Inverse variance weighted (fixed effects)
Inverse variance weighted (multiplicative random effects)
MR Egger
MR RAPS
Weighted median
Weighted mode
0.04
0.02
0.00
−0.02
−0.04
0.09
0.12
0.15
0.18
SNP effect on Lysozyme g−like protein 1
SNP effect on Gut microbiota abundance (genus Catenibacterium id.2153)

Batch 2−3628 : Gut microbiota abundance (genus Parasutterella id.2892) on Lysozyme g−like protein 1
with a threshold of 1e−05

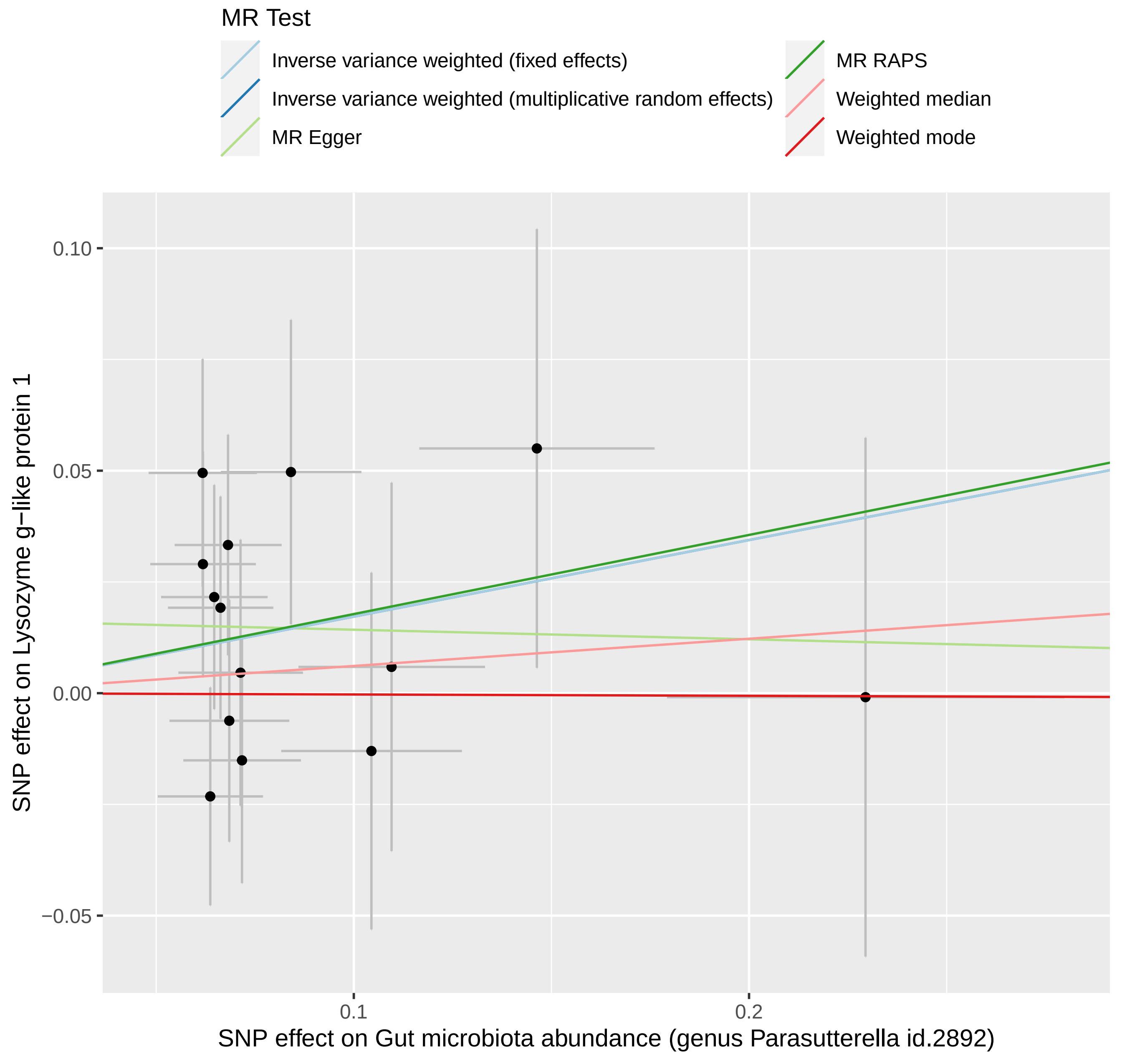

Batch 2−3629 : Gut microbiota abundance (genus Catenibacterium id.2153) on Tyrosine−protein kinase Lyn, isoform B
with a threshold of 1e−05

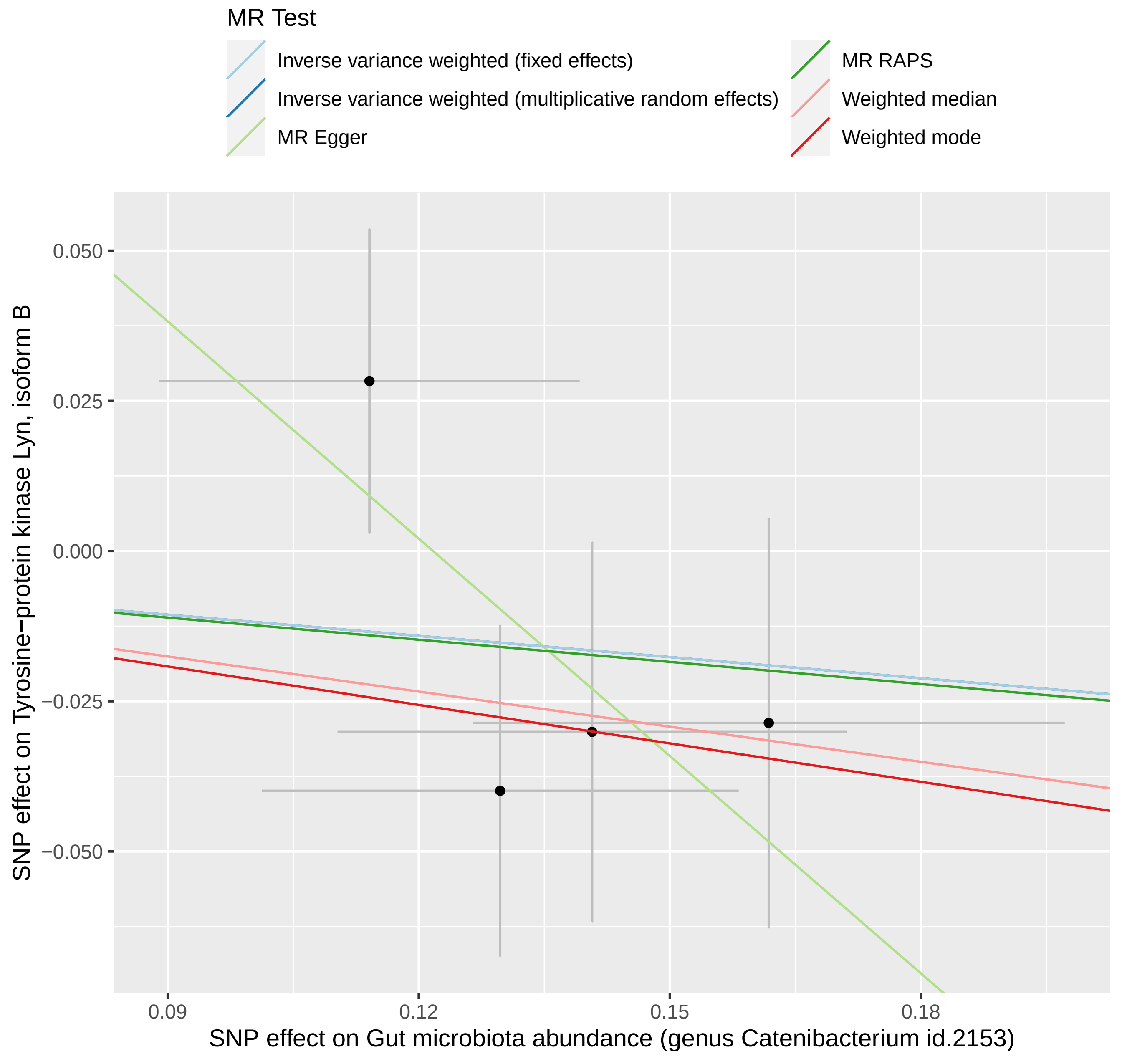

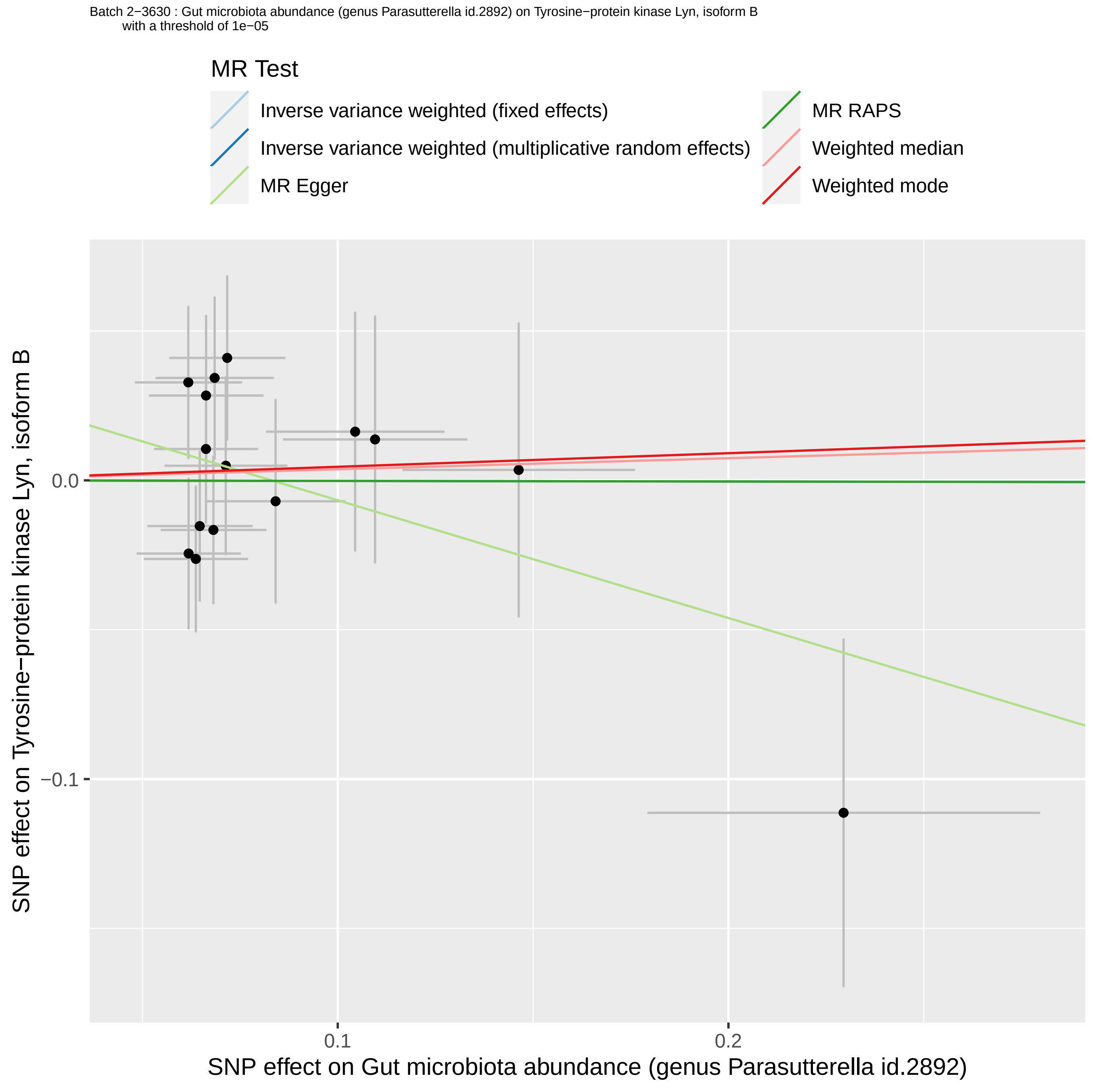
Batch 2−3630 : Gut microbiota abundance (genus Parasutterella id.2892) on Tyrosine−protein kinase Lyn, isoform B
with a threshold of 1e−05
MR Test
Inverse variance weighted (fixed effects)
Inverse variance weighted (multiplicative random effects)
MR Egger
MR RAPS
Weighted median
Weighted mode
SNP effect on Tyrosine−protein kinase Lyn, isoform B
0.0
−0.1
0.1
0.2
SNP effect on Gut microbiota abundance (genus Parasutterella id.2892)

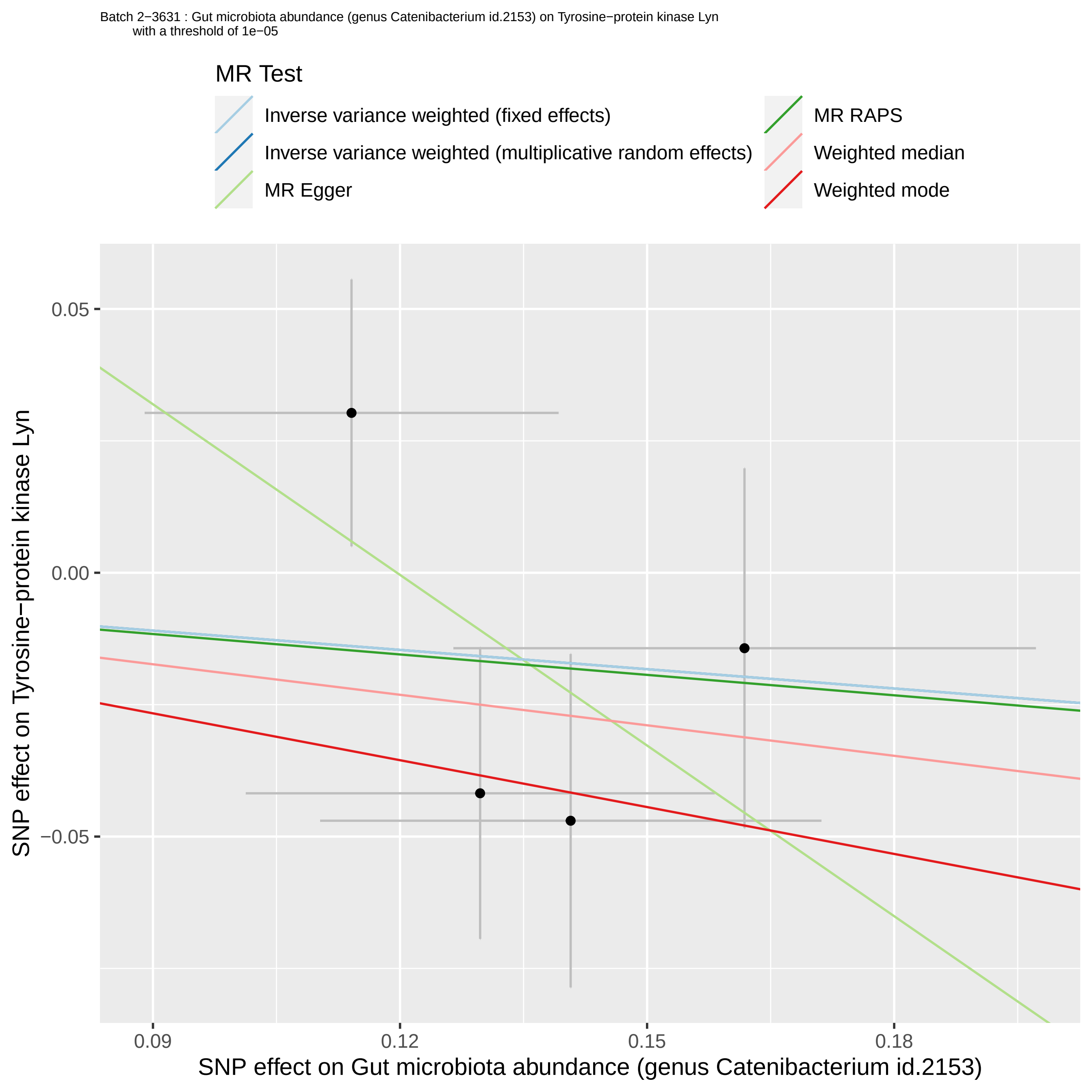
Batch 2−3631 : Gut microbiota abundance (genus Catenibacterium id.2153) on Tyrosine−protein kinase Lyn
with a threshold of 1e−05
MR Test
Inverse variance weighted (fixed effects)
Inverse variance weighted (multiplicative random effects)
MR Egger
MR RAPS
Weighted median
Weighted mode
0.05
0.00
−0.05
0.09
0.12
0.15
0.18
SNP effect on Tyrosine−protein kinase Lyn
SNP effect on Gut microbiota abundance (genus Catenibacterium id.2153)

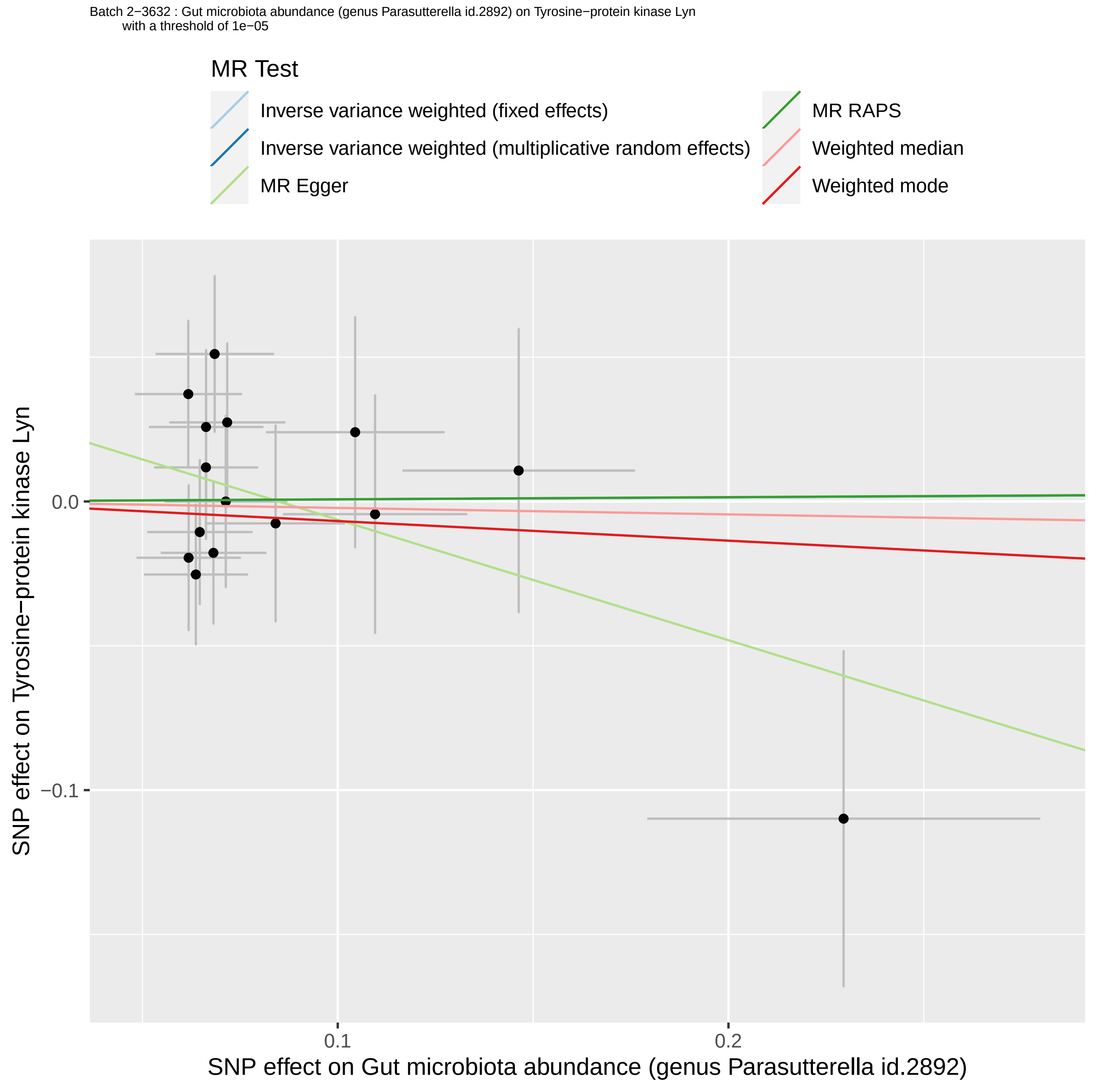

Batch 2−3632 : Gut microbiota abundance (genus Parasutterella id.2892) on Tyrosine−protein kinase Lyn
with a threshold of 1e−05
MR Test
Inverse variance weighted (fixed effects)
Inverse variance weighted (multiplicative random effects)
MR Egger
MR RAPS
Weighted median
Weighted mode
SNP effect on Tyrosine−protein kinase Lyn
0.0
−0.1
0.1
0.2
SNP effect on Gut microbiota abundance (genus Parasutterella id.2892)

Batch 2−3633 : Gut microbiota abundance (genus Catenibacterium id.2153) on Ly6/PLAUR domain−containing protein 3
with a threshold of 1e−05

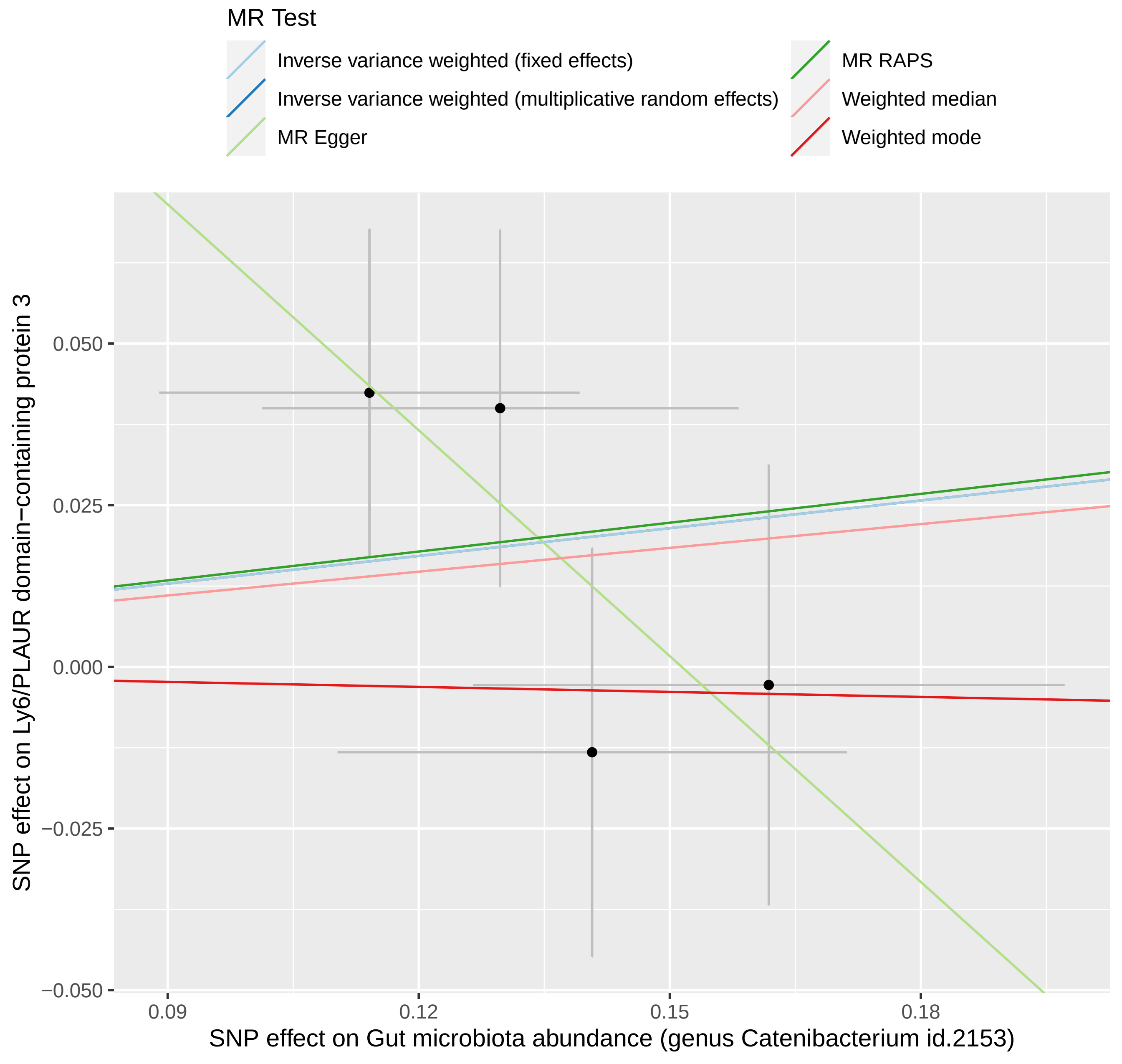

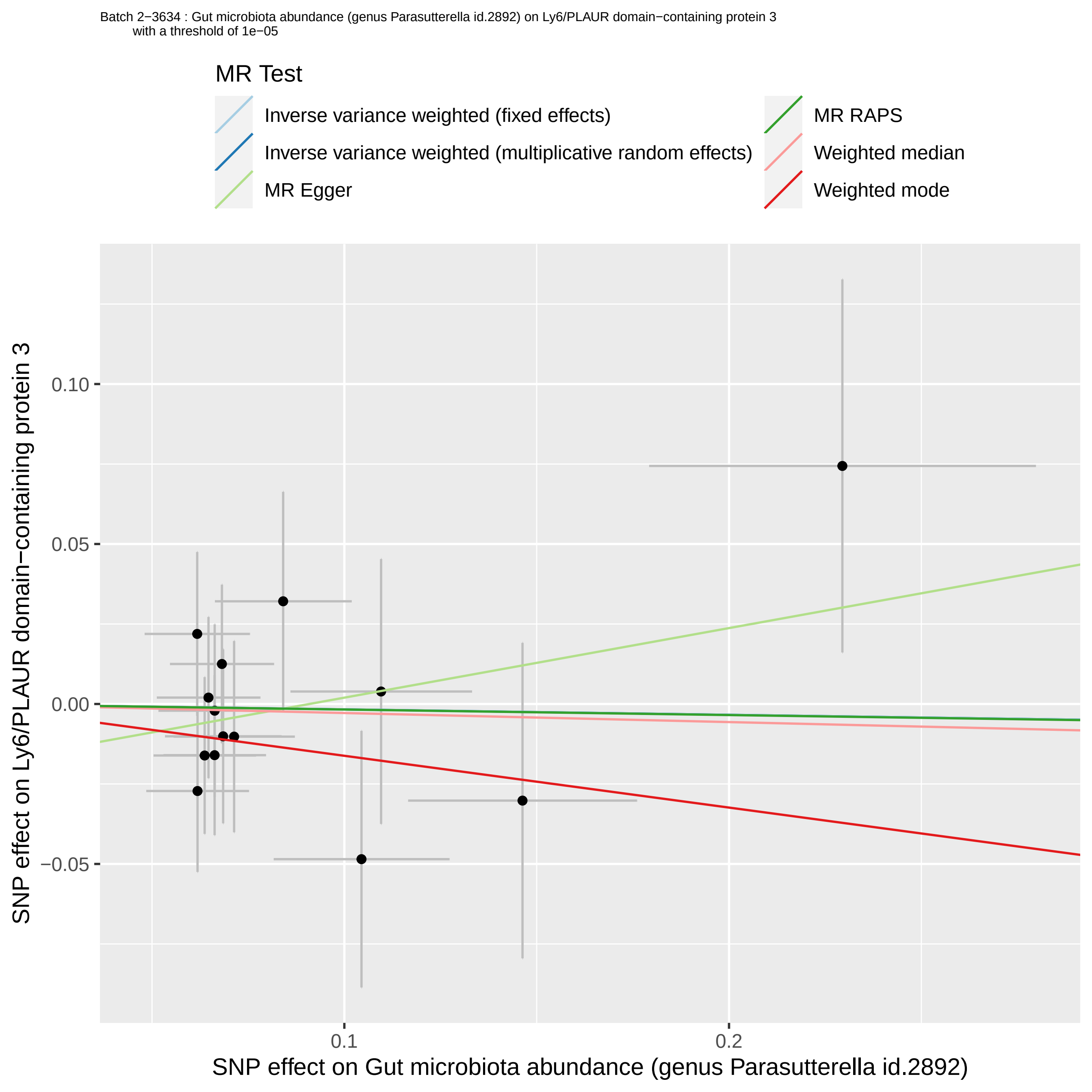

Batch 2−3634 : Gut microbiota abundance (genus Parasutterella id.2892) on Ly6/PLAUR domain−containing protein 3
with a threshold of 1e−05
MR Test
Inverse variance weighted (fixed effects)
Inverse variance weighted (multiplicative random effects)
MR Egger
MR RAPS
Weighted median
Weighted mode
0.10
0.05
0.00
−0.05
0.1
0.2
SNP effect on Ly6/PLAUR domain−containing protein 3
SNP effect on Gut microbiota abundance (genus Parasutterella id.2892)

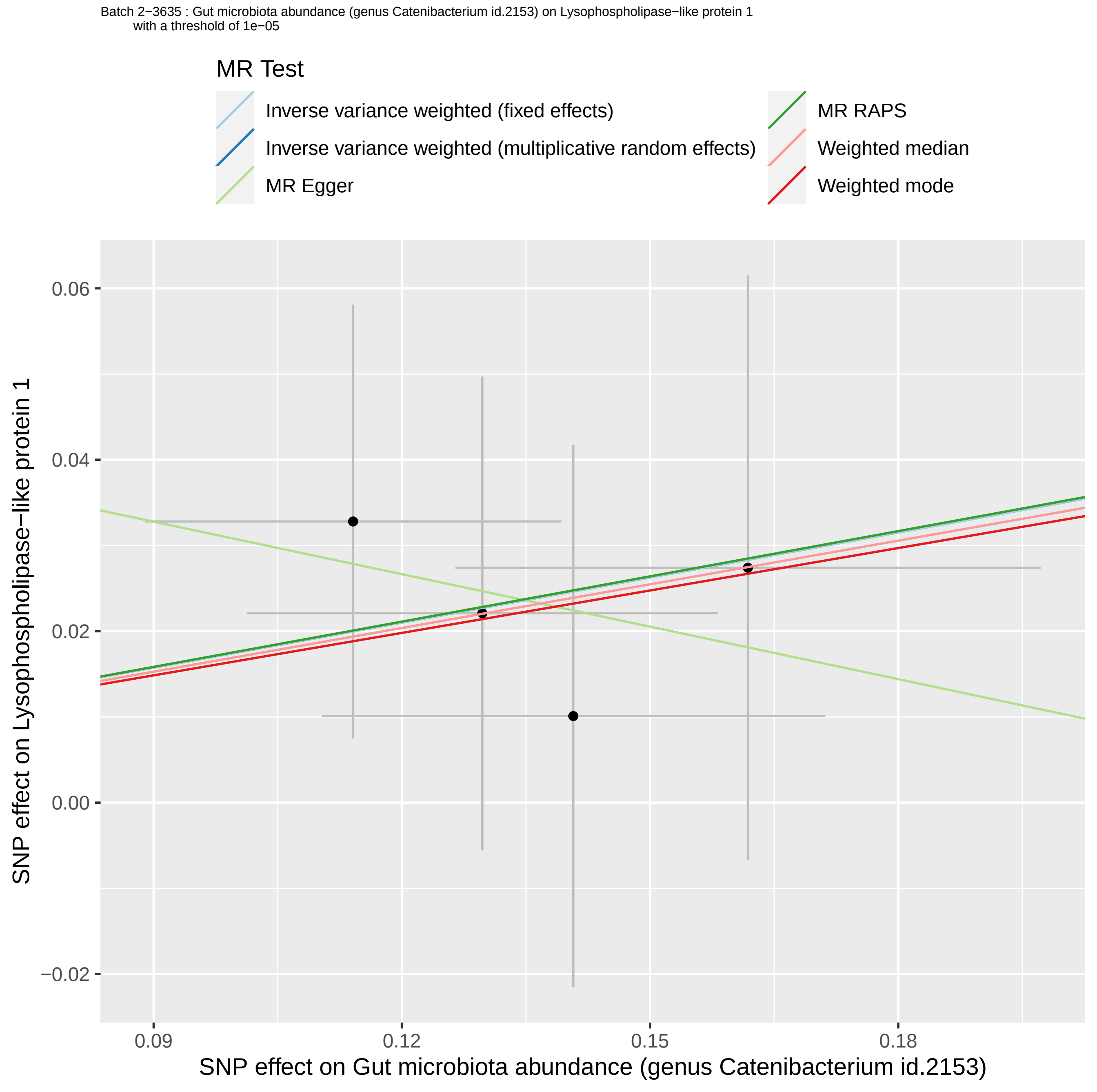
Batch 2−3635 : Gut microbiota abundance (genus Catenibacterium id.2153) on Lysophospholipase−like protein 1
with a threshold of 1e−05
MR Test
Inverse variance weighted (fixed effects)
Inverse variance weighted (multiplicative random effects)
MR Egger
MR RAPS
Weighted median
Weighted mode
0.06
0.04
0.02
0.00
−0.02
0.09
0.12
0.15
0.18
SNP effect on Lysophospholipase−like protein 1
SNP effect on Gut microbiota abundance (genus Catenibacterium id.2153)

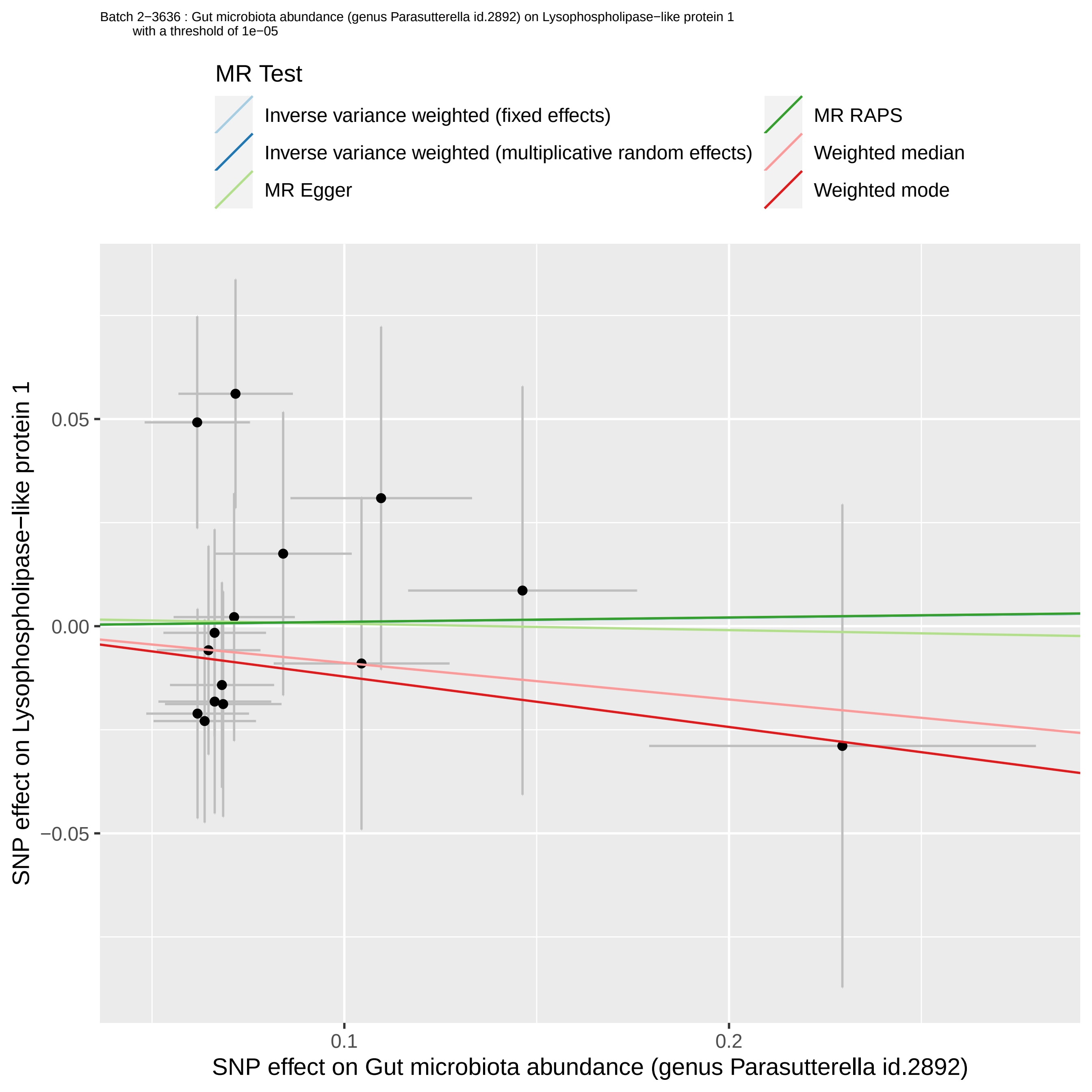
Batch 2−3636 : Gut microbiota abundance (genus Parasutterella id.2892) on Lysophospholipase−like protein 1
with a threshold of 1e−05
MR Test
Inverse variance weighted (fixed effects)
Inverse variance weighted (multiplicative random effects)
MR Egger
MR RAPS
Weighted median
Weighted mode
0.05
0.00
−0.05
0.1
0.2
SNP effect on Lysophospholipase−like protein 1
SNP effect on Gut microbiota abundance (genus Parasutterella id.2892)

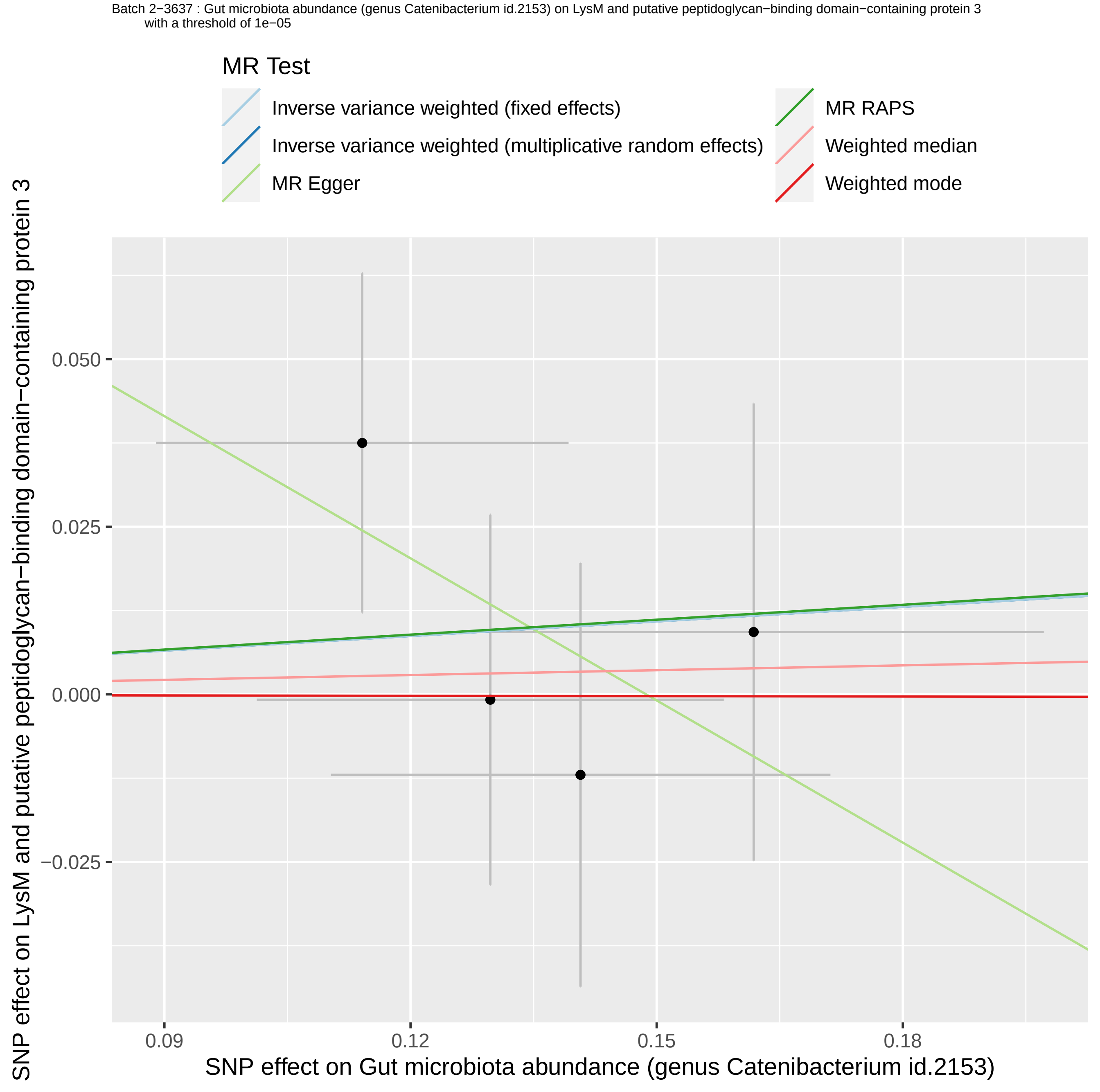
Batch 2−3637 : Gut microbiota abundance (genus Catenibacterium id.2153) on LysM and putative peptidoglycan−binding domain−containing protein 3 with a threshold of 1e−05
MR Test
Inverse variance weighted (fixed effects)
Inverse variance weighted (multiplicative random effects)
MR Egger
MR RAPS
Weighted median
Weighted mode
0.050
0.025
0.000
−0.025
0.09
0.12
0.15
0.18
SNP effect on Gut microbiota abundance (genus Catenibacterium id.2153)
SNP effect on LysM and putative peptidoglycan−binding domain−containing protein 3

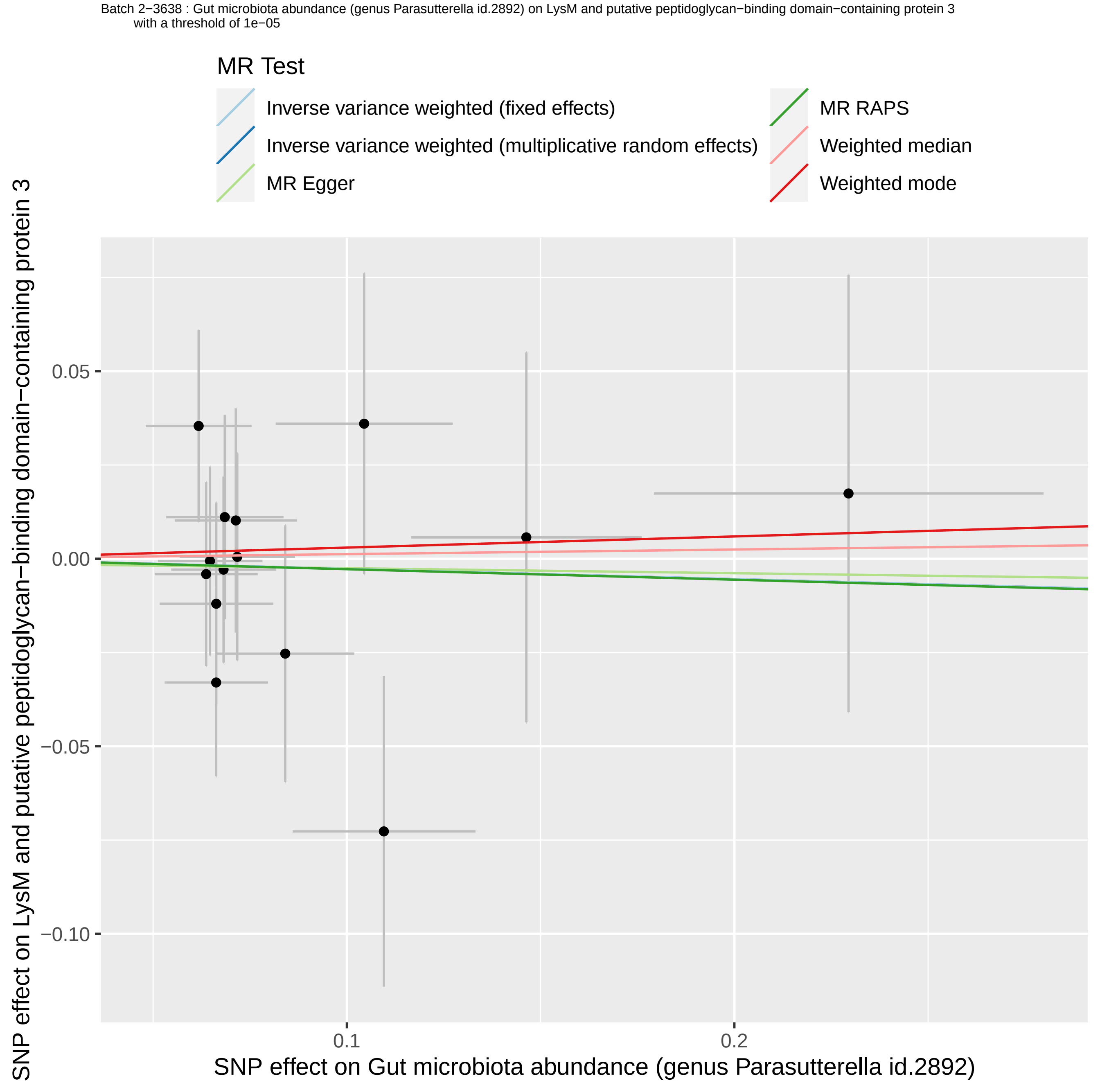
Batch 2−3638 : Gut microbiota abundance (genus Parasutterella id.2892) on LysM and putative peptidoglycan−binding domain−containing protein 3
with a threshold of 1e−05
MR Test
Inverse variance weighted (fixed effects)
Inverse variance weighted (multiplicative random effects)
MR Egger
MR RAPS
Weighted median
Weighted mode
SNP effect on LysM and putative peptidoglycan−binding domain−containing protein 3
0.05
0.00
−0.05
−0.10
0.1
0.2
SNP effect on Gut microbiota abundance (genus Parasutterella id.2892)

Batch 2−3639 : Gut microbiota abundance (genus Catenibacterium id.2153) on LysM and putative peptidoglycan−binding domain−containing protein 4 with a threshold of 1e−05

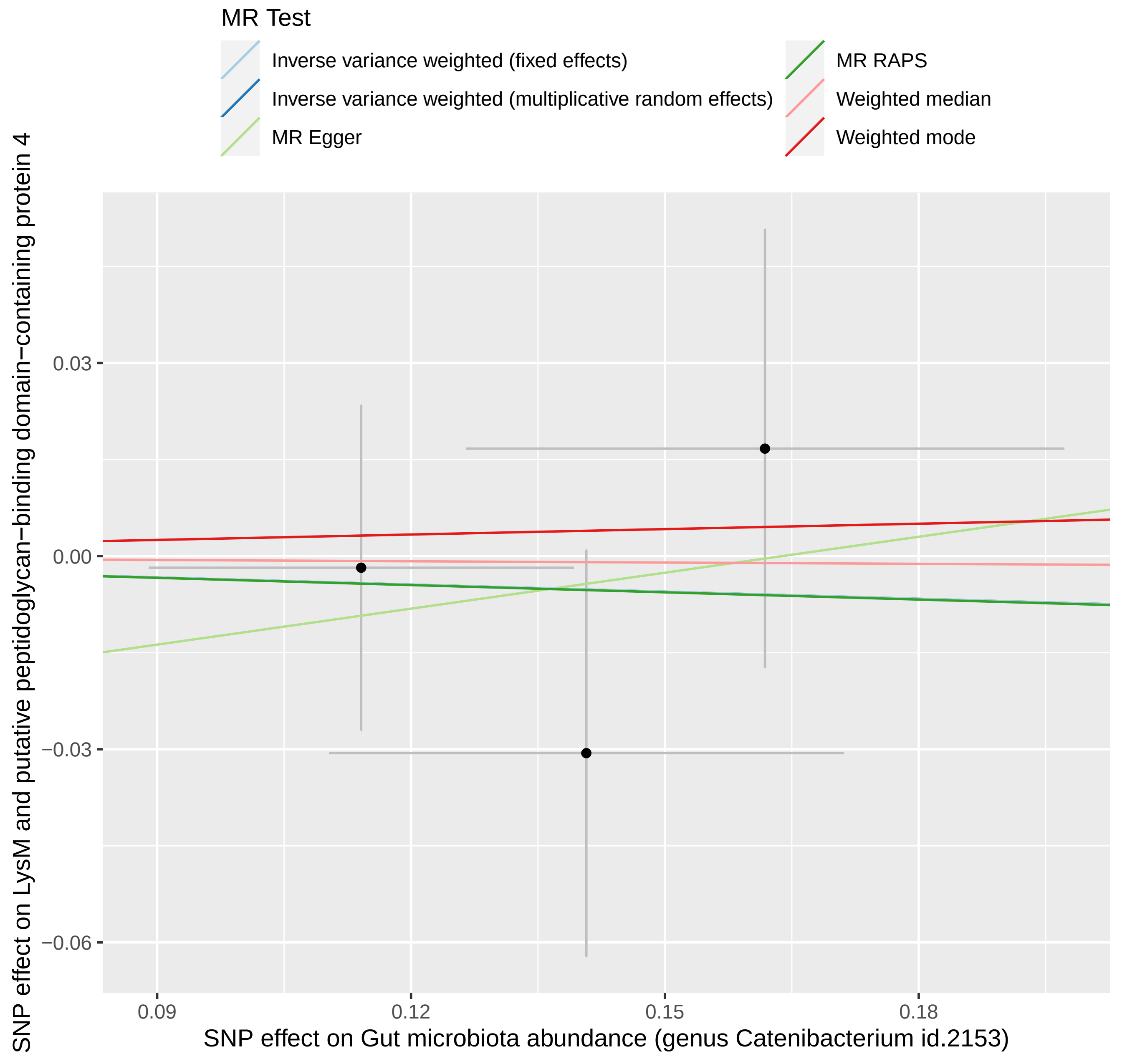

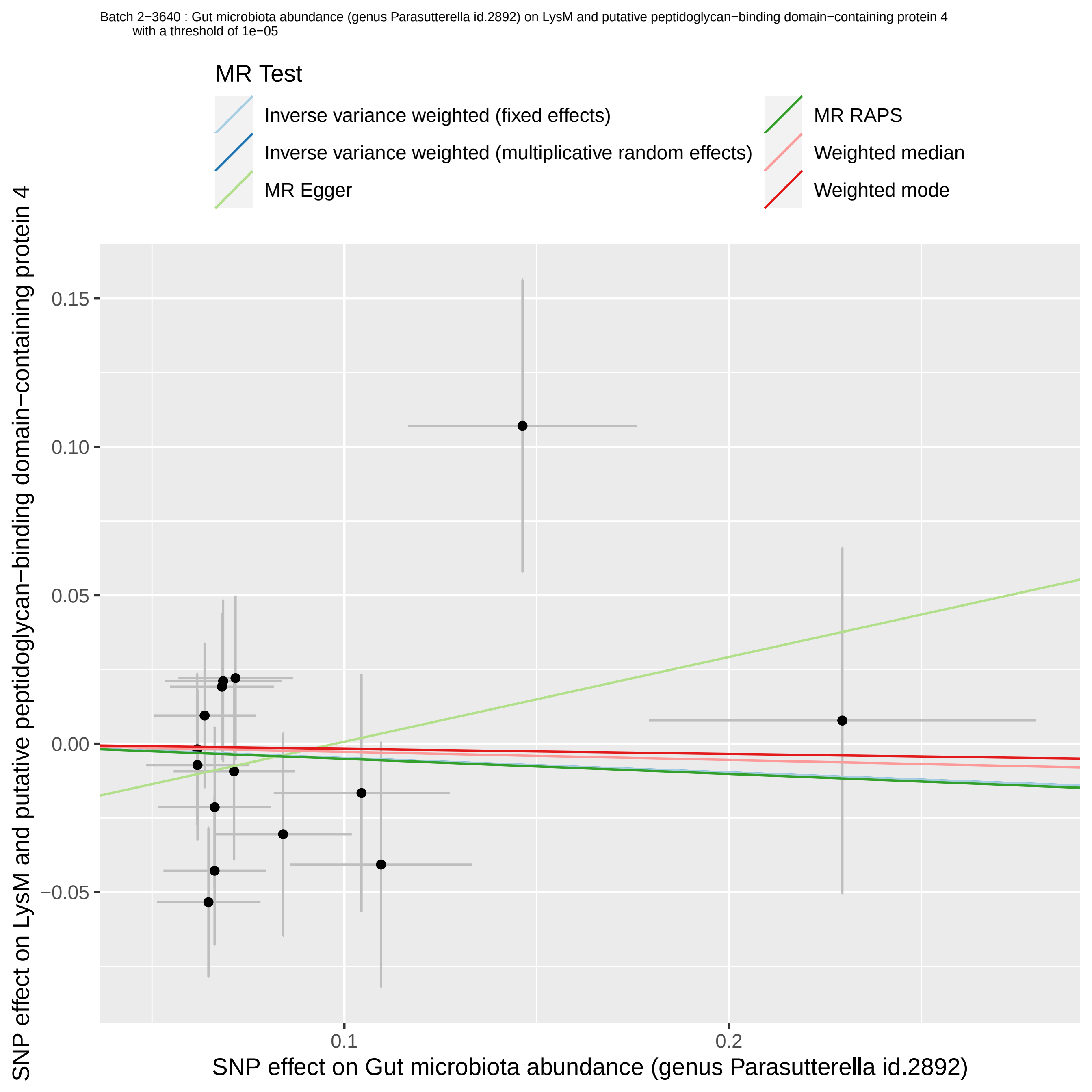
Batch 2−3640 : Gut microbiota abundance (genus Parasutterella id.2892) on LysM and putative peptidoglycan−binding domain−containing protein 4
with a threshold of 1e−05
MR Test
Inverse variance weighted (fixed effects)
Inverse variance weighted (multiplicative random effects)
MR Egger
MR RAPS
Weighted median
Weighted mode
0.15
0.10
0.05
0.00
−0.05
0.1
0.2
SNP effect on LysM and putative peptidoglycan−binding domain−containing protein 4
SNP effect on Gut microbiota abundance (genus Parasutterella id.2892)

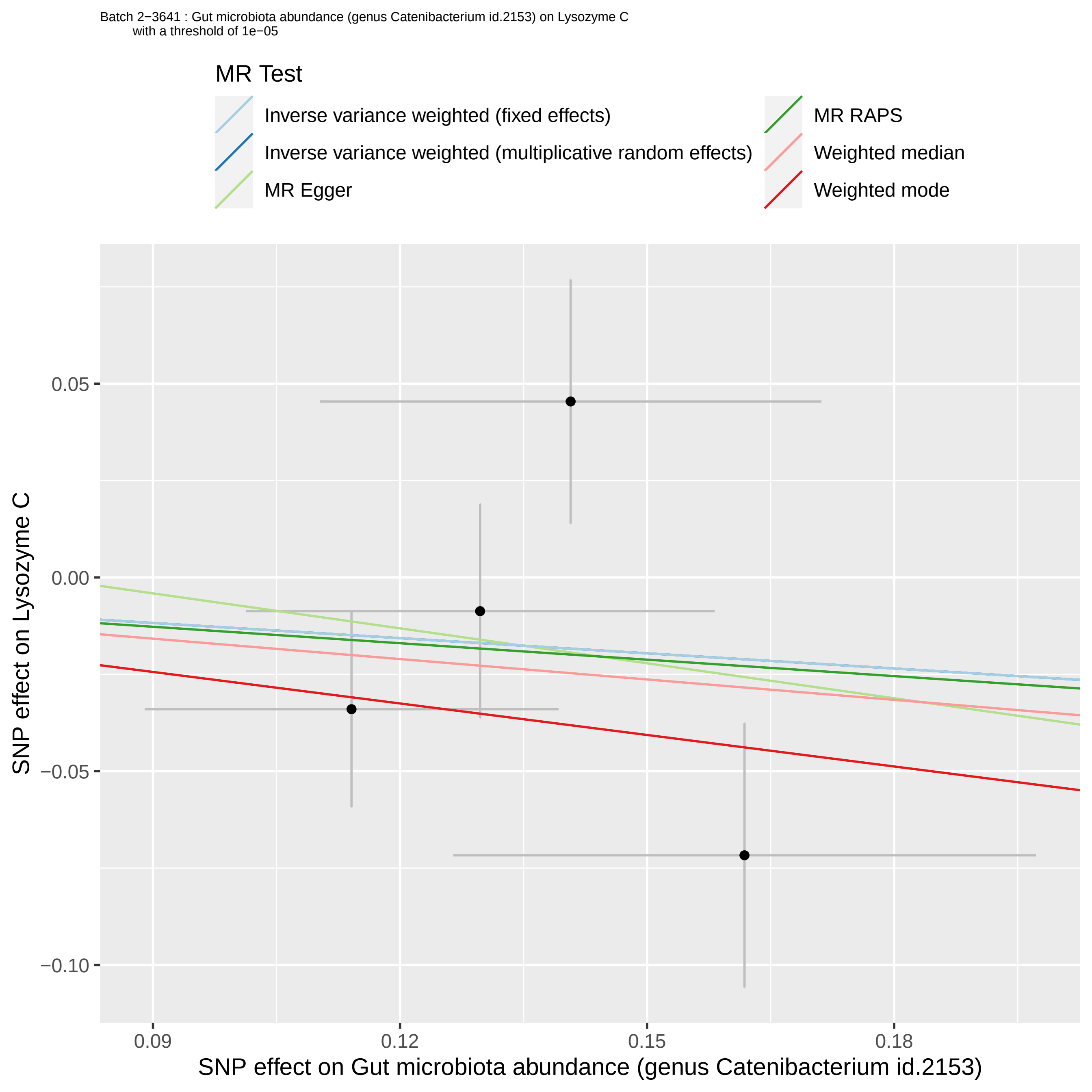

Batch 2−3641 : Gut microbiota abundance (genus Catenibacterium id.2153) on Lysozyme C
with a threshold of 1e−05
MR Test
Inverse variance weighted (fixed effects)
Inverse variance weighted (multiplicative random effects)
MR Egger
MR RAPS
Weighted median
Weighted mode
0.05
0.00
−0.05
−0.10
0.09
0.12
0.15
0.18
SNP effect on Lysozyme C
SNP effect on Gut microbiota abundance (genus Catenibacterium id.2153)

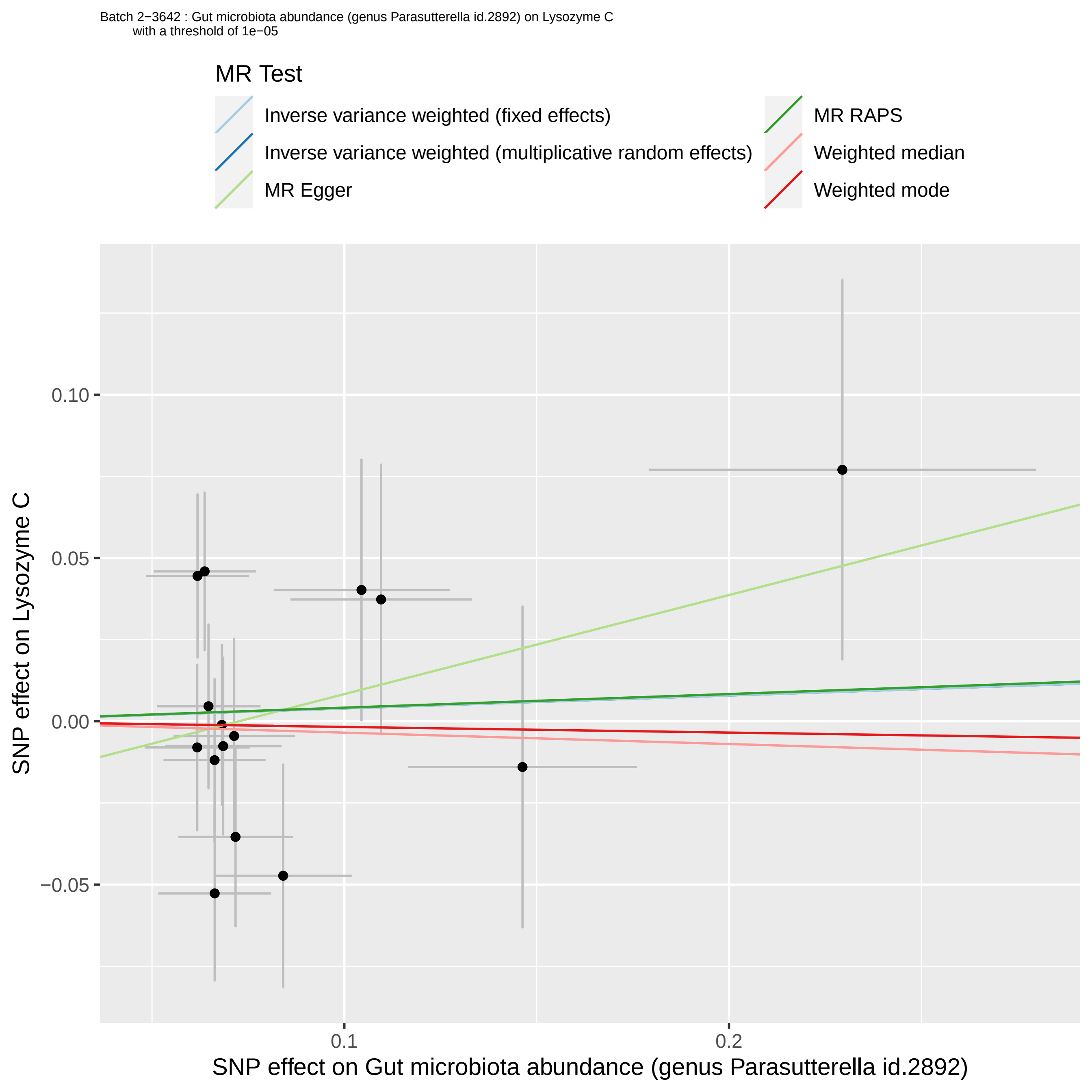
Batch 2−3642 : Gut microbiota abundance (genus Parasutterella id.2892) on Lysozyme C
with a threshold of 1e−05
MR Test
Inverse variance weighted (fixed effects)
Inverse variance weighted (multiplicative random effects)
MR Egger
MR RAPS
Weighted median
Weighted mode
0.10
0.05
0.00
−0.05
0.1
0.2
SNP effect on Lysozyme C
SNP effect on Gut microbiota abundance (genus Parasutterella id.2892)

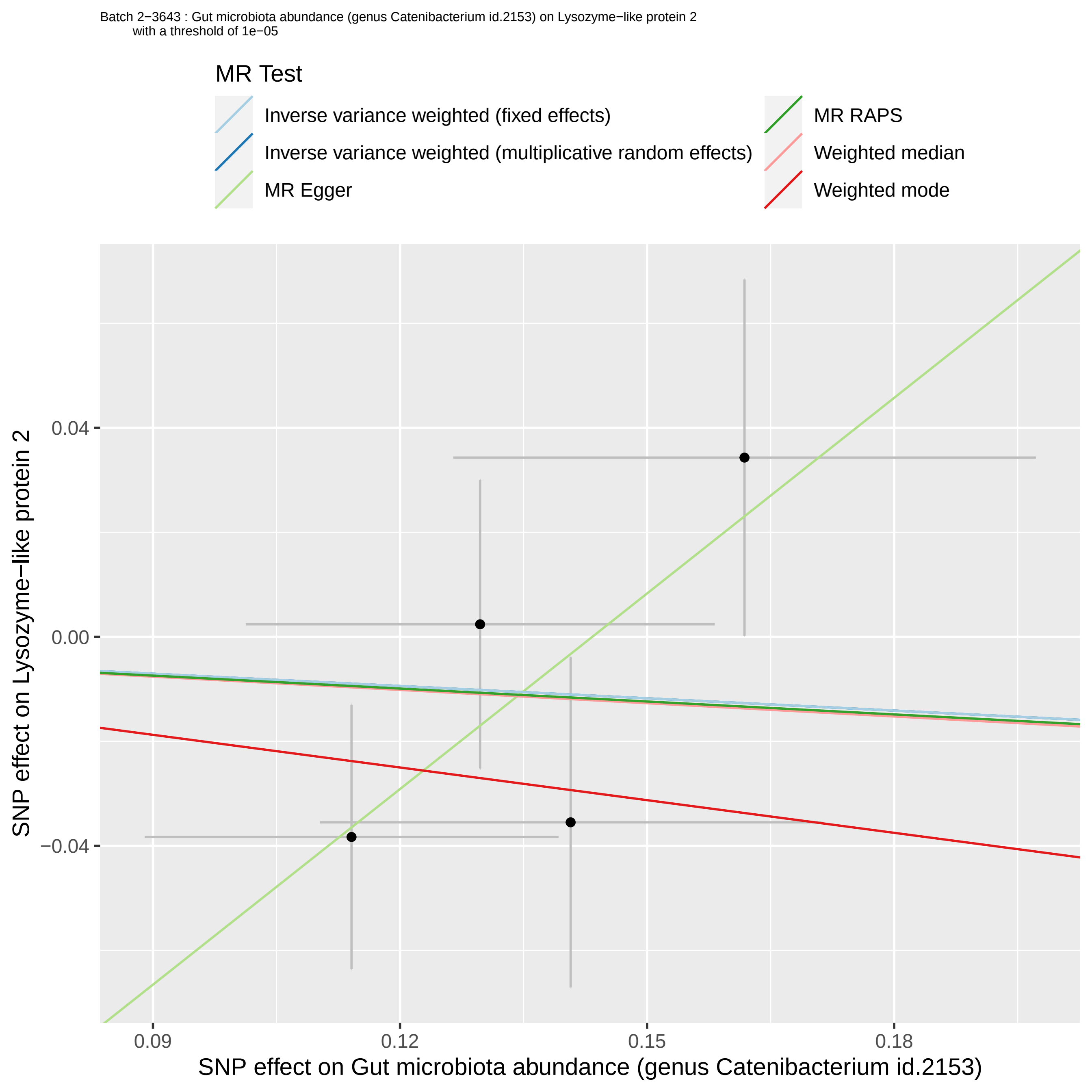
Batch 2−3643 : Gut microbiota abundance (genus Catenibacterium id.2153) on Lysozyme−like protein 2
with a threshold of 1e−05
MR Test
Inverse variance weighted (fixed effects)
Inverse variance weighted (multiplicative random effects)
MR Egger
MR RAPS
Weighted median
Weighted mode
0.04
0.00
−0.04
0.09
0.12
0.15
0.18
SNP effect on Lysozyme−like protein 2
SNP effect on Gut microbiota abundance (genus Catenibacterium id.2153)

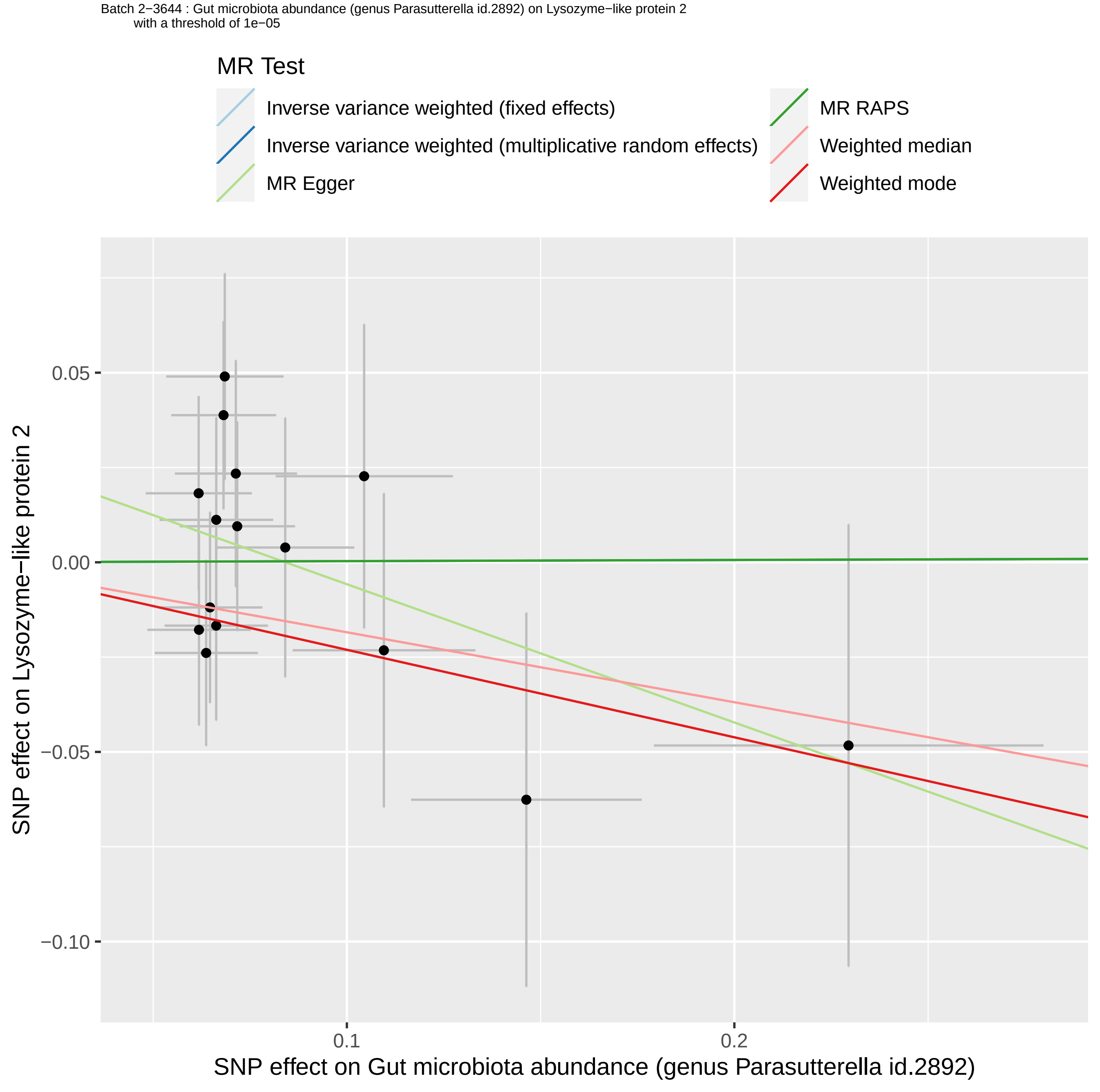

Batch 2−3644 : Gut microbiota abundance (genus Parasutterella id.2892) on Lysozyme−like protein 2
with a threshold of 1e−05
MR Test
Inverse variance weighted (fixed effects)
Inverse variance weighted (multiplicative random effects)
MR Egger
MR RAPS
Weighted median
Weighted mode
0.05
0.00
−0.05
−0.10
0.1
0.2
SNP effect on Lysozyme−like protein 2
SNP effect on Gut microbiota abundance (genus Parasutterella id.2892)

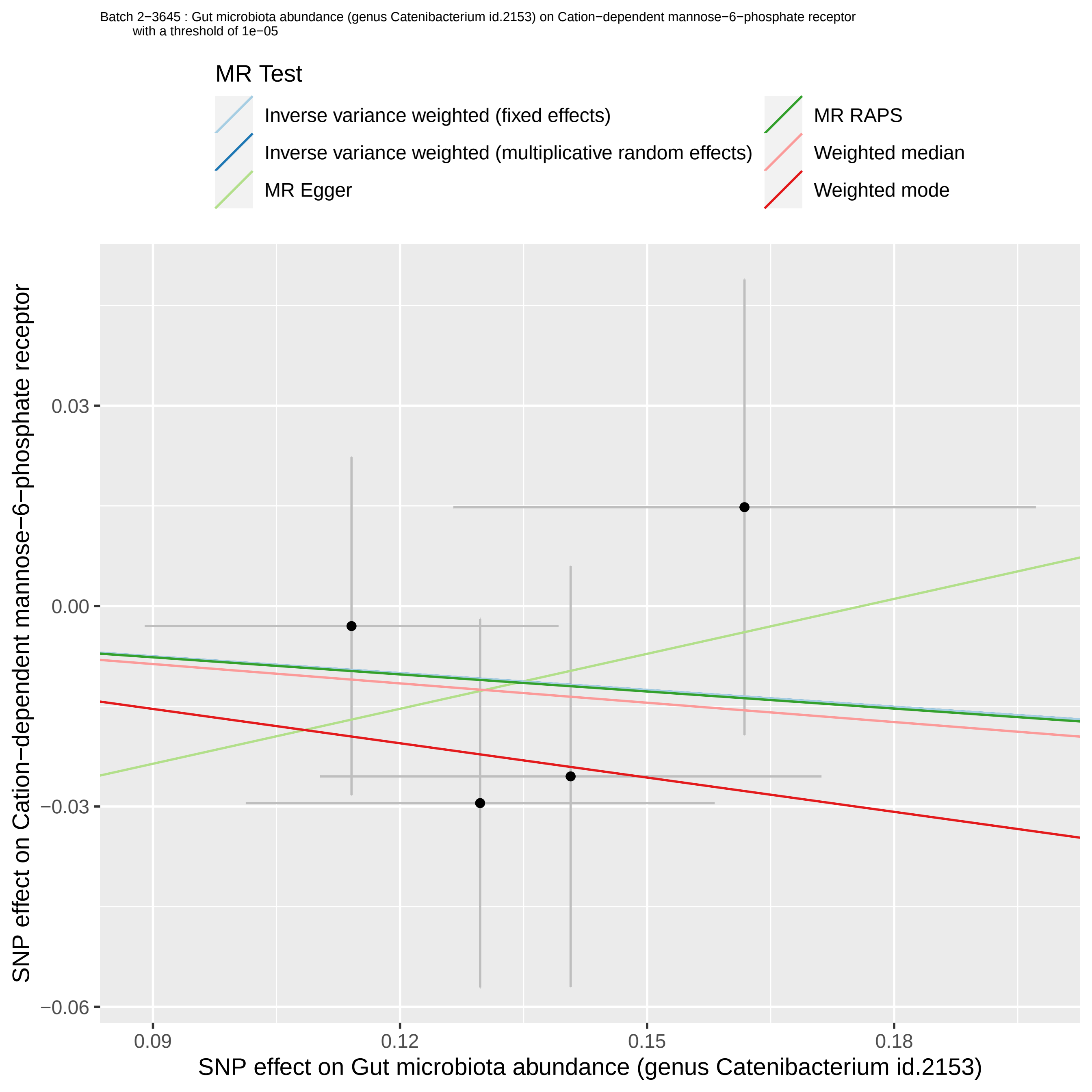

Batch 2−3645 : Gut microbiota abundance (genus Catenibacterium id.2153) on Cation−dependent mannose−6−phosphate receptor
with a threshold of 1e−05
MR Test
Inverse variance weighted (fixed effects)
Inverse variance weighted (multiplicative random effects)
MR Egger
MR RAPS
Weighted median
Weighted mode
SNP effect on Cation−dependent mannose−6−phosphate receptor
0.03
0.00
−0.03
−0.06
0.09
0.12
0.15
0.18
SNP effect on Gut microbiota abundance (genus Catenibacterium id.2153)

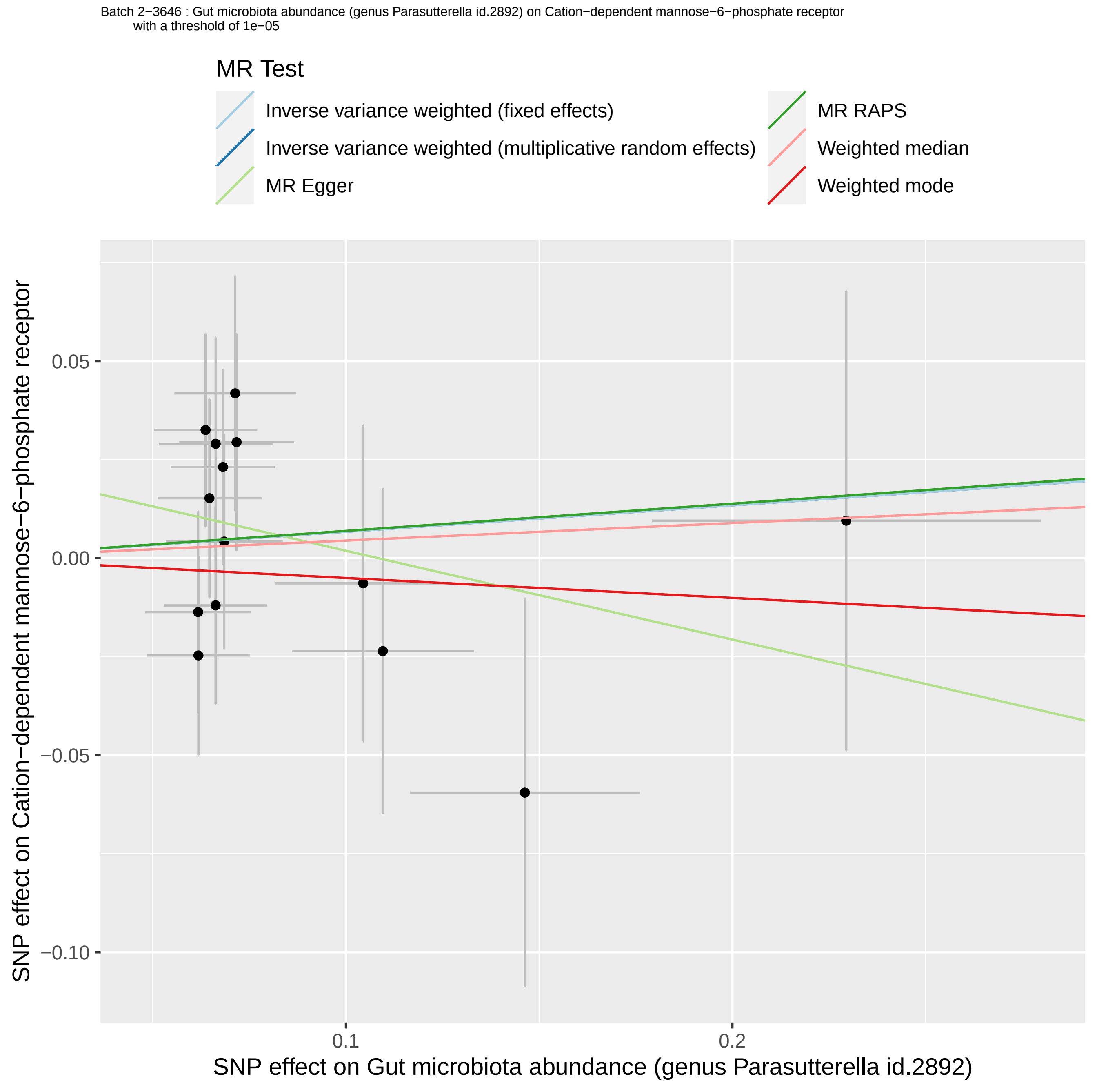
Batch 2−3646 : Gut microbiota abundance (genus Parasutterella id.2892) on Cation−dependent mannose−6−phosphate receptor
with a threshold of 1e−05
MR Test
Inverse variance weighted (fixed effects)
Inverse variance weighted (multiplicative random effects)
MR Egger
MR RAPS
Weighted median
Weighted mode
0.05
0.00
−0.05
−0.10
0.1
0.2
SNP effect on Cation−dependent mannose−6−phosphate receptor
SNP effect on Gut microbiota abundance (genus Parasutterella id.2892)

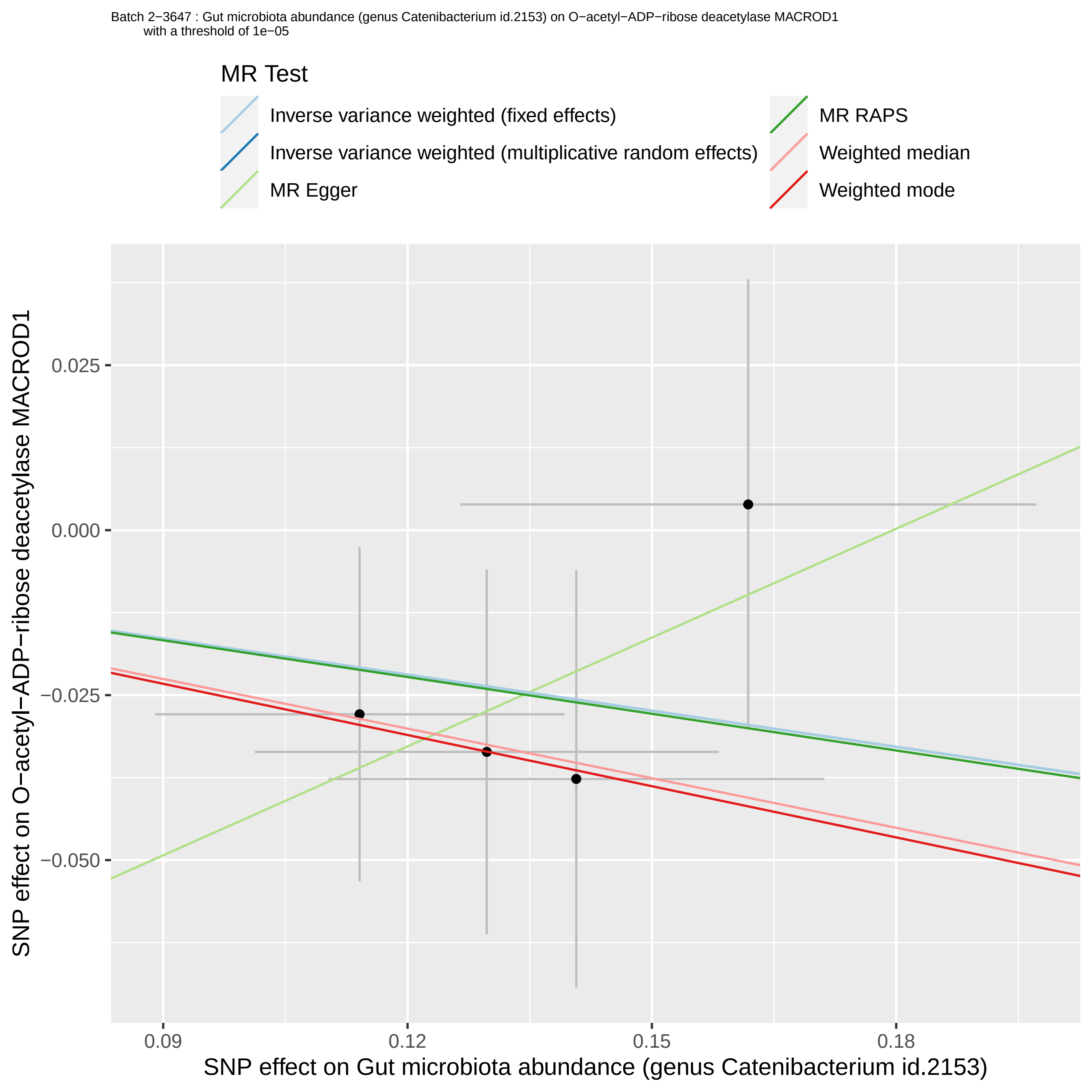

Batch 2−3647 : Gut microbiota abundance (genus Catenibacterium id.2153) on O−acetyl−ADP−ribose deacetylase MACROD1
with a threshold of 1e−05
MR Test
Inverse variance weighted (fixed effects)
Inverse variance weighted (multiplicative random effects)
MR Egger
MR RAPS
Weighted median
Weighted mode
0.025
0.000
−0.025
−0.050
0.09
0.12
0.15
0.18
SNP effect on Gut microbiota abundance (genus Catenibacterium id.2153)
SNP effect on O−acetyl−ADP−ribose deacetylase MACROD1

Batch 2−3648 : Gut microbiota abundance (genus Parasutterella id.2892) on O−acetyl−ADP−ribose deacetylase MACROD1
with a threshold of 1e−05

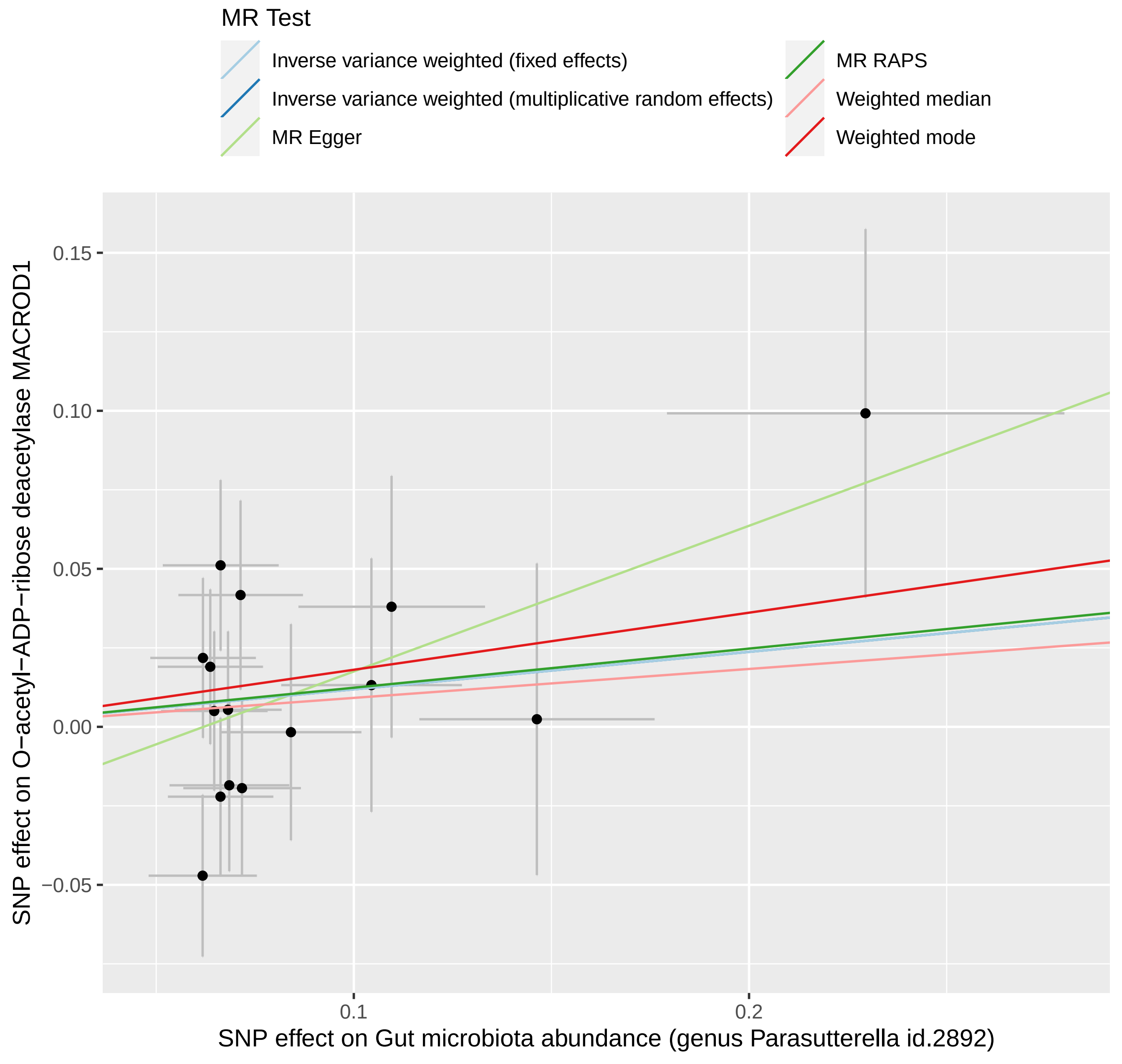

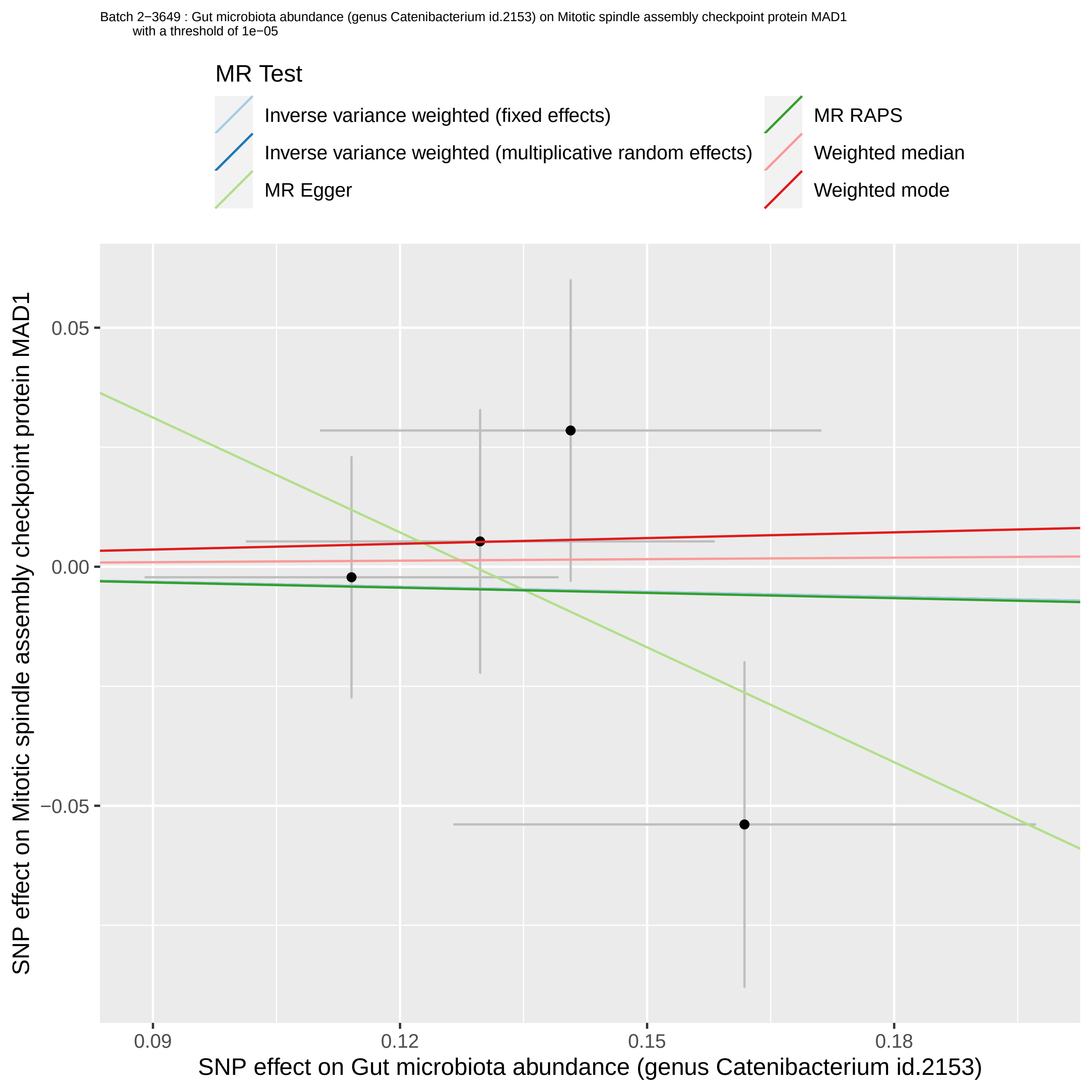

Batch 2−3649 : Gut microbiota abundance (genus Catenibacterium id.2153) on Mitotic spindle assembly checkpoint protein MAD1
with a threshold of 1e−05
MR Test
Inverse variance weighted (fixed effects)
Inverse variance weighted (multiplicative random effects)
MR Egger
MR RAPS
Weighted median
Weighted mode
0.05
0.00
−0.05
0.09
0.12
0.15
0.18
SNP effect on Mitotic spindle assembly checkpoint protein MAD1
SNP effect on Gut microbiota abundance (genus Catenibacterium id.2153)

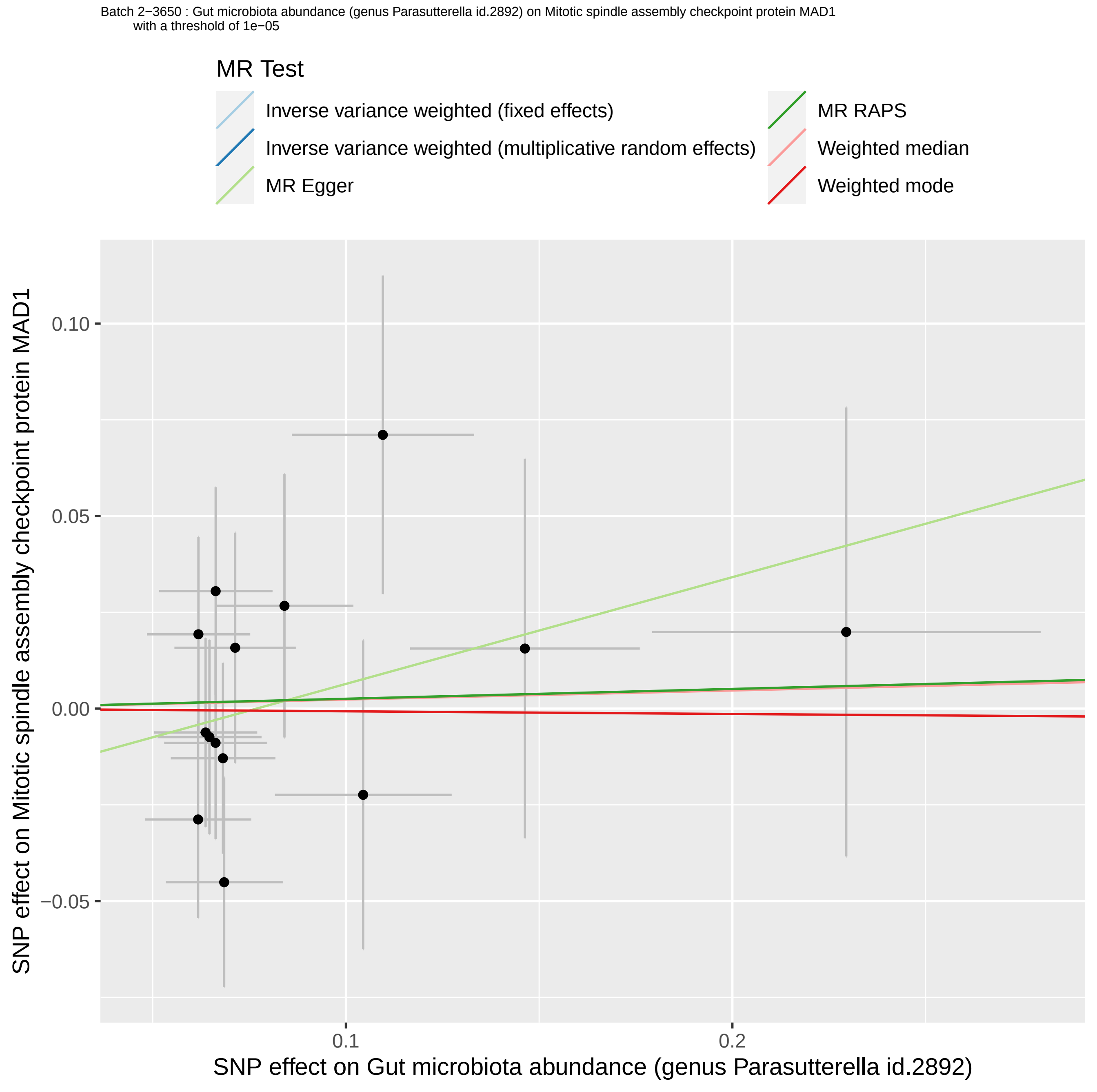
Batch 2−3650 : Gut microbiota abundance (genus Parasutterella id.2892) on Mitotic spindle assembly checkpoint protein MAD1
with a threshold of 1e−05
MR Test
Inverse variance weighted (fixed effects)
Inverse variance weighted (multiplicative random effects)
MR Egger
MR RAPS
Weighted median
Weighted mode
0.10
0.05
0.00
−0.05
0.1
0.2
SNP effect on Mitotic spindle assembly checkpoint protein MAD1
SNP effect on Gut microbiota abundance (genus Parasutterella id.2892)

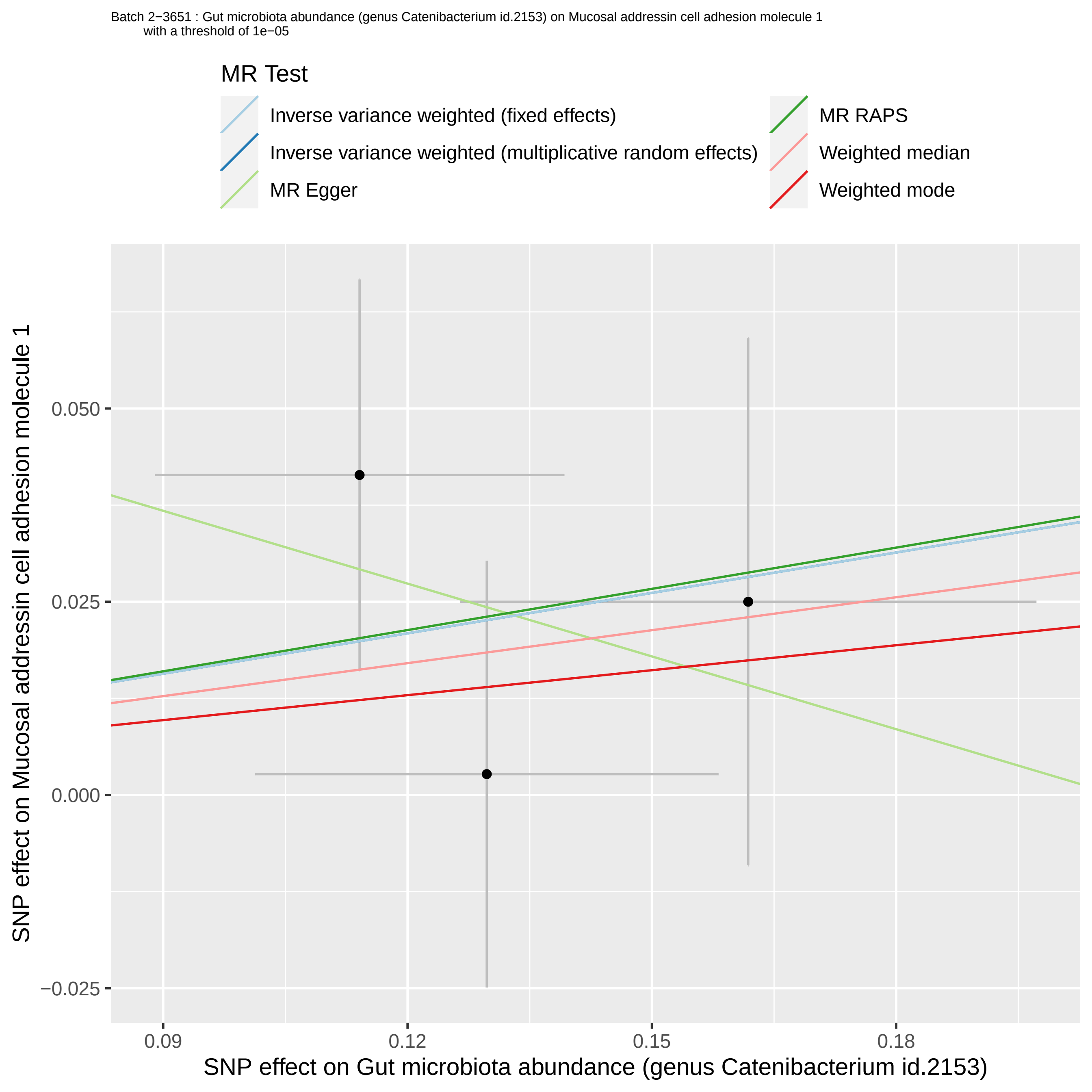
Batch 2−3651 : Gut microbiota abundance (genus Catenibacterium id.2153) on Mucosal addressin cell adhesion molecule 1
with a threshold of 1e−05
MR Test
Inverse variance weighted (fixed effects)
Inverse variance weighted (multiplicative random effects)
MR Egger
MR RAPS
Weighted median
Weighted mode
SNP effect on Mucosal addressin cell adhesion molecule 1
0.050
0.025
0.000
−0.025
0.09
0.12
0.15
0.18
SNP effect on Gut microbiota abundance (genus Catenibacterium id.2153)

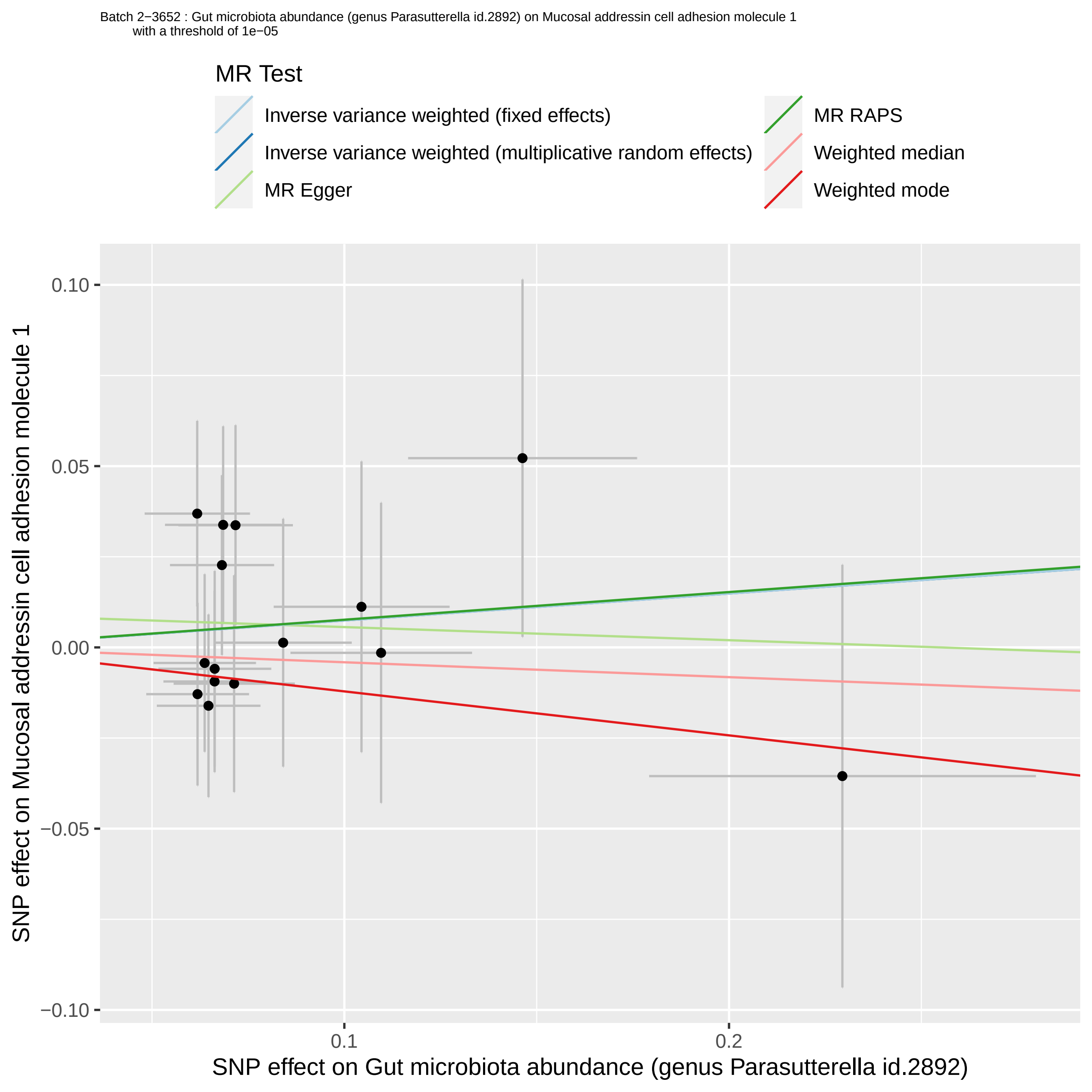

Batch 2−3652 : Gut microbiota abundance (genus Parasutterella id.2892) on Mucosal addressin cell adhesion molecule 1
with a threshold of 1e−05
MR Test
Inverse variance weighted (fixed effects)
Inverse variance weighted (multiplicative random effects)
MR Egger
MR RAPS
Weighted median
Weighted mode
0.10
0.05
0.00
−0.05
−0.10
0.1
0.2
SNP effect on Mucosal addressin cell adhesion molecule 1
SNP effect on Gut microbiota abundance (genus Parasutterella id.2892)

Batch 2−3653 : Gut microbiota abundance (genus Catenibacterium id.2153) on Melanoma−associated antigen 10
with a threshold of 1e−05

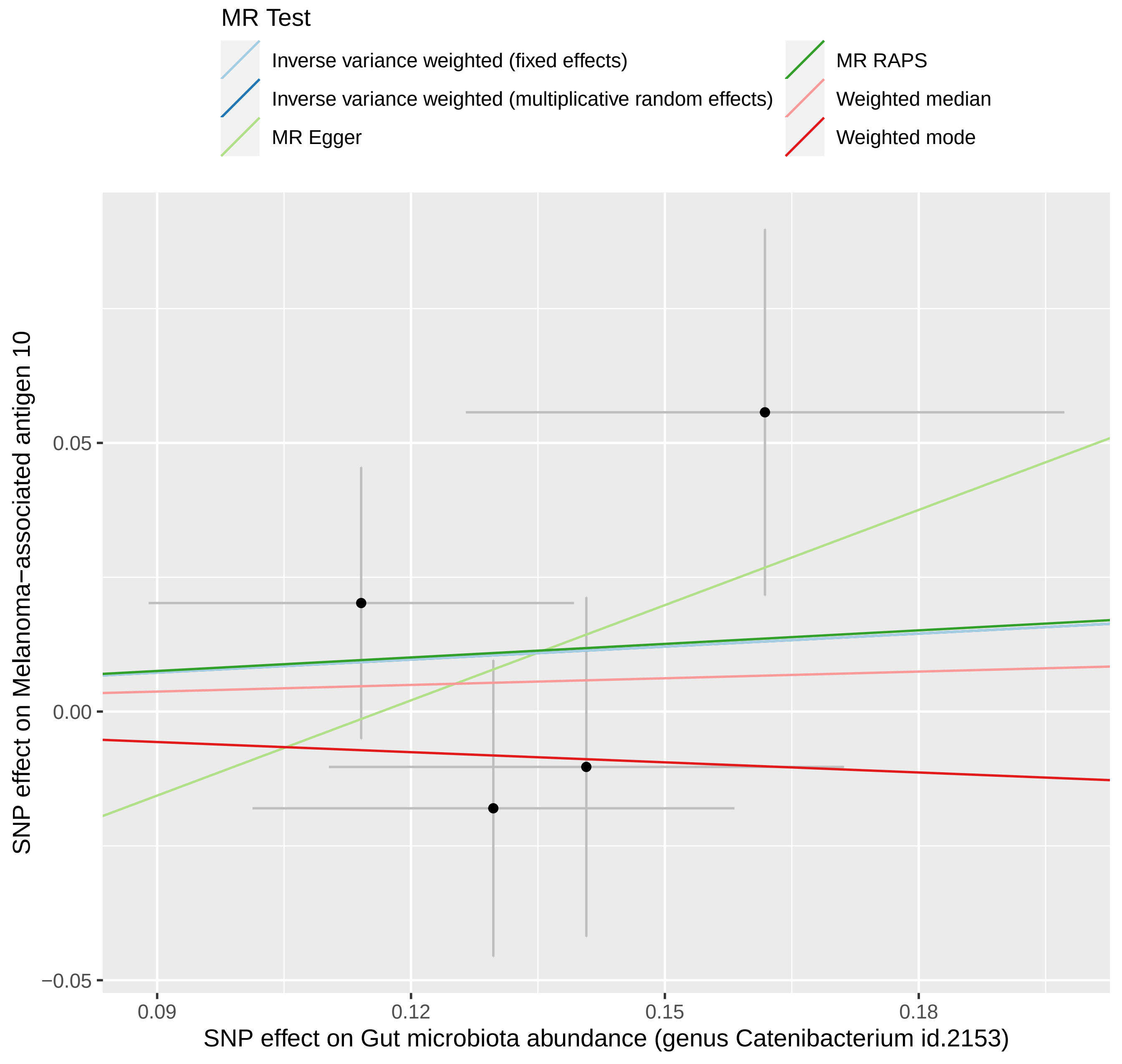

Batch 2−3654 : Gut microbiota abundance (genus Parasutterella id.2892) on Melanoma−associated antigen 10
with a threshold of 1e−05

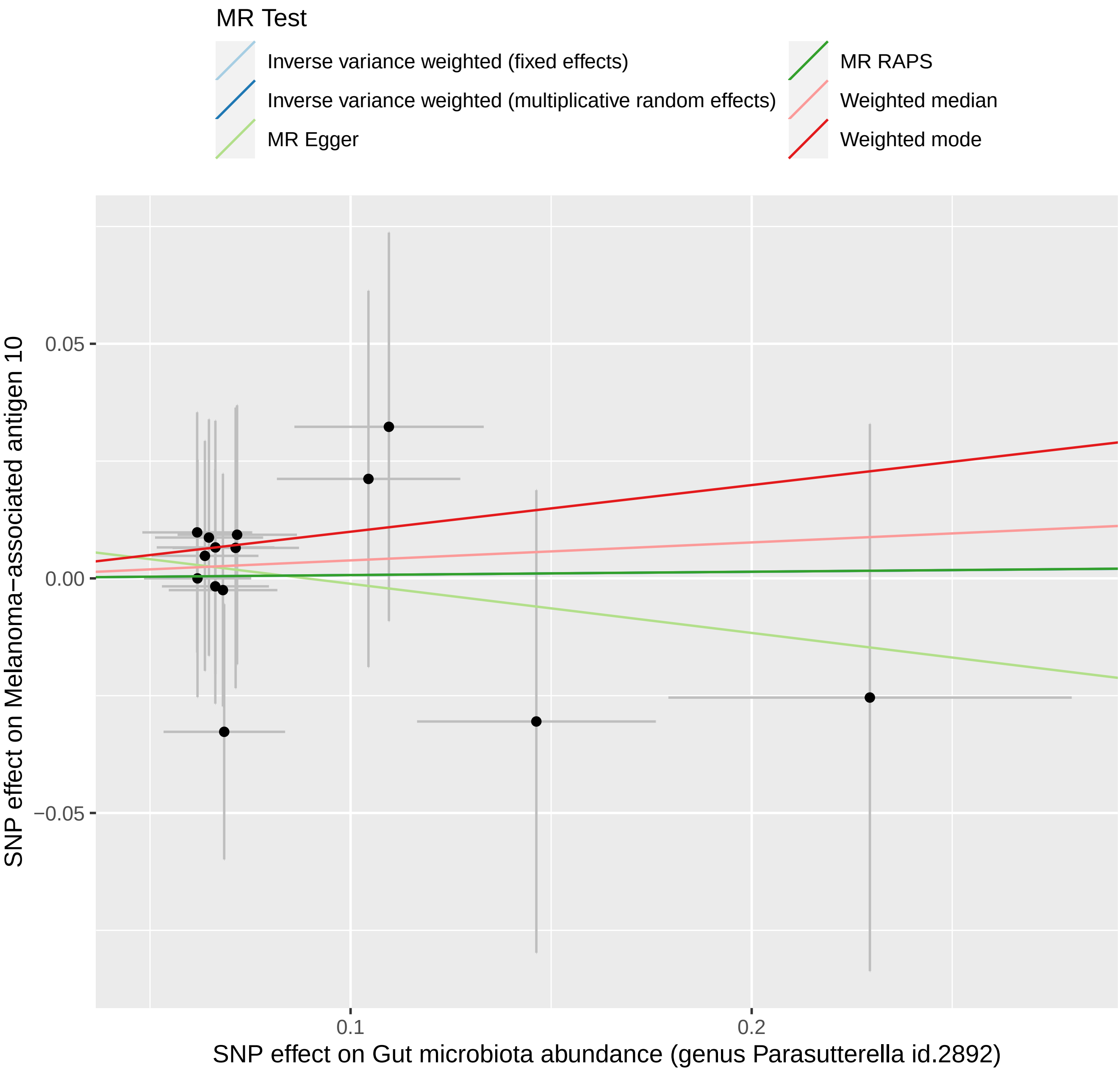

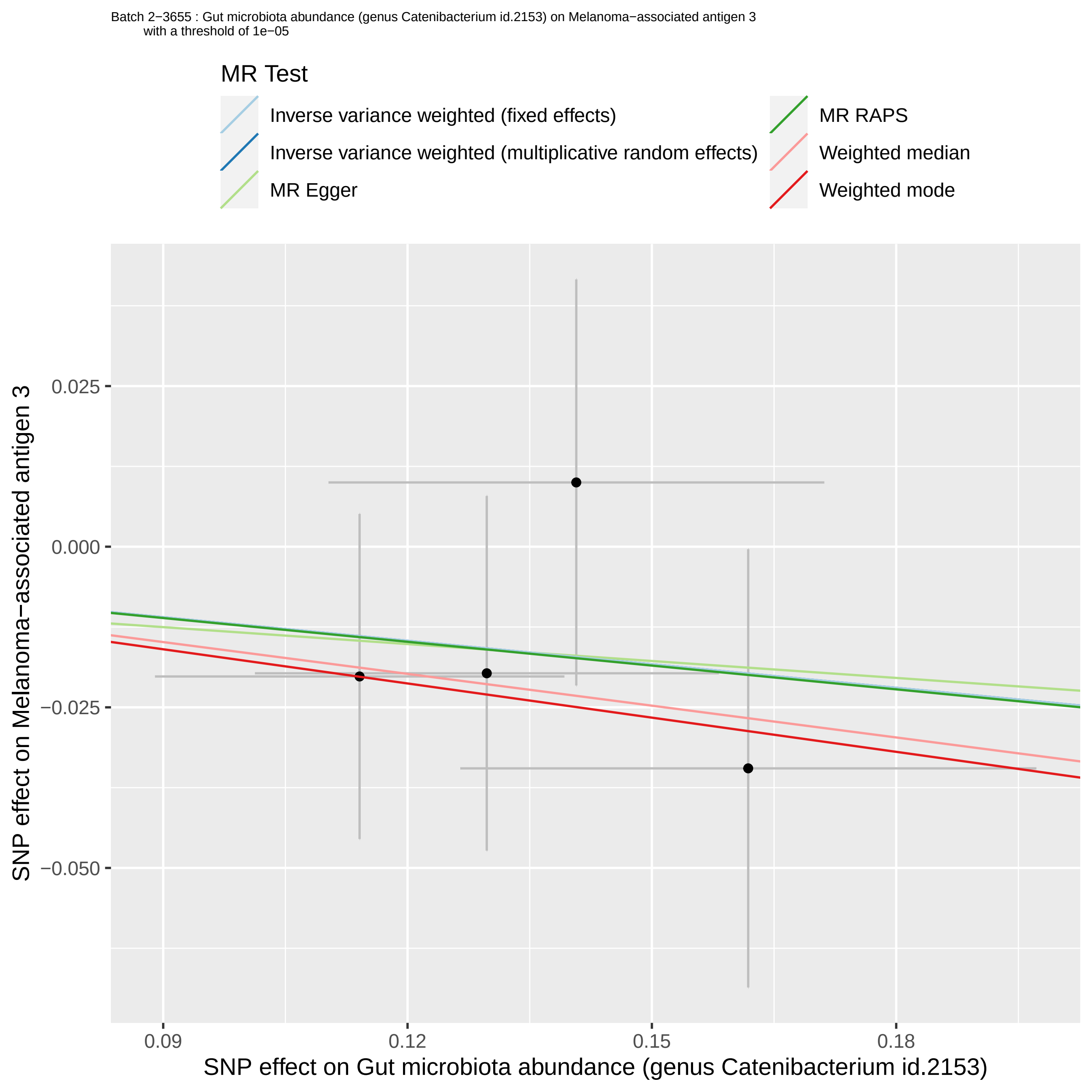

Batch 2−3655 : Gut microbiota abundance (genus Catenibacterium id.2153) on Melanoma−associated antigen 3
with a threshold of 1e−05
MR Test
Inverse variance weighted (fixed effects)
Inverse variance weighted (multiplicative random effects)
MR Egger
MR RAPS
Weighted median
Weighted mode
0.025
0.000
−0.025
−0.050
0.09
0.12
0.15
0.18
SNP effect on Melanoma−associated antigen 3
SNP effect on Gut microbiota abundance (genus Catenibacterium id.2153)

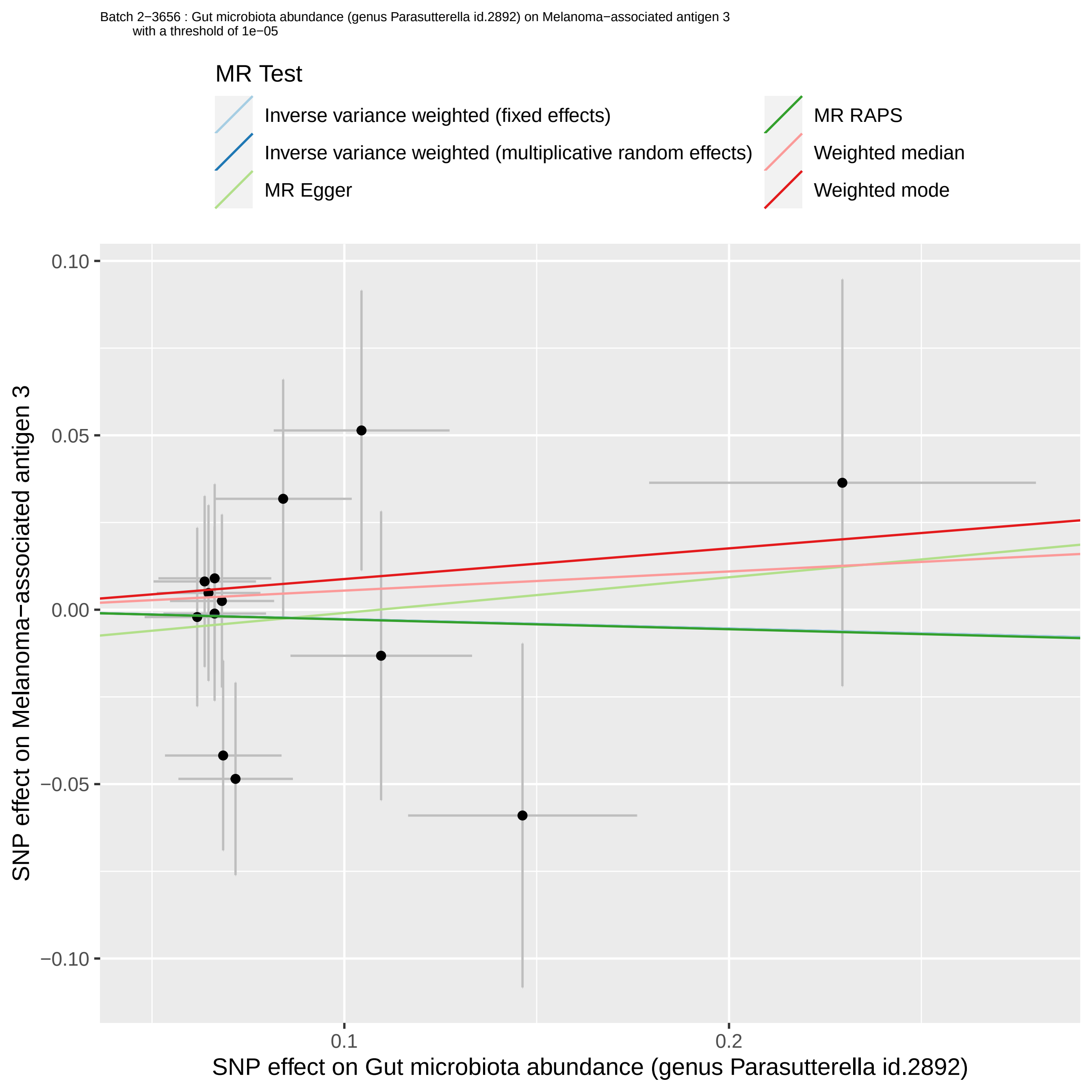

Batch 2−3656 : Gut microbiota abundance (genus Parasutterella id.2892) on Melanoma−associated antigen 3
with a threshold of 1e−05
MR Test
Inverse variance weighted (fixed effects)
Inverse variance weighted (multiplicative random effects)
MR Egger
MR RAPS
Weighted median
Weighted mode
0.10
0.05
0.00
−0.05
−0.10
0.1
0.2
SNP effect on Melanoma−associated antigen 3
SNP effect on Gut microbiota abundance (genus Parasutterella id.2892)

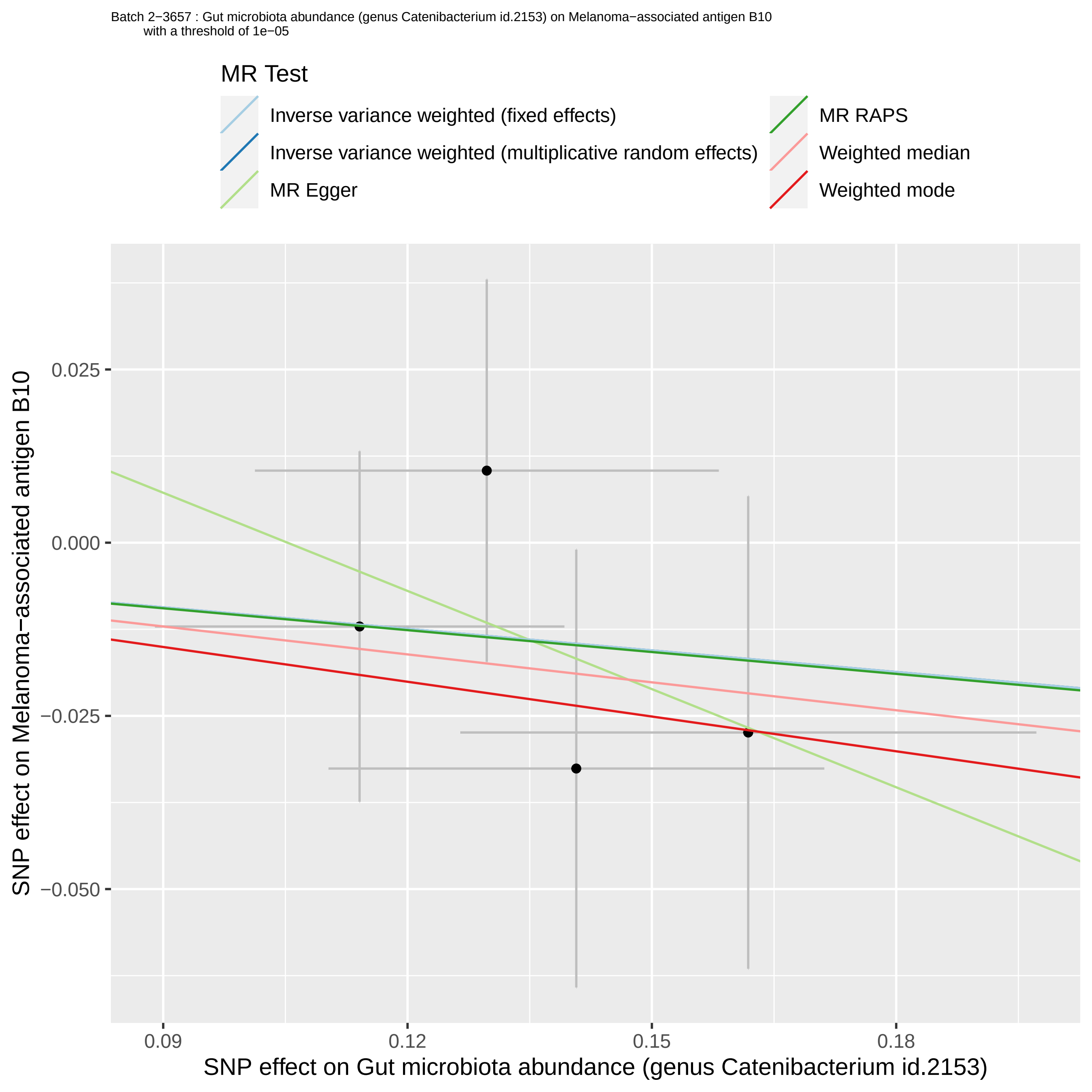

Batch 2−3657 : Gut microbiota abundance (genus Catenibacterium id.2153) on Melanoma−associated antigen B10
with a threshold of 1e−05
MR Test
Inverse variance weighted (fixed effects)
Inverse variance weighted (multiplicative random effects)
MR Egger
MR RAPS
Weighted median
Weighted mode
0.025
0.000
−0.025
−0.050
0.09
0.12
0.15
0.18
SNP effect on Melanoma−associated antigen B10
SNP effect on Gut microbiota abundance (genus Catenibacterium id.2153)

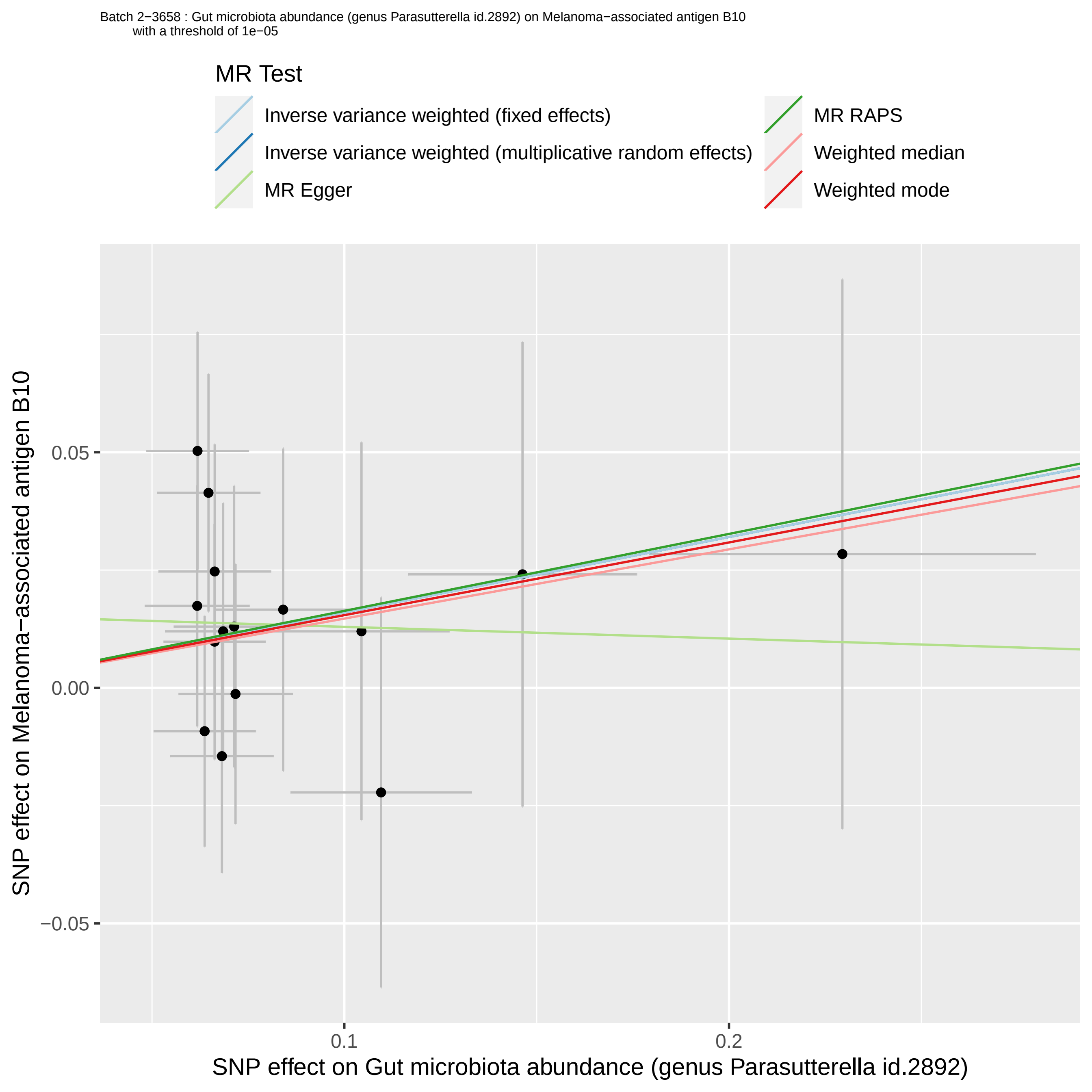
Batch 2−3658 : Gut microbiota abundance (genus Parasutterella id.2892) on Melanoma−associated antigen B10
with a threshold of 1e−05
MR Test
Inverse variance weighted (fixed effects)
Inverse variance weighted (multiplicative random effects)
MR Egger
MR RAPS
Weighted median
Weighted mode
SNP effect on Melanoma−associated antigen B10
0.05
0.00
−0.05
0.1
0.2
SNP effect on Gut microbiota abundance (genus Parasutterella id.2892)

Batch 2−3659 : Gut microbiota abundance (genus Catenibacterium id.2153) on Membrane−associated guanylate kinase, WW and PDZ domain−containing protein 2 with a threshold of 1e−05

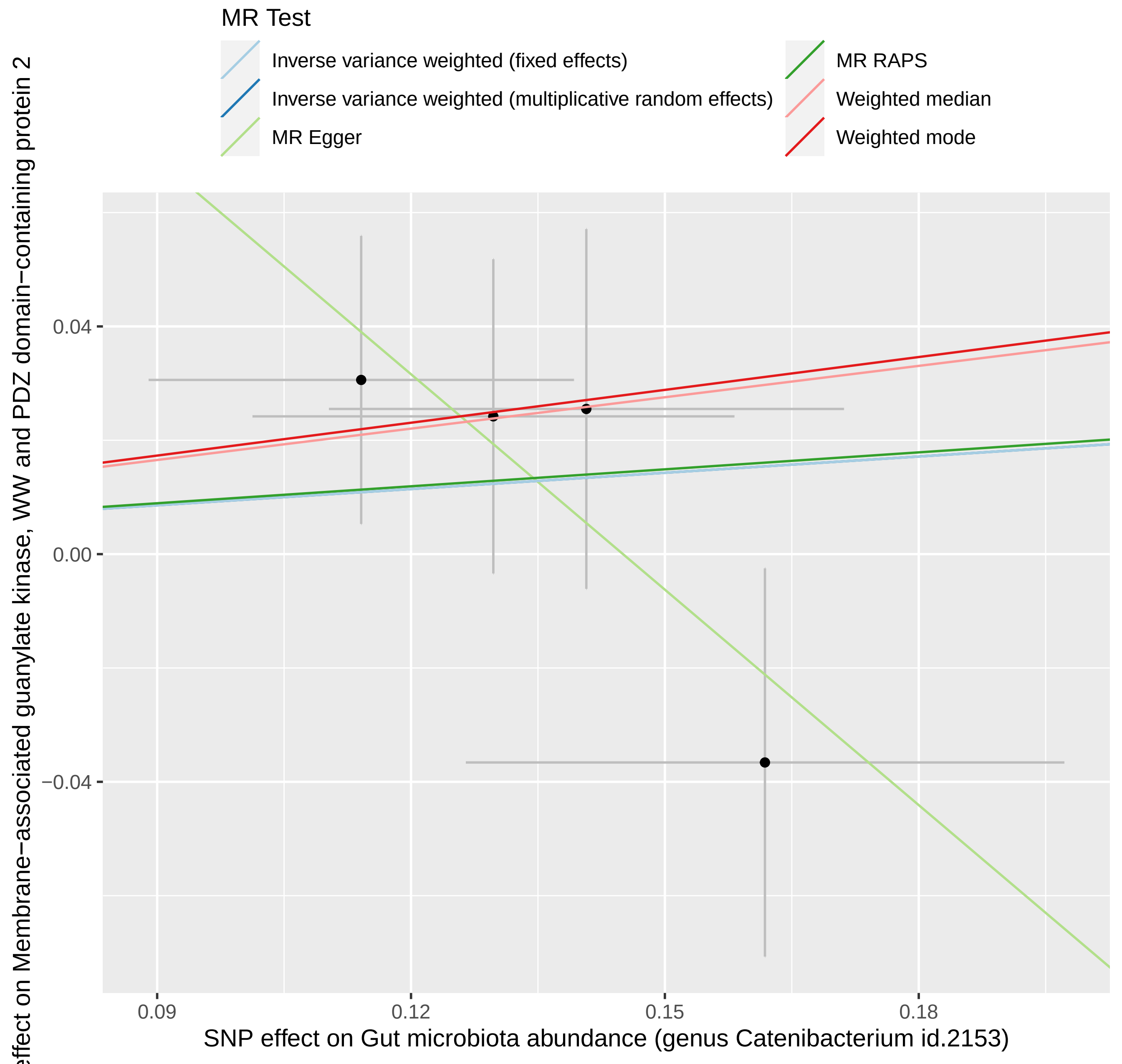

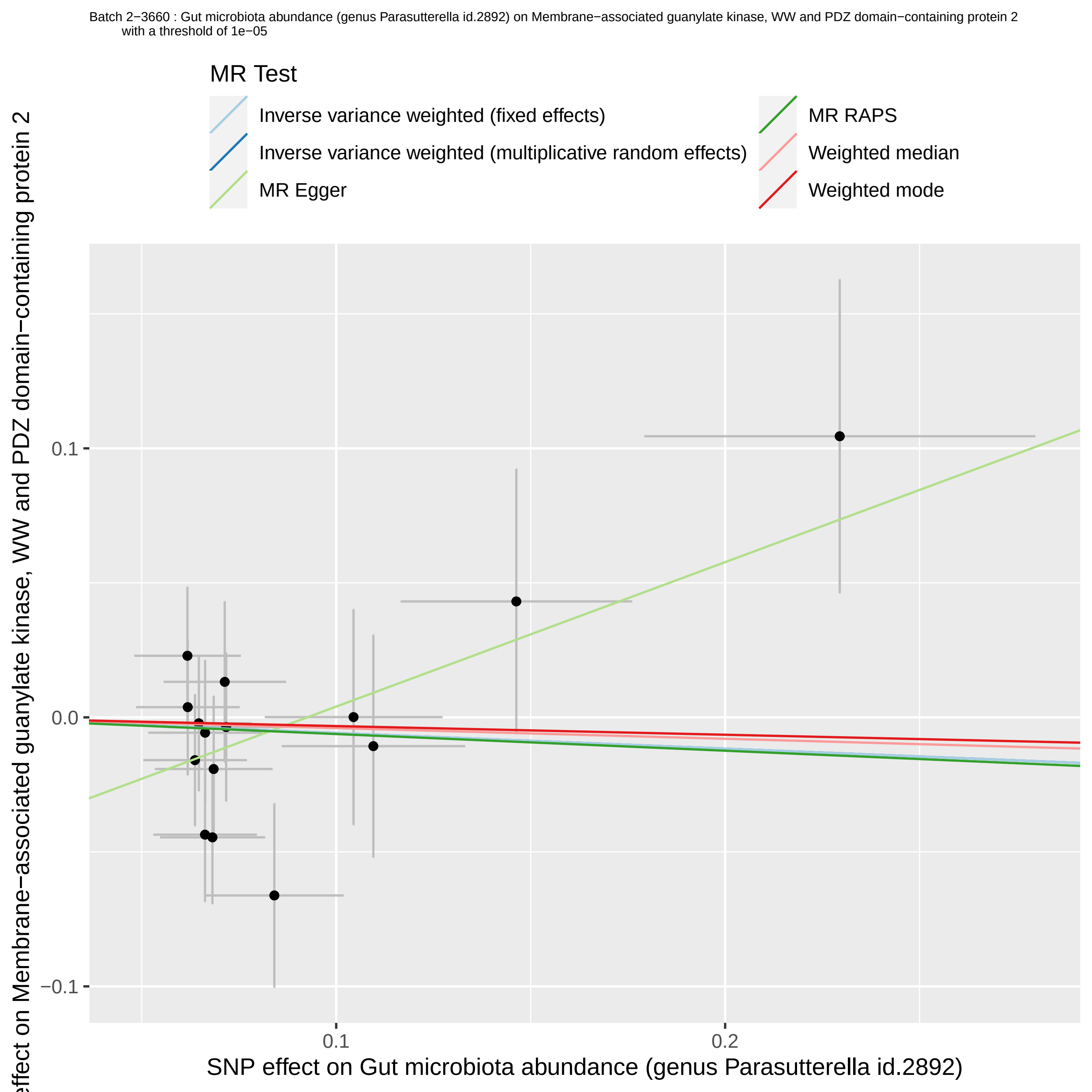

Batch 2−3660 : Gut microbiota abundance (genus Parasutterella id.2892) on Membrane−associated guanylate kinase, WW and PDZ domain−containing protein 2
with a threshold of 1e−05
MR Test
Inverse variance weighted (fixed effects)
Inverse variance weighted (multiplicative random effects)
MR Egger
MR RAPS
Weighted median
Weighted mode
0.1
0.0
−0.1
0.1
0.2
SNP effect on Gut microbiota abundance (genus Parasutterella id.2892)
effect on Membrane−associated guanylate kinase, WW and PDZ domain−containing protein 2

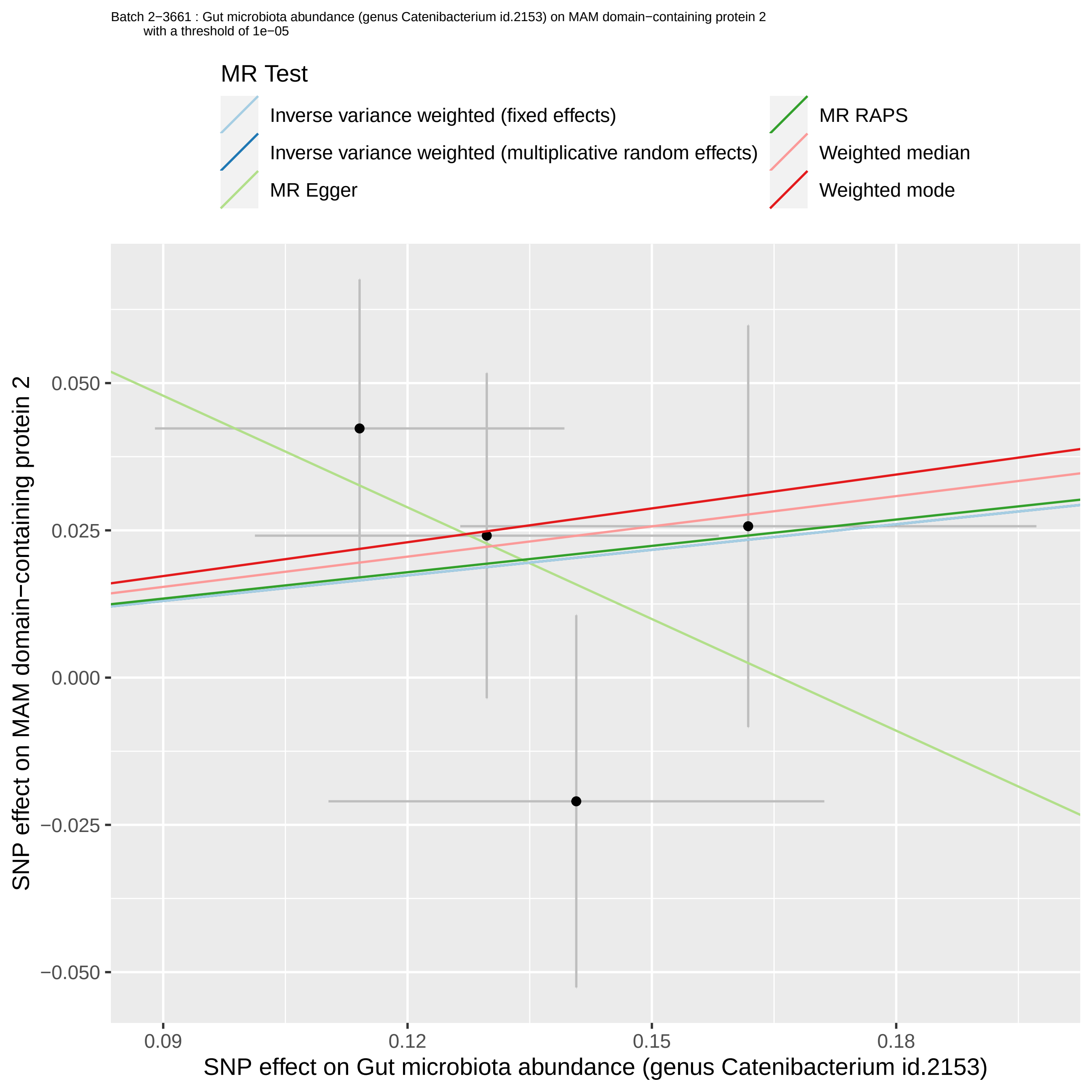
Batch 2−3661 : Gut microbiota abundance (genus Catenibacterium id.2153) on MAM domain−containing protein 2
with a threshold of 1e−05
MR Test
Inverse variance weighted (fixed effects)
Inverse variance weighted (multiplicative random effects)
MR Egger
MR RAPS
Weighted median
Weighted mode
SNP effect on MAM domain−containing protein 2
0.050
0.025
0.000
−0.025
−0.050
0.09
0.12
0.15
0.18
SNP effect on Gut microbiota abundance (genus Catenibacterium id.2153)

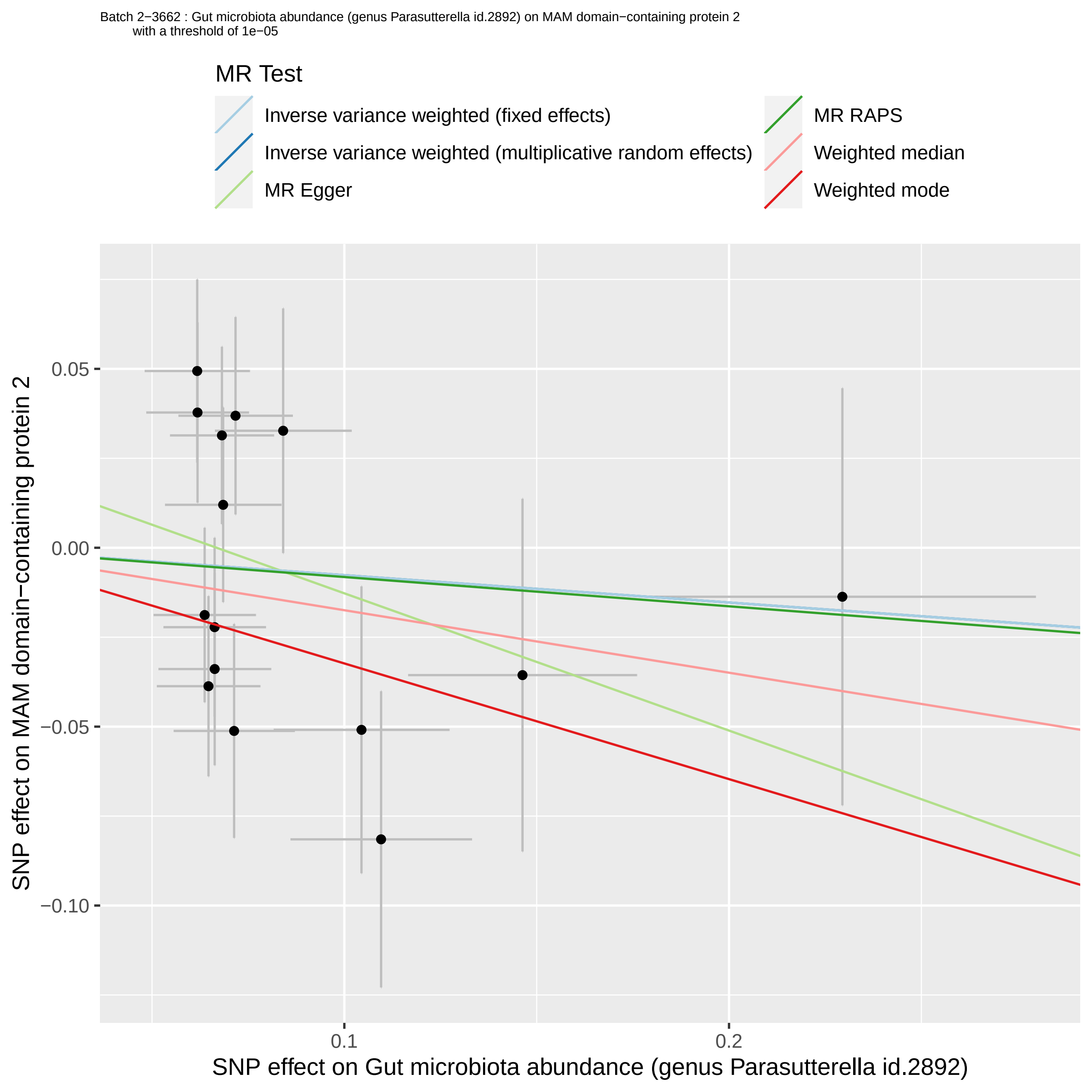
Batch 2−3662 : Gut microbiota abundance (genus Parasutterella id.2892) on MAM domain−containing protein 2
with a threshold of 1e−05
MR Test
Inverse variance weighted (fixed effects)
Inverse variance weighted (multiplicative random effects)
MR Egger
MR RAPS
Weighted median
Weighted mode
0.05
0.00
−0.05
−0.10
0.1
0.2
SNP effect on MAM domain−containing protein 2
SNP effect on Gut microbiota abundance (genus Parasutterella id.2892)

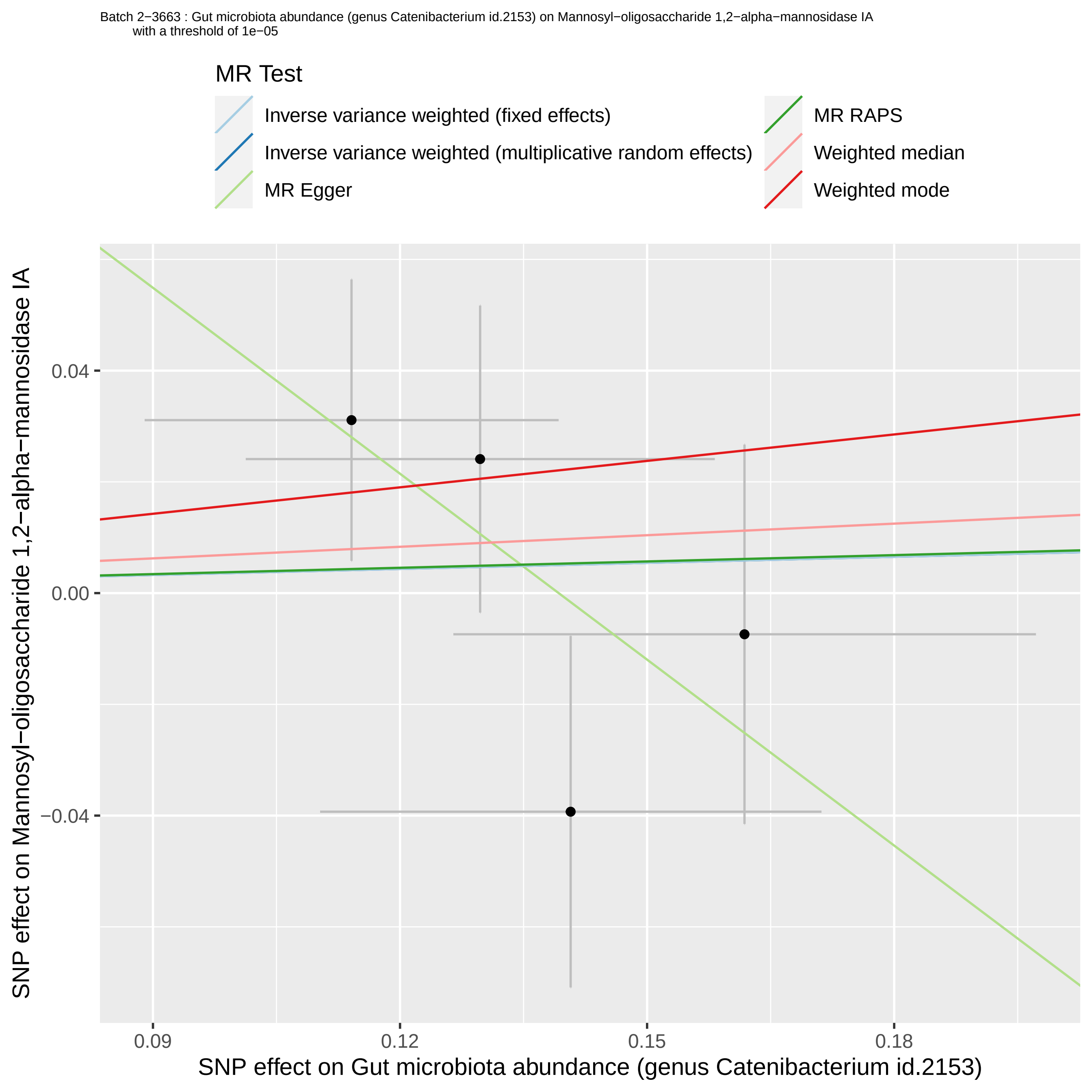
Batch 2−3663 : Gut microbiota abundance (genus Catenibacterium id.2153) on Mannosyl−oligosaccharide 1,2−alpha−mannosidase IA
with a threshold of 1e−05
MR Test
Inverse variance weighted (fixed effects)
Inverse variance weighted (multiplicative random effects)
MR Egger
MR RAPS
Weighted median
Weighted mode
0.04
0.00
−0.04
0.09
0.12
0.15
0.18
SNP effect on Mannosyl−oligosaccharide 1,2−alpha−mannosidase IA
SNP effect on Gut microbiota abundance (genus Catenibacterium id.2153)

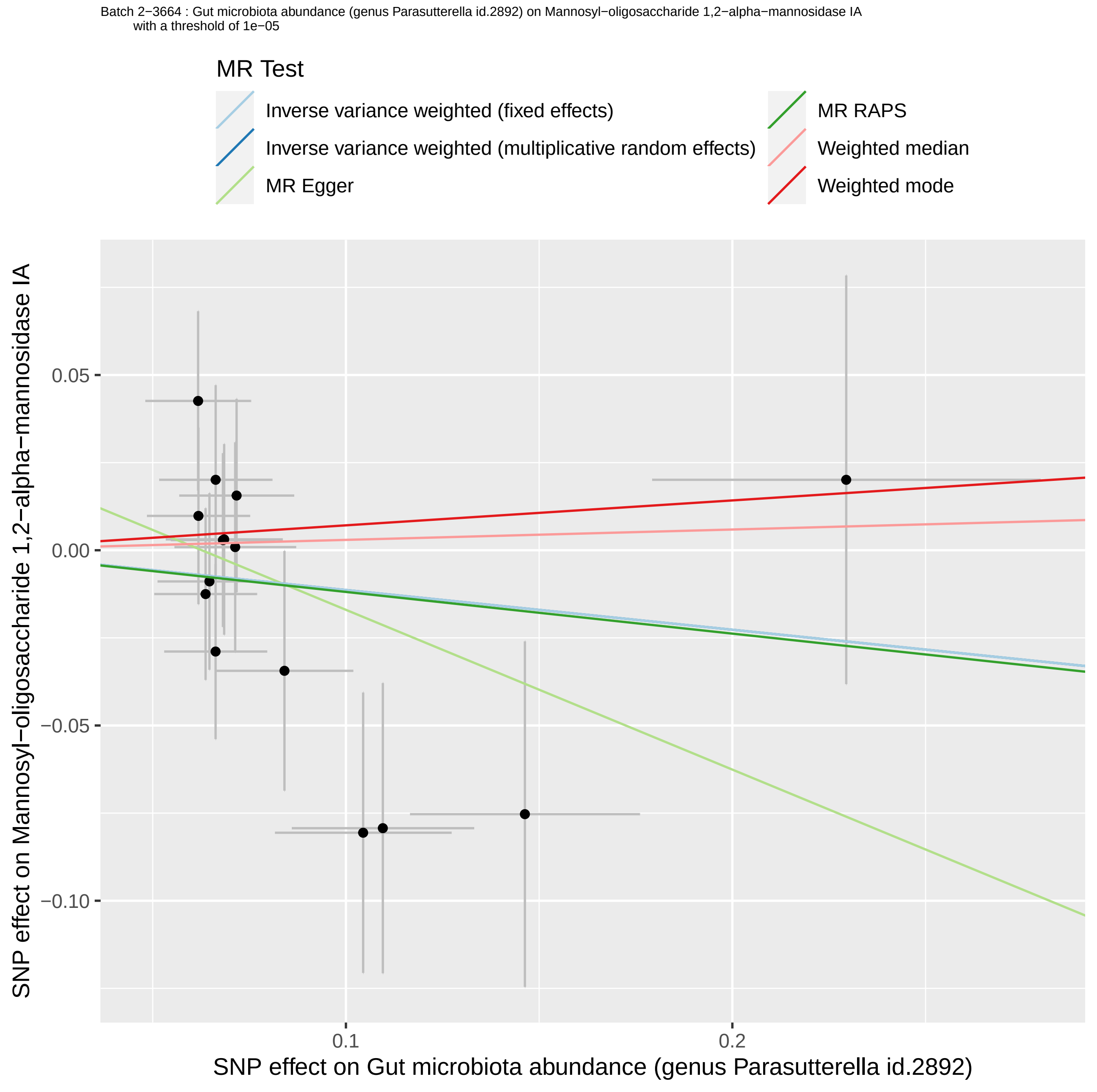

Batch 2−3664 : Gut microbiota abundance (genus Parasutterella id.2892) on Mannosyl−oligosaccharide 1,2−alpha−mannosidase IA
with a threshold of 1e−05
MR Test
Inverse variance weighted (fixed effects)
Inverse variance weighted (multiplicative random effects)
MR Egger
MR RAPS
Weighted median
Weighted mode
0.05
0.00
−0.05
−0.10
0.1
0.2
SNP effect on Mannosyl−oligosaccharide 1,2−alpha−mannosidase IA
SNP effect on Gut microbiota abundance (genus Parasutterella id.2892)

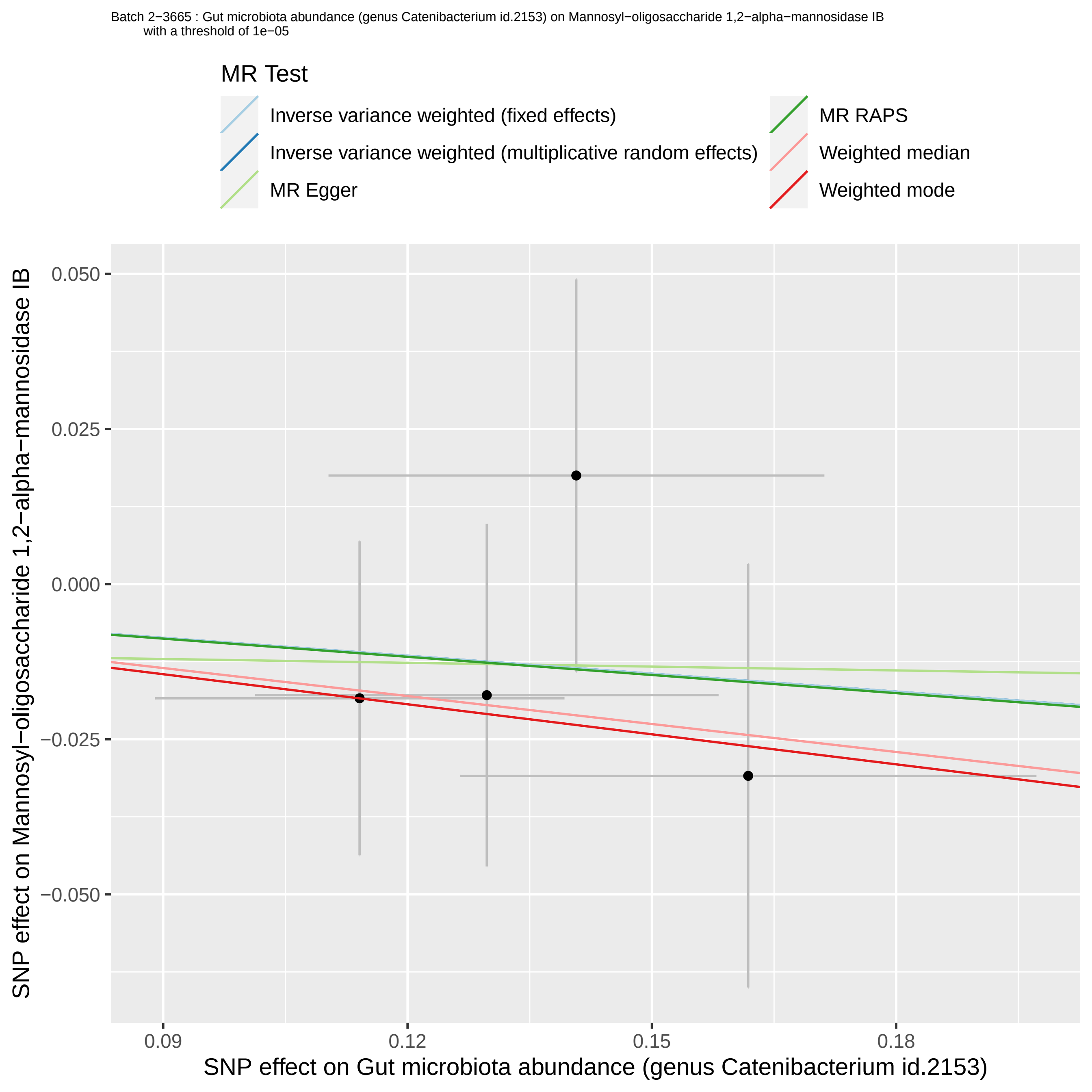
Batch 2−3665 : Gut microbiota abundance (genus Catenibacterium id.2153) on Mannosyl−oligosaccharide 1,2−alpha−mannosidase IB
with a threshold of 1e−05
MR Test
Inverse variance weighted (fixed effects)
Inverse variance weighted (multiplicative random effects)
MR Egger
MR RAPS
Weighted median
Weighted mode
0.050
0.025
0.000
−0.025
−0.050
0.09
0.12
0.15
0.18
SNP effect on Mannosyl−oligosaccharide 1,2−alpha−mannosidase IB
SNP effect on Gut microbiota abundance (genus Catenibacterium id.2153)

Batch 2−3666 : Gut microbiota abundance (genus Parasutterella id.2892) on Mannosyl−oligosaccharide 1,2−alpha−mannosidase IB
with a threshold of 1e−05

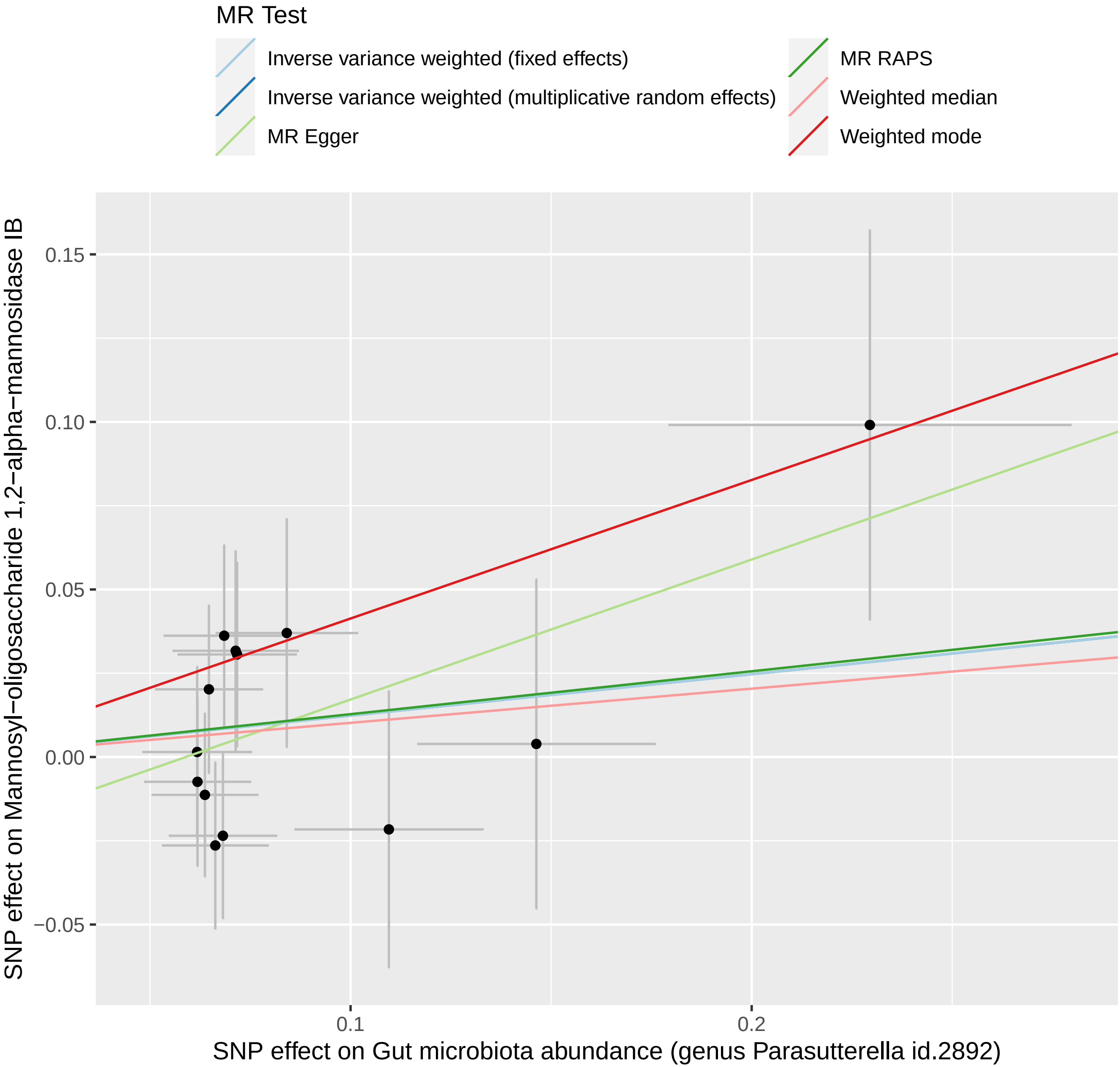

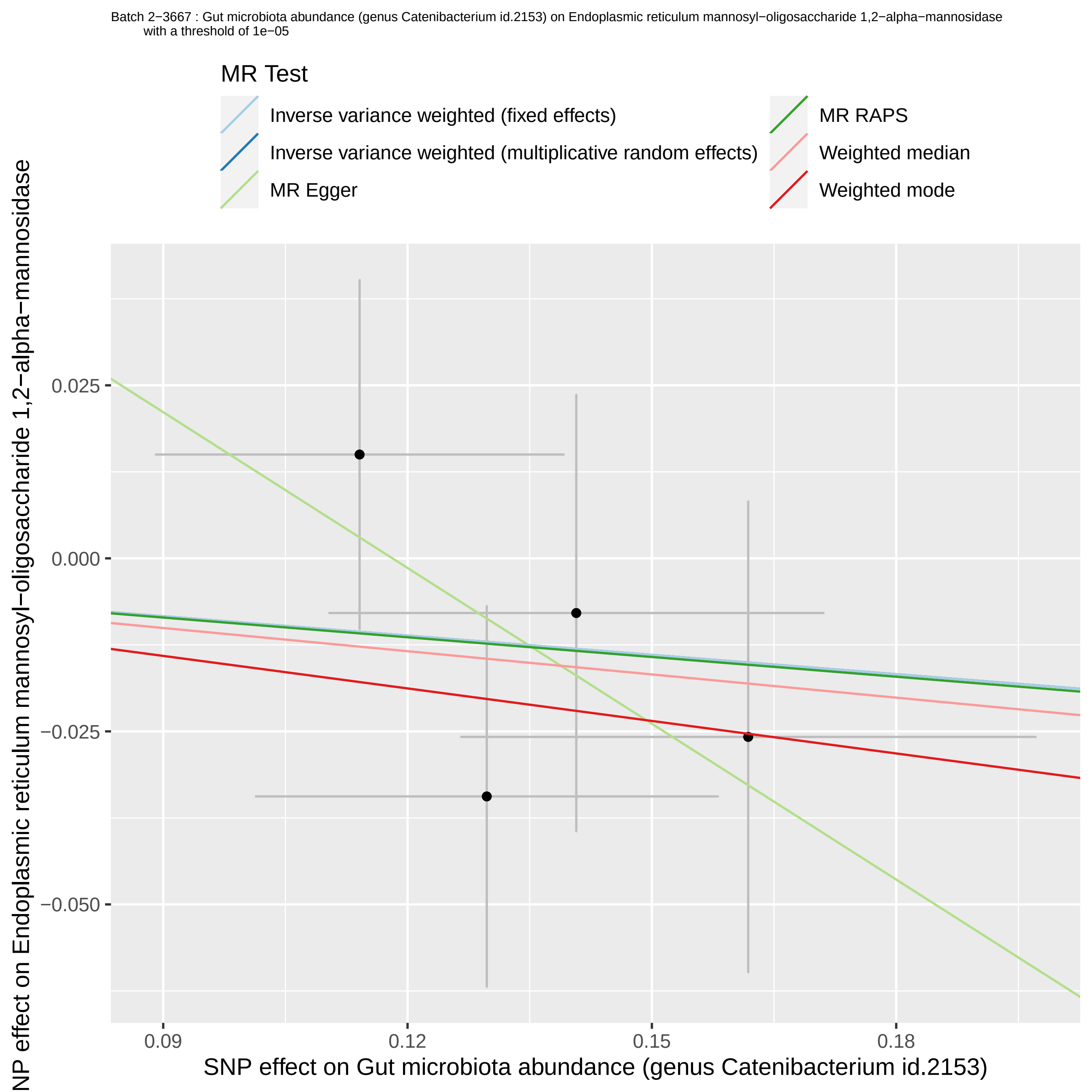
Batch 2−3667 : Gut microbiota abundance (genus Catenibacterium id.2153) on Endoplasmic reticulum mannosyl−oligosaccharide 1,2−alpha−mannosidase with a threshold of 1e−05
MR Test
Inverse variance weighted (fixed effects)
Inverse variance weighted (multiplicative random effects)
MR Egger
MR RAPS
Weighted median
Weighted mode
SNP effect on Endoplasmic reticulum mannosyl−oligosaccharide 1,2−alpha−mannosidase
0.025
0.000
−0.025
−0.050
0.09
0.12
0.15
0.18
SNP effect on Gut microbiota abundance (genus Catenibacterium id.2153)

Batch 2−3668 : Gut microbiota abundance (genus Parasutterella id.2892) on Endoplasmic reticulum mannosyl−oligosaccharide 1,2−alpha−mannosidase
with a threshold of 1e−05

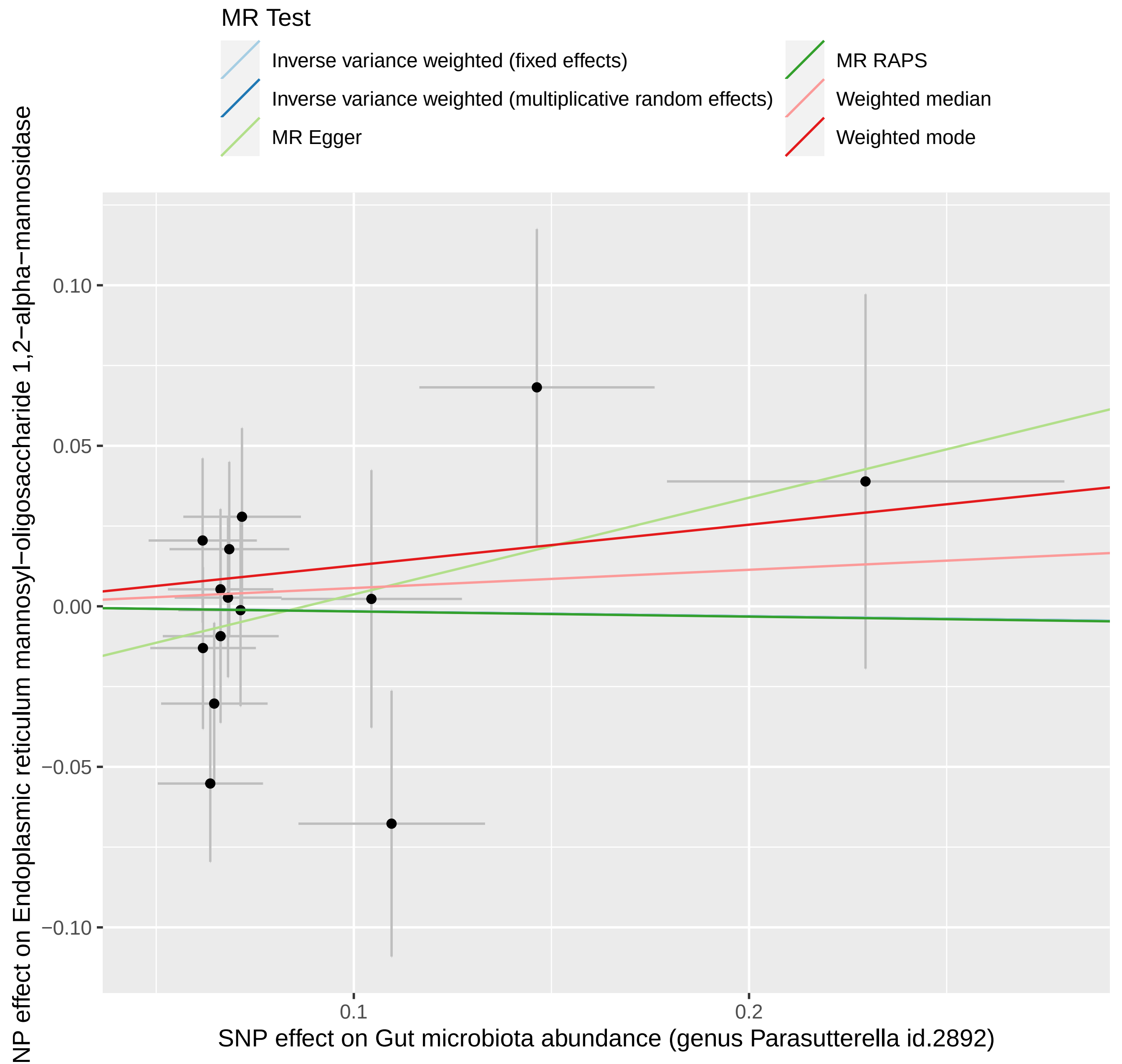

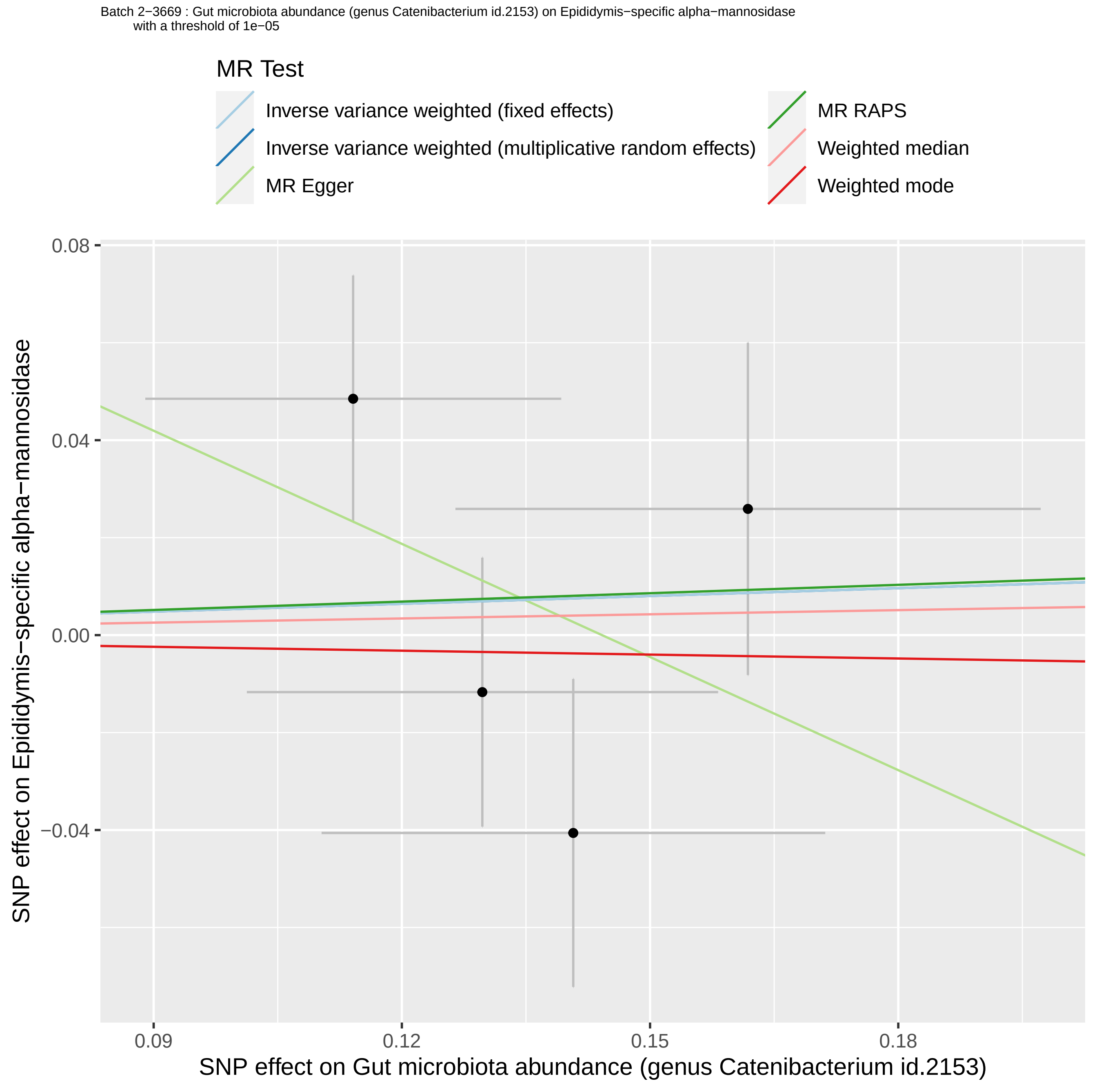

Batch 2−3669 : Gut microbiota abundance (genus Catenibacterium id.2153) on Epididymis−specific alpha−mannosidase
with a threshold of 1e−05
MR Test
Inverse variance weighted (fixed effects)
Inverse variance weighted (multiplicative random effects)
MR Egger
MR RAPS
Weighted median
Weighted mode
0.08
0.04
0.00
−0.04
0.09
0.12
0.15
0.18
SNP effect on Epididymis−specific alpha−mannosidase
SNP effect on Gut microbiota abundance (genus Catenibacterium id.2153)

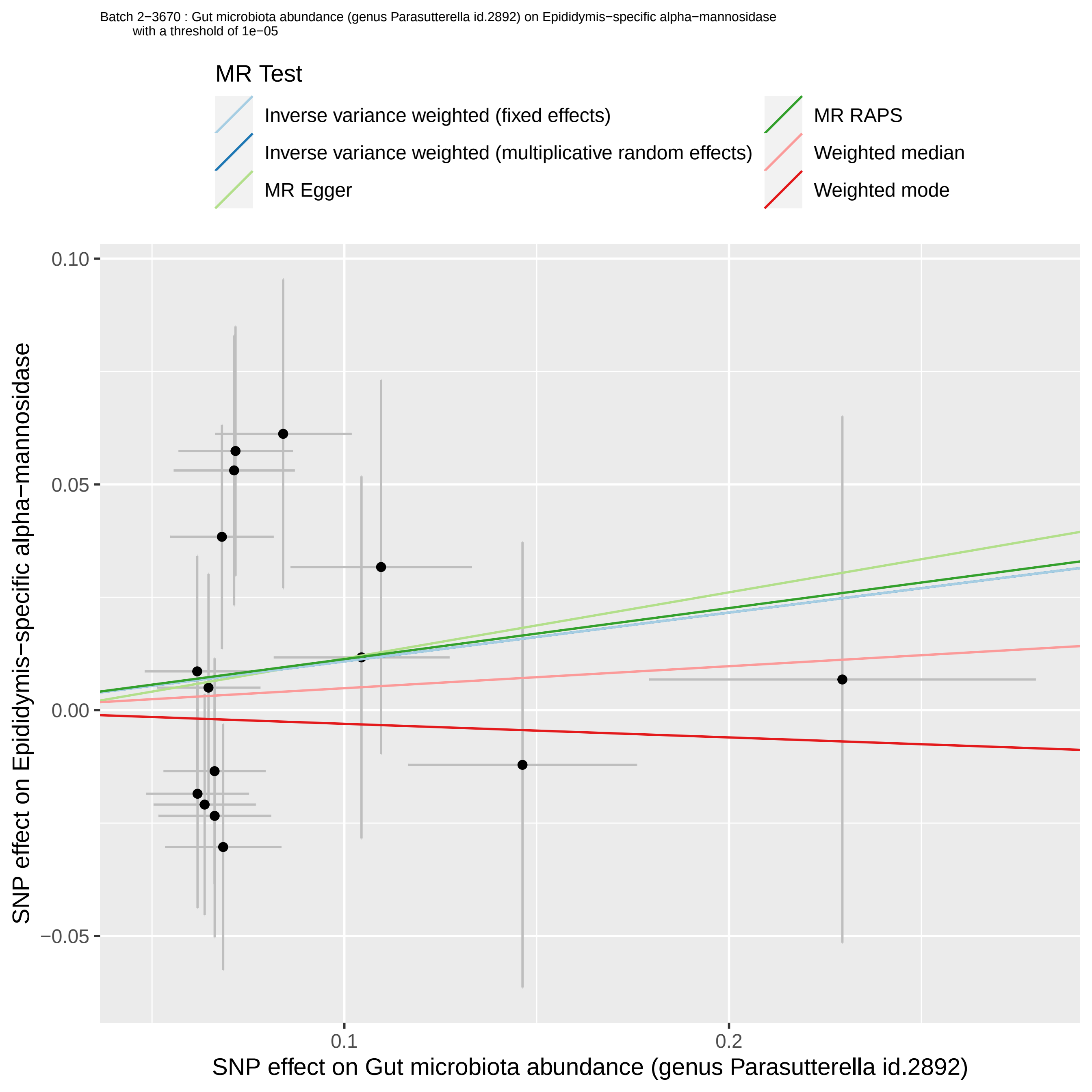

Batch 2−3670 : Gut microbiota abundance (genus Parasutterella id.2892) on Epididymis−specific alpha−mannosidase
with a threshold of 1e−05
MR Test
Inverse variance weighted (fixed effects)
Inverse variance weighted (multiplicative random effects)
MR Egger
MR RAPS
Weighted median
Weighted mode
0.10
0.05
0.00
−0.05
0.1
0.2
SNP effect on Epididymis−specific alpha−mannosidase
SNP effect on Gut microbiota abundance (genus Parasutterella id.2892)

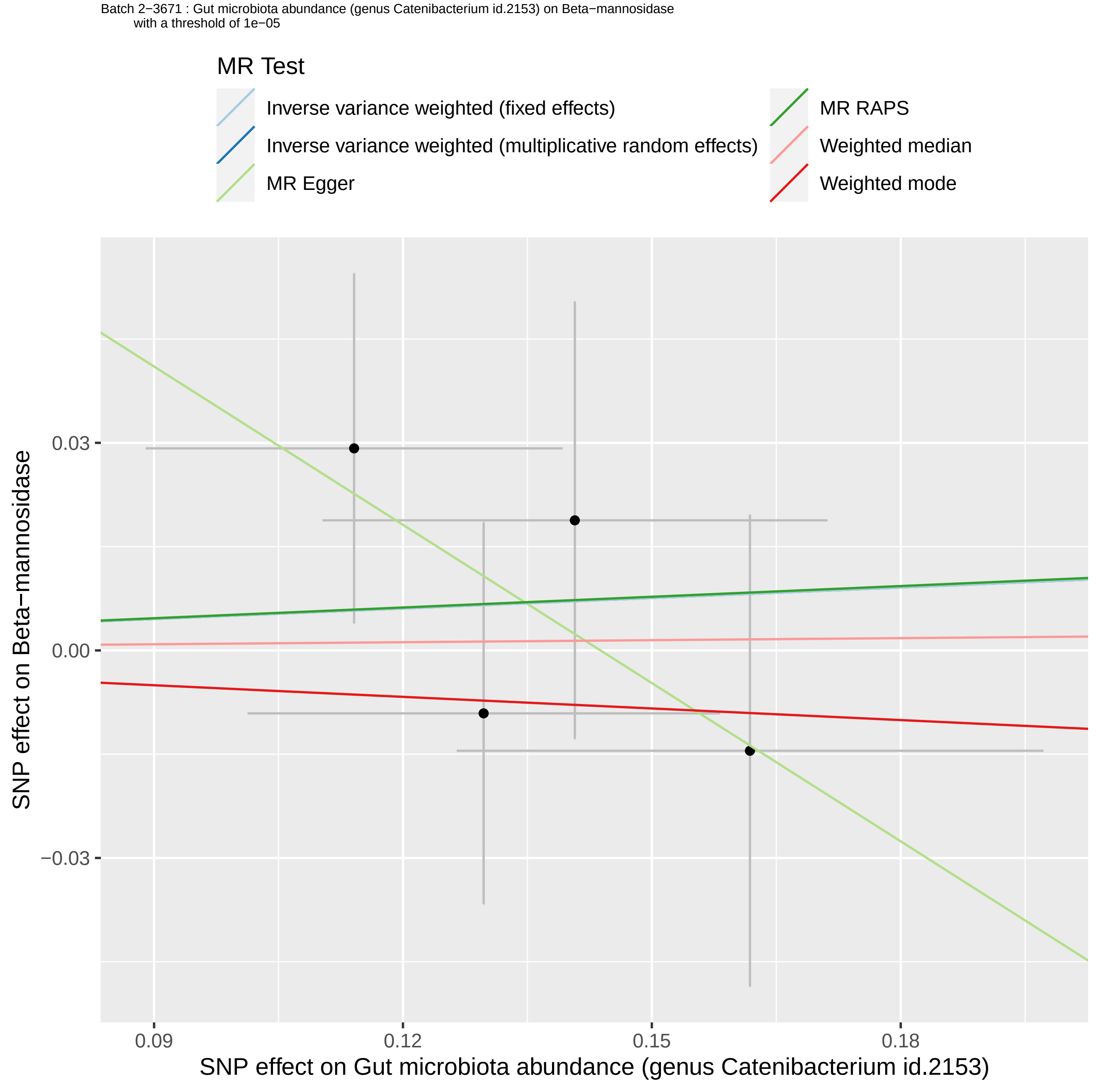

Batch 2−3671 : Gut microbiota abundance (genus Catenibacterium id.2153) on Beta−mannosidase
with a threshold of 1e−05
MR Test
Inverse variance weighted (fixed effects)
Inverse variance weighted (multiplicative random effects)
MR Egger
MR RAPS
Weighted median
Weighted mode
SNP effect on Beta−mannosidase
0.03
0.00
−0.03
0.09
0.12
0.15
0.18
SNP effect on Gut microbiota abundance (genus Catenibacterium id.2153)

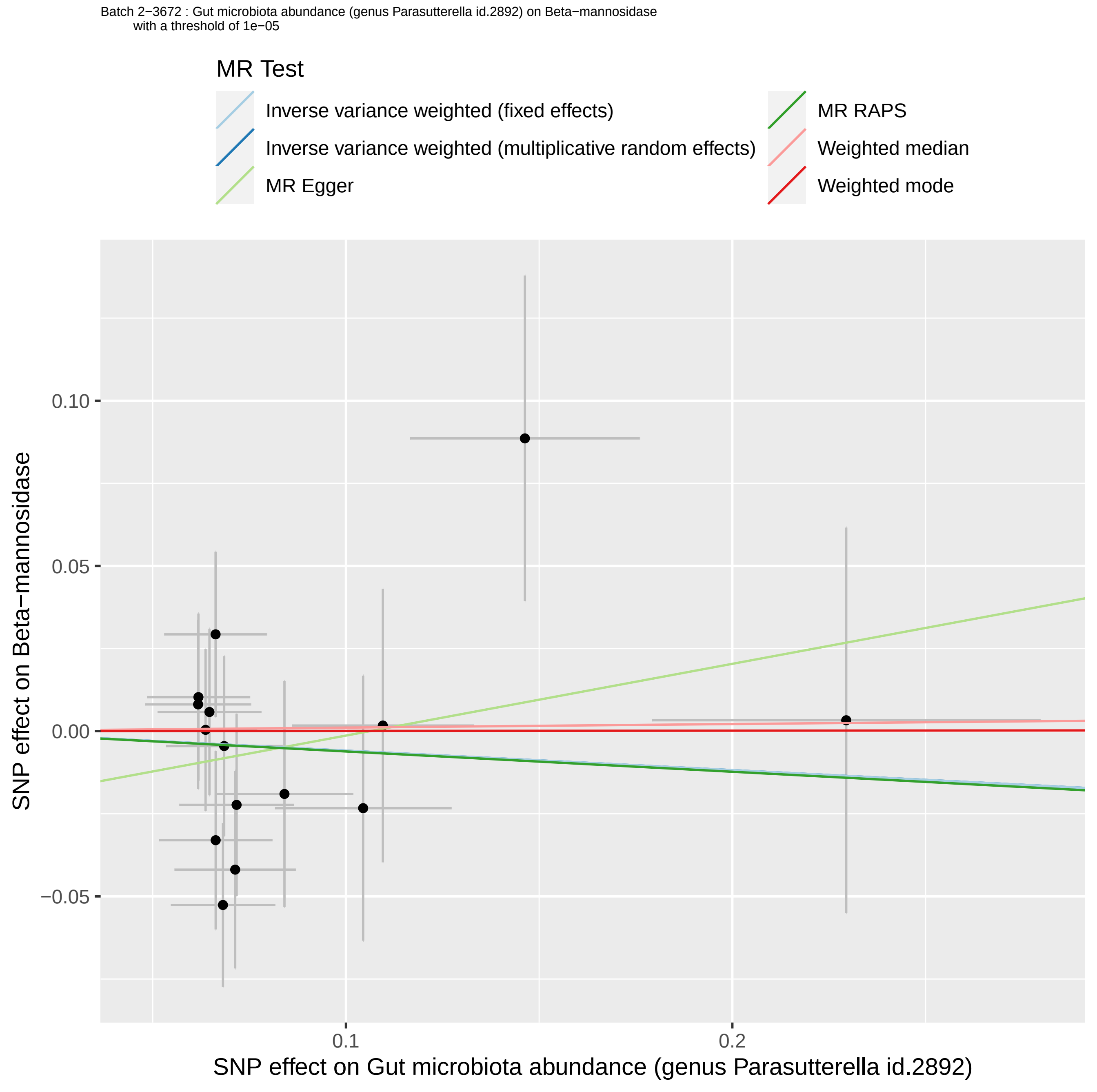
Batch 2−3672 : Gut microbiota abundance (genus Parasutterella id.2892) on Beta−mannosidase
with a threshold of 1e−05
MR Test
Inverse variance weighted (fixed effects)
Inverse variance weighted (multiplicative random effects)
MR Egger
MR RAPS
Weighted median
Weighted mode
SNP effect on Beta−mannosidase
0.10
0.05
0.00
−0.05
0.1
0.2
SNP effect on Gut microbiota abundance (genus Parasutterella id.2892)

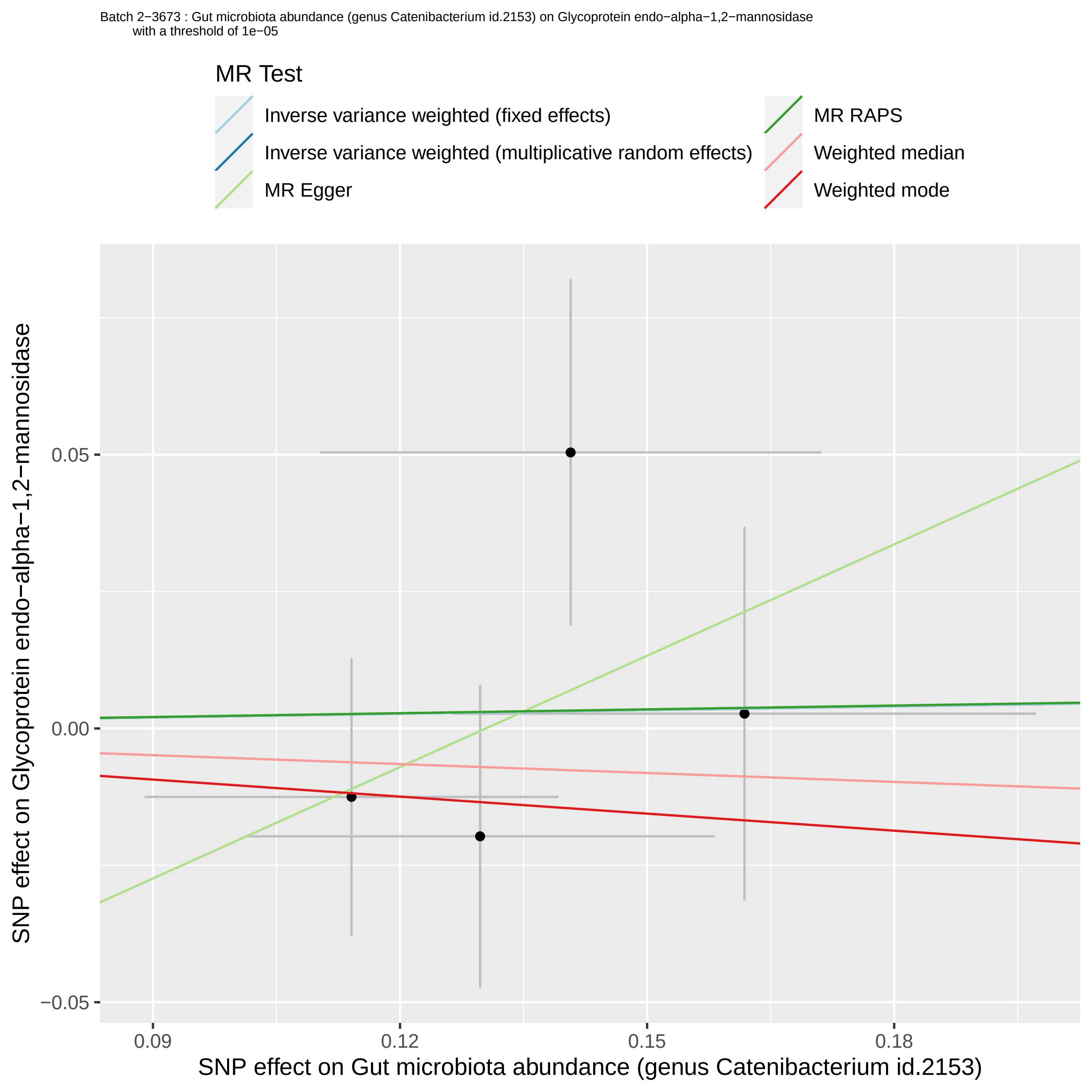

Batch 2−3673 : Gut microbiota abundance (genus Catenibacterium id.2153) on Glycoprotein endo−alpha−1,2−mannosidase
with a threshold of 1e−05
MR Test
Inverse variance weighted (fixed effects)
Inverse variance weighted (multiplicative random effects)
MR Egger
MR RAPS
Weighted median
Weighted mode
0.05
0.00
−0.05
0.09
0.12
0.15
0.18
SNP effect on Glycoprotein endo−alpha−1,2−mannosidase
SNP effect on Gut microbiota abundance (genus Catenibacterium id.2153)

Batch 2−3674 : Gut microbiota abundance (genus Parasutterella id.2892) on Glycoprotein endo−alpha−1,2−mannosidase
with a threshold of 1e−05

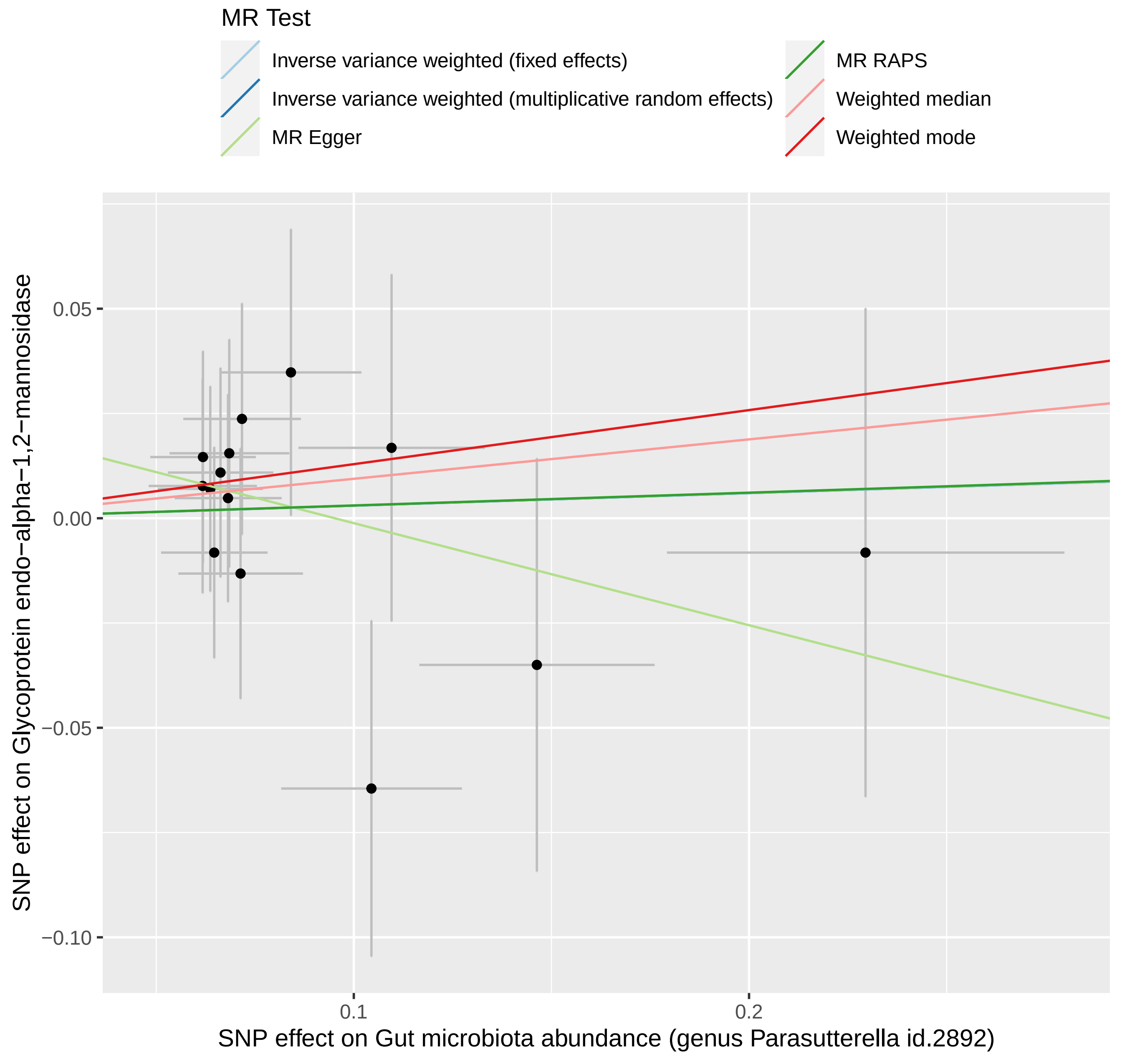

Batch 2−3675 : Gut microbiota abundance (genus Catenibacterium id.2153) on MANSC domain−containing protein 1
with a threshold of 1e−05

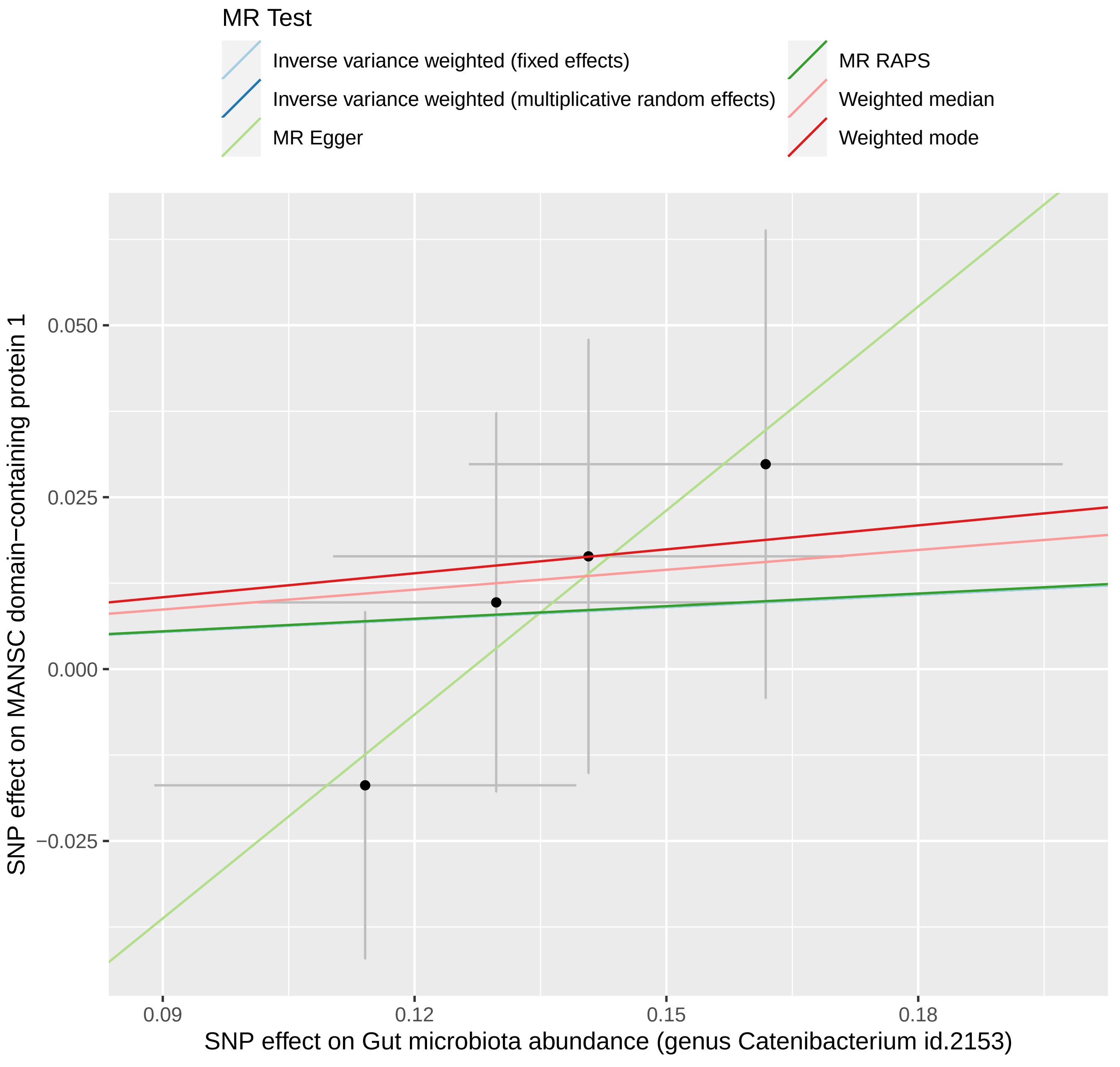

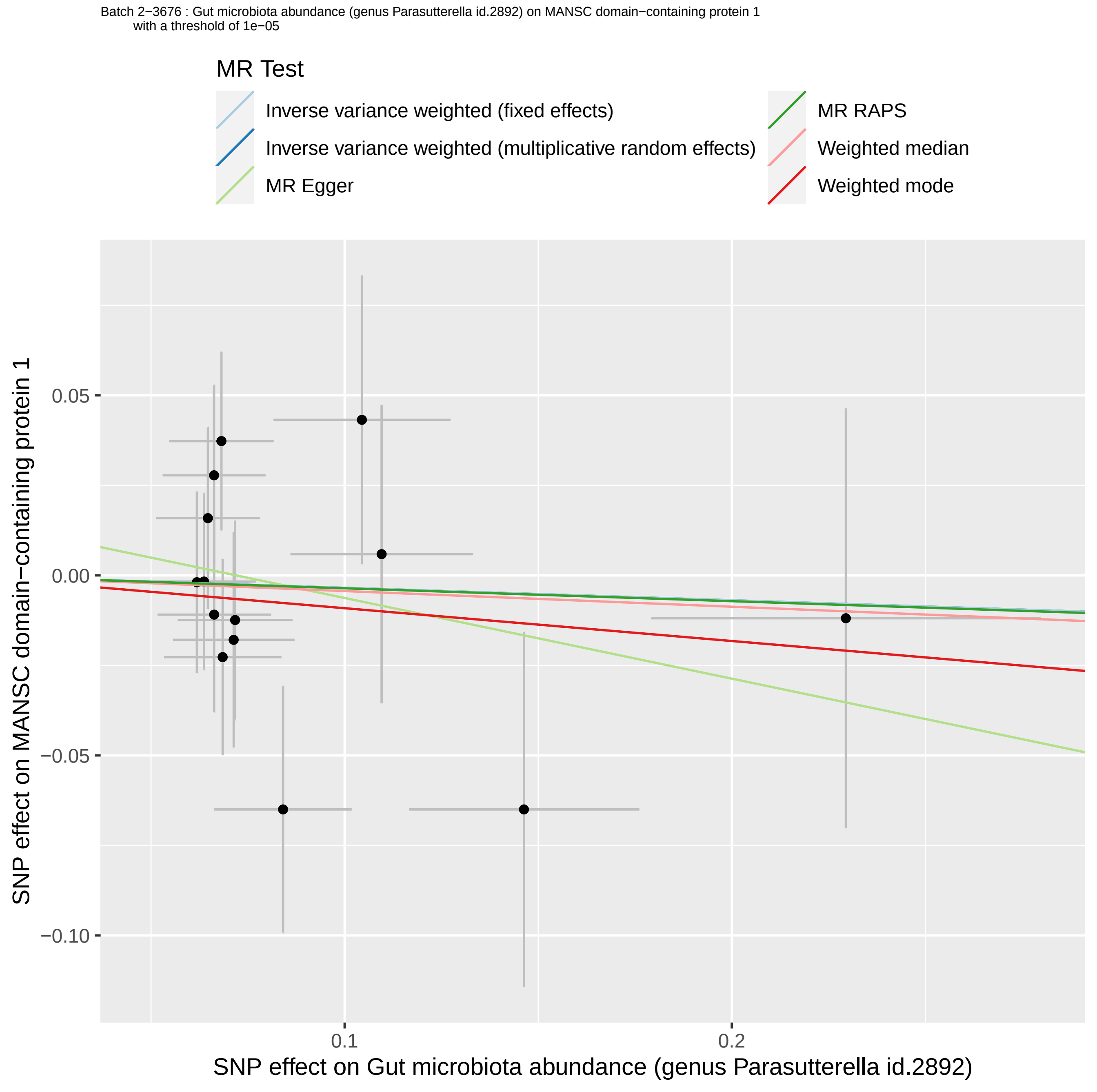

Batch 2−3676 : Gut microbiota abundance (genus Parasutterella id.2892) on MANSC domain−containing protein 1
with a threshold of 1e−05
MR Test
Inverse variance weighted (fixed effects)
Inverse variance weighted (multiplicative random effects)
MR Egger
MR RAPS
Weighted median
Weighted mode
0.05
0.00
−0.05
−0.10
0.1
0.2
SNP effect on MANSC domain−containing protein 1
SNP effect on Gut microbiota abundance (genus Parasutterella id.2892)

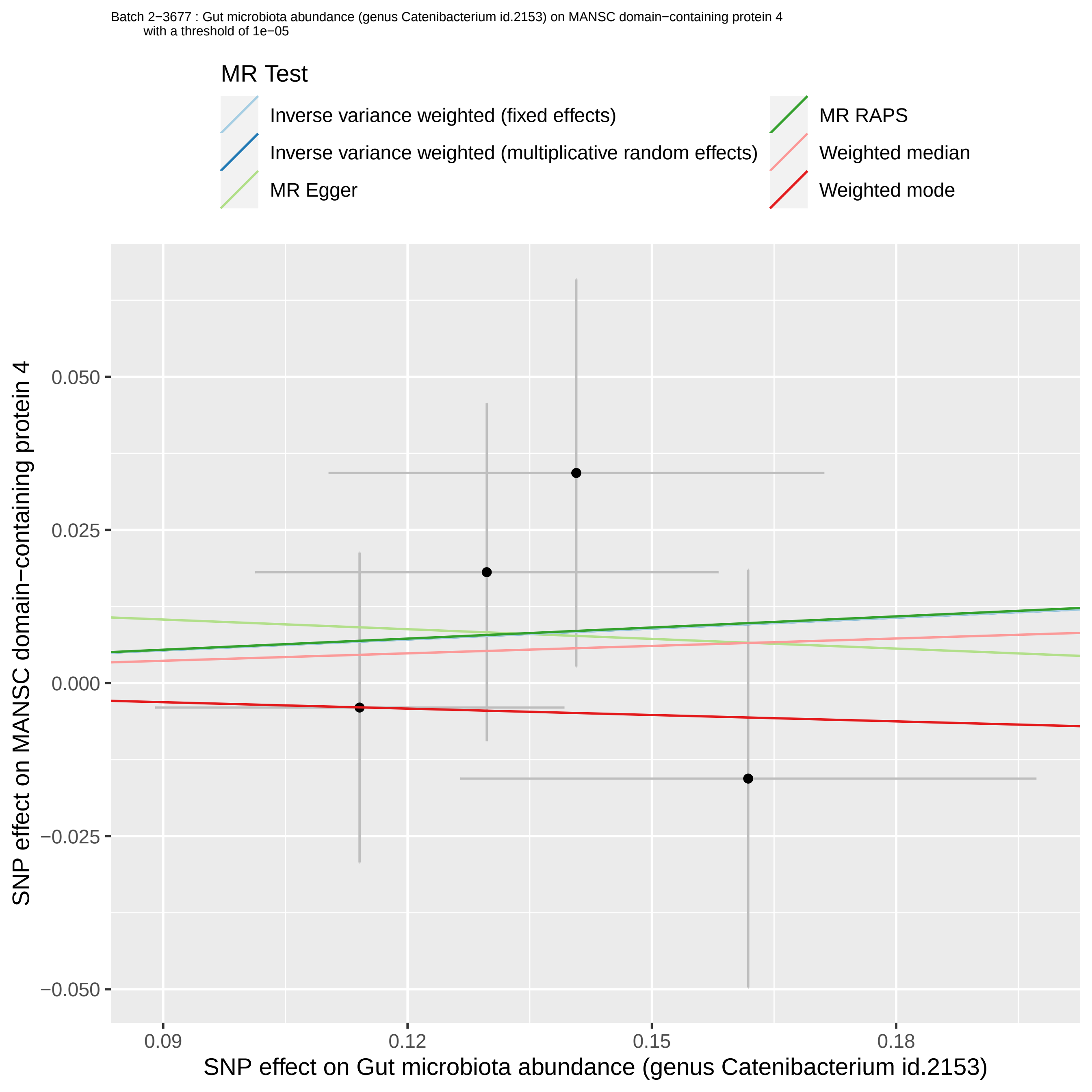

Batch 2−3677 : Gut microbiota abundance (genus Catenibacterium id.2153) on MANSC domain−containing protein 4 with a threshold of 1e−05
MR Test
Inverse variance weighted (fixed effects)
Inverse variance weighted (multiplicative random effects)
MR Egger
MR RAPS
Weighted median
Weighted mode
SNP effect on MANSC domain−containing protein 4
0.050
0.025
0.000
−0.025
−0.050
0.09
0.12
0.15
0.18
SNP effect on Gut microbiota abundance (genus Catenibacterium id.2153)

Batch 2−3678 : Gut microbiota abundance (genus Parasutterella id.2892) on MANSC domain−containing protein 4
with a threshold of 1e−05

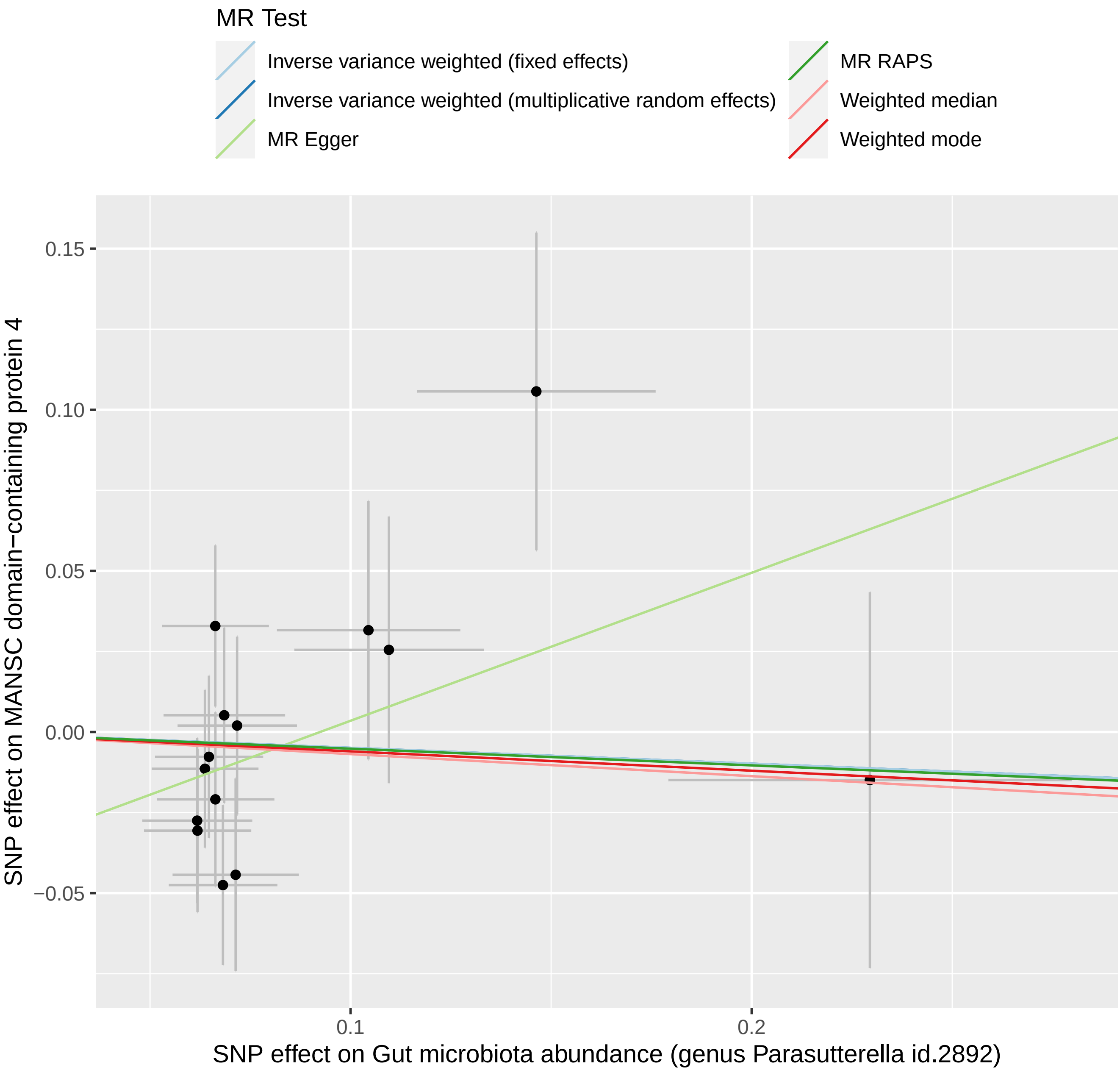

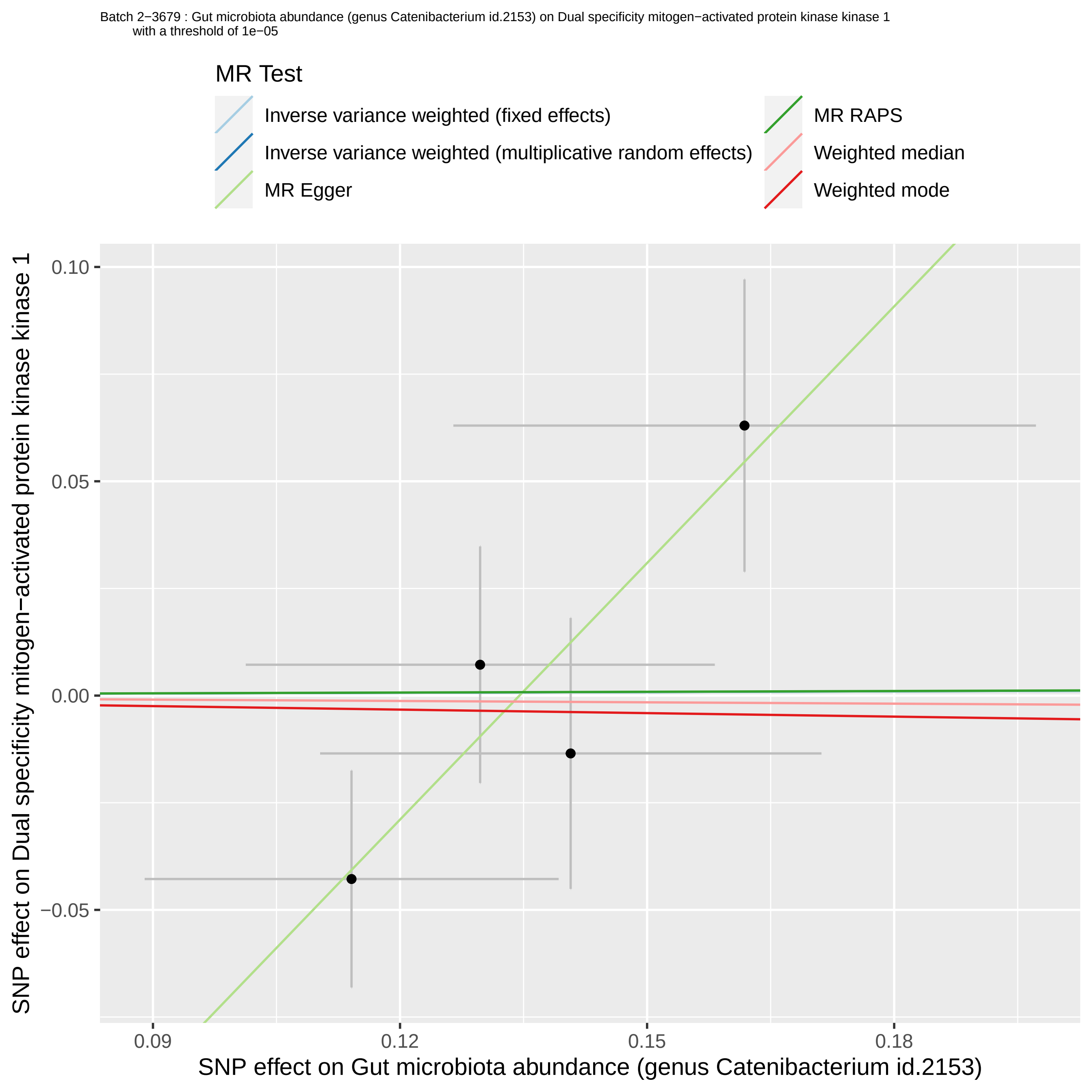

Batch 2−3679 : Gut microbiota abundance (genus Catenibacterium id.2153) on Dual specificity mitogen−activated protein kinase kinase 1
with a threshold of 1e−05
MR Test
Inverse variance weighted (fixed effects)
Inverse variance weighted (multiplicative random effects)
MR Egger
MR RAPS
Weighted median
Weighted mode
0.10
0.05
0.00
−0.05
0.09
0.12
0.15
0.18
SNP effect on Dual specificity mitogen−activated protein kinase kinase 1
SNP effect on Gut microbiota abundance (genus Catenibacterium id.2153)

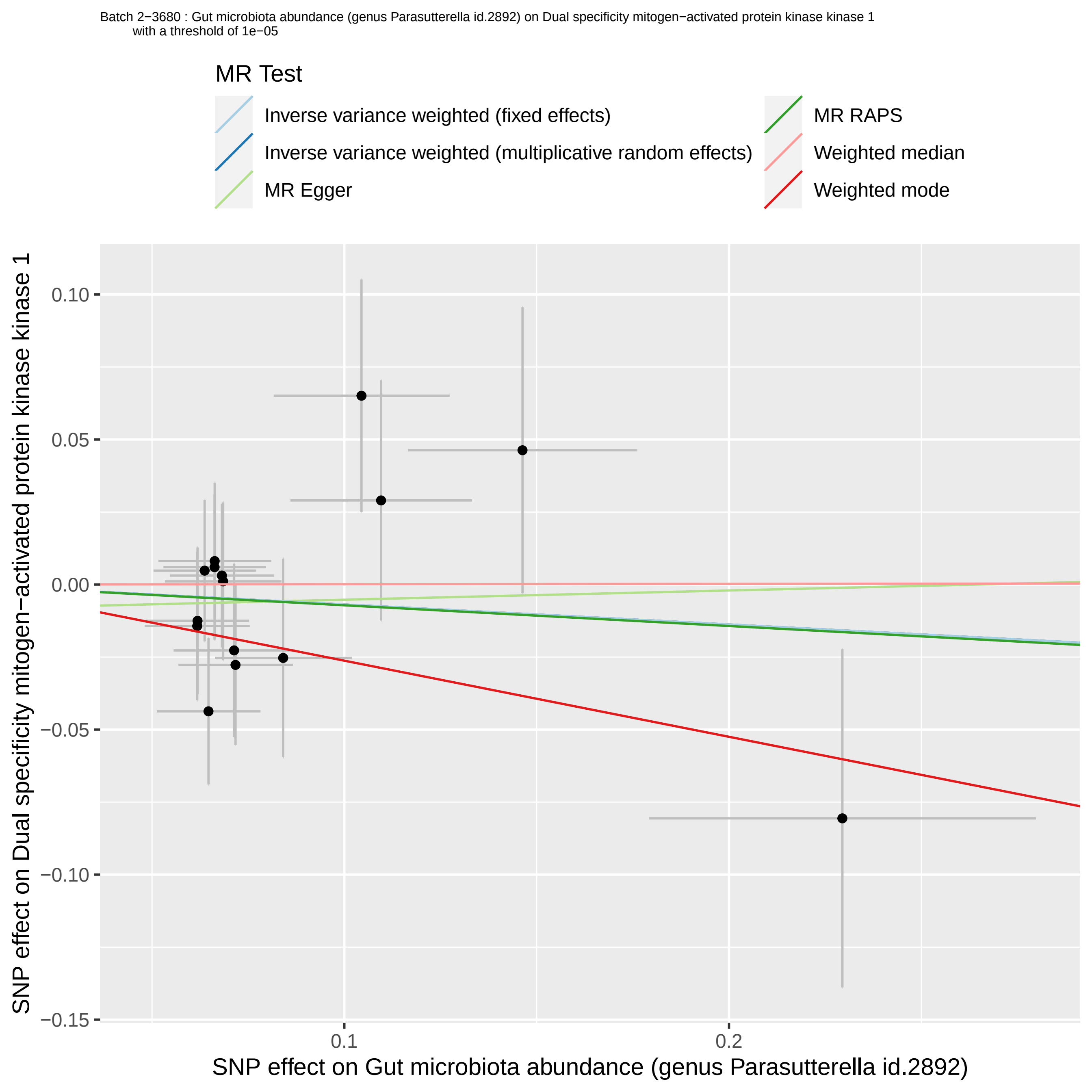
Batch 2−3680 : Gut microbiota abundance (genus Parasutterella id.2892) on Dual specificity mitogen−activated protein kinase kinase 1
with a threshold of 1e−05
MR Test
Inverse variance weighted (fixed effects)
Inverse variance weighted (multiplicative random effects)
MR Egger
MR RAPS
Weighted median
Weighted mode
SNP effect on Dual specificity mitogen−activated protein kinase kinase 1
0.10
0.05
0.00
−0.05
−0.10
−0.15
0.1
0.2
SNP effect on Gut microbiota abundance (genus Parasutterella id.2892)

Batch 2−3681 : Gut microbiota abundance (genus Catenibacterium id.2153) on Dual specificity mitogen−activated protein kinase kinase 3
with a threshold of 1e−05

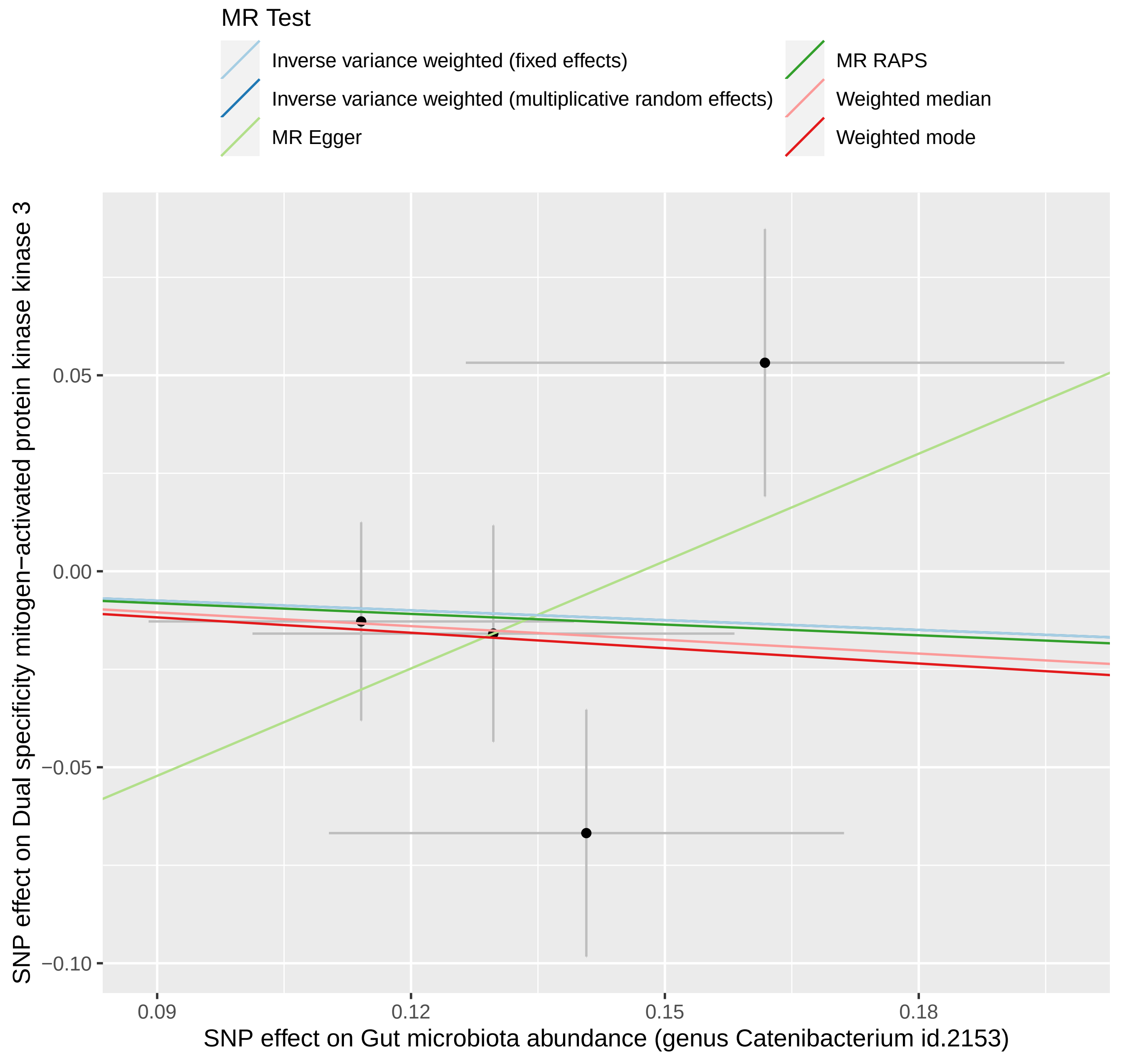

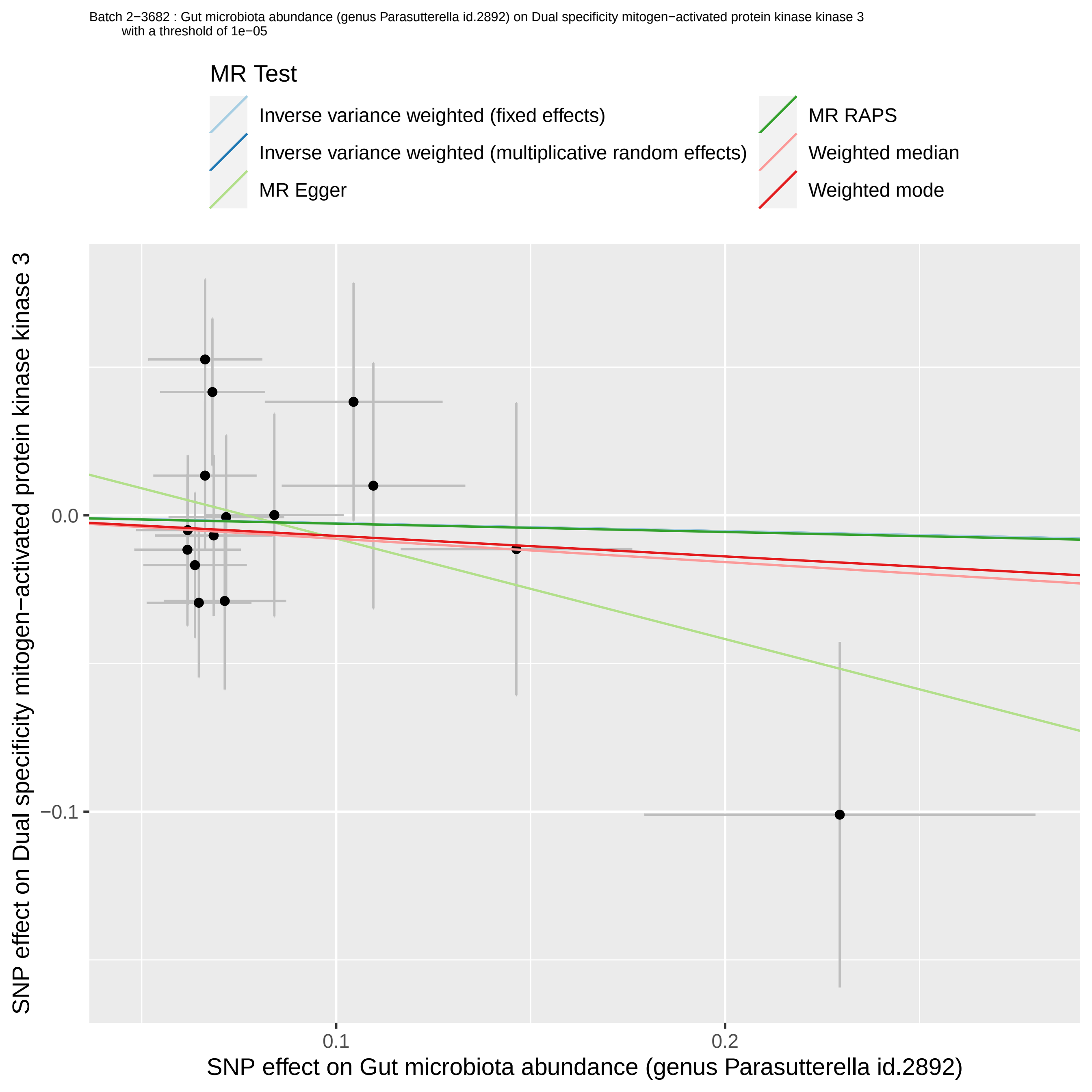

Batch 2−3682 : Gut microbiota abundance (genus Parasutterella id.2892) on Dual specificity mitogen−activated protein kinase kinase 3
with a threshold of 1e−05
MR Test
Inverse variance weighted (fixed effects)
Inverse variance weighted (multiplicative random effects)
MR Egger
MR RAPS
Weighted median
Weighted mode
0.0
−0.1
0.1
0.2
SNP effect on Dual specificity mitogen−activated protein kinase kinase 3
SNP effect on Gut microbiota abundance (genus Parasutterella id.2892)

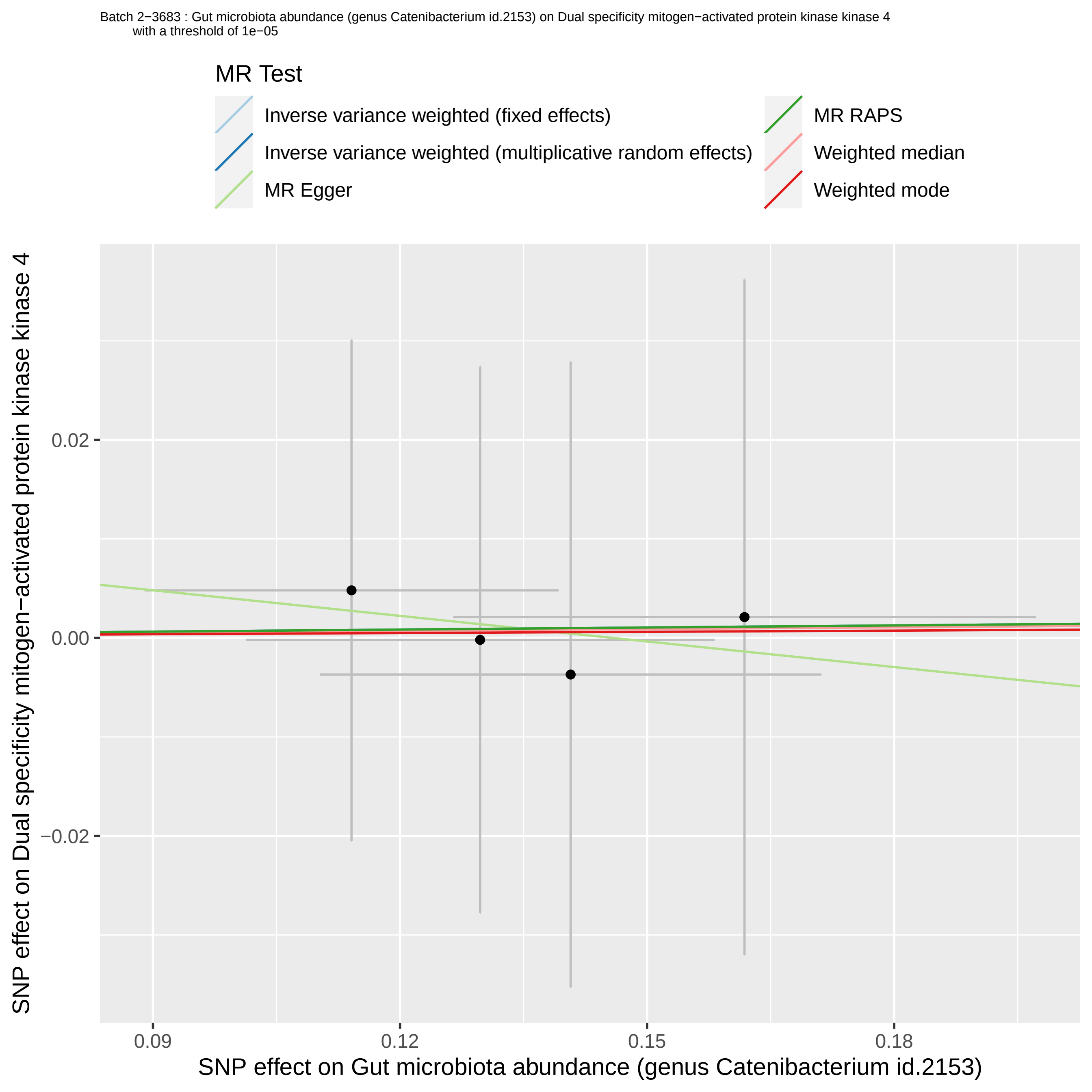

Batch 2−3683 : Gut microbiota abundance (genus Catenibacterium id.2153) on Dual specificity mitogen−activated protein kinase kinase 4
with a threshold of 1e−05
MR Test
Inverse variance weighted (fixed effects)
Inverse variance weighted (multiplicative random effects)
MR Egger
MR RAPS
Weighted median
Weighted mode
0.02
0.00
−0.02
0.09
0.12
0.15
0.18
SNP effect on Dual specificity mitogen−activated protein kinase kinase 4
SNP effect on Gut microbiota abundance (genus Catenibacterium id.2153)

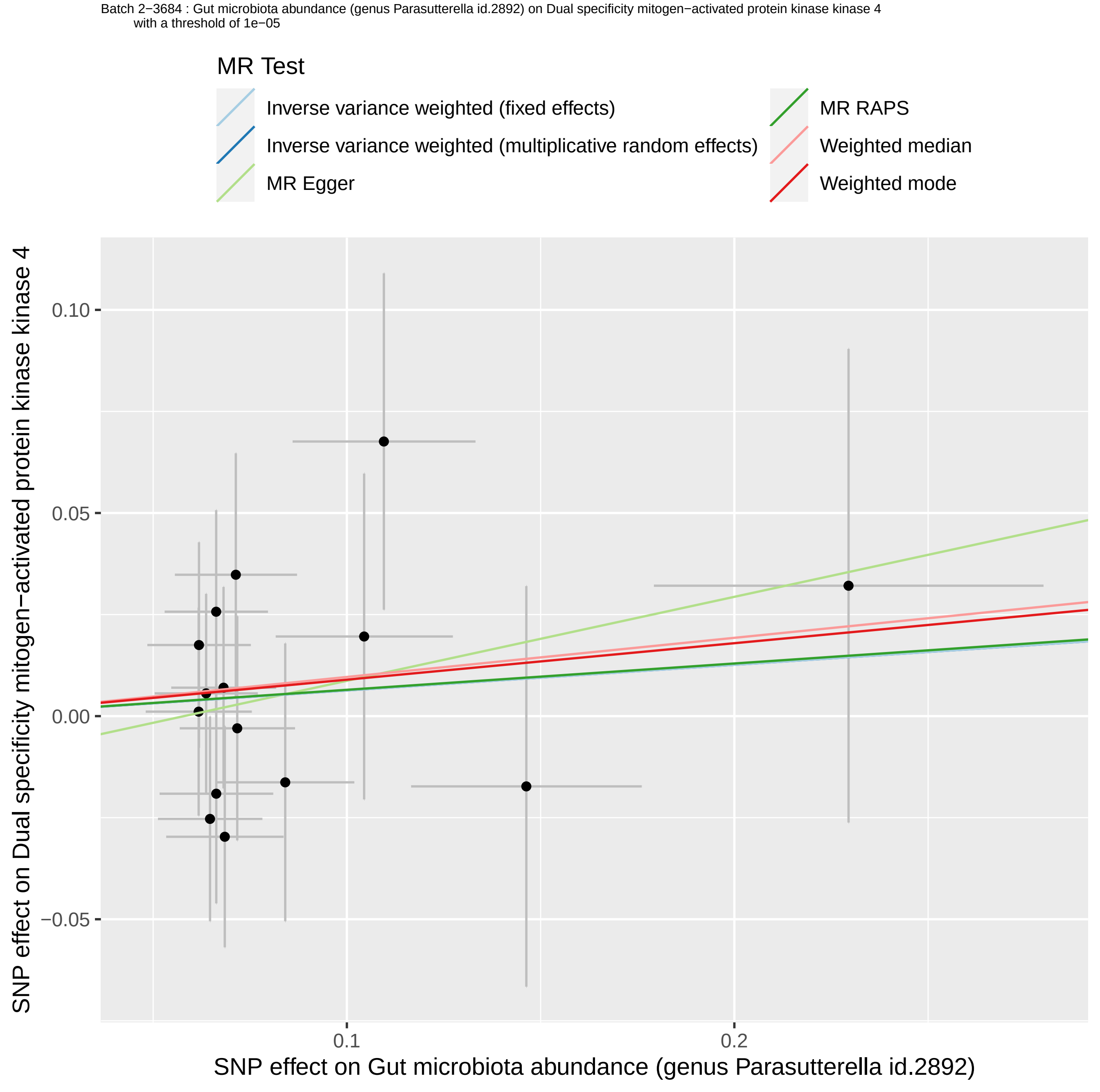
Batch 2−3684 : Gut microbiota abundance (genus Parasutterella id.2892) on Dual specificity mitogen−activated protein kinase kinase 4
with a threshold of 1e−05
MR Test
Inverse variance weighted (fixed effects)
Inverse variance weighted (multiplicative random effects)
MR Egger
MR RAPS
Weighted median
Weighted mode
SNP effect on Dual specificity mitogen−activated protein kinase kinase 4
0.10
0.05
0.00
−0.05
0.1
0.2
SNP effect on Gut microbiota abundance (genus Parasutterella id.2892)

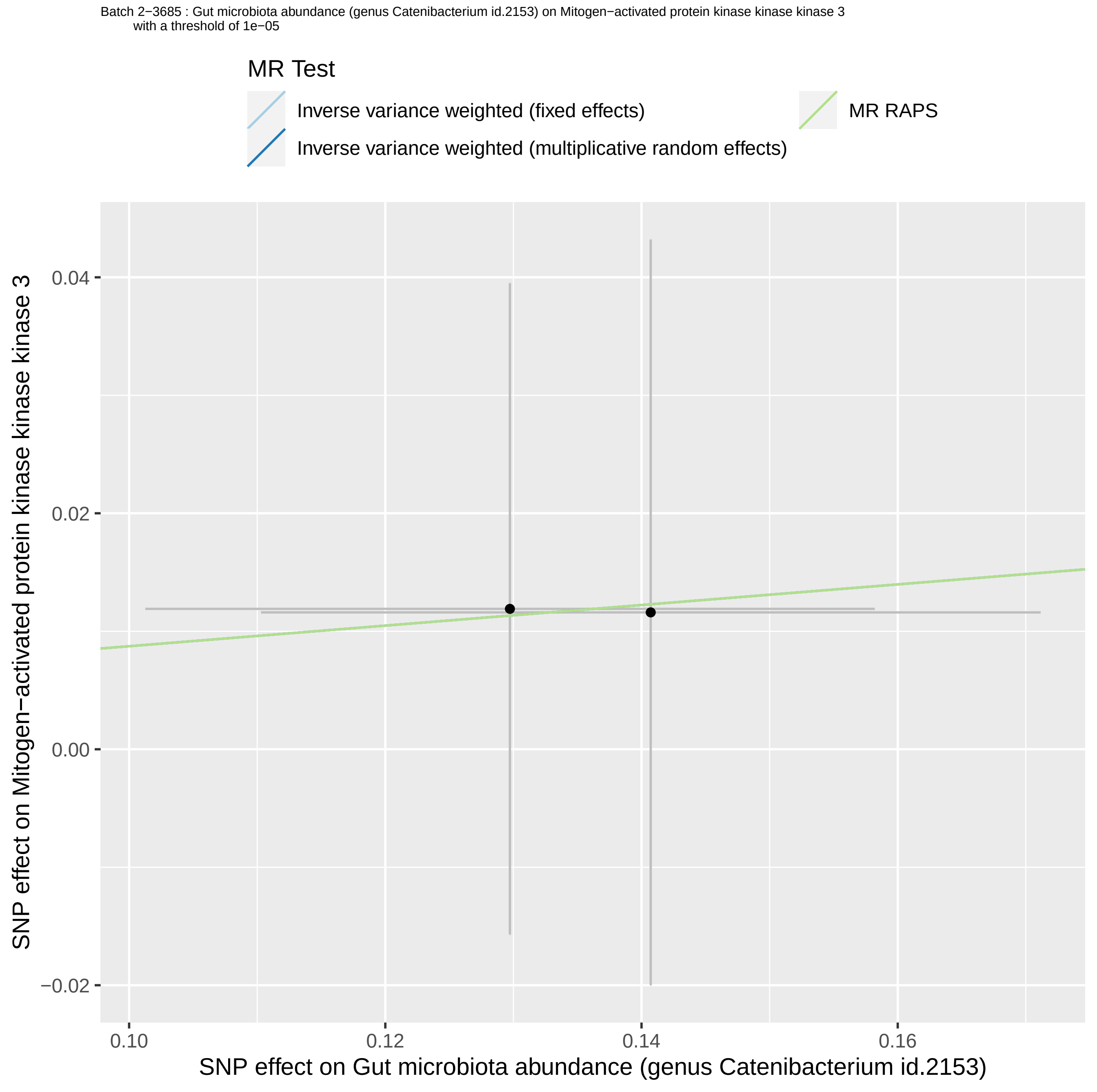

Batch 2−3685 : Gut microbiota abundance (genus Catenibacterium id.2153) on Mitogen−activated protein kinase kinase kinase 3
with a threshold of 1e−05
MR Test
Inverse variance weighted (fixed effects)
Inverse variance weighted (multiplicative random effects)
MR RAPS
0.04
0.02
0.00
−0.02
0.10
0.12
0.14
0.16
SNP effect on Mitogen−activated protein kinase kinase kinase 3
SNP effect on Gut microbiota abundance (genus Catenibacterium id.2153)

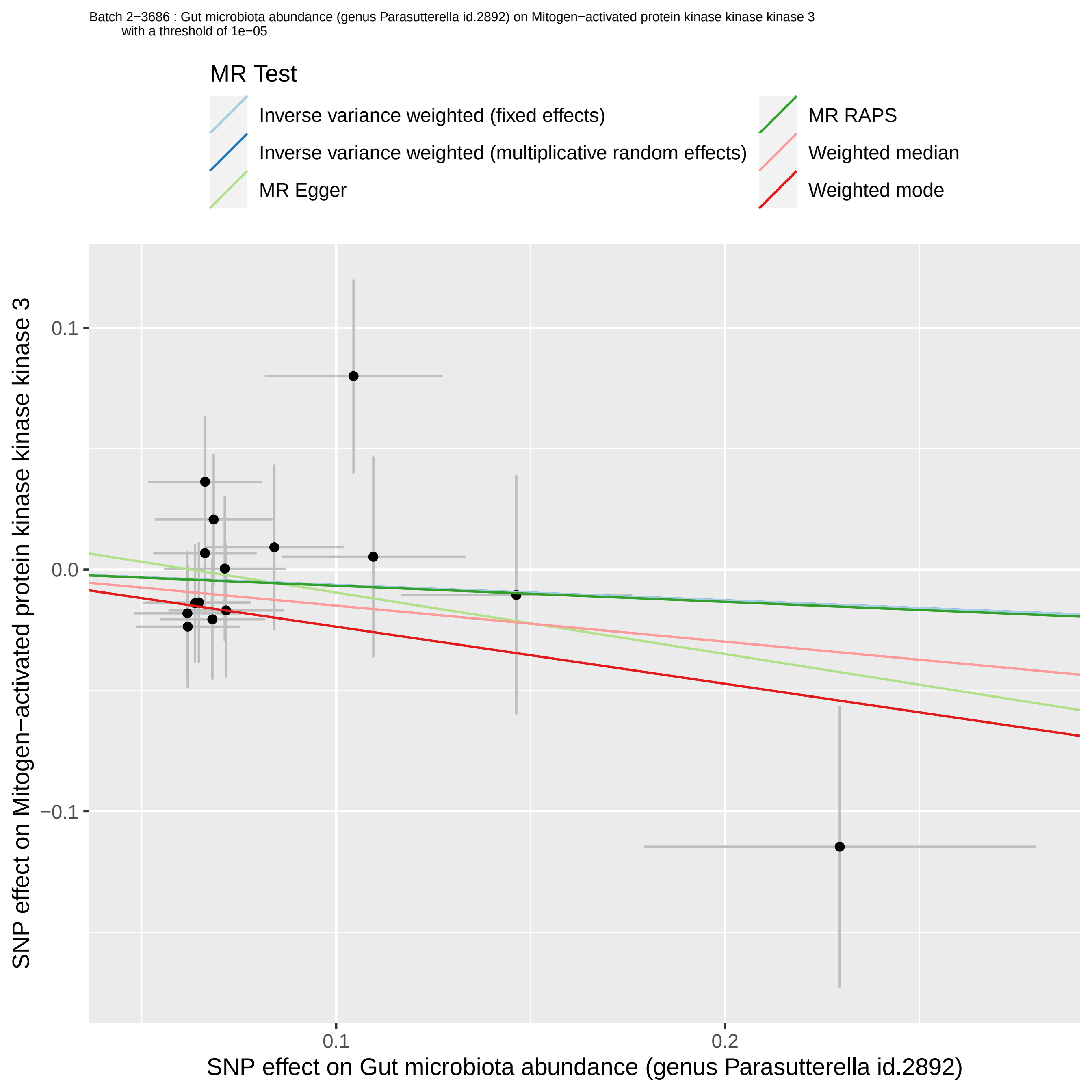
Batch 2−3686 : Gut microbiota abundance (genus Parasutterella id.2892) on Mitogen−activated protein kinase kinase kinase 3
with a threshold of 1e−05
MR Test
Inverse variance weighted (fixed effects)
Inverse variance weighted (multiplicative random effects)
MR Egger
MR RAPS
Weighted median
Weighted mode
0.1
0.0
−0.1
0.1
0.2
SNP effect on Mitogen−activated protein kinase kinase kinase 3
SNP effect on Gut microbiota abundance (genus Parasutterella id.2892)

Batch 2−3687 : Gut microbiota abundance (genus Catenibacterium id.2153) on Mitogen−activated protein kinase kinase kinase 7:TGF−beta−activated kinase 1 and MAP3K7
with a threshold of 1e−05

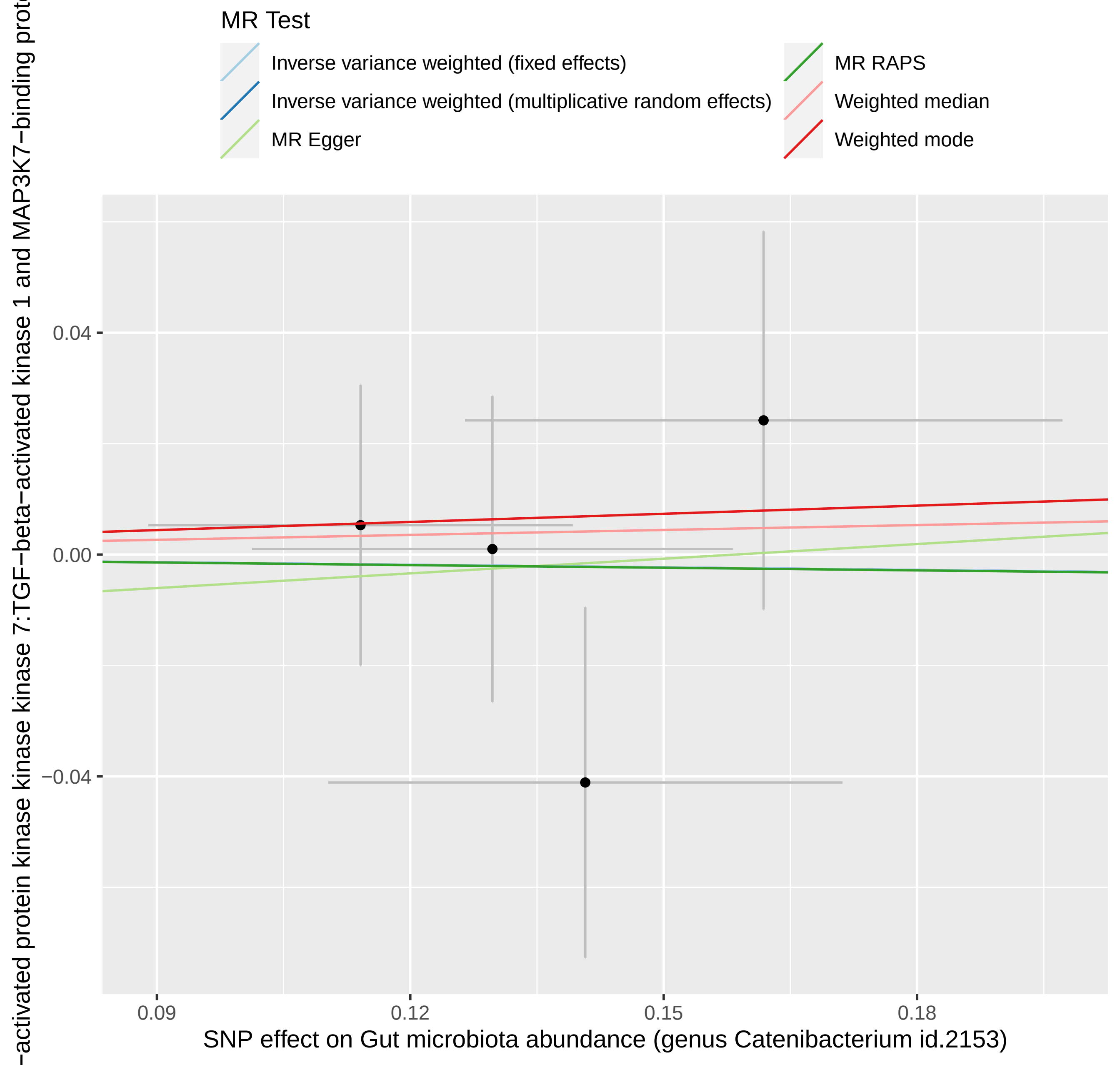

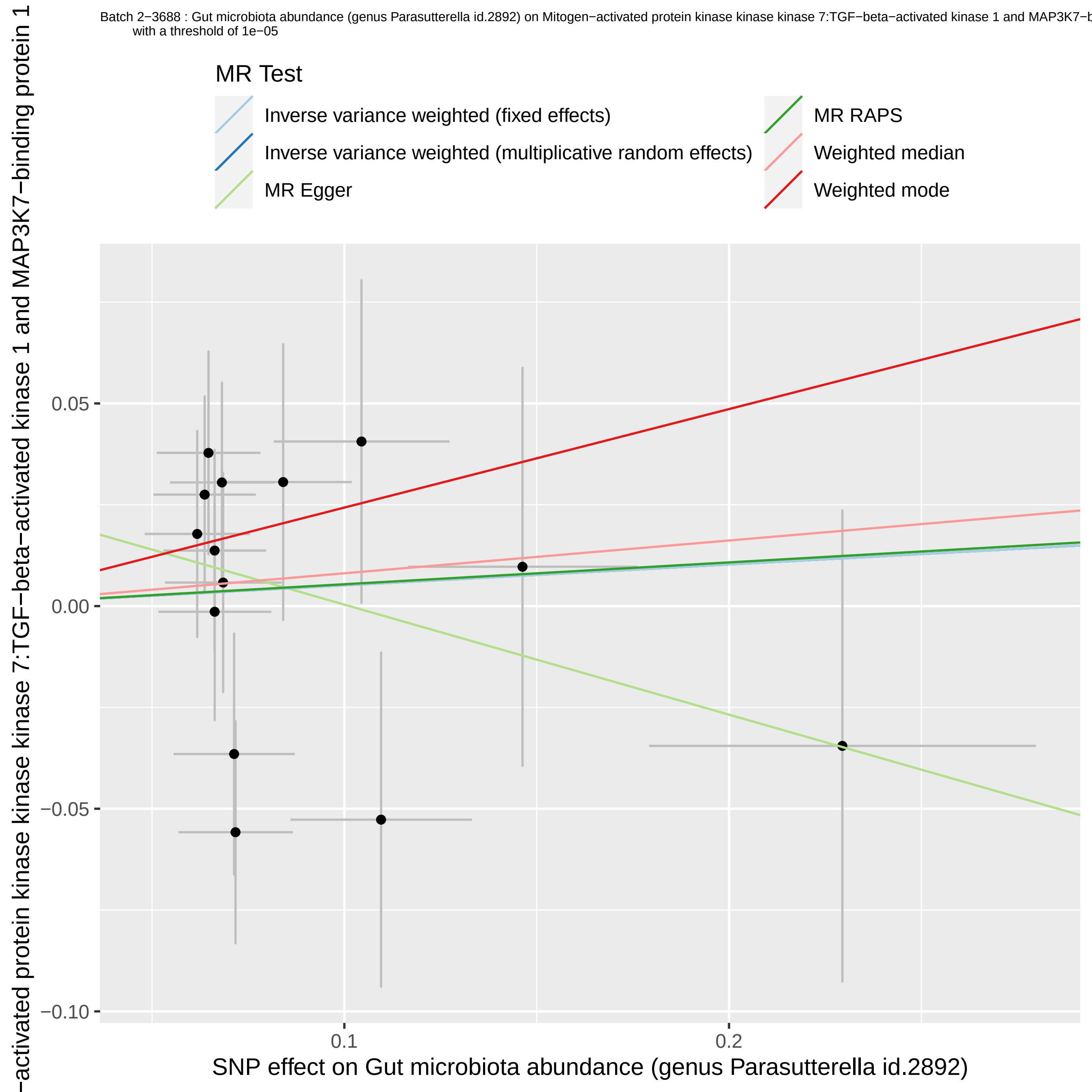
Batch 2−3688 : Gut microbiota abundance (genus Parasutterella id.2892) on Mitogen−activated protein kinase kinase kinase 7:TGF−beta−activated kinase 1 and MAP3K7−b
with a threshold of 1e−05
MR Test
Inverse variance weighted (fixed effects)
Inverse variance weighted (multiplicative random effects)
MR Egger
MR RAPS
Weighted median
Weighted mode
−activated protein kinase kinase kinase 7:TGF−beta−activated kinase 1 and MAP3K7−binding protein 1
0.05
0.00
−0.05
−0.10
0.1
0.2
SNP effect on Gut microbiota abundance (genus Parasutterella id.2892)

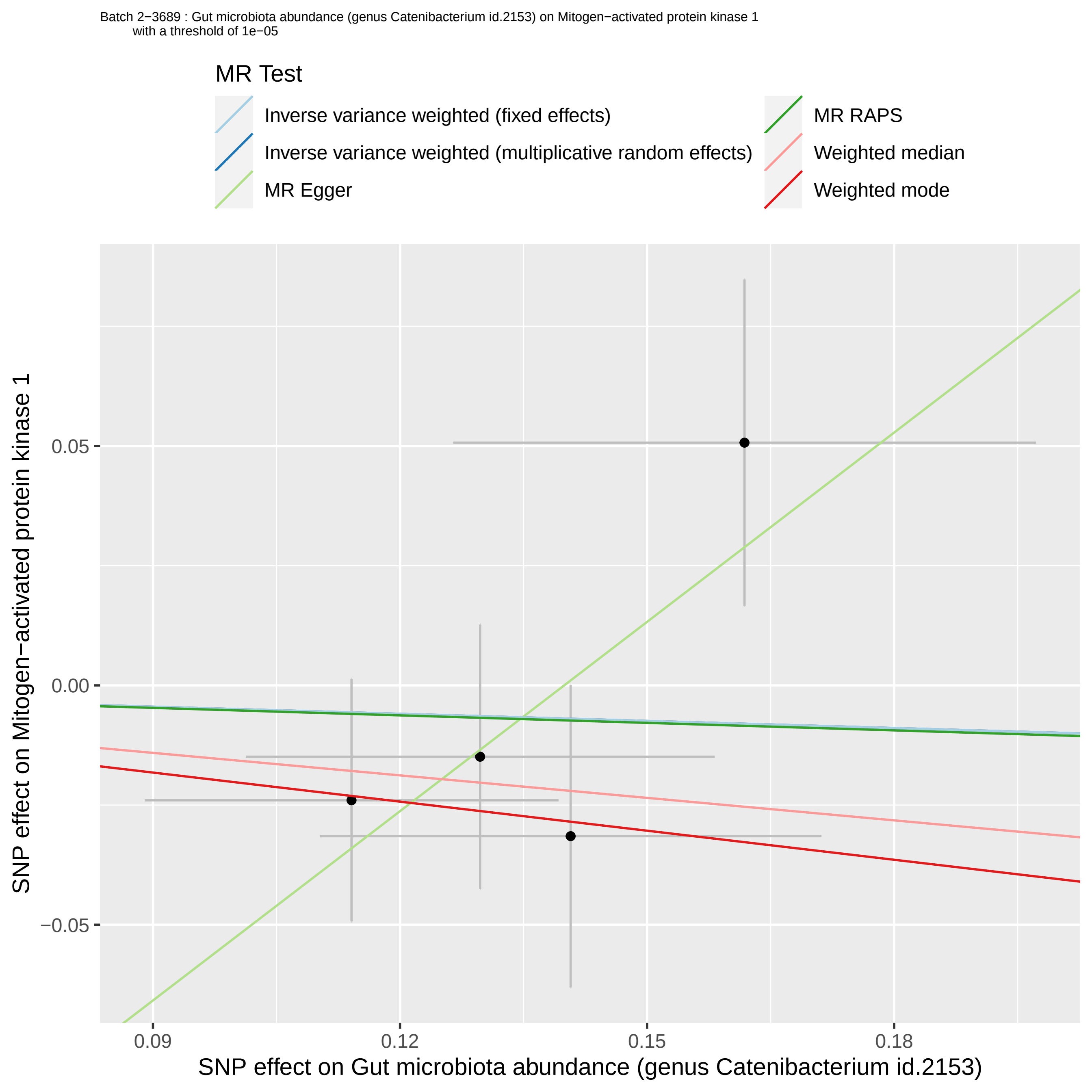

Batch 2−3689 : Gut microbiota abundance (genus Catenibacterium id.2153) on Mitogen−activated protein kinase 1
with a threshold of 1e−05
MR Test
Inverse variance weighted (fixed effects)
Inverse variance weighted (multiplicative random effects)
MR Egger
MR RAPS
Weighted median
Weighted mode
SNP effect on Mitogen−activated protein kinase 1
0.05
0.00
−0.05
0.09
0.12
0.15
0.18
SNP effect on Gut microbiota abundance (genus Catenibacterium id.2153)

Batch 2−3690 : Gut microbiota abundance (genus Parasutterella id.2892) on Mitogen−activated protein kinase 1 with a threshold of 1e−05

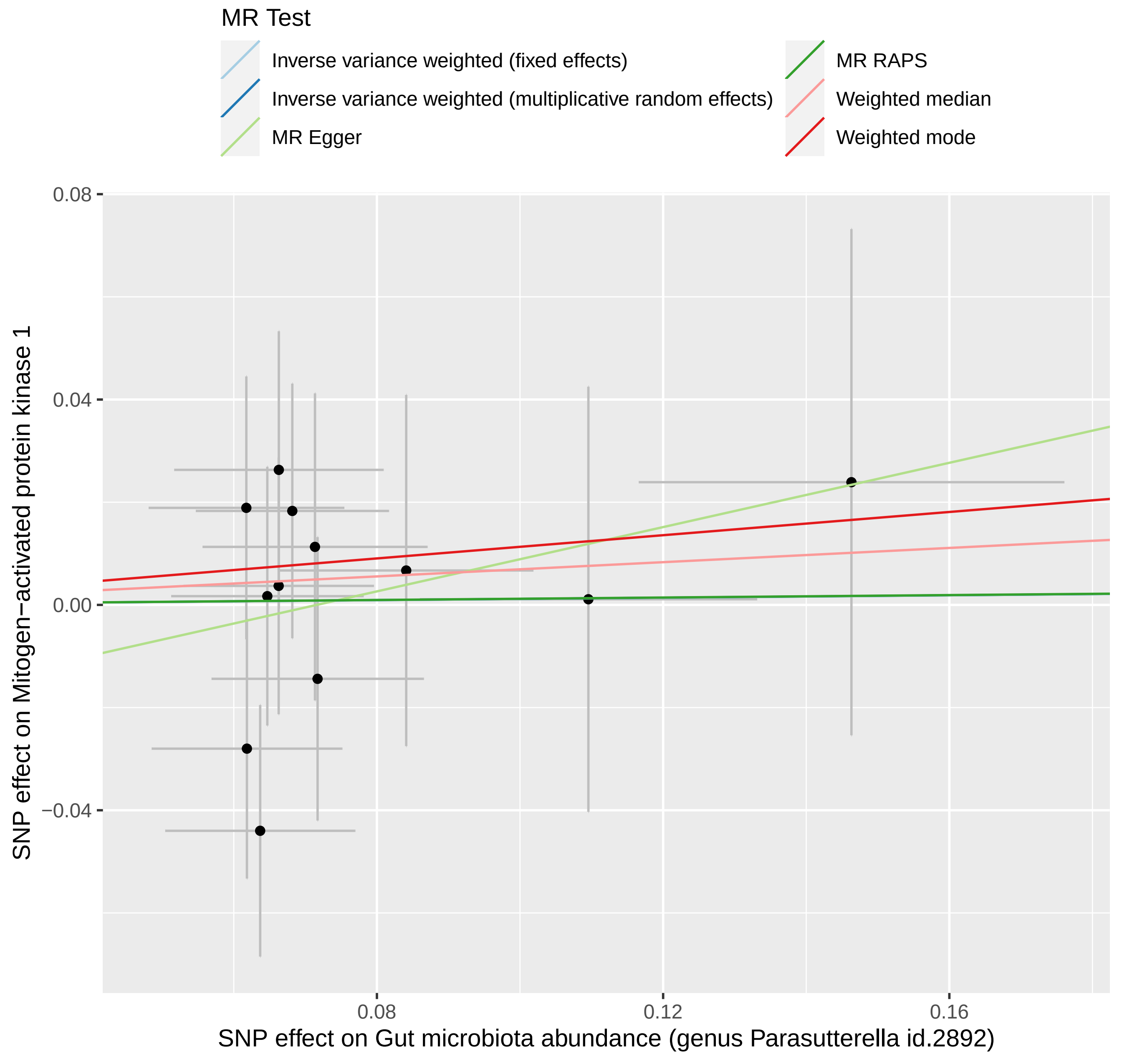

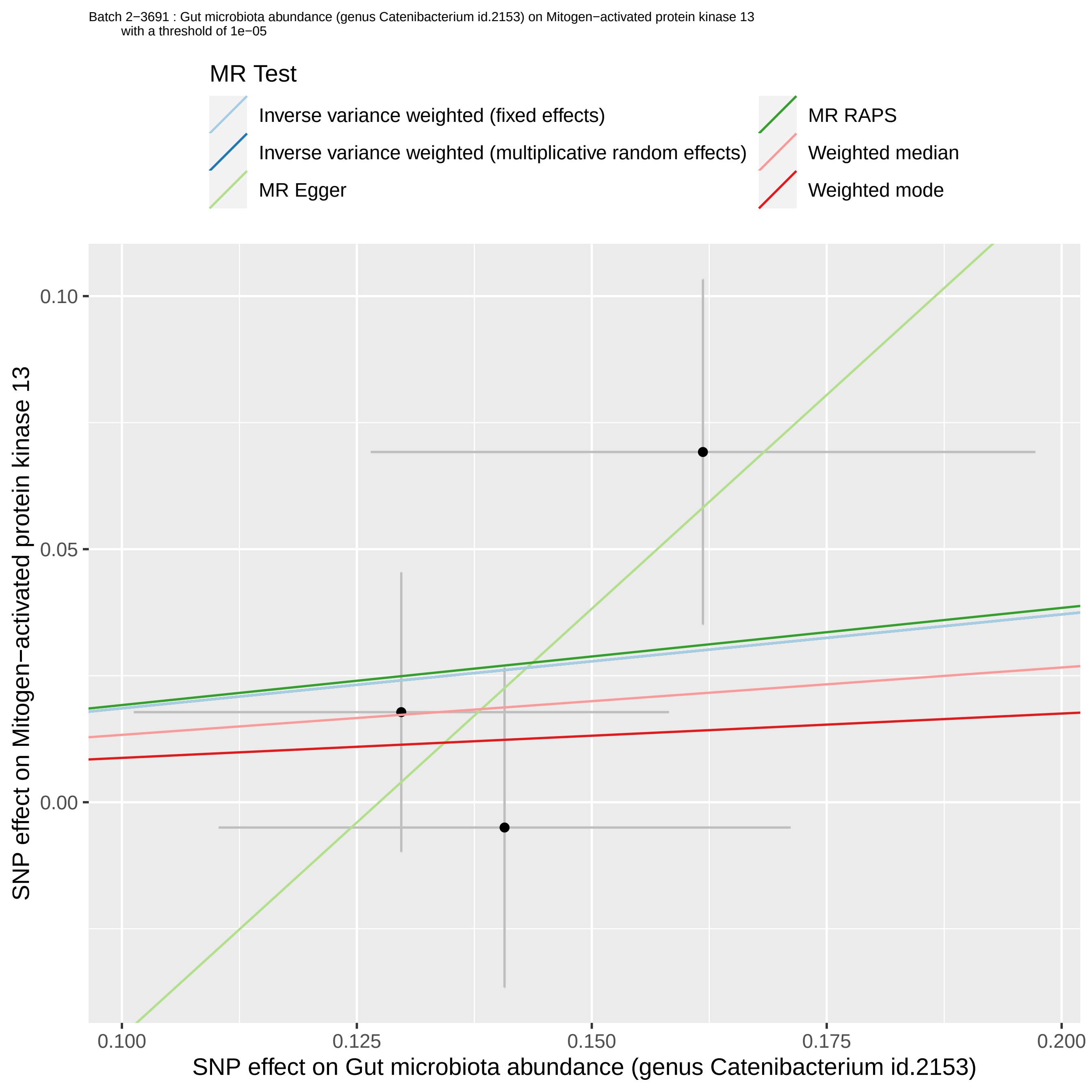

Batch 2−3691 : Gut microbiota abundance (genus Catenibacterium id.2153) on Mitogen−activated protein kinase 13
with a threshold of 1e−05
MR Test
Inverse variance weighted (fixed effects)
Inverse variance weighted (multiplicative random effects)
MR Egger
MR RAPS
Weighted median
Weighted mode
0.10
0.05
0.00
0.100
0.125
0.150
0.175
0.200
SNP effect on Mitogen−activated protein kinase 13
SNP effect on Gut microbiota abundance (genus Catenibacterium id.2153)

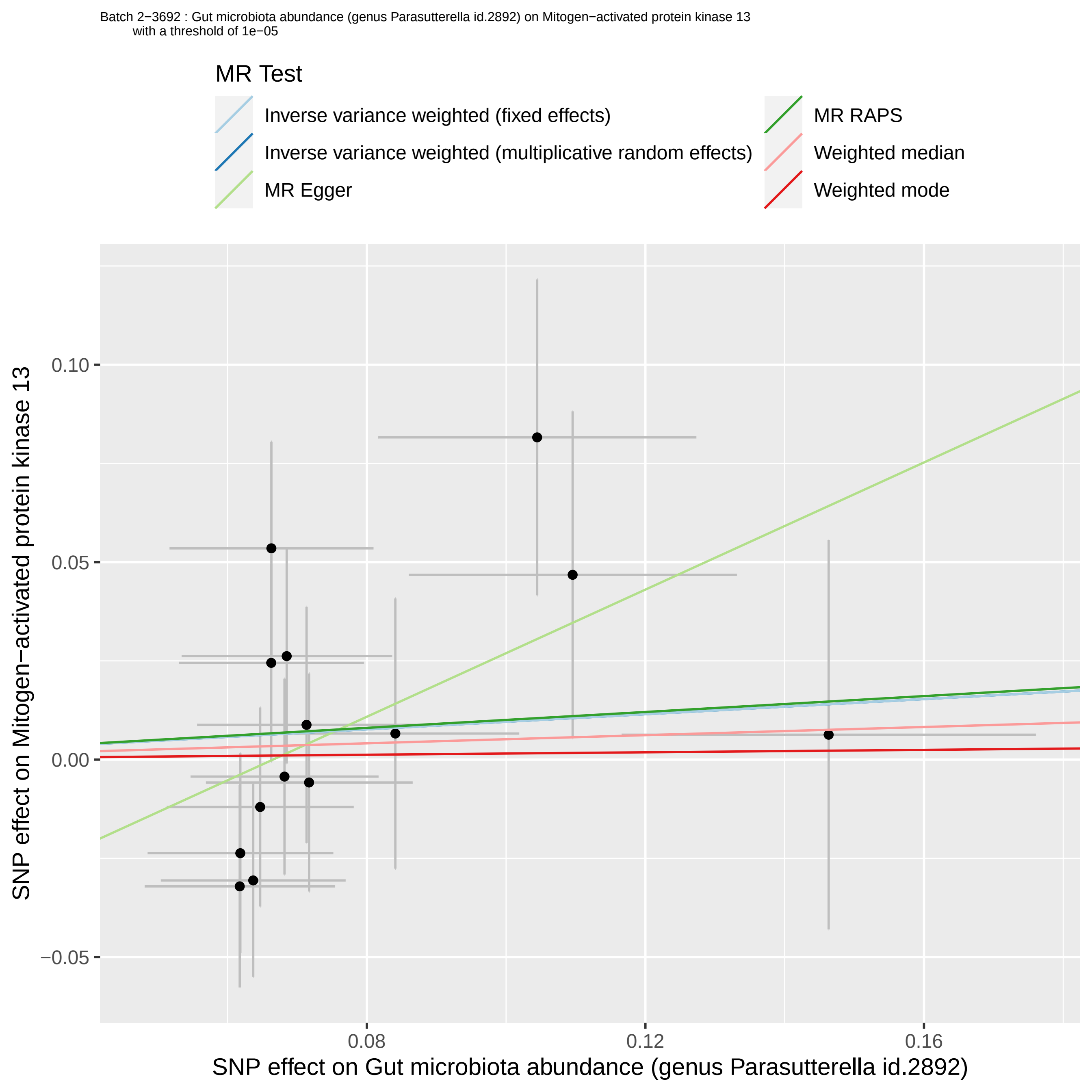
Batch 2−3692 : Gut microbiota abundance (genus Parasutterella id.2892) on Mitogen−activated protein kinase 13
with a threshold of 1e−05
MR Test
Inverse variance weighted (fixed effects)
Inverse variance weighted (multiplicative random effects)
MR Egger
MR RAPS
Weighted median
Weighted mode
0.10
0.05
0.00
−0.05
0.08
0.12
0.16
SNP effect on Mitogen−activated protein kinase 13
SNP effect on Gut microbiota abundance (genus Parasutterella id.2892)

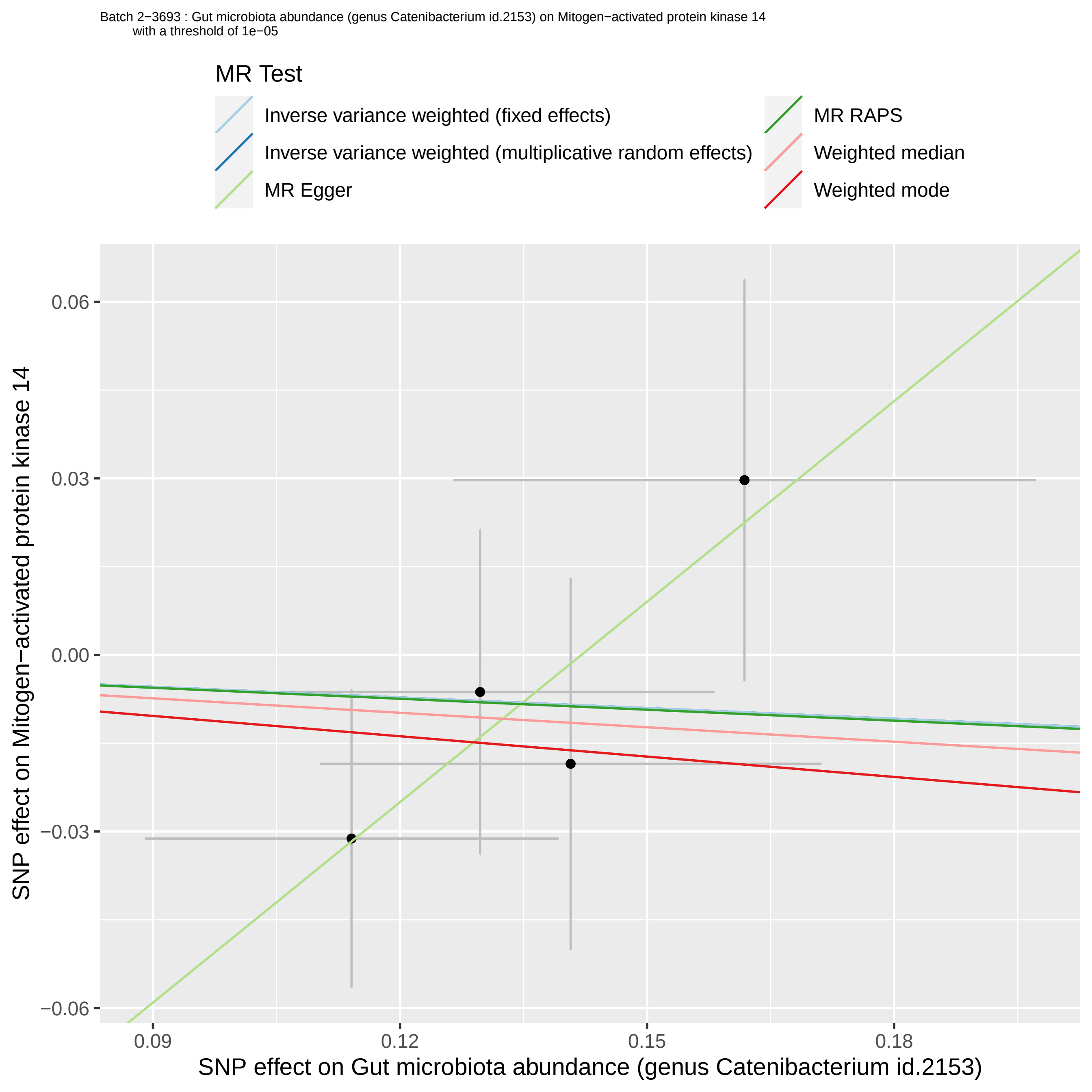
Batch 2−3693 : Gut microbiota abundance (genus Catenibacterium id.2153) on Mitogen−activated protein kinase 14
with a threshold of 1e−05
MR Test
Inverse variance weighted (fixed effects)
Inverse variance weighted (multiplicative random effects)
MR Egger
MR RAPS
Weighted median
Weighted mode
0.06
0.03
0.00
−0.03
−0.06
0.09
0.12
0.15
0.18
SNP effect on Mitogen−activated protein kinase 14
SNP effect on Gut microbiota abundance (genus Catenibacterium id.2153)

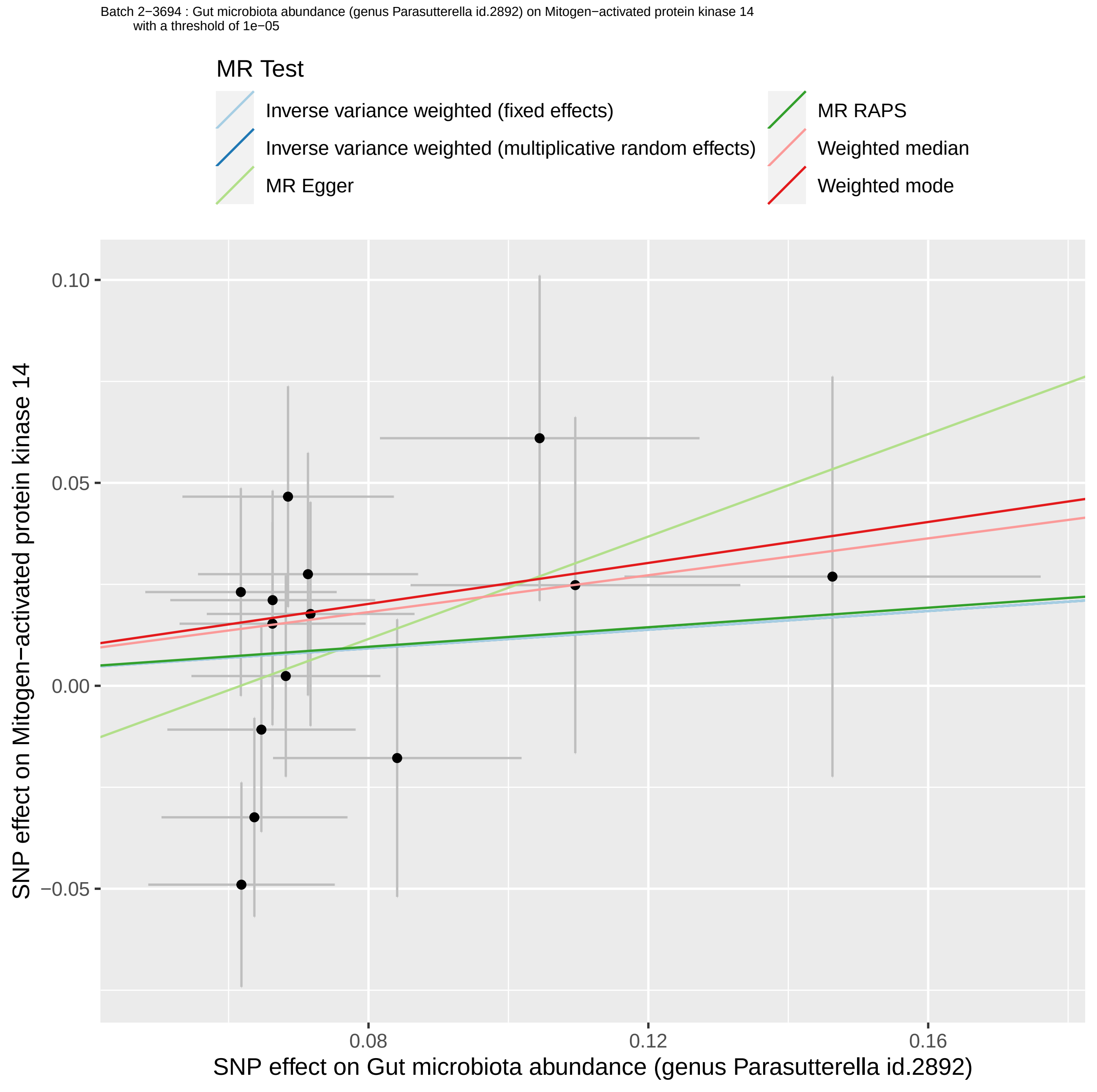

Batch 2−3694 : Gut microbiota abundance (genus Parasutterella id.2892) on Mitogen−activated protein kinase 14
with a threshold of 1e−05
MR Test
Inverse variance weighted (fixed effects)
Inverse variance weighted (multiplicative random effects)
MR Egger
MR RAPS
Weighted median
Weighted mode
SNP effect on Mitogen−activated protein kinase 14
0.10
0.05
0.00
−0.05
0.08
0.12
0.16
SNP effect on Gut microbiota abundance (genus Parasutterella id.2892)

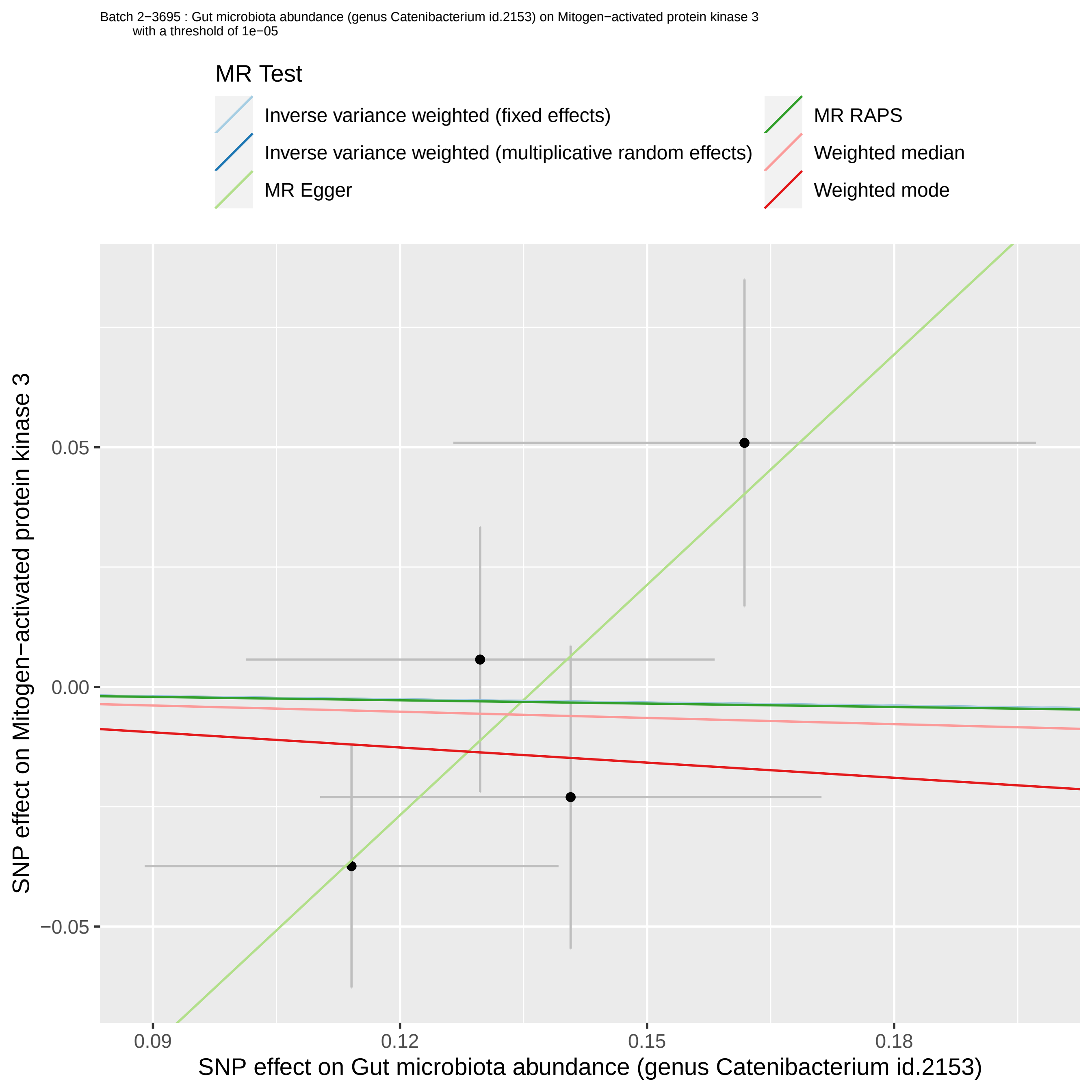
Batch 2−3695 : Gut microbiota abundance (genus Catenibacterium id.2153) on Mitogen−activated protein kinase 3
with a threshold of 1e−05
MR Test
Inverse variance weighted (fixed effects)
Inverse variance weighted (multiplicative random effects)
MR Egger
MR RAPS
Weighted median
Weighted mode
0.05
0.00
−0.05
0.09
0.12
0.15
0.18
SNP effect on Mitogen−activated protein kinase 3
SNP effect on Gut microbiota abundance (genus Catenibacterium id.2153)

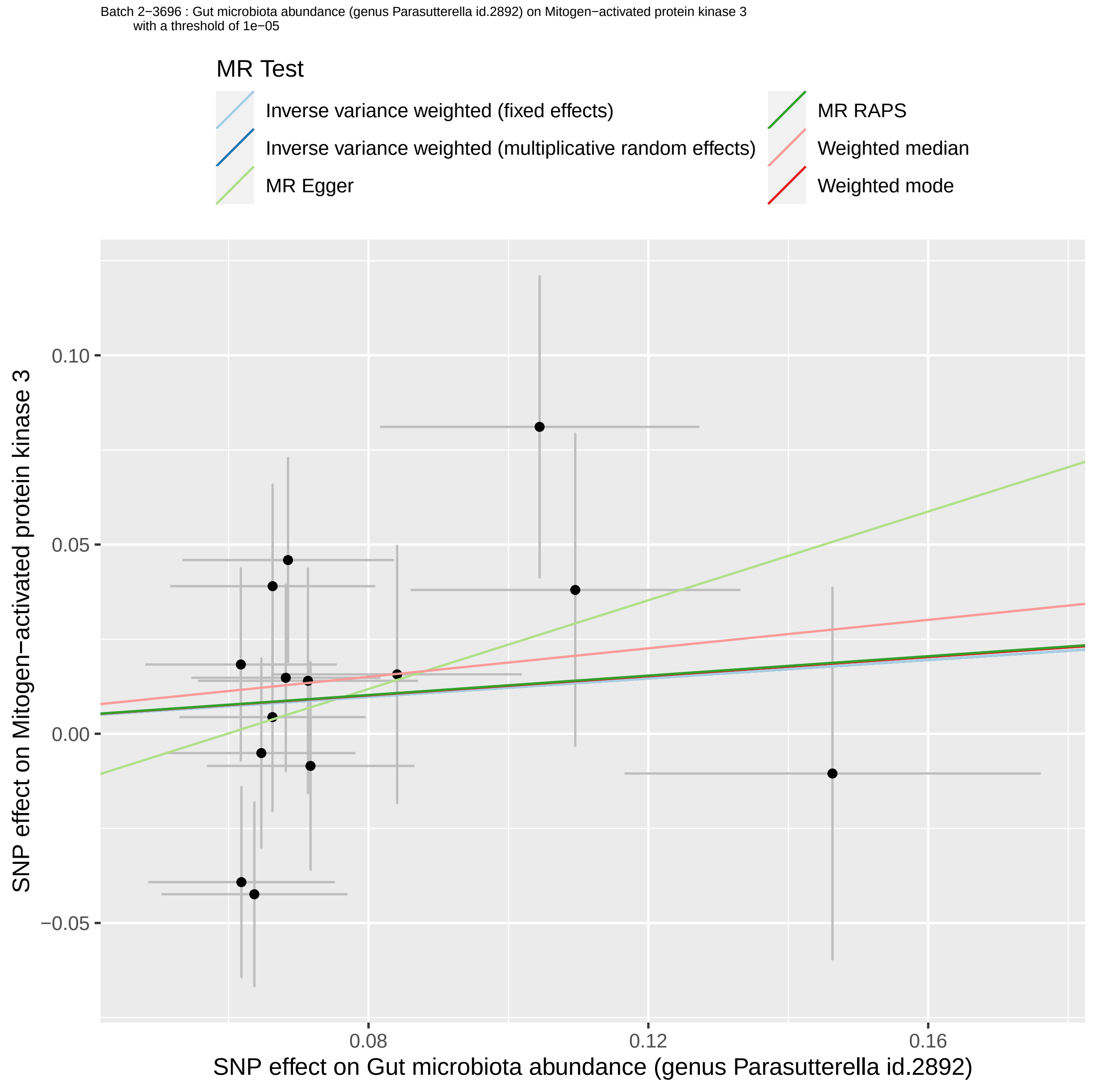
Batch 2−3696 : Gut microbiota abundance (genus Parasutterella id.2892) on Mitogen−activated protein kinase 3
with a threshold of 1e−05
MR Test
Inverse variance weighted (fixed effects)
Inverse variance weighted (multiplicative random effects)
MR Egger
MR RAPS
Weighted median
Weighted mode
0.10
0.05
0.00
−0.05
0.08
0.12
0.16
SNP effect on Mitogen−activated protein kinase 3
SNP effect on Gut microbiota abundance (genus Parasutterella id.2892)

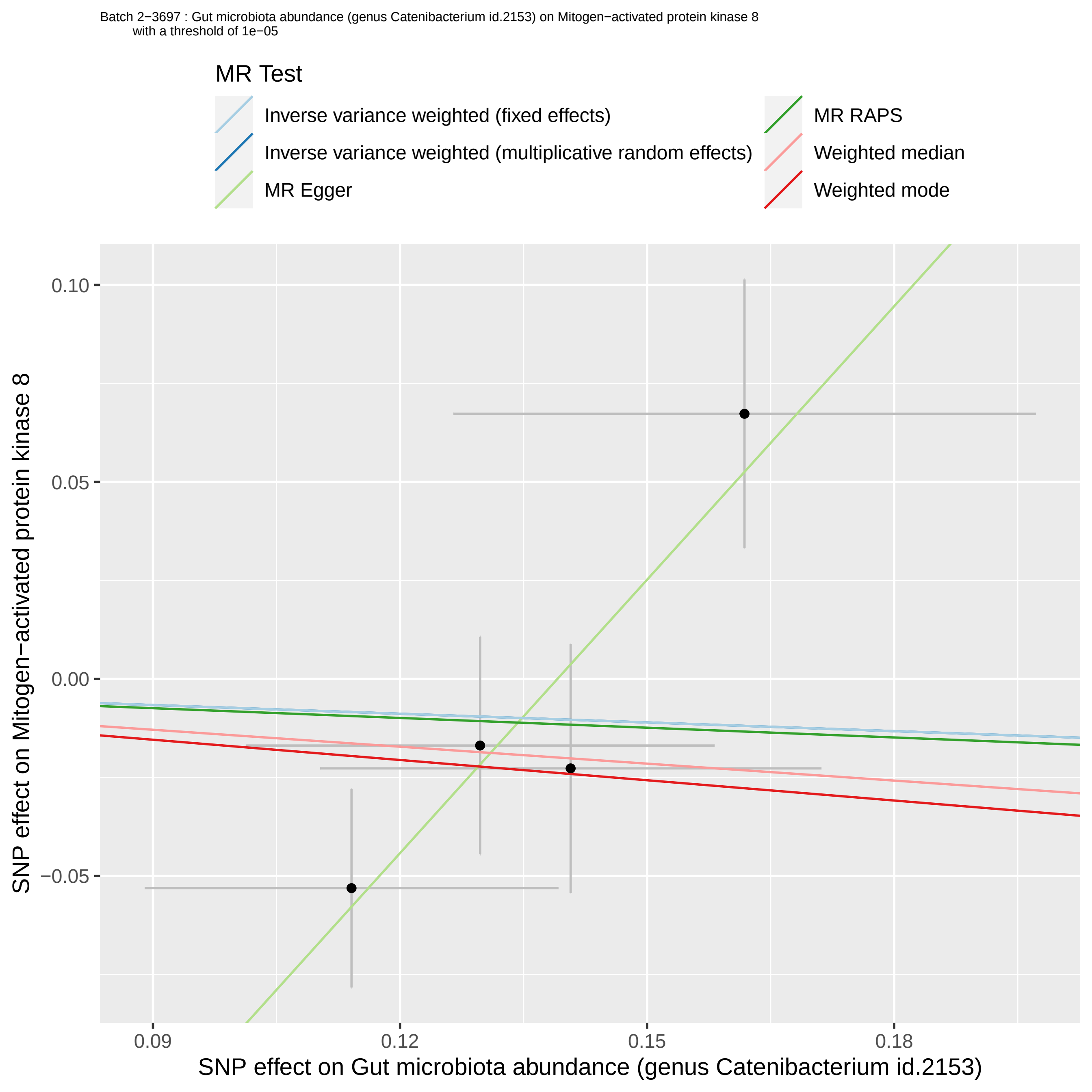

Batch 2−3697 : Gut microbiota abundance (genus Catenibacterium id.2153) on Mitogen−activated protein kinase 8
with a threshold of 1e−05
MR Test
Inverse variance weighted (fixed effects)
Inverse variance weighted (multiplicative random effects)
MR Egger
MR RAPS
Weighted median
Weighted mode
0.10
0.05
0.00
−0.05
SNP effect on Mitogen−activated protein kinase 8
0.09
0.12
0.15
0.18
SNP effect on Gut microbiota abundance (genus Catenibacterium id.2153)

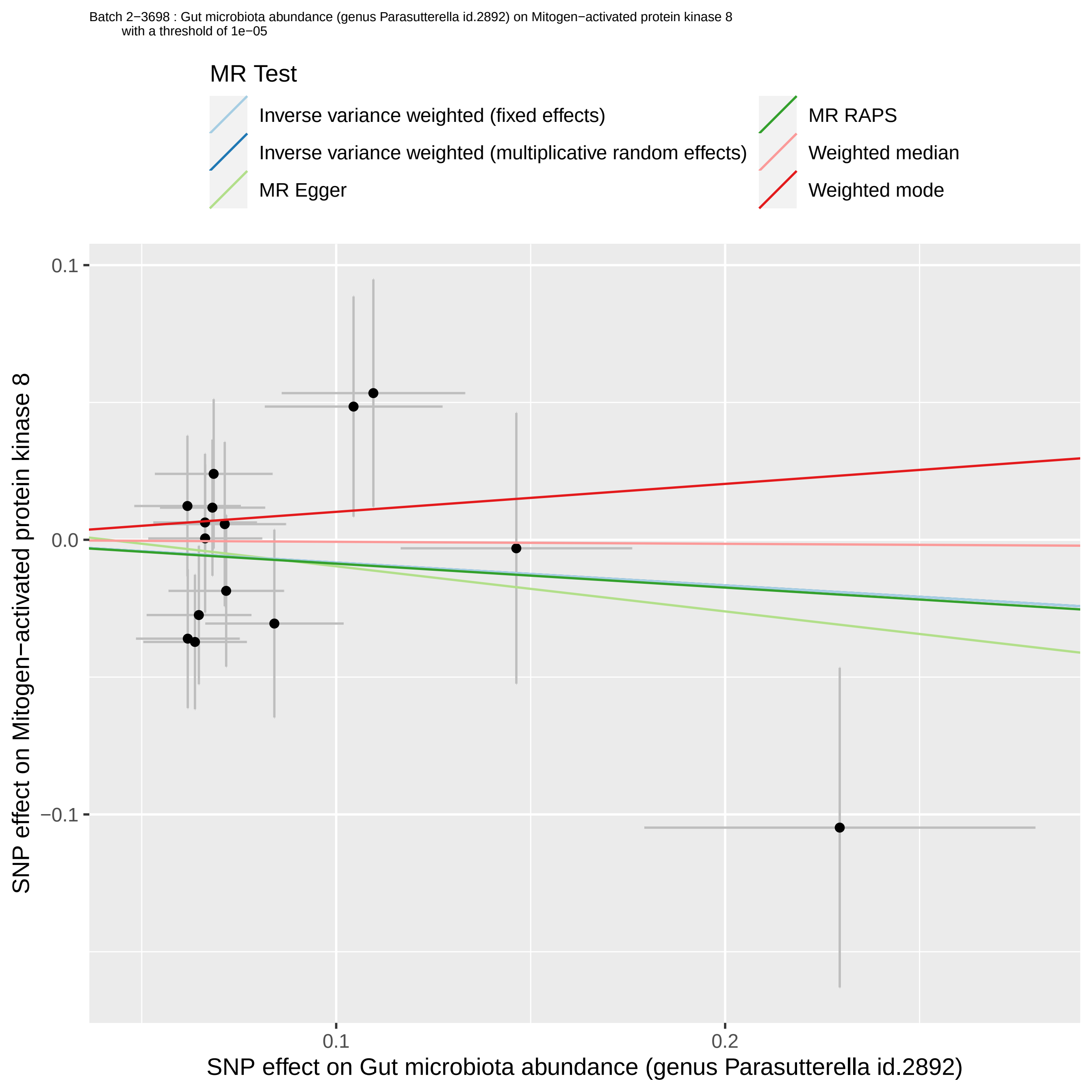

Batch 2−3698 : Gut microbiota abundance (genus Parasutterella id.2892) on Mitogen−activated protein kinase 8
with a threshold of 1e−05
MR Test
Inverse variance weighted (fixed effects)
Inverse variance weighted (multiplicative random effects)
MR Egger
MR RAPS
Weighted median
Weighted mode
0.1
0.0
−0.1
0.1
0.2
SNP effect on Mitogen−activated protein kinase 8
SNP effect on Gut microbiota abundance (genus Parasutterella id.2892)

Batch 2−3699 : Gut microbiota abundance (genus Catenibacterium id.2153) on Mitogen−activated protein kinase 9
with a threshold of 1e−05

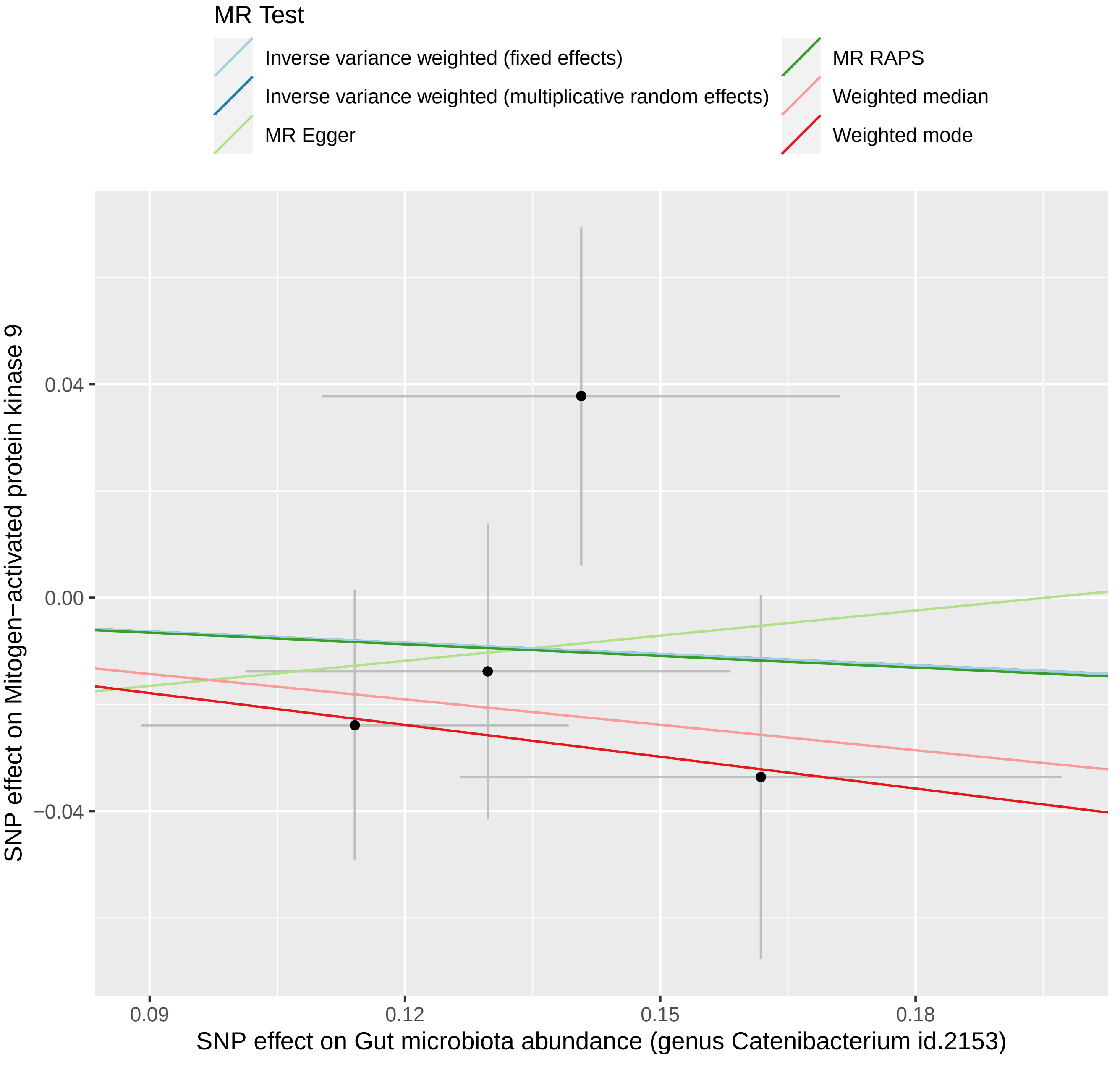

Batch 2−3700 : Gut microbiota abundance (genus Parasutterella id.2892) on Mitogen−activated protein kinase 9
with a threshold of 1e−05

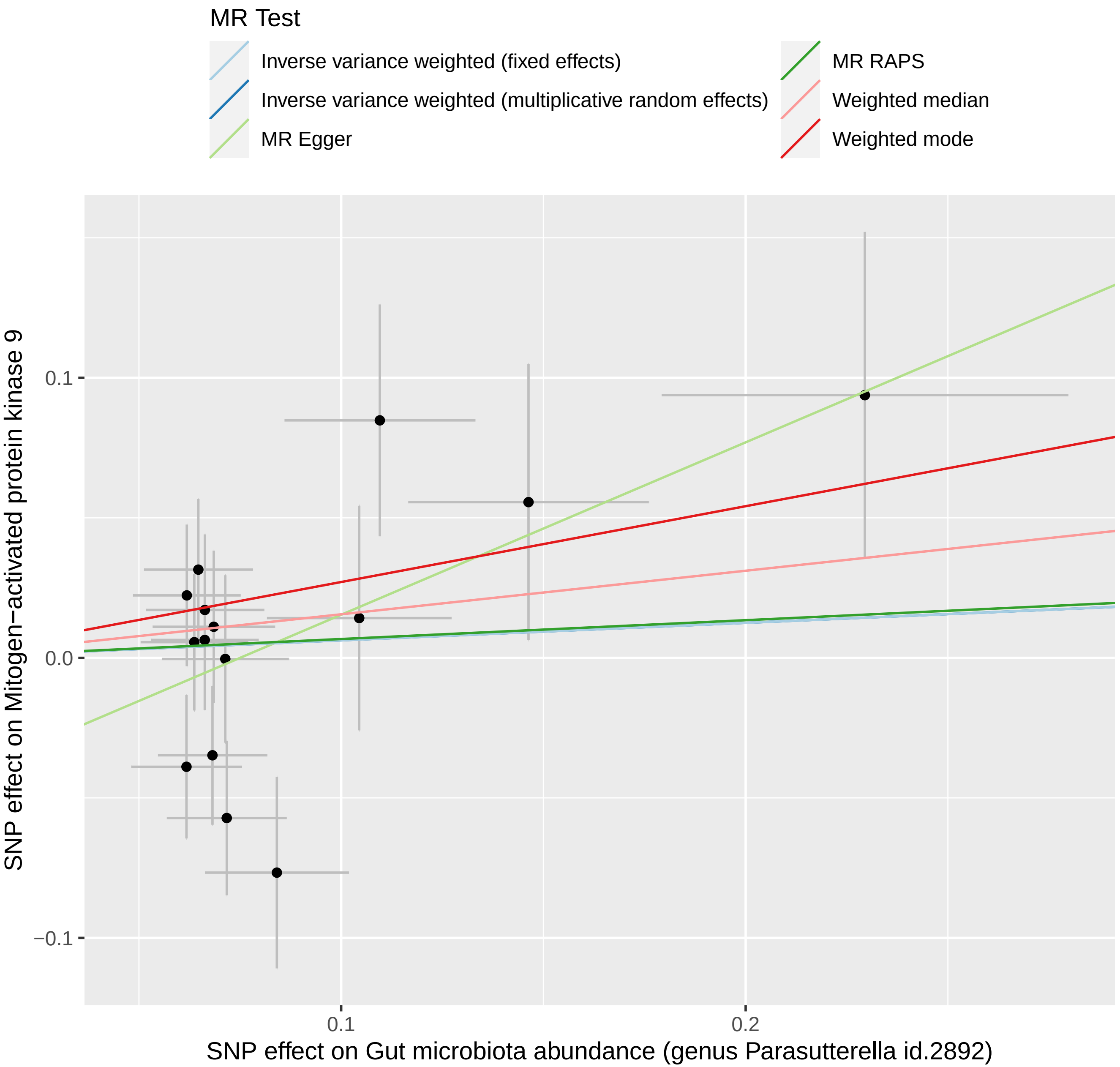

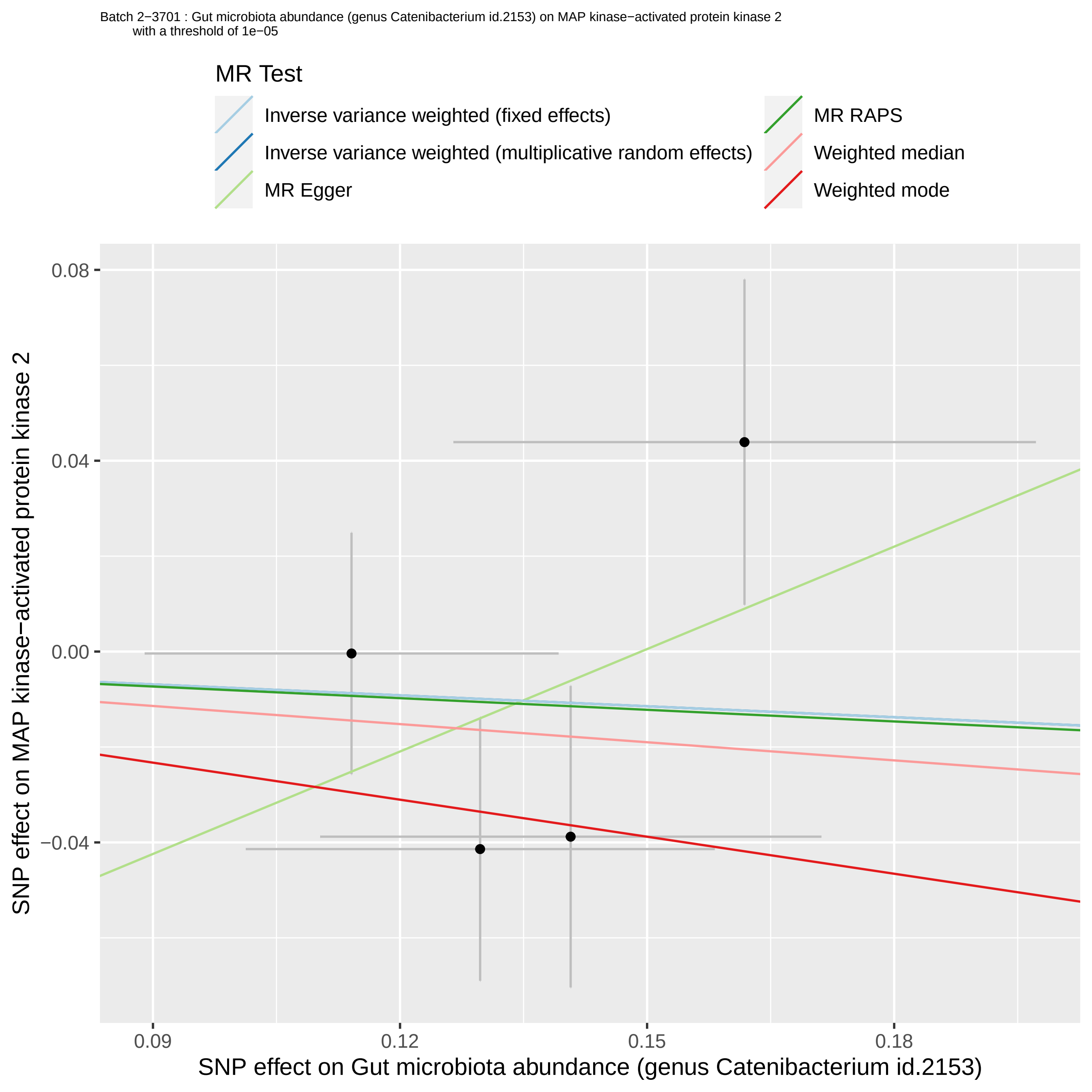
Batch 2−3701 : Gut microbiota abundance (genus Catenibacterium id.2153) on MAP kinase−activated protein kinase 2
with a threshold of 1e−05
MR Test
Inverse variance weighted (fixed effects)
Inverse variance weighted (multiplicative random effects)
MR Egger
MR RAPS
Weighted median
Weighted mode
0.08
0.04
0.00
−0.04
0.09
0.12
0.15
0.18
SNP effect on MAP kinase−activated protein kinase 2
SNP effect on Gut microbiota abundance (genus Catenibacterium id.2153)

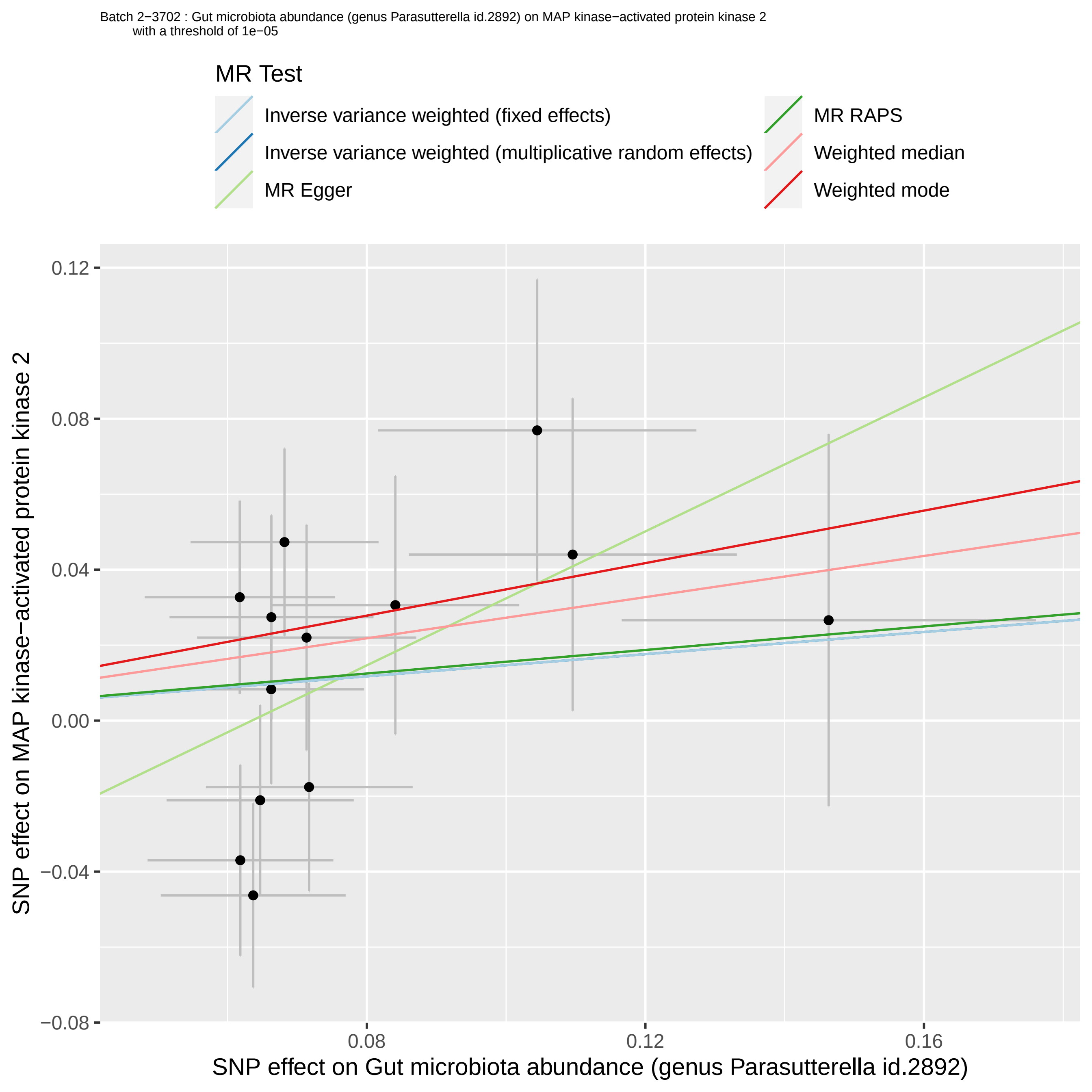
Batch 2−3702 : Gut microbiota abundance (genus Parasutterella id.2892) on MAP kinase−activated protein kinase 2
with a threshold of 1e−05
MR Test
Inverse variance weighted (fixed effects)
Inverse variance weighted (multiplicative random effects)
MR Egger
MR RAPS
Weighted median
Weighted mode
0.12
0.08
0.04
0.00
−0.04
−0.08
0.08
0.12
0.16
SNP effect on MAP kinase−activated protein kinase 2
SNP effect on Gut microbiota abundance (genus Parasutterella id.2892)

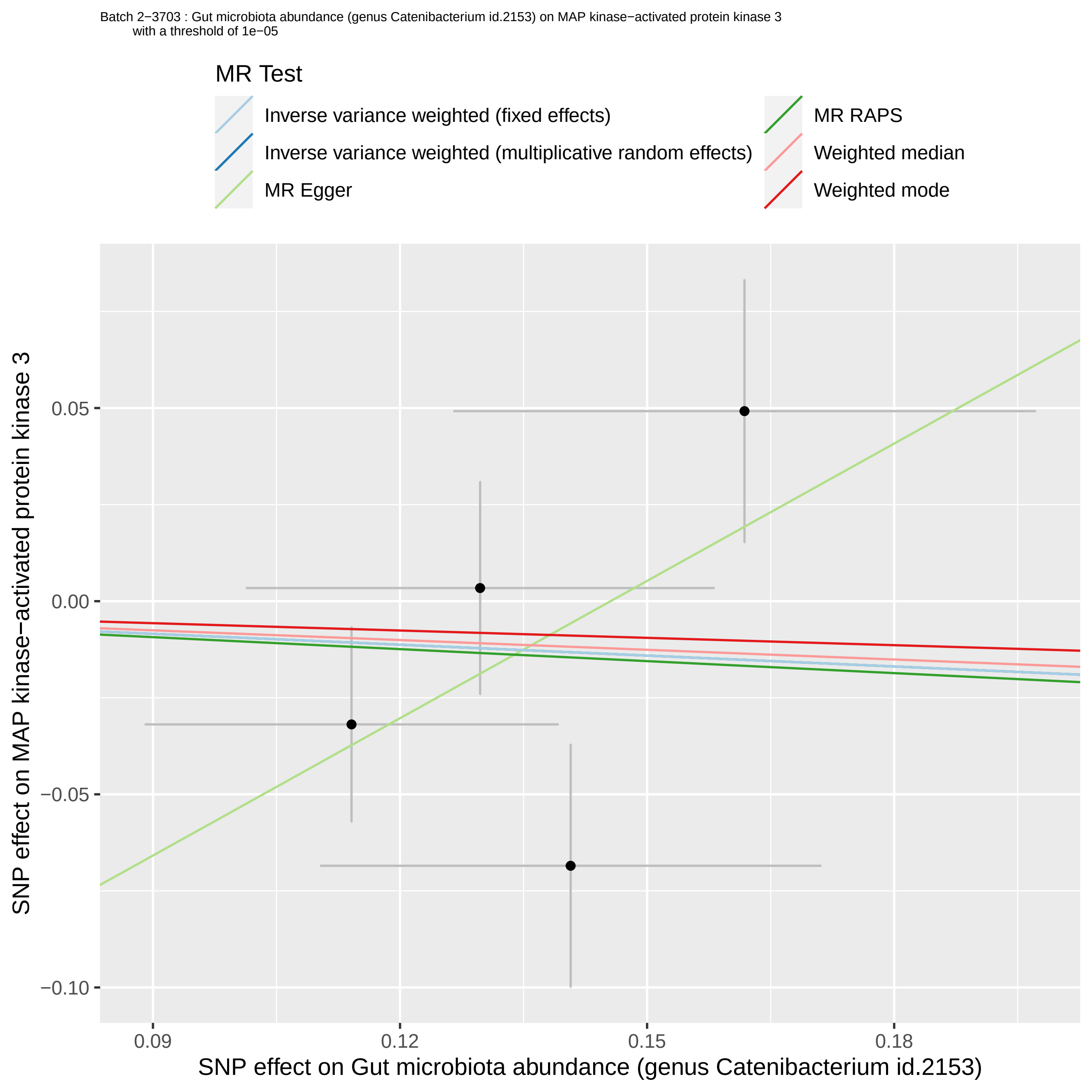

Batch 2−3703 : Gut microbiota abundance (genus Catenibacterium id.2153) on MAP kinase−activated protein kinase 3
with a threshold of 1e−05
MR Test
Inverse variance weighted (fixed effects)
Inverse variance weighted (multiplicative random effects)
MR Egger
MR RAPS
Weighted median
Weighted mode
0.05
0.00
−0.05
−0.10
0.09
0.12
0.15
0.18
SNP effect on MAP kinase−activated protein kinase 3
SNP effect on Gut microbiota abundance (genus Catenibacterium id.2153)

Batch 2−3704 : Gut microbiota abundance (genus Parasutterella id.2892) on MAP kinase−activated protein kinase 3
with a threshold of 1e−05

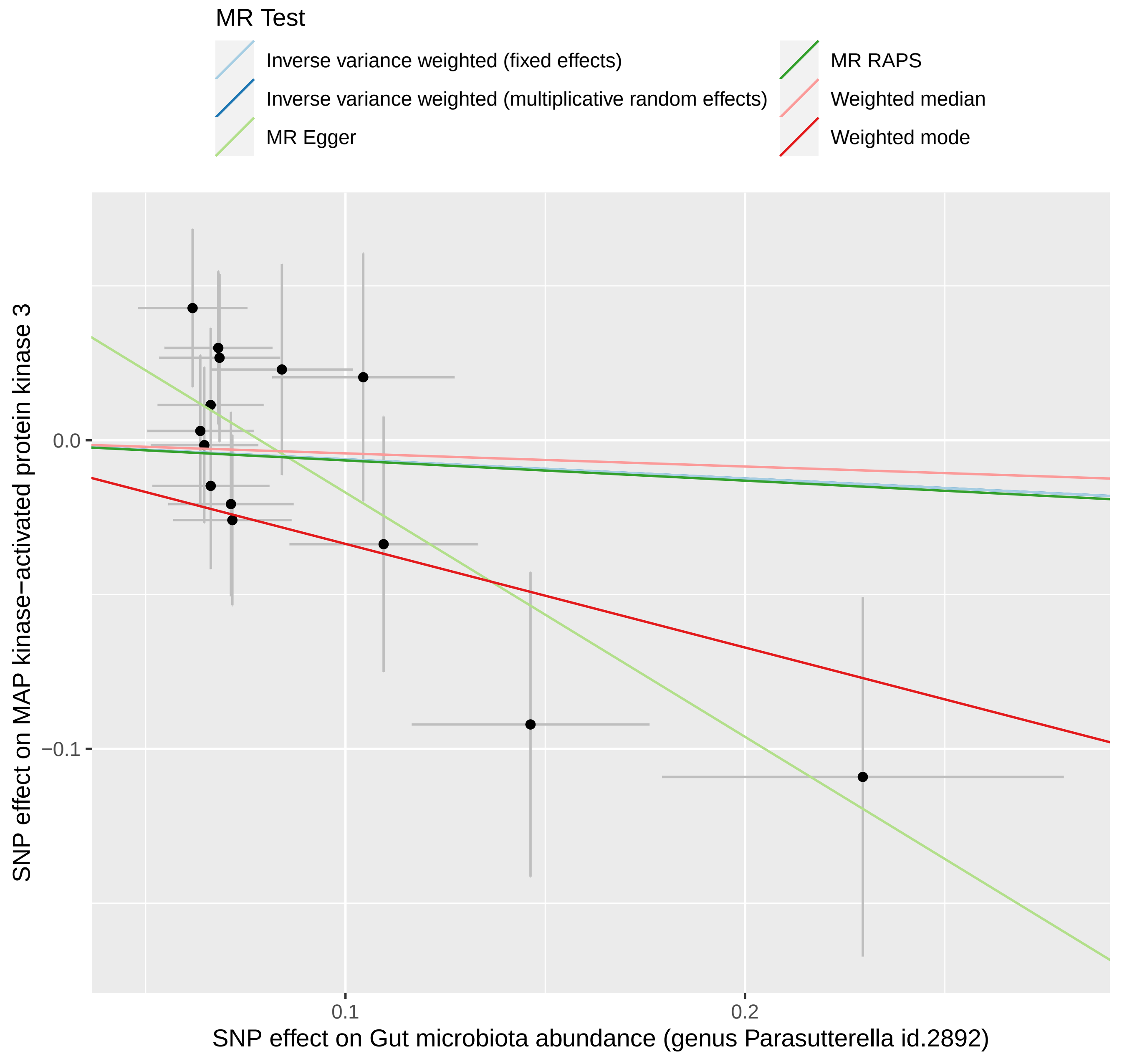

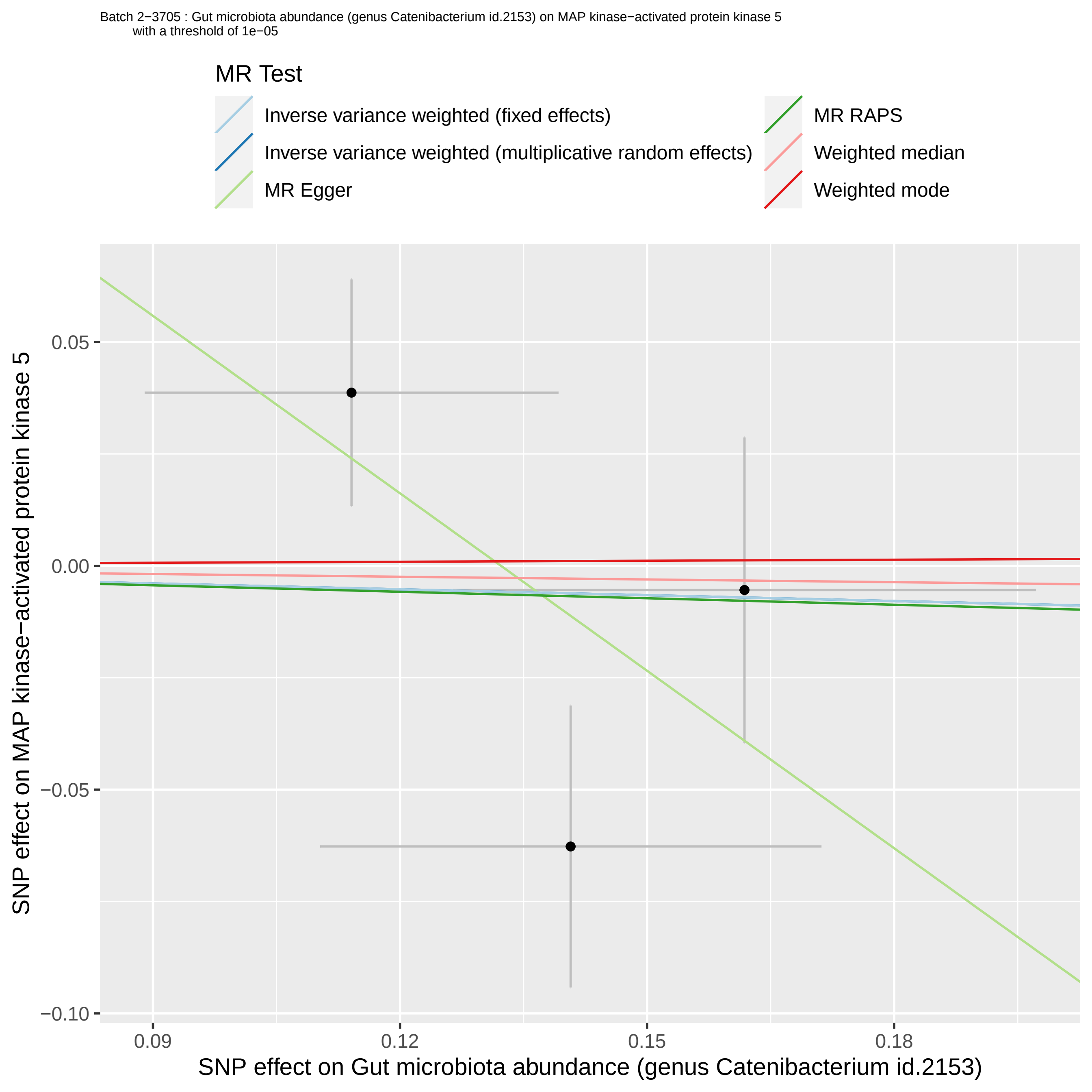
Batch 2−3705 : Gut microbiota abundance (genus Catenibacterium id.2153) on MAP kinase−activated protein kinase 5
with a threshold of 1e−05
MR Test
Inverse variance weighted (fixed effects)
Inverse variance weighted (multiplicative random effects)
MR Egger
MR RAPS
Weighted median
Weighted mode
0.05
0.00
−0.05
−0.10
0.09
0.12
0.15
0.18
SNP effect on MAP kinase−activated protein kinase 5
SNP effect on Gut microbiota abundance (genus Catenibacterium id.2153)

Batch 2−3706 : Gut microbiota abundance (genus Parasutterella id.2892) on MAP kinase−activated protein kinase 5
with a threshold of 1e−05

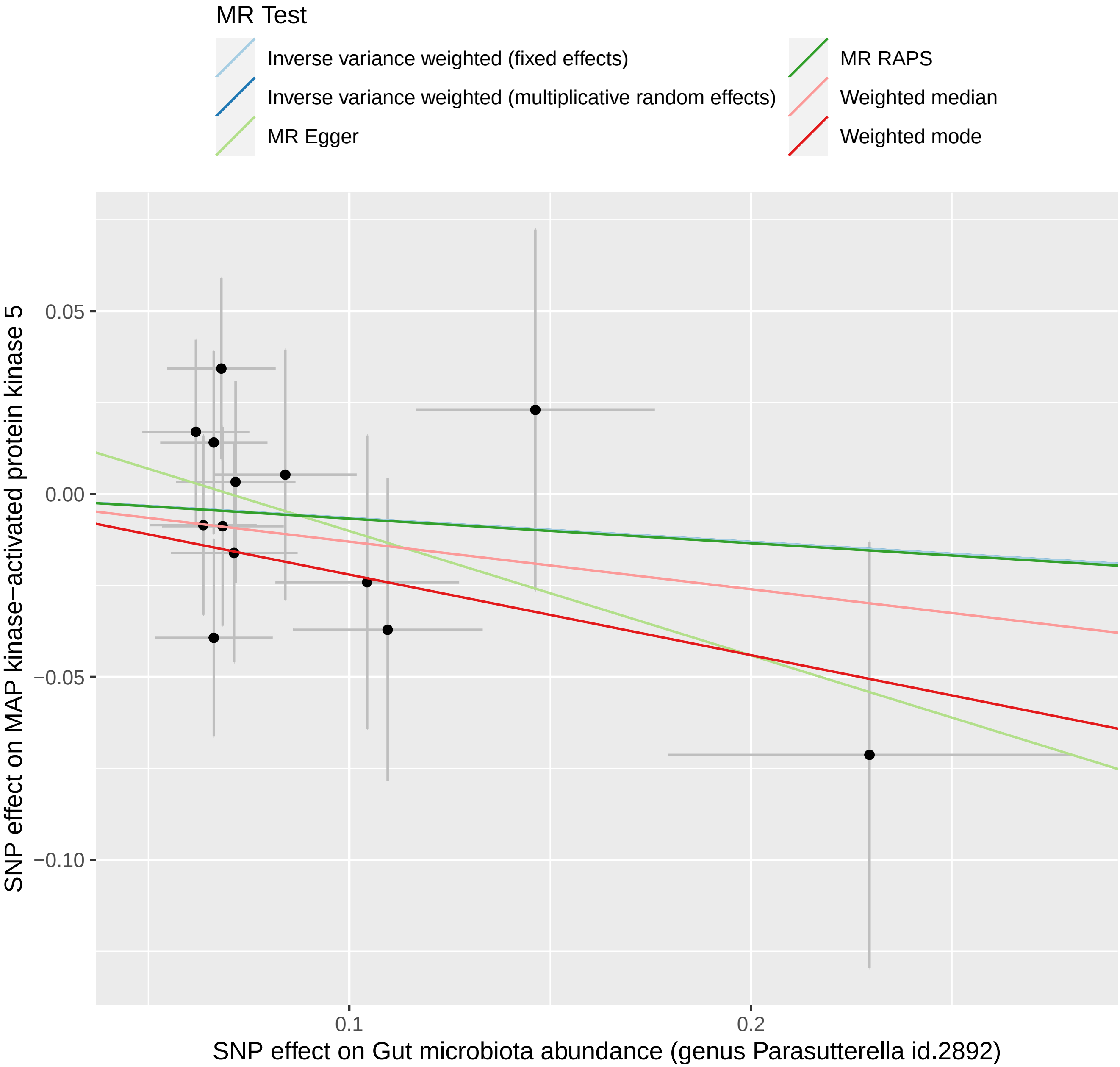

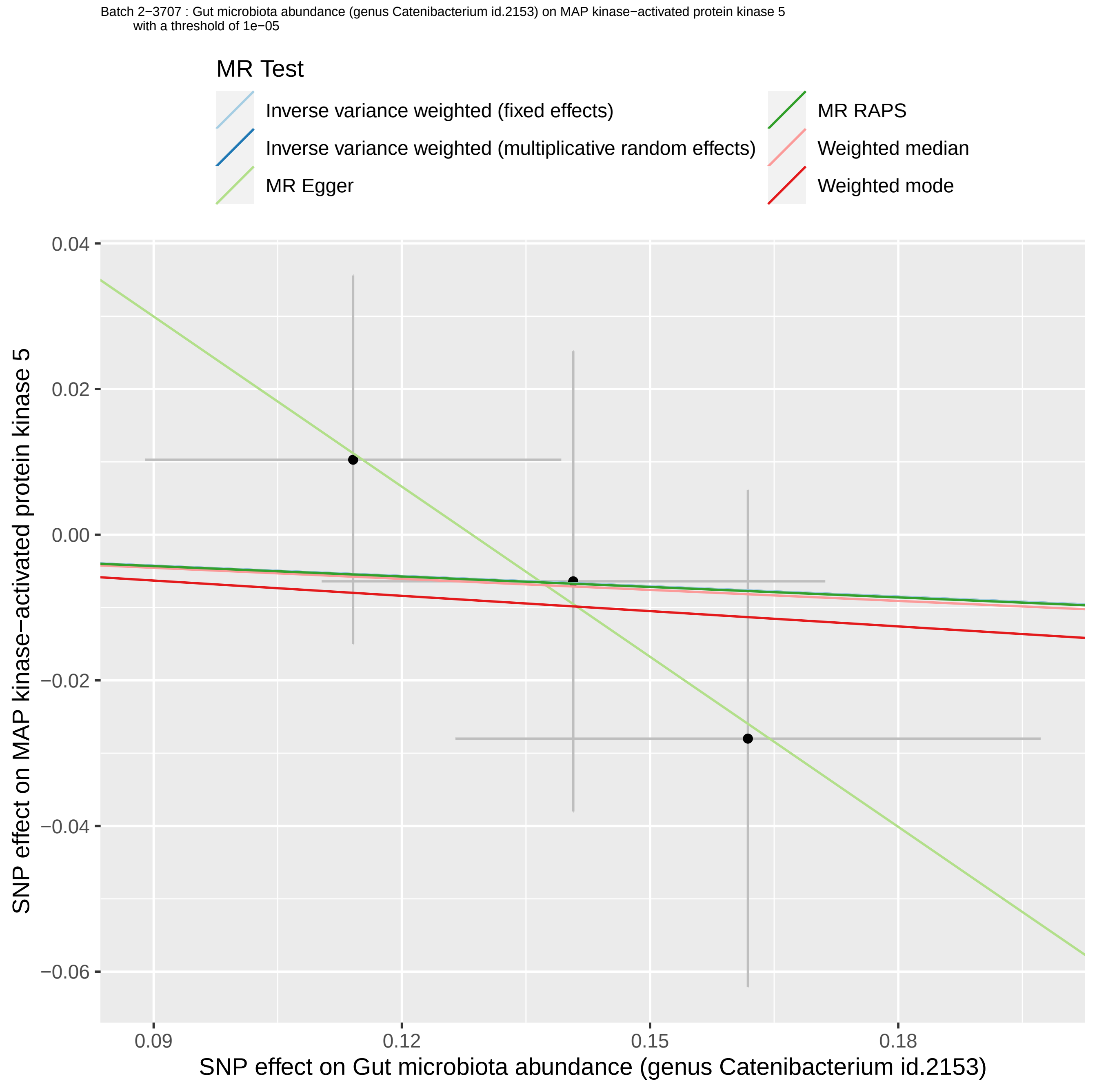

Batch 2−3707 : Gut microbiota abundance (genus Catenibacterium id.2153) on MAP kinase−activated protein kinase 5
with a threshold of 1e−05
MR Test
Inverse variance weighted (fixed effects)
Inverse variance weighted (multiplicative random effects)
MR Egger
MR RAPS
Weighted median
Weighted mode
0.04
0.02
0.00
−0.02
−0.04
−0.06
0.09
0.12
0.15
0.18
SNP effect on MAP kinase−activated protein kinase 5
SNP effect on Gut microbiota abundance (genus Catenibacterium id.2153)

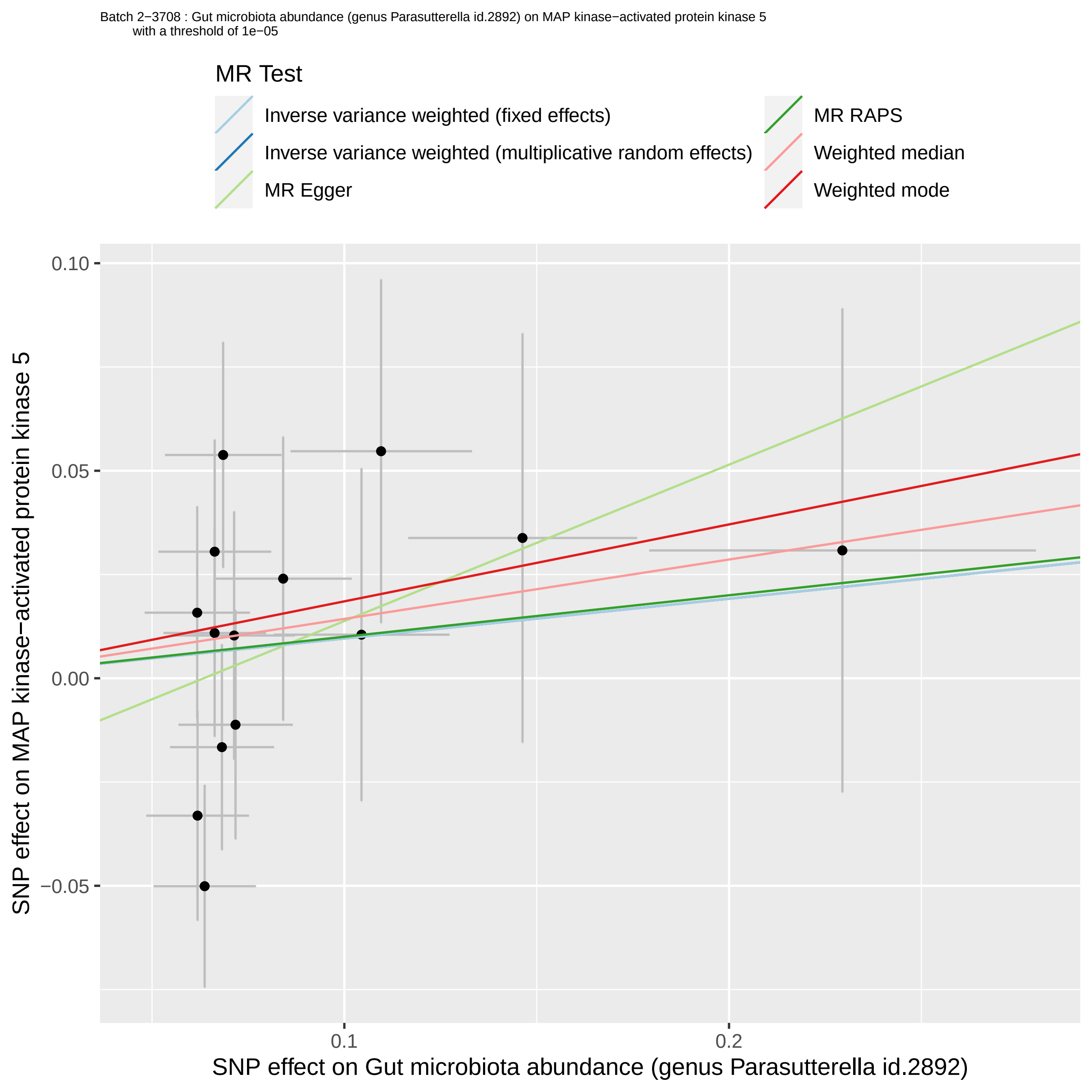

Batch 2−3708 : Gut microbiota abundance (genus Parasutterella id.2892) on MAP kinase−activated protein kinase 5
with a threshold of 1e−05
MR Test
Inverse variance weighted (fixed effects)
Inverse variance weighted (multiplicative random effects)
MR Egger
MR RAPS
Weighted median
Weighted mode
0.10
0.05
0.00
−0.05
0.1
0.2
SNP effect on MAP kinase−activated protein kinase 5
SNP effect on Gut microbiota abundance (genus Parasutterella id.2892)

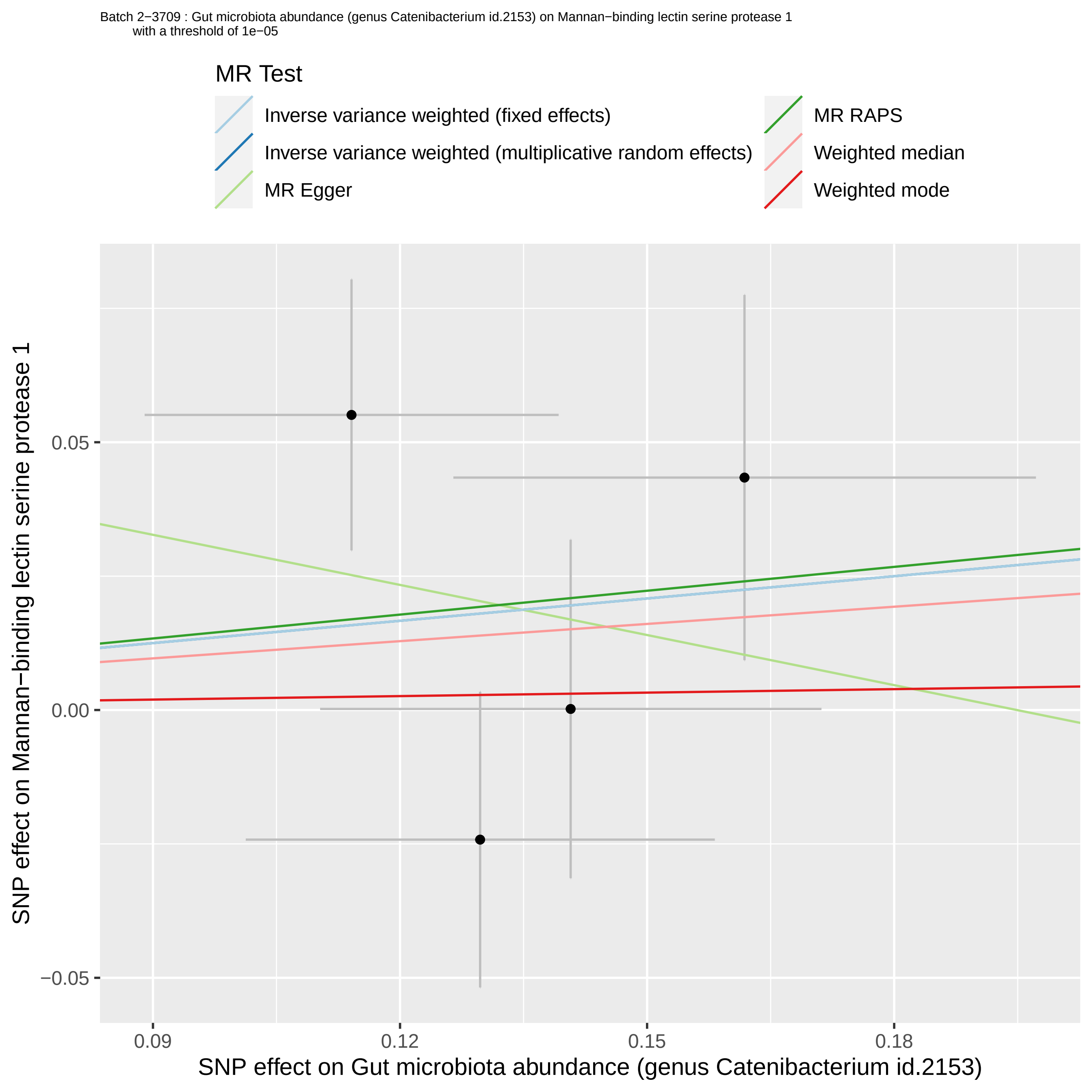

Batch 2−3709 : Gut microbiota abundance (genus Catenibacterium id.2153) on Mannan−binding lectin serine protease 1
with a threshold of 1e−05
MR Test
Inverse variance weighted (fixed effects)
Inverse variance weighted (multiplicative random effects)
MR Egger
MR RAPS
Weighted median
Weighted mode
SNP effect on Mannan−binding lectin serine protease 1
0.05
0.00
−0.05
0.09
0.12
0.15
0.18
SNP effect on Gut microbiota abundance (genus Catenibacterium id.2153)

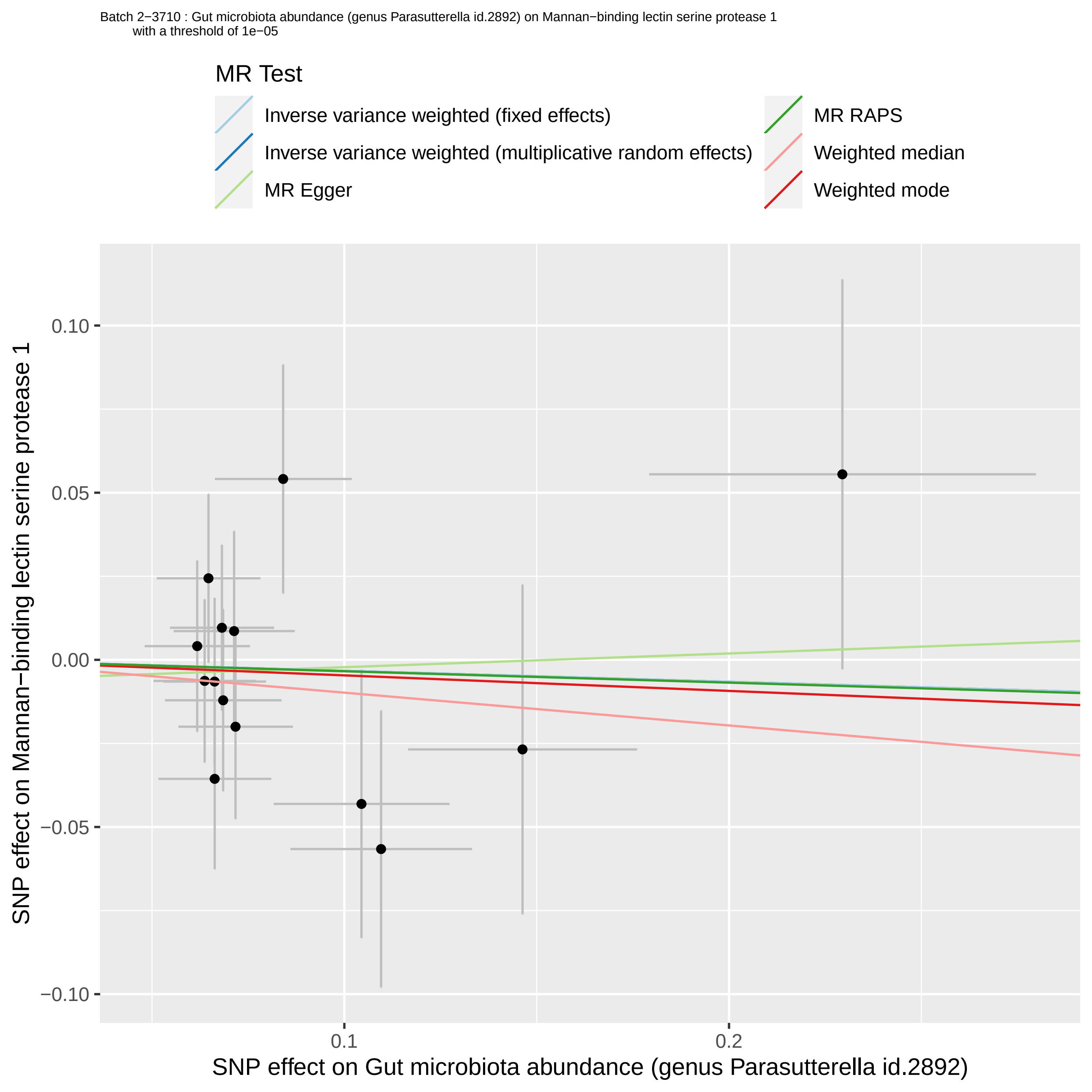
Batch 2−3710 : Gut microbiota abundance (genus Parasutterella id.2892) on Mannan−binding lectin serine protease 1
with a threshold of 1e−05
MR Test
Inverse variance weighted (fixed effects)
Inverse variance weighted (multiplicative random effects)
MR Egger
MR RAPS
Weighted median
Weighted mode
0.10
0.05
0.00
−0.05
−0.10
0.1
0.2
SNP effect on Mannan−binding lectin serine protease 1
SNP effect on Gut microbiota abundance (genus Parasutterella id.2892)

Batch 2−3711 : Gut microbiota abundance (genus Catenibacterium id.2153) on Megakaryocyte−associated tyrosine−protein kinase
with a threshold of 1e−05

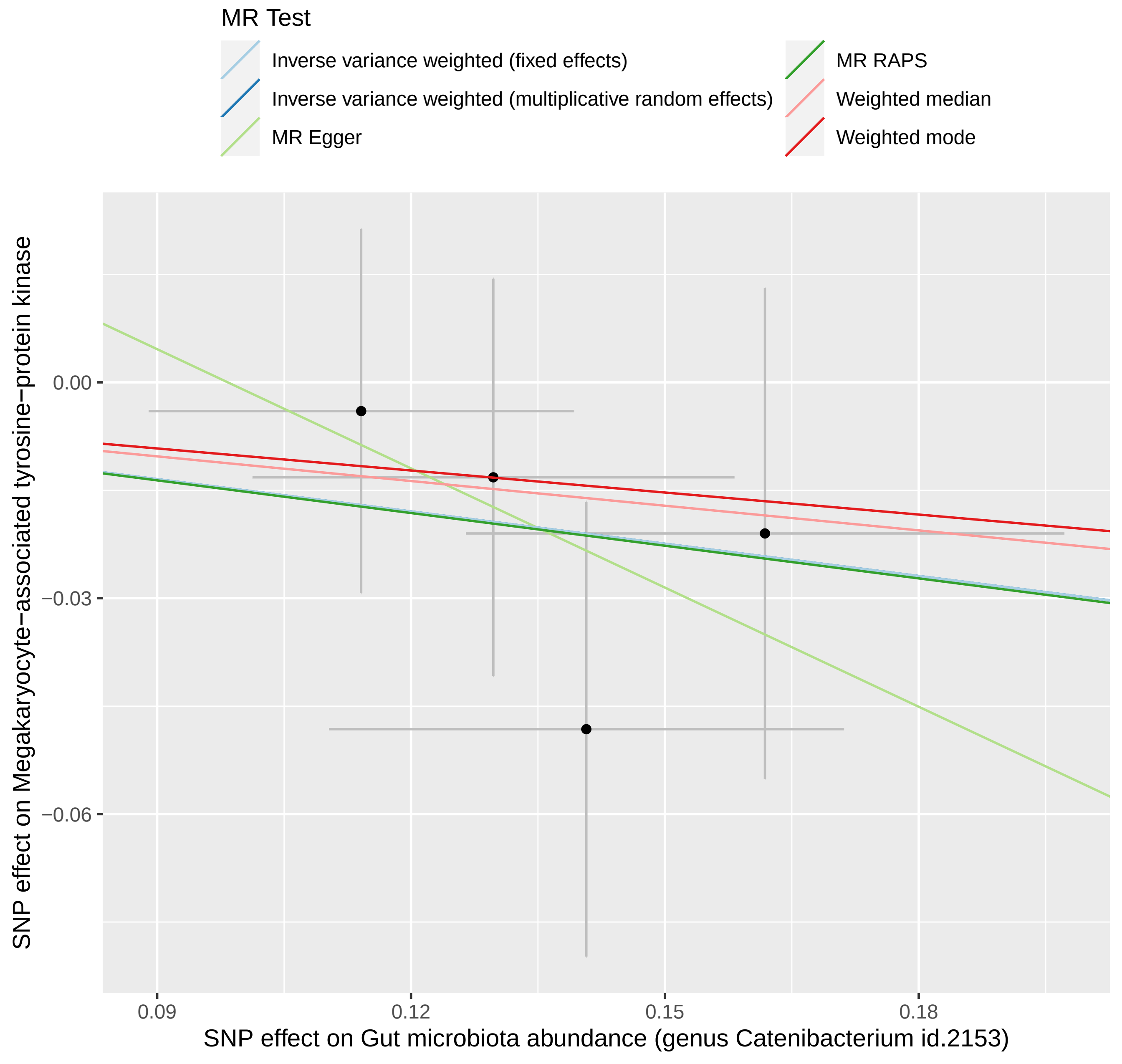

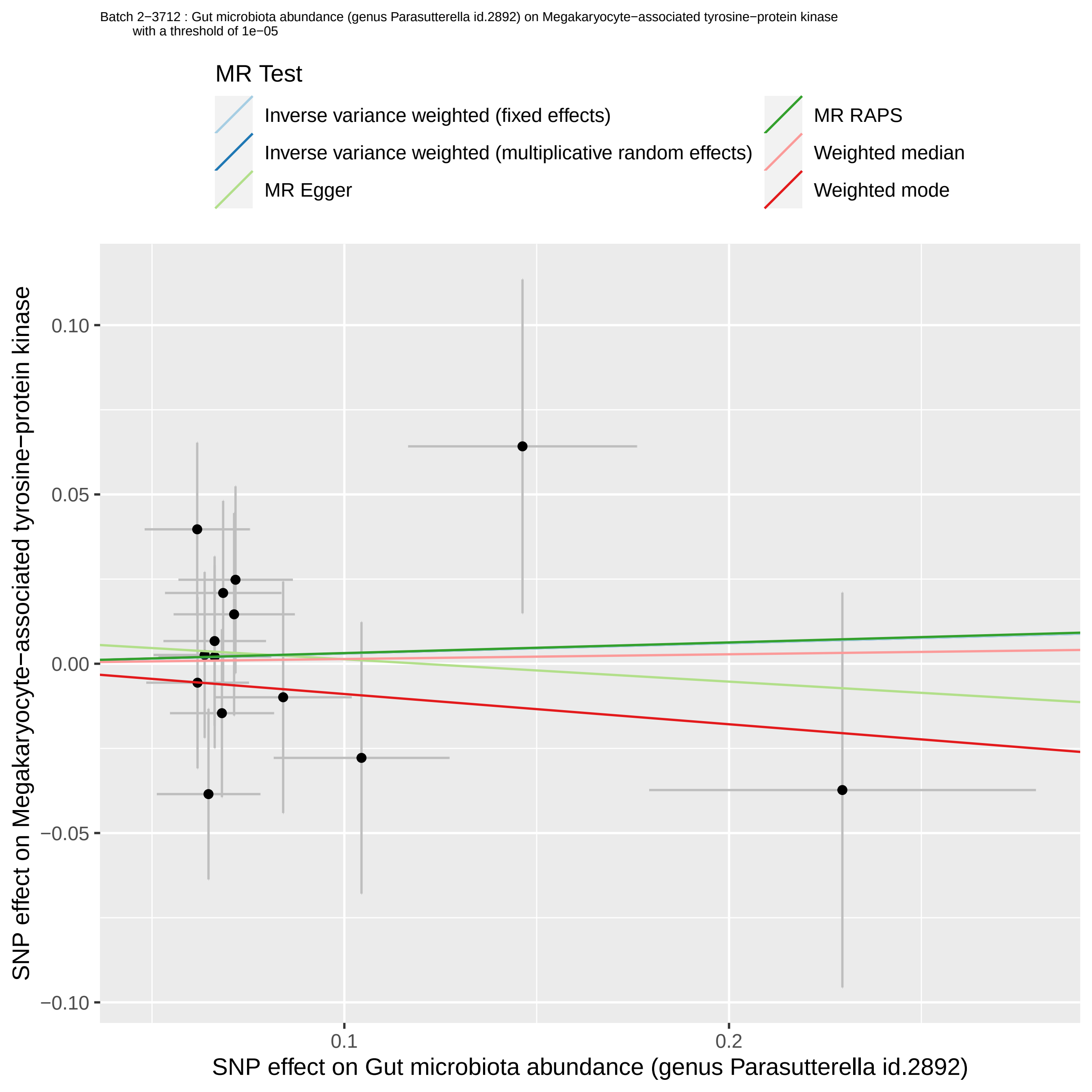
Batch 2−3712 : Gut microbiota abundance (genus Parasutterella id.2892) on Megakaryocyte−associated tyrosine−protein kinase
with a threshold of 1e−05
MR Test
Inverse variance weighted (fixed effects)
Inverse variance weighted (multiplicative random effects)
MR Egger
MR RAPS
Weighted median
Weighted mode
SNP effect on Megakaryocyte−associated tyrosine−protein kinase
0.10
0.05
0.00
−0.05
−0.10
0.1
0.2
SNP effect on Gut microbiota abundance (genus Parasutterella id.2892)

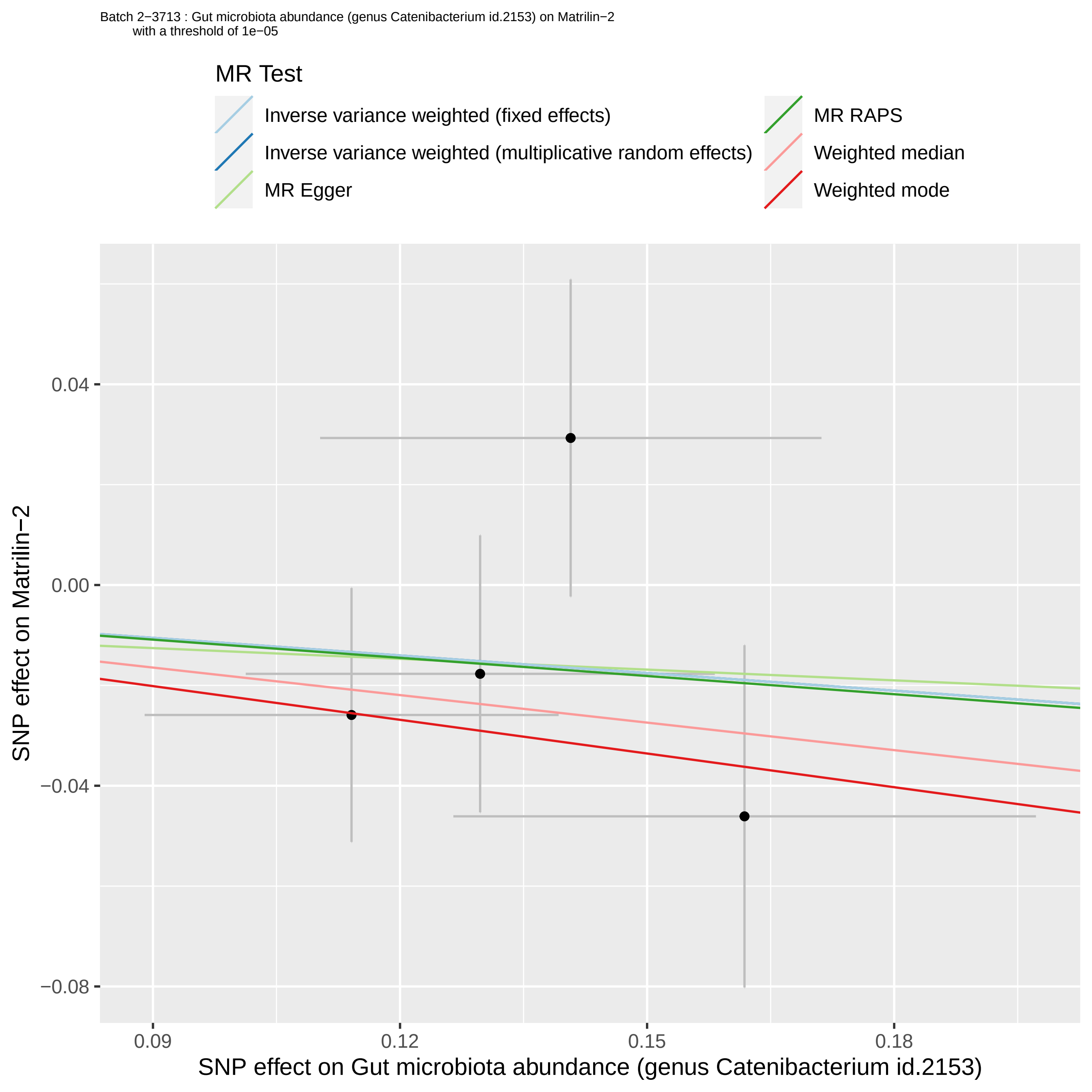

Batch 2−3713 : Gut microbiota abundance (genus Catenibacterium id.2153) on Matrilin−2
with a threshold of 1e−05
MR Test
Inverse variance weighted (fixed effects)
Inverse variance weighted (multiplicative random effects)
MR Egger
MR RAPS
Weighted median
Weighted mode
0.04
0.00
−0.04
−0.08
SNP effect on Matrilin−2
0.09
0.12
0.15
0.18
SNP effect on Gut microbiota abundance (genus Catenibacterium id.2153)

Batch 2−3714 : Gut microbiota abundance (genus Parasutterella id.2892) on Matrilin−2
with a threshold of 1e−05

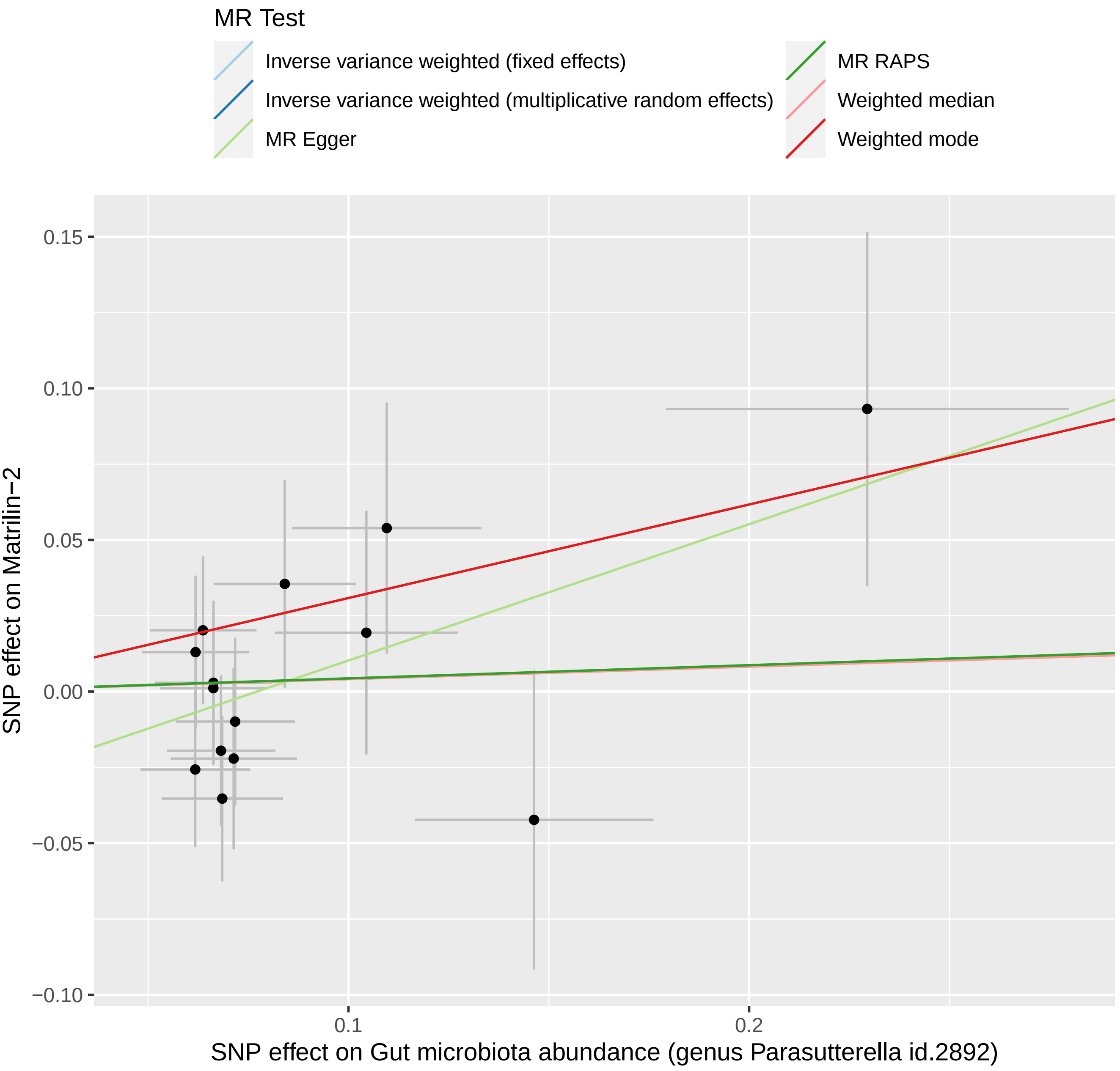

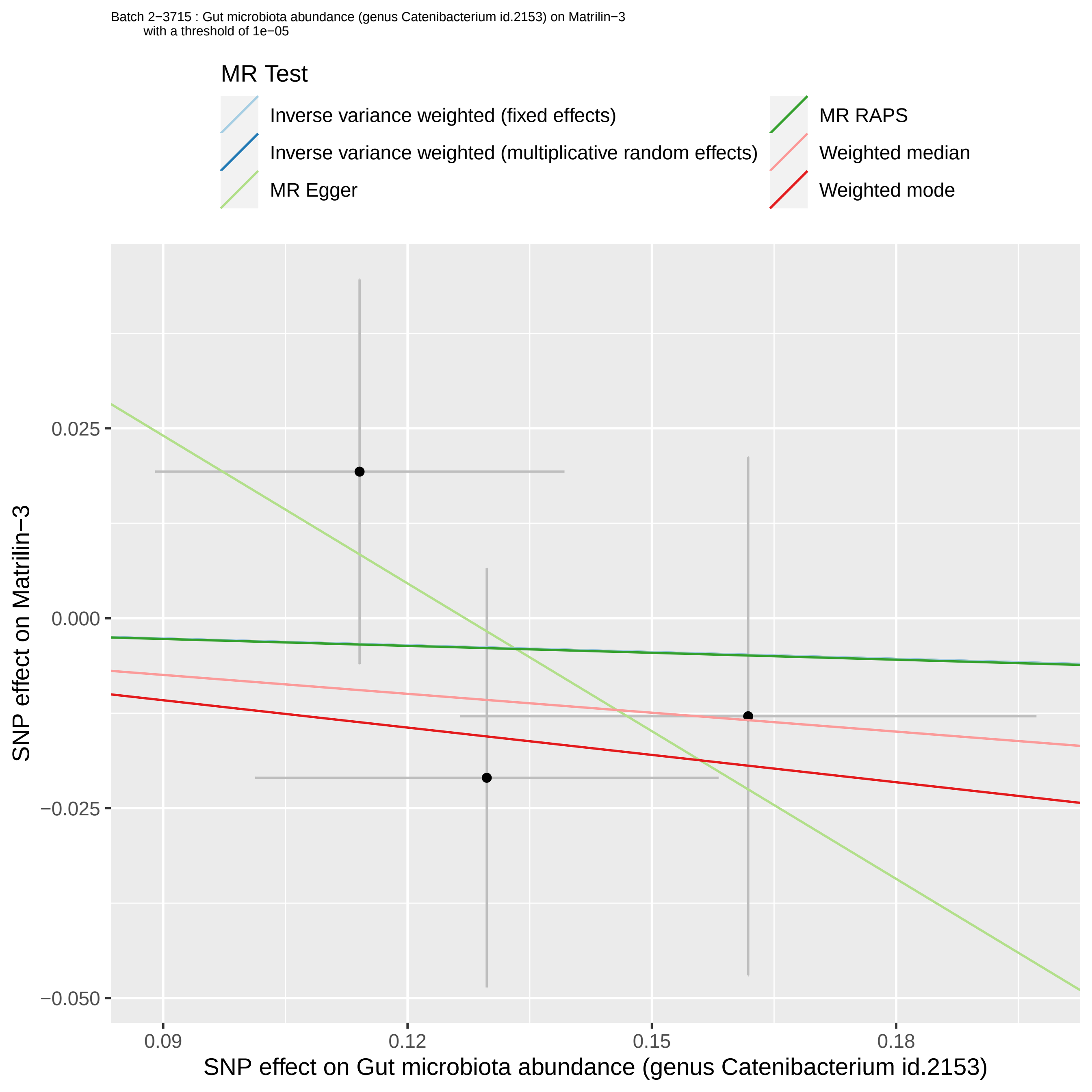

Batch 2−3715 : Gut microbiota abundance (genus Catenibacterium id.2153) on Matrilin−3
with a threshold of 1e−05
MR Test
Inverse variance weighted (fixed effects)
Inverse variance weighted (multiplicative random effects)
MR Egger
MR RAPS
Weighted median
Weighted mode
0.025
0.000
−0.025
−0.050
0.09
0.12
0.15
0.18
SNP effect on Matrilin−3
SNP effect on Gut microbiota abundance (genus Catenibacterium id.2153)

Batch 2−3716 : Gut microbiota abundance (genus Parasutterella id.2892) on Matrilin−3
with a threshold of 1e−05

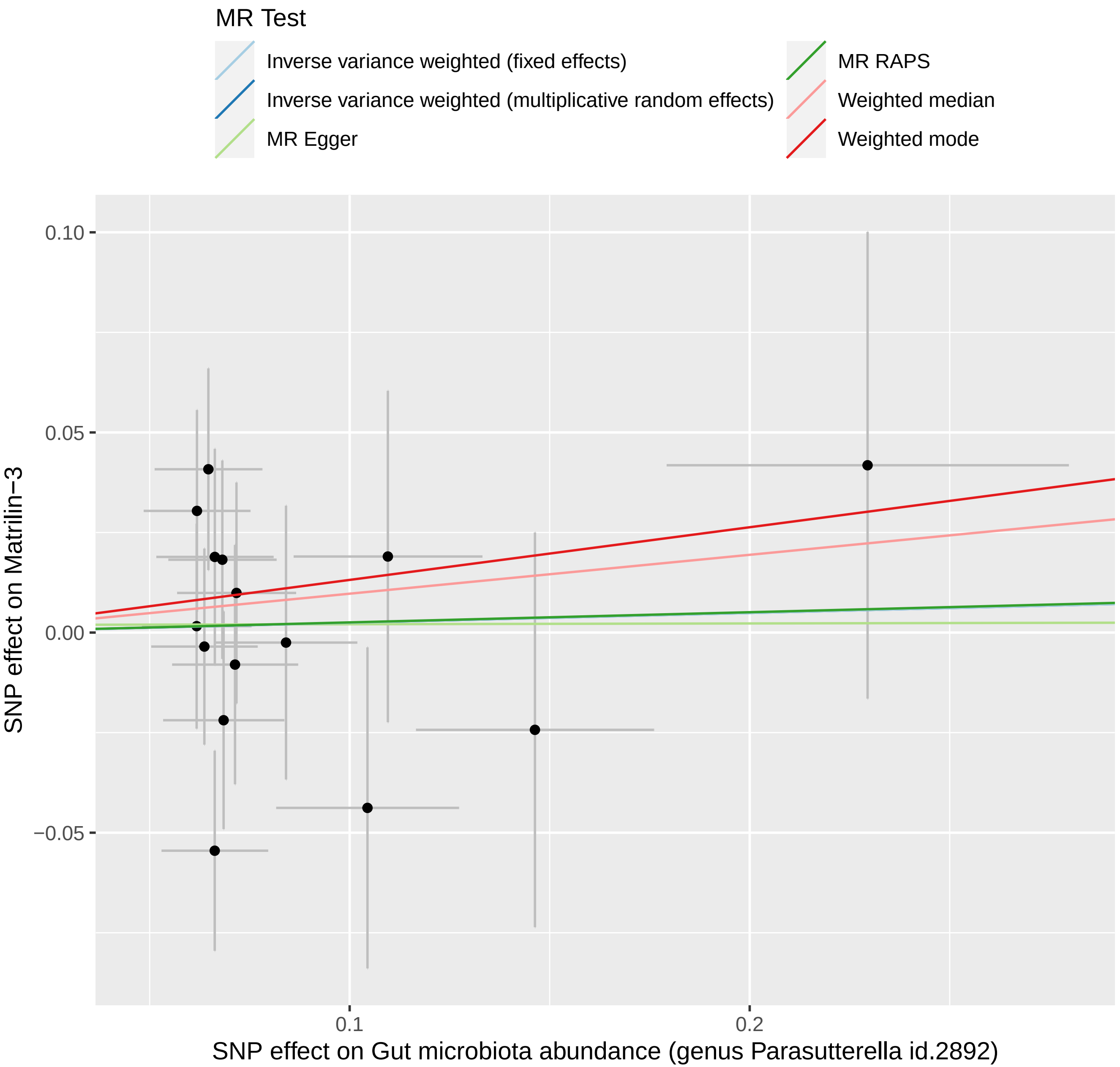

Batch 2−3717 : Gut microbiota abundance (genus Catenibacterium id.2153) on Matrilin−4
with a threshold of 1e−05

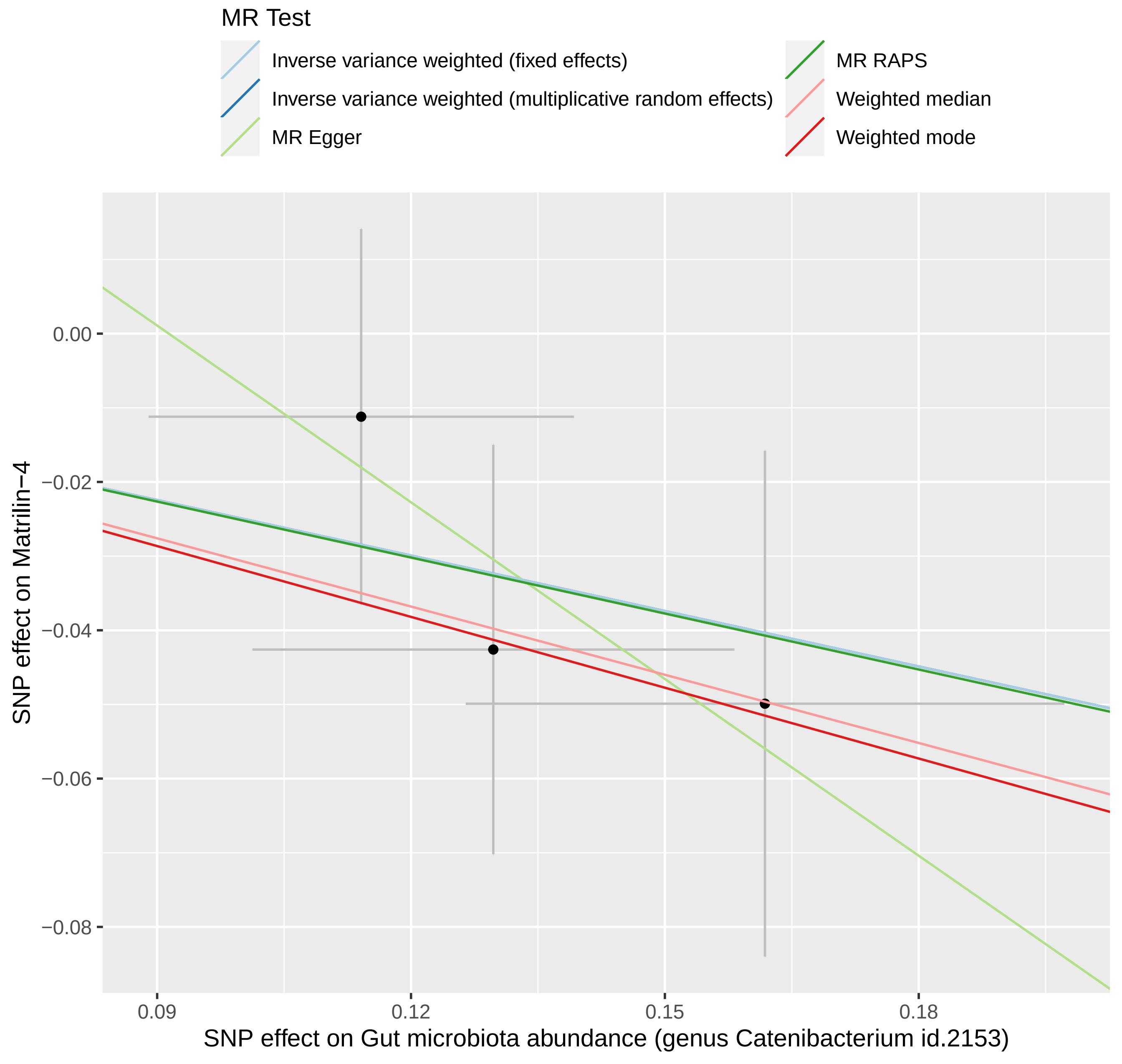

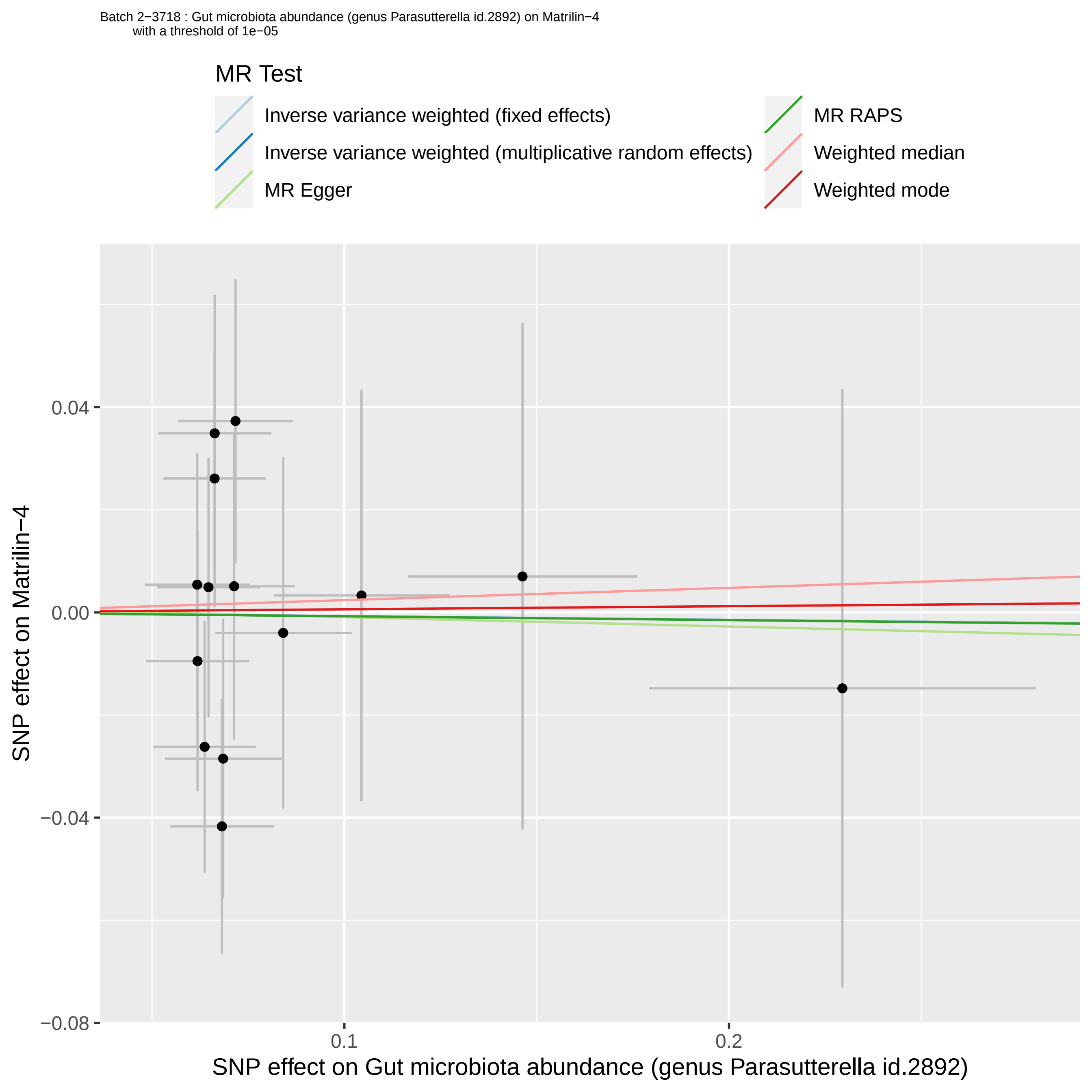
Batch 2−3718 : Gut microbiota abundance (genus Parasutterella id.2892) on Matrilin−4
with a threshold of 1e−05
MR Test
Inverse variance weighted (fixed effects)
Inverse variance weighted (multiplicative random effects)
MR Egger
MR RAPS
Weighted median
Weighted mode
0.04
0.00
−0.04
−0.08
0.1
0.2
SNP effect on Matrilin−4
SNP effect on Gut microbiota abundance (genus Parasutterella id.2892)

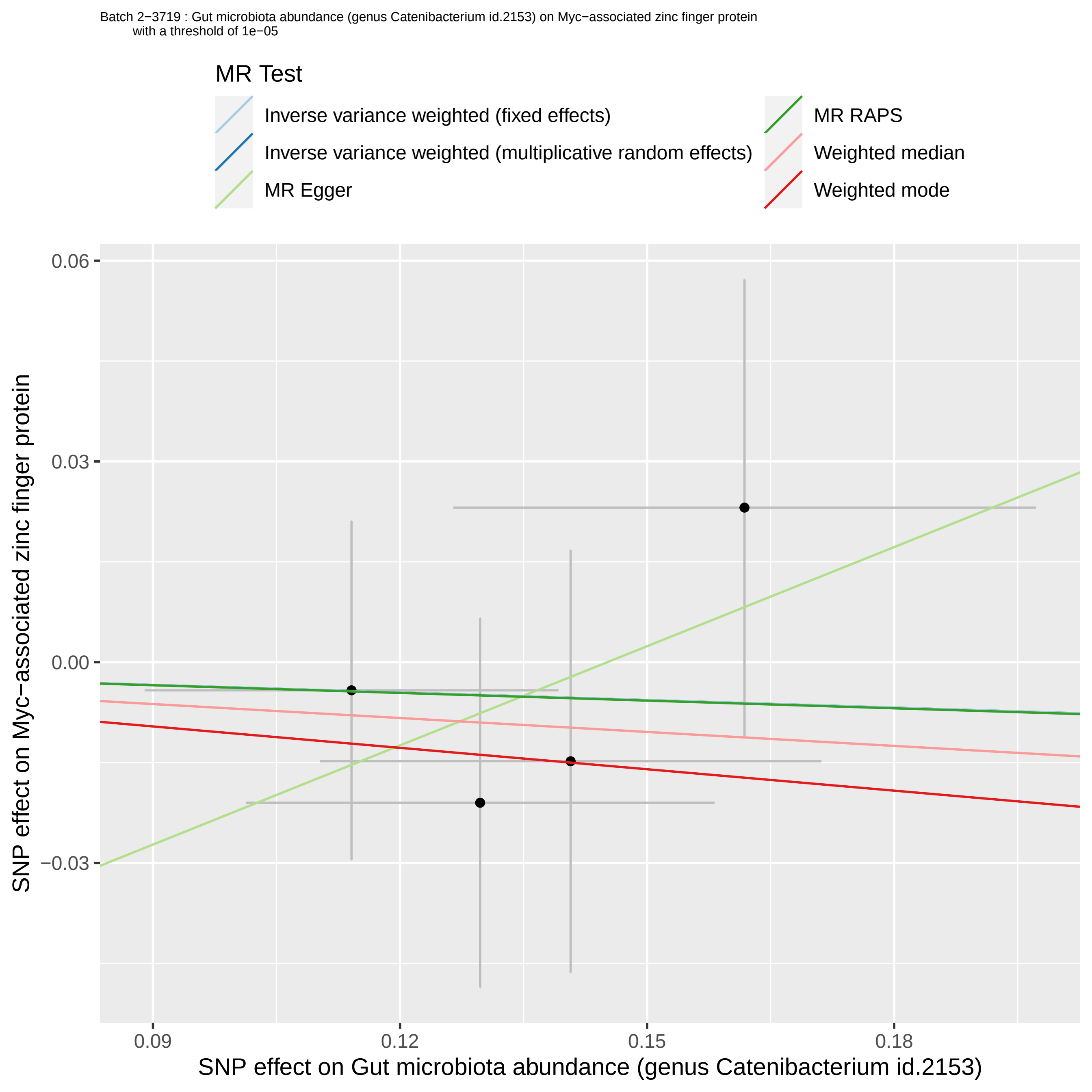

Batch 2−3719 : Gut microbiota abundance (genus Catenibacterium id.2153) on Myc−associated zinc finger protein
with a threshold of 1e−05
MR Test
Inverse variance weighted (fixed effects)
Inverse variance weighted (multiplicative random effects)
MR Egger
MR RAPS
Weighted median
Weighted mode
0.06
0.03
0.00
−0.03
0.09
0.12
0.15
0.18
SNP effect on Myc−associated zinc finger protein
SNP effect on Gut microbiota abundance (genus Catenibacterium id.2153)

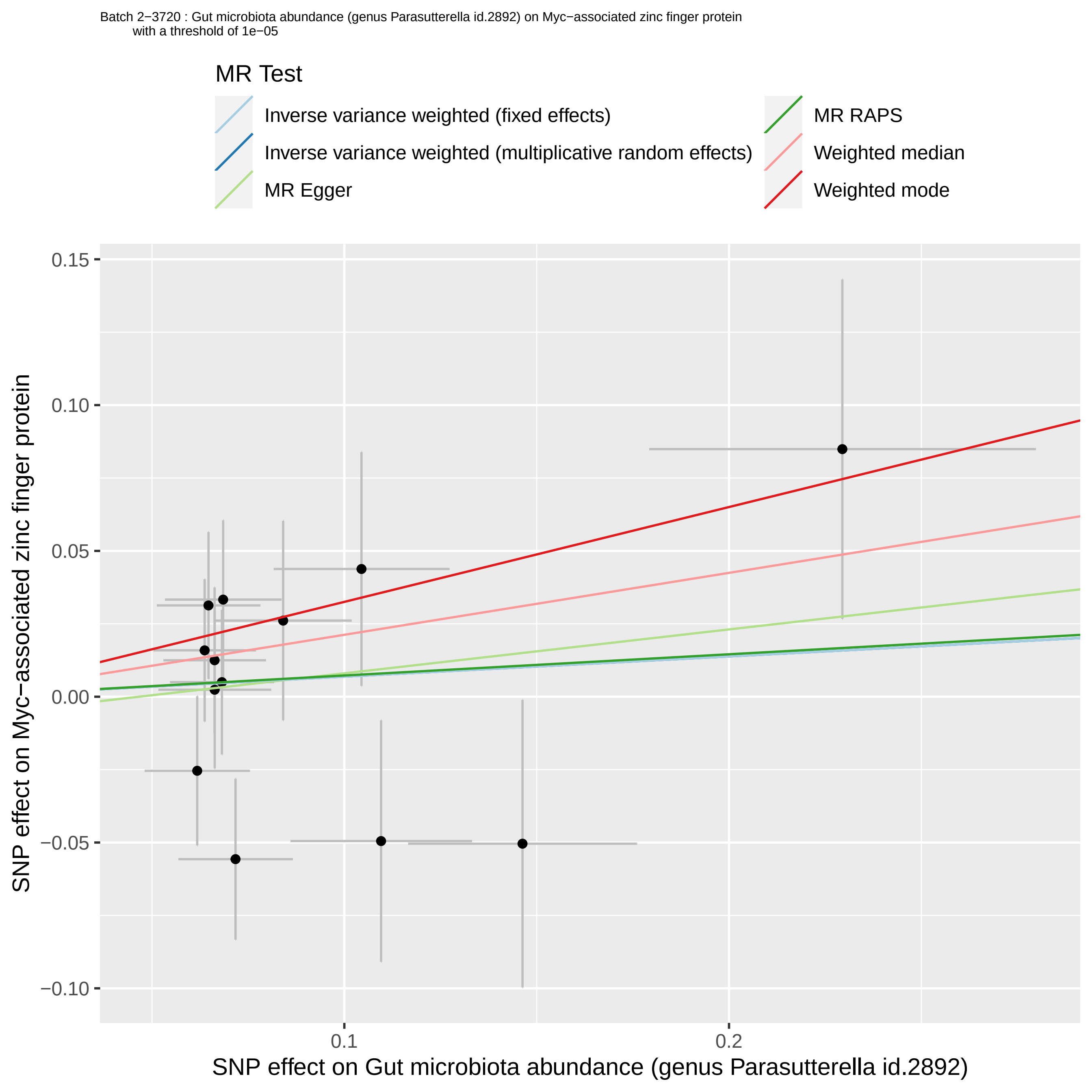

Batch 2−3720 : Gut microbiota abundance (genus Parasutterella id.2892) on Myc−associated zinc finger protein
with a threshold of 1e−05
MR Test
Inverse variance weighted (fixed effects)
Inverse variance weighted (multiplicative random effects)
MR Egger
MR RAPS
Weighted median
Weighted mode
0.15
0.10
0.05
0.00
−0.05
−0.10
0.1
0.2
SNP effect on Myc−associated zinc finger protein
SNP effect on Gut microbiota abundance (genus Parasutterella id.2892)

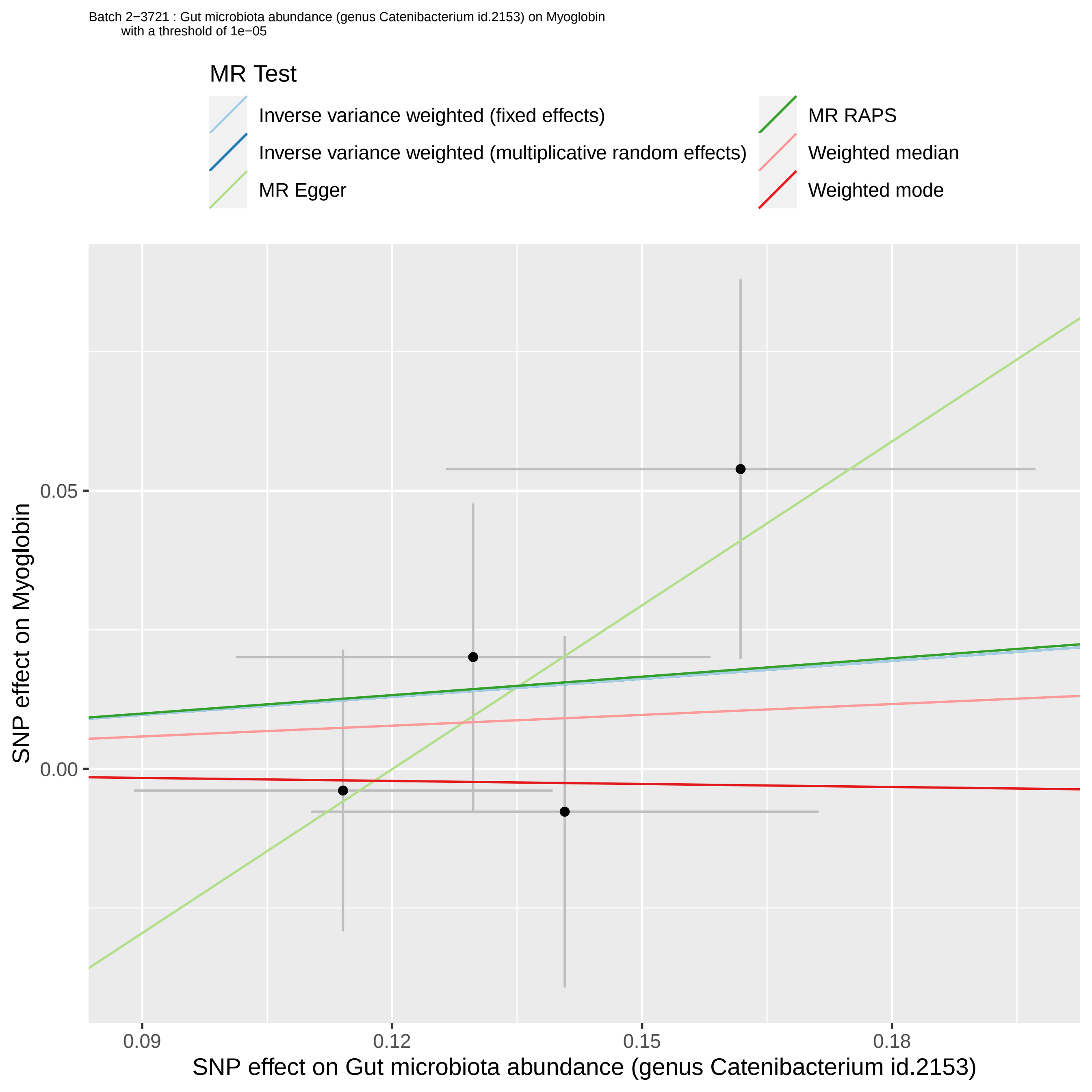
Batch 2−3721 : Gut microbiota abundance (genus Catenibacterium id.2153) on Myoglobin
with a threshold of 1e−05
MR Test
Inverse variance weighted (fixed effects)
Inverse variance weighted (multiplicative random effects)
MR Egger
MR RAPS
Weighted median
Weighted mode
0.05
0.00
0.09
0.12
0.15
0.18
SNP effect on Myoglobin
SNP effect on Gut microbiota abundance (genus Catenibacterium id.2153)

Batch 2−3722 : Gut microbiota abundance (genus Parasutterella id.2892) on Myoglobin
with a threshold of 1e−05

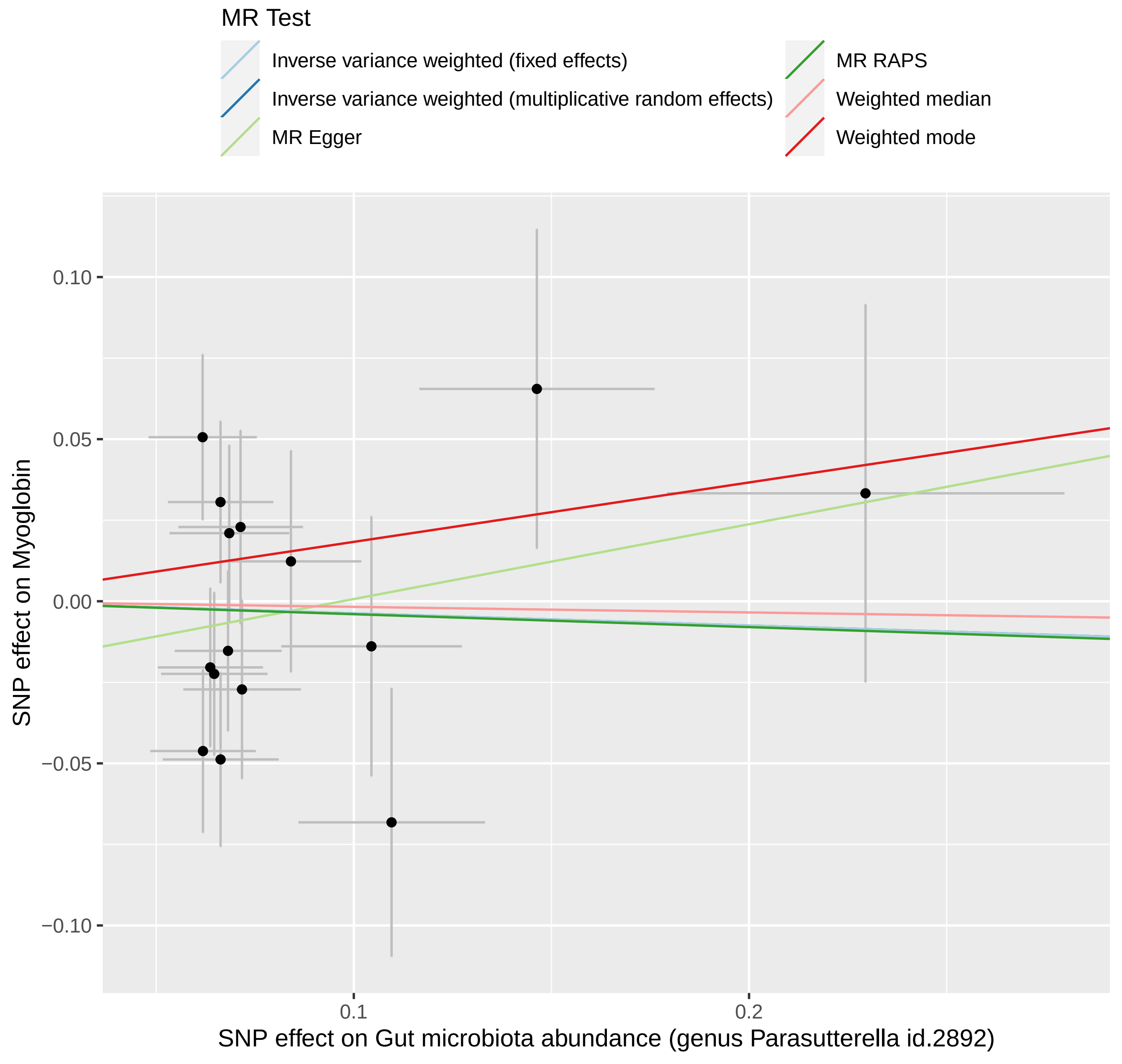

Batch 2−3723 : Gut microbiota abundance (genus Catenibacterium id.2153) on Methyl−CpG−binding domain protein 4
with a threshold of 1e−05

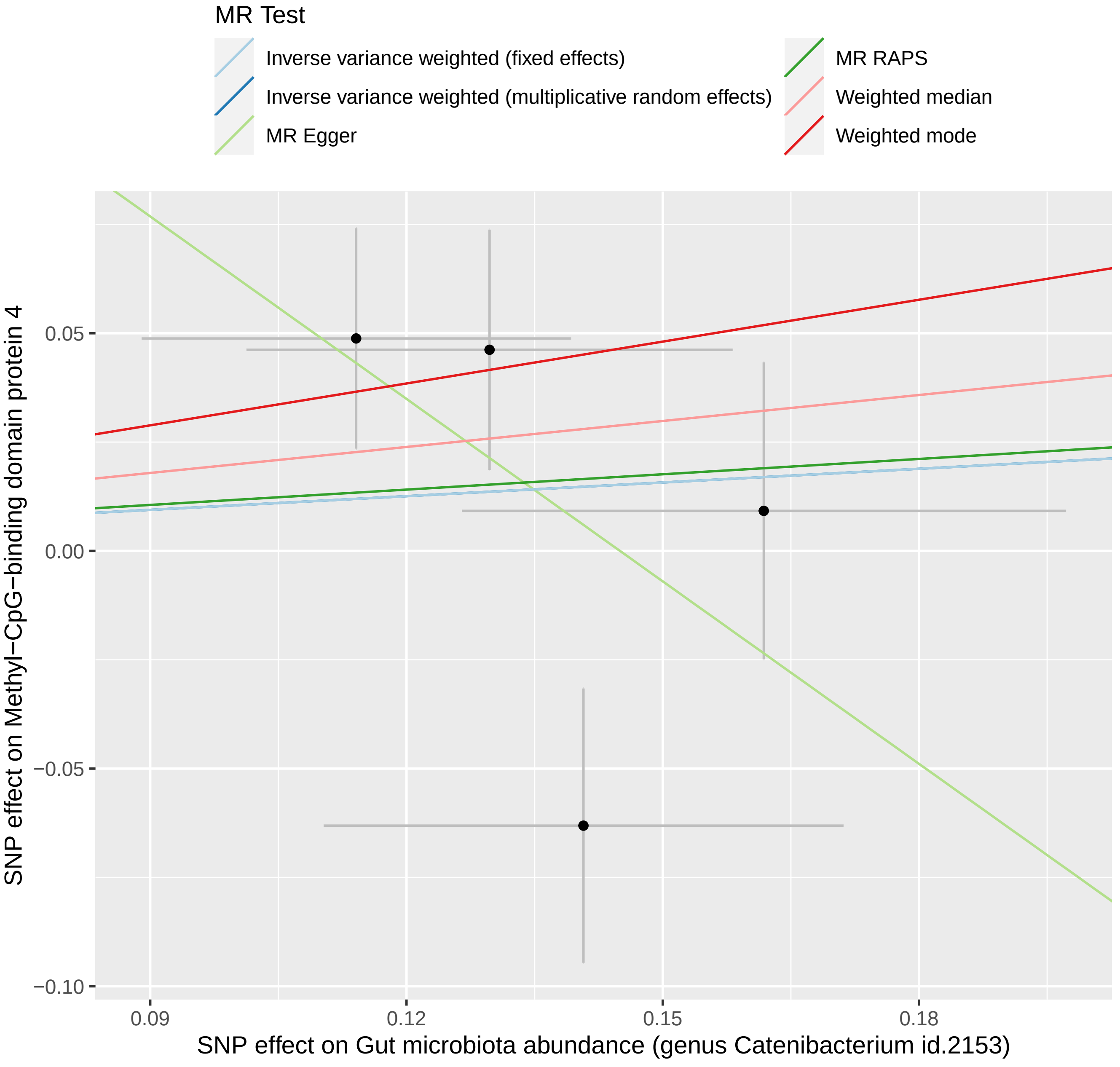

Batch 2−3724 : Gut microbiota abundance (genus Parasutterella id.2892) on Methyl−CpG−binding domain protein 4
with a threshold of 1e−05

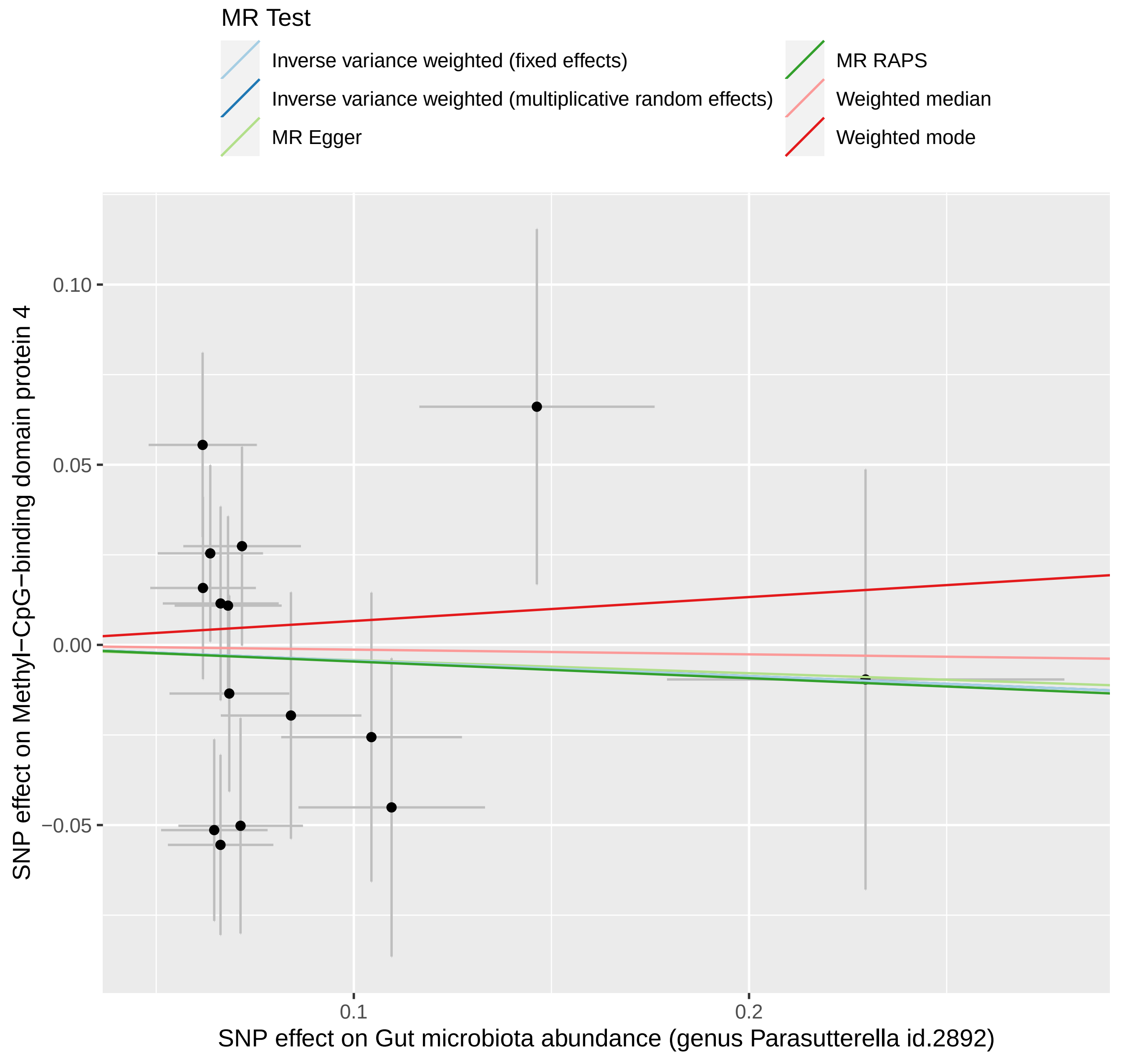

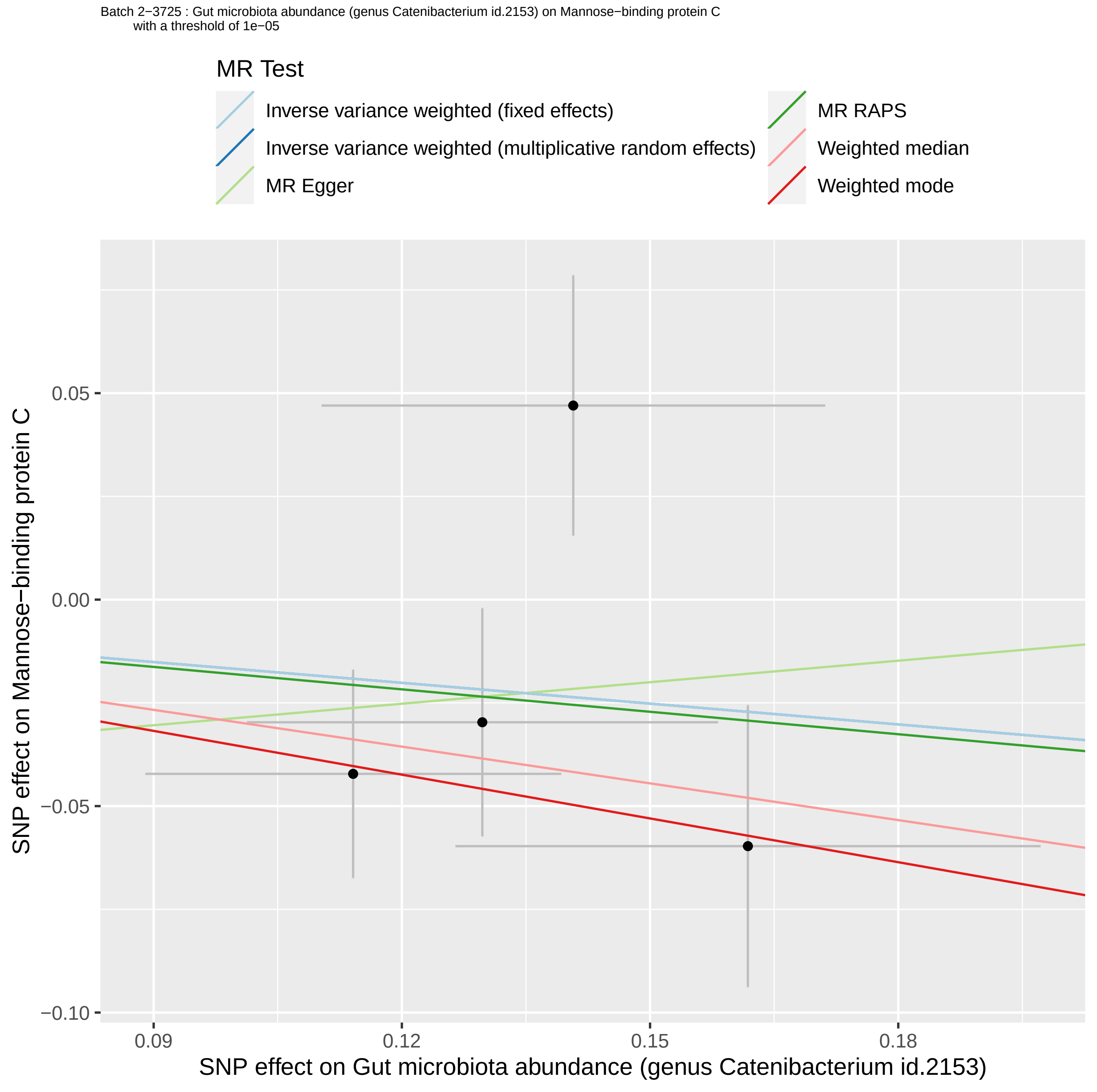
Batch 2−3725 : Gut microbiota abundance (genus Catenibacterium id.2153) on Mannose−binding protein C
with a threshold of 1e−05
MR Test
Inverse variance weighted (fixed effects)
Inverse variance weighted (multiplicative random effects)
MR Egger
MR RAPS
Weighted median
Weighted mode
0.05
0.00
−0.05
−0.10
0.09
0.12
0.15
0.18
SNP effect on Mannose−binding protein C
SNP effect on Gut microbiota abundance (genus Catenibacterium id.2153)

Batch 2−3726 : Gut microbiota abundance (genus Parasutterella id.2892) on Mannose−binding protein C
with a threshold of 1e−05

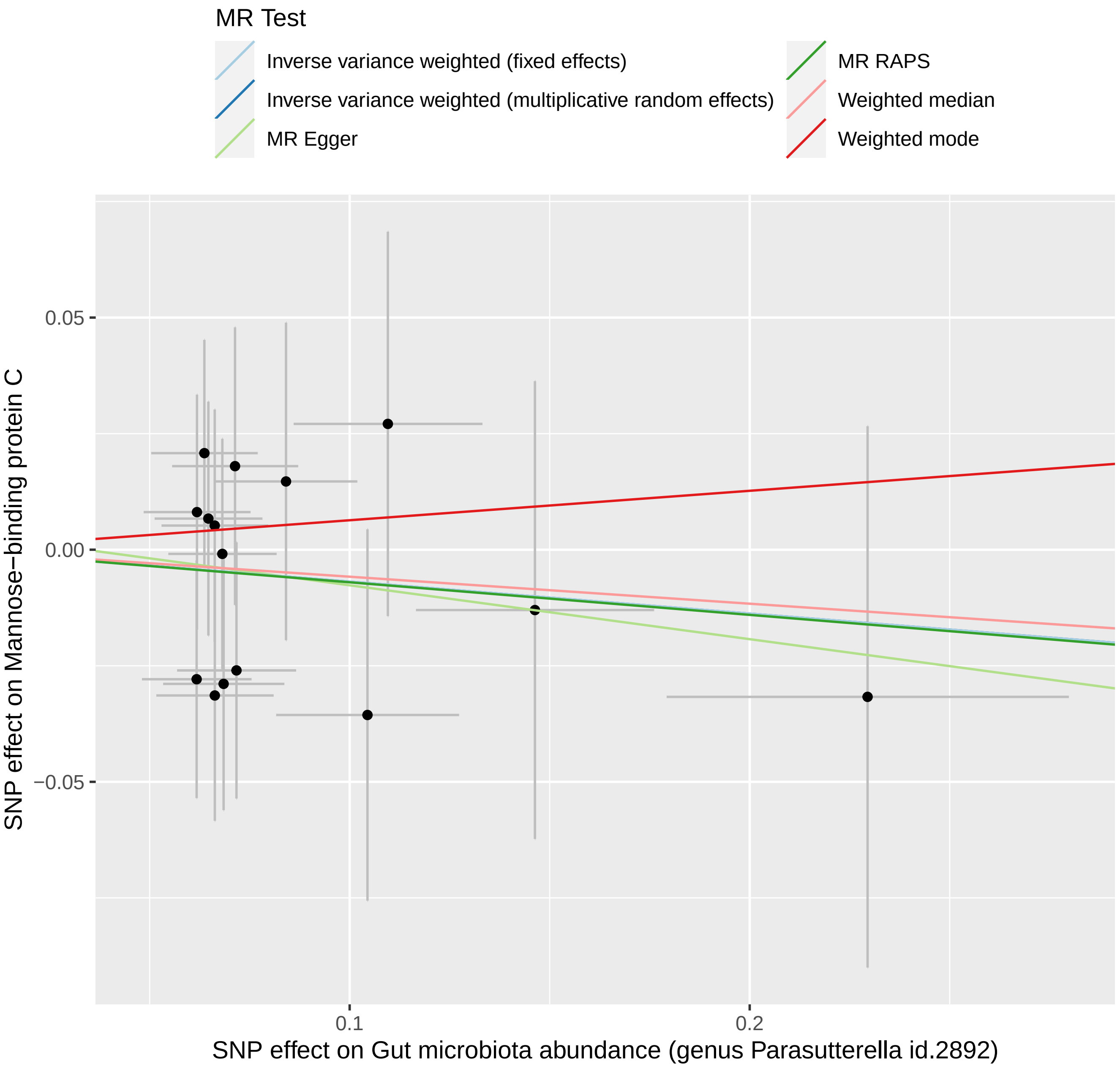

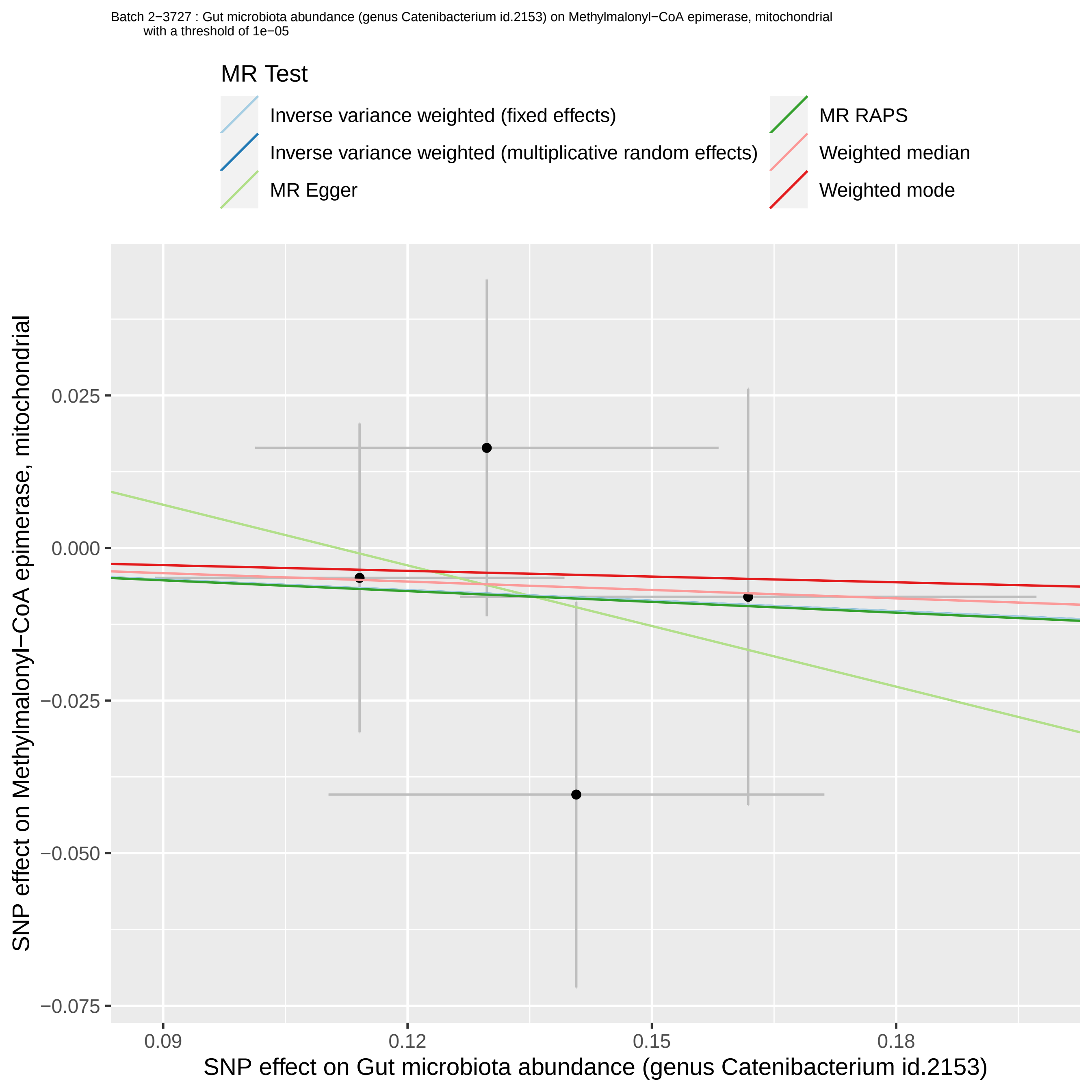

Batch 2−3727 : Gut microbiota abundance (genus Catenibacterium id.2153) on Methylmalonyl−CoA epimerase, mitochondrial
with a threshold of 1e−05
MR Test
Inverse variance weighted (fixed effects)
Inverse variance weighted (multiplicative random effects)
MR Egger
MR RAPS
Weighted median
Weighted mode
0.025
0.000
−0.025
−0.050
−0.075
0.09
0.12
0.15
0.18
SNP effect on Methylmalonyl−CoA epimerase, mitochondrial
SNP effect on Gut microbiota abundance (genus Catenibacterium id.2153)

Batch 2−3728 : Gut microbiota abundance (genus Parasutterella id.2892) on Methylmalonyl−CoA epimerase, mitochondrial
with a threshold of 1e−05

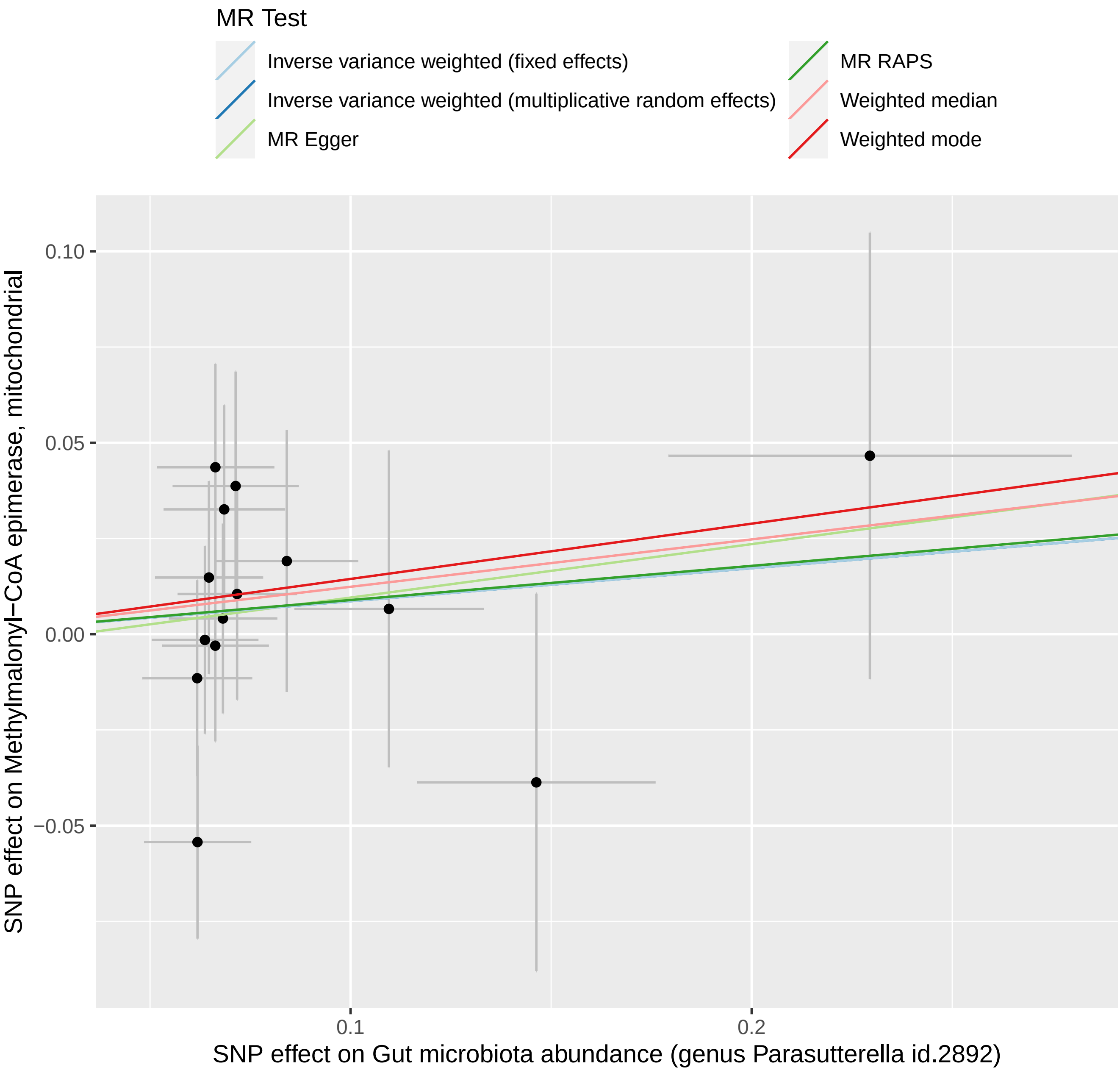

Batch 2−3729 : Gut microbiota abundance (genus Catenibacterium id.2153) on Mast cell−expressed membrane protein 1
with a threshold of 1e−05

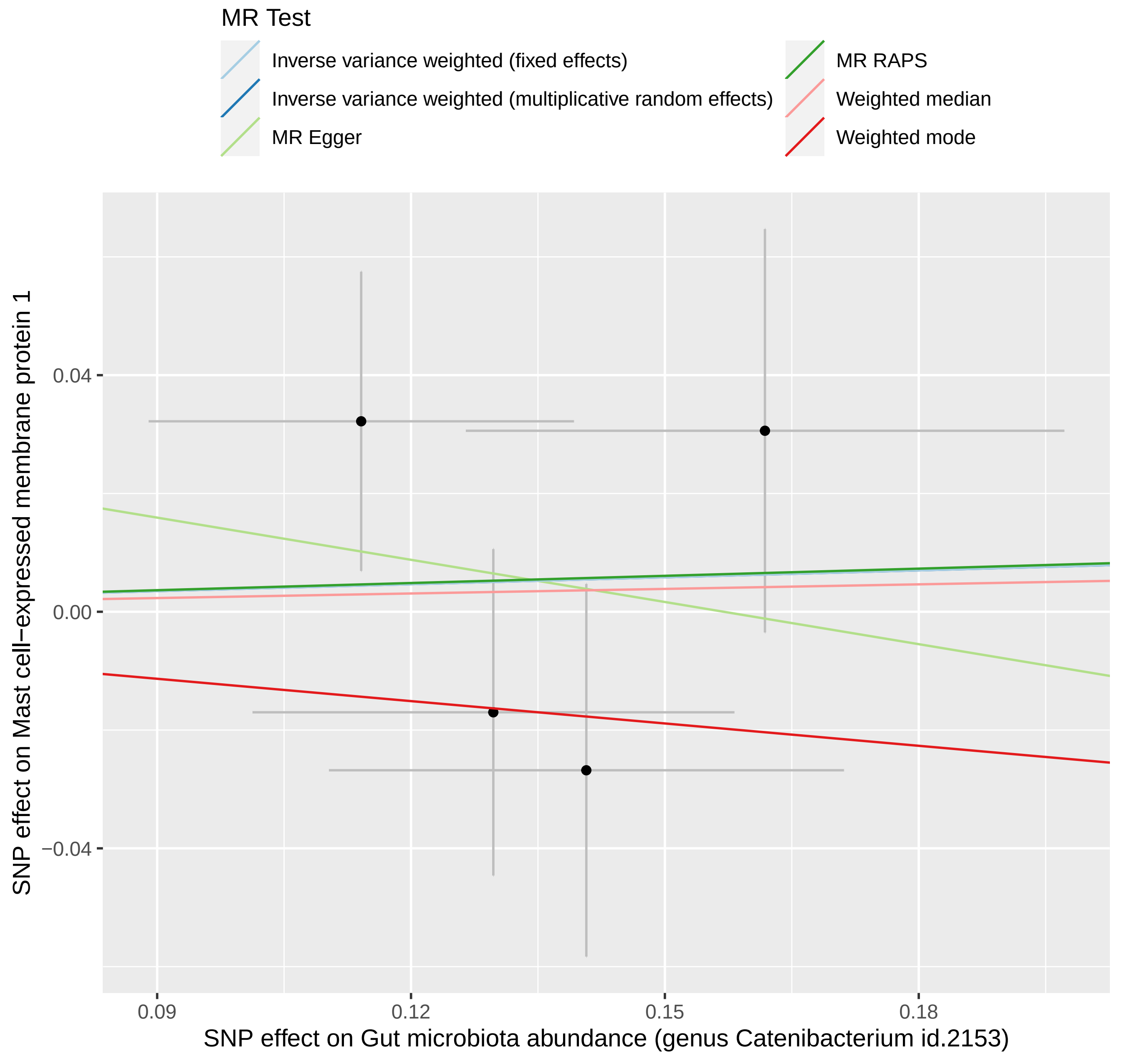

Batch 2−3730 : Gut microbiota abundance (genus Parasutterella id.2892) on Mast cell−expressed membrane protein 1
with a threshold of 1e−05

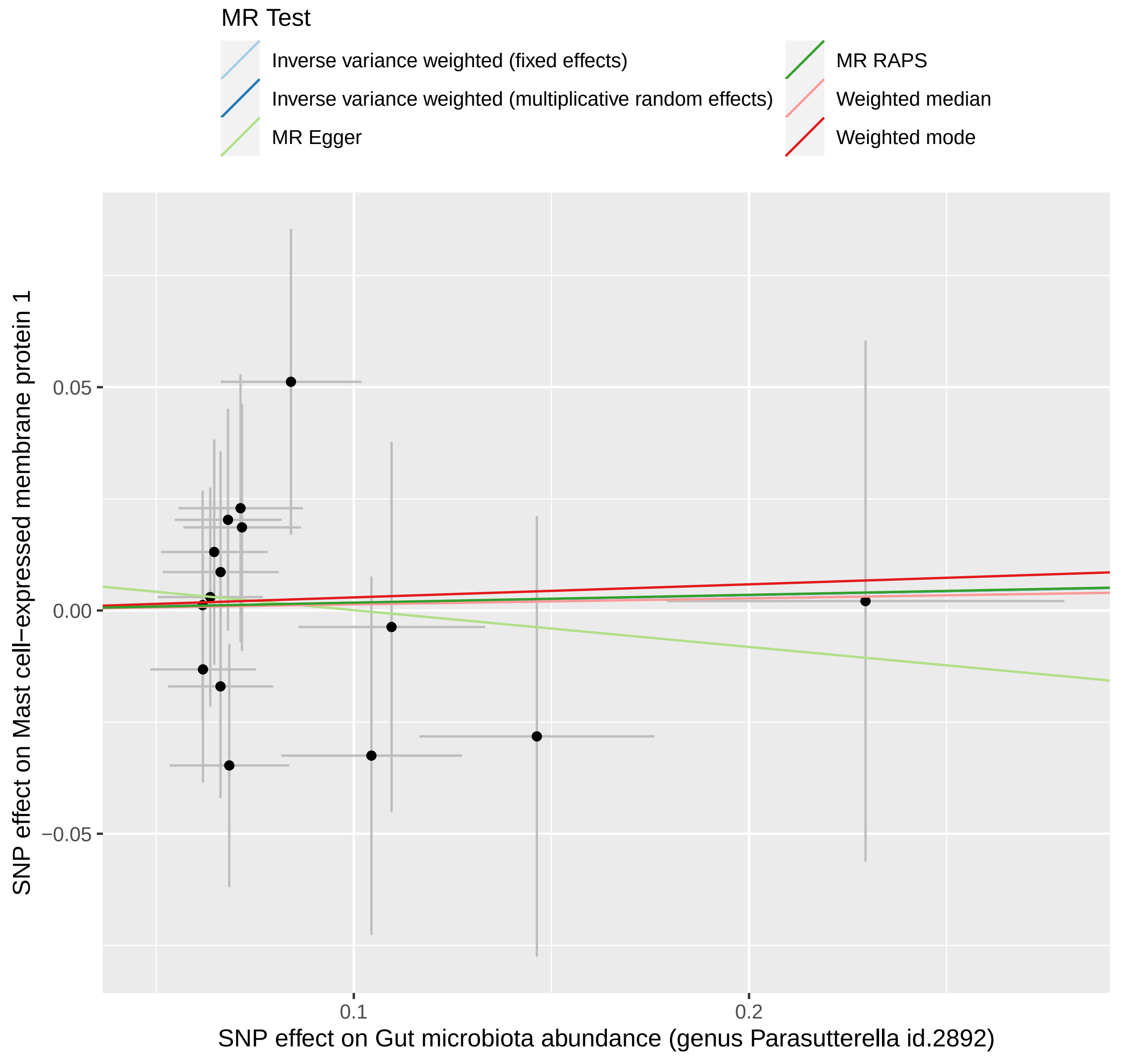

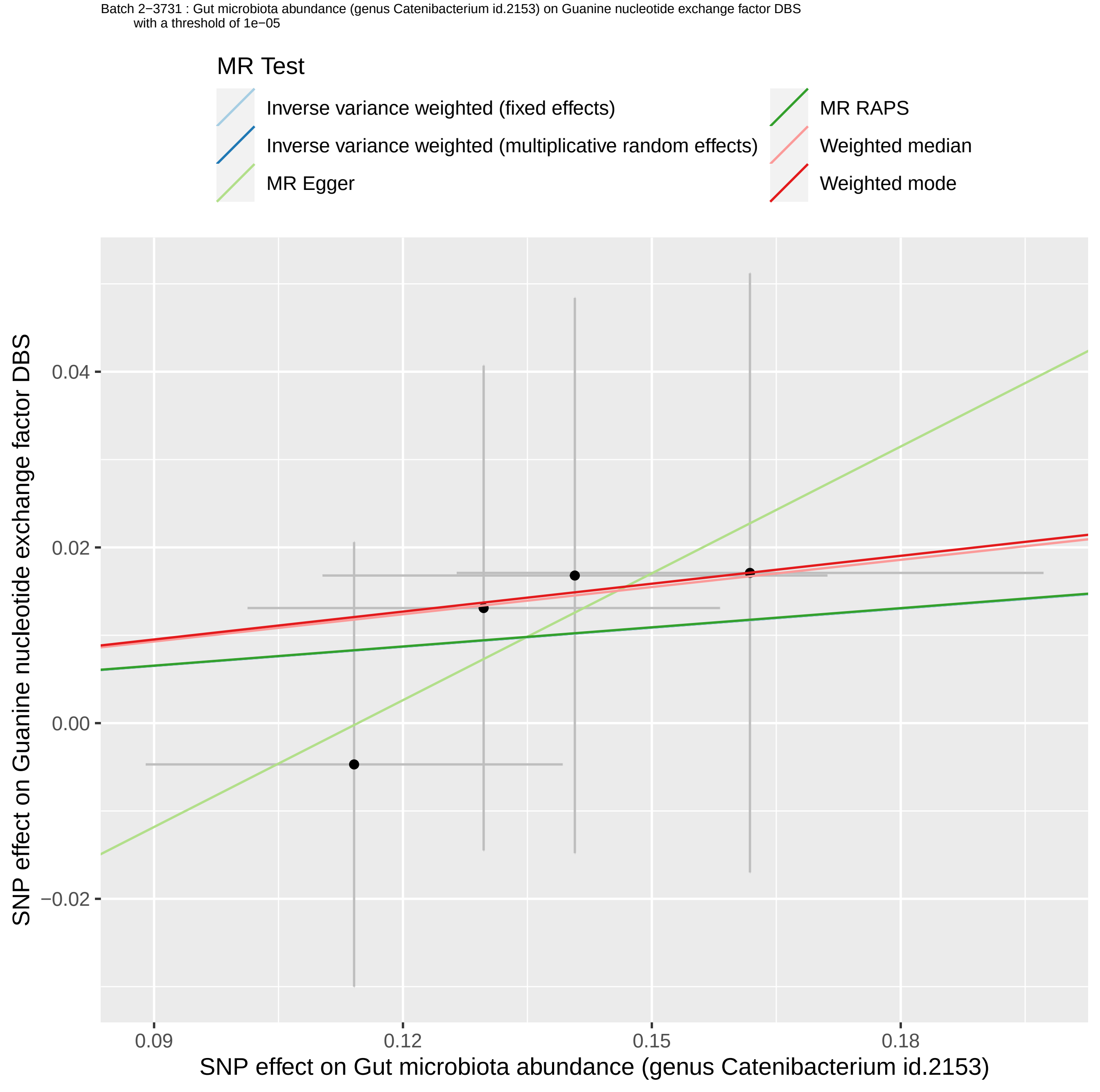
Batch 2−3731 : Gut microbiota abundance (genus Catenibacterium id.2153) on Guanine nucleotide exchange factor DBS
with a threshold of 1e−05
MR Test
Inverse variance weighted (fixed effects)
Inverse variance weighted (multiplicative random effects)
MR Egger
MR RAPS
Weighted median
Weighted mode
0.04
0.02
0.00
−0.02
0.09
0.12
0.15
0.18
SNP effect on Guanine nucleotide exchange factor DBS
SNP effect on Gut microbiota abundance (genus Catenibacterium id.2153)

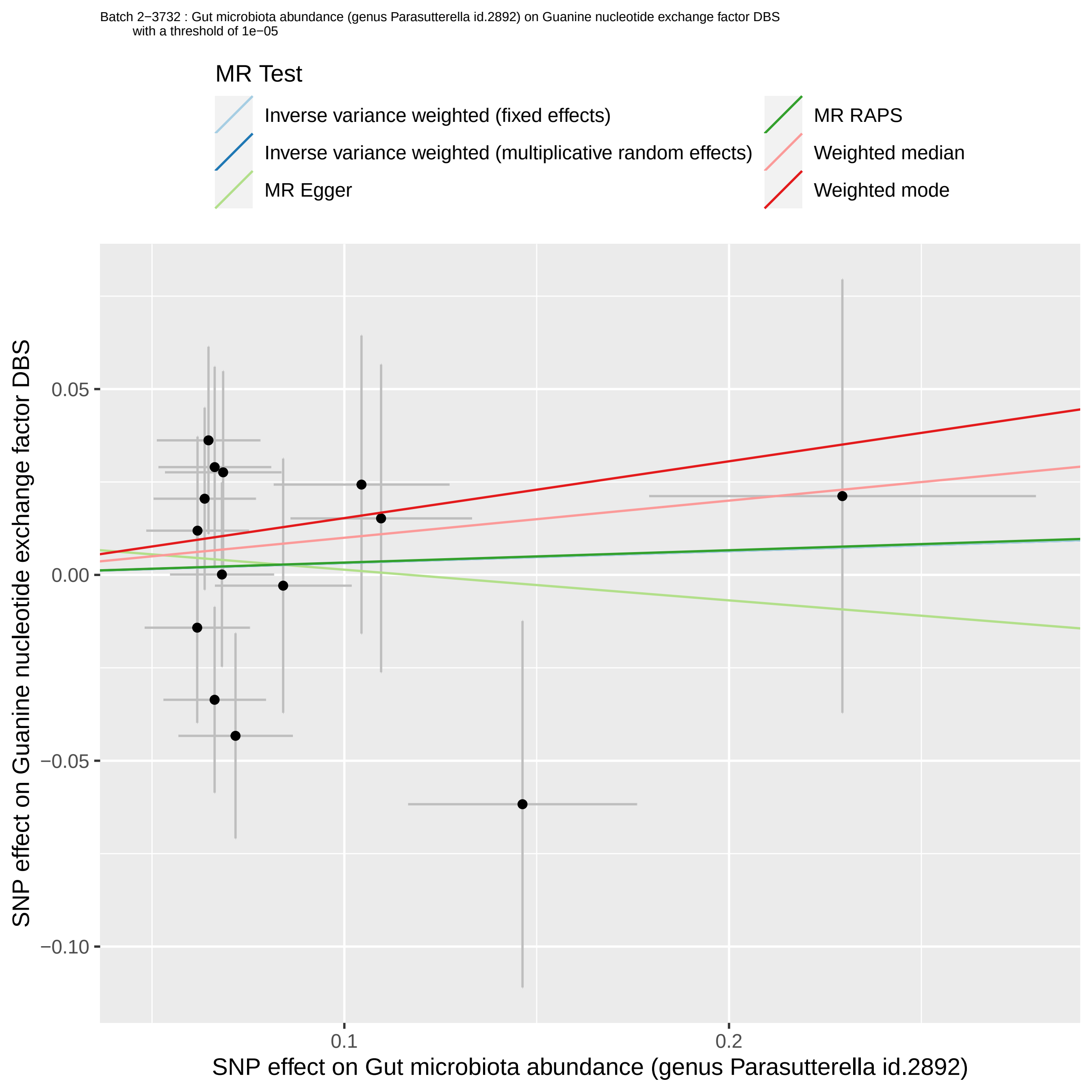
Batch 2−3732 : Gut microbiota abundance (genus Parasutterella id.2892) on Guanine nucleotide exchange factor DBS
with a threshold of 1e−05
MR Test
Inverse variance weighted (fixed effects)
Inverse variance weighted (multiplicative random effects)
MR Egger
MR RAPS
Weighted median
Weighted mode
0.05
0.00
−0.05
−0.10
0.1
0.2
SNP effect on Guanine nucleotide exchange factor DBS
SNP effect on Gut microbiota abundance (genus Parasutterella id.2892)

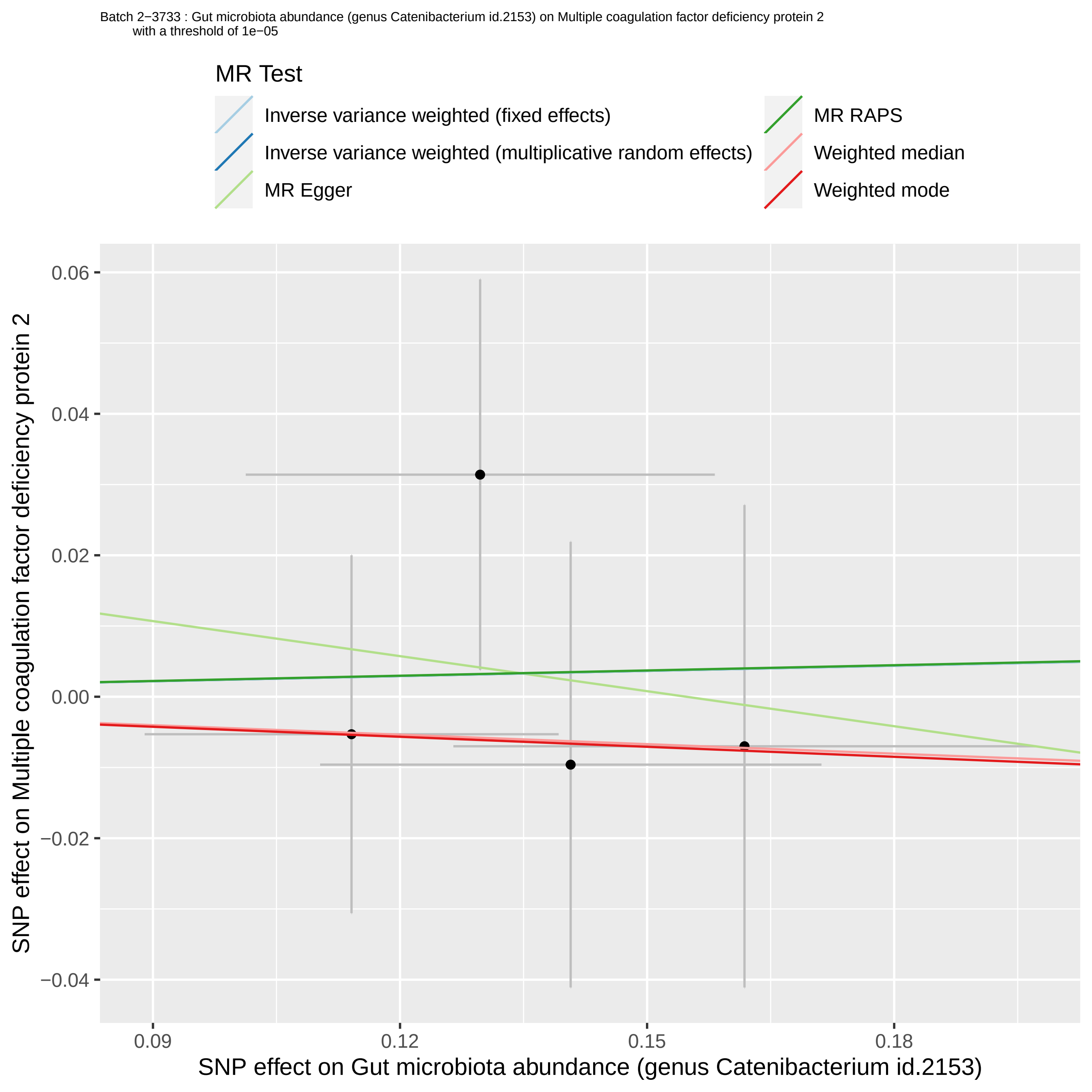
Batch 2−3733 : Gut microbiota abundance (genus Catenibacterium id.2153) on Multiple coagulation factor deficiency protein 2
with a threshold of 1e−05
MR Test
Inverse variance weighted (fixed effects)
Inverse variance weighted (multiplicative random effects)
MR Egger
MR RAPS
Weighted median
Weighted mode
SNP effect on Multiple coagulation factor deficiency protein 2
0.06
0.04
0.02
0.00
−0.02
−0.04
0.09
0.12
0.15
0.18
SNP effect on Gut microbiota abundance (genus Catenibacterium id.2153)

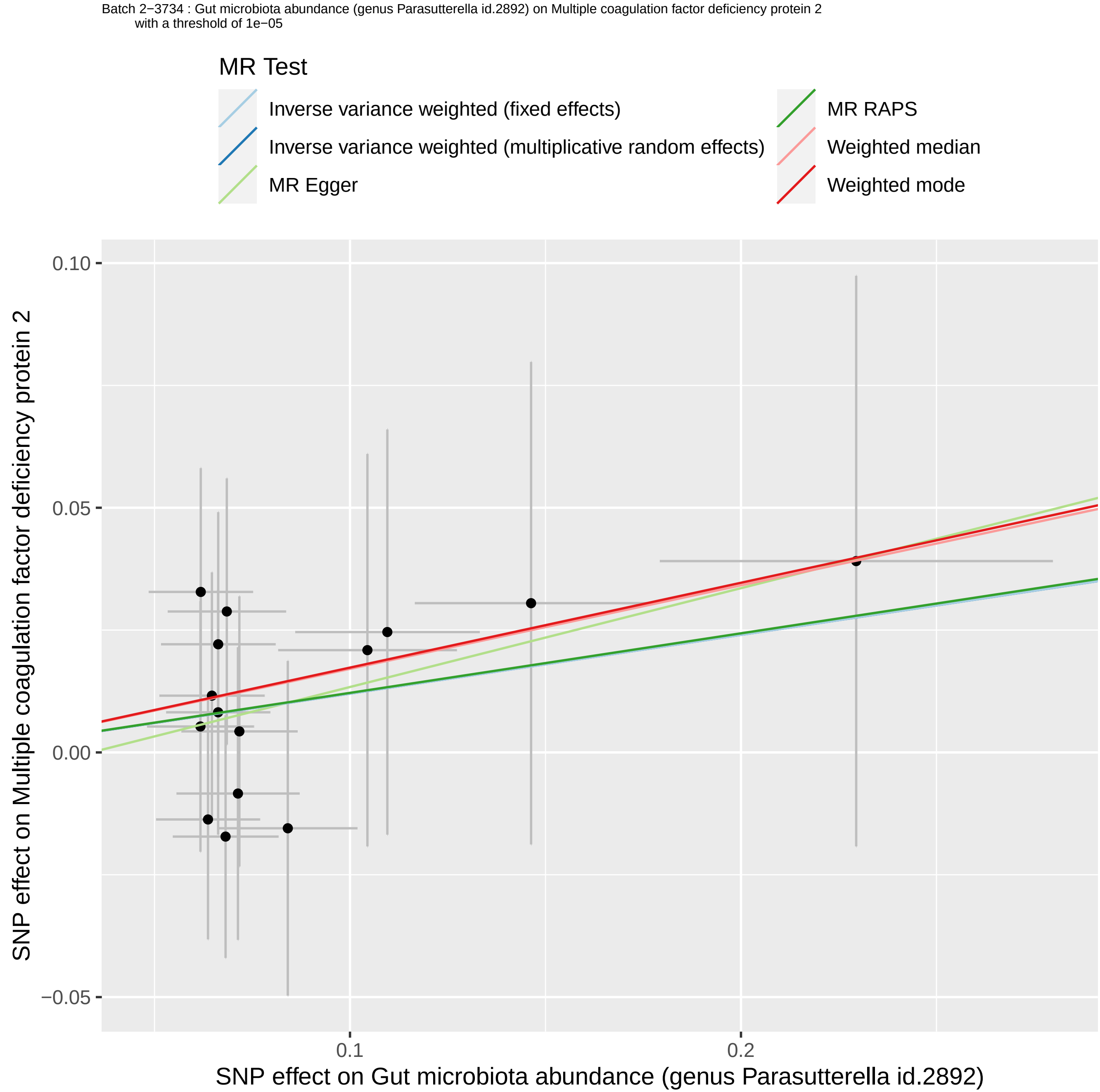

Batch 2−3734 : Gut microbiota abundance (genus Parasutterella id.2892) on Multiple coagulation factor deficiency protein 2
with a threshold of 1e−05
MR Test
Inverse variance weighted (fixed effects)
Inverse variance weighted (multiplicative random effects)
MR Egger
MR RAPS
Weighted median
Weighted mode
0.10
0.05
0.00
−0.05
0.1
0.2
SNP effect on Multiple coagulation factor deficiency protein 2
SNP effect on Gut microbiota abundance (genus Parasutterella id.2892)

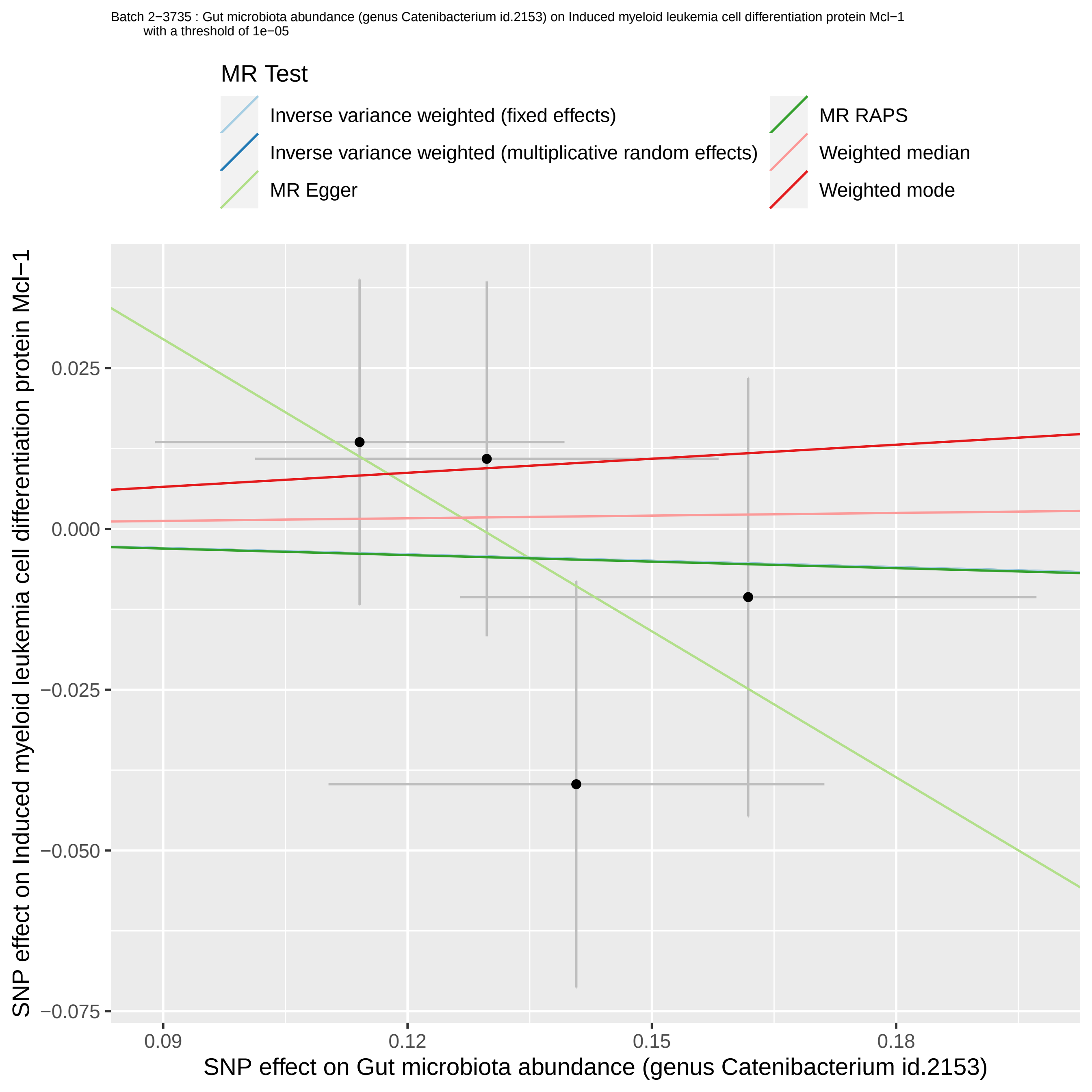
Batch 2−3735 : Gut microbiota abundance (genus Catenibacterium id.2153) on Induced myeloid leukemia cell differentiation protein Mcl−1
with a threshold of 1e−05
MR Test
Inverse variance weighted (fixed effects)
Inverse variance weighted (multiplicative random effects)
MR Egger
MR RAPS
Weighted median
Weighted mode
SNP effect on Induced myeloid leukemia cell differentiation protein Mcl−1
0.025
0.000
−0.025
−0.050
−0.075
0.09
0.12
0.15
0.18
SNP effect on Gut microbiota abundance (genus Catenibacterium id.2153)

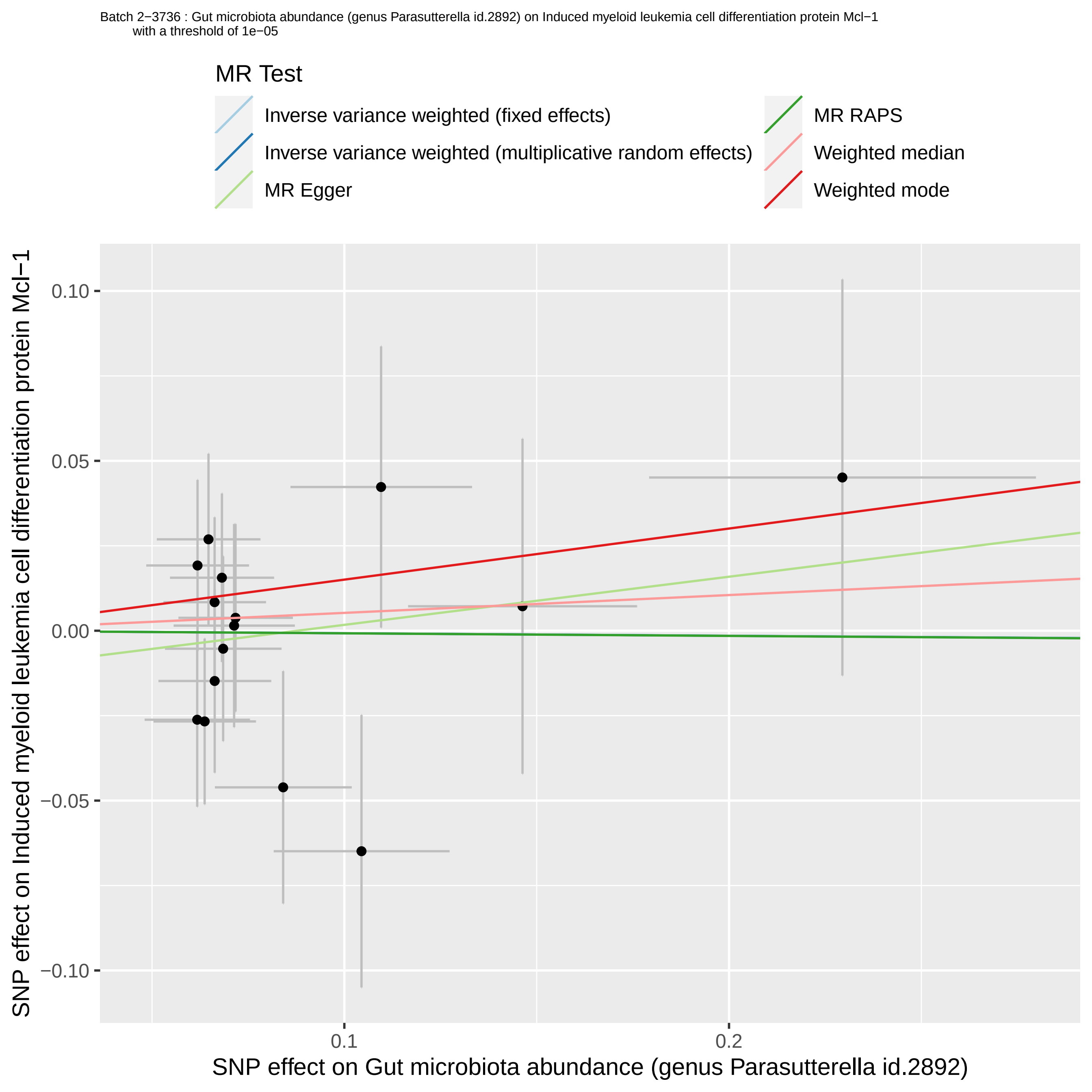
Batch 2−3736 : Gut microbiota abundance (genus Parasutterella id.2892) on Induced myeloid leukemia cell differentiation protein Mcl−1
with a threshold of 1e−05
MR Test
Inverse variance weighted (fixed effects)
Inverse variance weighted (multiplicative random effects)
MR Egger
MR RAPS
Weighted median
Weighted mode
0.10
0.05
0.00
−0.05
−0.10
0.1
0.2
SNP effect on Induced myeloid leukemia cell differentiation protein Mcl−1
SNP effect on Gut microbiota abundance (genus Parasutterella id.2892)

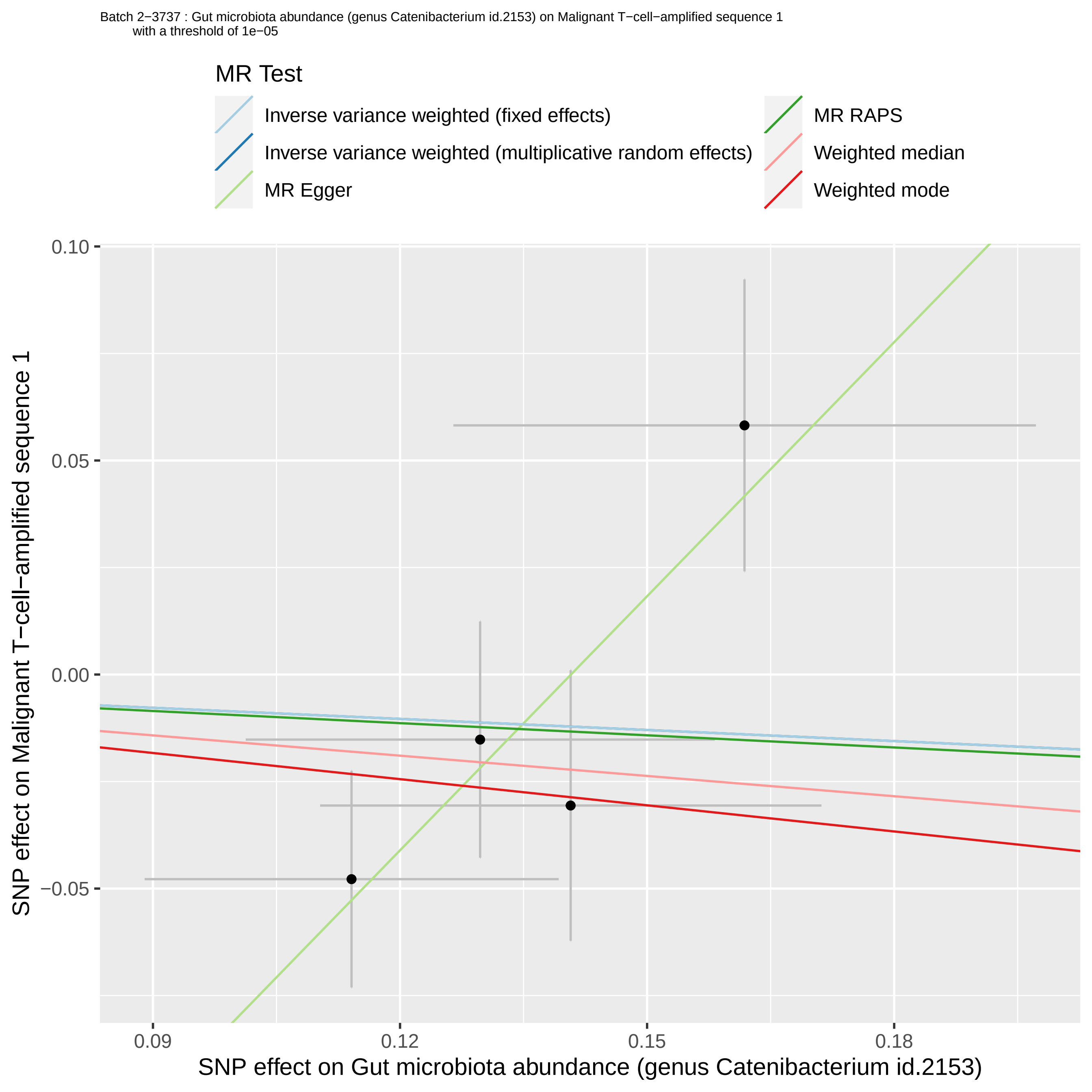
Batch 2−3737 : Gut microbiota abundance (genus Catenibacterium id.2153) on Malignant T−cell−amplified sequence 1
with a threshold of 1e−05
MR Test
Inverse variance weighted (fixed effects)
Inverse variance weighted (multiplicative random effects)
MR Egger
MR RAPS
Weighted median
Weighted mode
0.10
0.05
0.00
−0.05
0.09
0.12
0.15
0.18
SNP effect on Malignant T−cell−amplified sequence 1
SNP effect on Gut microbiota abundance (genus Catenibacterium id.2153)

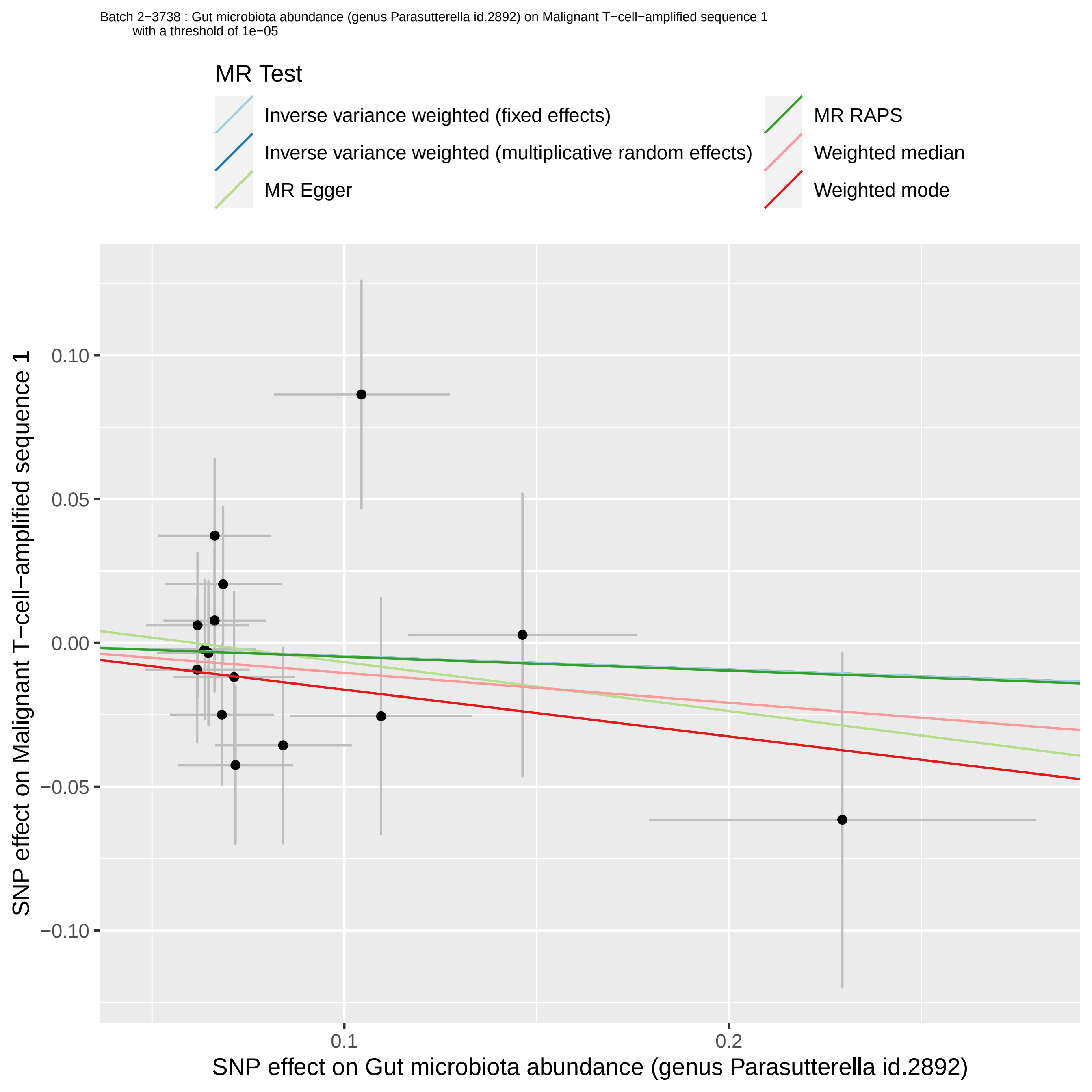

Batch 2−3738 : Gut microbiota abundance (genus Parasutterella id.2892) on Malignant T−cell−amplified sequence 1
with a threshold of 1e−05
MR Test
Inverse variance weighted (fixed effects)
Inverse variance weighted (multiplicative random effects)
MR Egger
MR RAPS
Weighted median
Weighted mode
0.10
0.05
0.00
−0.05
−0.10
0.1
0.2
SNP effect on Malignant T−cell−amplified sequence 1
SNP effect on Gut microbiota abundance (genus Parasutterella id.2892)

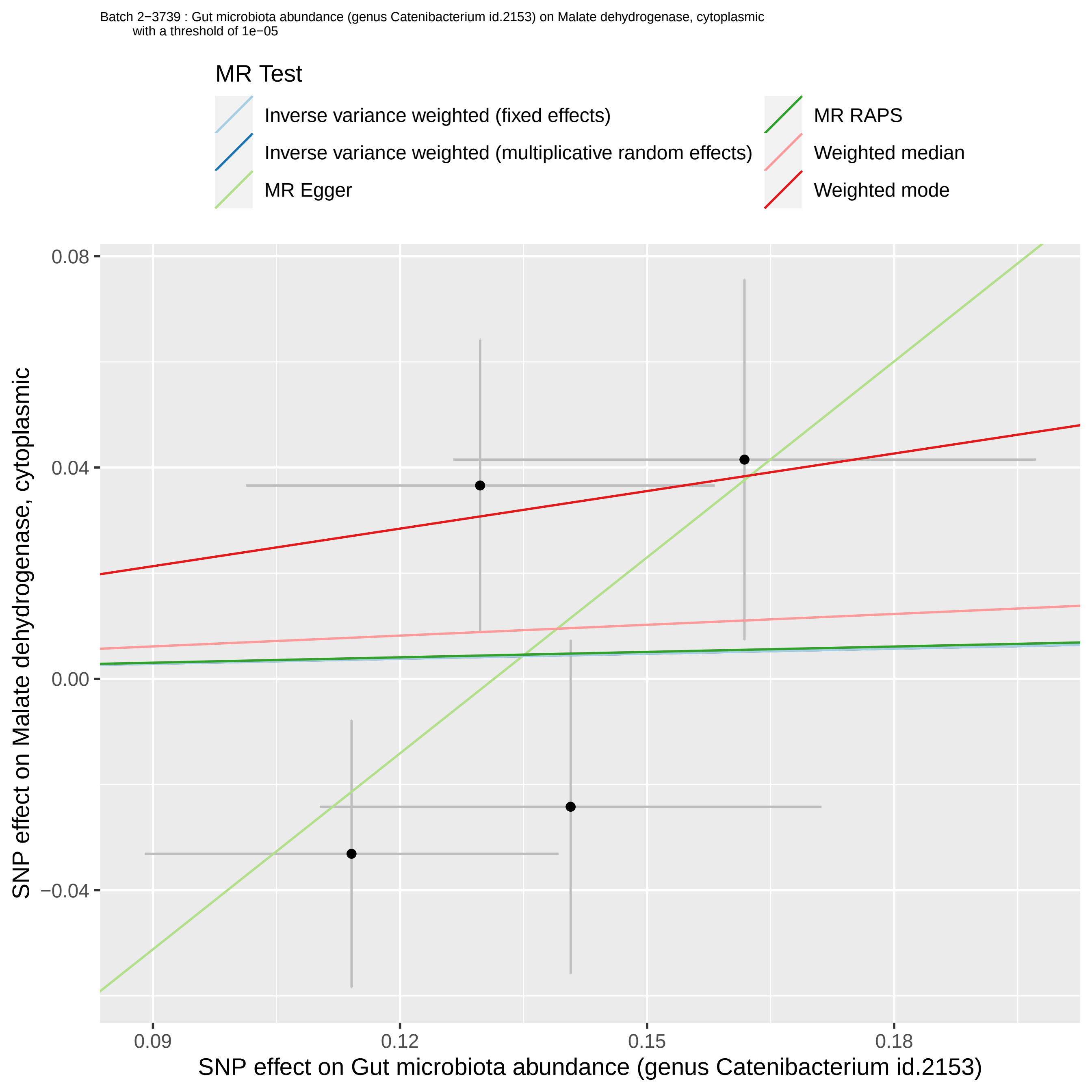

Batch 2−3739 : Gut microbiota abundance (genus Catenibacterium id.2153) on Malate dehydrogenase, cytoplasmic
with a threshold of 1e−05
MR Test
Inverse variance weighted (fixed effects)
Inverse variance weighted (multiplicative random effects)
MR Egger
MR RAPS
Weighted median
Weighted mode
SNP effect on Malate dehydrogenase, cytoplasmic
0.08
0.04
0.00
−0.04
0.09
0.12
0.15
0.18
SNP effect on Gut microbiota abundance (genus Catenibacterium id.2153)

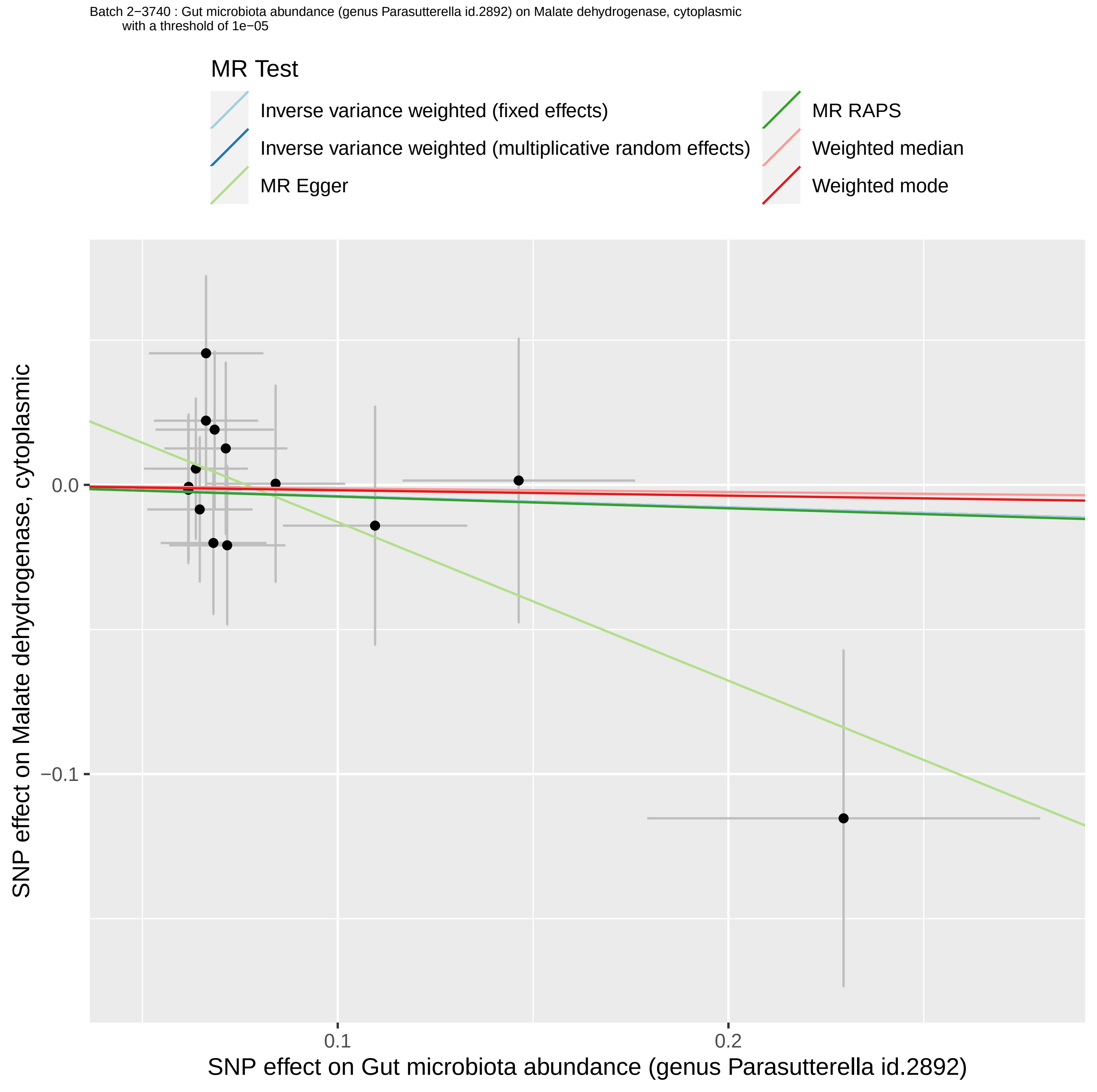

Batch 2−3740 : Gut microbiota abundance (genus Parasutterella id.2892) on Malate dehydrogenase, cytoplasmic
with a threshold of 1e−05
MR Test
Inverse variance weighted (fixed effects)
Inverse variance weighted (multiplicative random effects)
MR Egger
MR RAPS
Weighted median
Weighted mode
SNP effect on Malate dehydrogenase, cytoplasmic
0.0
−0.1
0.1
0.2
SNP effect on Gut microbiota abundance (genus Parasutterella id.2892)

Batch 2−3741 : Gut microbiota abundance (genus Catenibacterium id.2153) on Midkine
with a threshold of 1e−05

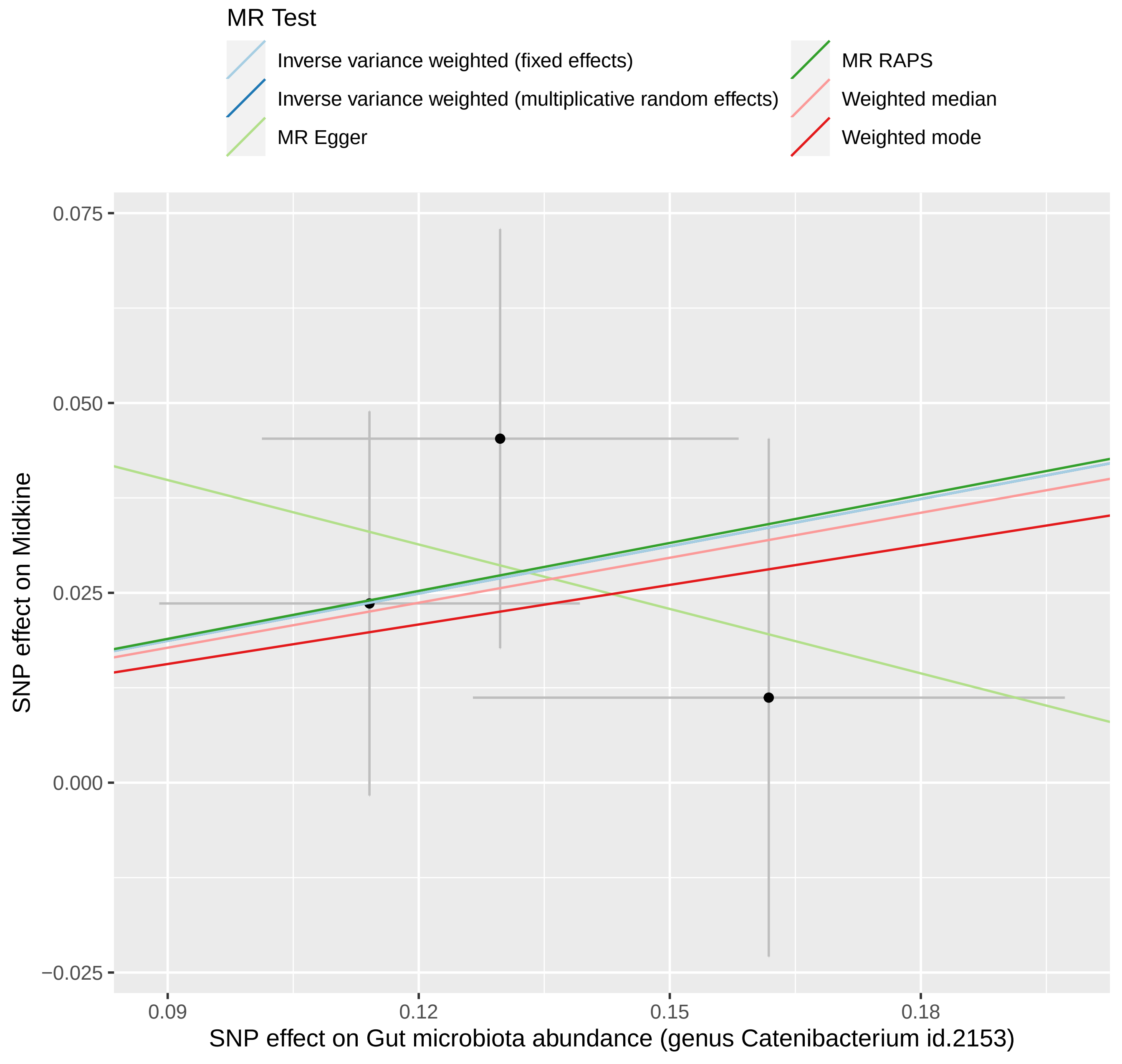

Batch 2−3742 : Gut microbiota abundance (genus Parasutterella id.2892) on Midkine
with a threshold of 1e−05

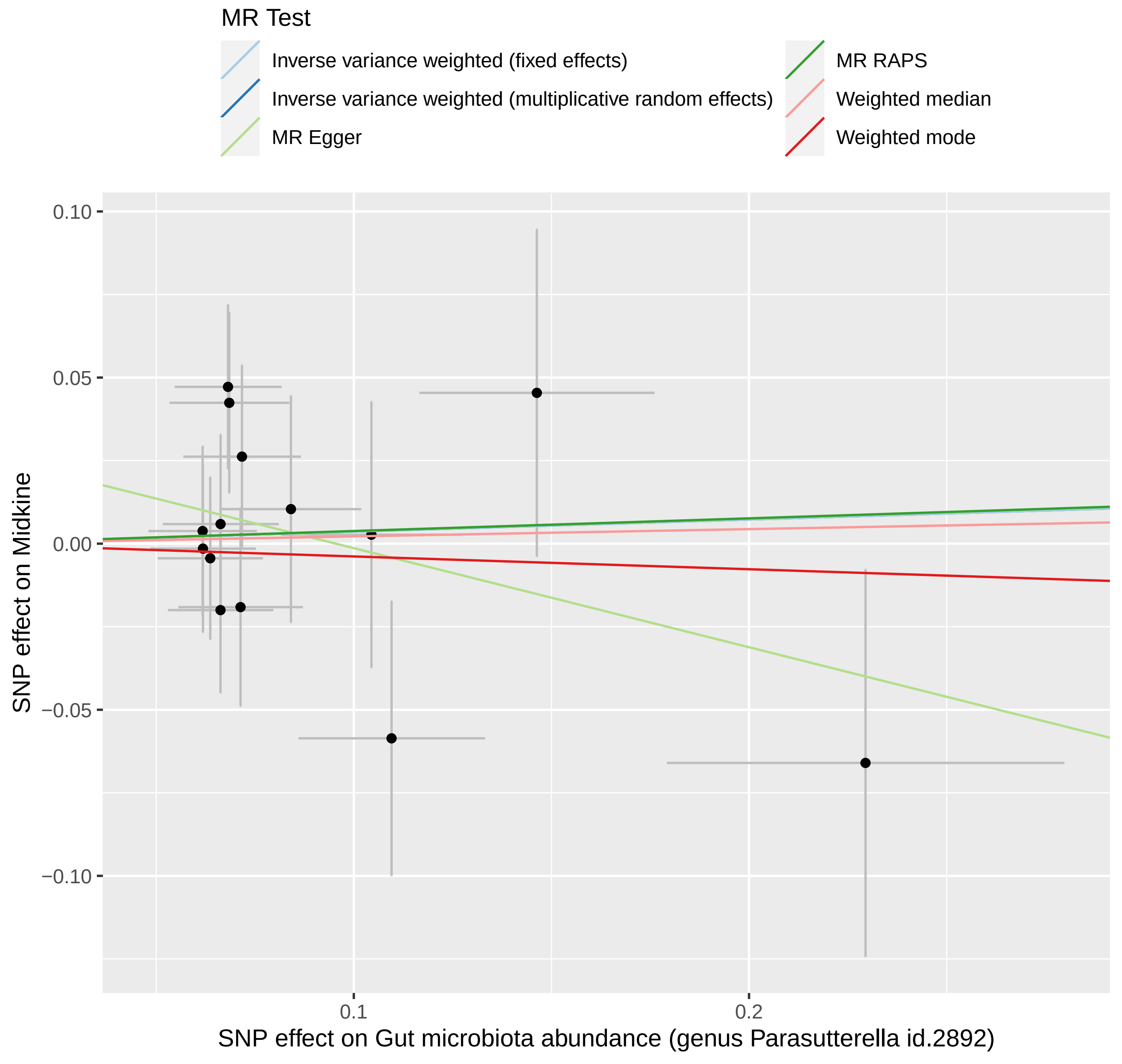

Batch 2−3743 : Gut microbiota abundance (genus Catenibacterium id.2153) on Nuclear protein MDM1
with a threshold of 1e−05

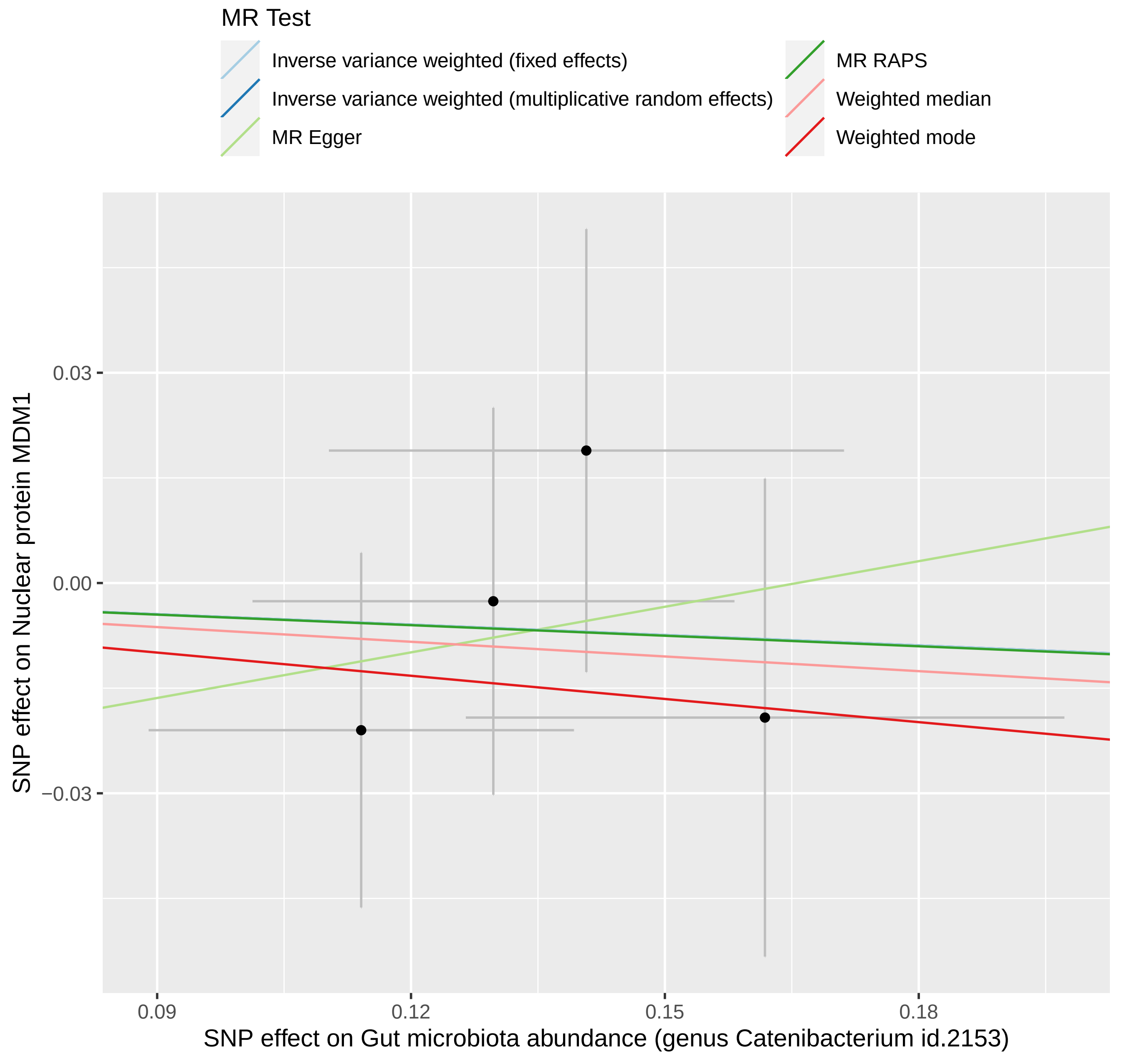

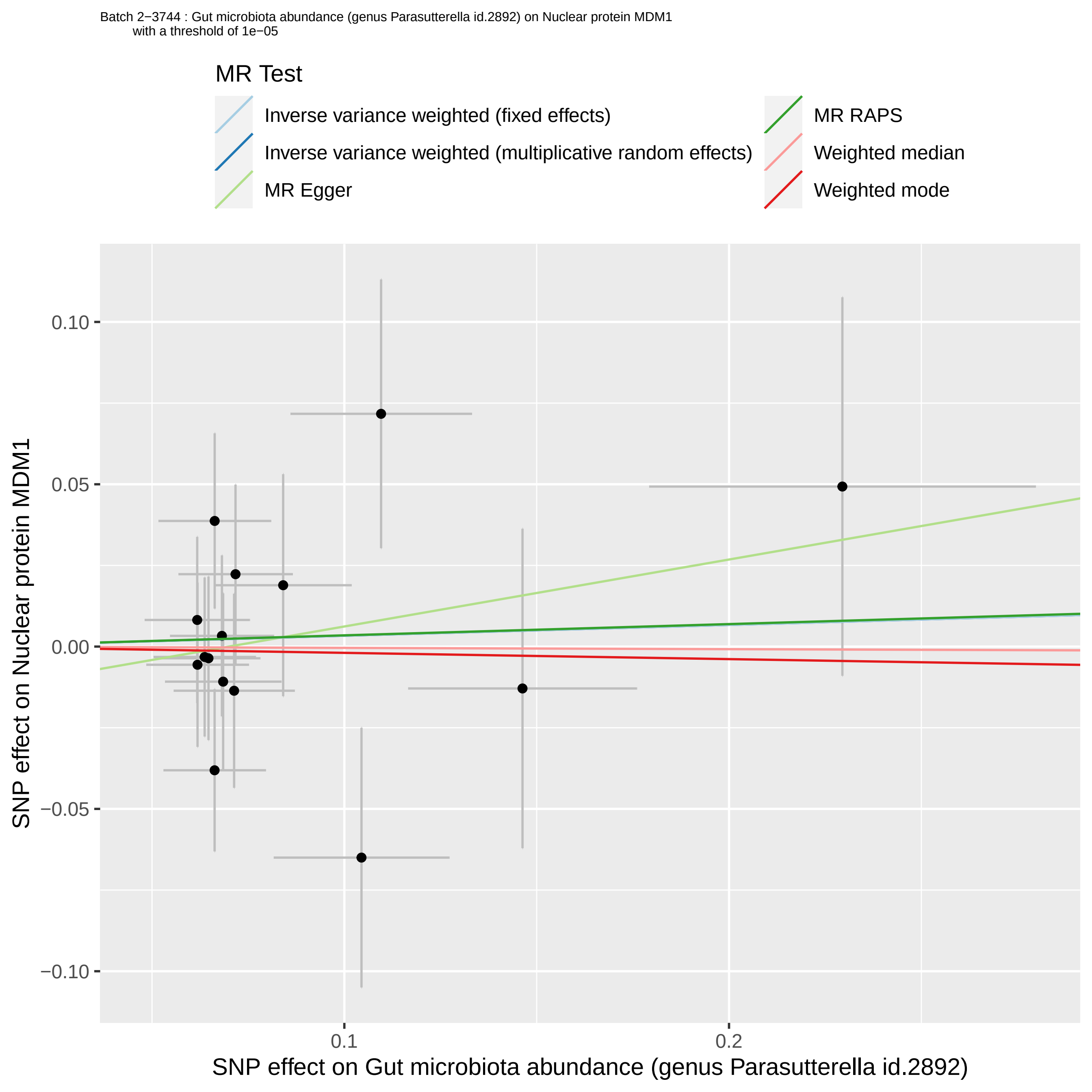
Batch 2−3744 : Gut microbiota abundance (genus Parasutterella id.2892) on Nuclear protein MDM1
with a threshold of 1e−05
MR Test
Inverse variance weighted (fixed effects)
Inverse variance weighted (multiplicative random effects)
MR Egger
MR RAPS
Weighted median
Weighted mode
0.10
0.05
0.00
−0.05
−0.10
0.1
0.2
SNP effect on Nuclear protein MDM1
SNP effect on Gut microbiota abundance (genus Parasutterella id.2892)

Batch 2−3745 : Gut microbiota abundance (genus Catenibacterium id.2153) on E3 ubiquitin−protein ligase Mdm2
with a threshold of 1e−05

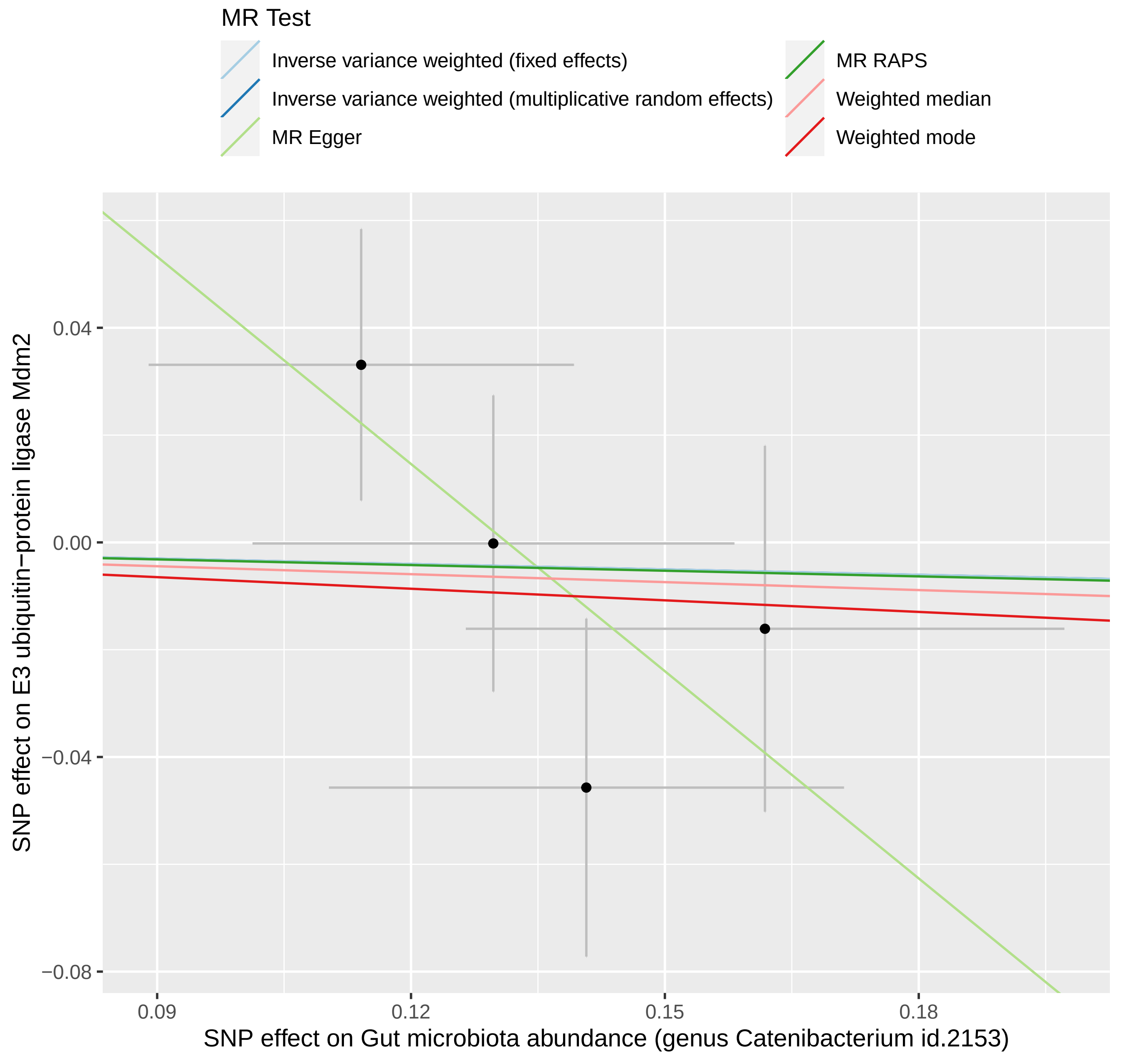

Batch 2−3746 : Gut microbiota abundance (genus Parasutterella id.2892) on E3 ubiquitin−protein ligase Mdm2
with a threshold of 1e−05

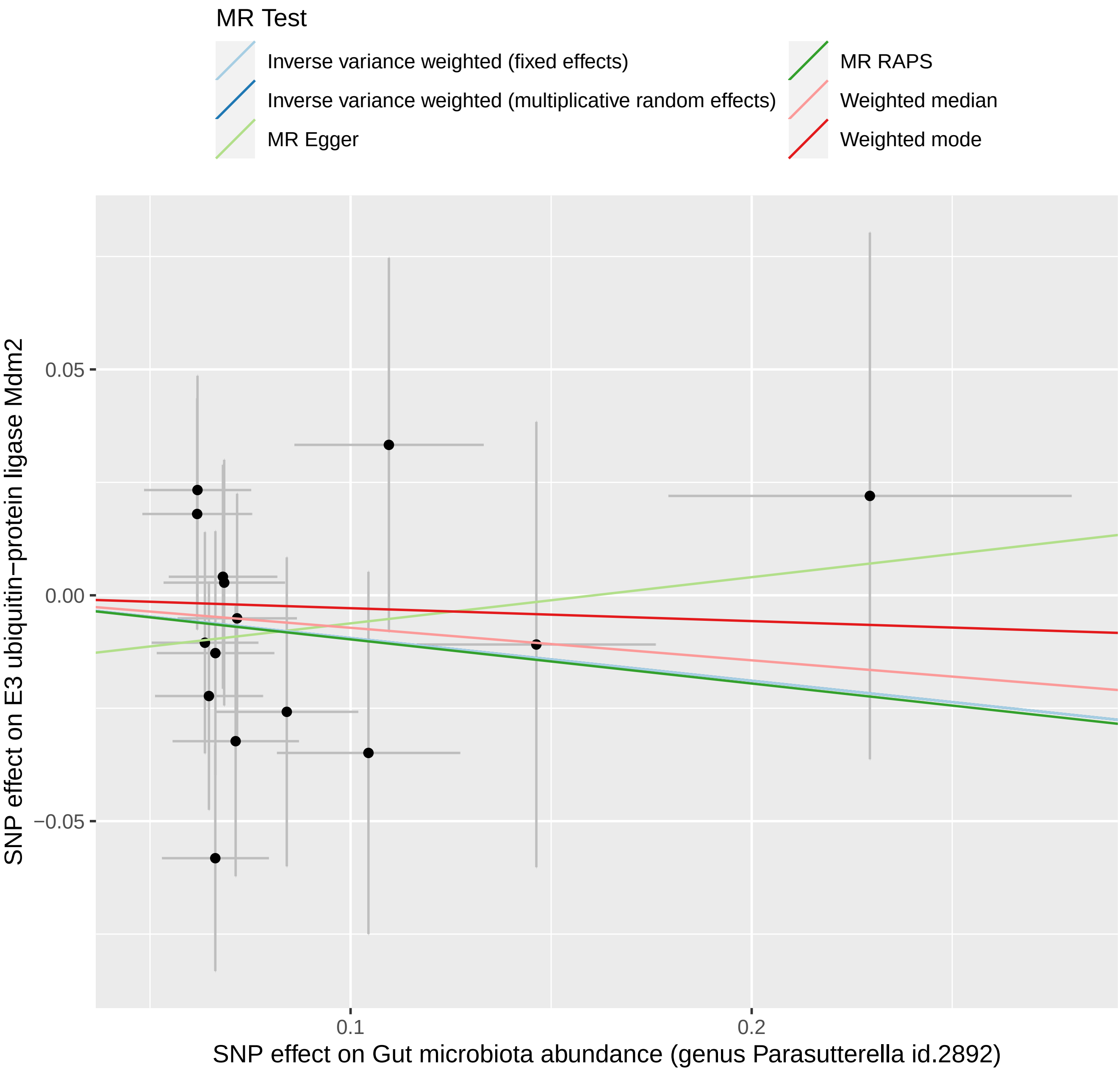

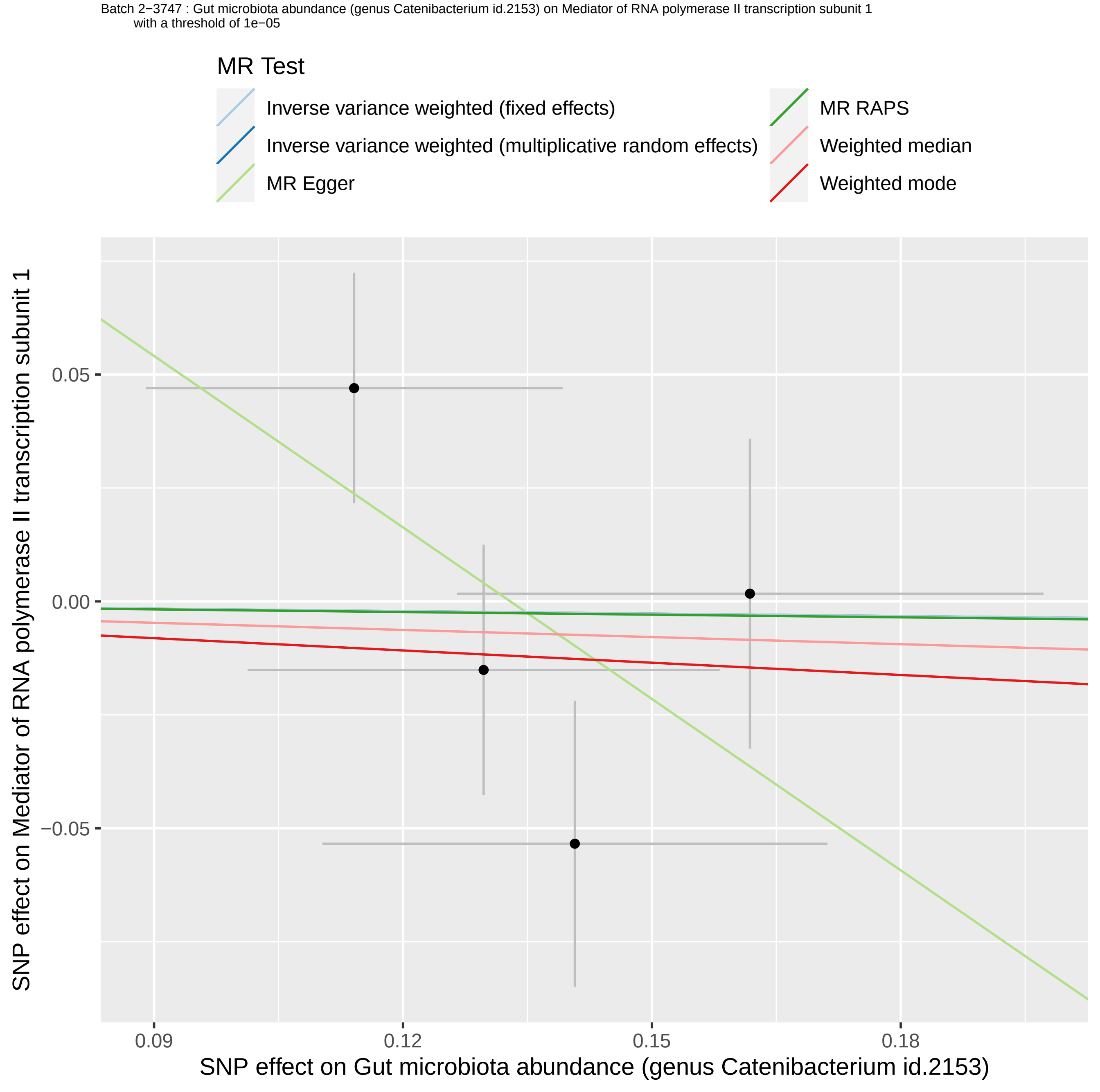

Batch 2−3747 : Gut microbiota abundance (genus Catenibacterium id.2153) on Mediator of RNA polymerase II transcription subunit 1
with a threshold of 1e−05
MR Test
Inverse variance weighted (fixed effects)
Inverse variance weighted (multiplicative random effects)
MR Egger
MR RAPS
Weighted median
Weighted mode
0.05
0.00
−0.05
0.09
0.12
0.15
0.18
SNP effect on Mediator of RNA polymerase II transcription subunit 1
SNP effect on Gut microbiota abundance (genus Catenibacterium id.2153)

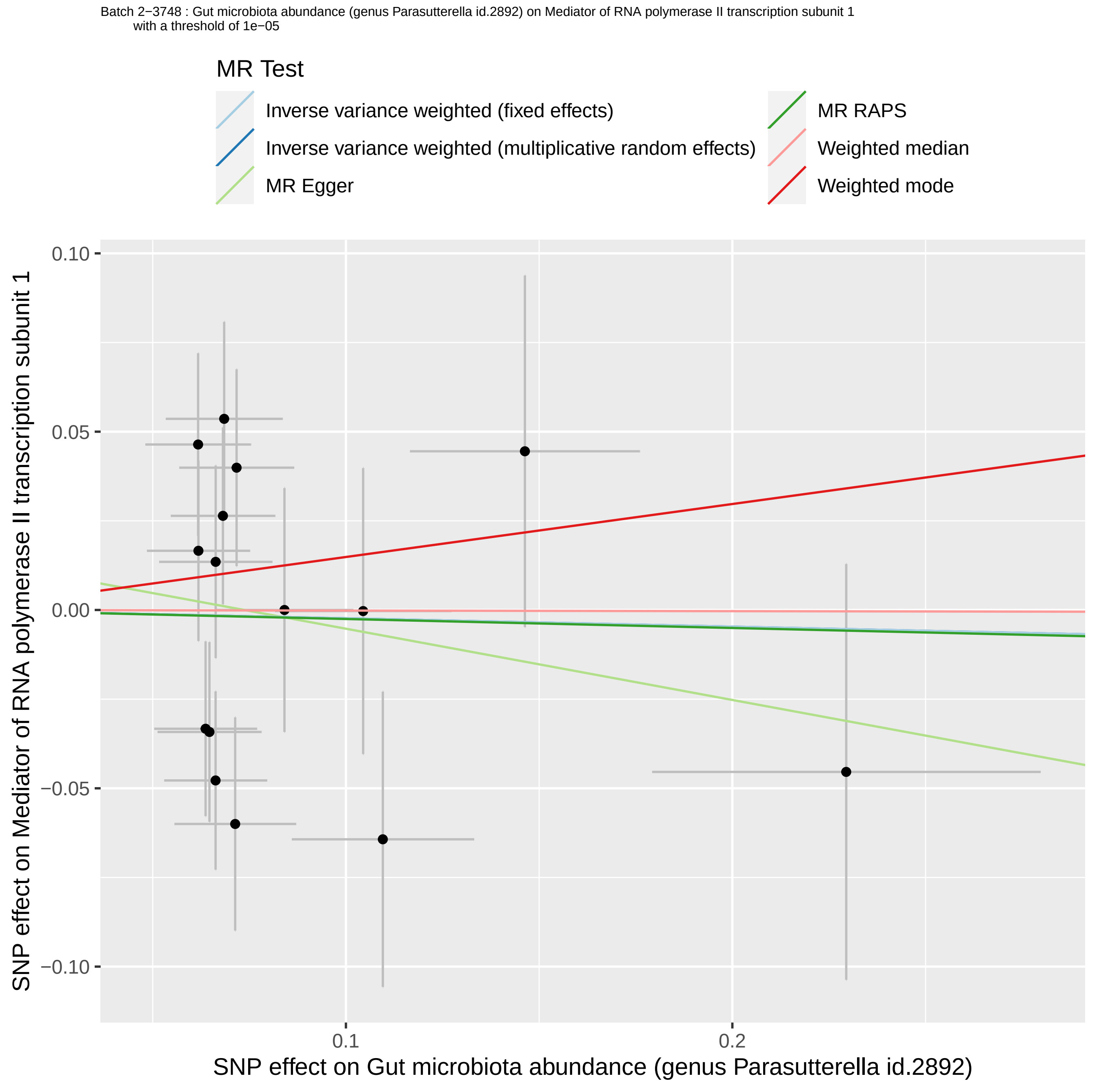
Batch 2−3748 : Gut microbiota abundance (genus Parasutterella id.2892) on Mediator of RNA polymerase II transcription subunit 1
with a threshold of 1e−05
MR Test
Inverse variance weighted (fixed effects)
Inverse variance weighted (multiplicative random effects)
MR Egger
MR RAPS
Weighted median
Weighted mode
SNP effect on Mediator of RNA polymerase II transcription subunit 1
0.10
0.05
0.00
−0.05
−0.10
0.1
0.2
SNP effect on Gut microbiota abundance (genus Parasutterella id.2892)

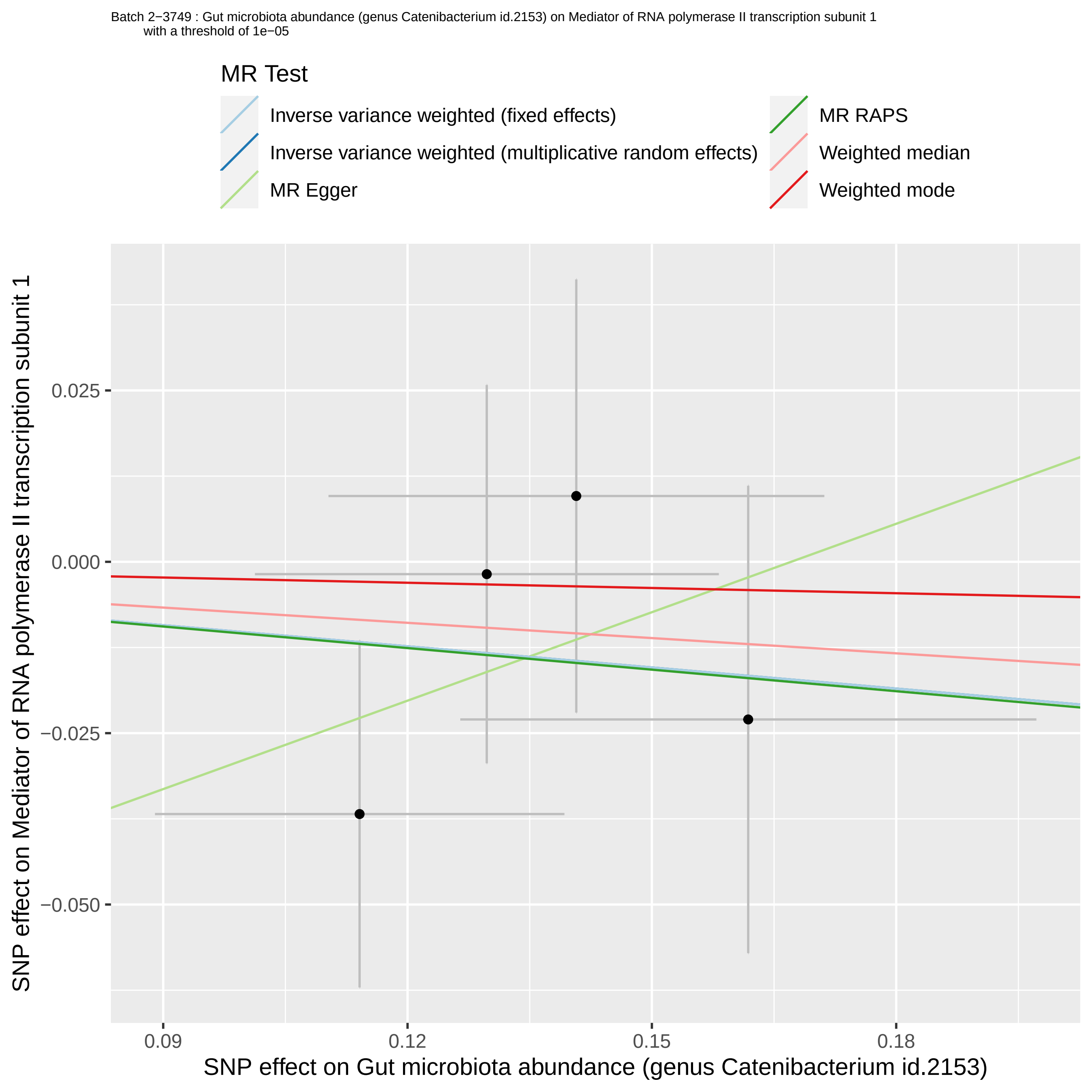

Batch 2−3749 : Gut microbiota abundance (genus Catenibacterium id.2153) on Mediator of RNA polymerase II transcription subunit 1
with a threshold of 1e−05
MR Test
Inverse variance weighted (fixed effects)
Inverse variance weighted (multiplicative random effects)
MR Egger
MR RAPS
Weighted median
Weighted mode
0.025
0.000
−0.025
−0.050
0.09
0.12
0.15
0.18
SNP effect on Mediator of RNA polymerase II transcription subunit 1
SNP effect on Gut microbiota abundance (genus Catenibacterium id.2153)

Batch 2−3750 : Gut microbiota abundance (genus Parasutterella id.2892) on Mediator of RNA polymerase II transcription subunit 1
with a threshold of 1e−05

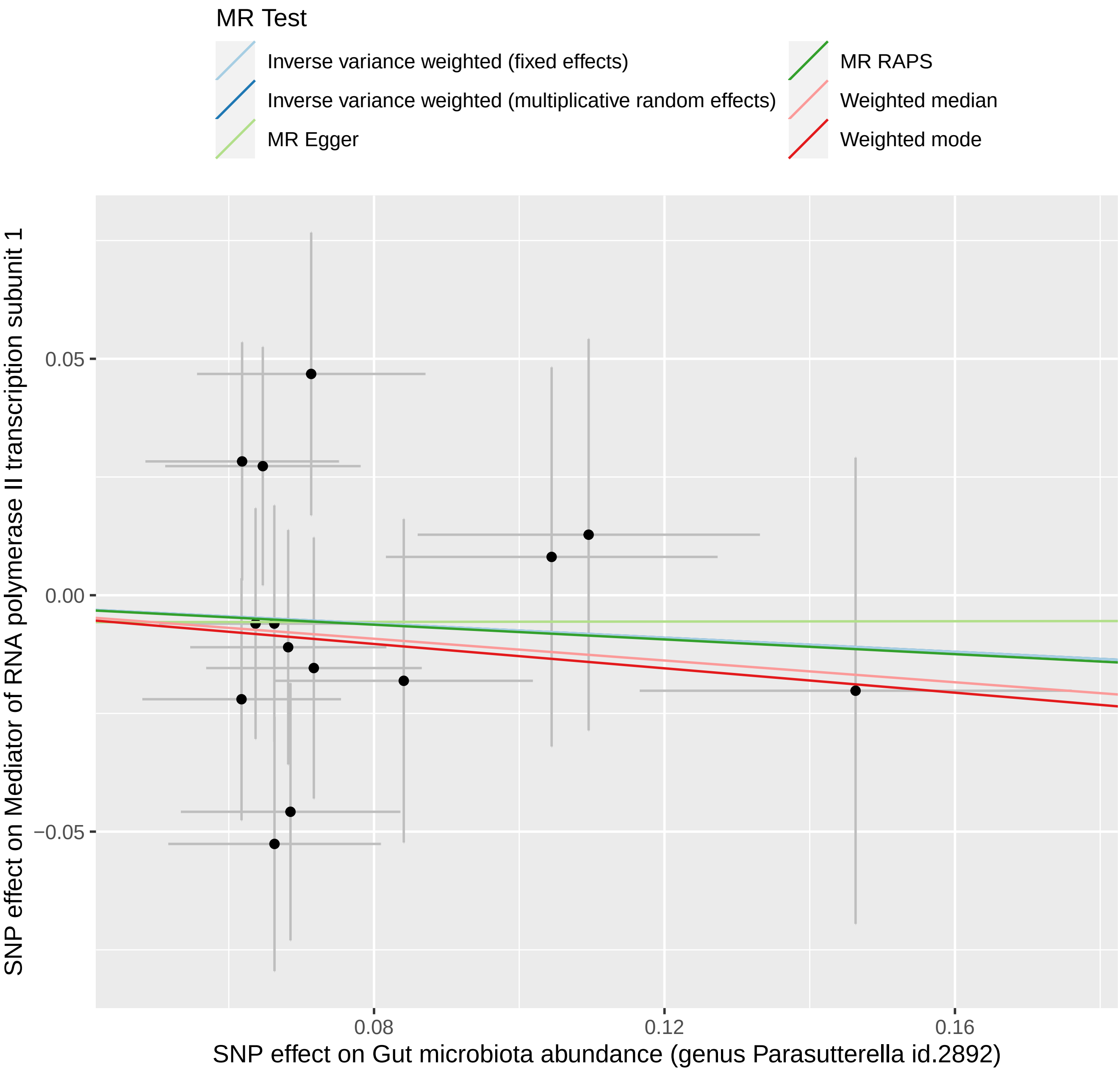

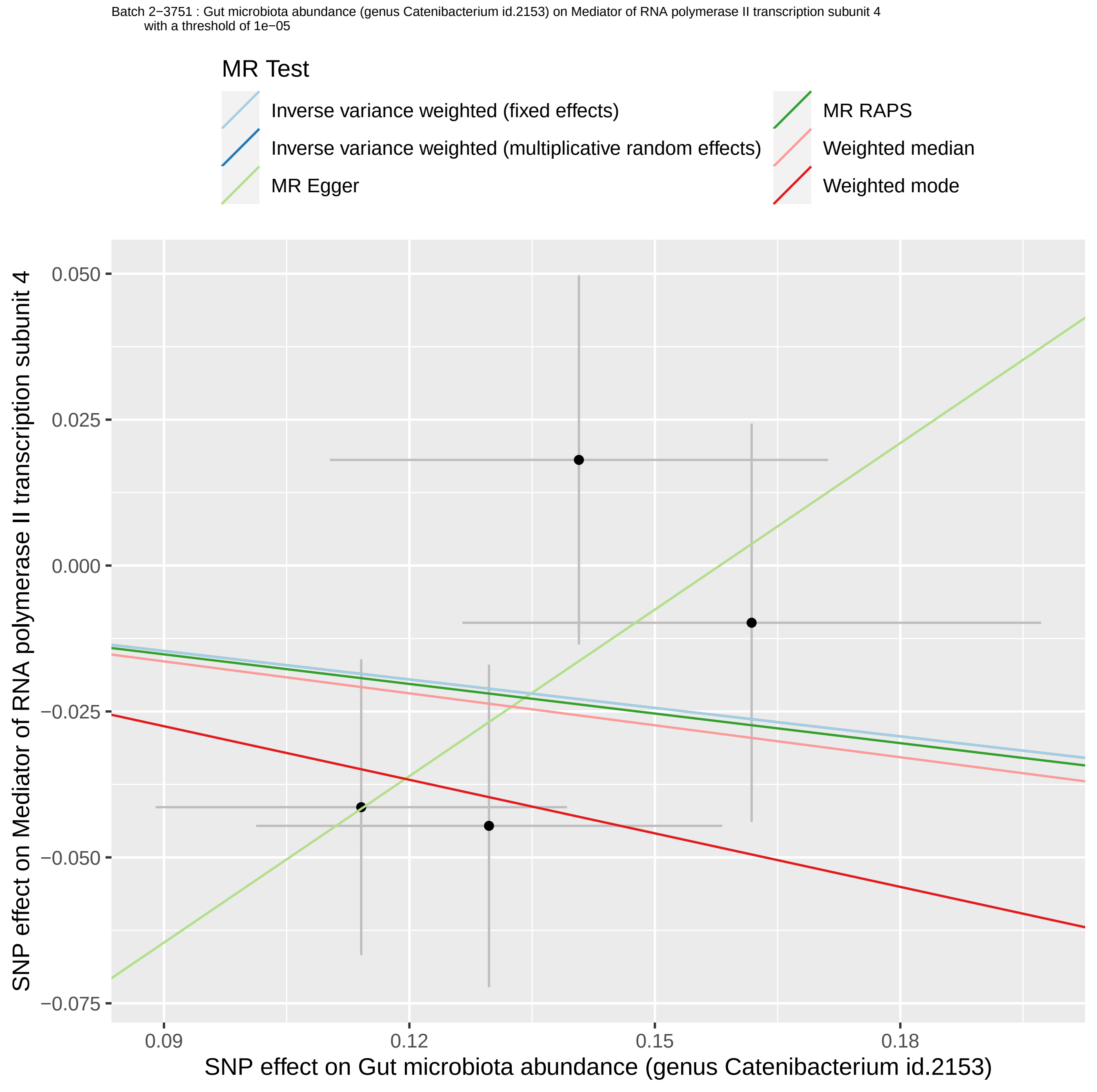
Batch 2−3751 : Gut microbiota abundance (genus Catenibacterium id.2153) on Mediator of RNA polymerase II transcription subunit 4
with a threshold of 1e−05
MR Test
Inverse variance weighted (fixed effects)
Inverse variance weighted (multiplicative random effects)
MR Egger
MR RAPS
Weighted median
Weighted mode
0.050
0.025
0.000
−0.025
−0.050
−0.075
0.09
0.12
0.15
0.18
SNP effect on Gut microbiota abundance (genus Catenibacterium id.2153)
SNP effect on Mediator of RNA polymerase II transcription subunit 4

Batch 2−3752 : Gut microbiota abundance (genus Parasutterella id.2892) on Mediator of RNA polymerase II transcription subunit 4
with a threshold of 1e−05

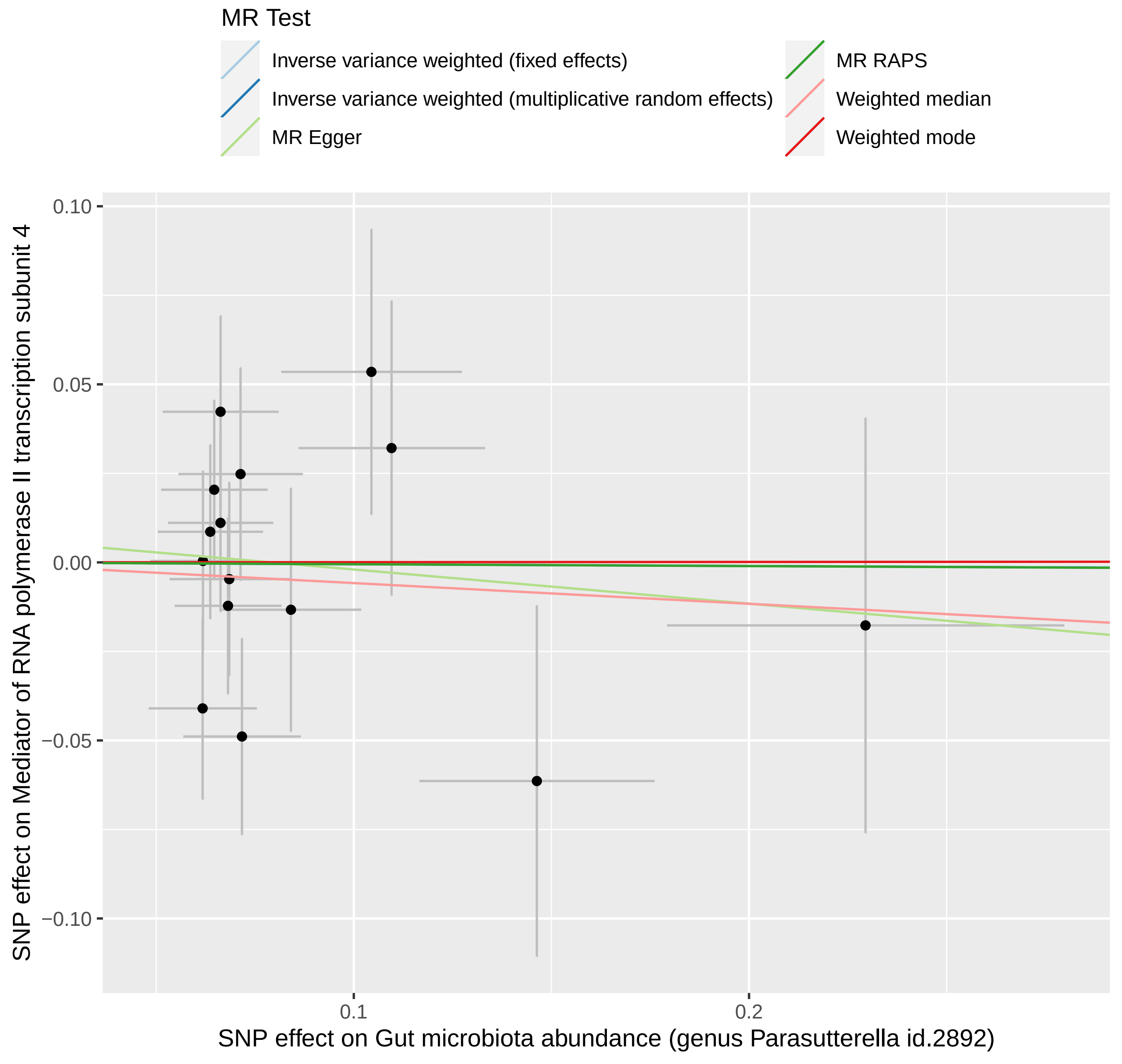

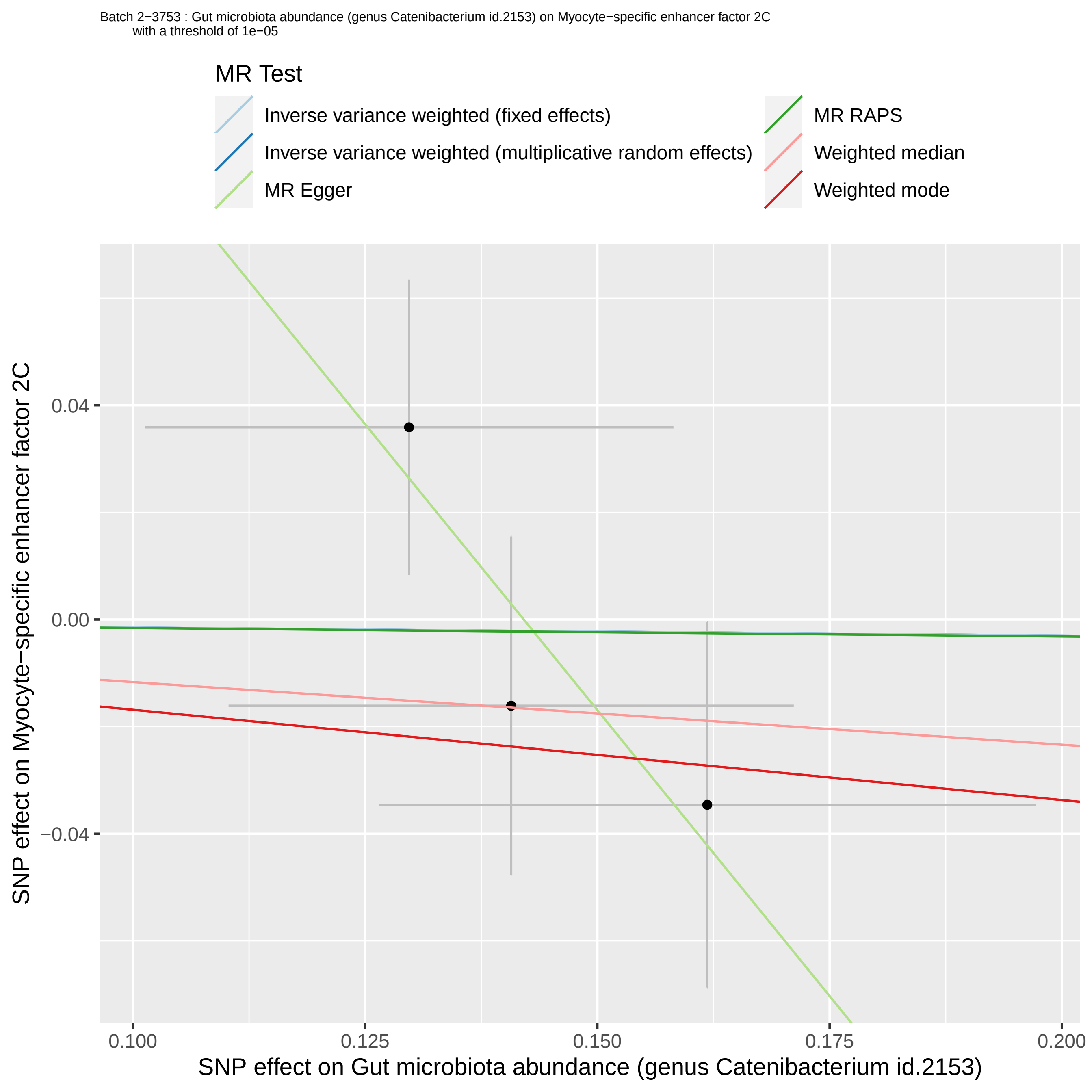
Batch 2−3753 : Gut microbiota abundance (genus Catenibacterium id.2153) on Myocyte−specific enhancer factor 2C
with a threshold of 1e−05
MR Test
Inverse variance weighted (fixed effects)
Inverse variance weighted (multiplicative random effects)
MR Egger
MR RAPS
Weighted median
Weighted mode
0.04
0.00
−0.04
0.100
0.125
0.150
0.175
0.200
SNP effect on Myocyte−specific enhancer factor 2C
SNP effect on Gut microbiota abundance (genus Catenibacterium id.2153)

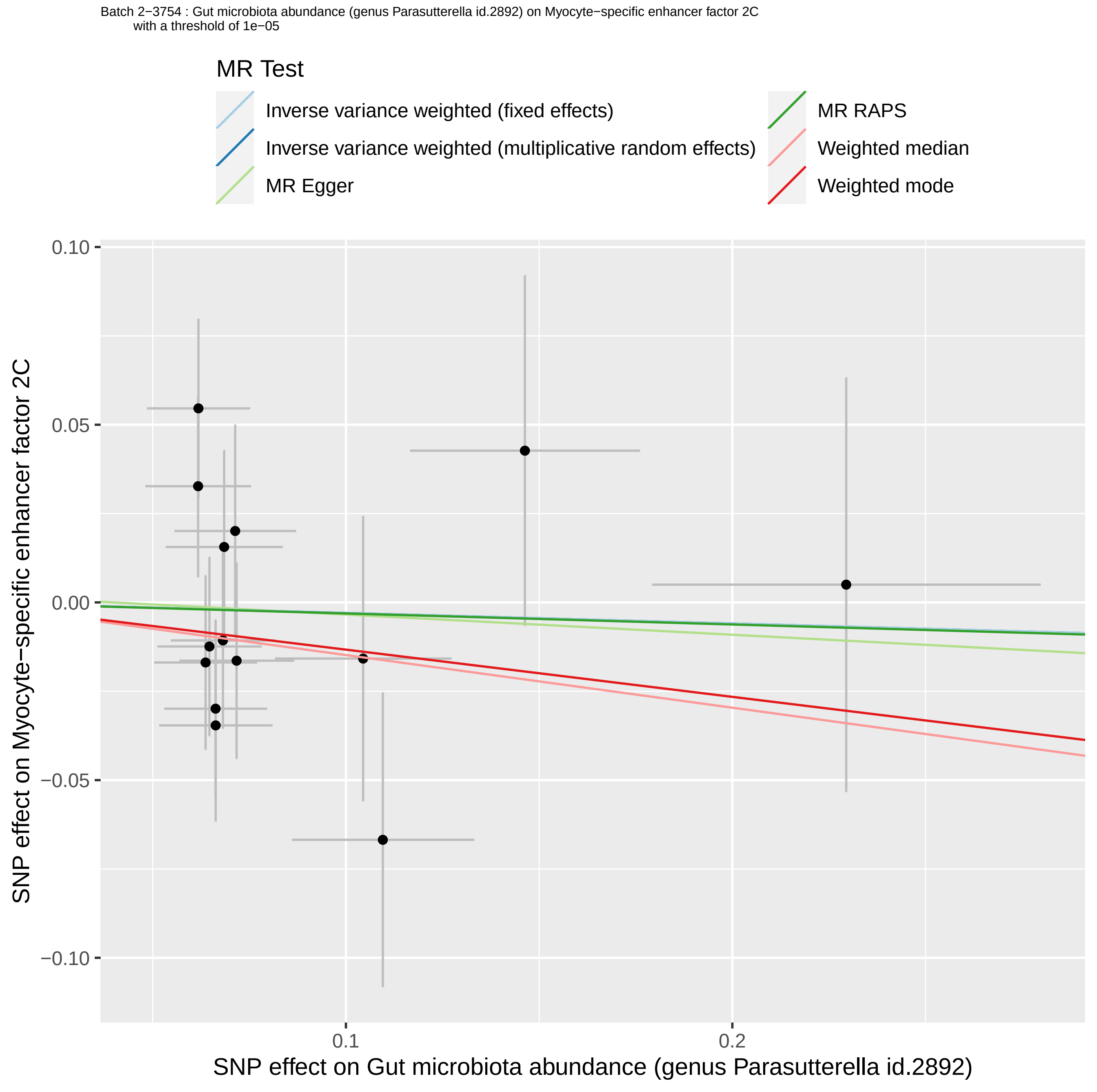

Batch 2−3754 : Gut microbiota abundance (genus Parasutterella id.2892) on Myocyte−specific enhancer factor 2C
with a threshold of 1e−05
MR Test
Inverse variance weighted (fixed effects)
Inverse variance weighted (multiplicative random effects)
MR Egger
MR RAPS
Weighted median
Weighted mode
0.10
0.05
0.00
−0.05
−0.10
0.1
0.2
SNP effect on Myocyte−specific enhancer factor 2C
SNP effect on Gut microbiota abundance (genus Parasutterella id.2892)

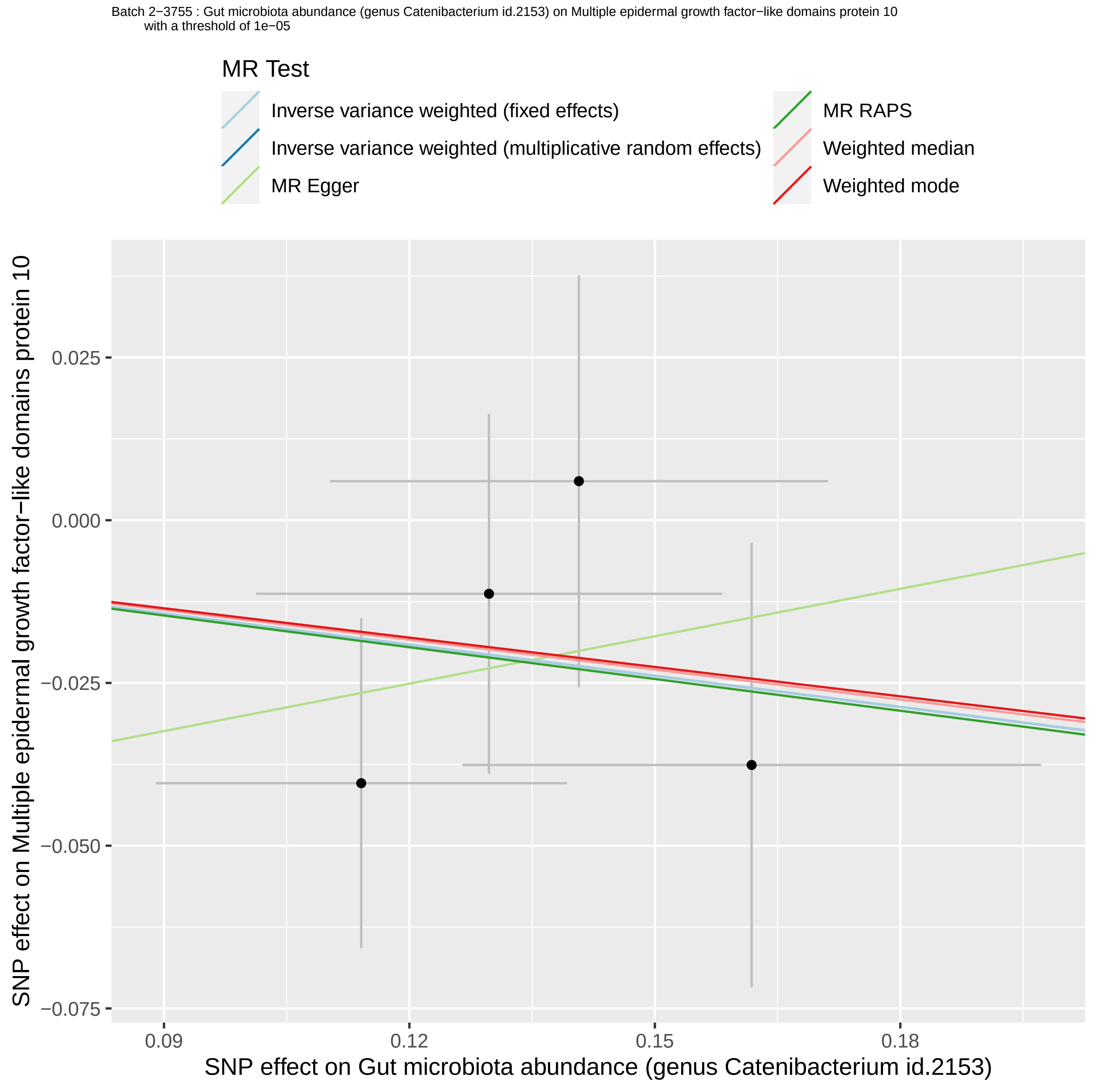

Batch 2−3755 : Gut microbiota abundance (genus Catenibacterium id.2153) on Multiple epidermal growth factor−like domains protein 10 with a threshold of 1e−05
MR Test
Inverse variance weighted (fixed effects)
Inverse variance weighted (multiplicative random effects)
MR Egger
MR RAPS
Weighted median
Weighted mode
0.025
0.000
−0.025
−0.050
−0.075
0.09
0.12
0.15
0.18
SNP effect on Multiple epidermal growth factor−like domains protein 10
SNP effect on Gut microbiota abundance (genus Catenibacterium id.2153)

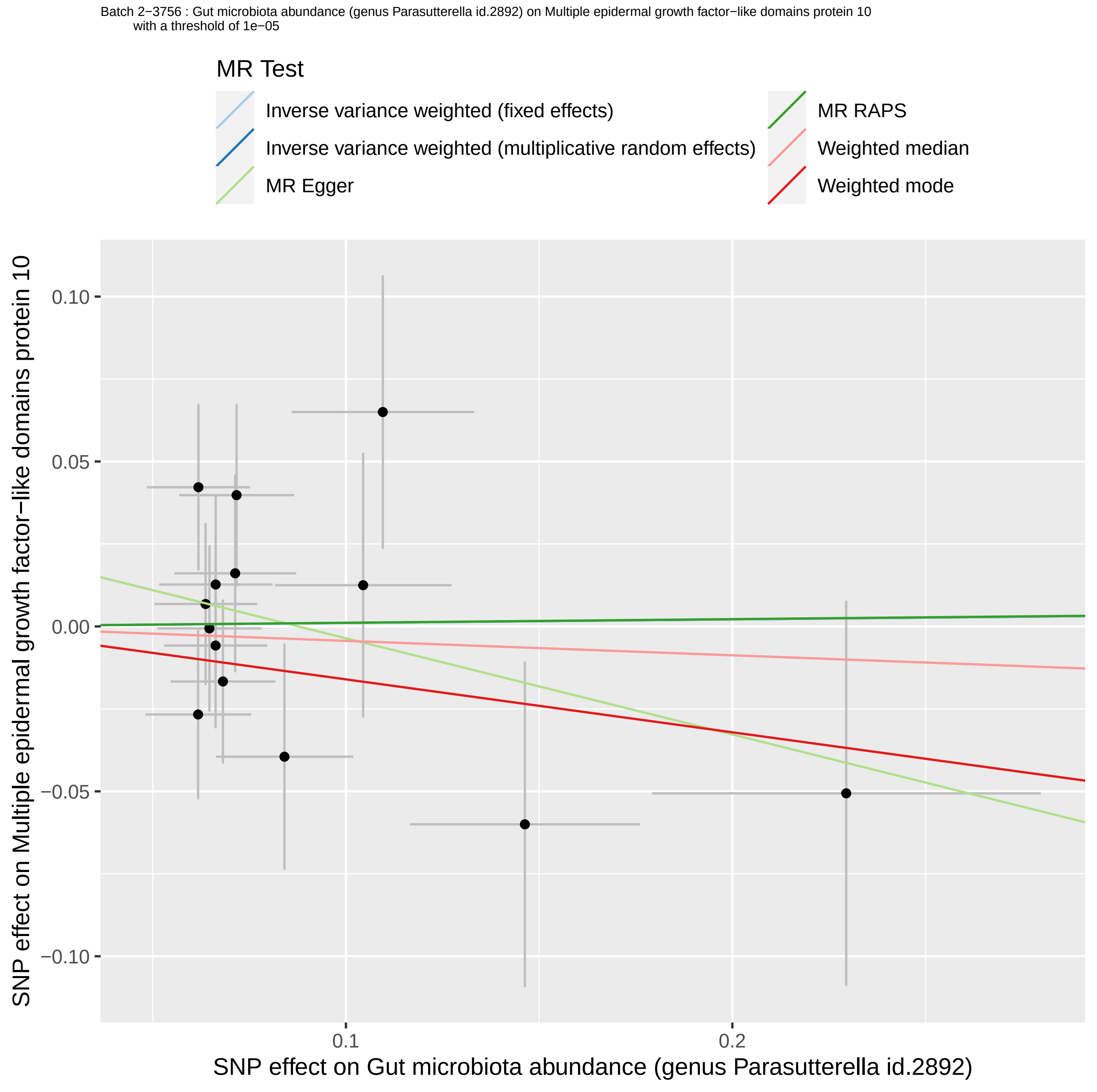
Batch 2−3756 : Gut microbiota abundance (genus Parasutterella id.2892) on Multiple epidermal growth factor−like domains protein 10
with a threshold of 1e−05
MR Test
Inverse variance weighted (fixed effects)
Inverse variance weighted (multiplicative random effects)
MR Egger
MR RAPS
Weighted median
Weighted mode
SNP effect on Multiple epidermal growth factor−like domains protein 10
0.10
0.05
0.00
−0.05
−0.10
0.1
0.2
SNP effect on Gut microbiota abundance (genus Parasutterella id.2892)

Batch 2−3757 : Gut microbiota abundance (genus Catenibacterium id.2153) on Protein MENT
with a threshold of 1e−05

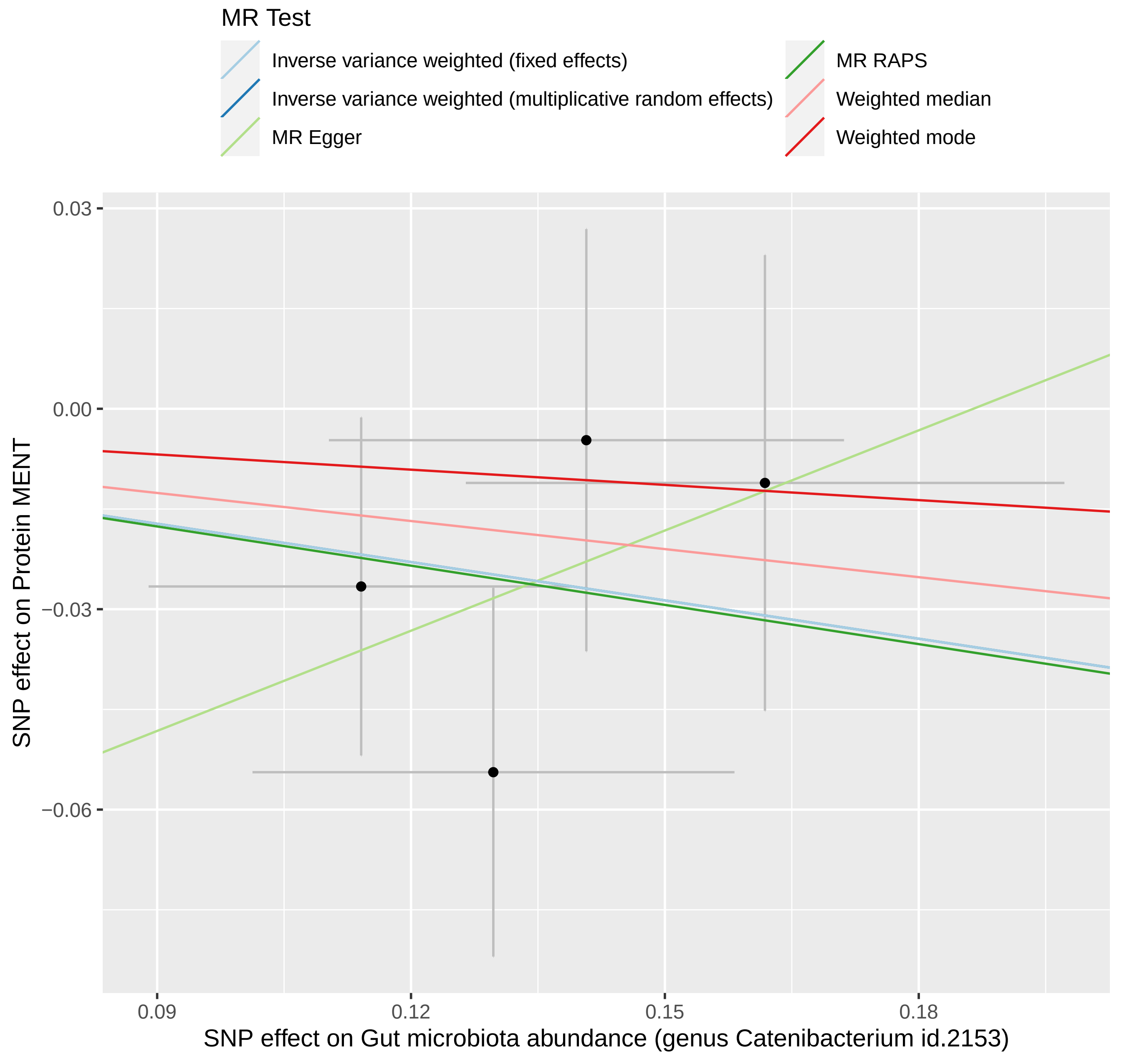

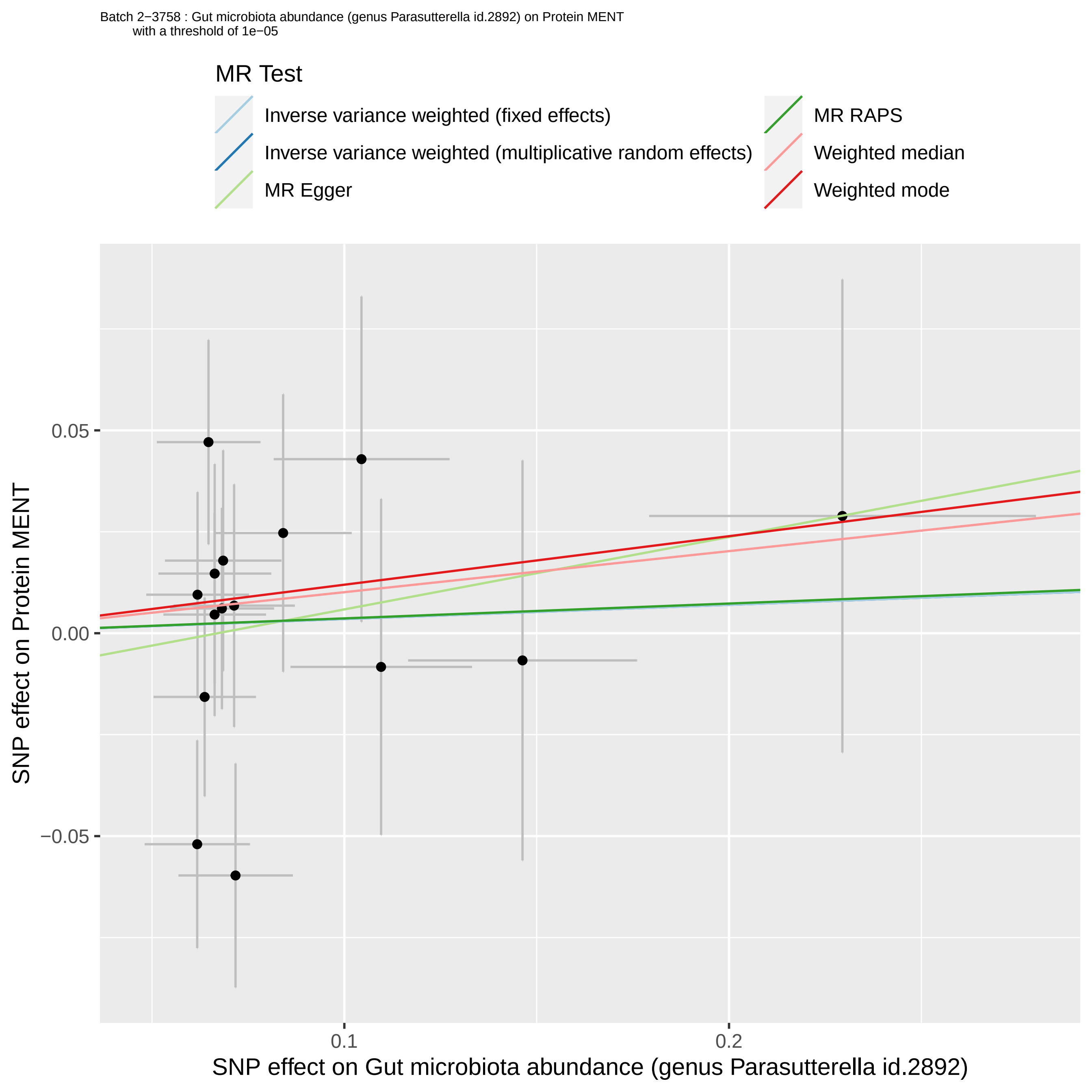

Batch 2−3758 : Gut microbiota abundance (genus Parasutterella id.2892) on Protein MENT
with a threshold of 1e−05
MR Test
Inverse variance weighted (fixed effects)
Inverse variance weighted (multiplicative random effects)
MR Egger
MR RAPS
Weighted median
Weighted mode
0.05
0.00
−0.05
0.1
0.2
SNP effect on Protein MENT
SNP effect on Gut microbiota abundance (genus Parasutterella id.2892)

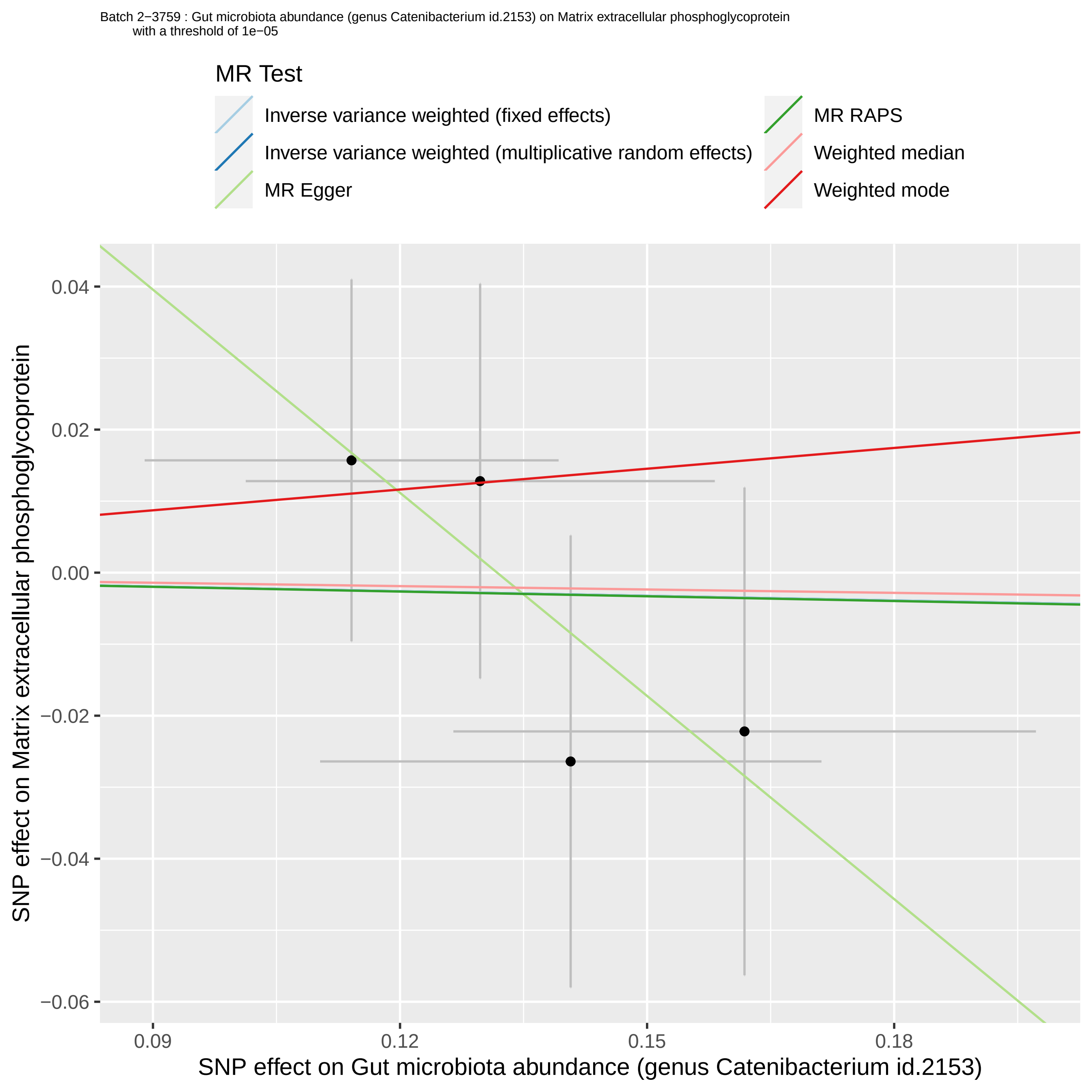
Batch 2−3759 : Gut microbiota abundance (genus Catenibacterium id.2153) on Matrix extracellular phosphoglycoprotein
with a threshold of 1e−05
MR Test
Inverse variance weighted (fixed effects)
Inverse variance weighted (multiplicative random effects)
MR Egger
MR RAPS
Weighted median
Weighted mode
0.04
0.02
0.00
−0.02
−0.04
−0.06
0.09
0.12
0.15
0.18
SNP effect on Matrix extracellular phosphoglycoprotein
SNP effect on Gut microbiota abundance (genus Catenibacterium id.2153)

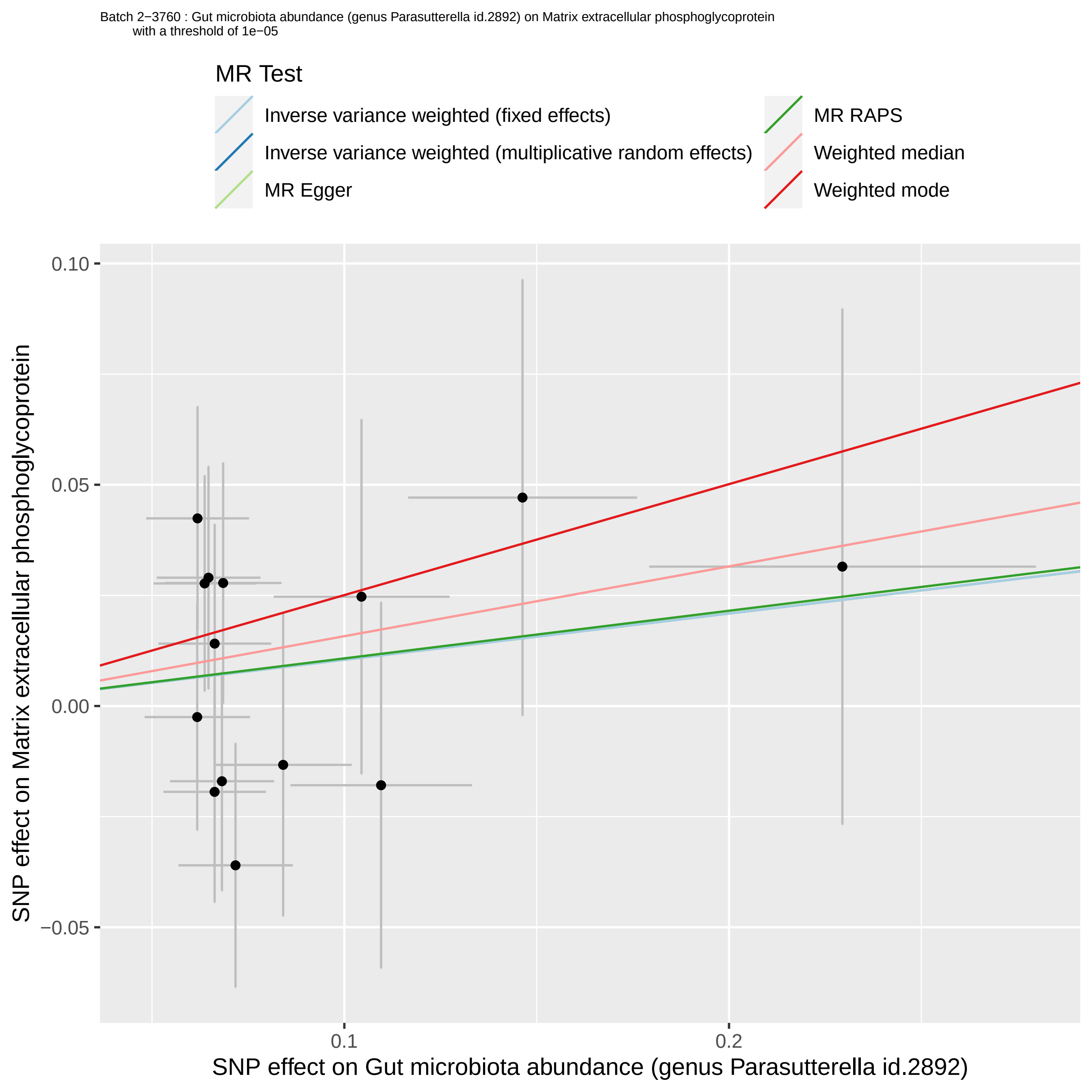
Batch 2−3760 : Gut microbiota abundance (genus Parasutterella id.2892) on Matrix extracellular phosphoglycoprotein
with a threshold of 1e−05
MR Test
Inverse variance weighted (fixed effects)
Inverse variance weighted (multiplicative random effects)
MR Egger
MR RAPS
Weighted median
Weighted mode
0.10
0.05
0.00
−0.05
0.1
0.2
SNP effect on Matrix extracellular phosphoglycoprotein
SNP effect on Gut microbiota abundance (genus Parasutterella id.2892)

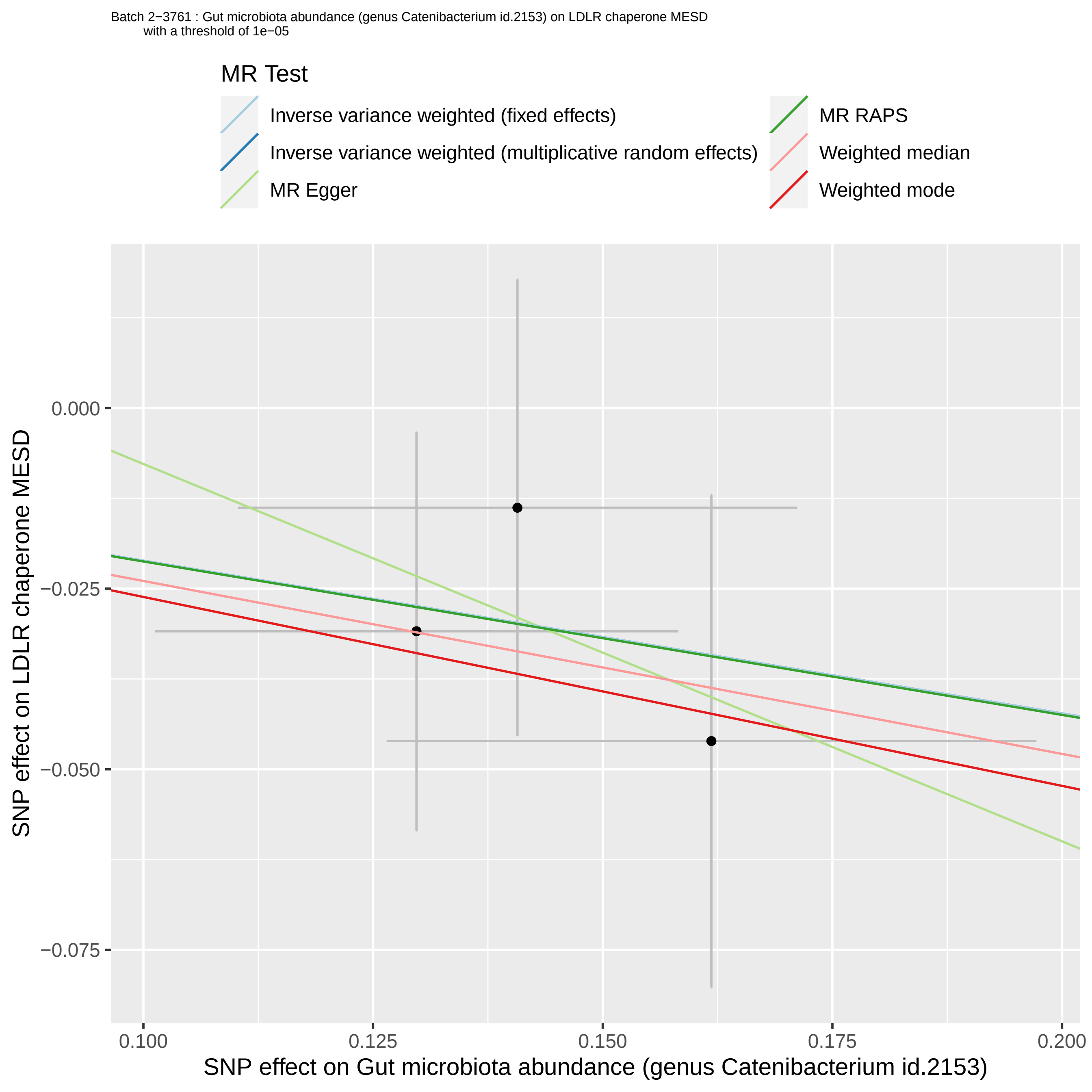

Batch 2−3761 : Gut microbiota abundance (genus Catenibacterium id.2153) on LDLR chaperone MESD
with a threshold of 1e−05
MR Test
Inverse variance weighted (fixed effects)
Inverse variance weighted (multiplicative random effects)
MR Egger
MR RAPS
Weighted median
Weighted mode
0.000
−0.025
−0.050
−0.075
0.100
0.125
0.150
0.175
0.200
SNP effect on LDLR chaperone MESD
SNP effect on Gut microbiota abundance (genus Catenibacterium id.2153)

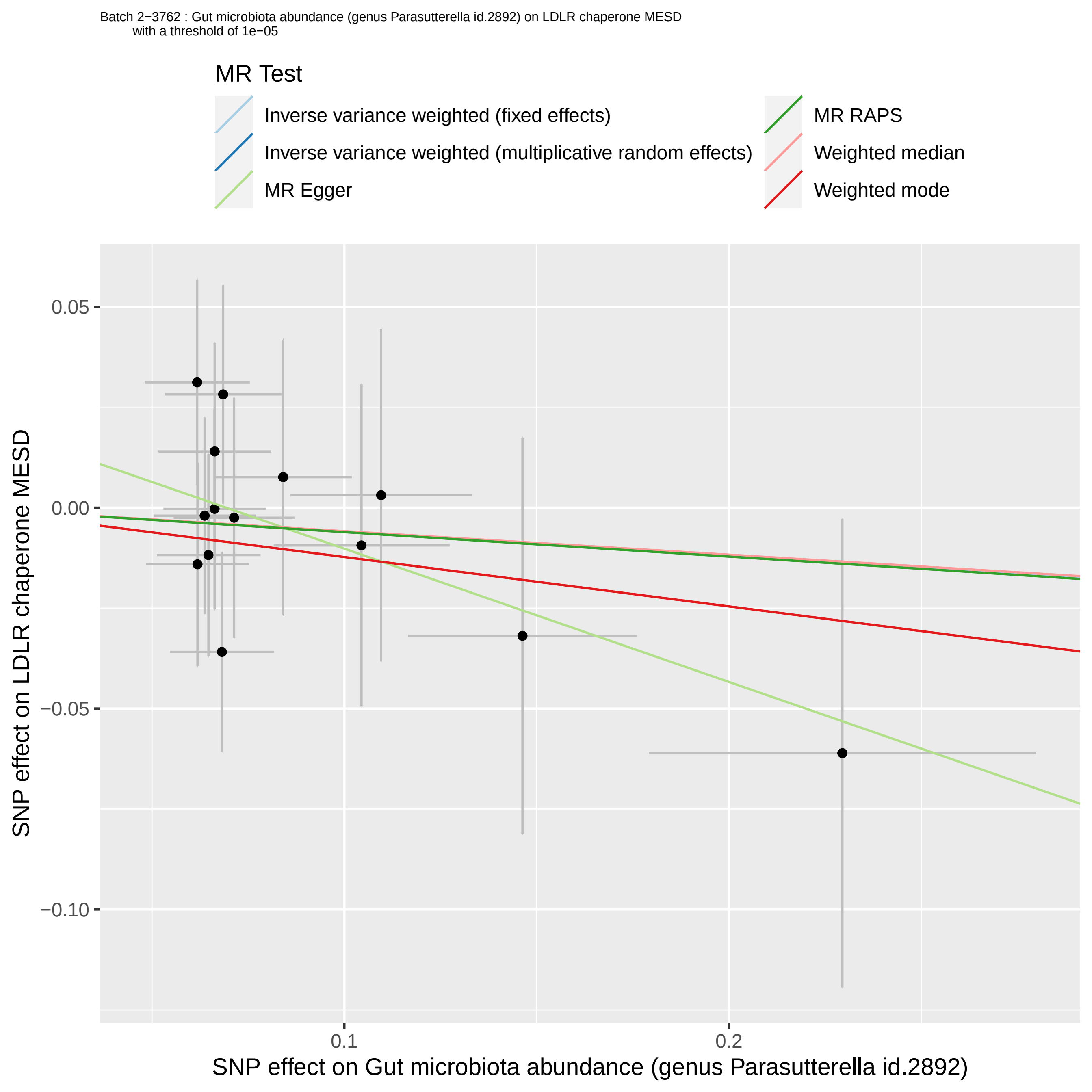
Batch 2−3762 : Gut microbiota abundance (genus Parasutterella id.2892) on LDLR chaperone MESD
with a threshold of 1e−05
MR Test
Inverse variance weighted (fixed effects)
Inverse variance weighted (multiplicative random effects)
MR Egger
MR RAPS
Weighted median
Weighted mode
0.05
0.00
−0.05
−0.10
0.1
0.2
SNP effect on LDLR chaperone MESD
SNP effect on Gut microbiota abundance (genus Parasutterella id.2892)

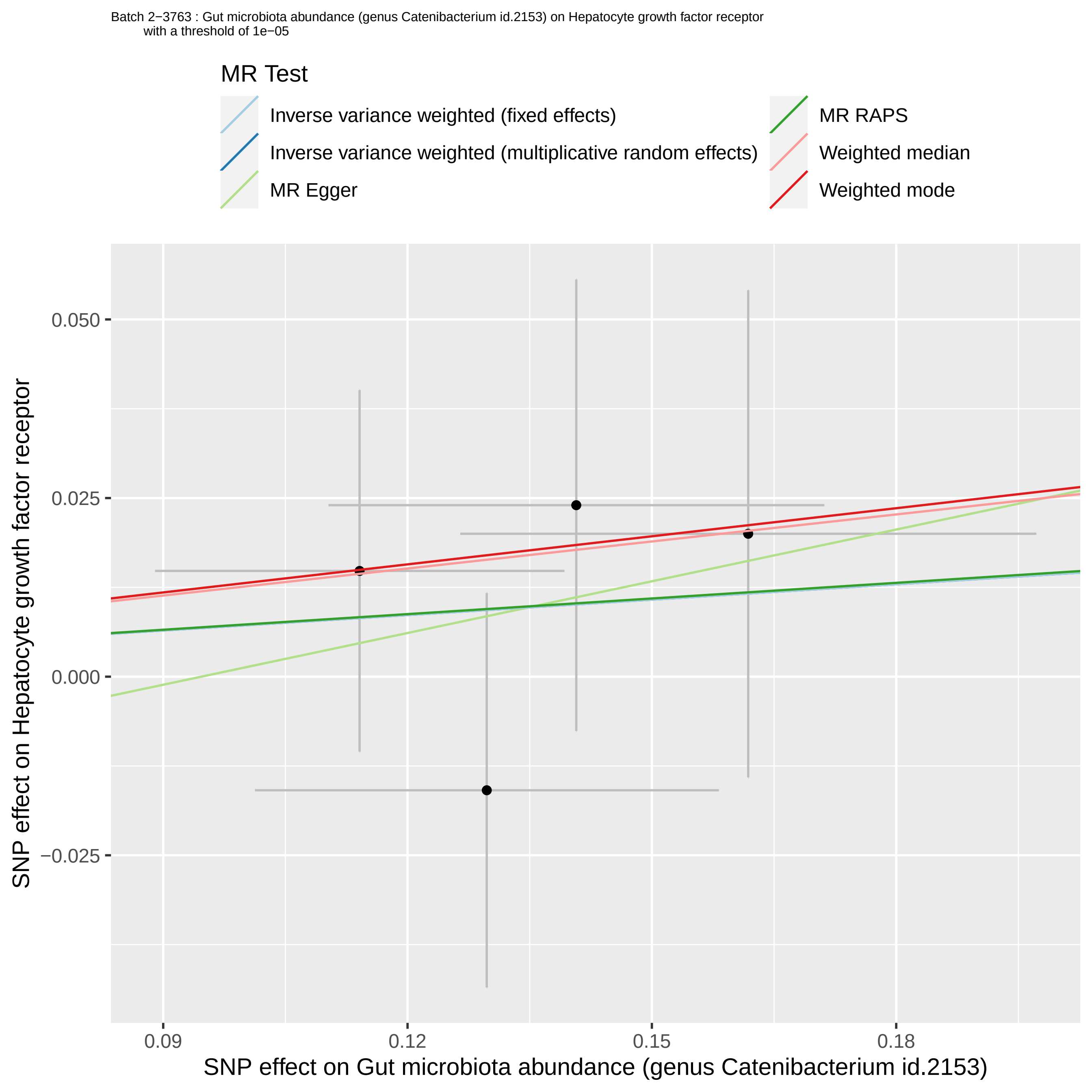

Batch 2−3763 : Gut microbiota abundance (genus Catenibacterium id.2153) on Hepatocyte growth factor receptor
with a threshold of 1e−05
MR Test
Inverse variance weighted (fixed effects)
Inverse variance weighted (multiplicative random effects)
MR Egger
MR RAPS
Weighted median
Weighted mode
0.050
0.025
0.000
−0.025
0.09
0.12
0.15
0.18
SNP effect on Gut microbiota abundance (genus Catenibacterium id.2153)
SNP effect on Hepatocyte growth factor receptor

Batch 2−3764 : Gut microbiota abundance (genus Parasutterella id.2892) on Hepatocyte growth factor receptor
with a threshold of 1e−05

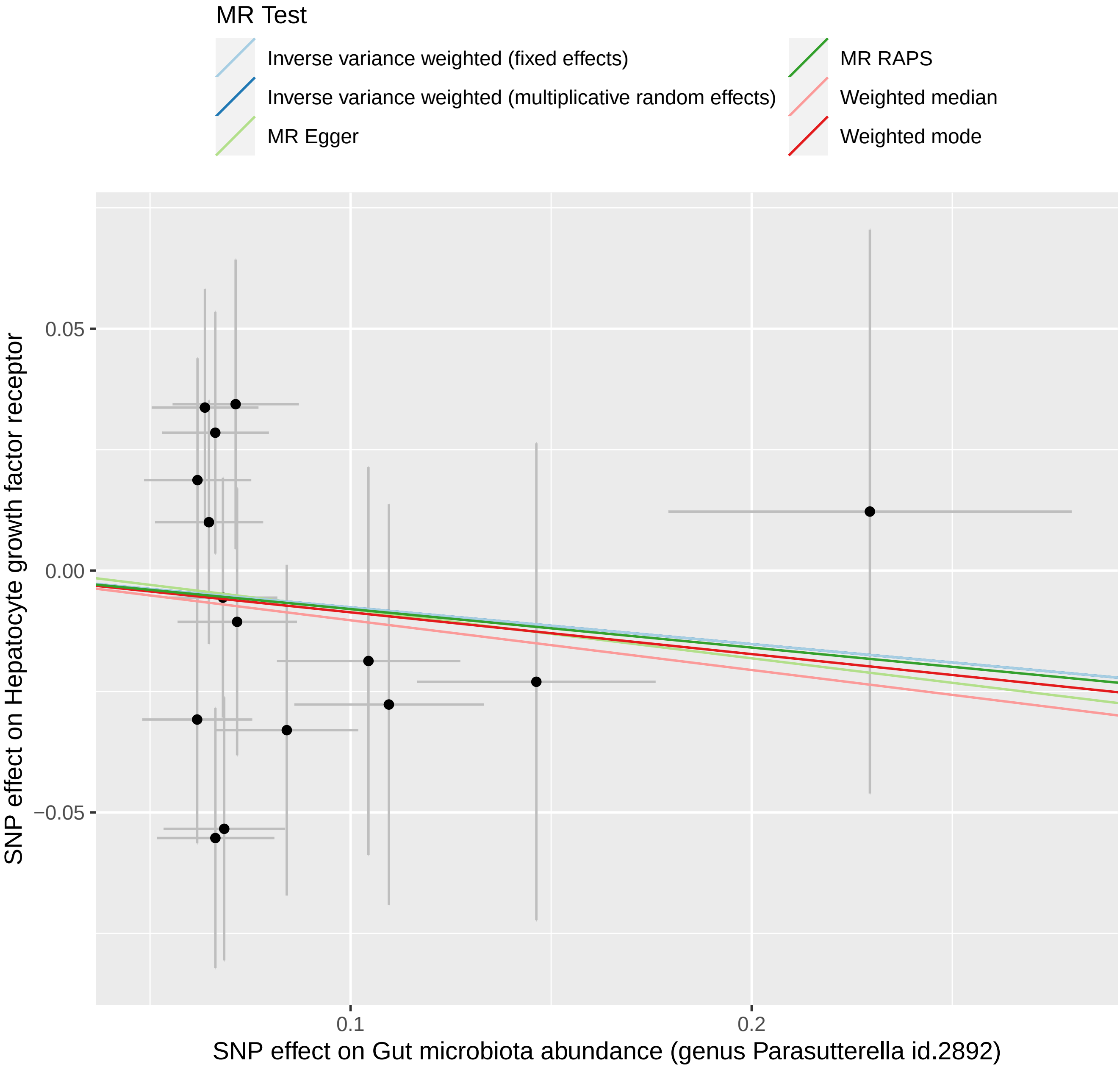

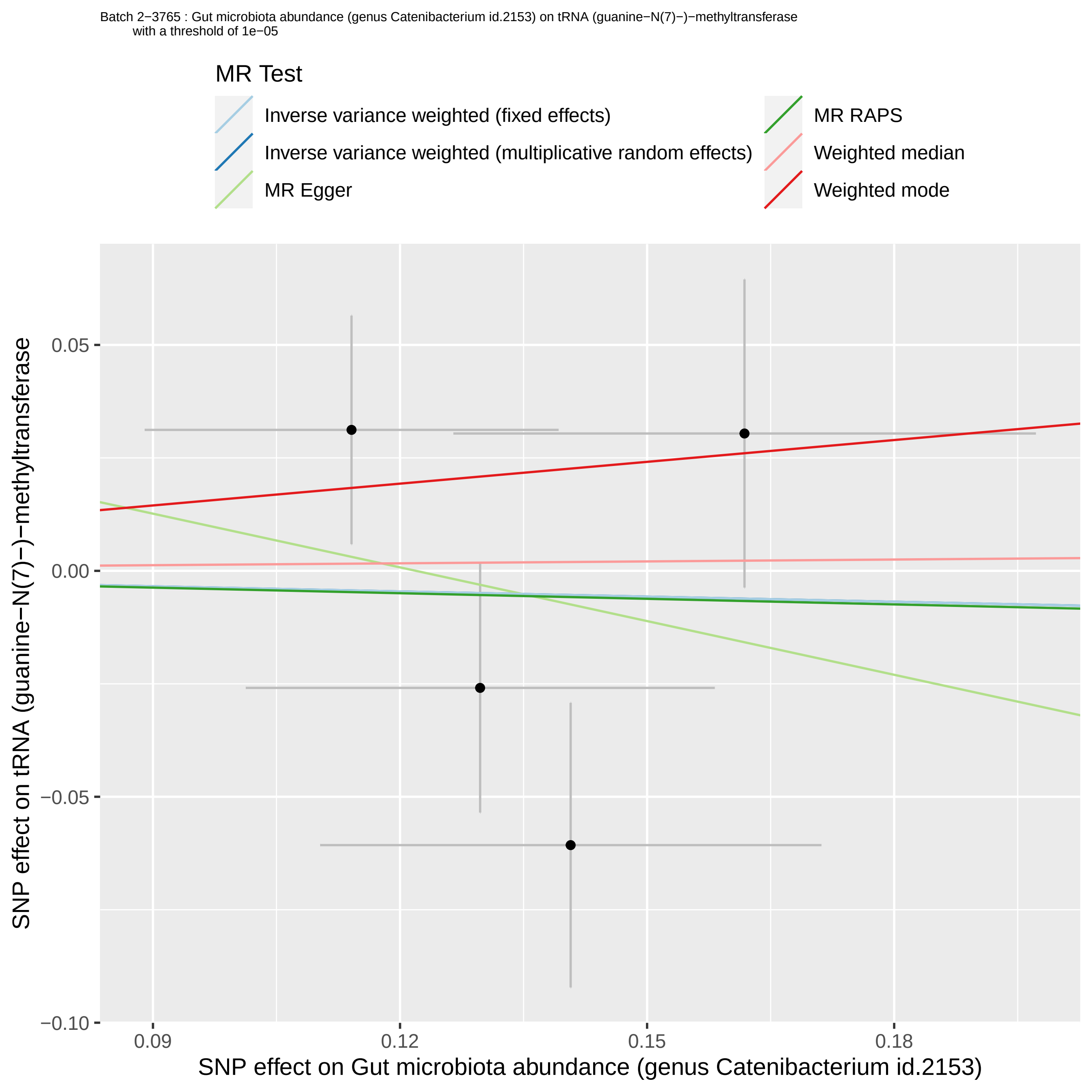
Batch 2−3765 : Gut microbiota abundance (genus Catenibacterium id.2153) on tRNA (guanine−N(7)−)−methyltransferase
with a threshold of 1e−05
MR Test
Inverse variance weighted (fixed effects)
Inverse variance weighted (multiplicative random effects)
MR Egger
MR RAPS
Weighted median
Weighted mode
0.05
0.00
−0.05
−0.10
0.09
0.12
0.15
0.18
SNP effect on tRNA (guanine−N(7)−)−methyltransferase
SNP effect on Gut microbiota abundance (genus Catenibacterium id.2153)

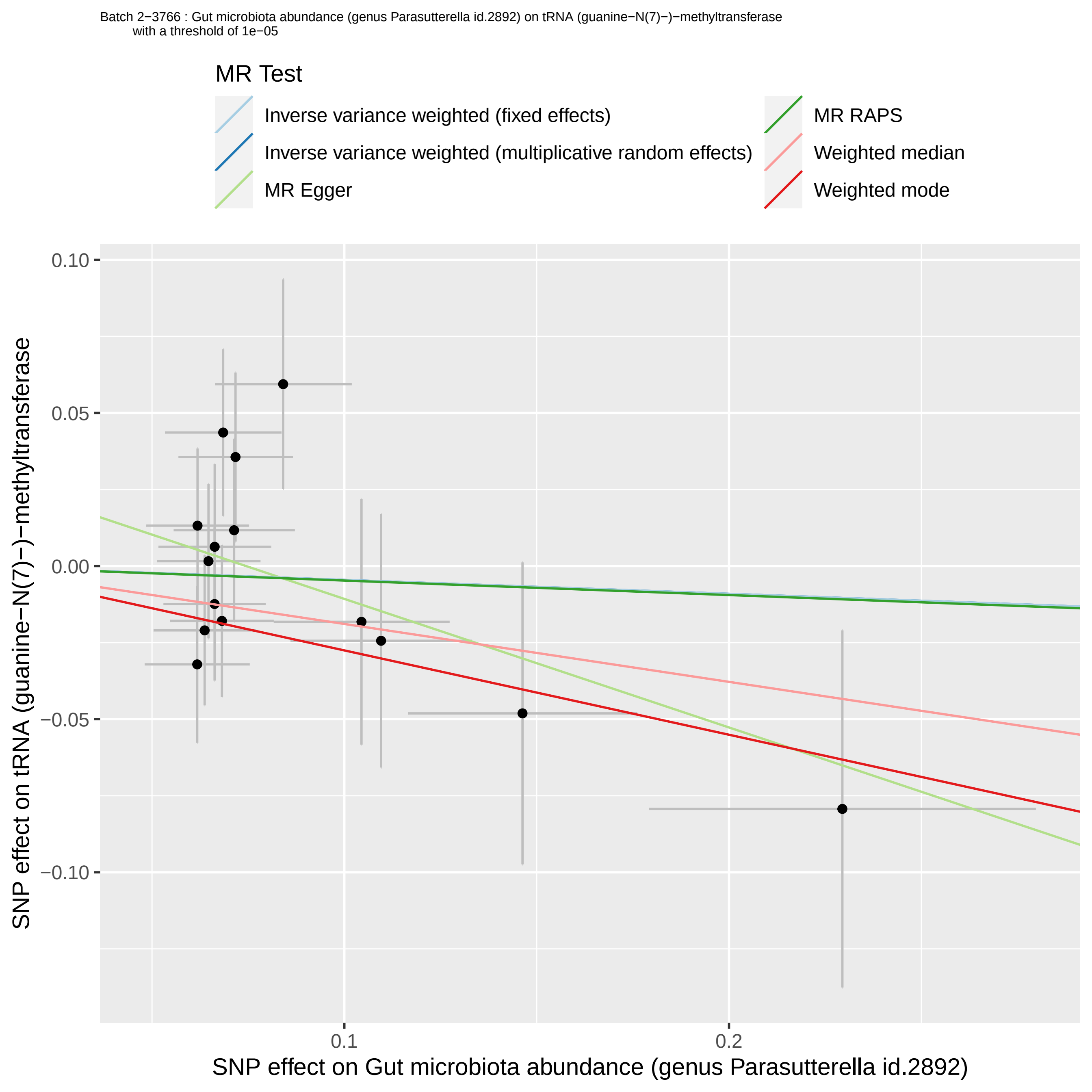

Batch 2−3766 : Gut microbiota abundance (genus Parasutterella id.2892) on tRNA (guanine−N(7)−)−methyltransferase
with a threshold of 1e−05
MR Test
Inverse variance weighted (fixed effects)
Inverse variance weighted (multiplicative random effects)
MR Egger
MR RAPS
Weighted median
Weighted mode
0.10
0.05
0.00
−0.05
−0.10
0.1
0.2
SNP effect on tRNA (guanine−N(7)−)−methyltransferase
SNP effect on Gut microbiota abundance (genus Parasutterella id.2892)

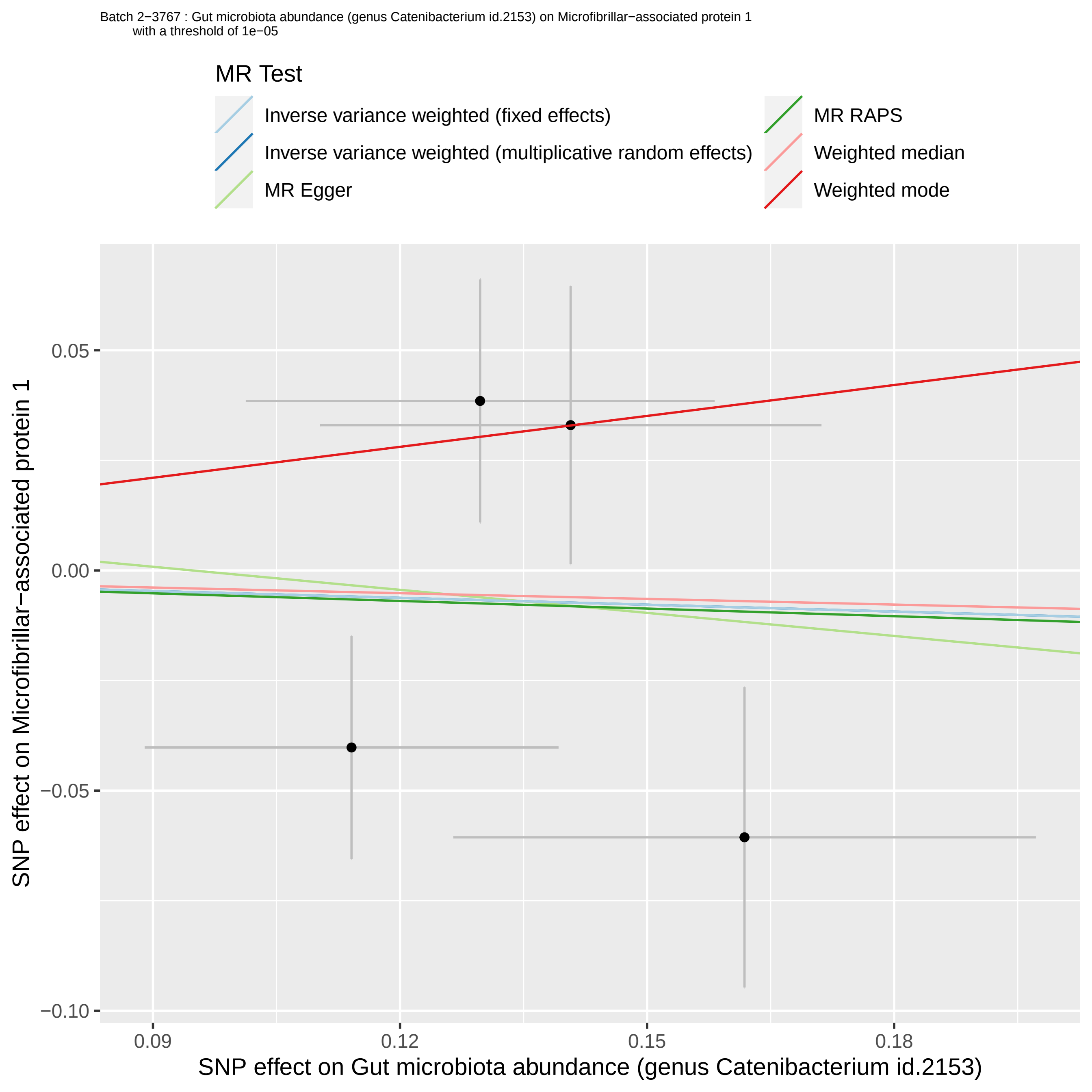

Batch 2−3767 : Gut microbiota abundance (genus Catenibacterium id.2153) on Microfibrillar−associated protein 1
with a threshold of 1e−05
MR Test
Inverse variance weighted (fixed effects)
Inverse variance weighted (multiplicative random effects)
MR Egger
MR RAPS
Weighted median
Weighted mode
0.05
0.00
−0.05
−0.10
0.09
0.12
0.15
0.18
SNP effect on Microfibrillar−associated protein 1
SNP effect on Gut microbiota abundance (genus Catenibacterium id.2153)

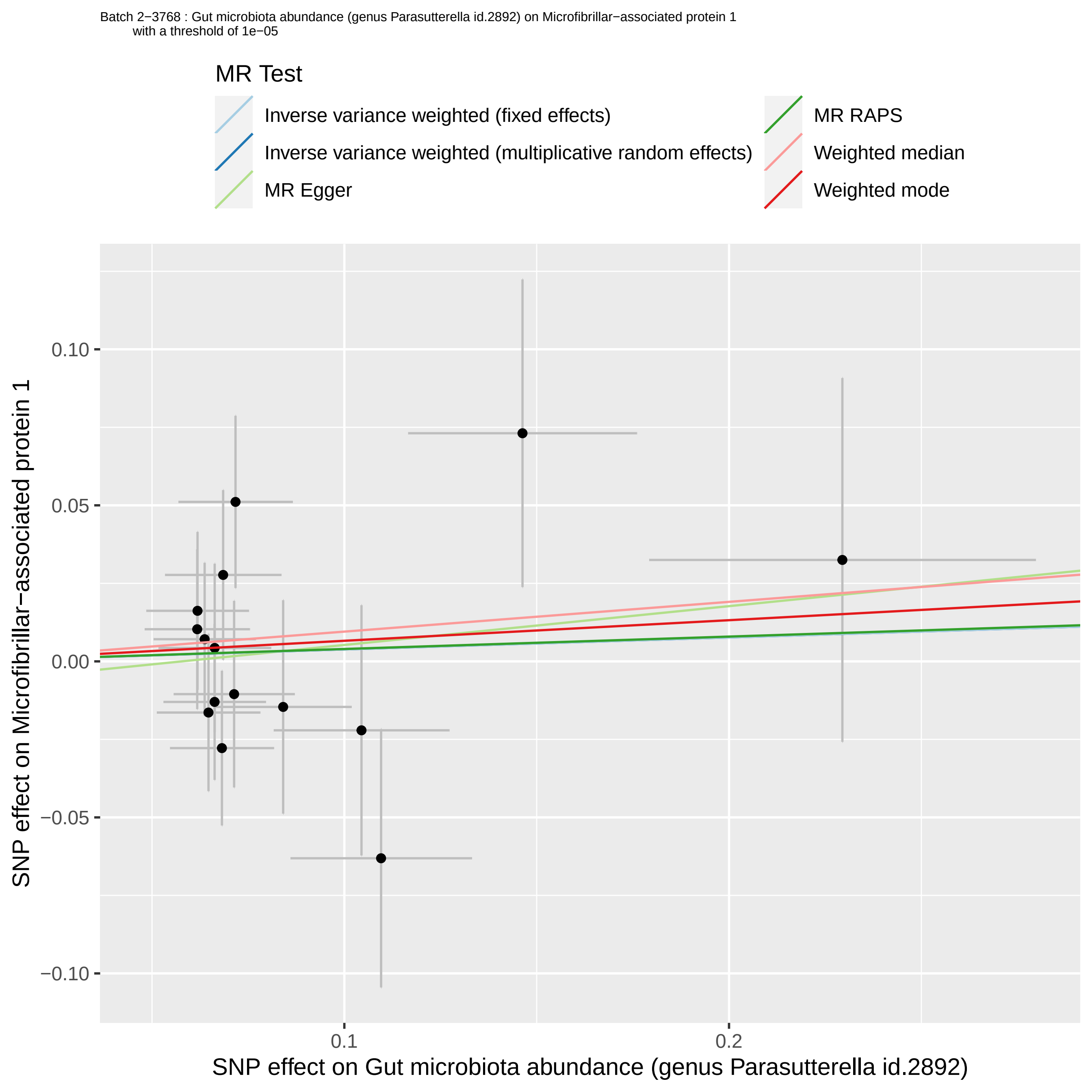
Batch 2−3768 : Gut microbiota abundance (genus Parasutterella id.2892) on Microfibrillar−associated protein 1
with a threshold of 1e−05
MR Test
Inverse variance weighted (fixed effects)
Inverse variance weighted (multiplicative random effects)
MR Egger
MR RAPS
Weighted median
Weighted mode
0.10
0.05
0.00
−0.05
−0.10
0.1
0.2
SNP effect on Microfibrillar−associated protein 1
SNP effect on Gut microbiota abundance (genus Parasutterella id.2892)

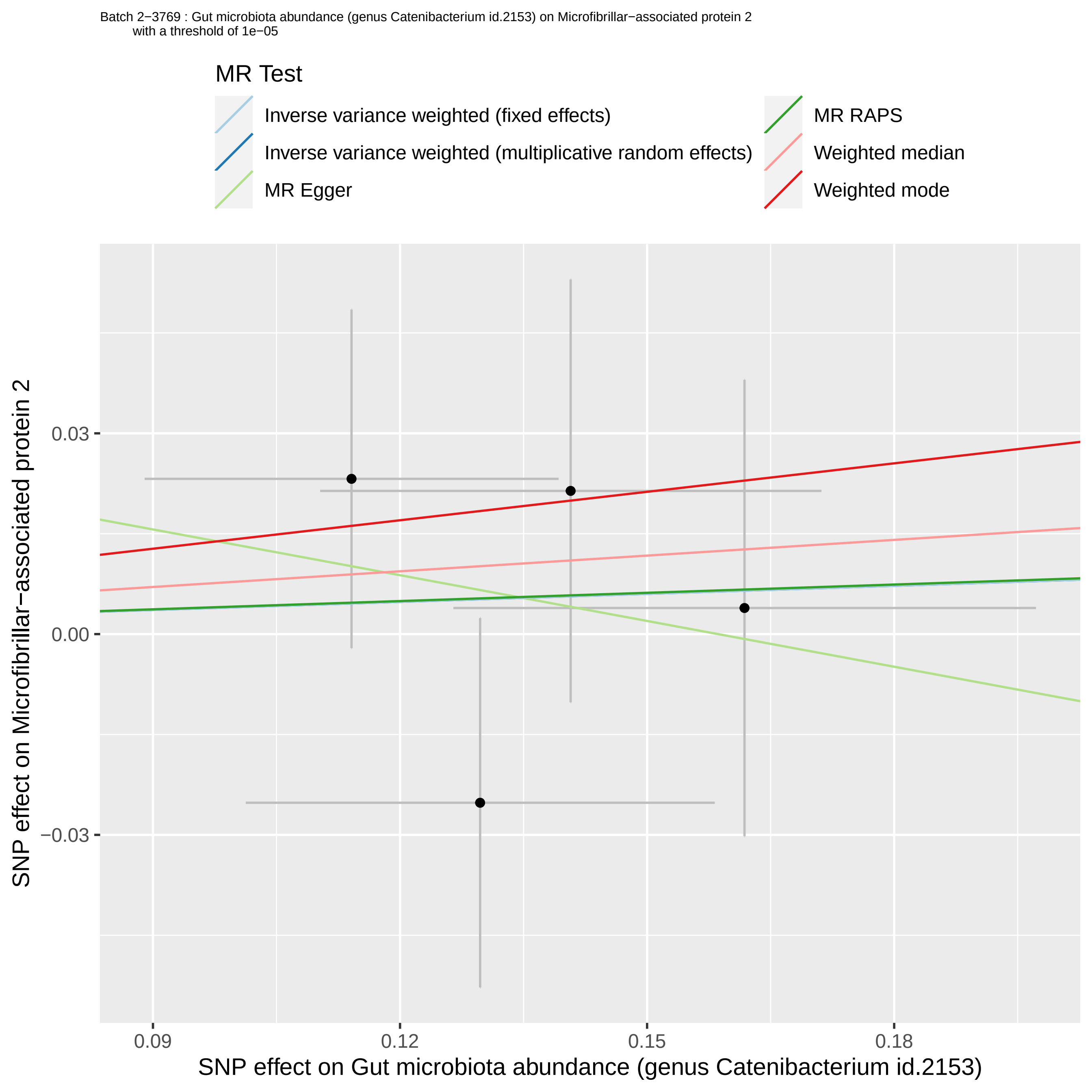
Batch 2−3769 : Gut microbiota abundance (genus Catenibacterium id.2153) on Microfibrillar−associated protein 2
with a threshold of 1e−05
MR Test
Inverse variance weighted (fixed effects)
Inverse variance weighted (multiplicative random effects)
MR Egger
MR RAPS
Weighted median
Weighted mode
0.03
0.00
−0.03
0.09
0.12
0.15
0.18
SNP effect on Microfibrillar−associated protein 2
SNP effect on Gut microbiota abundance (genus Catenibacterium id.2153)

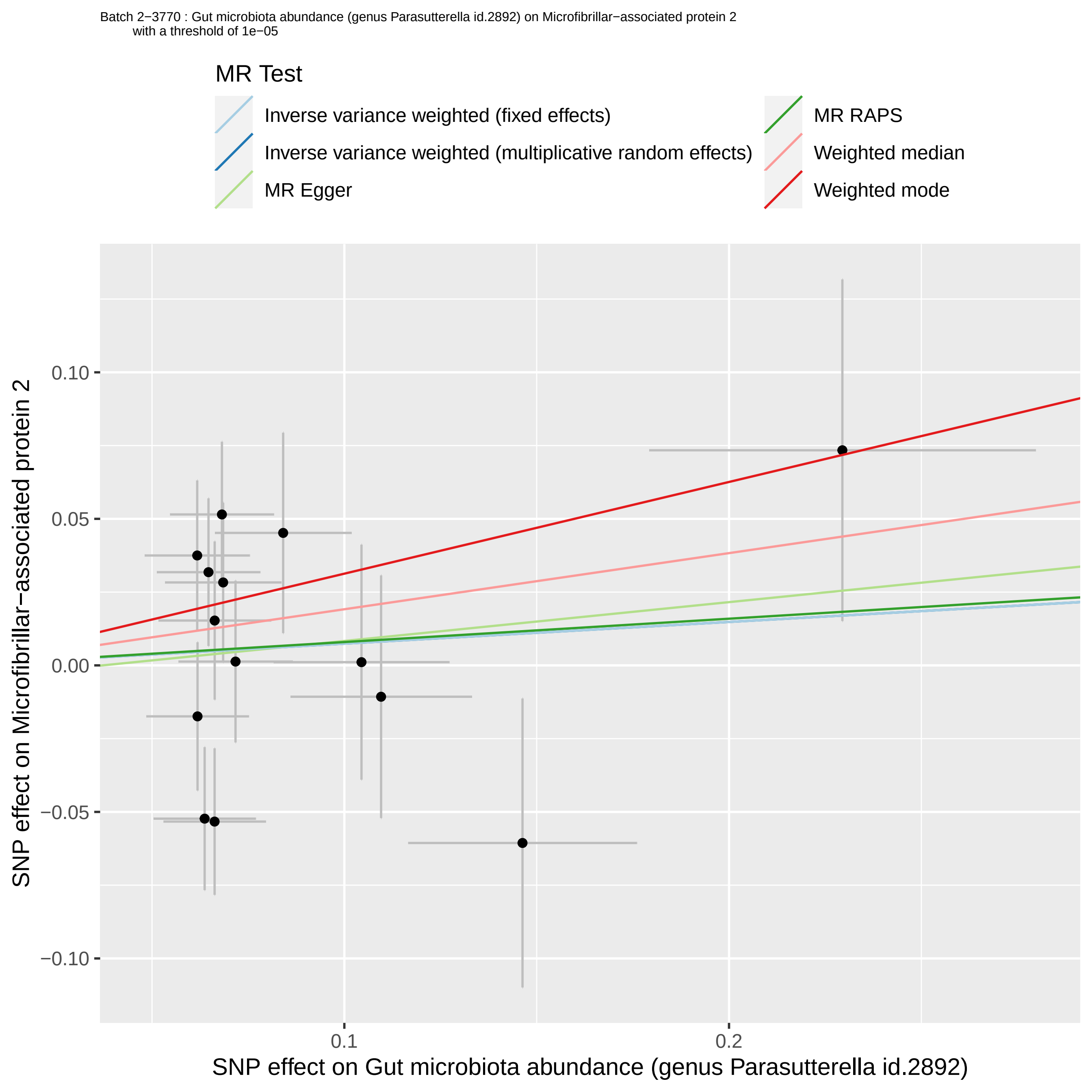
Batch 2−3770 : Gut microbiota abundance (genus Parasutterella id.2892) on Microfibrillar−associated protein 2
with a threshold of 1e−05
MR Test
Inverse variance weighted (fixed effects)
Inverse variance weighted (multiplicative random effects)
MR Egger
MR RAPS
Weighted median
Weighted mode
0.10
0.05
0.00
−0.05
−0.10
0.1
0.2
SNP effect on Microfibrillar−associated protein 2
SNP effect on Gut microbiota abundance (genus Parasutterella id.2892)

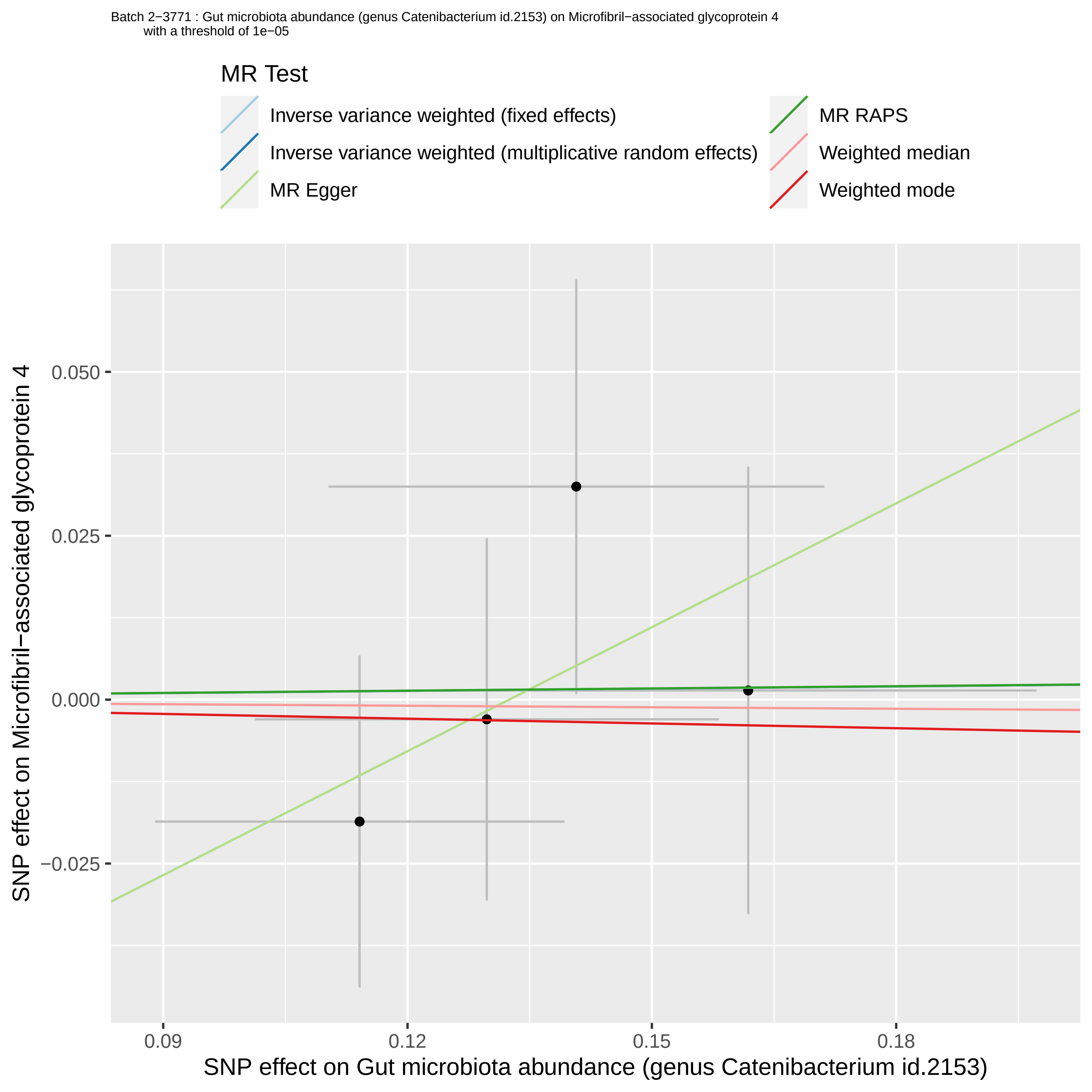

Batch 2−3771 : Gut microbiota abundance (genus Catenibacterium id.2153) on Microfibril−associated glycoprotein 4
with a threshold of 1e−05
MR Test
Inverse variance weighted (fixed effects)
Inverse variance weighted (multiplicative random effects)
MR Egger
MR RAPS
Weighted median
Weighted mode
0.050
0.025
0.000
−0.025
0.09
0.12
0.15
0.18
SNP effect on Microfibril−associated glycoprotein 4
SNP effect on Gut microbiota abundance (genus Catenibacterium id.2153)

Batch 2−3772 : Gut microbiota abundance (genus Parasutterella id.2892) on Microfibril−associated glycoprotein 4
with a threshold of 1e−05

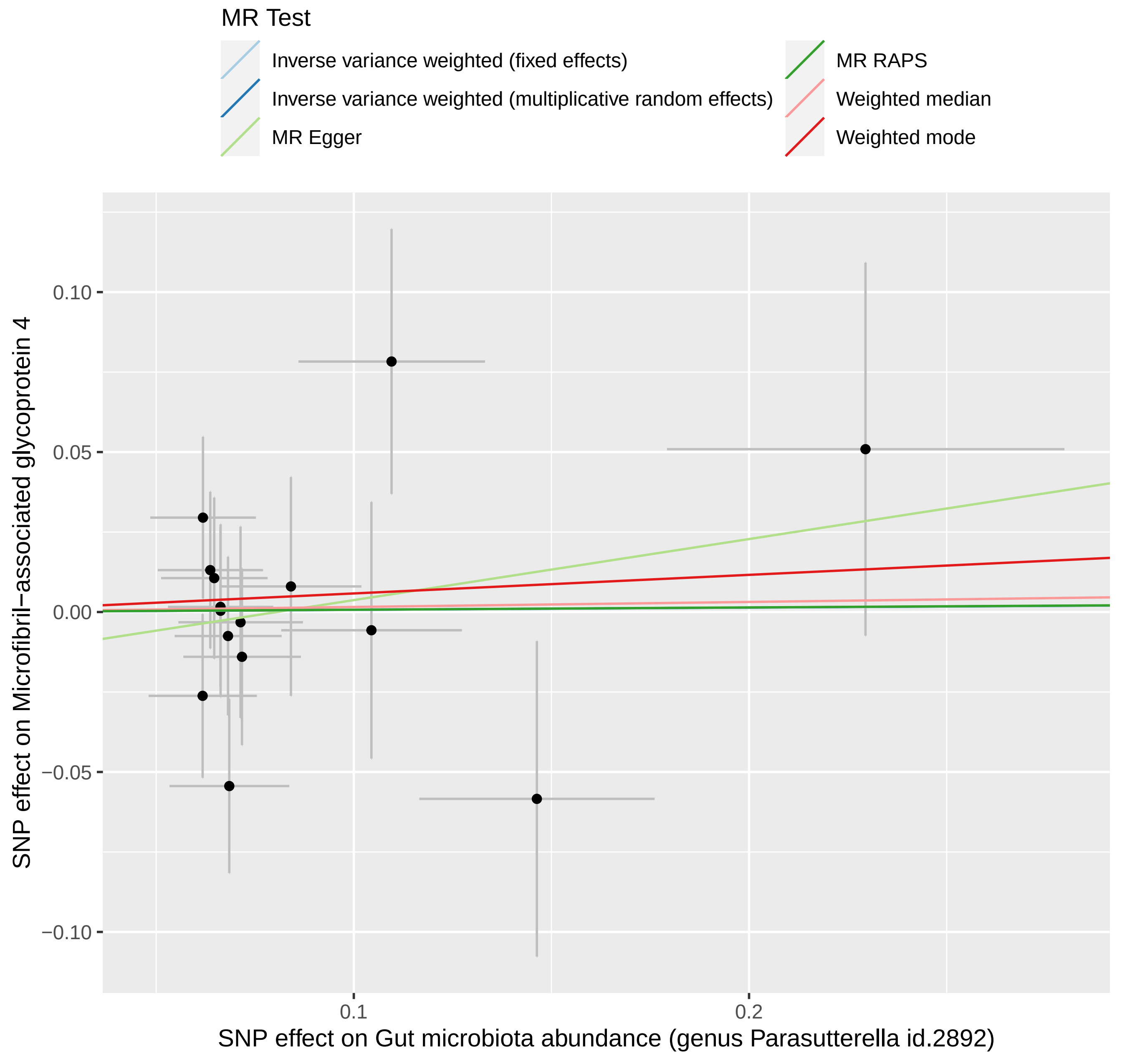

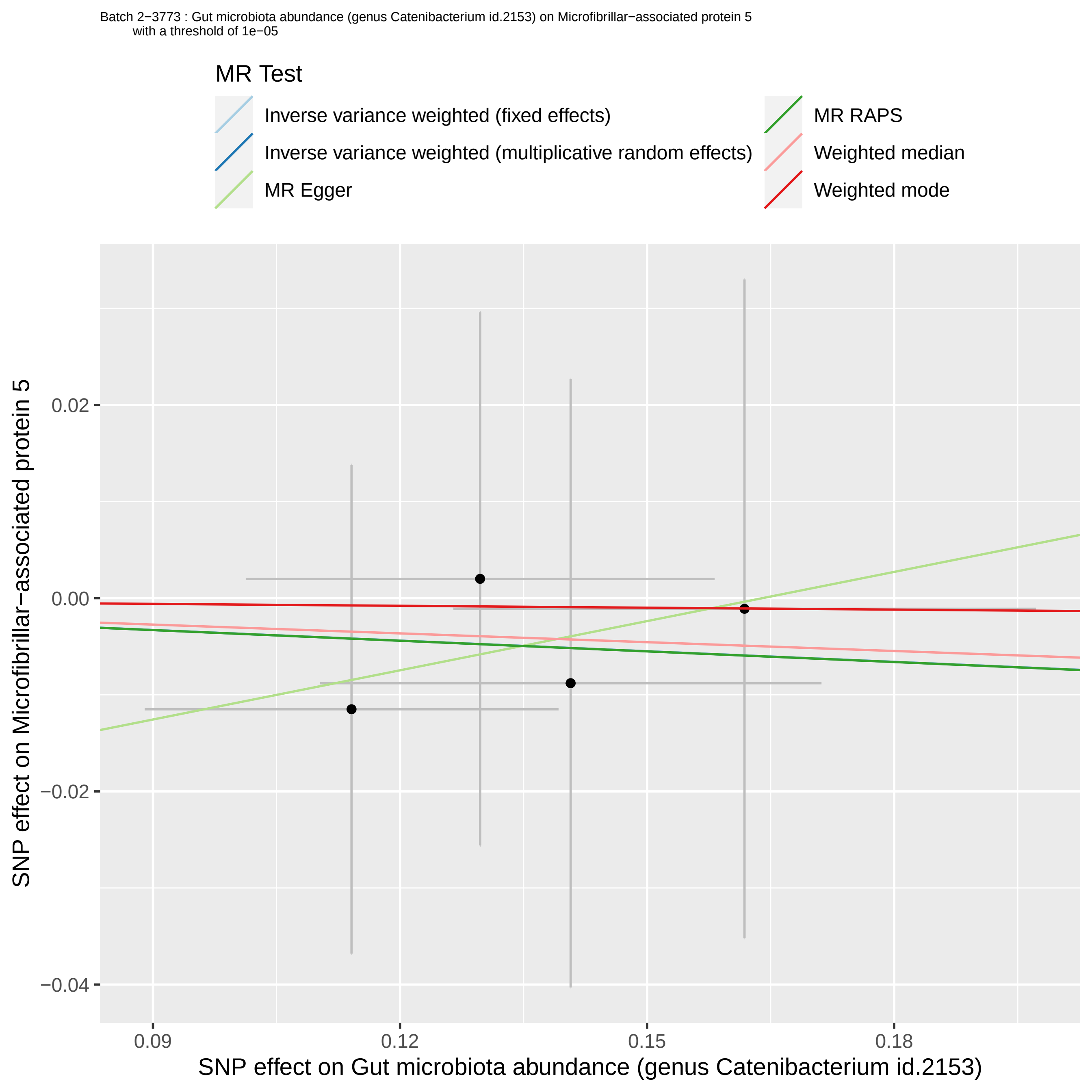
Batch 2−3773 : Gut microbiota abundance (genus Catenibacterium id.2153) on Microfibrillar−associated protein 5
with a threshold of 1e−05
MR Test
Inverse variance weighted (fixed effects)
Inverse variance weighted (multiplicative random effects)
MR Egger
MR RAPS
Weighted median
Weighted mode
SNP effect on Microfibrillar−associated protein 5
0.02
0.00
−0.02
−0.04
0.09
0.12
0.15
0.18
SNP effect on Gut microbiota abundance (genus Catenibacterium id.2153)

Batch 2−3774 : Gut microbiota abundance (genus Parasutterella id.2892) on Microfibrillar−associated protein 5
with a threshold of 1e−05

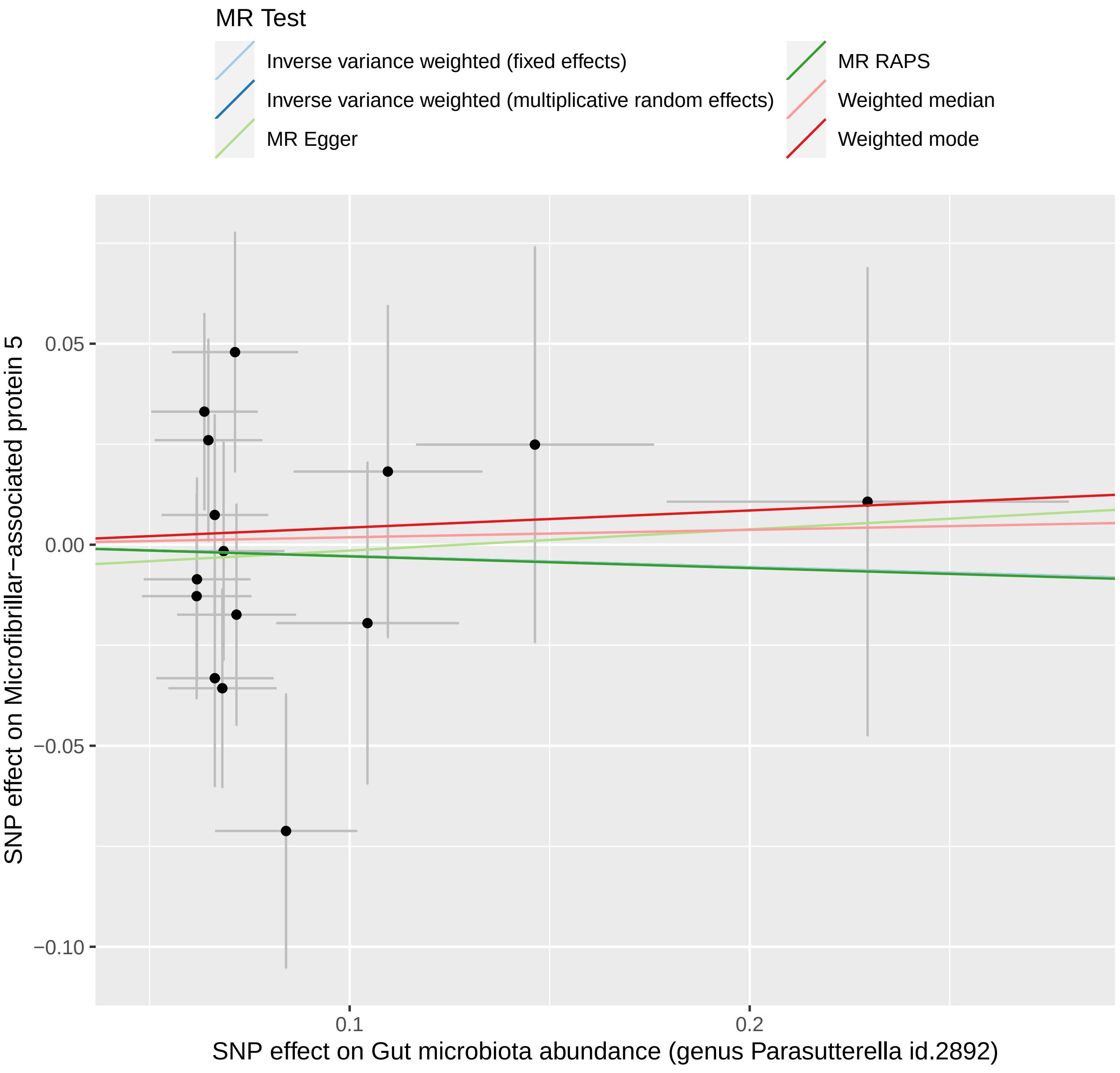

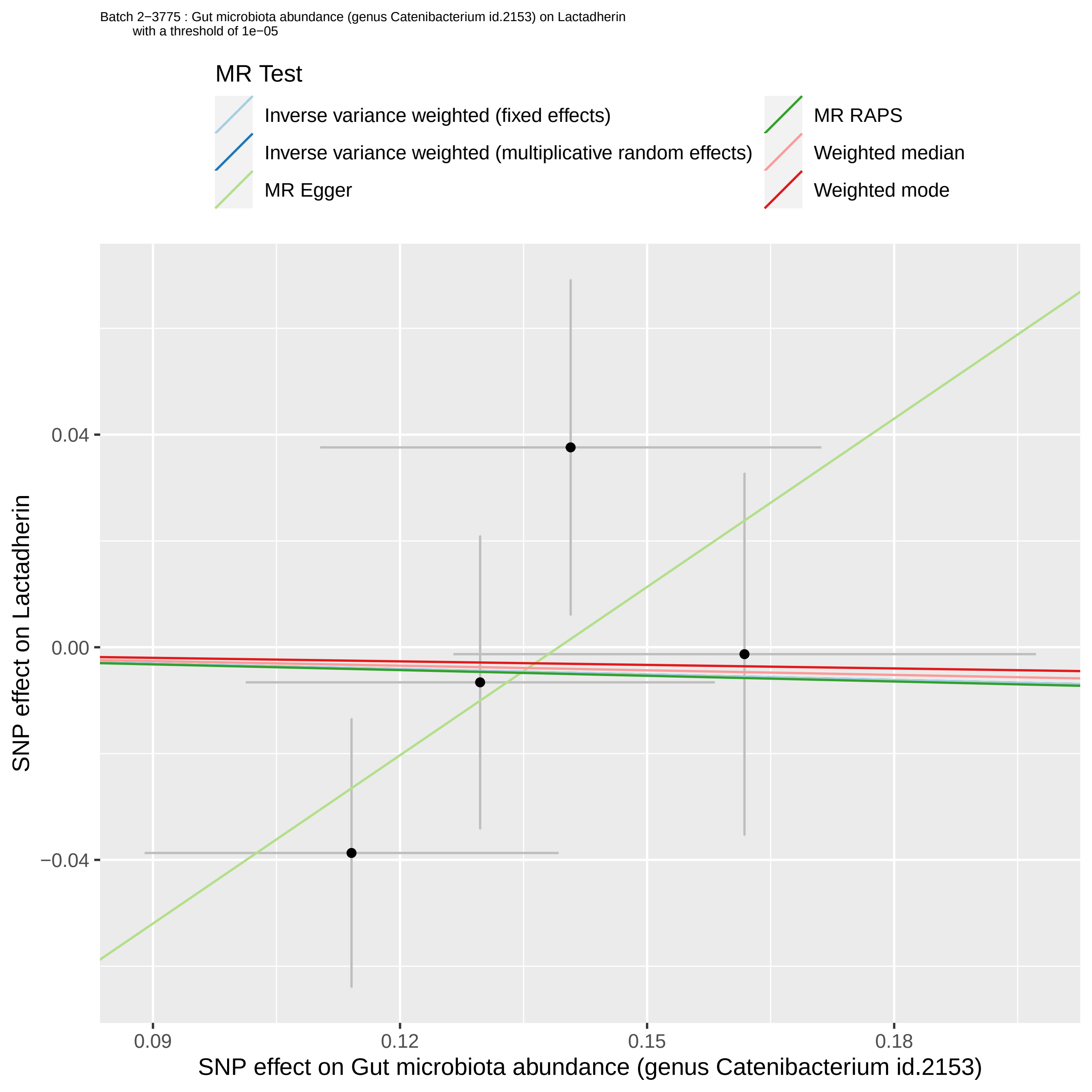
Batch 2−3775 : Gut microbiota abundance (genus Catenibacterium id.2153) on Lactadherin
with a threshold of 1e−05
MR Test
Inverse variance weighted (fixed effects)
Inverse variance weighted (multiplicative random effects)
MR Egger
MR RAPS
Weighted median
Weighted mode
0.04
0.00
−0.04
0.09
0.12
0.15
0.18
SNP effect on Lactadherin
SNP effect on Gut microbiota abundance (genus Catenibacterium id.2153)

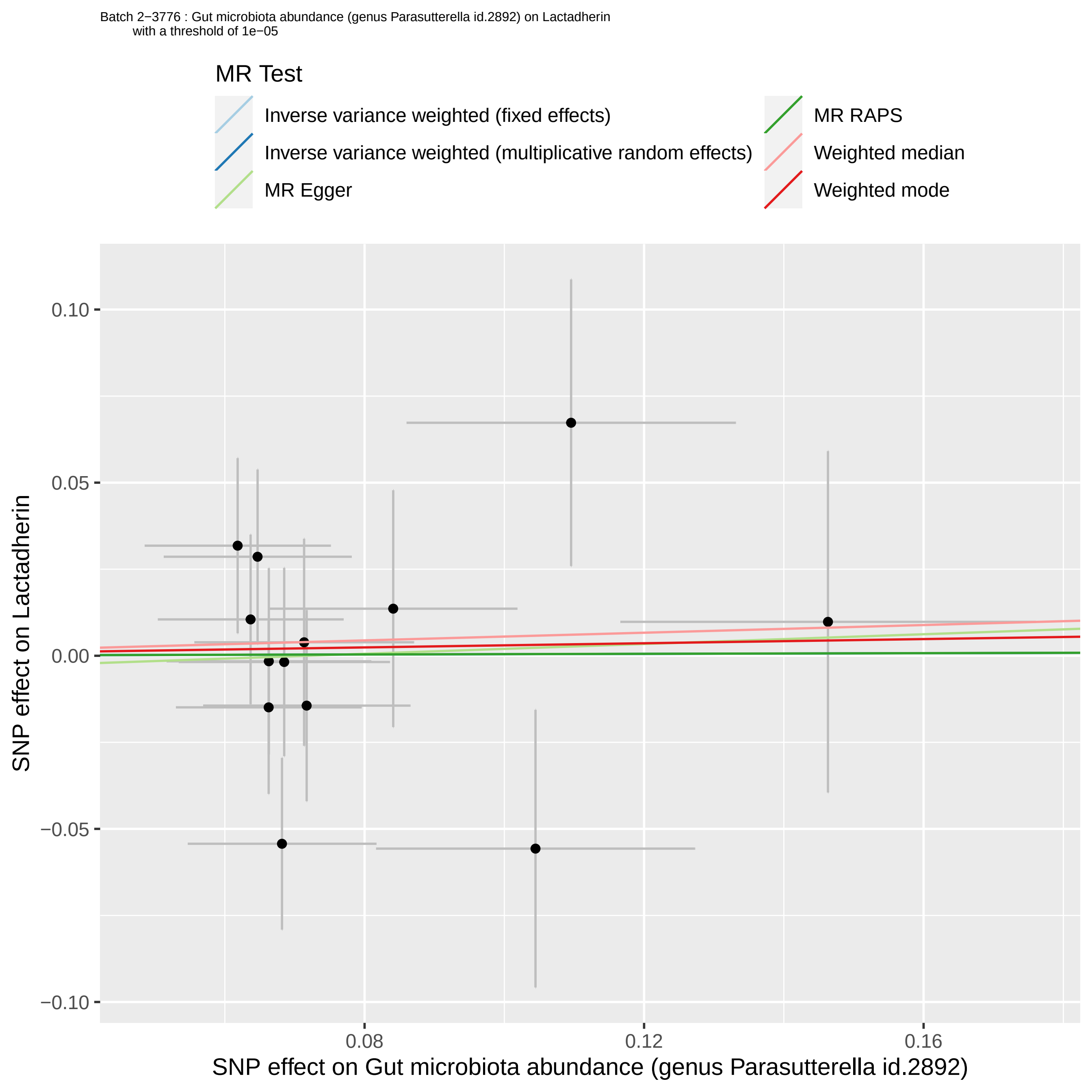
Batch 2−3776 : Gut microbiota abundance (genus Parasutterella id.2892) on Lactadherin
with a threshold of 1e−05
MR Test
Inverse variance weighted (fixed effects)
Inverse variance weighted (multiplicative random effects)
MR Egger
MR RAPS
Weighted median
Weighted mode
0.10
0.05
0.00
−0.05
−0.10
SNP effect on Lactadherin
0.08
0.12
0.16
SNP effect on Gut microbiota abundance (genus Parasutterella id.2892)

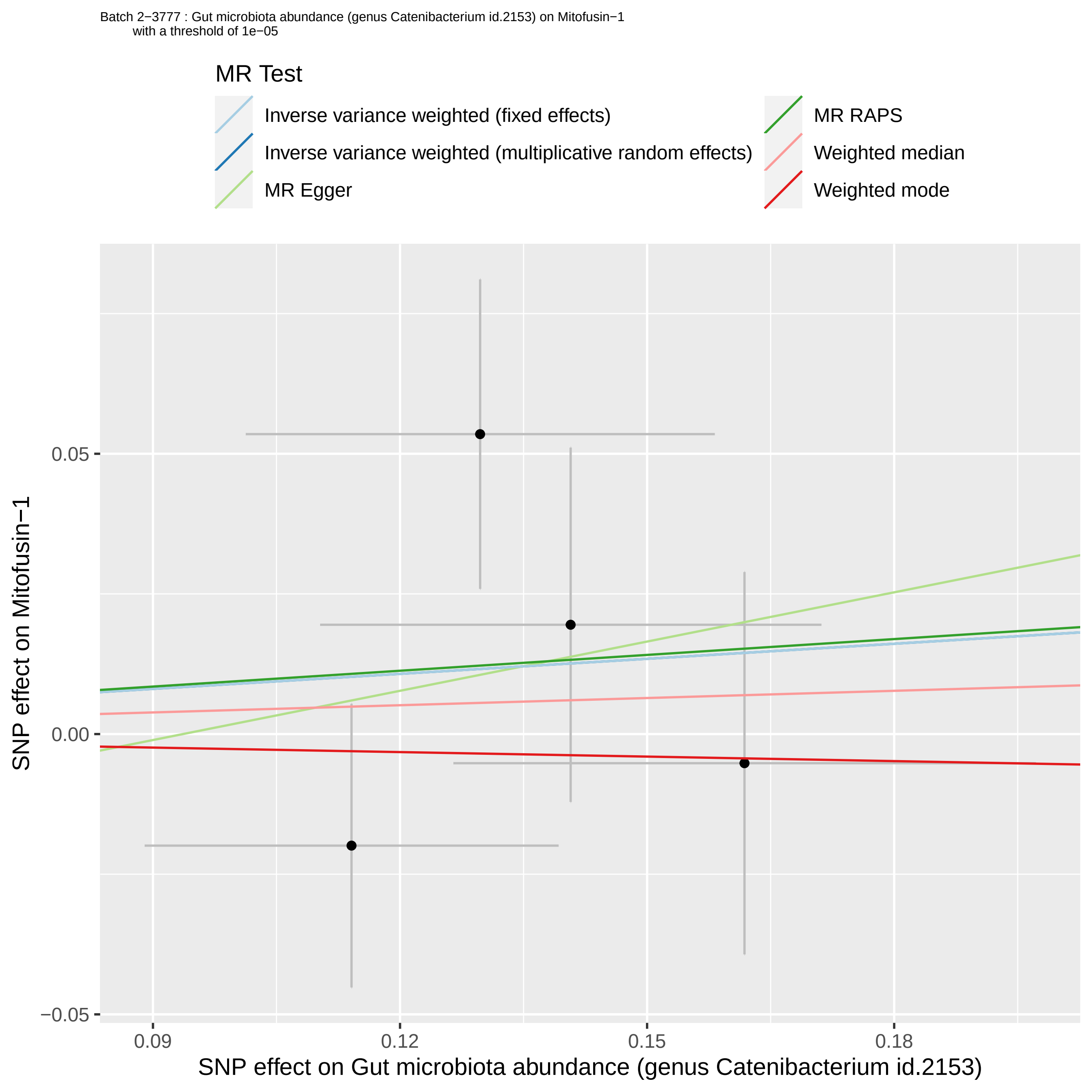

Batch 2−3777 : Gut microbiota abundance (genus Catenibacterium id.2153) on Mitofusin−1
with a threshold of 1e−05
MR Test
Inverse variance weighted (fixed effects)
Inverse variance weighted (multiplicative random effects)
MR Egger
MR RAPS
Weighted median
Weighted mode
0.05
0.00
−0.05
0.09
0.12
0.15
0.18
SNP effect on Mitofusin−1
SNP effect on Gut microbiota abundance (genus Catenibacterium id.2153)

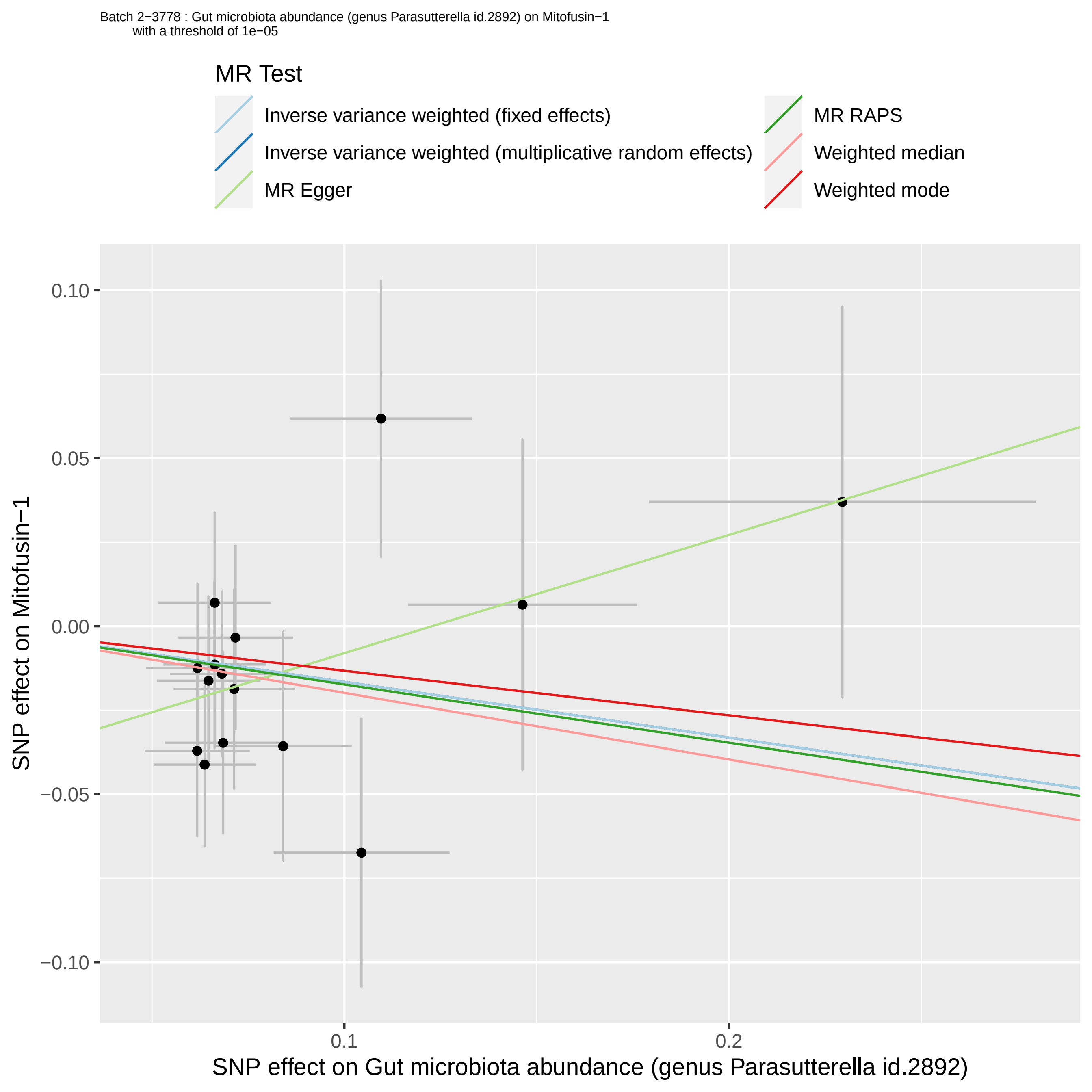
Batch 2−3778 : Gut microbiota abundance (genus Parasutterella id.2892) on Mitofusin−1
with a threshold of 1e−05
MR Test
Inverse variance weighted (fixed effects)
Inverse variance weighted (multiplicative random effects)
MR Egger
MR RAPS
Weighted median
Weighted mode
0.10
0.05
0.00
−0.05
−0.10
0.1
0.2
SNP effect on Mitofusin−1
SNP effect on Gut microbiota abundance (genus Parasutterella id.2892)

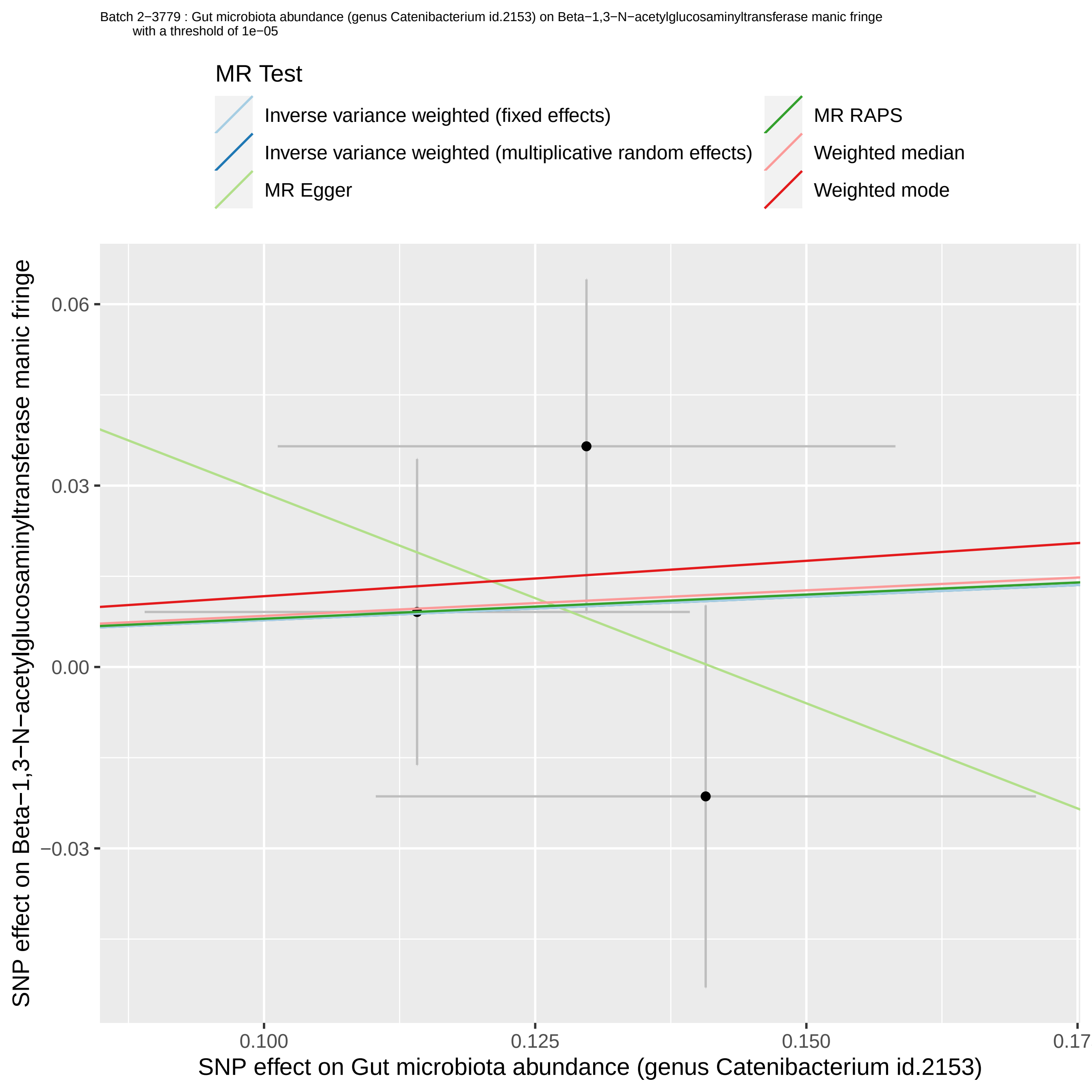

Batch 2−3779 : Gut microbiota abundance (genus Catenibacterium id.2153) on Beta−1,3−N−acetylglucosaminyltransferase manic fringe with a threshold of 1e−05
MR Test
Inverse variance weighted (fixed effects)
Inverse variance weighted (multiplicative random effects)
MR Egger
MR RAPS
Weighted median
Weighted mode
0.06
0.03
0.00
−0.03
0.100
0.125
0.150
0.17
SNP effect on Beta−1,3−N−acetylglucosaminyltransferase manic fringe
SNP effect on Gut microbiota abundance (genus Catenibacterium id.2153)

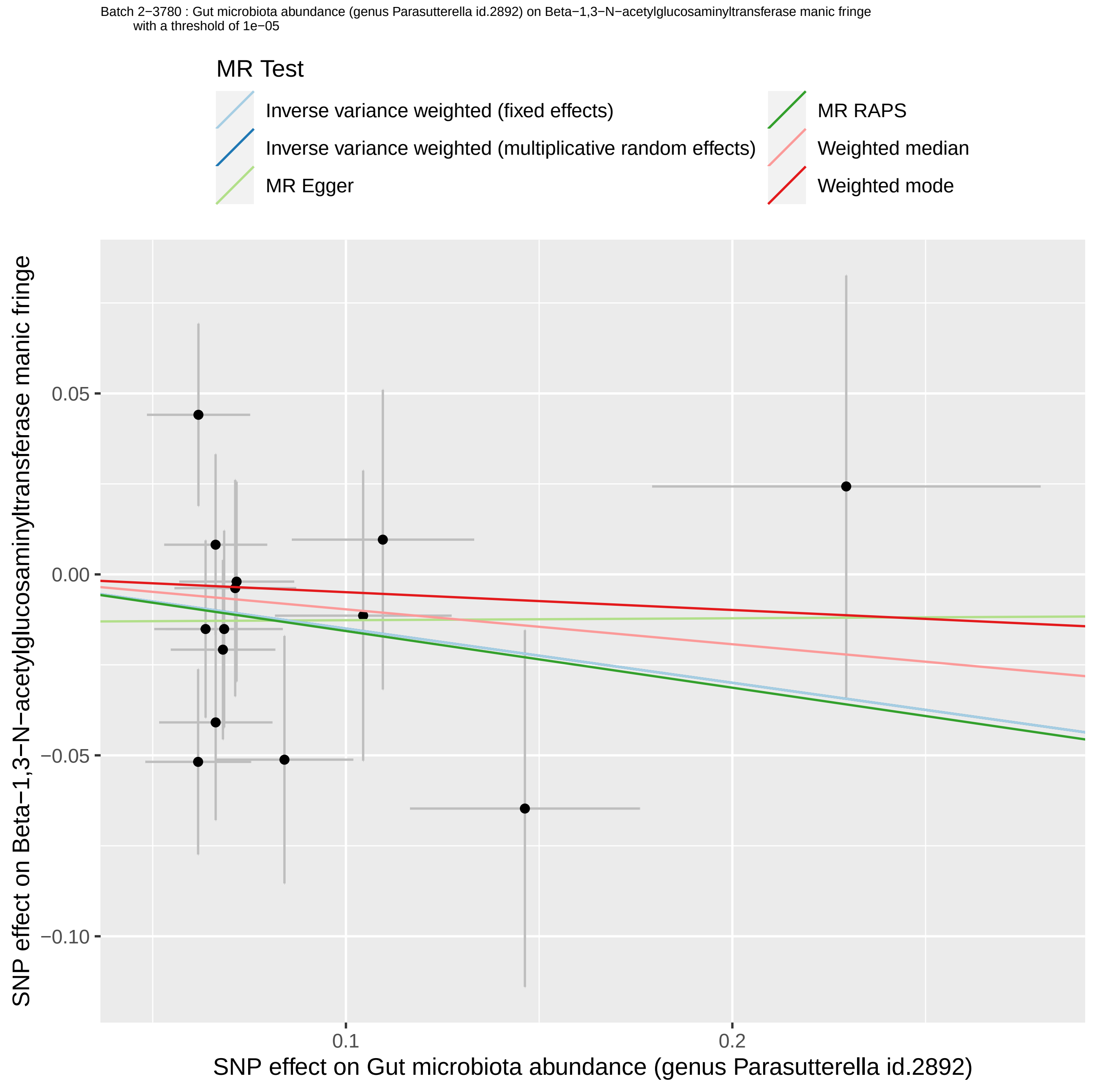
Batch 2−3780 : Gut microbiota abundance (genus Parasutterella id.2892) on Beta−1,3−N−acetylglucosaminyltransferase manic fringe
with a threshold of 1e−05
MR Test
Inverse variance weighted (fixed effects)
Inverse variance weighted (multiplicative random effects)
MR Egger
MR RAPS
Weighted median
Weighted mode
0.05
0.00
−0.05
−0.10
0.1
0.2
SNP effect on Beta−1,3−N−acetylglucosaminyltransferase manic fringe
SNP effect on Gut microbiota abundance (genus Parasutterella id.2892)

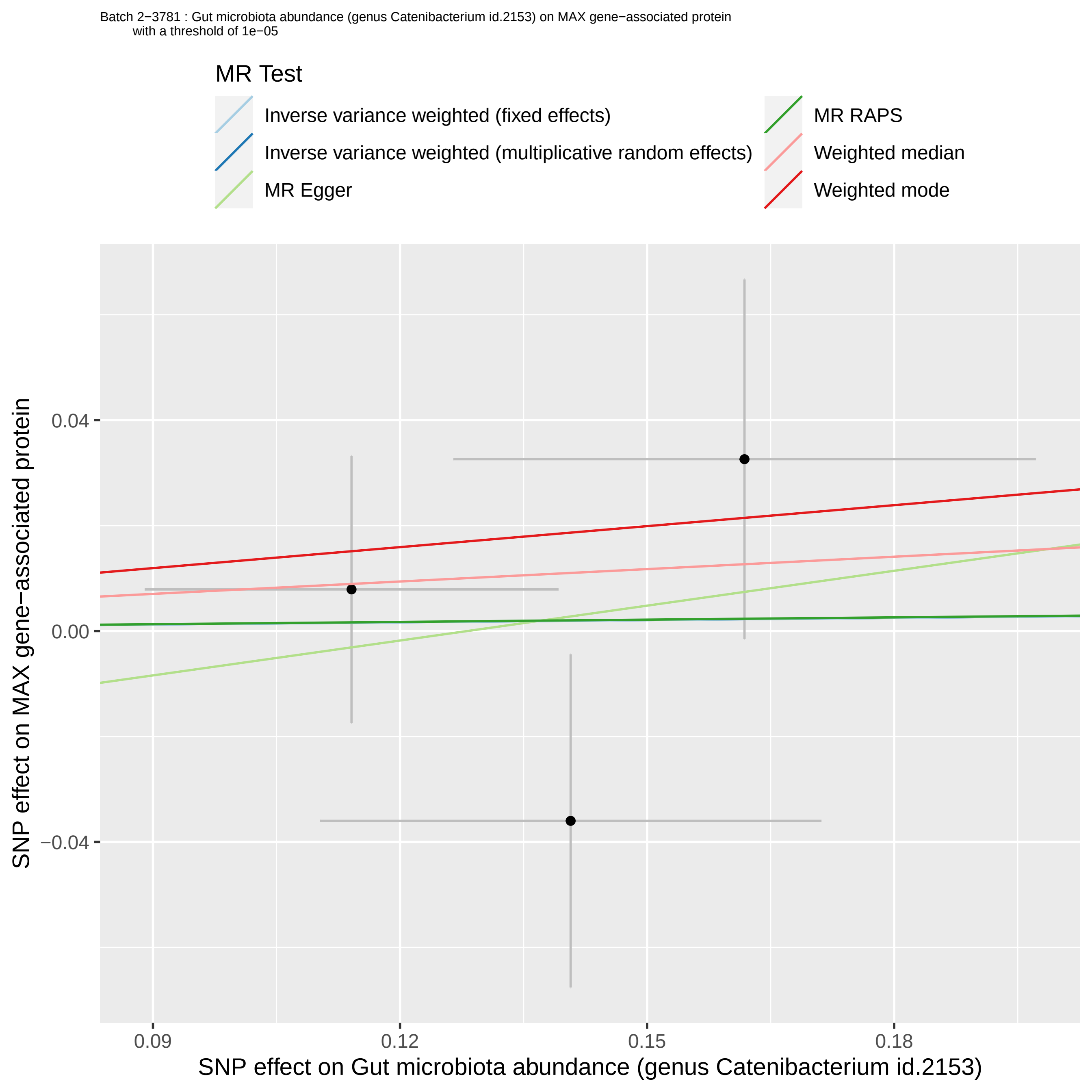

Batch 2−3781 : Gut microbiota abundance (genus Catenibacterium id.2153) on MAX gene−associated protein
with a threshold of 1e−05
MR Test
Inverse variance weighted (fixed effects)
Inverse variance weighted (multiplicative random effects)
MR Egger
MR RAPS
Weighted median
Weighted mode
0.04
0.00
−0.04
0.09
0.12
0.15
0.18
SNP effect on MAX gene−associated protein
SNP effect on Gut microbiota abundance (genus Catenibacterium id.2153)

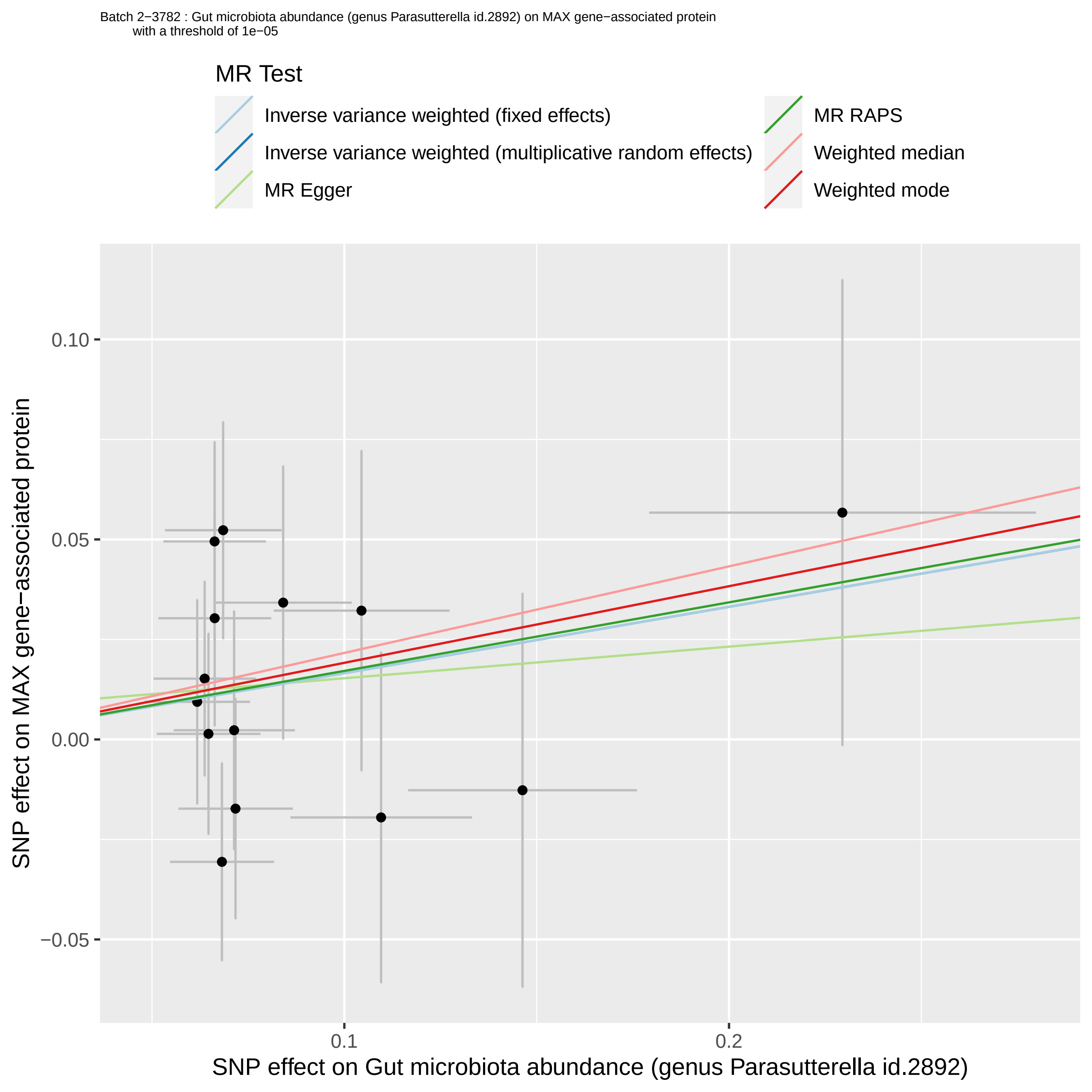

Batch 2−3782 : Gut microbiota abundance (genus Parasutterella id.2892) on MAX gene−associated protein
with a threshold of 1e−05
MR Test
Inverse variance weighted (fixed effects)
Inverse variance weighted (multiplicative random effects)
MR Egger
MR RAPS
Weighted median
Weighted mode
0.10
0.05
0.00
−0.05
0.1
0.2
SNP effect on MAX gene−associated protein
SNP effect on Gut microbiota abundance (genus Parasutterella id.2892)

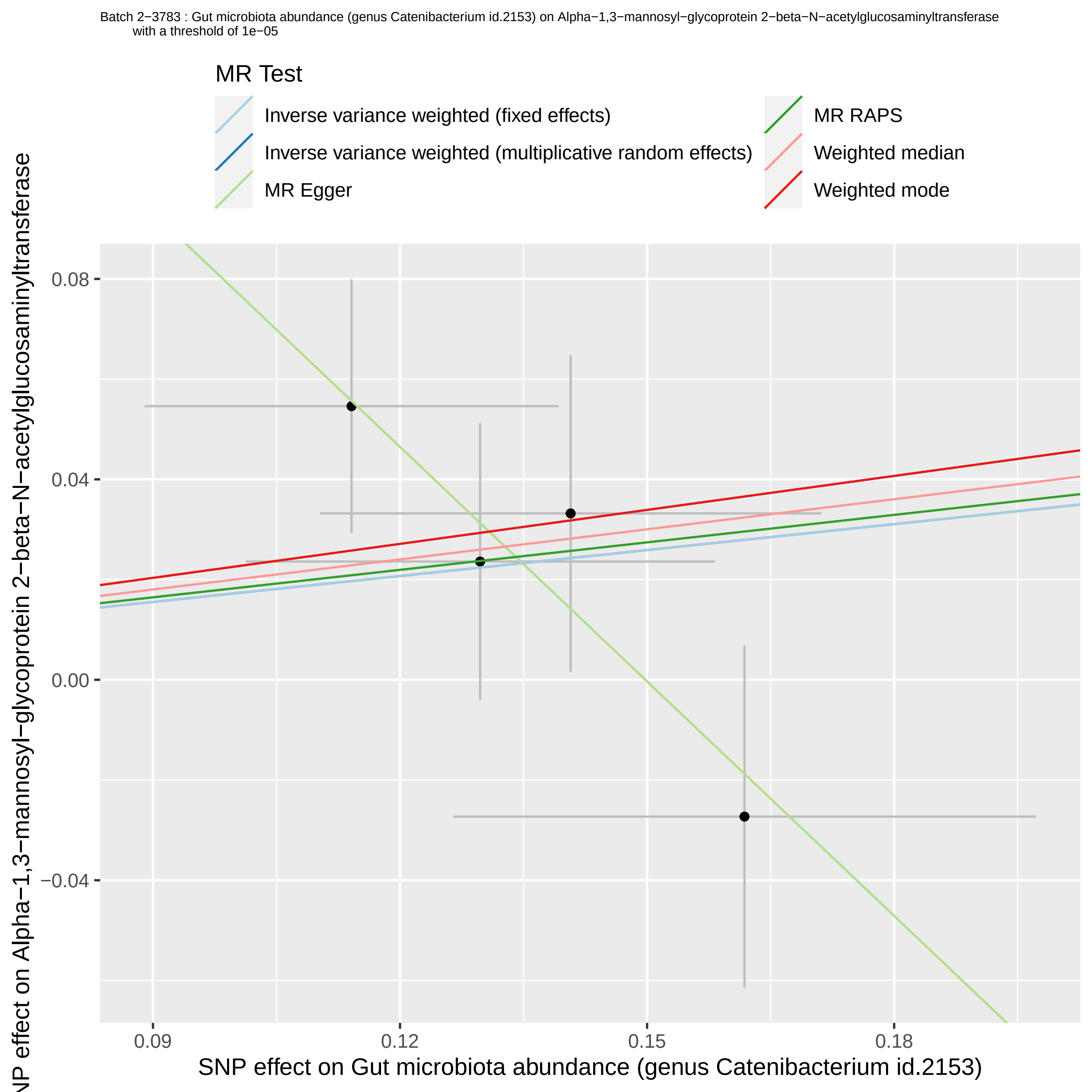

Batch 2−3783 : Gut microbiota abundance (genus Catenibacterium id.2153) on Alpha−1,3−mannosyl−glycoprotein 2−beta−N−acetylglucosaminyltransferase
with a threshold of 1e−05
MR Test
Inverse variance weighted (fixed effects)
Inverse variance weighted (multiplicative random effects)
MR Egger
MR RAPS
Weighted median
Weighted mode
0.08
0.04
0.00
−0.04
0.09
0.12
0.15
0.18
SNP effect on Gut microbiota abundance (genus Catenibacterium id.2153)
SNP effect on Alpha−1,3−mannosyl−glycoprotein 2−beta−N−acetylglucosaminyltransferase

Batch 2−3784 : Gut microbiota abundance (genus Parasutterella id.2892) on Alpha−1,3−mannosyl−glycoprotein 2−beta−N−acetylglucosaminyltransferase with a threshold of 1e−05

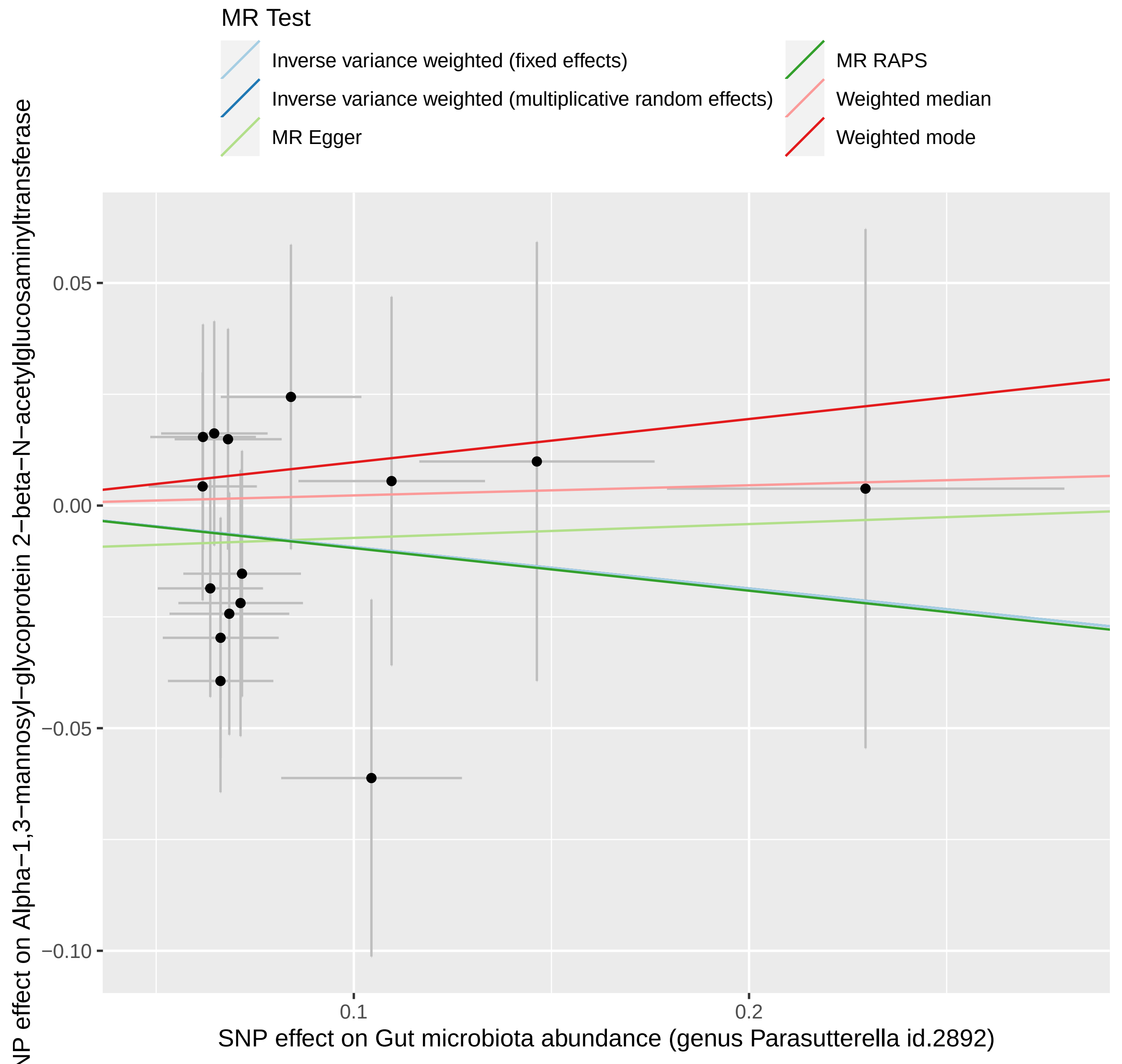

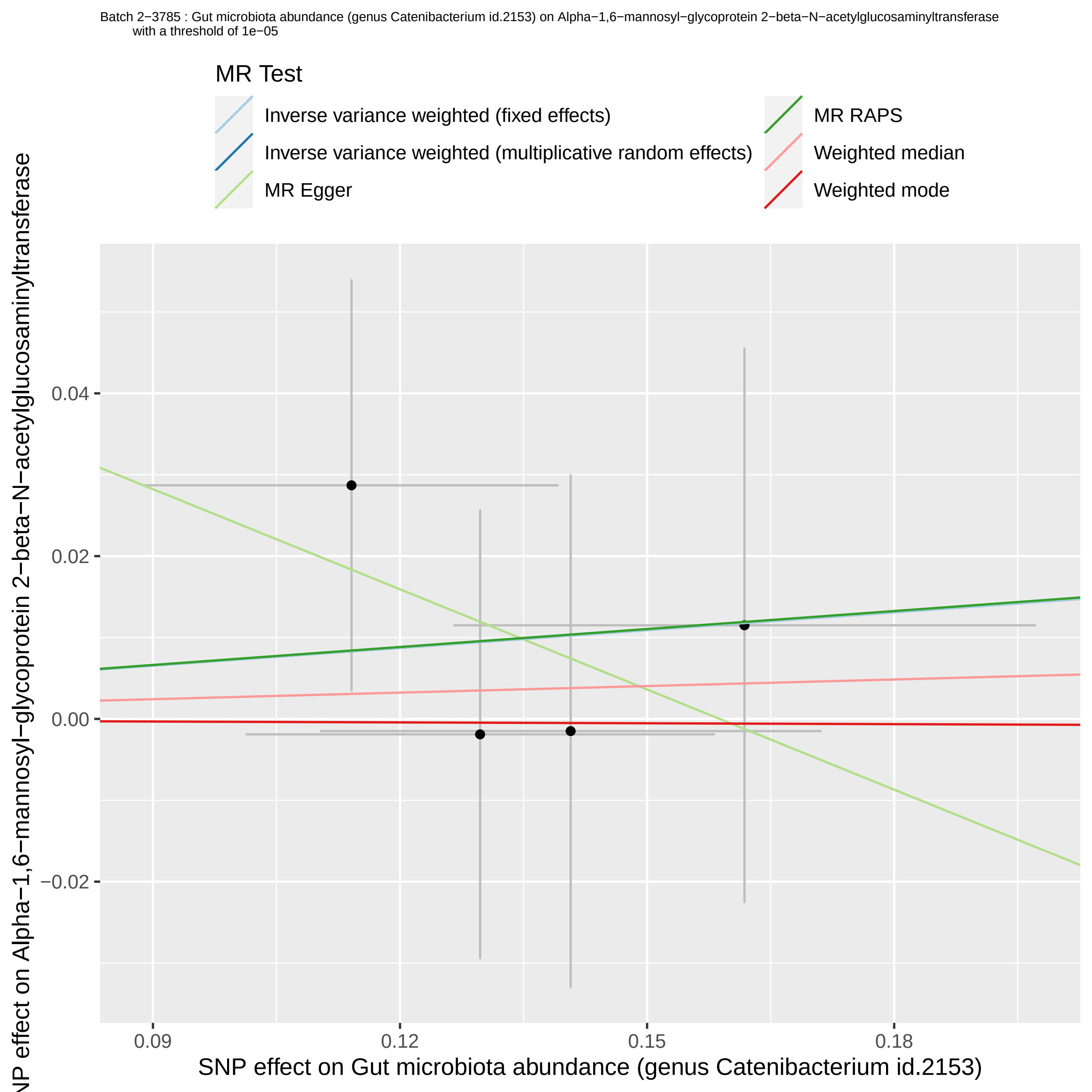

Batch 2−3785 : Gut microbiota abundance (genus Catenibacterium id.2153) on Alpha−1,6−mannosyl−glycoprotein 2−beta−N−acetylglucosaminyltransferase
with a threshold of 1e−05
MR Test
Inverse variance weighted (fixed effects)
Inverse variance weighted (multiplicative random effects)
MR Egger
MR RAPS
Weighted median
Weighted mode
0.04
0.02
0.00
−0.02
0.09
0.12
0.15
0.18
SNP effect on Gut microbiota abundance (genus Catenibacterium id.2153)
SNP effect on Alpha−1,6−mannosyl−glycoprotein 2−beta−N−acetylglucosaminyltransferase

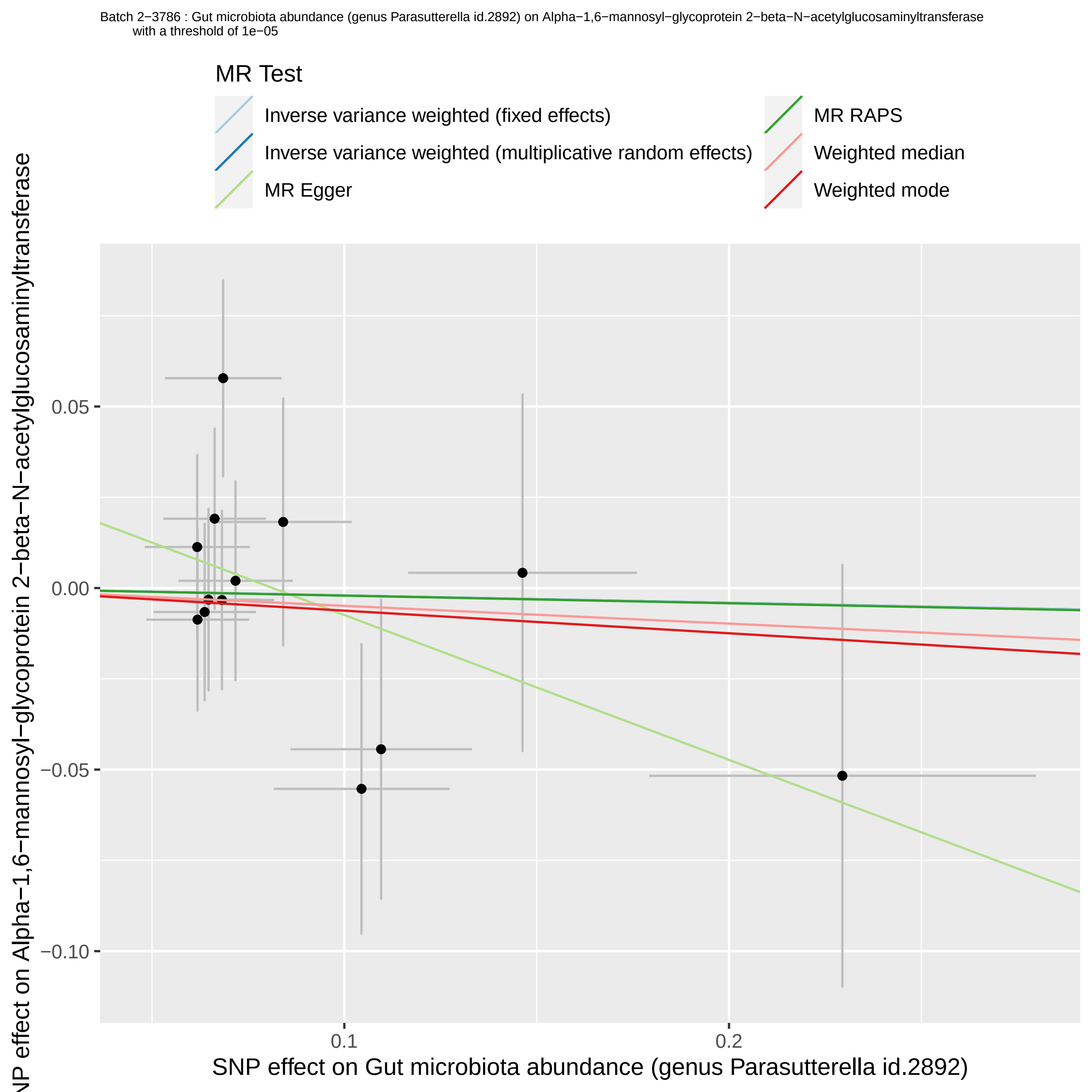
Batch 2−3786 : Gut microbiota abundance (genus Parasutterella id.2892) on Alpha−1,6−mannosyl−glycoprotein 2−beta−N−acetylglucosaminyltransferase
with a threshold of 1e−05
MR Test
Inverse variance weighted (fixed effects)
Inverse variance weighted (multiplicative random effects)
MR Egger
MR RAPS
Weighted median
Weighted mode
0.05
0.00
−0.05
−0.10
0.1
0.2
SNP effect on Gut microbiota abundance (genus Parasutterella id.2892)
SNP effect on Alpha−1,6−mannosyl−glycoprotein 2−beta−N−acetylglucosaminyltransferase

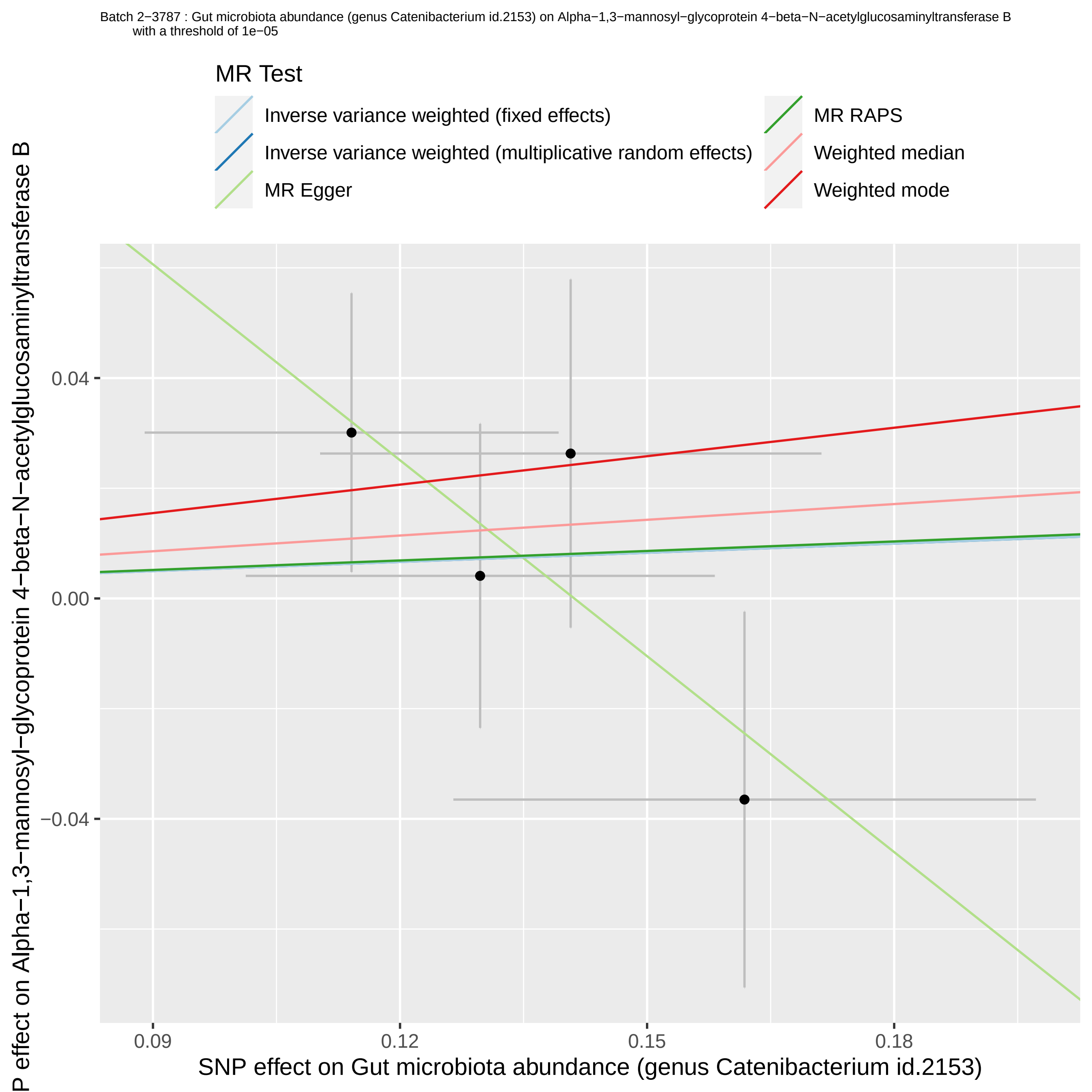

Batch 2−3787 : Gut microbiota abundance (genus Catenibacterium id.2153) on Alpha−1,3−mannosyl−glycoprotein 4−beta−N−acetylglucosaminyltransferase B
with a threshold of 1e−05
MR Test
Inverse variance weighted (fixed effects)
Inverse variance weighted (multiplicative random effects)
MR Egger
MR RAPS
Weighted median
Weighted mode
0.04
0.00
−0.04
0.09
0.12
0.15
0.18
SNP effect on Gut microbiota abundance (genus Catenibacterium id.2153)
P effect on Alpha−1,3−mannosyl−glycoprotein 4−beta−N−acetylglucosaminyltransferase B

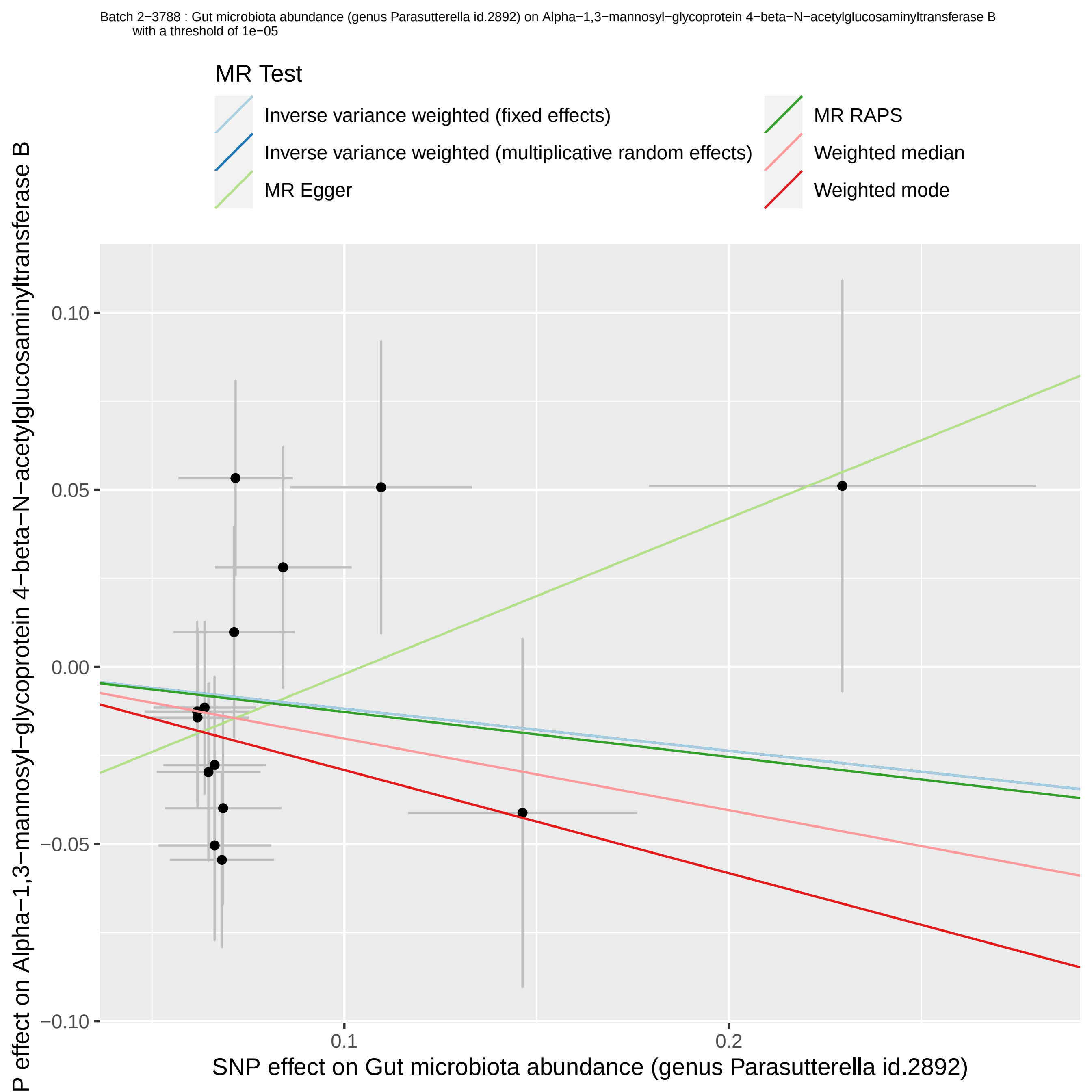

Batch 2−3788 : Gut microbiota abundance (genus Parasutterella id.2892) on Alpha−1,3−mannosyl−glycoprotein 4−beta−N−acetylglucosaminyltransferase B with a threshold of 1e−05
MR Test
Inverse variance weighted (fixed effects)
Inverse variance weighted (multiplicative random effects)
MR Egger
MR RAPS
Weighted median
Weighted mode
0.10
0.05
0.00
−0.05
−0.10
0.1
0.2
SNP effect on Gut microbiota abundance (genus Parasutterella id.2892)
P effect on Alpha−1,3−mannosyl−glycoprotein 4−beta−N−acetylglucosaminyltransferase B

Batch 2−3789 : Gut microbiota abundance (genus Catenibacterium id.2153) on Alpha−1,3−mannosyl−glycoprotein 4−beta−N−acetylglucosaminyltransferase C
with a threshold of 1e−05

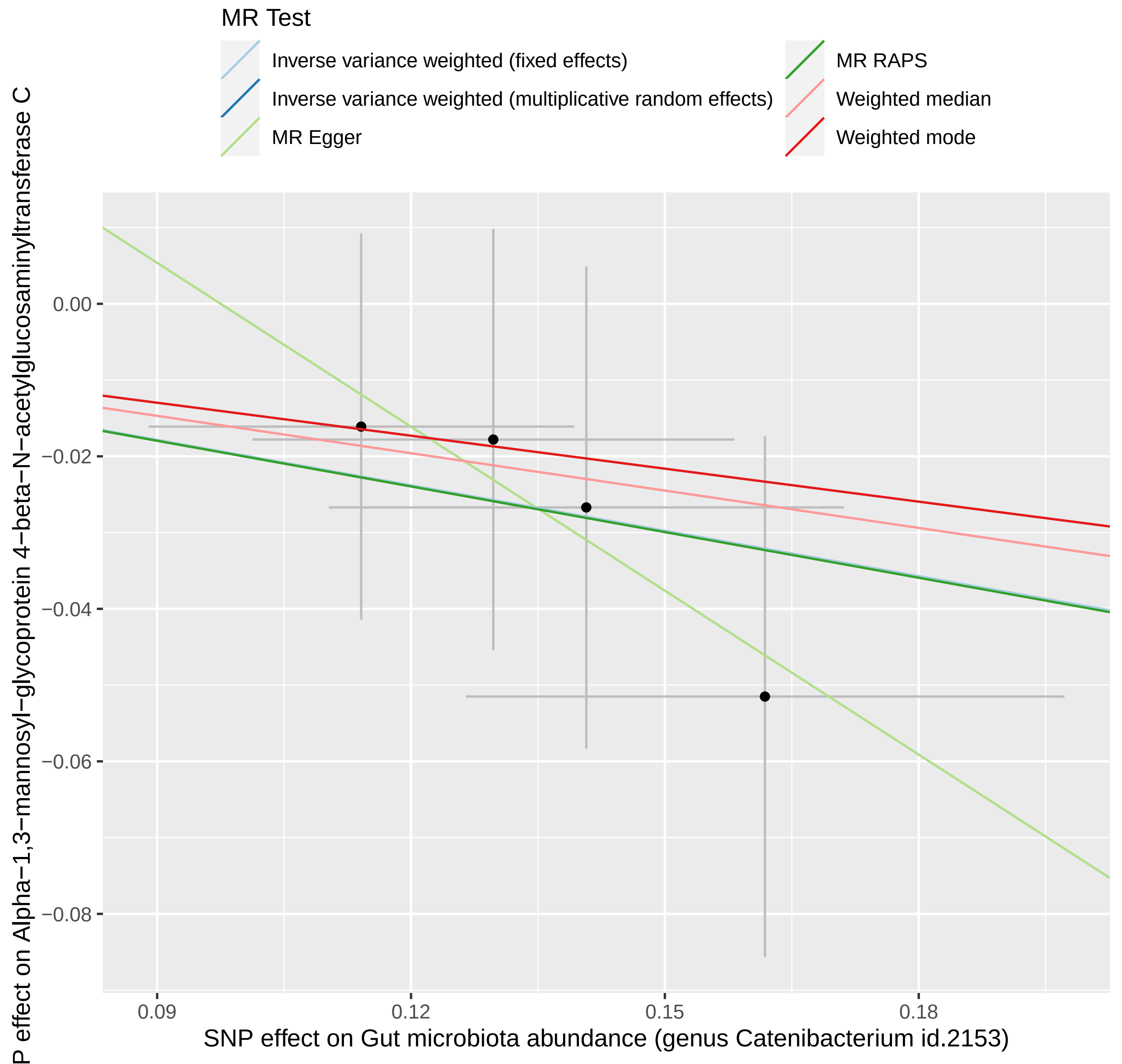

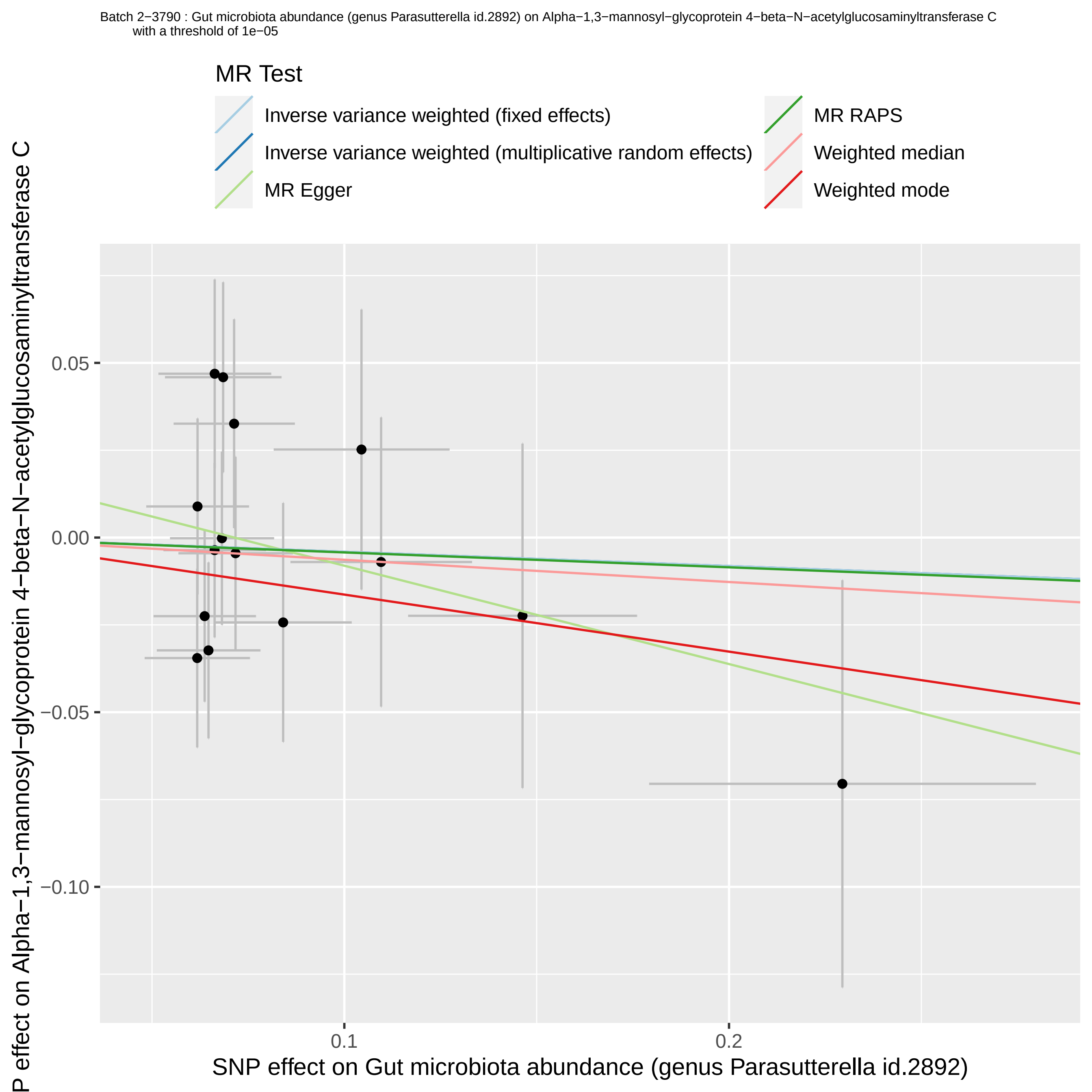
Batch 2−3790 : Gut microbiota abundance (genus Parasutterella id.2892) on Alpha−1,3−mannosyl−glycoprotein 4−beta−N−acetylglucosaminyltransferase C
with a threshold of 1e−05
MR Test
Inverse variance weighted (fixed effects)
Inverse variance weighted (multiplicative random effects)
MR Egger
MR RAPS
Weighted median
Weighted mode
0.05
0.00
−0.05
−0.10
0.1
0.2
SNP effect on Gut microbiota abundance (genus Parasutterella id.2892)
P effect on Alpha−1,3−mannosyl−glycoprotein 4−beta−N−acetylglucosaminyltransferase C

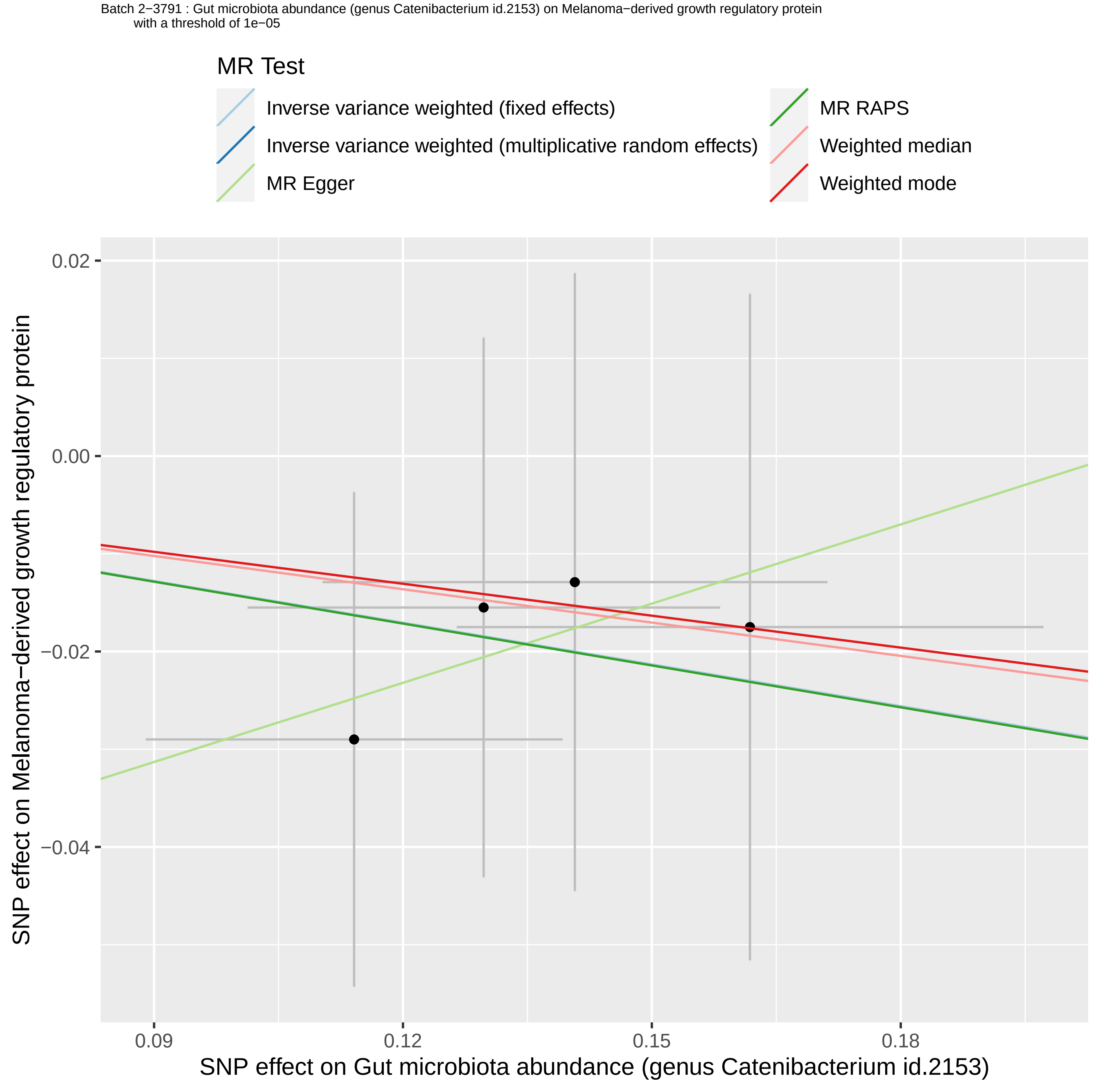

Batch 2−3791 : Gut microbiota abundance (genus Catenibacterium id.2153) on Melanoma−derived growth regulatory protein
with a threshold of 1e−05
MR Test
Inverse variance weighted (fixed effects)
Inverse variance weighted (multiplicative random effects)
MR Egger
MR RAPS
Weighted median
Weighted mode
0.02
0.00
−0.02
−0.04
0.09
0.12
0.15
0.18
SNP effect on Melanoma−derived growth regulatory protein
SNP effect on Gut microbiota abundance (genus Catenibacterium id.2153)

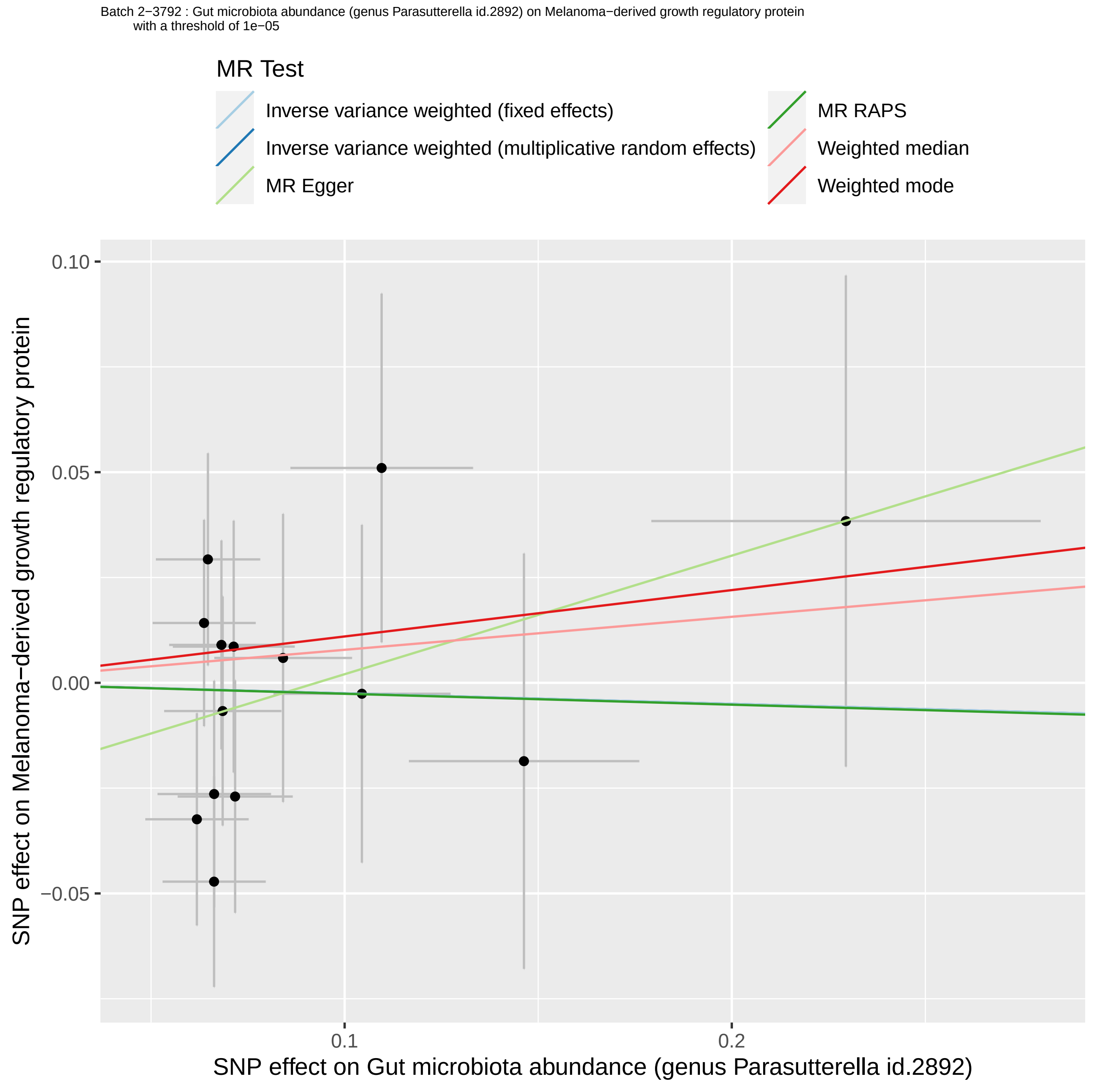

Batch 2−3792 : Gut microbiota abundance (genus Parasutterella id.2892) on Melanoma−derived growth regulatory protein
with a threshold of 1e−05
MR Test
Inverse variance weighted (fixed effects)
Inverse variance weighted (multiplicative random effects)
MR Egger
MR RAPS
Weighted median
Weighted mode
0.10
0.05
0.00
−0.05
0.1
0.2
SNP effect on Melanoma−derived growth regulatory protein
SNP effect on Gut microbiota abundance (genus Parasutterella id.2892)

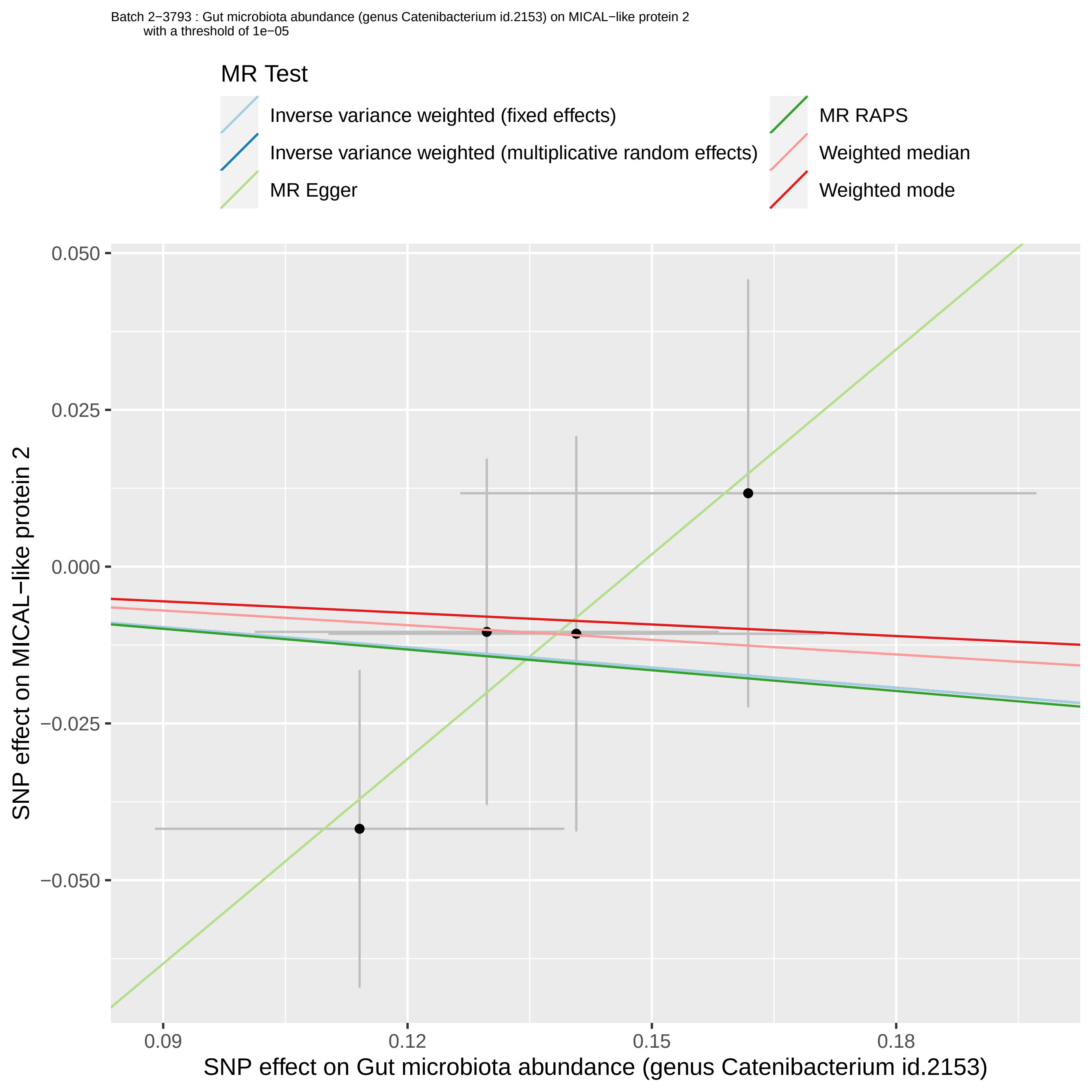
Batch 2−3793 : Gut microbiota abundance (genus Catenibacterium id.2153) on MICAL−like protein 2
with a threshold of 1e−05
MR Test
Inverse variance weighted (fixed effects)
Inverse variance weighted (multiplicative random effects)
MR Egger
MR RAPS
Weighted median
Weighted mode
0.050
0.025
0.000
−0.025
−0.050
0.09
0.12
0.15
0.18
SNP effect on MICAL−like protein 2
SNP effect on Gut microbiota abundance (genus Catenibacterium id.2153)

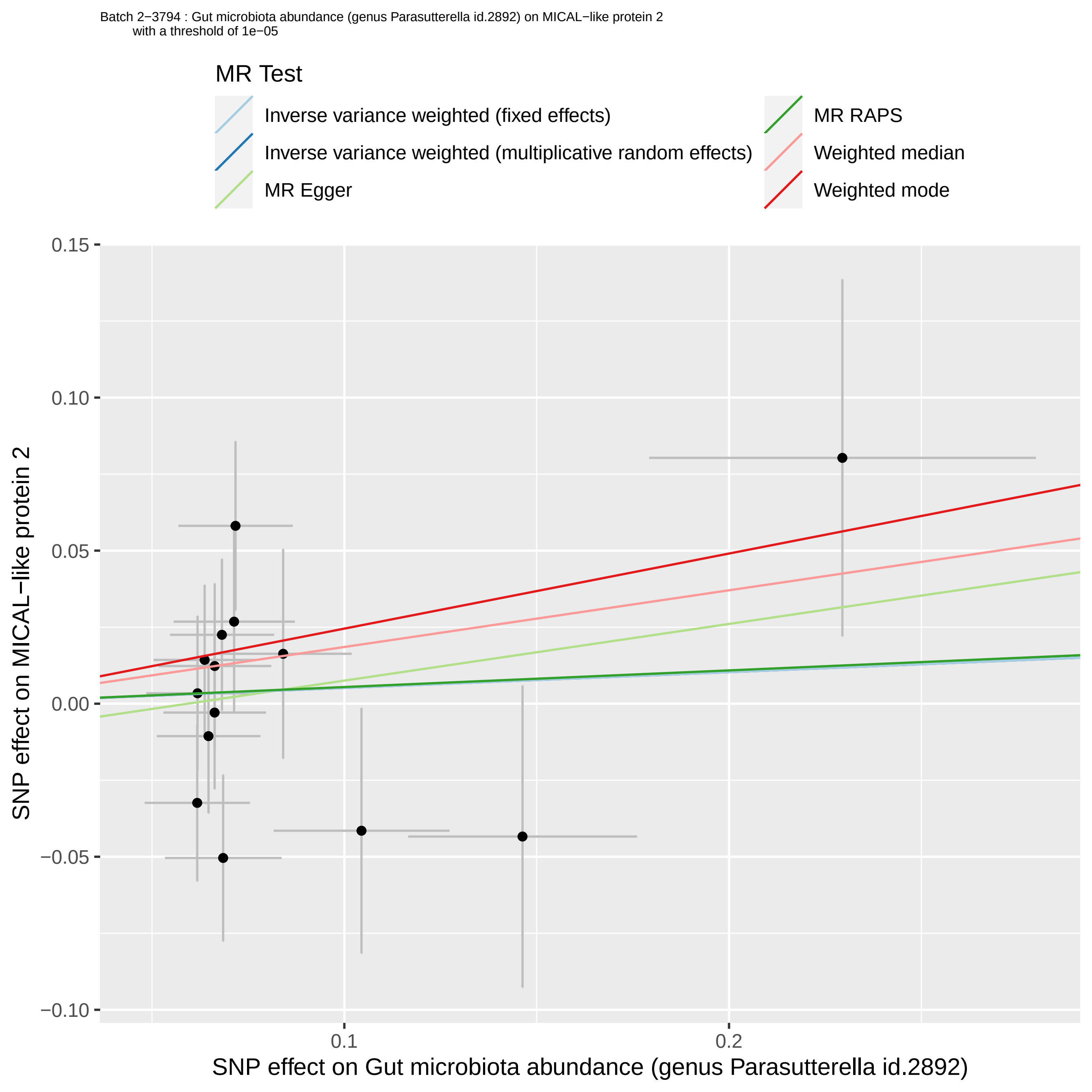
Batch 2−3794 : Gut microbiota abundance (genus Parasutterella id.2892) on MICAL−like protein 2
with a threshold of 1e−05
MR Test
Inverse variance weighted (fixed effects)
Inverse variance weighted (multiplicative random effects)
MR Egger
MR RAPS
Weighted median
Weighted mode
0.15
0.10
0.05
0.00
−0.05
−0.10
0.1
0.2
SNP effect on MICAL−like protein 2
SNP effect on Gut microbiota abundance (genus Parasutterella id.2892)

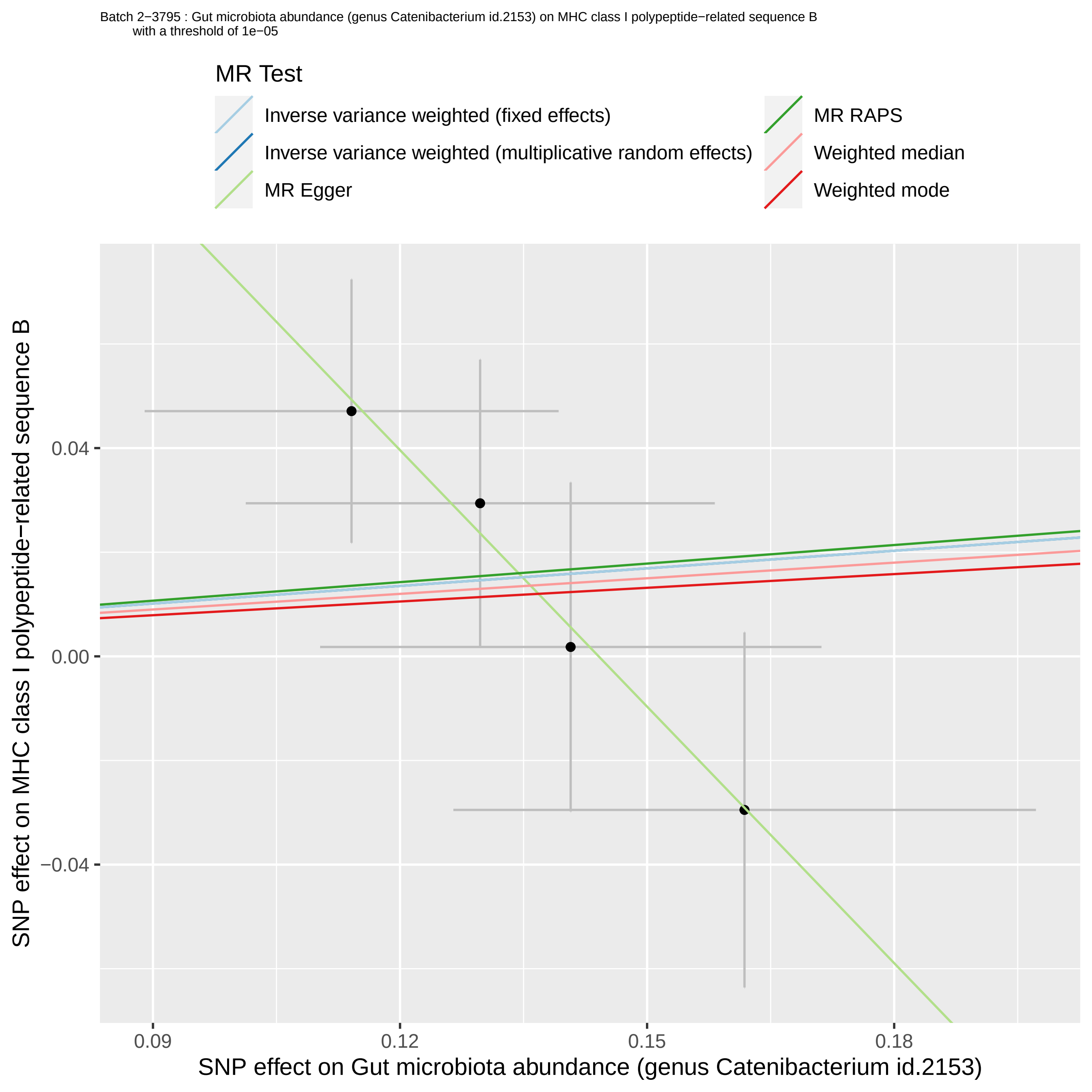
Batch 2−3795 : Gut microbiota abundance (genus Catenibacterium id.2153) on MHC class I polypeptide−related sequence B
with a threshold of 1e−05
MR Test
Inverse variance weighted (fixed effects)
Inverse variance weighted (multiplicative random effects)
MR Egger
MR RAPS
Weighted median
Weighted mode
SNP effect on MHC class I polypeptide−related sequence B
0.04
0.00
−0.04
0.09
0.12
0.15
0.18
SNP effect on Gut microbiota abundance (genus Catenibacterium id.2153)

Batch 2−3796 : Gut microbiota abundance (genus Parasutterella id.2892) on MHC class I polypeptide−related sequence B
with a threshold of 1e−05

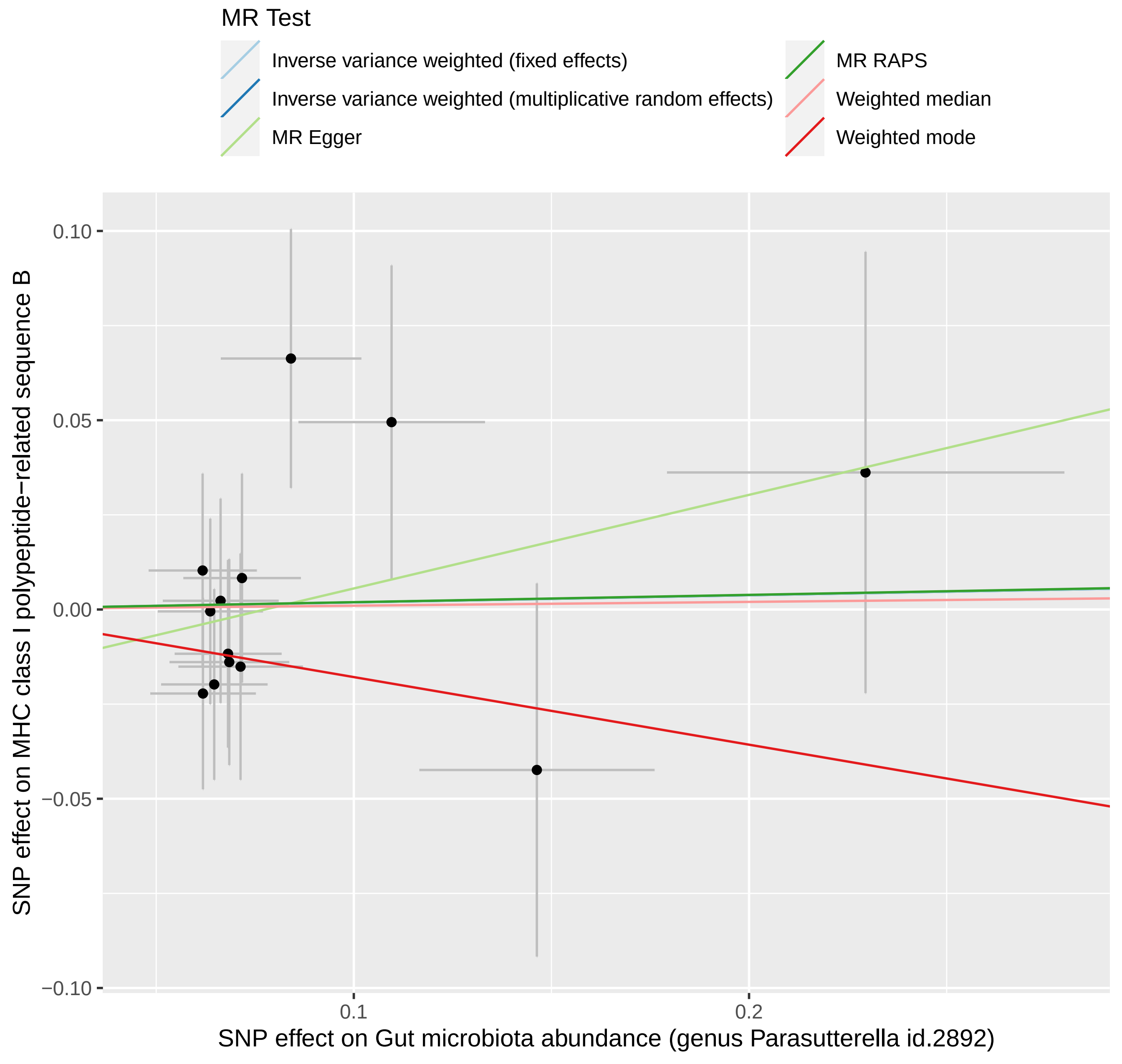

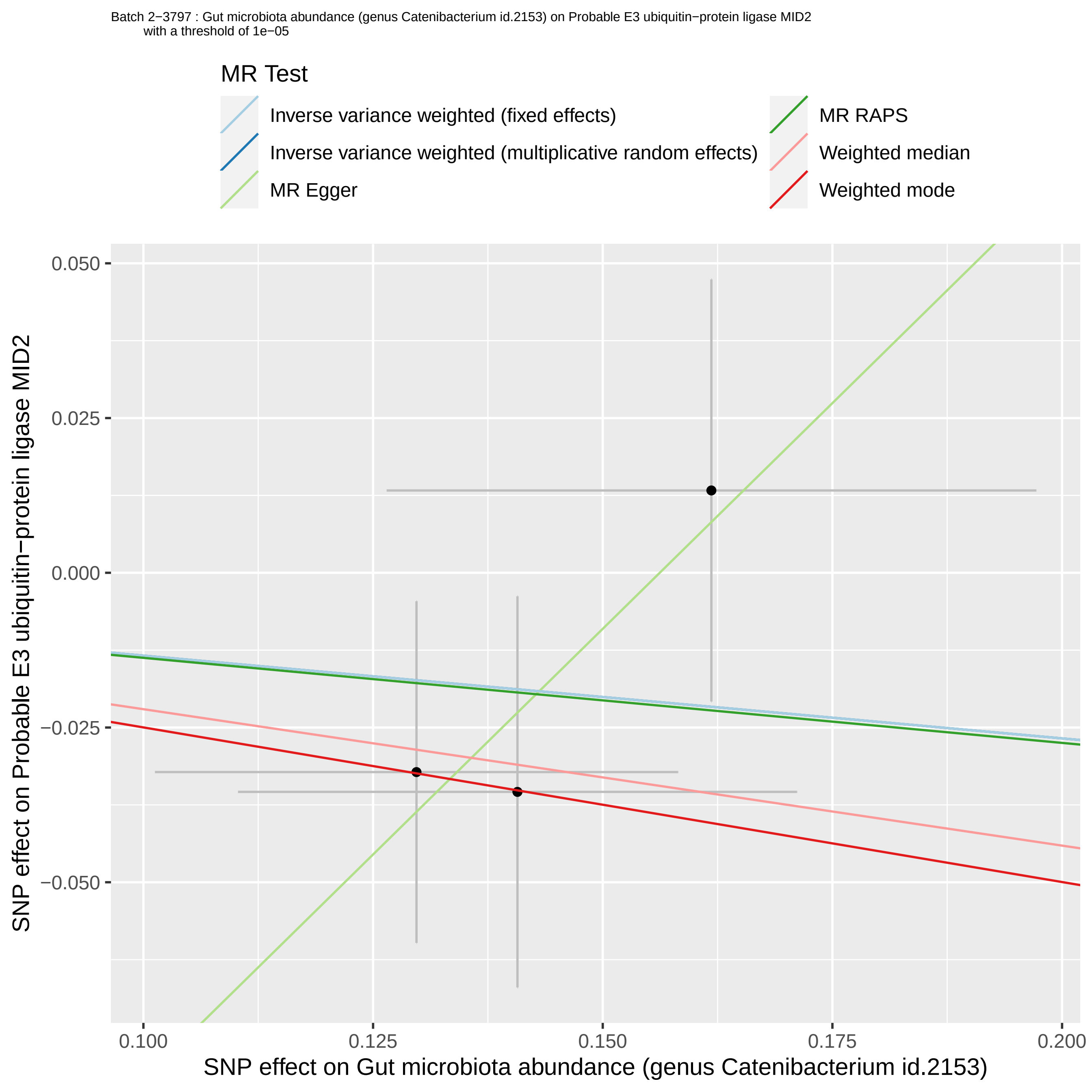

Batch 2−3797 : Gut microbiota abundance (genus Catenibacterium id.2153) on Probable E3 ubiquitin−protein ligase MID2
with a threshold of 1e−05
MR Test
Inverse variance weighted (fixed effects)
Inverse variance weighted (multiplicative random effects)
MR Egger
MR RAPS
Weighted median
Weighted mode
0.050
0.025
0.000
−0.025
−0.050
0.100
0.125
0.150
0.175
0.200
SNP effect on Probable E3 ubiquitin−protein ligase MID2
SNP effect on Gut microbiota abundance (genus Catenibacterium id.2153)

Batch 2−3798 : Gut microbiota abundance (genus Parasutterella id.2892) on Probable E3 ubiquitin−protein ligase MID2 with a threshold of 1e−05

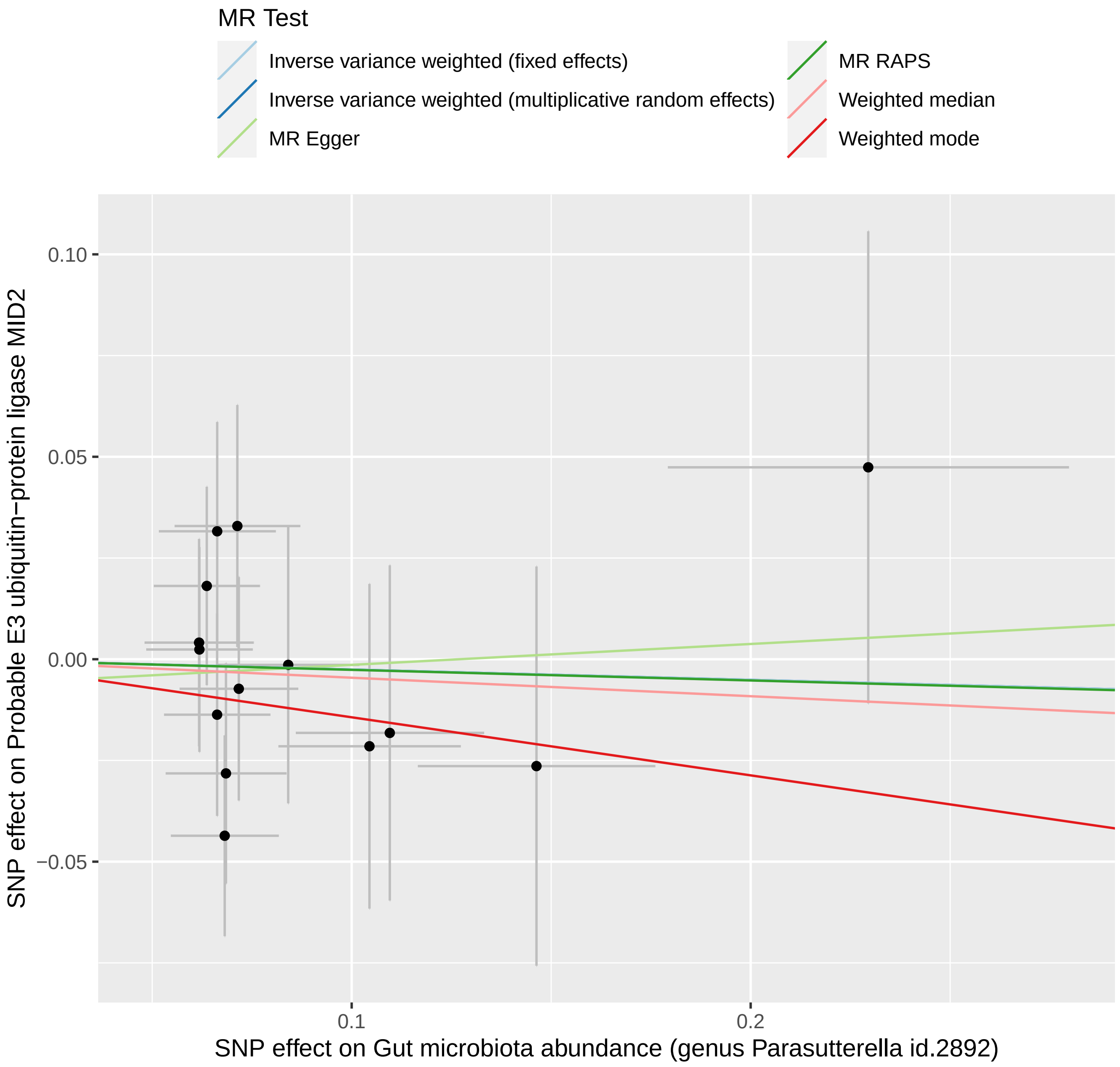

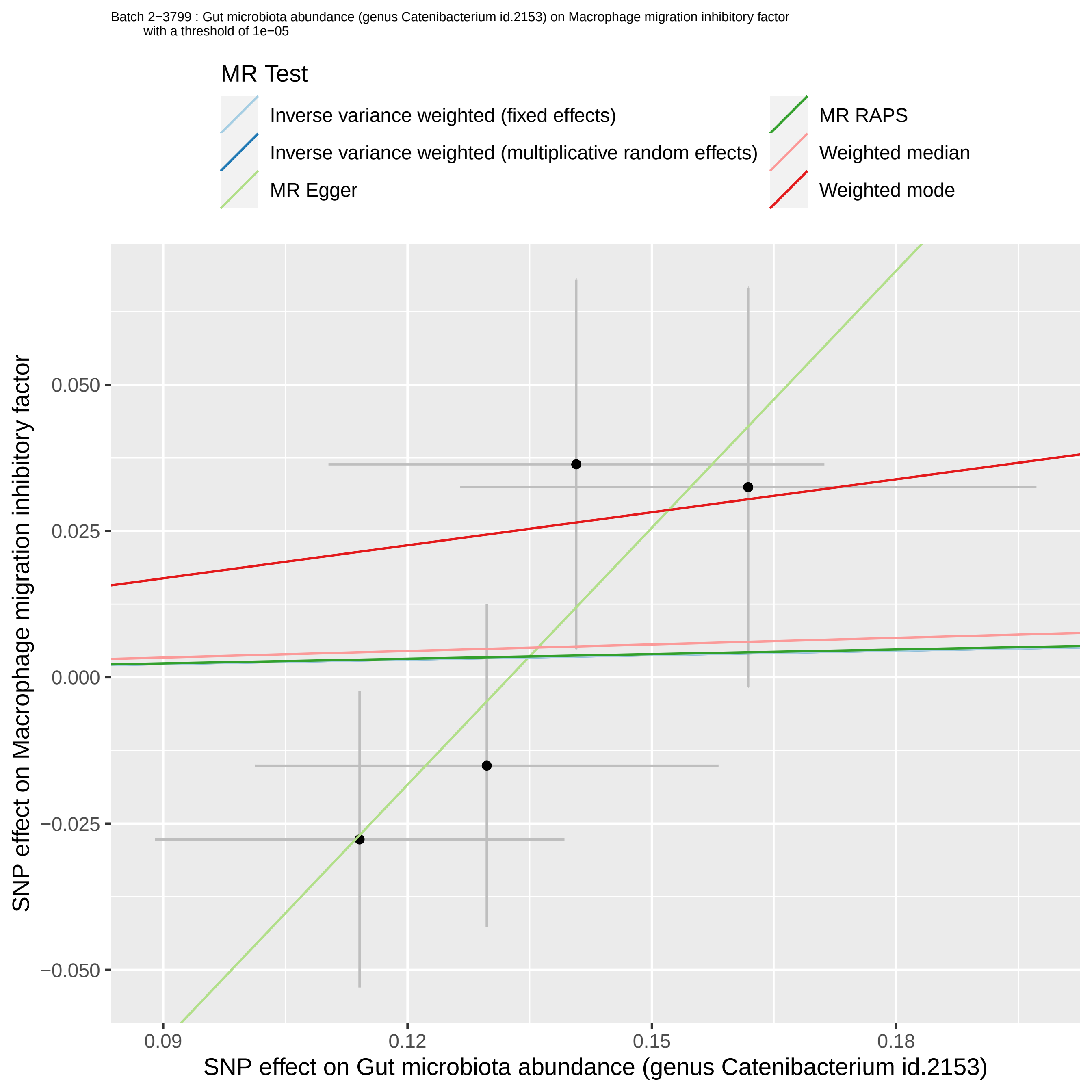
Batch 2−3799 : Gut microbiota abundance (genus Catenibacterium id.2153) on Macrophage migration inhibitory factor
with a threshold of 1e−05
MR Test
Inverse variance weighted (fixed effects)
Inverse variance weighted (multiplicative random effects)
MR Egger
MR RAPS
Weighted median
Weighted mode
SNP effect on Macrophage migration inhibitory factor
0.050
0.025
0.000
−0.025
−0.050
0.09
0.12
0.15
0.18
SNP effect on Gut microbiota abundance (genus Catenibacterium id.2153)

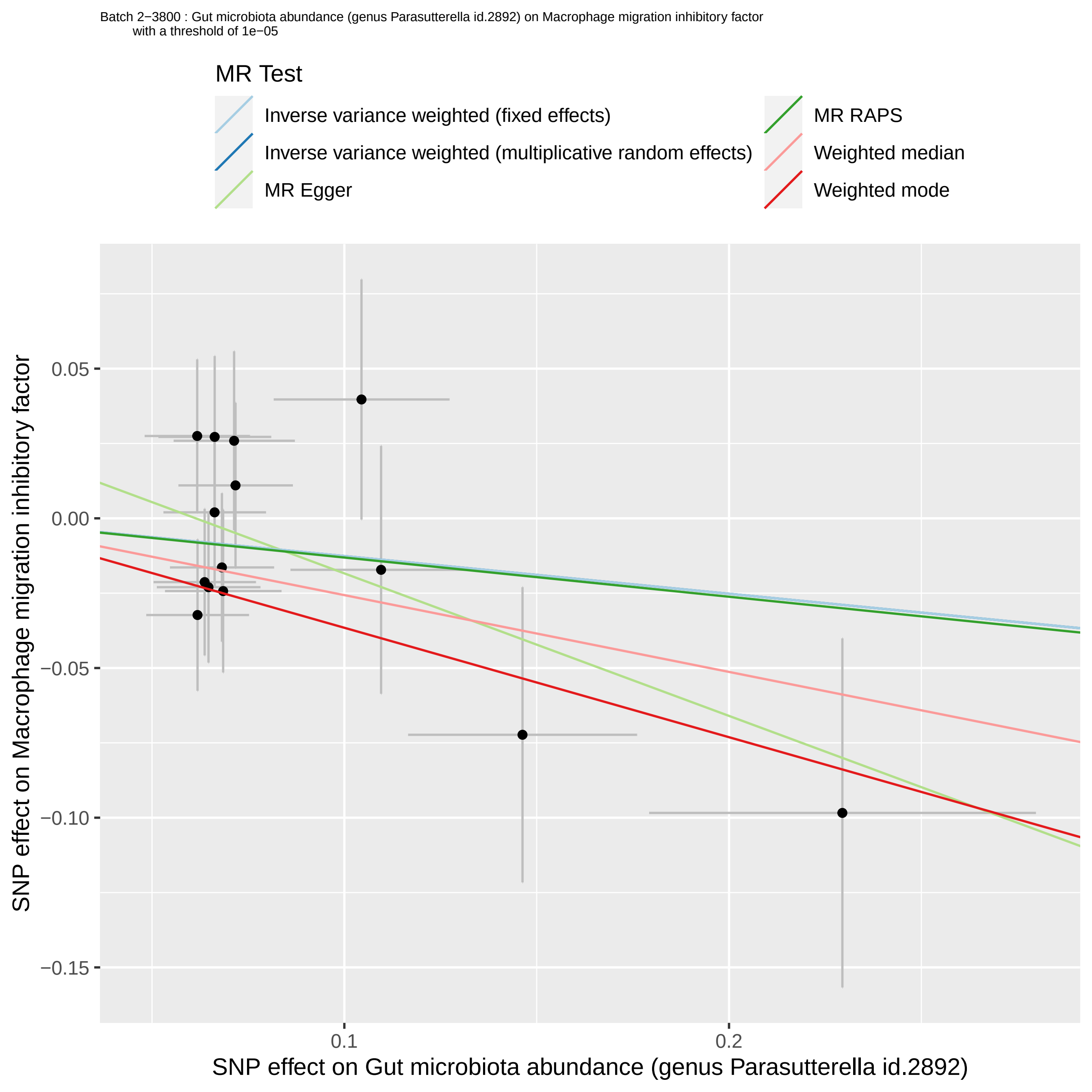

Batch 2−3800 : Gut microbiota abundance (genus Parasutterella id.2892) on Macrophage migration inhibitory factor
with a threshold of 1e−05
MR Test
Inverse variance weighted (fixed effects)
Inverse variance weighted (multiplicative random effects)
MR Egger
MR RAPS
Weighted median
Weighted mode
SNP effect on Macrophage migration inhibitory factor
0.05
0.00
−0.05
−0.10
−0.15
0.1
0.2
SNP effect on Gut microbiota abundance (genus Parasutterella id.2892)

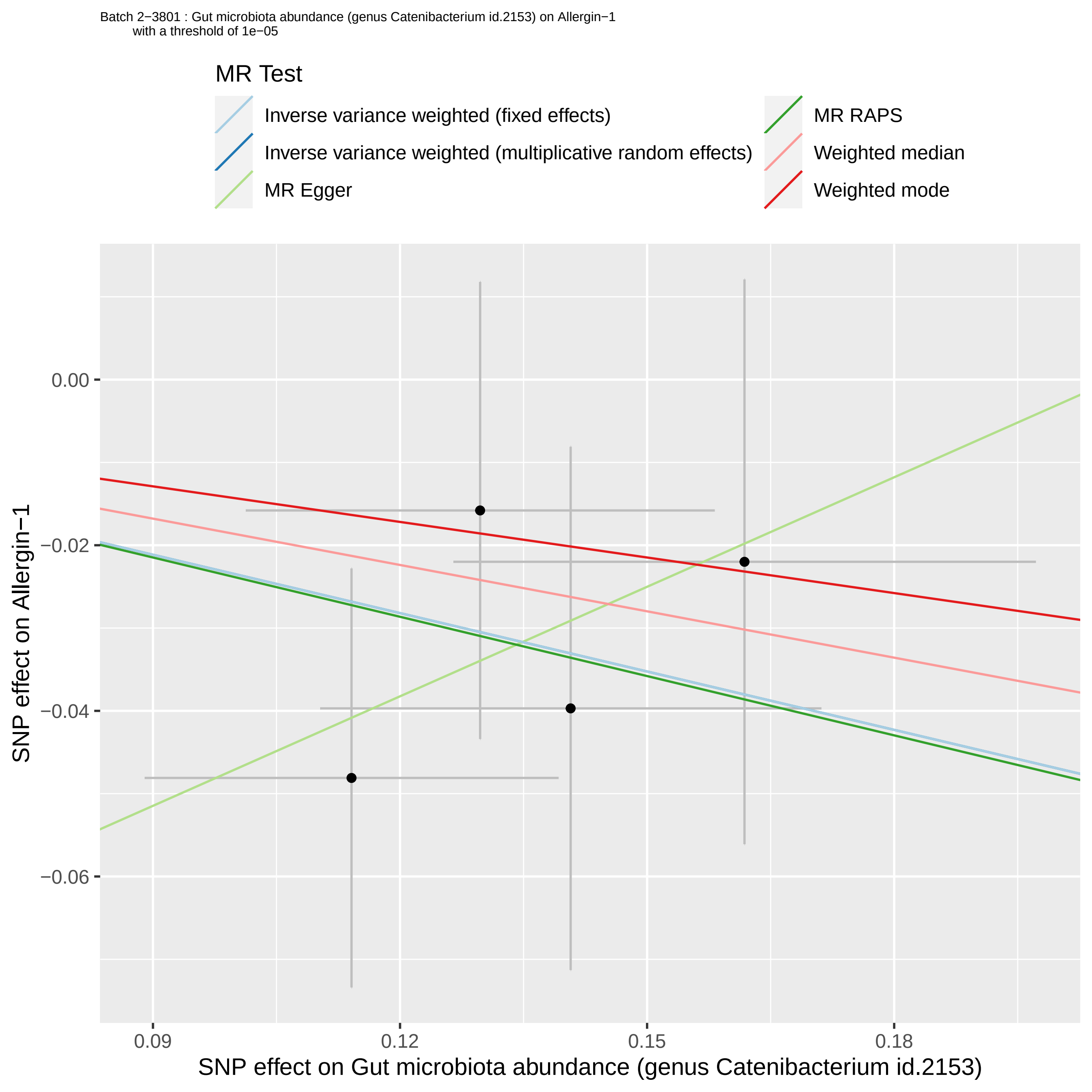
Batch 2−3801 : Gut microbiota abundance (genus Catenibacterium id.2153) on Allergin−1
with a threshold of 1e−05
MR Test
Inverse variance weighted (fixed effects)
Inverse variance weighted (multiplicative random effects)
MR Egger
MR RAPS
Weighted median
Weighted mode
0.00
−0.02
−0.04
−0.06
0.09
0.12
0.15
0.18
SNP effect on Allergin−1
SNP effect on Gut microbiota abundance (genus Catenibacterium id.2153)

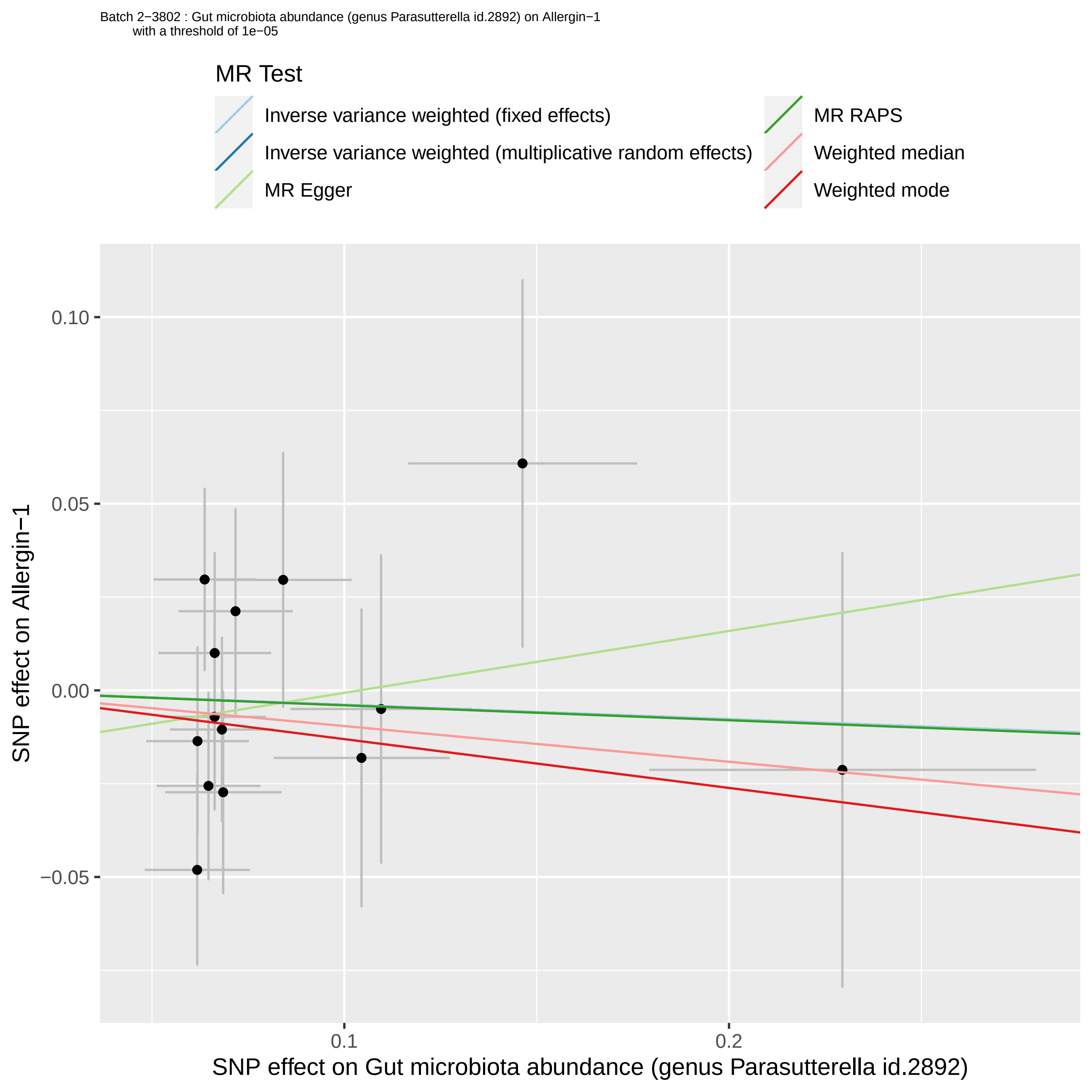
Batch 2−3802 : Gut microbiota abundance (genus Parasutterella id.2892) on Allergin−1
with a threshold of 1e−05
MR Test
Inverse variance weighted (fixed effects)
Inverse variance weighted (multiplicative random effects)
MR Egger
MR RAPS
Weighted median
Weighted mode
SNP effect on Allergin−1
0.10
0.05
0.00
−0.05
0.1
0.2
SNP effect on Gut microbiota abundance (genus Parasutterella id.2892)

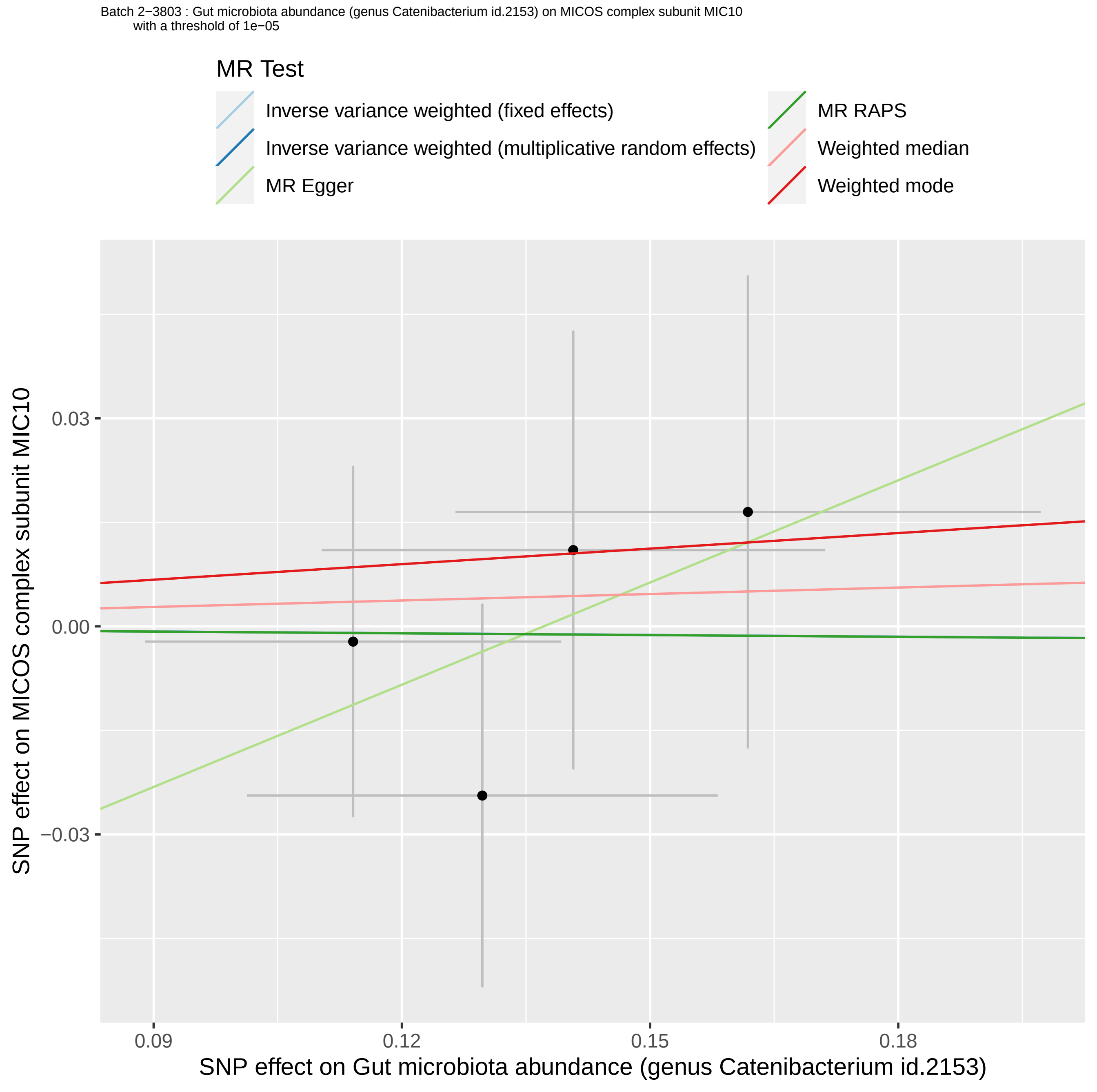
Batch 2−3803 : Gut microbiota abundance (genus Catenibacterium id.2153) on MICOS complex subunit MIC10
with a threshold of 1e−05
MR Test
Inverse variance weighted (fixed effects)
Inverse variance weighted (multiplicative random effects)
MR Egger
MR RAPS
Weighted median
Weighted mode
0.03
0.00
−0.03
0.09
0.12
0.15
0.18
SNP effect on MICOS complex subunit MIC10
SNP effect on Gut microbiota abundance (genus Catenibacterium id.2153)

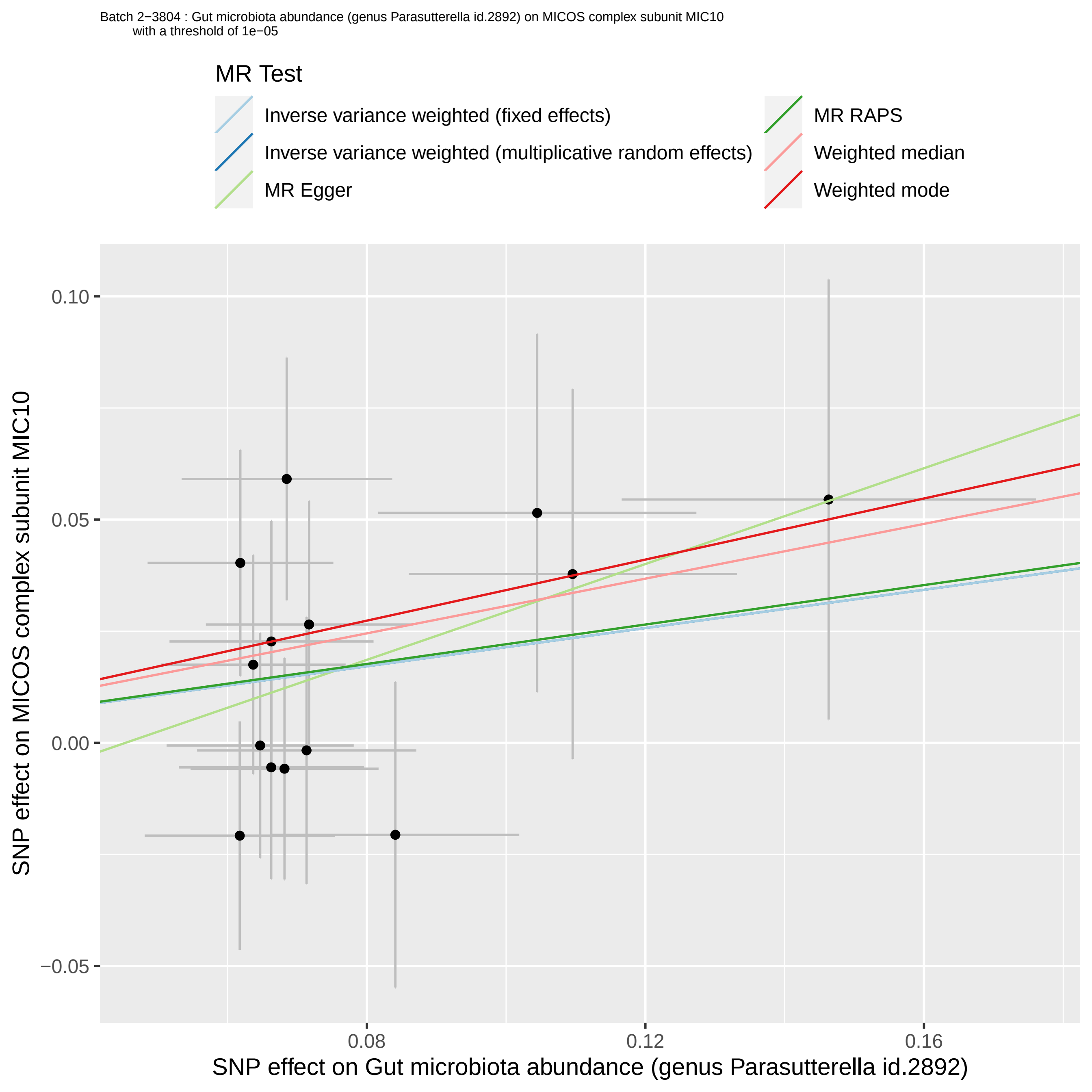
Batch 2−3804 : Gut microbiota abundance (genus Parasutterella id.2892) on MICOS complex subunit MIC10
with a threshold of 1e−05
MR Test
Inverse variance weighted (fixed effects)
Inverse variance weighted (multiplicative random effects)
MR Egger
MR RAPS
Weighted median
Weighted mode
0.10
0.05
0.00
−0.05
0.08
0.12
0.16
SNP effect on MICOS complex subunit MIC10
SNP effect on Gut microbiota abundance (genus Parasutterella id.2892)

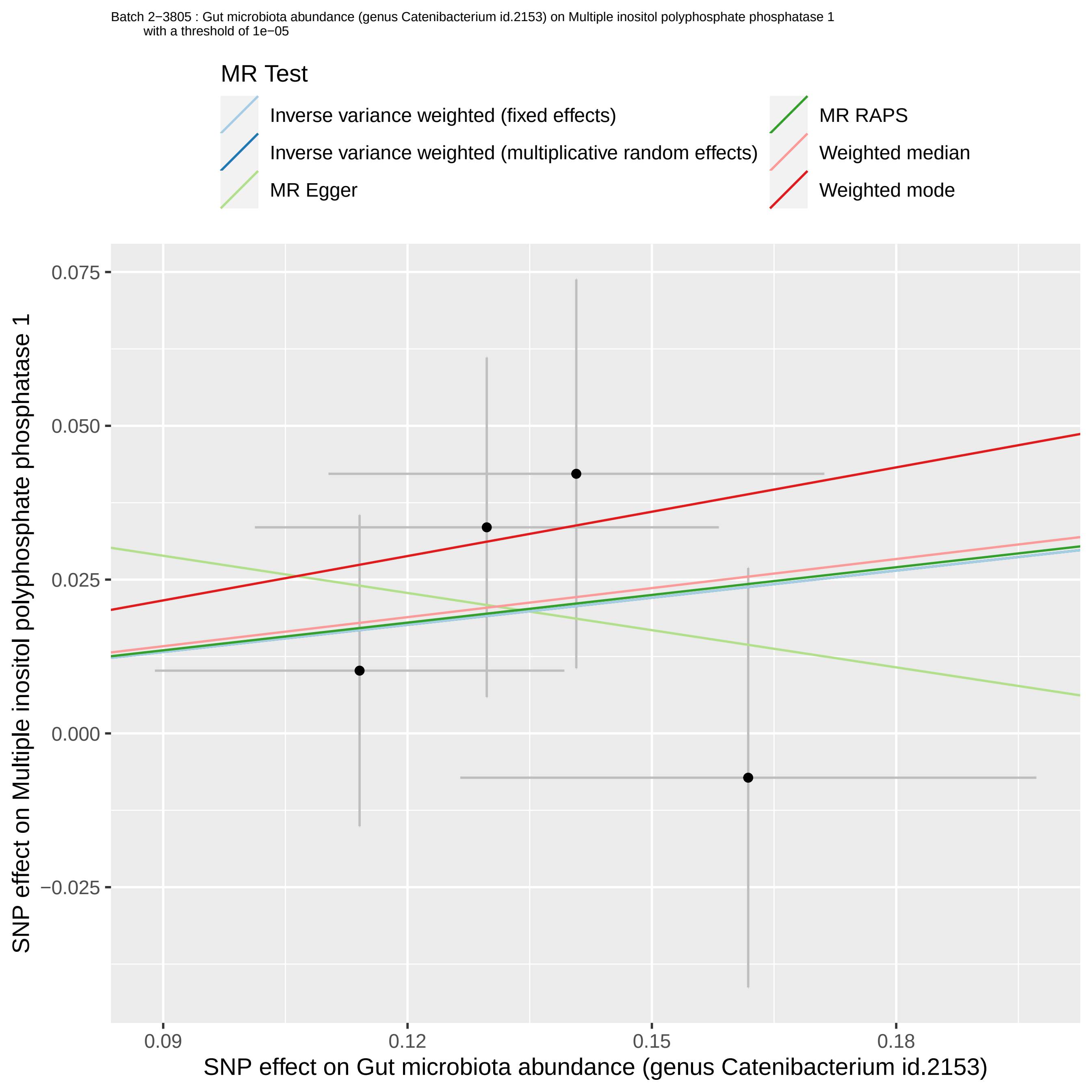
Batch 2−3805 : Gut microbiota abundance (genus Catenibacterium id.2153) on Multiple inositol polyphosphate phosphatase 1
with a threshold of 1e−05
MR Test
Inverse variance weighted (fixed effects)
Inverse variance weighted (multiplicative random effects)
MR Egger
MR RAPS
Weighted median
Weighted mode
0.075
0.050
0.025
0.000
−0.025
0.09
0.12
0.15
0.18
SNP effect on Multiple inositol polyphosphate phosphatase 1
SNP effect on Gut microbiota abundance (genus Catenibacterium id.2153)

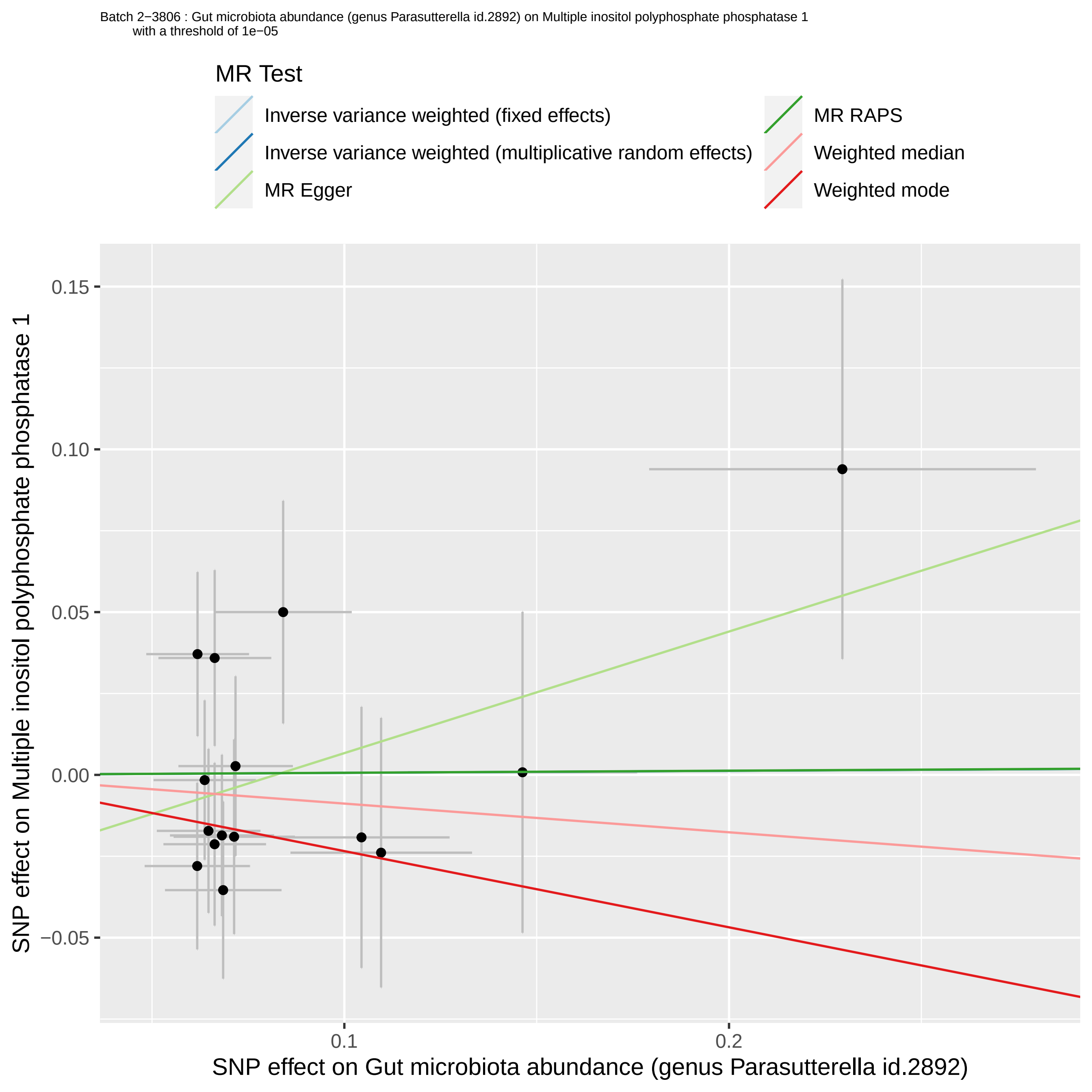
Batch 2−3806 : Gut microbiota abundance (genus Parasutterella id.2892) on Multiple inositol polyphosphate phosphatase 1
with a threshold of 1e−05
MR Test
Inverse variance weighted (fixed effects)
Inverse variance weighted (multiplicative random effects)
MR Egger
MR RAPS
Weighted median
Weighted mode
0.15
0.10
0.05
0.00
−0.05
0.1
0.2
SNP effect on Multiple inositol polyphosphate phosphatase 1
SNP effect on Gut microbiota abundance (genus Parasutterella id.2892)

Batch 2−3807 : Gut microbiota abundance (genus Catenibacterium id.2153) on Malectin
with a threshold of 1e−05

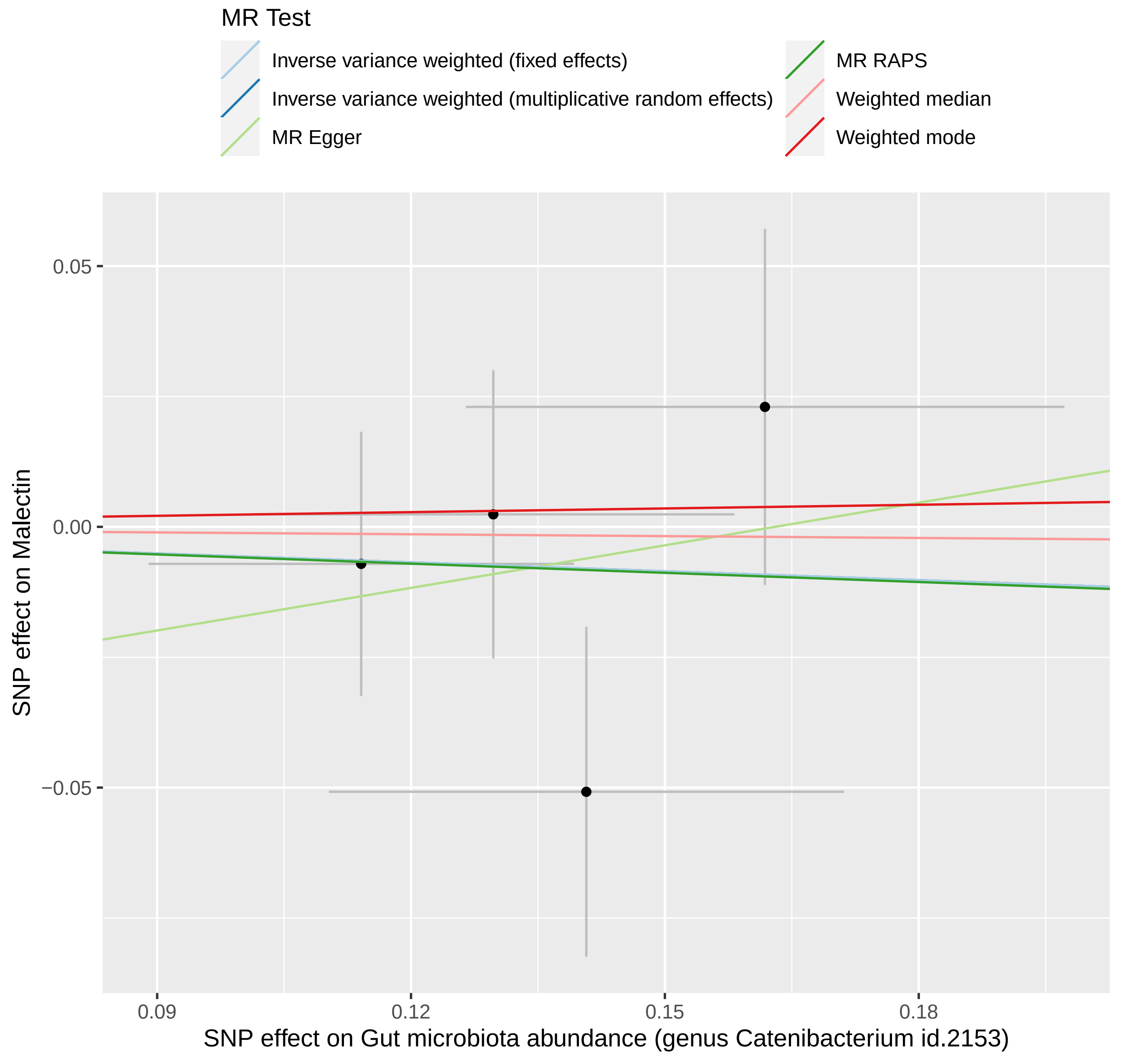

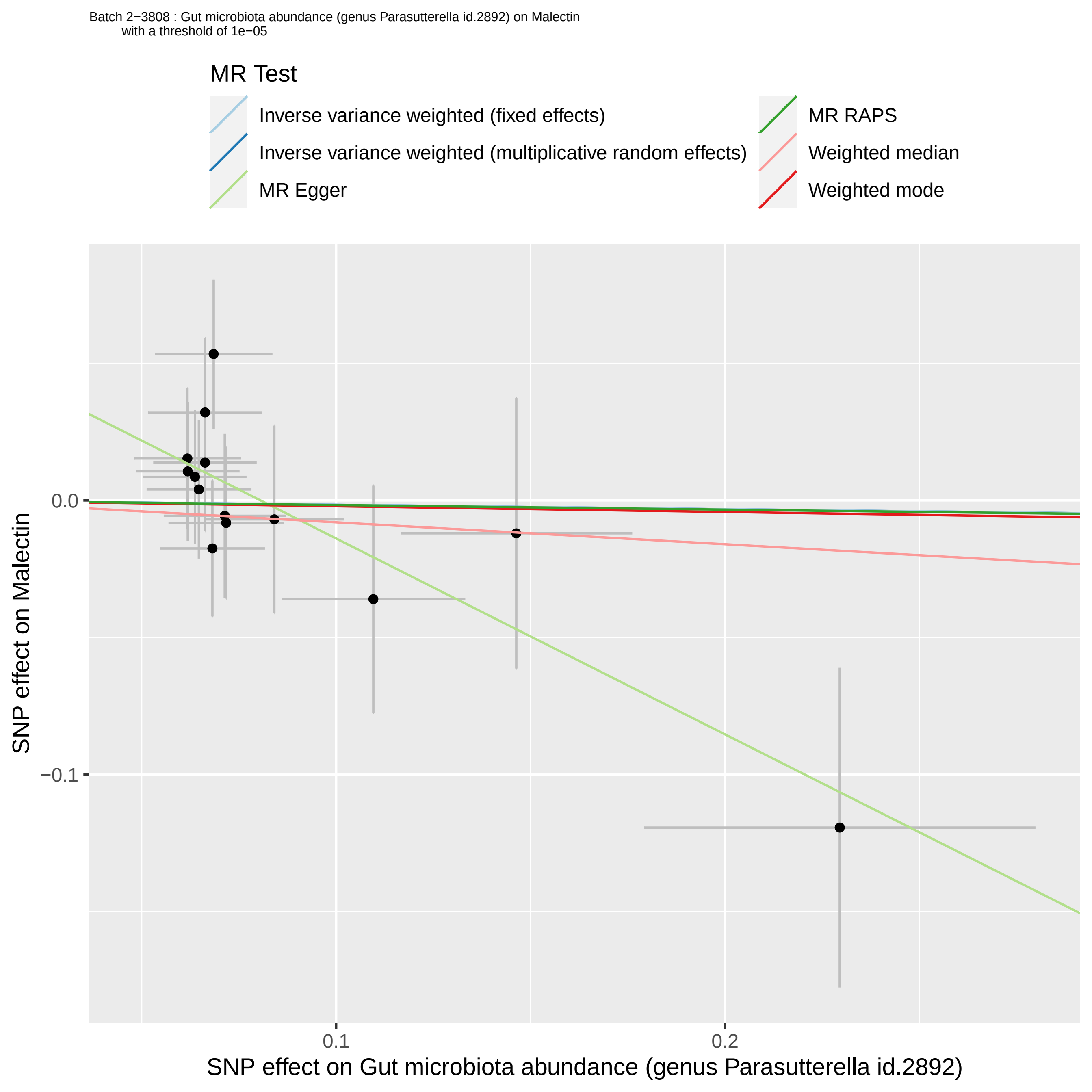
Batch 2−3808 : Gut microbiota abundance (genus Parasutterella id.2892) on Malectin
with a threshold of 1e−05
MR Test
Inverse variance weighted (fixed effects)
Inverse variance weighted (multiplicative random effects)
MR Egger
MR RAPS
Weighted median
Weighted mode
0.0
−0.1
0.1
0.2
SNP effect on Malectin
SNP effect on Gut microbiota abundance (genus Parasutterella id.2892)

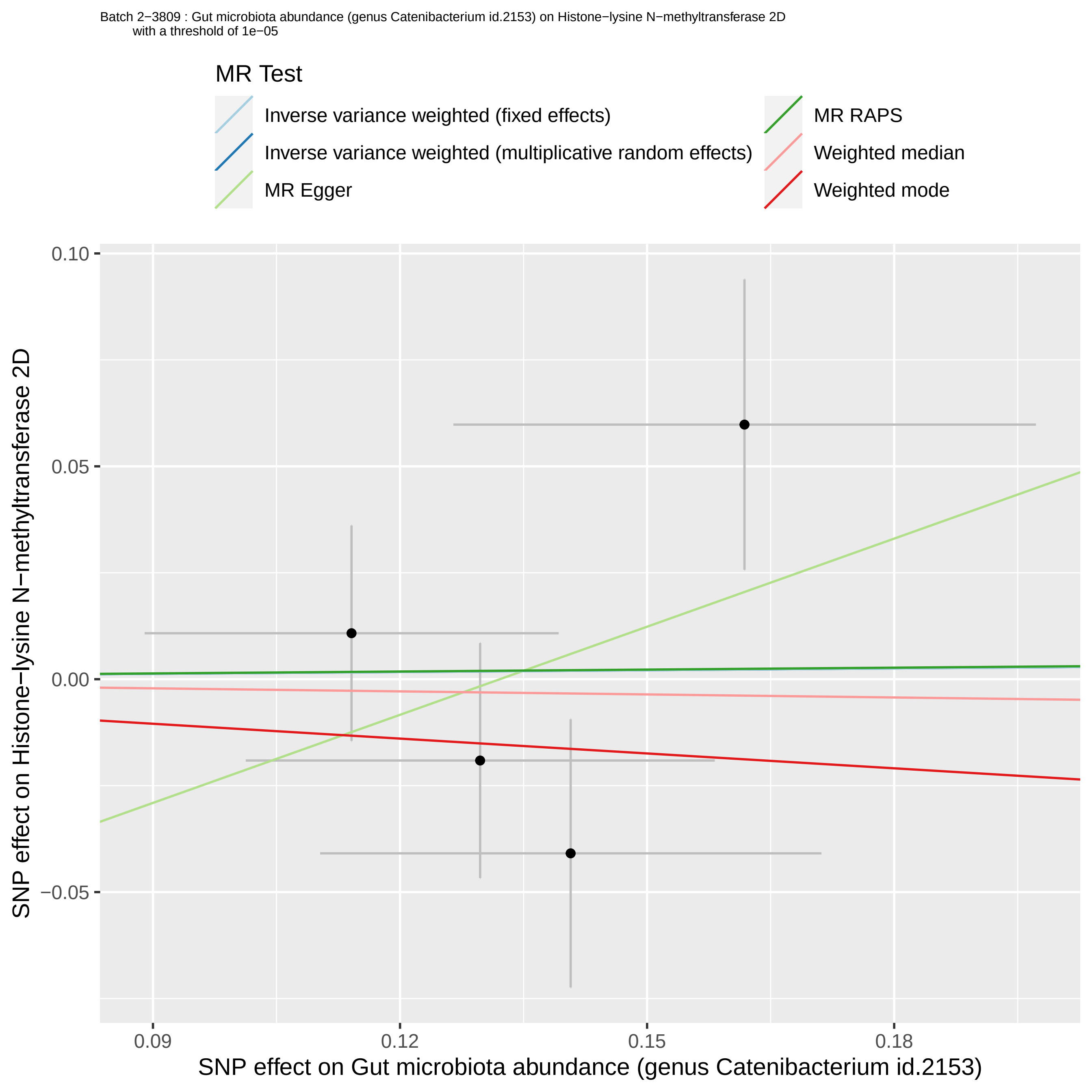
Batch 2−3809 : Gut microbiota abundance (genus Catenibacterium id.2153) on Histone−lysine N−methyltransferase 2D
with a threshold of 1e−05
MR Test
Inverse variance weighted (fixed effects)
Inverse variance weighted (multiplicative random effects)
MR Egger
MR RAPS
Weighted median
Weighted mode
0.10
0.05
0.00
−0.05
0.09
0.12
0.15
0.18
SNP effect on Histone−lysine N−methyltransferase 2D
SNP effect on Gut microbiota abundance (genus Catenibacterium id.2153)

Batch 2−3810 : Gut microbiota abundance (genus Parasutterella id.2892) on Histone−lysine N−methyltransferase 2D
with a threshold of 1e−05

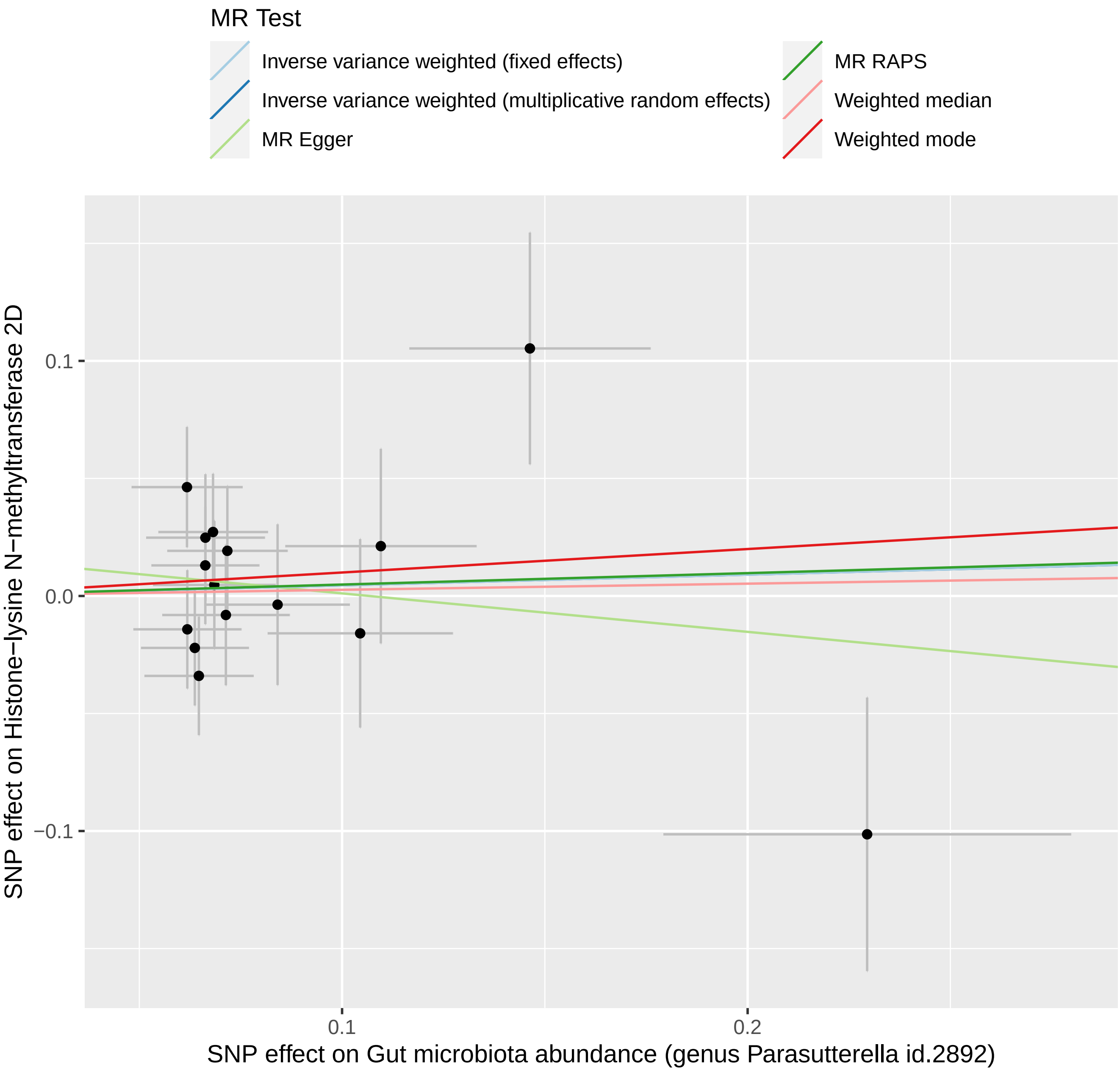

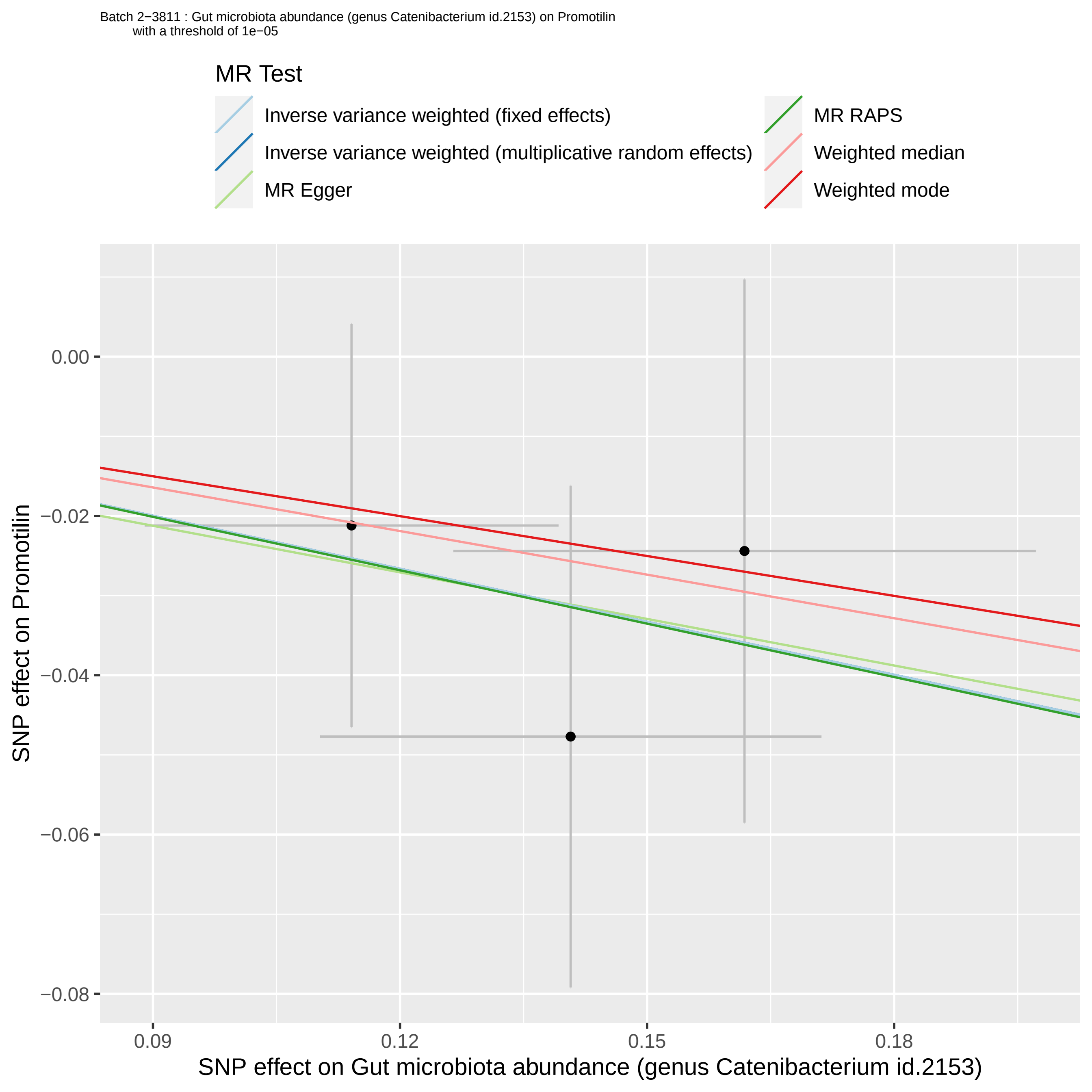
Batch 2−3811 : Gut microbiota abundance (genus Catenibacterium id.2153) on Promotilin
with a threshold of 1e−05
MR Test
Inverse variance weighted (fixed effects)
Inverse variance weighted (multiplicative random effects)
MR Egger
MR RAPS
Weighted median
Weighted mode
0.00
−0.02
−0.04
−0.06
−0.08
0.09
0.12
0.15
0.18
SNP effect on Promotilin
SNP effect on Gut microbiota abundance (genus Catenibacterium id.2153)

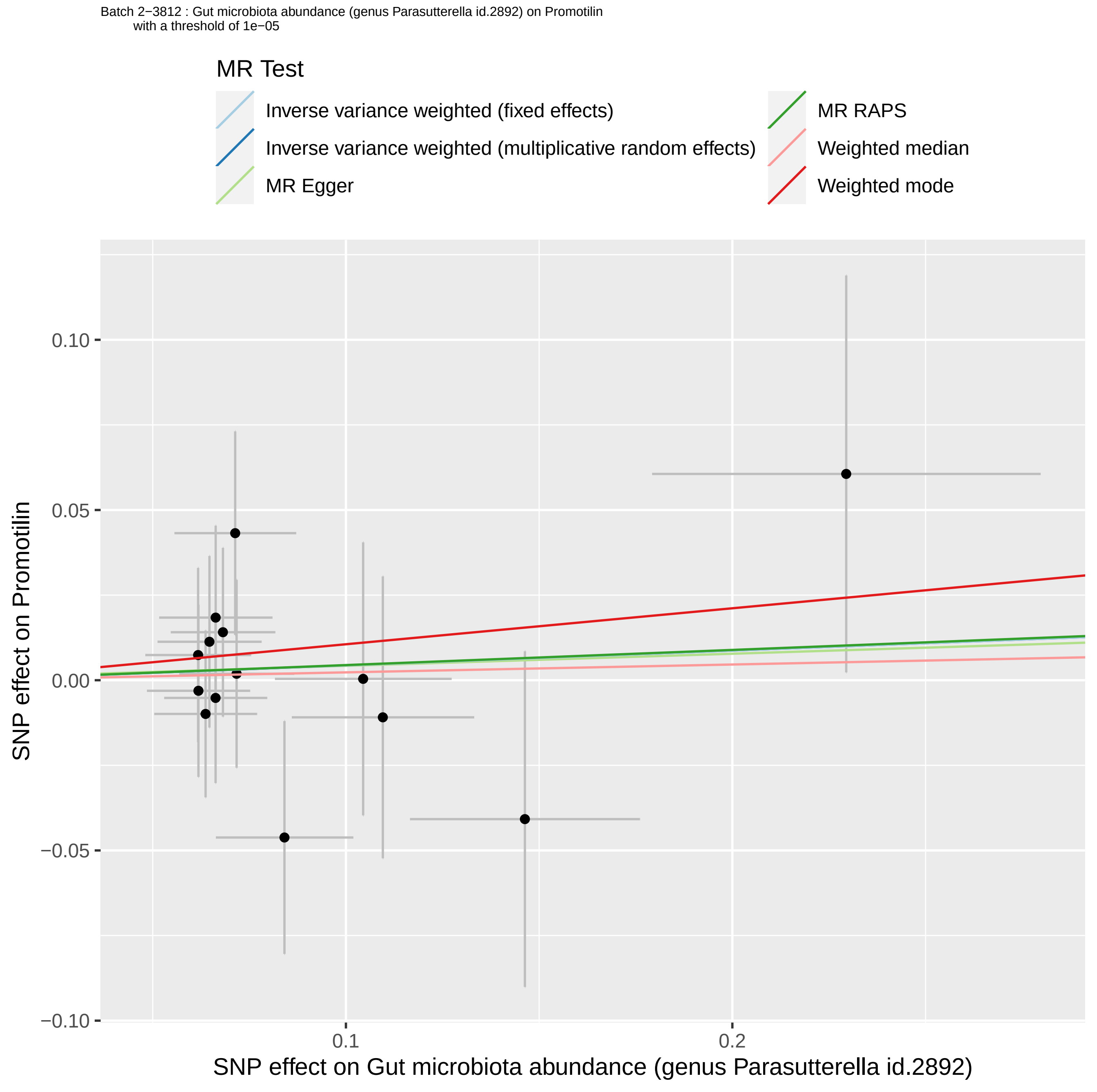
Batch 2−3812 : Gut microbiota abundance (genus Parasutterella id.2892) on Promotilin
with a threshold of 1e−05
MR Test
Inverse variance weighted (fixed effects)
Inverse variance weighted (multiplicative random effects)
MR Egger
MR RAPS
Weighted median
Weighted mode
0.10
0.05
0.00
−0.05
−0.10
0.1
0.2
SNP effect on Promotilin
SNP effect on Gut microbiota abundance (genus Parasutterella id.2892)

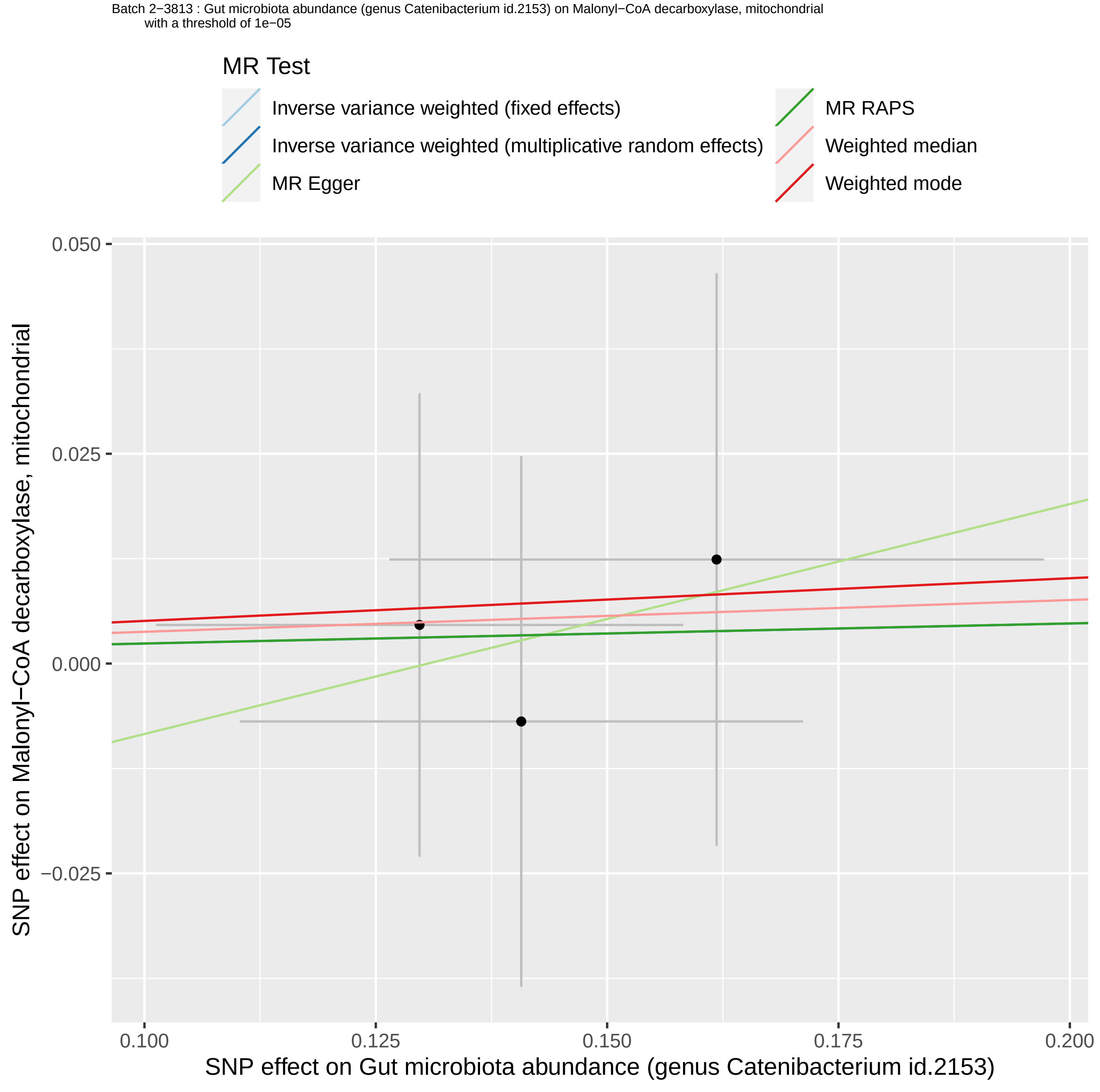

Batch 2−3813 : Gut microbiota abundance (genus Catenibacterium id.2153) on Malonyl−CoA decarboxylase, mitochondrial
with a threshold of 1e−05
MR Test
Inverse variance weighted (fixed effects)
Inverse variance weighted (multiplicative random effects)
MR Egger
MR RAPS
Weighted median
Weighted mode
0.050
0.025
0.000
−0.025
0.100
0.125
0.150
0.175
0.200
SNP effect on Malonyl−CoA decarboxylase, mitochondrial
SNP effect on Gut microbiota abundance (genus Catenibacterium id.2153)

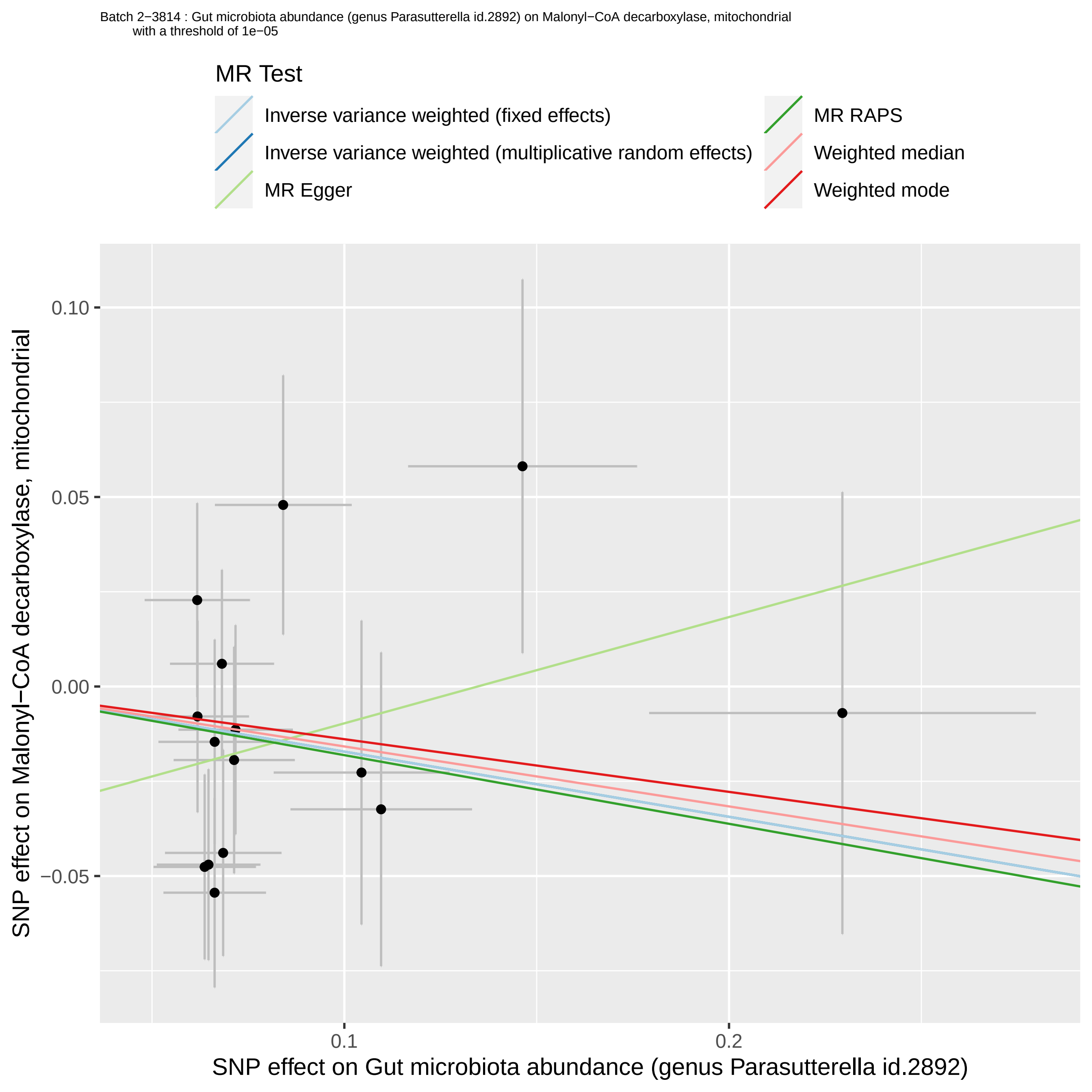
Batch 2−3814 : Gut microbiota abundance (genus Parasutterella id.2892) on Malonyl−CoA decarboxylase, mitochondrial
with a threshold of 1e−05
MR Test
Inverse variance weighted (fixed effects)
Inverse variance weighted (multiplicative random effects)
MR Egger
MR RAPS
Weighted median
Weighted mode
0.10
0.05
0.00
−0.05
0.1
0.2
SNP effect on Malonyl−CoA decarboxylase, mitochondrial
SNP effect on Gut microbiota abundance (genus Parasutterella id.2892)

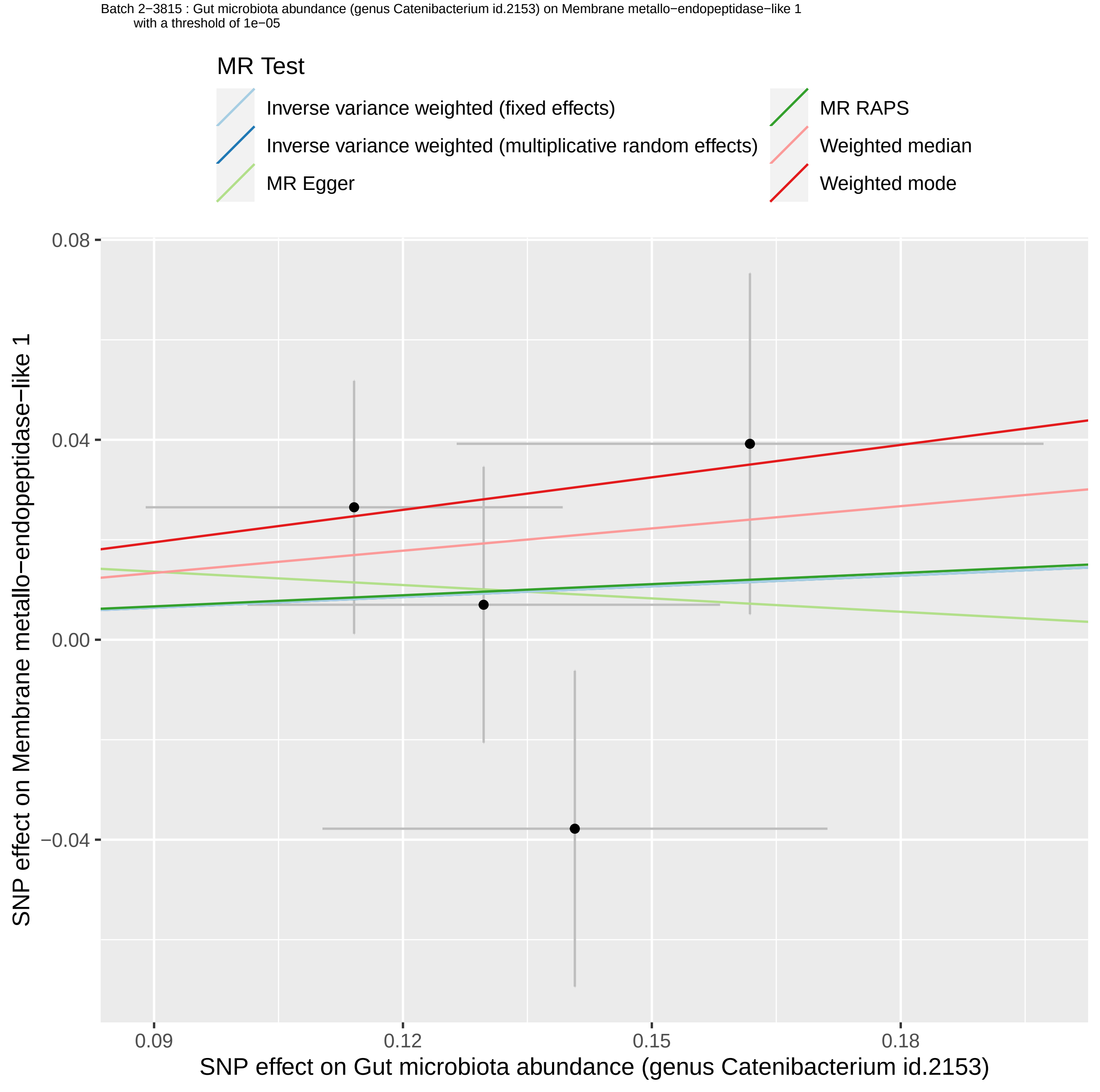
Batch 2−3815 : Gut microbiota abundance (genus Catenibacterium id.2153) on Membrane metallo−endopeptidase−like 1
with a threshold of 1e−05
MR Test
Inverse variance weighted (fixed effects)
Inverse variance weighted (multiplicative random effects)
MR Egger
MR RAPS
Weighted median
Weighted mode
0.08
0.04
0.00
−0.04
0.09
0.12
0.15
0.18
SNP effect on Membrane metallo−endopeptidase−like 1
SNP effect on Gut microbiota abundance (genus Catenibacterium id.2153)

Batch 2−3816 : Gut microbiota abundance (genus Parasutterella id.2892) on Membrane metallo−endopeptidase−like 1
with a threshold of 1e−05

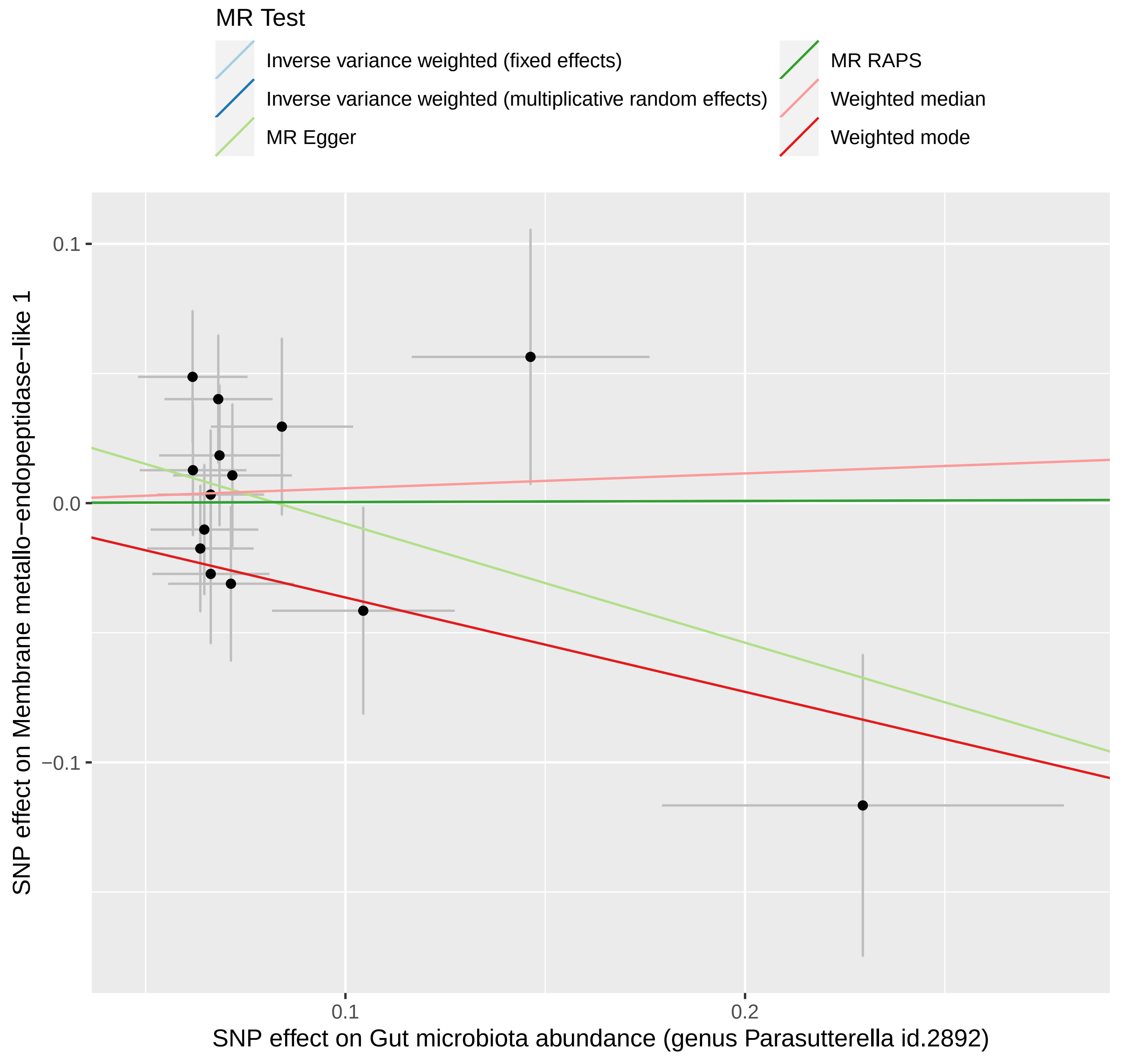

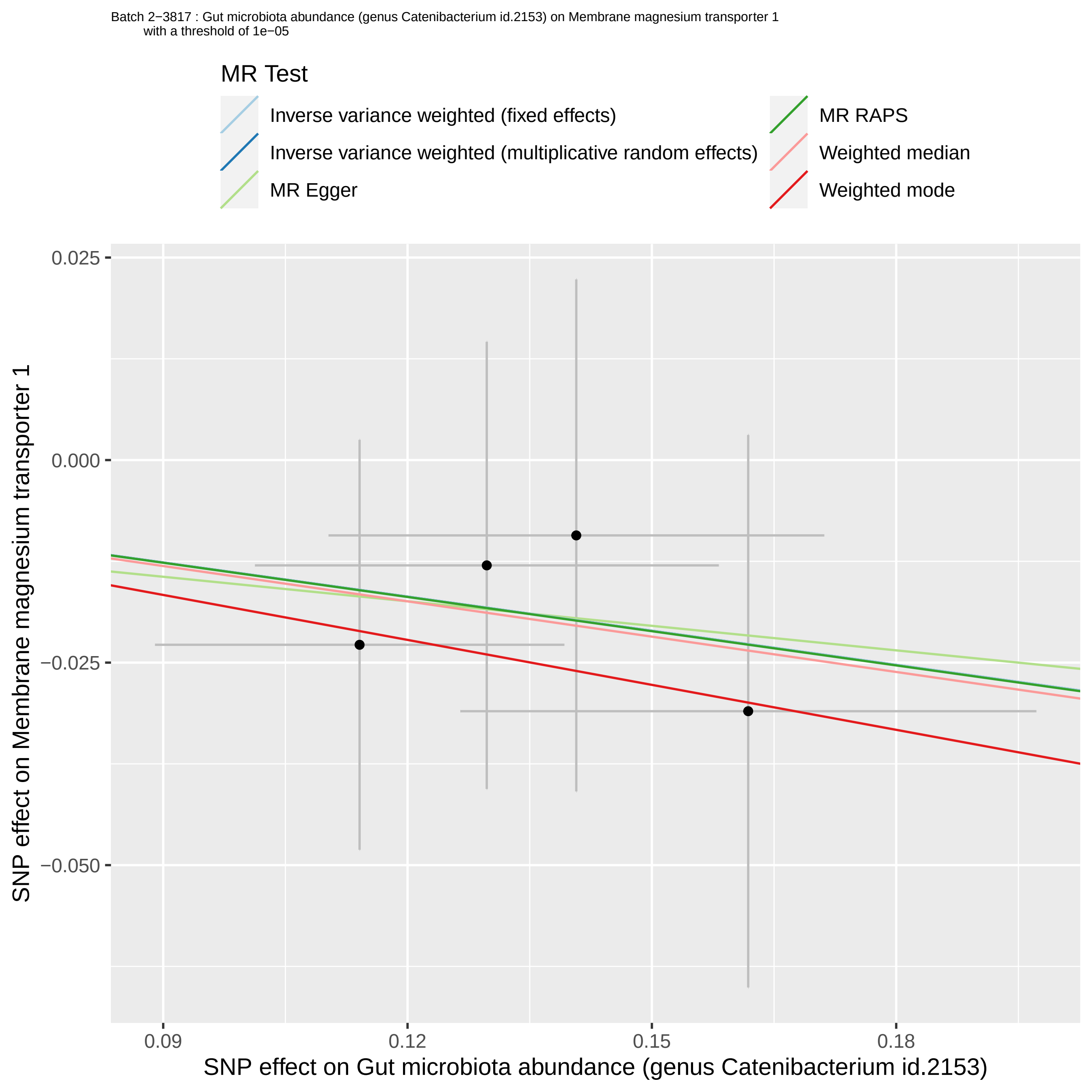

Batch 2−3817 : Gut microbiota abundance (genus Catenibacterium id.2153) on Membrane magnesium transporter 1
with a threshold of 1e−05
MR Test
Inverse variance weighted (fixed effects)
Inverse variance weighted (multiplicative random effects)
MR Egger
MR RAPS
Weighted median
Weighted mode
0.025
0.000
−0.025
−0.050
SNP effect on Membrane magnesium transporter 1
0.09
0.12
0.15
0.18
SNP effect on Gut microbiota abundance (genus Catenibacterium id.2153)

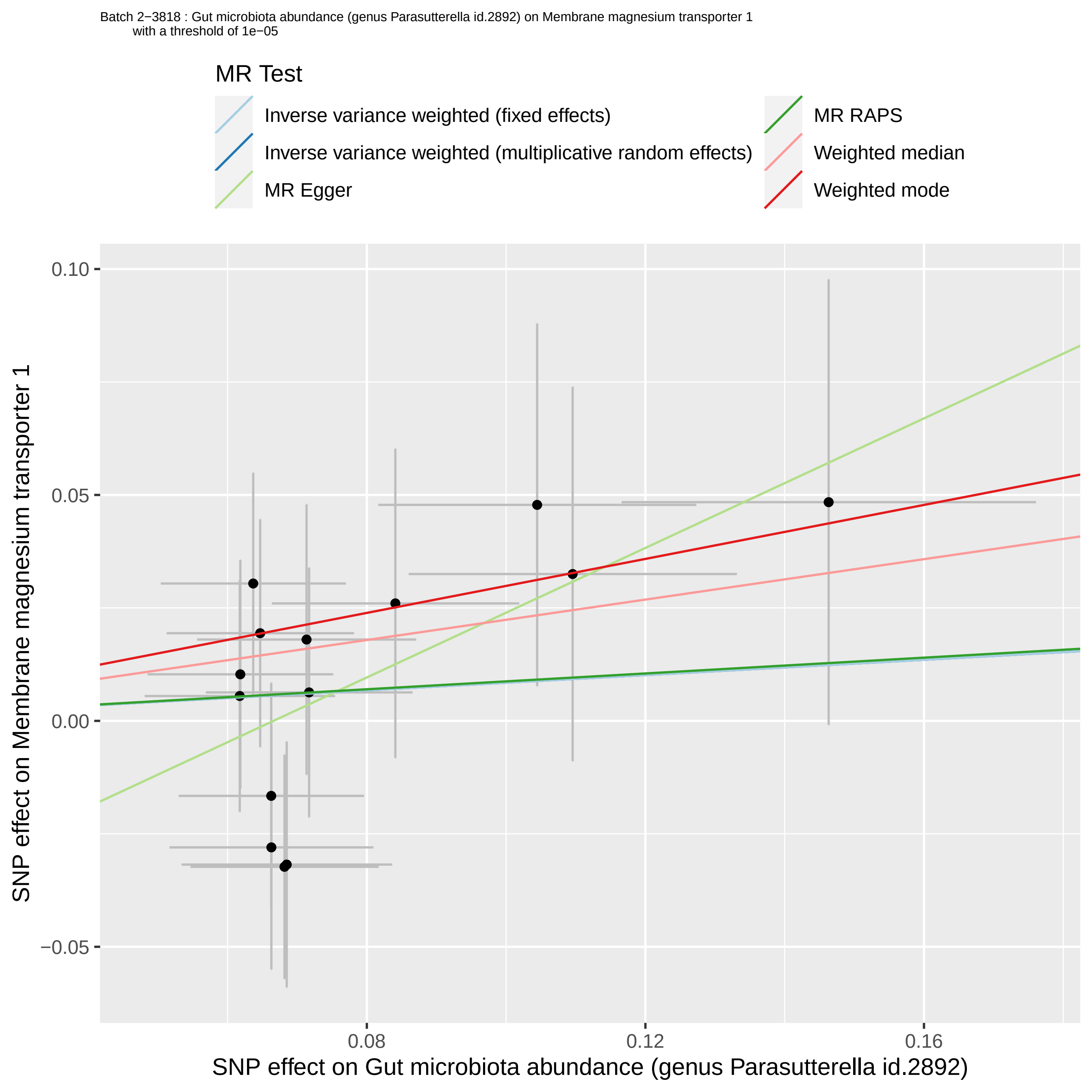

Batch 2−3818 : Gut microbiota abundance (genus Parasutterella id.2892) on Membrane magnesium transporter 1
with a threshold of 1e−05
MR Test
Inverse variance weighted (fixed effects)
Inverse variance weighted (multiplicative random effects)
MR Egger
MR RAPS
Weighted median
Weighted mode
0.10
0.05
0.00
−0.05
0.08
0.12
0.16
SNP effect on Membrane magnesium transporter 1
SNP effect on Gut microbiota abundance (genus Parasutterella id.2892)

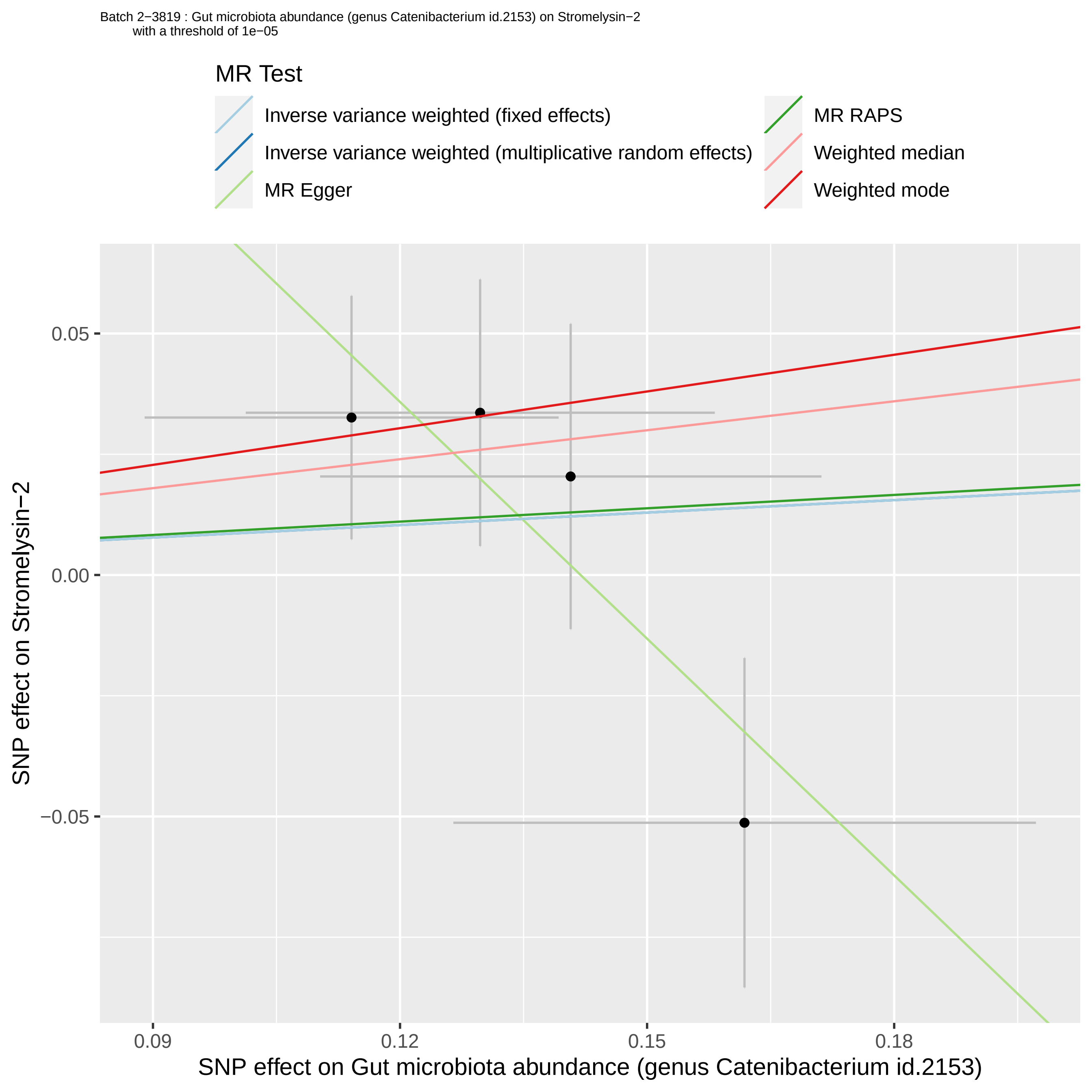
Batch 2−3819 : Gut microbiota abundance (genus Catenibacterium id.2153) on Stromelysin−2
with a threshold of 1e−05
MR Test
Inverse variance weighted (fixed effects)
Inverse variance weighted (multiplicative random effects)
MR Egger
MR RAPS
Weighted median
Weighted mode
0.05
0.00
−0.05
0.09
0.12
0.15
0.18
SNP effect on Stromelysin−2
SNP effect on Gut microbiota abundance (genus Catenibacterium id.2153)

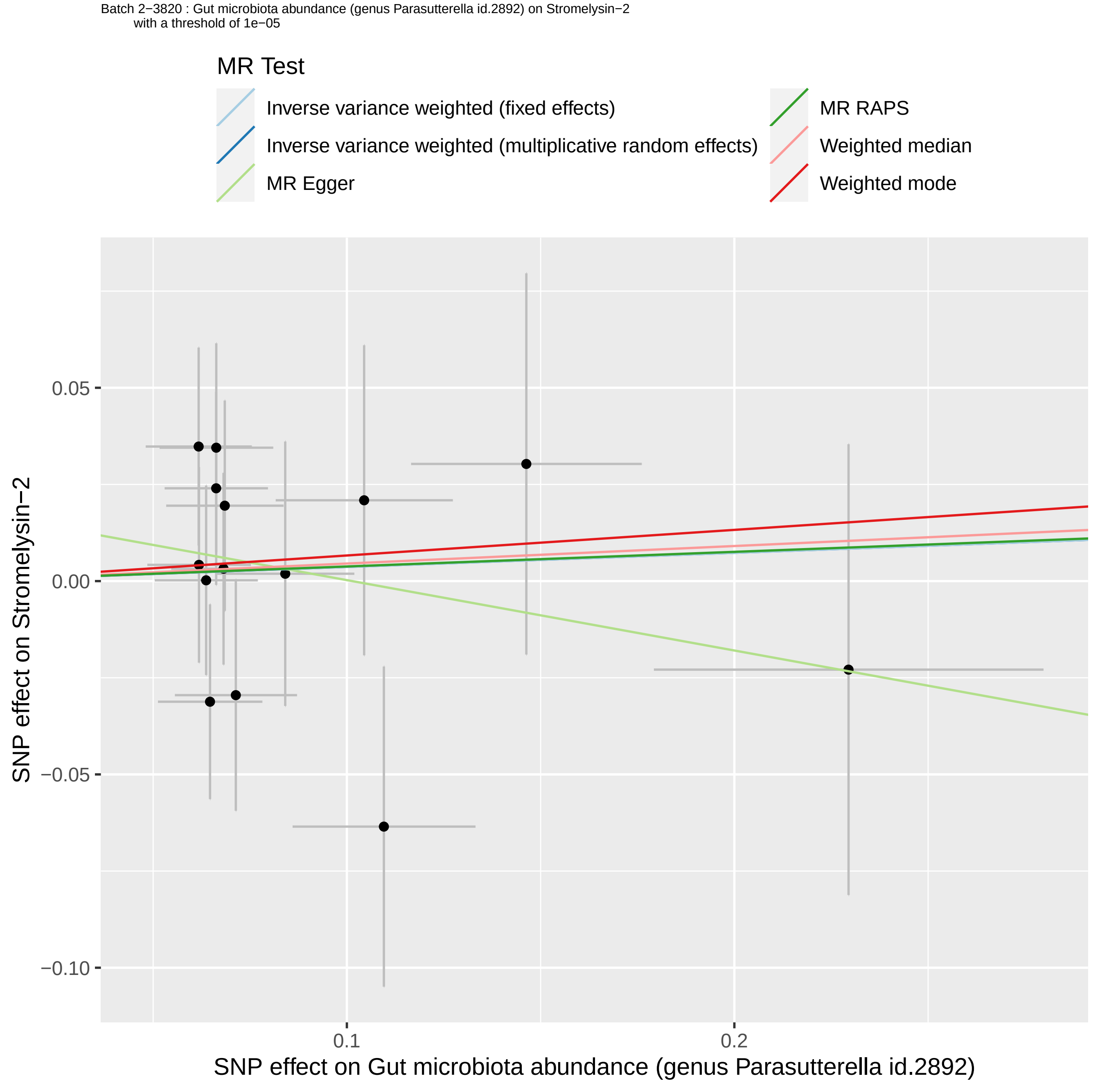

Batch 2−3820 : Gut microbiota abundance (genus Parasutterella id.2892) on Stromelysin−2
with a threshold of 1e−05
MR Test
Inverse variance weighted (fixed effects)
Inverse variance weighted (multiplicative random effects)
MR Egger
MR RAPS
Weighted median
Weighted mode
0.05
0.00
−0.05
−0.10
0.1
0.2
SNP effect on Stromelysin−2
SNP effect on Gut microbiota abundance (genus Parasutterella id.2892)

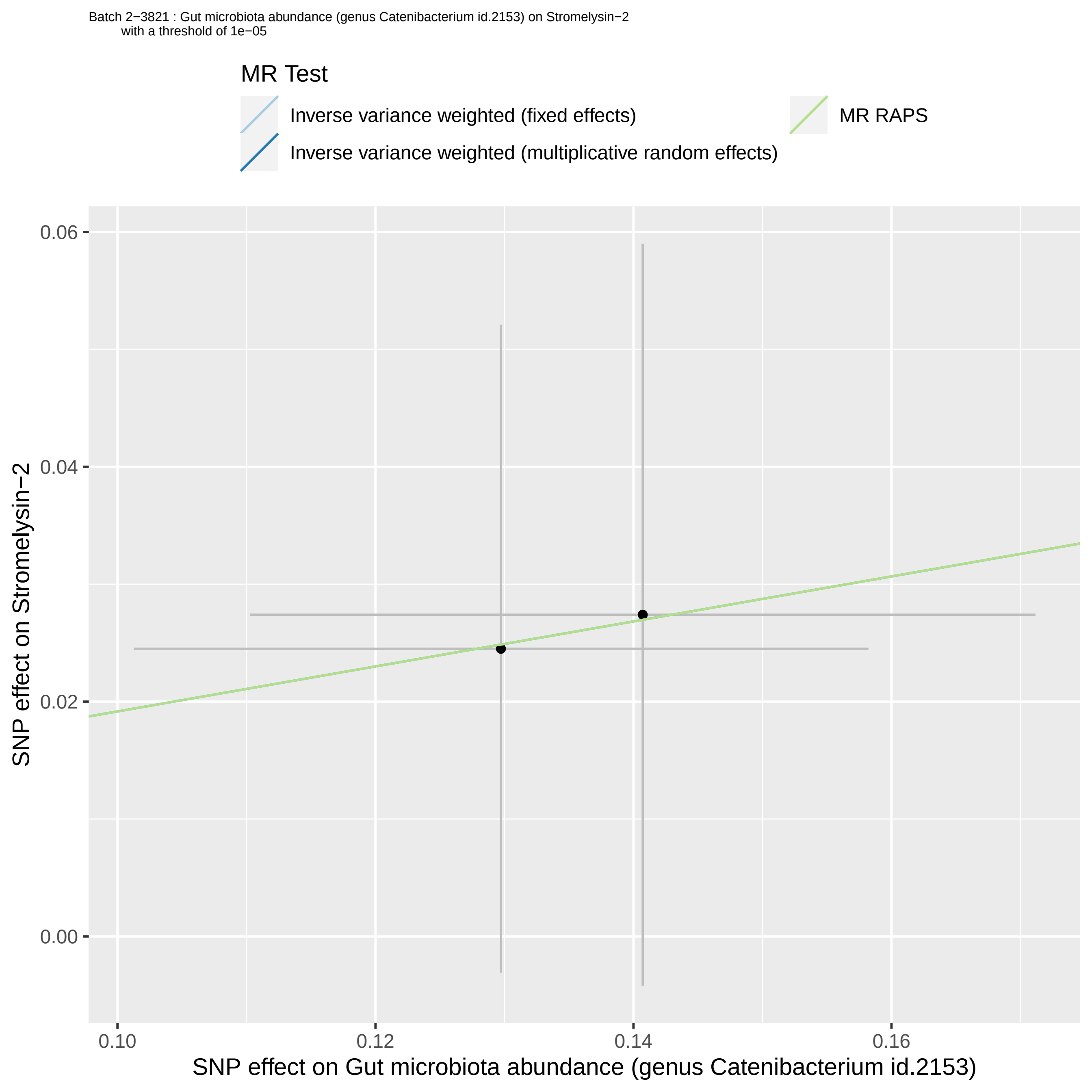
Batch 2−3821 : Gut microbiota abundance (genus Catenibacterium id.2153) on Stromelysin−2
with a threshold of 1e−05
MR Test
Inverse variance weighted (fixed effects)
Inverse variance weighted (multiplicative random effects)
MR RAPS
0.06
0.04
0.02
0.00
0.10
0.12
0.14
0.16
SNP effect on Stromelysin−2
SNP effect on Gut microbiota abundance (genus Catenibacterium id.2153)

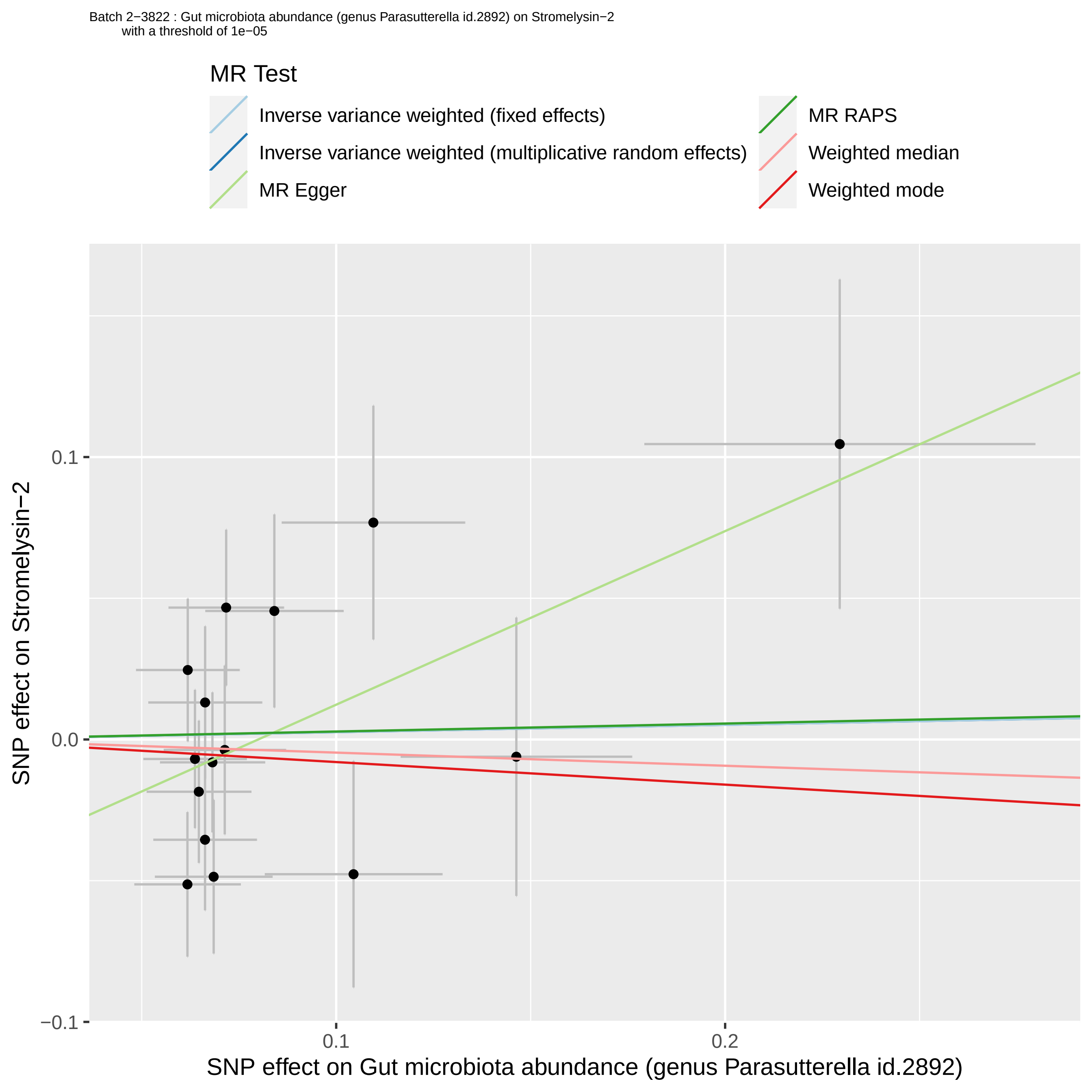
Batch 2−3822 : Gut microbiota abundance (genus Parasutterella id.2892) on Stromelysin−2
with a threshold of 1e−05
MR Test
Inverse variance weighted (fixed effects)
Inverse variance weighted (multiplicative random effects)
MR Egger
MR RAPS
Weighted median
Weighted mode
SNP effect on Stromelysin−2
0.1
0.0
−0.1
0.1
0.2
SNP effect on Gut microbiota abundance (genus Parasutterella id.2892)

Batch 2−3823 : Gut microbiota abundance (genus Catenibacterium id.2153) on Macrophage metalloelastase
with a threshold of 1e−05

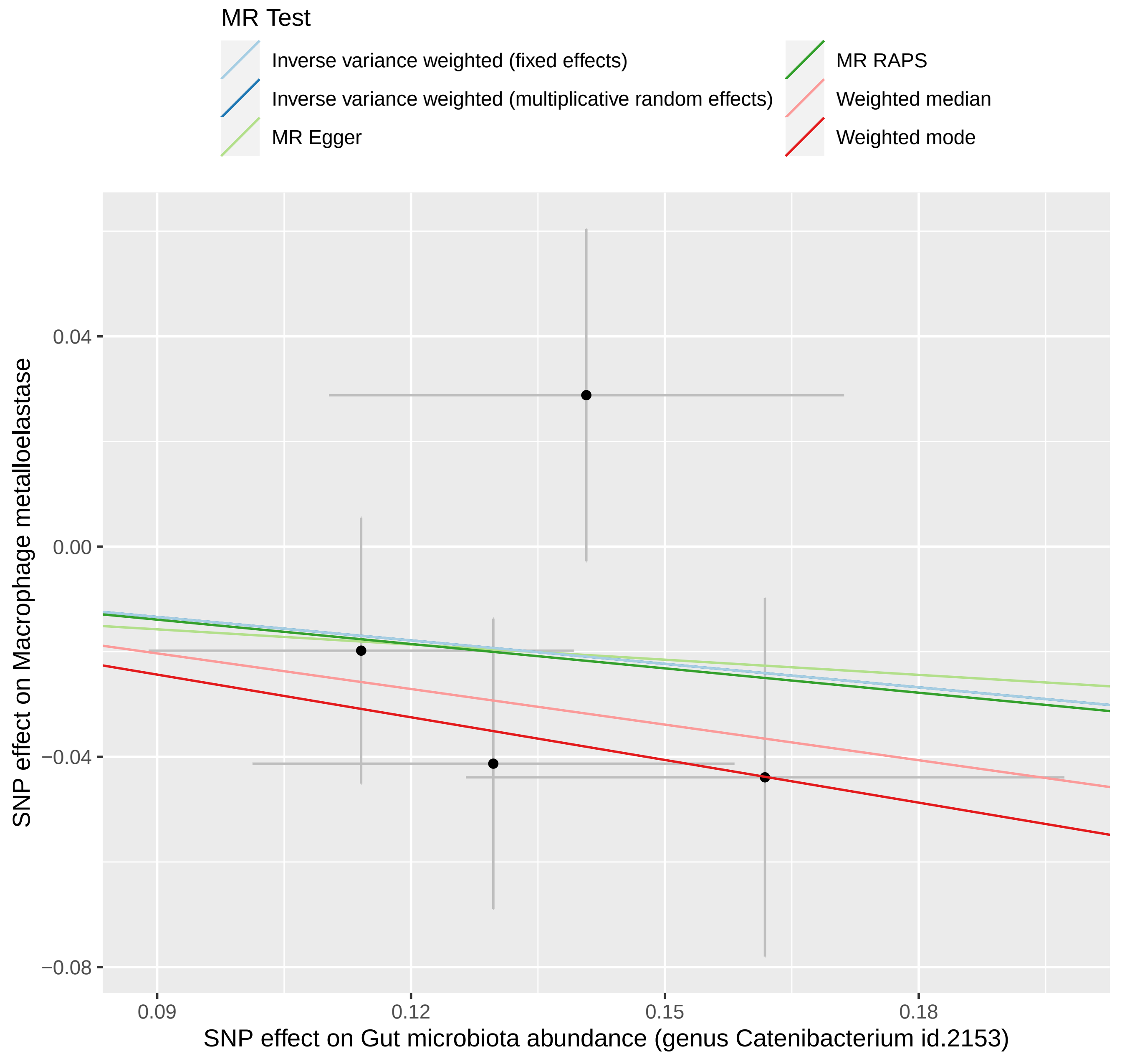

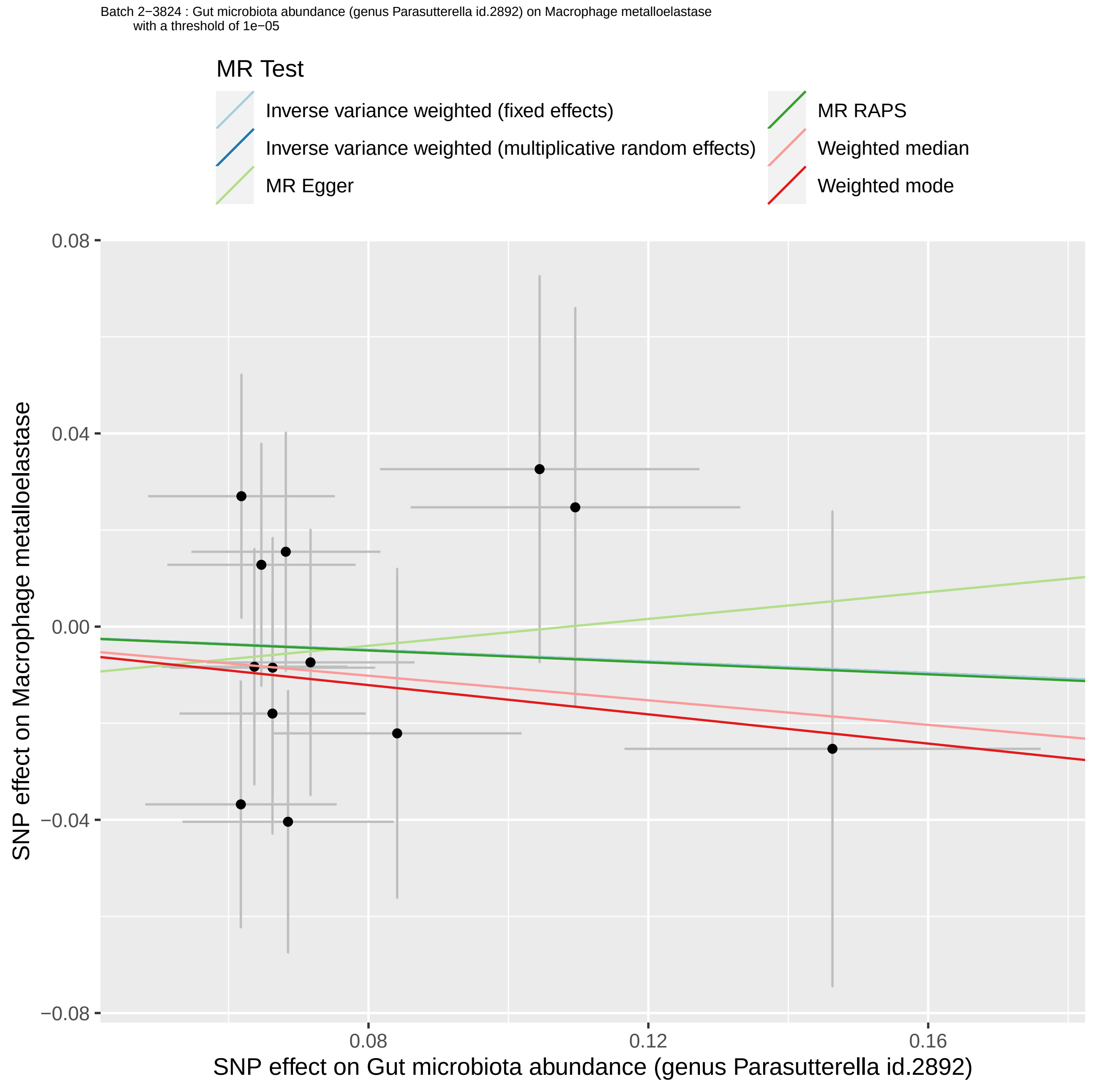

Batch 2−3824 : Gut microbiota abundance (genus Parasutterella id.2892) on Macrophage metalloelastase
with a threshold of 1e−05
MR Test
Inverse variance weighted (fixed effects)
Inverse variance weighted (multiplicative random effects)
MR Egger
MR RAPS
Weighted median
Weighted mode
0.08
0.04
0.00
−0.04
−0.08
0.08
0.12
0.16
SNP effect on Macrophage metalloelastase
SNP effect on Gut microbiota abundance (genus Parasutterella id.2892)

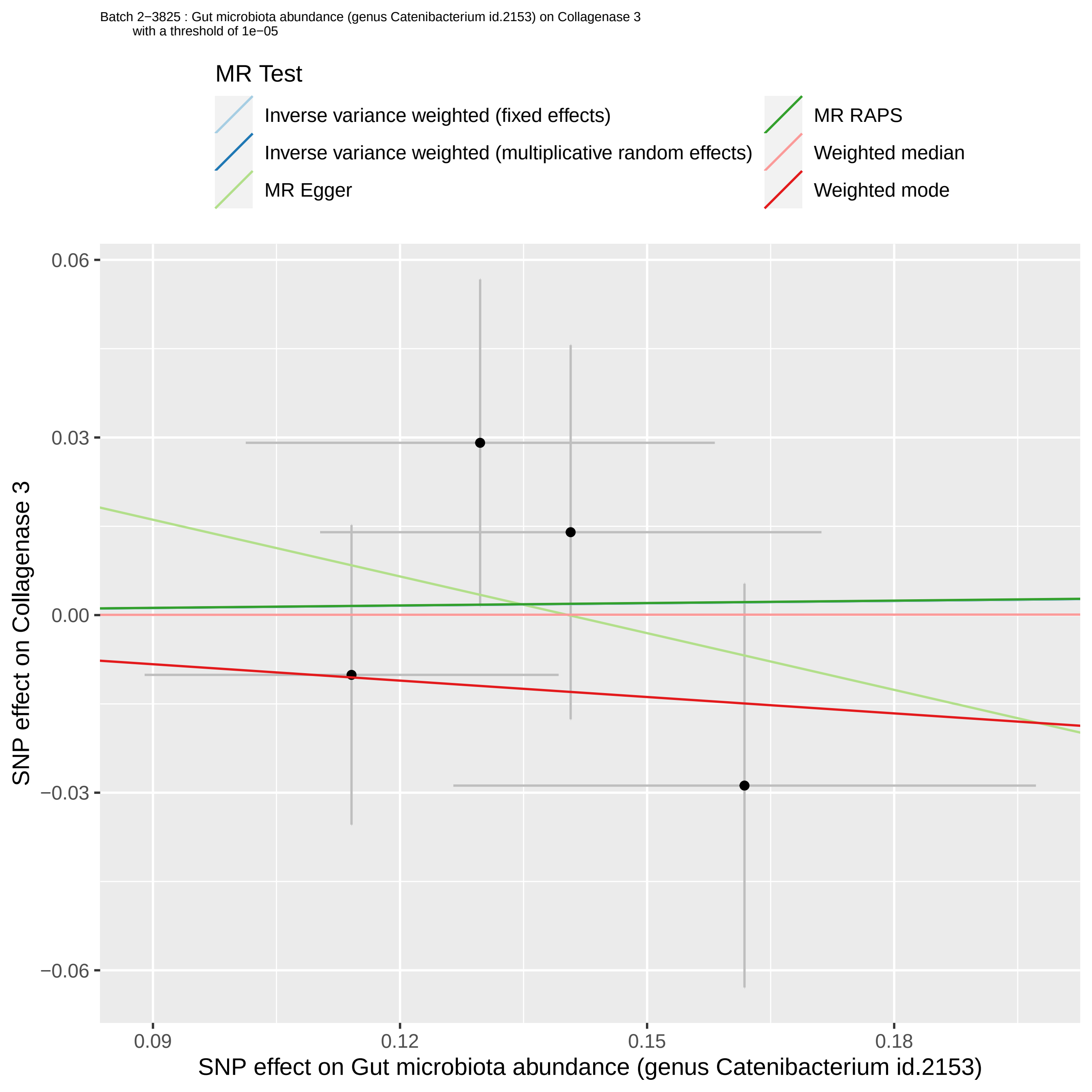
Batch 2−3825 : Gut microbiota abundance (genus Catenibacterium id.2153) on Collagenase 3
with a threshold of 1e−05
MR Test
Inverse variance weighted (fixed effects)
Inverse variance weighted (multiplicative random effects)
MR Egger
MR RAPS
Weighted median
Weighted mode
0.06
0.03
0.00
−0.03
−0.06
SNP effect on Collagenase 3
0.09
0.12
0.15
0.18
SNP effect on Gut microbiota abundance (genus Catenibacterium id.2153)

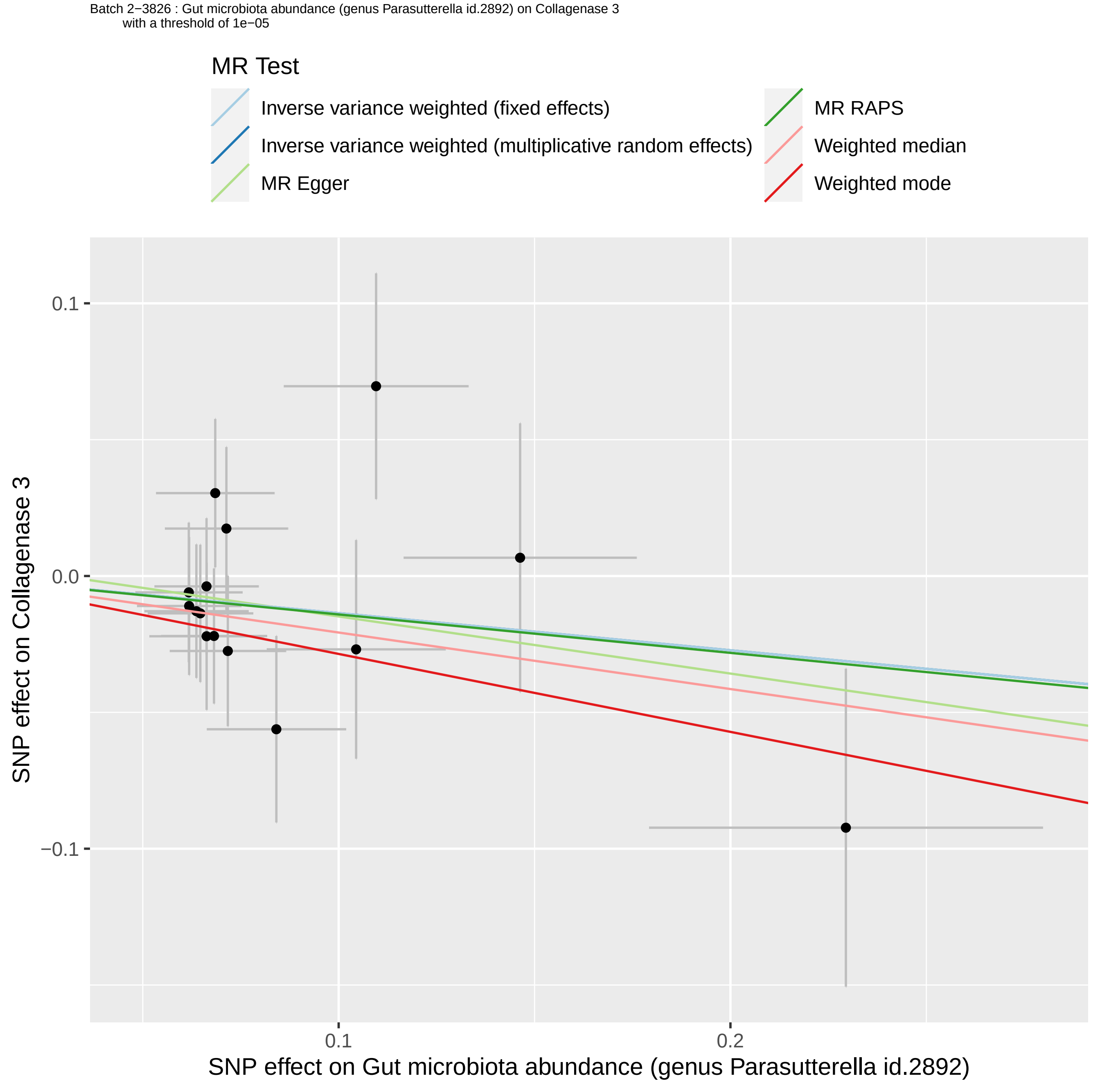

Batch 2−3826 : Gut microbiota abundance (genus Parasutterella id.2892) on Collagenase 3
with a threshold of 1e−05
MR Test
Inverse variance weighted (fixed effects)
Inverse variance weighted (multiplicative random effects)
MR Egger
MR RAPS
Weighted median
Weighted mode
0.1
0.0
−0.1
0.1
0.2
SNP effect on Collagenase 3
SNP effect on Gut microbiota abundance (genus Parasutterella id.2892)

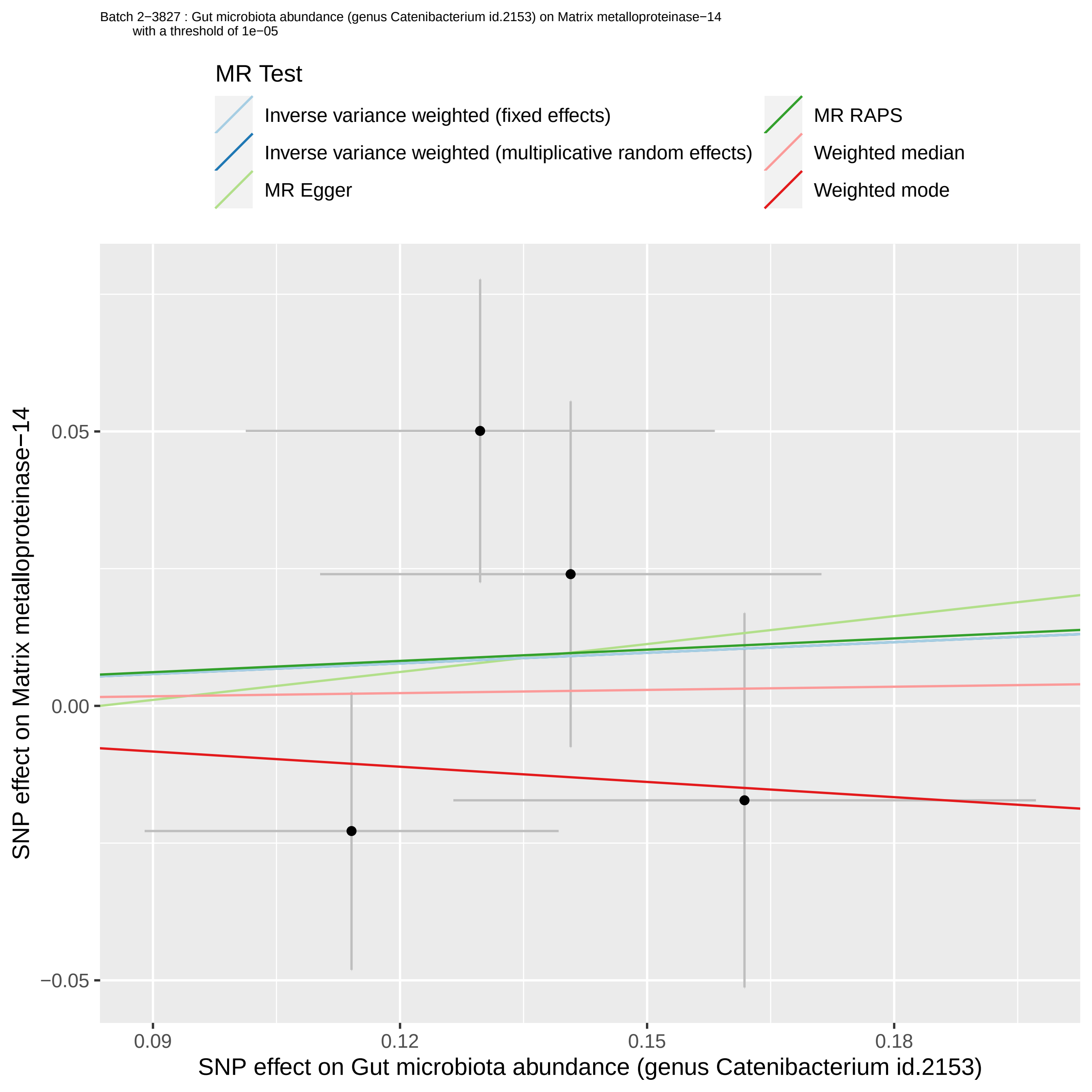

Batch 2−3827 : Gut microbiota abundance (genus Catenibacterium id.2153) on Matrix metalloproteinase−14
with a threshold of 1e−05
MR Test
Inverse variance weighted (fixed effects)
Inverse variance weighted (multiplicative random effects)
MR Egger
MR RAPS
Weighted median
Weighted mode
SNP effect on Matrix metalloproteinase−14
0.05
0.00
−0.05
0.09
0.12
0.15
0.18
SNP effect on Gut microbiota abundance (genus Catenibacterium id.2153)

Batch 2−3828 : Gut microbiota abundance (genus Parasutterella id.2892) on Matrix metalloproteinase−14
with a threshold of 1e−05

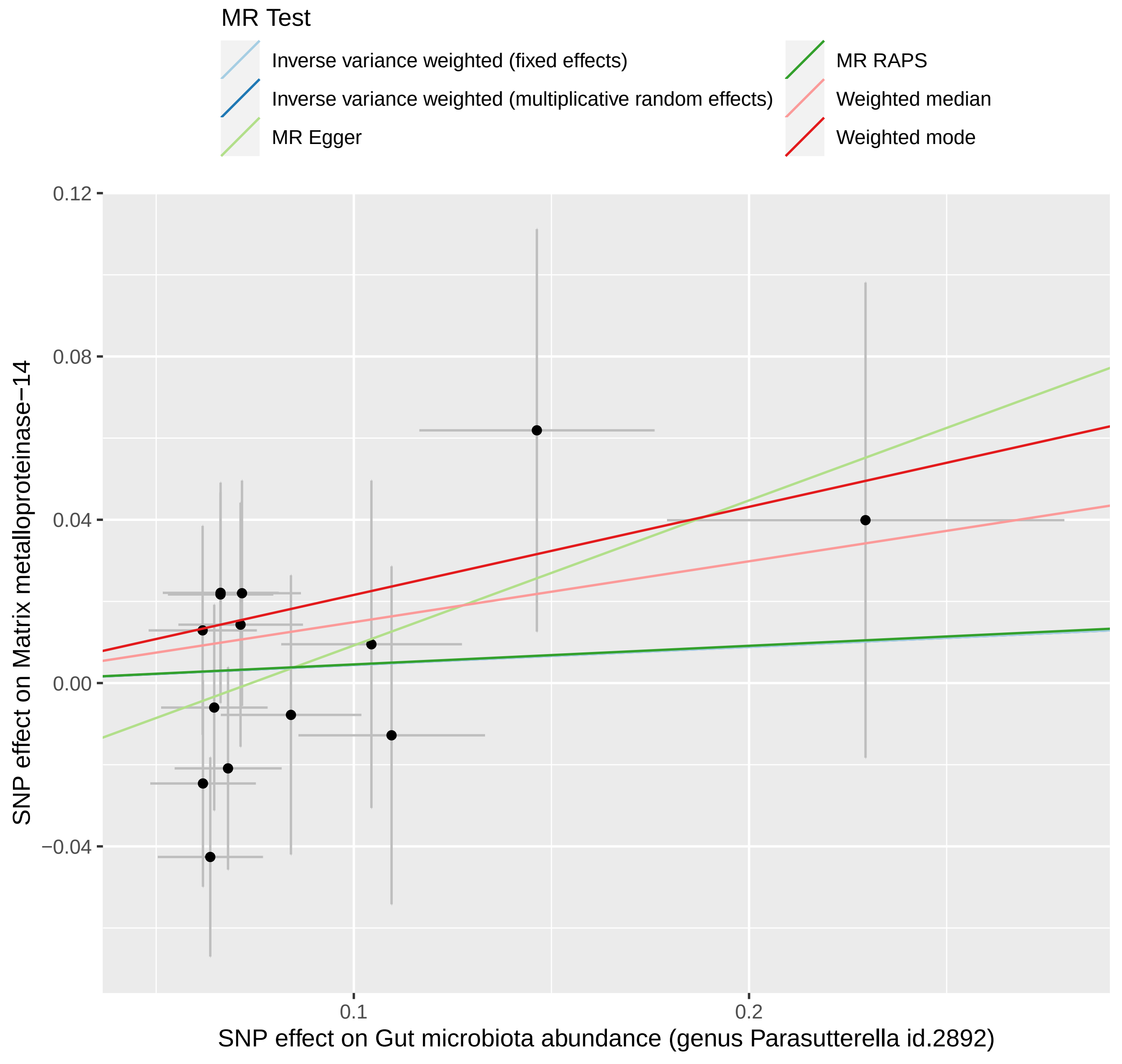

Batch 2−3829 : Gut microbiota abundance (genus Catenibacterium id.2153) on Matrix metalloproteinase−16
with a threshold of 1e−05

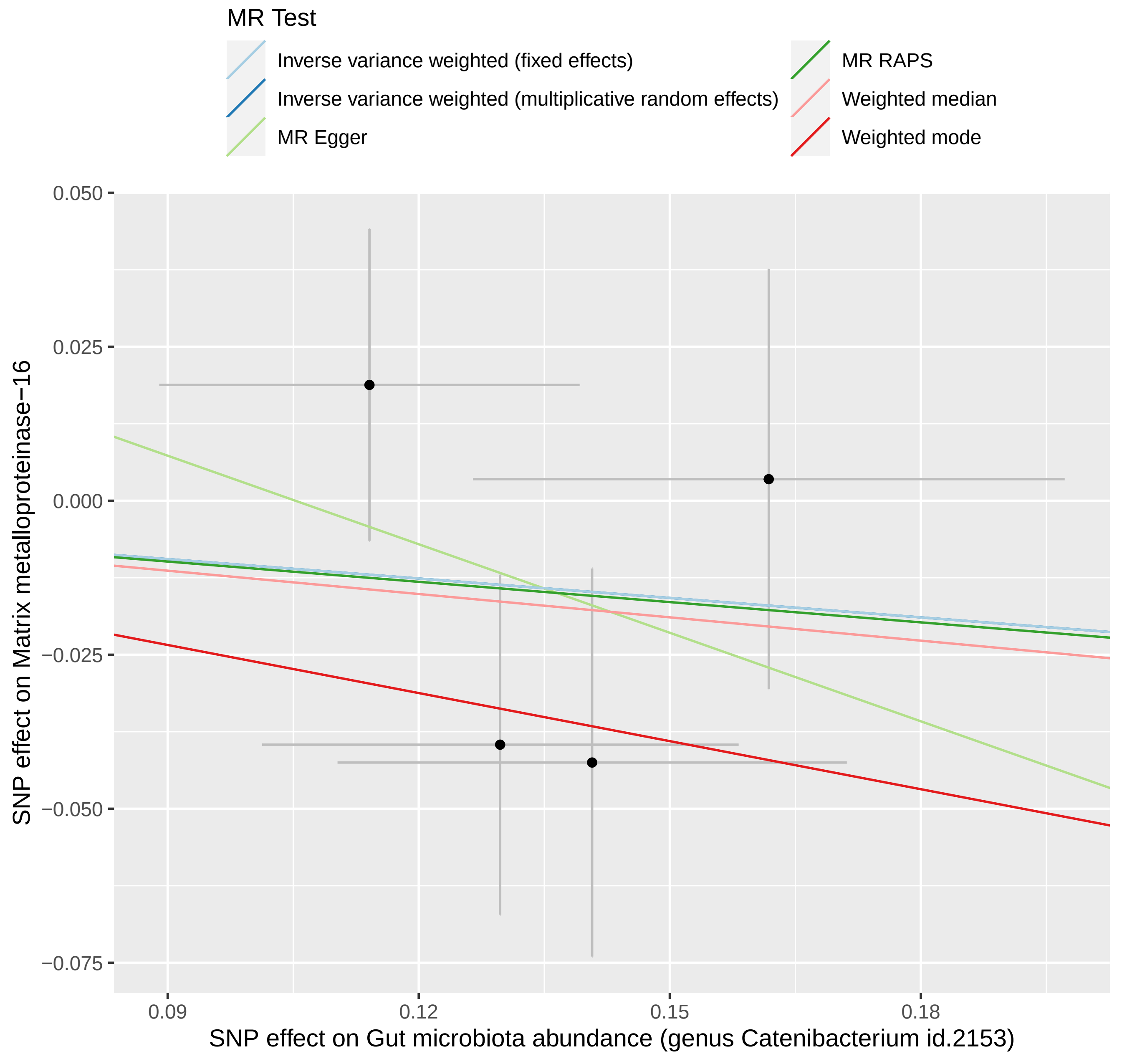

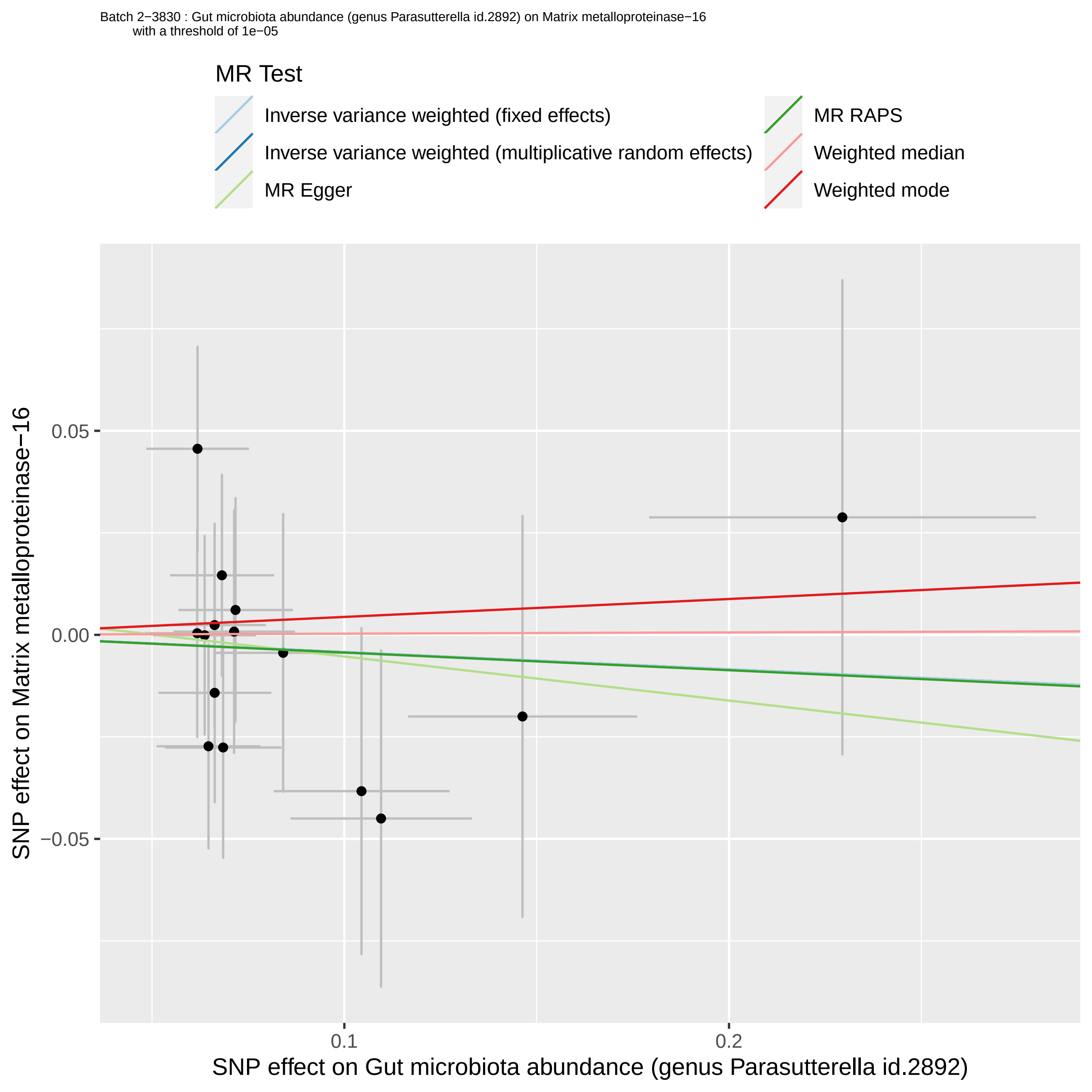
Batch 2−3830 : Gut microbiota abundance (genus Parasutterella id.2892) on Matrix metalloproteinase−16
with a threshold of 1e−05
MR Test
Inverse variance weighted (fixed effects)
Inverse variance weighted (multiplicative random effects)
MR Egger
MR RAPS
Weighted median
Weighted mode
0.05
0.00
−0.05
0.1
0.2
SNP effect on Matrix metalloproteinase−16
SNP effect on Gut microbiota abundance (genus Parasutterella id.2892)

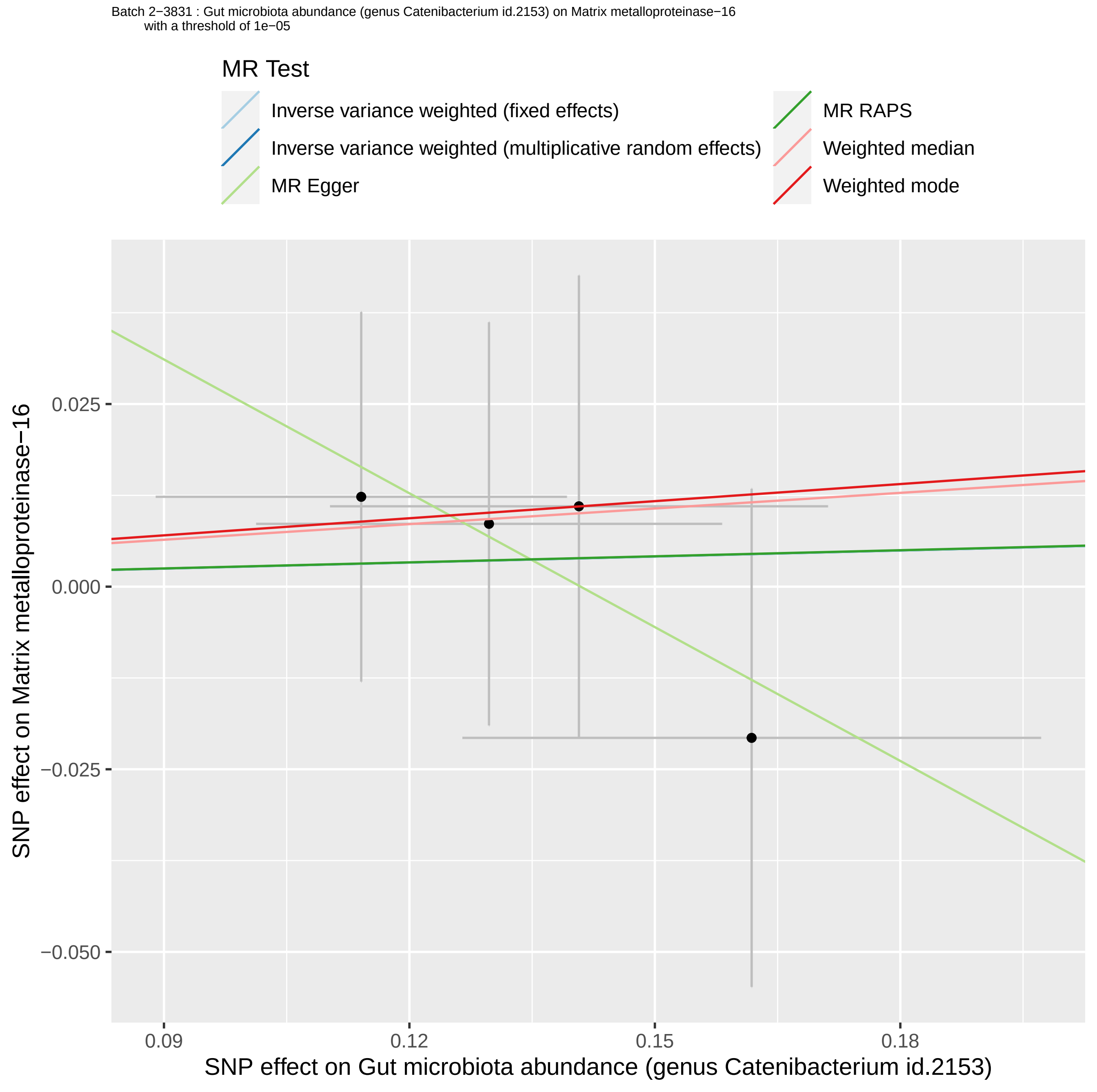

Batch 2−3831 : Gut microbiota abundance (genus Catenibacterium id.2153) on Matrix metalloproteinase−16
with a threshold of 1e−05
MR Test
Inverse variance weighted (fixed effects)
Inverse variance weighted (multiplicative random effects)
MR Egger
MR RAPS
Weighted median
Weighted mode
0.025
0.000
−0.025
−0.050
0.09
0.12
0.15
0.18
SNP effect on Matrix metalloproteinase−16
SNP effect on Gut microbiota abundance (genus Catenibacterium id.2153)

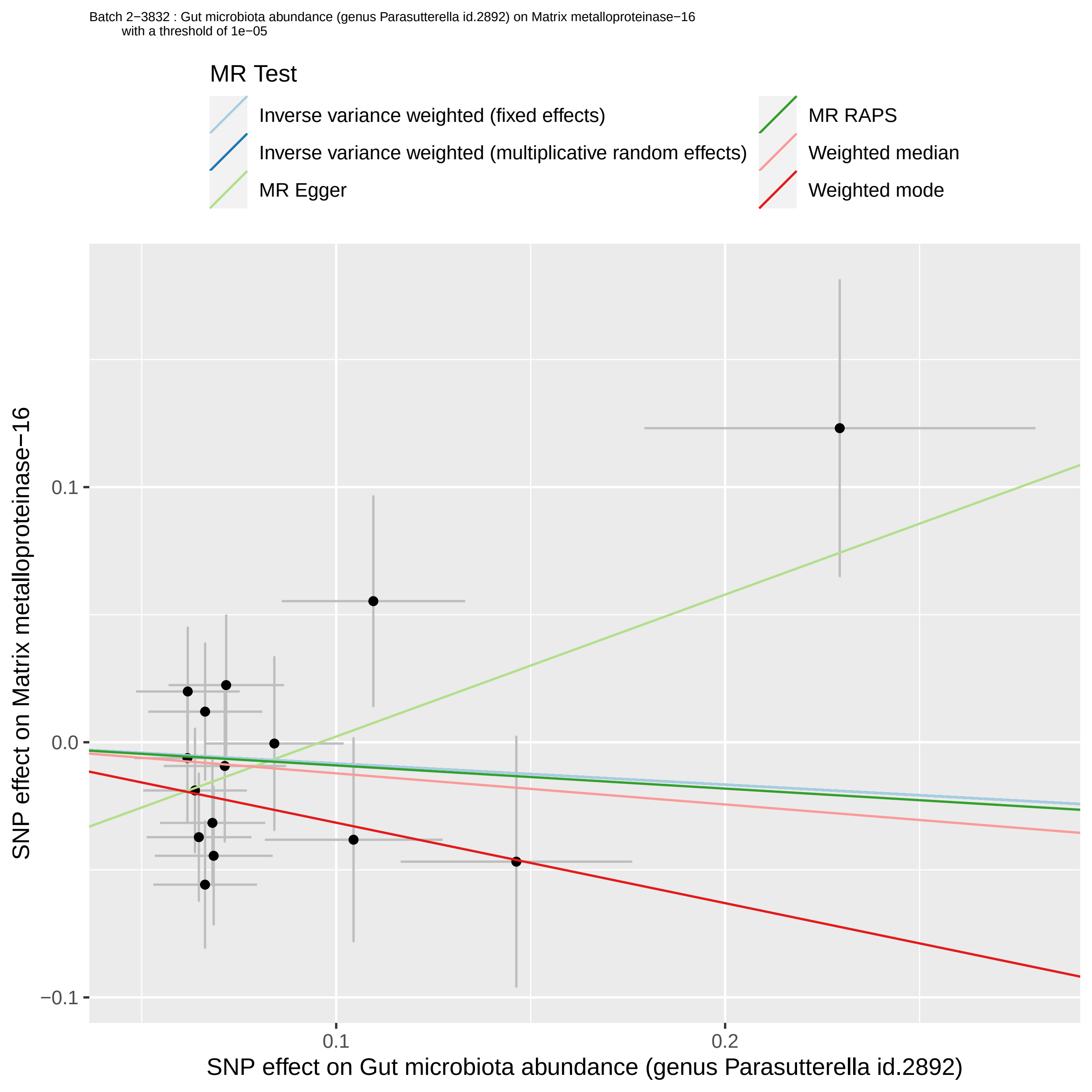

Batch 2−3832 : Gut microbiota abundance (genus Parasutterella id.2892) on Matrix metalloproteinase−16
with a threshold of 1e−05
MR Test
Inverse variance weighted (fixed effects)
Inverse variance weighted (multiplicative random effects)
MR Egger
MR RAPS
Weighted median
Weighted mode
0.1
0.0
−0.1
0.1
0.2
SNP effect on Matrix metalloproteinase−16
SNP effect on Gut microbiota abundance (genus Parasutterella id.2892)

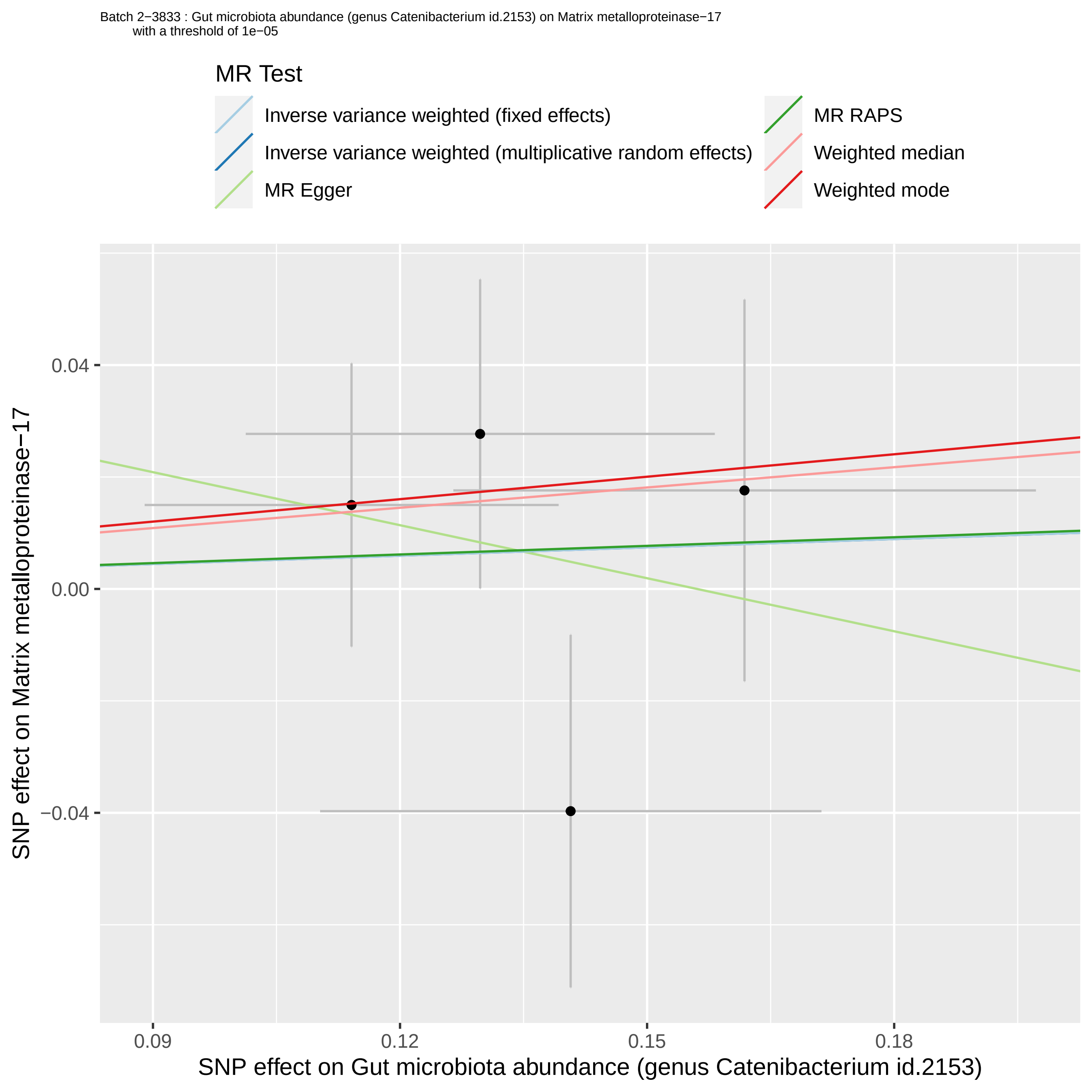
Batch 2−3833 : Gut microbiota abundance (genus Catenibacterium id.2153) on Matrix metalloproteinase−17
with a threshold of 1e−05
MR Test
Inverse variance weighted (fixed effects)
Inverse variance weighted (multiplicative random effects)
MR Egger
MR RAPS
Weighted median
Weighted mode
0.04
0.00
−0.04
0.09
0.12
0.15
0.18
SNP effect on Matrix metalloproteinase−17
SNP effect on Gut microbiota abundance (genus Catenibacterium id.2153)

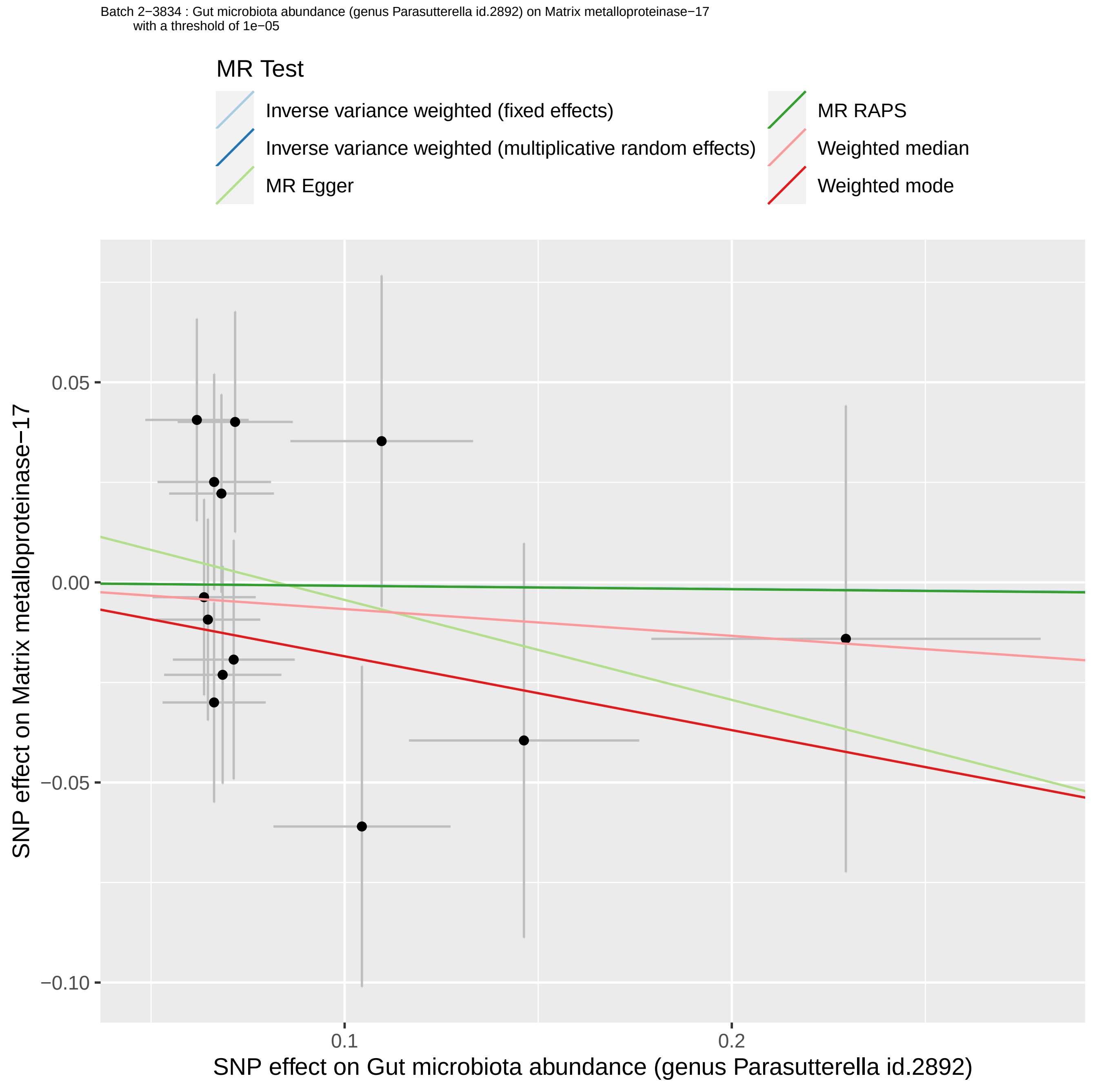

Batch 2−3834 : Gut microbiota abundance (genus Parasutterella id.2892) on Matrix metalloproteinase−17
with a threshold of 1e−05
MR Test
Inverse variance weighted (fixed effects)
Inverse variance weighted (multiplicative random effects)
MR Egger
MR RAPS
Weighted median
Weighted mode
0.05
0.00
−0.05
−0.10
0.1
0.2
SNP effect on Matrix metalloproteinase−17
SNP effect on Gut microbiota abundance (genus Parasutterella id.2892)

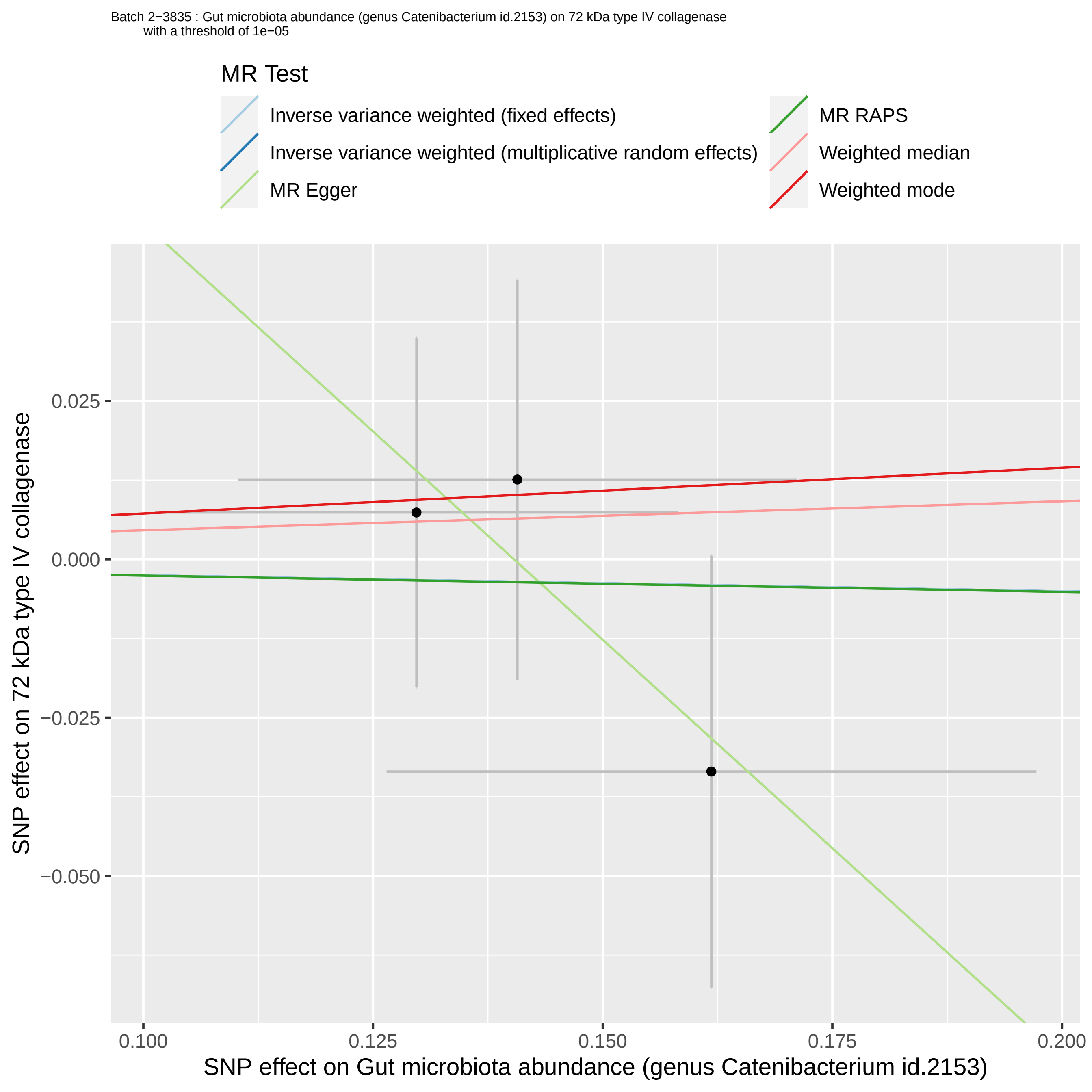
Batch 2−3835 : Gut microbiota abundance (genus Catenibacterium id.2153) on 72 kDa type IV collagenase
with a threshold of 1e−05
MR Test
Inverse variance weighted (fixed effects)
Inverse variance weighted (multiplicative random effects)
MR Egger
MR RAPS
Weighted median
Weighted mode
0.025
0.000
−0.025
−0.050
0.100
0.125
0.150
0.175
0.200
SNP effect on 72 kDa type IV collagenase
SNP effect on Gut microbiota abundance (genus Catenibacterium id.2153)

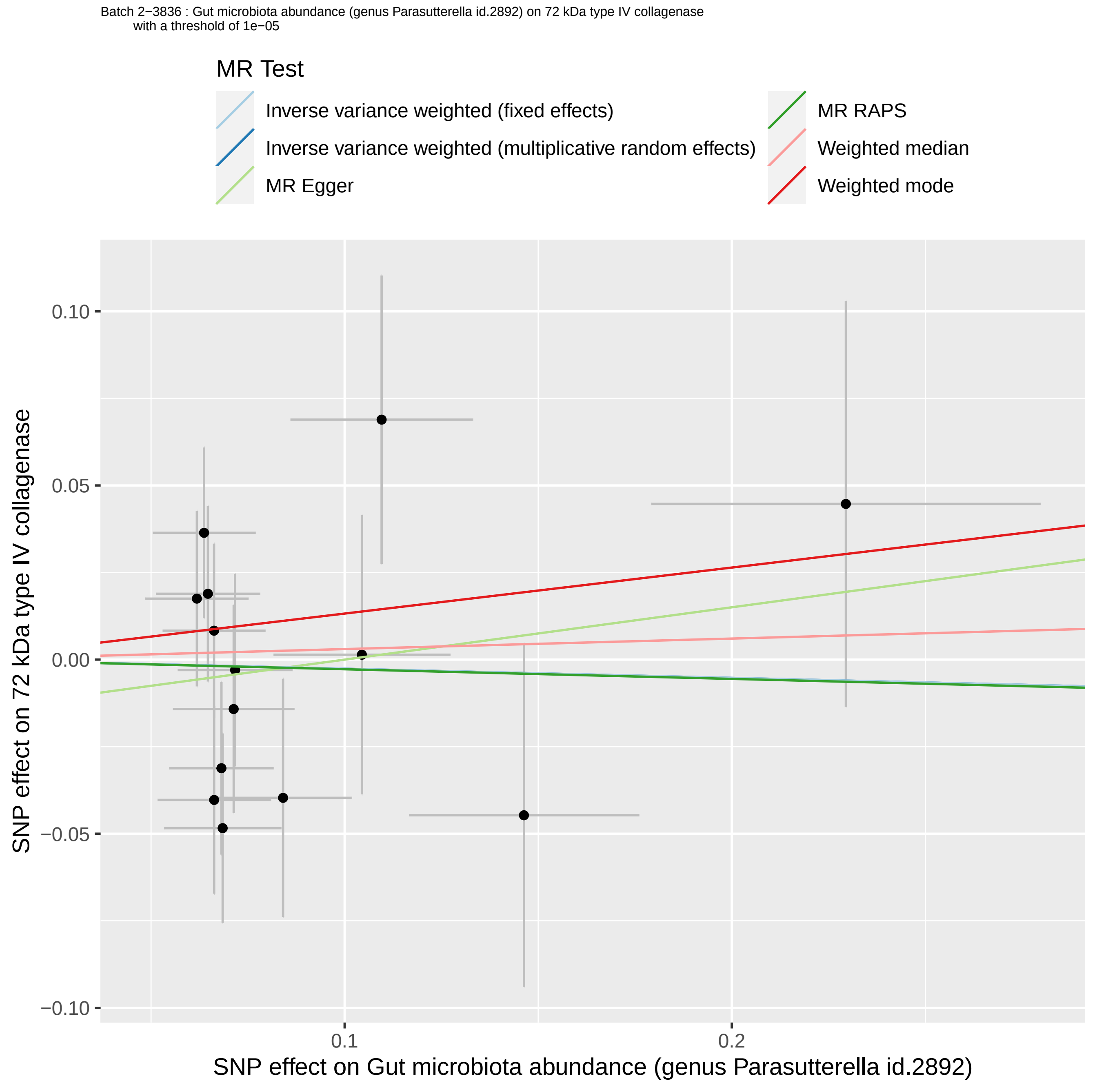
Batch 2−3836 : Gut microbiota abundance (genus Parasutterella id.2892) on 72 kDa type IV collagenase
with a threshold of 1e−05
MR Test
Inverse variance weighted (fixed effects)
Inverse variance weighted (multiplicative random effects)
MR Egger
MR RAPS
Weighted median
Weighted mode
SNP effect on 72 kDa type IV collagenase
0.10
0.05
0.00
−0.05
−0.10
0.1
0.2
SNP effect on Gut microbiota abundance (genus Parasutterella id.2892)

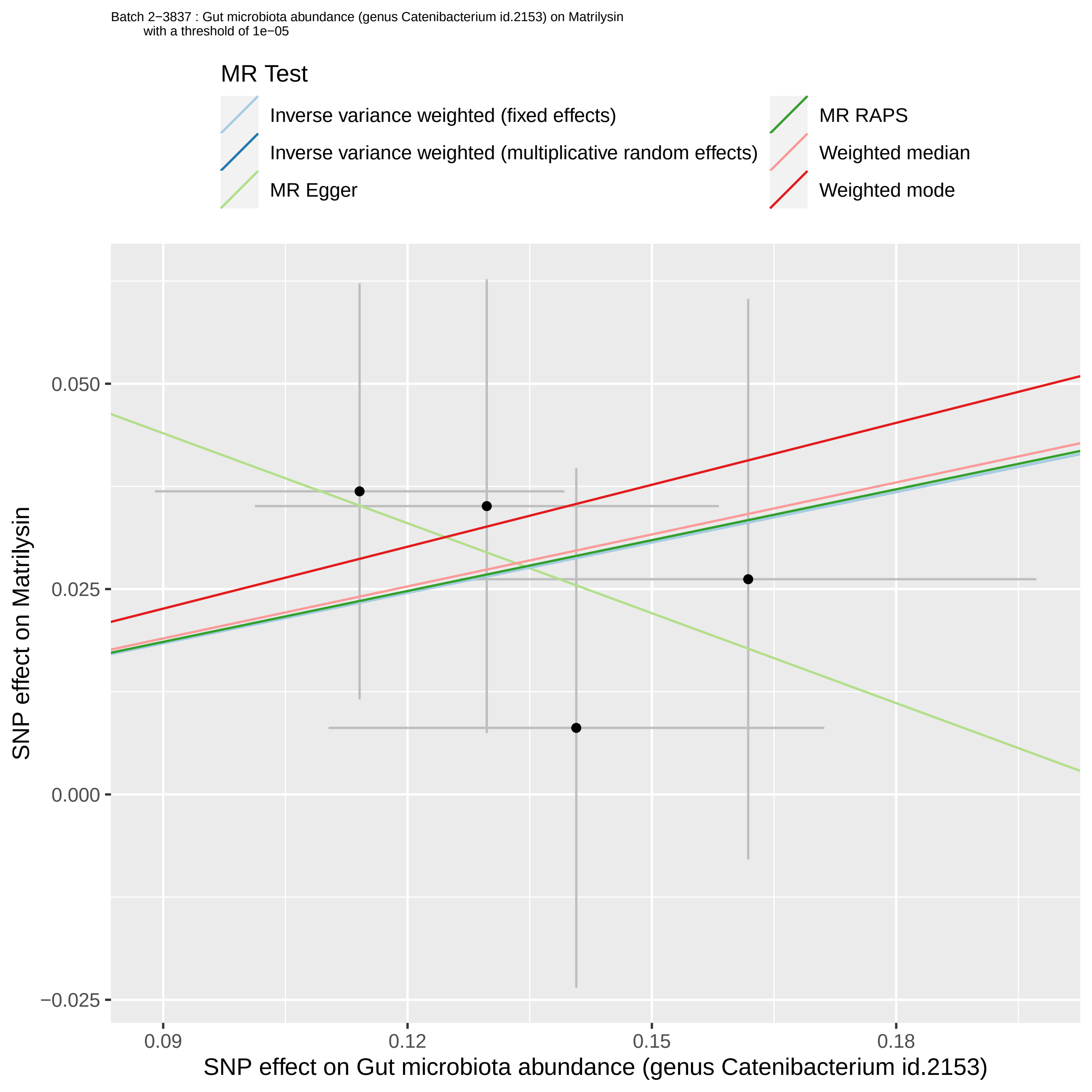
Batch 2−3837 : Gut microbiota abundance (genus Catenibacterium id.2153) on Matrilysin
with a threshold of 1e−05
MR Test
Inverse variance weighted (fixed effects)
Inverse variance weighted (multiplicative random effects)
MR Egger
MR RAPS
Weighted median
Weighted mode
SNP effect on Matrilysin
0.050
0.025
0.000
−0.025
0.09
0.12
0.15
0.18
SNP effect on Gut microbiota abundance (genus Catenibacterium id.2153)

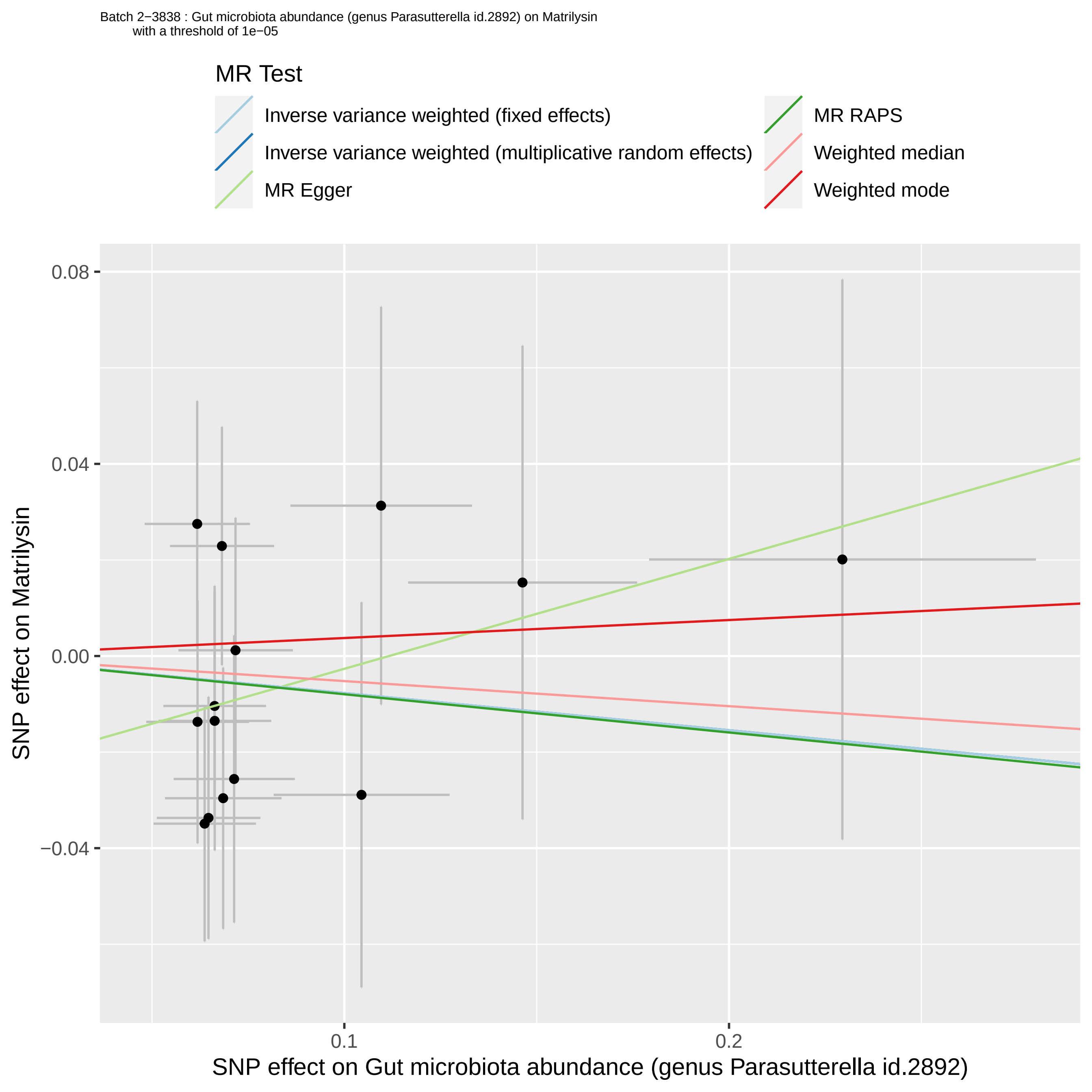

Batch 2−3838 : Gut microbiota abundance (genus Parasutterella id.2892) on Matrilysin
with a threshold of 1e−05
MR Test
Inverse variance weighted (fixed effects)
Inverse variance weighted (multiplicative random effects)
MR Egger
MR RAPS
Weighted median
Weighted mode
0.08
0.04
0.00
−0.04
0.1
0.2
SNP effect on Matrilysin
SNP effect on Gut microbiota abundance (genus Parasutterella id.2892)

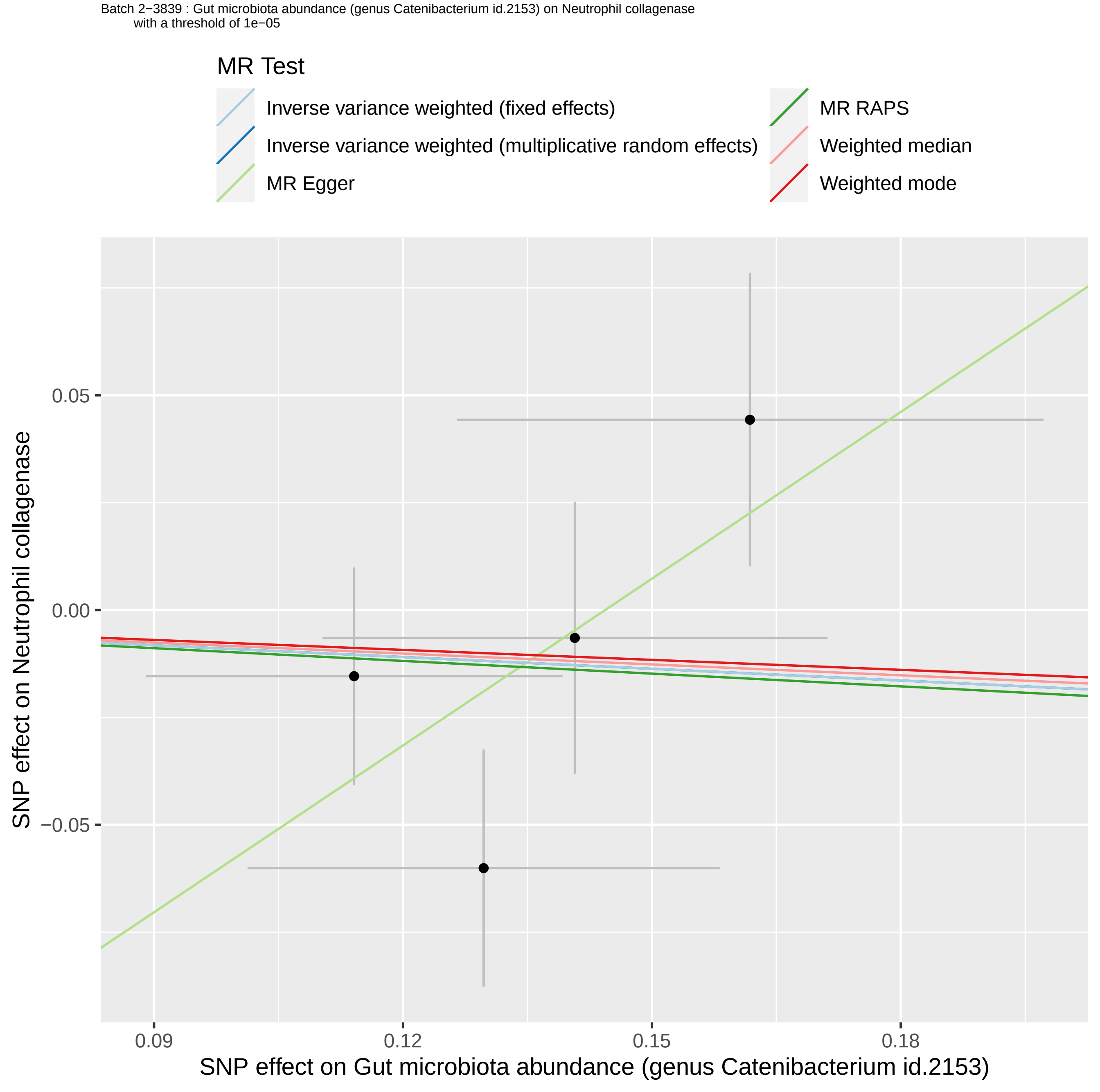

Batch 2−3839 : Gut microbiota abundance (genus Catenibacterium id.2153) on Neutrophil collagenase
with a threshold of 1e−05
MR Test
Inverse variance weighted (fixed effects)
Inverse variance weighted (multiplicative random effects)
MR Egger
MR RAPS
Weighted median
Weighted mode
0.05
0.00
−0.05
0.09
0.12
0.15
0.18
SNP effect on Neutrophil collagenase
SNP effect on Gut microbiota abundance (genus Catenibacterium id.2153)

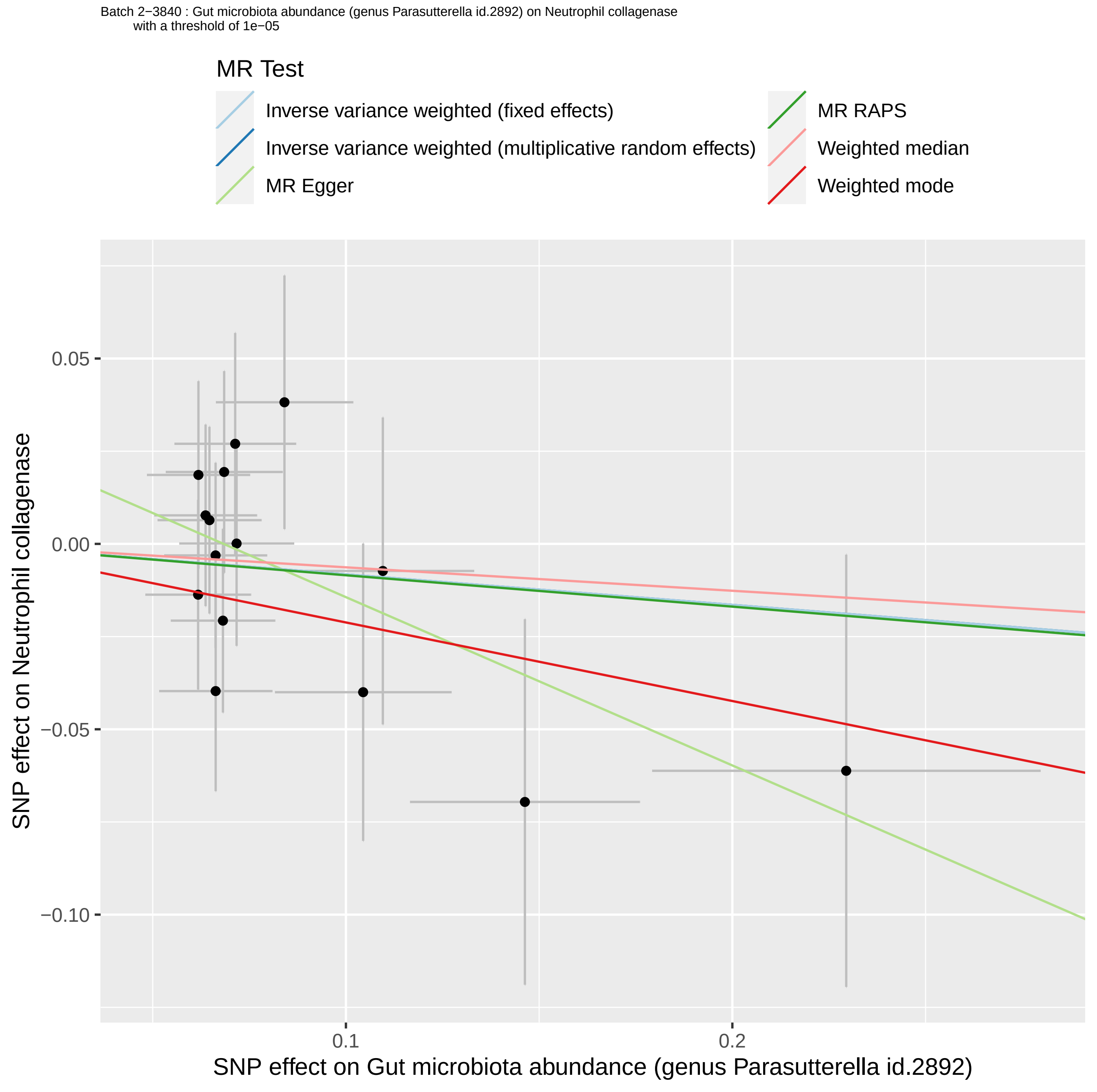

Batch 2−3840 : Gut microbiota abundance (genus Parasutterella id.2892) on Neutrophil collagenase
with a threshold of 1e−05
MR Test
Inverse variance weighted (fixed effects)
Inverse variance weighted (multiplicative random effects)
MR Egger
MR RAPS
Weighted median
Weighted mode
0.05
0.00
−0.05
−0.10
0.1
0.2
SNP effect on Neutrophil collagenase
SNP effect on Gut microbiota abundance (genus Parasutterella id.2892)

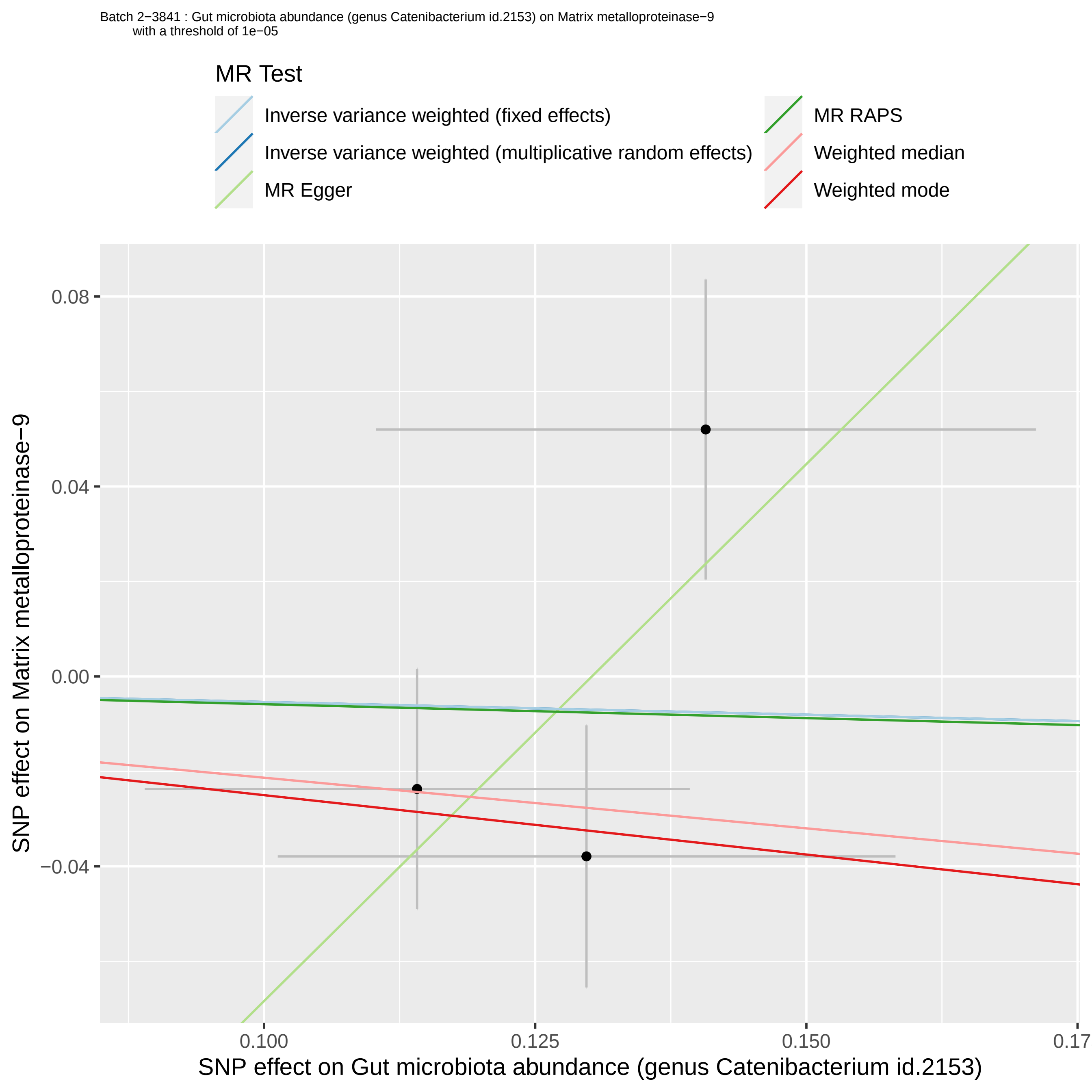
Batch 2−3841 : Gut microbiota abundance (genus Catenibacterium id.2153) on Matrix metalloproteinase−9
with a threshold of 1e−05
MR Test
Inverse variance weighted (fixed effects)
Inverse variance weighted (multiplicative random effects)
MR Egger
MR RAPS
Weighted median
Weighted mode
0.08
0.04
0.00
−0.04
0.100
0.125
0.150
0.17
SNP effect on Matrix metalloproteinase−9
SNP effect on Gut microbiota abundance (genus Catenibacterium id.2153)

Batch 2−3842 : Gut microbiota abundance (genus Parasutterella id.2892) on Matrix metalloproteinase−9
with a threshold of 1e−05

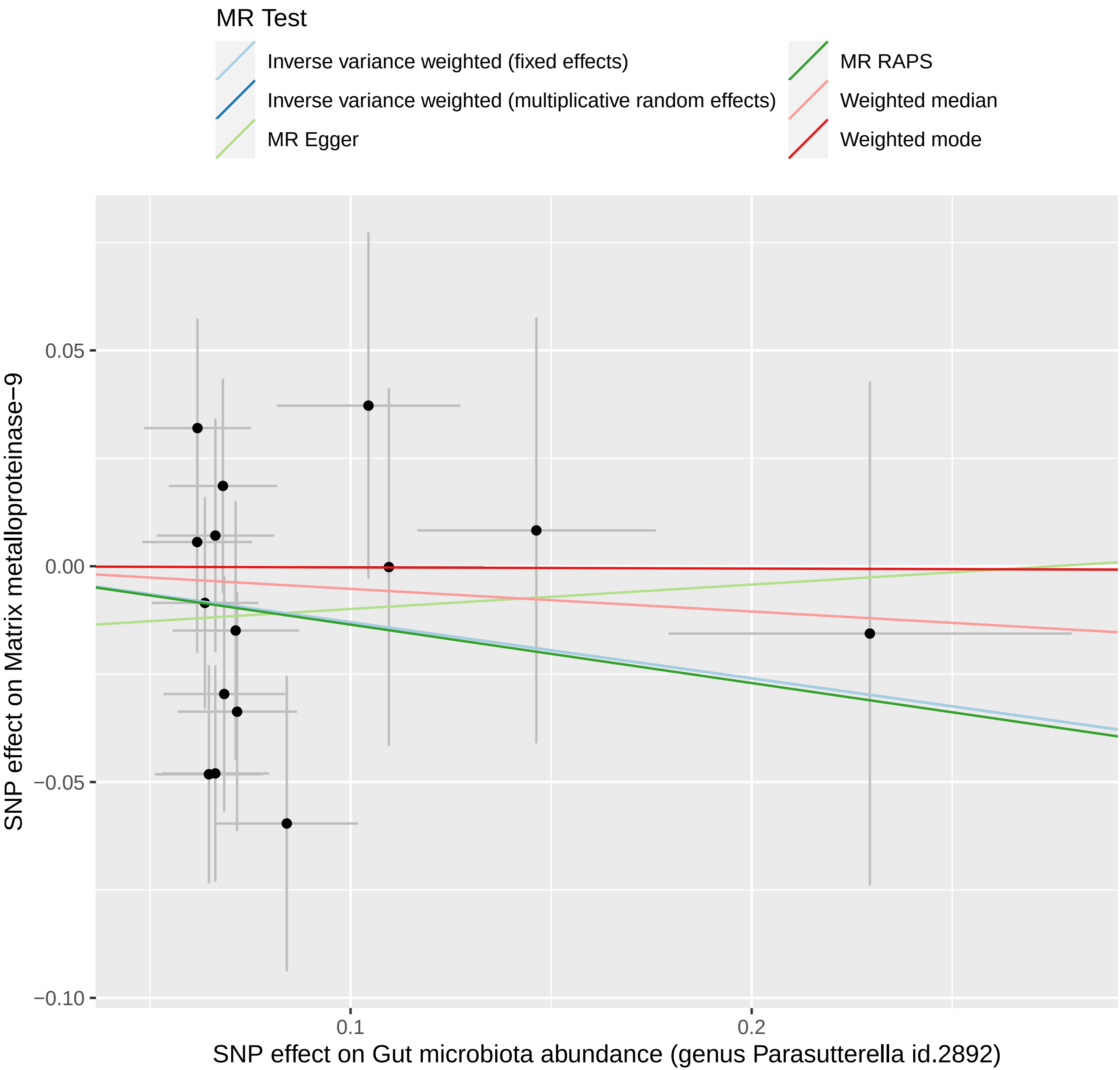

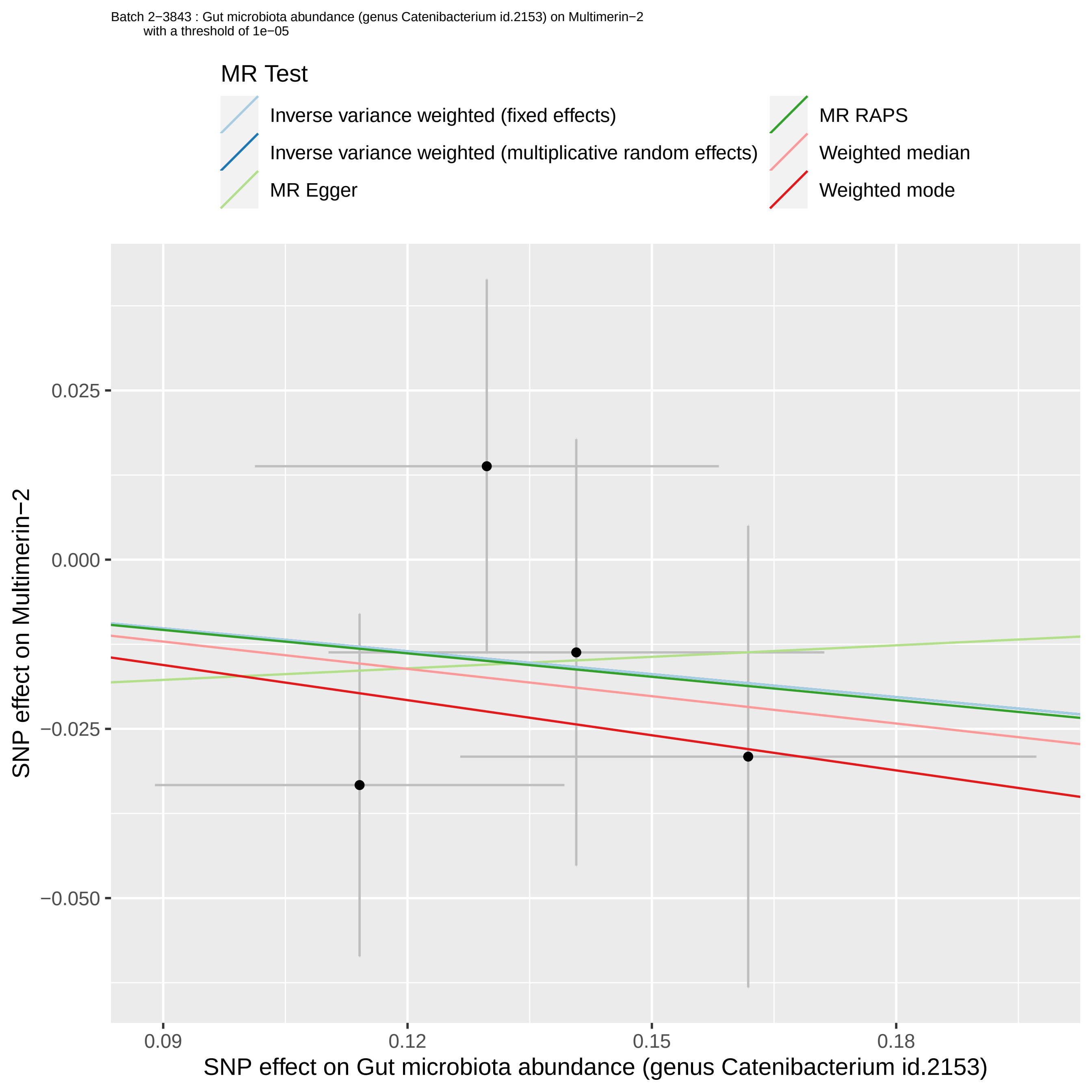

Batch 2−3843 : Gut microbiota abundance (genus Catenibacterium id.2153) on Multimerin−2
with a threshold of 1e−05
MR Test
Inverse variance weighted (fixed effects)
Inverse variance weighted (multiplicative random effects)
MR Egger
MR RAPS
Weighted median
Weighted mode
0.025
0.000
−0.025
−0.050
0.09
0.12
0.15
0.18
SNP effect on Multimerin−2
SNP effect on Gut microbiota abundance (genus Catenibacterium id.2153)

Batch 2−3844 : Gut microbiota abundance (genus Parasutterella id.2892) on Multimerin−2
with a threshold of 1e−05

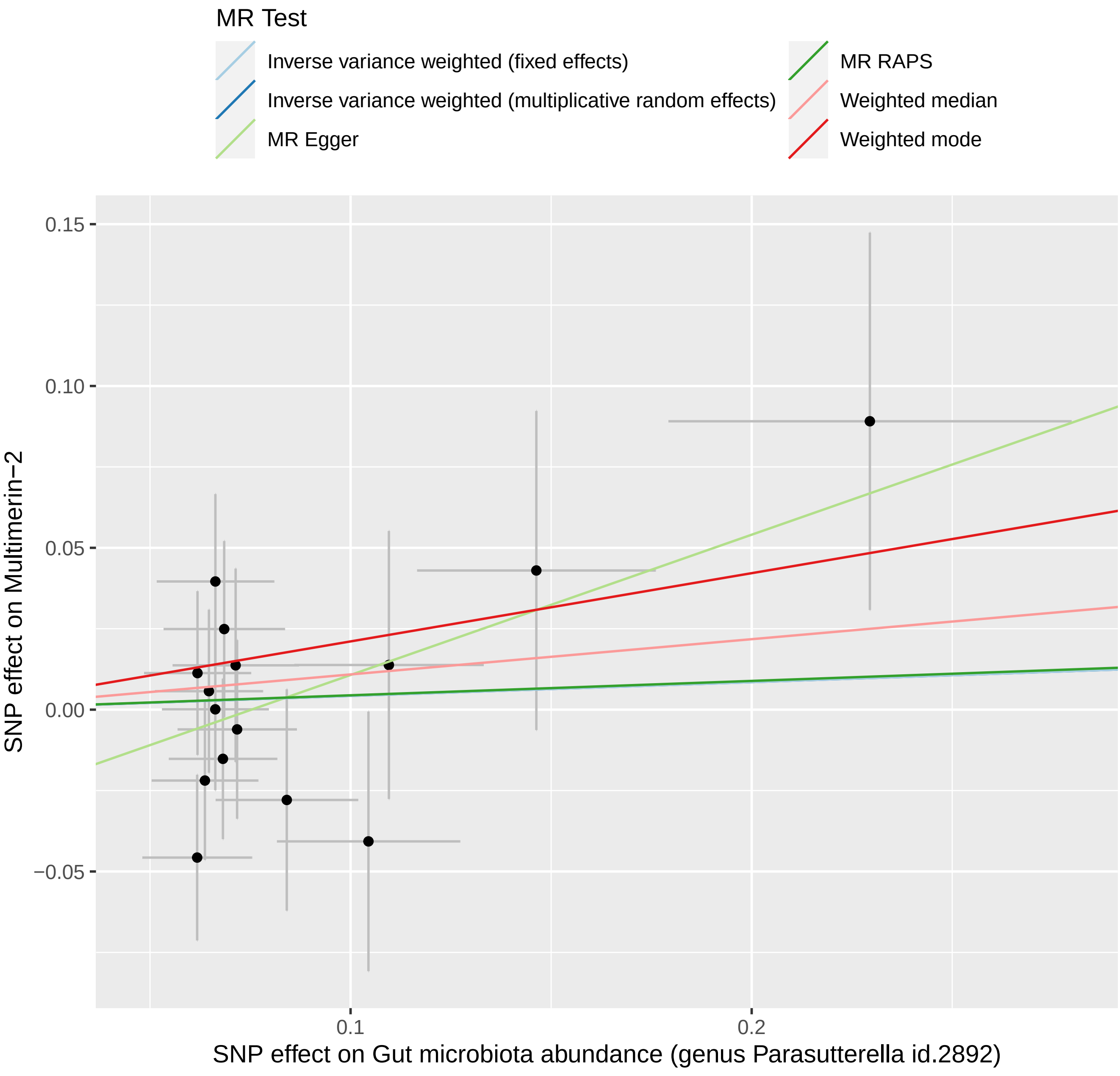

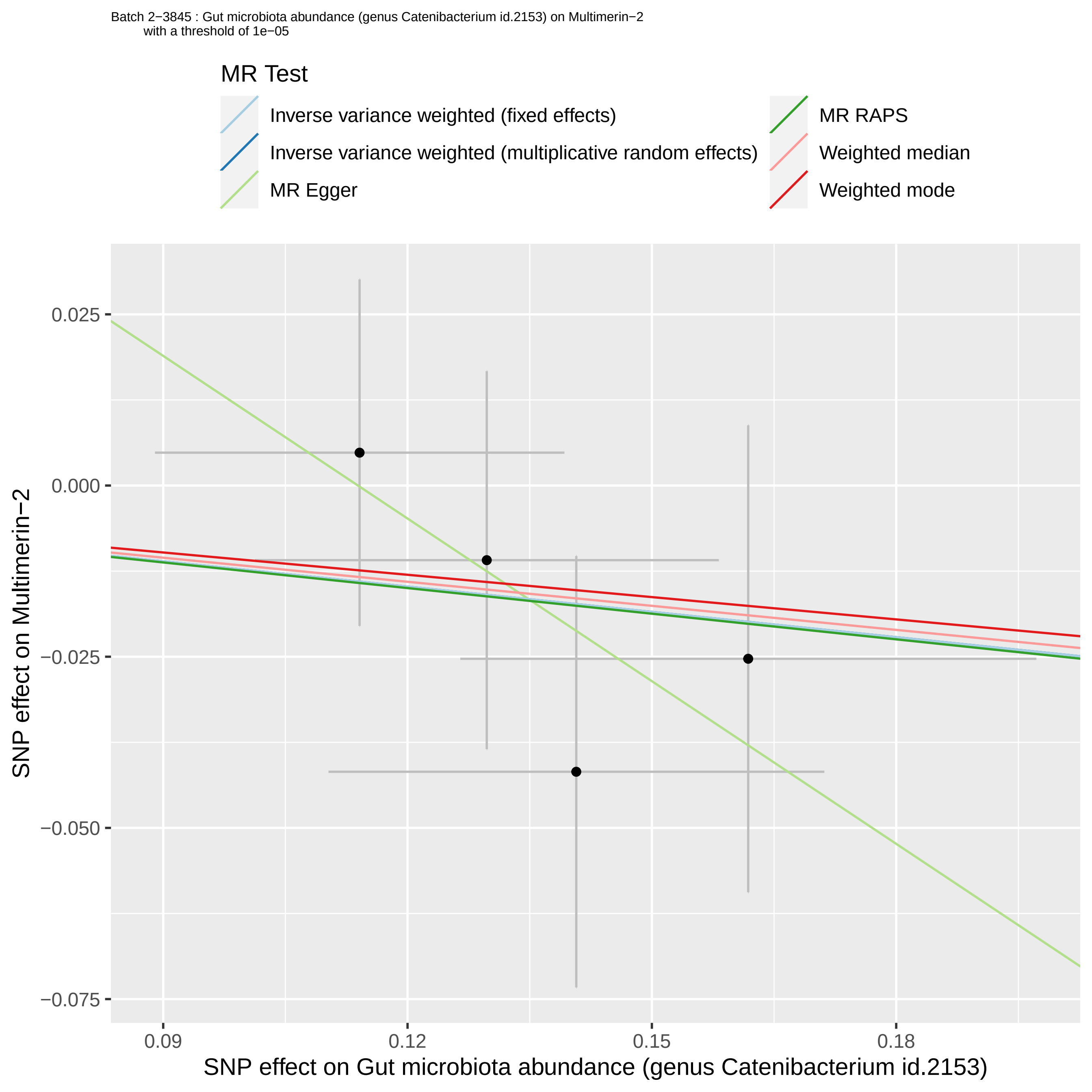

Batch 2−3845 : Gut microbiota abundance (genus Catenibacterium id.2153) on Multimerin−2
with a threshold of 1e−05
MR Test
Inverse variance weighted (fixed effects)
Inverse variance weighted (multiplicative random effects)
MR Egger
MR RAPS
Weighted median
Weighted mode
0.025
0.000
−0.025
−0.050
−0.075
0.09
0.12
0.15
0.18
SNP effect on Multimerin−2
SNP effect on Gut microbiota abundance (genus Catenibacterium id.2153)

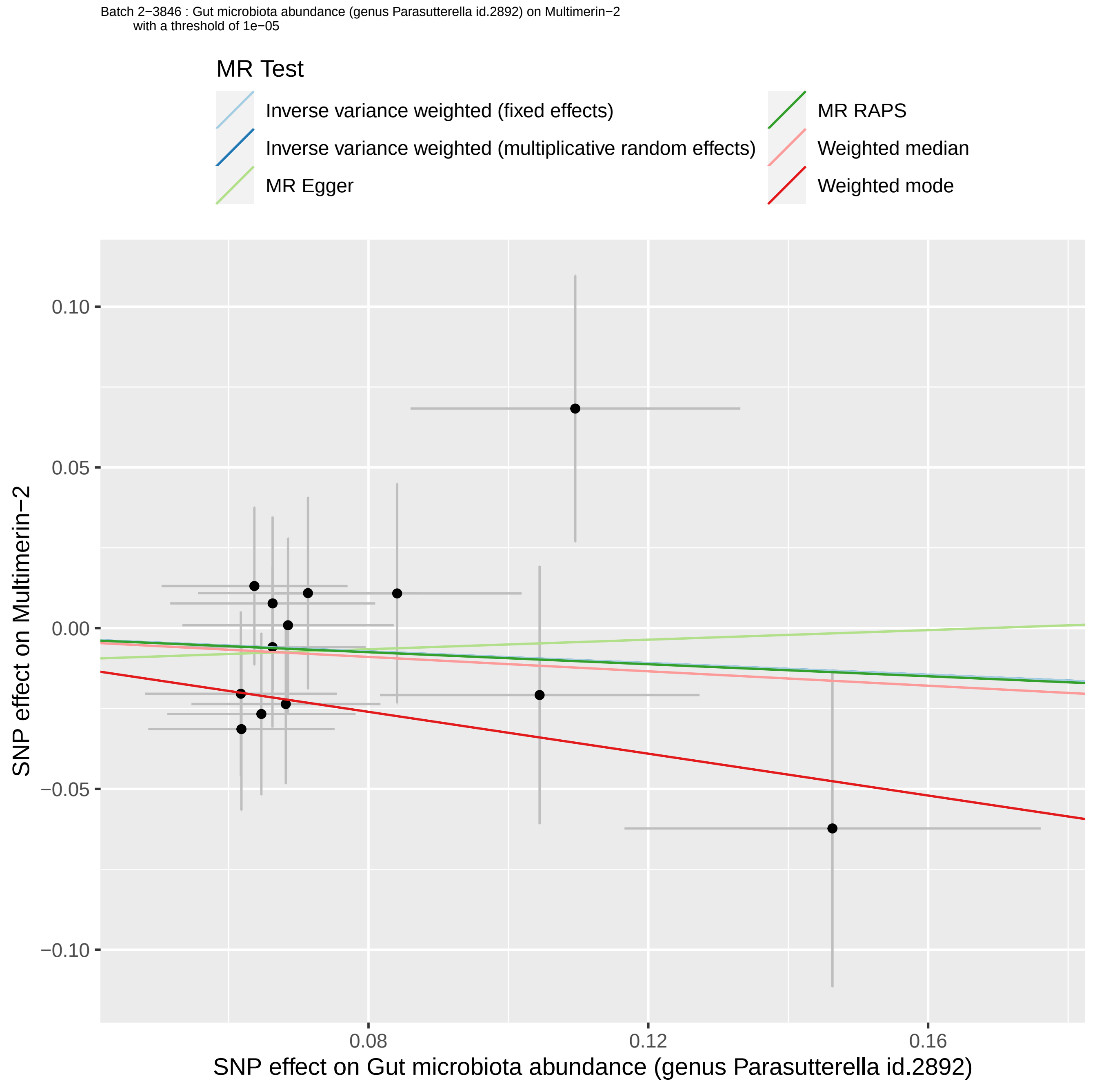
Batch 2−3846 : Gut microbiota abundance (genus Parasutterella id.2892) on Multimerin−2
with a threshold of 1e−05
MR Test
Inverse variance weighted (fixed effects)
Inverse variance weighted (multiplicative random effects)
MR Egger
MR RAPS
Weighted median
Weighted mode
0.10
0.05
0.00
−0.05
−0.10
0.08
0.12
0.16
SNP effect on Multimerin−2
SNP effect on Gut microbiota abundance (genus Parasutterella id.2892)

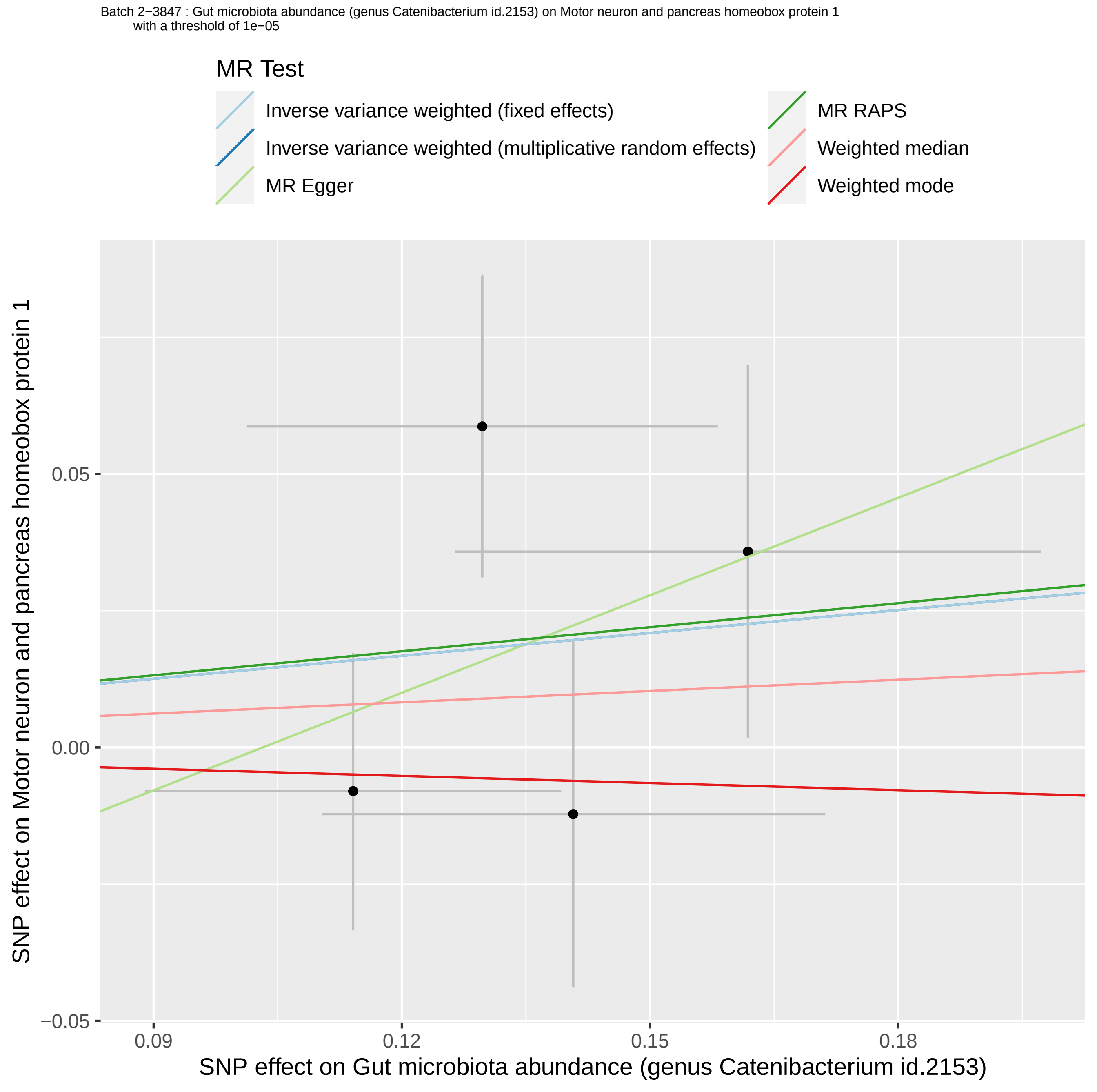

Batch 2−3847 : Gut microbiota abundance (genus Catenibacterium id.2153) on Motor neuron and pancreas homeobox protein 1
with a threshold of 1e−05
MR Test
Inverse variance weighted (fixed effects)
Inverse variance weighted (multiplicative random effects)
MR Egger
MR RAPS
Weighted median
Weighted mode
SNP effect on Motor neuron and pancreas homeobox protein 1
0.05
0.00
−0.05
0.09
0.12
0.15
0.18
SNP effect on Gut microbiota abundance (genus Catenibacterium id.2153)

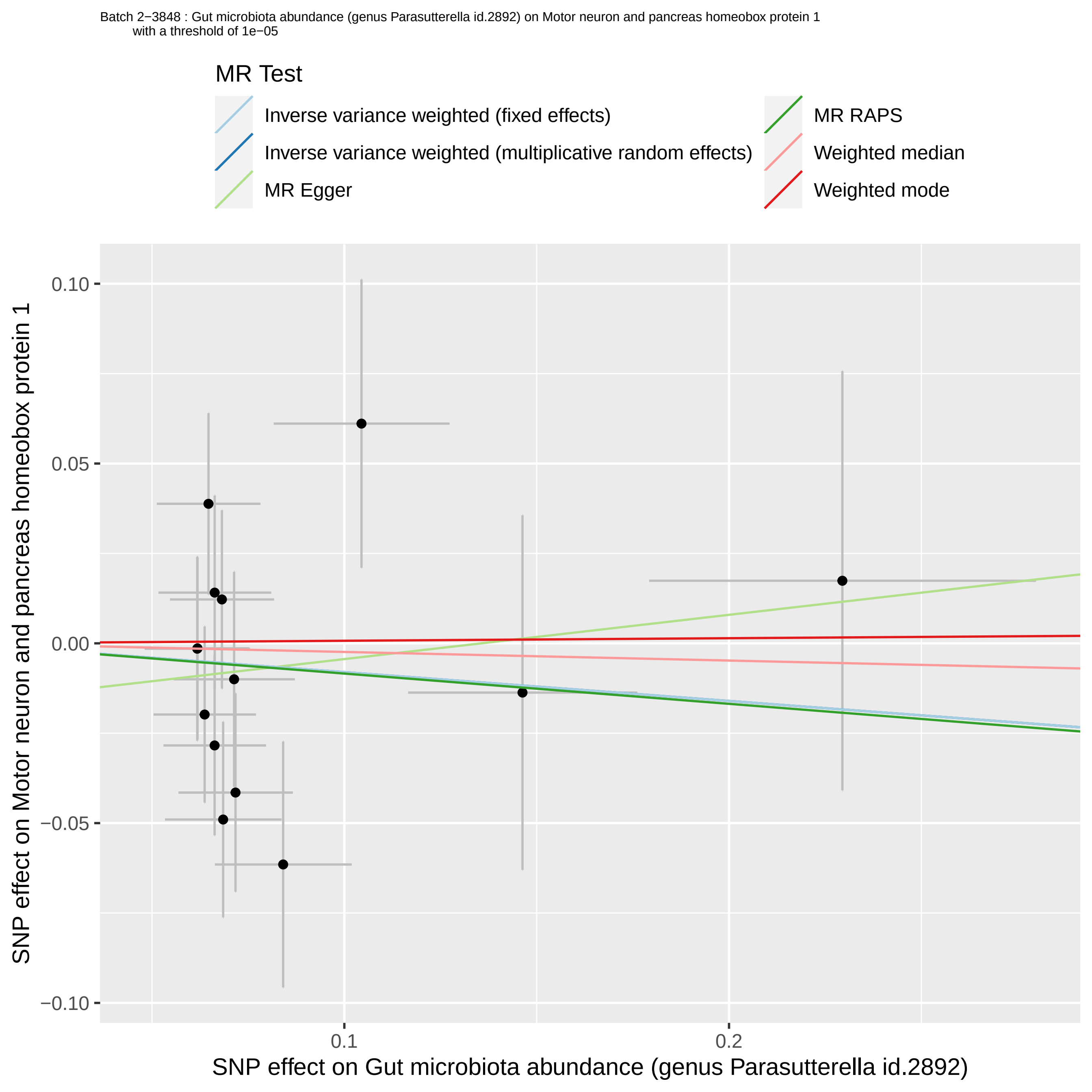

Batch 2−3848 : Gut microbiota abundance (genus Parasutterella id.2892) on Motor neuron and pancreas homeobox protein 1
with a threshold of 1e−05
MR Test
Inverse variance weighted (fixed effects)
Inverse variance weighted (multiplicative random effects)
MR Egger
MR RAPS
Weighted median
Weighted mode
0.10
0.05
0.00
−0.05
−0.10
0.1
0.2
SNP effect on Motor neuron and pancreas homeobox protein 1
SNP effect on Gut microbiota abundance (genus Parasutterella id.2892)

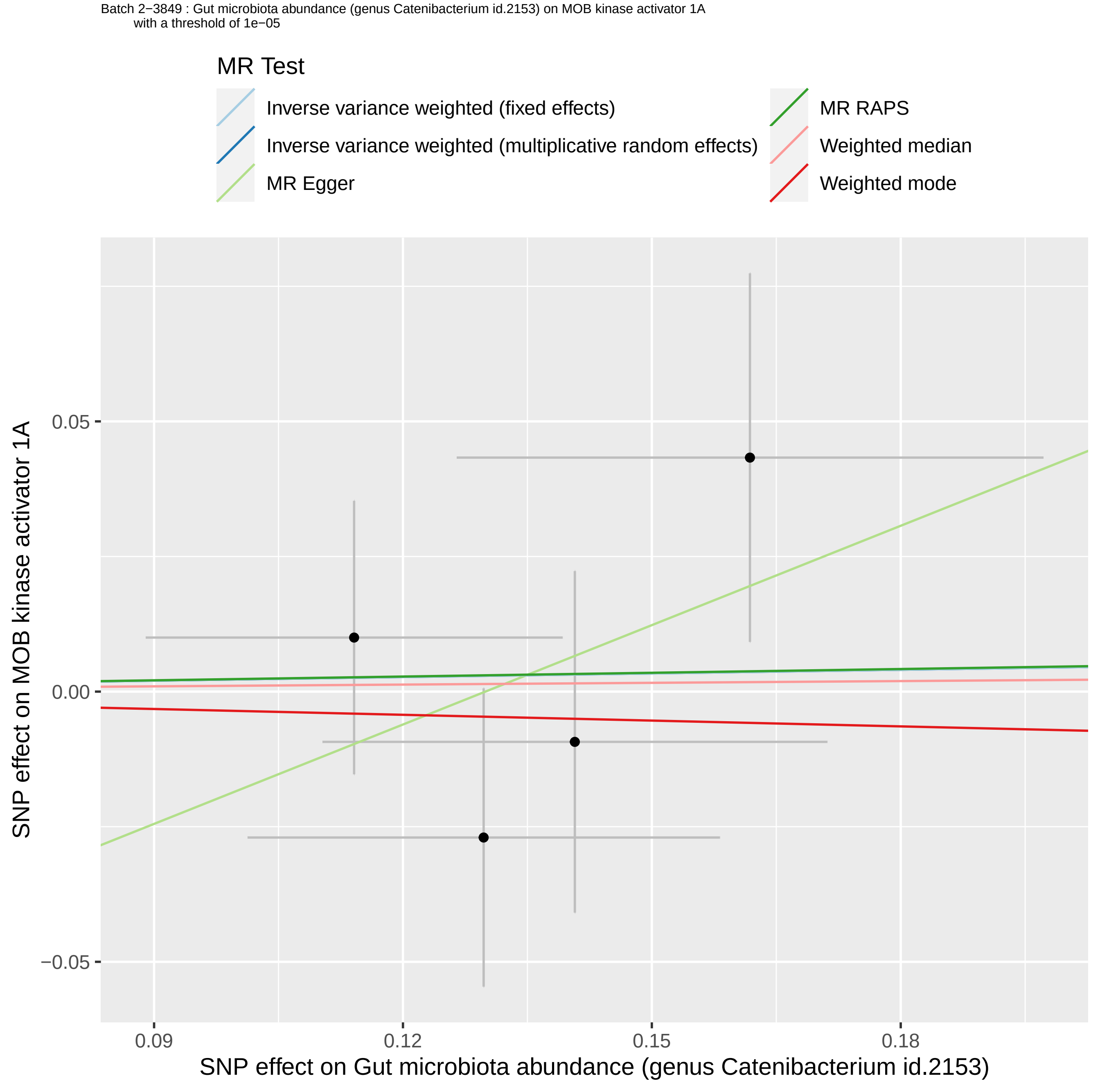

Batch 2−3849 : Gut microbiota abundance (genus Catenibacterium id.2153) on MOB kinase activator 1A
with a threshold of 1e−05
MR Test
Inverse variance weighted (fixed effects)
Inverse variance weighted (multiplicative random effects)
MR Egger
MR RAPS
Weighted median
Weighted mode
0.05
0.00
−0.05
0.09
0.12
0.15
0.18
SNP effect on MOB kinase activator 1A
SNP effect on Gut microbiota abundance (genus Catenibacterium id.2153)

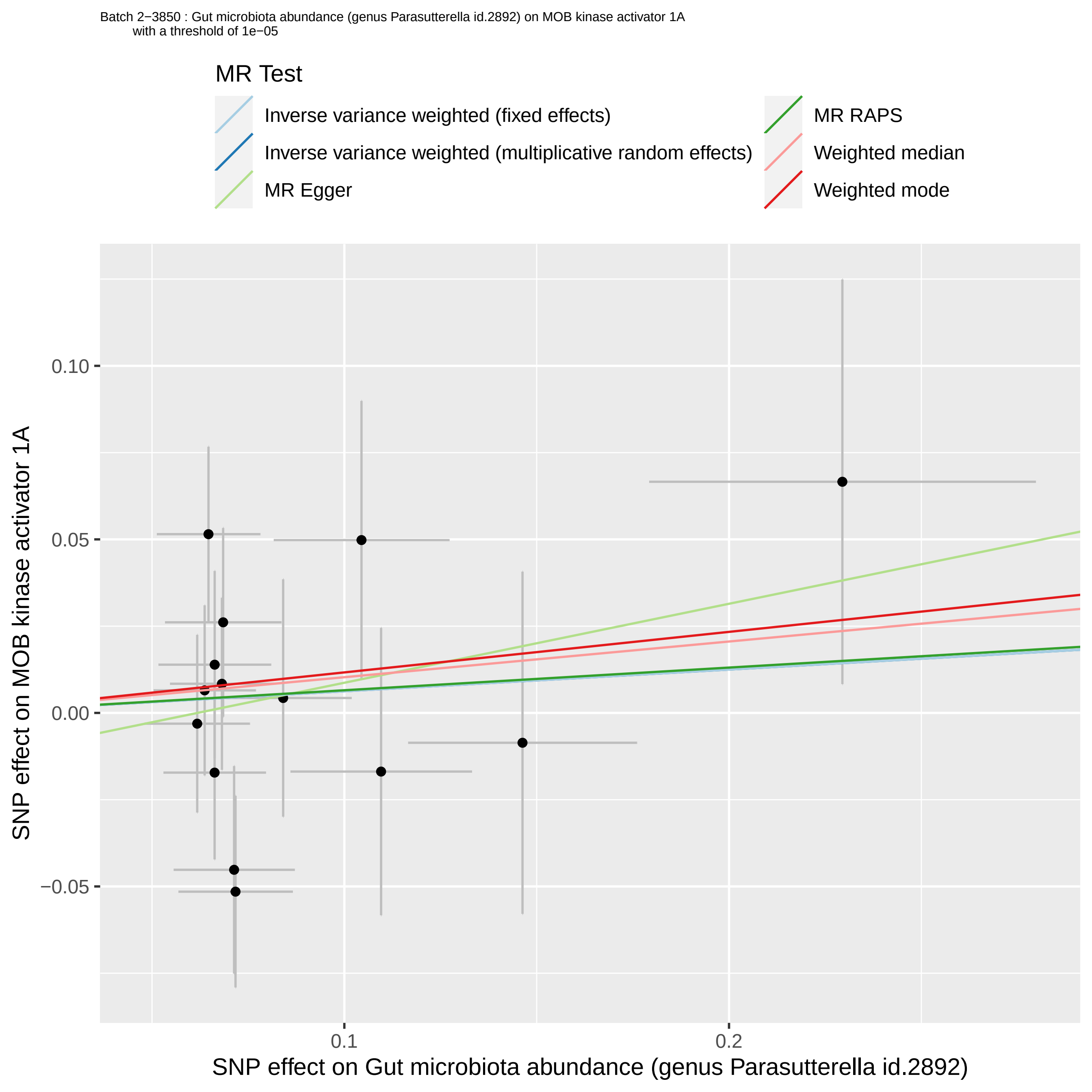
Batch 2−3850 : Gut microbiota abundance (genus Parasutterella id.2892) on MOB kinase activator 1A
with a threshold of 1e−05
MR Test
Inverse variance weighted (fixed effects)
Inverse variance weighted (multiplicative random effects)
MR Egger
MR RAPS
Weighted median
Weighted mode
0.10
0.05
0.00
−0.05
0.1
0.2
SNP effect on MOB kinase activator 1A
SNP effect on Gut microbiota abundance (genus Parasutterella id.2892)

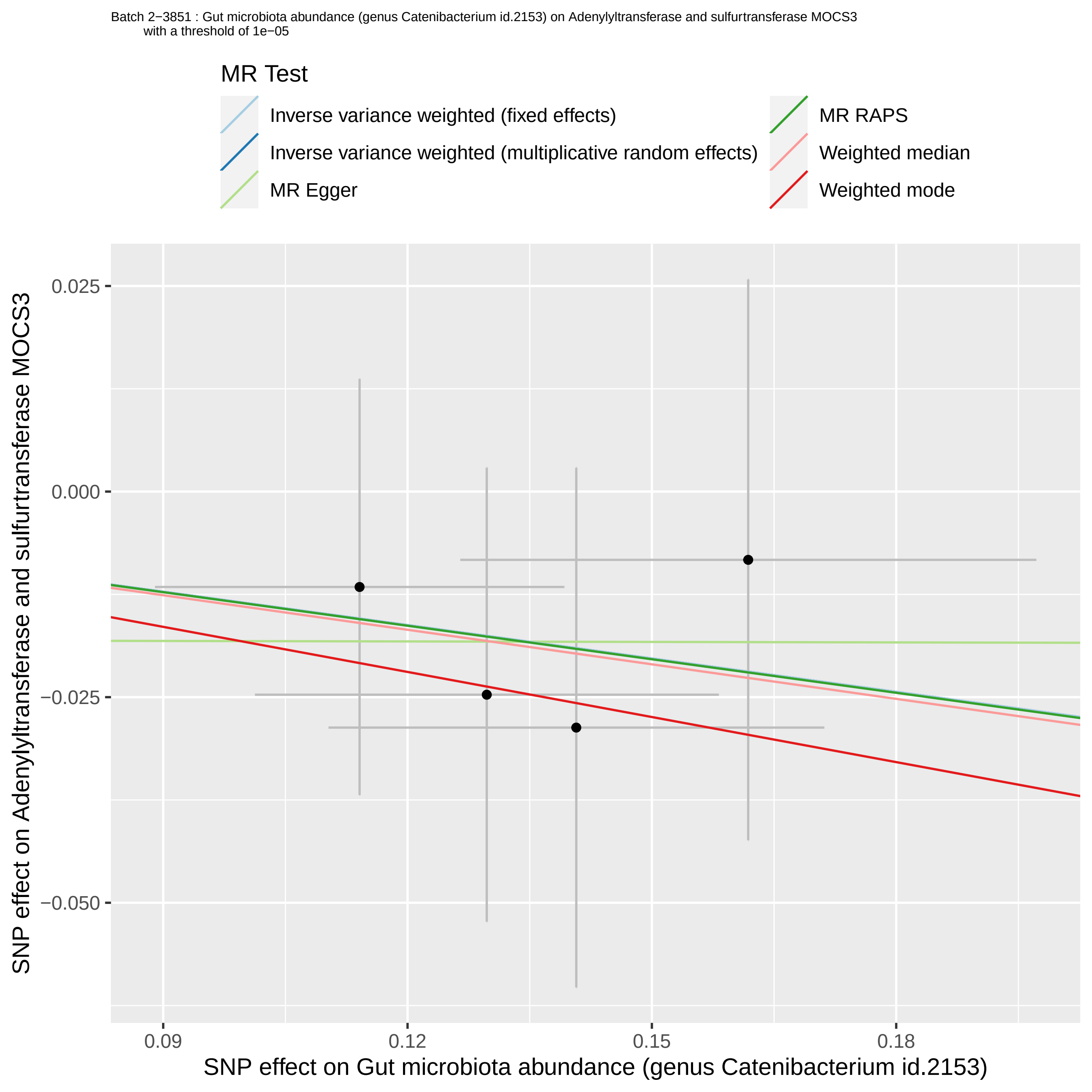

Batch 2−3851 : Gut microbiota abundance (genus Catenibacterium id.2153) on Adenylyltransferase and sulfurtransferase MOCS3
with a threshold of 1e−05
MR Test
Inverse variance weighted (fixed effects)
Inverse variance weighted (multiplicative random effects)
MR Egger
MR RAPS
Weighted median
Weighted mode
0.025
0.000
−0.025
−0.050
0.09
0.12
0.15
0.18
SNP effect on Gut microbiota abundance (genus Catenibacterium id.2153)
SNP effect on Adenylyltransferase and sulfurtransferase MOCS3

Batch 2−3852 : Gut microbiota abundance (genus Parasutterella id.2892) on Adenylyltransferase and sulfurtransferase MOCS3
with a threshold of 1e−05

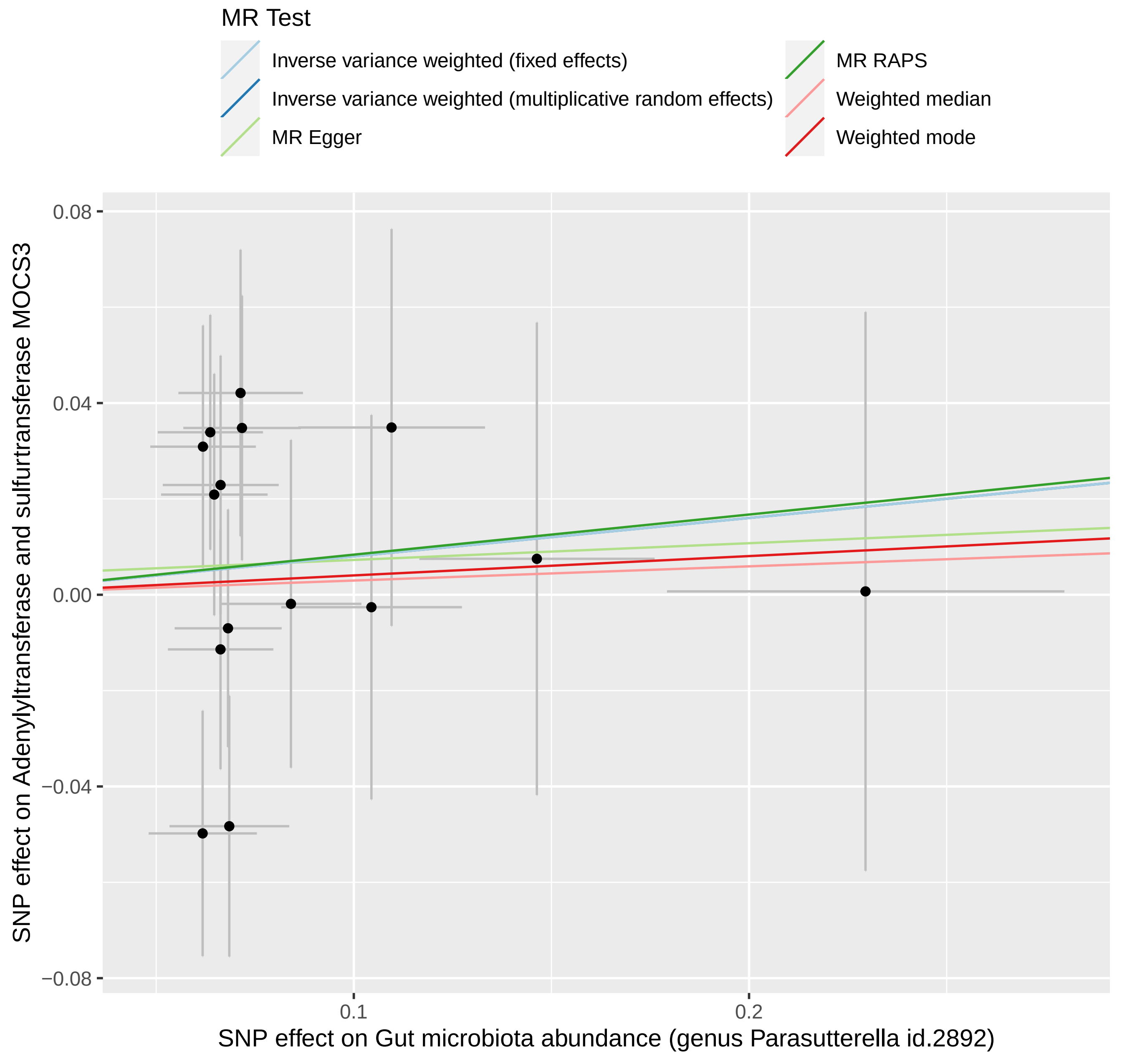

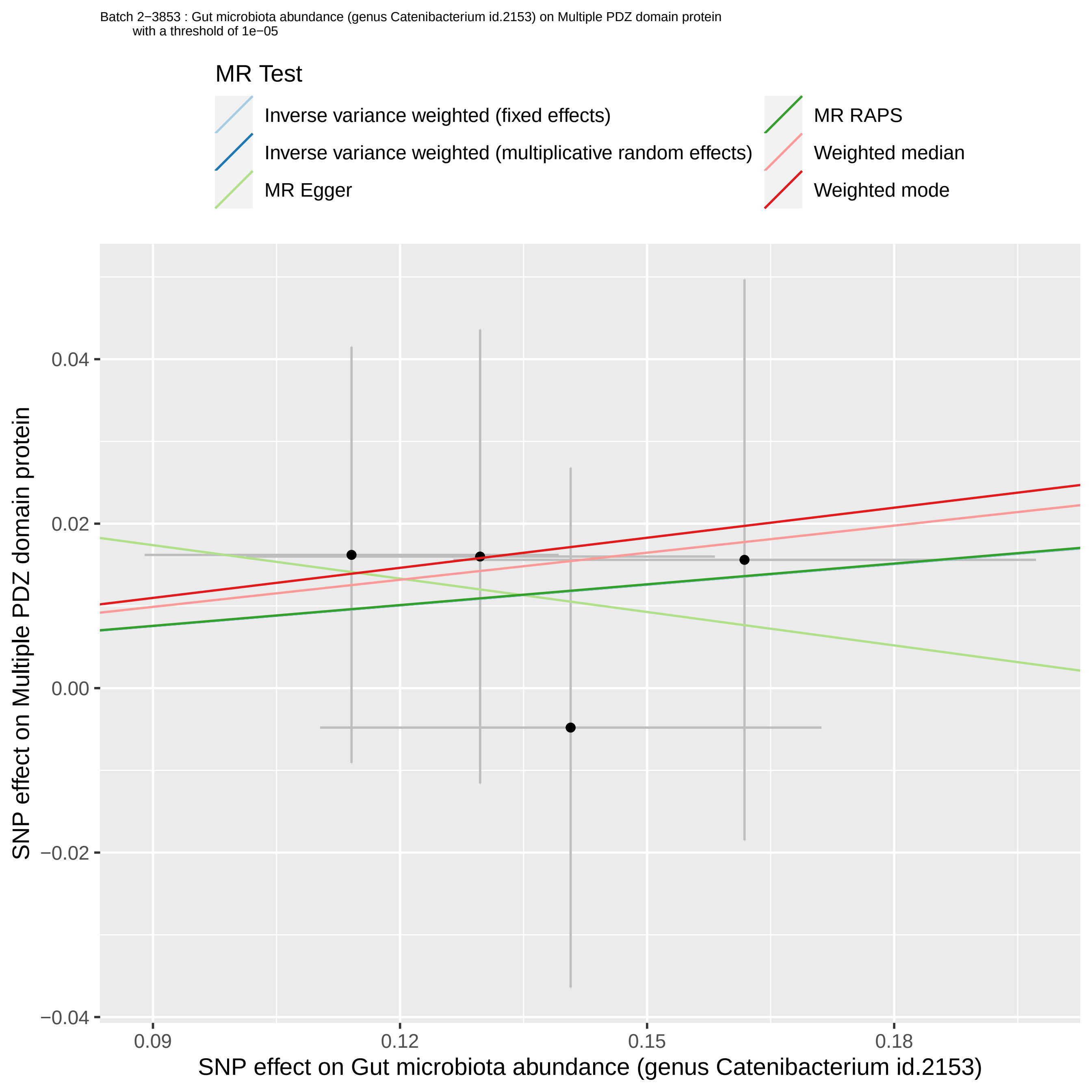
Batch 2−3853 : Gut microbiota abundance (genus Catenibacterium id.2153) on Multiple PDZ domain protein
with a threshold of 1e−05
MR Test
Inverse variance weighted (fixed effects)
Inverse variance weighted (multiplicative random effects)
MR Egger
MR RAPS
Weighted median
Weighted mode
0.04
0.02
0.00
−0.02
−0.04
0.09
0.12
0.15
0.18
SNP effect on Multiple PDZ domain protein
SNP effect on Gut microbiota abundance (genus Catenibacterium id.2153)

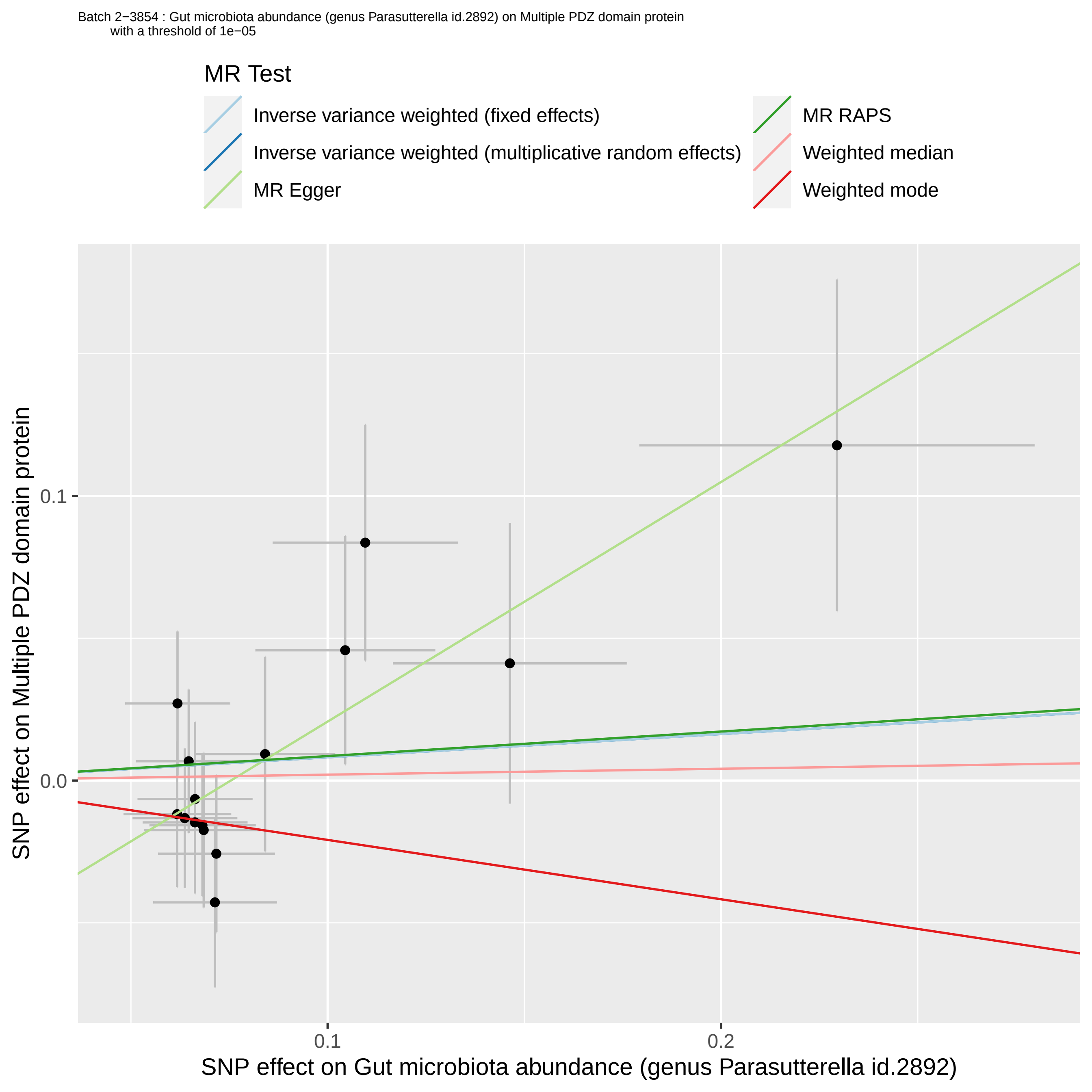
Batch 2−3854 : Gut microbiota abundance (genus Parasutterella id.2892) on Multiple PDZ domain protein
with a threshold of 1e−05
MR Test
Inverse variance weighted (fixed effects)
Inverse variance weighted (multiplicative random effects)
MR Egger
MR RAPS
Weighted median
Weighted mode
0.1
0.0
0.1
0.2
SNP effect on Multiple PDZ domain protein
SNP effect on Gut microbiota abundance (genus Parasutterella id.2892)

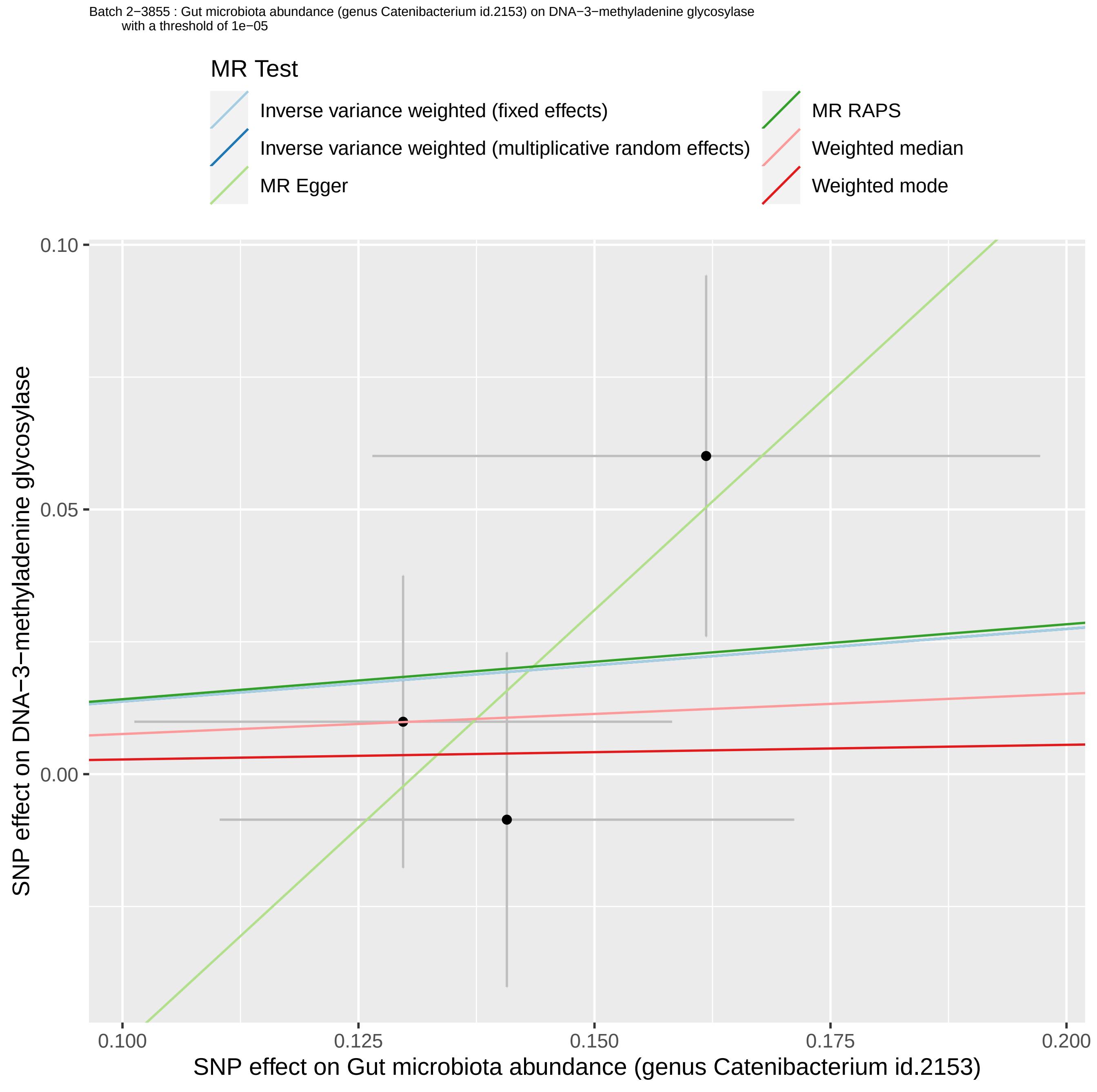
Batch 2−3855 : Gut microbiota abundance (genus Catenibacterium id.2153) on DNA−3−methyladenine glycosylase
with a threshold of 1e−05
MR Test
Inverse variance weighted (fixed effects)
Inverse variance weighted (multiplicative random effects)
MR Egger
MR RAPS
Weighted median
Weighted mode
0.10
0.05
0.00
0.100
0.125
0.150
0.175
0.200
SNP effect on DNA−3−methyladenine glycosylase
SNP effect on Gut microbiota abundance (genus Catenibacterium id.2153)

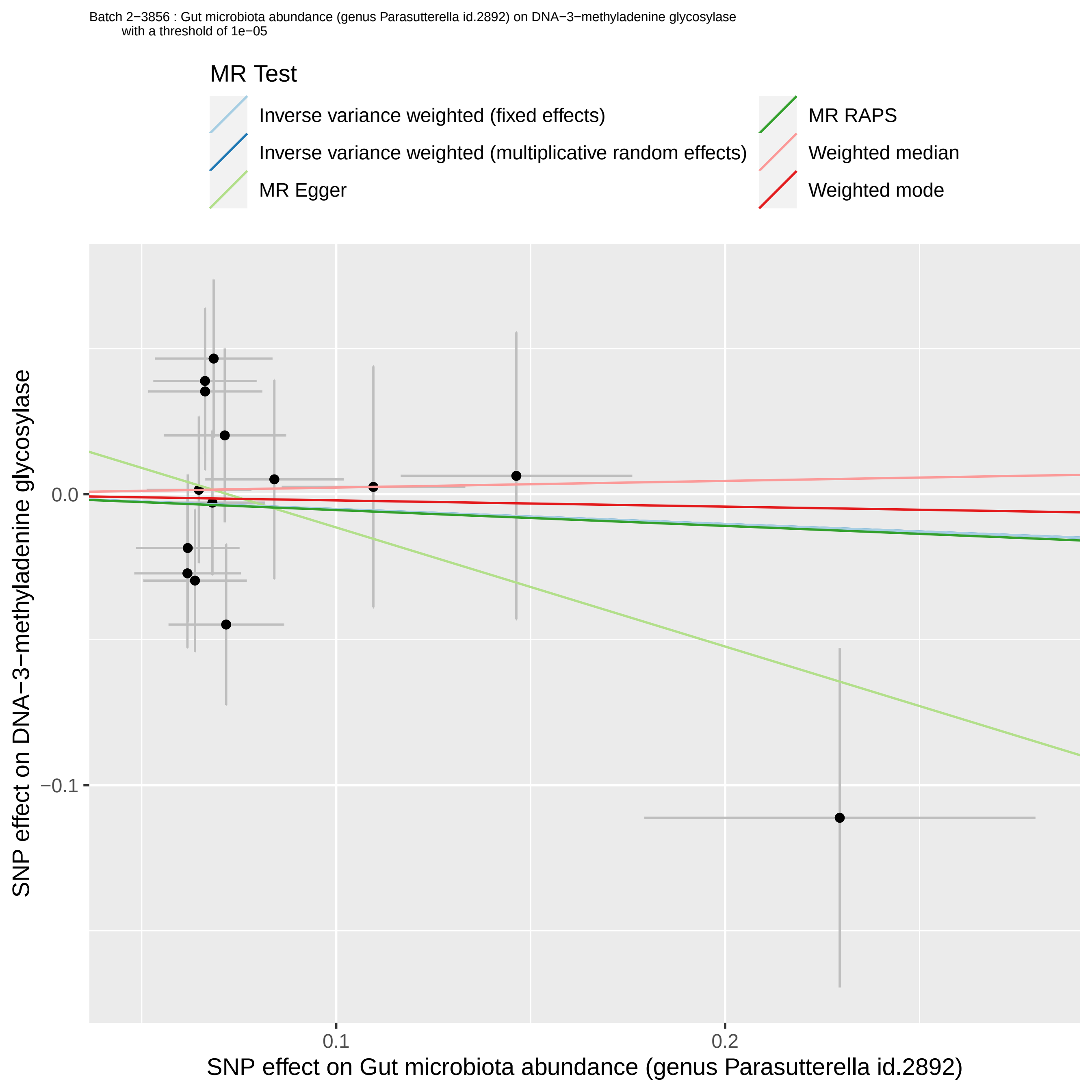

Batch 2−3856 : Gut microbiota abundance (genus Parasutterella id.2892) on DNA−3−methyladenine glycosylase
with a threshold of 1e−05
MR Test
Inverse variance weighted (fixed effects)
Inverse variance weighted (multiplicative random effects)
MR Egger
MR RAPS
Weighted median
Weighted mode
0.0
−0.1
0.1
0.2
SNP effect on DNA−3−methyladenine glycosylase
SNP effect on Gut microbiota abundance (genus Parasutterella id.2892)

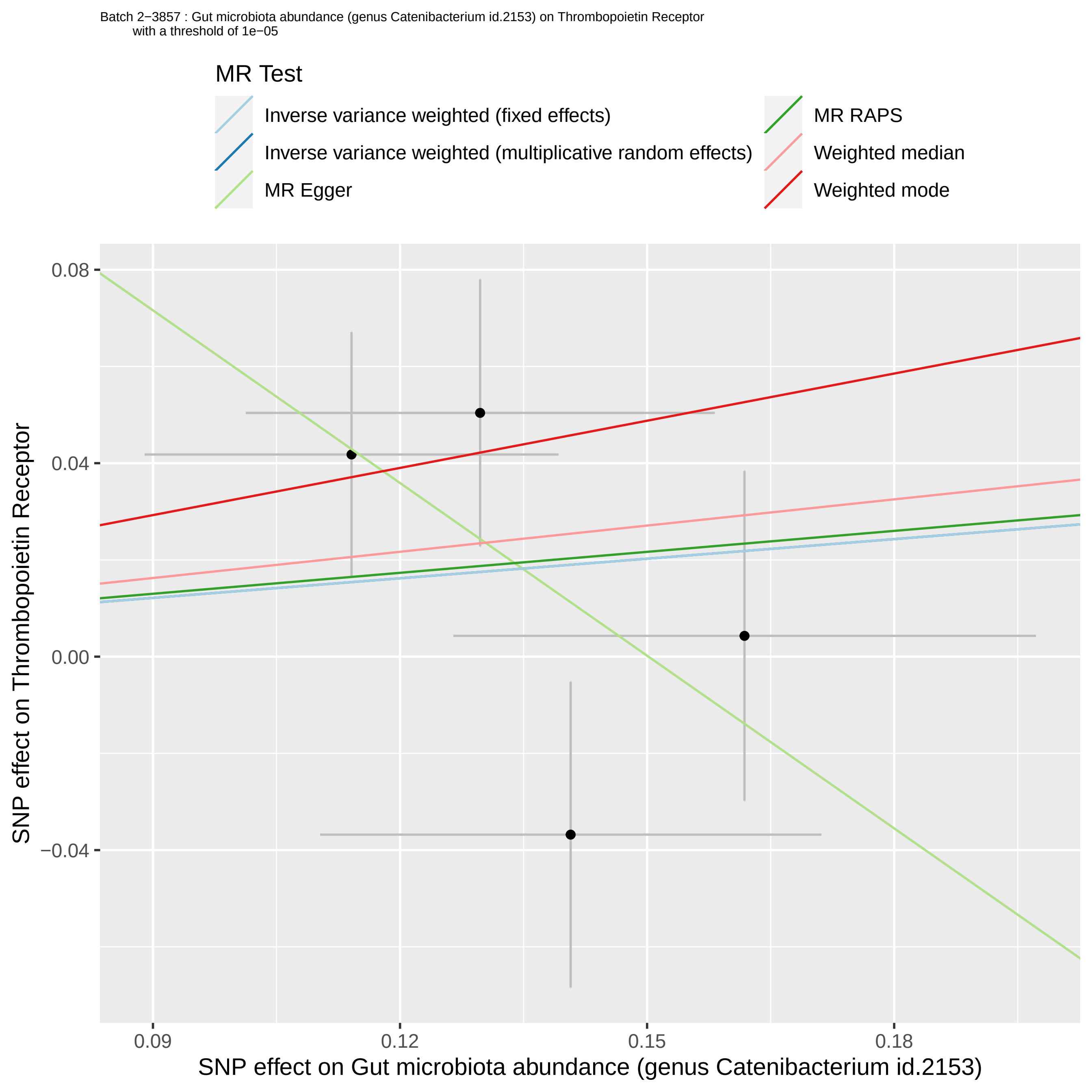
Batch 2−3857 : Gut microbiota abundance (genus Catenibacterium id.2153) on Thrombopoietin Receptor
with a threshold of 1e−05
MR Test
Inverse variance weighted (fixed effects)
Inverse variance weighted (multiplicative random effects)
MR Egger
MR RAPS
Weighted median
Weighted mode
0.08
0.04
0.00
−0.04
0.09
0.12
0.15
0.18
SNP effect on Thrombopoietin Receptor
SNP effect on Gut microbiota abundance (genus Catenibacterium id.2153)

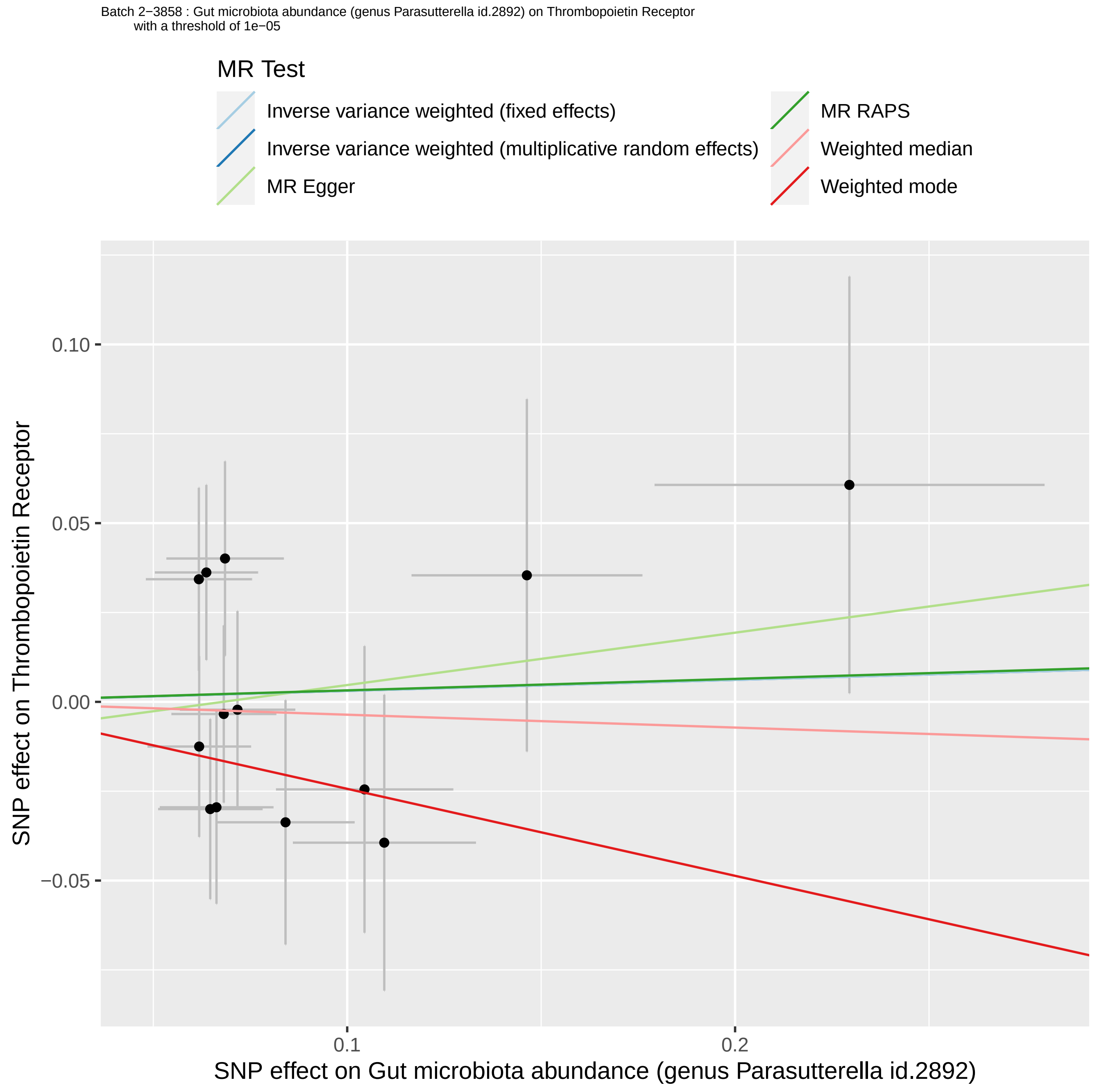

Batch 2−3858 : Gut microbiota abundance (genus Parasutterella id.2892) on Thrombopoietin Receptor
with a threshold of 1e−05
MR Test
Inverse variance weighted (fixed effects)
Inverse variance weighted (multiplicative random effects)
MR Egger
MR RAPS
Weighted median
Weighted mode
0.10
0.05
0.00
−0.05
0.1
0.2
SNP effect on Thrombopoietin Receptor
SNP effect on Gut microbiota abundance (genus Parasutterella id.2892)

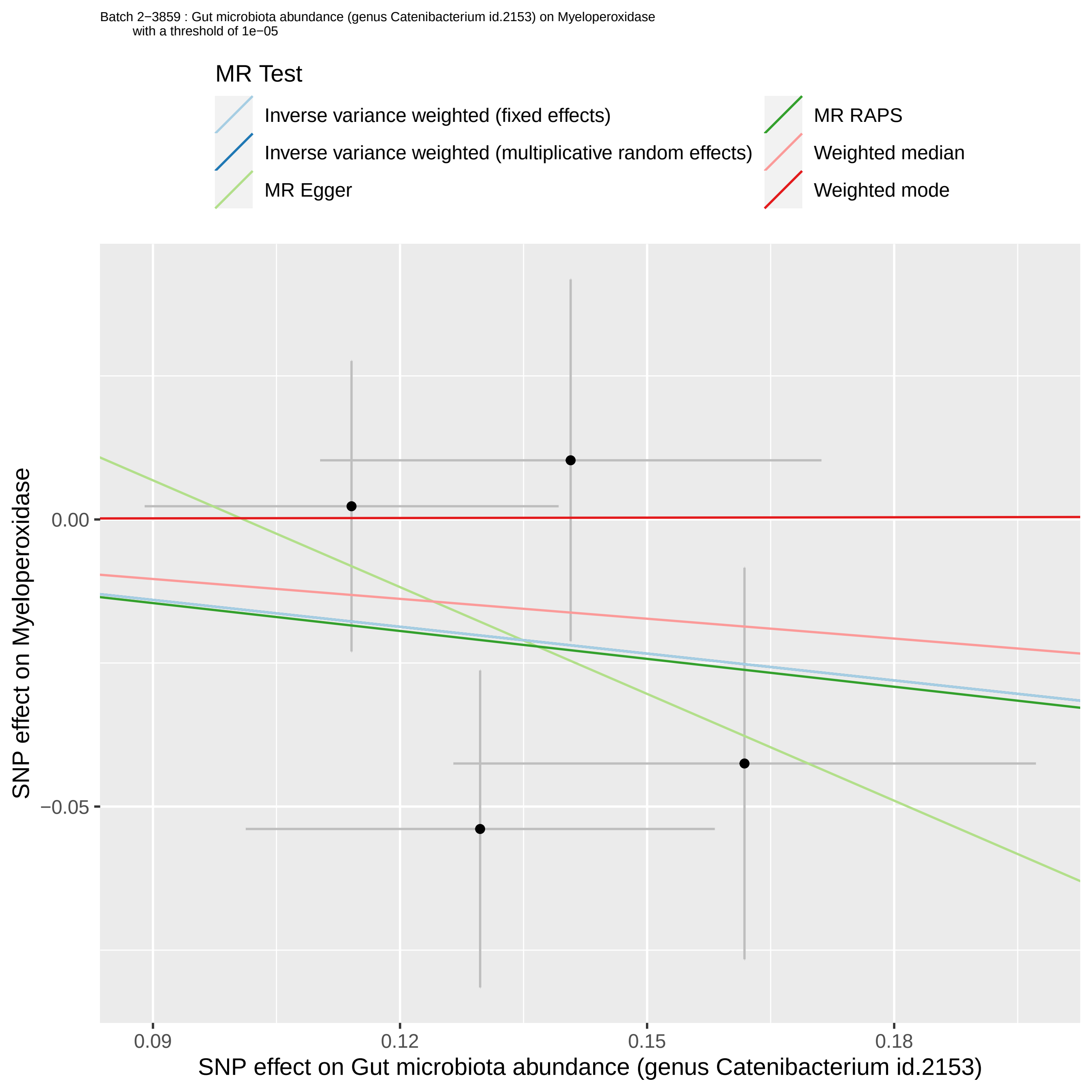
Batch 2−3859 : Gut microbiota abundance (genus Catenibacterium id.2153) on Myeloperoxidase
with a threshold of 1e−05
MR Test
Inverse variance weighted (fixed effects)
Inverse variance weighted (multiplicative random effects)
MR Egger
MR RAPS
Weighted median
Weighted mode
0.00
−0.05
0.09
0.12
0.15
0.18
SNP effect on Myeloperoxidase
SNP effect on Gut microbiota abundance (genus Catenibacterium id.2153)

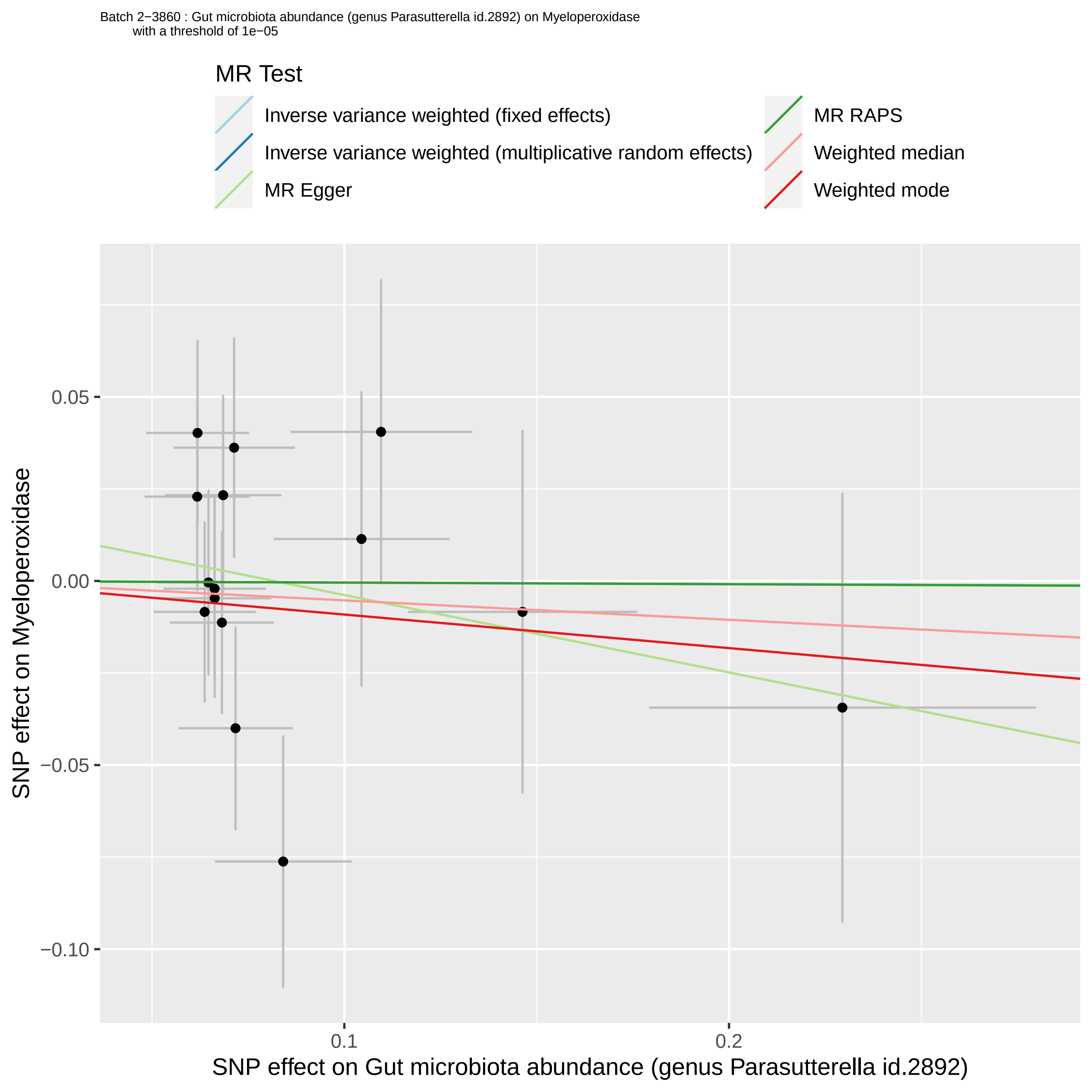
Batch 2−3860 : Gut microbiota abundance (genus Parasutterella id.2892) on Myeloperoxidase
with a threshold of 1e−05
MR Test
Inverse variance weighted (fixed effects)
Inverse variance weighted (multiplicative random effects)
MR Egger
MR RAPS
Weighted median
Weighted mode
0.05
0.00
−0.05
−0.10
0.1
0.2
SNP effect on Myeloperoxidase
SNP effect on Gut microbiota abundance (genus Parasutterella id.2892)

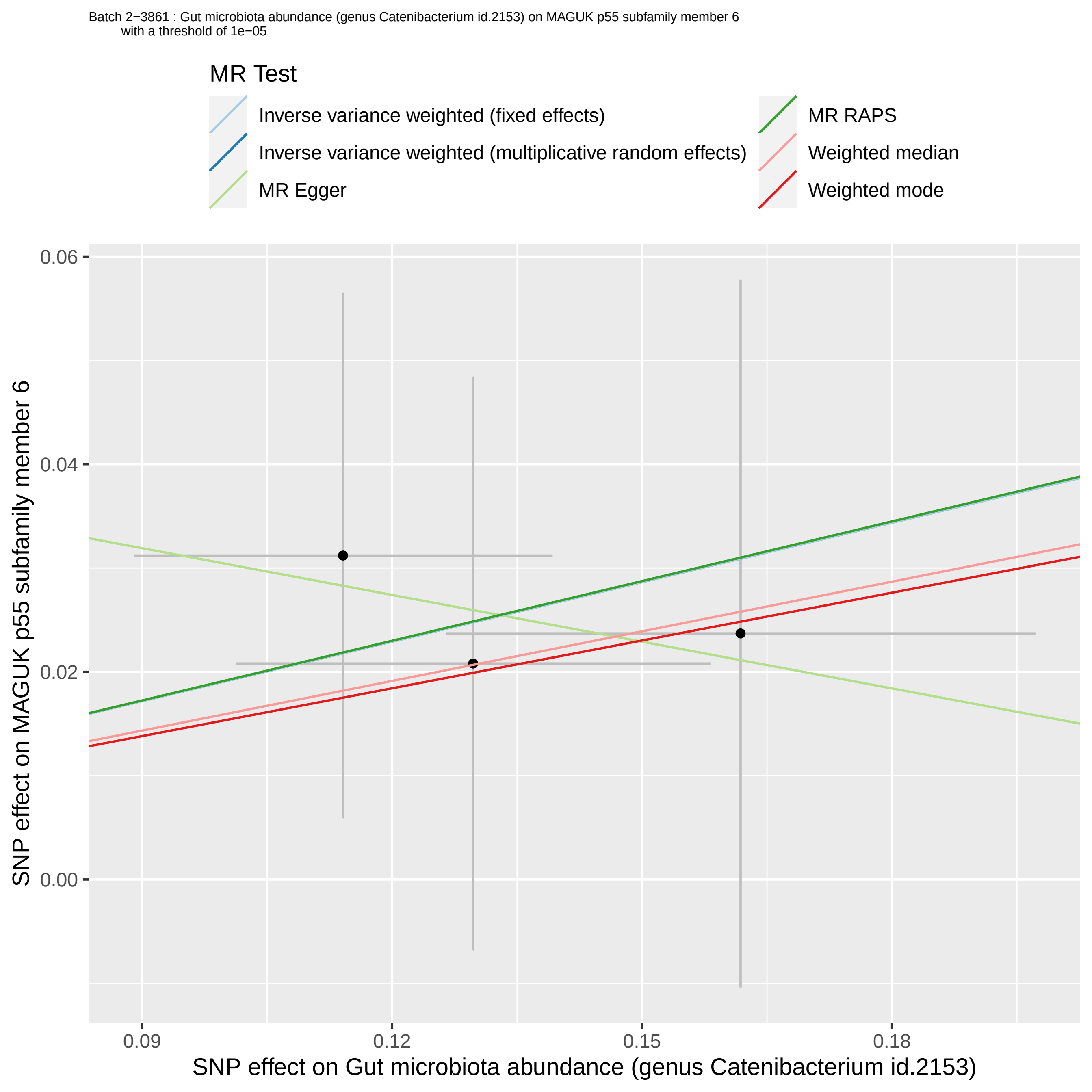

Batch 2−3861 : Gut microbiota abundance (genus Catenibacterium id.2153) on MAGUK p55 subfamily member 6
with a threshold of 1e−05
MR Test
Inverse variance weighted (fixed effects)
Inverse variance weighted (multiplicative random effects)
MR Egger
MR RAPS
Weighted median
Weighted mode
0.06
0.04
0.02
0.00
0.09
0.12
0.15
0.18
SNP effect on MAGUK p55 subfamily member 6
SNP effect on Gut microbiota abundance (genus Catenibacterium id.2153)

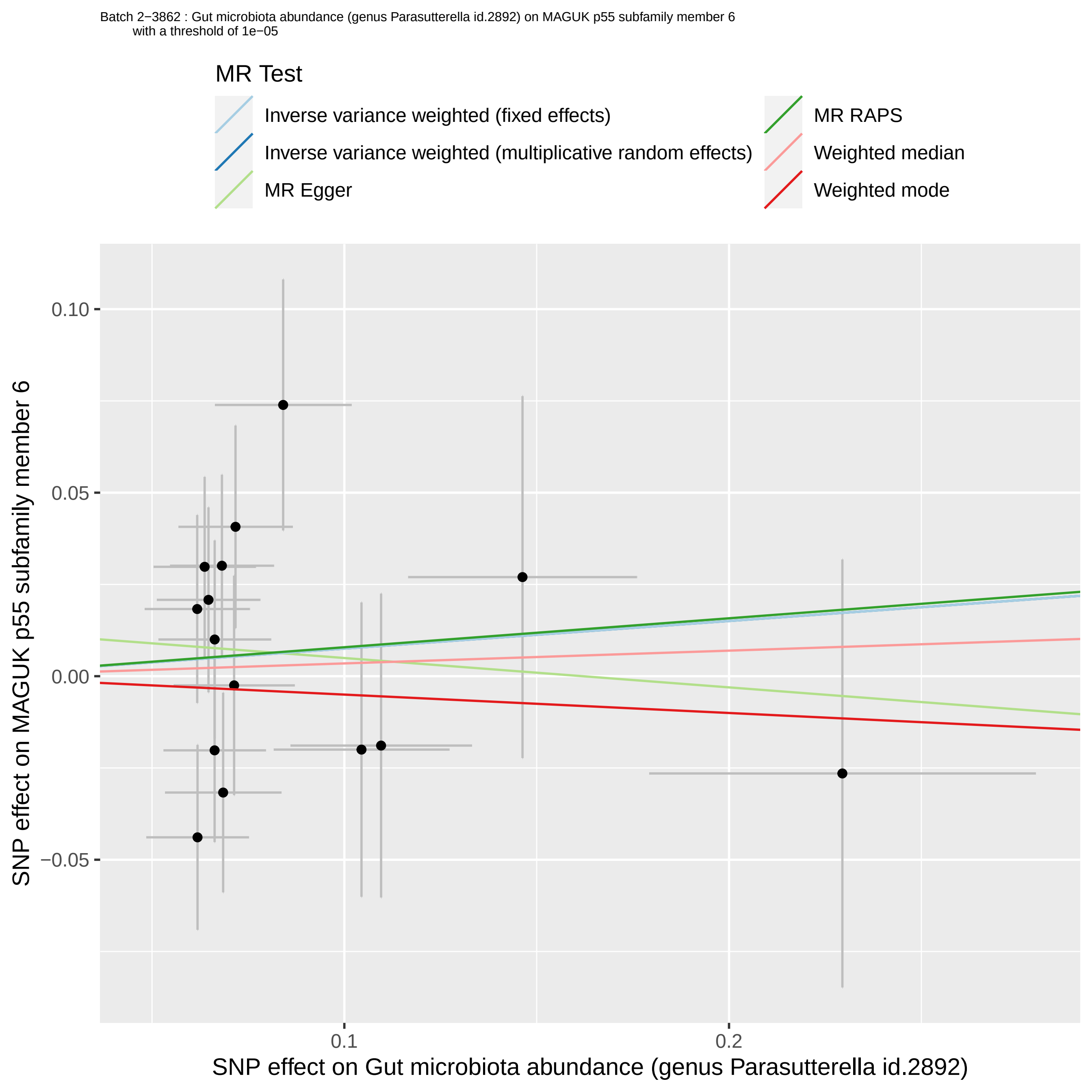

Batch 2−3862 : Gut microbiota abundance (genus Parasutterella id.2892) on MAGUK p55 subfamily member 6
with a threshold of 1e−05
MR Test
Inverse variance weighted (fixed effects)
Inverse variance weighted (multiplicative random effects)
MR Egger
MR RAPS
Weighted median
Weighted mode
0.10
0.05
0.00
−0.05
0.1
0.2
SNP effect on MAGUK p55 subfamily member 6
SNP effect on Gut microbiota abundance (genus Parasutterella id.2892)

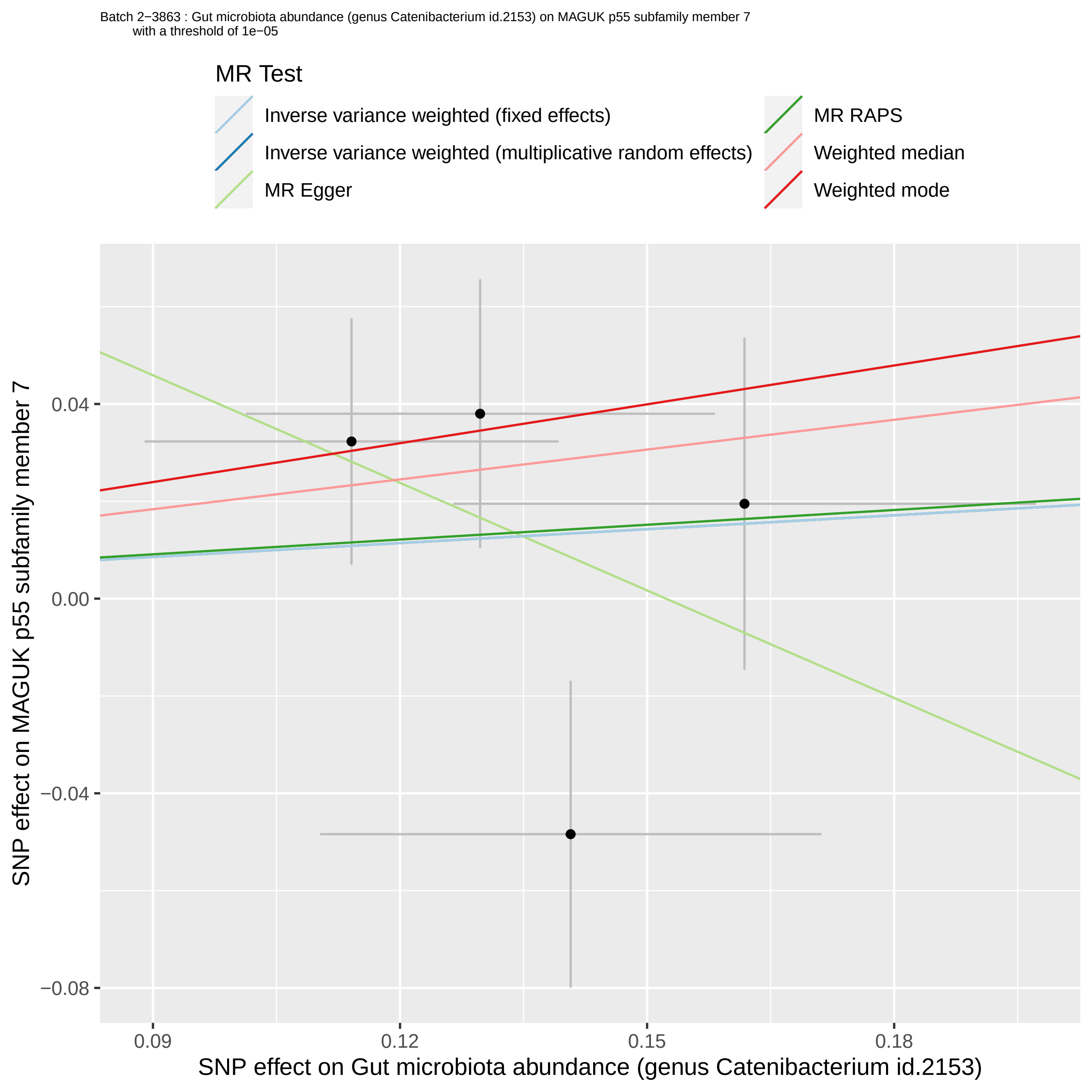
Batch 2−3863 : Gut microbiota abundance (genus Catenibacterium id.2153) on MAGUK p55 subfamily member 7
with a threshold of 1e−05
MR Test
Inverse variance weighted (fixed effects)
Inverse variance weighted (multiplicative random effects)
MR Egger
MR RAPS
Weighted median
Weighted mode
SNP effect on MAGUK p55 subfamily member 7
0.04
0.00
−0.04
−0.08
0.09
0.12
0.15
0.18
SNP effect on Gut microbiota abundance (genus Catenibacterium id.2153)

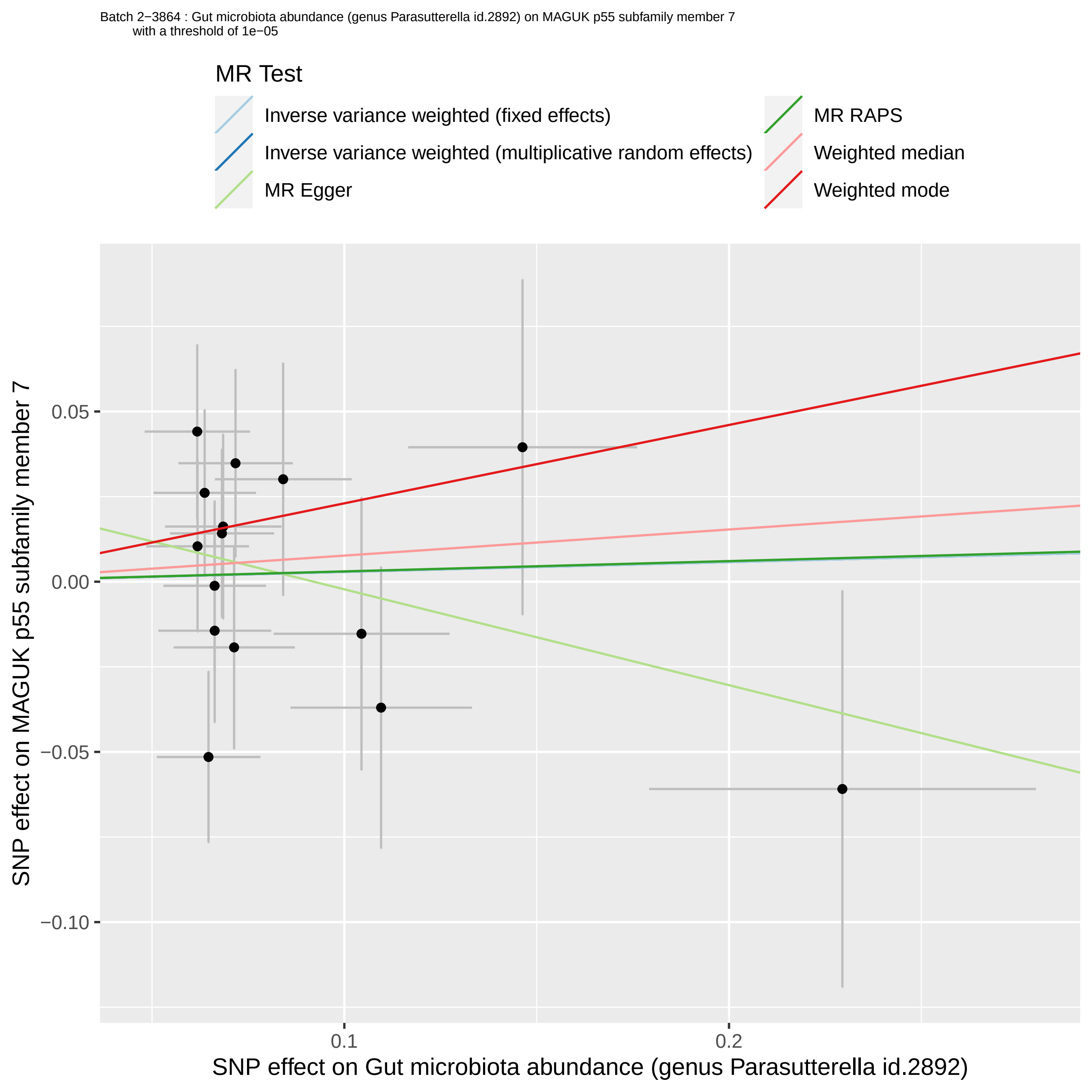
Batch 2−3864 : Gut microbiota abundance (genus Parasutterella id.2892) on MAGUK p55 subfamily member 7
with a threshold of 1e−05
MR Test
Inverse variance weighted (fixed effects)
Inverse variance weighted (multiplicative random effects)
MR Egger
MR RAPS
Weighted median
Weighted mode
SNP effect on MAGUK p55 subfamily member 7
0.05
0.00
−0.05
−0.10
0.1
0.2
SNP effect on Gut microbiota abundance (genus Parasutterella id.2892)

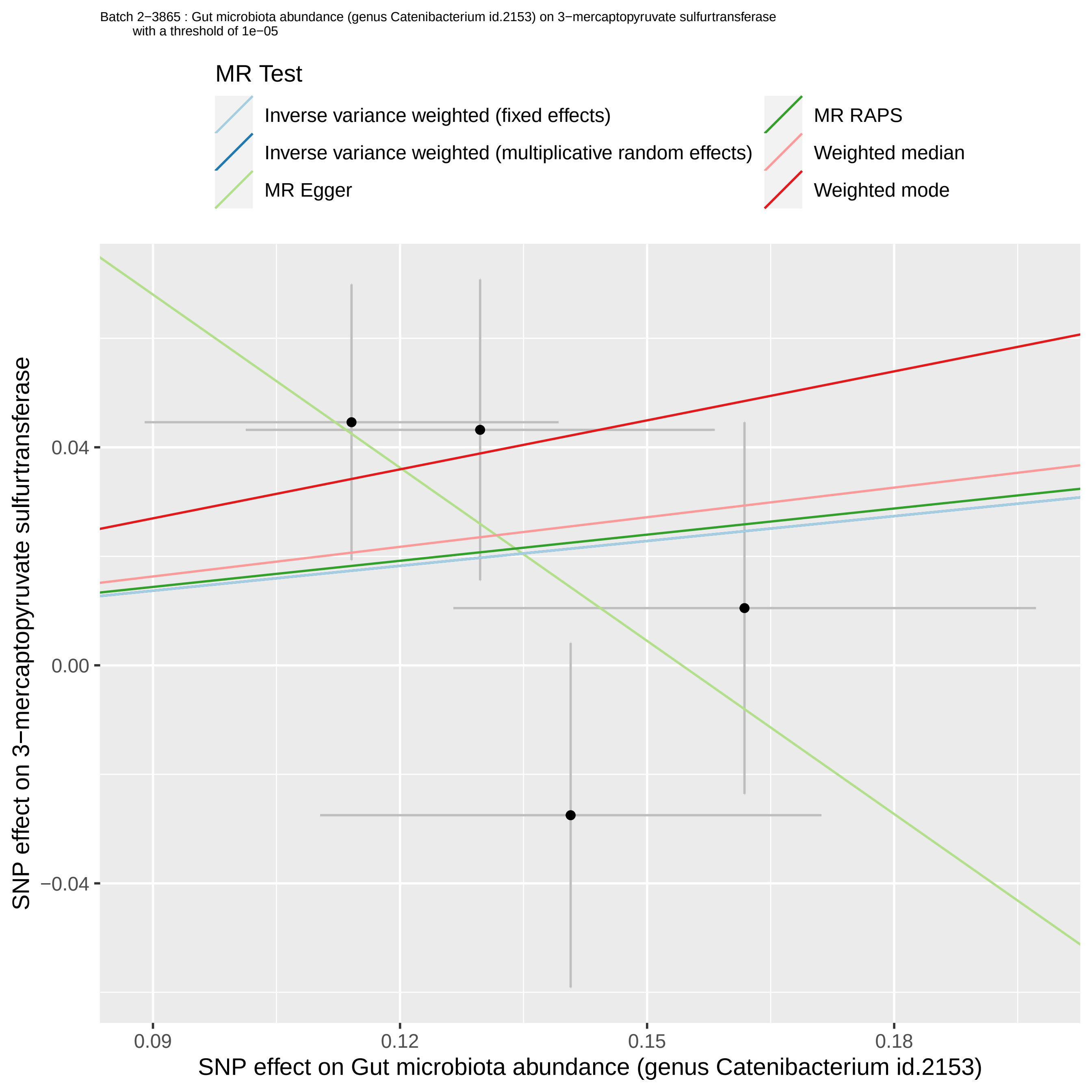

Batch 2−3865 : Gut microbiota abundance (genus Catenibacterium id.2153) on 3−mercaptopyruvate sulfurtransferase
with a threshold of 1e−05
MR Test
Inverse variance weighted (fixed effects)
Inverse variance weighted (multiplicative random effects)
MR Egger
MR RAPS
Weighted median
Weighted mode
0.04
0.00
−0.04
0.09
0.12
0.15
0.18
SNP effect on 3−mercaptopyruvate sulfurtransferase
SNP effect on Gut microbiota abundance (genus Catenibacterium id.2153)

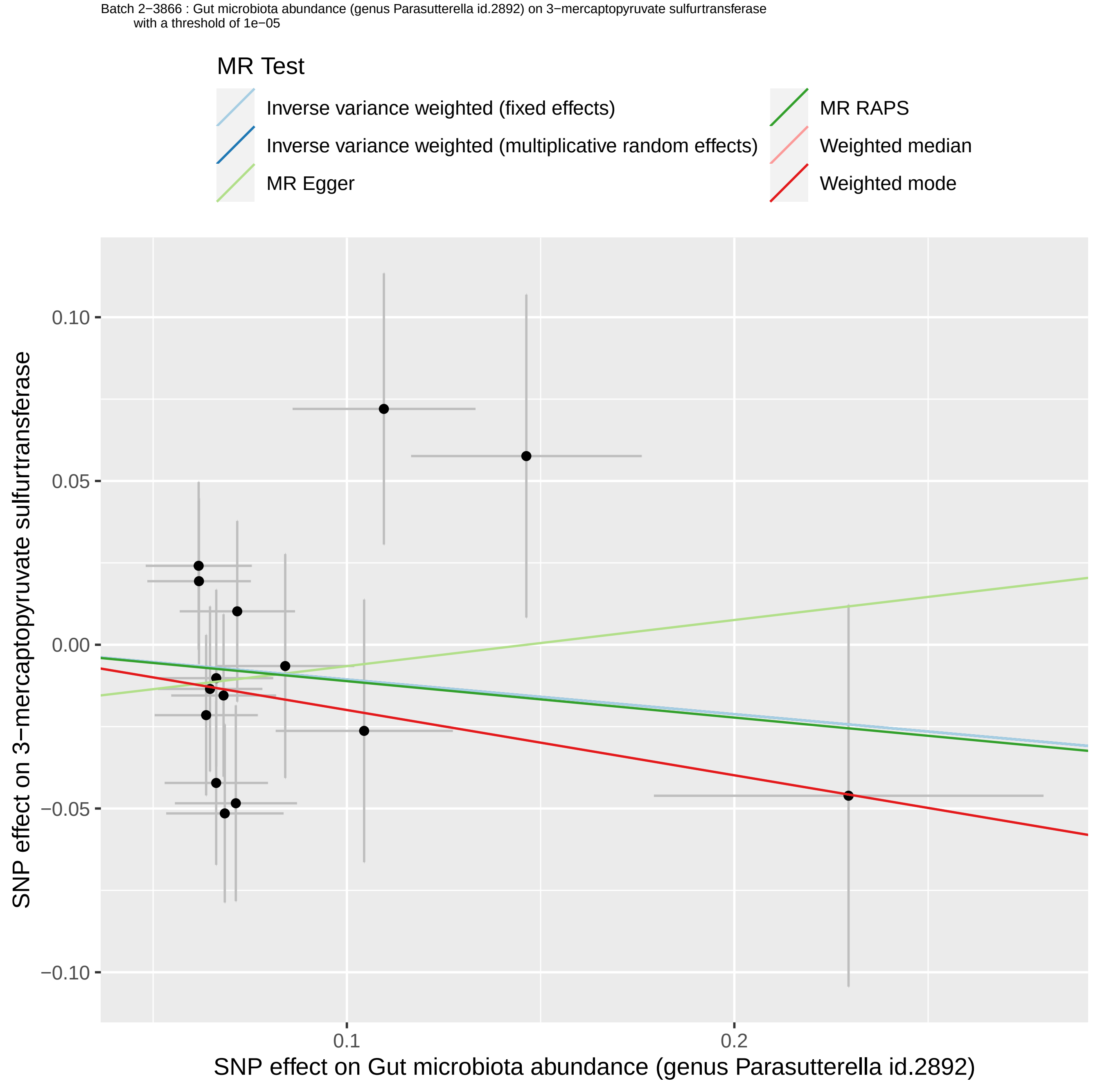

Batch 2−3866 : Gut microbiota abundance (genus Parasutterella id.2892) on 3−mercaptopyruvate sulfurtransferase
with a threshold of 1e−05
MR Test
Inverse variance weighted (fixed effects)
Inverse variance weighted (multiplicative random effects)
MR Egger
MR RAPS
Weighted median
Weighted mode
0.10
0.05
0.00
−0.05
−0.10
0.1
0.2
SNP effect on 3−mercaptopyruvate sulfurtransferase
SNP effect on Gut microbiota abundance (genus Parasutterella id.2892)

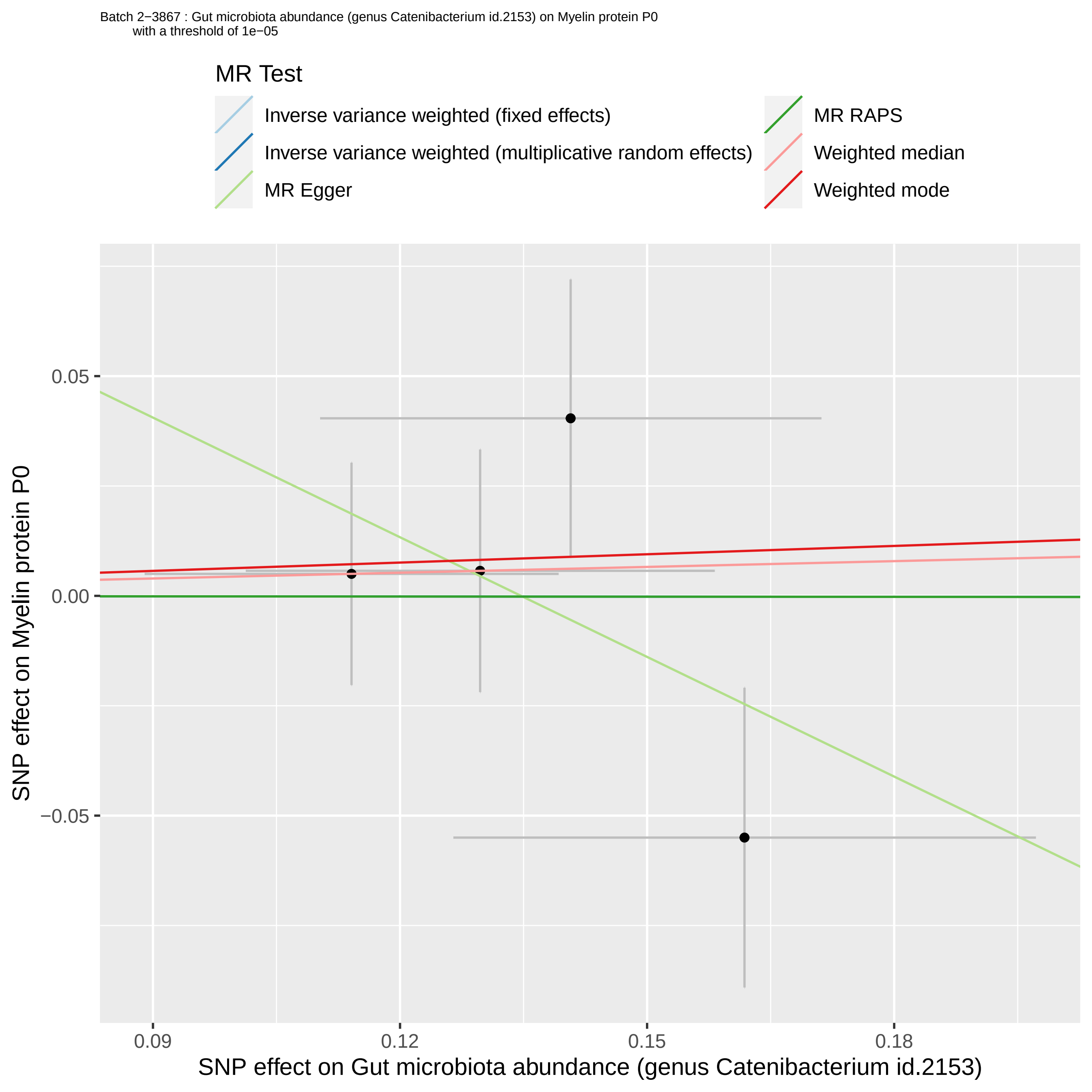
Batch 2−3867 : Gut microbiota abundance (genus Catenibacterium id.2153) on Myelin protein P0
with a threshold of 1e−05
MR Test
Inverse variance weighted (fixed effects)
Inverse variance weighted (multiplicative random effects)
MR Egger
MR RAPS
Weighted median
Weighted mode
0.05
0.00
−0.05
0.09
0.12
0.15
0.18
SNP effect on Myelin protein P0
SNP effect on Gut microbiota abundance (genus Catenibacterium id.2153)

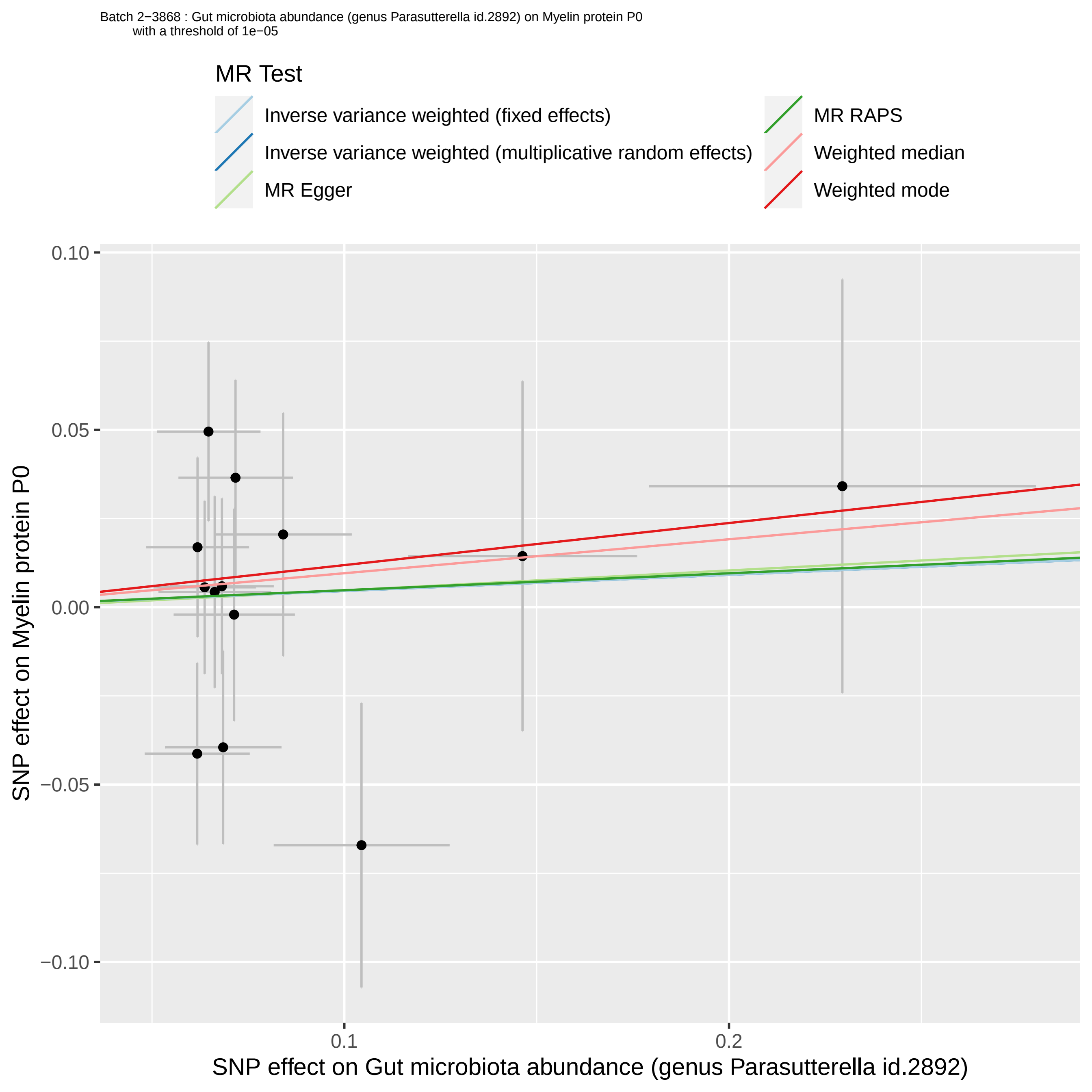

Batch 2−3868 : Gut microbiota abundance (genus Parasutterella id.2892) on Myelin protein P0
with a threshold of 1e−05
MR Test
Inverse variance weighted (fixed effects)
Inverse variance weighted (multiplicative random effects)
MR Egger
MR RAPS
Weighted median
Weighted mode
0.10
0.05
0.00
−0.05
−0.10
0.1
0.2
SNP effect on Myelin protein P0
SNP effect on Gut microbiota abundance (genus Parasutterella id.2892)

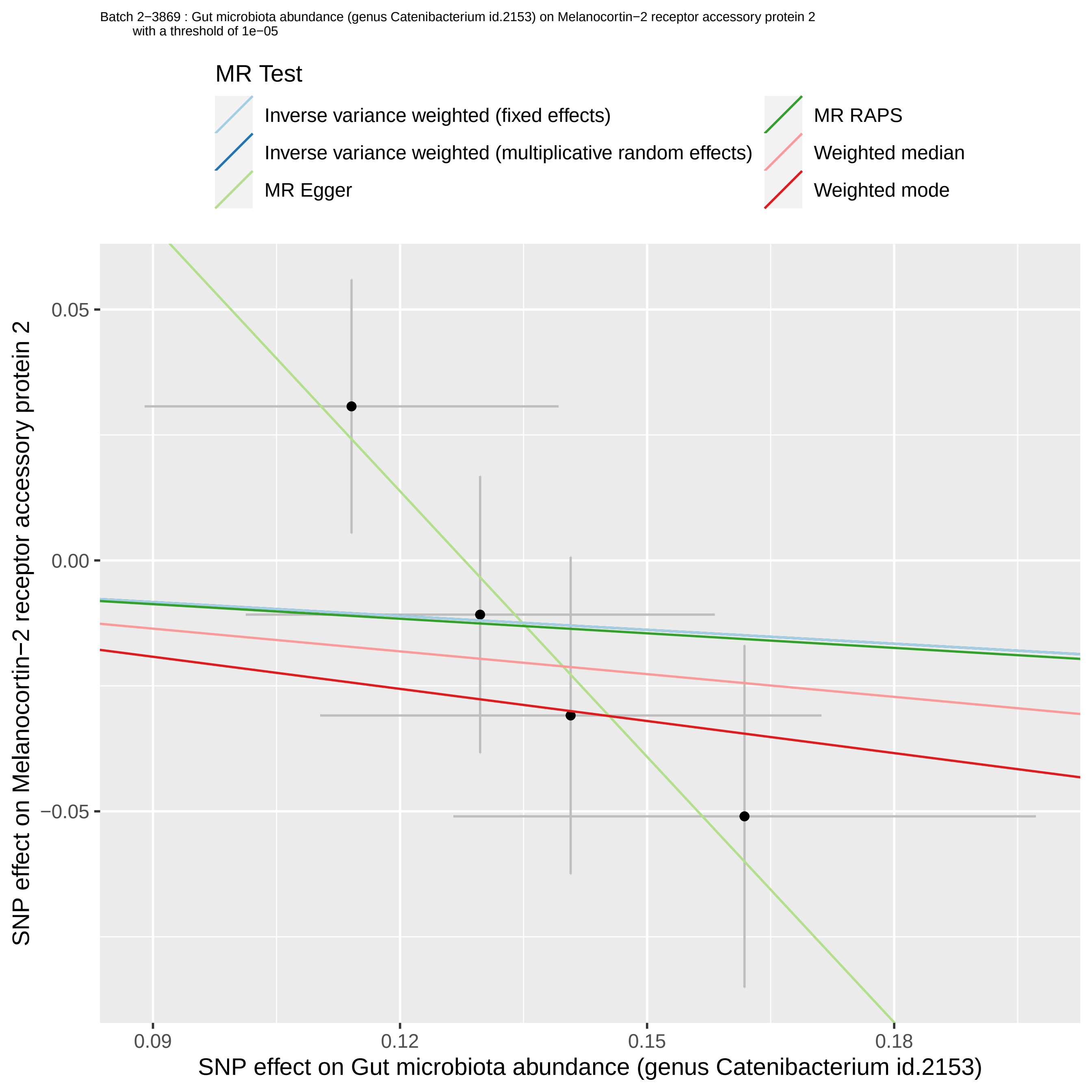
Batch 2−3869 : Gut microbiota abundance (genus Catenibacterium id.2153) on Melanocortin−2 receptor accessory protein 2
with a threshold of 1e−05
MR Test
Inverse variance weighted (fixed effects)
Inverse variance weighted (multiplicative random effects)
MR Egger
MR RAPS
Weighted median
Weighted mode
0.05
0.00
−0.05
0.09
0.12
0.15
0.18
SNP effect on Melanocortin−2 receptor accessory protein 2
SNP effect on Gut microbiota abundance (genus Catenibacterium id.2153)

Batch 2−3870 : Gut microbiota abundance (genus Parasutterella id.2892) on Melanocortin−2 receptor accessory protein 2
with a threshold of 1e−05

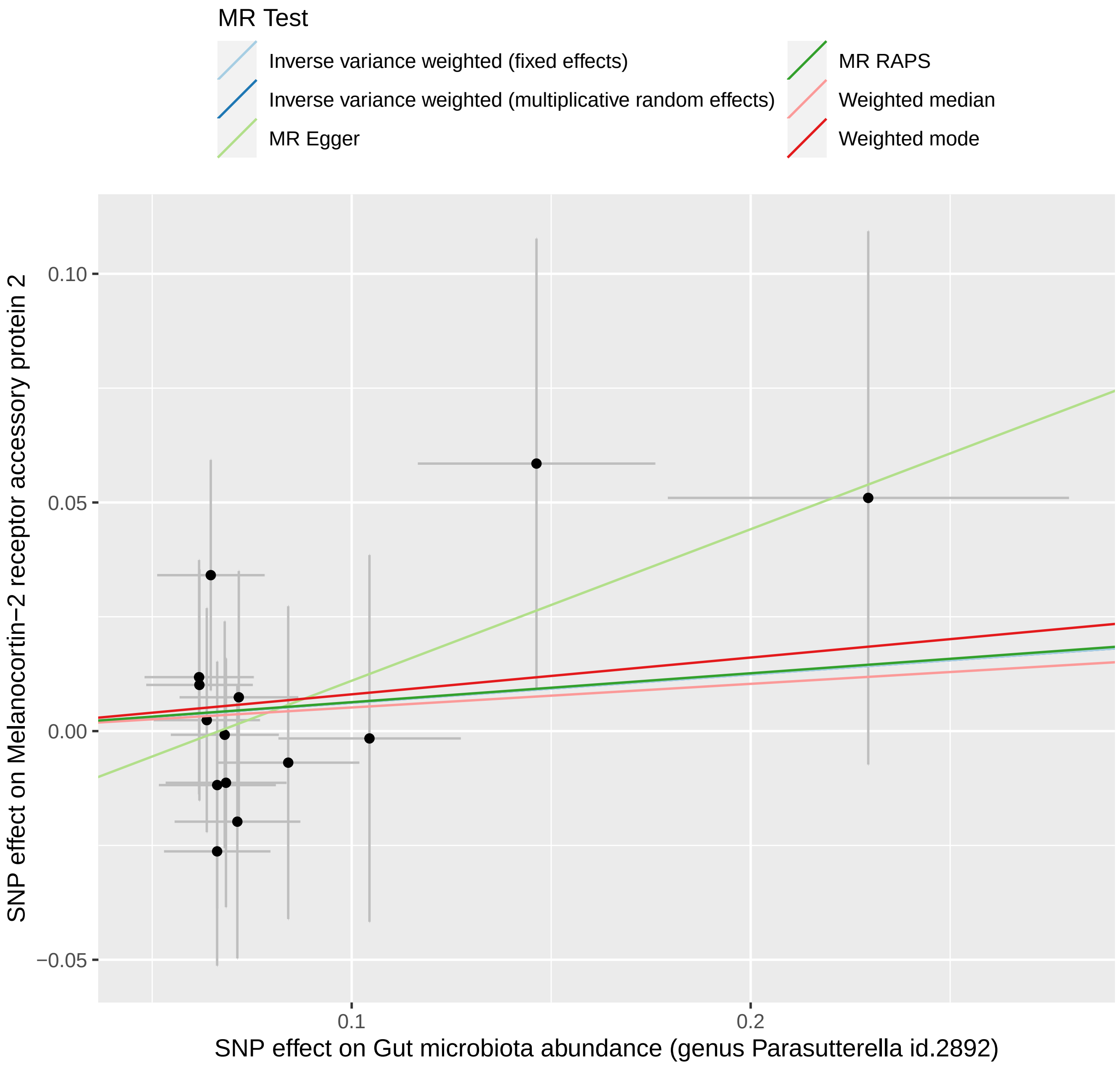

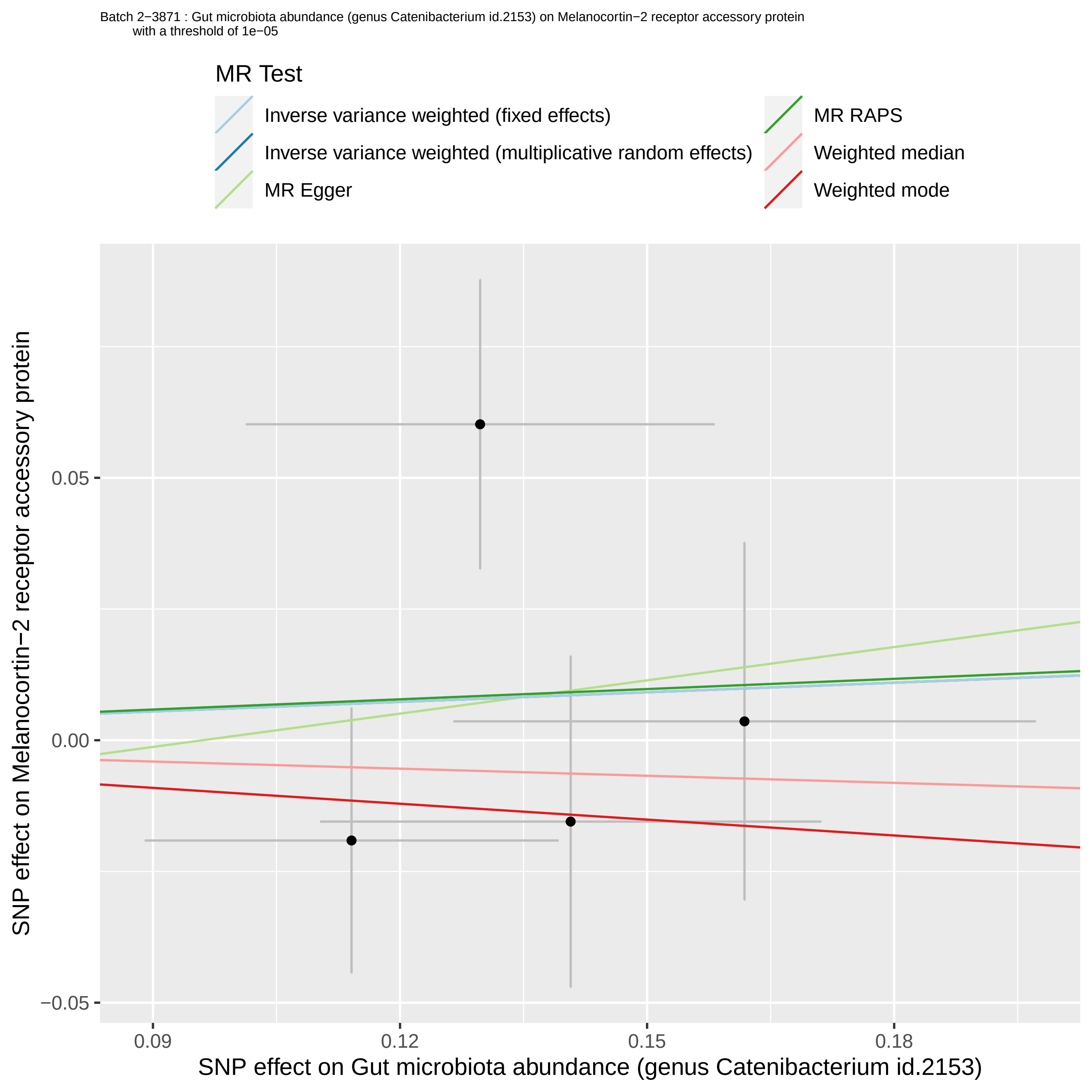
Batch 2−3871 : Gut microbiota abundance (genus Catenibacterium id.2153) on Melanocortin−2 receptor accessory protein with a threshold of 1e−05
MR Test
Inverse variance weighted (fixed effects)
Inverse variance weighted (multiplicative random effects)
MR Egger
MR RAPS
Weighted median
Weighted mode
SNP effect on Melanocortin−2 receptor accessory protein
0.05
0.00
−0.05
0.09
0.12
0.15
0.18
SNP effect on Gut microbiota abundance (genus Catenibacterium id.2153)

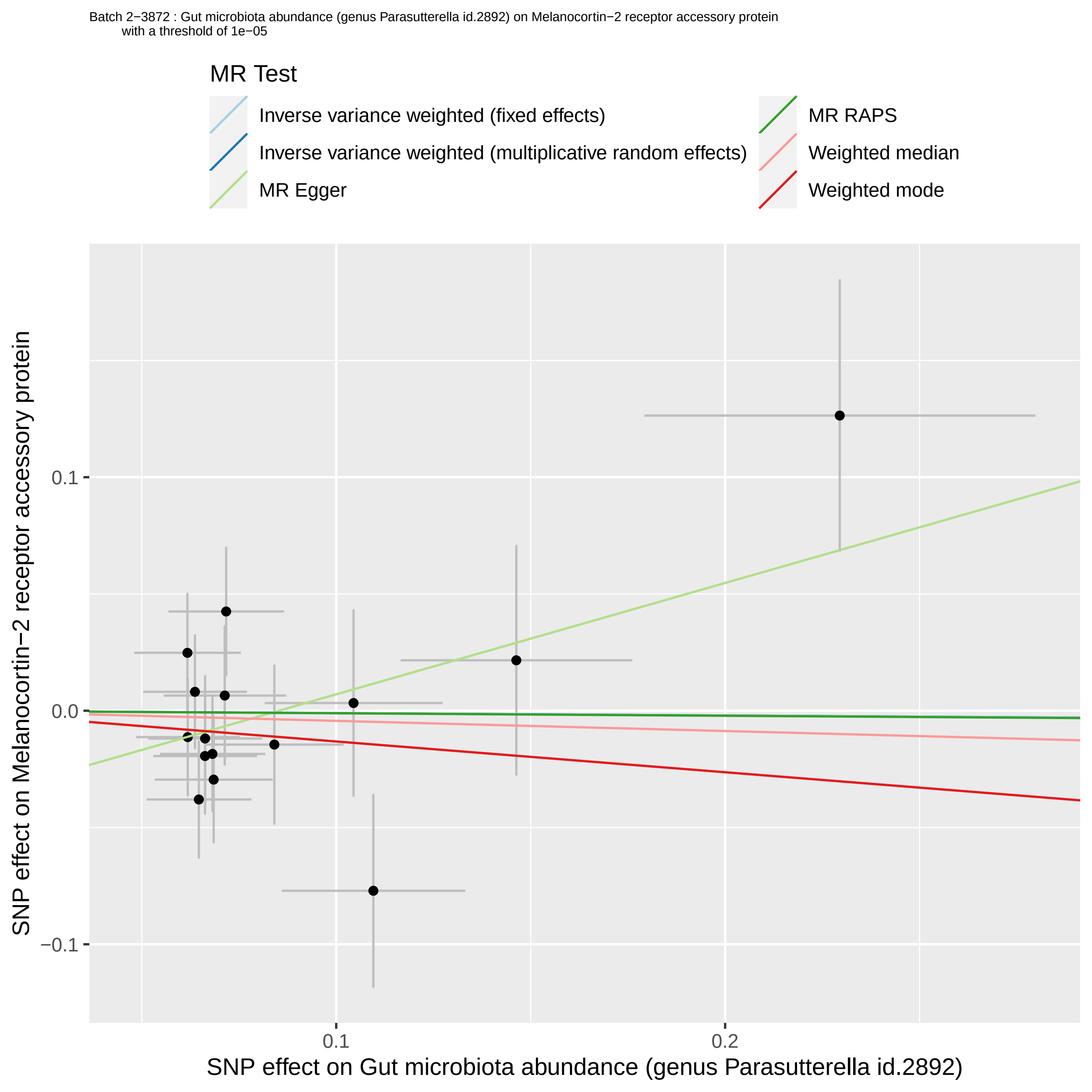
Batch 2−3872 : Gut microbiota abundance (genus Parasutterella id.2892) on Melanocortin−2 receptor accessory protein with a threshold of 1e−05
MR Test
Inverse variance weighted (fixed effects)
Inverse variance weighted (multiplicative random effects)
MR Egger
MR RAPS
Weighted median
Weighted mode
SNP effect on Melanocortin−2 receptor accessory protein
0.1
0.0
−0.1
0.1
0.2
SNP effect on Gut microbiota abundance (genus Parasutterella id.2892)

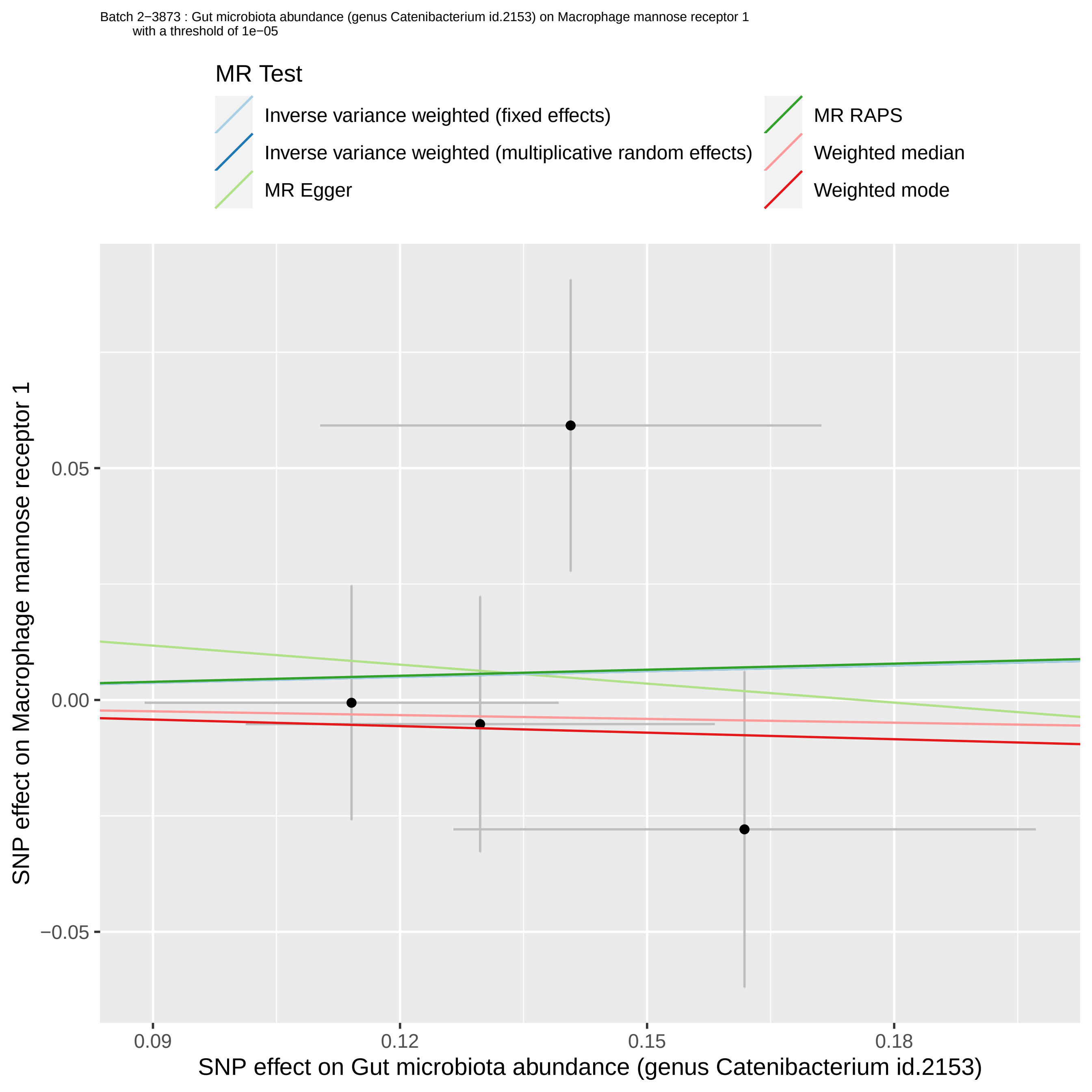
Batch 2−3873 : Gut microbiota abundance (genus Catenibacterium id.2153) on Macrophage mannose receptor 1
with a threshold of 1e−05
MR Test
Inverse variance weighted (fixed effects)
Inverse variance weighted (multiplicative random effects)
MR Egger
MR RAPS
Weighted median
Weighted mode
SNP effect on Macrophage mannose receptor 1
0.05
0.00
−0.05
0.09
0.12
0.15
0.18
SNP effect on Gut microbiota abundance (genus Catenibacterium id.2153)

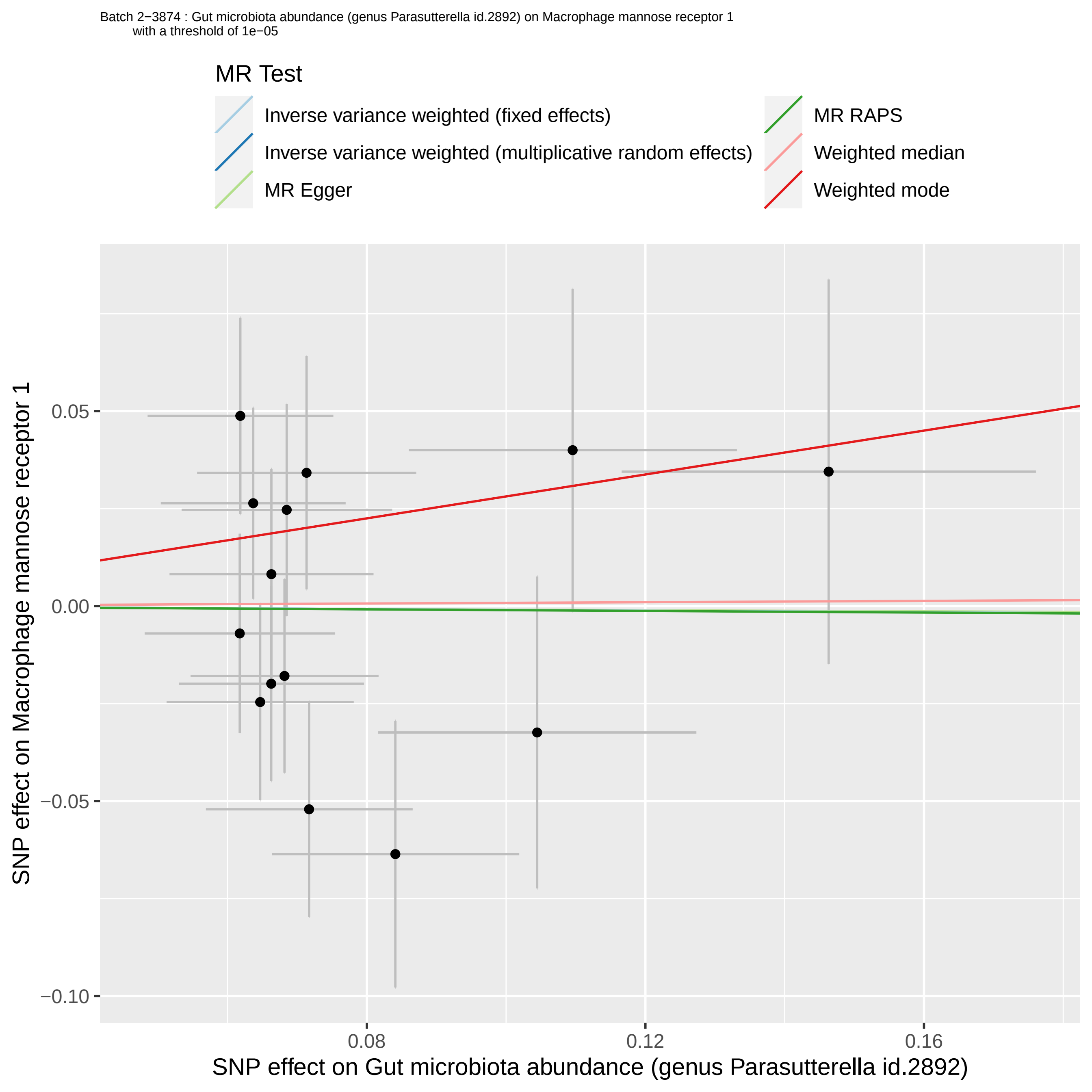
Batch 2−3874 : Gut microbiota abundance (genus Parasutterella id.2892) on Macrophage mannose receptor 1
with a threshold of 1e−05
MR Test
Inverse variance weighted (fixed effects)
Inverse variance weighted (multiplicative random effects)
MR Egger
MR RAPS
Weighted median
Weighted mode
SNP effect on Macrophage mannose receptor 1
0.05
0.00
−0.05
−0.10
0.08
0.12
0.16
SNP effect on Gut microbiota abundance (genus Parasutterella id.2892)

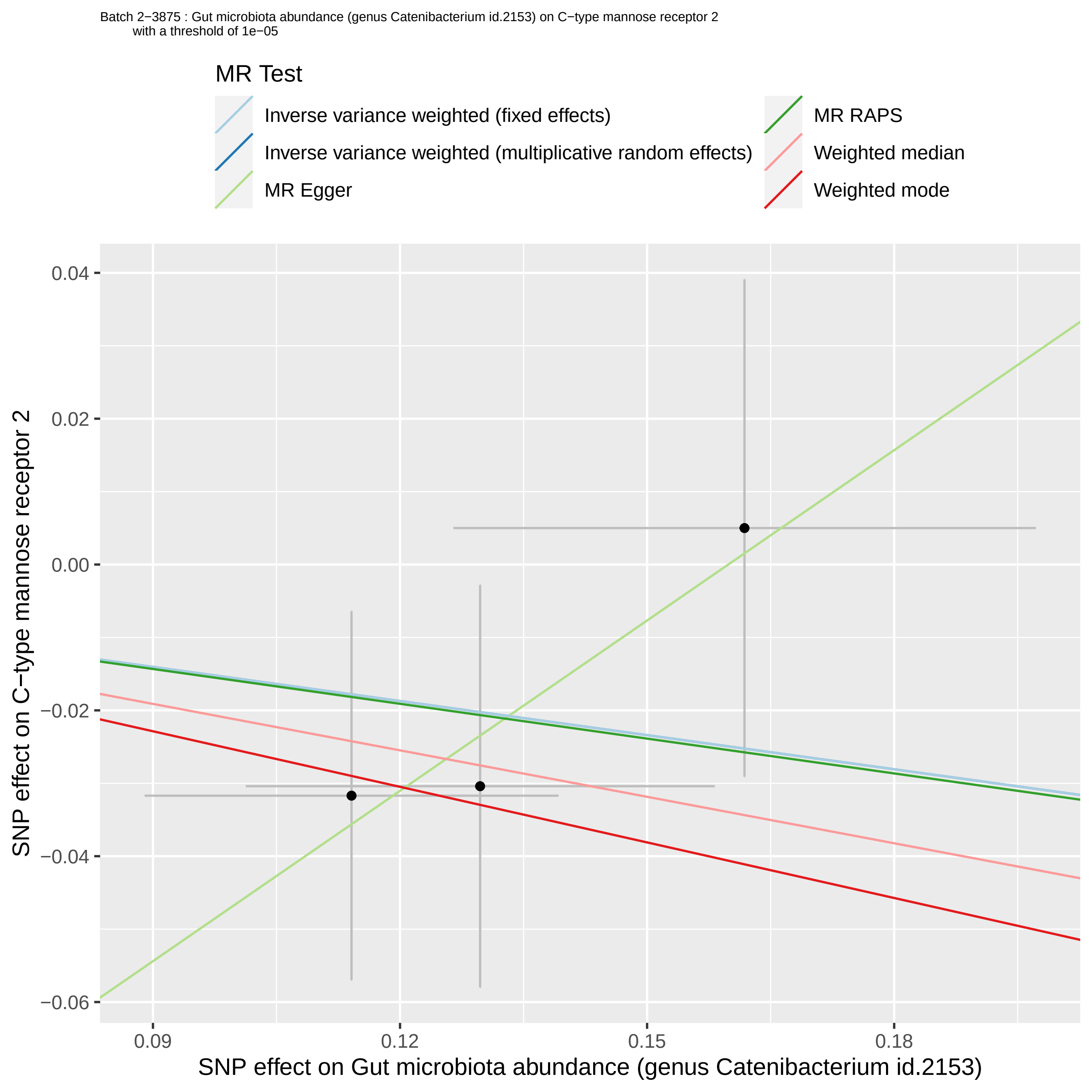
Batch 2−3875 : Gut microbiota abundance (genus Catenibacterium id.2153) on C−type mannose receptor 2
with a threshold of 1e−05
MR Test
Inverse variance weighted (fixed effects)
Inverse variance weighted (multiplicative random effects)
MR Egger
MR RAPS
Weighted median
Weighted mode
0.04
0.02
0.00
−0.02
−0.04
−0.06
0.09
0.12
0.15
0.18
SNP effect on C−type mannose receptor 2
SNP effect on Gut microbiota abundance (genus Catenibacterium id.2153)

Batch 2−3876 : Gut microbiota abundance (genus Parasutterella id.2892) on C−type mannose receptor 2
with a threshold of 1e−05

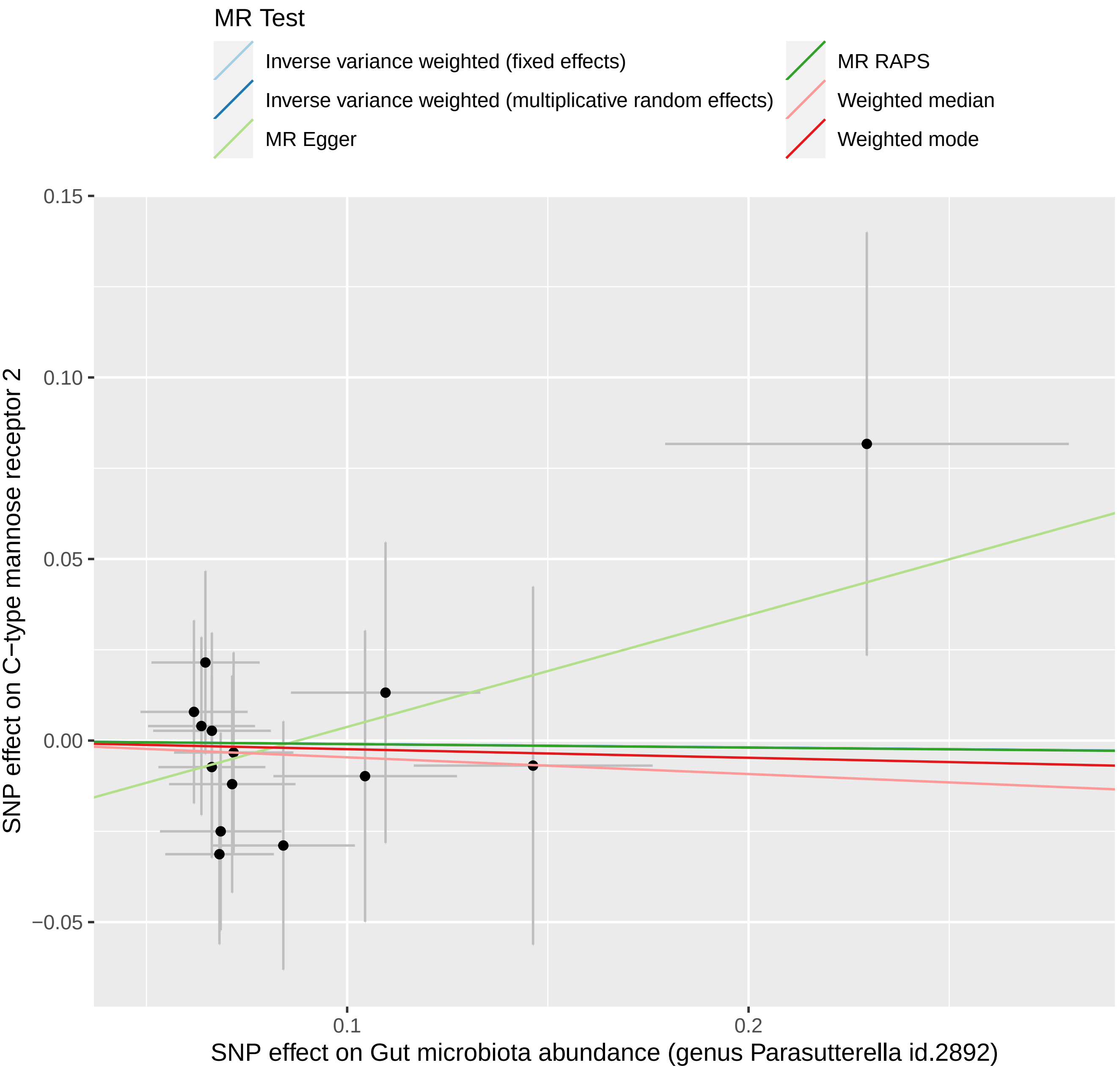

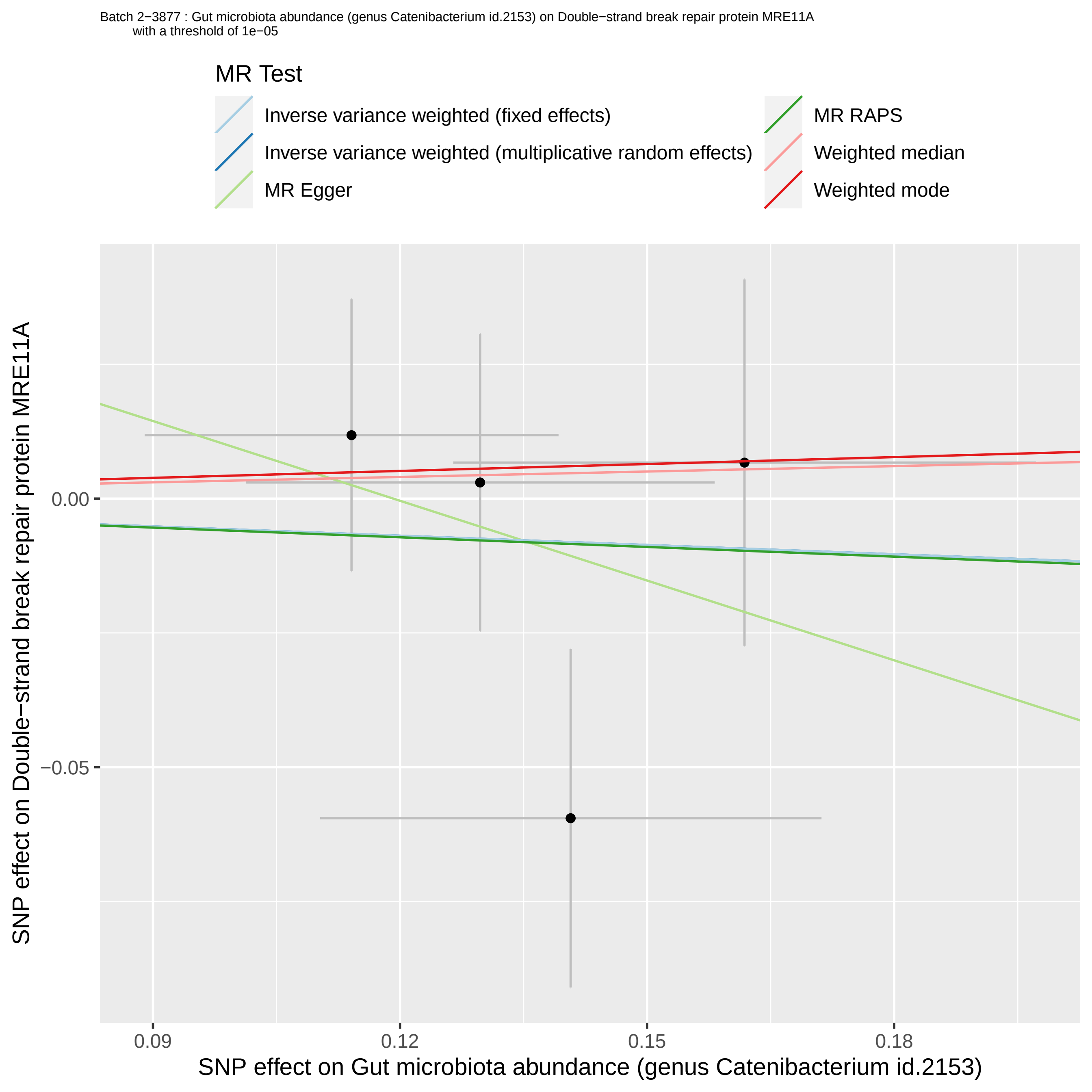

Batch 2−3877 : Gut microbiota abundance (genus Catenibacterium id.2153) on Double−strand break repair protein MRE11A
with a threshold of 1e−05
MR Test
Inverse variance weighted (fixed effects)
Inverse variance weighted (multiplicative random effects)
MR Egger
MR RAPS
Weighted median
Weighted mode
SNP effect on Double−strand break repair protein MRE11A
0.00
−0.05
0.09
0.12
0.15
0.18
SNP effect on Gut microbiota abundance (genus Catenibacterium id.2153)

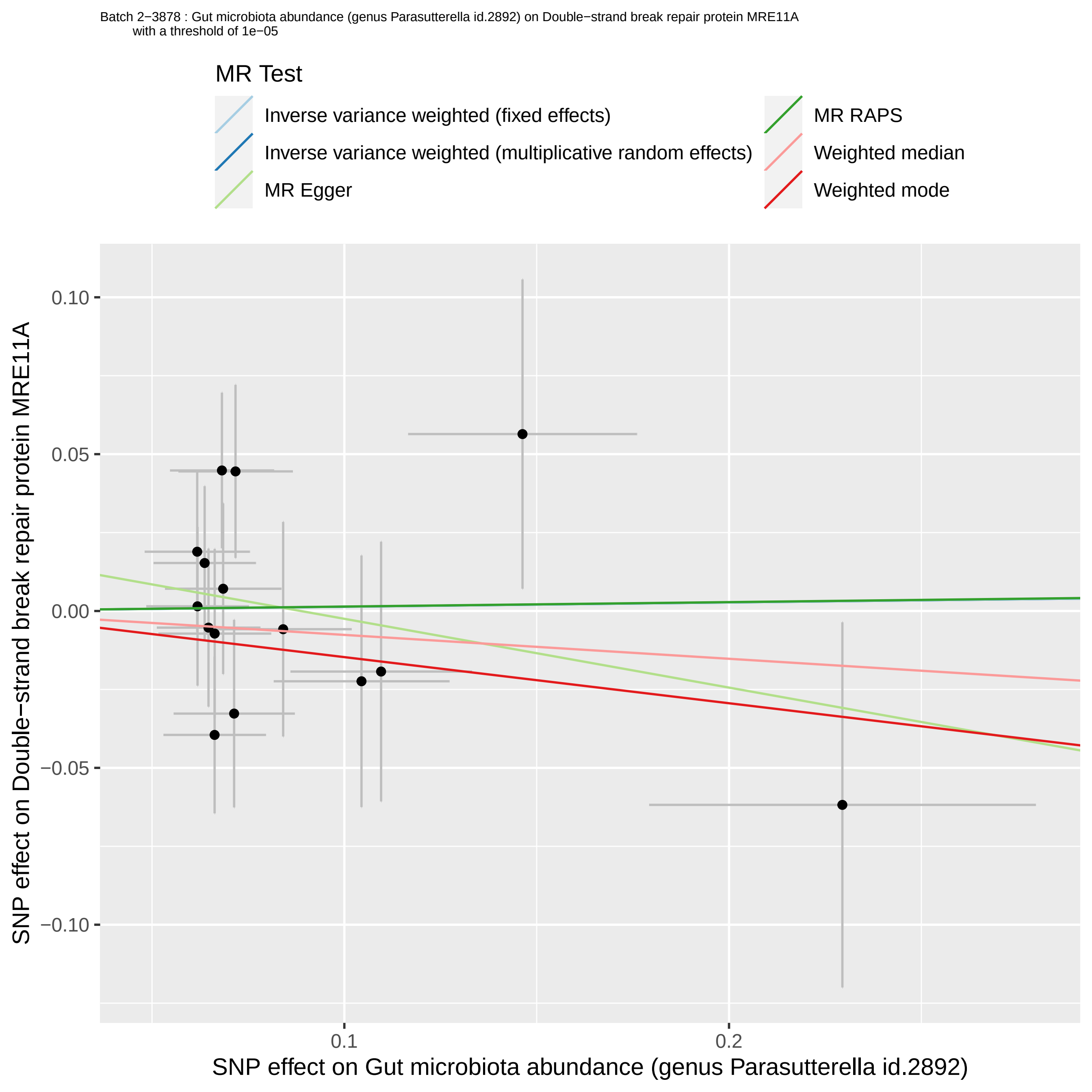

Batch 2−3878 : Gut microbiota abundance (genus Parasutterella id.2892) on Double−strand break repair protein MRE11A
with a threshold of 1e−05
MR Test
Inverse variance weighted (fixed effects)
Inverse variance weighted (multiplicative random effects)
MR Egger
MR RAPS
Weighted median
Weighted mode
0.10
0.05
0.00
−0.05
−0.10
0.1
0.2
SNP effect on Double−strand break repair protein MRE11A
SNP effect on Gut microbiota abundance (genus Parasutterella id.2892)

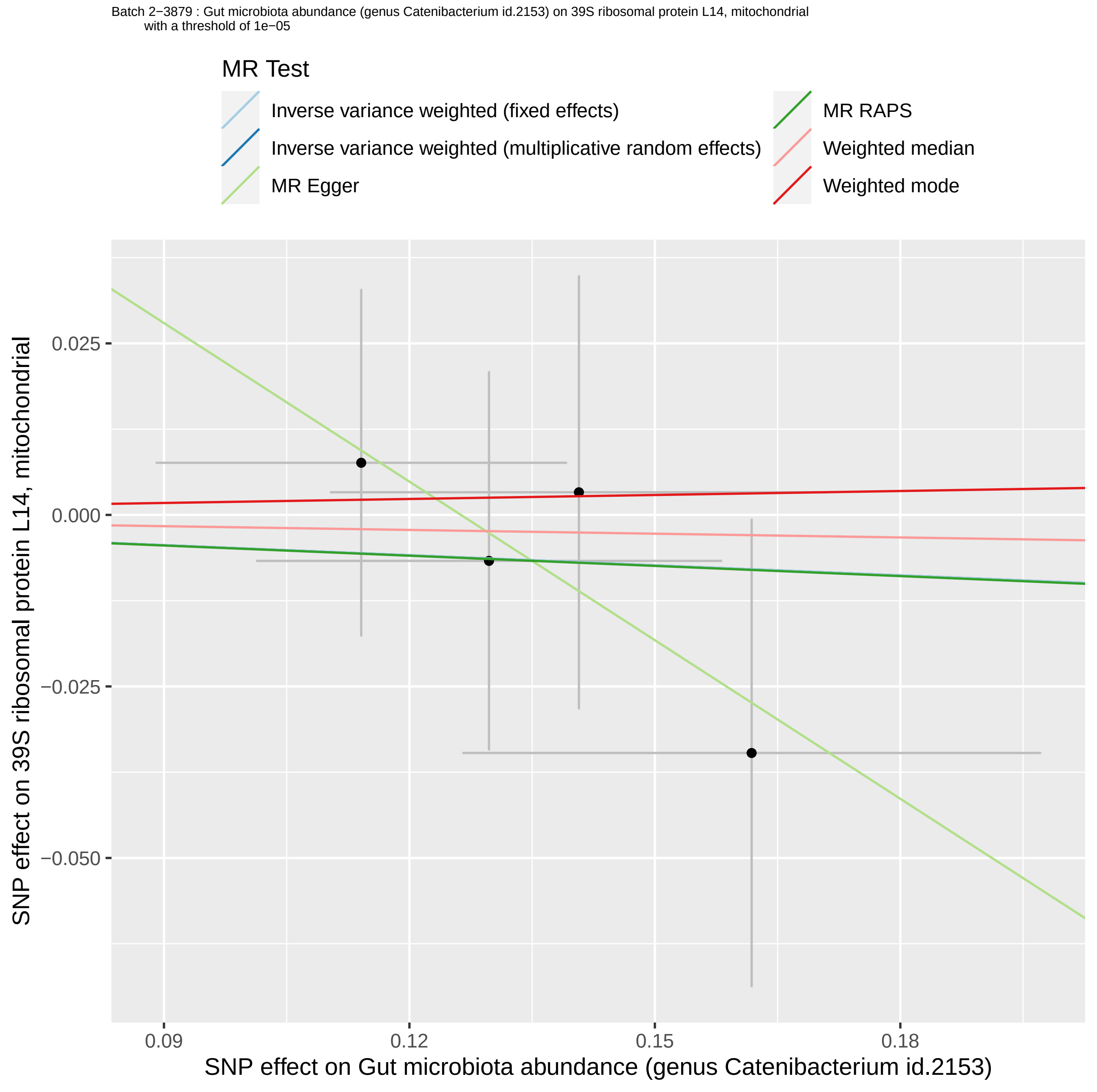

Batch 2−3879 : Gut microbiota abundance (genus Catenibacterium id.2153) on 39S ribosomal protein L14, mitochondrial
with a threshold of 1e−05
MR Test
Inverse variance weighted (fixed effects)
Inverse variance weighted (multiplicative random effects)
MR Egger
MR RAPS
Weighted median
Weighted mode
0.025
0.000
−0.025
−0.050
0.09
0.12
0.15
0.18
SNP effect on 39S ribosomal protein L14, mitochondrial
SNP effect on Gut microbiota abundance (genus Catenibacterium id.2153)

Batch 2−3880 : Gut microbiota abundance (genus Parasutterella id.2892) on 39S ribosomal protein L14, mitochondrial
with a threshold of 1e−05

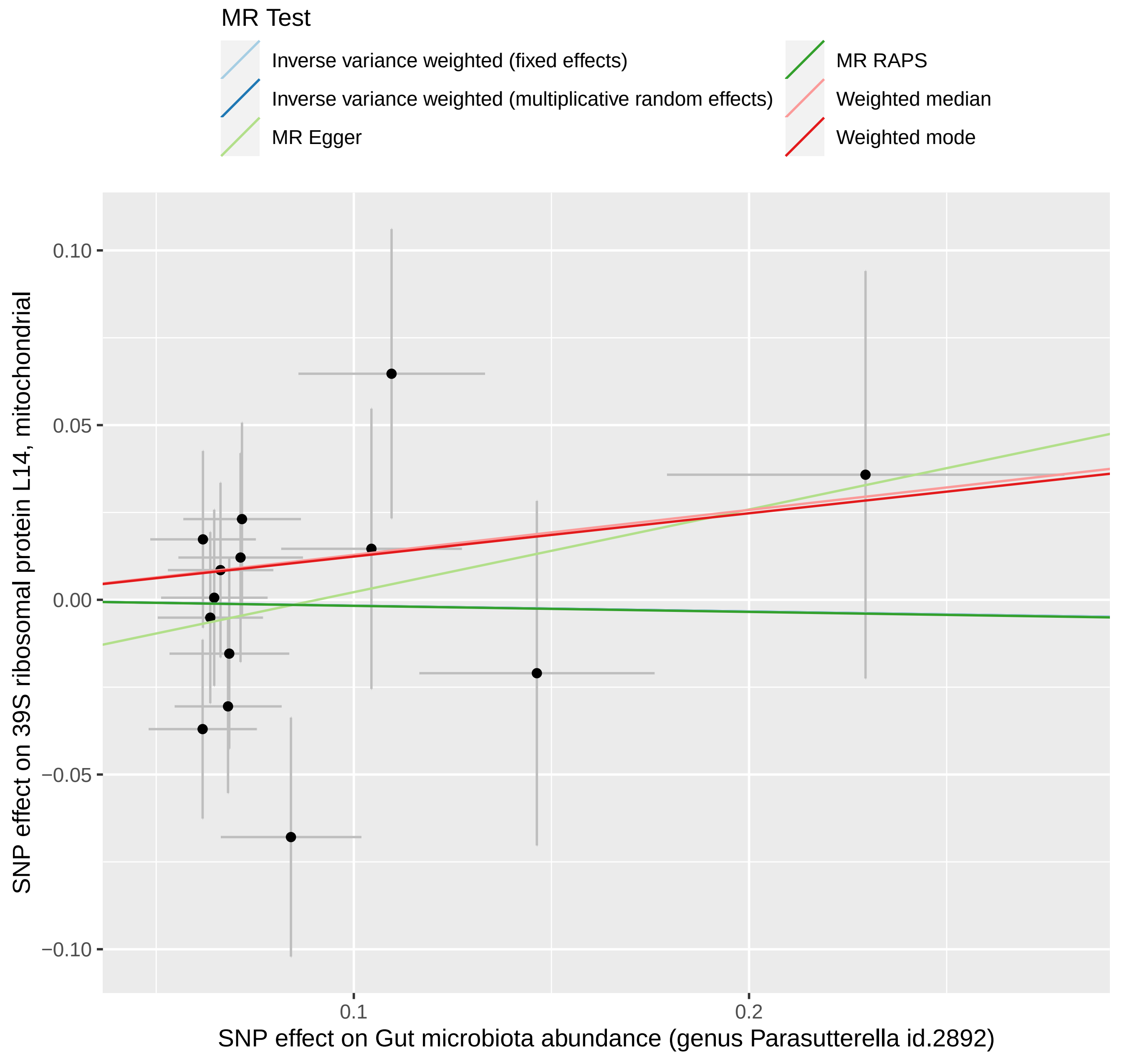

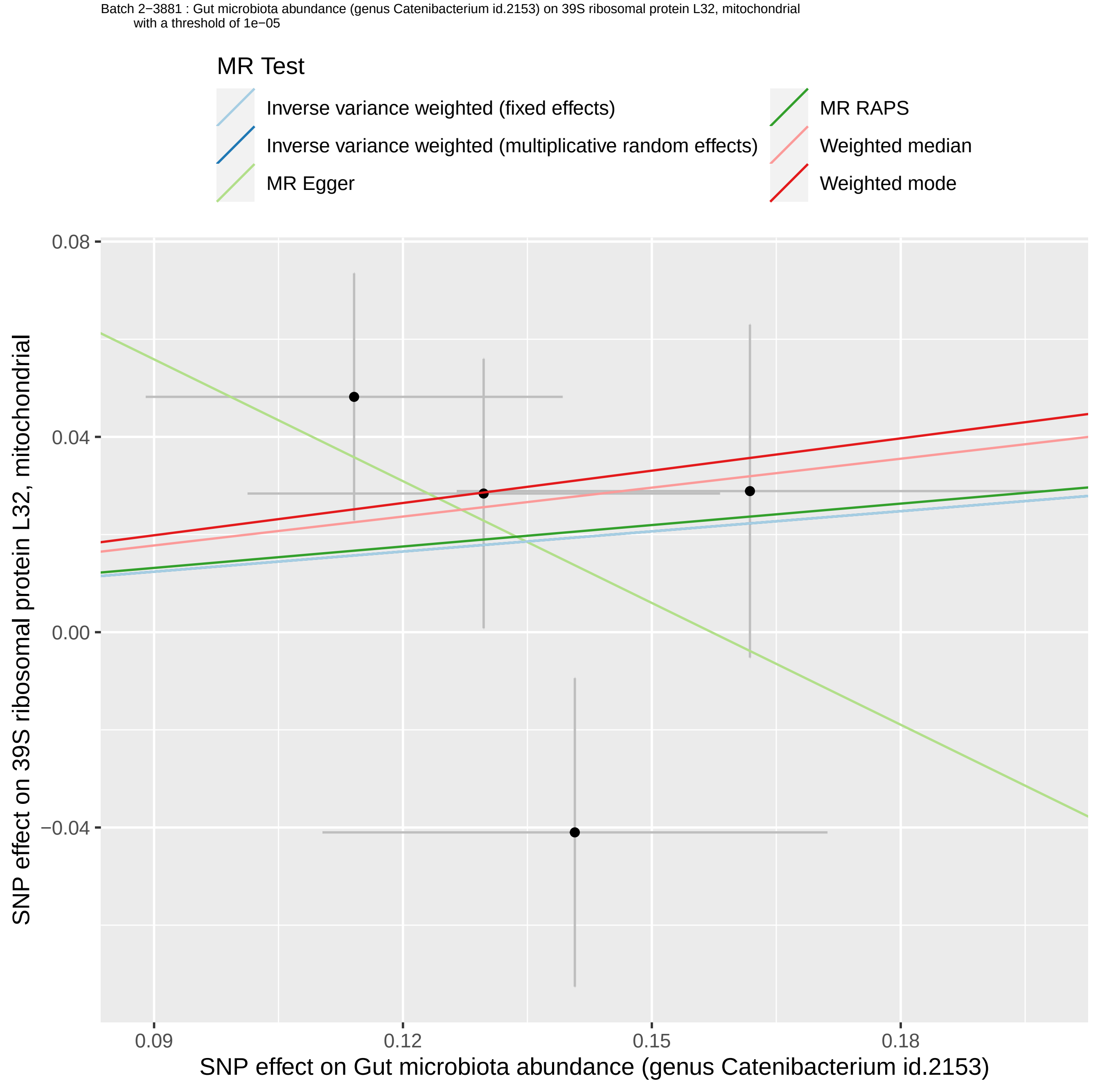
Batch 2−3881 : Gut microbiota abundance (genus Catenibacterium id.2153) on 39S ribosomal protein L32, mitochondrial
with a threshold of 1e−05
MR Test
Inverse variance weighted (fixed effects)
Inverse variance weighted (multiplicative random effects)
MR Egger
MR RAPS
Weighted median
Weighted mode
0.08
0.04
0.00
−0.04
0.09
0.12
0.15
0.18
SNP effect on 39S ribosomal protein L32, mitochondrial
SNP effect on Gut microbiota abundance (genus Catenibacterium id.2153)

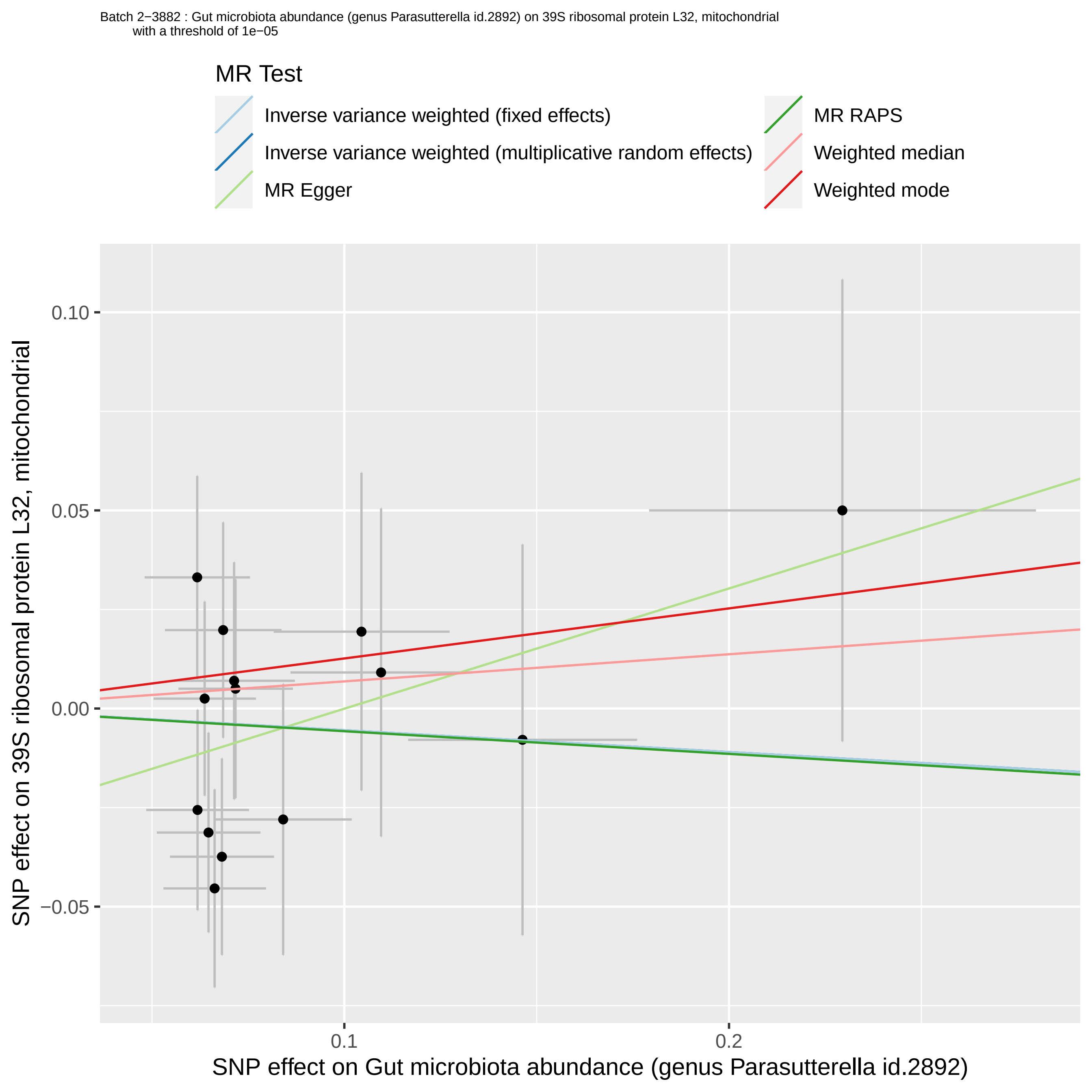
Batch 2−3882 : Gut microbiota abundance (genus Parasutterella id.2892) on 39S ribosomal protein L32, mitochondrial
with a threshold of 1e−05
MR Test
Inverse variance weighted (fixed effects)
Inverse variance weighted (multiplicative random effects)
MR Egger
MR RAPS
Weighted median
Weighted mode
0.10
0.05
0.00
−0.05
0.1
0.2
SNP effect on 39S ribosomal protein L32, mitochondrial
SNP effect on Gut microbiota abundance (genus Parasutterella id.2892)

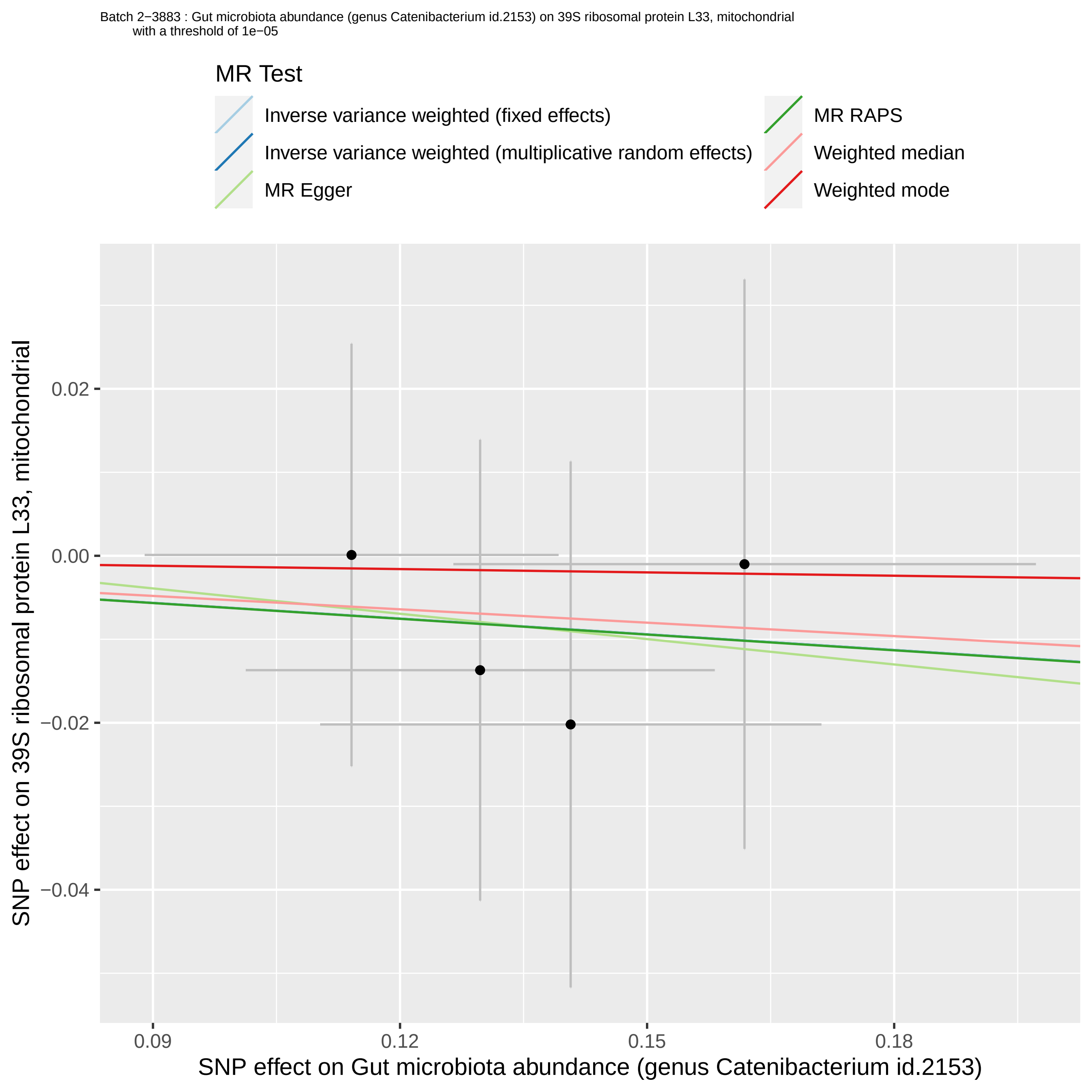
Batch 2−3883 : Gut microbiota abundance (genus Catenibacterium id.2153) on 39S ribosomal protein L33, mitochondrial
with a threshold of 1e−05
MR Test
Inverse variance weighted (fixed effects)
Inverse variance weighted (multiplicative random effects)
MR Egger
MR RAPS
Weighted median
Weighted mode
0.02
0.00
−0.02
−0.04
0.09
0.12
0.15
0.18
SNP effect on 39S ribosomal protein L33, mitochondrial
SNP effect on Gut microbiota abundance (genus Catenibacterium id.2153)

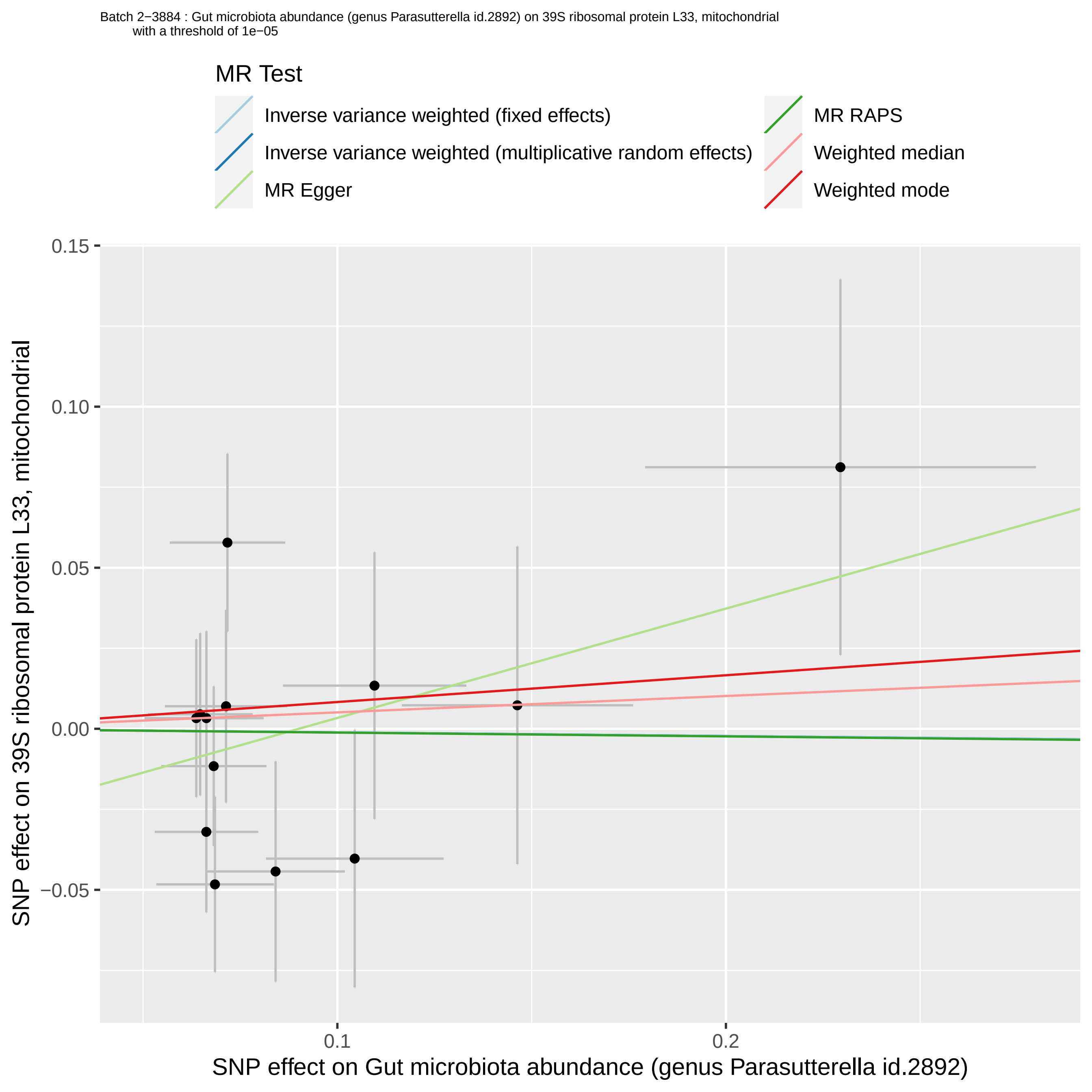

Batch 2−3884 : Gut microbiota abundance (genus Parasutterella id.2892) on 39S ribosomal protein L33, mitochondrial
with a threshold of 1e−05
MR Test
Inverse variance weighted (fixed effects)
Inverse variance weighted (multiplicative random effects)
MR Egger
MR RAPS
Weighted median
Weighted mode
0.15
0.10
0.05
0.00
−0.05
0.1
0.2
SNP effect on 39S ribosomal protein L33, mitochondrial
SNP effect on Gut microbiota abundance (genus Parasutterella id.2892)

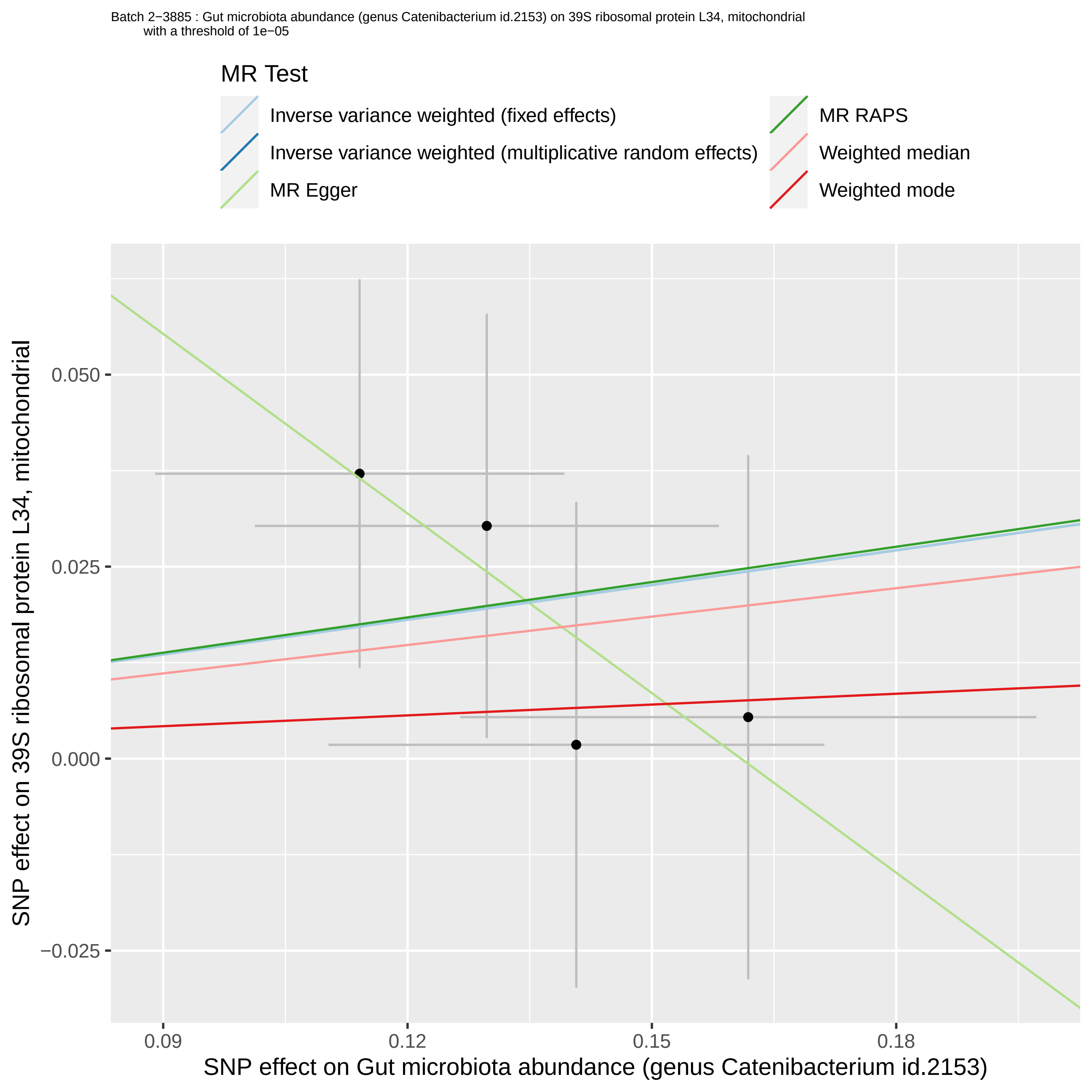
Batch 2−3885 : Gut microbiota abundance (genus Catenibacterium id.2153) on 39S ribosomal protein L34, mitochondrial
with a threshold of 1e−05
MR Test
Inverse variance weighted (fixed effects)
Inverse variance weighted (multiplicative random effects)
MR Egger
MR RAPS
Weighted median
Weighted mode
0.050
0.025
0.000
−0.025
0.09
0.12
0.15
0.18
SNP effect on 39S ribosomal protein L34, mitochondrial
SNP effect on Gut microbiota abundance (genus Catenibacterium id.2153)

Batch 2−3886 : Gut microbiota abundance (genus Parasutterella id.2892) on 39S ribosomal protein L34, mitochondrial
with a threshold of 1e−05

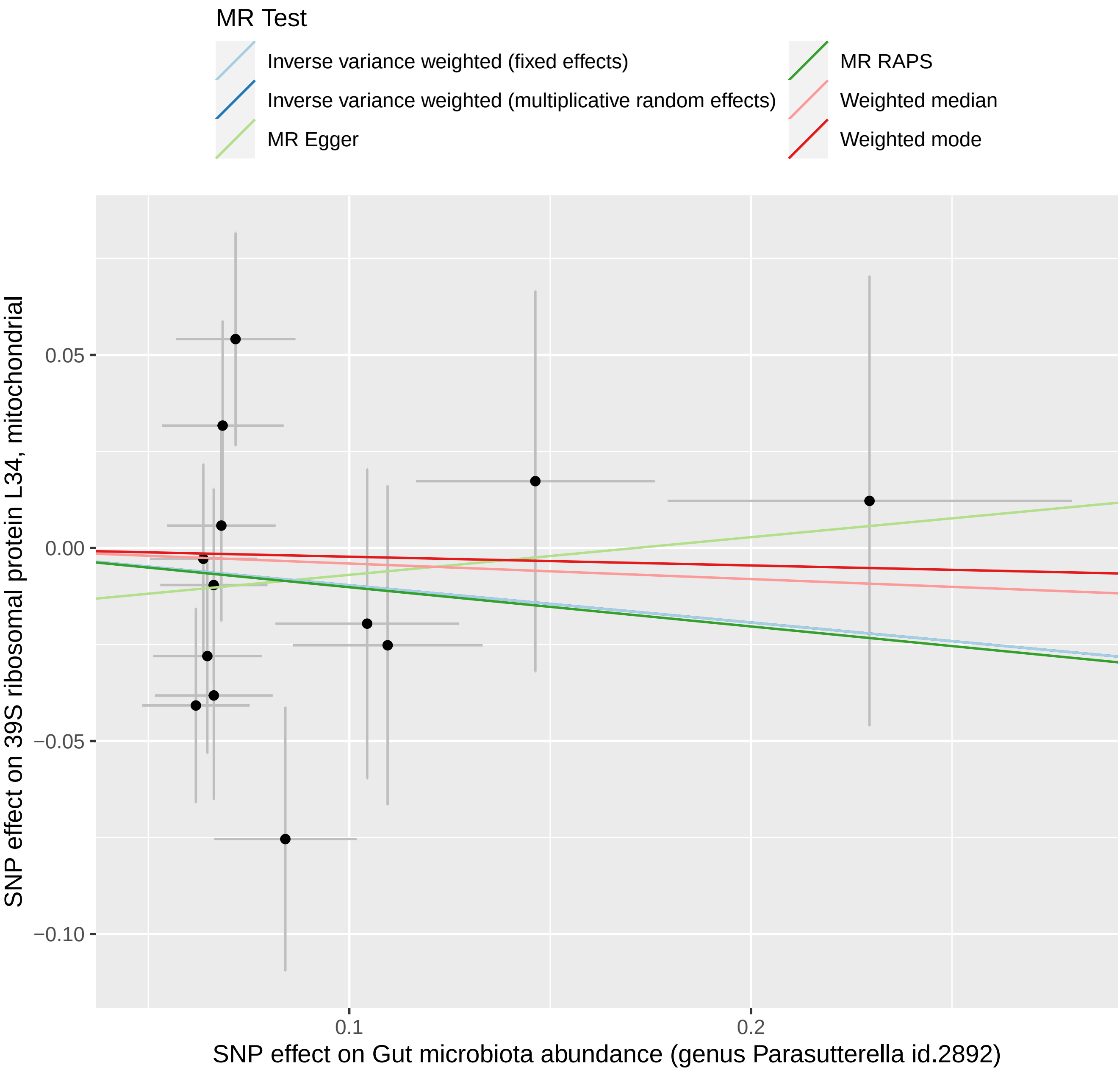

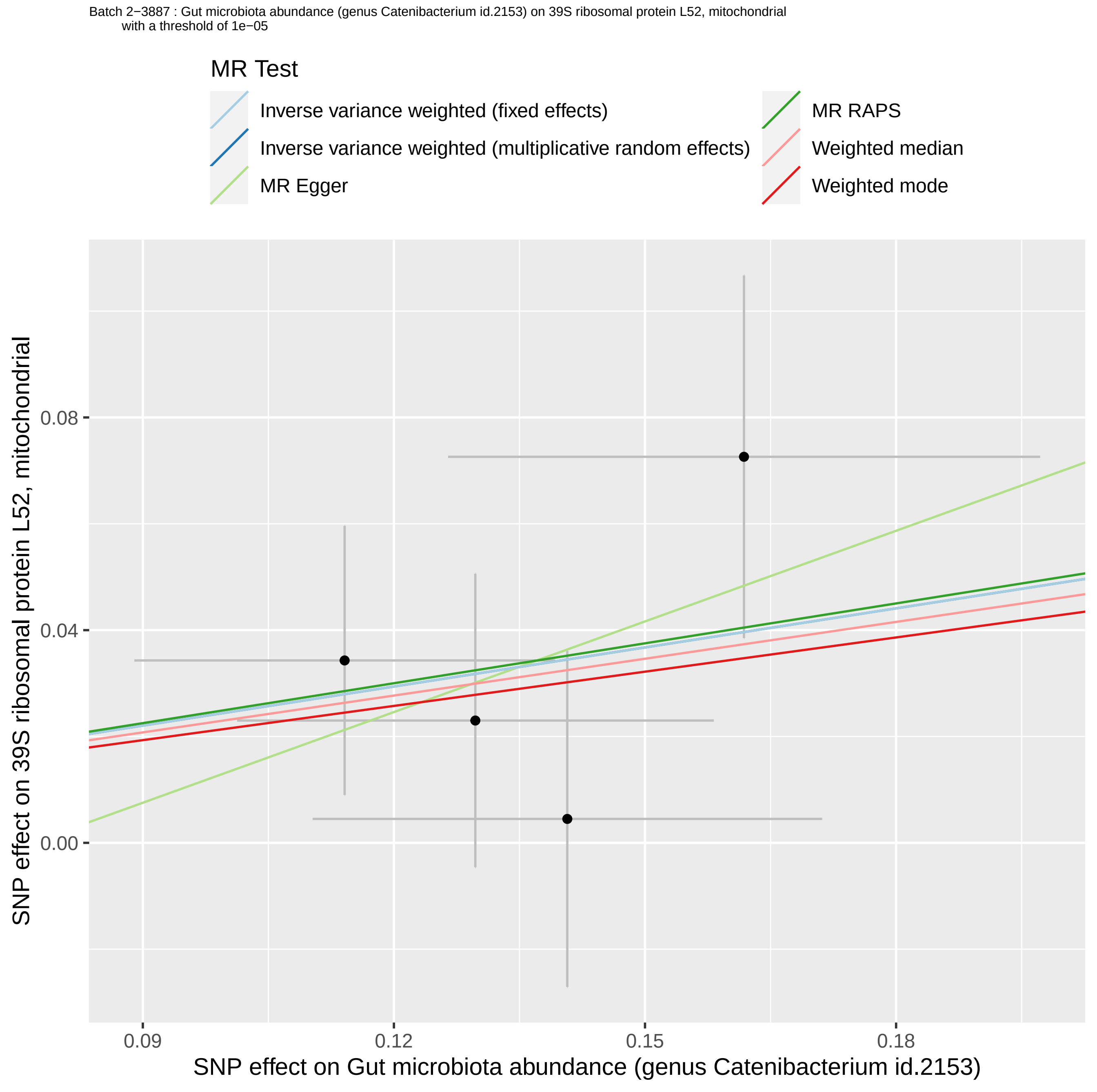
Batch 2−3887 : Gut microbiota abundance (genus Catenibacterium id.2153) on 39S ribosomal protein L52, mitochondrial
with a threshold of 1e−05
MR Test
Inverse variance weighted (fixed effects)
Inverse variance weighted (multiplicative random effects)
MR Egger
MR RAPS
Weighted median
Weighted mode
0.08
0.04
0.00
0.09
0.12
0.15
0.18
SNP effect on 39S ribosomal protein L52, mitochondrial
SNP effect on Gut microbiota abundance (genus Catenibacterium id.2153)

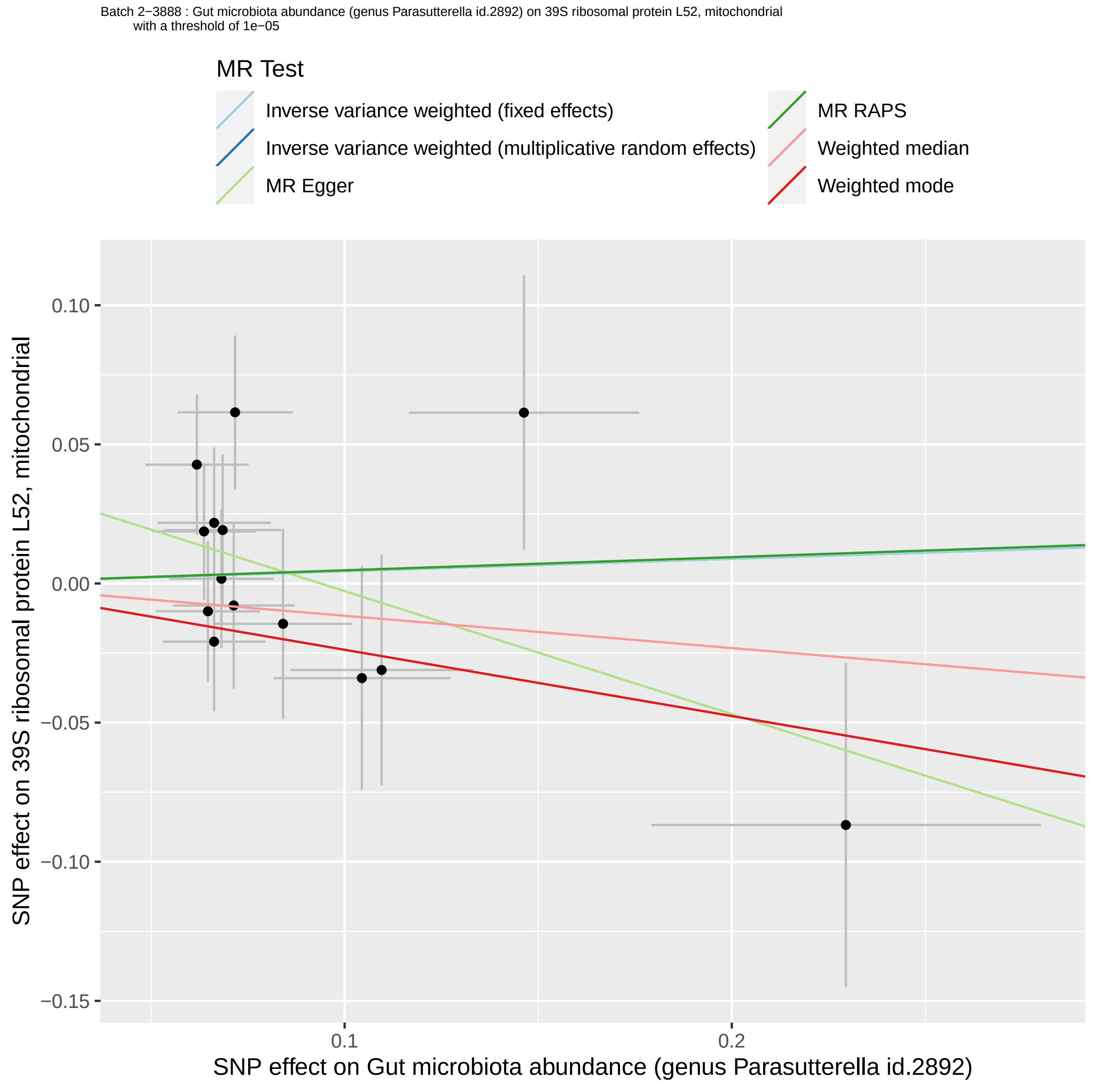
Batch 2−3888 : Gut microbiota abundance (genus Parasutterella id.2892) on 39S ribosomal protein L52, mitochondrial
with a threshold of 1e−05
MR Test
Inverse variance weighted (fixed effects)
Inverse variance weighted (multiplicative random effects)
MR Egger
MR RAPS
Weighted median
Weighted mode
0.10
0.05
0.00
−0.05
−0.10
−0.15
0.1
0.2
SNP effect on 39S ribosomal protein L52, mitochondrial
SNP effect on Gut microbiota abundance (genus Parasutterella id.2892)

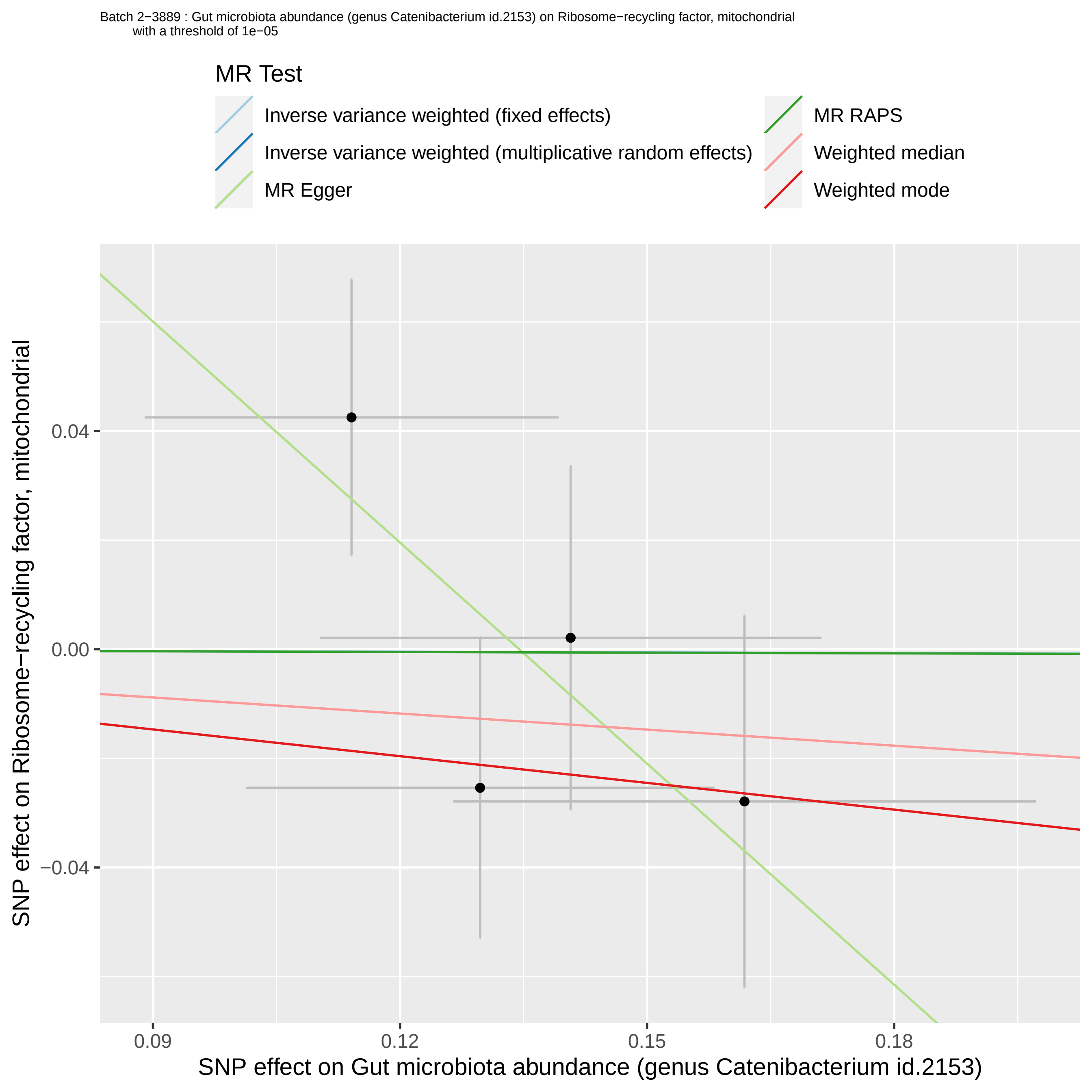
Batch 2−3889 : Gut microbiota abundance (genus Catenibacterium id.2153) on Ribosome−recycling factor, mitochondrial
with a threshold of 1e−05
MR Test
Inverse variance weighted (fixed effects)
Inverse variance weighted (multiplicative random effects)
MR Egger
MR RAPS
Weighted median
Weighted mode
0.04
0.00
−0.04
0.09
0.12
0.15
0.18
SNP effect on Ribosome−recycling factor, mitochondrial
SNP effect on Gut microbiota abundance (genus Catenibacterium id.2153)

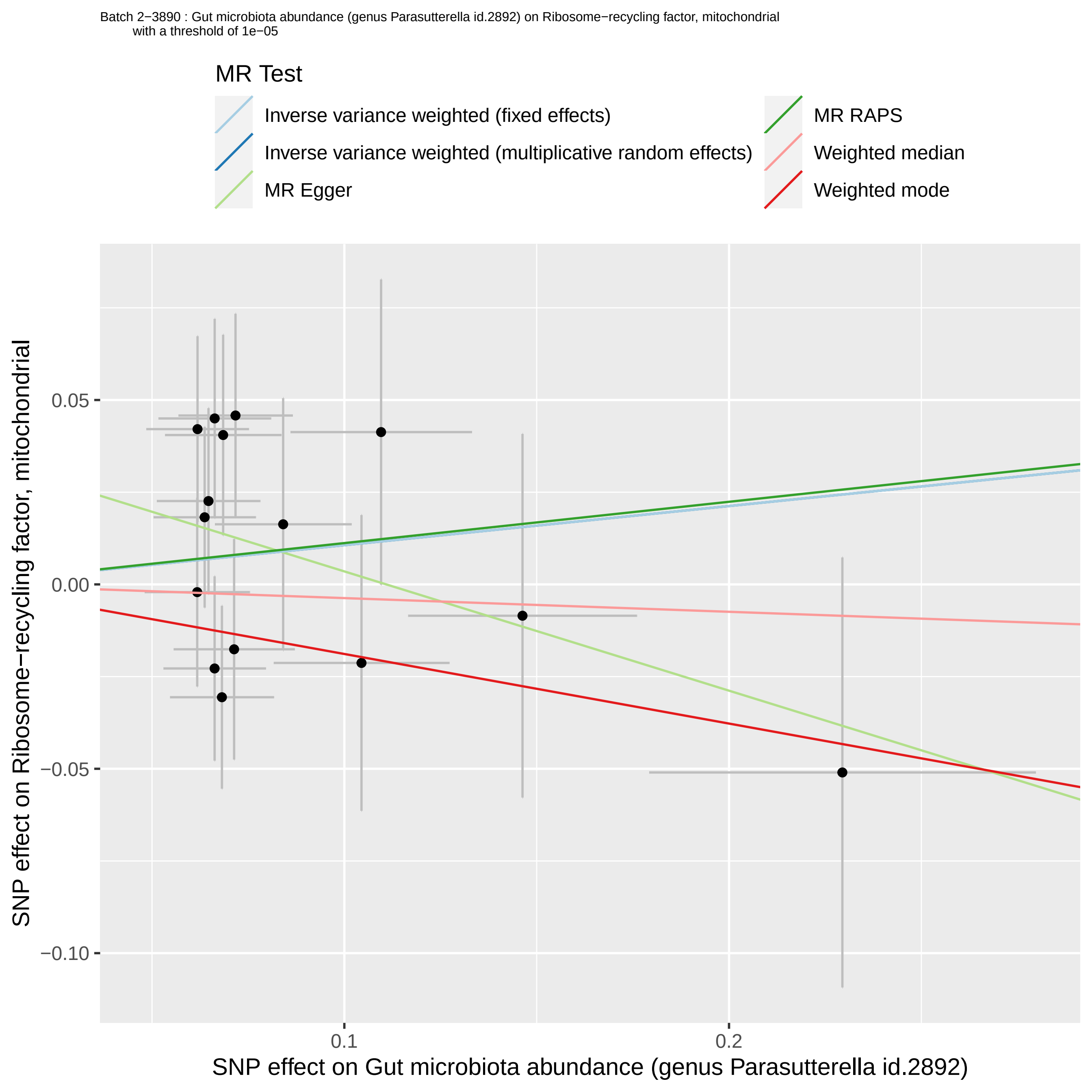

Batch 2−3890 : Gut microbiota abundance (genus Parasutterella id.2892) on Ribosome−recycling factor, mitochondrial
with a threshold of 1e−05
MR Test
Inverse variance weighted (fixed effects)
Inverse variance weighted (multiplicative random effects)
MR Egger
MR RAPS
Weighted median
Weighted mode
0.05
0.00
−0.05
−0.10
0.1
0.2
SNP effect on Ribosome−recycling factor, mitochondrial
SNP effect on Gut microbiota abundance (genus Parasutterella id.2892)

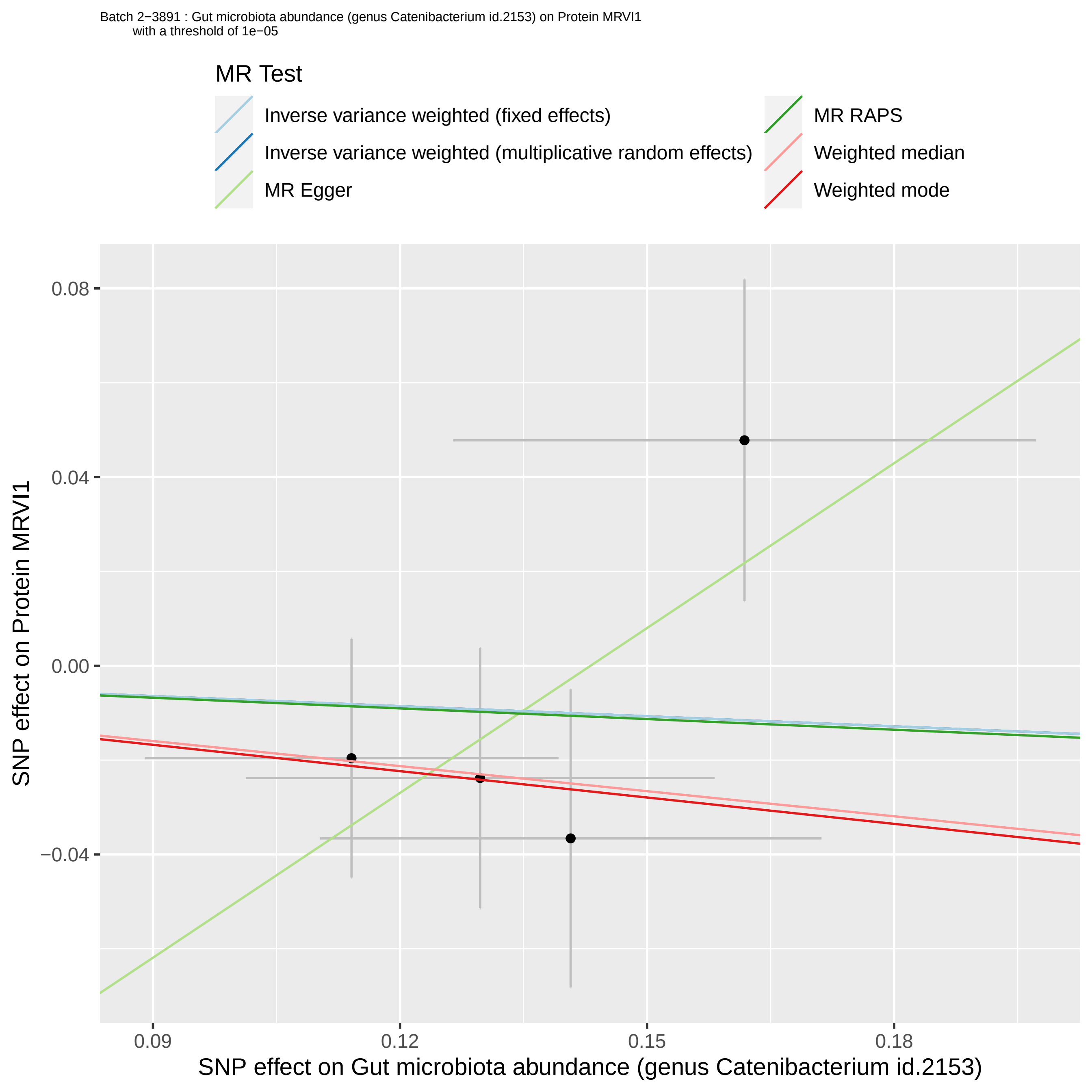

Batch 2−3891 : Gut microbiota abundance (genus Catenibacterium id.2153) on Protein MRVI1
with a threshold of 1e−05
MR Test
Inverse variance weighted (fixed effects)
Inverse variance weighted (multiplicative random effects)
MR Egger
MR RAPS
Weighted median
Weighted mode
0.08
0.04
0.00
−0.04
0.09
0.12
0.15
0.18
SNP effect on Protein MRVI1
SNP effect on Gut microbiota abundance (genus Catenibacterium id.2153)

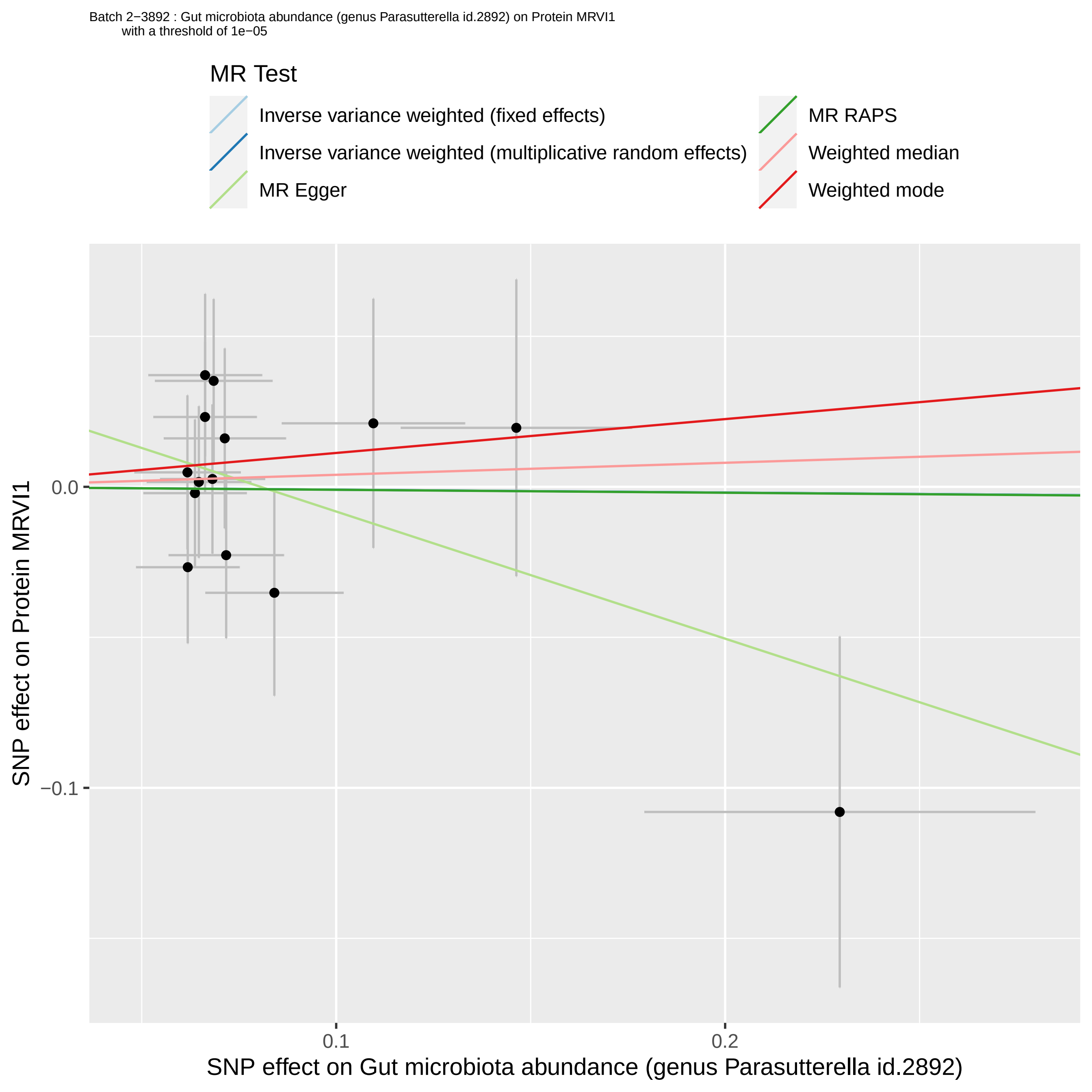

Batch 2−3892 : Gut microbiota abundance (genus Parasutterella id.2892) on Protein MRVI1
with a threshold of 1e−05
MR Test
Inverse variance weighted (fixed effects)
Inverse variance weighted (multiplicative random effects)
MR Egger
MR RAPS
Weighted median
Weighted mode
0.0
−0.1
0.1
0.2
SNP effect on Protein MRVI1
SNP effect on Gut microbiota abundance (genus Parasutterella id.2892)

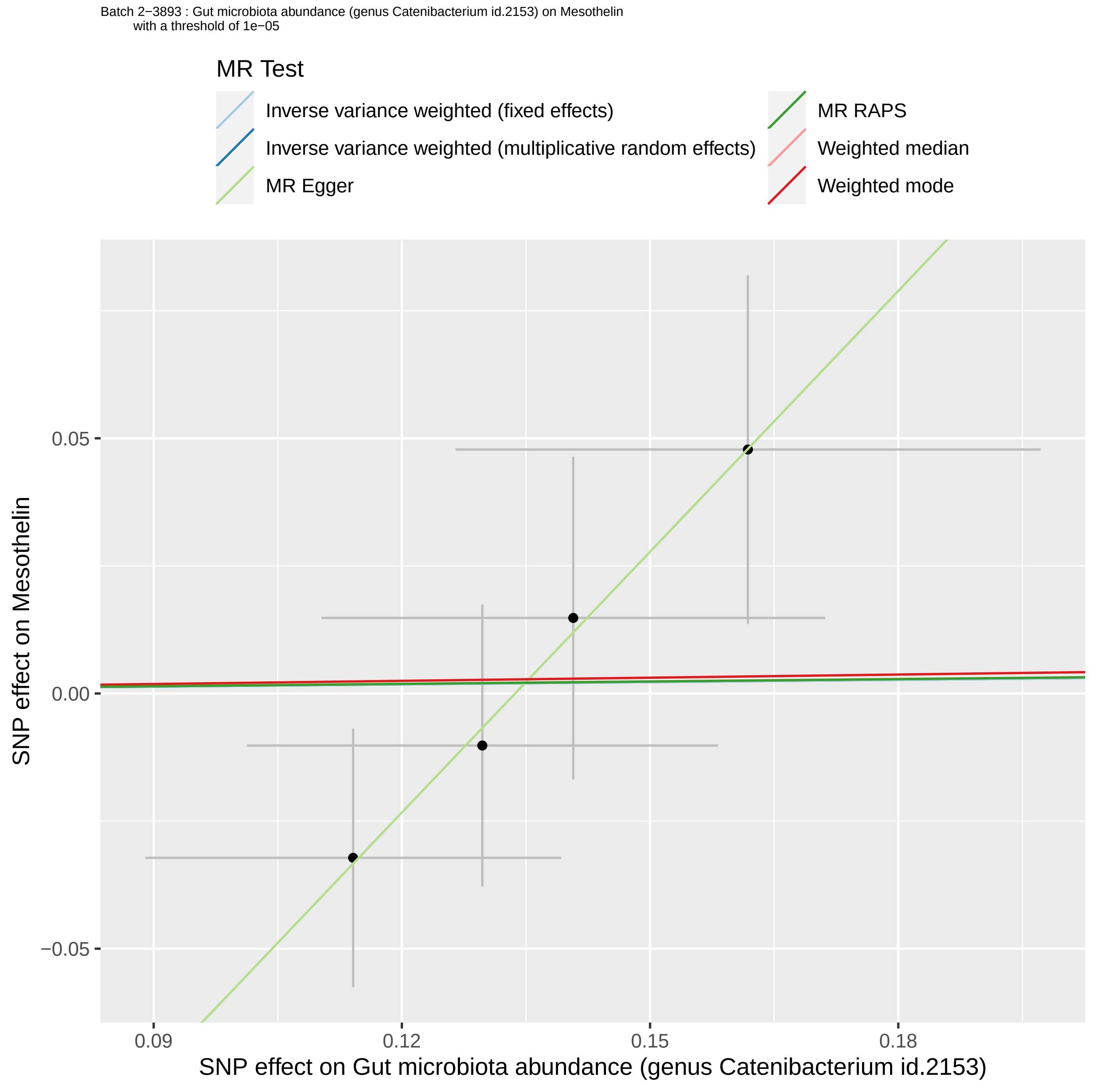

Batch 2−3893 : Gut microbiota abundance (genus Catenibacterium id.2153) on Mesothelin
with a threshold of 1e−05
MR Test
Inverse variance weighted (fixed effects)
Inverse variance weighted (multiplicative random effects)
MR Egger
MR RAPS
Weighted median
Weighted mode
0.05
0.00
−0.05
0.09
0.12
0.15
0.18
SNP effect on Mesothelin
SNP effect on Gut microbiota abundance (genus Catenibacterium id.2153)

Batch 2−3894 : Gut microbiota abundance (genus Parasutterella id.2892) on Mesothelin
with a threshold of 1e−05

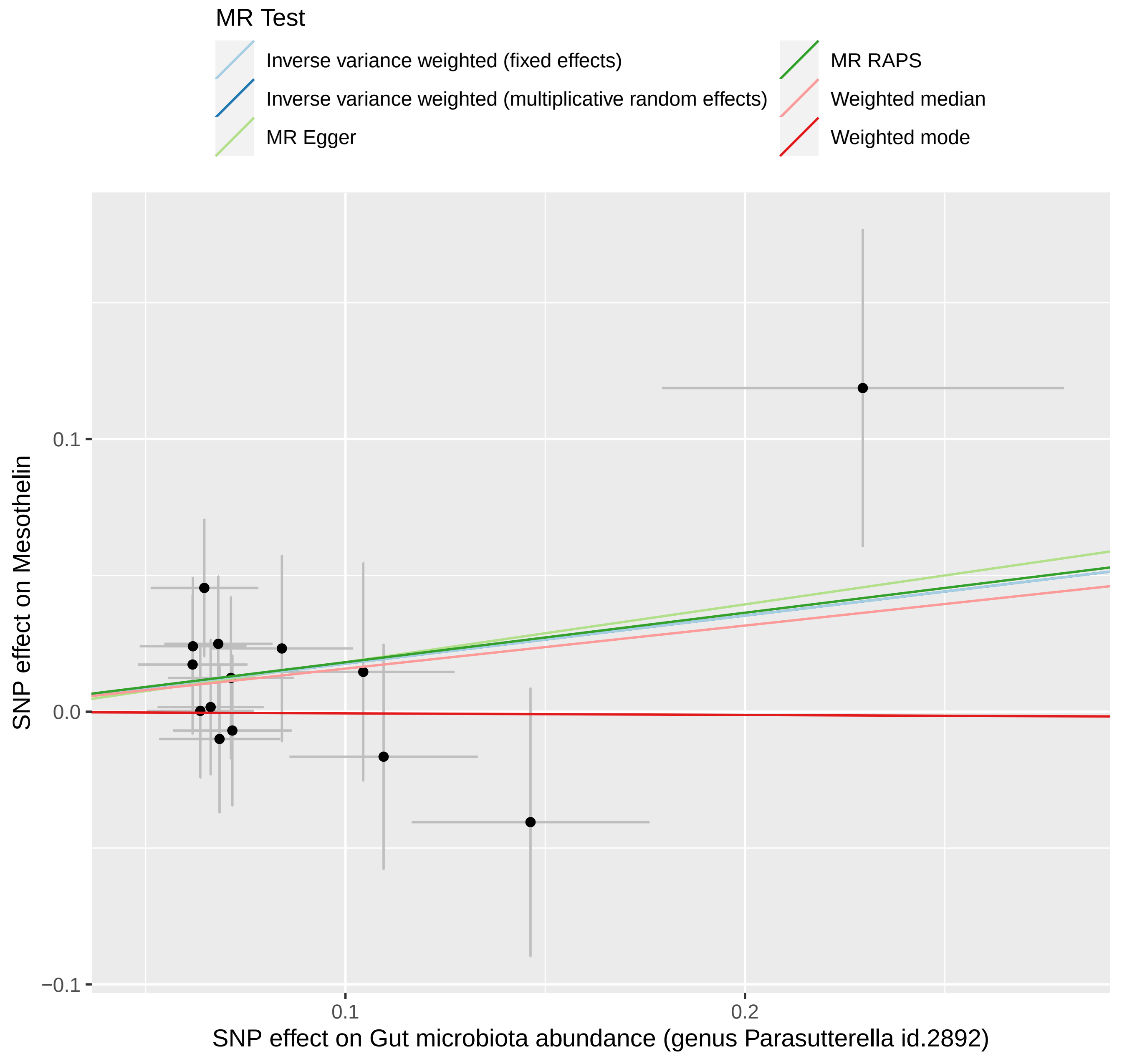

Batch 2−3895 : Gut microbiota abundance (genus Catenibacterium id.2153) on Mesothelin
with a threshold of 1e−05

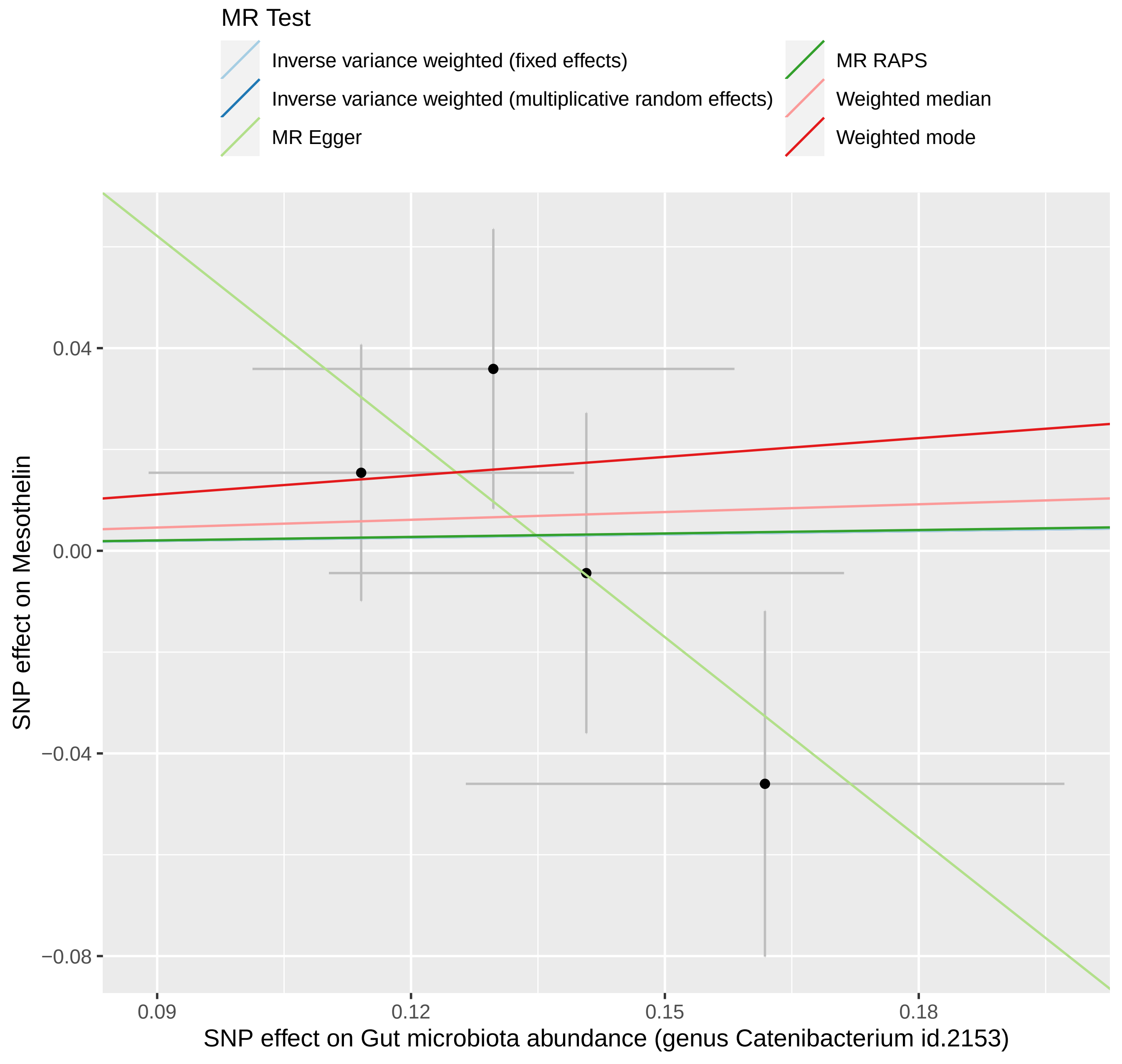

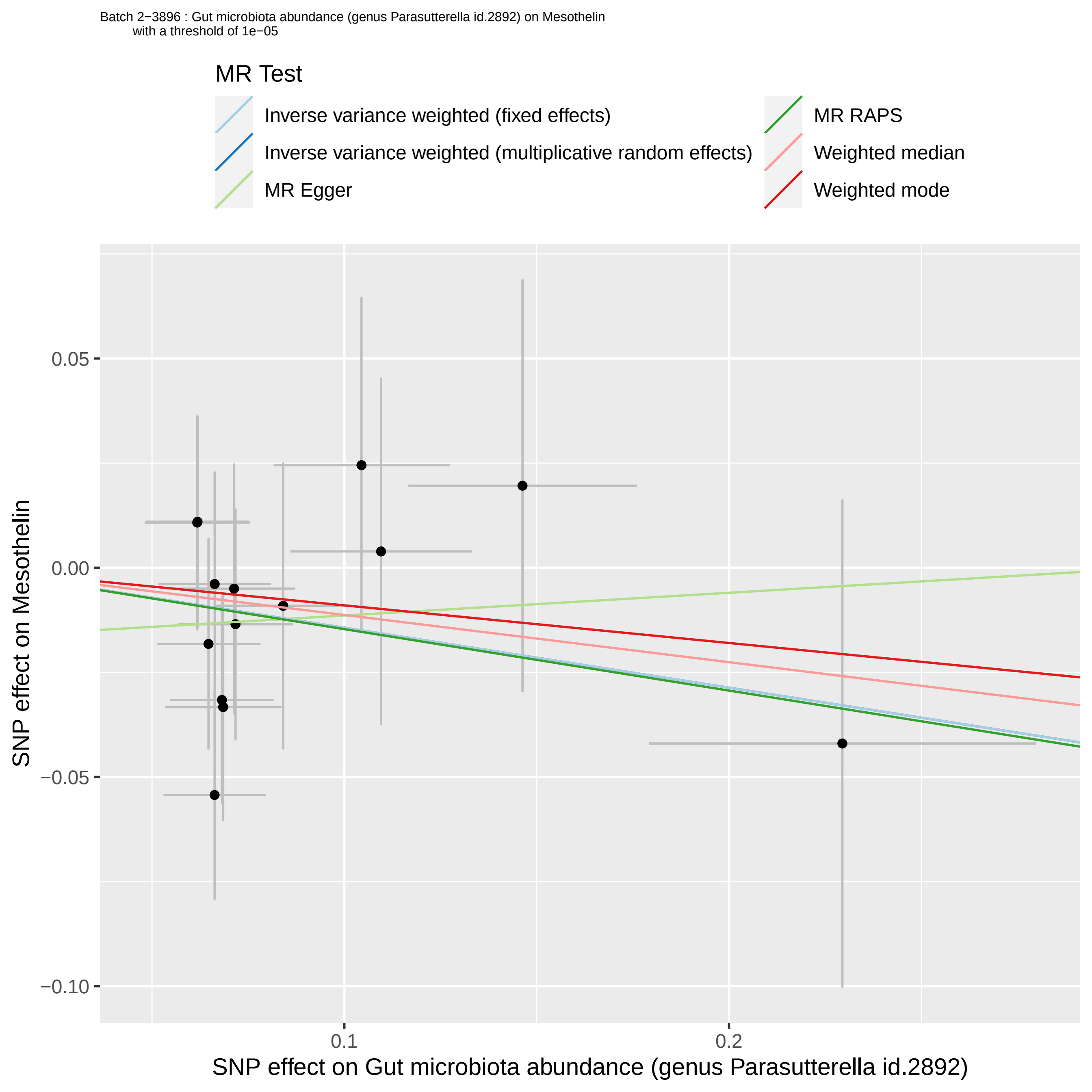
Batch 2−3896 : Gut microbiota abundance (genus Parasutterella id.2892) on Mesothelin
with a threshold of 1e−05
MR Test
Inverse variance weighted (fixed effects)
Inverse variance weighted (multiplicative random effects)
MR Egger
MR RAPS
Weighted median
Weighted mode
0.05
0.00
−0.05
−0.10
0.1
0.2
SNP effect on Mesothelin
SNP effect on Gut microbiota abundance (genus Parasutterella id.2892)

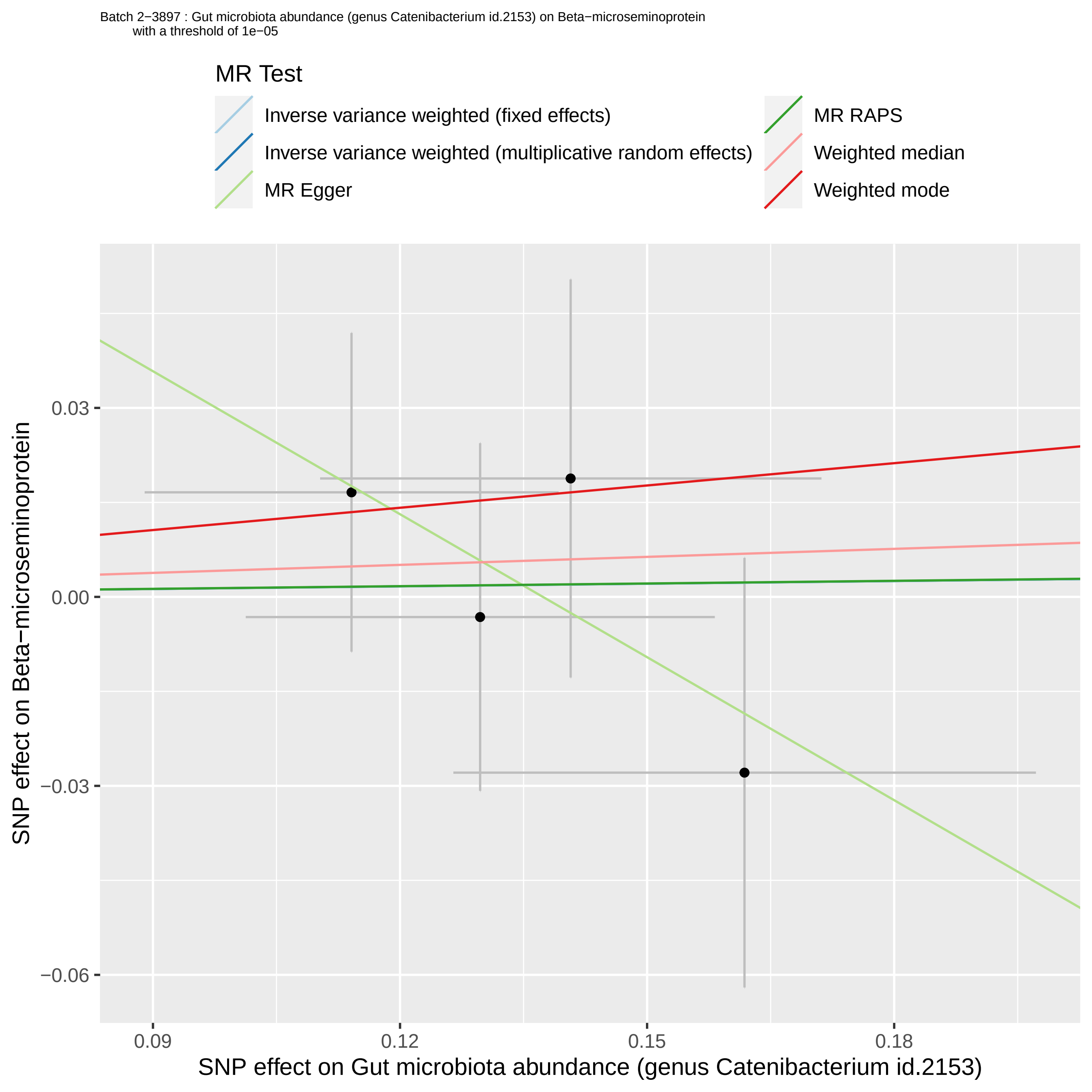

Batch 2−3897 : Gut microbiota abundance (genus Catenibacterium id.2153) on Beta−microseminoprotein
with a threshold of 1e−05
MR Test
Inverse variance weighted (fixed effects)
Inverse variance weighted (multiplicative random effects)
MR Egger
MR RAPS
Weighted median
Weighted mode
0.03
0.00
−0.03
−0.06
0.09
0.12
0.15
0.18
SNP effect on Beta−microseminoprotein
SNP effect on Gut microbiota abundance (genus Catenibacterium id.2153)

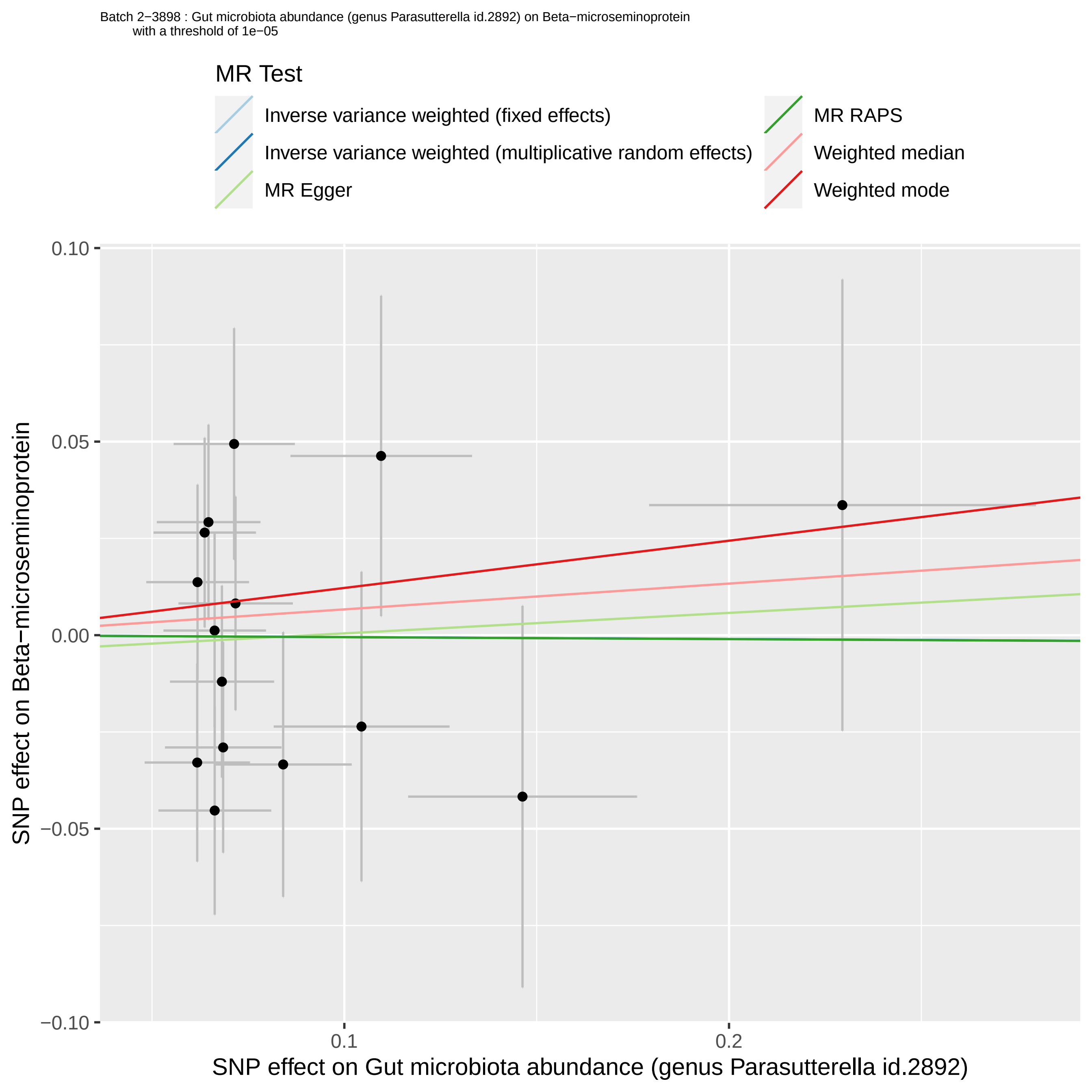

Batch 2−3898 : Gut microbiota abundance (genus Parasutterella id.2892) on Beta−microseminoprotein
with a threshold of 1e−05
MR Test
Inverse variance weighted (fixed effects)
Inverse variance weighted (multiplicative random effects)
MR Egger
MR RAPS
Weighted median
Weighted mode
0.10
0.05
0.00
−0.05
−0.10
0.1
0.2
SNP effect on Beta−microseminoprotein
SNP effect on Gut microbiota abundance (genus Parasutterella id.2892)

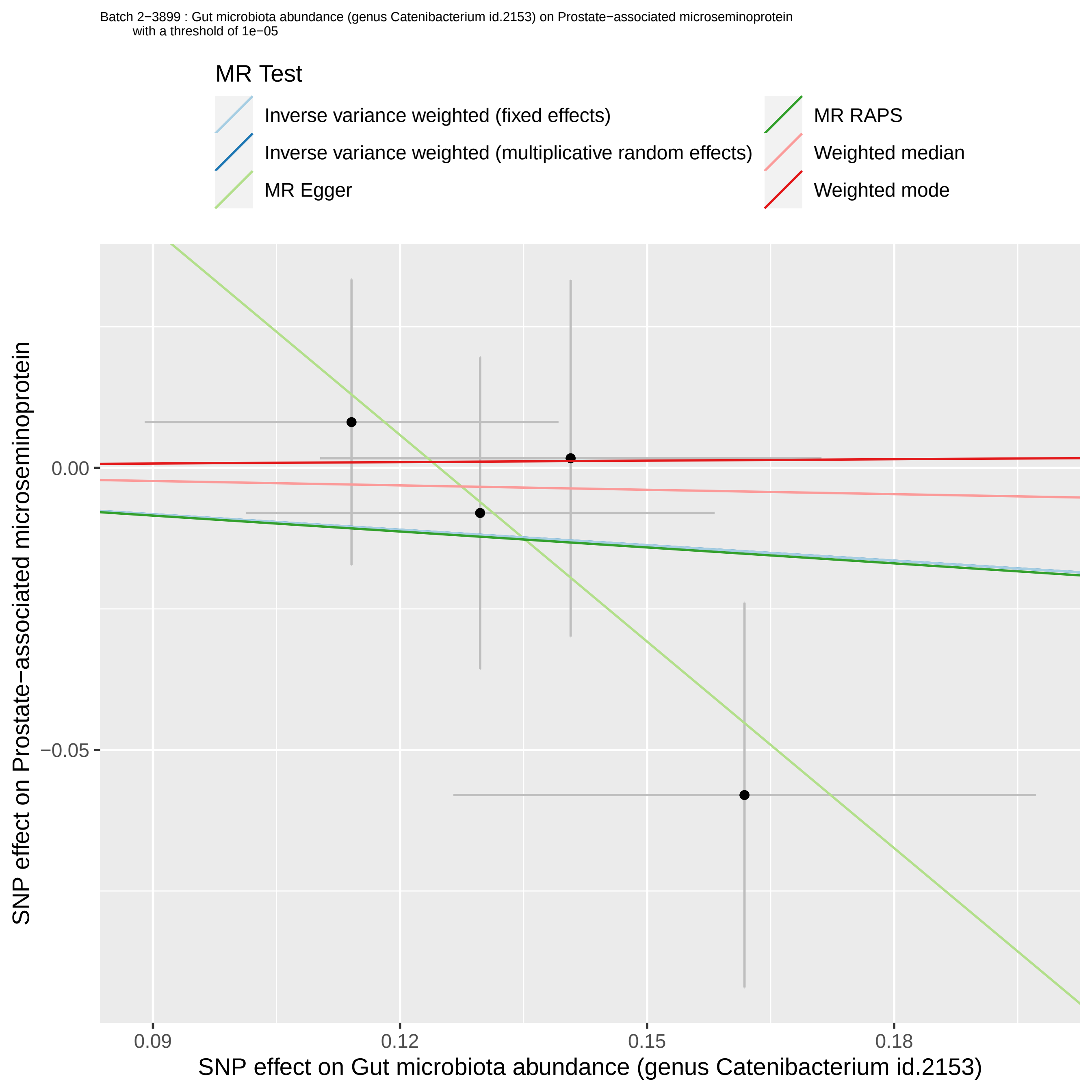

Batch 2−3899 : Gut microbiota abundance (genus Catenibacterium id.2153) on Prostate−associated microseminoprotein
with a threshold of 1e−05
MR Test
Inverse variance weighted (fixed effects)
Inverse variance weighted (multiplicative random effects)
MR Egger
MR RAPS
Weighted median
Weighted mode
0.00
−0.05
0.09
0.12
0.15
0.18
SNP effect on Gut microbiota abundance (genus Catenibacterium id.2153)
SNP effect on Prostate−associated microseminoprotein

Batch 2−3900 : Gut microbiota abundance (genus Parasutterella id.2892) on Prostate−associated microseminoprotein
with a threshold of 1e−05

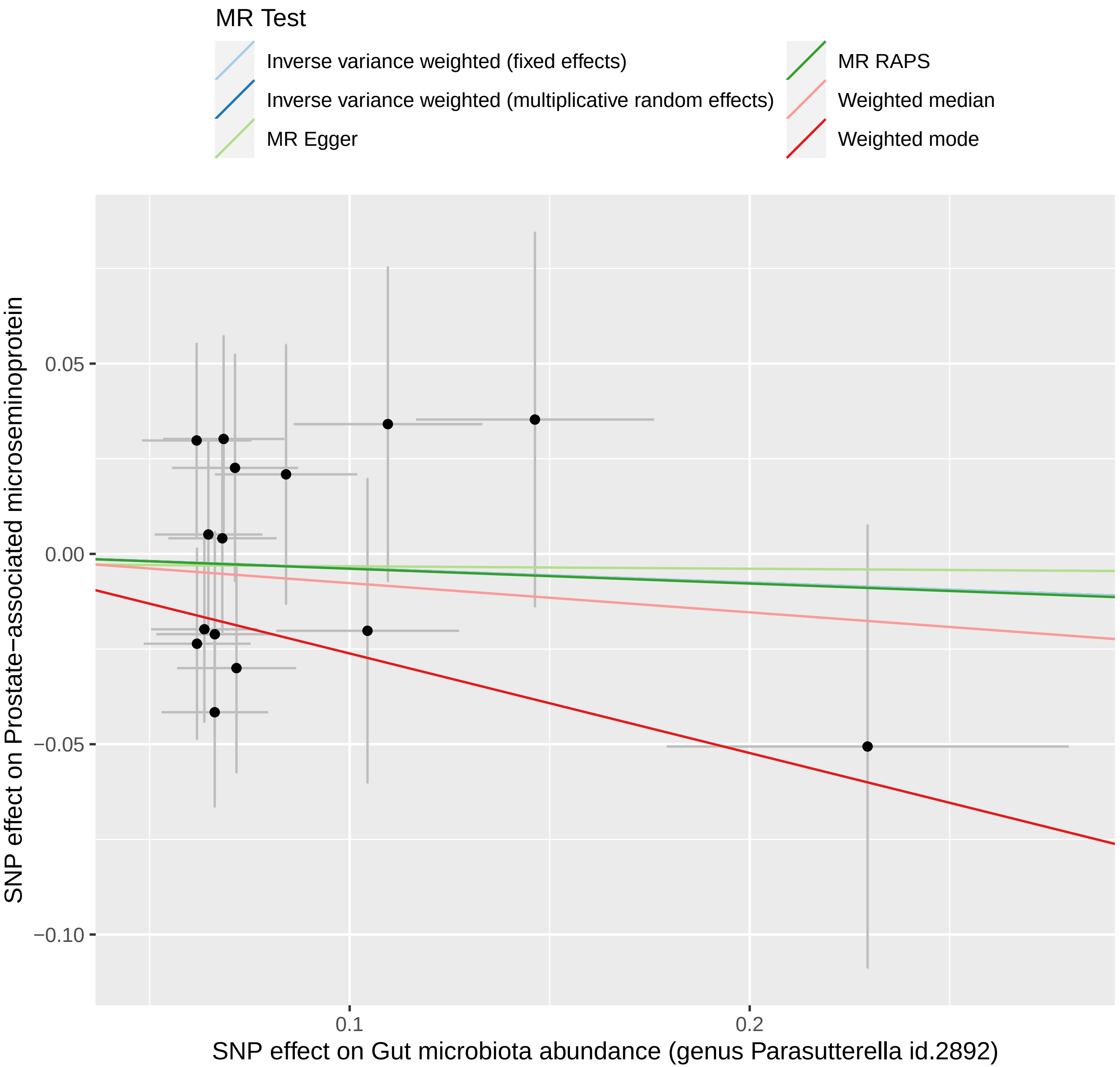

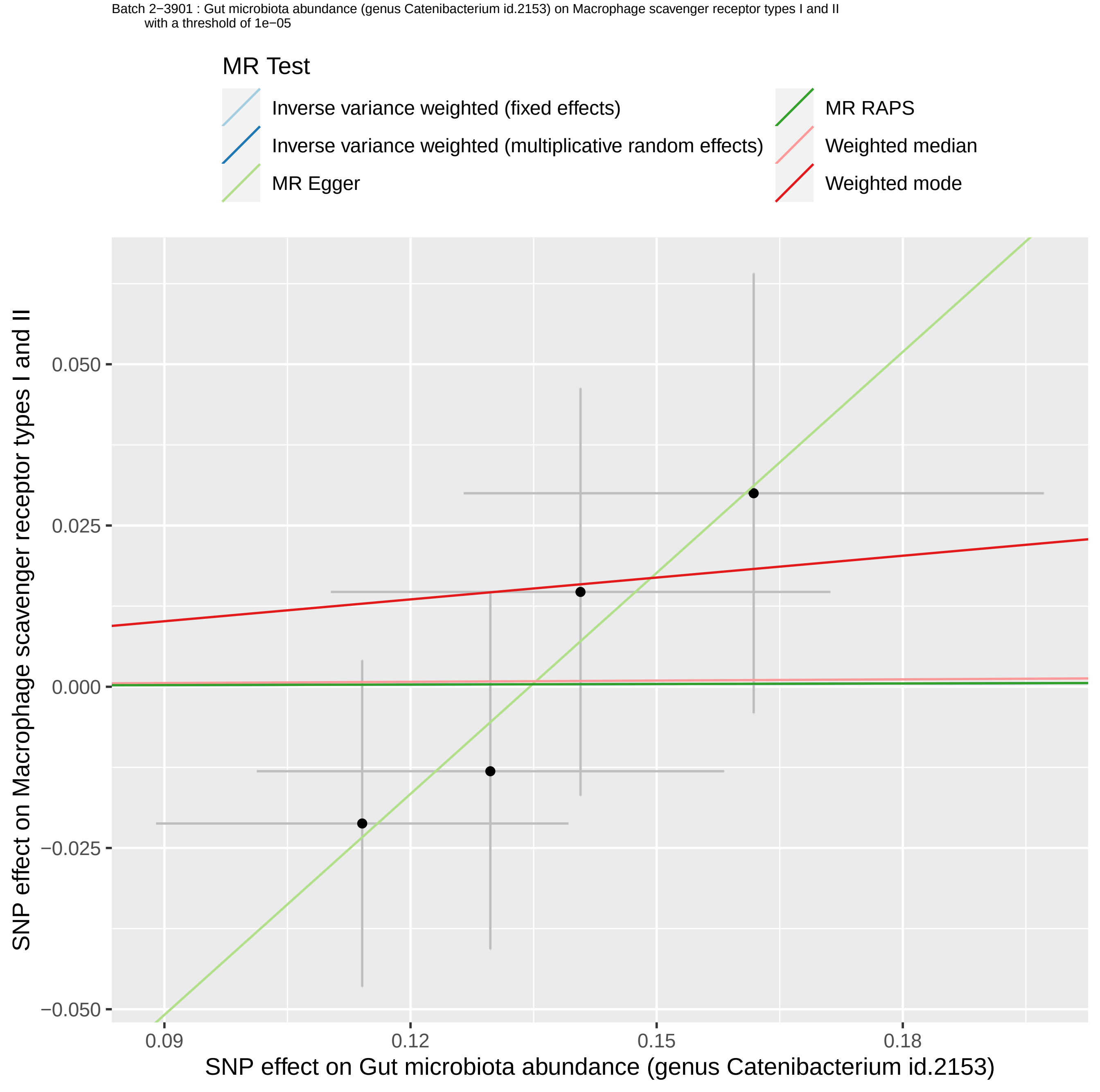

Batch 2−3901 : Gut microbiota abundance (genus Catenibacterium id.2153) on Macrophage scavenger receptor types I and II
with a threshold of 1e−05
MR Test
Inverse variance weighted (fixed effects)
Inverse variance weighted (multiplicative random effects)
MR Egger
MR RAPS
Weighted median
Weighted mode
0.050
0.025
0.000
−0.025
−0.050
0.09
0.12
0.15
0.18
SNP effect on Macrophage scavenger receptor types I and II
SNP effect on Gut microbiota abundance (genus Catenibacterium id.2153)

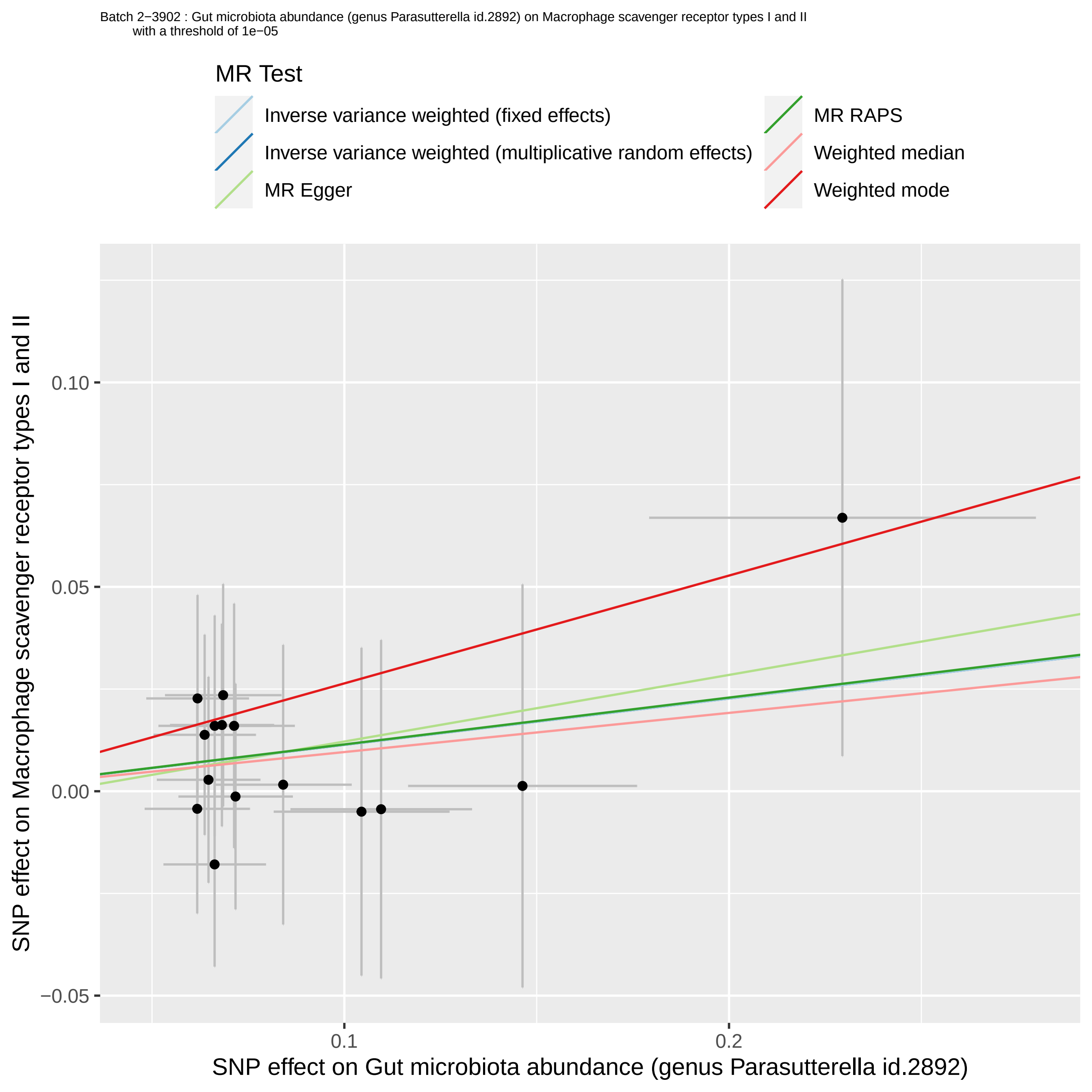
Batch 2−3902 : Gut microbiota abundance (genus Parasutterella id.2892) on Macrophage scavenger receptor types I and II
with a threshold of 1e−05
MR Test
Inverse variance weighted (fixed effects)
Inverse variance weighted (multiplicative random effects)
MR Egger
MR RAPS
Weighted median
Weighted mode
SNP effect on Macrophage scavenger receptor types I and II
0.10
0.05
0.00
−0.05
0.1
0.2
SNP effect on Gut microbiota abundance (genus Parasutterella id.2892)

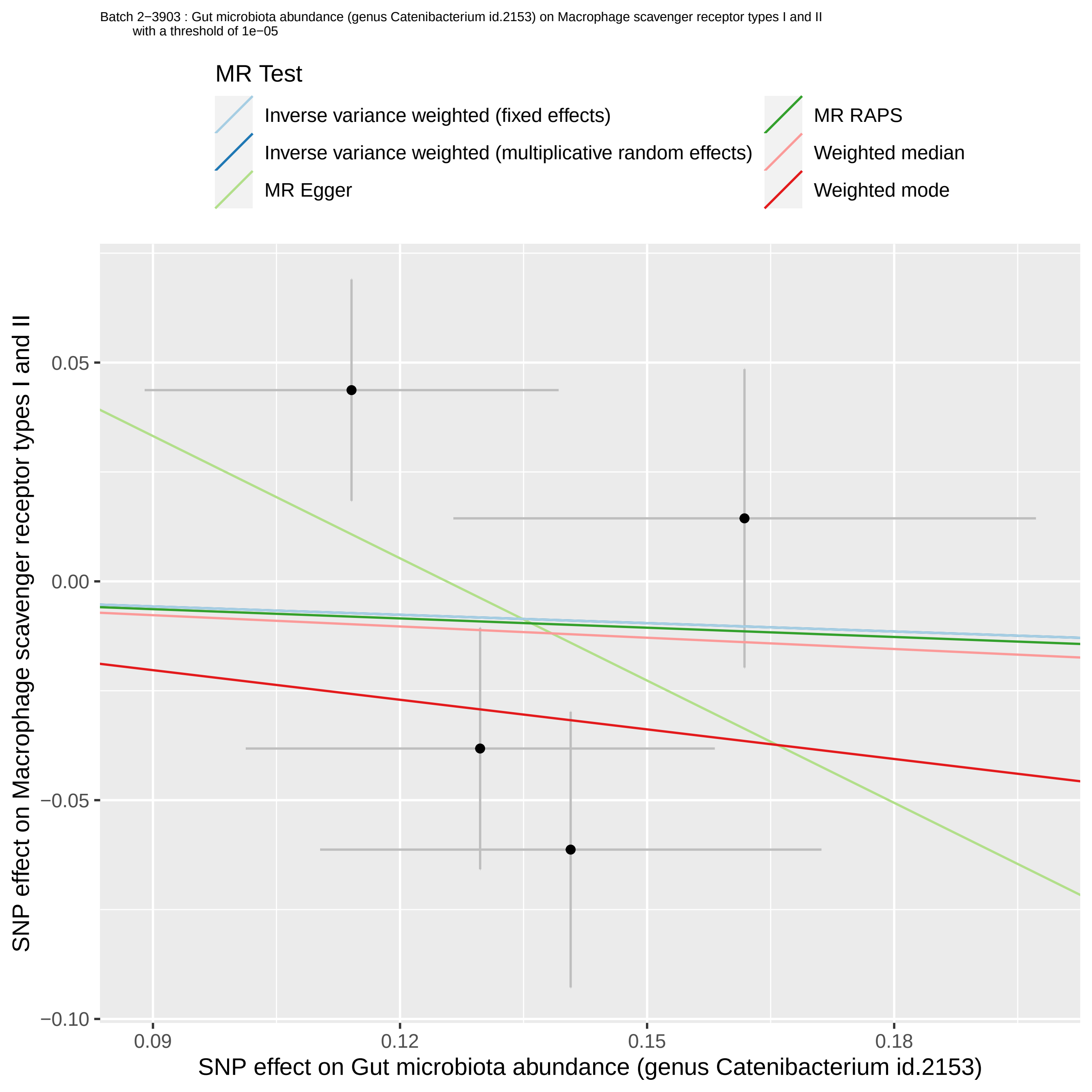
Batch 2−3903 : Gut microbiota abundance (genus Catenibacterium id.2153) on Macrophage scavenger receptor types I and II
with a threshold of 1e−05
MR Test
Inverse variance weighted (fixed effects)
Inverse variance weighted (multiplicative random effects)
MR Egger
MR RAPS
Weighted median
Weighted mode
0.05
0.00
−0.05
−0.10
0.09
0.12
0.15
0.18
SNP effect on Macrophage scavenger receptor types I and II
SNP effect on Gut microbiota abundance (genus Catenibacterium id.2153)

Batch 2−3904 : Gut microbiota abundance (genus Parasutterella id.2892) on Macrophage scavenger receptor types I and II
with a threshold of 1e−05

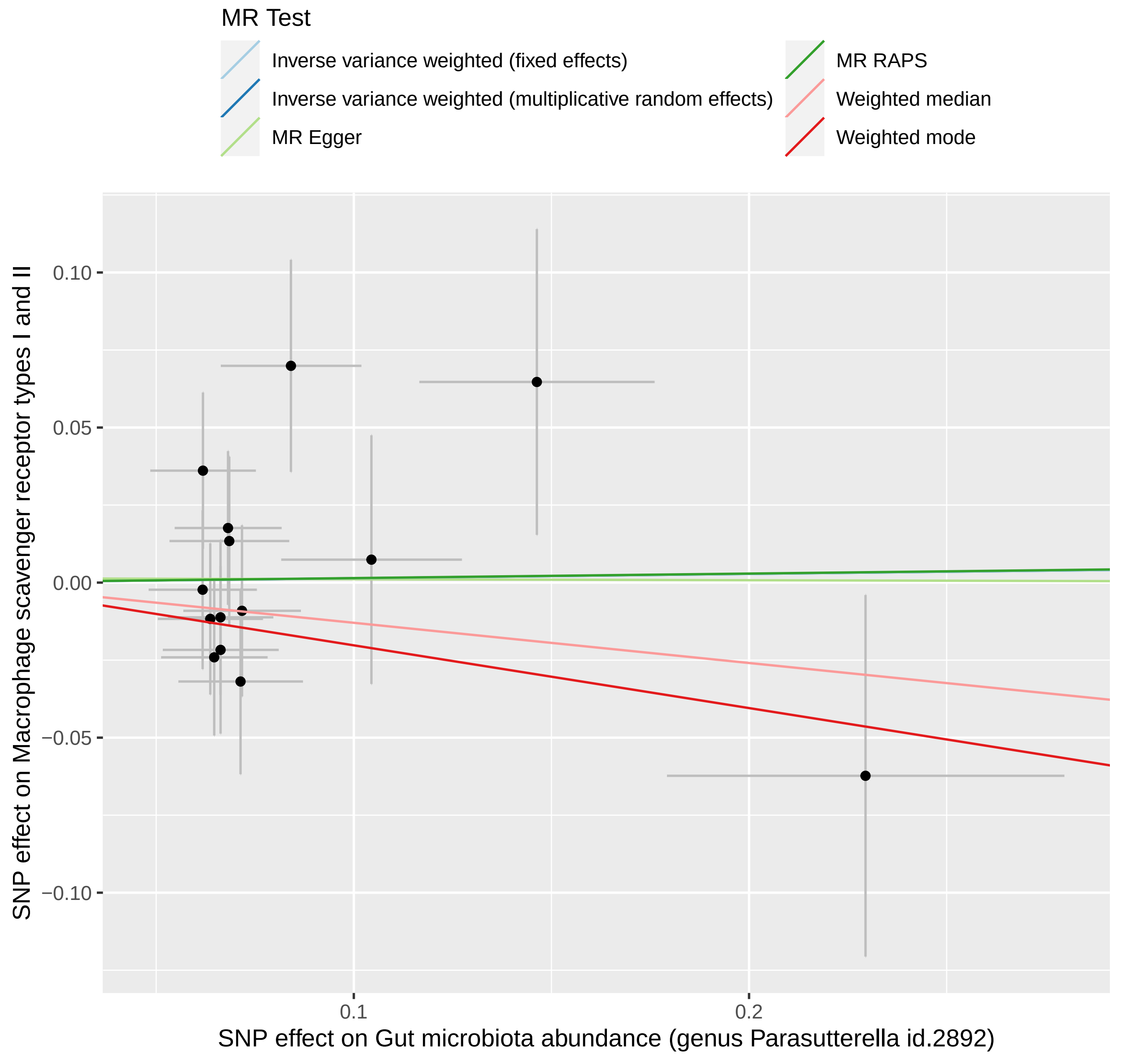

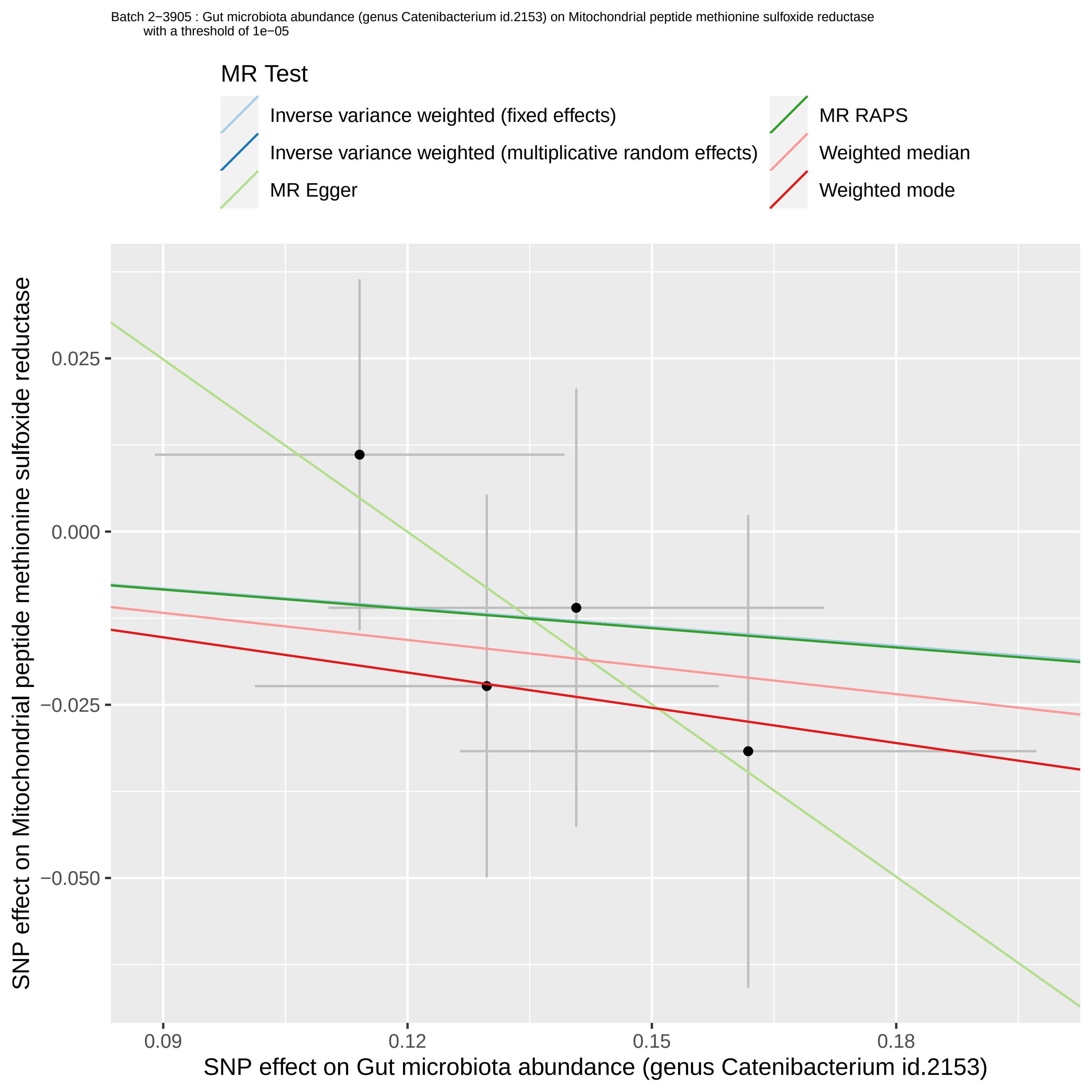
Batch 2−3905 : Gut microbiota abundance (genus Catenibacterium id.2153) on Mitochondrial peptide methionine sulfoxide reductase
with a threshold of 1e−05
MR Test
Inverse variance weighted (fixed effects)
Inverse variance weighted (multiplicative random effects)
MR Egger
MR RAPS
Weighted median
Weighted mode
0.025
0.000
−0.025
−0.050
0.09
0.12
0.15
0.18
SNP effect on Mitochondrial peptide methionine sulfoxide reductase
SNP effect on Gut microbiota abundance (genus Catenibacterium id.2153)

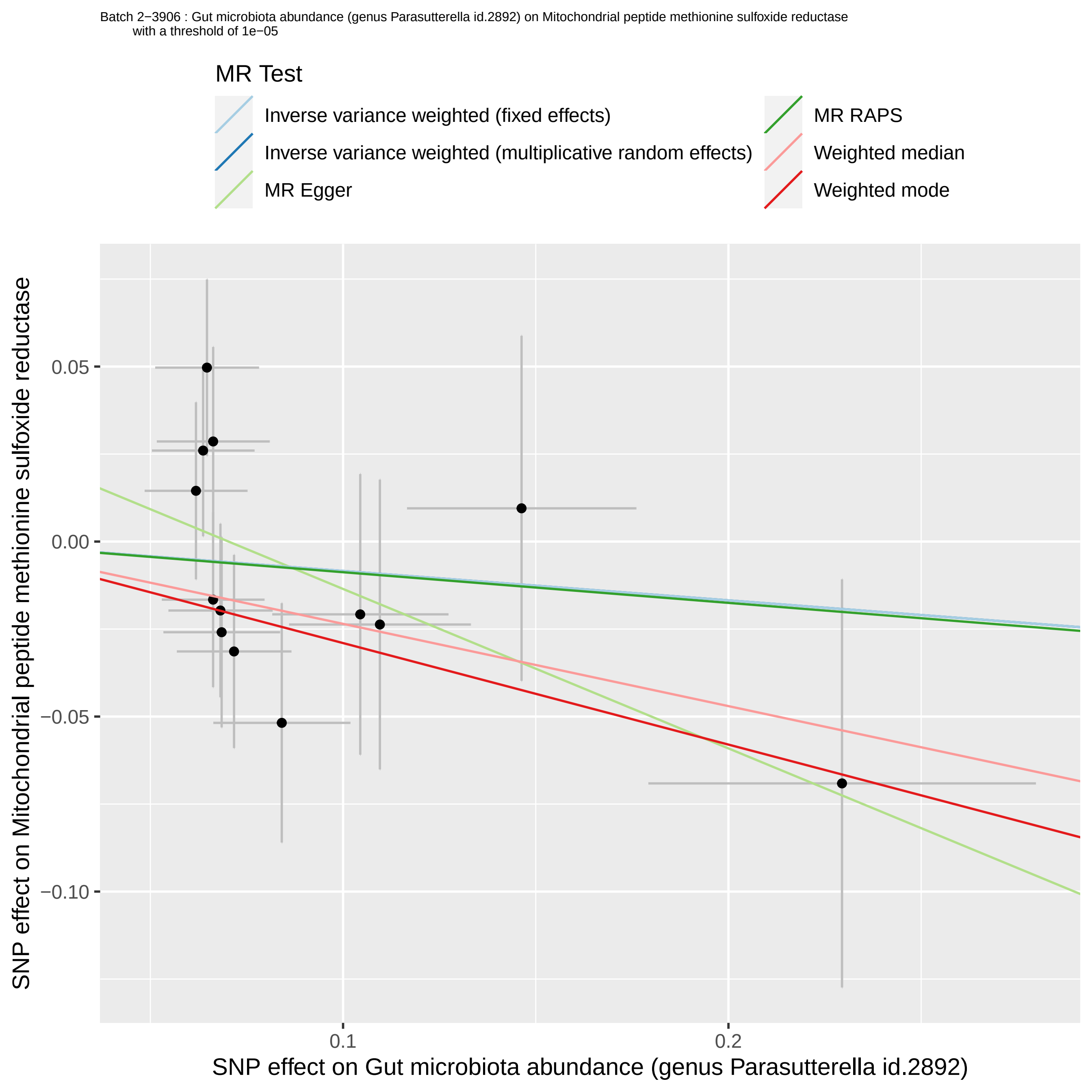
Batch 2−3906 : Gut microbiota abundance (genus Parasutterella id.2892) on Mitochondrial peptide methionine sulfoxide reductase
with a threshold of 1e−05
MR Test
Inverse variance weighted (fixed effects)
Inverse variance weighted (multiplicative random effects)
MR Egger
MR RAPS
Weighted median
Weighted mode
0.05
0.00
−0.05
−0.10
0.1
0.2
SNP effect on Mitochondrial peptide methionine sulfoxide reductase
SNP effect on Gut microbiota abundance (genus Parasutterella id.2892)

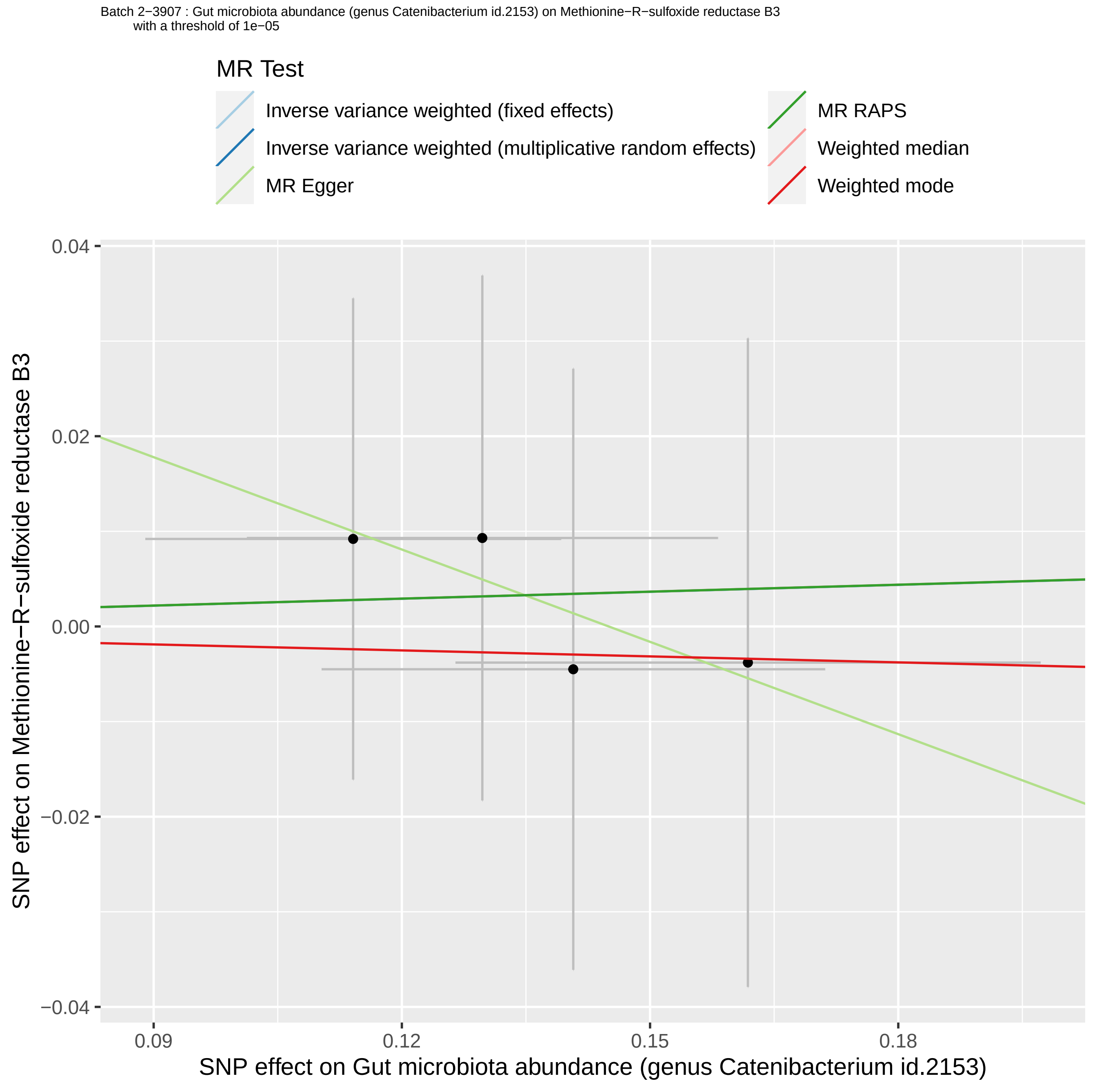

Batch 2−3907 : Gut microbiota abundance (genus Catenibacterium id.2153) on Methionine−R−sulfoxide reductase B3
with a threshold of 1e−05
MR Test
Inverse variance weighted (fixed effects)
Inverse variance weighted (multiplicative random effects)
MR Egger
MR RAPS
Weighted median
Weighted mode
0.04
0.02
0.00
−0.02
−0.04
0.09
0.12
0.15
0.18
SNP effect on Methionine−R−sulfoxide reductase B3
SNP effect on Gut microbiota abundance (genus Catenibacterium id.2153)

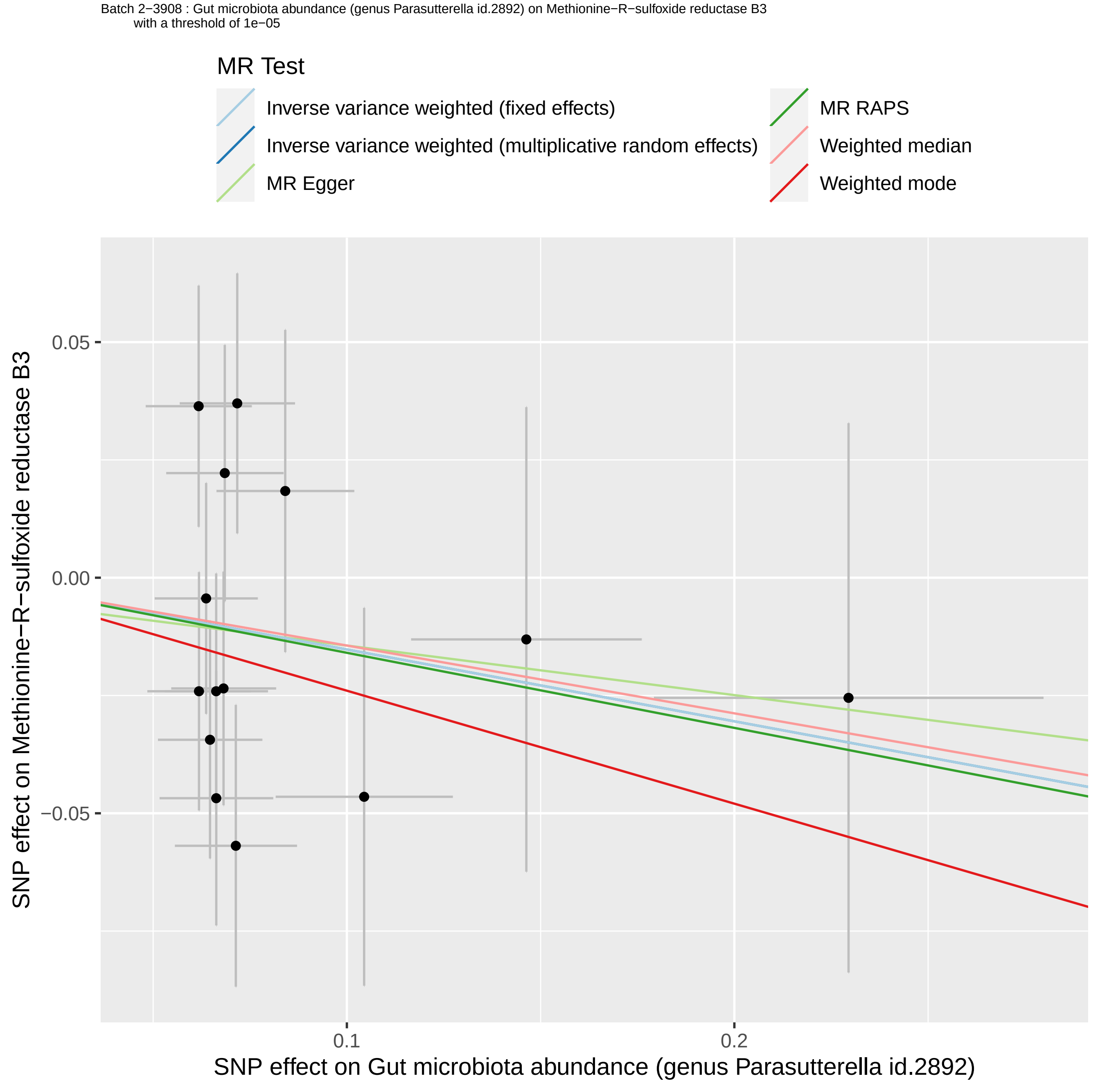
Batch 2−3908 : Gut microbiota abundance (genus Parasutterella id.2892) on Methionine−R−sulfoxide reductase B3
with a threshold of 1e−05
MR Test
Inverse variance weighted (fixed effects)
Inverse variance weighted (multiplicative random effects)
MR Egger
MR RAPS
Weighted median
Weighted mode
0.05
0.00
−0.05
0.1
0.2
SNP effect on Methionine−R−sulfoxide reductase B3
SNP effect on Gut microbiota abundance (genus Parasutterella id.2892)

Batch 2−3909 : Gut microbiota abundance (genus Catenibacterium id.2153) on Macrophage−stimulating protein receptor
with a threshold of 1e−05

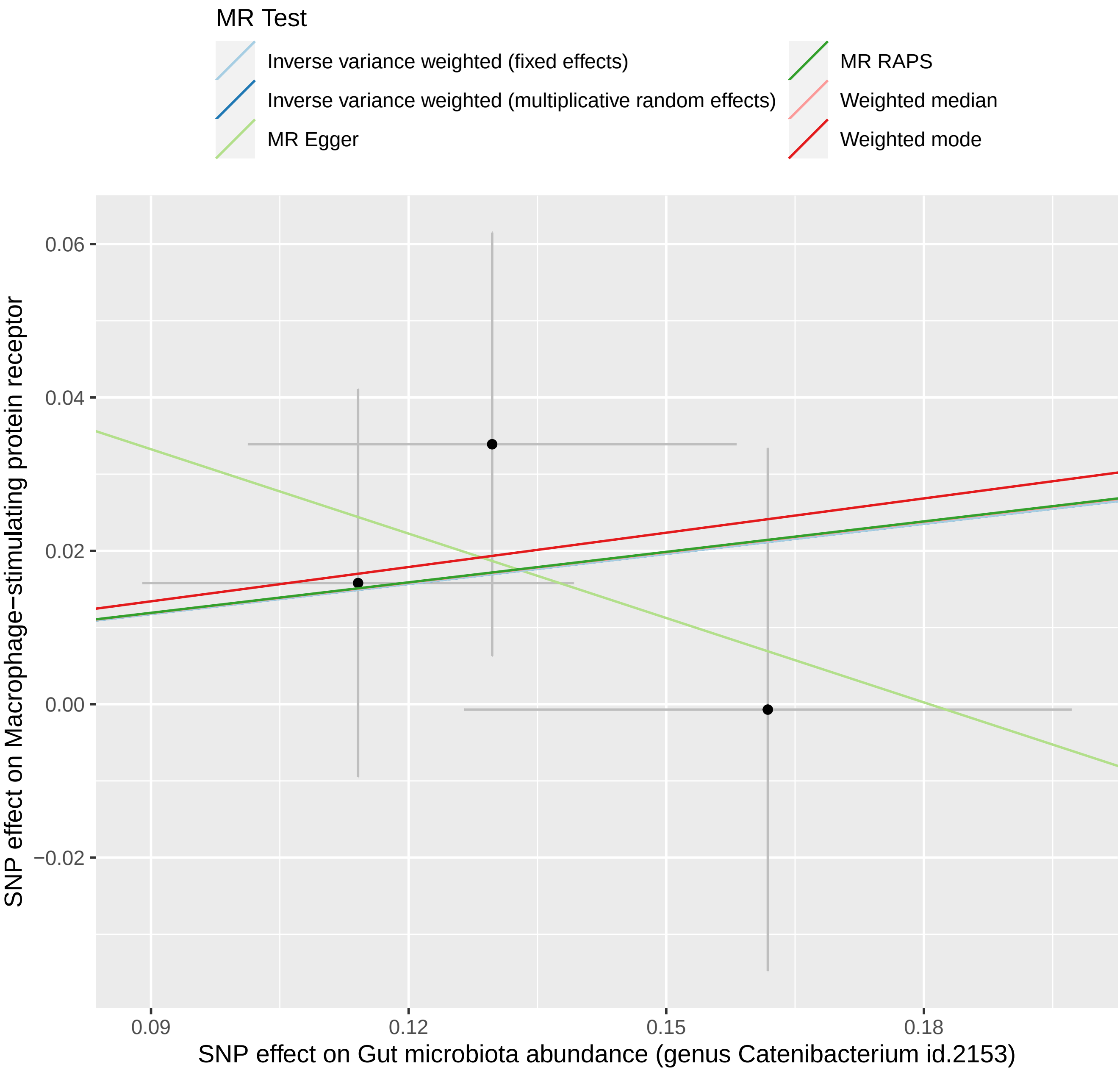

Batch 2−3910 : Gut microbiota abundance (genus Parasutterella id.2892) on Macrophage−stimulating protein receptor
with a threshold of 1e−05

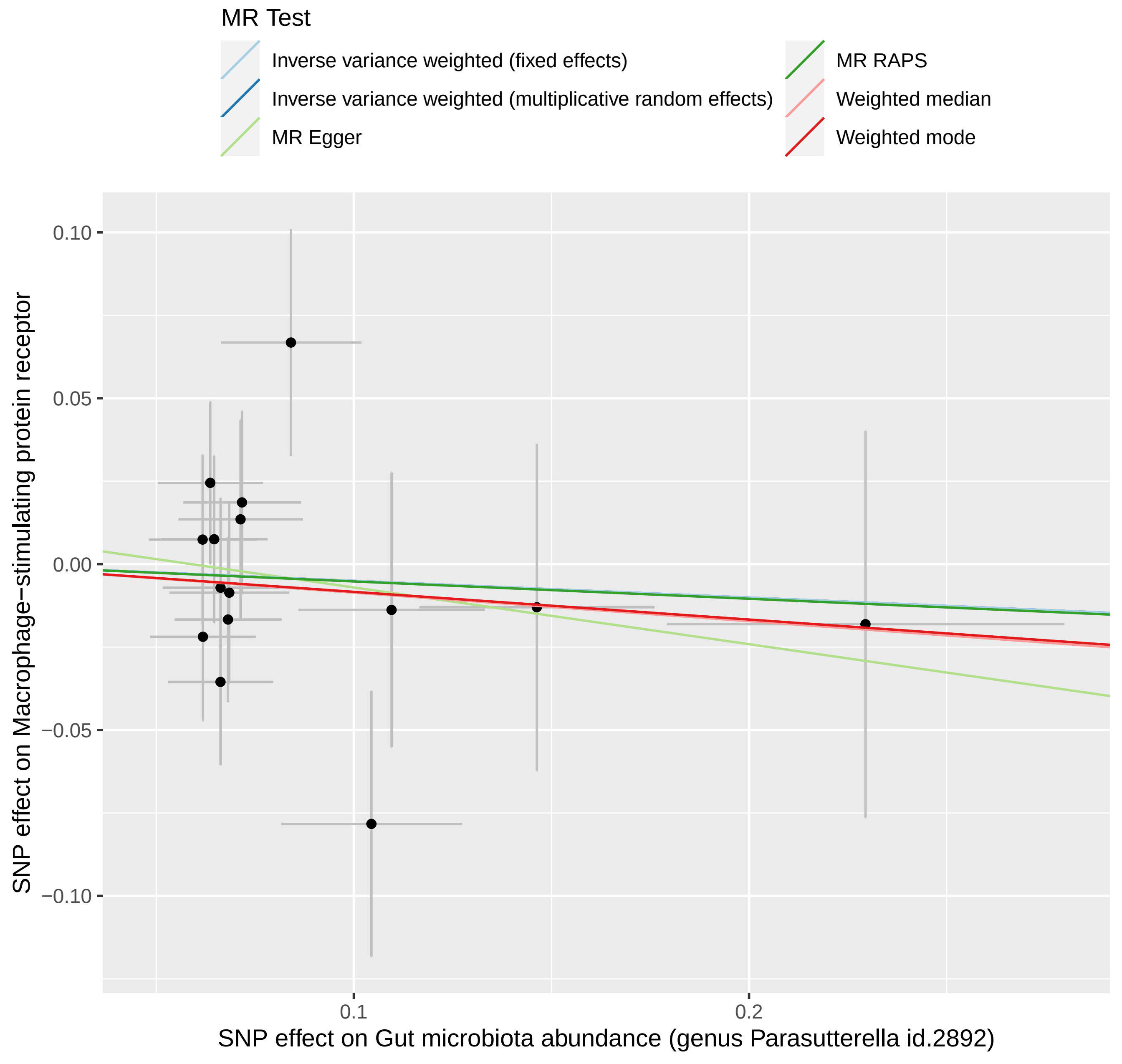

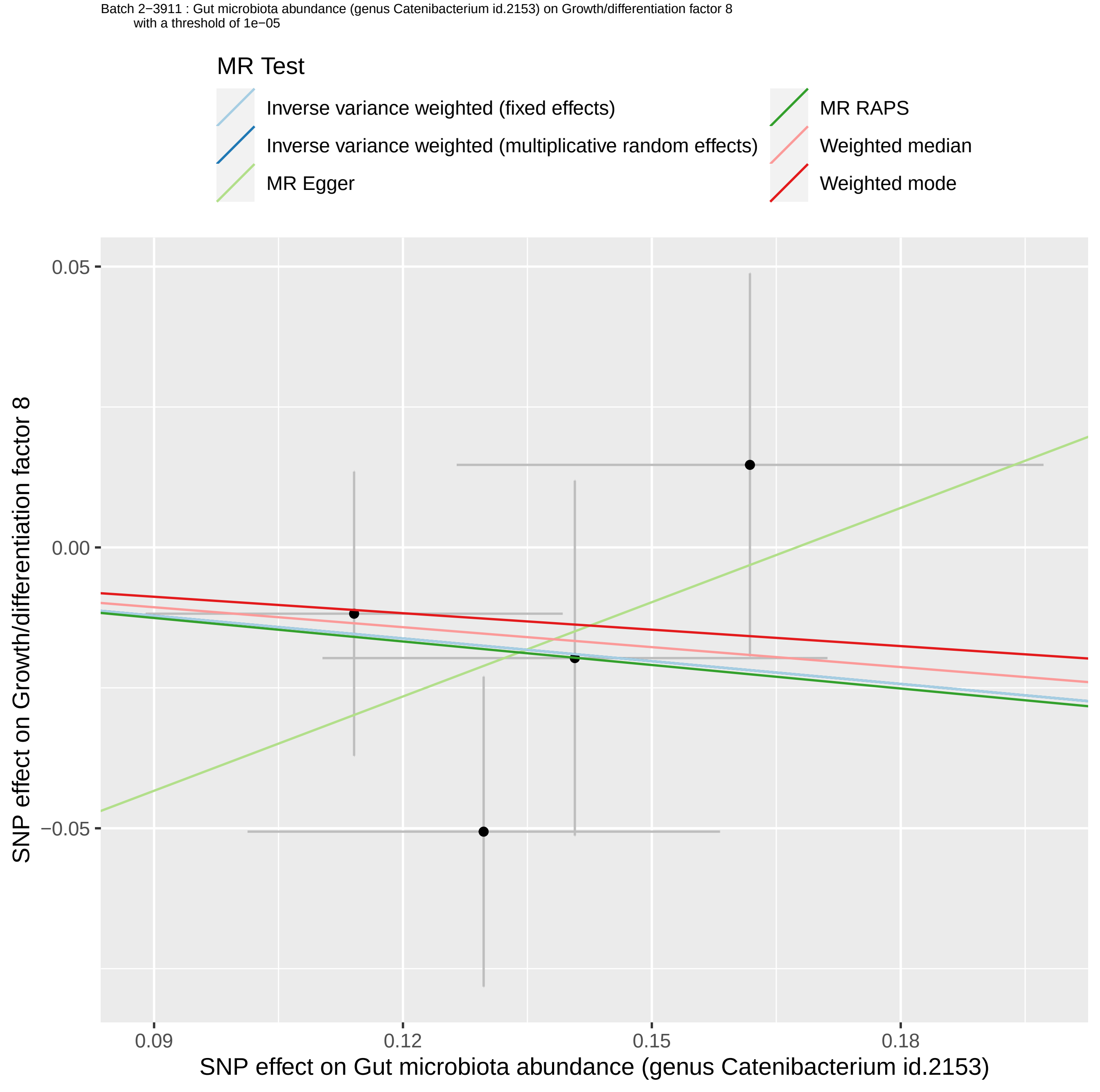
Batch 2−3911 : Gut microbiota abundance (genus Catenibacterium id.2153) on Growth/differentiation factor 8
with a threshold of 1e−05
MR Test
Inverse variance weighted (fixed effects)
Inverse variance weighted (multiplicative random effects)
MR Egger
MR RAPS
Weighted median
Weighted mode
0.05
0.00
−0.05
0.09
0.12
0.15
0.18
SNP effect on Growth/differentiation factor 8
SNP effect on Gut microbiota abundance (genus Catenibacterium id.2153)

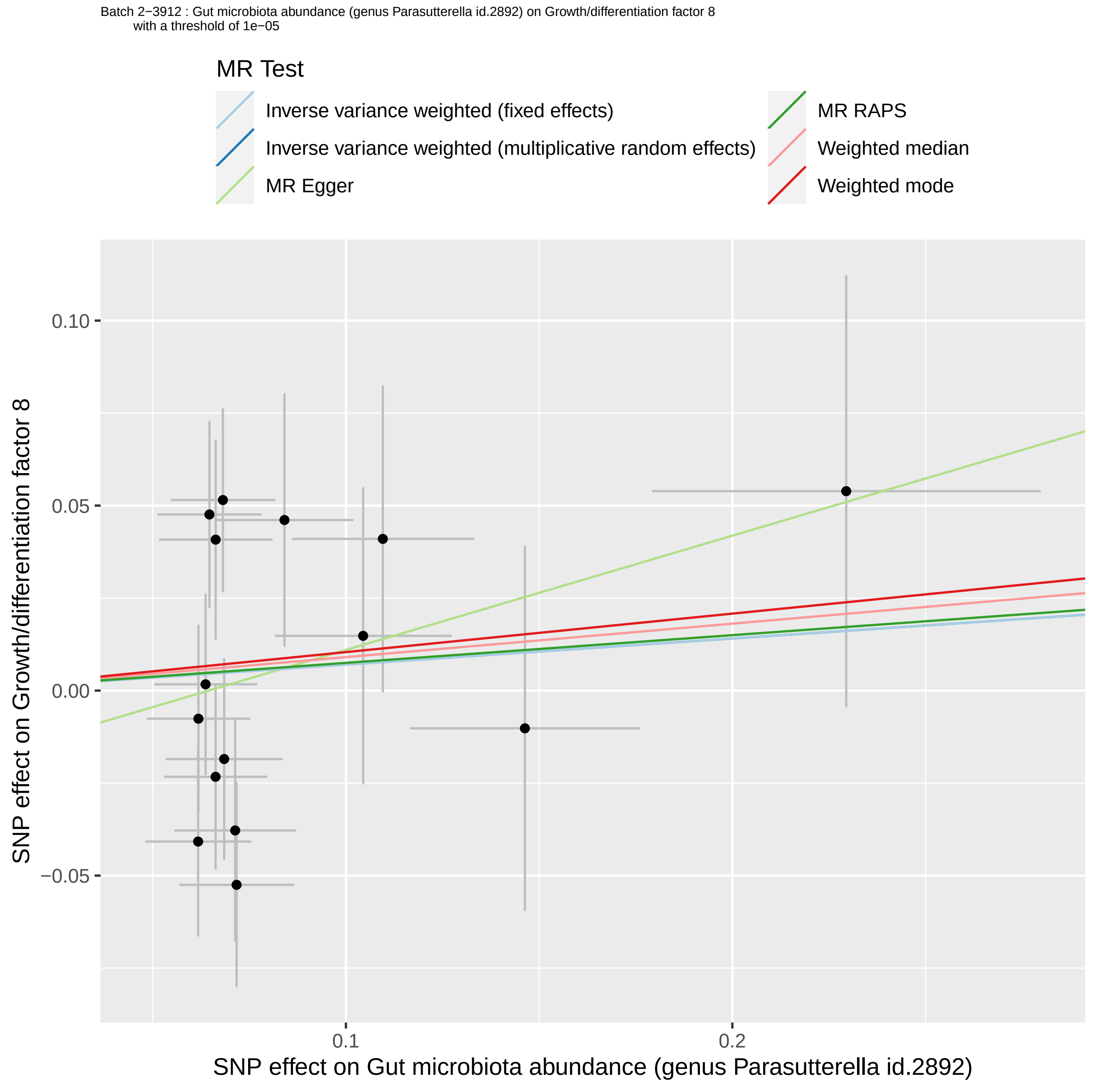

Batch 2−3912 : Gut microbiota abundance (genus Parasutterella id.2892) on Growth/differentiation factor 8
with a threshold of 1e−05
MR Test
Inverse variance weighted (fixed effects)
Inverse variance weighted (multiplicative random effects)
MR Egger
MR RAPS
Weighted median
Weighted mode
0.10
0.05
0.00
−0.05
0.1
0.2
SNP effect on Growth/differentiation factor 8
SNP effect on Gut microbiota abundance (genus Parasutterella id.2892)

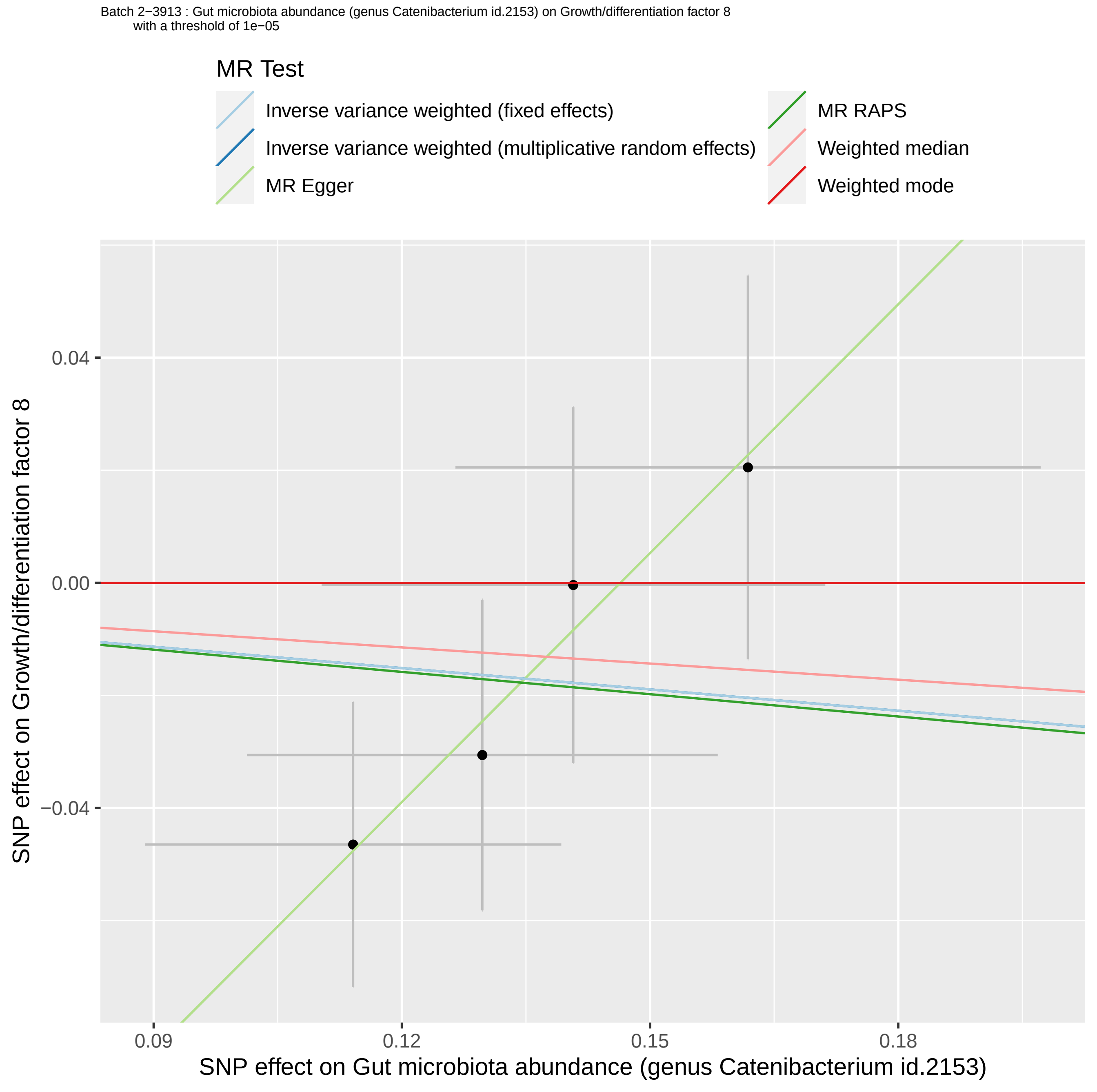
Batch 2−3913 : Gut microbiota abundance (genus Catenibacterium id.2153) on Growth/differentiation factor 8
with a threshold of 1e−05
MR Test
Inverse variance weighted (fixed effects)
Inverse variance weighted (multiplicative random effects)
MR Egger
MR RAPS
Weighted median
Weighted mode
0.04
0.00
−0.04
0.09
0.12
0.15
0.18
SNP effect on Growth/differentiation factor 8
SNP effect on Gut microbiota abundance (genus Catenibacterium id.2153)

Batch 2−3914 : Gut microbiota abundance (genus Parasutterella id.2892) on Growth/differentiation factor 8
with a threshold of 1e−05

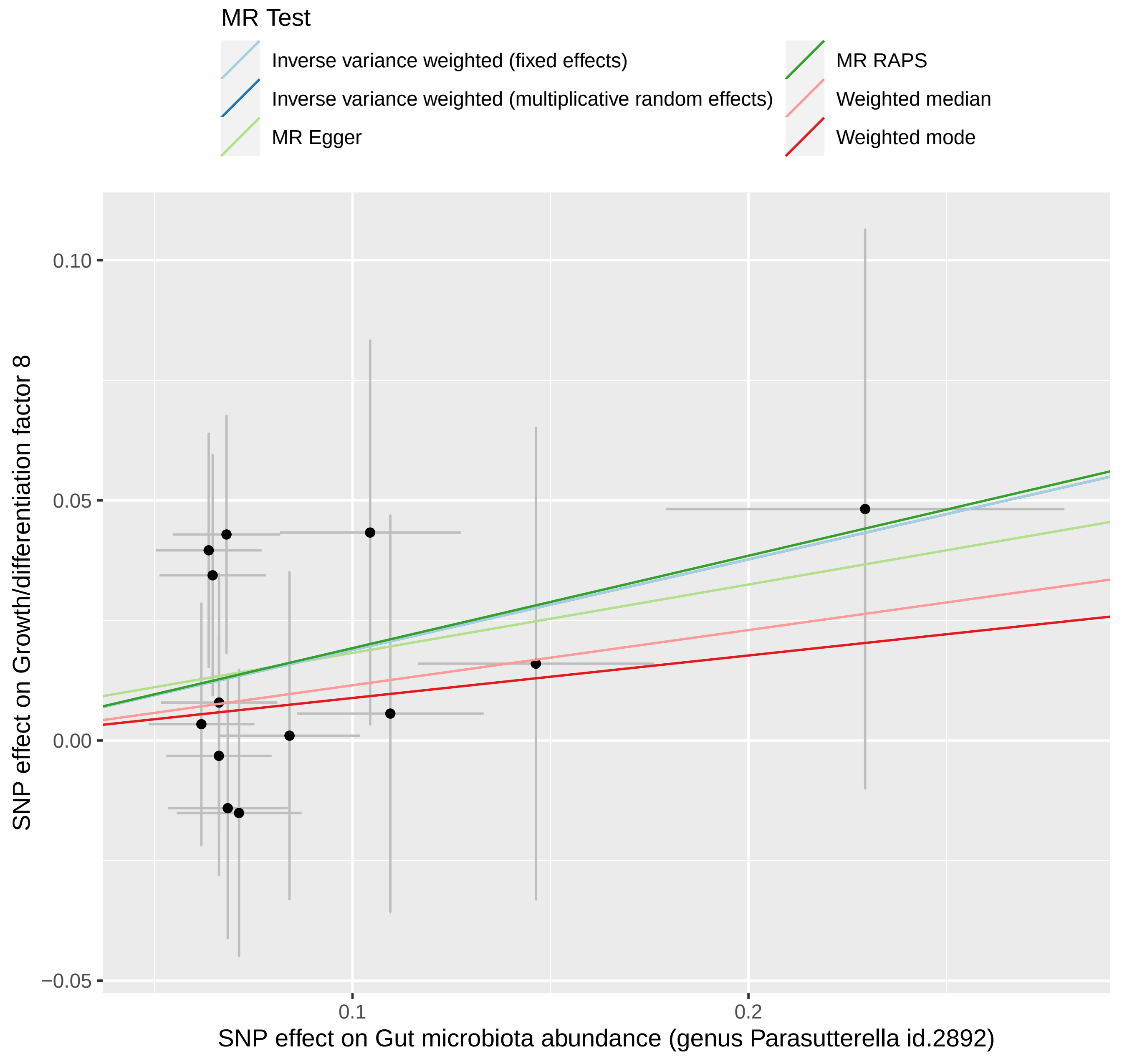

Batch 2−3915 : Gut microbiota abundance (genus Catenibacterium id.2153) on Metallothionein−1F
with a threshold of 1e−05

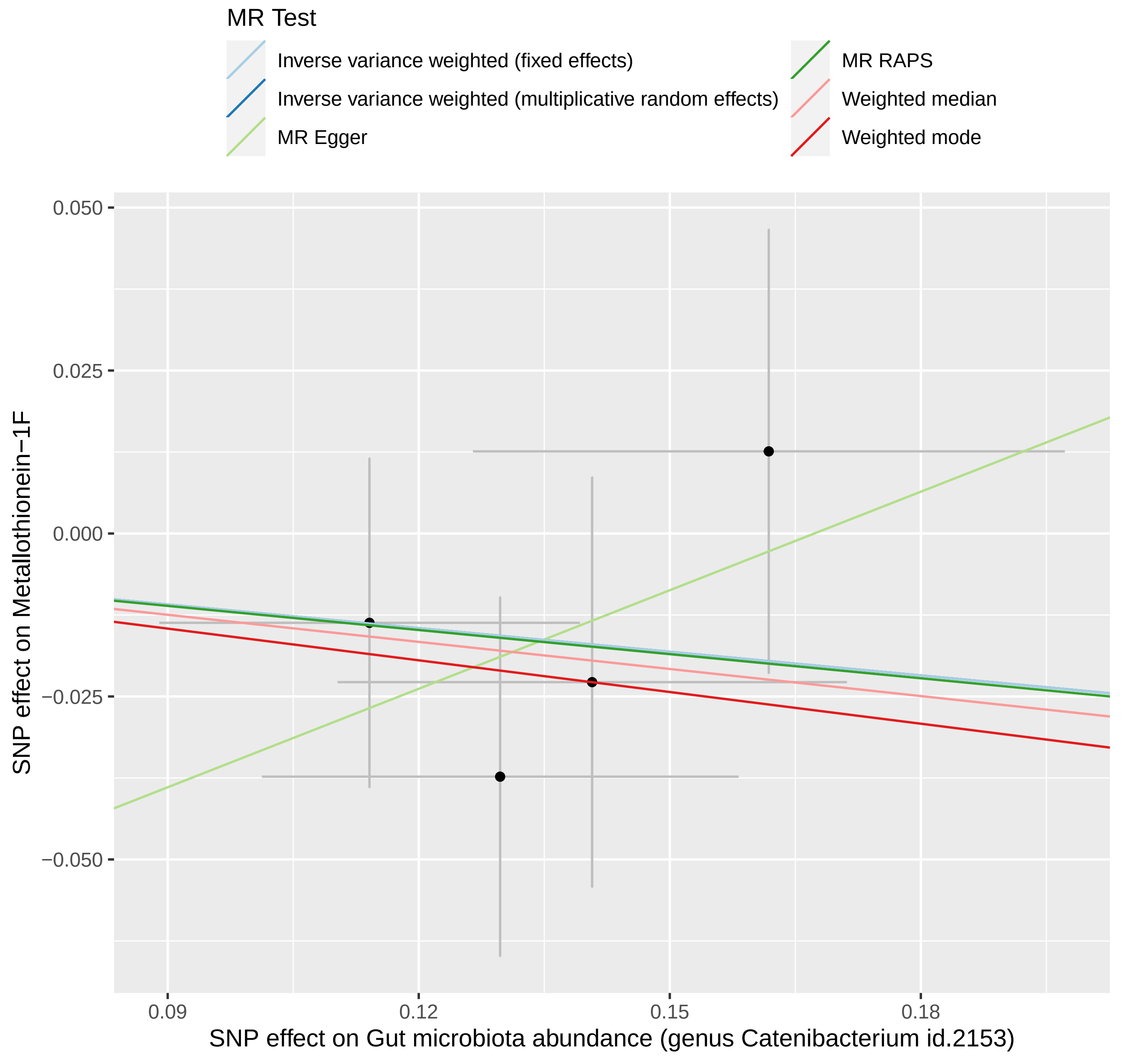

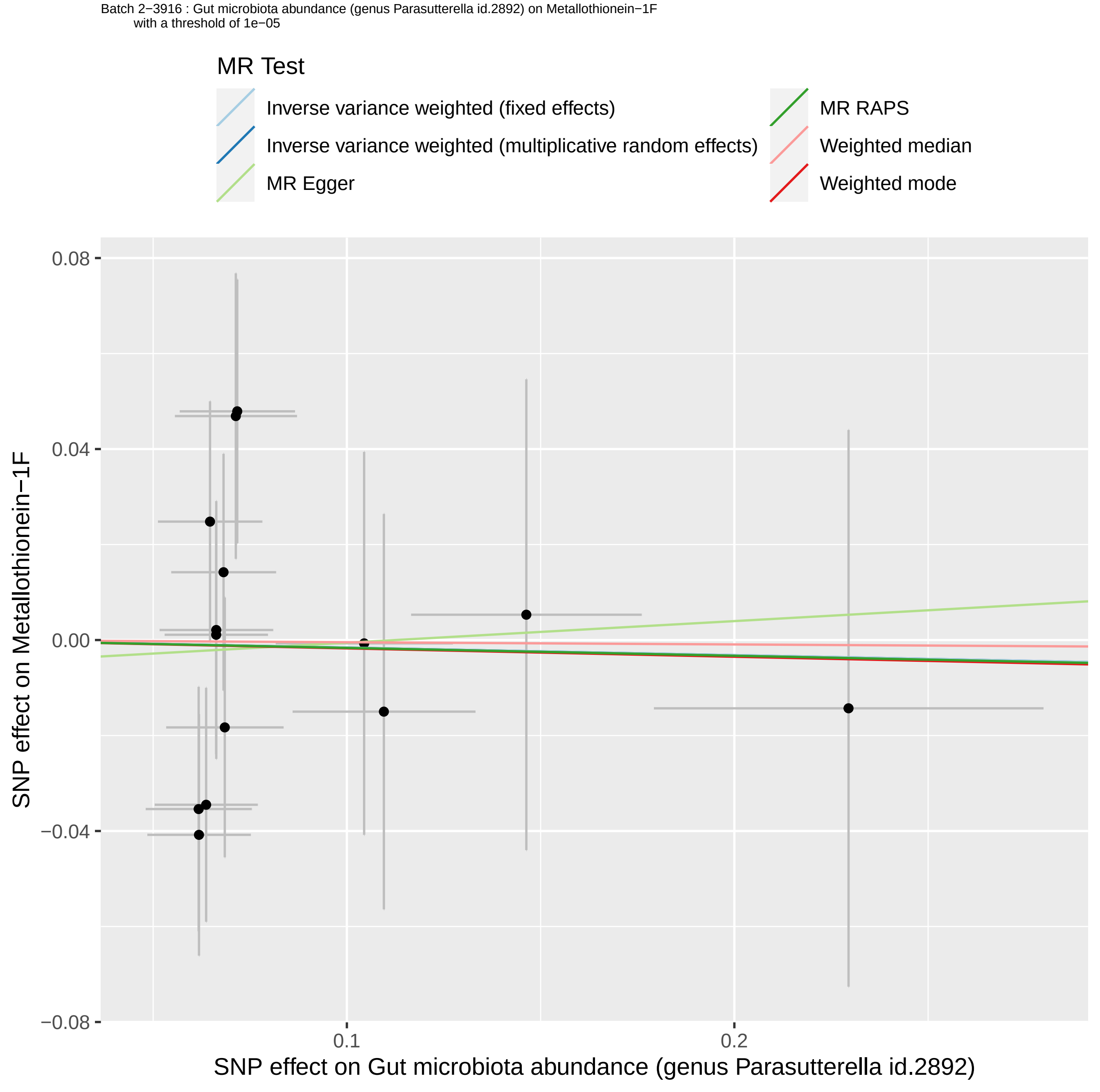

Batch 2−3916 : Gut microbiota abundance (genus Parasutterella id.2892) on Metallothionein−1F
with a threshold of 1e−05
MR Test
Inverse variance weighted (fixed effects)
Inverse variance weighted (multiplicative random effects)
MR Egger
MR RAPS
Weighted median
Weighted mode
0.08
0.04
0.00
−0.04
−0.08
0.1
0.2
SNP effect on Metallothionein−1F
SNP effect on Gut microbiota abundance (genus Parasutterella id.2892)

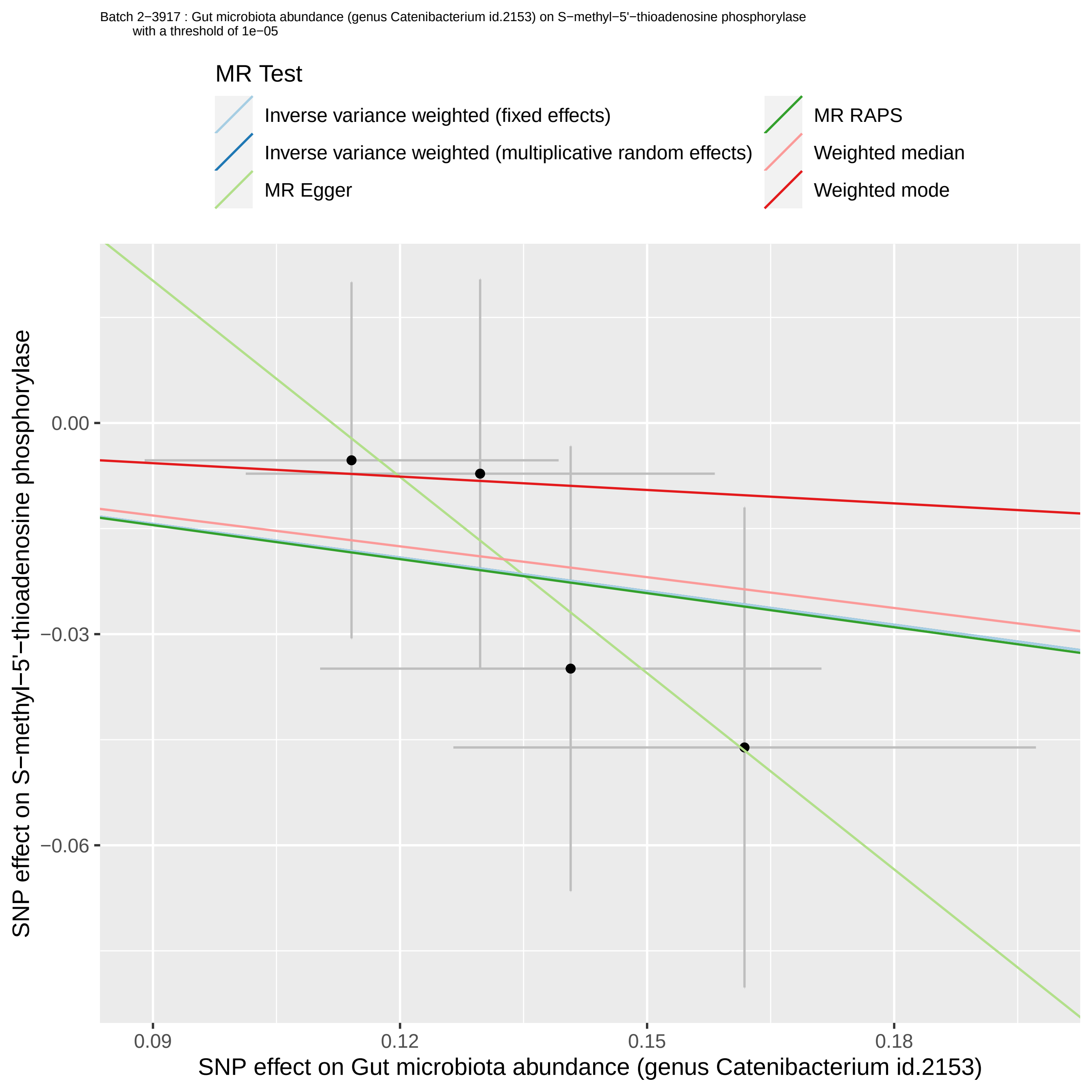
Batch 2−3917 : Gut microbiota abundance (genus Catenibacterium id.2153) on S−methyl−5'−thioadenosine phosphorylase
with a threshold of 1e−05
MR Test
Inverse variance weighted (fixed effects)
Inverse variance weighted (multiplicative random effects)
MR Egger
MR RAPS
Weighted median
Weighted mode
0.00
−0.03
−0.06
0.09
0.12
0.15
0.18
SNP effect on S−methyl−5'−thioadenosine phosphorylase
SNP effect on Gut microbiota abundance (genus Catenibacterium id.2153)

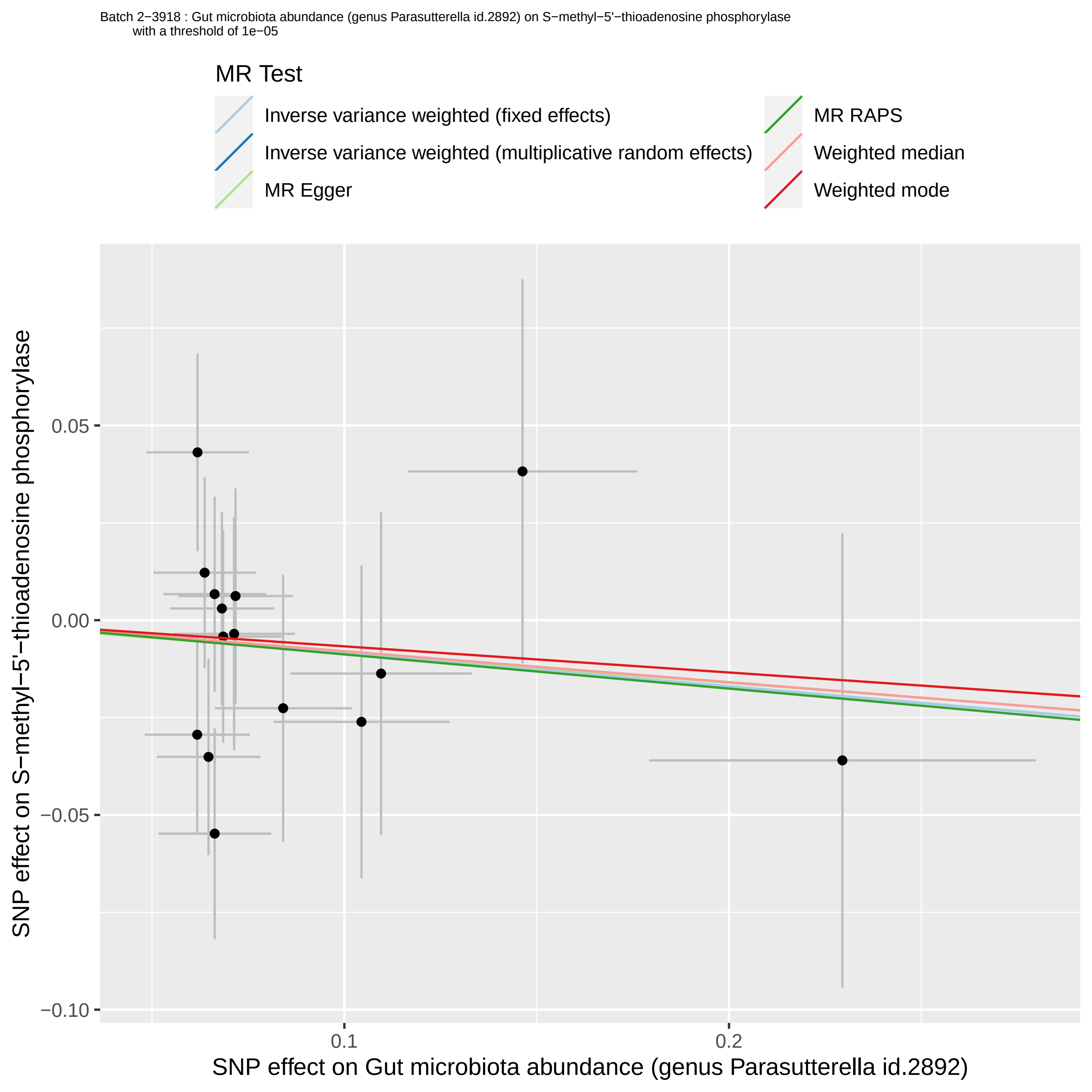

Batch 2−3918 : Gut microbiota abundance (genus Parasutterella id.2892) on S−methyl−5'−thioadenosine phosphorylase
with a threshold of 1e−05
MR Test
Inverse variance weighted (fixed effects)
Inverse variance weighted (multiplicative random effects)
MR Egger
MR RAPS
Weighted median
Weighted mode
SNP effect on S−methyl−5'−thioadenosine phosphorylase
0.05
0.00
−0.05
−0.10
0.1
0.2
SNP effect on Gut microbiota abundance (genus Parasutterella id.2892)

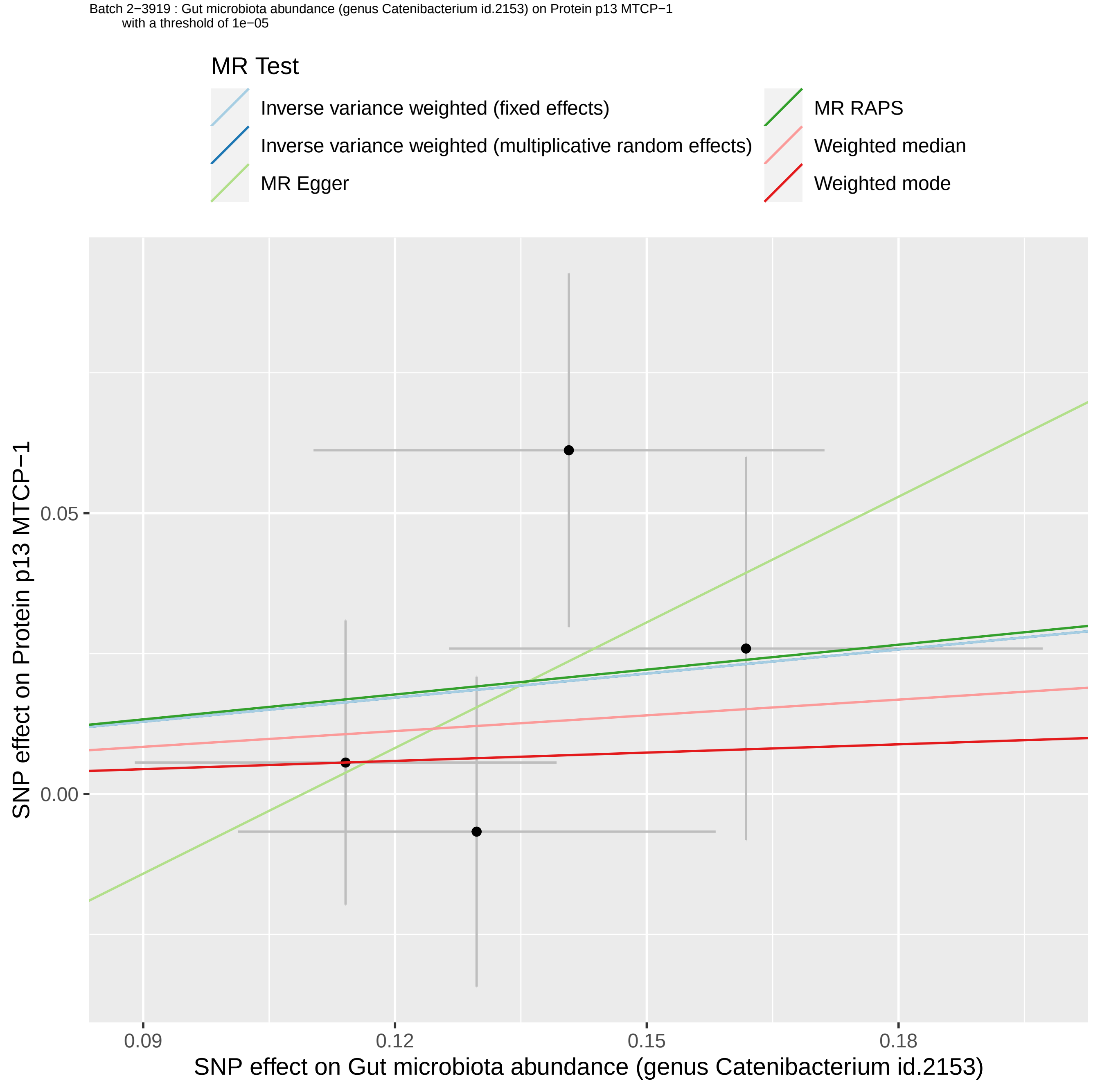
Batch 2−3919 : Gut microbiota abundance (genus Catenibacterium id.2153) on Protein p13 MTCP−1
with a threshold of 1e−05
MR Test
Inverse variance weighted (fixed effects)
Inverse variance weighted (multiplicative random effects)
MR Egger
MR RAPS
Weighted median
Weighted mode
0.05
0.00
0.09
0.12
0.15
0.18
SNP effect on Protein p13 MTCP−1
SNP effect on Gut microbiota abundance (genus Catenibacterium id.2153)

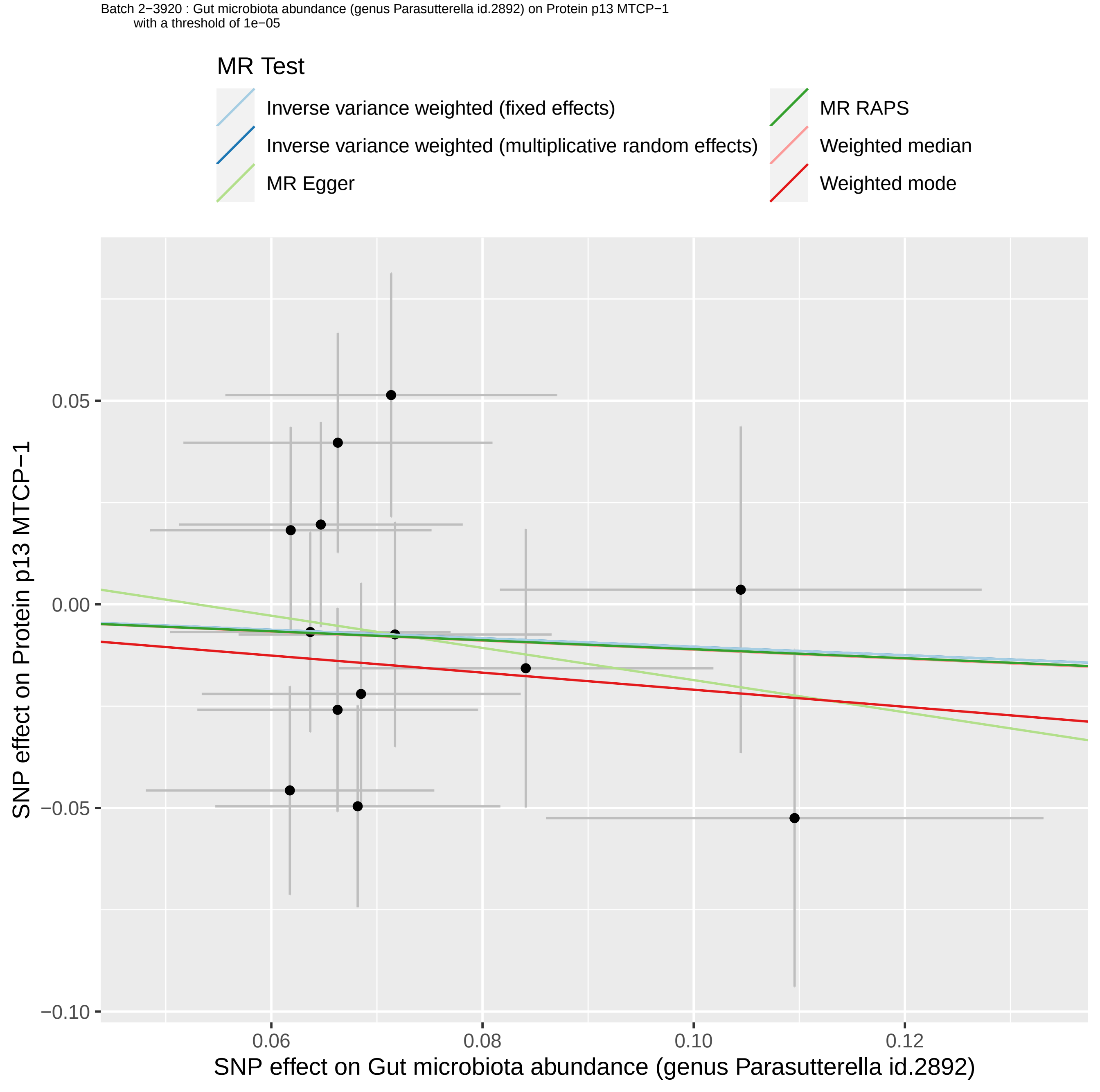
Batch 2−3920 : Gut microbiota abundance (genus Parasutterella id.2892) on Protein p13 MTCP−1
with a threshold of 1e−05
MR Test
Inverse variance weighted (fixed effects)
Inverse variance weighted (multiplicative random effects)
MR Egger
MR RAPS
Weighted median
Weighted mode
SNP effect on Protein p13 MTCP−1
0.05
0.00
−0.05
−0.10
0.06
0.08
0.10
0.12
SNP effect on Gut microbiota abundance (genus Parasutterella id.2892)

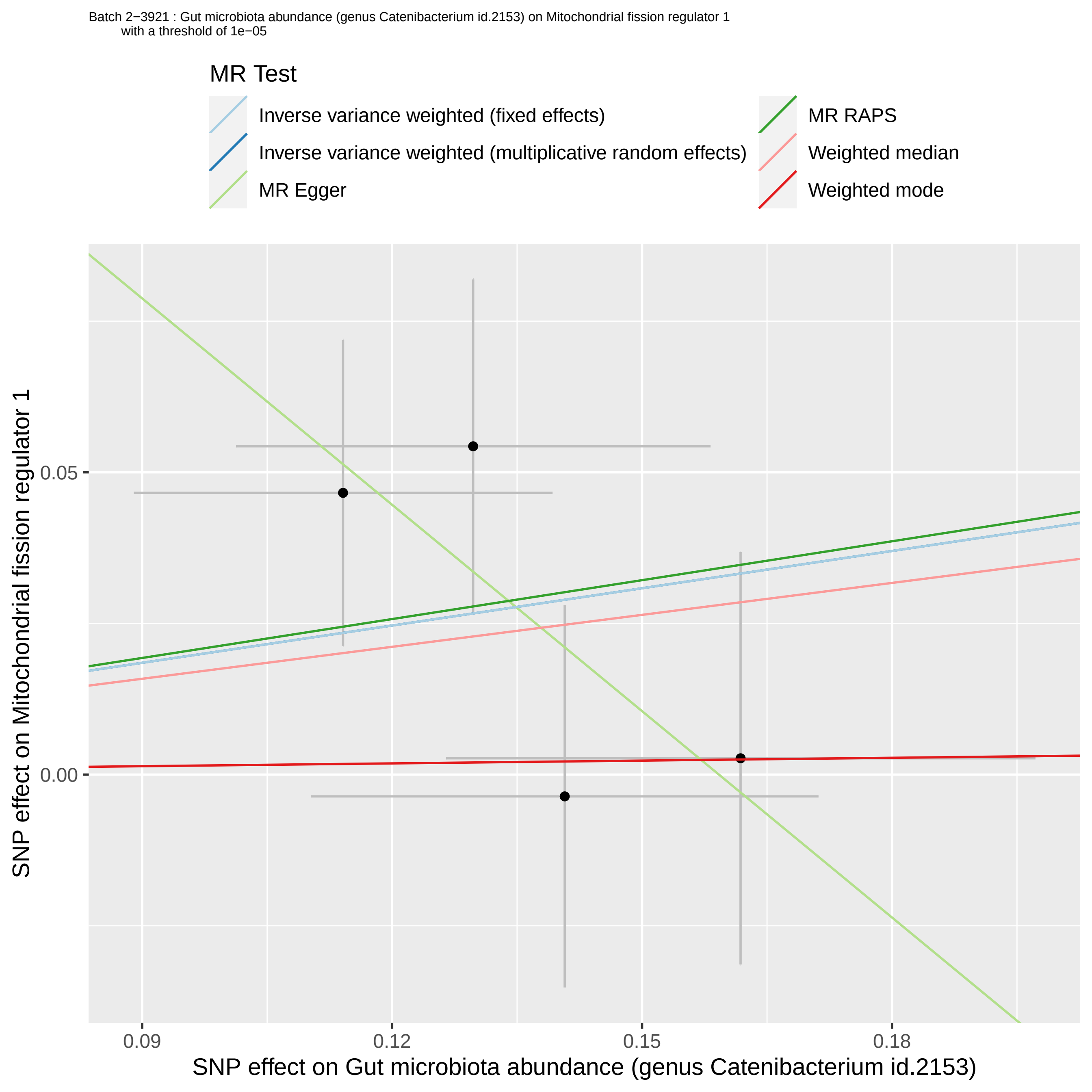
Batch 2−3921 : Gut microbiota abundance (genus Catenibacterium id.2153) on Mitochondrial fission regulator 1
with a threshold of 1e−05
MR Test
Inverse variance weighted (fixed effects)
Inverse variance weighted (multiplicative random effects)
MR Egger
MR RAPS
Weighted median
Weighted mode
SNP effect on Mitochondrial fission regulator 1
0.05
0.00
0.09
0.12
0.15
0.18
SNP effect on Gut microbiota abundance (genus Catenibacterium id.2153)

Batch 2−3922 : Gut microbiota abundance (genus Parasutterella id.2892) on Mitochondrial fission regulator 1
with a threshold of 1e−05

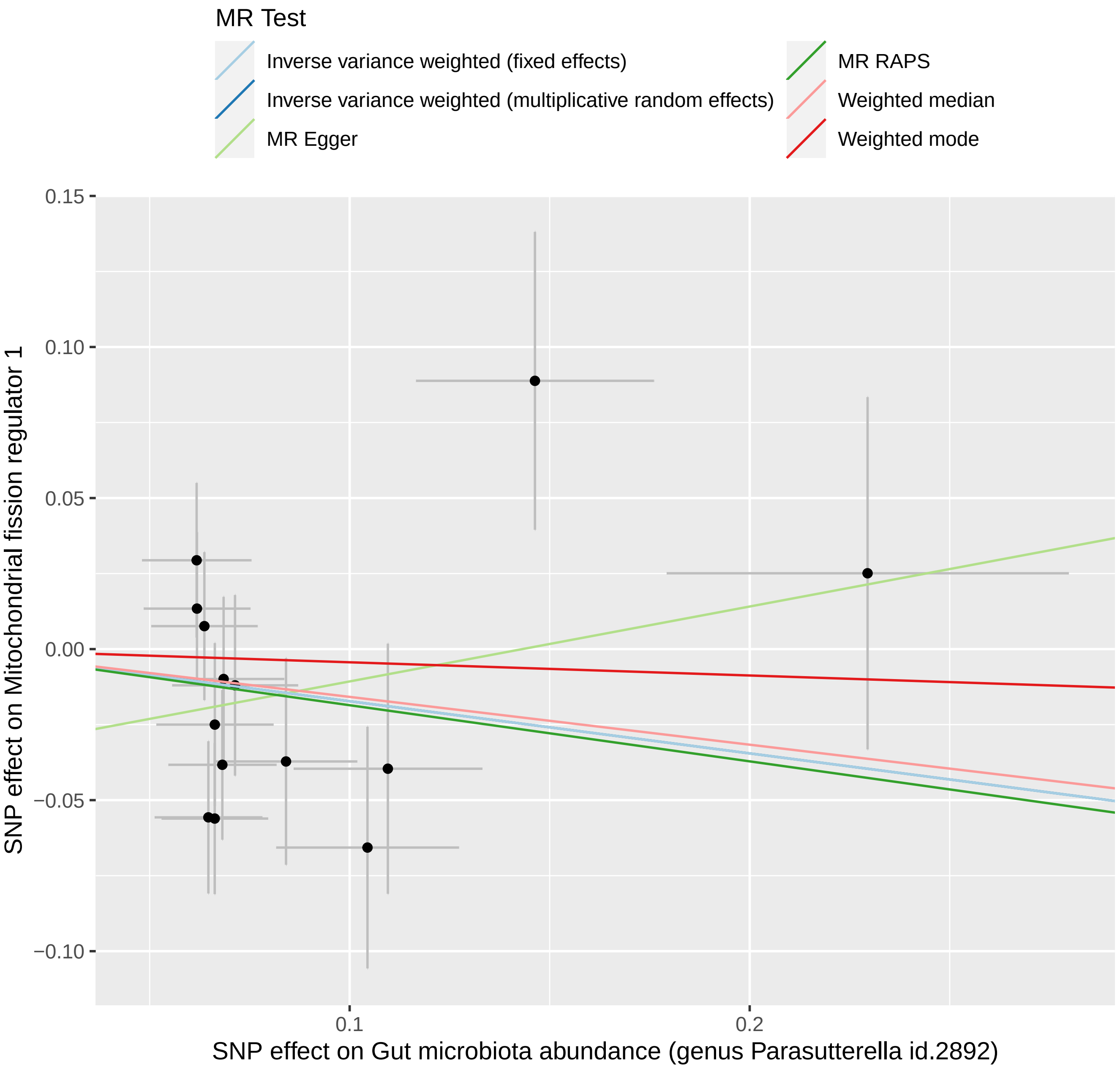

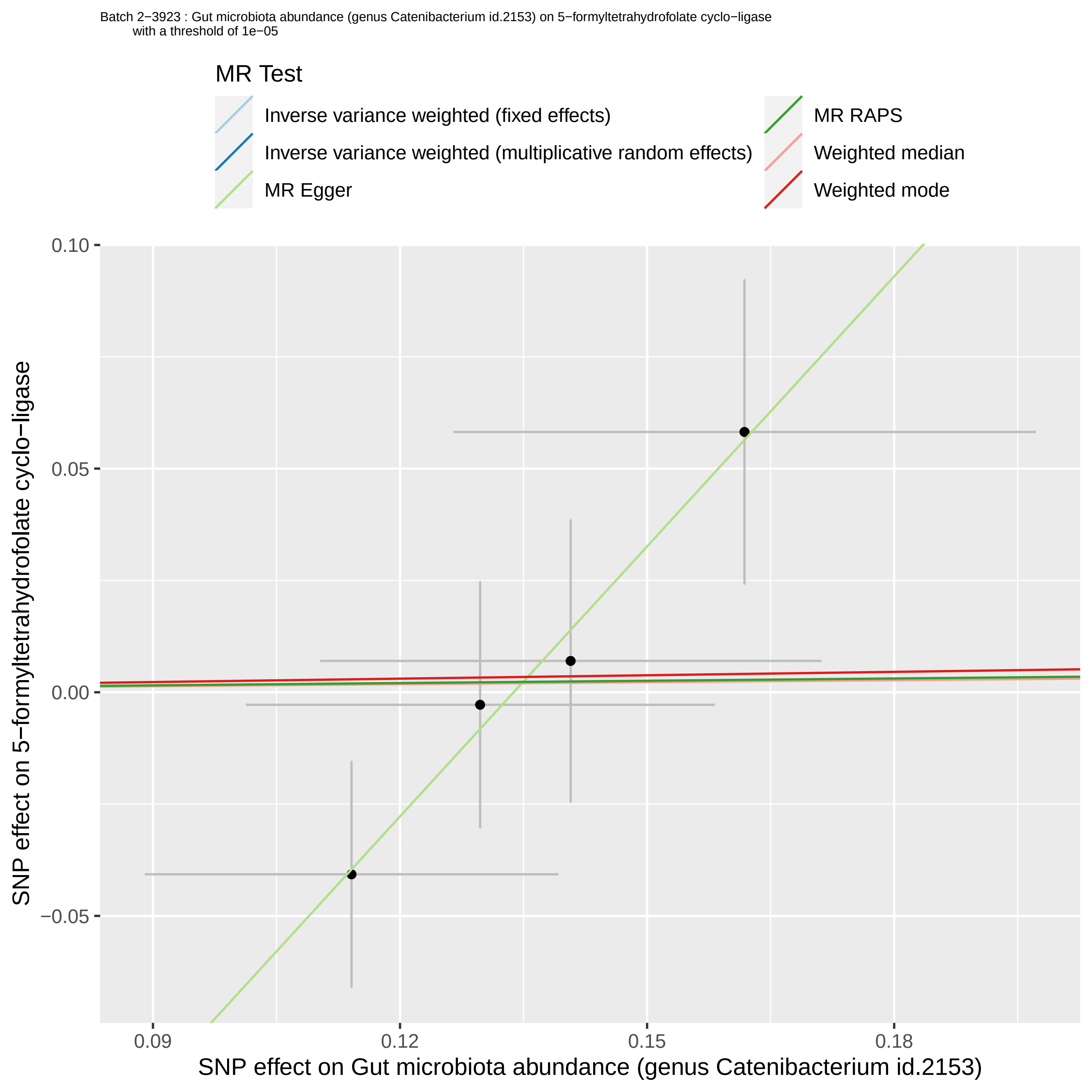
Batch 2−3923 : Gut microbiota abundance (genus Catenibacterium id.2153) on 5−formyltetrahydrofolate cyclo−ligase
with a threshold of 1e−05
MR Test
Inverse variance weighted (fixed effects)
Inverse variance weighted (multiplicative random effects)
MR Egger
MR RAPS
Weighted median
Weighted mode
0.10
0.05
0.00
−0.05
0.09
0.12
0.15
0.18
SNP effect on Gut microbiota abundance (genus Catenibacterium id.2153)
SNP effect on 5−formyltetrahydrofolate cyclo−ligase

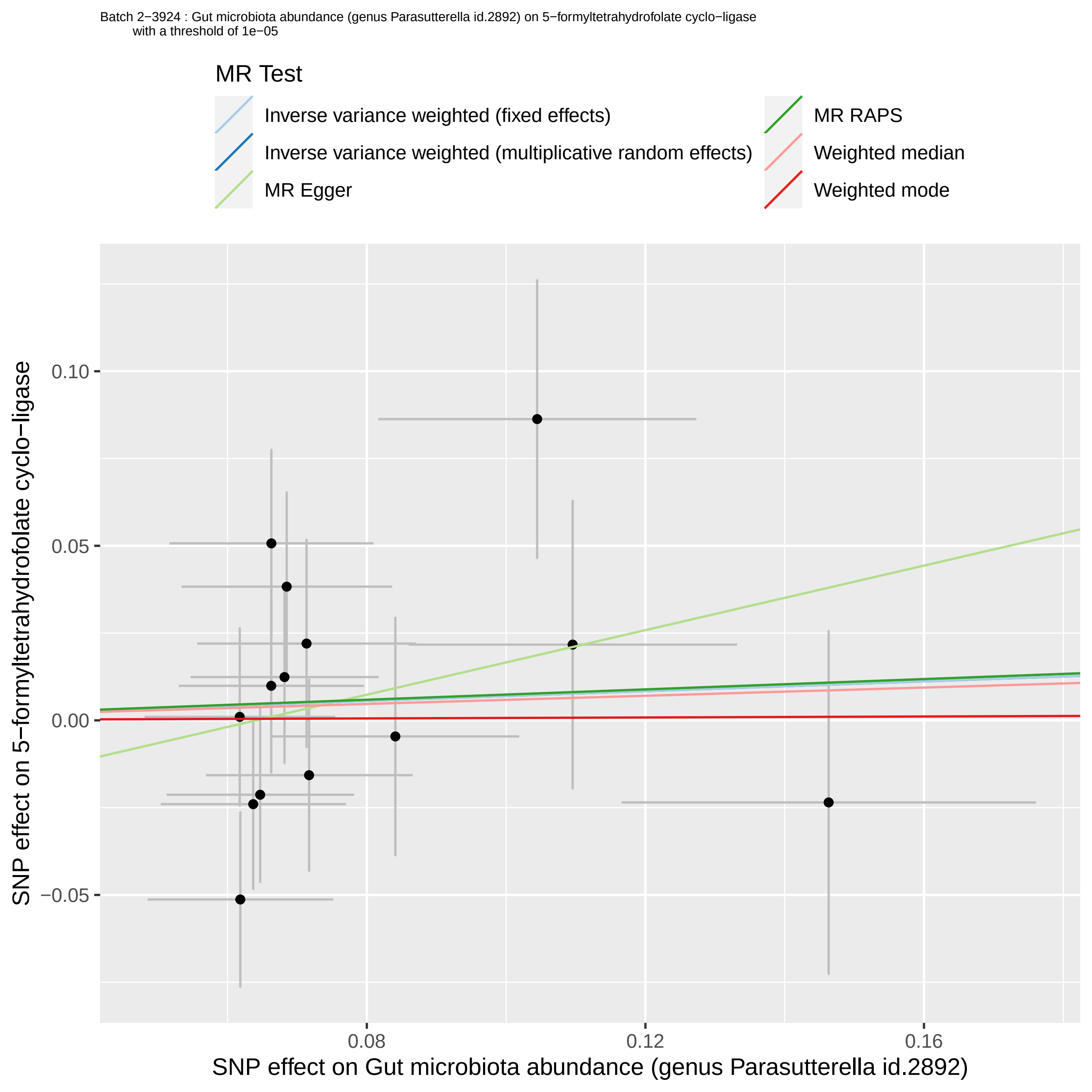

Batch 2−3924 : Gut microbiota abundance (genus Parasutterella id.2892) on 5−formyltetrahydrofolate cyclo−ligase
with a threshold of 1e−05
MR Test
Inverse variance weighted (fixed effects)
Inverse variance weighted (multiplicative random effects)
MR Egger
MR RAPS
Weighted median
Weighted mode
SNP effect on 5−formyltetrahydrofolate cyclo−ligase
0.10
0.05
0.00
−0.05
0.08
0.12
0.16
SNP effect on Gut microbiota abundance (genus Parasutterella id.2892)

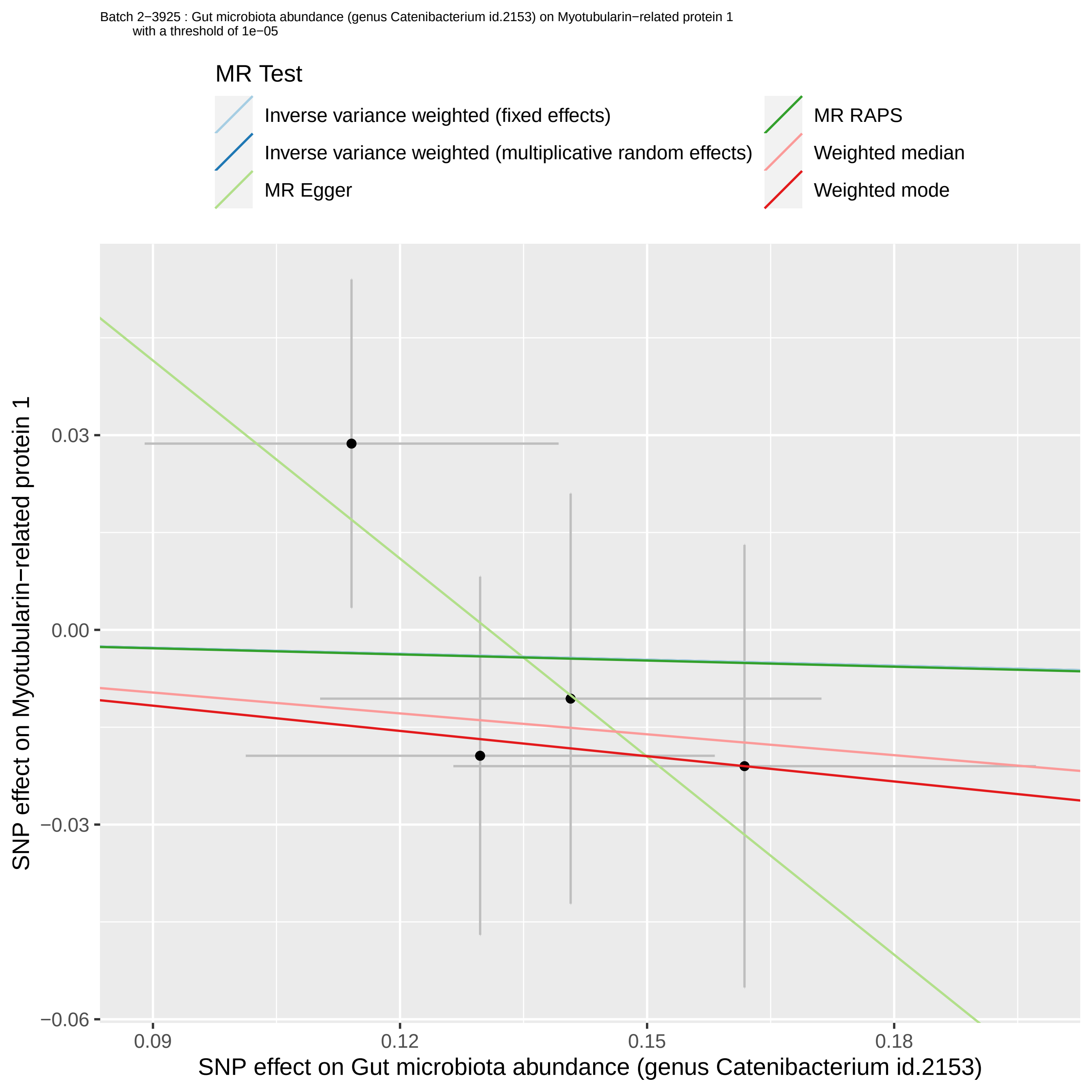
Batch 2−3925 : Gut microbiota abundance (genus Catenibacterium id.2153) on Myotubularin−related protein 1
with a threshold of 1e−05
MR Test
Inverse variance weighted (fixed effects)
Inverse variance weighted (multiplicative random effects)
MR Egger
MR RAPS
Weighted median
Weighted mode
0.03
0.00
−0.03
−0.06
0.09
0.12
0.15
0.18
SNP effect on Myotubularin−related protein 1
SNP effect on Gut microbiota abundance (genus Catenibacterium id.2153)

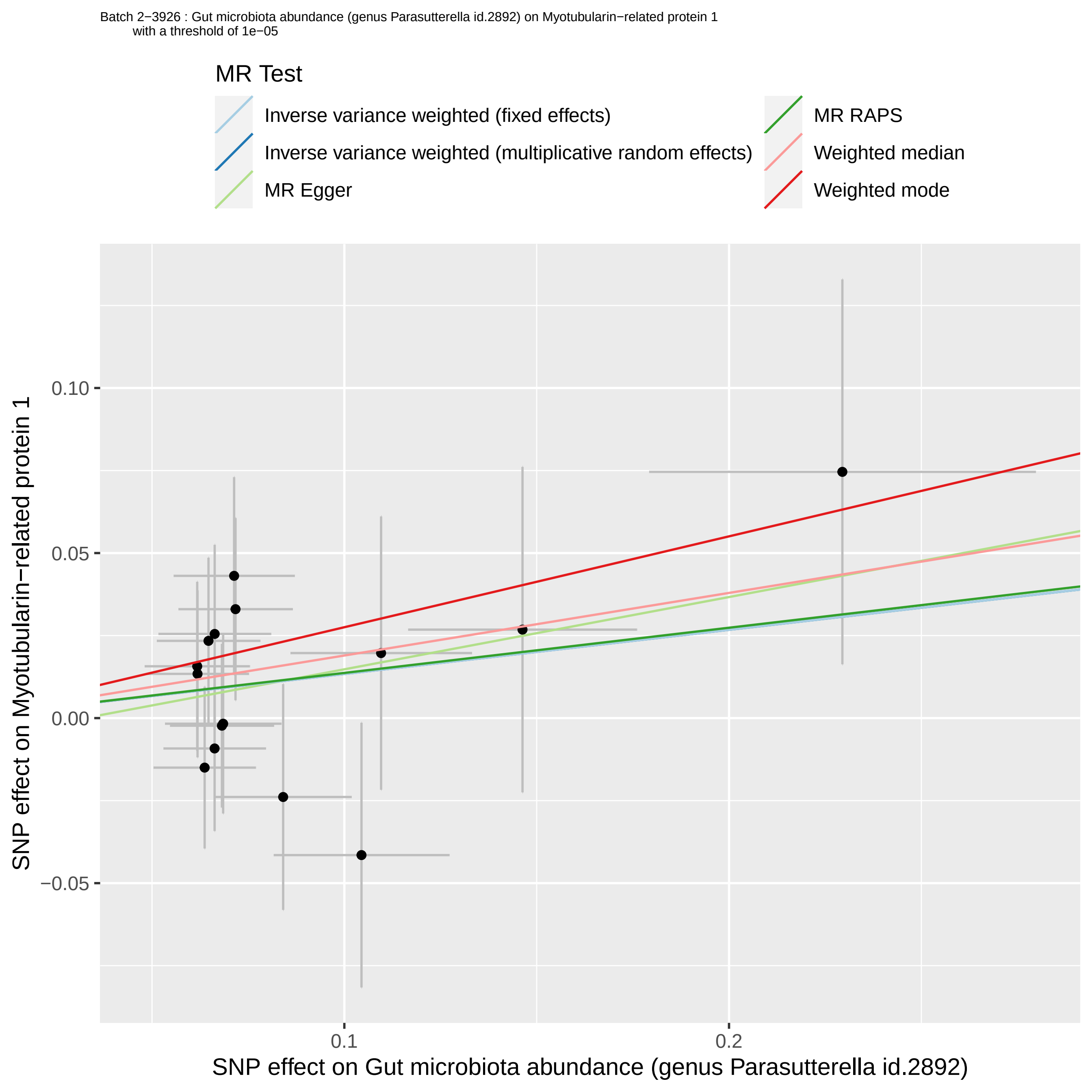

Batch 2−3926 : Gut microbiota abundance (genus Parasutterella id.2892) on Myotubularin−related protein 1
with a threshold of 1e−05
MR Test
Inverse variance weighted (fixed effects)
Inverse variance weighted (multiplicative random effects)
MR Egger
MR RAPS
Weighted median
Weighted mode
0.10
0.05
0.00
−0.05
0.1
0.2
SNP effect on Myotubularin−related protein 1
SNP effect on Gut microbiota abundance (genus Parasutterella id.2892)

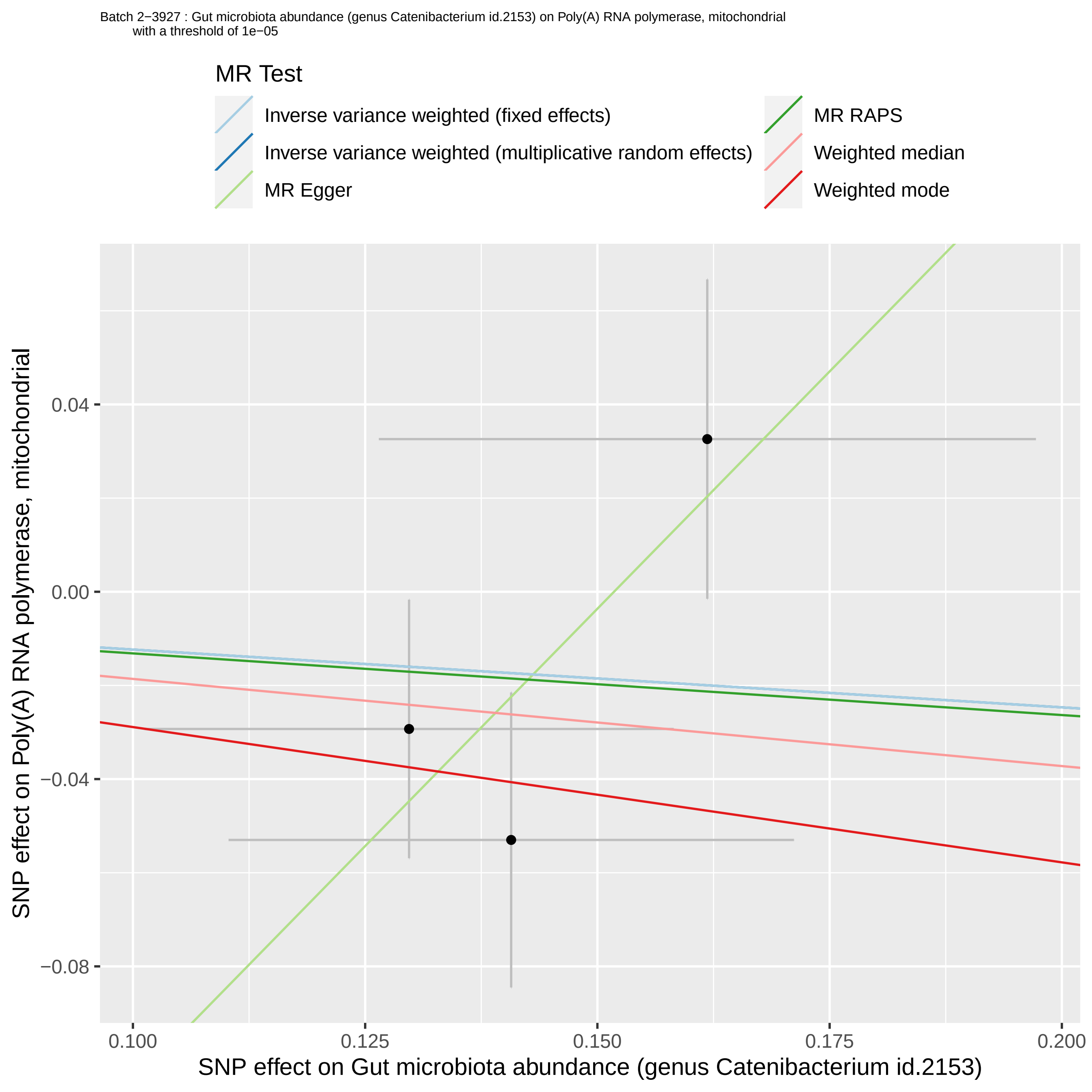
Batch 2−3927 : Gut microbiota abundance (genus Catenibacterium id.2153) on Poly(A) RNA polymerase, mitochondrial
with a threshold of 1e−05
MR Test
Inverse variance weighted (fixed effects)
Inverse variance weighted (multiplicative random effects)
MR Egger
MR RAPS
Weighted median
Weighted mode
SNP effect on Poly(A) RNA polymerase, mitochondrial
0.04
0.00
−0.04
−0.08
0.100
0.125
0.150
0.175
0.200
SNP effect on Gut microbiota abundance (genus Catenibacterium id.2153)

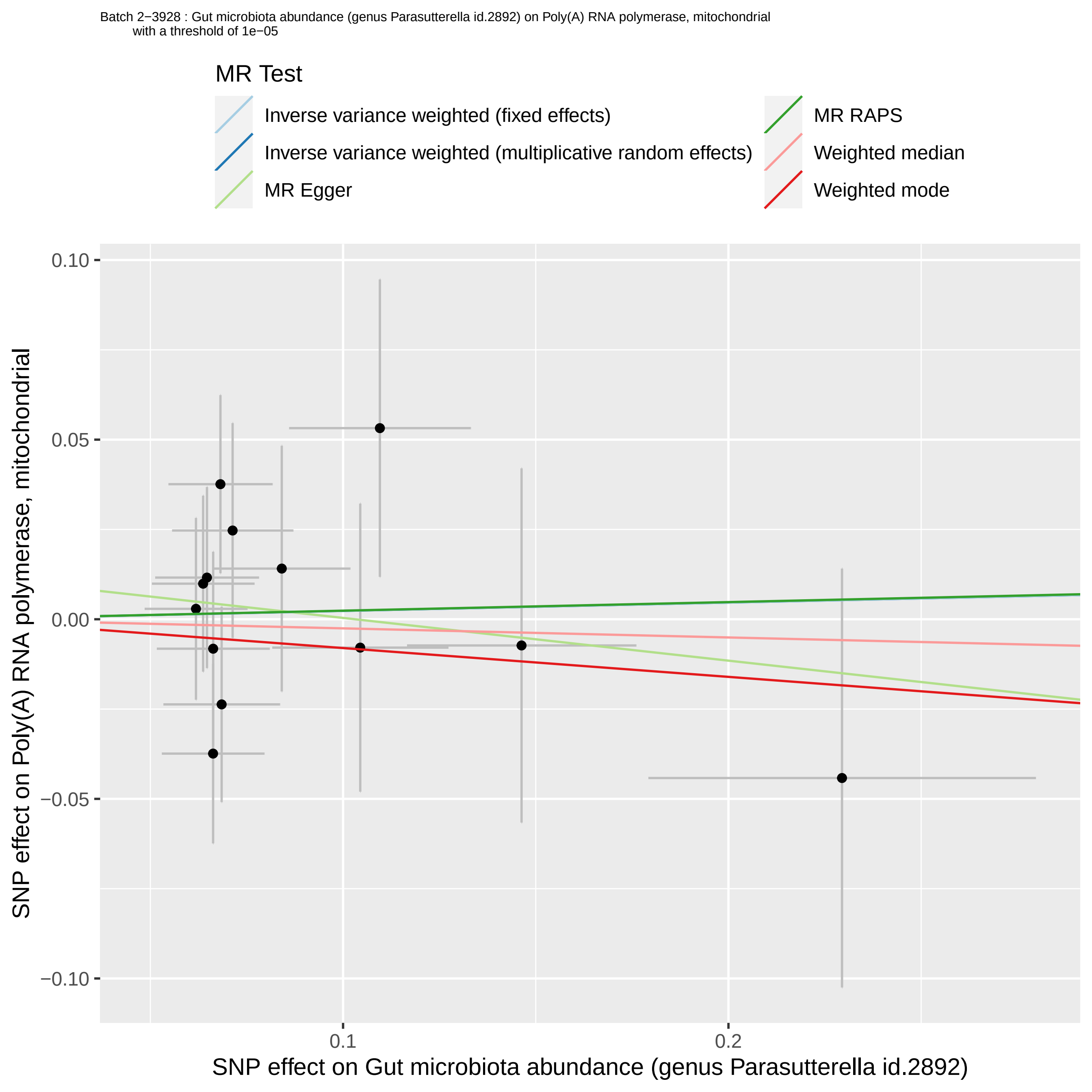

Batch 2−3928 : Gut microbiota abundance (genus Parasutterella id.2892) on Poly(A) RNA polymerase, mitochondrial
with a threshold of 1e−05
MR Test
Inverse variance weighted (fixed effects)
Inverse variance weighted (multiplicative random effects)
MR Egger
MR RAPS
Weighted median
Weighted mode
0.10
0.05
0.00
−0.05
−0.10
0.1
0.2
SNP effect on Poly(A) RNA polymerase, mitochondrial
SNP effect on Gut microbiota abundance (genus Parasutterella id.2892)

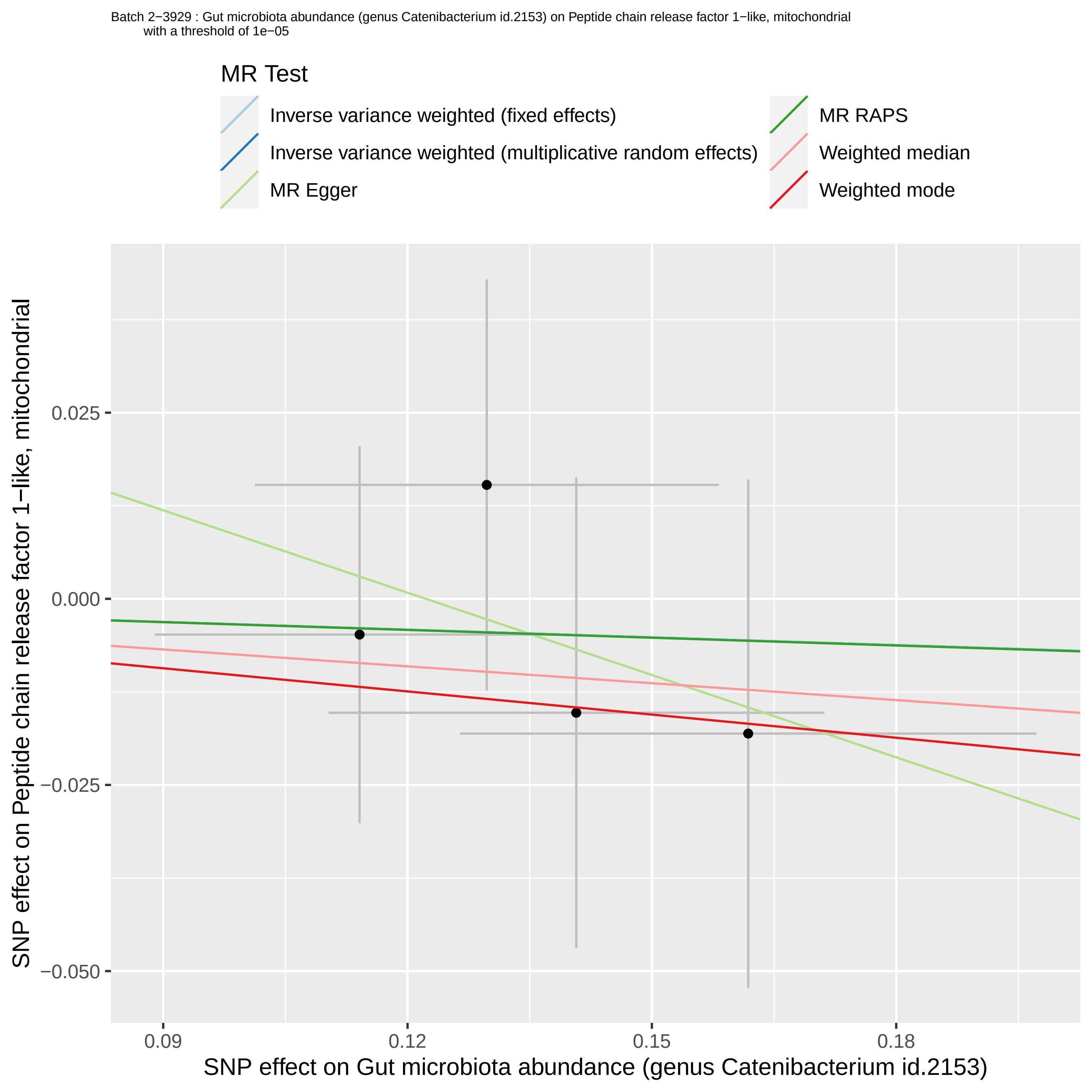

Batch 2−3929 : Gut microbiota abundance (genus Catenibacterium id.2153) on Peptide chain release factor 1−like, mitochondrial
with a threshold of 1e−05
MR Test
Inverse variance weighted (fixed effects)
Inverse variance weighted (multiplicative random effects)
MR Egger
MR RAPS
Weighted median
Weighted mode
0.025
0.000
−0.025
−0.050
0.09
0.12
0.15
0.18
SNP effect on Gut microbiota abundance (genus Catenibacterium id.2153)
SNP effect on Peptide chain release factor 1−like, mitochondrial

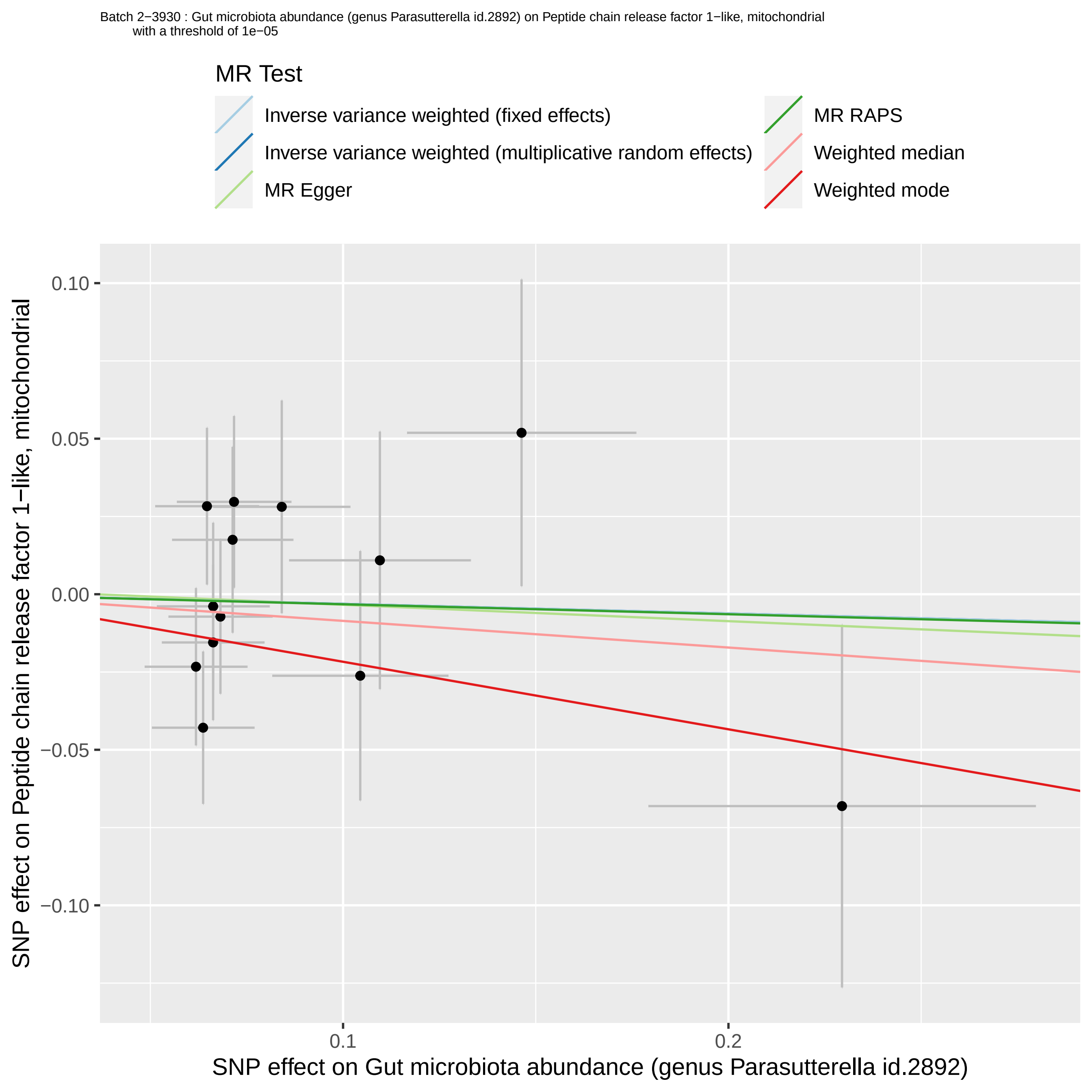

Batch 2−3930 : Gut microbiota abundance (genus Parasutterella id.2892) on Peptide chain release factor 1−like, mitochondrial
with a threshold of 1e−05
MR Test
Inverse variance weighted (fixed effects)
Inverse variance weighted (multiplicative random effects)
MR Egger
MR RAPS
Weighted median
Weighted mode
0.10
0.05
0.00
−0.05
−0.10
0.1
0.2
SNP effect on Peptide chain release factor 1−like, mitochondrial
SNP effect on Gut microbiota abundance (genus Parasutterella id.2892)

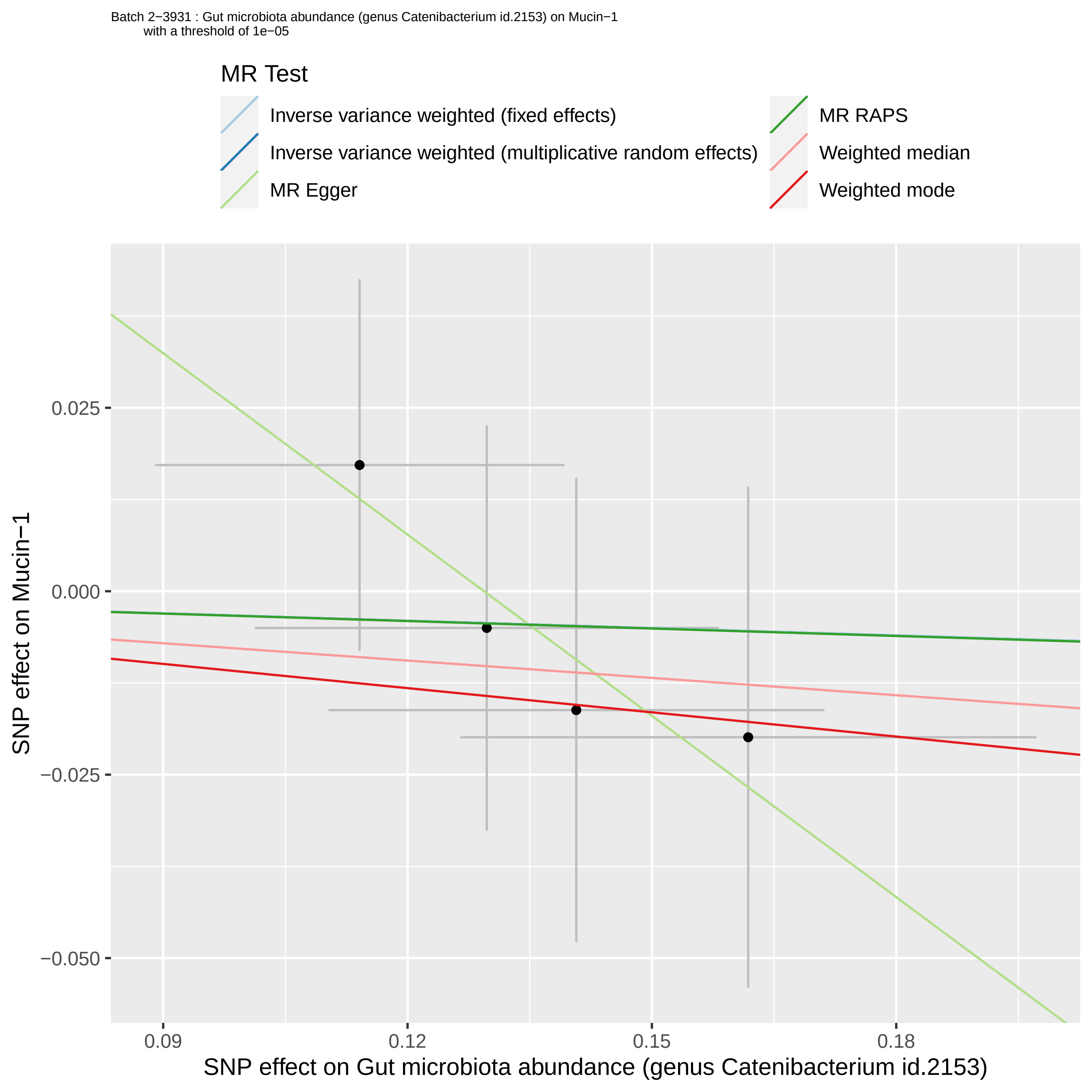

Batch 2−3931 : Gut microbiota abundance (genus Catenibacterium id.2153) on Mucin−1
with a threshold of 1e−05
MR Test
Inverse variance weighted (fixed effects)
Inverse variance weighted (multiplicative random effects)
MR Egger
MR RAPS
Weighted median
Weighted mode
0.025
0.000
−0.025
−0.050
0.09
0.12
0.15
0.18
SNP effect on Mucin−1
SNP effect on Gut microbiota abundance (genus Catenibacterium id.2153)

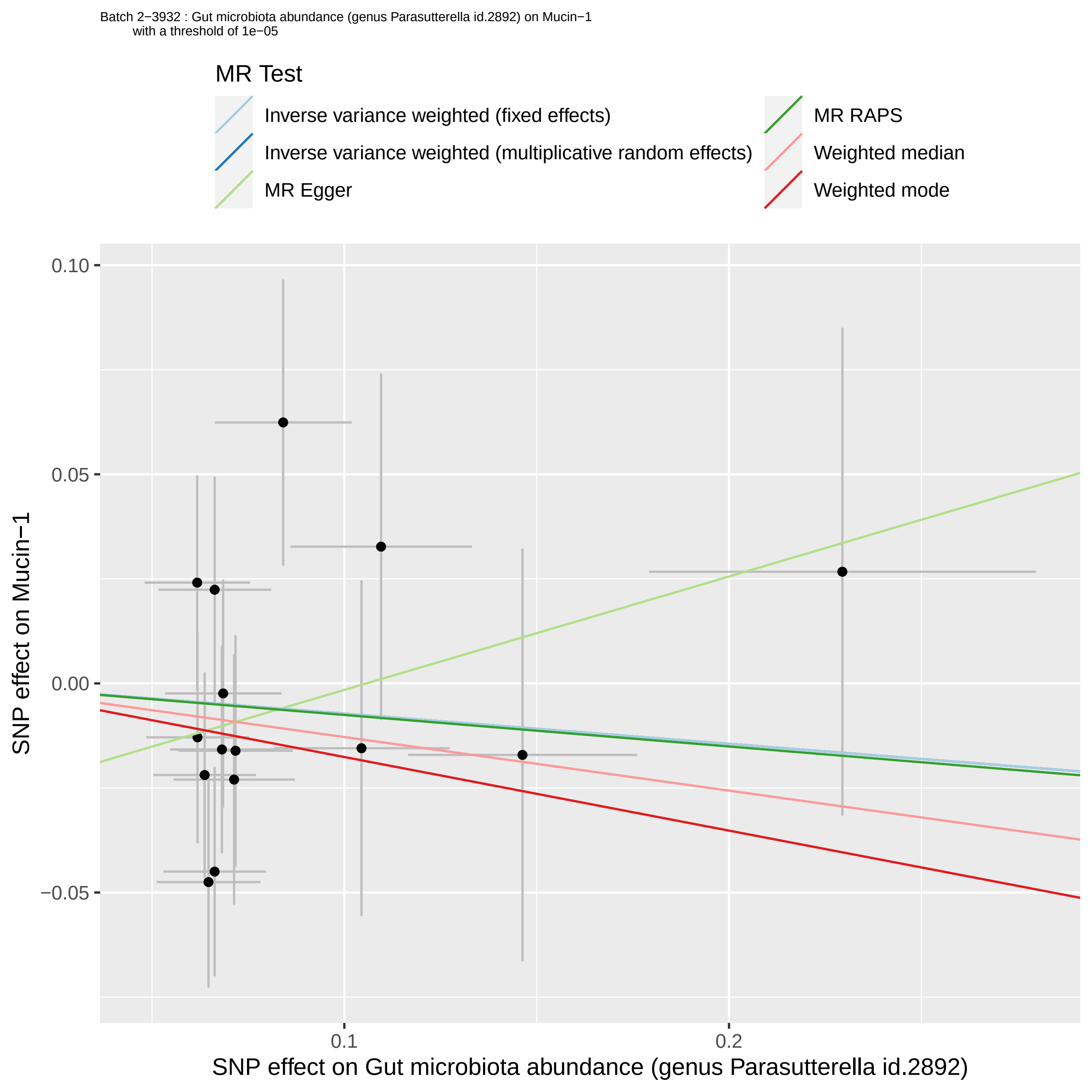
Batch 2−3932 : Gut microbiota abundance (genus Parasutterella id.2892) on Mucin−1
with a threshold of 1e−05
MR Test
Inverse variance weighted (fixed effects)
Inverse variance weighted (multiplicative random effects)
MR Egger
MR RAPS
Weighted median
Weighted mode
0.10
0.05
0.00
−0.05
SNP effect on Mucin−1
0.1
0.2
SNP effect on Gut microbiota abundance (genus Parasutterella id.2892)

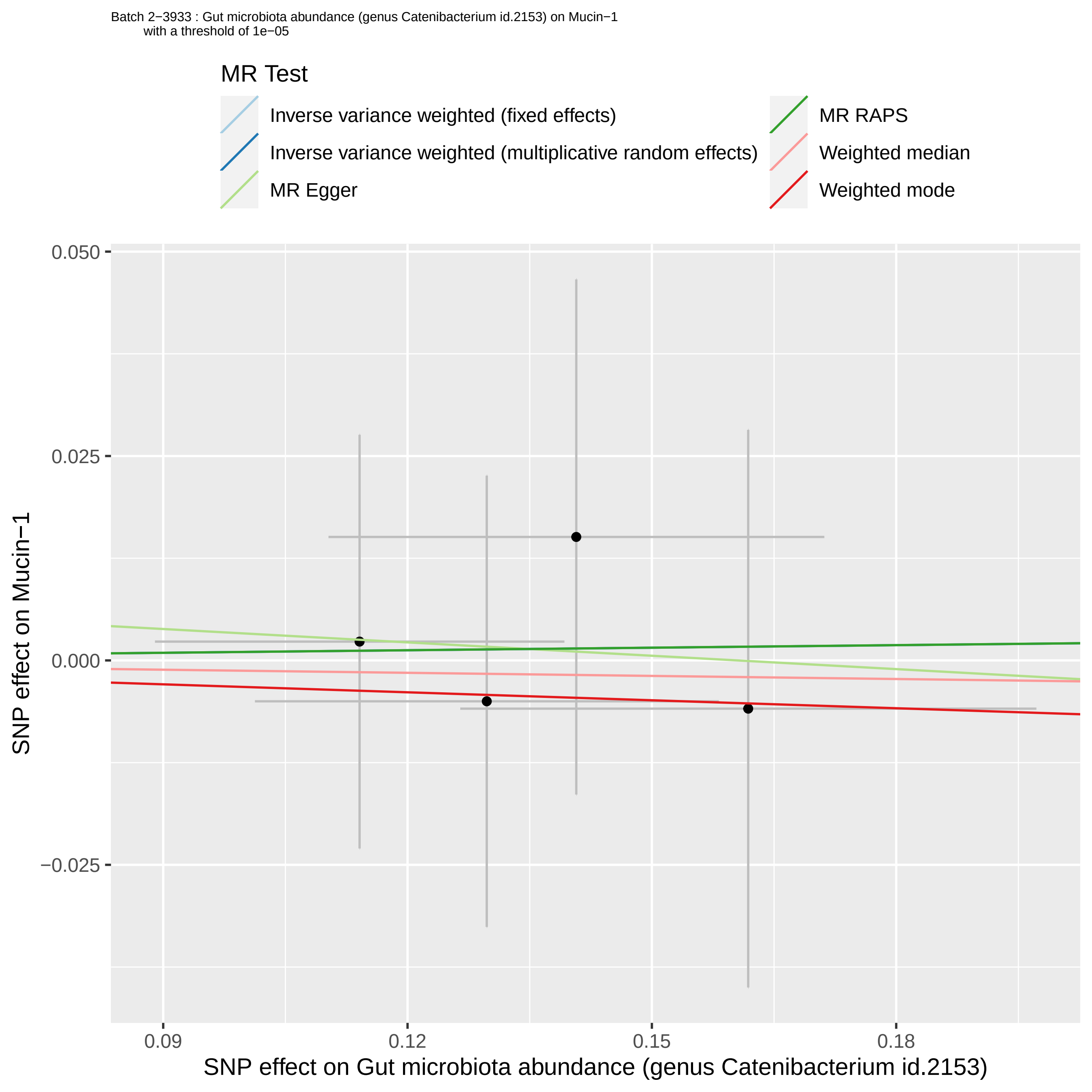
Batch 2−3933 : Gut microbiota abundance (genus Catenibacterium id.2153) on Mucin−1
with a threshold of 1e−05
MR Test
Inverse variance weighted (fixed effects)
Inverse variance weighted (multiplicative random effects)
MR Egger
MR RAPS
Weighted median
Weighted mode
0.050
0.025
0.000
−0.025
0.09
0.12
0.15
0.18
SNP effect on Mucin−1
SNP effect on Gut microbiota abundance (genus Catenibacterium id.2153)

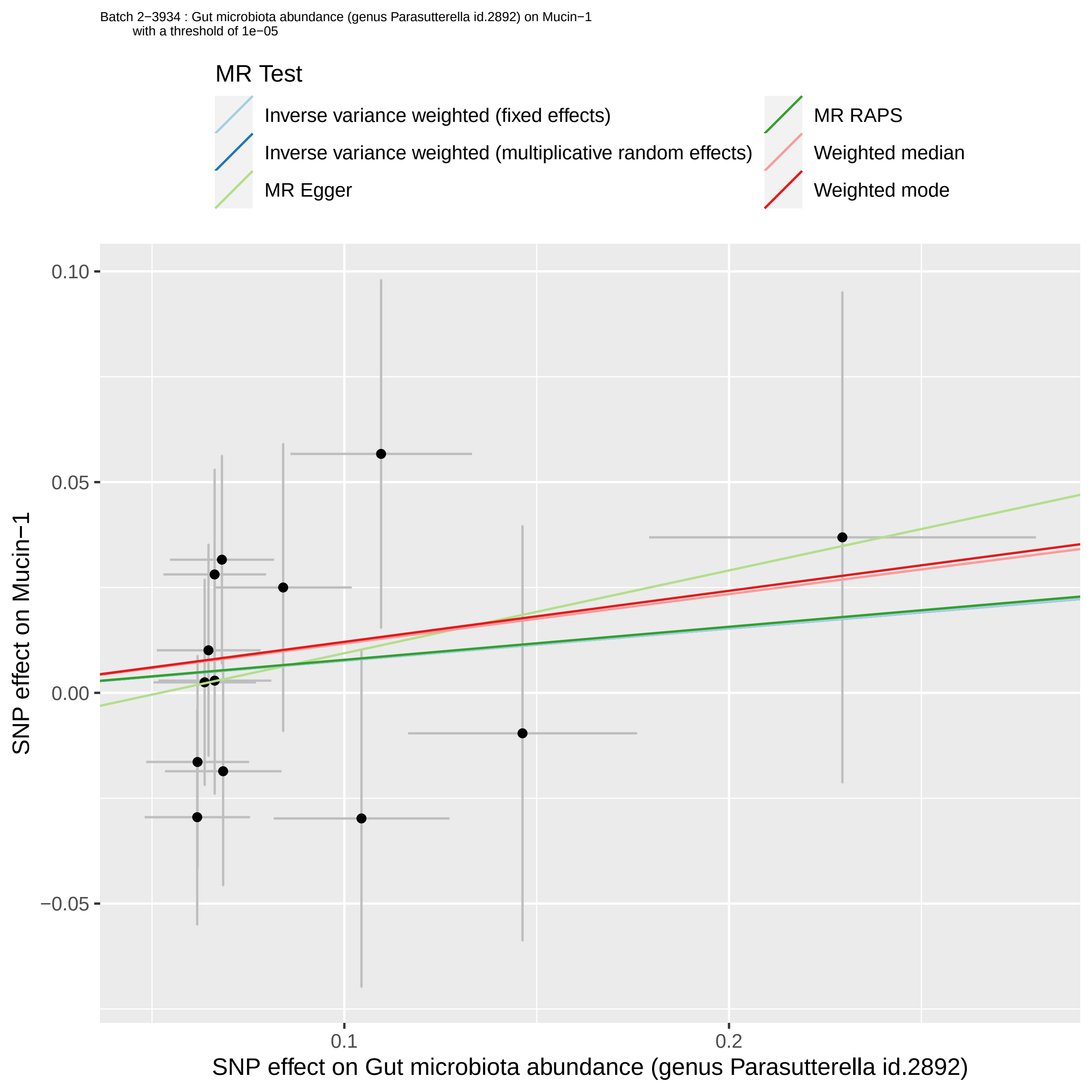

Batch 2−3934 : Gut microbiota abundance (genus Parasutterella id.2892) on Mucin−1
with a threshold of 1e−05
MR Test
Inverse variance weighted (fixed effects)
Inverse variance weighted (multiplicative random effects)
MR Egger
MR RAPS
Weighted median
Weighted mode
0.10
0.05
0.00
−0.05
0.1
0.2
SNP effect on Mucin−1
SNP effect on Gut microbiota abundance (genus Parasutterella id.2892)

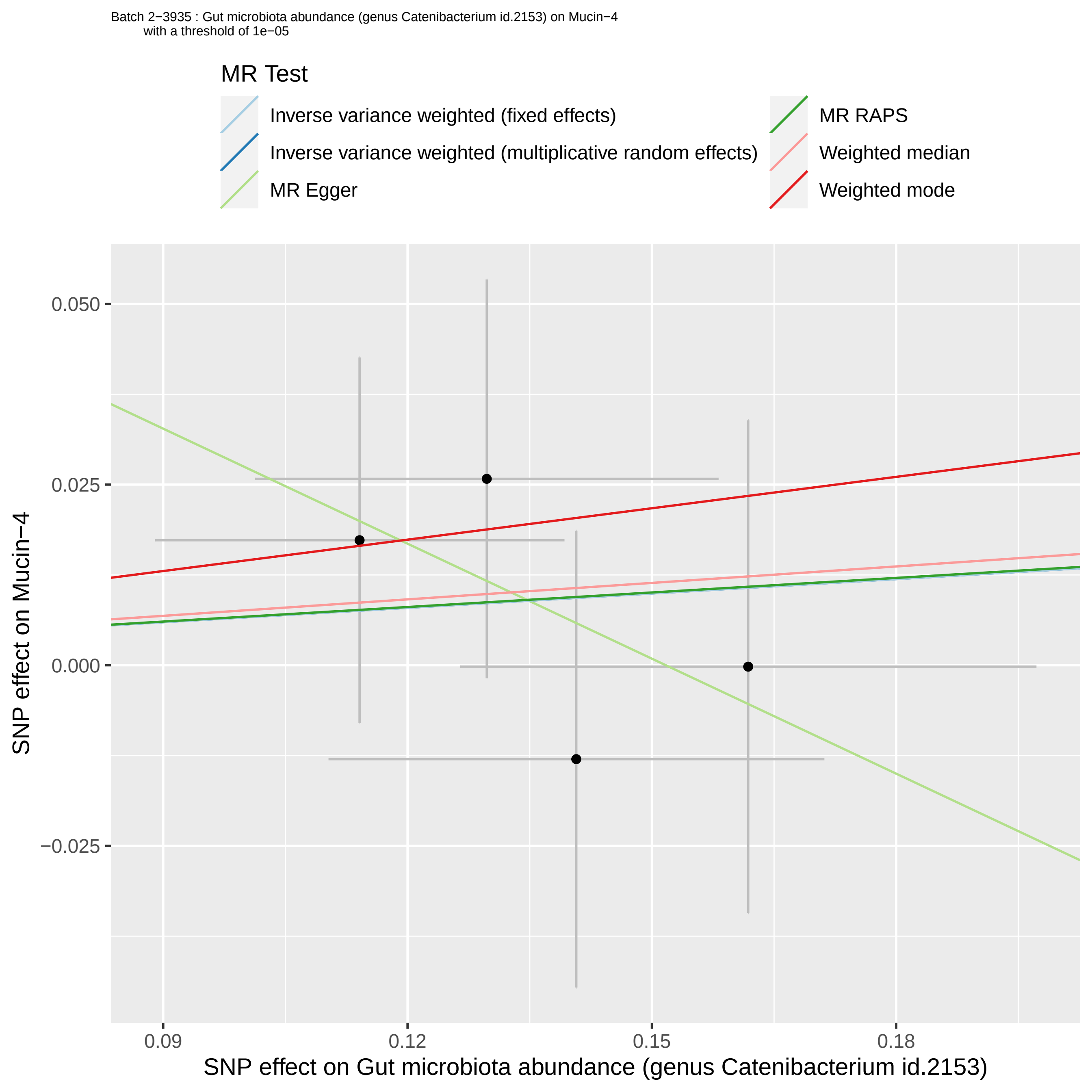

Batch 2−3935 : Gut microbiota abundance (genus Catenibacterium id.2153) on Mucin−4
with a threshold of 1e−05
MR Test
Inverse variance weighted (fixed effects)
Inverse variance weighted (multiplicative random effects)
MR Egger
MR RAPS
Weighted median
Weighted mode
0.050
0.025
0.000
−0.025
0.09
0.12
0.15
0.18
SNP effect on Mucin−4
SNP effect on Gut microbiota abundance (genus Catenibacterium id.2153)

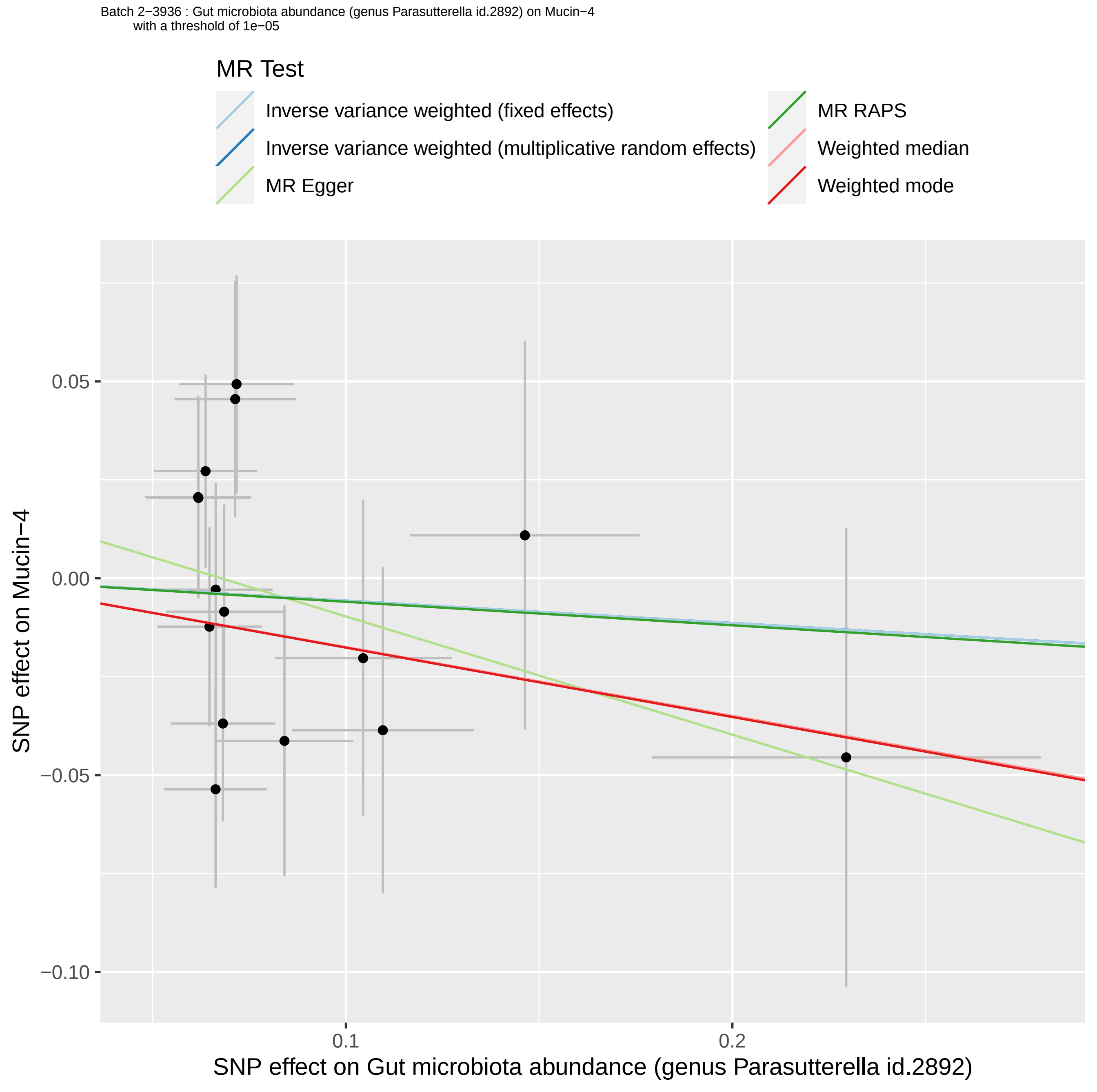
Batch 2−3936 : Gut microbiota abundance (genus Parasutterella id.2892) on Mucin−4
with a threshold of 1e−05
MR Test
Inverse variance weighted (fixed effects)
Inverse variance weighted (multiplicative random effects)
MR Egger
MR RAPS
Weighted median
Weighted mode
0.05
0.00
−0.05
−0.10
0.1
0.2
SNP effect on Mucin−4
SNP effect on Gut microbiota abundance (genus Parasutterella id.2892)

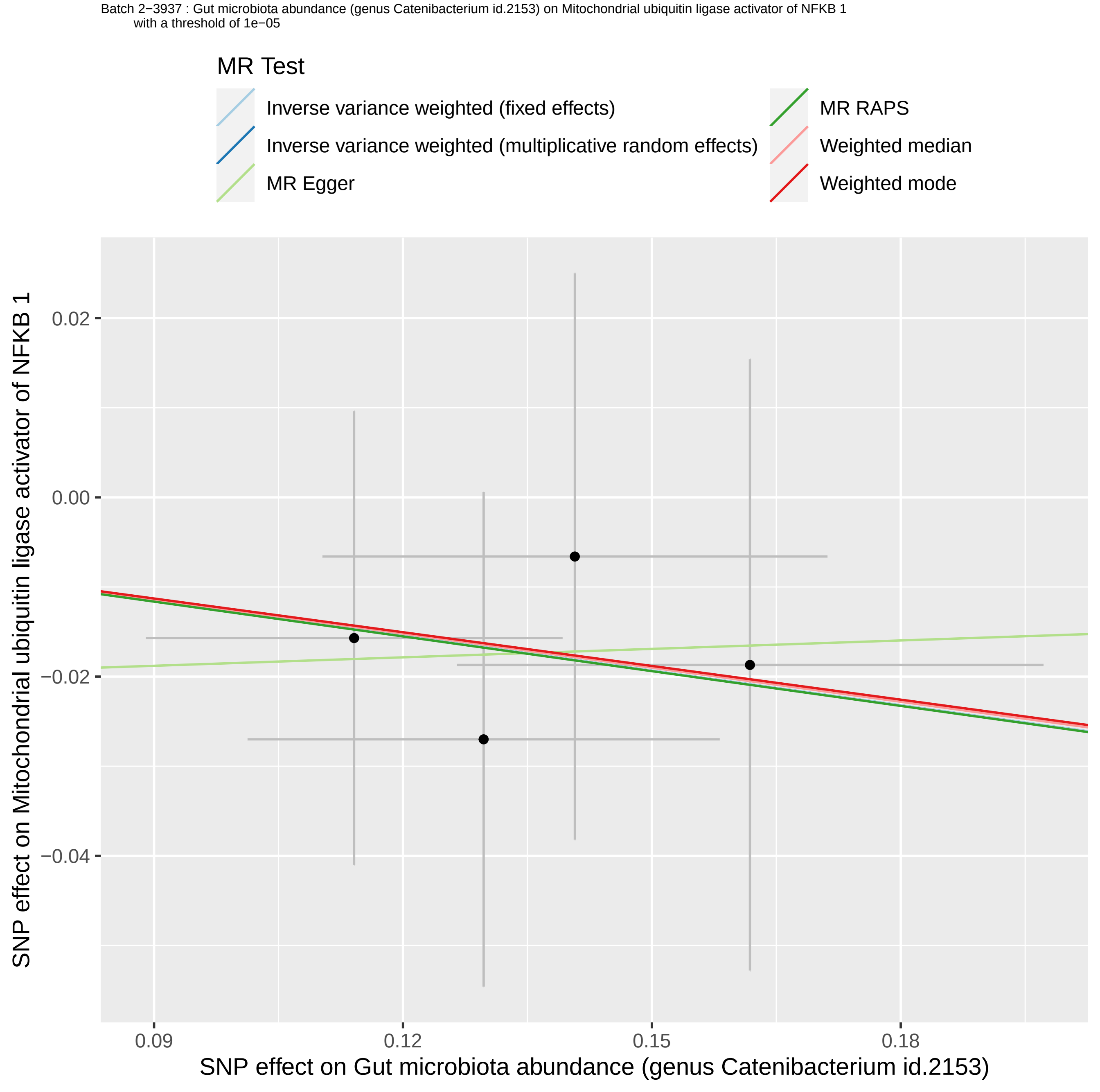
Batch 2−3937 : Gut microbiota abundance (genus Catenibacterium id.2153) on Mitochondrial ubiquitin ligase activator of NFKB 1
with a threshold of 1e−05
MR Test
Inverse variance weighted (fixed effects)
Inverse variance weighted (multiplicative random effects)
MR Egger
MR RAPS
Weighted median
Weighted mode
0.02
0.00
−0.02
−0.04
0.09
0.12
0.15
0.18
SNP effect on Mitochondrial ubiquitin ligase activator of NFKB 1
SNP effect on Gut microbiota abundance (genus Catenibacterium id.2153)

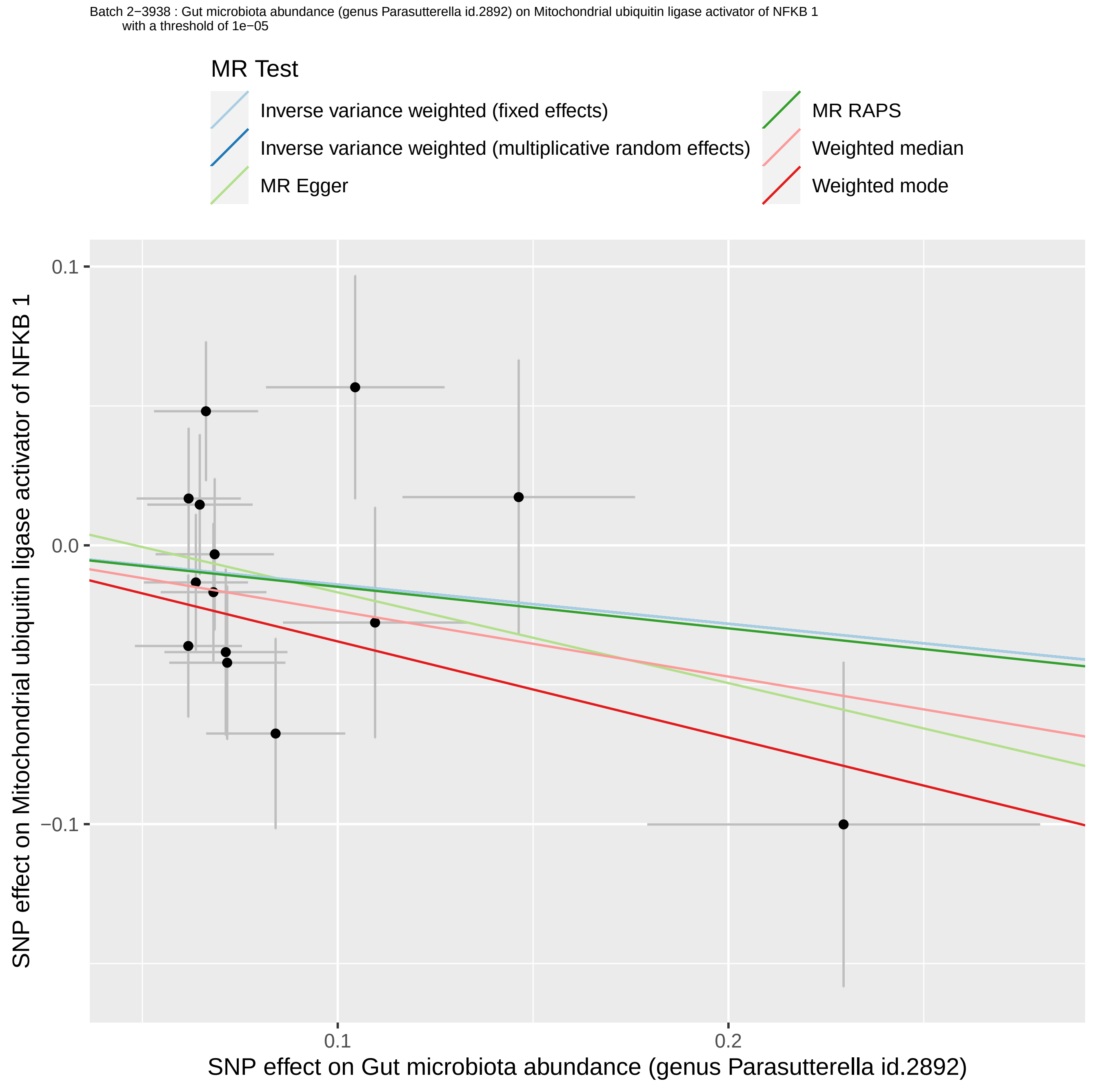
Batch 2−3938 : Gut microbiota abundance (genus Parasutterella id.2892) on Mitochondrial ubiquitin ligase activator of NFKB 1
with a threshold of 1e−05
MR Test
Inverse variance weighted (fixed effects)
Inverse variance weighted (multiplicative random effects)
MR Egger
MR RAPS
Weighted median
Weighted mode
0.1
0.0
−0.1
0.1
0.2
SNP effect on Mitochondrial ubiquitin ligase activator of NFKB 1
SNP effect on Gut microbiota abundance (genus Parasutterella id.2892)

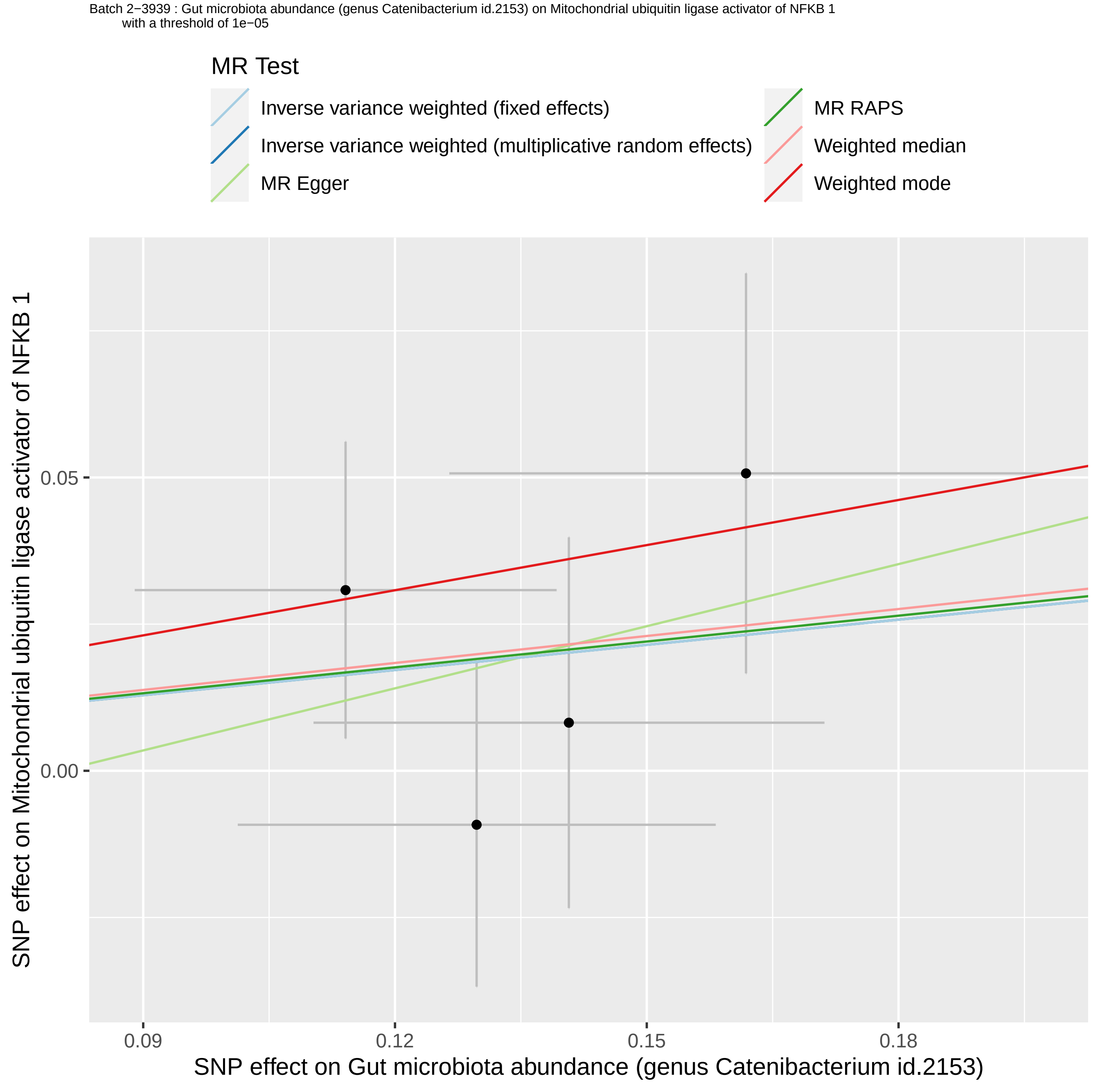

Batch 2−3939 : Gut microbiota abundance (genus Catenibacterium id.2153) on Mitochondrial ubiquitin ligase activator of NFKB 1
with a threshold of 1e−05
MR Test
Inverse variance weighted (fixed effects)
Inverse variance weighted (multiplicative random effects)
MR Egger
MR RAPS
Weighted median
Weighted mode
0.05
0.00
0.09
0.12
0.15
0.18
SNP effect on Gut microbiota abundance (genus Catenibacterium id.2153)
SNP effect on Mitochondrial ubiquitin ligase activator of NFKB 1

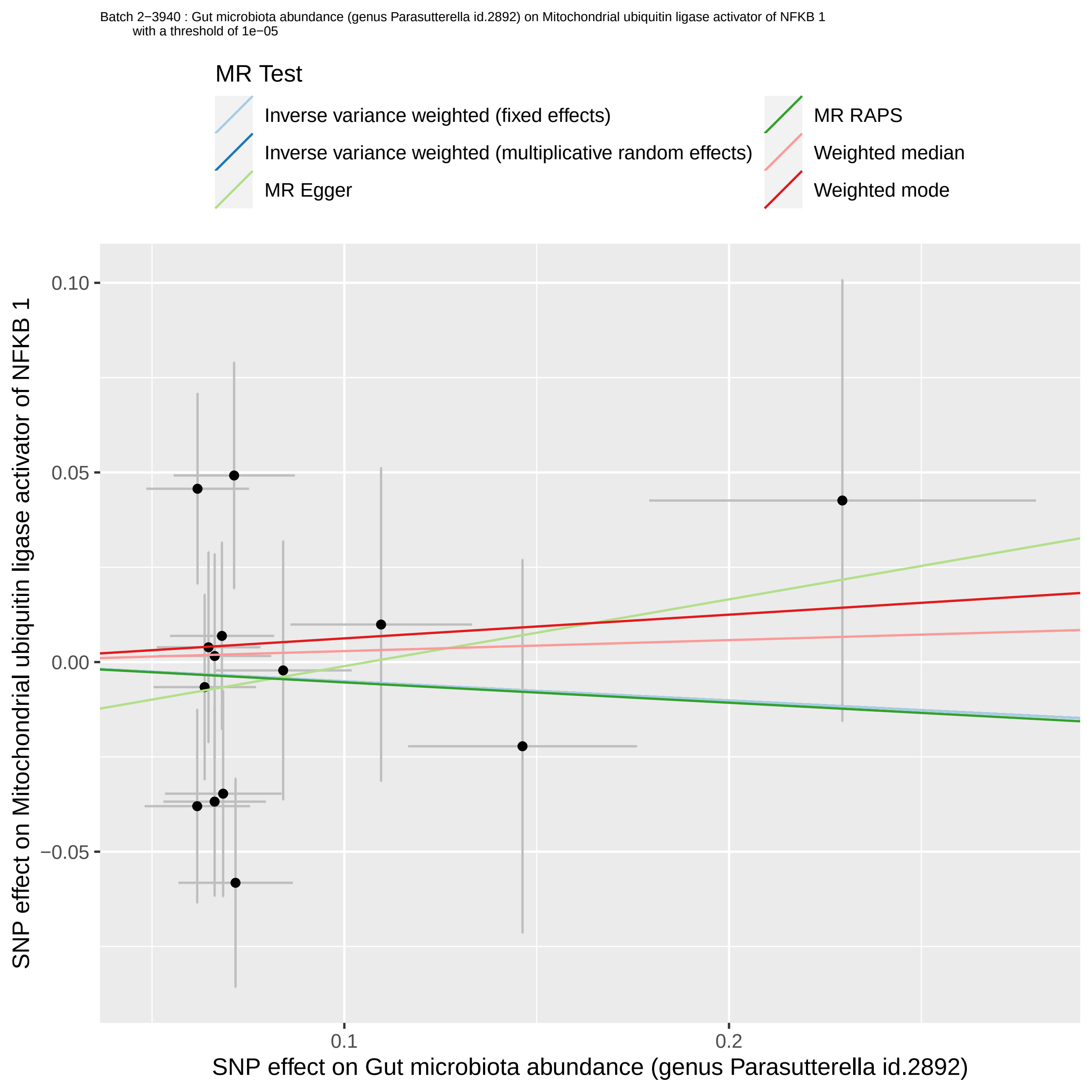

Batch 2−3940 : Gut microbiota abundance (genus Parasutterella id.2892) on Mitochondrial ubiquitin ligase activator of NFKB 1
with a threshold of 1e−05
MR Test
Inverse variance weighted (fixed effects)
Inverse variance weighted (multiplicative random effects)
MR Egger
MR RAPS
Weighted median
Weighted mode
0.10
0.05
0.00
−0.05
0.1
0.2
SNP effect on Mitochondrial ubiquitin ligase activator of NFKB 1
SNP effect on Gut microbiota abundance (genus Parasutterella id.2892)

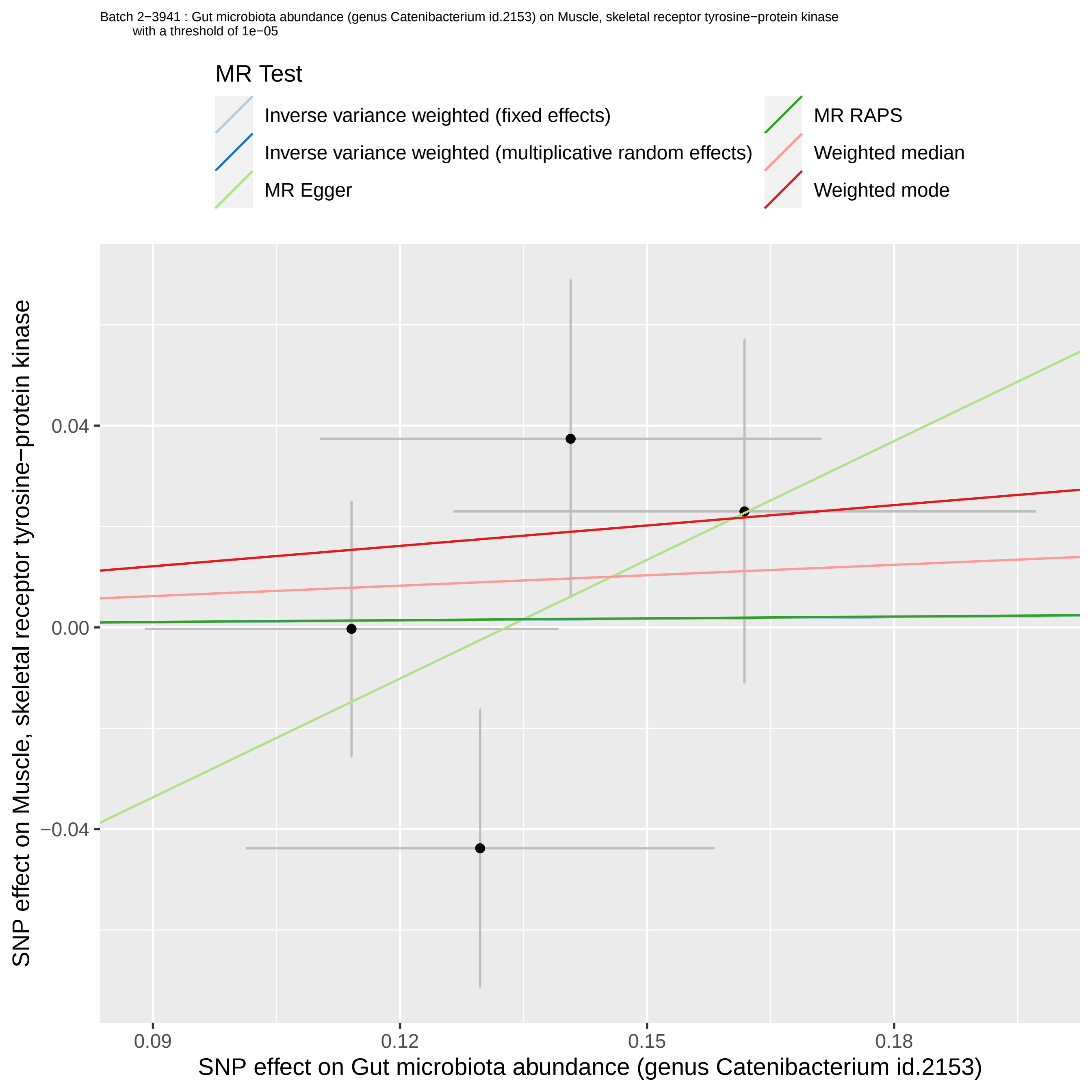
Batch 2−3941 : Gut microbiota abundance (genus Catenibacterium id.2153) on Muscle, skeletal receptor tyrosine−protein kinase
with a threshold of 1e−05
MR Test
Inverse variance weighted (fixed effects)
Inverse variance weighted (multiplicative random effects)
MR Egger
MR RAPS
Weighted median
Weighted mode
0.04
0.00
−0.04
0.09
0.12
0.15
0.18
SNP effect on Muscle, skeletal receptor tyrosine−protein kinase
SNP effect on Gut microbiota abundance (genus Catenibacterium id.2153)

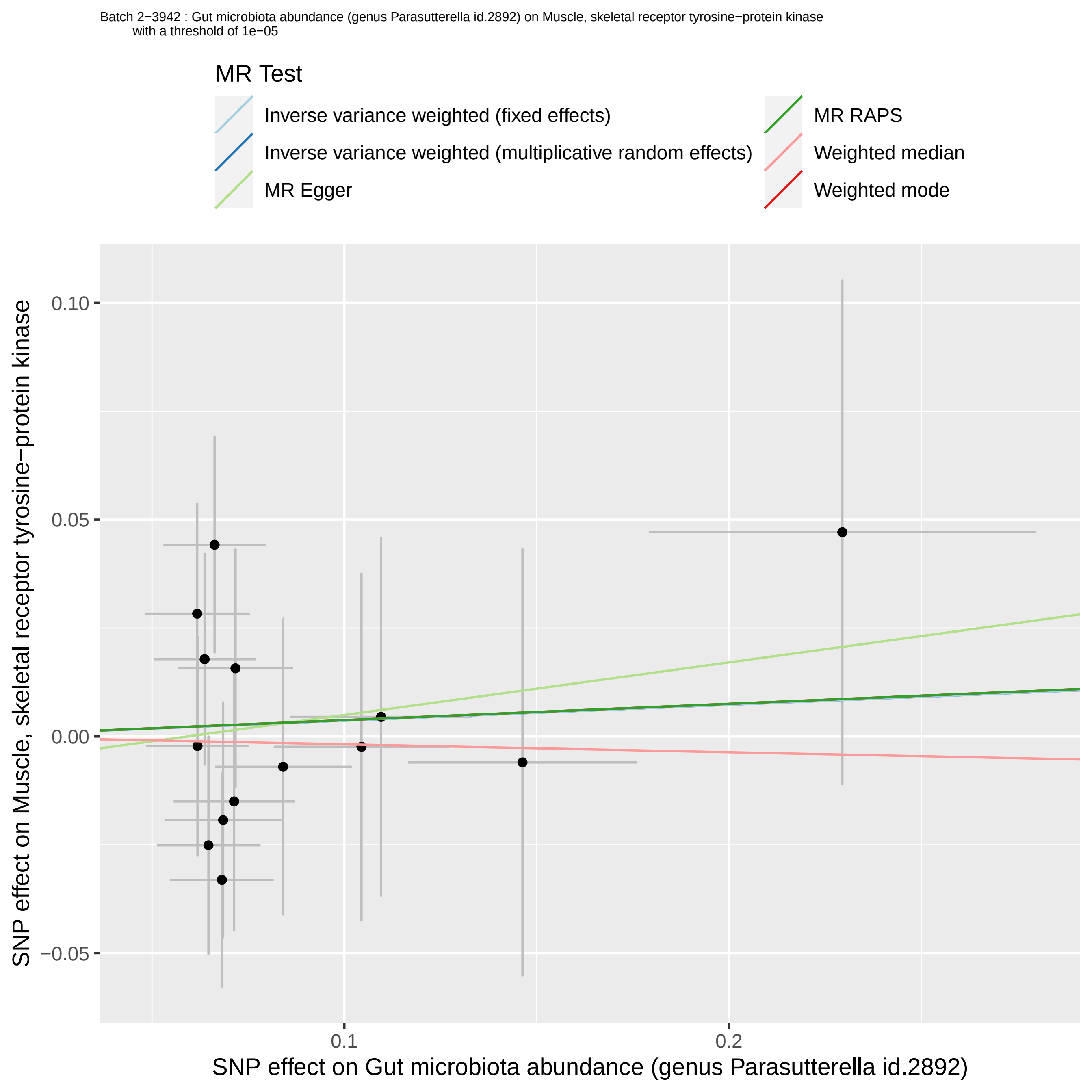

Batch 2−3942 : Gut microbiota abundance (genus Parasutterella id.2892) on Muscle, skeletal receptor tyrosine−protein kinase
with a threshold of 1e−05
MR Test
Inverse variance weighted (fixed effects)
Inverse variance weighted (multiplicative random effects)
MR Egger
MR RAPS
Weighted median
Weighted mode
SNP effect on Muscle, skeletal receptor tyrosine−protein kinase
0.10
0.05
0.00
−0.05
0.1
0.2
SNP effect on Gut microbiota abundance (genus Parasutterella id.2892)

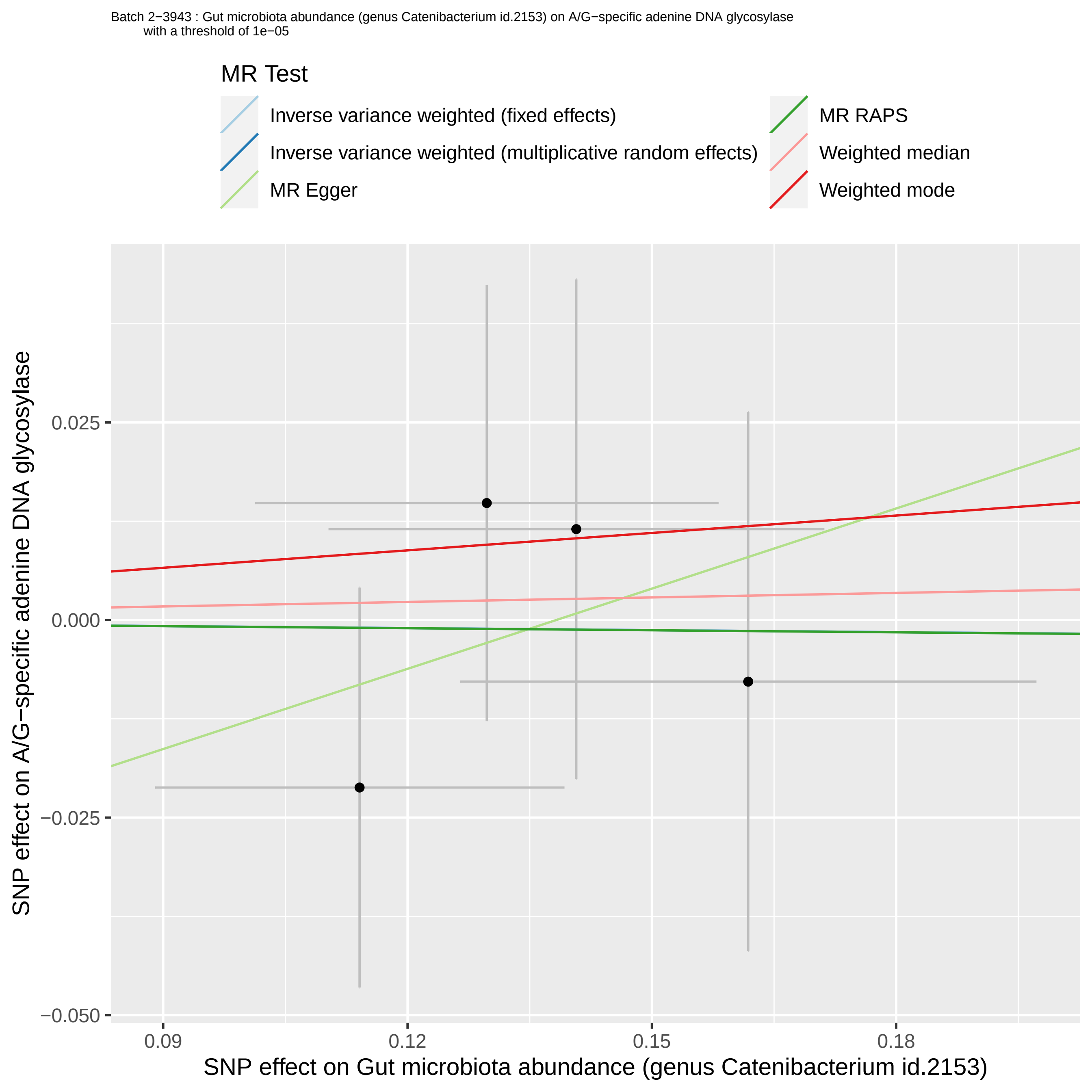
Batch 2−3943 : Gut microbiota abundance (genus Catenibacterium id.2153) on A/G−specific adenine DNA glycosylase with a threshold of 1e−05
MR Test
Inverse variance weighted (fixed effects)
Inverse variance weighted (multiplicative random effects)
MR Egger
MR RAPS
Weighted median
Weighted mode
SNP effect on A/G−specific adenine DNA glycosylase
0.025
0.000
−0.025
−0.050
0.09
0.12
0.15
0.18
SNP effect on Gut microbiota abundance (genus Catenibacterium id.2153)

Batch 2−3944 : Gut microbiota abundance (genus Parasutterella id.2892) on A/G−specific adenine DNA glycosylase
with a threshold of 1e−05

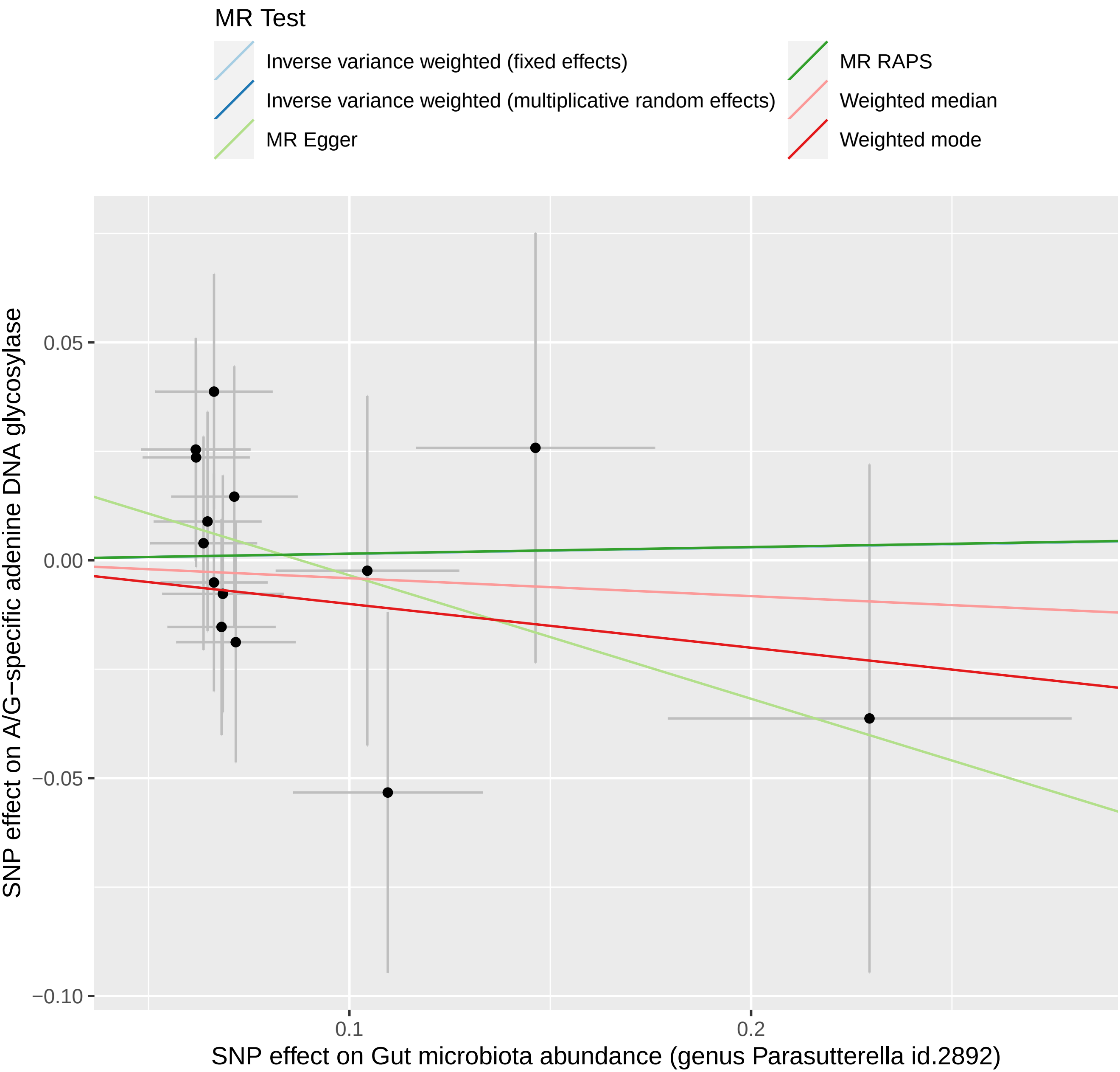

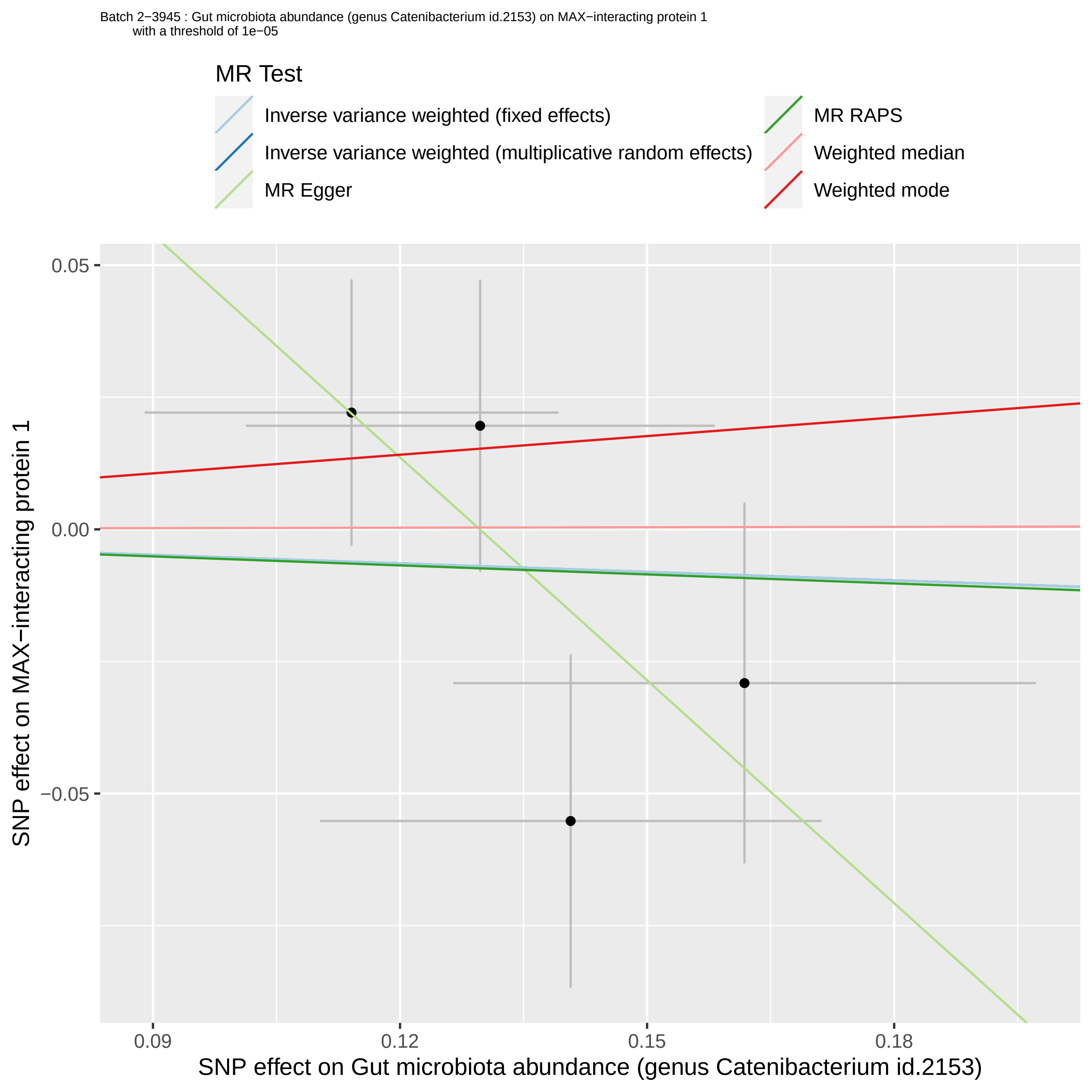

Batch 2−3945 : Gut microbiota abundance (genus Catenibacterium id.2153) on MAX−interacting protein 1
with a threshold of 1e−05
MR Test
Inverse variance weighted (fixed effects)
Inverse variance weighted (multiplicative random effects)
MR Egger
MR RAPS
Weighted median
Weighted mode
0.05
0.00
−0.05
0.09
0.12
0.15
0.18
SNP effect on MAX−interacting protein 1
SNP effect on Gut microbiota abundance (genus Catenibacterium id.2153)

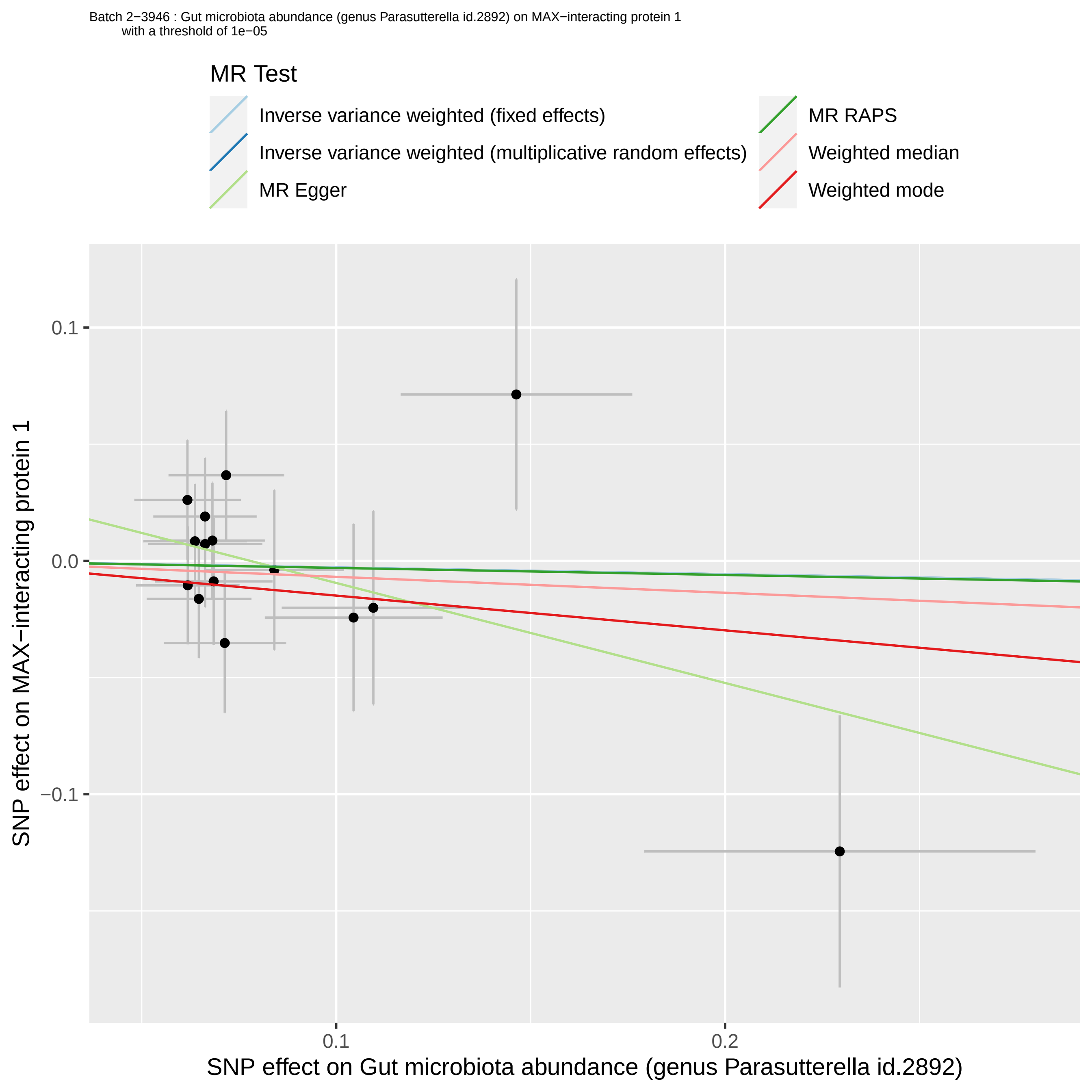
Batch 2−3946 : Gut microbiota abundance (genus Parasutterella id.2892) on MAX−interacting protein 1
with a threshold of 1e−05
MR Test
Inverse variance weighted (fixed effects)
Inverse variance weighted (multiplicative random effects)
MR Egger
MR RAPS
Weighted median
Weighted mode
0.1
0.0
−0.1
0.1
0.2
SNP effect on MAX−interacting protein 1
SNP effect on Gut microbiota abundance (genus Parasutterella id.2892)

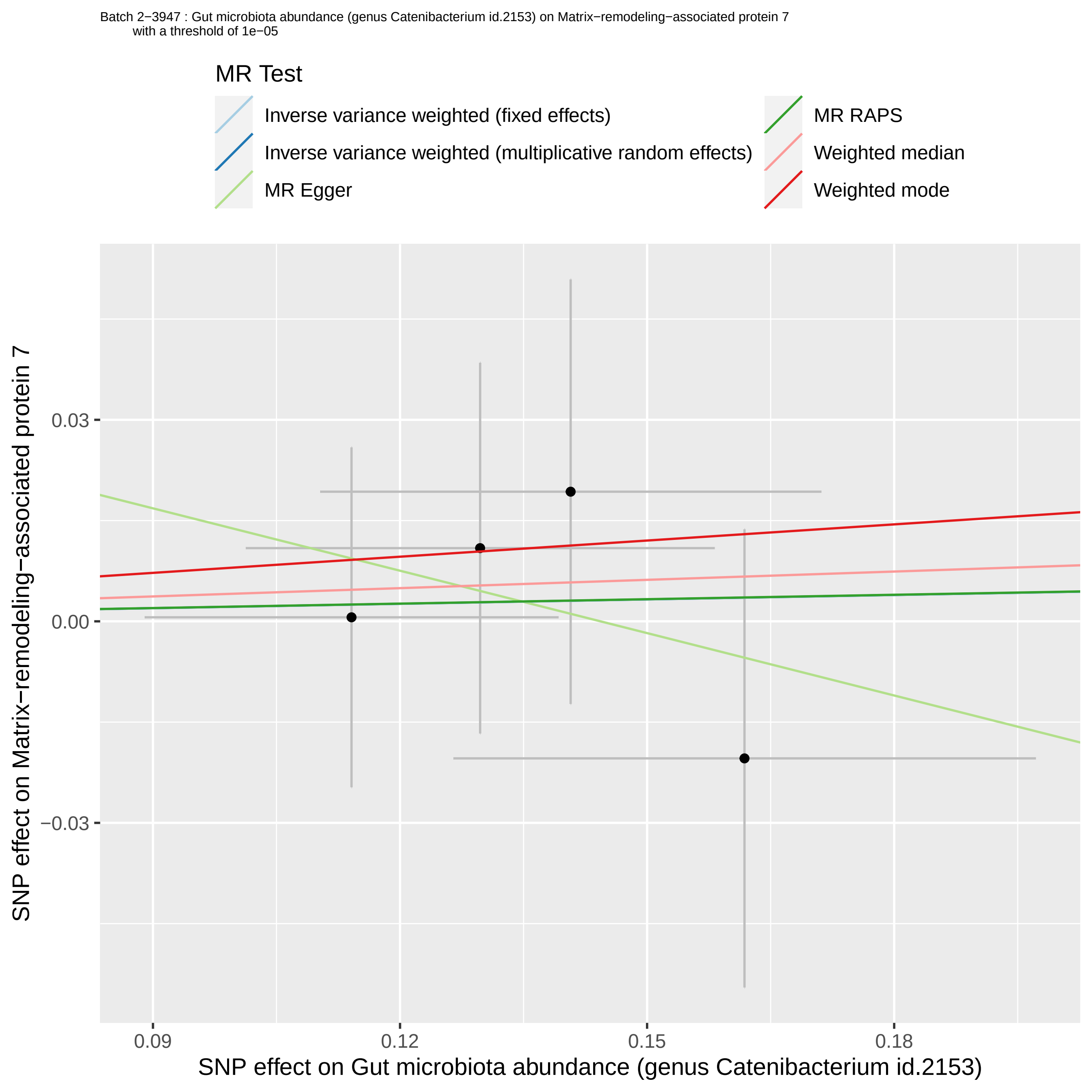
Batch 2−3947 : Gut microbiota abundance (genus Catenibacterium id.2153) on Matrix−remodeling−associated protein 7
with a threshold of 1e−05
MR Test
Inverse variance weighted (fixed effects)
Inverse variance weighted (multiplicative random effects)
MR Egger
MR RAPS
Weighted median
Weighted mode
0.03
0.00
−0.03
0.09
0.12
0.15
0.18
SNP effect on Matrix−remodeling−associated protein 7
SNP effect on Gut microbiota abundance (genus Catenibacterium id.2153)

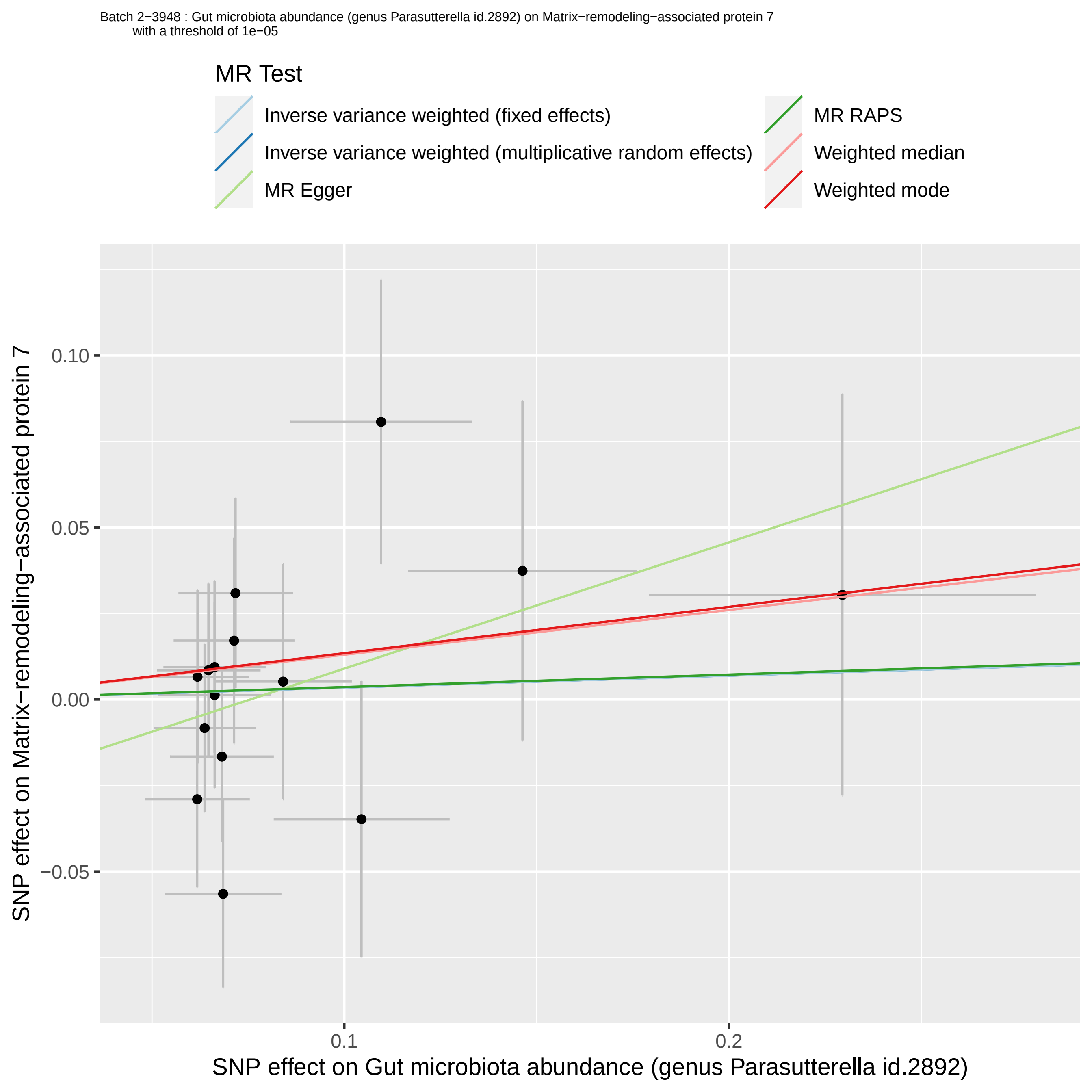
Batch 2−3948 : Gut microbiota abundance (genus Parasutterella id.2892) on Matrix−remodeling−associated protein 7
with a threshold of 1e−05
MR Test
Inverse variance weighted (fixed effects)
Inverse variance weighted (multiplicative random effects)
MR Egger
MR RAPS
Weighted median
Weighted mode
0.10
0.05
0.00
−0.05
0.1
0.2
SNP effect on Matrix−remodeling−associated protein 7
SNP effect on Gut microbiota abundance (genus Parasutterella id.2892)

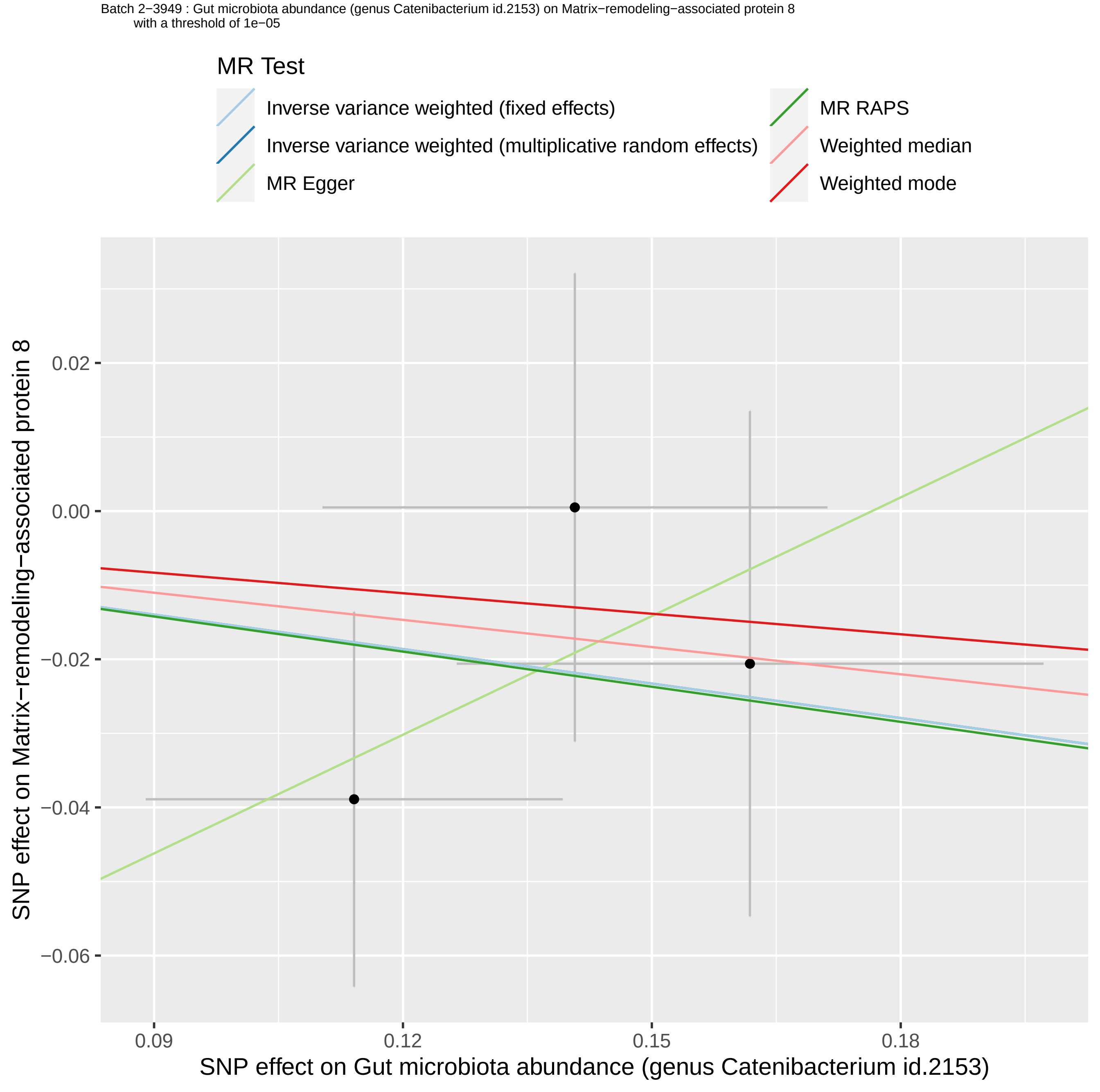

Batch 2−3949 : Gut microbiota abundance (genus Catenibacterium id.2153) on Matrix−remodeling−associated protein 8
with a threshold of 1e−05
MR Test
Inverse variance weighted (fixed effects)
Inverse variance weighted (multiplicative random effects)
MR Egger
MR RAPS
Weighted median
Weighted mode
0.02
0.00
−0.02
−0.04
−0.06
SNP effect on Matrix−remodeling−associated protein 8
0.09
0.12
0.15
0.18
SNP effect on Gut microbiota abundance (genus Catenibacterium id.2153)

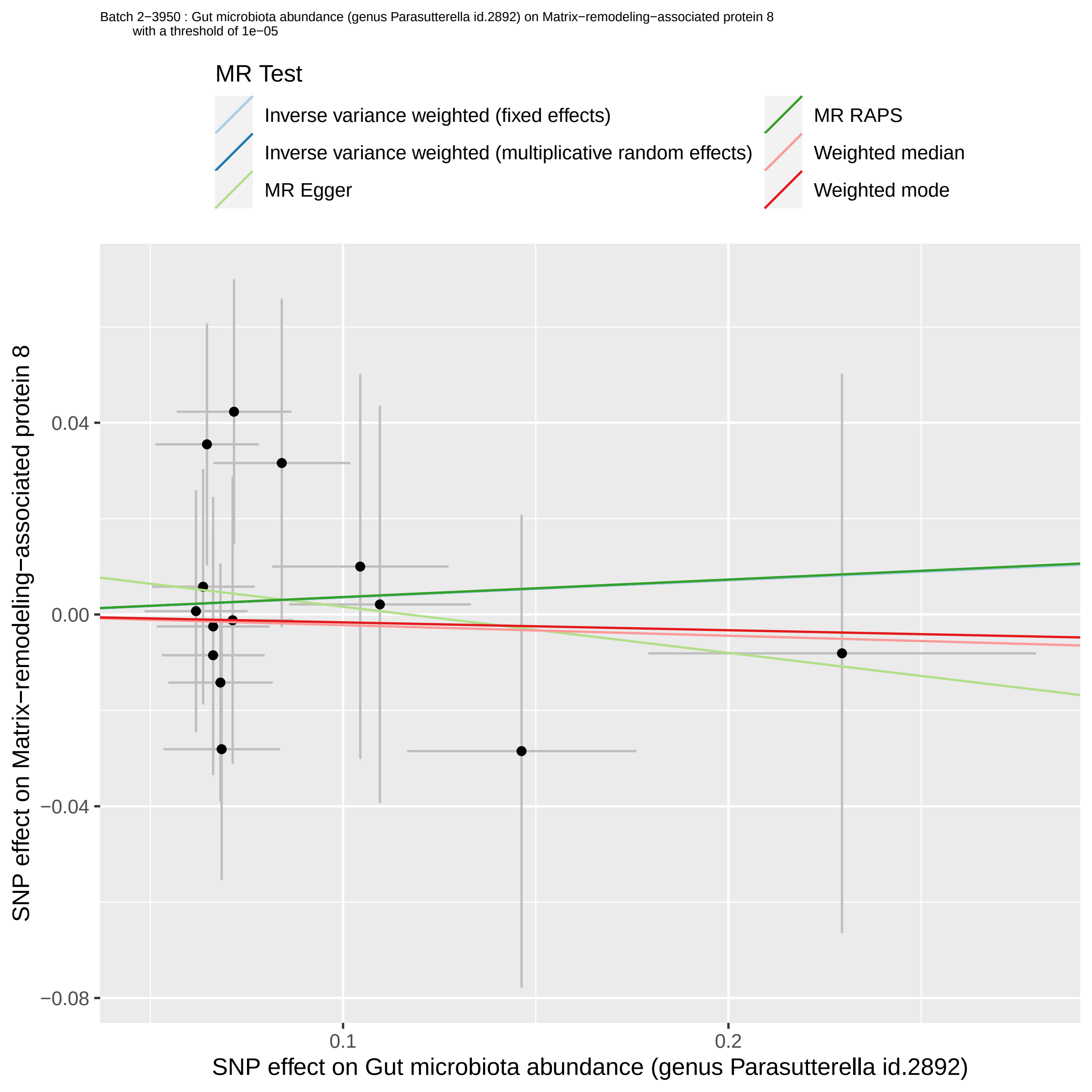

Batch 2−3950 : Gut microbiota abundance (genus Parasutterella id.2892) on Matrix−remodeling−associated protein 8
with a threshold of 1e−05
MR Test
Inverse variance weighted (fixed effects)
Inverse variance weighted (multiplicative random effects)
MR Egger
MR RAPS
Weighted median
Weighted mode
SNP effect on Matrix−remodeling−associated protein 8
0.04
0.00
−0.04
−0.08
0.1
0.2
SNP effect on Gut microbiota abundance (genus Parasutterella id.2892)

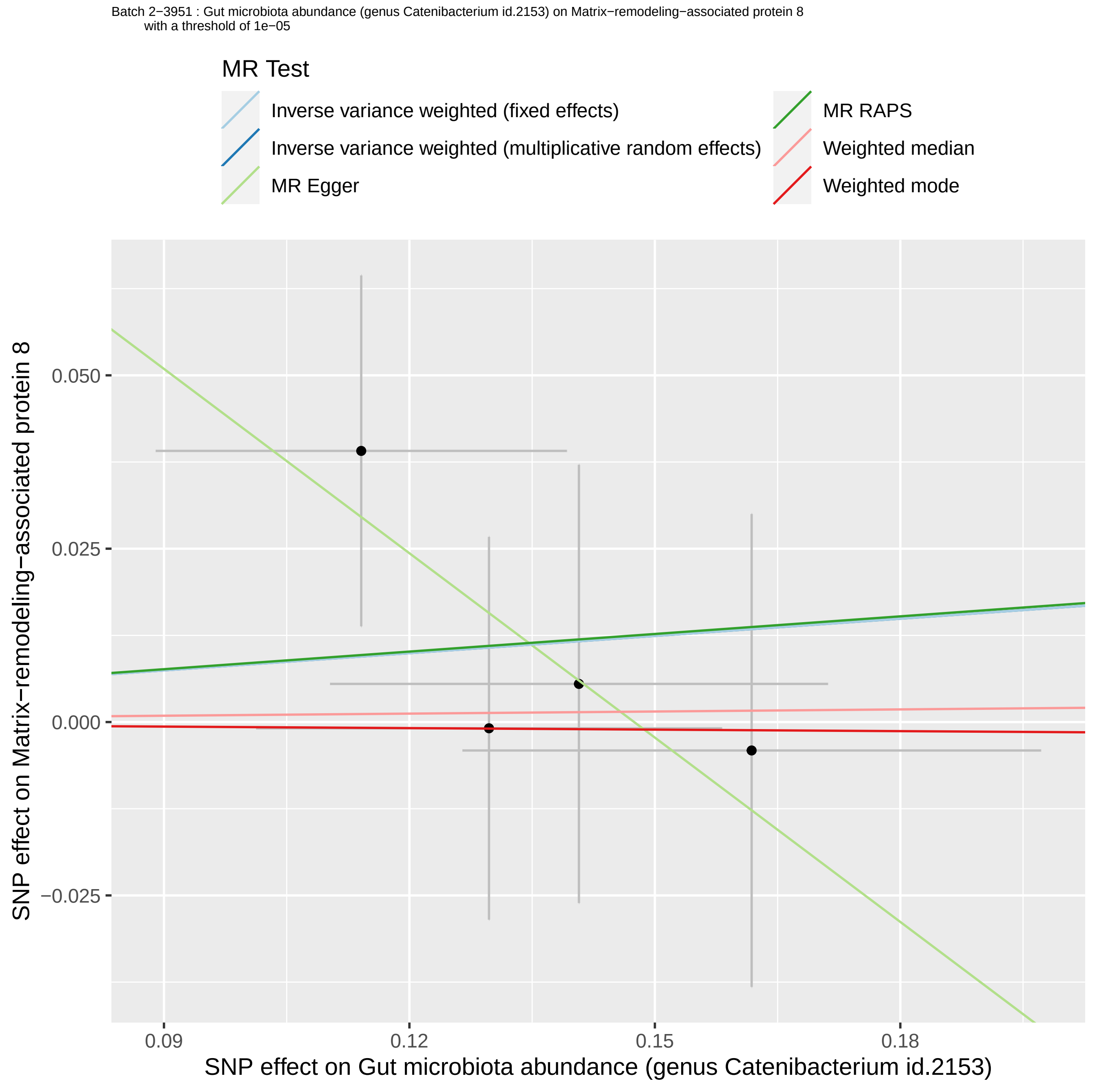
Batch 2−3951 : Gut microbiota abundance (genus Catenibacterium id.2153) on Matrix−remodeling−associated protein 8
with a threshold of 1e−05
MR Test
Inverse variance weighted (fixed effects)
Inverse variance weighted (multiplicative random effects)
MR Egger
MR RAPS
Weighted median
Weighted mode
SNP effect on Matrix−remodeling−associated protein 8
0.050
0.025
0.000
−0.025
0.09
0.12
0.15
0.18
SNP effect on Gut microbiota abundance (genus Catenibacterium id.2153)

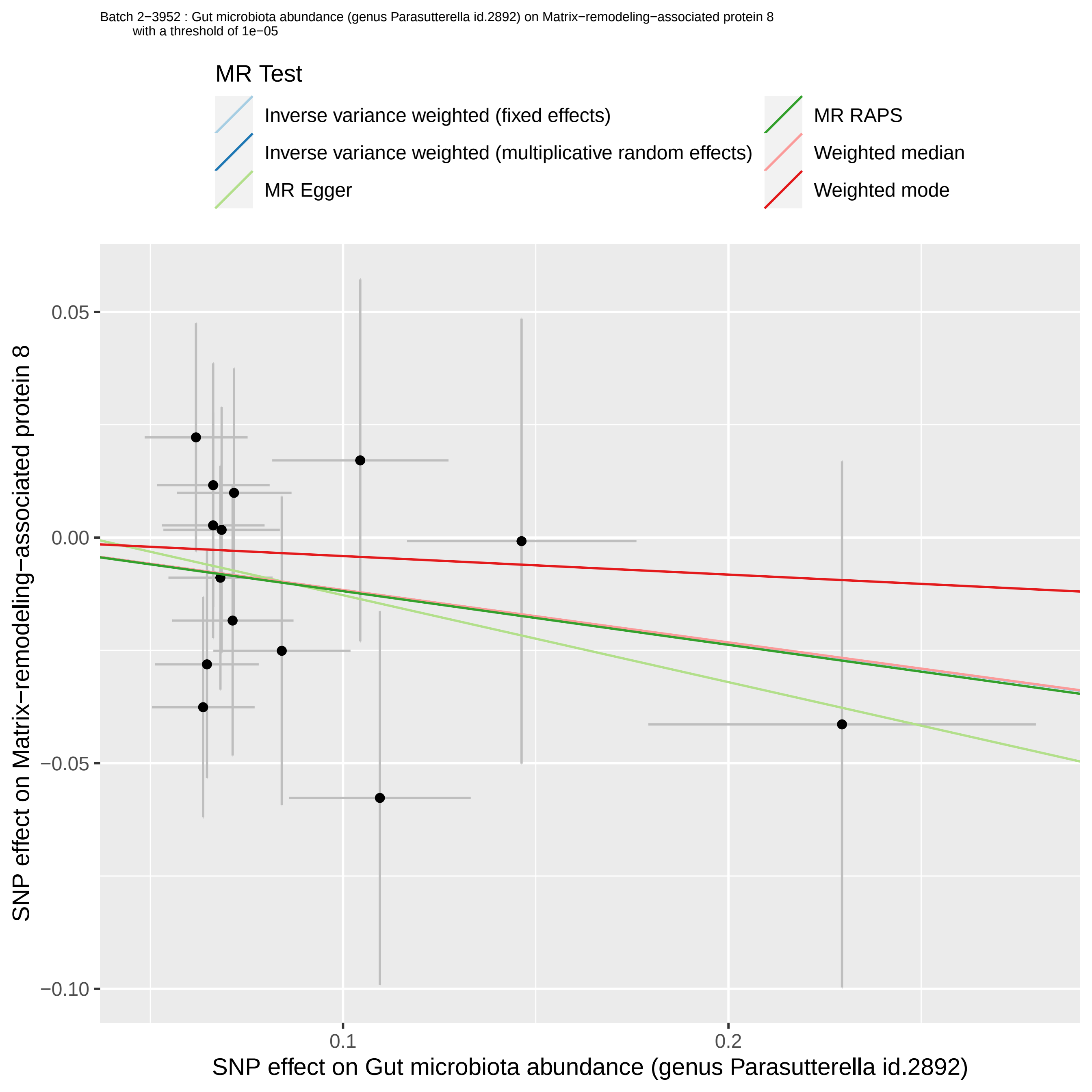

Batch 2−3952 : Gut microbiota abundance (genus Parasutterella id.2892) on Matrix−remodeling−associated protein 8
with a threshold of 1e−05
MR Test
Inverse variance weighted (fixed effects)
Inverse variance weighted (multiplicative random effects)
MR Egger
MR RAPS
Weighted median
Weighted mode
0.05
0.00
−0.05
−0.10
0.1
0.2
SNP effect on Matrix−remodeling−associated protein 8
SNP effect on Gut microbiota abundance (genus Parasutterella id.2892)

Batch 2−3953 : Gut microbiota abundance (genus Catenibacterium id.2153) on Myosin−binding protein C, slow−type
with a threshold of 1e−05

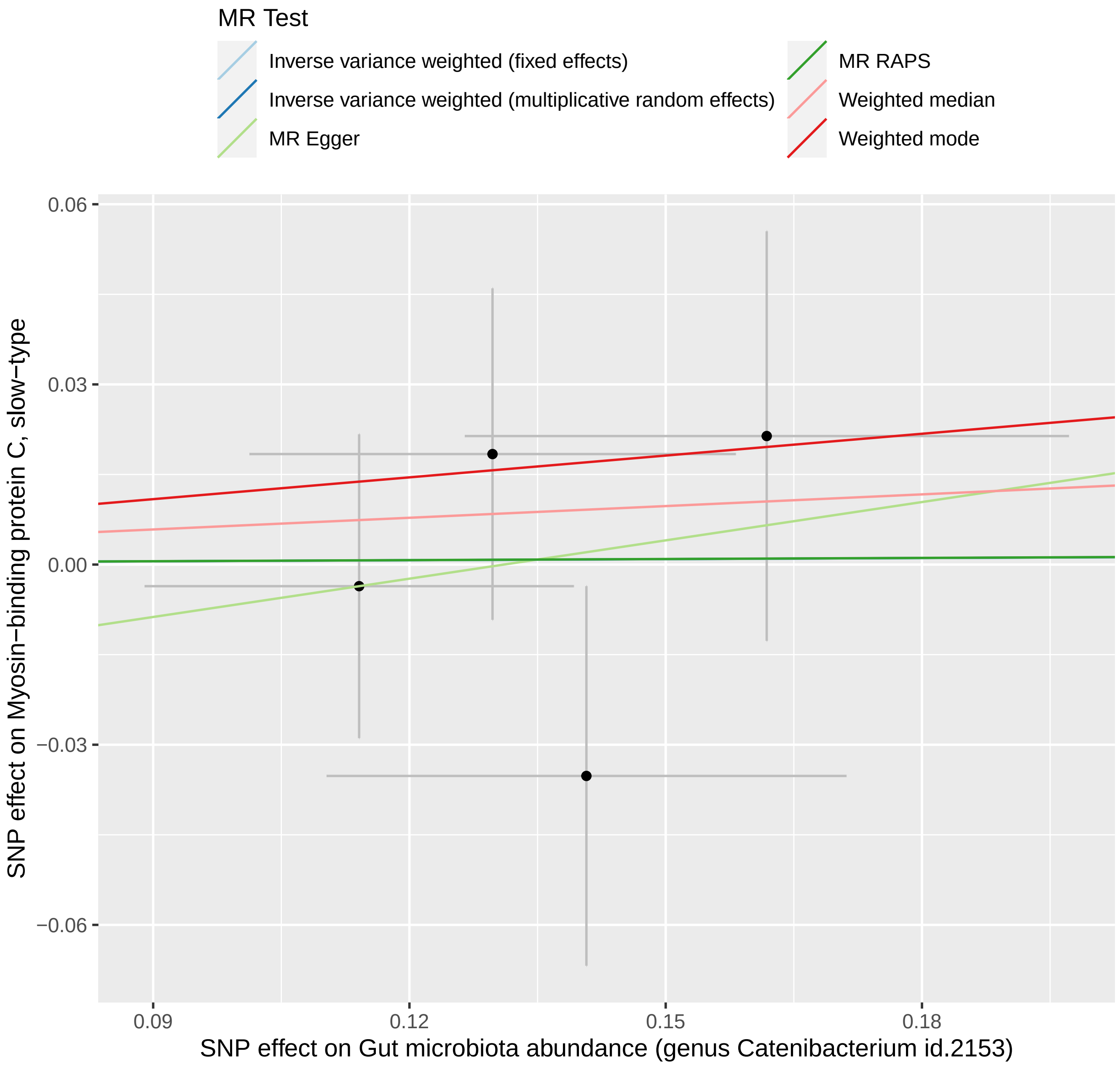

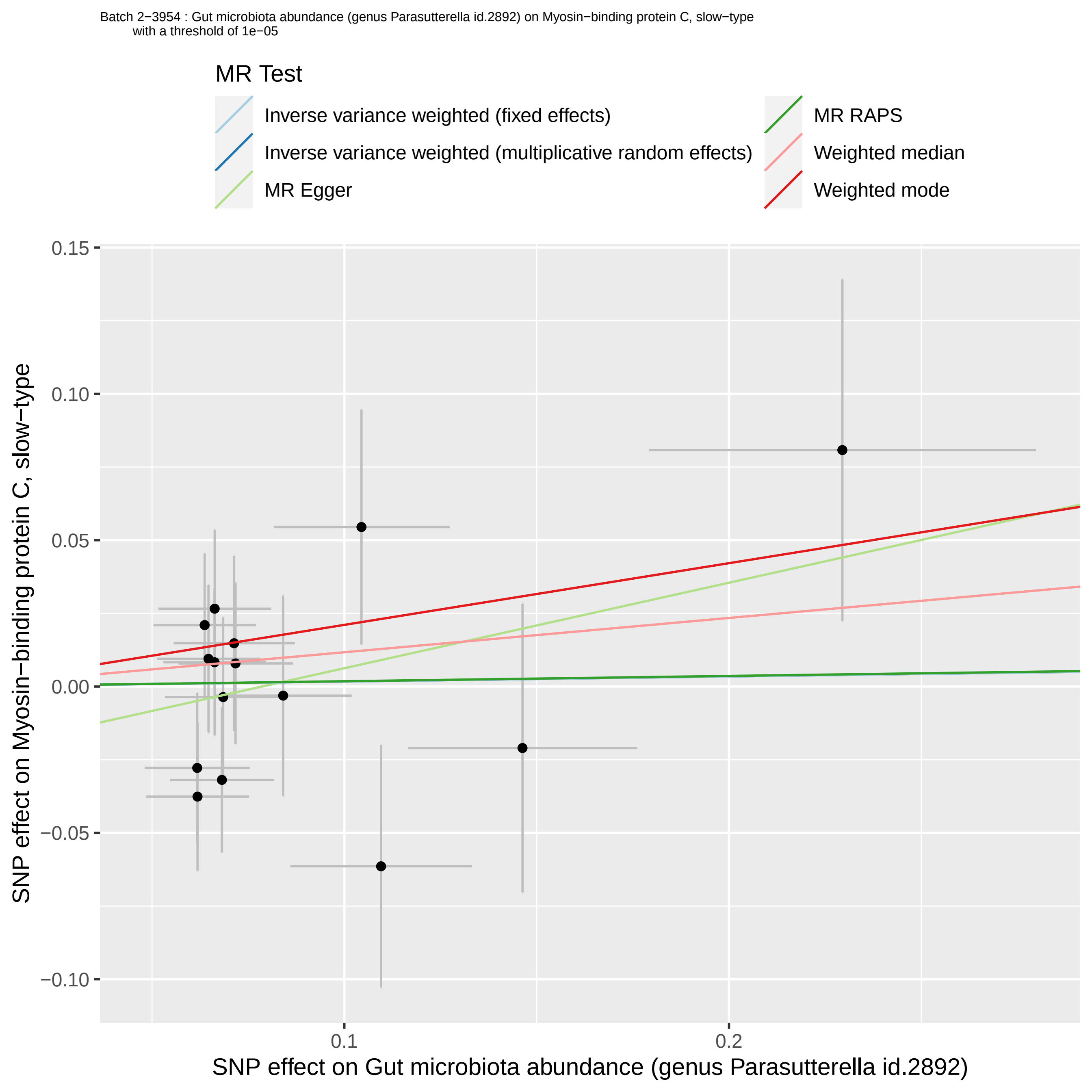

Batch 2−3954 : Gut microbiota abundance (genus Parasutterella id.2892) on Myosin−binding protein C, slow−type
with a threshold of 1e−05
MR Test
Inverse variance weighted (fixed effects)
Inverse variance weighted (multiplicative random effects)
MR Egger
MR RAPS
Weighted median
Weighted mode
0.15
0.10
0.05
0.00
−0.05
−0.10
0.1
0.2
SNP effect on Myosin−binding protein C, slow−type
SNP effect on Gut microbiota abundance (genus Parasutterella id.2892)

Batch 2−3955 : Gut microbiota abundance (genus Catenibacterium id.2153) on Myc proto−oncogene protein
with a threshold of 1e−05

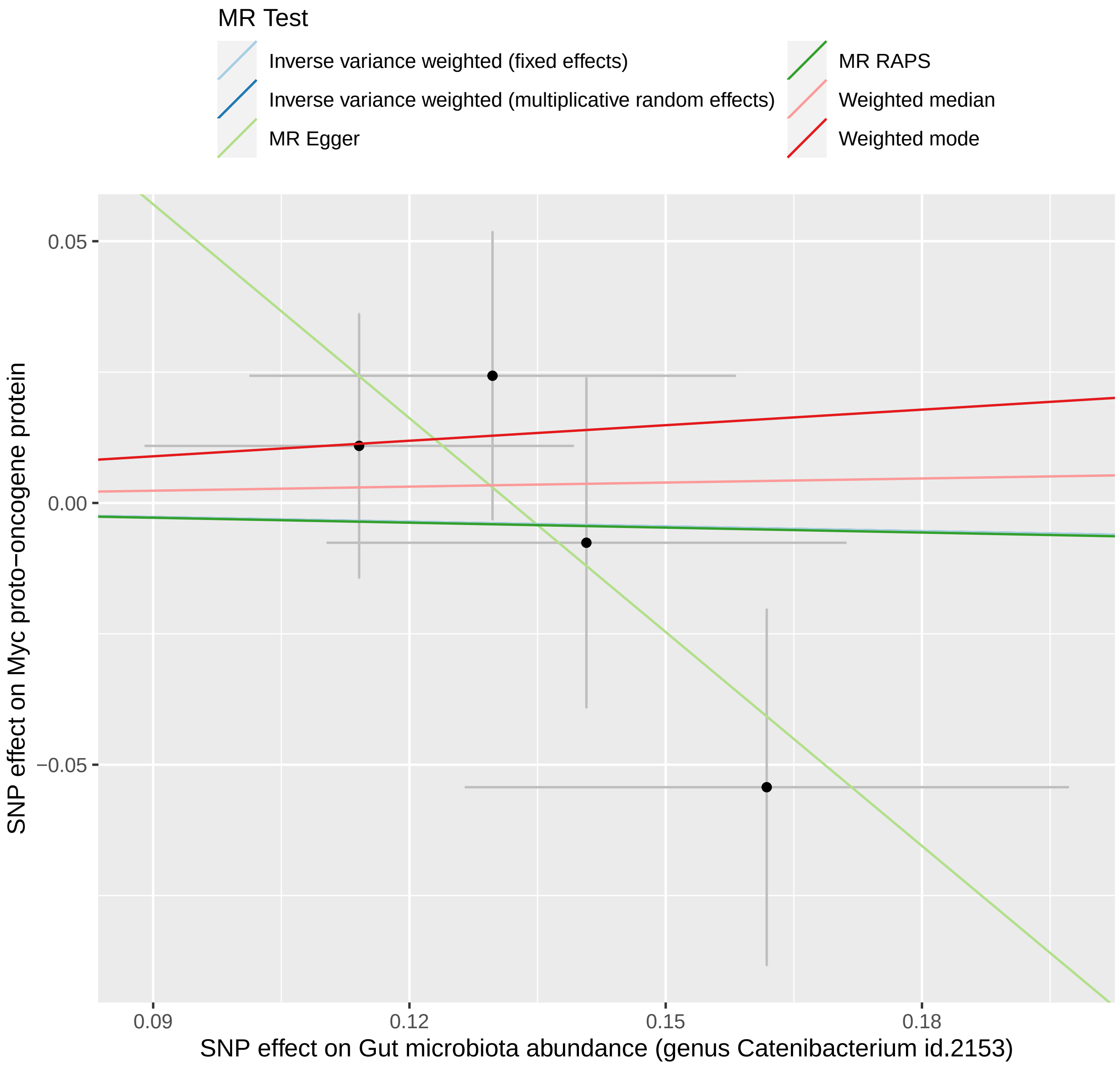

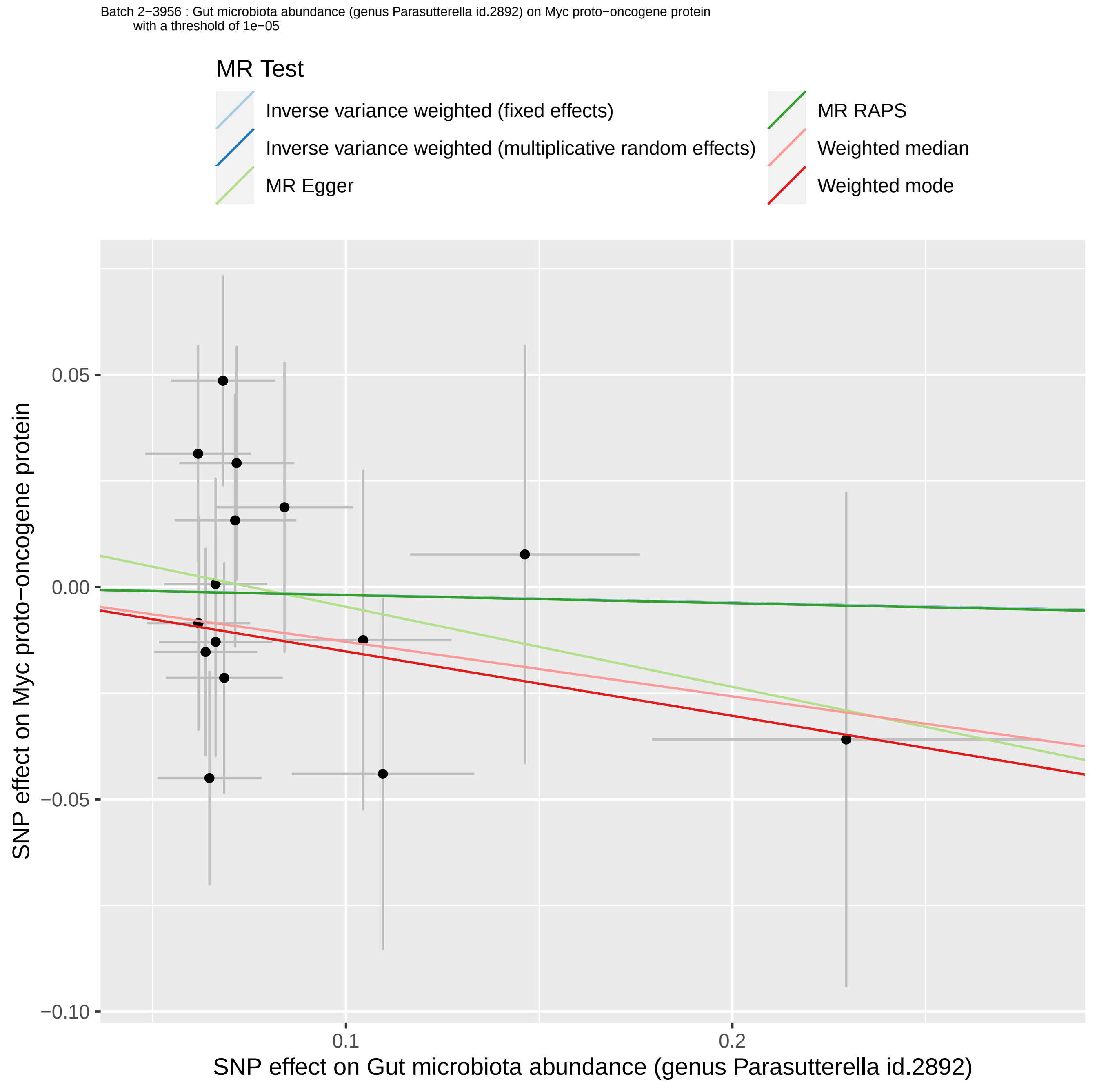
Batch 2−3956 : Gut microbiota abundance (genus Parasutterella id.2892) on Myc proto−oncogene protein
with a threshold of 1e−05
MR Test
Inverse variance weighted (fixed effects)
Inverse variance weighted (multiplicative random effects)
MR Egger
MR RAPS
Weighted median
Weighted mode
0.05
0.00
−0.05
−0.10
0.1
0.2
SNP effect on Myc proto−oncogene protein
SNP effect on Gut microbiota abundance (genus Parasutterella id.2892)

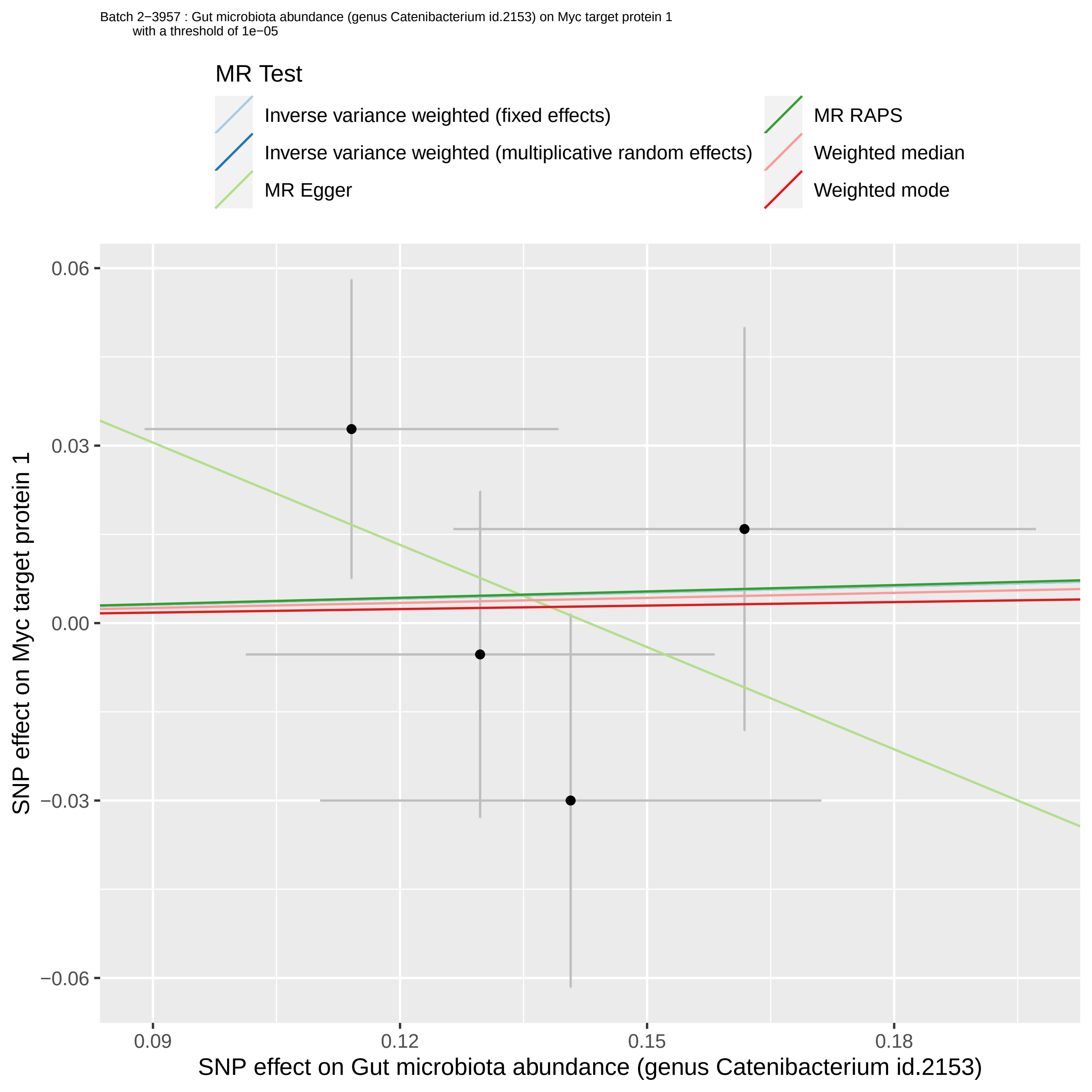

Batch 2−3957 : Gut microbiota abundance (genus Catenibacterium id.2153) on Myc target protein 1
with a threshold of 1e−05
MR Test
Inverse variance weighted (fixed effects)
Inverse variance weighted (multiplicative random effects)
MR Egger
MR RAPS
Weighted median
Weighted mode
0.06
0.03
0.00
−0.03
−0.06
0.09
0.12
0.15
0.18
SNP effect on Myc target protein 1
SNP effect on Gut microbiota abundance (genus Catenibacterium id.2153)

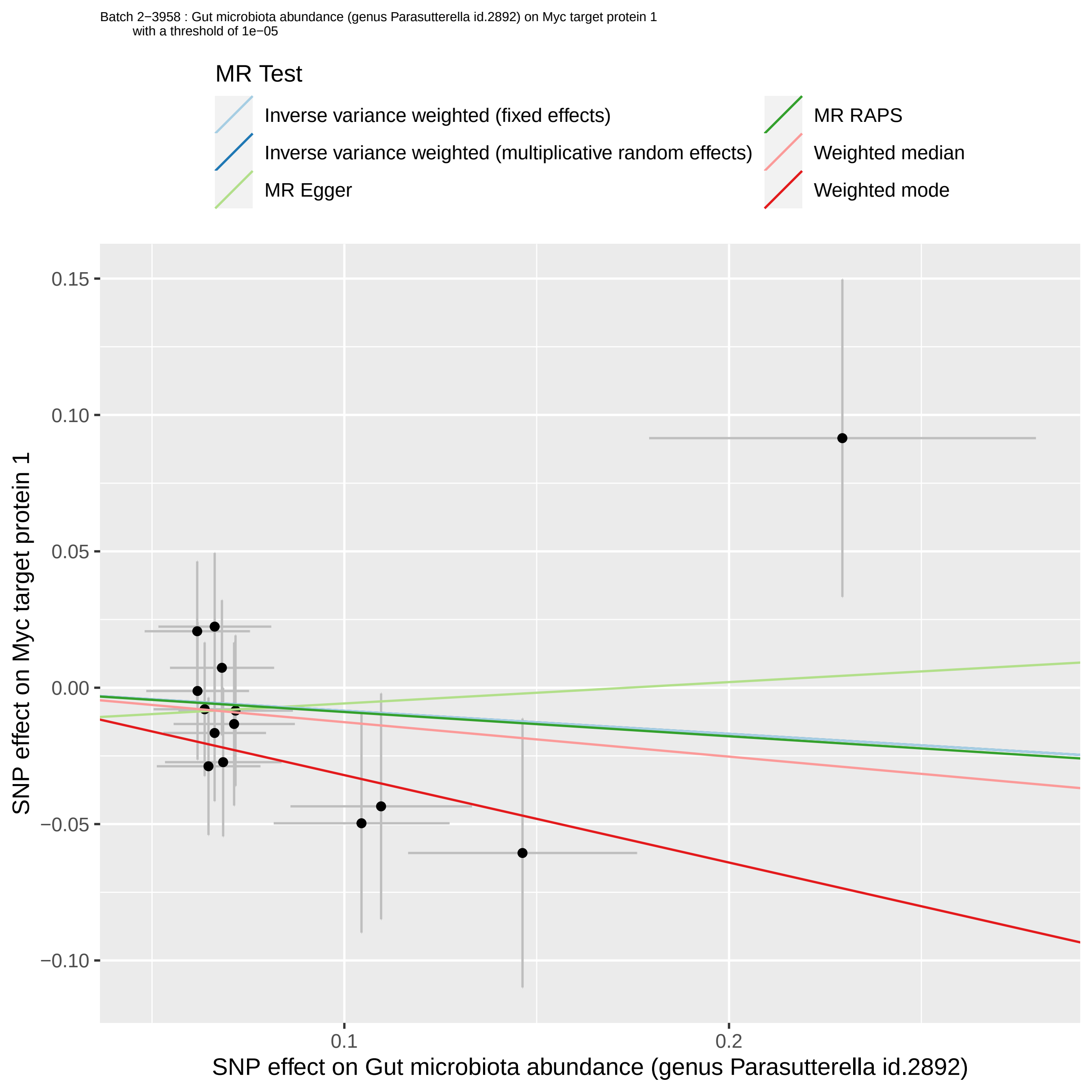
Batch 2−3958 : Gut microbiota abundance (genus Parasutterella id.2892) on Myc target protein 1
with a threshold of 1e−05
MR Test
Inverse variance weighted (fixed effects)
Inverse variance weighted (multiplicative random effects)
MR Egger
MR RAPS
Weighted median
Weighted mode
SNP effect on Myc target protein 1
0.15
0.10
0.05
0.00
−0.05
−0.10
0.1
0.2
SNP effect on Gut microbiota abundance (genus Parasutterella id.2892)

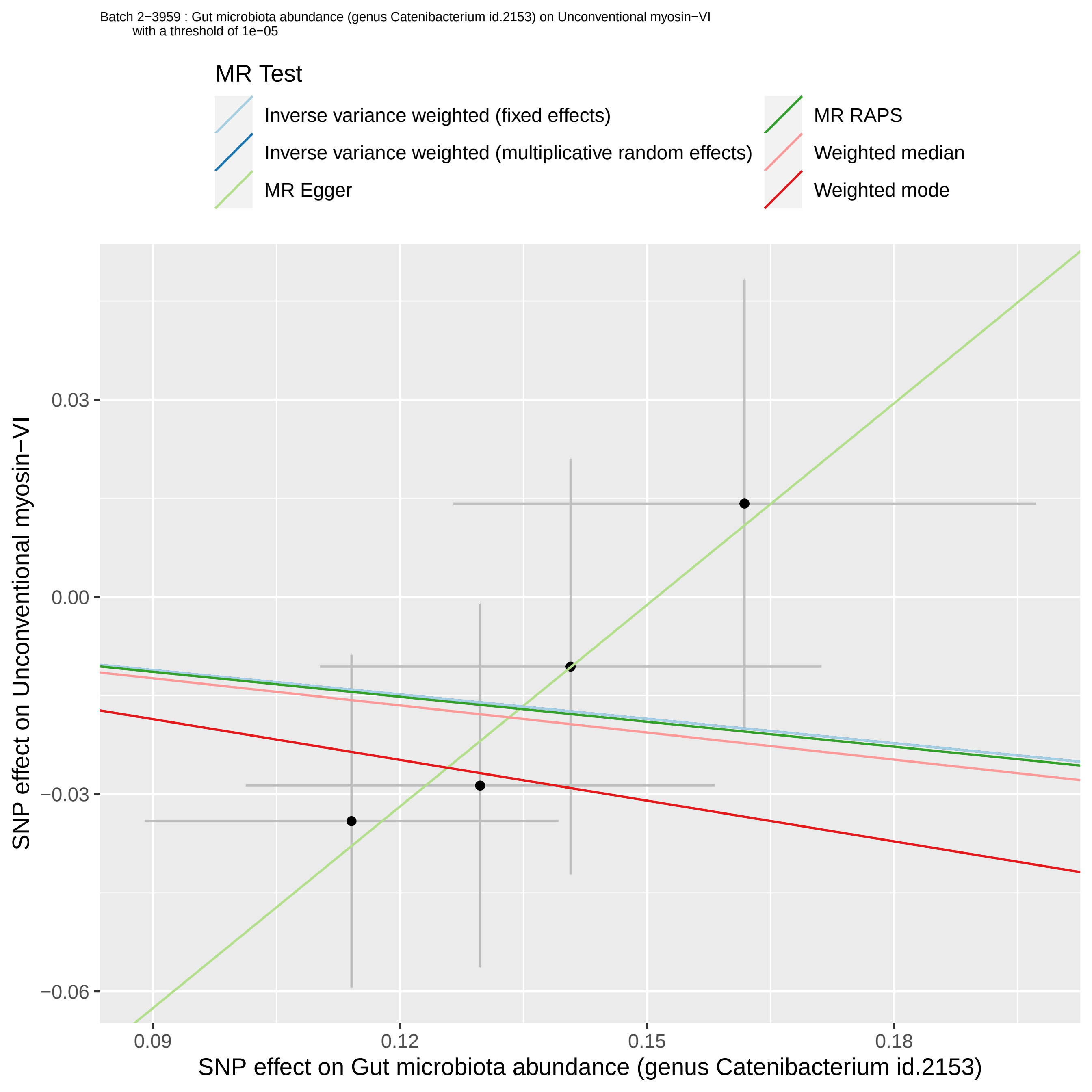

Batch 2−3959 : Gut microbiota abundance (genus Catenibacterium id.2153) on Unconventional myosin−VI
with a threshold of 1e−05
MR Test
Inverse variance weighted (fixed effects)
Inverse variance weighted (multiplicative random effects)
MR Egger
MR RAPS
Weighted median
Weighted mode
SNP effect on Unconventional myosin−VI
0.03
0.00
−0.03
−0.06
0.09
0.12
0.15
0.18
SNP effect on Gut microbiota abundance (genus Catenibacterium id.2153)

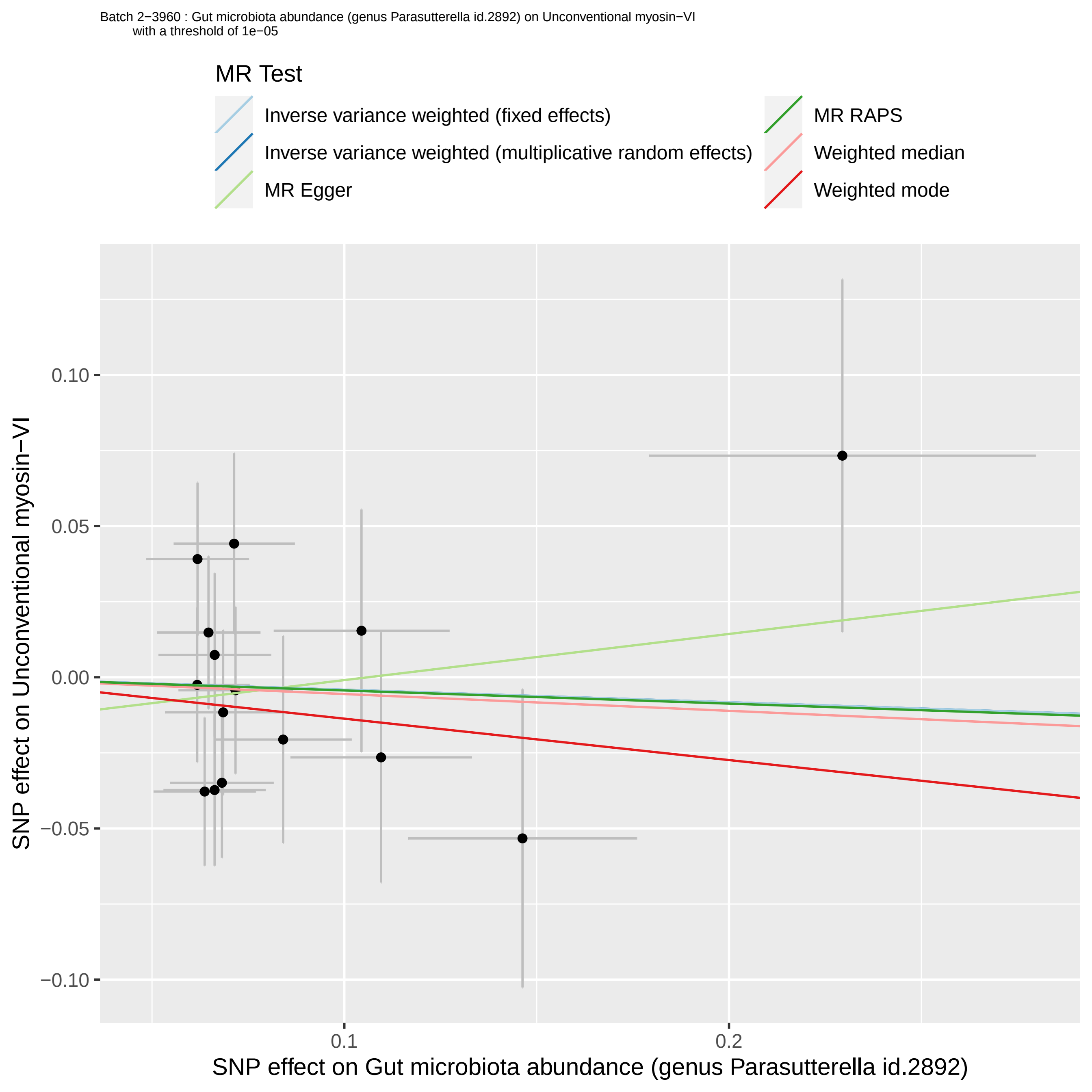
Batch 2−3960 : Gut microbiota abundance (genus Parasutterella id.2892) on Unconventional myosin−VI
with a threshold of 1e−05
MR Test
Inverse variance weighted (fixed effects)
Inverse variance weighted (multiplicative random effects)
MR Egger
MR RAPS
Weighted median
Weighted mode
0.10
0.05
0.00
−0.05
−0.10
0.1
0.2
SNP effect on Unconventional myosin−VI
SNP effect on Gut microbiota abundance (genus Parasutterella id.2892)

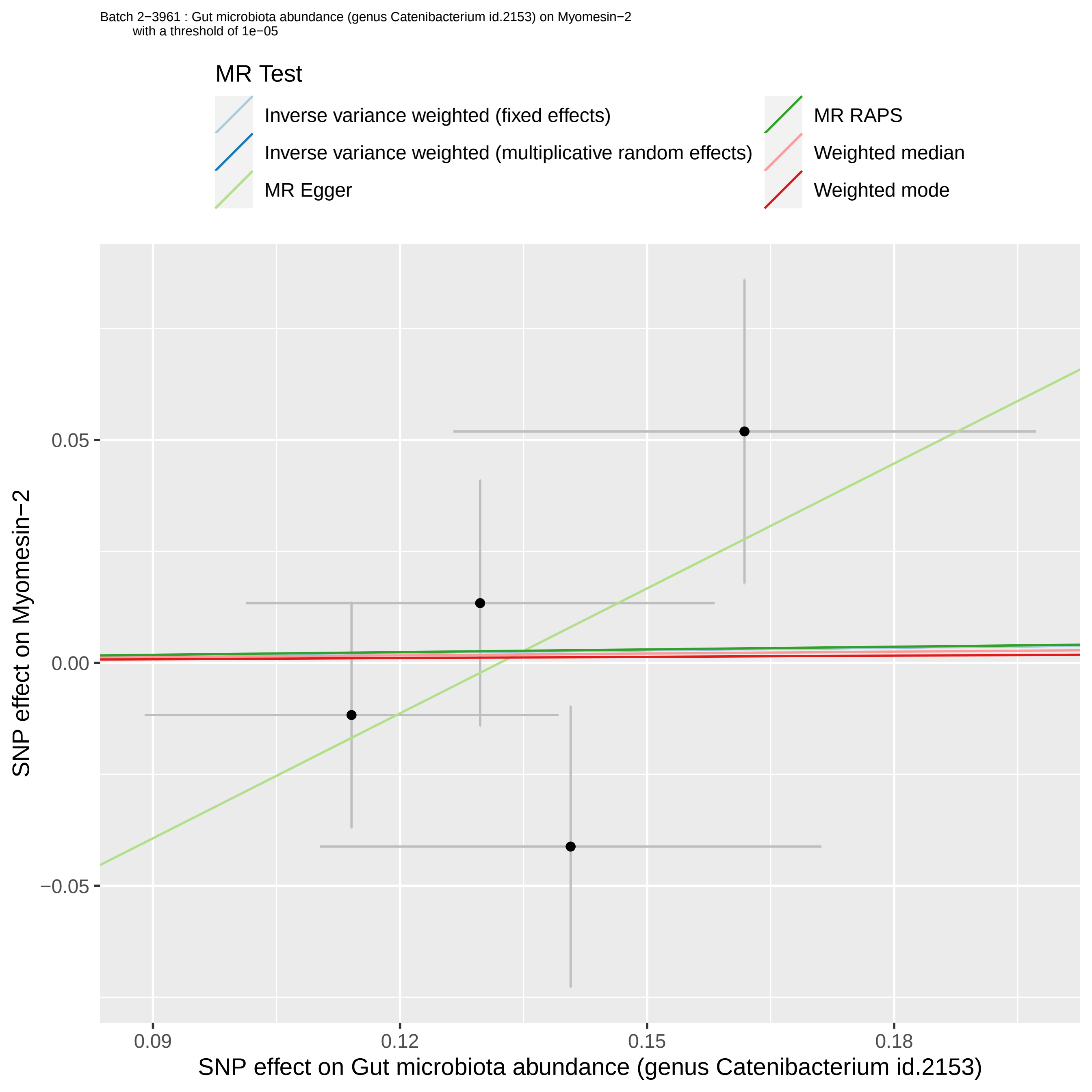
Batch 2−3961 : Gut microbiota abundance (genus Catenibacterium id.2153) on Myomesin−2
with a threshold of 1e−05
MR Test
Inverse variance weighted (fixed effects)
Inverse variance weighted (multiplicative random effects)
MR Egger
MR RAPS
Weighted median
Weighted mode
0.05
0.00
−0.05
0.09
0.12
0.15
0.18
SNP effect on Myomesin−2
SNP effect on Gut microbiota abundance (genus Catenibacterium id.2153)

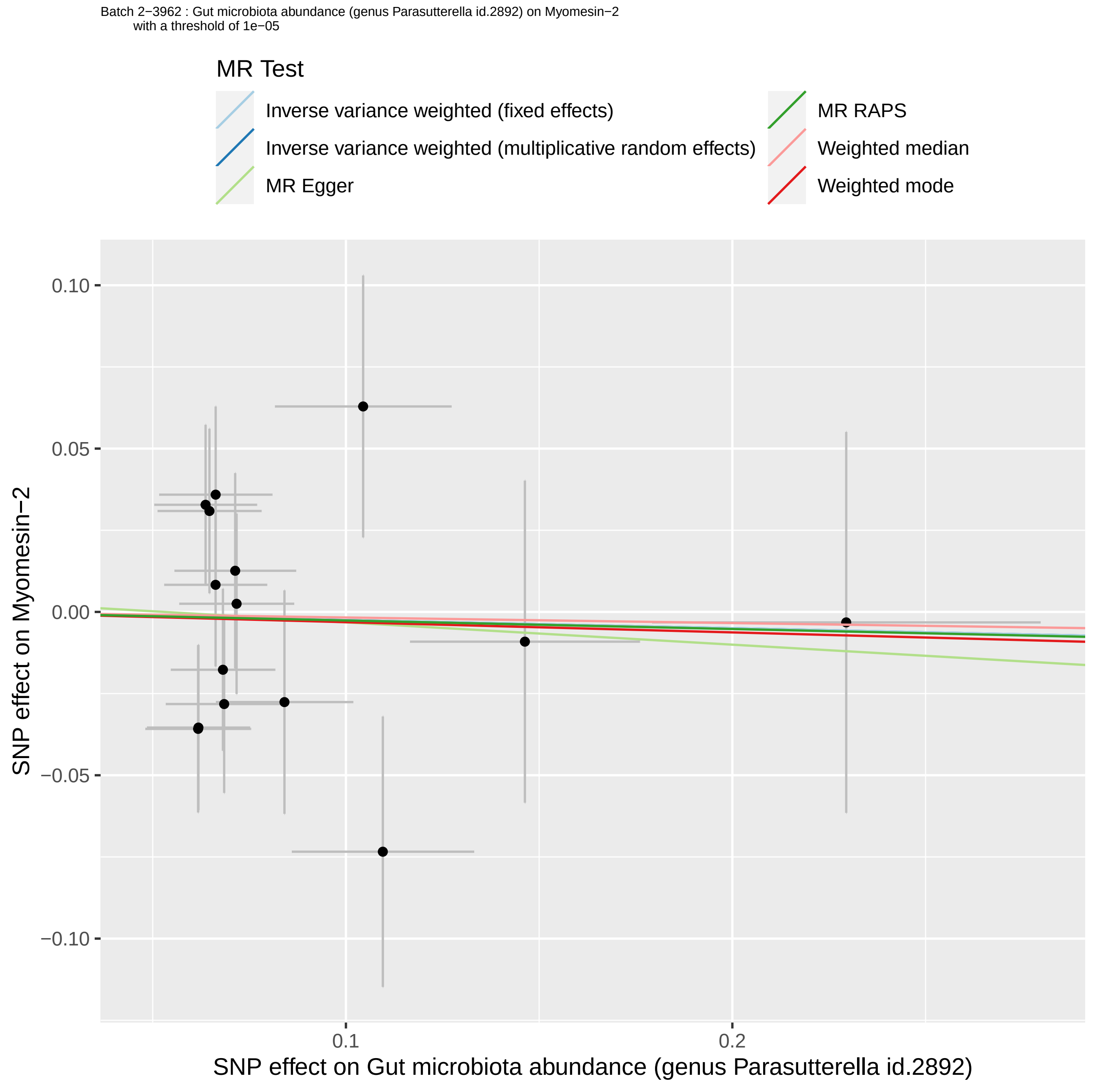
Batch 2−3962 : Gut microbiota abundance (genus Parasutterella id.2892) on Myomesin−2
with a threshold of 1e−05
MR Test
Inverse variance weighted (fixed effects)
Inverse variance weighted (multiplicative random effects)
MR Egger
MR RAPS
Weighted median
Weighted mode
0.10
0.05
0.00
−0.05
−0.10
0.1
0.2
SNP effect on Myomesin−2
SNP effect on Gut microbiota abundance (genus Parasutterella id.2892)

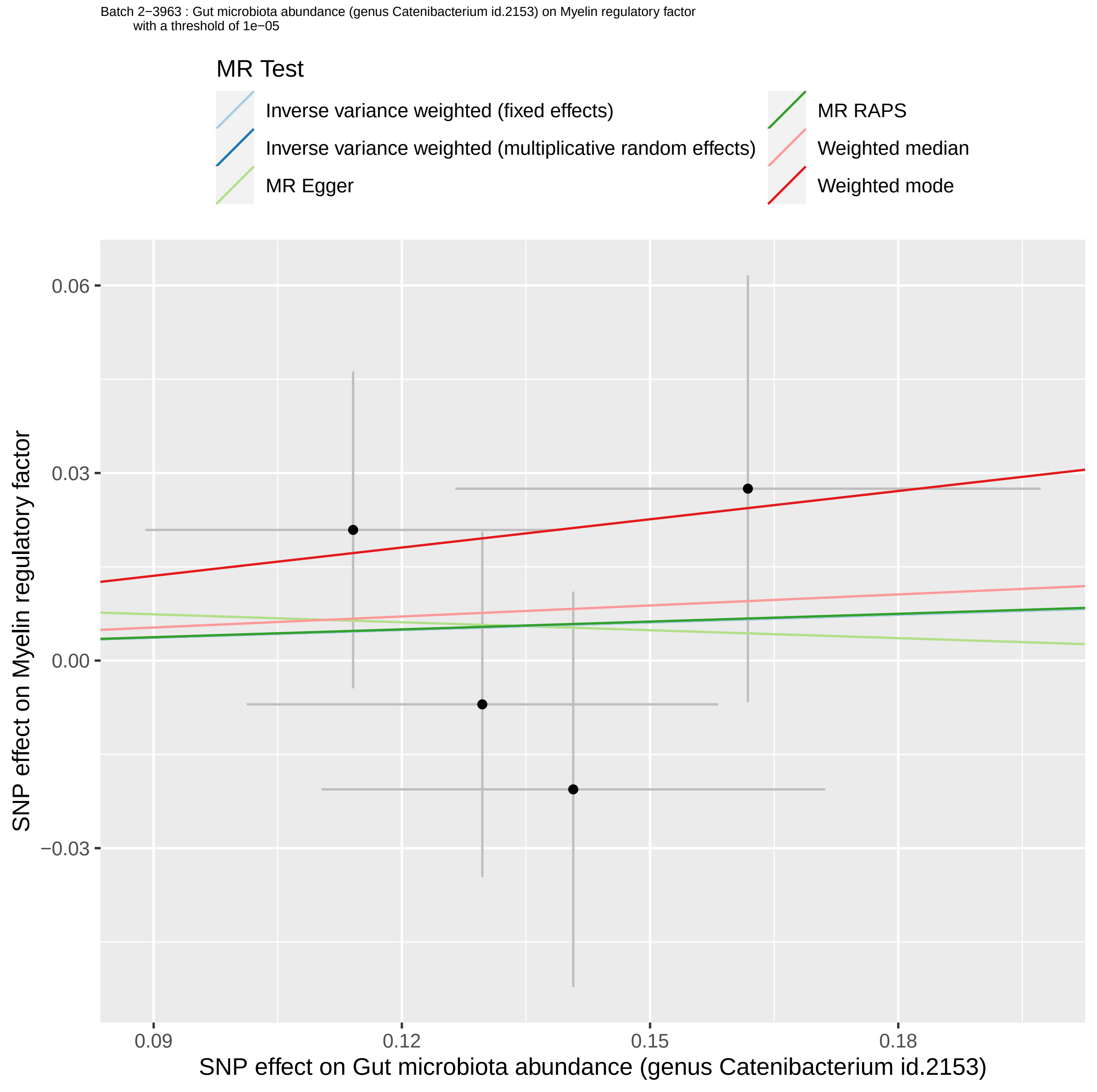
Batch 2−3963 : Gut microbiota abundance (genus Catenibacterium id.2153) on Myelin regulatory factor
with a threshold of 1e−05
MR Test
Inverse variance weighted (fixed effects)
Inverse variance weighted (multiplicative random effects)
MR Egger
MR RAPS
Weighted median
Weighted mode
0.06
0.03
0.00
−0.03
0.09
0.12
0.15
0.18
SNP effect on Myelin regulatory factor
SNP effect on Gut microbiota abundance (genus Catenibacterium id.2153)

Batch 2−3964 : Gut microbiota abundance (genus Parasutterella id.2892) on Myelin regulatory factor with a threshold of 1e−05

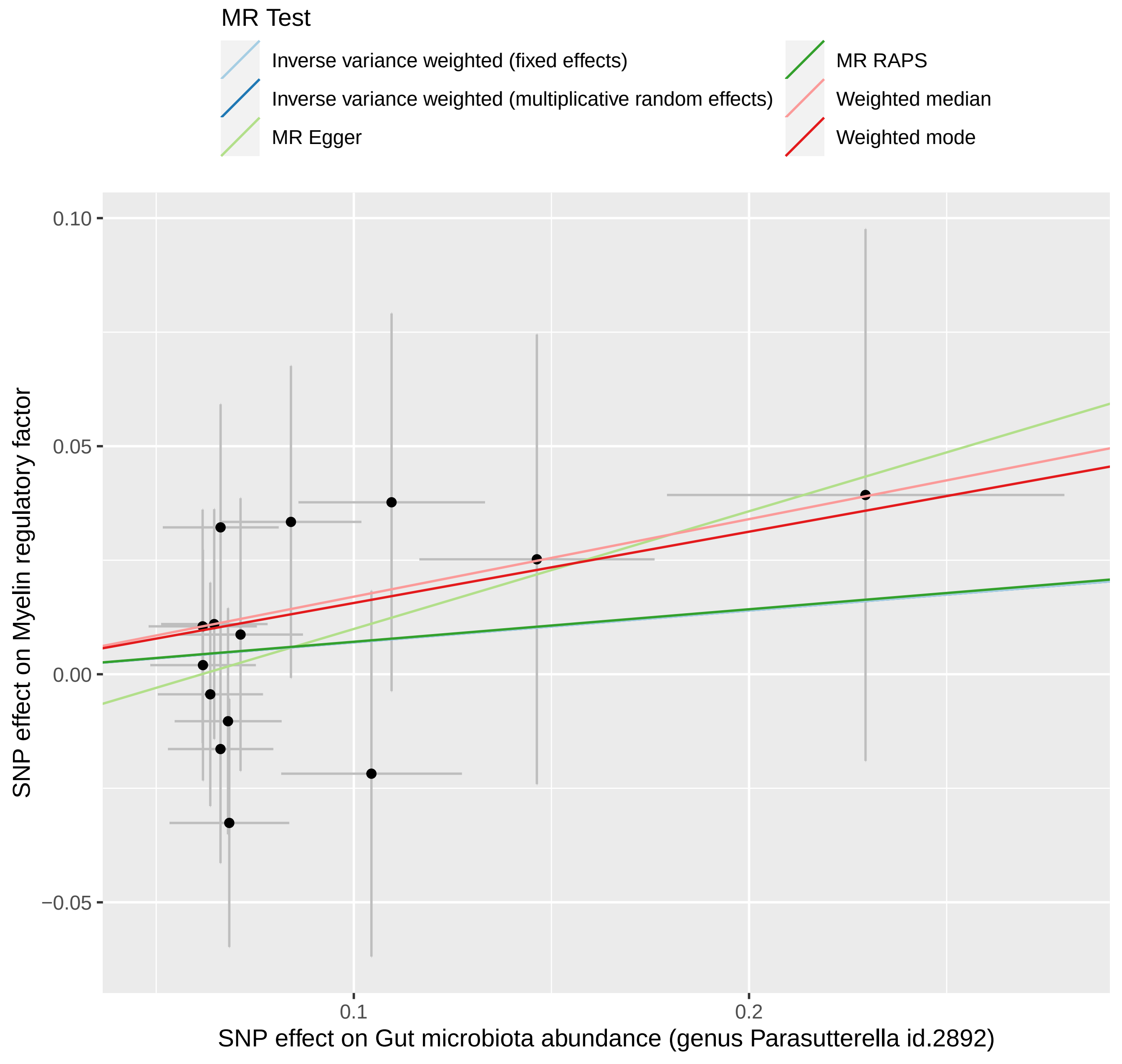

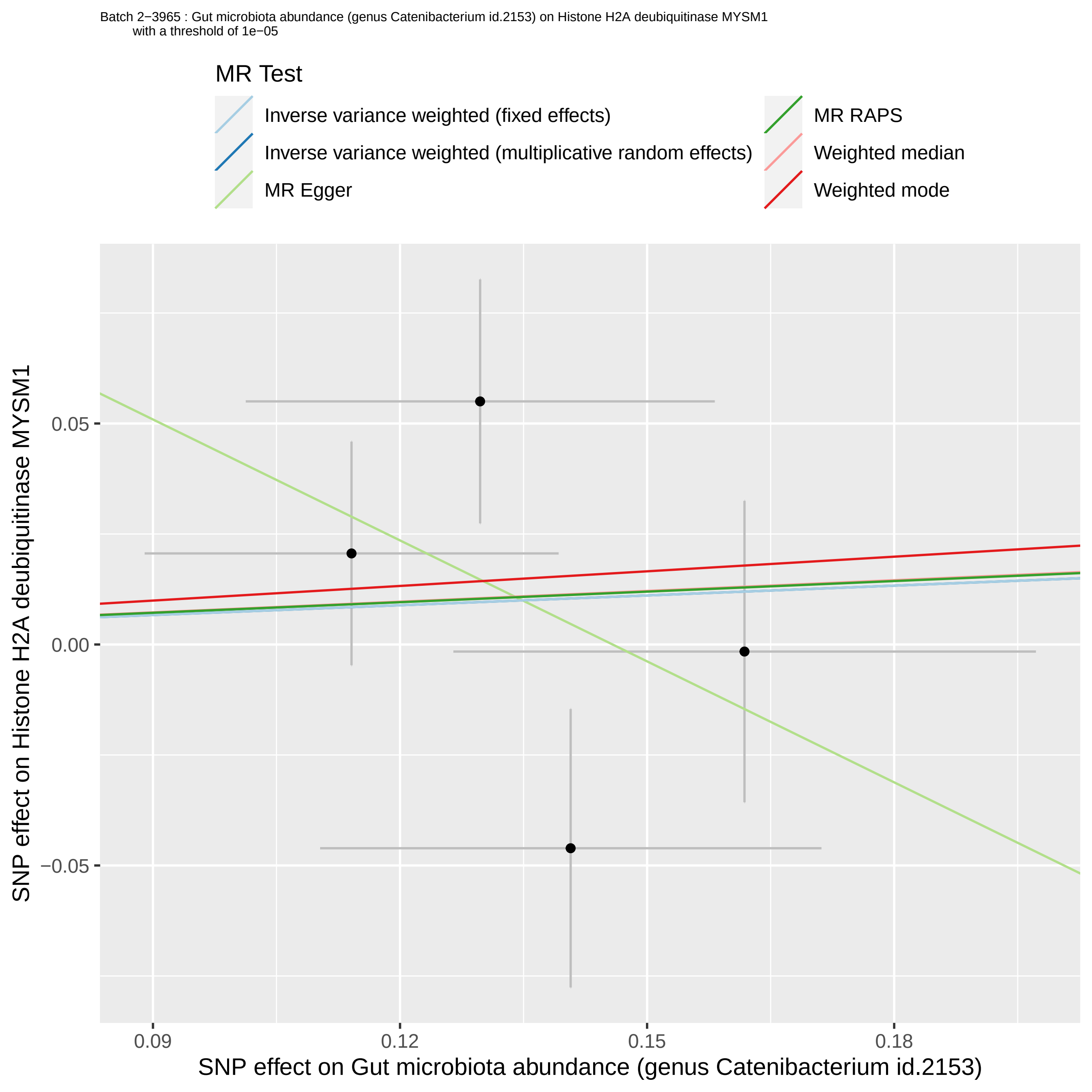
Batch 2−3965 : Gut microbiota abundance (genus Catenibacterium id.2153) on Histone H2A deubiquitinase MYSM1
with a threshold of 1e−05
MR Test
Inverse variance weighted (fixed effects)
Inverse variance weighted (multiplicative random effects)
MR Egger
MR RAPS
Weighted median
Weighted mode
SNP effect on Histone H2A deubiquitinase MYSM1
0.05
0.00
−0.05
0.09
0.12
0.15
0.18
SNP effect on Gut microbiota abundance (genus Catenibacterium id.2153)

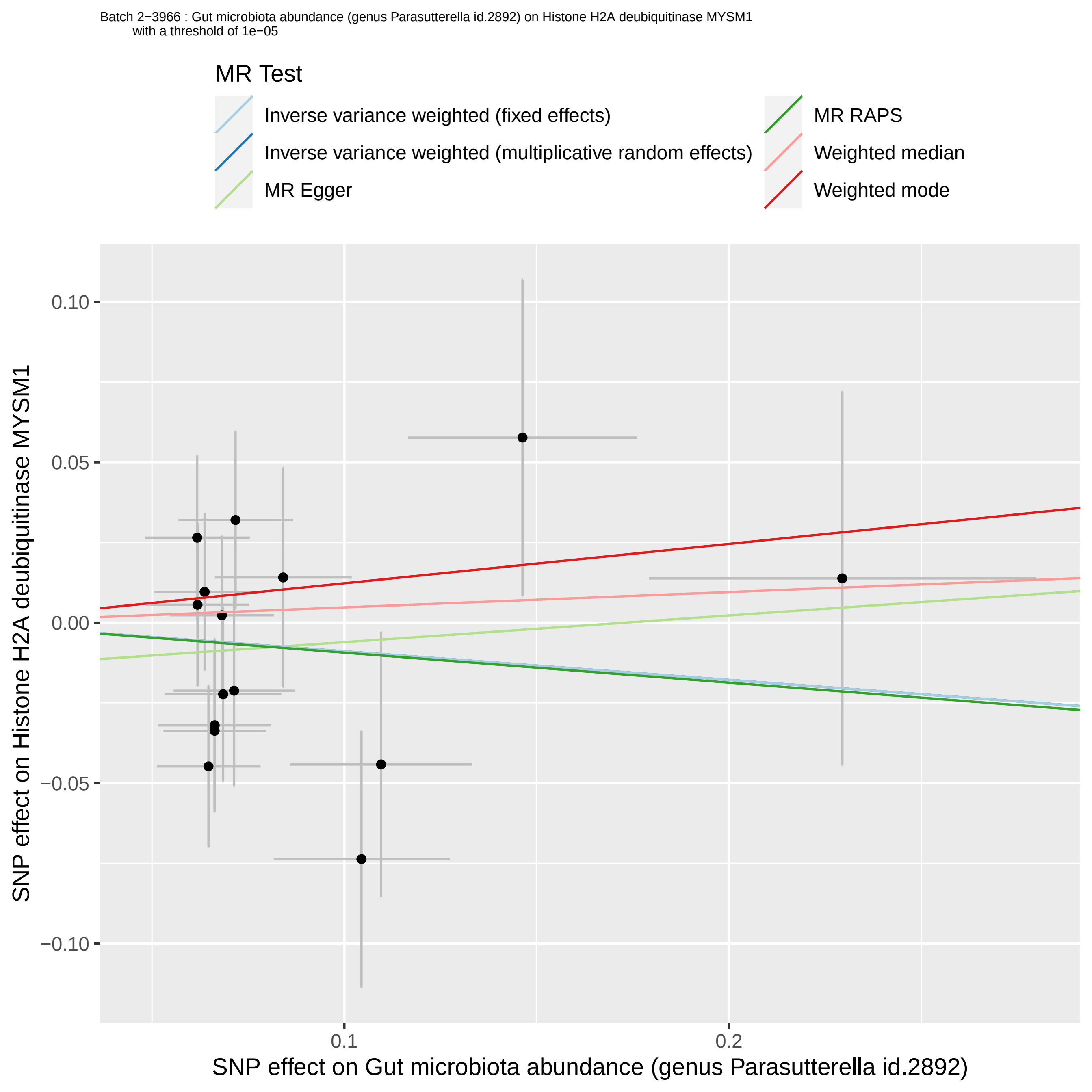
Batch 2−3966 : Gut microbiota abundance (genus Parasutterella id.2892) on Histone H2A deubiquitinase MYSM1
with a threshold of 1e−05
MR Test
Inverse variance weighted (fixed effects)
Inverse variance weighted (multiplicative random effects)
MR Egger
MR RAPS
Weighted median
Weighted mode
0.10
0.05
0.00
−0.05
−0.10
0.1
0.2
SNP effect on Histone H2A deubiquitinase MYSM1
SNP effect on Gut microbiota abundance (genus Parasutterella id.2892)

Batch 2−3967 : Gut microbiota abundance (genus Catenibacterium id.2153) on Myocardial zonula adherens protein
with a threshold of 1e−05

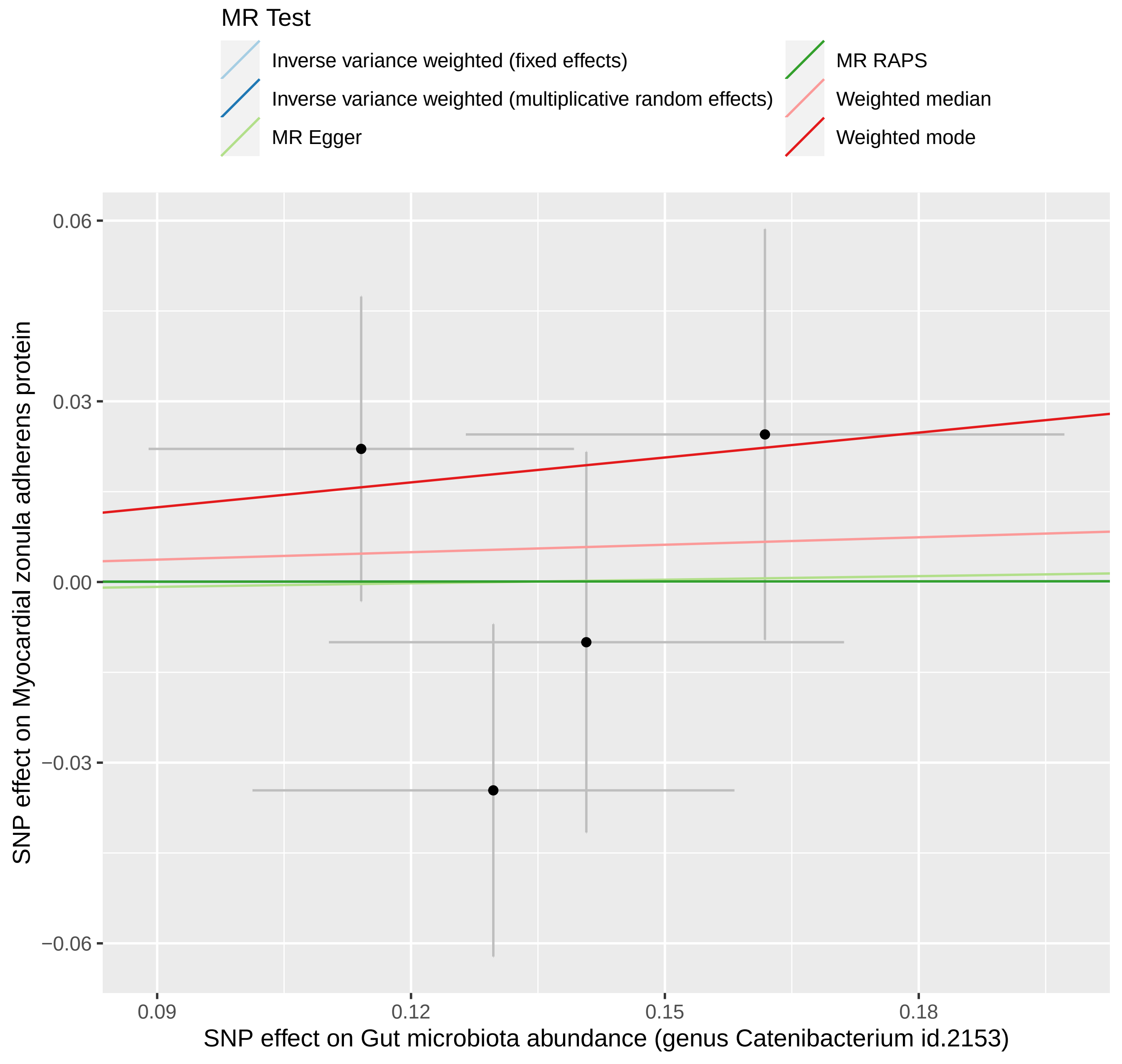

Batch 2−3968 : Gut microbiota abundance (genus Parasutterella id.2892) on Myocardial zonula adherens protein
with a threshold of 1e−05

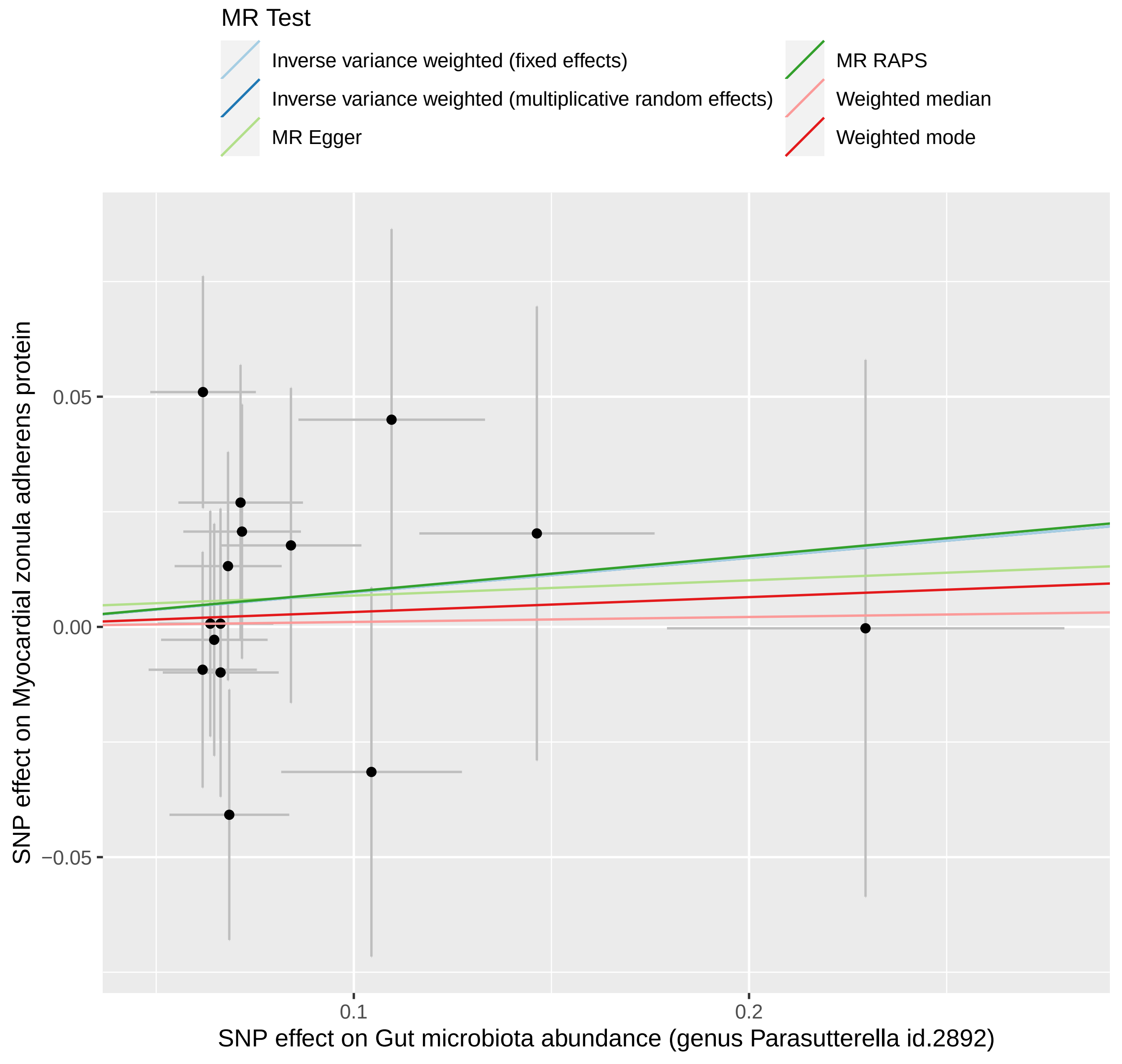

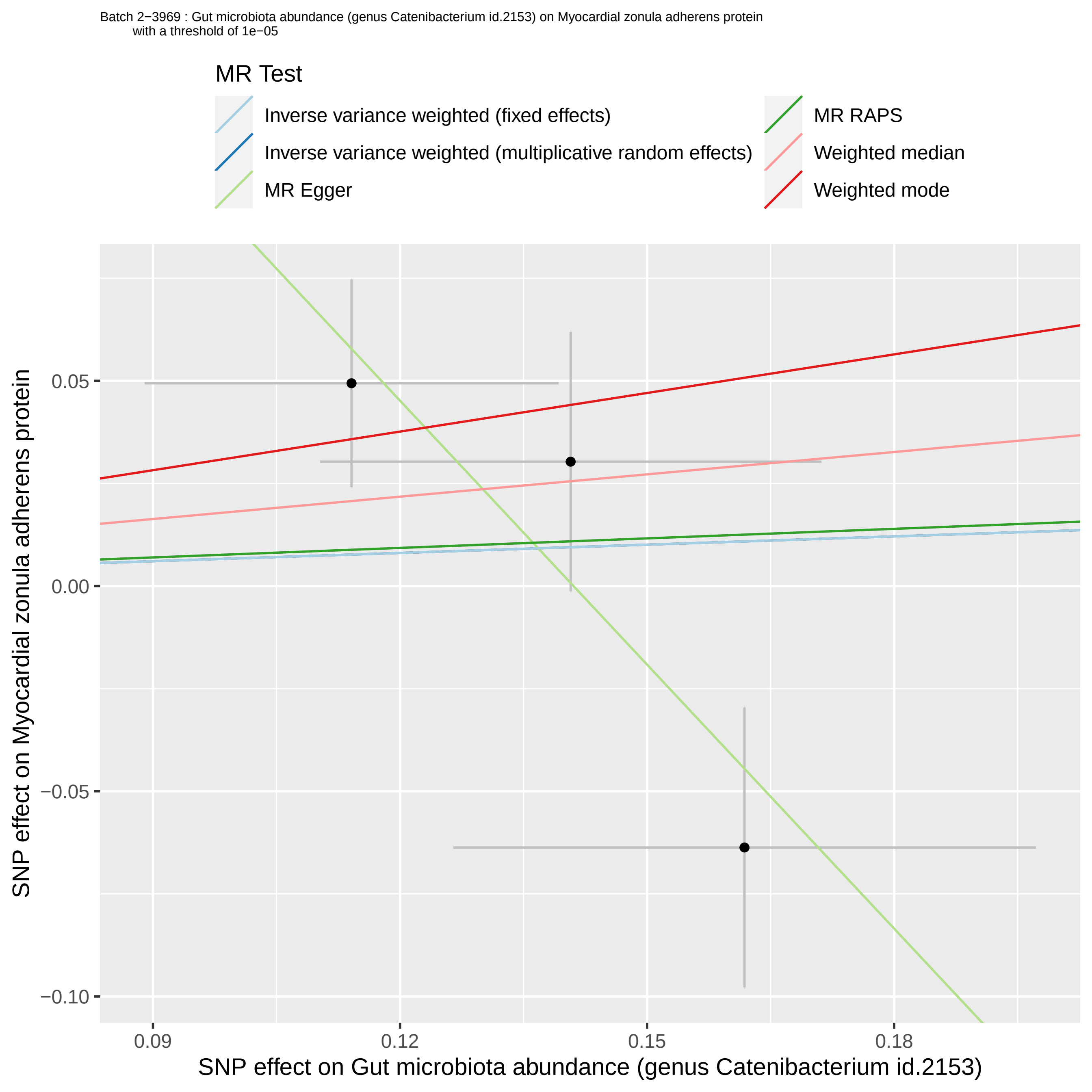
Batch 2−3969 : Gut microbiota abundance (genus Catenibacterium id.2153) on Myocardial zonula adherens protein
with a threshold of 1e−05
MR Test
Inverse variance weighted (fixed effects)
Inverse variance weighted (multiplicative random effects)
MR Egger
MR RAPS
Weighted median
Weighted mode
SNP effect on Myocardial zonula adherens protein
0.05
0.00
−0.05
−0.10
0.09
0.12
0.15
0.18
SNP effect on Gut microbiota abundance (genus Catenibacterium id.2153)

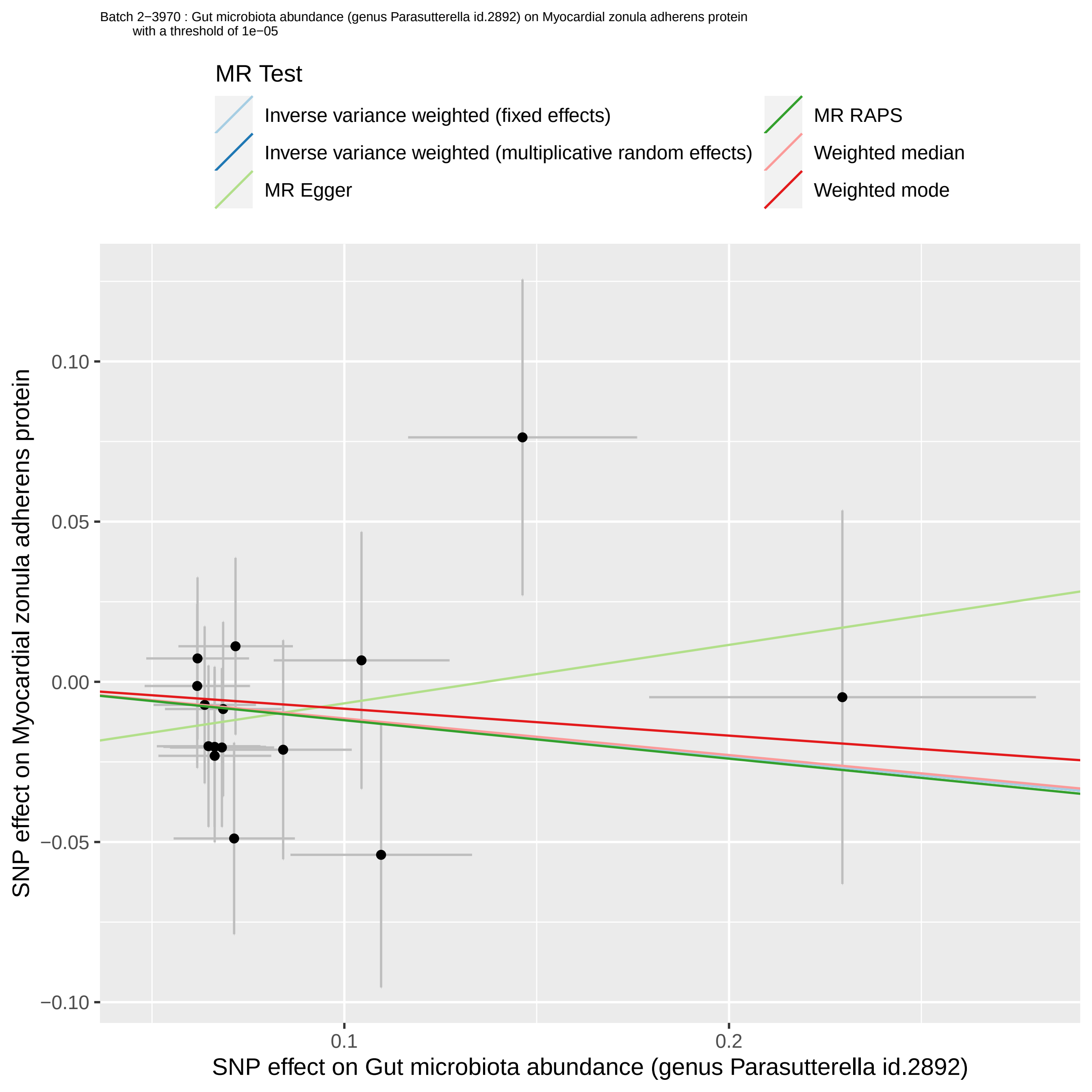
Batch 2−3970 : Gut microbiota abundance (genus Parasutterella id.2892) on Myocardial zonula adherens protein
with a threshold of 1e−05
MR Test
Inverse variance weighted (fixed effects)
Inverse variance weighted (multiplicative random effects)
MR Egger
MR RAPS
Weighted median
Weighted mode
0.10
0.05
0.00
−0.05
−0.10
0.1
0.2
SNP effect on Myocardial zonula adherens protein
SNP effect on Gut microbiota abundance (genus Parasutterella id.2892)

Batch 2−3971 : Gut microbiota abundance (genus Catenibacterium id.2153) on Myocardial zonula adherens protein
with a threshold of 1e−05

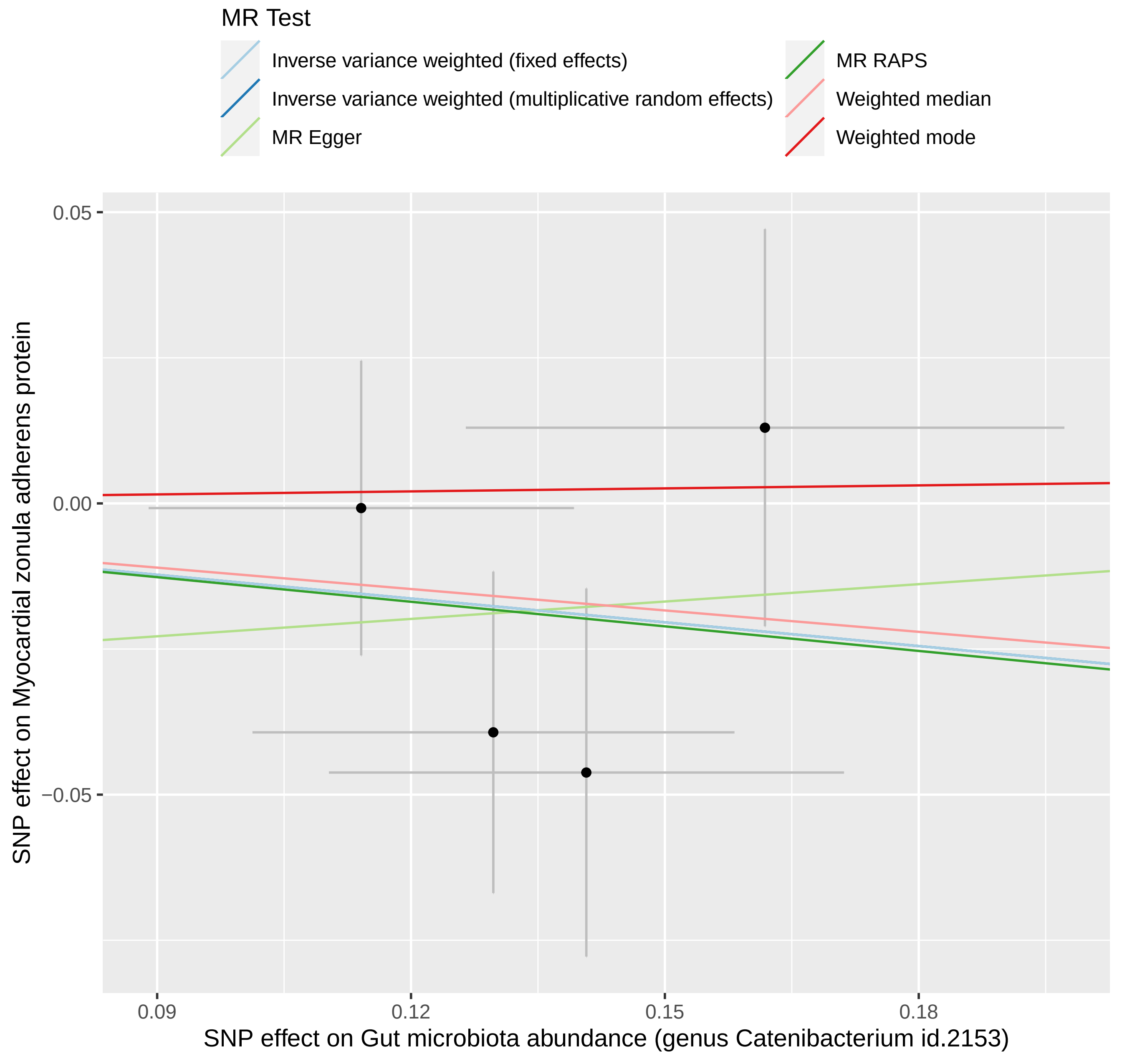

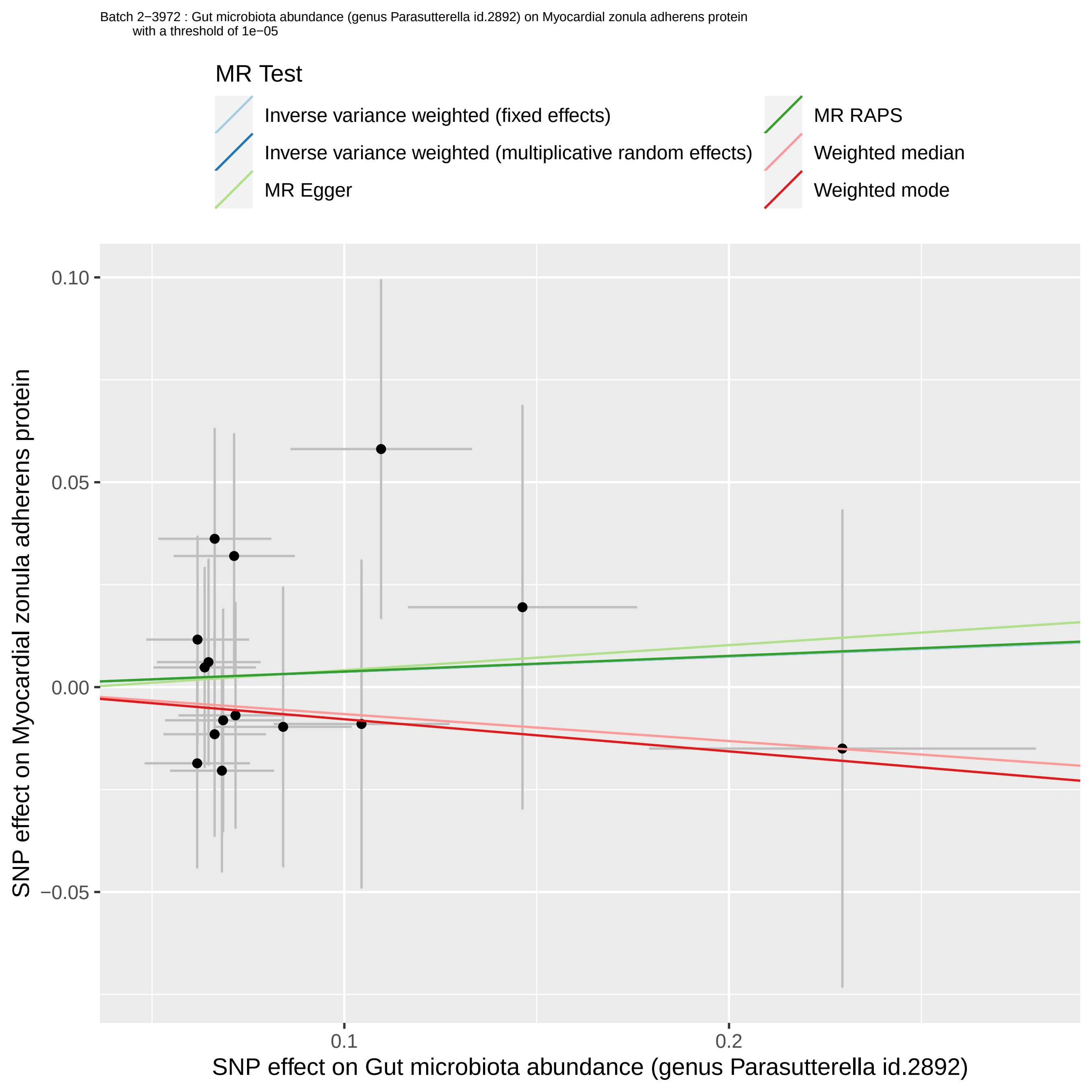
Batch 2−3972 : Gut microbiota abundance (genus Parasutterella id.2892) on Myocardial zonula adherens protein
with a threshold of 1e−05
MR Test
Inverse variance weighted (fixed effects)
Inverse variance weighted (multiplicative random effects)
MR Egger
MR RAPS
Weighted median
Weighted mode
0.10
0.05
0.00
−0.05
0.1
0.2
SNP effect on Myocardial zonula adherens protein
SNP effect on Gut microbiota abundance (genus Parasutterella id.2892)

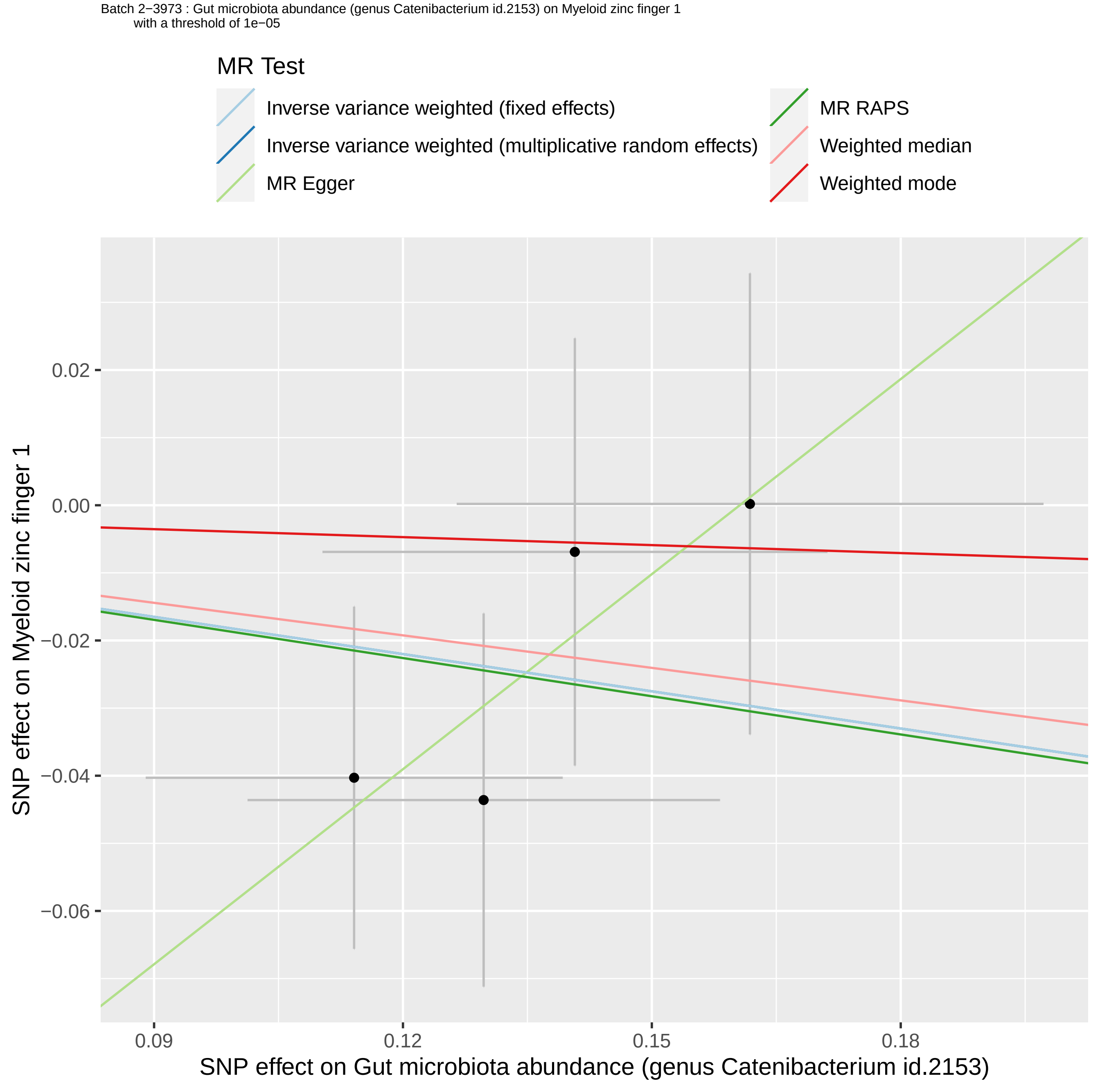

Batch 2−3973 : Gut microbiota abundance (genus Catenibacterium id.2153) on Myeloid zinc finger 1
with a threshold of 1e−05
MR Test
Inverse variance weighted (fixed effects)
Inverse variance weighted (multiplicative random effects)
MR Egger
MR RAPS
Weighted median
Weighted mode
0.02
0.00
−0.02
−0.04
−0.06
0.09
0.12
0.15
0.18
SNP effect on Myeloid zinc finger 1
SNP effect on Gut microbiota abundance (genus Catenibacterium id.2153)

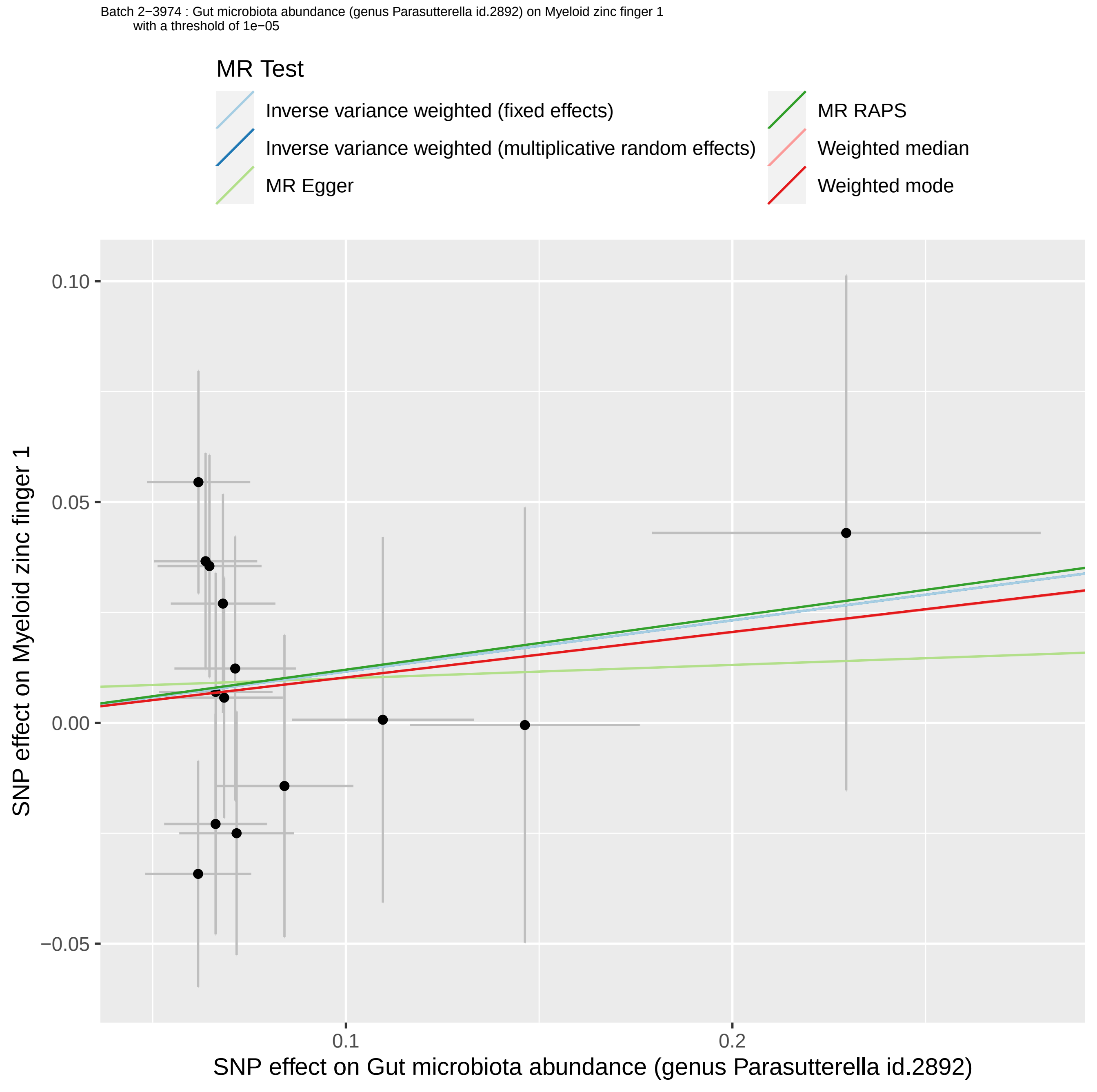

Batch 2−3974 : Gut microbiota abundance (genus Parasutterella id.2892) on Myeloid zinc finger 1
with a threshold of 1e−05
MR Test
Inverse variance weighted (fixed effects)
Inverse variance weighted (multiplicative random effects)
MR Egger
MR RAPS
Weighted median
Weighted mode
0.10
0.05
0.00
−0.05
0.1
0.2
SNP effect on Myeloid zinc finger 1
SNP effect on Gut microbiota abundance (genus Parasutterella id.2892)

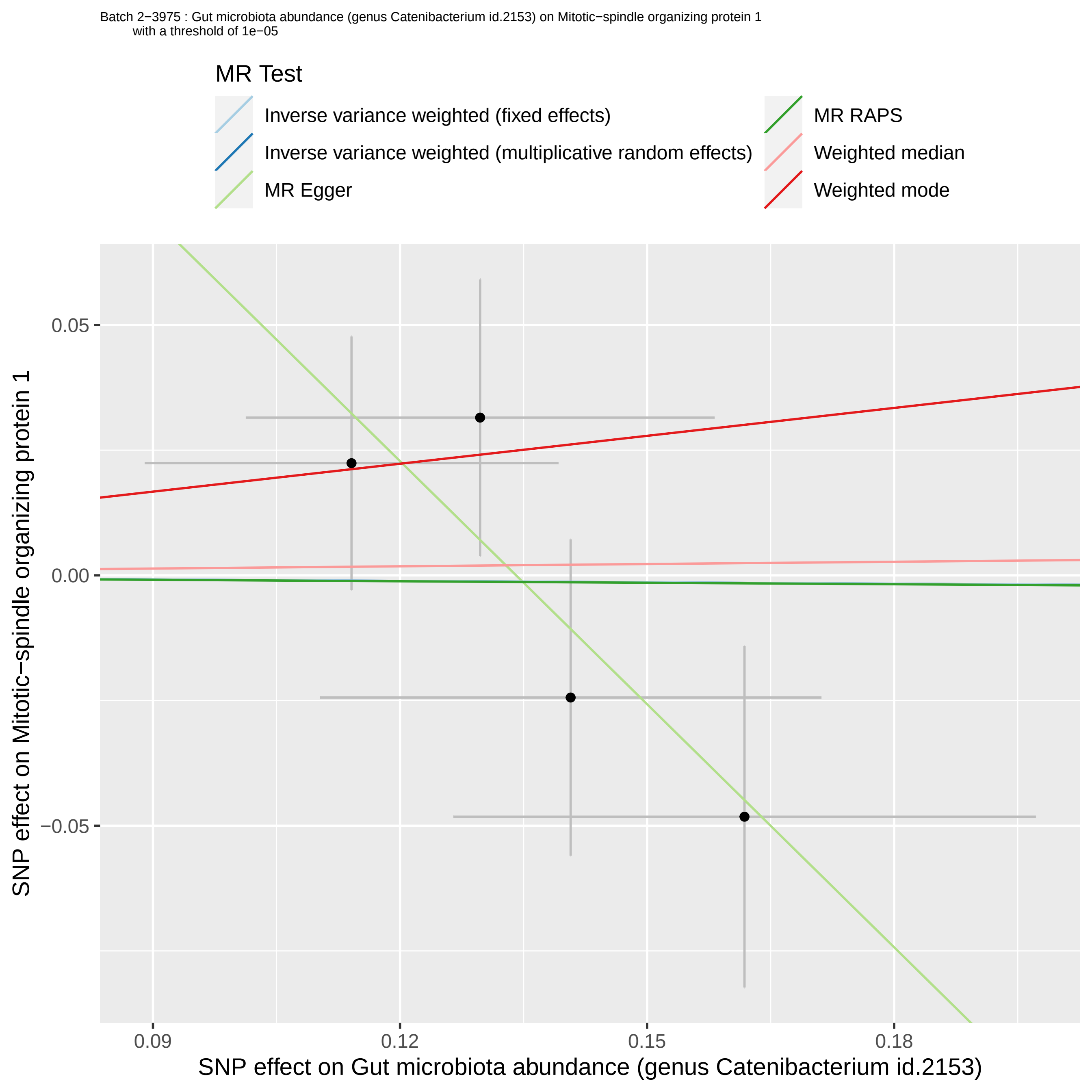
Batch 2−3975 : Gut microbiota abundance (genus Catenibacterium id.2153) on Mitotic−spindle organizing protein 1
with a threshold of 1e−05
MR Test
Inverse variance weighted (fixed effects)
Inverse variance weighted (multiplicative random effects)
MR Egger
MR RAPS
Weighted median
Weighted mode
0.05
0.00
−0.05
0.09
0.12
0.15
0.18
SNP effect on Mitotic−spindle organizing protein 1
SNP effect on Gut microbiota abundance (genus Catenibacterium id.2153)

Batch 2−3976 : Gut microbiota abundance (genus Parasutterella id.2892) on Mitotic−spindle organizing protein 1
with a threshold of 1e−05

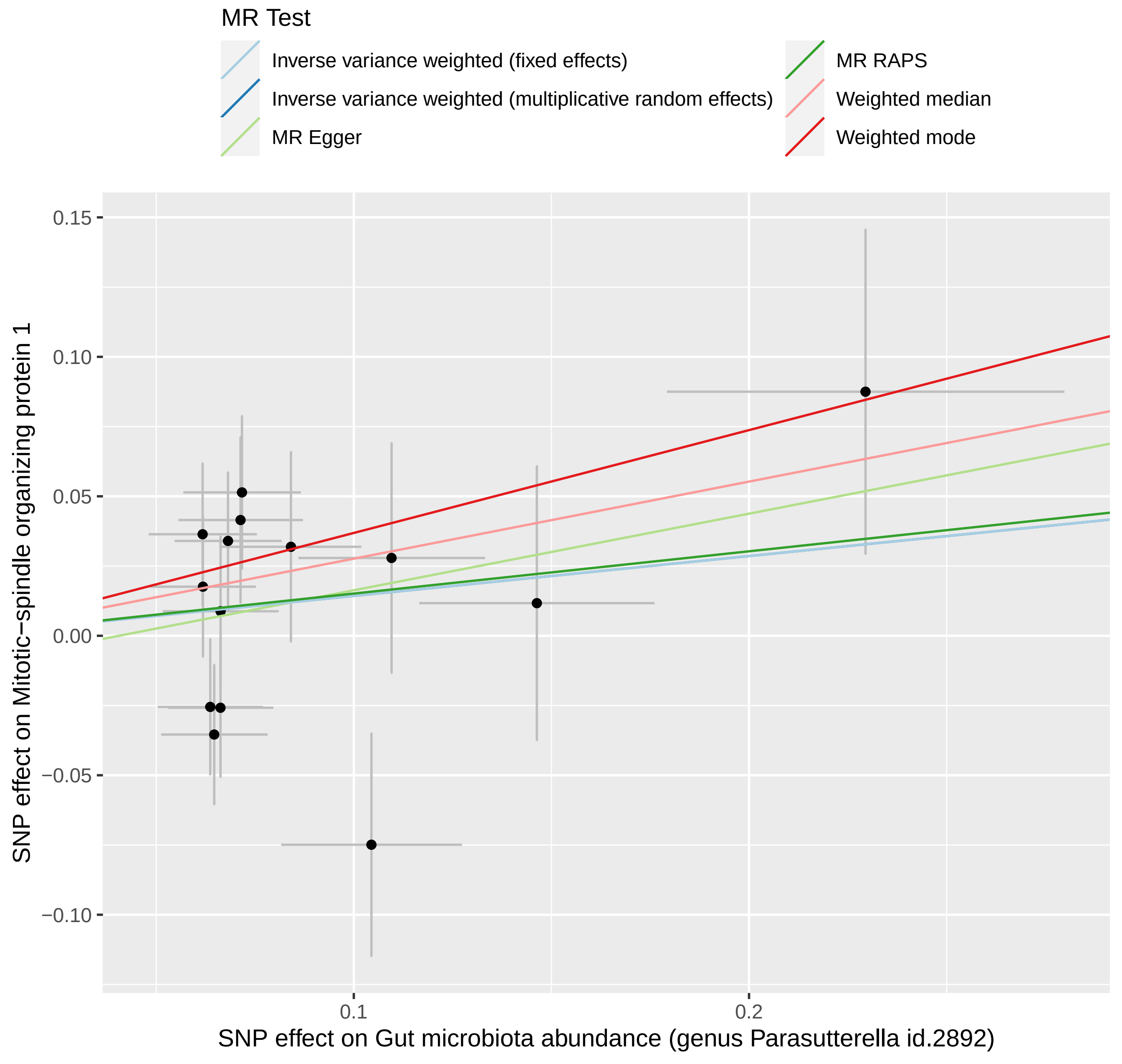

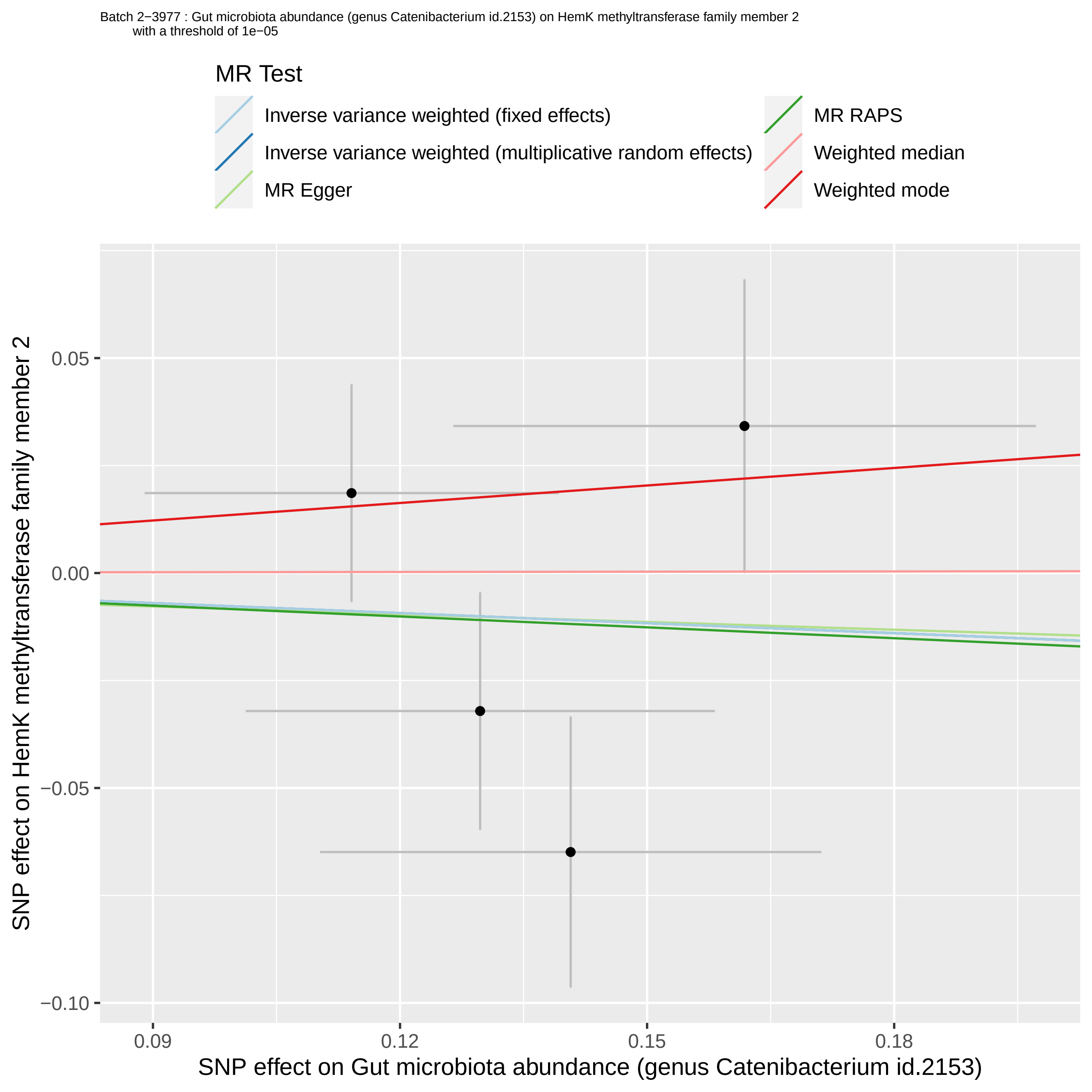
Batch 2−3977 : Gut microbiota abundance (genus Catenibacterium id.2153) on HemK methyltransferase family member 2
with a threshold of 1e−05
MR Test
Inverse variance weighted (fixed effects)
Inverse variance weighted (multiplicative random effects)
MR Egger
MR RAPS
Weighted median
Weighted mode
0.05
0.00
−0.05
−0.10
0.09
0.12
0.15
0.18
SNP effect on HemK methyltransferase family member 2
SNP effect on Gut microbiota abundance (genus Catenibacterium id.2153)

Batch 2−3978 : Gut microbiota abundance (genus Parasutterella id.2892) on HemK methyltransferase family member 2
with a threshold of 1e−05

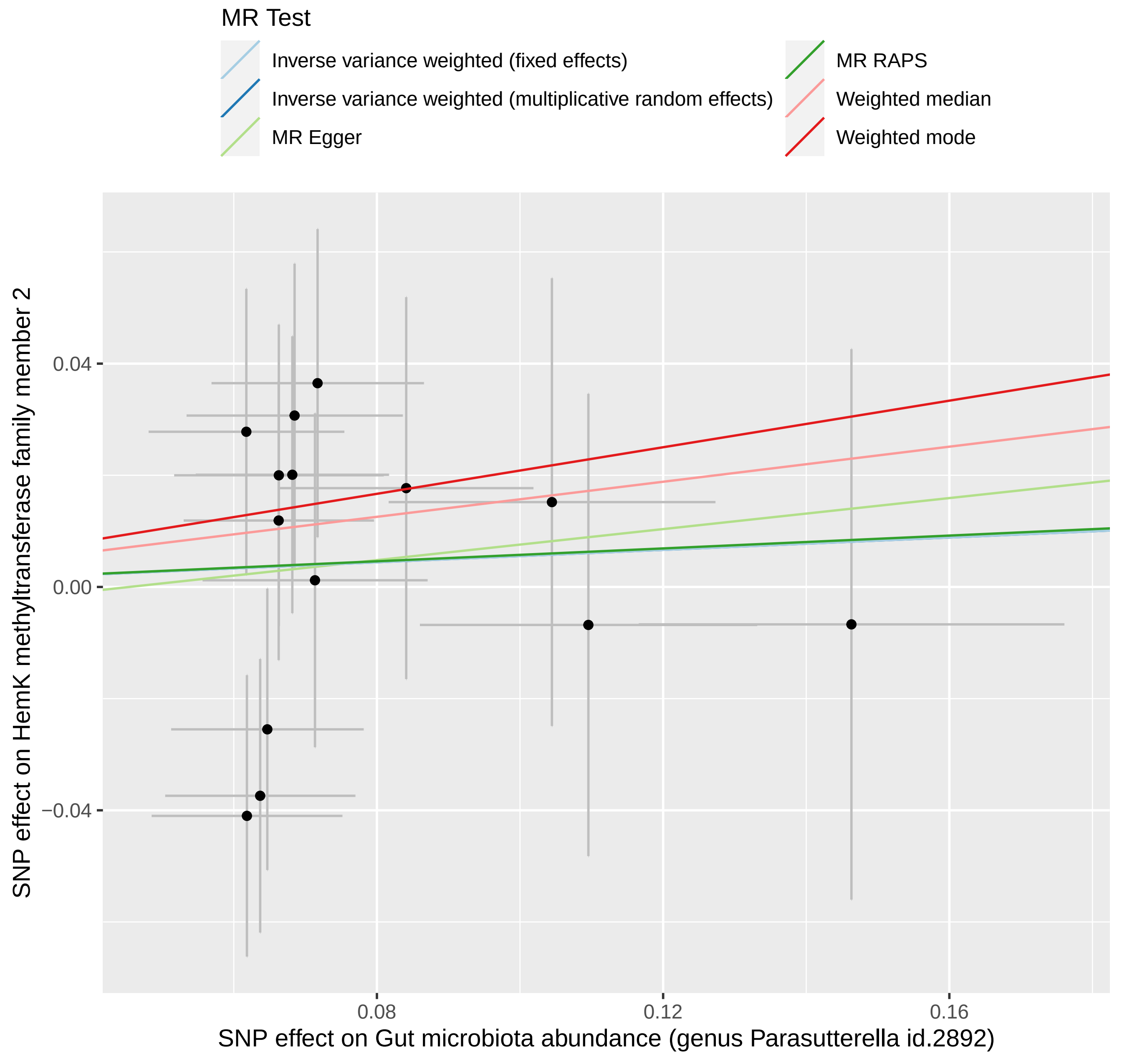

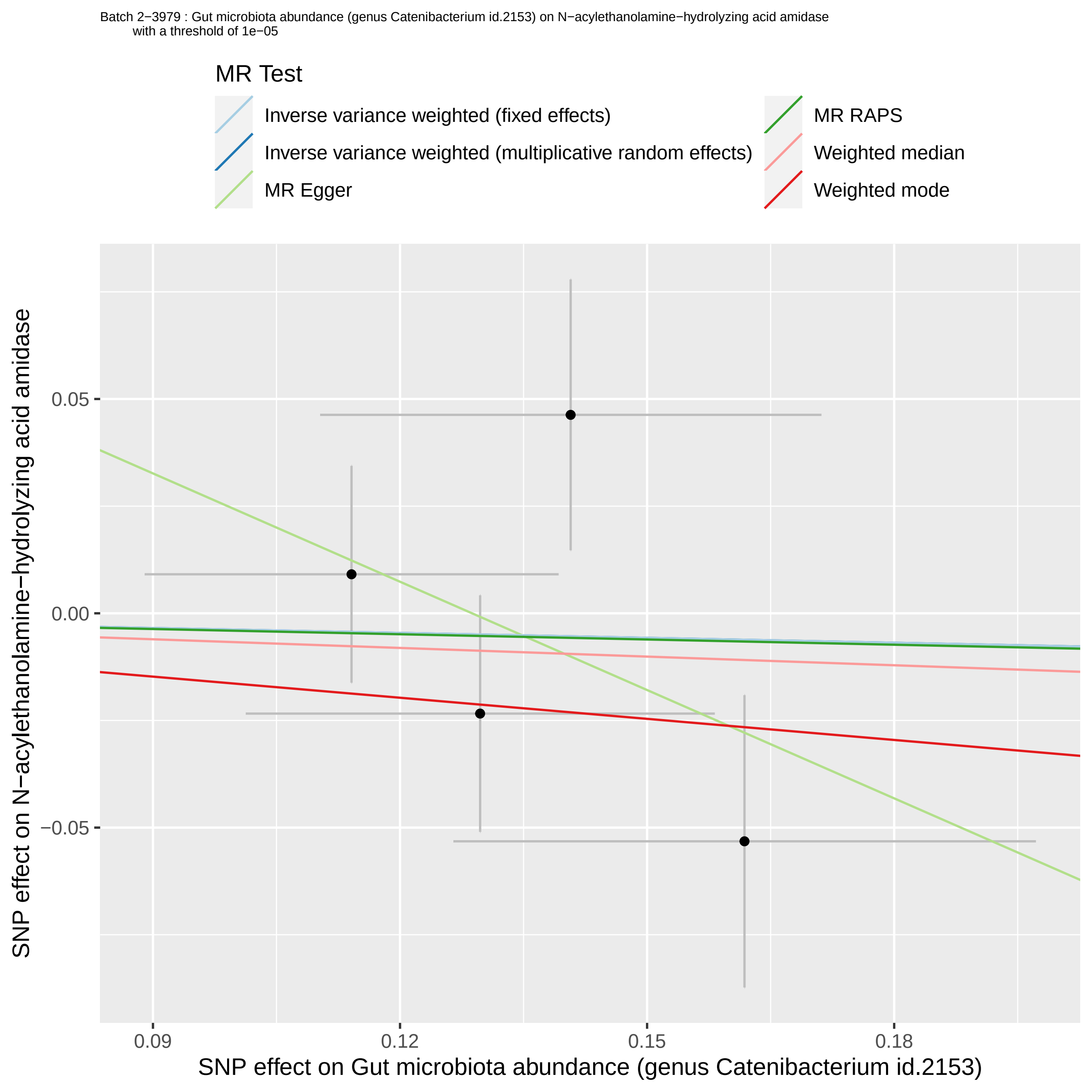
Batch 2−3979 : Gut microbiota abundance (genus Catenibacterium id.2153) on N−acylethanolamine−hydrolyzing acid amidase
with a threshold of 1e−05
MR Test
Inverse variance weighted (fixed effects)
Inverse variance weighted (multiplicative random effects)
MR Egger
MR RAPS
Weighted median
Weighted mode
SNP effect on N−acylethanolamine−hydrolyzing acid amidase
0.05
0.00
−0.05
0.09
0.12
0.15
0.18
SNP effect on Gut microbiota abundance (genus Catenibacterium id.2153)

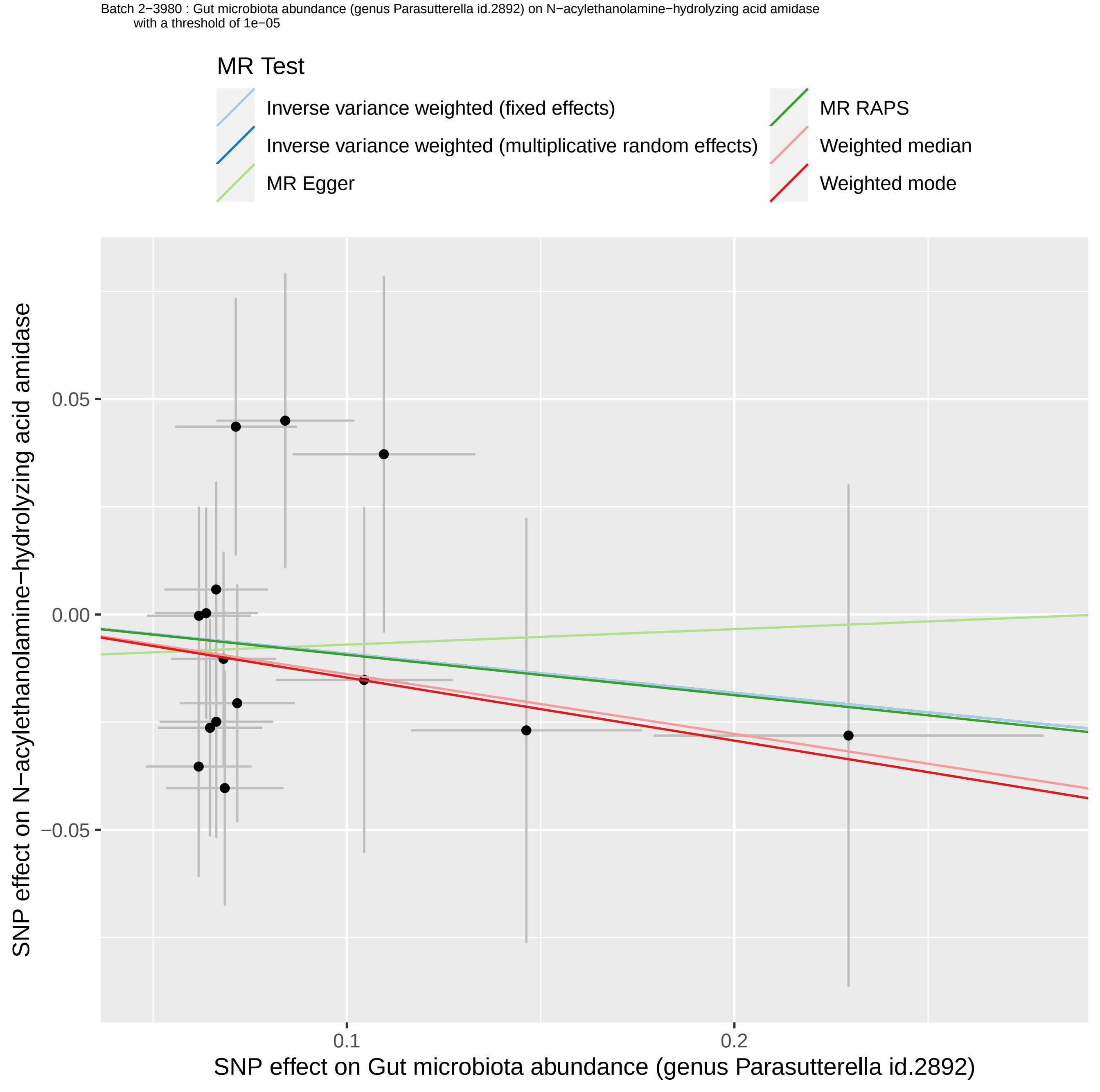

Batch 2−3980 : Gut microbiota abundance (genus Parasutterella id.2892) on N−acylethanolamine−hydrolyzing acid amidase
with a threshold of 1e−05
MR Test
Inverse variance weighted (fixed effects)
Inverse variance weighted (multiplicative random effects)
MR Egger
MR RAPS
Weighted median
Weighted mode
0.05
0.00
−0.05
0.1
0.2
SNP effect on N−acylethanolamine−hydrolyzing acid amidase
SNP effect on Gut microbiota abundance (genus Parasutterella id.2892)

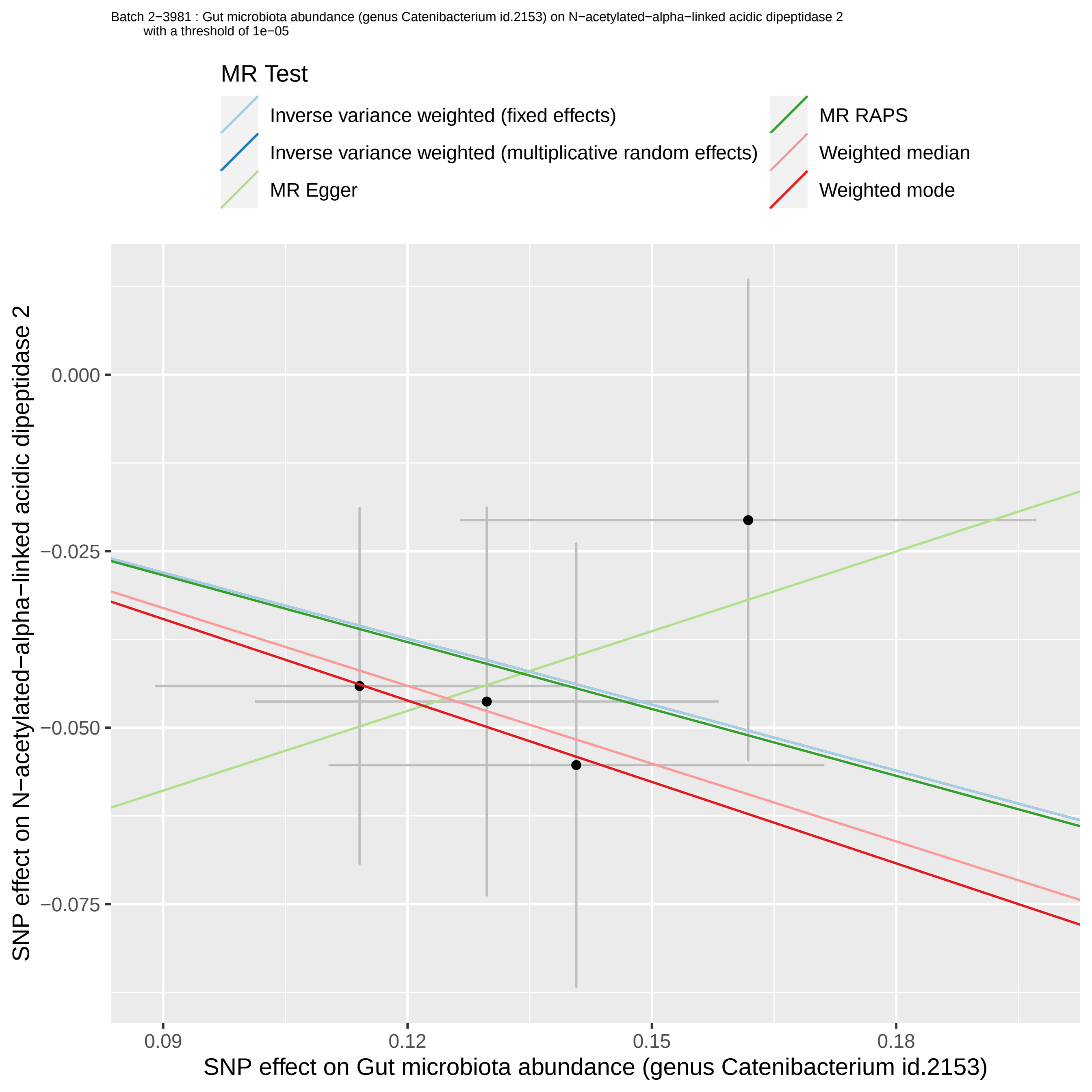

Batch 2−3981 : Gut microbiota abundance (genus Catenibacterium id.2153) on N−acetylated−alpha−linked acidic dipeptidase 2
with a threshold of 1e−05
MR Test
Inverse variance weighted (fixed effects)
Inverse variance weighted (multiplicative random effects)
MR Egger
MR RAPS
Weighted median
Weighted mode
0.000
−0.025
−0.050
−0.075
0.09
0.12
0.15
0.18
SNP effect on N−acetylated−alpha−linked acidic dipeptidase 2
SNP effect on Gut microbiota abundance (genus Catenibacterium id.2153)

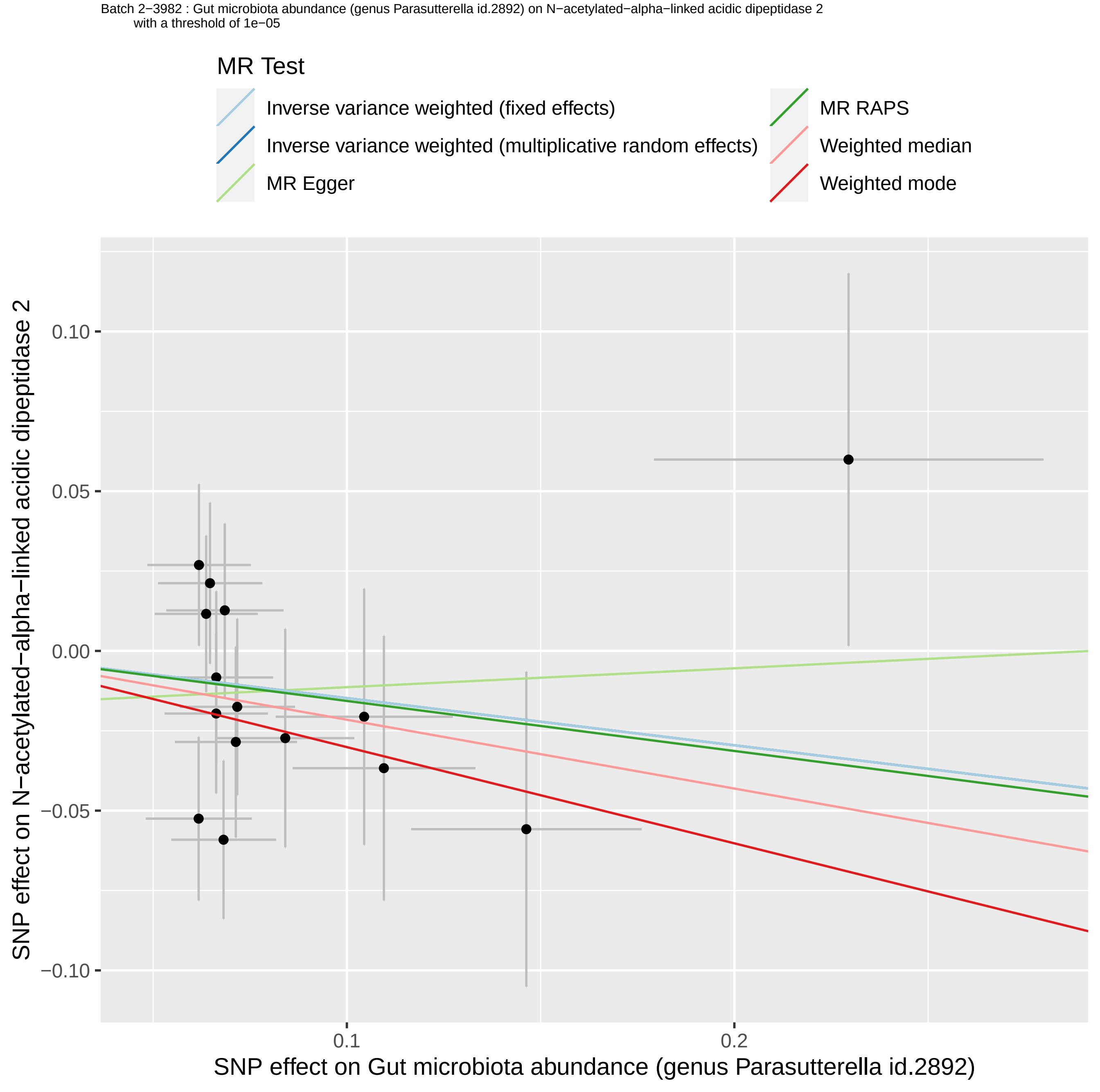
Batch 2−3982 : Gut microbiota abundance (genus Parasutterella id.2892) on N−acetylated−alpha−linked acidic dipeptidase 2
with a threshold of 1e−05
MR Test
Inverse variance weighted (fixed effects)
Inverse variance weighted (multiplicative random effects)
MR Egger
MR RAPS
Weighted median
Weighted mode
SNP effect on N−acetylated−alpha−linked acidic dipeptidase 2
0.10
0.05
0.00
−0.05
−0.10
0.1
0.2
SNP effect on Gut microbiota abundance (genus Parasutterella id.2892)

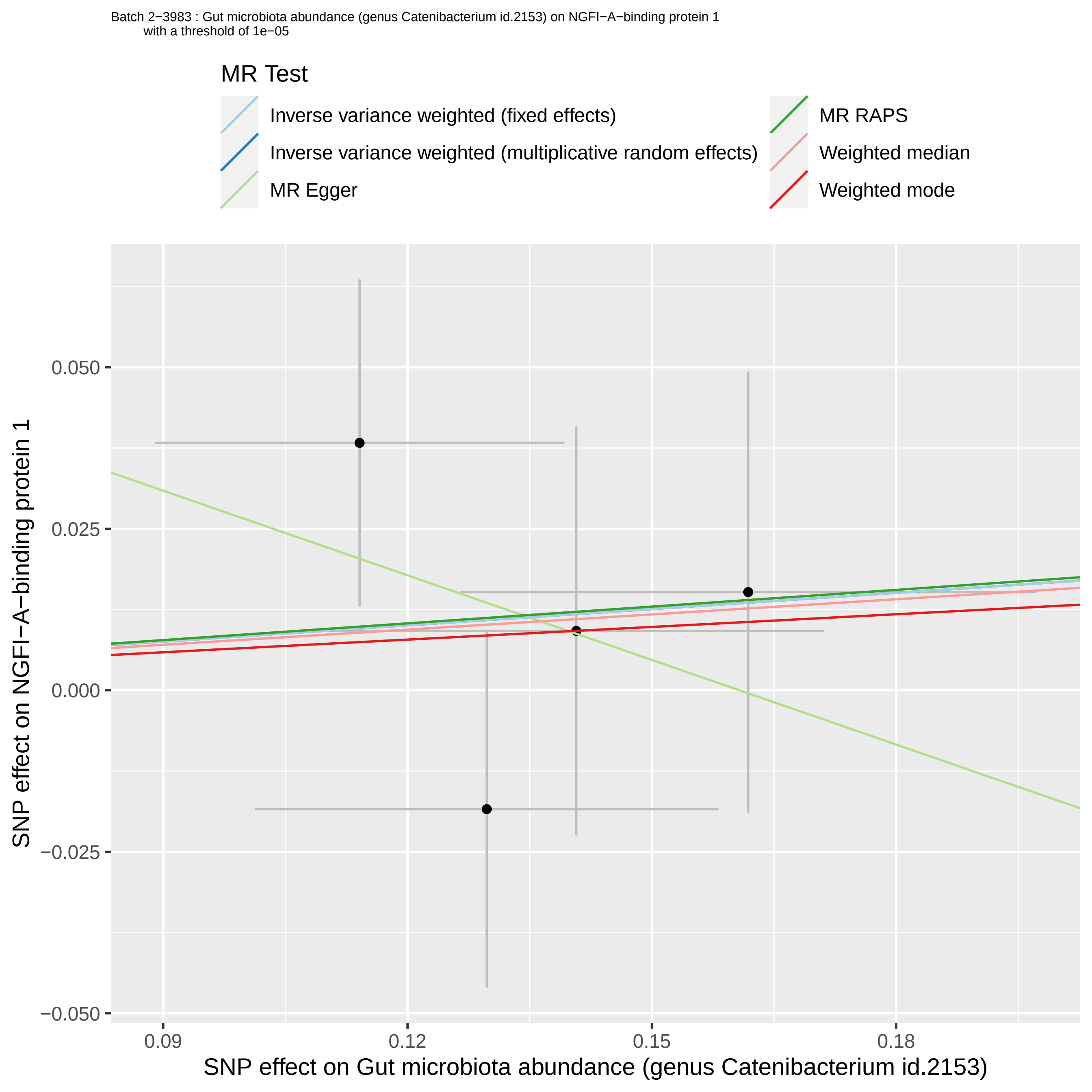
Batch 2−3983 : Gut microbiota abundance (genus Catenibacterium id.2153) on NGFI−A−binding protein 1
with a threshold of 1e−05
MR Test
Inverse variance weighted (fixed effects)
Inverse variance weighted (multiplicative random effects)
MR Egger
MR RAPS
Weighted median
Weighted mode
SNP effect on NGFI−A−binding protein 1
0.050
0.025
0.000
−0.025
−0.050
0.09
0.12
0.15
0.18
SNP effect on Gut microbiota abundance (genus Catenibacterium id.2153)

Batch 2−3984 : Gut microbiota abundance (genus Parasutterella id.2892) on NGFI−A−binding protein 1
with a threshold of 1e−05

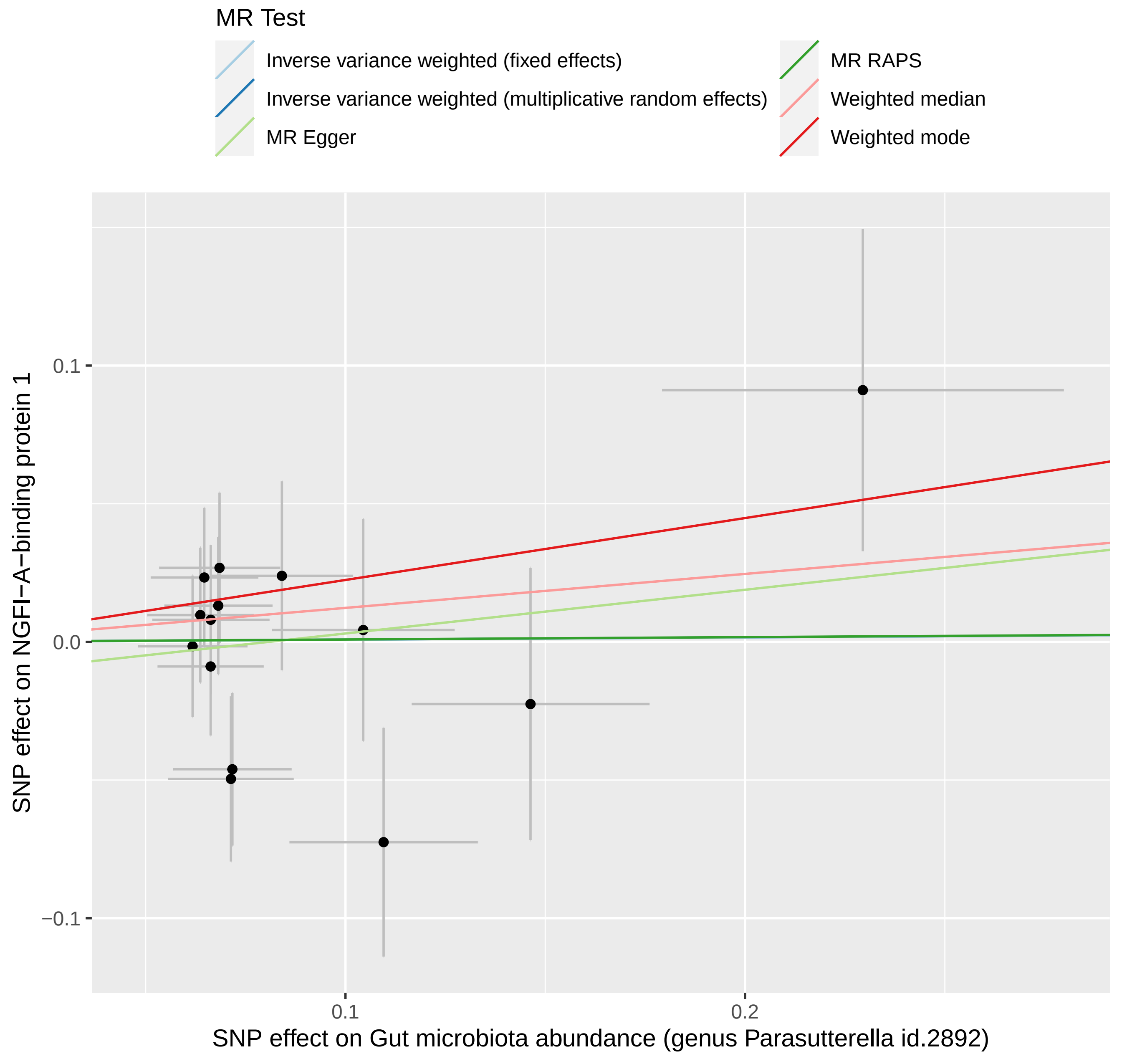

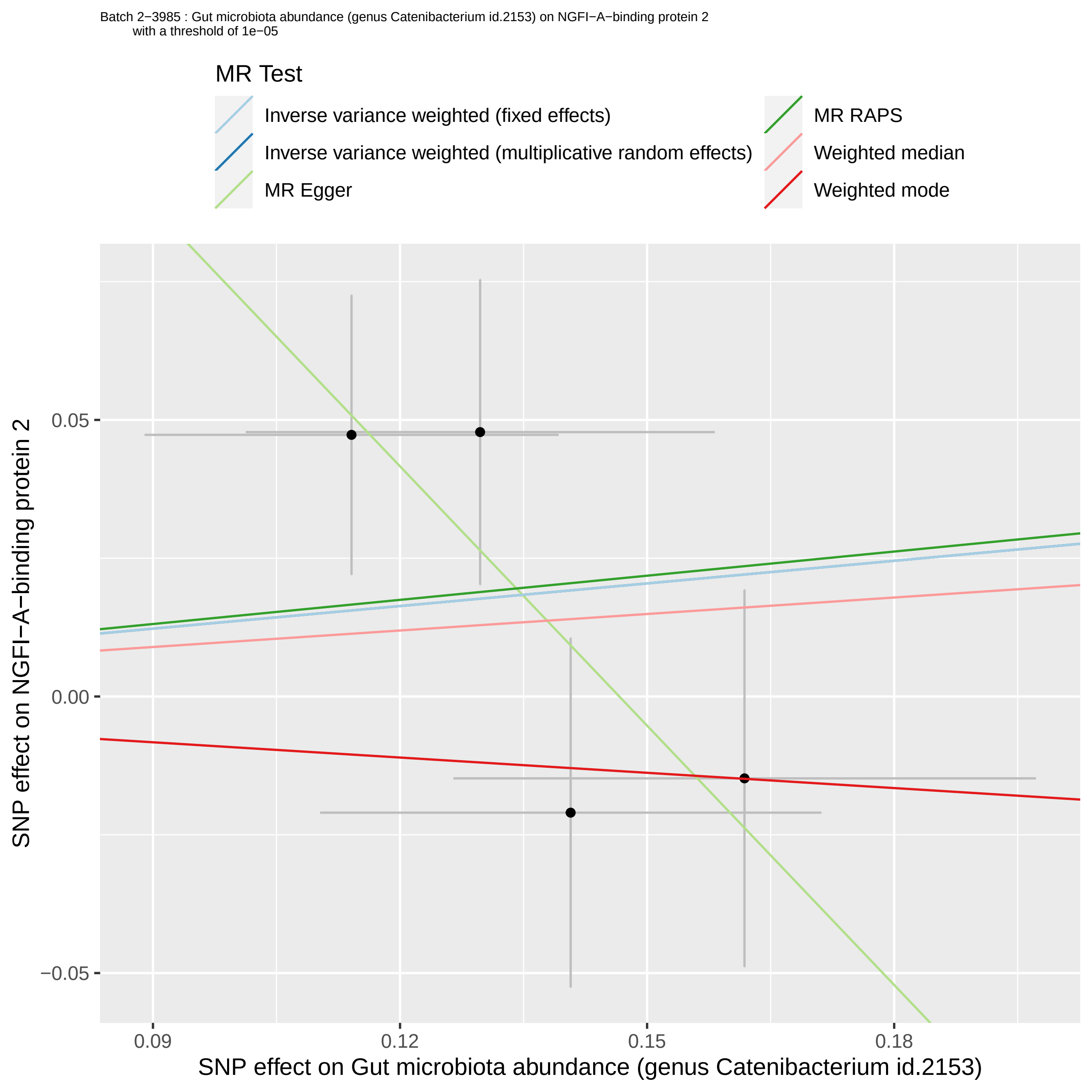
Batch 2−3985 : Gut microbiota abundance (genus Catenibacterium id.2153) on NGFI−A−binding protein 2
with a threshold of 1e−05
MR Test
Inverse variance weighted (fixed effects)
Inverse variance weighted (multiplicative random effects)
MR Egger
MR RAPS
Weighted median
Weighted mode
0.05
0.00
−0.05
0.09
0.12
0.15
0.18
SNP effect on NGFI−A−binding protein 2
SNP effect on Gut microbiota abundance (genus Catenibacterium id.2153)

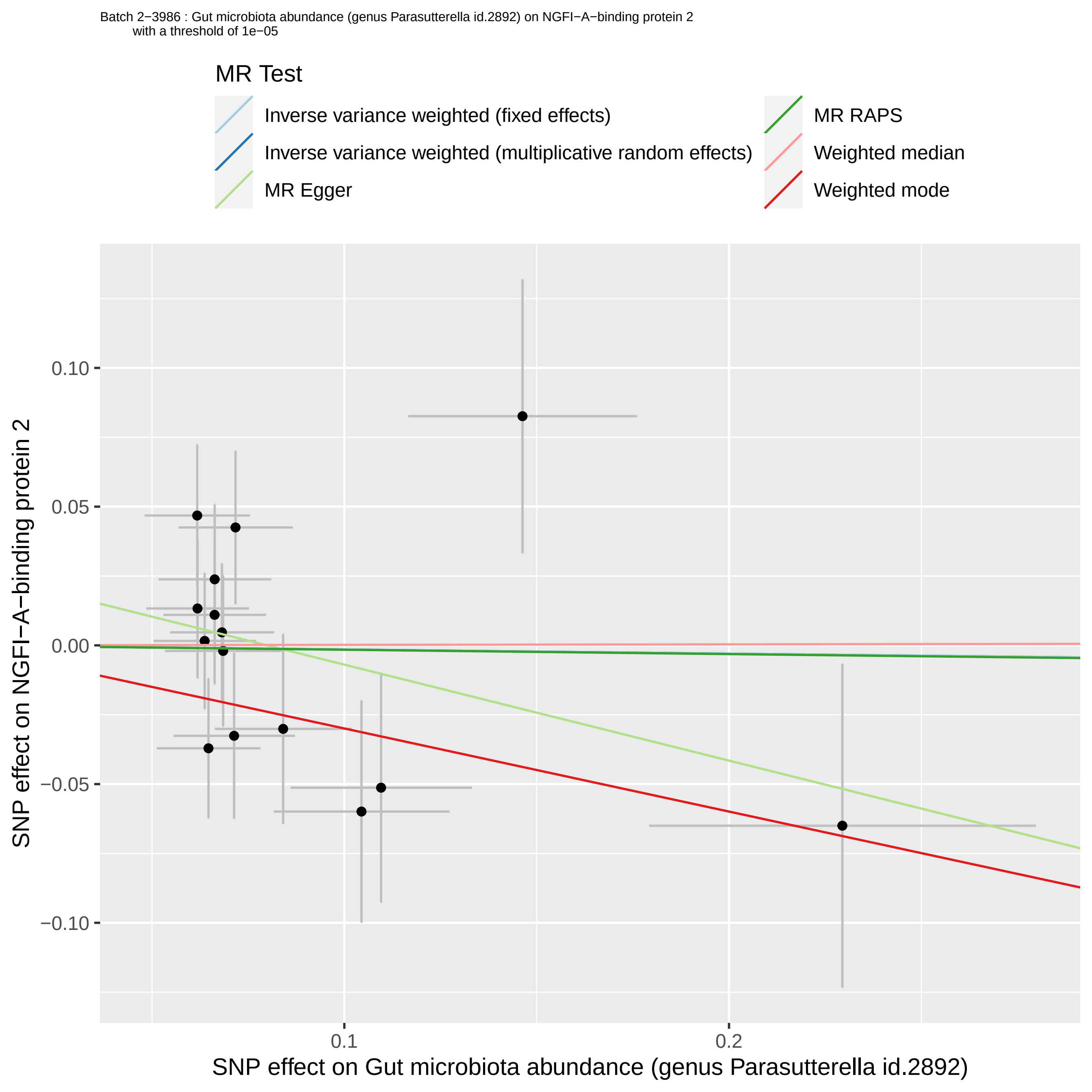
Batch 2−3986 : Gut microbiota abundance (genus Parasutterella id.2892) on NGFI−A−binding protein 2
with a threshold of 1e−05
MR Test
Inverse variance weighted (fixed effects)
Inverse variance weighted (multiplicative random effects)
MR Egger
MR RAPS
Weighted median
Weighted mode
SNP effect on NGFI−A−binding protein 2
0.10
0.05
0.00
−0.05
−0.10
0.1
0.2
SNP effect on Gut microbiota abundance (genus Parasutterella id.2892)

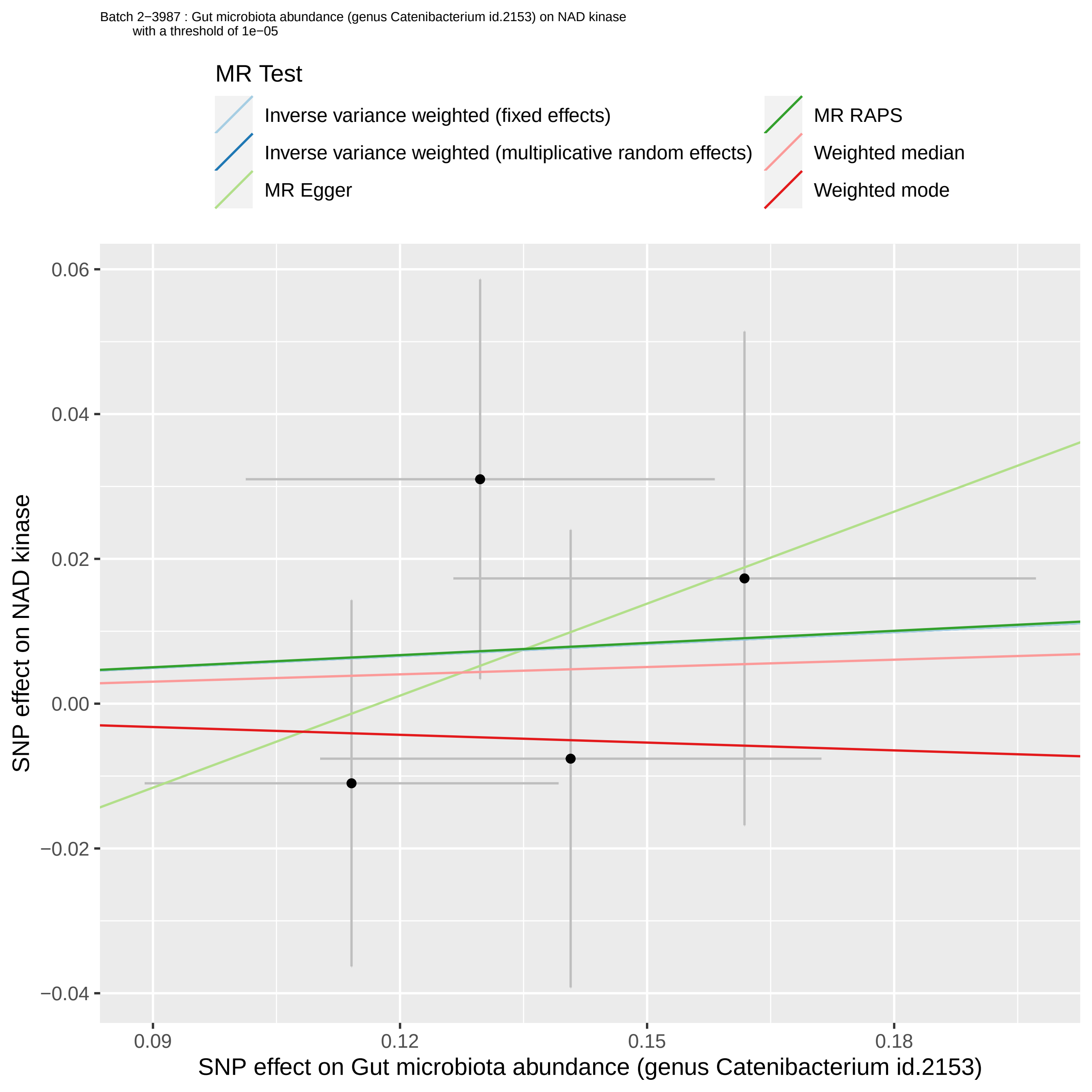
Batch 2−3987 : Gut microbiota abundance (genus Catenibacterium id.2153) on NAD kinase
with a threshold of 1e−05
MR Test
Inverse variance weighted (fixed effects)
Inverse variance weighted (multiplicative random effects)
MR Egger
MR RAPS
Weighted median
Weighted mode
0.06
0.04
0.02
0.00
−0.02
−0.04
SNP effect on NAD kinase
0.09
0.12
0.15
0.18
SNP effect on Gut microbiota abundance (genus Catenibacterium id.2153)

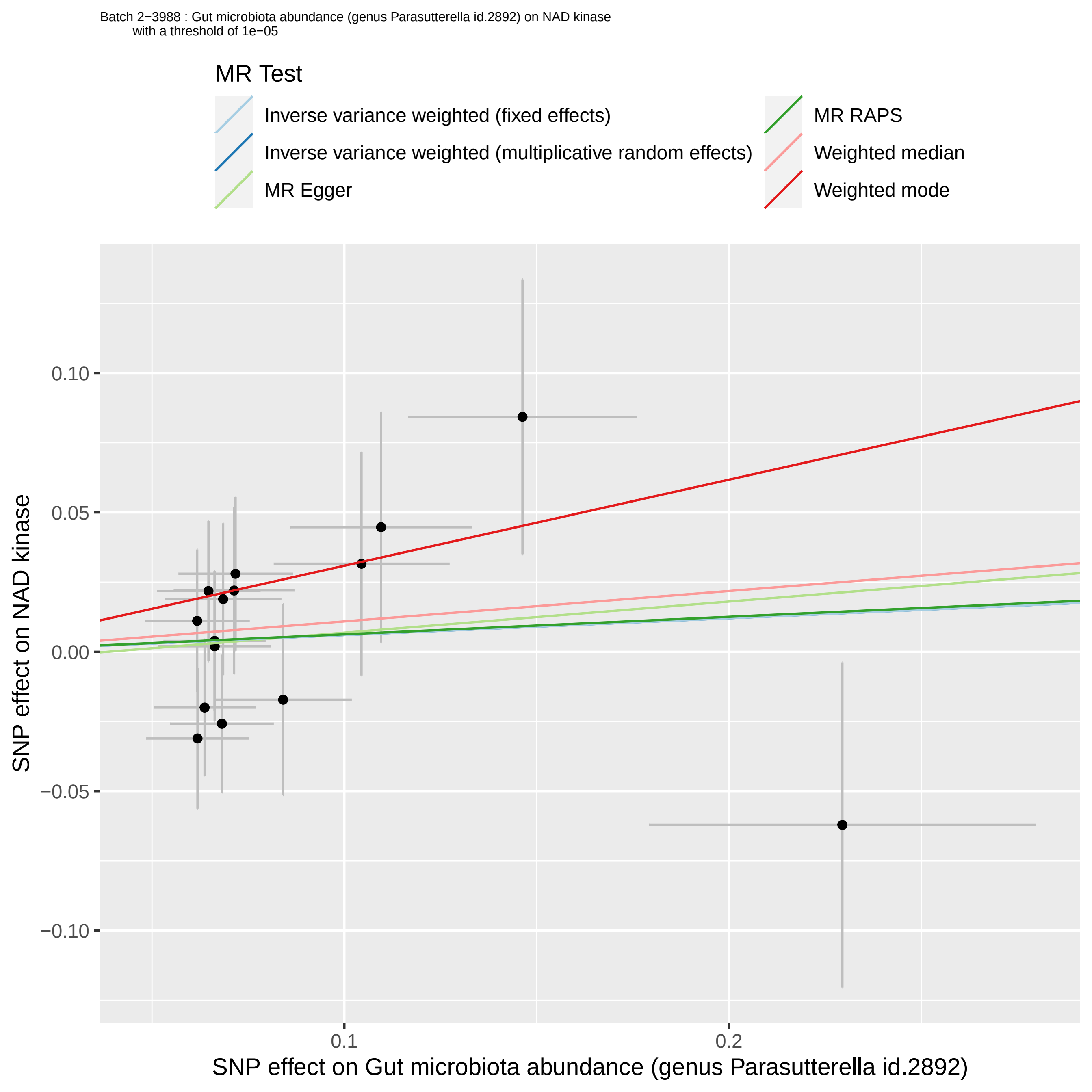
Batch 2−3988 : Gut microbiota abundance (genus Parasutterella id.2892) on NAD kinase
with a threshold of 1e−05
MR Test
Inverse variance weighted (fixed effects)
Inverse variance weighted (multiplicative random effects)
MR Egger
MR RAPS
Weighted median
Weighted mode
0.10
0.05
0.00
−0.05
−0.10
SNP effect on NAD kinase
0.1
0.2
SNP effect on Gut microbiota abundance (genus Parasutterella id.2892)

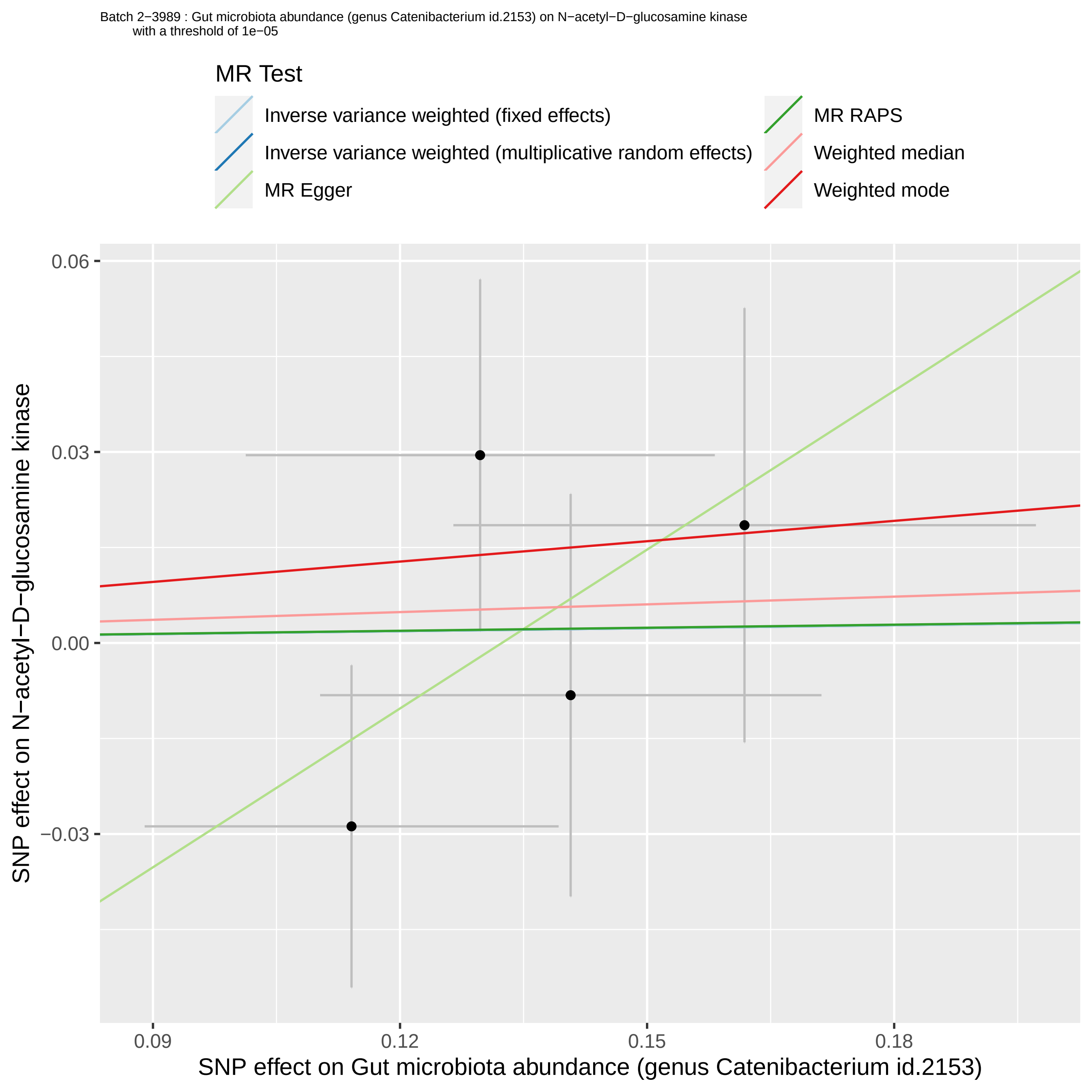
Batch 2−3989 : Gut microbiota abundance (genus Catenibacterium id.2153) on N−acetyl−D−glucosamine kinase
with a threshold of 1e−05
MR Test
Inverse variance weighted (fixed effects)
Inverse variance weighted (multiplicative random effects)
MR Egger
MR RAPS
Weighted median
Weighted mode
0.06
0.03
0.00
−0.03
0.09
0.12
0.15
0.18
SNP effect on N−acetyl−D−glucosamine kinase
SNP effect on Gut microbiota abundance (genus Catenibacterium id.2153)

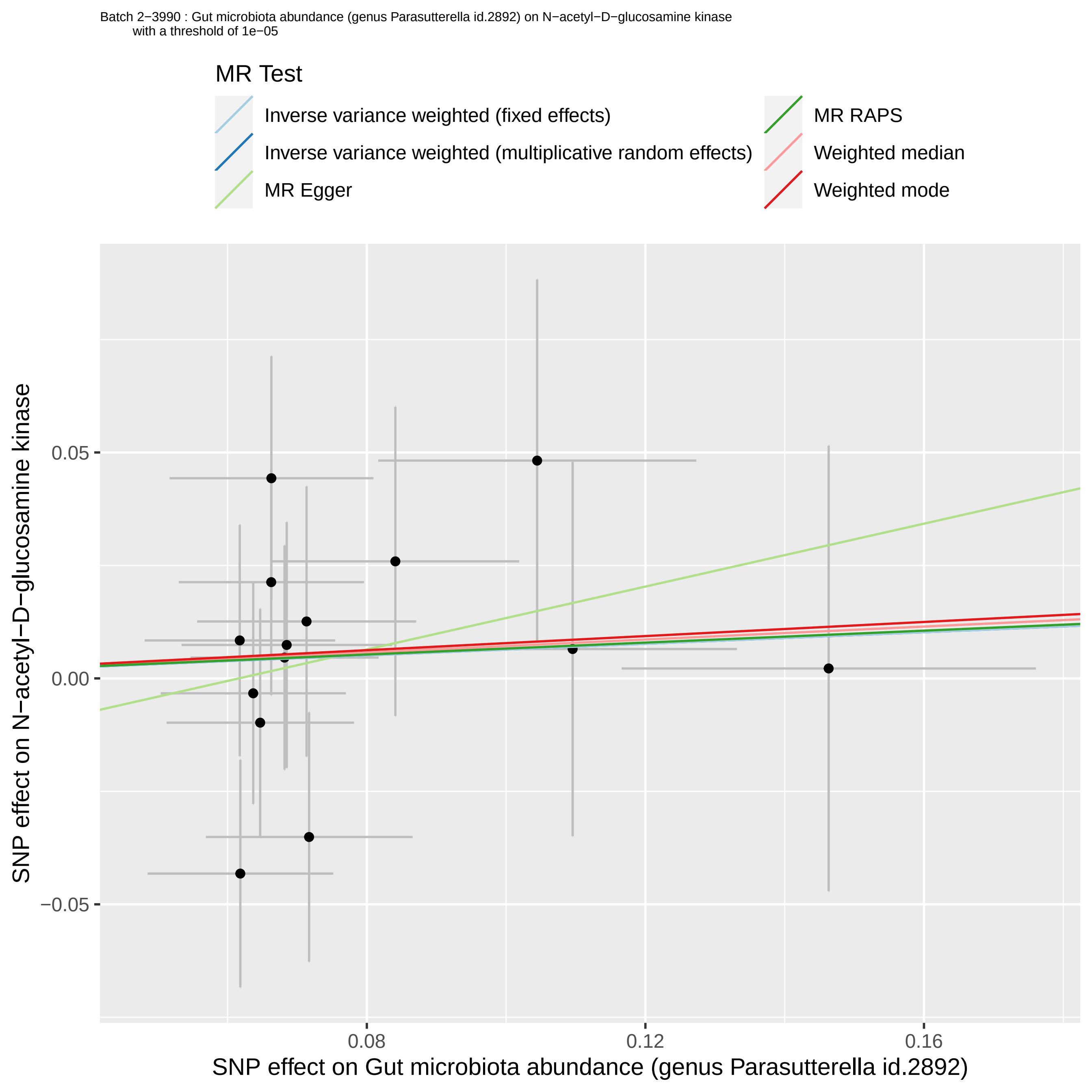

Batch 2−3990 : Gut microbiota abundance (genus Parasutterella id.2892) on N−acetyl−D−glucosamine kinase
with a threshold of 1e−05
MR Test
Inverse variance weighted (fixed effects)
Inverse variance weighted (multiplicative random effects)
MR Egger
MR RAPS
Weighted median
Weighted mode
0.05
0.00
−0.05
0.08
0.12
0.16
SNP effect on N−acetyl−D−glucosamine kinase
SNP effect on Gut microbiota abundance (genus Parasutterella id.2892)

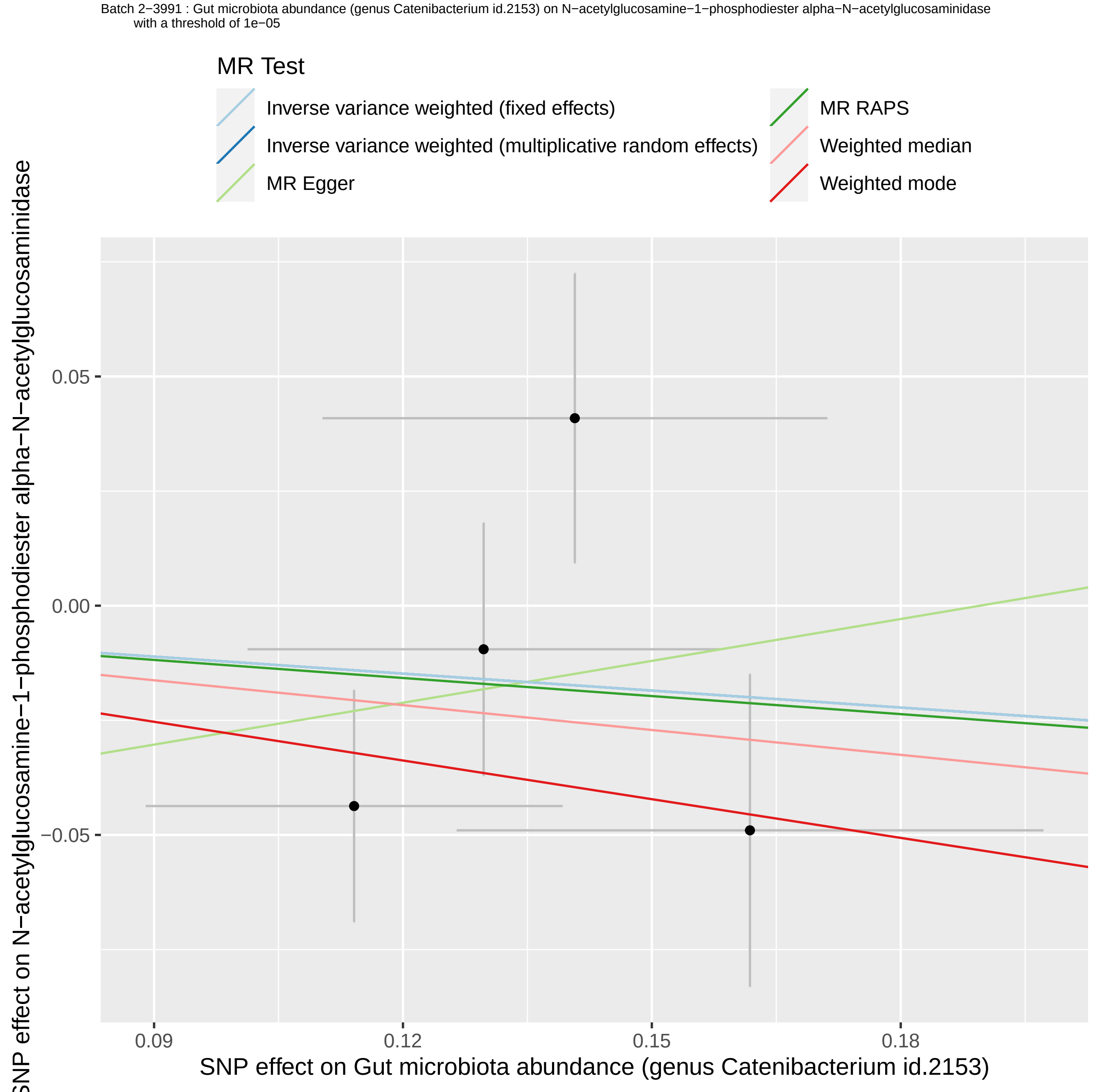

Batch 2−3991 : Gut microbiota abundance (genus Catenibacterium id.2153) on N−acetylglucosamine−1−phosphodiester alpha−N−acetylglucosaminidase
with a threshold of 1e−05
MR Test
Inverse variance weighted (fixed effects)
Inverse variance weighted (multiplicative random effects)
MR Egger
MR RAPS
Weighted median
Weighted mode
0.05
0.00
−0.05
0.09
0.12
0.15
0.18
SNP effect on Gut microbiota abundance (genus Catenibacterium id.2153)
SNP effect on N−acetylglucosamine−1−phosphodiester alpha−N−acetylglucosaminidase

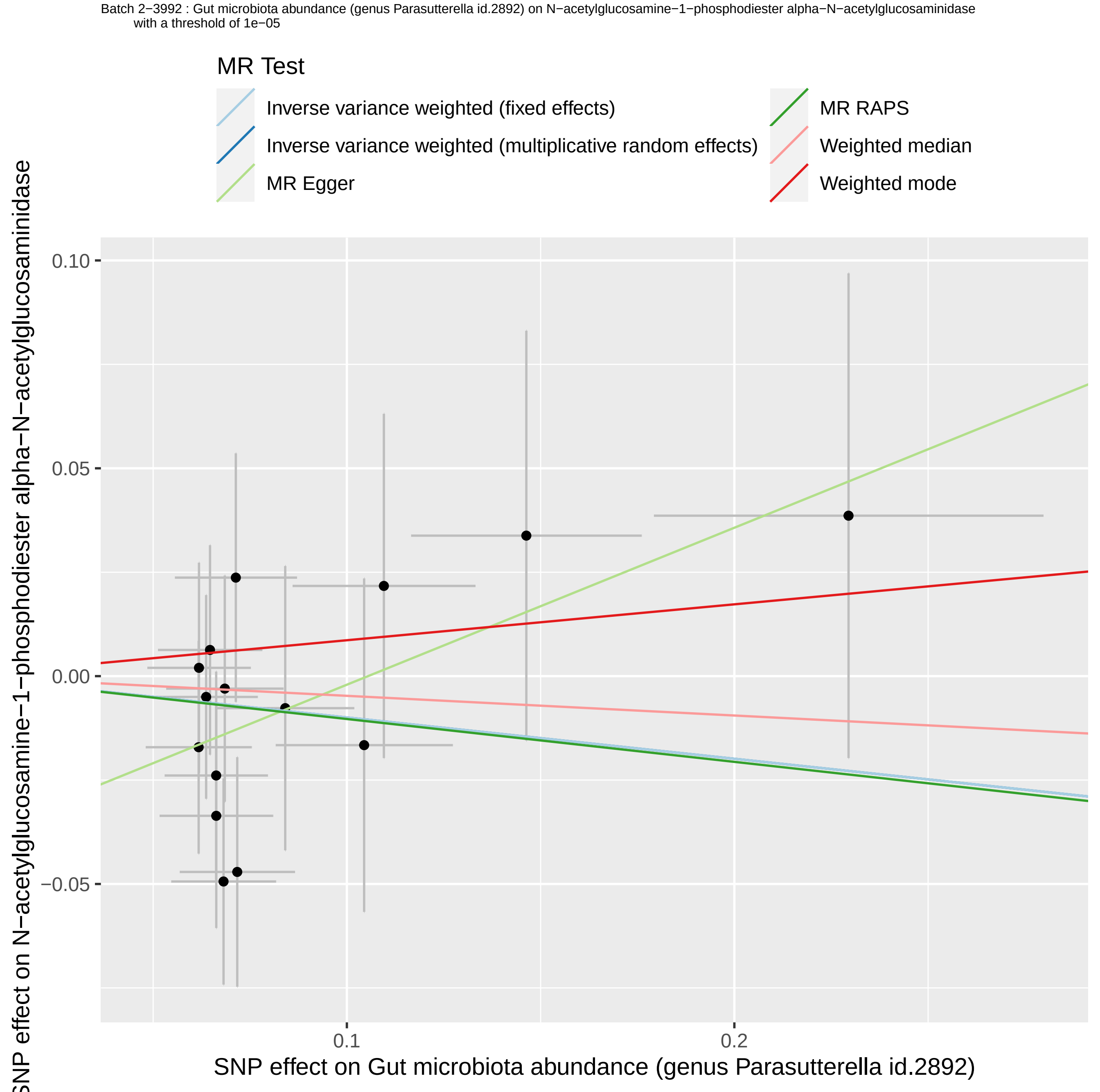
Batch 2−3992 : Gut microbiota abundance (genus Parasutterella id.2892) on N−acetylglucosamine−1−phosphodiester alpha−N−acetylglucosaminidase
with a threshold of 1e−05
MR Test
Inverse variance weighted (fixed effects)
Inverse variance weighted (multiplicative random effects)
MR Egger
MR RAPS
Weighted median
Weighted mode
0.10
0.05
0.00
−0.05
0.1
0.2
SNP effect on Gut microbiota abundance (genus Parasutterella id.2892)
SNP effect on N−acetylglucosamine−1−phosphodiester alpha−N−acetylglucosaminidase

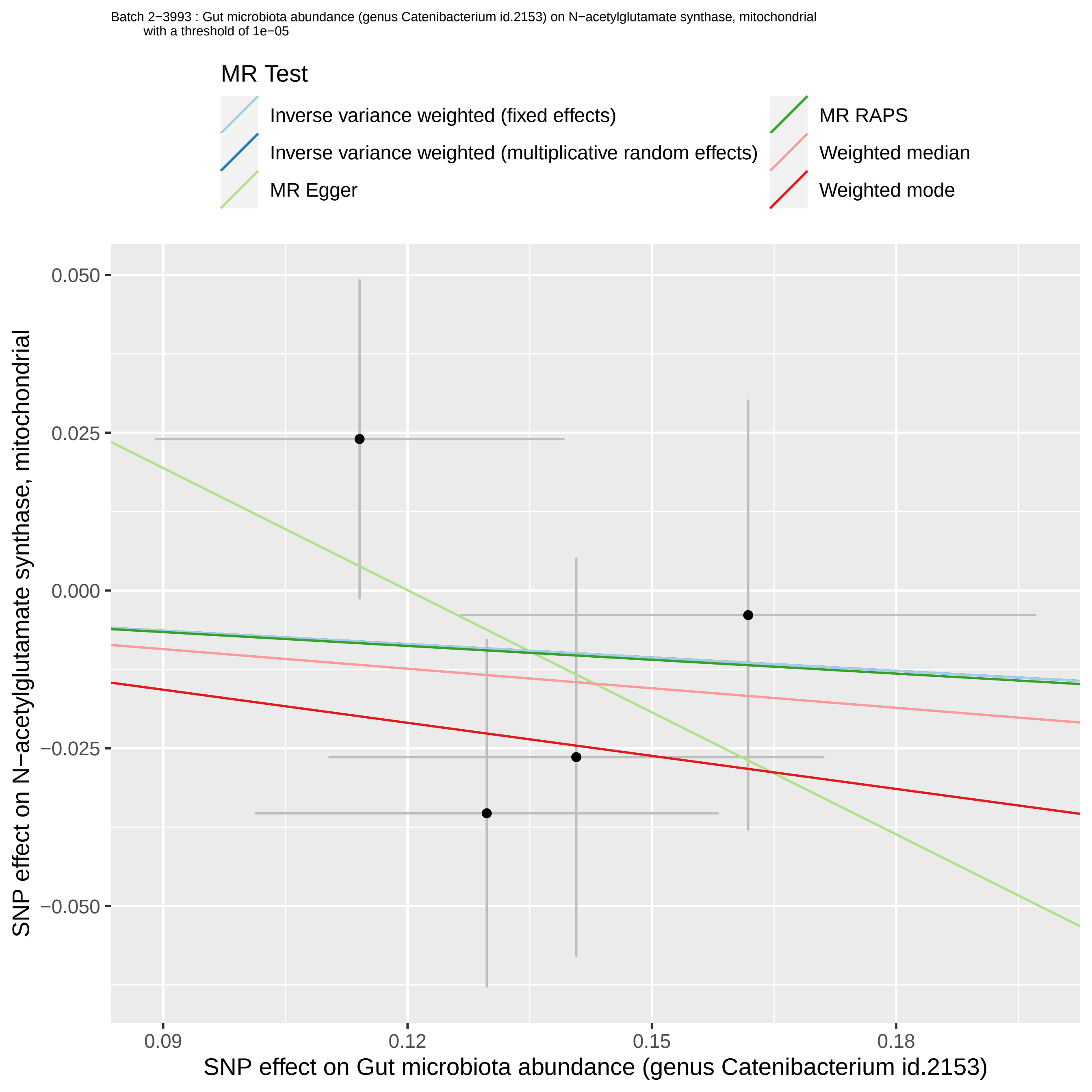
Batch 2−3993 : Gut microbiota abundance (genus Catenibacterium id.2153) on N−acetylglutamate synthase, mitochondrial
with a threshold of 1e−05
MR Test
Inverse variance weighted (fixed effects)
Inverse variance weighted (multiplicative random effects)
MR Egger
MR RAPS
Weighted median
Weighted mode
0.050
0.025
0.000
−0.025
−0.050
SNP effect on N−acetylglutamate synthase, mitochondrial
0.09
0.12
0.15
0.18
SNP effect on Gut microbiota abundance (genus Catenibacterium id.2153)

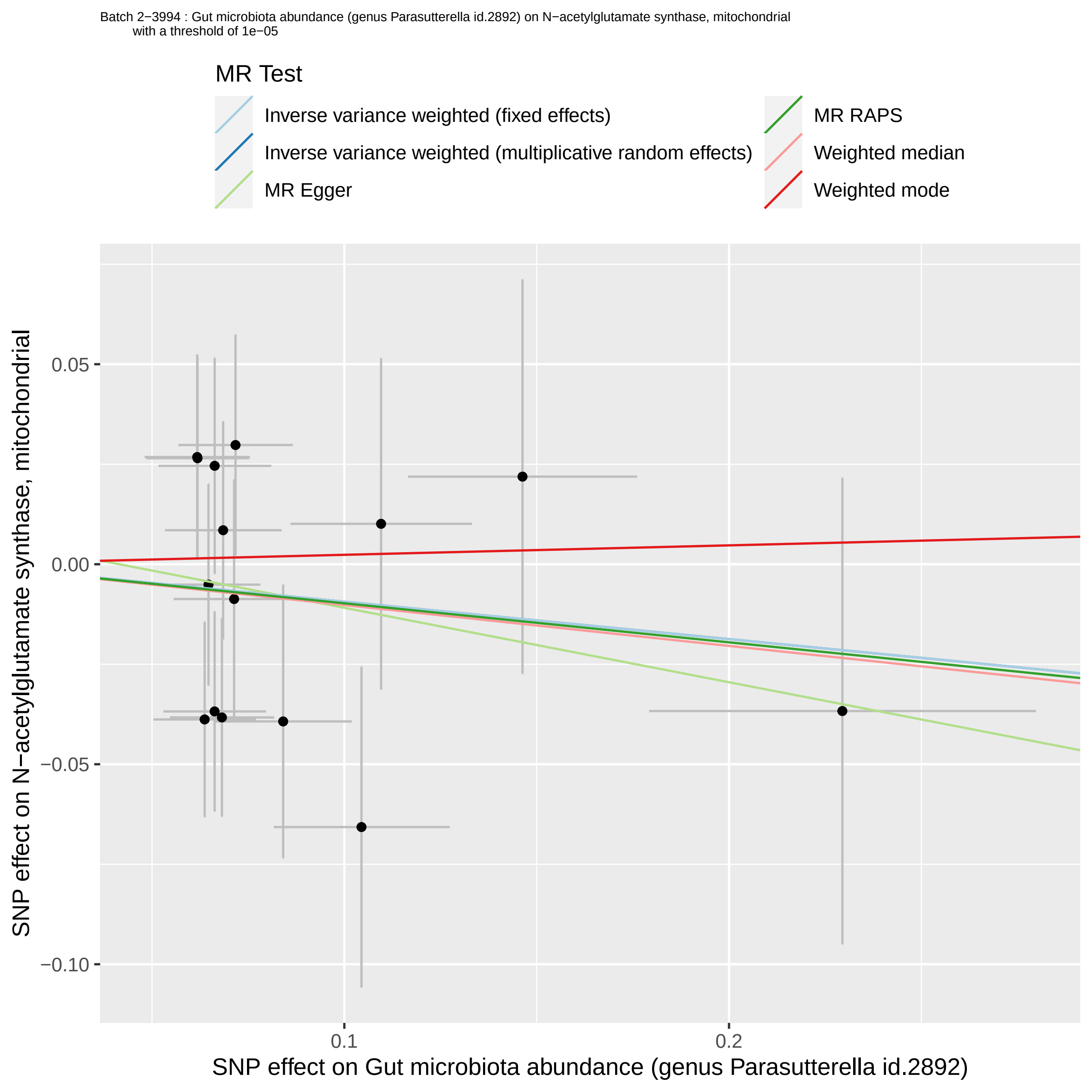

Batch 2−3994 : Gut microbiota abundance (genus Parasutterella id.2892) on N−acetylglutamate synthase, mitochondrial
with a threshold of 1e−05
MR Test
Inverse variance weighted (fixed effects)
Inverse variance weighted (multiplicative random effects)
MR Egger
MR RAPS
Weighted median
Weighted mode
0.05
0.00
−0.05
−0.10
0.1
0.2
SNP effect on N−acetylglutamate synthase, mitochondrial
SNP effect on Gut microbiota abundance (genus Parasutterella id.2892)

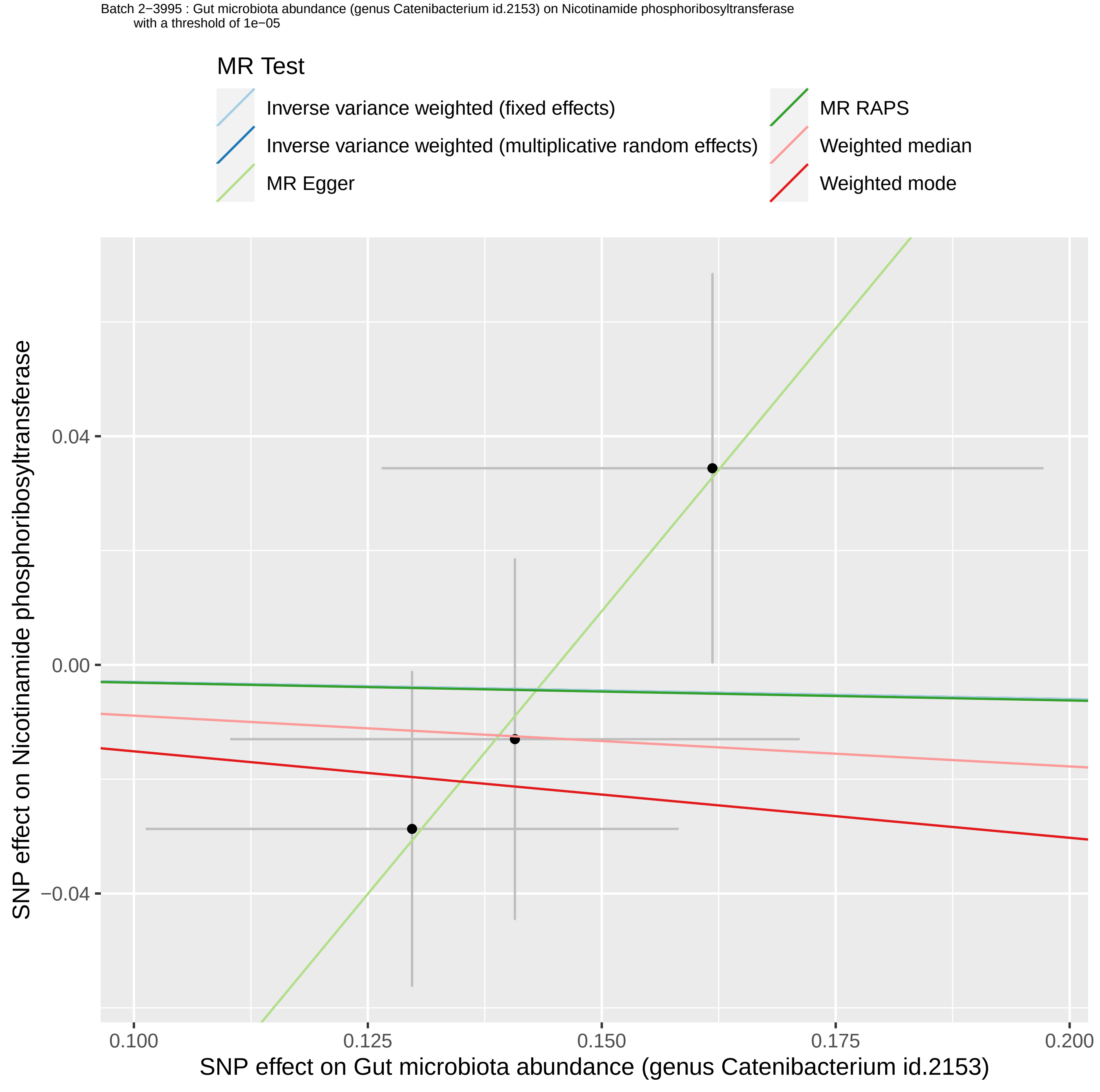

Batch 2−3995 : Gut microbiota abundance (genus Catenibacterium id.2153) on Nicotinamide phosphoribosyltransferase
with a threshold of 1e−05
MR Test
Inverse variance weighted (fixed effects)
Inverse variance weighted (multiplicative random effects)
MR Egger
MR RAPS
Weighted median
Weighted mode
0.04
0.00
−0.04
0.100
0.125
0.150
0.175
0.200
SNP effect on Nicotinamide phosphoribosyltransferase
SNP effect on Gut microbiota abundance (genus Catenibacterium id.2153)

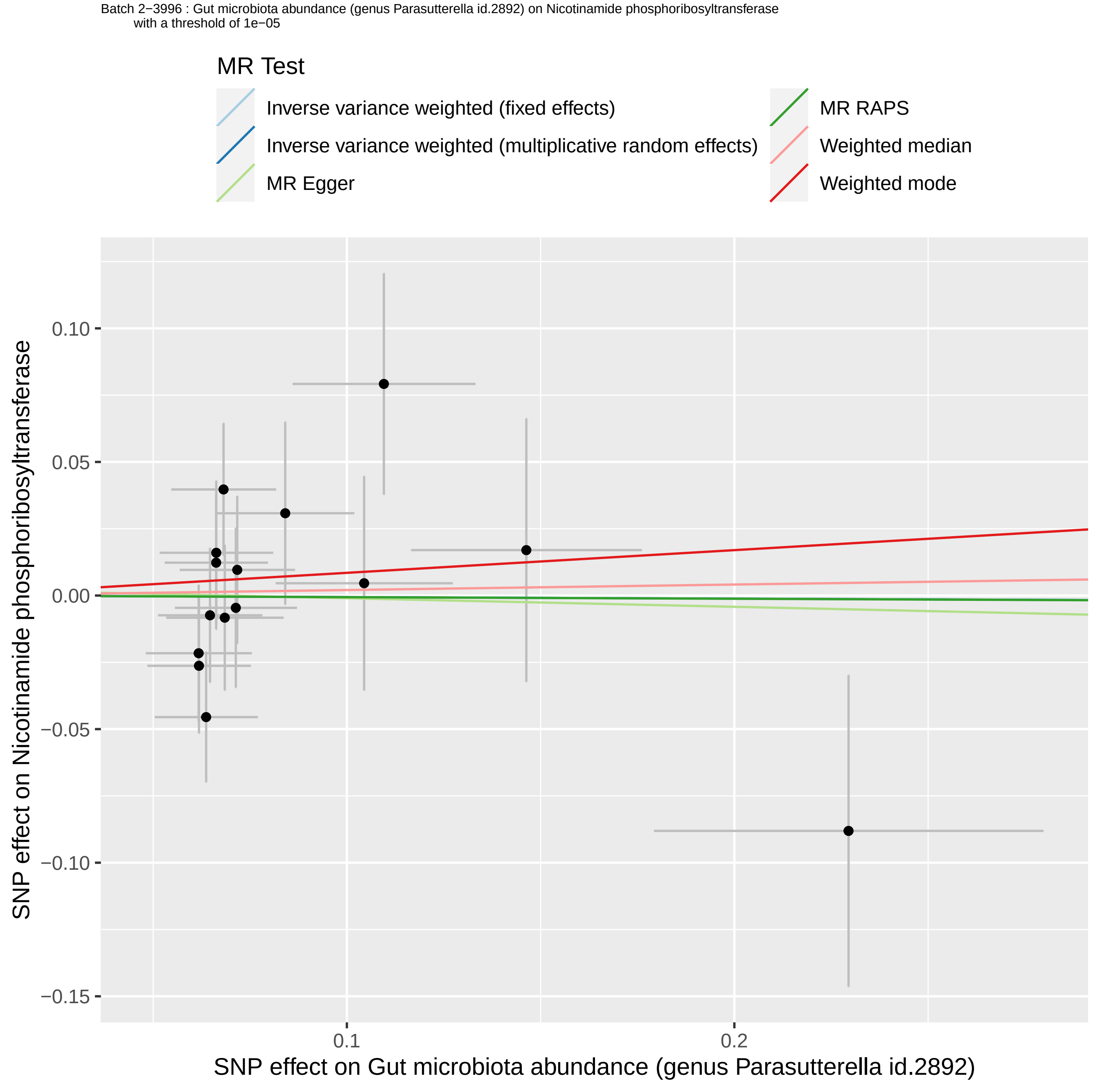

Batch 2−3996 : Gut microbiota abundance (genus Parasutterella id.2892) on Nicotinamide phosphoribosyltransferase
with a threshold of 1e−05
MR Test
Inverse variance weighted (fixed effects)
Inverse variance weighted (multiplicative random effects)
MR Egger
MR RAPS
Weighted median
Weighted mode
0.10
0.05
0.00
−0.05
−0.10
−0.15
0.1
0.2
SNP effect on Nicotinamide phosphoribosyltransferase
SNP effect on Gut microbiota abundance (genus Parasutterella id.2892)

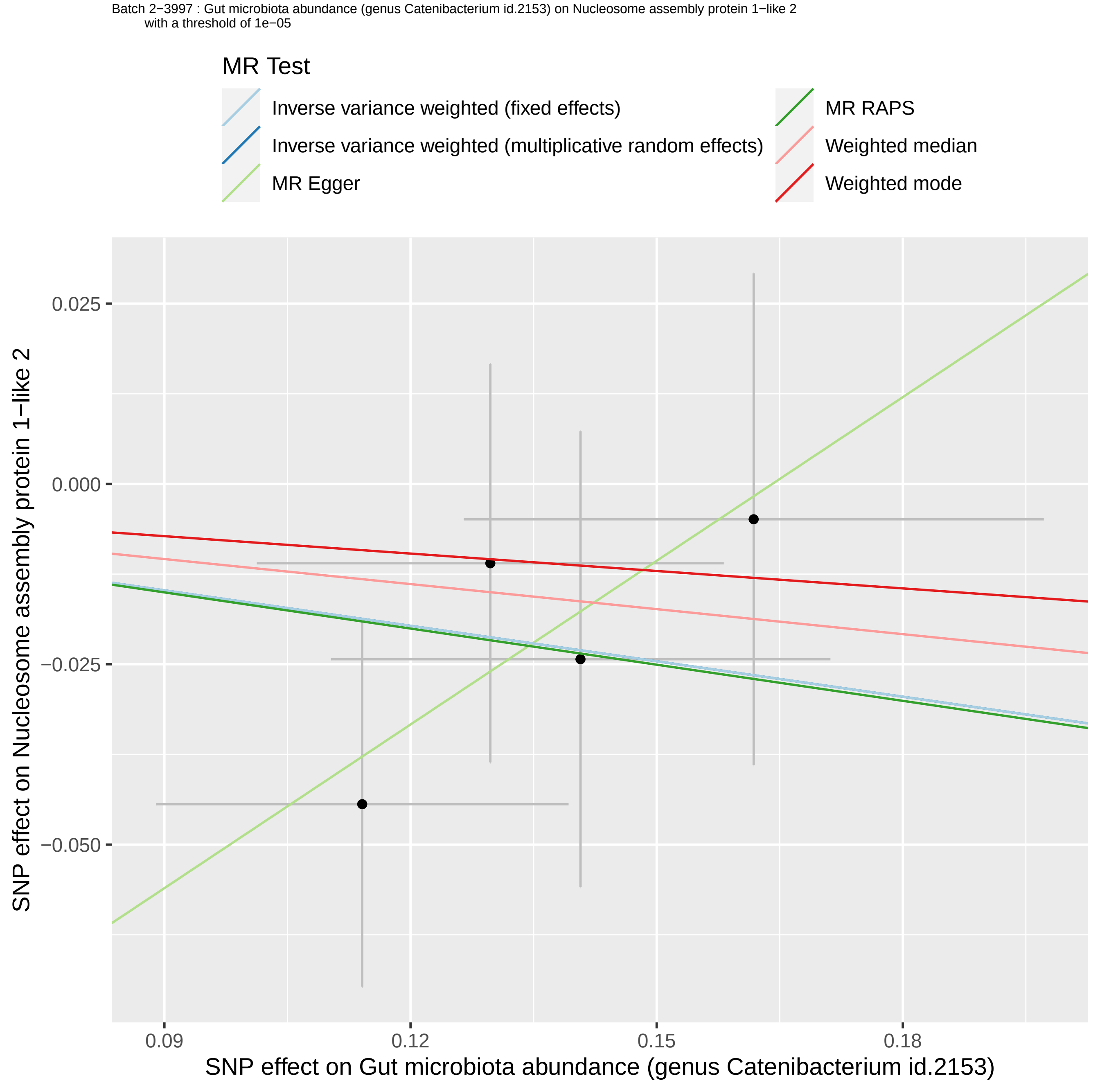

Batch 2−3997 : Gut microbiota abundance (genus Catenibacterium id.2153) on Nucleosome assembly protein 1−like 2
with a threshold of 1e−05
MR Test
Inverse variance weighted (fixed effects)
Inverse variance weighted (multiplicative random effects)
MR Egger
MR RAPS
Weighted median
Weighted mode
0.025
0.000
−0.025
−0.050
0.09
0.12
0.15
0.18
SNP effect on Nucleosome assembly protein 1−like 2
SNP effect on Gut microbiota abundance (genus Catenibacterium id.2153)

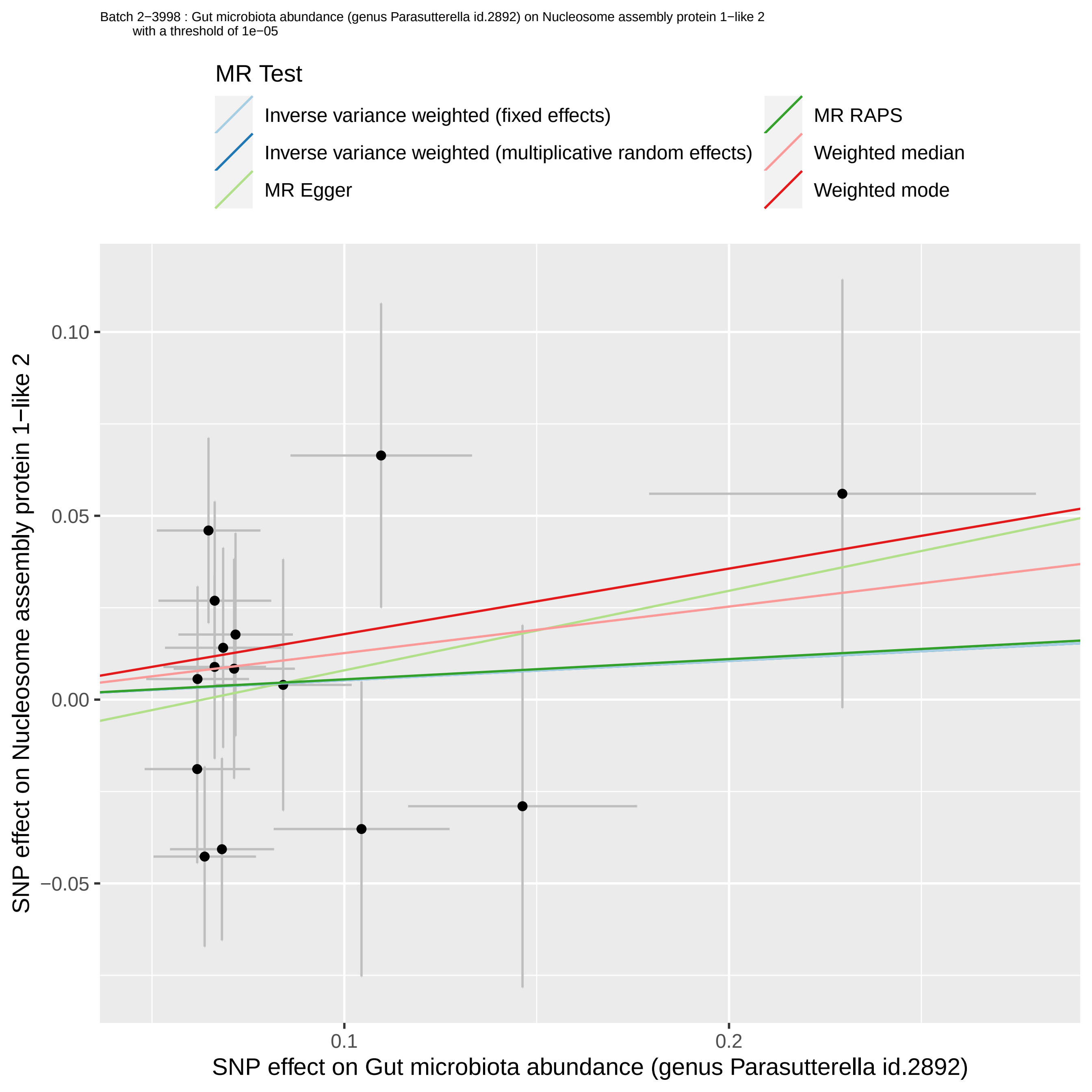

Batch 2−3998 : Gut microbiota abundance (genus Parasutterella id.2892) on Nucleosome assembly protein 1−like 2
with a threshold of 1e−05
MR Test
Inverse variance weighted (fixed effects)
Inverse variance weighted (multiplicative random effects)
MR Egger
MR RAPS
Weighted median
Weighted mode
0.10
0.05
0.00
−0.05
0.1
0.2
SNP effect on Nucleosome assembly protein 1−like 2
SNP effect on Gut microbiota abundance (genus Parasutterella id.2892)

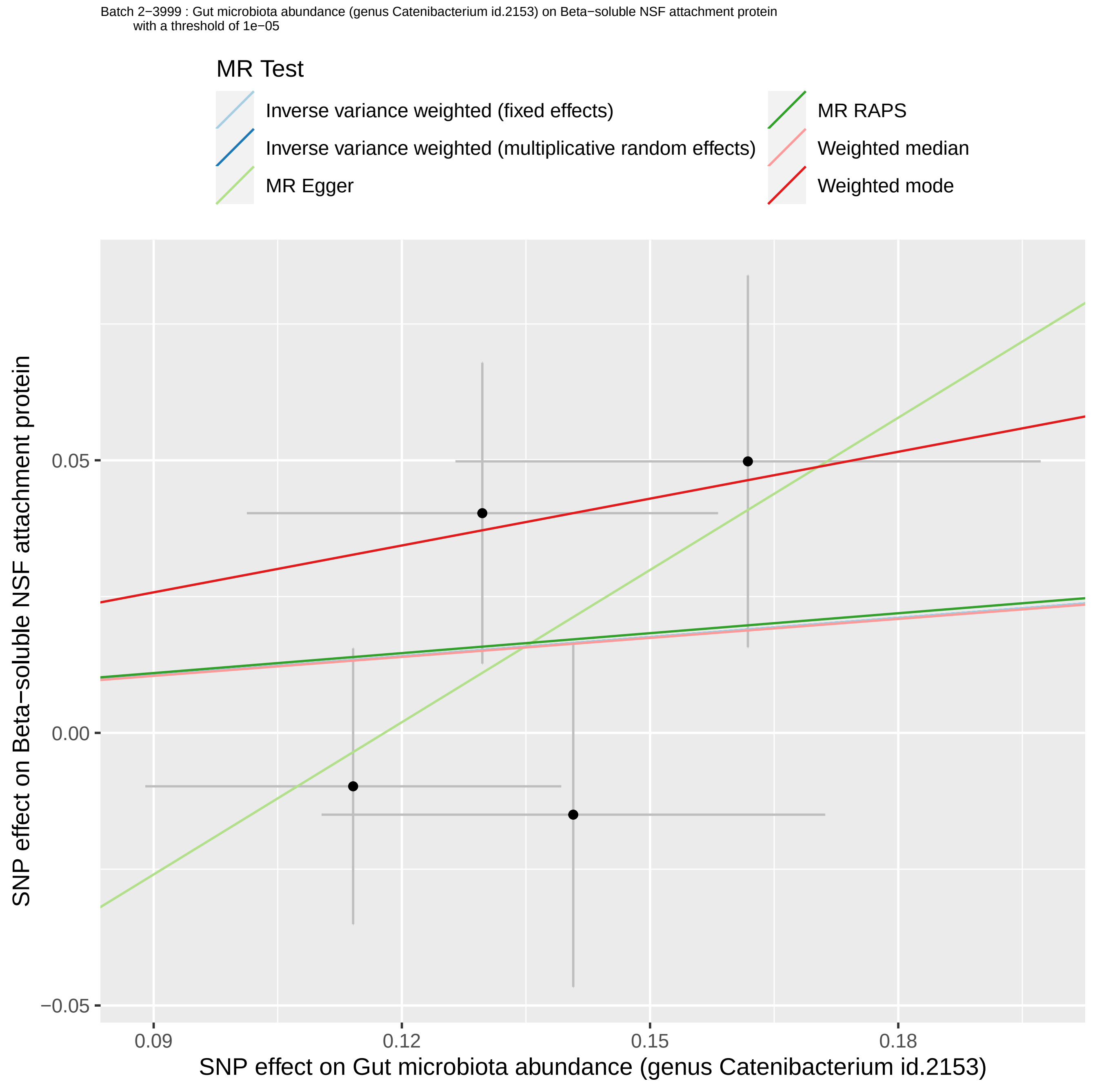
Batch 2−3999 : Gut microbiota abundance (genus Catenibacterium id.2153) on Beta−soluble NSF attachment protein
with a threshold of 1e−05
MR Test
Inverse variance weighted (fixed effects)
Inverse variance weighted (multiplicative random effects)
MR Egger
MR RAPS
Weighted median
Weighted mode
SNP effect on Beta−soluble NSF attachment protein
0.05
0.00
−0.05
0.09
0.12
0.15
0.18
SNP effect on Gut microbiota abundance (genus Catenibacterium id.2153)

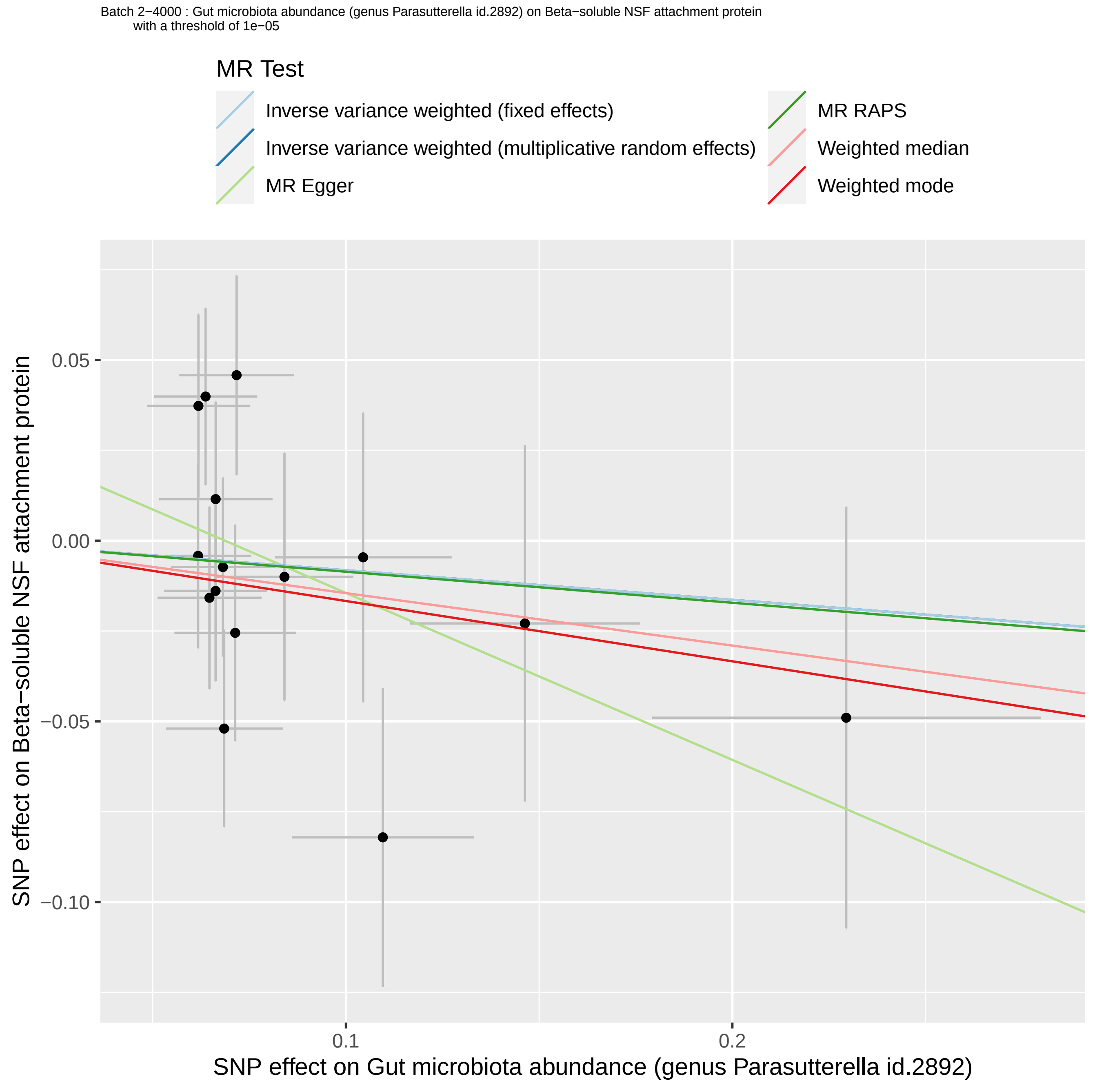

Batch 2−4000 : Gut microbiota abundance (genus Parasutterella id.2892) on Beta−soluble NSF attachment protein
with a threshold of 1e−05
MR Test
Inverse variance weighted (fixed effects)
Inverse variance weighted (multiplicative random effects)
MR Egger
MR RAPS
Weighted median
Weighted mode
0.05
0.00
−0.05
−0.10
0.1
0.2
SNP effect on Beta−soluble NSF attachment protein
SNP effect on Gut microbiota abundance (genus Parasutterella id.2892)

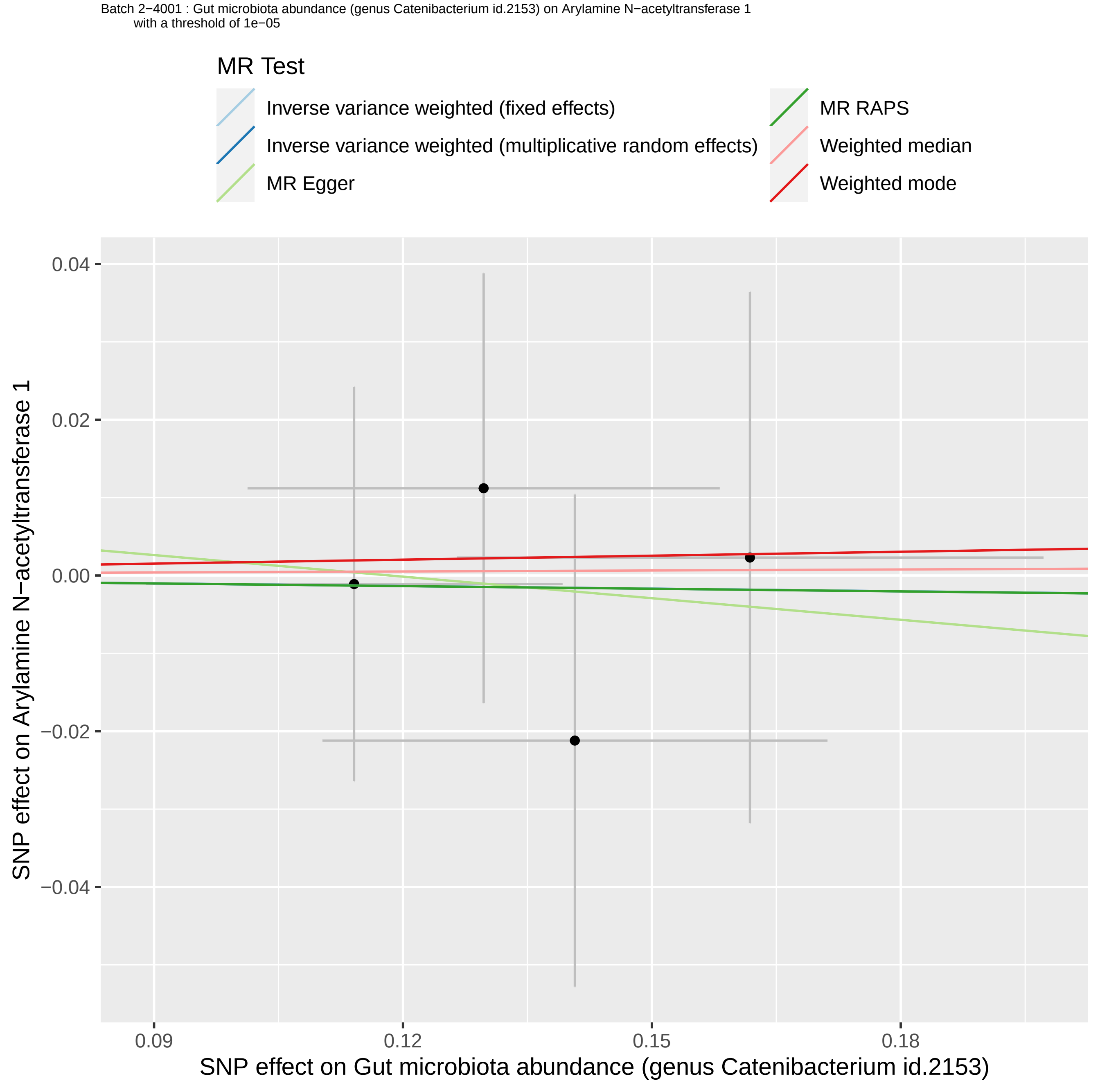
Batch 2−4001 : Gut microbiota abundance (genus Catenibacterium id.2153) on Arylamine N−acetyltransferase 1
with a threshold of 1e−05
MR Test
Inverse variance weighted (fixed effects)
Inverse variance weighted (multiplicative random effects)
MR Egger
MR RAPS
Weighted median
Weighted mode
0.04
0.02
0.00
−0.02
−0.04
0.09
0.12
0.15
0.18
SNP effect on Arylamine N−acetyltransferase 1
SNP effect on Gut microbiota abundance (genus Catenibacterium id.2153)

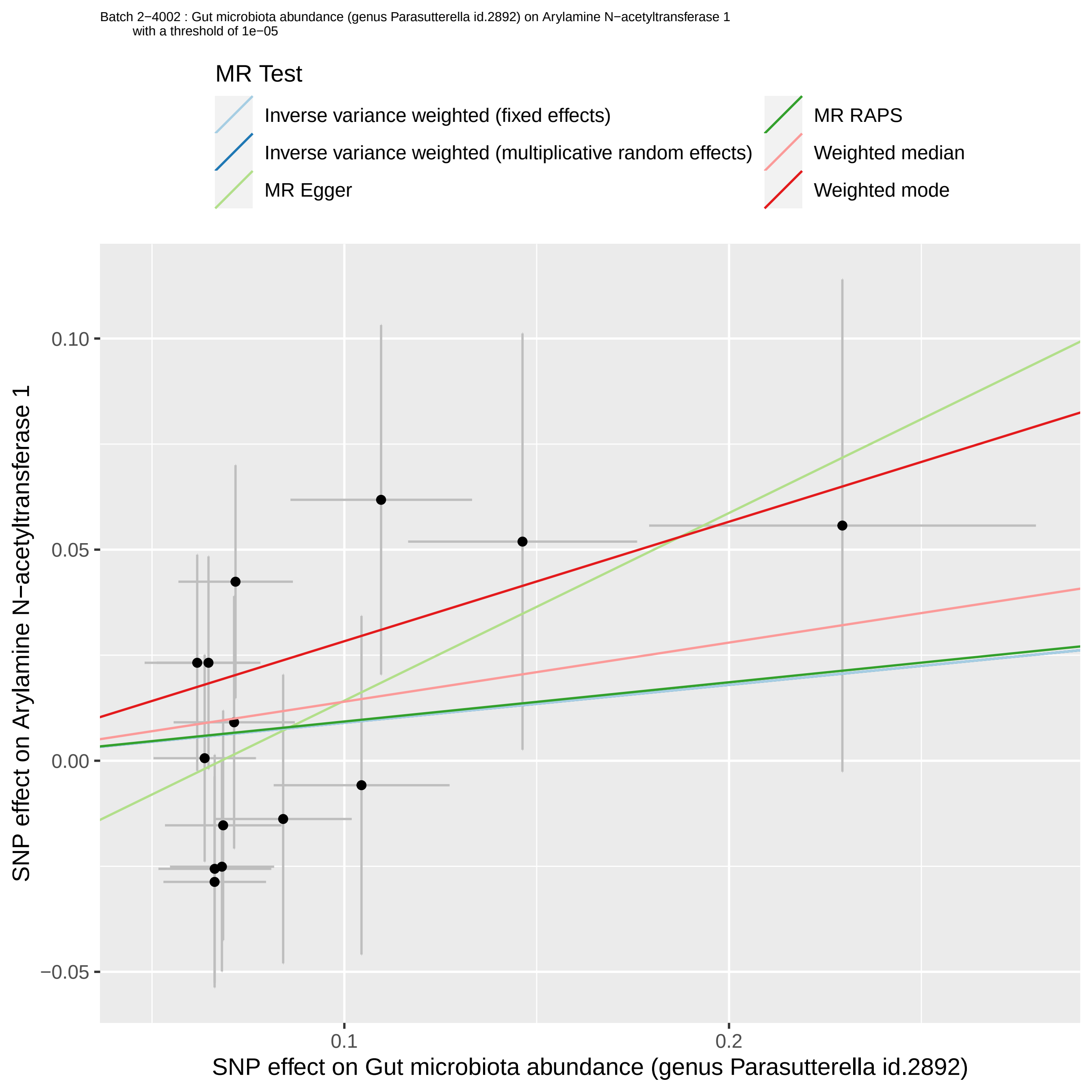
Batch 2−4002 : Gut microbiota abundance (genus Parasutterella id.2892) on Arylamine N−acetyltransferase 1
with a threshold of 1e−05
MR Test
Inverse variance weighted (fixed effects)
Inverse variance weighted (multiplicative random effects)
MR Egger
MR RAPS
Weighted median
Weighted mode
0.10
0.05
0.00
−0.05
0.1
0.2
SNP effect on Arylamine N−acetyltransferase 1
SNP effect on Gut microbiota abundance (genus Parasutterella id.2892)

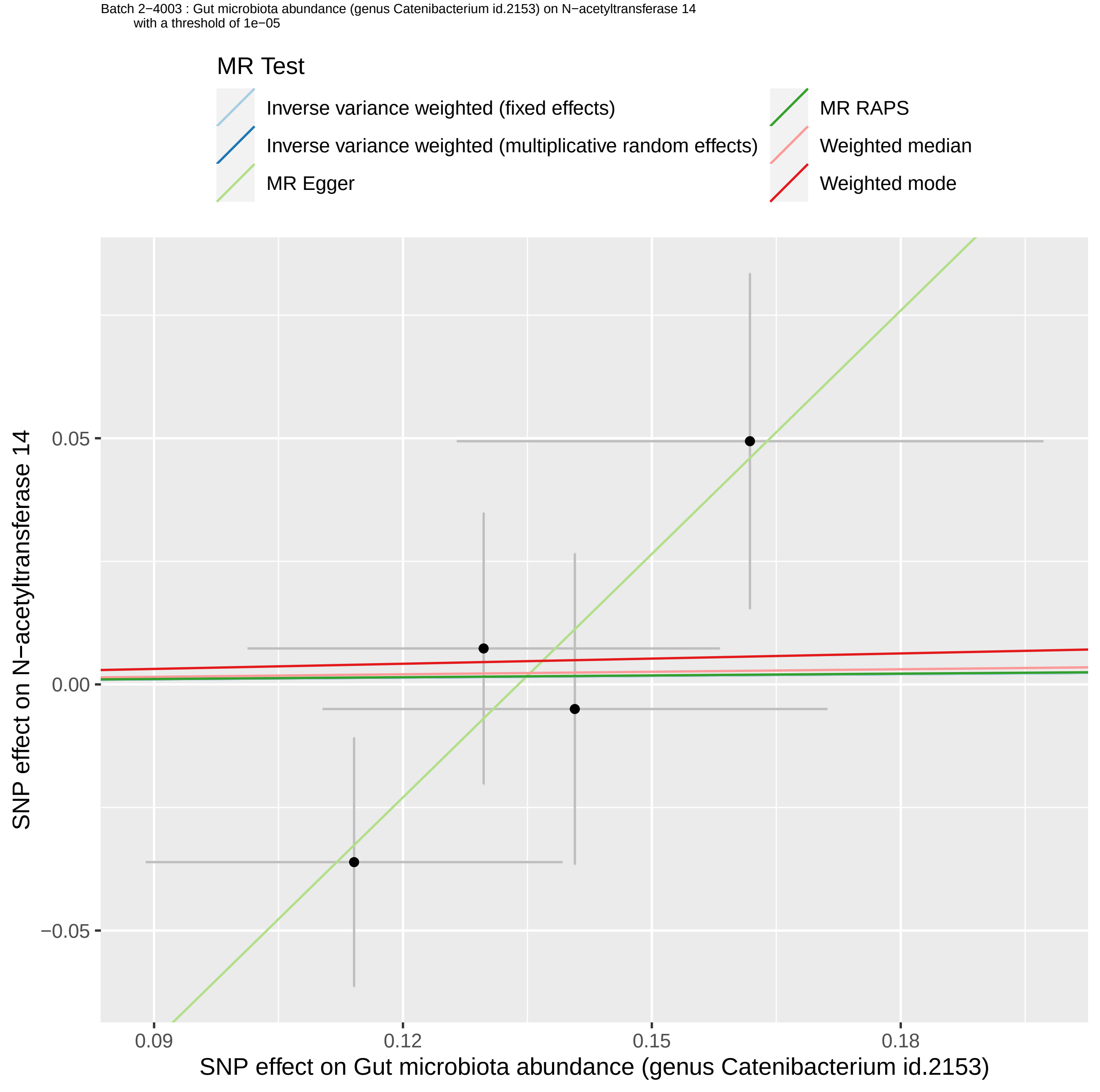
Batch 2−4003 : Gut microbiota abundance (genus Catenibacterium id.2153) on N−acetyltransferase 14
with a threshold of 1e−05
MR Test
Inverse variance weighted (fixed effects)
Inverse variance weighted (multiplicative random effects)
MR Egger
MR RAPS
Weighted median
Weighted mode
0.05
0.00
−0.05
0.09
0.12
0.15
0.18
SNP effect on N−acetyltransferase 14
SNP effect on Gut microbiota abundance (genus Catenibacterium id.2153)

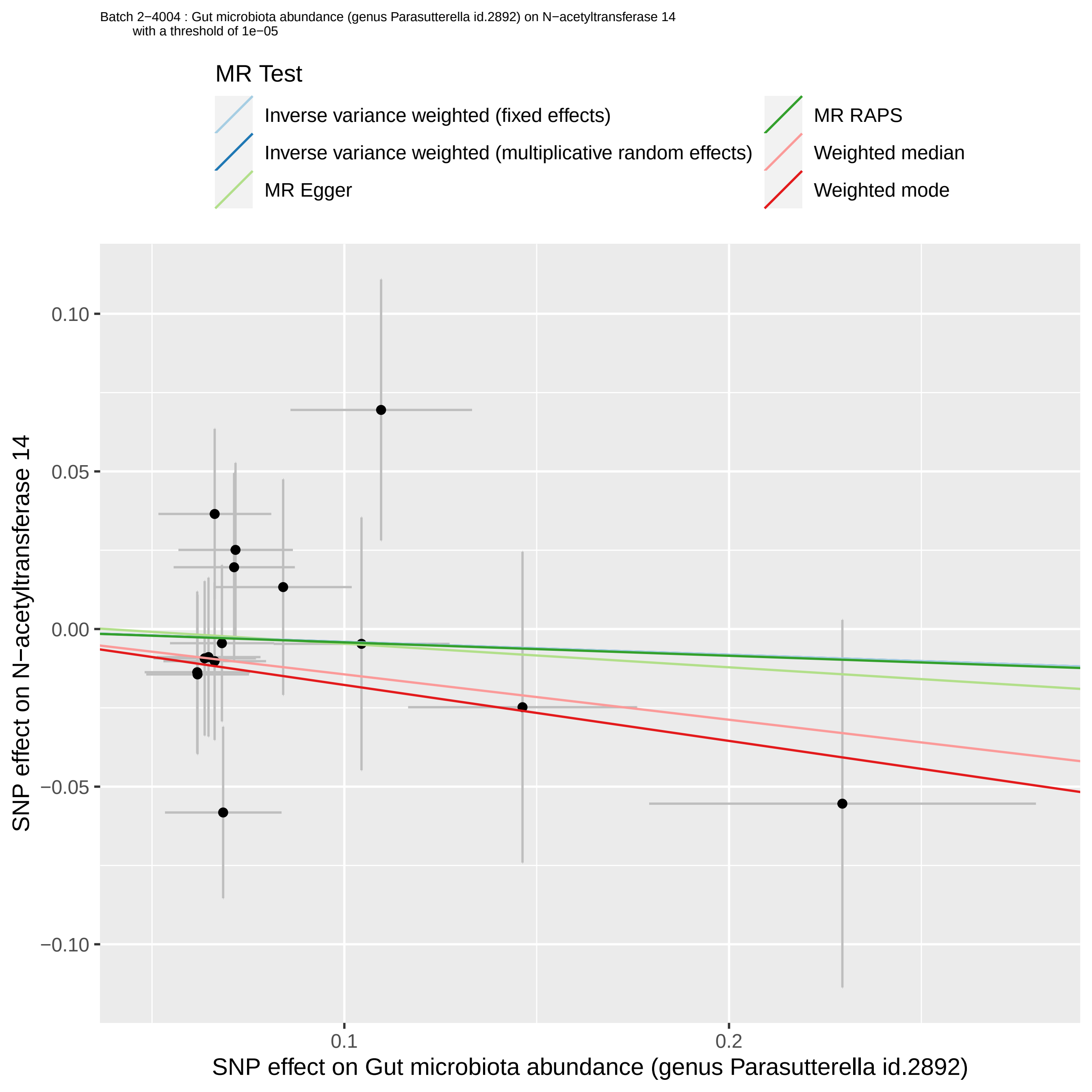

Batch 2−4004 : Gut microbiota abundance (genus Parasutterella id.2892) on N−acetyltransferase 14
with a threshold of 1e−05
MR Test
Inverse variance weighted (fixed effects)
Inverse variance weighted (multiplicative random effects)
MR Egger
MR RAPS
Weighted median
Weighted mode
0.10
0.05
0.00
−0.05
−0.10
0.1
0.2
SNP effect on N−acetyltransferase 14
SNP effect on Gut microbiota abundance (genus Parasutterella id.2892)

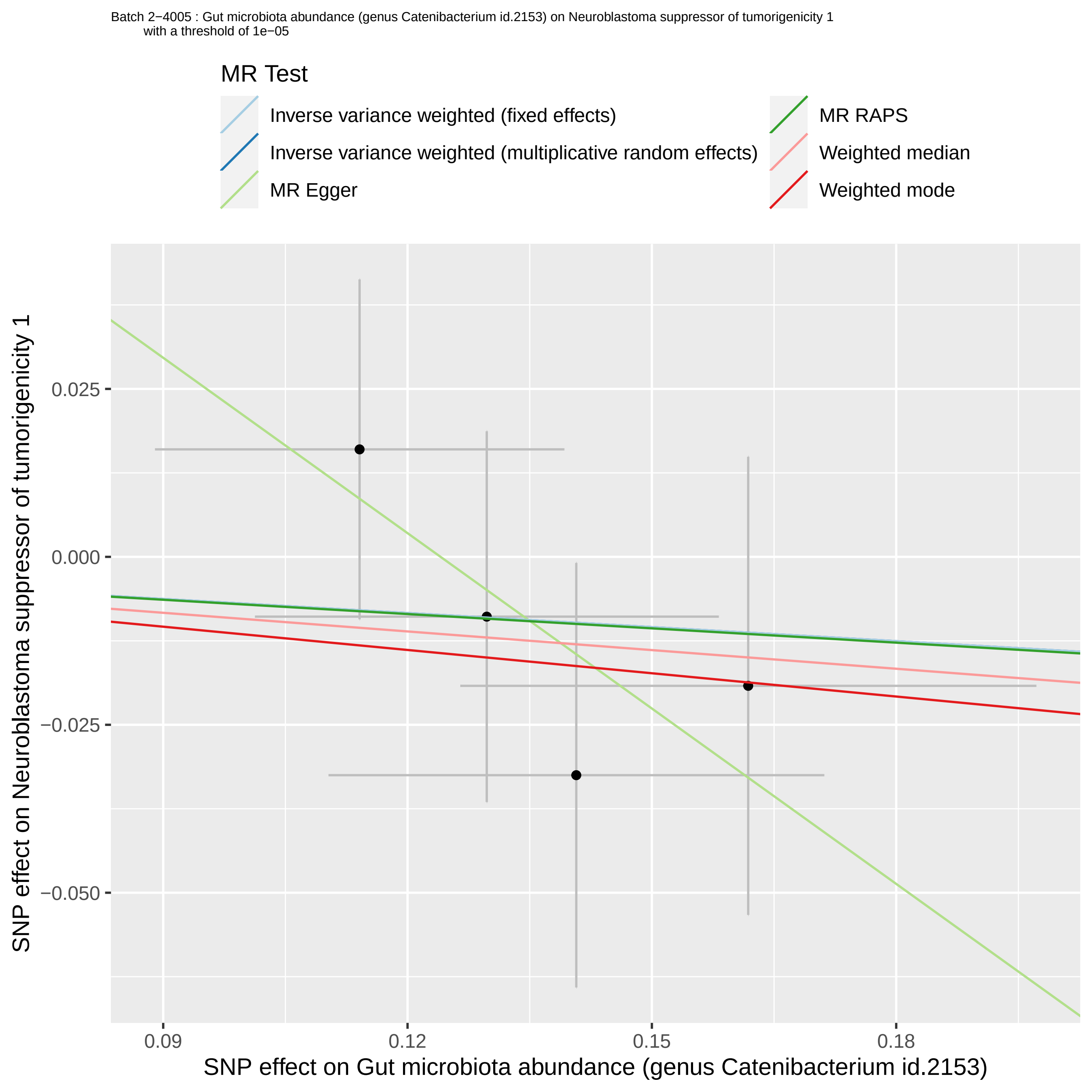
Batch 2−4005 : Gut microbiota abundance (genus Catenibacterium id.2153) on Neuroblastoma suppressor of tumorigenicity 1
with a threshold of 1e−05
MR Test
Inverse variance weighted (fixed effects)
Inverse variance weighted (multiplicative random effects)
MR Egger
MR RAPS
Weighted median
Weighted mode
0.025
0.000
−0.025
−0.050
0.09
0.12
0.15
0.18
SNP effect on Neuroblastoma suppressor of tumorigenicity 1
SNP effect on Gut microbiota abundance (genus Catenibacterium id.2153)

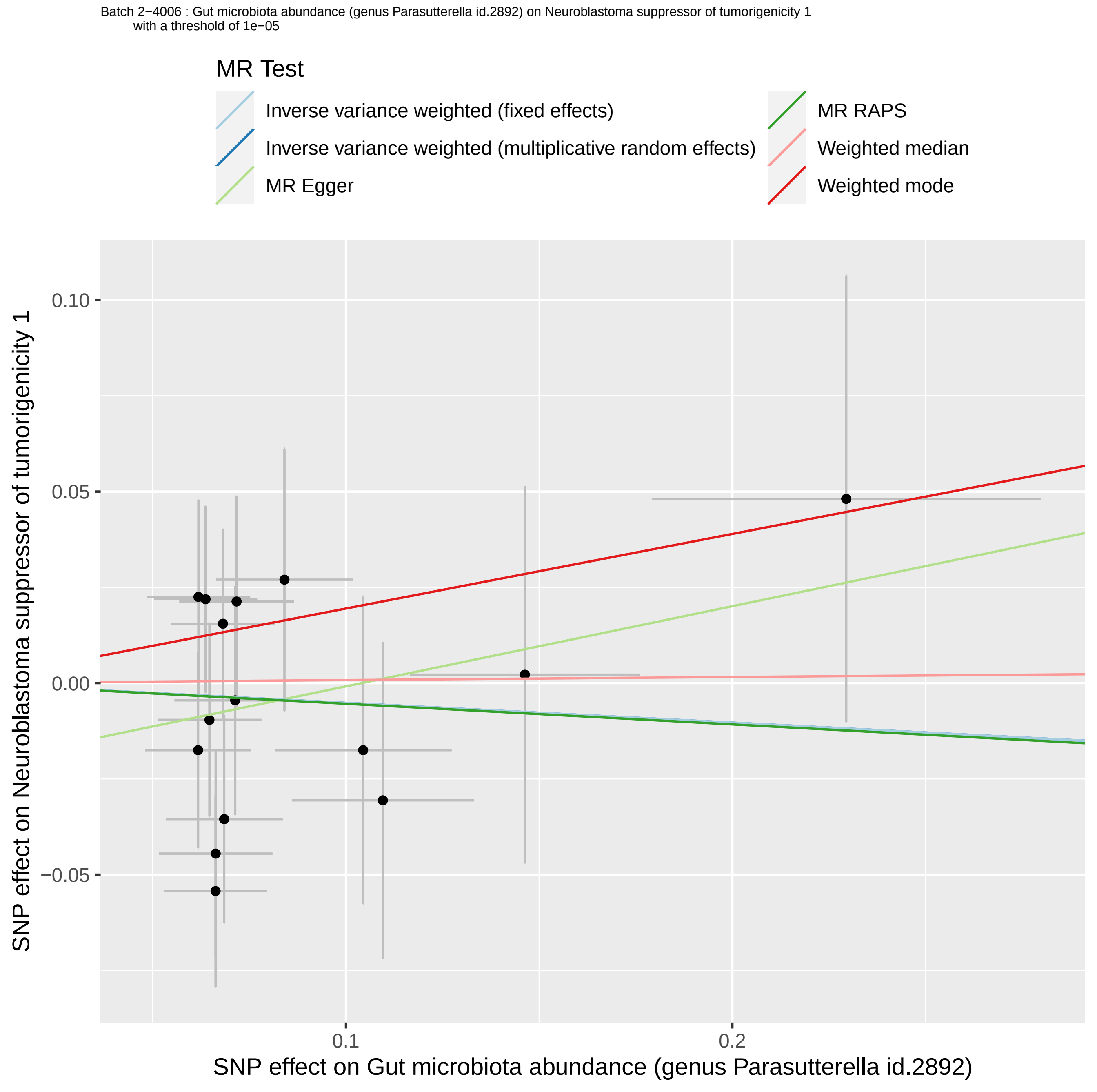

Batch 2−4006 : Gut microbiota abundance (genus Parasutterella id.2892) on Neuroblastoma suppressor of tumorigenicity 1
with a threshold of 1e−05
MR Test
Inverse variance weighted (fixed effects)
Inverse variance weighted (multiplicative random effects)
MR Egger
MR RAPS
Weighted median
Weighted mode
0.10
0.05
0.00
−0.05
0.1
0.2
SNP effect on Neuroblastoma suppressor of tumorigenicity 1
SNP effect on Gut microbiota abundance (genus Parasutterella id.2892)

Batch 2−4007 : Gut microbiota abundance (genus Catenibacterium id.2153) on Next to BRCA1 gene 1 protein
with a threshold of 1e−05

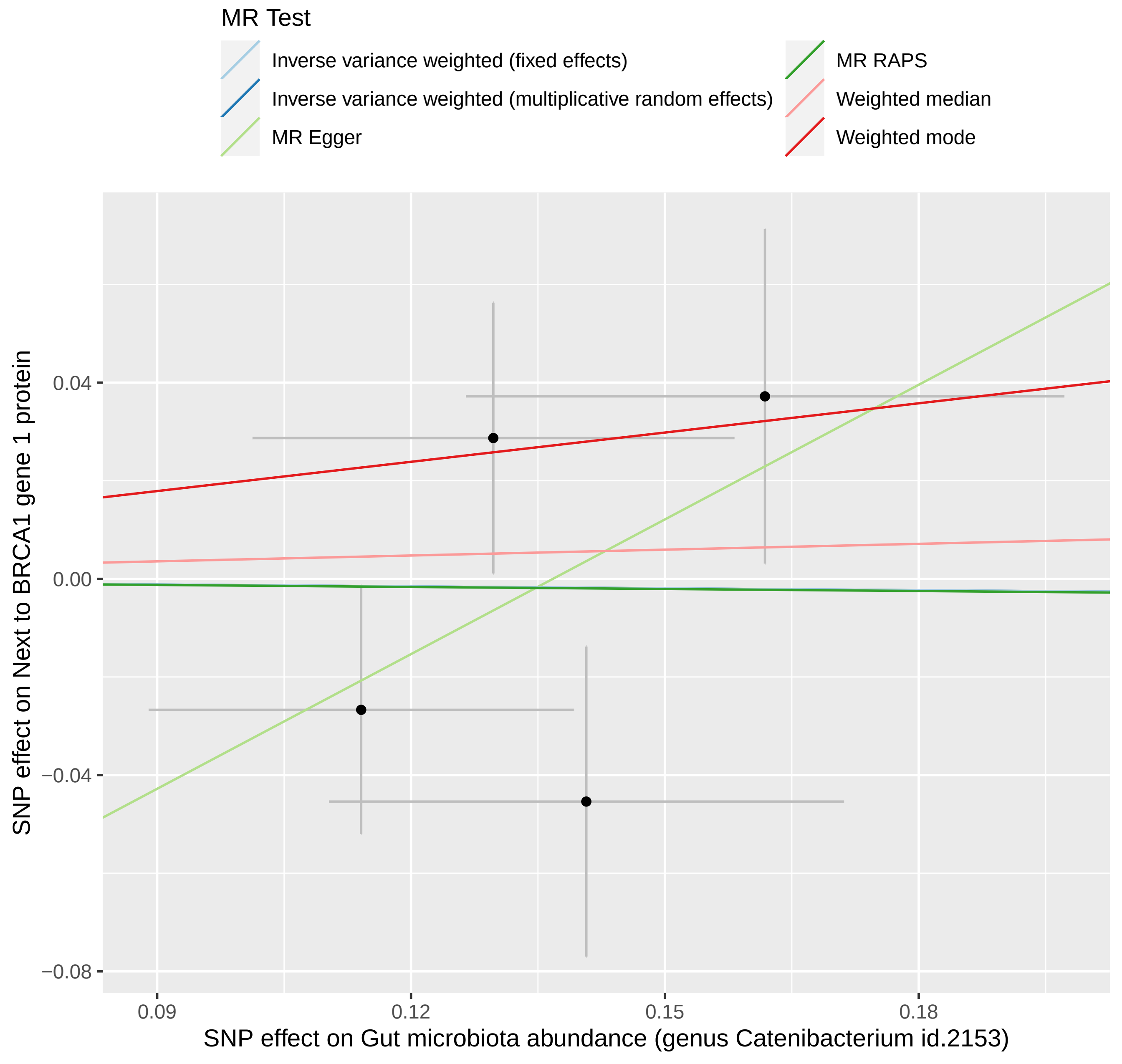

Batch 2−4008 : Gut microbiota abundance (genus Parasutterella id.2892) on Next to BRCA1 gene 1 protein
with a threshold of 1e−05

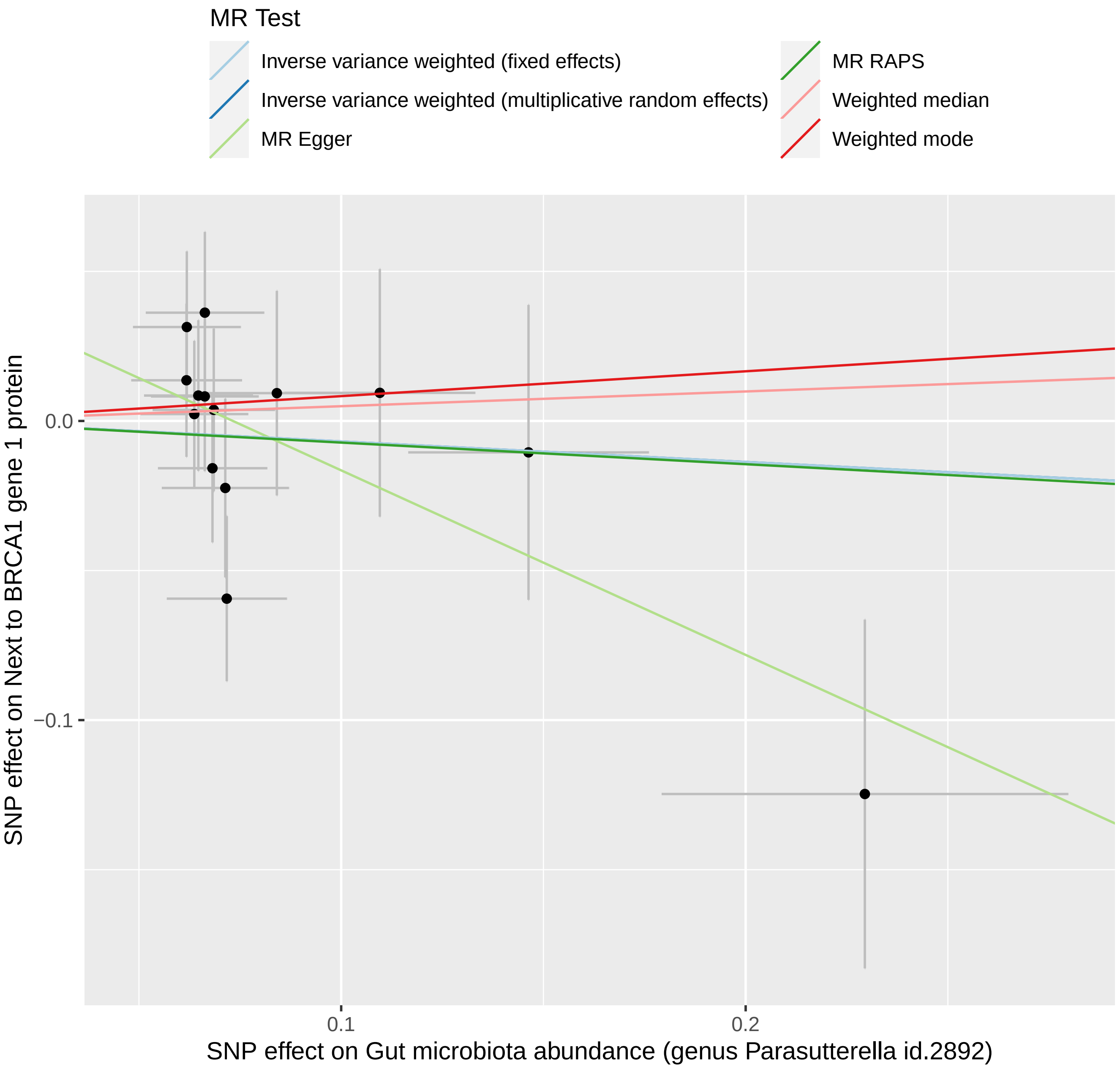

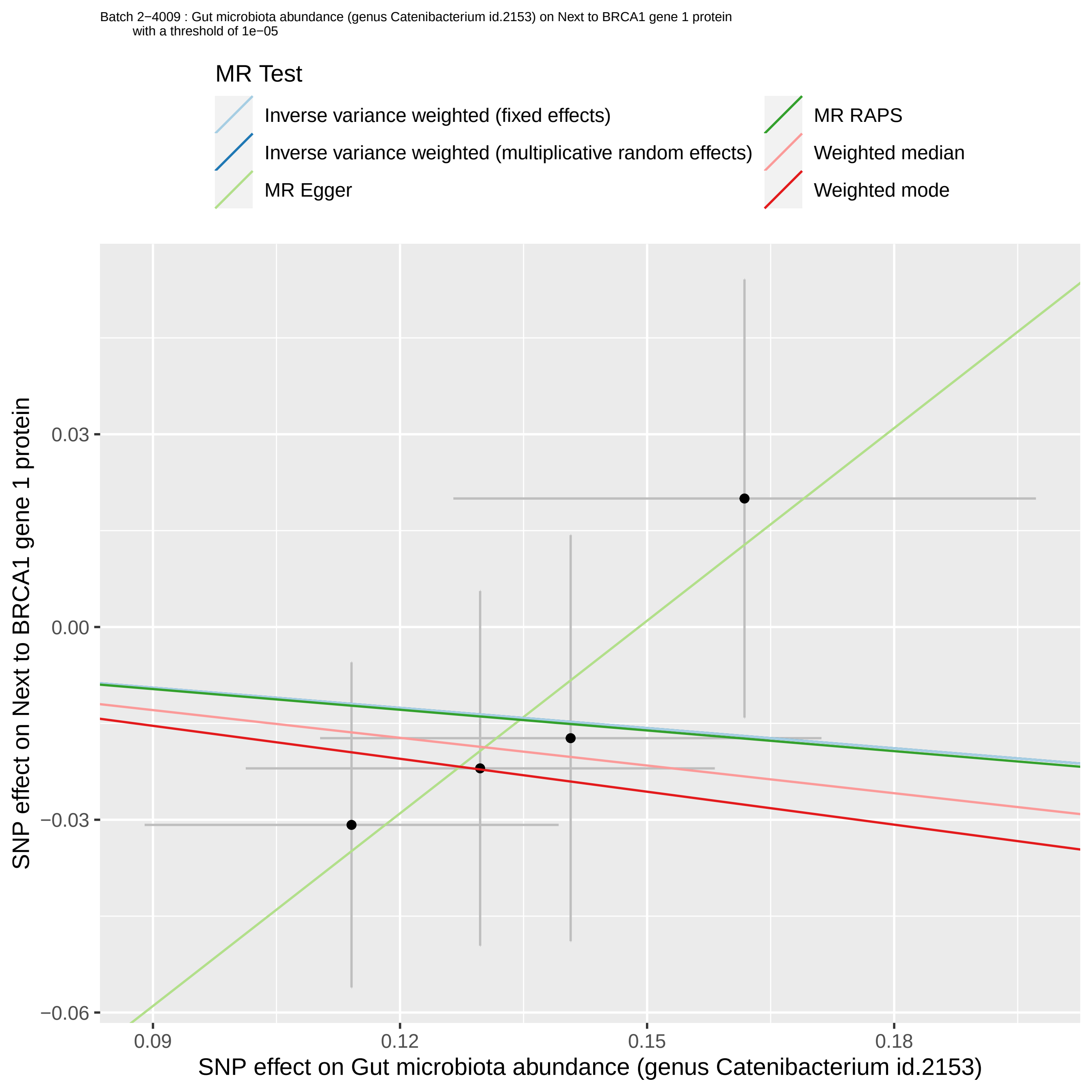
Batch 2−4009 : Gut microbiota abundance (genus Catenibacterium id.2153) on Next to BRCA1 gene 1 protein
with a threshold of 1e−05
MR Test
Inverse variance weighted (fixed effects)
Inverse variance weighted (multiplicative random effects)
MR Egger
MR RAPS
Weighted median
Weighted mode
0.03
0.00
−0.03
−0.06
0.09
0.12
0.15
0.18
SNP effect on Next to BRCA1 gene 1 protein
SNP effect on Gut microbiota abundance (genus Catenibacterium id.2153)

Batch 2−4010 : Gut microbiota abundance (genus Parasutterella id.2892) on Next to BRCA1 gene 1 protein
with a threshold of 1e−05

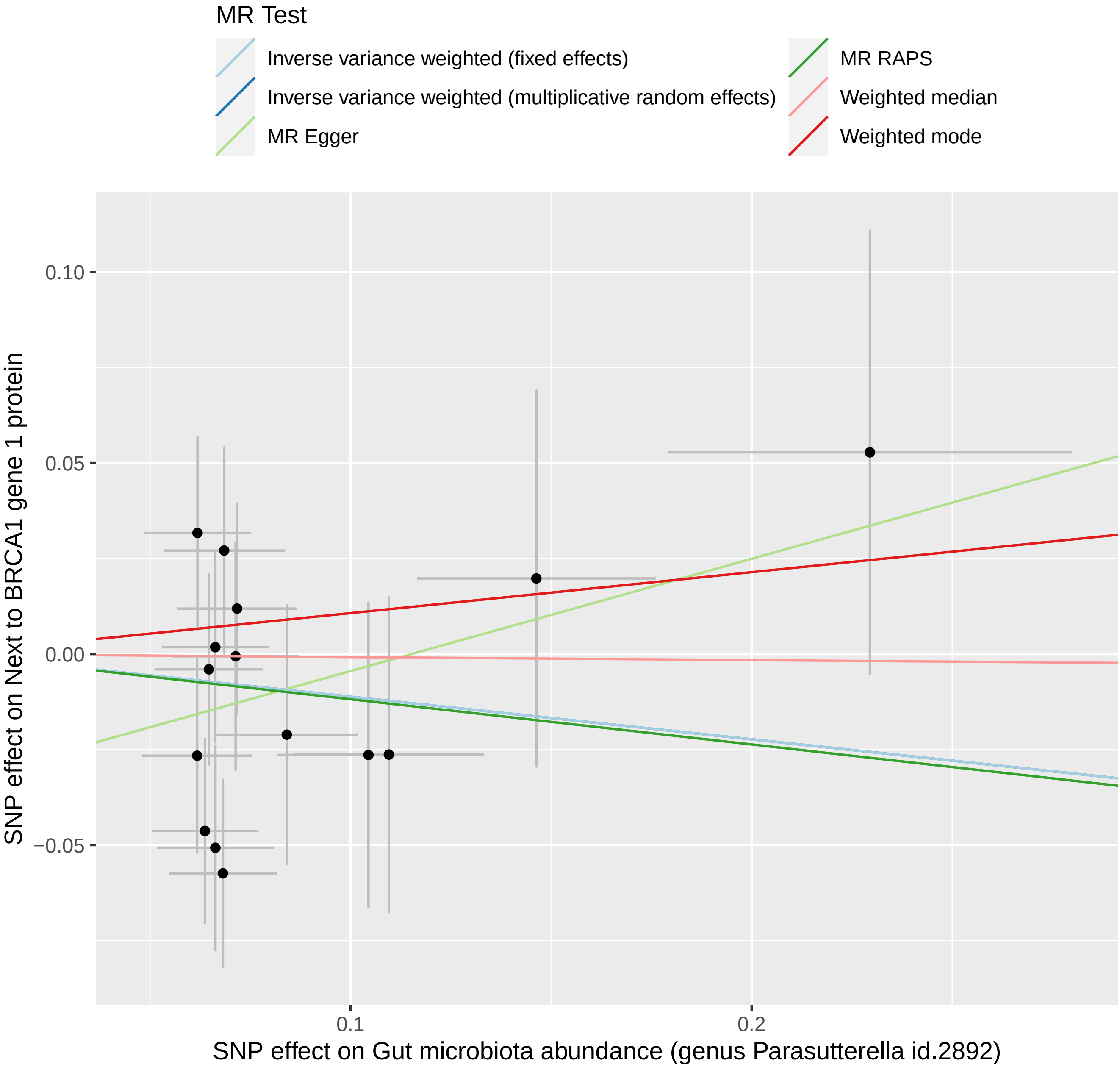

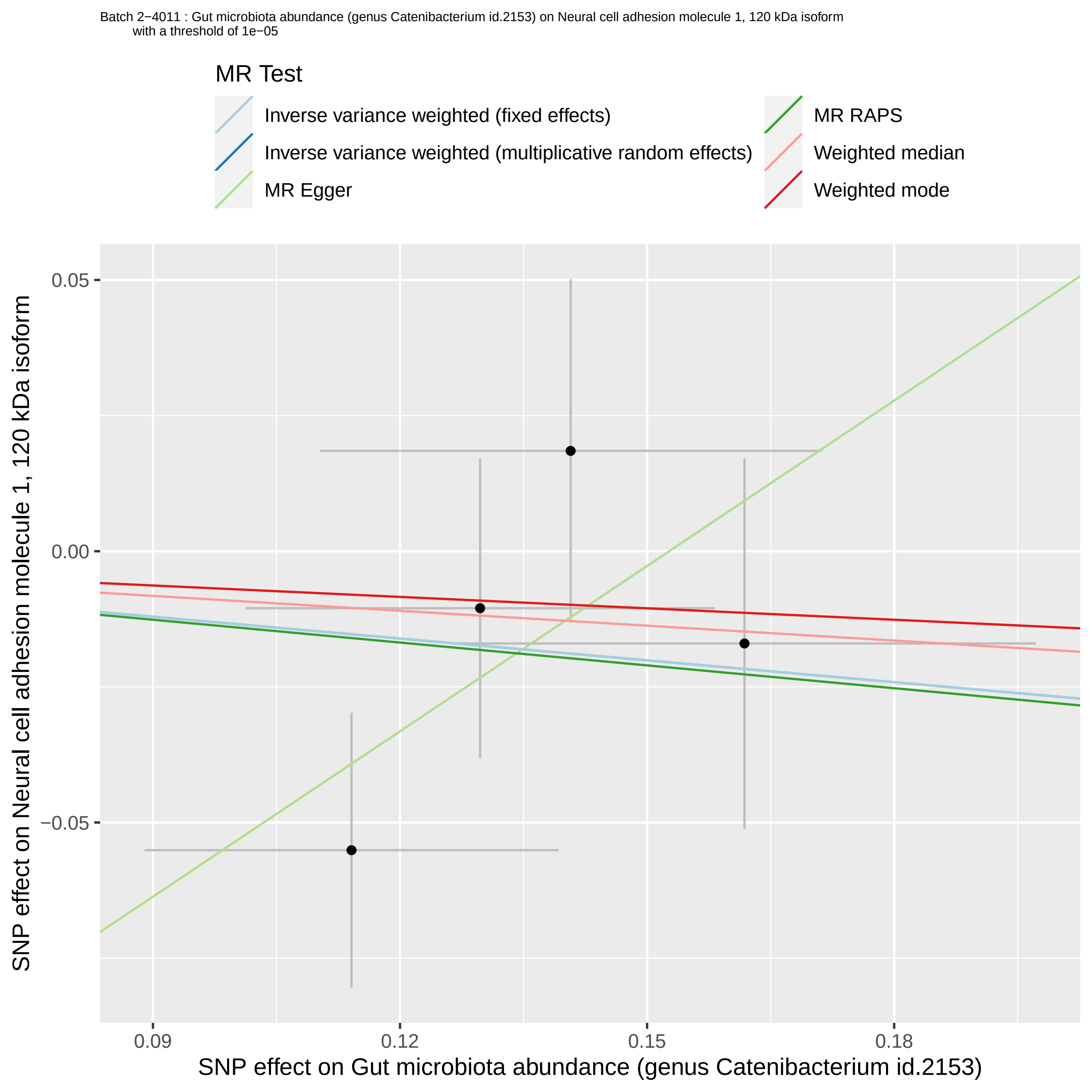

Batch 2−4011 : Gut microbiota abundance (genus Catenibacterium id.2153) on Neural cell adhesion molecule 1, 120 kDa isoform
with a threshold of 1e−05
MR Test
Inverse variance weighted (fixed effects)
Inverse variance weighted (multiplicative random effects)
MR Egger
MR RAPS
Weighted median
Weighted mode
0.05
0.00
−0.05
0.09
0.12
0.15
0.18
SNP effect on Neural cell adhesion molecule 1, 120 kDa isoform
SNP effect on Gut microbiota abundance (genus Catenibacterium id.2153)

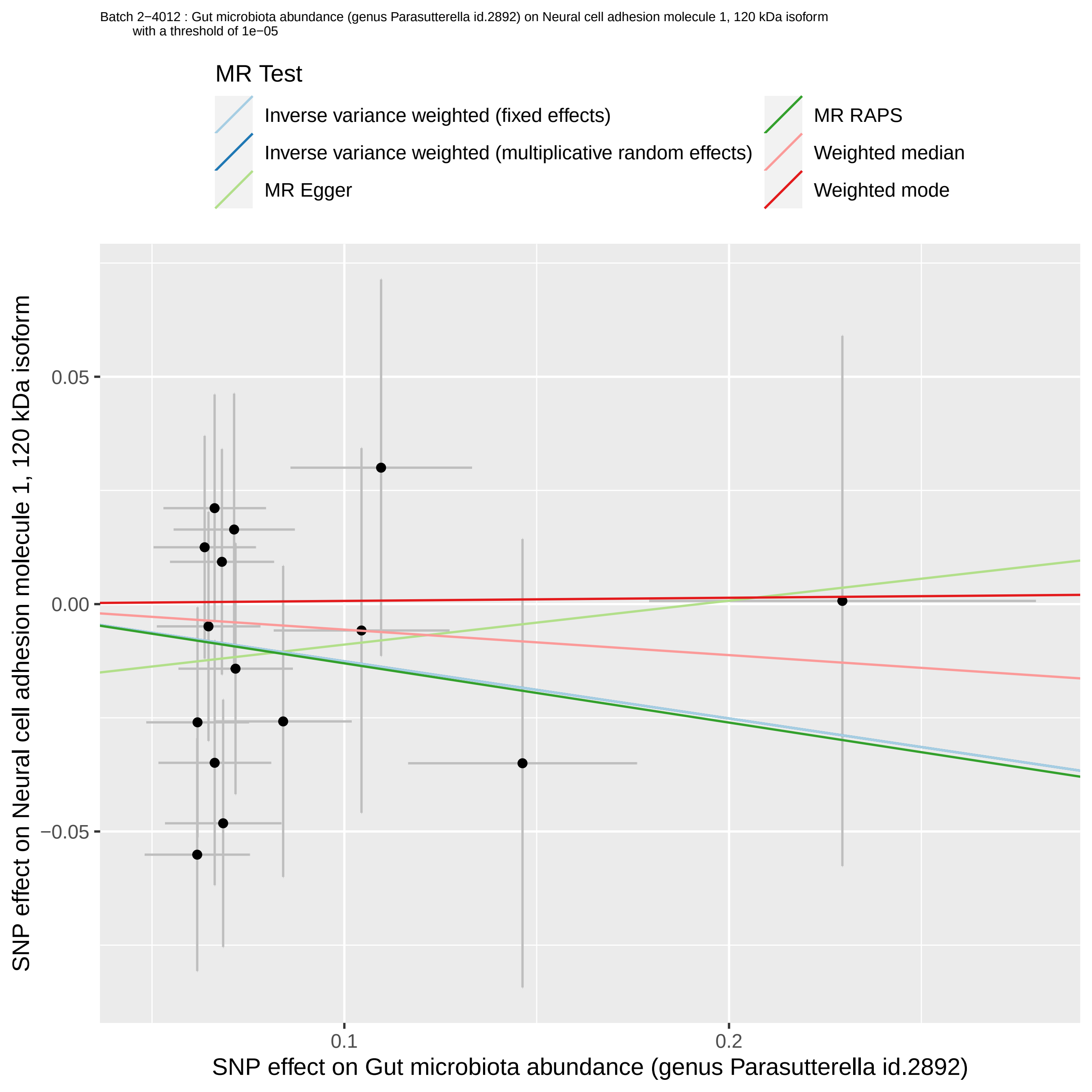
Batch 2−4012 : Gut microbiota abundance (genus Parasutterella id.2892) on Neural cell adhesion molecule 1, 120 kDa isoform
with a threshold of 1e−05
MR Test
Inverse variance weighted (fixed effects)
Inverse variance weighted (multiplicative random effects)
MR Egger
MR RAPS
Weighted median
Weighted mode
0.05
0.00
−0.05
0.1
0.2
SNP effect on Neural cell adhesion molecule 1, 120 kDa isoform
SNP effect on Gut microbiota abundance (genus Parasutterella id.2892)

Batch 2−4013 : Gut microbiota abundance (genus Catenibacterium id.2153) on Neural cell adhesion molecule 1
with a threshold of 1e−05

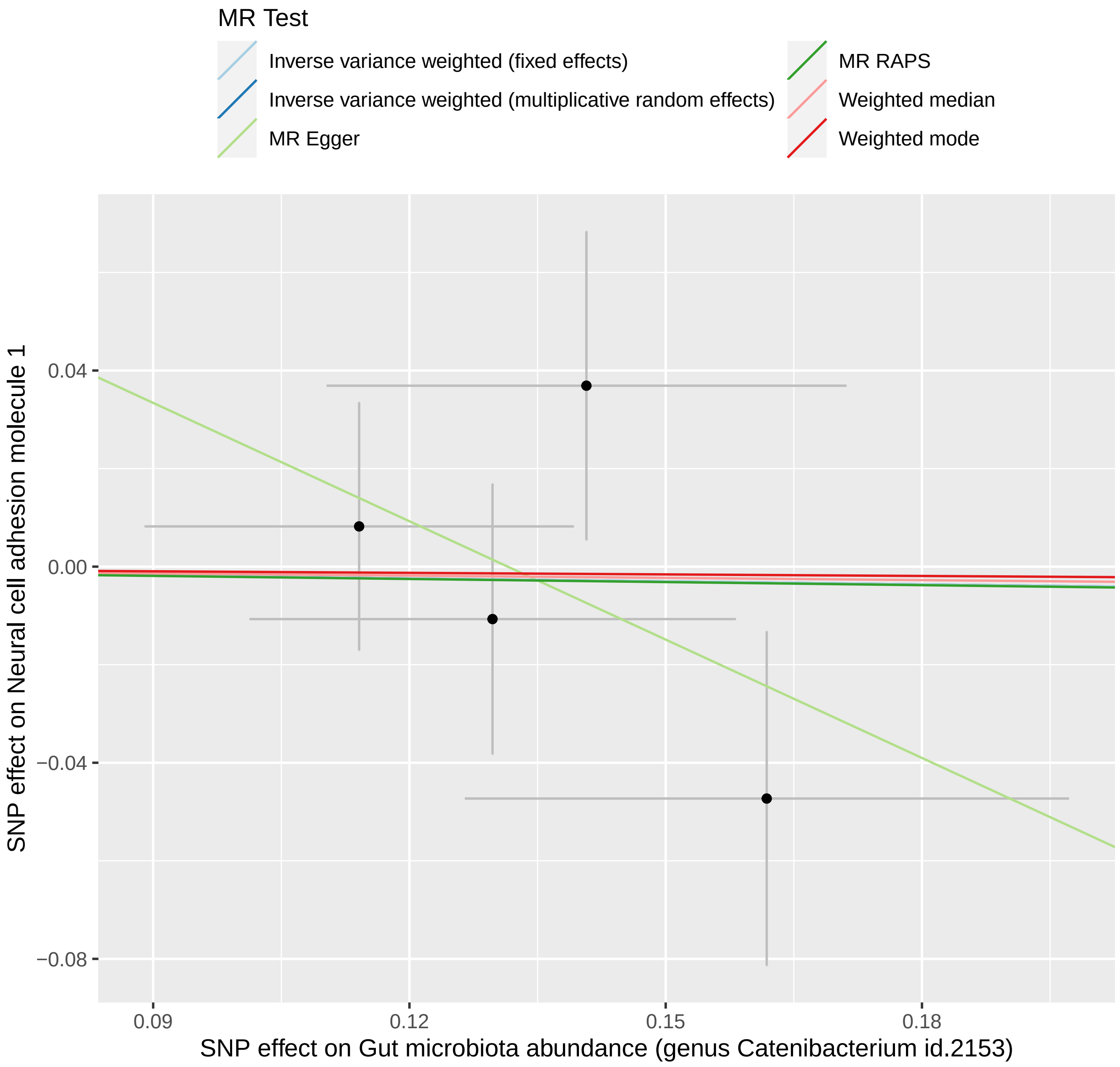

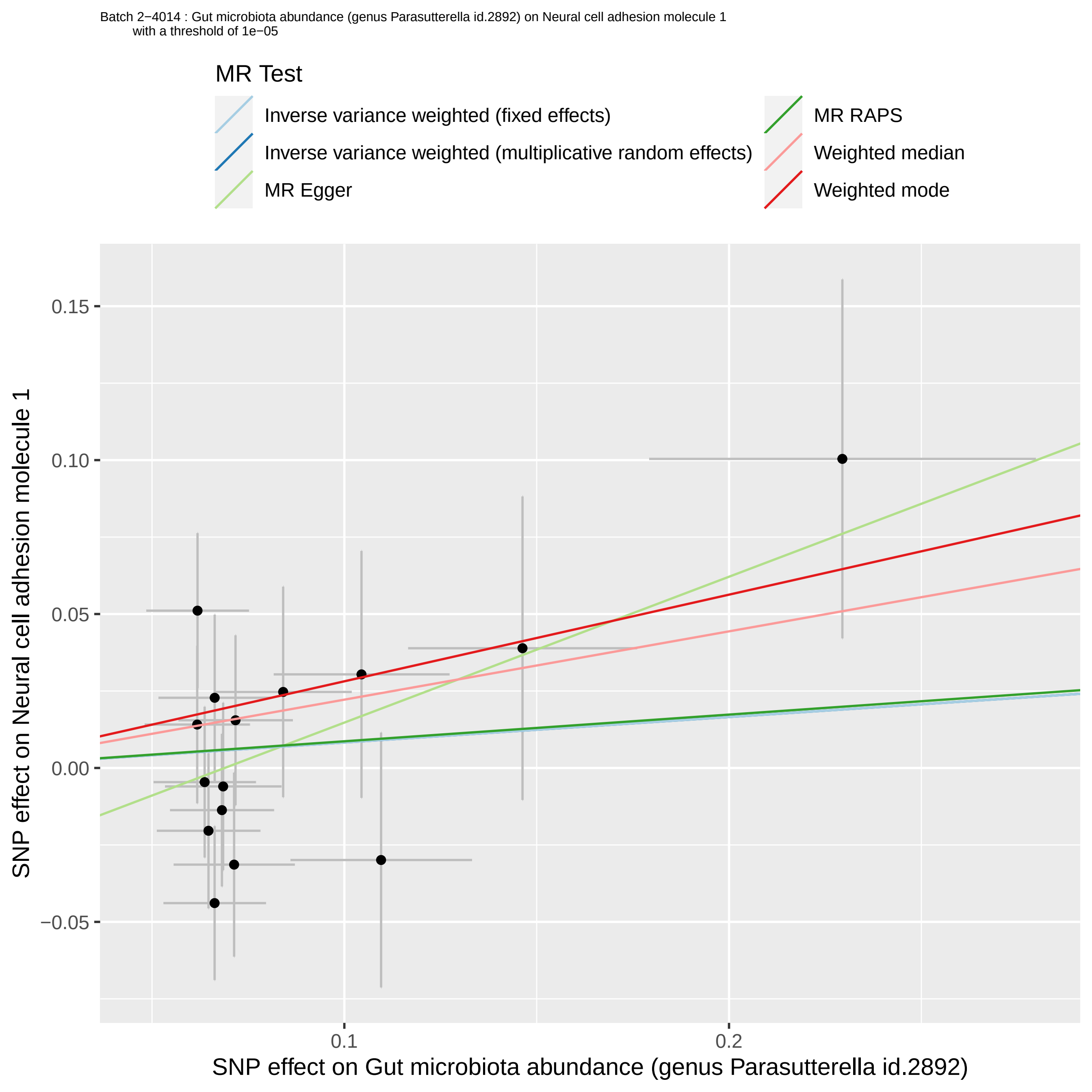

Batch 2−4014 : Gut microbiota abundance (genus Parasutterella id.2892) on Neural cell adhesion molecule 1
with a threshold of 1e−05
MR Test
Inverse variance weighted (fixed effects)
Inverse variance weighted (multiplicative random effects)
MR Egger
MR RAPS
Weighted median
Weighted mode
0.15
0.10
0.05
0.00
−0.05
0.1
0.2
SNP effect on Neural cell adhesion molecule 1
SNP effect on Gut microbiota abundance (genus Parasutterella id.2892)

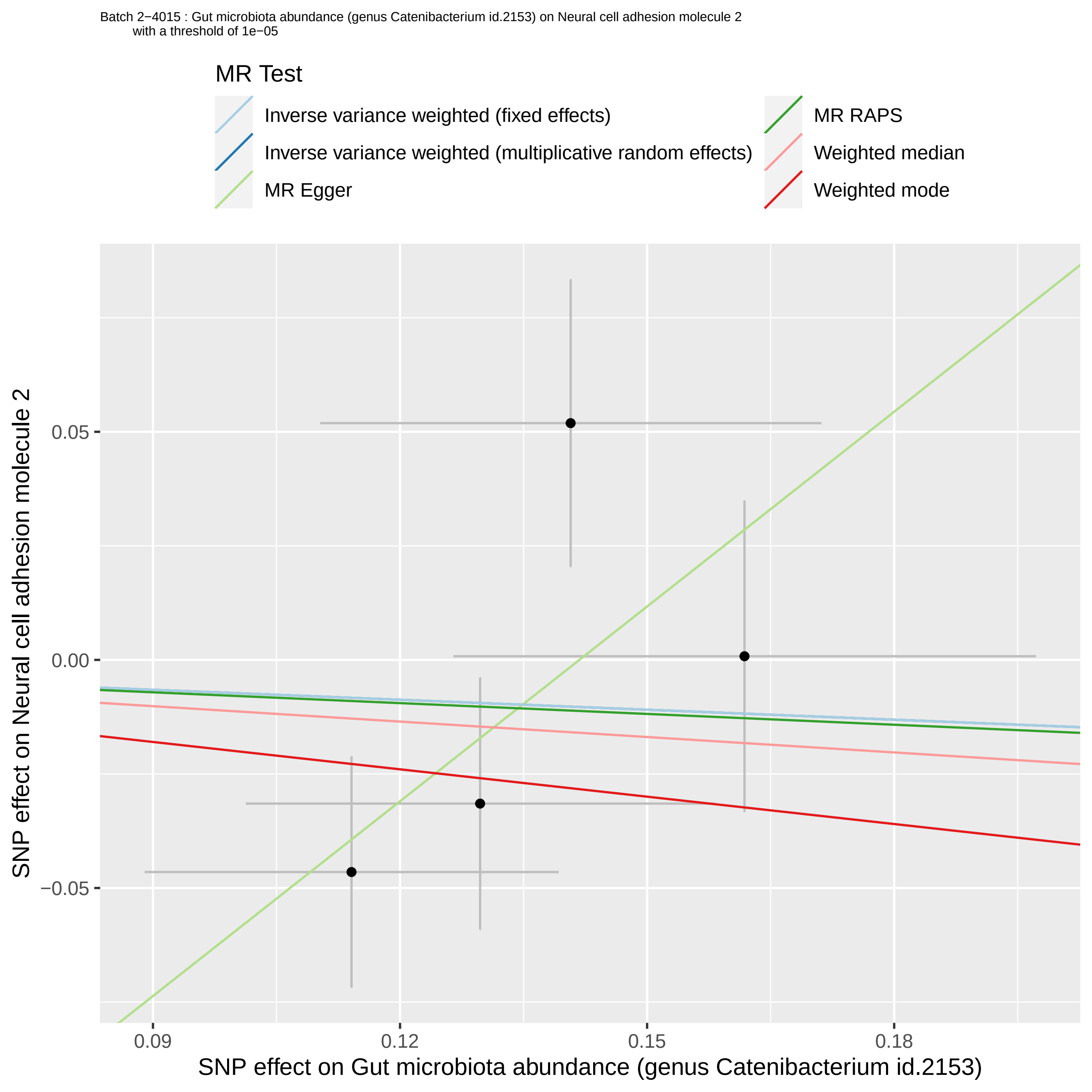
Batch 2−4015 : Gut microbiota abundance (genus Catenibacterium id.2153) on Neural cell adhesion molecule 2
with a threshold of 1e−05
MR Test
Inverse variance weighted (fixed effects)
Inverse variance weighted (multiplicative random effects)
MR Egger
MR RAPS
Weighted median
Weighted mode
0.05
0.00
−0.05
0.09
0.12
0.15
0.18
SNP effect on Neural cell adhesion molecule 2
SNP effect on Gut microbiota abundance (genus Catenibacterium id.2153)

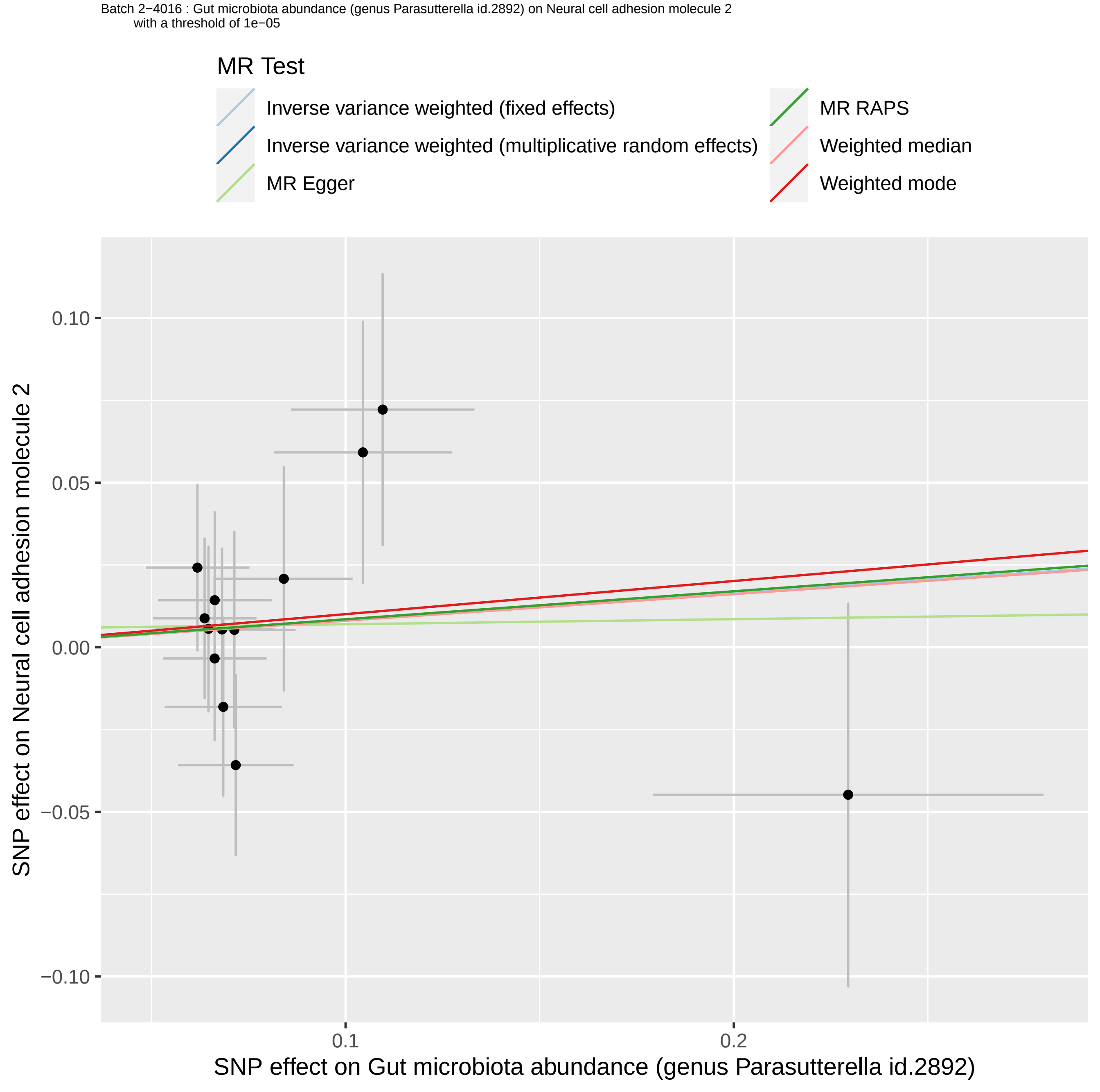

Batch 2−4016 : Gut microbiota abundance (genus Parasutterella id.2892) on Neural cell adhesion molecule 2
with a threshold of 1e−05
MR Test
Inverse variance weighted (fixed effects)
Inverse variance weighted (multiplicative random effects)
MR Egger
MR RAPS
Weighted median
Weighted mode
0.10
0.05
0.00
−0.05
−0.10
0.1
0.2
SNP effect on Neural cell adhesion molecule 2
SNP effect on Gut microbiota abundance (genus Parasutterella id.2892)

Batch 2−4017 : Gut microbiota abundance (genus Catenibacterium id.2153) on Neutrophil cytosol factor 2
with a threshold of 1e−05

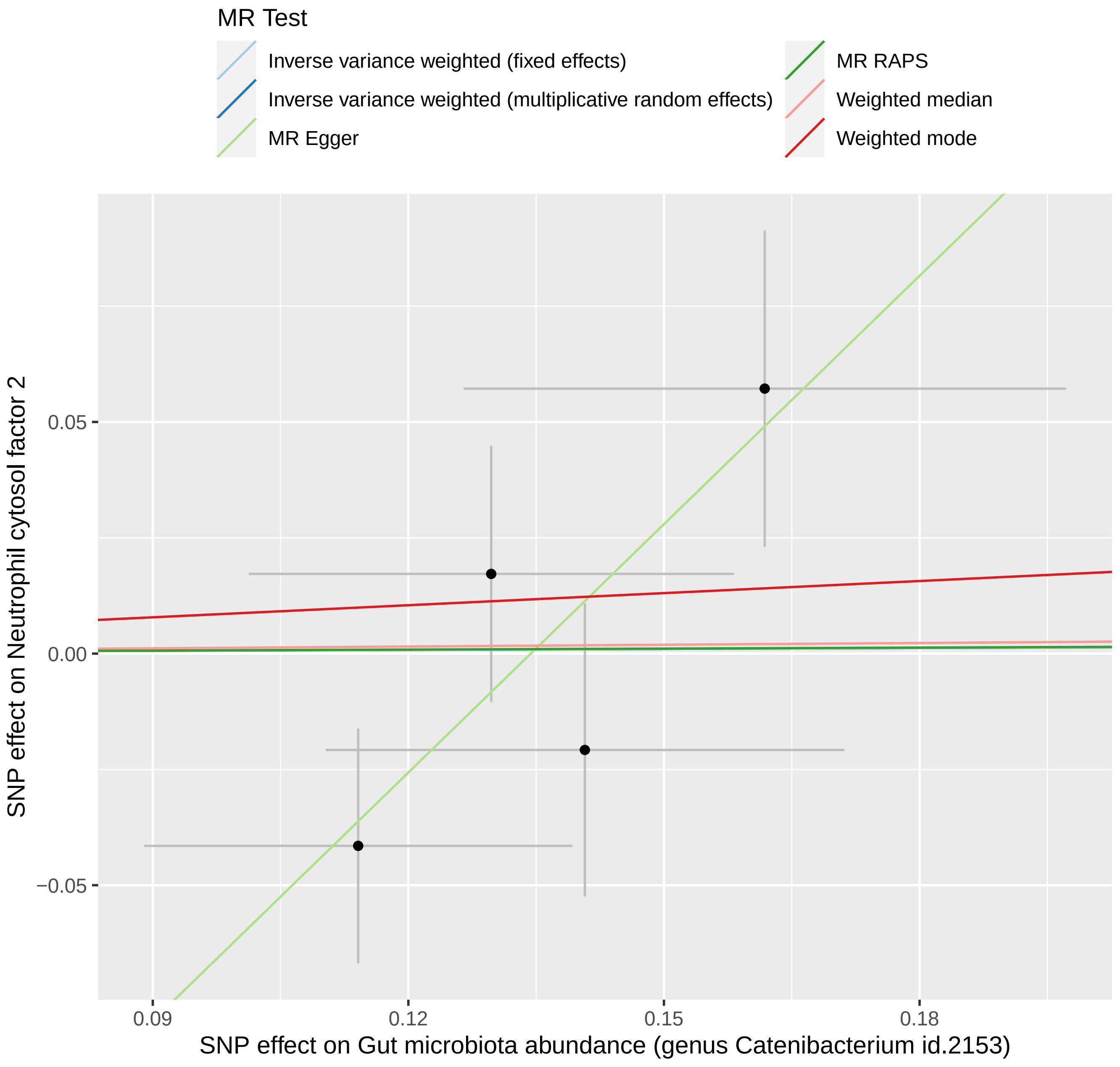

Batch 2−4018 : Gut microbiota abundance (genus Parasutterella id.2892) on Neutrophil cytosol factor 2
with a threshold of 1e−05

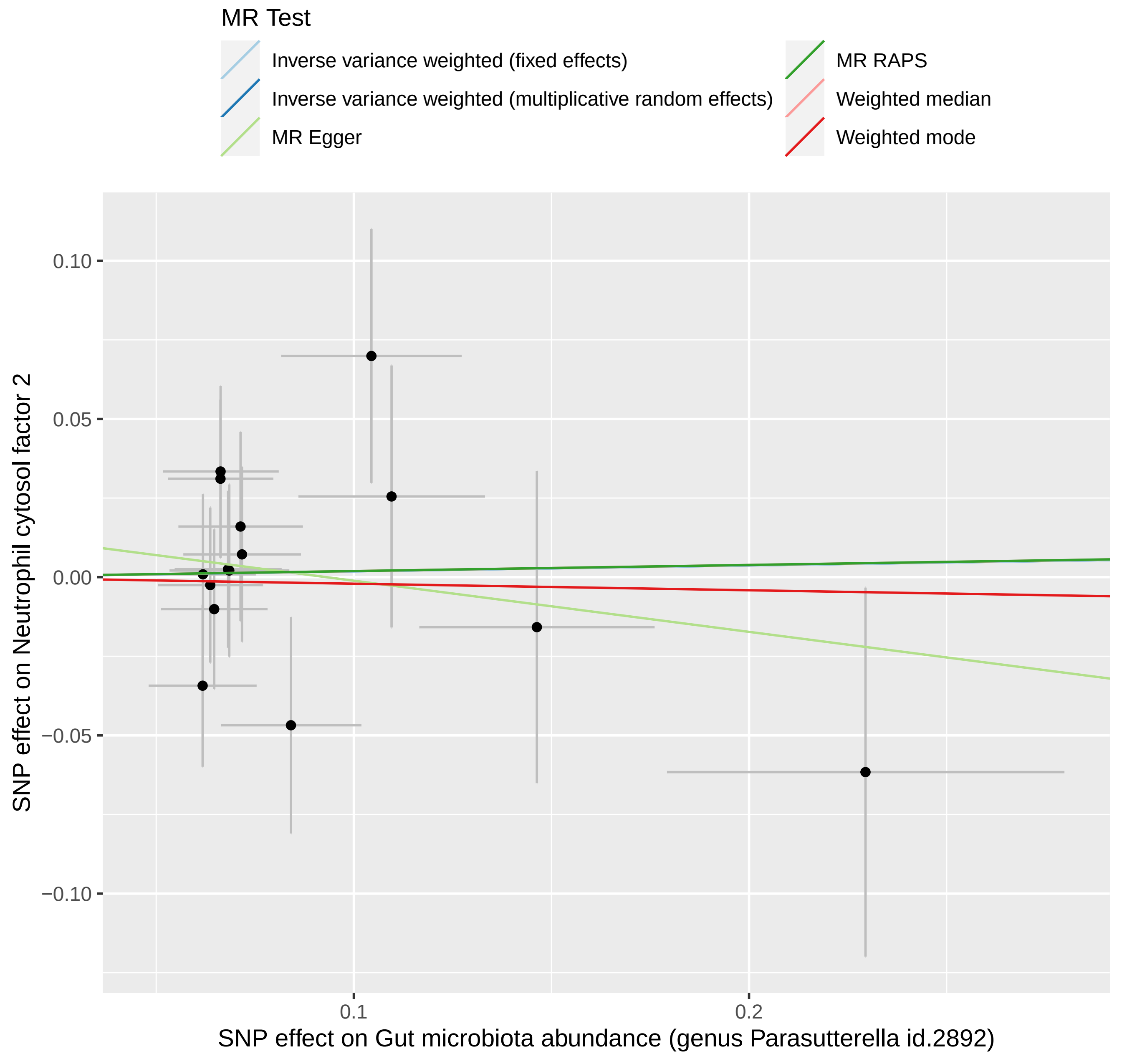

Batch 2−4019 : Gut microbiota abundance (genus Catenibacterium id.2153) on Nucleolin
with a threshold of 1e−05

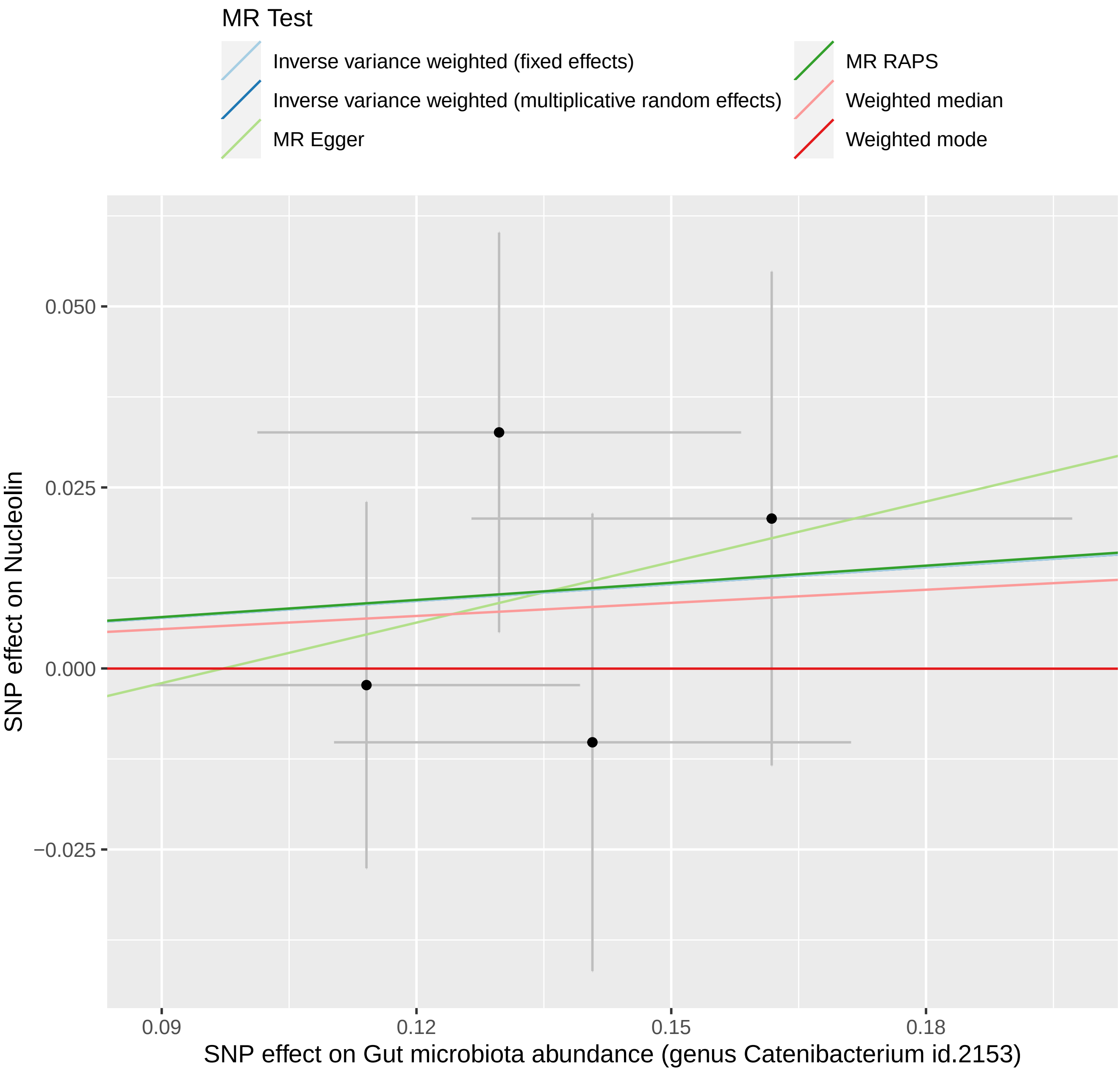

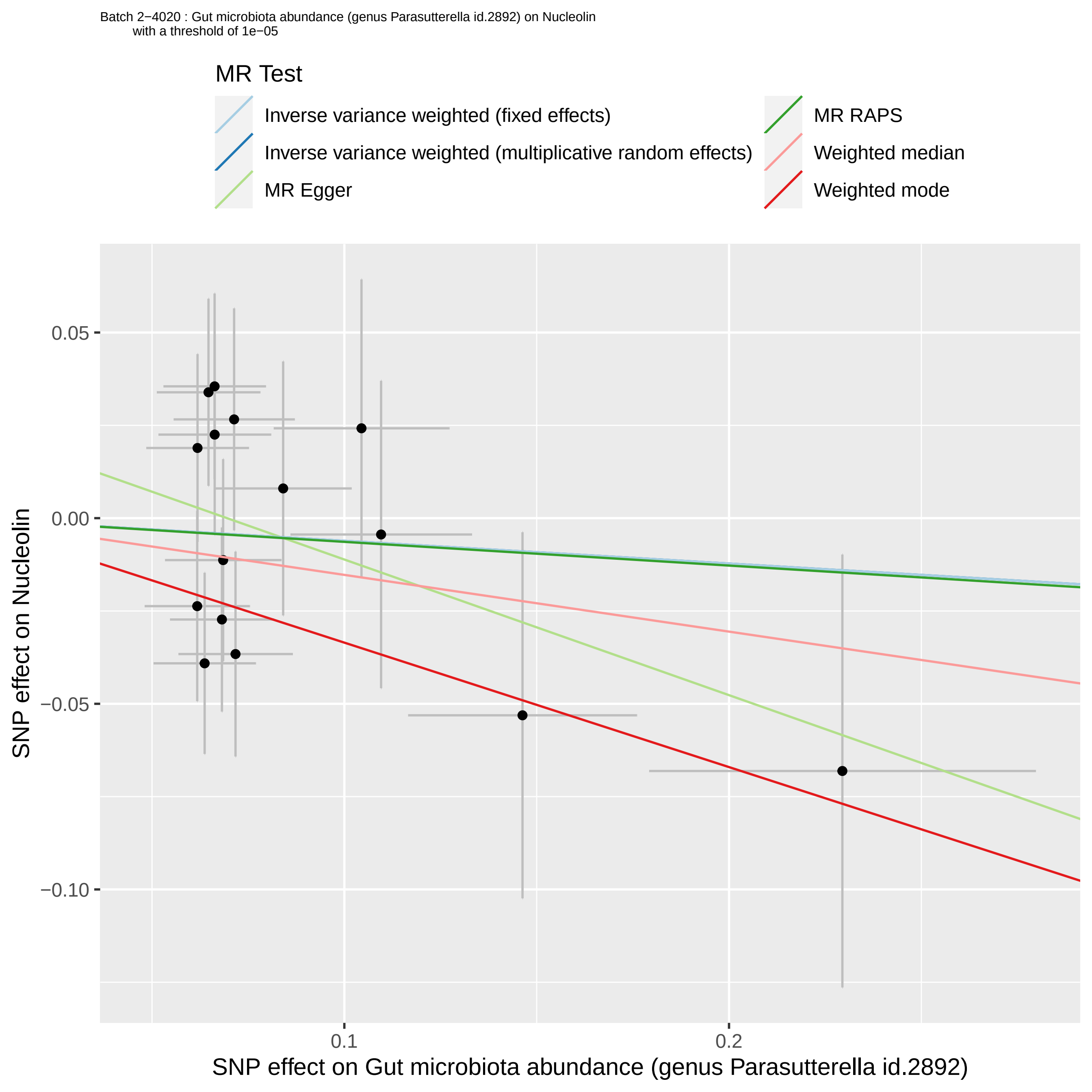

Batch 2−4020 : Gut microbiota abundance (genus Parasutterella id.2892) on Nucleolin
with a threshold of 1e−05
MR Test
Inverse variance weighted (fixed effects)
Inverse variance weighted (multiplicative random effects)
MR Egger
MR RAPS
Weighted median
Weighted mode
0.05
0.00
−0.05
−0.10
0.1
0.2
SNP effect on Nucleolin
SNP effect on Gut microbiota abundance (genus Parasutterella id.2892)

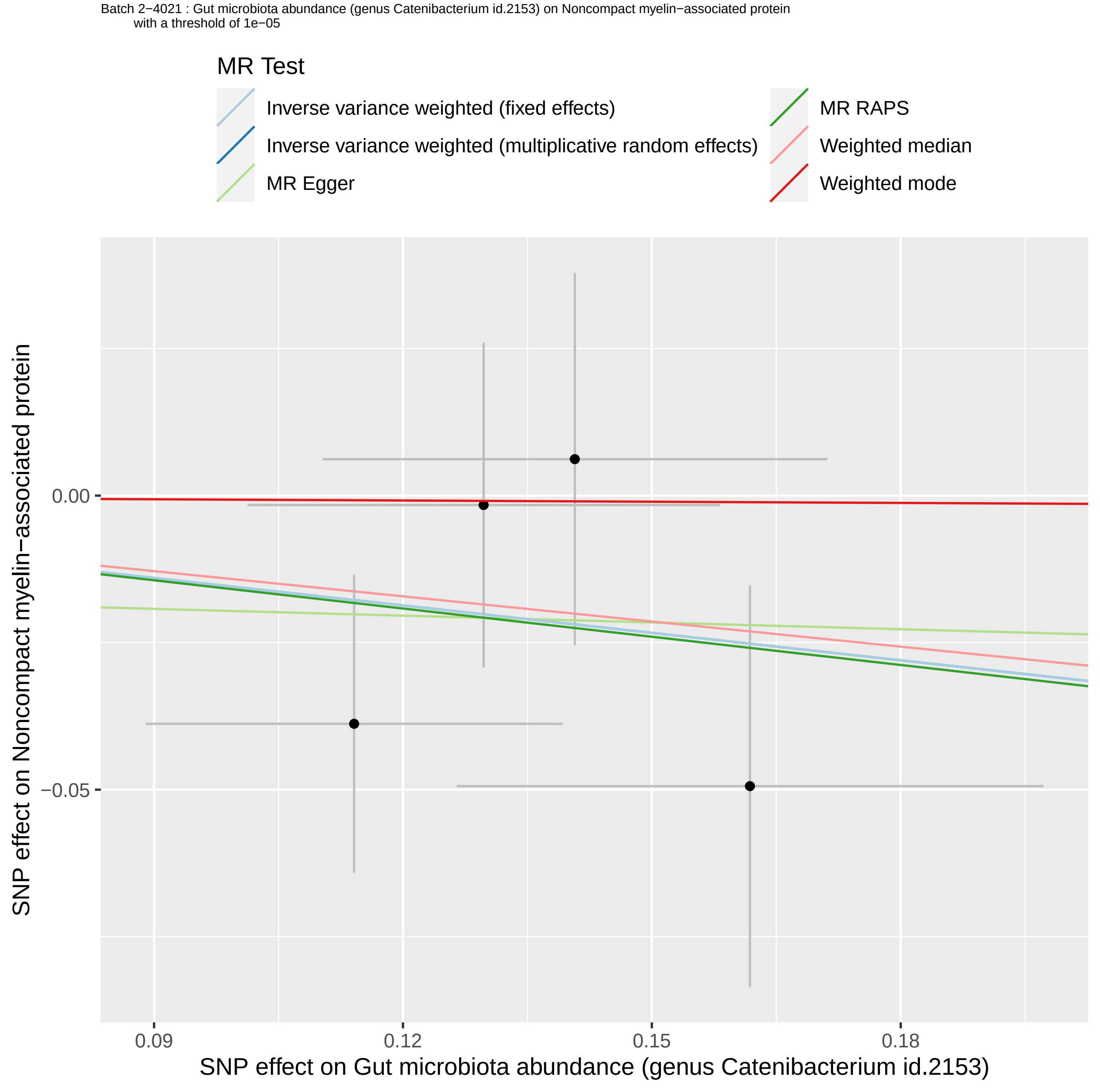
Batch 2−4021 : Gut microbiota abundance (genus Catenibacterium id.2153) on Noncompact myelin−associated protein
with a threshold of 1e−05
MR Test
Inverse variance weighted (fixed effects)
Inverse variance weighted (multiplicative random effects)
MR Egger
MR RAPS
Weighted median
Weighted mode
0.00
−0.05
0.09
0.12
0.15
0.18
SNP effect on Noncompact myelin−associated protein
SNP effect on Gut microbiota abundance (genus Catenibacterium id.2153)

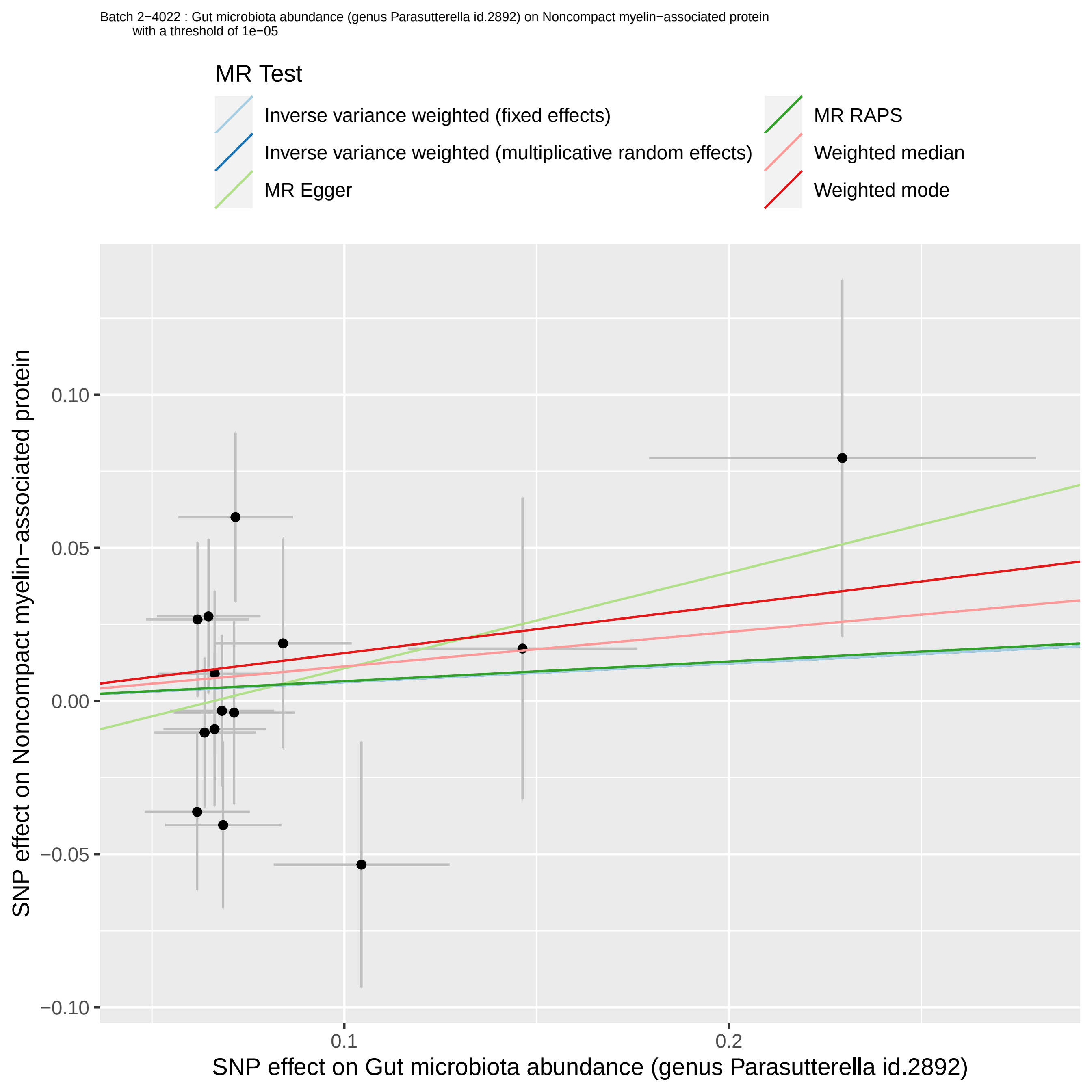

Batch 2−4022 : Gut microbiota abundance (genus Parasutterella id.2892) on Noncompact myelin−associated protein
with a threshold of 1e−05
MR Test
Inverse variance weighted (fixed effects)
Inverse variance weighted (multiplicative random effects)
MR Egger
MR RAPS
Weighted median
Weighted mode
0.10
0.05
0.00
−0.05
−0.10
0.1
0.2
SNP effect on Noncompact myelin−associated protein
SNP effect on Gut microbiota abundance (genus Parasutterella id.2892)

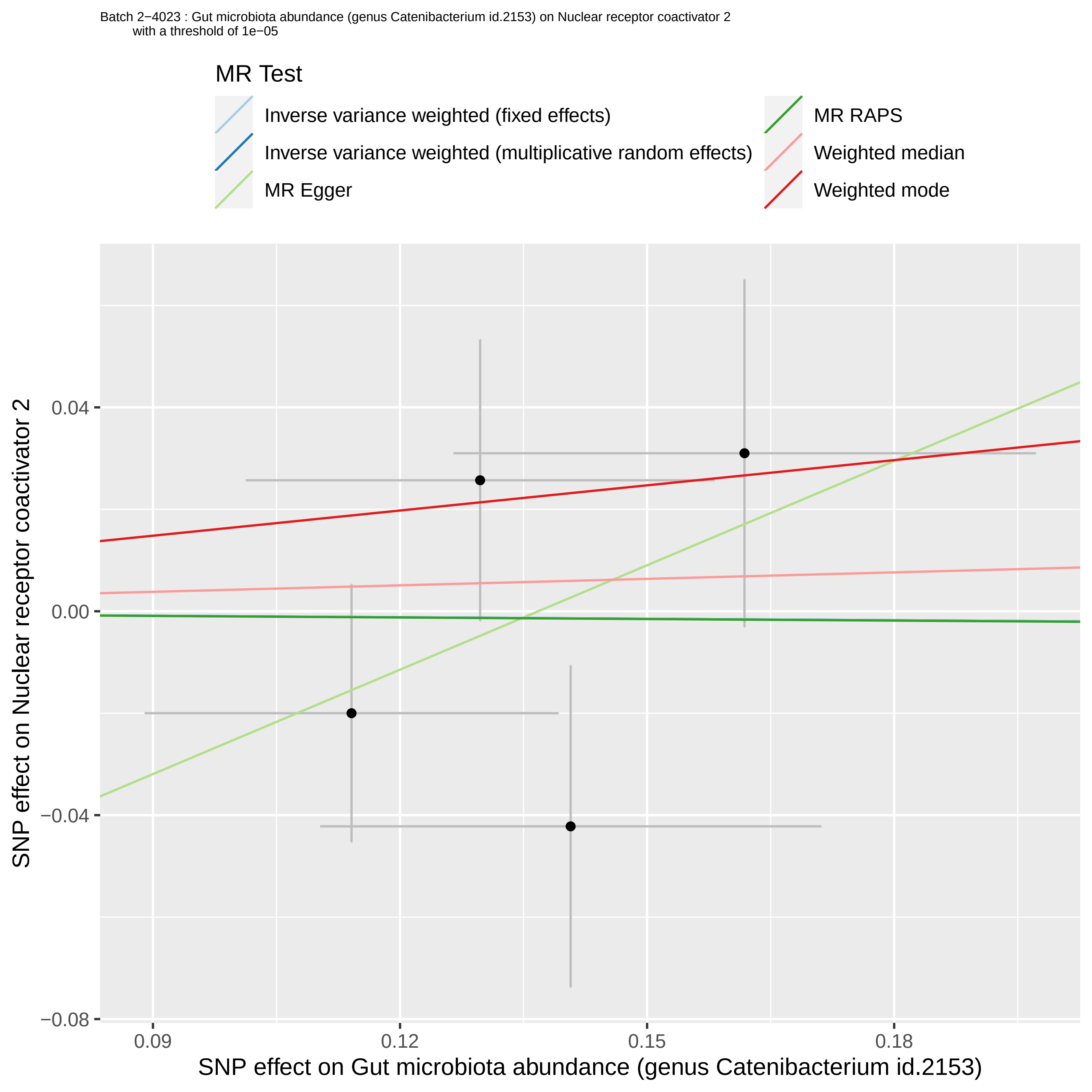

Batch 2−4023 : Gut microbiota abundance (genus Catenibacterium id.2153) on Nuclear receptor coactivator 2
with a threshold of 1e−05
MR Test
Inverse variance weighted (fixed effects)
Inverse variance weighted (multiplicative random effects)
MR Egger
MR RAPS
Weighted median
Weighted mode
SNP effect on Nuclear receptor coactivator 2
0.04
0.00
−0.04
−0.08
0.09
0.12
0.15
0.18
SNP effect on Gut microbiota abundance (genus Catenibacterium id.2153)

Batch 2−4024 : Gut microbiota abundance (genus Parasutterella id.2892) on Nuclear receptor coactivator 2
with a threshold of 1e−05

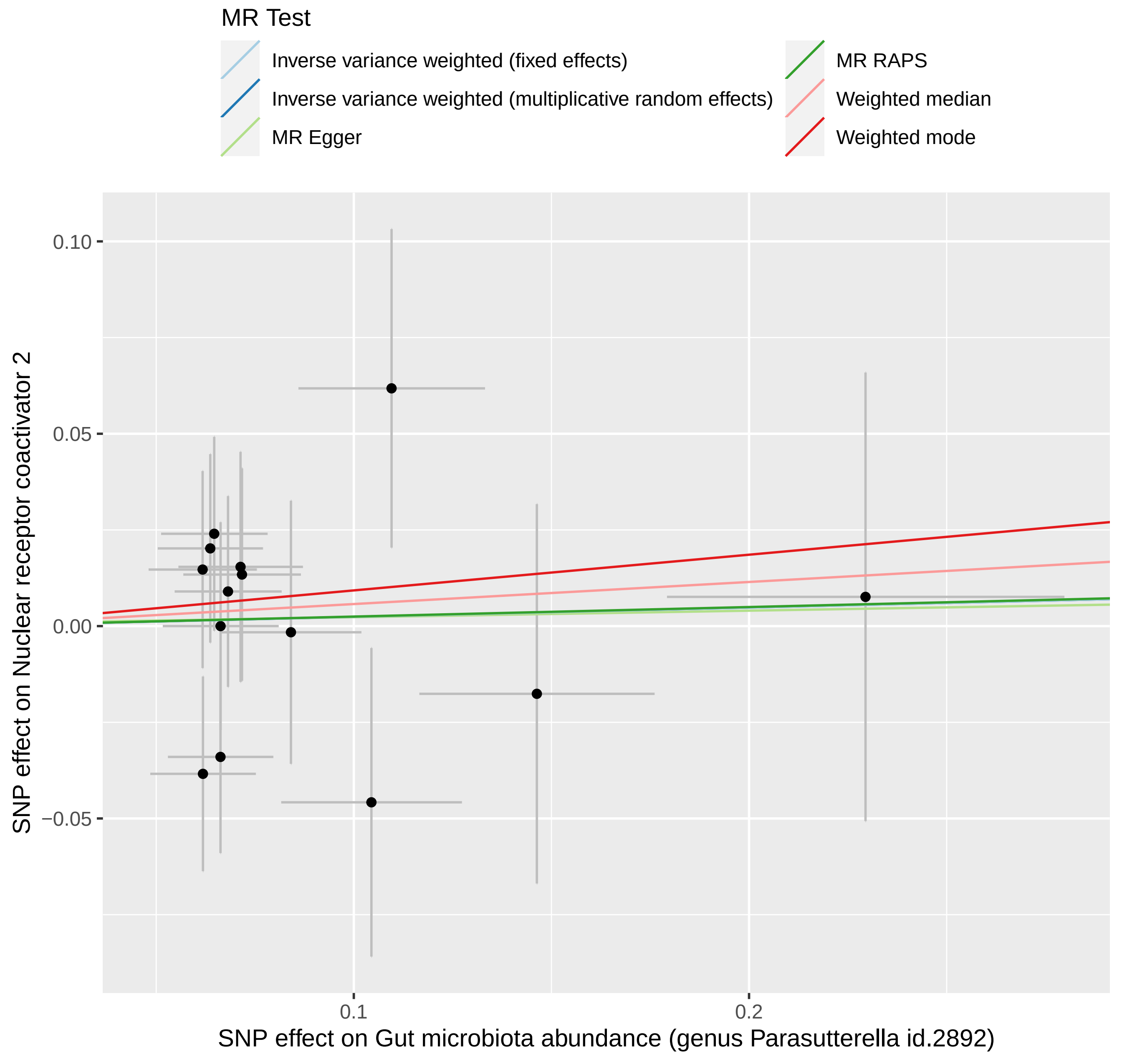

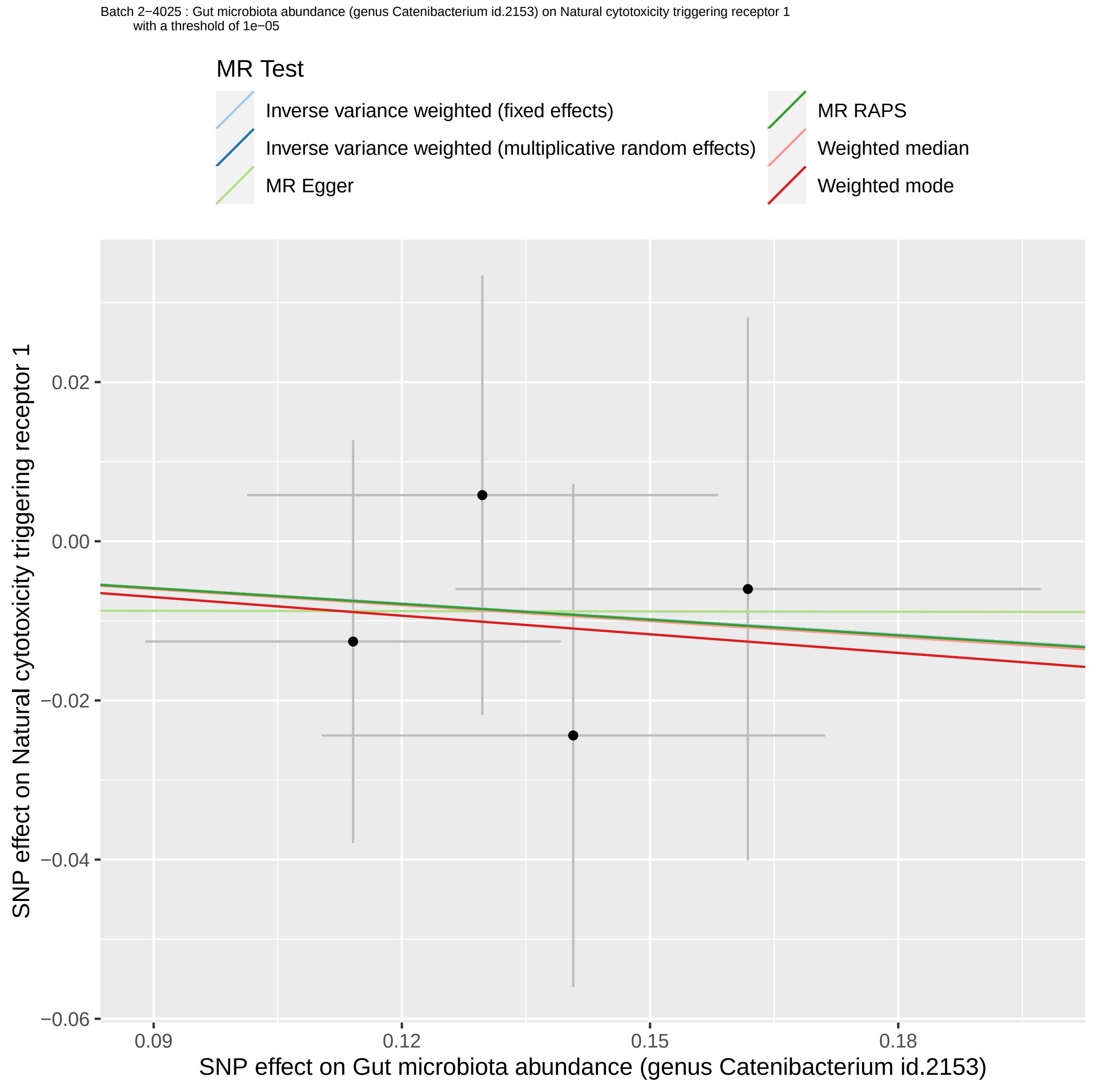

Batch 2−4025 : Gut microbiota abundance (genus Catenibacterium id.2153) on Natural cytotoxicity triggering receptor 1
with a threshold of 1e−05
MR Test
Inverse variance weighted (fixed effects)
Inverse variance weighted (multiplicative random effects)
MR Egger
MR RAPS
Weighted median
Weighted mode
0.02
0.00
−0.02
−0.04
−0.06
0.09
0.12
0.15
0.18
SNP effect on Natural cytotoxicity triggering receptor 1
SNP effect on Gut microbiota abundance (genus Catenibacterium id.2153)

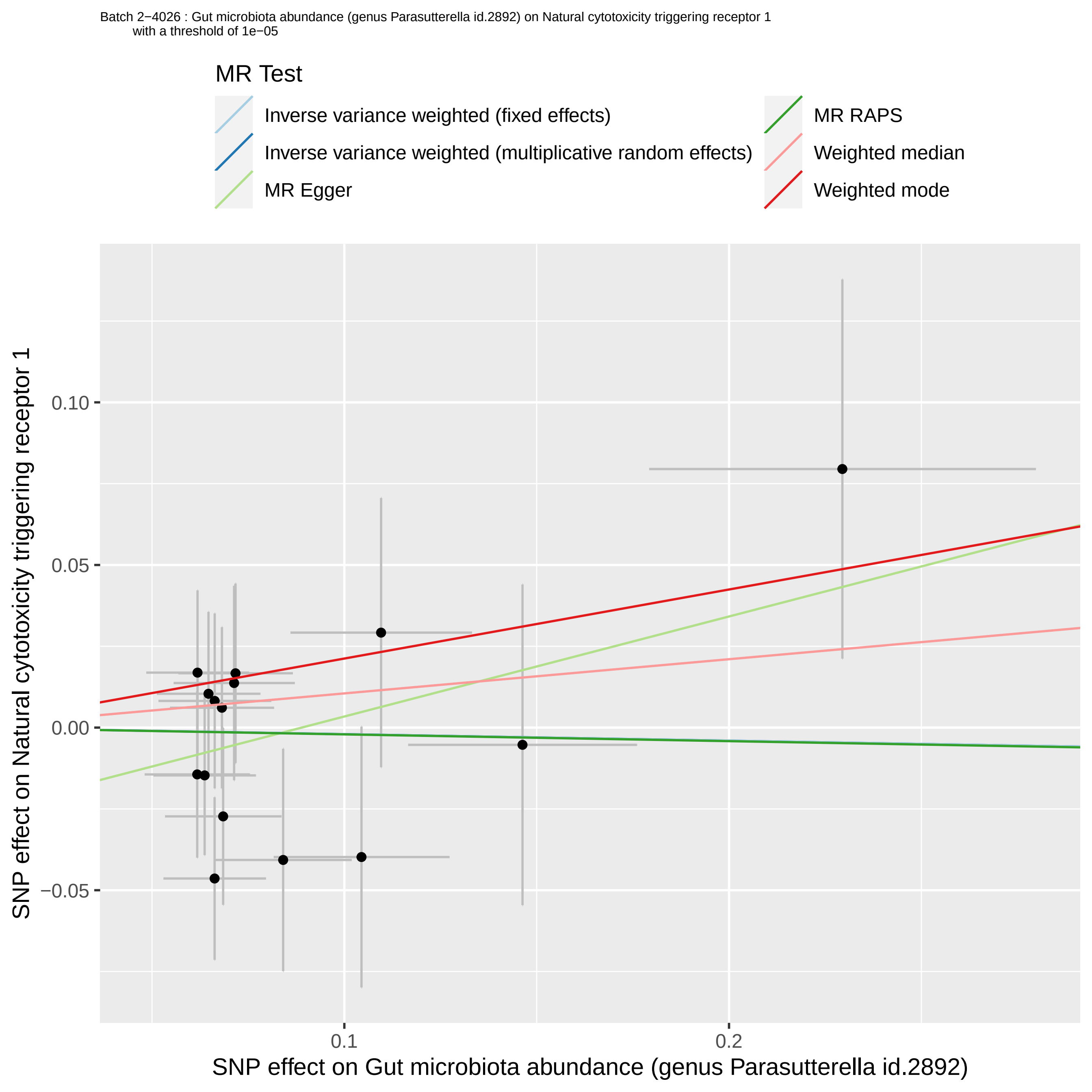

Batch 2−4026 : Gut microbiota abundance (genus Parasutterella id.2892) on Natural cytotoxicity triggering receptor 1
with a threshold of 1e−05
MR Test
Inverse variance weighted (fixed effects)
Inverse variance weighted (multiplicative random effects)
MR Egger
MR RAPS
Weighted median
Weighted mode
SNP effect on Natural cytotoxicity triggering receptor 1
0.10
0.05
0.00
−0.05
0.1
0.2
SNP effect on Gut microbiota abundance (genus Parasutterella id.2892)

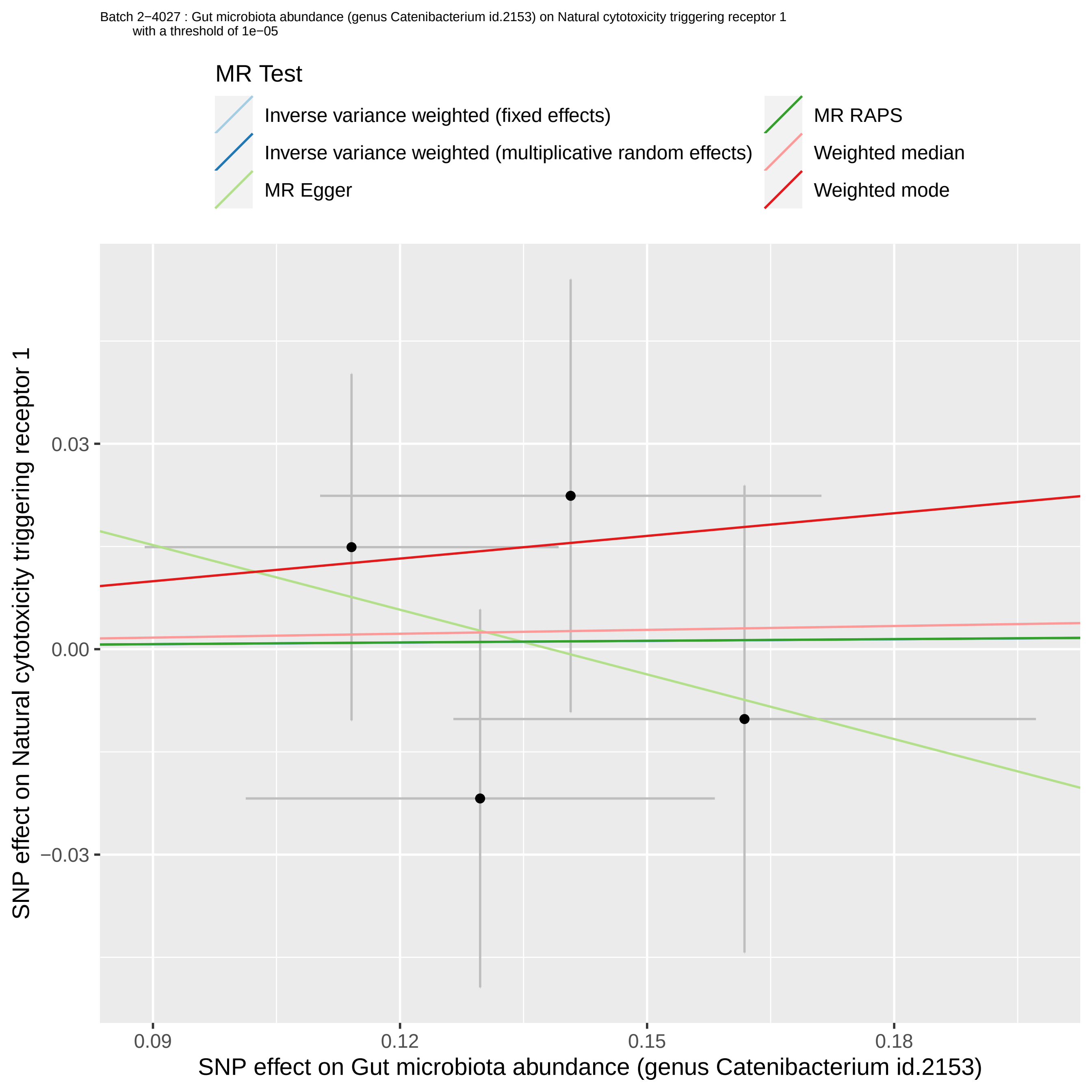
Batch 2−4027 : Gut microbiota abundance (genus Catenibacterium id.2153) on Natural cytotoxicity triggering receptor 1
with a threshold of 1e−05
MR Test
Inverse variance weighted (fixed effects)
Inverse variance weighted (multiplicative random effects)
MR Egger
MR RAPS
Weighted median
Weighted mode
SNP effect on Natural cytotoxicity triggering receptor 1
0.03
0.00
−0.03
0.09
0.12
0.15
0.18
SNP effect on Gut microbiota abundance (genus Catenibacterium id.2153)

Batch 2−4028 : Gut microbiota abundance (genus Parasutterella id.2892) on Natural cytotoxicity triggering receptor 1
with a threshold of 1e−05

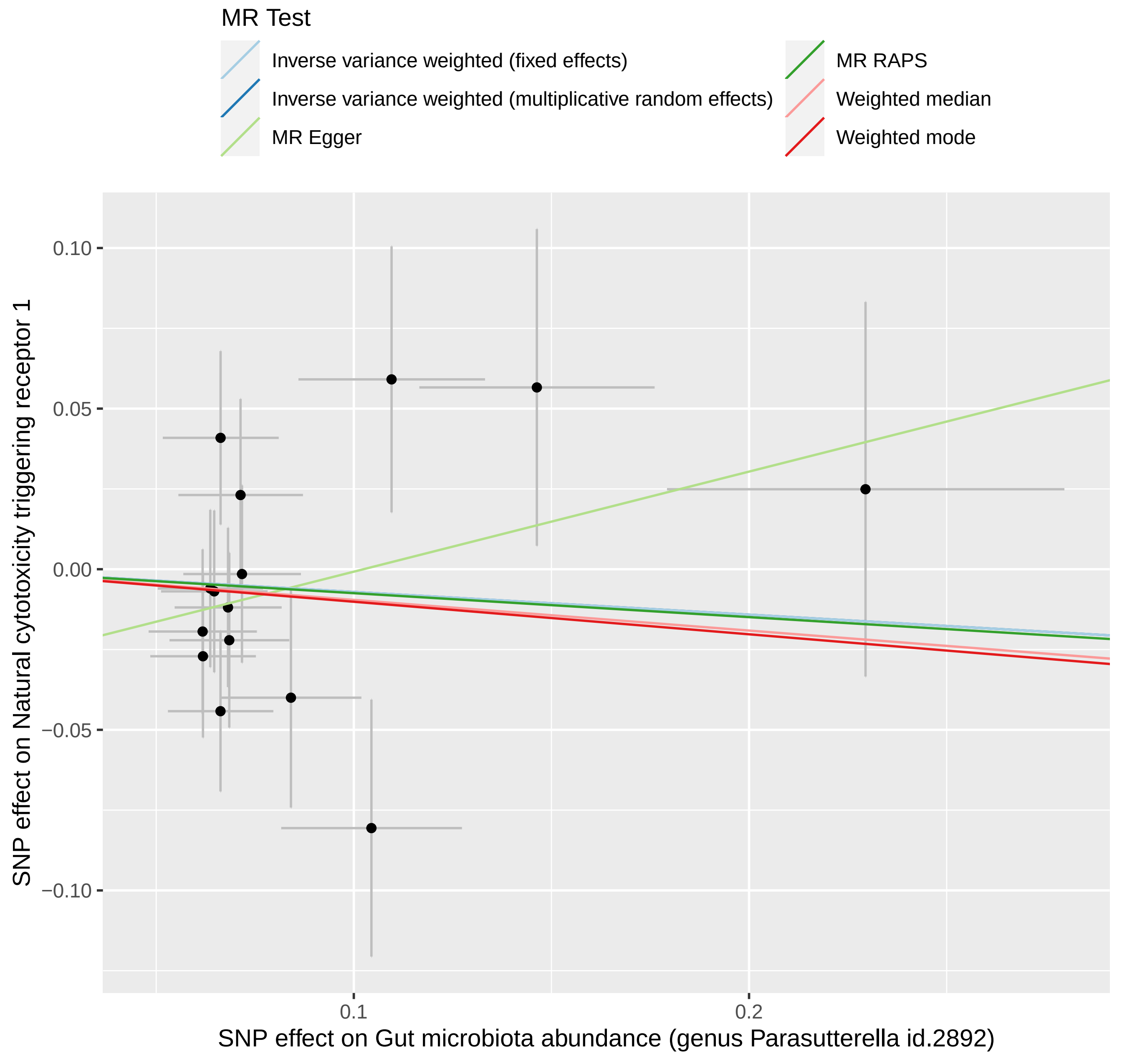

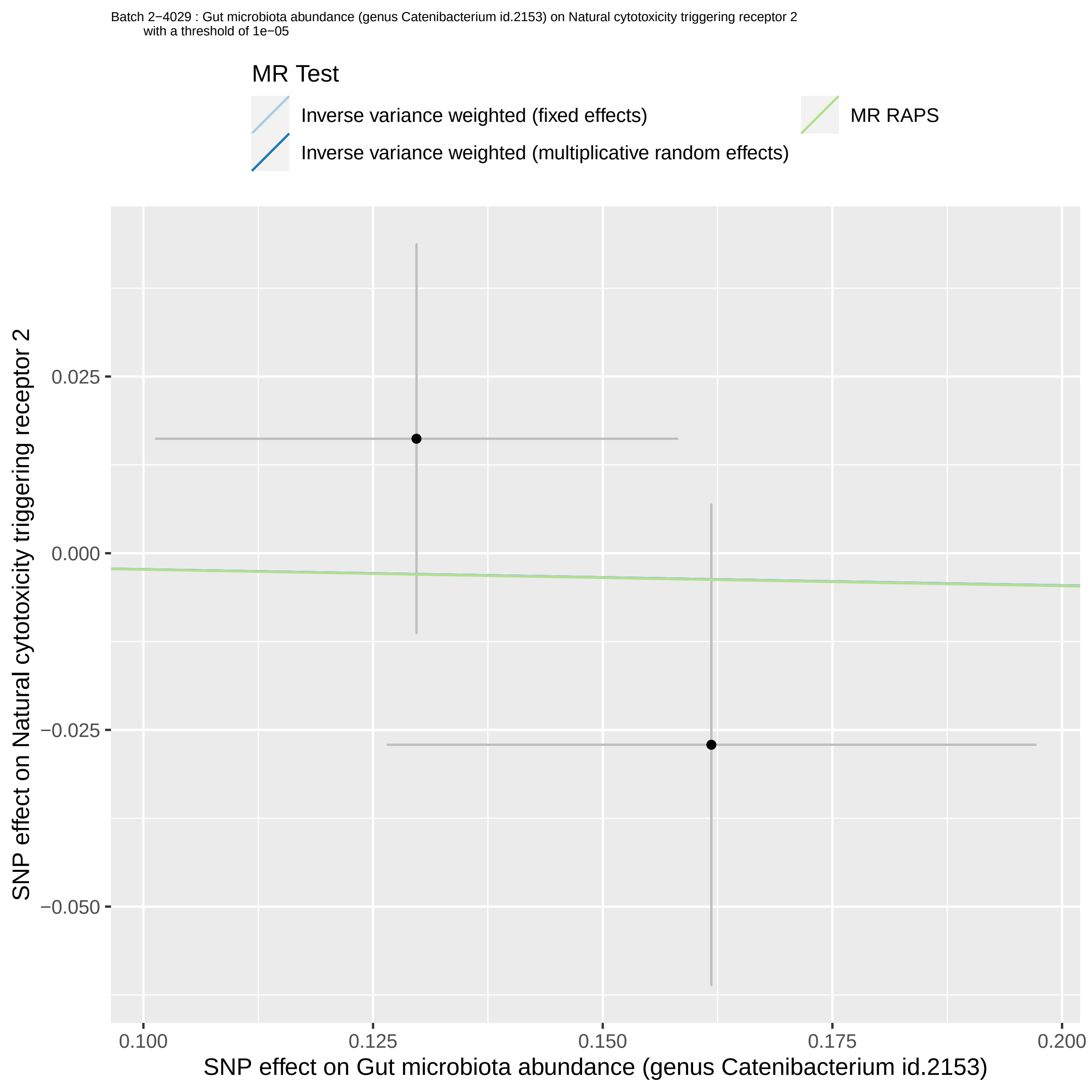

Batch 2−4029 : Gut microbiota abundance (genus Catenibacterium id.2153) on Natural cytotoxicity triggering receptor 2
with a threshold of 1e−05
MR Test
Inverse variance weighted (fixed effects)
Inverse variance weighted (multiplicative random effects)
MR RAPS
SNP effect on Natural cytotoxicity triggering receptor 2
0.025
0.000
−0.025
−0.050
0.100
0.125
0.150
0.175
0.200
SNP effect on Gut microbiota abundance (genus Catenibacterium id.2153)

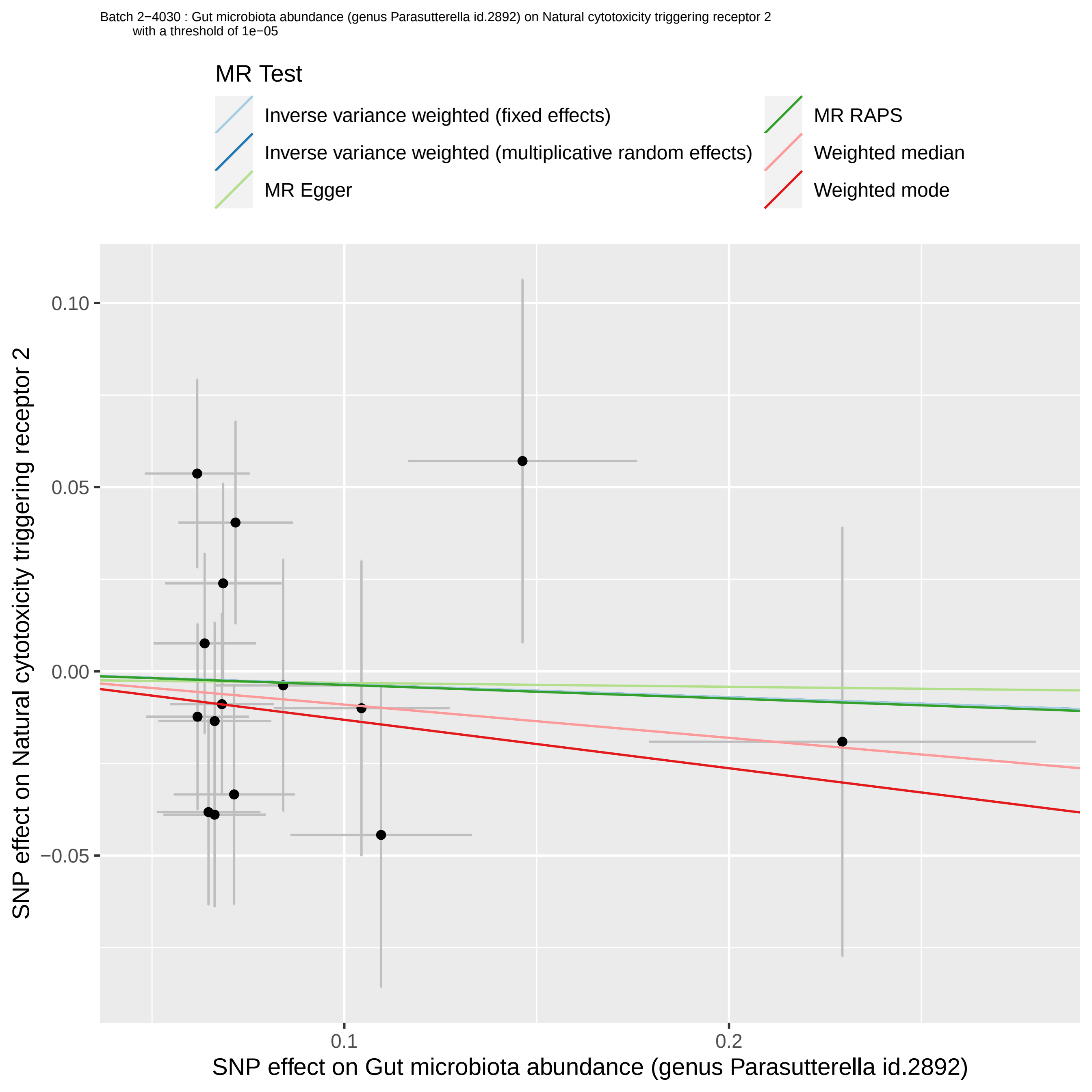
Batch 2−4030 : Gut microbiota abundance (genus Parasutterella id.2892) on Natural cytotoxicity triggering receptor 2
with a threshold of 1e−05
MR Test
Inverse variance weighted (fixed effects)
Inverse variance weighted (multiplicative random effects)
MR Egger
MR RAPS
Weighted median
Weighted mode
0.10
0.05
0.00
−0.05
0.1
0.2
SNP effect on Natural cytotoxicity triggering receptor 2
SNP effect on Gut microbiota abundance (genus Parasutterella id.2892)

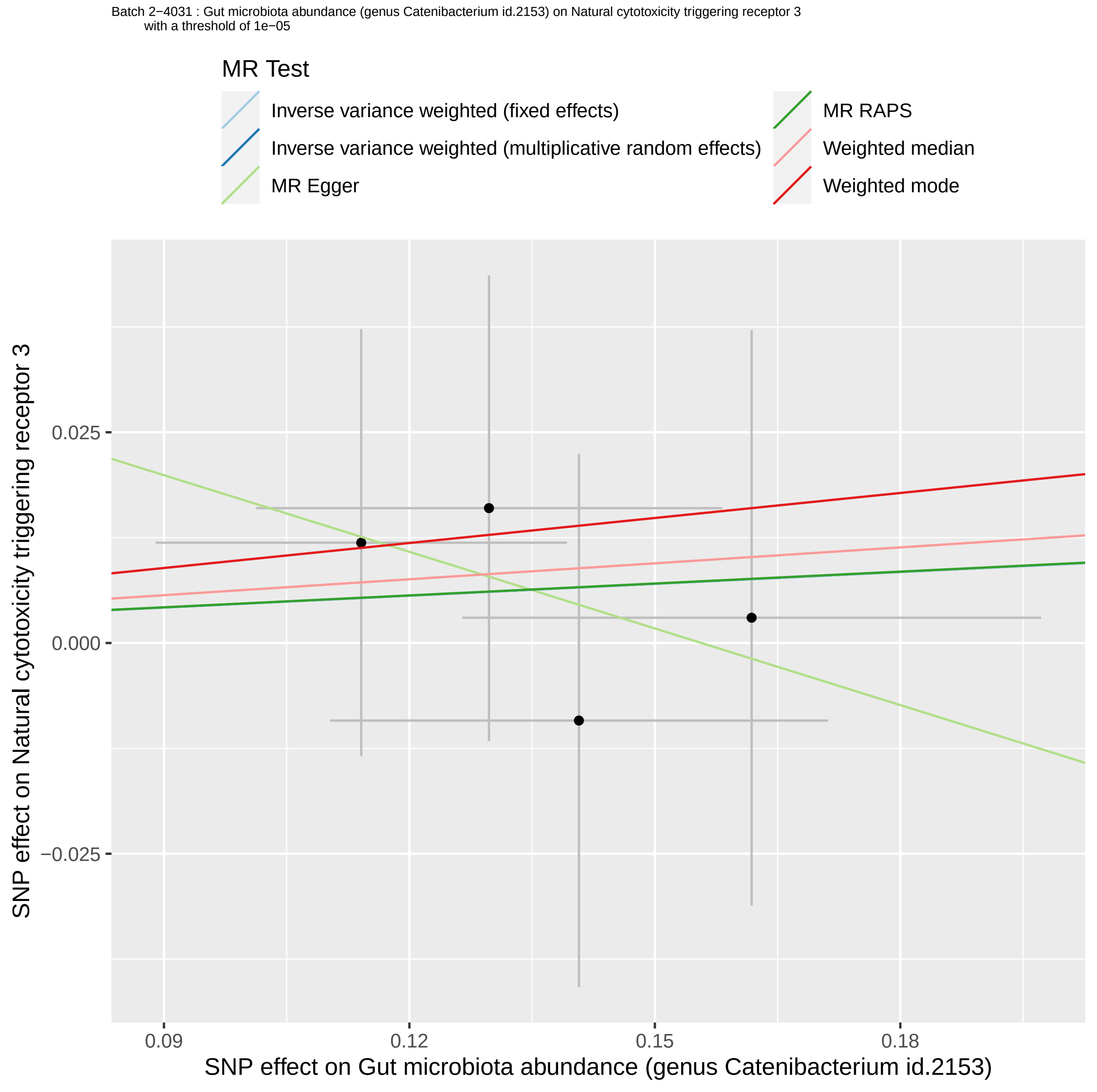
Batch 2−4031 : Gut microbiota abundance (genus Catenibacterium id.2153) on Natural cytotoxicity triggering receptor 3
with a threshold of 1e−05
MR Test
Inverse variance weighted (fixed effects)
Inverse variance weighted (multiplicative random effects)
MR Egger
MR RAPS
Weighted median
Weighted mode
0.025
0.000
−0.025
0.09
0.12
0.15
0.18
SNP effect on Natural cytotoxicity triggering receptor 3
SNP effect on Gut microbiota abundance (genus Catenibacterium id.2153)

Batch 2−4032 : Gut microbiota abundance (genus Parasutterella id.2892) on Natural cytotoxicity triggering receptor 3
with a threshold of 1e−05

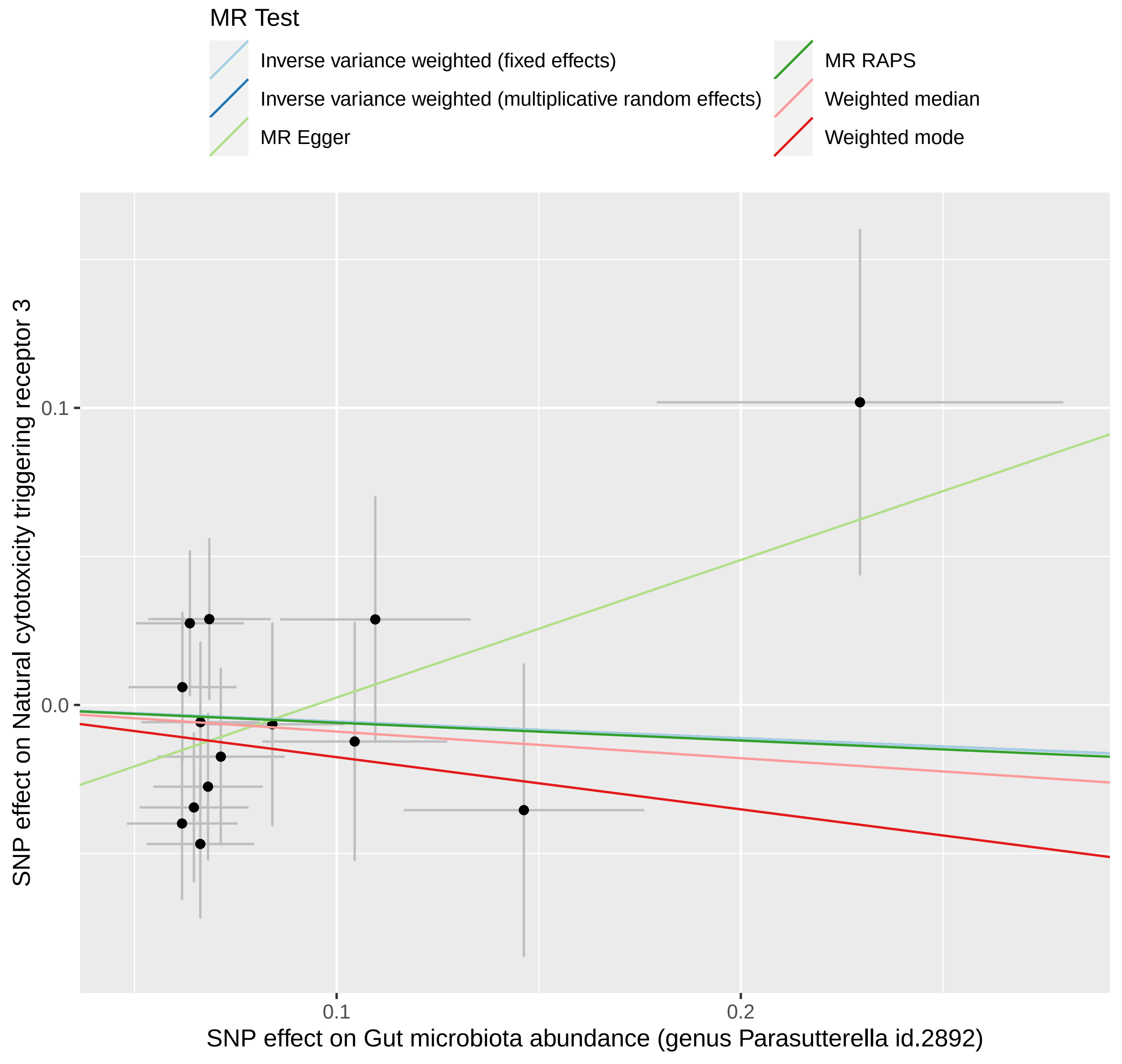

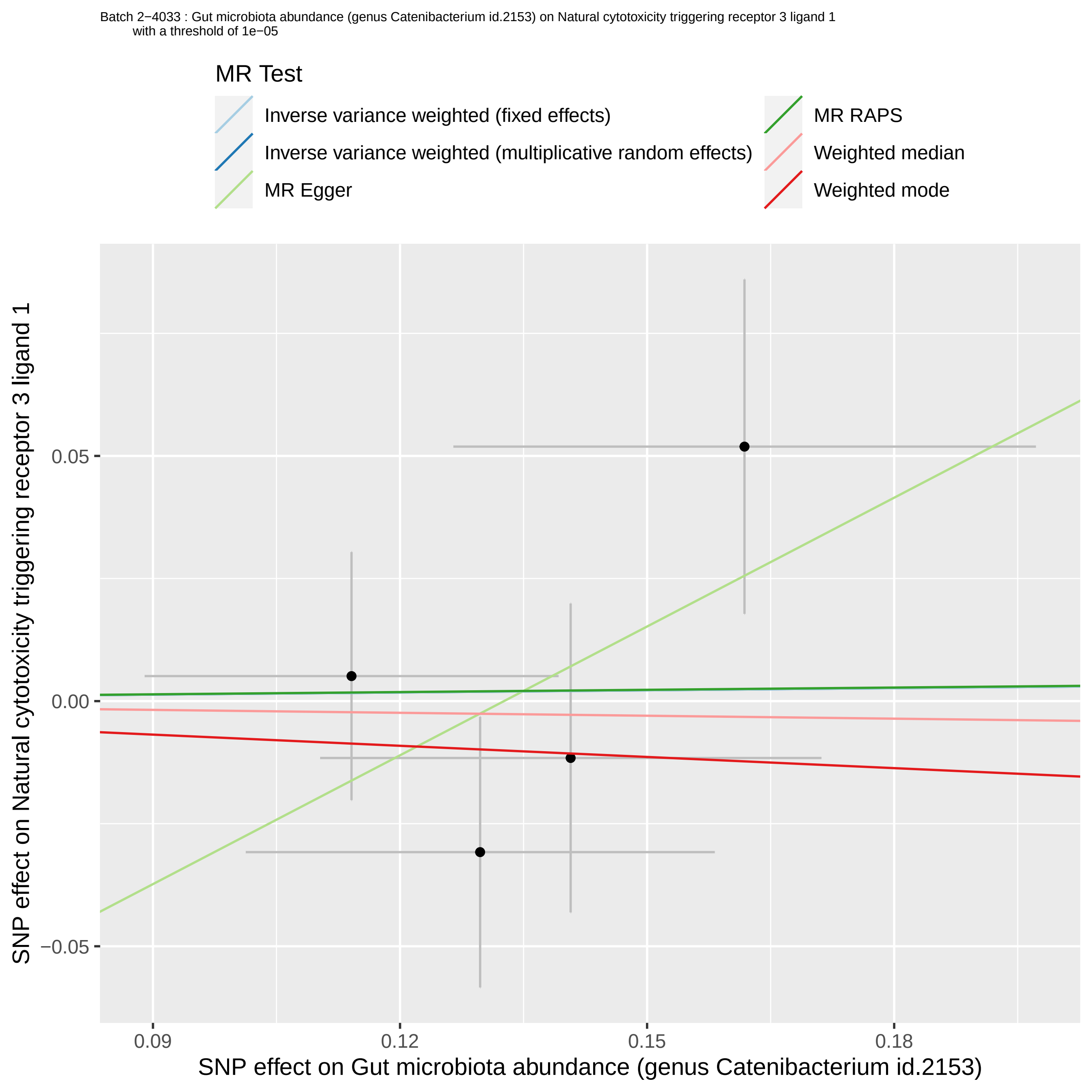
Batch 2−4033 : Gut microbiota abundance (genus Catenibacterium id.2153) on Natural cytotoxicity triggering receptor 3 ligand 1
with a threshold of 1e−05
MR Test
Inverse variance weighted (fixed effects)
Inverse variance weighted (multiplicative random effects)
MR Egger
MR RAPS
Weighted median
Weighted mode
SNP effect on Natural cytotoxicity triggering receptor 3 ligand 1
0.05
0.00
−0.05
0.09
0.12
0.15
0.18
SNP effect on Gut microbiota abundance (genus Catenibacterium id.2153)

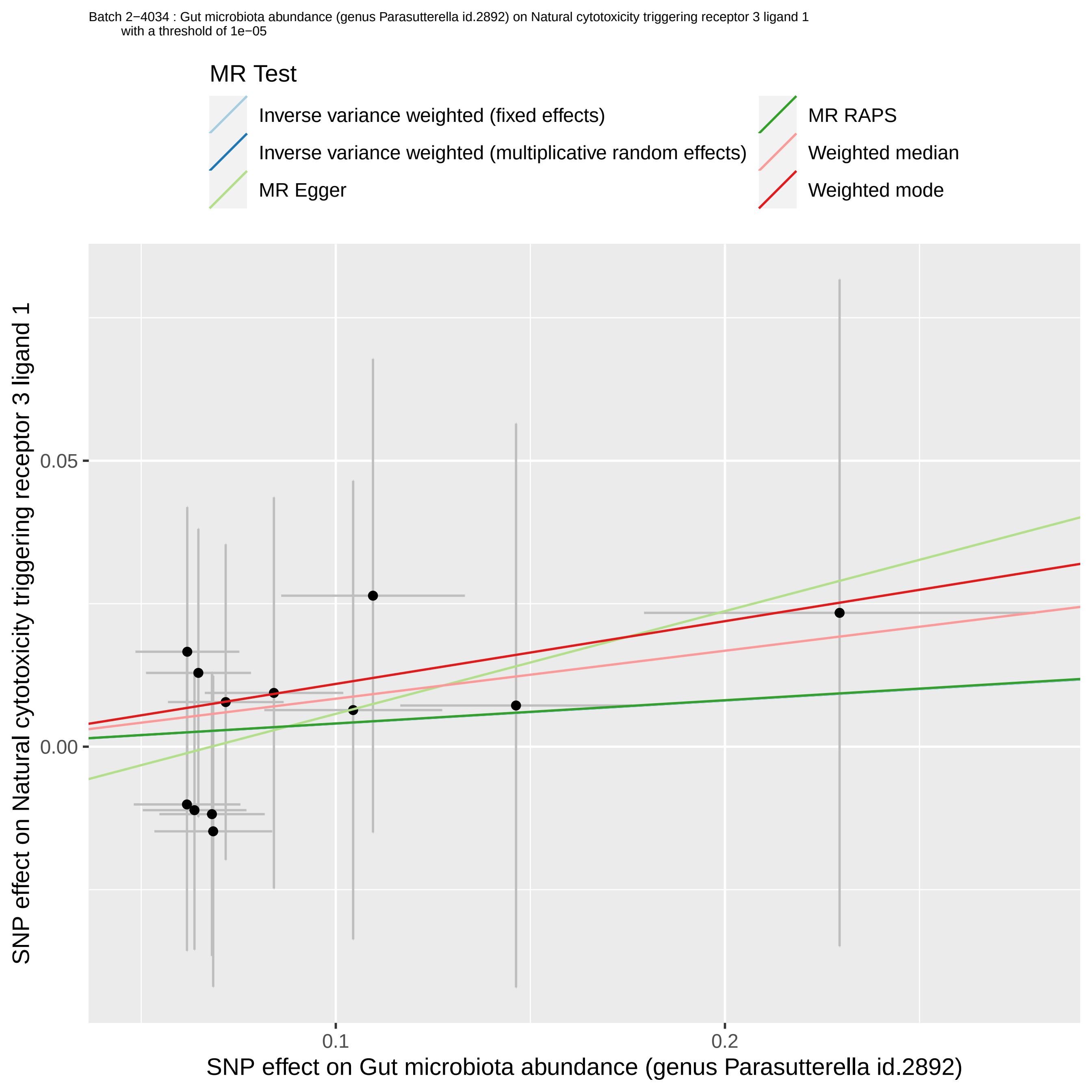

Batch 2−4034 : Gut microbiota abundance (genus Parasutterella id.2892) on Natural cytotoxicity triggering receptor 3 ligand 1
with a threshold of 1e−05
MR Test
Inverse variance weighted (fixed effects)
Inverse variance weighted (multiplicative random effects)
MR Egger
MR RAPS
Weighted median
Weighted mode
0.05
0.00
0.1
0.2
SNP effect on Natural cytotoxicity triggering receptor 3 ligand 1
SNP effect on Gut microbiota abundance (genus Parasutterella id.2892)

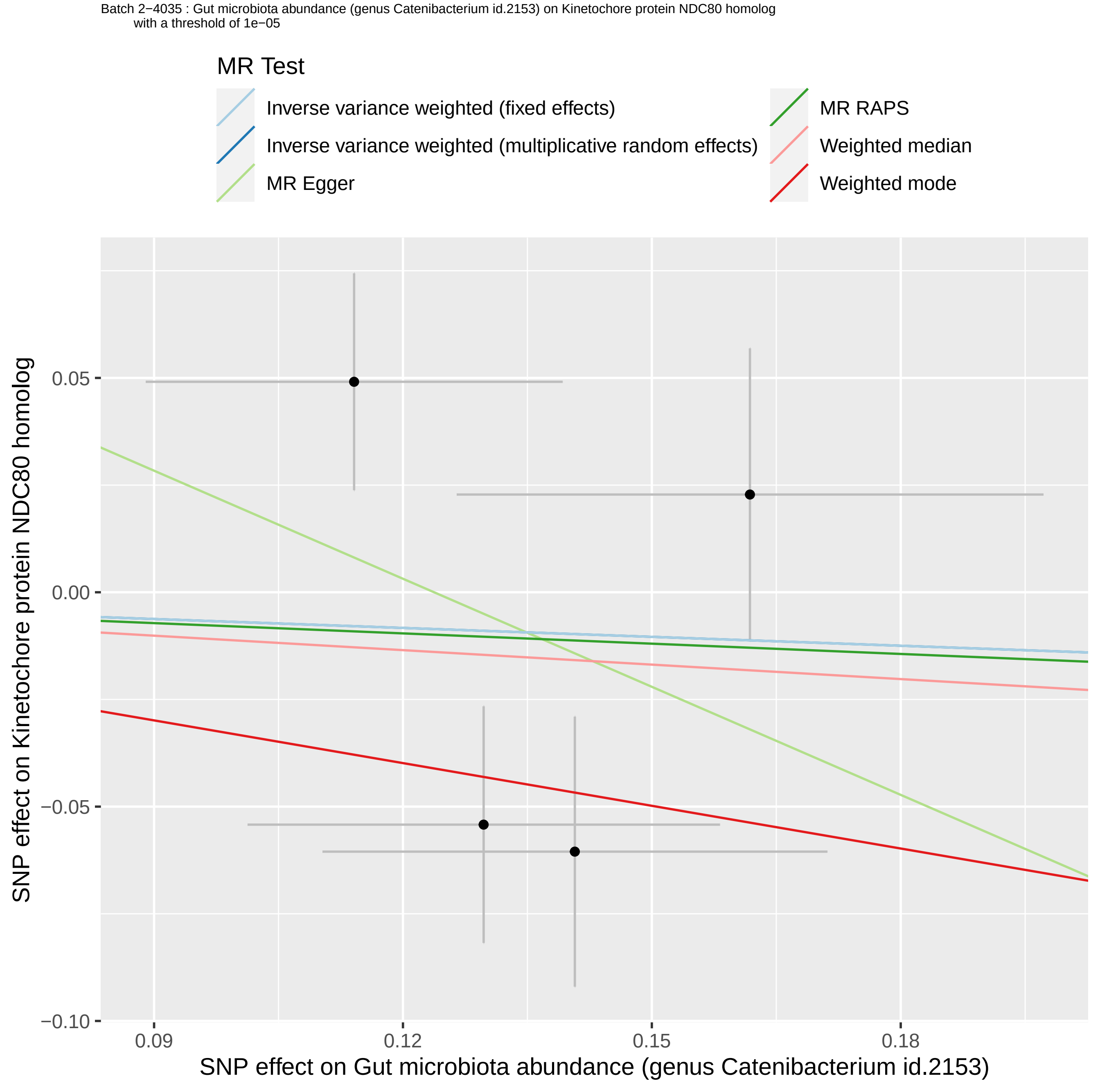

Batch 2−4035 : Gut microbiota abundance (genus Catenibacterium id.2153) on Kinetochore protein NDC80 homolog
with a threshold of 1e−05
MR Test
Inverse variance weighted (fixed effects)
Inverse variance weighted (multiplicative random effects)
MR Egger
MR RAPS
Weighted median
Weighted mode
0.05
0.00
−0.05
−0.10
0.09
0.12
0.15
0.18
SNP effect on Kinetochore protein NDC80 homolog
SNP effect on Gut microbiota abundance (genus Catenibacterium id.2153)

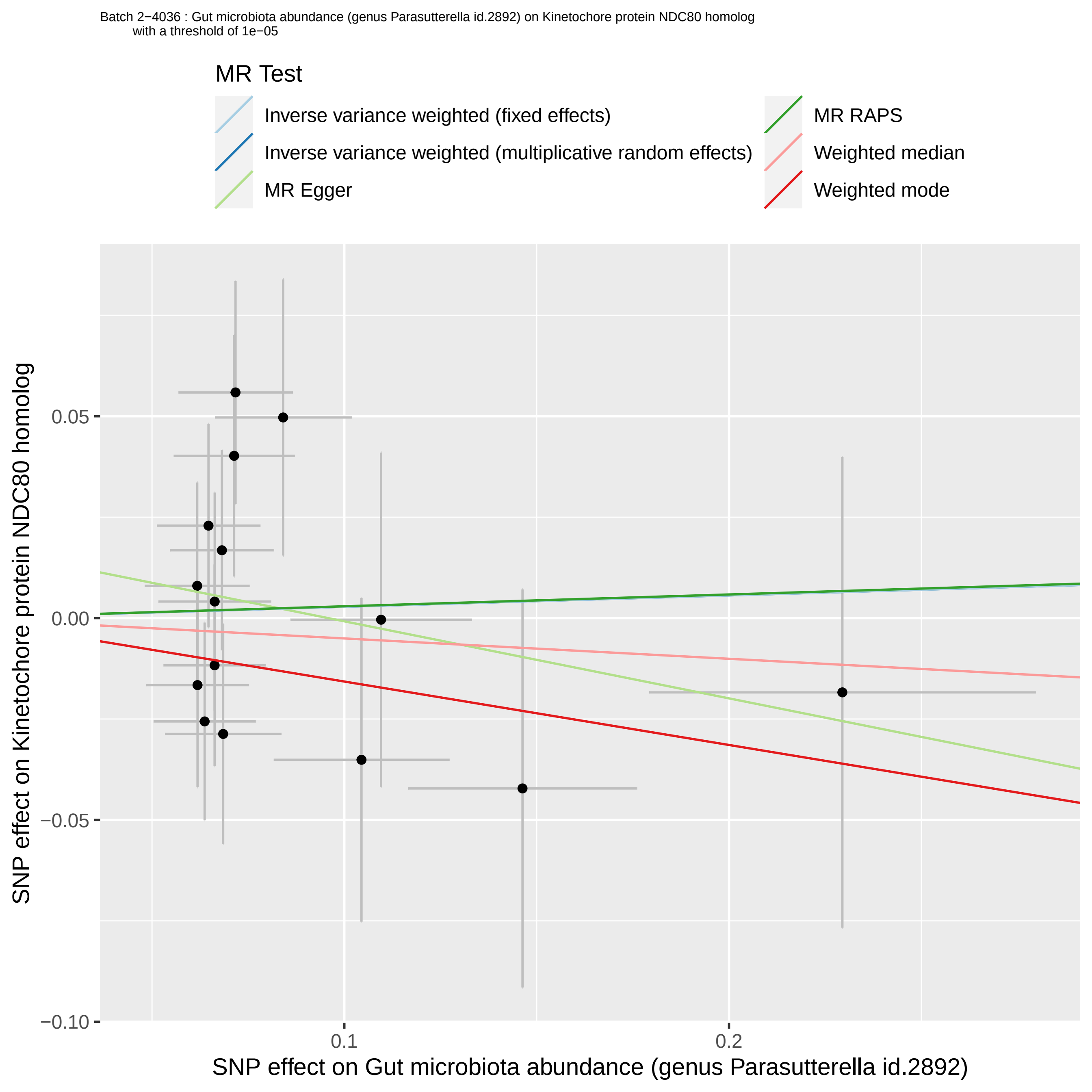

Batch 2−4036 : Gut microbiota abundance (genus Parasutterella id.2892) on Kinetochore protein NDC80 homolog
with a threshold of 1e−05
MR Test
Inverse variance weighted (fixed effects)
Inverse variance weighted (multiplicative random effects)
MR Egger
MR RAPS
Weighted median
Weighted mode
SNP effect on Kinetochore protein NDC80 homolog
0.05
0.00
−0.05
−0.10
0.1
0.2
SNP effect on Gut microbiota abundance (genus Parasutterella id.2892)

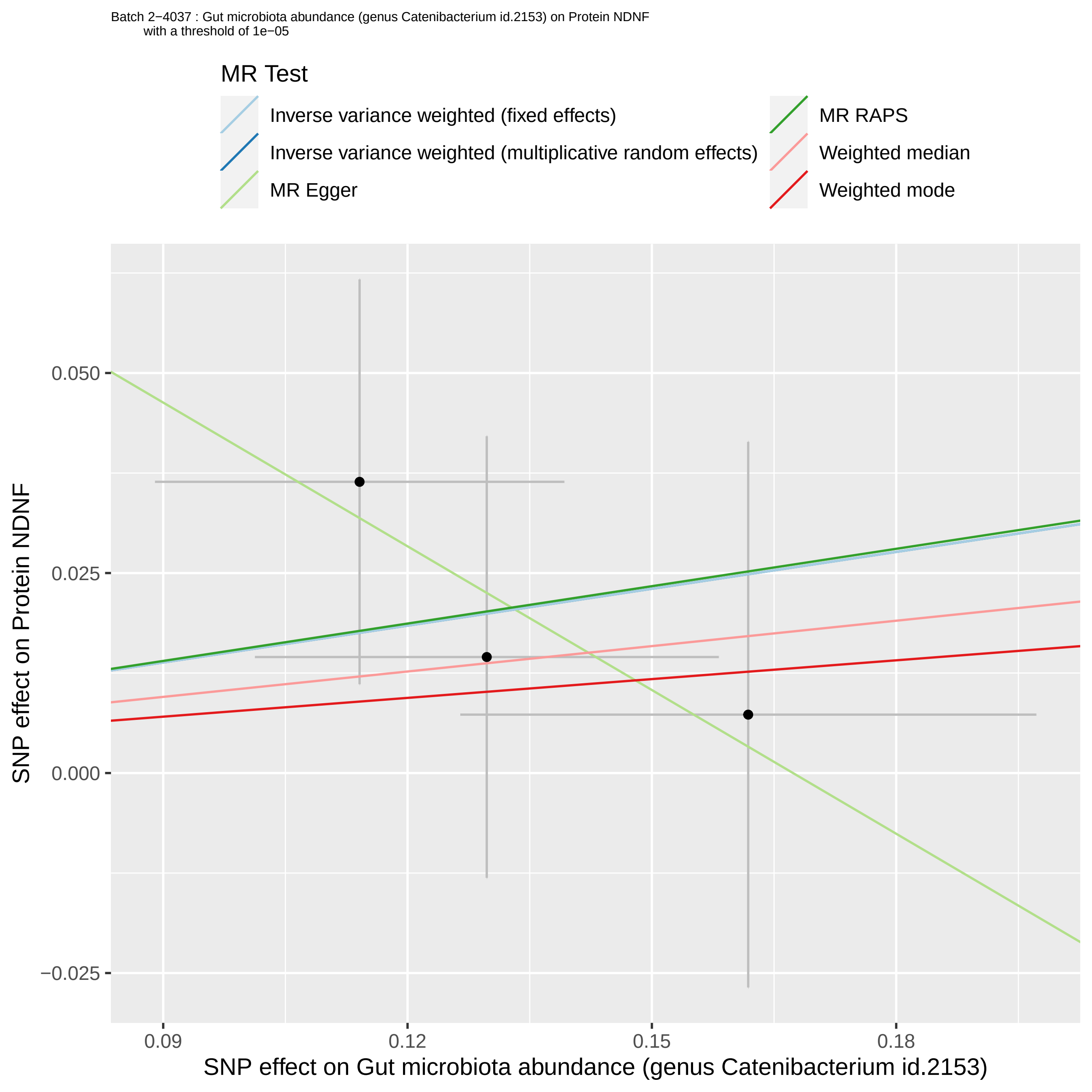

Batch 2−4037 : Gut microbiota abundance (genus Catenibacterium id.2153) on Protein NDNF
with a threshold of 1e−05
MR Test
Inverse variance weighted (fixed effects)
Inverse variance weighted (multiplicative random effects)
MR Egger
MR RAPS
Weighted median
Weighted mode
0.050
0.025
0.000
−0.025
0.09
0.12
0.15
0.18
SNP effect on Protein NDNF
SNP effect on Gut microbiota abundance (genus Catenibacterium id.2153)

Batch 2−4038 : Gut microbiota abundance (genus Parasutterella id.2892) on Protein NDNF
with a threshold of 1e−05

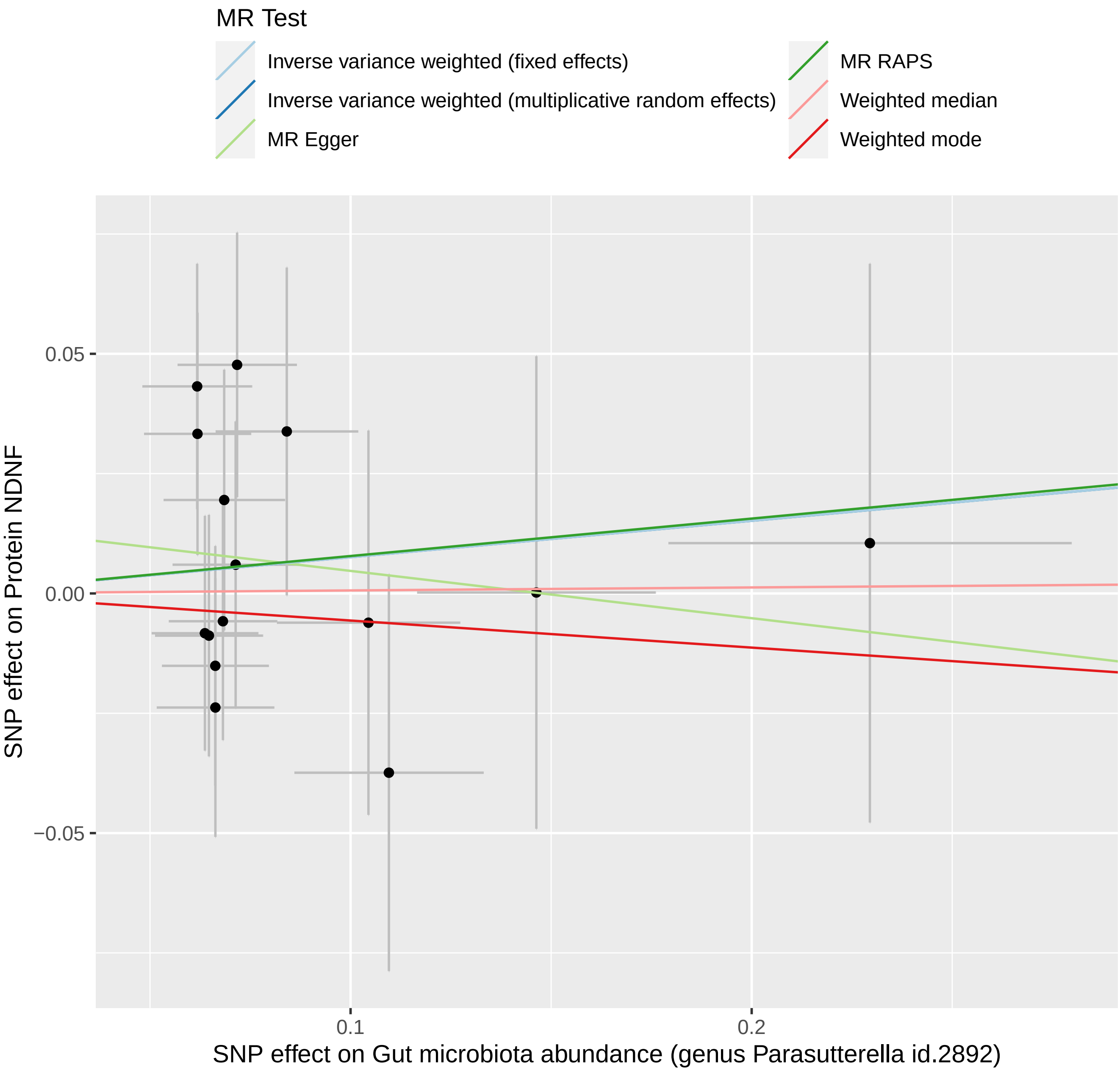

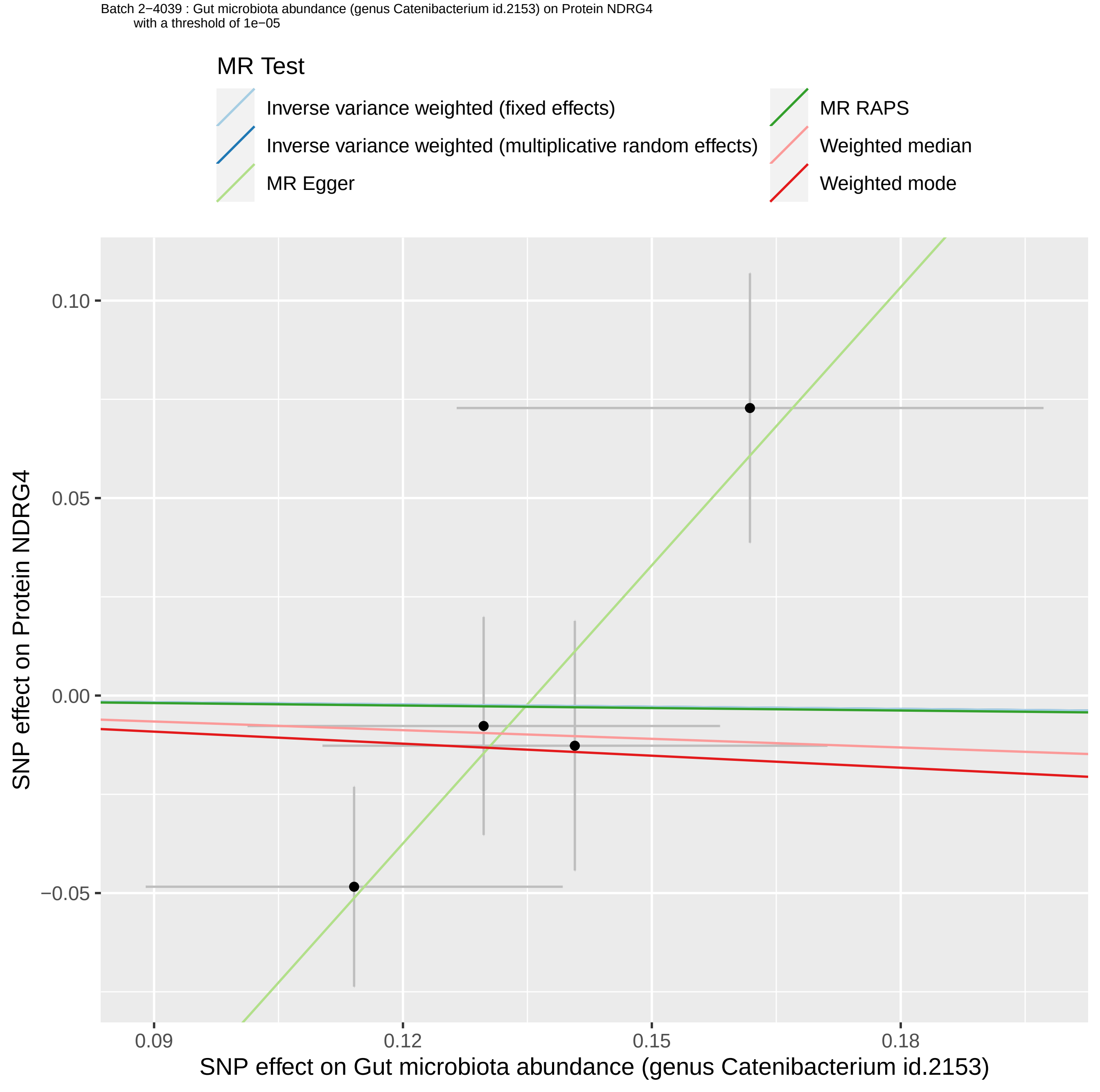
Batch 2−4039 : Gut microbiota abundance (genus Catenibacterium id.2153) on Protein NDRG4
with a threshold of 1e−05
MR Test
Inverse variance weighted (fixed effects)
Inverse variance weighted (multiplicative random effects)
MR Egger
MR RAPS
Weighted median
Weighted mode
0.10
0.05
0.00
−0.05
0.09
0.12
0.15
0.18
SNP effect on Protein NDRG4
SNP effect on Gut microbiota abundance (genus Catenibacterium id.2153)

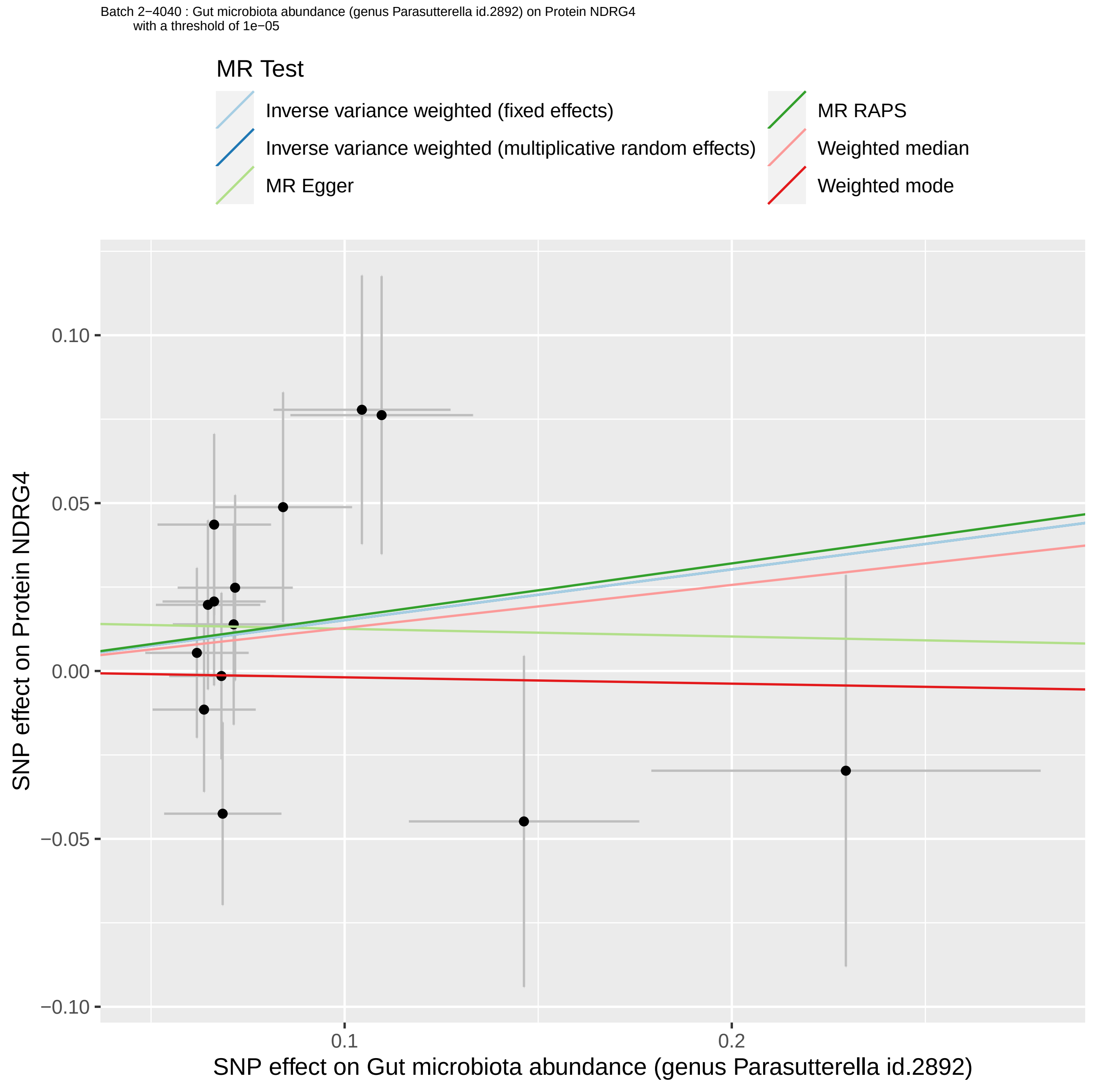
Batch 2−4040 : Gut microbiota abundance (genus Parasutterella id.2892) on Protein NDRG4
with a threshold of 1e−05
MR Test
Inverse variance weighted (fixed effects)
Inverse variance weighted (multiplicative random effects)
MR Egger
MR RAPS
Weighted median
Weighted mode
0.10
0.05
0.00
−0.05
−0.10
0.1
0.2
SNP effect on Protein NDRG4
SNP effect on Gut microbiota abundance (genus Parasutterella id.2892)

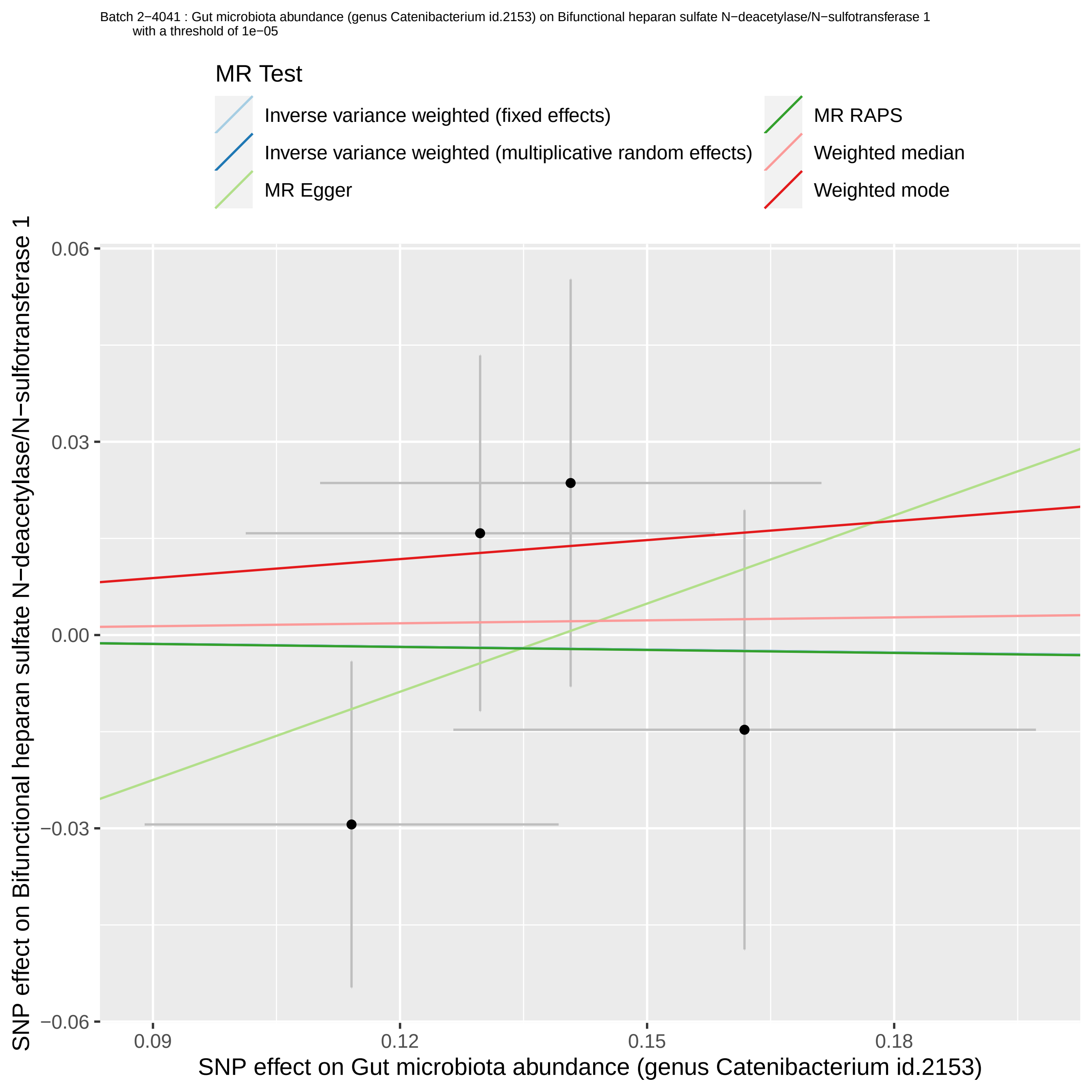

Batch 2−4041 : Gut microbiota abundance (genus Catenibacterium id.2153) on Bifunctional heparan sulfate N−deacetylase/N−sulfotransferase 1 with a threshold of 1e−05
MR Test
Inverse variance weighted (fixed effects)
Inverse variance weighted (multiplicative random effects)
MR Egger
MR RAPS
Weighted median
Weighted mode
0.06
0.03
0.00
−0.03
−0.06
0.09
0.12
0.15
0.18
SNP effect on Bifunctional heparan sulfate N−deacetylase/N−sulfotransferase 1
SNP effect on Gut microbiota abundance (genus Catenibacterium id.2153)

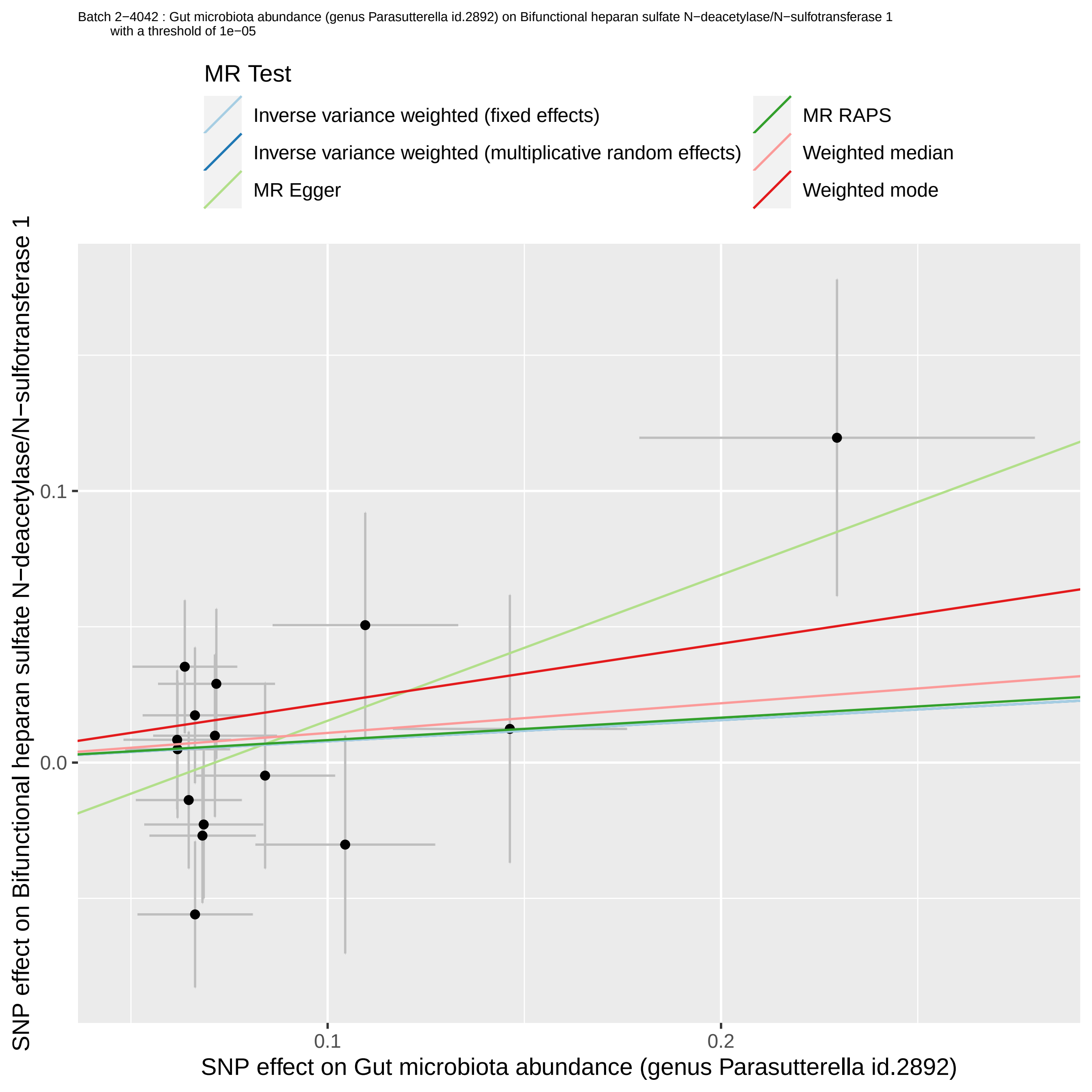
Batch 2−4042 : Gut microbiota abundance (genus Parasutterella id.2892) on Bifunctional heparan sulfate N−deacetylase/N−sulfotransferase 1
with a threshold of 1e−05
MR Test
Inverse variance weighted (fixed effects)
Inverse variance weighted (multiplicative random effects)
MR Egger
MR RAPS
Weighted median
Weighted mode
SNP effect on Bifunctional heparan sulfate N−deacetylase/N−sulfotransferase 1
0.1
0.0
0.1
0.2
SNP effect on Gut microbiota abundance (genus Parasutterella id.2892)

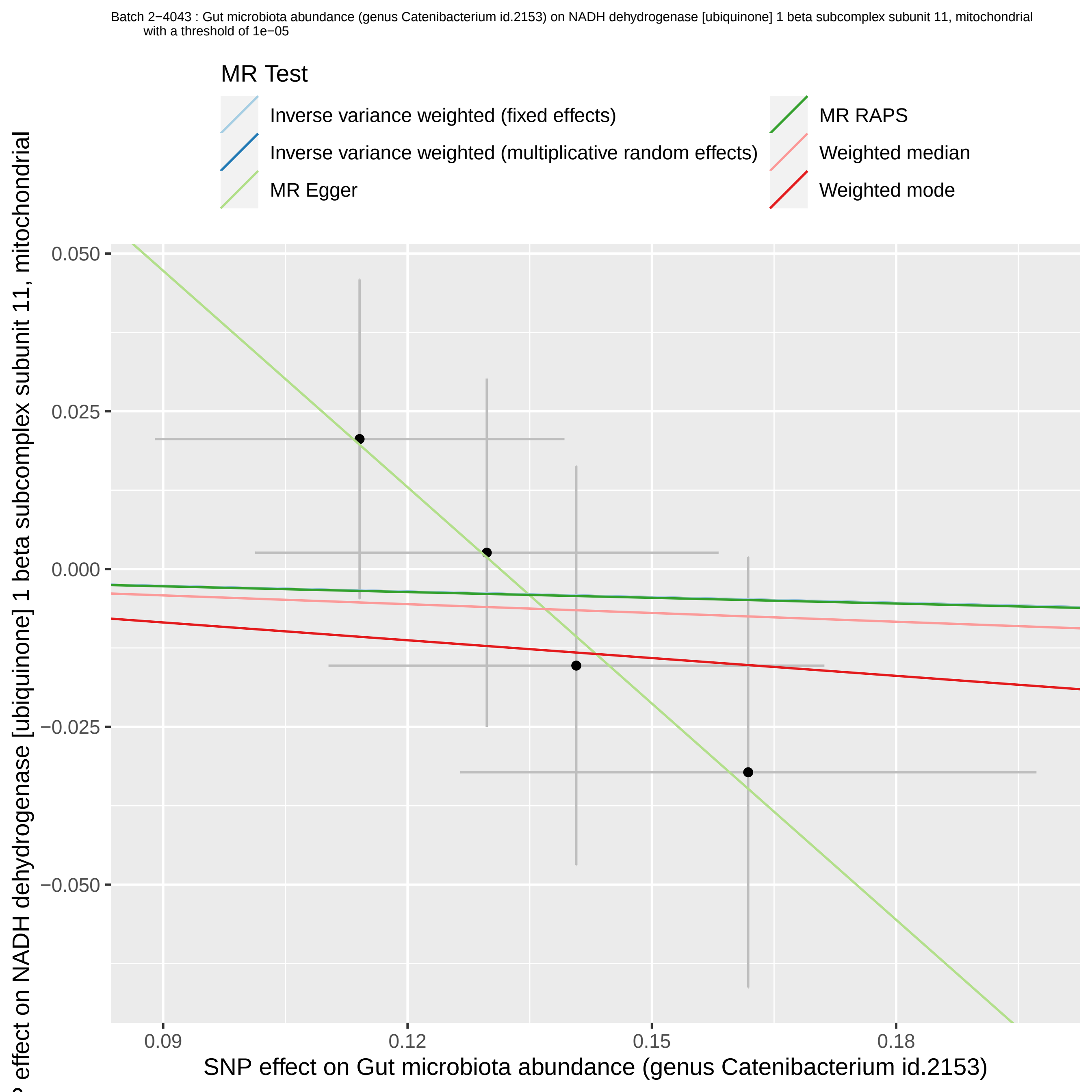
Batch 2−4043 : Gut microbiota abundance (genus Catenibacterium id.2153) on NADH dehydrogenase [ubiquinone] 1 beta subcomplex subunit 11, mitochondrial with a threshold of 1e−05
MR Test
Inverse variance weighted (fixed effects)
Inverse variance weighted (multiplicative random effects)
MR Egger
MR RAPS
Weighted median
Weighted mode
0.050
0.025
0.000
−0.025
−0.050
0.09
0.12
0.15
0.18
SNP effect on Gut microbiota abundance (genus Catenibacterium id.2153)
effect on NADH dehydrogenase [ubiquinone] 1 beta subcomplex subunit 11, mitochondrial

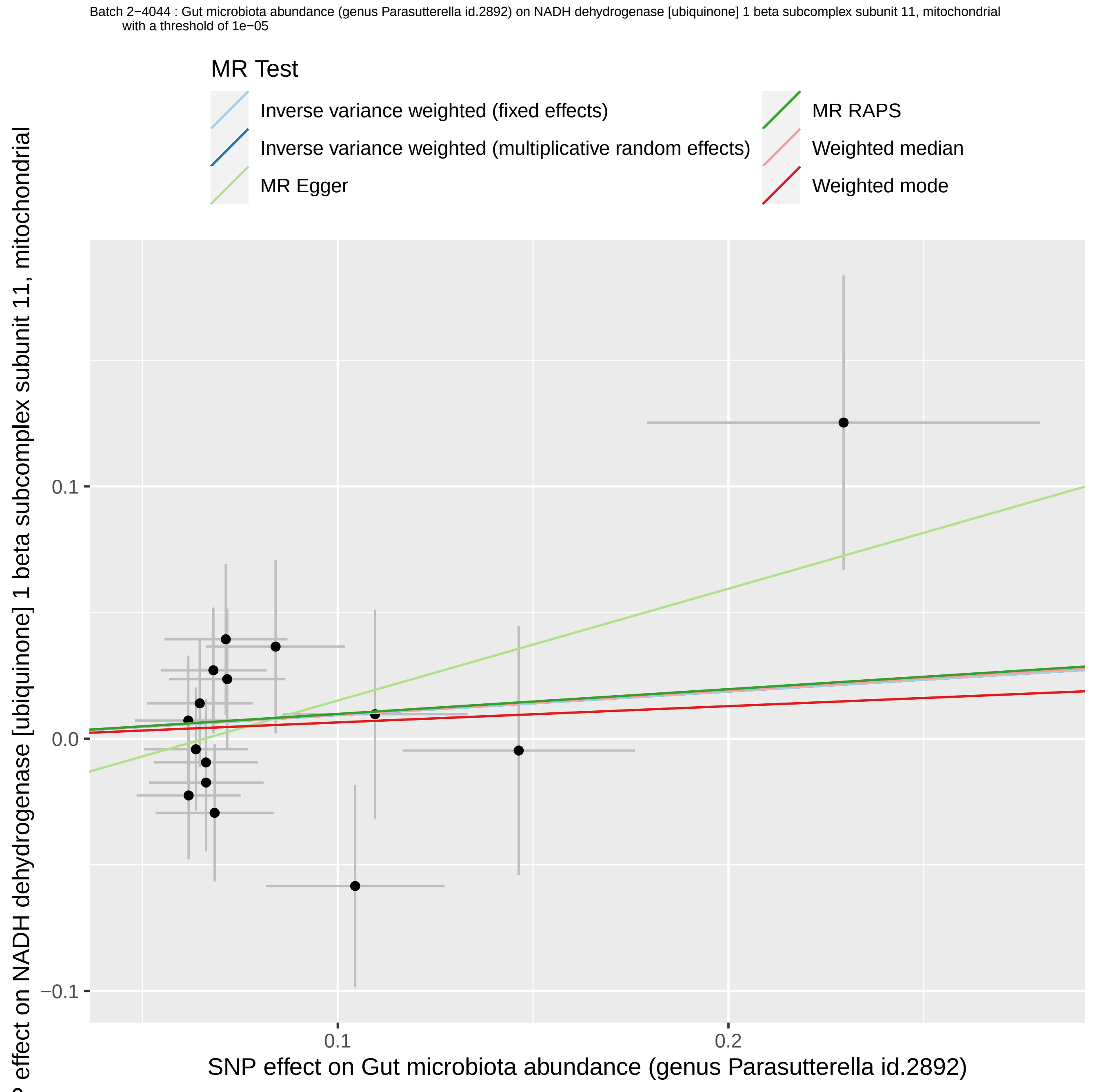

Batch 2−4044 : Gut microbiota abundance (genus Parasutterella id.2892) on NADH dehydrogenase [ubiquinone] 1 beta subcomplex subunit 11, mitochondrial with a threshold of 1e−05
MR Test
Inverse variance weighted (fixed effects)
Inverse variance weighted (multiplicative random effects)
MR Egger
MR RAPS
Weighted median
Weighted mode
0.1
0.0
−0.1
0.1
0.2
SNP effect on Gut microbiota abundance (genus Parasutterella id.2892)
SNP effect on NADH dehydrogenase [ubiquinone] 1 beta subcomplex subunit 11, mitochondrial

Batch 2−4045 : Gut microbiota abundance (genus Catenibacterium id.2153) on NADH dehydrogenase [ubiquinone] 1 beta subcomplex subunit 4 with a threshold of 1e−05

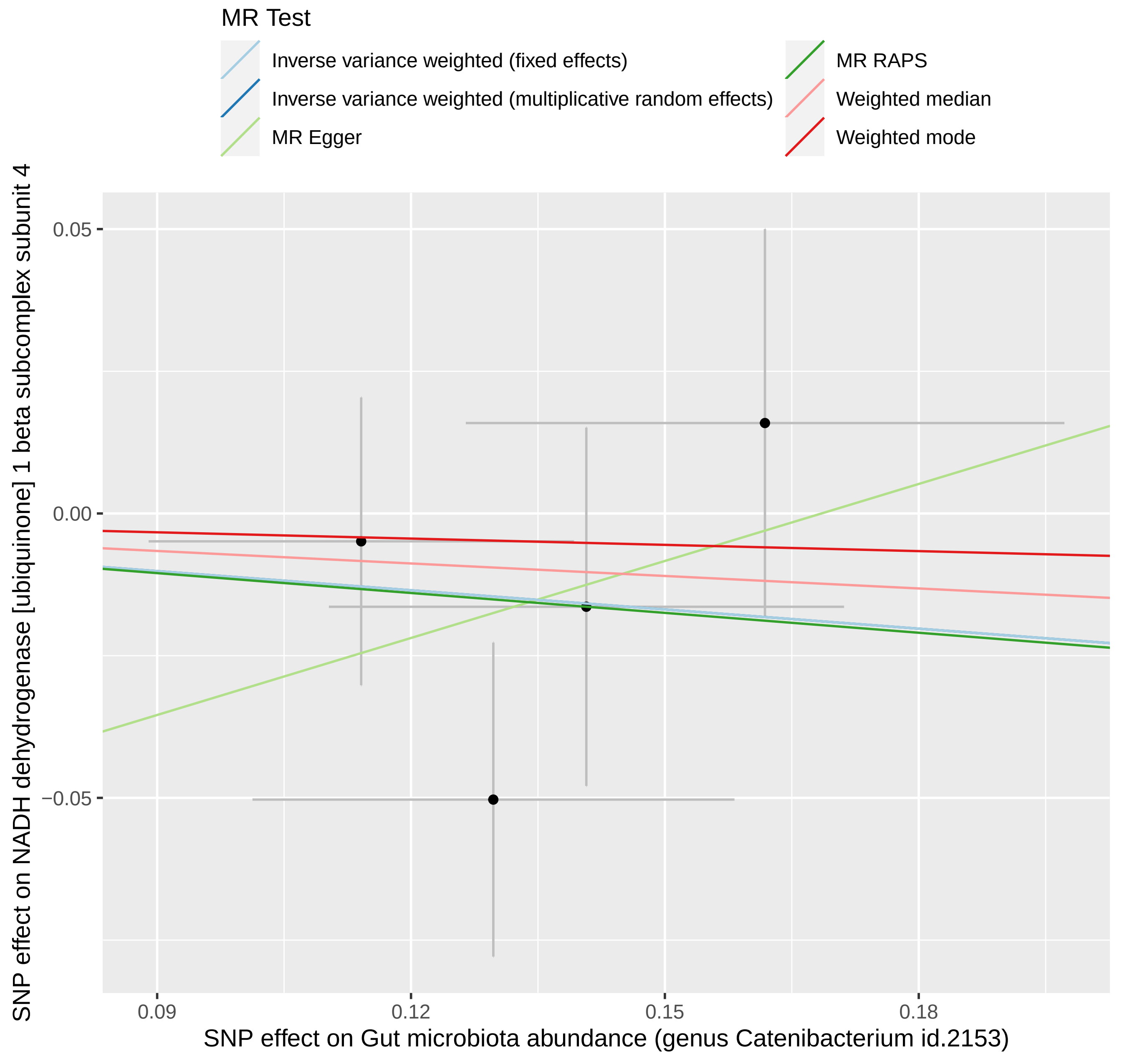

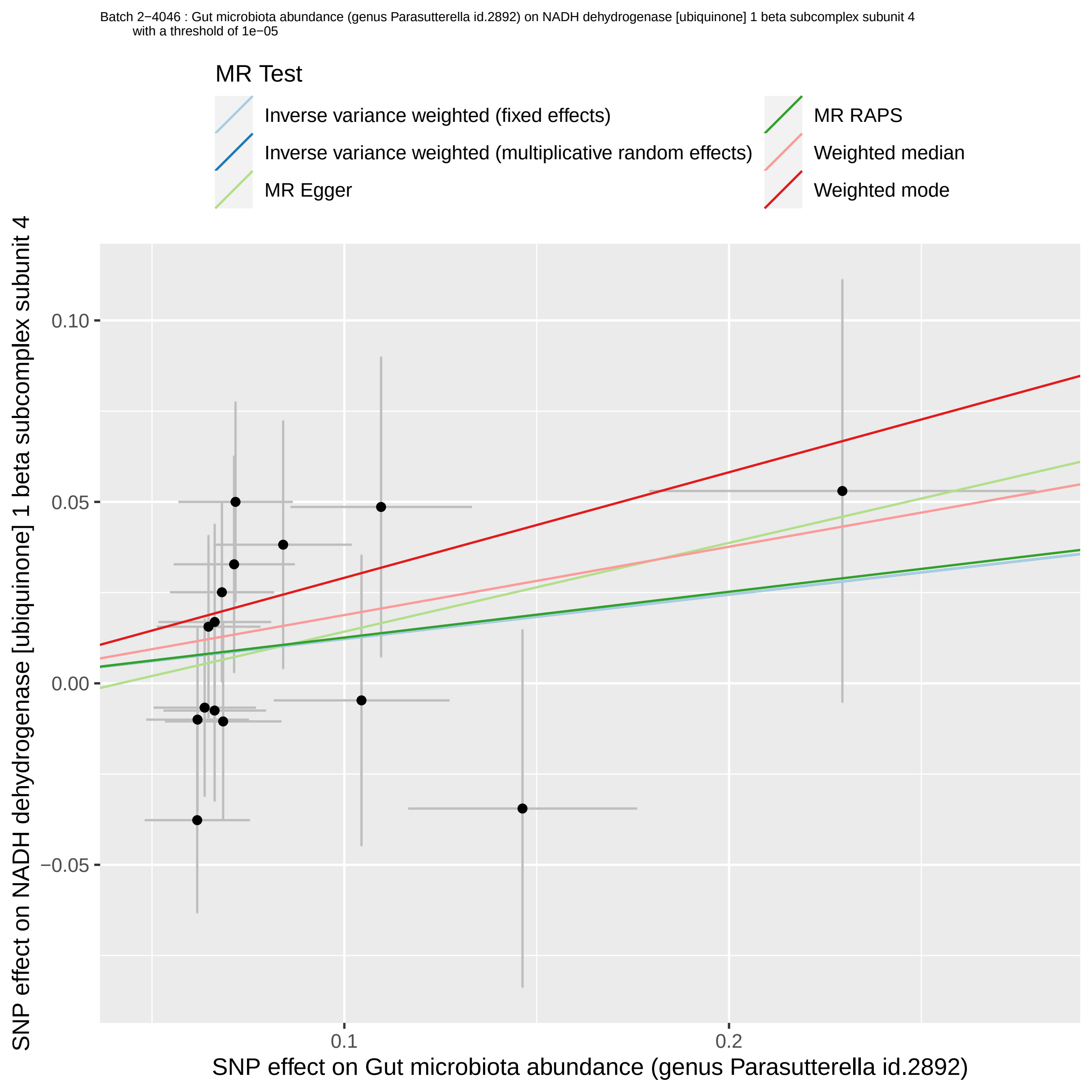
Batch 2−4046 : Gut microbiota abundance (genus Parasutterella id.2892) on NADH dehydrogenase [ubiquinone] 1 beta subcomplex subunit 4
with a threshold of 1e−05
MR Test
Inverse variance weighted (fixed effects)
Inverse variance weighted (multiplicative random effects)
MR Egger
MR RAPS
Weighted median
Weighted mode
0.10
0.05
0.00
−0.05
0.1
0.2
SNP effect on Gut microbiota abundance (genus Parasutterella id.2892)
SNP effect on NADH dehydrogenase [ubiquinone] 1 beta subcomplex subunit 4

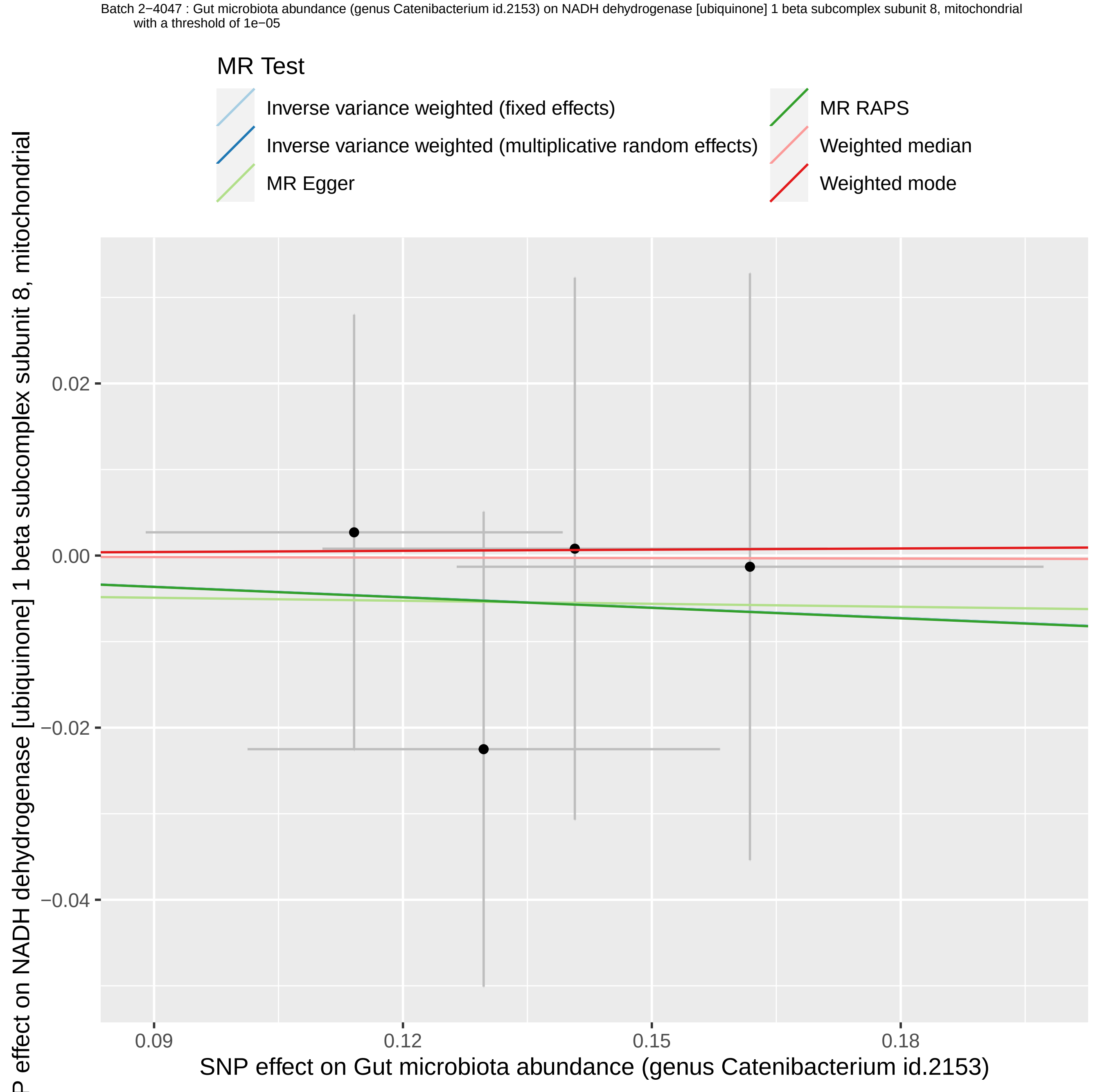

Batch 2−4047 : Gut microbiota abundance (genus Catenibacterium id.2153) on NADH dehydrogenase [ubiquinone] 1 beta subcomplex subunit 8, mitochondrial with a threshold of 1e−05
MR Test
Inverse variance weighted (fixed effects)
Inverse variance weighted (multiplicative random effects)
MR Egger
MR RAPS
Weighted median
Weighted mode
0.02
0.00
−0.02
−0.04
0.09
0.12
0.15
0.18
SNP effect on Gut microbiota abundance (genus Catenibacterium id.2153)
P effect on NADH dehydrogenase [ubiquinone] 1 beta subcomplex subunit 8, mitochondrial

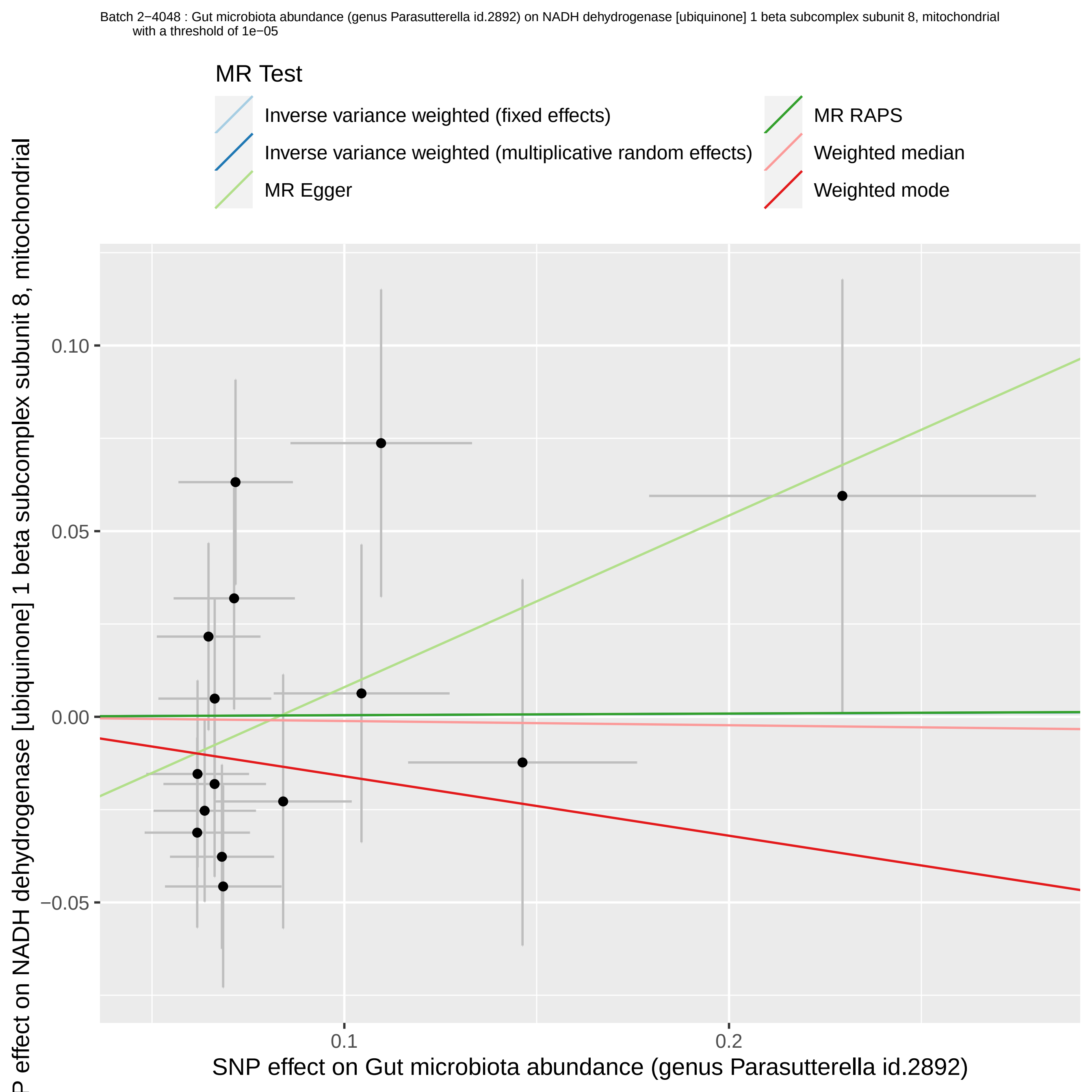

Batch 2−4048 : Gut microbiota abundance (genus Parasutterella id.2892) on NADH dehydrogenase [ubiquinone] 1 beta subcomplex subunit 8, mitochondrial with a threshold of 1e−05
MR Test
Inverse variance weighted (fixed effects)
Inverse variance weighted (multiplicative random effects)
MR Egger
MR RAPS
Weighted median
Weighted mode
0.10
0.05
0.00
−0.05
0.1
0.2
SNP effect on Gut microbiota abundance (genus Parasutterella id.2892)
P effect on NADH dehydrogenase [ubiquinone] 1 beta subcomplex subunit 8, mitochondrial

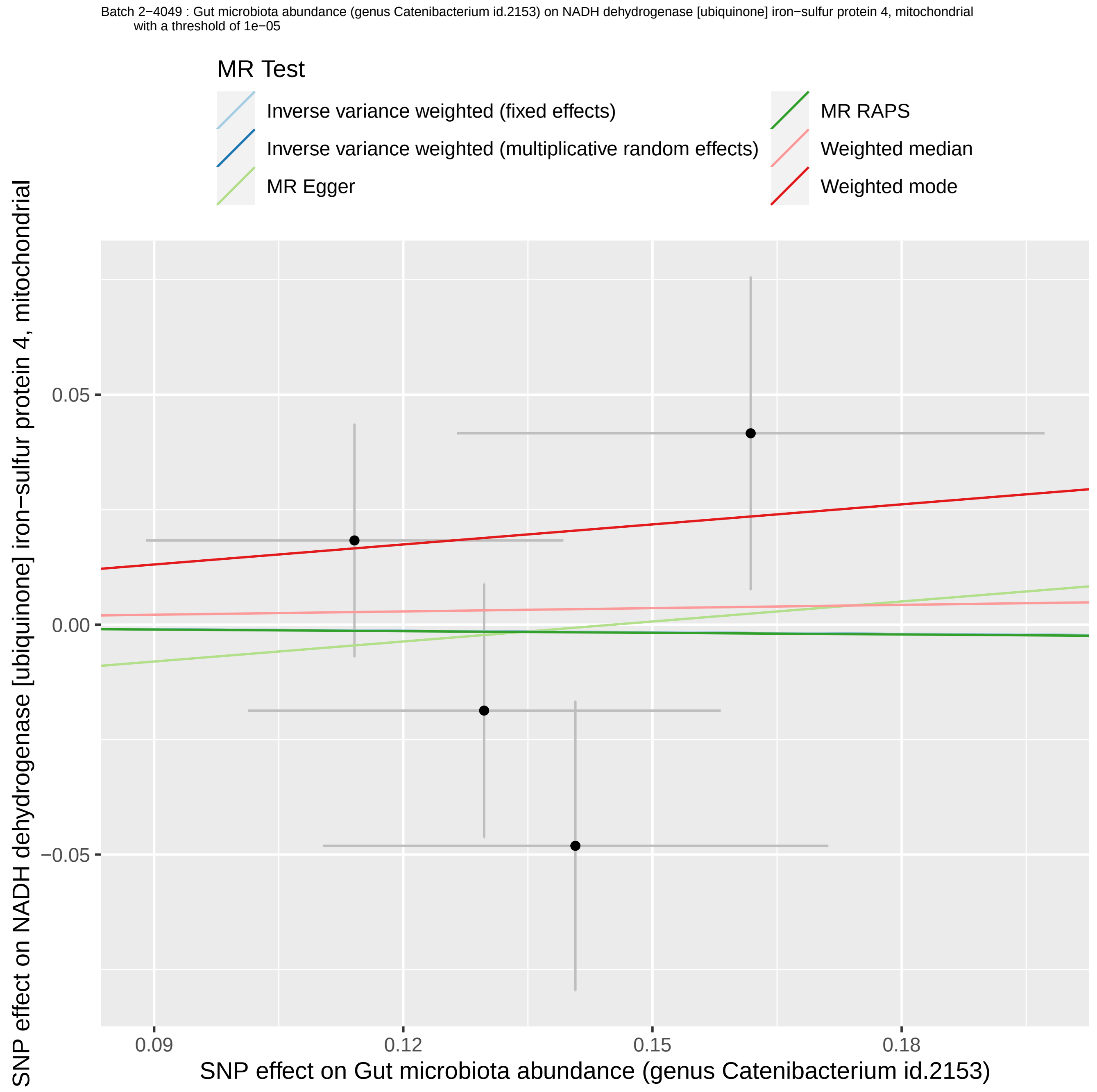
Batch 2−4049 : Gut microbiota abundance (genus Catenibacterium id.2153) on NADH dehydrogenase [ubiquinone] iron−sulfur protein 4, mitochondrial
with a threshold of 1e−05
MR Test
Inverse variance weighted (fixed effects)
Inverse variance weighted (multiplicative random effects)
MR Egger
MR RAPS
Weighted median
Weighted mode
0.05
0.00
−0.05
0.09
0.12
0.15
0.18
SNP effect on Gut microbiota abundance (genus Catenibacterium id.2153)
SNP effect on NADH dehydrogenase [ubiquinone] iron−sulfur protein 4, mitochondrial

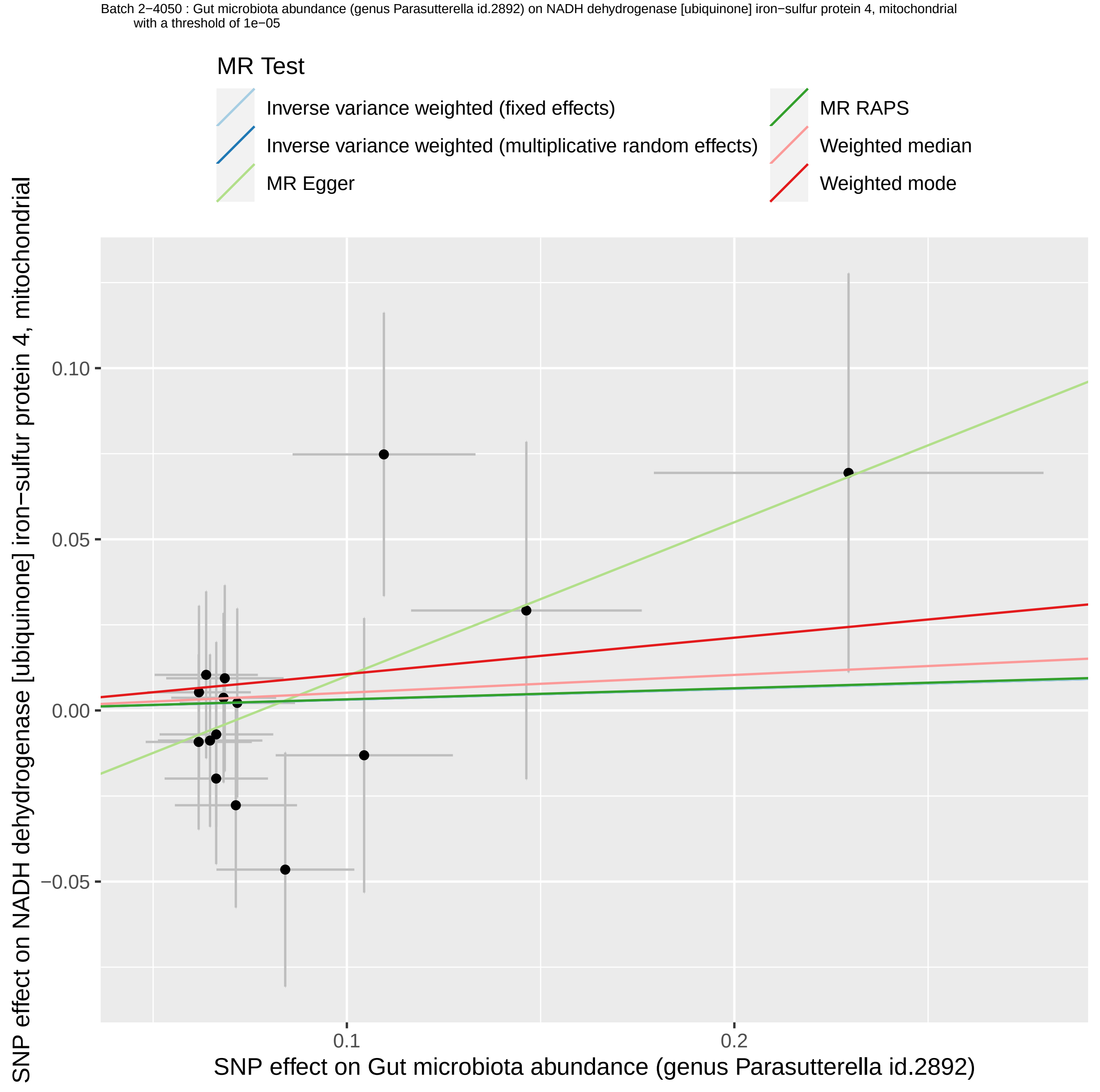
Batch 2−4050 : Gut microbiota abundance (genus Parasutterella id.2892) on NADH dehydrogenase [ubiquinone] iron−sulfur protein 4, mitochondrial with a threshold of 1e−05
MR Test
Inverse variance weighted (fixed effects)
Inverse variance weighted (multiplicative random effects)
MR Egger
MR RAPS
Weighted median
Weighted mode
0.10
0.05
0.00
−0.05
0.1
0.2
SNP effect on NADH dehydrogenase [ubiquinone] iron−sulfur protein 4, mitochondrial
SNP effect on Gut microbiota abundance (genus Parasutterella id.2892)

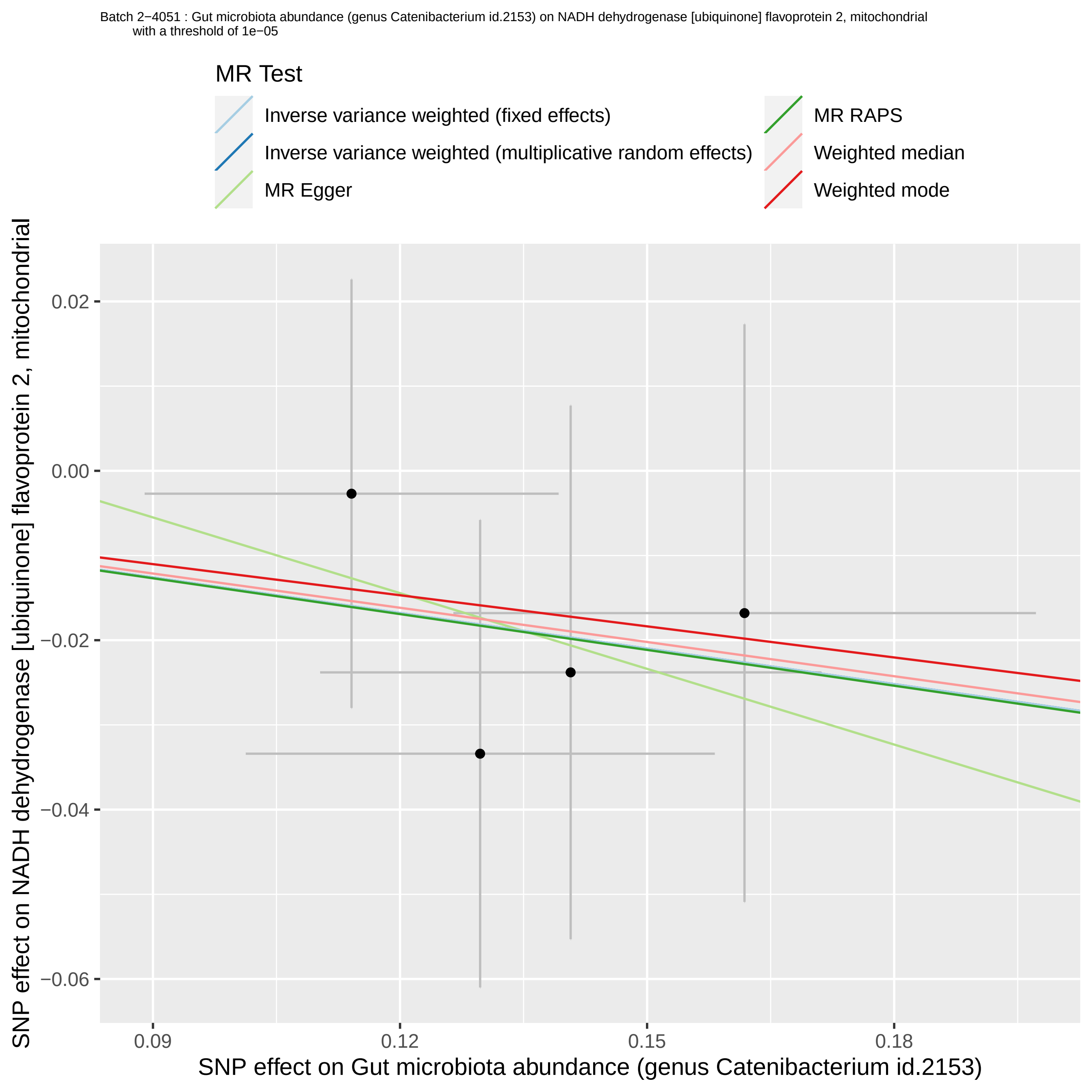
Batch 2−4051 : Gut microbiota abundance (genus Catenibacterium id.2153) on NADH dehydrogenase [ubiquinone] flavoprotein 2, mitochondrial
with a threshold of 1e−05
MR Test
Inverse variance weighted (fixed effects)
Inverse variance weighted (multiplicative random effects)
MR Egger
MR RAPS
Weighted median
Weighted mode
0.02
0.00
−0.02
−0.04
−0.06
0.09
0.12
0.15
0.18
SNP effect on NADH dehydrogenase [ubiquinone] flavoprotein 2, mitochondrial
SNP effect on Gut microbiota abundance (genus Catenibacterium id.2153)

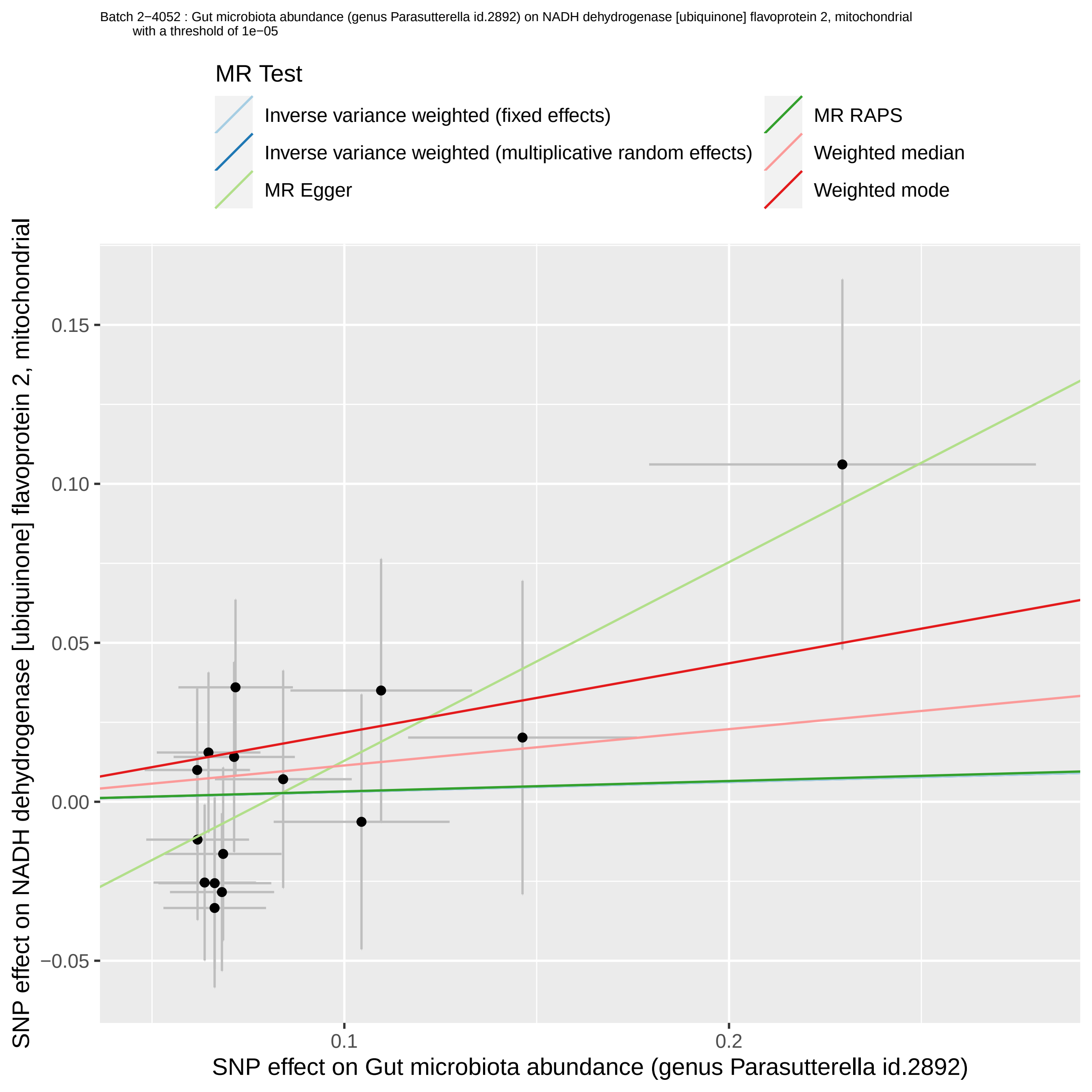
Batch 2−4052 : Gut microbiota abundance (genus Parasutterella id.2892) on NADH dehydrogenase [ubiquinone] flavoprotein 2, mitochondrial
with a threshold of 1e−05
MR Test
Inverse variance weighted (fixed effects)
Inverse variance weighted (multiplicative random effects)
MR Egger
MR RAPS
Weighted median
Weighted mode
0.15
0.10
0.05
0.00
−0.05
0.1
0.2
SNP effect on Gut microbiota abundance (genus Parasutterella id.2892)
SNP effect on NADH dehydrogenase [ubiquinone] flavoprotein 2, mitochondrial

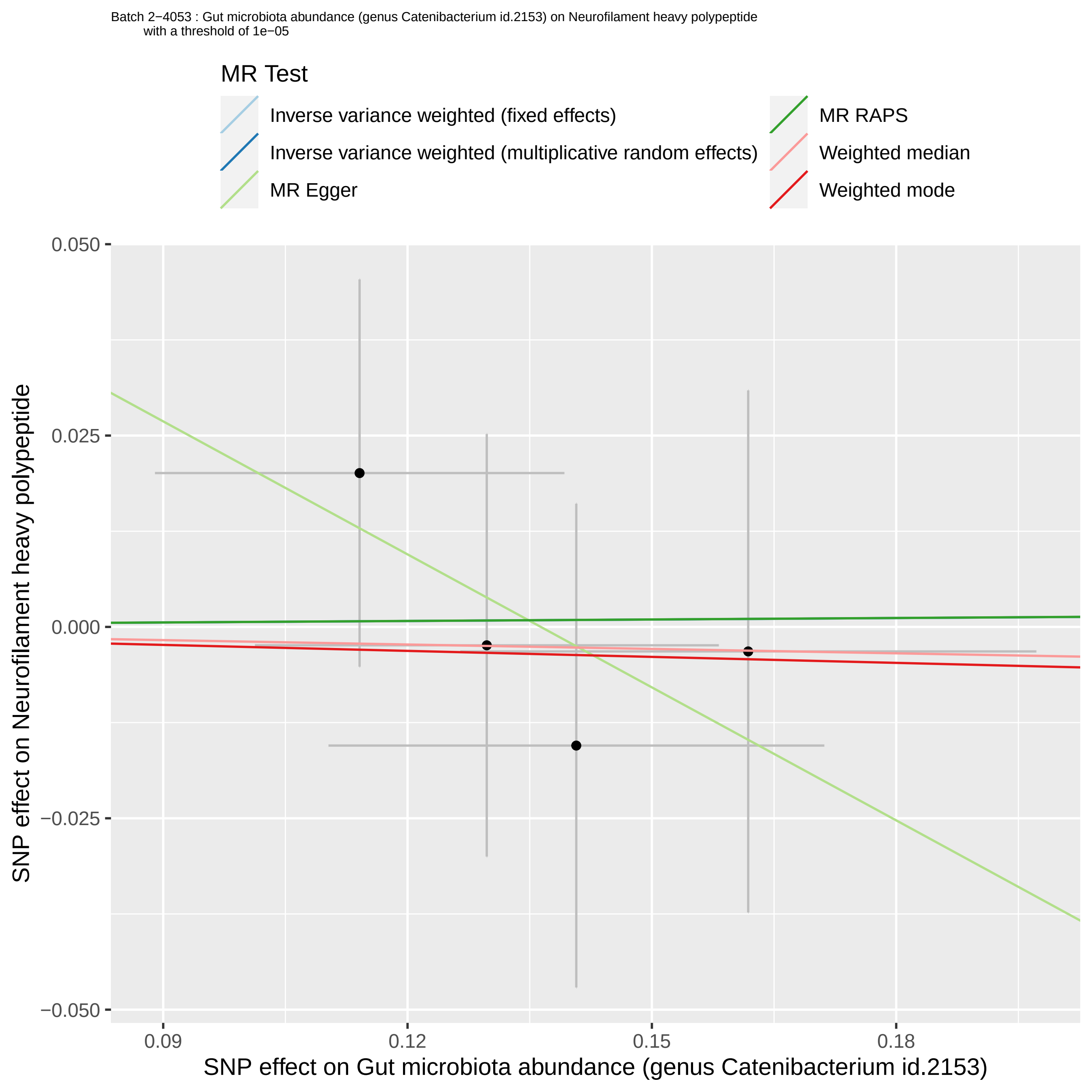
Batch 2−4053 : Gut microbiota abundance (genus Catenibacterium id.2153) on Neurofilament heavy polypeptide
with a threshold of 1e−05
MR Test
Inverse variance weighted (fixed effects)
Inverse variance weighted (multiplicative random effects)
MR Egger
MR RAPS
Weighted median
Weighted mode
0.050
0.025
0.000
−0.025
−0.050
0.09
0.12
0.15
0.18
SNP effect on Neurofilament heavy polypeptide
SNP effect on Gut microbiota abundance (genus Catenibacterium id.2153)

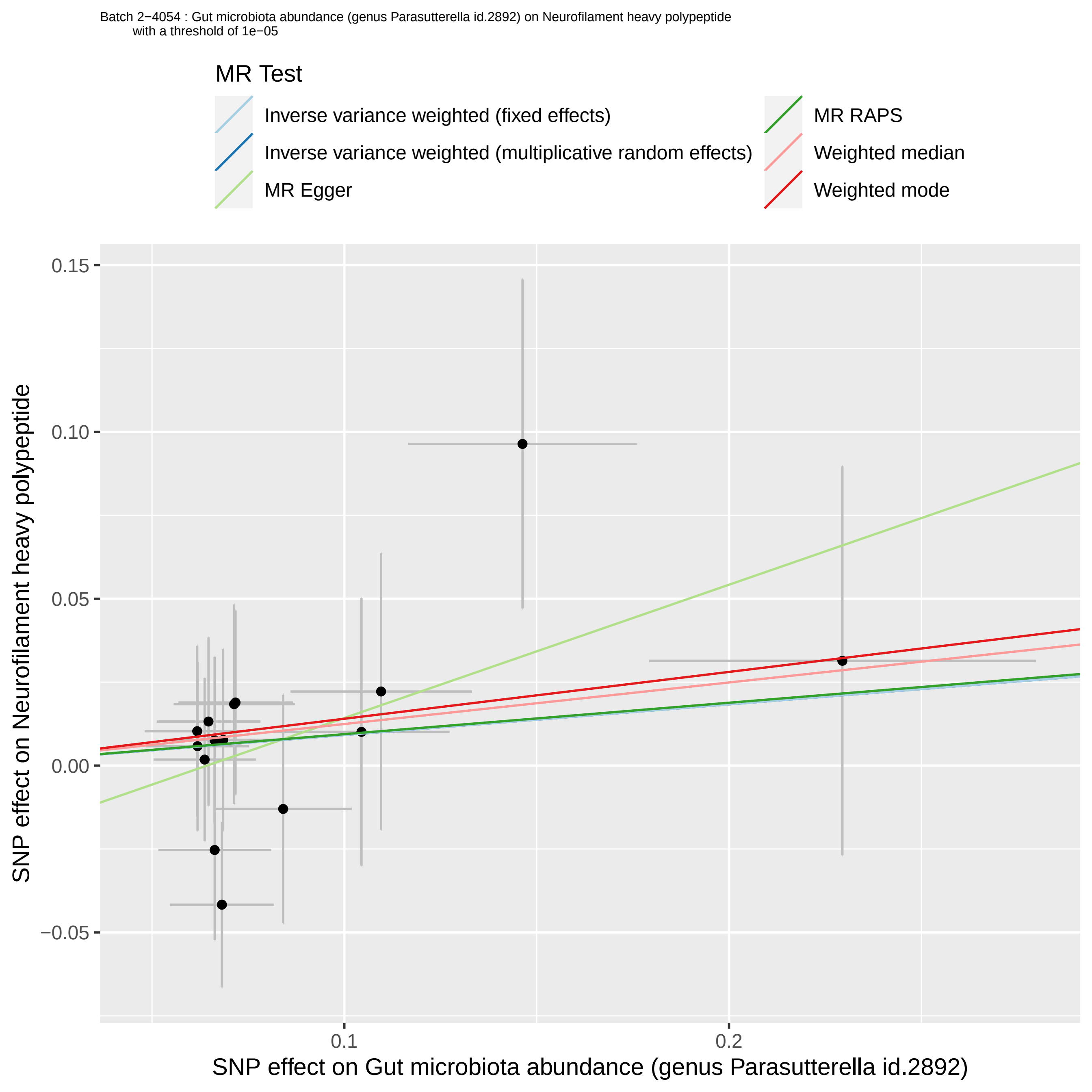
Batch 2−4054 : Gut microbiota abundance (genus Parasutterella id.2892) on Neurofilament heavy polypeptide
with a threshold of 1e−05
MR Test
Inverse variance weighted (fixed effects)
Inverse variance weighted (multiplicative random effects)
MR Egger
MR RAPS
Weighted median
Weighted mode
0.15
0.10
0.05
0.00
−0.05
0.1
0.2
SNP effect on Neurofilament heavy polypeptide
SNP effect on Gut microbiota abundance (genus Parasutterella id.2892)

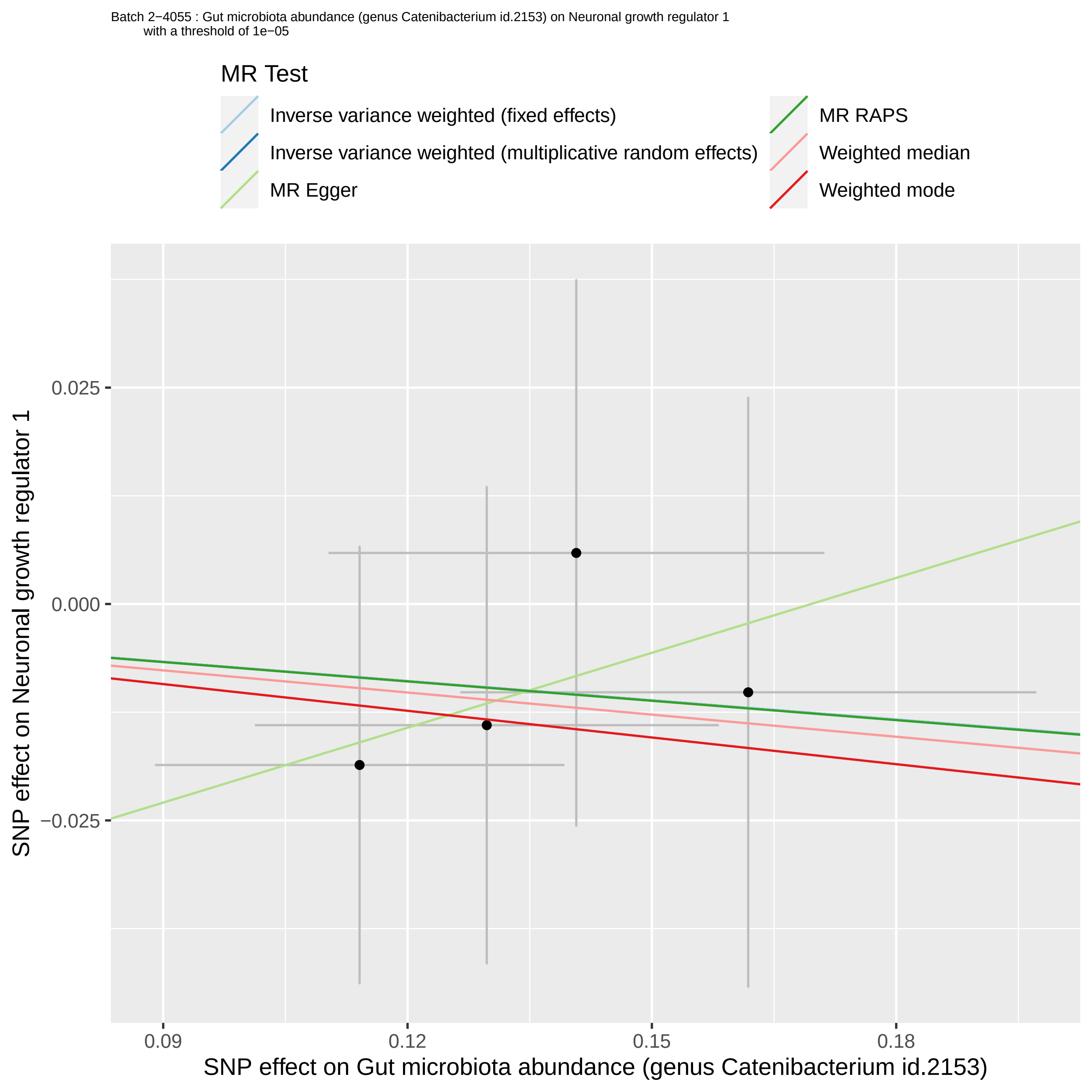

Batch 2−4055 : Gut microbiota abundance (genus Catenibacterium id.2153) on Neuronal growth regulator 1
with a threshold of 1e−05
MR Test
Inverse variance weighted (fixed effects)
Inverse variance weighted (multiplicative random effects)
MR Egger
MR RAPS
Weighted median
Weighted mode
0.025
0.000
−0.025
0.09
0.12
0.15
0.18
SNP effect on Neuronal growth regulator 1
SNP effect on Gut microbiota abundance (genus Catenibacterium id.2153)

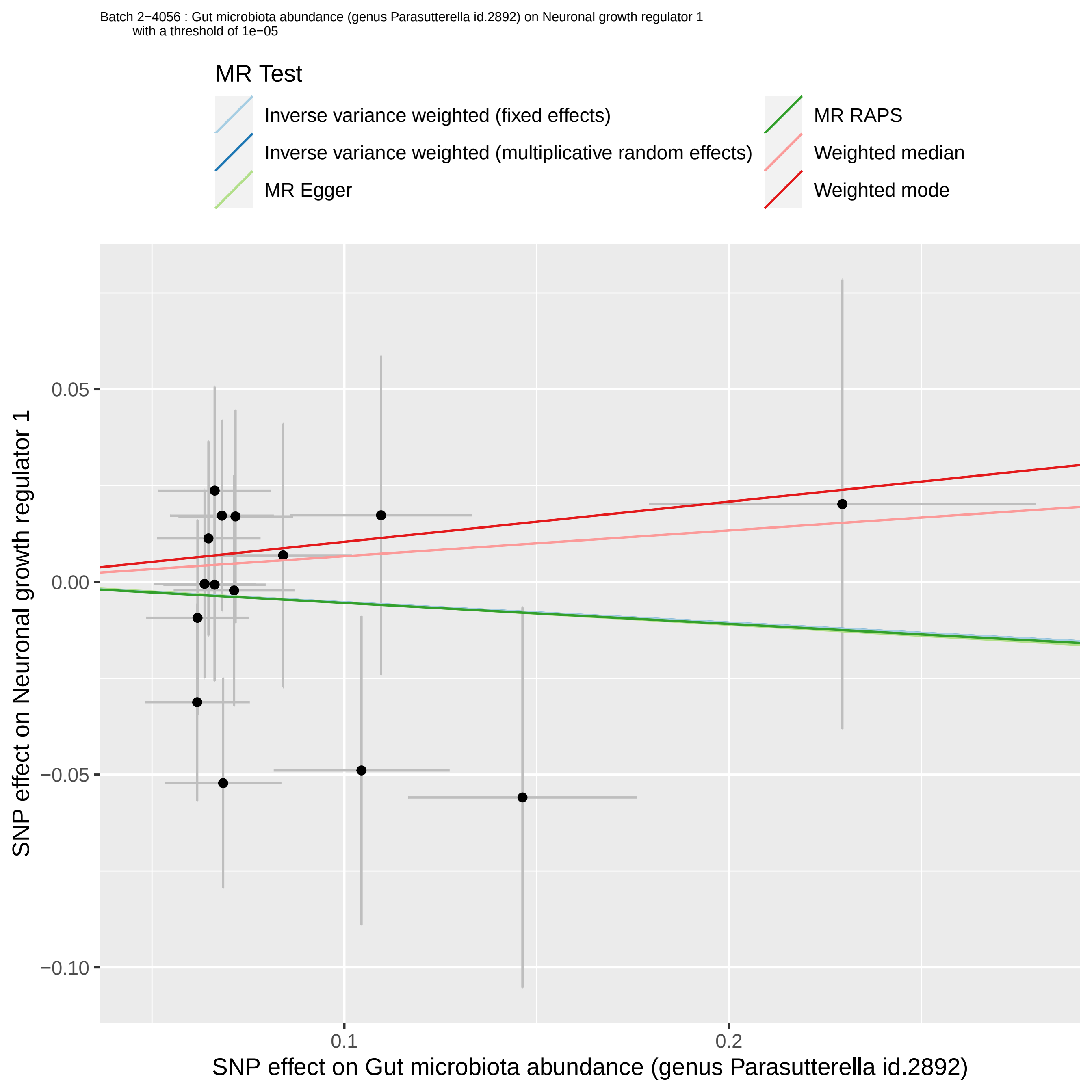

Batch 2−4056 : Gut microbiota abundance (genus Parasutterella id.2892) on Neuronal growth regulator 1
with a threshold of 1e−05
MR Test
Inverse variance weighted (fixed effects)
Inverse variance weighted (multiplicative random effects)
MR Egger
MR RAPS
Weighted median
Weighted mode
0.05
0.00
−0.05
−0.10
0.1
0.2
SNP effect on Neuronal growth regulator 1
SNP effect on Gut microbiota abundance (genus Parasutterella id.2892)

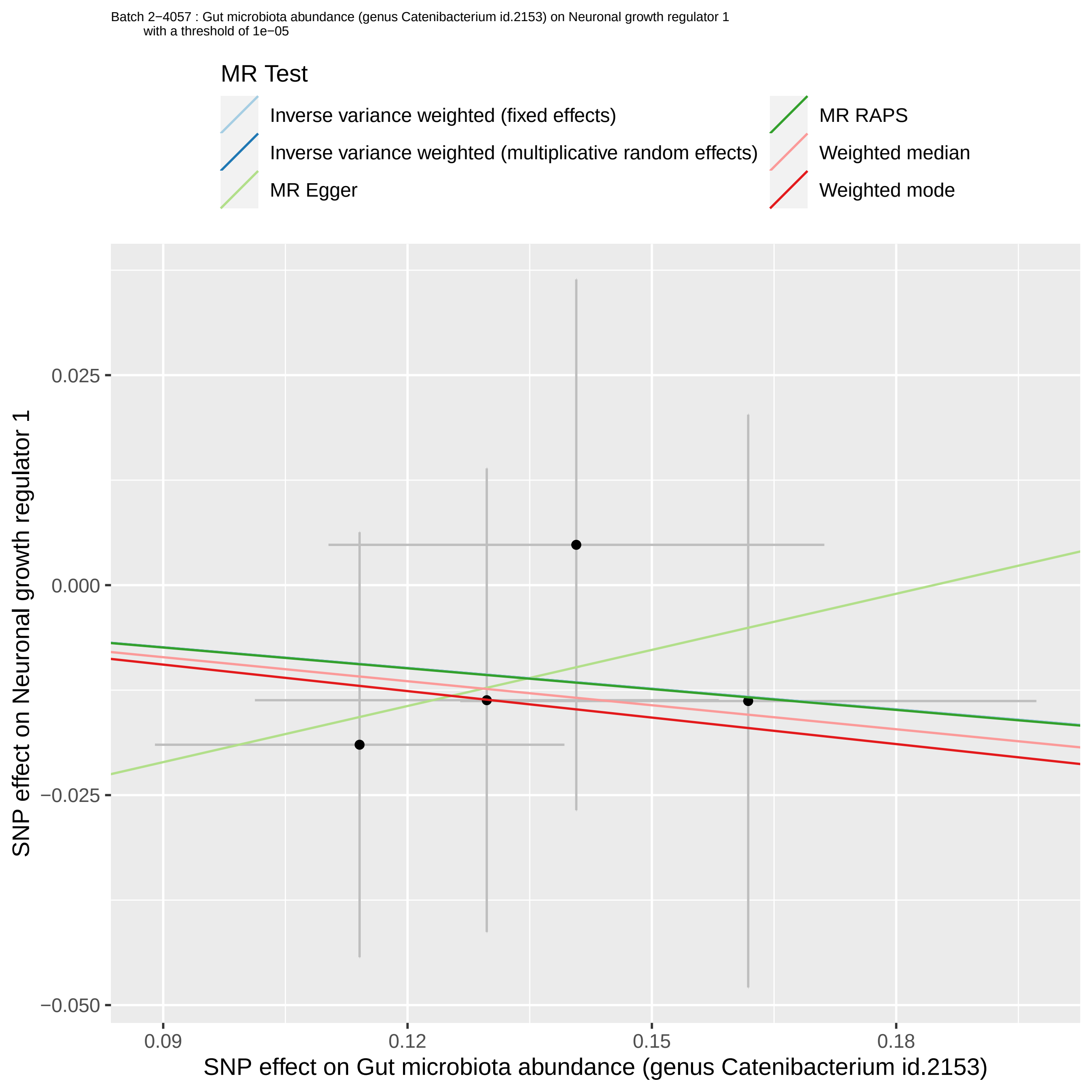

Batch 2−4057 : Gut microbiota abundance (genus Catenibacterium id.2153) on Neuronal growth regulator 1
with a threshold of 1e−05
MR Test
Inverse variance weighted (fixed effects)
Inverse variance weighted (multiplicative random effects)
MR Egger
MR RAPS
Weighted median
Weighted mode
0.025
0.000
−0.025
−0.050
0.09
0.12
0.15
0.18
SNP effect on Neuronal growth regulator 1
SNP effect on Gut microbiota abundance (genus Catenibacterium id.2153)

Batch 2−4058 : Gut microbiota abundance (genus Parasutterella id.2892) on Neuronal growth regulator 1
with a threshold of 1e−05

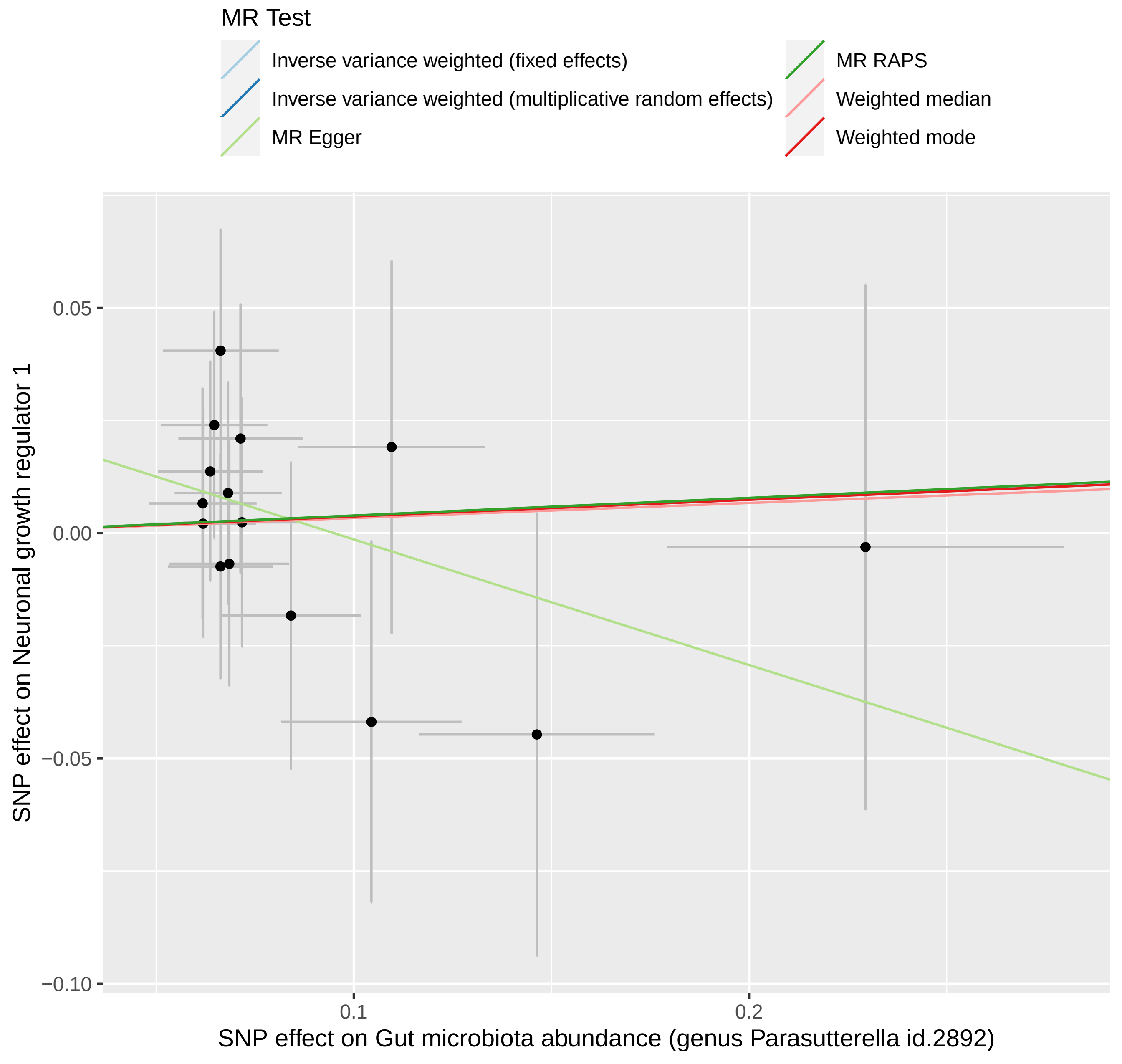

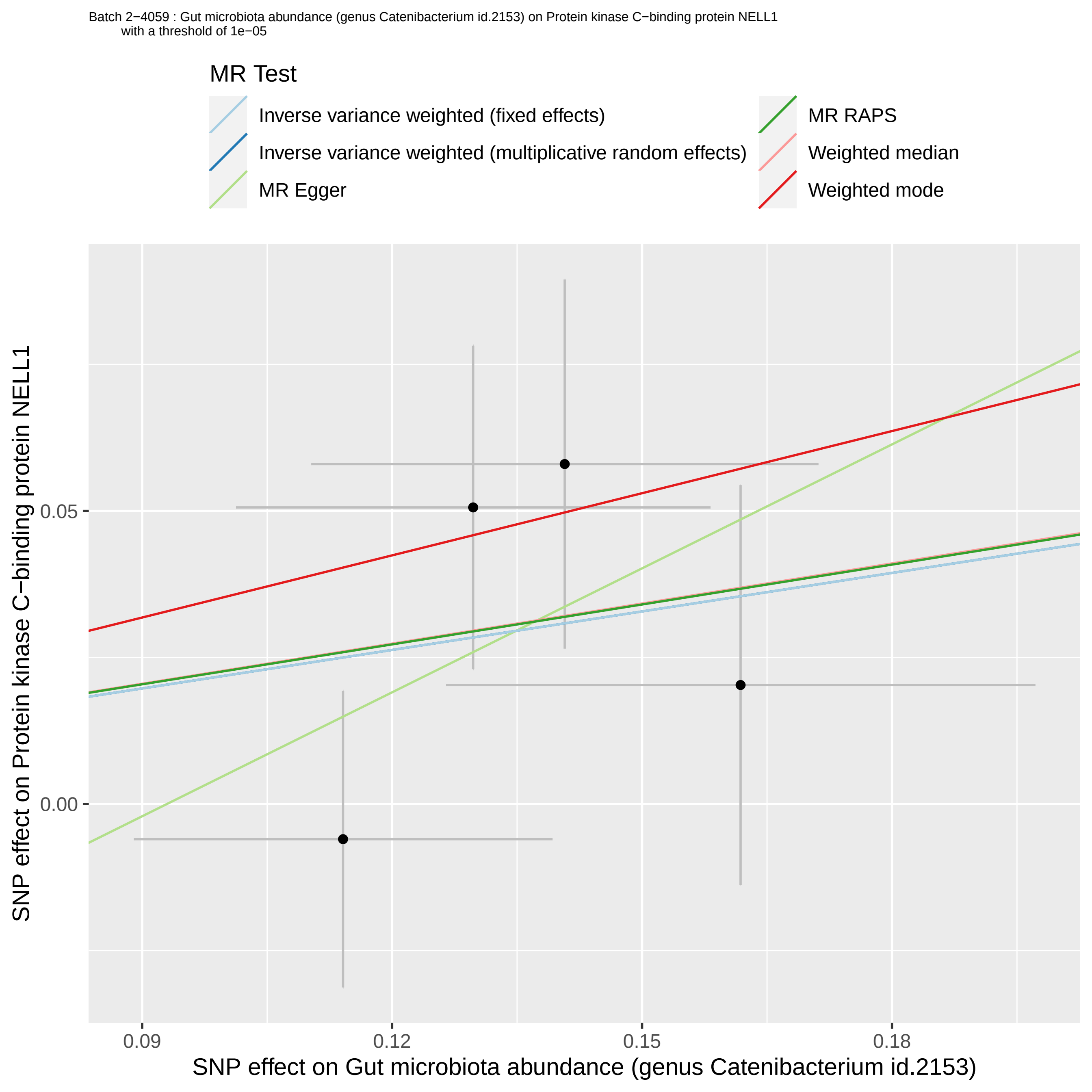
Batch 2−4059 : Gut microbiota abundance (genus Catenibacterium id.2153) on Protein kinase C−binding protein NELL1
with a threshold of 1e−05
MR Test
Inverse variance weighted (fixed effects)
Inverse variance weighted (multiplicative random effects)
MR Egger
MR RAPS
Weighted median
Weighted mode
0.05
0.00
0.09
0.12
0.15
0.18
SNP effect on Gut microbiota abundance (genus Catenibacterium id.2153)
SNP effect on Protein kinase C−binding protein NELL1

Batch 2−4060 : Gut microbiota abundance (genus Parasutterella id.2892) on Protein kinase C−binding protein NELL1
with a threshold of 1e−05

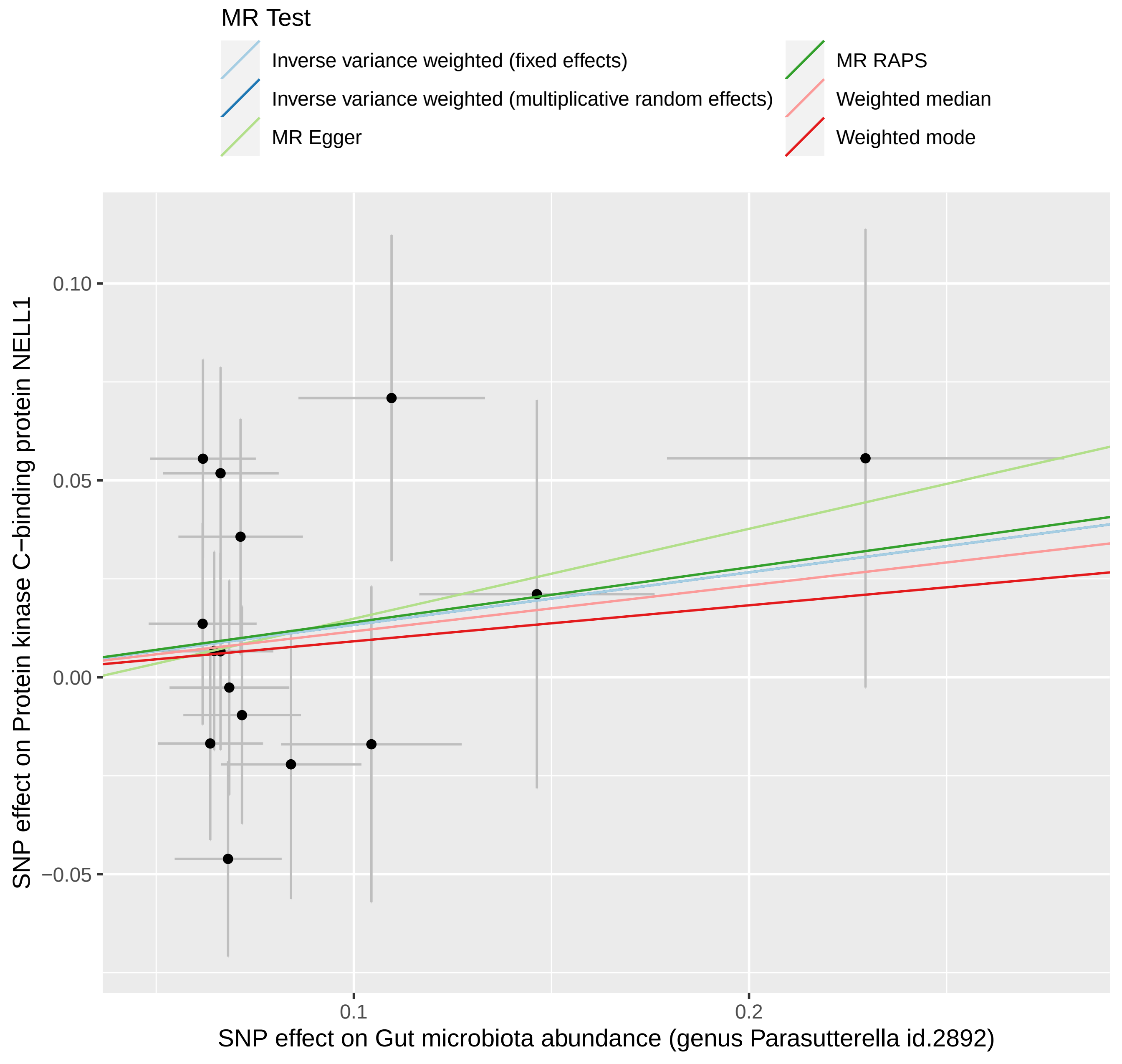

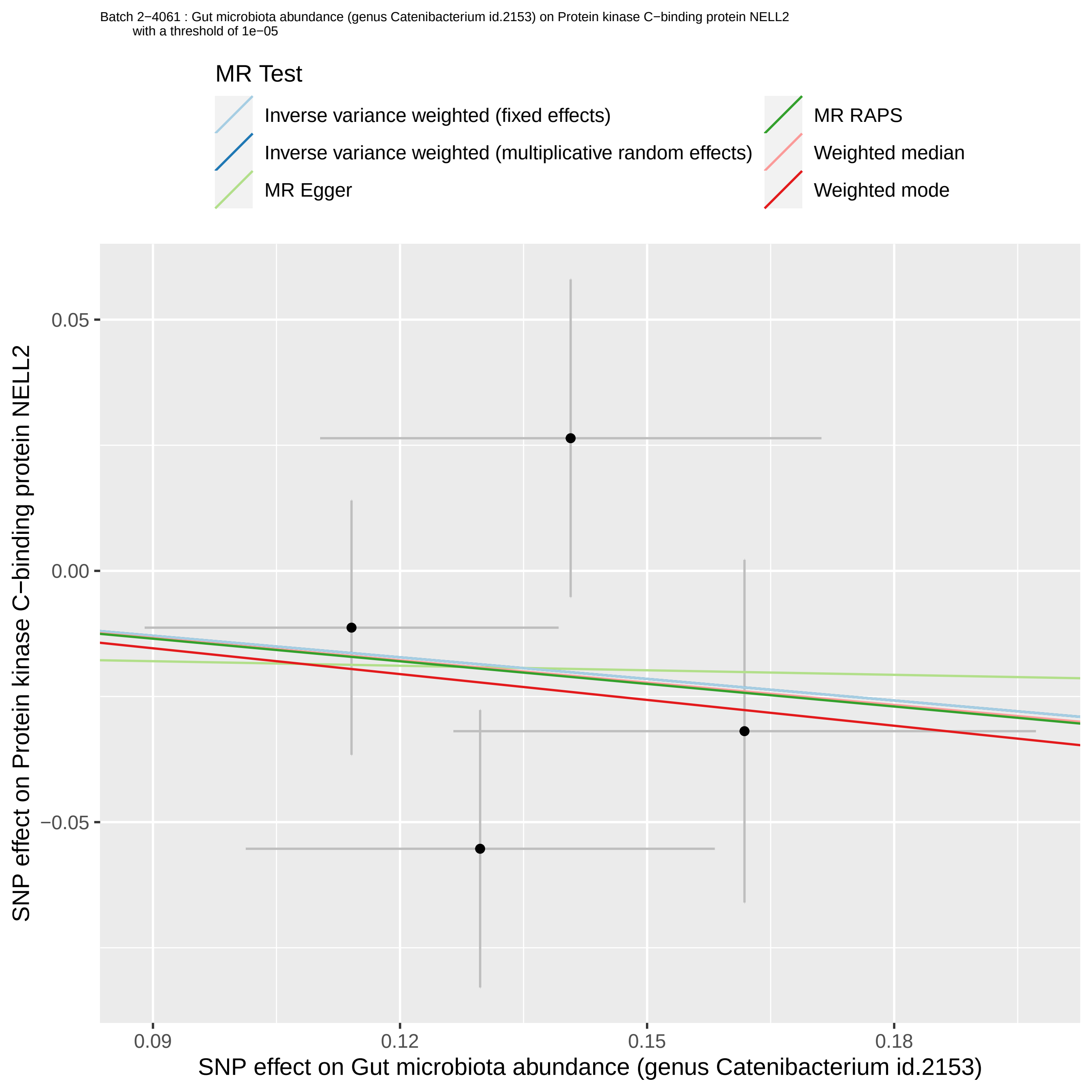
Batch 2−4061 : Gut microbiota abundance (genus Catenibacterium id.2153) on Protein kinase C−binding protein NELL2
with a threshold of 1e−05
MR Test
Inverse variance weighted (fixed effects)
Inverse variance weighted (multiplicative random effects)
MR Egger
MR RAPS
Weighted median
Weighted mode
0.05
0.00
−0.05
0.09
0.12
0.15
0.18
SNP effect on Gut microbiota abundance (genus Catenibacterium id.2153)
SNP effect on Protein kinase C−binding protein NELL2

Batch 2−4062 : Gut microbiota abundance (genus Parasutterella id.2892) on Protein kinase C−binding protein NELL2
with a threshold of 1e−05

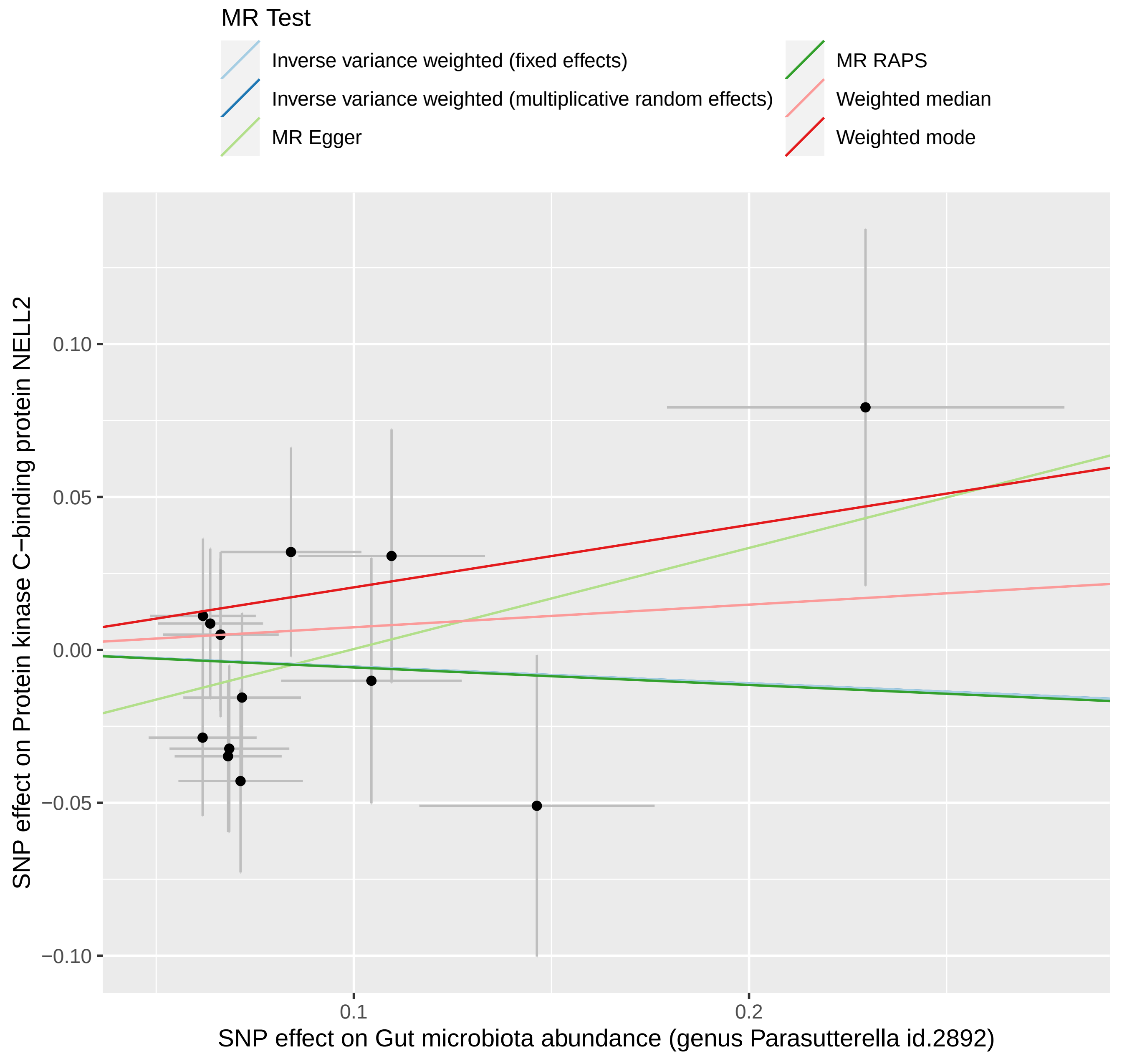

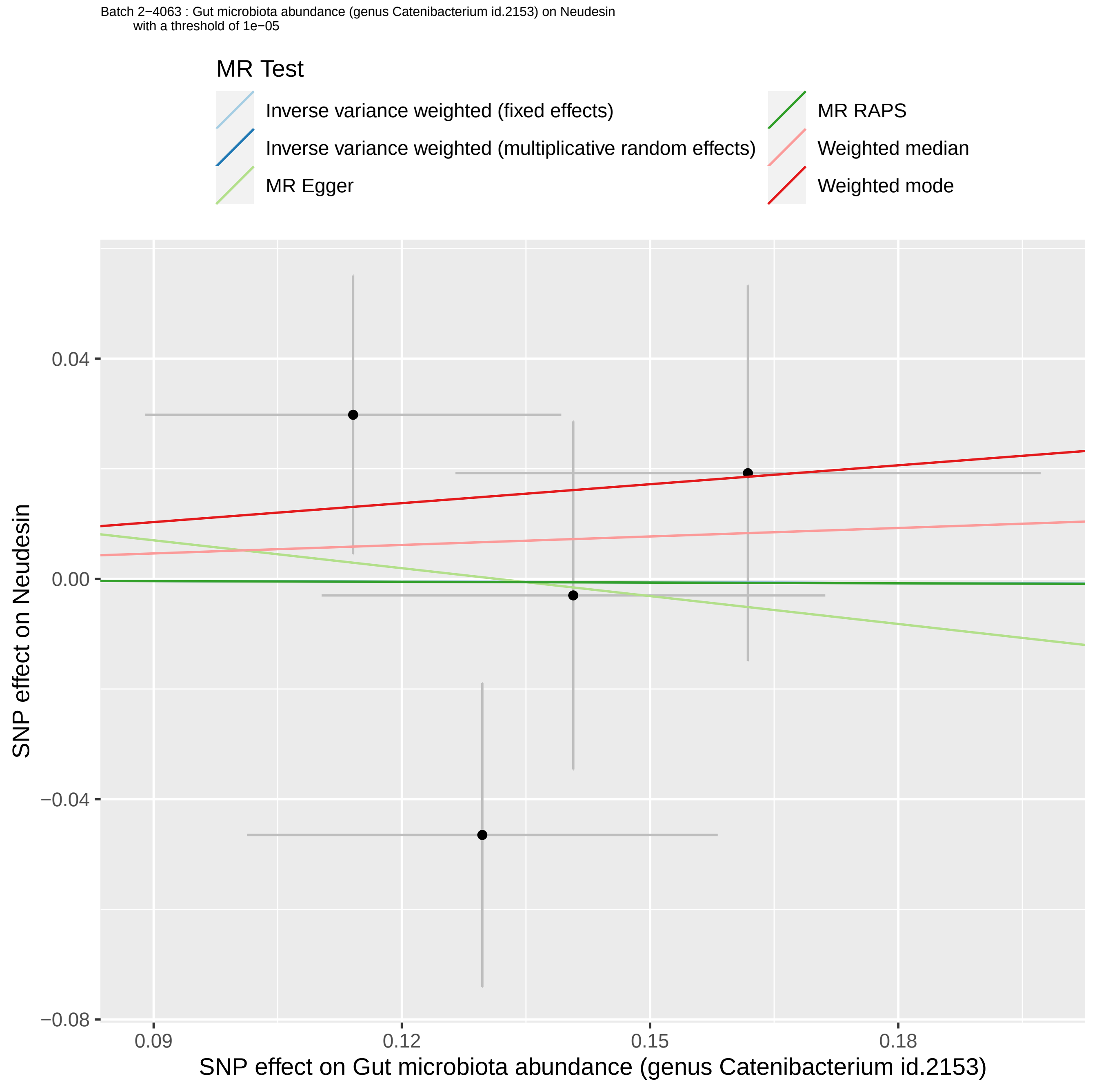

Batch 2−4063 : Gut microbiota abundance (genus Catenibacterium id.2153) on Neudesin
with a threshold of 1e−05
MR Test
Inverse variance weighted (fixed effects)
Inverse variance weighted (multiplicative random effects)
MR Egger
MR RAPS
Weighted median
Weighted mode
0.04
0.00
−0.04
−0.08
0.09
0.12
0.15
0.18
SNP effect on Neudesin
SNP effect on Gut microbiota abundance (genus Catenibacterium id.2153)

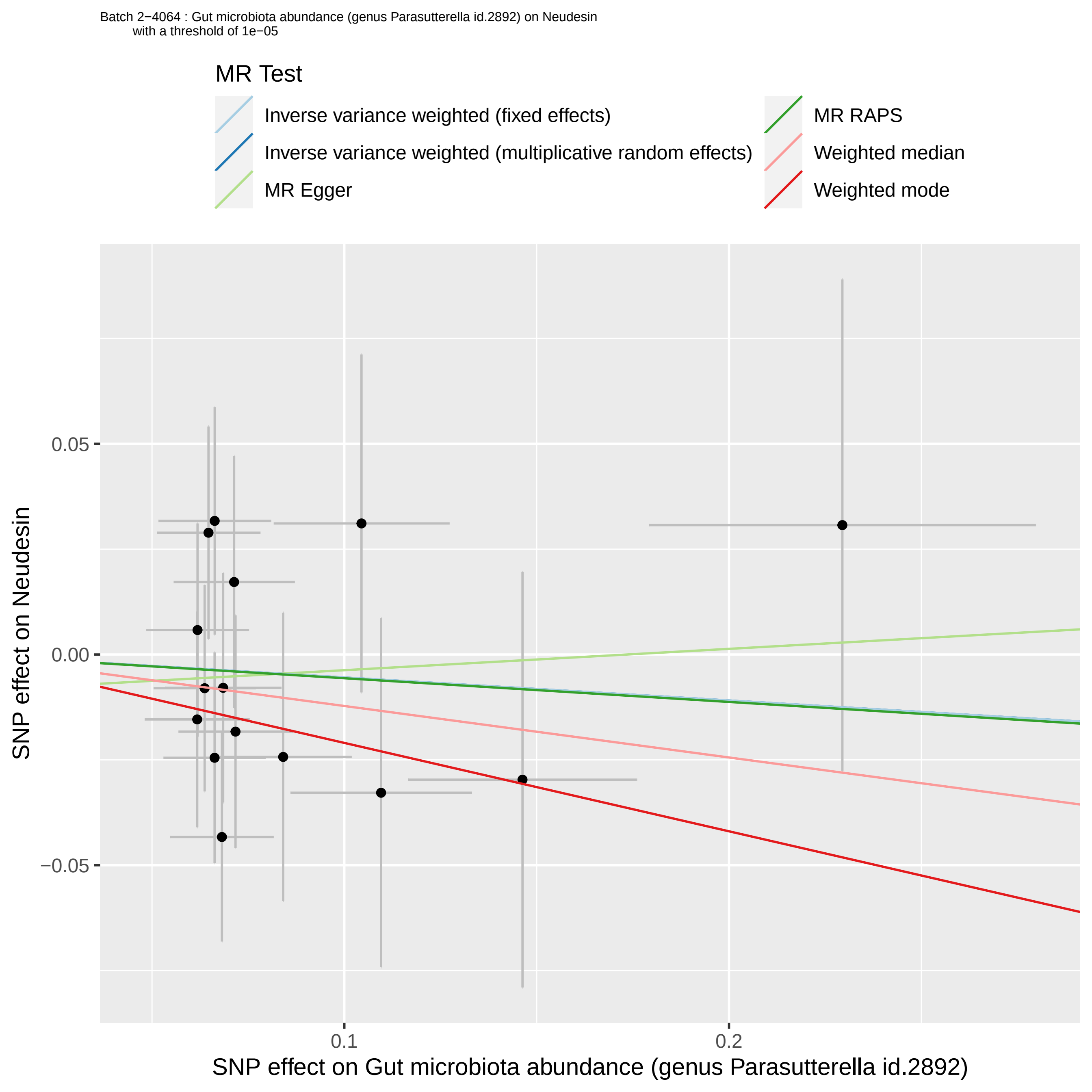
Batch 2−4064 : Gut microbiota abundance (genus Parasutterella id.2892) on Neudesin
with a threshold of 1e−05
MR Test
Inverse variance weighted (fixed effects)
Inverse variance weighted (multiplicative random effects)
MR Egger
MR RAPS
Weighted median
Weighted mode
0.05
0.00
−0.05
0.1
0.2
SNP effect on Neudesin
SNP effect on Gut microbiota abundance (genus Parasutterella id.2892)

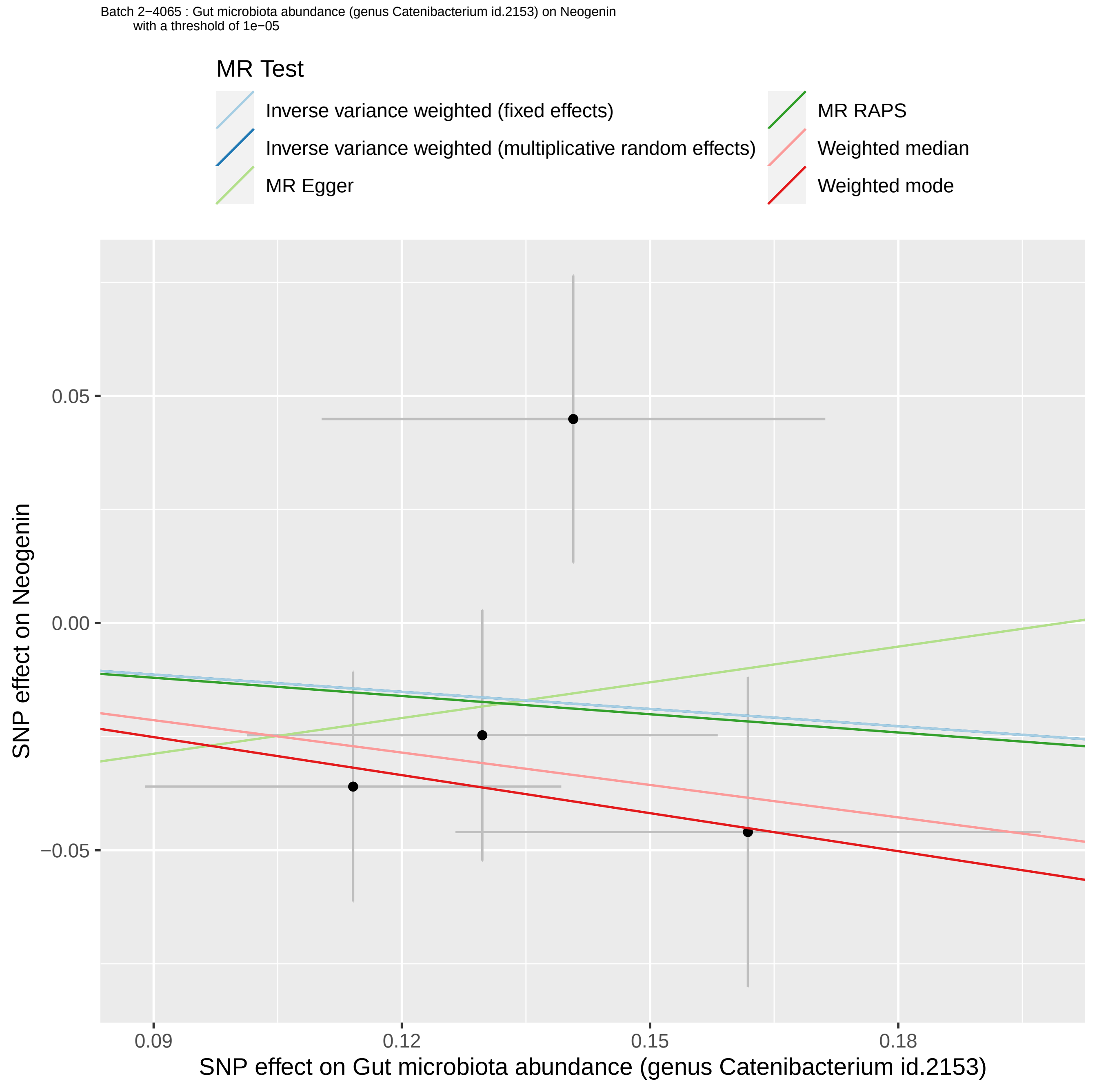
Batch 2−4065 : Gut microbiota abundance (genus Catenibacterium id.2153) on Neogenin
with a threshold of 1e−05
MR Test
Inverse variance weighted (fixed effects)
Inverse variance weighted (multiplicative random effects)
MR Egger
MR RAPS
Weighted median
Weighted mode
0.05
0.00
−0.05
0.09
0.12
0.15
0.18
SNP effect on Neogenin
SNP effect on Gut microbiota abundance (genus Catenibacterium id.2153)

Batch 2−4066 : Gut microbiota abundance (genus Parasutterella id.2892) on Neogenin
with a threshold of 1e−05

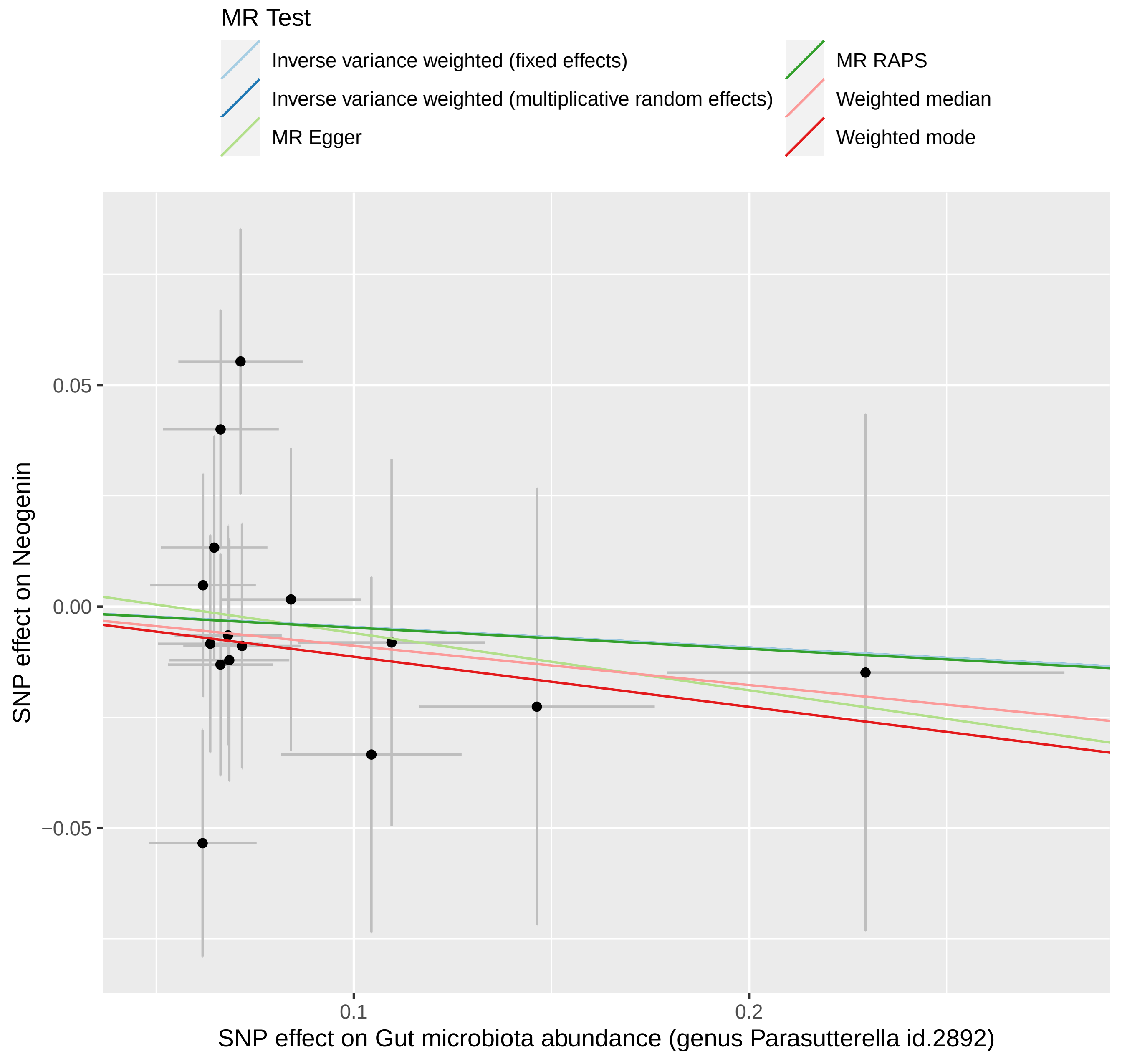

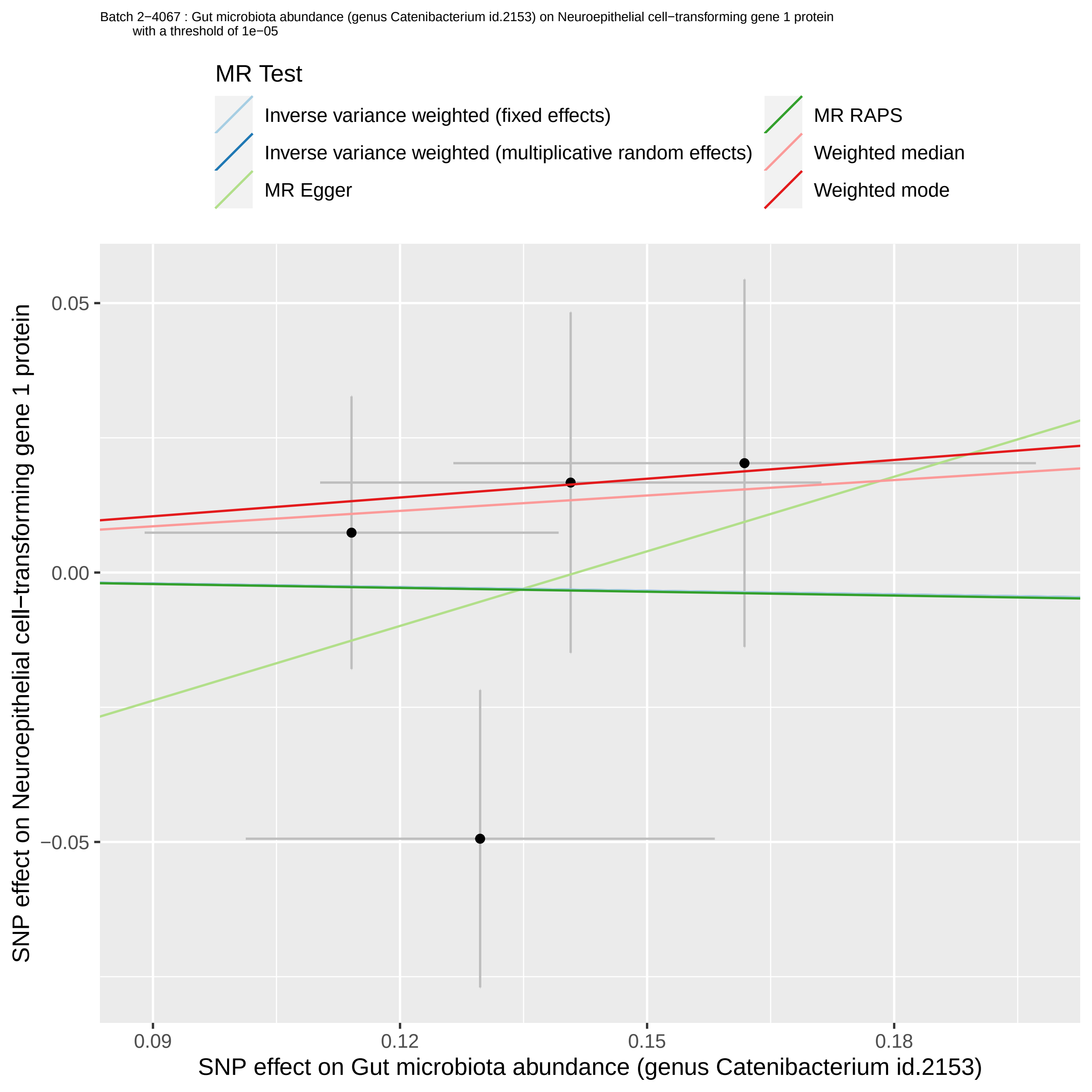
Batch 2−4067 : Gut microbiota abundance (genus Catenibacterium id.2153) on Neuroepithelial cell−transforming gene 1 protein
with a threshold of 1e−05
MR Test
Inverse variance weighted (fixed effects)
Inverse variance weighted (multiplicative random effects)
MR Egger
MR RAPS
Weighted median
Weighted mode
0.05
0.00
−0.05
0.09
0.12
0.15
0.18
SNP effect on Neuroepithelial cell−transforming gene 1 protein
SNP effect on Gut microbiota abundance (genus Catenibacterium id.2153)

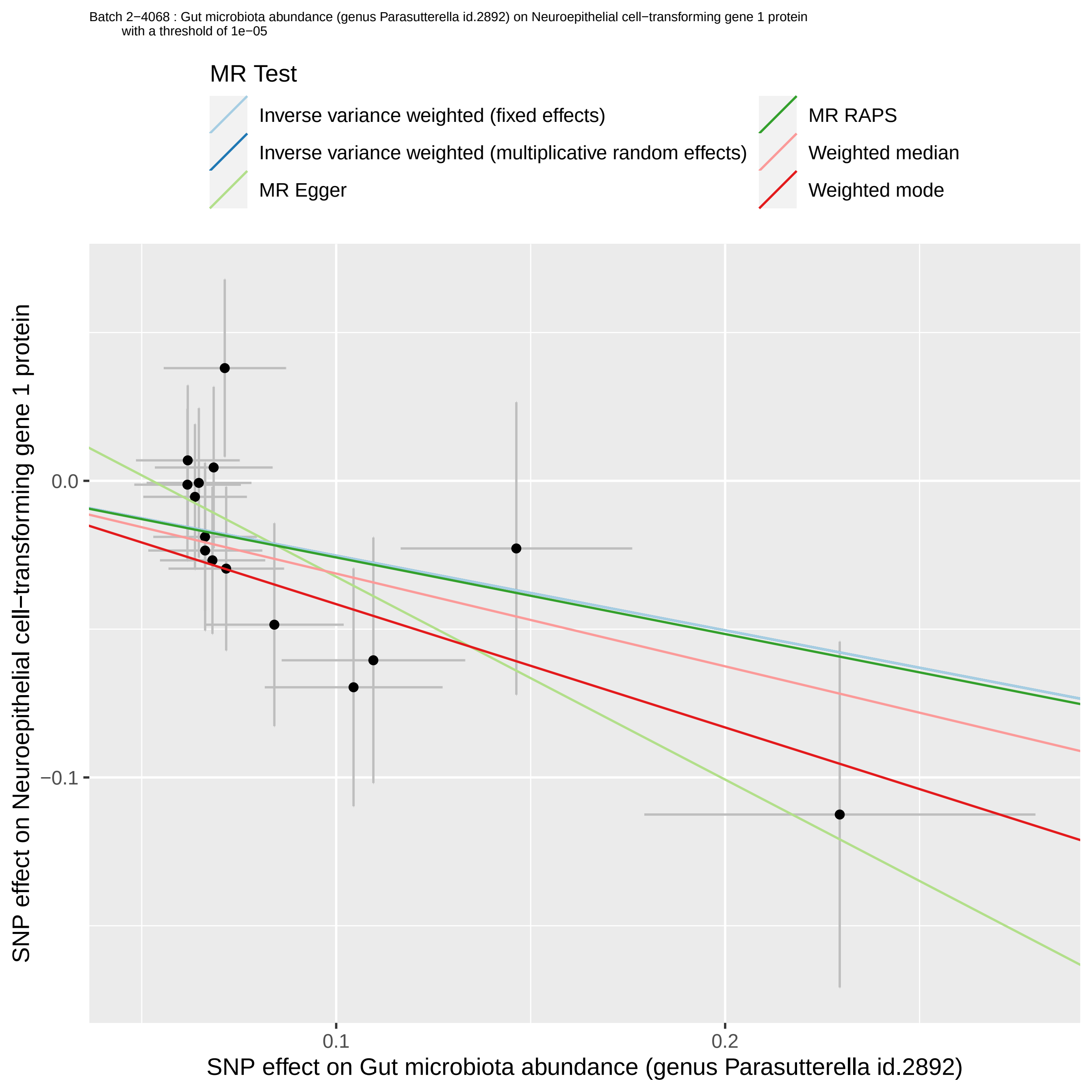
Batch 2−4068 : Gut microbiota abundance (genus Parasutterella id.2892) on Neuroepithelial cell−transforming gene 1 protein
with a threshold of 1e−05
MR Test
Inverse variance weighted (fixed effects)
Inverse variance weighted (multiplicative random effects)
MR Egger
MR RAPS
Weighted median
Weighted mode
0.0
−0.1
0.1
0.2
SNP effect on Neuroepithelial cell−transforming gene 1 protein
SNP effect on Gut microbiota abundance (genus Parasutterella id.2892)

Batch 2−4069 : Gut microbiota abundance (genus Catenibacterium id.2153) on Neuropilin and tolloid−like protein 1
with a threshold of 1e−05

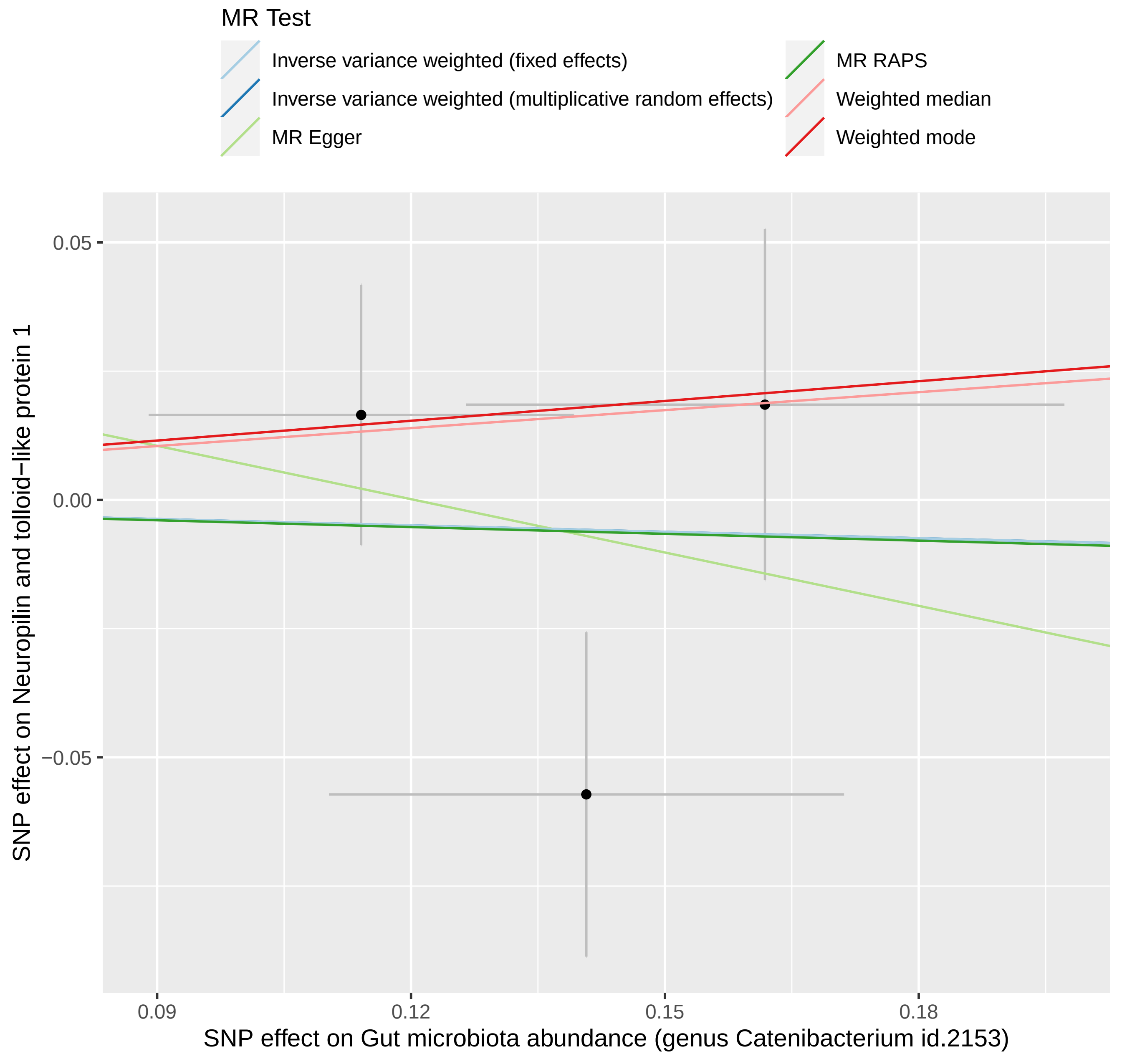

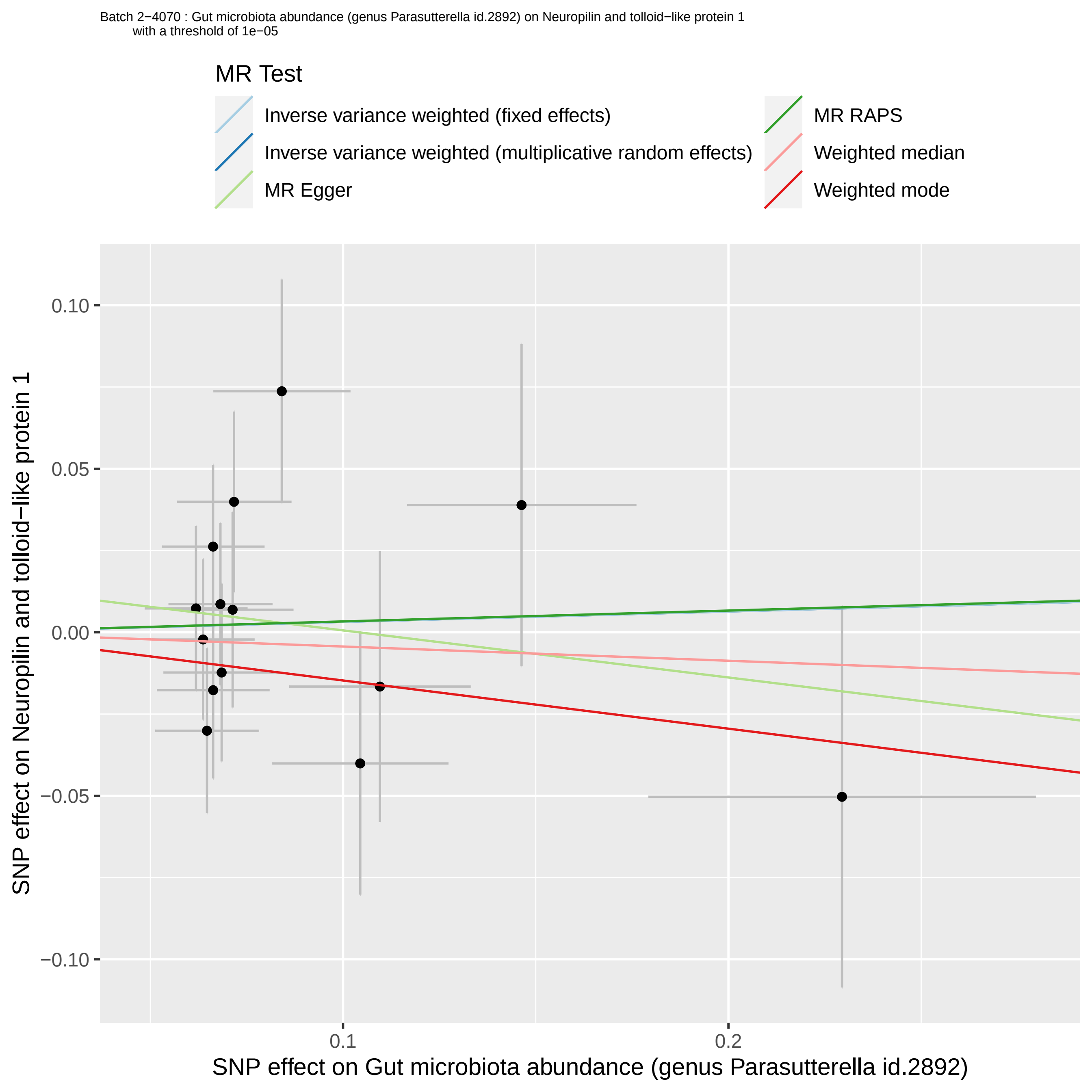

Batch 2−4070 : Gut microbiota abundance (genus Parasutterella id.2892) on Neuropilin and tolloid−like protein 1
with a threshold of 1e−05
MR Test
Inverse variance weighted (fixed effects)
Inverse variance weighted (multiplicative random effects)
MR Egger
MR RAPS
Weighted median
Weighted mode
0.10
0.05
0.00
−0.05
−0.10
0.1
0.2
SNP effect on Neuropilin and tolloid−like protein 1
SNP effect on Gut microbiota abundance (genus Parasutterella id.2892)

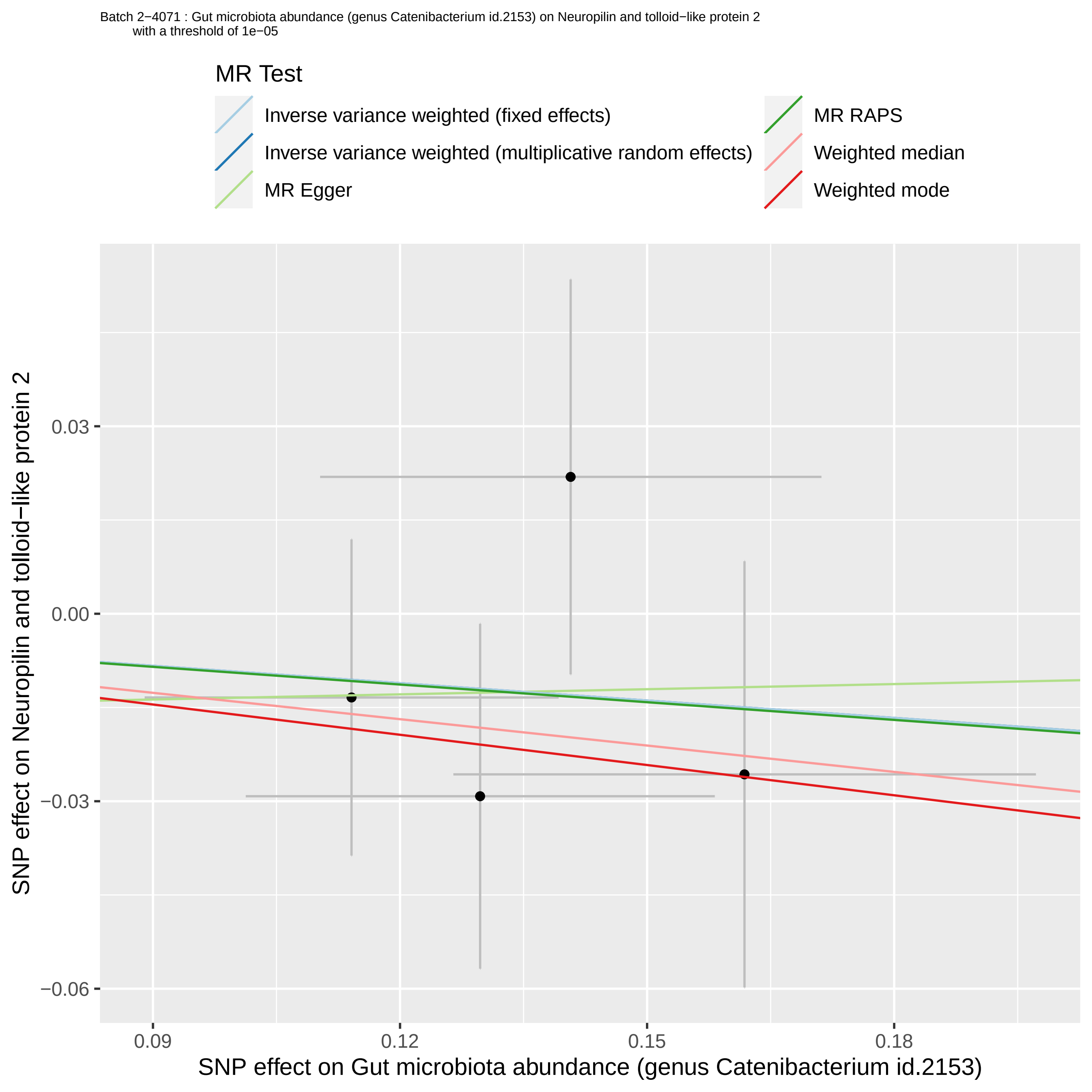

Batch 2−4071 : Gut microbiota abundance (genus Catenibacterium id.2153) on Neuropilin and tolloid−like protein 2
with a threshold of 1e−05
MR Test
Inverse variance weighted (fixed effects)
Inverse variance weighted (multiplicative random effects)
MR Egger
MR RAPS
Weighted median
Weighted mode
0.03
0.00
−0.03
−0.06
0.09
0.12
0.15
0.18
SNP effect on Neuropilin and tolloid−like protein 2
SNP effect on Gut microbiota abundance (genus Catenibacterium id.2153)

Batch 2−4072 : Gut microbiota abundance (genus Parasutterella id.2892) on Neuropilin and tolloid−like protein 2
with a threshold of 1e−05

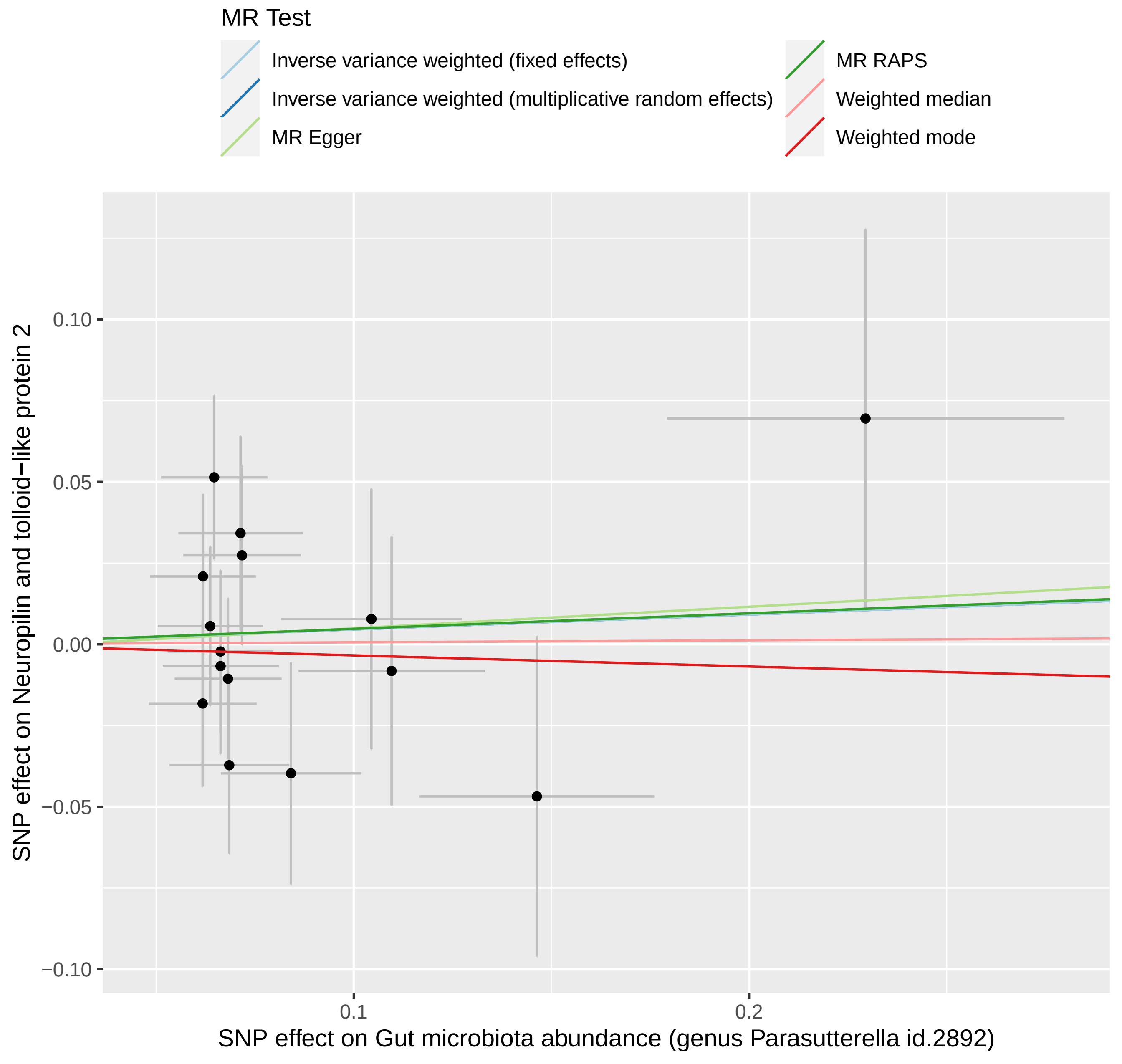

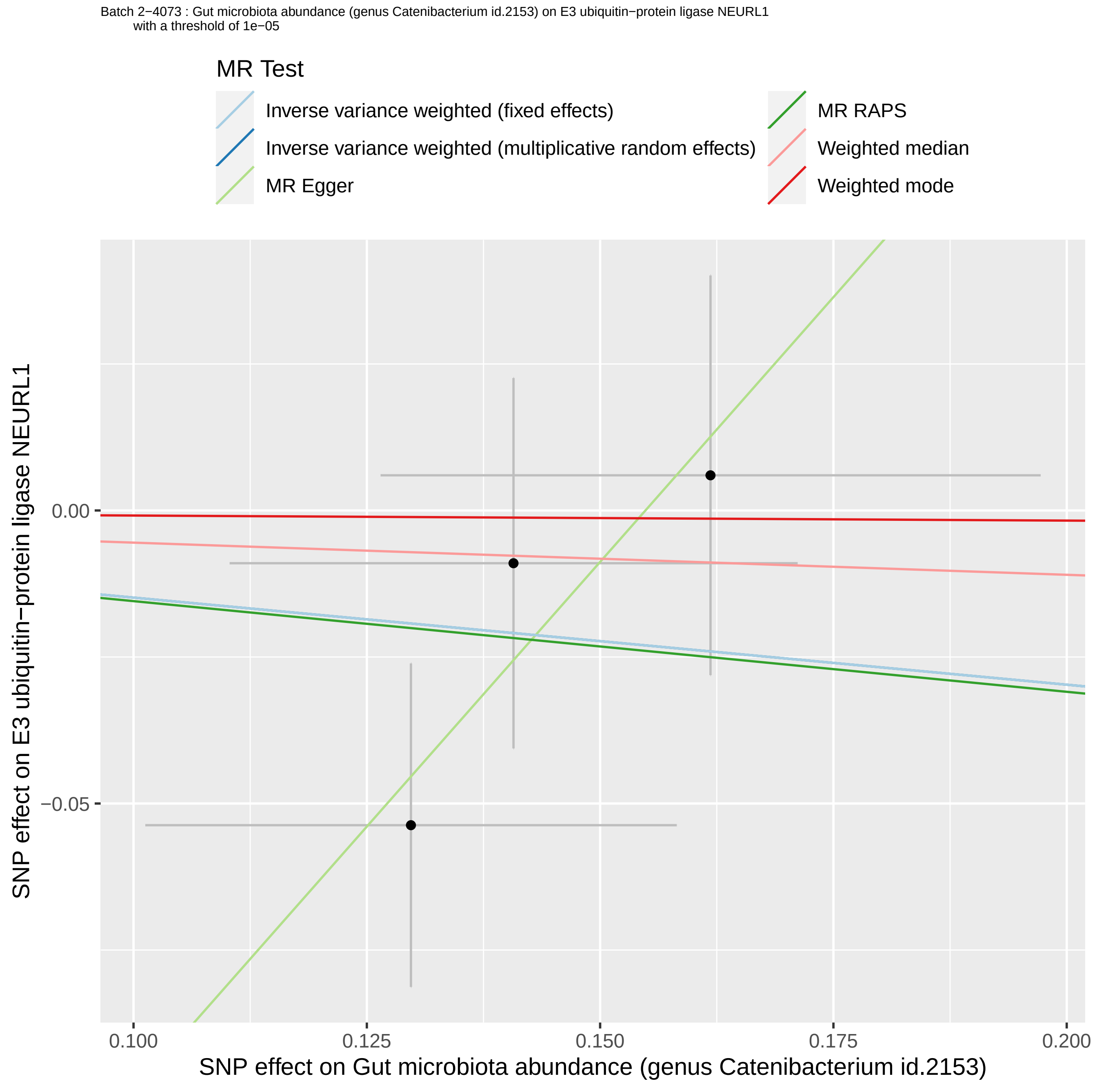

Batch 2−4073 : Gut microbiota abundance (genus Catenibacterium id.2153) on E3 ubiquitin−protein ligase NEURL1
with a threshold of 1e−05
MR Test
Inverse variance weighted (fixed effects)
Inverse variance weighted (multiplicative random effects)
MR Egger
MR RAPS
Weighted median
Weighted mode
0.00
−0.05
0.100
0.125
0.150
0.175
0.200
SNP effect on Gut microbiota abundance (genus Catenibacterium id.2153)
SNP effect on E3 ubiquitin−protein ligase NEURL1

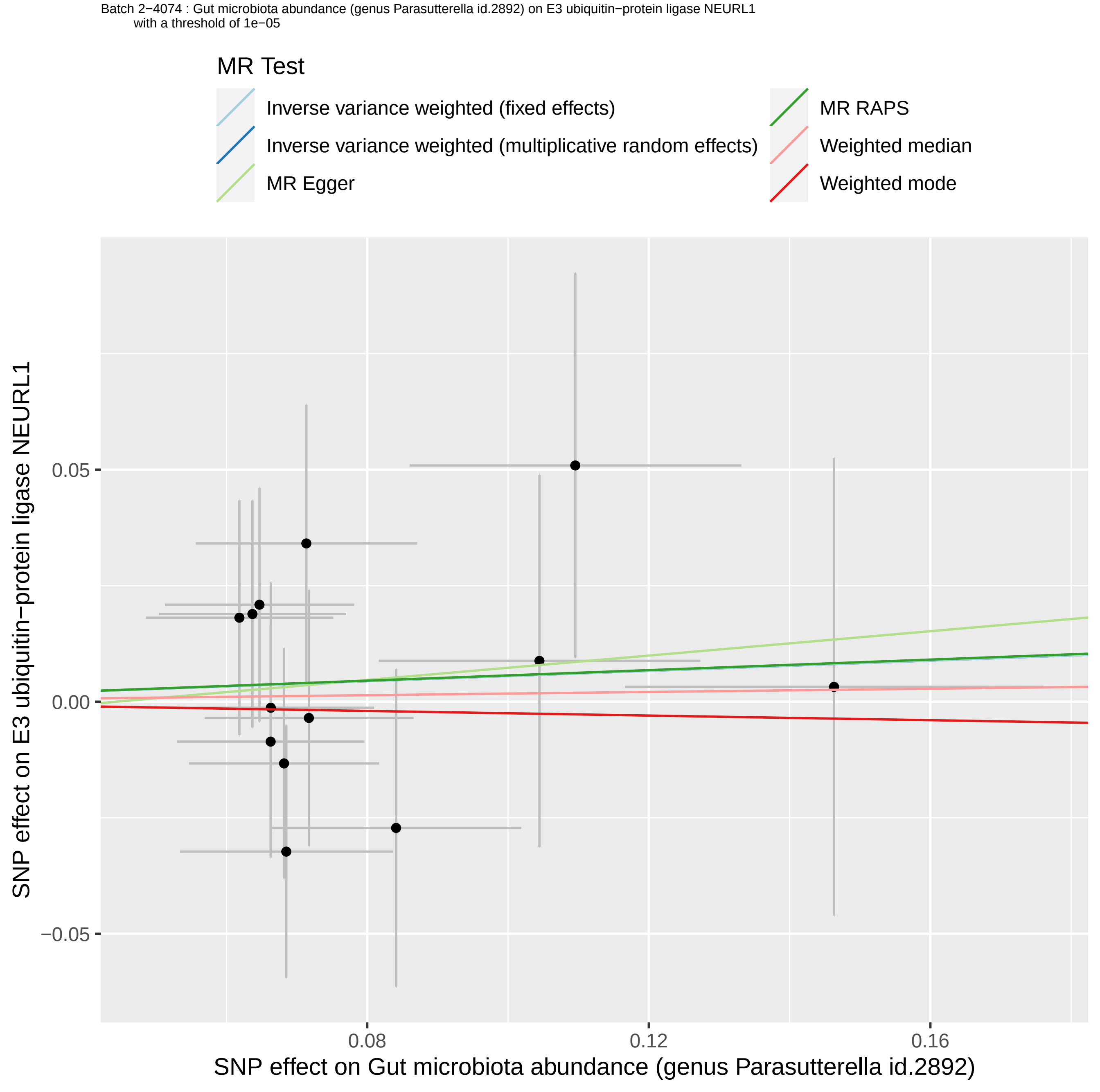

Batch 2−4074 : Gut microbiota abundance (genus Parasutterella id.2892) on E3 ubiquitin−protein ligase NEURL1
with a threshold of 1e−05
MR Test
Inverse variance weighted (fixed effects)
Inverse variance weighted (multiplicative random effects)
MR Egger
MR RAPS
Weighted median
Weighted mode
SNP effect on E3 ubiquitin−protein ligase NEURL1
0.05
0.00
−0.05
0.08
0.12
0.16
SNP effect on Gut microbiota abundance (genus Parasutterella id.2892)

Batch 2−4075 : Gut microbiota abundance (genus Catenibacterium id.2153) on Neuralized−like protein 4 with a threshold of 1e−05

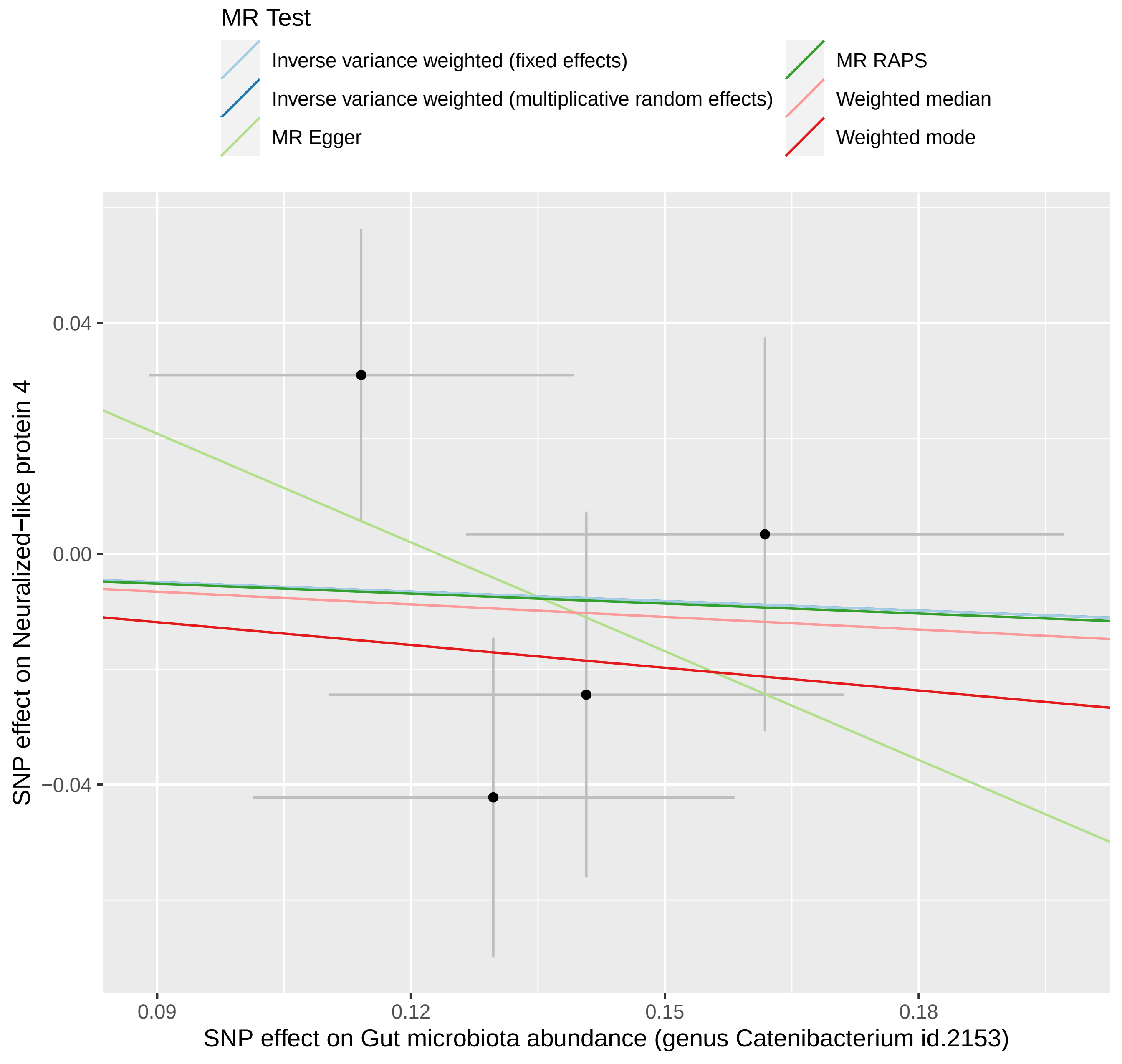

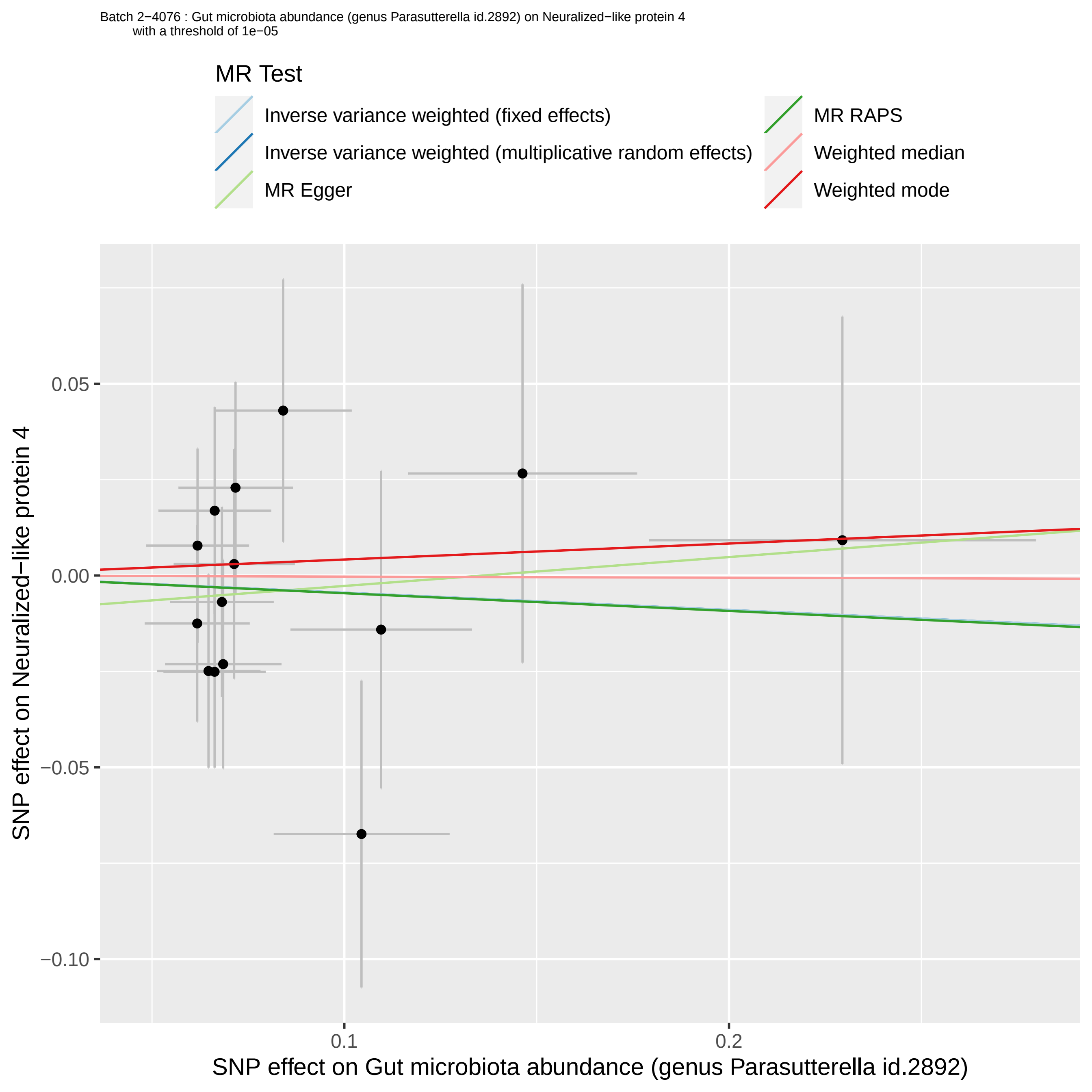
Batch 2−4076 : Gut microbiota abundance (genus Parasutterella id.2892) on Neuralized−like protein 4
with a threshold of 1e−05
MR Test
Inverse variance weighted (fixed effects)
Inverse variance weighted (multiplicative random effects)
MR Egger
MR RAPS
Weighted median
Weighted mode
0.05
0.00
−0.05
−0.10
0.1
0.2
SNP effect on Neuralized−like protein 4
SNP effect on Gut microbiota abundance (genus Parasutterella id.2892)

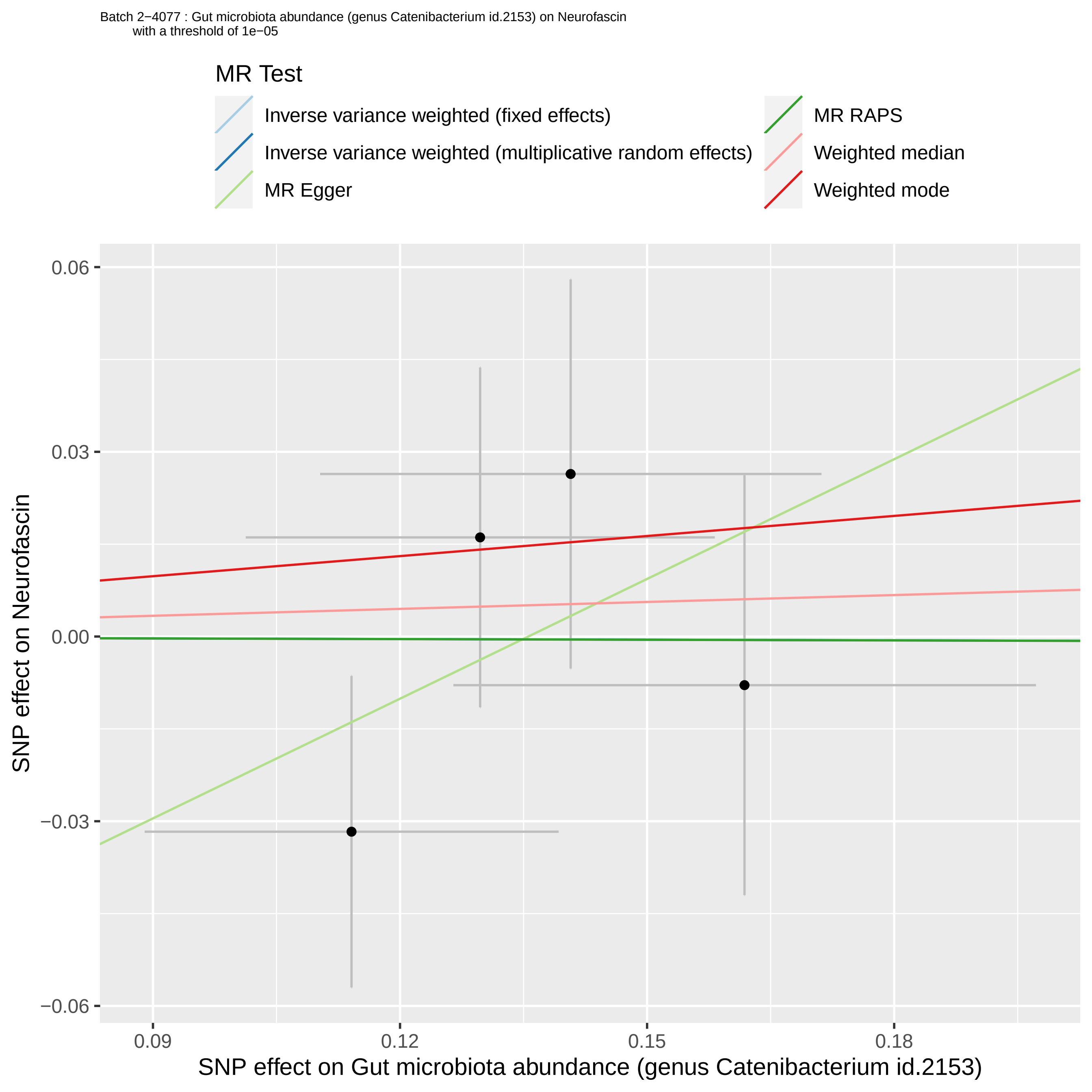
Batch 2−4077 : Gut microbiota abundance (genus Catenibacterium id.2153) on Neurofascin
with a threshold of 1e−05
MR Test
Inverse variance weighted (fixed effects)
Inverse variance weighted (multiplicative random effects)
MR Egger
MR RAPS
Weighted median
Weighted mode
0.06
0.03
0.00
−0.03
−0.06
0.09
0.12
0.15
0.18
SNP effect on Neurofascin
SNP effect on Gut microbiota abundance (genus Catenibacterium id.2153)

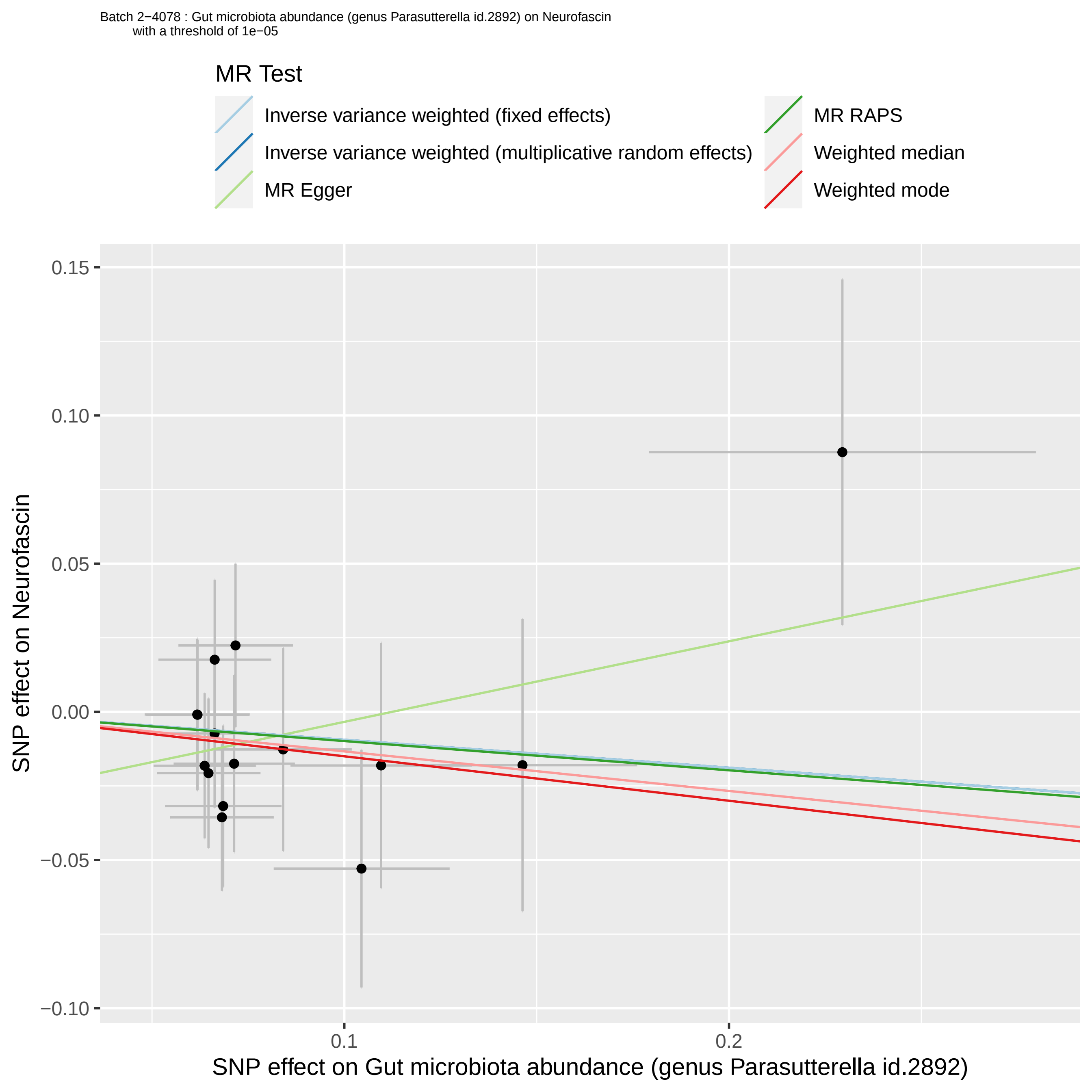
Batch 2−4078 : Gut microbiota abundance (genus Parasutterella id.2892) on Neurofascin
with a threshold of 1e−05
MR Test
Inverse variance weighted (fixed effects)
Inverse variance weighted (multiplicative random effects)
MR Egger
MR RAPS
Weighted median
Weighted mode
0.15
0.10
0.05
0.00
−0.05
−0.10
0.1
0.2
SNP effect on Neurofascin
SNP effect on Gut microbiota abundance (genus Parasutterella id.2892)

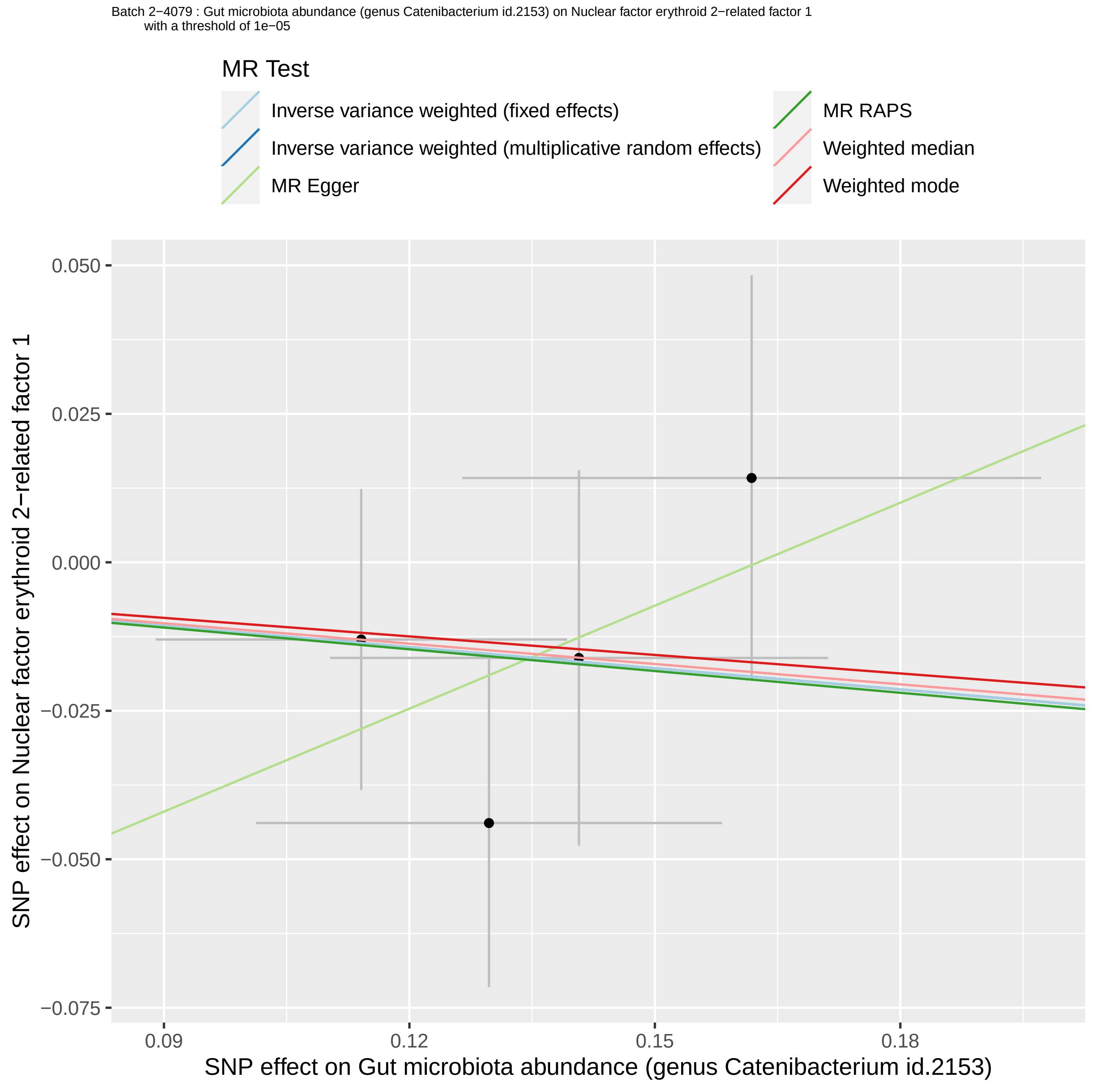
Batch 2−4079 : Gut microbiota abundance (genus Catenibacterium id.2153) on Nuclear factor erythroid 2−related factor 1
with a threshold of 1e−05
MR Test
Inverse variance weighted (fixed effects)
Inverse variance weighted (multiplicative random effects)
MR Egger
MR RAPS
Weighted median
Weighted mode
0.050
0.025
0.000
−0.025
−0.050
−0.075
0.09
0.12
0.15
0.18
SNP effect on Nuclear factor erythroid 2−related factor 1
SNP effect on Gut microbiota abundance (genus Catenibacterium id.2153)

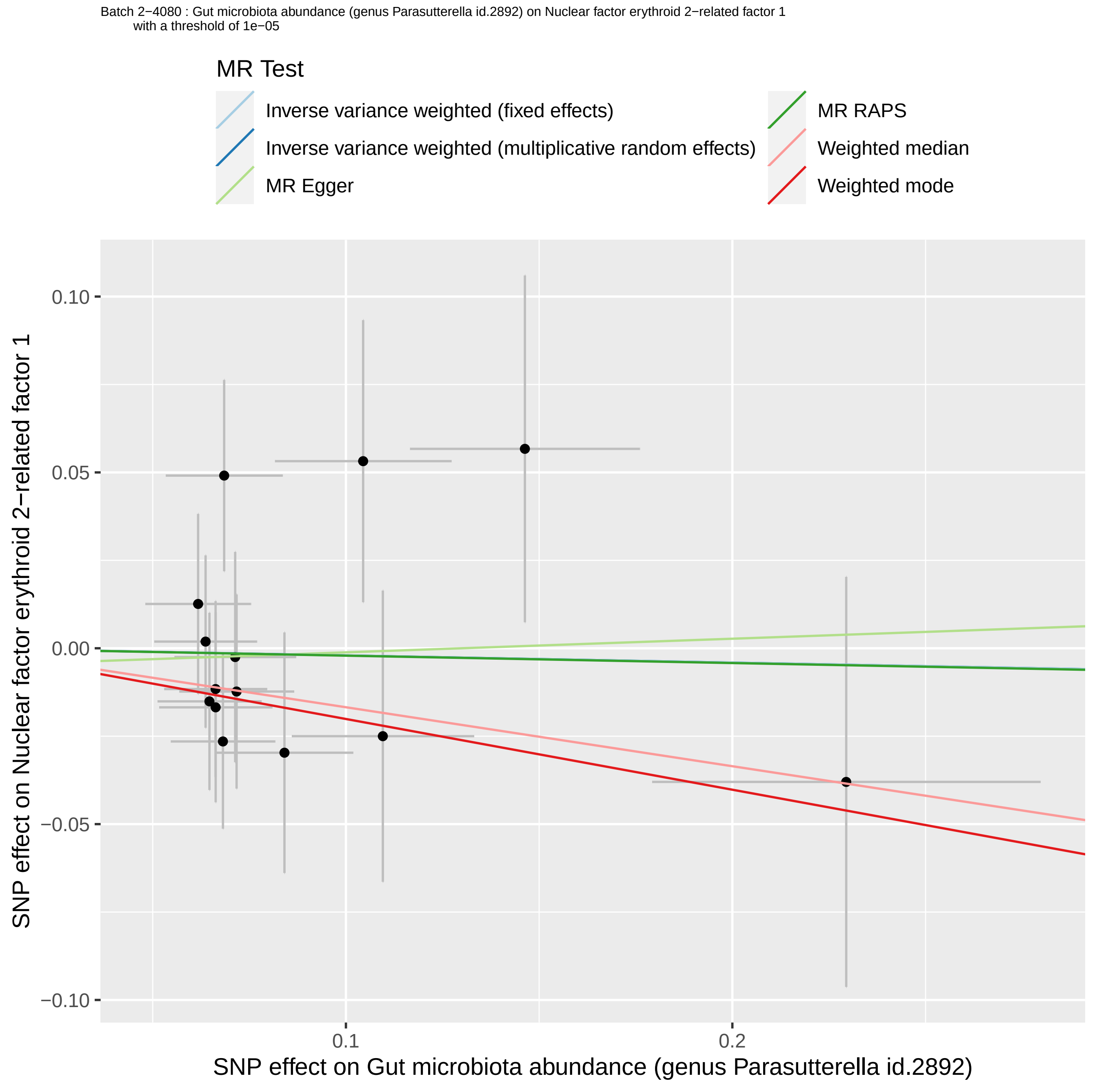

Batch 2−4080 : Gut microbiota abundance (genus Parasutterella id.2892) on Nuclear factor erythroid 2−related factor 1
with a threshold of 1e−05
MR Test
Inverse variance weighted (fixed effects)
Inverse variance weighted (multiplicative random effects)
MR Egger
MR RAPS
Weighted median
Weighted mode
0.10
0.05
0.00
−0.05
−0.10
0.1
0.2
SNP effect on Nuclear factor erythroid 2−related factor 1
SNP effect on Gut microbiota abundance (genus Parasutterella id.2892)

Batch 2−4081 : Gut microbiota abundance (genus Catenibacterium id.2153) on NFU1 iron−sulfur cluster scaffold homolog, mitochondrial
with a threshold of 1e−05

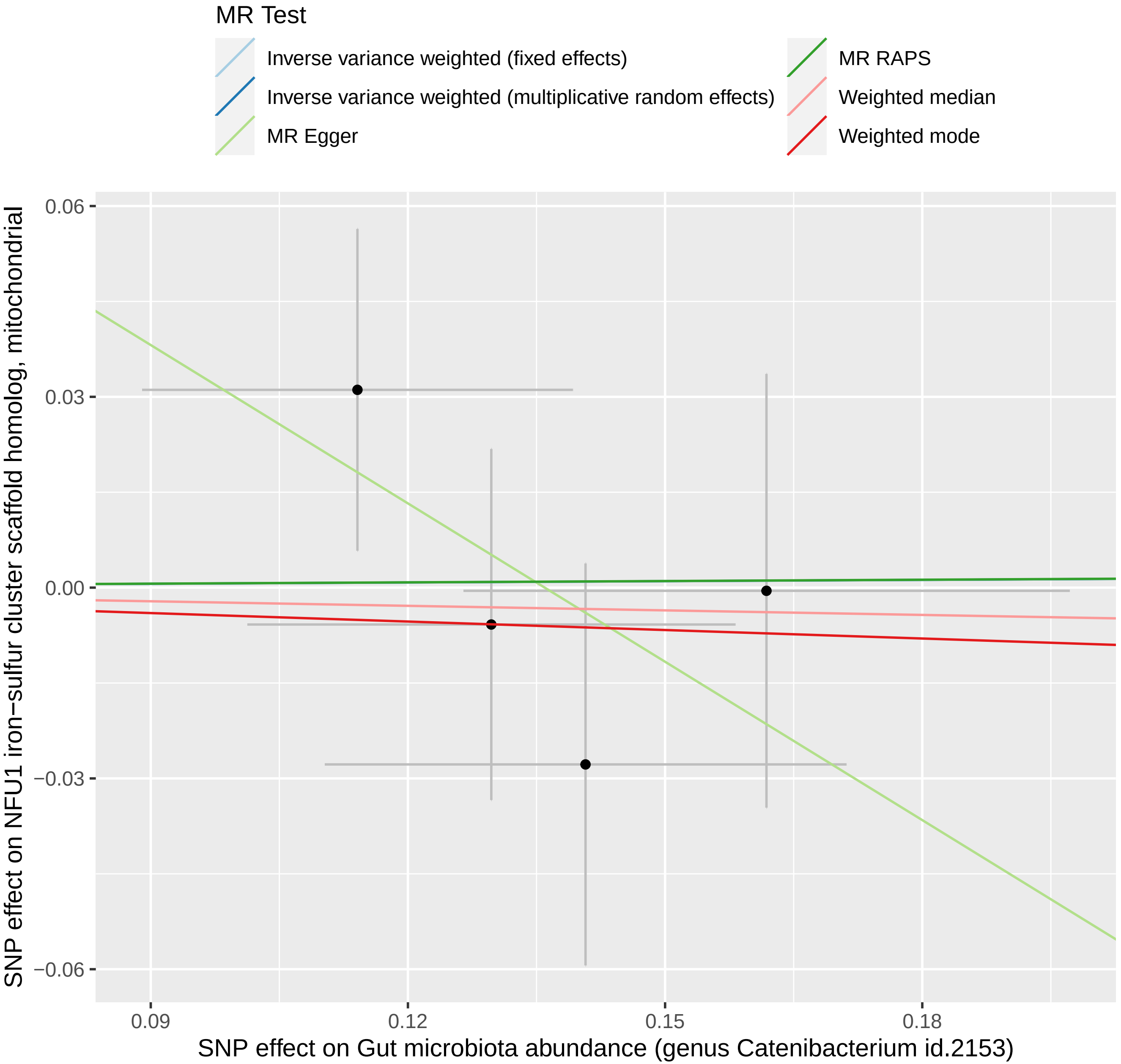

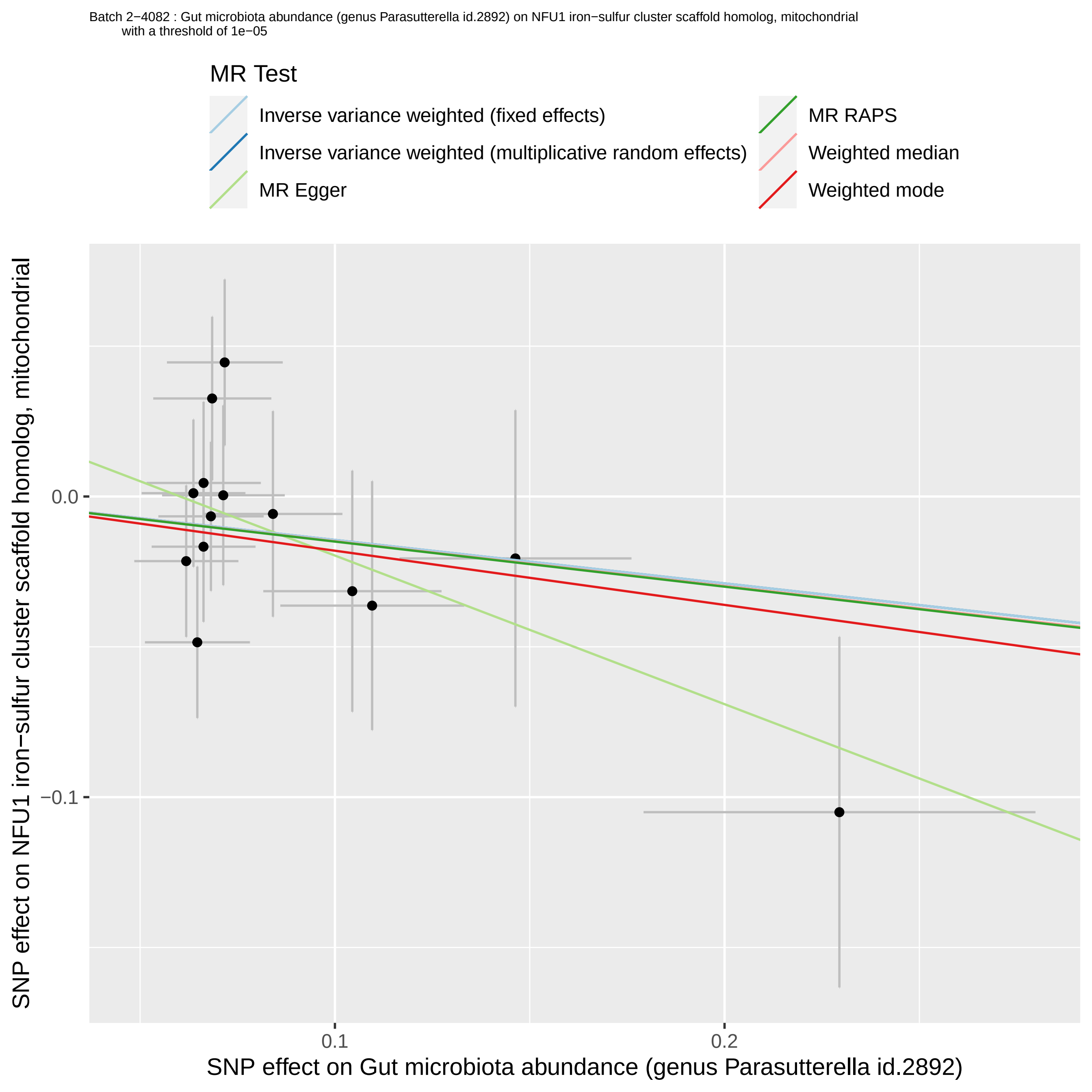
Batch 2−4082 : Gut microbiota abundance (genus Parasutterella id.2892) on NFU1 iron−sulfur cluster scaffold homolog, mitochondrial
with a threshold of 1e−05
MR Test
Inverse variance weighted (fixed effects)
Inverse variance weighted (multiplicative random effects)
MR Egger
MR RAPS
Weighted median
Weighted mode
0.0
−0.1
0.1
0.2
SNP effect on NFU1 iron−sulfur cluster scaffold homolog, mitochondrial
SNP effect on Gut microbiota abundance (genus Parasutterella id.2892)

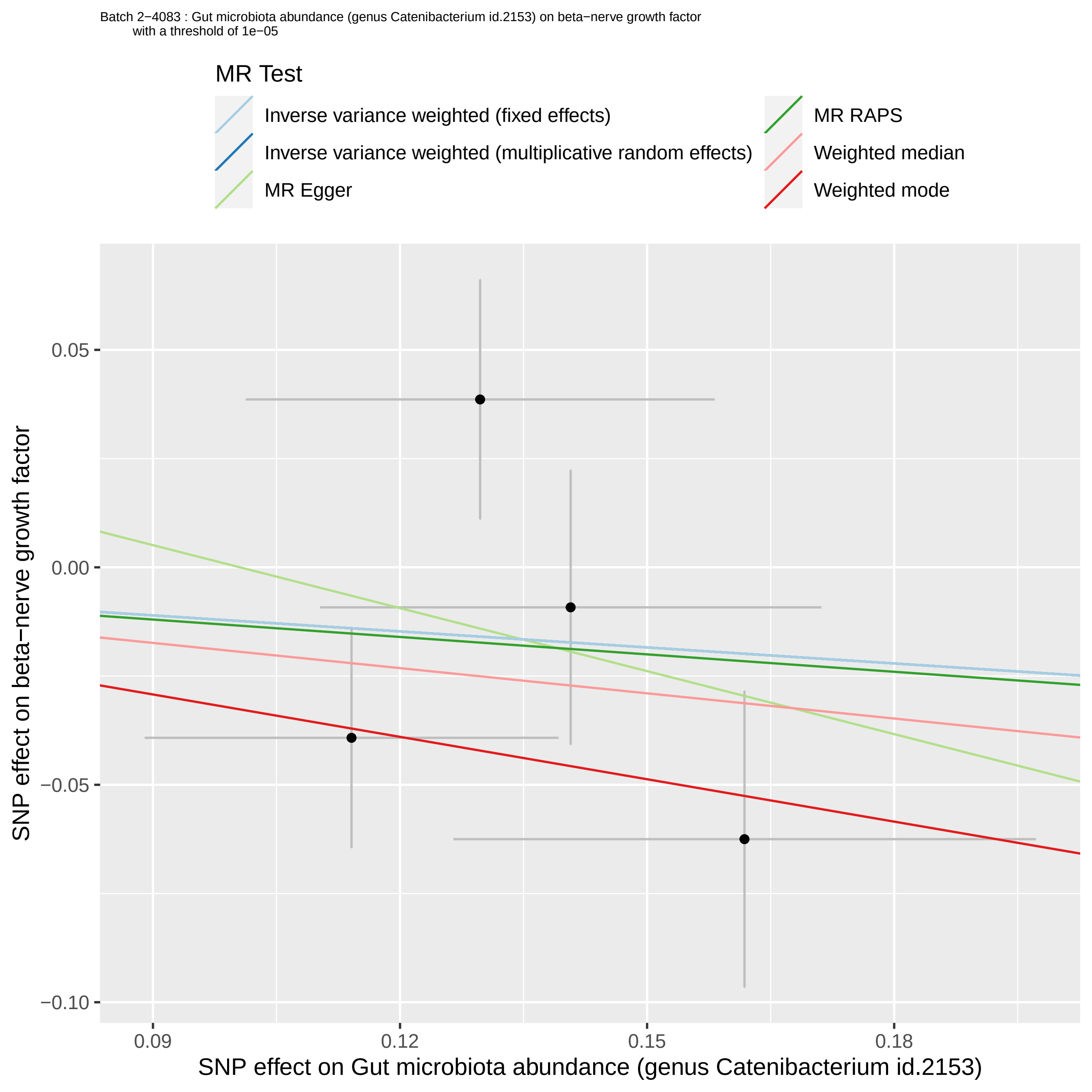
Batch 2−4083 : Gut microbiota abundance (genus Catenibacterium id.2153) on beta−nerve growth factor
with a threshold of 1e−05
MR Test
Inverse variance weighted (fixed effects)
Inverse variance weighted (multiplicative random effects)
MR Egger
MR RAPS
Weighted median
Weighted mode
SNP effect on beta−nerve growth factor
0.05
0.00
−0.05
−0.10
0.09
0.12
0.15
0.18
SNP effect on Gut microbiota abundance (genus Catenibacterium id.2153)

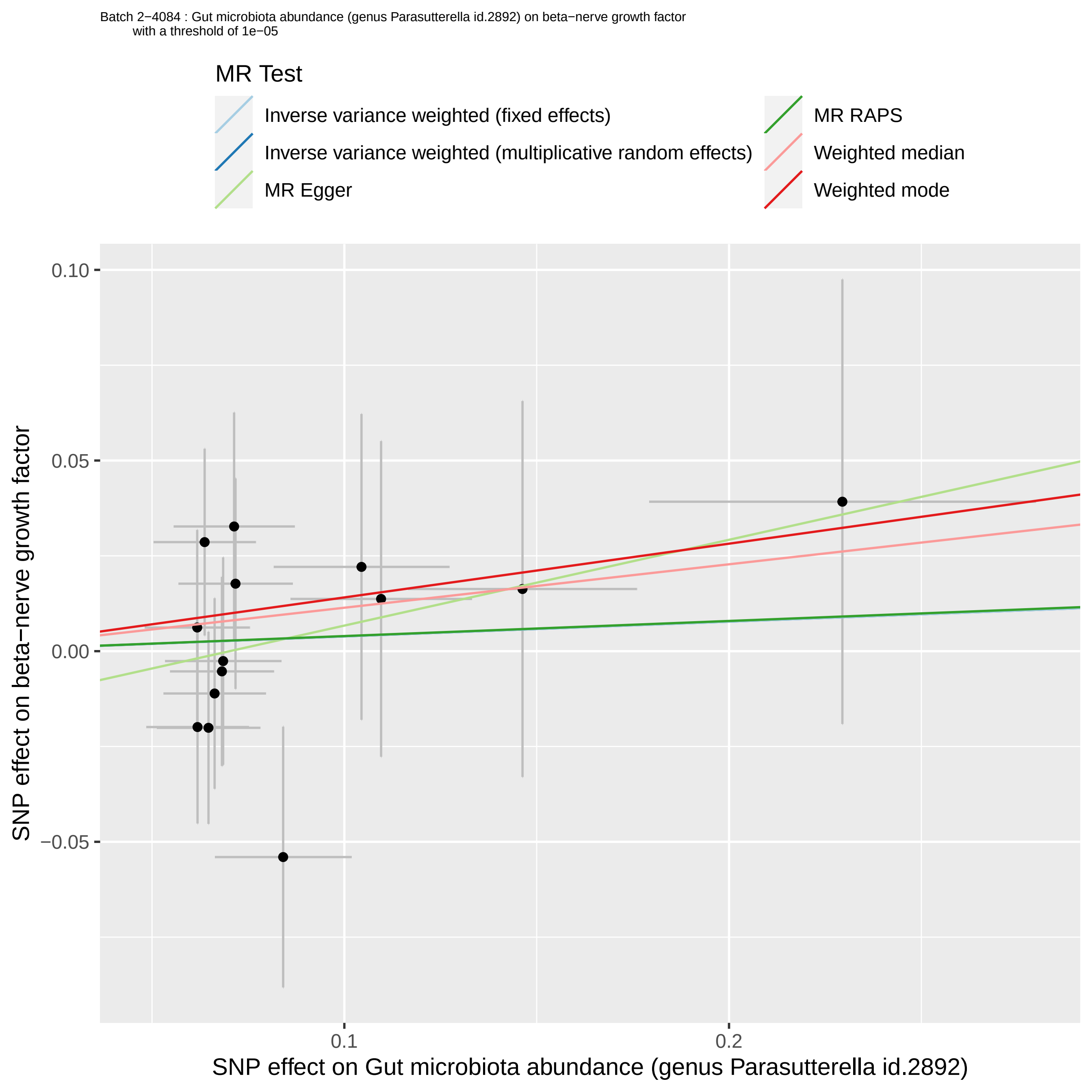

Batch 2−4084 : Gut microbiota abundance (genus Parasutterella id.2892) on beta−nerve growth factor
with a threshold of 1e−05
MR Test
Inverse variance weighted (fixed effects)
Inverse variance weighted (multiplicative random effects)
MR Egger
MR RAPS
Weighted median
Weighted mode
0.10
0.05
0.00
−0.05
0.1
0.2
SNP effect on beta−nerve growth factor
SNP effect on Gut microbiota abundance (genus Parasutterella id.2892)

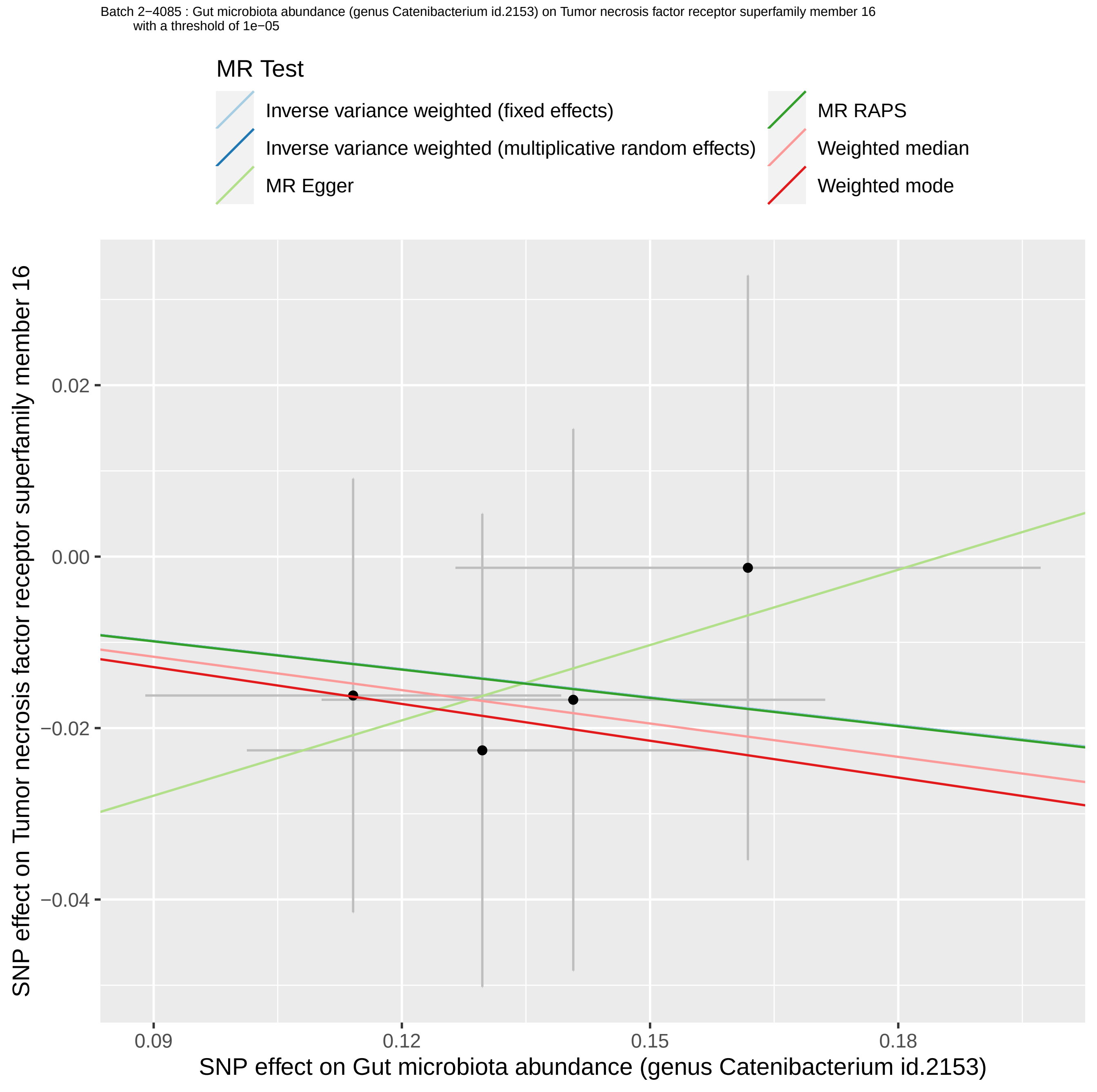

Batch 2−4085 : Gut microbiota abundance (genus Catenibacterium id.2153) on Tumor necrosis factor receptor superfamily member 16
with a threshold of 1e−05
MR Test
Inverse variance weighted (fixed effects)
Inverse variance weighted (multiplicative random effects)
MR Egger
MR RAPS
Weighted median
Weighted mode
SNP effect on Tumor necrosis factor receptor superfamily member 16
0.02
0.00
−0.02
−0.04
0.09
0.12
0.15
0.18
SNP effect on Gut microbiota abundance (genus Catenibacterium id.2153)

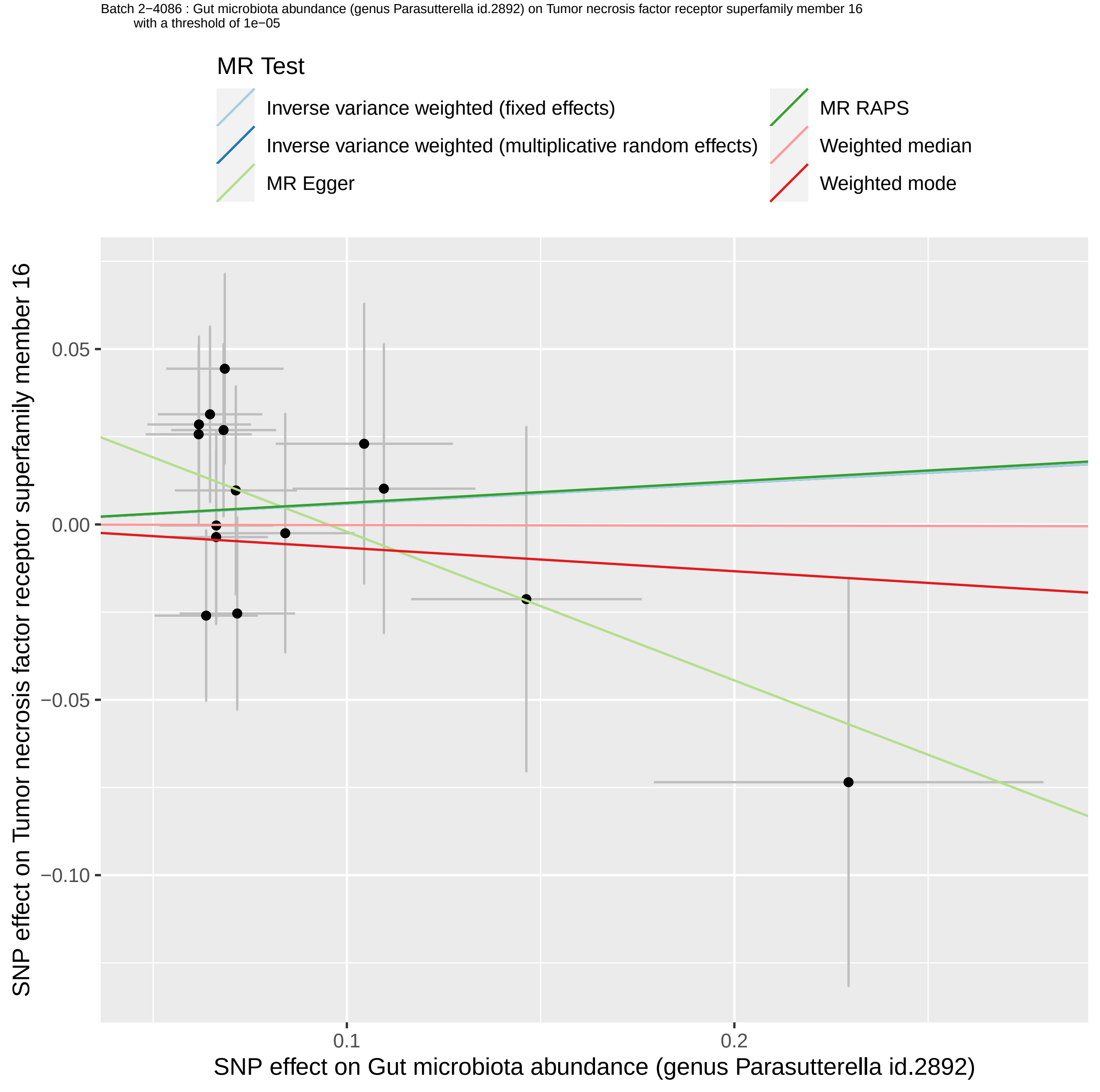
Batch 2−4086 : Gut microbiota abundance (genus Parasutterella id.2892) on Tumor necrosis factor receptor superfamily member 16
with a threshold of 1e−05
MR Test
Inverse variance weighted (fixed effects)
Inverse variance weighted (multiplicative random effects)
MR Egger
MR RAPS
Weighted median
Weighted mode
0.05
0.00
−0.05
−0.10
0.1
0.2
SNP effect on Tumor necrosis factor receptor superfamily member 16
SNP effect on Gut microbiota abundance (genus Parasutterella id.2892)

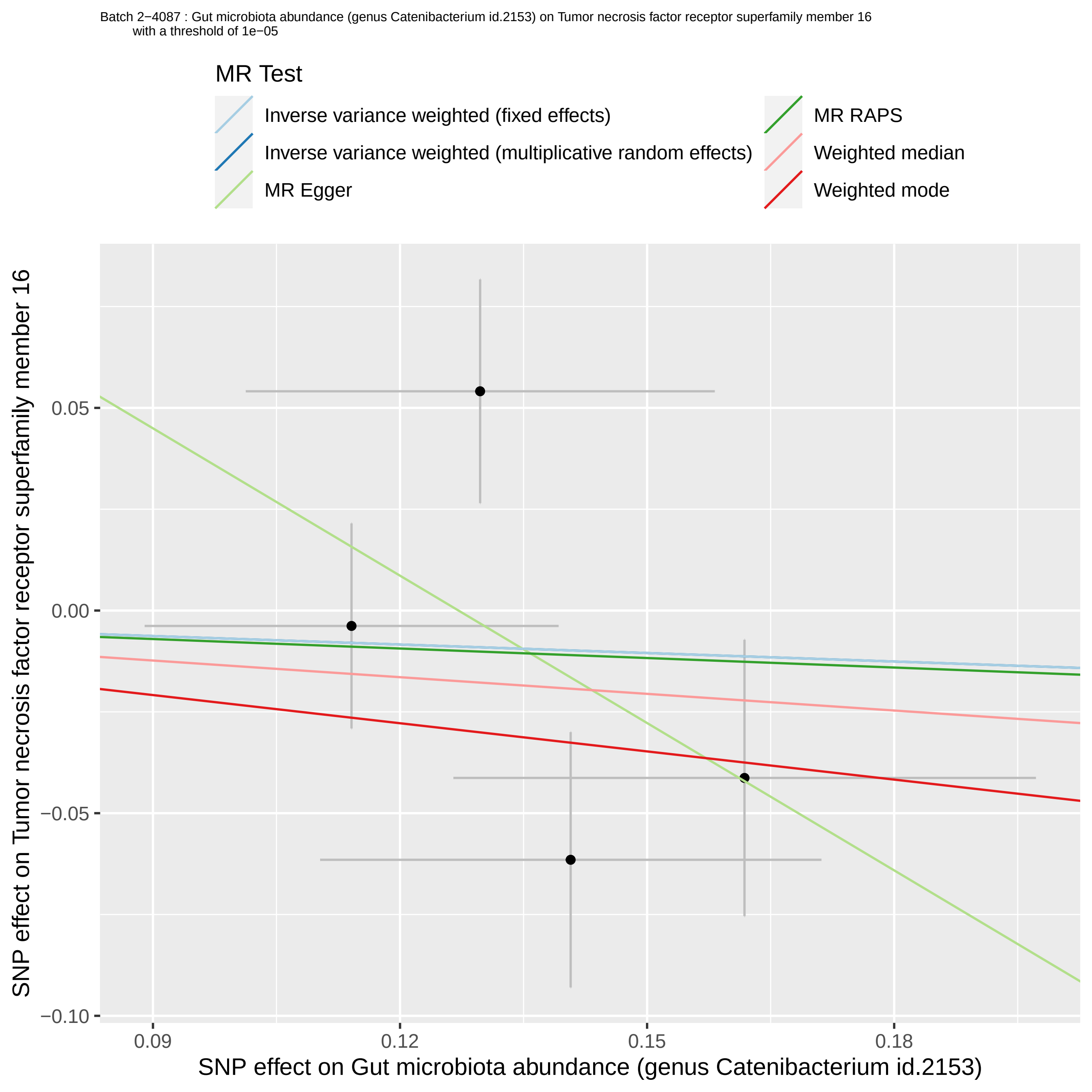
Batch 2−4087 : Gut microbiota abundance (genus Catenibacterium id.2153) on Tumor necrosis factor receptor superfamily member 16
with a threshold of 1e−05
MR Test
Inverse variance weighted (fixed effects)
Inverse variance weighted (multiplicative random effects)
MR Egger
MR RAPS
Weighted median
Weighted mode
SNP effect on Tumor necrosis factor receptor superfamily member 16
0.05
0.00
−0.05
−0.10
0.09
0.12
0.15
0.18
SNP effect on Gut microbiota abundance (genus Catenibacterium id.2153)

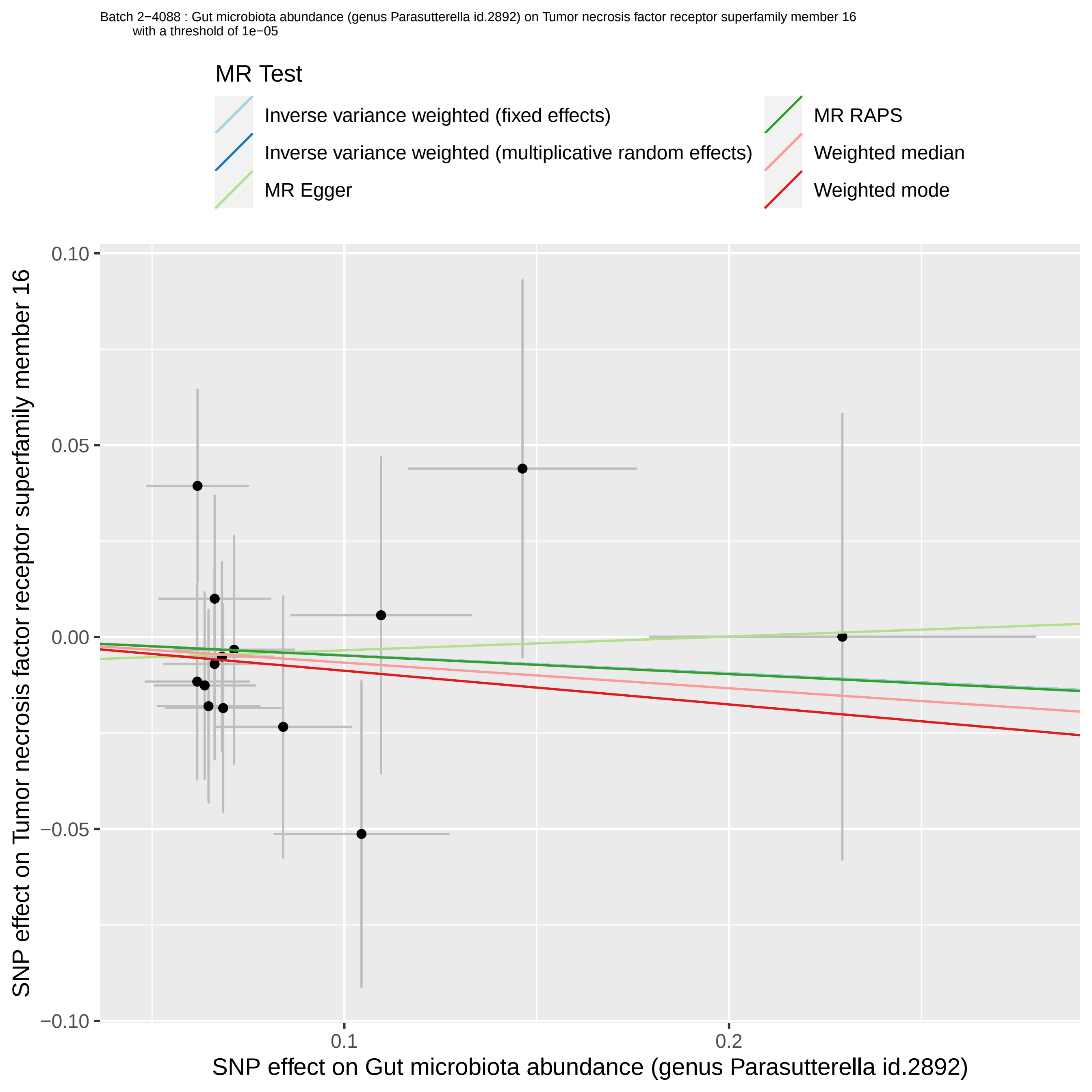
Batch 2−4088 : Gut microbiota abundance (genus Parasutterella id.2892) on Tumor necrosis factor receptor superfamily member 16
with a threshold of 1e−05
MR Test
Inverse variance weighted (fixed effects)
Inverse variance weighted (multiplicative random effects)
MR Egger
MR RAPS
Weighted median
Weighted mode
0.10
0.05
0.00
−0.05
−0.10
0.1
0.2
SNP effect on Tumor necrosis factor receptor superfamily member 16
SNP effect on Gut microbiota abundance (genus Parasutterella id.2892)

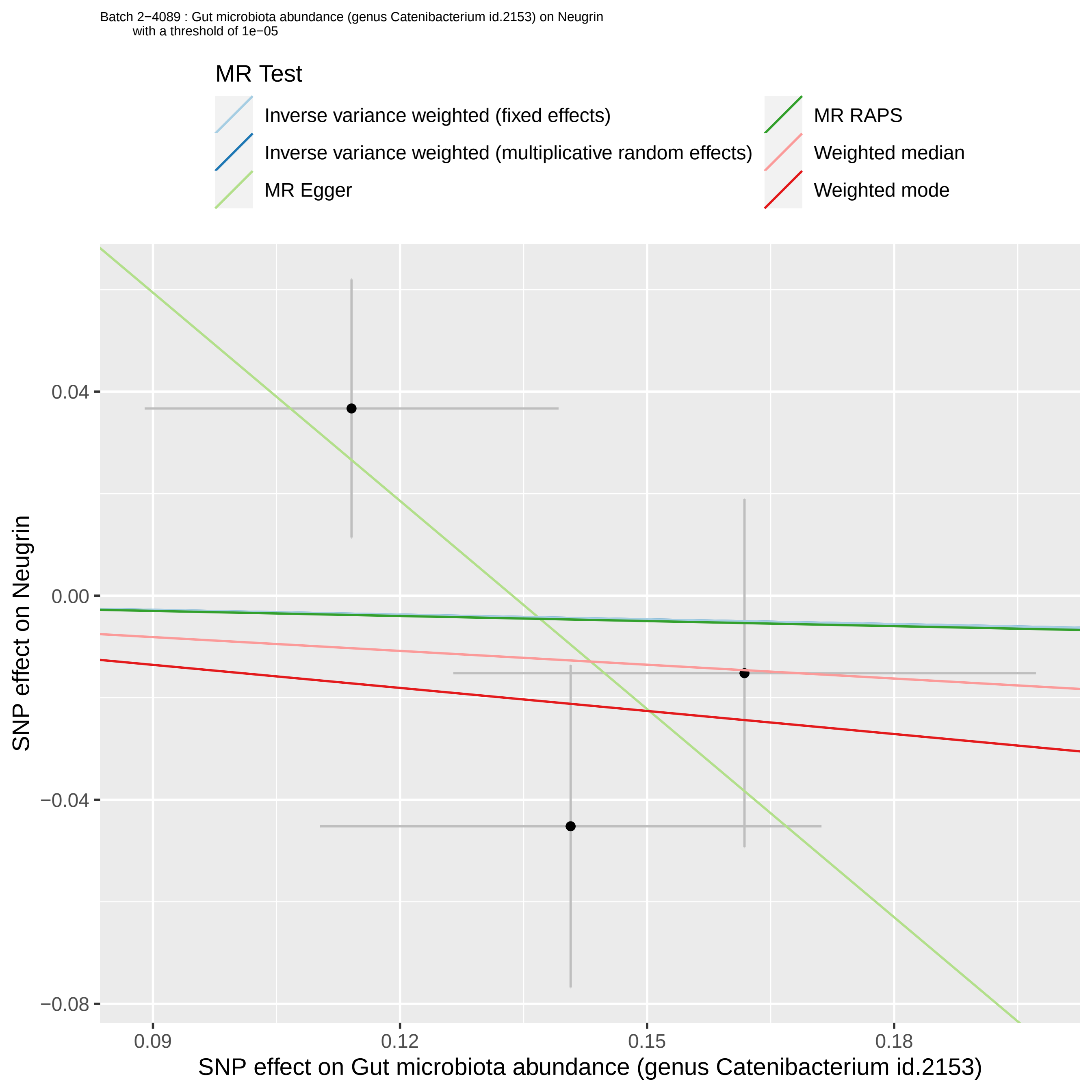
Batch 2−4089 : Gut microbiota abundance (genus Catenibacterium id.2153) on Neugrin
with a threshold of 1e−05
MR Test
Inverse variance weighted (fixed effects)
Inverse variance weighted (multiplicative random effects)
MR Egger
MR RAPS
Weighted median
Weighted mode
SNP effect on Neugrin
0.04
0.00
−0.04
−0.08
0.09
0.12
0.15
0.18
SNP effect on Gut microbiota abundance (genus Catenibacterium id.2153)

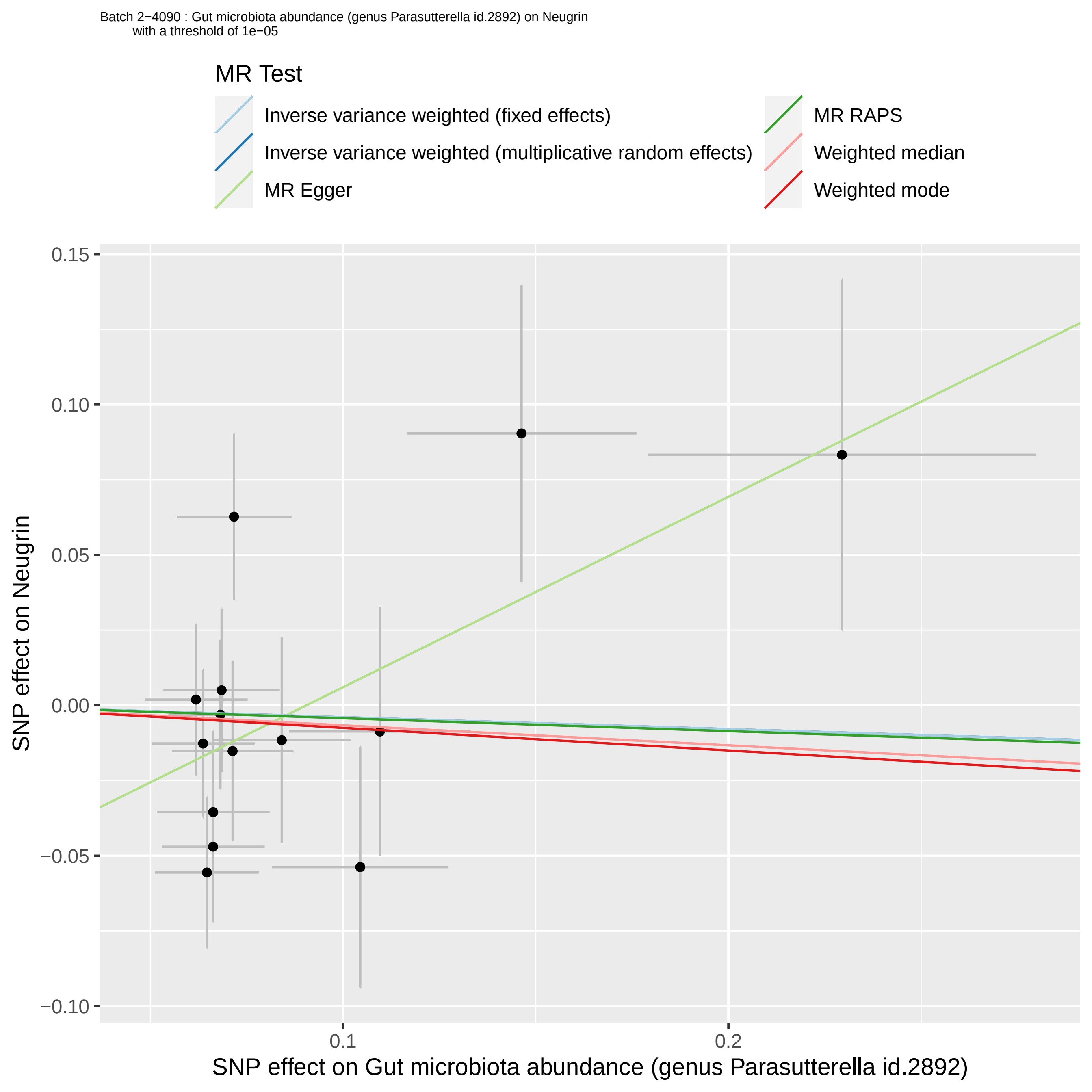
Batch 2−4090 : Gut microbiota abundance (genus Parasutterella id.2892) on Neugrin
with a threshold of 1e−05
MR Test
Inverse variance weighted (fixed effects)
Inverse variance weighted (multiplicative random effects)
MR Egger
MR RAPS
Weighted median
Weighted mode
0.15
0.10
0.05
0.00
−0.05
−0.10
0.1
0.2
SNP effect on Neugrin
SNP effect on Gut microbiota abundance (genus Parasutterella id.2892)

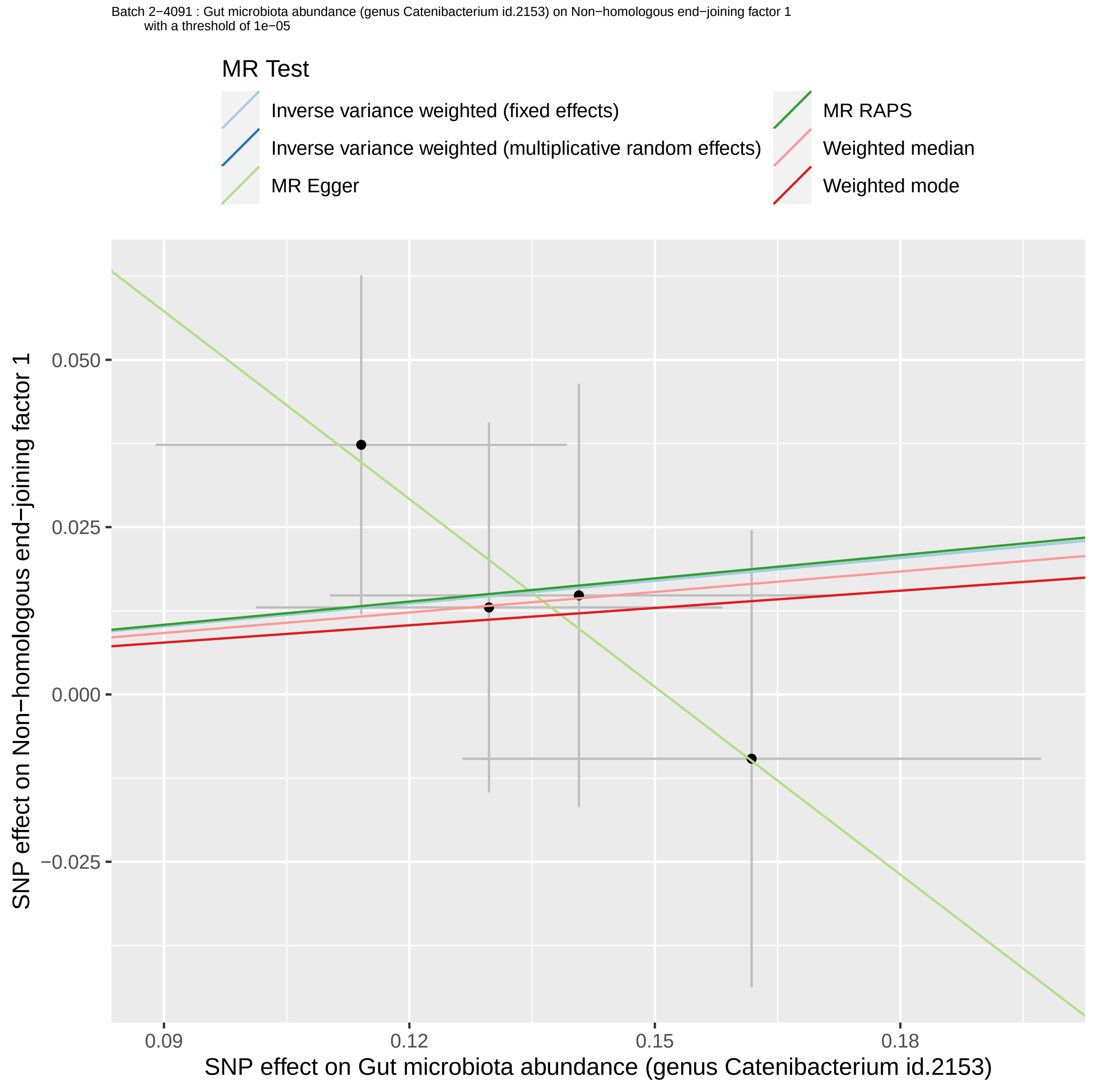

Batch 2−4091 : Gut microbiota abundance (genus Catenibacterium id.2153) on Non−homologous end−joining factor 1
with a threshold of 1e−05
MR Test
Inverse variance weighted (fixed effects)
Inverse variance weighted (multiplicative random effects)
MR Egger
MR RAPS
Weighted median
Weighted mode
0.050
0.025
0.000
−0.025
0.09
0.12
0.15
0.18
SNP effect on Non−homologous end−joining factor 1
SNP effect on Gut microbiota abundance (genus Catenibacterium id.2153)

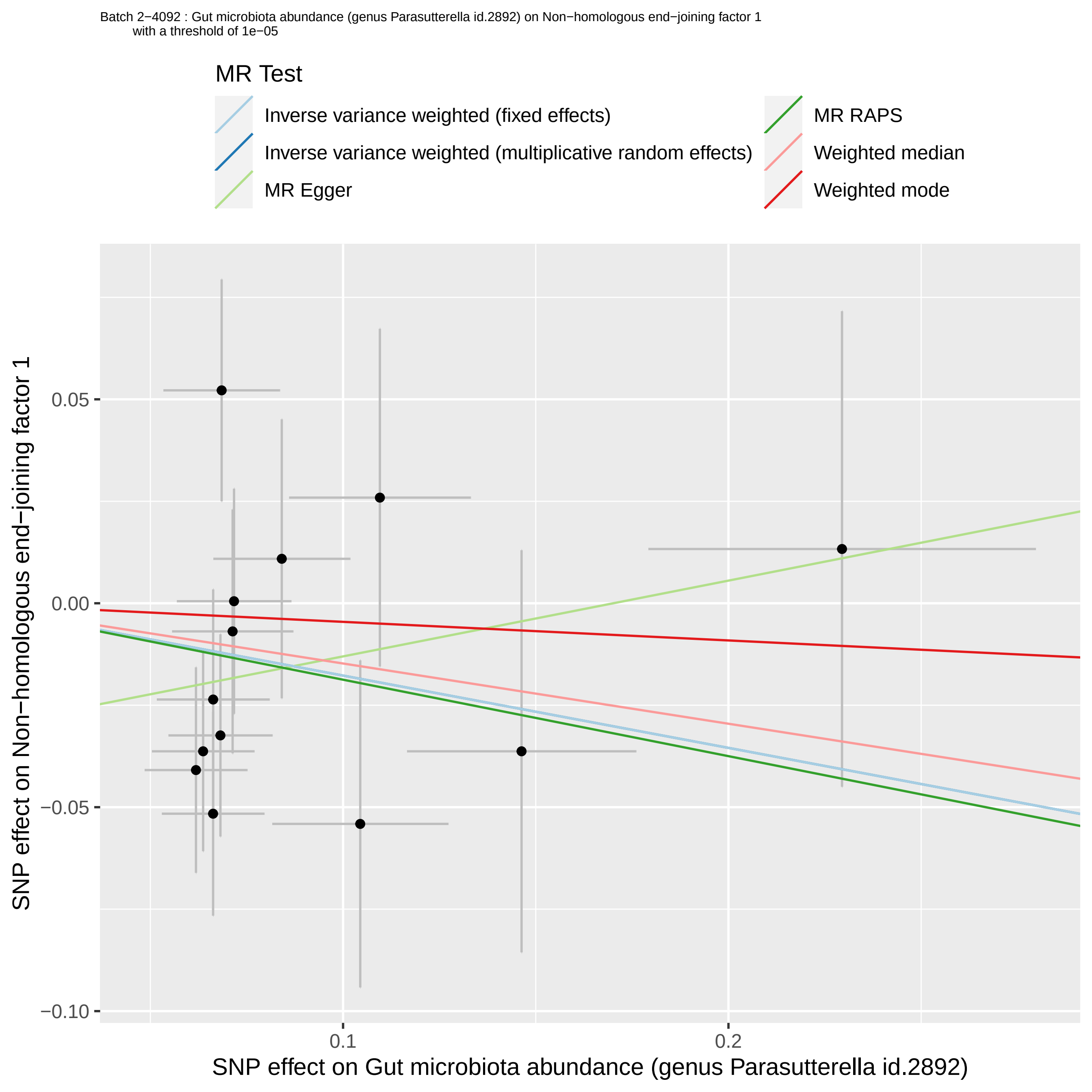
Batch 2−4092 : Gut microbiota abundance (genus Parasutterella id.2892) on Non−homologous end−joining factor 1
with a threshold of 1e−05
MR Test
Inverse variance weighted (fixed effects)
Inverse variance weighted (multiplicative random effects)
MR Egger
MR RAPS
Weighted median
Weighted mode
0.05
0.00
−0.05
−0.10
0.1
0.2
SNP effect on Non−homologous end−joining factor 1
SNP effect on Gut microbiota abundance (genus Parasutterella id.2892)

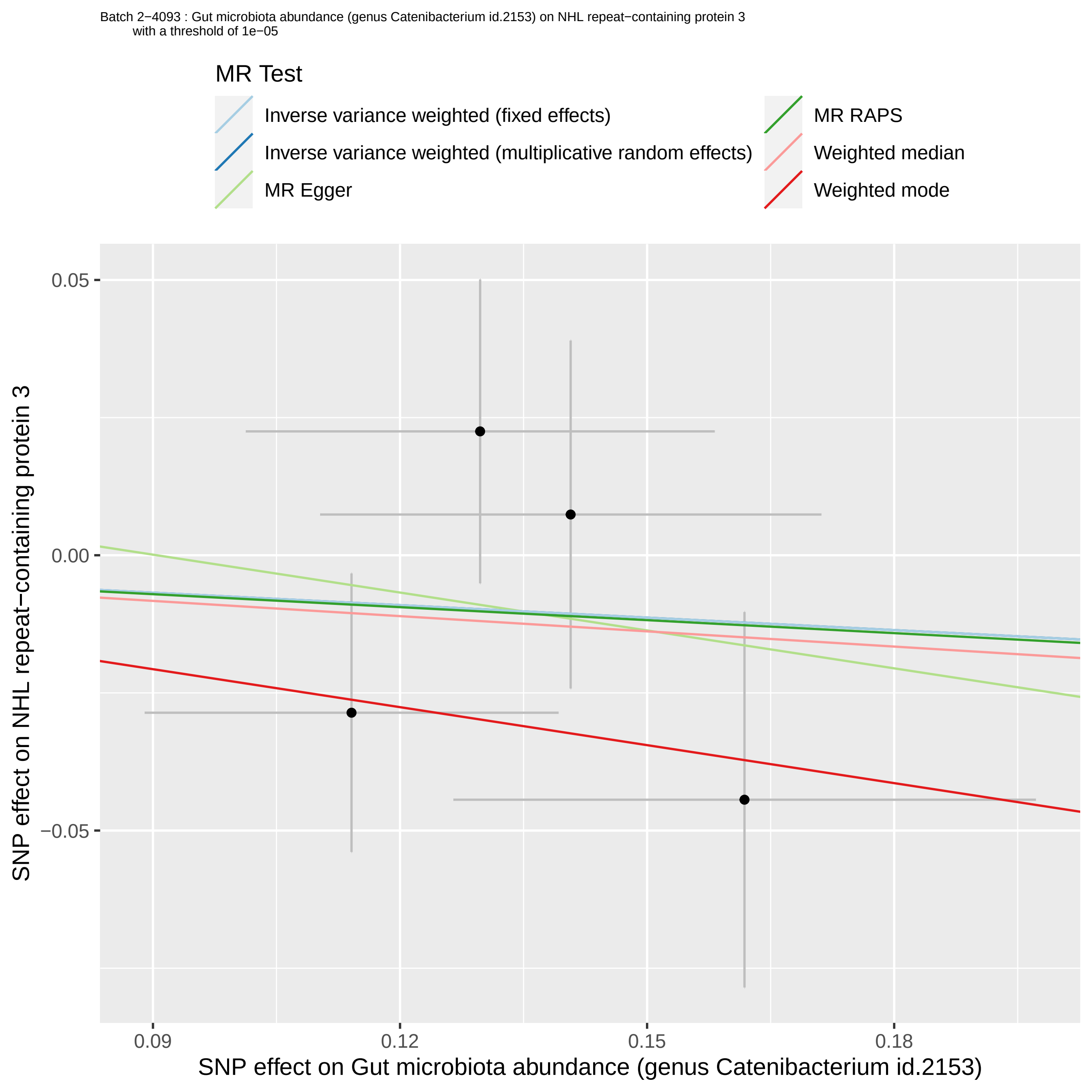
Batch 2−4093 : Gut microbiota abundance (genus Catenibacterium id.2153) on NHL repeat−containing protein 3
with a threshold of 1e−05
MR Test
Inverse variance weighted (fixed effects)
Inverse variance weighted (multiplicative random effects)
MR Egger
MR RAPS
Weighted median
Weighted mode
0.05
0.00
−0.05
0.09
0.12
0.15
0.18
SNP effect on NHL repeat−containing protein 3
SNP effect on Gut microbiota abundance (genus Catenibacterium id.2153)

Batch 2−4094 : Gut microbiota abundance (genus Parasutterella id.2892) on NHL repeat−containing protein 3
with a threshold of 1e−05

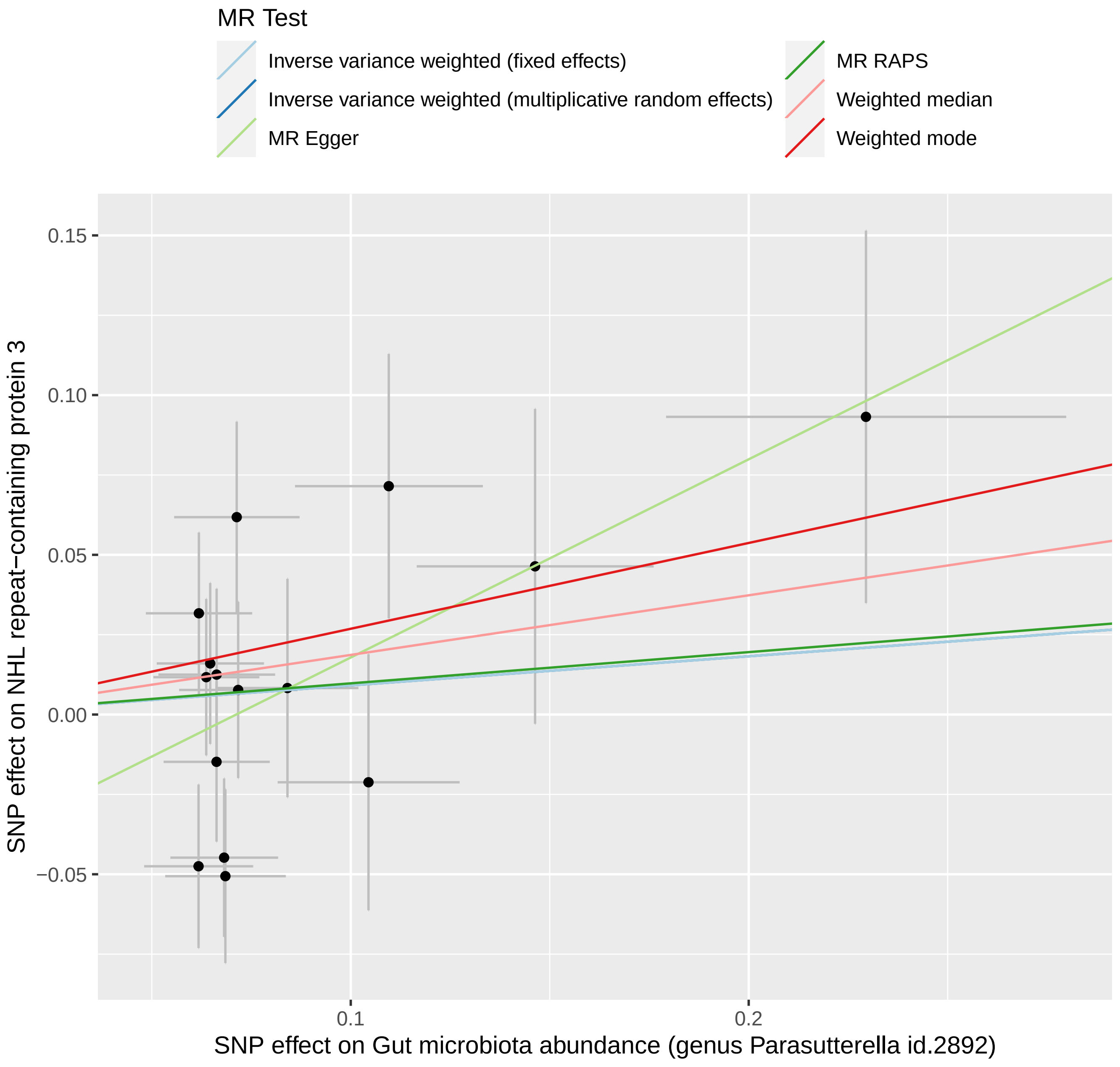

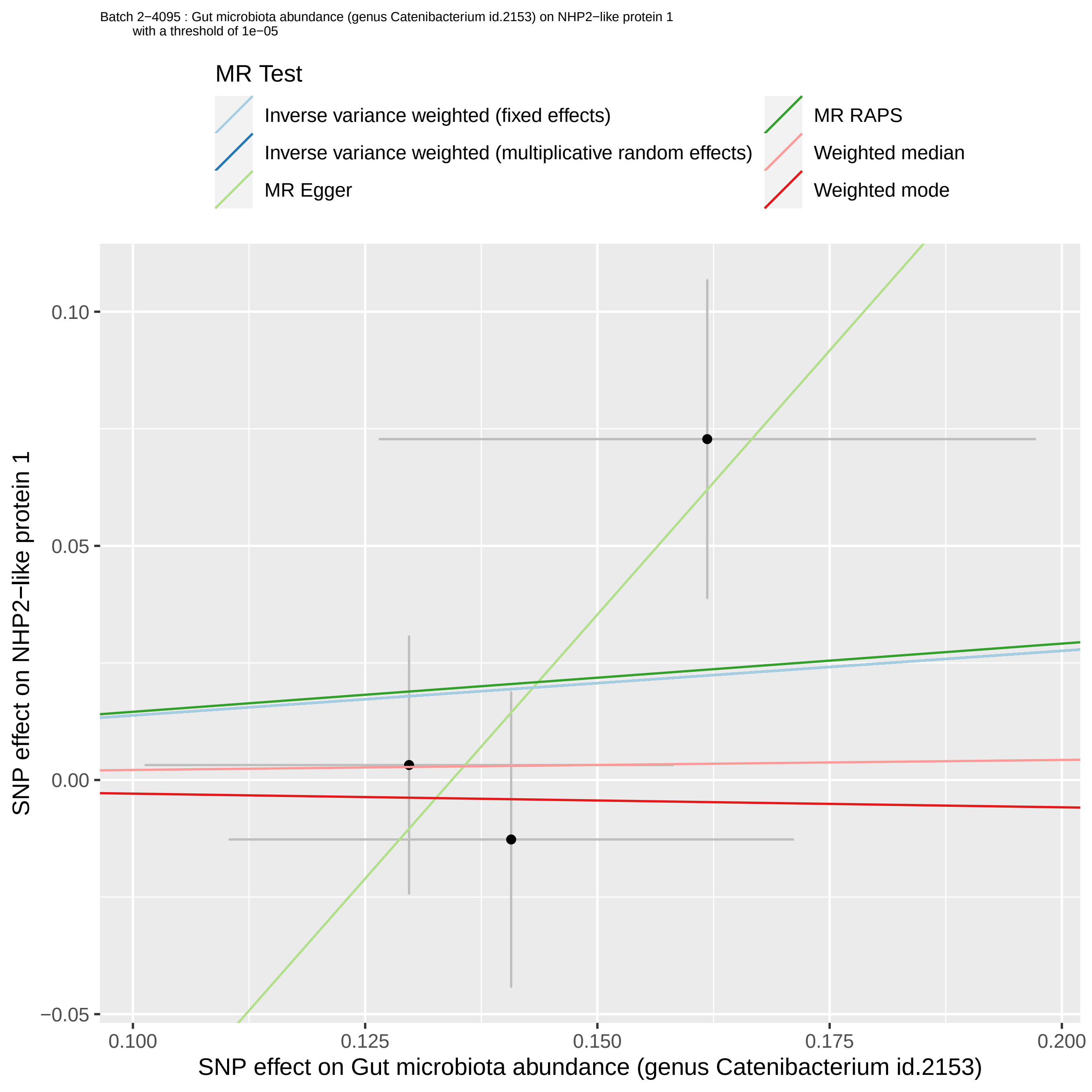

Batch 2−4095 : Gut microbiota abundance (genus Catenibacterium id.2153) on NHP2−like protein 1
with a threshold of 1e−05
MR Test
Inverse variance weighted (fixed effects)
Inverse variance weighted (multiplicative random effects)
MR Egger
MR RAPS
Weighted median
Weighted mode
0.10
0.05
0.00
−0.05
0.100
0.125
0.150
0.175
0.200
SNP effect on NHP2−like protein 1
SNP effect on Gut microbiota abundance (genus Catenibacterium id.2153)

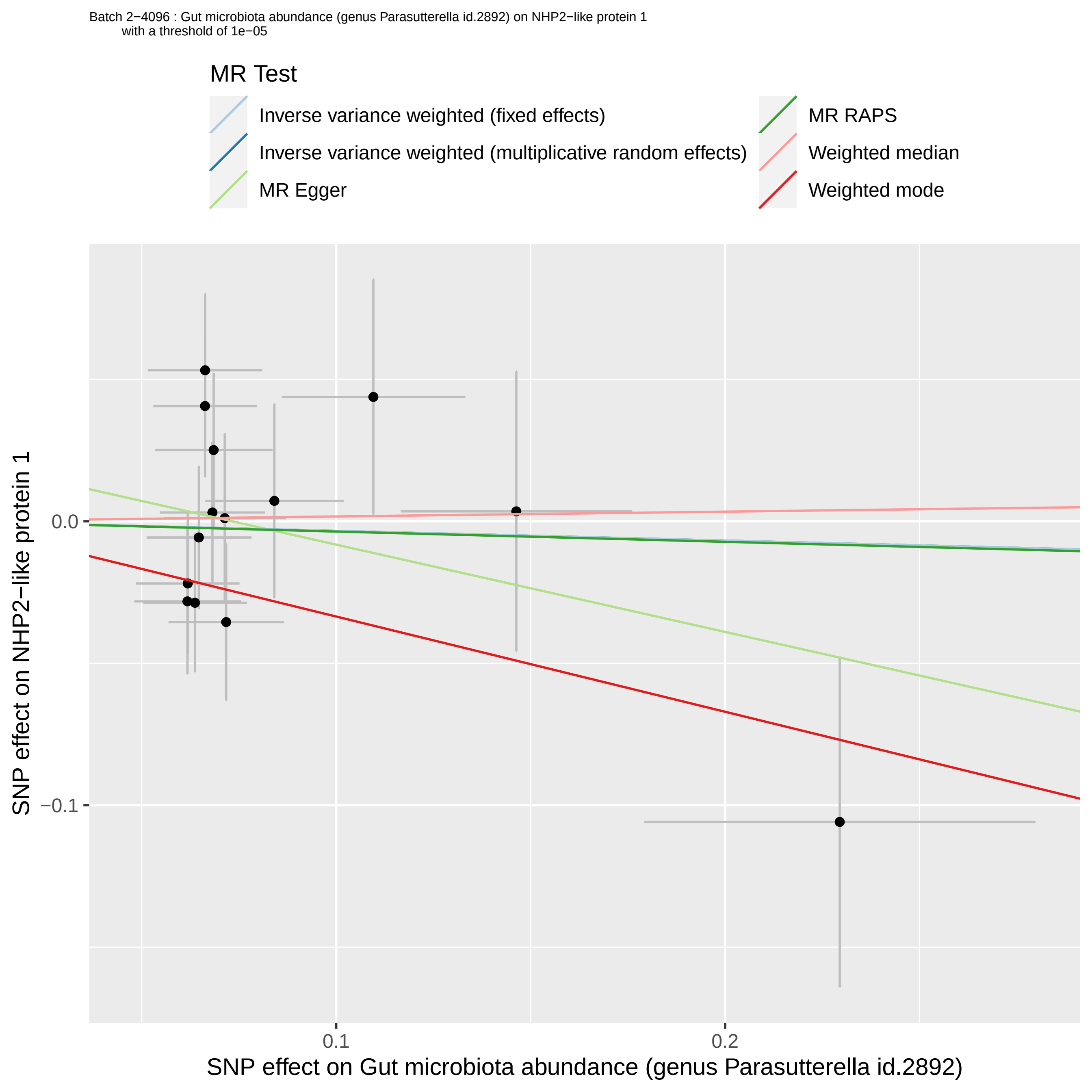
Batch 2−4096 : Gut microbiota abundance (genus Parasutterella id.2892) on NHP2−like protein 1
with a threshold of 1e−05
MR Test
Inverse variance weighted (fixed effects)
Inverse variance weighted (multiplicative random effects)
MR Egger
MR RAPS
Weighted median
Weighted mode
SNP effect on NHP2−like protein 1
0.0
−0.1
0.1
0.2
SNP effect on Gut microbiota abundance (genus Parasutterella id.2892)

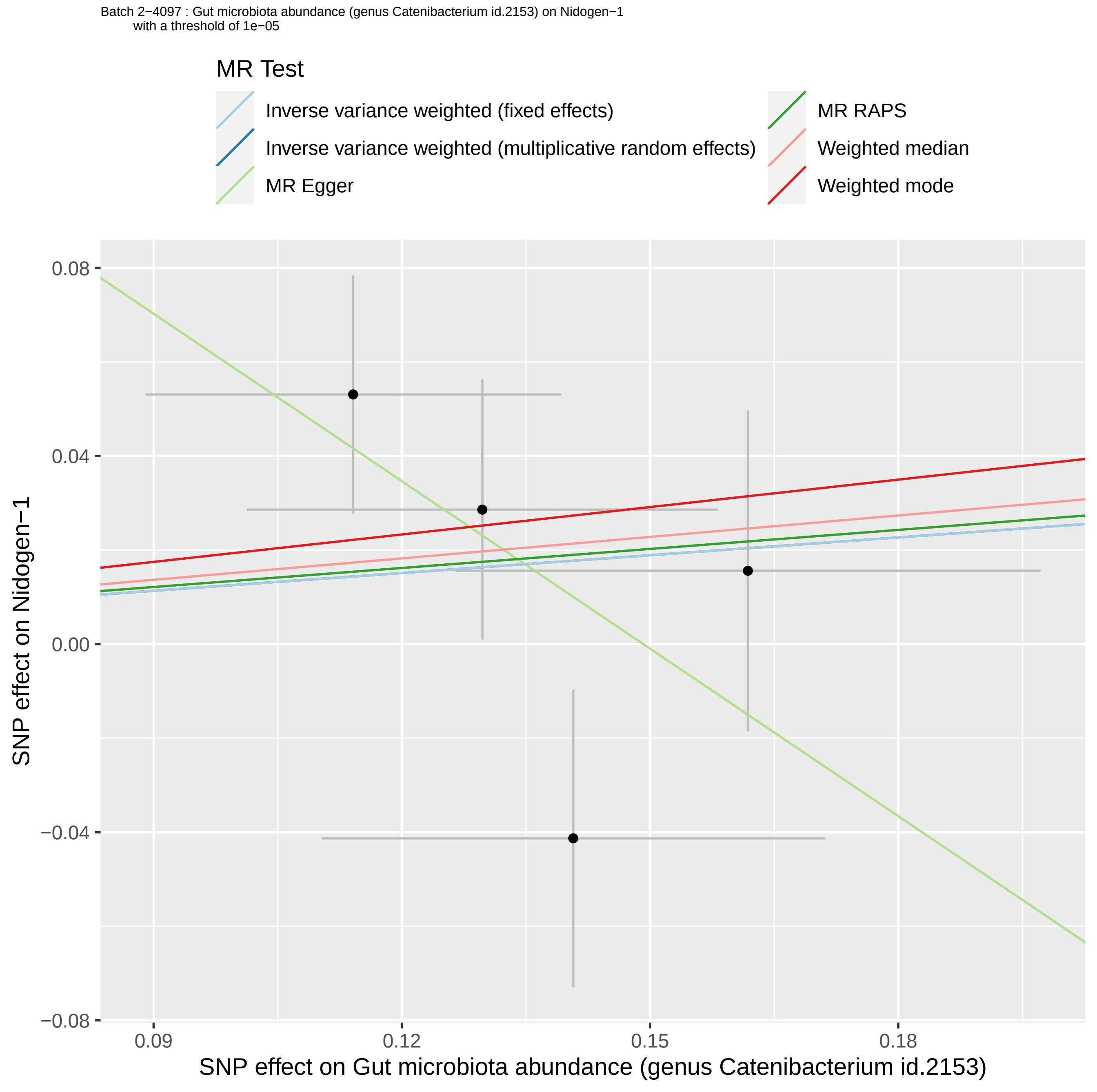
Batch 2−4097 : Gut microbiota abundance (genus Catenibacterium id.2153) on Nidogen−1
with a threshold of 1e−05
MR Test
Inverse variance weighted (fixed effects)
Inverse variance weighted (multiplicative random effects)
MR Egger
MR RAPS
Weighted median
Weighted mode
0.08
0.04
0.00
−0.04
−0.08
0.09
0.12
0.15
0.18
SNP effect on Nidogen−1
SNP effect on Gut microbiota abundance (genus Catenibacterium id.2153)

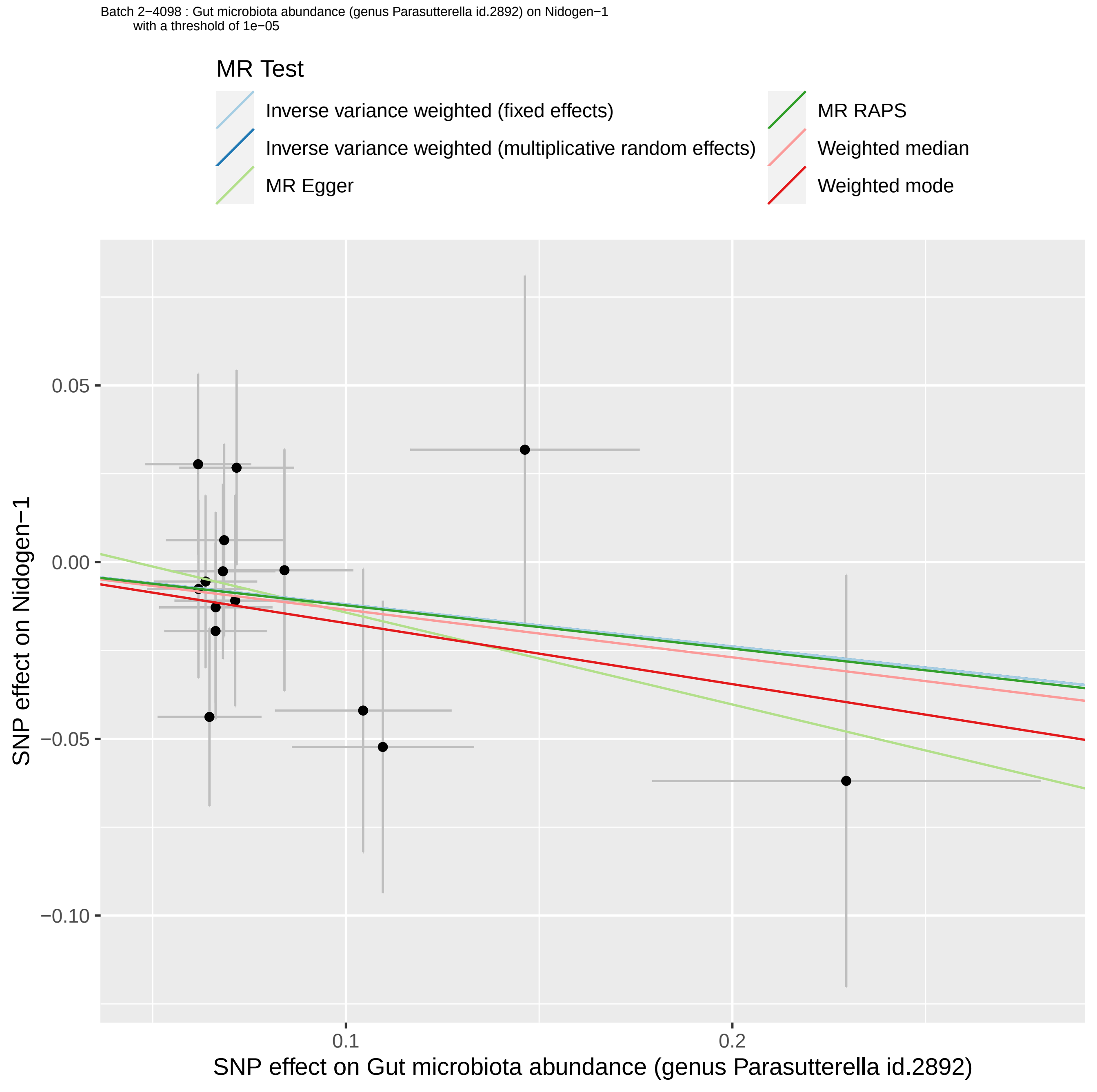
Batch 2−4098 : Gut microbiota abundance (genus Parasutterella id.2892) on Nidogen−1
with a threshold of 1e−05
MR Test
Inverse variance weighted (fixed effects)
Inverse variance weighted (multiplicative random effects)
MR Egger
MR RAPS
Weighted median
Weighted mode
0.05
0.00
−0.05
−0.10
0.1
0.2
SNP effect on Nidogen−1
SNP effect on Gut microbiota abundance (genus Parasutterella id.2892)

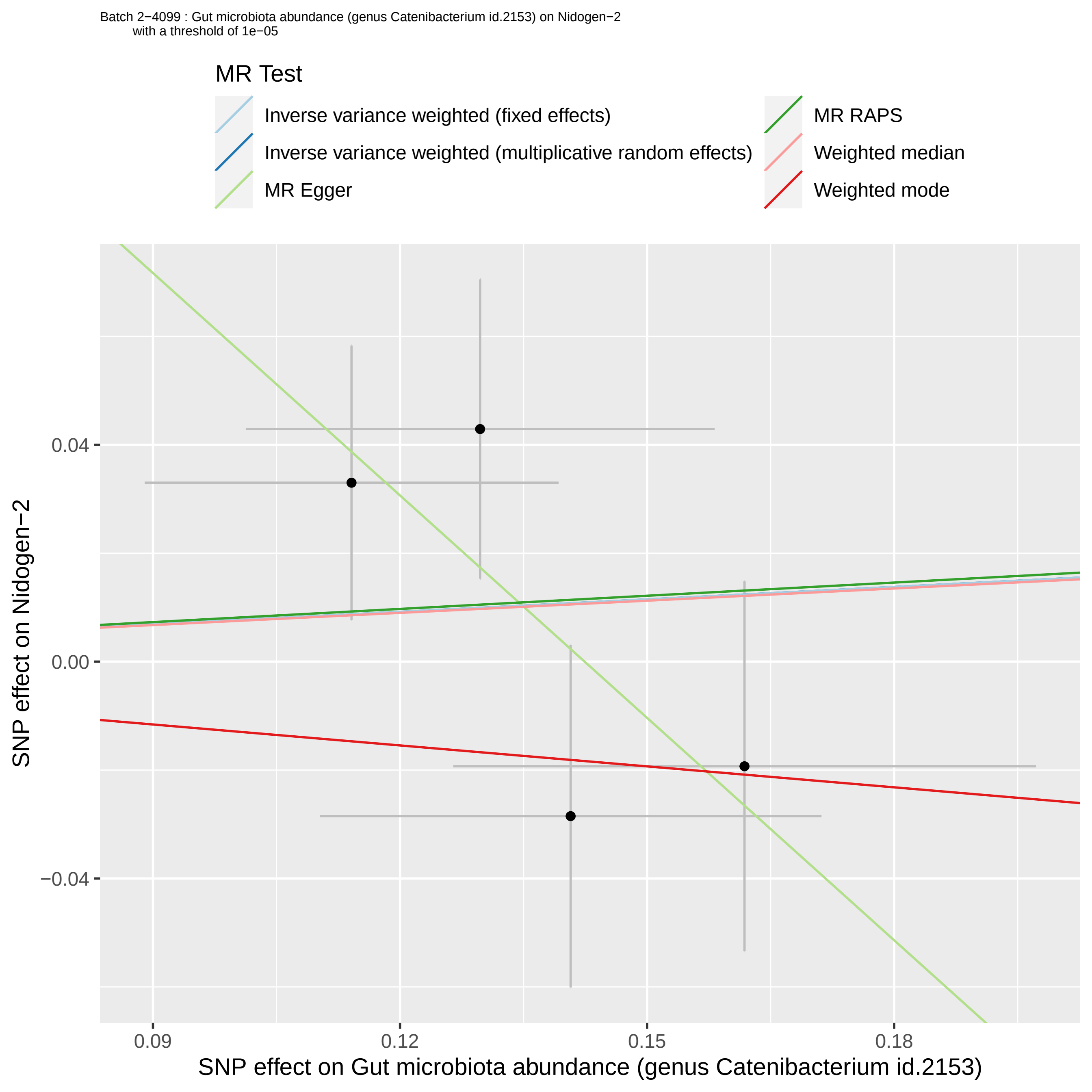
Batch 2−4099 : Gut microbiota abundance (genus Catenibacterium id.2153) on Nidogen−2
with a threshold of 1e−05
MR Test
Inverse variance weighted (fixed effects)
Inverse variance weighted (multiplicative random effects)
MR Egger
MR RAPS
Weighted median
Weighted mode
0.04
0.00
−0.04
0.09
0.12
0.15
0.18
SNP effect on Nidogen−2
SNP effect on Gut microbiota abundance (genus Catenibacterium id.2153)

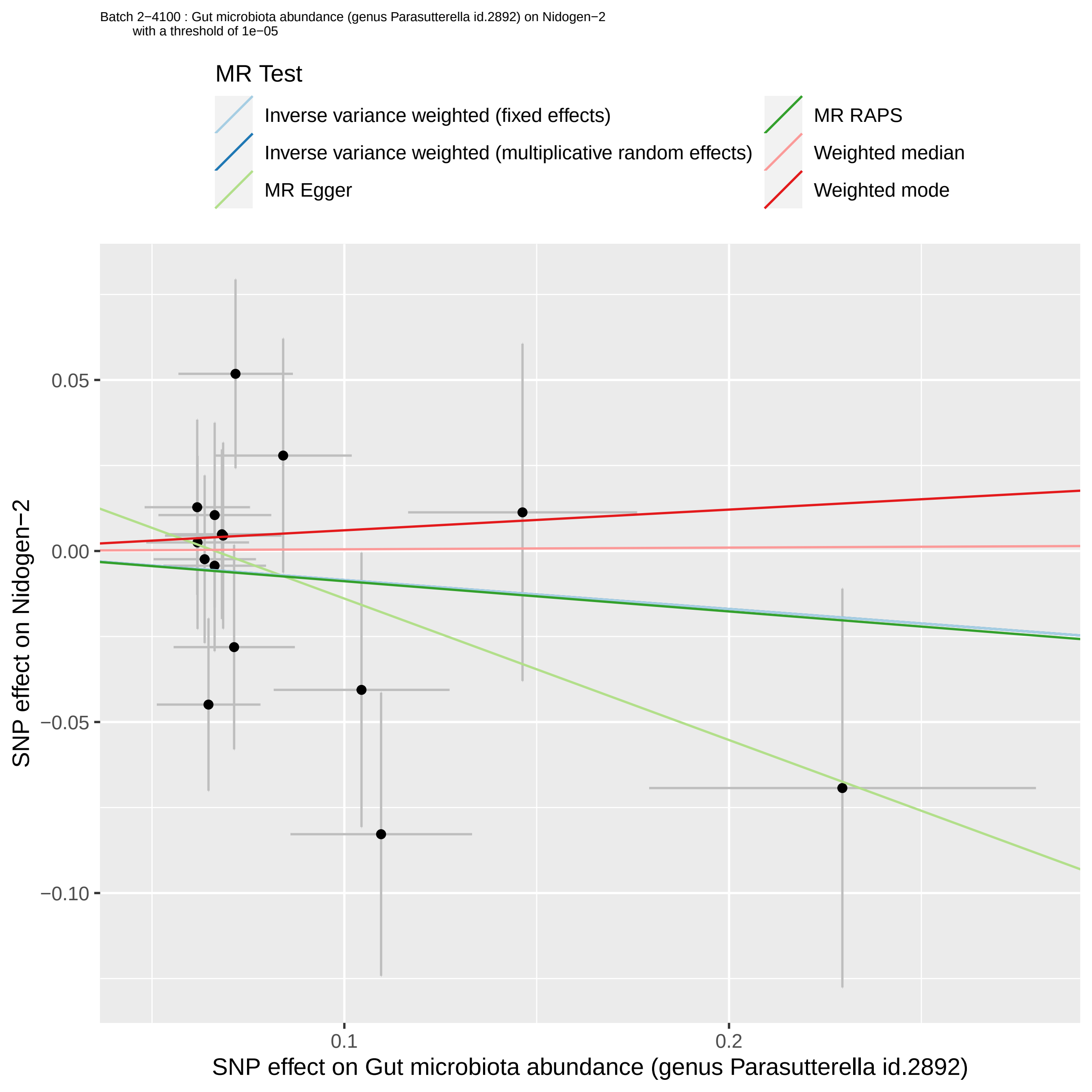

Batch 2−4100 : Gut microbiota abundance (genus Parasutterella id.2892) on Nidogen−2
with a threshold of 1e−05
MR Test
Inverse variance weighted (fixed effects)
Inverse variance weighted (multiplicative random effects)
MR Egger
MR RAPS
Weighted median
Weighted mode
0.05
0.00
−0.05
−0.10
0.1
0.2
SNP effect on Nidogen−2
SNP effect on Gut microbiota abundance (genus Parasutterella id.2892)

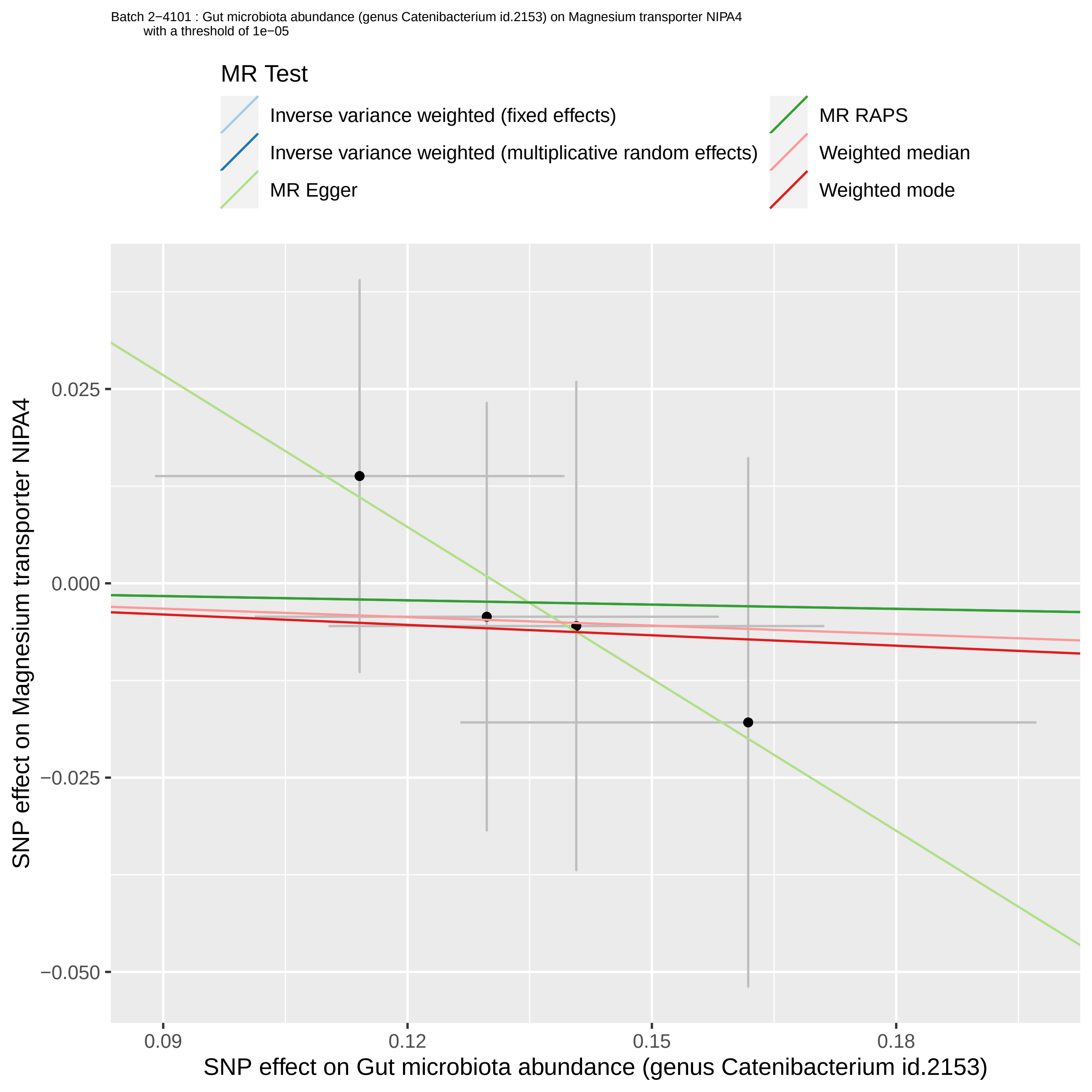
Batch 2−4101 : Gut microbiota abundance (genus Catenibacterium id.2153) on Magnesium transporter NIPA4
with a threshold of 1e−05
MR Test
Inverse variance weighted (fixed effects)
Inverse variance weighted (multiplicative random effects)
MR Egger
MR RAPS
Weighted median
Weighted mode
SNP effect on Magnesium transporter NIPA4
0.025
0.000
−0.025
−0.050
0.09
0.12
0.15
0.18
SNP effect on Gut microbiota abundance (genus Catenibacterium id.2153)

Batch 2−4102 : Gut microbiota abundance (genus Parasutterella id.2892) on Magnesium transporter NIPA4
with a threshold of 1e−05

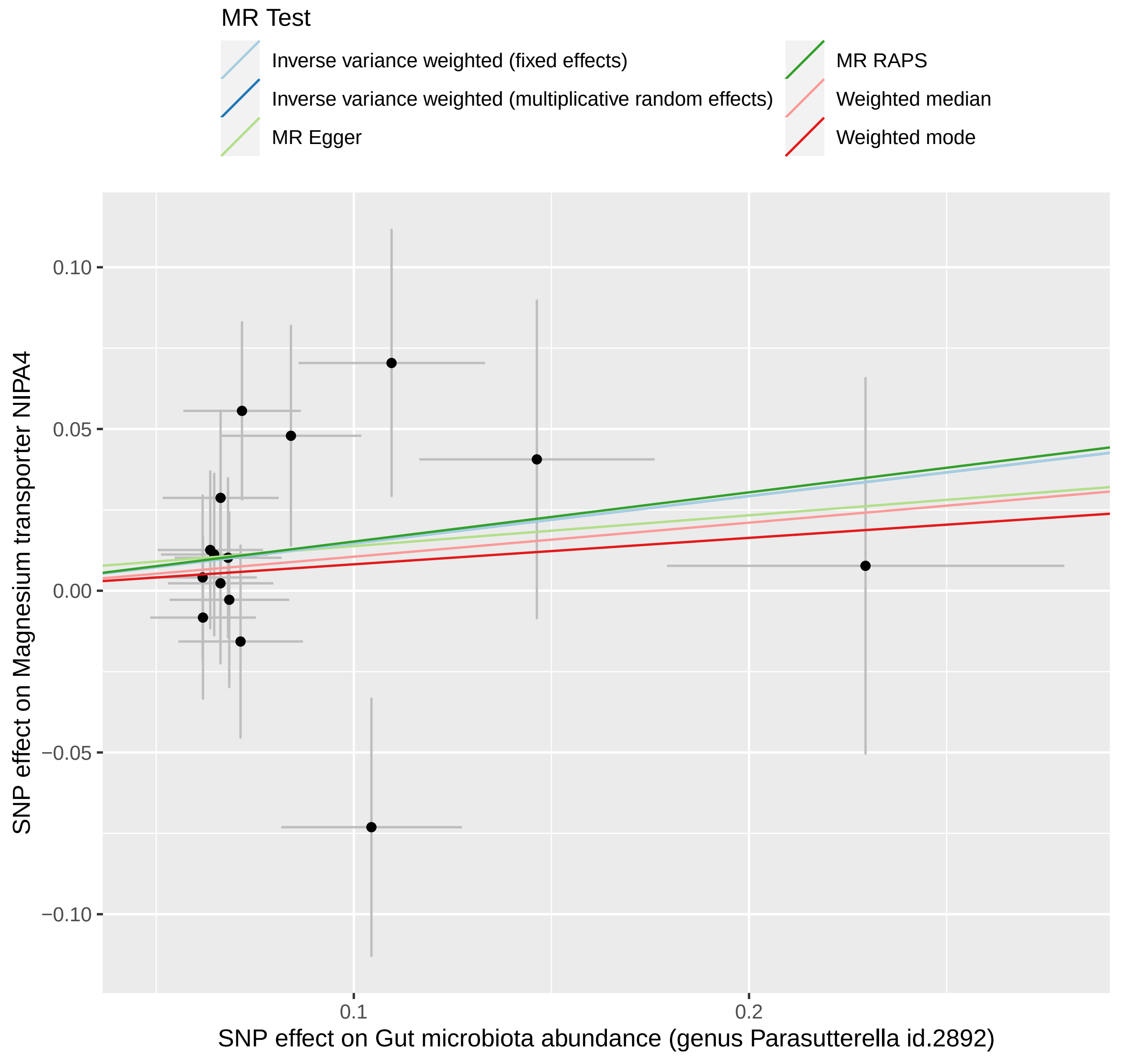

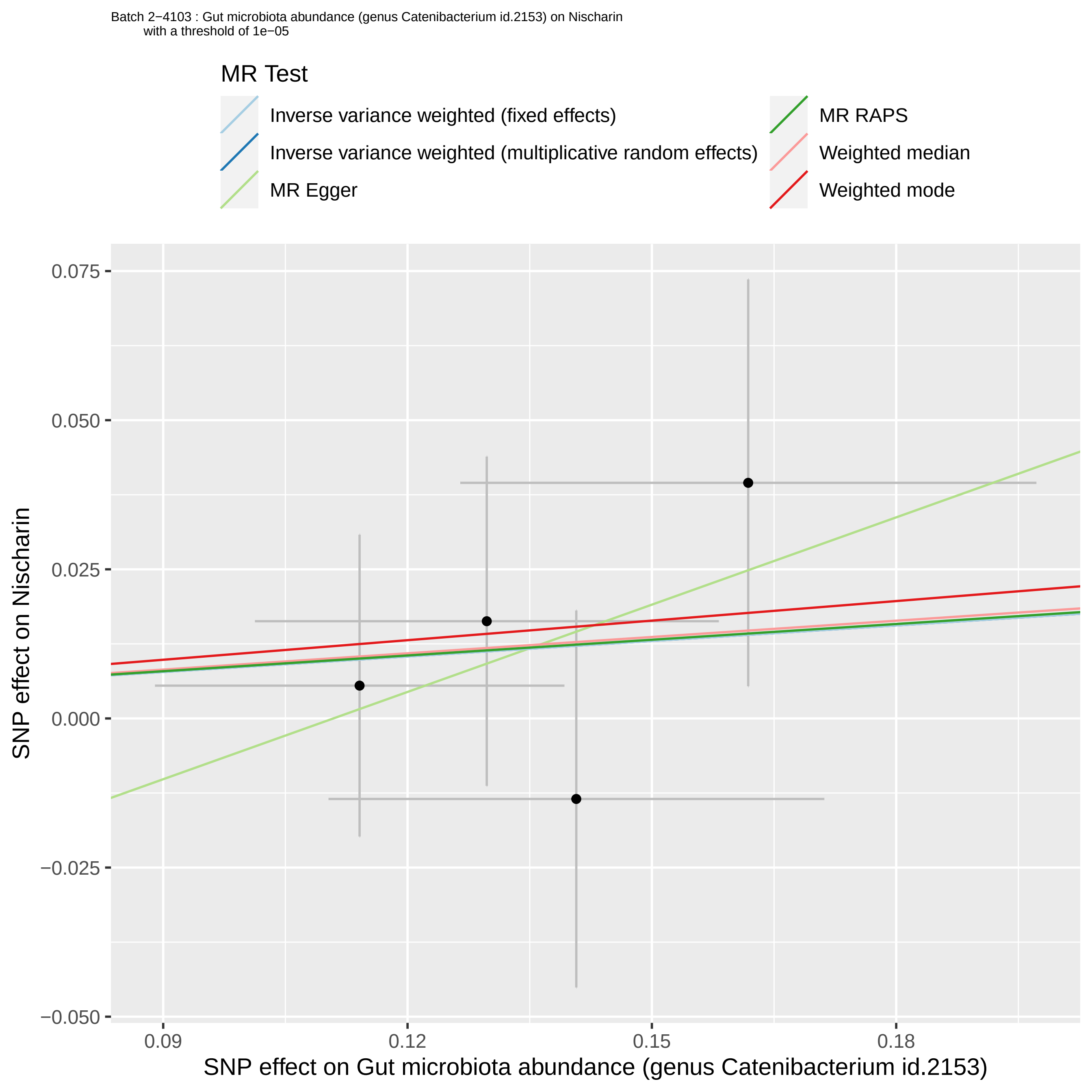

Batch 2−4103 : Gut microbiota abundance (genus Catenibacterium id.2153) on Nischarin
with a threshold of 1e−05
MR Test
Inverse variance weighted (fixed effects)
Inverse variance weighted (multiplicative random effects)
MR Egger
MR RAPS
Weighted median
Weighted mode
0.075
0.050
0.025
0.000
−0.025
−0.050
SNP effect on Nischarin
0.09
0.12
0.15
0.18
SNP effect on Gut microbiota abundance (genus Catenibacterium id.2153)

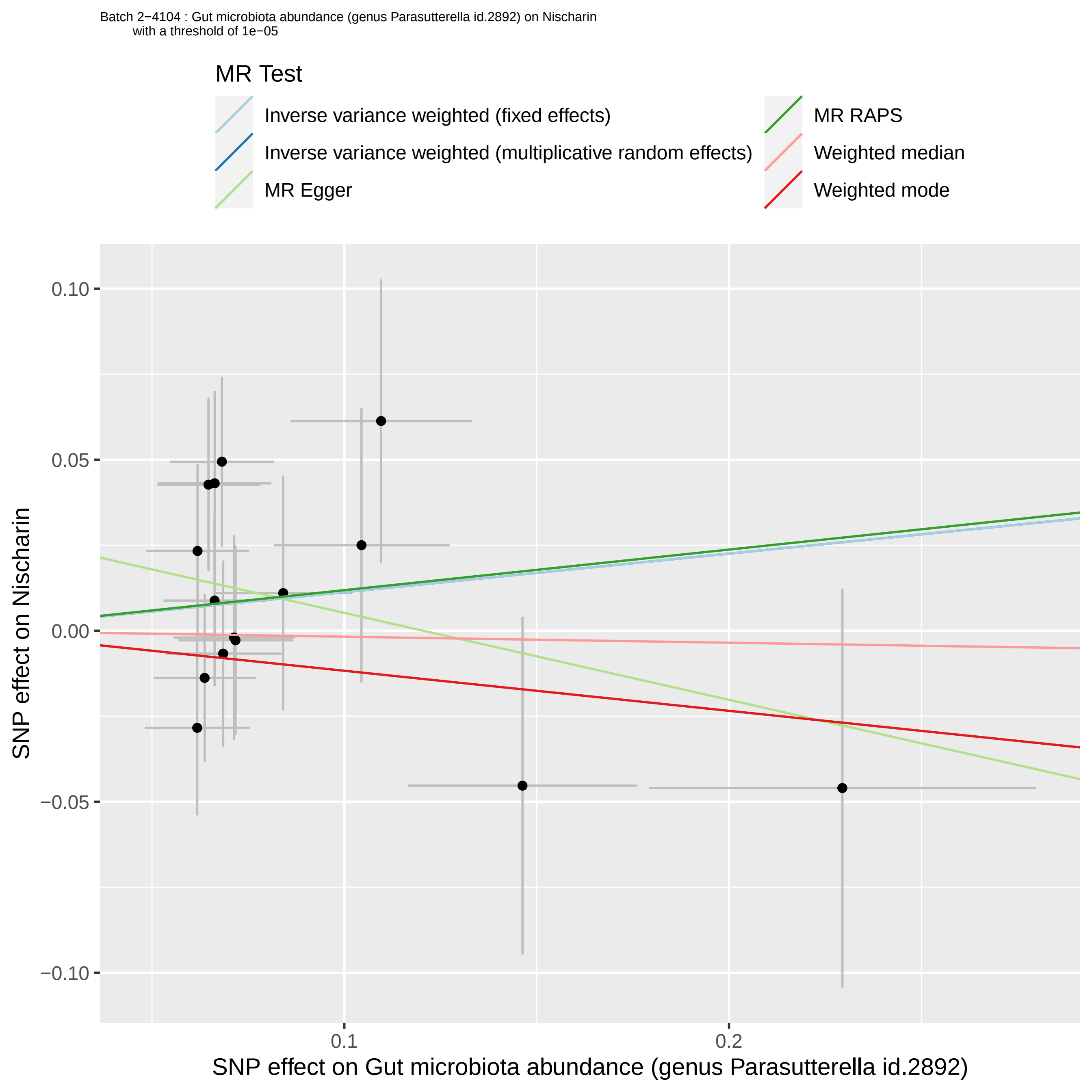

Batch 2−4104 : Gut microbiota abundance (genus Parasutterella id.2892) on Nischarin
with a threshold of 1e−05
MR Test
Inverse variance weighted (fixed effects)
Inverse variance weighted (multiplicative random effects)
MR Egger
MR RAPS
Weighted median
Weighted mode
SNP effect on Nischarin
0.10
0.05
0.00
−0.05
−0.10
0.1
0.2
SNP effect on Gut microbiota abundance (genus Parasutterella id.2892)

Batch 2−4105 : Gut microbiota abundance (genus Catenibacterium id.2153) on Neuroligin−1
with a threshold of 1e−05

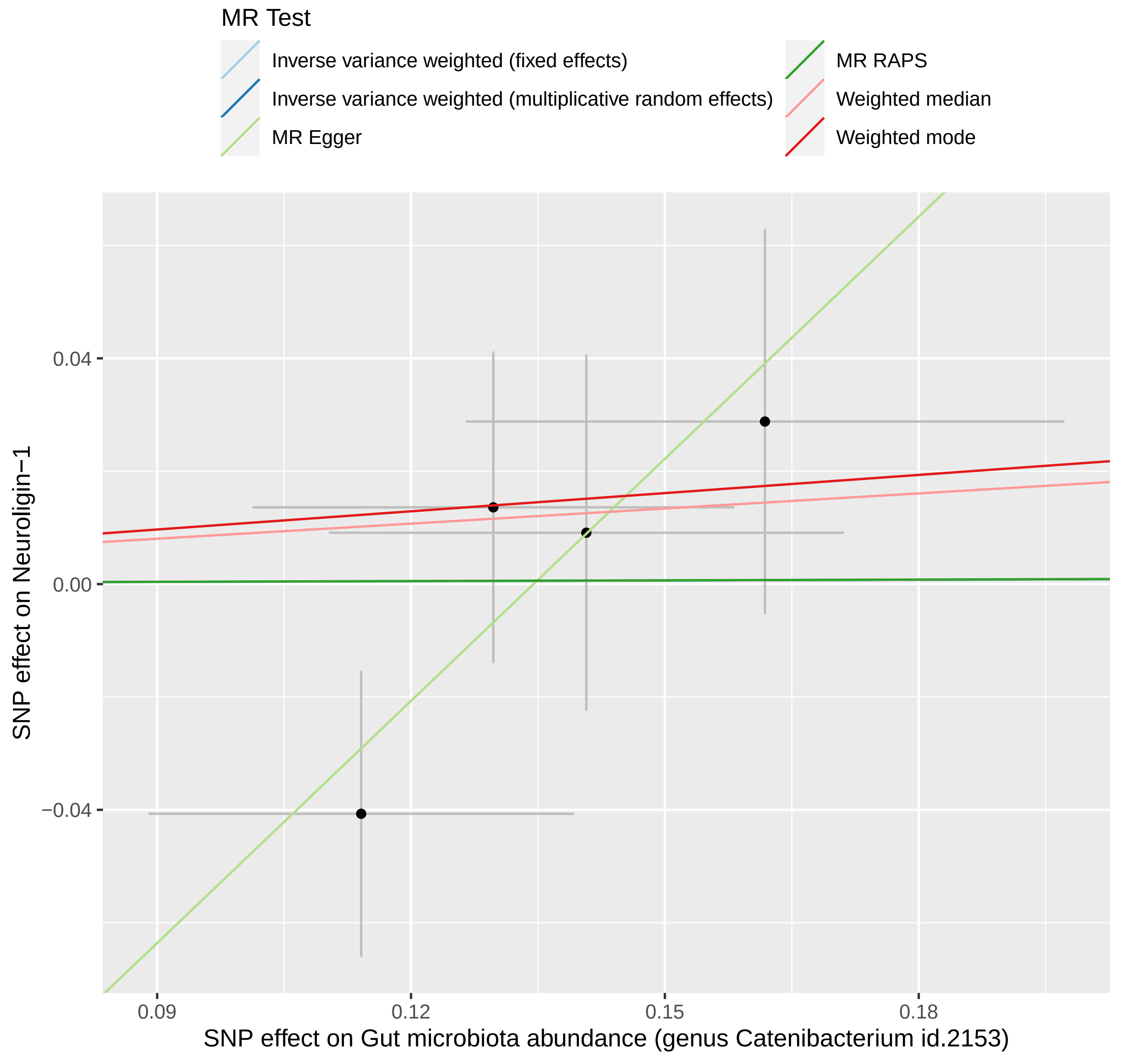

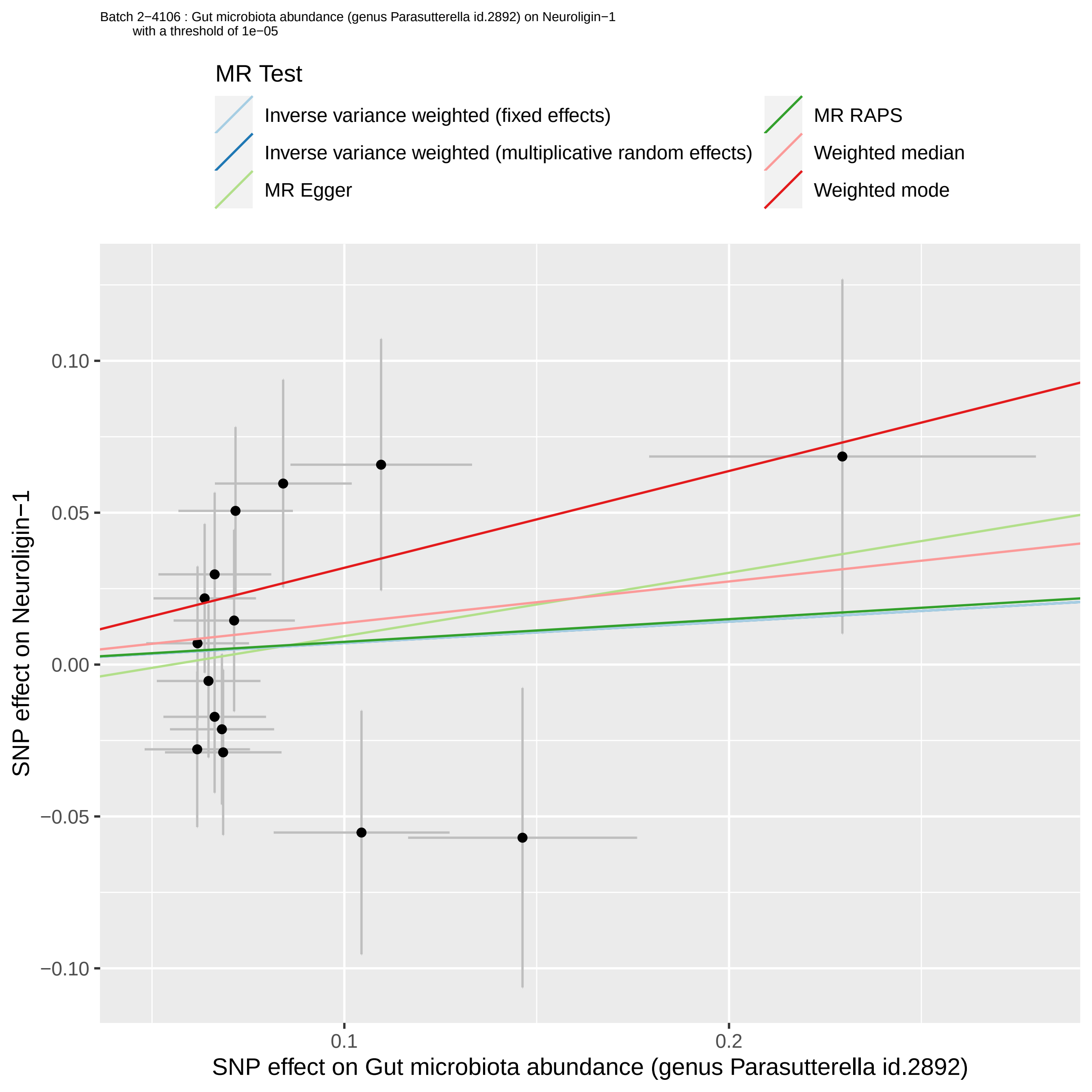
Batch 2−4106 : Gut microbiota abundance (genus Parasutterella id.2892) on Neuroligin−1
with a threshold of 1e−05
MR Test
Inverse variance weighted (fixed effects)
Inverse variance weighted (multiplicative random effects)
MR Egger
MR RAPS
Weighted median
Weighted mode
SNP effect on Neuroligin−1
0.10
0.05
0.00
−0.05
−0.10
0.1
0.2
SNP effect on Gut microbiota abundance (genus Parasutterella id.2892)

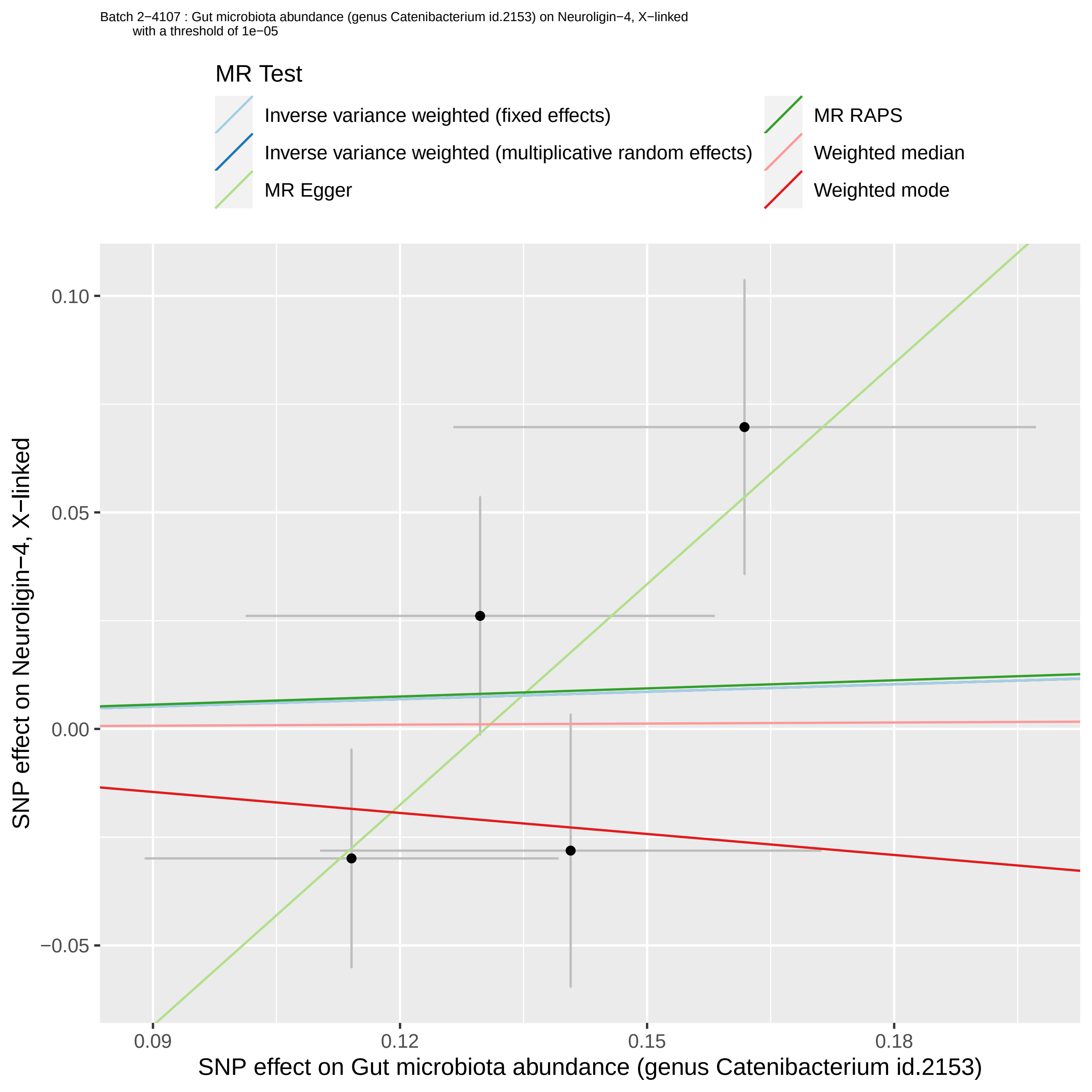

Batch 2−4107 : Gut microbiota abundance (genus Catenibacterium id.2153) on Neuroligin−4, X−linked
with a threshold of 1e−05
MR Test
Inverse variance weighted (fixed effects)
Inverse variance weighted (multiplicative random effects)
MR Egger
MR RAPS
Weighted median
Weighted mode
0.10
0.05
0.00
−0.05
0.09
0.12
0.15
0.18
SNP effect on Neuroligin−4, X−linked
SNP effect on Gut microbiota abundance (genus Catenibacterium id.2153)

Batch 2−4108 : Gut microbiota abundance (genus Parasutterella id.2892) on Neuroligin−4, X−linked
with a threshold of 1e−05

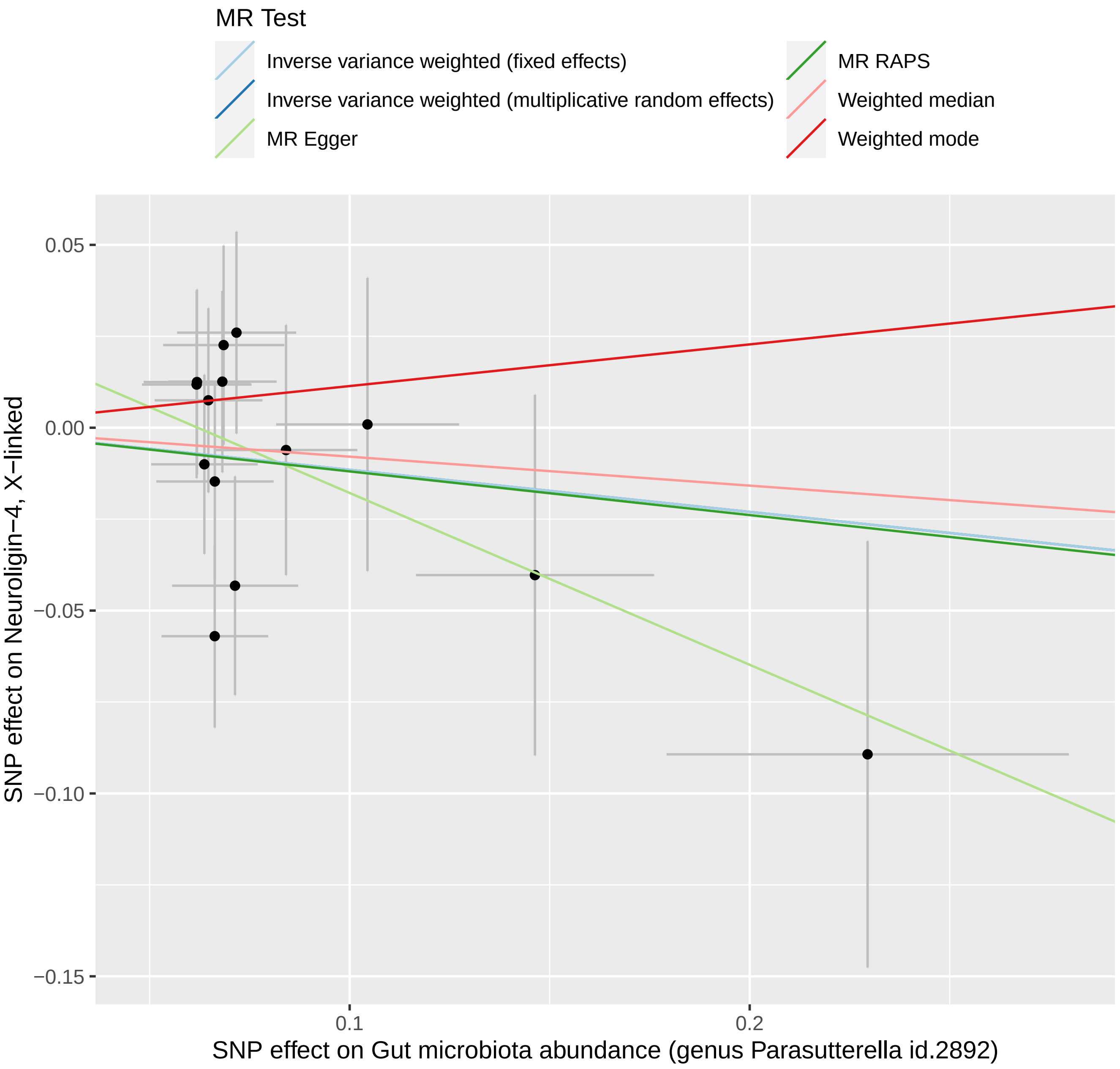

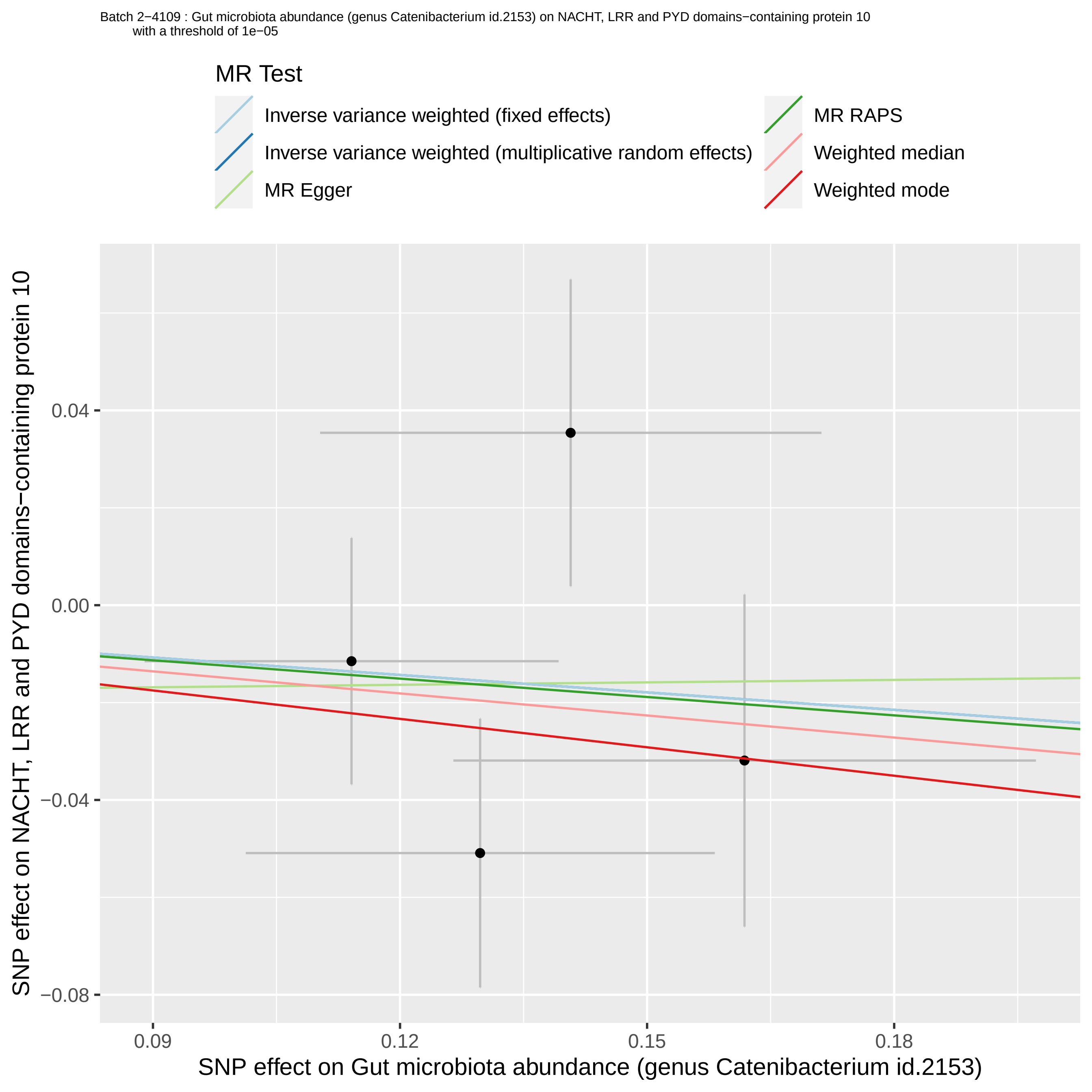

Batch 2−4109 : Gut microbiota abundance (genus Catenibacterium id.2153) on NACHT, LRR and PYD domains−containing protein 10
with a threshold of 1e−05
MR Test
Inverse variance weighted (fixed effects)
Inverse variance weighted (multiplicative random effects)
MR Egger
MR RAPS
Weighted median
Weighted mode
0.04
0.00
−0.04
−0.08
0.09
0.12
0.15
0.18
SNP effect on NACHT, LRR and PYD domains−containing protein 10
SNP effect on Gut microbiota abundance (genus Catenibacterium id.2153)

Batch 2−4110 : Gut microbiota abundance (genus Parasutterella id.2892) on NACHT, LRR and PYD domains−containing protein 10
with a threshold of 1e−05

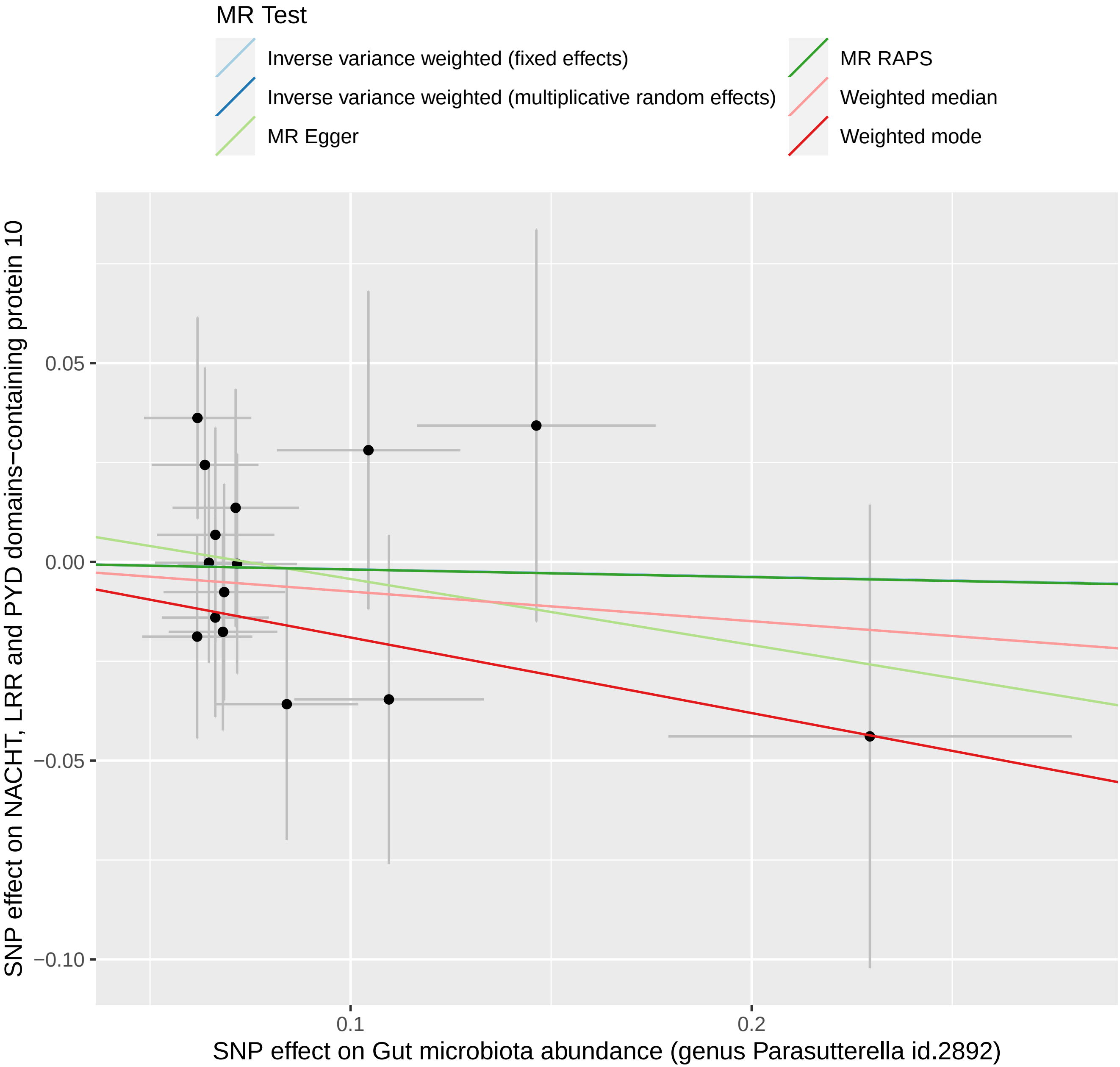

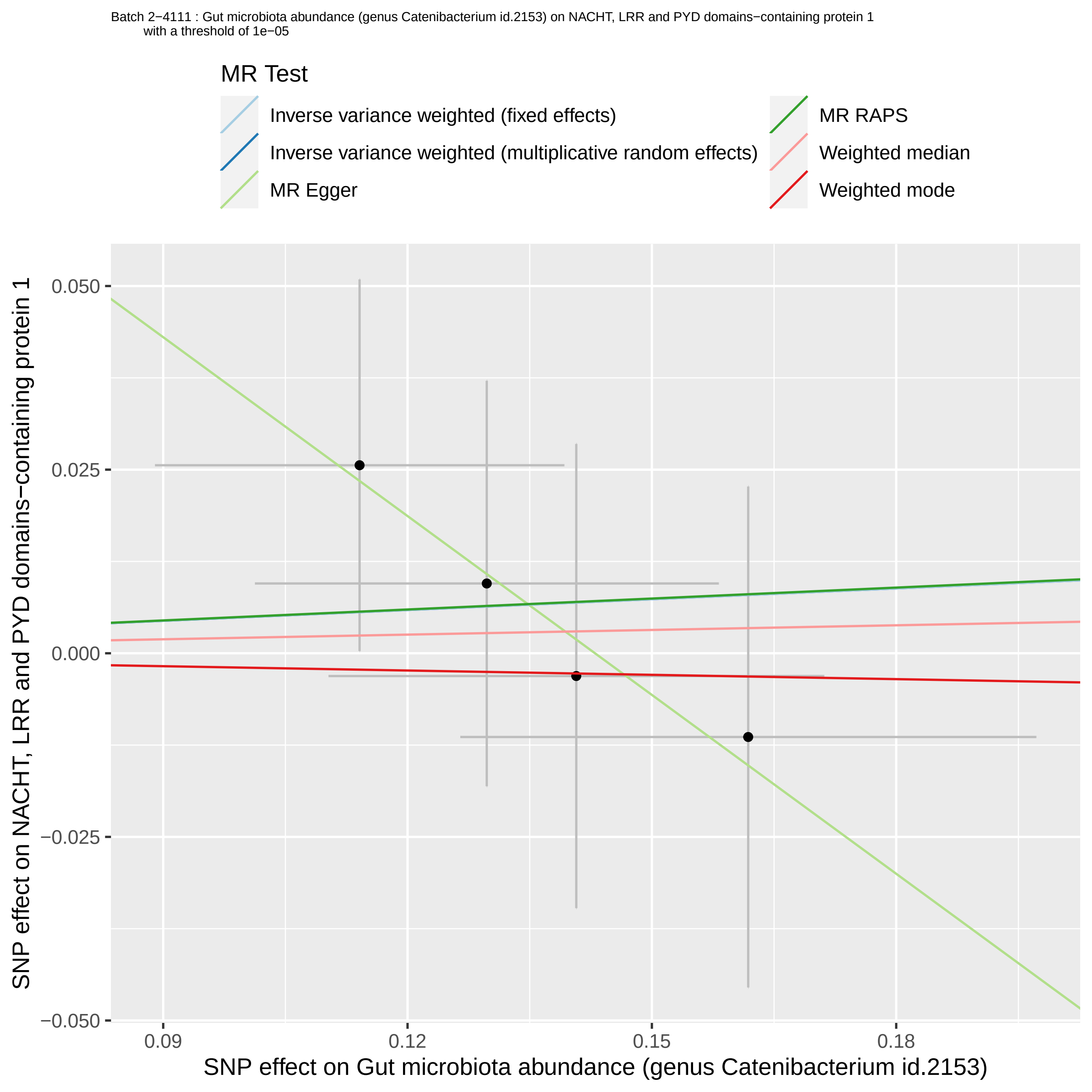
Batch 2−4111 : Gut microbiota abundance (genus Catenibacterium id.2153) on NACHT, LRR and PYD domains−containing protein 1 with a threshold of 1e−05
MR Test
Inverse variance weighted (fixed effects)
Inverse variance weighted (multiplicative random effects)
MR Egger
MR RAPS
Weighted median
Weighted mode
0.050
0.025
0.000
−0.025
−0.050
0.09
0.12
0.15
0.18
SNP effect on NACHT, LRR and PYD domains−containing protein 1
SNP effect on Gut microbiota abundance (genus Catenibacterium id.2153)

Batch 2−4112 : Gut microbiota abundance (genus Parasutterella id.2892) on NACHT, LRR and PYD domains−containing protein 1
with a threshold of 1e−05

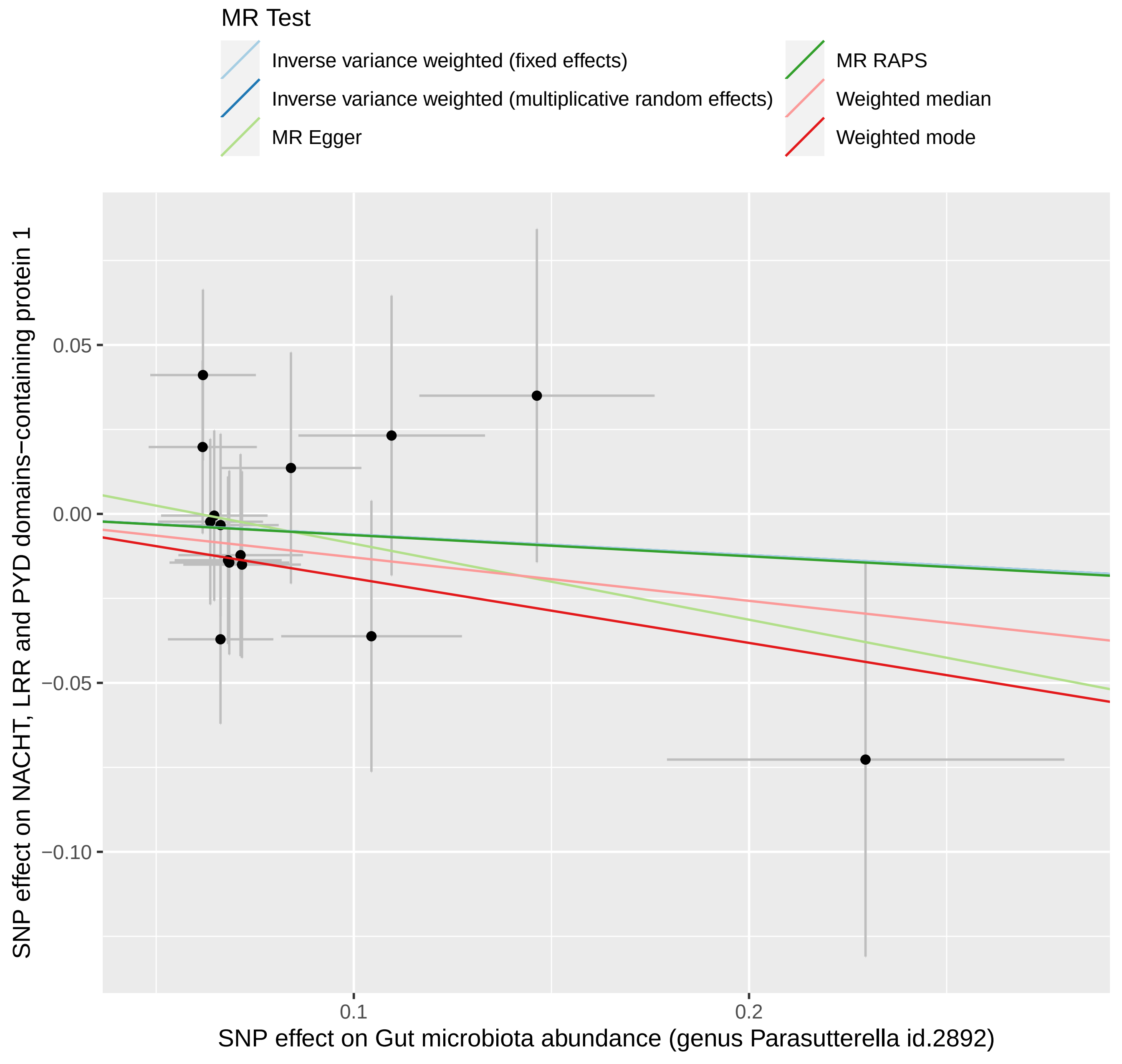

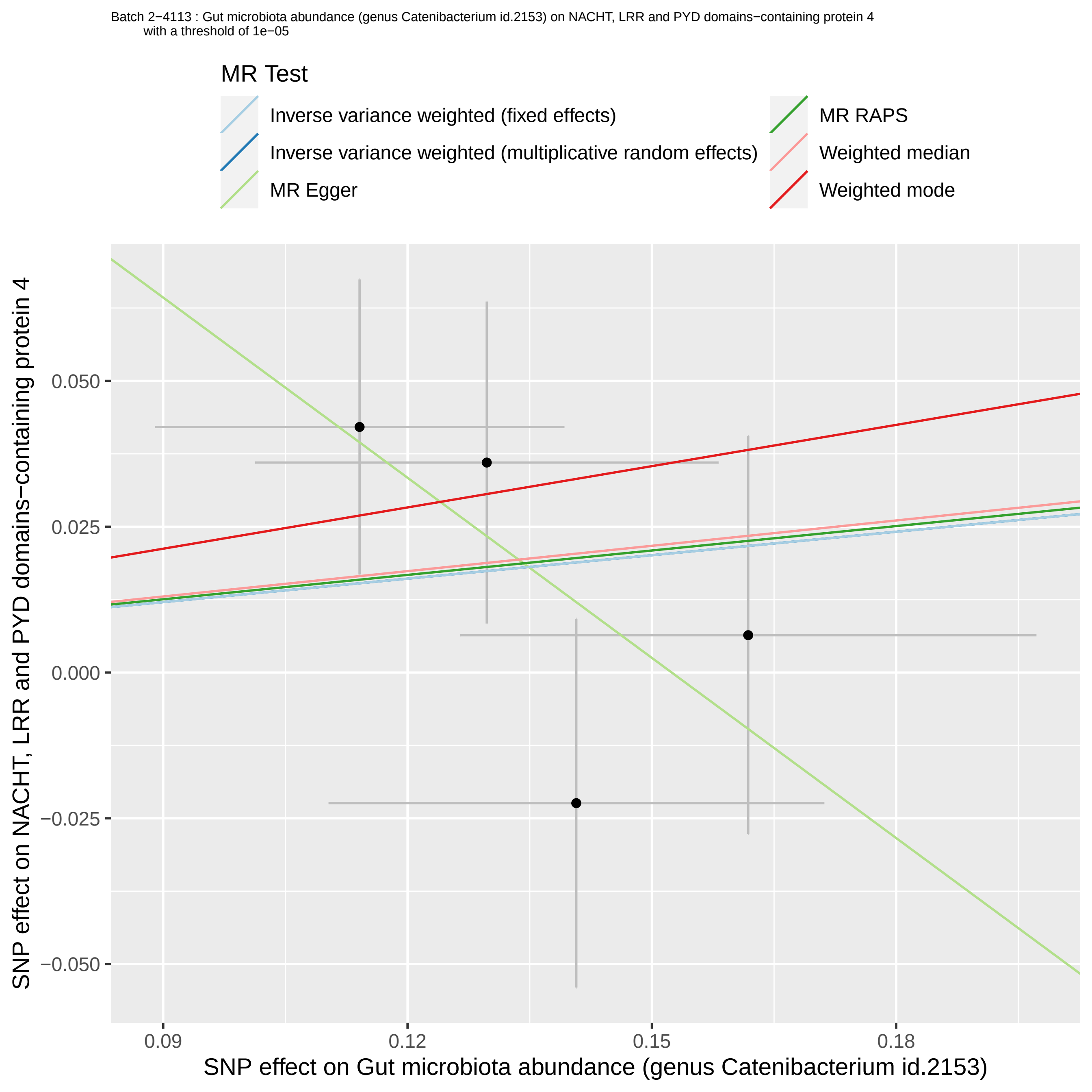
Batch 2−4113 : Gut microbiota abundance (genus Catenibacterium id.2153) on NACHT, LRR and PYD domains−containing protein 4
with a threshold of 1e−05
MR Test
Inverse variance weighted (fixed effects)
Inverse variance weighted (multiplicative random effects)
MR Egger
MR RAPS
Weighted median
Weighted mode
0.050
0.025
0.000
−0.025
−0.050
0.09
0.12
0.15
0.18
SNP effect on NACHT, LRR and PYD domains−containing protein 4
SNP effect on Gut microbiota abundance (genus Catenibacterium id.2153)

Batch 2−4114 : Gut microbiota abundance (genus Parasutterella id.2892) on NACHT, LRR and PYD domains−containing protein 4
with a threshold of 1e−05

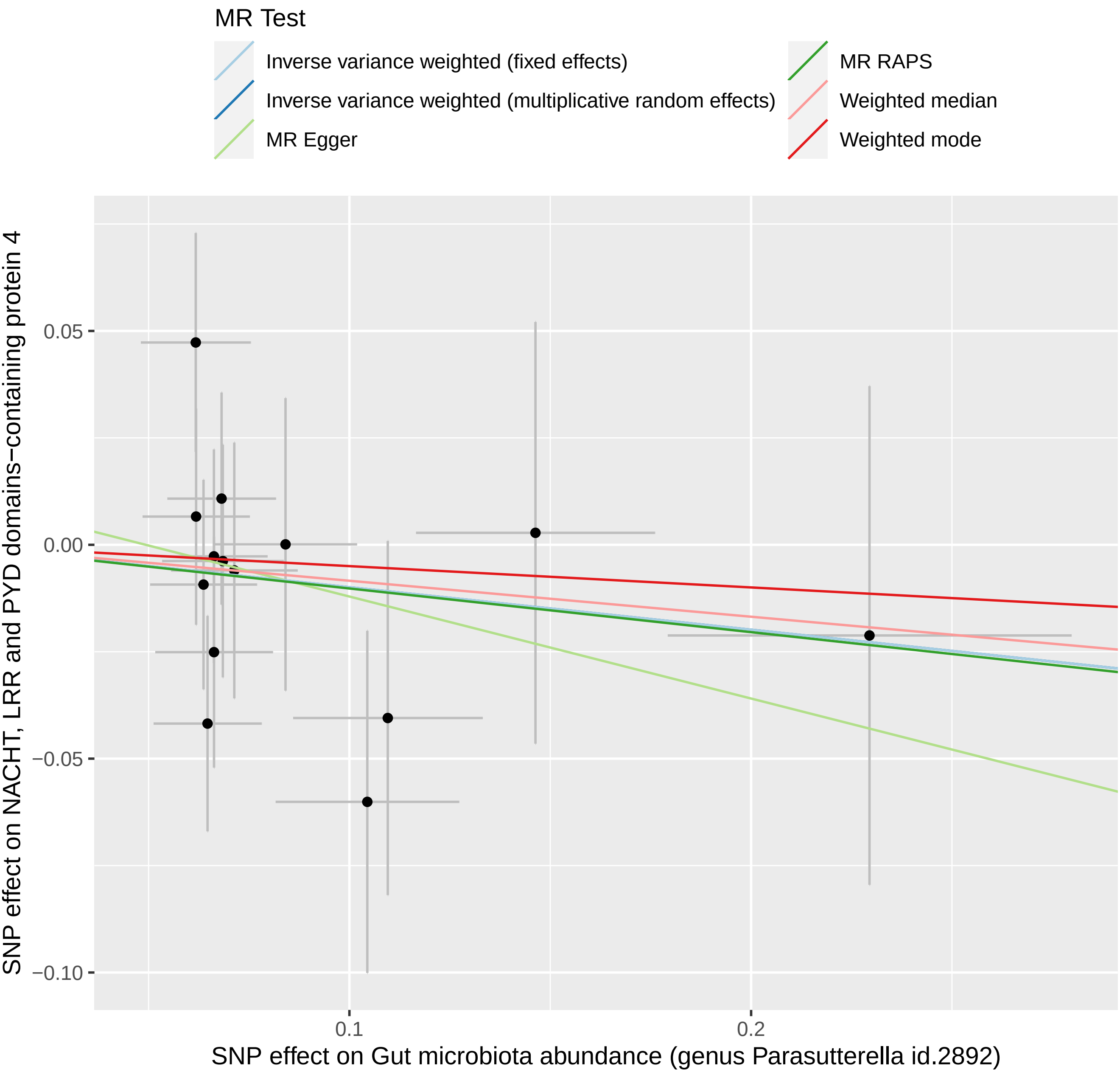

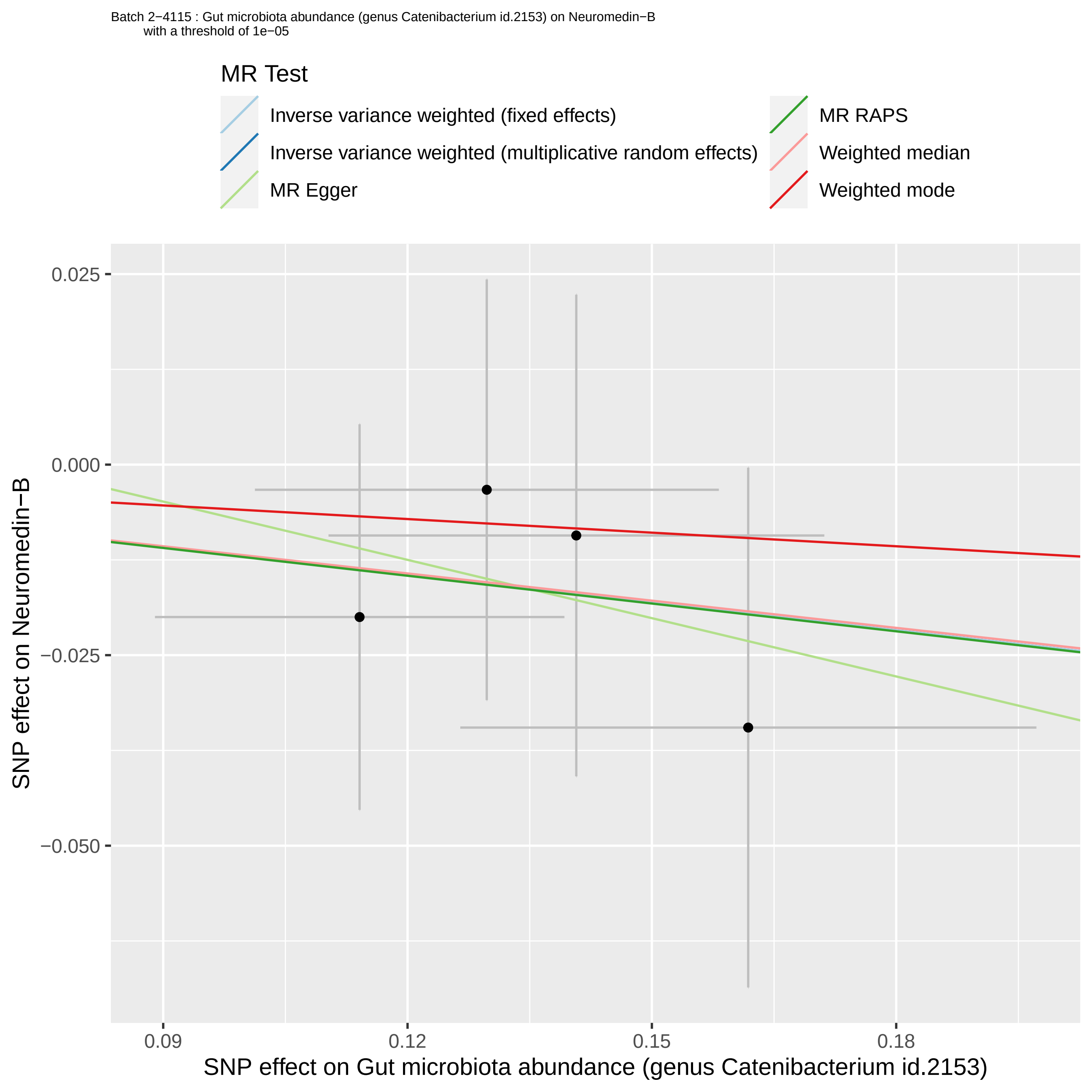

Batch 2−4115 : Gut microbiota abundance (genus Catenibacterium id.2153) on Neuromedin−B
with a threshold of 1e−05
MR Test
Inverse variance weighted (fixed effects)
Inverse variance weighted (multiplicative random effects)
MR Egger
MR RAPS
Weighted median
Weighted mode
0.025
0.000
−0.025
−0.050
SNP effect on Neuromedin−B
0.09
0.12
0.15
0.18
SNP effect on Gut microbiota abundance (genus Catenibacterium id.2153)

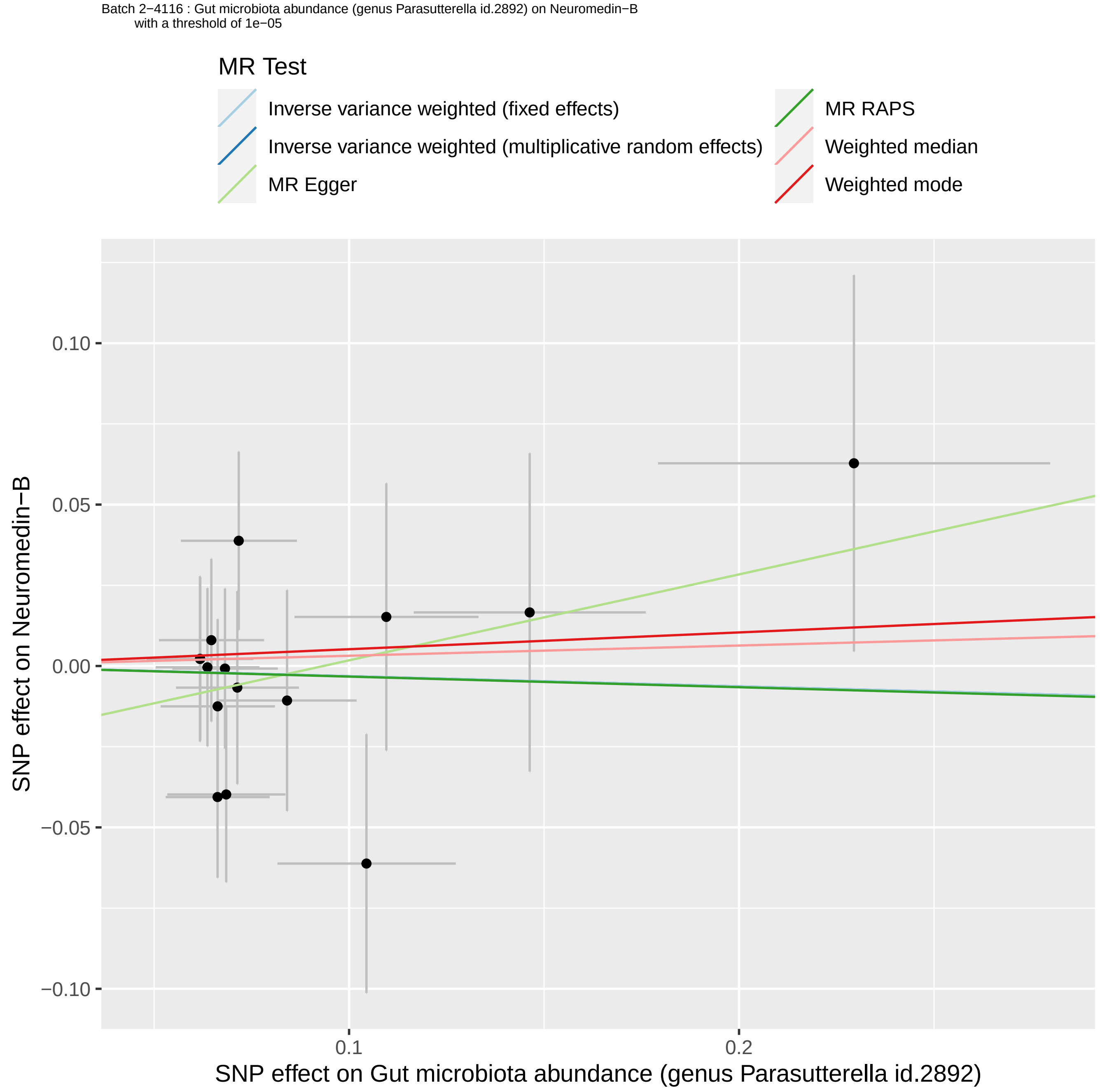
Batch 2−4116 : Gut microbiota abundance (genus Parasutterella id.2892) on Neuromedin−B
with a threshold of 1e−05
MR Test
Inverse variance weighted (fixed effects)
Inverse variance weighted (multiplicative random effects)
MR Egger
MR RAPS
Weighted median
Weighted mode
0.10
0.05
0.00
−0.05
−0.10
0.1
0.2
SNP effect on Neuromedin−B
SNP effect on Gut microbiota abundance (genus Parasutterella id.2892)

Batch 2−4117 : Gut microbiota abundance (genus Catenibacterium id.2153) on Nucleoside diphosphate kinase A
with a threshold of 1e−05

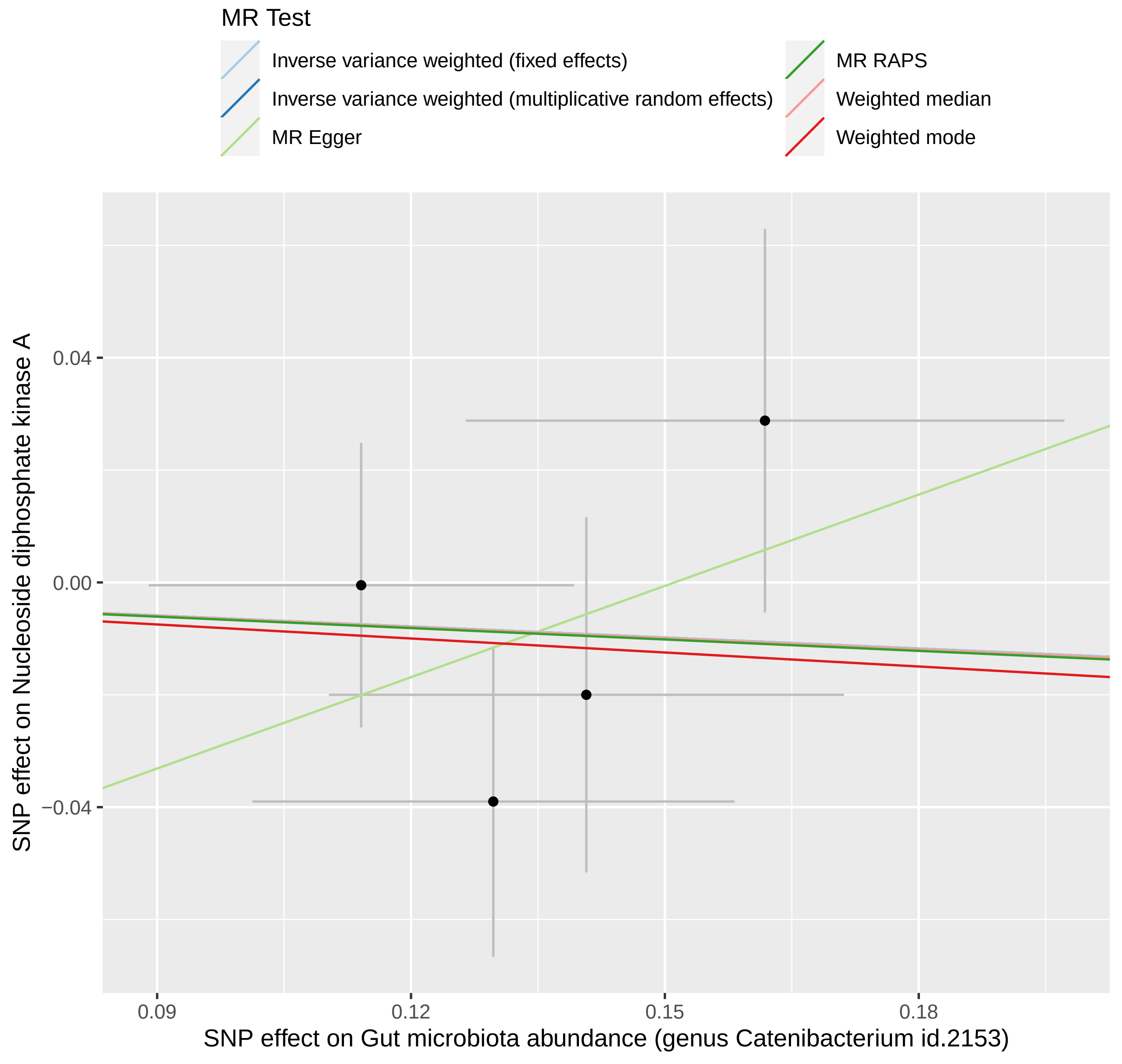

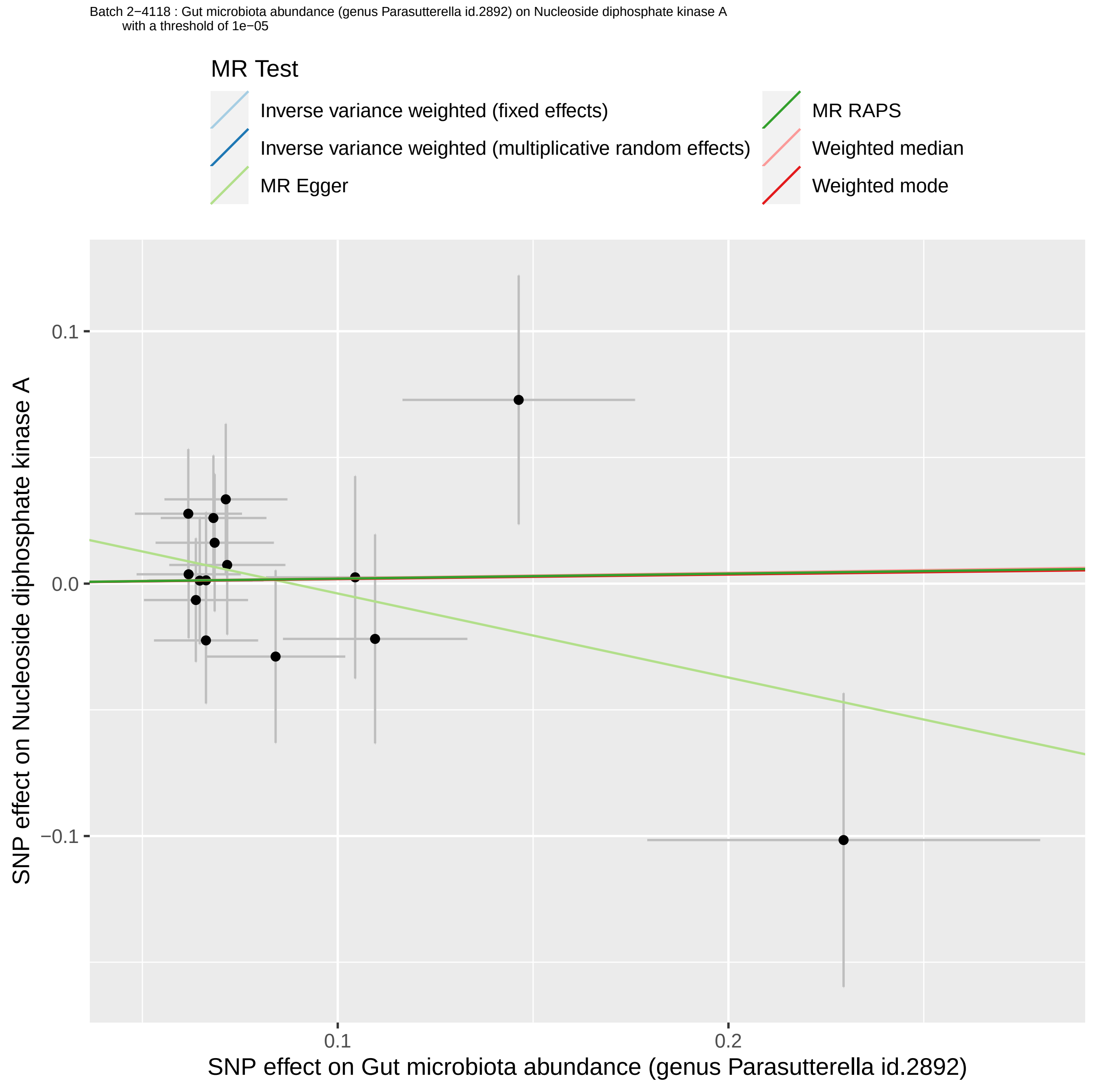
Batch 2−4118 : Gut microbiota abundance (genus Parasutterella id.2892) on Nucleoside diphosphate kinase A
with a threshold of 1e−05
MR Test
Inverse variance weighted (fixed effects)
Inverse variance weighted (multiplicative random effects)
MR Egger
MR RAPS
Weighted median
Weighted mode
0.1
0.0
−0.1
0.1
0.2
SNP effect on Nucleoside diphosphate kinase A
SNP effect on Gut microbiota abundance (genus Parasutterella id.2892)

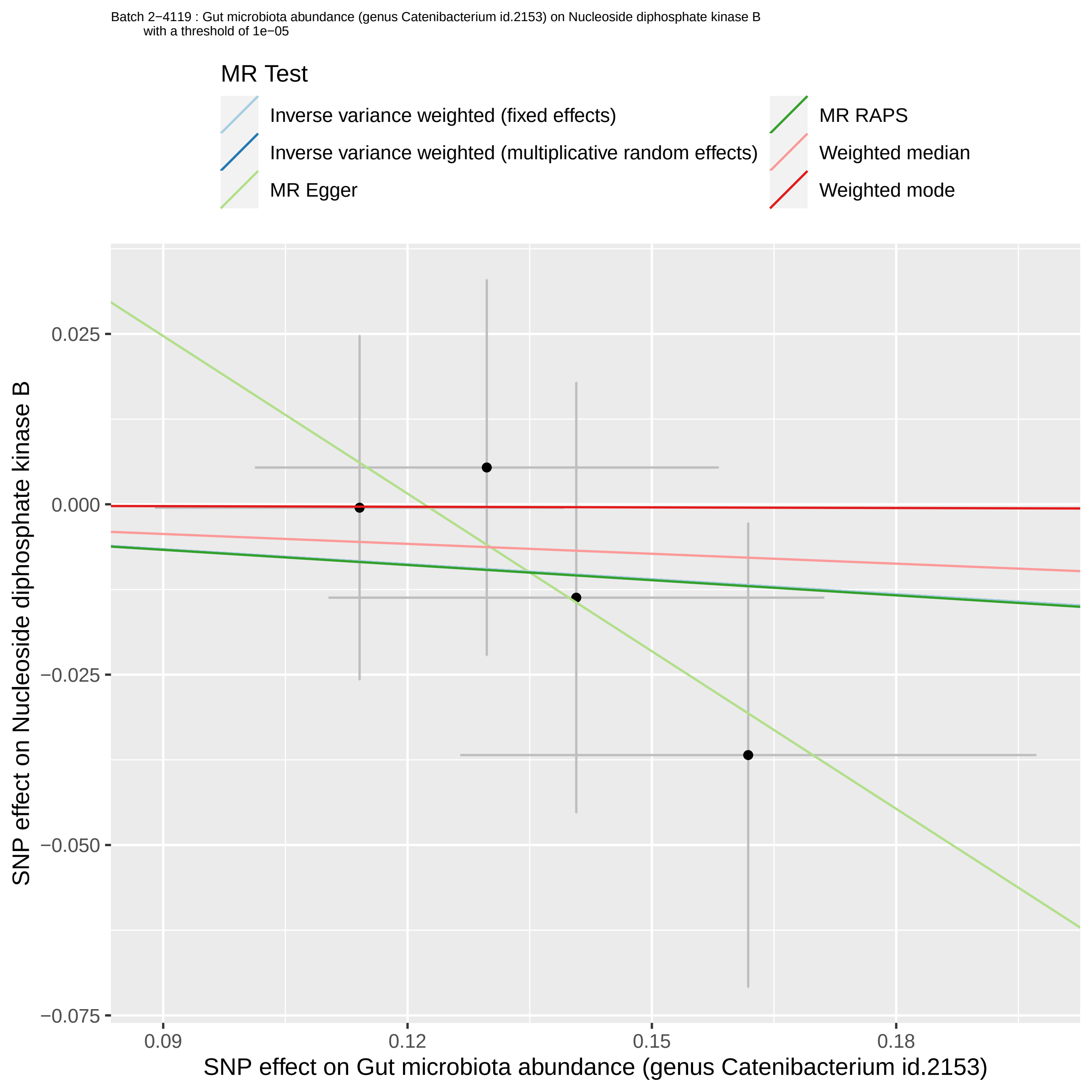

Batch 2−4119 : Gut microbiota abundance (genus Catenibacterium id.2153) on Nucleoside diphosphate kinase B
with a threshold of 1e−05
MR Test
Inverse variance weighted (fixed effects)
Inverse variance weighted (multiplicative random effects)
MR Egger
MR RAPS
Weighted median
Weighted mode
0.025
0.000
−0.025
−0.050
−0.075
0.09
0.12
0.15
0.18
SNP effect on Nucleoside diphosphate kinase B
SNP effect on Gut microbiota abundance (genus Catenibacterium id.2153)

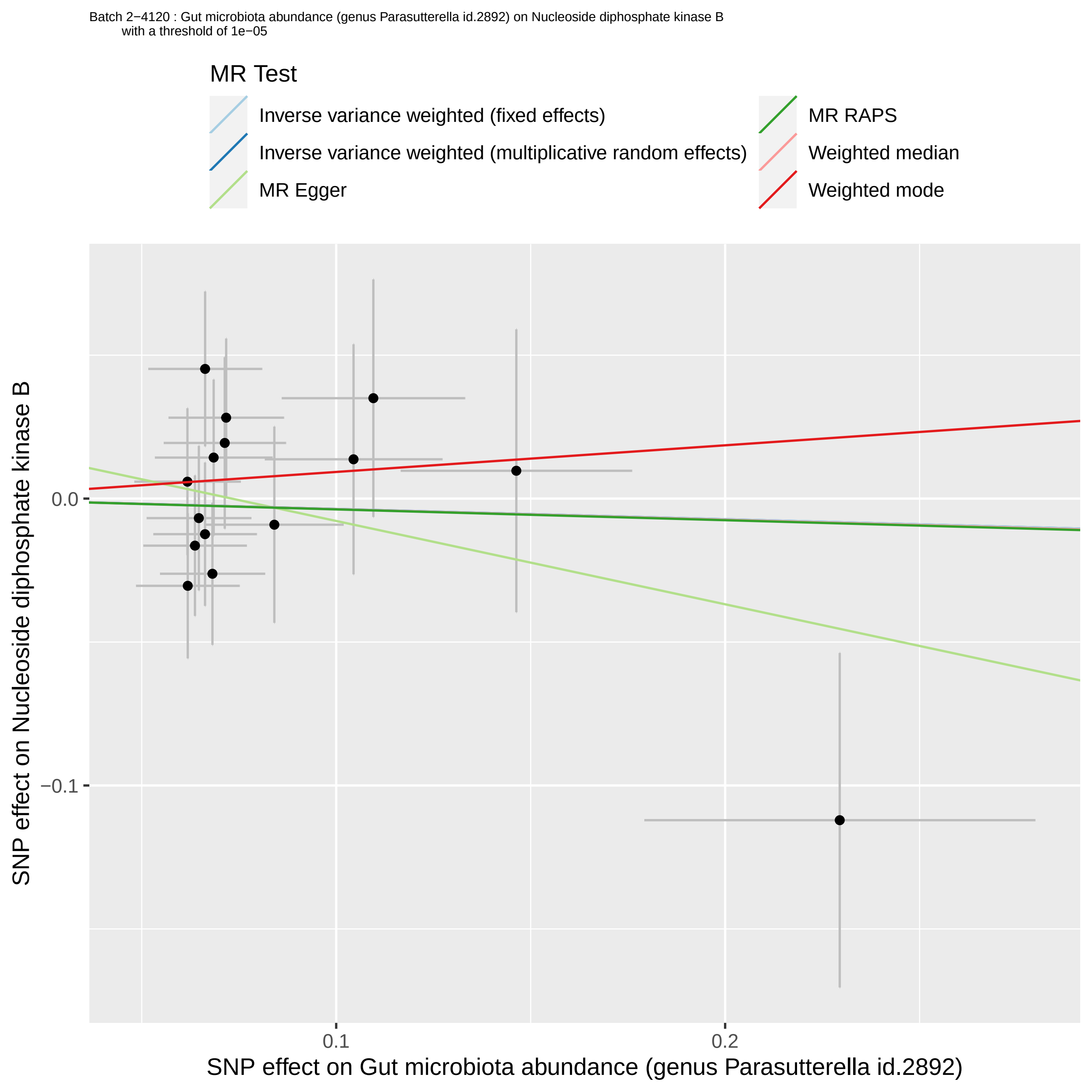
Batch 2−4120 : Gut microbiota abundance (genus Parasutterella id.2892) on Nucleoside diphosphate kinase B
with a threshold of 1e−05
MR Test
Inverse variance weighted (fixed effects)
Inverse variance weighted (multiplicative random effects)
MR Egger
MR RAPS
Weighted median
Weighted mode
0.0
−0.1
0.1
0.2
SNP effect on Nucleoside diphosphate kinase B
SNP effect on Gut microbiota abundance (genus Parasutterella id.2892)

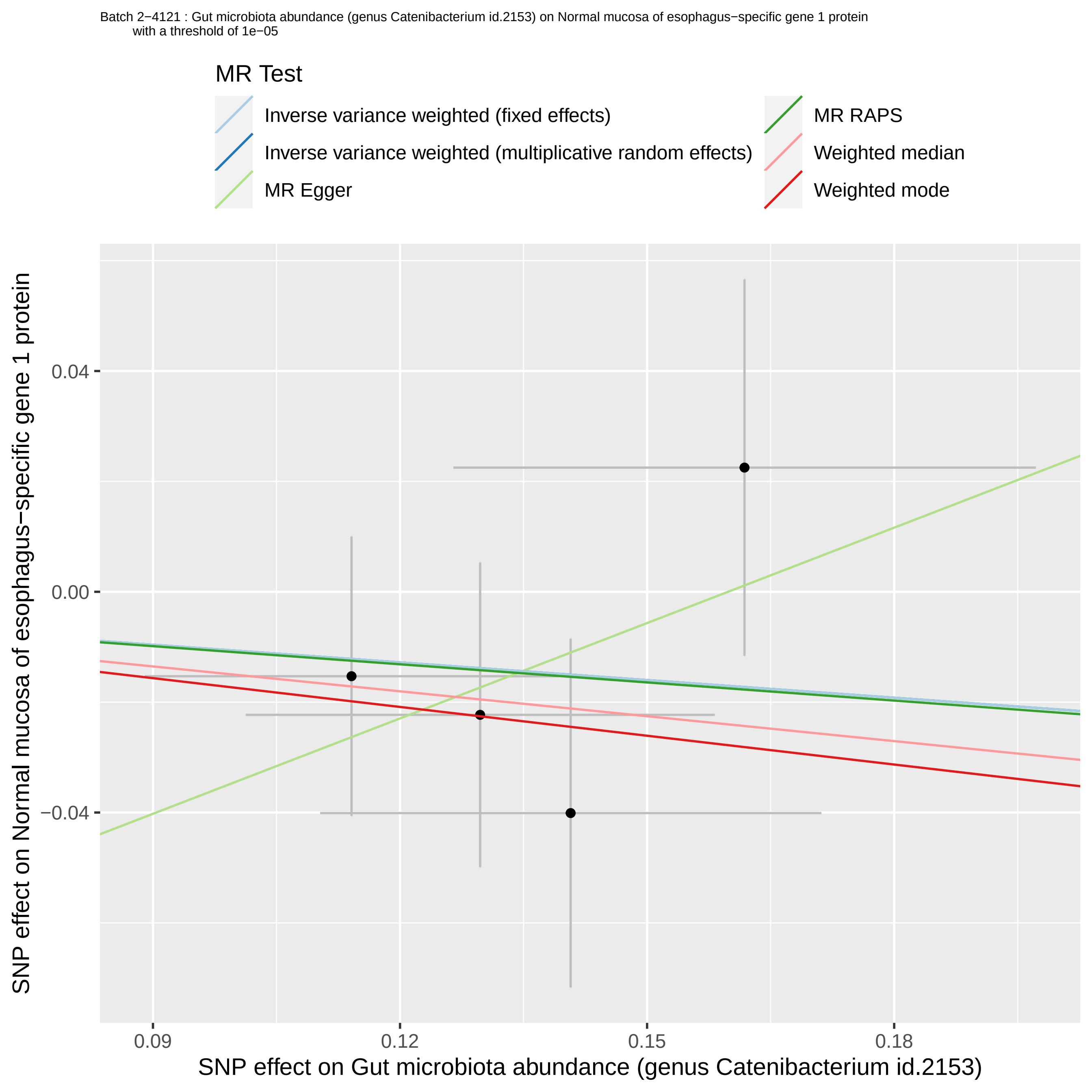
Batch 2−4121 : Gut microbiota abundance (genus Catenibacterium id.2153) on Normal mucosa of esophagus−specific gene 1 protein with a threshold of 1e−05
MR Test
Inverse variance weighted (fixed effects)
Inverse variance weighted (multiplicative random effects)
MR Egger
MR RAPS
Weighted median
Weighted mode
0.04
0.00
−0.04
0.09
0.12
0.15
0.18
SNP effect on Normal mucosa of esophagus−specific gene 1 protein
SNP effect on Gut microbiota abundance (genus Catenibacterium id.2153)

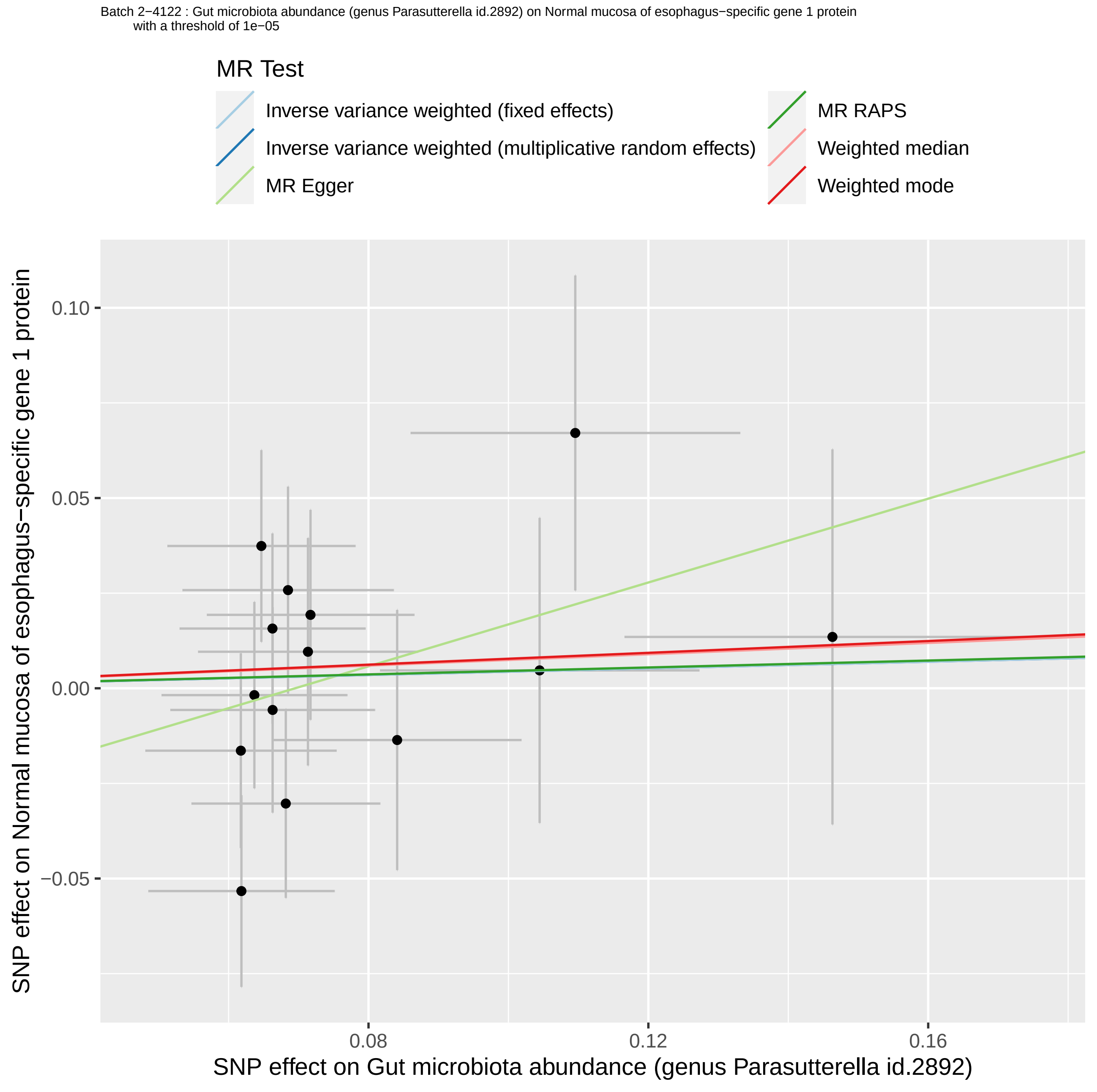
Batch 2−4122 : Gut microbiota abundance (genus Parasutterella id.2892) on Normal mucosa of esophagus−specific gene 1 protein
with a threshold of 1e−05
MR Test
Inverse variance weighted (fixed effects)
Inverse variance weighted (multiplicative random effects)
MR Egger
MR RAPS
Weighted median
Weighted mode
0.10
0.05
0.00
−0.05
0.08
0.12
0.16
SNP effect on Normal mucosa of esophagus−specific gene 1 protein
SNP effect on Gut microbiota abundance (genus Parasutterella id.2892)

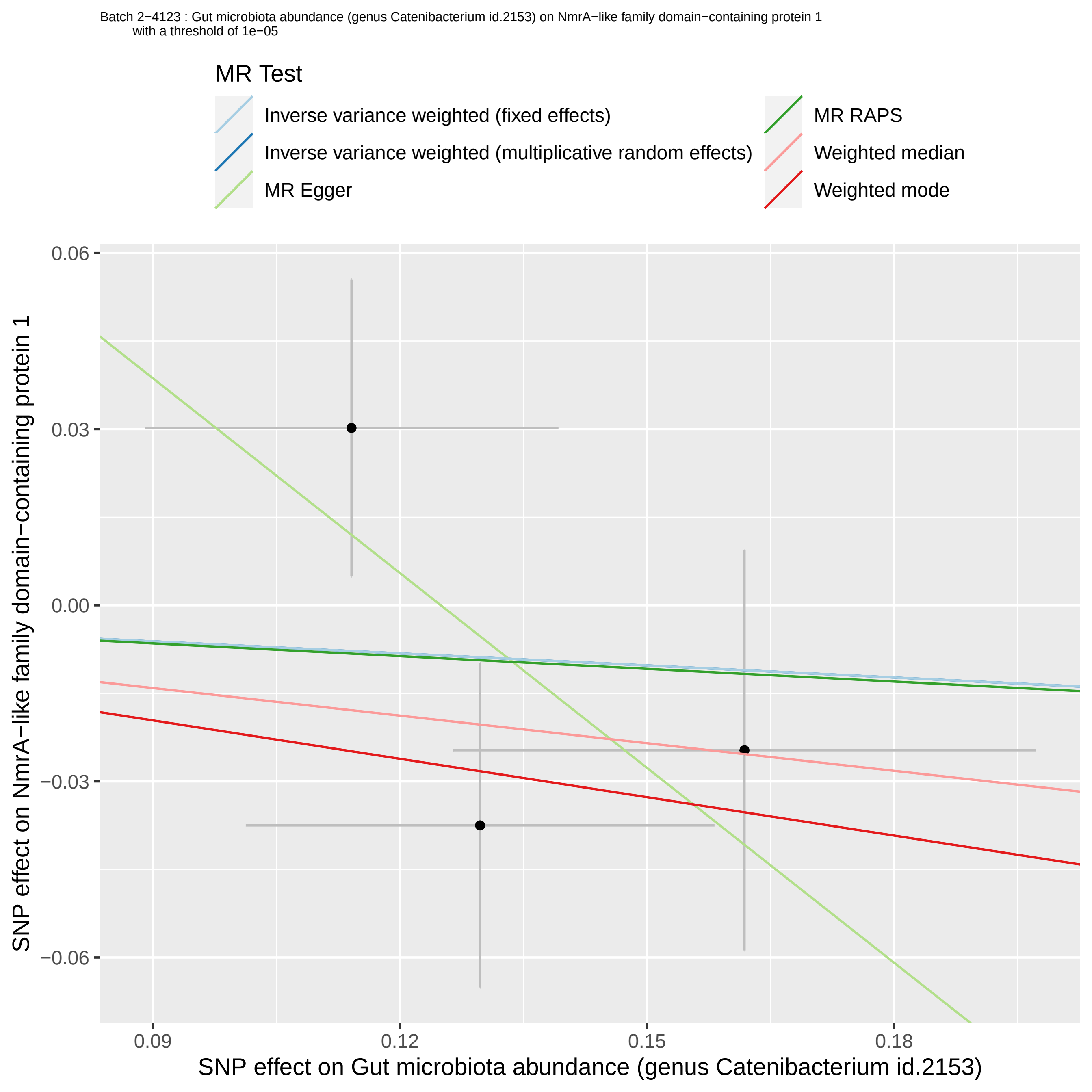
Batch 2−4123 : Gut microbiota abundance (genus Catenibacterium id.2153) on NmrA−like family domain−containing protein 1
with a threshold of 1e−05
MR Test
Inverse variance weighted (fixed effects)
Inverse variance weighted (multiplicative random effects)
MR Egger
MR RAPS
Weighted median
Weighted mode
0.06
0.03
0.00
−0.03
−0.06
0.09
0.12
0.15
0.18
SNP effect on NmrA−like family domain−containing protein 1
SNP effect on Gut microbiota abundance (genus Catenibacterium id.2153)

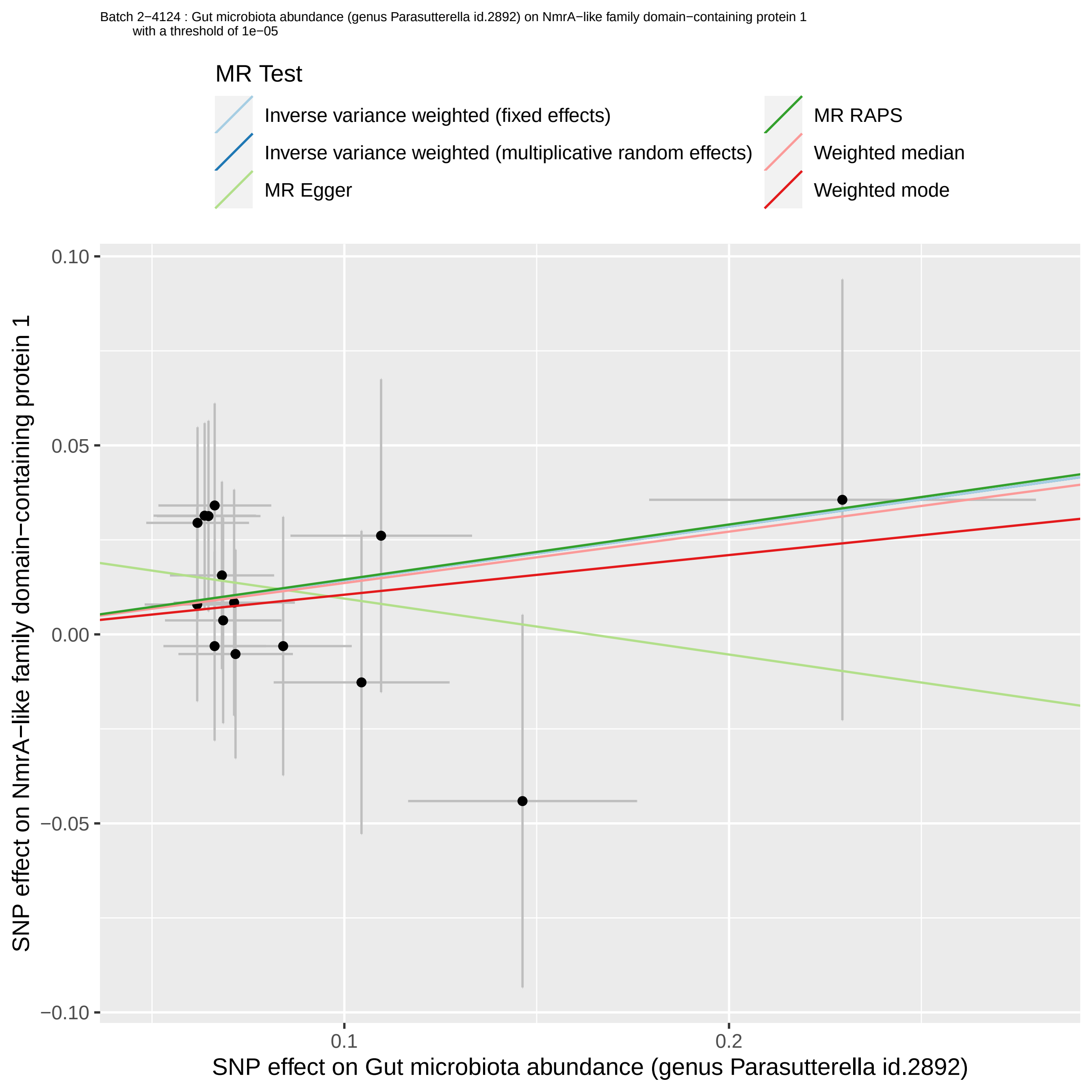
Batch 2−4124 : Gut microbiota abundance (genus Parasutterella id.2892) on NmrA−like family domain−containing protein 1
with a threshold of 1e−05
MR Test
Inverse variance weighted (fixed effects)
Inverse variance weighted (multiplicative random effects)
MR Egger
MR RAPS
Weighted median
Weighted mode
0.10
0.05
0.00
−0.05
−0.10
0.1
0.2
SNP effect on NmrA−like family domain−containing protein 1
SNP effect on Gut microbiota abundance (genus Parasutterella id.2892)

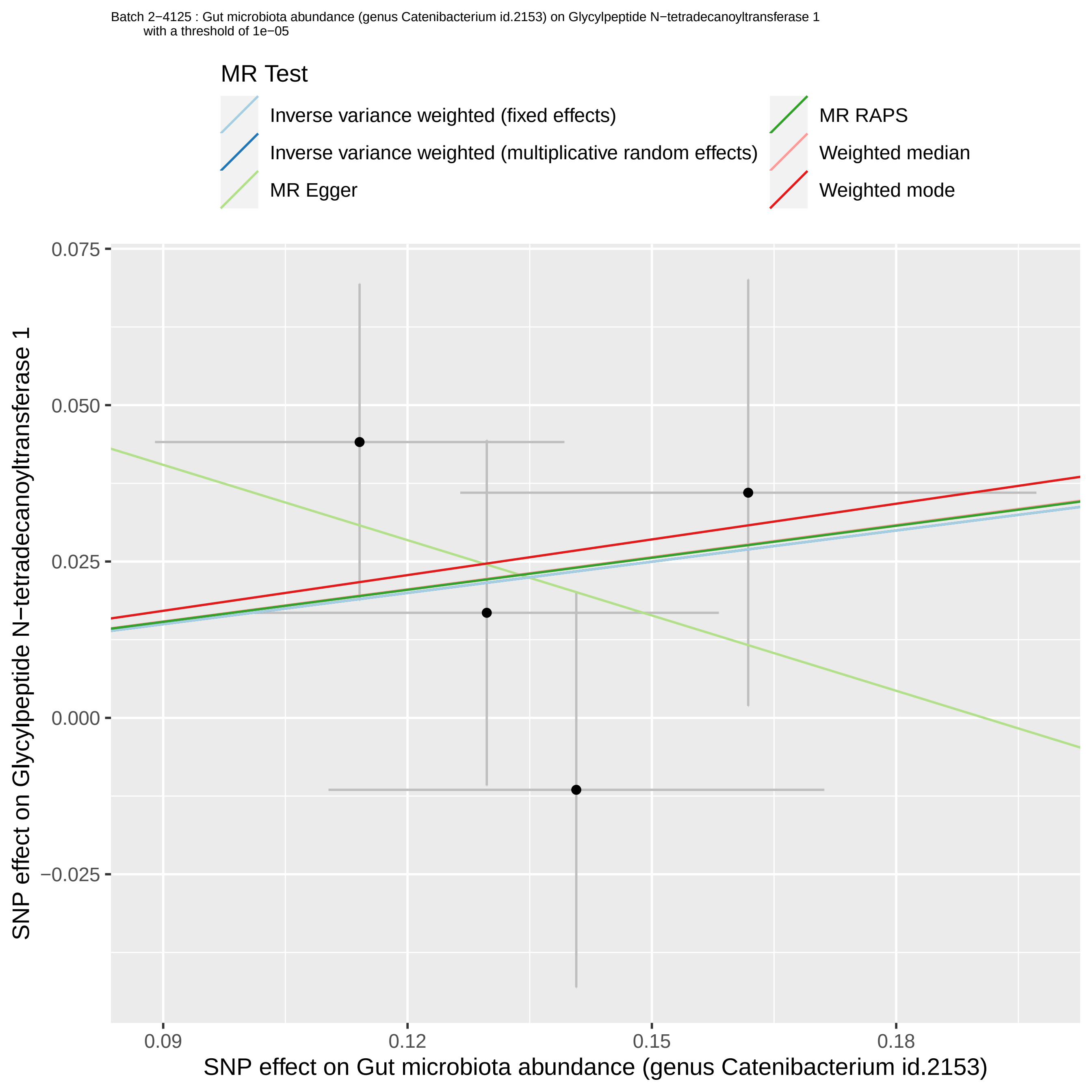
Batch 2−4125 : Gut microbiota abundance (genus Catenibacterium id.2153) on Glycylpeptide N−tetradecanoyltransferase 1
with a threshold of 1e−05
MR Test
Inverse variance weighted (fixed effects)
Inverse variance weighted (multiplicative random effects)
MR Egger
MR RAPS
Weighted median
Weighted mode
0.075
0.050
0.025
0.000
−0.025
0.09
0.12
0.15
0.18
SNP effect on Glycylpeptide N−tetradecanoyltransferase 1
SNP effect on Gut microbiota abundance (genus Catenibacterium id.2153)

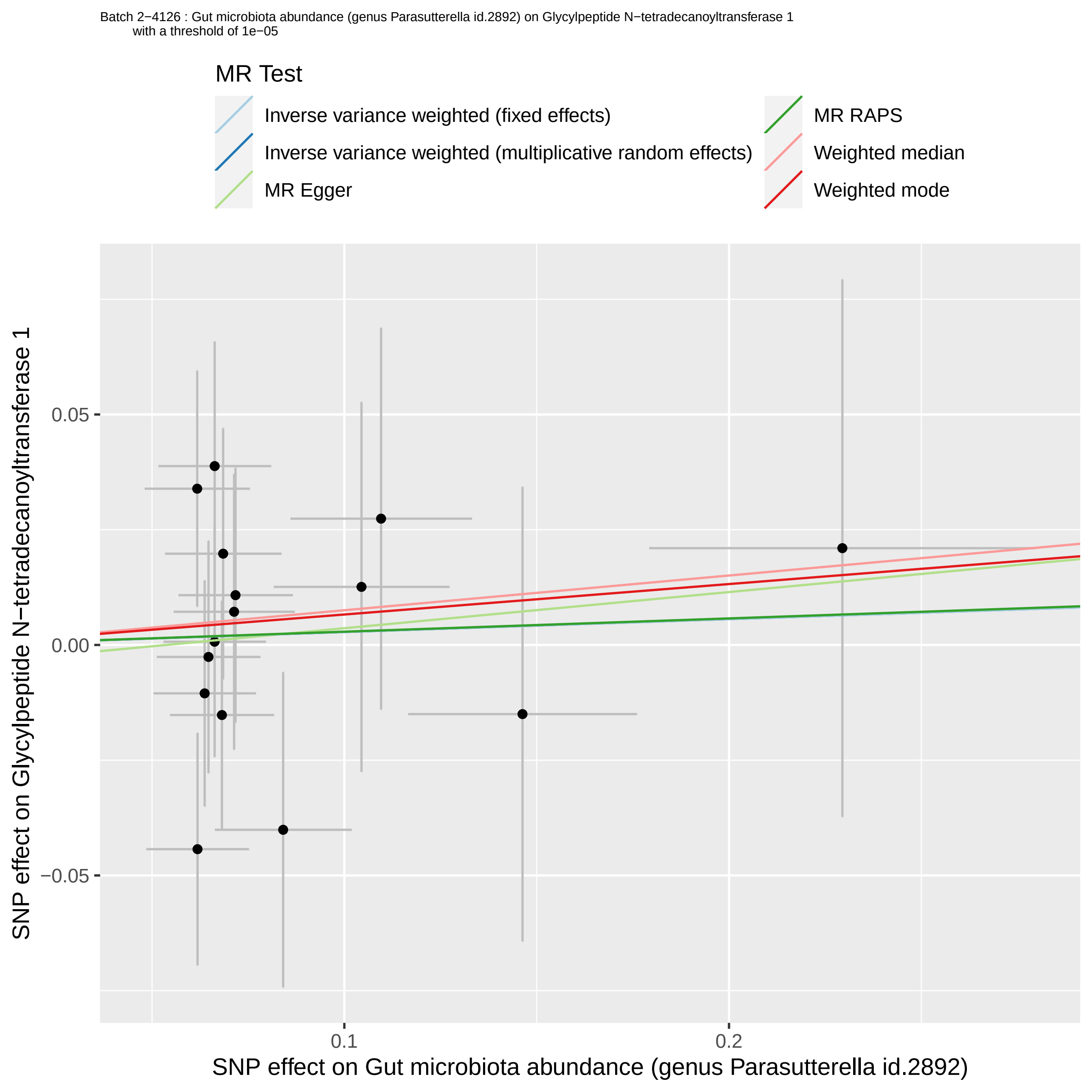
Batch 2−4126 : Gut microbiota abundance (genus Parasutterella id.2892) on Glycylpeptide N−tetradecanoyltransferase 1
with a threshold of 1e−05
MR Test
Inverse variance weighted (fixed effects)
Inverse variance weighted (multiplicative random effects)
MR Egger
MR RAPS
Weighted median
Weighted mode
0.05
0.00
−0.05
0.1
0.2
SNP effect on Glycylpeptide N−tetradecanoyltransferase 1
SNP effect on Gut microbiota abundance (genus Parasutterella id.2892)

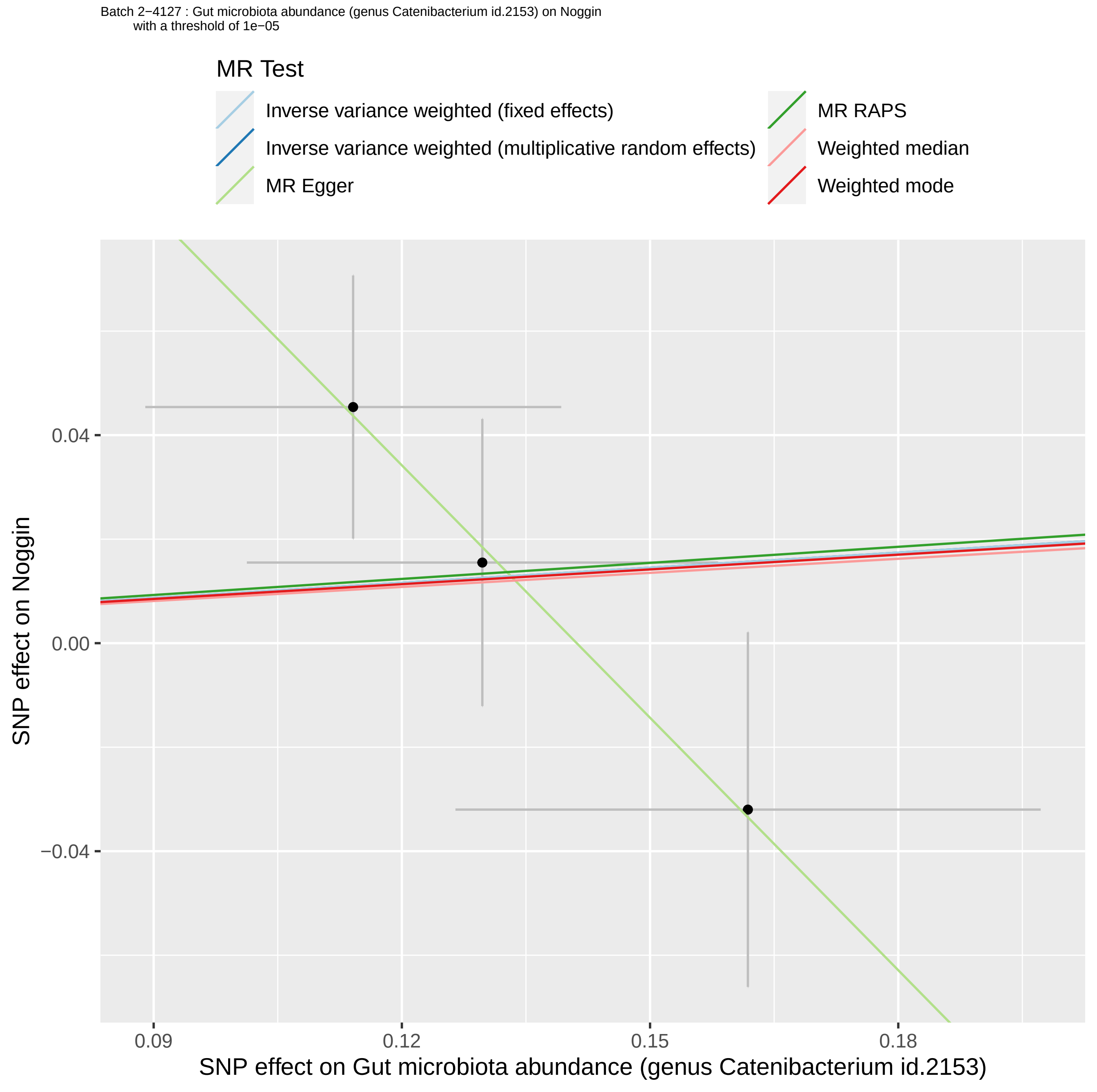
Batch 2−4127 : Gut microbiota abundance (genus Catenibacterium id.2153) on Noggin
with a threshold of 1e−05
MR Test
Inverse variance weighted (fixed effects)
Inverse variance weighted (multiplicative random effects)
MR Egger
MR RAPS
Weighted median
Weighted mode
0.04
0.00
−0.04
0.09
0.12
0.15
0.18
SNP effect on Noggin
SNP effect on Gut microbiota abundance (genus Catenibacterium id.2153)

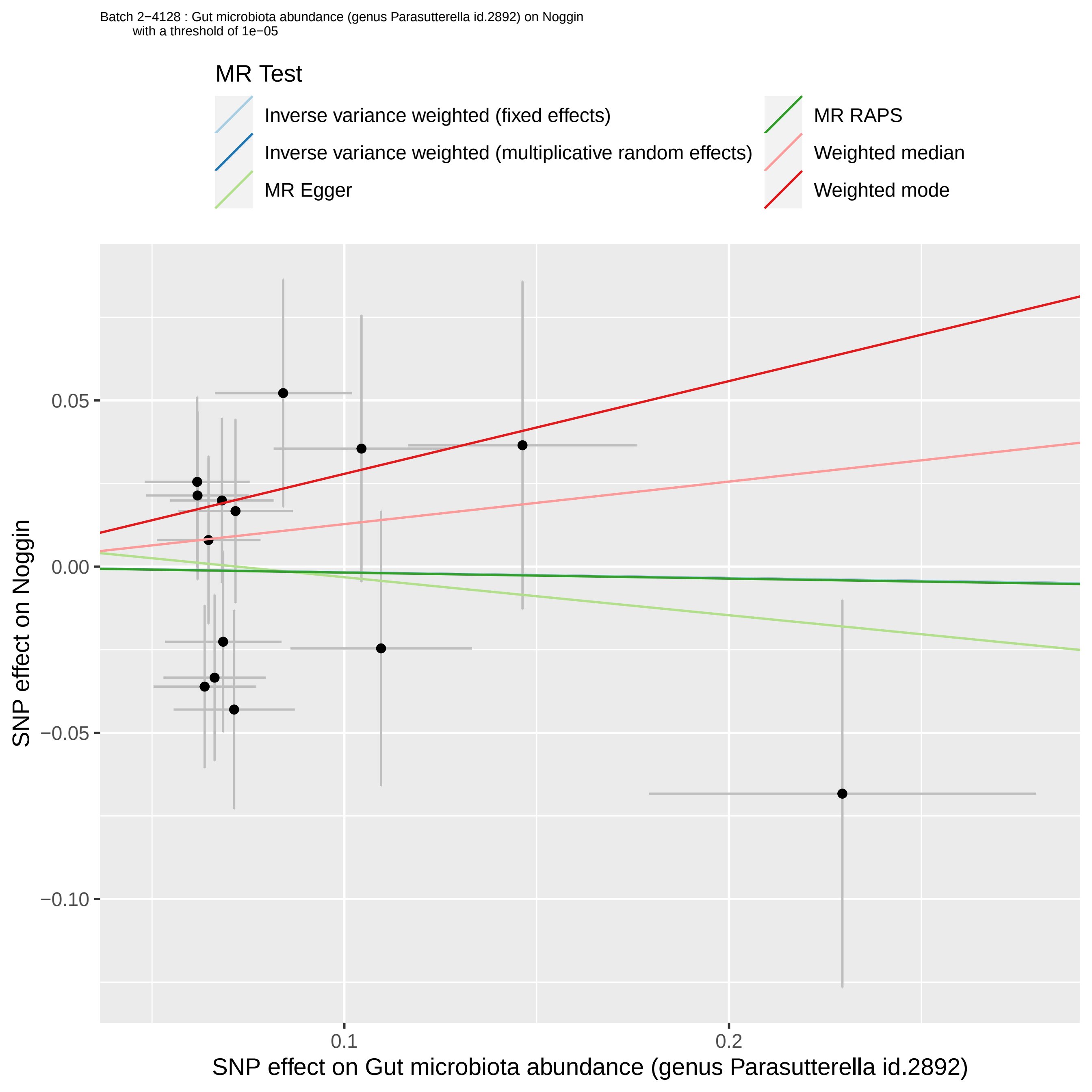
Batch 2−4128 : Gut microbiota abundance (genus Parasutterella id.2892) on Noggin
with a threshold of 1e−05
MR Test
Inverse variance weighted (fixed effects)
Inverse variance weighted (multiplicative random effects)
MR Egger
MR RAPS
Weighted median
Weighted mode
0.05
0.00
−0.05
−0.10
0.1
0.2
SNP effect on Noggin
SNP effect on Gut microbiota abundance (genus Parasutterella id.2892)

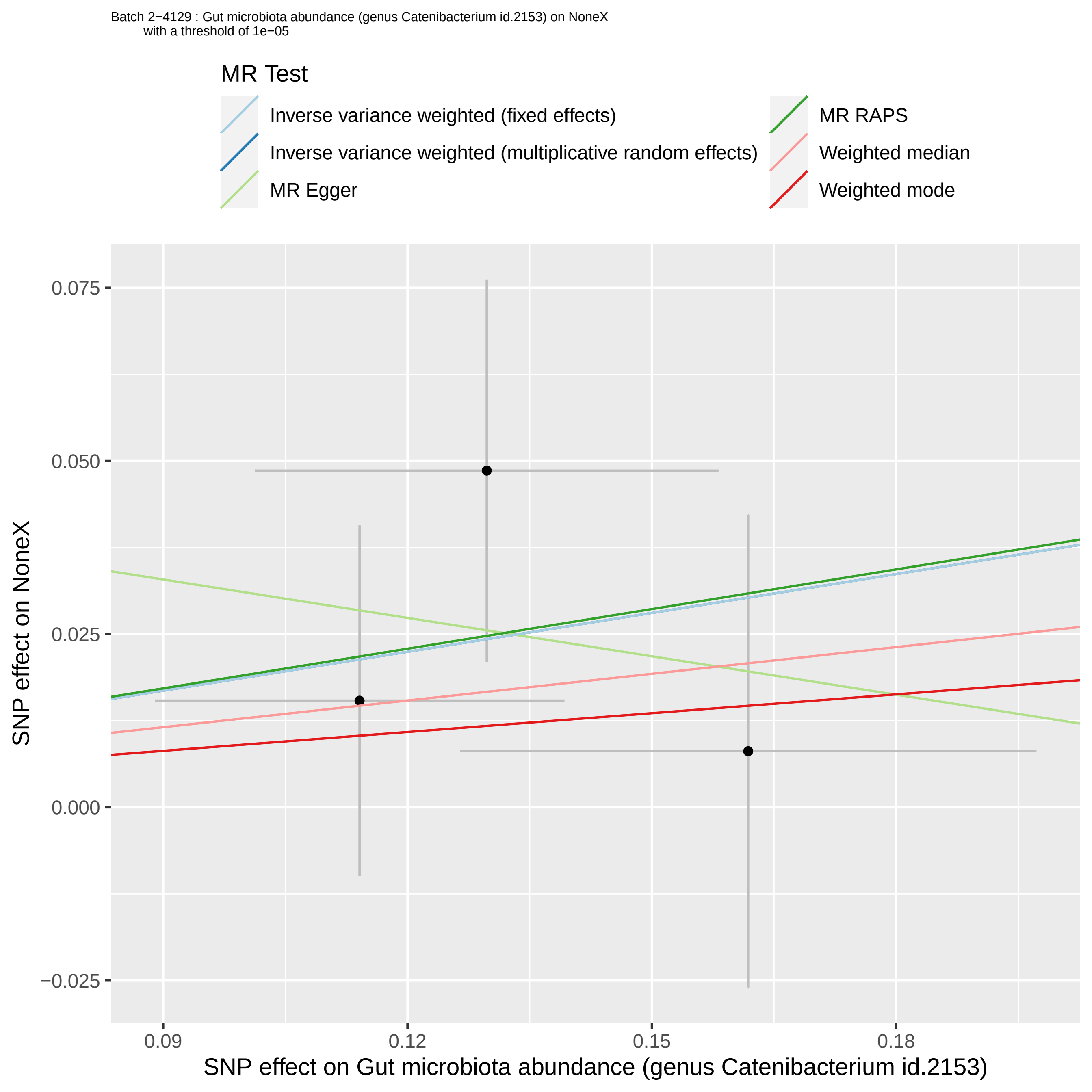
Batch 2−4129 : Gut microbiota abundance (genus Catenibacterium id.2153) on NoneX
with a threshold of 1e−05
MR Test
Inverse variance weighted (fixed effects)
Inverse variance weighted (multiplicative random effects)
MR Egger
MR RAPS
Weighted median
Weighted mode
SNP effect on NoneX
0.075
0.050
0.025
0.000
−0.025
0.09
0.12
0.15
0.18
SNP effect on Gut microbiota abundance (genus Catenibacterium id.2153)

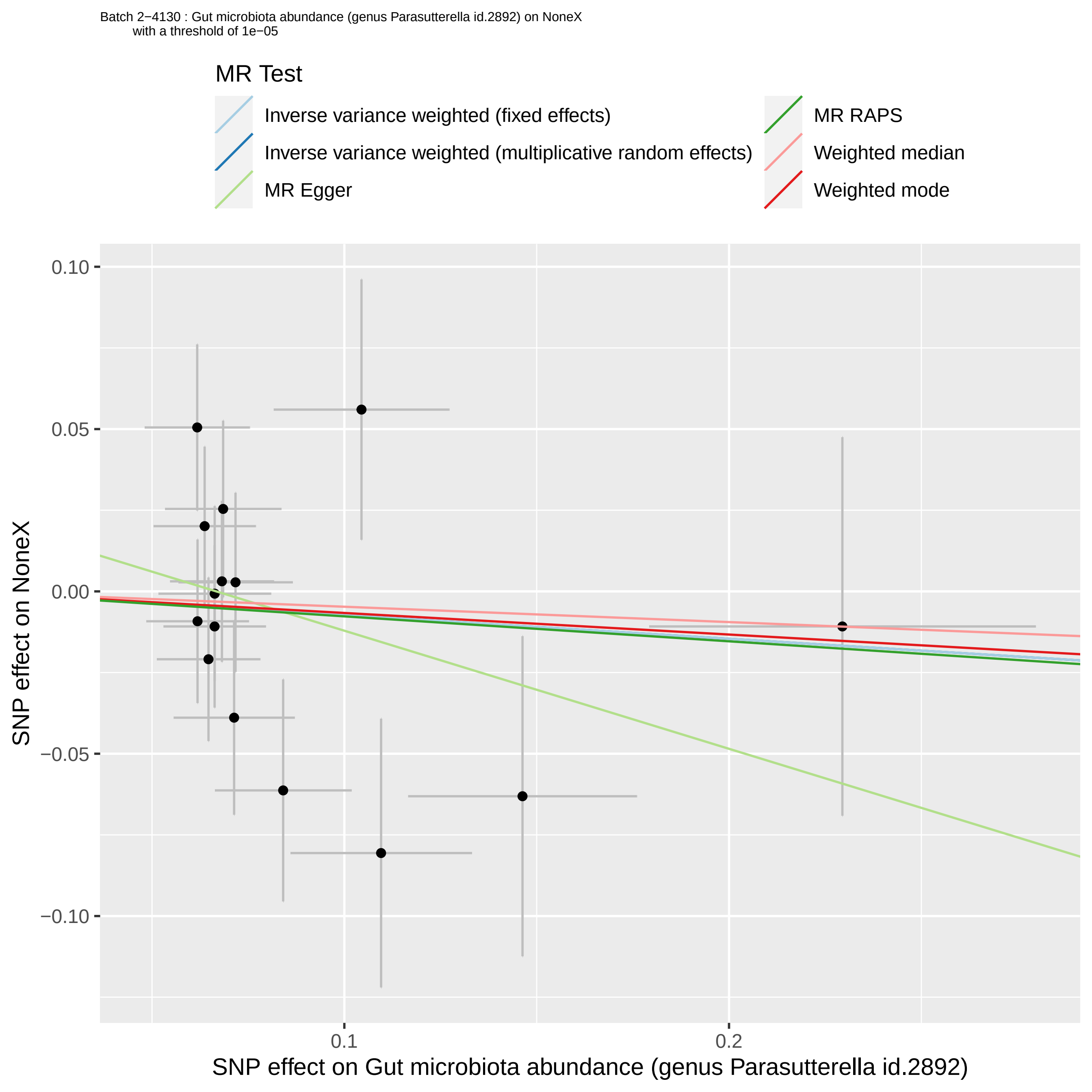
Batch 2−4130 : Gut microbiota abundance (genus Parasutterella id.2892) on NoneX
with a threshold of 1e−05
MR Test
Inverse variance weighted (fixed effects)
Inverse variance weighted (multiplicative random effects)
MR Egger
MR RAPS
Weighted median
Weighted mode
0.10
0.05
0.00
−0.05
−0.10
SNP effect on NoneX
0.1
0.2
SNP effect on Gut microbiota abundance (genus Parasutterella id.2892)

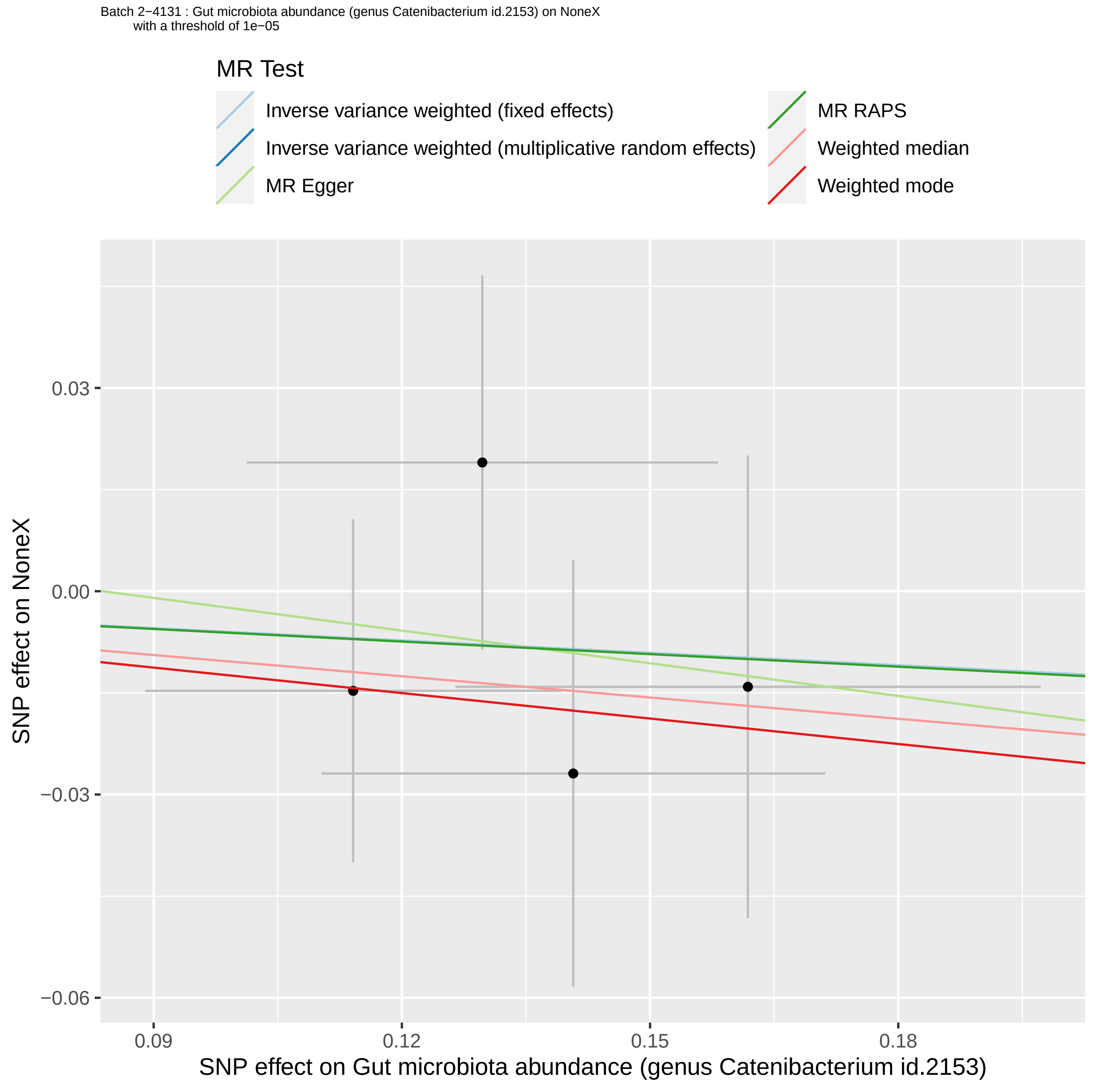

Batch 2−4131 : Gut microbiota abundance (genus Catenibacterium id.2153) on NoneX
with a threshold of 1e−05
MR Test
Inverse variance weighted (fixed effects)
Inverse variance weighted (multiplicative random effects)
MR Egger
MR RAPS
Weighted median
Weighted mode
SNP effect on NoneX
0.03
0.00
−0.03
−0.06
0.09
0.12
0.15
0.18
SNP effect on Gut microbiota abundance (genus Catenibacterium id.2153)

Batch 2−4132 : Gut microbiota abundance (genus Parasutterella id.2892) on NoneX
with a threshold of 1e−05

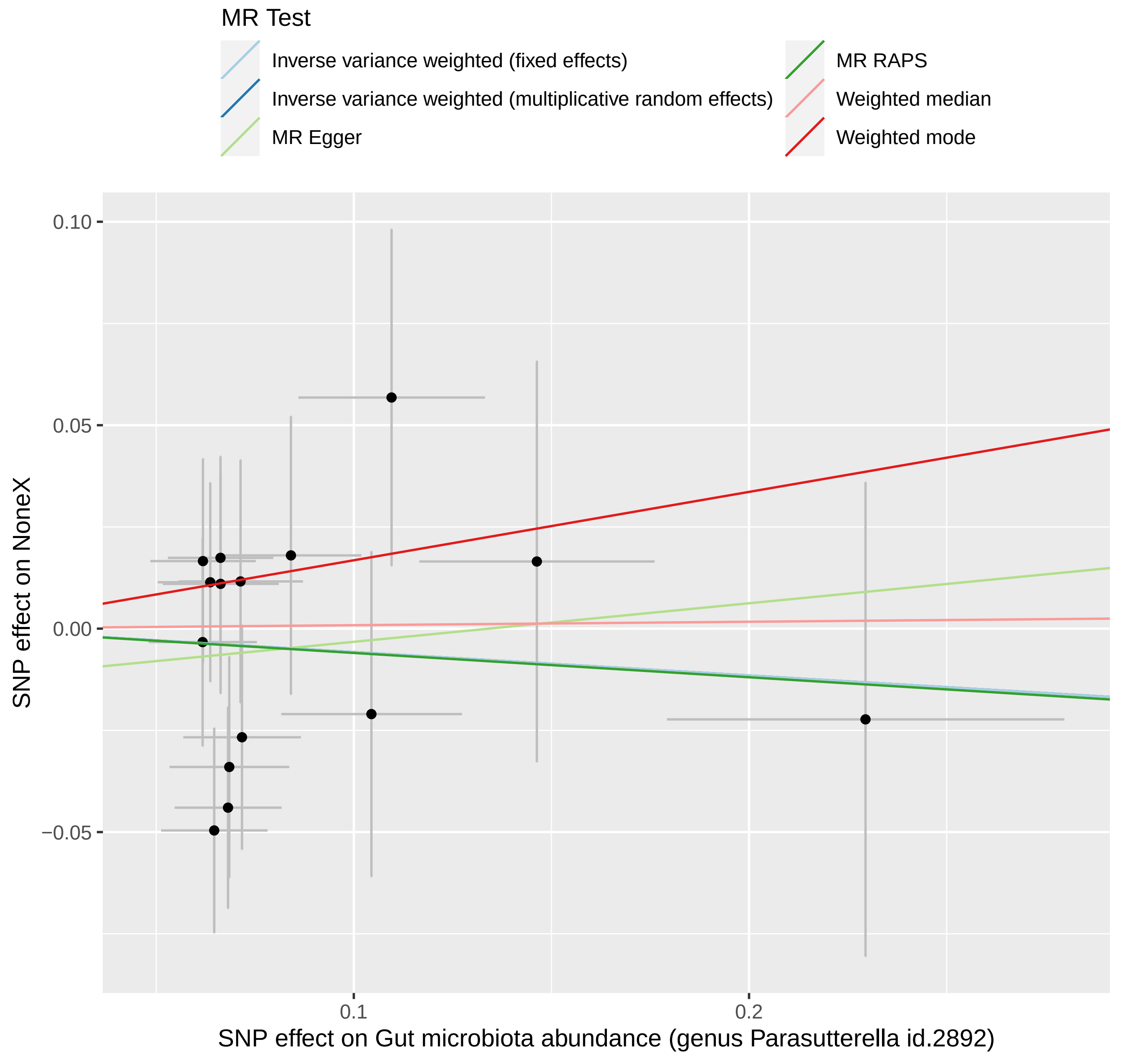

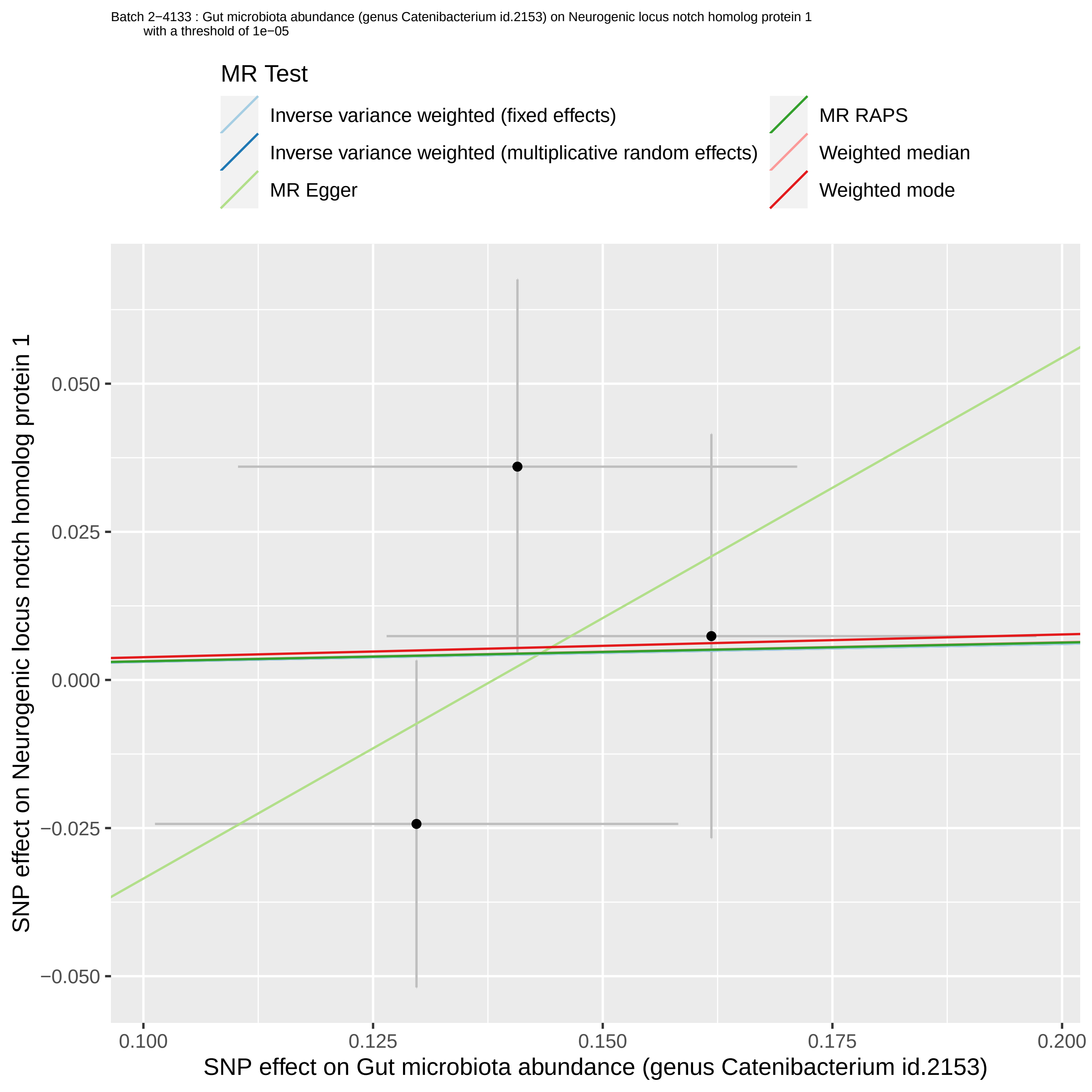
Batch 2−4133 : Gut microbiota abundance (genus Catenibacterium id.2153) on Neurogenic locus notch homolog protein 1
with a threshold of 1e−05
MR Test
Inverse variance weighted (fixed effects)
Inverse variance weighted (multiplicative random effects)
MR Egger
MR RAPS
Weighted median
Weighted mode
0.050
0.025
0.000
−0.025
−0.050
0.100
0.125
0.150
0.175
0.200
SNP effect on Neurogenic locus notch homolog protein 1
SNP effect on Gut microbiota abundance (genus Catenibacterium id.2153)

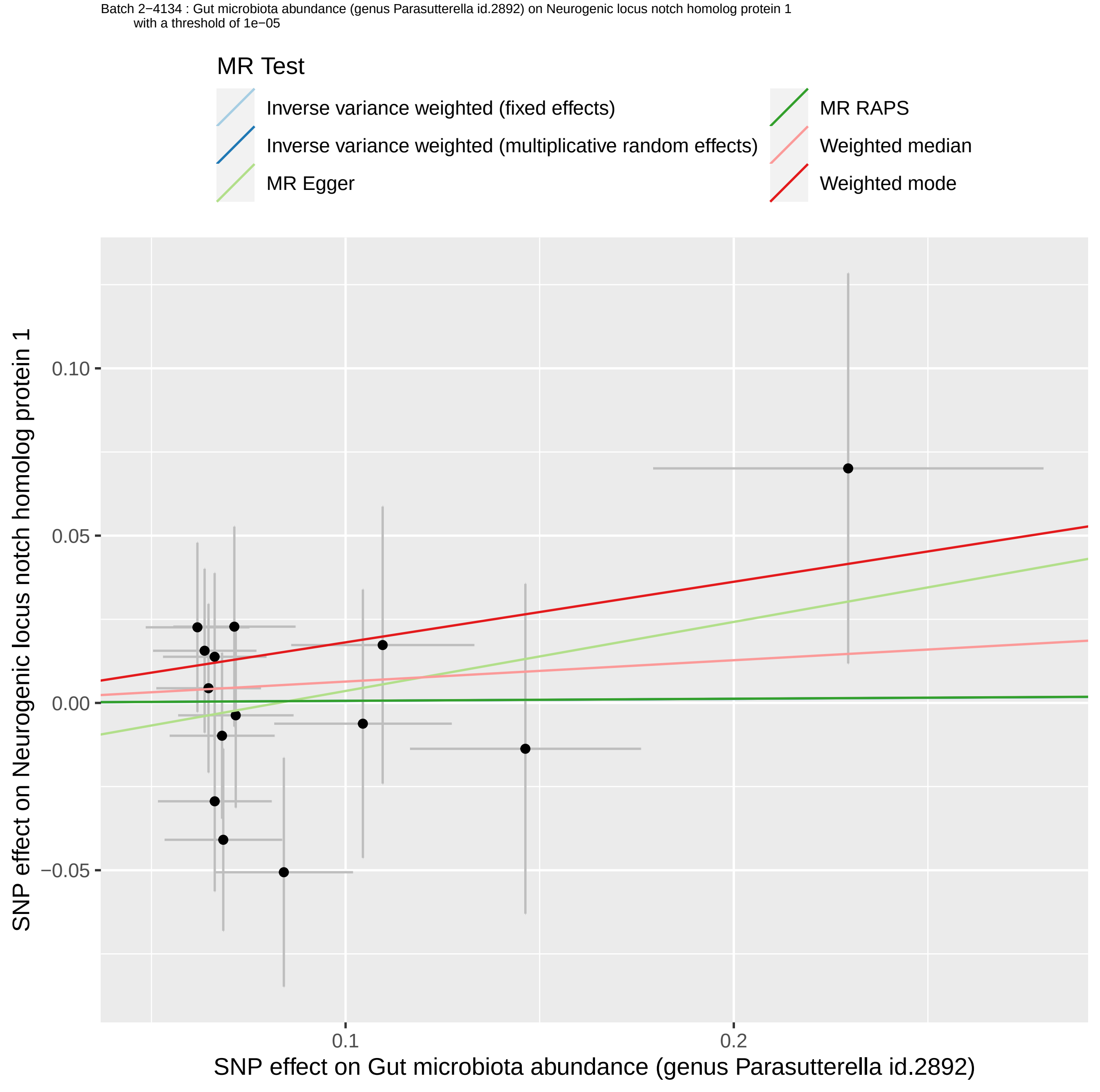

Batch 2−4134 : Gut microbiota abundance (genus Parasutterella id.2892) on Neurogenic locus notch homolog protein 1
with a threshold of 1e−05
MR Test
Inverse variance weighted (fixed effects)
Inverse variance weighted (multiplicative random effects)
MR Egger
MR RAPS
Weighted median
Weighted mode
0.10
0.05
0.00
−0.05
0.1
0.2
SNP effect on Neurogenic locus notch homolog protein 1
SNP effect on Gut microbiota abundance (genus Parasutterella id.2892)

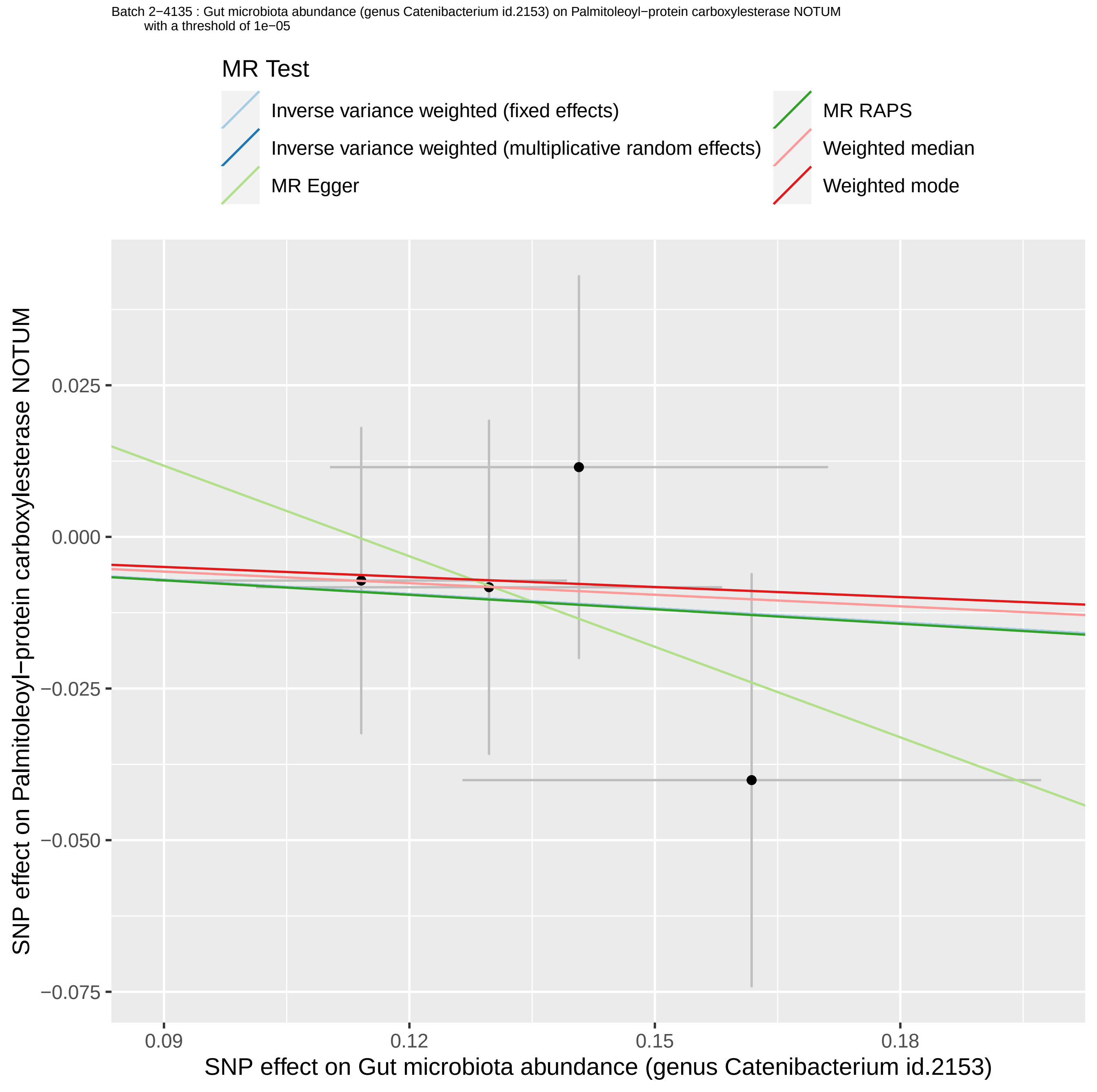
Batch 2−4135 : Gut microbiota abundance (genus Catenibacterium id.2153) on Palmitoleoyl−protein carboxylesterase NOTUM
with a threshold of 1e−05
MR Test
Inverse variance weighted (fixed effects)
Inverse variance weighted (multiplicative random effects)
MR Egger
MR RAPS
Weighted median
Weighted mode
0.025
0.000
−0.025
−0.050
−0.075
0.09
0.12
0.15
0.18
SNP effect on Palmitoleoyl−protein carboxylesterase NOTUM
SNP effect on Gut microbiota abundance (genus Catenibacterium id.2153)

Batch 2−4136 : Gut microbiota abundance (genus Parasutterella id.2892) on Palmitoleoyl−protein carboxylesterase NOTUM
with a threshold of 1e−05

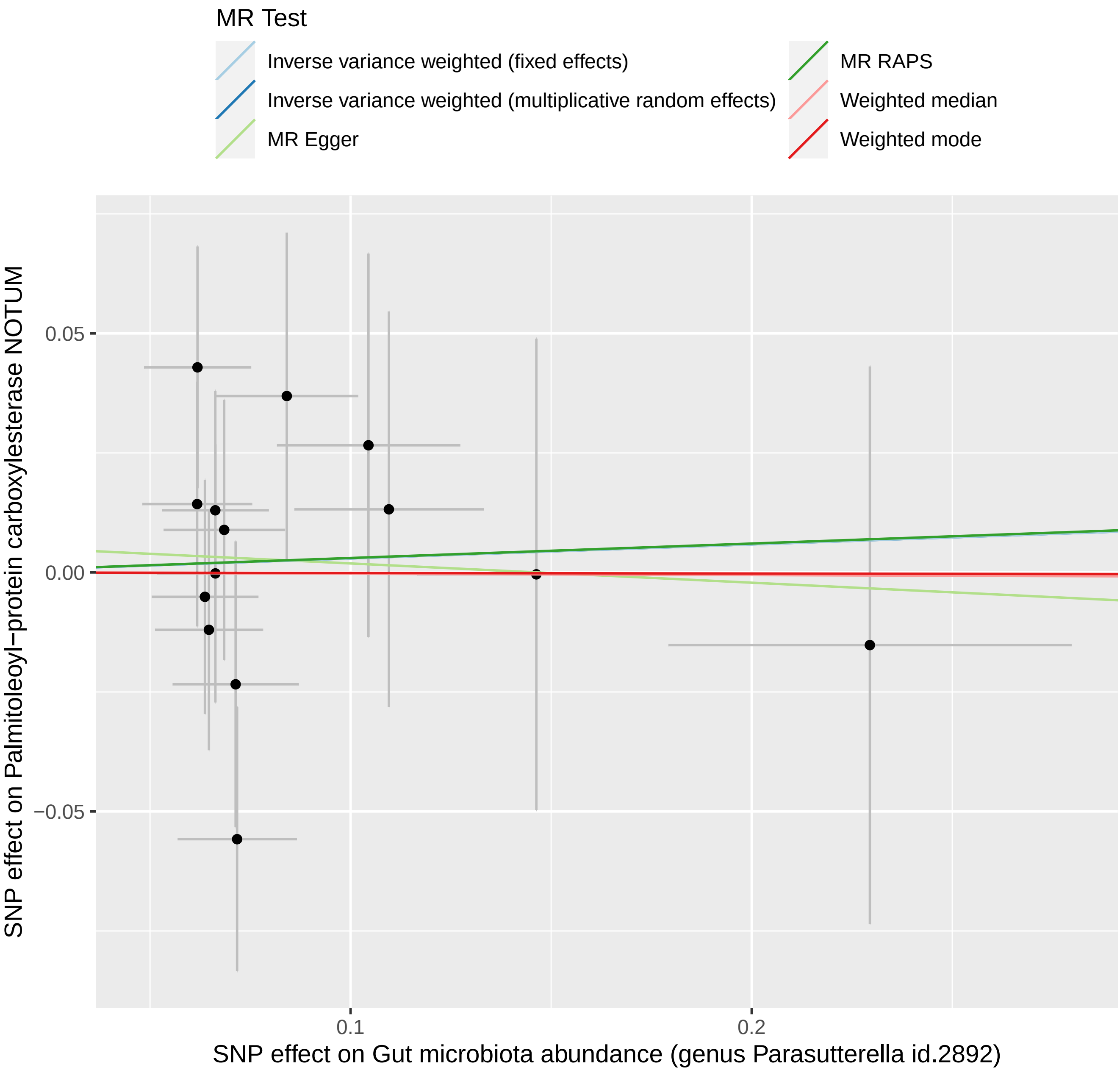

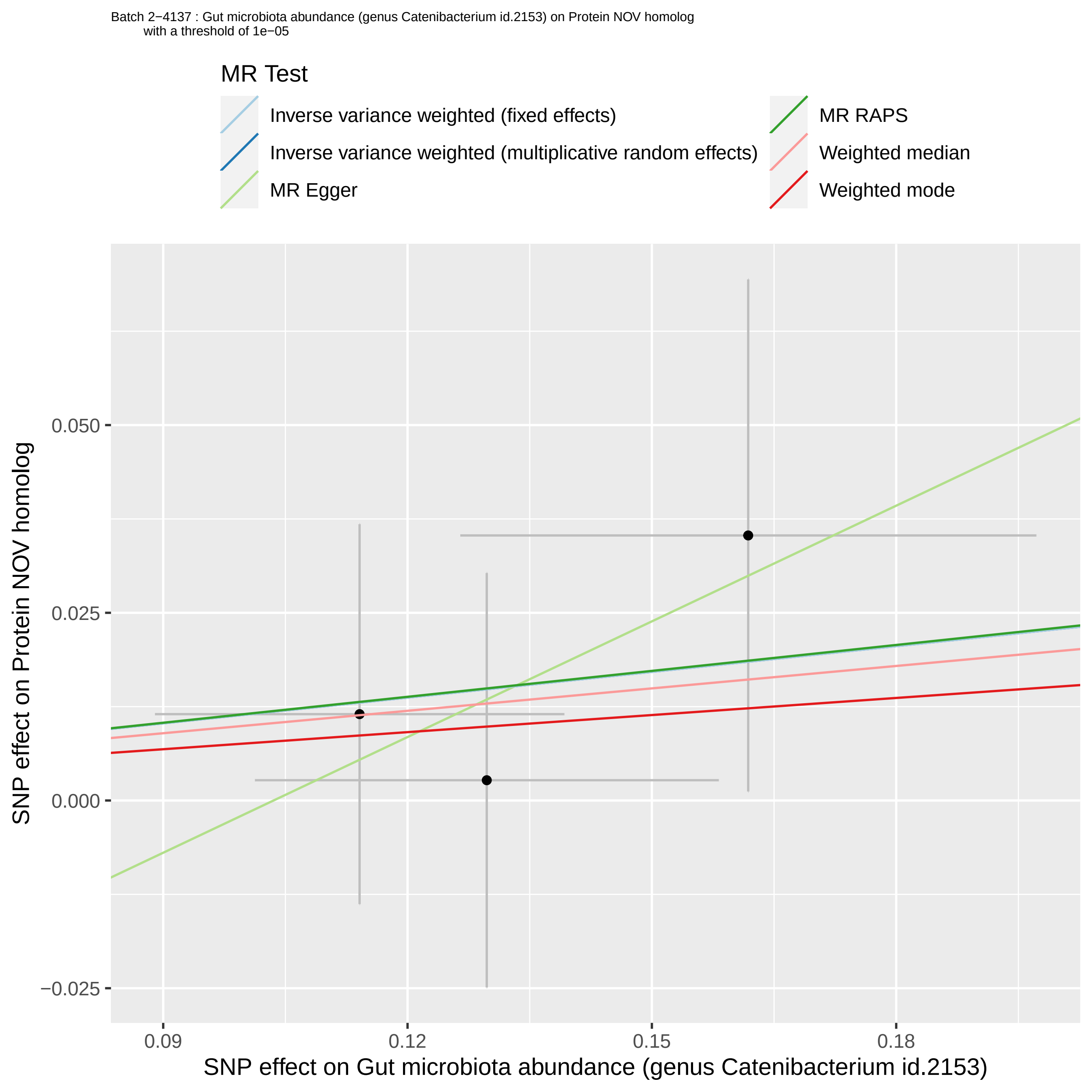
Batch 2−4137 : Gut microbiota abundance (genus Catenibacterium id.2153) on Protein NOV homolog
with a threshold of 1e−05
MR Test
Inverse variance weighted (fixed effects)
Inverse variance weighted (multiplicative random effects)
MR Egger
MR RAPS
Weighted median
Weighted mode
0.050
0.025
0.000
−0.025
0.09
0.12
0.15
0.18
SNP effect on Protein NOV homolog
SNP effect on Gut microbiota abundance (genus Catenibacterium id.2153)

Batch 2−4138 : Gut microbiota abundance (genus Parasutterella id.2892) on Protein NOV homolog
with a threshold of 1e−05

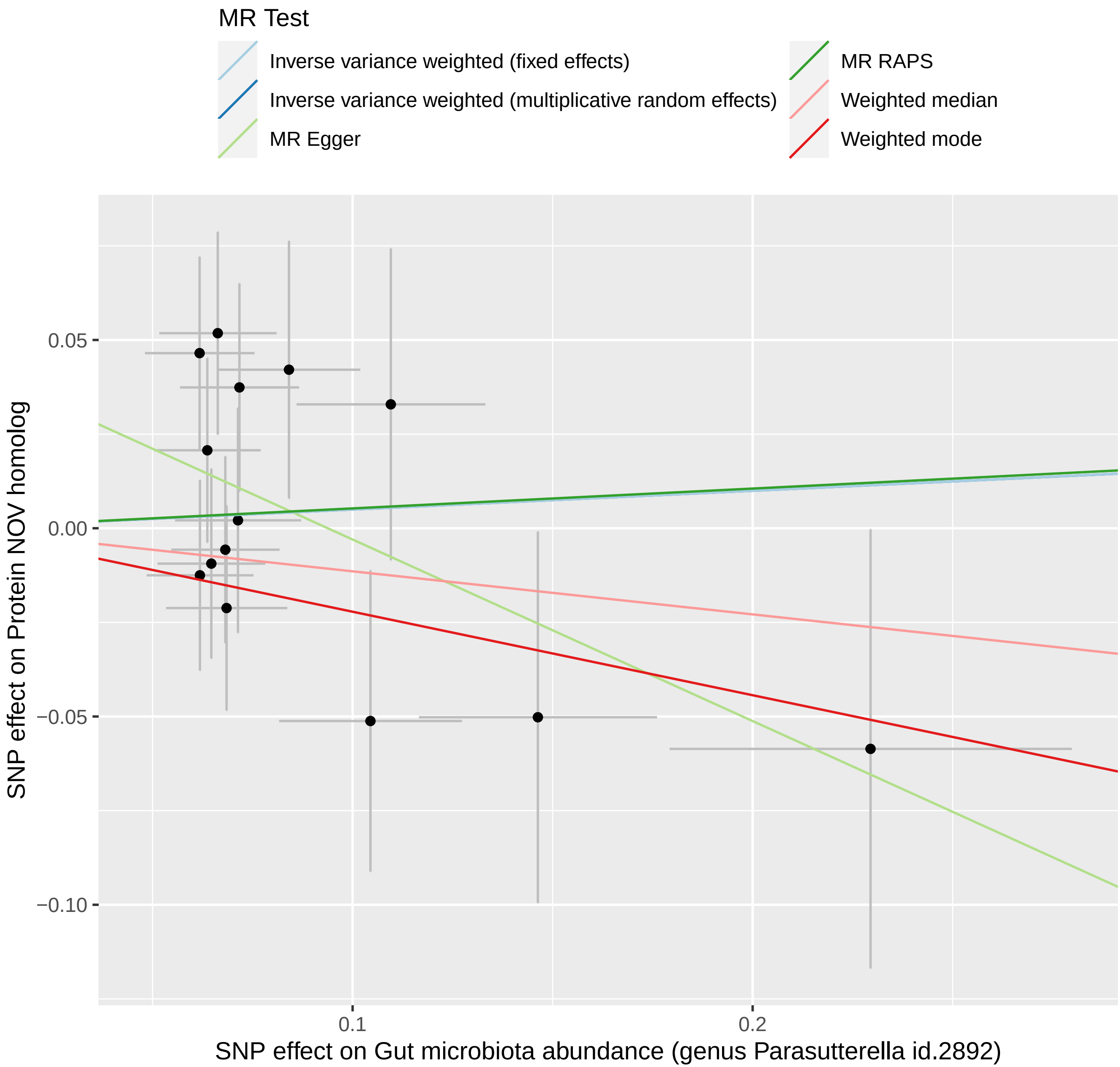

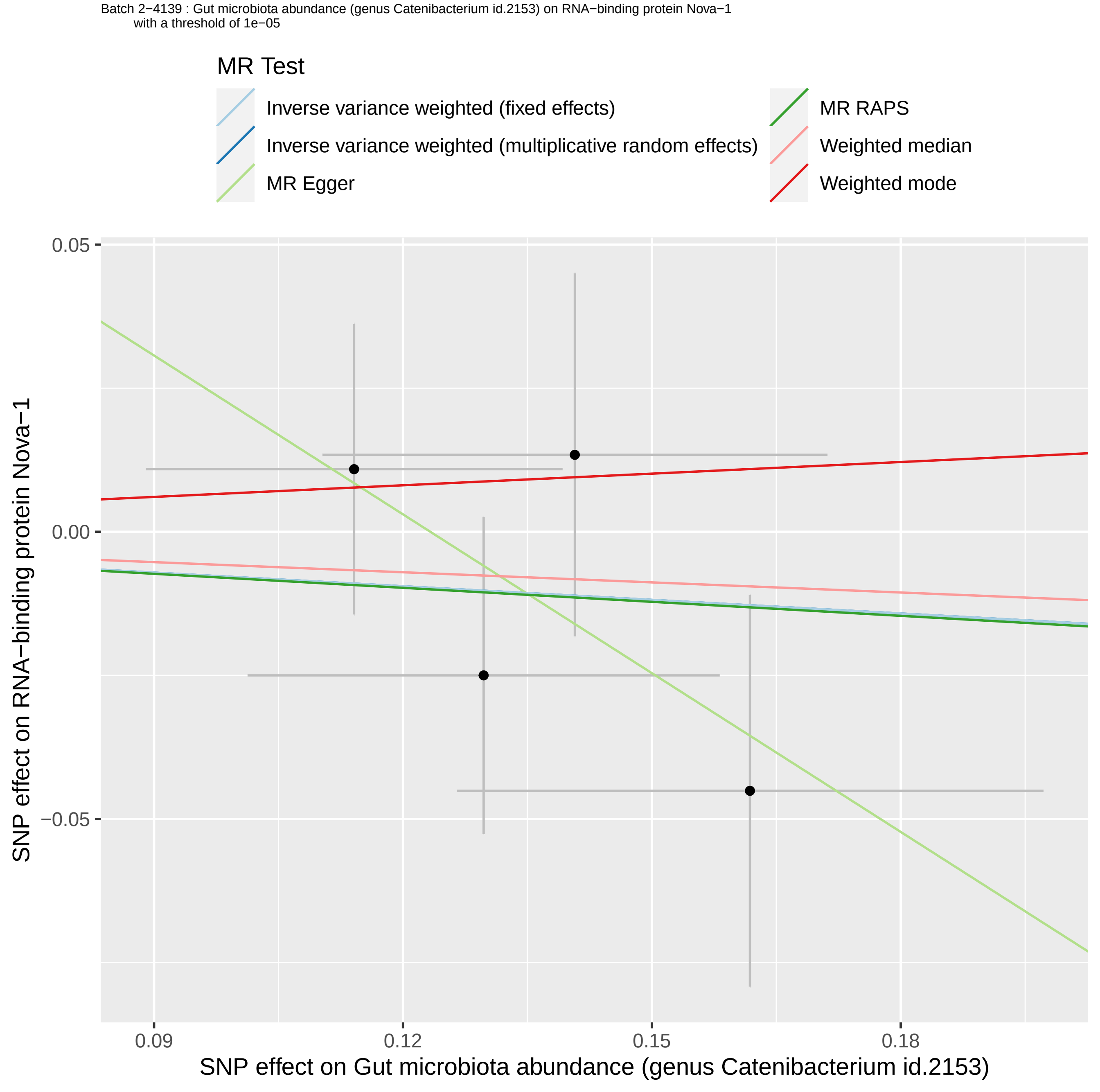
Batch 2−4139 : Gut microbiota abundance (genus Catenibacterium id.2153) on RNA−binding protein Nova−1
with a threshold of 1e−05
MR Test
Inverse variance weighted (fixed effects)
Inverse variance weighted (multiplicative random effects)
MR Egger
MR RAPS
Weighted median
Weighted mode
0.05
0.00
−0.05
0.09
0.12
0.15
0.18
SNP effect on RNA−binding protein Nova−1
SNP effect on Gut microbiota abundance (genus Catenibacterium id.2153)

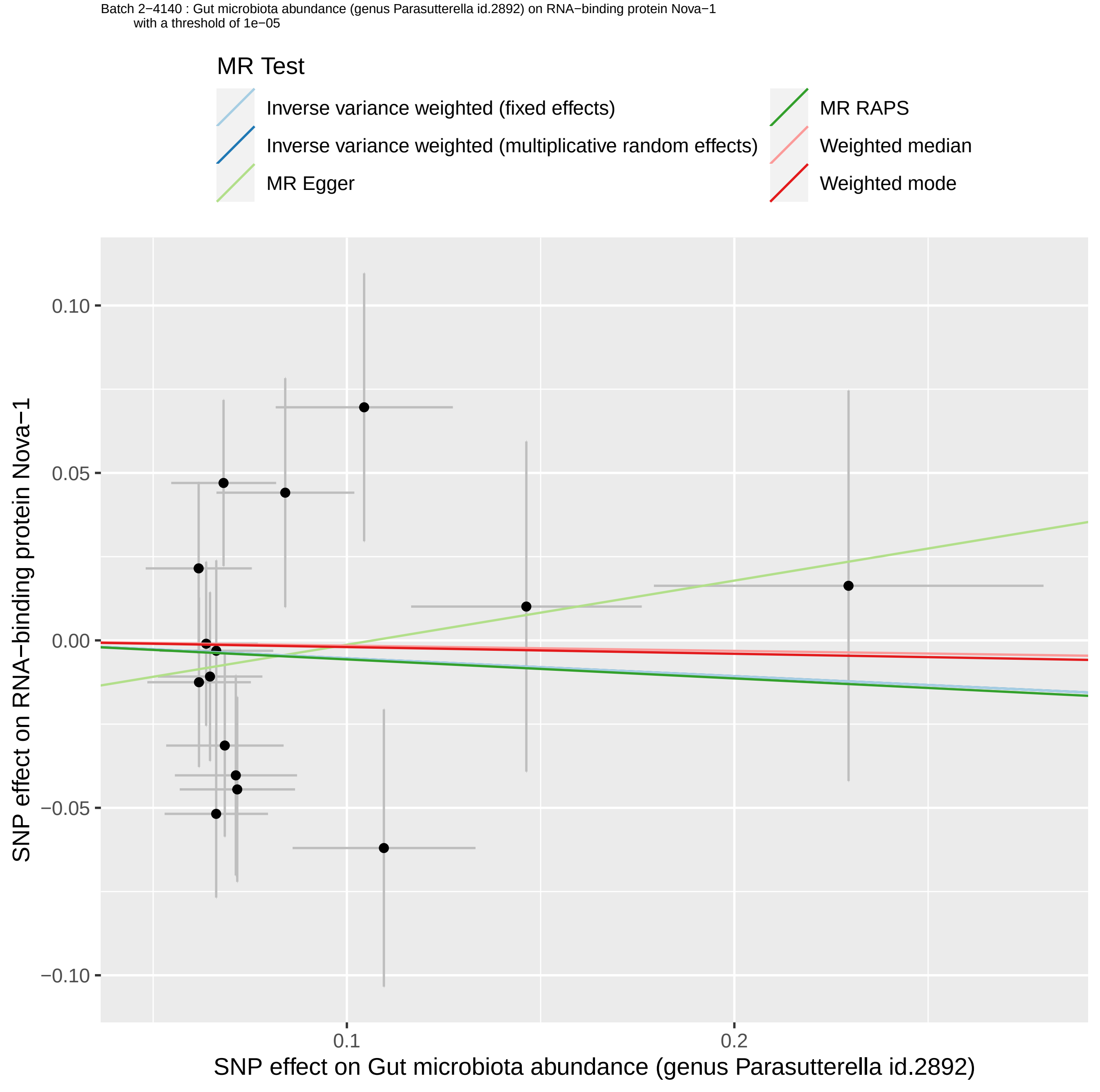

Batch 2−4140 : Gut microbiota abundance (genus Parasutterella id.2892) on RNA−binding protein Nova−1
with a threshold of 1e−05
MR Test
Inverse variance weighted (fixed effects)
Inverse variance weighted (multiplicative random effects)
MR Egger
MR RAPS
Weighted median
Weighted mode
0.10
0.05
0.00
−0.05
−0.10
0.1
0.2
SNP effect on RNA−binding protein Nova−1
SNP effect on Gut microbiota abundance (genus Parasutterella id.2892)

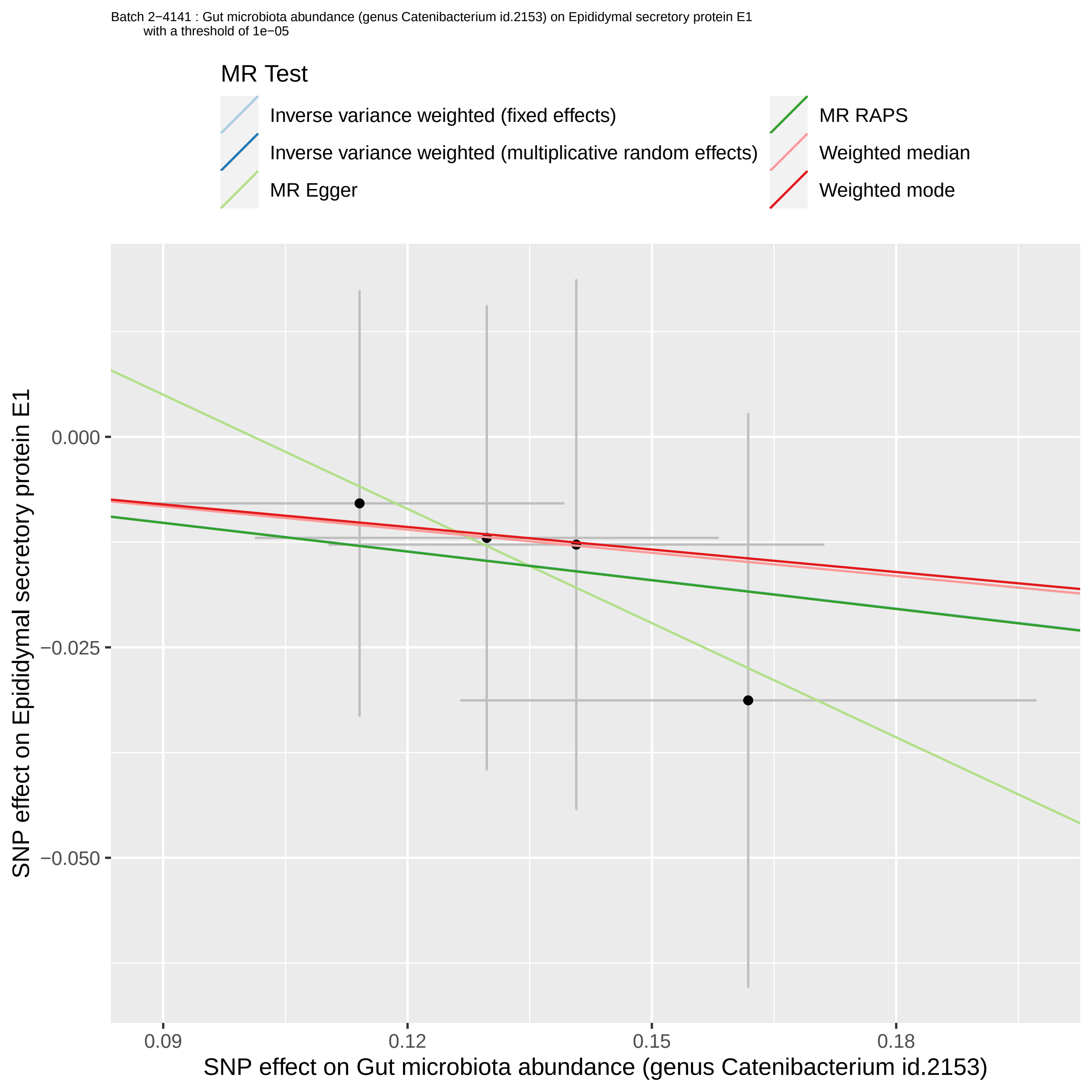

Batch 2−4141 : Gut microbiota abundance (genus Catenibacterium id.2153) on Epididymal secretory protein E1
with a threshold of 1e−05
MR Test
Inverse variance weighted (fixed effects)
Inverse variance weighted (multiplicative random effects)
MR Egger
MR RAPS
Weighted median
Weighted mode
0.000
−0.025
−0.050
0.09
0.12
0.15
0.18
SNP effect on Epididymal secretory protein E1
SNP effect on Gut microbiota abundance (genus Catenibacterium id.2153)

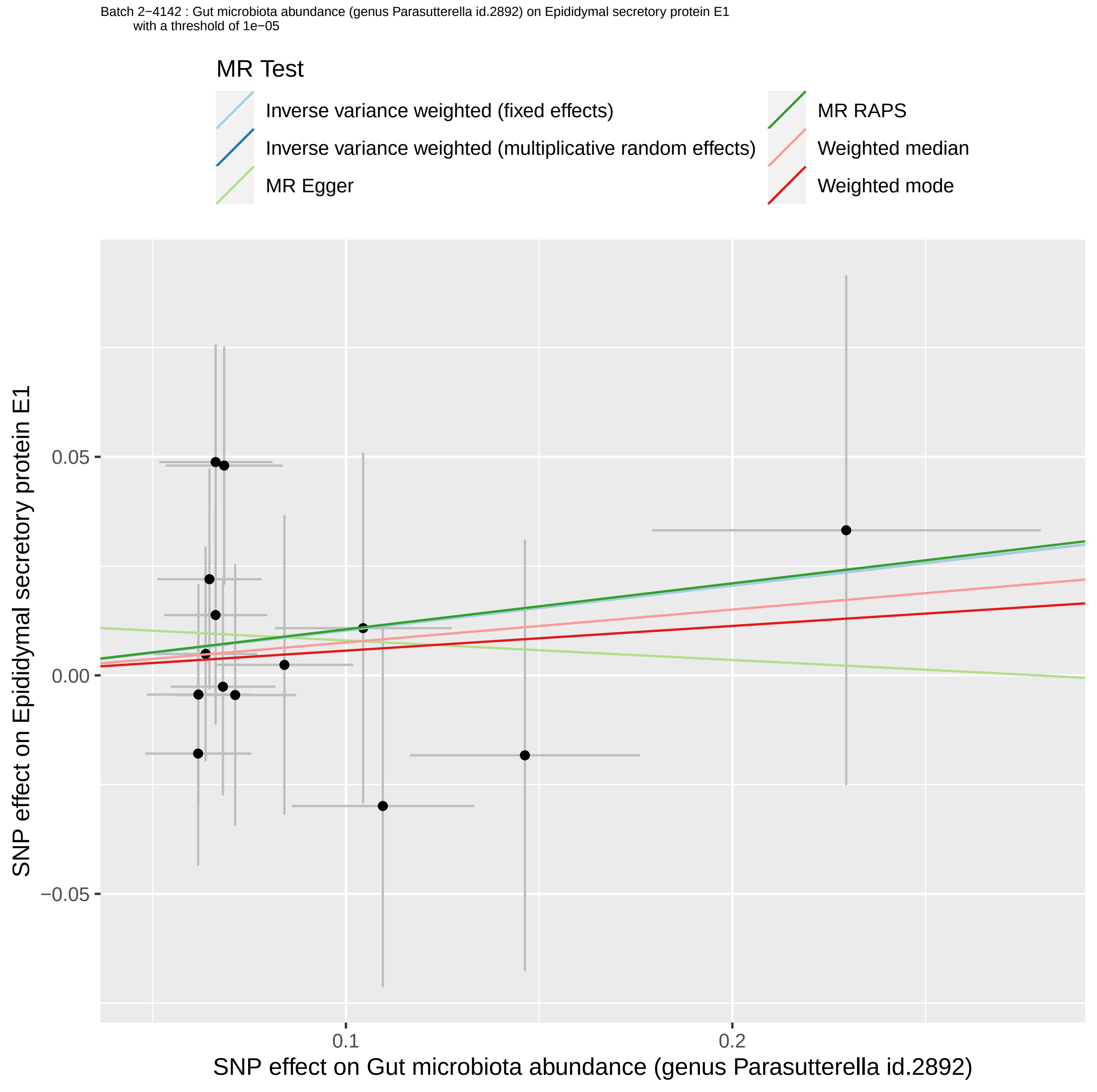
Batch 2−4142 : Gut microbiota abundance (genus Parasutterella id.2892) on Epididymal secretory protein E1
with a threshold of 1e−05
MR Test
Inverse variance weighted (fixed effects)
Inverse variance weighted (multiplicative random effects)
MR Egger
MR RAPS
Weighted median
Weighted mode
SNP effect on Epididymal secretory protein E1
0.05
0.00
−0.05
0.1
0.2
SNP effect on Gut microbiota abundance (genus Parasutterella id.2892)

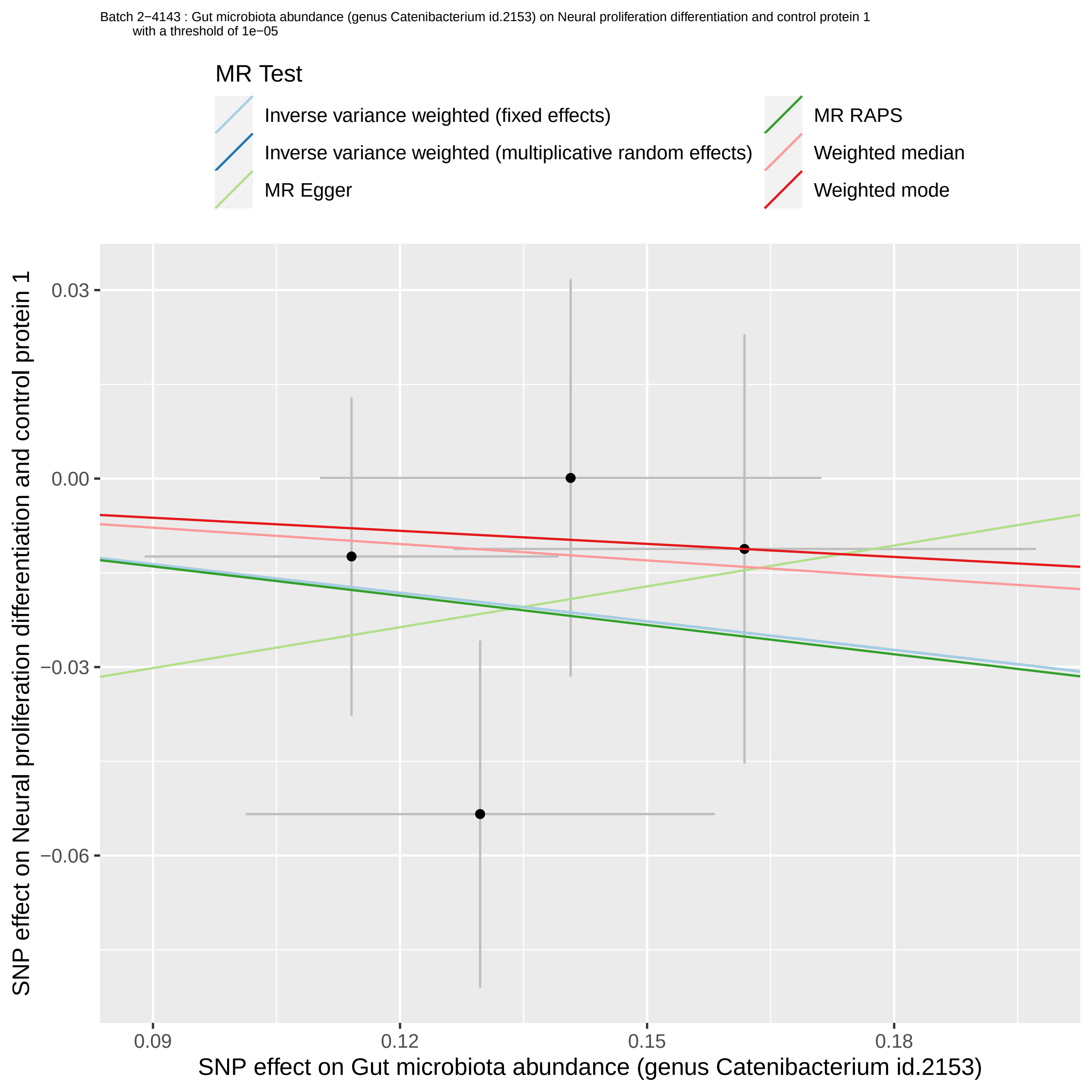
Batch 2−4143 : Gut microbiota abundance (genus Catenibacterium id.2153) on Neural proliferation differentiation and control protein 1
with a threshold of 1e−05
MR Test
Inverse variance weighted (fixed effects)
Inverse variance weighted (multiplicative random effects)
MR Egger
MR RAPS
Weighted median
Weighted mode
0.03
0.00
−0.03
−0.06
0.09
0.12
0.15
0.18
SNP effect on Neural proliferation differentiation and control protein 1
SNP effect on Gut microbiota abundance (genus Catenibacterium id.2153)

Batch 2−4144 : Gut microbiota abundance (genus Parasutterella id.2892) on Neural proliferation differentiation and control protein 1
with a threshold of 1e−05

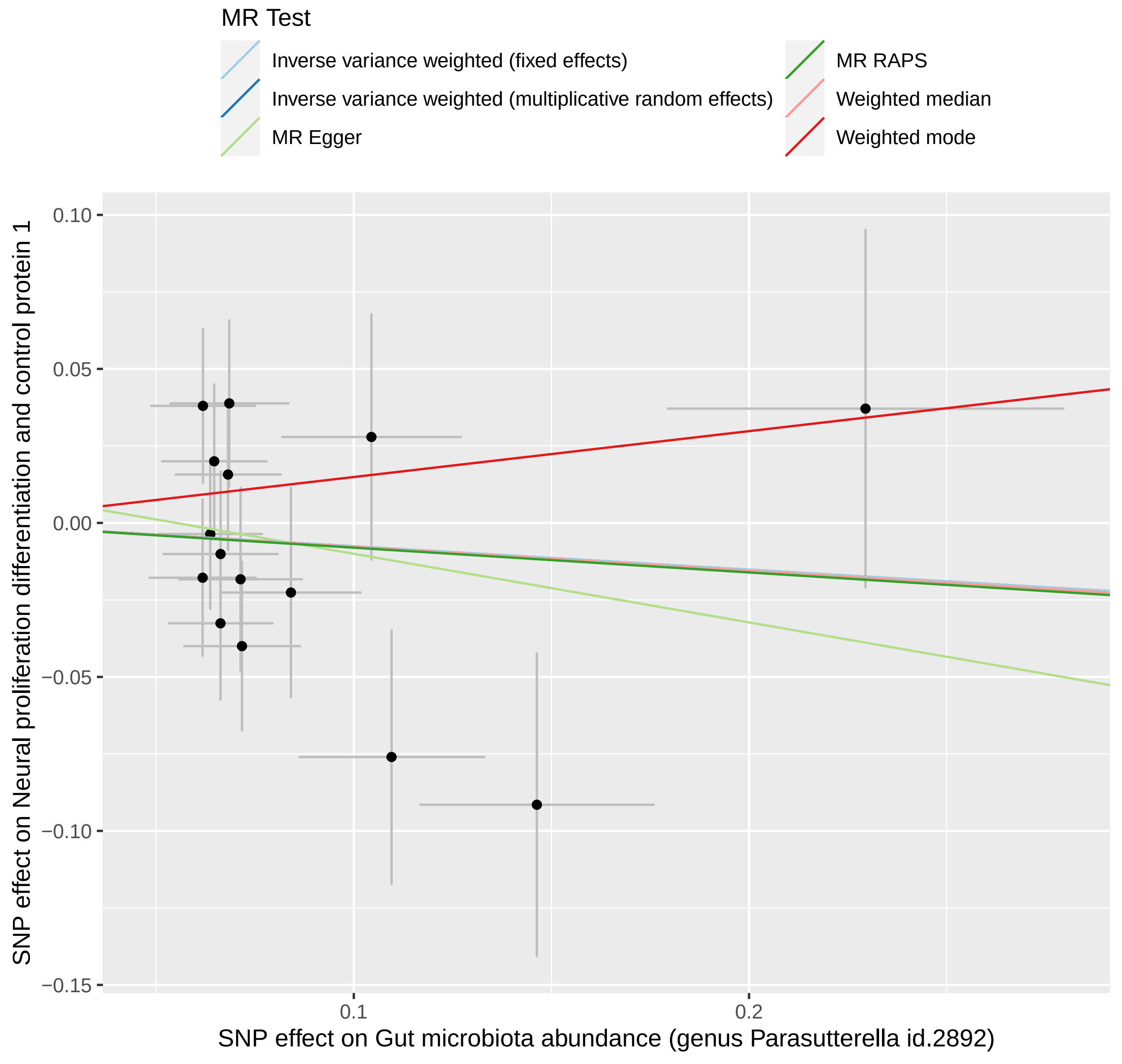

Batch 2−4145 : Gut microbiota abundance (genus Catenibacterium id.2153) on Pro−FMRFamide−related neuropeptide FF
with a threshold of 1e−05

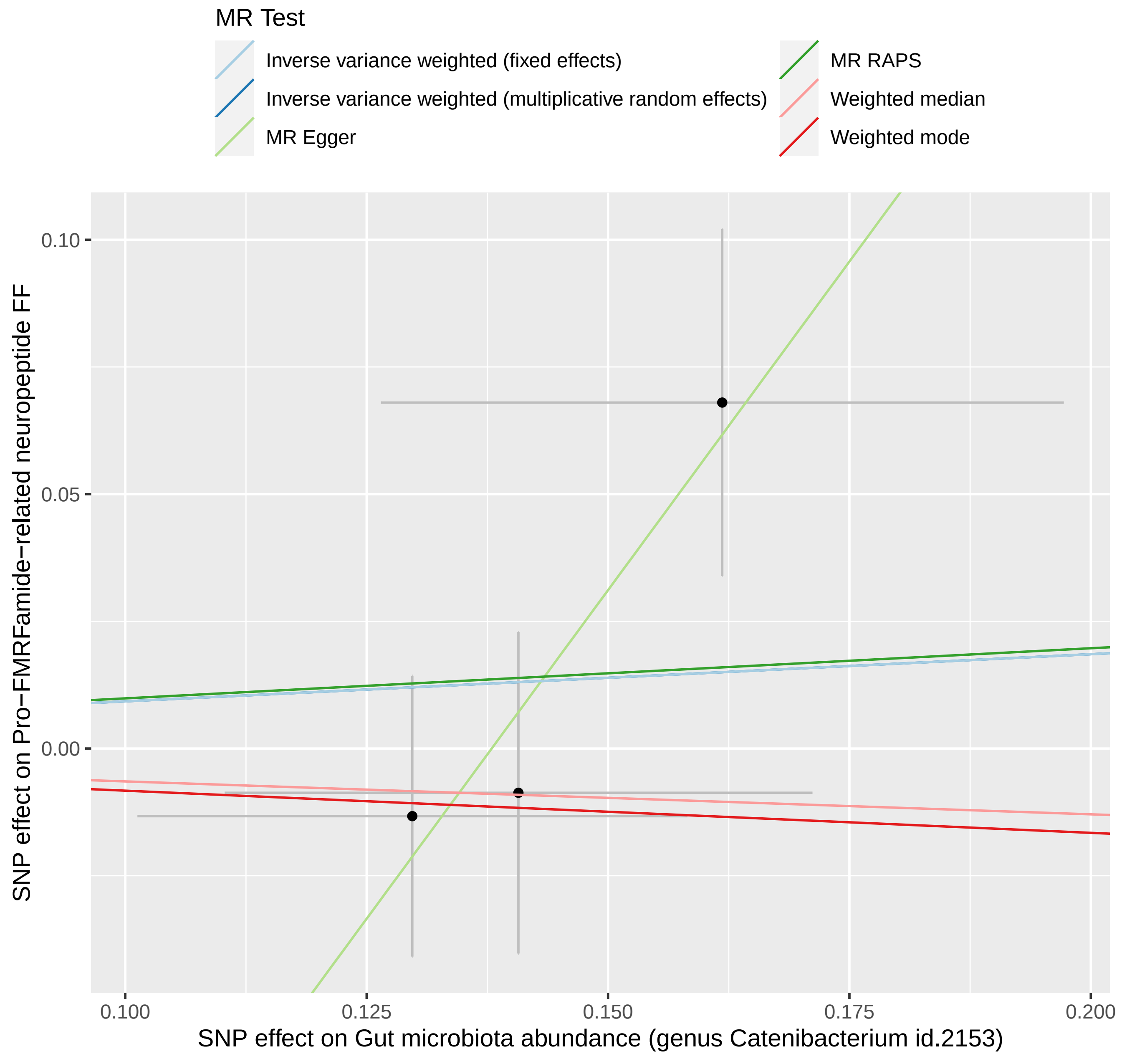

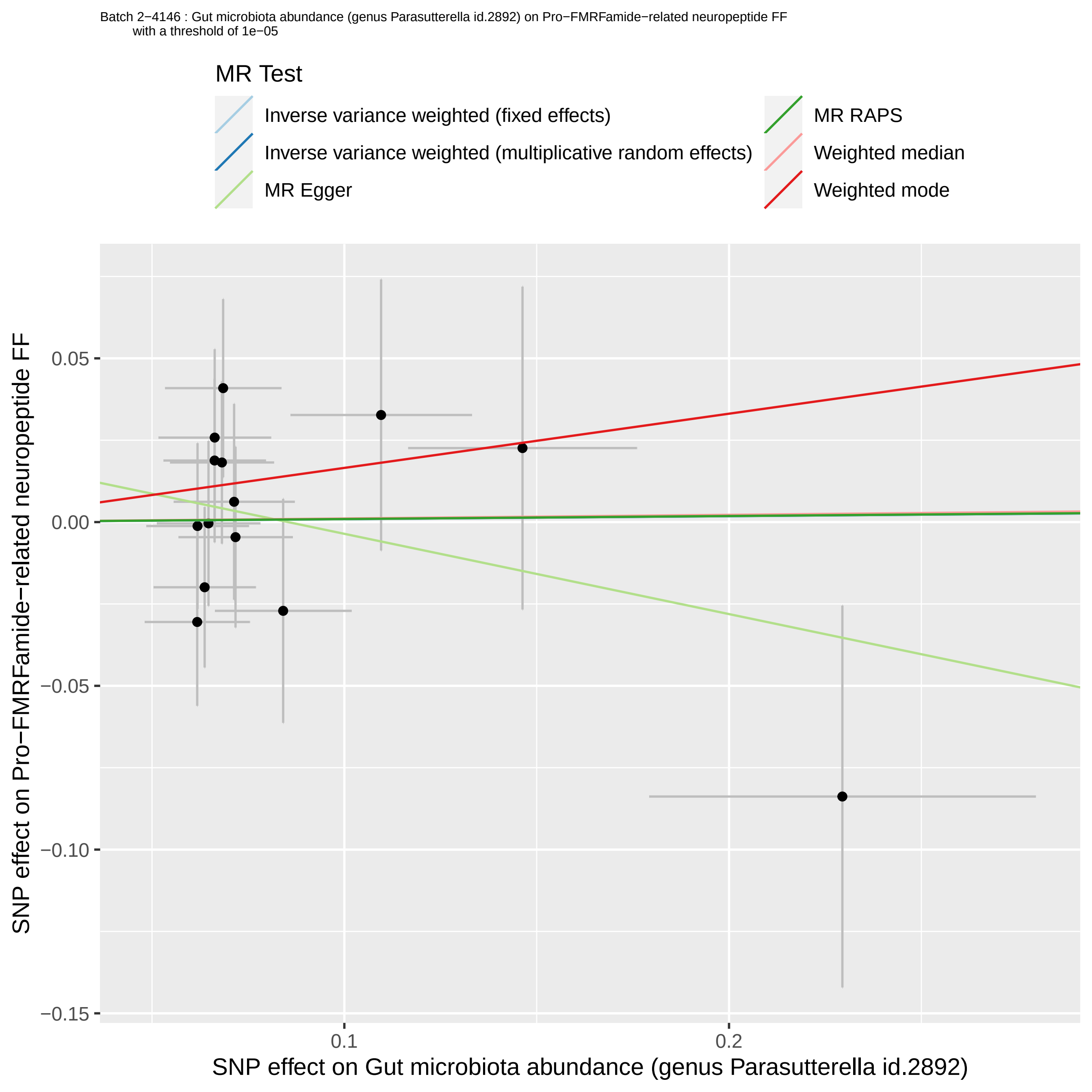
Batch 2−4146 : Gut microbiota abundance (genus Parasutterella id.2892) on Pro−FMRFamide−related neuropeptide FF
with a threshold of 1e−05
MR Test
Inverse variance weighted (fixed effects)
Inverse variance weighted (multiplicative random effects)
MR Egger
MR RAPS
Weighted median
Weighted mode
0.05
0.00
−0.05
−0.10
−0.15
0.1
0.2
SNP effect on Pro−FMRFamide−related neuropeptide FF
SNP effect on Gut microbiota abundance (genus Parasutterella id.2892)

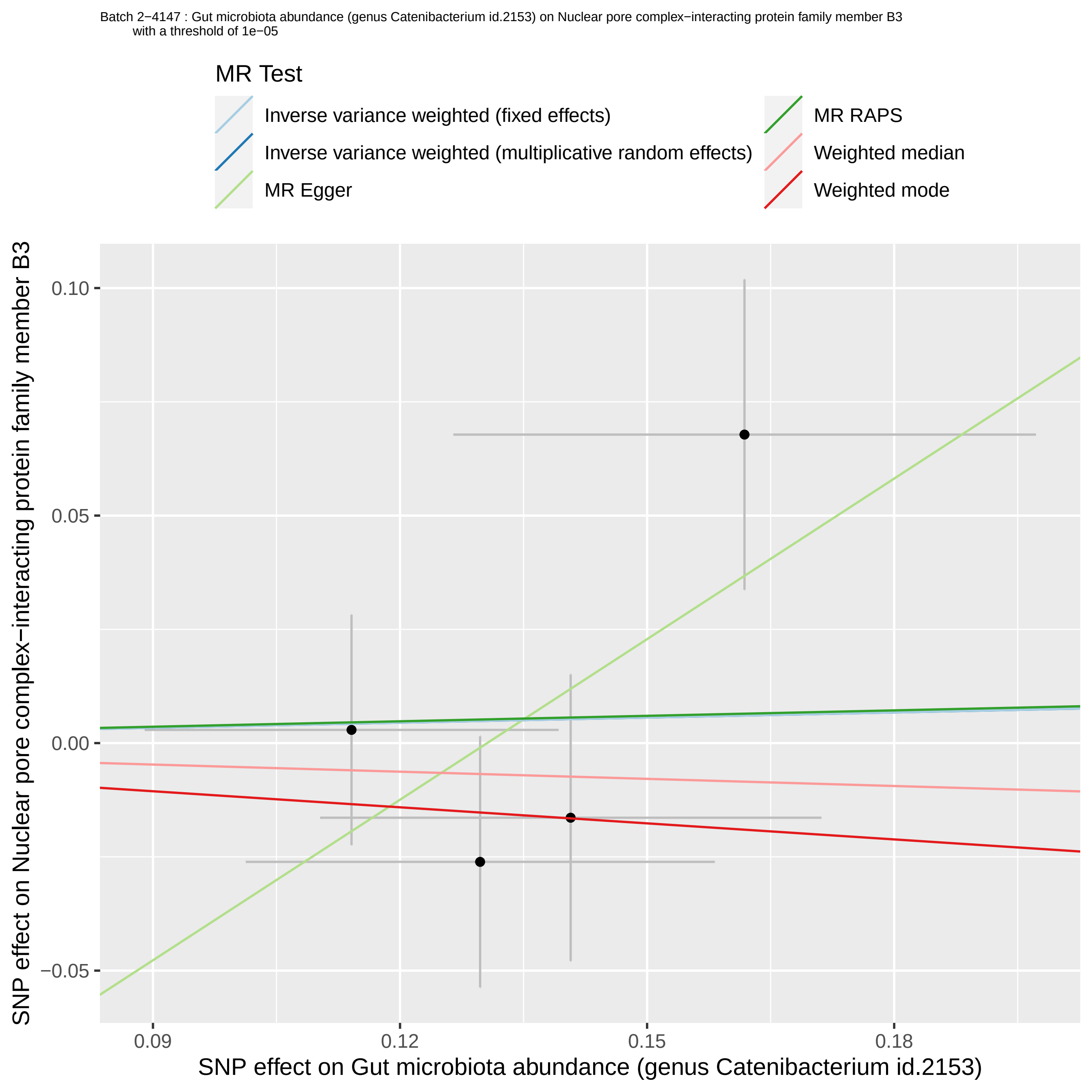
Batch 2−4147 : Gut microbiota abundance (genus Catenibacterium id.2153) on Nuclear pore complex−interacting protein family member B3
with a threshold of 1e−05
MR Test
Inverse variance weighted (fixed effects)
Inverse variance weighted (multiplicative random effects)
MR Egger
MR RAPS
Weighted median
Weighted mode
SNP effect on Nuclear pore complex−interacting protein family member B3
0.10
0.05
0.00
−0.05
0.09
0.12
0.15
0.18
SNP effect on Gut microbiota abundance (genus Catenibacterium id.2153)

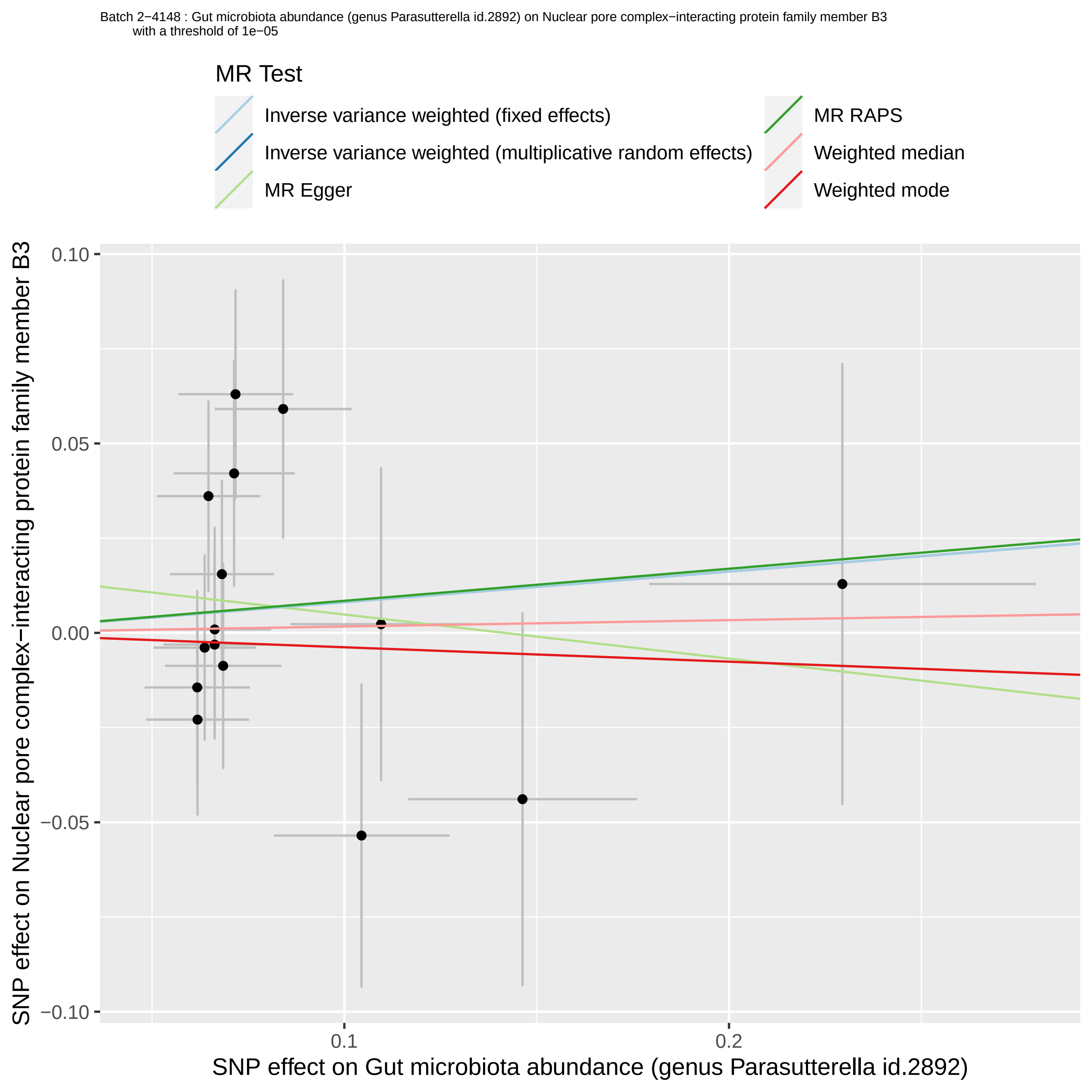

Batch 2−4148 : Gut microbiota abundance (genus Parasutterella id.2892) on Nuclear pore complex−interacting protein family member B3
with a threshold of 1e−05
MR Test
Inverse variance weighted (fixed effects)
Inverse variance weighted (multiplicative random effects)
MR Egger
MR RAPS
Weighted median
Weighted mode
0.10
0.05
0.00
−0.05
−0.10
0.1
0.2
SNP effect on Nuclear pore complex−interacting protein family member B3
SNP effect on Gut microbiota abundance (genus Parasutterella id.2892)

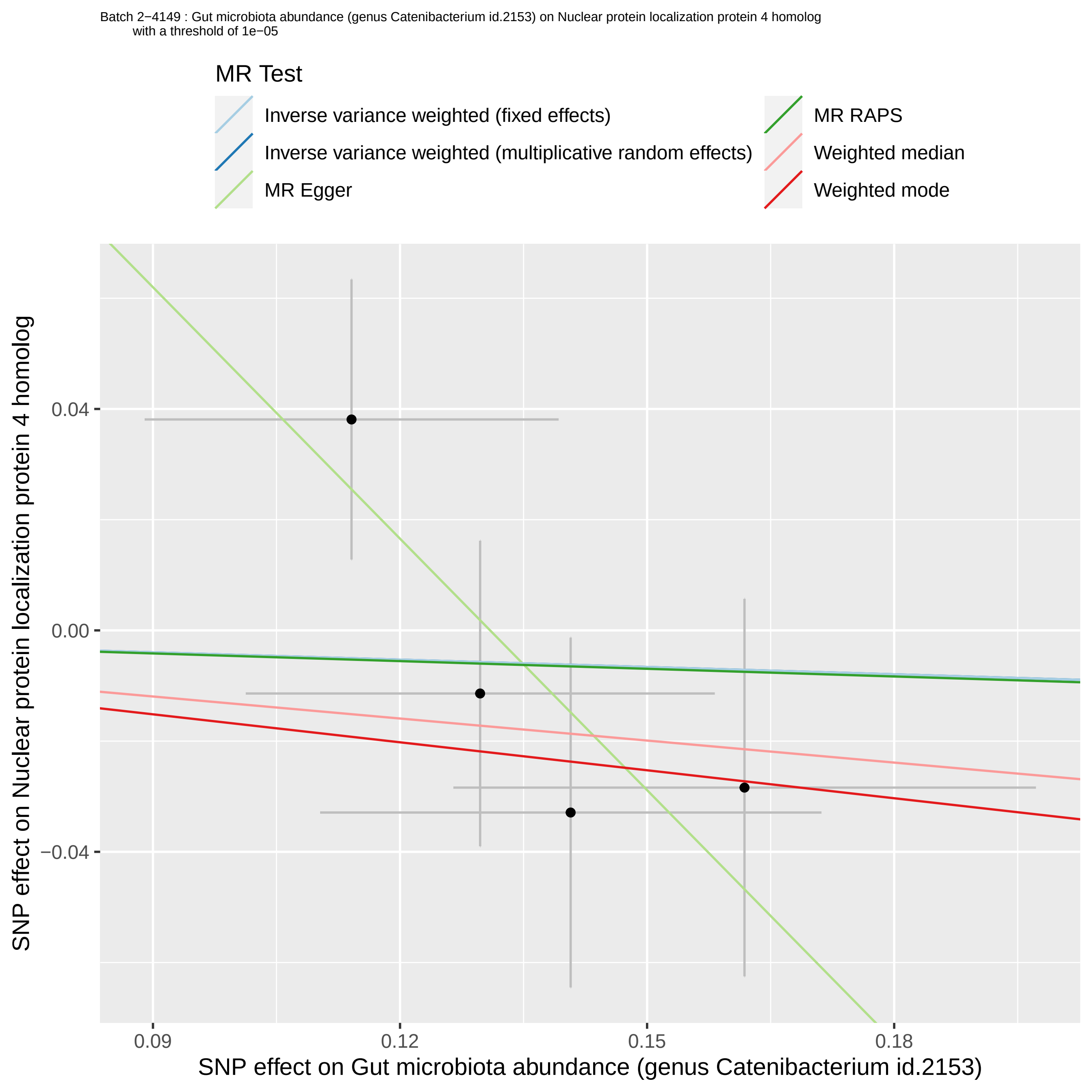
Batch 2−4149 : Gut microbiota abundance (genus Catenibacterium id.2153) on Nuclear protein localization protein 4 homolog
with a threshold of 1e−05
MR Test
Inverse variance weighted (fixed effects)
Inverse variance weighted (multiplicative random effects)
MR Egger
MR RAPS
Weighted median
Weighted mode
0.04
0.00
−0.04
0.09
0.12
0.15
0.18
SNP effect on Nuclear protein localization protein 4 homolog
SNP effect on Gut microbiota abundance (genus Catenibacterium id.2153)

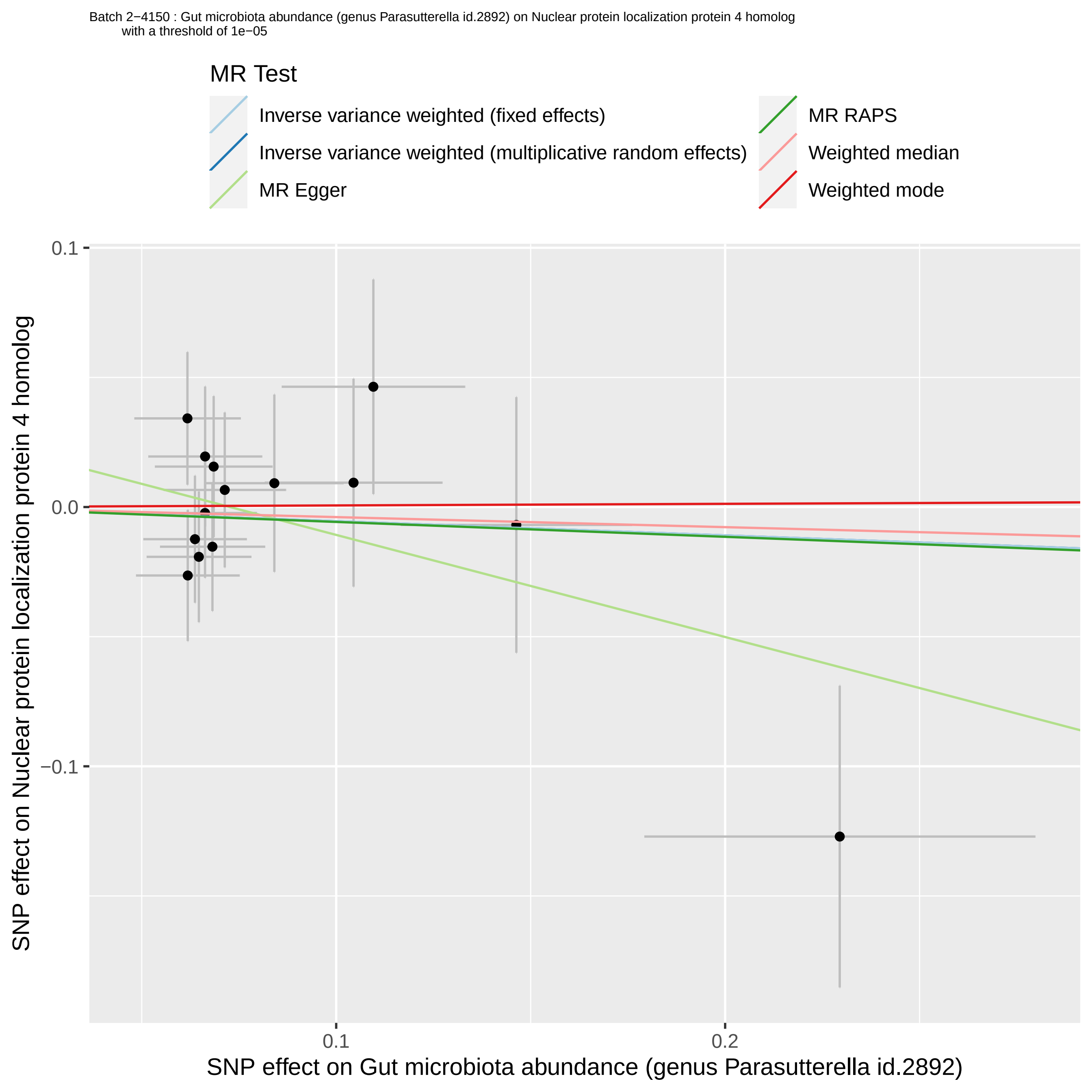

Batch 2−4150 : Gut microbiota abundance (genus Parasutterella id.2892) on Nuclear protein localization protein 4 homolog
with a threshold of 1e−05
MR Test
Inverse variance weighted (fixed effects)
Inverse variance weighted (multiplicative random effects)
MR Egger
MR RAPS
Weighted median
Weighted mode
0.1
0.0
−0.1
0.1
0.2
SNP effect on Nuclear protein localization protein 4 homolog
SNP effect on Gut microbiota abundance (genus Parasutterella id.2892)

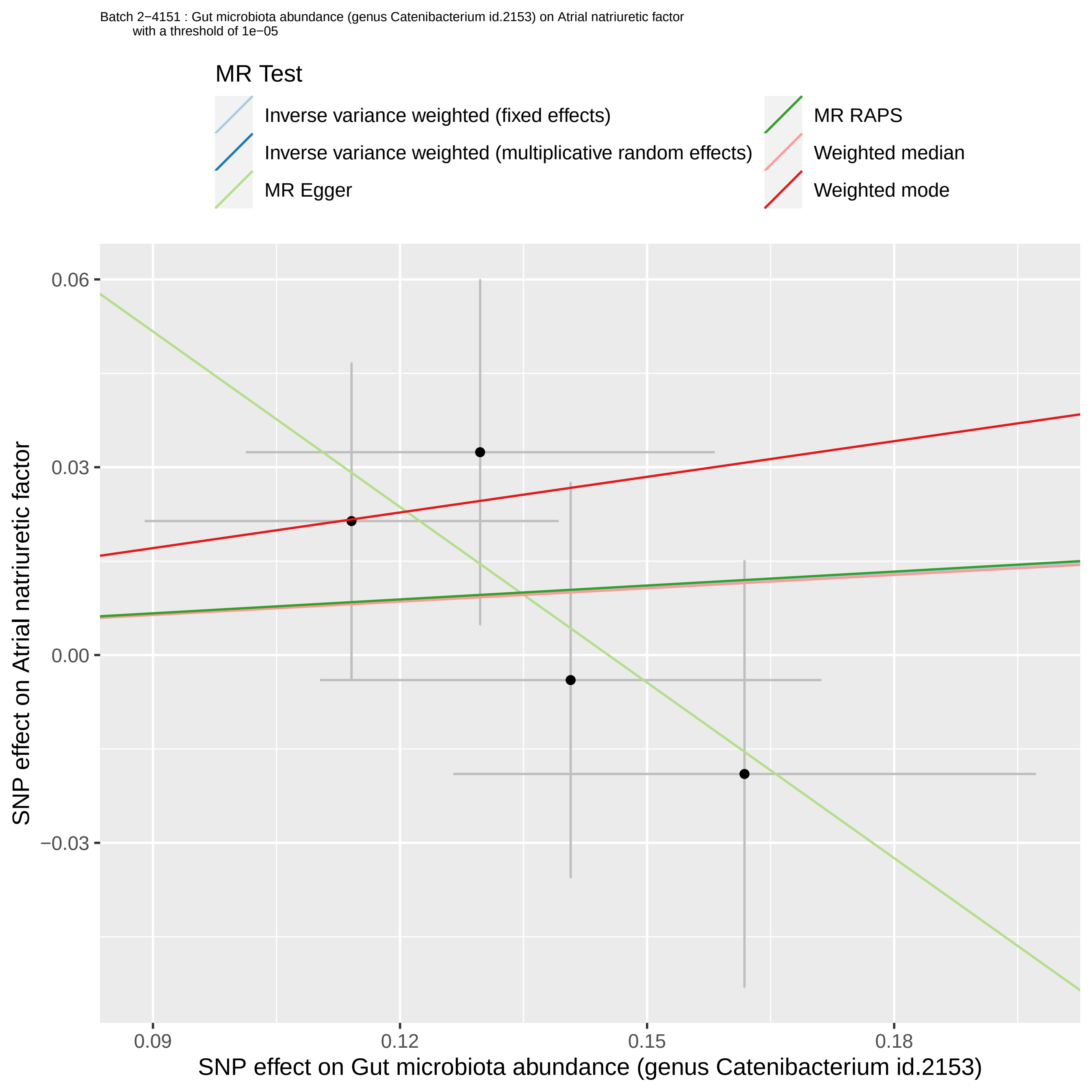
Batch 2−4151 : Gut microbiota abundance (genus Catenibacterium id.2153) on Atrial natriuretic factor
with a threshold of 1e−05
MR Test
Inverse variance weighted (fixed effects)
Inverse variance weighted (multiplicative random effects)
MR Egger
MR RAPS
Weighted median
Weighted mode
0.06
0.03
0.00
−0.03
0.09
0.12
0.15
0.18
SNP effect on Atrial natriuretic factor
SNP effect on Gut microbiota abundance (genus Catenibacterium id.2153)

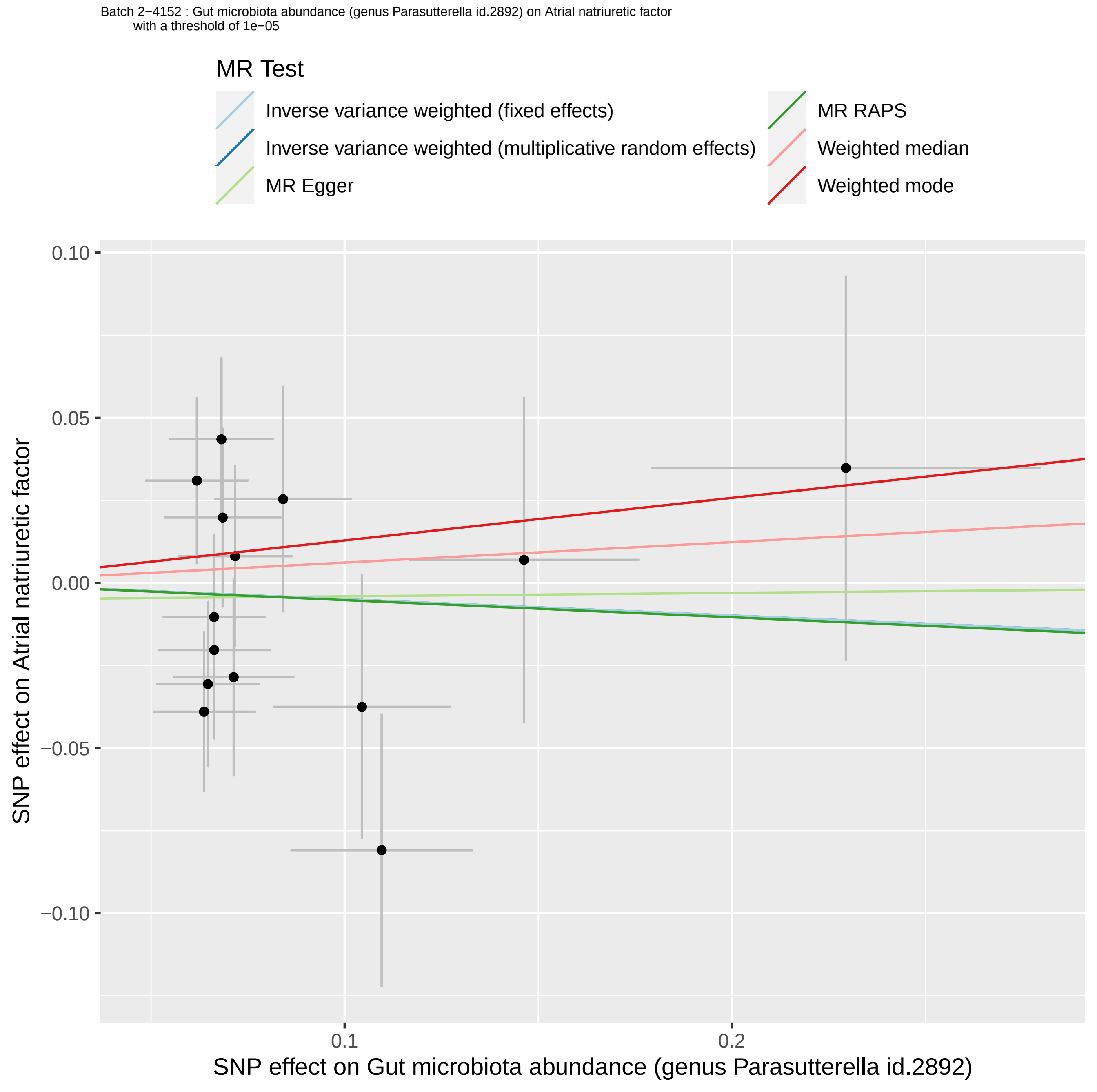
Batch 2−4152 : Gut microbiota abundance (genus Parasutterella id.2892) on Atrial natriuretic factor
with a threshold of 1e−05
MR Test
Inverse variance weighted (fixed effects)
Inverse variance weighted (multiplicative random effects)
MR Egger
MR RAPS
Weighted median
Weighted mode
0.10
0.05
0.00
−0.05
−0.10
0.1
0.2
SNP effect on Atrial natriuretic factor
SNP effect on Gut microbiota abundance (genus Parasutterella id.2892)

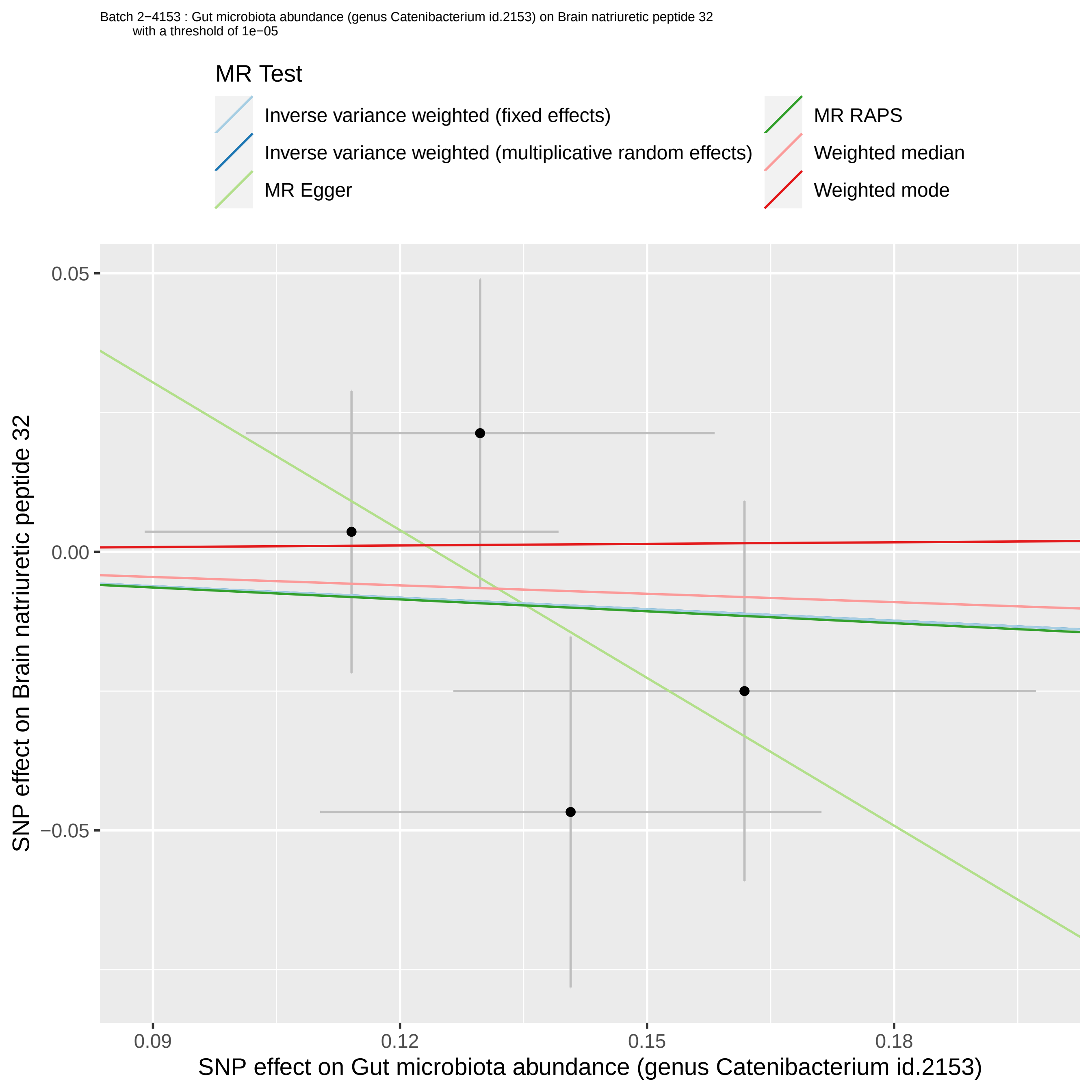
Batch 2−4153 : Gut microbiota abundance (genus Catenibacterium id.2153) on Brain natriuretic peptide 32
with a threshold of 1e−05
MR Test
Inverse variance weighted (fixed effects)
Inverse variance weighted (multiplicative random effects)
MR Egger
MR RAPS
Weighted median
Weighted mode
0.05
0.00
−0.05
0.09
0.12
0.15
0.18
SNP effect on Brain natriuretic peptide 32
SNP effect on Gut microbiota abundance (genus Catenibacterium id.2153)

Batch 2−4154 : Gut microbiota abundance (genus Parasutterella id.2892) on Brain natriuretic peptide 32
with a threshold of 1e−05

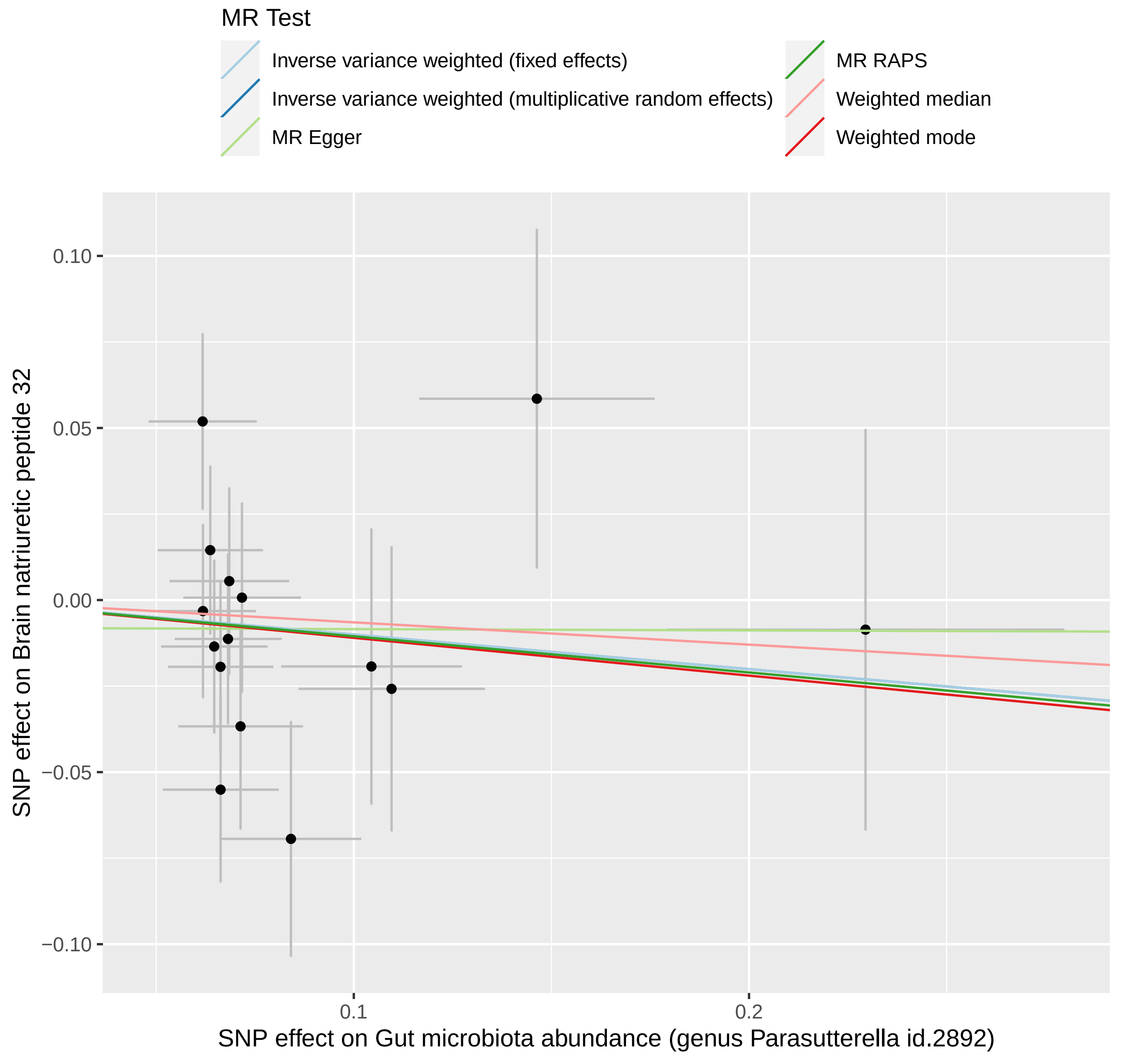

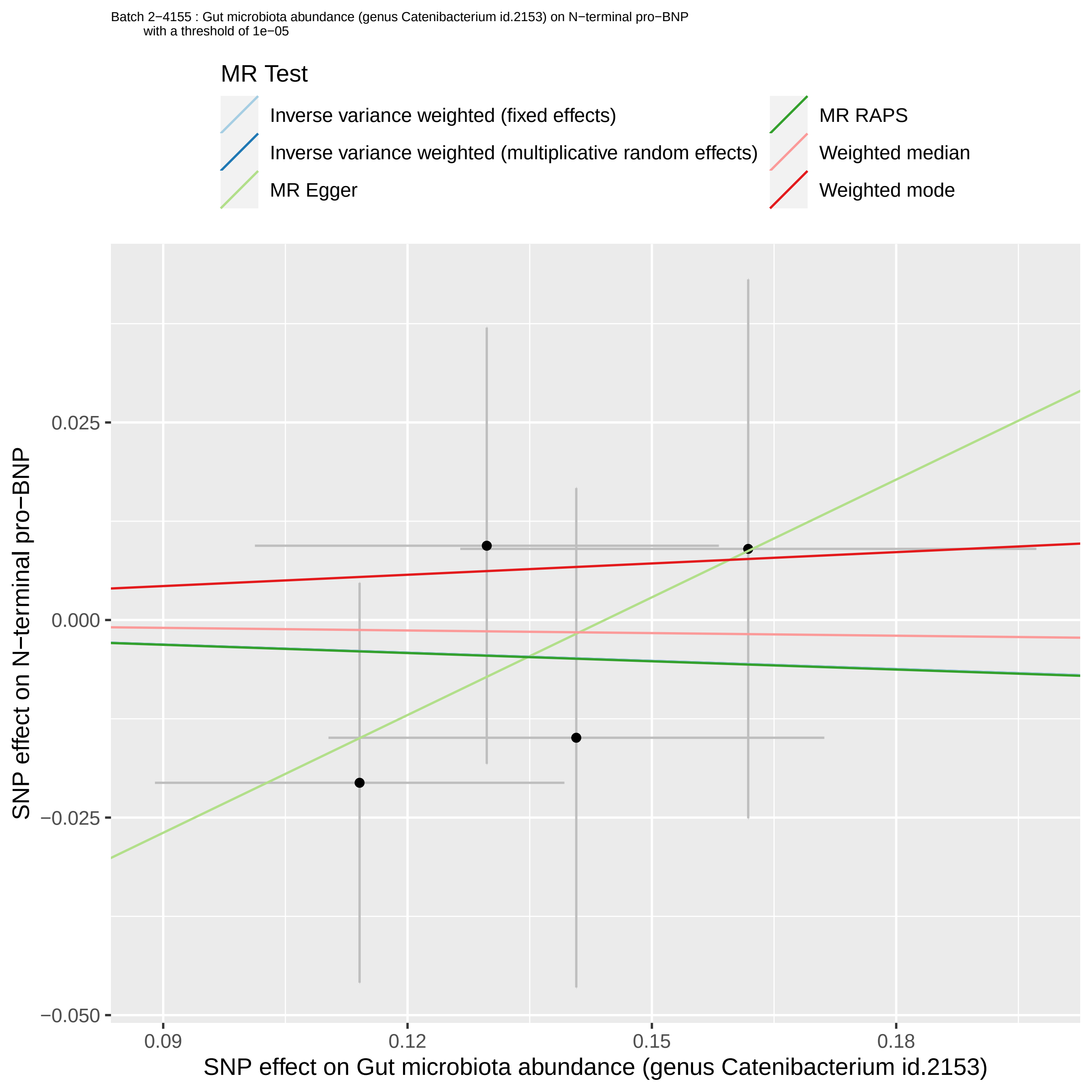
Batch 2−4155 : Gut microbiota abundance (genus Catenibacterium id.2153) on N−terminal pro−BNP
with a threshold of 1e−05
MR Test
Inverse variance weighted (fixed effects)
Inverse variance weighted (multiplicative random effects)
MR Egger
MR RAPS
Weighted median
Weighted mode
0.025
0.000
−0.025
−0.050
0.09
0.12
0.15
0.18
SNP effect on N−terminal pro−BNP
SNP effect on Gut microbiota abundance (genus Catenibacterium id.2153)

Batch 2−4156 : Gut microbiota abundance (genus Parasutterella id.2892) on N−terminal pro−BNP
with a threshold of 1e−05

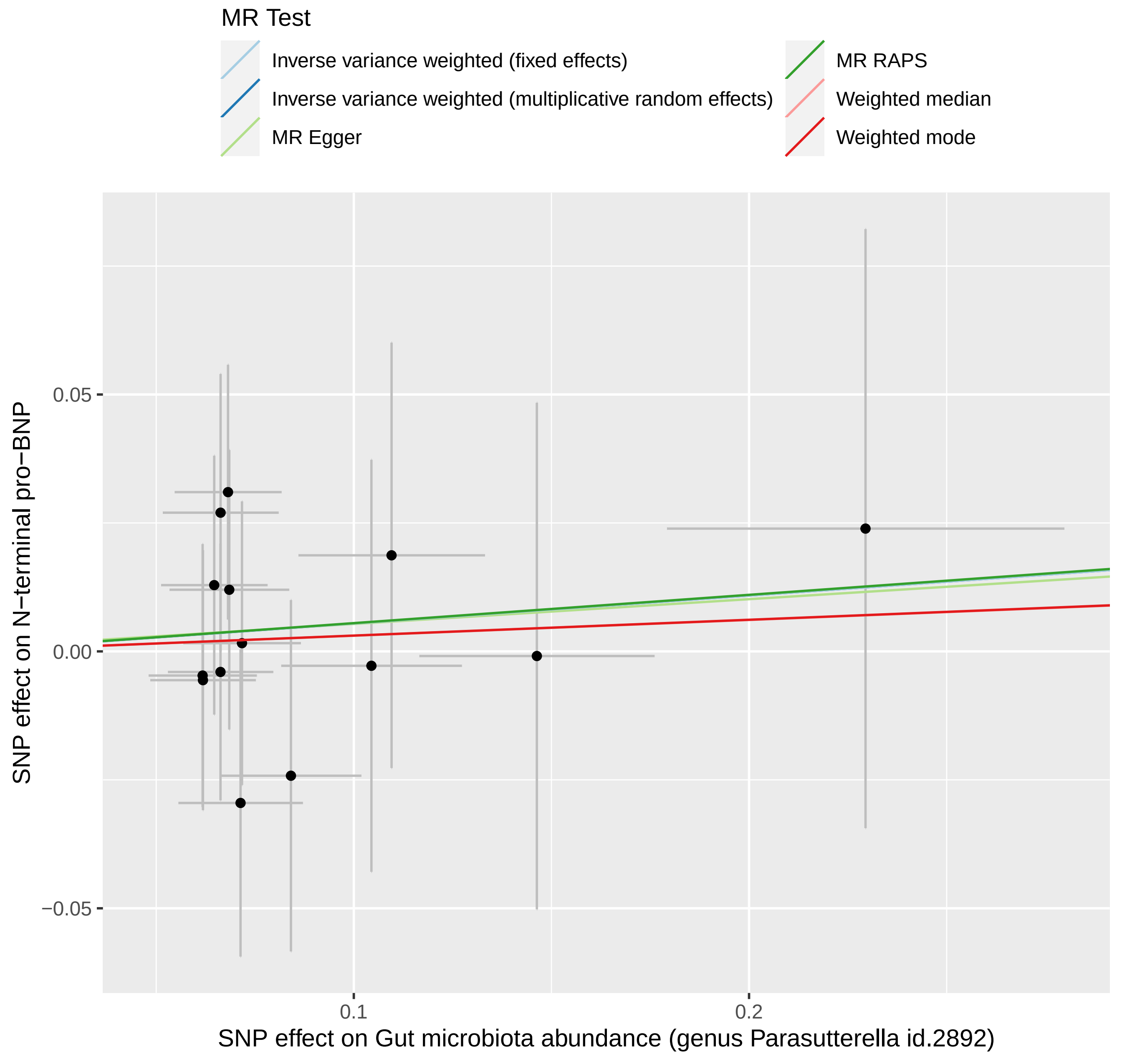

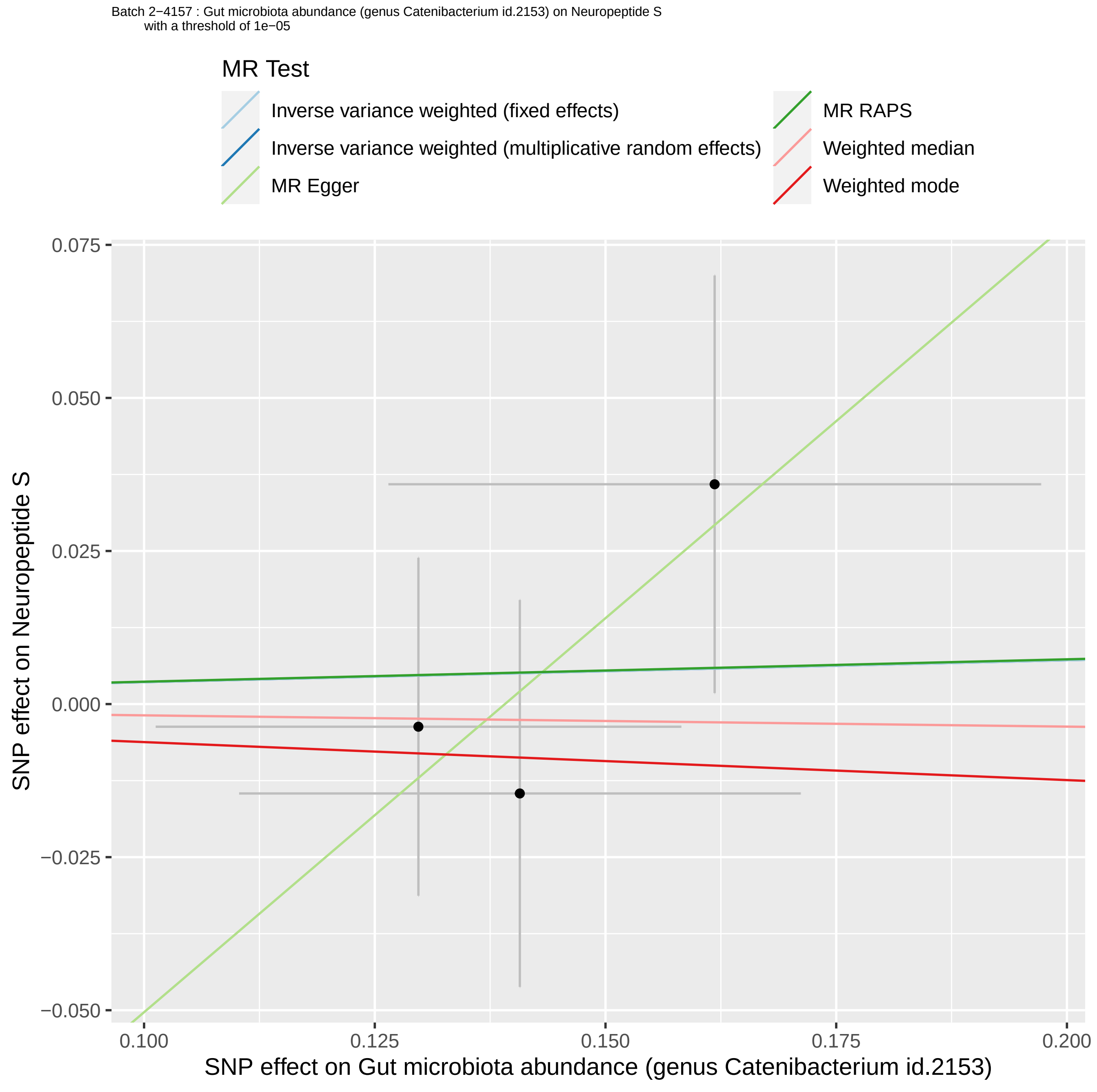

Batch 2−4157 : Gut microbiota abundance (genus Catenibacterium id.2153) on Neuropeptide S
with a threshold of 1e−05
MR Test
Inverse variance weighted (fixed effects)
Inverse variance weighted (multiplicative random effects)
MR Egger
MR RAPS
Weighted median
Weighted mode
SNP effect on Neuropeptide S
0.075
0.050
0.025
0.000
−0.025
−0.050
0.100
0.125
0.150
0.175
0.200
SNP effect on Gut microbiota abundance (genus Catenibacterium id.2153)

Batch 2−4158 : Gut microbiota abundance (genus Parasutterella id.2892) on Neuropeptide S
with a threshold of 1e−05

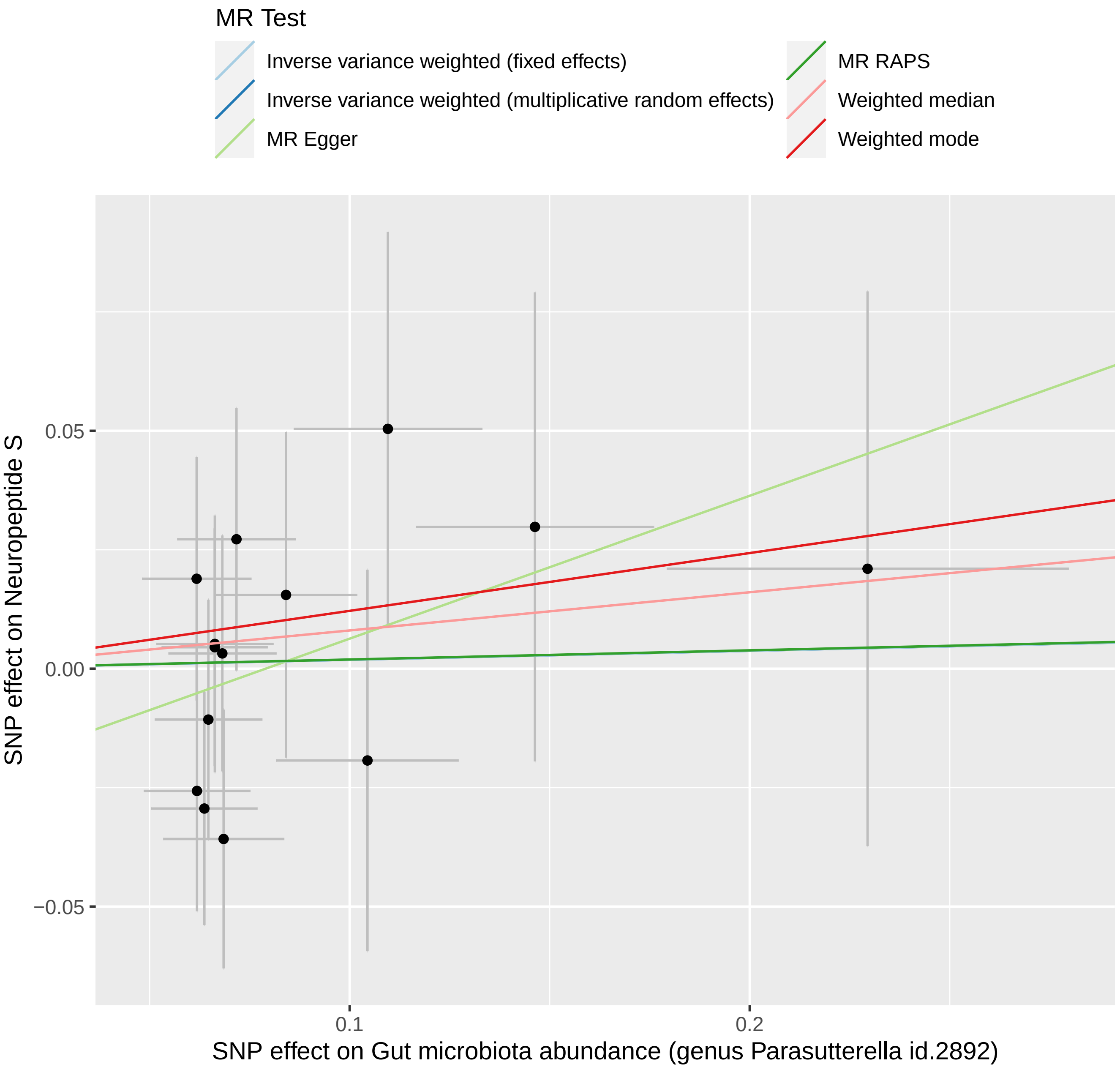

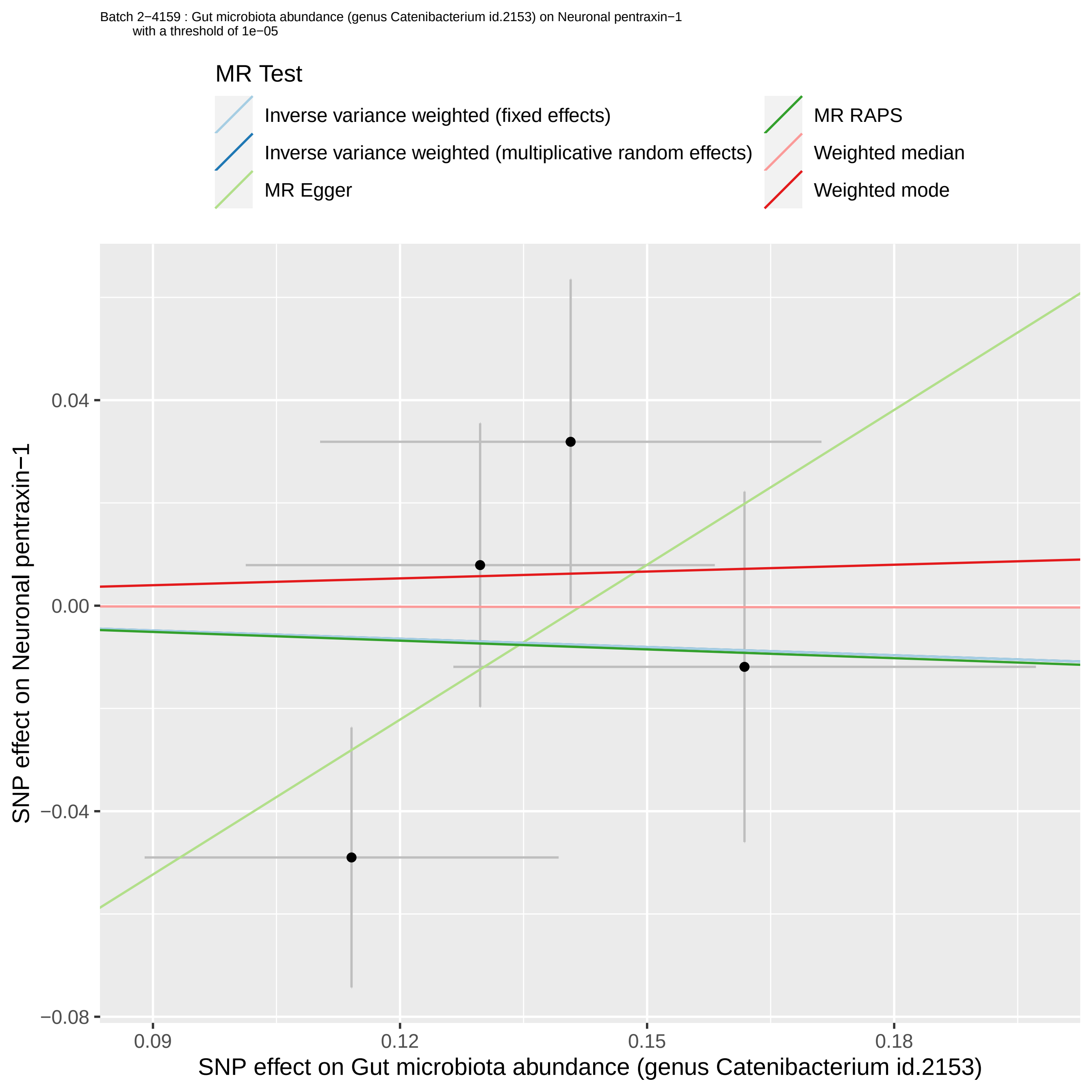
Batch 2−4159 : Gut microbiota abundance (genus Catenibacterium id.2153) on Neuronal pentraxin−1
with a threshold of 1e−05
MR Test
Inverse variance weighted (fixed effects)
Inverse variance weighted (multiplicative random effects)
MR Egger
MR RAPS
Weighted median
Weighted mode
0.04
0.00
−0.04
−0.08
0.09
0.12
0.15
0.18
SNP effect on Neuronal pentraxin−1
SNP effect on Gut microbiota abundance (genus Catenibacterium id.2153)

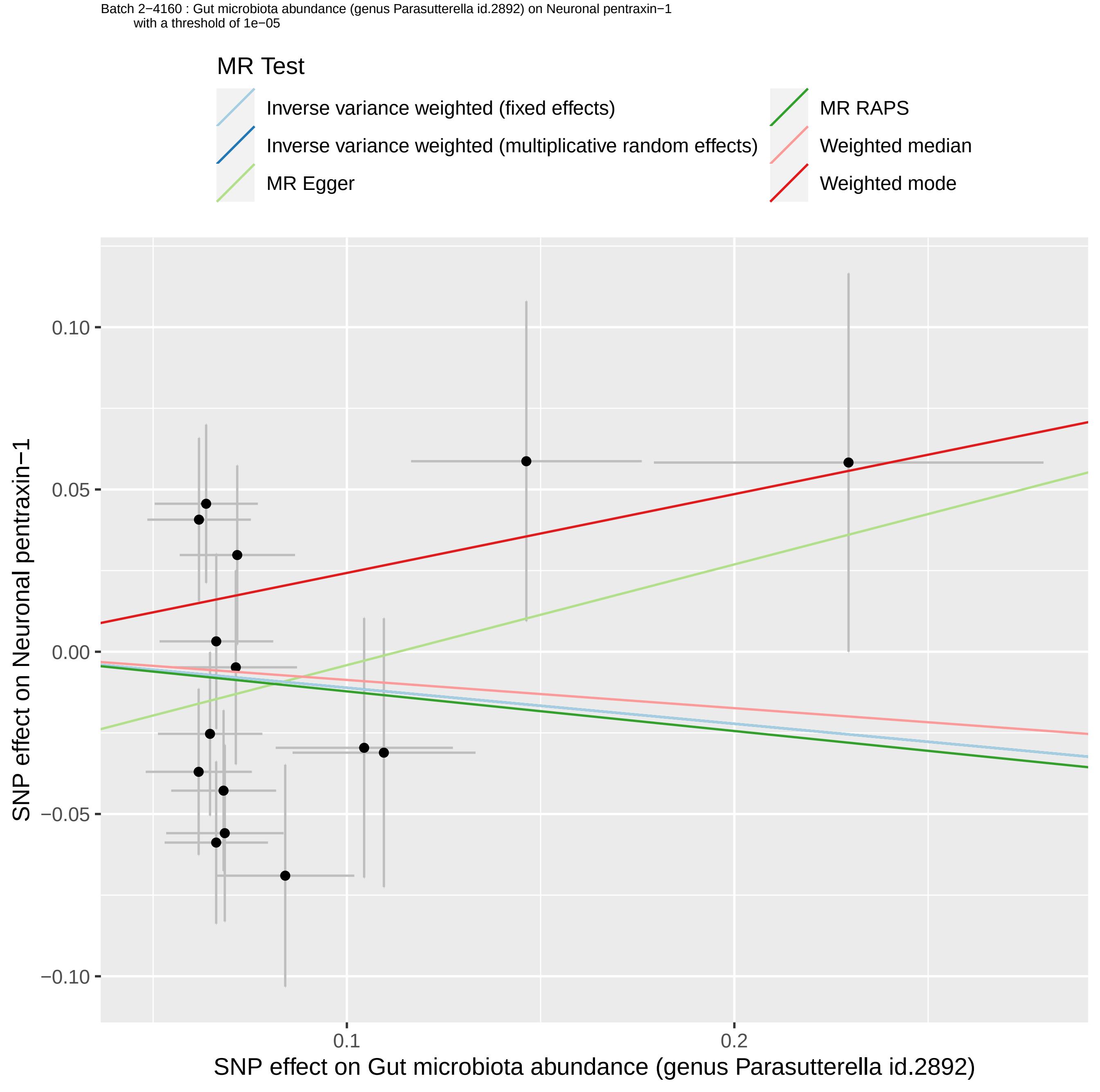

Batch 2−4160 : Gut microbiota abundance (genus Parasutterella id.2892) on Neuronal pentraxin−1
with a threshold of 1e−05
MR Test
Inverse variance weighted (fixed effects)
Inverse variance weighted (multiplicative random effects)
MR Egger
MR RAPS
Weighted median
Weighted mode
0.10
0.05
0.00
−0.05
−0.10
0.1
0.2
SNP effect on Neuronal pentraxin−1
SNP effect on Gut microbiota abundance (genus Parasutterella id.2892)

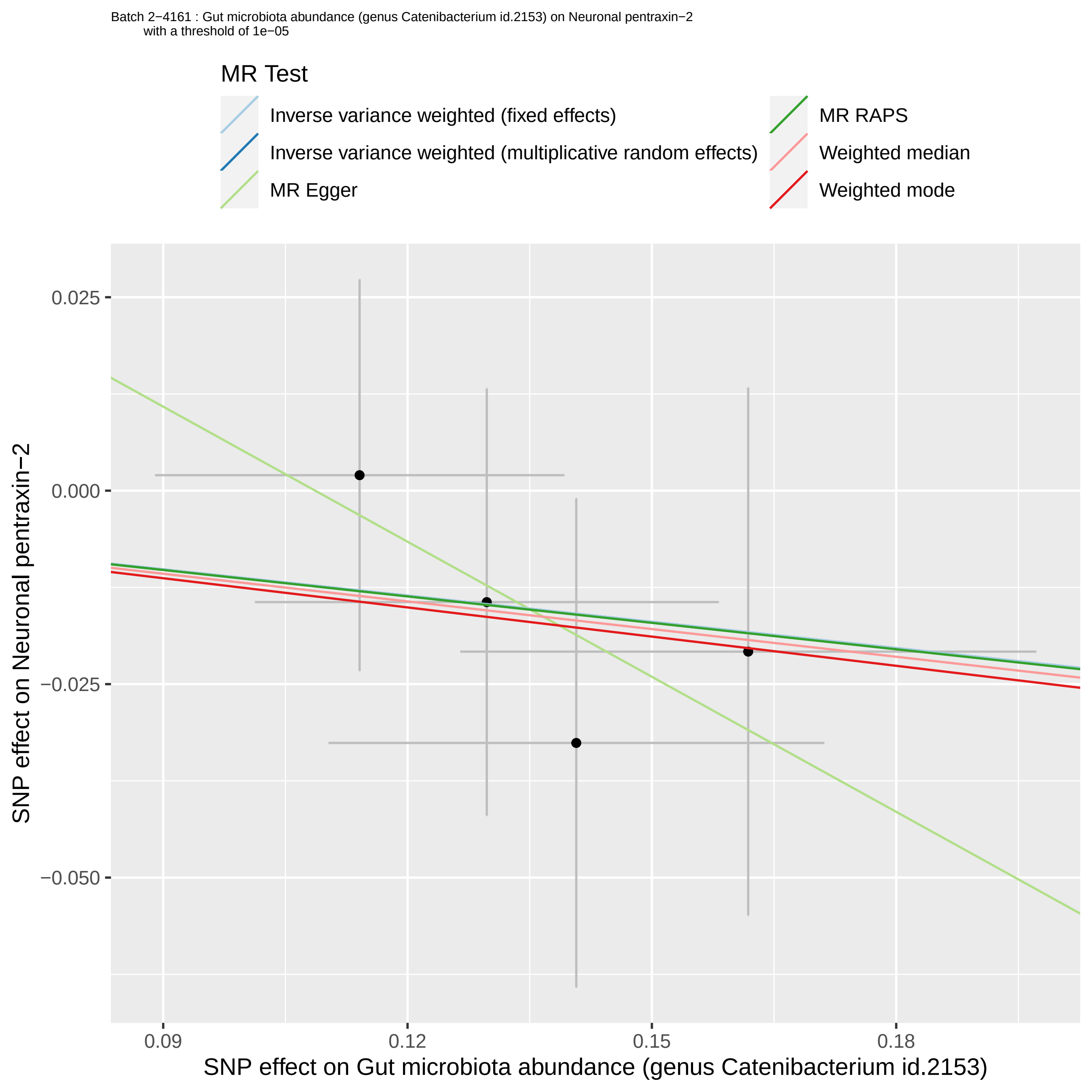

Batch 2−4161 : Gut microbiota abundance (genus Catenibacterium id.2153) on Neuronal pentraxin−2
with a threshold of 1e−05
MR Test
Inverse variance weighted (fixed effects)
Inverse variance weighted (multiplicative random effects)
MR Egger
MR RAPS
Weighted median
Weighted mode
0.025
0.000
−0.025
−0.050
0.09
0.12
0.15
0.18
SNP effect on Neuronal pentraxin−2
SNP effect on Gut microbiota abundance (genus Catenibacterium id.2153)

Batch 2−4162 : Gut microbiota abundance (genus Parasutterella id.2892) on Neuronal pentraxin−2
with a threshold of 1e−05

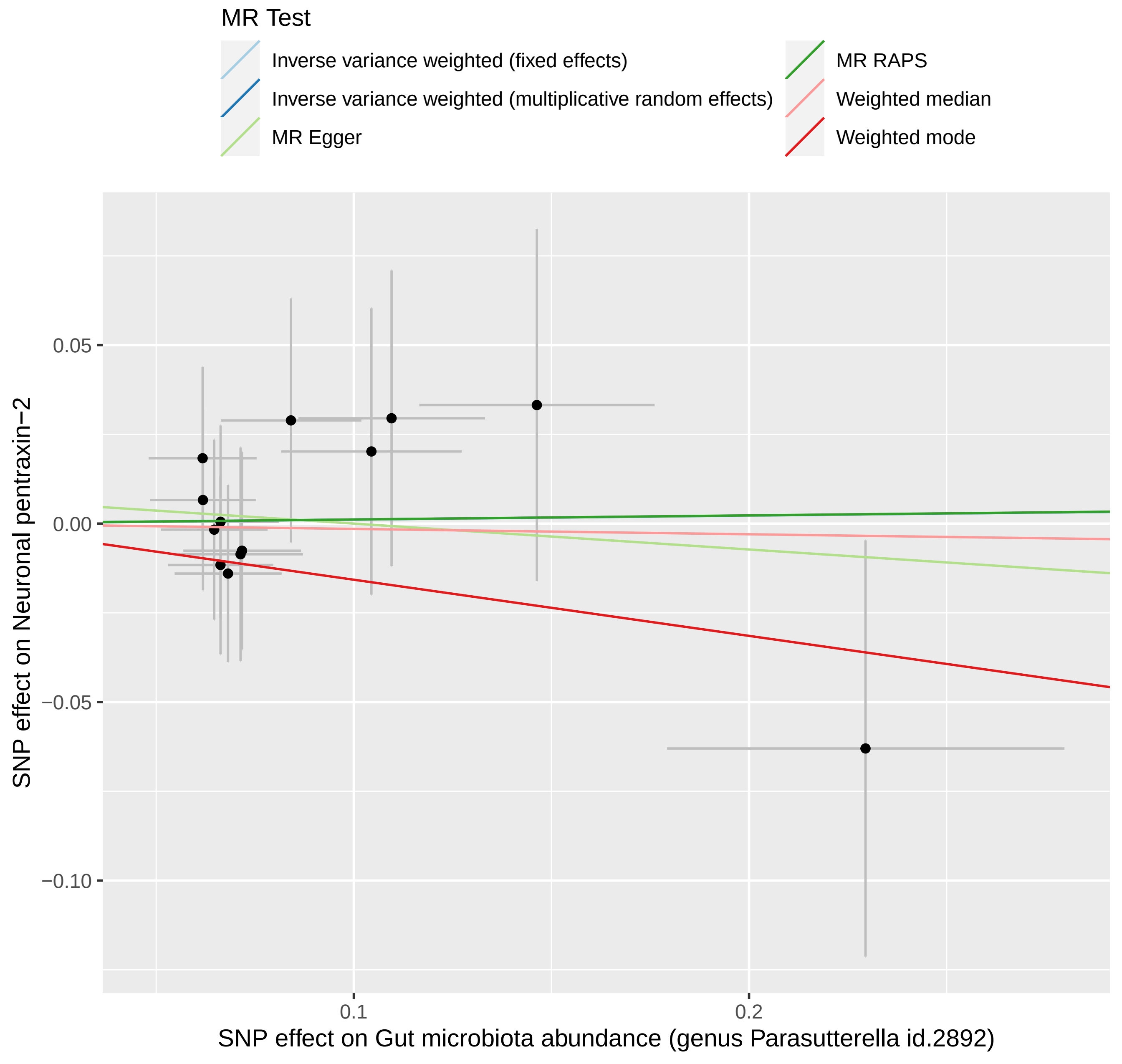

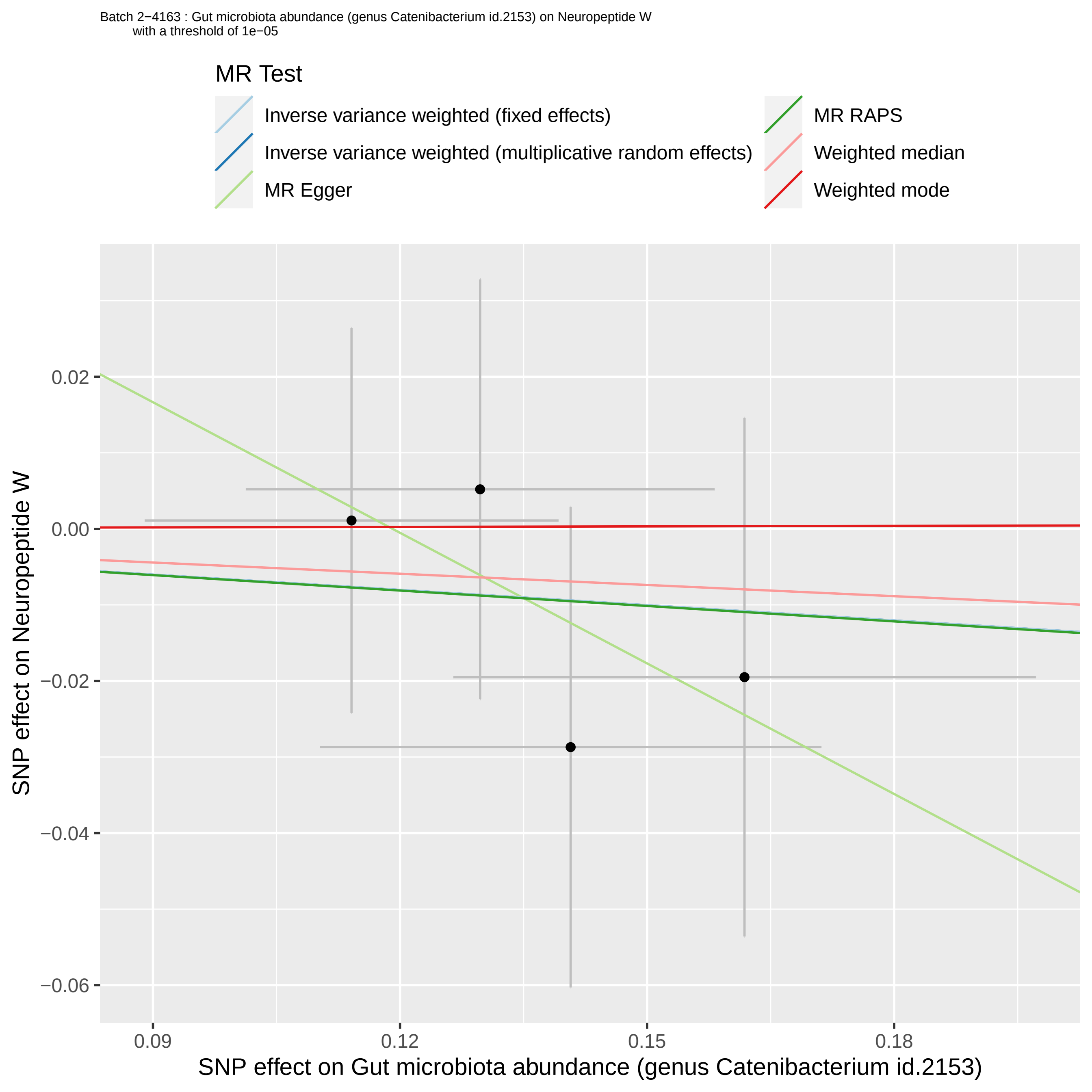
Batch 2−4163 : Gut microbiota abundance (genus Catenibacterium id.2153) on Neuropeptide W
with a threshold of 1e−05
MR Test
Inverse variance weighted (fixed effects)
Inverse variance weighted (multiplicative random effects)
MR Egger
MR RAPS
Weighted median
Weighted mode
0.02
0.00
−0.02
−0.04
−0.06
0.09
0.12
0.15
0.18
SNP effect on Neuropeptide W
SNP effect on Gut microbiota abundance (genus Catenibacterium id.2153)

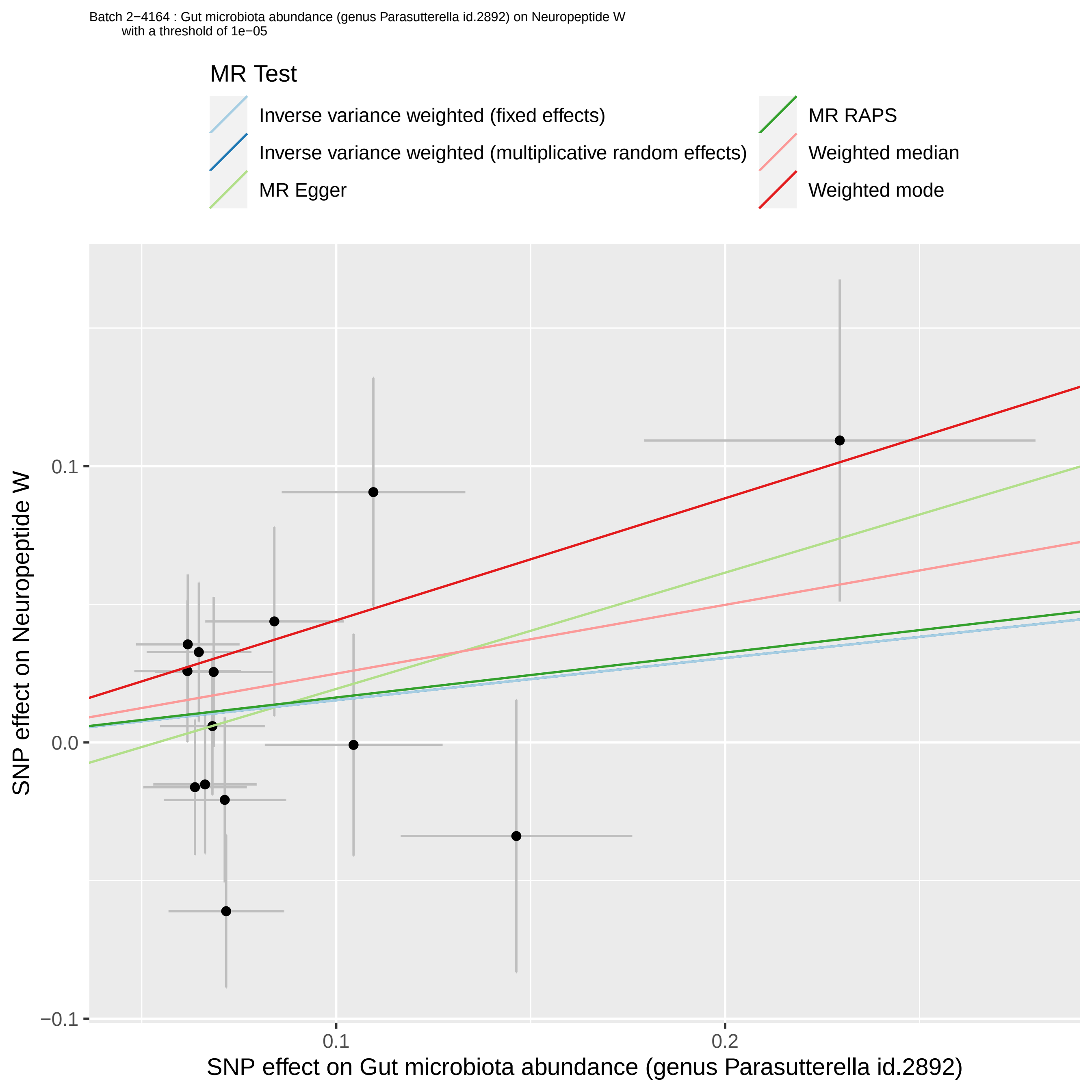
Batch 2−4164 : Gut microbiota abundance (genus Parasutterella id.2892) on Neuropeptide W
with a threshold of 1e−05
MR Test
Inverse variance weighted (fixed effects)
Inverse variance weighted (multiplicative random effects)
MR Egger
MR RAPS
Weighted median
Weighted mode
0.1
0.0
−0.1
SNP effect on Neuropeptide W
0.1
0.2
SNP effect on Gut microbiota abundance (genus Parasutterella id.2892)

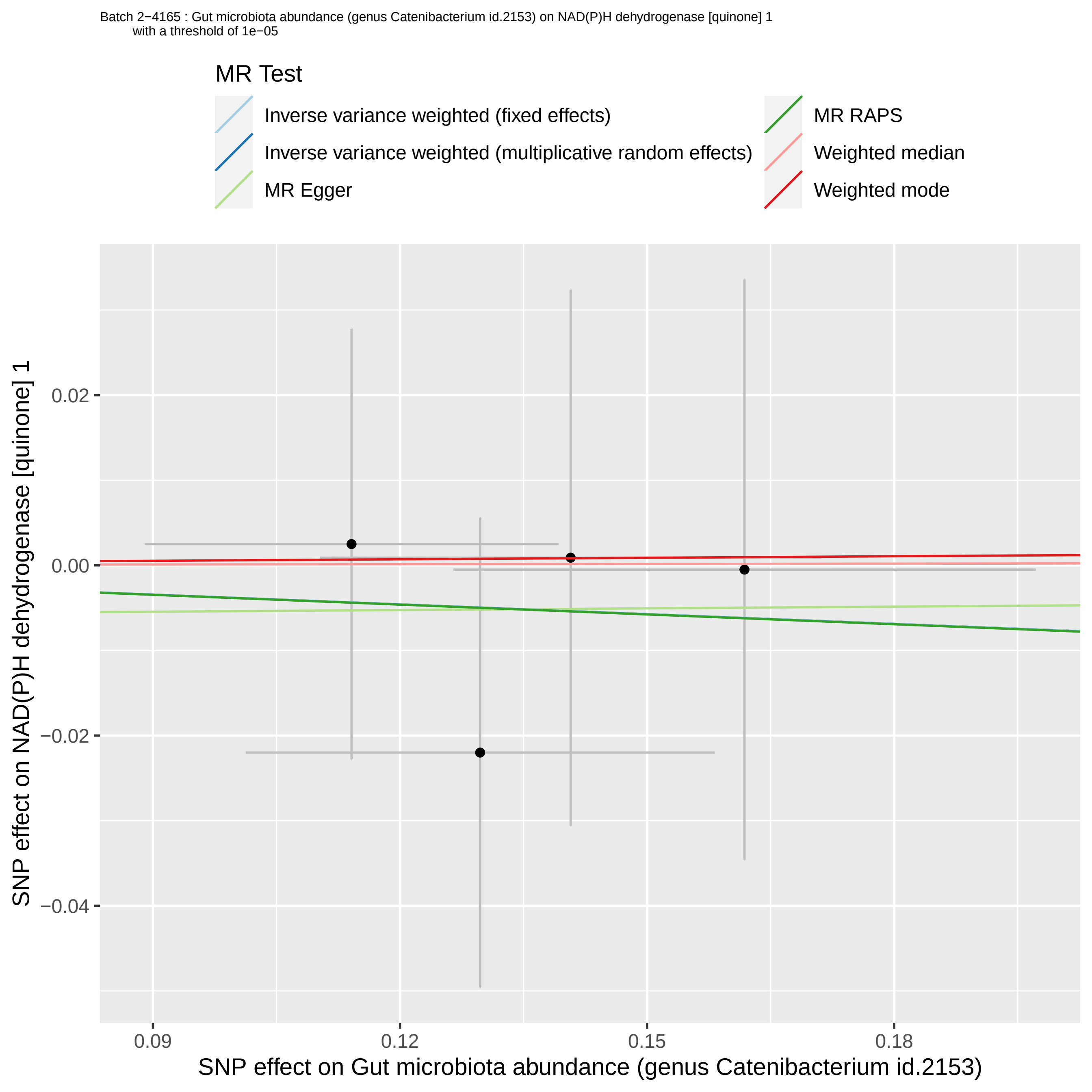

Batch 2−4165 : Gut microbiota abundance (genus Catenibacterium id.2153) on NAD(P)H dehydrogenase [quinone] 1
with a threshold of 1e−05
MR Test
Inverse variance weighted (fixed effects)
Inverse variance weighted (multiplicative random effects)
MR Egger
MR RAPS
Weighted median
Weighted mode
0.02
0.00
−0.02
−0.04
0.09
0.12
0.15
0.18
SNP effect on NAD(P)H dehydrogenase [quinone] 1
SNP effect on Gut microbiota abundance (genus Catenibacterium id.2153)

Batch 2−4166 : Gut microbiota abundance (genus Parasutterella id.2892) on NAD(P)H dehydrogenase [quinone] 1
with a threshold of 1e−05

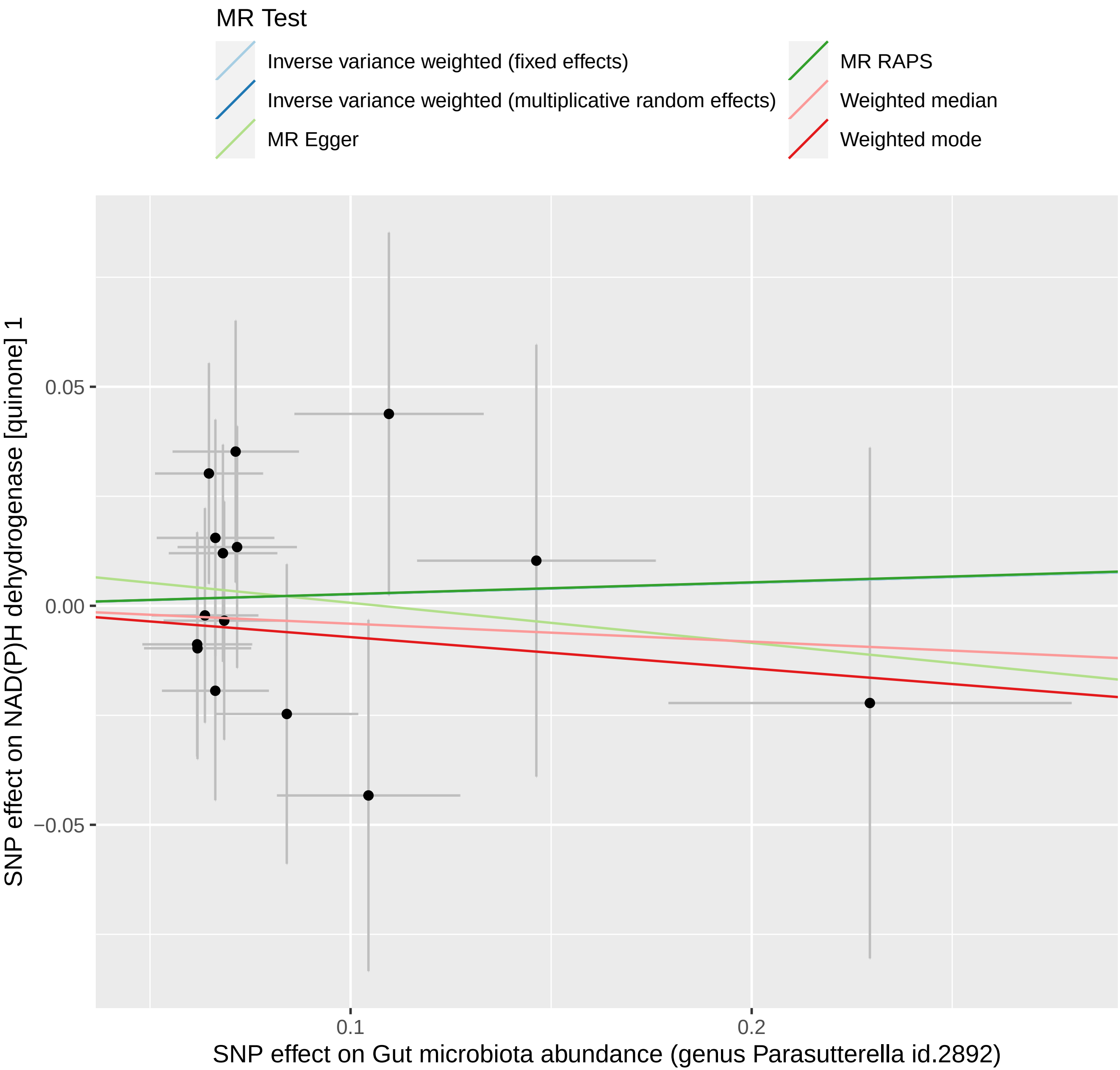

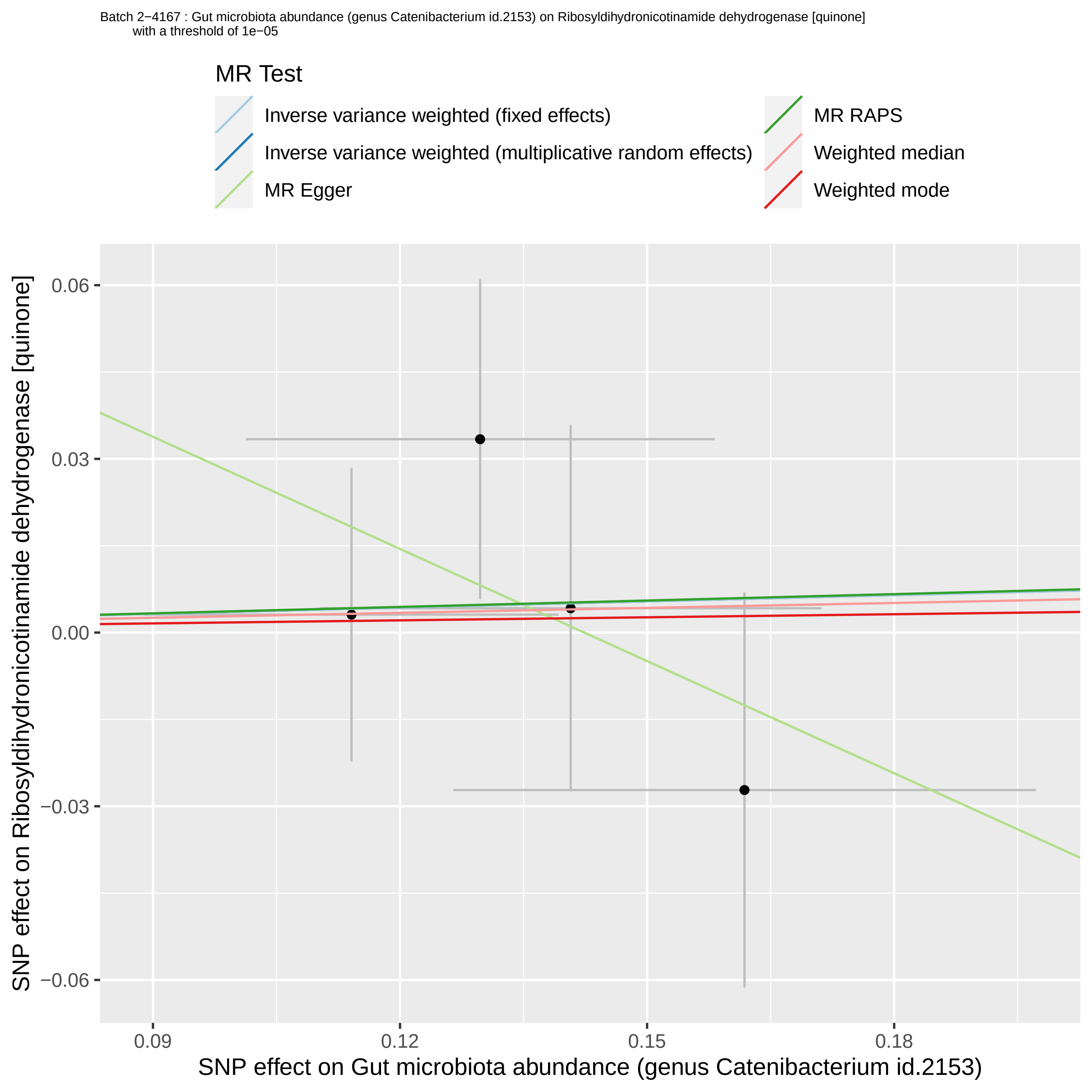

Batch 2−4167 : Gut microbiota abundance (genus Catenibacterium id.2153) on Ribosyldihydronicotinamide dehydrogenase [quinone]
with a threshold of 1e−05
MR Test
Inverse variance weighted (fixed effects)
Inverse variance weighted (multiplicative random effects)
MR Egger
MR RAPS
Weighted median
Weighted mode
0.06
0.03
0.00
−0.03
−0.06
0.09
0.12
0.15
0.18
SNP effect on Ribosyldihydronicotinamide dehydrogenase [quinone]
SNP effect on Gut microbiota abundance (genus Catenibacterium id.2153)

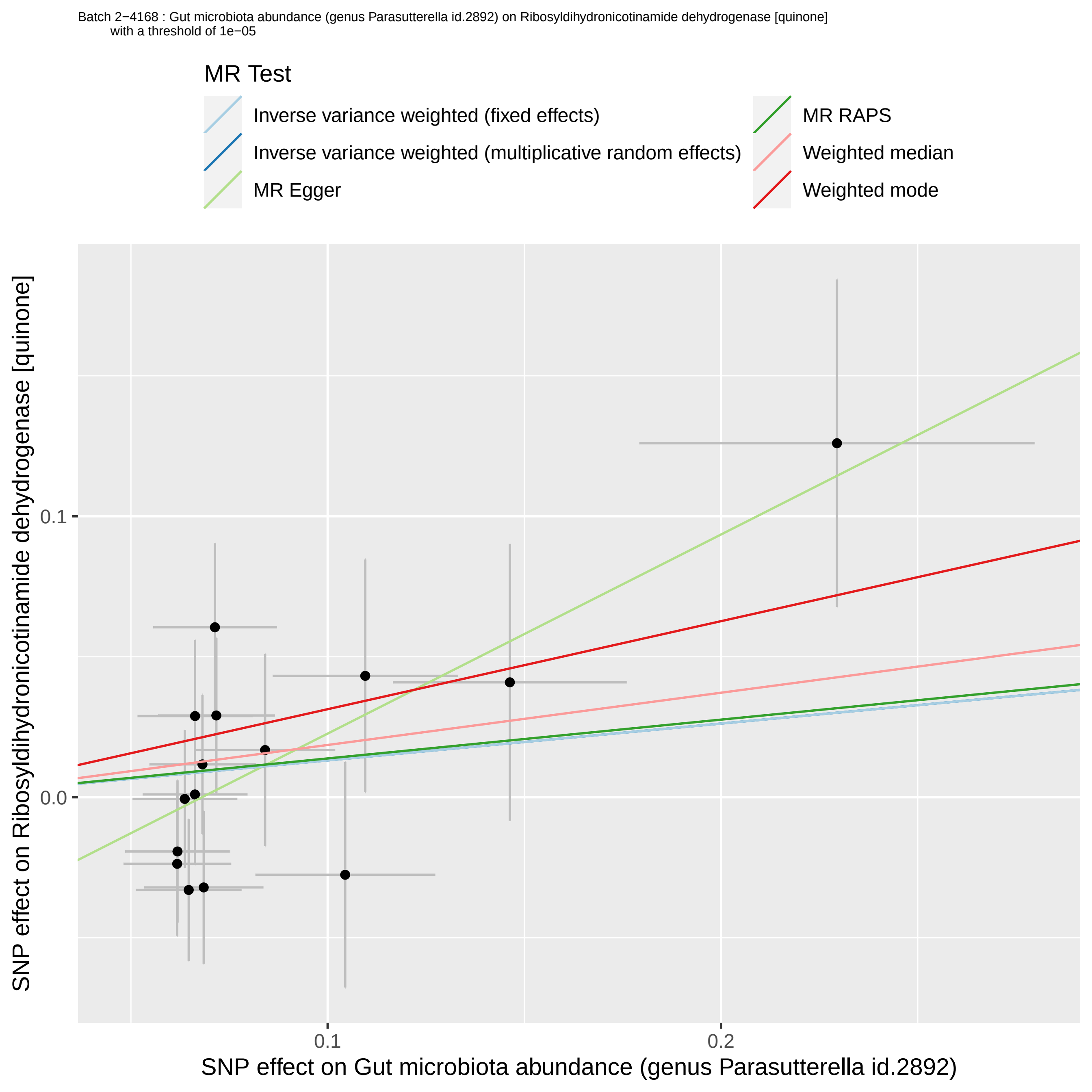
Batch 2−4168 : Gut microbiota abundance (genus Parasutterella id.2892) on Ribosyldihydronicotinamide dehydrogenase [quinone]
with a threshold of 1e−05
MR Test
Inverse variance weighted (fixed effects)
Inverse variance weighted (multiplicative random effects)
MR Egger
MR RAPS
Weighted median
Weighted mode
0.1
0.0
0.1
0.2
SNP effect on Ribosyldihydronicotinamide dehydrogenase [quinone]
SNP effect on Gut microbiota abundance (genus Parasutterella id.2892)

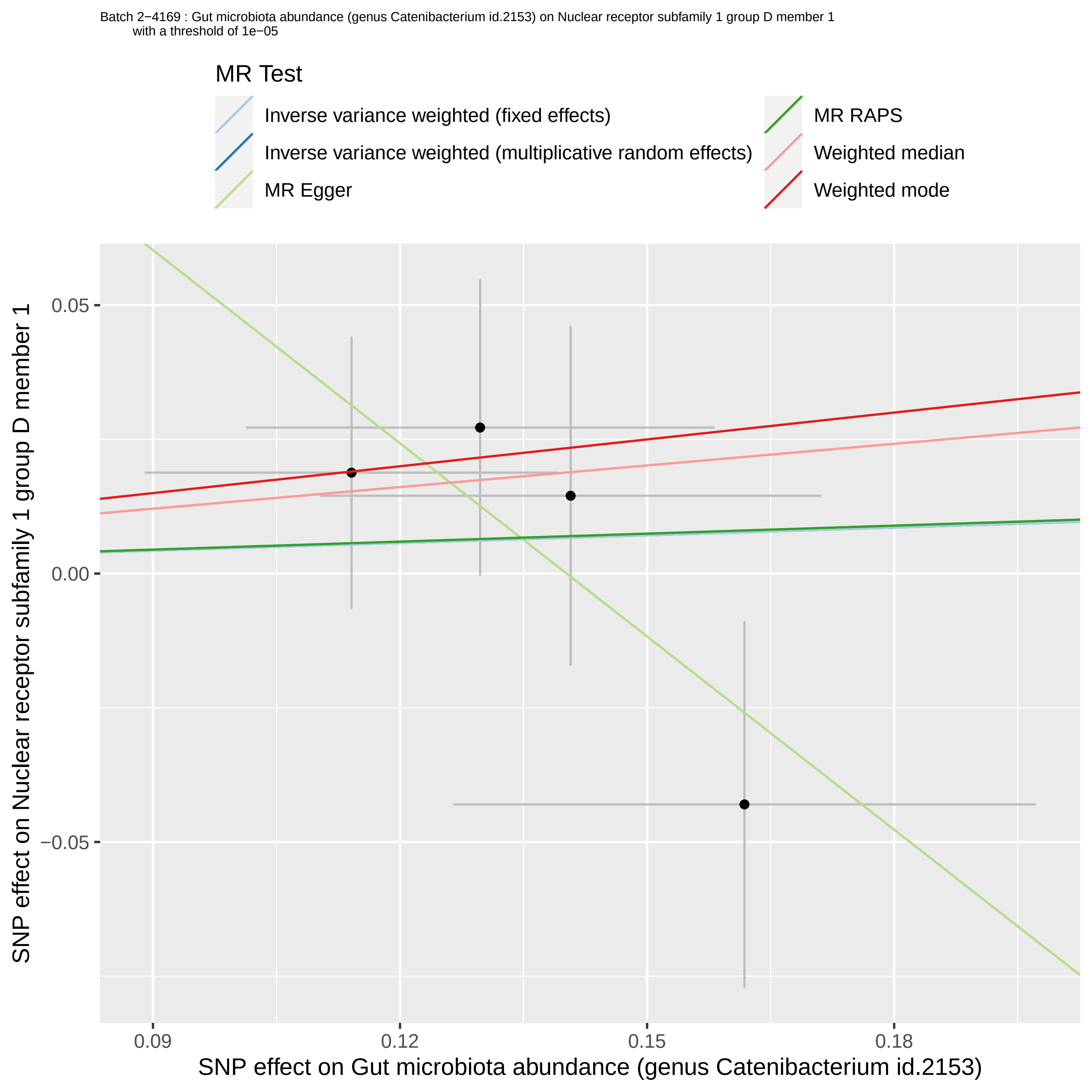
Batch 2−4169 : Gut microbiota abundance (genus Catenibacterium id.2153) on Nuclear receptor subfamily 1 group D member 1
with a threshold of 1e−05
MR Test
Inverse variance weighted (fixed effects)
Inverse variance weighted (multiplicative random effects)
MR Egger
MR RAPS
Weighted median
Weighted mode
0.05
0.00
−0.05
0.09
0.12
0.15
0.18
SNP effect on Nuclear receptor subfamily 1 group D member 1
SNP effect on Gut microbiota abundance (genus Catenibacterium id.2153)

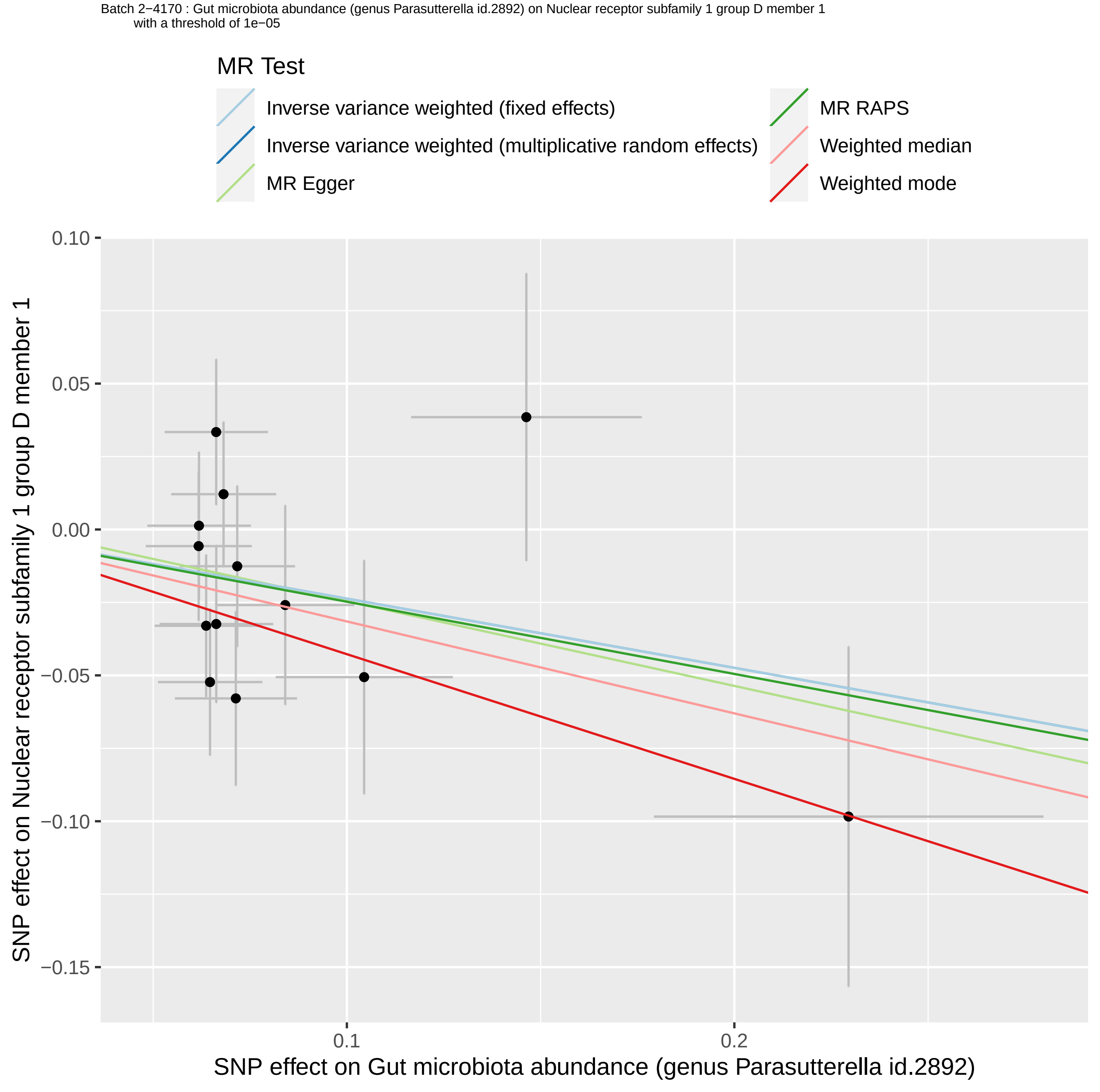

Batch 2−4170 : Gut microbiota abundance (genus Parasutterella id.2892) on Nuclear receptor subfamily 1 group D member 1
with a threshold of 1e−05
MR Test
Inverse variance weighted (fixed effects)
Inverse variance weighted (multiplicative random effects)
MR Egger
MR RAPS
Weighted median
Weighted mode
0.10
0.05
0.00
−0.05
−0.10
−0.15
0.1
0.2
SNP effect on Nuclear receptor subfamily 1 group D member 1
SNP effect on Gut microbiota abundance (genus Parasutterella id.2892)

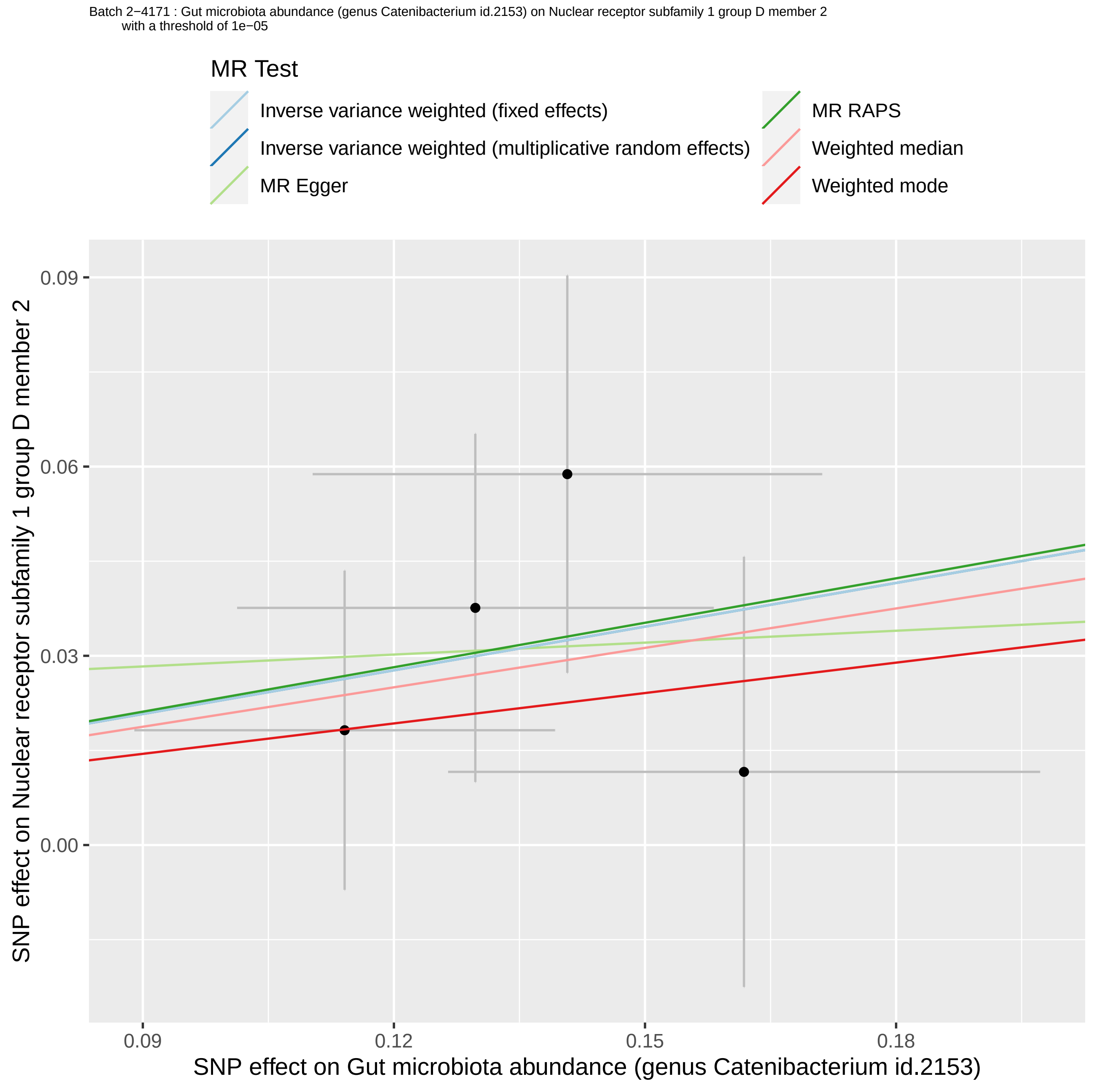

Batch 2−4171 : Gut microbiota abundance (genus Catenibacterium id.2153) on Nuclear receptor subfamily 1 group D member 2
with a threshold of 1e−05
MR Test
Inverse variance weighted (fixed effects)
Inverse variance weighted (multiplicative random effects)
MR Egger
MR RAPS
Weighted median
Weighted mode
0.09
0.06
0.03
0.00
0.09
0.12
0.15
0.18
SNP effect on Nuclear receptor subfamily 1 group D member 2
SNP effect on Gut microbiota abundance (genus Catenibacterium id.2153)

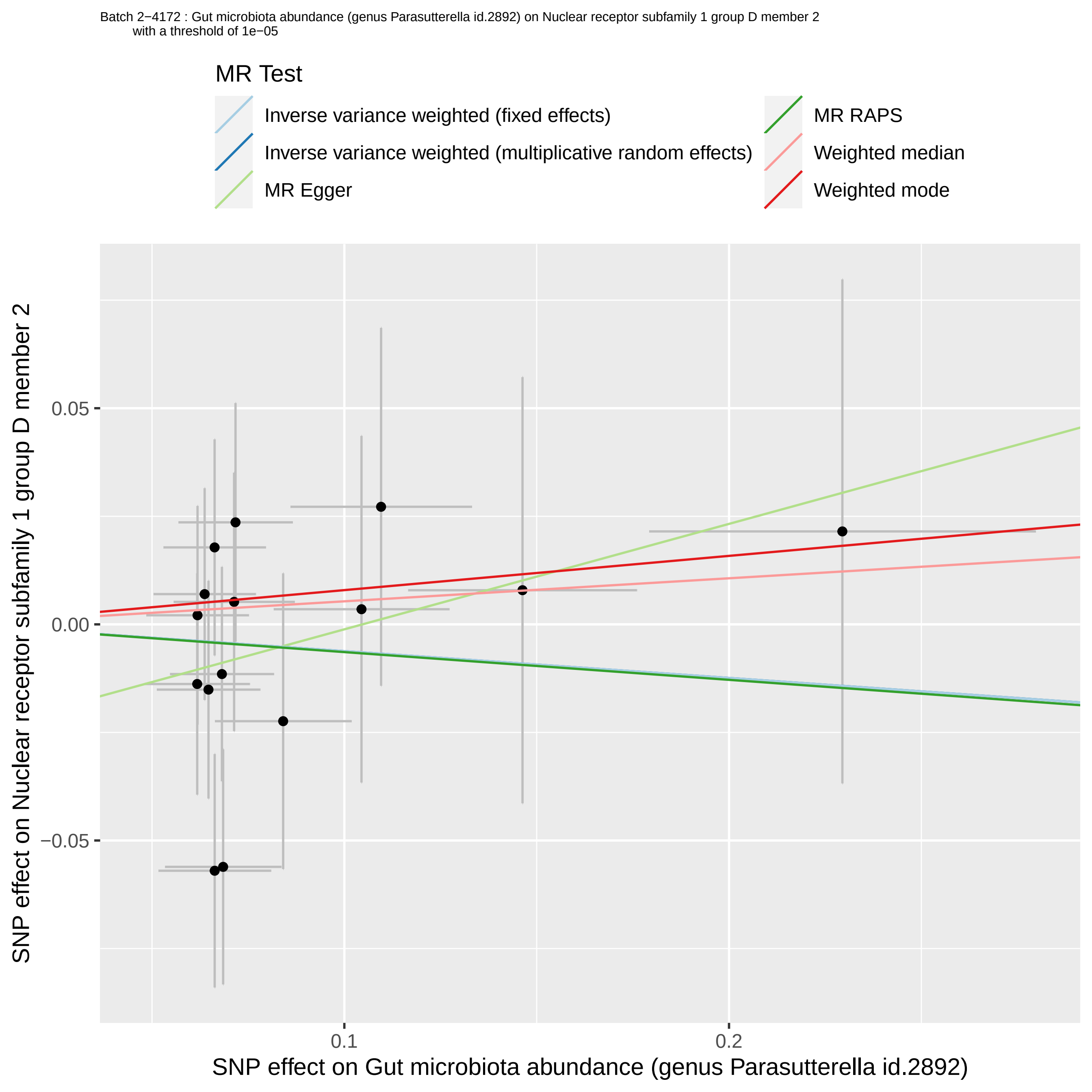
Batch 2−4172 : Gut microbiota abundance (genus Parasutterella id.2892) on Nuclear receptor subfamily 1 group D member 2
with a threshold of 1e−05
MR Test
Inverse variance weighted (fixed effects)
Inverse variance weighted (multiplicative random effects)
MR Egger
MR RAPS
Weighted median
Weighted mode
0.05
0.00
−0.05
0.1
0.2
SNP effect on Nuclear receptor subfamily 1 group D member 2
SNP effect on Gut microbiota abundance (genus Parasutterella id.2892)

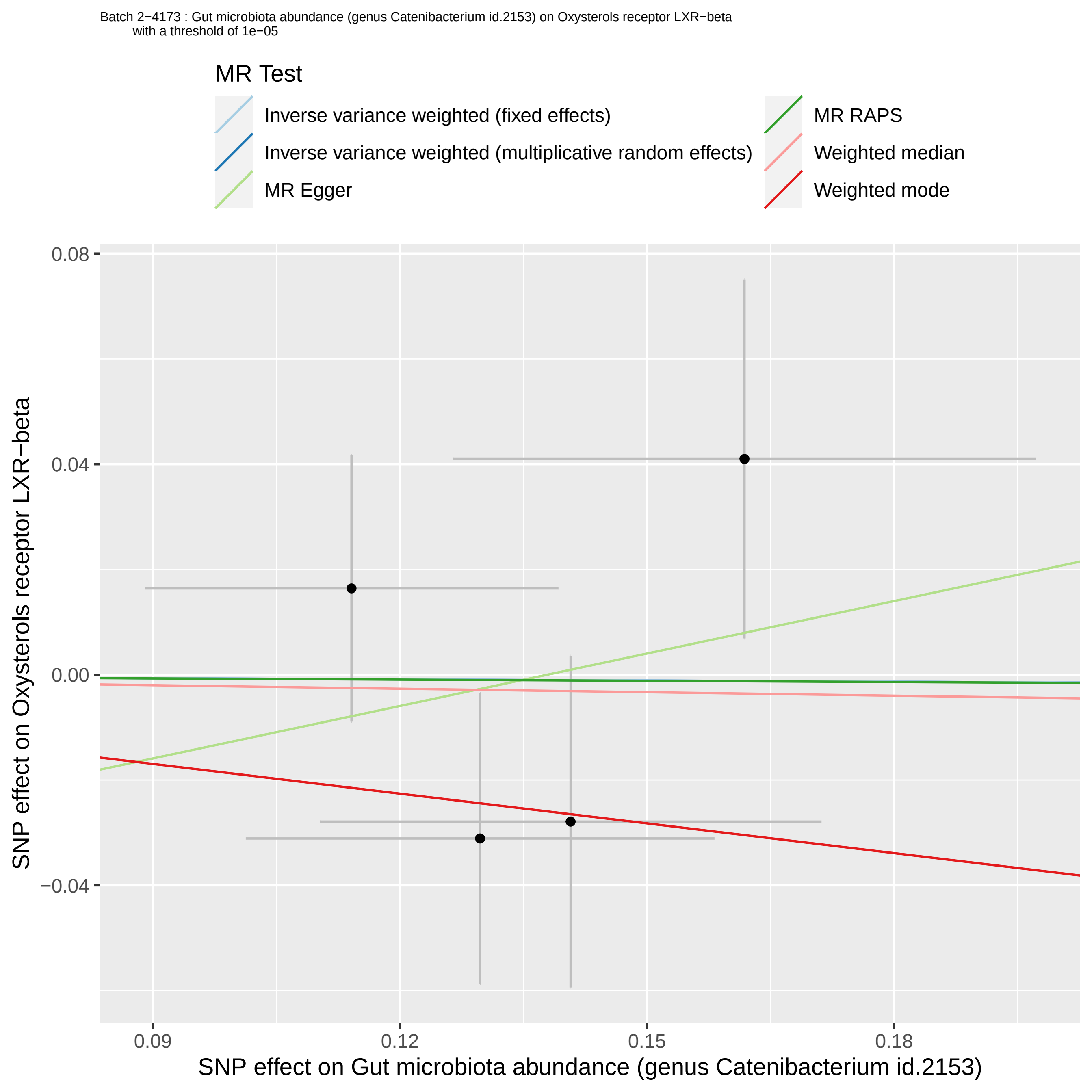
Batch 2−4173 : Gut microbiota abundance (genus Catenibacterium id.2153) on Oxysterols receptor LXR−beta
with a threshold of 1e−05
MR Test
Inverse variance weighted (fixed effects)
Inverse variance weighted (multiplicative random effects)
MR Egger
MR RAPS
Weighted median
Weighted mode
0.08
0.04
0.00
−0.04
0.09
0.12
0.15
0.18
SNP effect on Oxysterols receptor LXR−beta
SNP effect on Gut microbiota abundance (genus Catenibacterium id.2153)

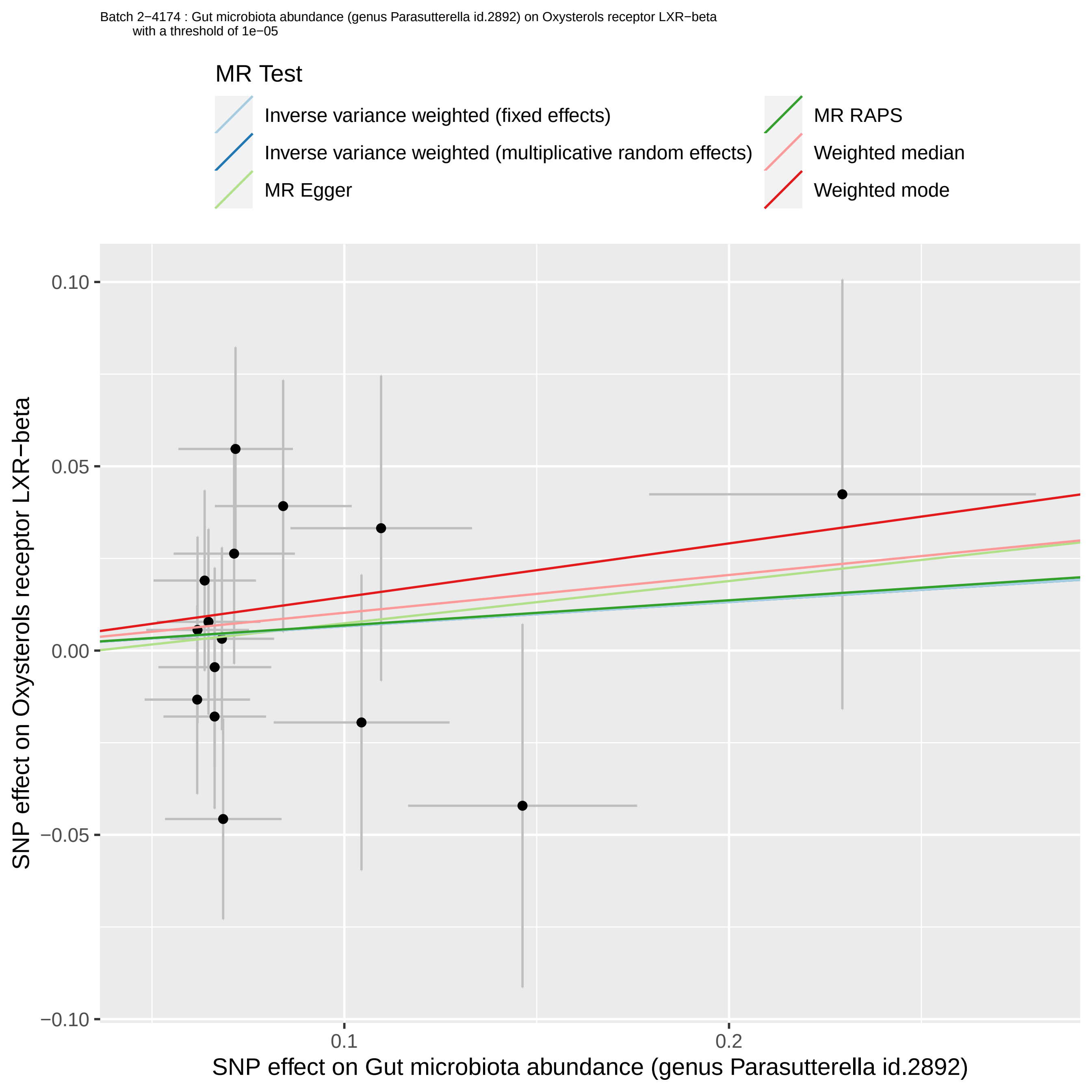
Batch 2−4174 : Gut microbiota abundance (genus Parasutterella id.2892) on Oxysterols receptor LXR−beta
with a threshold of 1e−05
MR Test
Inverse variance weighted (fixed effects)
Inverse variance weighted (multiplicative random effects)
MR Egger
MR RAPS
Weighted median
Weighted mode
0.10
0.05
0.00
−0.05
−0.10
0.1
0.2
SNP effect on Oxysterols receptor LXR−beta
SNP effect on Gut microbiota abundance (genus Parasutterella id.2892)

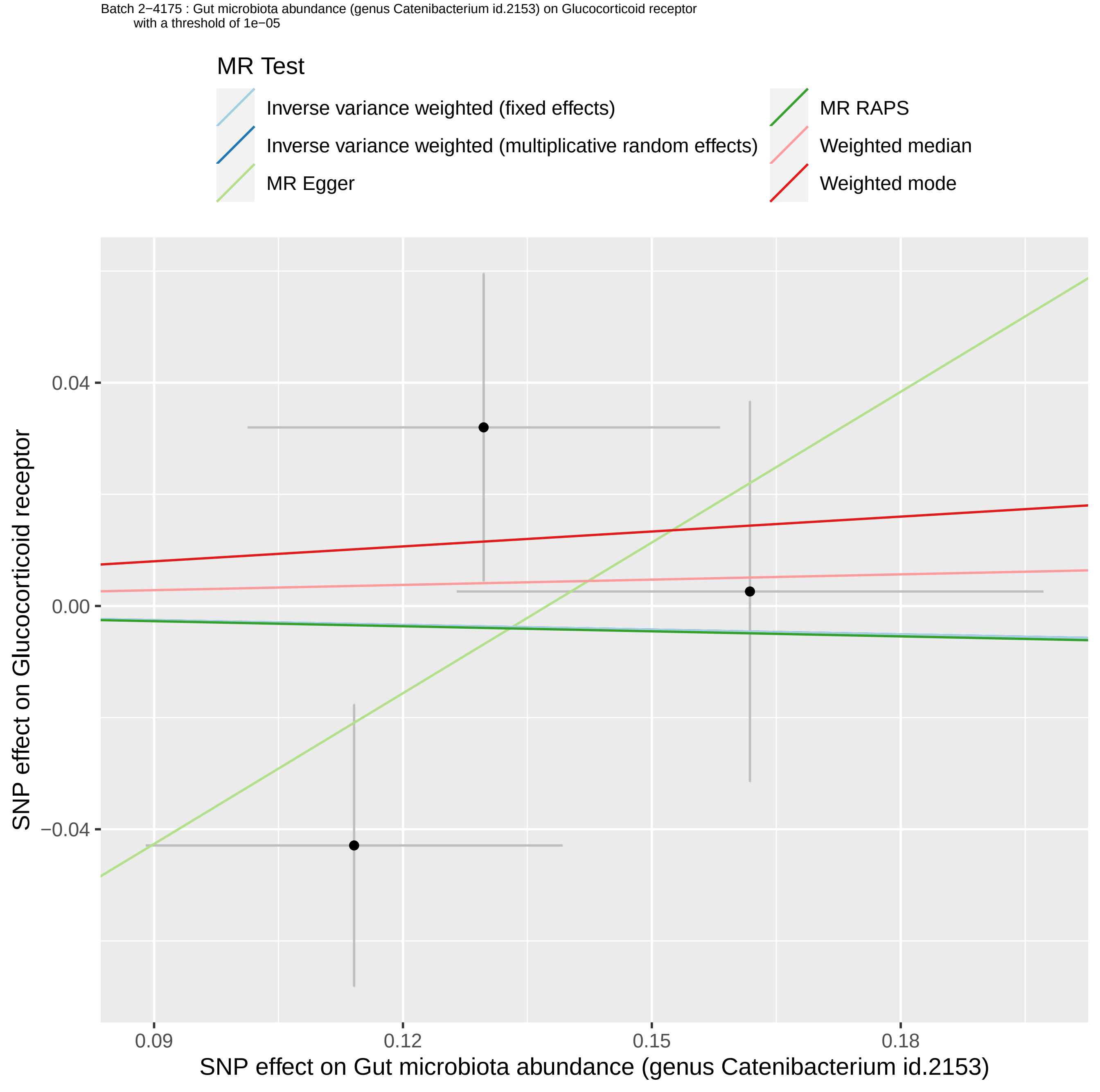

Batch 2−4175 : Gut microbiota abundance (genus Catenibacterium id.2153) on Glucocorticoid receptor
with a threshold of 1e−05
MR Test
Inverse variance weighted (fixed effects)
Inverse variance weighted (multiplicative random effects)
MR Egger
MR RAPS
Weighted median
Weighted mode
0.04
0.00
−0.04
0.09
0.12
0.15
0.18
SNP effect on Glucocorticoid receptor
SNP effect on Gut microbiota abundance (genus Catenibacterium id.2153)

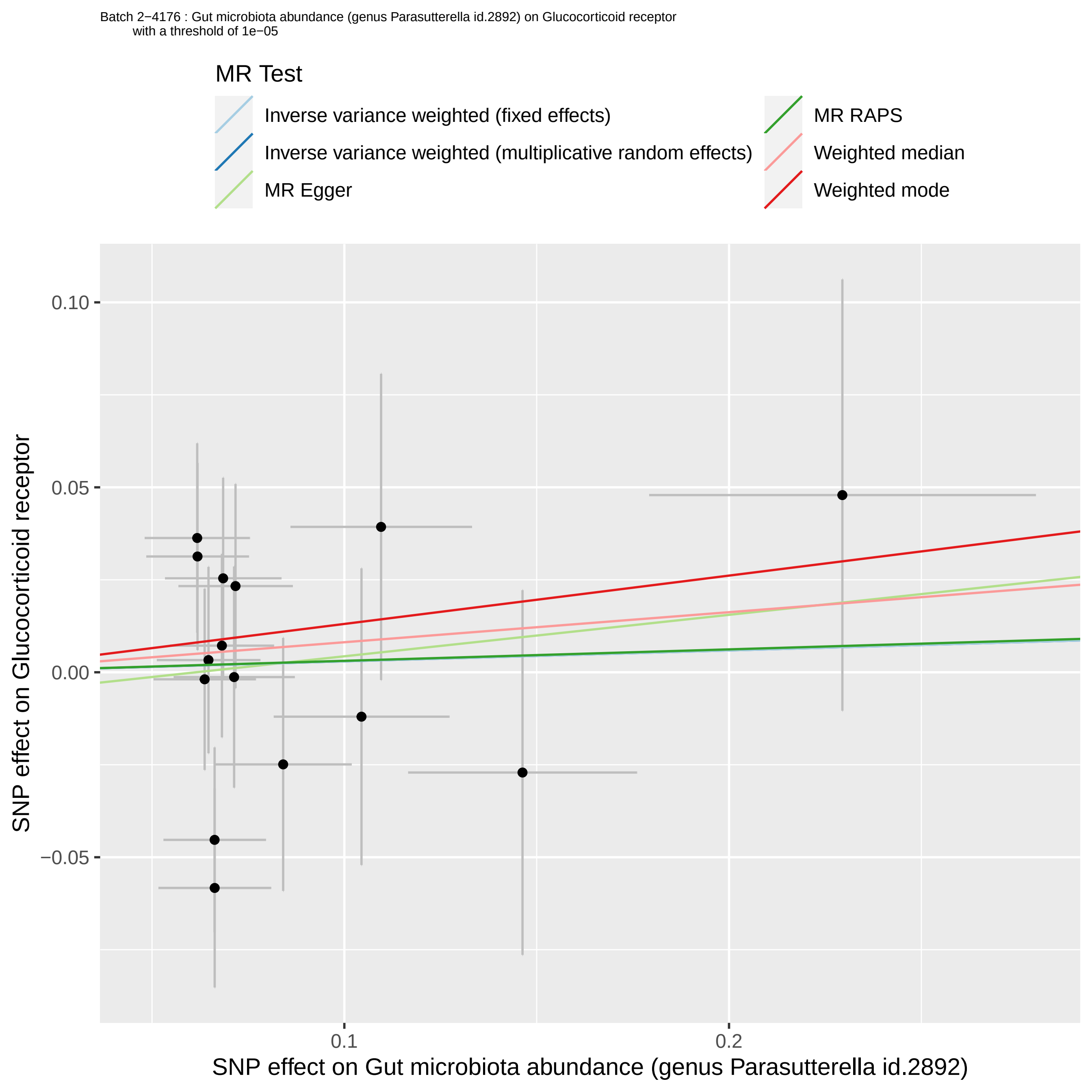

Batch 2−4176 : Gut microbiota abundance (genus Parasutterella id.2892) on Glucocorticoid receptor
with a threshold of 1e−05
MR Test
Inverse variance weighted (fixed effects)
Inverse variance weighted (multiplicative random effects)
MR Egger
MR RAPS
Weighted median
Weighted mode
0.10
0.05
0.00
−0.05
0.1
0.2
SNP effect on Glucocorticoid receptor
SNP effect on Gut microbiota abundance (genus Parasutterella id.2892)

Batch 2−4177 : Gut microbiota abundance (genus Catenibacterium id.2153) on Mineralocorticoid receptor
with a threshold of 1e−05

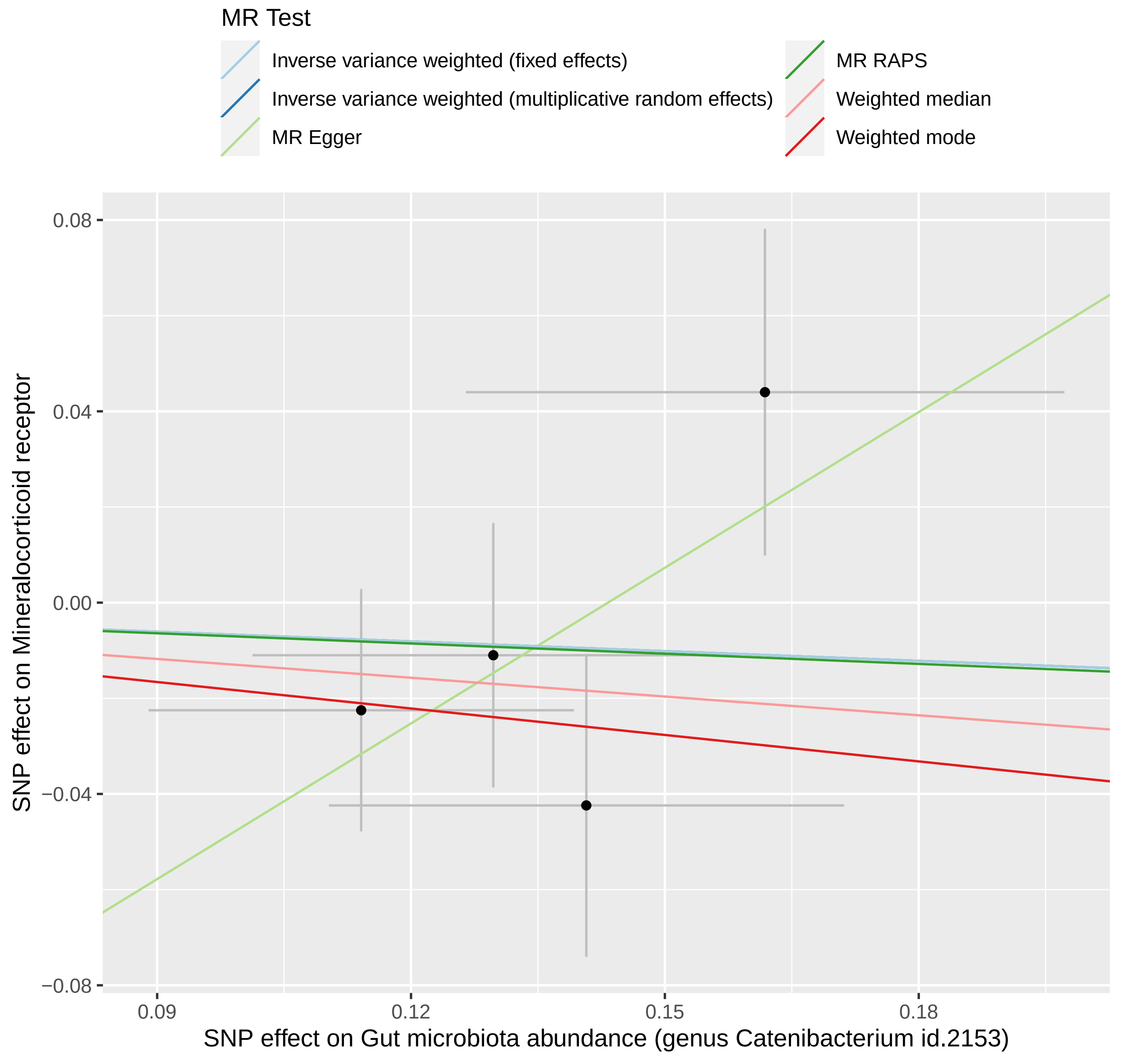

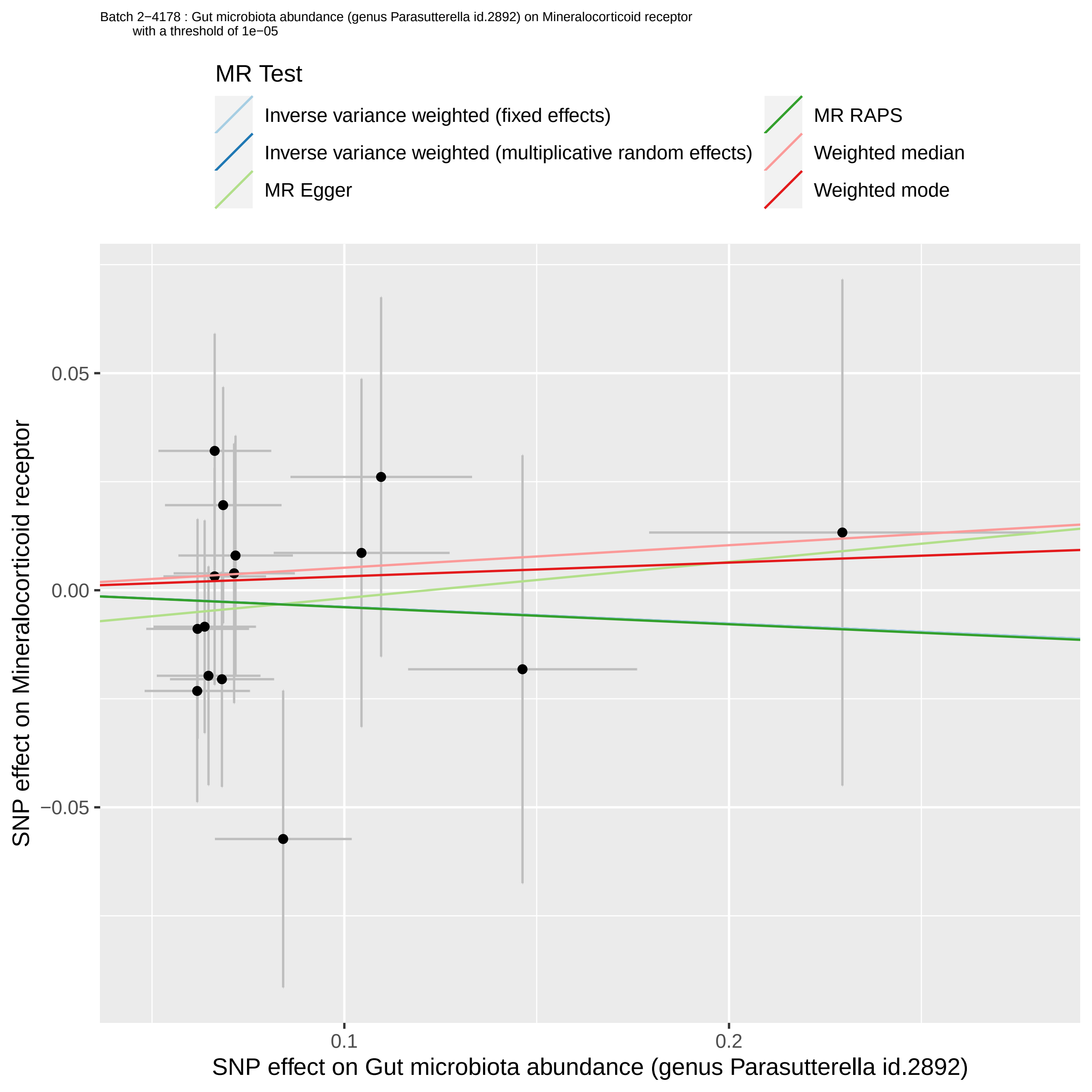
Batch 2−4178 : Gut microbiota abundance (genus Parasutterella id.2892) on Mineralocorticoid receptor
with a threshold of 1e−05
MR Test
Inverse variance weighted (fixed effects)
Inverse variance weighted (multiplicative random effects)
MR Egger
MR RAPS
Weighted median
Weighted mode
0.05
0.00
−0.05
0.1
0.2
SNP effect on Mineralocorticoid receptor
SNP effect on Gut microbiota abundance (genus Parasutterella id.2892)

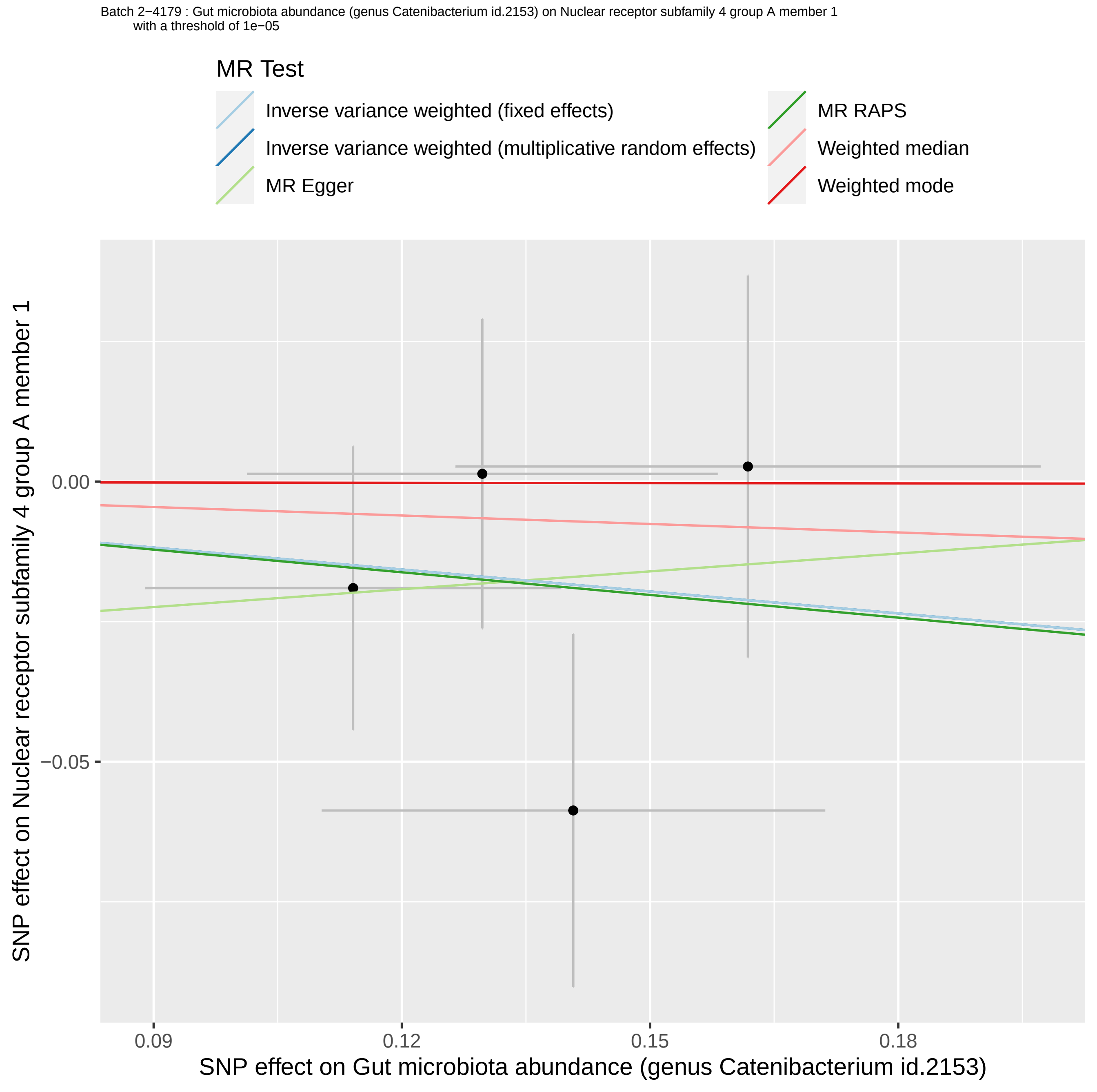
Batch 2−4179 : Gut microbiota abundance (genus Catenibacterium id.2153) on Nuclear receptor subfamily 4 group A member 1
with a threshold of 1e−05
MR Test
Inverse variance weighted (fixed effects)
Inverse variance weighted (multiplicative random effects)
MR Egger
MR RAPS
Weighted median
Weighted mode
0.00
−0.05
0.09
0.12
0.15
0.18
SNP effect on Nuclear receptor subfamily 4 group A member 1
SNP effect on Gut microbiota abundance (genus Catenibacterium id.2153)

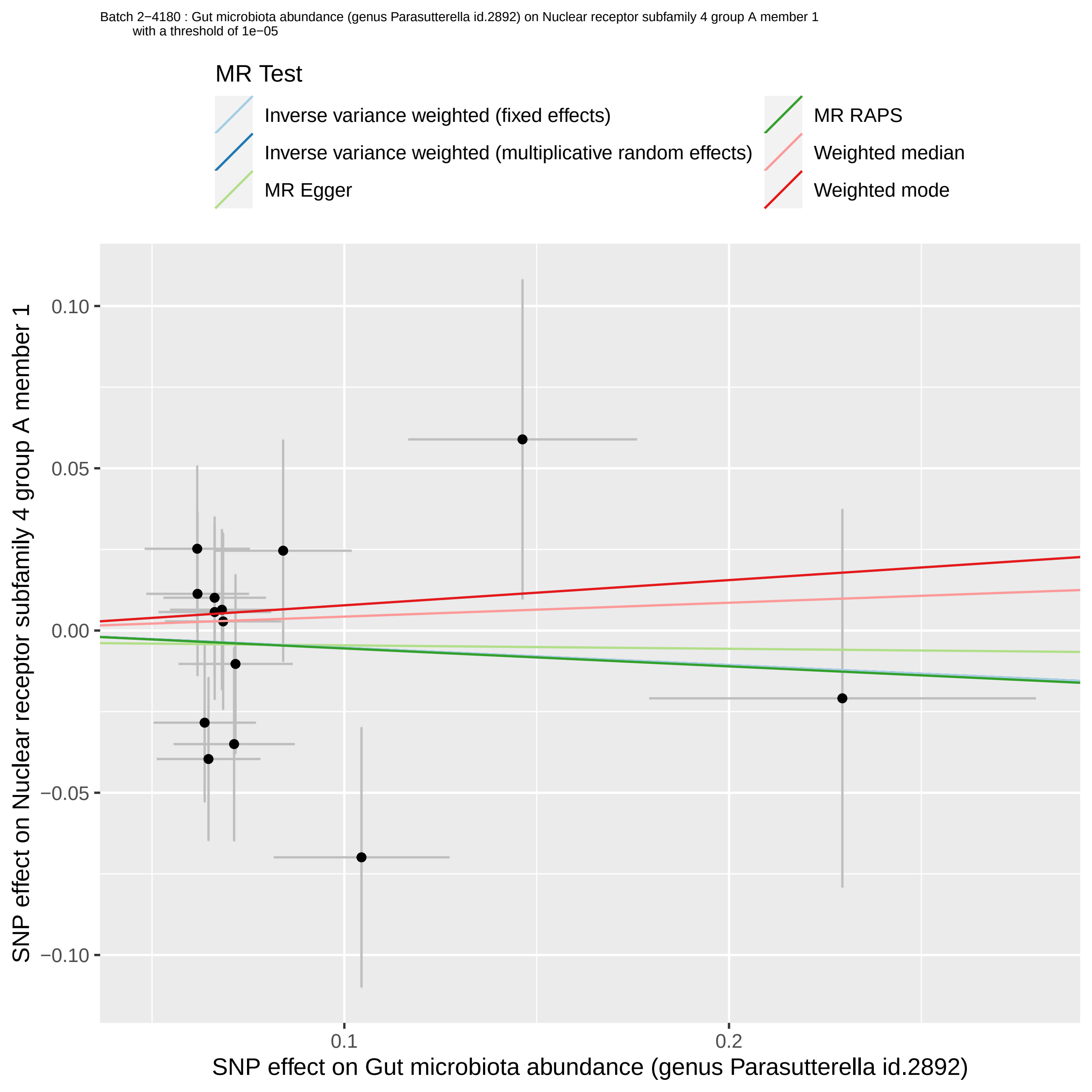
Batch 2−4180 : Gut microbiota abundance (genus Parasutterella id.2892) on Nuclear receptor subfamily 4 group A member 1
with a threshold of 1e−05
MR Test
Inverse variance weighted (fixed effects)
Inverse variance weighted (multiplicative random effects)
MR Egger
MR RAPS
Weighted median
Weighted mode
0.10
0.05
0.00
−0.05
−0.10
0.1
0.2
SNP effect on Nuclear receptor subfamily 4 group A member 1
SNP effect on Gut microbiota abundance (genus Parasutterella id.2892)

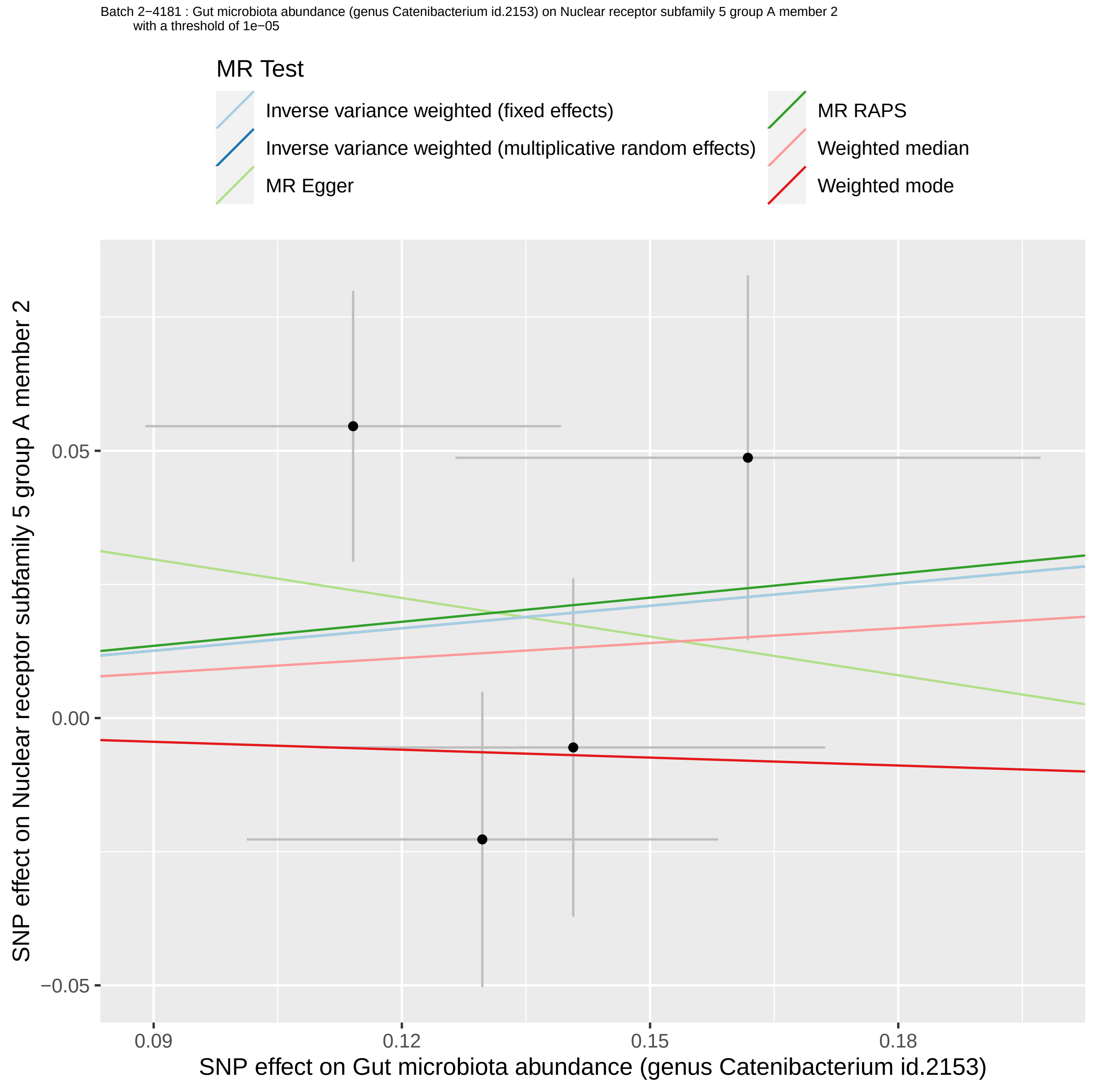
Batch 2−4181 : Gut microbiota abundance (genus Catenibacterium id.2153) on Nuclear receptor subfamily 5 group A member 2
with a threshold of 1e−05
MR Test
Inverse variance weighted (fixed effects)
Inverse variance weighted (multiplicative random effects)
MR Egger
MR RAPS
Weighted median
Weighted mode
0.05
0.00
−0.05
0.09
0.12
0.15
0.18
SNP effect on Nuclear receptor subfamily 5 group A member 2
SNP effect on Gut microbiota abundance (genus Catenibacterium id.2153)

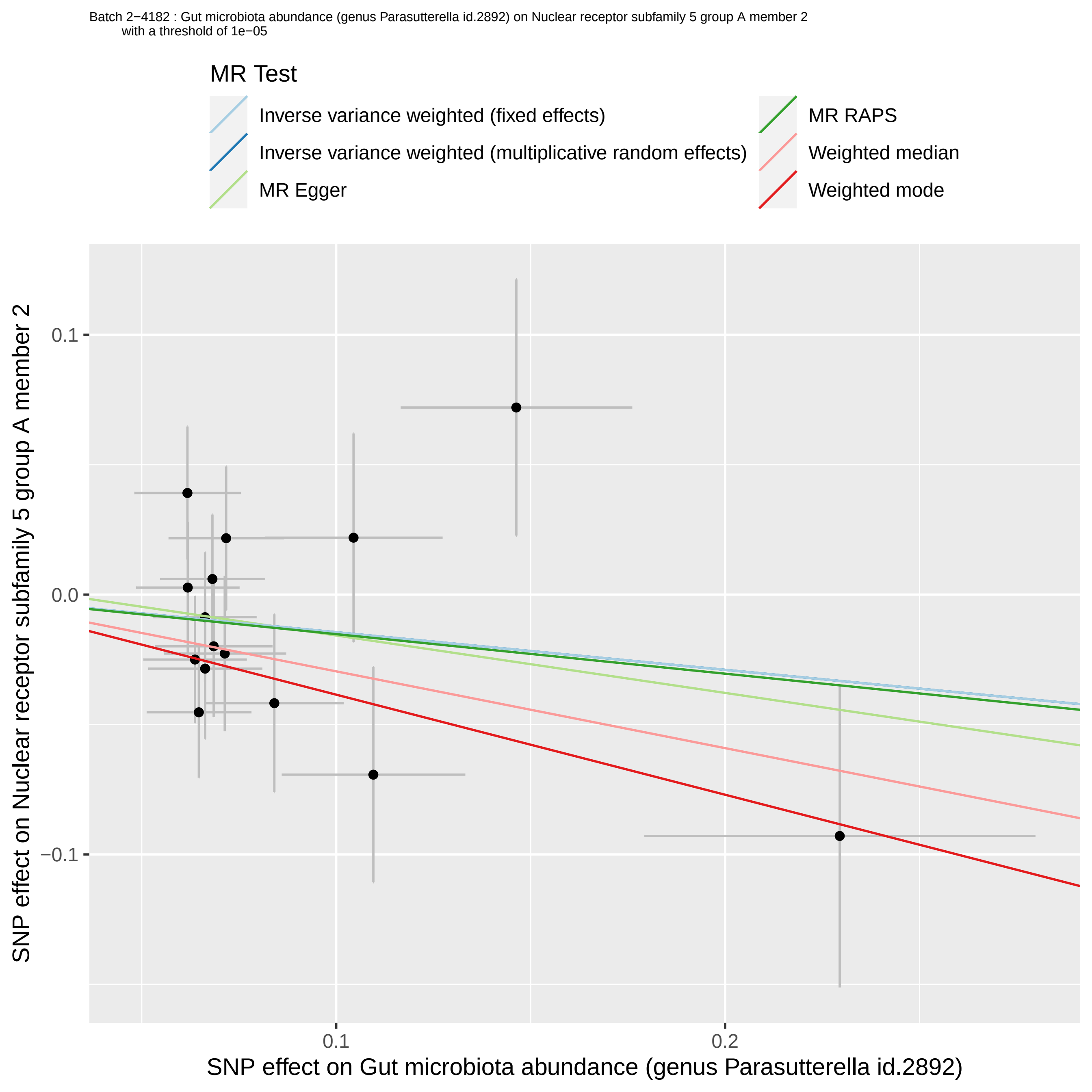

Batch 2−4182 : Gut microbiota abundance (genus Parasutterella id.2892) on Nuclear receptor subfamily 5 group A member 2
with a threshold of 1e−05
MR Test
Inverse variance weighted (fixed effects)
Inverse variance weighted (multiplicative random effects)
MR Egger
MR RAPS
Weighted median
Weighted mode
0.1
0.0
−0.1
0.1
0.2
SNP effect on Nuclear receptor subfamily 5 group A member 2
SNP effect on Gut microbiota abundance (genus Parasutterella id.2892)

Batch 2−4183 : Gut microbiota abundance (genus Catenibacterium id.2153) on Nutritionally−regulated adipose and cardiac enriched protein homolog
with a threshold of 1e−05

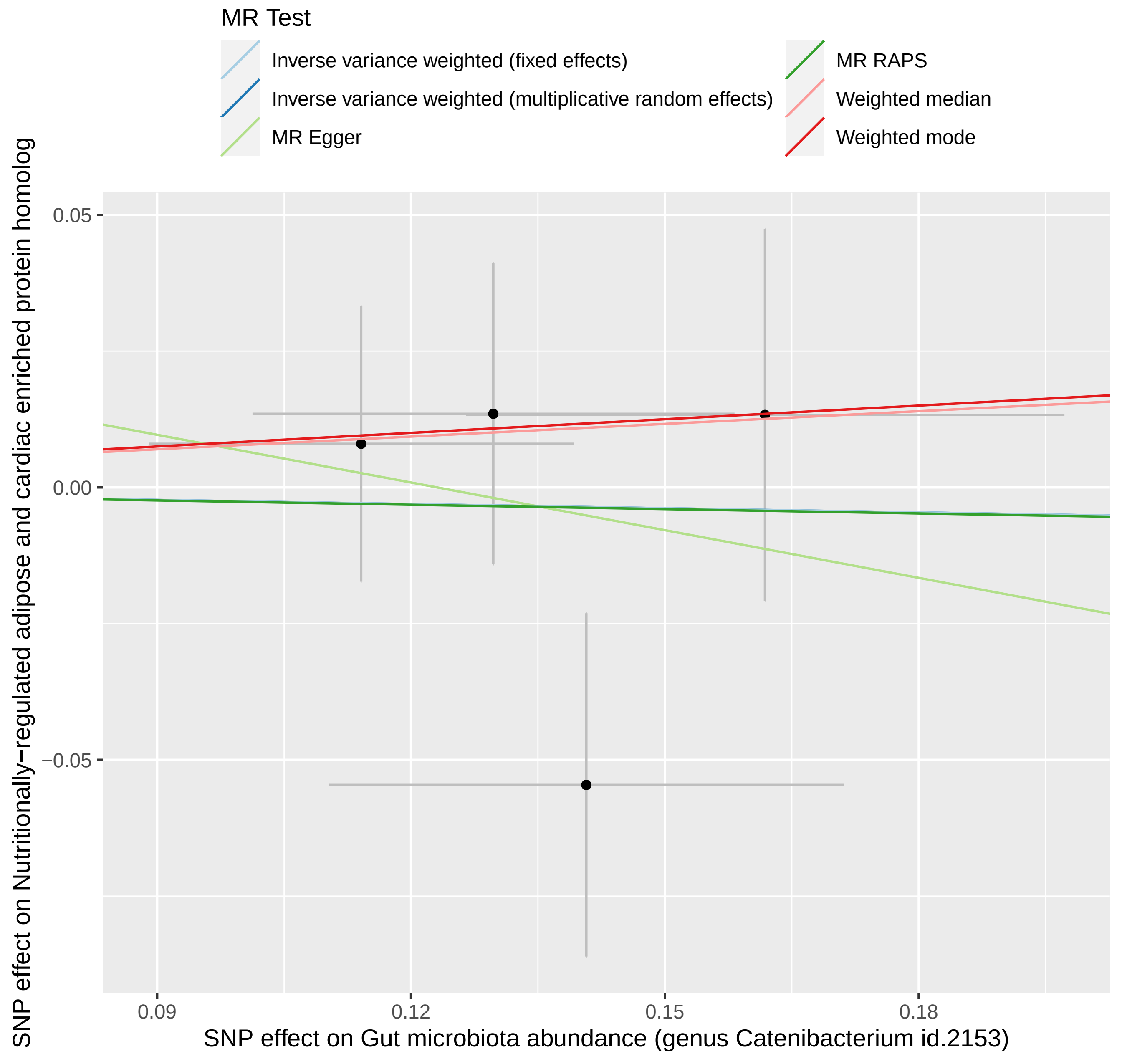

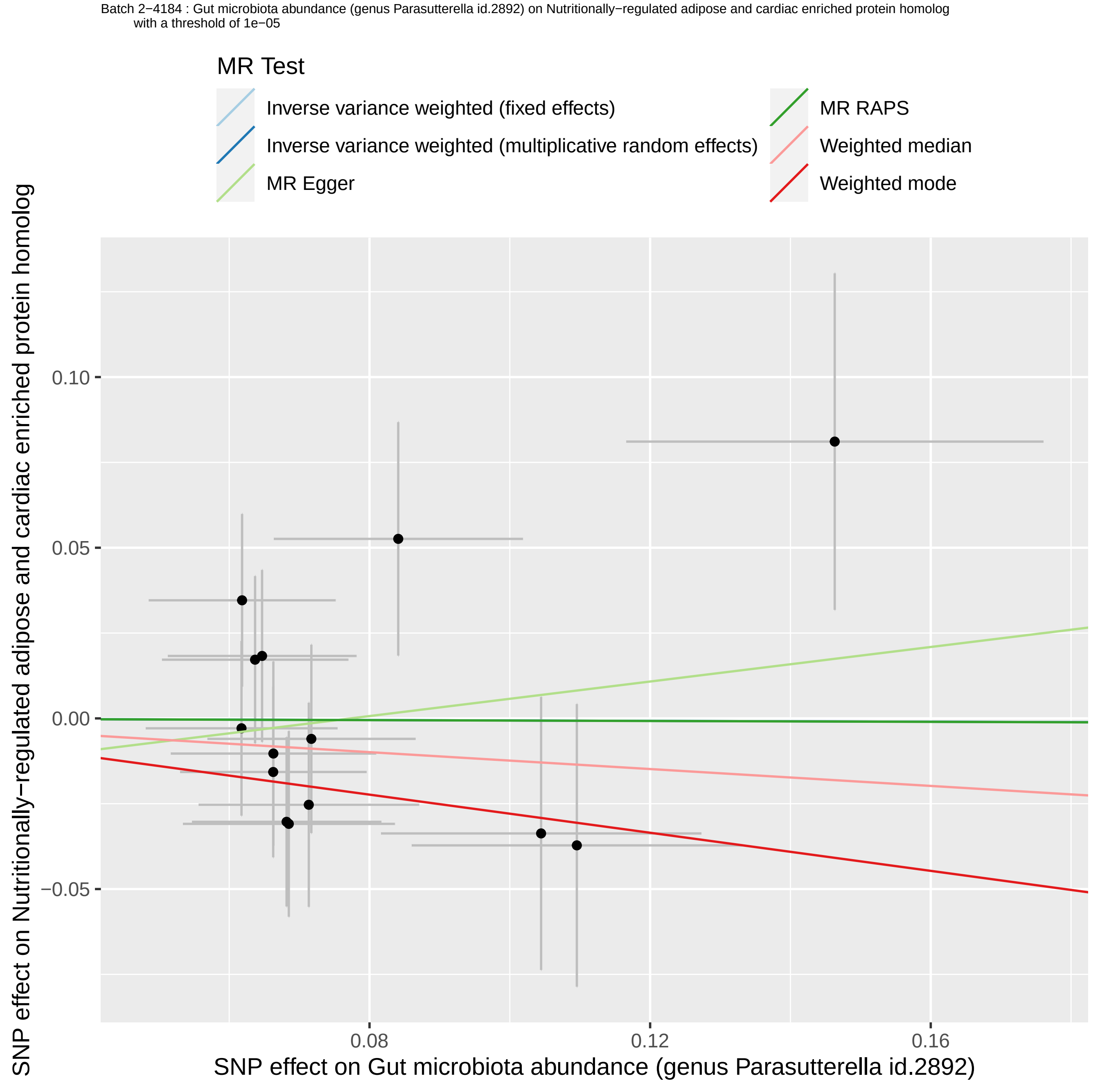
Batch 2−4184 : Gut microbiota abundance (genus Parasutterella id.2892) on Nutritionally−regulated adipose and cardiac enriched protein homolog
with a threshold of 1e−05
MR Test
Inverse variance weighted (fixed effects)
Inverse variance weighted (multiplicative random effects)
MR Egger
MR RAPS
Weighted median
Weighted mode
0.10
0.05
0.00
−0.05
0.08
0.12
0.16
SNP effect on Gut microbiota abundance (genus Parasutterella id.2892)
SNP effect on Nutritionally−regulated adipose and cardiac enriched protein homolog

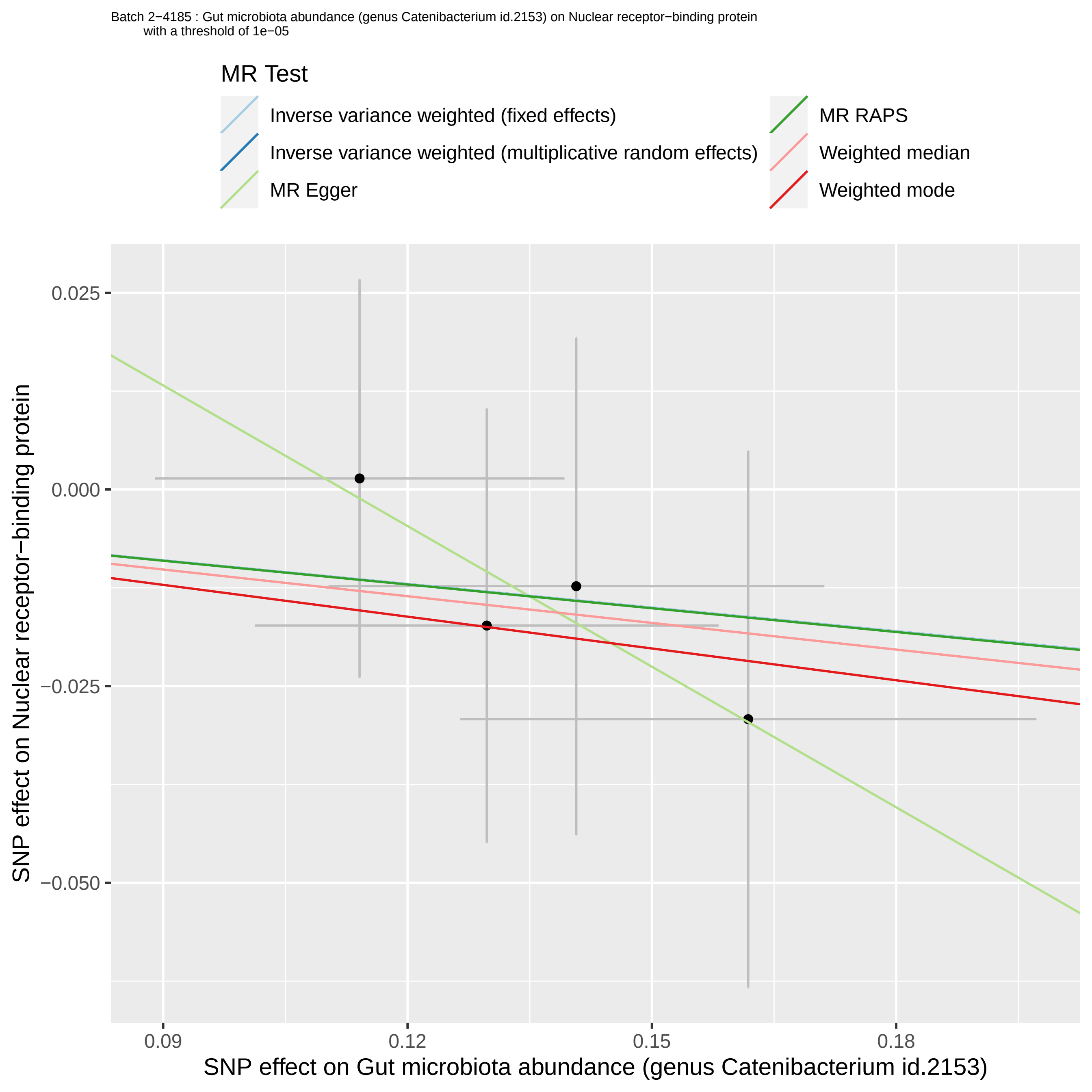
Batch 2−4185 : Gut microbiota abundance (genus Catenibacterium id.2153) on Nuclear receptor−binding protein
with a threshold of 1e−05
MR Test
Inverse variance weighted (fixed effects)
Inverse variance weighted (multiplicative random effects)
MR Egger
MR RAPS
Weighted median
Weighted mode
0.025
0.000
−0.025
−0.050
0.09
0.12
0.15
0.18
SNP effect on Nuclear receptor−binding protein
SNP effect on Gut microbiota abundance (genus Catenibacterium id.2153)

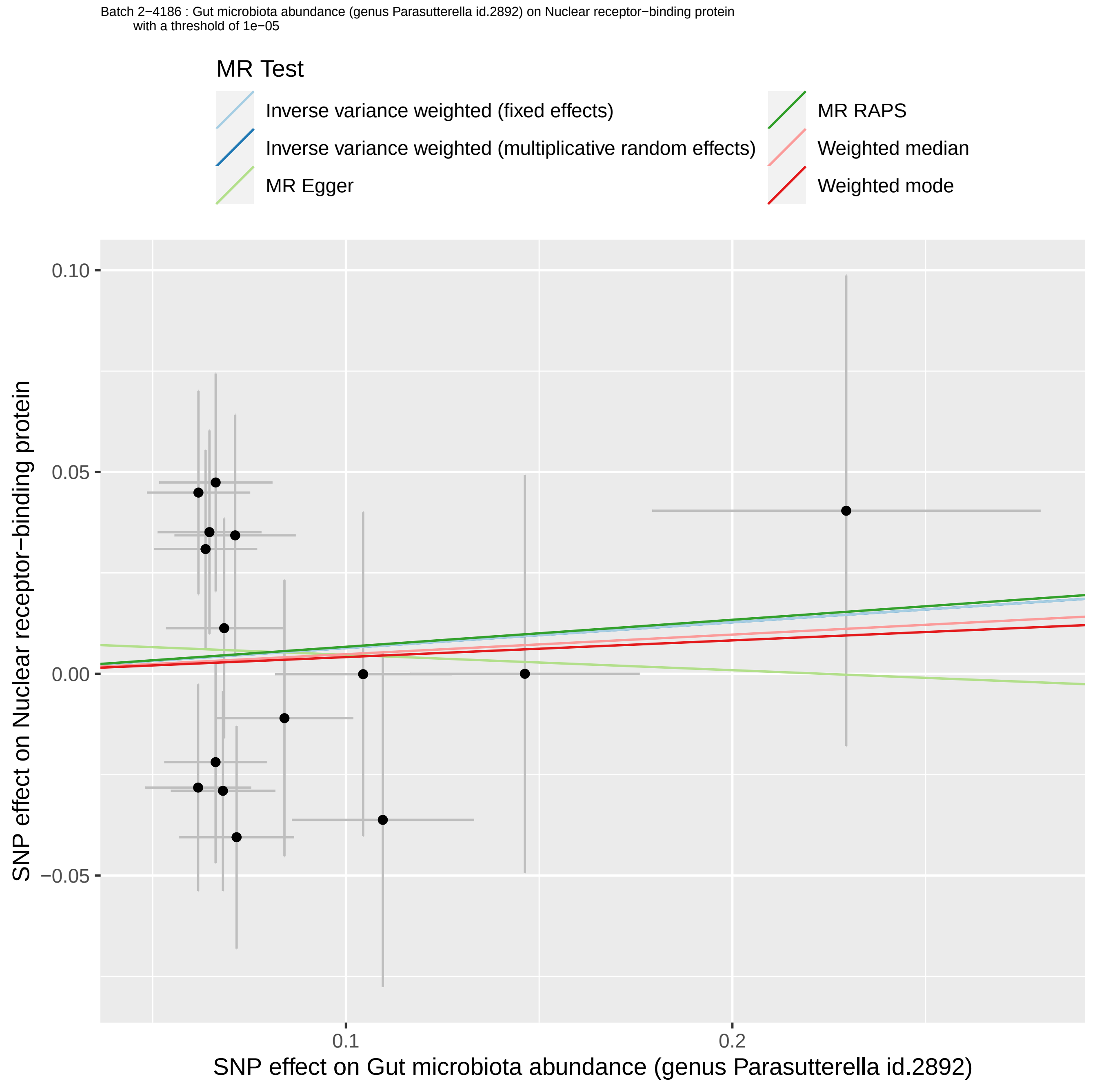

Batch 2−4186 : Gut microbiota abundance (genus Parasutterella id.2892) on Nuclear receptor−binding protein
with a threshold of 1e−05
MR Test
Inverse variance weighted (fixed effects)
Inverse variance weighted (multiplicative random effects)
MR Egger
MR RAPS
Weighted median
Weighted mode
0.10
0.05
0.00
−0.05
0.1
0.2
SNP effect on Nuclear receptor−binding protein
SNP effect on Gut microbiota abundance (genus Parasutterella id.2892)

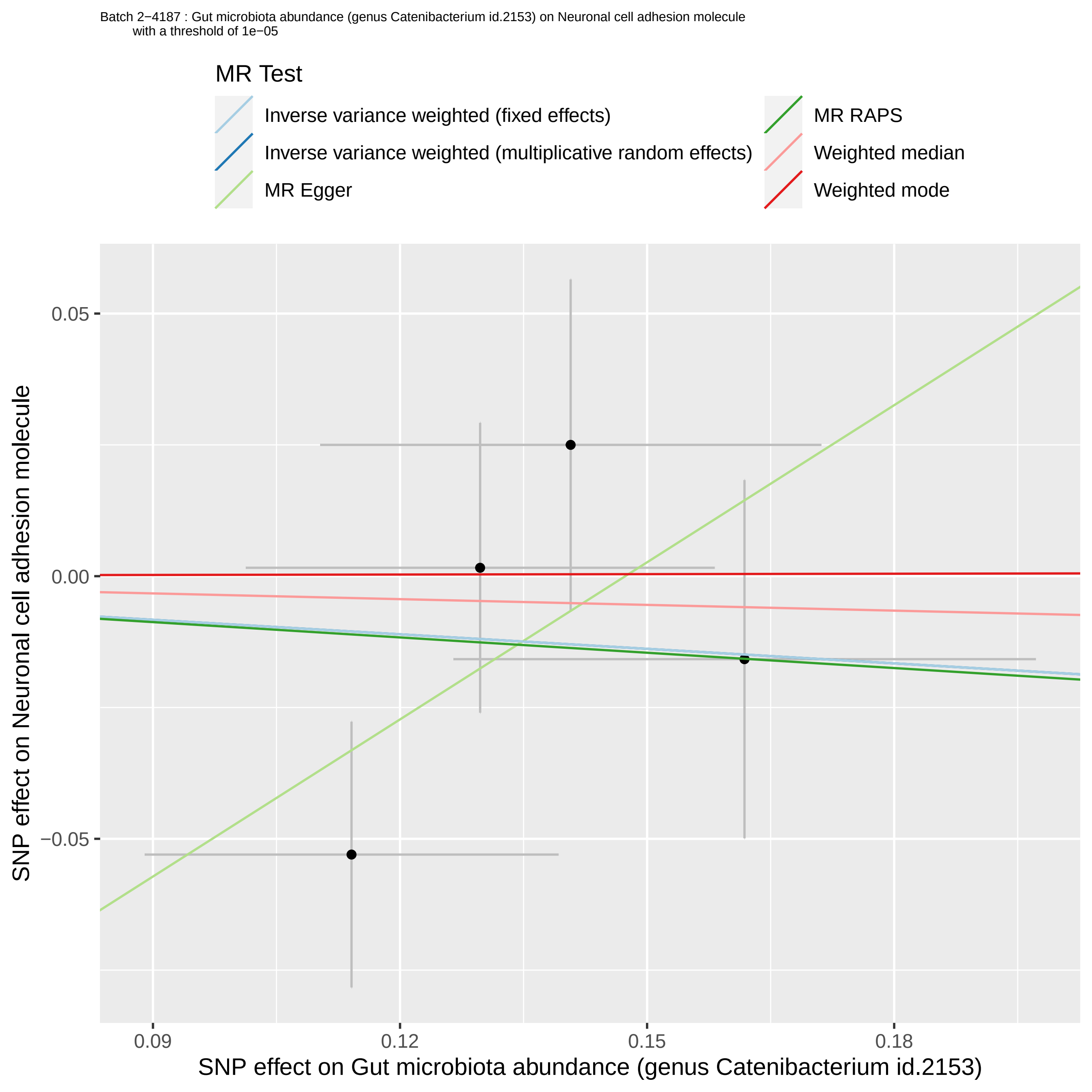

Batch 2−4187 : Gut microbiota abundance (genus Catenibacterium id.2153) on Neuronal cell adhesion molecule
with a threshold of 1e−05
MR Test
Inverse variance weighted (fixed effects)
Inverse variance weighted (multiplicative random effects)
MR Egger
MR RAPS
Weighted median
Weighted mode
0.05
0.00
−0.05
0.09
0.12
0.15
0.18
SNP effect on Neuronal cell adhesion molecule
SNP effect on Gut microbiota abundance (genus Catenibacterium id.2153)

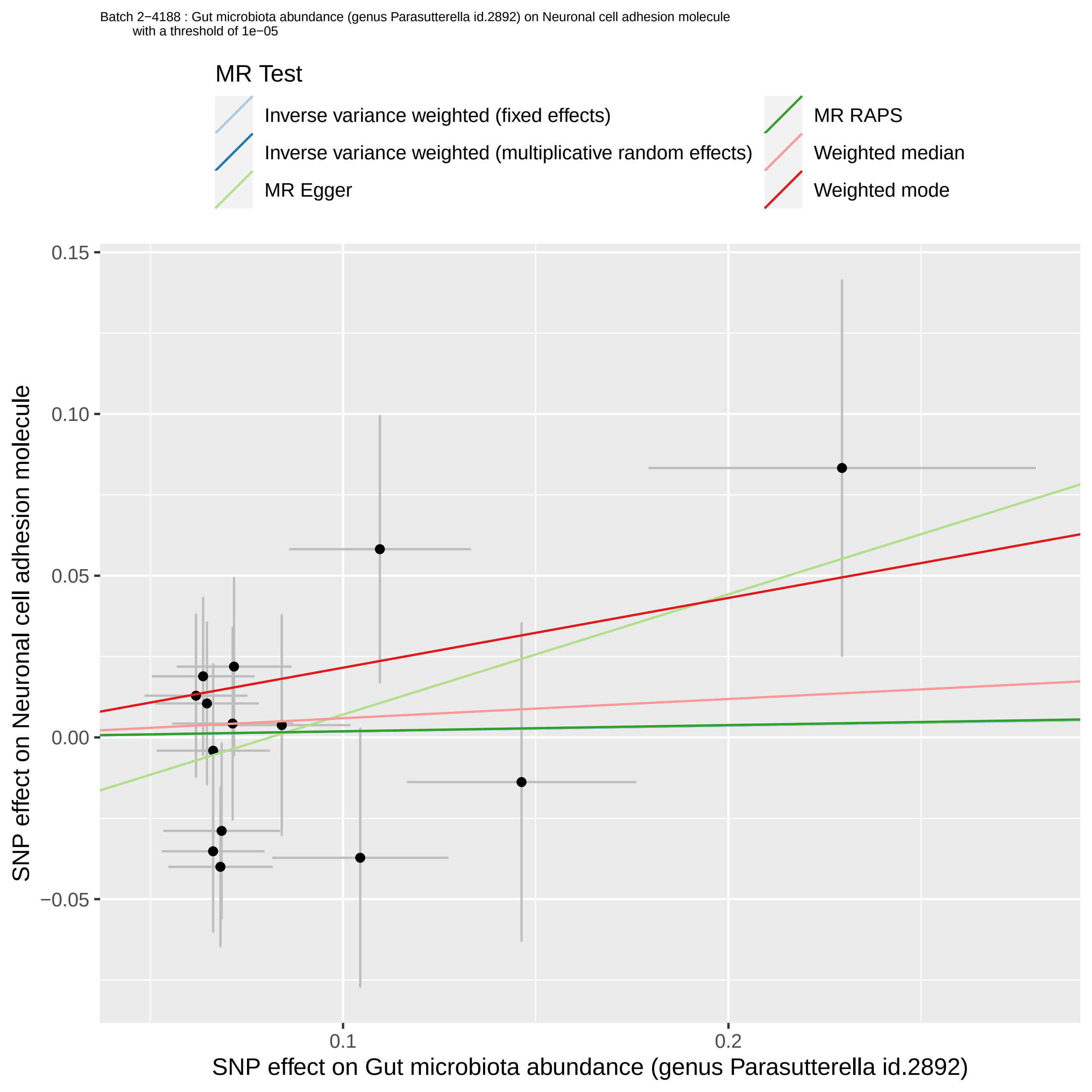
Batch 2−4188 : Gut microbiota abundance (genus Parasutterella id.2892) on Neuronal cell adhesion molecule
with a threshold of 1e−05
MR Test
Inverse variance weighted (fixed effects)
Inverse variance weighted (multiplicative random effects)
MR Egger
MR RAPS
Weighted median
Weighted mode
0.15
0.10
0.05
0.00
−0.05
0.1
0.2
SNP effect on Neuronal cell adhesion molecule
SNP effect on Gut microbiota abundance (genus Parasutterella id.2892)

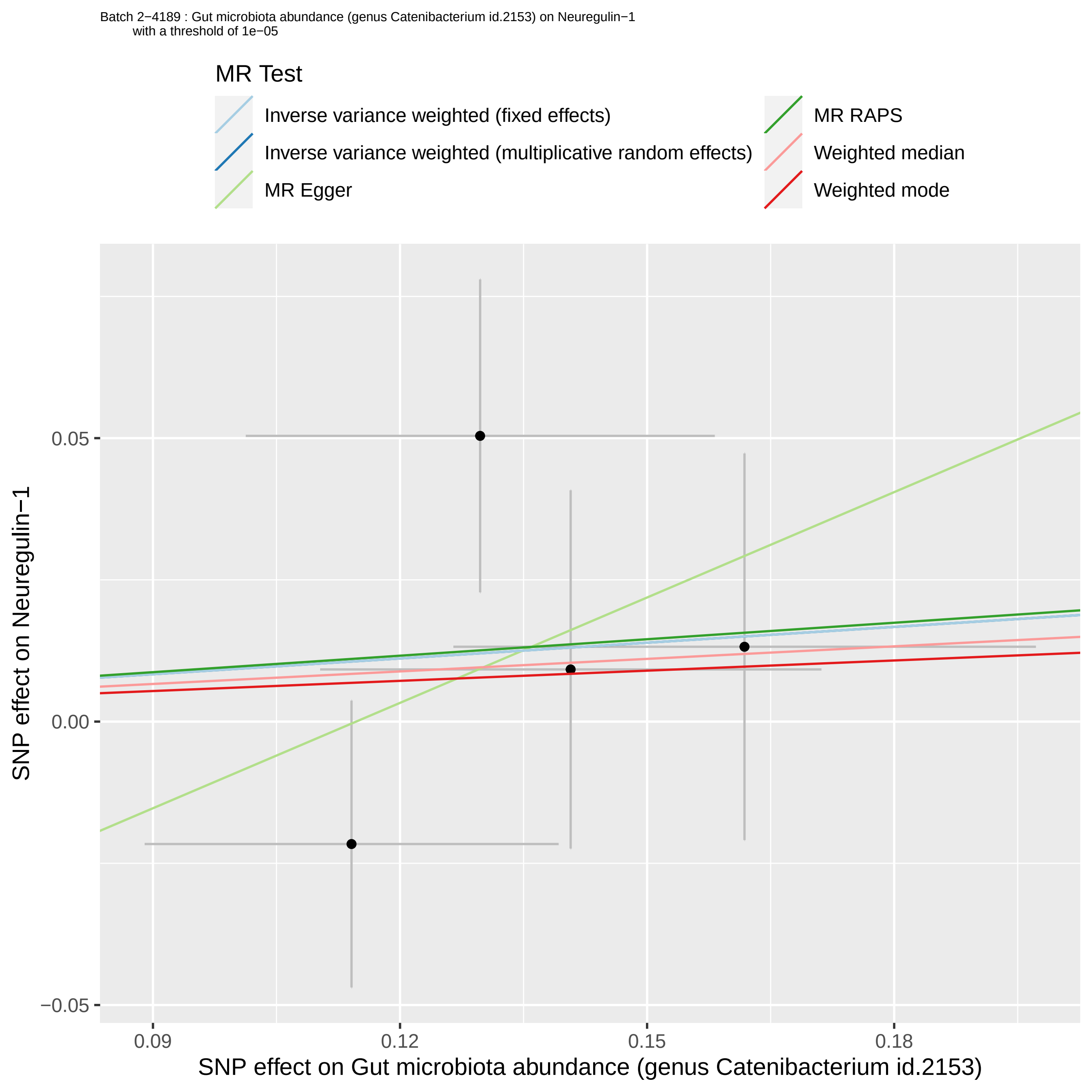

Batch 2−4189 : Gut microbiota abundance (genus Catenibacterium id.2153) on Neuregulin−1
with a threshold of 1e−05
MR Test
Inverse variance weighted (fixed effects)
Inverse variance weighted (multiplicative random effects)
MR Egger
MR RAPS
Weighted median
Weighted mode
0.05
0.00
−0.05
0.09
0.12
0.15
0.18
SNP effect on Neuregulin−1
SNP effect on Gut microbiota abundance (genus Catenibacterium id.2153)

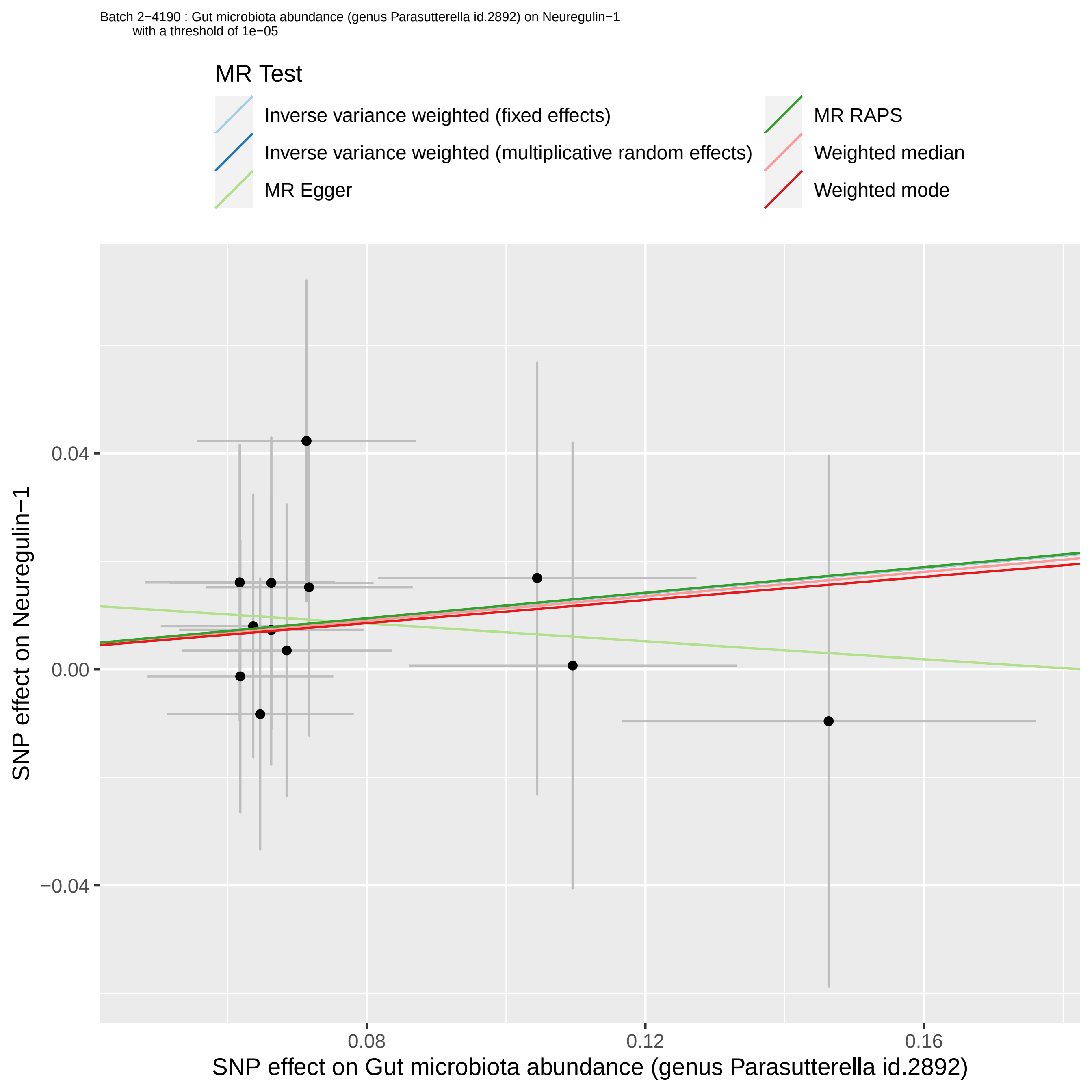

Batch 2−4190 : Gut microbiota abundance (genus Parasutterella id.2892) on Neuregulin−1
with a threshold of 1e−05
MR Test
Inverse variance weighted (fixed effects)
Inverse variance weighted (multiplicative random effects)
MR Egger
MR RAPS
Weighted median
Weighted mode
0.04
0.00
−0.04
0.08
0.12
0.16
SNP effect on Neuregulin−1
SNP effect on Gut microbiota abundance (genus Parasutterella id.2892)

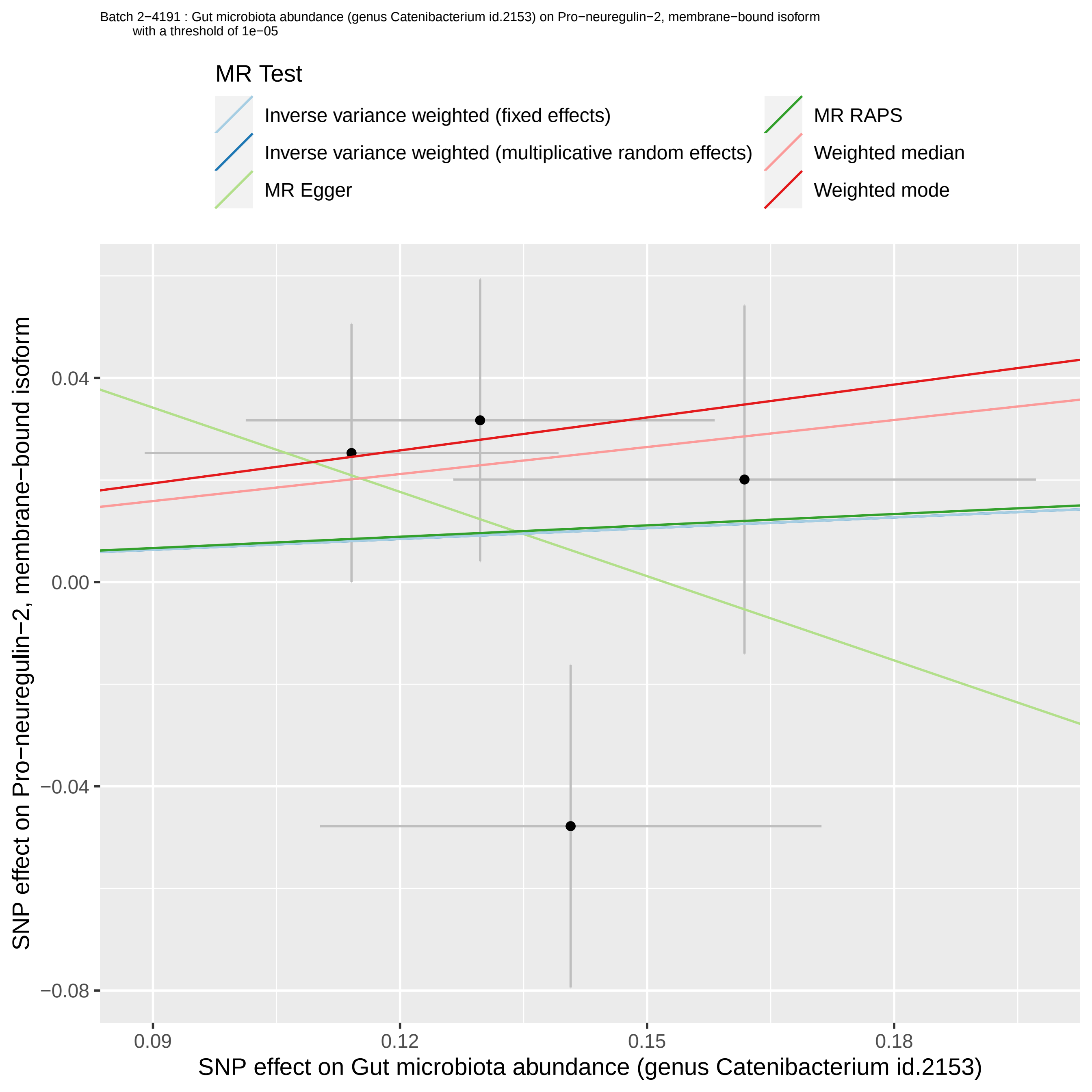
Batch 2−4191 : Gut microbiota abundance (genus Catenibacterium id.2153) on Pro−neuregulin−2, membrane−bound isoform
with a threshold of 1e−05
MR Test
Inverse variance weighted (fixed effects)
Inverse variance weighted (multiplicative random effects)
MR Egger
MR RAPS
Weighted median
Weighted mode
0.04
0.00
−0.04
−0.08
0.09
0.12
0.15
0.18
SNP effect on Pro−neuregulin−2, membrane−bound isoform
SNP effect on Gut microbiota abundance (genus Catenibacterium id.2153)

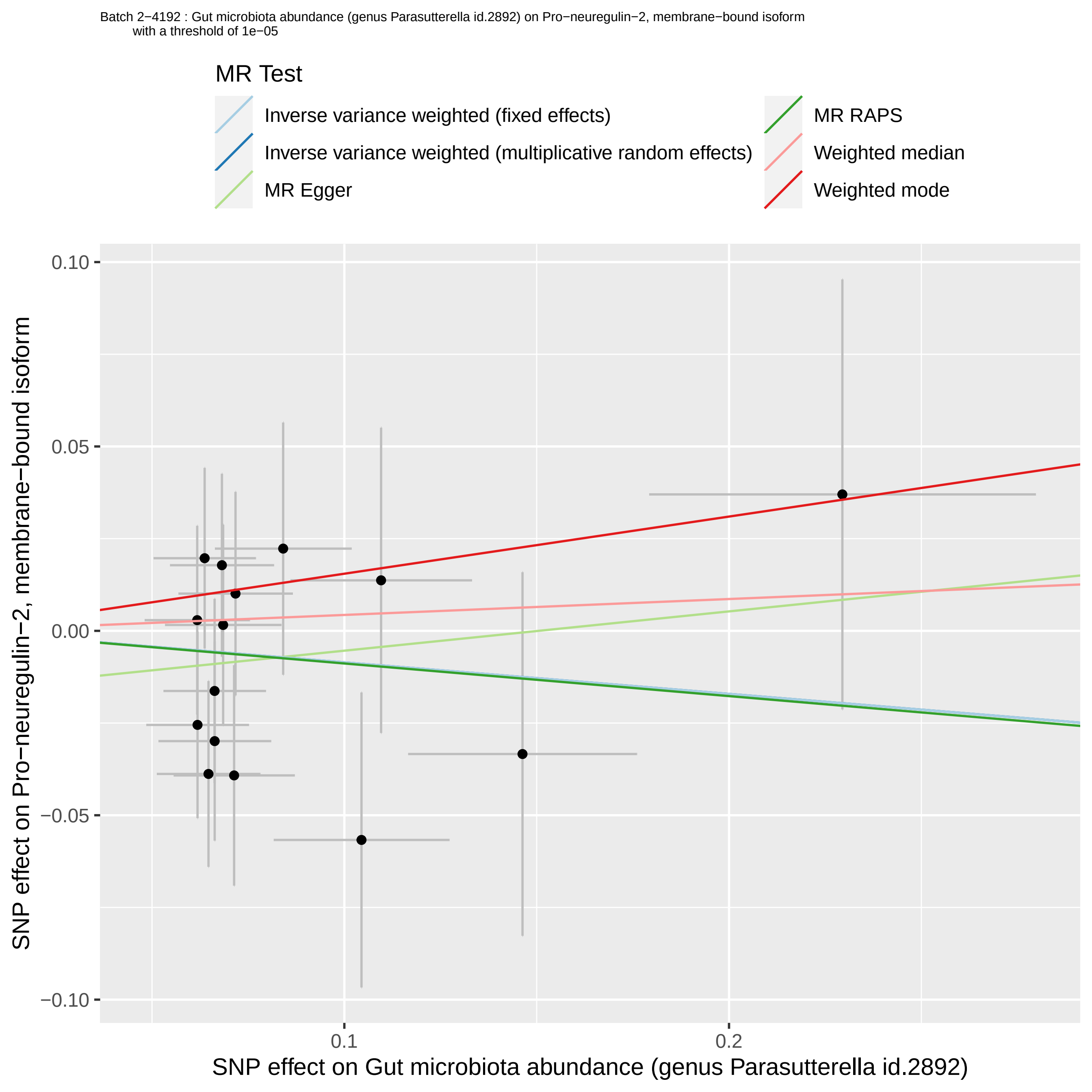

Batch 2−4192 : Gut microbiota abundance (genus Parasutterella id.2892) on Pro−neuregulin−2, membrane−bound isoform
with a threshold of 1e−05
MR Test
Inverse variance weighted (fixed effects)
Inverse variance weighted (multiplicative random effects)
MR Egger
MR RAPS
Weighted median
Weighted mode
0.10
0.05
0.00
−0.05
−0.10
0.1
0.2
SNP effect on Pro−neuregulin−2, membrane−bound isoform
SNP effect on Gut microbiota abundance (genus Parasutterella id.2892)

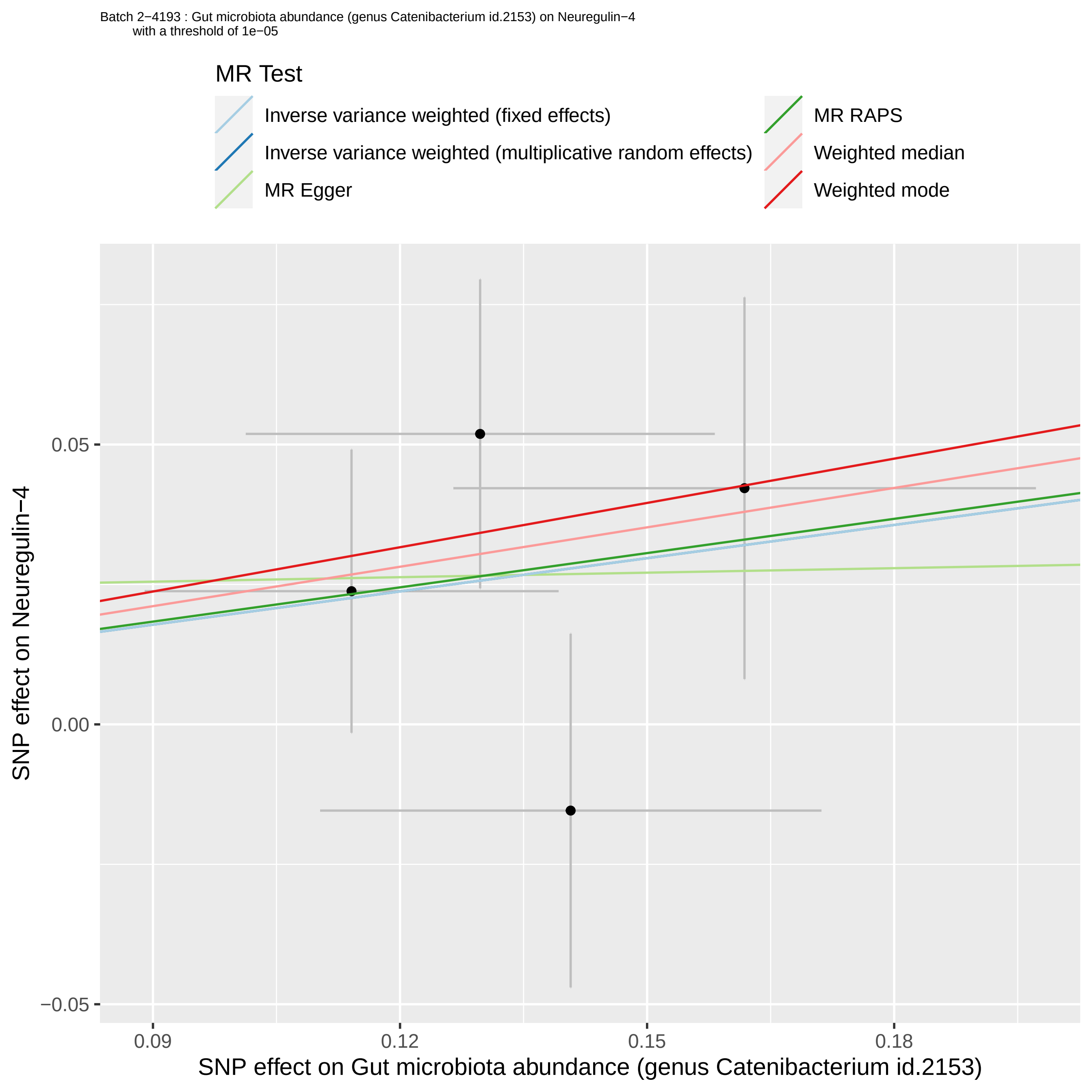

Batch 2−4193 : Gut microbiota abundance (genus Catenibacterium id.2153) on Neuregulin−4
with a threshold of 1e−05
MR Test
Inverse variance weighted (fixed effects)
Inverse variance weighted (multiplicative random effects)
MR Egger
MR RAPS
Weighted median
Weighted mode
0.05
0.00
−0.05
0.09
0.12
0.15
0.18
SNP effect on Neuregulin−4
SNP effect on Gut microbiota abundance (genus Catenibacterium id.2153)

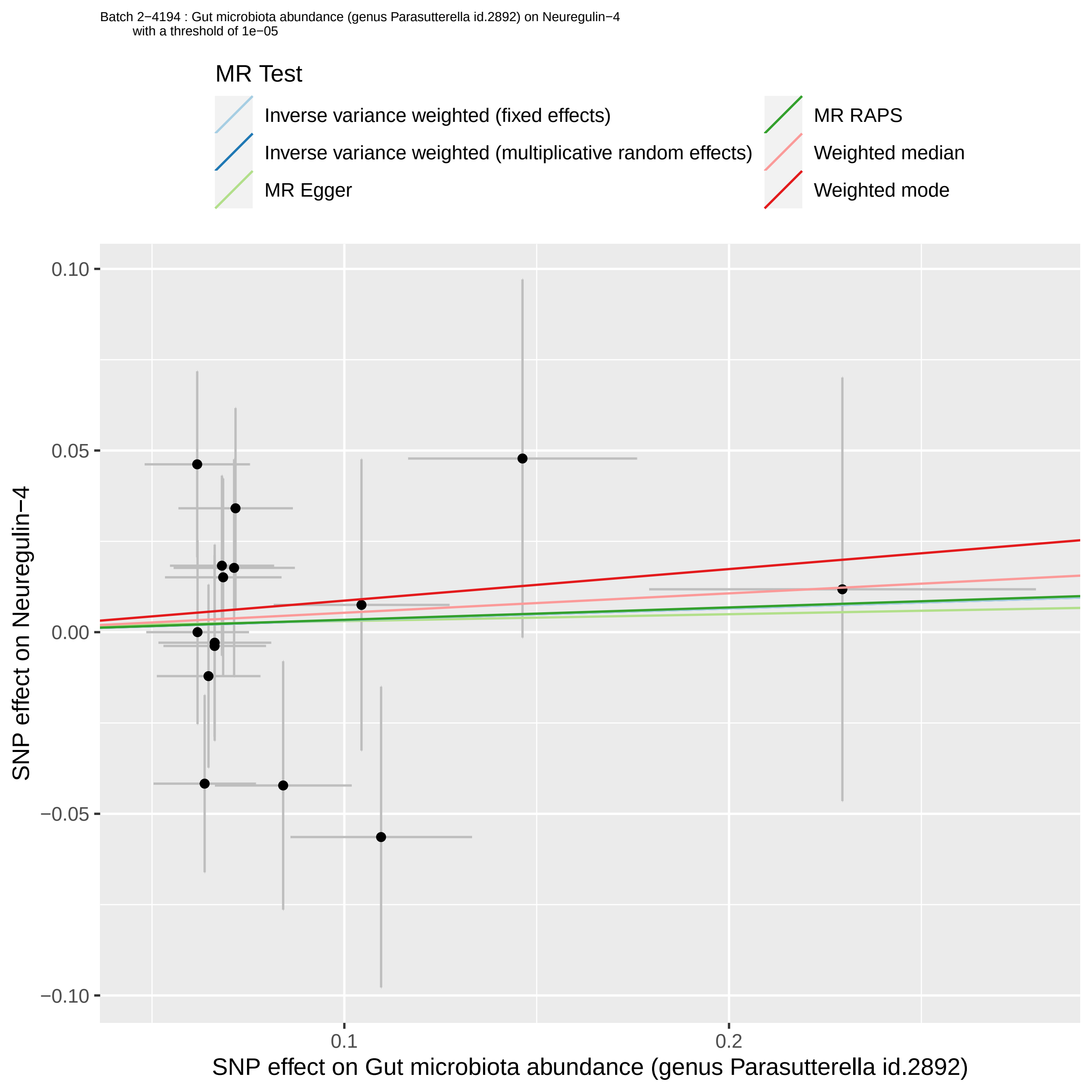

Batch 2−4194 : Gut microbiota abundance (genus Parasutterella id.2892) on Neuregulin−4
with a threshold of 1e−05
MR Test
Inverse variance weighted (fixed effects)
Inverse variance weighted (multiplicative random effects)
MR Egger
MR RAPS
Weighted median
Weighted mode
0.10
0.05
0.00
−0.05
−0.10
0.1
0.2
SNP effect on Neuregulin−4
SNP effect on Gut microbiota abundance (genus Parasutterella id.2892)

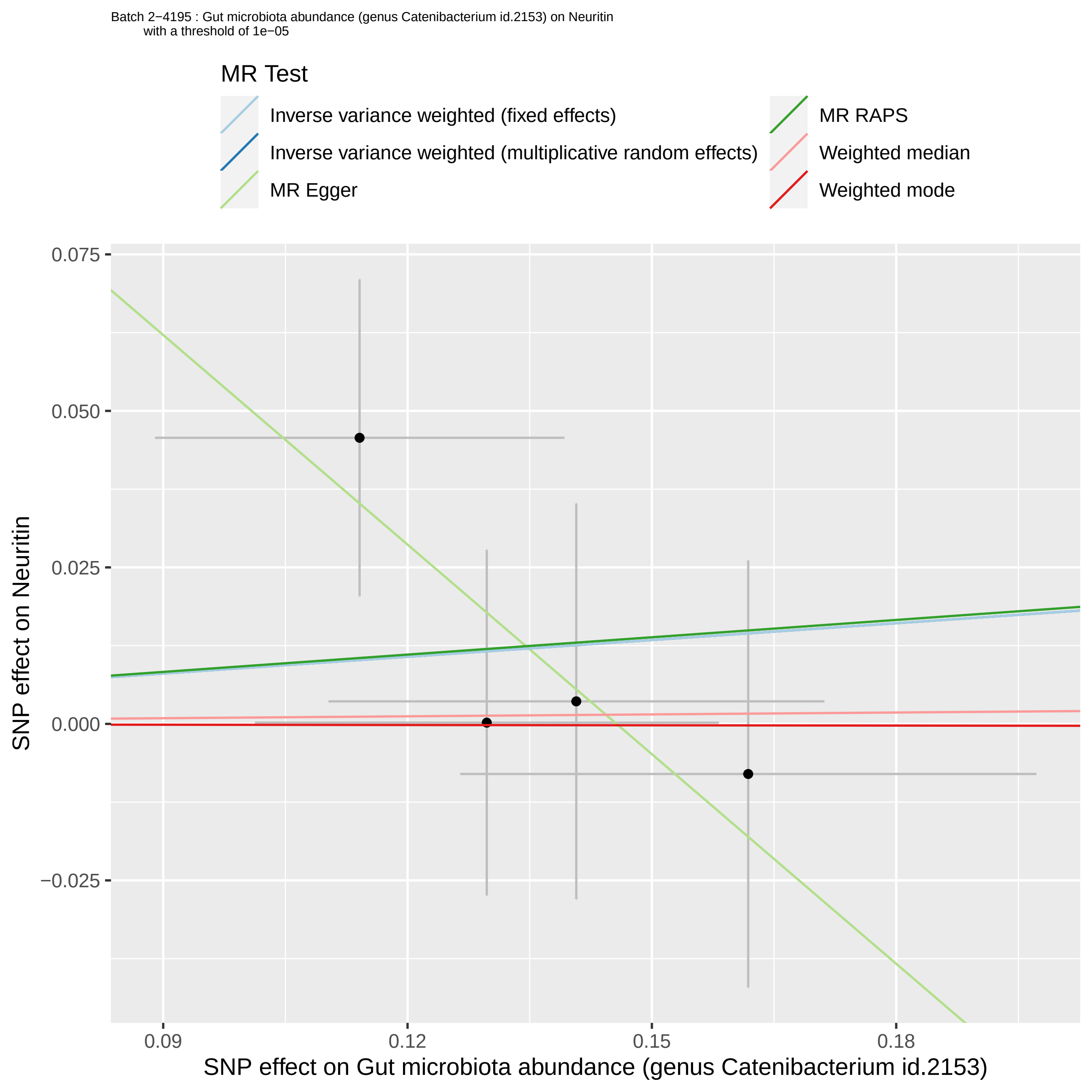
Batch 2−4195 : Gut microbiota abundance (genus Catenibacterium id.2153) on Neuritin
with a threshold of 1e−05
MR Test
Inverse variance weighted (fixed effects)
Inverse variance weighted (multiplicative random effects)
MR Egger
MR RAPS
Weighted median
Weighted mode
0.075
0.050
0.025
0.000
−0.025
0.09
0.12
0.15
0.18
SNP effect on Neuritin
SNP effect on Gut microbiota abundance (genus Catenibacterium id.2153)

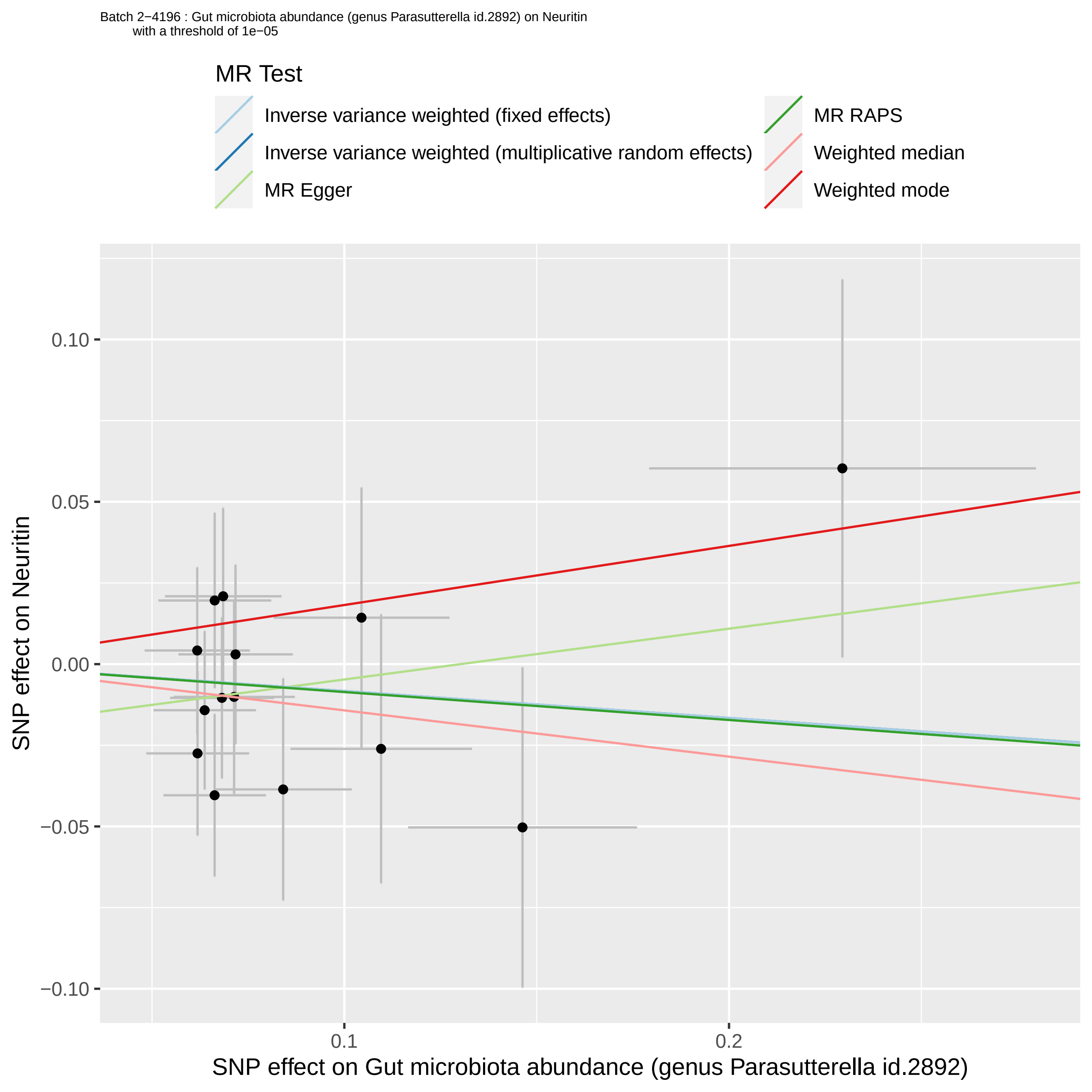
Batch 2−4196 : Gut microbiota abundance (genus Parasutterella id.2892) on Neuritin
with a threshold of 1e−05
MR Test
Inverse variance weighted (fixed effects)
Inverse variance weighted (multiplicative random effects)
MR Egger
MR RAPS
Weighted median
Weighted mode
0.10
0.05
0.00
−0.05
−0.10
SNP effect on Neuritin
0.1
0.2
SNP effect on Gut microbiota abundance (genus Parasutterella id.2892)

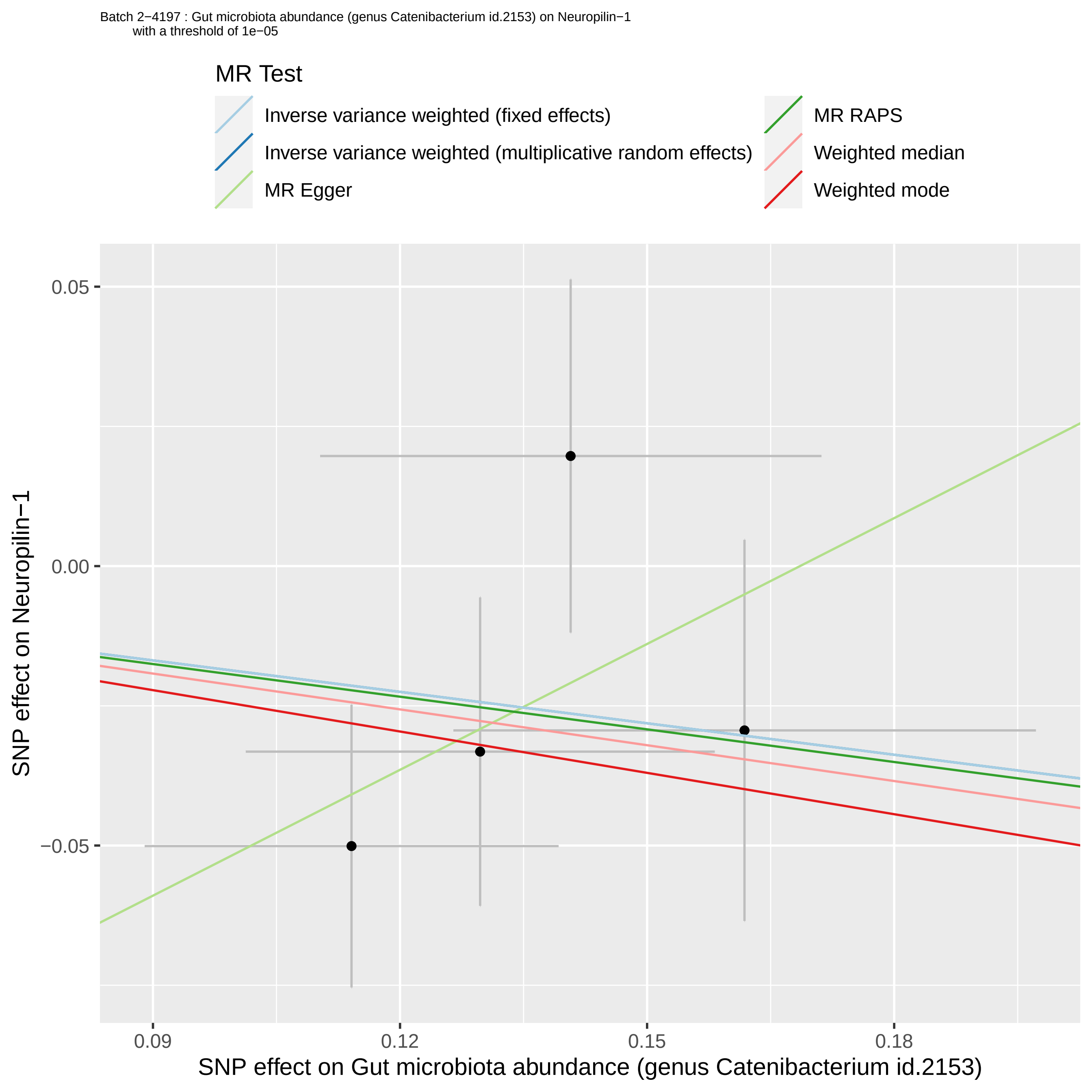
Batch 2−4197 : Gut microbiota abundance (genus Catenibacterium id.2153) on Neuropilin−1
with a threshold of 1e−05
MR Test
Inverse variance weighted (fixed effects)
Inverse variance weighted (multiplicative random effects)
MR Egger
MR RAPS
Weighted median
Weighted mode
0.05
0.00
−0.05
0.09
0.12
0.15
0.18
SNP effect on Neuropilin−1
SNP effect on Gut microbiota abundance (genus Catenibacterium id.2153)

Batch 2−4198 : Gut microbiota abundance (genus Parasutterella id.2892) on Neuropilin−1
with a threshold of 1e−05

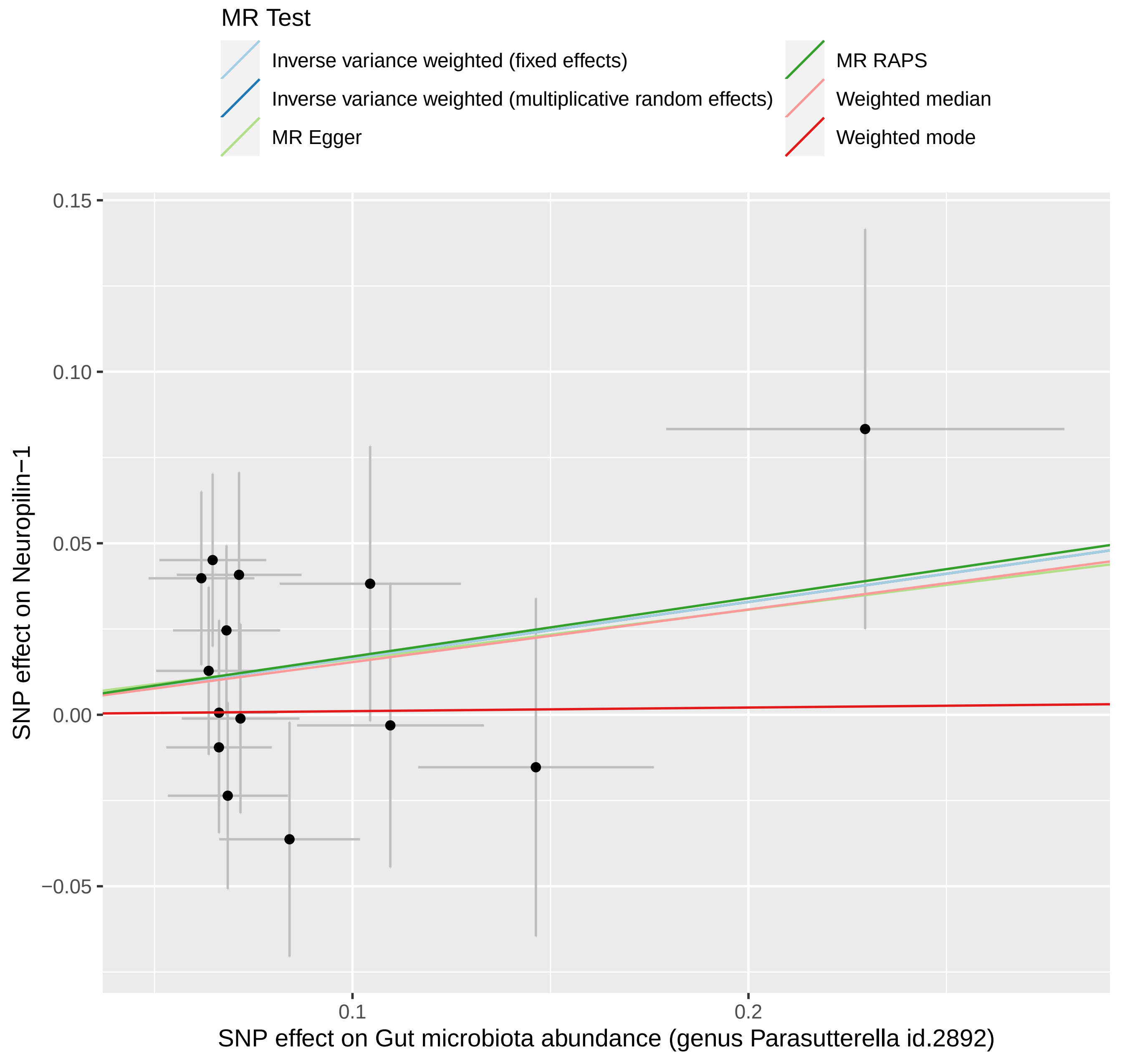

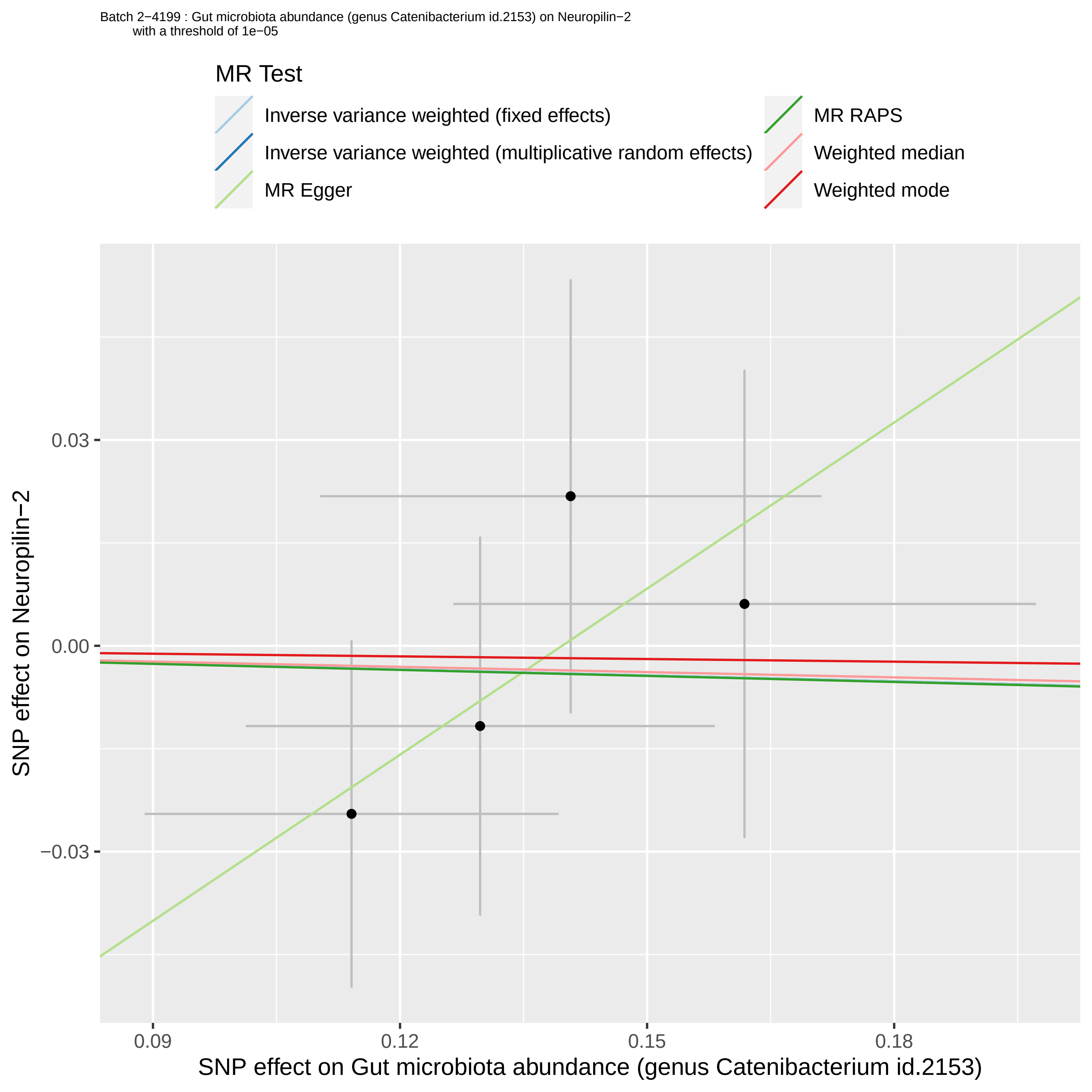

Batch 2−4199 : Gut microbiota abundance (genus Catenibacterium id.2153) on Neuropilin−2
with a threshold of 1e−05
MR Test
Inverse variance weighted (fixed effects)
Inverse variance weighted (multiplicative random effects)
MR Egger
MR RAPS
Weighted median
Weighted mode
0.03
0.00
−0.03
0.09
0.12
0.15
0.18
SNP effect on Neuropilin−2
SNP effect on Gut microbiota abundance (genus Catenibacterium id.2153)

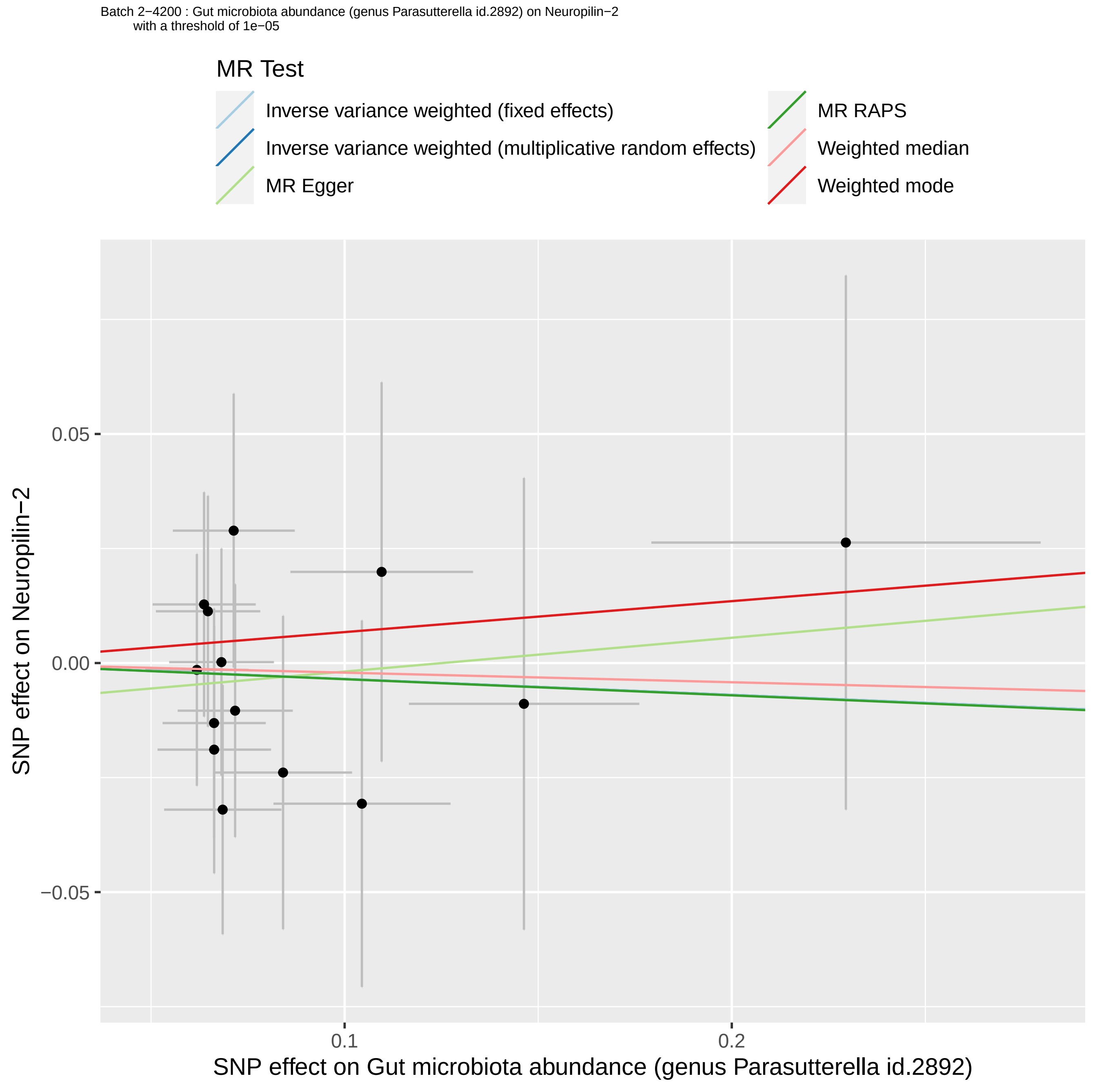

Batch 2−4200 : Gut microbiota abundance (genus Parasutterella id.2892) on Neuropilin−2
with a threshold of 1e−05
MR Test
Inverse variance weighted (fixed effects)
Inverse variance weighted (multiplicative random effects)
MR Egger
MR RAPS
Weighted median
Weighted mode
0.05
0.00
−0.05
0.1
0.2
SNP effect on Neuropilin−2
SNP effect on Gut microbiota abundance (genus Parasutterella id.2892)

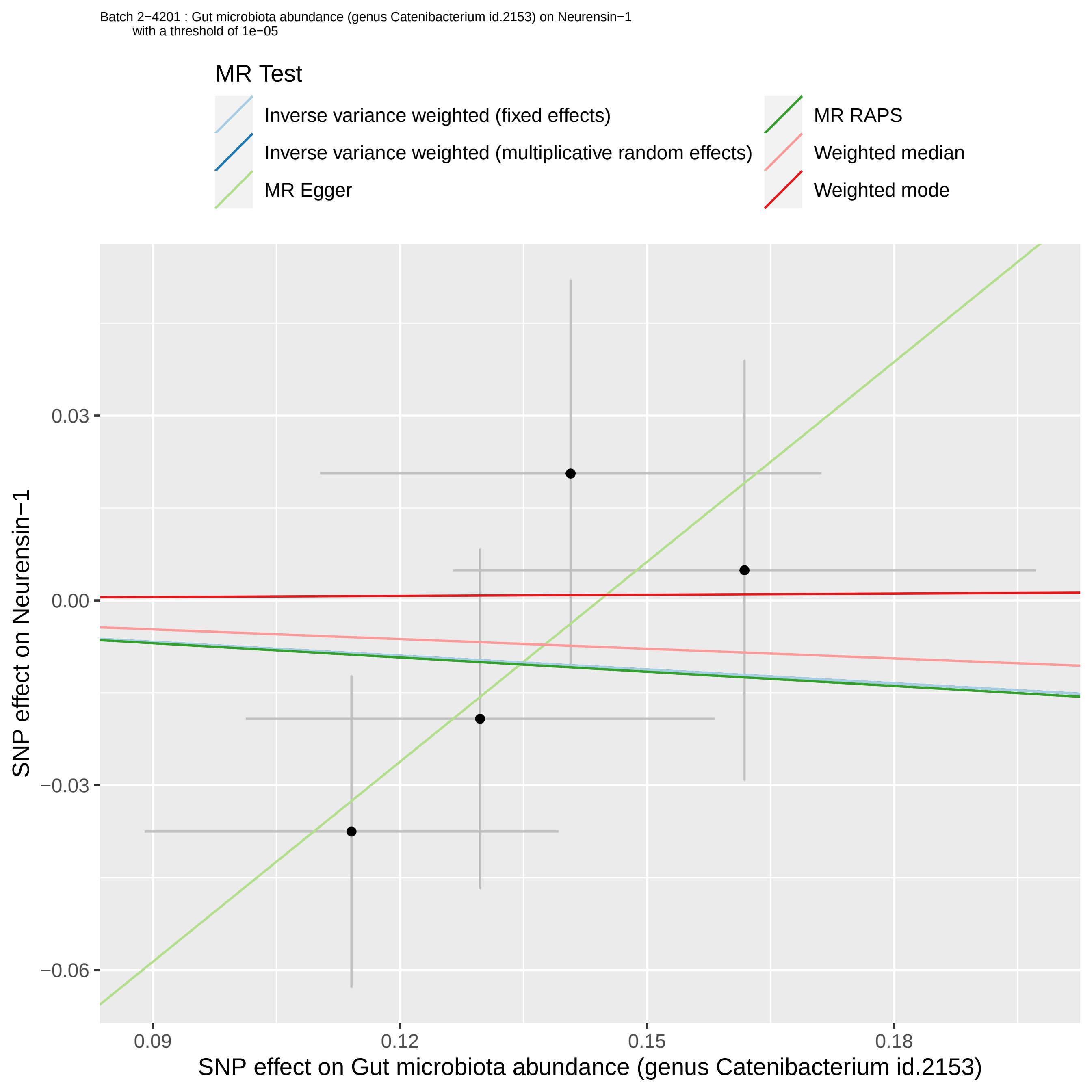
Batch 2−4201 : Gut microbiota abundance (genus Catenibacterium id.2153) on Neurensin−1
with a threshold of 1e−05
MR Test
Inverse variance weighted (fixed effects)
Inverse variance weighted (multiplicative random effects)
MR Egger
MR RAPS
Weighted median
Weighted mode
0.03
0.00
−0.03
−0.06
0.09
0.12
0.15
0.18
SNP effect on Neurensin−1
SNP effect on Gut microbiota abundance (genus Catenibacterium id.2153)

Batch 2−4202 : Gut microbiota abundance (genus Parasutterella id.2892) on Neurensin−1
with a threshold of 1e−05

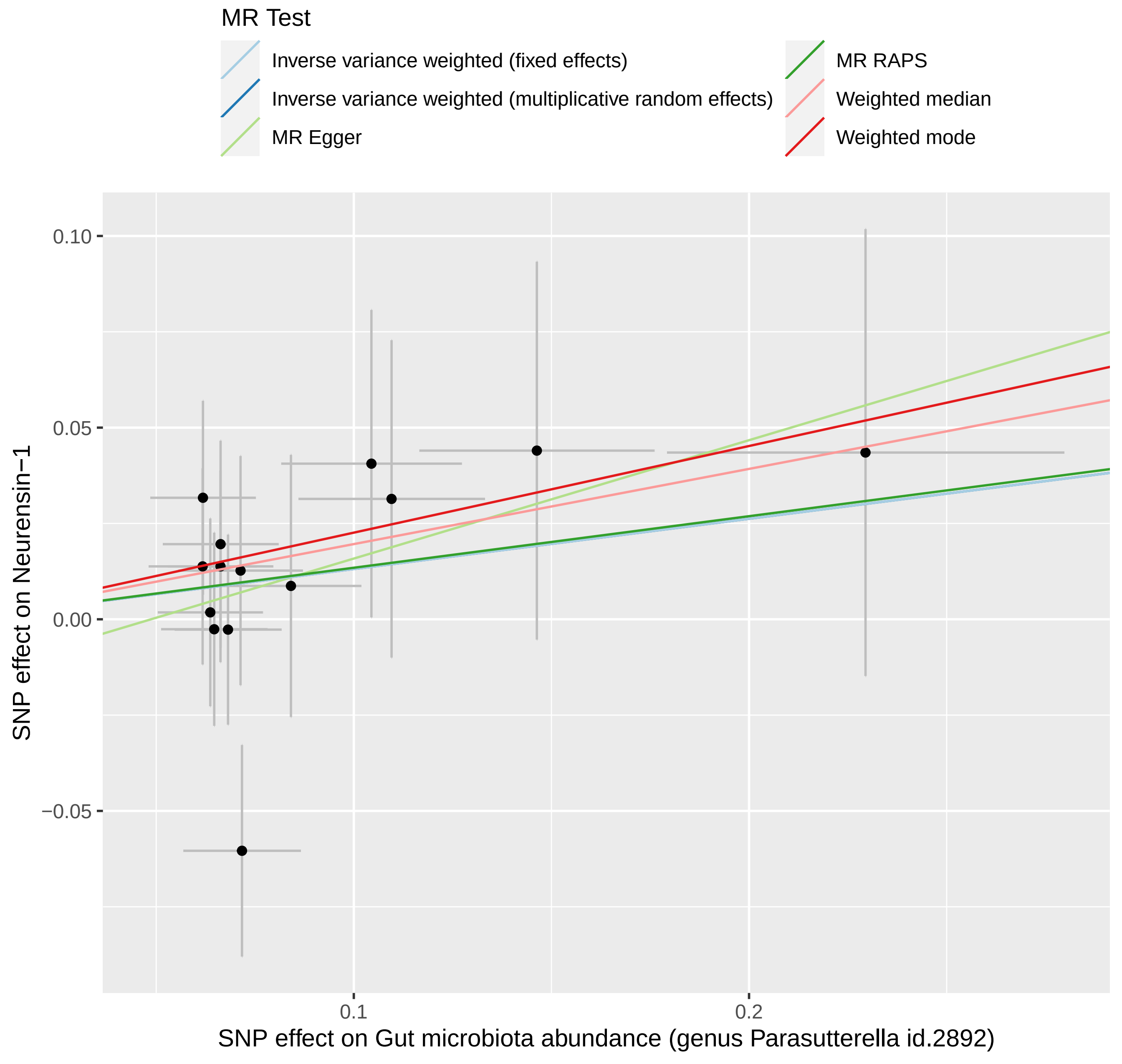

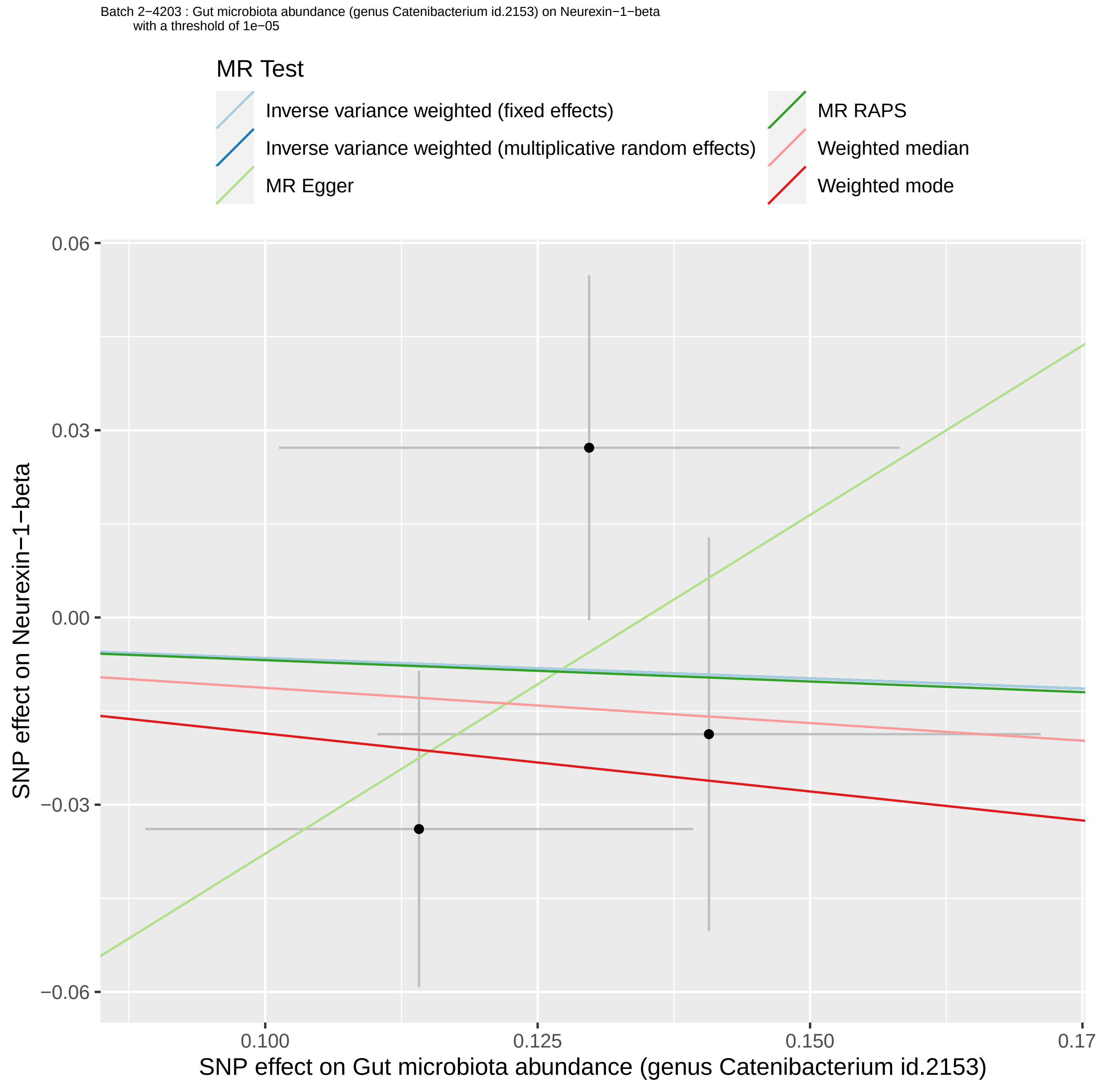

Batch 2−4203 : Gut microbiota abundance (genus Catenibacterium id.2153) on Neurexin−1−beta
with a threshold of 1e−05
MR Test
Inverse variance weighted (fixed effects)
Inverse variance weighted (multiplicative random effects)
MR Egger
MR RAPS
Weighted median
Weighted mode
0.06
0.03
0.00
−0.03
−0.06
0.100
0.125
0.150
0.17
SNP effect on Neurexin−1−beta
SNP effect on Gut microbiota abundance (genus Catenibacterium id.2153)

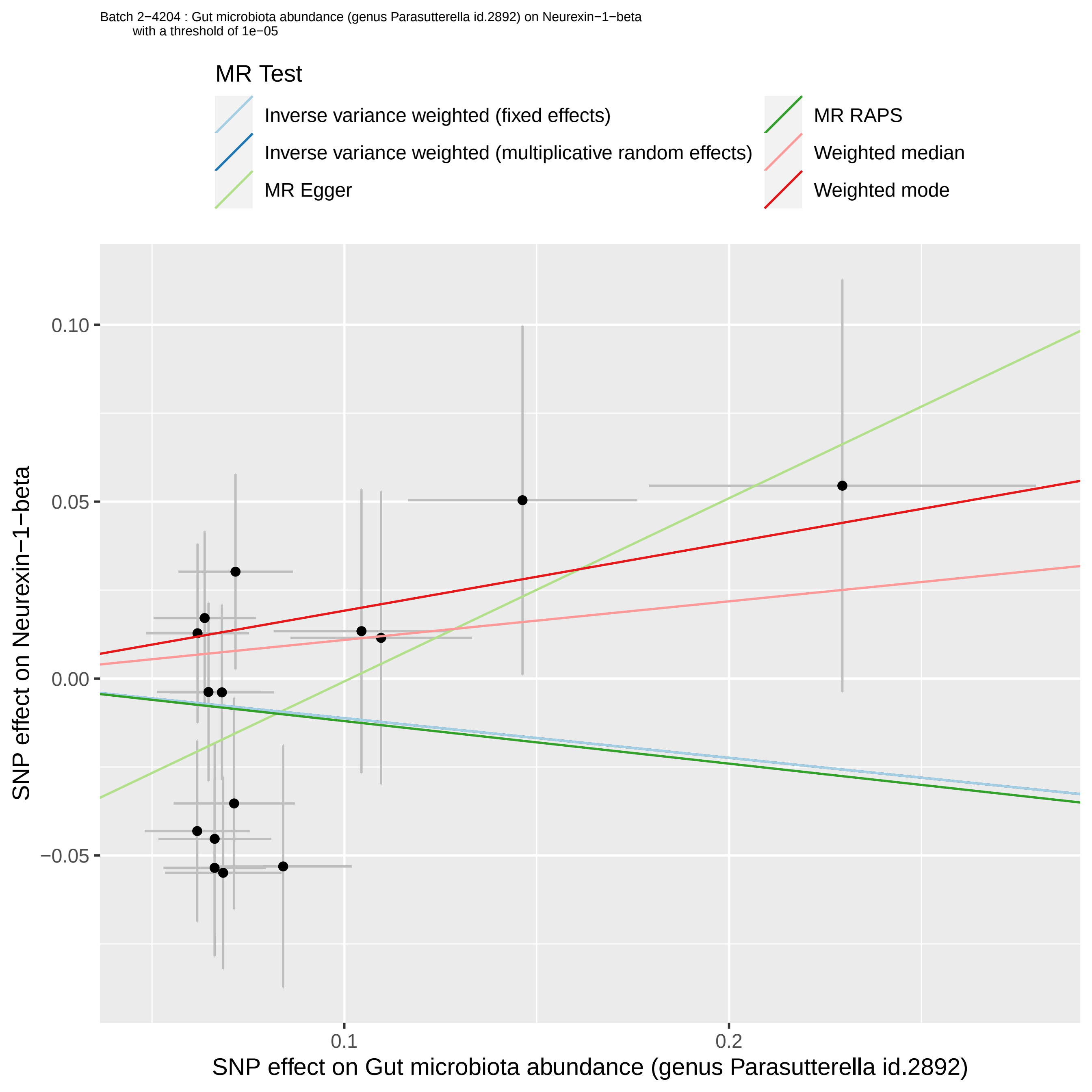

Batch 2−4204 : Gut microbiota abundance (genus Parasutterella id.2892) on Neurexin−1−beta
with a threshold of 1e−05
MR Test
Inverse variance weighted (fixed effects)
Inverse variance weighted (multiplicative random effects)
MR Egger
MR RAPS
Weighted median
Weighted mode
0.10
0.05
0.00
−0.05
0.1
0.2
SNP effect on Neurexin−1−beta
SNP effect on Gut microbiota abundance (genus Parasutterella id.2892)

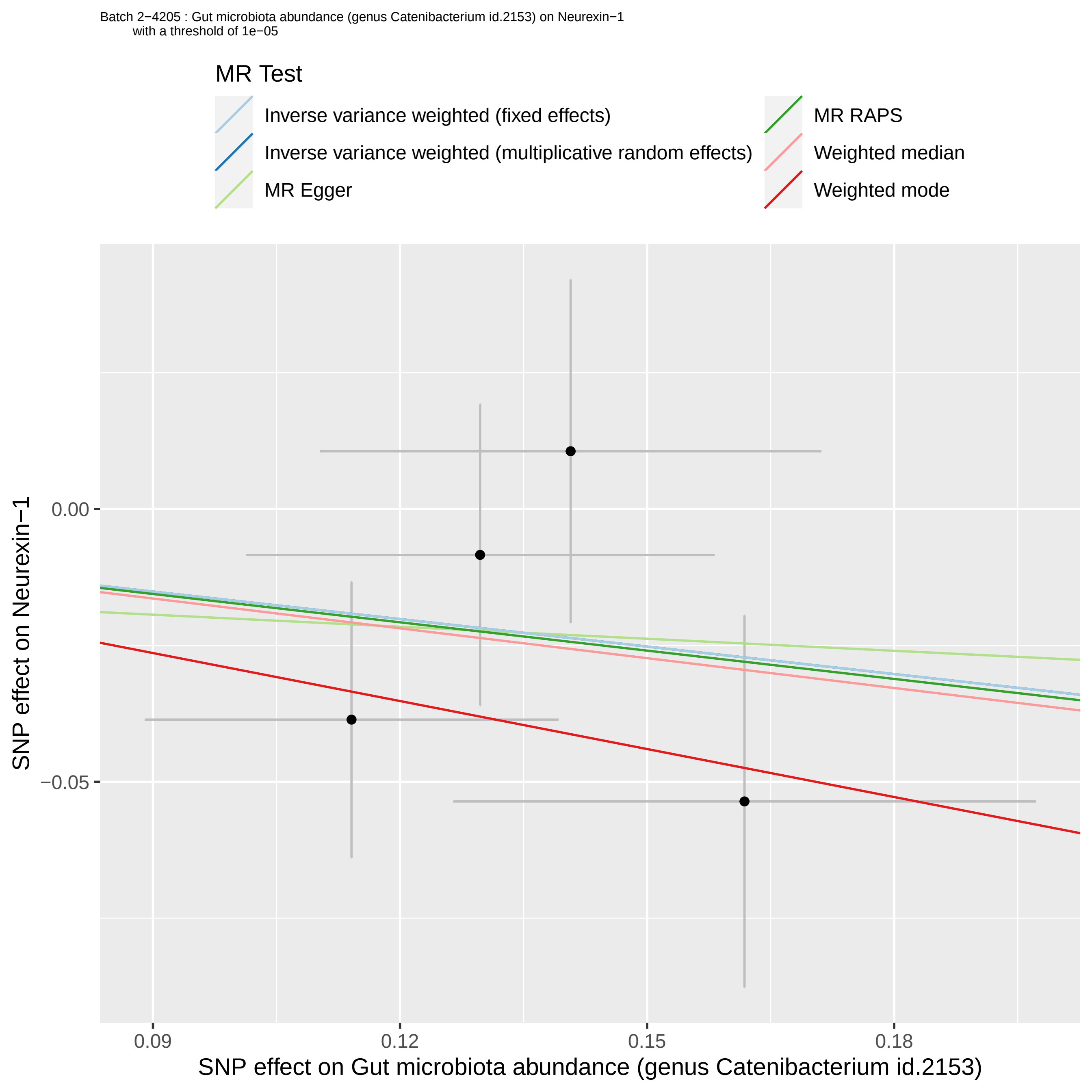

Batch 2−4205 : Gut microbiota abundance (genus Catenibacterium id.2153) on Neurexin−1
with a threshold of 1e−05
MR Test
Inverse variance weighted (fixed effects)
Inverse variance weighted (multiplicative random effects)
MR Egger
MR RAPS
Weighted median
Weighted mode
SNP effect on Neurexin−1
0.00
−0.05
0.09
0.12
0.15
0.18
SNP effect on Gut microbiota abundance (genus Catenibacterium id.2153)

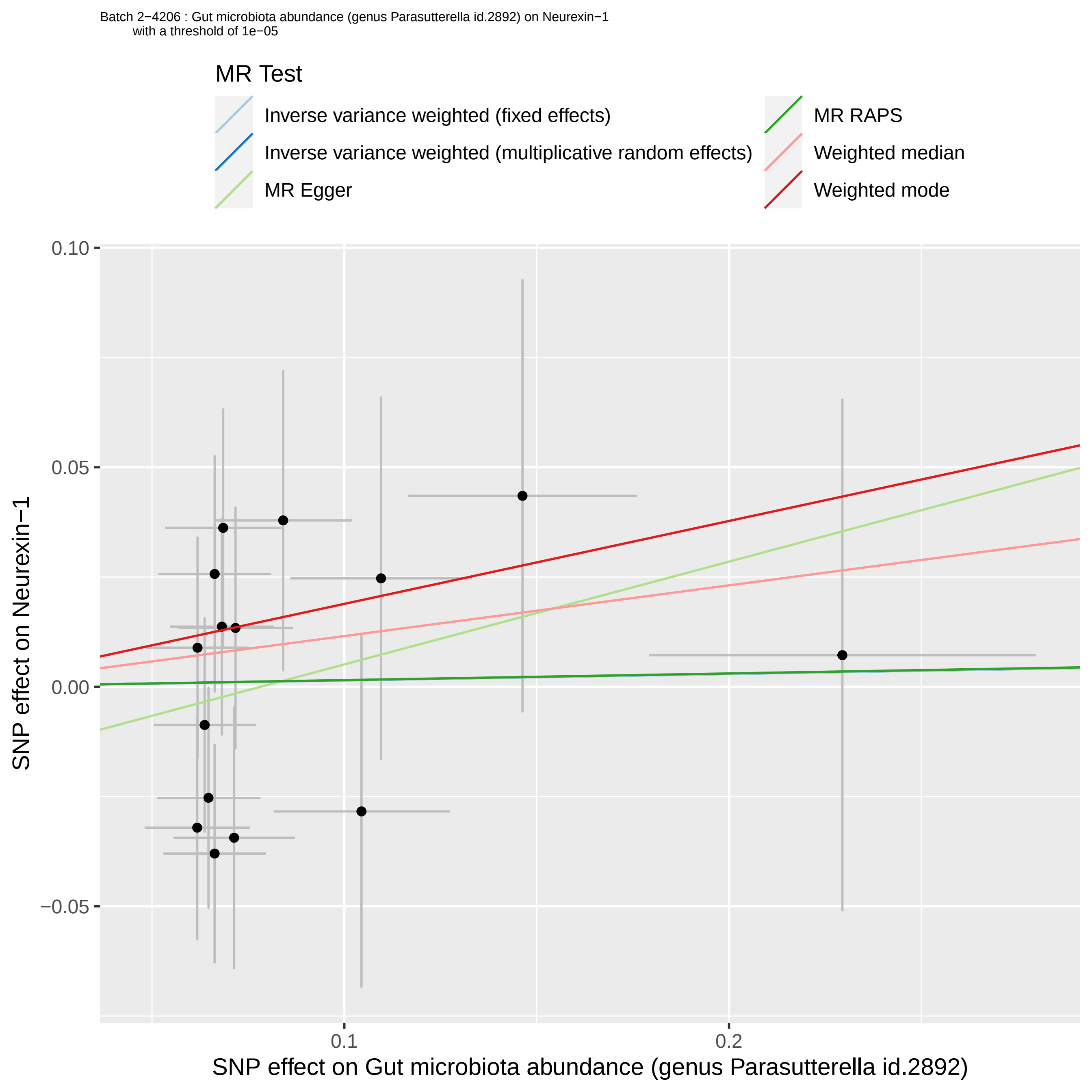
Batch 2−4206 : Gut microbiota abundance (genus Parasutterella id.2892) on Neurexin−1
with a threshold of 1e−05
MR Test
Inverse variance weighted (fixed effects)
Inverse variance weighted (multiplicative random effects)
MR Egger
MR RAPS
Weighted median
Weighted mode
0.10
0.05
0.00
−0.05
0.1
0.2
SNP effect on Neurexin−1
SNP effect on Gut microbiota abundance (genus Parasutterella id.2892)

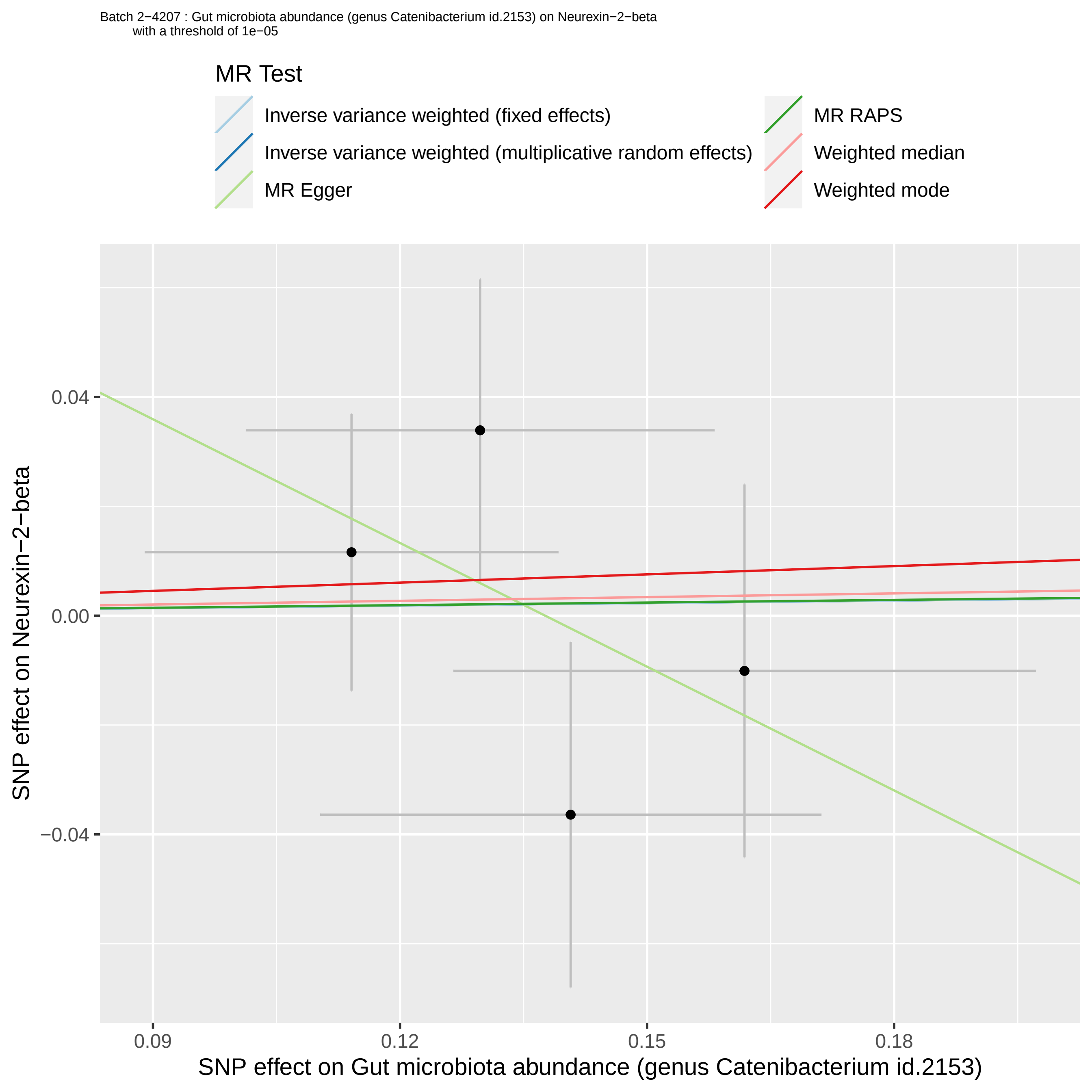

Batch 2−4207 : Gut microbiota abundance (genus Catenibacterium id.2153) on Neurexin−2−beta
with a threshold of 1e−05
MR Test
Inverse variance weighted (fixed effects)
Inverse variance weighted (multiplicative random effects)
MR Egger
MR RAPS
Weighted median
Weighted mode
SNP effect on Neurexin−2−beta
0.04
0.00
−0.04
0.09
0.12
0.15
0.18
SNP effect on Gut microbiota abundance (genus Catenibacterium id.2153)

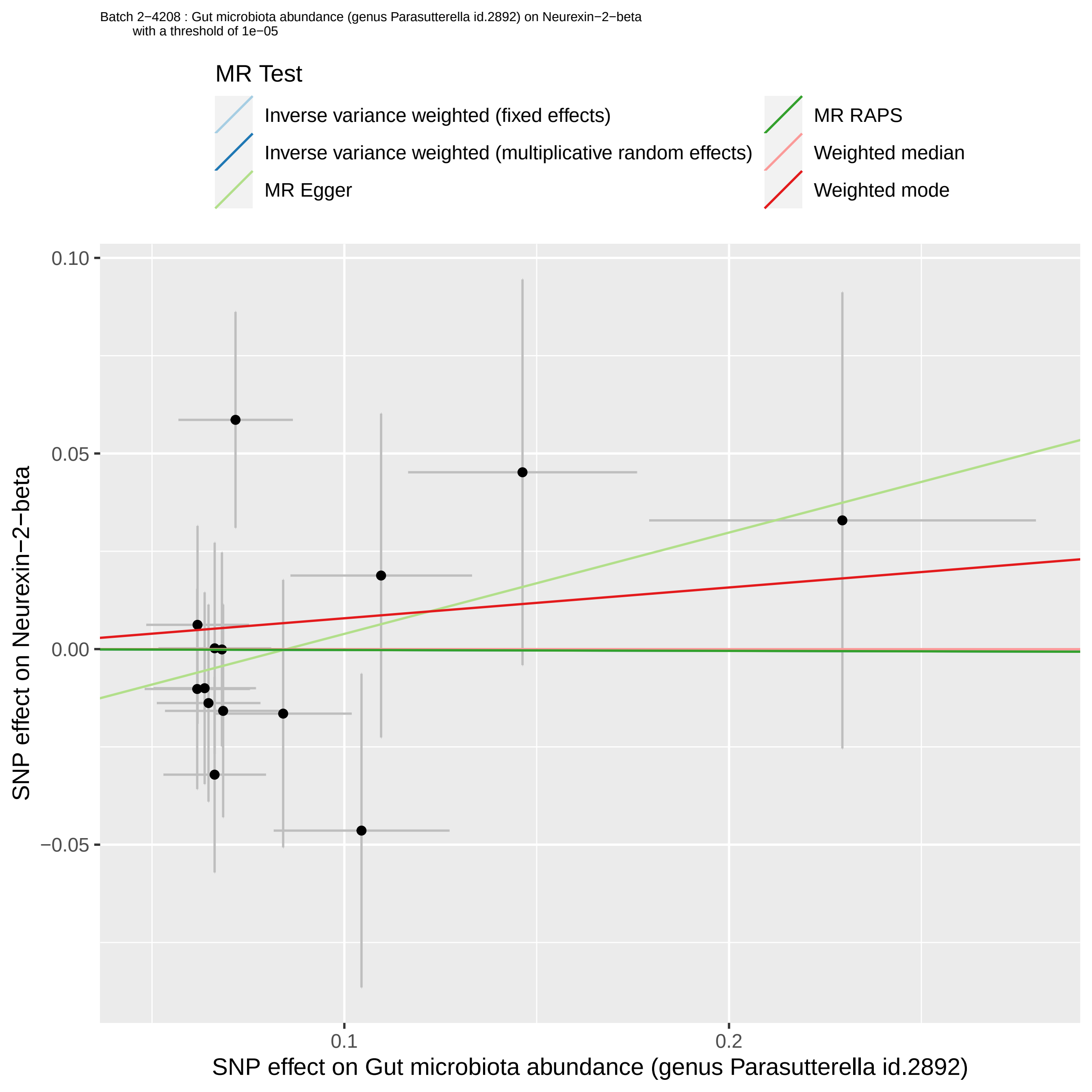
Batch 2−4208 : Gut microbiota abundance (genus Parasutterella id.2892) on Neurexin−2−beta
with a threshold of 1e−05
MR Test
Inverse variance weighted (fixed effects)
Inverse variance weighted (multiplicative random effects)
MR Egger
MR RAPS
Weighted median
Weighted mode
0.10
0.05
0.00
−0.05
0.1
0.2
SNP effect on Neurexin−2−beta
SNP effect on Gut microbiota abundance (genus Parasutterella id.2892)

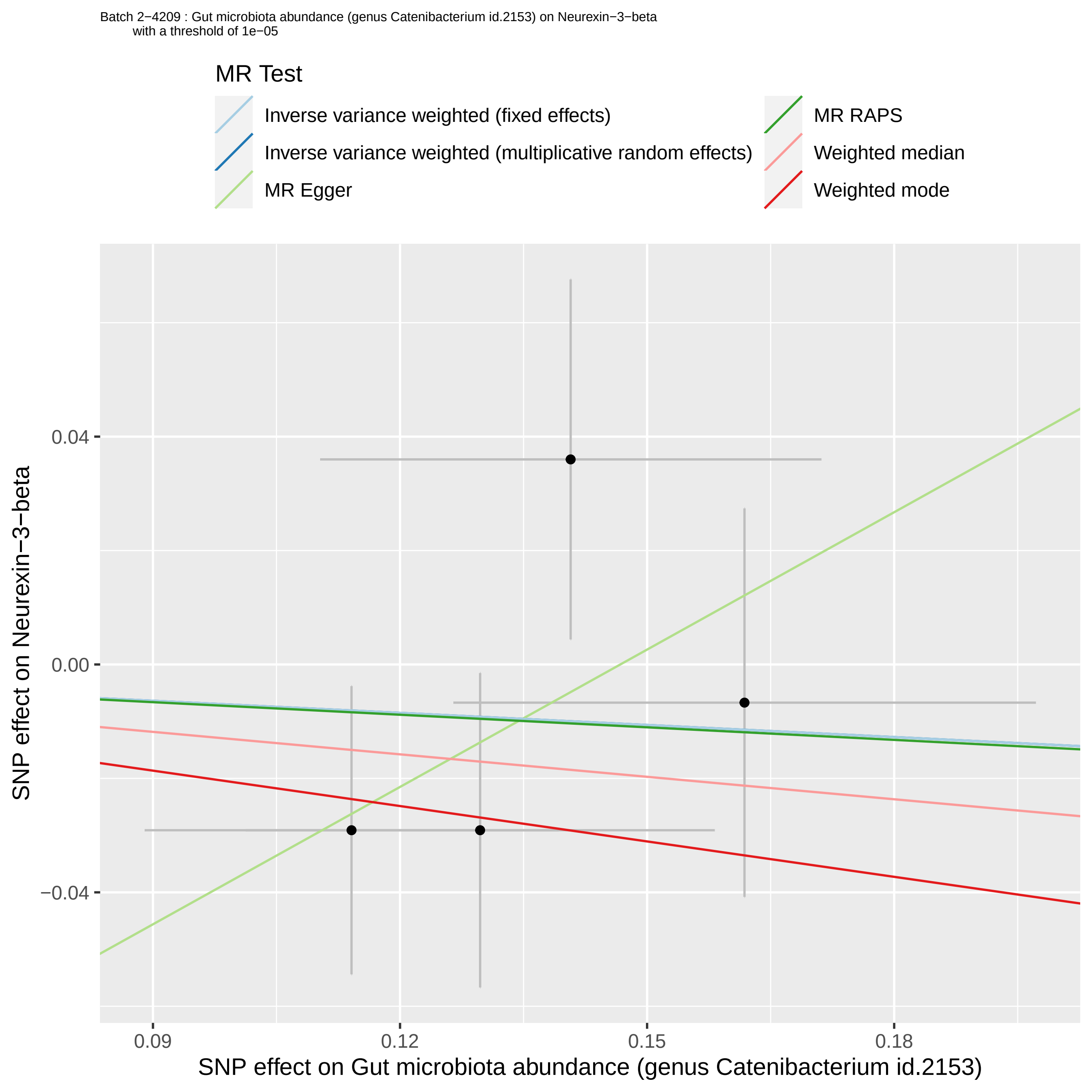

Batch 2−4209 : Gut microbiota abundance (genus Catenibacterium id.2153) on Neurexin−3−beta
with a threshold of 1e−05
MR Test
Inverse variance weighted (fixed effects)
Inverse variance weighted (multiplicative random effects)
MR Egger
MR RAPS
Weighted median
Weighted mode
0.04
0.00
−0.04
0.09
0.12
0.15
0.18
SNP effect on Neurexin−3−beta
SNP effect on Gut microbiota abundance (genus Catenibacterium id.2153)

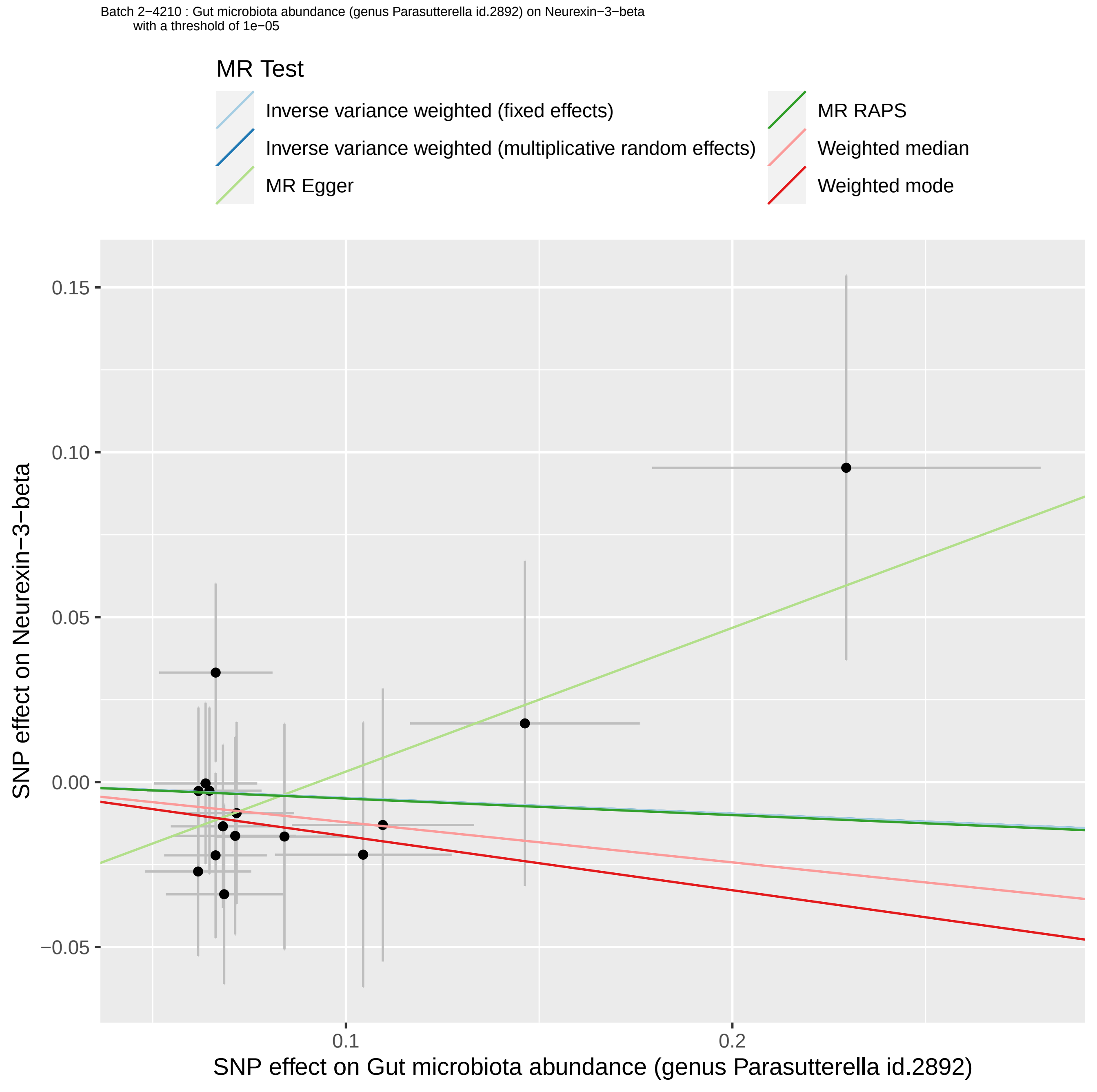

Batch 2−4210 : Gut microbiota abundance (genus Parasutterella id.2892) on Neurexin−3−beta
with a threshold of 1e−05
MR Test
Inverse variance weighted (fixed effects)
Inverse variance weighted (multiplicative random effects)
MR Egger
MR RAPS
Weighted median
Weighted mode
0.15
0.10
0.05
0.00
−0.05
0.1
0.2
SNP effect on Neurexin−3−beta
SNP effect on Gut microbiota abundance (genus Parasutterella id.2892)

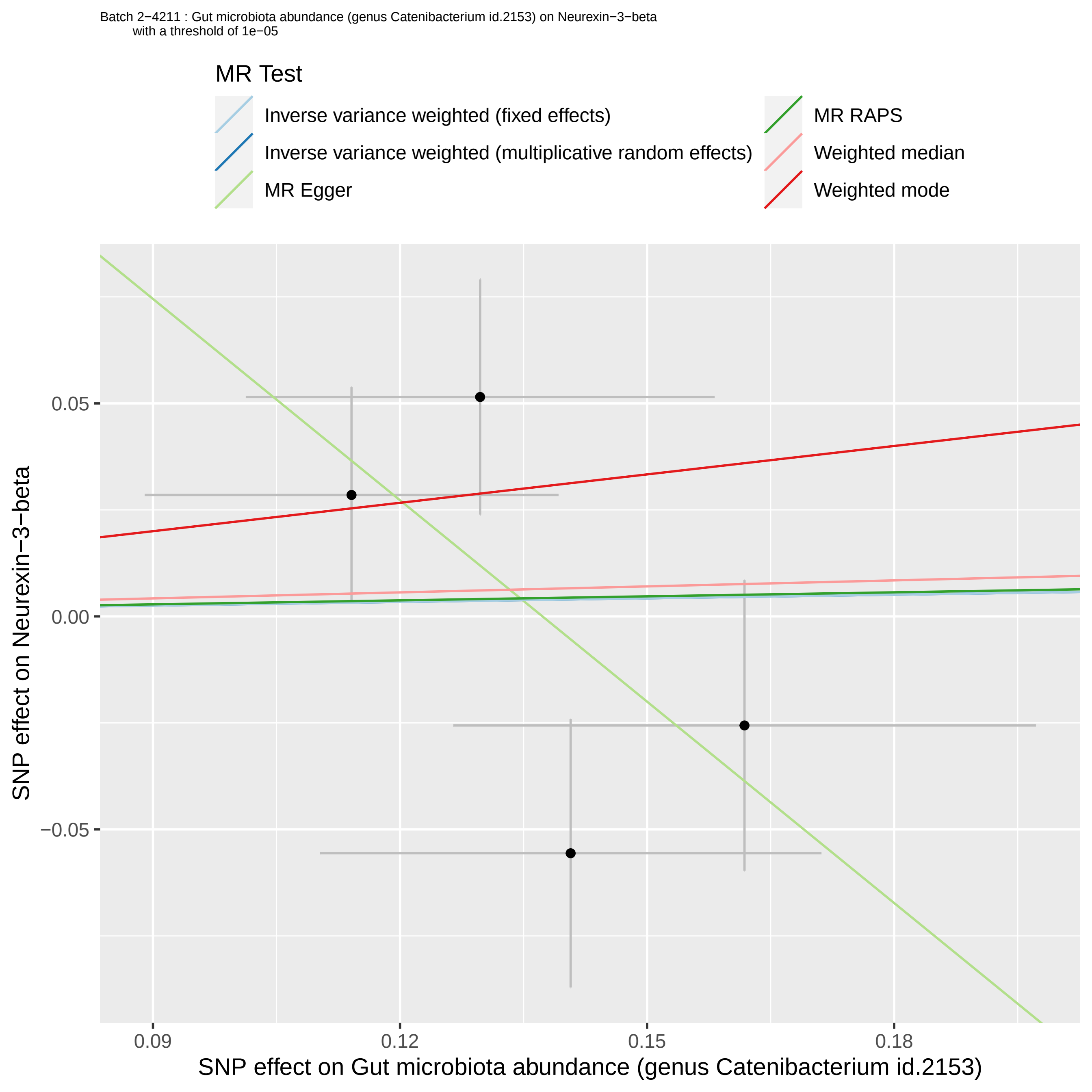

Batch 2−4211 : Gut microbiota abundance (genus Catenibacterium id.2153) on Neurexin−3−beta
with a threshold of 1e−05
MR Test
Inverse variance weighted (fixed effects)
Inverse variance weighted (multiplicative random effects)
MR Egger
MR RAPS
Weighted median
Weighted mode
0.05
0.00
−0.05
0.09
0.12
0.15
0.18
SNP effect on Neurexin−3−beta
SNP effect on Gut microbiota abundance (genus Catenibacterium id.2153)

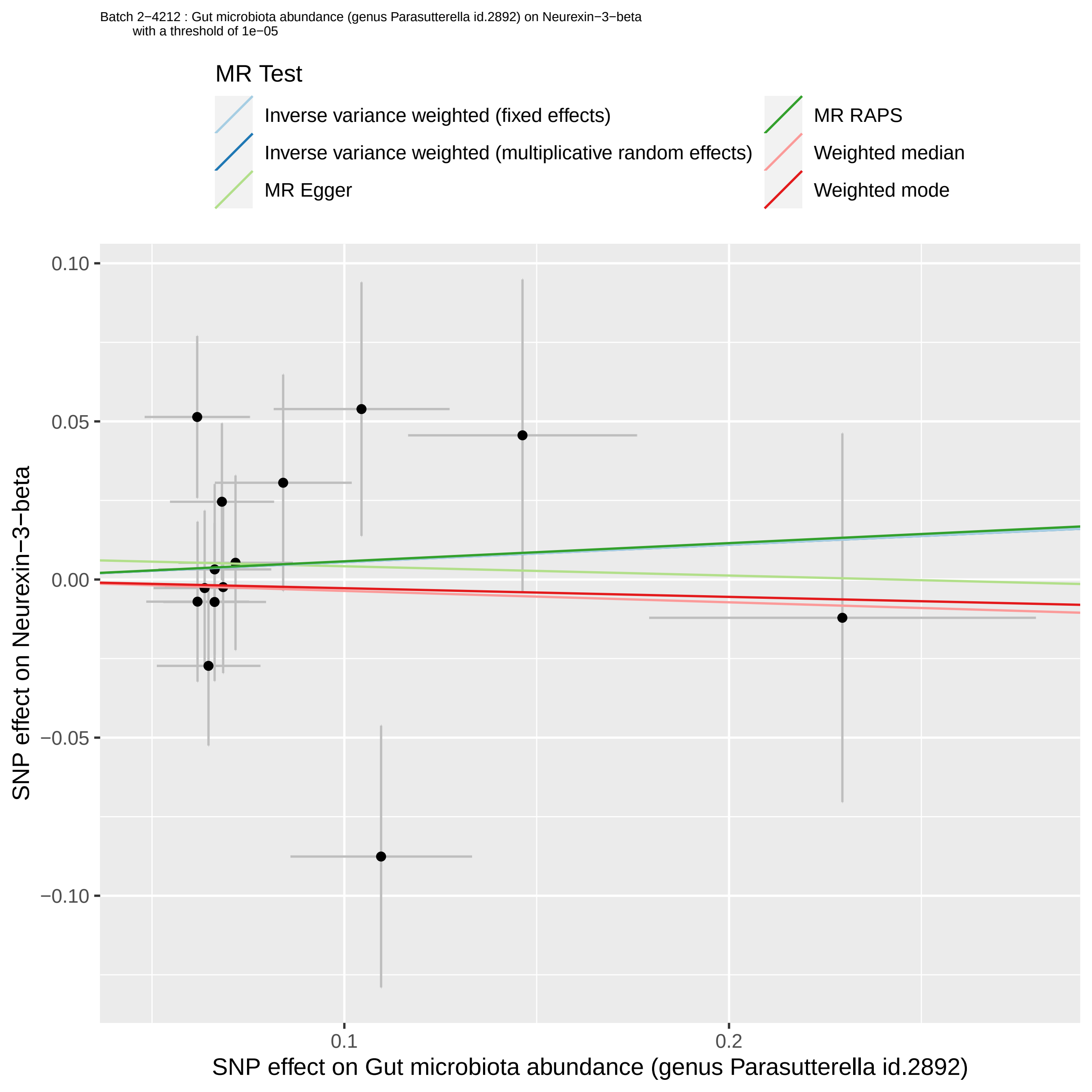

Batch 2−4212 : Gut microbiota abundance (genus Parasutterella id.2892) on Neurexin−3−beta
with a threshold of 1e−05
MR Test
Inverse variance weighted (fixed effects)
Inverse variance weighted (multiplicative random effects)
MR Egger
MR RAPS
Weighted median
Weighted mode
0.10
0.05
0.00
−0.05
−0.10
0.1
0.2
SNP effect on Neurexin−3−beta
SNP effect on Gut microbiota abundance (genus Parasutterella id.2892)

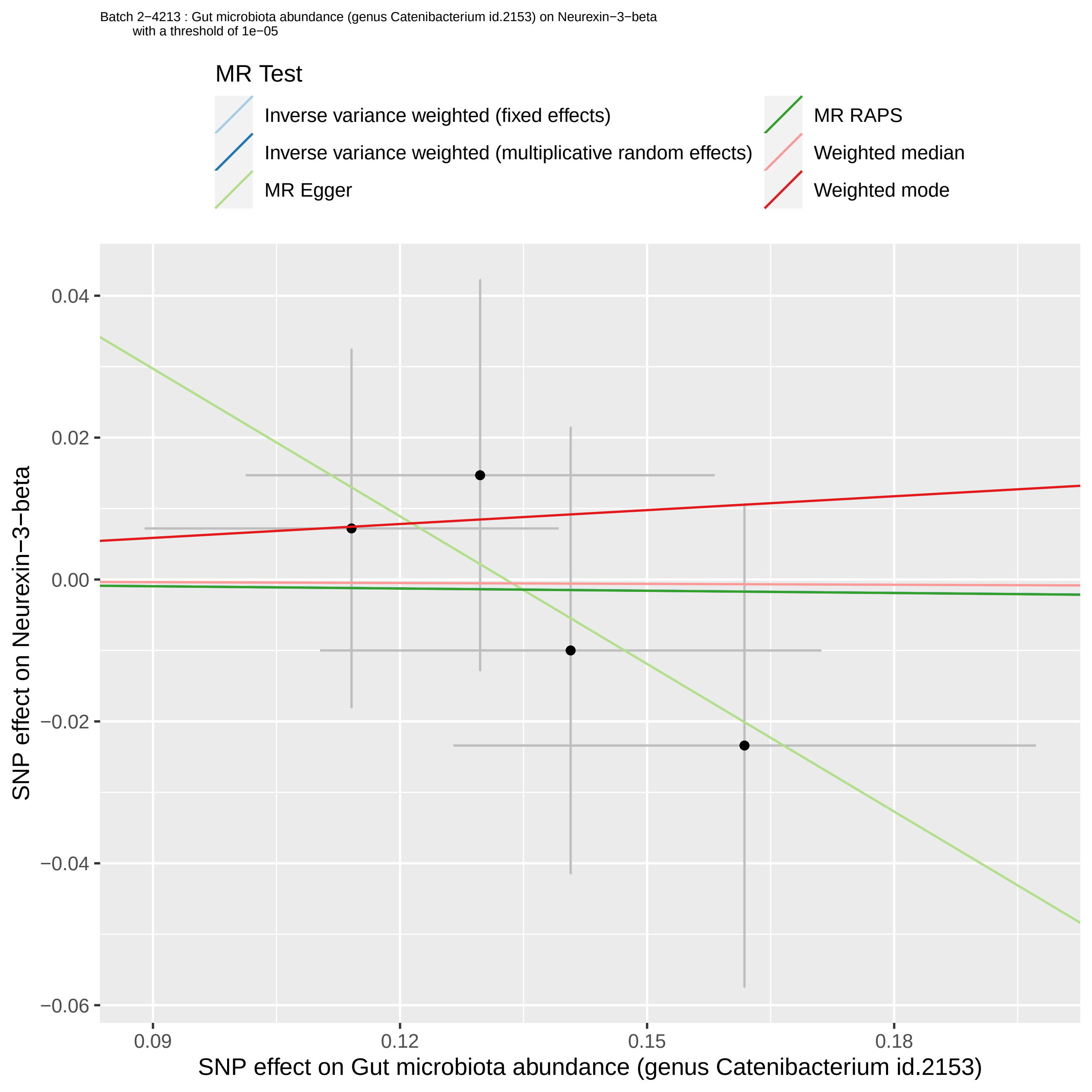
Batch 2−4213 : Gut microbiota abundance (genus Catenibacterium id.2153) on Neurexin−3−beta
with a threshold of 1e−05
MR Test
Inverse variance weighted (fixed effects)
Inverse variance weighted (multiplicative random effects)
MR Egger
MR RAPS
Weighted median
Weighted mode
0.04
0.02
0.00
−0.02
−0.04
−0.06
0.09
0.12
0.15
0.18
SNP effect on Neurexin−3−beta
SNP effect on Gut microbiota abundance (genus Catenibacterium id.2153)

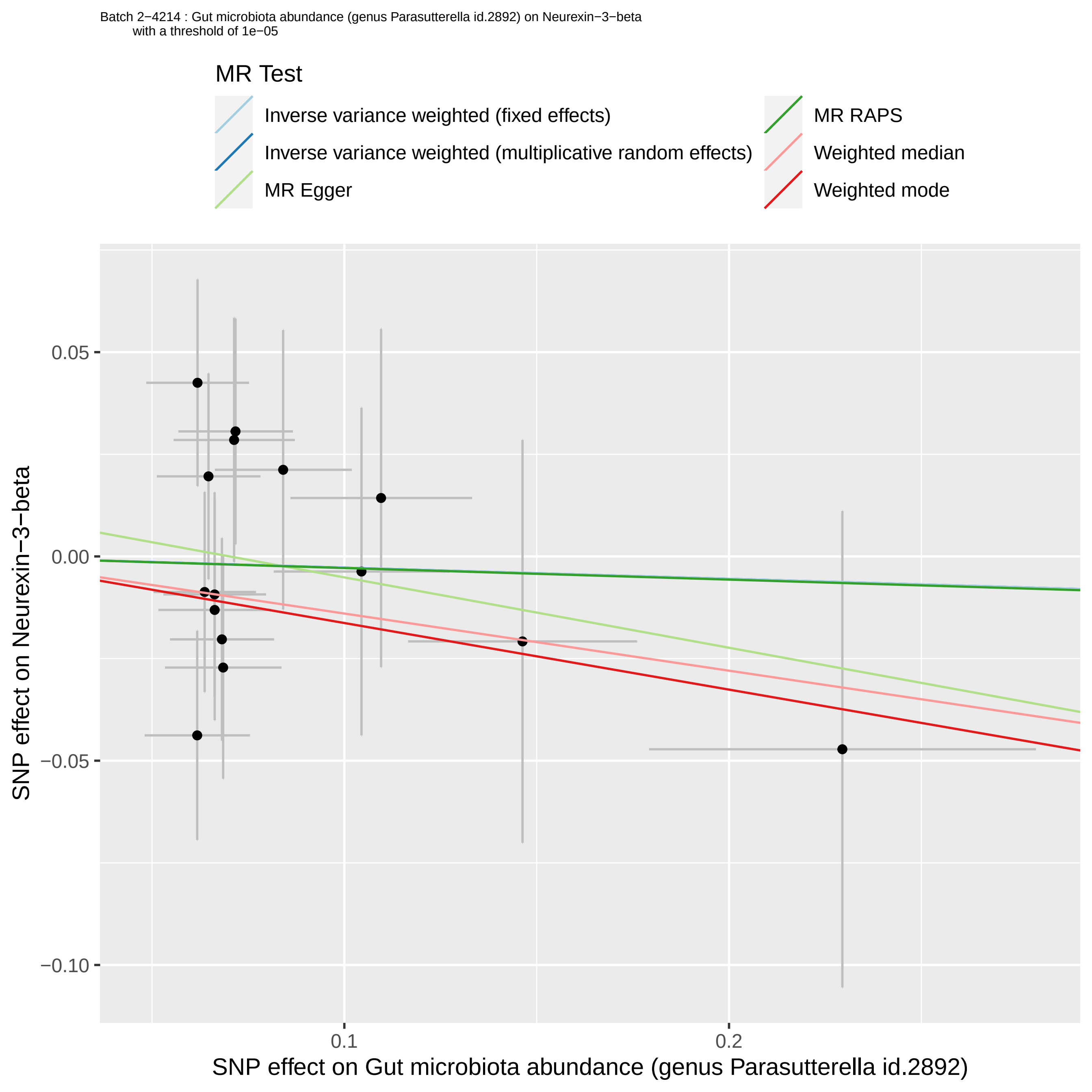

Batch 2−4214 : Gut microbiota abundance (genus Parasutterella id.2892) on Neurexin−3−beta
with a threshold of 1e−05
MR Test
Inverse variance weighted (fixed effects)
Inverse variance weighted (multiplicative random effects)
MR Egger
MR RAPS
Weighted median
Weighted mode
SNP effect on Neurexin−3−beta
0.05
0.00
−0.05
−0.10
0.1
0.2
SNP effect on Gut microbiota abundance (genus Parasutterella id.2892)

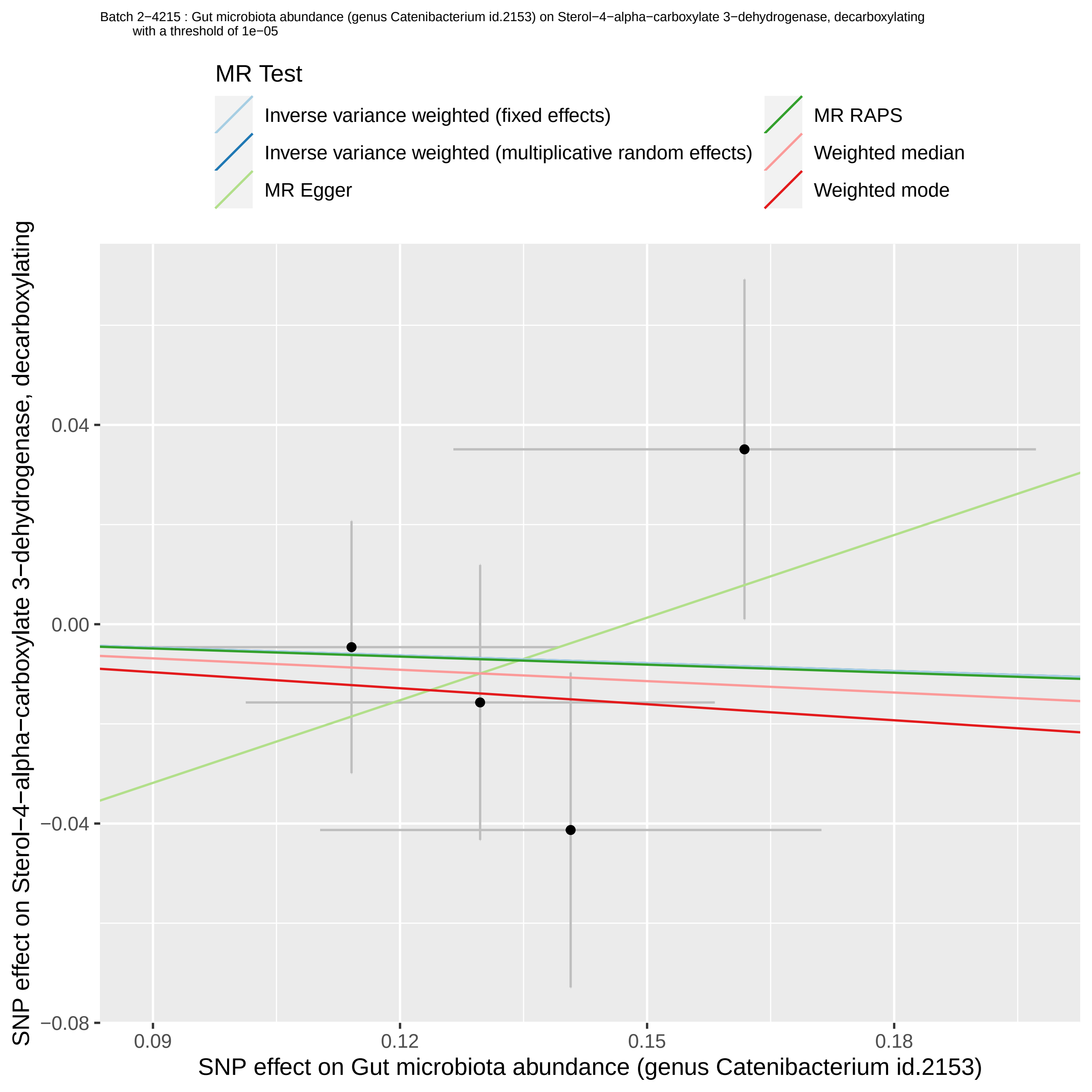

Batch 2−4215 : Gut microbiota abundance (genus Catenibacterium id.2153) on Sterol−4−alpha−carboxylate 3−dehydrogenase, decarboxylating
with a threshold of 1e−05
MR Test
Inverse variance weighted (fixed effects)
Inverse variance weighted (multiplicative random effects)
MR Egger
MR RAPS
Weighted median
Weighted mode
0.04
0.00
−0.04
−0.08
0.09
0.12
0.15
0.18
SNP effect on Sterol−4−alpha−carboxylate 3−dehydrogenase, decarboxylating
SNP effect on Gut microbiota abundance (genus Catenibacterium id.2153)

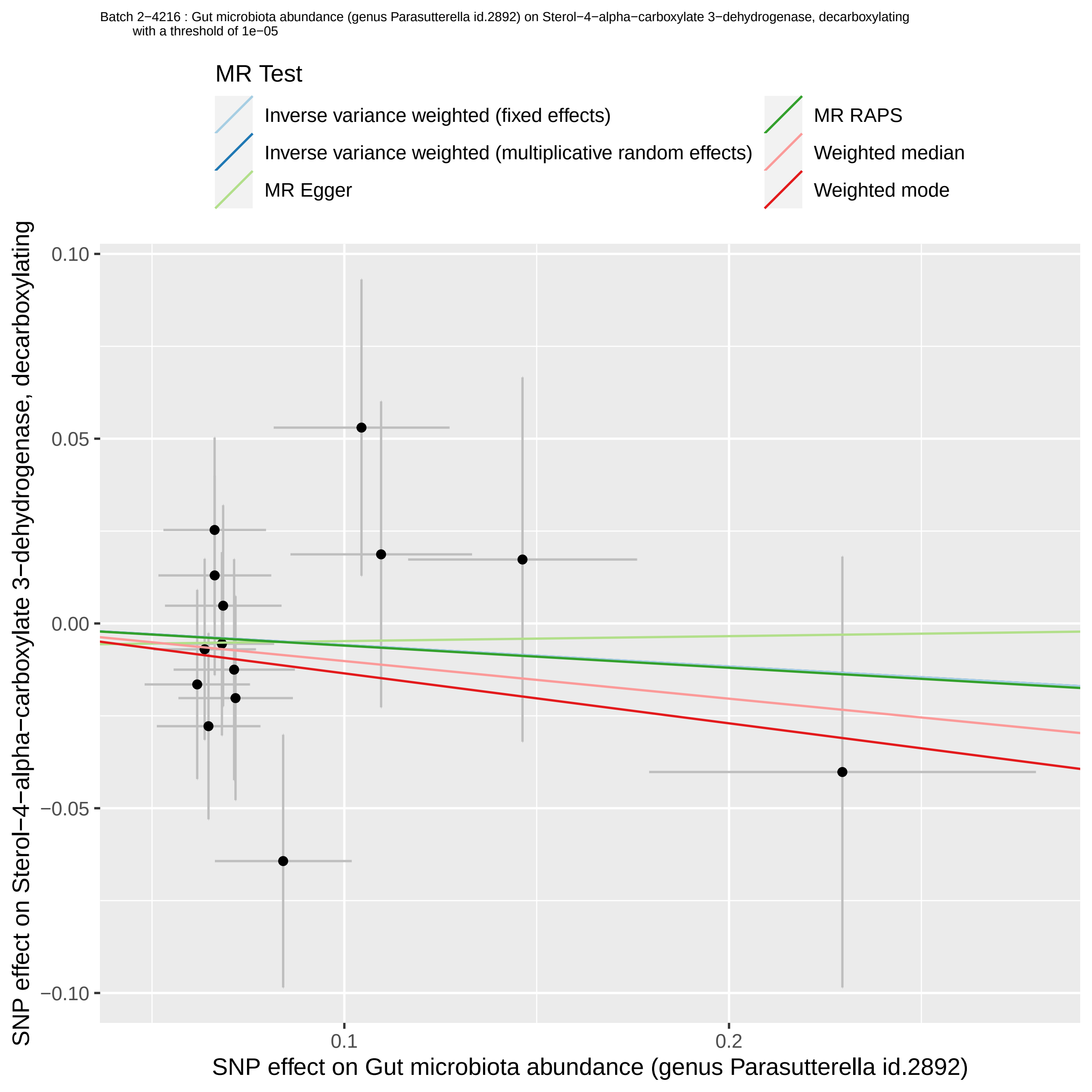
Batch 2−4216 : Gut microbiota abundance (genus Parasutterella id.2892) on Sterol−4−alpha−carboxylate 3−dehydrogenase, decarboxylating
with a threshold of 1e−05
MR Test
Inverse variance weighted (fixed effects)
Inverse variance weighted (multiplicative random effects)
MR Egger
MR RAPS
Weighted median
Weighted mode
SNP effect on Sterol−4−alpha−carboxylate 3−dehydrogenase, decarboxylating
0.10
0.05
0.00
−0.05
−0.10
0.1
0.2
SNP effect on Gut microbiota abundance (genus Parasutterella id.2892)

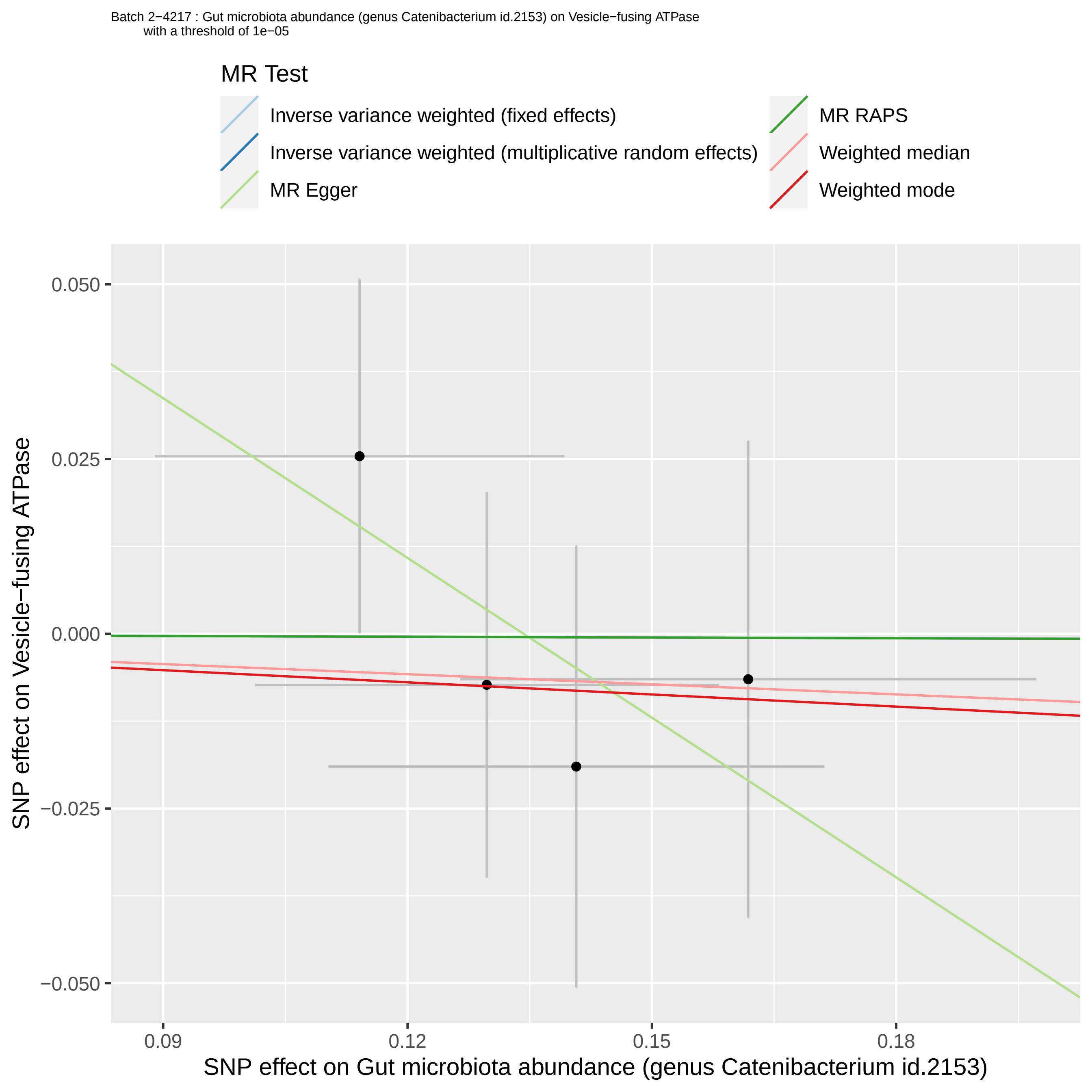
Batch 2−4217 : Gut microbiota abundance (genus Catenibacterium id.2153) on Vesicle−fusing ATPase
with a threshold of 1e−05
MR Test
Inverse variance weighted (fixed effects)
Inverse variance weighted (multiplicative random effects)
MR Egger
MR RAPS
Weighted median
Weighted mode
0.050
0.025
0.000
−0.025
−0.050
0.09
0.12
0.15
0.18
SNP effect on Vesicle−fusing ATPase
SNP effect on Gut microbiota abundance (genus Catenibacterium id.2153)

Batch 2−4218 : Gut microbiota abundance (genus Parasutterella id.2892) on Vesicle−fusing ATPase
with a threshold of 1e−05

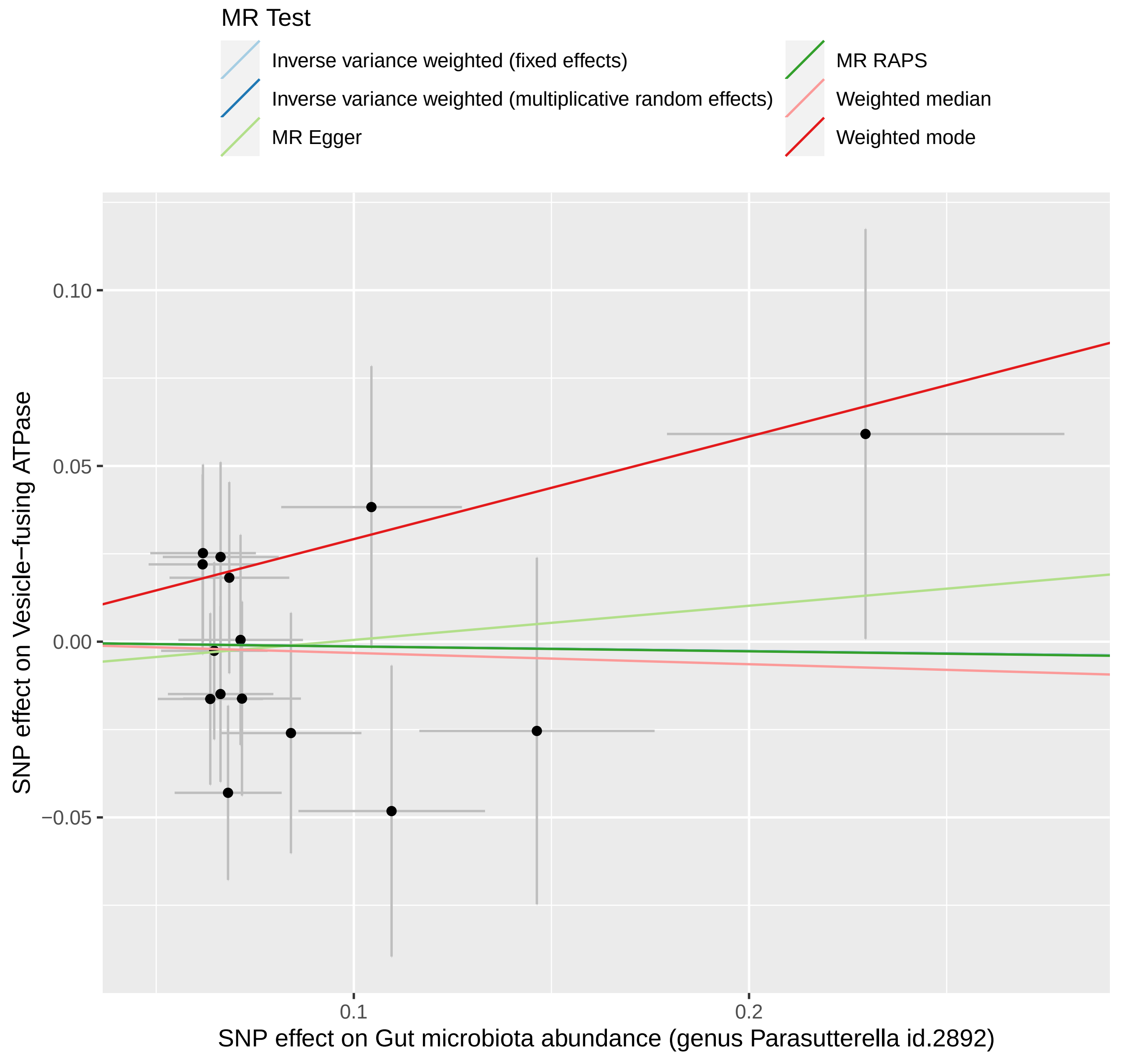

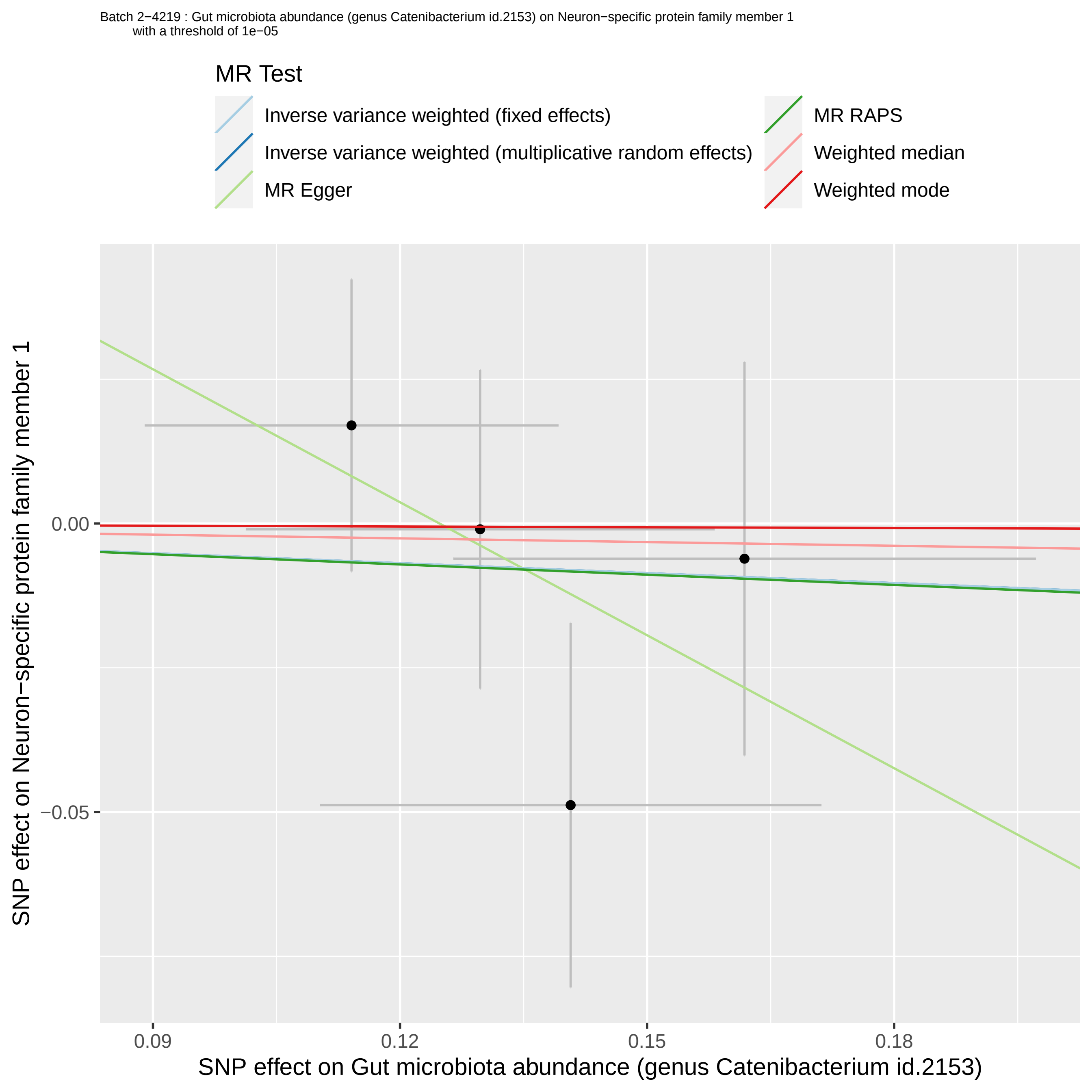
Batch 2−4219 : Gut microbiota abundance (genus Catenibacterium id.2153) on Neuron−specific protein family member 1
with a threshold of 1e−05
MR Test
Inverse variance weighted (fixed effects)
Inverse variance weighted (multiplicative random effects)
MR Egger
MR RAPS
Weighted median
Weighted mode
0.00
−0.05
0.09
0.12
0.15
0.18
SNP effect on Gut microbiota abundance (genus Catenibacterium id.2153)
SNP effect on Neuron−specific protein family member 1

Batch 2−4220 : Gut microbiota abundance (genus Parasutterella id.2892) on Neuron−specific protein family member 1
with a threshold of 1e−05

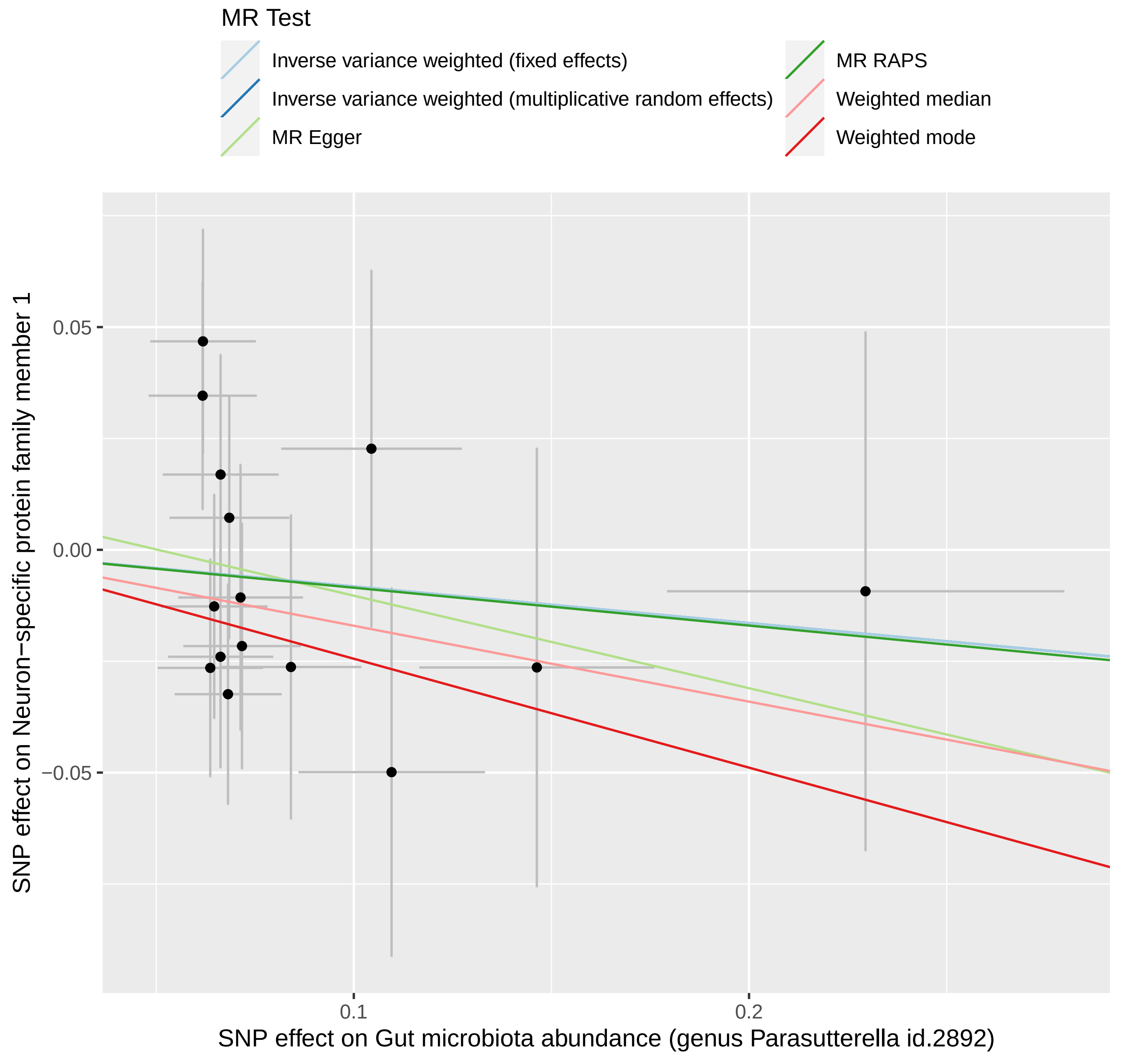

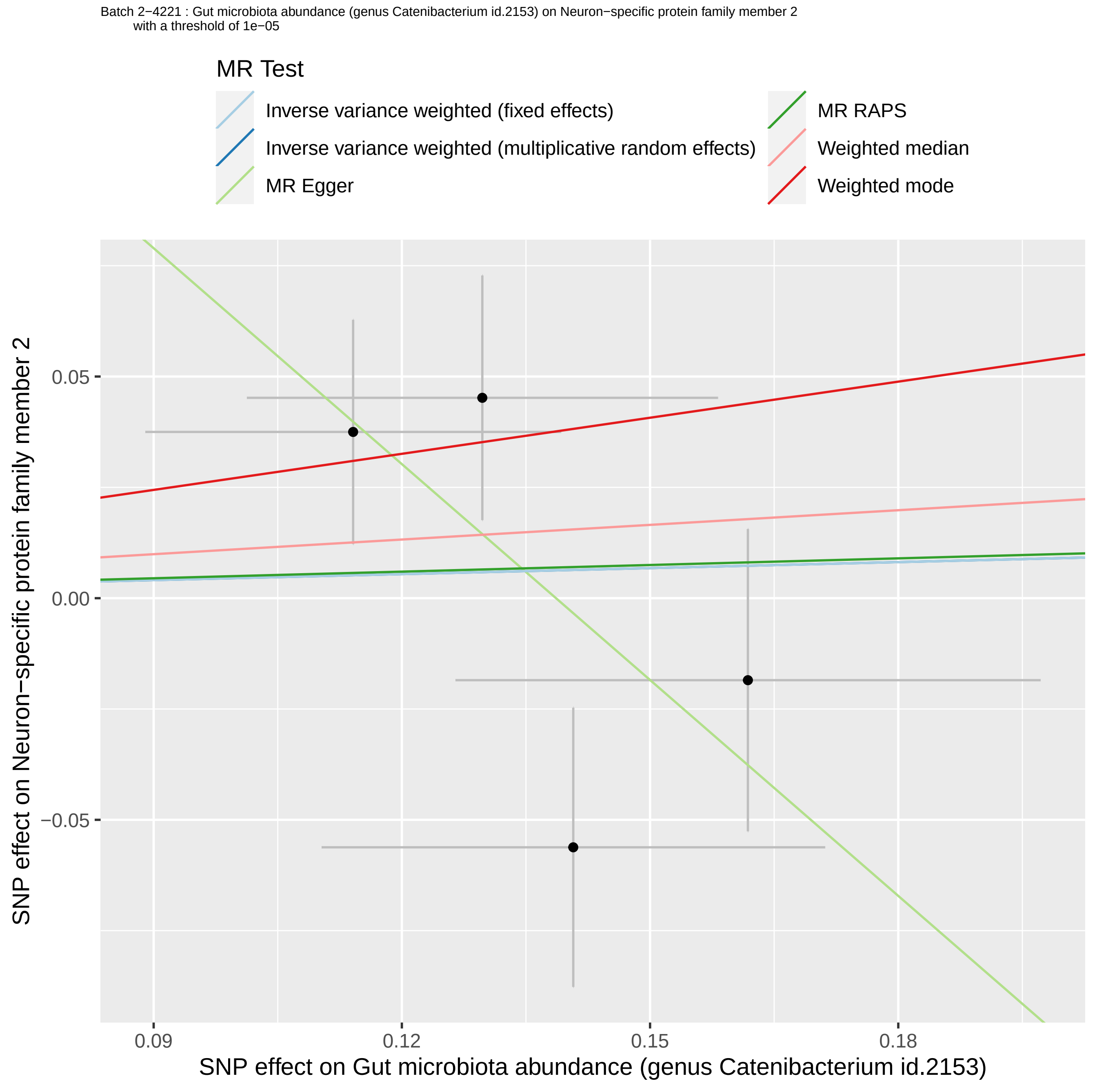
Batch 2−4221 : Gut microbiota abundance (genus Catenibacterium id.2153) on Neuron−specific protein family member 2
with a threshold of 1e−05
MR Test
Inverse variance weighted (fixed effects)
Inverse variance weighted (multiplicative random effects)
MR Egger
MR RAPS
Weighted median
Weighted mode
0.05
0.00
−0.05
0.09
0.12
0.15
0.18
SNP effect on Neuron−specific protein family member 2
SNP effect on Gut microbiota abundance (genus Catenibacterium id.2153)

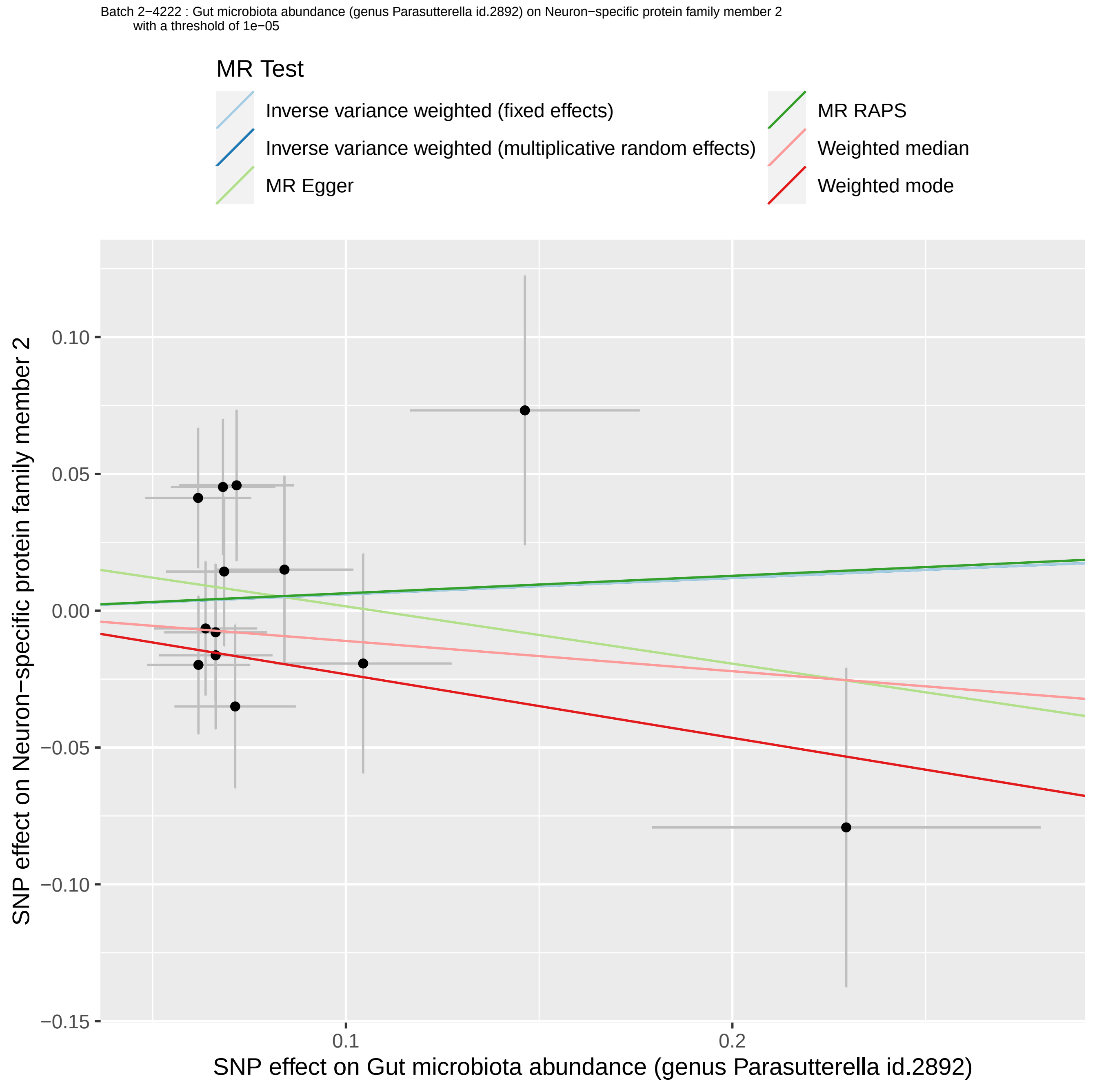
Batch 2−4222 : Gut microbiota abundance (genus Parasutterella id.2892) on Neuron−specific protein family member 2
with a threshold of 1e−05
MR Test
Inverse variance weighted (fixed effects)
Inverse variance weighted (multiplicative random effects)
MR Egger
MR RAPS
Weighted median
Weighted mode
0.10
0.05
0.00
−0.05
−0.10
−0.15
0.1
0.2
SNP effect on Neuron−specific protein family member 2
SNP effect on Gut microbiota abundance (genus Parasutterella id.2892)

Batch 2−4223 : Gut microbiota abundance (genus Catenibacterium id.2153) on 5'(3')−deoxyribonucleotidase, cytosolic type with a threshold of 1e−05

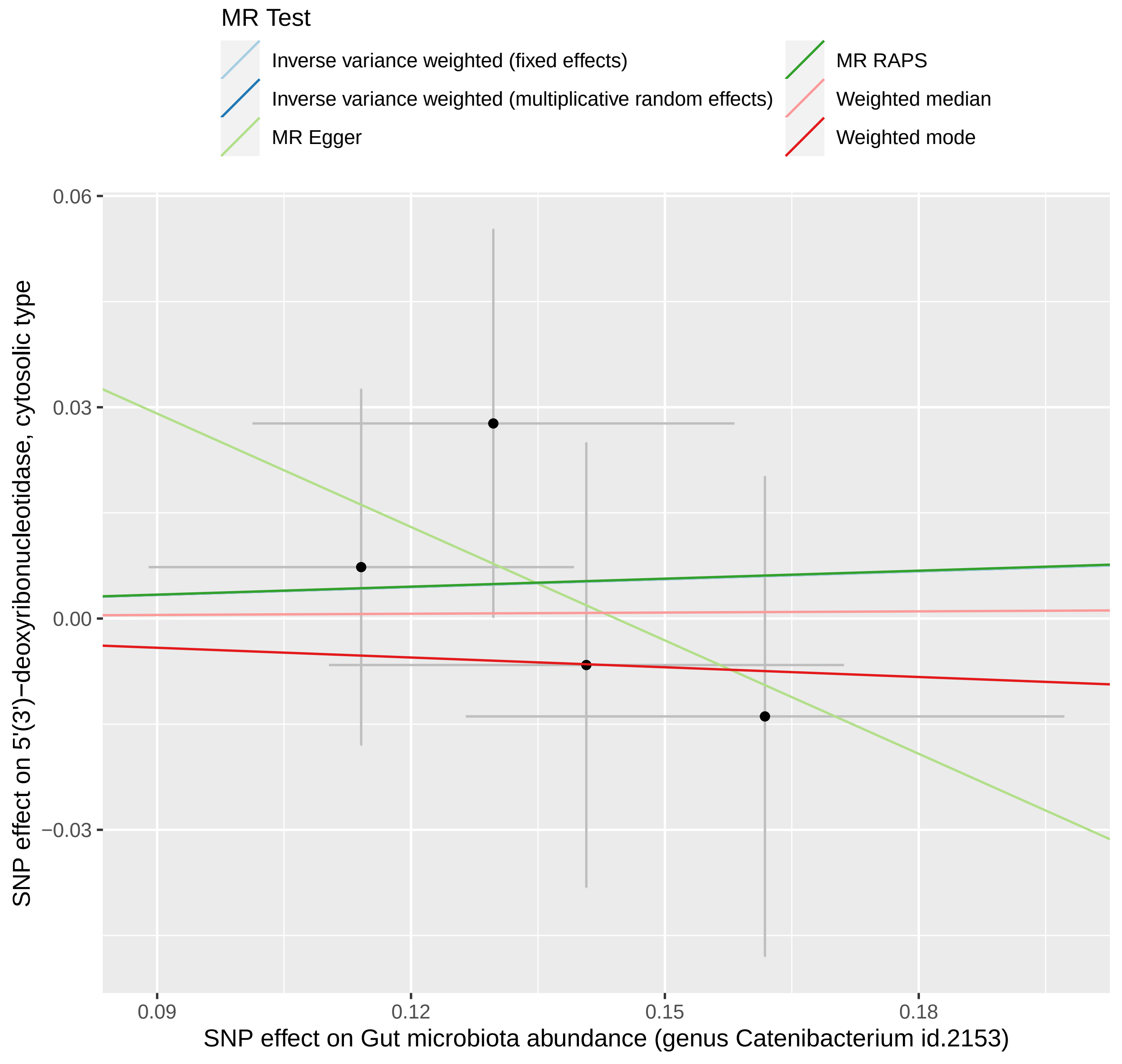

Batch 2−4224 : Gut microbiota abundance (genus Parasutterella id.2892) on 5'(3')−deoxyribonucleotidase, cytosolic type with a threshold of 1e−05

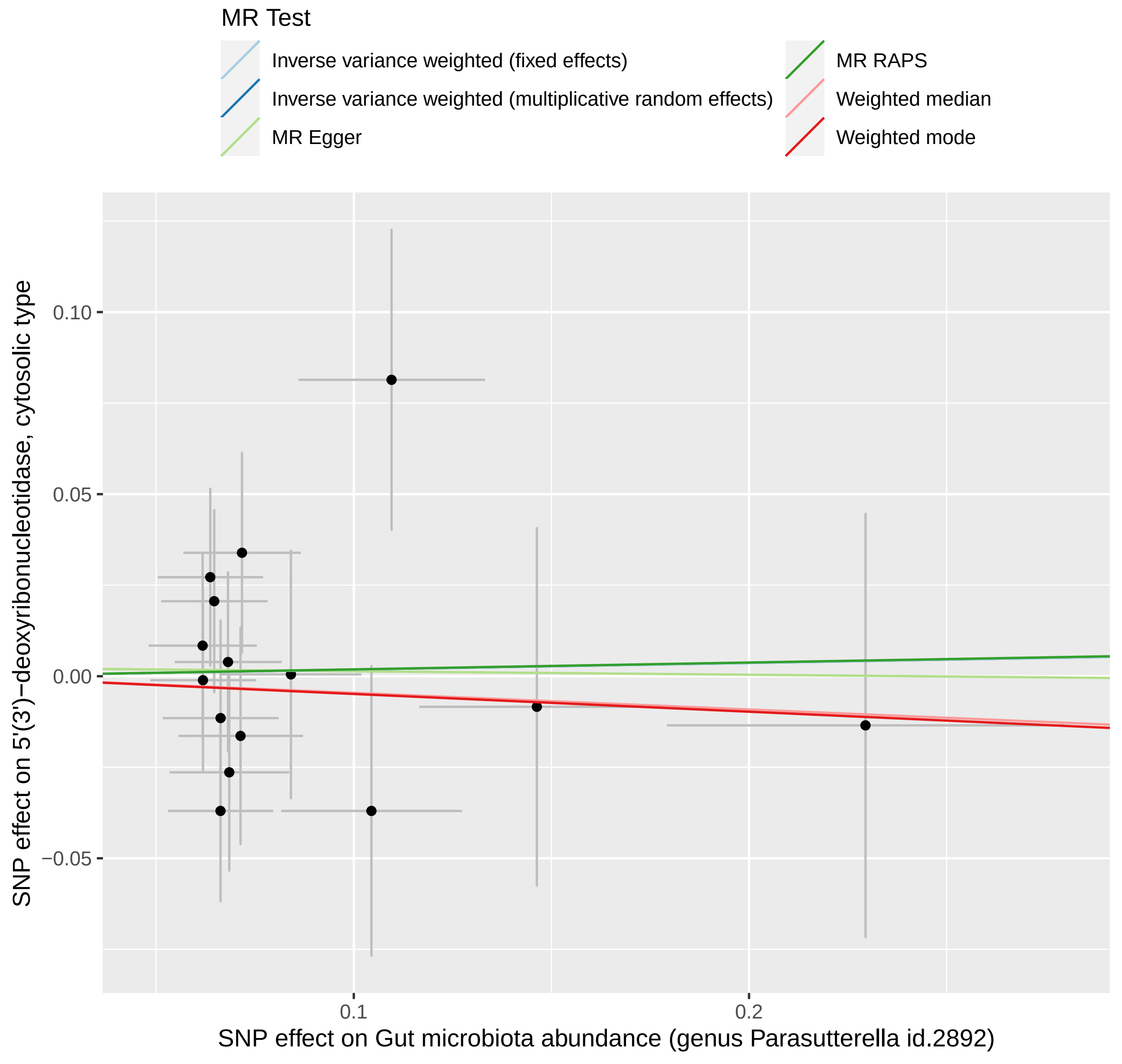

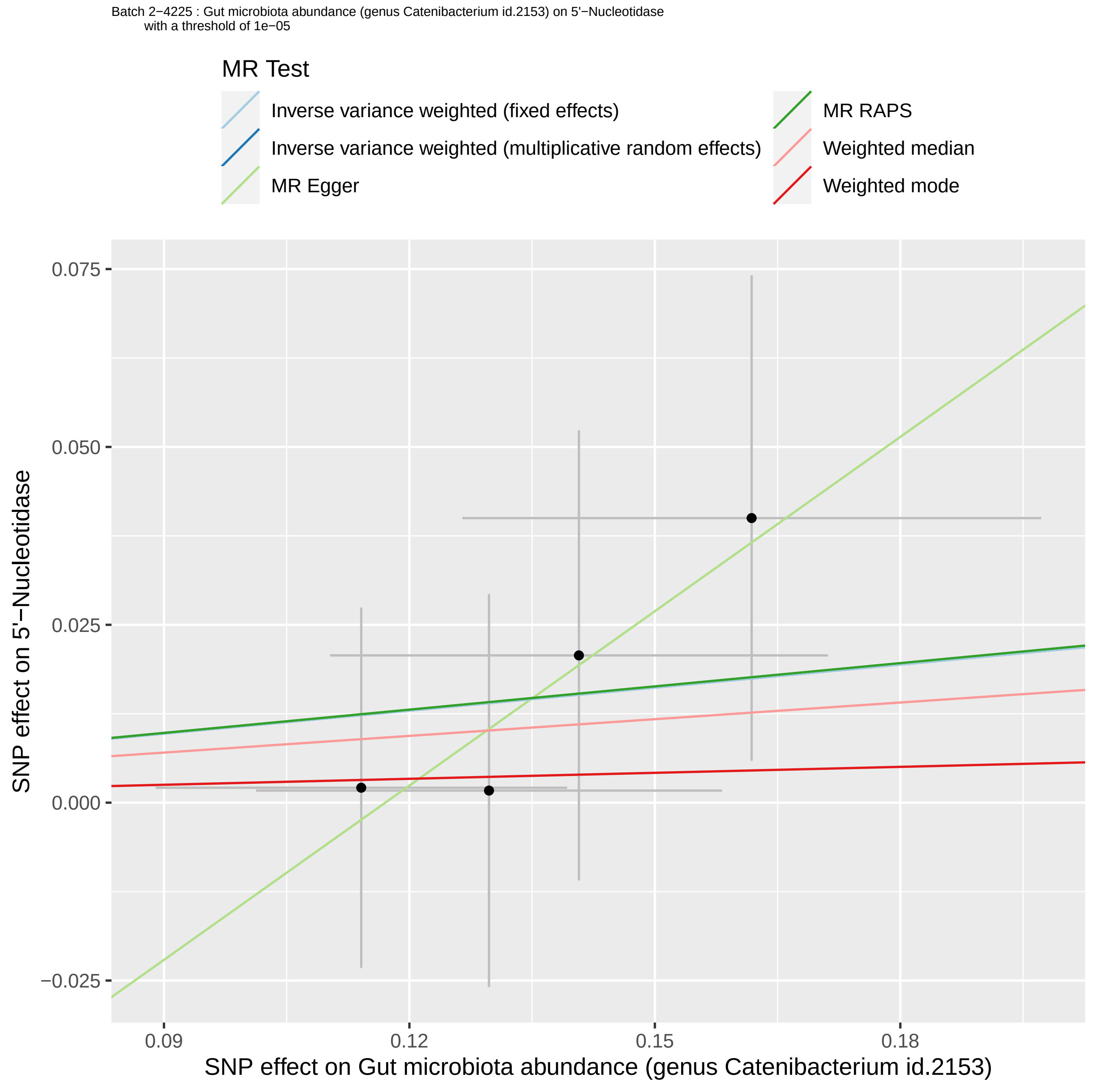
Batch 2−4225 : Gut microbiota abundance (genus Catenibacterium id.2153) on 5'−Nucleotidase
with a threshold of 1e−05
MR Test
Inverse variance weighted (fixed effects)
Inverse variance weighted (multiplicative random effects)
MR Egger
MR RAPS
Weighted median
Weighted mode
0.075
0.050
0.025
0.000
−0.025
0.09
0.12
0.15
0.18
SNP effect on 5'−Nucleotidase
SNP effect on Gut microbiota abundance (genus Catenibacterium id.2153)

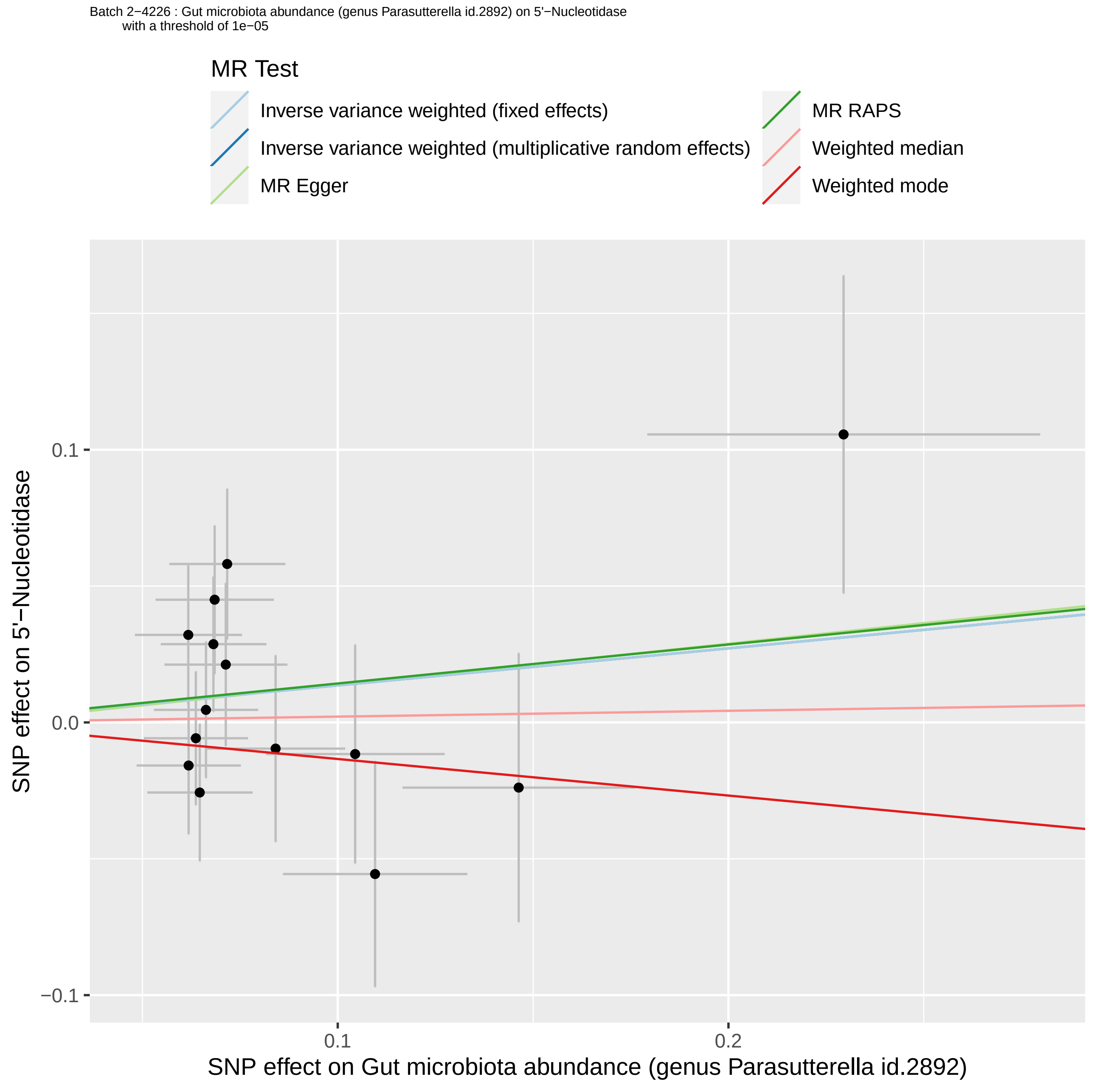

Batch 2−4226 : Gut microbiota abundance (genus Parasutterella id.2892) on 5'−Nucleotidase
with a threshold of 1e−05
MR Test
Inverse variance weighted (fixed effects)
Inverse variance weighted (multiplicative random effects)
MR Egger
MR RAPS
Weighted median
Weighted mode
0.1
0.0
−0.1
0.1
0.2
SNP effect on 5'−Nucleotidase
SNP effect on Gut microbiota abundance (genus Parasutterella id.2892)

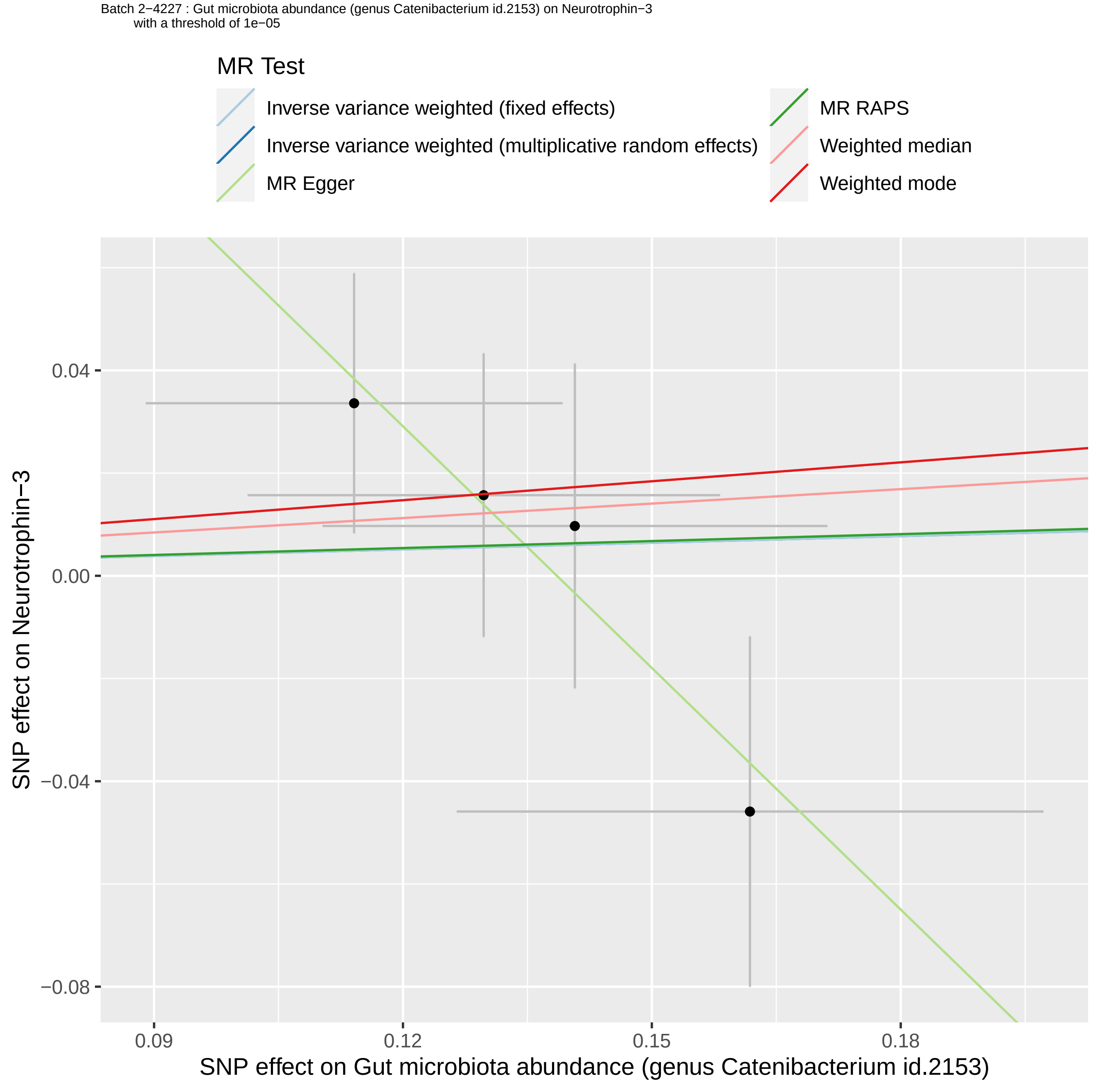

Batch 2−4227 : Gut microbiota abundance (genus Catenibacterium id.2153) on Neurotrophin−3
with a threshold of 1e−05
MR Test
Inverse variance weighted (fixed effects)
Inverse variance weighted (multiplicative random effects)
MR Egger
MR RAPS
Weighted median
Weighted mode
SNP effect on Neurotrophin−3
0.04
0.00
−0.04
−0.08
0.09
0.12
0.15
0.18
SNP effect on Gut microbiota abundance (genus Catenibacterium id.2153)

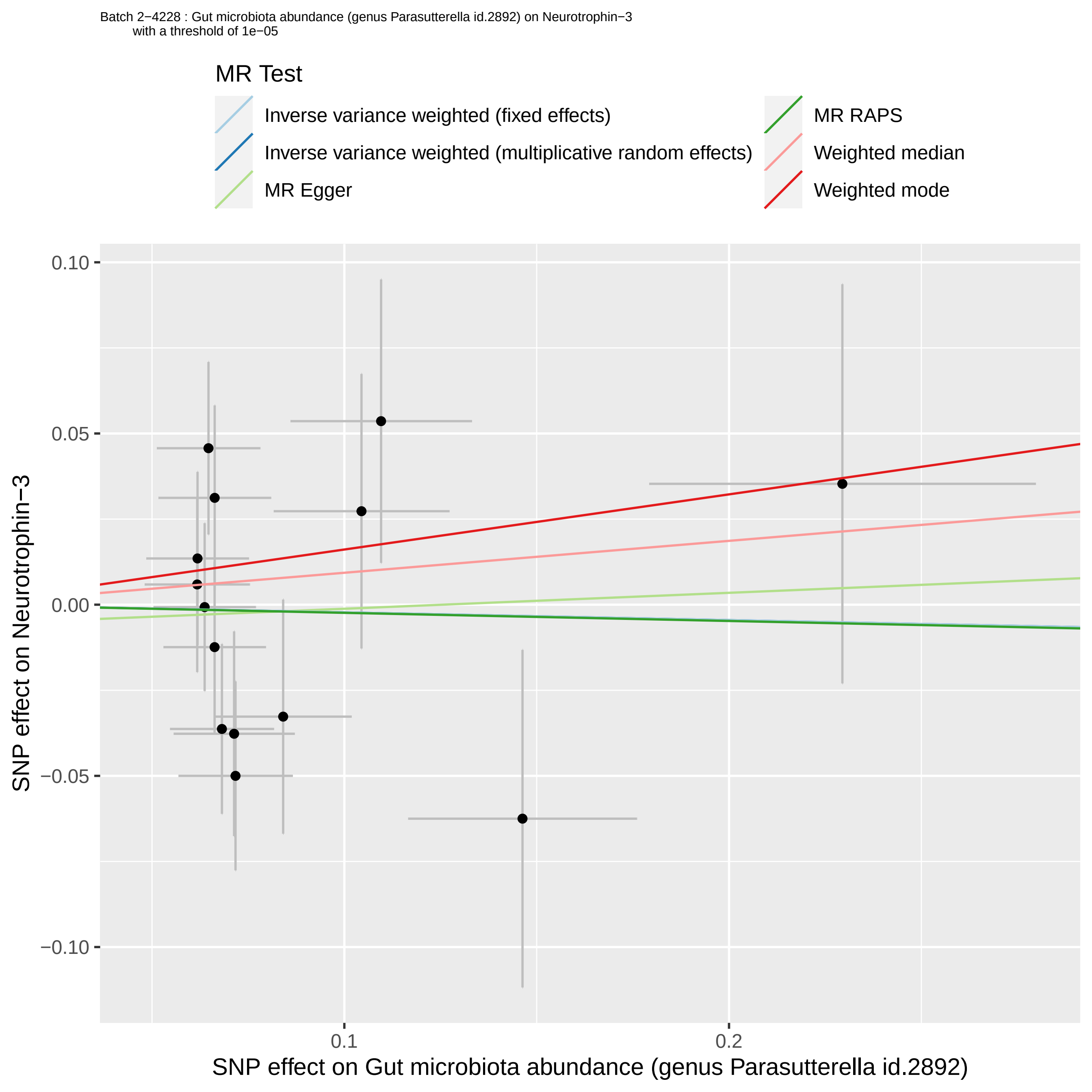
Batch 2−4228 : Gut microbiota abundance (genus Parasutterella id.2892) on Neurotrophin−3
with a threshold of 1e−05
MR Test
Inverse variance weighted (fixed effects)
Inverse variance weighted (multiplicative random effects)
MR Egger
MR RAPS
Weighted median
Weighted mode
SNP effect on Neurotrophin−3
0.10
0.05
0.00
−0.05
−0.10
0.1
0.2
SNP effect on Gut microbiota abundance (genus Parasutterella id.2892)

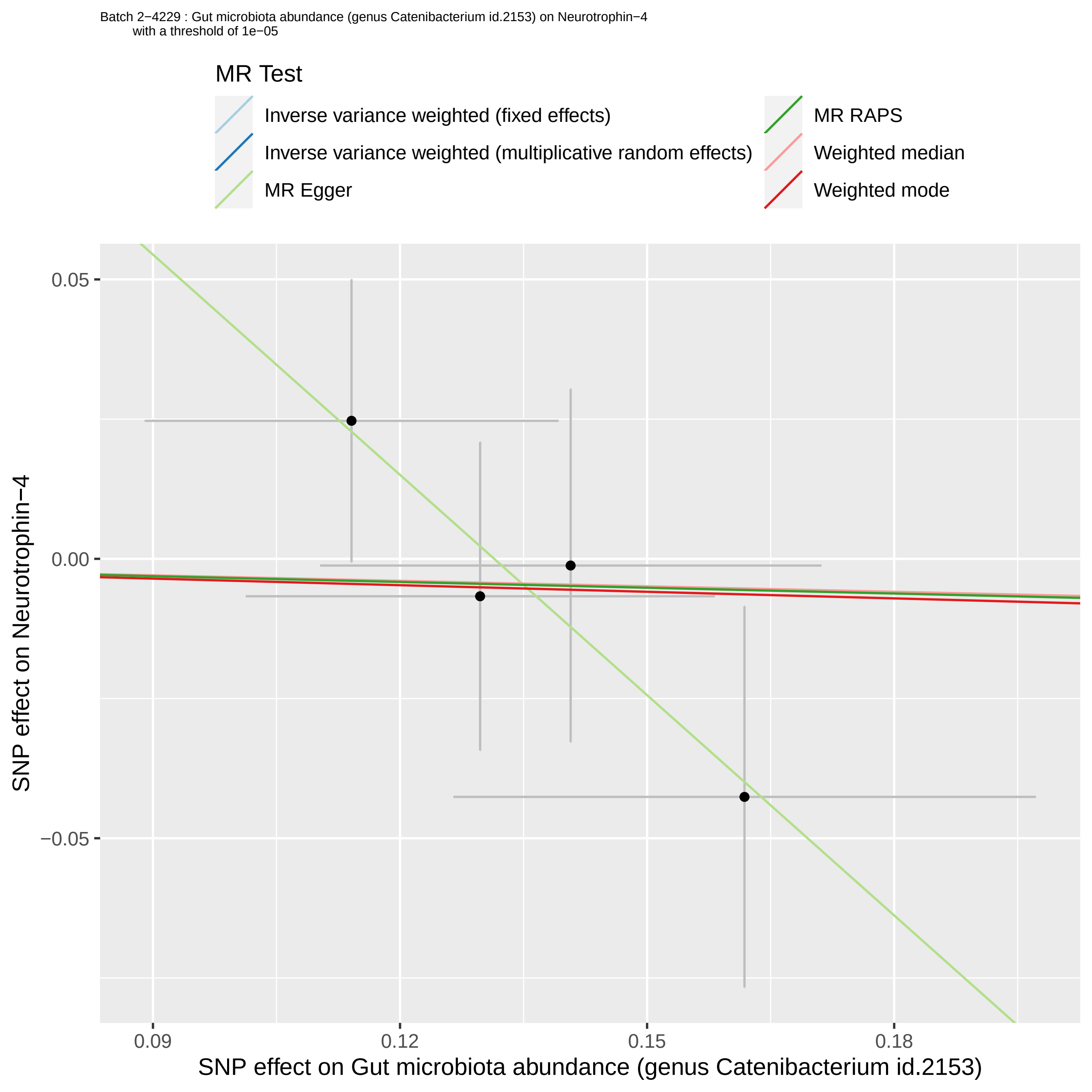
Batch 2−4229 : Gut microbiota abundance (genus Catenibacterium id.2153) on Neurotrophin−4
with a threshold of 1e−05
MR Test
Inverse variance weighted (fixed effects)
Inverse variance weighted (multiplicative random effects)
MR Egger
MR RAPS
Weighted median
Weighted mode
0.05
0.00
−0.05
SNP effect on Neurotrophin−4
0.09
0.12
0.15
0.18
SNP effect on Gut microbiota abundance (genus Catenibacterium id.2153)

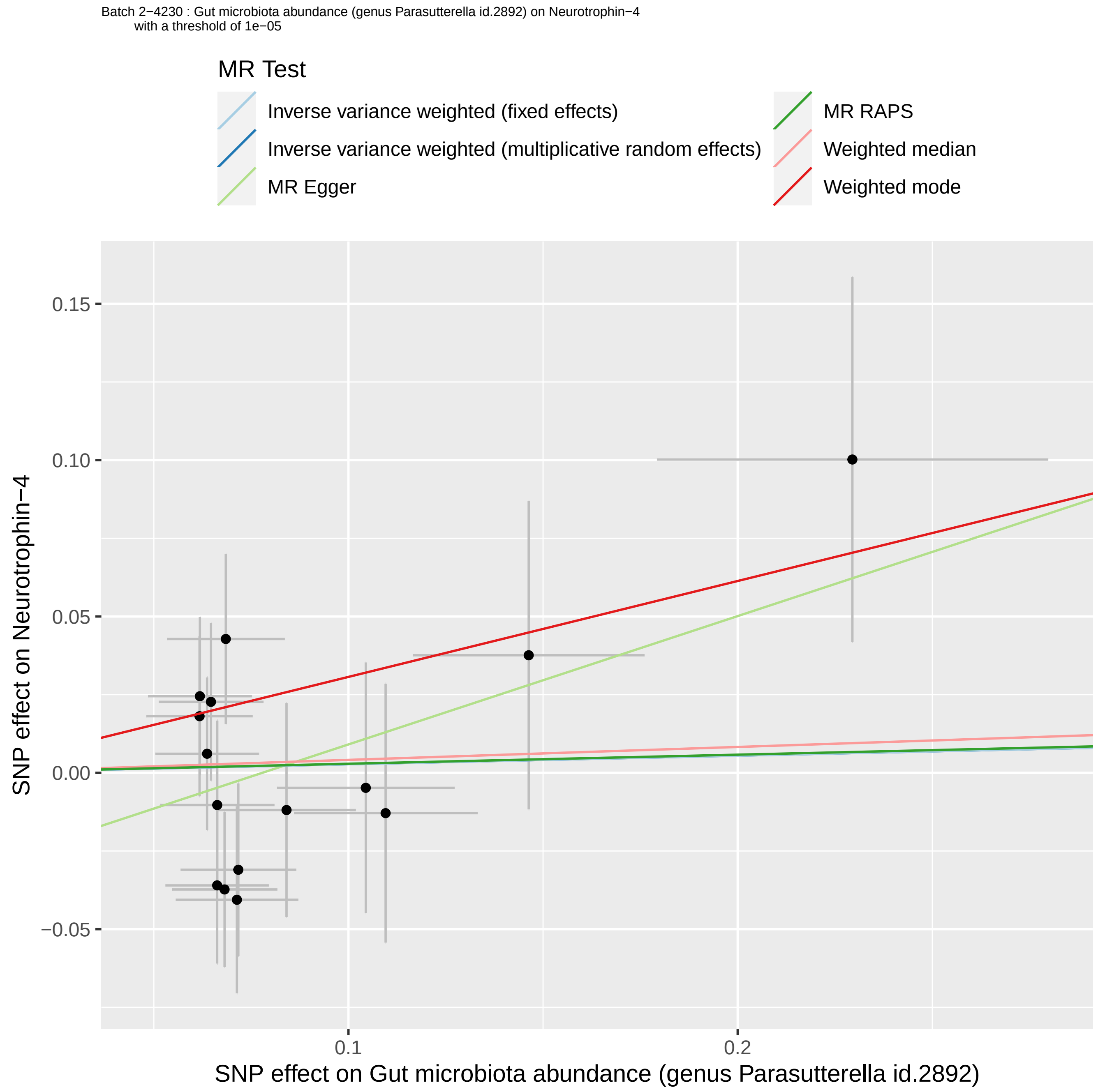

Batch 2−4230 : Gut microbiota abundance (genus Parasutterella id.2892) on Neurotrophin−4
with a threshold of 1e−05
MR Test
Inverse variance weighted (fixed effects)
Inverse variance weighted (multiplicative random effects)
MR Egger
MR RAPS
Weighted median
Weighted mode
SNP effect on Neurotrophin−4
0.15
0.10
0.05
0.00
−0.05
0.1
0.2
SNP effect on Gut microbiota abundance (genus Parasutterella id.2892)

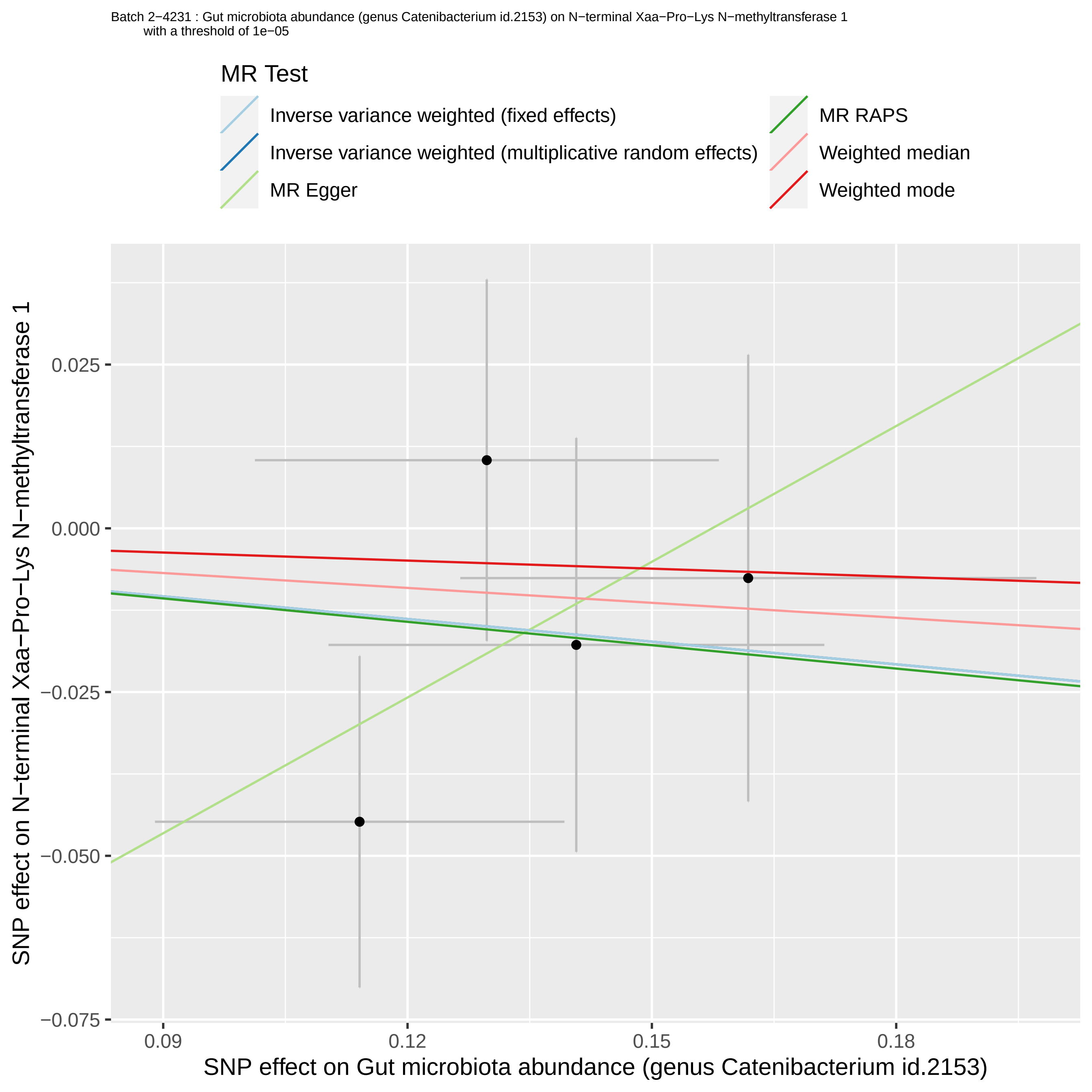
Batch 2−4231 : Gut microbiota abundance (genus Catenibacterium id.2153) on N−terminal Xaa−Pro−Lys N−methyltransferase 1
with a threshold of 1e−05
MR Test
Inverse variance weighted (fixed effects)
Inverse variance weighted (multiplicative random effects)
MR Egger
MR RAPS
Weighted median
Weighted mode
SNP effect on N−terminal Xaa−Pro−Lys N−methyltransferase 1
0.025
0.000
−0.025
−0.050
−0.075
0.09
0.12
0.15
0.18
SNP effect on Gut microbiota abundance (genus Catenibacterium id.2153)

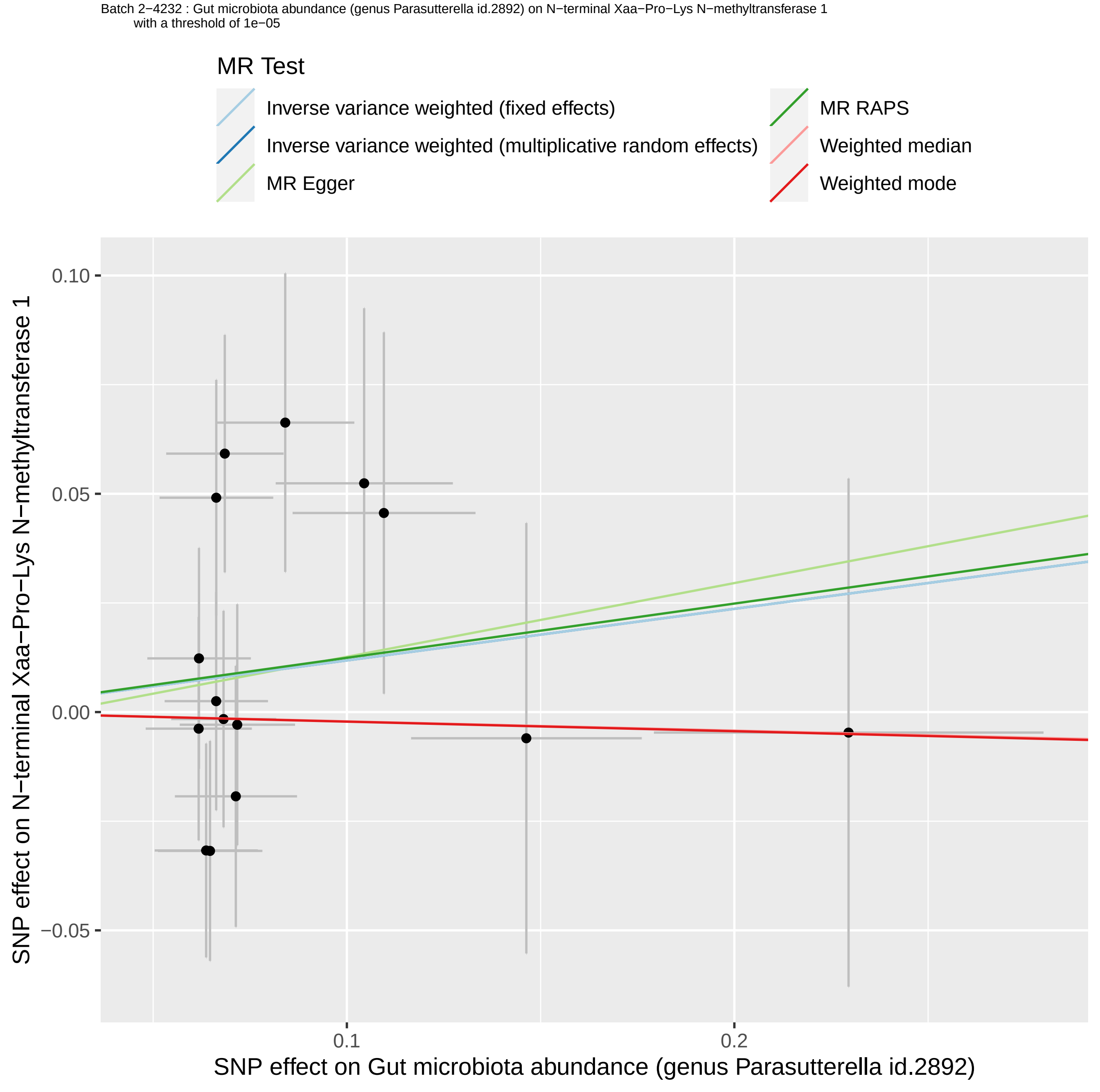

Batch 2−4232 : Gut microbiota abundance (genus Parasutterella id.2892) on N−terminal Xaa−Pro−Lys N−methyltransferase 1
with a threshold of 1e−05
MR Test
Inverse variance weighted (fixed effects)
Inverse variance weighted (multiplicative random effects)
MR Egger
MR RAPS
Weighted median
Weighted mode
0.10
0.05
0.00
−0.05
0.1
0.2
SNP effect on N−terminal Xaa−Pro−Lys N−methyltransferase 1
SNP effect on Gut microbiota abundance (genus Parasutterella id.2892)

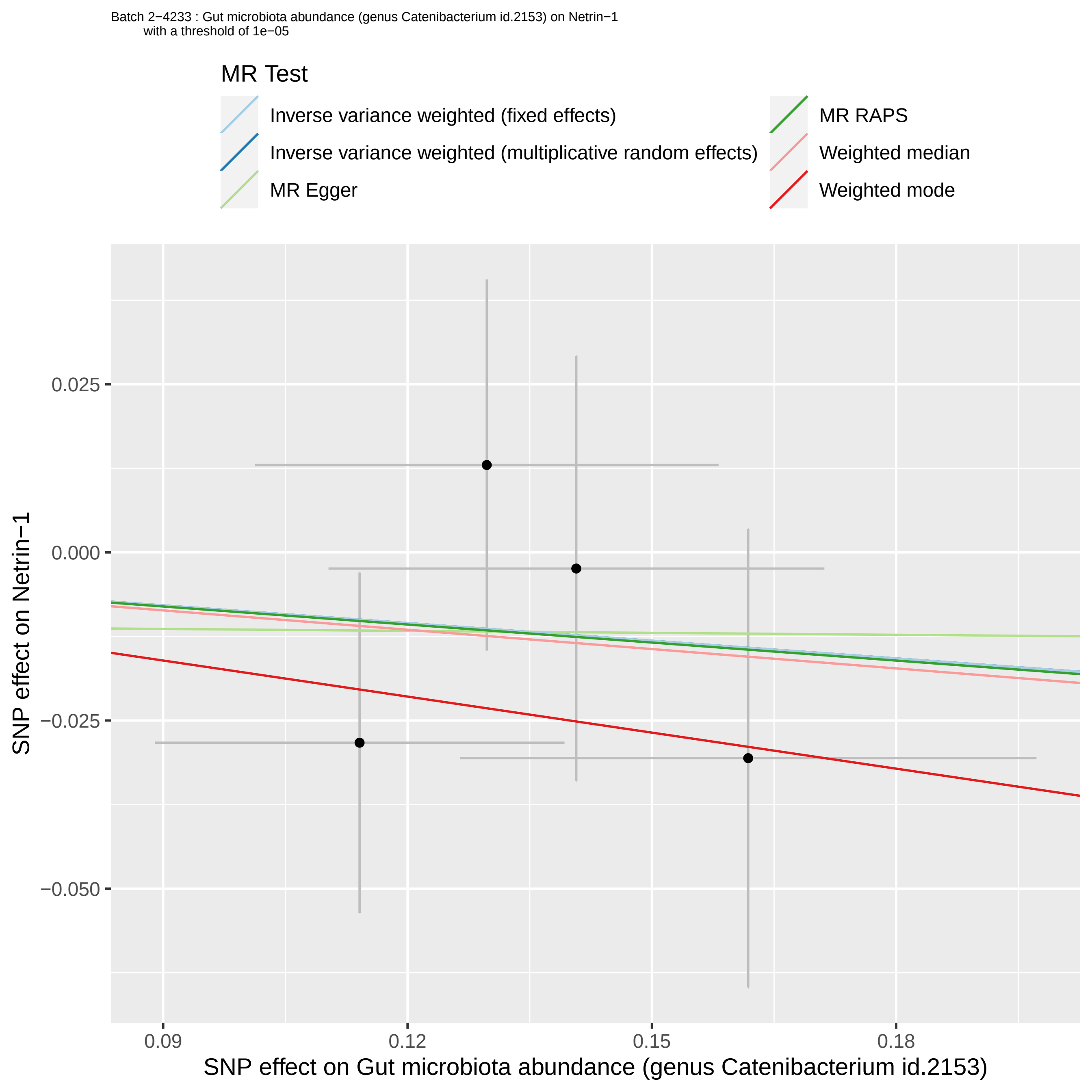
Batch 2−4233 : Gut microbiota abundance (genus Catenibacterium id.2153) on Netrin−1
with a threshold of 1e−05
MR Test
Inverse variance weighted (fixed effects)
Inverse variance weighted (multiplicative random effects)
MR Egger
MR RAPS
Weighted median
Weighted mode
0.025
0.000
−0.025
−0.050
0.09
0.12
0.15
0.18
SNP effect on Netrin−1
SNP effect on Gut microbiota abundance (genus Catenibacterium id.2153)

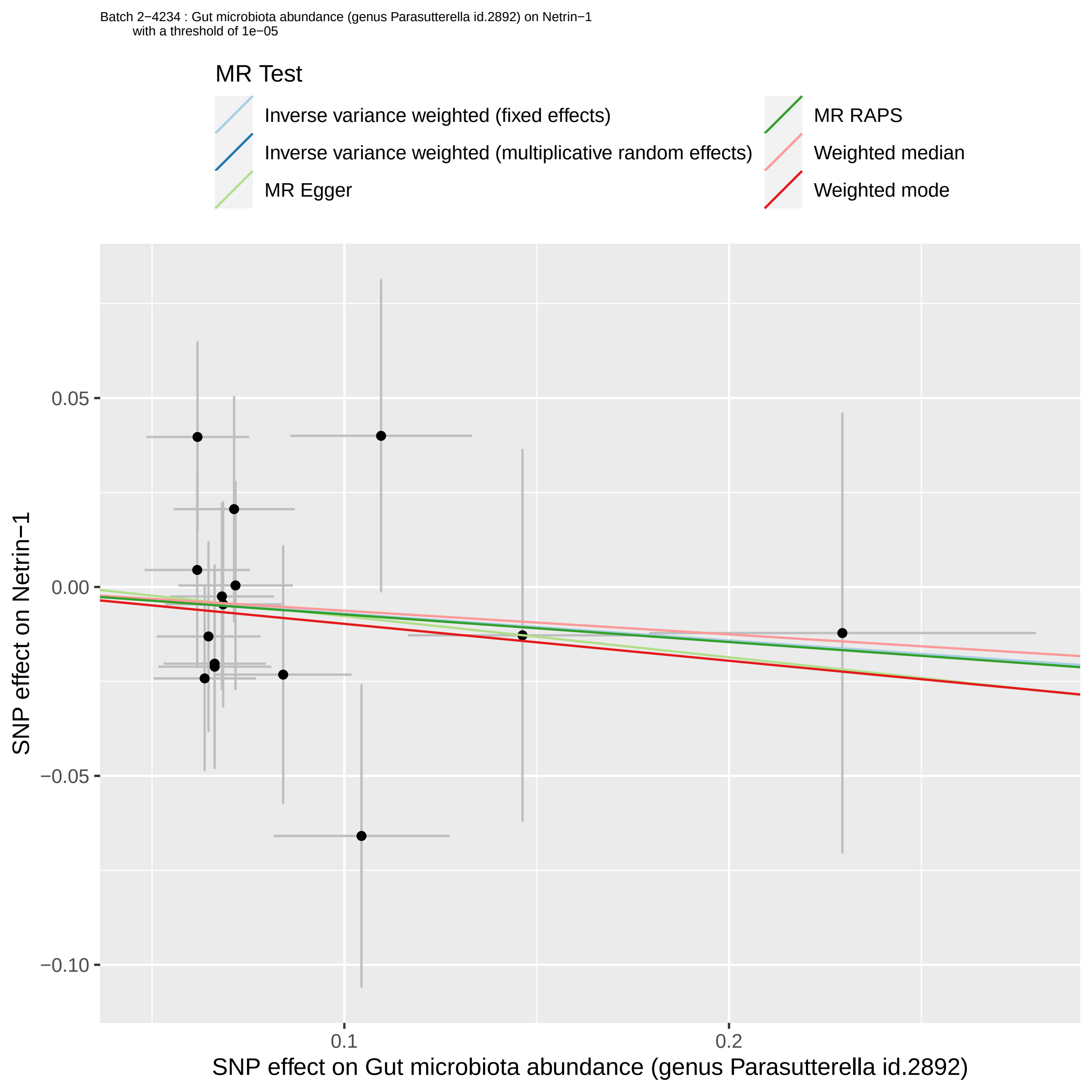

Batch 2−4234 : Gut microbiota abundance (genus Parasutterella id.2892) on Netrin−1
with a threshold of 1e−05
MR Test
Inverse variance weighted (fixed effects)
Inverse variance weighted (multiplicative random effects)
MR Egger
MR RAPS
Weighted median
Weighted mode
0.05
0.00
−0.05
−0.10
0.1
0.2
SNP effect on Netrin−1
SNP effect on Gut microbiota abundance (genus Parasutterella id.2892)

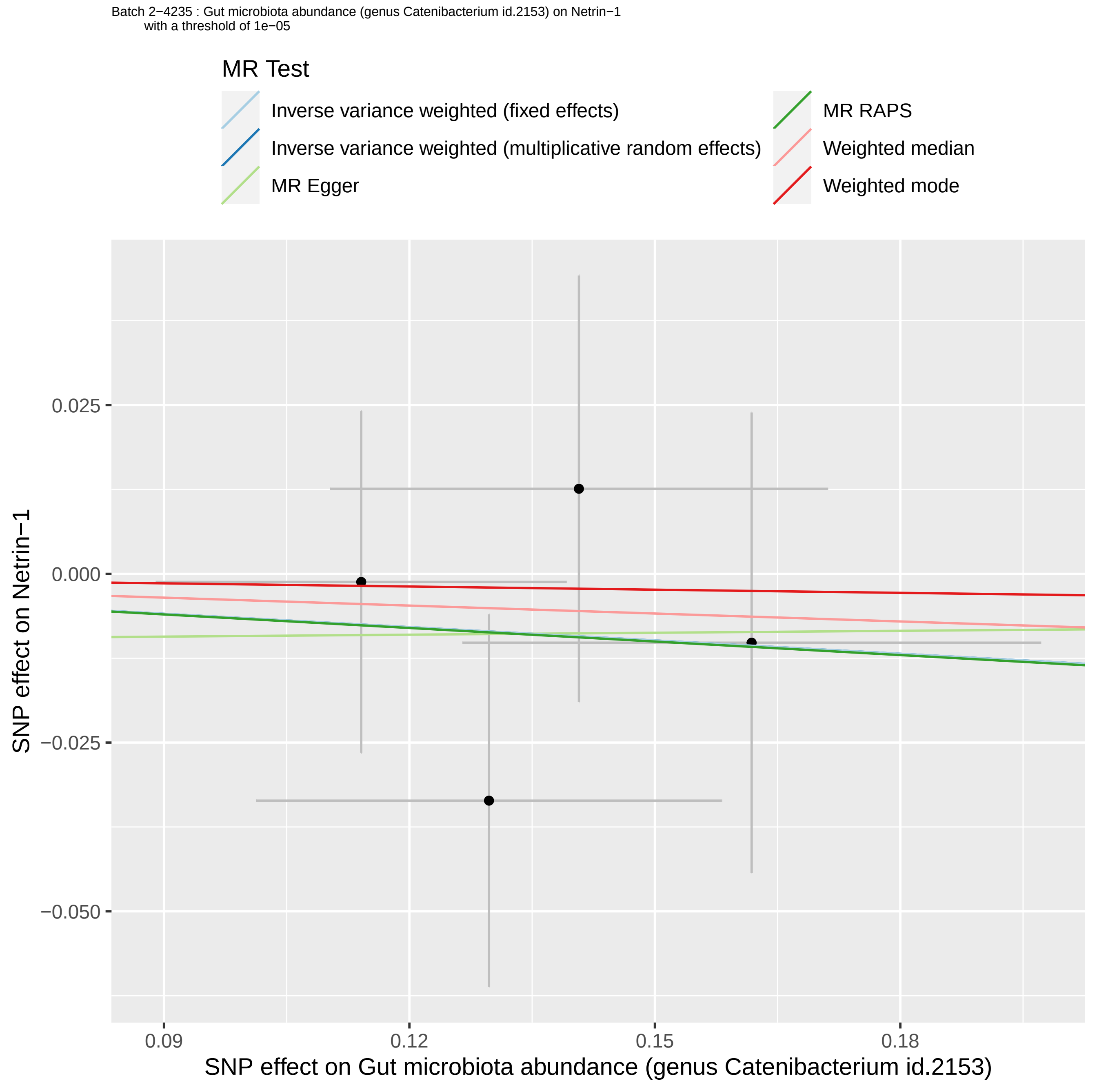
Batch 2−4235 : Gut microbiota abundance (genus Catenibacterium id.2153) on Netrin−1
with a threshold of 1e−05
MR Test
Inverse variance weighted (fixed effects)
Inverse variance weighted (multiplicative random effects)
MR Egger
MR RAPS
Weighted median
Weighted mode
0.025
0.000
−0.025
−0.050
0.09
0.12
0.15
0.18
SNP effect on Netrin−1
SNP effect on Gut microbiota abundance (genus Catenibacterium id.2153)

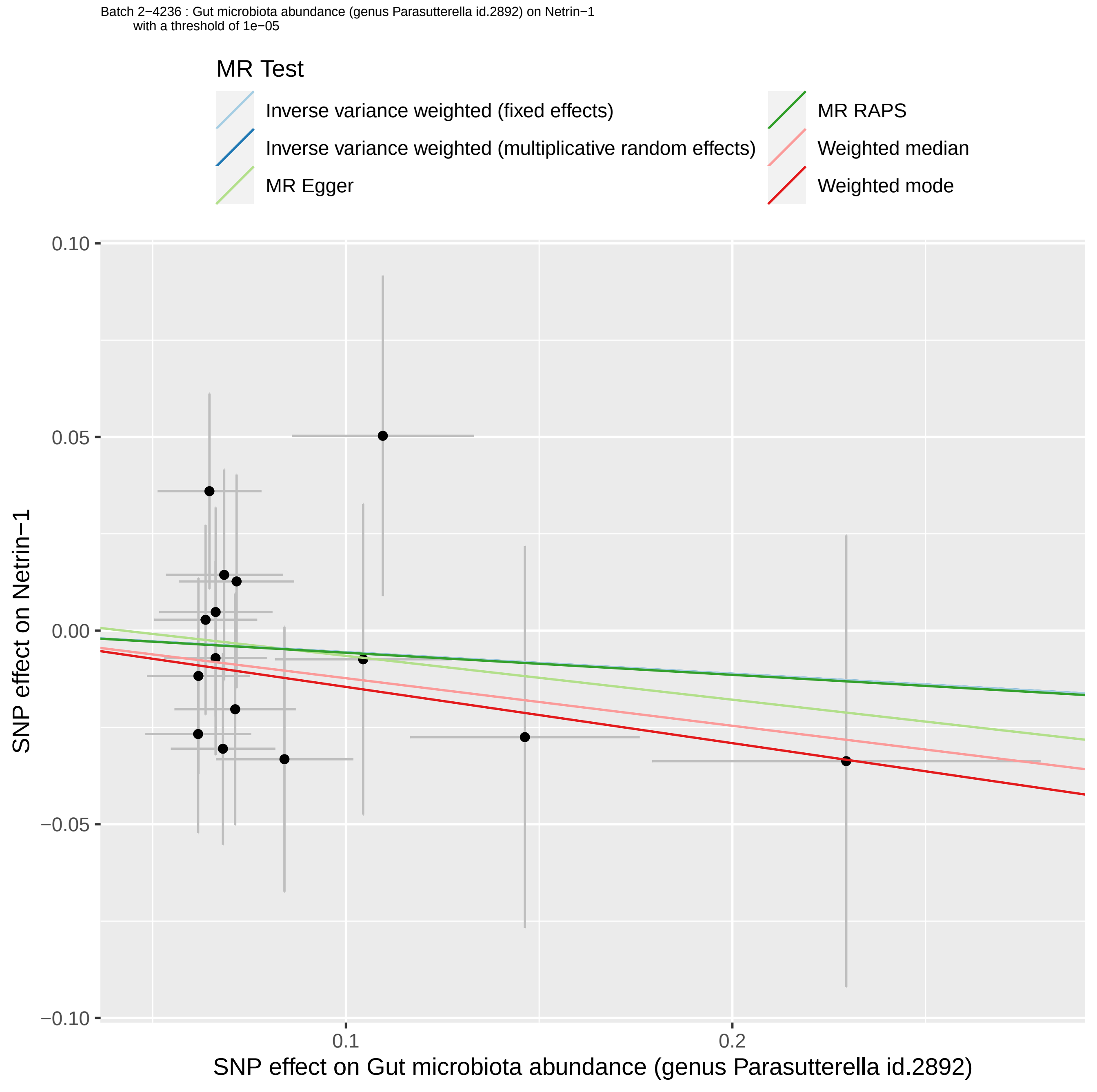

Batch 2−4236 : Gut microbiota abundance (genus Parasutterella id.2892) on Netrin−1
with a threshold of 1e−05
MR Test
Inverse variance weighted (fixed effects)
Inverse variance weighted (multiplicative random effects)
MR Egger
MR RAPS
Weighted median
Weighted mode
0.10
0.05
0.00
−0.05
−0.10
0.1
0.2
SNP effect on Netrin−1
SNP effect on Gut microbiota abundance (genus Parasutterella id.2892)

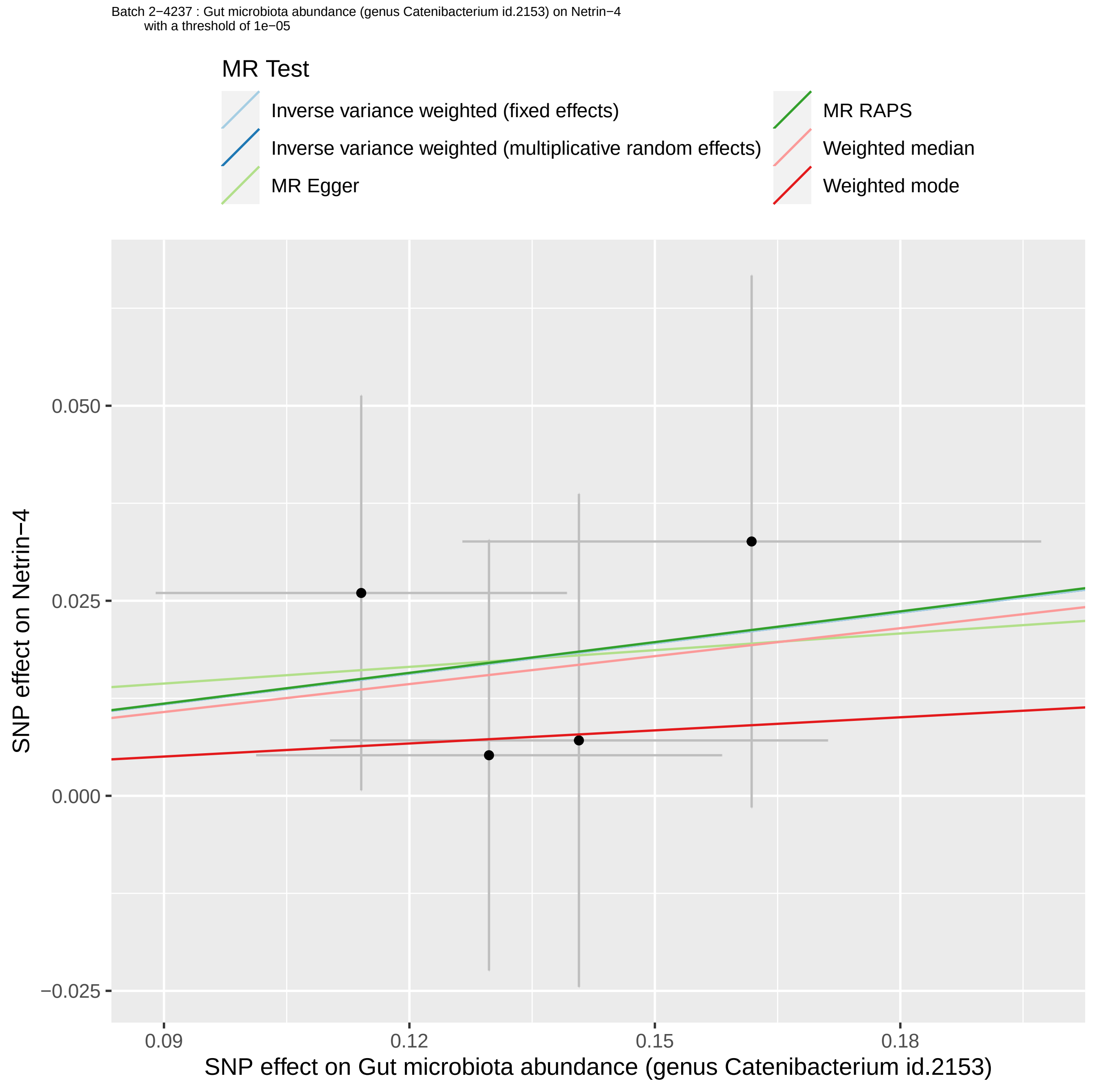
Batch 2−4237 : Gut microbiota abundance (genus Catenibacterium id.2153) on Netrin−4
with a threshold of 1e−05
MR Test
Inverse variance weighted (fixed effects)
Inverse variance weighted (multiplicative random effects)
MR Egger
MR RAPS
Weighted median
Weighted mode
0.050
0.025
0.000
−0.025
0.09
0.12
0.15
0.18
SNP effect on Netrin−4
SNP effect on Gut microbiota abundance (genus Catenibacterium id.2153)

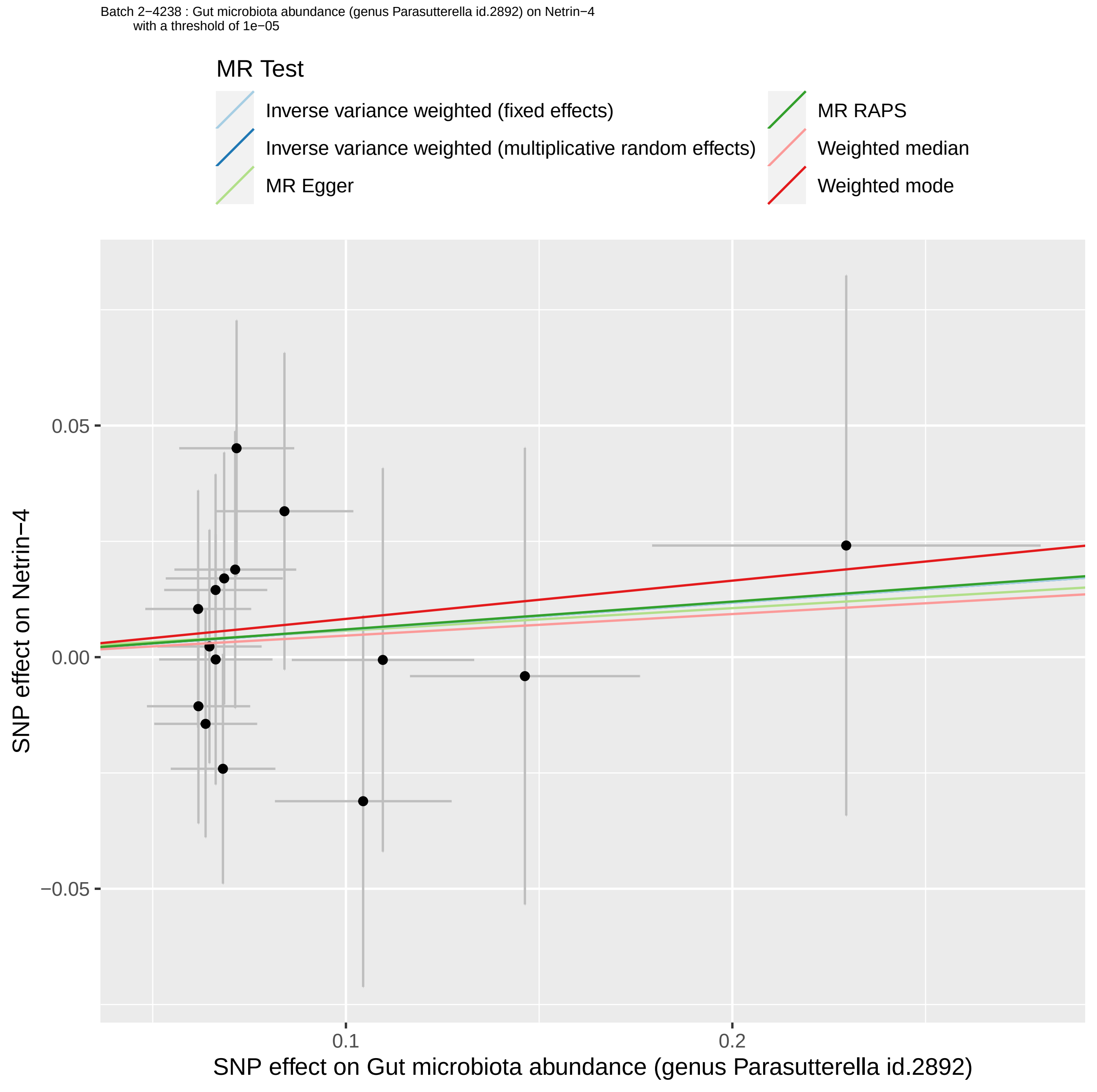

Batch 2−4238 : Gut microbiota abundance (genus Parasutterella id.2892) on Netrin−4
with a threshold of 1e−05
MR Test
Inverse variance weighted (fixed effects)
Inverse variance weighted (multiplicative random effects)
MR Egger
MR RAPS
Weighted median
Weighted mode
0.05
0.00
−0.05
0.1
0.2
SNP effect on Netrin−4
SNP effect on Gut microbiota abundance (genus Parasutterella id.2892)

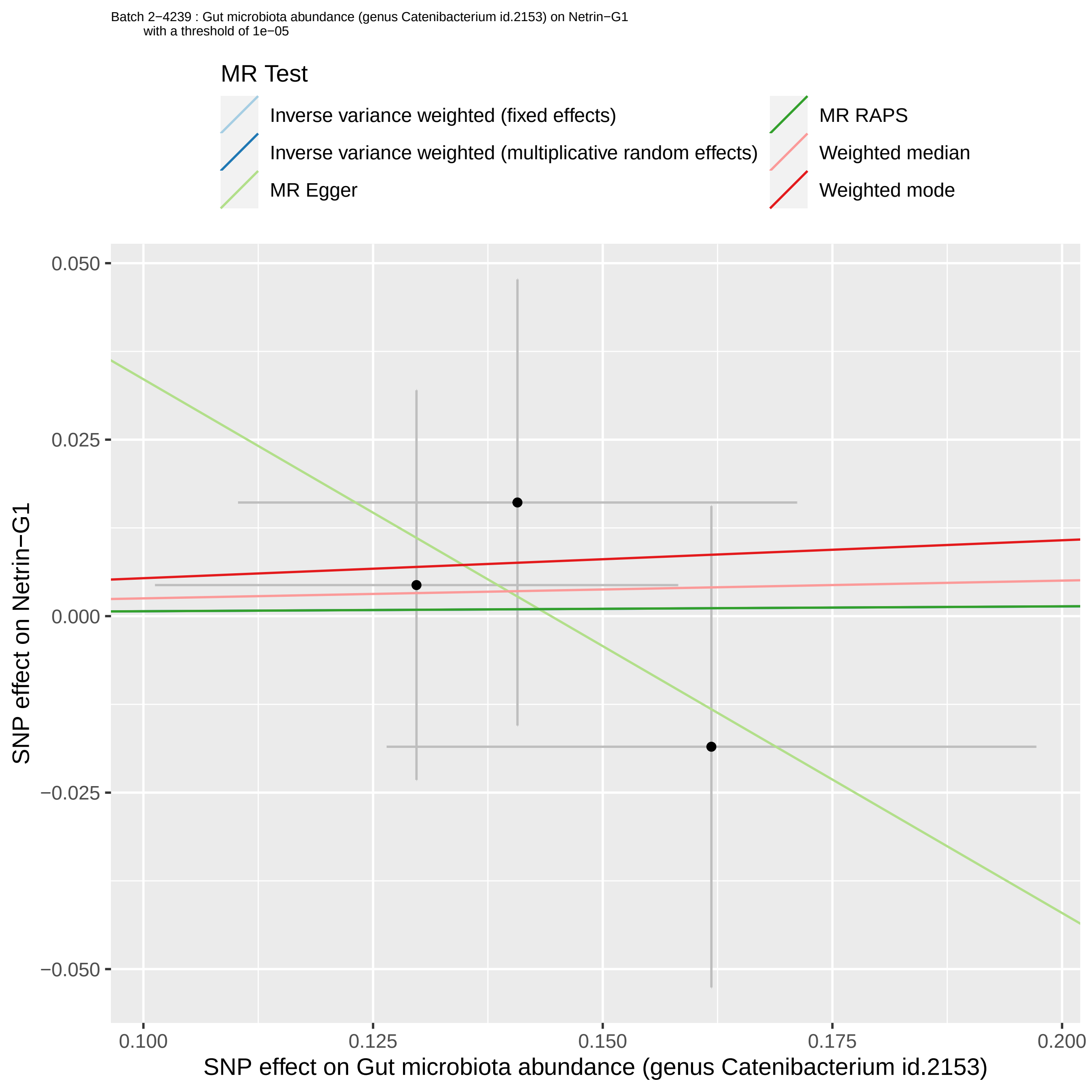

Batch 2−4239 : Gut microbiota abundance (genus Catenibacterium id.2153) on Netrin−G1
with a threshold of 1e−05
MR Test
Inverse variance weighted (fixed effects)
Inverse variance weighted (multiplicative random effects)
MR Egger
MR RAPS
Weighted median
Weighted mode
0.050
0.025
0.000
−0.025
−0.050
SNP effect on Netrin−G1
0.100
0.125
0.150
0.175
0.200
SNP effect on Gut microbiota abundance (genus Catenibacterium id.2153)

Batch 2−4240 : Gut microbiota abundance (genus Parasutterella id.2892) on Netrin−G1
with a threshold of 1e−05

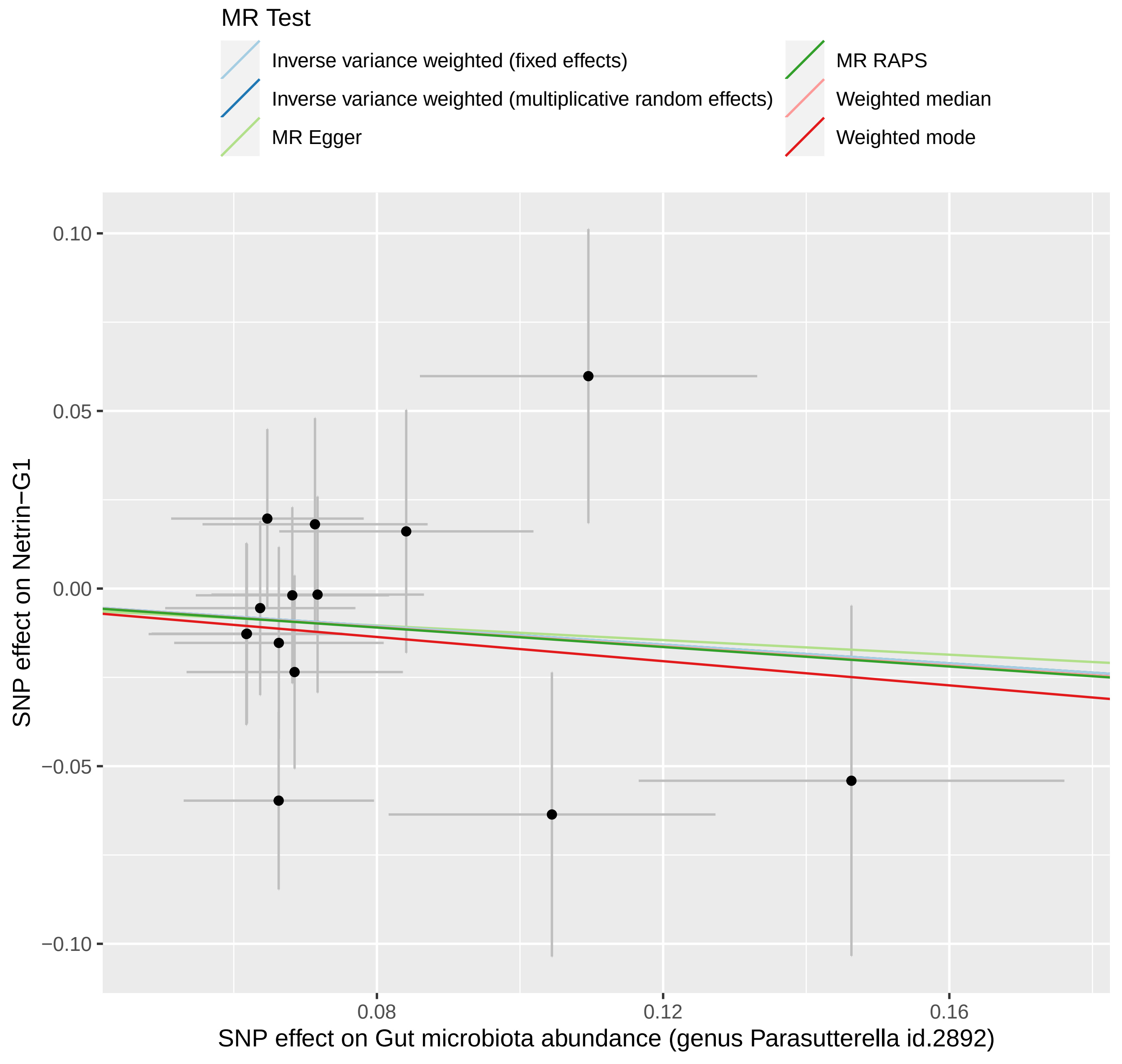

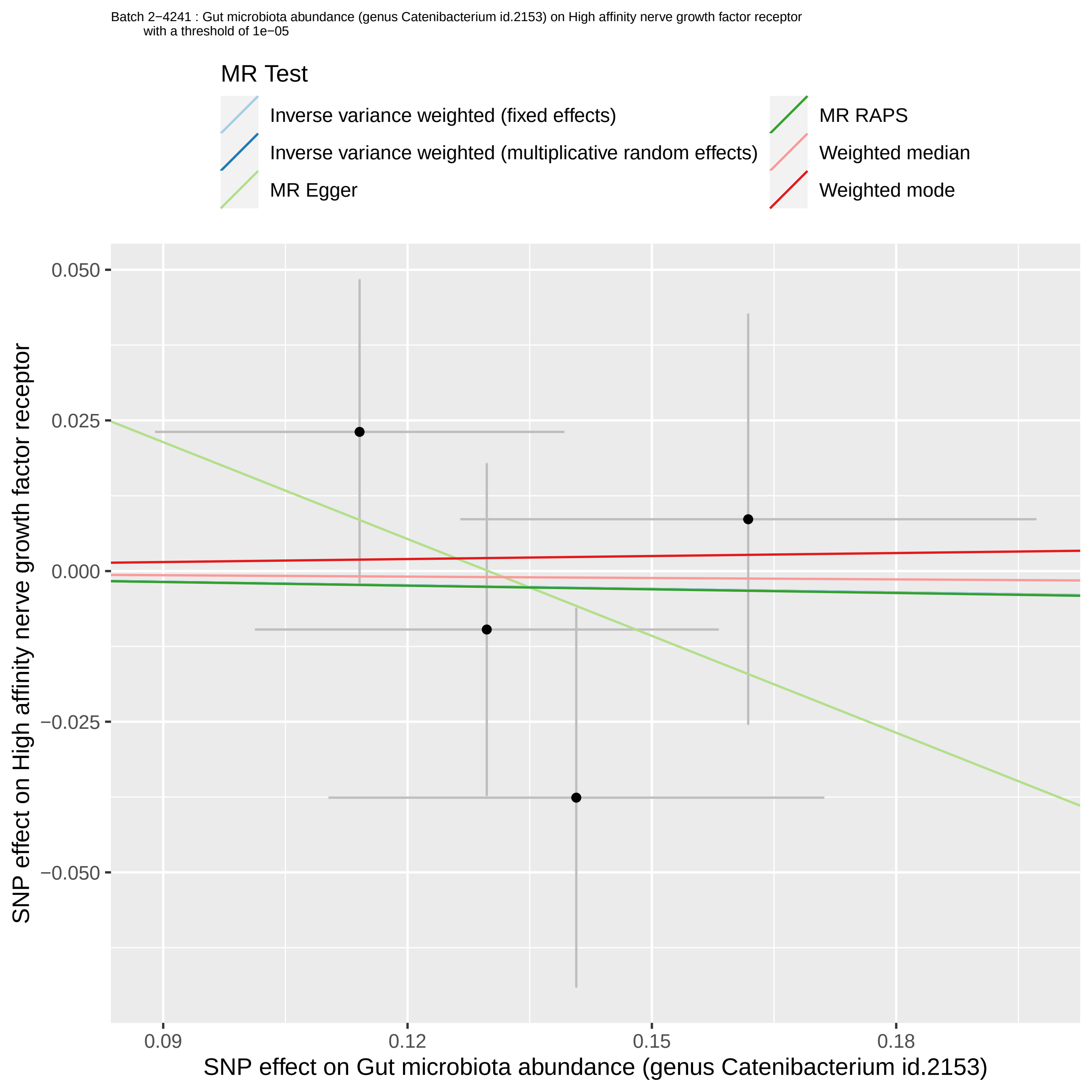
Batch 2−4241 : Gut microbiota abundance (genus Catenibacterium id.2153) on High affinity nerve growth factor receptor
with a threshold of 1e−05
MR Test
Inverse variance weighted (fixed effects)
Inverse variance weighted (multiplicative random effects)
MR Egger
MR RAPS
Weighted median
Weighted mode
0.050
0.025
0.000
−0.025
−0.050
0.09
0.12
0.15
0.18
SNP effect on High affinity nerve growth factor receptor
SNP effect on Gut microbiota abundance (genus Catenibacterium id.2153)

Batch 2−4242 : Gut microbiota abundance (genus Parasutterella id.2892) on High affinity nerve growth factor receptor
with a threshold of 1e−05

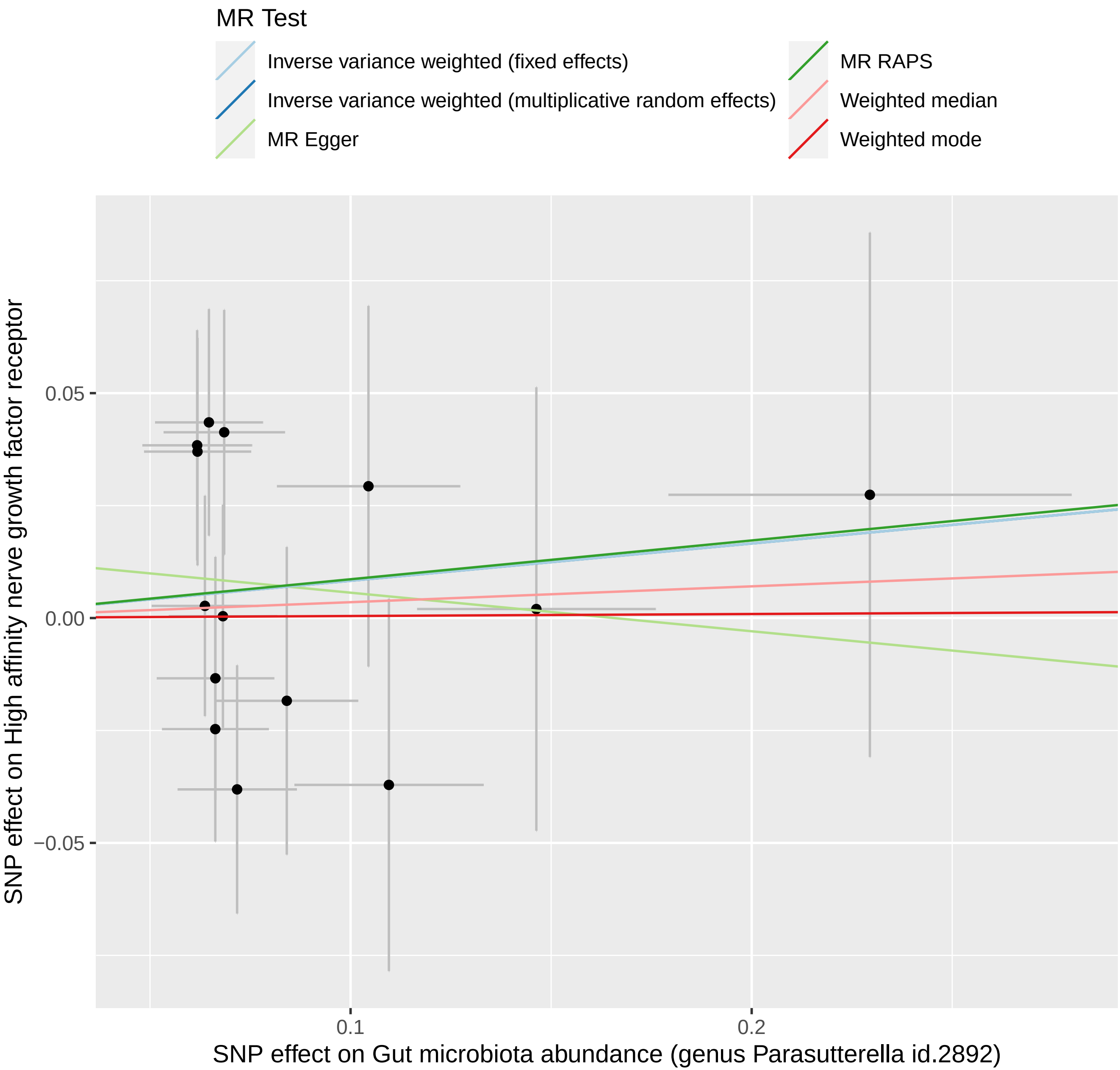

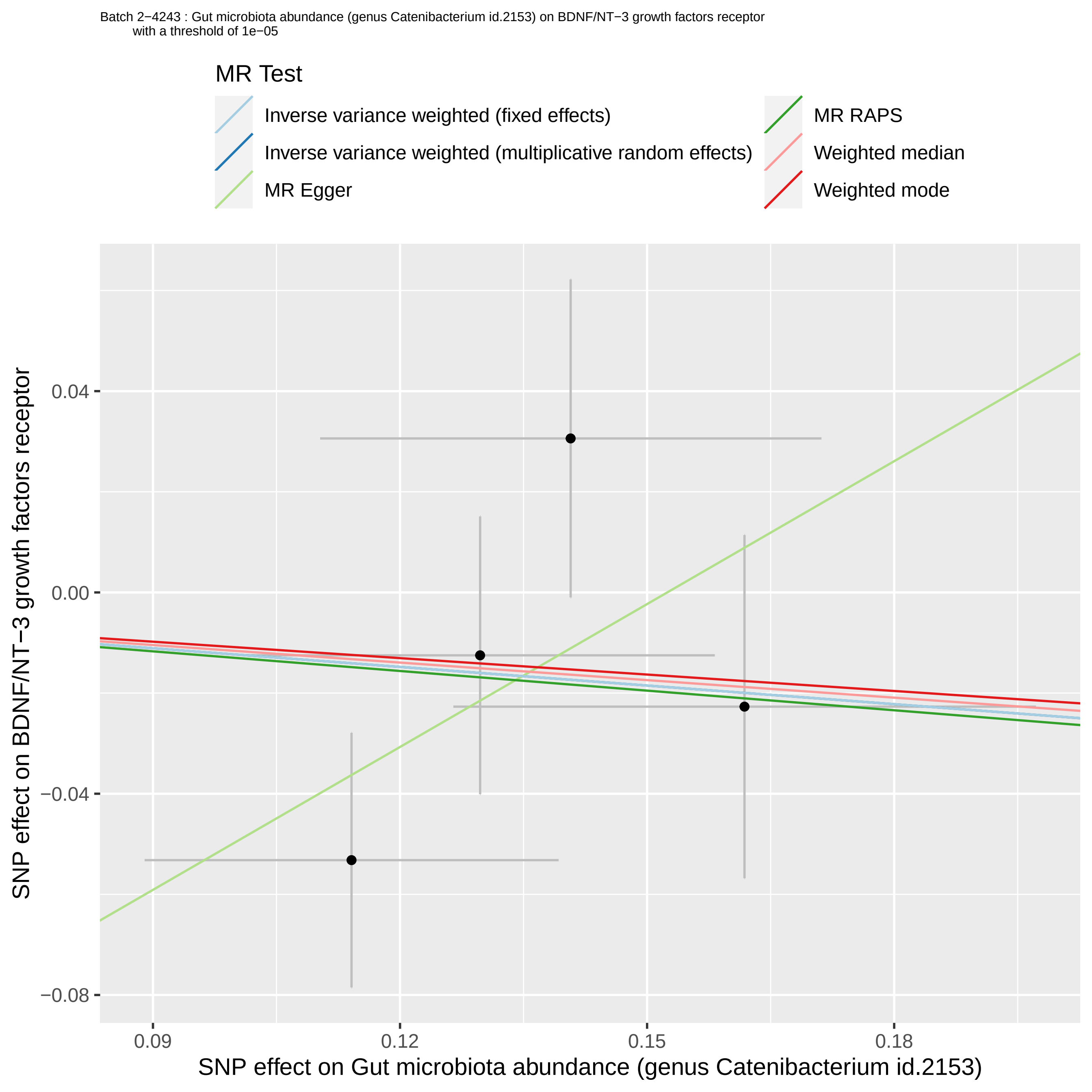
Batch 2−4243 : Gut microbiota abundance (genus Catenibacterium id.2153) on BDNF/NT−3 growth factors receptor
with a threshold of 1e−05
MR Test
Inverse variance weighted (fixed effects)
Inverse variance weighted (multiplicative random effects)
MR Egger
MR RAPS
Weighted median
Weighted mode
0.04
0.00
−0.04
−0.08
0.09
0.12
0.15
0.18
SNP effect on BDNF/NT−3 growth factors receptor
SNP effect on Gut microbiota abundance (genus Catenibacterium id.2153)

Batch 2−4244 : Gut microbiota abundance (genus Parasutterella id.2892) on BDNF/NT−3 growth factors receptor
with a threshold of 1e−05

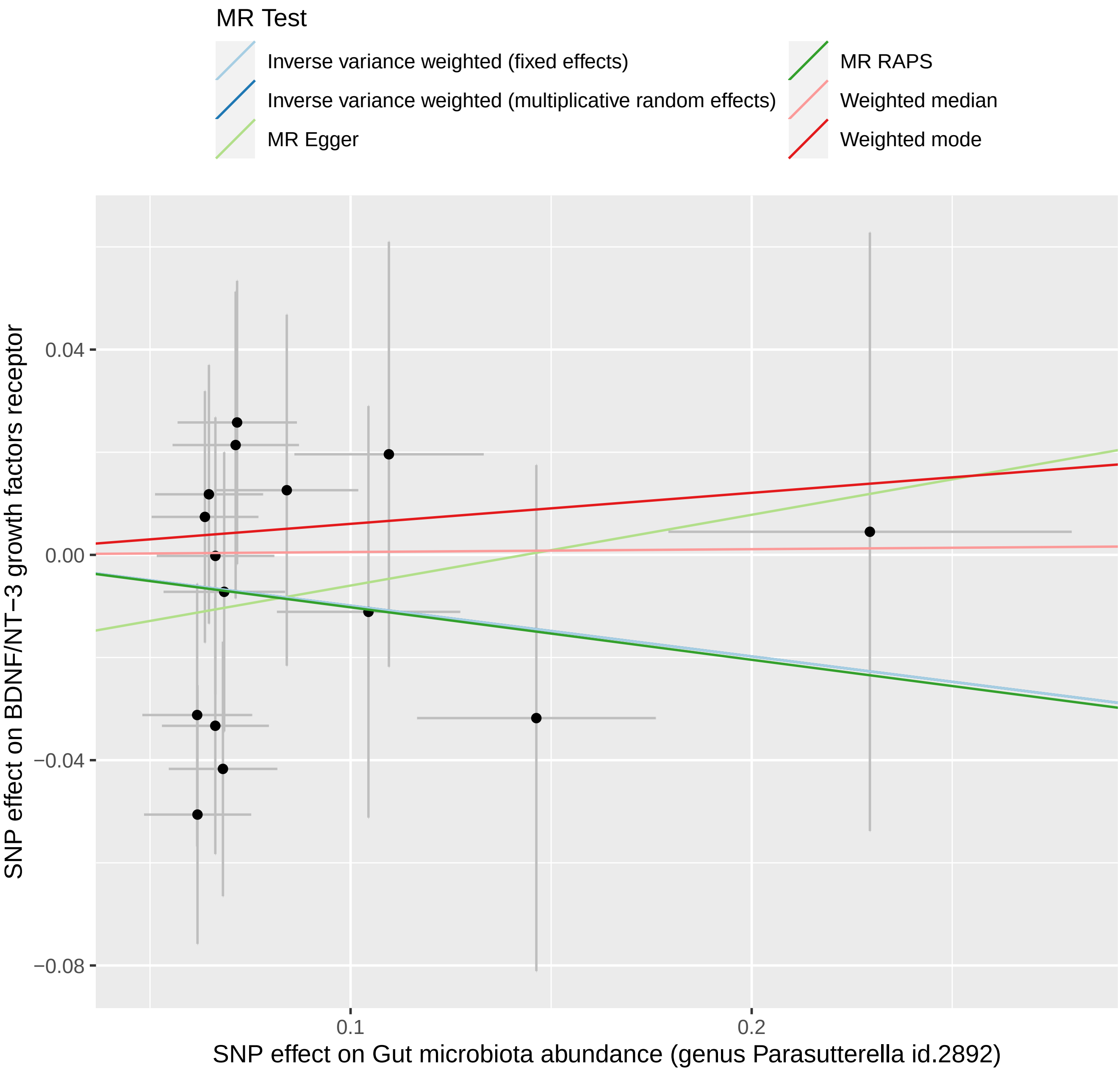

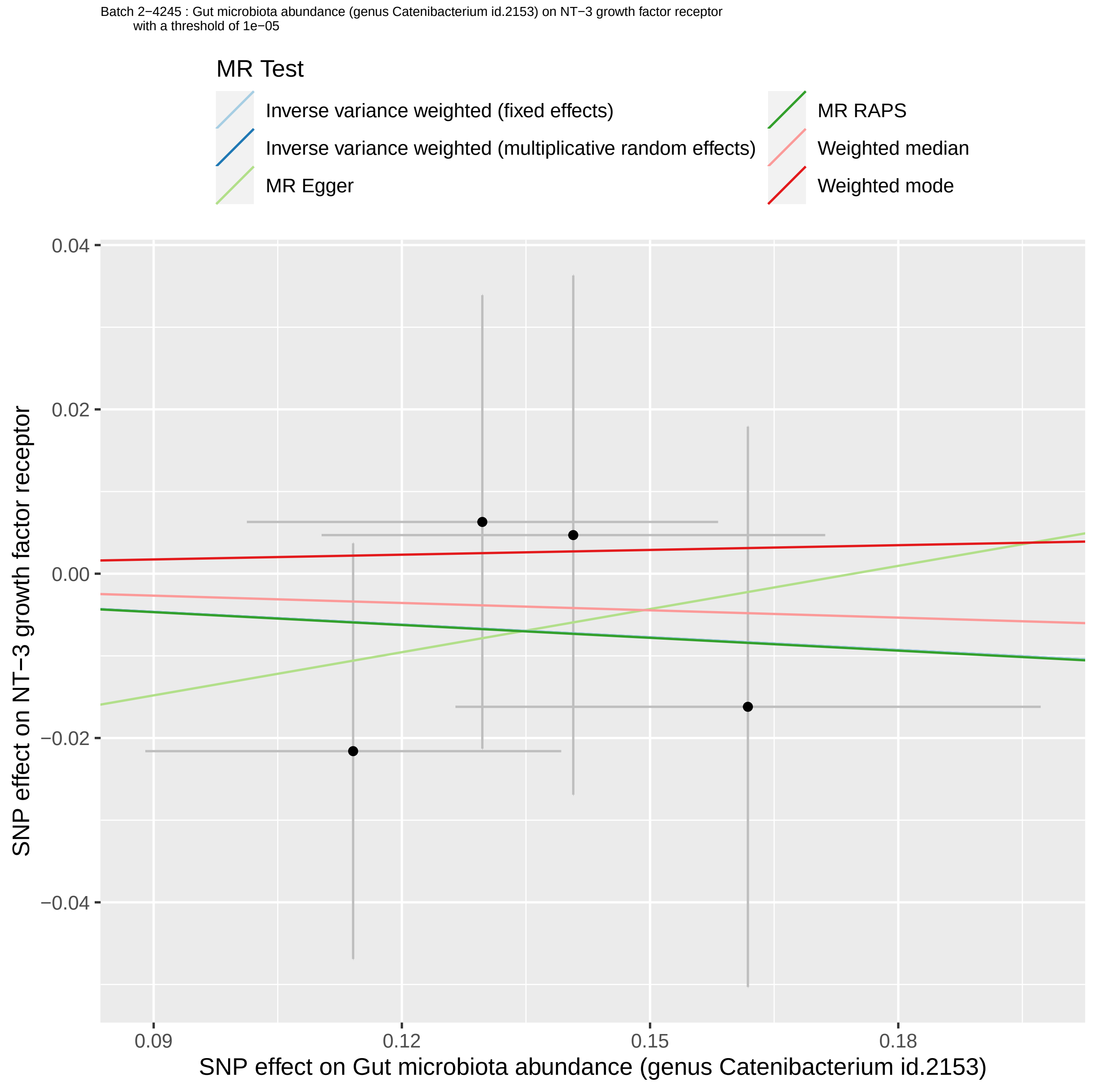

Batch 2−4245 : Gut microbiota abundance (genus Catenibacterium id.2153) on NT−3 growth factor receptor
with a threshold of 1e−05
MR Test
Inverse variance weighted (fixed effects)
Inverse variance weighted (multiplicative random effects)
MR Egger
MR RAPS
Weighted median
Weighted mode
0.04
0.02
0.00
−0.02
−0.04
0.09
0.12
0.15
0.18
SNP effect on NT−3 growth factor receptor
SNP effect on Gut microbiota abundance (genus Catenibacterium id.2153)

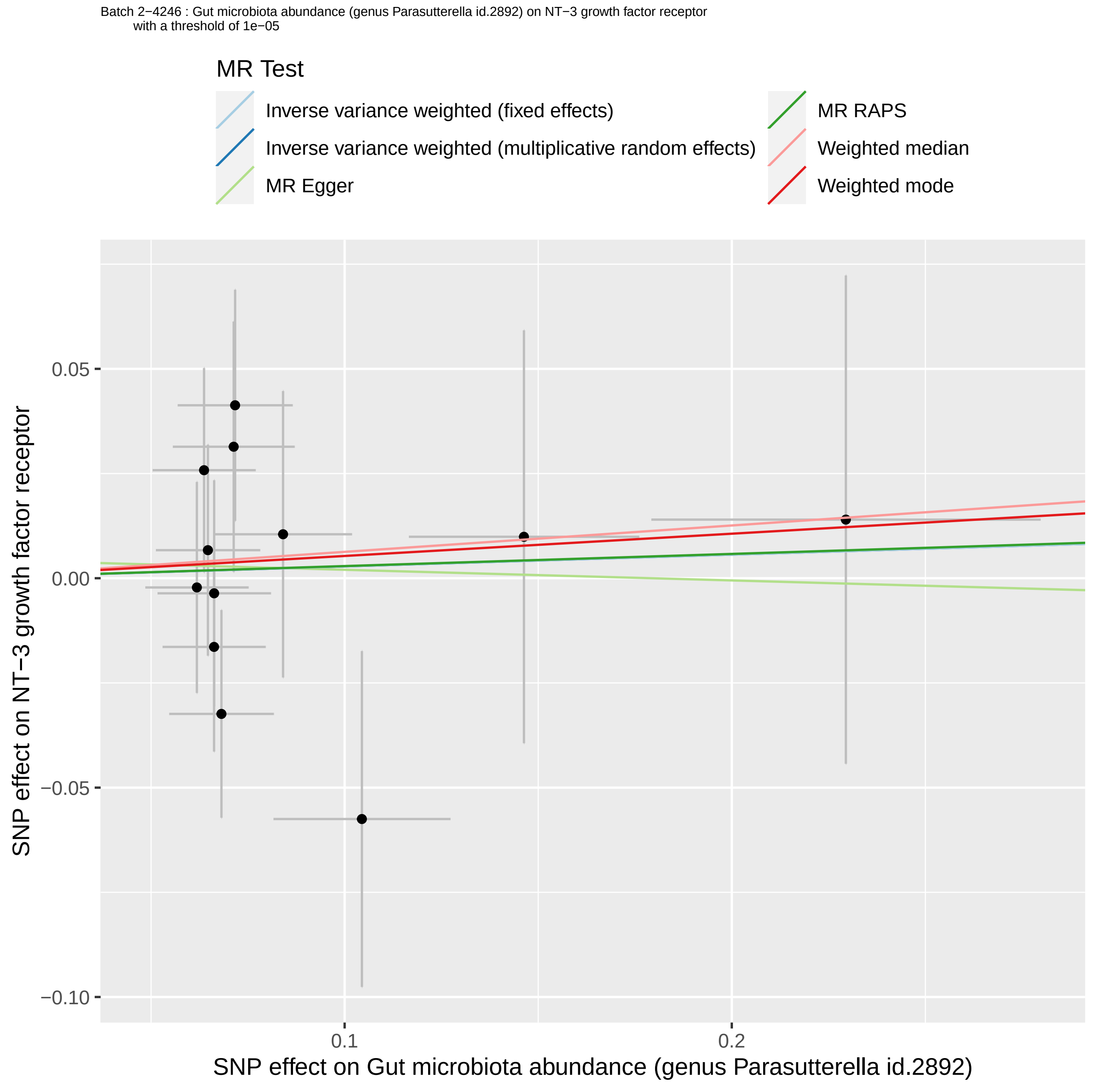
Batch 2−4246 : Gut microbiota abundance (genus Parasutterella id.2892) on NT−3 growth factor receptor
with a threshold of 1e−05
MR Test
Inverse variance weighted (fixed effects)
Inverse variance weighted (multiplicative random effects)
MR Egger
MR RAPS
Weighted median
Weighted mode
0.05
0.00
−0.05
−0.10
0.1
0.2
SNP effect on NT−3 growth factor receptor
SNP effect on Gut microbiota abundance (genus Parasutterella id.2892)

Batch 2−4247 : Gut microbiota abundance (genus Catenibacterium id.2153) on Neurotensin/neuromedin N
with a threshold of 1e−05

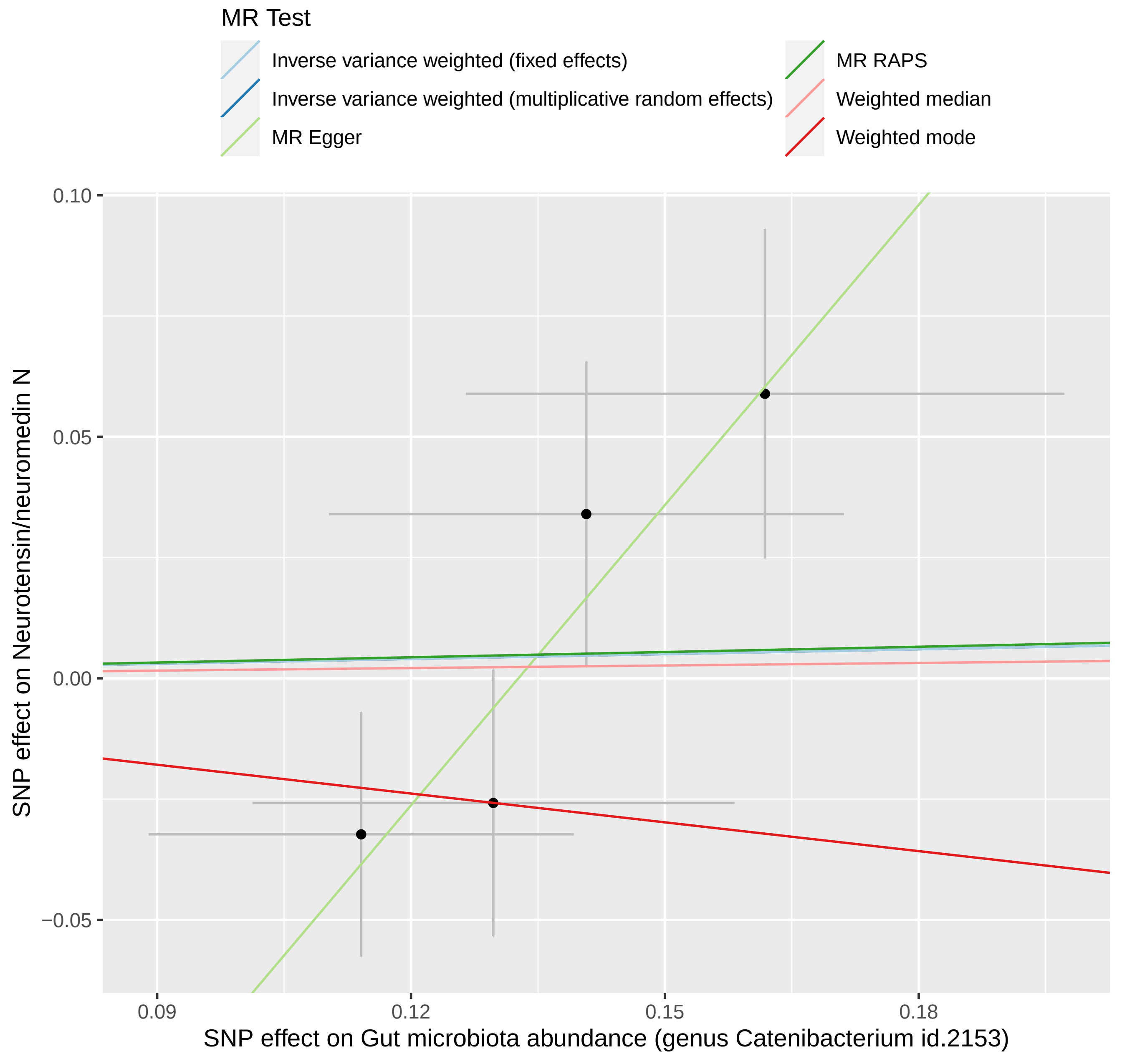

Batch 2−4248 : Gut microbiota abundance (genus Parasutterella id.2892) on Neurotensin/neuromedin N
with a threshold of 1e−05

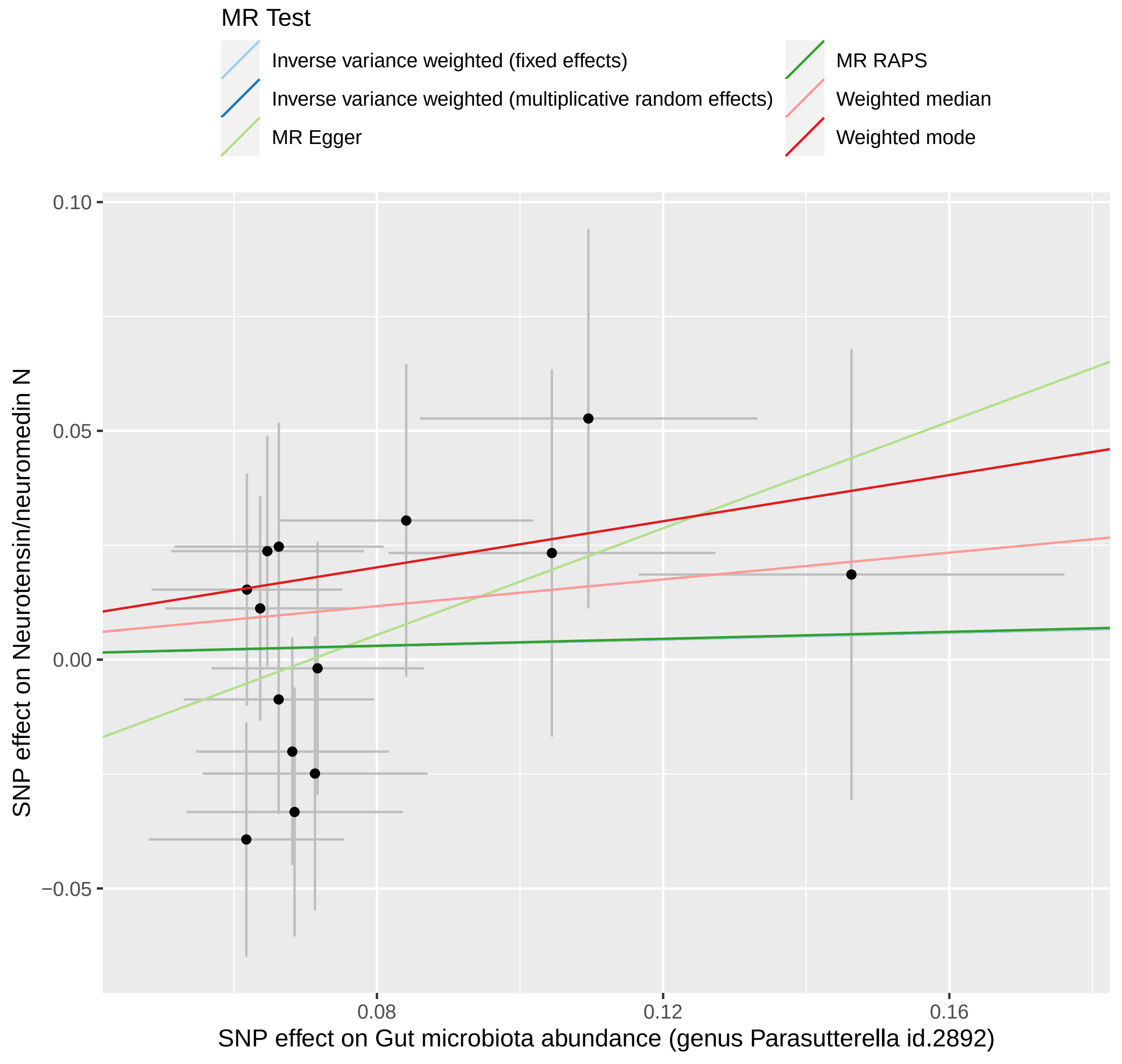

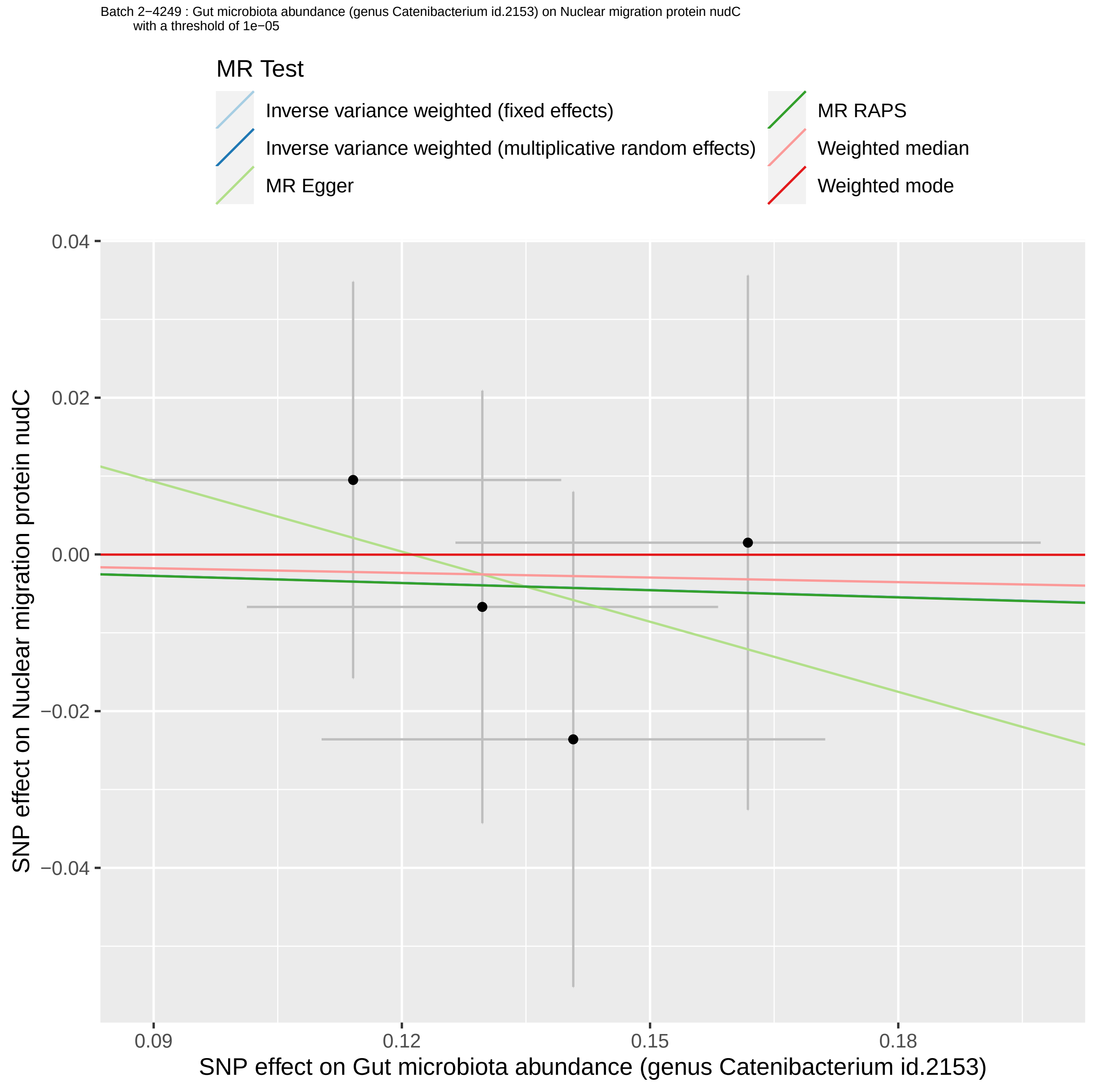

Batch 2−4249 : Gut microbiota abundance (genus Catenibacterium id.2153) on Nuclear migration protein nudC
with a threshold of 1e−05
MR Test
Inverse variance weighted (fixed effects)
Inverse variance weighted (multiplicative random effects)
MR Egger
MR RAPS
Weighted median
Weighted mode
0.04
0.02
0.00
−0.02
−0.04
0.09
0.12
0.15
0.18
SNP effect on Nuclear migration protein nudC
SNP effect on Gut microbiota abundance (genus Catenibacterium id.2153)

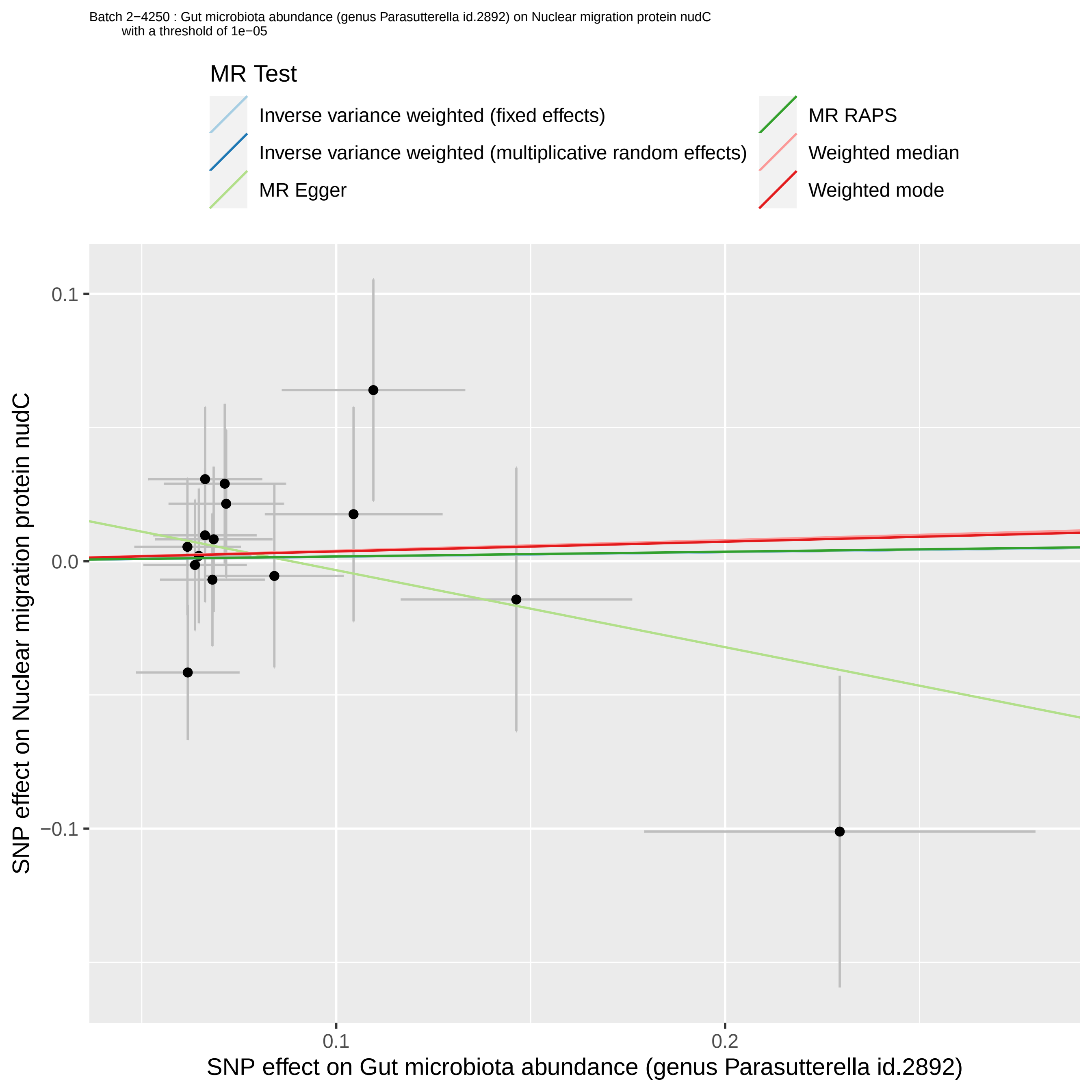
Batch 2−4250 : Gut microbiota abundance (genus Parasutterella id.2892) on Nuclear migration protein nudC
with a threshold of 1e−05
MR Test
Inverse variance weighted (fixed effects)
Inverse variance weighted (multiplicative random effects)
MR Egger
MR RAPS
Weighted median
Weighted mode
0.1
0.0
−0.1
0.1
0.2
SNP effect on Nuclear migration protein nudC
SNP effect on Gut microbiota abundance (genus Parasutterella id.2892)

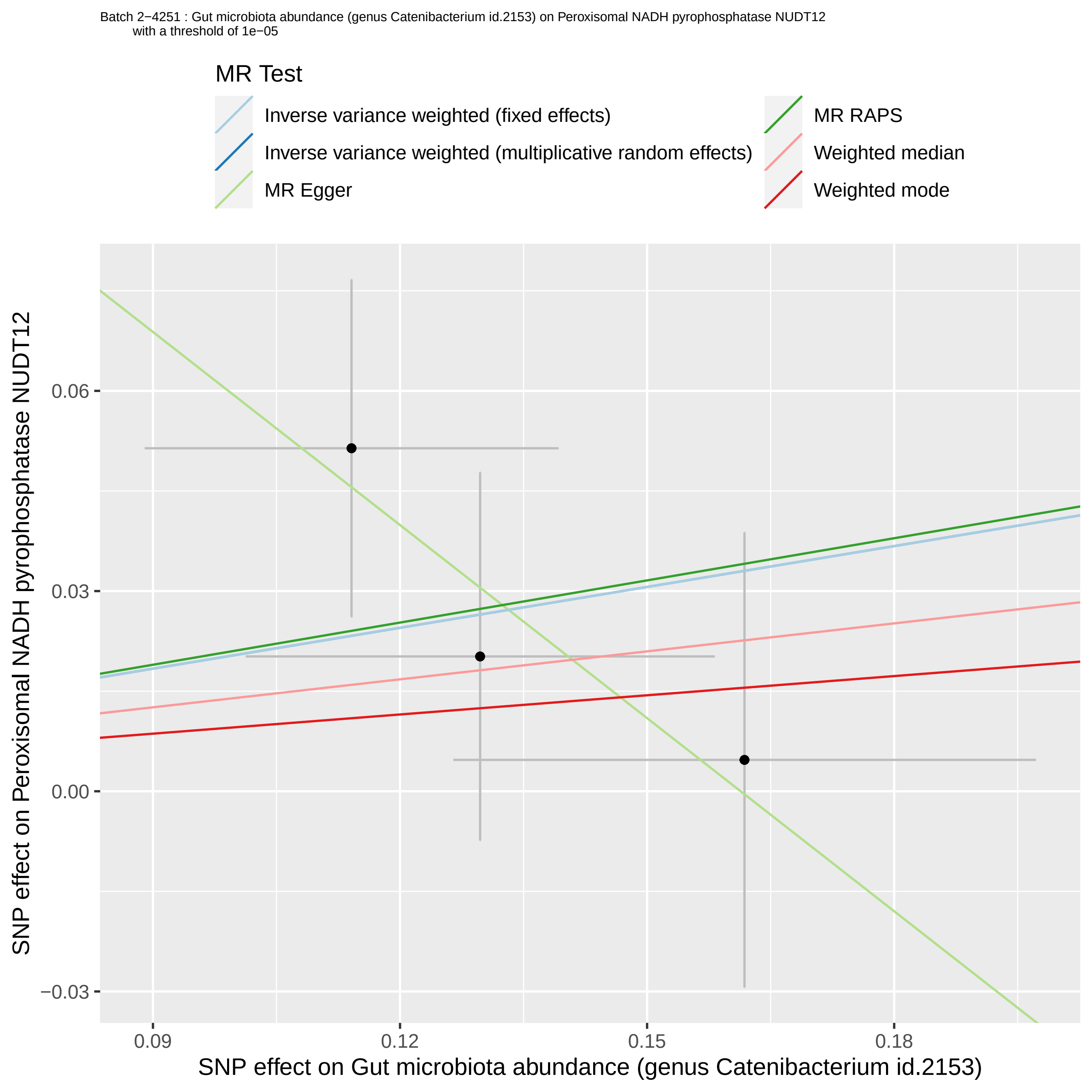
Batch 2−4251 : Gut microbiota abundance (genus Catenibacterium id.2153) on Peroxisomal NADH pyrophosphatase NUDT12
with a threshold of 1e−05
MR Test
Inverse variance weighted (fixed effects)
Inverse variance weighted (multiplicative random effects)
MR Egger
MR RAPS
Weighted median
Weighted mode
0.06
0.03
0.00
−0.03
0.09
0.12
0.15
0.18
SNP effect on Peroxisomal NADH pyrophosphatase NUDT12
SNP effect on Gut microbiota abundance (genus Catenibacterium id.2153)

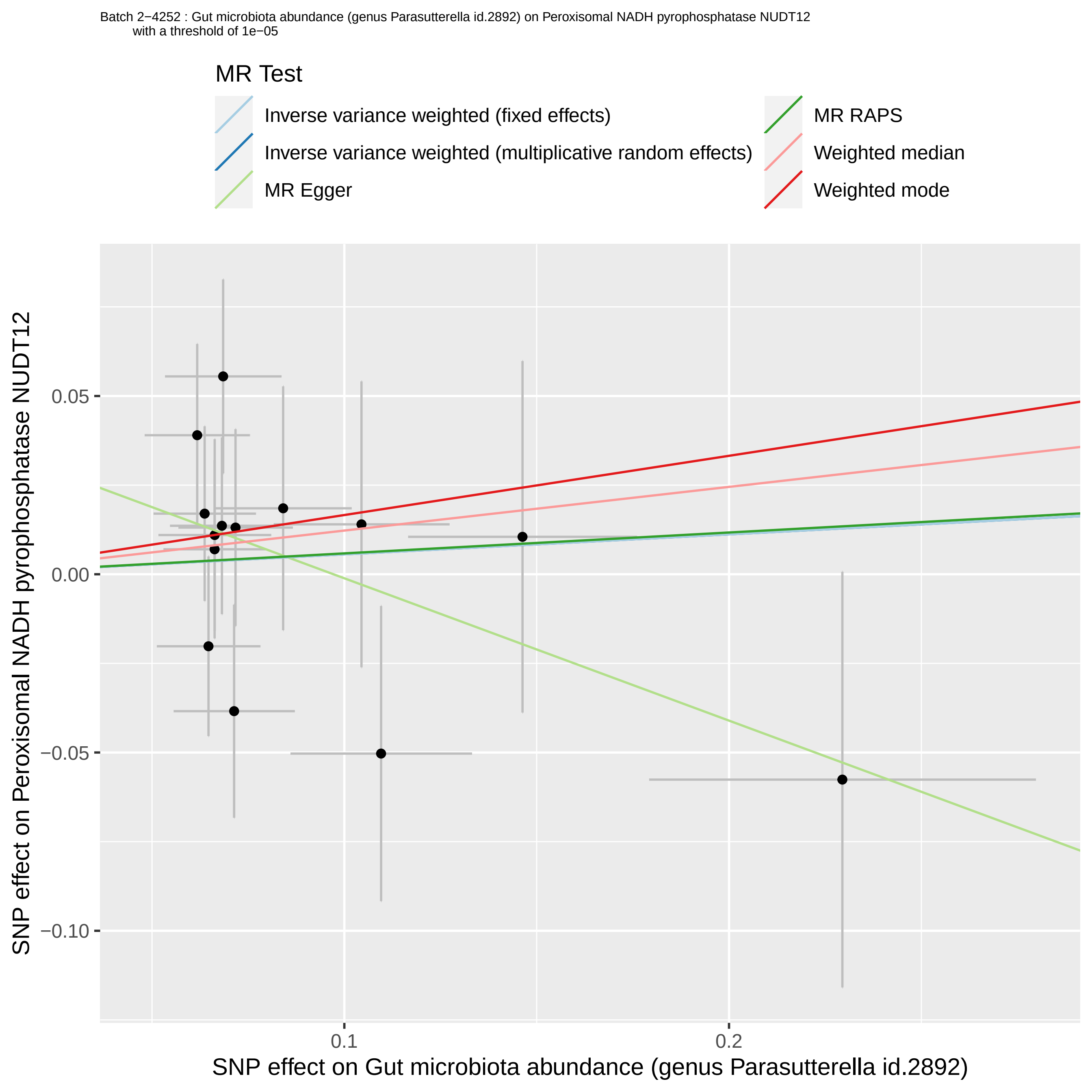
Batch 2−4252 : Gut microbiota abundance (genus Parasutterella id.2892) on Peroxisomal NADH pyrophosphatase NUDT12
with a threshold of 1e−05
MR Test
Inverse variance weighted (fixed effects)
Inverse variance weighted (multiplicative random effects)
MR Egger
MR RAPS
Weighted median
Weighted mode
0.05
0.00
−0.05
−0.10
0.1
0.2
SNP effect on Peroxisomal NADH pyrophosphatase NUDT12
SNP effect on Gut microbiota abundance (genus Parasutterella id.2892)

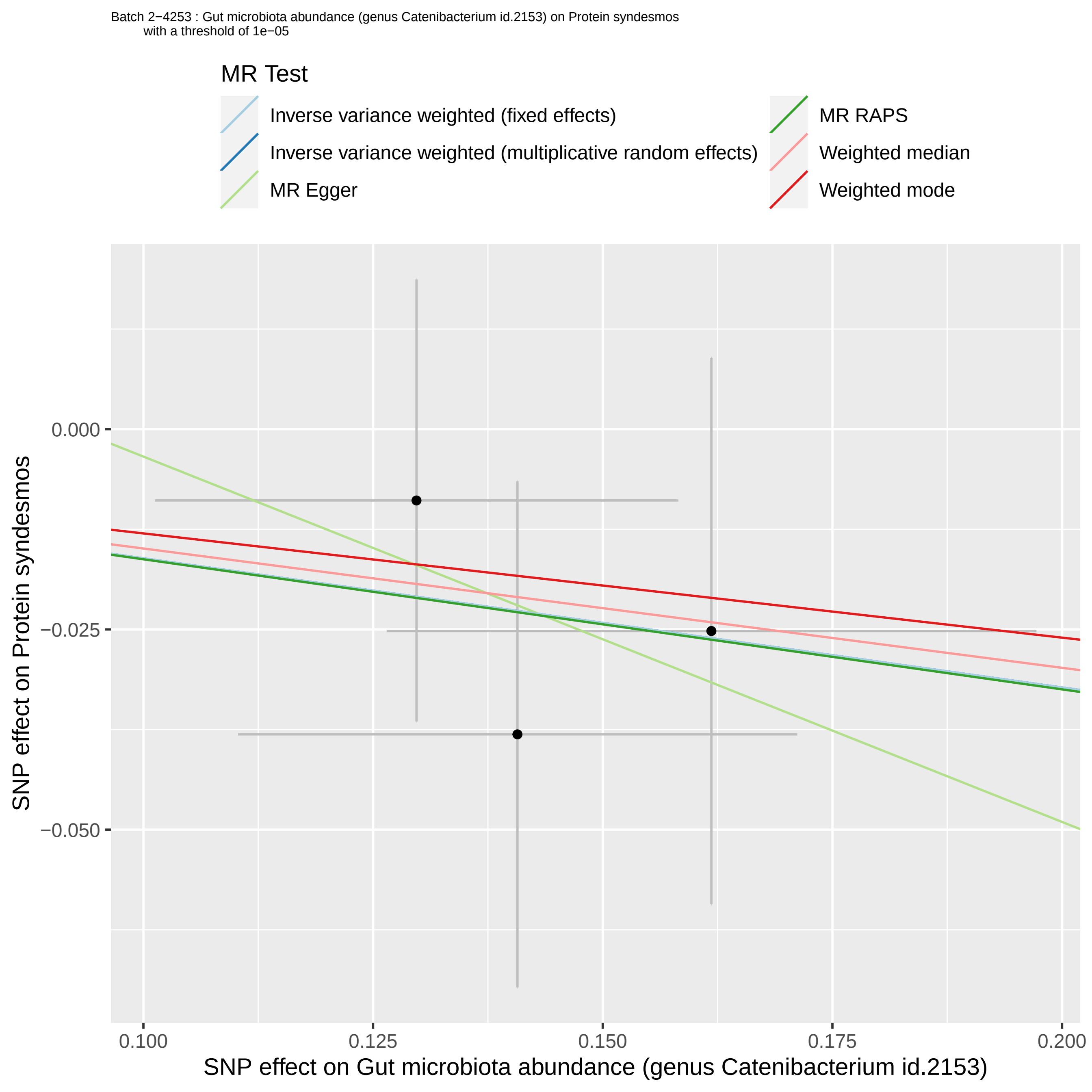
Batch 2−4253 : Gut microbiota abundance (genus Catenibacterium id.2153) on Protein syndesmos
with a threshold of 1e−05
MR Test
Inverse variance weighted (fixed effects)
Inverse variance weighted (multiplicative random effects)
MR Egger
MR RAPS
Weighted median
Weighted mode
0.000
−0.025
−0.050
SNP effect on Protein syndesmos
0.100
0.125
0.150
0.175
0.200
SNP effect on Gut microbiota abundance (genus Catenibacterium id.2153)

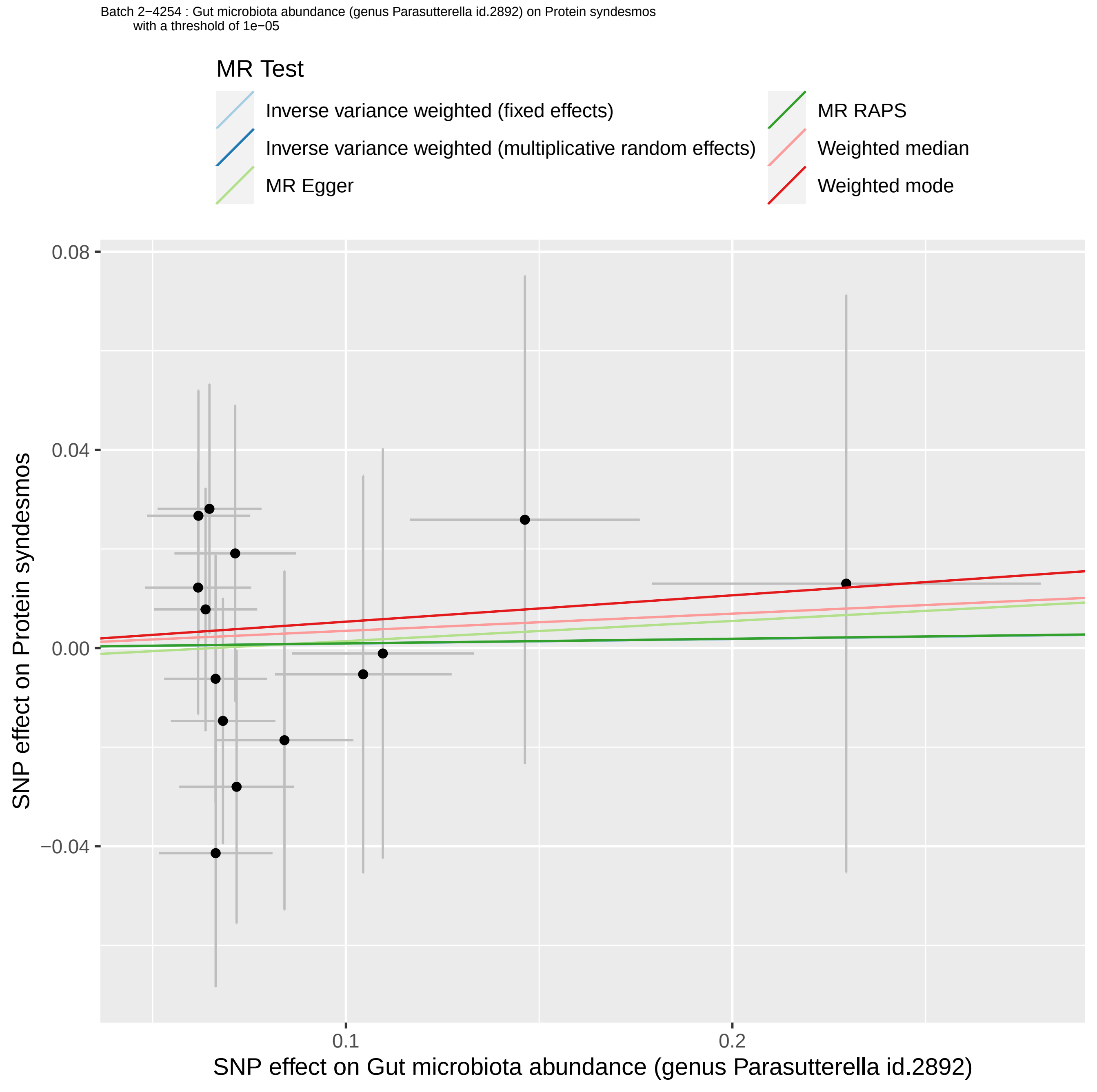
Batch 2−4254 : Gut microbiota abundance (genus Parasutterella id.2892) on Protein syndesmos
with a threshold of 1e−05
MR Test
Inverse variance weighted (fixed effects)
Inverse variance weighted (multiplicative random effects)
MR Egger
MR RAPS
Weighted median
Weighted mode
0.08
0.04
0.00
−0.04
0.1
0.2
SNP effect on Protein syndesmos
SNP effect on Gut microbiota abundance (genus Parasutterella id.2892)

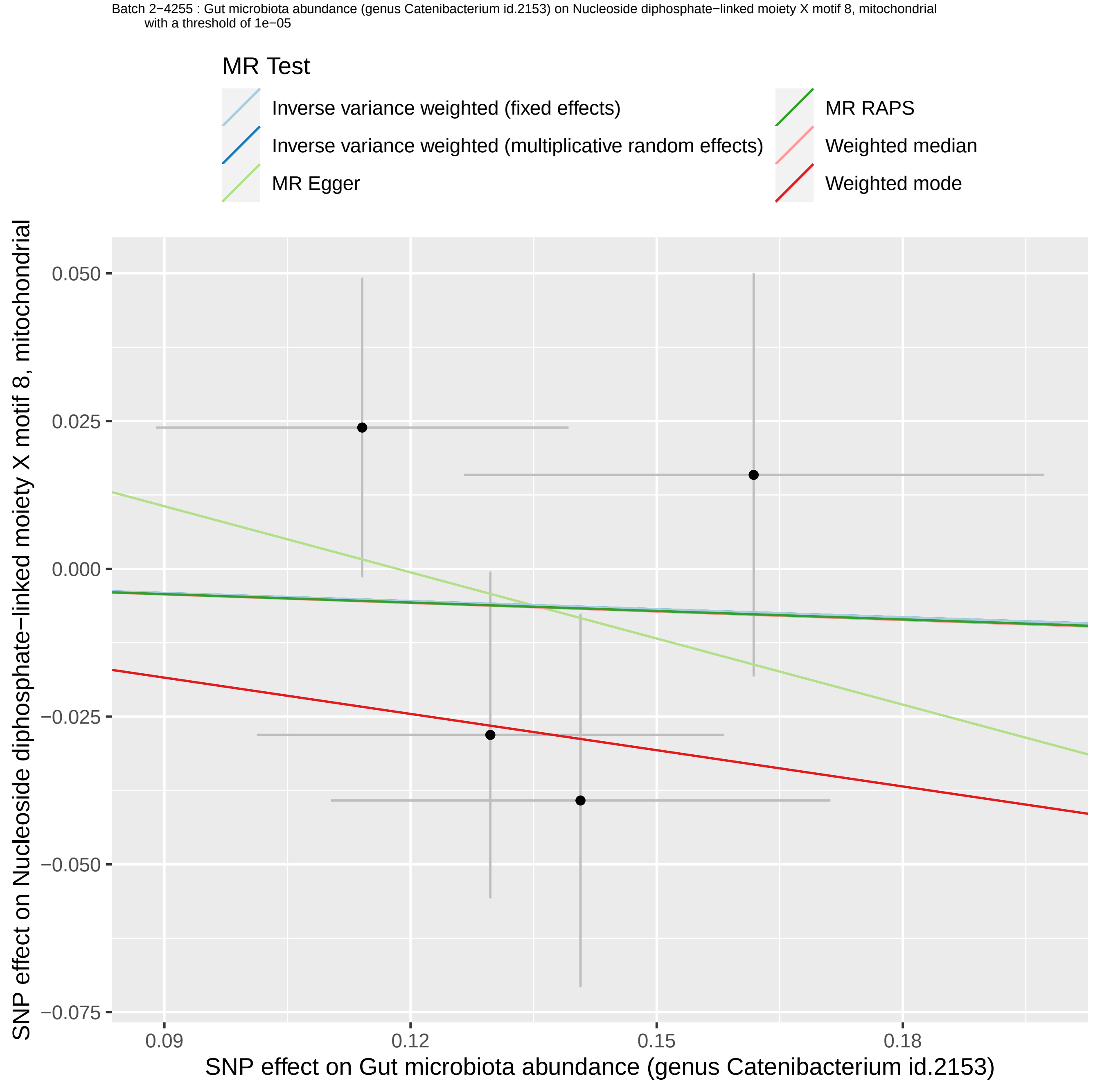

Batch 2−4255 : Gut microbiota abundance (genus Catenibacterium id.2153) on Nucleoside diphosphate−linked moiety X motif 8, mitochondrial with a threshold of 1e−05
MR Test
Inverse variance weighted (fixed effects)
Inverse variance weighted (multiplicative random effects)
MR Egger
MR RAPS
Weighted median
Weighted mode
0.050
0.025
0.000
−0.025
−0.050
−0.075
0.09
0.12
0.15
0.18
SNP effect on Nucleoside diphosphate−linked moiety X motif 8, mitochondrial
SNP effect on Gut microbiota abundance (genus Catenibacterium id.2153)

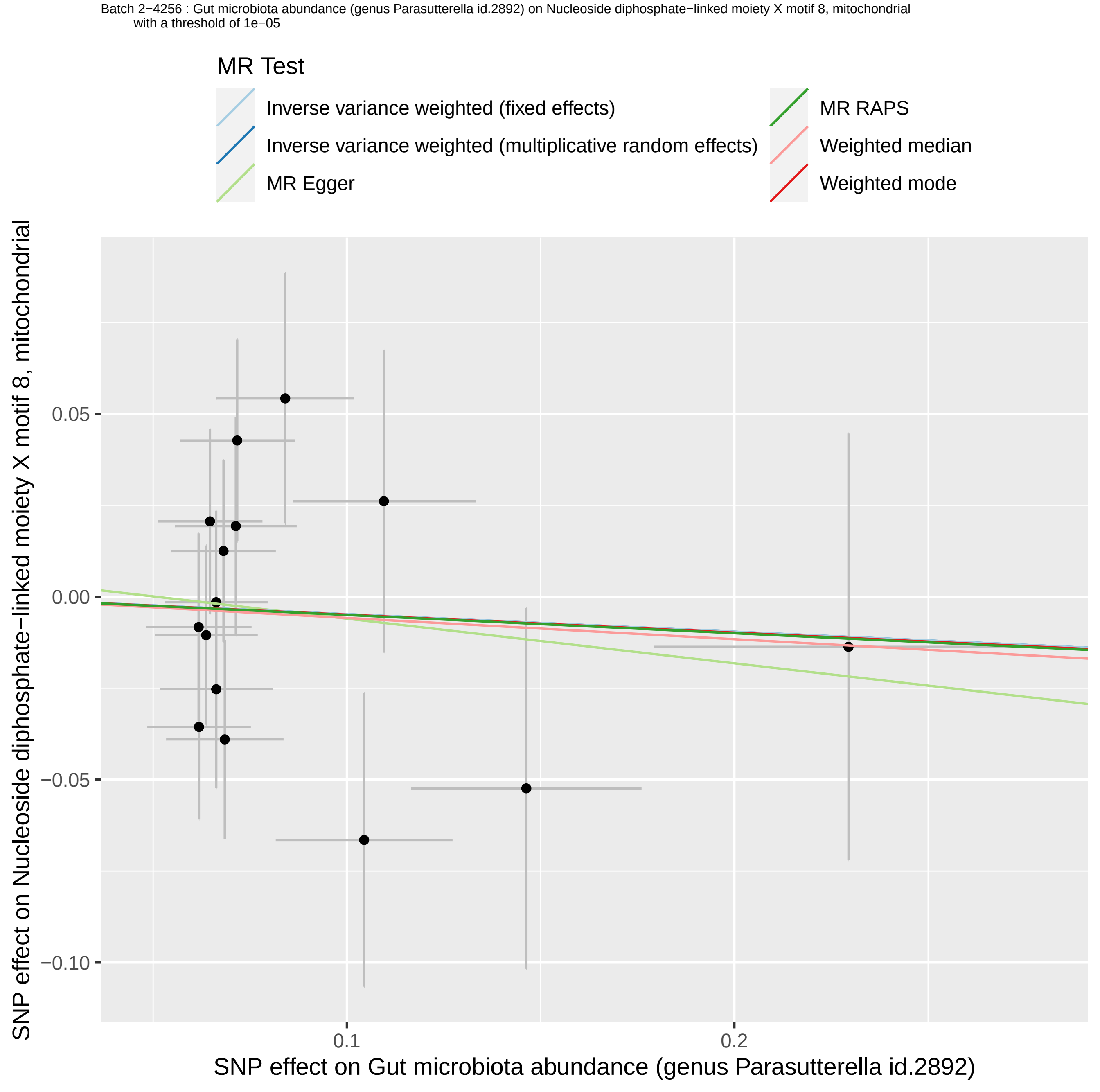

Batch 2−4256 : Gut microbiota abundance (genus Parasutterella id.2892) on Nucleoside diphosphate−linked moiety X motif 8, mitochondrial
with a threshold of 1e−05
MR Test
Inverse variance weighted (fixed effects)
Inverse variance weighted (multiplicative random effects)
MR Egger
MR RAPS
Weighted median
Weighted mode
0.05
0.00
−0.05
−0.10
0.1
0.2
SNP effect on Nucleoside diphosphate−linked moiety X motif 8, mitochondrial
SNP effect on Gut microbiota abundance (genus Parasutterella id.2892)

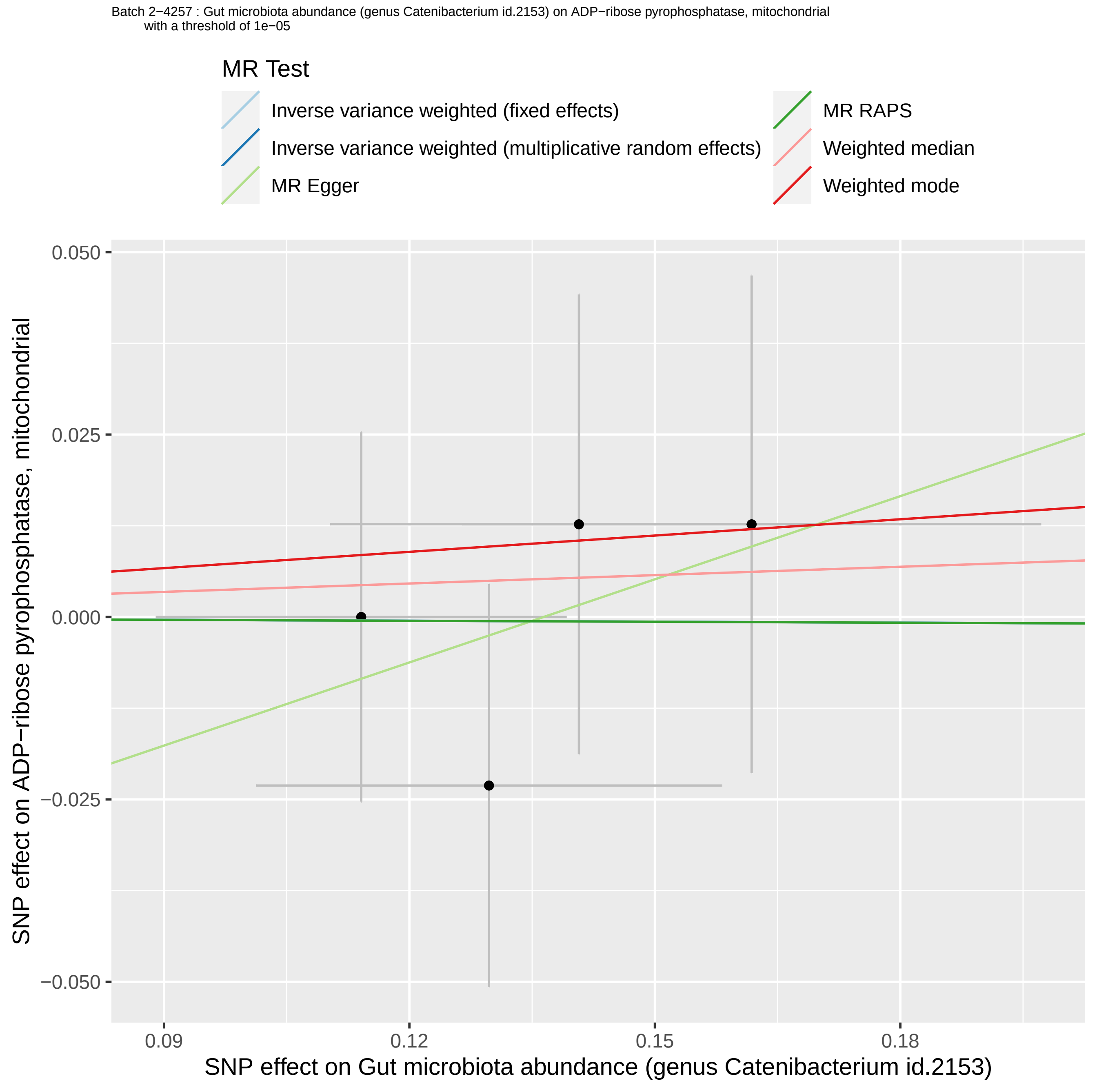

Batch 2−4257 : Gut microbiota abundance (genus Catenibacterium id.2153) on ADP−ribose pyrophosphatase, mitochondrial
with a threshold of 1e−05
MR Test
Inverse variance weighted (fixed effects)
Inverse variance weighted (multiplicative random effects)
MR Egger
MR RAPS
Weighted median
Weighted mode
0.050
0.025
0.000
−0.025
−0.050
0.09
0.12
0.15
0.18
SNP effect on ADP−ribose pyrophosphatase, mitochondrial
SNP effect on Gut microbiota abundance (genus Catenibacterium id.2153)

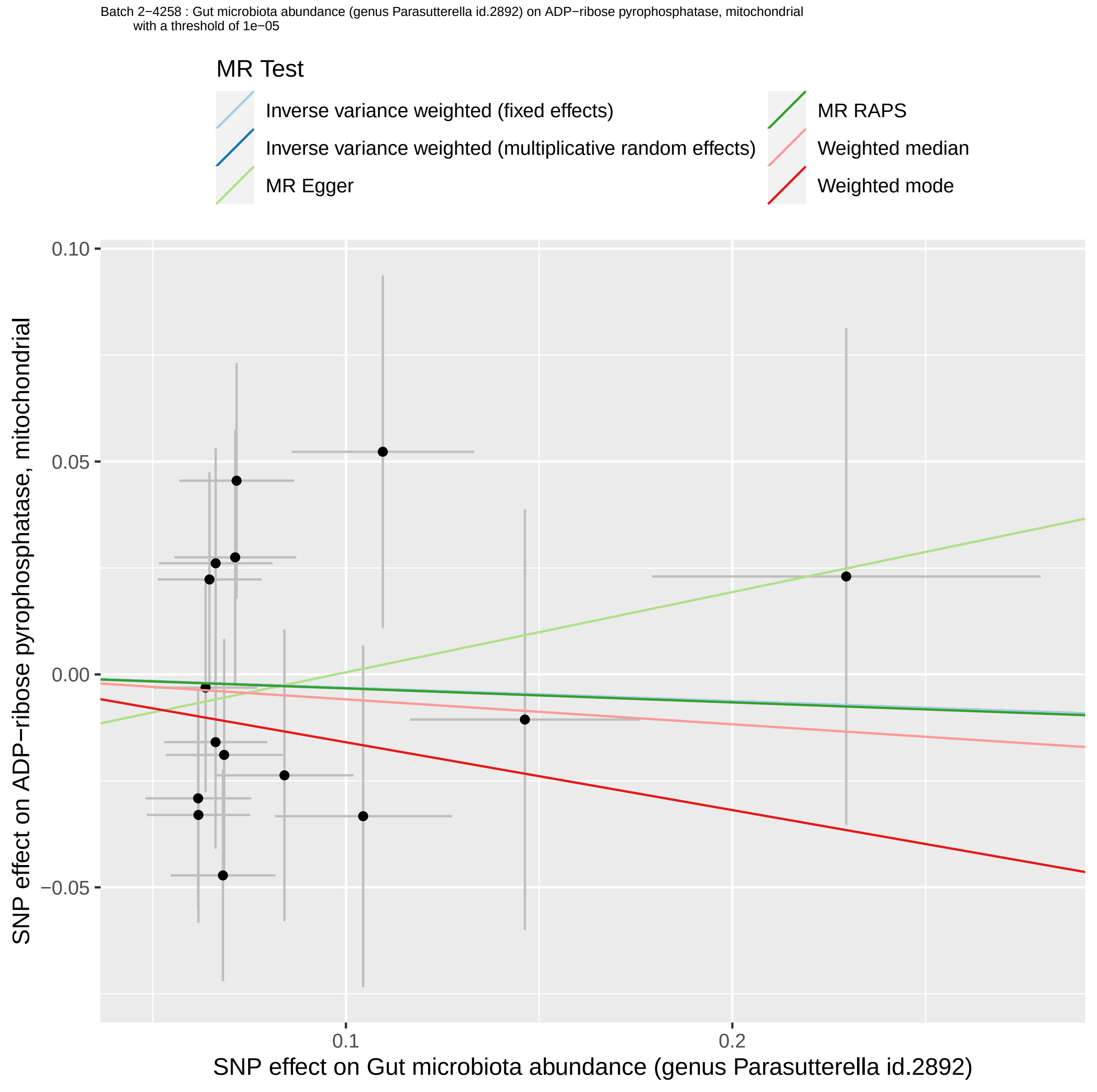

Batch 2−4258 : Gut microbiota abundance (genus Parasutterella id.2892) on ADP−ribose pyrophosphatase, mitochondrial
with a threshold of 1e−05
MR Test
Inverse variance weighted (fixed effects)
Inverse variance weighted (multiplicative random effects)
MR Egger
MR RAPS
Weighted median
Weighted mode
0.10
0.05
0.00
−0.05
0.1
0.2
SNP effect on ADP−ribose pyrophosphatase, mitochondrial
SNP effect on Gut microbiota abundance (genus Parasutterella id.2892)

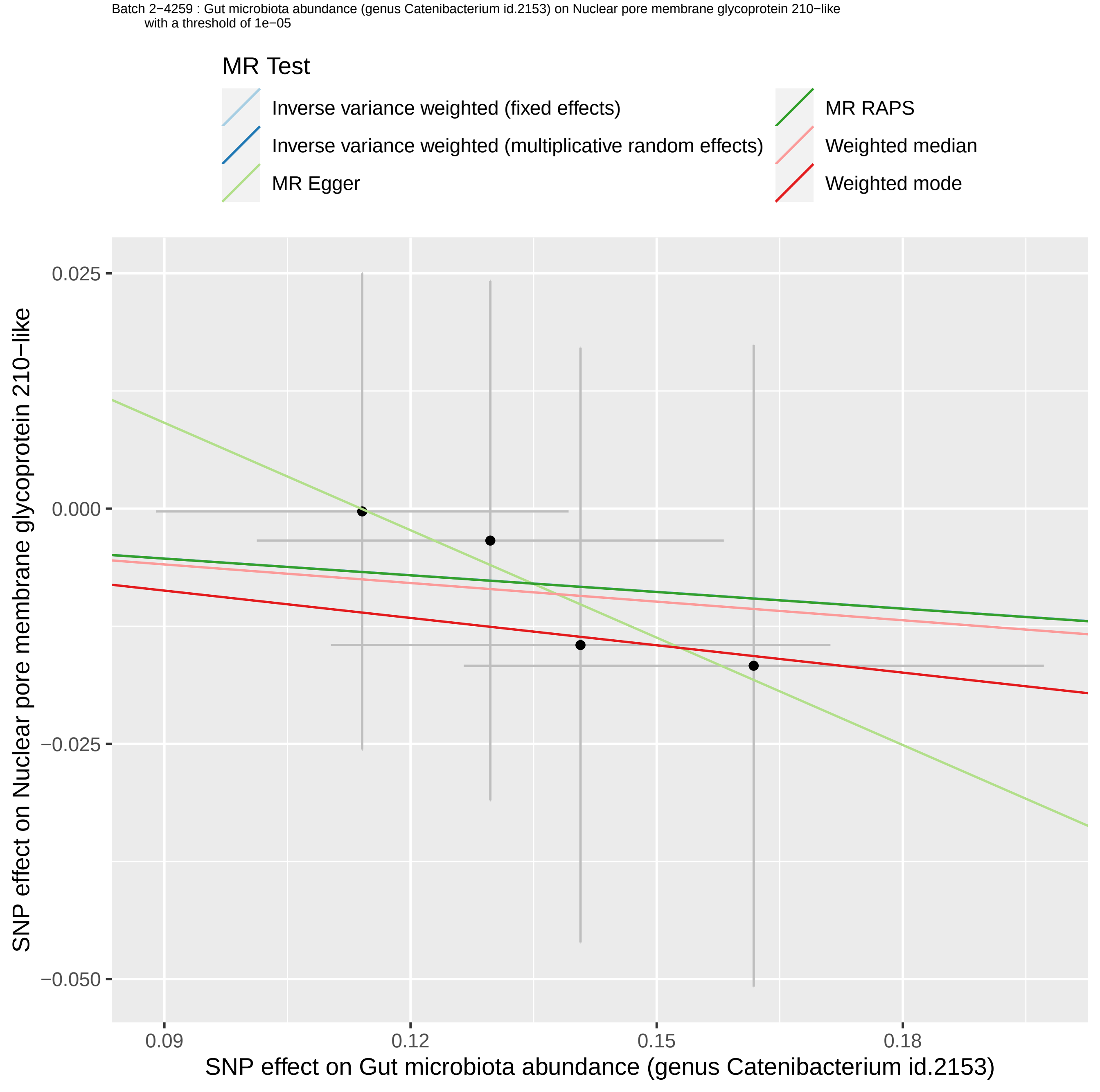

Batch 2−4259 : Gut microbiota abundance (genus Catenibacterium id.2153) on Nuclear pore membrane glycoprotein 210−like
with a threshold of 1e−05
MR Test
Inverse variance weighted (fixed effects)
Inverse variance weighted (multiplicative random effects)
MR Egger
MR RAPS
Weighted median
Weighted mode
0.025
0.000
−0.025
−0.050
0.09
0.12
0.15
0.18
SNP effect on Nuclear pore membrane glycoprotein 210−like
SNP effect on Gut microbiota abundance (genus Catenibacterium id.2153)

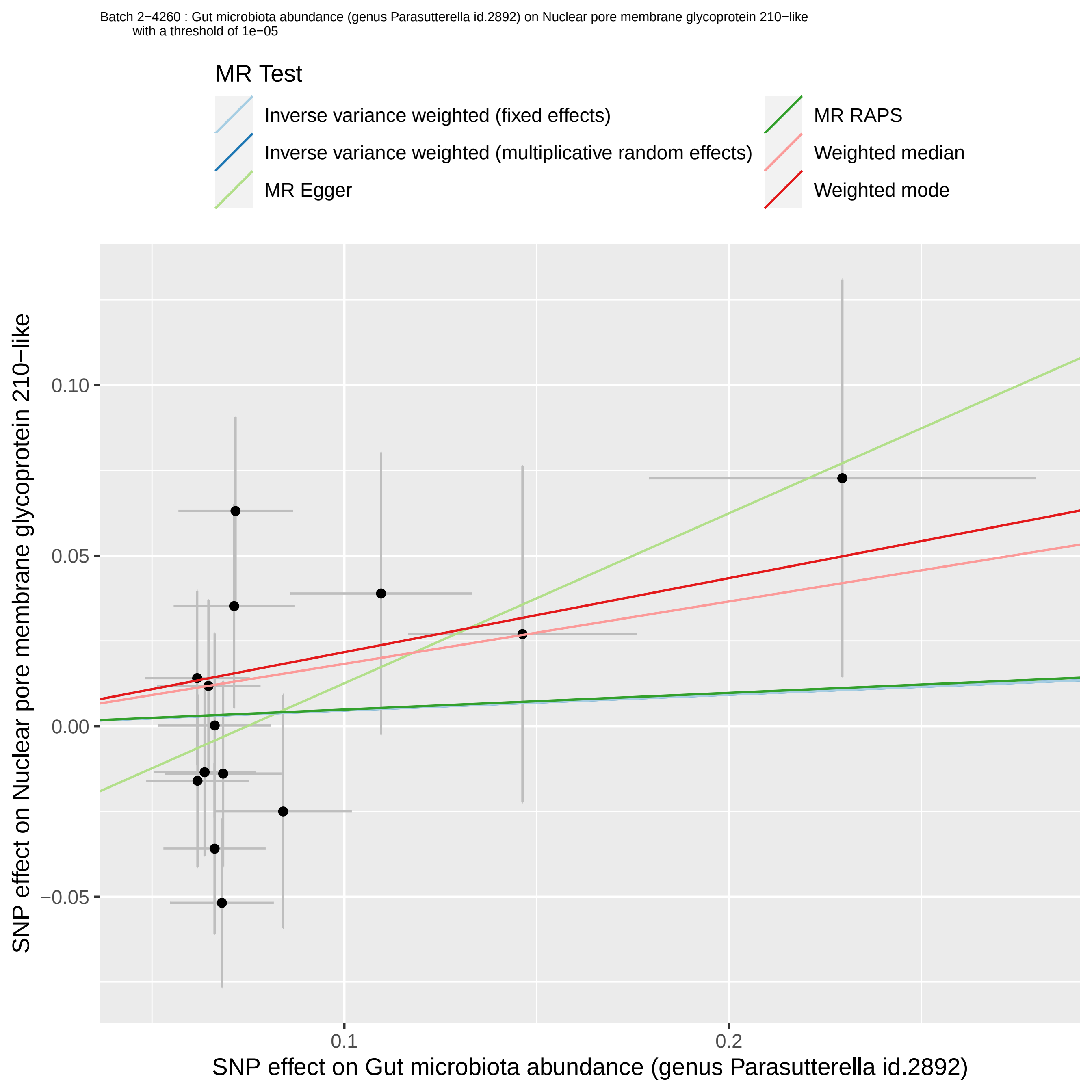
Batch 2−4260 : Gut microbiota abundance (genus Parasutterella id.2892) on Nuclear pore membrane glycoprotein 210−like
with a threshold of 1e−05
MR Test
Inverse variance weighted (fixed effects)
Inverse variance weighted (multiplicative random effects)
MR Egger
MR RAPS
Weighted median
Weighted mode
0.10
0.05
0.00
−0.05
0.1
0.2
SNP effect on Nuclear pore membrane glycoprotein 210−like
SNP effect on Gut microbiota abundance (genus Parasutterella id.2892)

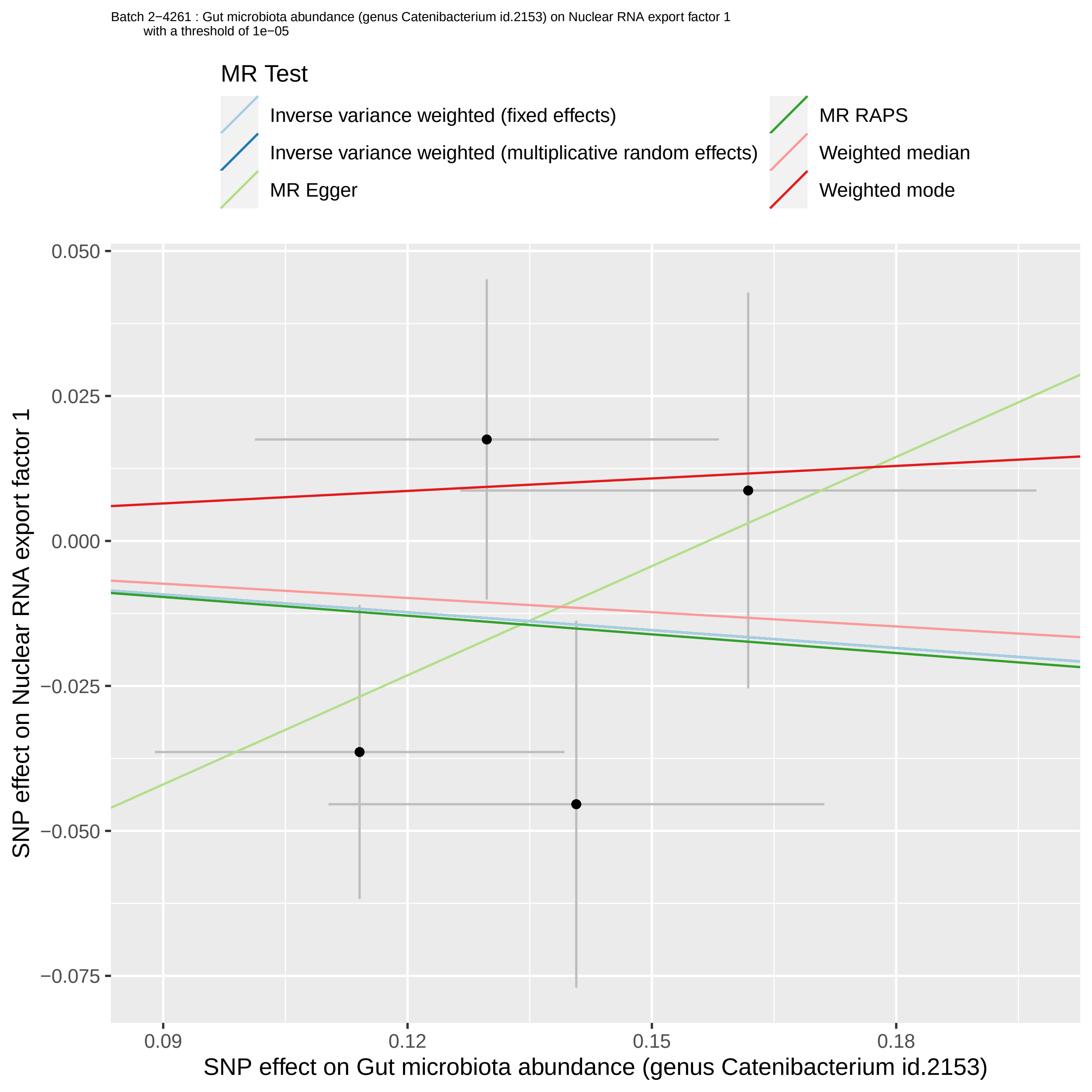
Batch 2−4261 : Gut microbiota abundance (genus Catenibacterium id.2153) on Nuclear RNA export factor 1
with a threshold of 1e−05
MR Test
Inverse variance weighted (fixed effects)
Inverse variance weighted (multiplicative random effects)
MR Egger
MR RAPS
Weighted median
Weighted mode
0.050
0.025
0.000
−0.025
−0.050
−0.075
0.09
0.12
0.15
0.18
SNP effect on Nuclear RNA export factor 1
SNP effect on Gut microbiota abundance (genus Catenibacterium id.2153)

Batch 2−4262 : Gut microbiota abundance (genus Parasutterella id.2892) on Nuclear RNA export factor 1
with a threshold of 1e−05

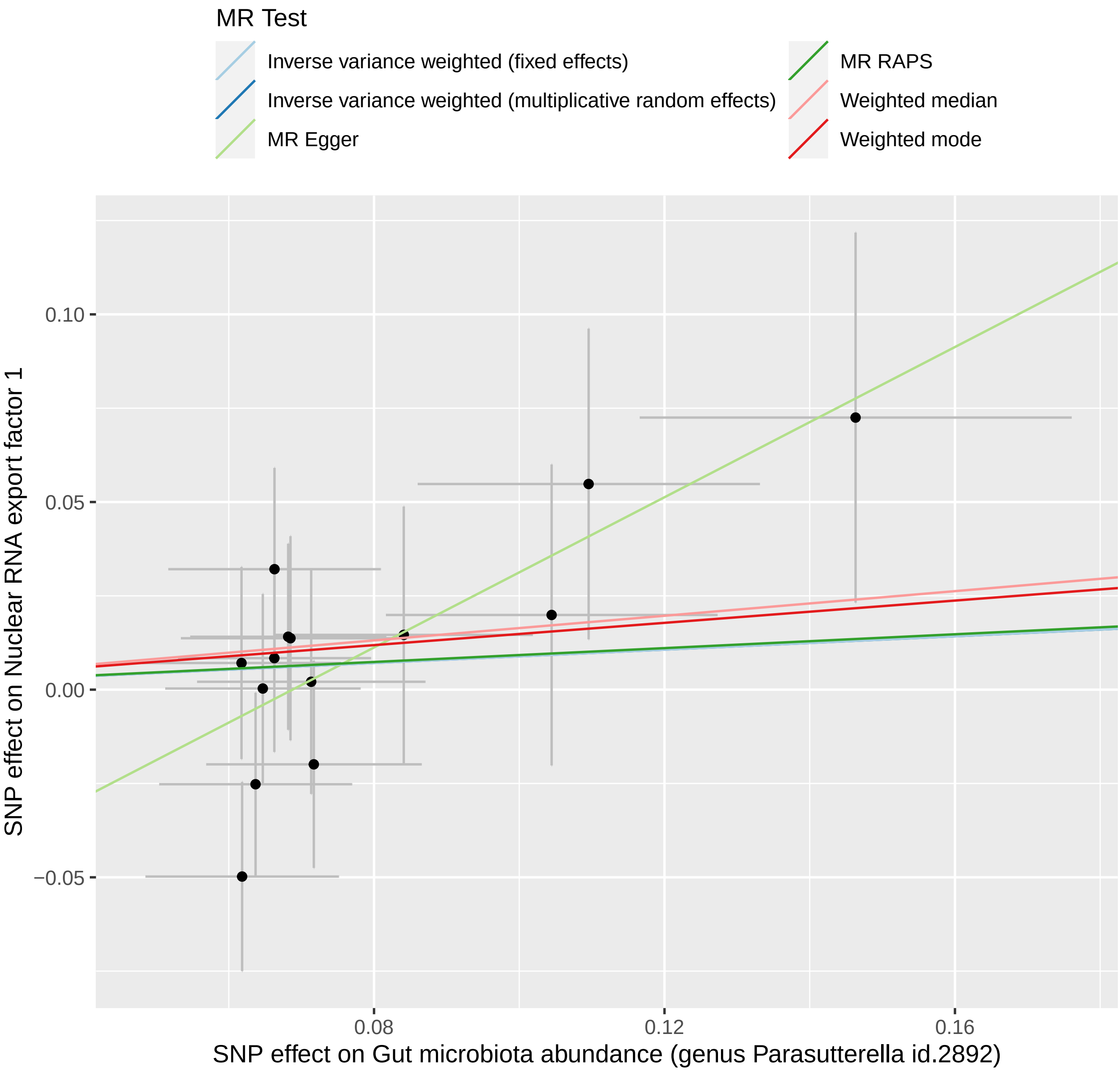

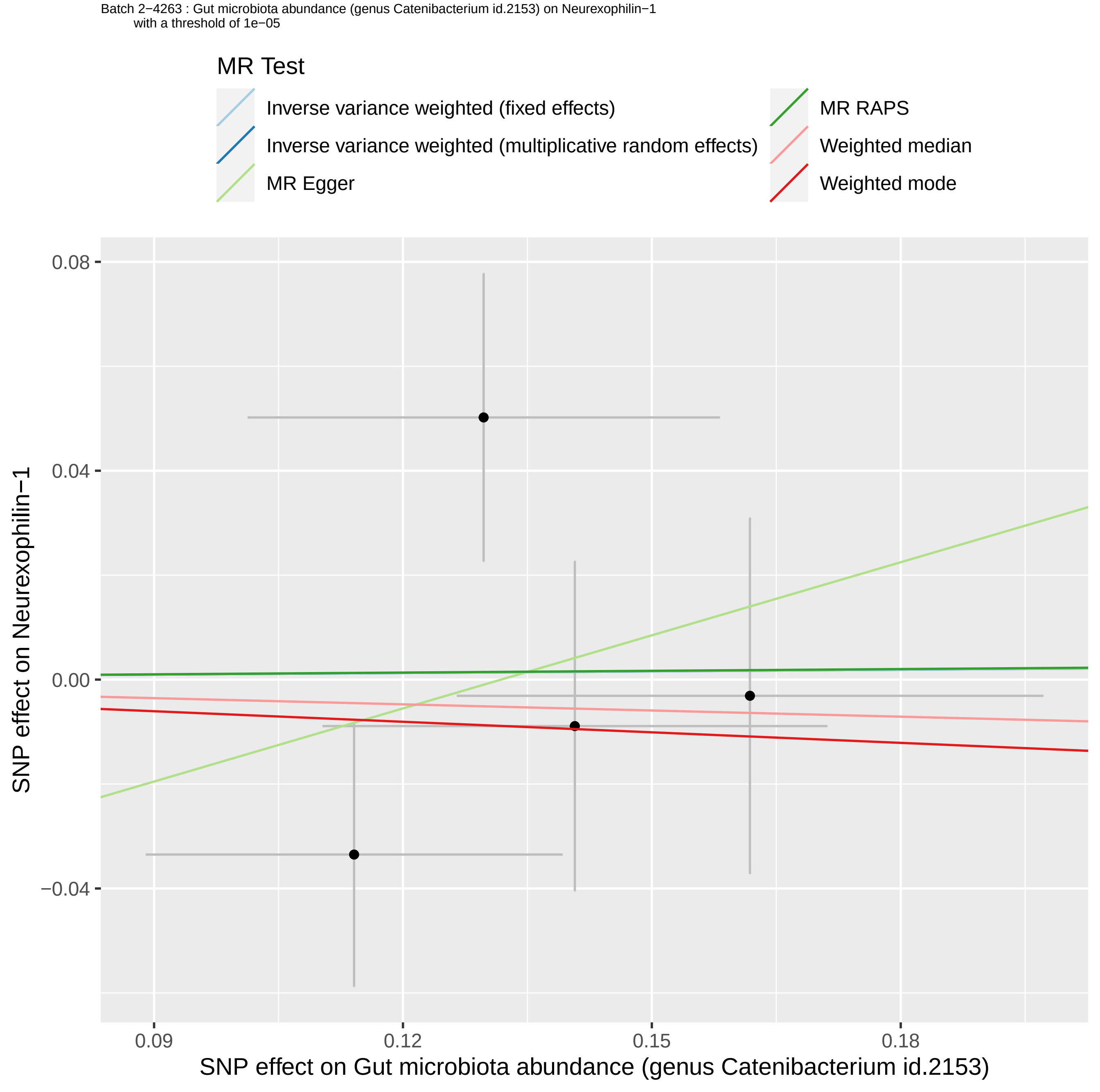

Batch 2−4263 : Gut microbiota abundance (genus Catenibacterium id.2153) on Neurexophilin−1
with a threshold of 1e−05
MR Test
Inverse variance weighted (fixed effects)
Inverse variance weighted (multiplicative random effects)
MR Egger
MR RAPS
Weighted median
Weighted mode
0.08
0.04
0.00
−0.04
0.09
0.12
0.15
0.18
SNP effect on Neurexophilin−1
SNP effect on Gut microbiota abundance (genus Catenibacterium id.2153)

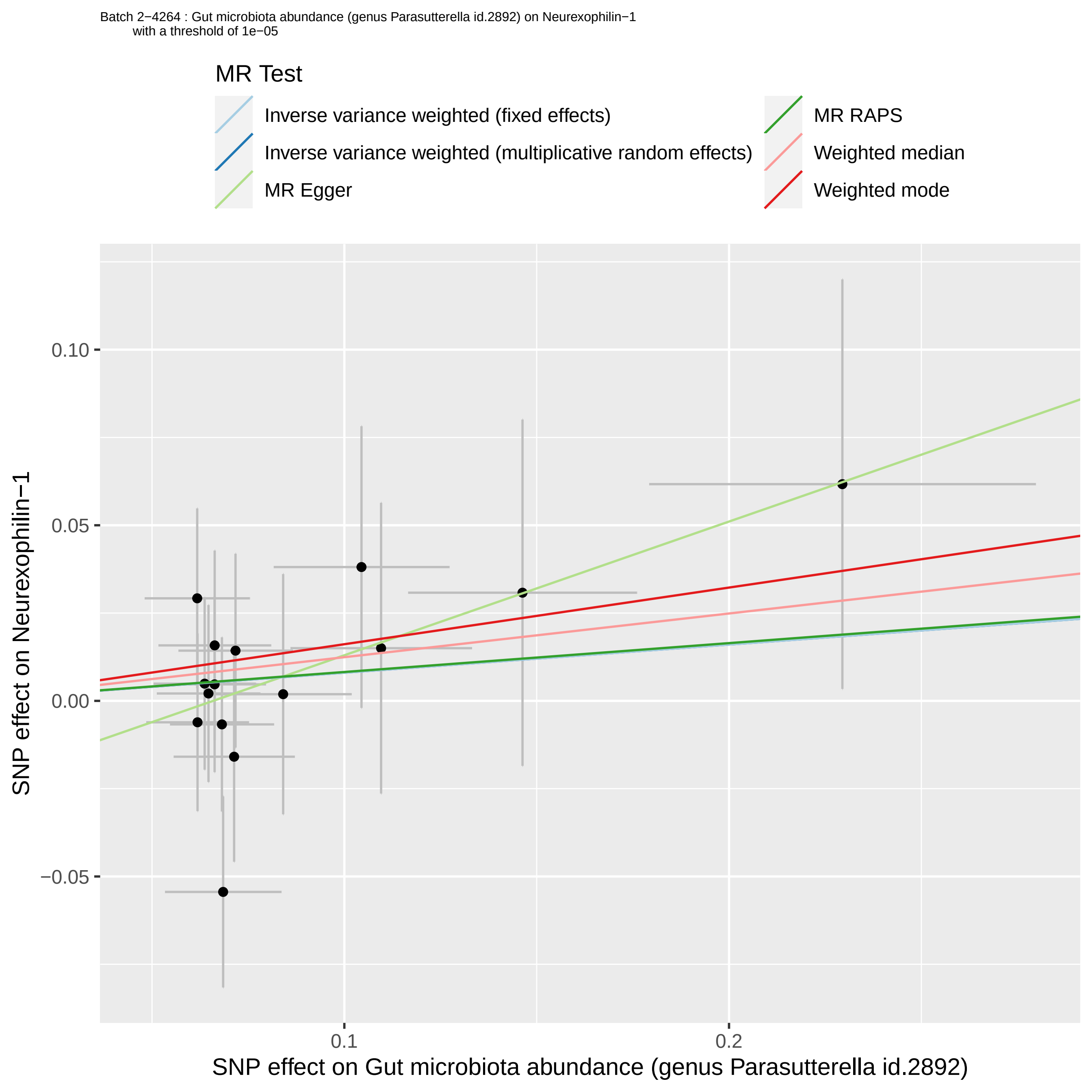

Batch 2−4264 : Gut microbiota abundance (genus Parasutterella id.2892) on Neurexophilin−1
with a threshold of 1e−05
MR Test
Inverse variance weighted (fixed effects)
Inverse variance weighted (multiplicative random effects)
MR Egger
MR RAPS
Weighted median
Weighted mode
0.10
0.05
0.00
−0.05
0.1
0.2
SNP effect on Neurexophilin−1
SNP effect on Gut microbiota abundance (genus Parasutterella id.2892)

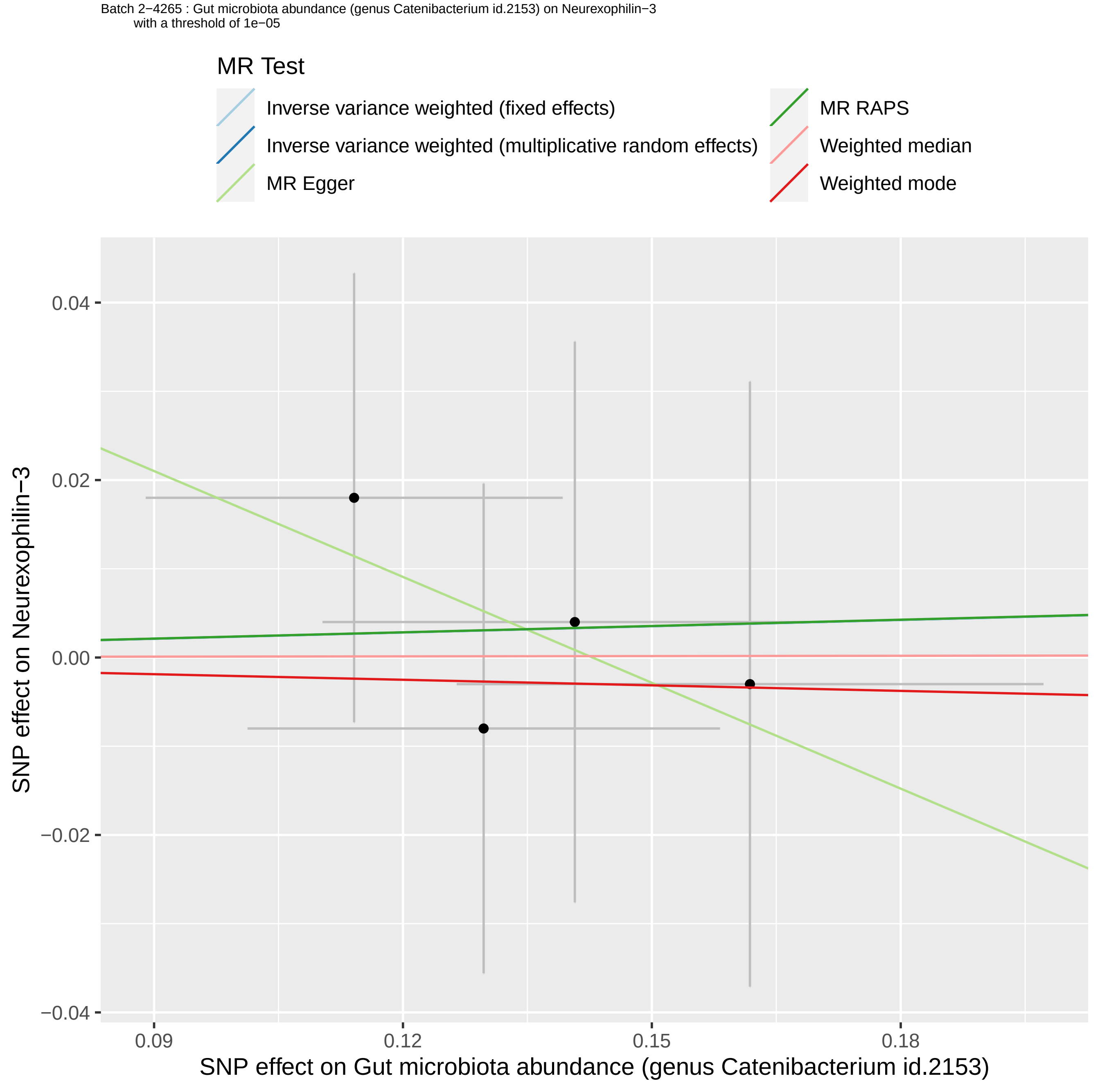

Batch 2−4265 : Gut microbiota abundance (genus Catenibacterium id.2153) on Neurexophilin−3
with a threshold of 1e−05
MR Test
Inverse variance weighted (fixed effects)
Inverse variance weighted (multiplicative random effects)
MR Egger
MR RAPS
Weighted median
Weighted mode
SNP effect on Neurexophilin−3
0.04
0.02
0.00
−0.02
−0.04
0.09
0.12
0.15
0.18
SNP effect on Gut microbiota abundance (genus Catenibacterium id.2153)

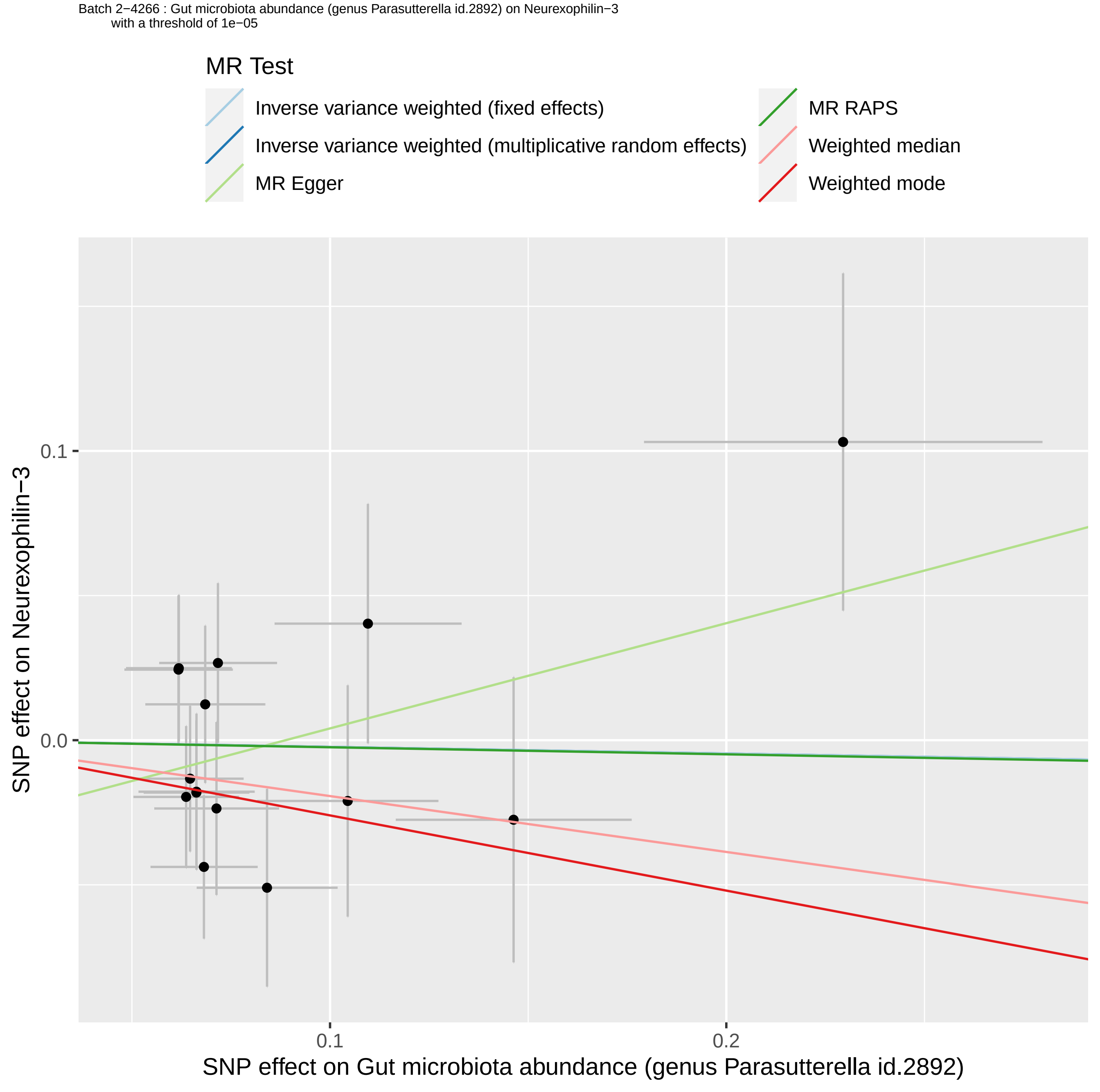

Batch 2−4266 : Gut microbiota abundance (genus Parasutterella id.2892) on Neurexophilin−3
with a threshold of 1e−05
MR Test
Inverse variance weighted (fixed effects)
Inverse variance weighted (multiplicative random effects)
MR Egger
MR RAPS
Weighted median
Weighted mode
0.1
0.0
0.1
0.2
SNP effect on Neurexophilin−3
SNP effect on Gut microbiota abundance (genus Parasutterella id.2892)

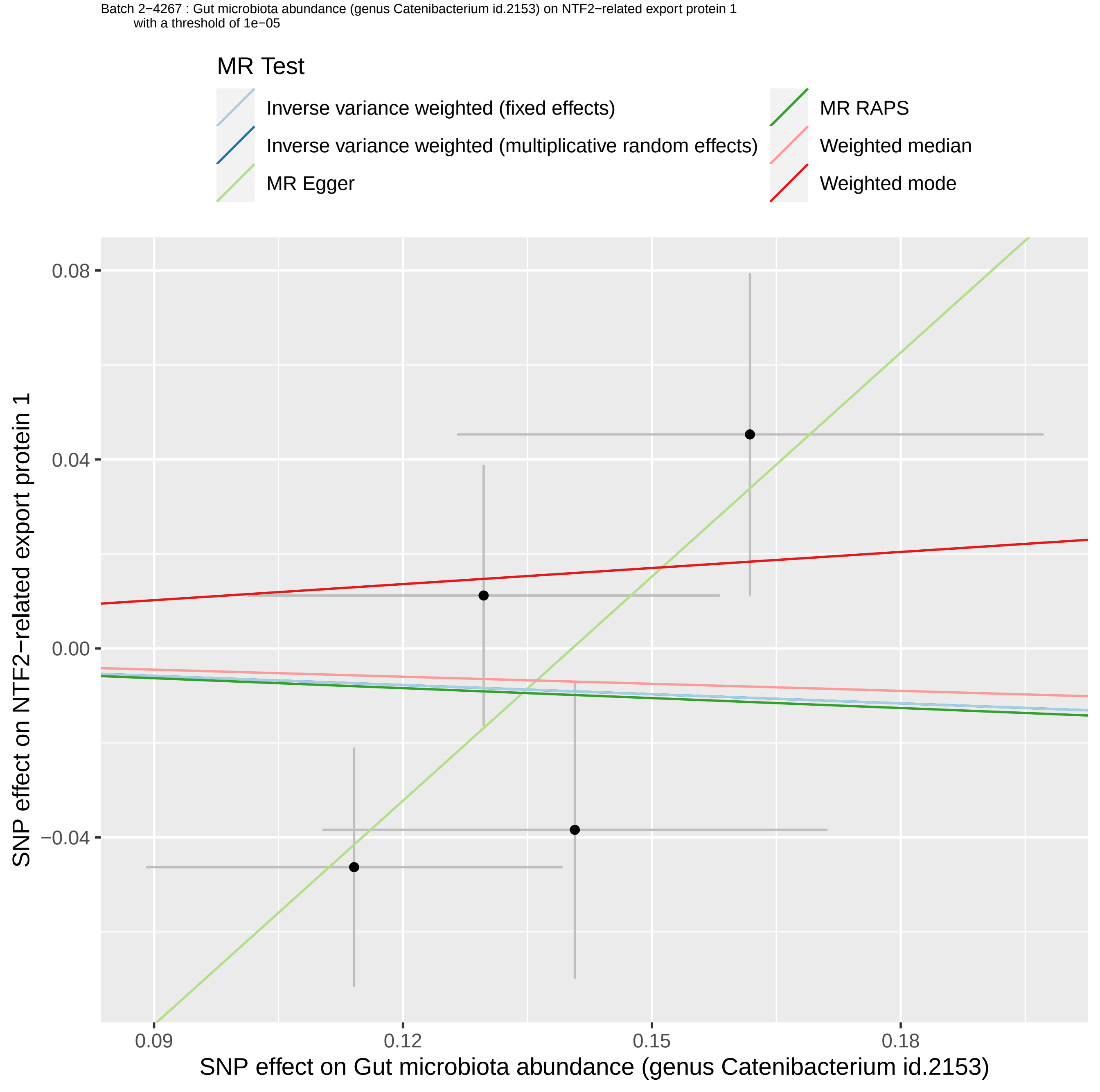
Batch 2−4267 : Gut microbiota abundance (genus Catenibacterium id.2153) on NTF2−related export protein 1
with a threshold of 1e−05
MR Test
Inverse variance weighted (fixed effects)
Inverse variance weighted (multiplicative random effects)
MR Egger
MR RAPS
Weighted median
Weighted mode
0.08
0.04
0.00
−0.04
0.09
0.12
0.15
0.18
SNP effect on NTF2−related export protein 1
SNP effect on Gut microbiota abundance (genus Catenibacterium id.2153)

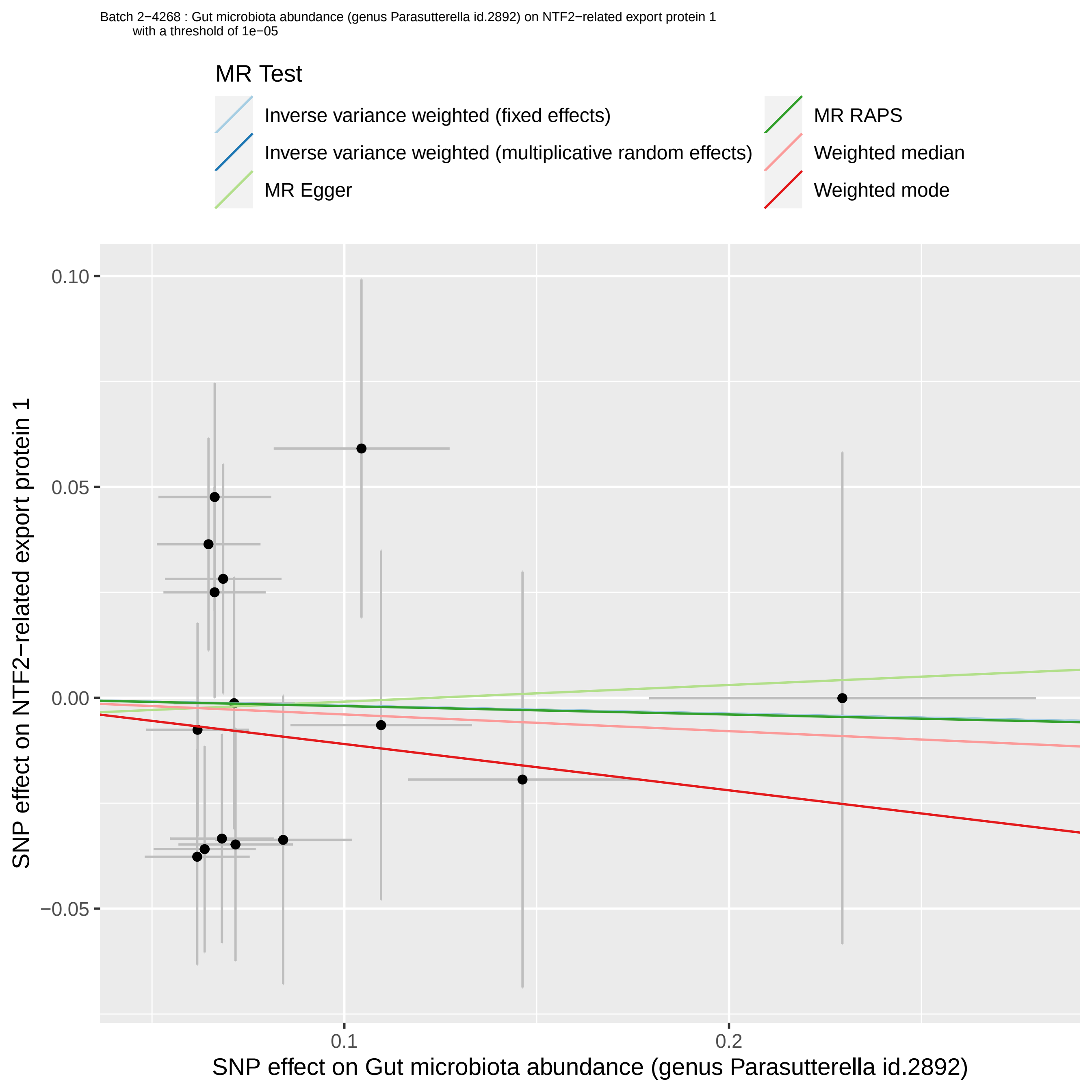
Batch 2−4268 : Gut microbiota abundance (genus Parasutterella id.2892) on NTF2−related export protein 1
with a threshold of 1e−05
MR Test
Inverse variance weighted (fixed effects)
Inverse variance weighted (multiplicative random effects)
MR Egger
MR RAPS
Weighted median
Weighted mode
0.10
0.05
0.00
−0.05
0.1
0.2
SNP effect on NTF2−related export protein 1
SNP effect on Gut microbiota abundance (genus Parasutterella id.2892)

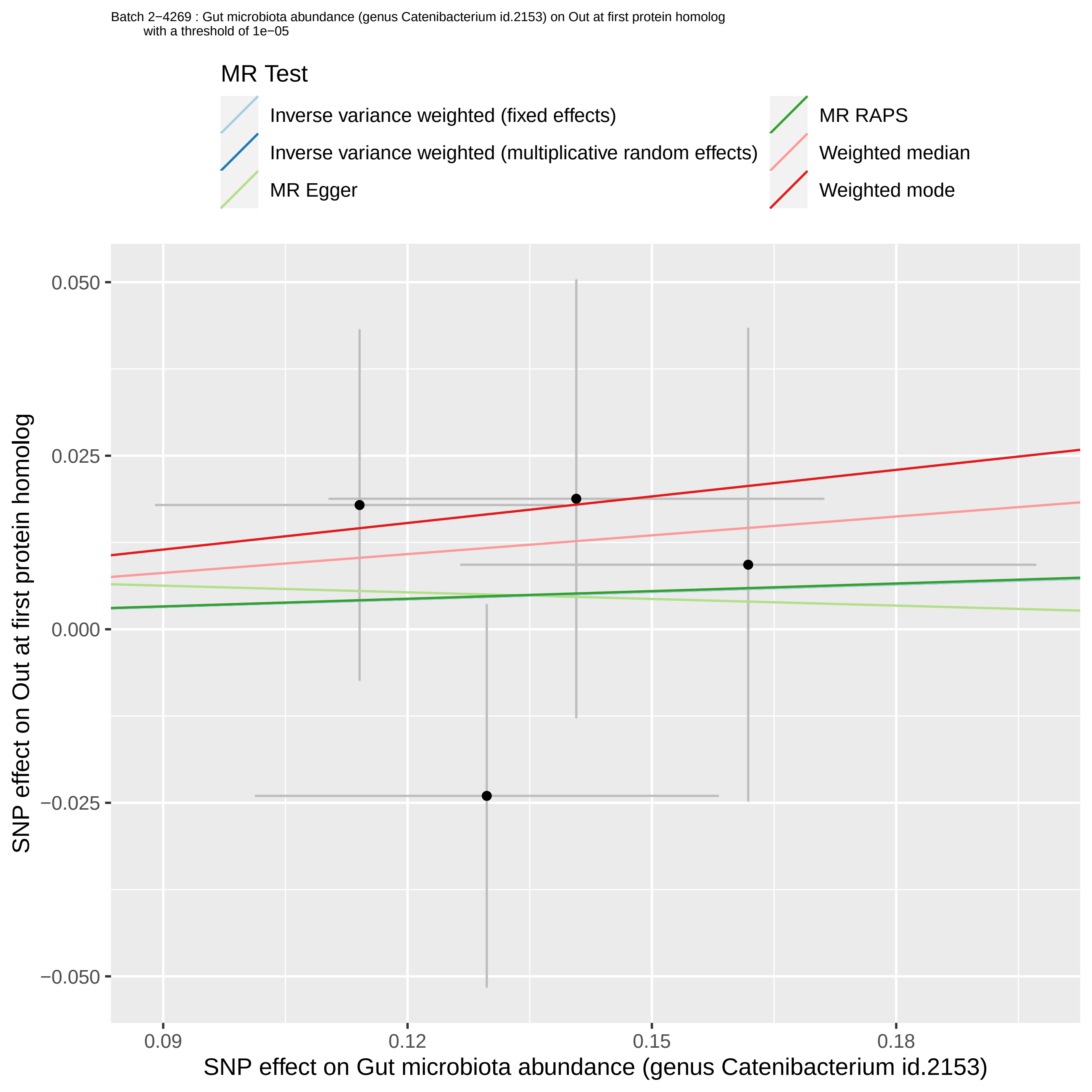
Batch 2−4269 : Gut microbiota abundance (genus Catenibacterium id.2153) on Out at first protein homolog
with a threshold of 1e−05
MR Test
Inverse variance weighted (fixed effects)
Inverse variance weighted (multiplicative random effects)
MR Egger
MR RAPS
Weighted median
Weighted mode
0.050
0.025
0.000
−0.025
−0.050
0.09
0.12
0.15
0.18
SNP effect on Out at first protein homolog
SNP effect on Gut microbiota abundance (genus Catenibacterium id.2153)

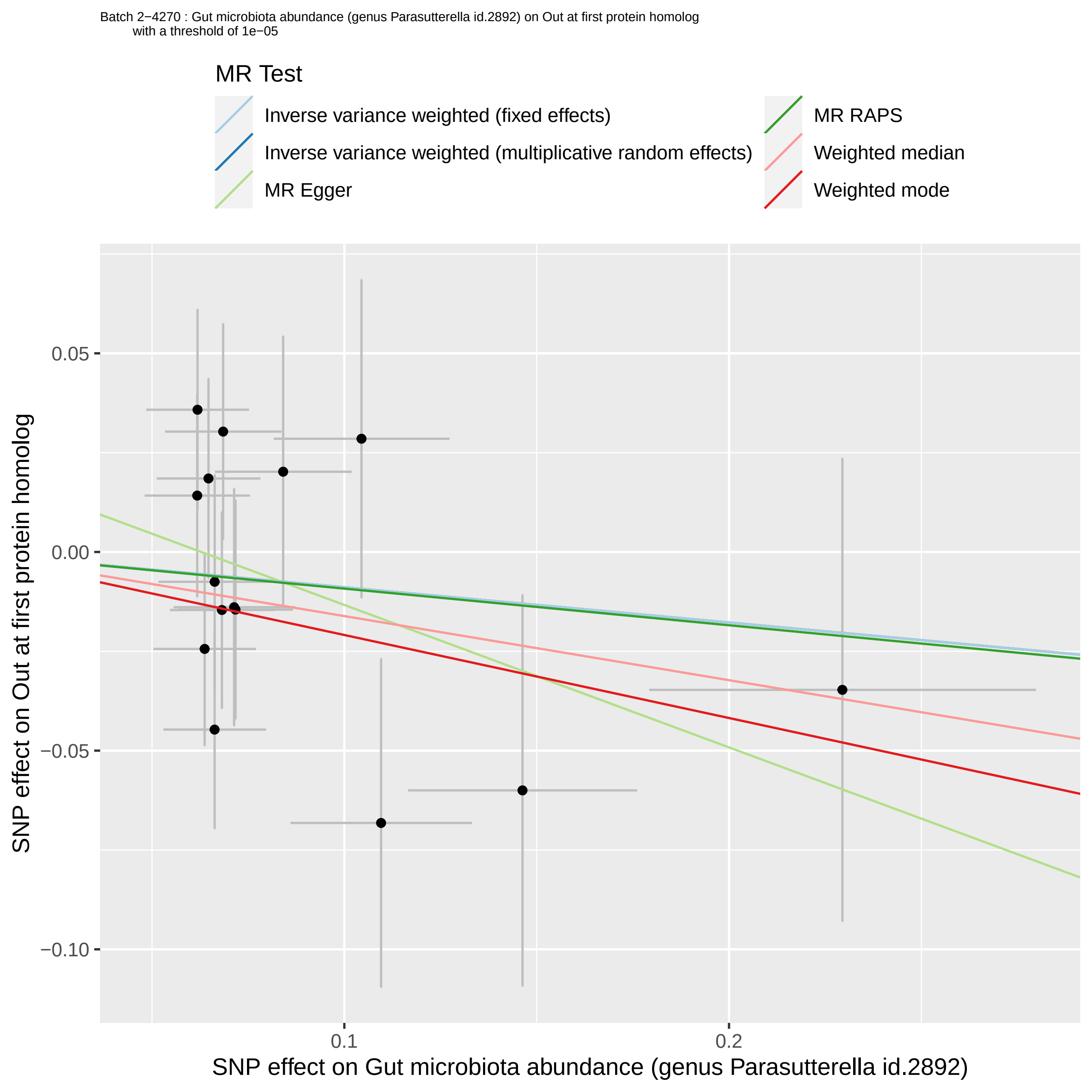
Batch 2−4270 : Gut microbiota abundance (genus Parasutterella id.2892) on Out at first protein homolog
with a threshold of 1e−05
MR Test
Inverse variance weighted (fixed effects)
Inverse variance weighted (multiplicative random effects)
MR Egger
MR RAPS
Weighted median
Weighted mode
0.05
0.00
−0.05
−0.10
0.1
0.2
SNP effect on Out at first protein homolog
SNP effect on Gut microbiota abundance (genus Parasutterella id.2892)

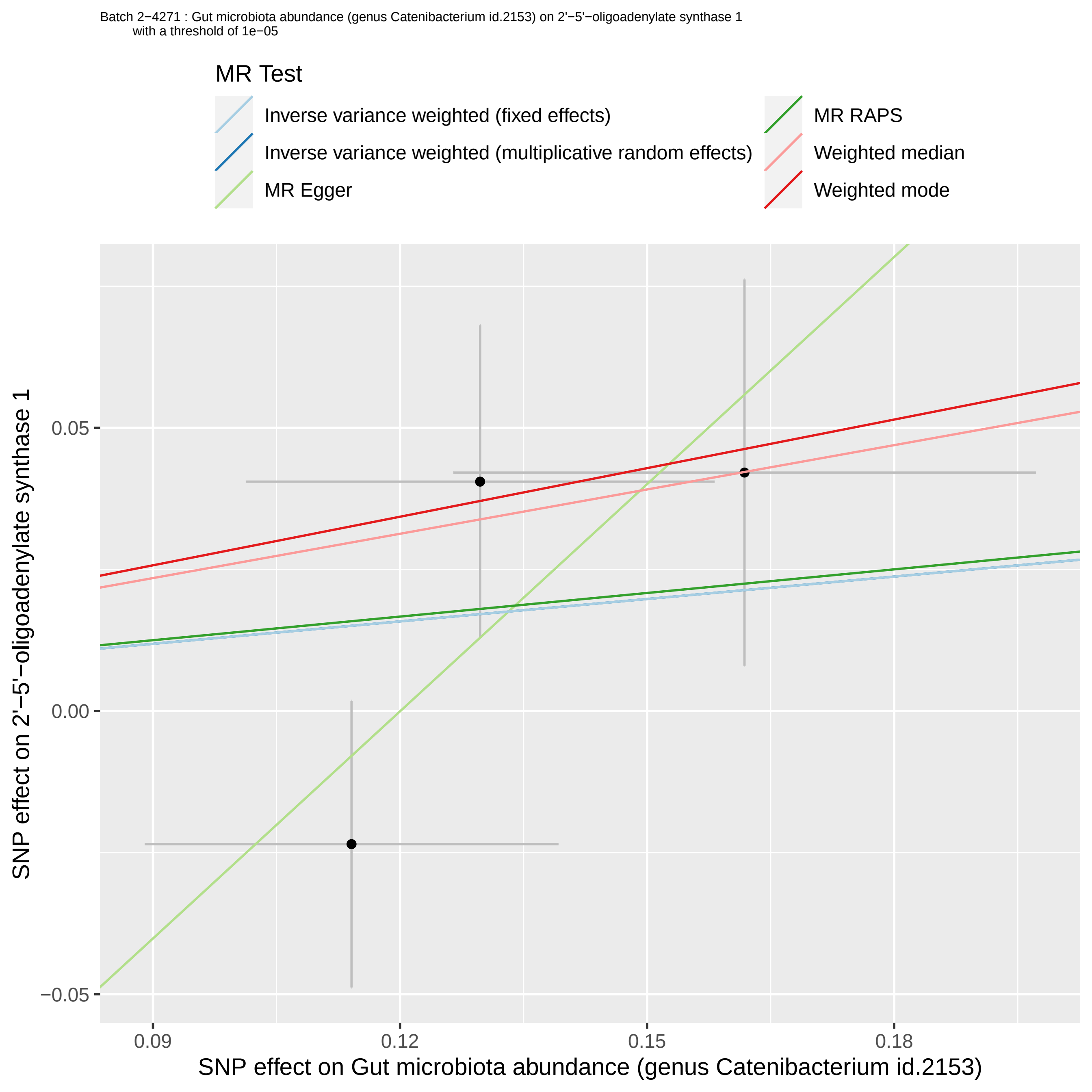
Batch 2−4271 : Gut microbiota abundance (genus Catenibacterium id.2153) on 2'−5'−oligoadenylate synthase 1
with a threshold of 1e−05
MR Test
Inverse variance weighted (fixed effects)
Inverse variance weighted (multiplicative random effects)
MR Egger
MR RAPS
Weighted median
Weighted mode
0.05
0.00
−0.05
0.09
0.12
0.15
0.18
SNP effect on 2'−5'−oligoadenylate synthase 1
SNP effect on Gut microbiota abundance (genus Catenibacterium id.2153)

Batch 2−4272 : Gut microbiota abundance (genus Parasutterella id.2892) on 2'−5'−oligoadenylate synthase 1
with a threshold of 1e−05

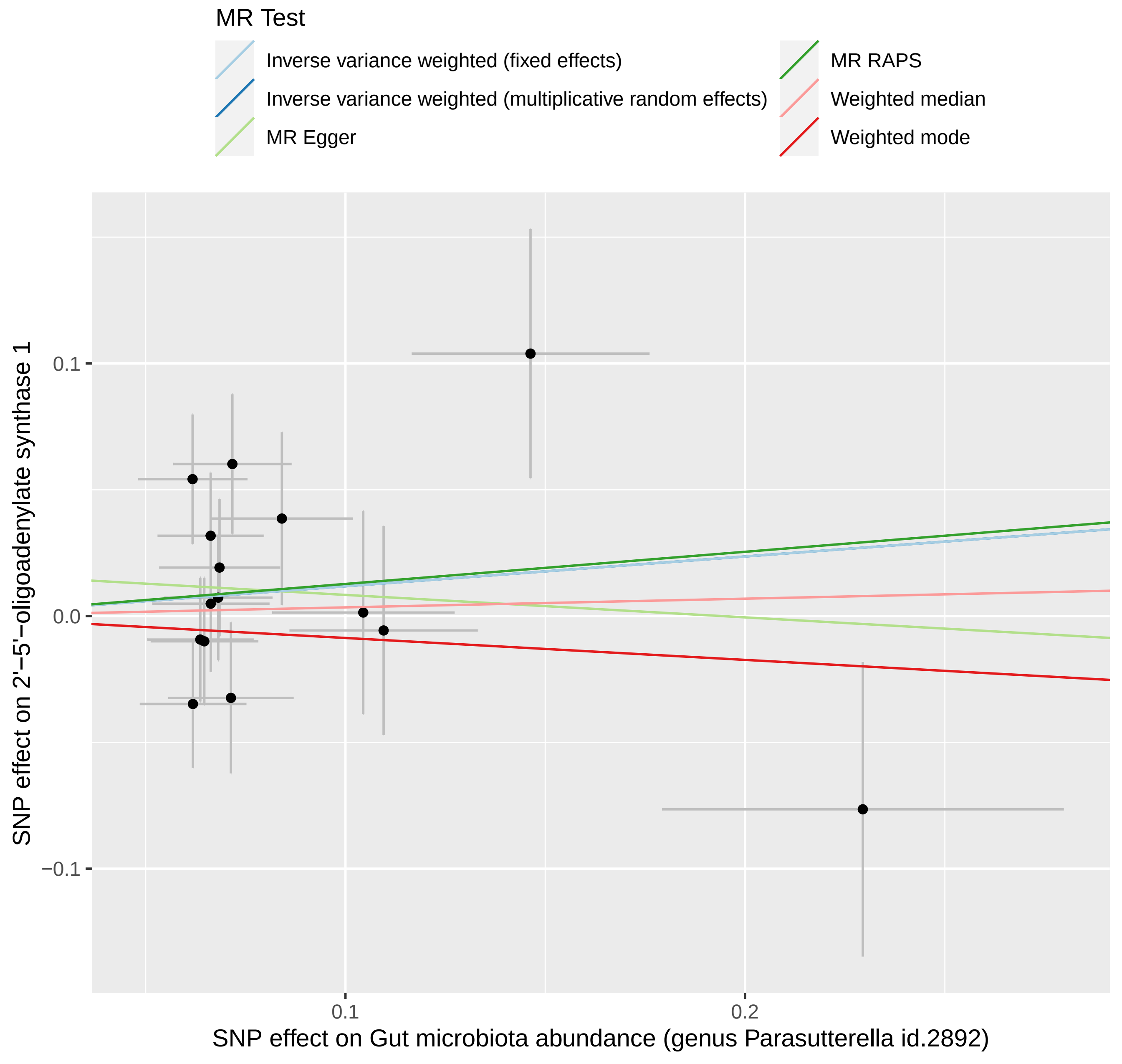

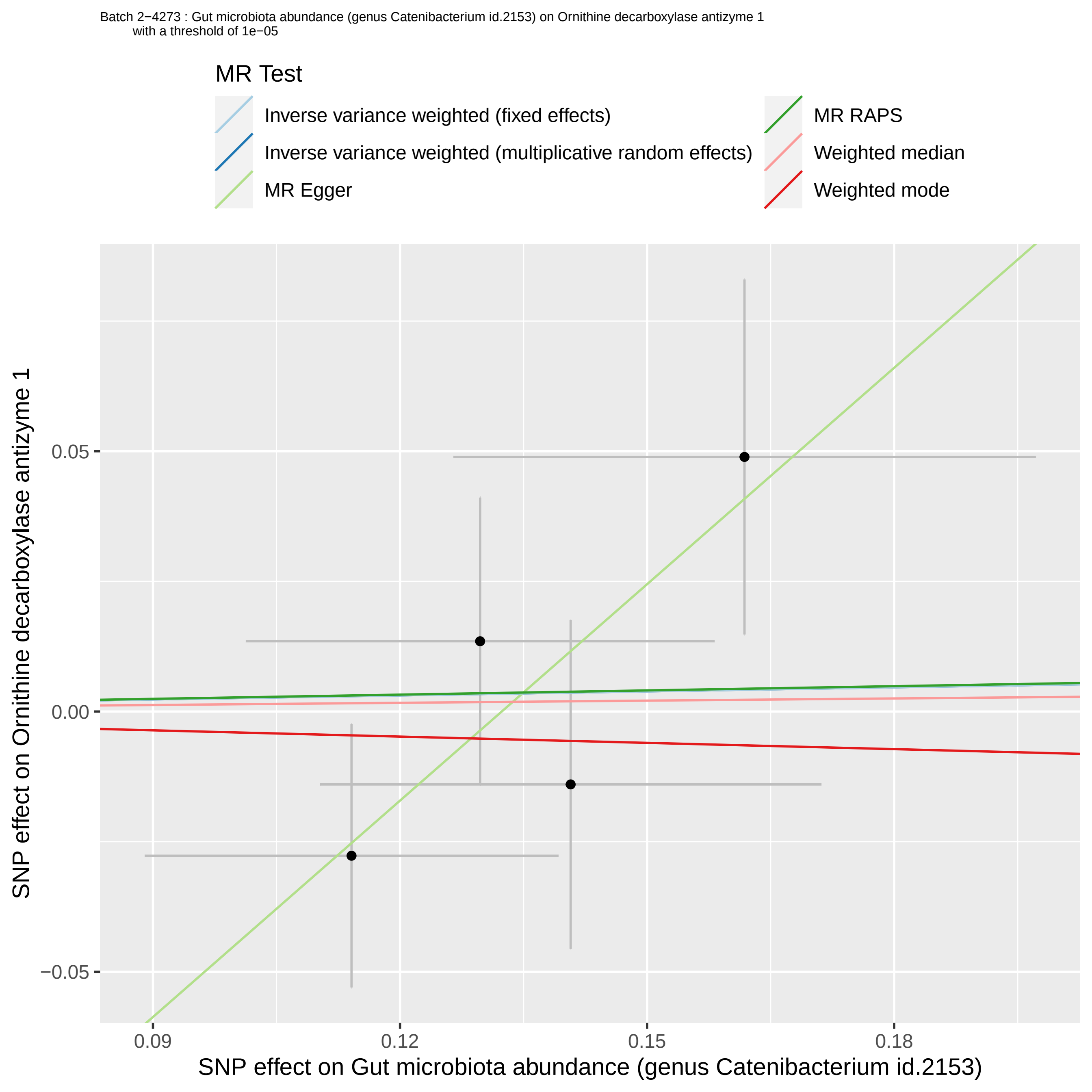
Batch 2−4273 : Gut microbiota abundance (genus Catenibacterium id.2153) on Ornithine decarboxylase antizyme 1
with a threshold of 1e−05
MR Test
Inverse variance weighted (fixed effects)
Inverse variance weighted (multiplicative random effects)
MR Egger
MR RAPS
Weighted median
Weighted mode
0.05
0.00
−0.05
0.09
0.12
0.15
0.18
SNP effect on Ornithine decarboxylase antizyme 1
SNP effect on Gut microbiota abundance (genus Catenibacterium id.2153)

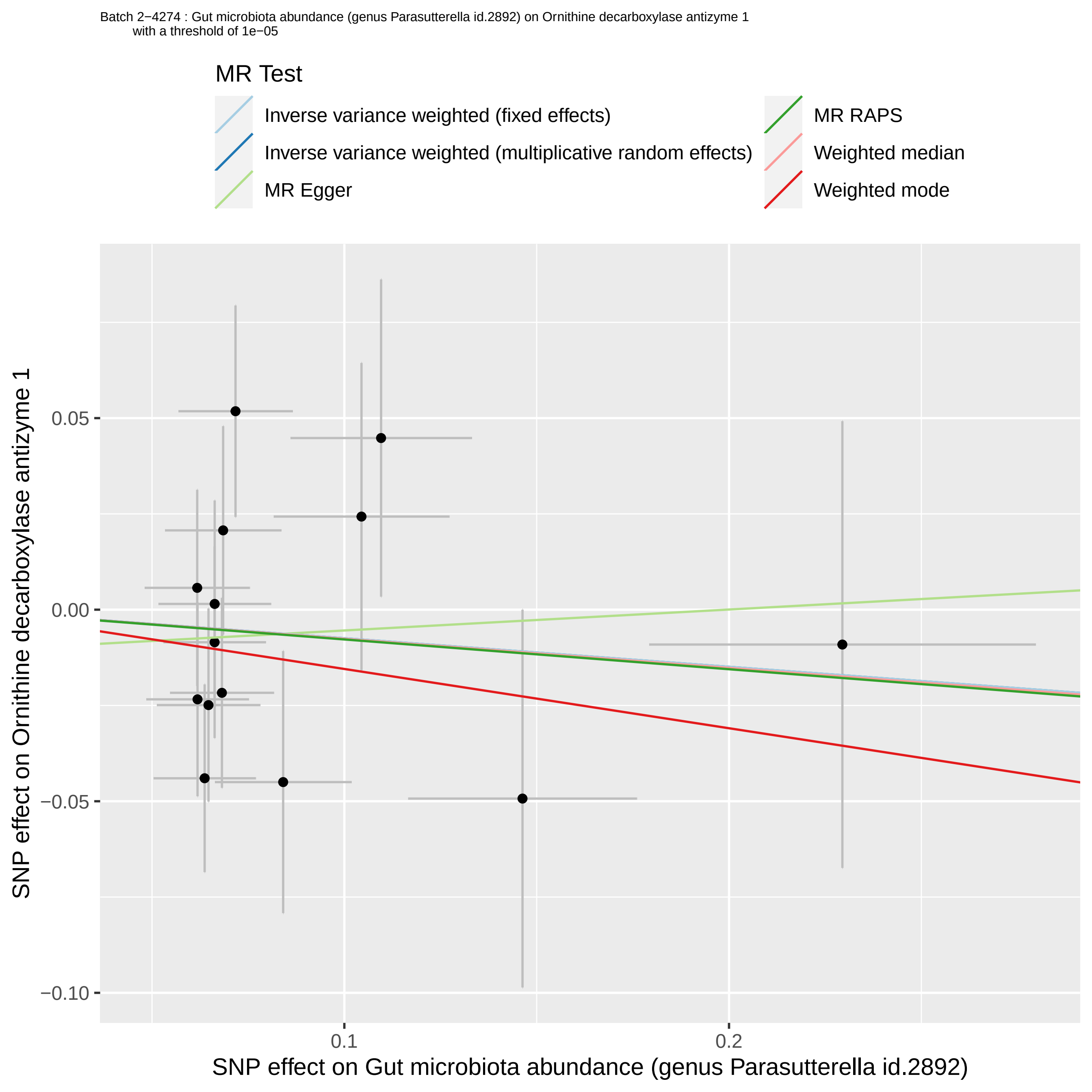

Batch 2−4274 : Gut microbiota abundance (genus Parasutterella id.2892) on Ornithine decarboxylase antizyme 1
with a threshold of 1e−05
MR Test
Inverse variance weighted (fixed effects)
Inverse variance weighted (multiplicative random effects)
MR Egger
MR RAPS
Weighted median
Weighted mode
0.05
0.00
−0.05
−0.10
0.1
0.2
SNP effect on Ornithine decarboxylase antizyme 1
SNP effect on Gut microbiota abundance (genus Parasutterella id.2892)

Batch 2−4275 : Gut microbiota abundance (genus Catenibacterium id.2153) on Odorant−binding protein 2a
with a threshold of 1e−05

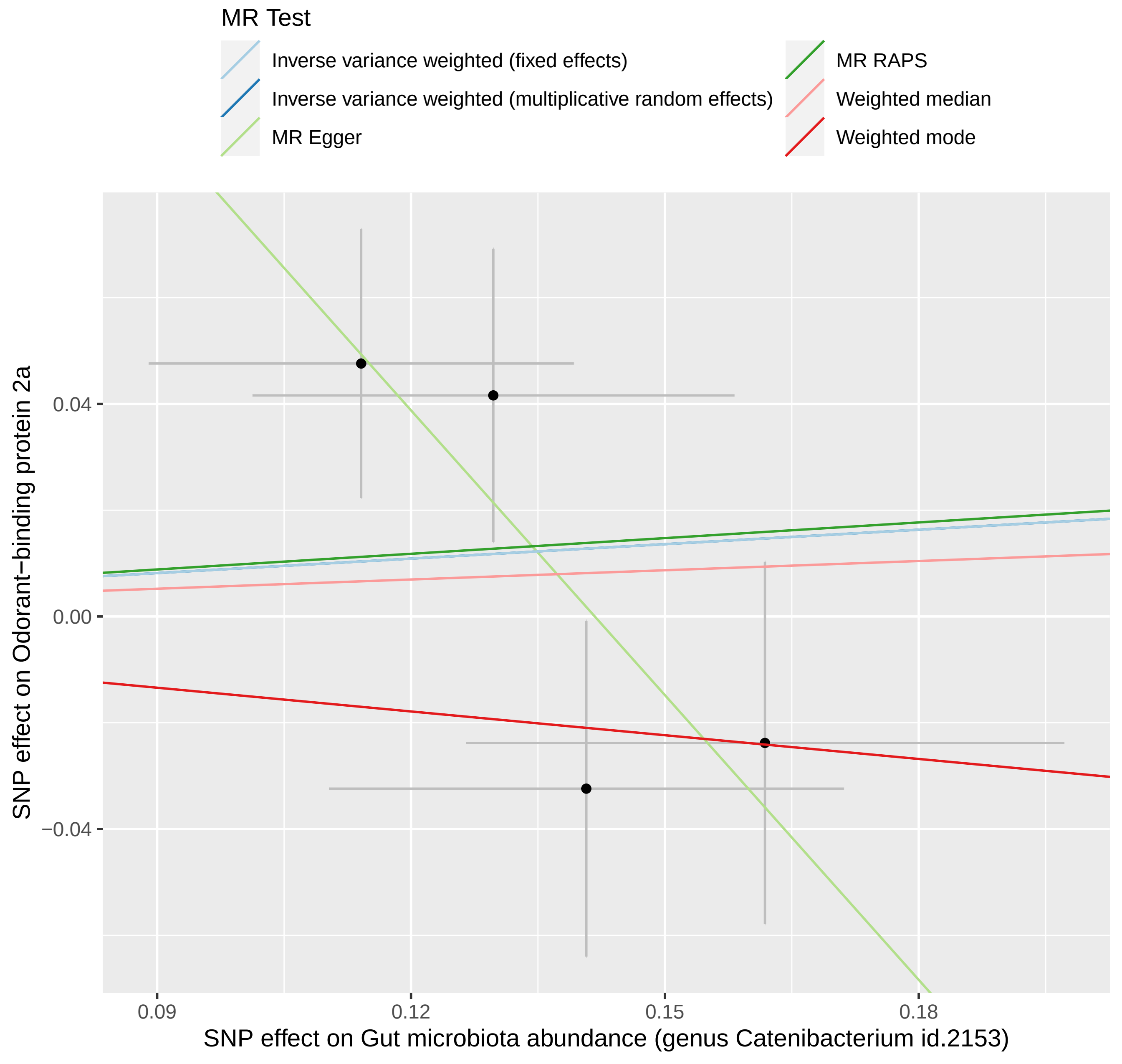

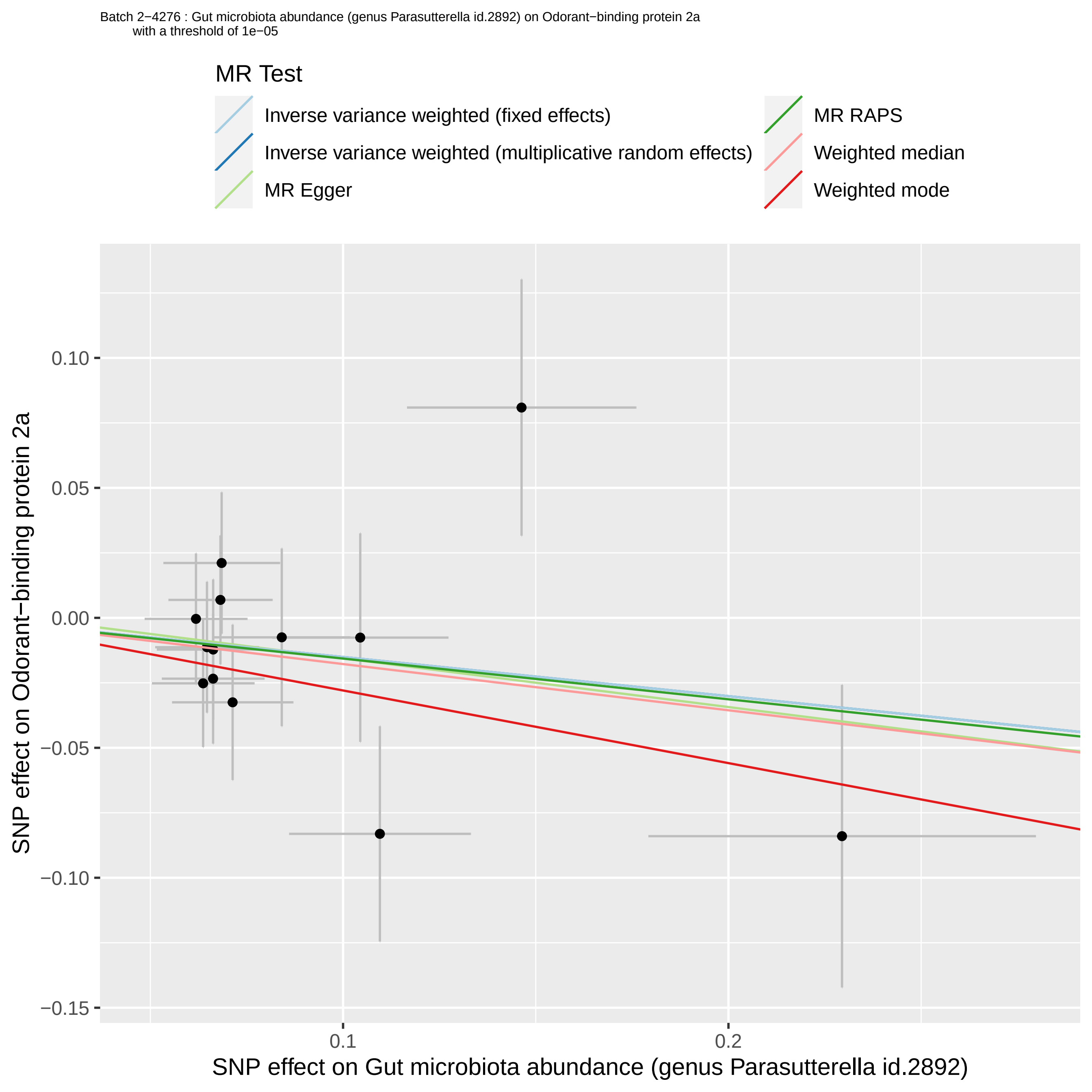
Batch 2−4276 : Gut microbiota abundance (genus Parasutterella id.2892) on Odorant−binding protein 2a
with a threshold of 1e−05
MR Test
Inverse variance weighted (fixed effects)
Inverse variance weighted (multiplicative random effects)
MR Egger
MR RAPS
Weighted median
Weighted mode
0.10
0.05
0.00
−0.05
−0.10
−0.15
0.1
0.2
SNP effect on Odorant−binding protein 2a
SNP effect on Gut microbiota abundance (genus Parasutterella id.2892)

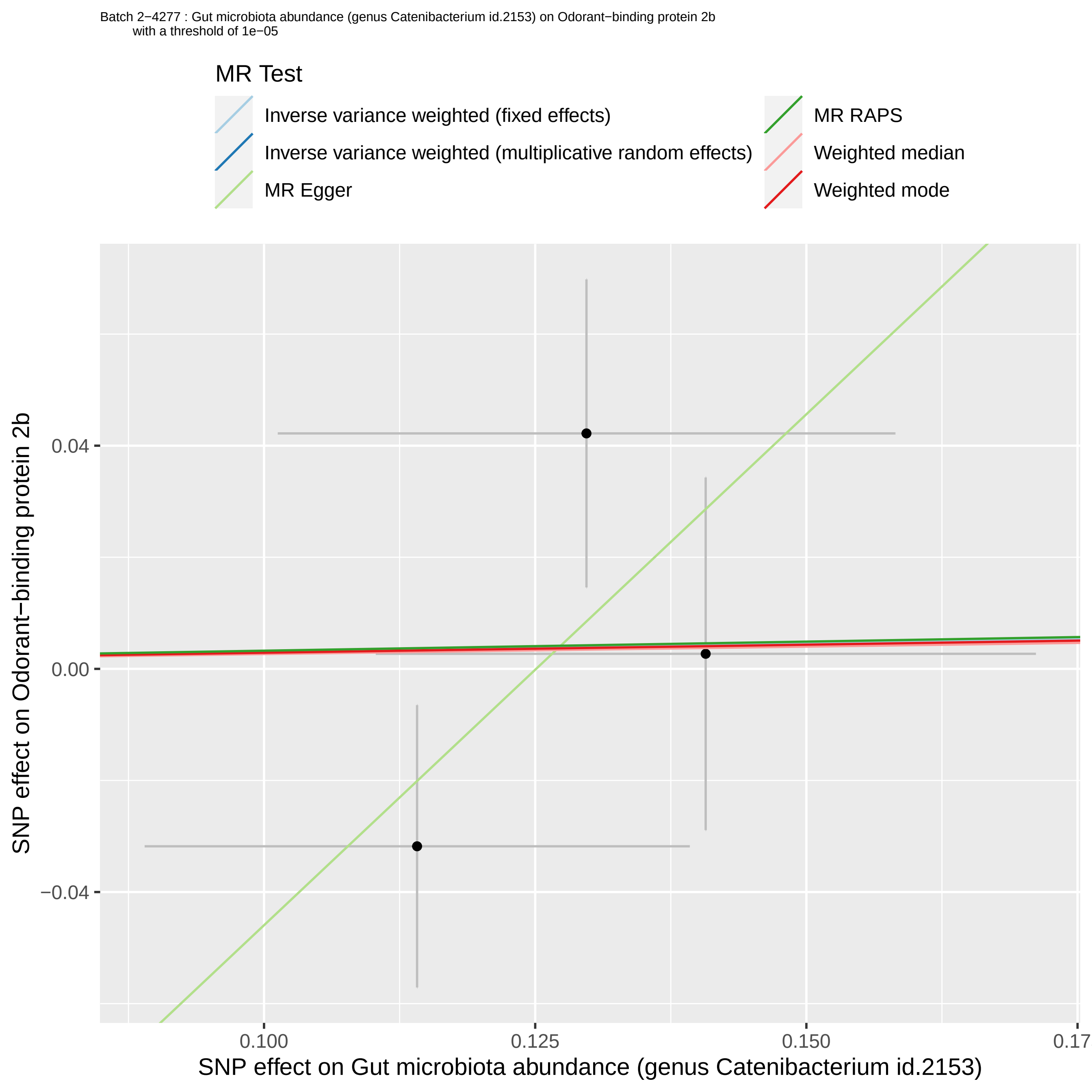
Batch 2−4277 : Gut microbiota abundance (genus Catenibacterium id.2153) on Odorant−binding protein 2b
with a threshold of 1e−05
MR Test
Inverse variance weighted (fixed effects)
Inverse variance weighted (multiplicative random effects)
MR Egger
MR RAPS
Weighted median
Weighted mode
SNP effect on Odorant−binding protein 2b
0.04
0.00
−0.04
0.100
0.125
0.150
0.17
SNP effect on Gut microbiota abundance (genus Catenibacterium id.2153)

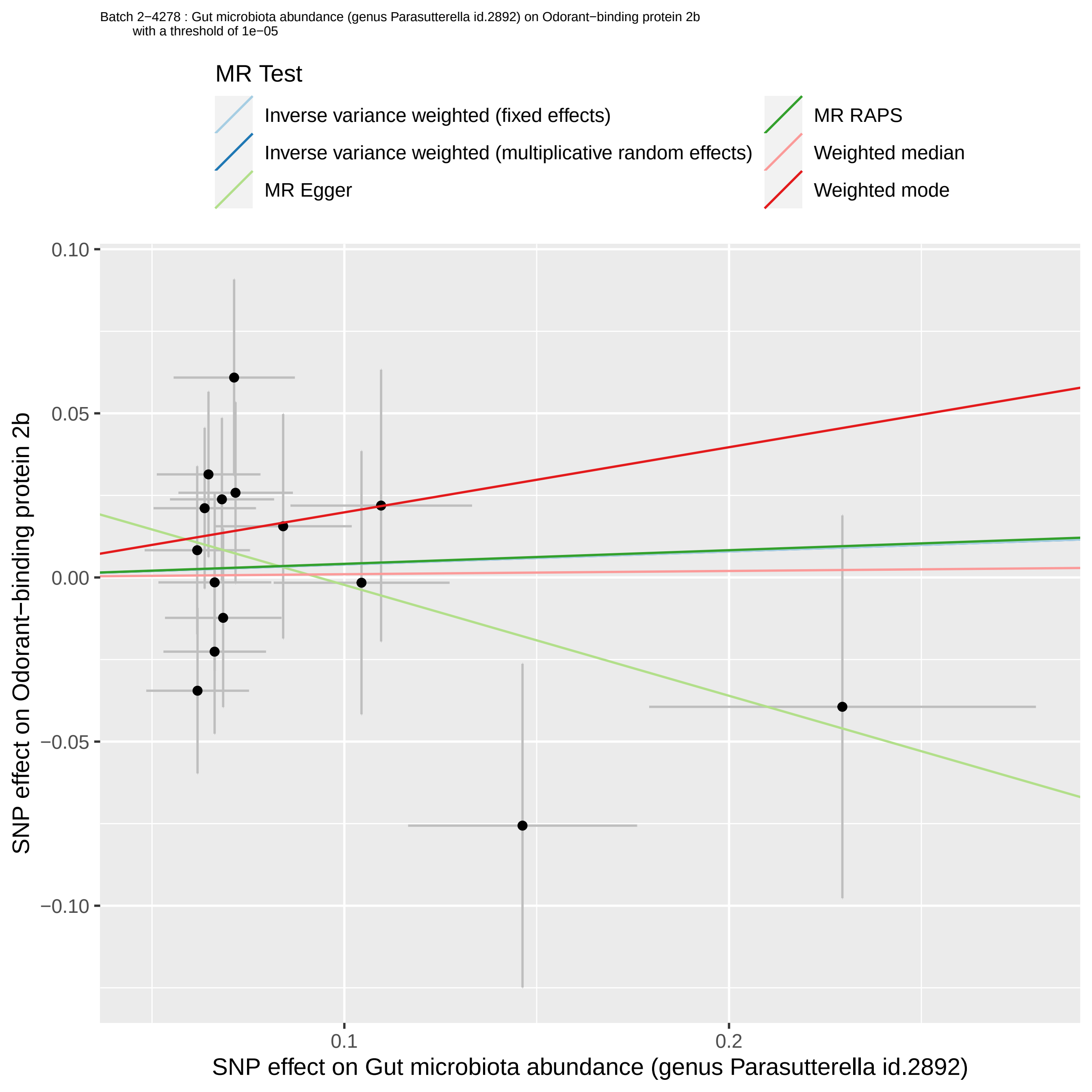
Batch 2−4278 : Gut microbiota abundance (genus Parasutterella id.2892) on Odorant−binding protein 2b
with a threshold of 1e−05
MR Test
Inverse variance weighted (fixed effects)
Inverse variance weighted (multiplicative random effects)
MR Egger
MR RAPS
Weighted median
Weighted mode
0.10
0.05
0.00
−0.05
−0.10
SNP effect on Odorant−binding protein 2b
0.1
0.2
SNP effect on Gut microbiota abundance (genus Parasutterella id.2892)

Batch 2−4279 : Gut microbiota abundance (genus Catenibacterium id.2153) on OCIA domain−containing protein 1
with a threshold of 1e−05

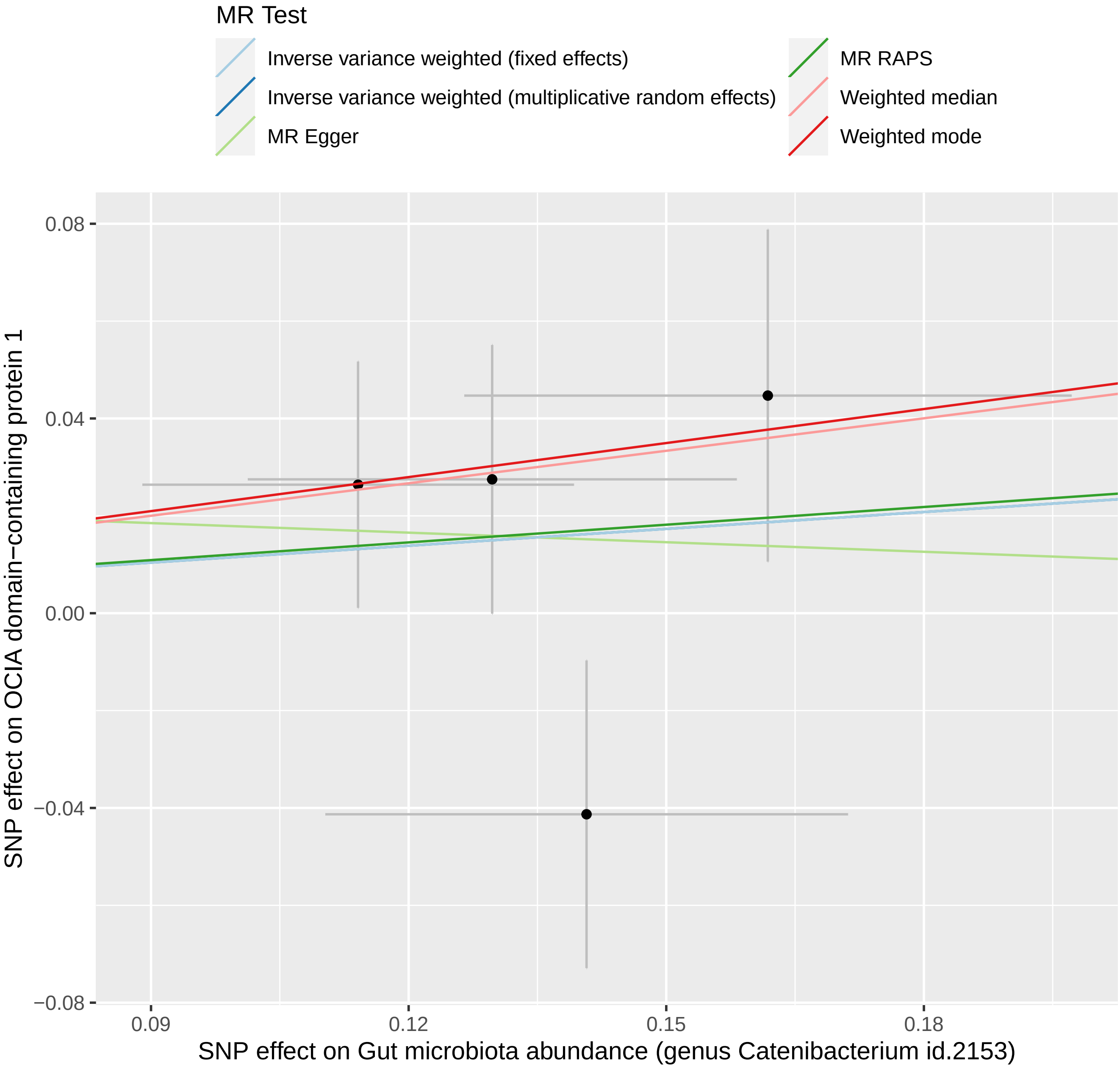

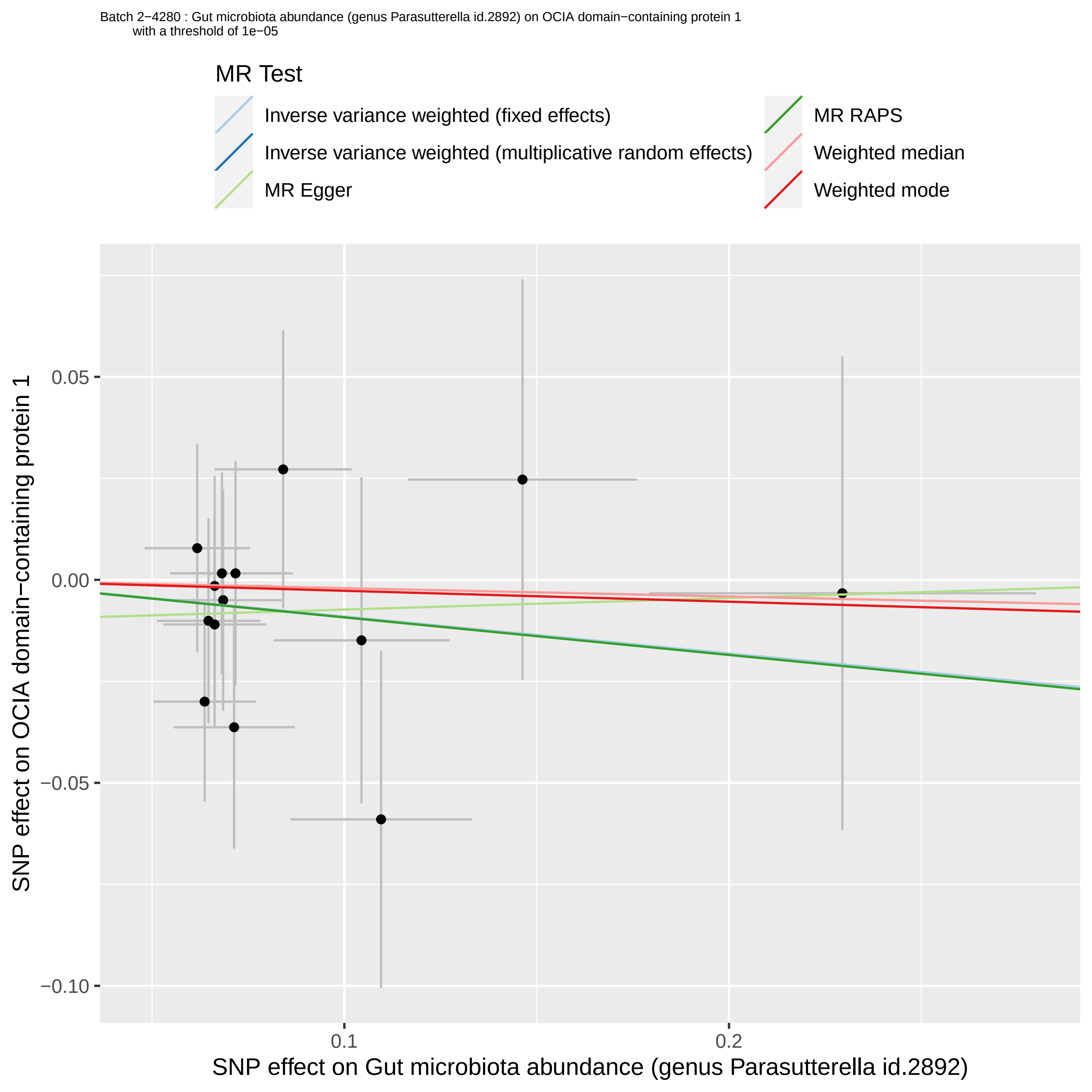

Batch 2−4280 : Gut microbiota abundance (genus Parasutterella id.2892) on OCIA domain−containing protein 1
with a threshold of 1e−05
MR Test
Inverse variance weighted (fixed effects)
Inverse variance weighted (multiplicative random effects)
MR Egger
MR RAPS
Weighted median
Weighted mode
SNP effect on OCIA domain−containing protein 1
0.05
0.00
−0.05
−0.10
0.1
0.2
SNP effect on Gut microbiota abundance (genus Parasutterella id.2892)

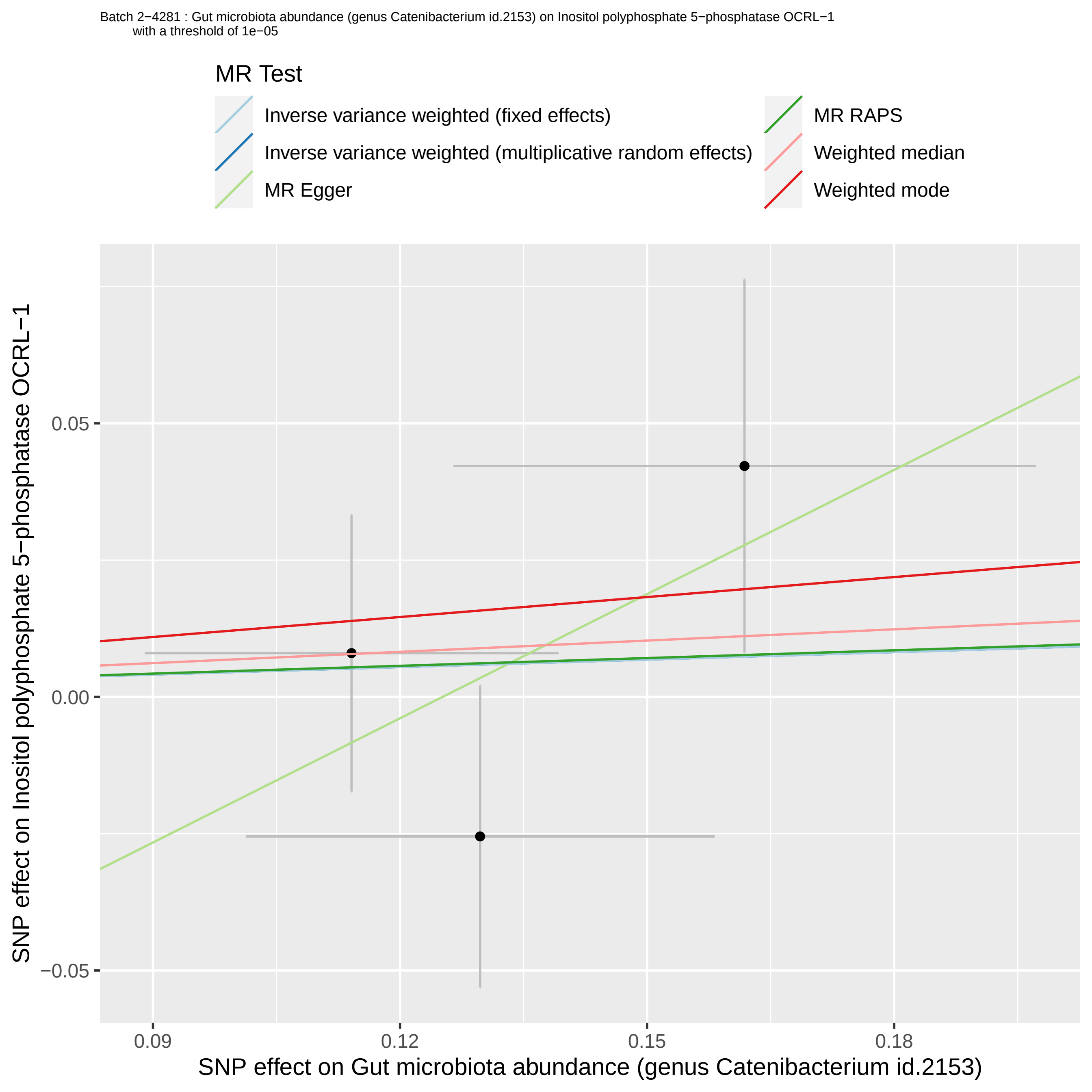
Batch 2−4281 : Gut microbiota abundance (genus Catenibacterium id.2153) on Inositol polyphosphate 5−phosphatase OCRL−1
with a threshold of 1e−05
MR Test
Inverse variance weighted (fixed effects)
Inverse variance weighted (multiplicative random effects)
MR Egger
MR RAPS
Weighted median
Weighted mode
0.05
0.00
−0.05
0.09
0.12
0.15
0.18
SNP effect on Inositol polyphosphate 5−phosphatase OCRL−1
SNP effect on Gut microbiota abundance (genus Catenibacterium id.2153)

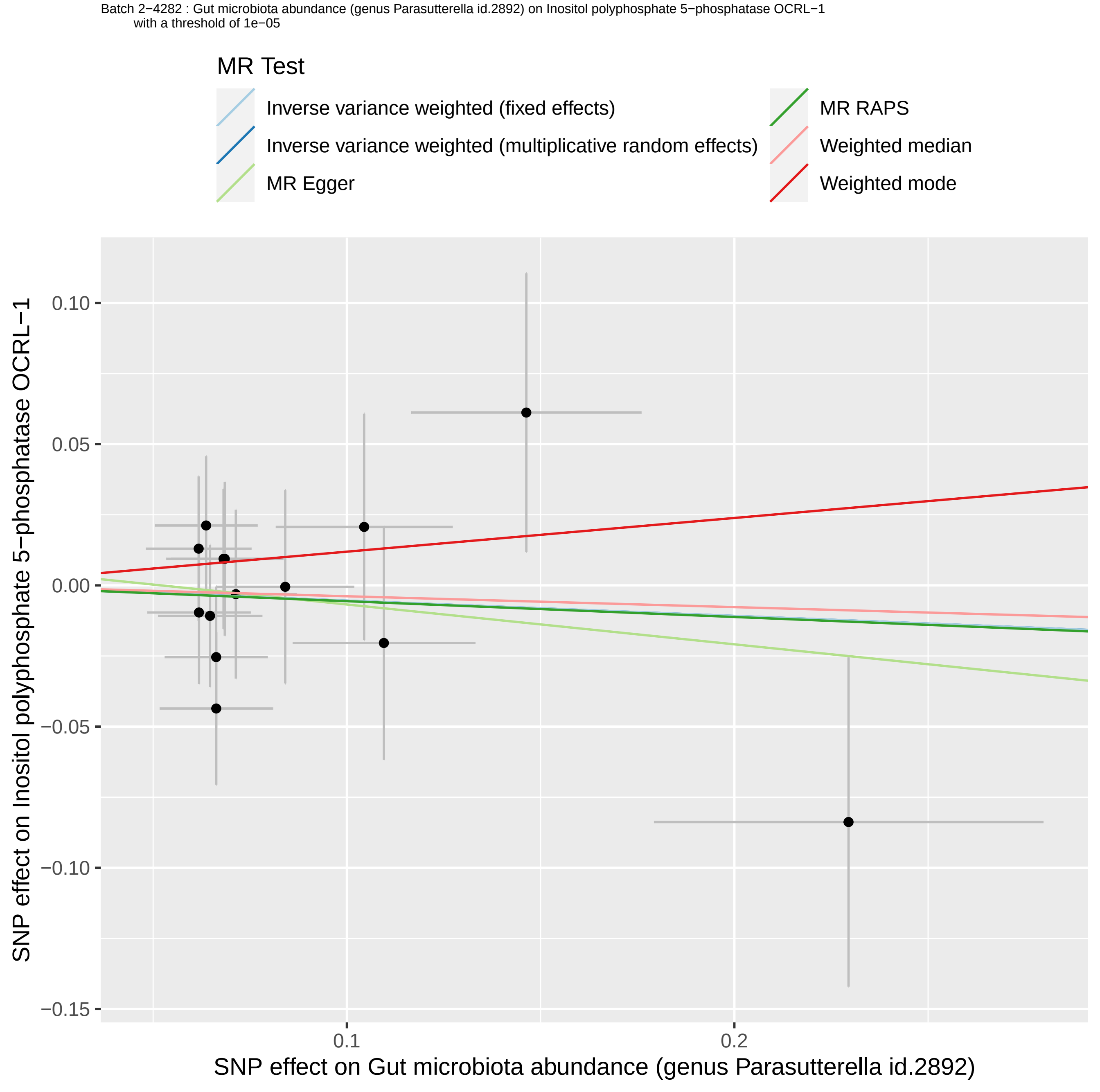

Batch 2−4282 : Gut microbiota abundance (genus Parasutterella id.2892) on Inositol polyphosphate 5−phosphatase OCRL−1
with a threshold of 1e−05
MR Test
Inverse variance weighted (fixed effects)
Inverse variance weighted (multiplicative random effects)
MR Egger
MR RAPS
Weighted median
Weighted mode
0.10
0.05
0.00
−0.05
−0.10
−0.15
0.1
0.2
SNP effect on Inositol polyphosphate 5−phosphatase OCRL−1
SNP effect on Gut microbiota abundance (genus Parasutterella id.2892)

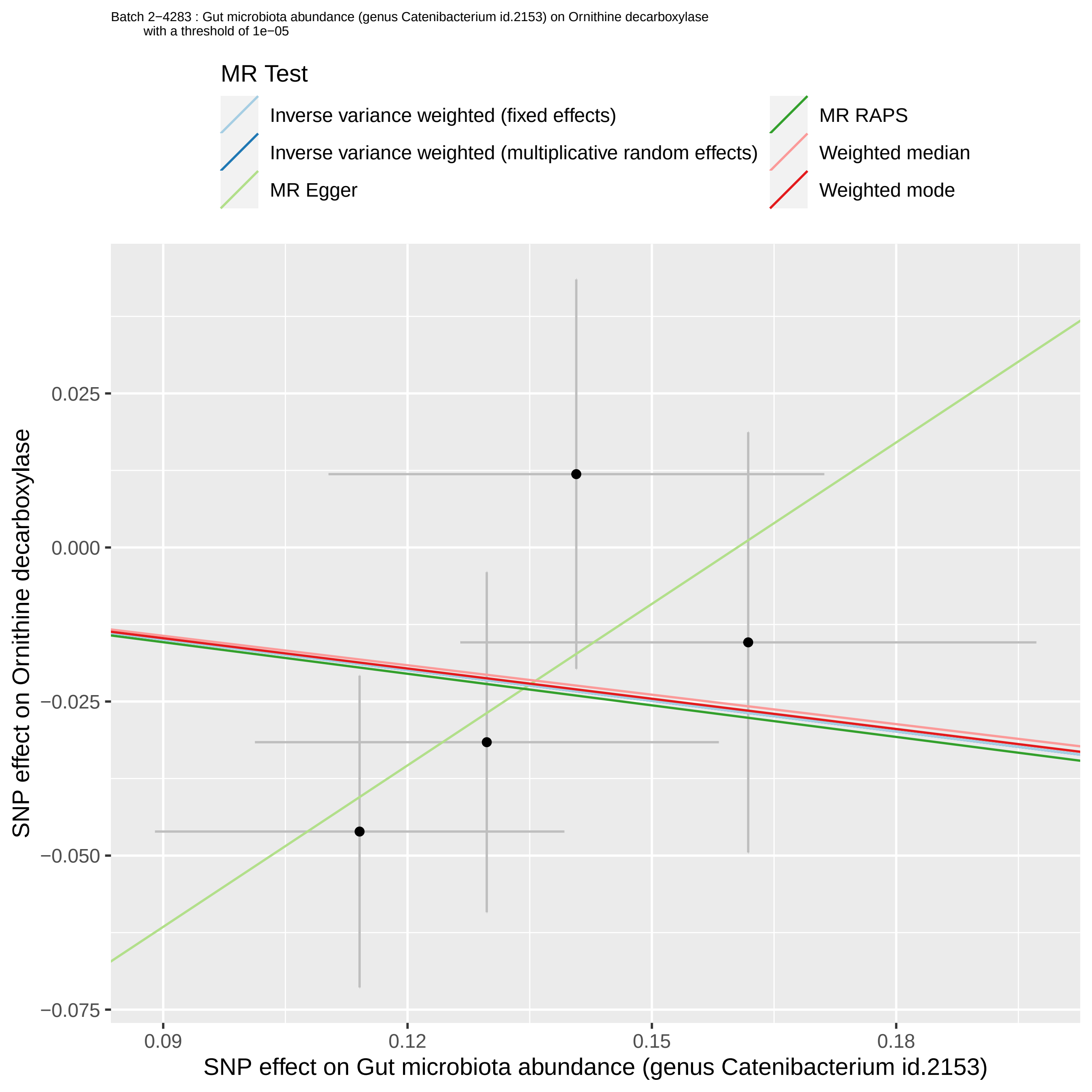

Batch 2−4283 : Gut microbiota abundance (genus Catenibacterium id.2153) on Ornithine decarboxylase with a threshold of 1e−05
MR Test
Inverse variance weighted (fixed effects)
Inverse variance weighted (multiplicative random effects)
MR Egger
MR RAPS
Weighted median
Weighted mode
0.025
0.000
−0.025
−0.050
−0.075
SNP effect on Ornithine decarboxylase
0.09
0.12
0.15
0.18
SNP effect on Gut microbiota abundance (genus Catenibacterium id.2153)

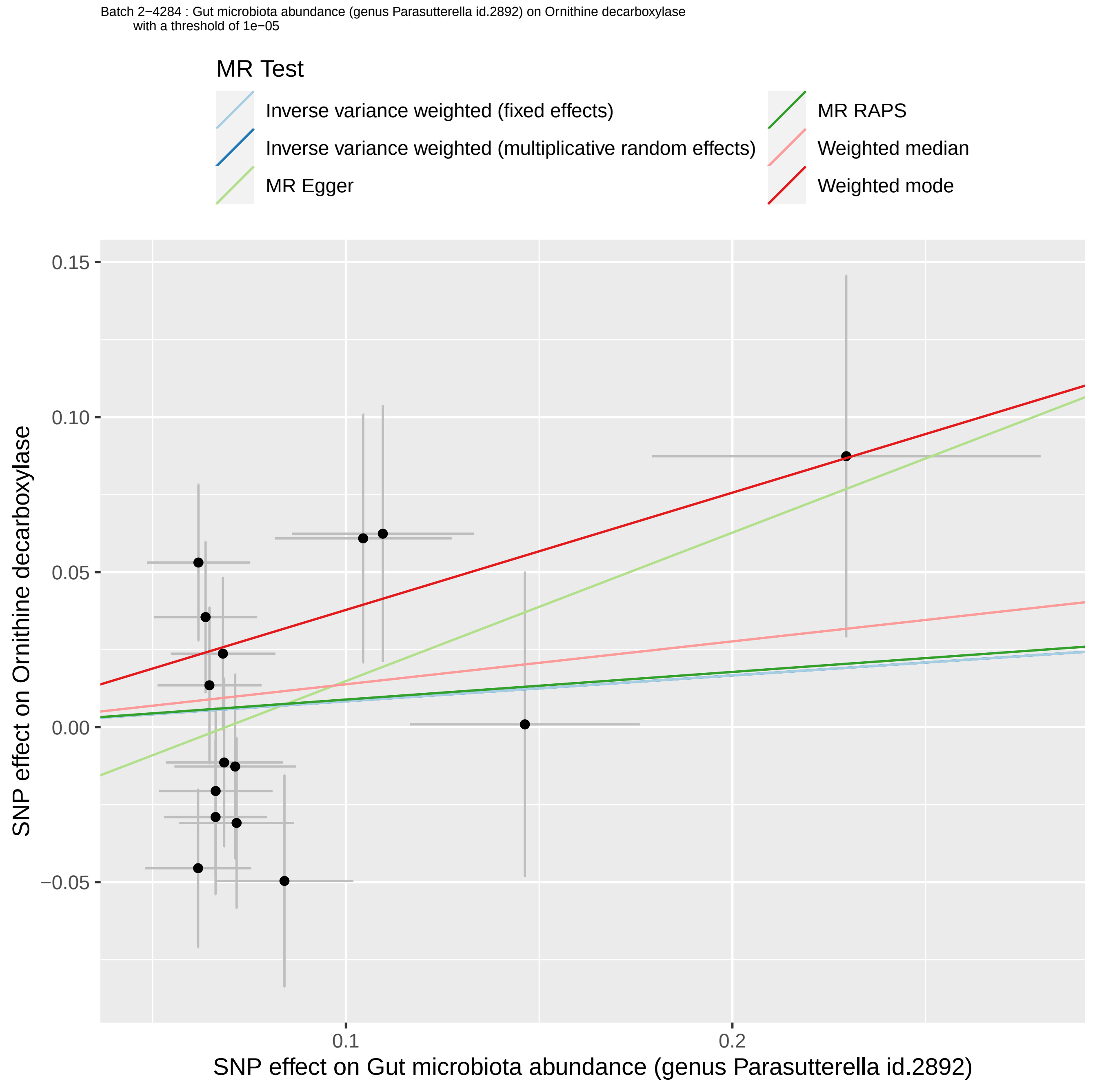

Batch 2−4284 : Gut microbiota abundance (genus Parasutterella id.2892) on Ornithine decarboxylase
with a threshold of 1e−05
MR Test
Inverse variance weighted (fixed effects)
Inverse variance weighted (multiplicative random effects)
MR Egger
MR RAPS
Weighted median
Weighted mode
0.15
0.10
0.05
0.00
−0.05
0.1
0.2
SNP effect on Ornithine decarboxylase
SNP effect on Gut microbiota abundance (genus Parasutterella id.2892)

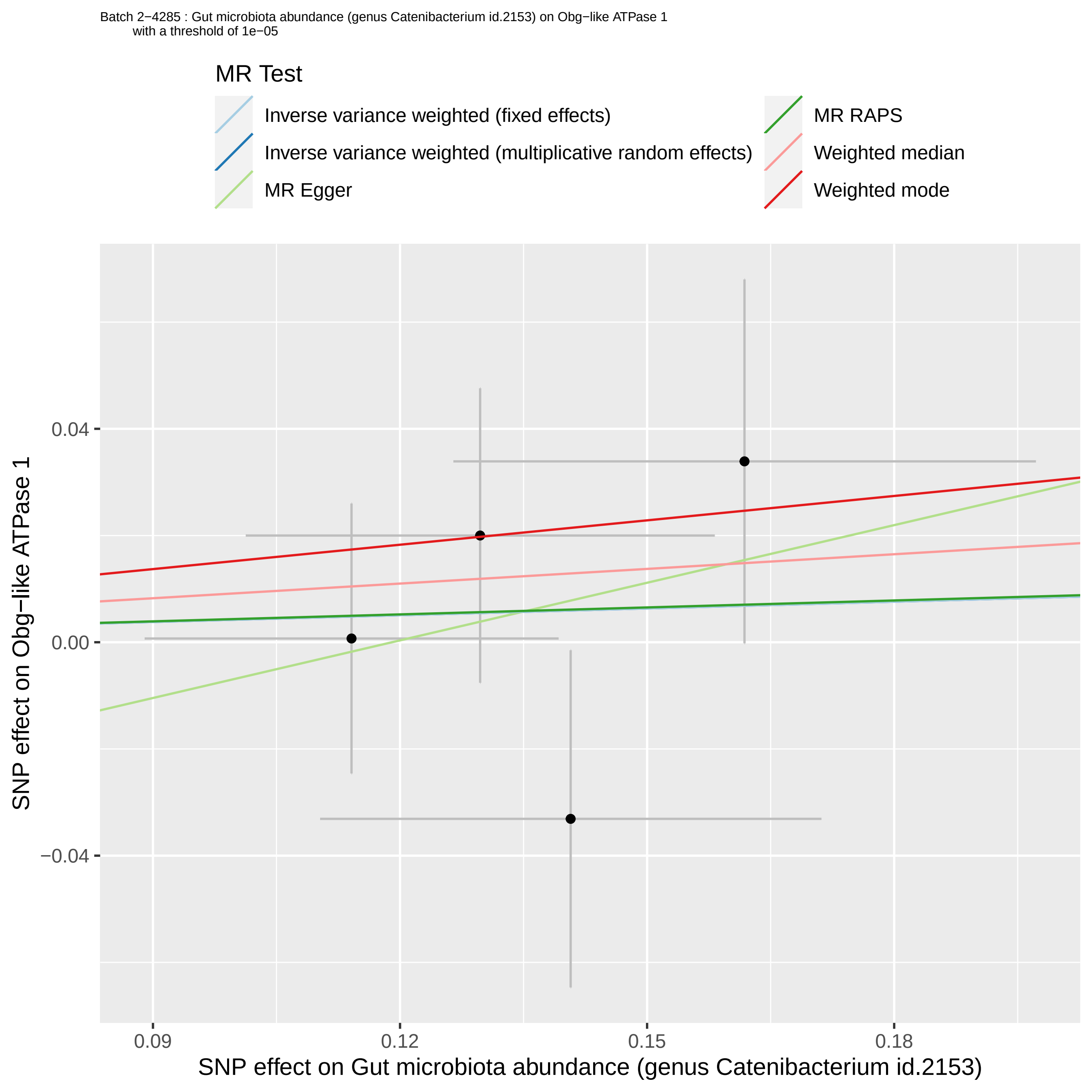
Batch 2−4285 : Gut microbiota abundance (genus Catenibacterium id.2153) on Obg−like ATPase 1
with a threshold of 1e−05
MR Test
Inverse variance weighted (fixed effects)
Inverse variance weighted (multiplicative random effects)
MR Egger
MR RAPS
Weighted median
Weighted mode
0.04
0.00
−0.04
0.09
0.12
0.15
0.18
SNP effect on Obg−like ATPase 1
SNP effect on Gut microbiota abundance (genus Catenibacterium id.2153)

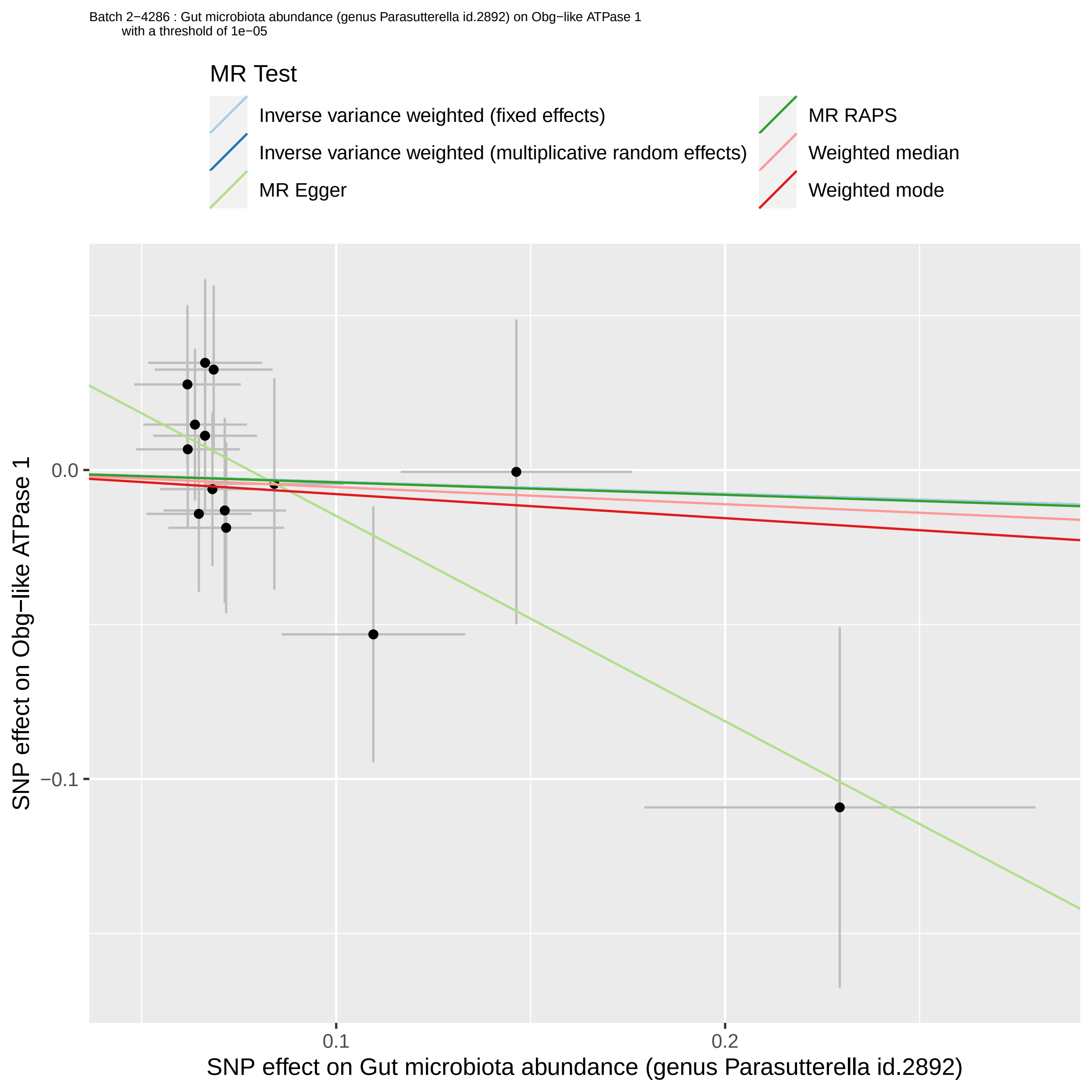

Batch 2−4286 : Gut microbiota abundance (genus Parasutterella id.2892) on Obg−like ATPase 1
with a threshold of 1e−05
MR Test
Inverse variance weighted (fixed effects)
Inverse variance weighted (multiplicative random effects)
MR Egger
MR RAPS
Weighted median
Weighted mode
0.0
−0.1
0.1
0.2
SNP effect on Obg−like ATPase 1
SNP effect on Gut microbiota abundance (genus Parasutterella id.2892)

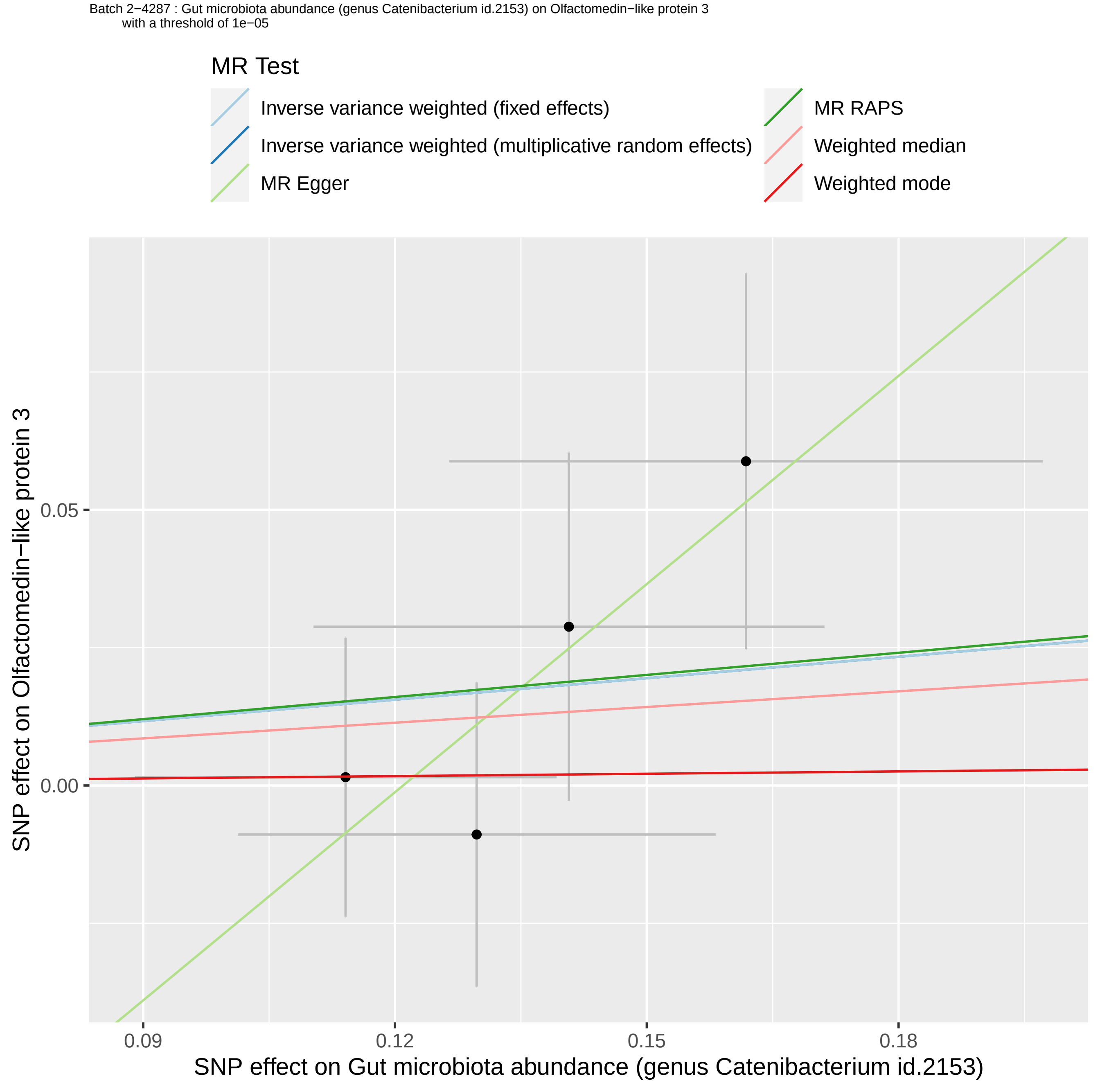

Batch 2−4287 : Gut microbiota abundance (genus Catenibacterium id.2153) on Olfactomedin−like protein 3
with a threshold of 1e−05
MR Test
Inverse variance weighted (fixed effects)
Inverse variance weighted (multiplicative random effects)
MR Egger
MR RAPS
Weighted median
Weighted mode
SNP effect on Olfactomedin−like protein 3
0.05
0.00
0.09
0.12
0.15
0.18
SNP effect on Gut microbiota abundance (genus Catenibacterium id.2153)

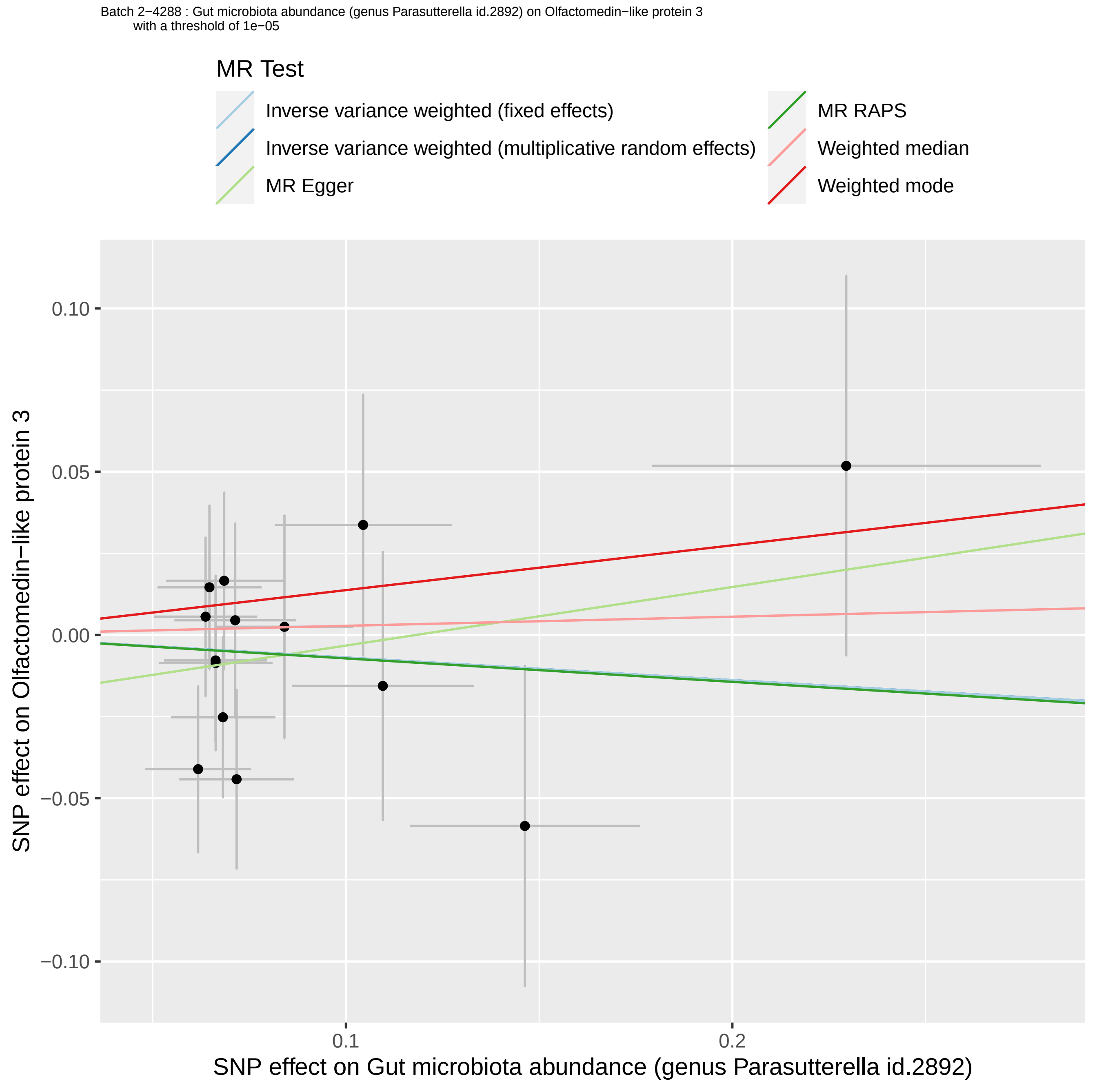

Batch 2−4288 : Gut microbiota abundance (genus Parasutterella id.2892) on Olfactomedin−like protein 3
with a threshold of 1e−05
MR Test
Inverse variance weighted (fixed effects)
Inverse variance weighted (multiplicative random effects)
MR Egger
MR RAPS
Weighted median
Weighted mode
0.10
0.05
0.00
−0.05
−0.10
0.1
0.2
SNP effect on Olfactomedin−like protein 3
SNP effect on Gut microbiota abundance (genus Parasutterella id.2892)

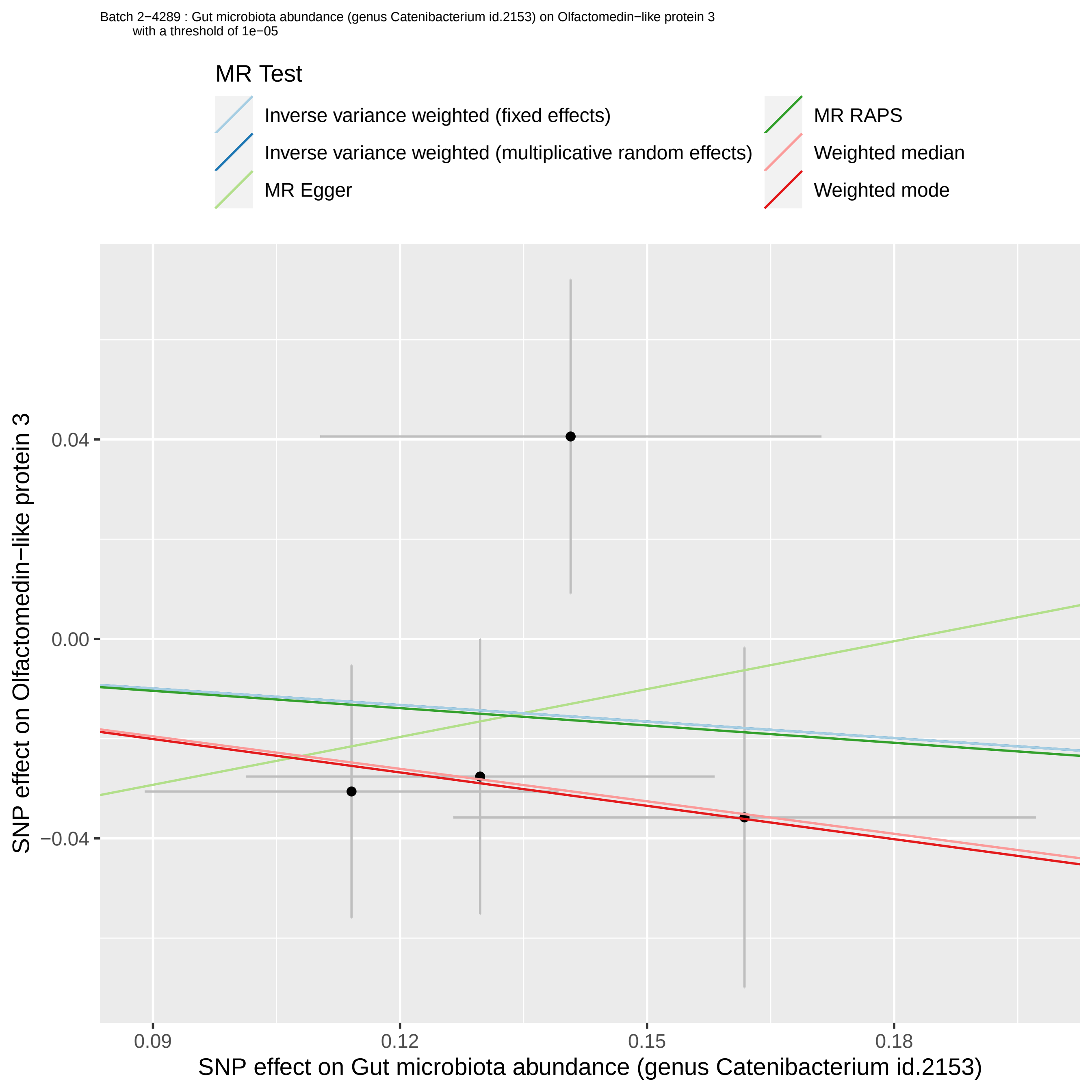
Batch 2−4289 : Gut microbiota abundance (genus Catenibacterium id.2153) on Olfactomedin−like protein 3
with a threshold of 1e−05
MR Test
Inverse variance weighted (fixed effects)
Inverse variance weighted (multiplicative random effects)
MR Egger
MR RAPS
Weighted median
Weighted mode
0.04
0.00
−0.04
0.09
0.12
0.15
0.18
SNP effect on Olfactomedin−like protein 3
SNP effect on Gut microbiota abundance (genus Catenibacterium id.2153)

Batch 2−4290 : Gut microbiota abundance (genus Parasutterella id.2892) on Olfactomedin−like protein 3
with a threshold of 1e−05

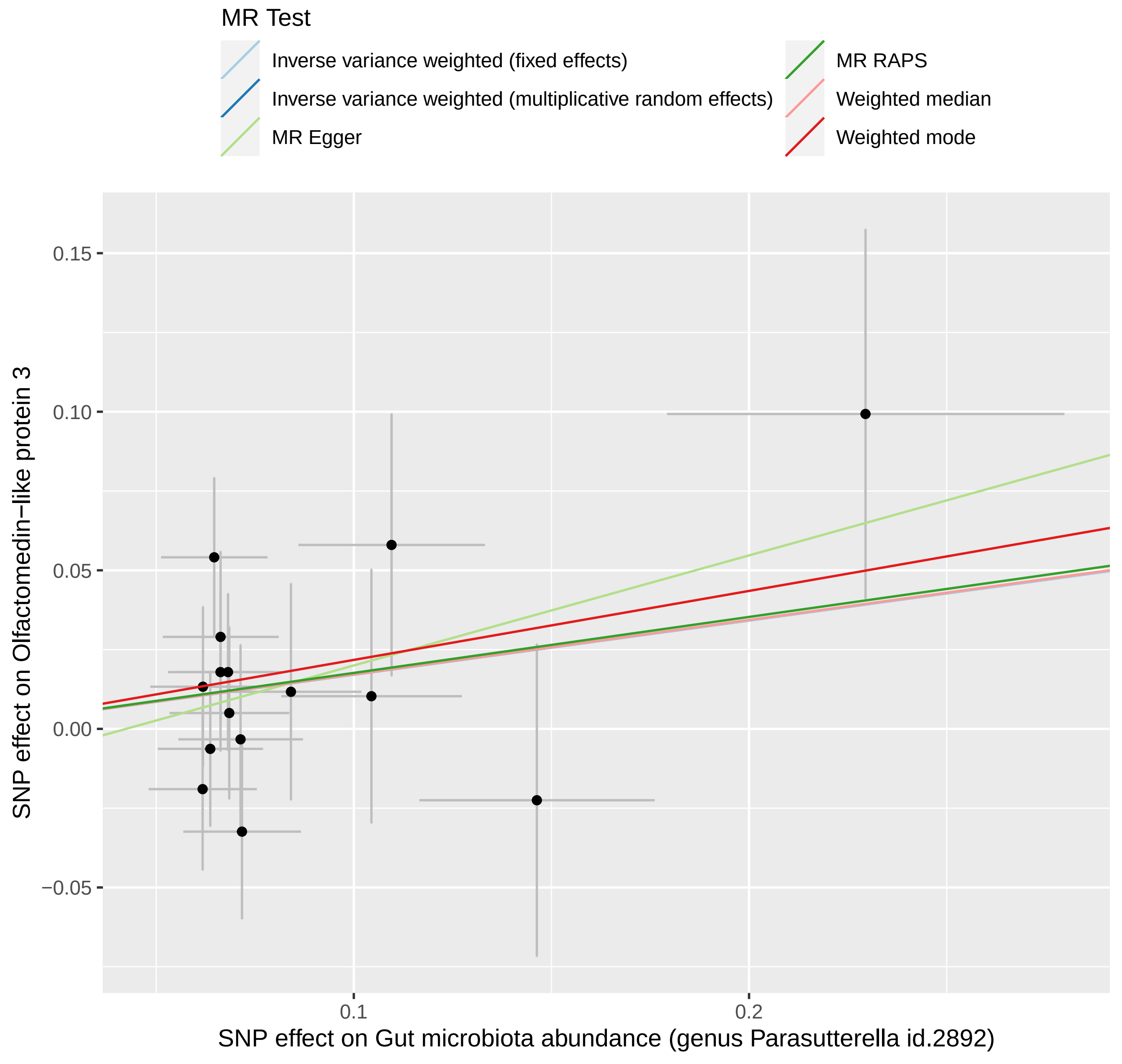

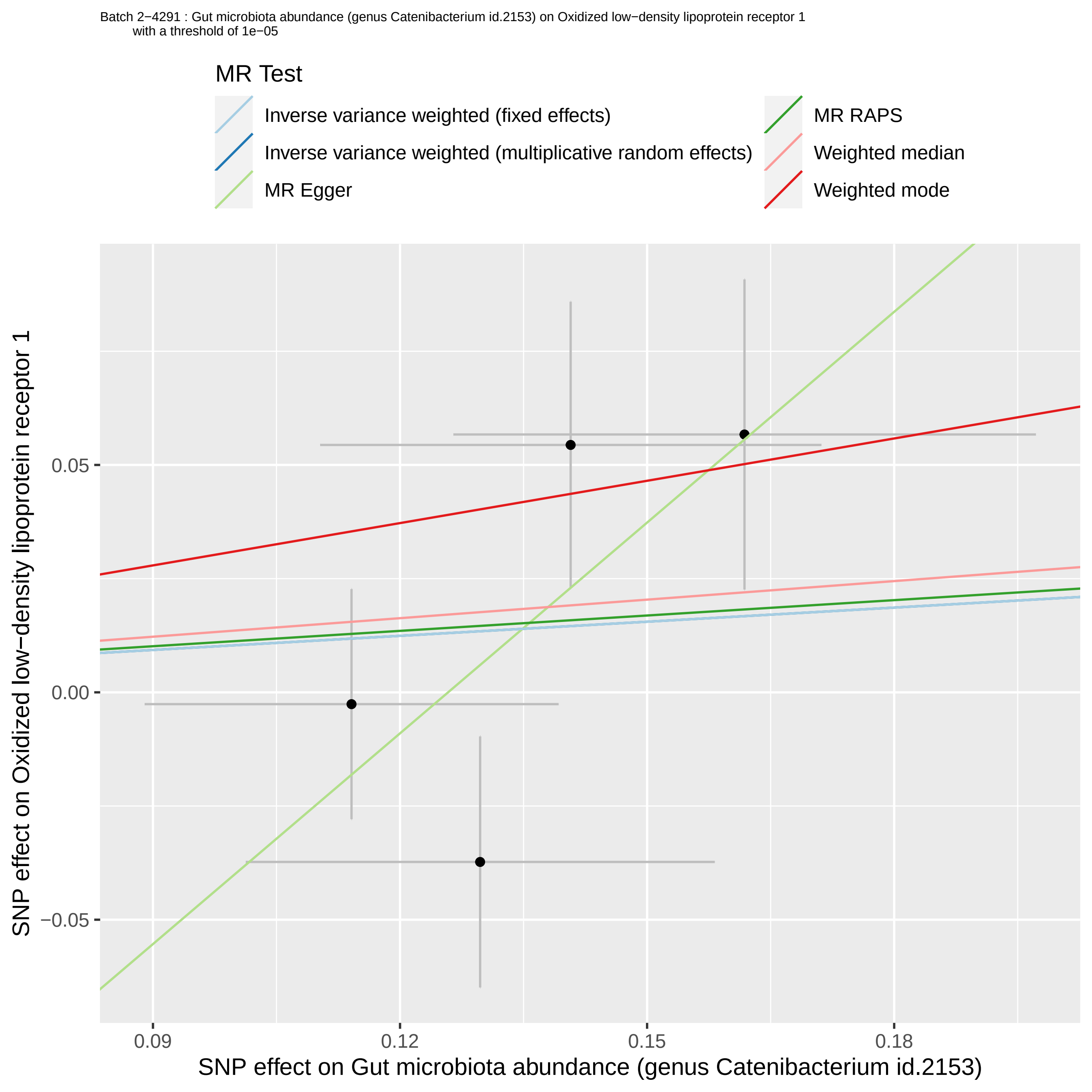
Batch 2−4291 : Gut microbiota abundance (genus Catenibacterium id.2153) on Oxidized low−density lipoprotein receptor 1
with a threshold of 1e−05
MR Test
Inverse variance weighted (fixed effects)
Inverse variance weighted (multiplicative random effects)
MR Egger
MR RAPS
Weighted median
Weighted mode
0.05
0.00
−0.05
0.09
0.12
0.15
0.18
SNP effect on Oxidized low−density lipoprotein receptor 1
SNP effect on Gut microbiota abundance (genus Catenibacterium id.2153)

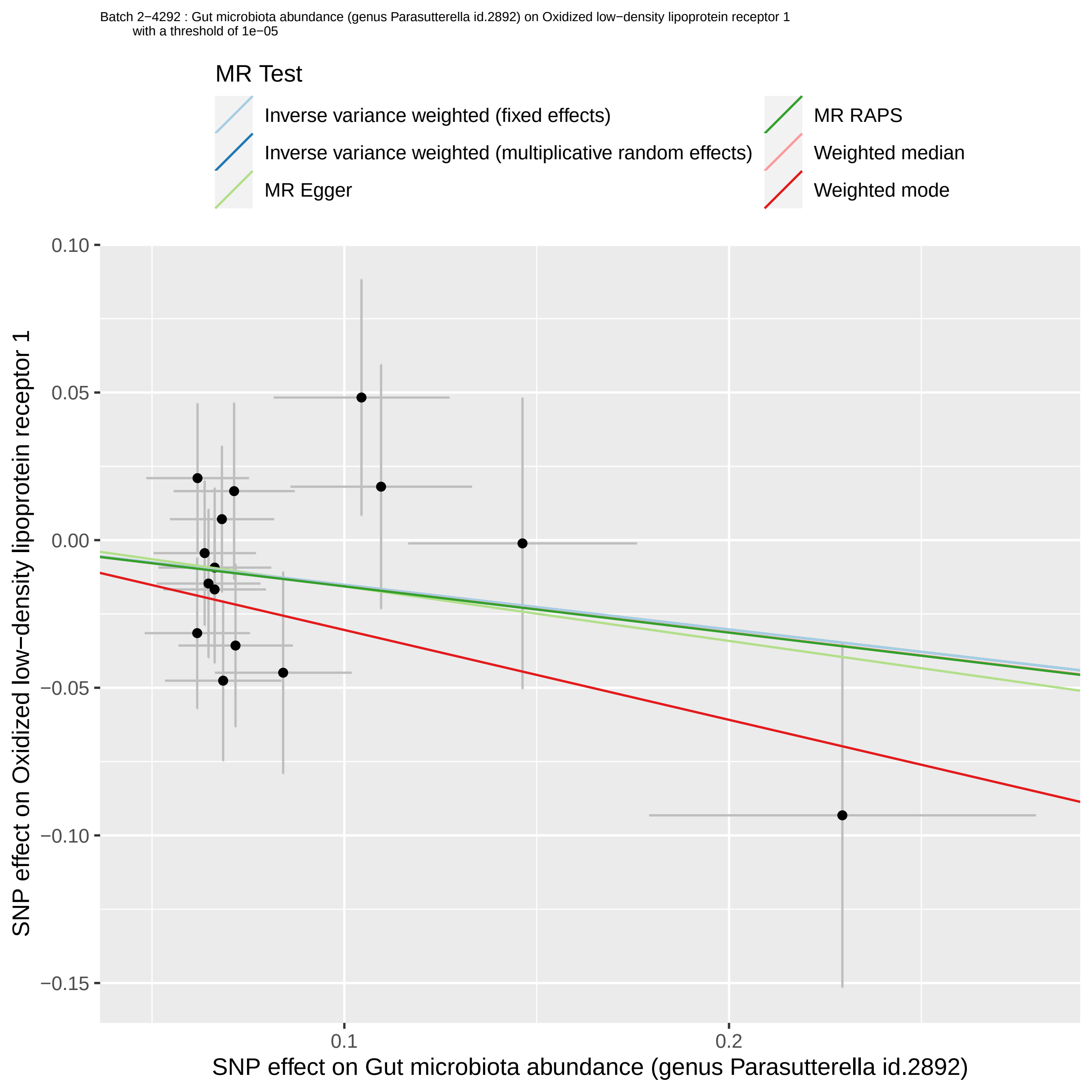
Batch 2−4292 : Gut microbiota abundance (genus Parasutterella id.2892) on Oxidized low−density lipoprotein receptor 1
with a threshold of 1e−05
MR Test
Inverse variance weighted (fixed effects)
Inverse variance weighted (multiplicative random effects)
MR Egger
MR RAPS
Weighted median
Weighted mode
0.10
0.05
0.00
−0.05
−0.10
−0.15
0.1
0.2
SNP effect on Oxidized low−density lipoprotein receptor 1
SNP effect on Gut microbiota abundance (genus Parasutterella id.2892)

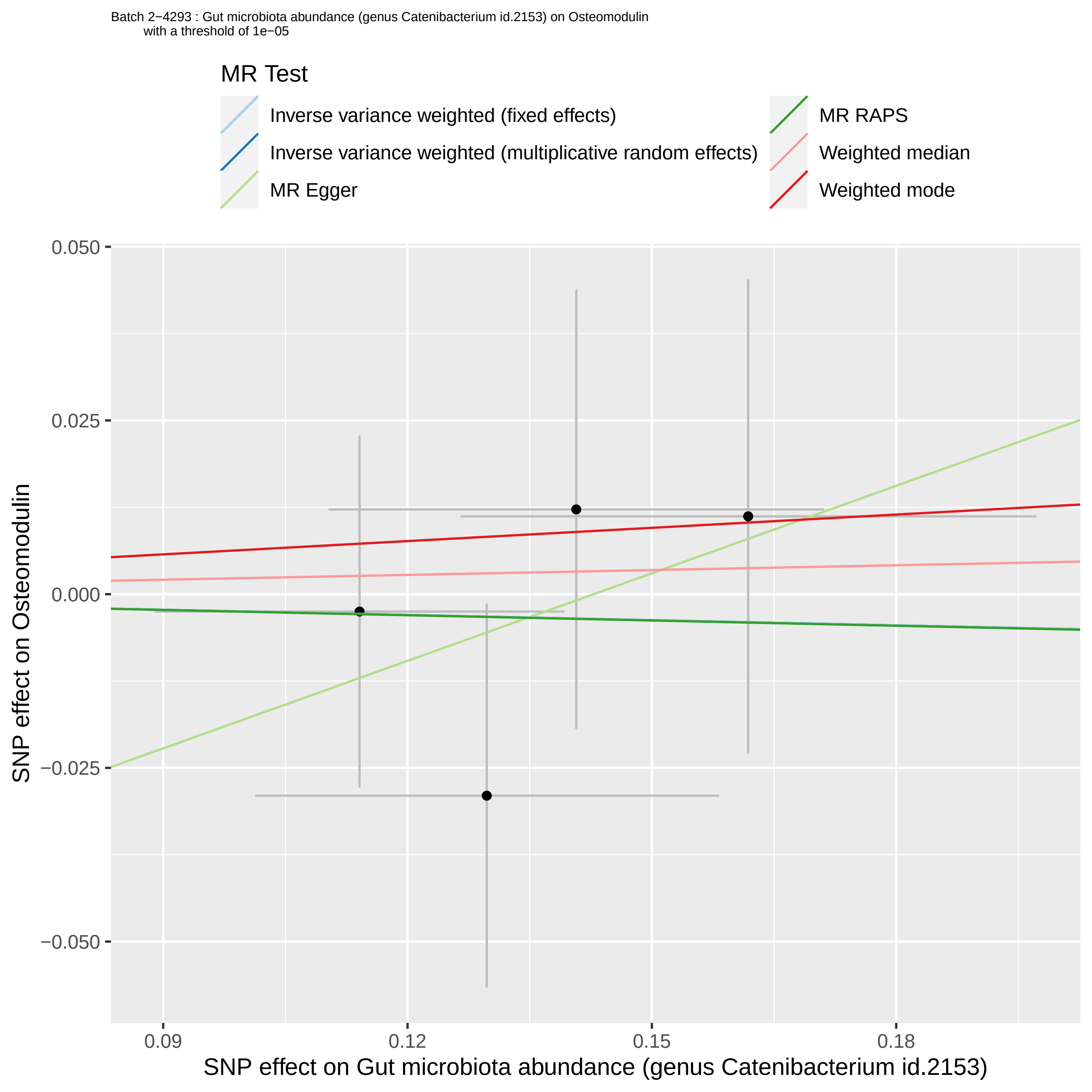

Batch 2−4293 : Gut microbiota abundance (genus Catenibacterium id.2153) on Osteomodulin
with a threshold of 1e−05
MR Test
Inverse variance weighted (fixed effects)
Inverse variance weighted (multiplicative random effects)
MR Egger
MR RAPS
Weighted median
Weighted mode
0.050
0.025
0.000
−0.025
−0.050
0.09
0.12
0.15
0.18
SNP effect on Osteomodulin
SNP effect on Gut microbiota abundance (genus Catenibacterium id.2153)

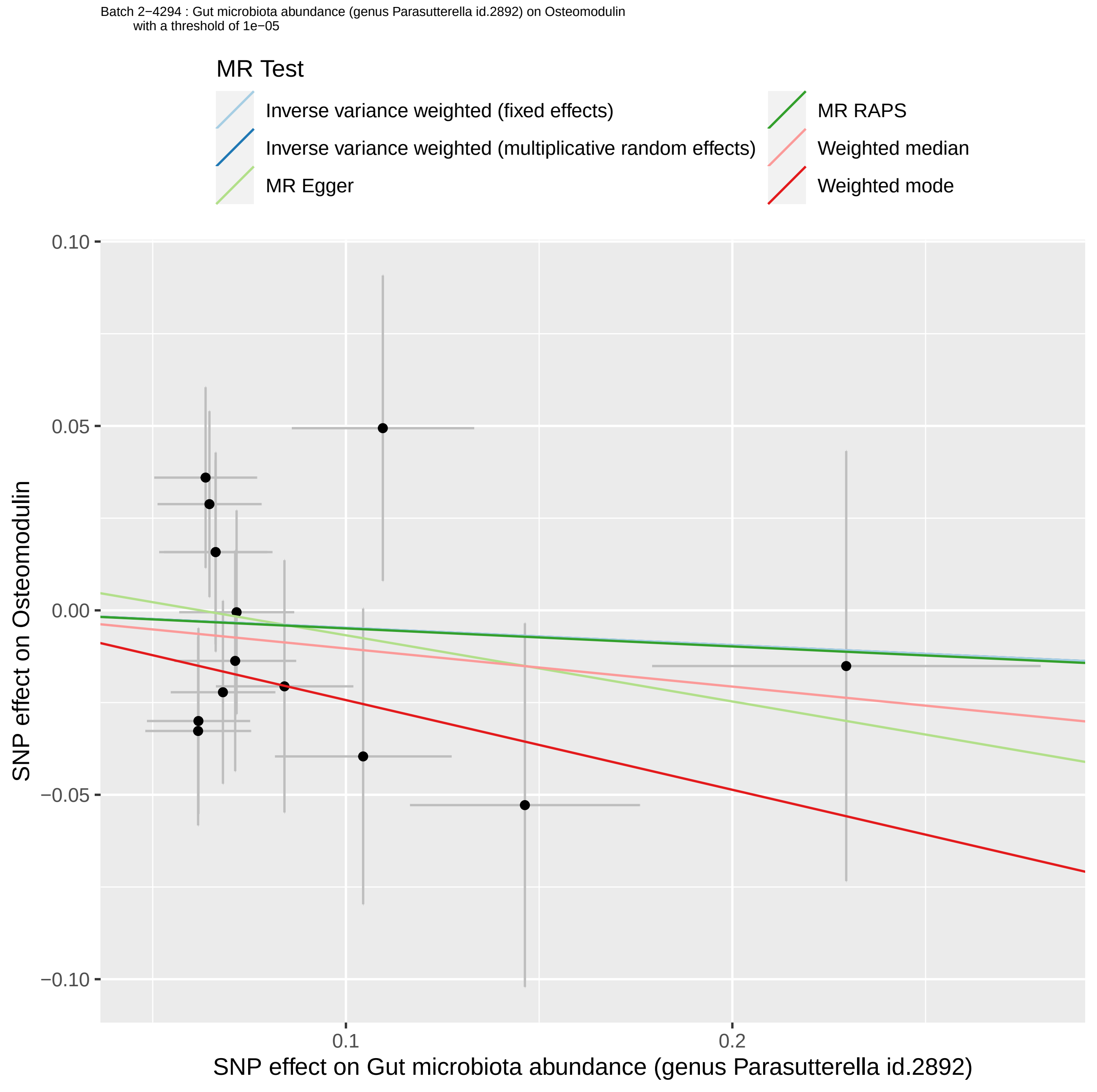
Batch 2−4294 : Gut microbiota abundance (genus Parasutterella id.2892) on Osteomodulin
with a threshold of 1e−05
MR Test
Inverse variance weighted (fixed effects)
Inverse variance weighted (multiplicative random effects)
MR Egger
MR RAPS
Weighted median
Weighted mode
0.10
0.05
0.00
−0.05
−0.10
0.1
0.2
SNP effect on Osteomodulin
SNP effect on Gut microbiota abundance (genus Parasutterella id.2892)

Batch 2−4295 : Gut microbiota abundance (genus Catenibacterium id.2153) on Opalin
with a threshold of 1e−05

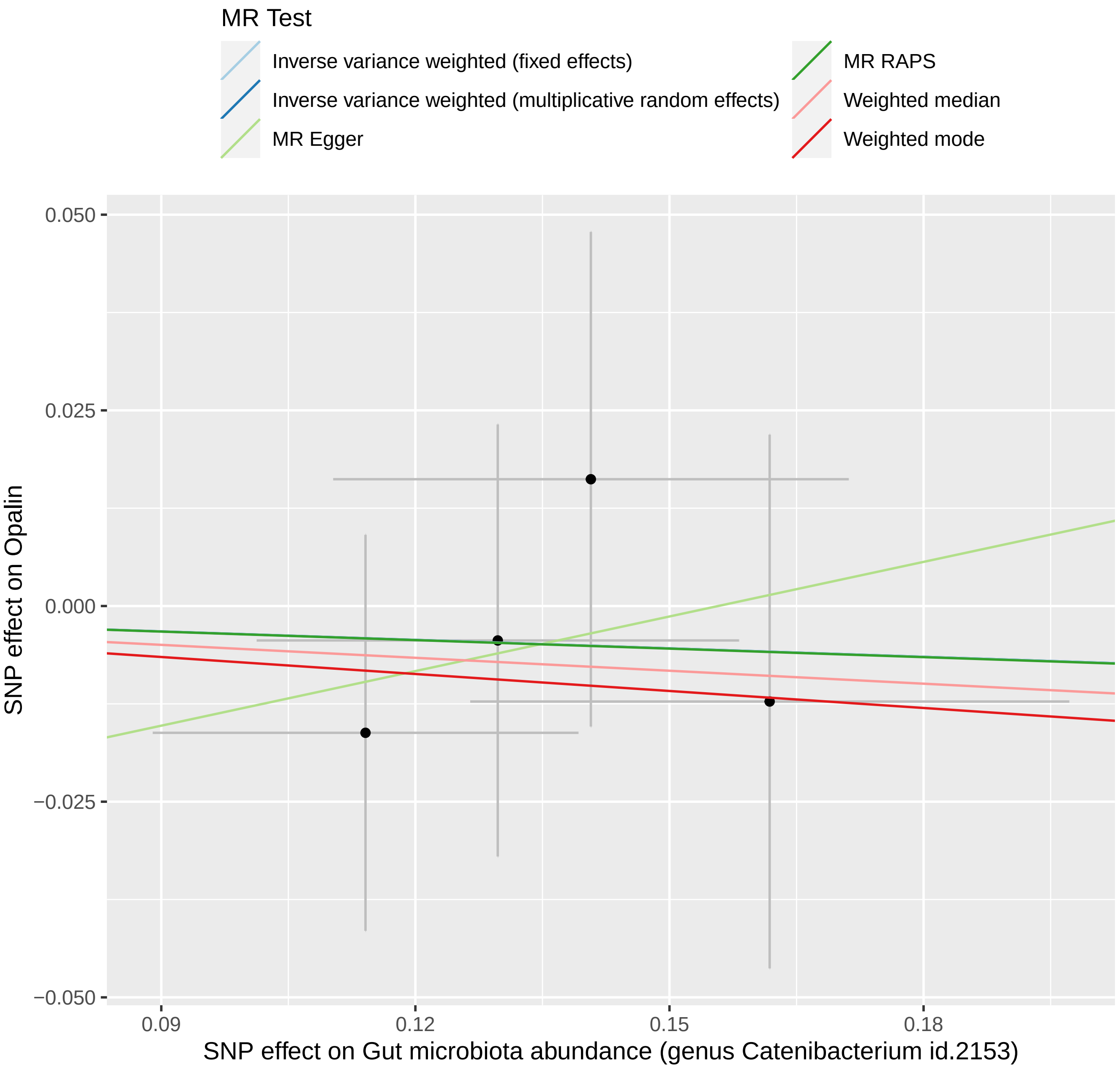

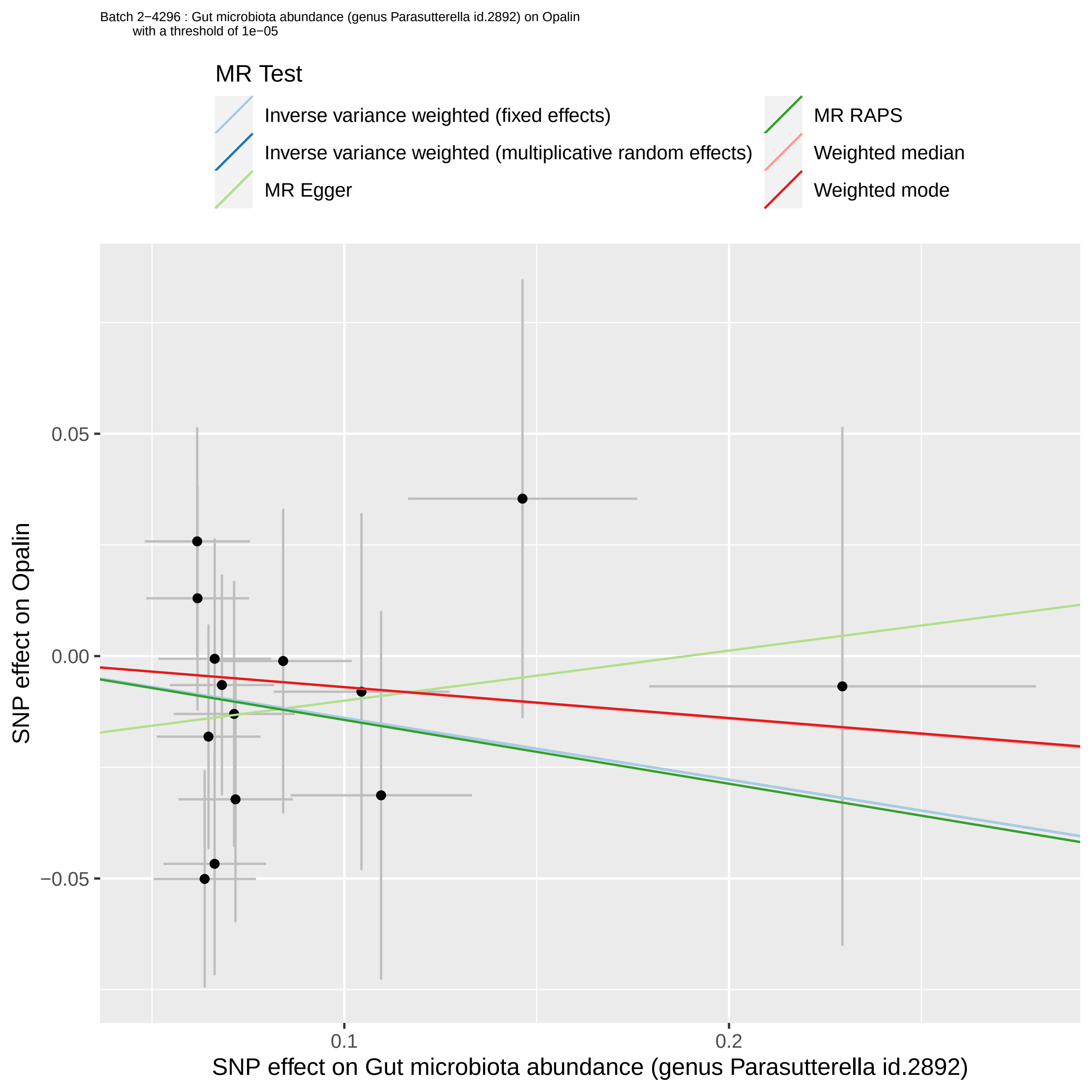
Batch 2−4296 : Gut microbiota abundance (genus Parasutterella id.2892) on Opalin
with a threshold of 1e−05
MR Test
Inverse variance weighted (fixed effects)
Inverse variance weighted (multiplicative random effects)
MR Egger
MR RAPS
Weighted median
Weighted mode
0.05
0.00
−0.05
0.1
0.2
SNP effect on Opalin
SNP effect on Gut microbiota abundance (genus Parasutterella id.2892)

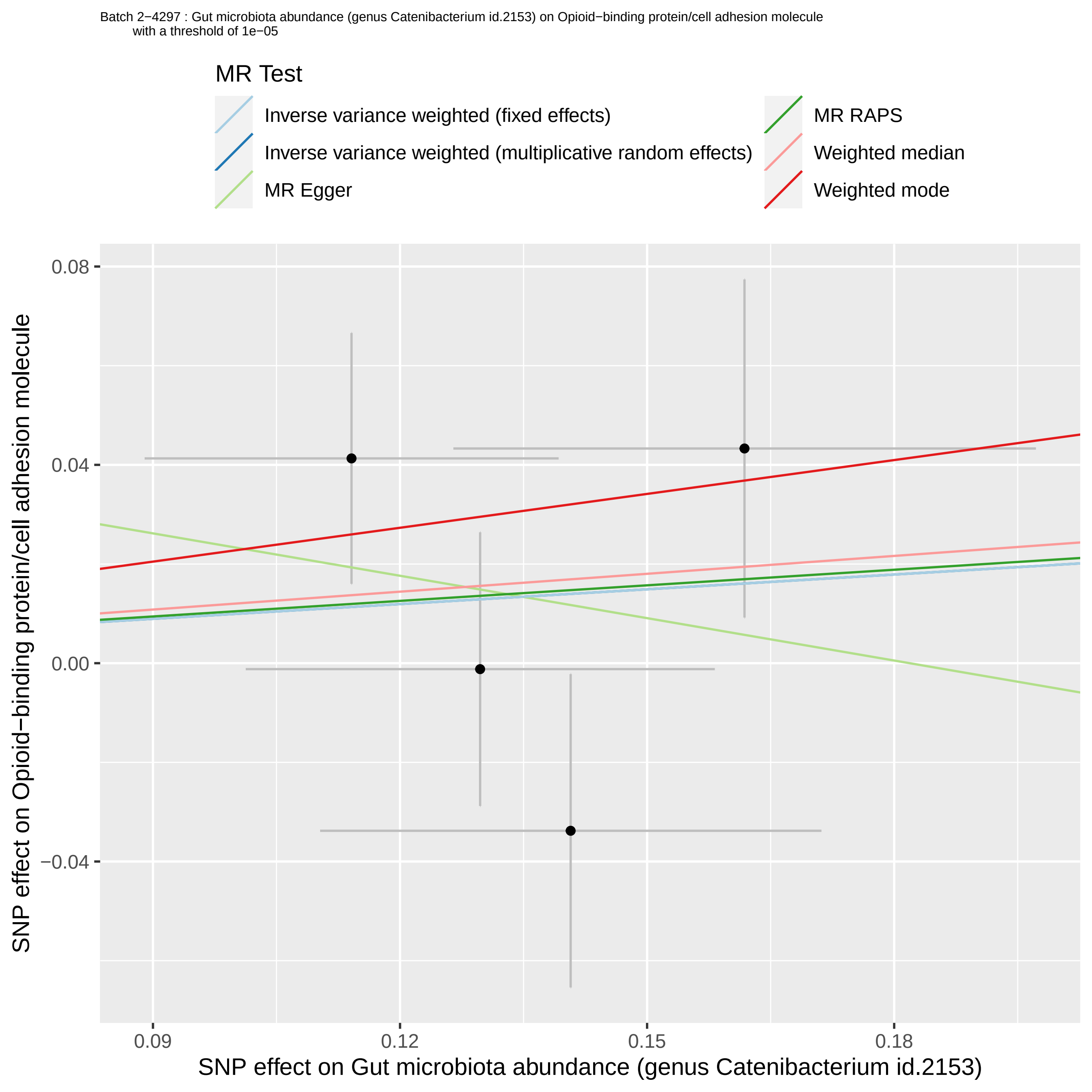
Batch 2−4297 : Gut microbiota abundance (genus Catenibacterium id.2153) on Opioid−binding protein/cell adhesion molecule
with a threshold of 1e−05
MR Test
Inverse variance weighted (fixed effects)
Inverse variance weighted (multiplicative random effects)
MR Egger
MR RAPS
Weighted median
Weighted mode
0.08
0.04
0.00
−0.04
0.09
0.12
0.15
0.18
SNP effect on Opioid−binding protein/cell adhesion molecule
SNP effect on Gut microbiota abundance (genus Catenibacterium id.2153)

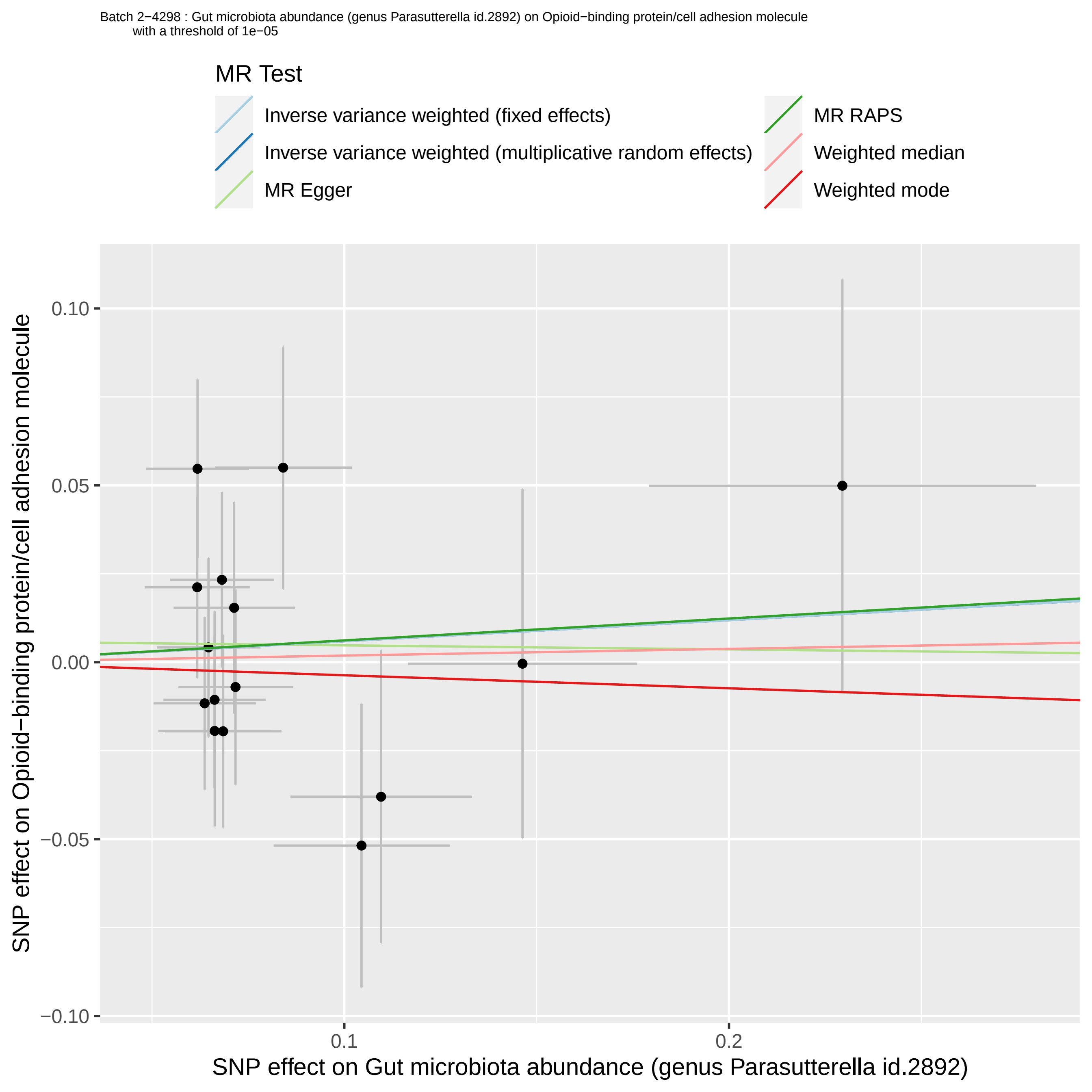

Batch 2−4298 : Gut microbiota abundance (genus Parasutterella id.2892) on Opioid−binding protein/cell adhesion molecule
with a threshold of 1e−05
MR Test
Inverse variance weighted (fixed effects)
Inverse variance weighted (multiplicative random effects)
MR Egger
MR RAPS
Weighted median
Weighted mode
0.10
0.05
0.00
−0.05
−0.10
0.1
0.2
SNP effect on Opioid−binding protein/cell adhesion molecule
SNP effect on Gut microbiota abundance (genus Parasutterella id.2892)

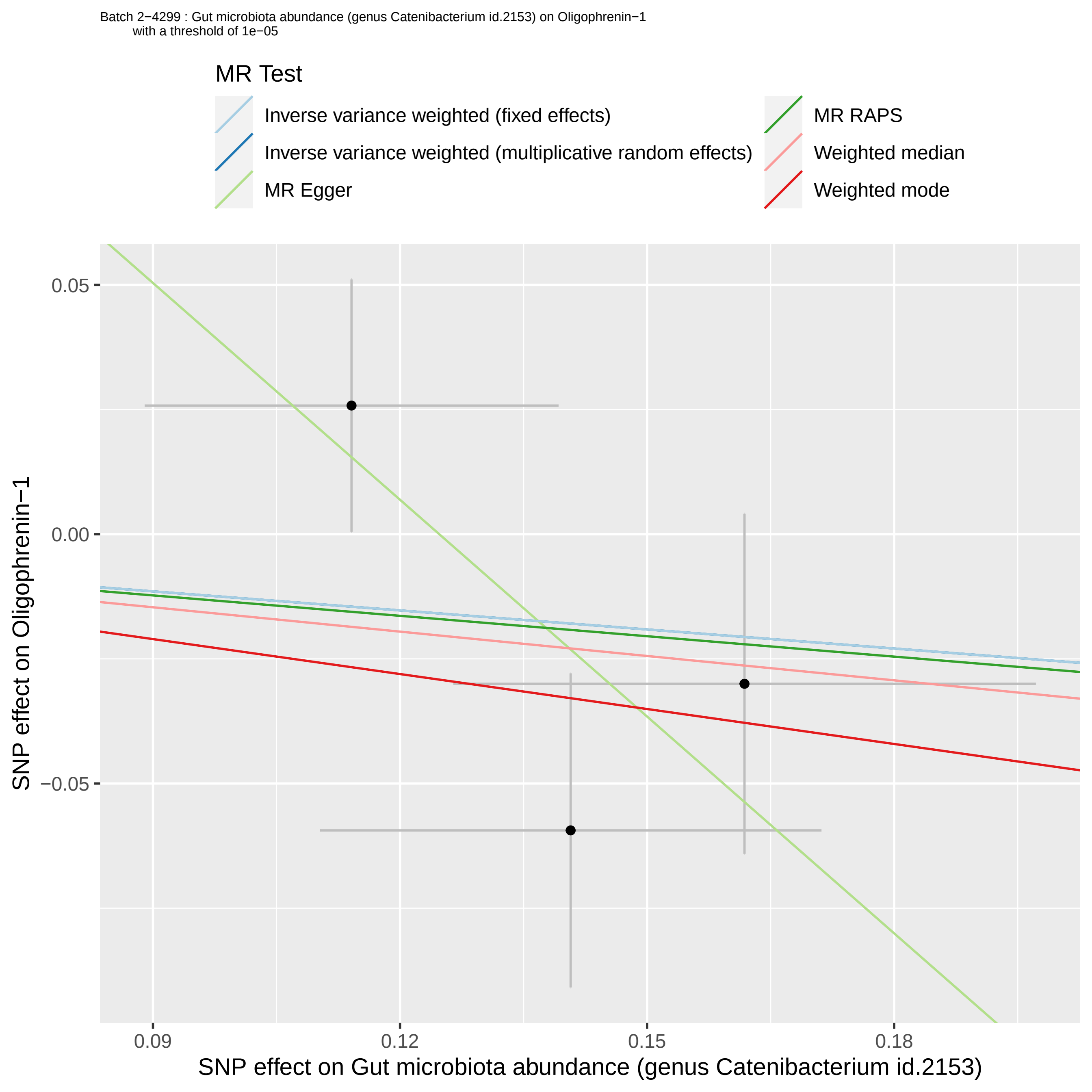
Batch 2−4299 : Gut microbiota abundance (genus Catenibacterium id.2153) on Oligophrenin−1
with a threshold of 1e−05
MR Test
Inverse variance weighted (fixed effects)
Inverse variance weighted (multiplicative random effects)
MR Egger
MR RAPS
Weighted median
Weighted mode
0.05
0.00
−0.05
0.09
0.12
0.15
0.18
SNP effect on Oligophrenin−1
SNP effect on Gut microbiota abundance (genus Catenibacterium id.2153)

Batch 2−4300 : Gut microbiota abundance (genus Parasutterella id.2892) on Oligophrenin−1
with a threshold of 1e−05

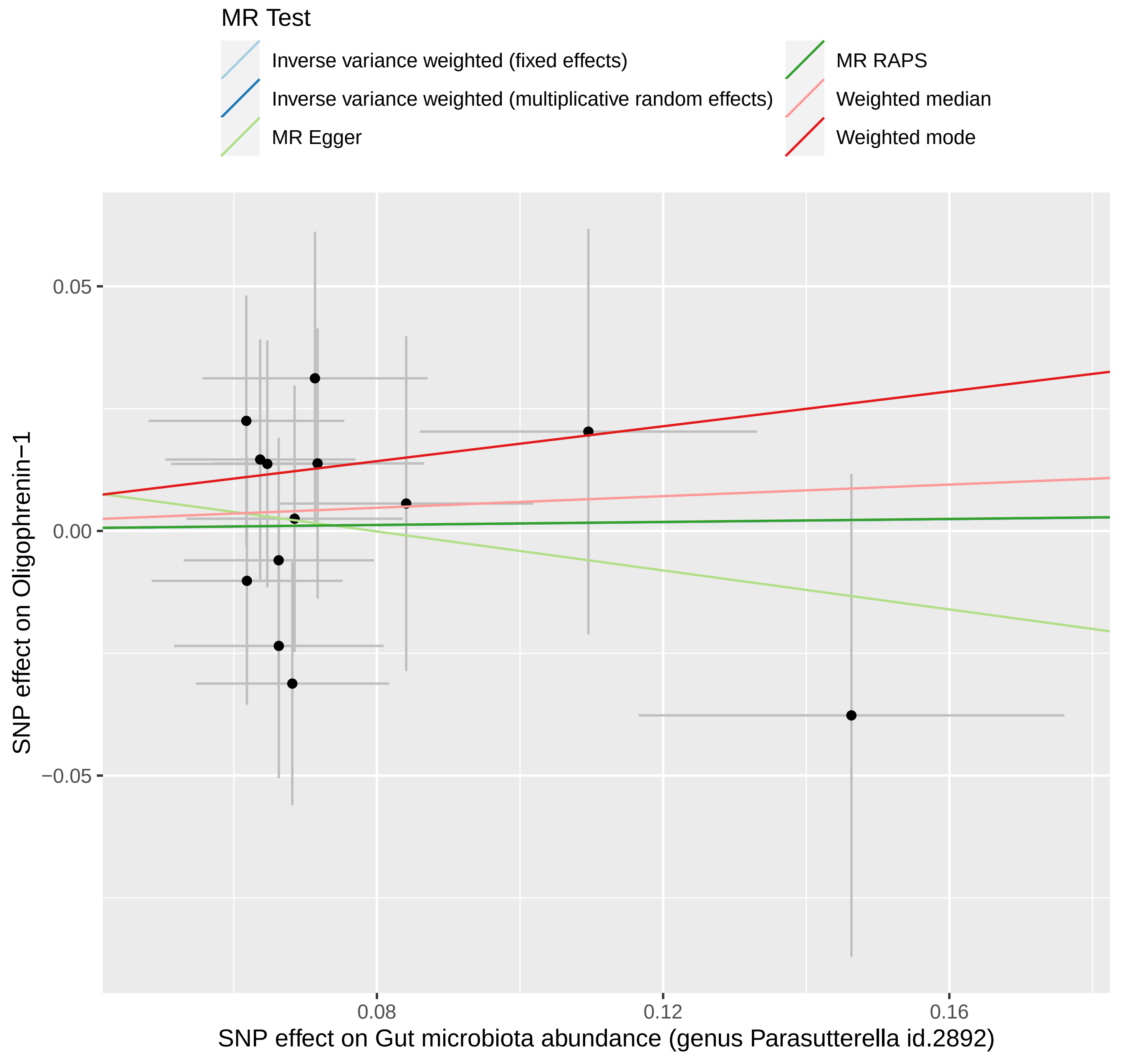

Batch 2−4301 : Gut microbiota abundance (genus Catenibacterium id.2153) on Origin recognition complex subunit 6
with a threshold of 1e−05

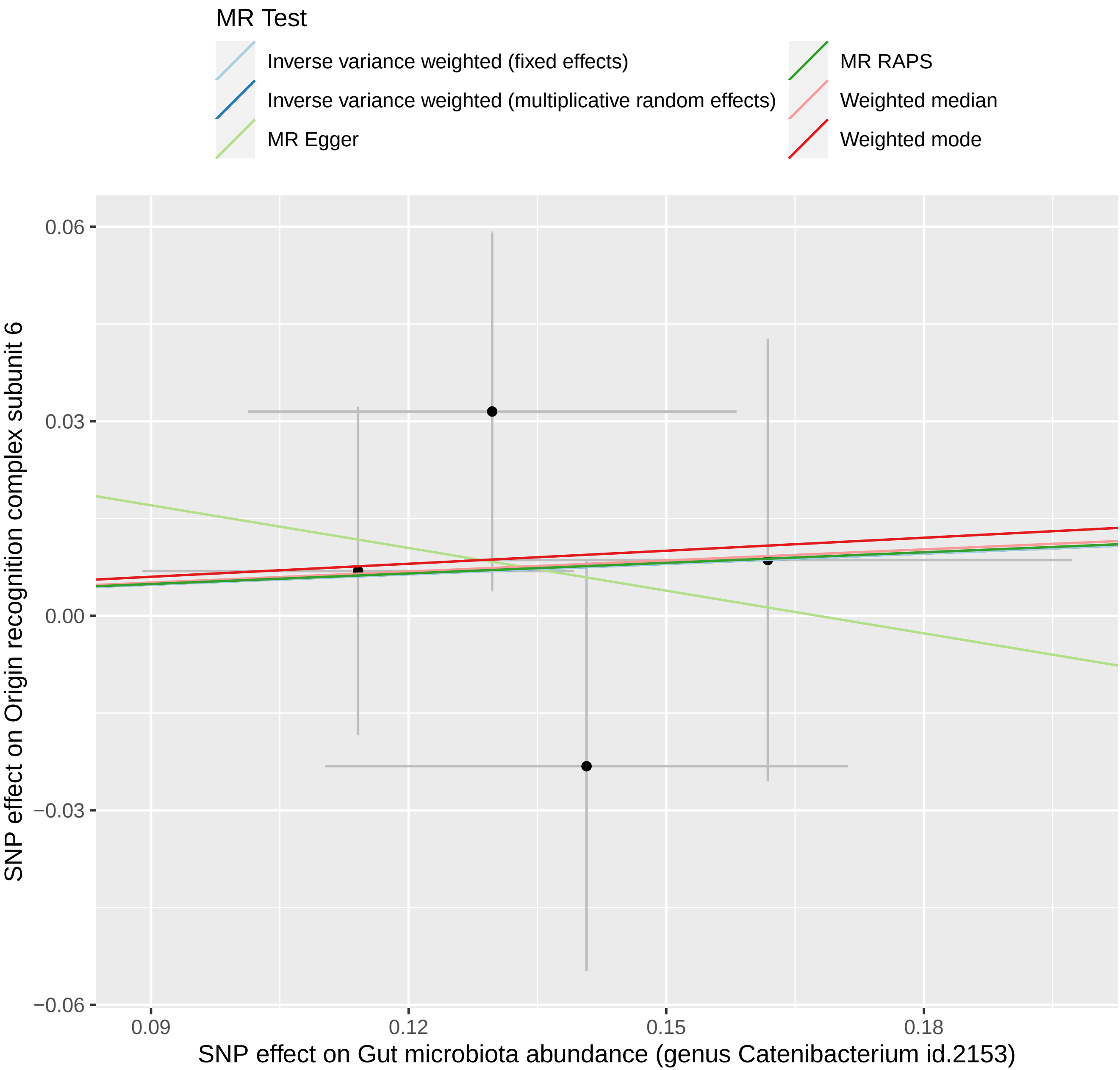

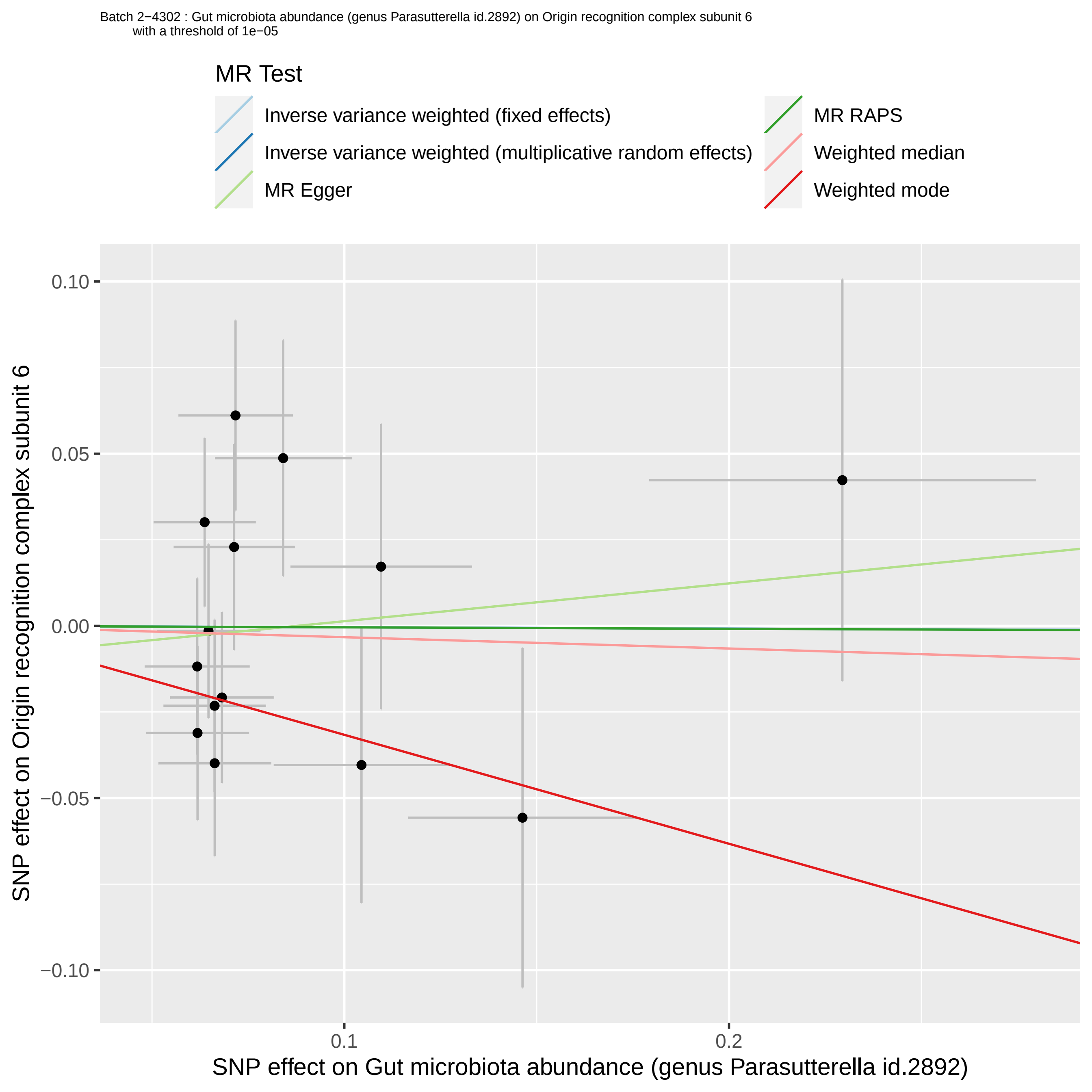
Batch 2−4302 : Gut microbiota abundance (genus Parasutterella id.2892) on Origin recognition complex subunit 6
with a threshold of 1e−05
MR Test
Inverse variance weighted (fixed effects)
Inverse variance weighted (multiplicative random effects)
MR Egger
MR RAPS
Weighted median
Weighted mode
0.10
0.05
0.00
−0.05
−0.10
0.1
0.2
SNP effect on Origin recognition complex subunit 6
SNP effect on Gut microbiota abundance (genus Parasutterella id.2892)

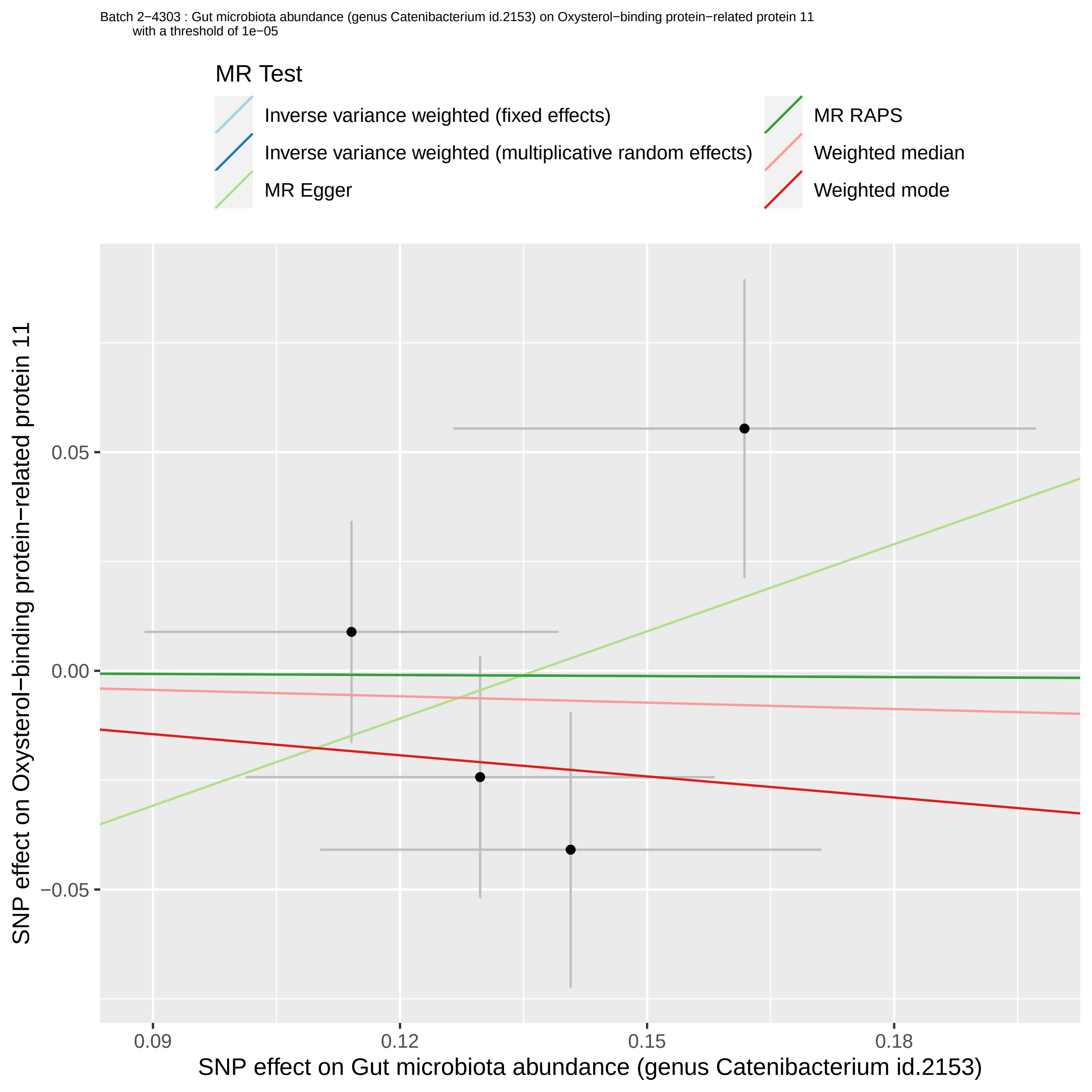
Batch 2−4303 : Gut microbiota abundance (genus Catenibacterium id.2153) on Oxysterol−binding protein−related protein 11
with a threshold of 1e−05
MR Test
Inverse variance weighted (fixed effects)
Inverse variance weighted (multiplicative random effects)
MR Egger
MR RAPS
Weighted median
Weighted mode
0.05
0.00
−0.05
0.09
0.12
0.15
0.18
SNP effect on Oxysterol−binding protein−related protein 11
SNP effect on Gut microbiota abundance (genus Catenibacterium id.2153)

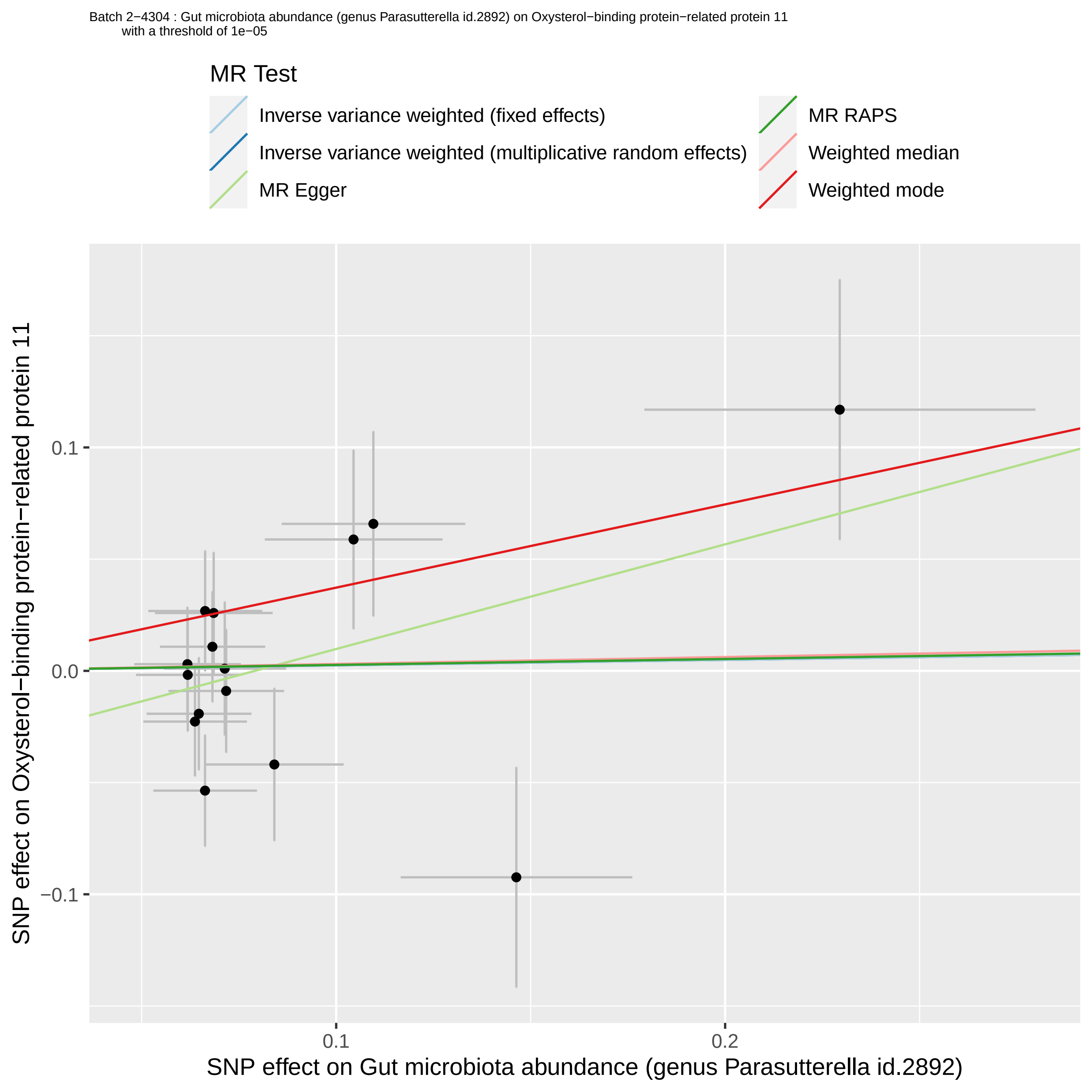

Batch 2−4304 : Gut microbiota abundance (genus Parasutterella id.2892) on Oxysterol−binding protein−related protein 11
with a threshold of 1e−05
MR Test
Inverse variance weighted (fixed effects)
Inverse variance weighted (multiplicative random effects)
MR Egger
MR RAPS
Weighted median
Weighted mode
SNP effect on Oxysterol−binding protein−related protein 11
0.1
0.0
−0.1
0.1
0.2
SNP effect on Gut microbiota abundance (genus Parasutterella id.2892)

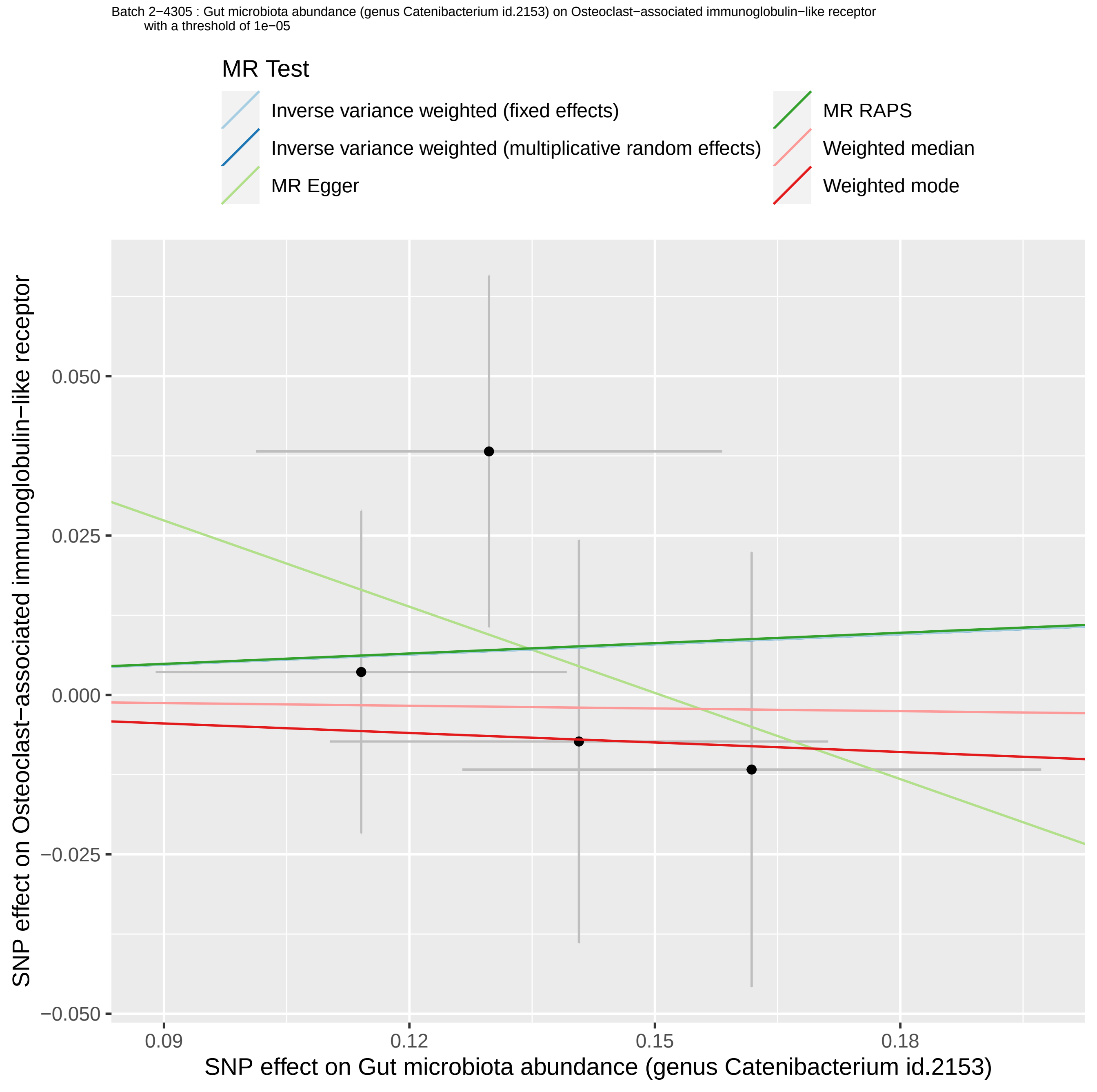
Batch 2−4305 : Gut microbiota abundance (genus Catenibacterium id.2153) on Osteoclast−associated immunoglobulin−like receptor
with a threshold of 1e−05
MR Test
Inverse variance weighted (fixed effects)
Inverse variance weighted (multiplicative random effects)
MR Egger
MR RAPS
Weighted median
Weighted mode
0.050
0.025
0.000
−0.025
−0.050
0.09
0.12
0.15
0.18
SNP effect on Osteoclast−associated immunoglobulin−like receptor
SNP effect on Gut microbiota abundance (genus Catenibacterium id.2153)

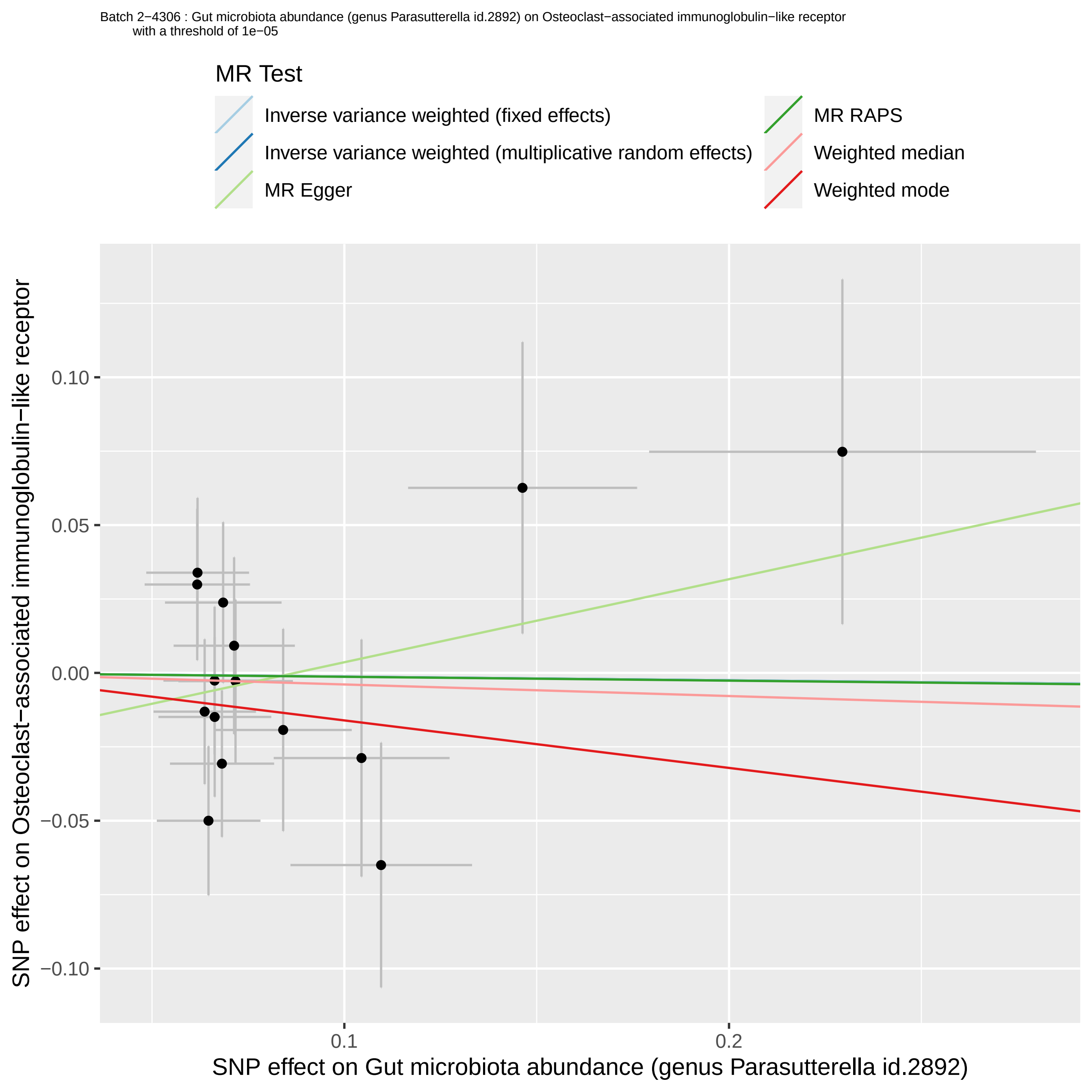

Batch 2−4306 : Gut microbiota abundance (genus Parasutterella id.2892) on Osteoclast−associated immunoglobulin−like receptor
with a threshold of 1e−05
MR Test
Inverse variance weighted (fixed effects)
Inverse variance weighted (multiplicative random effects)
MR Egger
MR RAPS
Weighted median
Weighted mode
0.10
0.05
0.00
−0.05
−0.10
0.1
0.2
SNP effect on Osteoclast−associated immunoglobulin−like receptor
SNP effect on Gut microbiota abundance (genus Parasutterella id.2892)

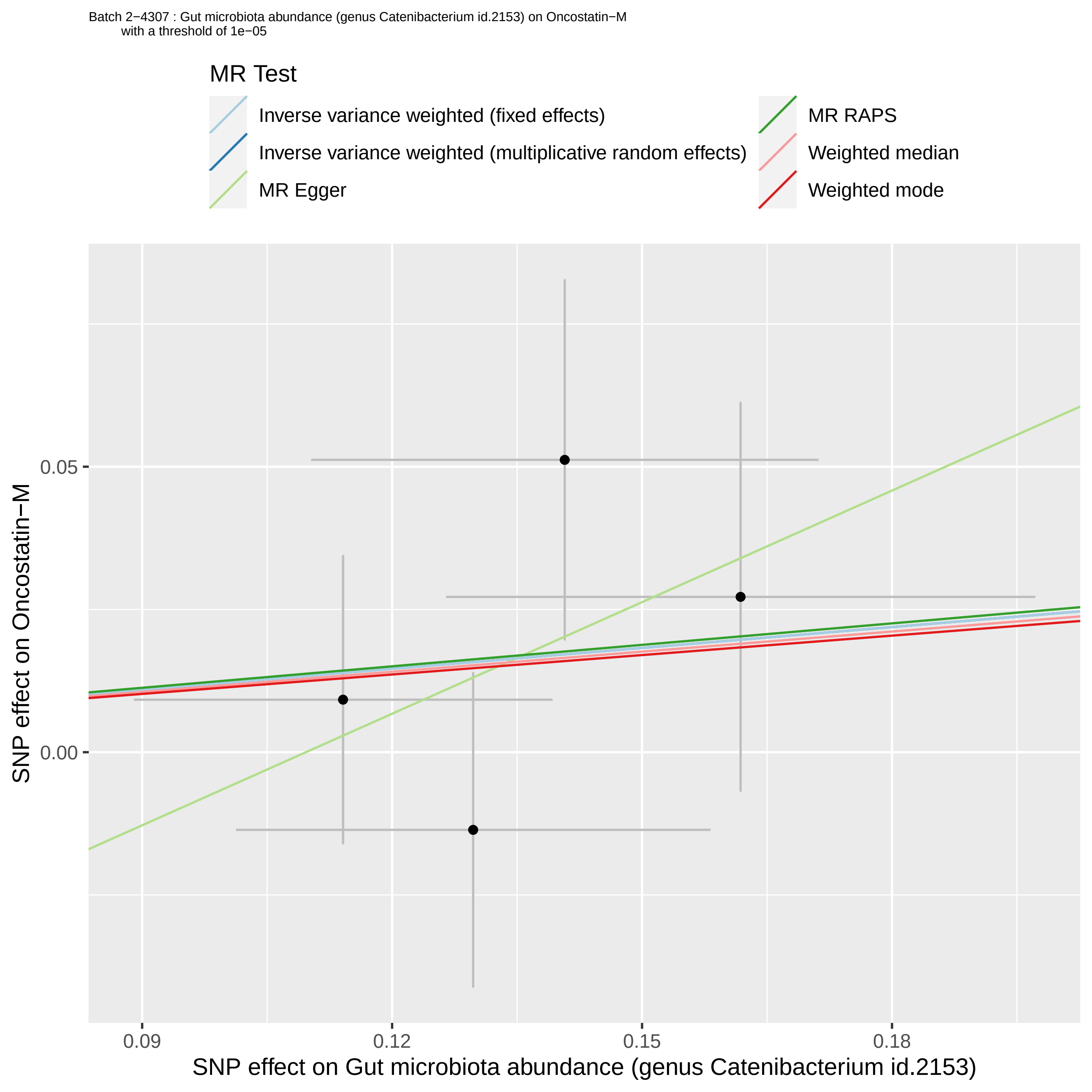

Batch 2−4307 : Gut microbiota abundance (genus Catenibacterium id.2153) on Oncostatin−M
with a threshold of 1e−05
MR Test
Inverse variance weighted (fixed effects)
Inverse variance weighted (multiplicative random effects)
MR Egger
MR RAPS
Weighted median
Weighted mode
0.05
0.00
SNP effect on Oncostatin−M
0.09
0.12
0.15
0.18
SNP effect on Gut microbiota abundance (genus Catenibacterium id.2153)

Batch 2−4308 : Gut microbiota abundance (genus Parasutterella id.2892) on Oncostatin−M
with a threshold of 1e−05

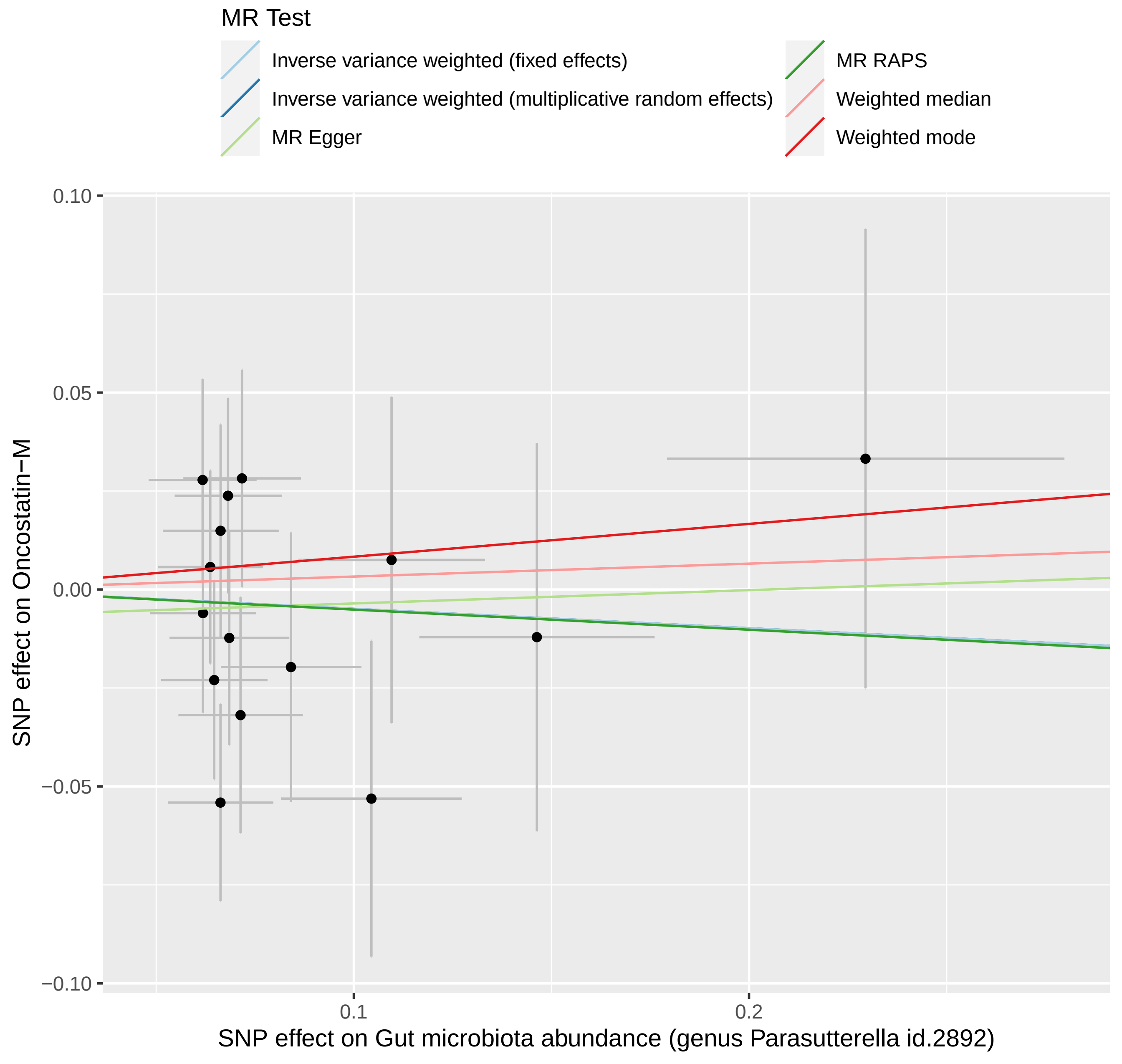

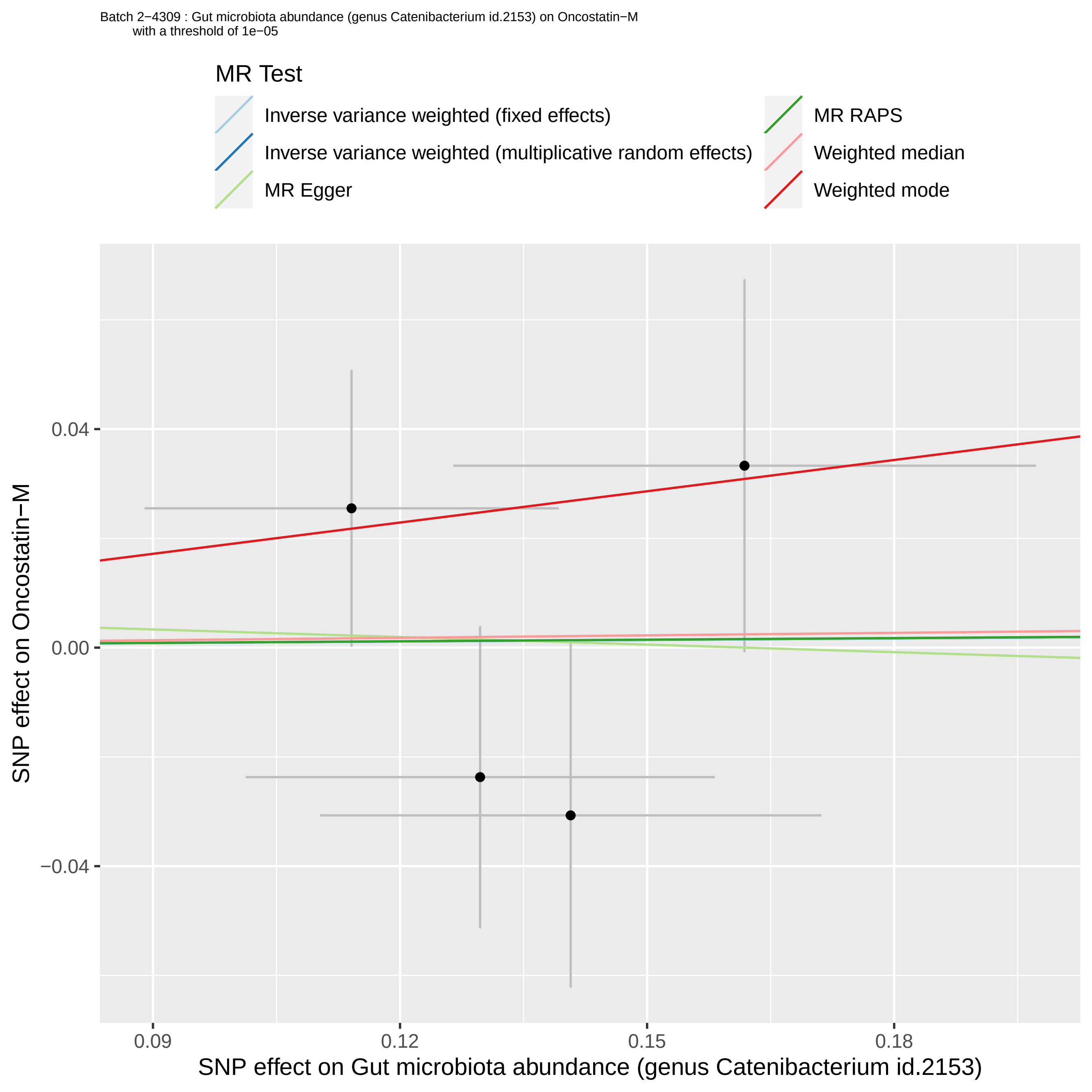

Batch 2−4309 : Gut microbiota abundance (genus Catenibacterium id.2153) on Oncostatin−M
with a threshold of 1e−05
MR Test
Inverse variance weighted (fixed effects)
Inverse variance weighted (multiplicative random effects)
MR Egger
MR RAPS
Weighted median
Weighted mode
0.04
0.00
−0.04
0.09
0.12
0.15
0.18
SNP effect on Oncostatin−M
SNP effect on Gut microbiota abundance (genus Catenibacterium id.2153)

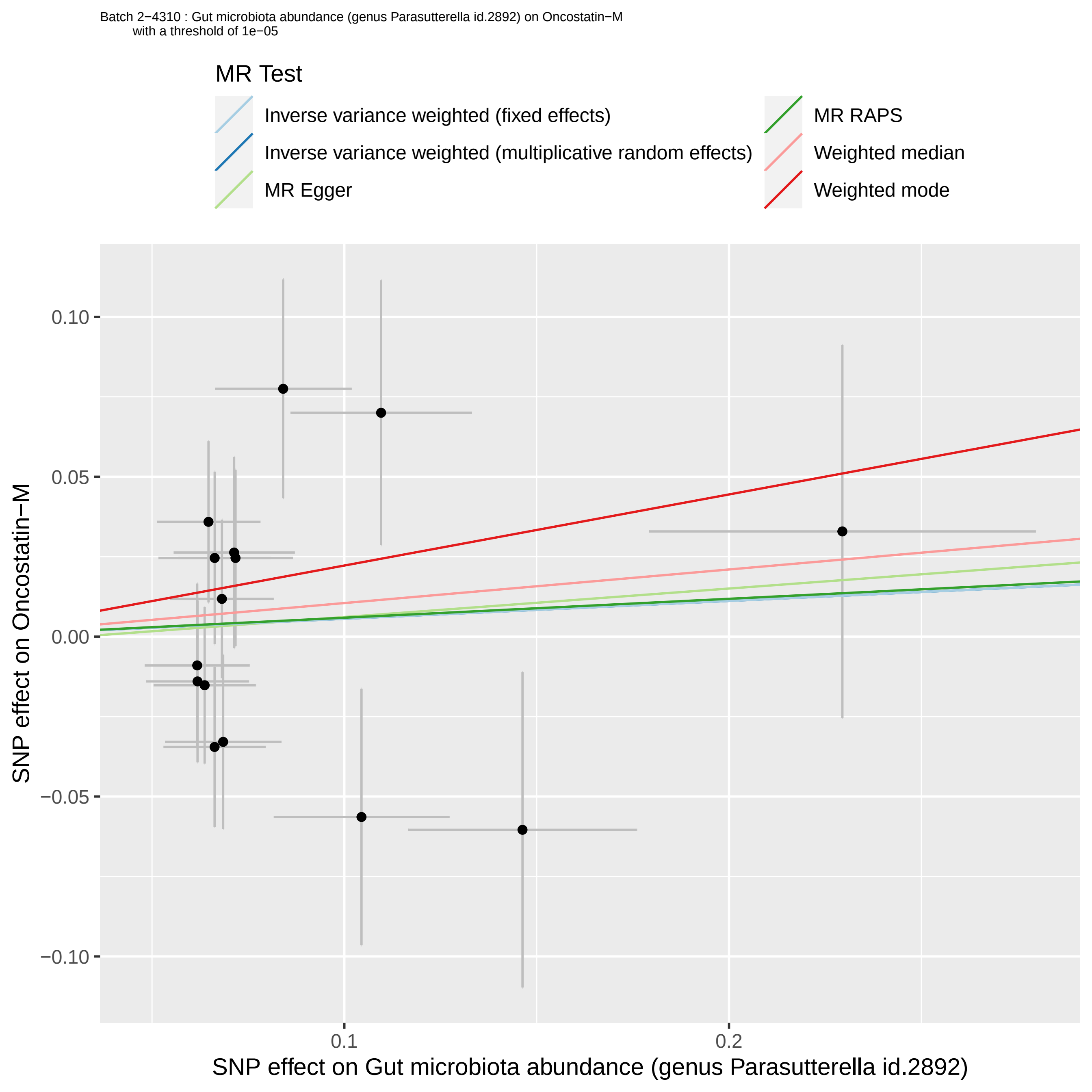

Batch 2−4310 : Gut microbiota abundance (genus Parasutterella id.2892) on Oncostatin−M
with a threshold of 1e−05
MR Test
Inverse variance weighted (fixed effects)
Inverse variance weighted (multiplicative random effects)
MR Egger
MR RAPS
Weighted median
Weighted mode
0.10
0.05
0.00
−0.05
−0.10
SNP effect on Oncostatin−M
0.1
0.2
SNP effect on Gut microbiota abundance (genus Parasutterella id.2892)

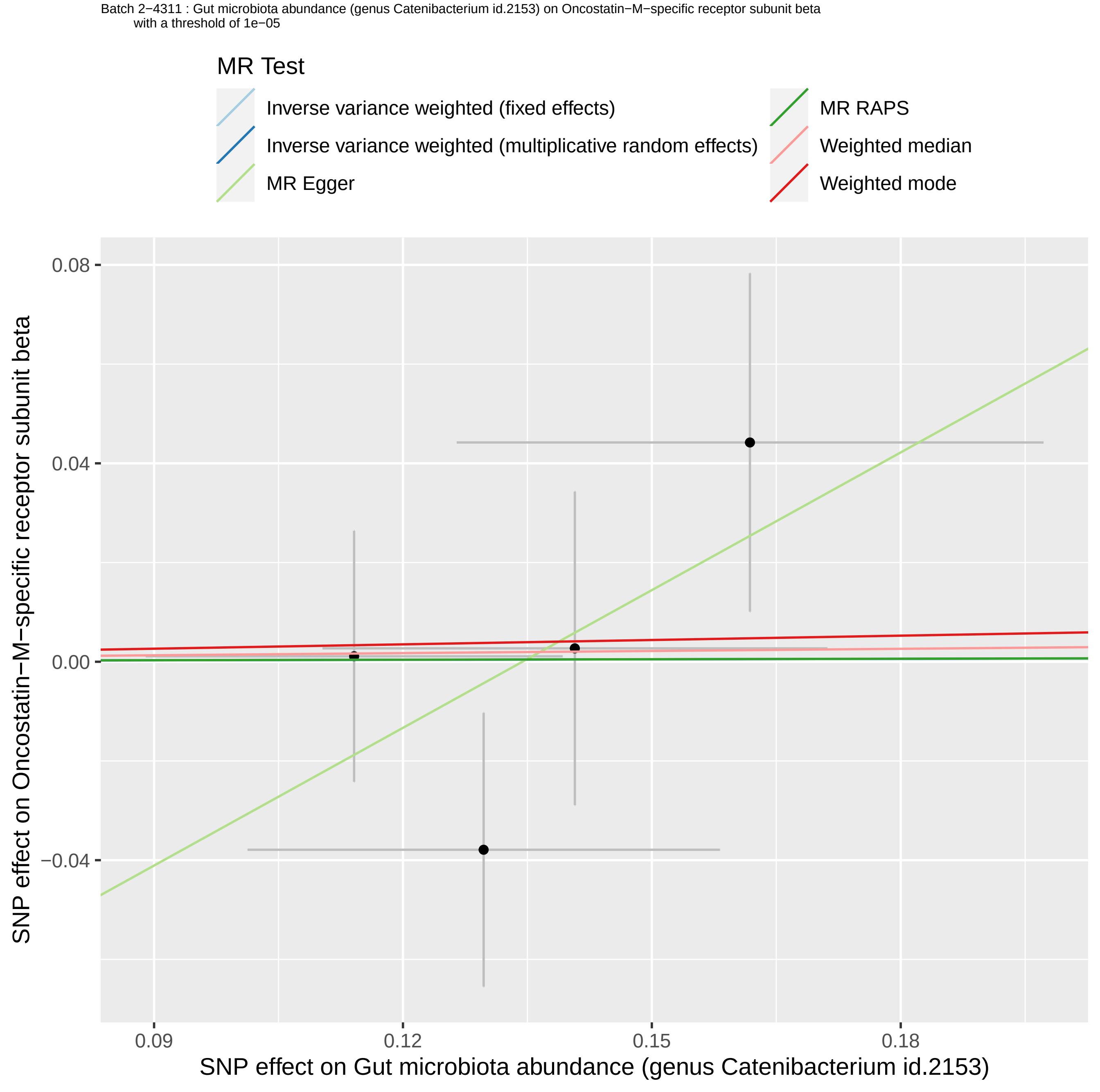
Batch 2−4311 : Gut microbiota abundance (genus Catenibacterium id.2153) on Oncostatin−M−specific receptor subunit beta
with a threshold of 1e−05
MR Test
Inverse variance weighted (fixed effects)
Inverse variance weighted (multiplicative random effects)
MR Egger
MR RAPS
Weighted median
Weighted mode
0.08
0.04
0.00
−0.04
0.09
0.12
0.15
0.18
SNP effect on Oncostatin−M−specific receptor subunit beta
SNP effect on Gut microbiota abundance (genus Catenibacterium id.2153)

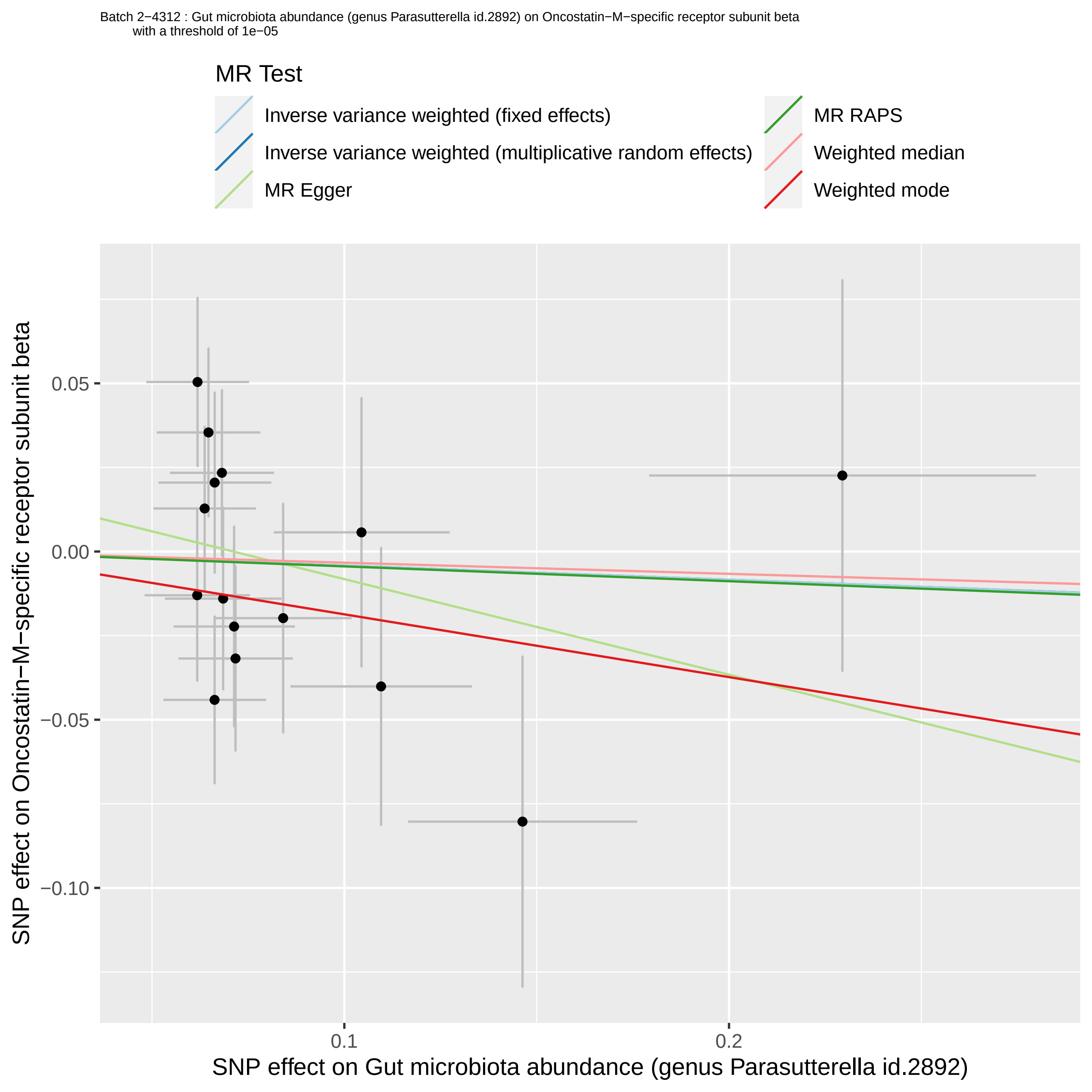
Batch 2−4312 : Gut microbiota abundance (genus Parasutterella id.2892) on Oncostatin−M−specific receptor subunit beta
with a threshold of 1e−05
MR Test
Inverse variance weighted (fixed effects)
Inverse variance weighted (multiplicative random effects)
MR Egger
MR RAPS
Weighted median
Weighted mode
0.05
0.00
−0.05
−0.10
0.1
0.2
SNP effect on Oncostatin−M−specific receptor subunit beta
SNP effect on Gut microbiota abundance (genus Parasutterella id.2892)

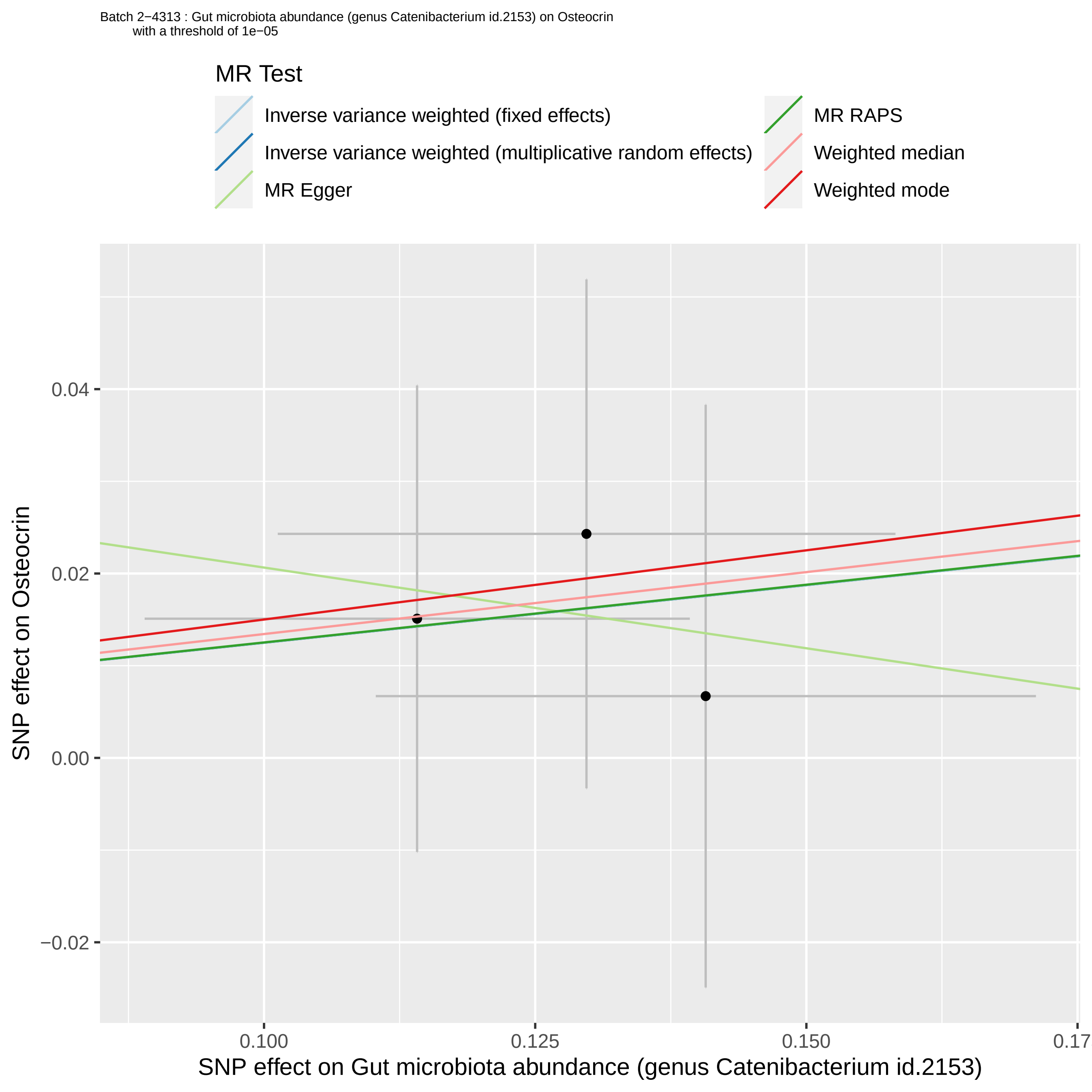

Batch 2−4313 : Gut microbiota abundance (genus Catenibacterium id.2153) on Osteocrin
with a threshold of 1e−05
MR Test
Inverse variance weighted (fixed effects)
Inverse variance weighted (multiplicative random effects)
MR Egger
MR RAPS
Weighted median
Weighted mode
SNP effect on Osteocrin
0.04
0.02
0.00
−0.02
0.100
0.125
0.150
0.17
SNP effect on Gut microbiota abundance (genus Catenibacterium id.2153)

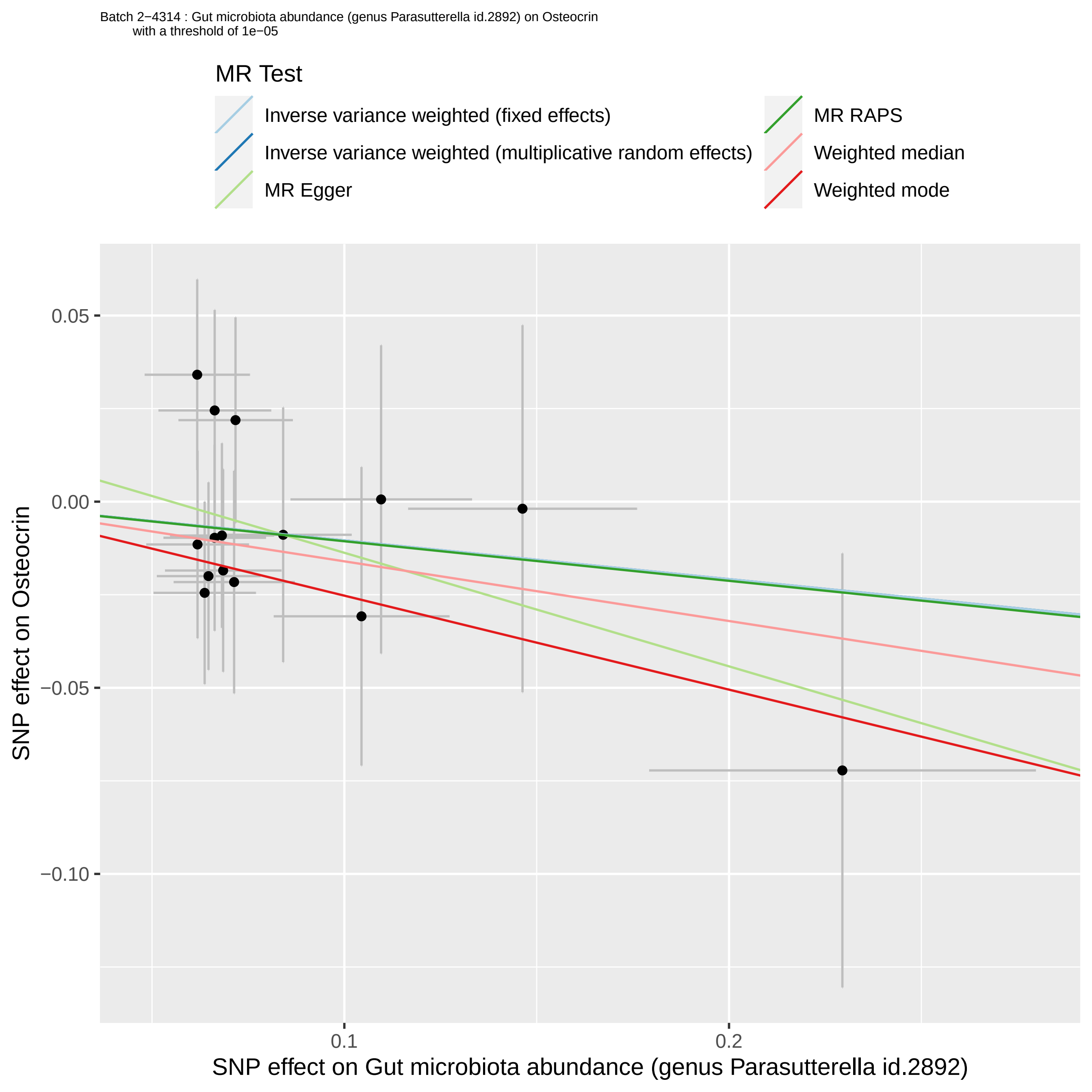
Batch 2−4314 : Gut microbiota abundance (genus Parasutterella id.2892) on Osteocrin
with a threshold of 1e−05
MR Test
Inverse variance weighted (fixed effects)
Inverse variance weighted (multiplicative random effects)
MR Egger
MR RAPS
Weighted median
Weighted mode
0.05
0.00
−0.05
−0.10
SNP effect on Osteocrin
0.1
0.2
SNP effect on Gut microbiota abundance (genus Parasutterella id.2892)

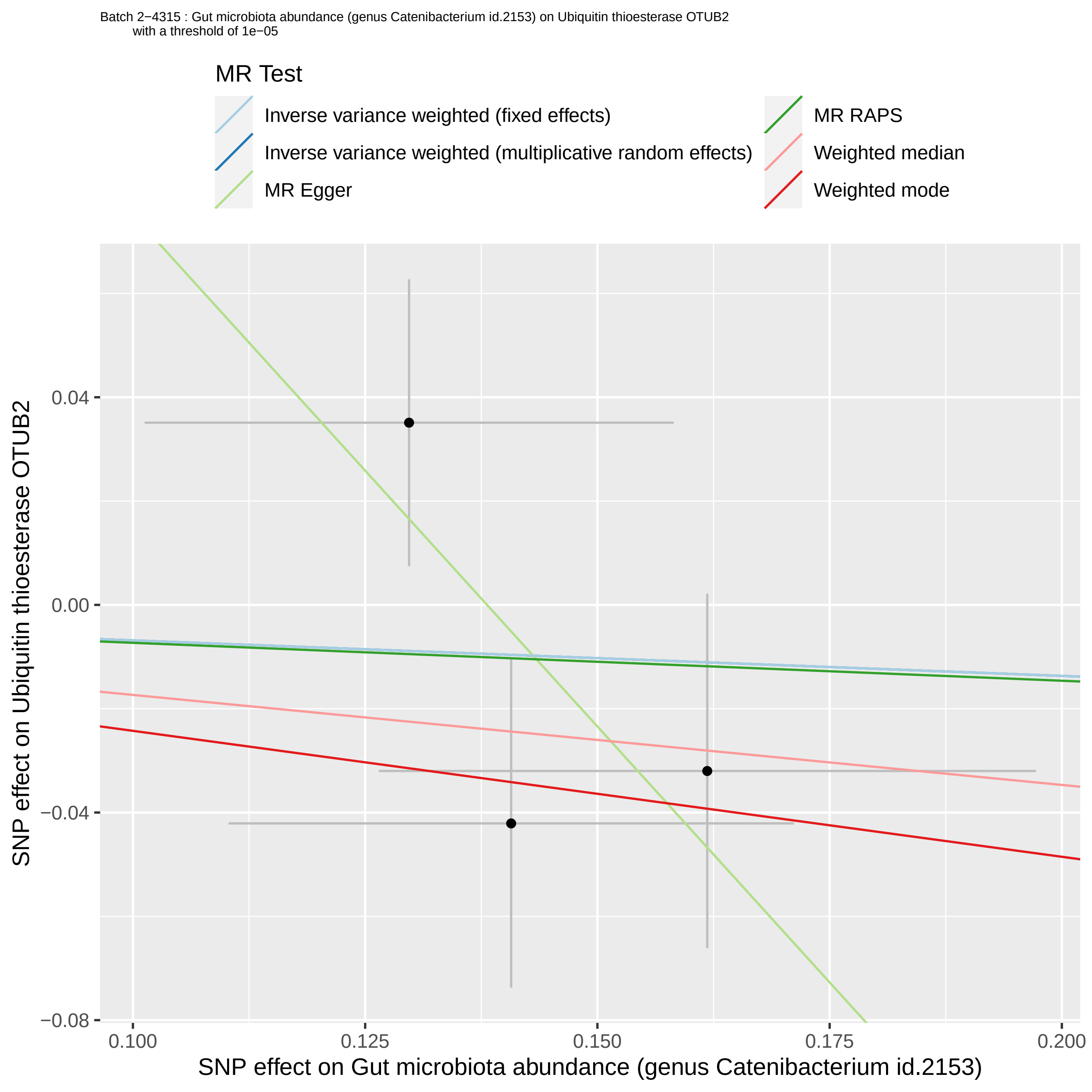

Batch 2−4315 : Gut microbiota abundance (genus Catenibacterium id.2153) on Ubiquitin thioesterase OTUB2
with a threshold of 1e−05
MR Test
Inverse variance weighted (fixed effects)
Inverse variance weighted (multiplicative random effects)
MR Egger
MR RAPS
Weighted median
Weighted mode
SNP effect on Ubiquitin thioesterase OTUB2
0.04
0.00
−0.04
−0.08
0.100
0.125
0.150
0.175
0.200
SNP effect on Gut microbiota abundance (genus Catenibacterium id.2153)

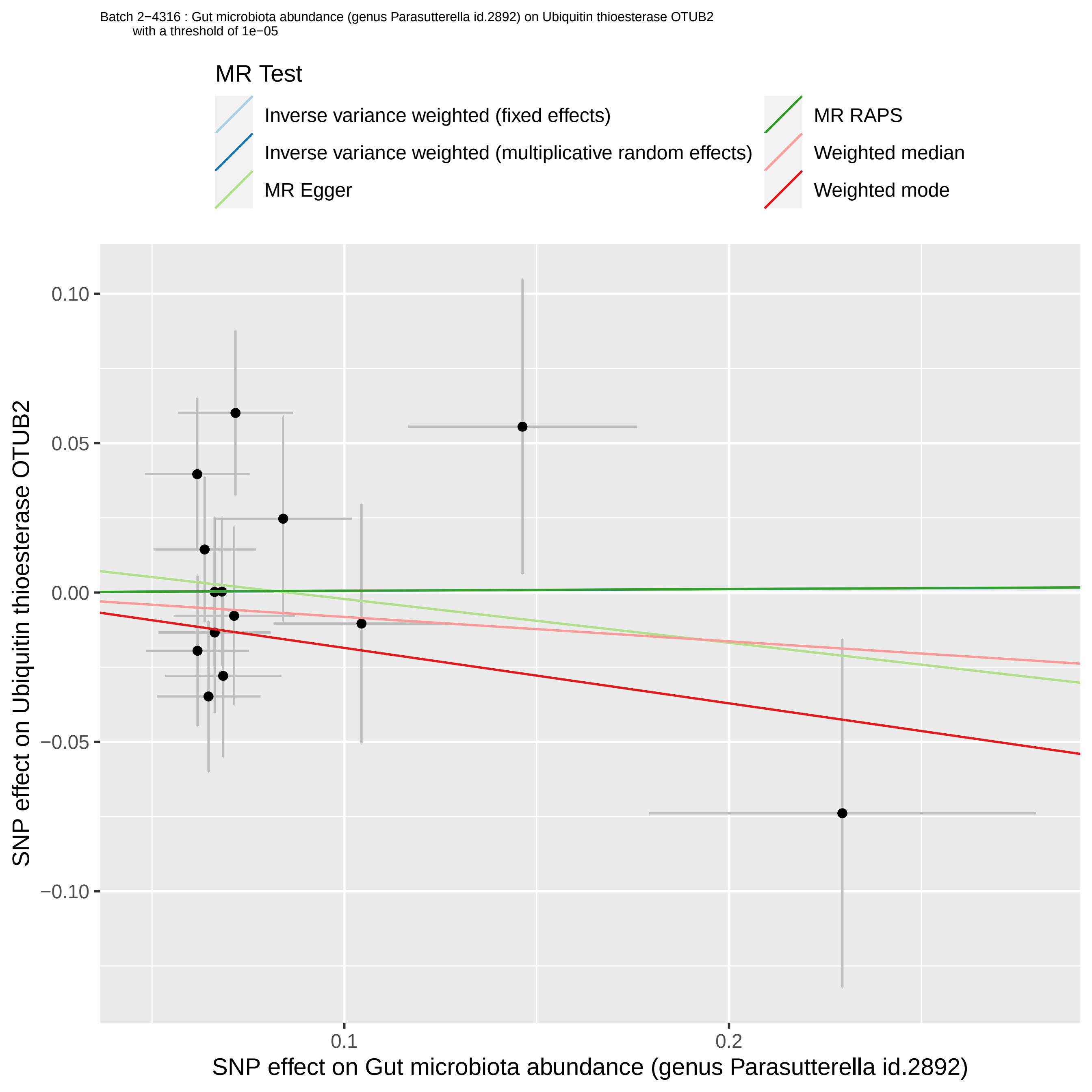
Batch 2−4316 : Gut microbiota abundance (genus Parasutterella id.2892) on Ubiquitin thioesterase OTUB2
with a threshold of 1e−05
MR Test
Inverse variance weighted (fixed effects)
Inverse variance weighted (multiplicative random effects)
MR Egger
MR RAPS
Weighted median
Weighted mode
0.10
0.05
0.00
−0.05
−0.10
0.1
0.2
SNP effect on Ubiquitin thioesterase OTUB2
SNP effect on Gut microbiota abundance (genus Parasutterella id.2892)

Batch 2−4317 : Gut microbiota abundance (genus Catenibacterium id.2153) on Oxytocin−neurophysin 1
with a threshold of 1e−05

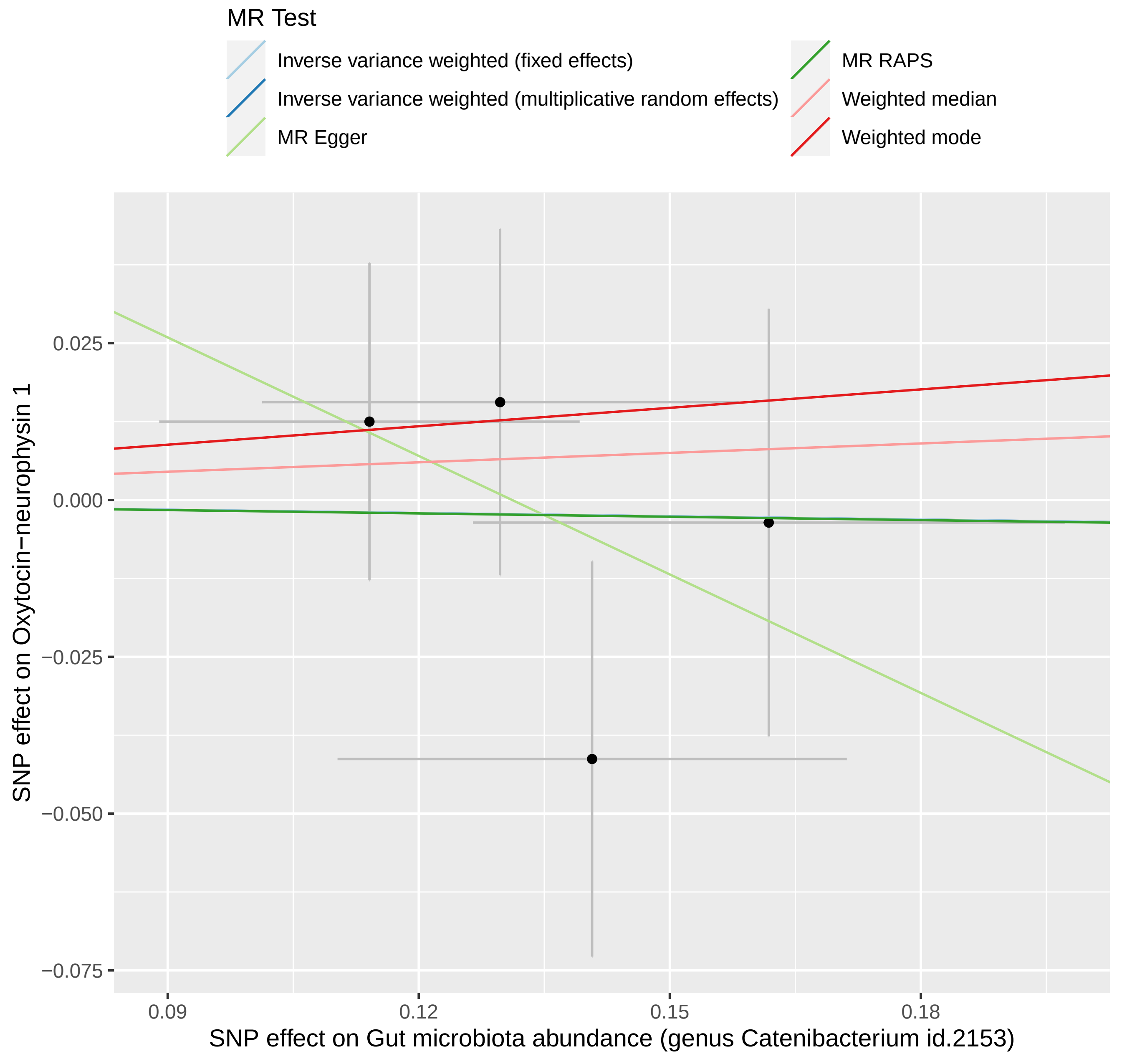

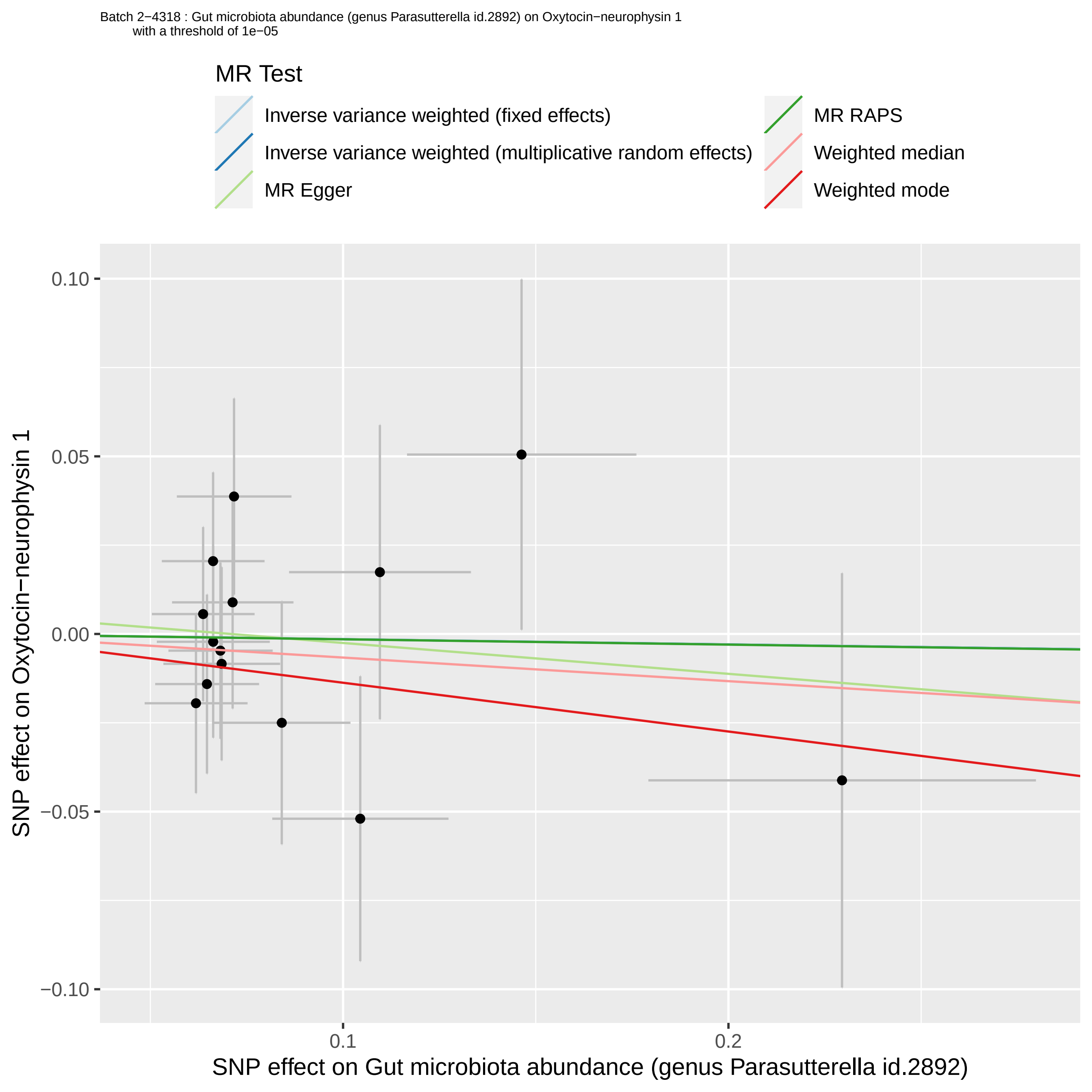
Batch 2−4318 : Gut microbiota abundance (genus Parasutterella id.2892) on Oxytocin−neurophysin 1
with a threshold of 1e−05
MR Test
Inverse variance weighted (fixed effects)
Inverse variance weighted (multiplicative random effects)
MR Egger
MR RAPS
Weighted median
Weighted mode
0.10
0.05
0.00
−0.05
−0.10
0.1
0.2
SNP effect on Oxytocin−neurophysin 1
SNP effect on Gut microbiota abundance (genus Parasutterella id.2892)

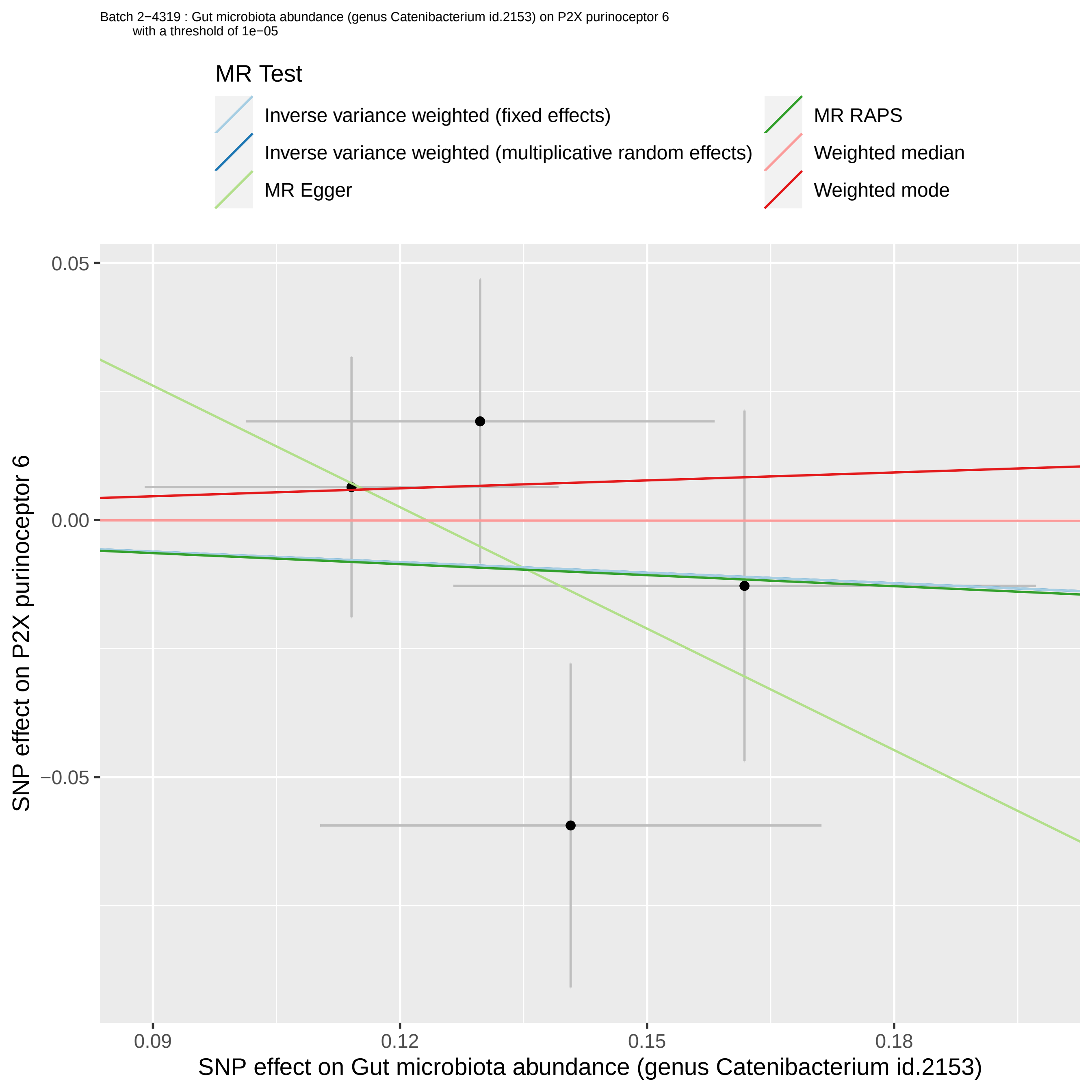
Batch 2−4319 : Gut microbiota abundance (genus Catenibacterium id.2153) on P2X purinoceptor 6
with a threshold of 1e−05
MR Test
Inverse variance weighted (fixed effects)
Inverse variance weighted (multiplicative random effects)
MR Egger
MR RAPS
Weighted median
Weighted mode
0.05
0.00
−0.05
0.09
0.12
0.15
0.18
SNP effect on P2X purinoceptor 6
SNP effect on Gut microbiota abundance (genus Catenibacterium id.2153)

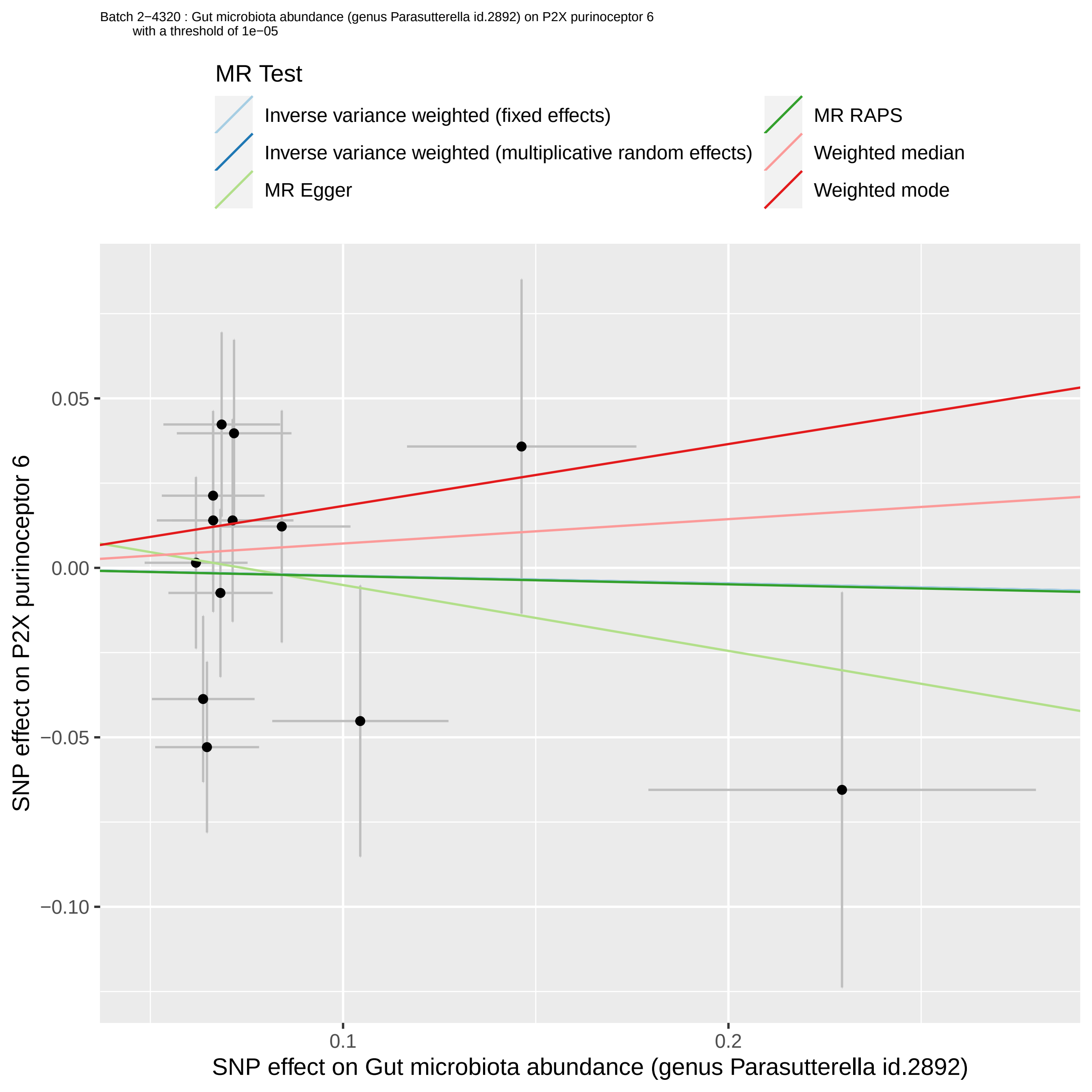
Batch 2−4320 : Gut microbiota abundance (genus Parasutterella id.2892) on P2X purinoceptor 6
with a threshold of 1e−05
MR Test
Inverse variance weighted (fixed effects)
Inverse variance weighted (multiplicative random effects)
MR Egger
MR RAPS
Weighted median
Weighted mode
0.05
0.00
−0.05
−0.10
0.1
0.2
SNP effect on P2X purinoceptor 6
SNP effect on Gut microbiota abundance (genus Parasutterella id.2892)

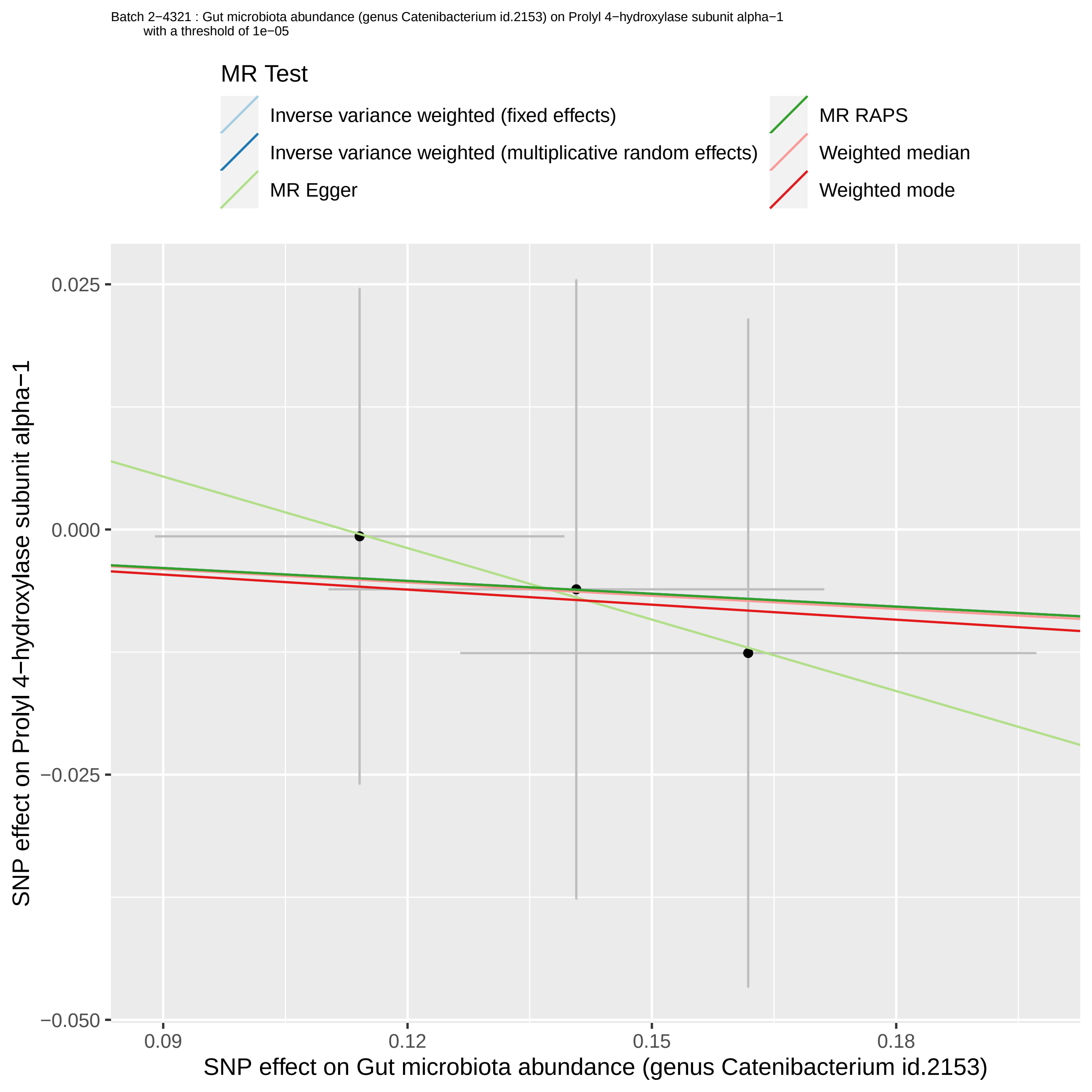
Batch 2−4321 : Gut microbiota abundance (genus Catenibacterium id.2153) on Prolyl 4−hydroxylase subunit alpha−1
with a threshold of 1e−05
MR Test
Inverse variance weighted (fixed effects)
Inverse variance weighted (multiplicative random effects)
MR Egger
MR RAPS
Weighted median
Weighted mode
0.025
0.000
−0.025
−0.050
0.09
0.12
0.15
0.18
SNP effect on Prolyl 4−hydroxylase subunit alpha−1
SNP effect on Gut microbiota abundance (genus Catenibacterium id.2153)

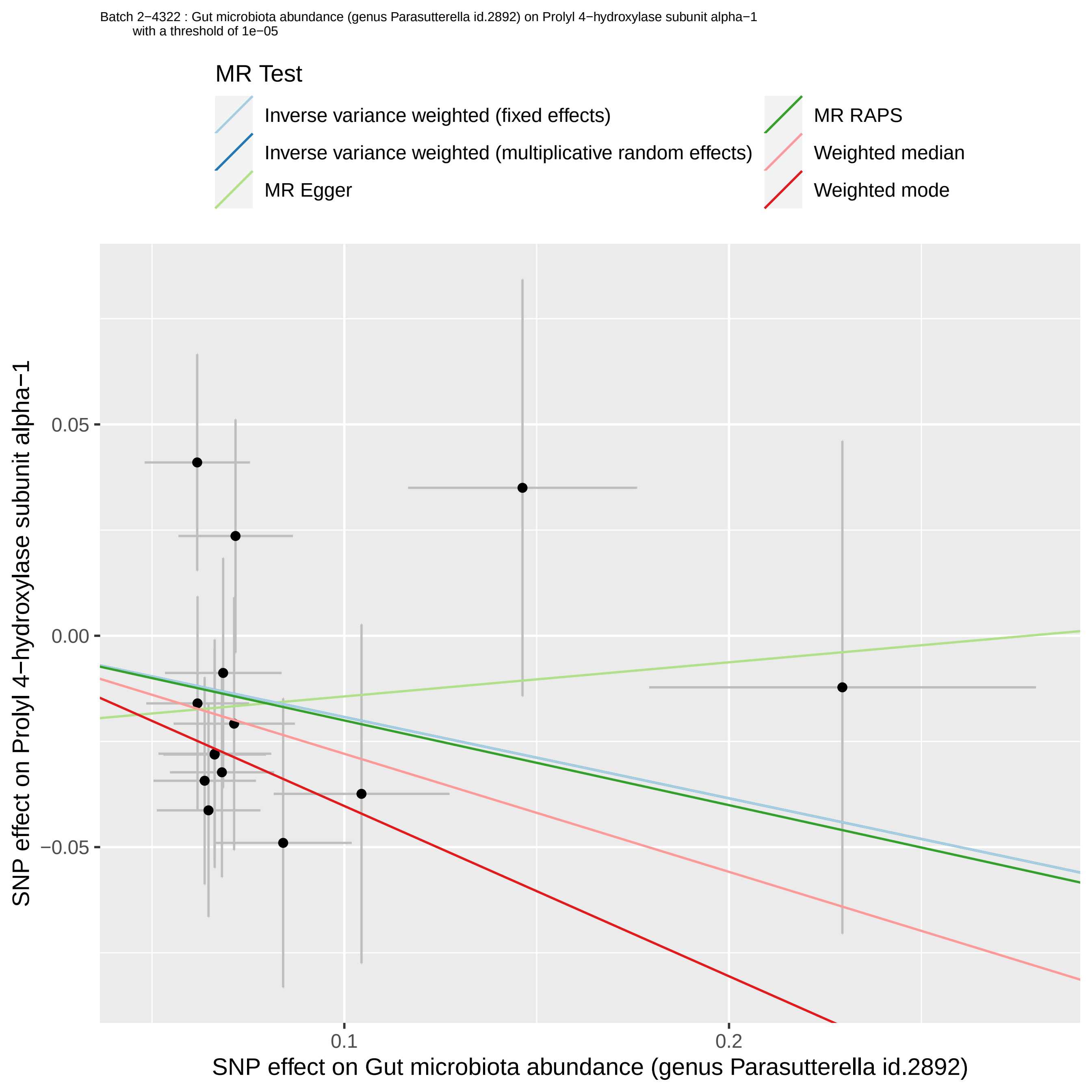

Batch 2−4322 : Gut microbiota abundance (genus Parasutterella id.2892) on Prolyl 4−hydroxylase subunit alpha−1
with a threshold of 1e−05
MR Test
Inverse variance weighted (fixed effects)
Inverse variance weighted (multiplicative random effects)
MR Egger
MR RAPS
Weighted median
Weighted mode
0.05
0.00
−0.05
0.1
0.2
SNP effect on Prolyl 4−hydroxylase subunit alpha−1
SNP effect on Gut microbiota abundance (genus Parasutterella id.2892)

Batch 2−4323 : Gut microbiota abundance (genus Catenibacterium id.2153) on Prolyl 4−hydroxylase subunit alpha−2
with a threshold of 1e−05

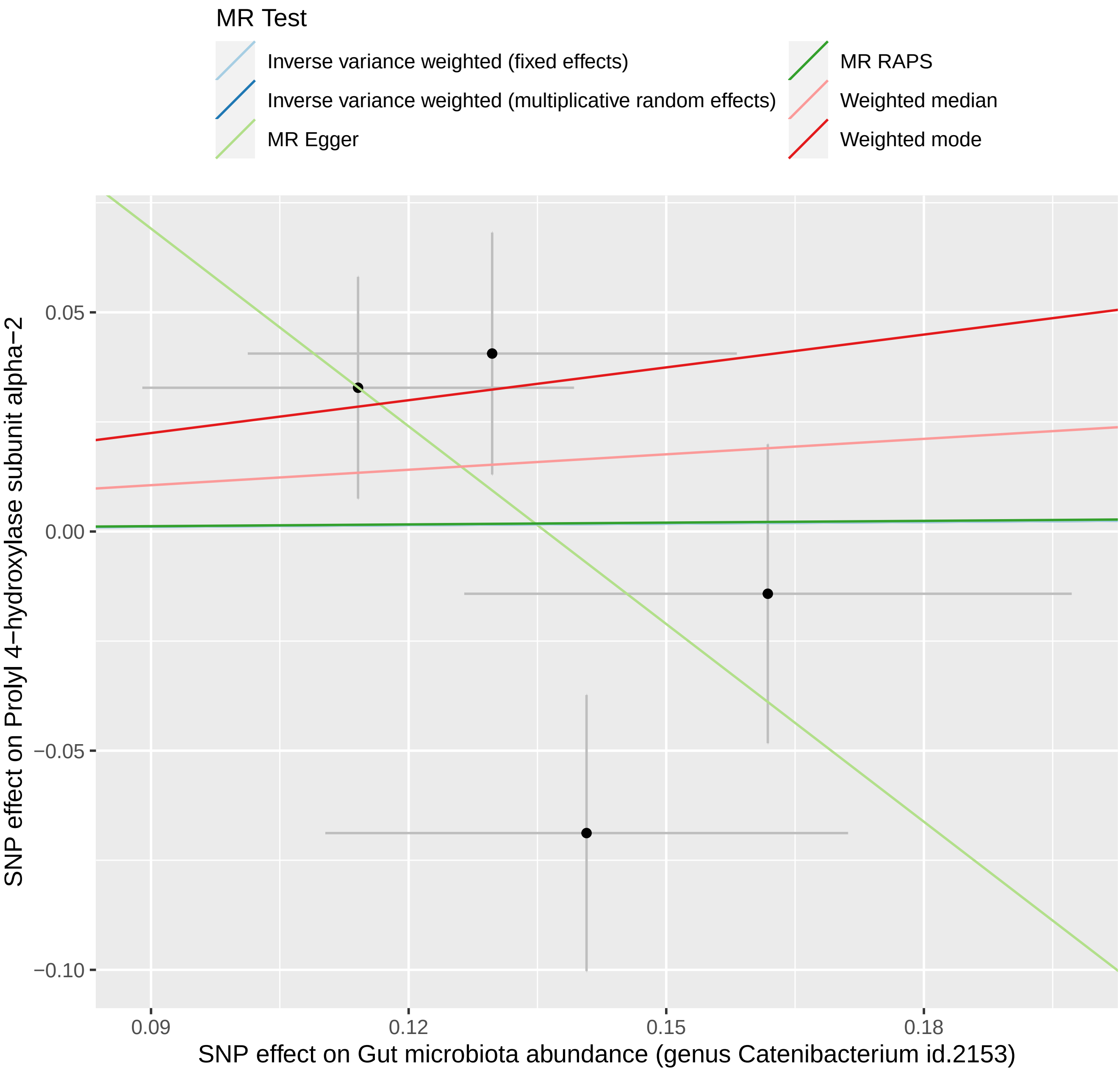

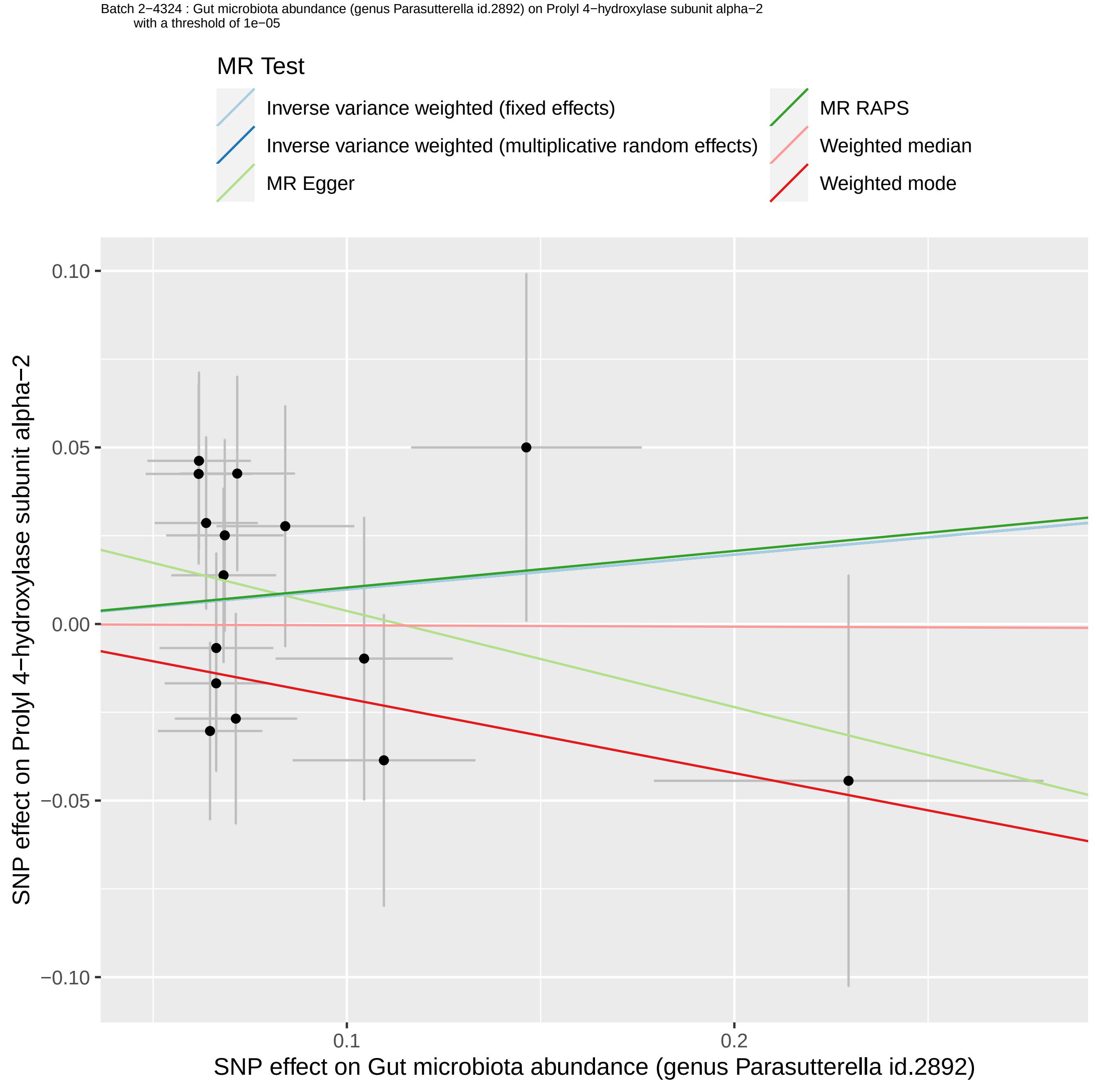
Batch 2−4324 : Gut microbiota abundance (genus Parasutterella id.2892) on Prolyl 4−hydroxylase subunit alpha−2
with a threshold of 1e−05
MR Test
Inverse variance weighted (fixed effects)
Inverse variance weighted (multiplicative random effects)
MR Egger
MR RAPS
Weighted median
Weighted mode
0.10
0.05
0.00
−0.05
−0.10
0.1
0.2
SNP effect on Prolyl 4−hydroxylase subunit alpha−2
SNP effect on Gut microbiota abundance (genus Parasutterella id.2892)

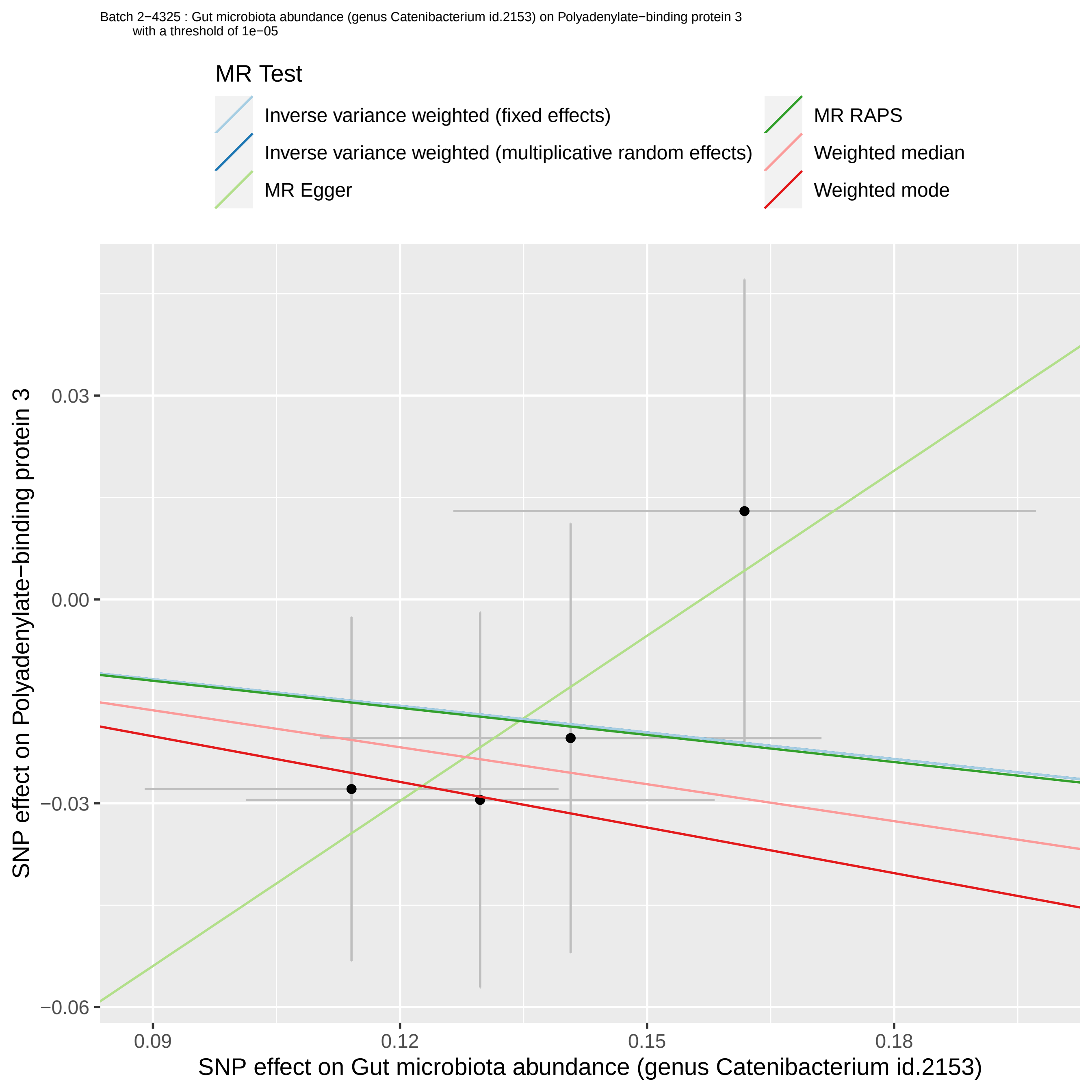

Batch 2−4325 : Gut microbiota abundance (genus Catenibacterium id.2153) on Polyadenylate−binding protein 3
with a threshold of 1e−05
MR Test
Inverse variance weighted (fixed effects)
Inverse variance weighted (multiplicative random effects)
MR Egger
MR RAPS
Weighted median
Weighted mode
0.03
0.00
−0.03
−0.06
0.09
0.12
0.15
0.18
SNP effect on Polyadenylate−binding protein 3
SNP effect on Gut microbiota abundance (genus Catenibacterium id.2153)

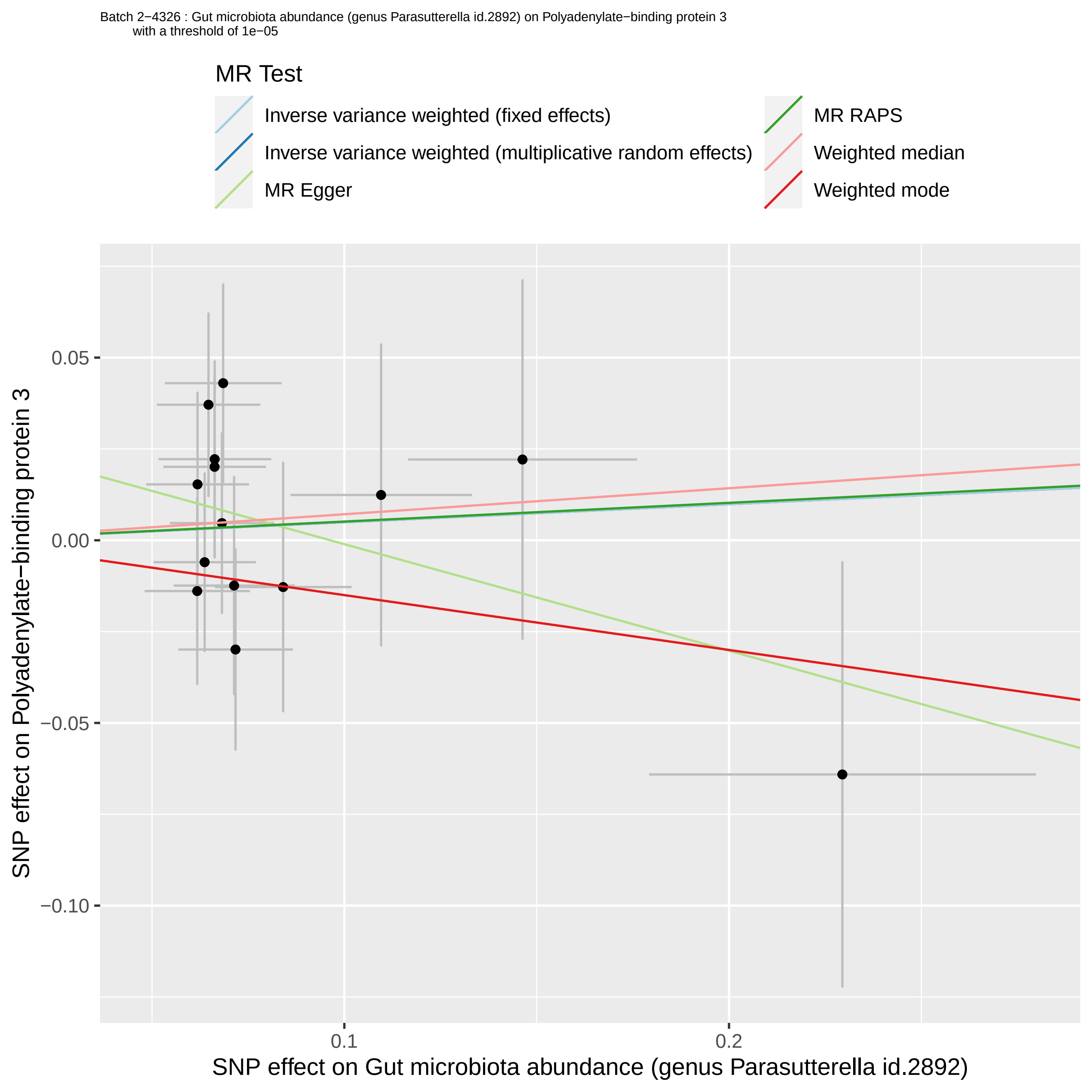

Batch 2−4326 : Gut microbiota abundance (genus Parasutterella id.2892) on Polyadenylate−binding protein 3
with a threshold of 1e−05
MR Test
Inverse variance weighted (fixed effects)
Inverse variance weighted (multiplicative random effects)
MR Egger
MR RAPS
Weighted median
Weighted mode
0.05
0.00
−0.05
−0.10
0.1
0.2
SNP effect on Polyadenylate−binding protein 3
SNP effect on Gut microbiota abundance (genus Parasutterella id.2892)

Batch 2−4327 : Gut microbiota abundance (genus Catenibacterium id.2153) on Polyadenylate−binding protein 4
with a threshold of 1e−05

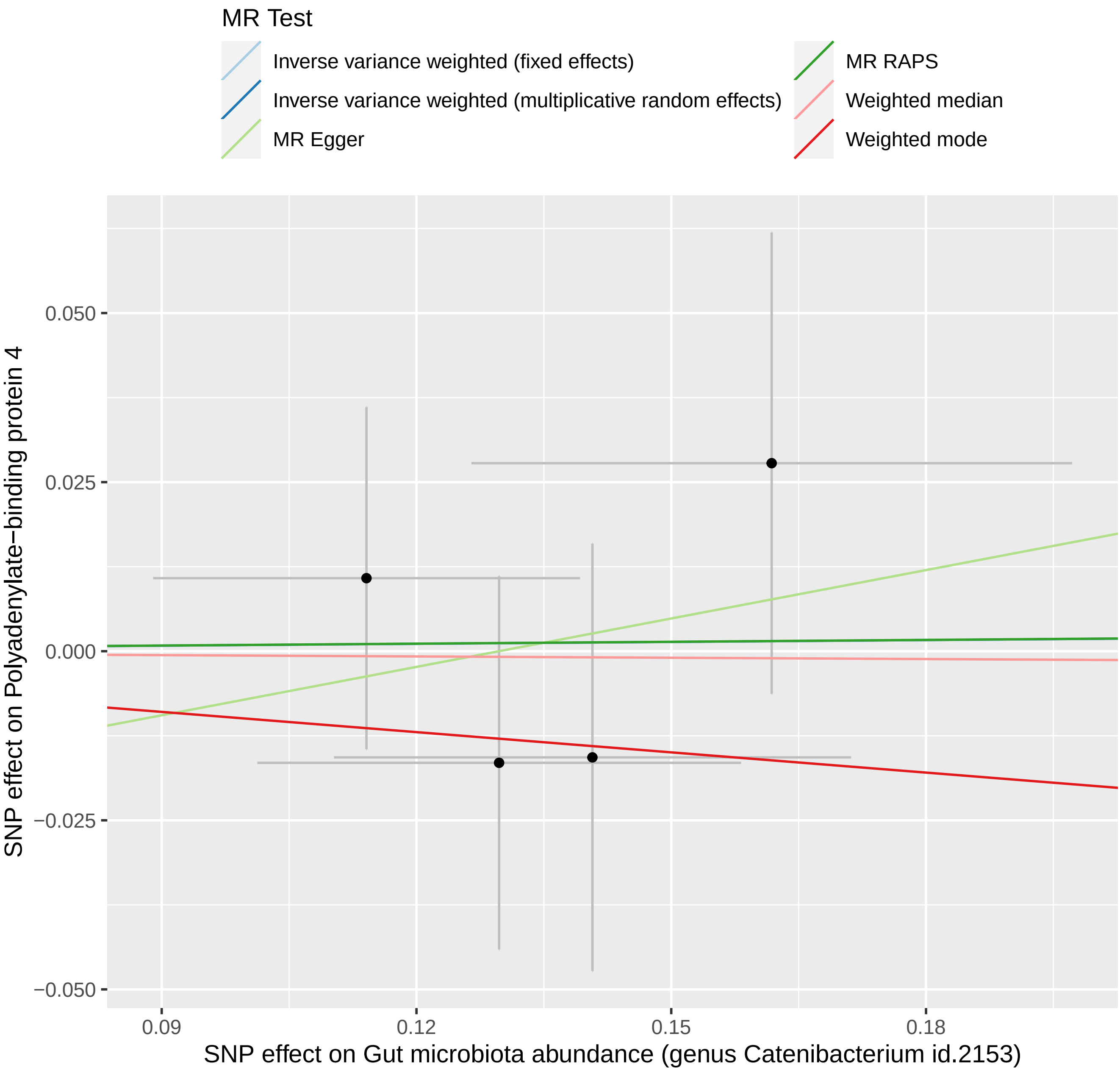

Batch 2−4328 : Gut microbiota abundance (genus Parasutterella id.2892) on Polyadenylate−binding protein 4
with a threshold of 1e−05

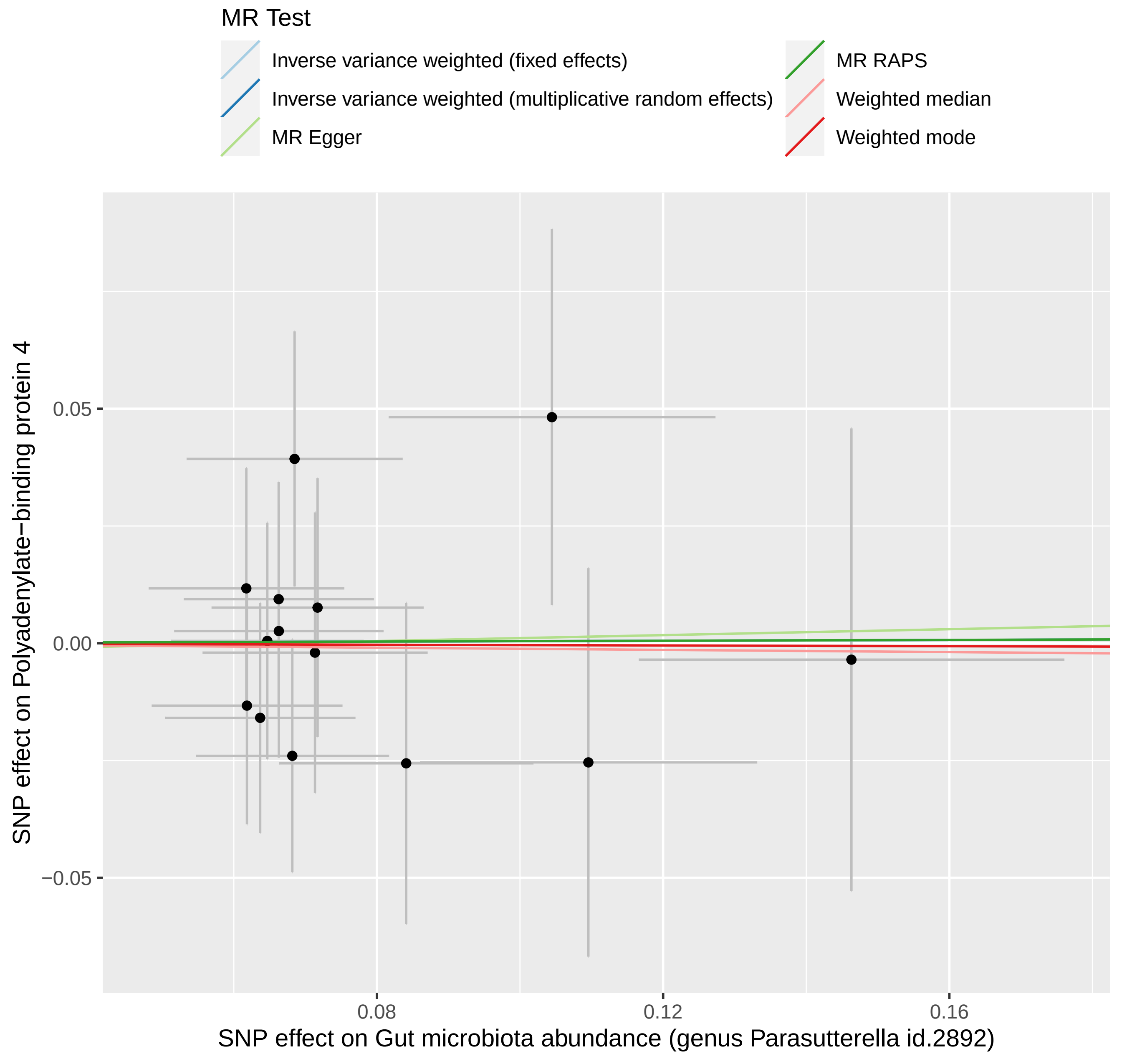

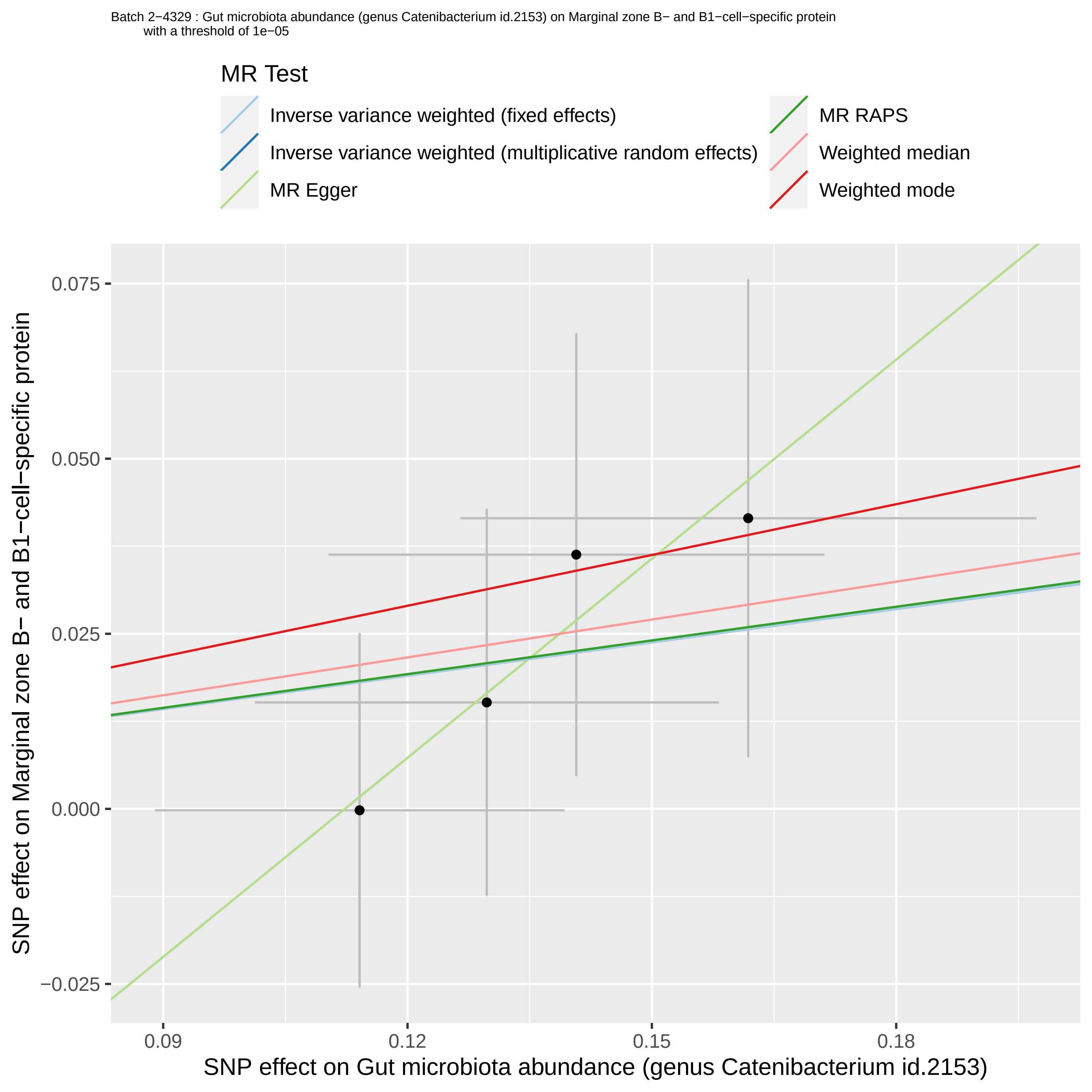

Batch 2−4329 : Gut microbiota abundance (genus Catenibacterium id.2153) on Marginal zone B− and B1−cell−specific protein
with a threshold of 1e−05
MR Test
Inverse variance weighted (fixed effects)
Inverse variance weighted (multiplicative random effects)
MR Egger
MR RAPS
Weighted median
Weighted mode
0.075
0.050
0.025
0.000
−0.025
0.09
0.12
0.15
0.18
SNP effect on Marginal zone B− and B1−cell−specific protein
SNP effect on Gut microbiota abundance (genus Catenibacterium id.2153)

Batch 2−4330 : Gut microbiota abundance (genus Parasutterella id.2892) on Marginal zone B− and B1−cell−specific protein
with a threshold of 1e−05

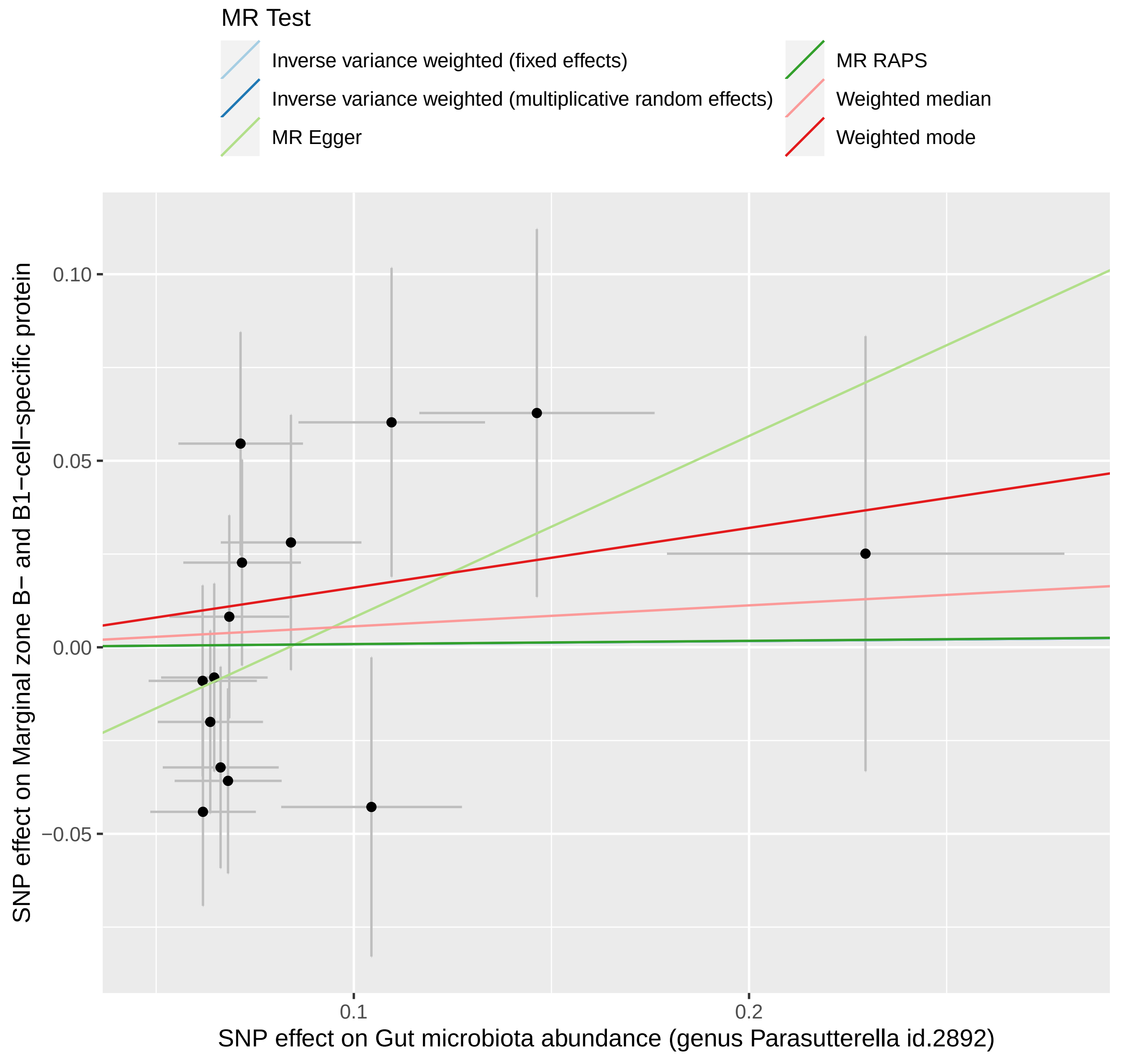

Batch 2−4331 : Gut microbiota abundance (genus Catenibacterium id.2153) on Platelet−activating factor acetylhydrolase IB subunit beta
with a threshold of 1e−05

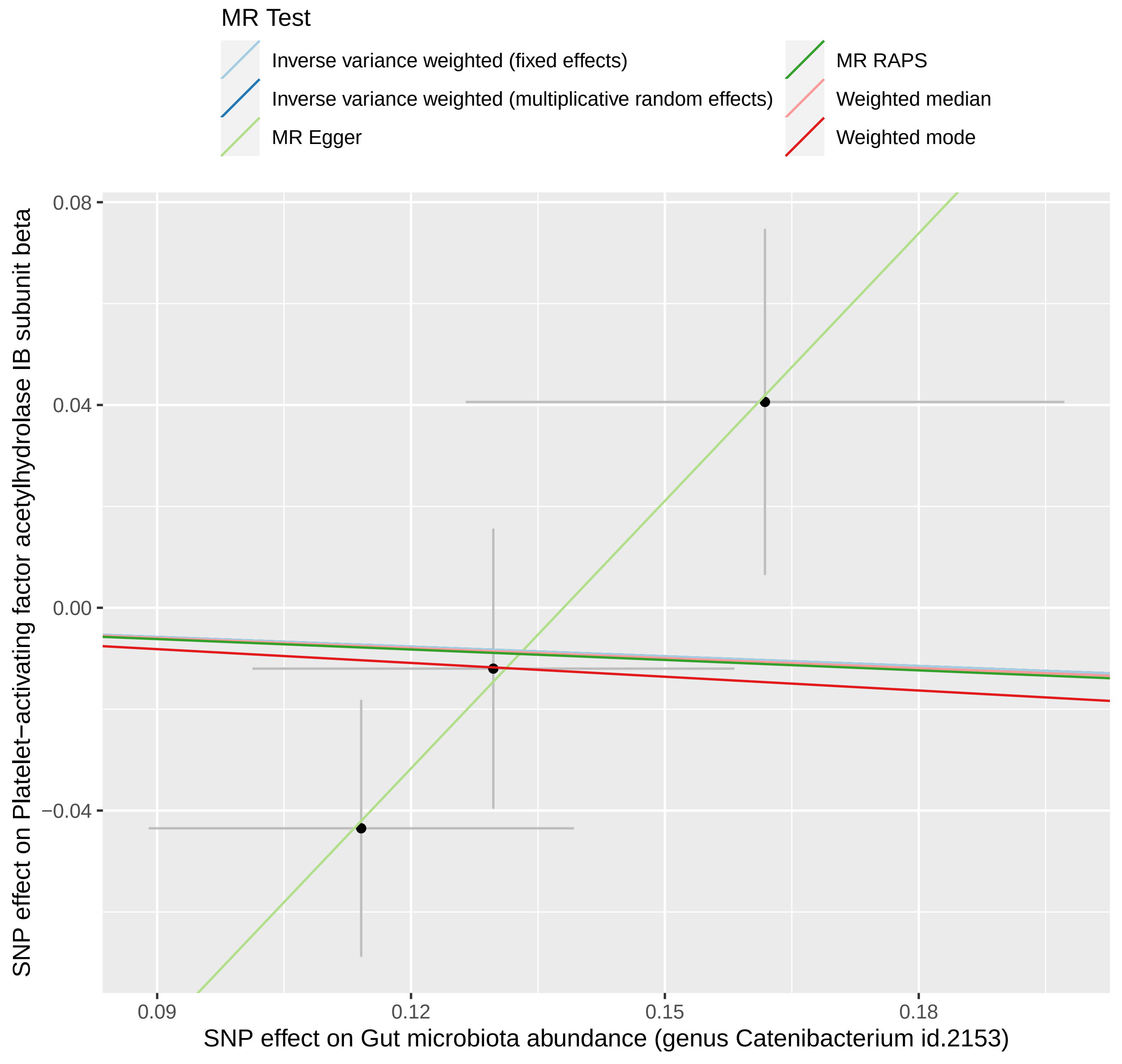

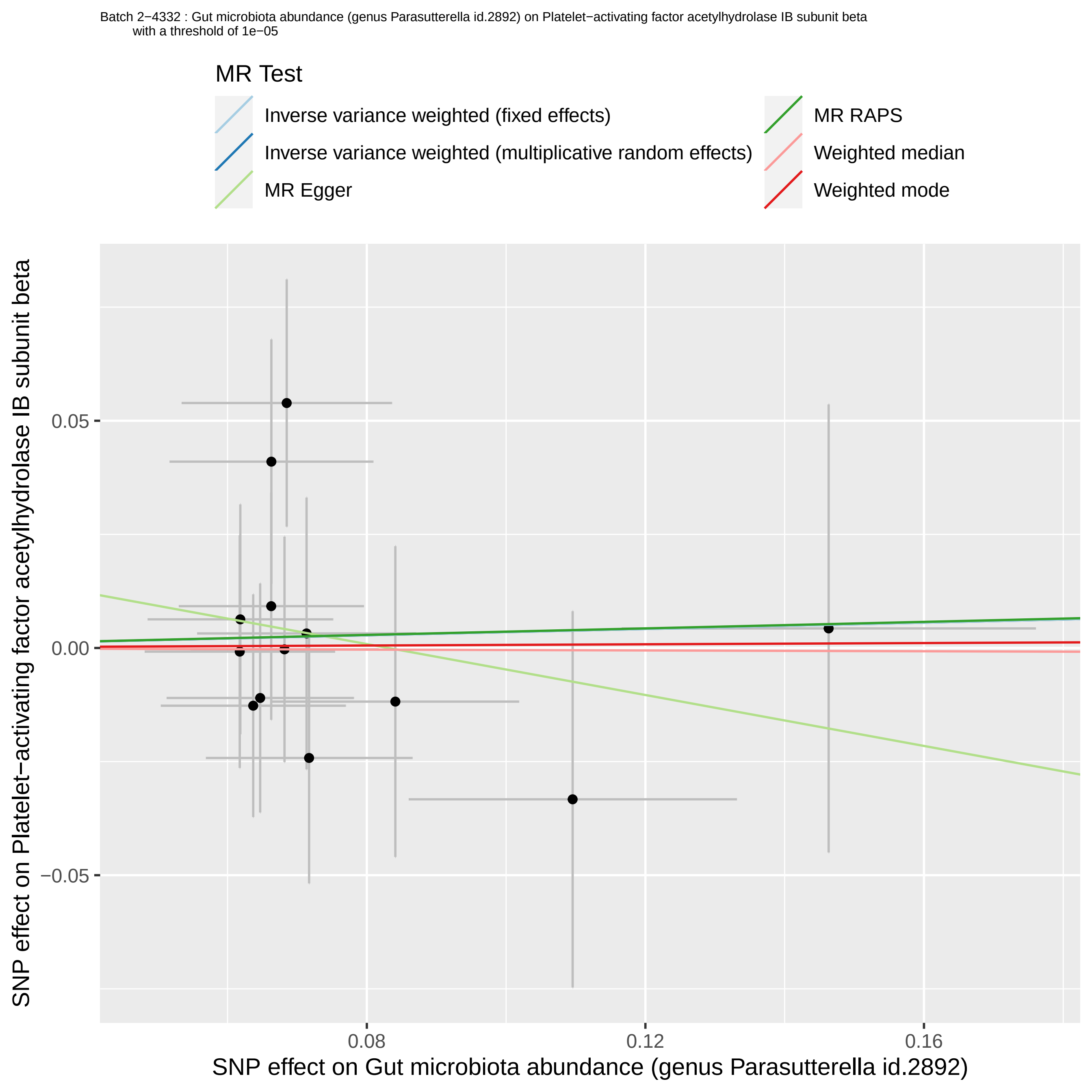
Batch 2−4332 : Gut microbiota abundance (genus Parasutterella id.2892) on Platelet−activating factor acetylhydrolase IB subunit beta
with a threshold of 1e−05
MR Test
Inverse variance weighted (fixed effects)
Inverse variance weighted (multiplicative random effects)
MR Egger
MR RAPS
Weighted median
Weighted mode
0.05
0.00
−0.05
0.08
0.12
0.16
SNP effect on Platelet−activating factor acetylhydrolase IB subunit beta
SNP effect on Gut microbiota abundance (genus Parasutterella id.2892)

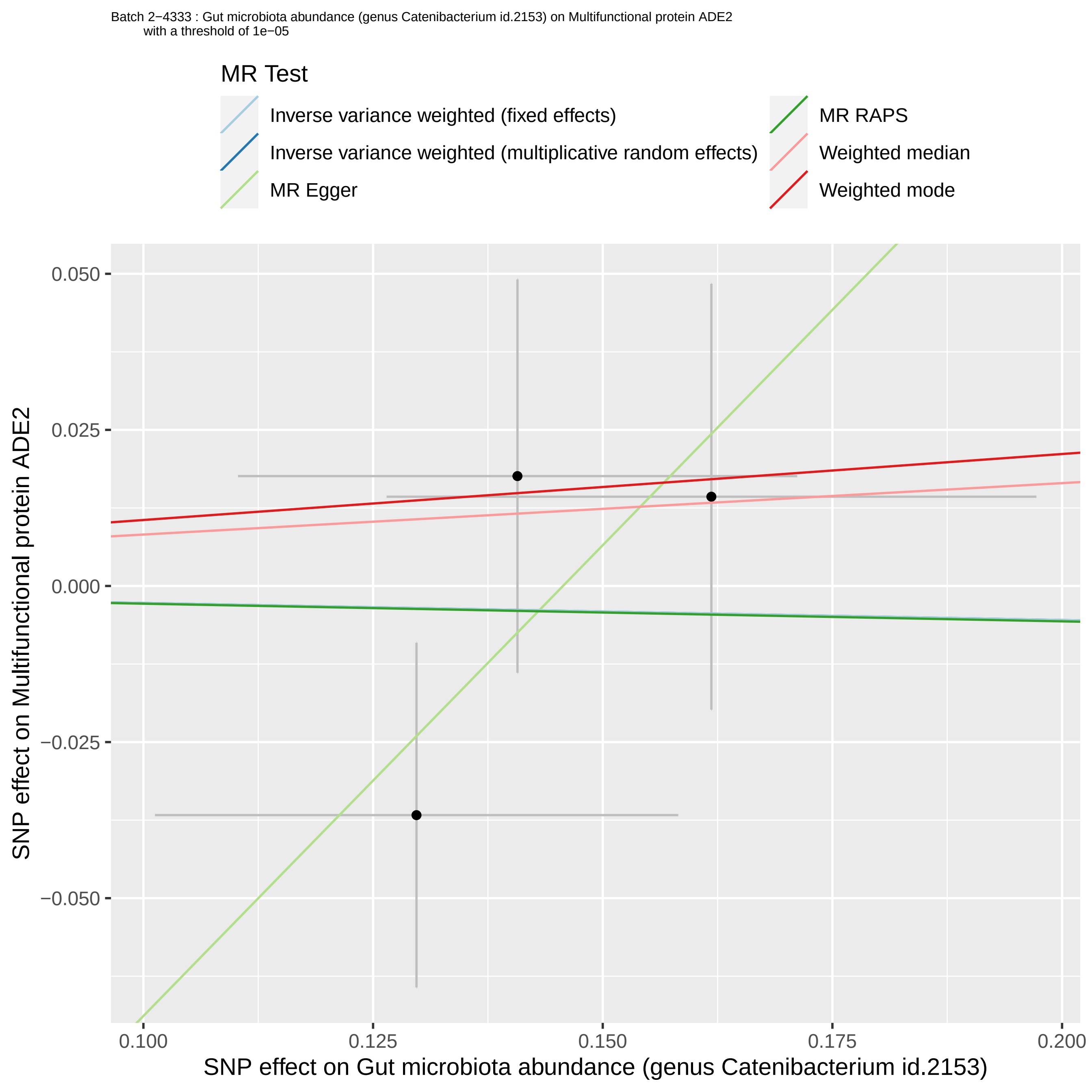
Batch 2−4333 : Gut microbiota abundance (genus Catenibacterium id.2153) on Multifunctional protein ADE2
with a threshold of 1e−05
MR Test
Inverse variance weighted (fixed effects)
Inverse variance weighted (multiplicative random effects)
MR Egger
MR RAPS
Weighted median
Weighted mode
0.050
0.025
0.000
−0.025
−0.050
0.100
0.125
0.150
0.175
0.200
SNP effect on Multifunctional protein ADE2
SNP effect on Gut microbiota abundance (genus Catenibacterium id.2153)

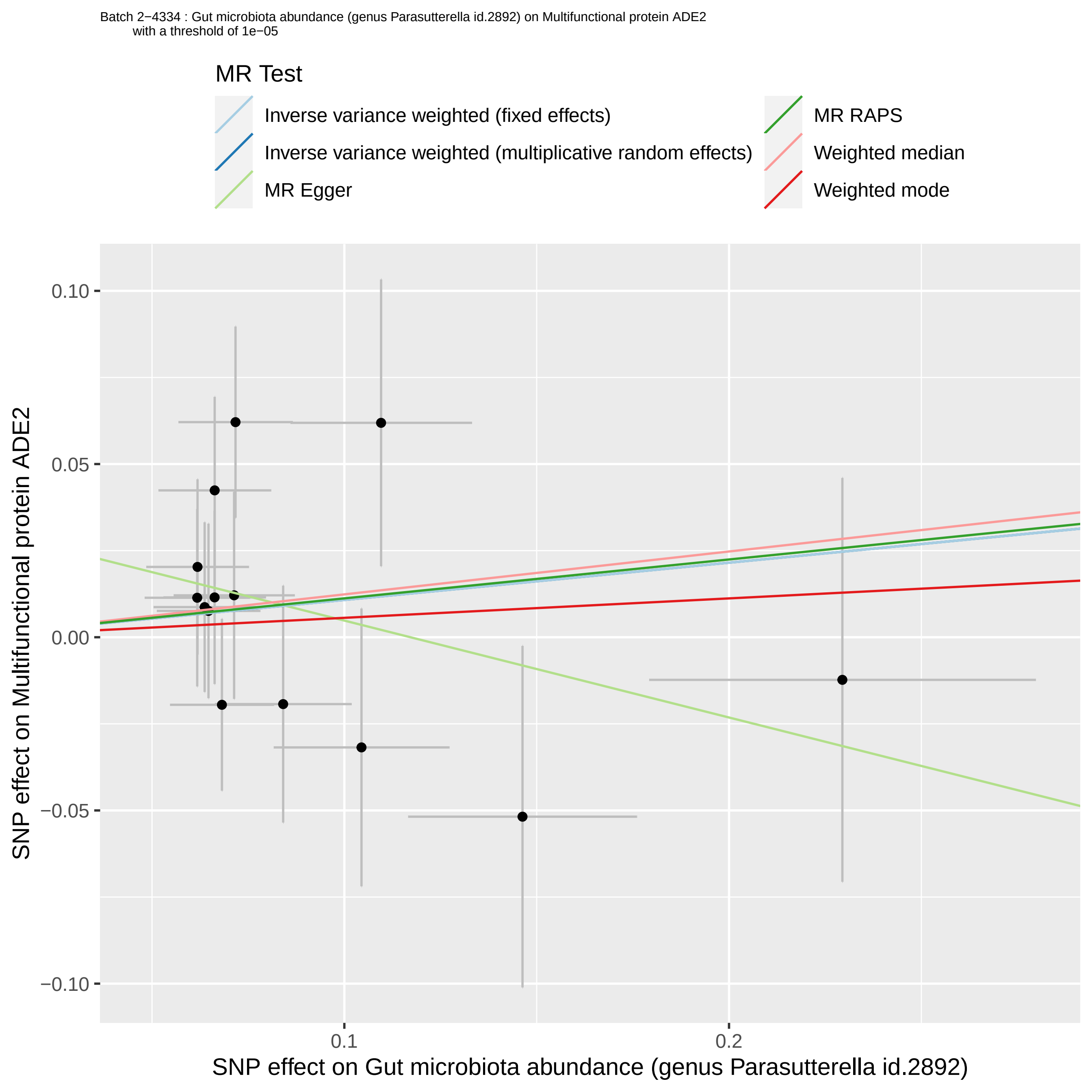

Batch 2−4334 : Gut microbiota abundance (genus Parasutterella id.2892) on Multifunctional protein ADE2
with a threshold of 1e−05
MR Test
Inverse variance weighted (fixed effects)
Inverse variance weighted (multiplicative random effects)
MR Egger
MR RAPS
Weighted median
Weighted mode
0.10
0.05
0.00
−0.05
−0.10
0.1
0.2
SNP effect on Multifunctional protein ADE2
SNP effect on Gut microbiota abundance (genus Parasutterella id.2892)

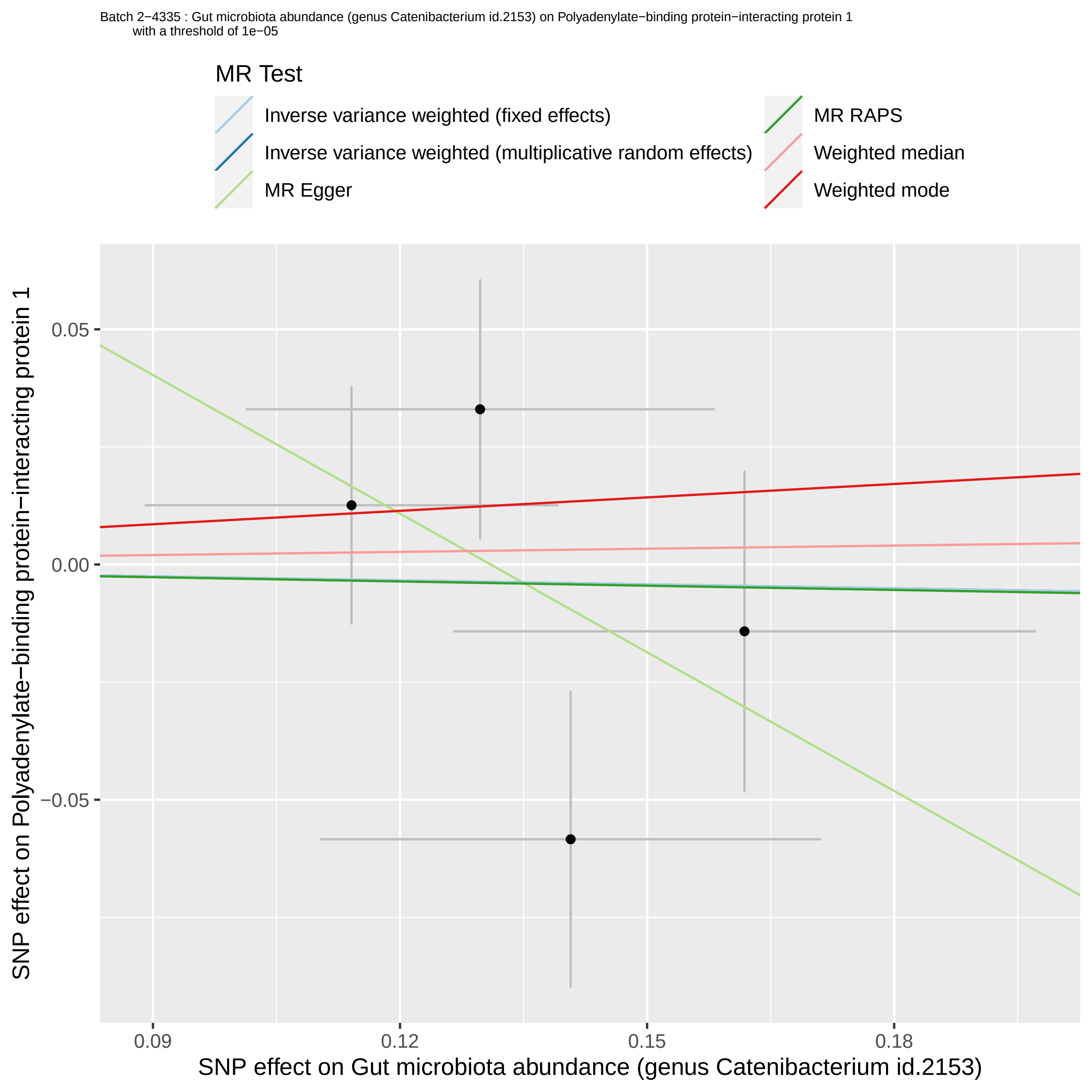

Batch 2−4335 : Gut microbiota abundance (genus Catenibacterium id.2153) on Polyadenylate−binding protein−interacting protein 1
with a threshold of 1e−05
MR Test
Inverse variance weighted (fixed effects)
Inverse variance weighted (multiplicative random effects)
MR Egger
MR RAPS
Weighted median
Weighted mode
0.05
0.00
−0.05
0.09
0.12
0.15
0.18
SNP effect on Polyadenylate−binding protein−interacting protein 1
SNP effect on Gut microbiota abundance (genus Catenibacterium id.2153)

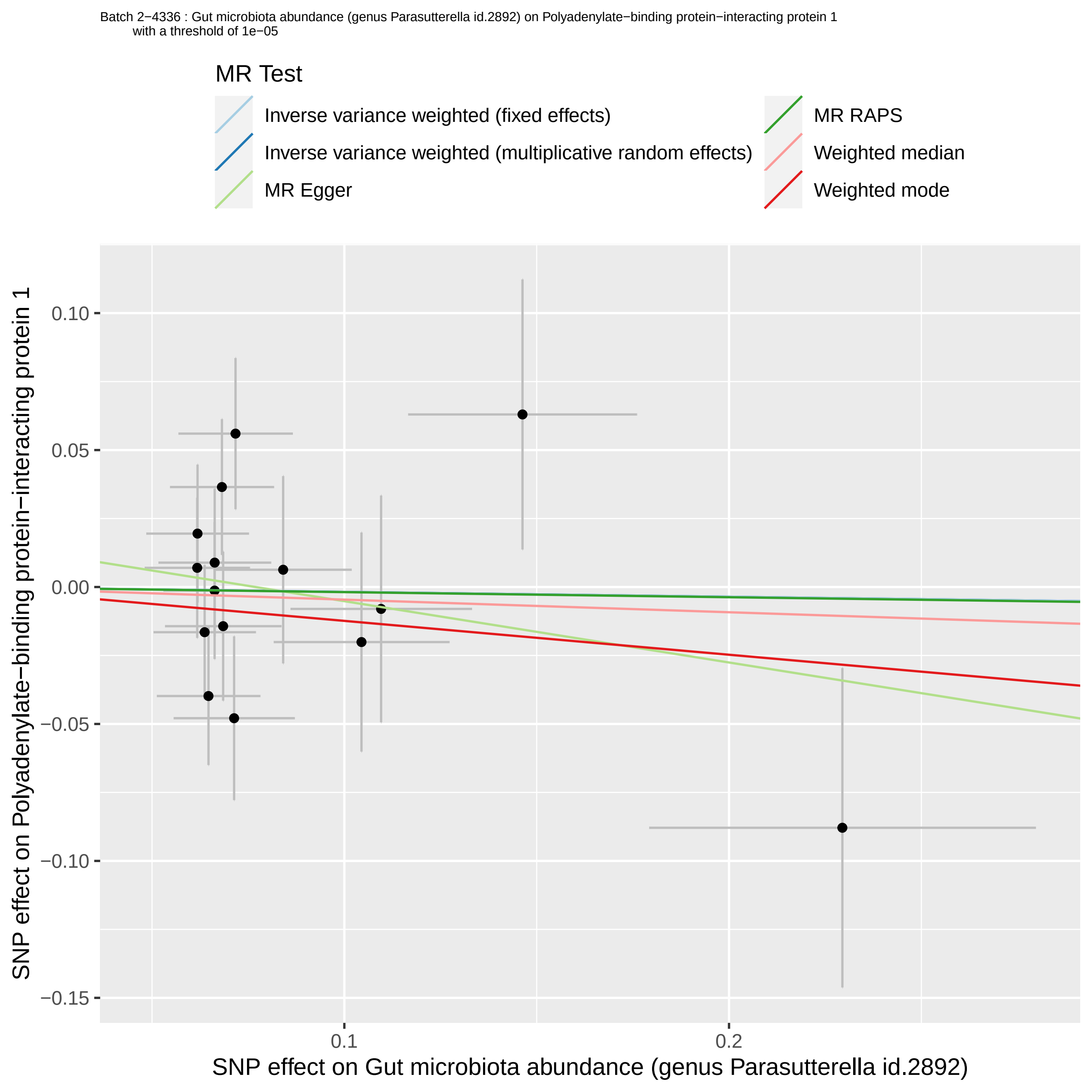

Batch 2−4336 : Gut microbiota abundance (genus Parasutterella id.2892) on Polyadenylate−binding protein−interacting protein 1
with a threshold of 1e−05
MR Test
Inverse variance weighted (fixed effects)
Inverse variance weighted (multiplicative random effects)
MR Egger
MR RAPS
Weighted median
Weighted mode
0.10
0.05
0.00
−0.05
−0.10
−0.15
0.1
0.2
SNP effect on Polyadenylate−binding protein−interacting protein 1
SNP effect on Gut microbiota abundance (genus Parasutterella id.2892)

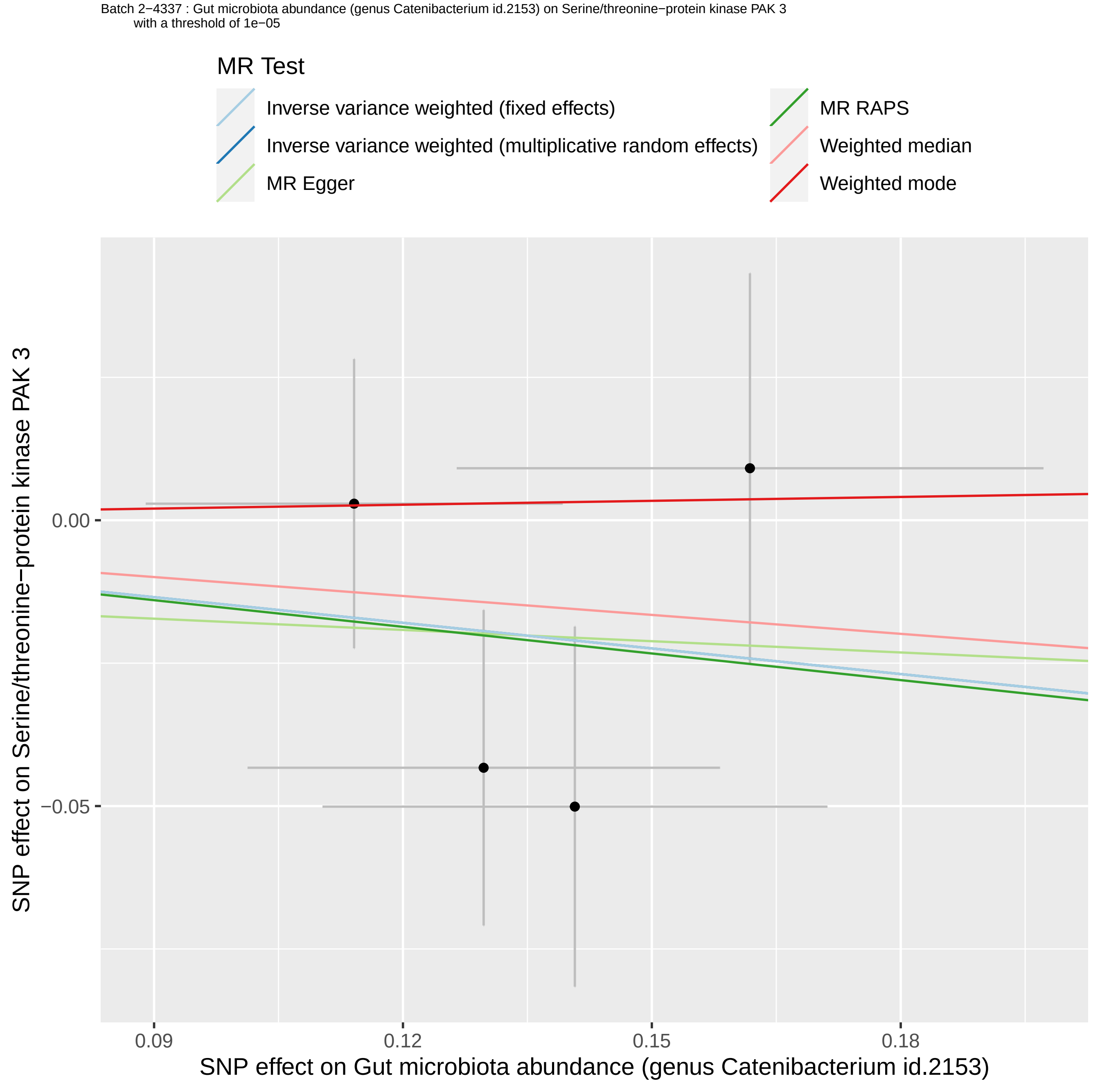

Batch 2−4337 : Gut microbiota abundance (genus Catenibacterium id.2153) on Serine/threonine−protein kinase PAK 3
with a threshold of 1e−05
MR Test
Inverse variance weighted (fixed effects)
Inverse variance weighted (multiplicative random effects)
MR Egger
MR RAPS
Weighted median
Weighted mode
0.00
−0.05
0.09
0.12
0.15
0.18
SNP effect on Serine/threonine−protein kinase PAK 3
SNP effect on Gut microbiota abundance (genus Catenibacterium id.2153)

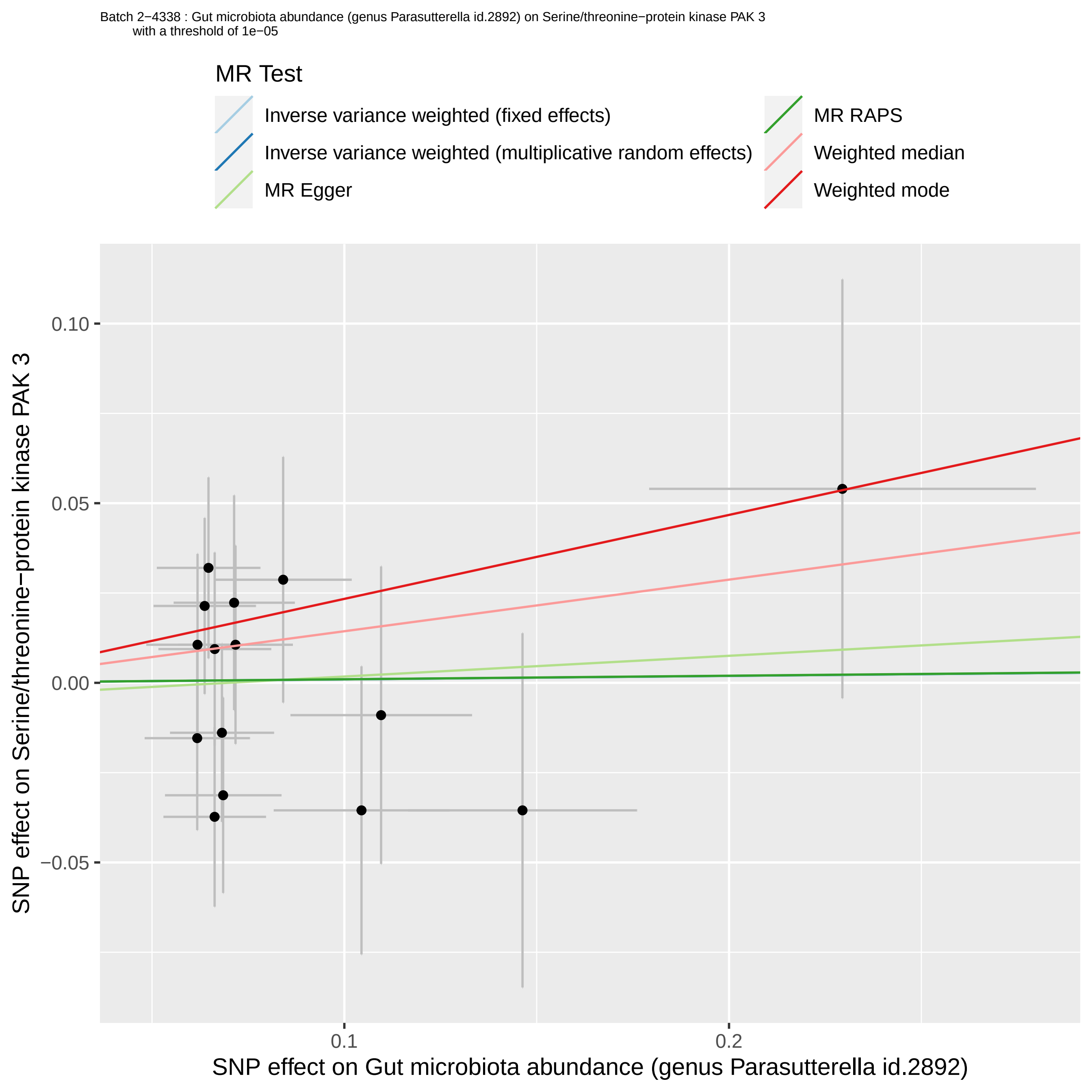
Batch 2−4338 : Gut microbiota abundance (genus Parasutterella id.2892) on Serine/threonine−protein kinase PAK 3
with a threshold of 1e−05
MR Test
Inverse variance weighted (fixed effects)
Inverse variance weighted (multiplicative random effects)
MR Egger
MR RAPS
Weighted median
Weighted mode
0.10
0.05
0.00
−0.05
0.1
0.2
SNP effect on Serine/threonine−protein kinase PAK 3
SNP effect on Gut microbiota abundance (genus Parasutterella id.2892)

Batch 2−4339 : Gut microbiota abundance (genus Catenibacterium id.2153) on Serine/threonine−protein kinase PAK 4
with a threshold of 1e−05

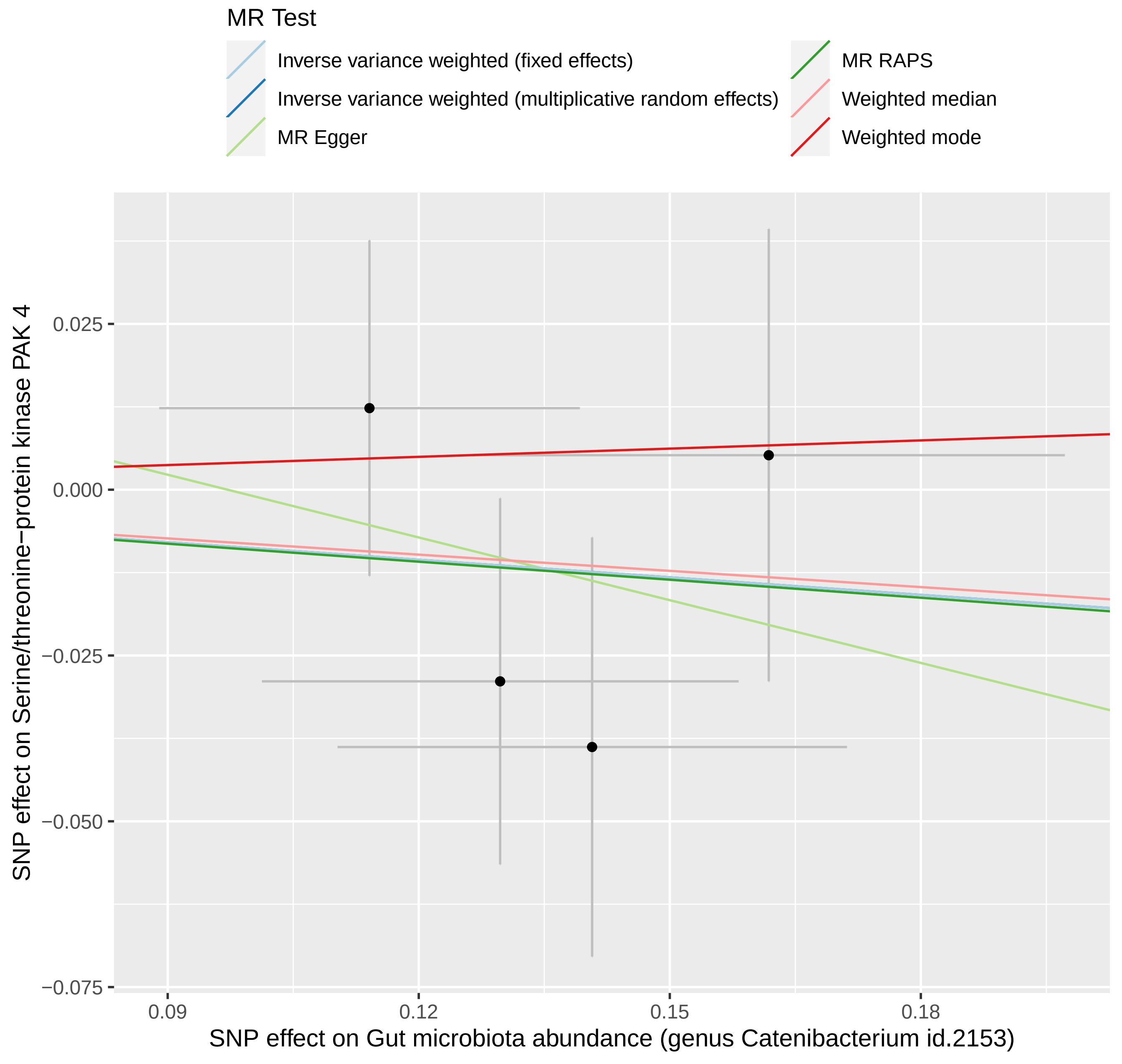

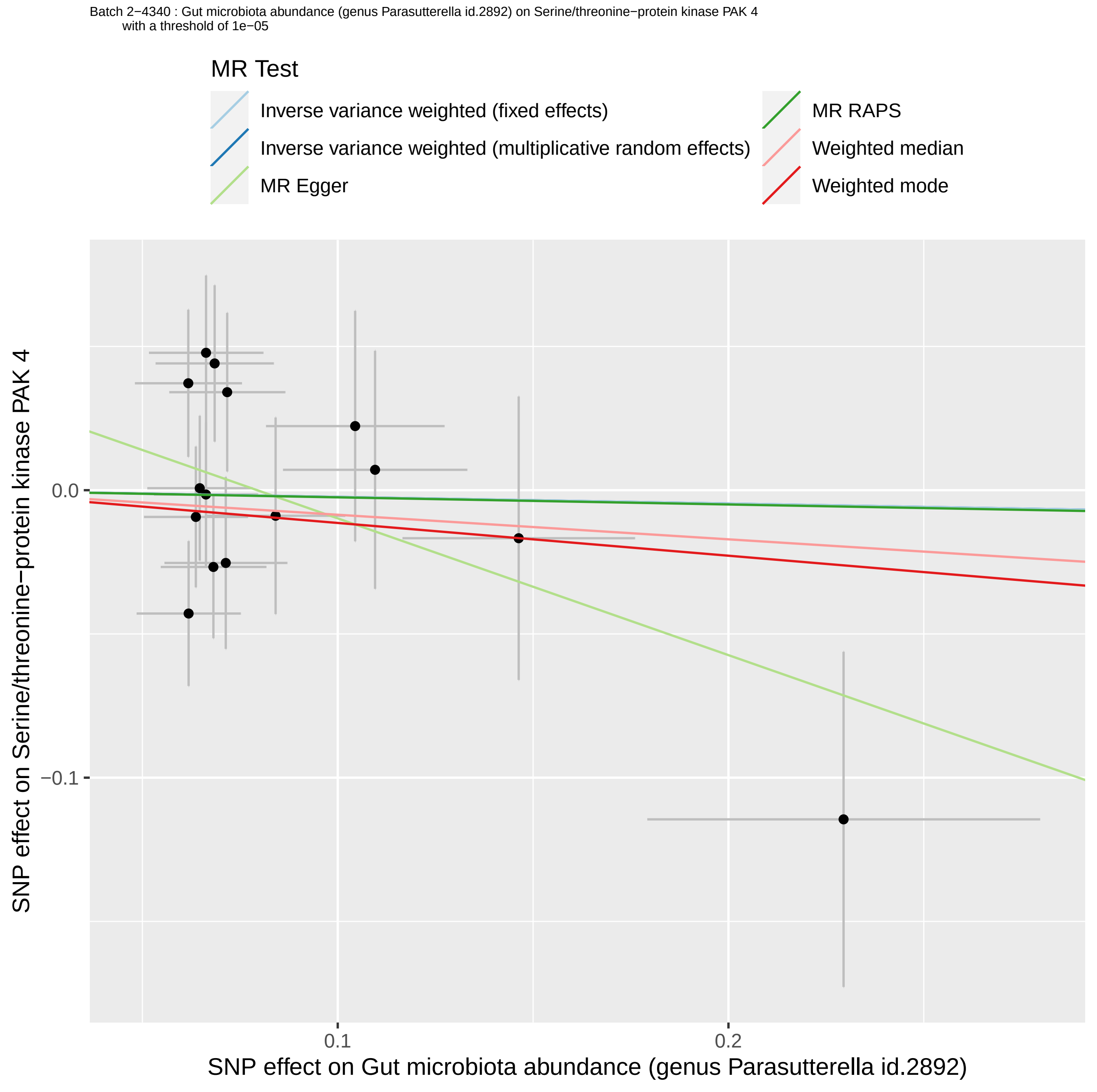

Batch 2−4340 : Gut microbiota abundance (genus Parasutterella id.2892) on Serine/threonine−protein kinase PAK 4
with a threshold of 1e−05
MR Test
Inverse variance weighted (fixed effects)
Inverse variance weighted (multiplicative random effects)
MR Egger
MR RAPS
Weighted median
Weighted mode
0.0
−0.1
0.1
0.2
SNP effect on Serine/threonine−protein kinase PAK 4
SNP effect on Gut microbiota abundance (genus Parasutterella id.2892)

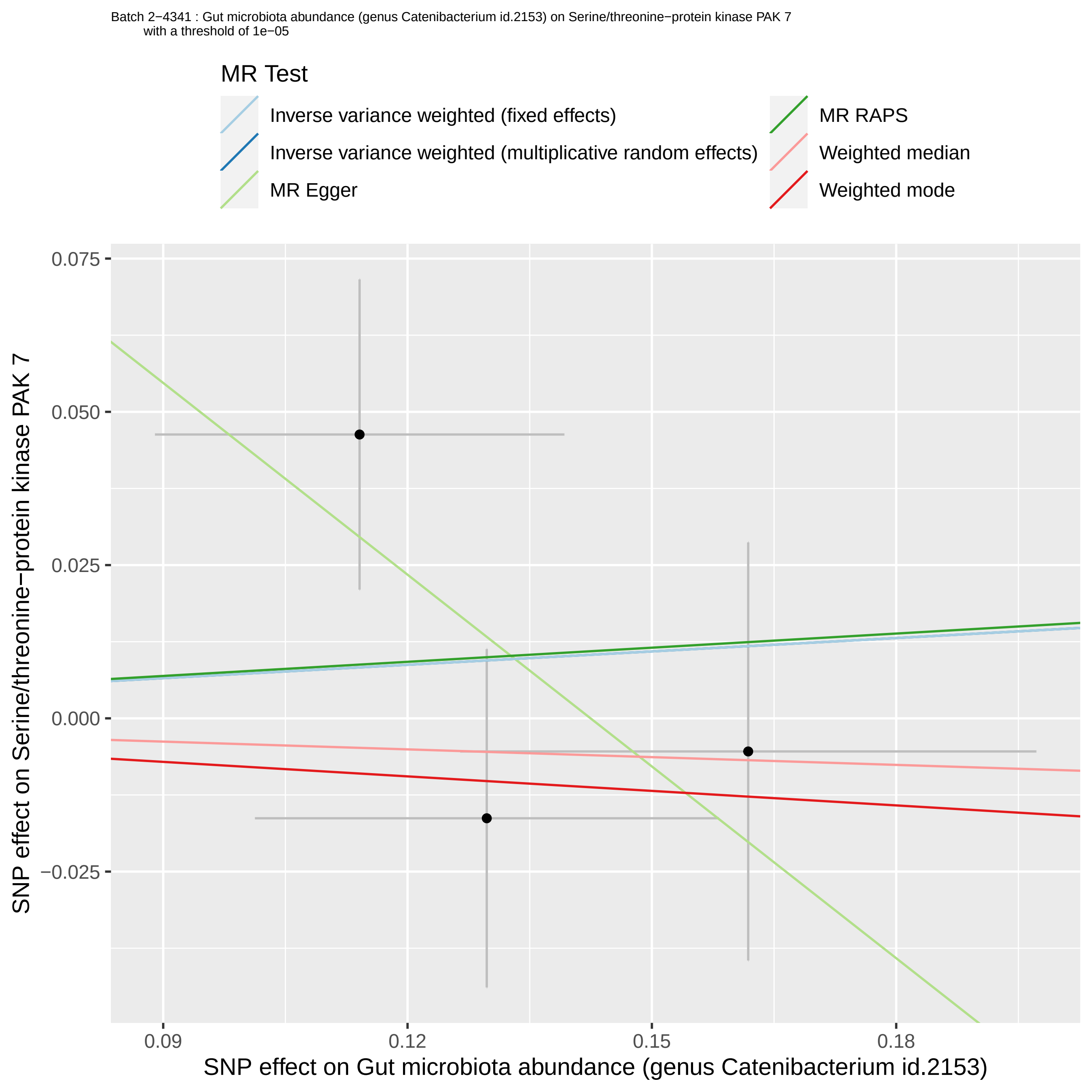

Batch 2−4341 : Gut microbiota abundance (genus Catenibacterium id.2153) on Serine/threonine−protein kinase PAK 7
with a threshold of 1e−05
MR Test
Inverse variance weighted (fixed effects)
Inverse variance weighted (multiplicative random effects)
MR Egger
MR RAPS
Weighted median
Weighted mode
0.075
0.050
0.025
0.000
−0.025
0.09
0.12
0.15
0.18
SNP effect on Serine/threonine−protein kinase PAK 7
SNP effect on Gut microbiota abundance (genus Catenibacterium id.2153)

Batch 2−4342 : Gut microbiota abundance (genus Parasutterella id.2892) on Serine/threonine−protein kinase PAK 7
with a threshold of 1e−05

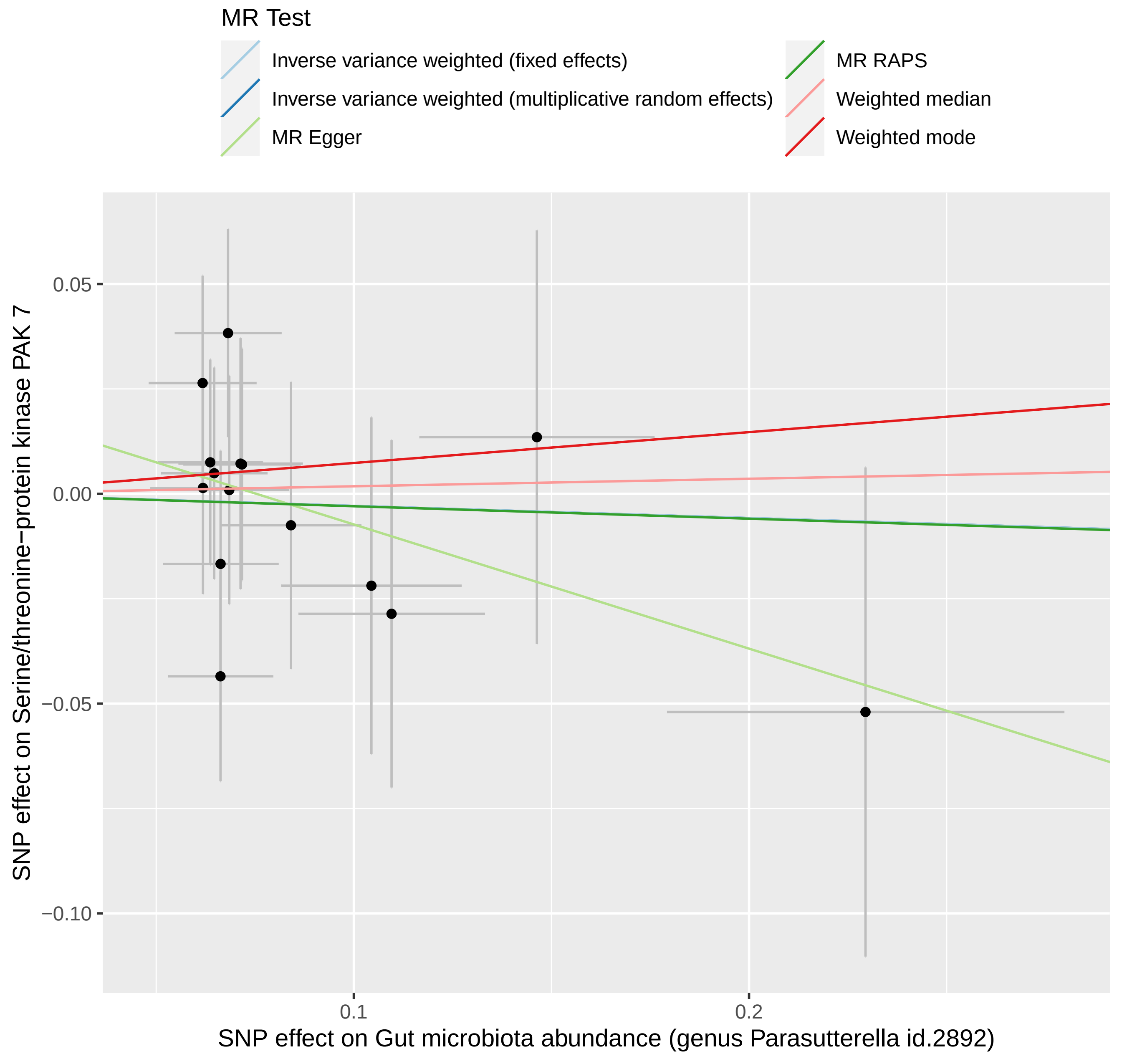

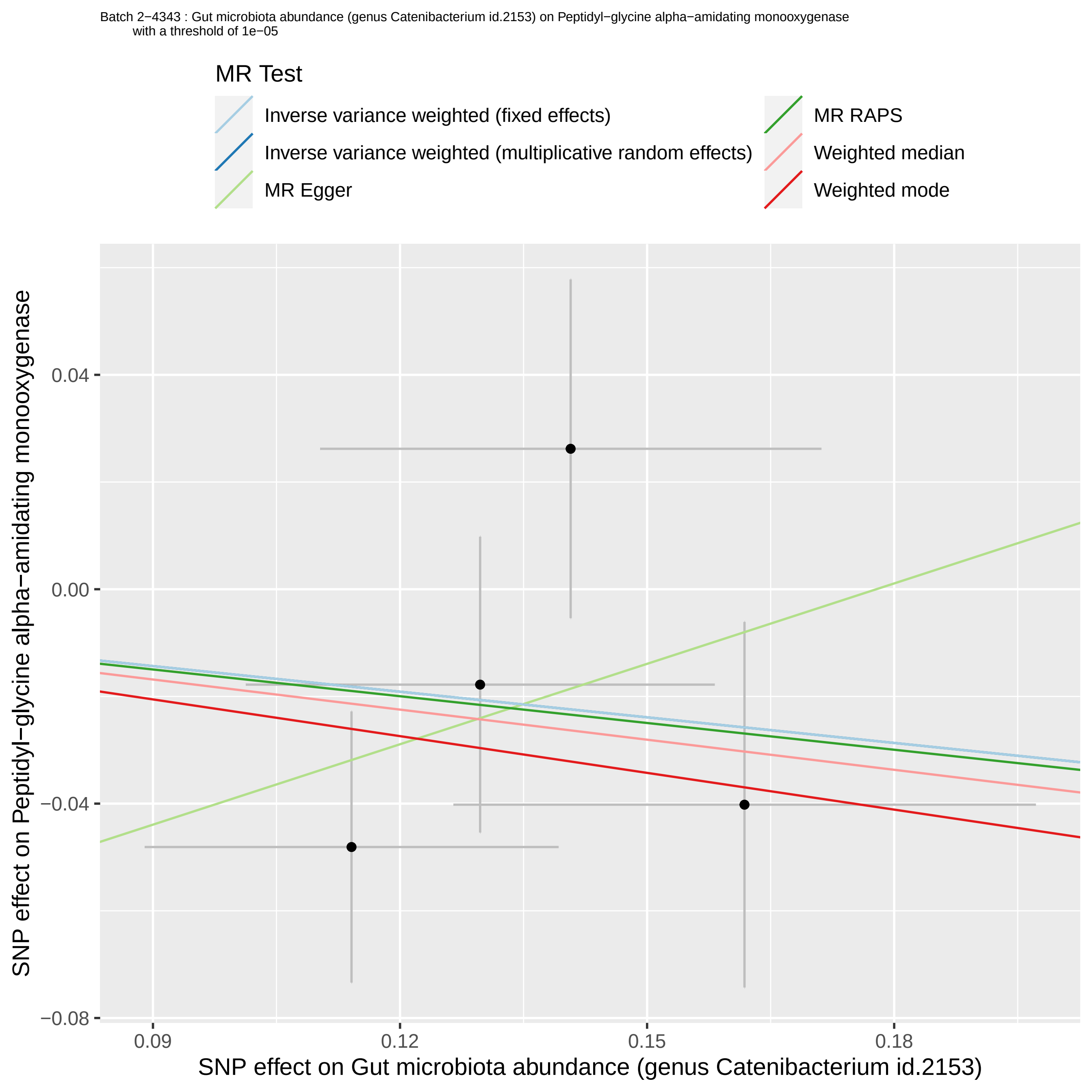
Batch 2−4343 : Gut microbiota abundance (genus Catenibacterium id.2153) on Peptidyl−glycine alpha−amidating monooxygenase
with a threshold of 1e−05
MR Test
Inverse variance weighted (fixed effects)
Inverse variance weighted (multiplicative random effects)
MR Egger
MR RAPS
Weighted median
Weighted mode
0.04
0.00
−0.04
−0.08
0.09
0.12
0.15
0.18
SNP effect on Peptidyl−glycine alpha−amidating monooxygenase
SNP effect on Gut microbiota abundance (genus Catenibacterium id.2153)

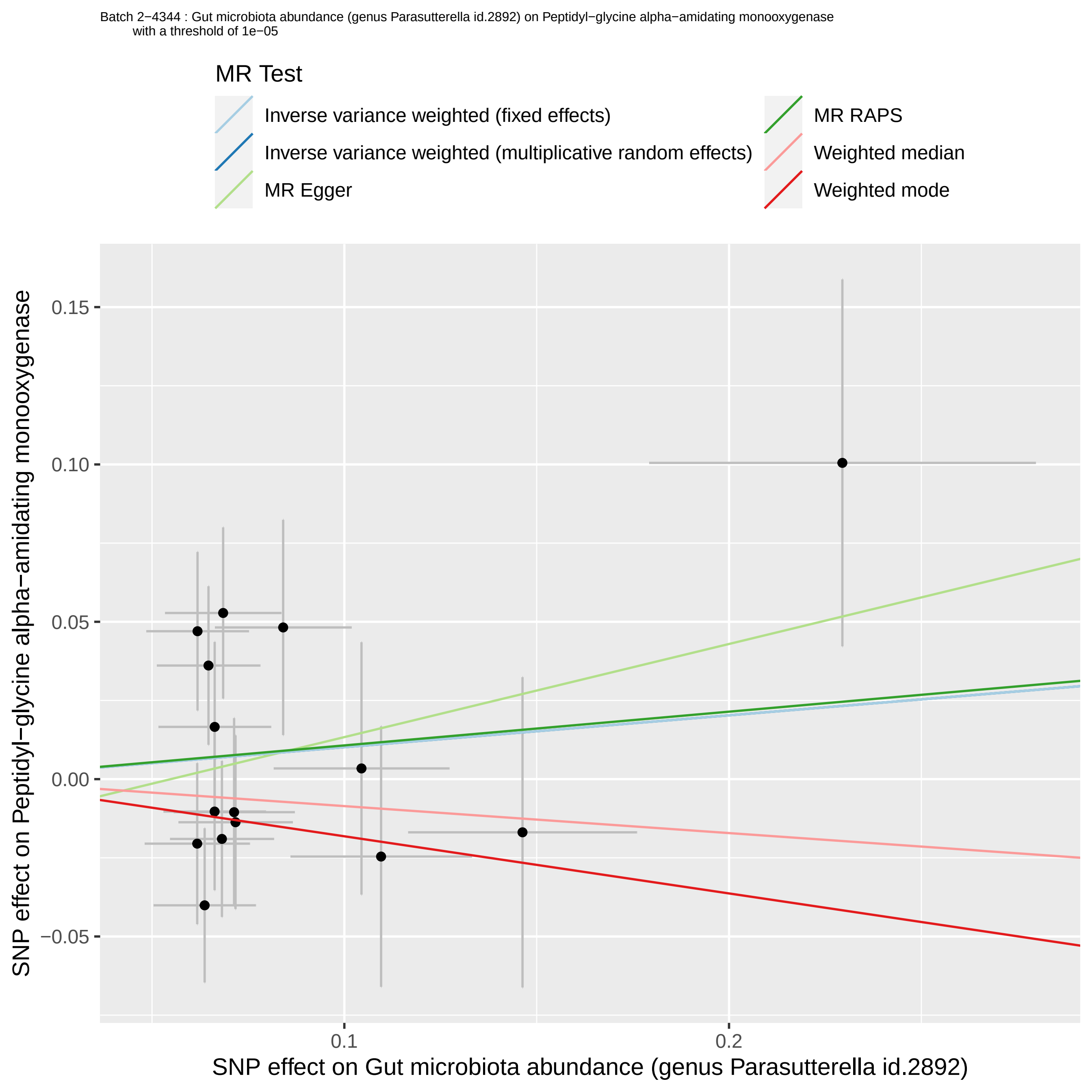

Batch 2−4344 : Gut microbiota abundance (genus Parasutterella id.2892) on Peptidyl−glycine alpha−amidating monooxygenase
with a threshold of 1e−05
MR Test
Inverse variance weighted (fixed effects)
Inverse variance weighted (multiplicative random effects)
MR Egger
MR RAPS
Weighted median
Weighted mode
0.15
0.10
0.05
0.00
−0.05
0.1
0.2
SNP effect on Peptidyl−glycine alpha−amidating monooxygenase
SNP effect on Gut microbiota abundance (genus Parasutterella id.2892)

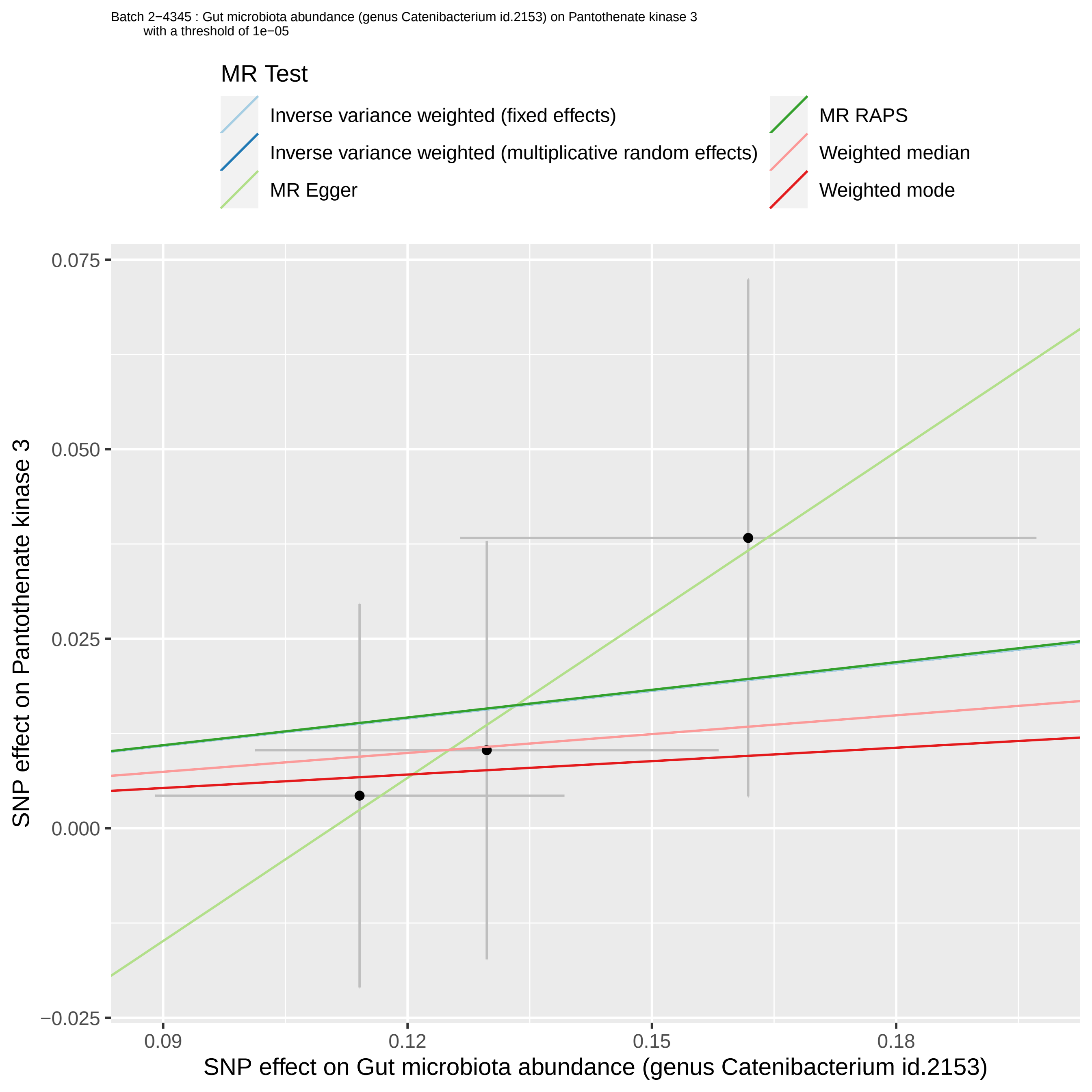
Batch 2−4345 : Gut microbiota abundance (genus Catenibacterium id.2153) on Pantothenate kinase 3
with a threshold of 1e−05
MR Test
Inverse variance weighted (fixed effects)
Inverse variance weighted (multiplicative random effects)
MR Egger
MR RAPS
Weighted median
Weighted mode
0.075
0.050
0.025
0.000
−0.025
0.09
0.12
0.15
0.18
SNP effect on Pantothenate kinase 3
SNP effect on Gut microbiota abundance (genus Catenibacterium id.2153)

Batch 2−4346 : Gut microbiota abundance (genus Parasutterella id.2892) on Pantothenate kinase 3
with a threshold of 1e−05

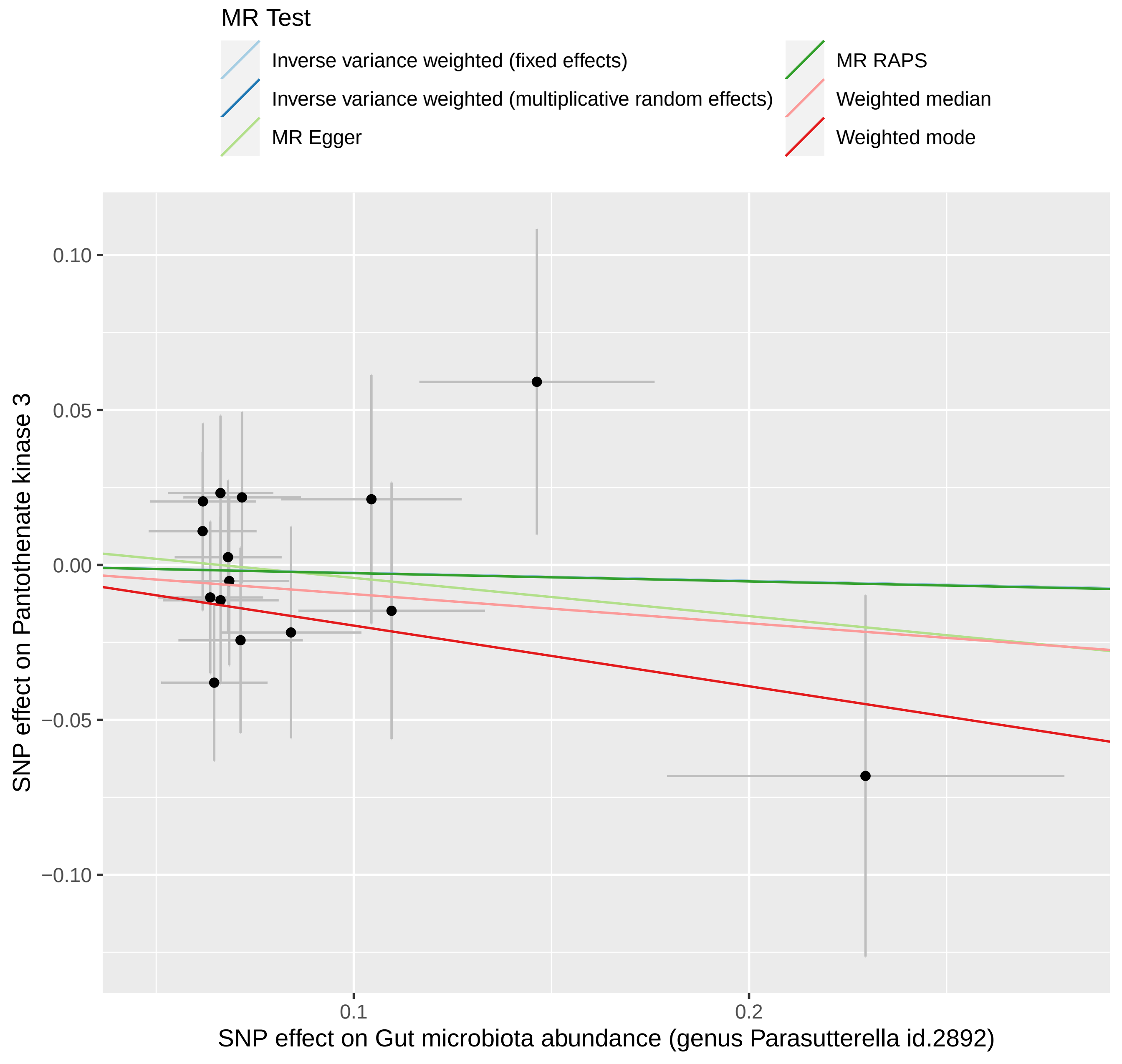

Batch 2−4347 : Gut microbiota abundance (genus Catenibacterium id.2153) on Papilin
with a threshold of 1e−05

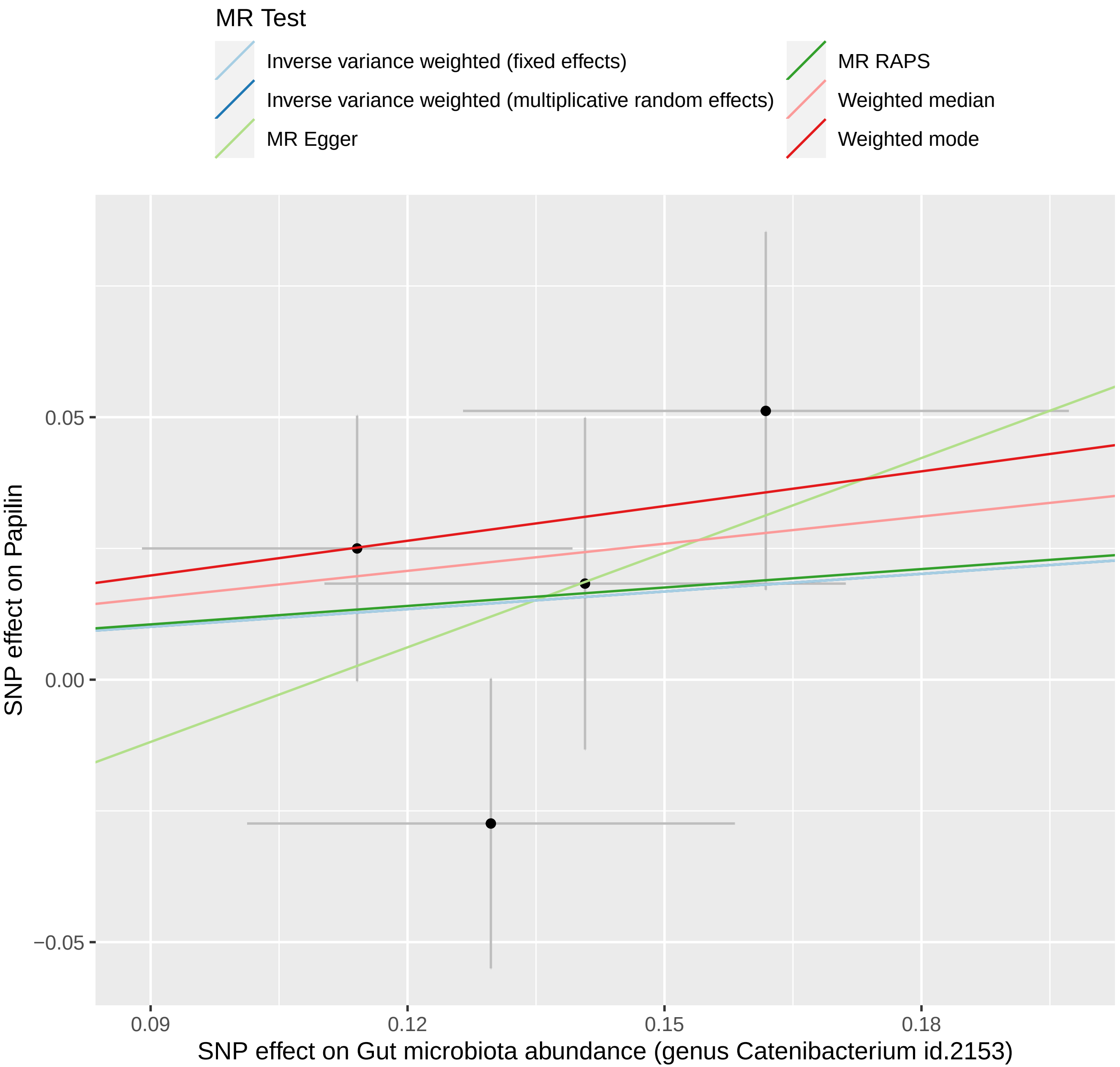

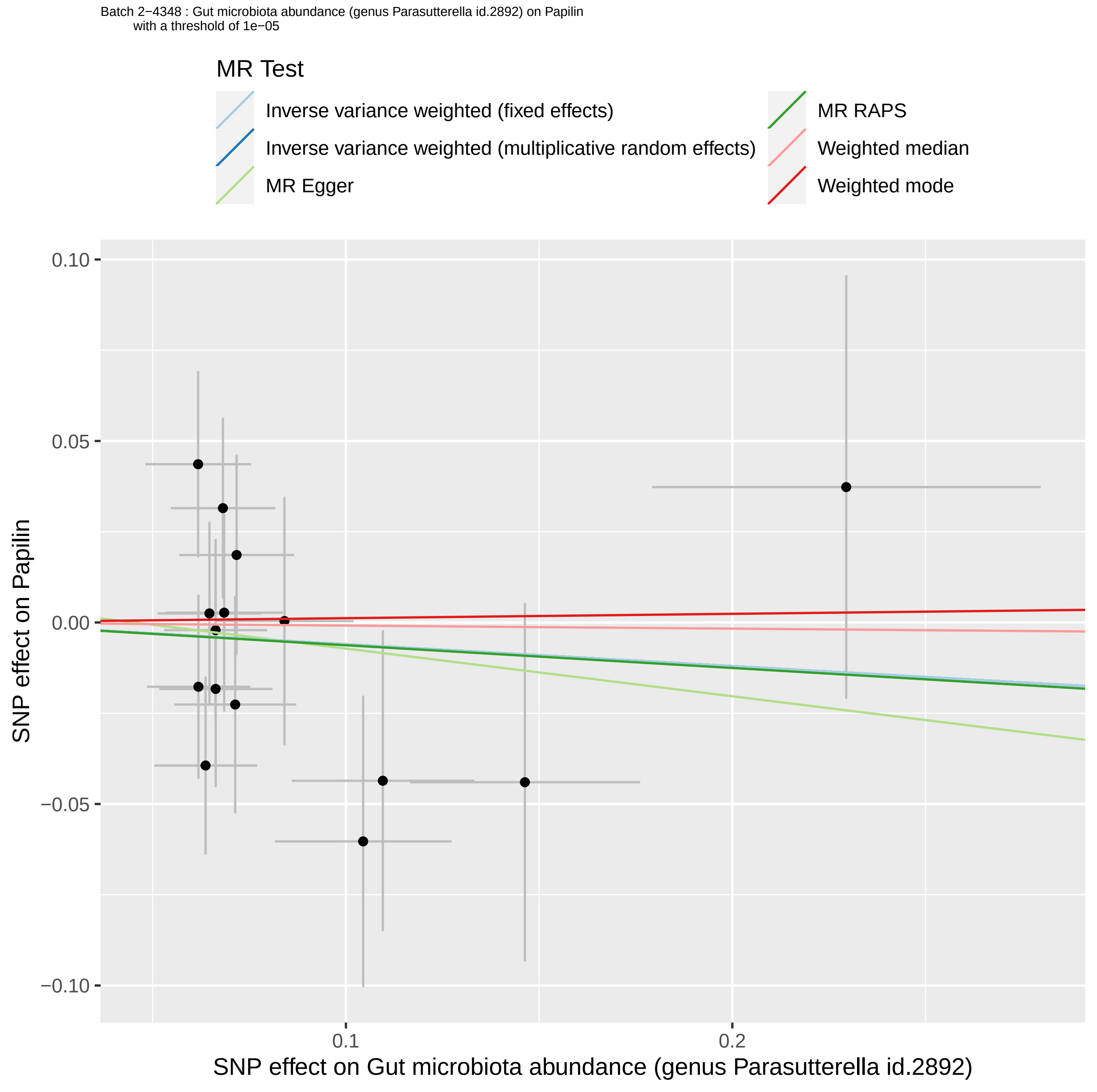
Batch 2−4348 : Gut microbiota abundance (genus Parasutterella id.2892) on Papilin
with a threshold of 1e−05
MR Test
Inverse variance weighted (fixed effects)
Inverse variance weighted (multiplicative random effects)
MR Egger
MR RAPS
Weighted median
Weighted mode
0.10
0.05
0.00
−0.05
−0.10
0.1
0.2
SNP effect on Papilin
SNP effect on Gut microbiota abundance (genus Parasutterella id.2892)

Batch 2−4349 : Gut microbiota abundance (genus Catenibacterium id.2153) on Poly(A) polymerase gamma
with a threshold of 1e−05

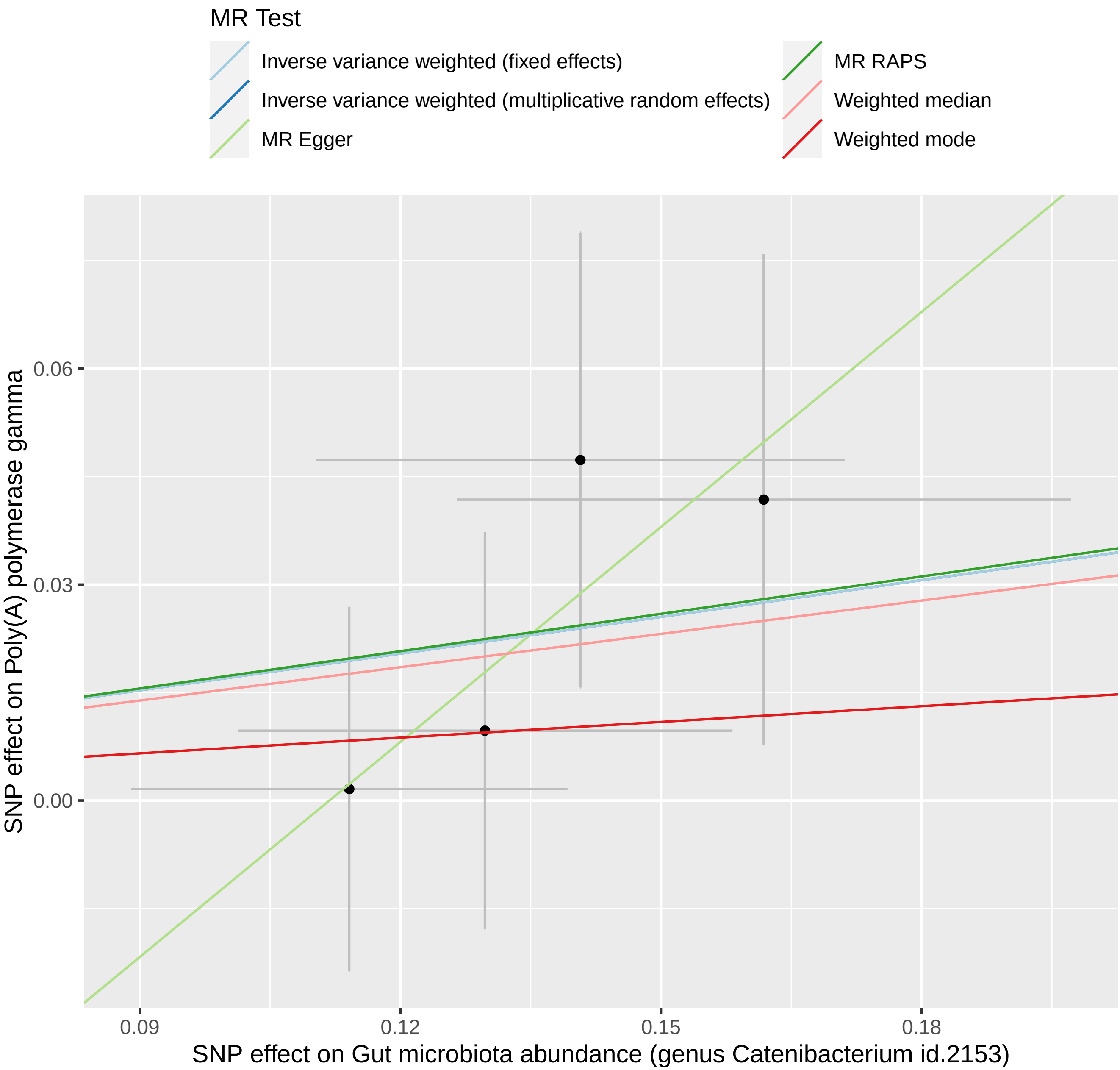

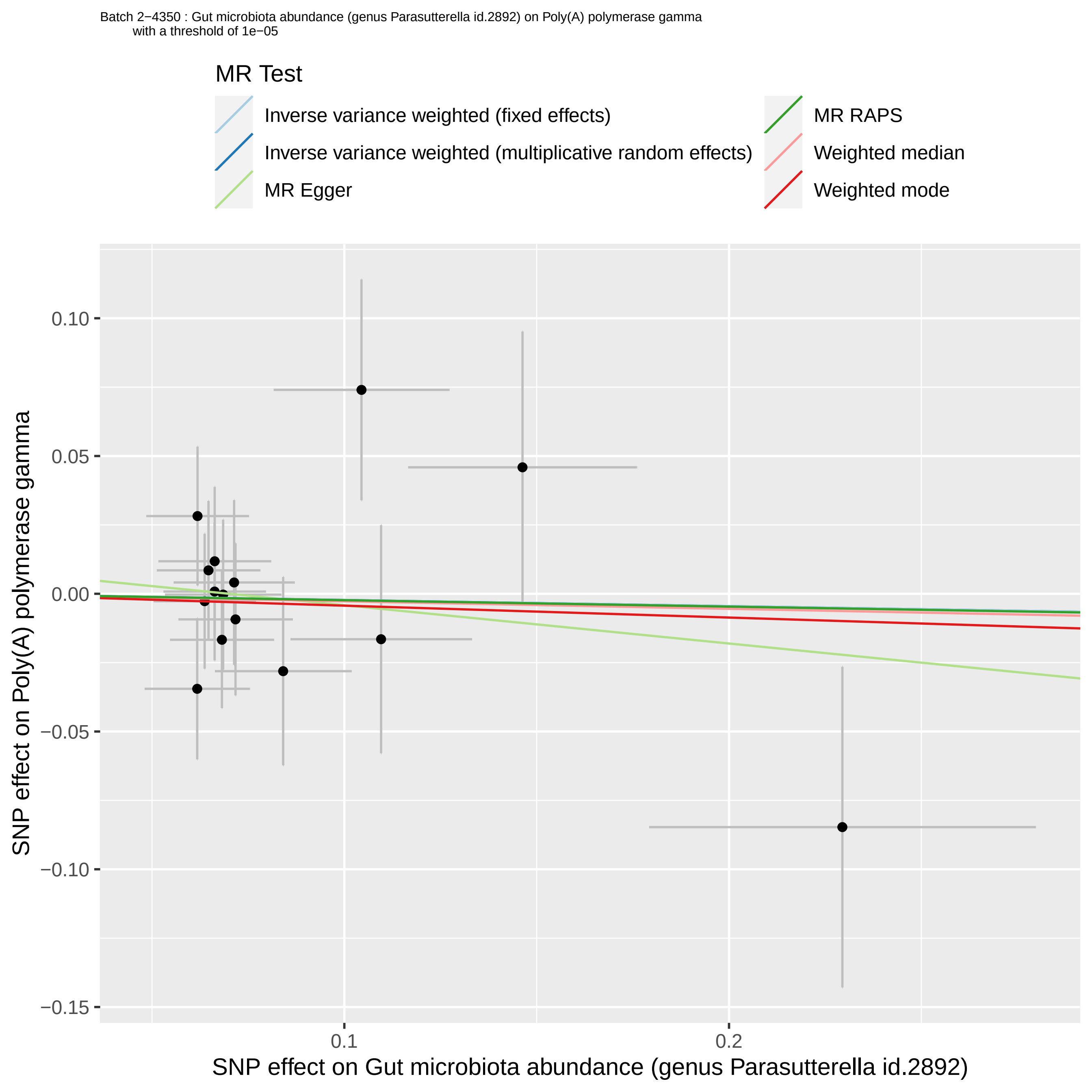
Batch 2−4350 : Gut microbiota abundance (genus Parasutterella id.2892) on Poly(A) polymerase gamma
with a threshold of 1e−05
MR Test
Inverse variance weighted (fixed effects)
Inverse variance weighted (multiplicative random effects)
MR Egger
MR RAPS
Weighted median
Weighted mode
0.10
0.05
0.00
−0.05
−0.10
−0.15
0.1
0.2
SNP effect on Poly(A) polymerase gamma
SNP effect on Gut microbiota abundance (genus Parasutterella id.2892)

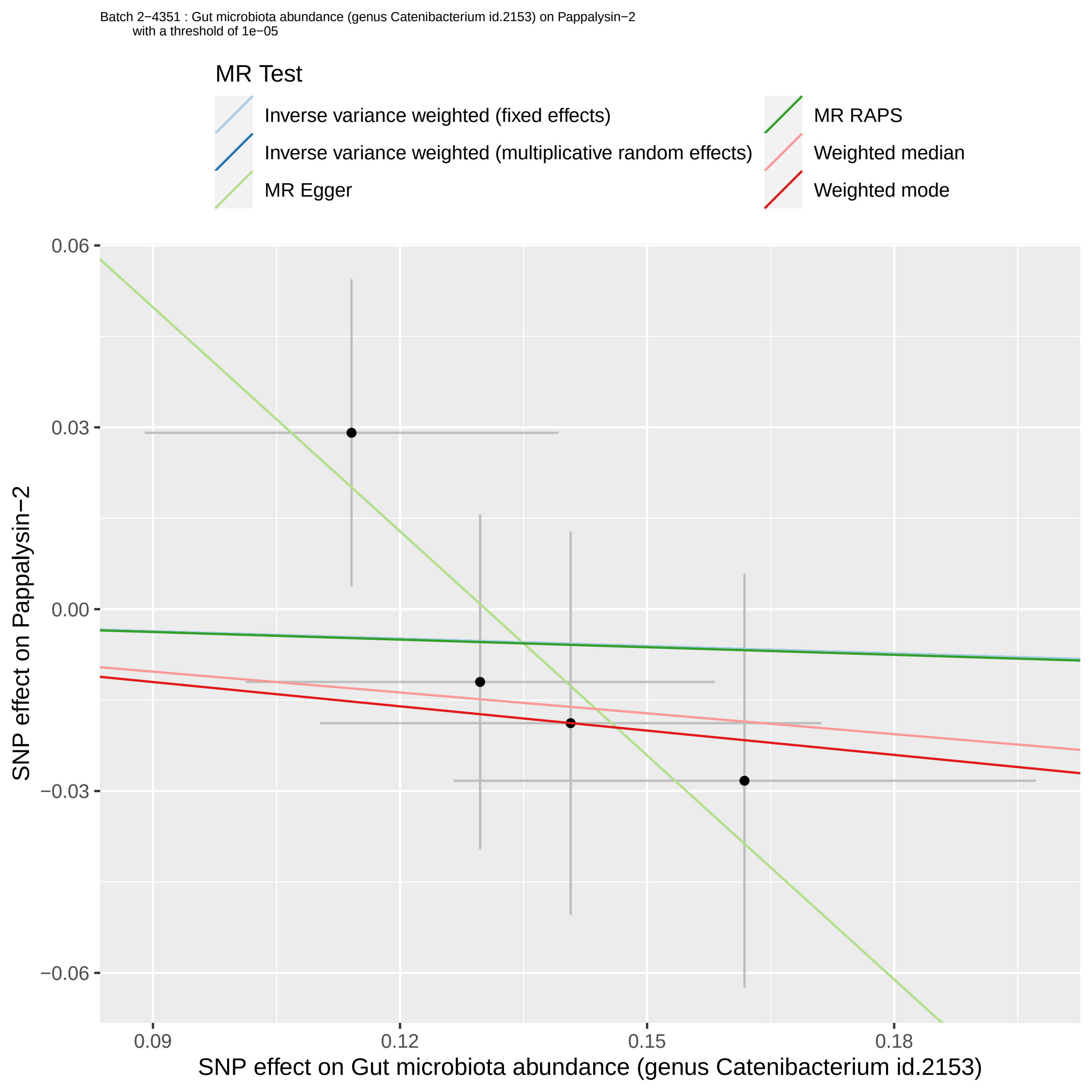
Batch 2−4351 : Gut microbiota abundance (genus Catenibacterium id.2153) on Pappalysin−2
with a threshold of 1e−05
MR Test
Inverse variance weighted (fixed effects)
Inverse variance weighted (multiplicative random effects)
MR Egger
MR RAPS
Weighted median
Weighted mode
0.06
0.03
0.00
−0.03
−0.06
0.09
0.12
0.15
0.18
SNP effect on Pappalysin−2
SNP effect on Gut microbiota abundance (genus Catenibacterium id.2153)

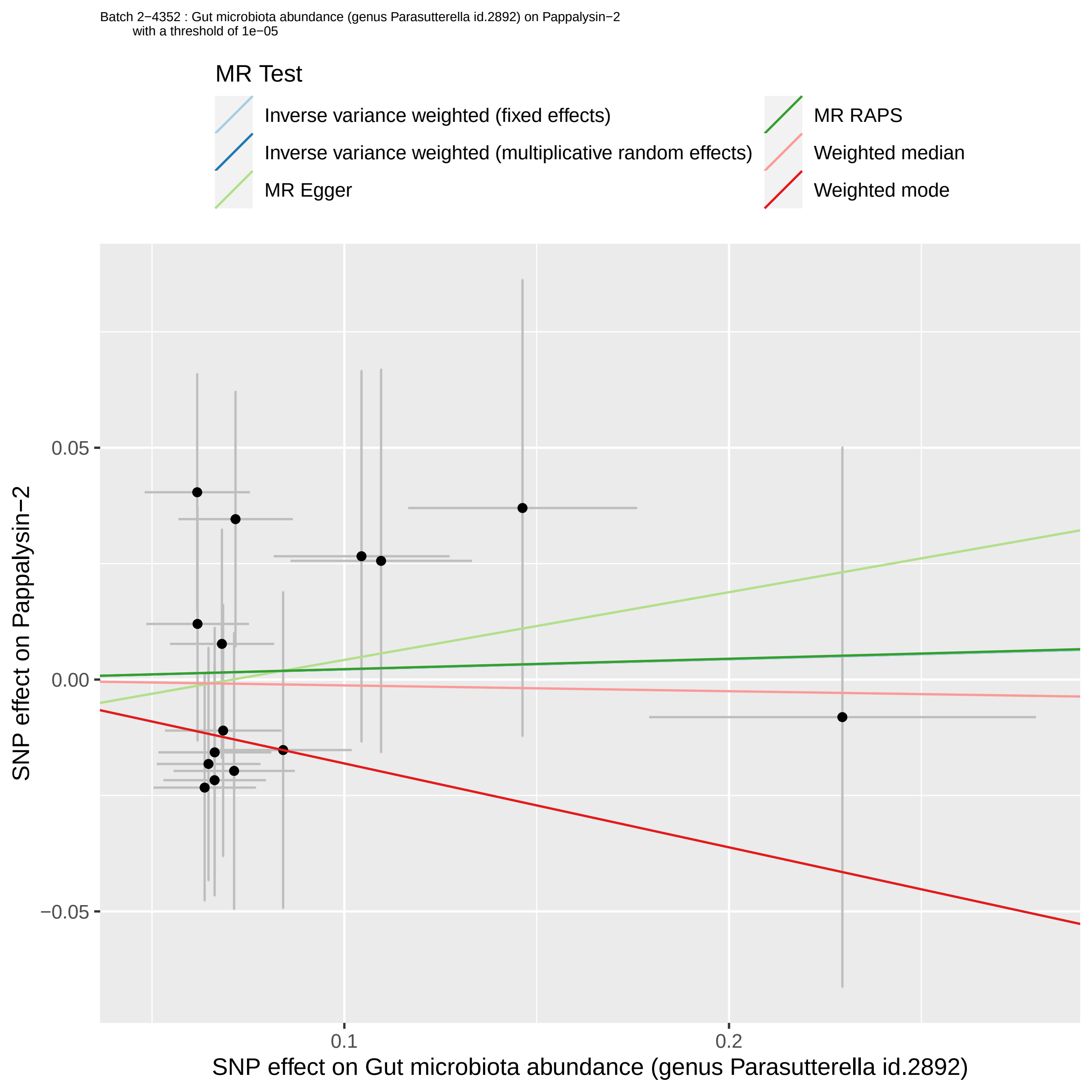
Batch 2−4352 : Gut microbiota abundance (genus Parasutterella id.2892) on Pappalysin−2
with a threshold of 1e−05
MR Test
Inverse variance weighted (fixed effects)
Inverse variance weighted (multiplicative random effects)
MR Egger
MR RAPS
Weighted median
Weighted mode
0.05
0.00
−0.05
0.1
0.2
SNP effect on Pappalysin−2
SNP effect on Gut microbiota abundance (genus Parasutterella id.2892)

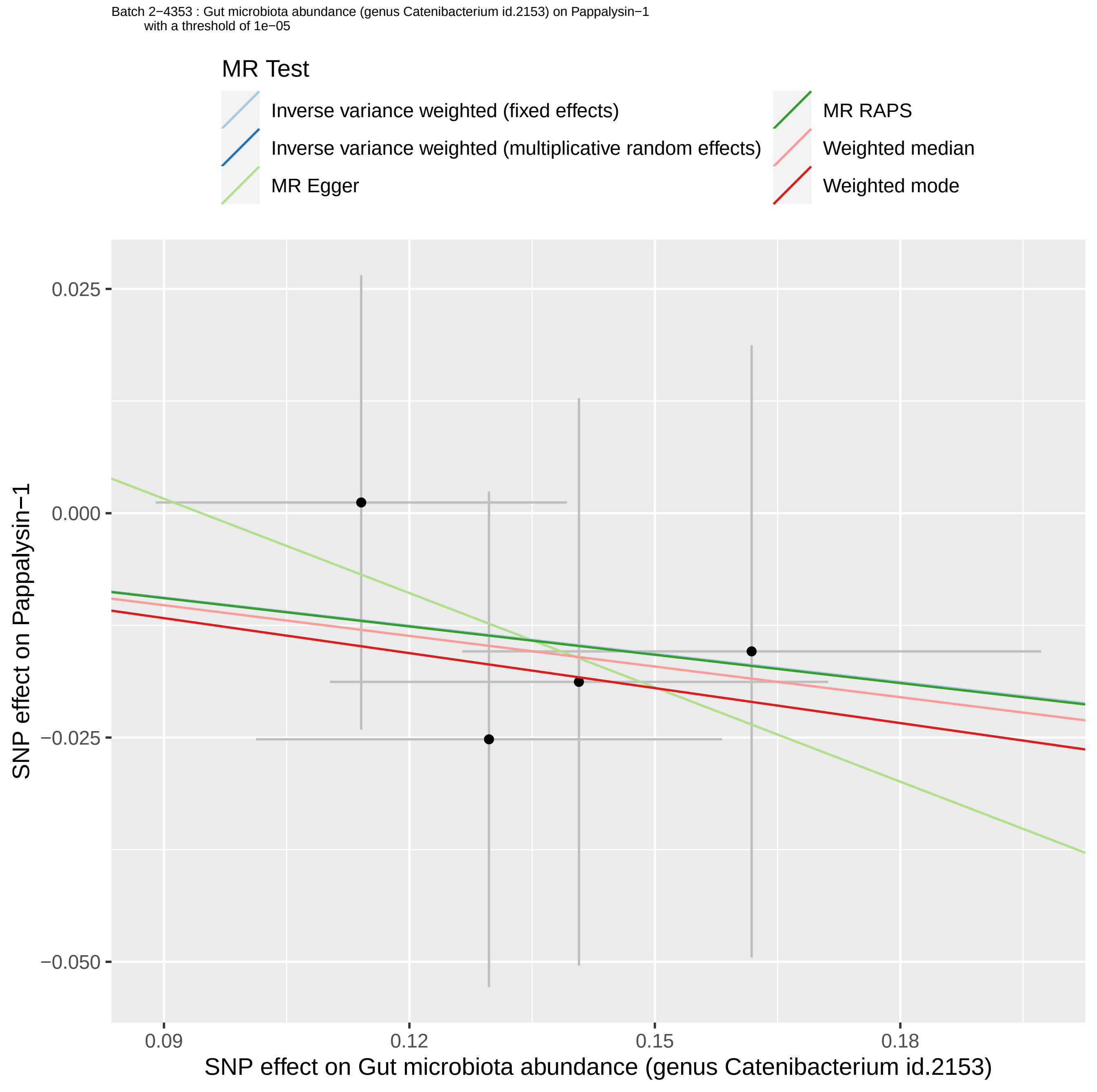
Batch 2−4353 : Gut microbiota abundance (genus Catenibacterium id.2153) on Pappalysin−1
with a threshold of 1e−05
MR Test
Inverse variance weighted (fixed effects)
Inverse variance weighted (multiplicative random effects)
MR Egger
MR RAPS
Weighted median
Weighted mode
SNP effect on Pappalysin−1
0.025
0.000
−0.025
−0.050
0.09
0.12
0.15
0.18
SNP effect on Gut microbiota abundance (genus Catenibacterium id.2153)

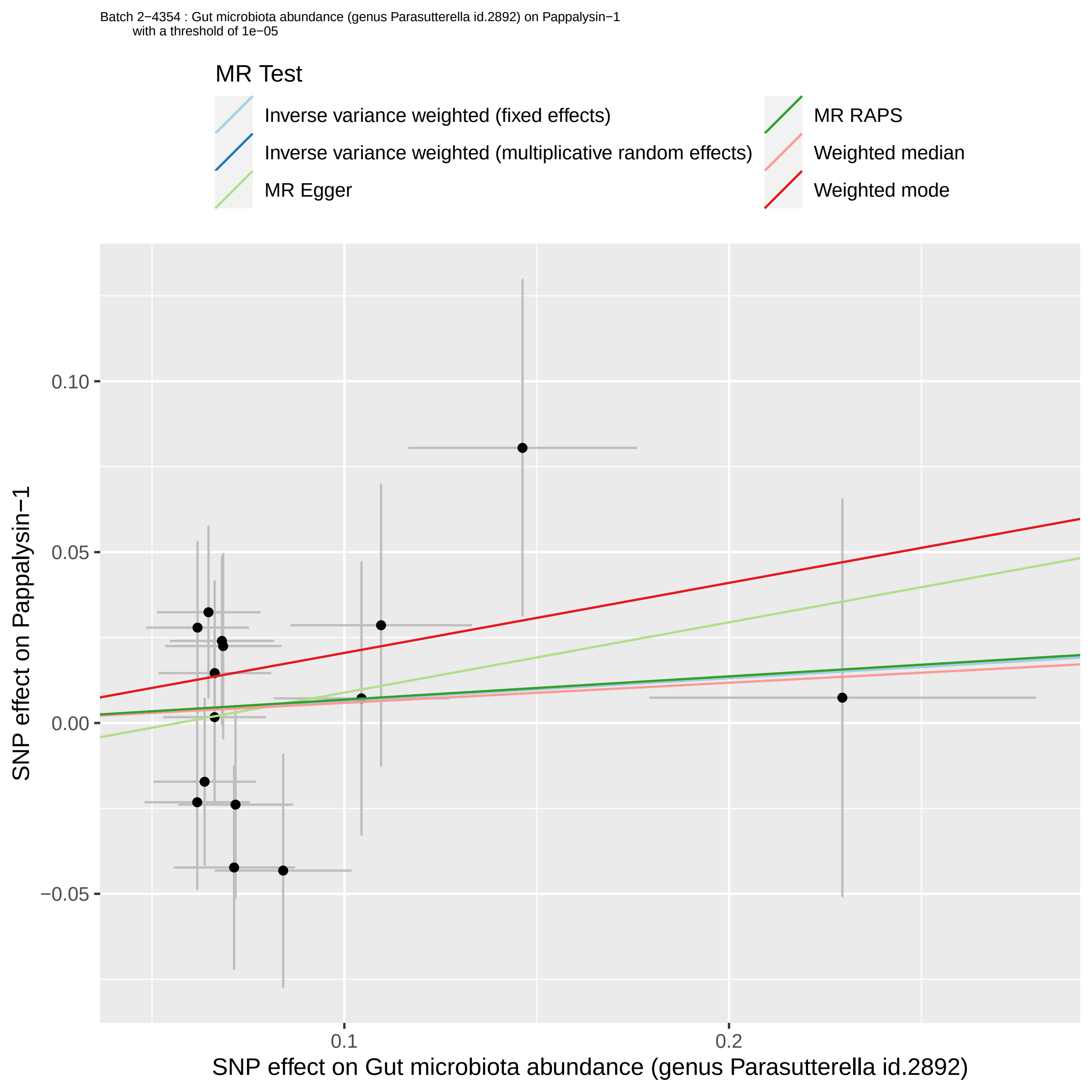
Batch 2−4354 : Gut microbiota abundance (genus Parasutterella id.2892) on Pappalysin−1
with a threshold of 1e−05
MR Test
Inverse variance weighted (fixed effects)
Inverse variance weighted (multiplicative random effects)
MR Egger
MR RAPS
Weighted median
Weighted mode
0.10
0.05
0.00
−0.05
0.1
0.2
SNP effect on Pappalysin−1
SNP effect on Gut microbiota abundance (genus Parasutterella id.2892)

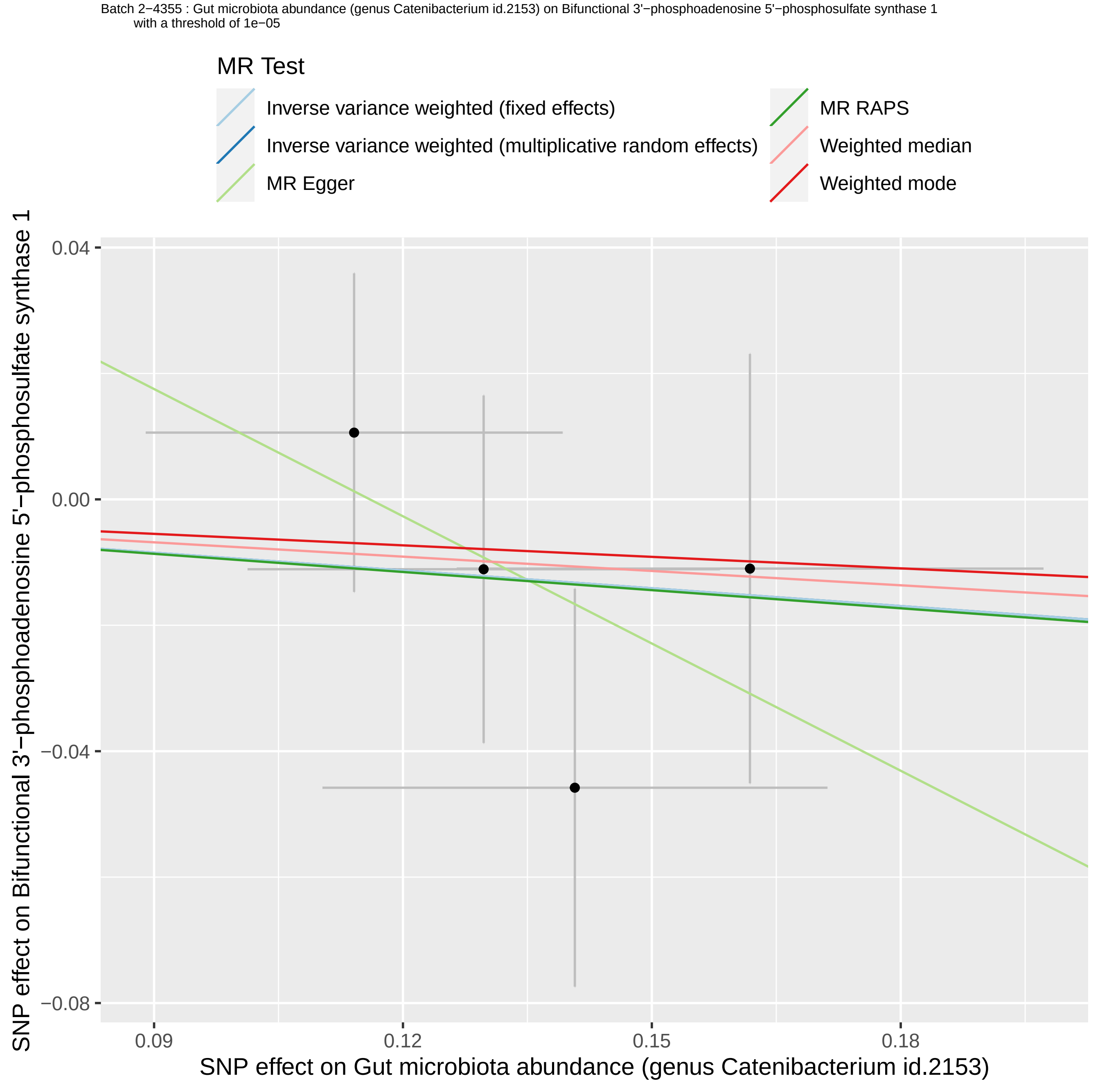

Batch 2−4355 : Gut microbiota abundance (genus Catenibacterium id.2153) on Bifunctional 3'−phosphoadenosine 5'−phosphosulfate synthase 1
with a threshold of 1e−05
MR Test
Inverse variance weighted (fixed effects)
Inverse variance weighted (multiplicative random effects)
MR Egger
MR RAPS
Weighted median
Weighted mode
0.04
0.00
−0.04
−0.08
0.09
0.12
0.15
0.18
SNP effect on Bifunctional 3'−phosphoadenosine 5'−phosphosulfate synthase 1
SNP effect on Gut microbiota abundance (genus Catenibacterium id.2153)

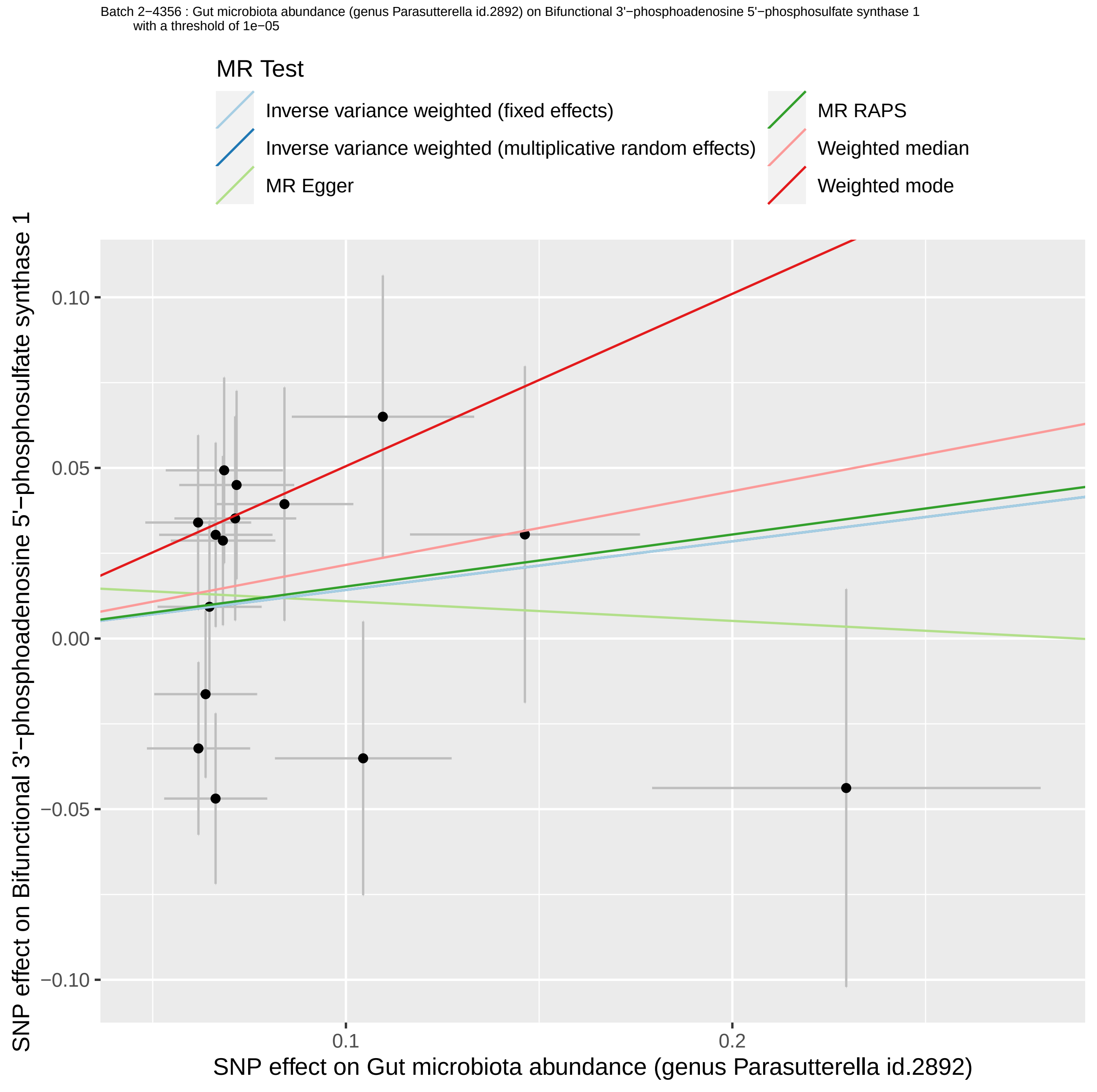

Batch 2−4356 : Gut microbiota abundance (genus Parasutterella id.2892) on Bifunctional 3'−phosphoadenosine 5'−phosphosulfate synthase 1
with a threshold of 1e−05
MR Test
Inverse variance weighted (fixed effects)
Inverse variance weighted (multiplicative random effects)
MR Egger
MR RAPS
Weighted median
Weighted mode
0.10
0.05
0.00
−0.05
−0.10
0.1
0.2
SNP effect on Bifunctional 3'−phosphoadenosine 5'−phosphosulfate synthase 1
SNP effect on Gut microbiota abundance (genus Parasutterella id.2892)

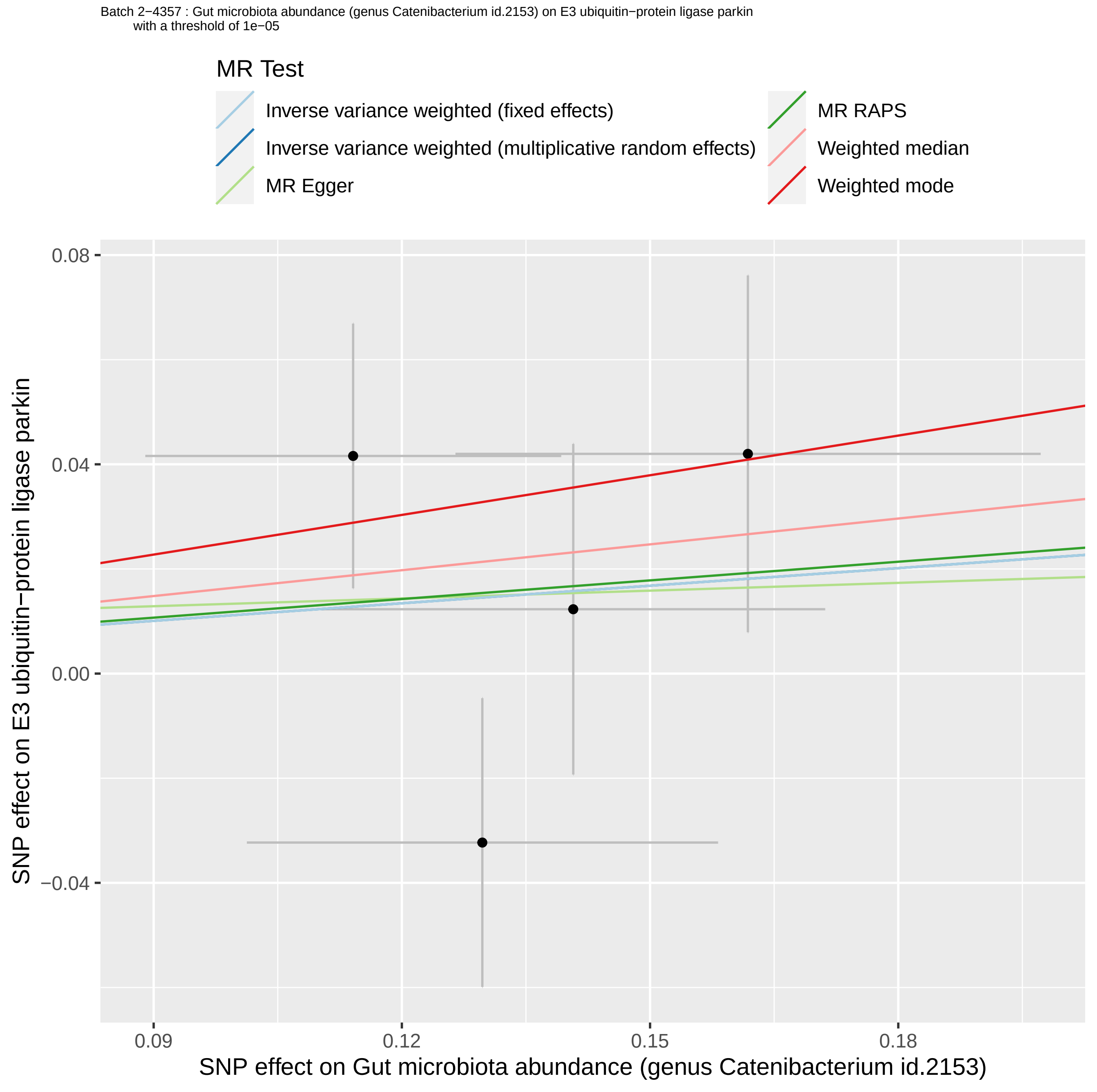
Batch 2−4357 : Gut microbiota abundance (genus Catenibacterium id.2153) on E3 ubiquitin−protein ligase parkin
with a threshold of 1e−05
MR Test
Inverse variance weighted (fixed effects)
Inverse variance weighted (multiplicative random effects)
MR Egger
MR RAPS
Weighted median
Weighted mode
0.08
0.04
0.00
−0.04
0.09
0.12
0.15
0.18
SNP effect on E3 ubiquitin−protein ligase parkin
SNP effect on Gut microbiota abundance (genus Catenibacterium id.2153)

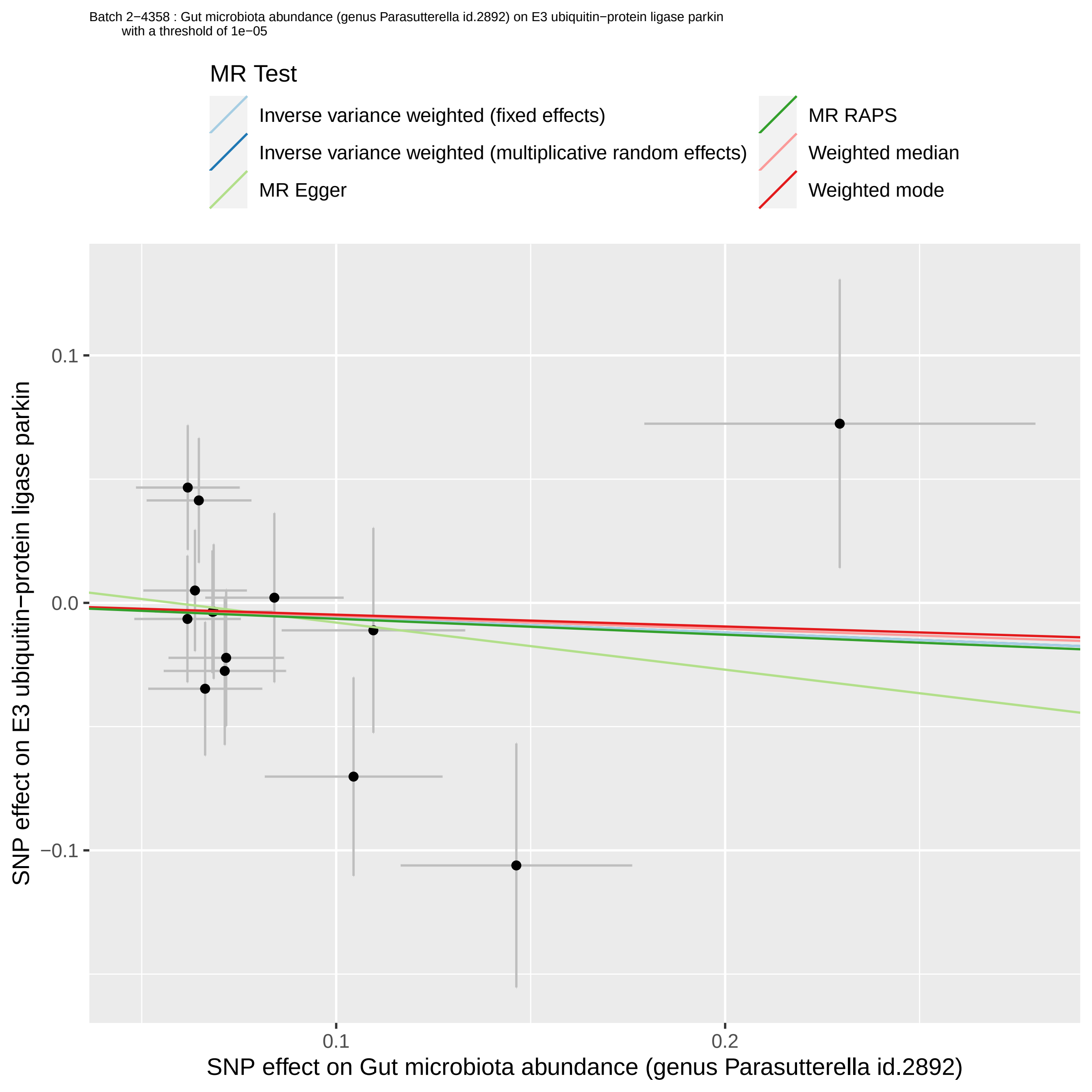

Batch 2−4358 : Gut microbiota abundance (genus Parasutterella id.2892) on E3 ubiquitin−protein ligase parkin
with a threshold of 1e−05
MR Test
Inverse variance weighted (fixed effects)
Inverse variance weighted (multiplicative random effects)
MR Egger
MR RAPS
Weighted median
Weighted mode
0.1
0.0
−0.1
0.1
0.2
SNP effect on E3 ubiquitin−protein ligase parkin
SNP effect on Gut microbiota abundance (genus Parasutterella id.2892)

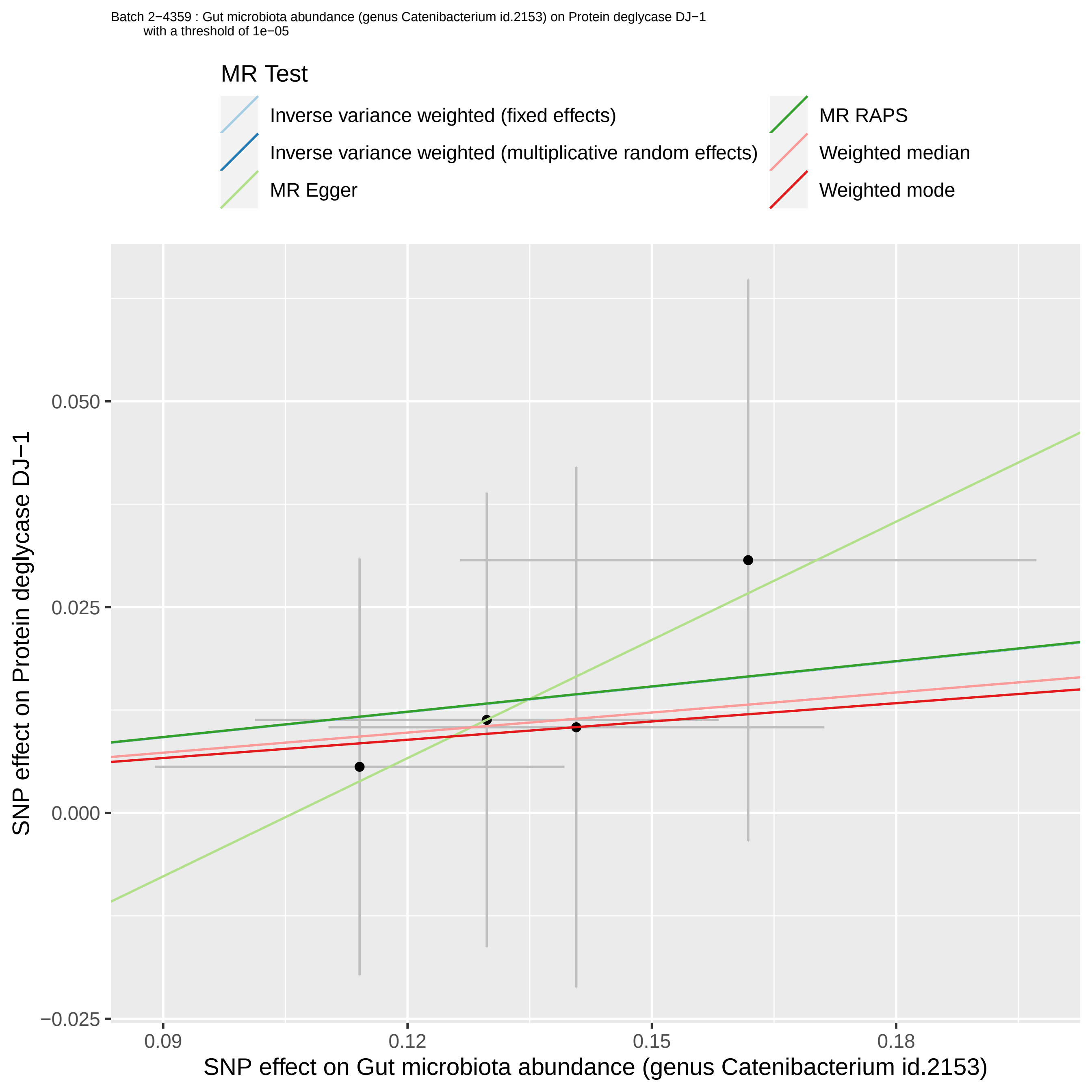
Batch 2−4359 : Gut microbiota abundance (genus Catenibacterium id.2153) on Protein deglycase DJ−1
with a threshold of 1e−05
MR Test
Inverse variance weighted (fixed effects)
Inverse variance weighted (multiplicative random effects)
MR Egger
MR RAPS
Weighted median
Weighted mode
SNP effect on Protein deglycase DJ−1
0.050
0.025
0.000
−0.025
0.09
0.12
0.15
0.18
SNP effect on Gut microbiota abundance (genus Catenibacterium id.2153)

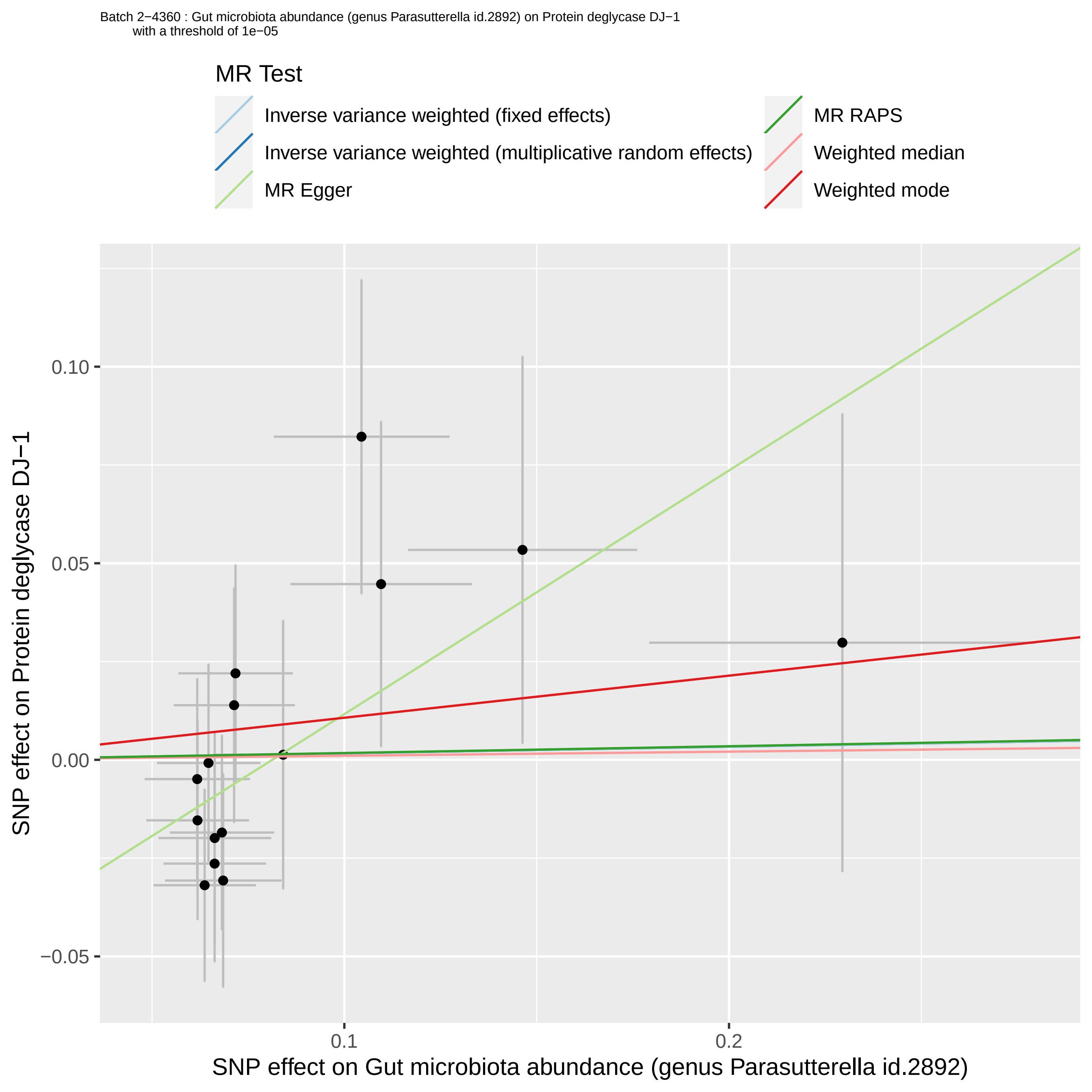

Batch 2−4360 : Gut microbiota abundance (genus Parasutterella id.2892) on Protein deglycase DJ−1
with a threshold of 1e−05
MR Test
Inverse variance weighted (fixed effects)
Inverse variance weighted (multiplicative random effects)
MR Egger
MR RAPS
Weighted median
Weighted mode
0.10
0.05
0.00
−0.05
0.1
0.2
SNP effect on Protein deglycase DJ−1
SNP effect on Gut microbiota abundance (genus Parasutterella id.2892)

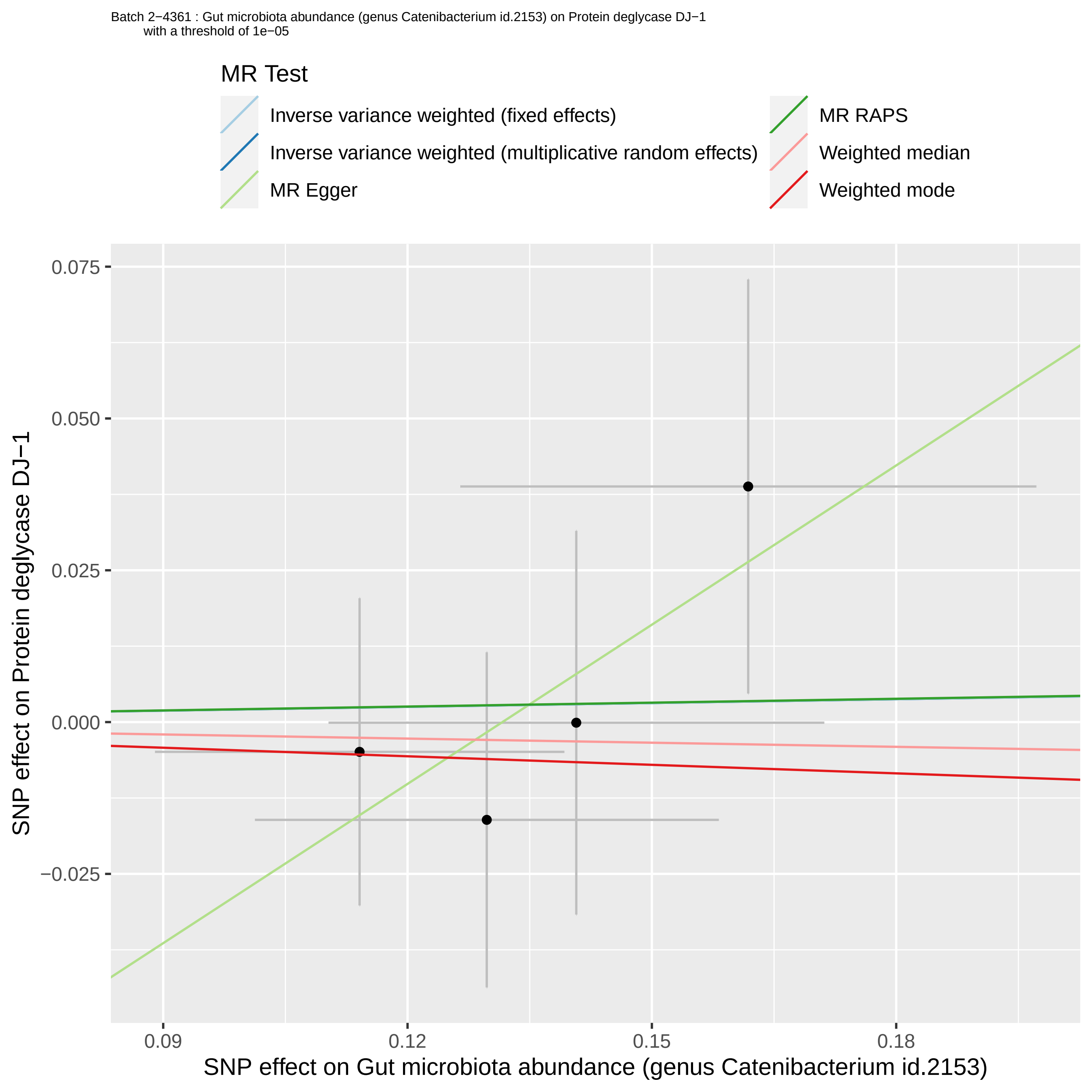

Batch 2−4361 : Gut microbiota abundance (genus Catenibacterium id.2153) on Protein deglycase DJ−1
with a threshold of 1e−05
MR Test
Inverse variance weighted (fixed effects)
Inverse variance weighted (multiplicative random effects)
MR Egger
MR RAPS
Weighted median
Weighted mode
0.075
0.050
0.025
0.000
−0.025
0.09
0.12
0.15
0.18
SNP effect on Protein deglycase DJ−1
SNP effect on Gut microbiota abundance (genus Catenibacterium id.2153)

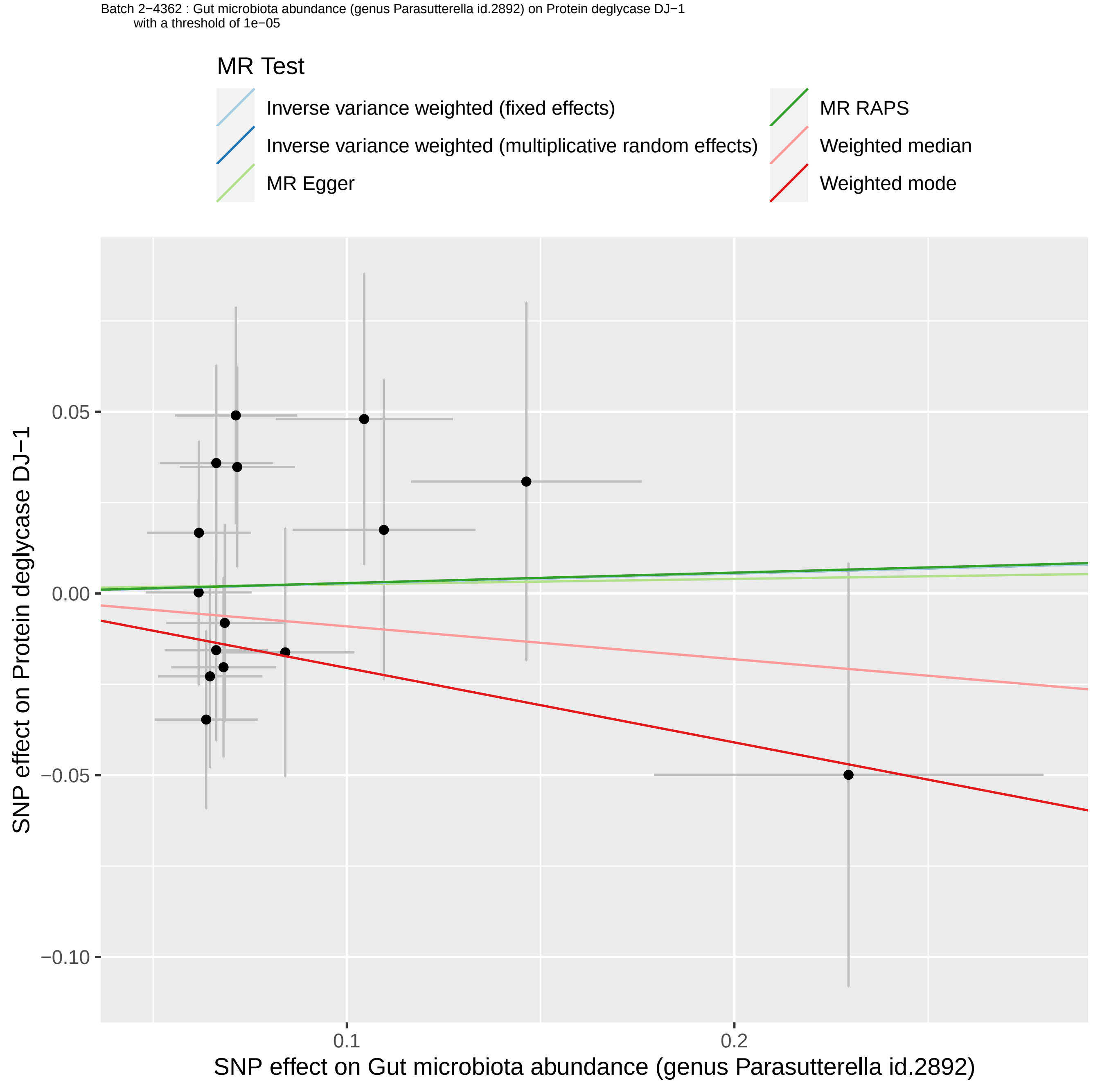
Batch 2−4362 : Gut microbiota abundance (genus Parasutterella id.2892) on Protein deglycase DJ−1
with a threshold of 1e−05
MR Test
Inverse variance weighted (fixed effects)
Inverse variance weighted (multiplicative random effects)
MR Egger
MR RAPS
Weighted median
Weighted mode
0.05
0.00
−0.05
−0.10
0.1
0.2
SNP effect on Protein deglycase DJ−1
SNP effect on Gut microbiota abundance (genus Parasutterella id.2892)

Batch 2−4363 : Gut microbiota abundance (genus Catenibacterium id.2153) on Poly [ADP−ribose] polymerase 1
with a threshold of 1e−05

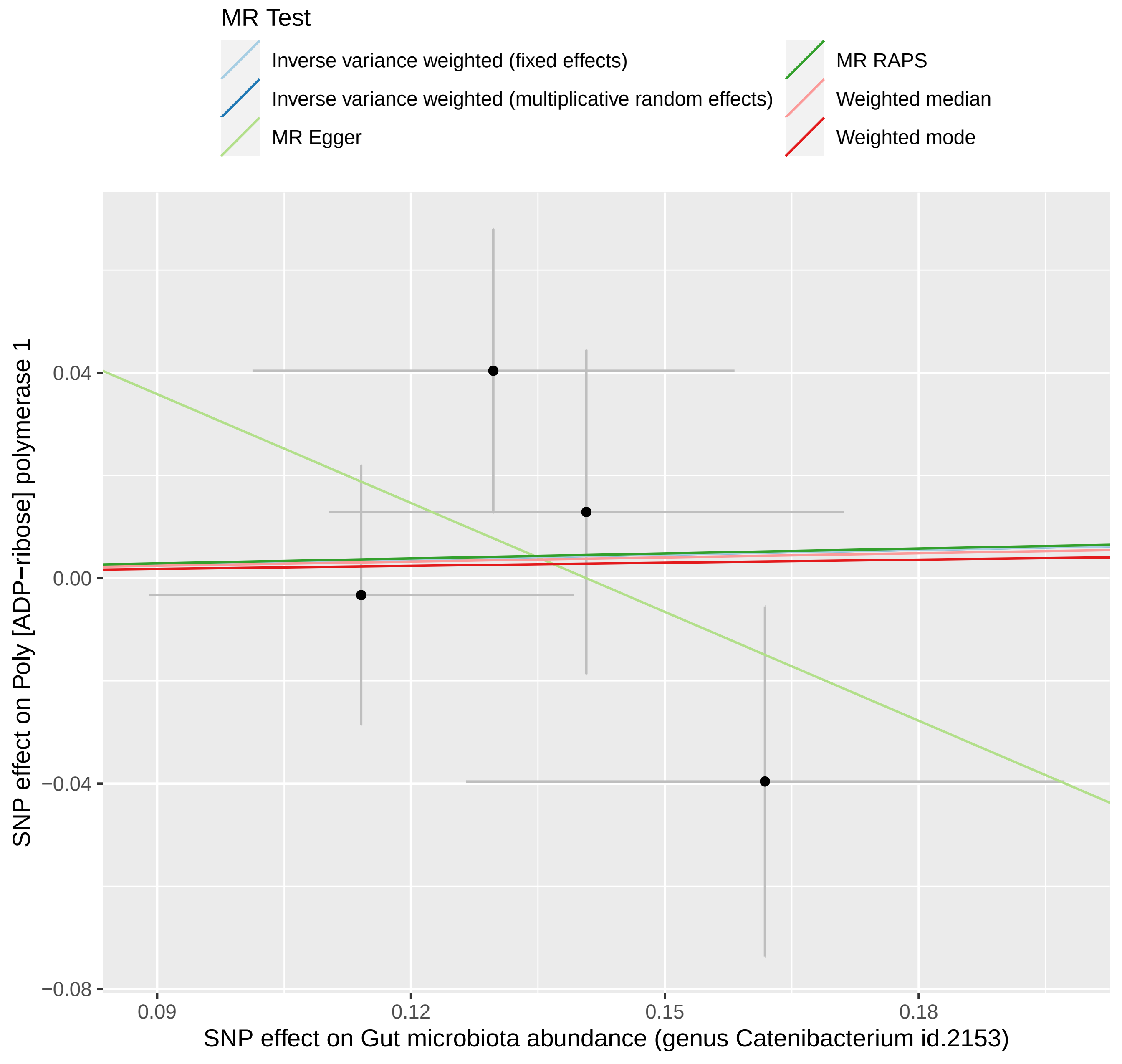

Batch 2−4364 : Gut microbiota abundance (genus Parasutterella id.2892) on Poly [ADP−ribose] polymerase 1 with a threshold of 1e−05

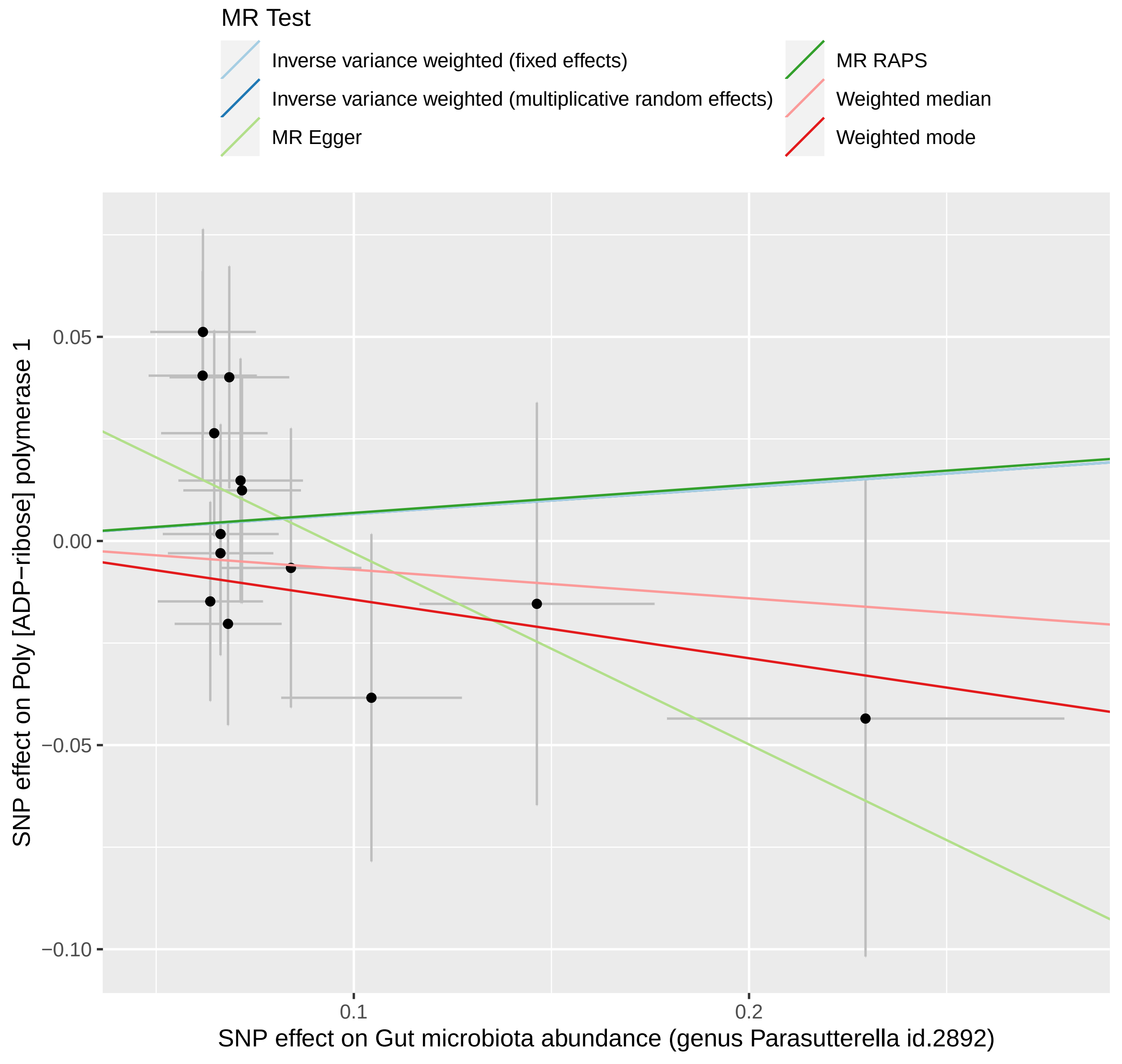

Batch 2−4365 : Gut microbiota abundance (genus Catenibacterium id.2153) on Poly [ADP−ribose] polymerase 11
with a threshold of 1e−05

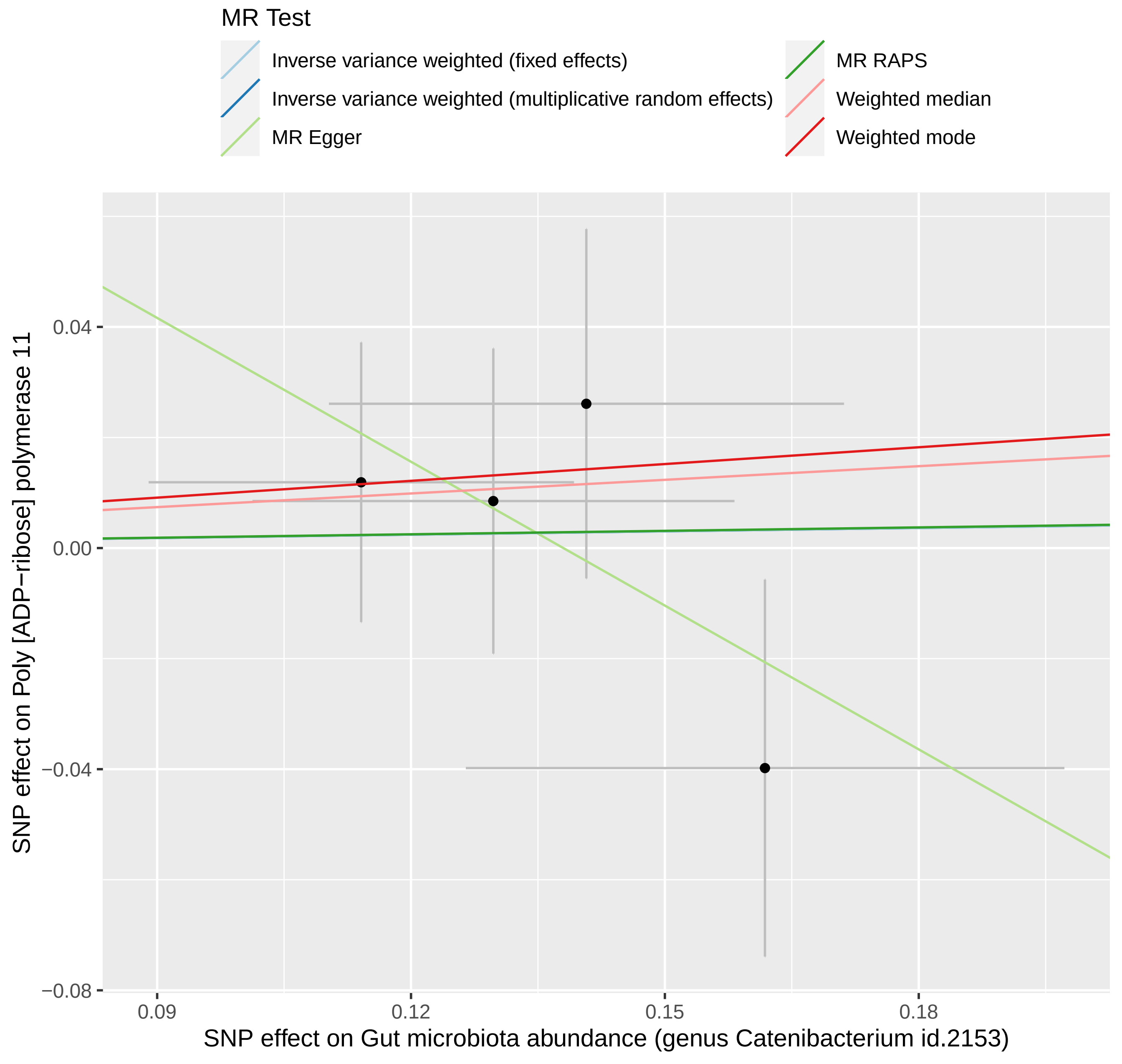

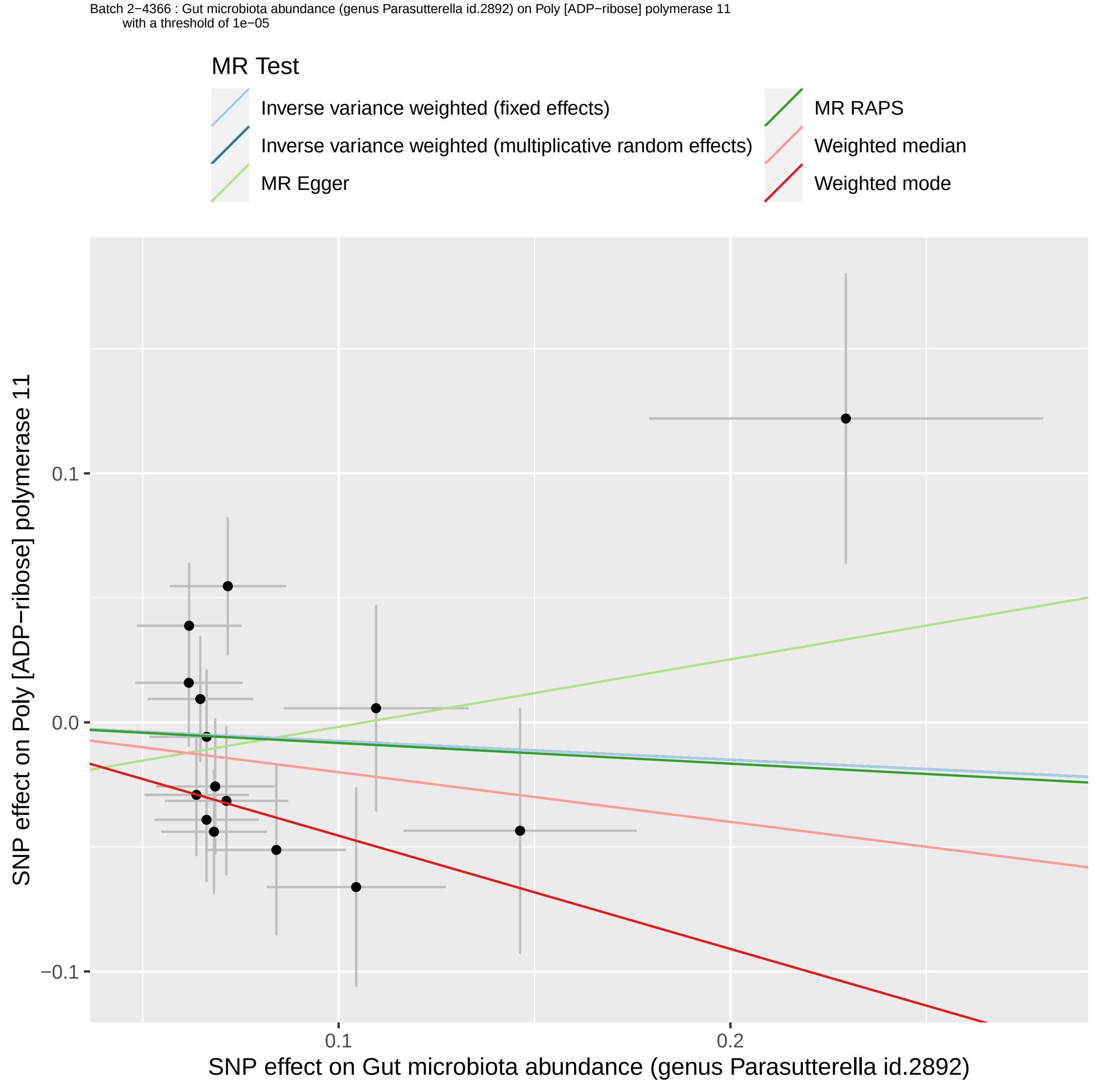
Batch 2−4366 : Gut microbiota abundance (genus Parasutterella id.2892) on Poly [ADP−ribose] polymerase 11
with a threshold of 1e−05
MR Test
Inverse variance weighted (fixed effects)
Inverse variance weighted (multiplicative random effects)
MR Egger
MR RAPS
Weighted median
Weighted mode
0.1
0.0
−0.1
0.1
0.2
SNP effect on Poly [ADP−ribose] polymerase 11
SNP effect on Gut microbiota abundance (genus Parasutterella id.2892)

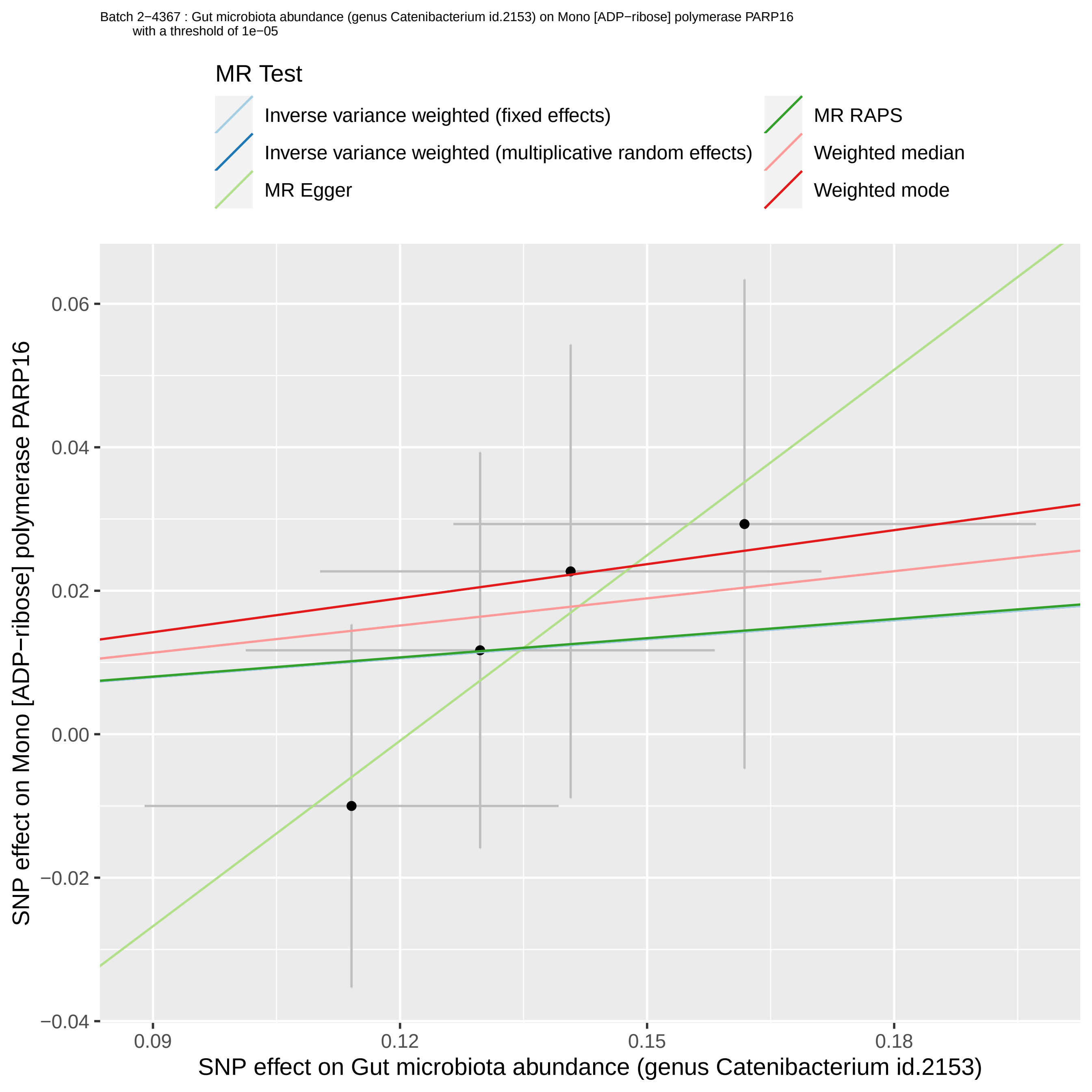

Batch 2−4367 : Gut microbiota abundance (genus Catenibacterium id.2153) on Mono [ADP−ribose] polymerase PARP16
with a threshold of 1e−05
MR Test
Inverse variance weighted (fixed effects)
Inverse variance weighted (multiplicative random effects)
MR Egger
MR RAPS
Weighted median
Weighted mode
0.06
0.04
0.02
0.00
−0.02
−0.04
SNP effect on Mono [ADP−ribose] polymerase PARP16
0.09
0.12
0.15
0.18
SNP effect on Gut microbiota abundance (genus Catenibacterium id.2153)

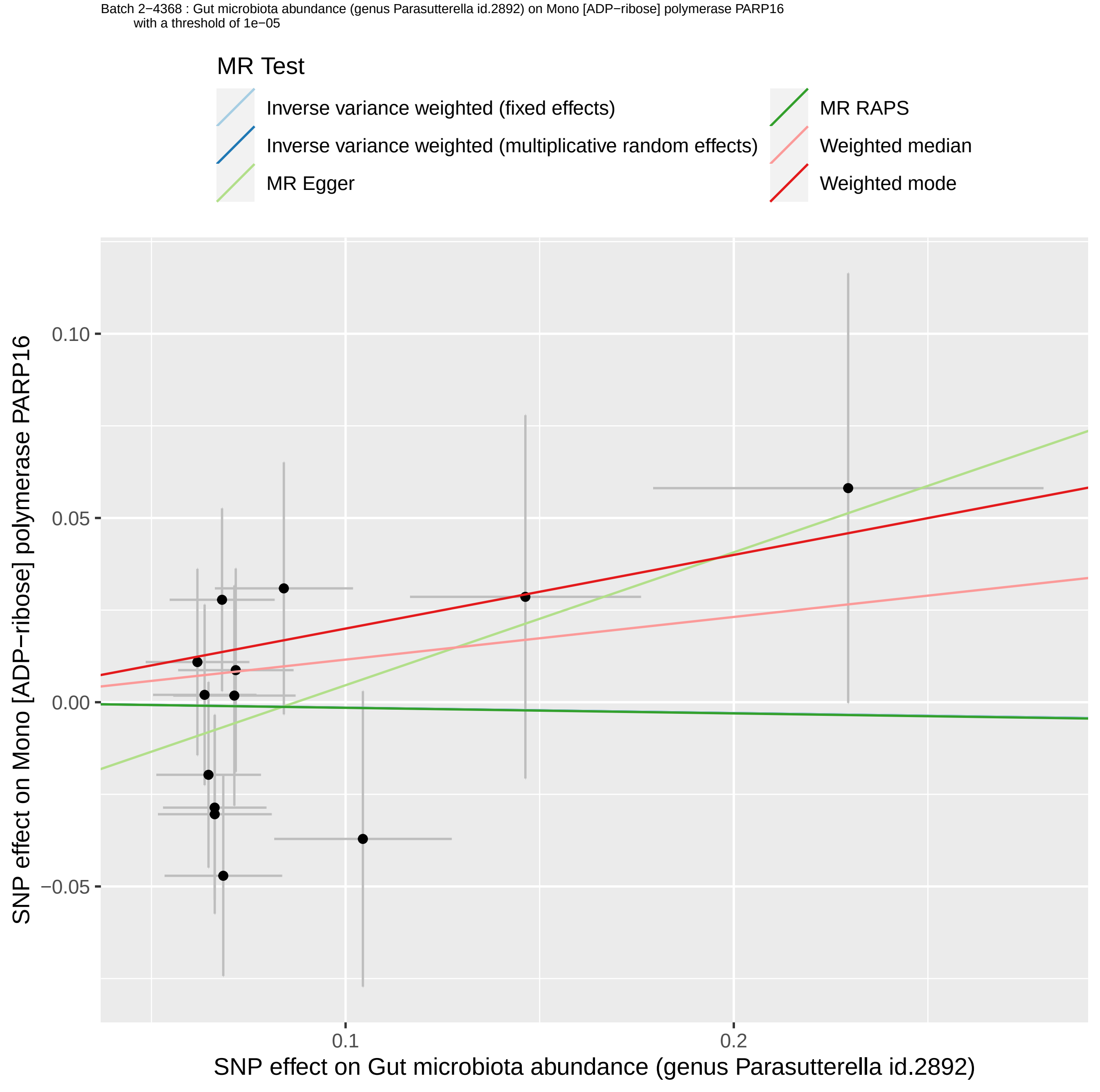
Batch 2−4368 : Gut microbiota abundance (genus Parasutterella id.2892) on Mono [ADP−ribose] polymerase PARP16
with a threshold of 1e−05
MR Test
Inverse variance weighted (fixed effects)
Inverse variance weighted (multiplicative random effects)
MR Egger
MR RAPS
Weighted median
Weighted mode
SNP effect on Mono [ADP−ribose] polymerase PARP16
0.10
0.05
0.00
−0.05
0.1
0.2
SNP effect on Gut microbiota abundance (genus Parasutterella id.2892)

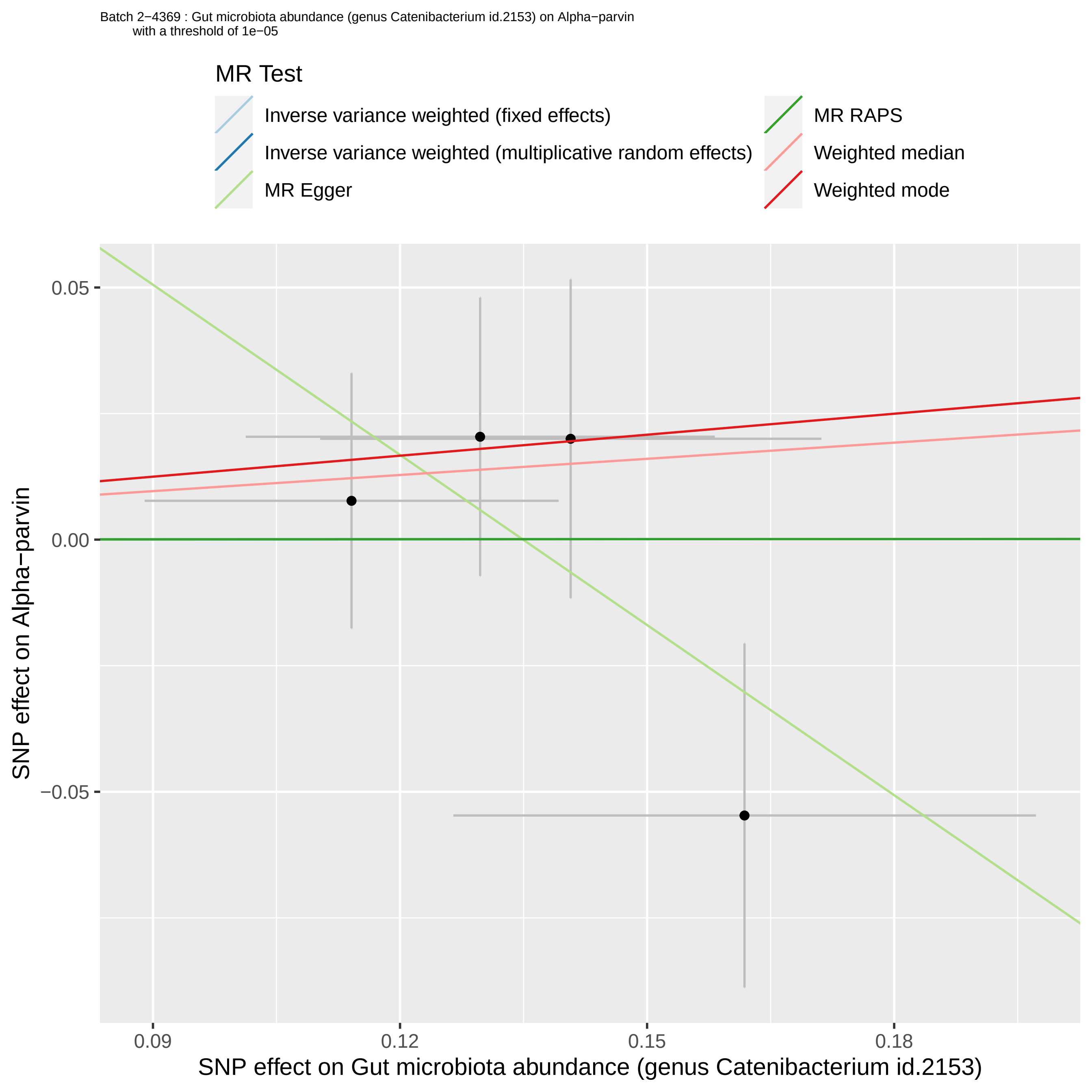
Batch 2−4369 : Gut microbiota abundance (genus Catenibacterium id.2153) on Alpha−parvin
with a threshold of 1e−05
MR Test
Inverse variance weighted (fixed effects)
Inverse variance weighted (multiplicative random effects)
MR Egger
MR RAPS
Weighted median
Weighted mode
0.05
0.00
−0.05
0.09
0.12
0.15
0.18
SNP effect on Alpha−parvin
SNP effect on Gut microbiota abundance (genus Catenibacterium id.2153)

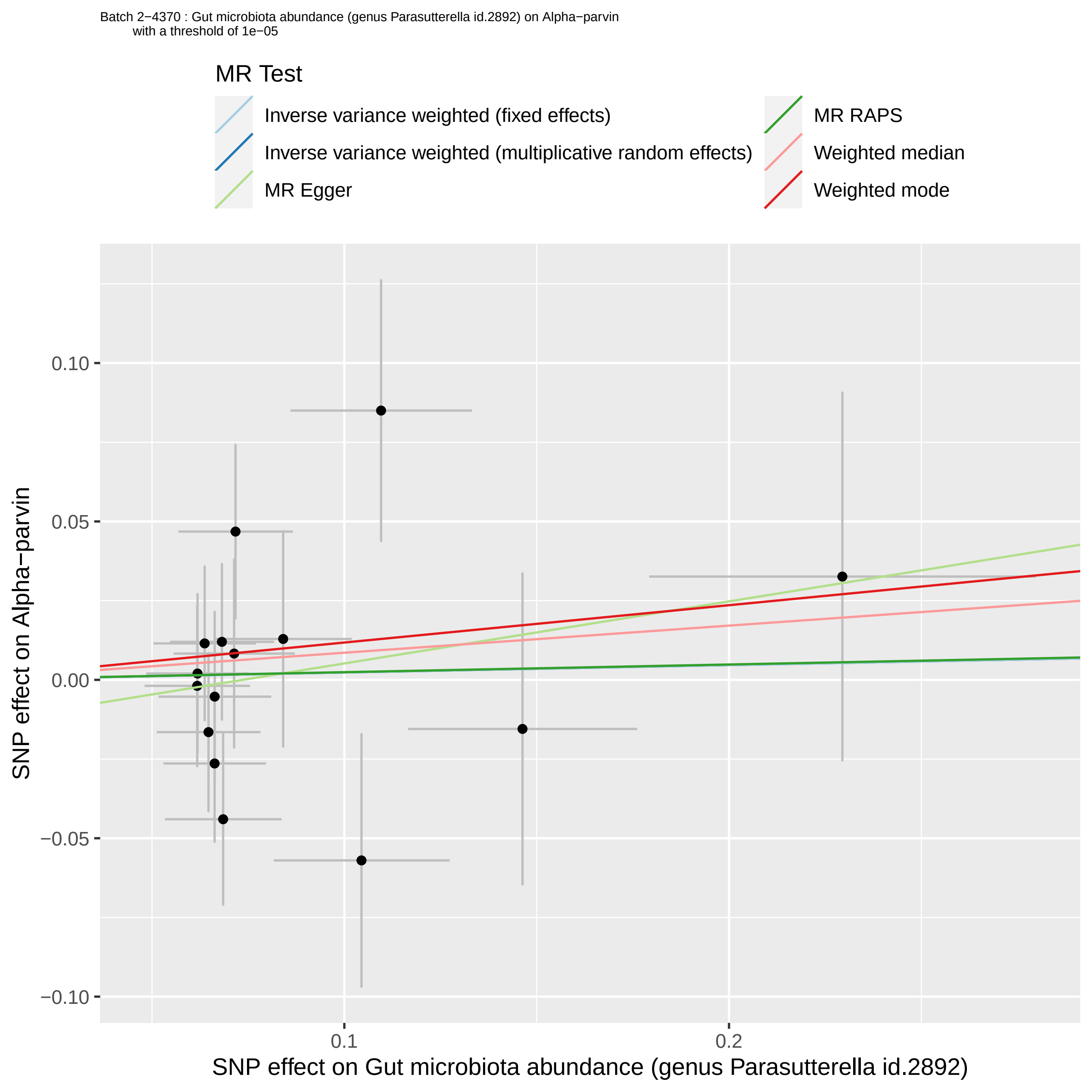

Batch 2−4370 : Gut microbiota abundance (genus Parasutterella id.2892) on Alpha−parvin
with a threshold of 1e−05
MR Test
Inverse variance weighted (fixed effects)
Inverse variance weighted (multiplicative random effects)
MR Egger
MR RAPS
Weighted median
Weighted mode
0.10
0.05
0.00
−0.05
−0.10
0.1
0.2
SNP effect on Alpha−parvin
SNP effect on Gut microbiota abundance (genus Parasutterella id.2892)

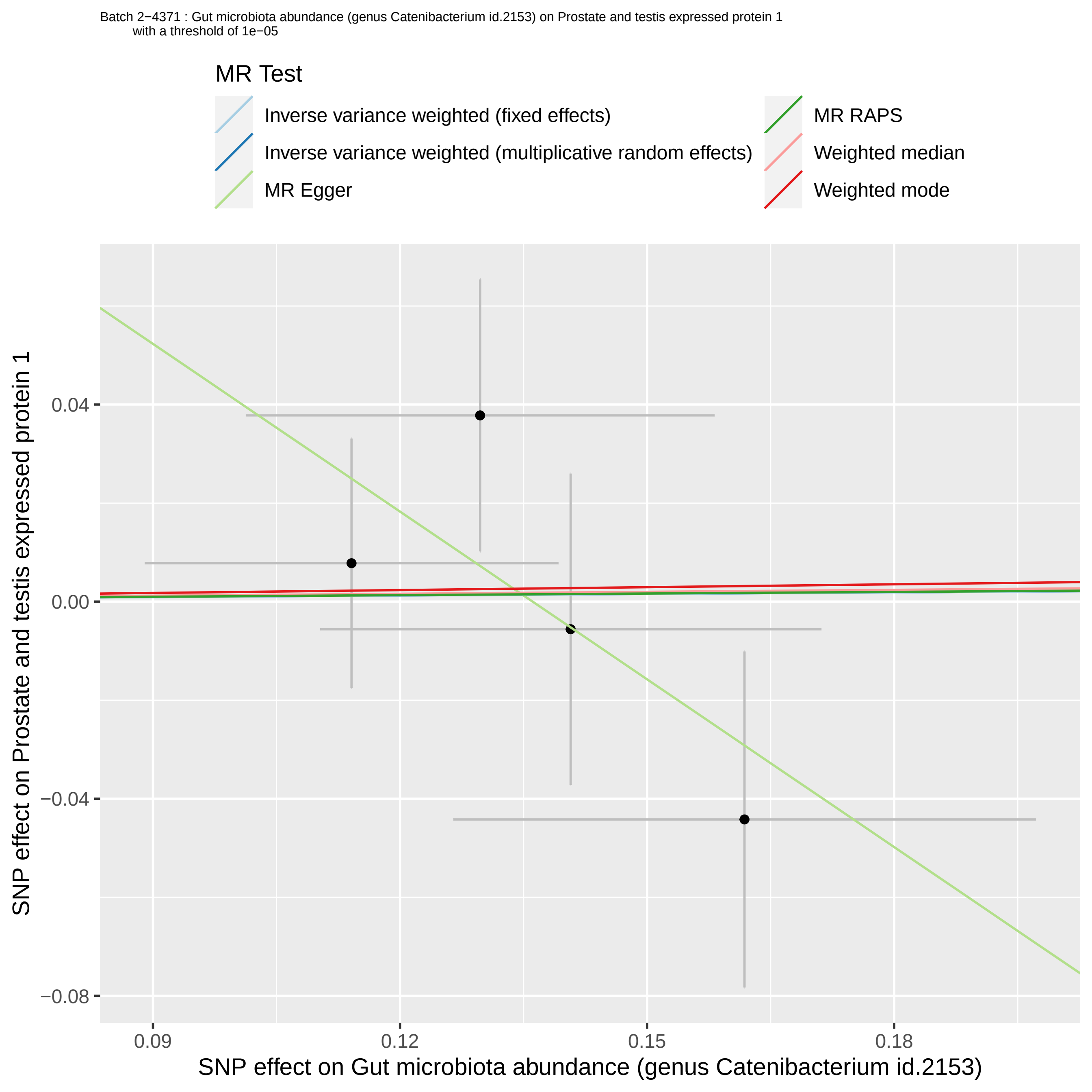
Batch 2−4371 : Gut microbiota abundance (genus Catenibacterium id.2153) on Prostate and testis expressed protein 1
with a threshold of 1e−05
MR Test
Inverse variance weighted (fixed effects)
Inverse variance weighted (multiplicative random effects)
MR Egger
MR RAPS
Weighted median
Weighted mode
SNP effect on Prostate and testis expressed protein 1
0.04
0.00
−0.04
−0.08
0.09
0.12
0.15
0.18
SNP effect on Gut microbiota abundance (genus Catenibacterium id.2153)

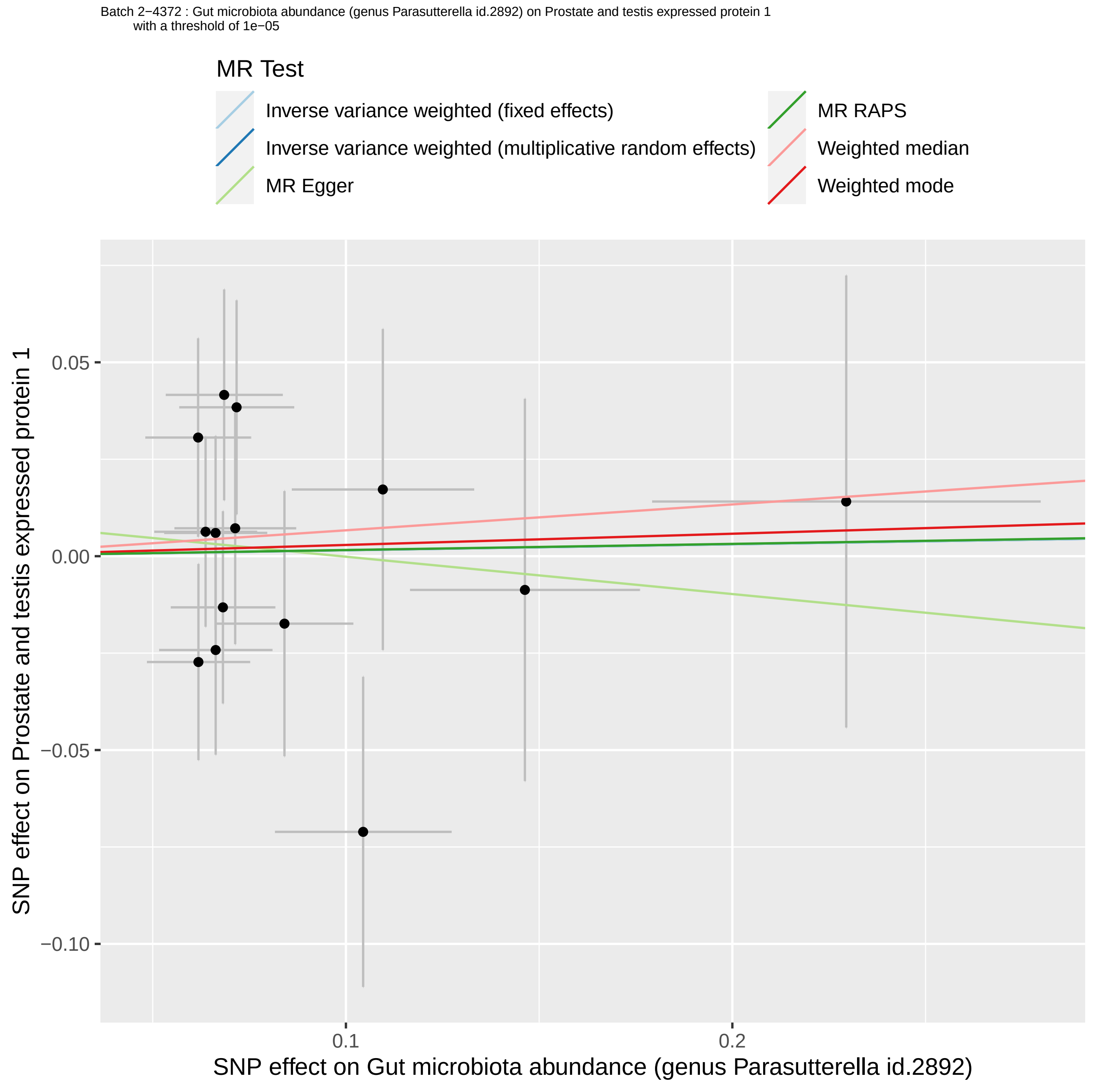

Batch 2−4372 : Gut microbiota abundance (genus Parasutterella id.2892) on Prostate and testis expressed protein 1
with a threshold of 1e−05
MR Test
Inverse variance weighted (fixed effects)
Inverse variance weighted (multiplicative random effects)
MR Egger
MR RAPS
Weighted median
Weighted mode
0.05
0.00
−0.05
−0.10
0.1
0.2
SNP effect on Prostate and testis expressed protein 1
SNP effect on Gut microbiota abundance (genus Parasutterella id.2892)

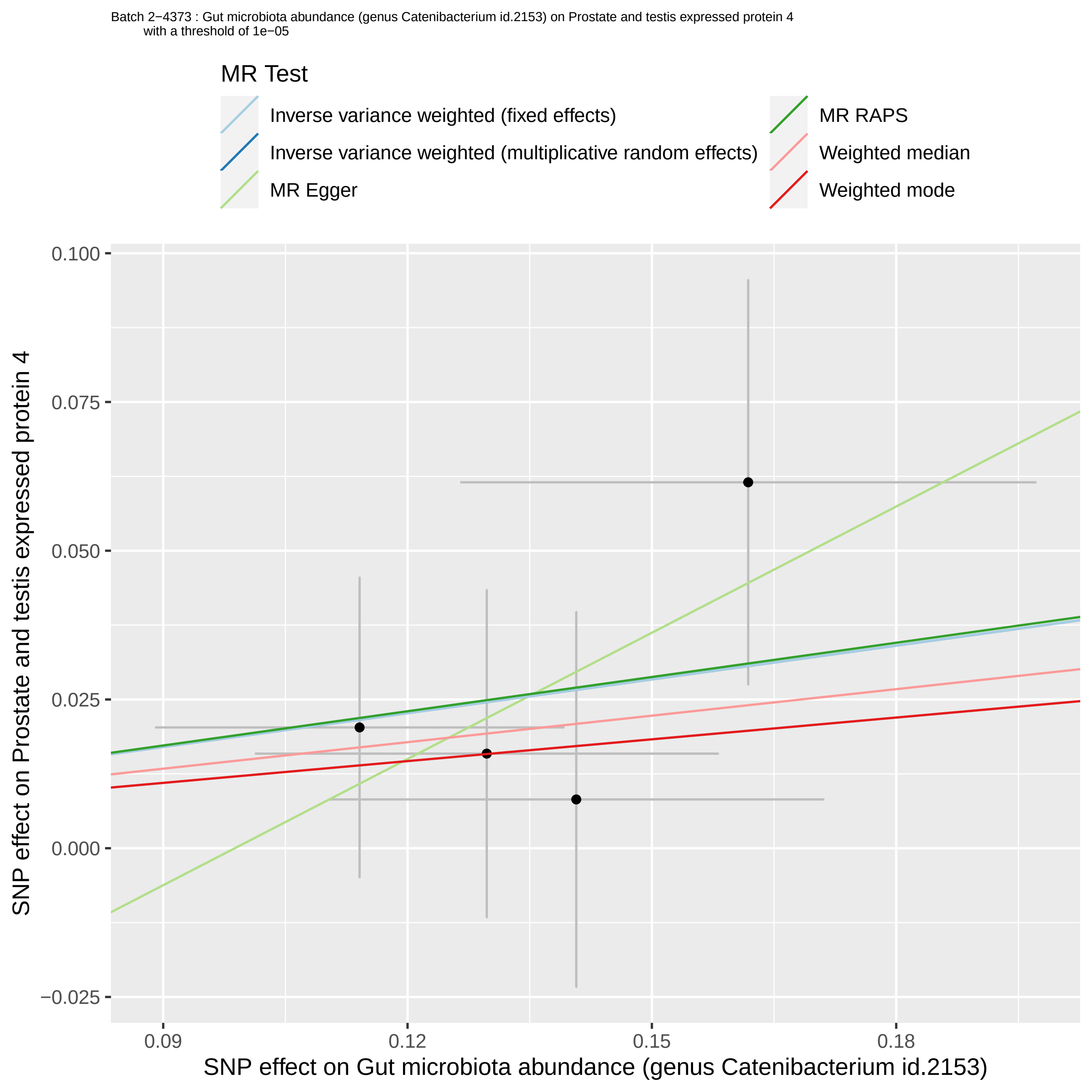
Batch 2−4373 : Gut microbiota abundance (genus Catenibacterium id.2153) on Prostate and testis expressed protein 4
with a threshold of 1e−05
MR Test
Inverse variance weighted (fixed effects)
Inverse variance weighted (multiplicative random effects)
MR Egger
MR RAPS
Weighted median
Weighted mode
0.100
0.075
0.050
0.025
0.000
−0.025
0.09
0.12
0.15
0.18
SNP effect on Gut microbiota abundance (genus Catenibacterium id.2153)
SNP effect on Prostate and testis expressed protein 4

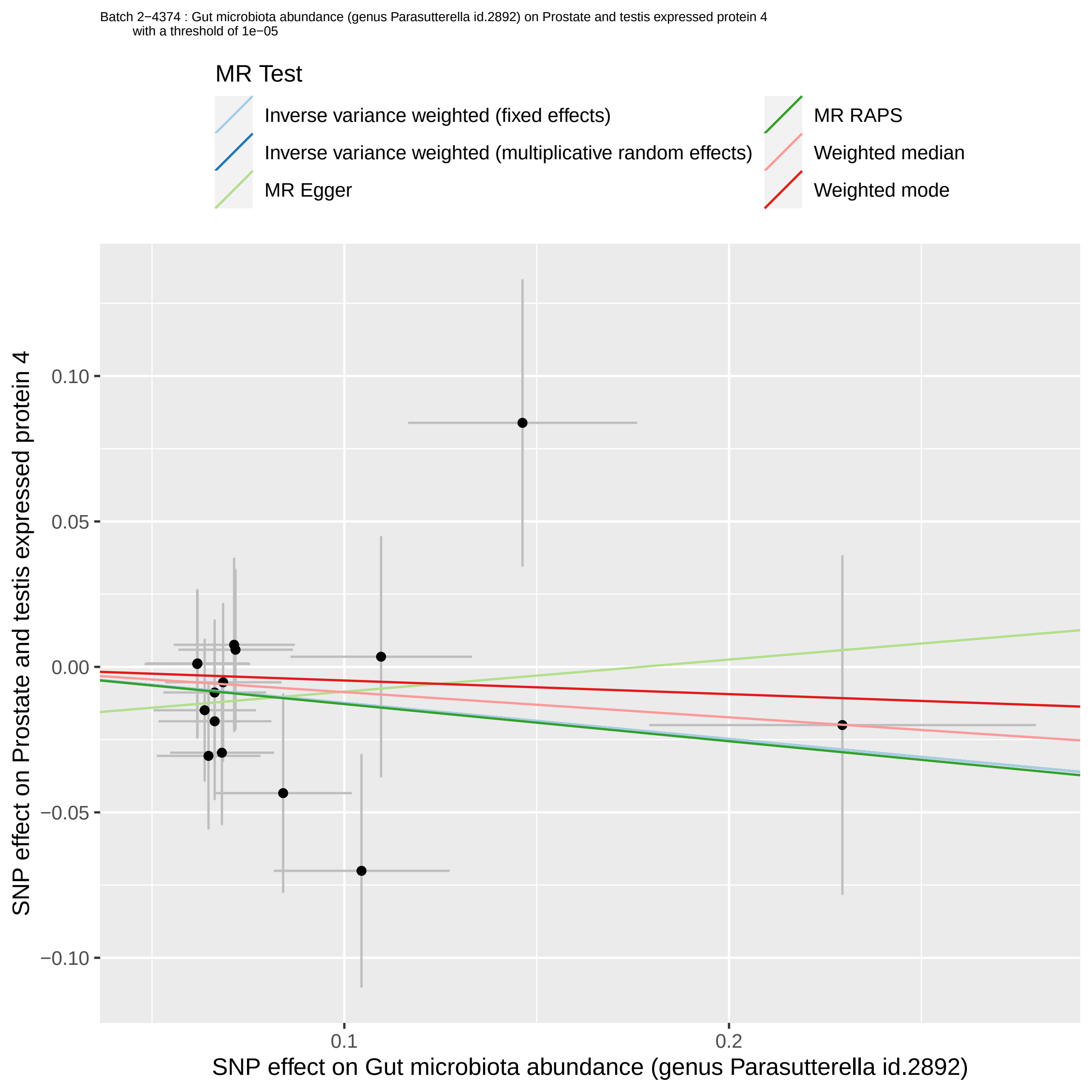

Batch 2−4374 : Gut microbiota abundance (genus Parasutterella id.2892) on Prostate and testis expressed protein 4
with a threshold of 1e−05
MR Test
Inverse variance weighted (fixed effects)
Inverse variance weighted (multiplicative random effects)
MR Egger
MR RAPS
Weighted median
Weighted mode
0.10
0.05
0.00
−0.05
−0.10
0.1
0.2
SNP effect on Prostate and testis expressed protein 4
SNP effect on Gut microbiota abundance (genus Parasutterella id.2892)

Batch 2−4375 : Gut microbiota abundance (genus Catenibacterium id.2153) on Paired box protein Pax−4
with a threshold of 1e−05

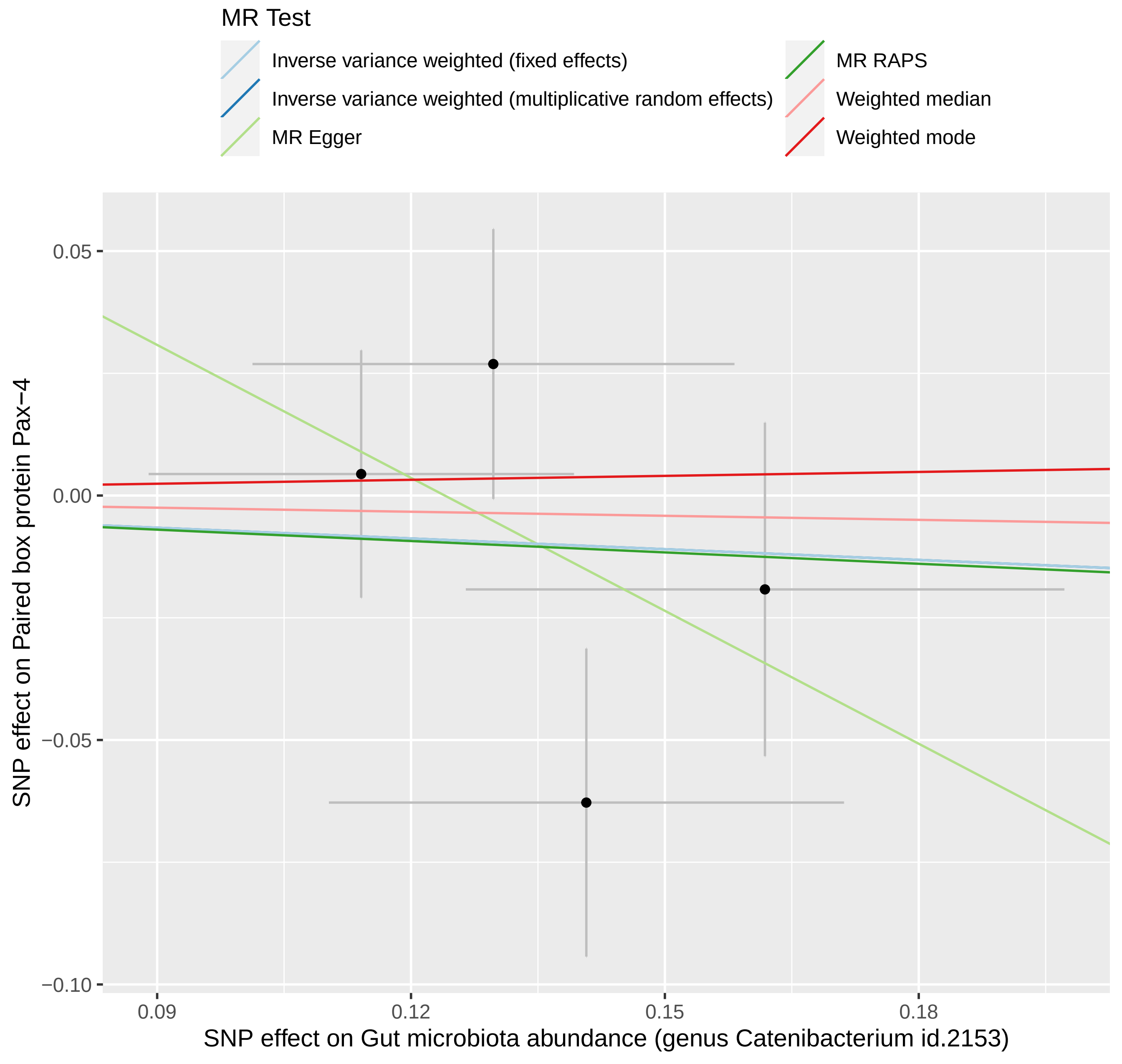

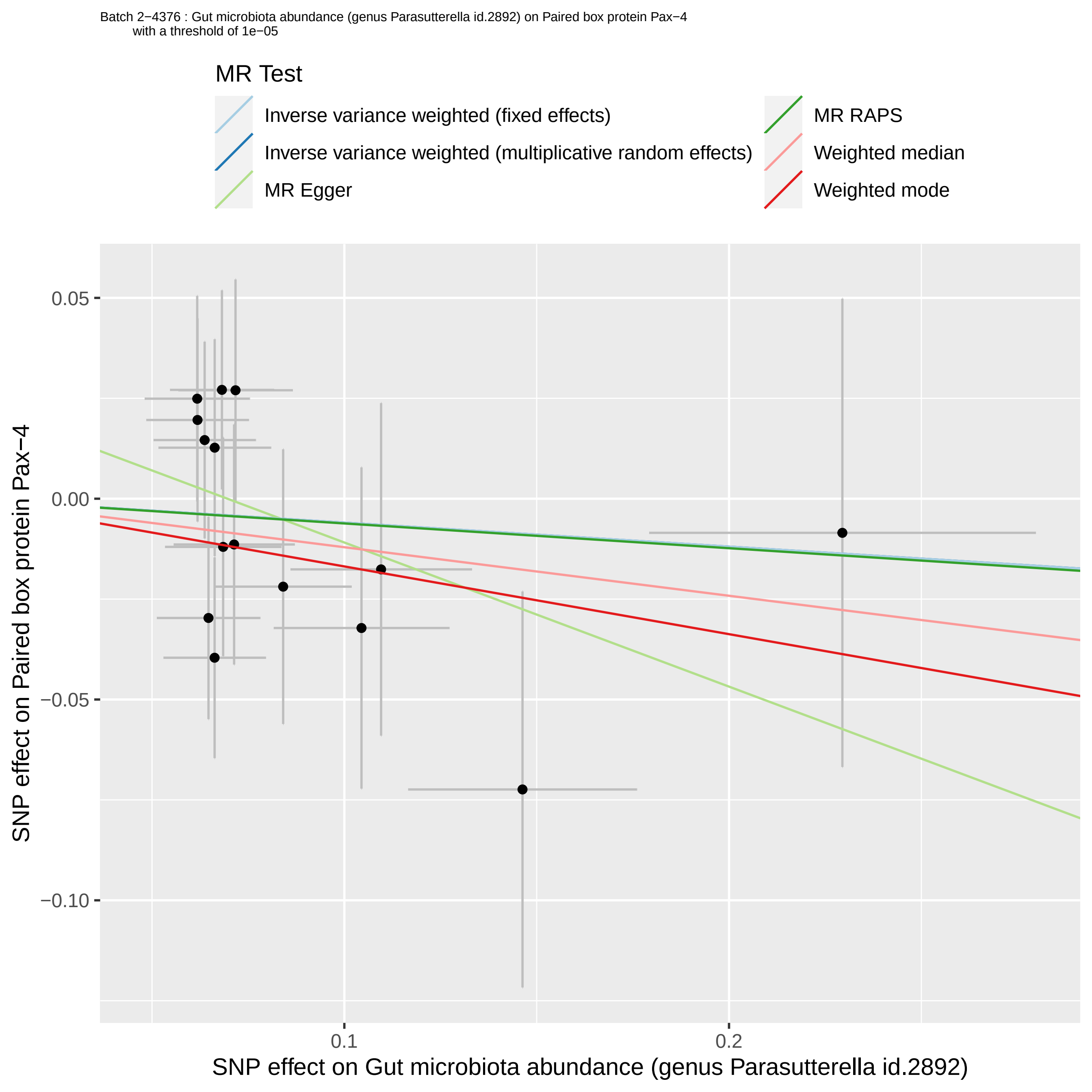
Batch 2−4376 : Gut microbiota abundance (genus Parasutterella id.2892) on Paired box protein Pax−4
with a threshold of 1e−05
MR Test
Inverse variance weighted (fixed effects)
Inverse variance weighted (multiplicative random effects)
MR Egger
MR RAPS
Weighted median
Weighted mode
0.05
0.00
−0.05
−0.10
0.1
0.2
SNP effect on Paired box protein Pax−4
SNP effect on Gut microbiota abundance (genus Parasutterella id.2892)

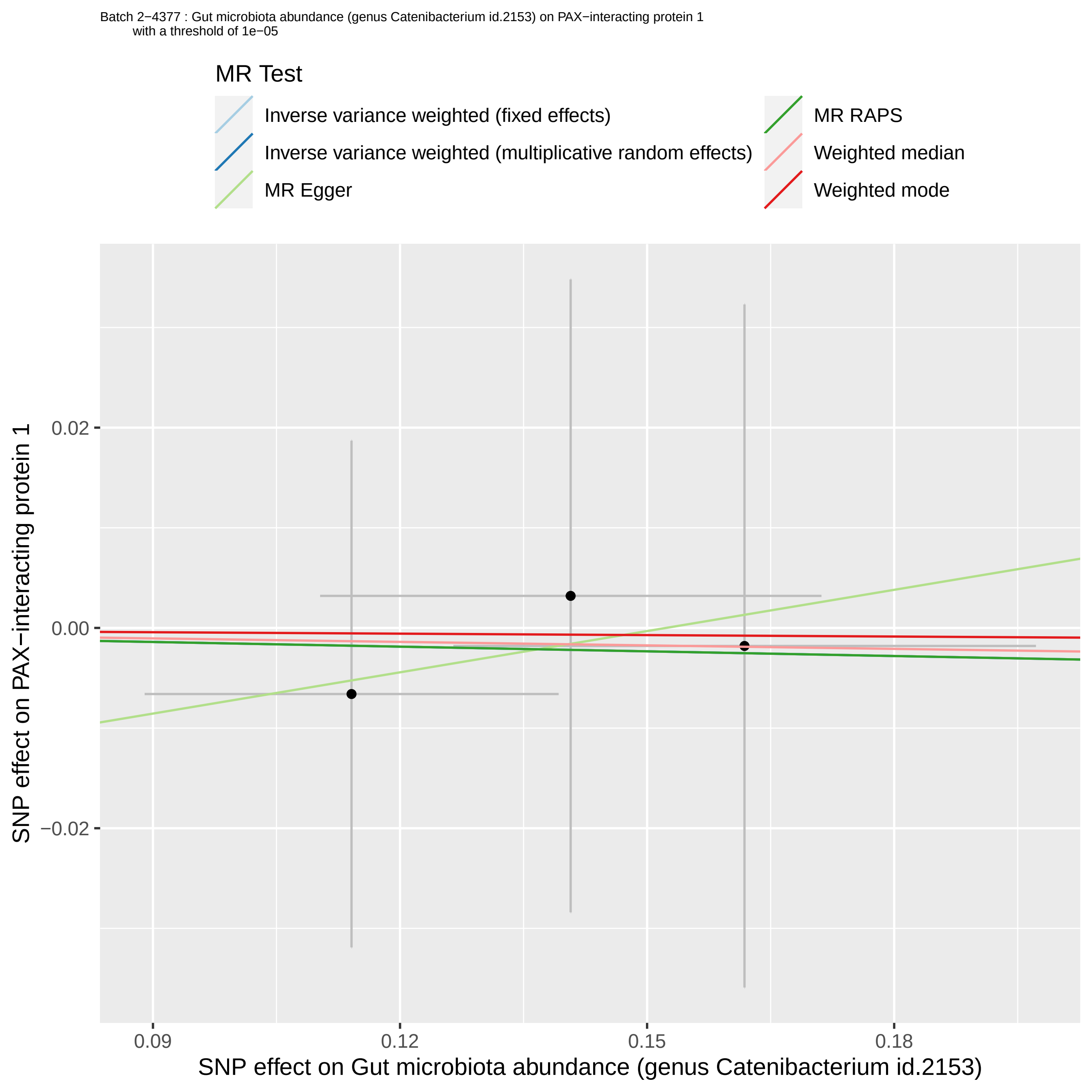

Batch 2−4377 : Gut microbiota abundance (genus Catenibacterium id.2153) on PAX−interacting protein 1
with a threshold of 1e−05
MR Test
Inverse variance weighted (fixed effects)
Inverse variance weighted (multiplicative random effects)
MR Egger
MR RAPS
Weighted median
Weighted mode
0.02
0.00
−0.02
0.09
0.12
0.15
0.18
SNP effect on PAX−interacting protein 1
SNP effect on Gut microbiota abundance (genus Catenibacterium id.2153)

Batch 2−4378 : Gut microbiota abundance (genus Parasutterella id.2892) on PAX−interacting protein 1
with a threshold of 1e−05

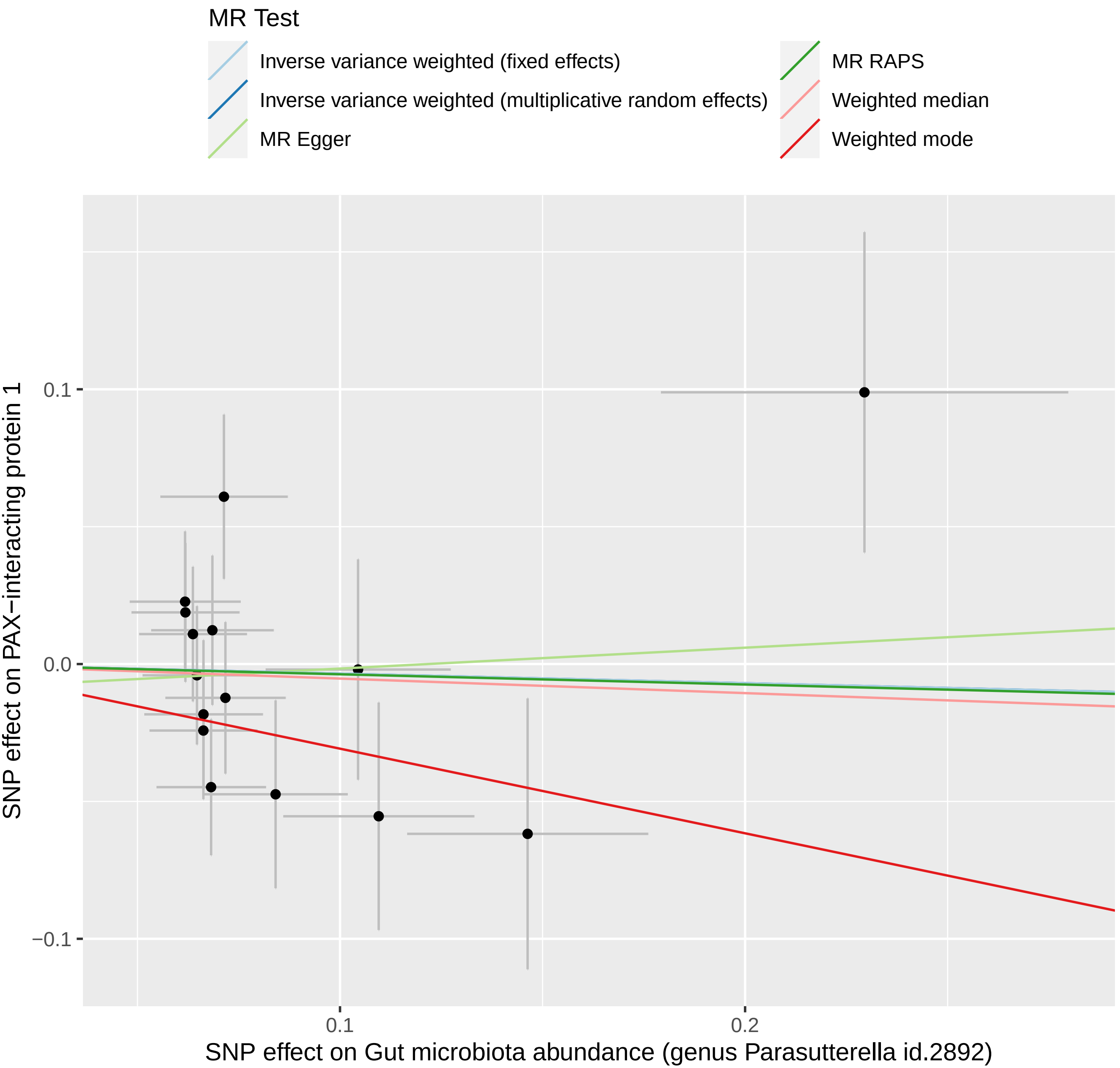

Batch 2−4379 : Gut microbiota abundance (genus Catenibacterium id.2153) on Pyruvate carboxylase, mitochondrial
with a threshold of 1e−05

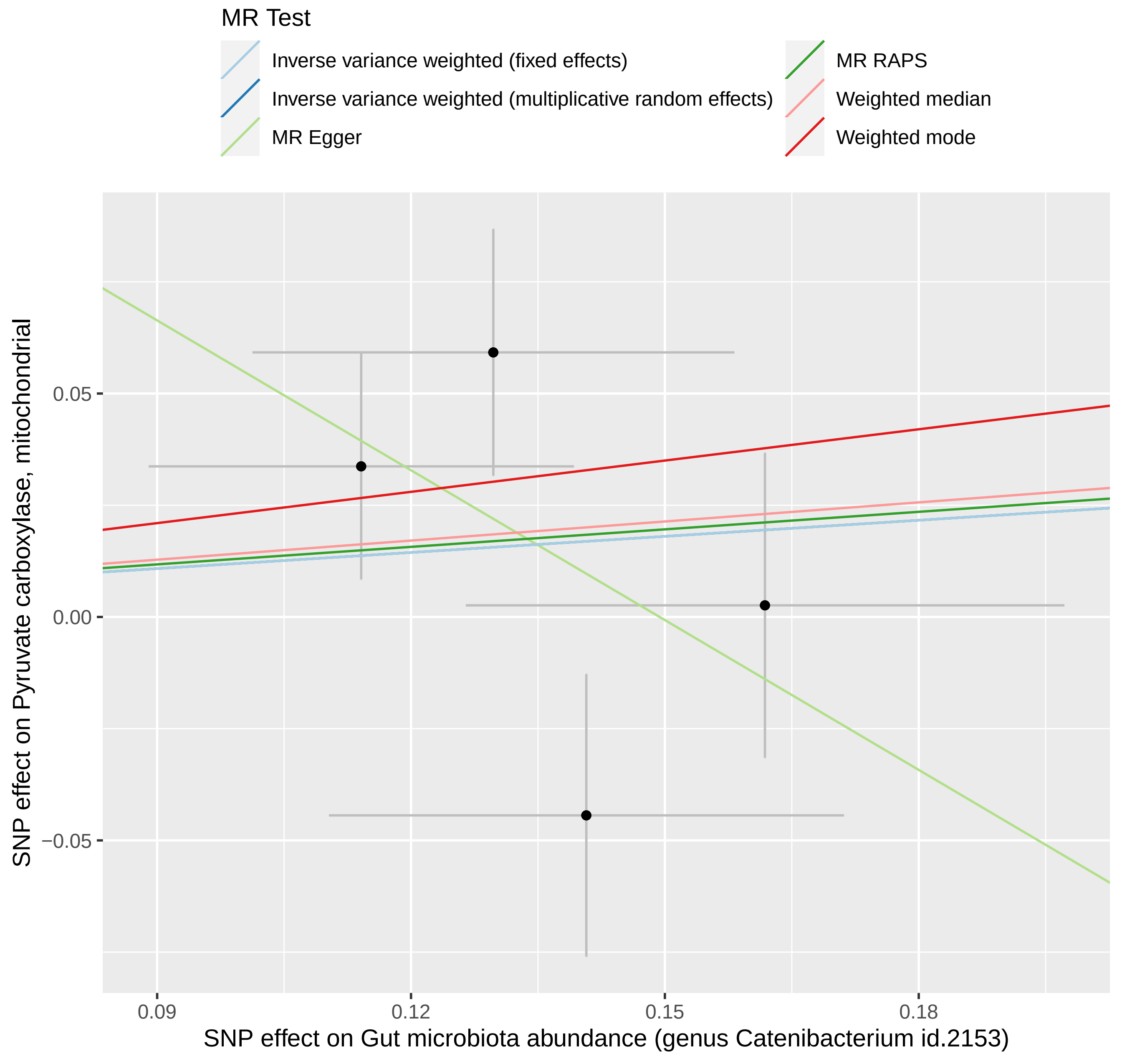

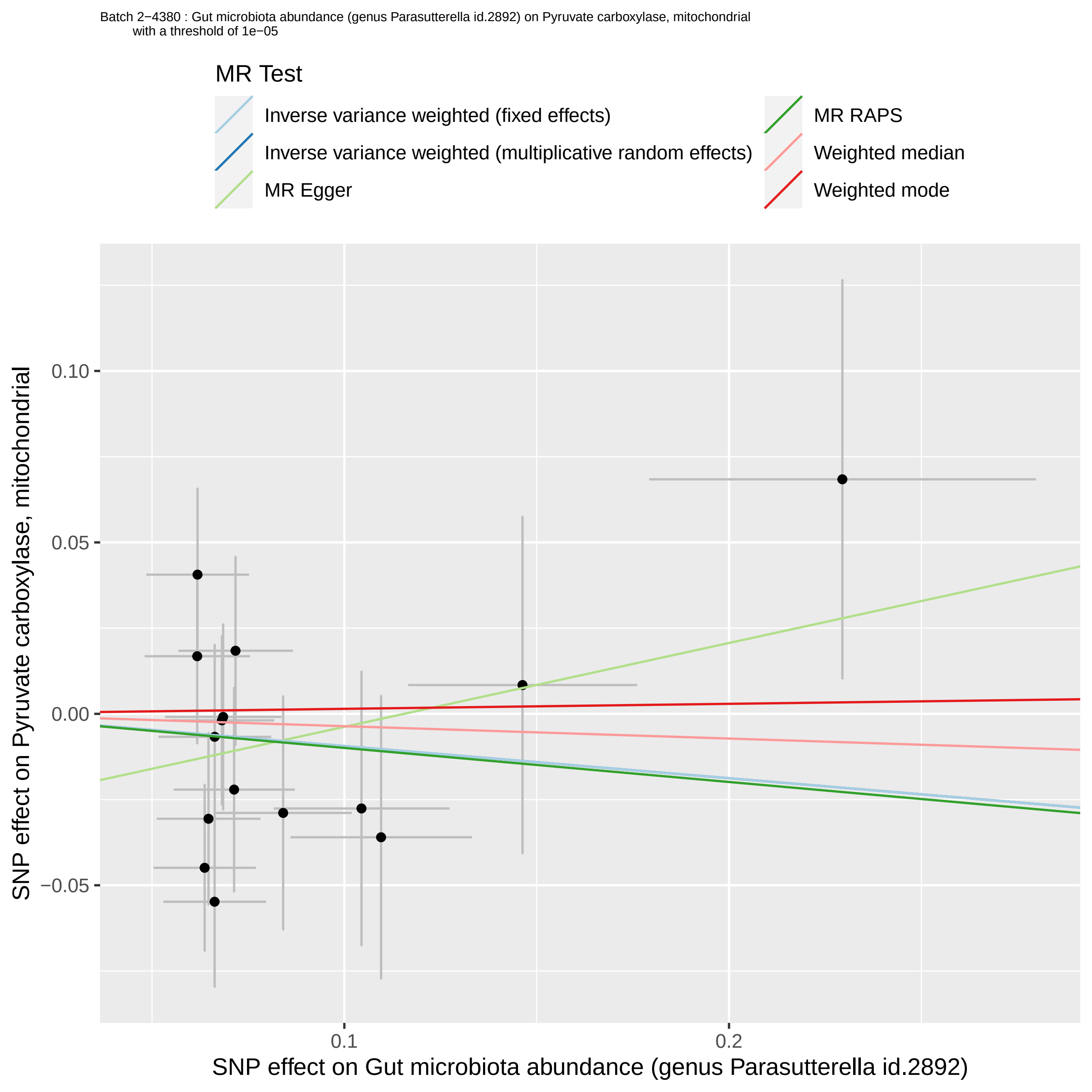
Batch 2−4380 : Gut microbiota abundance (genus Parasutterella id.2892) on Pyruvate carboxylase, mitochondrial
with a threshold of 1e−05
MR Test
Inverse variance weighted (fixed effects)
Inverse variance weighted (multiplicative random effects)
MR Egger
MR RAPS
Weighted median
Weighted mode
0.10
0.05
0.00
−0.05
0.1
0.2
SNP effect on Pyruvate carboxylase, mitochondrial
SNP effect on Gut microbiota abundance (genus Parasutterella id.2892)

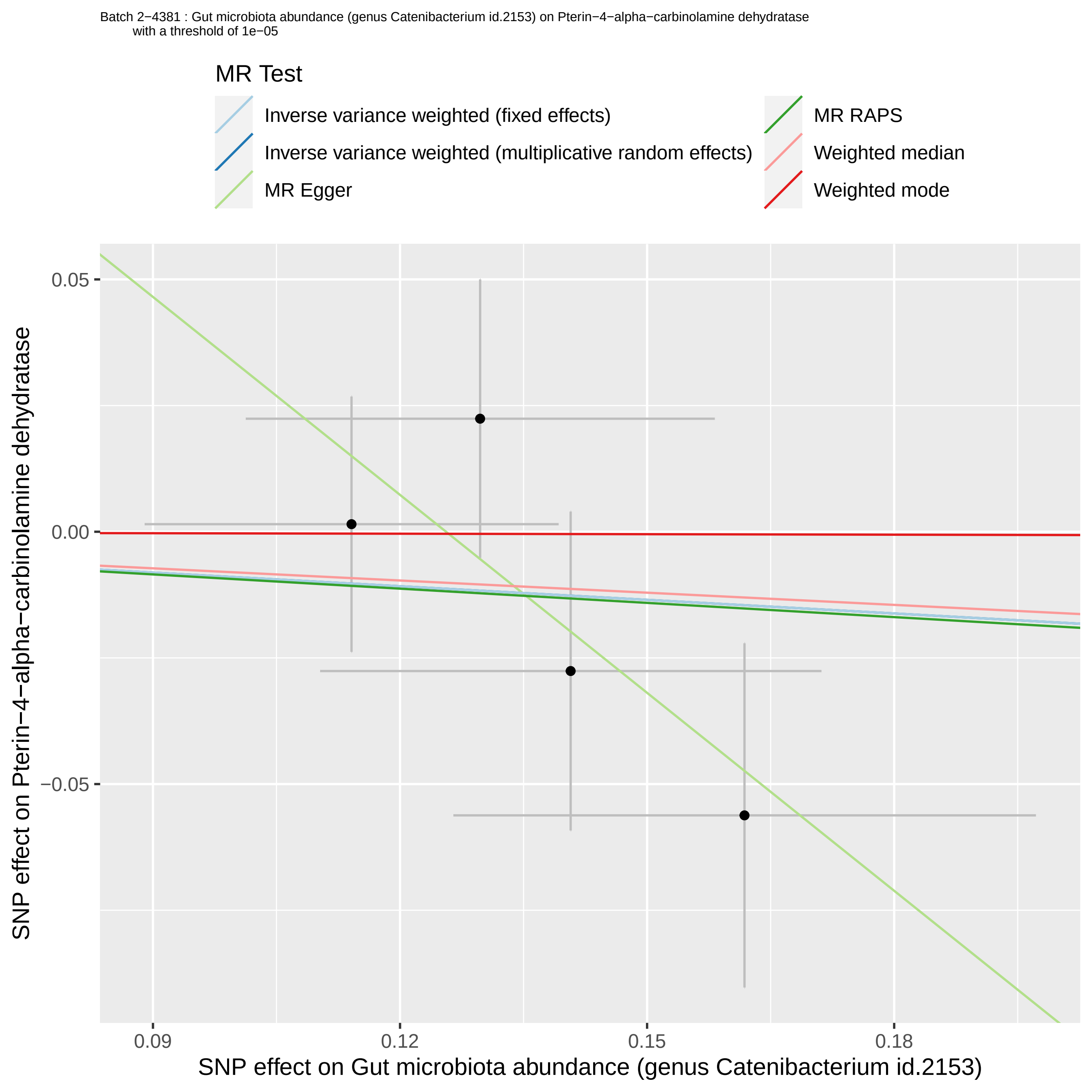
Batch 2−4381 : Gut microbiota abundance (genus Catenibacterium id.2153) on Pterin−4−alpha−carbinolamine dehydratase
with a threshold of 1e−05
MR Test
Inverse variance weighted (fixed effects)
Inverse variance weighted (multiplicative random effects)
MR Egger
MR RAPS
Weighted median
Weighted mode
0.05
0.00
−0.05
0.09
0.12
0.15
0.18
SNP effect on Pterin−4−alpha−carbinolamine dehydratase
SNP effect on Gut microbiota abundance (genus Catenibacterium id.2153)

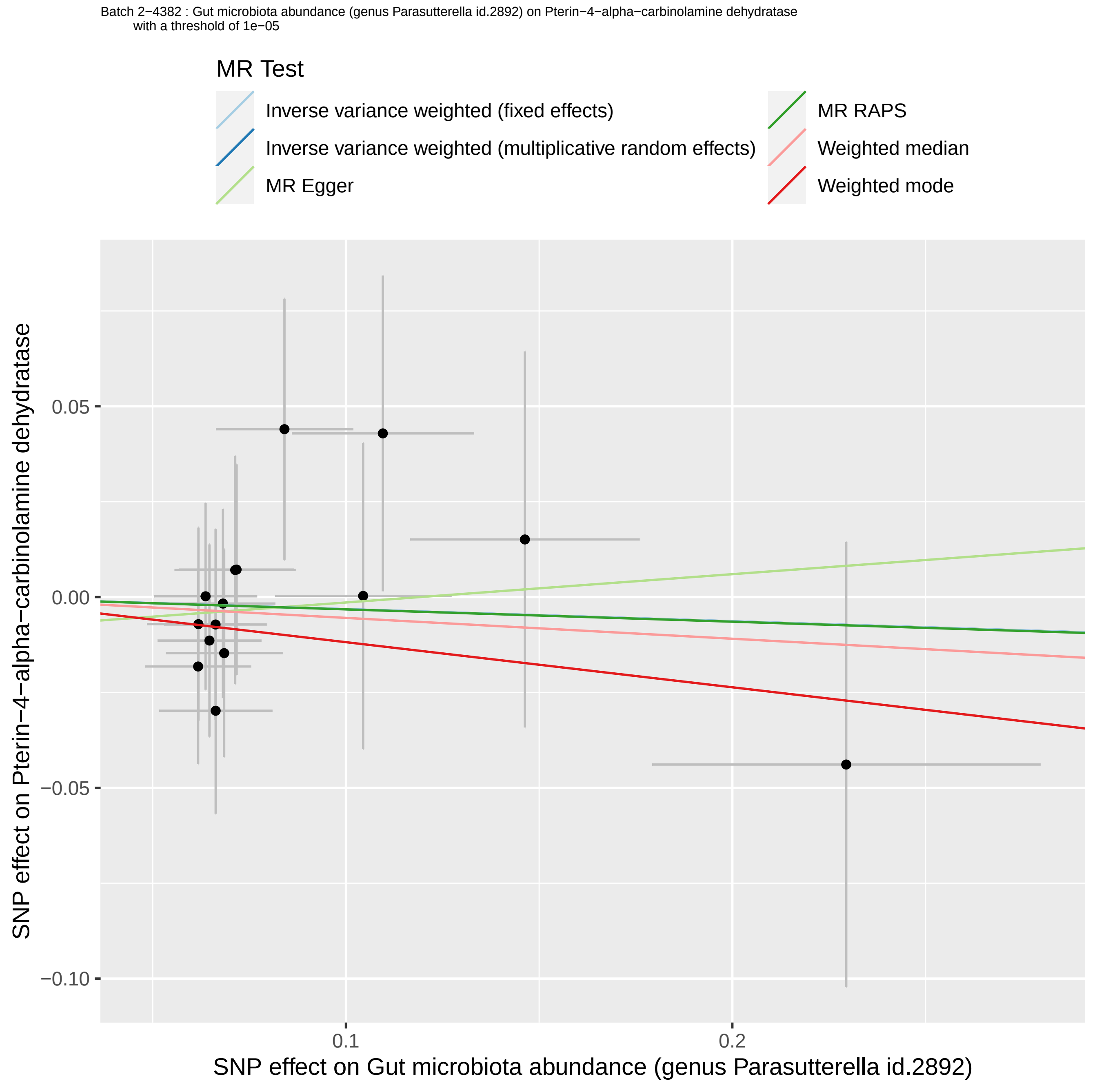

Batch 2−4382 : Gut microbiota abundance (genus Parasutterella id.2892) on Pterin−4−alpha−carbinolamine dehydratase
with a threshold of 1e−05
MR Test
Inverse variance weighted (fixed effects)
Inverse variance weighted (multiplicative random effects)
MR Egger
MR RAPS
Weighted median
Weighted mode
0.05
0.00
−0.05
−0.10
0.1
0.2
SNP effect on Pterin−4−alpha−carbinolamine dehydratase
SNP effect on Gut microbiota abundance (genus Parasutterella id.2892)

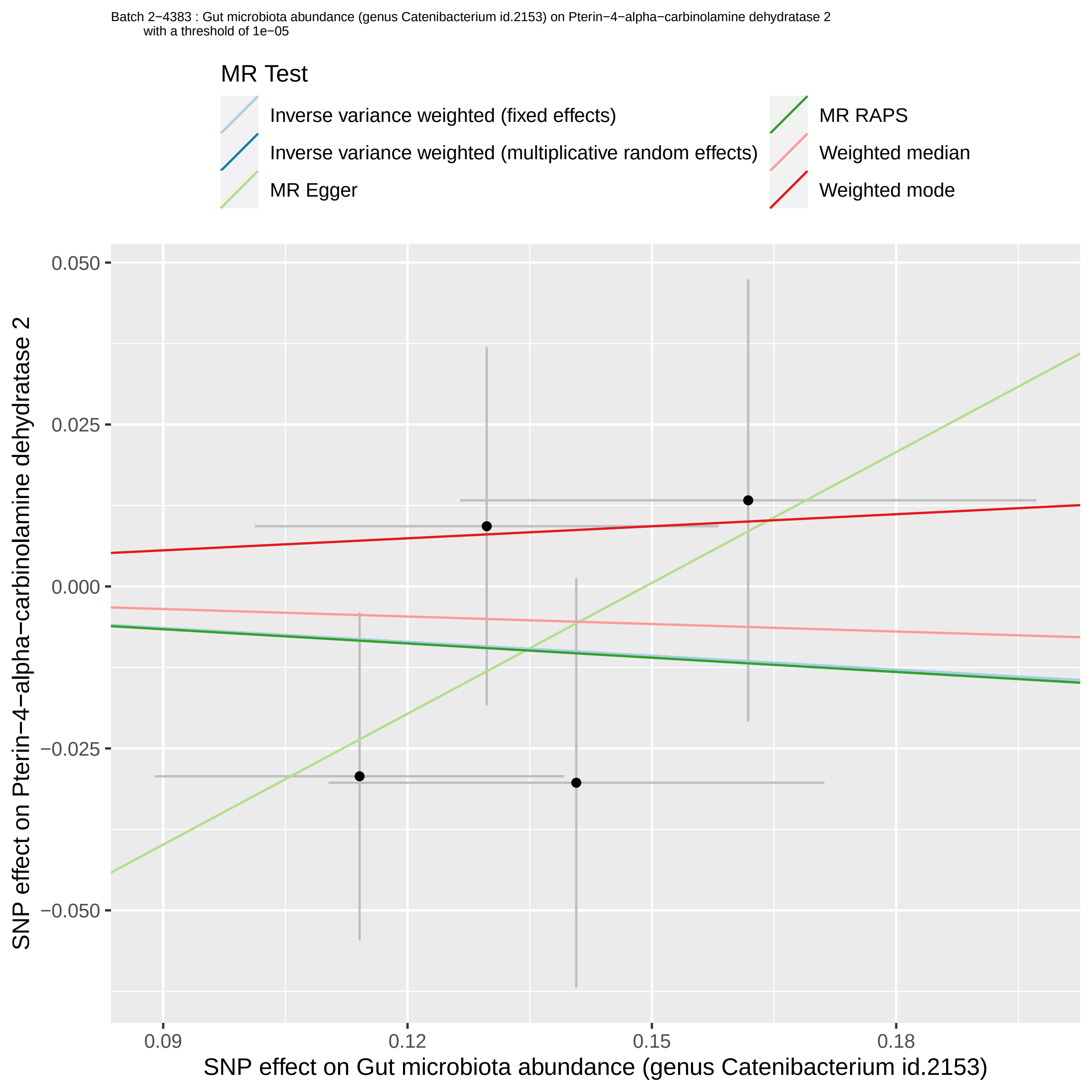
Batch 2−4383 : Gut microbiota abundance (genus Catenibacterium id.2153) on Pterin−4−alpha−carbinolamine dehydratase 2
with a threshold of 1e−05
MR Test
Inverse variance weighted (fixed effects)
Inverse variance weighted (multiplicative random effects)
MR Egger
MR RAPS
Weighted median
Weighted mode
0.050
0.025
0.000
−0.025
−0.050
0.09
0.12
0.15
0.18
SNP effect on Pterin−4−alpha−carbinolamine dehydratase 2
SNP effect on Gut microbiota abundance (genus Catenibacterium id.2153)

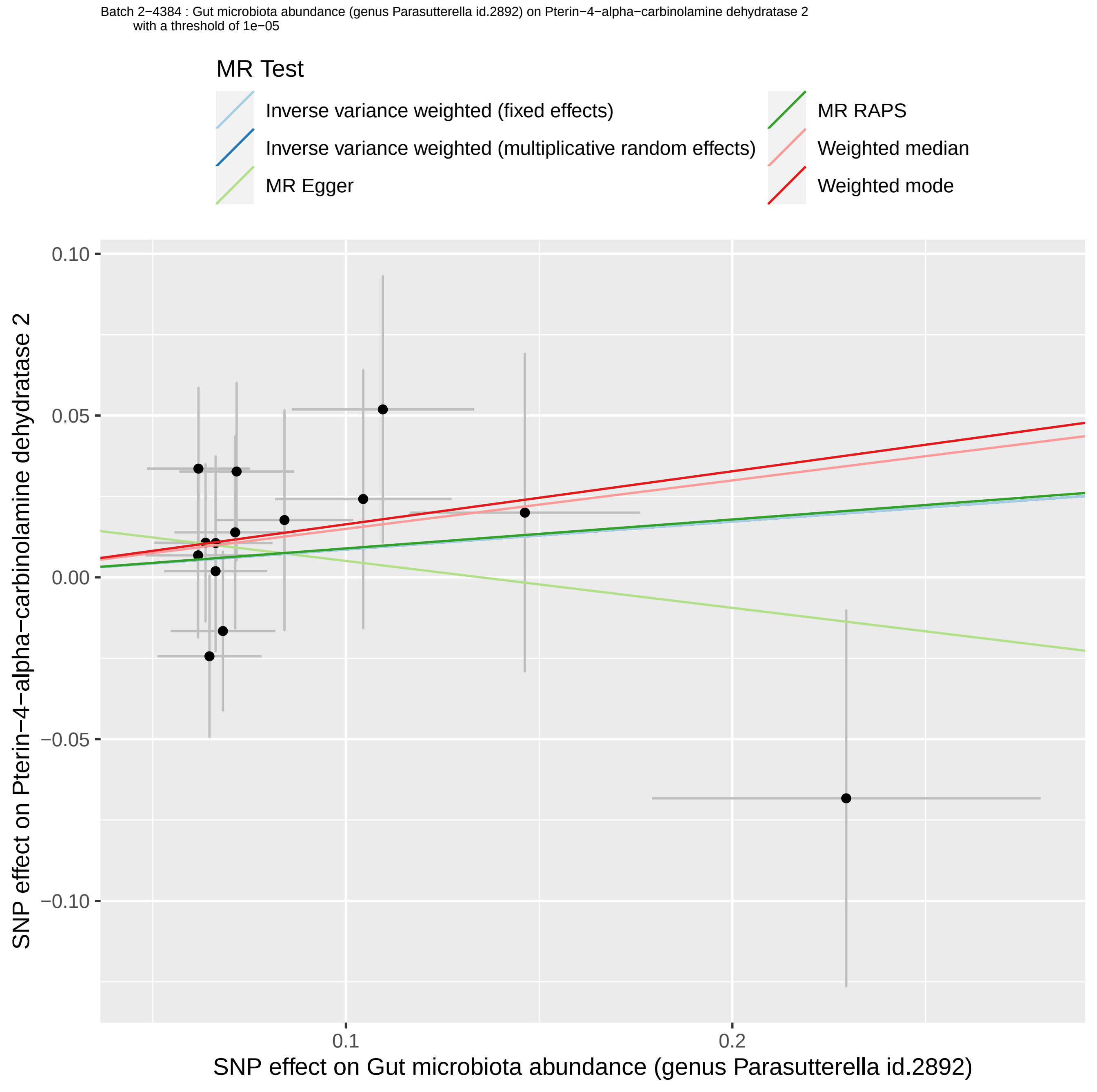

Batch 2−4384 : Gut microbiota abundance (genus Parasutterella id.2892) on Pterin−4−alpha−carbinolamine dehydratase 2
with a threshold of 1e−05
MR Test
Inverse variance weighted (fixed effects)
Inverse variance weighted (multiplicative random effects)
MR Egger
MR RAPS
Weighted median
Weighted mode
0.10
0.05
0.00
−0.05
−0.10
0.1
0.2
SNP effect on Pterin−4−alpha−carbinolamine dehydratase 2
SNP effect on Gut microbiota abundance (genus Parasutterella id.2892)

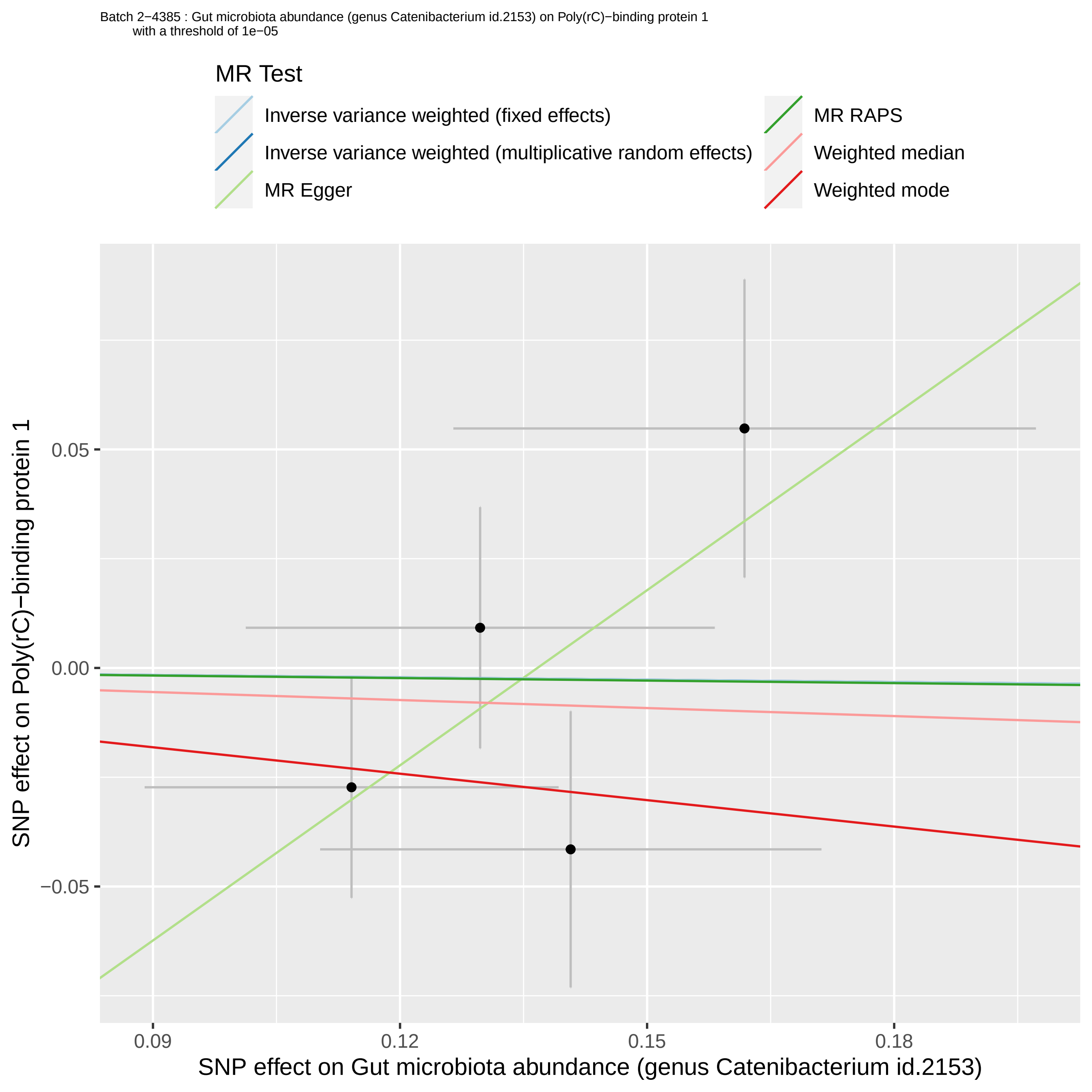

Batch 2−4385 : Gut microbiota abundance (genus Catenibacterium id.2153) on Poly(rC)−binding protein 1
with a threshold of 1e−05
MR Test
Inverse variance weighted (fixed effects)
Inverse variance weighted (multiplicative random effects)
MR Egger
MR RAPS
Weighted median
Weighted mode
SNP effect on Poly(rC)−binding protein 1
0.05
0.00
−0.05
0.09
0.12
0.15
0.18
SNP effect on Gut microbiota abundance (genus Catenibacterium id.2153)

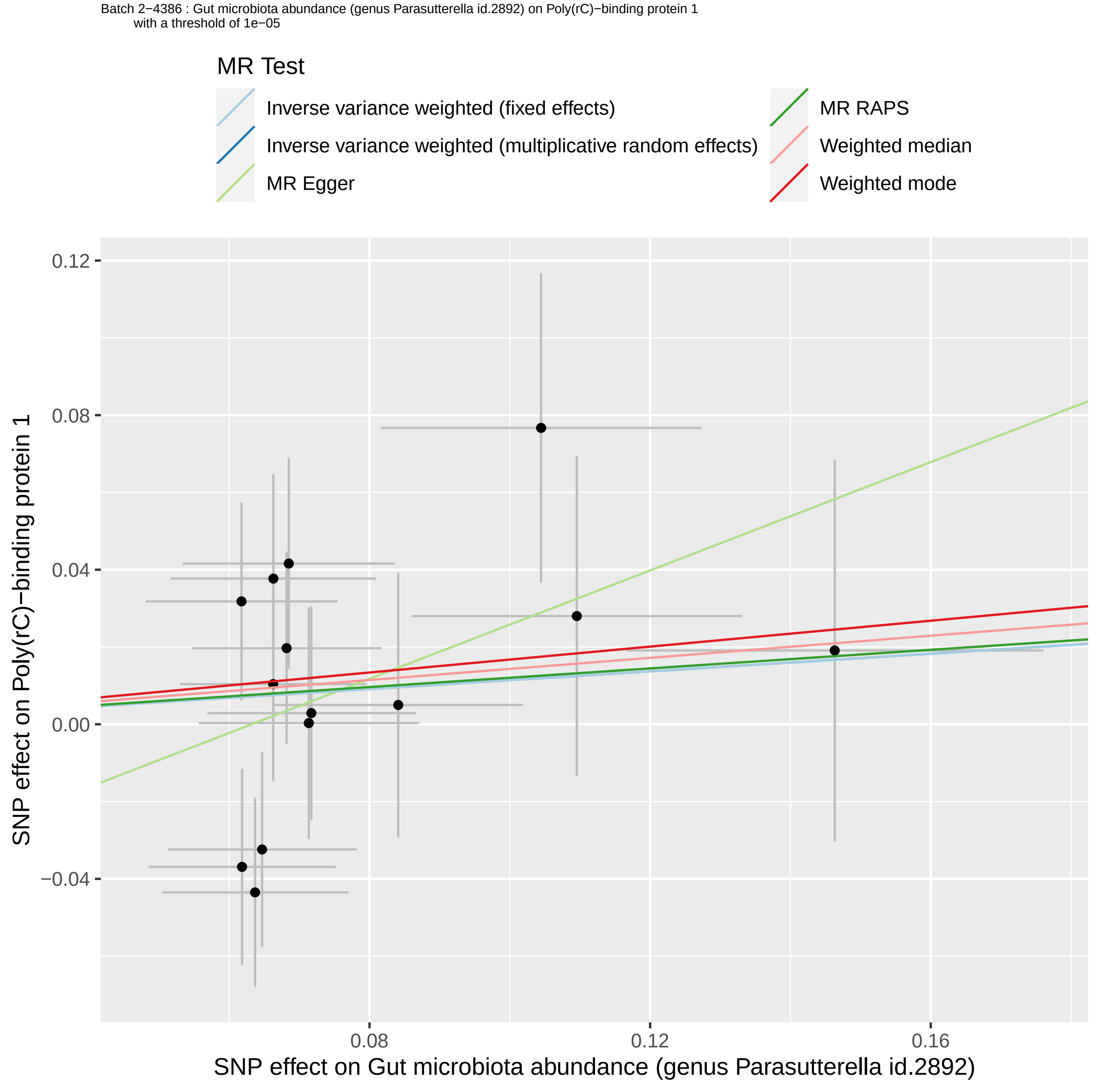

Batch 2−4386 : Gut microbiota abundance (genus Parasutterella id.2892) on Poly(rC)−binding protein 1
with a threshold of 1e−05
MR Test
Inverse variance weighted (fixed effects)
Inverse variance weighted (multiplicative random effects)
MR Egger
MR RAPS
Weighted median
Weighted mode
0.12
0.08
0.04
0.00
−0.04
0.08
0.12
0.16
SNP effect on Poly(rC)−binding protein 1
SNP effect on Gut microbiota abundance (genus Parasutterella id.2892)

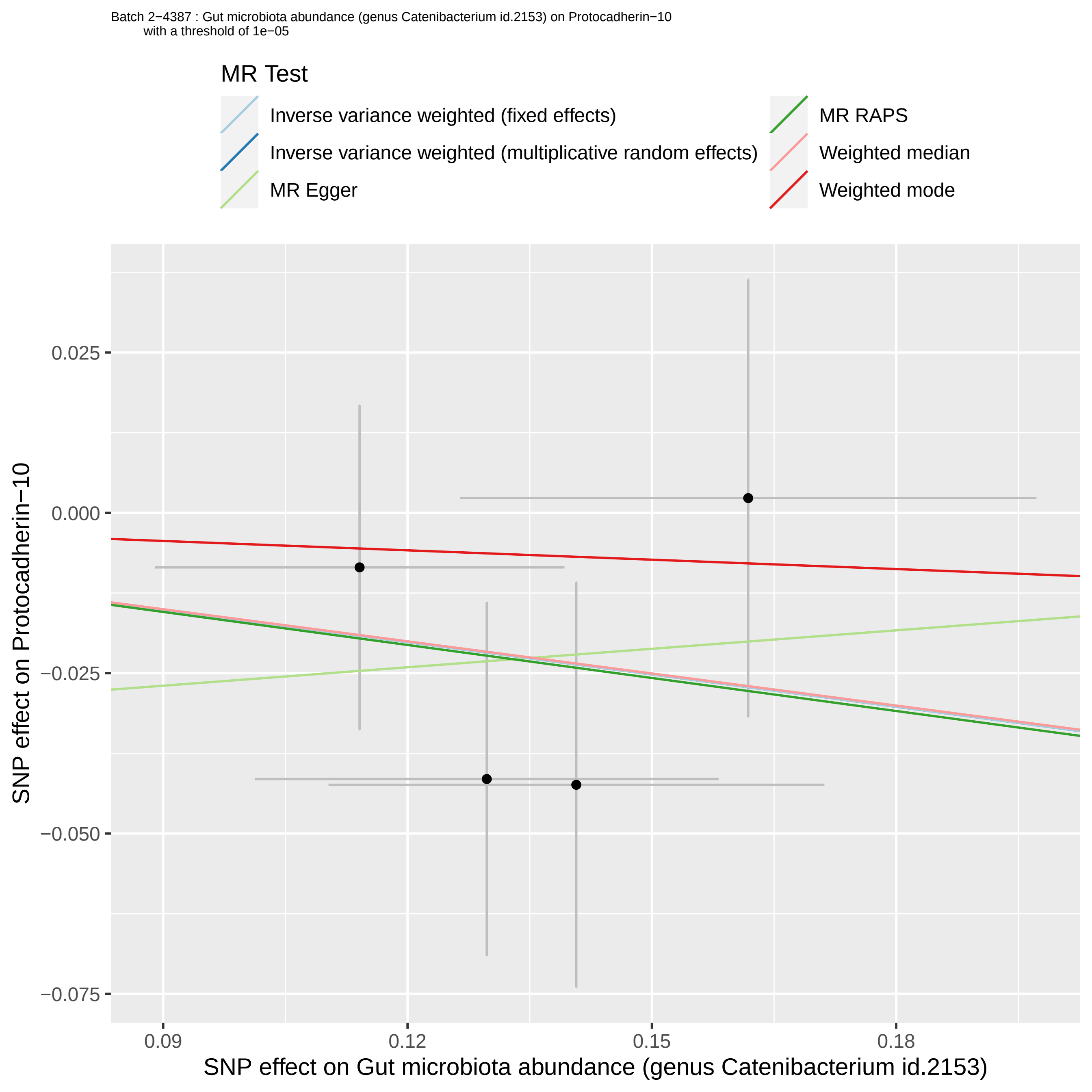

Batch 2−4387 : Gut microbiota abundance (genus Catenibacterium id.2153) on Protocadherin−10
with a threshold of 1e−05
MR Test
Inverse variance weighted (fixed effects)
Inverse variance weighted (multiplicative random effects)
MR Egger
MR RAPS
Weighted median
Weighted mode
0.025
0.000
−0.025
−0.050
−0.075
0.09
0.12
0.15
0.18
SNP effect on Protocadherin−10
SNP effect on Gut microbiota abundance (genus Catenibacterium id.2153)

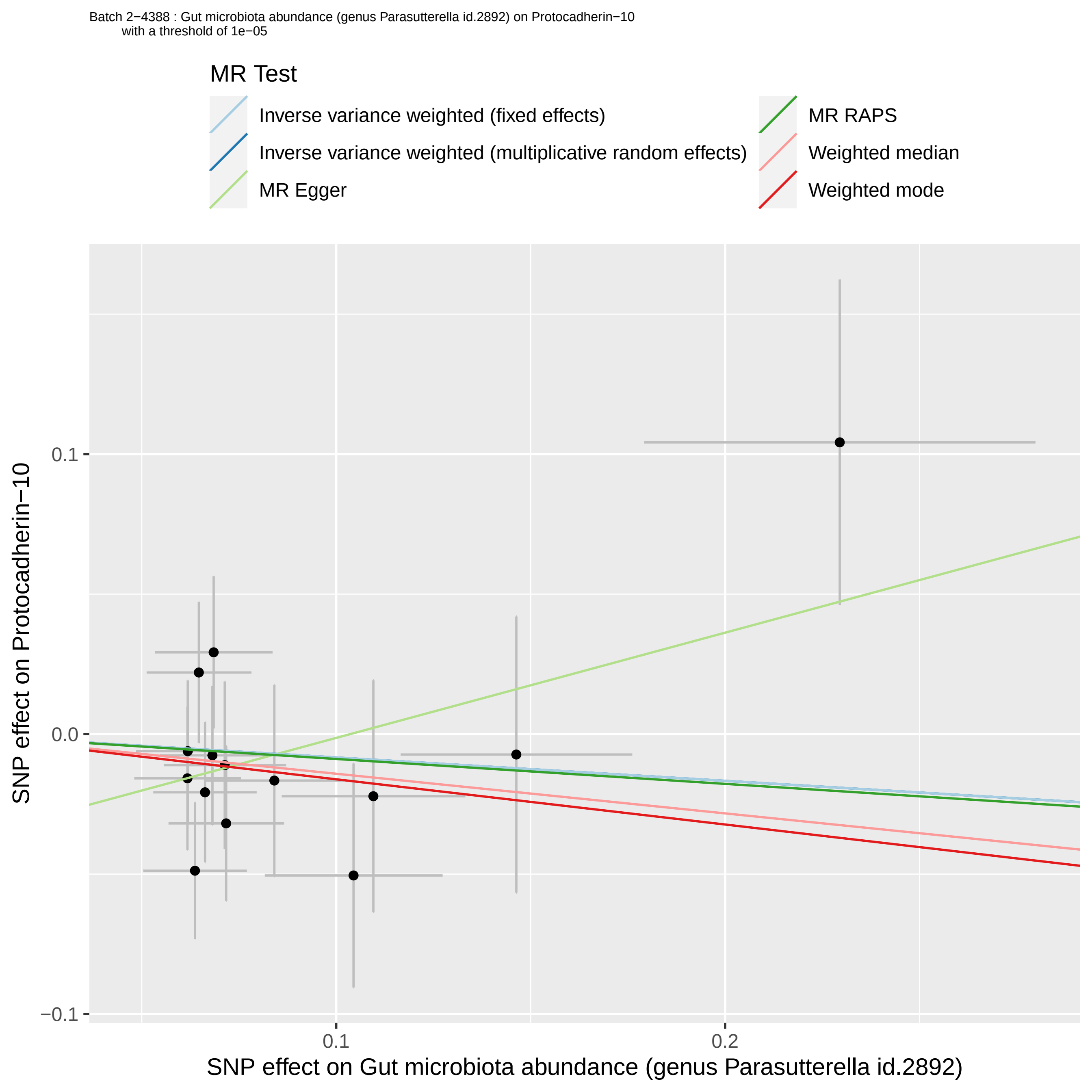
Batch 2−4388 : Gut microbiota abundance (genus Parasutterella id.2892) on Protocadherin−10
with a threshold of 1e−05
MR Test
Inverse variance weighted (fixed effects)
Inverse variance weighted (multiplicative random effects)
MR Egger
MR RAPS
Weighted median
Weighted mode
SNP effect on Protocadherin−10
0.1
0.0
−0.1
0.1
0.2
SNP effect on Gut microbiota abundance (genus Parasutterella id.2892)

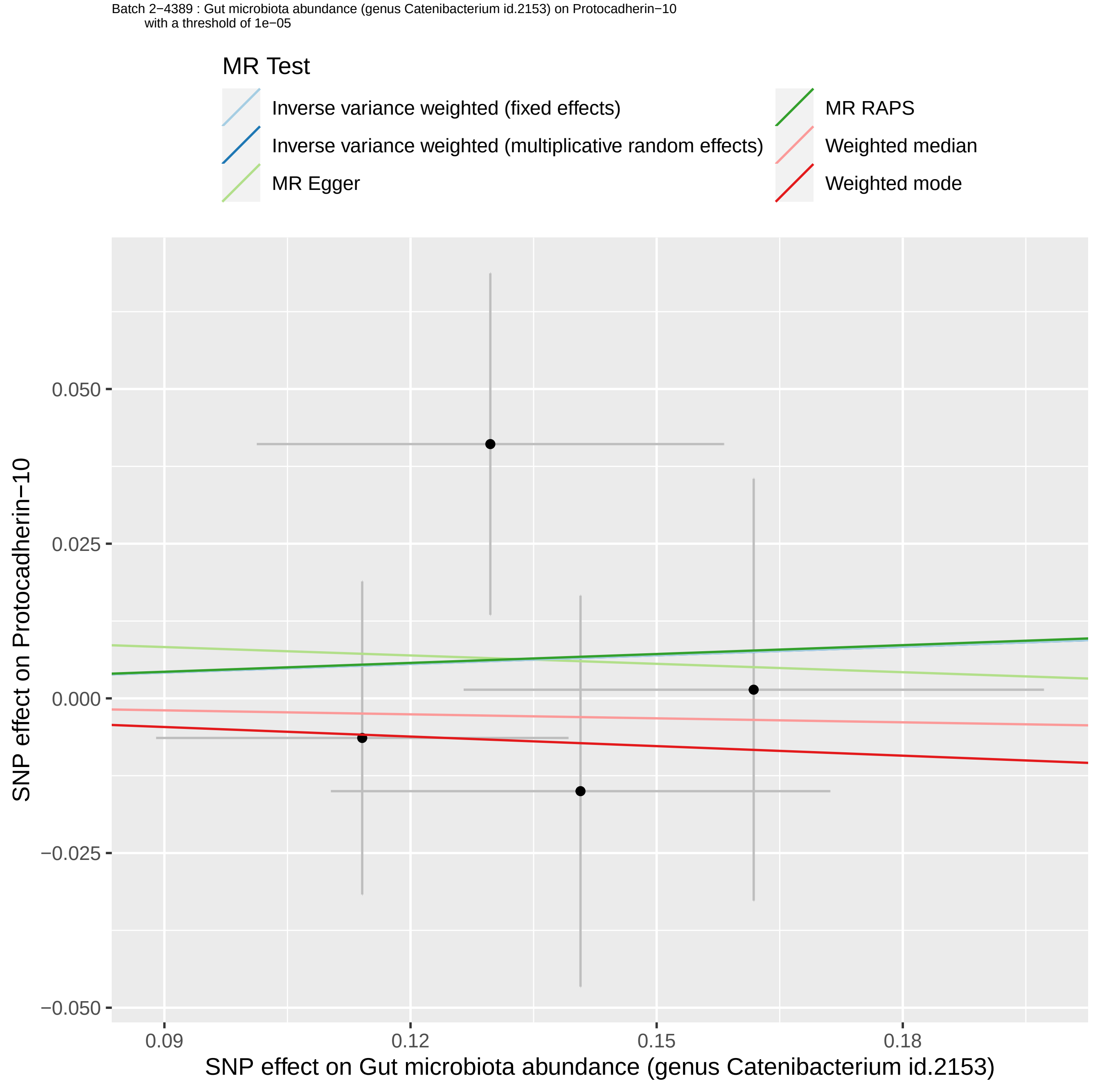

Batch 2−4389 : Gut microbiota abundance (genus Catenibacterium id.2153) on Protocadherin−10
with a threshold of 1e−05
MR Test
Inverse variance weighted (fixed effects)
Inverse variance weighted (multiplicative random effects)
MR Egger
MR RAPS
Weighted median
Weighted mode
0.050
0.025
0.000
−0.025
−0.050
0.09
0.12
0.15
0.18
SNP effect on Protocadherin−10
SNP effect on Gut microbiota abundance (genus Catenibacterium id.2153)

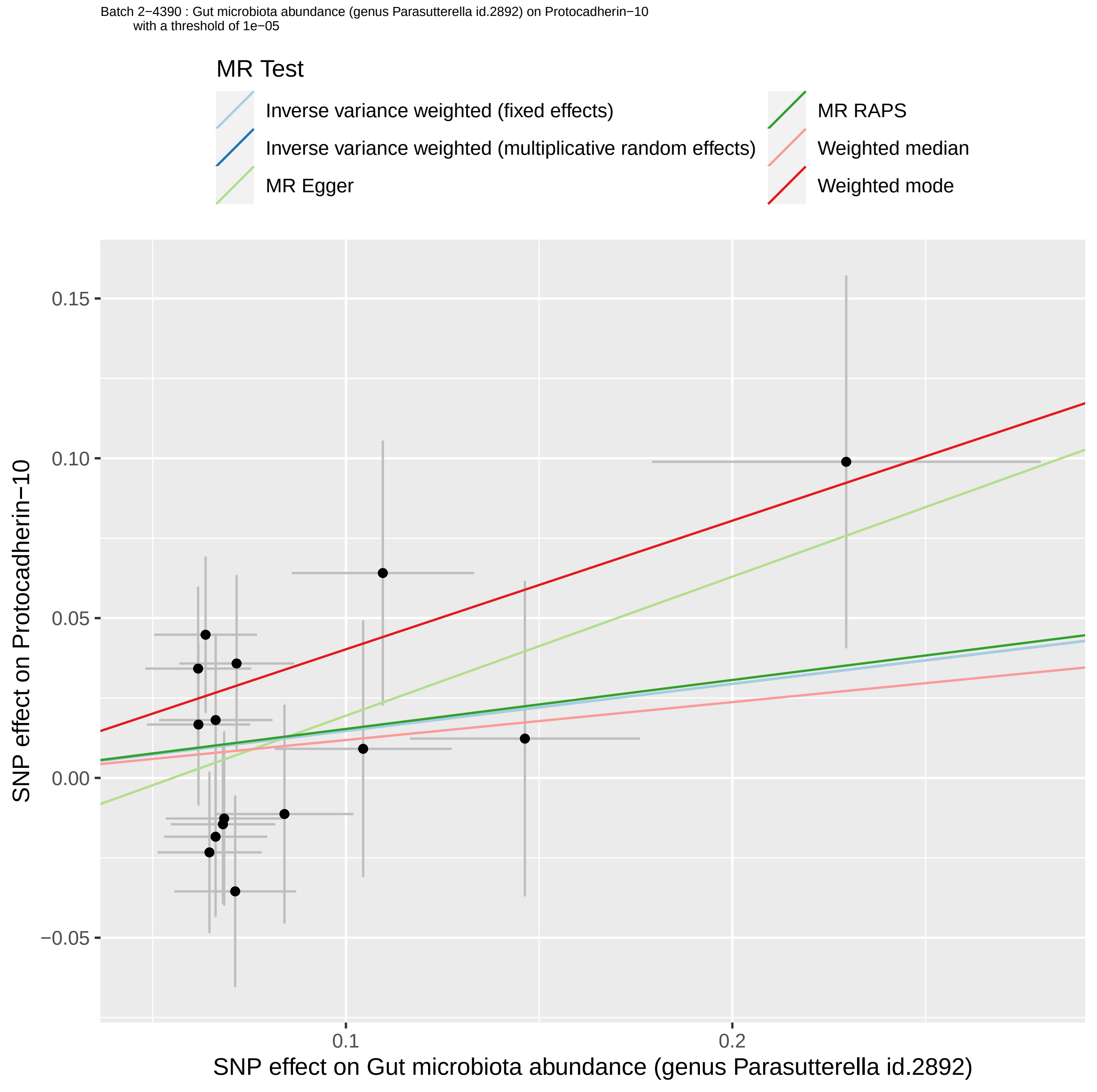
Batch 2−4390 : Gut microbiota abundance (genus Parasutterella id.2892) on Protocadherin−10
with a threshold of 1e−05
MR Test
Inverse variance weighted (fixed effects)
Inverse variance weighted (multiplicative random effects)
MR Egger
MR RAPS
Weighted median
Weighted mode
0.15
0.10
0.05
0.00
−0.05
0.1
0.2
SNP effect on Protocadherin−10
SNP effect on Gut microbiota abundance (genus Parasutterella id.2892)

Batch 2−4391 : Gut microbiota abundance (genus Catenibacterium id.2153) on Protocadherin−15
with a threshold of 1e−05

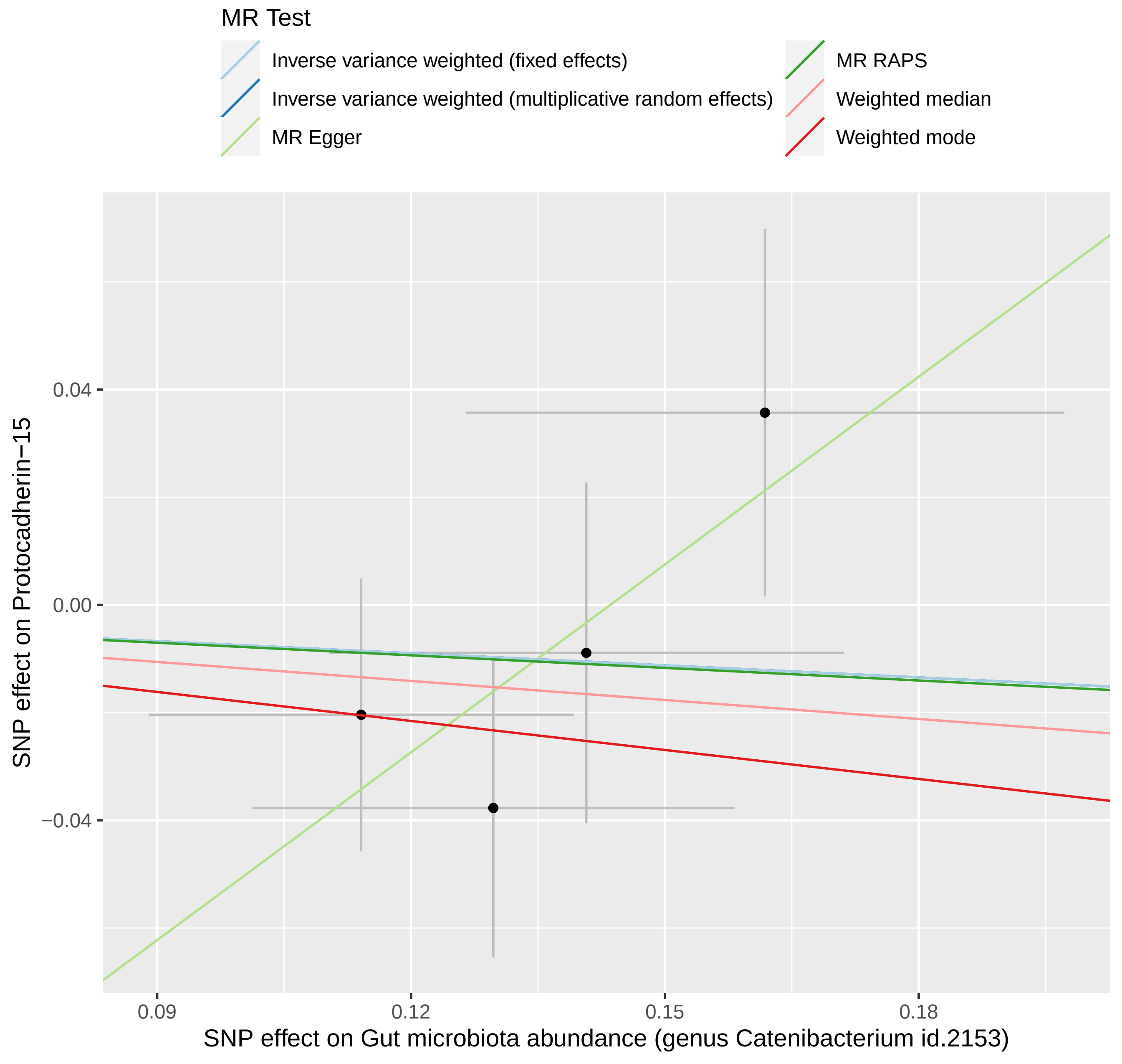

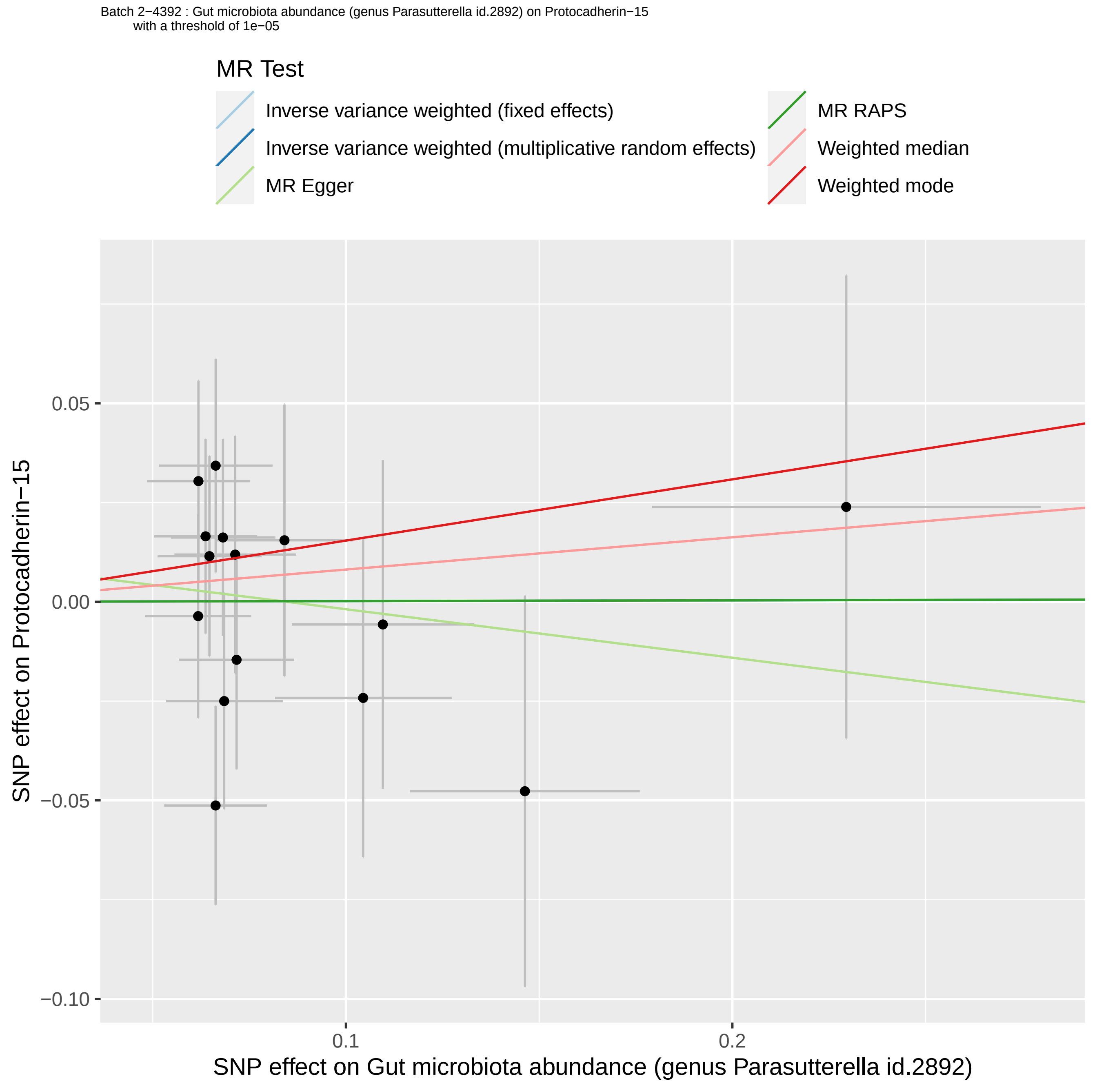

Batch 2−4392 : Gut microbiota abundance (genus Parasutterella id.2892) on Protocadherin−15
with a threshold of 1e−05
MR Test
Inverse variance weighted (fixed effects)
Inverse variance weighted (multiplicative random effects)
MR Egger
MR RAPS
Weighted median
Weighted mode
0.05
0.00
−0.05
−0.10
0.1
0.2
SNP effect on Protocadherin−15
SNP effect on Gut microbiota abundance (genus Parasutterella id.2892)

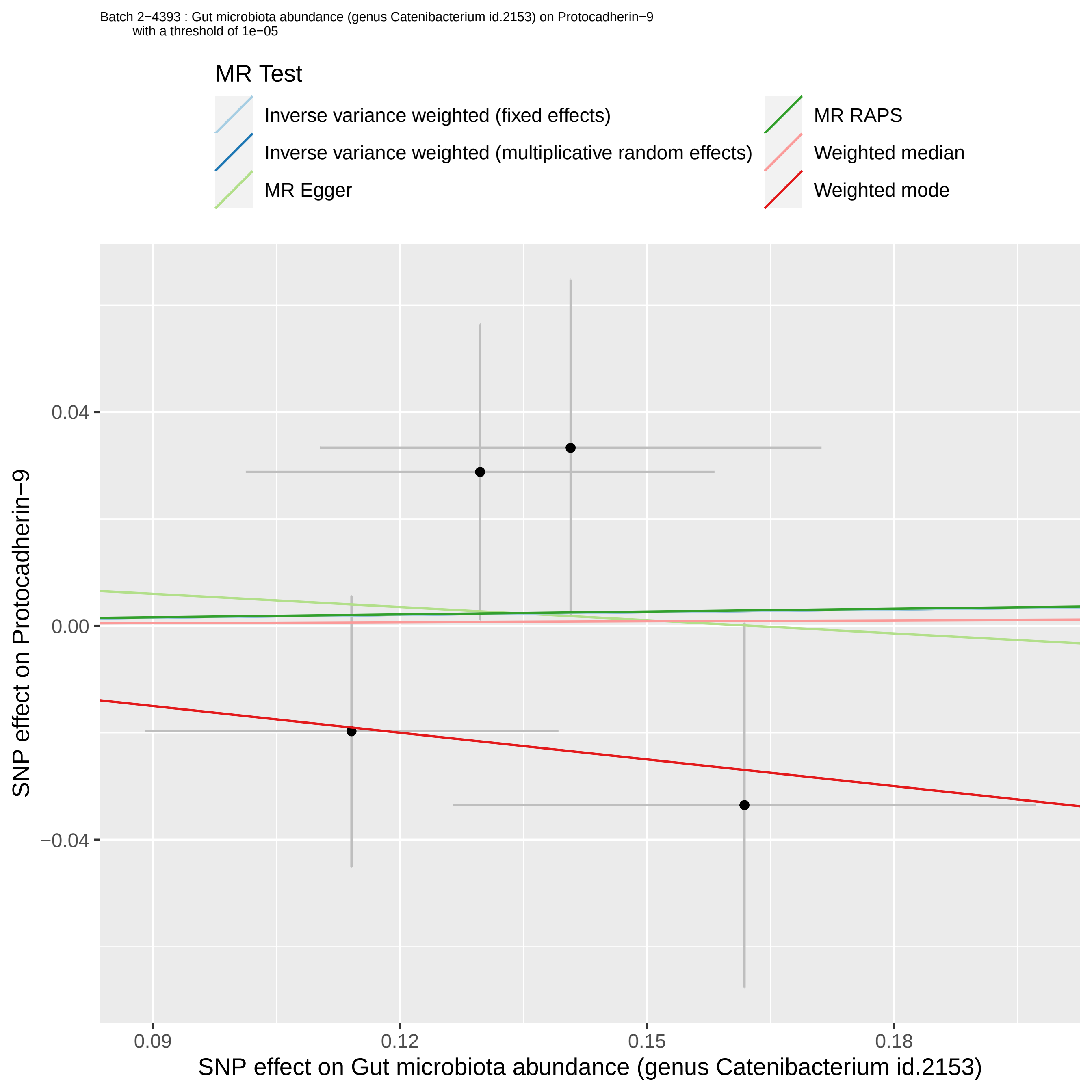
Batch 2−4393 : Gut microbiota abundance (genus Catenibacterium id.2153) on Protocadherin−9
with a threshold of 1e−05
MR Test
Inverse variance weighted (fixed effects)
Inverse variance weighted (multiplicative random effects)
MR Egger
MR RAPS
Weighted median
Weighted mode
0.04
0.00
−0.04
0.09
0.12
0.15
0.18
SNP effect on Protocadherin−9
SNP effect on Gut microbiota abundance (genus Catenibacterium id.2153)

Batch 2−4394 : Gut microbiota abundance (genus Parasutterella id.2892) on Protocadherin−9
with a threshold of 1e−05

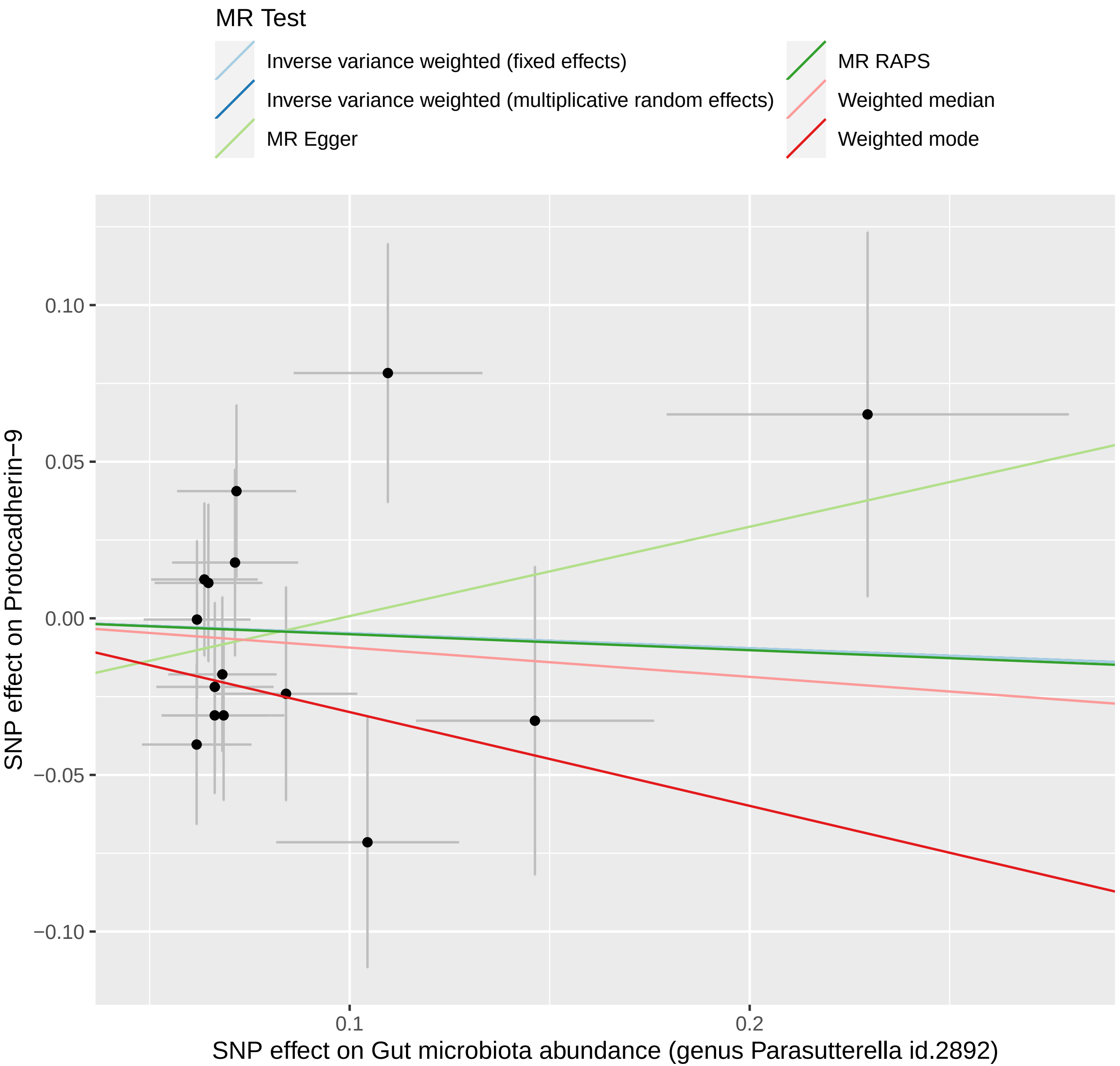

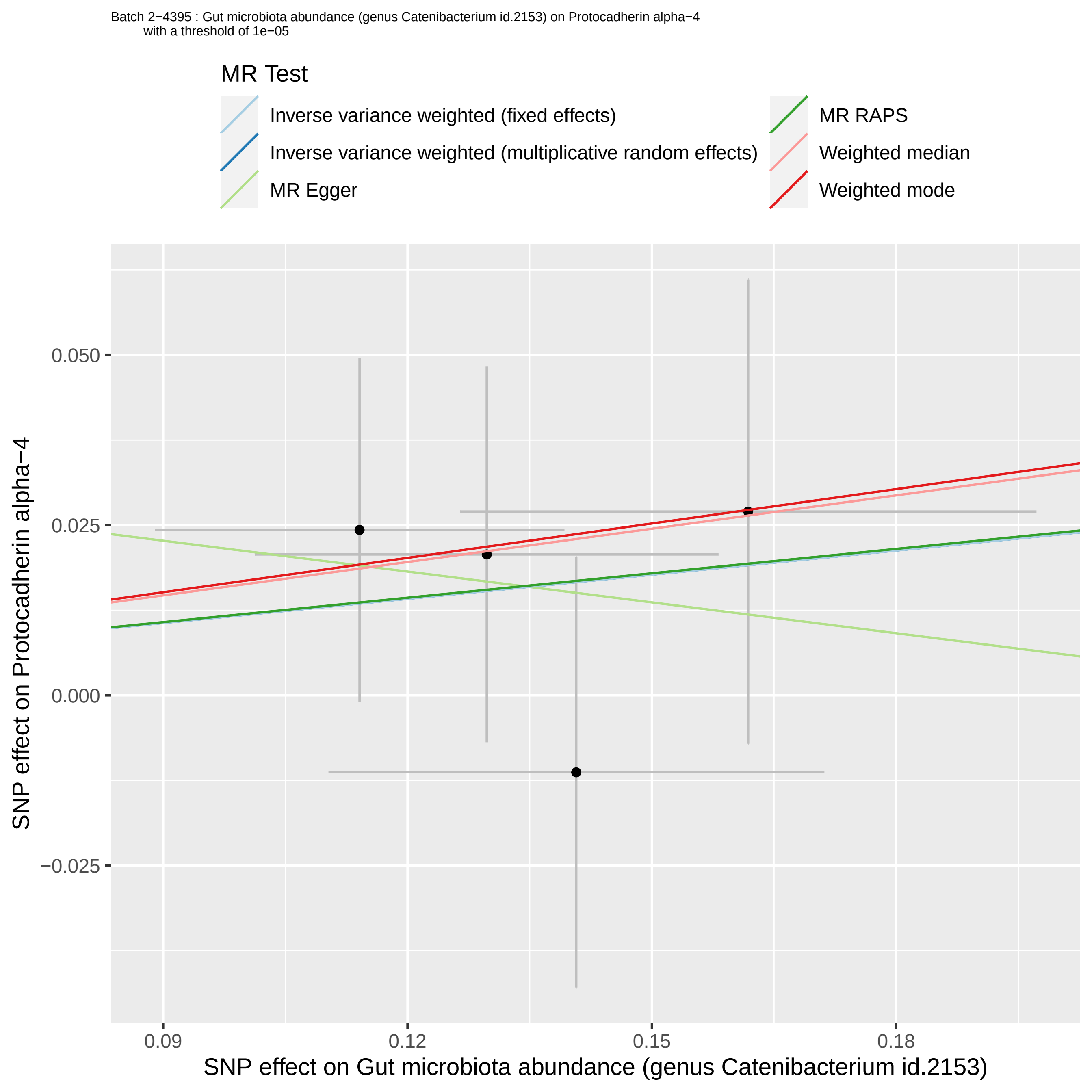

Batch 2−4395 : Gut microbiota abundance (genus Catenibacterium id.2153) on Protocadherin alpha−4
with a threshold of 1e−05
MR Test
Inverse variance weighted (fixed effects)
Inverse variance weighted (multiplicative random effects)
MR Egger
MR RAPS
Weighted median
Weighted mode
0.050
0.025
0.000
−0.025
0.09
0.12
0.15
0.18
SNP effect on Protocadherin alpha−4
SNP effect on Gut microbiota abundance (genus Catenibacterium id.2153)

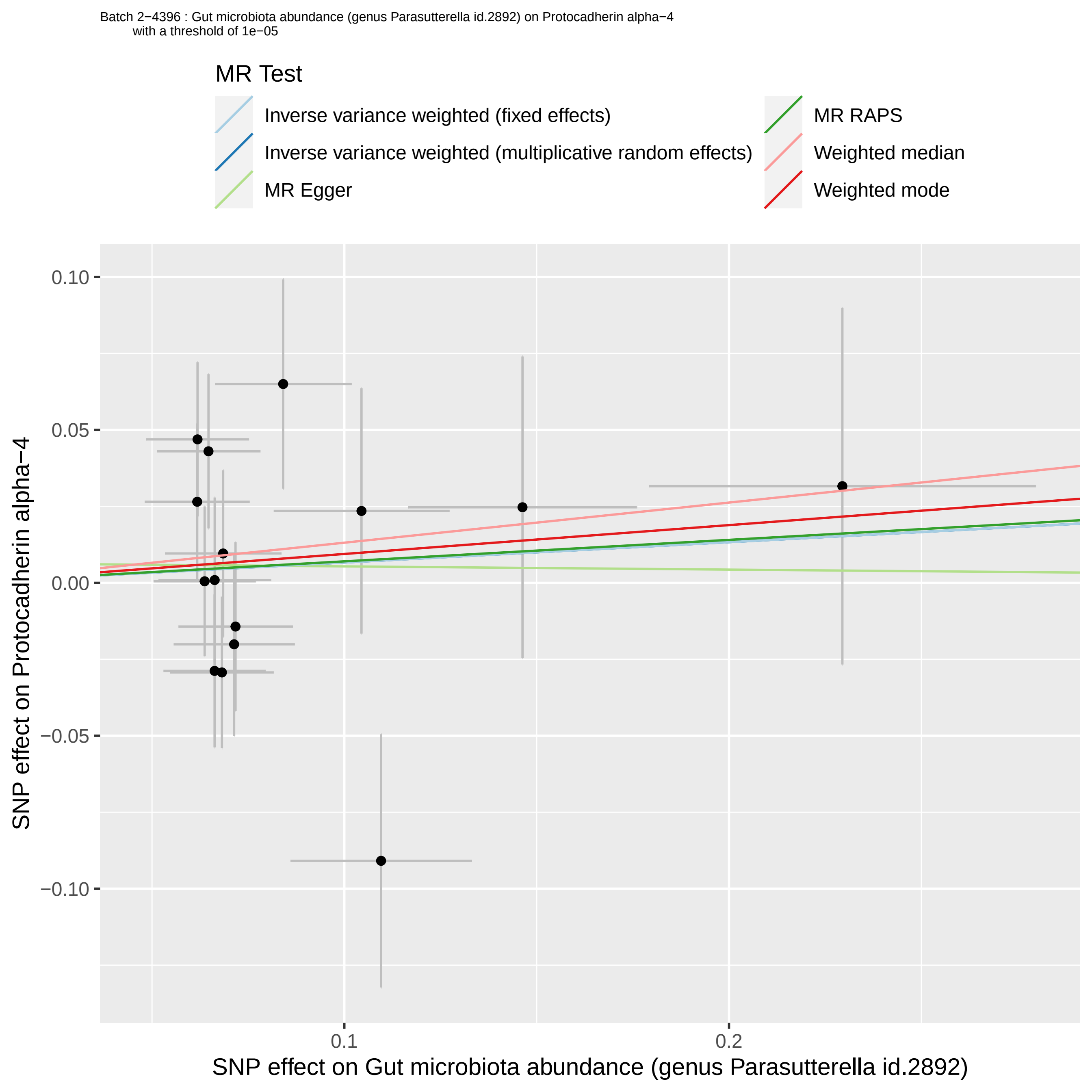

Batch 2−4396 : Gut microbiota abundance (genus Parasutterella id.2892) on Protocadherin alpha−4
with a threshold of 1e−05
MR Test
Inverse variance weighted (fixed effects)
Inverse variance weighted (multiplicative random effects)
MR Egger
MR RAPS
Weighted median
Weighted mode
0.10
0.05
0.00
−0.05
−0.10
0.1
0.2
SNP effect on Protocadherin alpha−4
SNP effect on Gut microbiota abundance (genus Parasutterella id.2892)

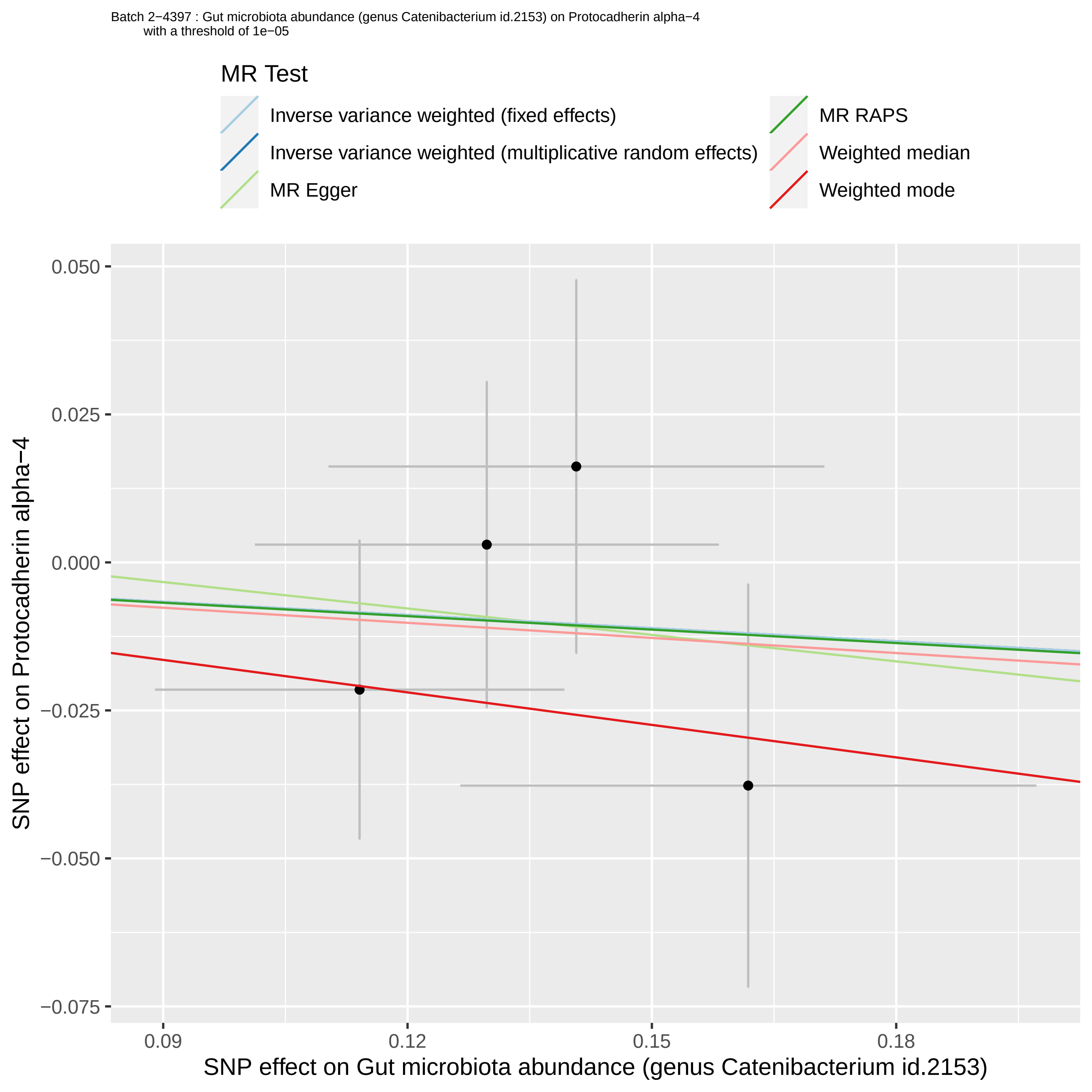
Batch 2−4397 : Gut microbiota abundance (genus Catenibacterium id.2153) on Protocadherin alpha−4
with a threshold of 1e−05
MR Test
Inverse variance weighted (fixed effects)
Inverse variance weighted (multiplicative random effects)
MR Egger
MR RAPS
Weighted median
Weighted mode
0.050
0.025
0.000
−0.025
−0.050
−0.075
0.09
0.12
0.15
0.18
SNP effect on Protocadherin alpha−4
SNP effect on Gut microbiota abundance (genus Catenibacterium id.2153)

Batch 2−4398 : Gut microbiota abundance (genus Parasutterella id.2892) on Protocadherin alpha−4
with a threshold of 1e−05

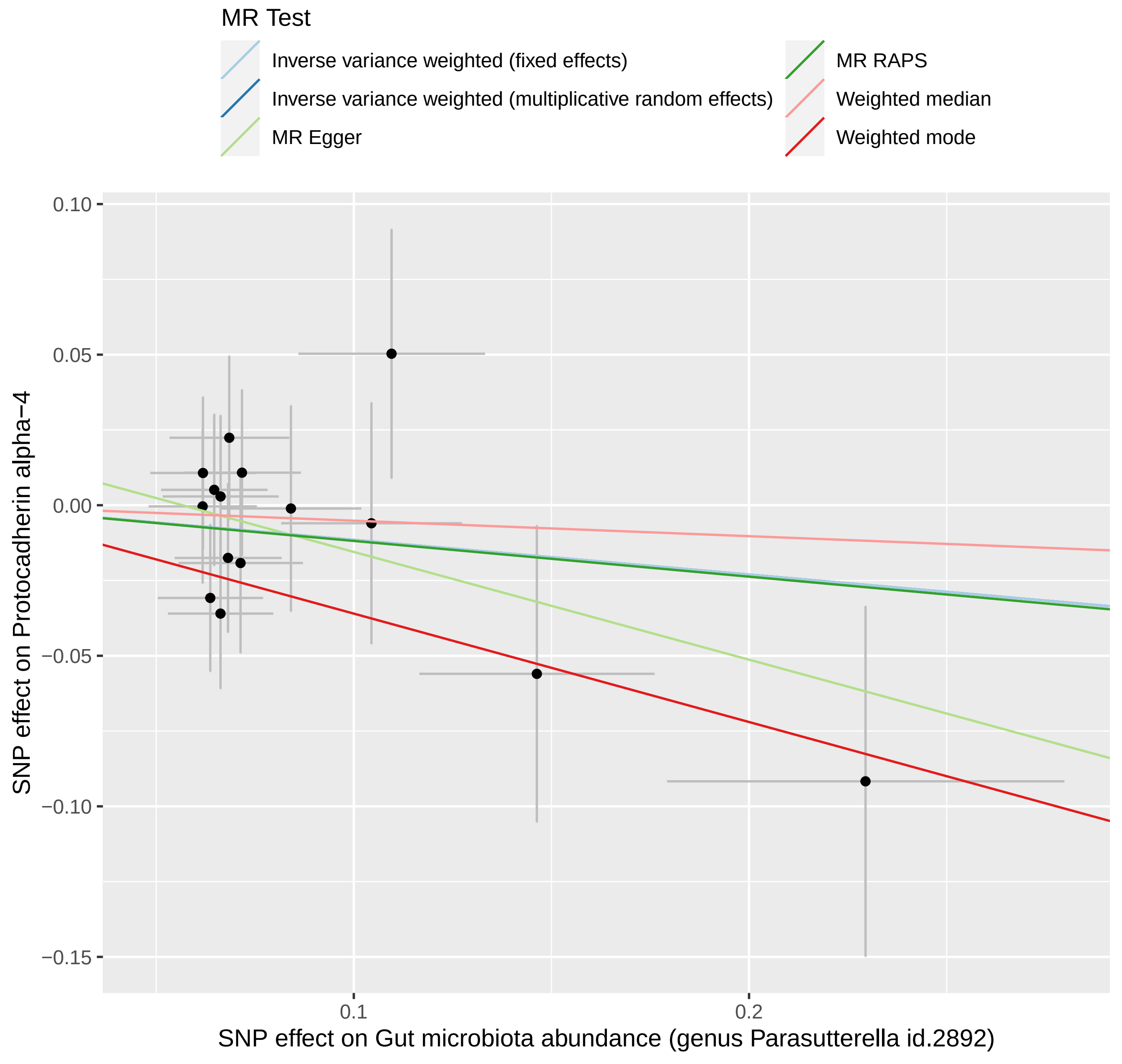

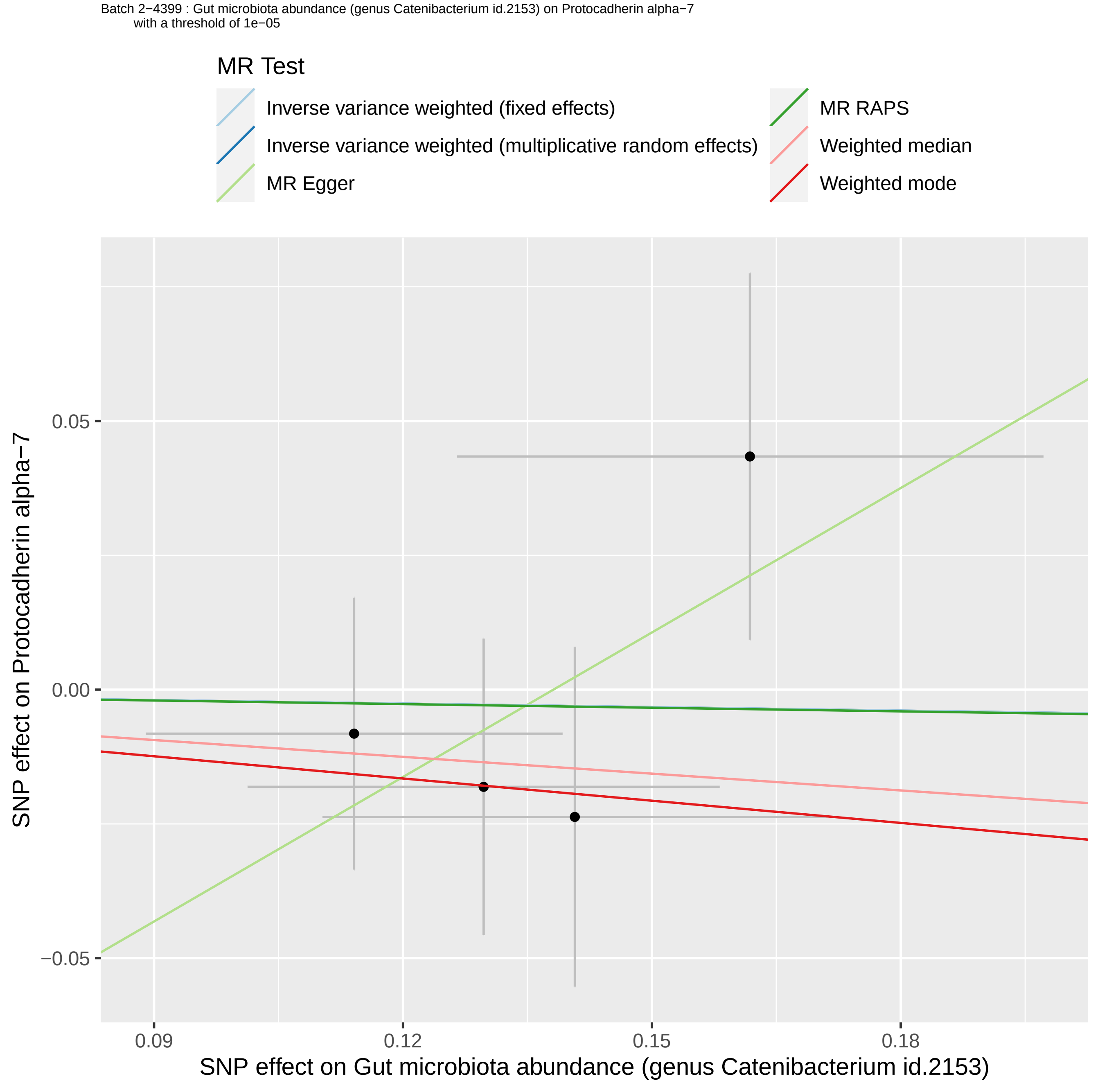

Batch 2−4399 : Gut microbiota abundance (genus Catenibacterium id.2153) on Protocadherin alpha−7
with a threshold of 1e−05
MR Test
Inverse variance weighted (fixed effects)
Inverse variance weighted (multiplicative random effects)
MR Egger
MR RAPS
Weighted median
Weighted mode
0.05
0.00
−0.05
0.09
0.12
0.15
0.18
SNP effect on Protocadherin alpha−7
SNP effect on Gut microbiota abundance (genus Catenibacterium id.2153)

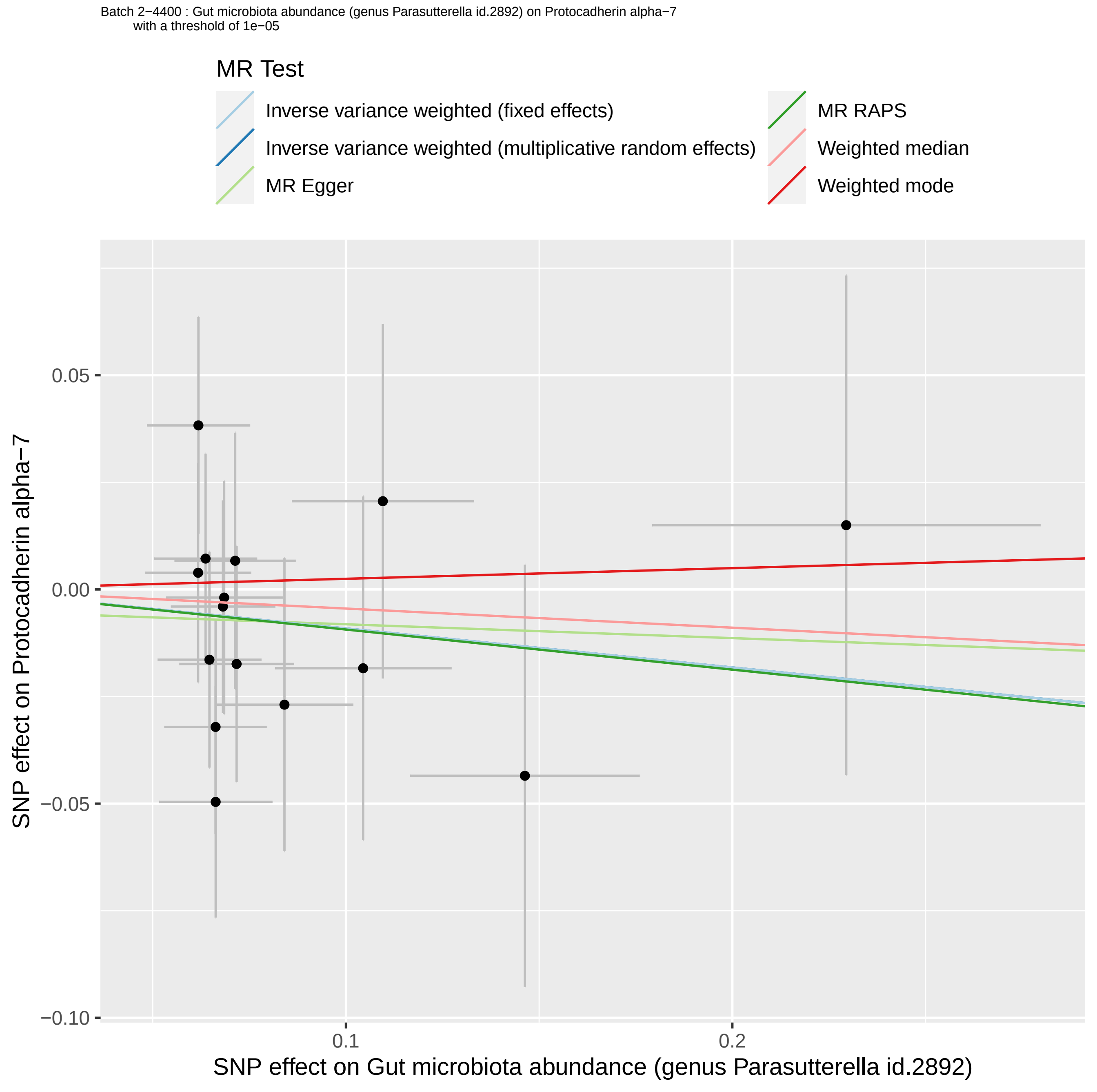

Batch 2−4400 : Gut microbiota abundance (genus Parasutterella id.2892) on Protocadherin alpha−7
with a threshold of 1e−05
MR Test
Inverse variance weighted (fixed effects)
Inverse variance weighted (multiplicative random effects)
MR Egger
MR RAPS
Weighted median
Weighted mode
0.05
0.00
−0.05
−0.10
0.1
0.2
SNP effect on Protocadherin alpha−7
SNP effect on Gut microbiota abundance (genus Parasutterella id.2892)

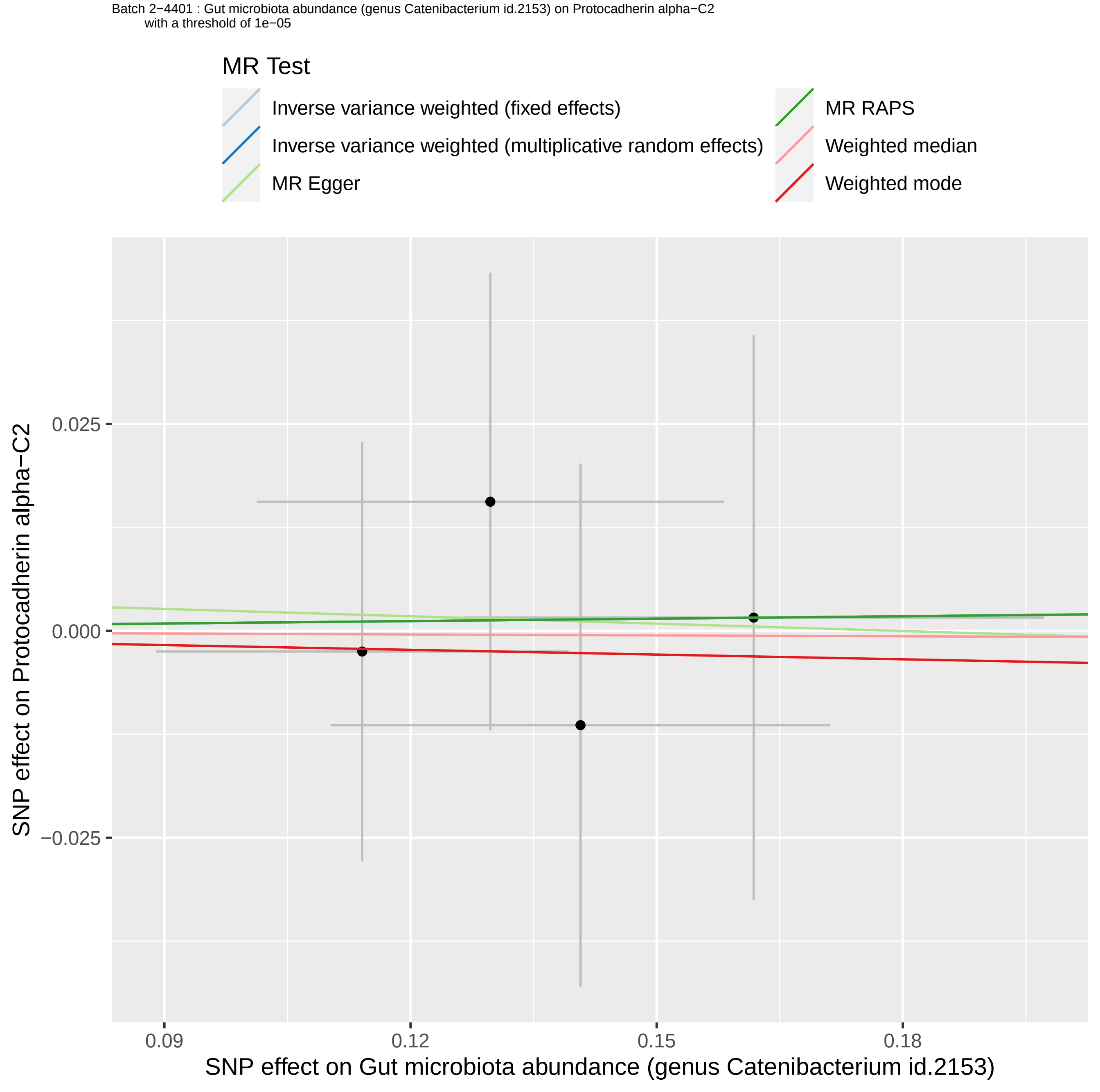

Batch 2−4401 : Gut microbiota abundance (genus Catenibacterium id.2153) on Protocadherin alpha−C2
with a threshold of 1e−05
MR Test
Inverse variance weighted (fixed effects)
Inverse variance weighted (multiplicative random effects)
MR Egger
MR RAPS
Weighted median
Weighted mode
0.025
0.000
−0.025
0.09
0.12
0.15
0.18
SNP effect on Protocadherin alpha−C2
SNP effect on Gut microbiota abundance (genus Catenibacterium id.2153)

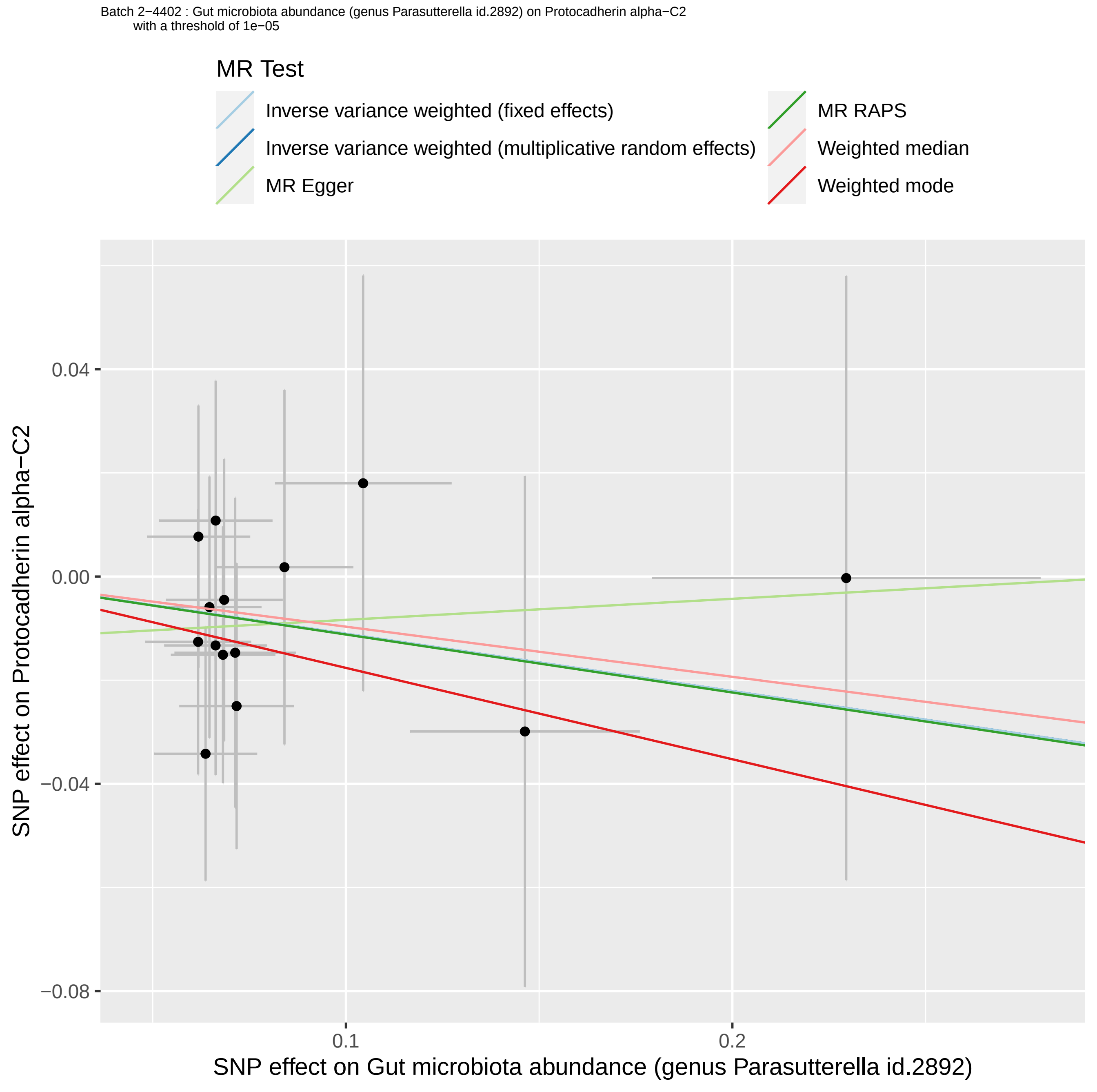
Batch 2−4402 : Gut microbiota abundance (genus Parasutterella id.2892) on Protocadherin alpha−C2
with a threshold of 1e−05
MR Test
Inverse variance weighted (fixed effects)
Inverse variance weighted (multiplicative random effects)
MR Egger
MR RAPS
Weighted median
Weighted mode
0.04
0.00
−0.04
−0.08
0.1
0.2
SNP effect on Protocadherin alpha−C2
SNP effect on Gut microbiota abundance (genus Parasutterella id.2892)

Batch 2−4403 : Gut microbiota abundance (genus Catenibacterium id.2153) on Protocadherin beta−10
with a threshold of 1e−05

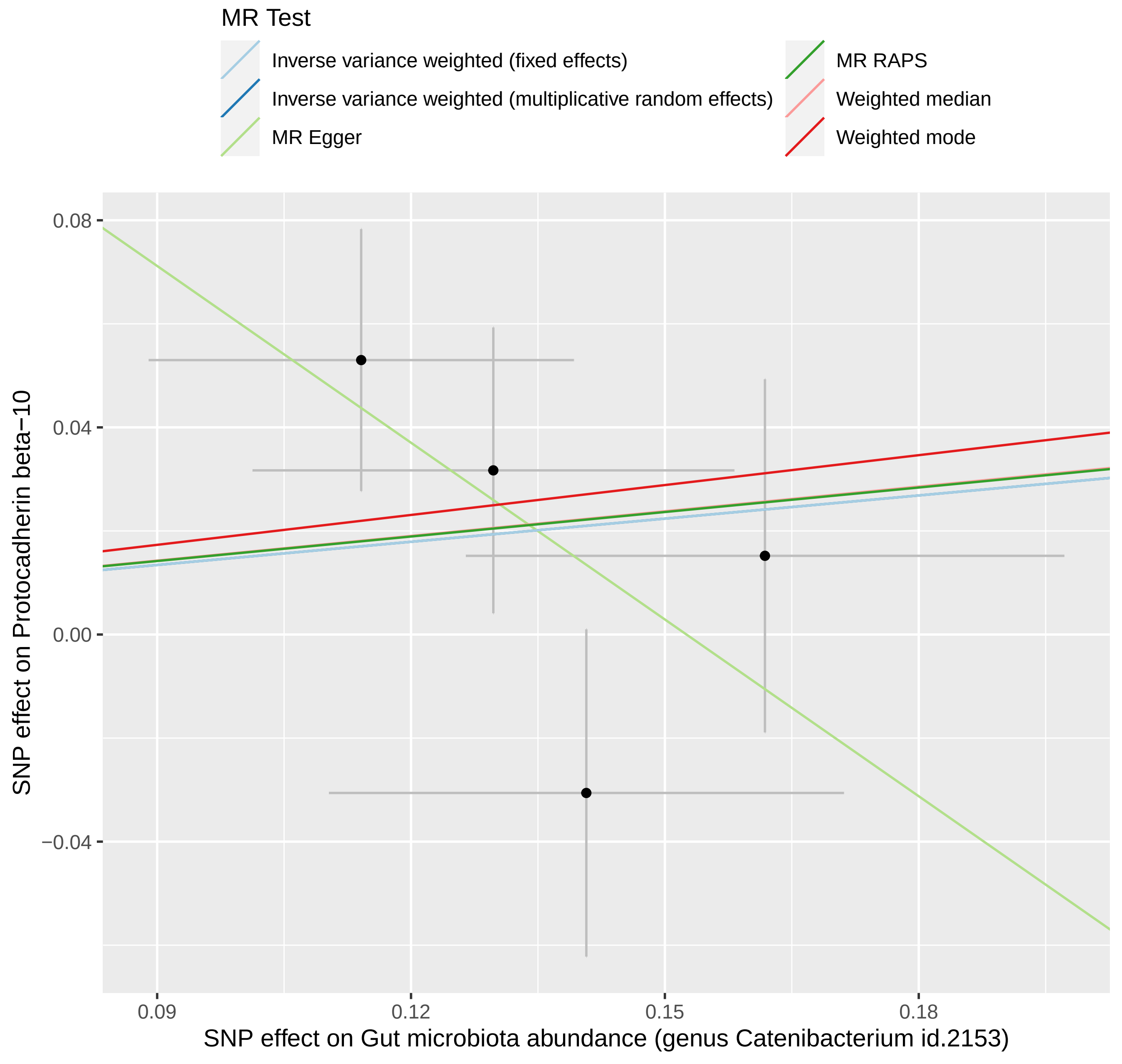

Batch 2−4404 : Gut microbiota abundance (genus Parasutterella id.2892) on Protocadherin beta−10
with a threshold of 1e−05

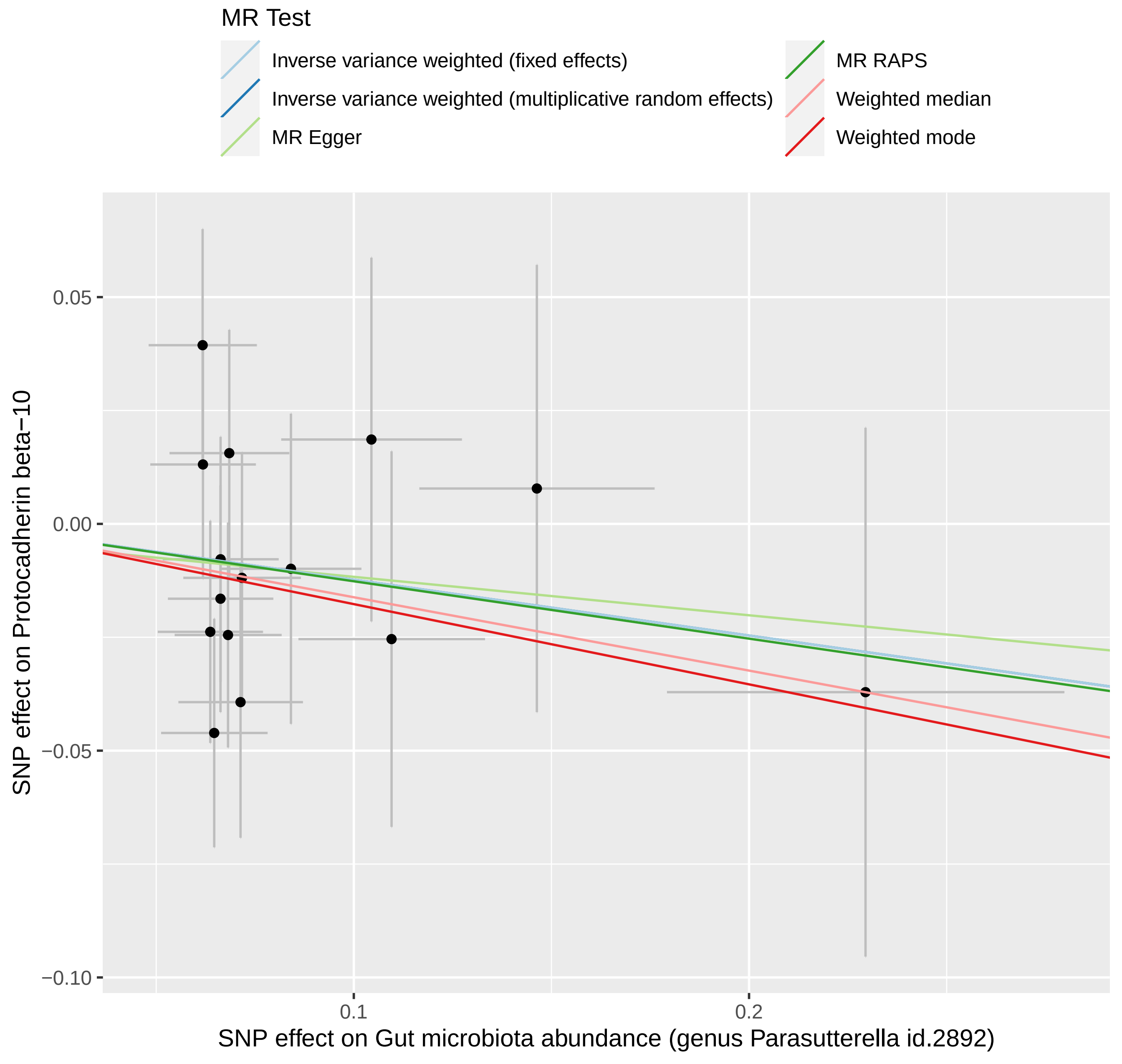

Batch 2−4405 : Gut microbiota abundance (genus Catenibacterium id.2153) on Protocadherin beta−1
with a threshold of 1e−05

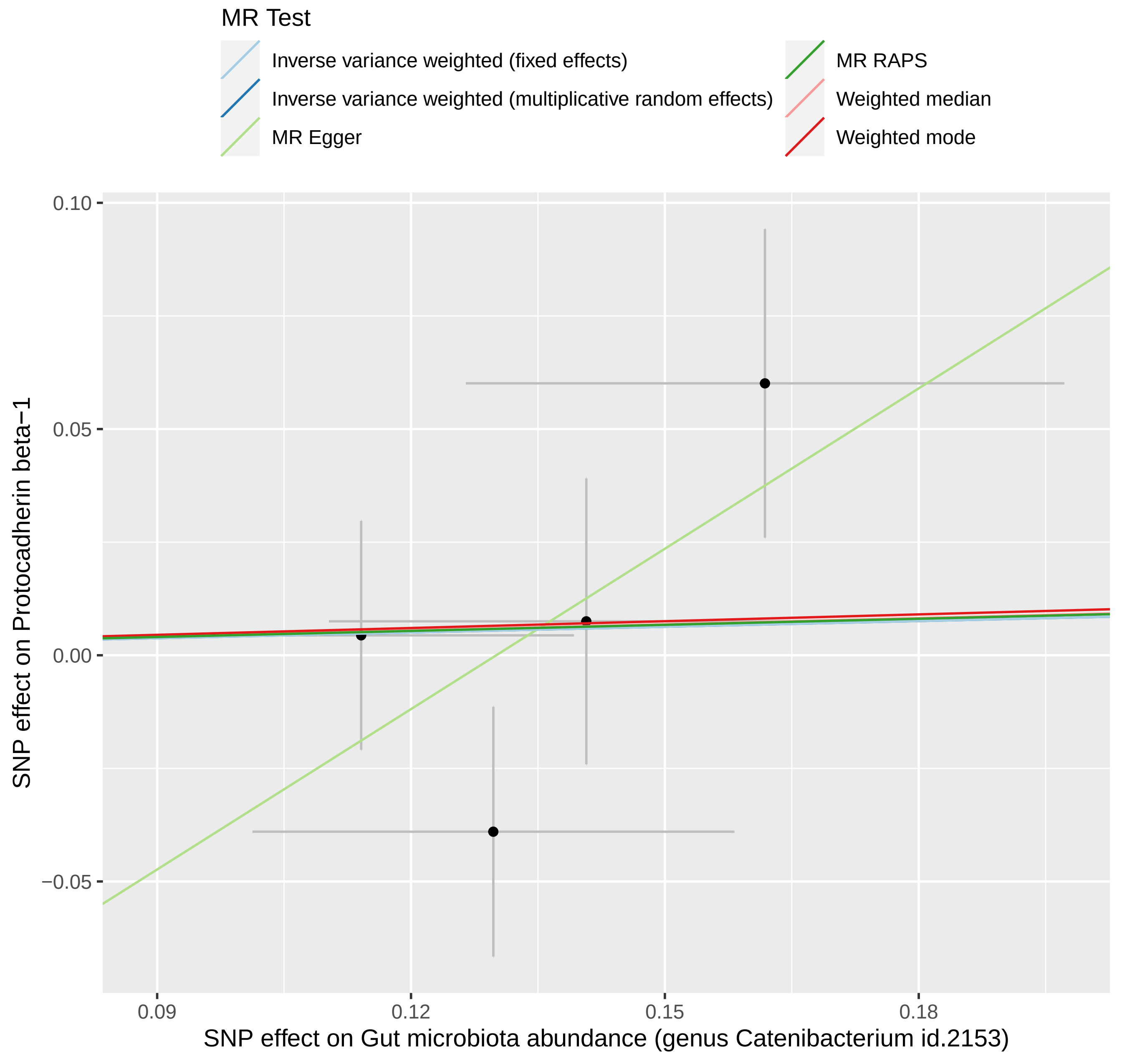

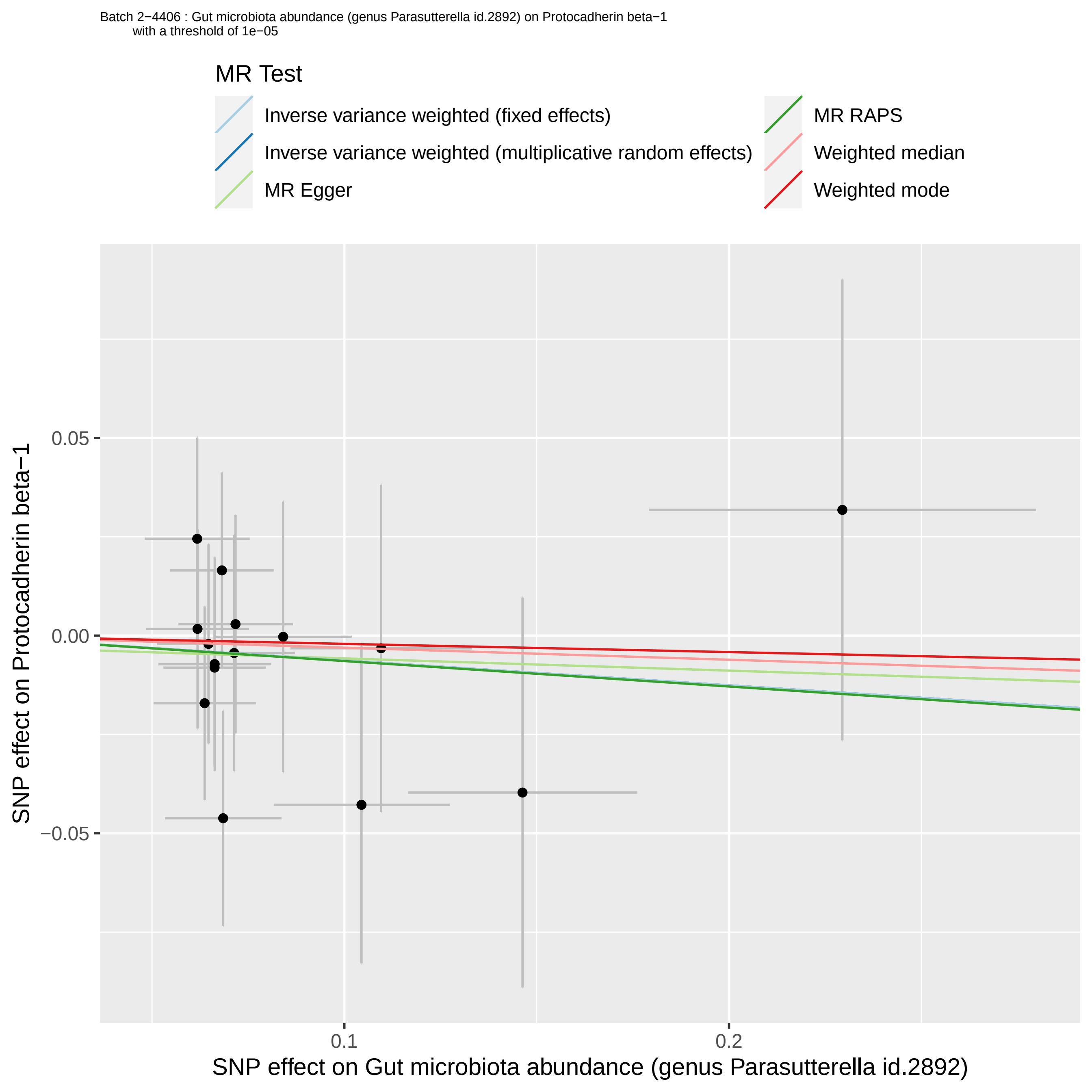

Batch 2−4406 : Gut microbiota abundance (genus Parasutterella id.2892) on Protocadherin beta−1
with a threshold of 1e−05
MR Test
Inverse variance weighted (fixed effects)
Inverse variance weighted (multiplicative random effects)
MR Egger
MR RAPS
Weighted median
Weighted mode
0.05
0.00
−0.05
0.1
0.2
SNP effect on Protocadherin beta−1
SNP effect on Gut microbiota abundance (genus Parasutterella id.2892)

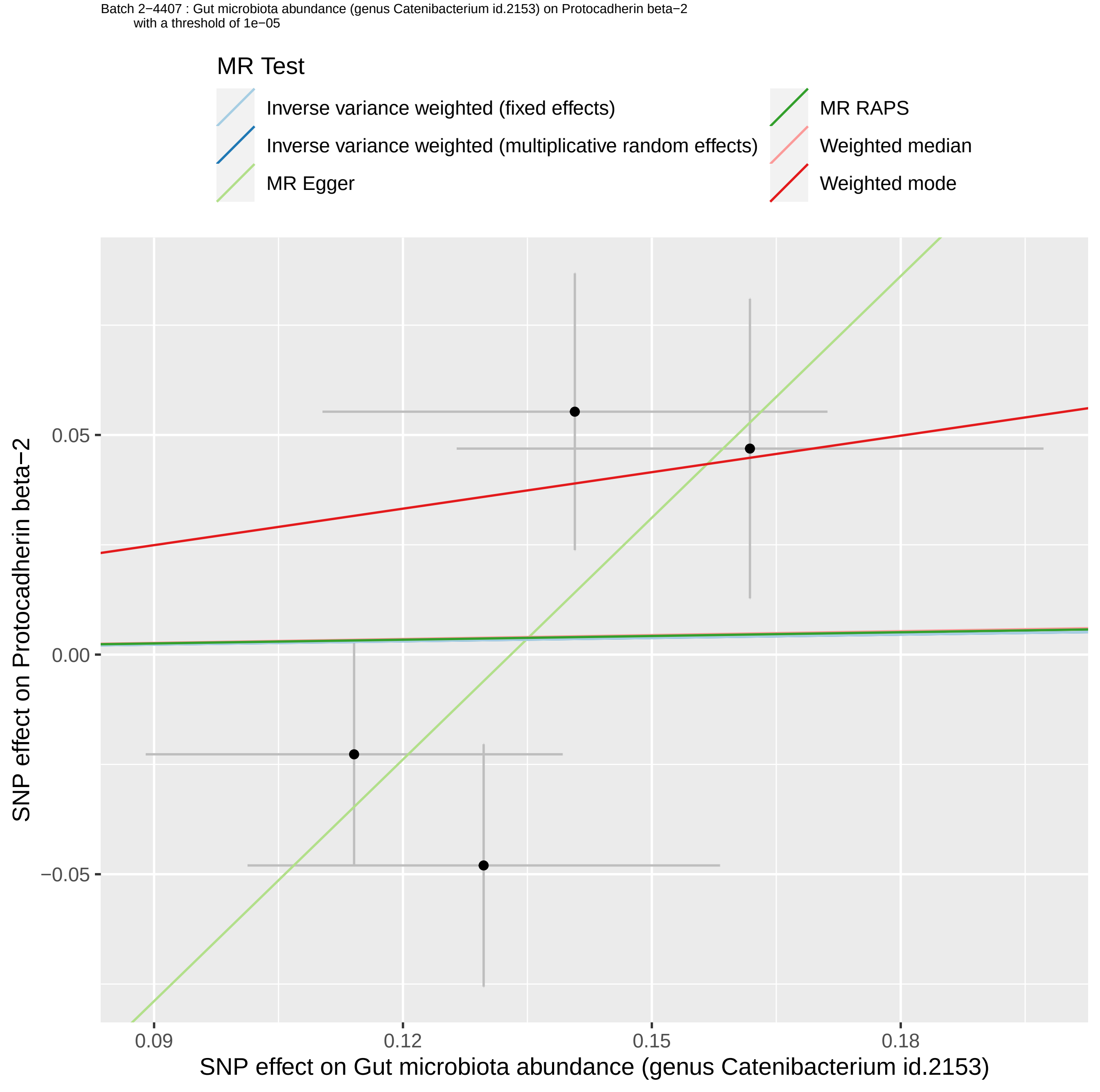
Batch 2−4407 : Gut microbiota abundance (genus Catenibacterium id.2153) on Protocadherin beta−2
with a threshold of 1e−05
MR Test
Inverse variance weighted (fixed effects)
Inverse variance weighted (multiplicative random effects)
MR Egger
MR RAPS
Weighted median
Weighted mode
0.05
0.00
−0.05
0.09
0.12
0.15
0.18
SNP effect on Protocadherin beta−2
SNP effect on Gut microbiota abundance (genus Catenibacterium id.2153)

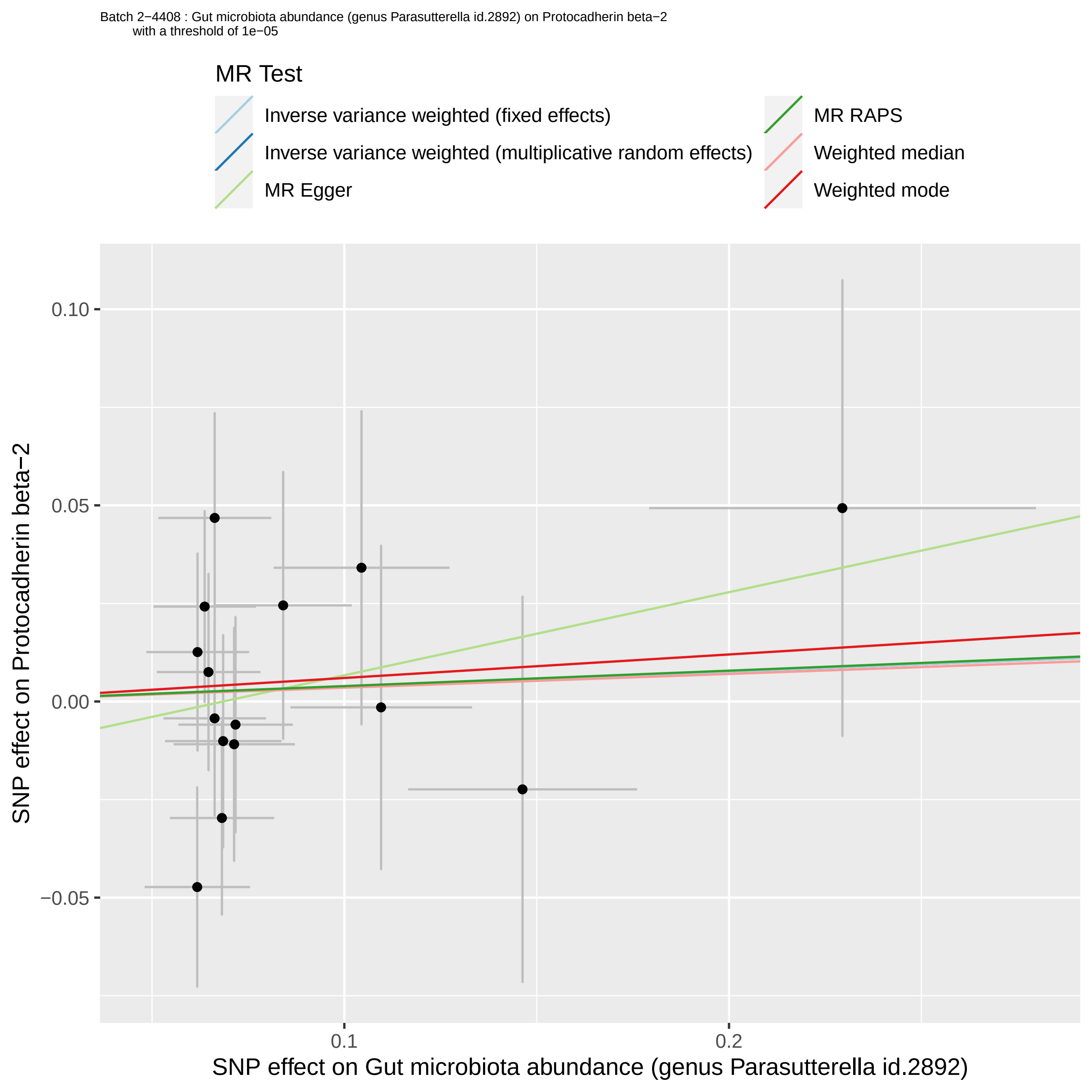
Batch 2−4408 : Gut microbiota abundance (genus Parasutterella id.2892) on Protocadherin beta−2
with a threshold of 1e−05
MR Test
Inverse variance weighted (fixed effects)
Inverse variance weighted (multiplicative random effects)
MR Egger
MR RAPS
Weighted median
Weighted mode
0.10
0.05
0.00
−0.05
0.1
0.2
SNP effect on Protocadherin beta−2
SNP effect on Gut microbiota abundance (genus Parasutterella id.2892)

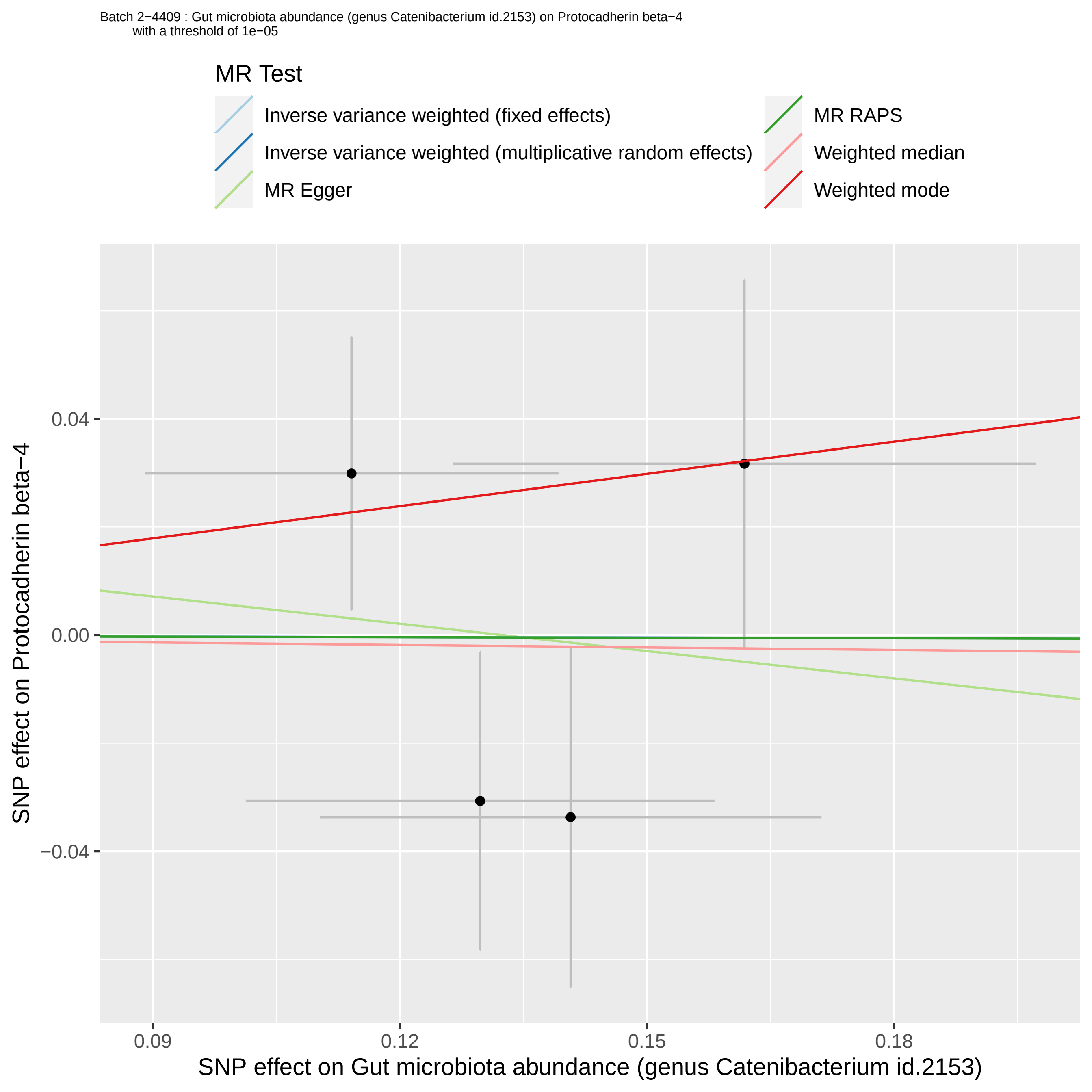
Batch 2−4409 : Gut microbiota abundance (genus Catenibacterium id.2153) on Protocadherin beta−4
with a threshold of 1e−05
MR Test
Inverse variance weighted (fixed effects)
Inverse variance weighted (multiplicative random effects)
MR Egger
MR RAPS
Weighted median
Weighted mode
0.04
0.00
−0.04
0.09
0.12
0.15
0.18
SNP effect on Protocadherin beta−4
SNP effect on Gut microbiota abundance (genus Catenibacterium id.2153)

Batch 2−4410 : Gut microbiota abundance (genus Parasutterella id.2892) on Protocadherin beta−4
with a threshold of 1e−05

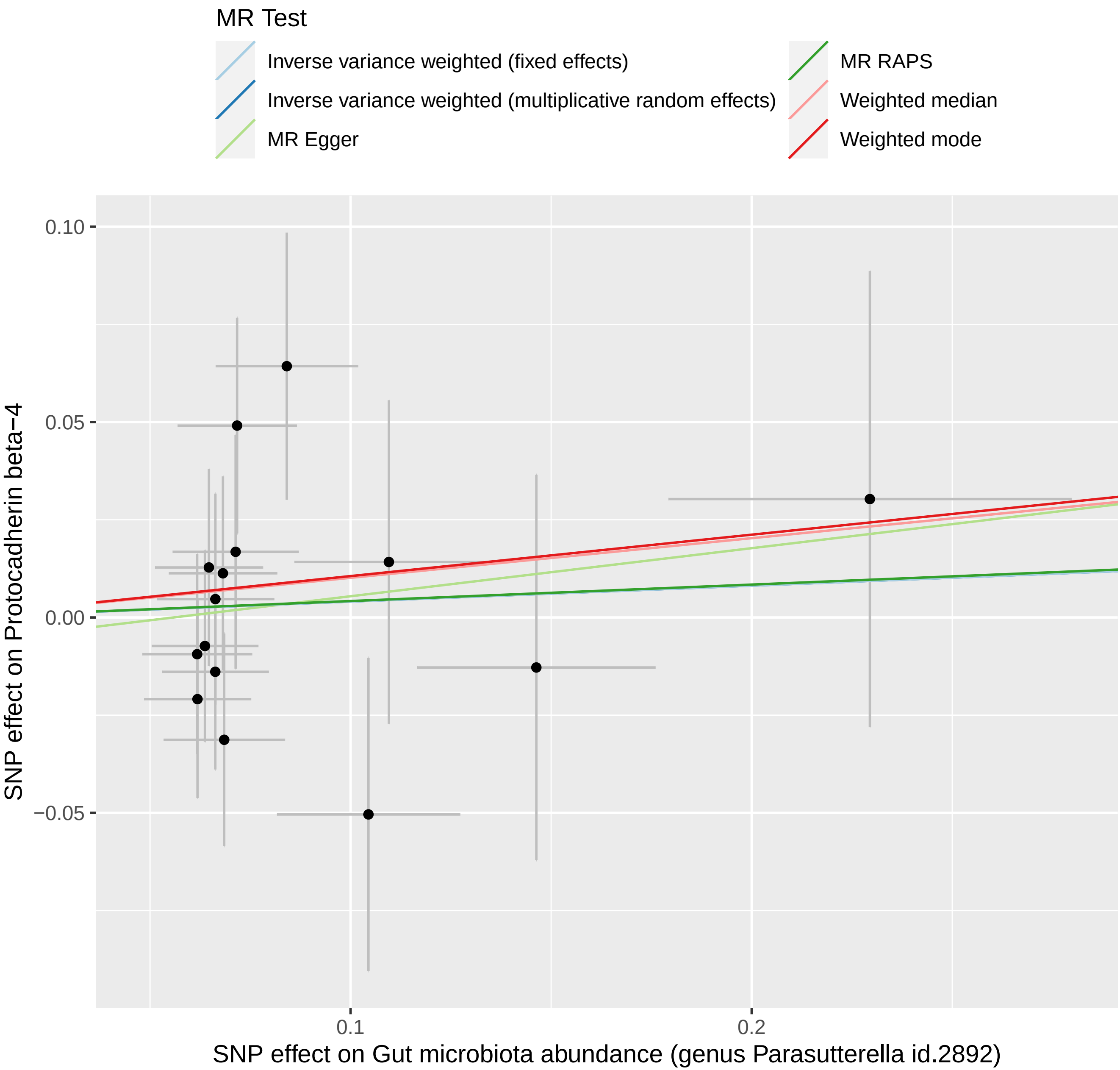

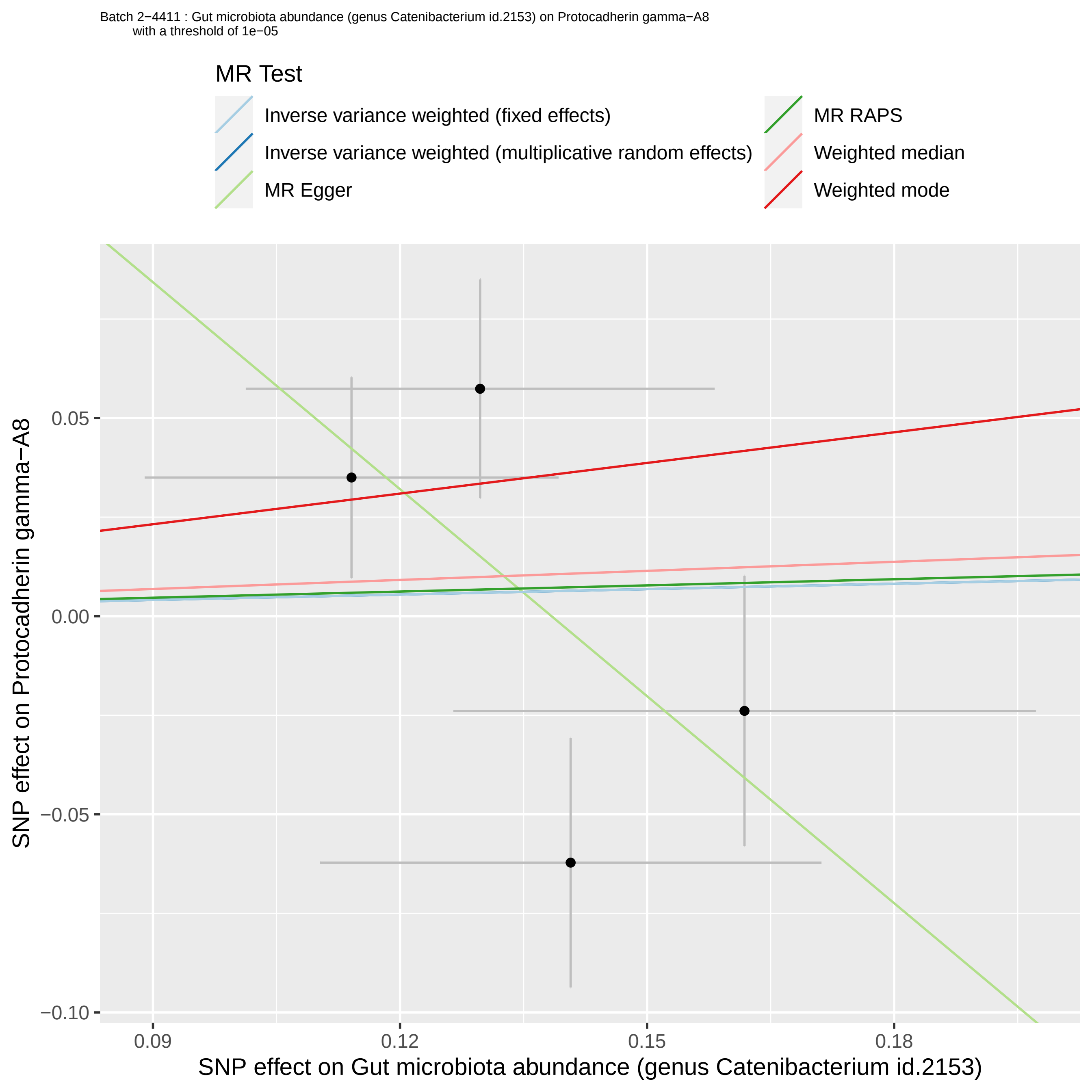

Batch 2−4411 : Gut microbiota abundance (genus Catenibacterium id.2153) on Protocadherin gamma−A8
with a threshold of 1e−05
MR Test
Inverse variance weighted (fixed effects)
Inverse variance weighted (multiplicative random effects)
MR Egger
MR RAPS
Weighted median
Weighted mode
0.05
0.00
−0.05
−0.10
0.09
0.12
0.15
0.18
SNP effect on Protocadherin gamma−A8
SNP effect on Gut microbiota abundance (genus Catenibacterium id.2153)

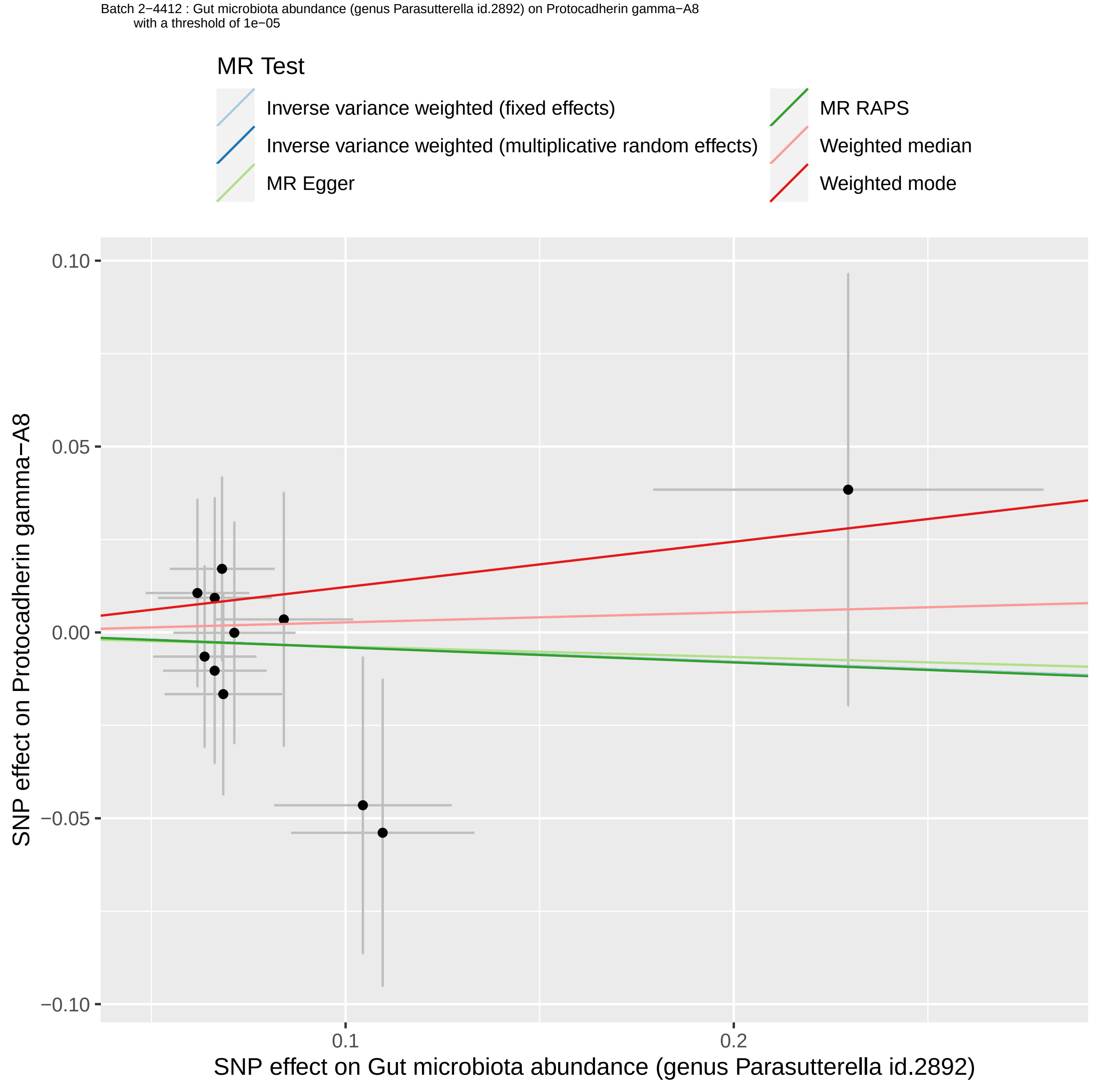
Batch 2−4412 : Gut microbiota abundance (genus Parasutterella id.2892) on Protocadherin gamma−A8
with a threshold of 1e−05
MR Test
Inverse variance weighted (fixed effects)
Inverse variance weighted (multiplicative random effects)
MR Egger
MR RAPS
Weighted median
Weighted mode
0.10
0.05
0.00
−0.05
−0.10
SNP effect on Protocadherin gamma−A8
0.1
0.2
SNP effect on Gut microbiota abundance (genus Parasutterella id.2892)

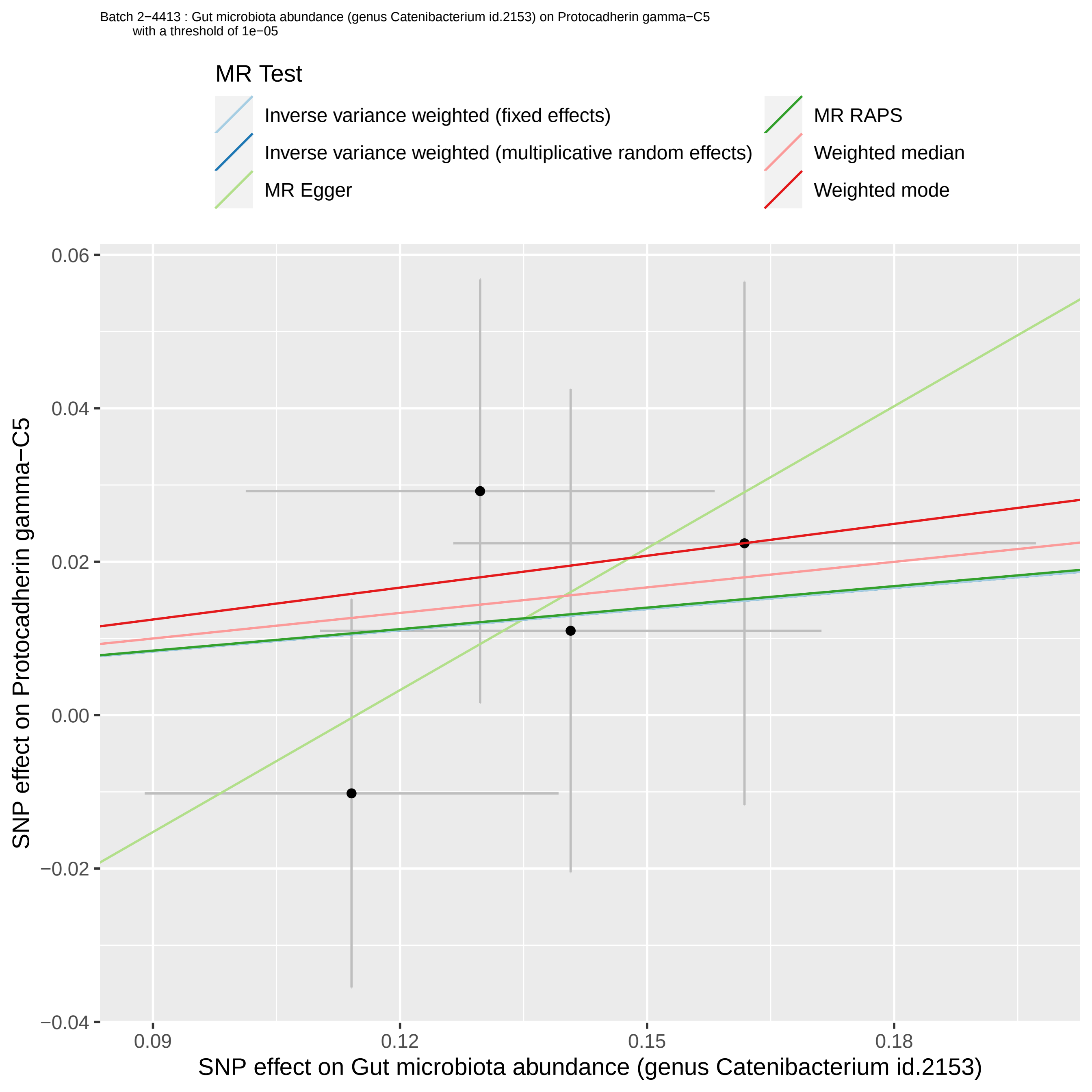
Batch 2−4413 : Gut microbiota abundance (genus Catenibacterium id.2153) on Protocadherin gamma−C5
with a threshold of 1e−05
MR Test
Inverse variance weighted (fixed effects)
Inverse variance weighted (multiplicative random effects)
MR Egger
MR RAPS
Weighted median
Weighted mode
0.06
0.04
0.02
0.00
−0.02
−0.04
0.09
0.12
0.15
0.18
SNP effect on Protocadherin gamma−C5
SNP effect on Gut microbiota abundance (genus Catenibacterium id.2153)

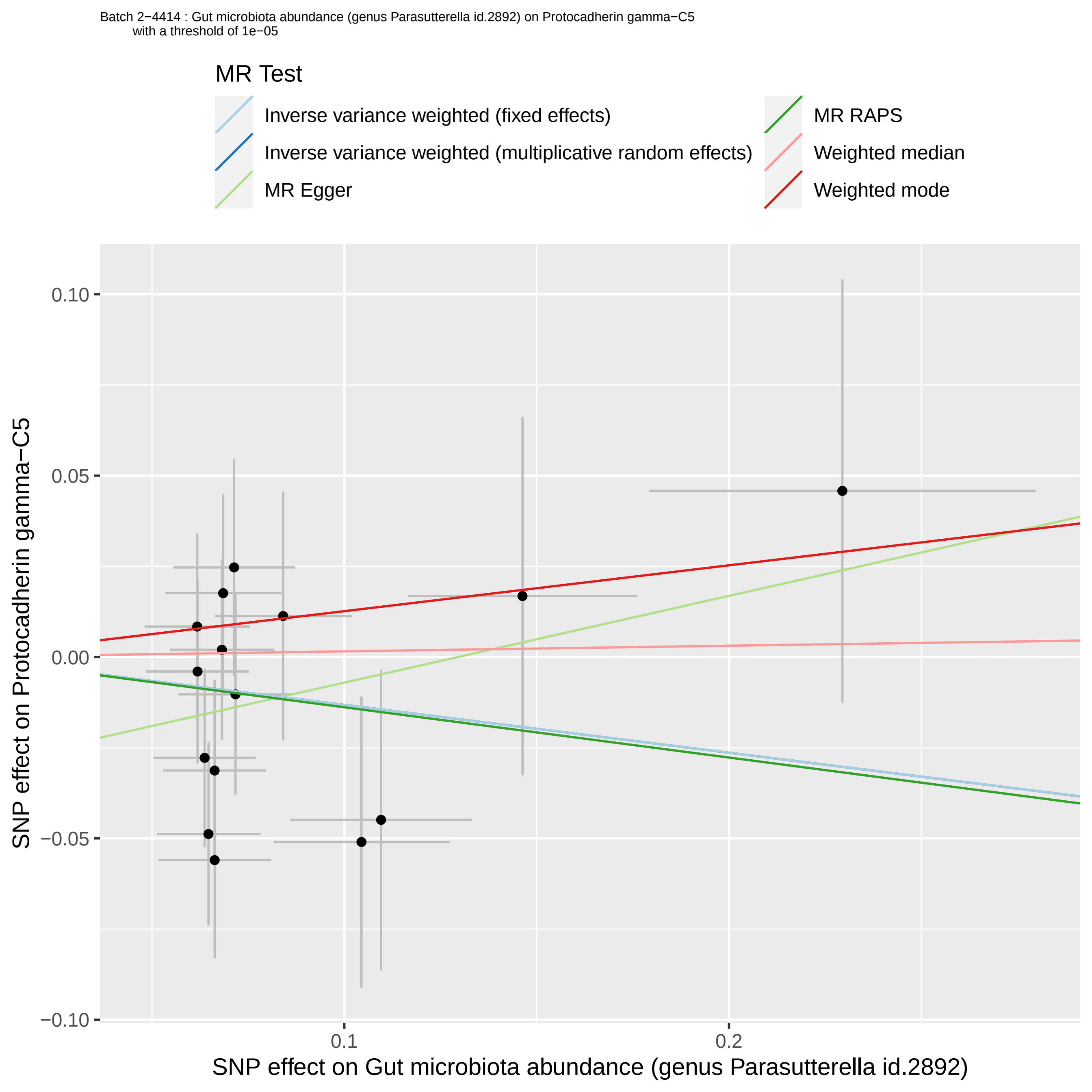
Batch 2−4414 : Gut microbiota abundance (genus Parasutterella id.2892) on Protocadherin gamma−C5
with a threshold of 1e−05
MR Test
Inverse variance weighted (fixed effects)
Inverse variance weighted (multiplicative random effects)
MR Egger
MR RAPS
Weighted median
Weighted mode
SNP effect on Protocadherin gamma−C5
0.10
0.05
0.00
−0.05
−0.10
0.1
0.2
SNP effect on Gut microbiota abundance (genus Parasutterella id.2892)

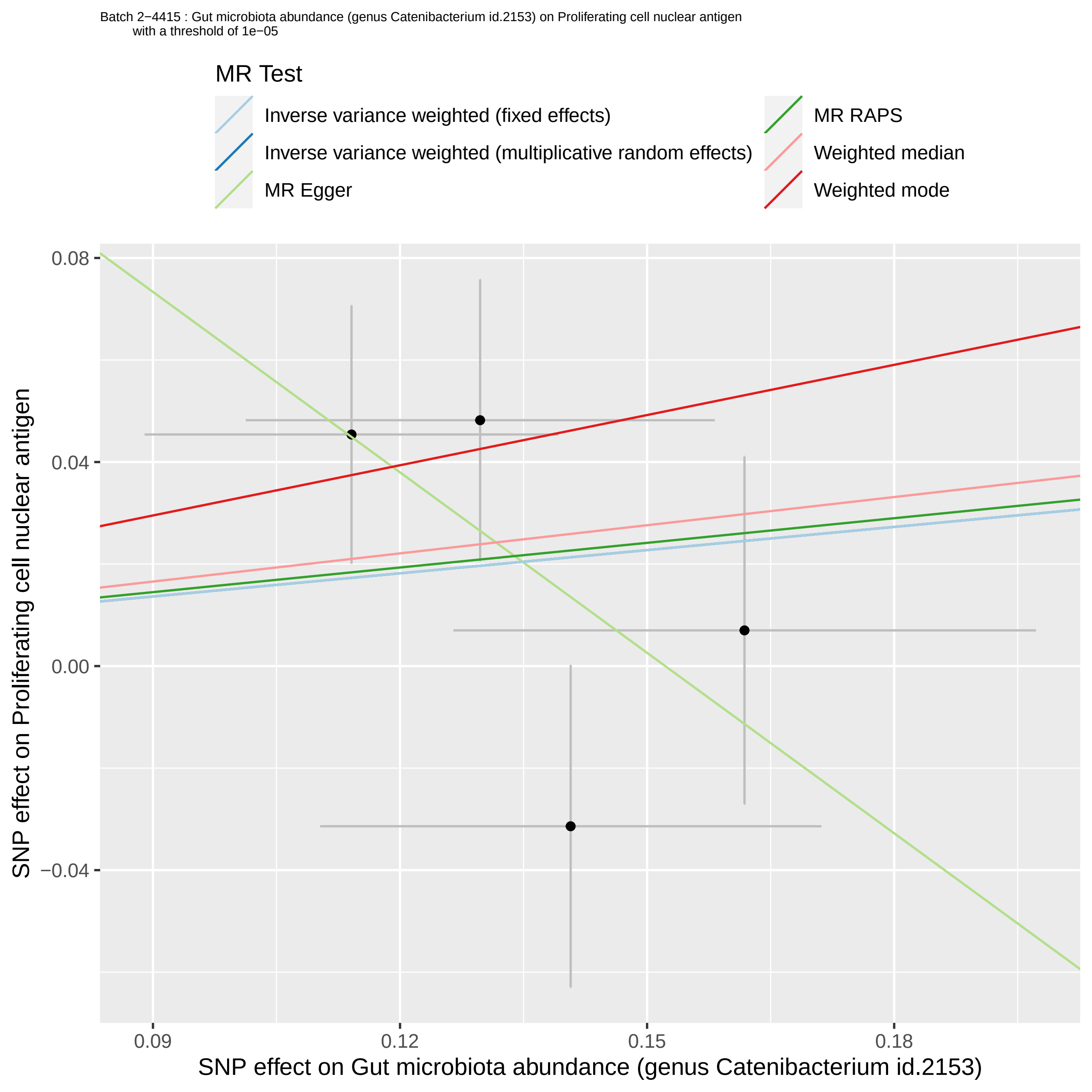
Batch 2−4415 : Gut microbiota abundance (genus Catenibacterium id.2153) on Proliferating cell nuclear antigen
with a threshold of 1e−05
MR Test
Inverse variance weighted (fixed effects)
Inverse variance weighted (multiplicative random effects)
MR Egger
MR RAPS
Weighted median
Weighted mode
0.08
0.04
0.00
−0.04
0.09
0.12
0.15
0.18
SNP effect on Proliferating cell nuclear antigen
SNP effect on Gut microbiota abundance (genus Catenibacterium id.2153)

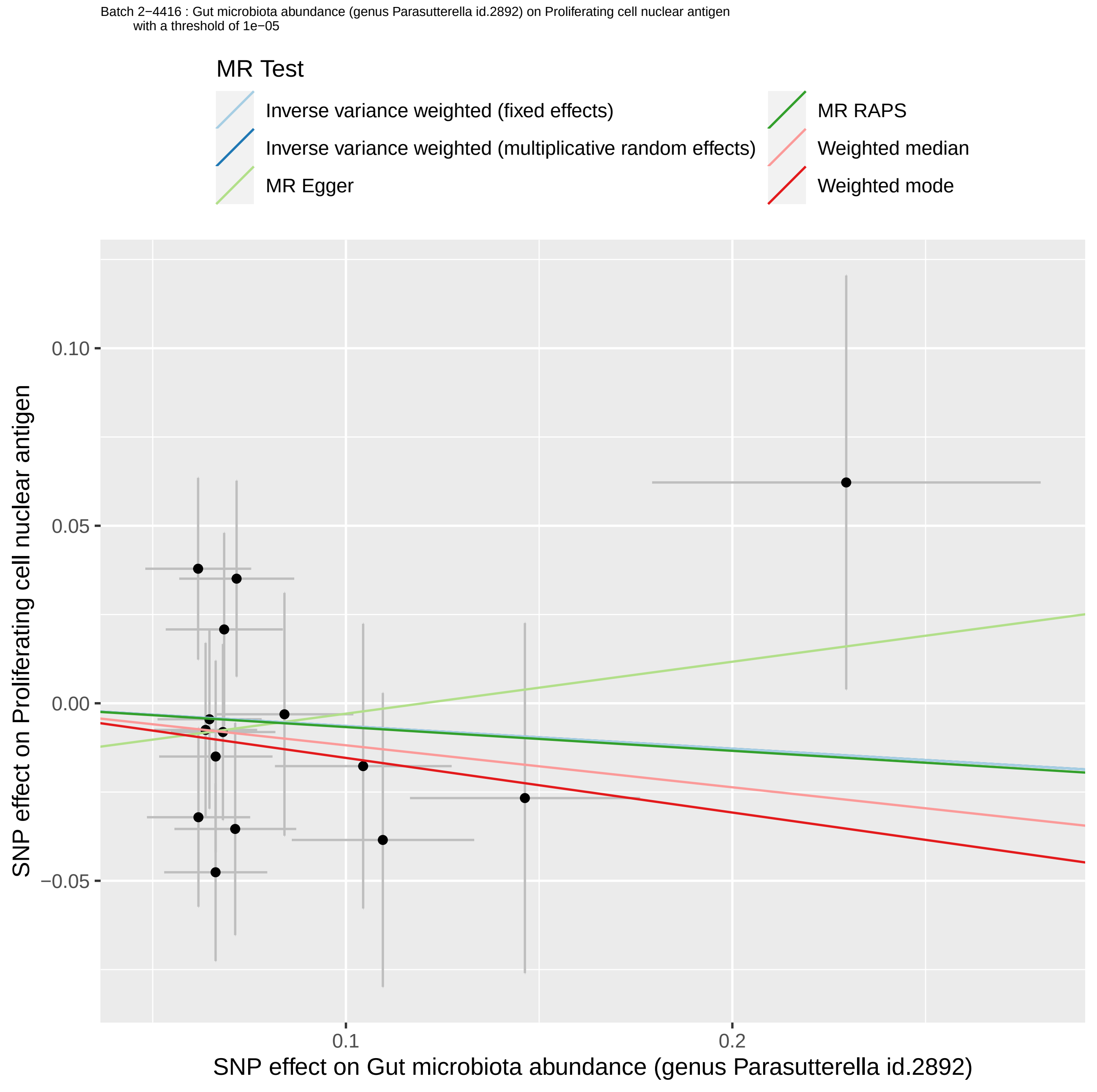

Batch 2−4416 : Gut microbiota abundance (genus Parasutterella id.2892) on Proliferating cell nuclear antigen
with a threshold of 1e−05
MR Test
Inverse variance weighted (fixed effects)
Inverse variance weighted (multiplicative random effects)
MR Egger
MR RAPS
Weighted median
Weighted mode
0.10
0.05
0.00
−0.05
0.1
0.2
SNP effect on Proliferating cell nuclear antigen
SNP effect on Gut microbiota abundance (genus Parasutterella id.2892)

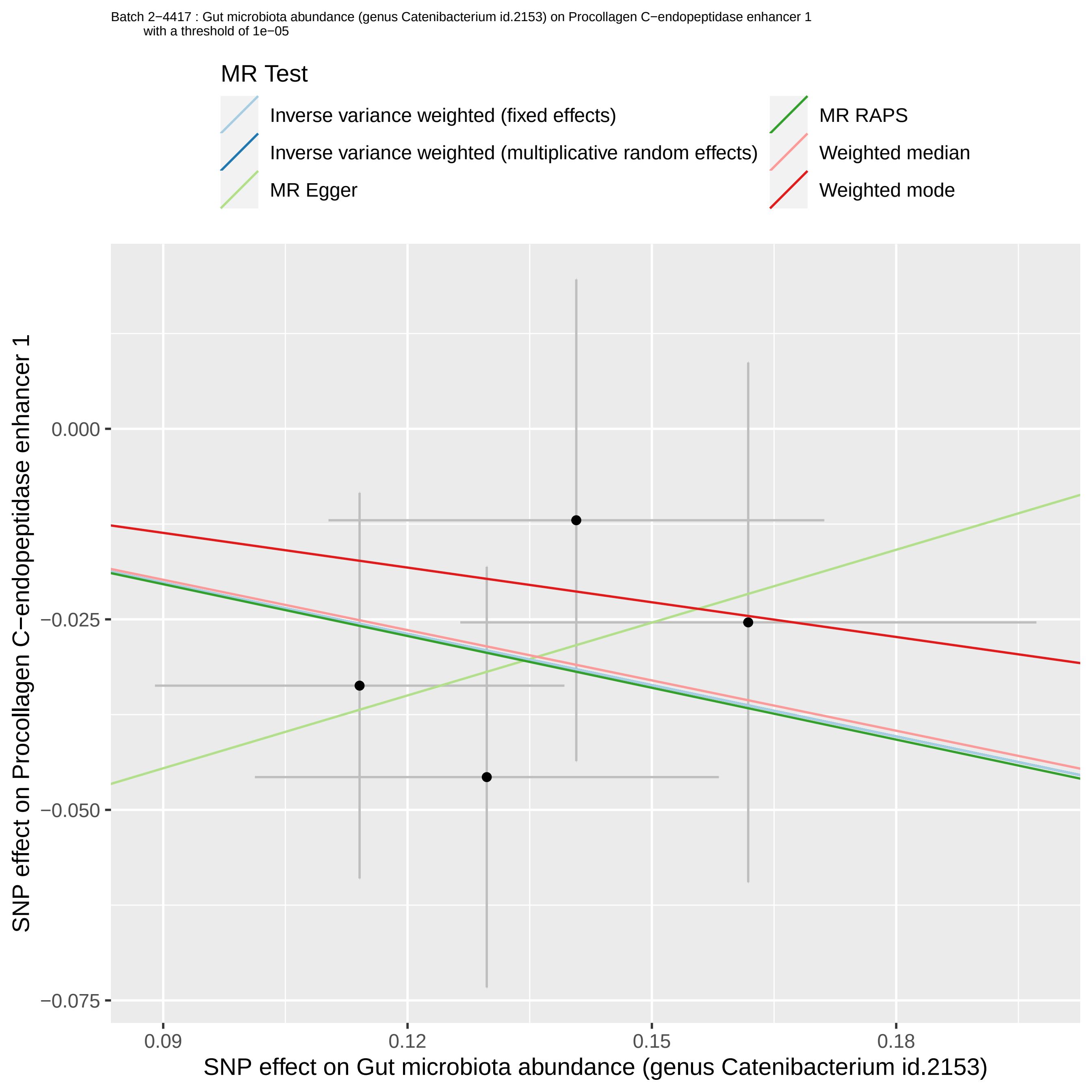
Batch 2−4417 : Gut microbiota abundance (genus Catenibacterium id.2153) on Procollagen C−endopeptidase enhancer 1
with a threshold of 1e−05
MR Test
Inverse variance weighted (fixed effects)
Inverse variance weighted (multiplicative random effects)
MR Egger
MR RAPS
Weighted median
Weighted mode
0.000
−0.025
−0.050
−0.075
0.09
0.12
0.15
0.18
SNP effect on Procollagen C−endopeptidase enhancer 1
SNP effect on Gut microbiota abundance (genus Catenibacterium id.2153)

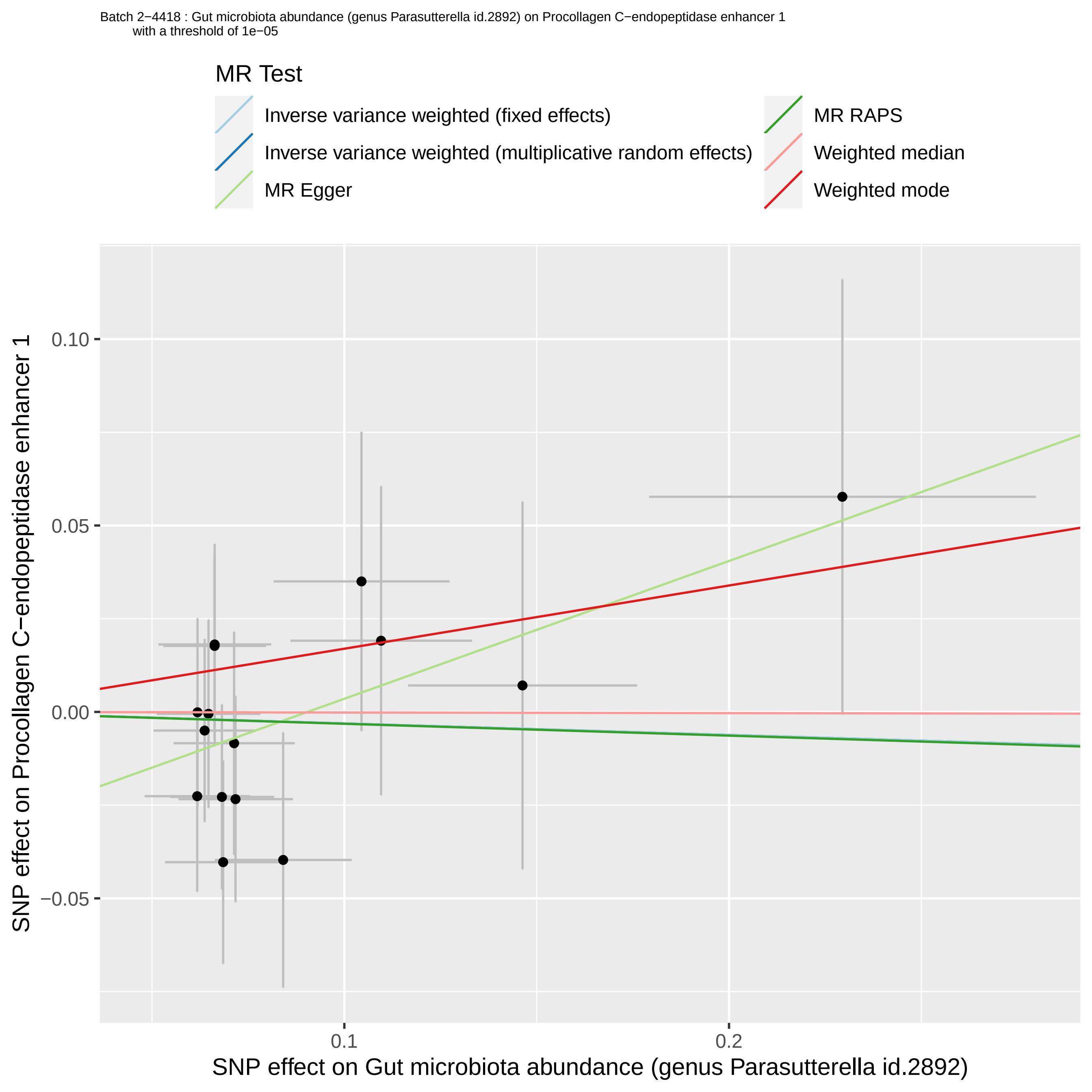
Batch 2−4418 : Gut microbiota abundance (genus Parasutterella id.2892) on Procollagen C−endopeptidase enhancer 1
with a threshold of 1e−05
MR Test
Inverse variance weighted (fixed effects)
Inverse variance weighted (multiplicative random effects)
MR Egger
MR RAPS
Weighted median
Weighted mode
0.10
0.05
0.00
−0.05
0.1
0.2
SNP effect on Procollagen C−endopeptidase enhancer 1
SNP effect on Gut microbiota abundance (genus Parasutterella id.2892)

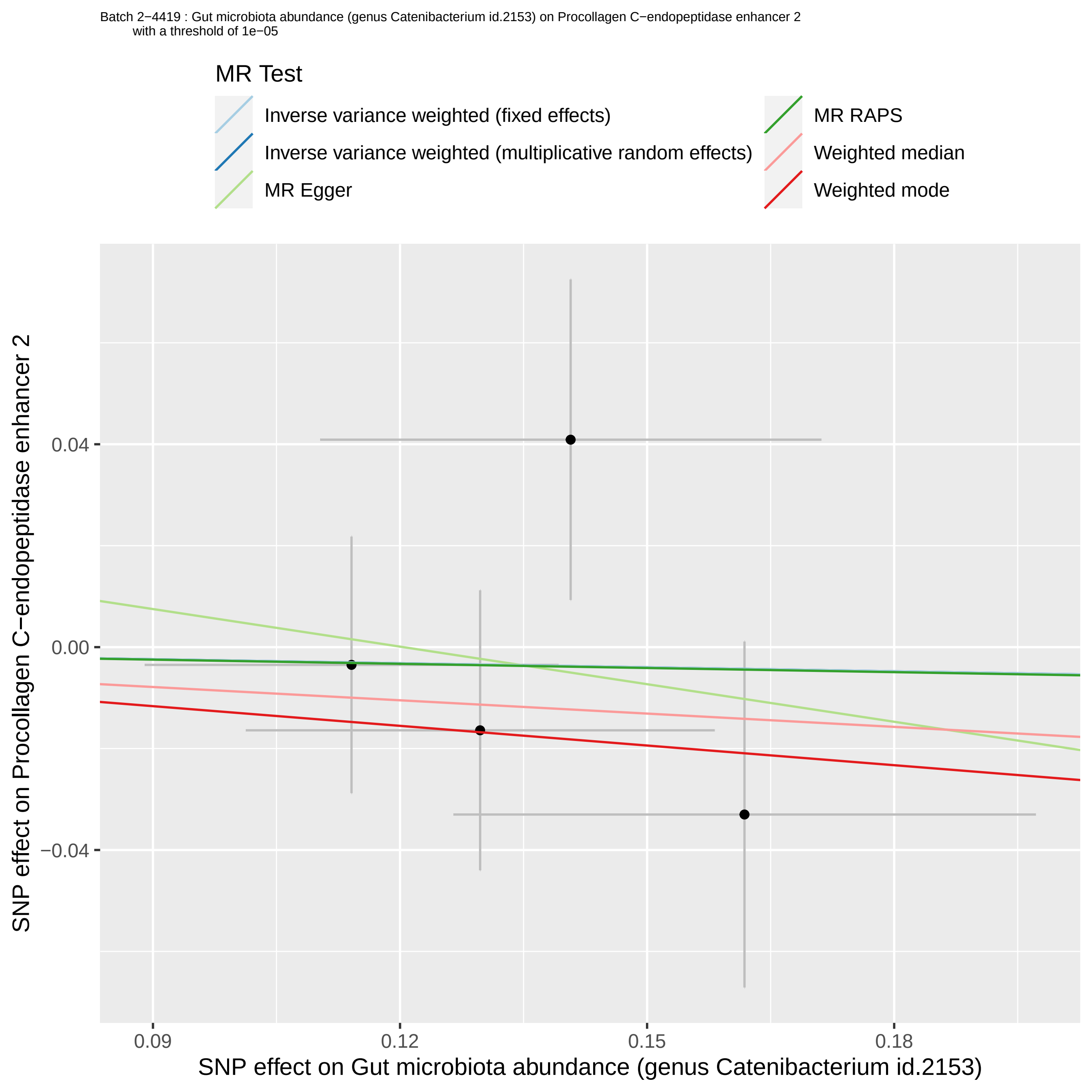

Batch 2−4419 : Gut microbiota abundance (genus Catenibacterium id.2153) on Procollagen C−endopeptidase enhancer 2
with a threshold of 1e−05
MR Test
Inverse variance weighted (fixed effects)
Inverse variance weighted (multiplicative random effects)
MR Egger
MR RAPS
Weighted median
Weighted mode
SNP effect on Procollagen C−endopeptidase enhancer 2
0.04
0.00
−0.04
0.09
0.12
0.15
0.18
SNP effect on Gut microbiota abundance (genus Catenibacterium id.2153)

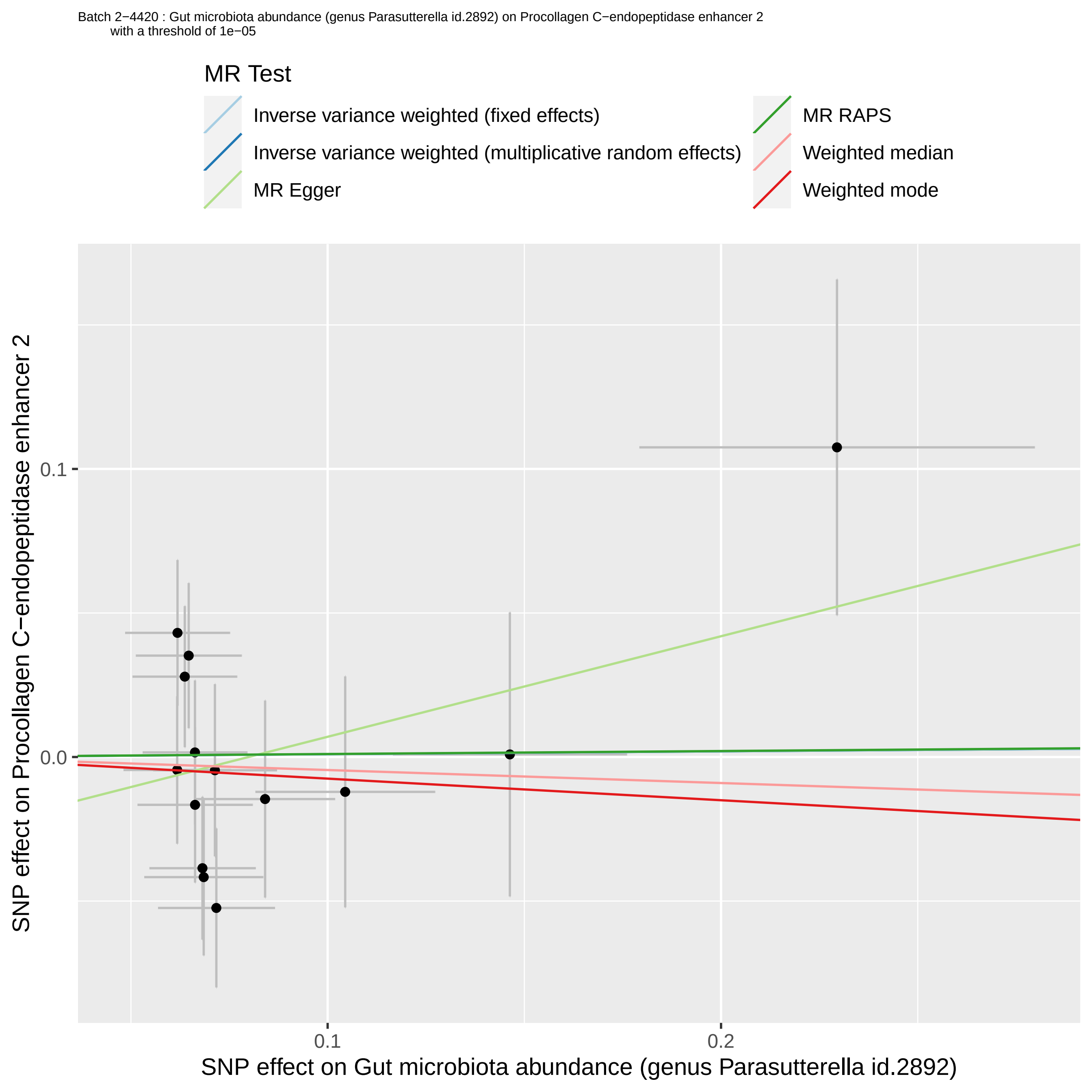

Batch 2−4420 : Gut microbiota abundance (genus Parasutterella id.2892) on Procollagen C−endopeptidase enhancer 2
with a threshold of 1e−05
MR Test
Inverse variance weighted (fixed effects)
Inverse variance weighted (multiplicative random effects)
MR Egger
MR RAPS
Weighted median
Weighted mode
SNP effect on Procollagen C−endopeptidase enhancer 2
0.1
0.0
0.1
0.2
SNP effect on Gut microbiota abundance (genus Parasutterella id.2892)

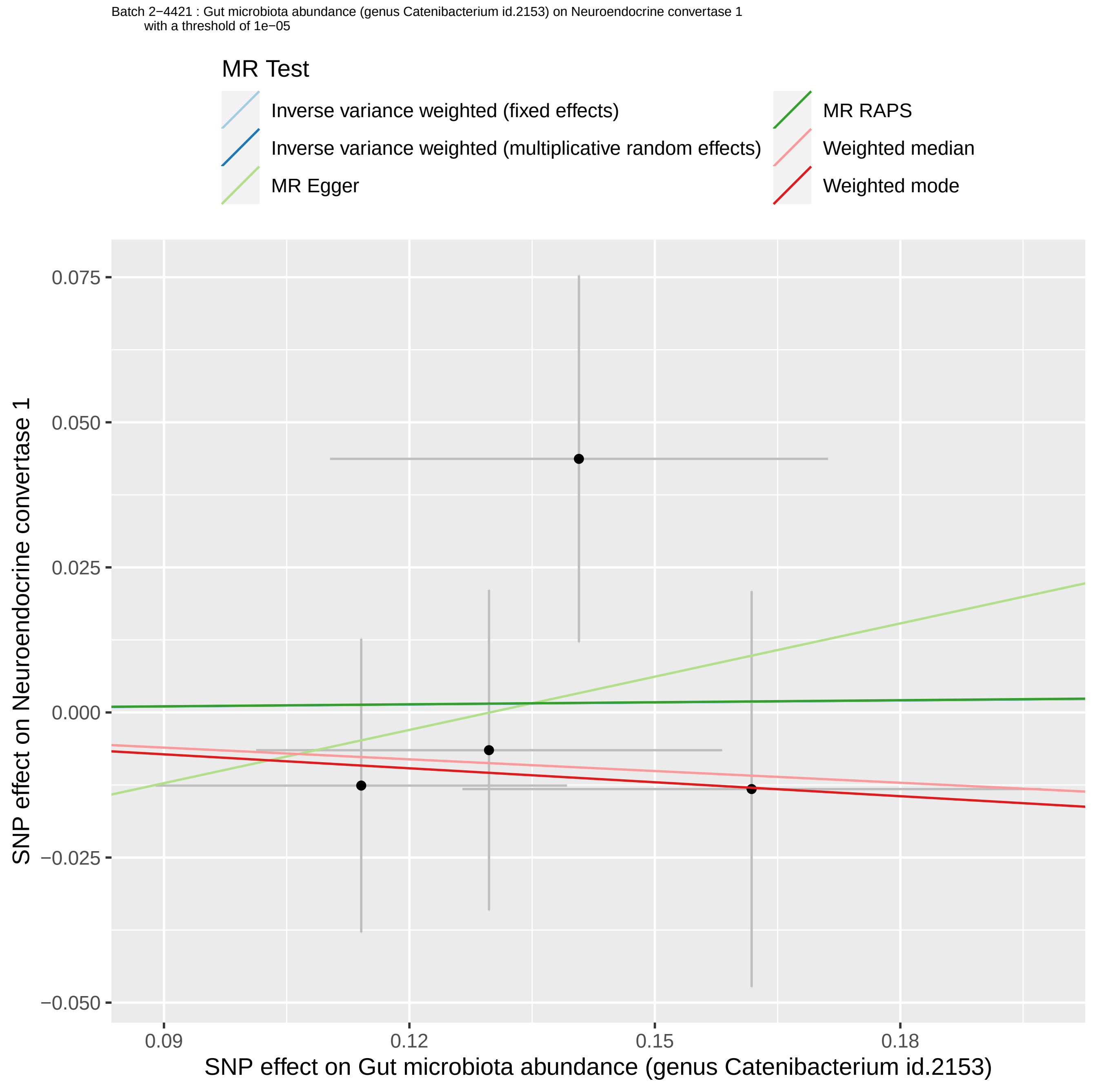

Batch 2−4421 : Gut microbiota abundance (genus Catenibacterium id.2153) on Neuroendocrine convertase 1
with a threshold of 1e−05
MR Test
Inverse variance weighted (fixed effects)
Inverse variance weighted (multiplicative random effects)
MR Egger
MR RAPS
Weighted median
Weighted mode
0.075
0.050
0.025
0.000
−0.025
−0.050
0.09
0.12
0.15
0.18
SNP effect on Neuroendocrine convertase 1
SNP effect on Gut microbiota abundance (genus Catenibacterium id.2153)

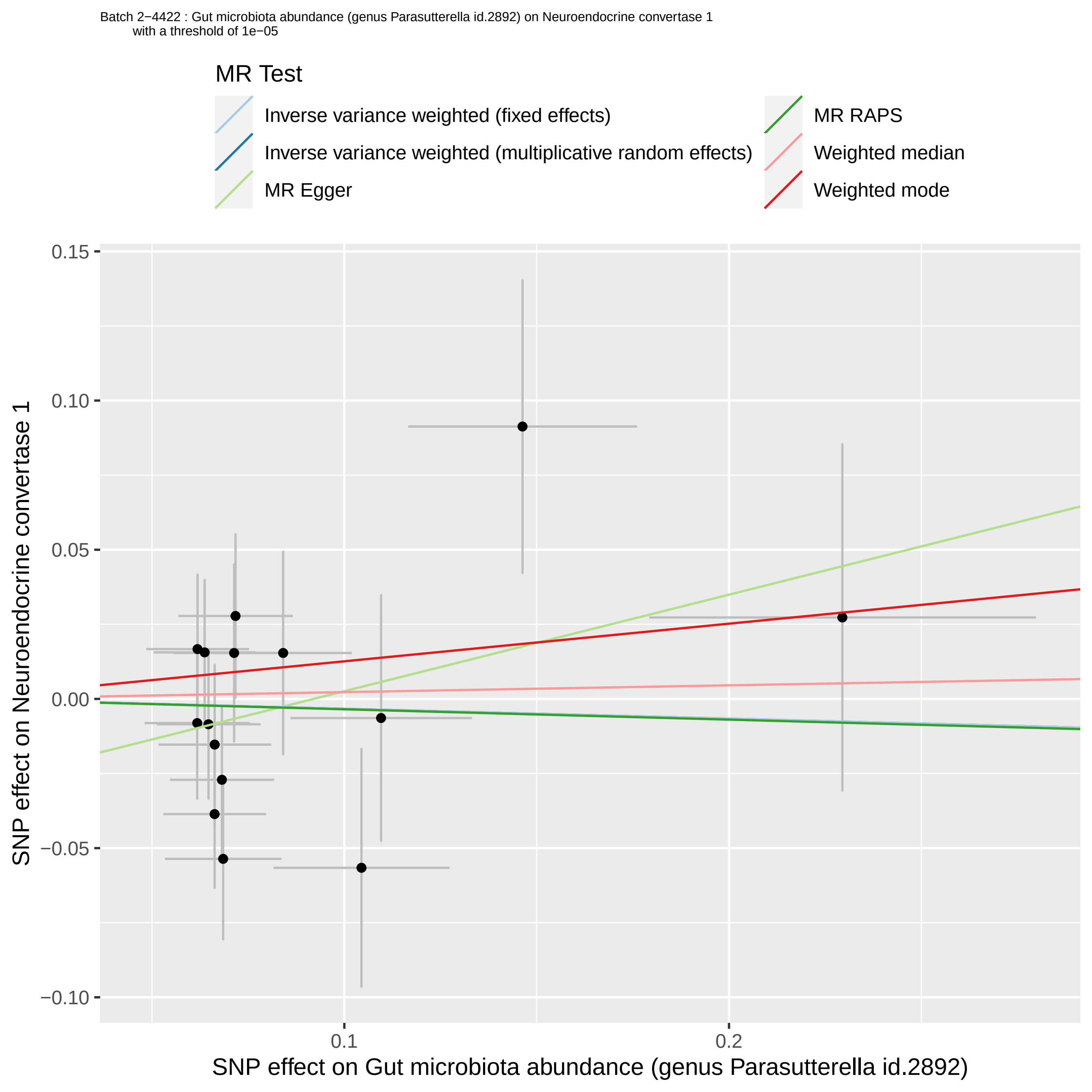
Batch 2−4422 : Gut microbiota abundance (genus Parasutterella id.2892) on Neuroendocrine convertase 1
with a threshold of 1e−05
MR Test
Inverse variance weighted (fixed effects)
Inverse variance weighted (multiplicative random effects)
MR Egger
MR RAPS
Weighted median
Weighted mode
0.15
0.10
0.05
0.00
−0.05
−0.10
0.1
0.2
SNP effect on Neuroendocrine convertase 1
SNP effect on Gut microbiota abundance (genus Parasutterella id.2892)

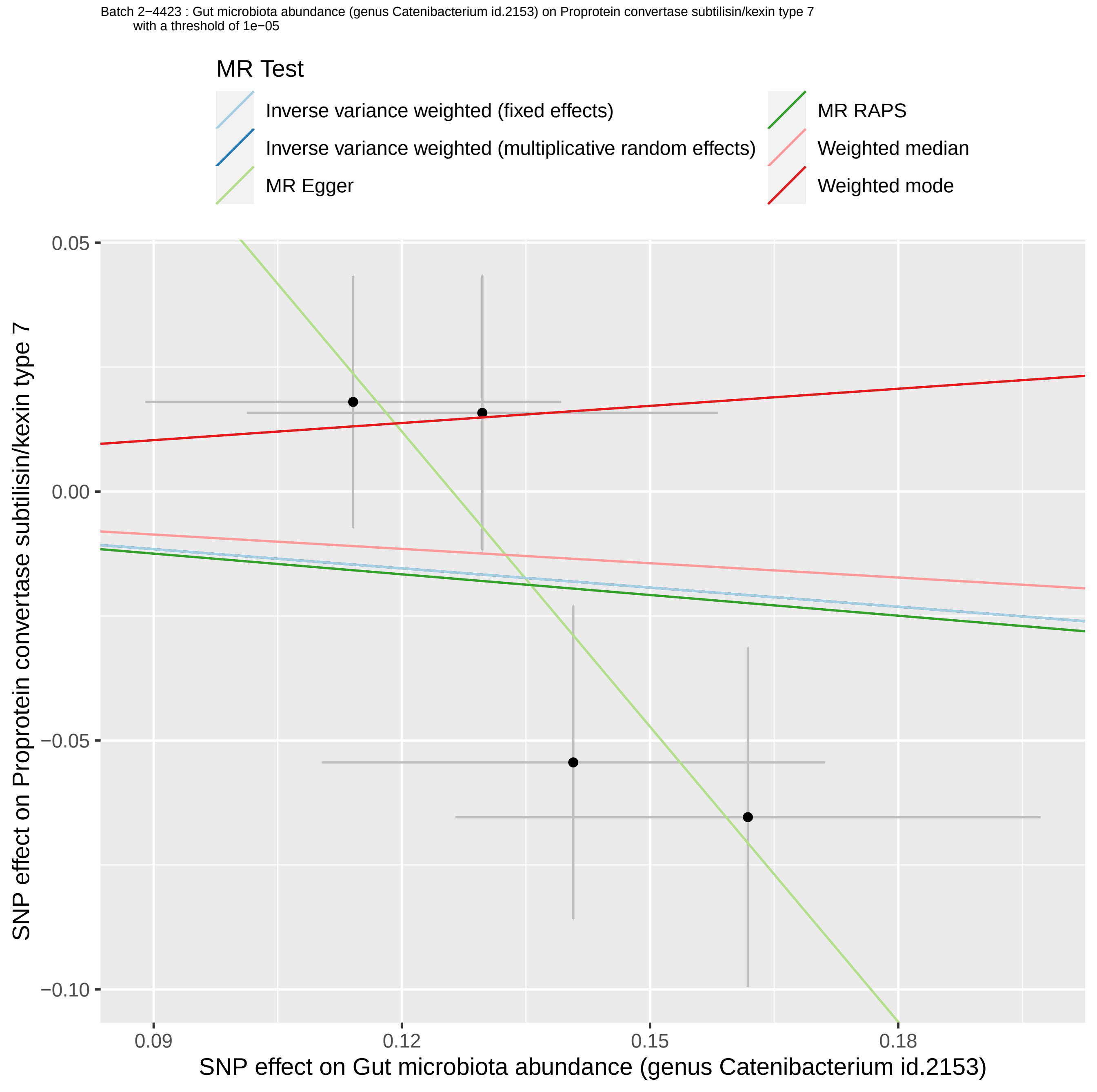

Batch 2−4423 : Gut microbiota abundance (genus Catenibacterium id.2153) on Proprotein convertase subtilisin/kexin type 7
with a threshold of 1e−05
MR Test
Inverse variance weighted (fixed effects)
Inverse variance weighted (multiplicative random effects)
MR Egger
MR RAPS
Weighted median
Weighted mode
0.05
0.00
−0.05
−0.10
0.09
0.12
0.15
0.18
SNP effect on Proprotein convertase subtilisin/kexin type 7
SNP effect on Gut microbiota abundance (genus Catenibacterium id.2153)

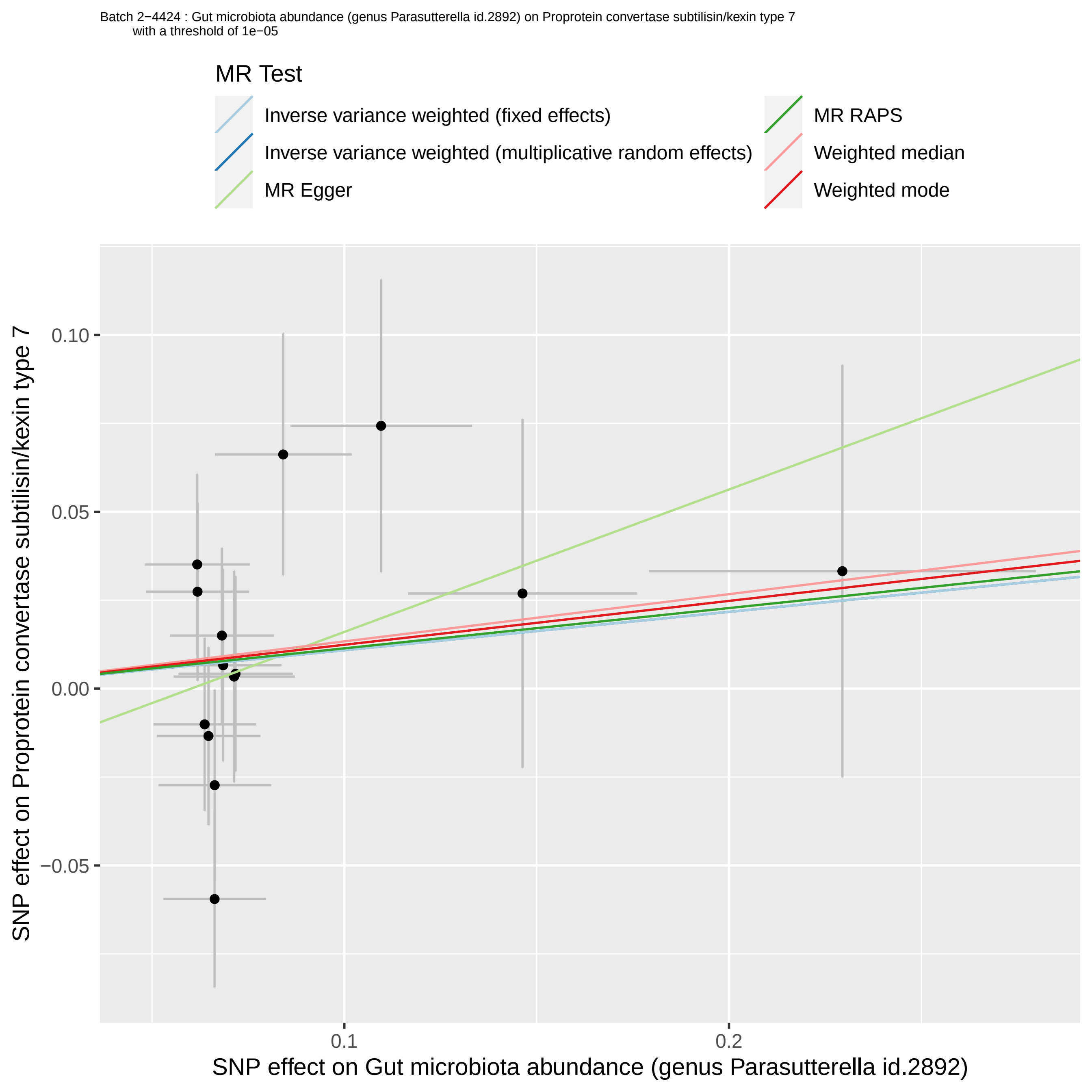

Batch 2−4424 : Gut microbiota abundance (genus Parasutterella id.2892) on Proprotein convertase subtilisin/kexin type 7
with a threshold of 1e−05
MR Test
Inverse variance weighted (fixed effects)
Inverse variance weighted (multiplicative random effects)
MR Egger
MR RAPS
Weighted median
Weighted mode
0.10
0.05
0.00
−0.05
0.1
0.2
SNP effect on Proprotein convertase subtilisin/kexin type 7
SNP effect on Gut microbiota abundance (genus Parasutterella id.2892)

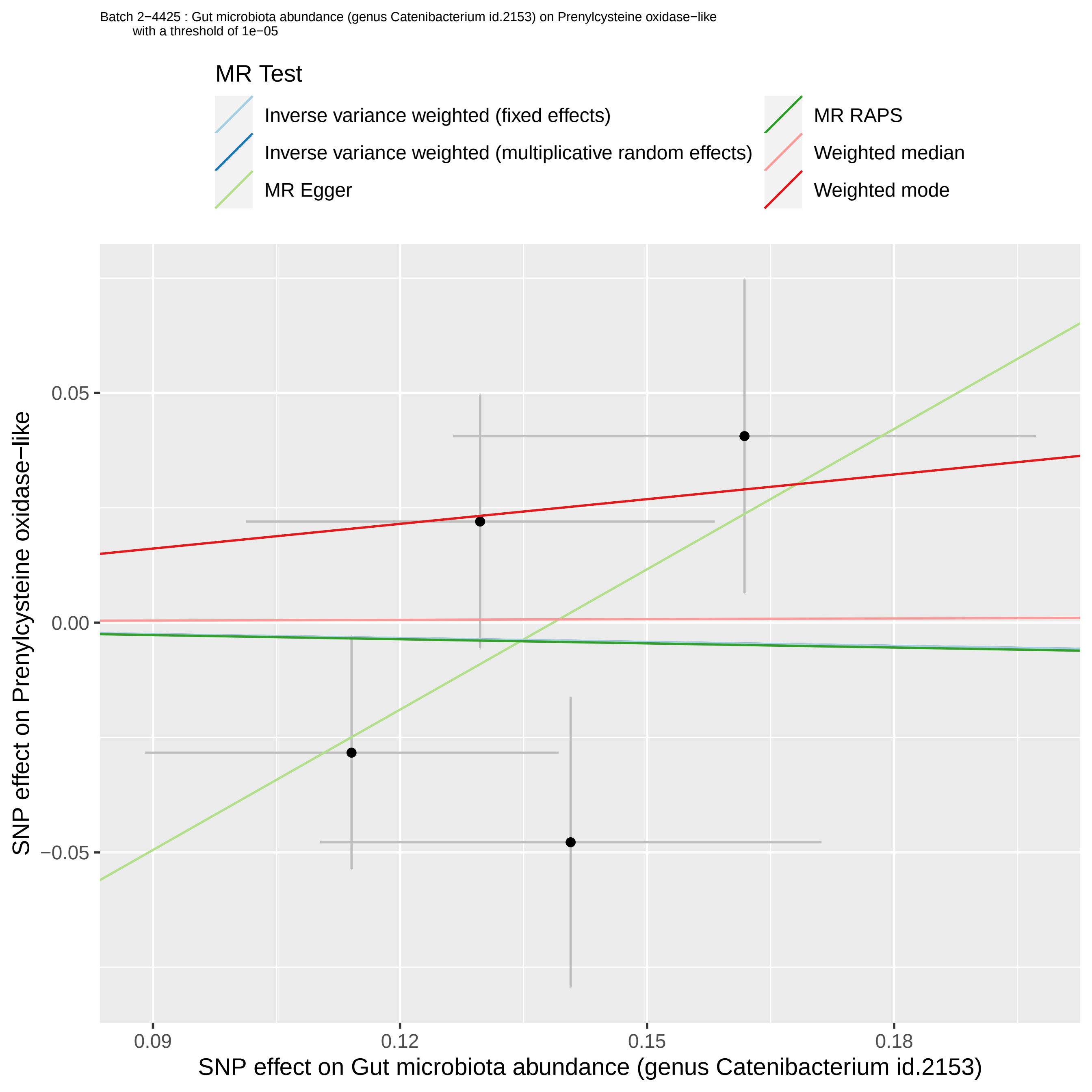
Batch 2−4425 : Gut microbiota abundance (genus Catenibacterium id.2153) on Prenylcysteine oxidase−like
with a threshold of 1e−05
MR Test
Inverse variance weighted (fixed effects)
Inverse variance weighted (multiplicative random effects)
MR Egger
MR RAPS
Weighted median
Weighted mode
0.05
0.00
−0.05
0.09
0.12
0.15
0.18
SNP effect on Prenylcysteine oxidase−like
SNP effect on Gut microbiota abundance (genus Catenibacterium id.2153)

Batch 2−4426 : Gut microbiota abundance (genus Parasutterella id.2892) on Prenylcysteine oxidase−like
with a threshold of 1e−05

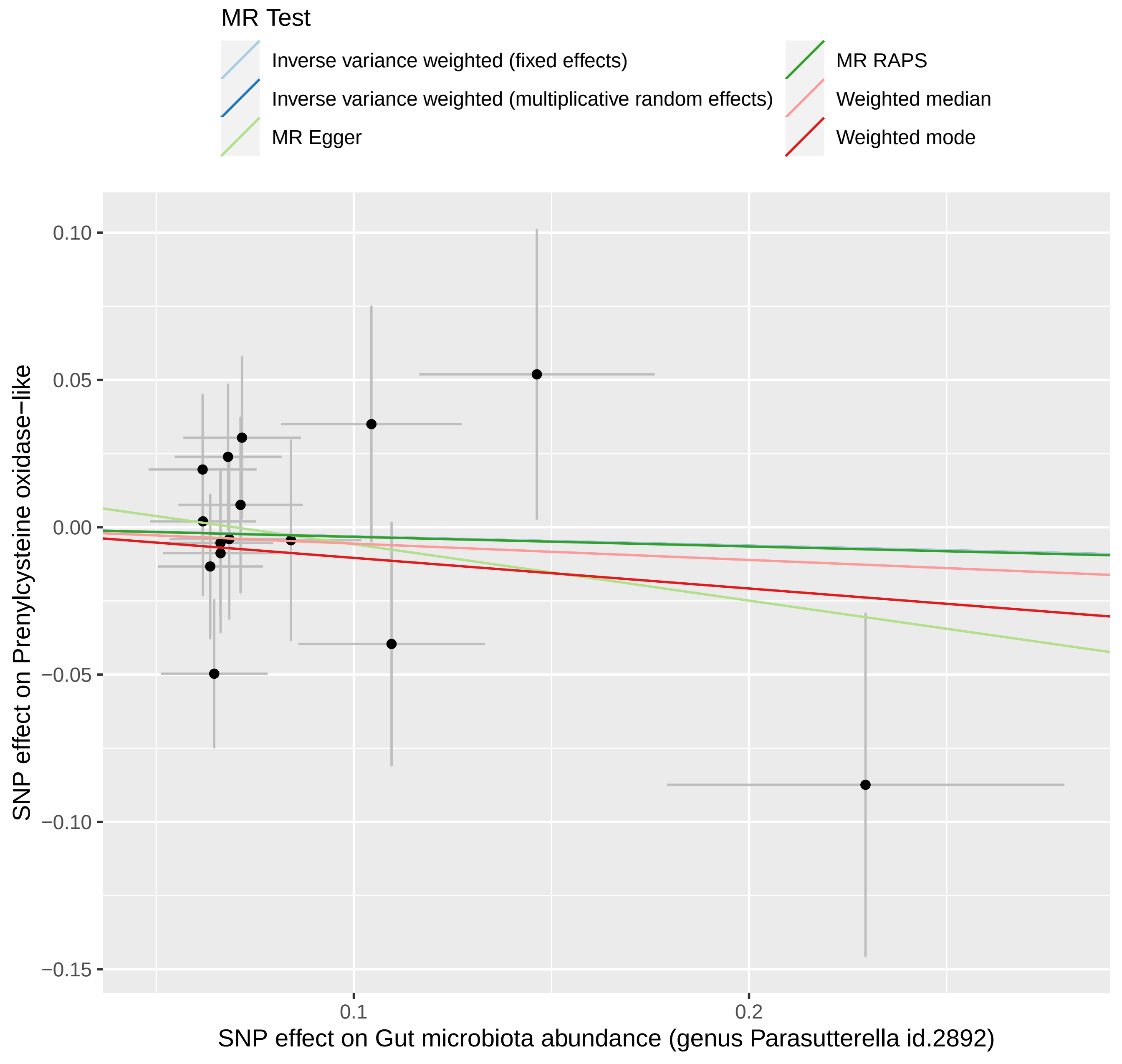

Batch 2−4427 : Gut microbiota abundance (genus Catenibacterium id.2153) on Programmed cell death protein 1
with a threshold of 1e−05

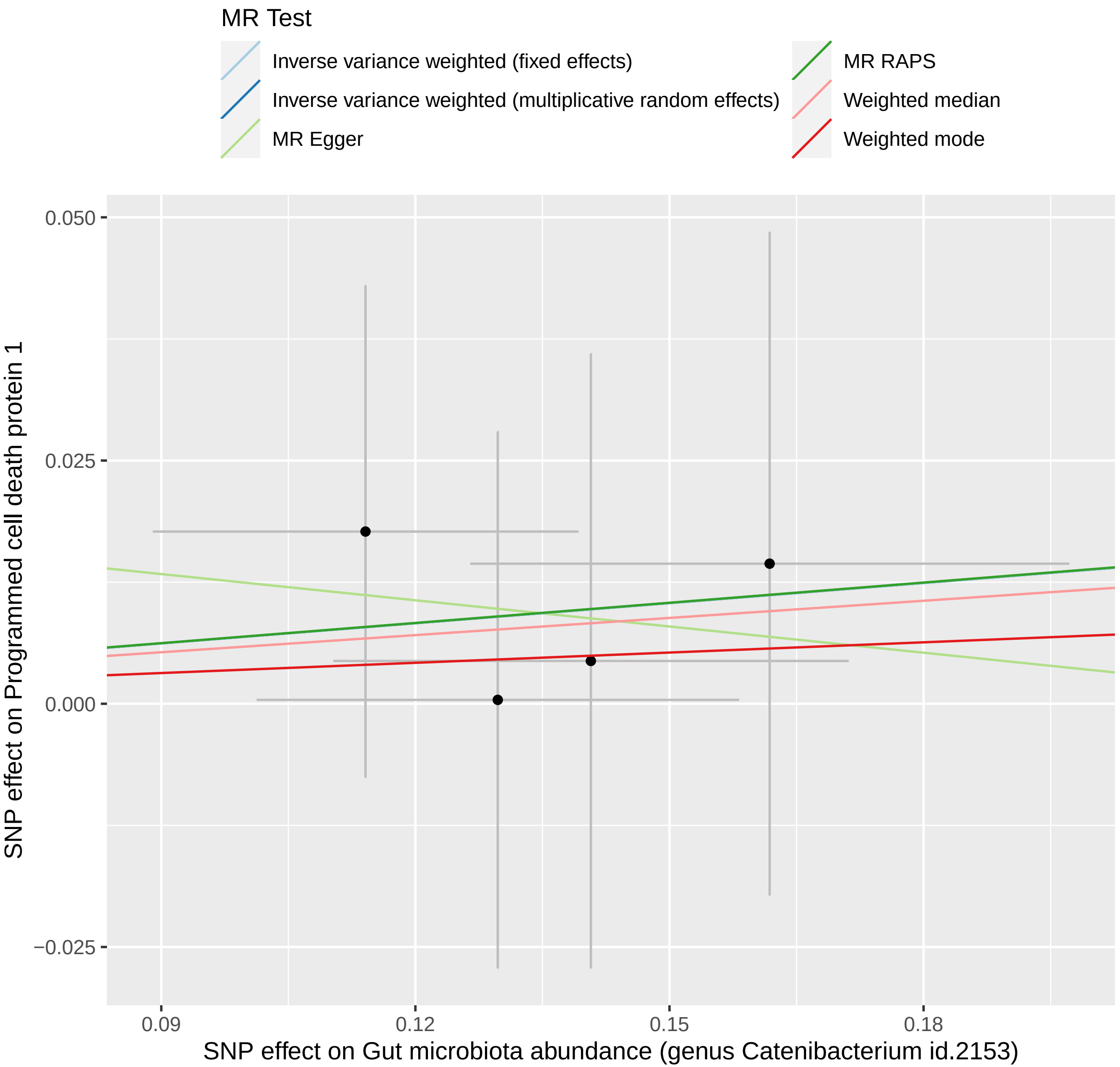

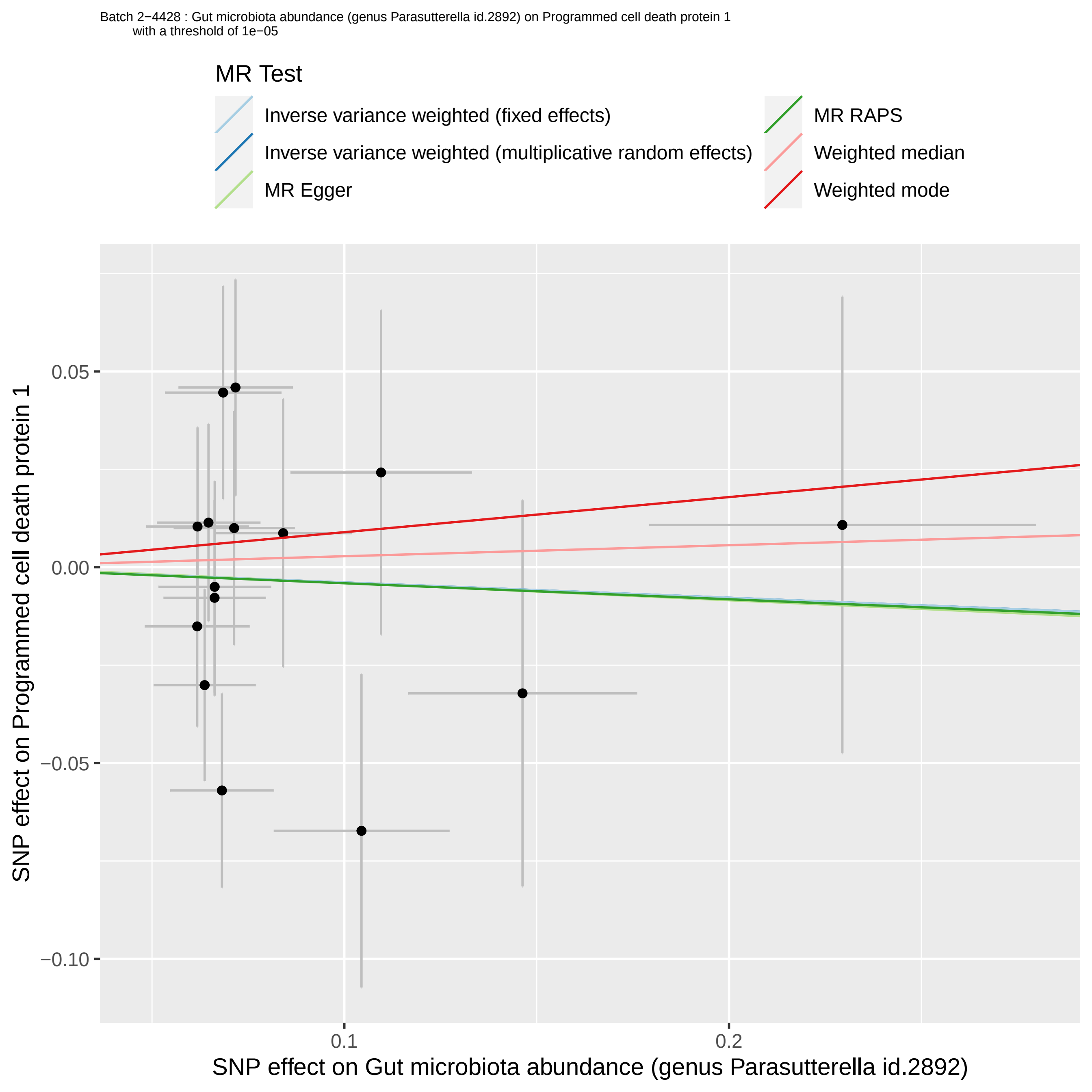

Batch 2−4428 : Gut microbiota abundance (genus Parasutterella id.2892) on Programmed cell death protein 1
with a threshold of 1e−05
MR Test
Inverse variance weighted (fixed effects)
Inverse variance weighted (multiplicative random effects)
MR Egger
MR RAPS
Weighted median
Weighted mode
0.05
0.00
−0.05
−0.10
0.1
0.2
SNP effect on Programmed cell death protein 1
SNP effect on Gut microbiota abundance (genus Parasutterella id.2892)

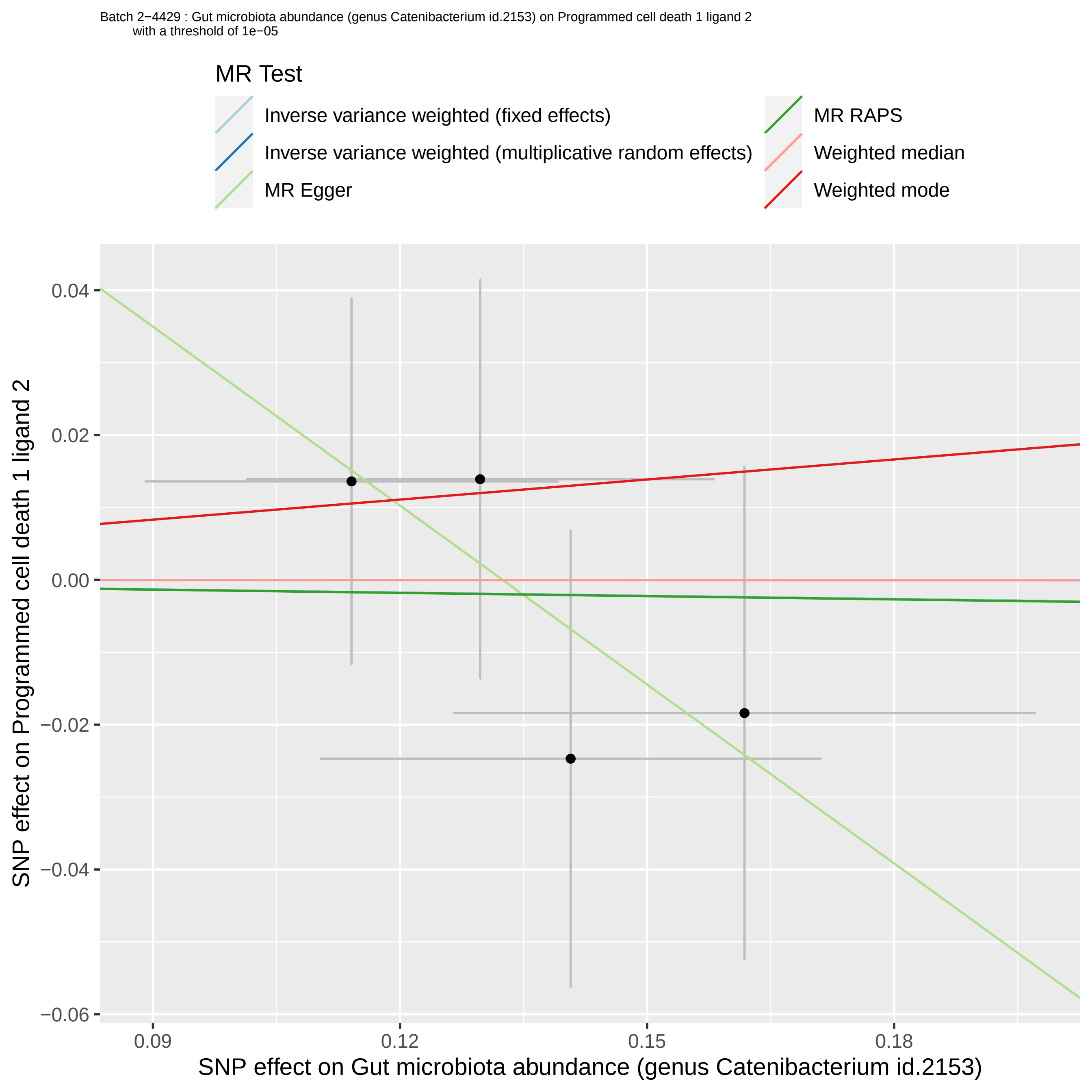
Batch 2−4429 : Gut microbiota abundance (genus Catenibacterium id.2153) on Programmed cell death 1 ligand 2
with a threshold of 1e−05
MR Test
Inverse variance weighted (fixed effects)
Inverse variance weighted (multiplicative random effects)
MR Egger
MR RAPS
Weighted median
Weighted mode
0.04
0.02
0.00
−0.02
−0.04
−0.06
0.09
0.12
0.15
0.18
SNP effect on Programmed cell death 1 ligand 2
SNP effect on Gut microbiota abundance (genus Catenibacterium id.2153)

Batch 2−4430 : Gut microbiota abundance (genus Parasutterella id.2892) on Programmed cell death 1 ligand 2
with a threshold of 1e−05

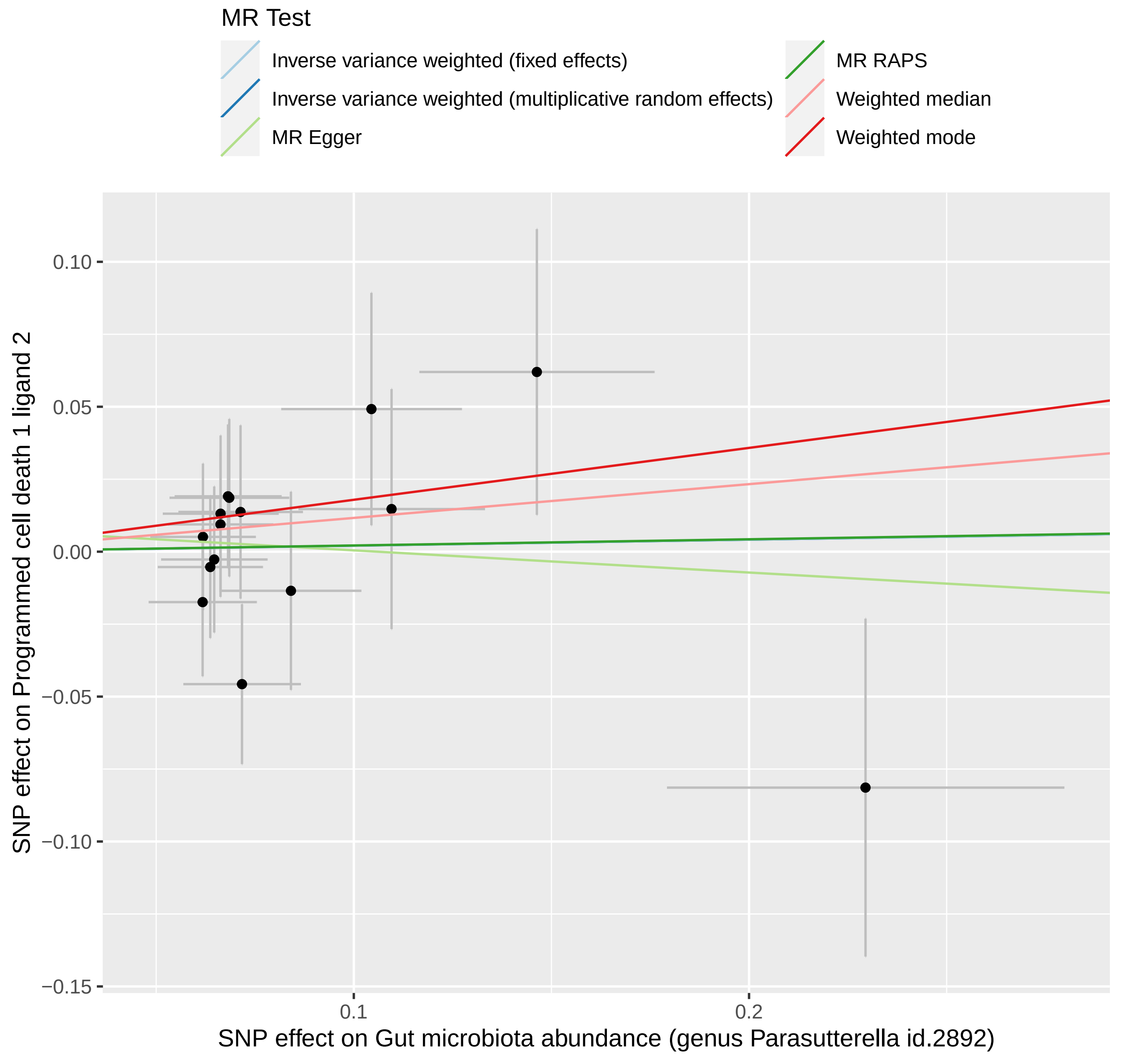

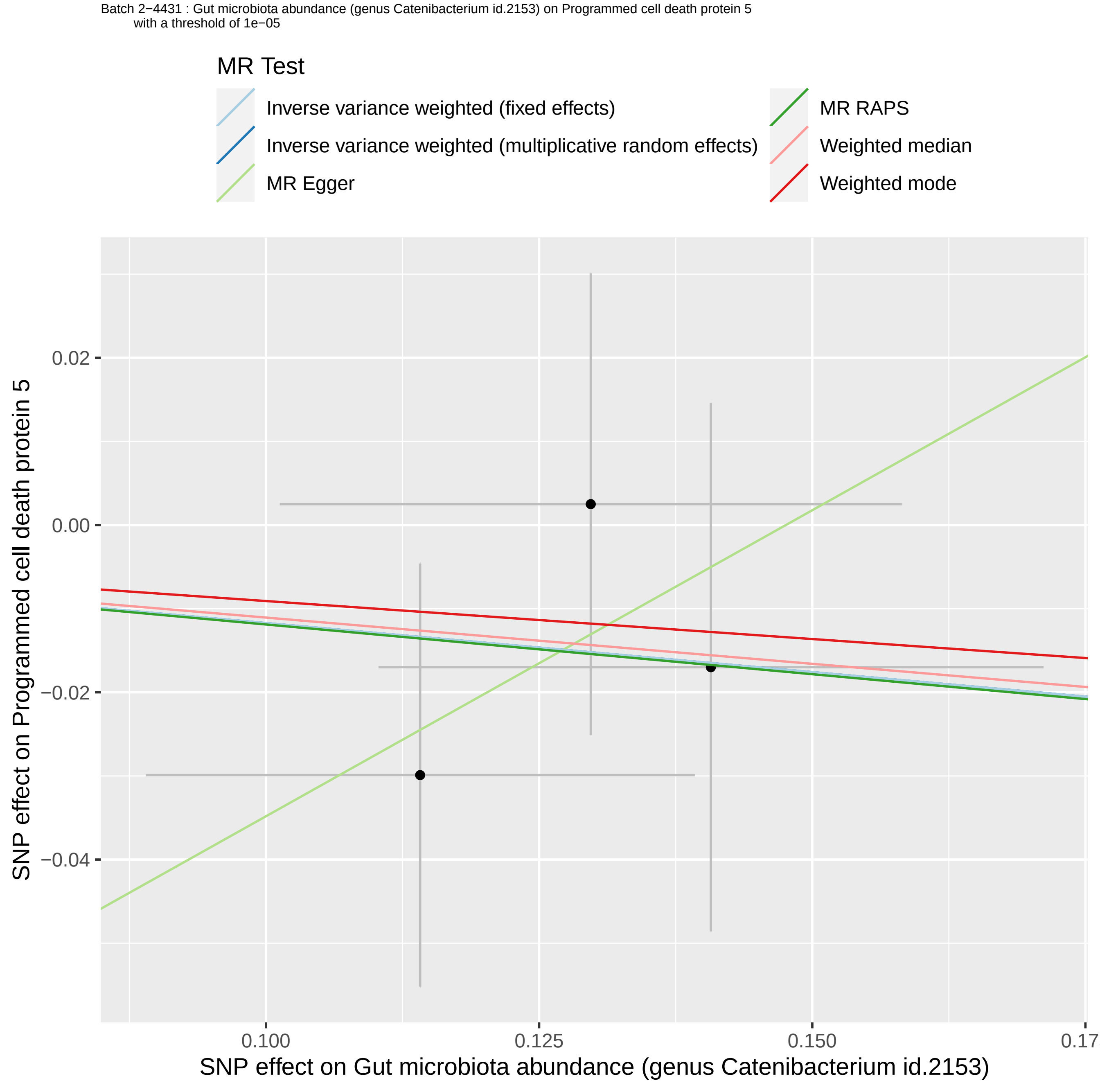

Batch 2−4431 : Gut microbiota abundance (genus Catenibacterium id.2153) on Programmed cell death protein 5
with a threshold of 1e−05
MR Test
Inverse variance weighted (fixed effects)
Inverse variance weighted (multiplicative random effects)
MR Egger
MR RAPS
Weighted median
Weighted mode
SNP effect on Programmed cell death protein 5
0.02
0.00
−0.02
−0.04
0.100
0.125
0.150
0.17
SNP effect on Gut microbiota abundance (genus Catenibacterium id.2153)

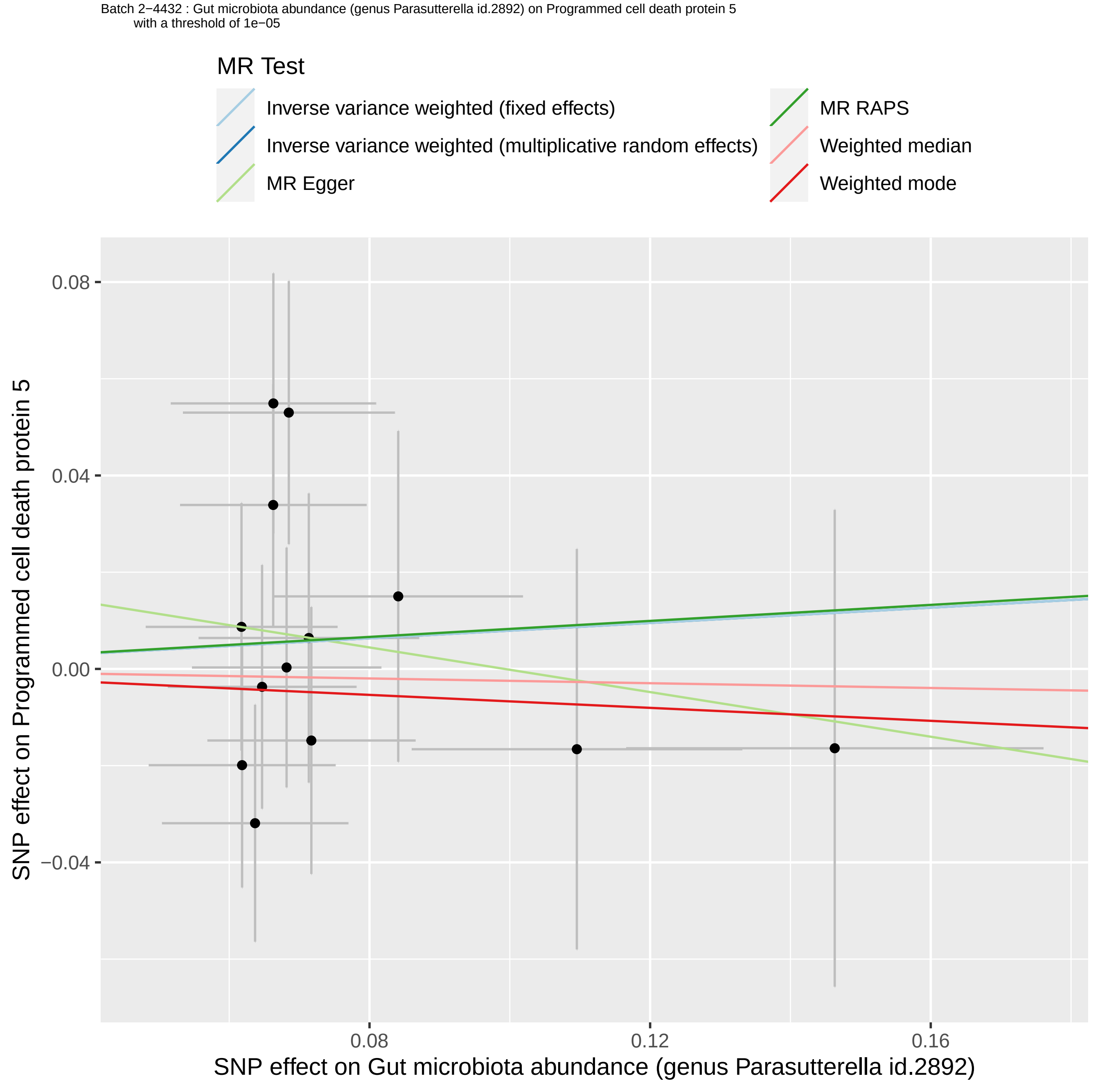
Batch 2−4432 : Gut microbiota abundance (genus Parasutterella id.2892) on Programmed cell death protein 5
with a threshold of 1e−05
MR Test
Inverse variance weighted (fixed effects)
Inverse variance weighted (multiplicative random effects)
MR Egger
MR RAPS
Weighted median
Weighted mode
0.08
0.04
0.00
−0.04
0.08
0.12
0.16
SNP effect on Programmed cell death protein 5
SNP effect on Gut microbiota abundance (genus Parasutterella id.2892)

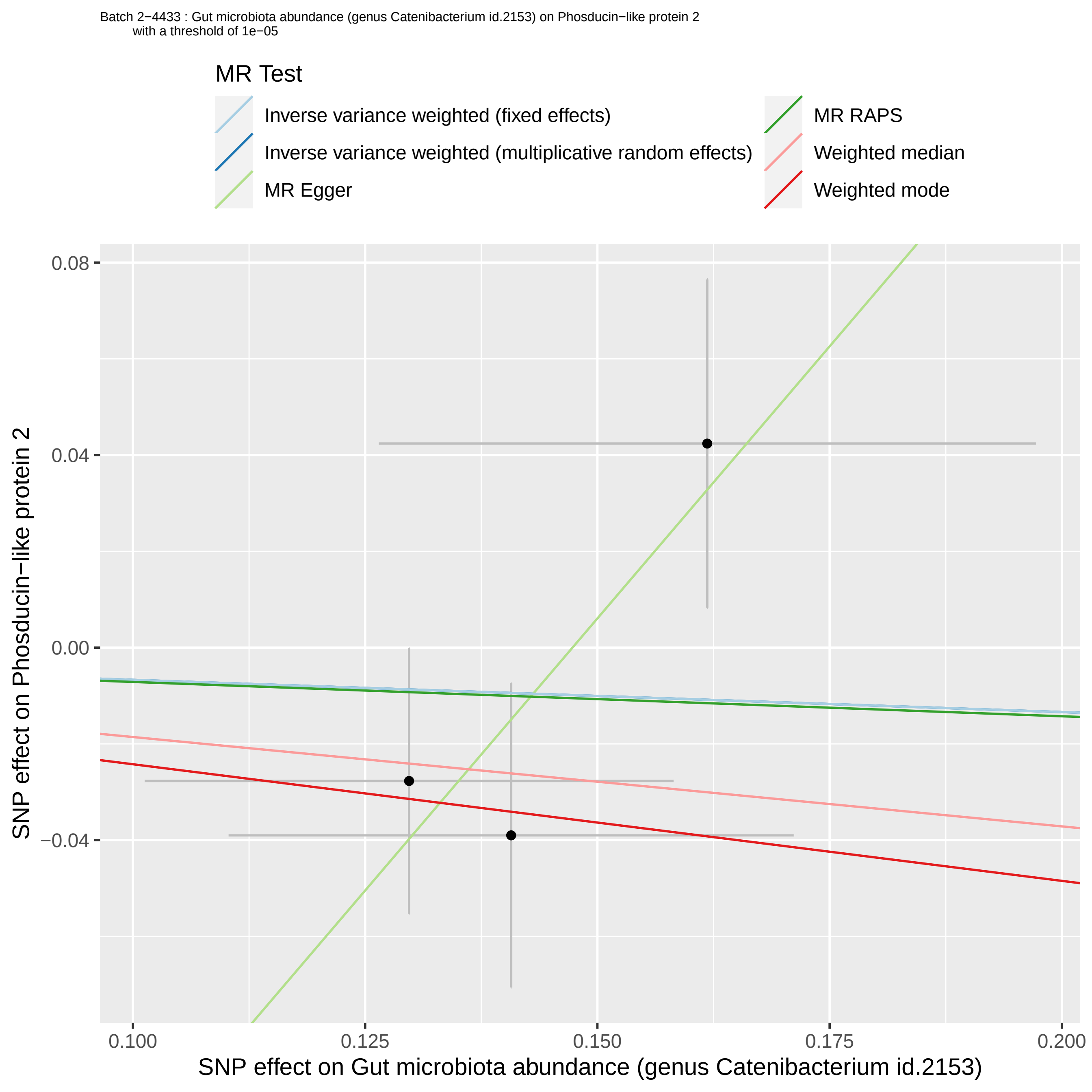

Batch 2−4433 : Gut microbiota abundance (genus Catenibacterium id.2153) on Phosducin−like protein 2
with a threshold of 1e−05
MR Test
Inverse variance weighted (fixed effects)
Inverse variance weighted (multiplicative random effects)
MR Egger
MR RAPS
Weighted median
Weighted mode
0.08
0.04
0.00
−0.04
0.100
0.125
0.150
0.175
0.200
SNP effect on Phosducin−like protein 2
SNP effect on Gut microbiota abundance (genus Catenibacterium id.2153)

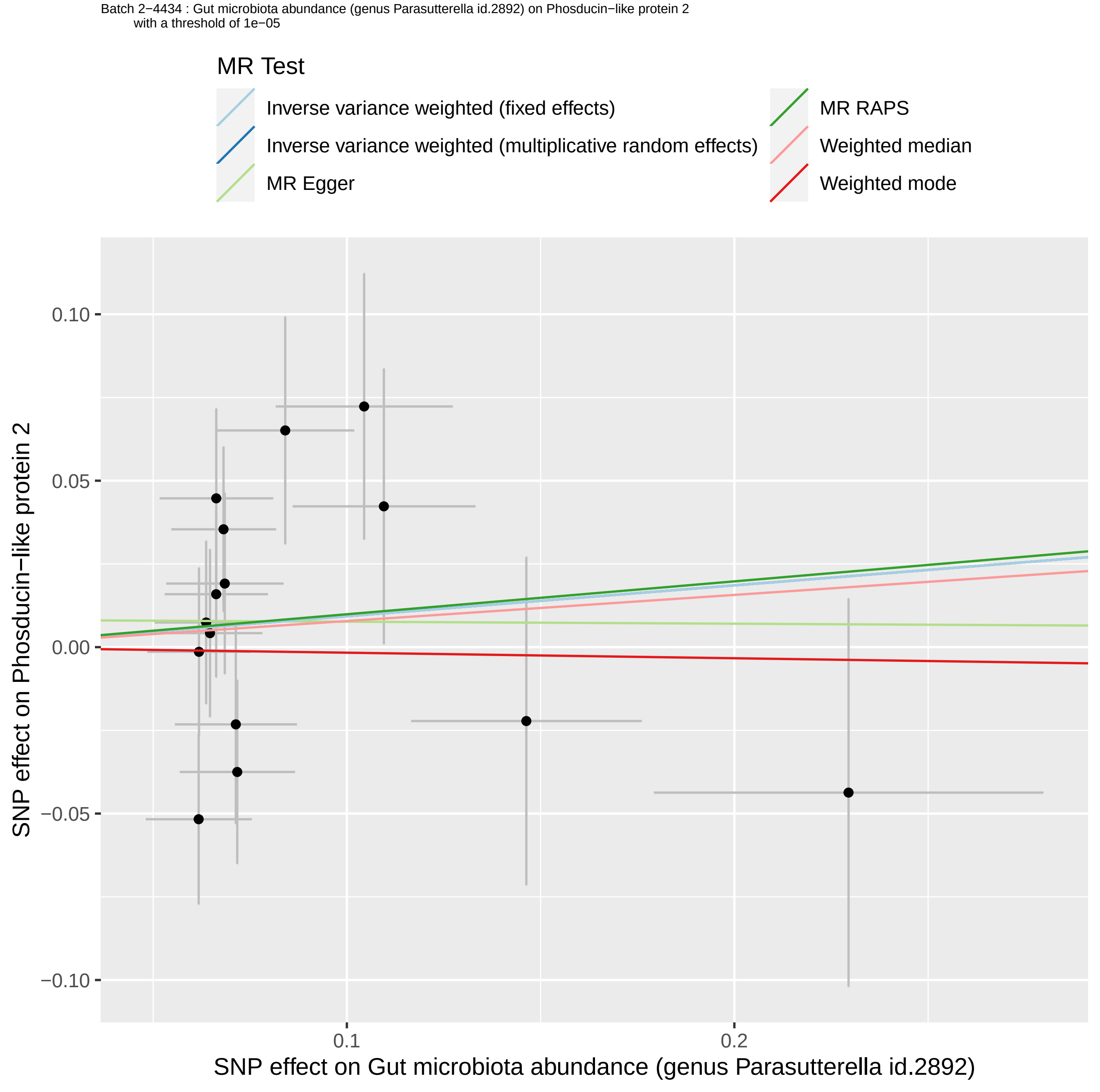
Batch 2−4434 : Gut microbiota abundance (genus Parasutterella id.2892) on Phosducin−like protein 2
with a threshold of 1e−05
MR Test
Inverse variance weighted (fixed effects)
Inverse variance weighted (multiplicative random effects)
MR Egger
MR RAPS
Weighted median
Weighted mode
SNP effect on Phosducin−like protein 2
0.10
0.05
0.00
−0.05
−0.10
0.1
0.2
SNP effect on Gut microbiota abundance (genus Parasutterella id.2892)

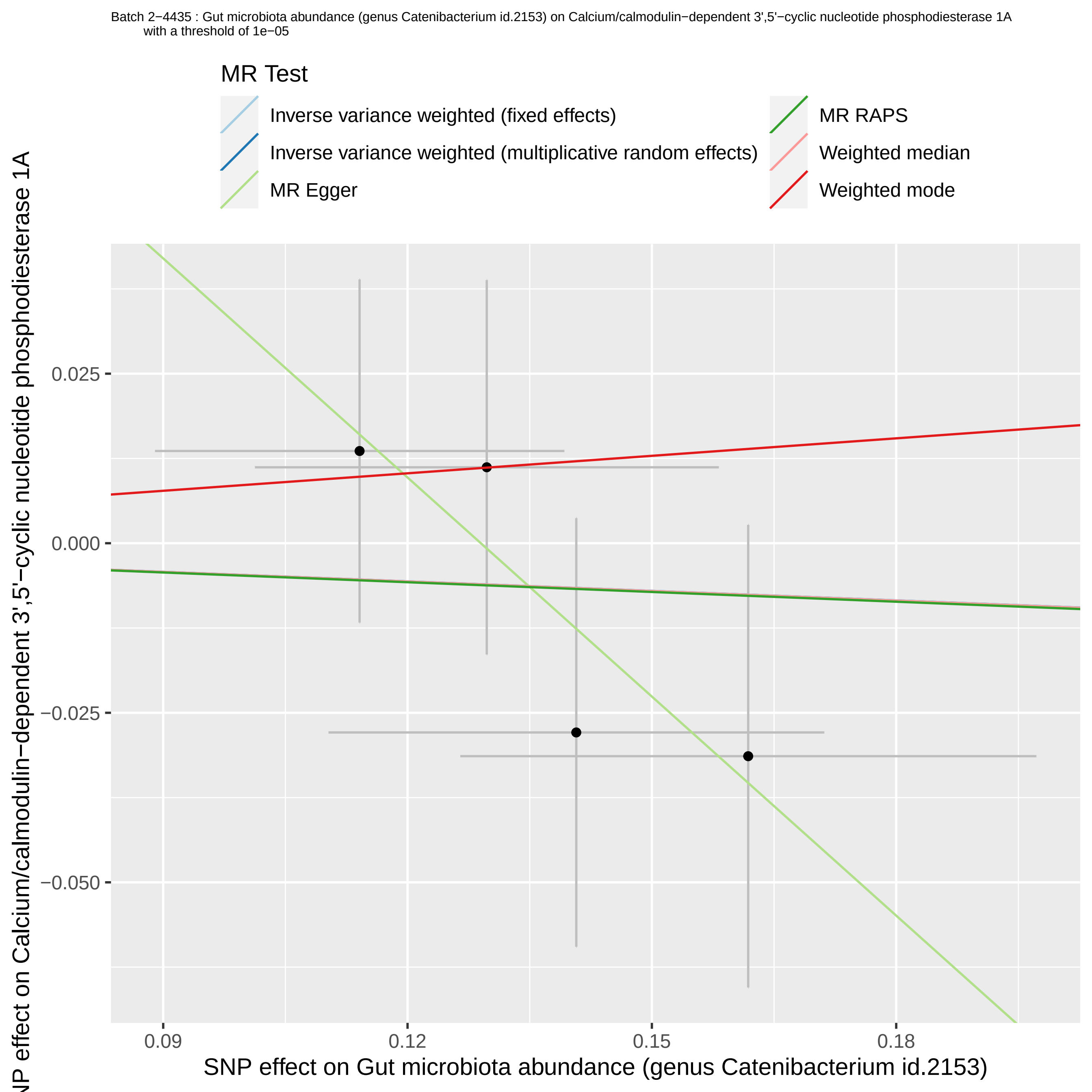
Batch 2−4435 : Gut microbiota abundance (genus Catenibacterium id.2153) on Calcium/calmodulin−dependent 3',5'−cyclic nucleotide phosphodiesterase 1A with a threshold of 1e−05
MR Test
Inverse variance weighted (fixed effects)
Inverse variance weighted (multiplicative random effects)
MR Egger
MR RAPS
Weighted median
Weighted mode
0.025
0.000
−0.025
−0.050
0.09
0.12
0.15
0.18
SNP effect on Gut microbiota abundance (genus Catenibacterium id.2153)
SNP effect on Calcium/calmodulin−dependent 3',5'−cyclic nucleotide phosphodiesterase 1A

Batch 2−4436 : Gut microbiota abundance (genus Parasutterella id.2892) on Calcium/calmodulin−dependent 3',5'−cyclic nucleotide phosphodiesterase 1A
with a threshold of 1e−05

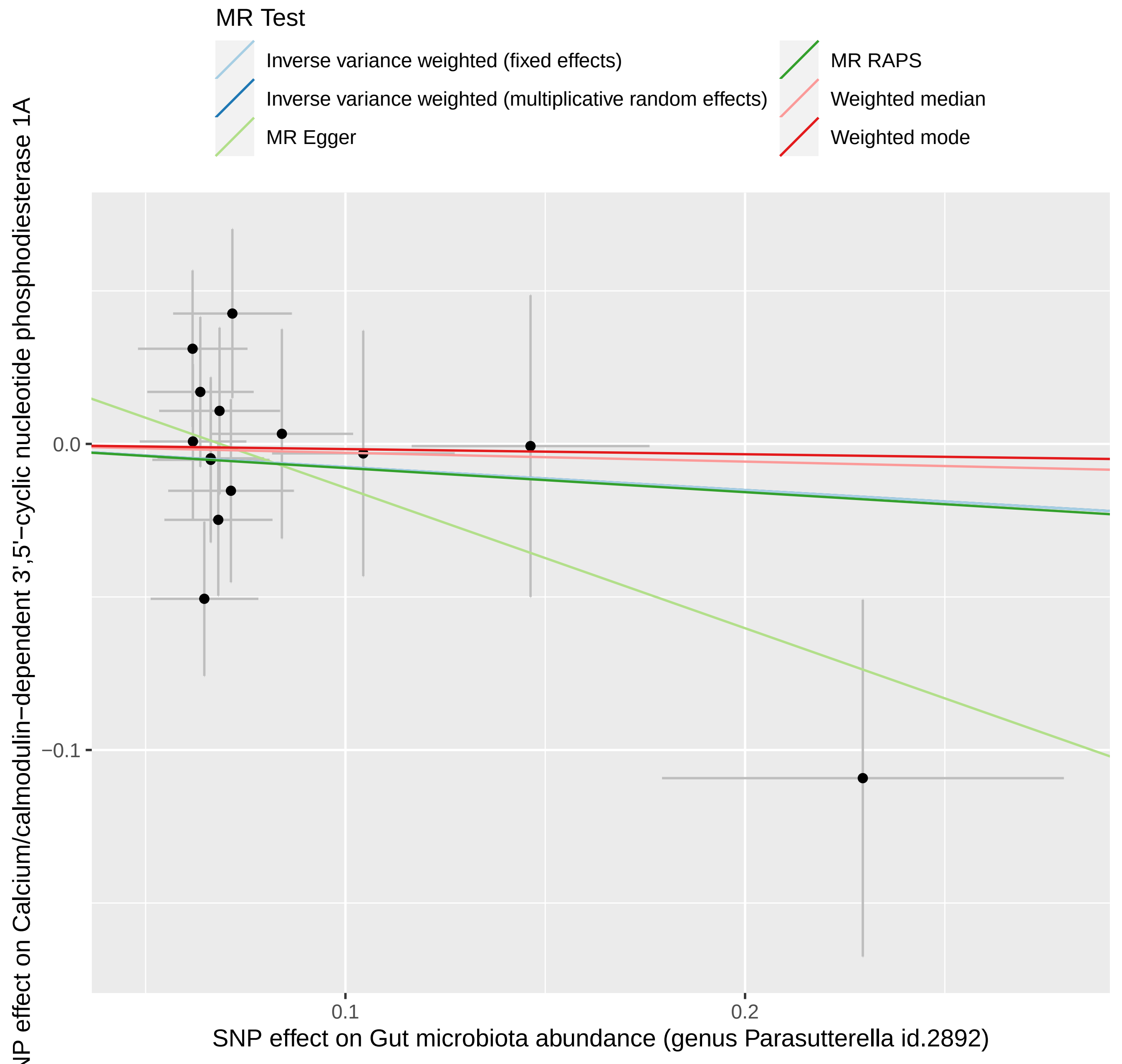

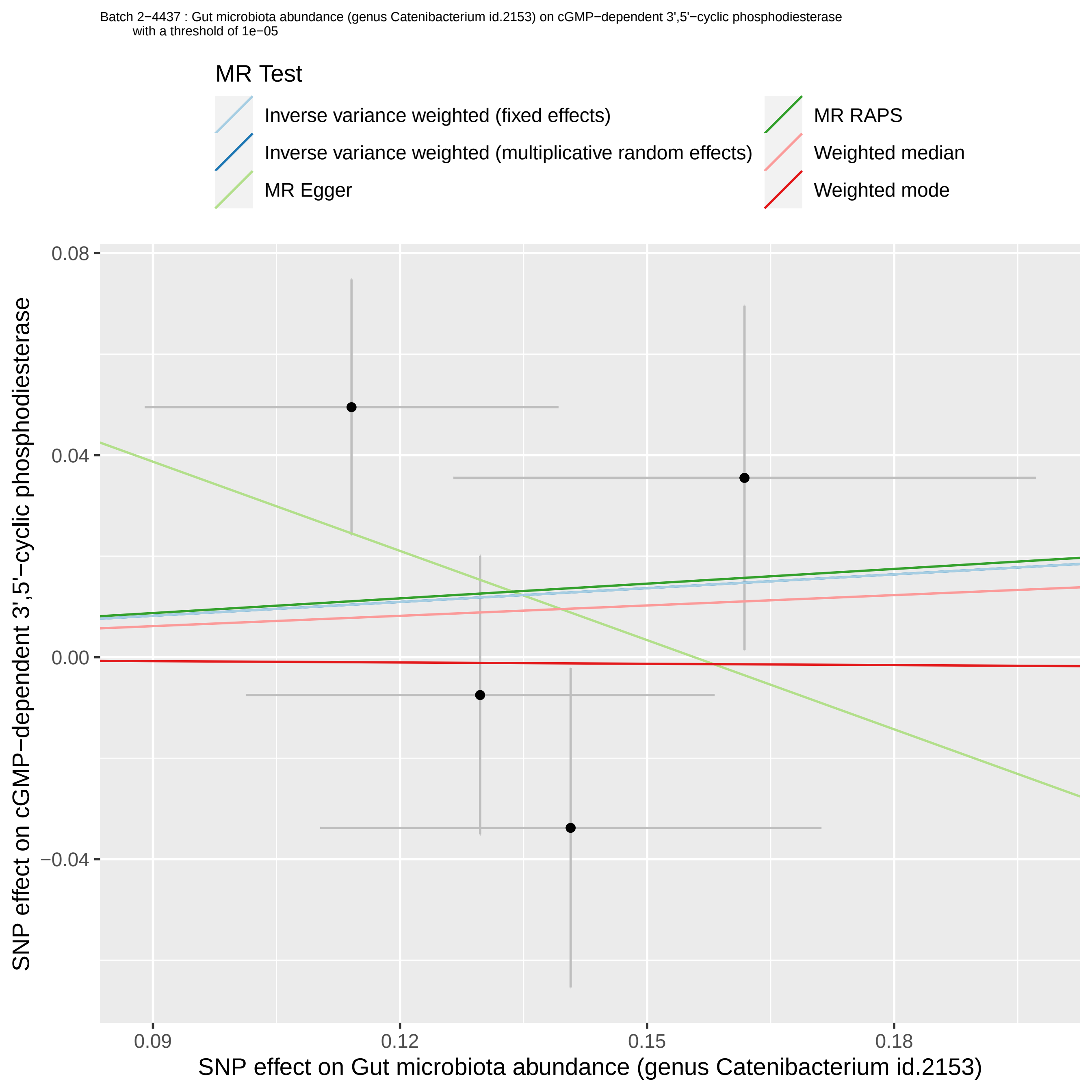
Batch 2−4437 : Gut microbiota abundance (genus Catenibacterium id.2153) on cGMP−dependent 3',5'−cyclic phosphodiesterase
with a threshold of 1e−05
MR Test
Inverse variance weighted (fixed effects)
Inverse variance weighted (multiplicative random effects)
MR Egger
MR RAPS
Weighted median
Weighted mode
0.08
0.04
0.00
−0.04
0.09
0.12
0.15
0.18
SNP effect on cGMP−dependent 3',5'−cyclic phosphodiesterase
SNP effect on Gut microbiota abundance (genus Catenibacterium id.2153)

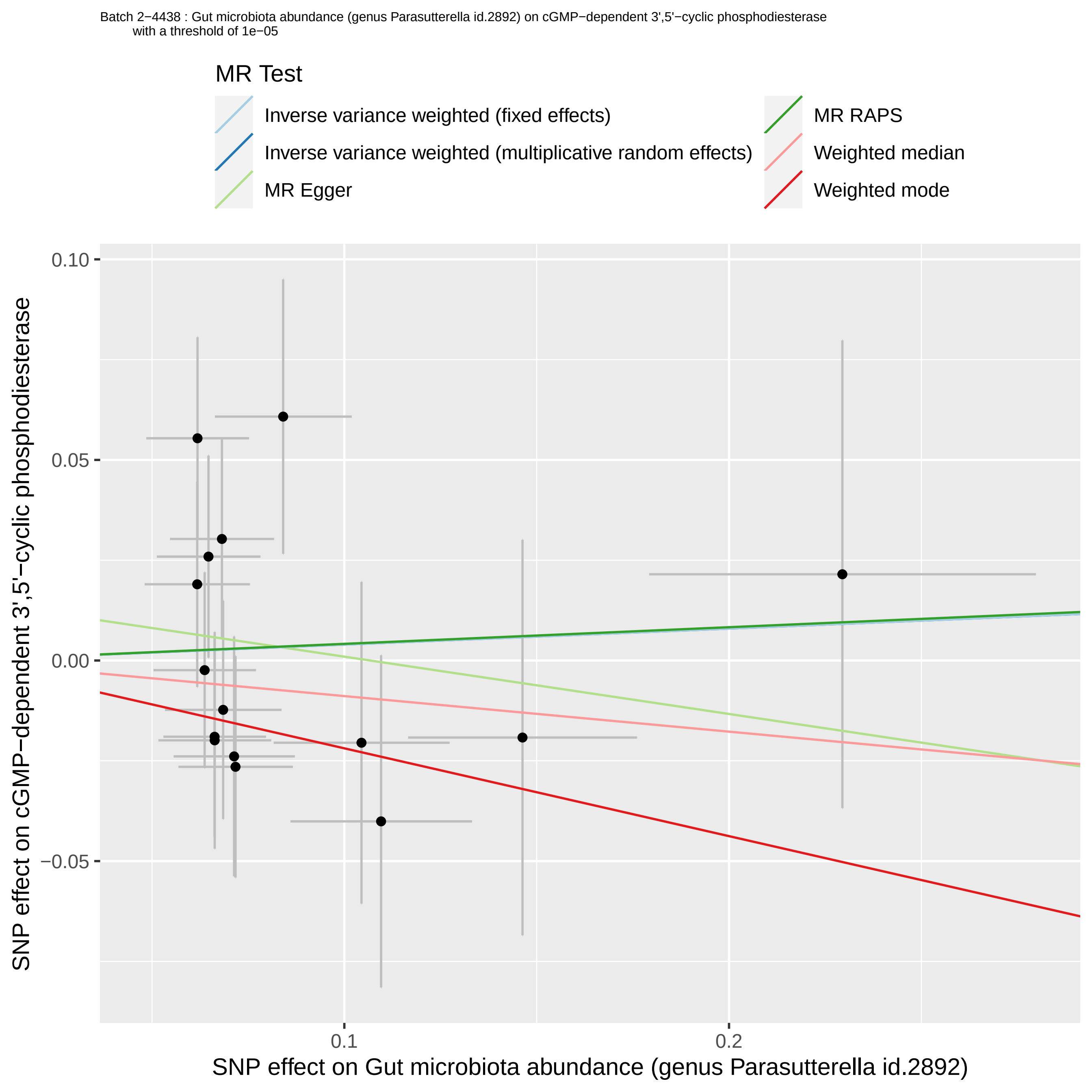

Batch 2−4438 : Gut microbiota abundance (genus Parasutterella id.2892) on cGMP−dependent 3',5'−cyclic phosphodiesterase
with a threshold of 1e−05
MR Test
Inverse variance weighted (fixed effects)
Inverse variance weighted (multiplicative random effects)
MR Egger
MR RAPS
Weighted median
Weighted mode
0.10
0.05
0.00
−0.05
0.1
0.2
SNP effect on cGMP−dependent 3',5'−cyclic phosphodiesterase
SNP effect on Gut microbiota abundance (genus Parasutterella id.2892)

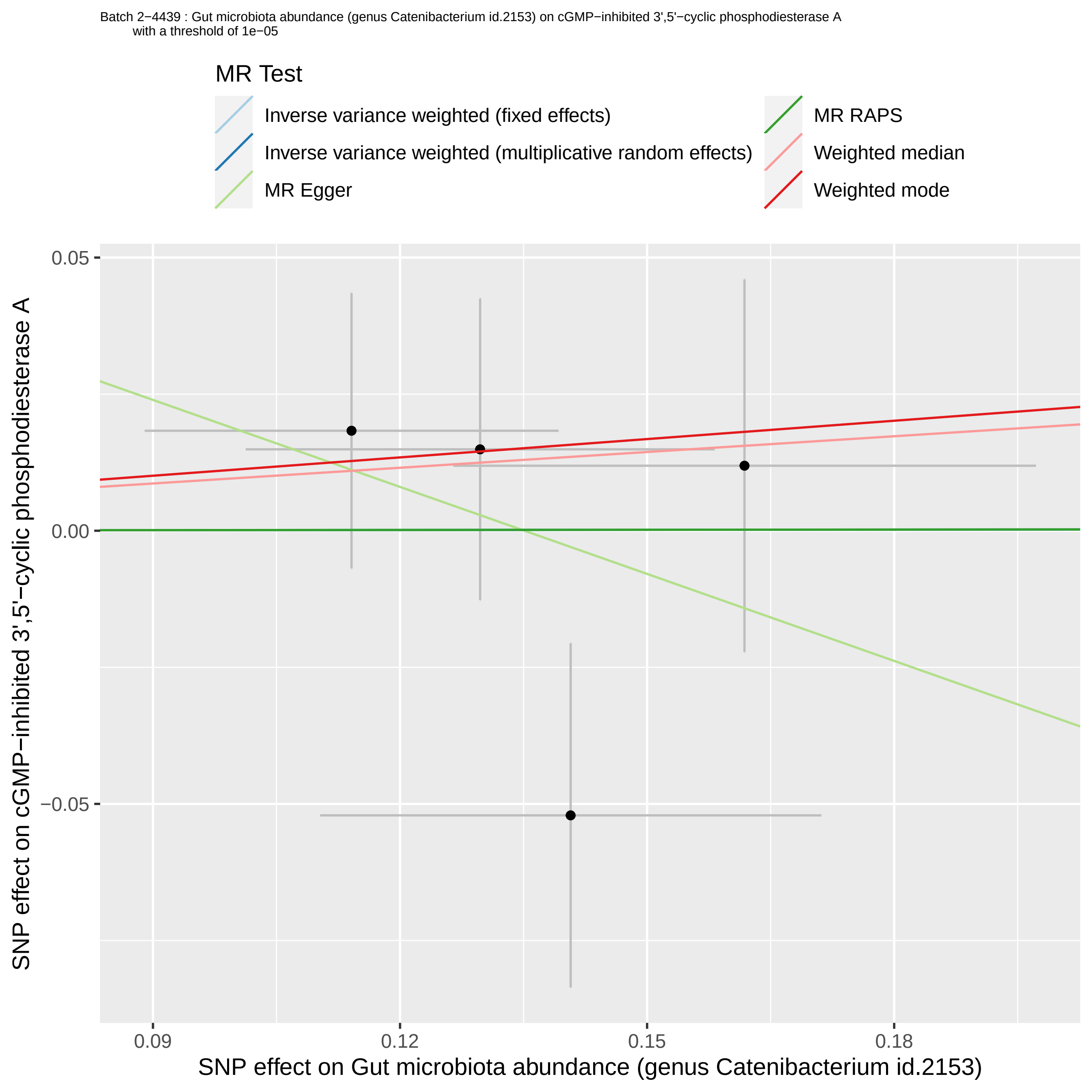
Batch 2−4439 : Gut microbiota abundance (genus Catenibacterium id.2153) on cGMP−inhibited 3',5'−cyclic phosphodiesterase A
with a threshold of 1e−05
MR Test
Inverse variance weighted (fixed effects)
Inverse variance weighted (multiplicative random effects)
MR Egger
MR RAPS
Weighted median
Weighted mode
0.05
0.00
−0.05
0.09
0.12
0.15
0.18
SNP effect on cGMP−inhibited 3',5'−cyclic phosphodiesterase A
SNP effect on Gut microbiota abundance (genus Catenibacterium id.2153)

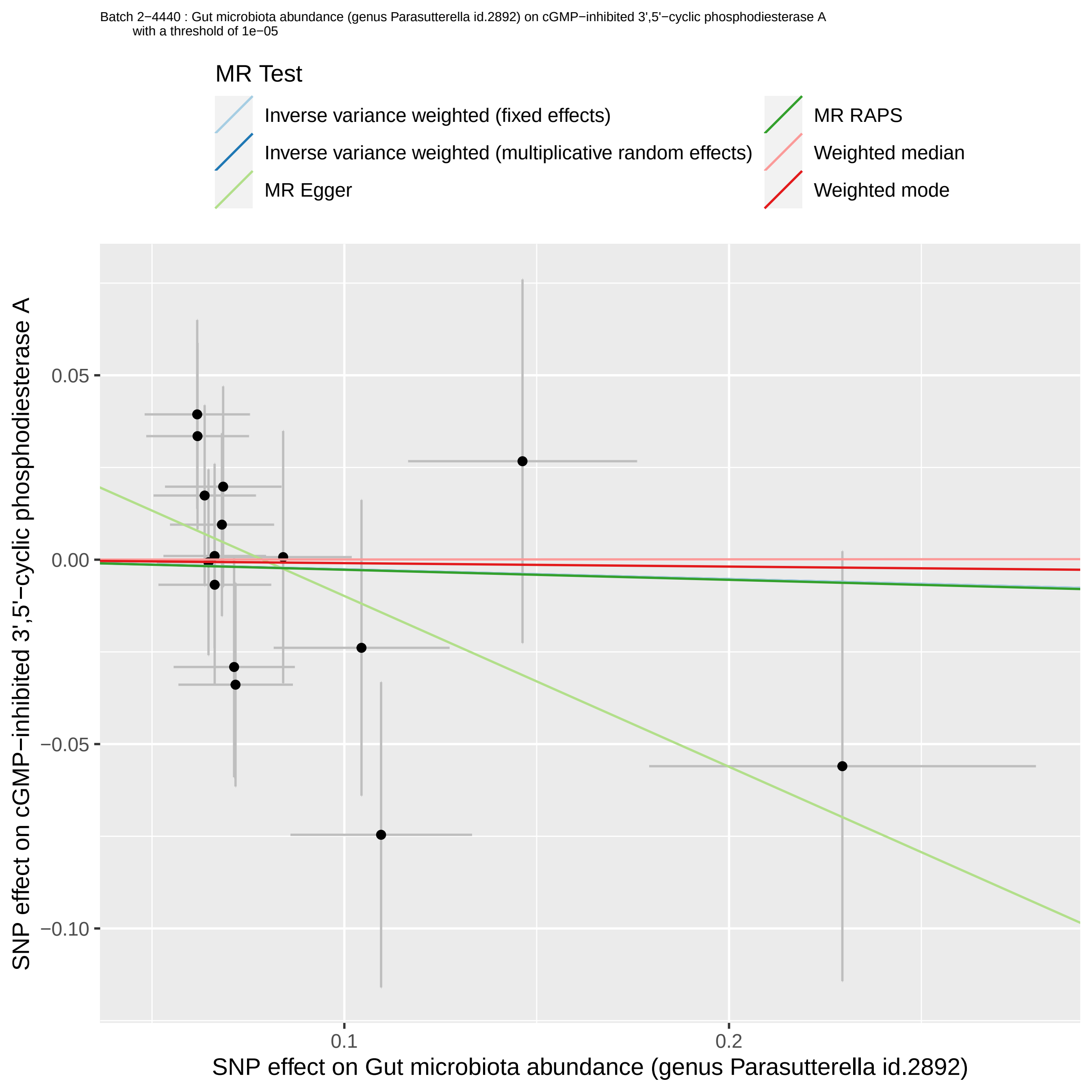

Batch 2−4440 : Gut microbiota abundance (genus Parasutterella id.2892) on cGMP−inhibited 3',5'−cyclic phosphodiesterase A
with a threshold of 1e−05
MR Test
Inverse variance weighted (fixed effects)
Inverse variance weighted (multiplicative random effects)
MR Egger
MR RAPS
Weighted median
Weighted mode
0.05
0.00
−0.05
−0.10
0.1
0.2
SNP effect on cGMP−inhibited 3',5'−cyclic phosphodiesterase A
SNP effect on Gut microbiota abundance (genus Parasutterella id.2892)

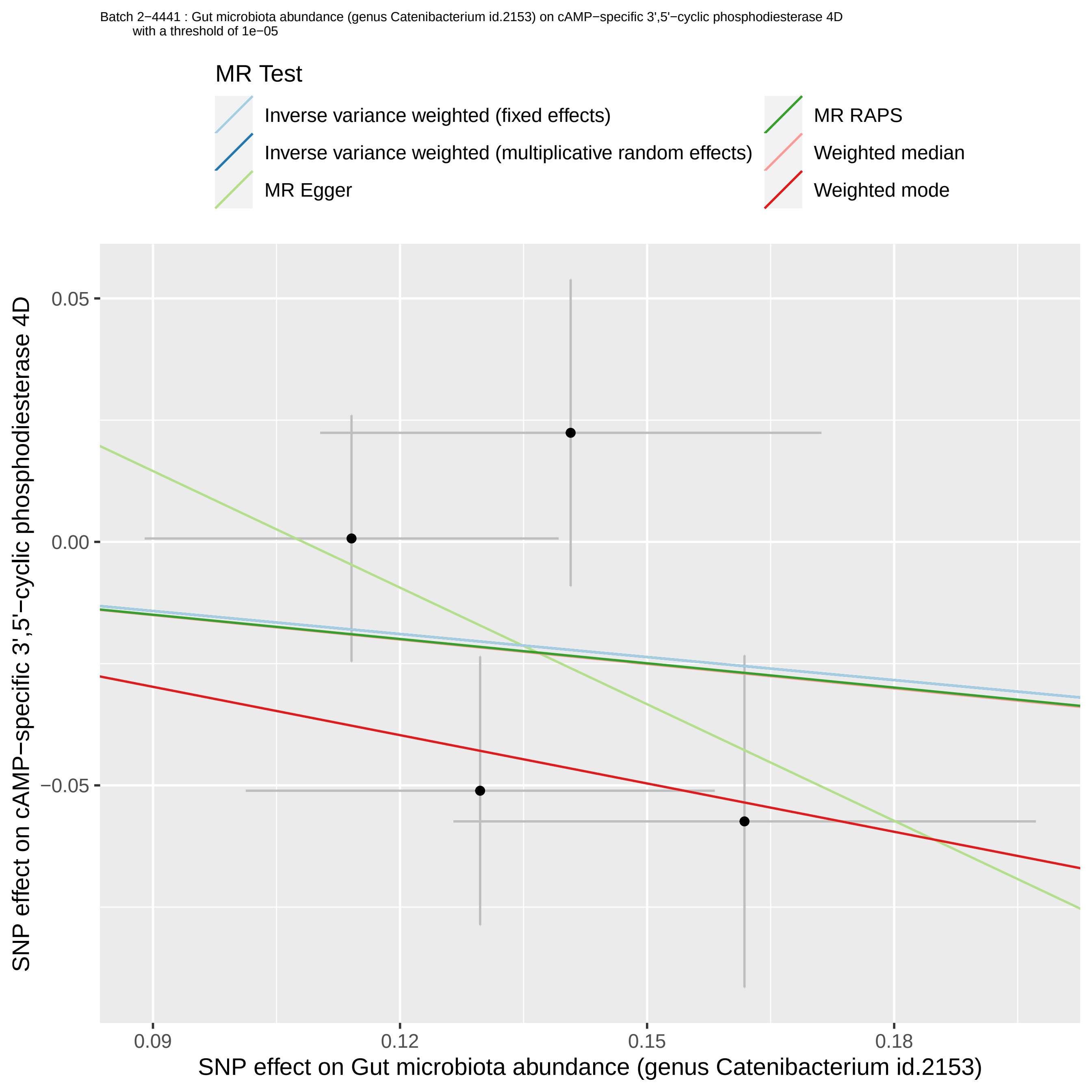
Batch 2−4441 : Gut microbiota abundance (genus Catenibacterium id.2153) on cAMP−specific 3',5'−cyclic phosphodiesterase 4D
with a threshold of 1e−05
MR Test
Inverse variance weighted (fixed effects)
Inverse variance weighted (multiplicative random effects)
MR Egger
MR RAPS
Weighted median
Weighted mode
0.05
0.00
−0.05
0.09
0.12
0.15
0.18
SNP effect on cAMP−specific 3',5'−cyclic phosphodiesterase 4D
SNP effect on Gut microbiota abundance (genus Catenibacterium id.2153)

Batch 2−4442 : Gut microbiota abundance (genus Parasutterella id.2892) on cAMP−specific 3',5'−cyclic phosphodiesterase 4D
with a threshold of 1e−05

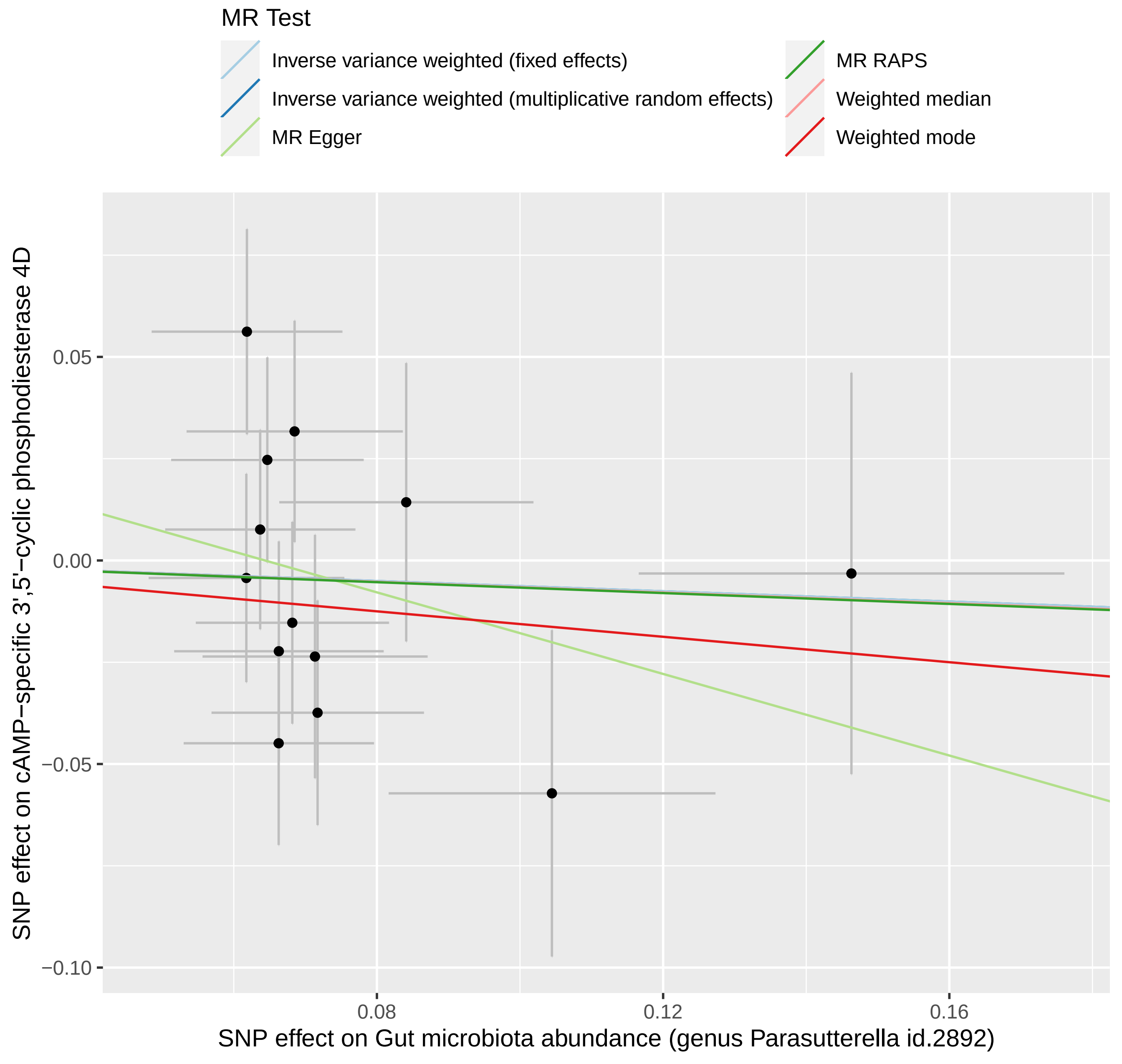

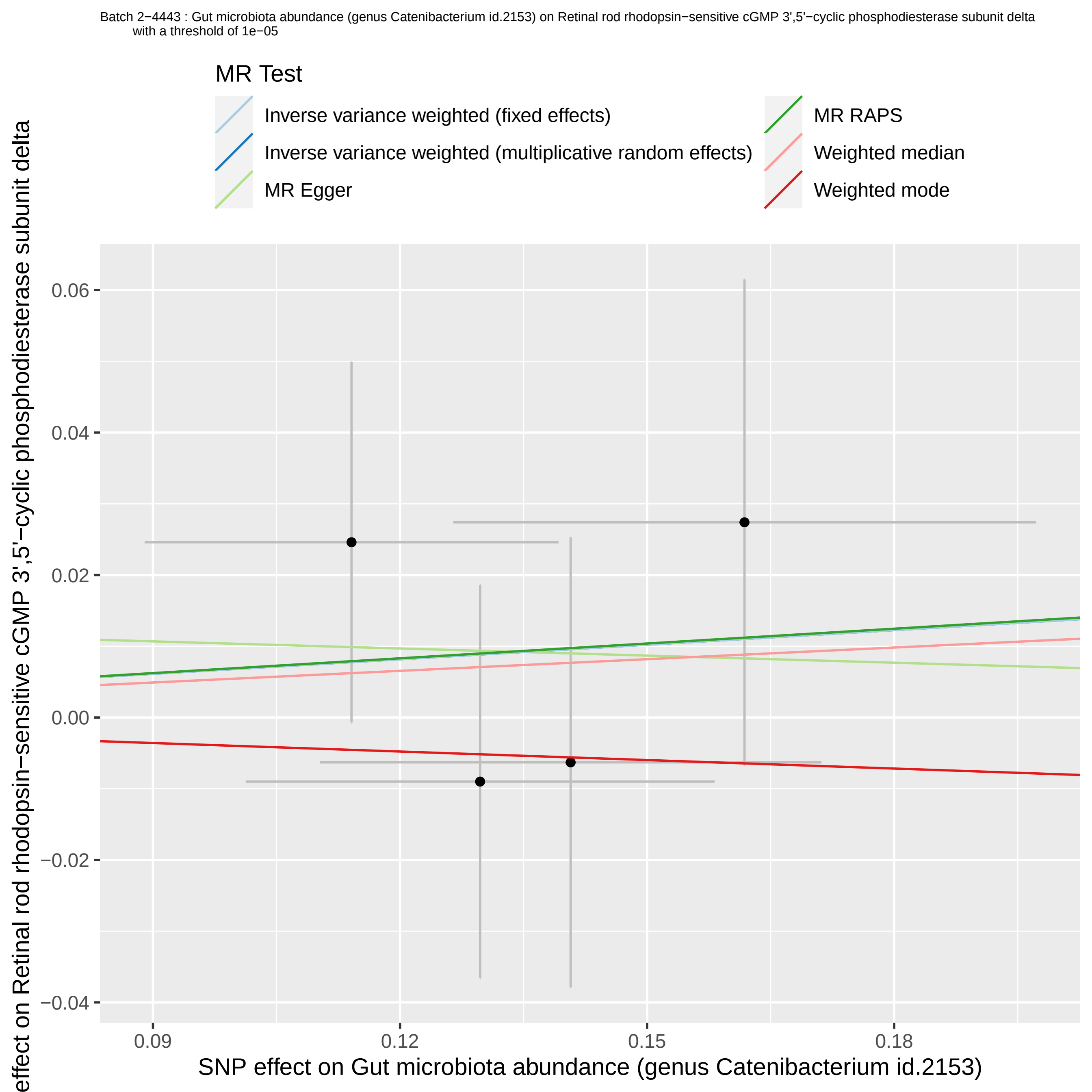
Batch 2−4443 : Gut microbiota abundance (genus Catenibacterium id.2153) on Retinal rod rhodopsin−sensitive cGMP 3',5'−cyclic phosphodiesterase subunit delta with a threshold of 1e−05
MR Test
Inverse variance weighted (fixed effects)
Inverse variance weighted (multiplicative random effects)
MR Egger
MR RAPS
Weighted median
Weighted mode
0.06
0.04
0.02
0.00
−0.02
−0.04
0.09
0.12
0.15
0.18
SNP effect on Gut microbiota abundance (genus Catenibacterium id.2153)
effect on Retinal rod rhodopsin−sensitive cGMP 3',5'−cyclic phosphodiesterase subunit delta

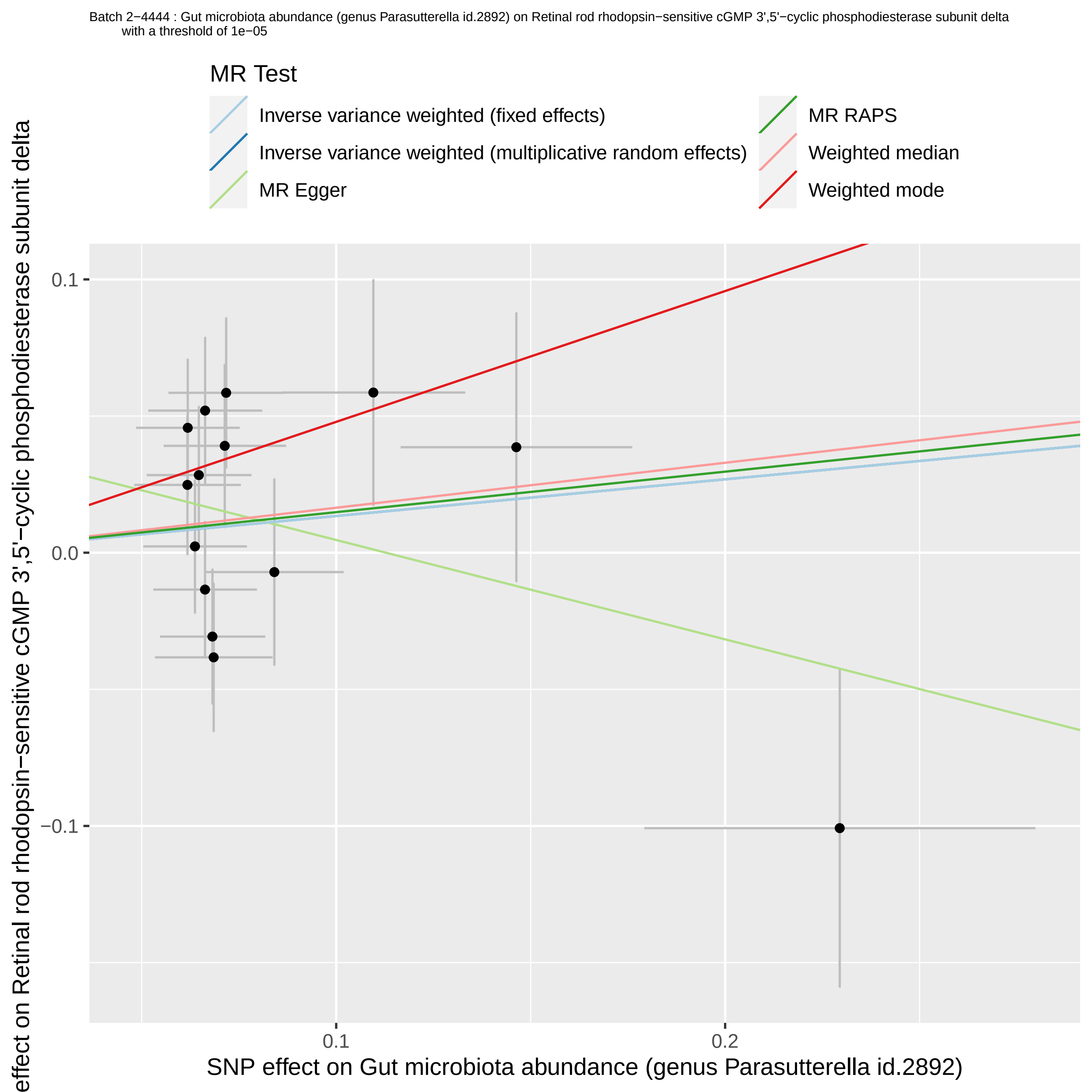

Batch 2−4444 : Gut microbiota abundance (genus Parasutterella id.2892) on Retinal rod rhodopsin−sensitive cGMP 3',5'−cyclic phosphodiesterase subunit delta
with a threshold of 1e−05
MR Test
Inverse variance weighted (fixed effects)
Inverse variance weighted (multiplicative random effects)
MR Egger
MR RAPS
Weighted median
Weighted mode
0.1
0.0
−0.1
0.1
0.2
SNP effect on Gut microbiota abundance (genus Parasutterella id.2892)
effect on Retinal rod rhodopsin−sensitive cGMP 3',5'−cyclic phosphodiesterase subunit delta

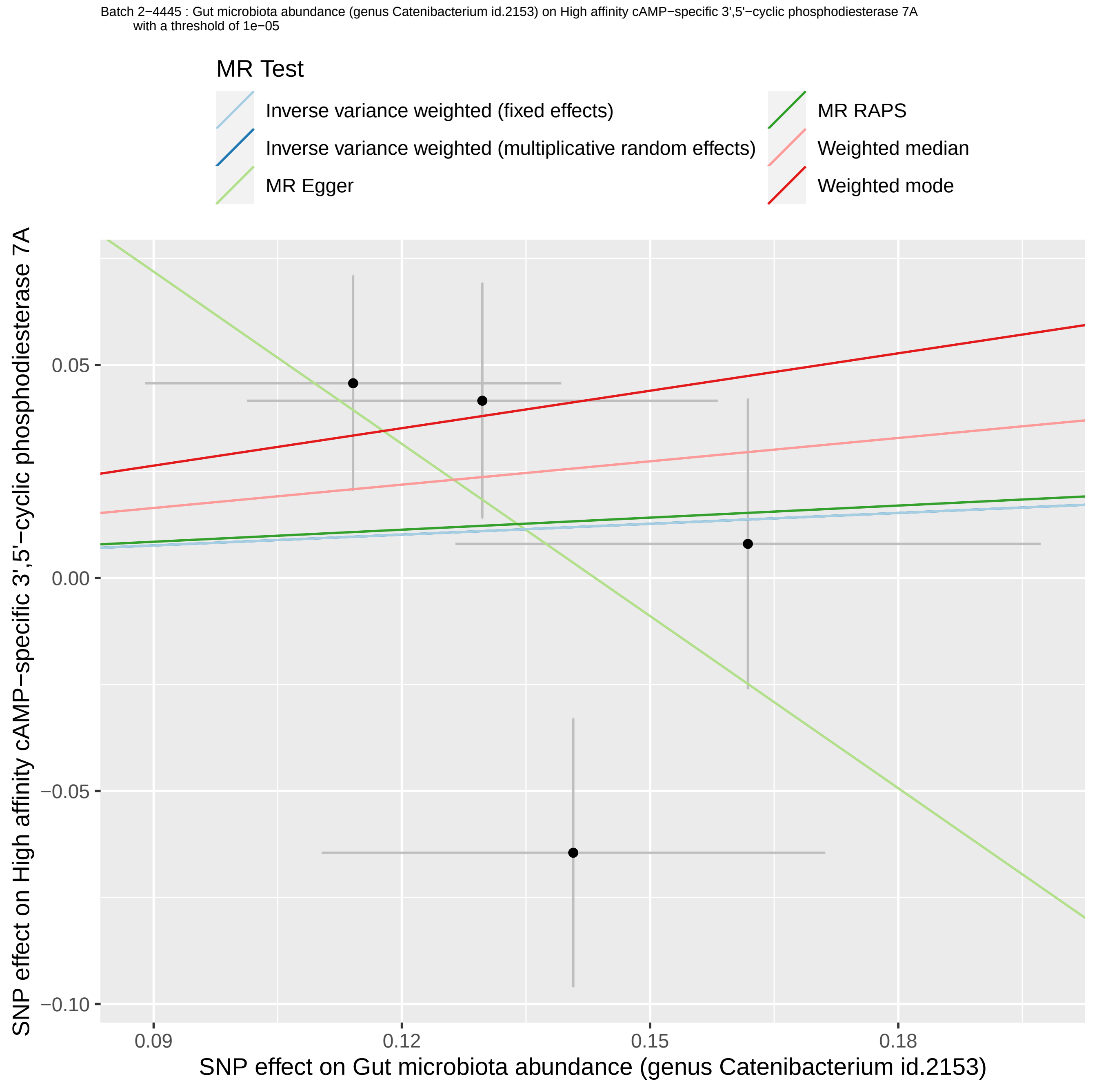

Batch 2−4445 : Gut microbiota abundance (genus Catenibacterium id.2153) on High affinity cAMP−specific 3',5'−cyclic phosphodiesterase 7A with a threshold of 1e−05
MR Test
Inverse variance weighted (fixed effects)
Inverse variance weighted (multiplicative random effects)
MR Egger
MR RAPS
Weighted median
Weighted mode
SNP effect on High affinity cAMP−specific 3',5'−cyclic phosphodiesterase 7A
0.05
0.00
−0.05
−0.10
0.09
0.12
0.15
0.18
SNP effect on Gut microbiota abundance (genus Catenibacterium id.2153)

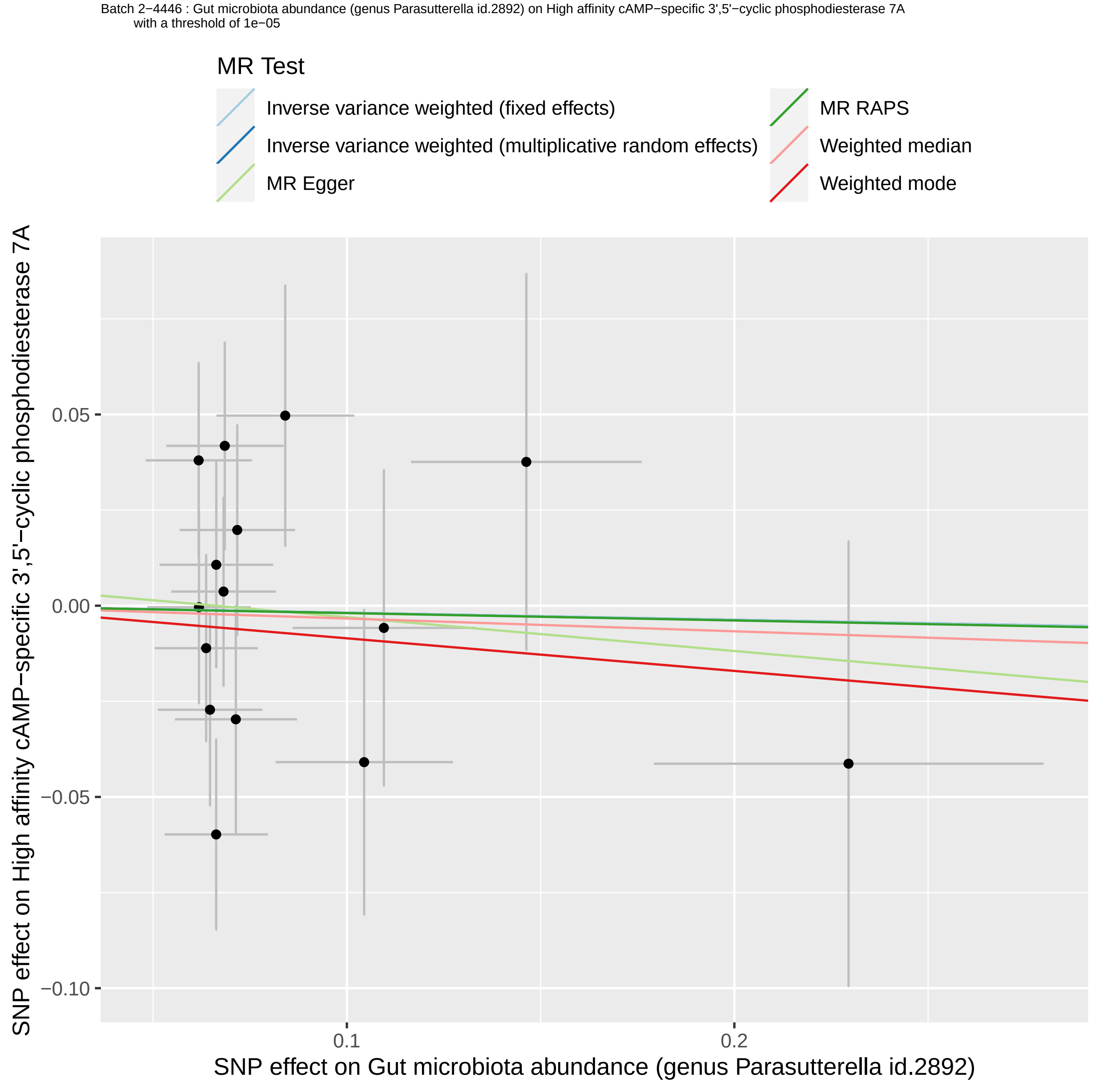
Batch 2−4446 : Gut microbiota abundance (genus Parasutterella id.2892) on High affinity cAMP−specific 3',5'−cyclic phosphodiesterase 7A
with a threshold of 1e−05
MR Test
Inverse variance weighted (fixed effects)
Inverse variance weighted (multiplicative random effects)
MR Egger
MR RAPS
Weighted median
Weighted mode
0.05
0.00
−0.05
−0.10
0.1
0.2
SNP effect on High affinity cAMP−specific 3',5'−cyclic phosphodiesterase 7A
SNP effect on Gut microbiota abundance (genus Parasutterella id.2892)

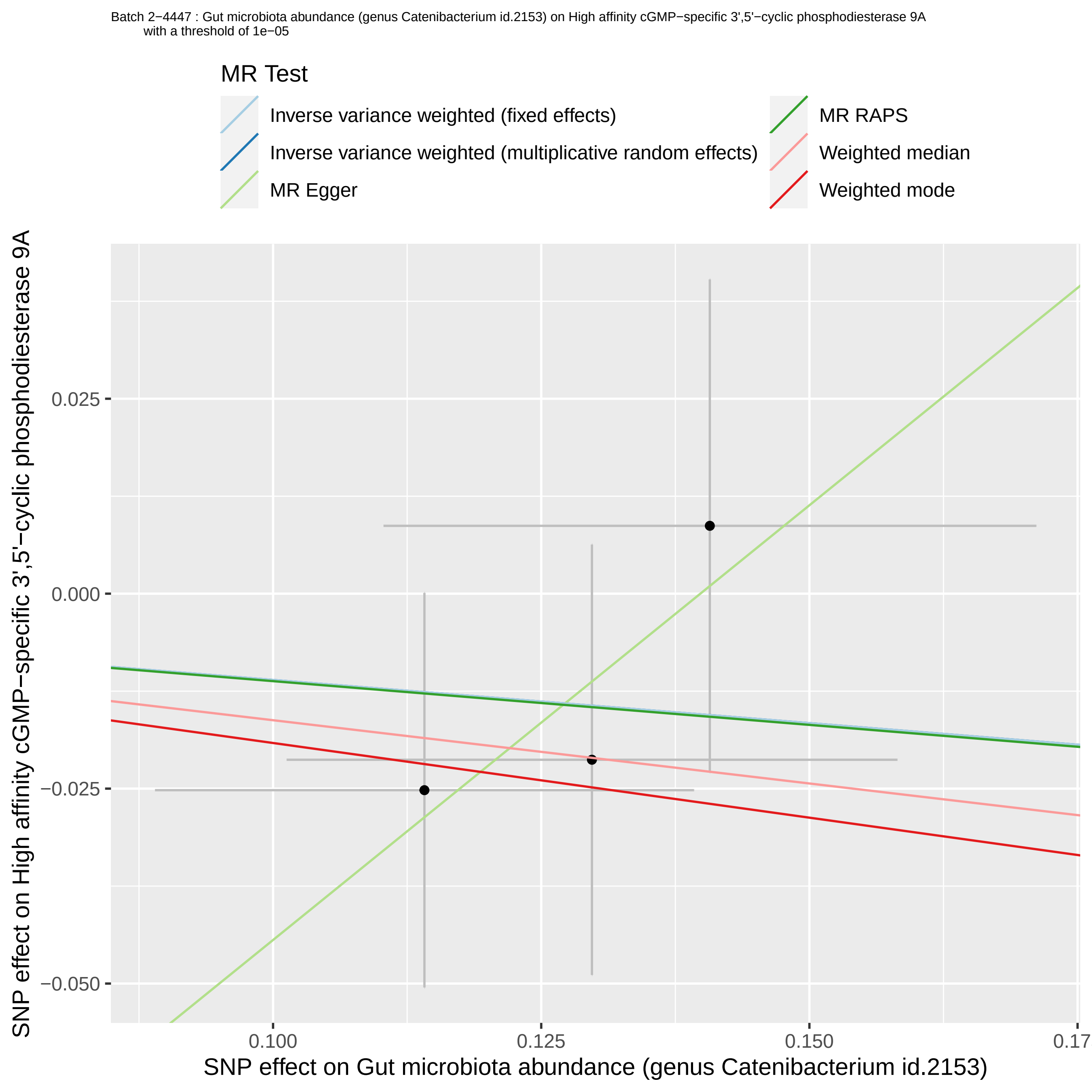

Batch 2−4447 : Gut microbiota abundance (genus Catenibacterium id.2153) on High affinity cGMP−specific 3',5'−cyclic phosphodiesterase 9A
with a threshold of 1e−05
MR Test
Inverse variance weighted (fixed effects)
Inverse variance weighted (multiplicative random effects)
MR Egger
MR RAPS
Weighted median
Weighted mode
SNP effect on High affinity cGMP−specific 3',5'−cyclic phosphodiesterase 9A
0.025
0.000
−0.025
−0.050
0.100
0.125
0.150
0.17
SNP effect on Gut microbiota abundance (genus Catenibacterium id.2153)

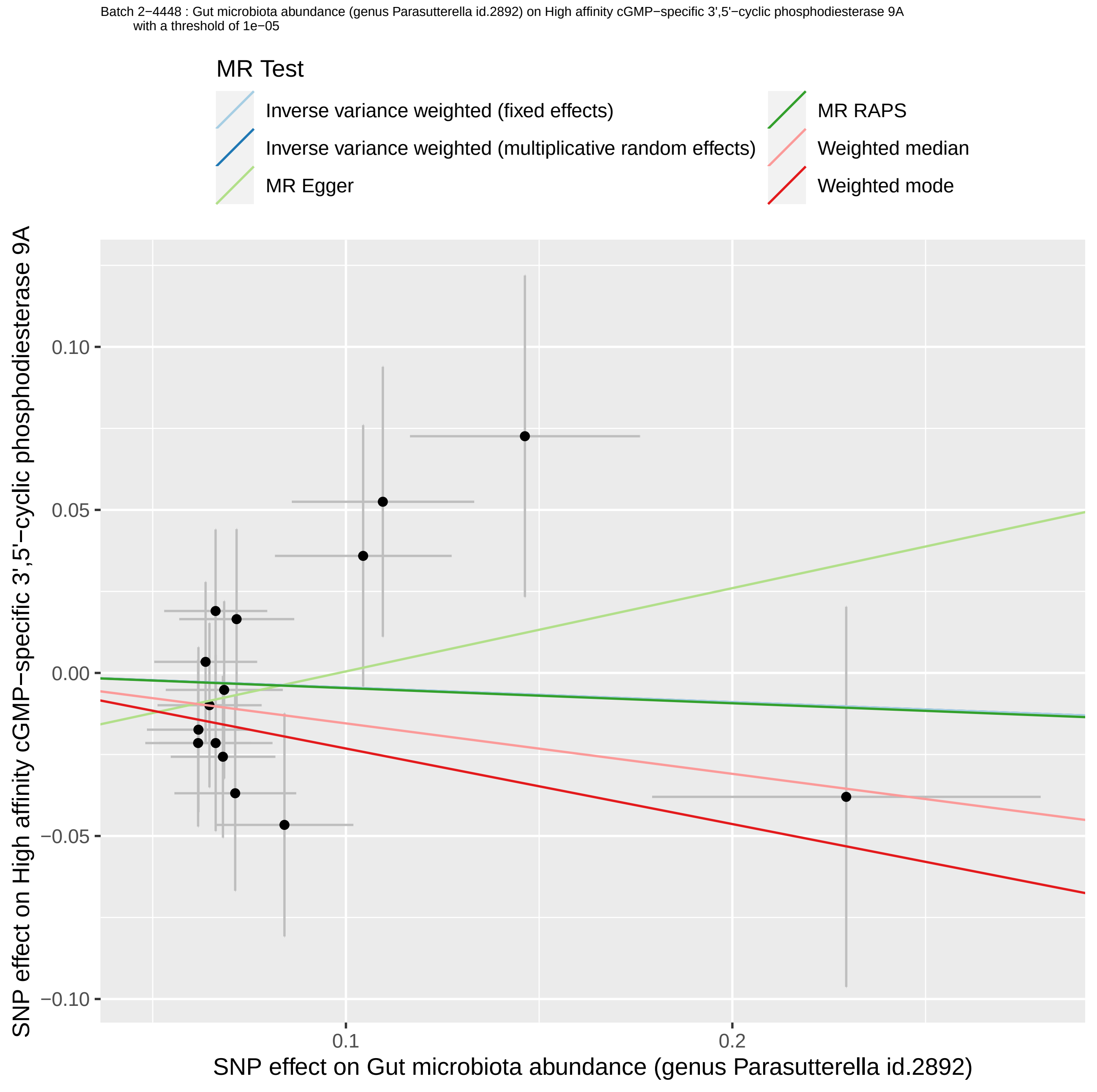
Batch 2−4448 : Gut microbiota abundance (genus Parasutterella id.2892) on High affinity cGMP−specific 3',5'−cyclic phosphodiesterase 9A
with a threshold of 1e−05
MR Test
Inverse variance weighted (fixed effects)
Inverse variance weighted (multiplicative random effects)
MR Egger
MR RAPS
Weighted median
Weighted mode
0.10
0.05
0.00
−0.05
−0.10
0.1
0.2
SNP effect on High affinity cGMP−specific 3',5'−cyclic phosphodiesterase 9A
SNP effect on Gut microbiota abundance (genus Parasutterella id.2892)

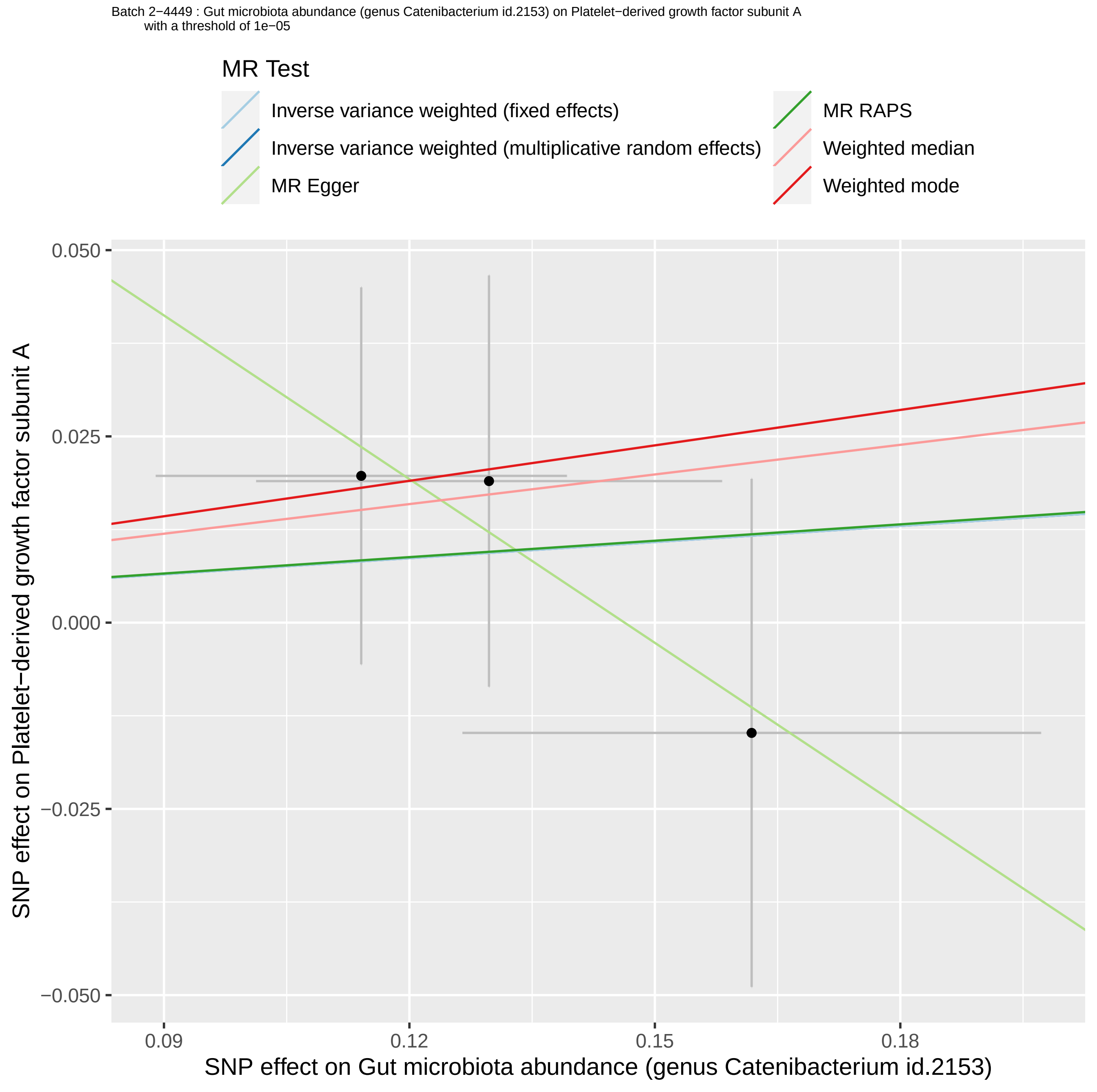

Batch 2−4449 : Gut microbiota abundance (genus Catenibacterium id.2153) on Platelet−derived growth factor subunit A
with a threshold of 1e−05
MR Test
Inverse variance weighted (fixed effects)
Inverse variance weighted (multiplicative random effects)
MR Egger
MR RAPS
Weighted median
Weighted mode
0.050
0.025
0.000
−0.025
−0.050
0.09
0.12
0.15
0.18
SNP effect on Platelet−derived growth factor subunit A
SNP effect on Gut microbiota abundance (genus Catenibacterium id.2153)

Batch 2−4450 : Gut microbiota abundance (genus Parasutterella id.2892) on Platelet−derived growth factor subunit A
with a threshold of 1e−05

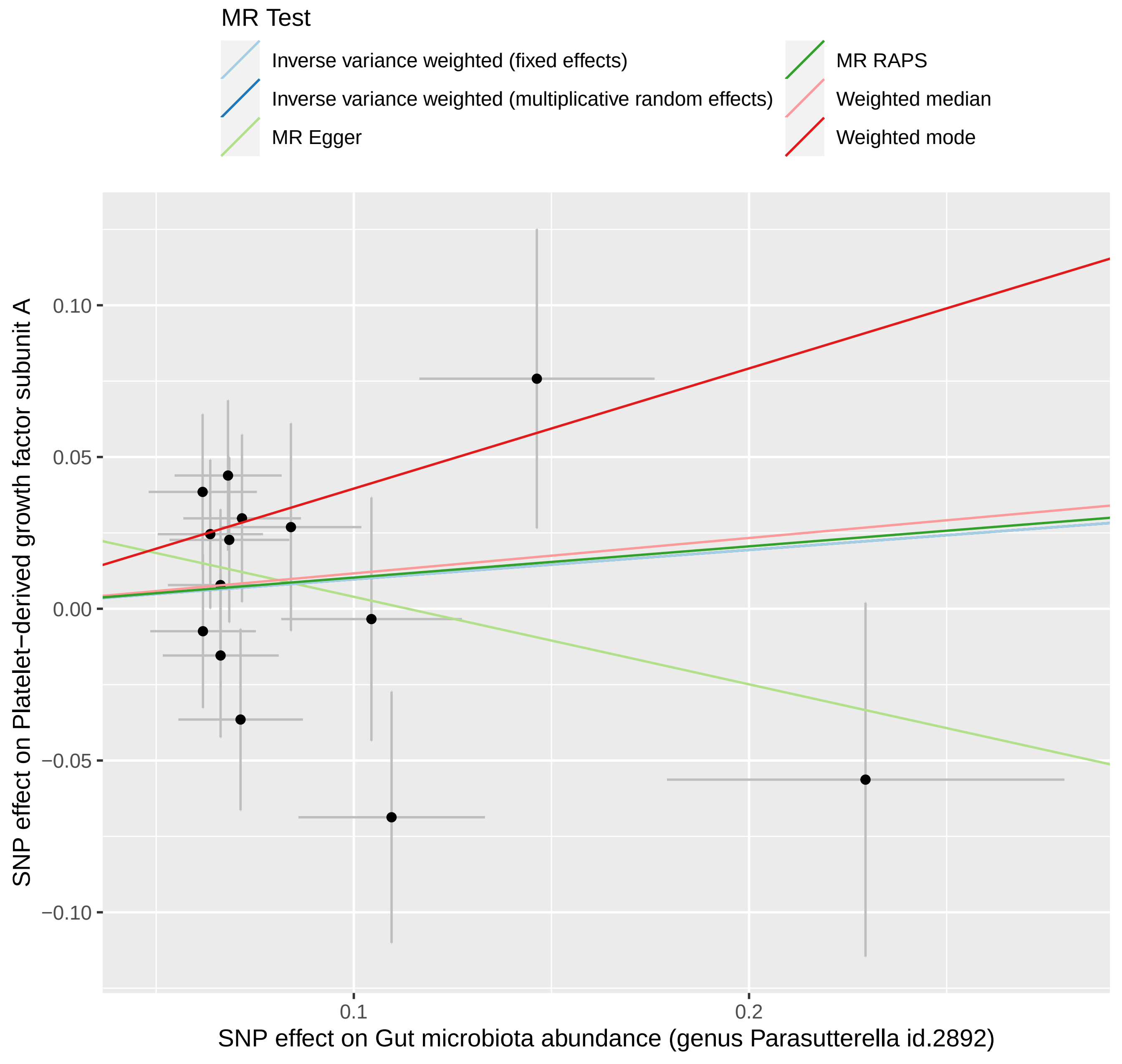

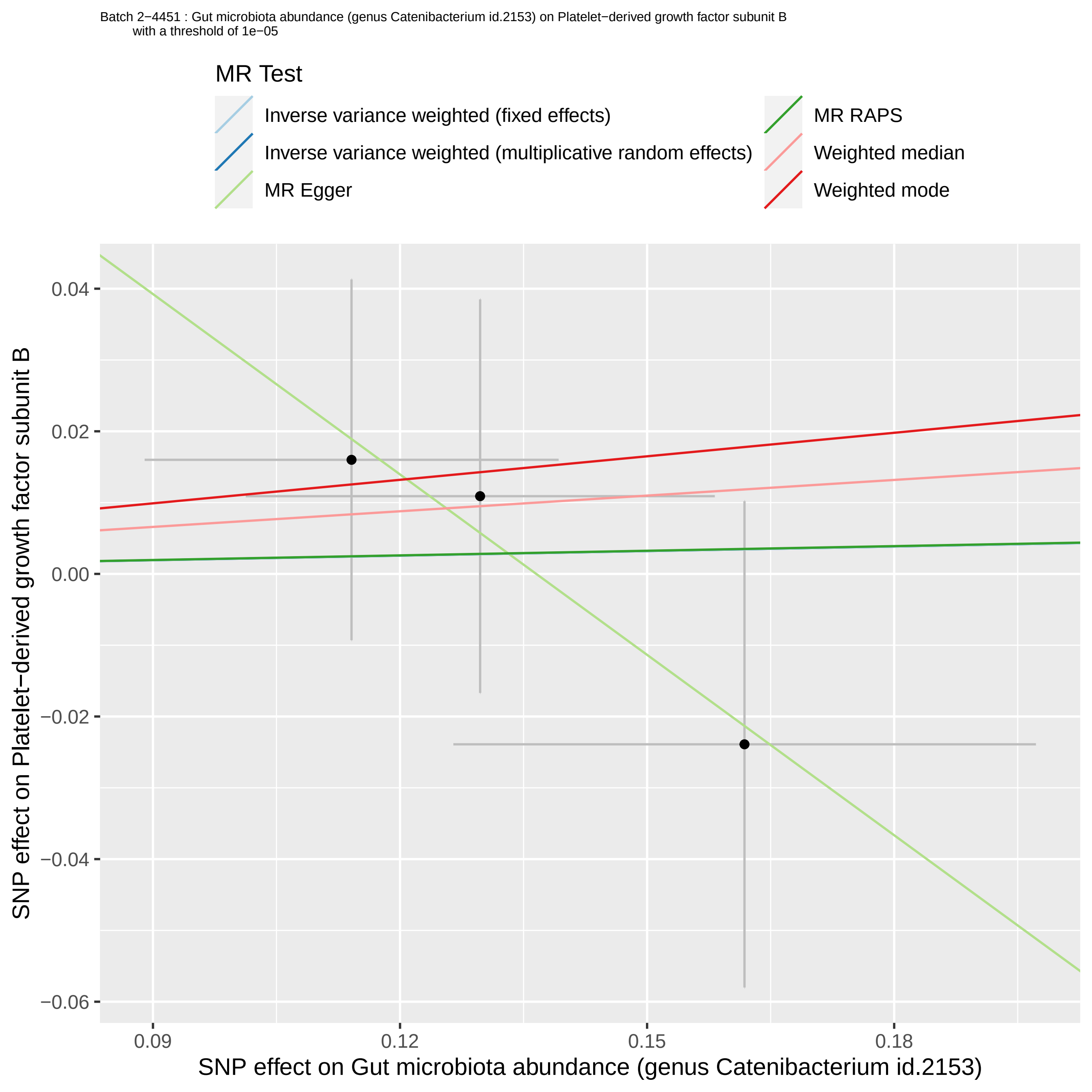
Batch 2−4451 : Gut microbiota abundance (genus Catenibacterium id.2153) on Platelet−derived growth factor subunit B
with a threshold of 1e−05
MR Test
Inverse variance weighted (fixed effects)
Inverse variance weighted (multiplicative random effects)
MR Egger
MR RAPS
Weighted median
Weighted mode
0.04
0.02
0.00
−0.02
−0.04
−0.06
0.09
0.12
0.15
0.18
SNP effect on Platelet−derived growth factor subunit B
SNP effect on Gut microbiota abundance (genus Catenibacterium id.2153)

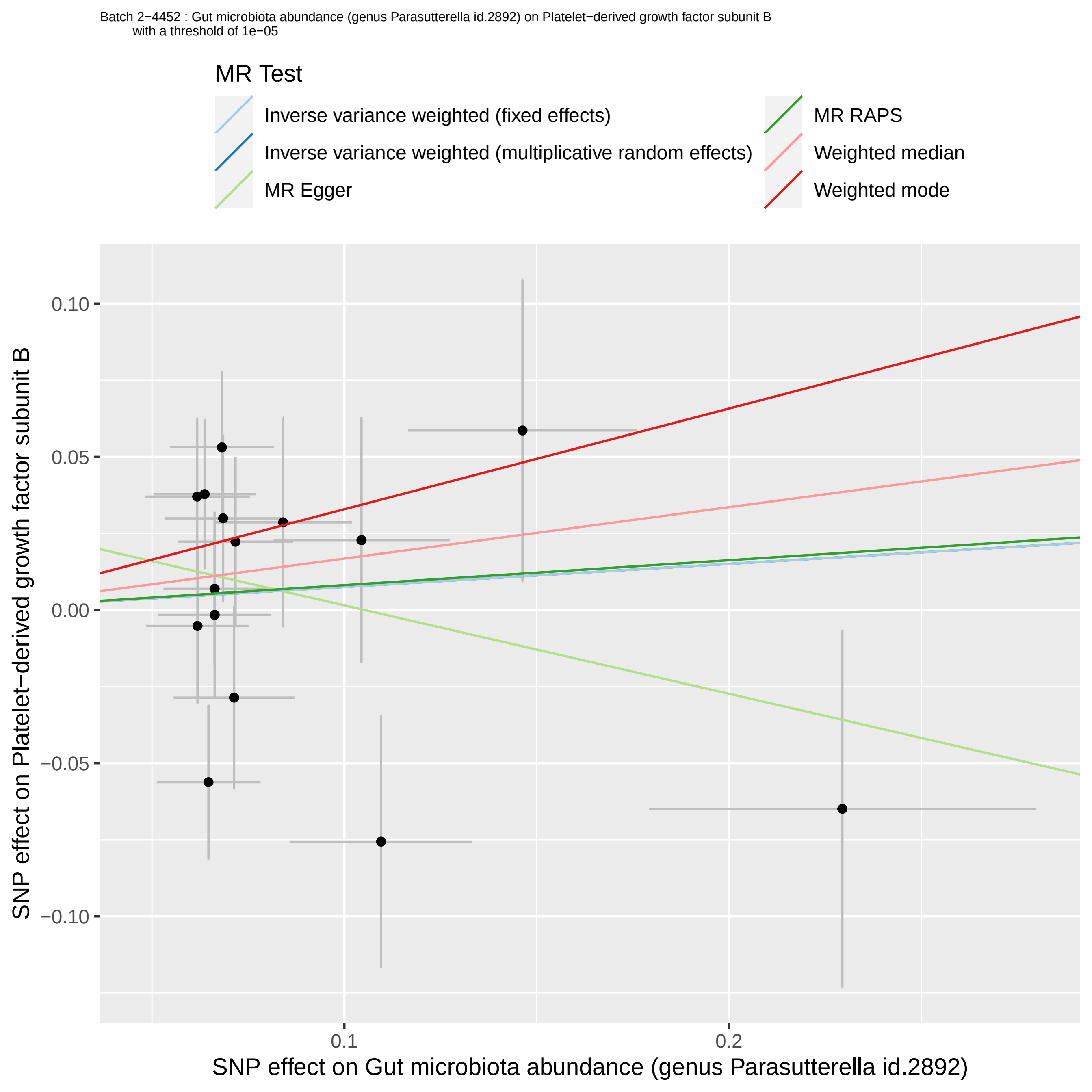

Batch 2−4452 : Gut microbiota abundance (genus Parasutterella id.2892) on Platelet−derived growth factor subunit B
with a threshold of 1e−05
MR Test
Inverse variance weighted (fixed effects)
Inverse variance weighted (multiplicative random effects)
MR Egger
MR RAPS
Weighted median
Weighted mode
0.10
0.05
0.00
−0.05
−0.10
0.1
0.2
SNP effect on Platelet−derived growth factor subunit B
SNP effect on Gut microbiota abundance (genus Parasutterella id.2892)

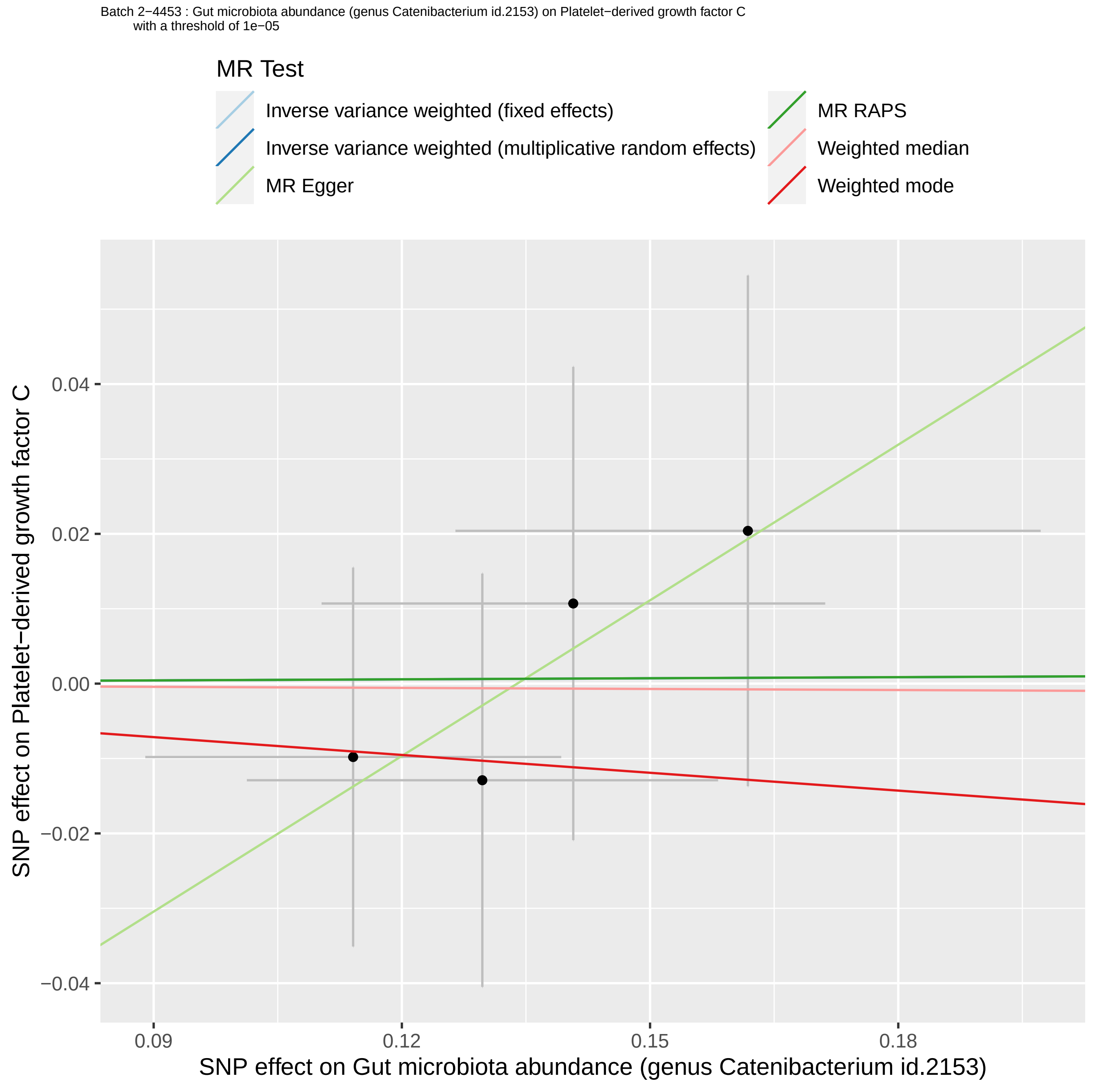
Batch 2−4453 : Gut microbiota abundance (genus Catenibacterium id.2153) on Platelet−derived growth factor C
with a threshold of 1e−05
MR Test
Inverse variance weighted (fixed effects)
Inverse variance weighted (multiplicative random effects)
MR Egger
MR RAPS
Weighted median
Weighted mode
SNP effect on Platelet−derived growth factor C
0.04
0.02
0.00
−0.02
−0.04
0.09
0.12
0.15
0.18
SNP effect on Gut microbiota abundance (genus Catenibacterium id.2153)

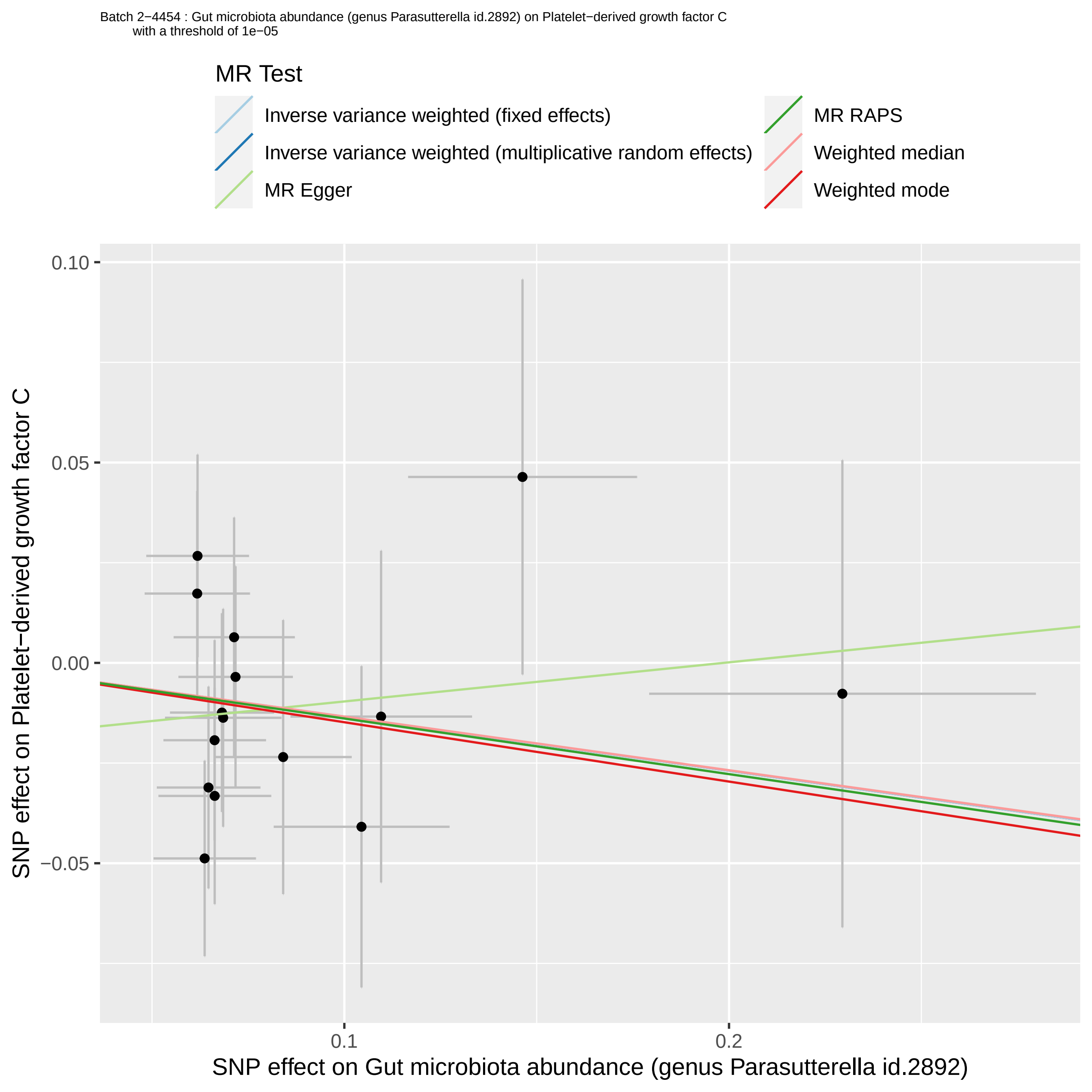

Batch 2−4454 : Gut microbiota abundance (genus Parasutterella id.2892) on Platelet−derived growth factor C
with a threshold of 1e−05
MR Test
Inverse variance weighted (fixed effects)
Inverse variance weighted (multiplicative random effects)
MR Egger
MR RAPS
Weighted median
Weighted mode
0.10
0.05
0.00
−0.05
0.1
0.2
SNP effect on Platelet−derived growth factor C
SNP effect on Gut microbiota abundance (genus Parasutterella id.2892)

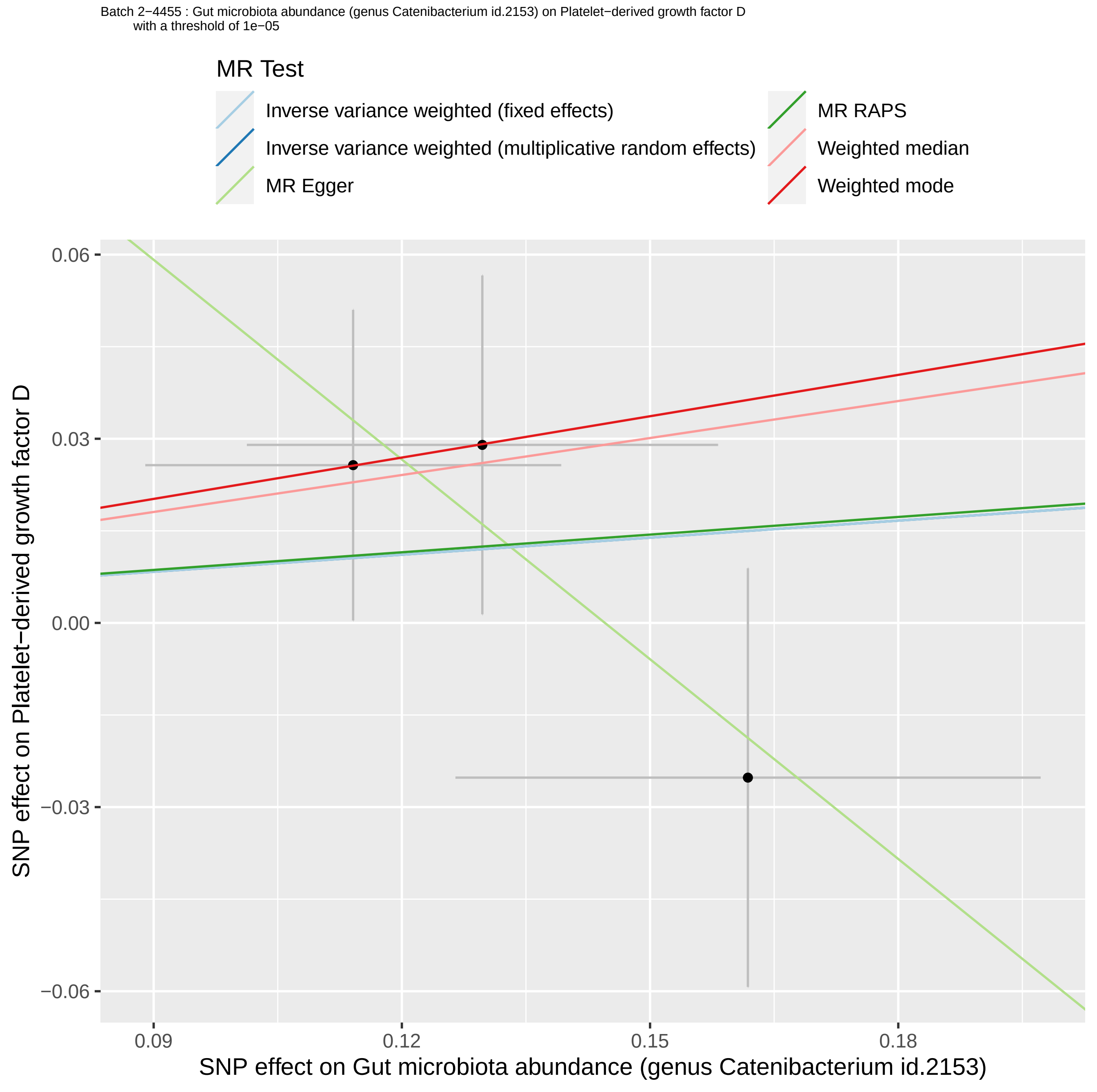

Batch 2−4455 : Gut microbiota abundance (genus Catenibacterium id.2153) on Platelet−derived growth factor D
with a threshold of 1e−05
MR Test
Inverse variance weighted (fixed effects)
Inverse variance weighted (multiplicative random effects)
MR Egger
MR RAPS
Weighted median
Weighted mode
0.06
0.03
0.00
−0.03
−0.06
0.09
0.12
0.15
0.18
SNP effect on Platelet−derived growth factor D
SNP effect on Gut microbiota abundance (genus Catenibacterium id.2153)

Batch 2−4456 : Gut microbiota abundance (genus Parasutterella id.2892) on Platelet−derived growth factor D
with a threshold of 1e−05

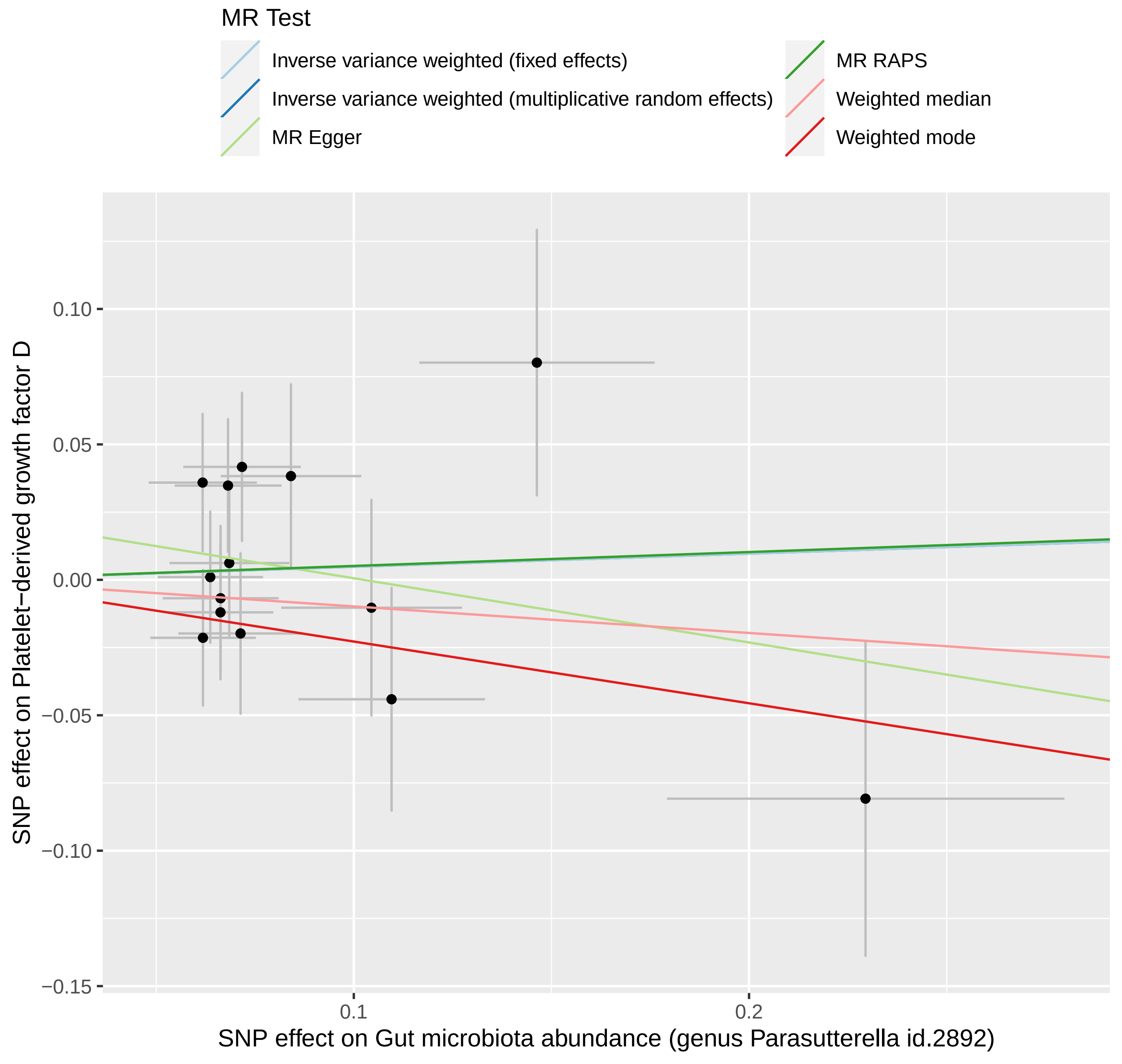

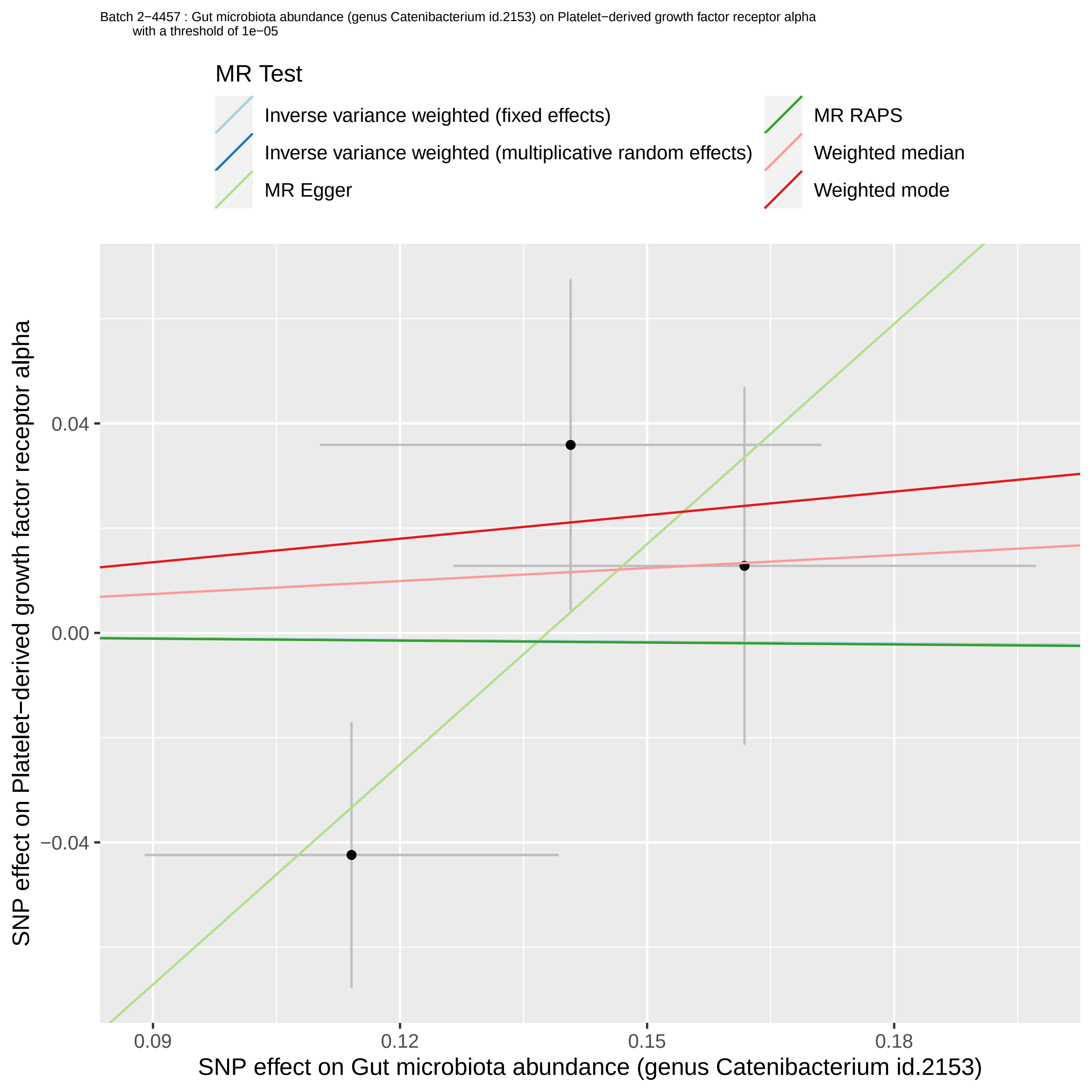
Batch 2−4457 : Gut microbiota abundance (genus Catenibacterium id.2153) on Platelet−derived growth factor receptor alpha
with a threshold of 1e−05
MR Test
Inverse variance weighted (fixed effects)
Inverse variance weighted (multiplicative random effects)
MR Egger
MR RAPS
Weighted median
Weighted mode
SNP effect on Platelet−derived growth factor receptor alpha
0.04
0.00
−0.04
0.09
0.12
0.15
0.18
SNP effect on Gut microbiota abundance (genus Catenibacterium id.2153)

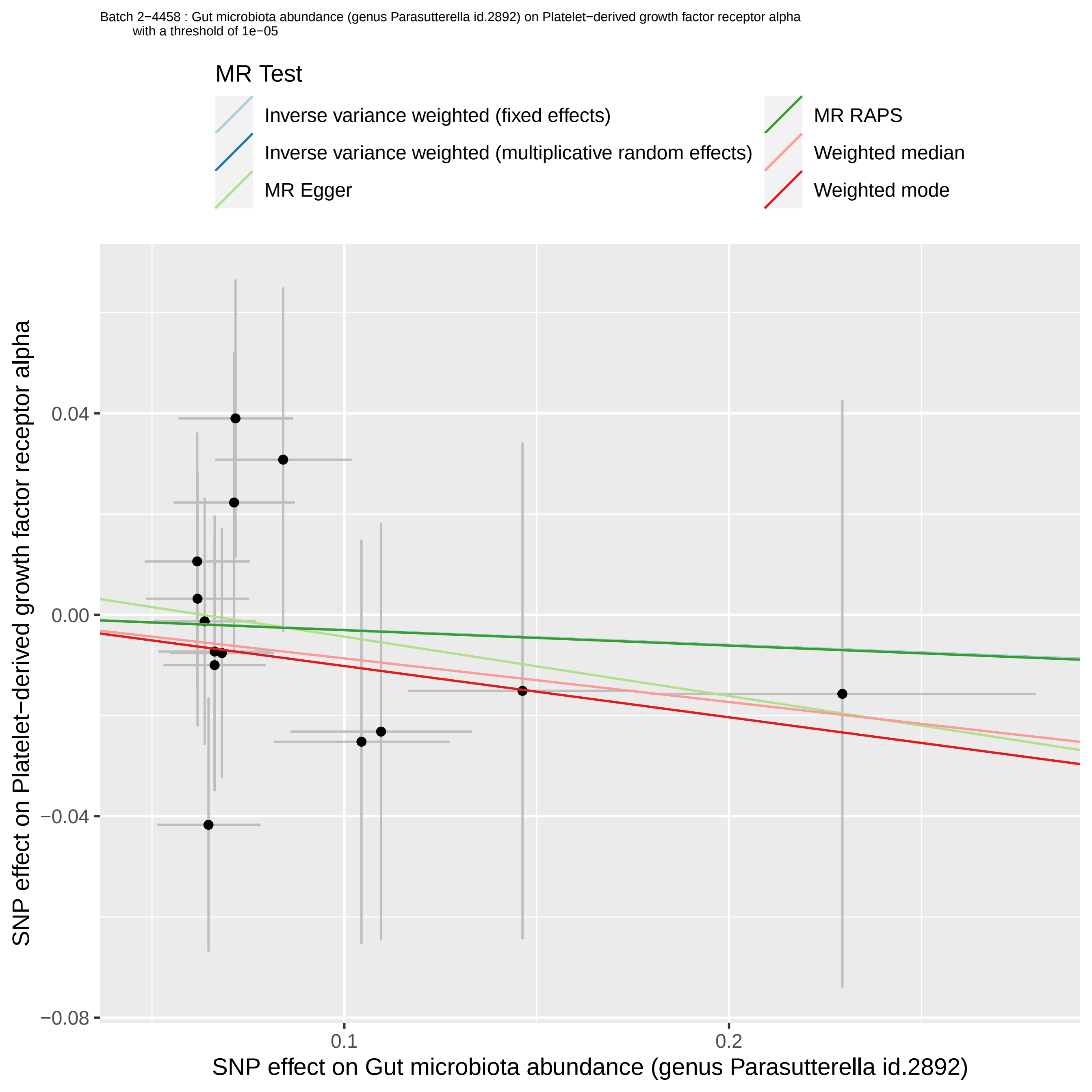

Batch 2−4458 : Gut microbiota abundance (genus Parasutterella id.2892) on Platelet−derived growth factor receptor alpha
with a threshold of 1e−05
MR Test
Inverse variance weighted (fixed effects)
Inverse variance weighted (multiplicative random effects)
MR Egger
MR RAPS
Weighted median
Weighted mode
0.04
0.00
−0.04
−0.08
0.1
0.2
SNP effect on Platelet−derived growth factor receptor alpha
SNP effect on Gut microbiota abundance (genus Parasutterella id.2892)

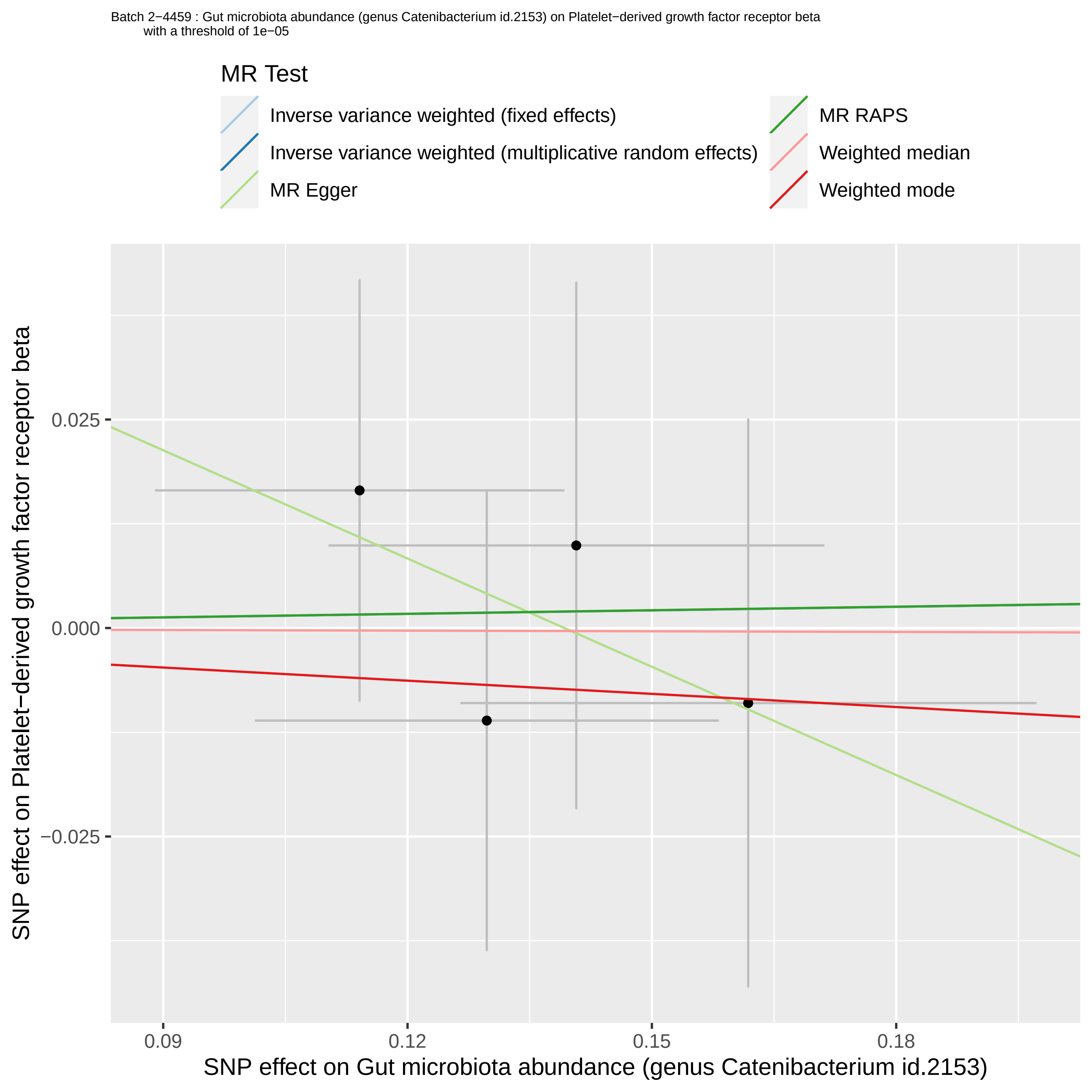
Batch 2−4459 : Gut microbiota abundance (genus Catenibacterium id.2153) on Platelet−derived growth factor receptor beta
with a threshold of 1e−05
MR Test
Inverse variance weighted (fixed effects)
Inverse variance weighted (multiplicative random effects)
MR Egger
MR RAPS
Weighted median
Weighted mode
0.025
0.000
−0.025
0.09
0.12
0.15
0.18
SNP effect on Platelet−derived growth factor receptor beta
SNP effect on Gut microbiota abundance (genus Catenibacterium id.2153)

Batch 2−4460 : Gut microbiota abundance (genus Parasutterella id.2892) on Platelet−derived growth factor receptor beta
with a threshold of 1e−05

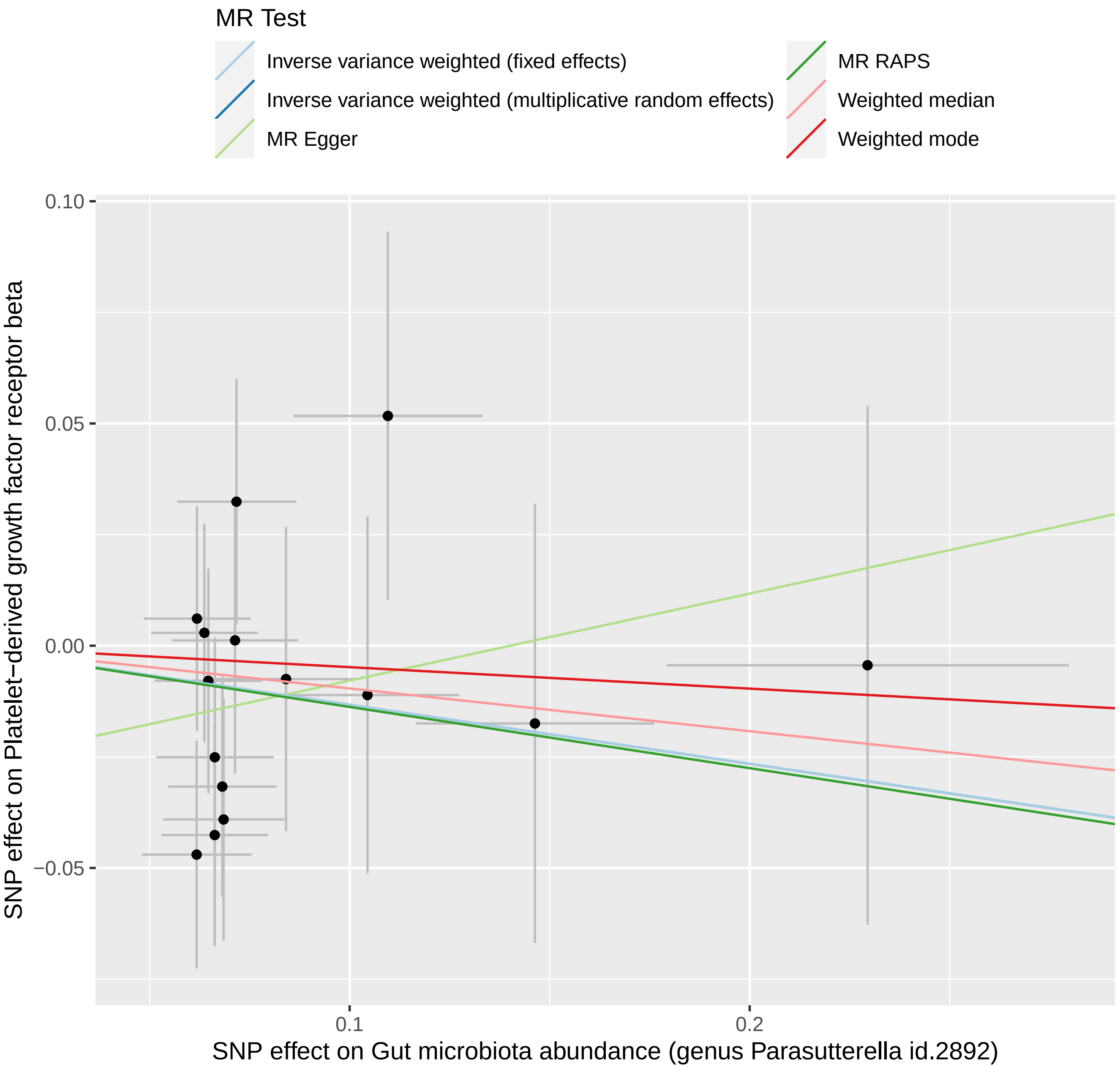

Batch 2−4461 : Gut microbiota abundance (genus Catenibacterium id.2153) on Platelet−derived growth factor receptor−like protein
with a threshold of 1e−05

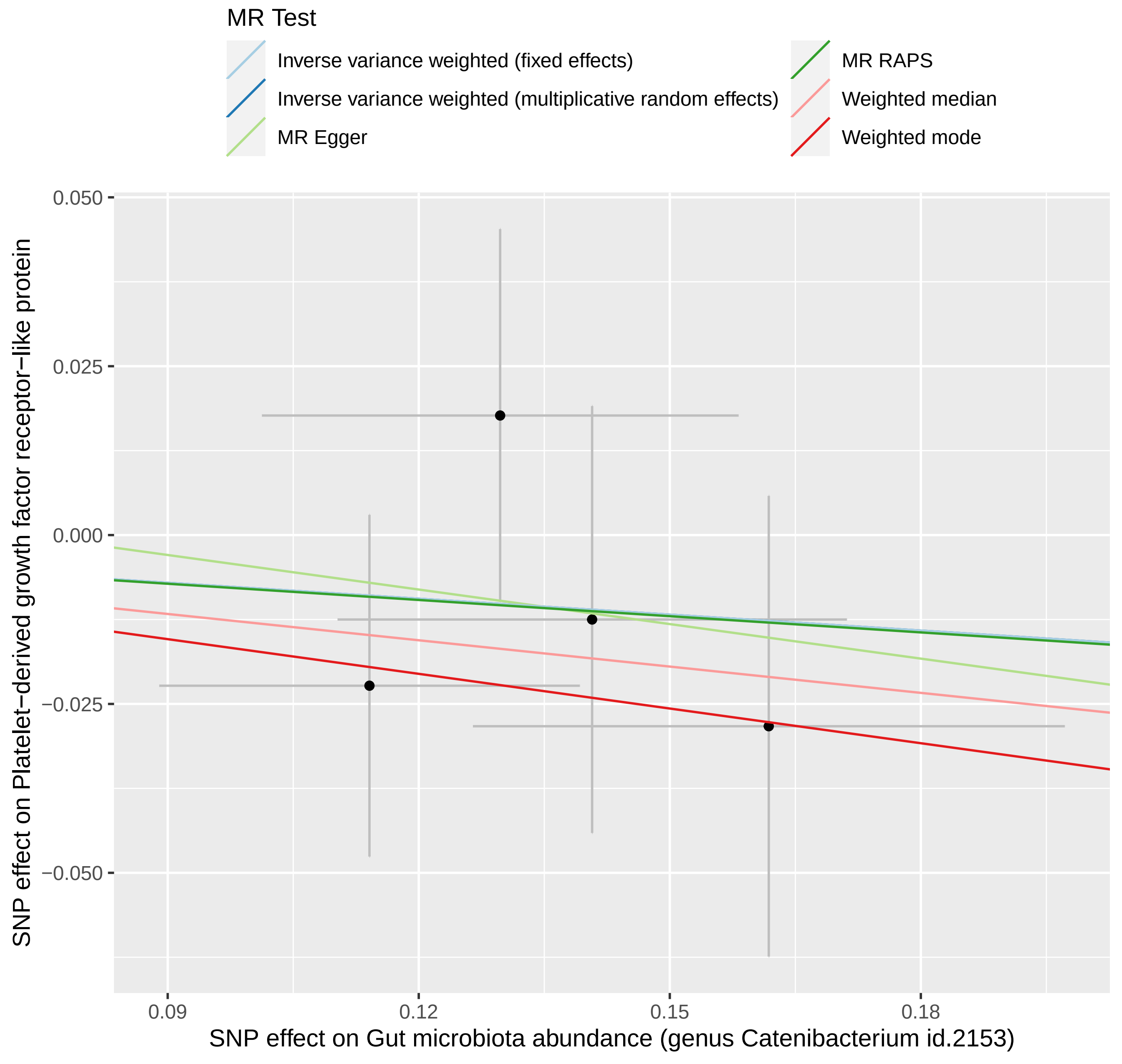

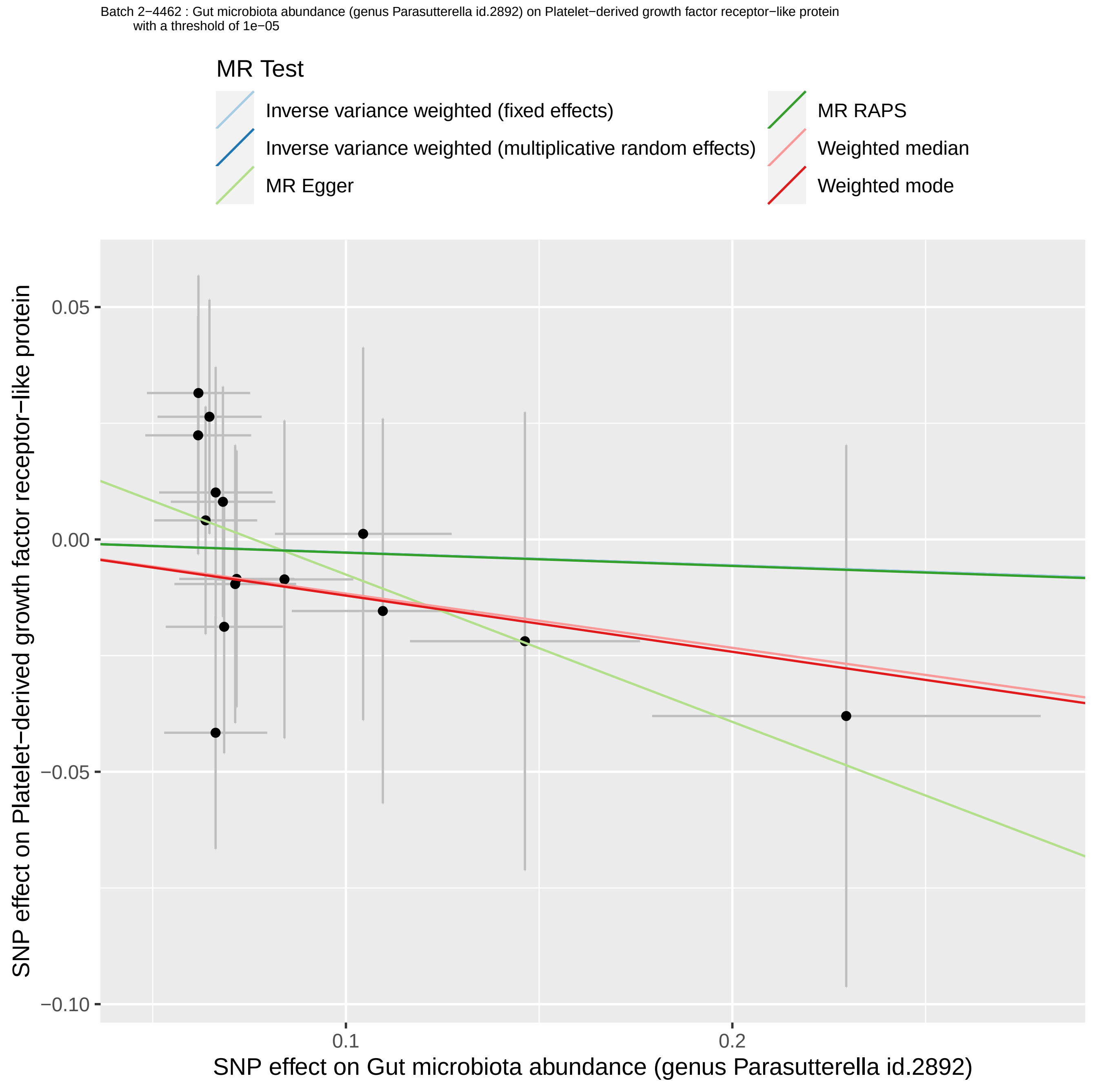

Batch 2−4462 : Gut microbiota abundance (genus Parasutterella id.2892) on Platelet−derived growth factor receptor−like protein
with a threshold of 1e−05
MR Test
Inverse variance weighted (fixed effects)
Inverse variance weighted (multiplicative random effects)
MR Egger
MR RAPS
Weighted median
Weighted mode
0.05
0.00
−0.05
−0.10
0.1
0.2
SNP effect on Platelet−derived growth factor receptor−like protein
SNP effect on Gut microbiota abundance (genus Parasutterella id.2892)

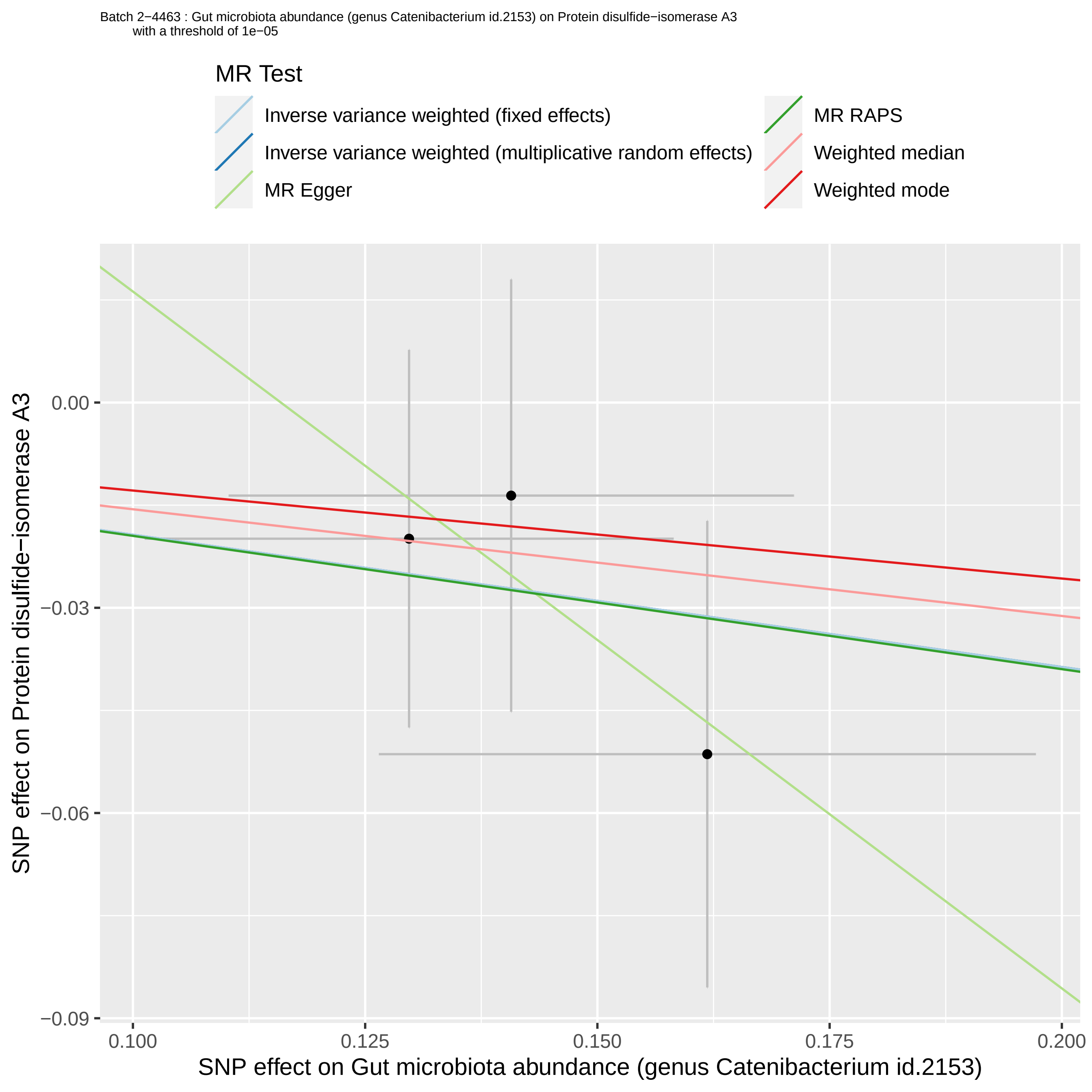
Batch 2−4463 : Gut microbiota abundance (genus Catenibacterium id.2153) on Protein disulfide−isomerase A3
with a threshold of 1e−05
MR Test
Inverse variance weighted (fixed effects)
Inverse variance weighted (multiplicative random effects)
MR Egger
MR RAPS
Weighted median
Weighted mode
0.00
−0.03
−0.06
−0.09
0.100
0.125
0.150
0.175
0.200
SNP effect on Protein disulfide−isomerase A3
SNP effect on Gut microbiota abundance (genus Catenibacterium id.2153)

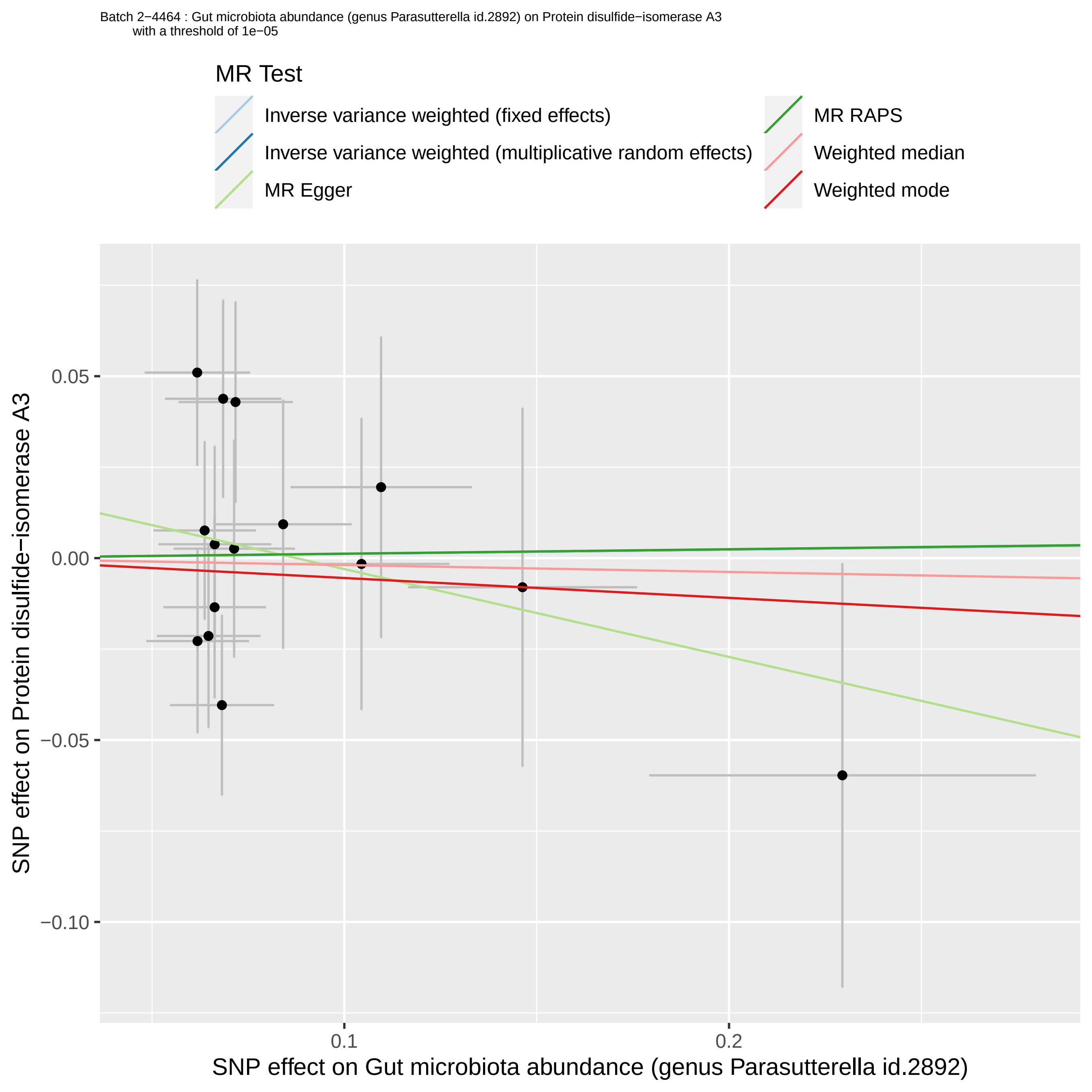
Batch 2−4464 : Gut microbiota abundance (genus Parasutterella id.2892) on Protein disulfide−isomerase A3
with a threshold of 1e−05
MR Test
Inverse variance weighted (fixed effects)
Inverse variance weighted (multiplicative random effects)
MR Egger
MR RAPS
Weighted median
Weighted mode
0.05
0.00
−0.05
−0.10
0.1
0.2
SNP effect on Protein disulfide−isomerase A3
SNP effect on Gut microbiota abundance (genus Parasutterella id.2892)

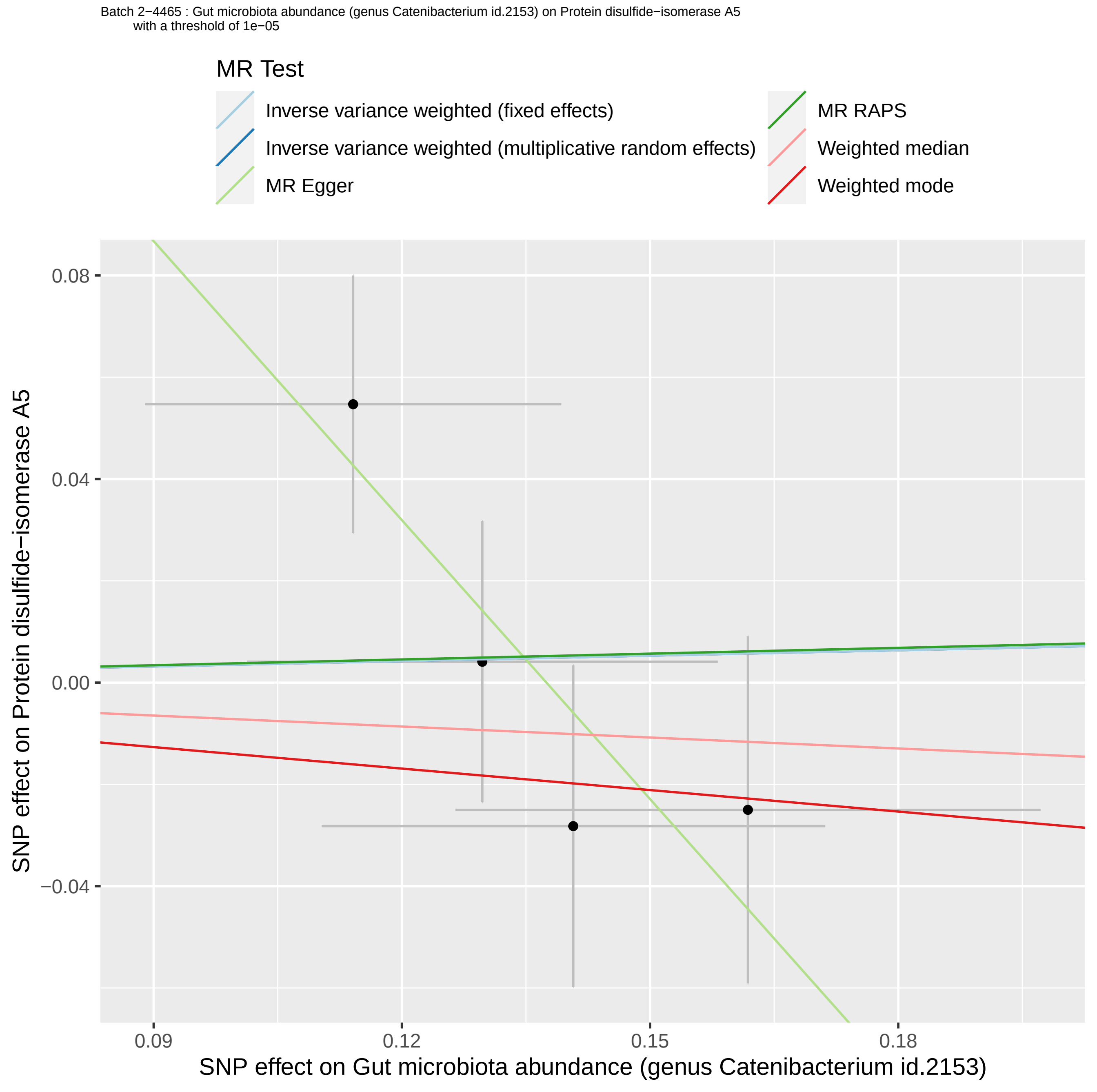
Batch 2−4465 : Gut microbiota abundance (genus Catenibacterium id.2153) on Protein disulfide−isomerase A5
with a threshold of 1e−05
MR Test
Inverse variance weighted (fixed effects)
Inverse variance weighted (multiplicative random effects)
MR Egger
MR RAPS
Weighted median
Weighted mode
0.08
0.04
0.00
−0.04
0.09
0.12
0.15
0.18
SNP effect on Protein disulfide−isomerase A5
SNP effect on Gut microbiota abundance (genus Catenibacterium id.2153)

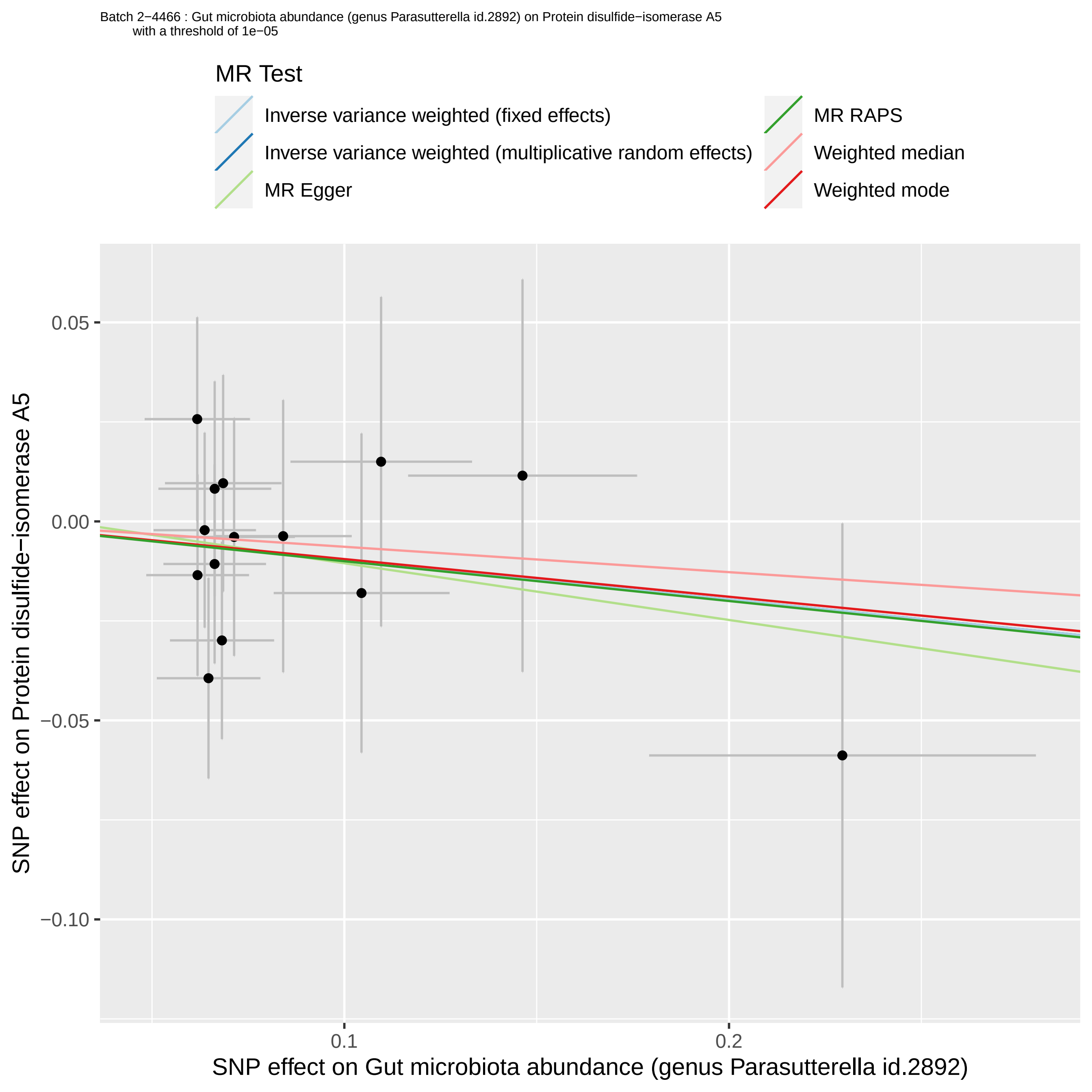

Batch 2−4466 : Gut microbiota abundance (genus Parasutterella id.2892) on Protein disulfide−isomerase A5
with a threshold of 1e−05
MR Test
Inverse variance weighted (fixed effects)
Inverse variance weighted (multiplicative random effects)
MR Egger
MR RAPS
Weighted median
Weighted mode
0.05
0.00
−0.05
−0.10
0.1
0.2
SNP effect on Protein disulfide−isomerase A5
SNP effect on Gut microbiota abundance (genus Parasutterella id.2892)

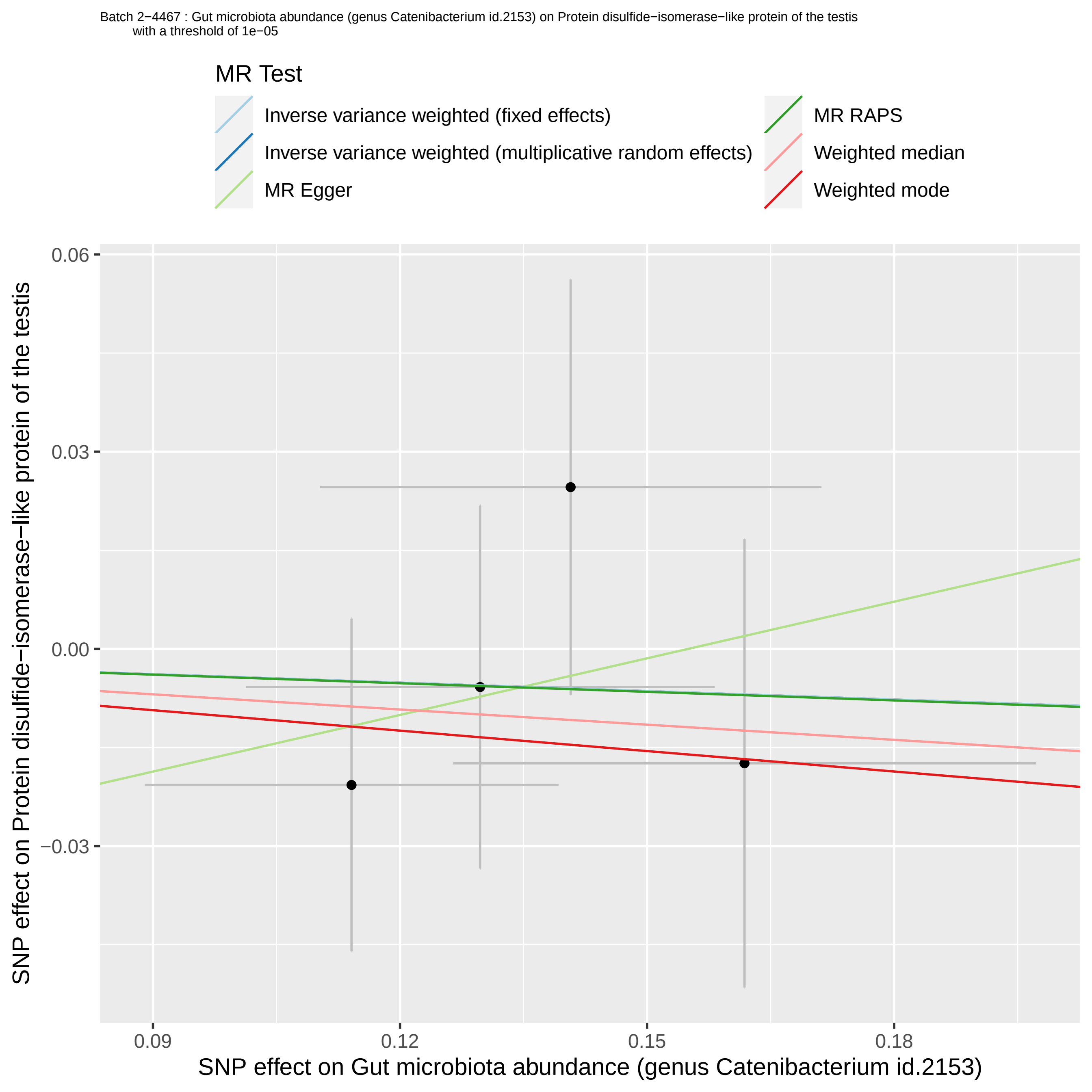
Batch 2−4467 : Gut microbiota abundance (genus Catenibacterium id.2153) on Protein disulfide−isomerase−like protein of the testis
with a threshold of 1e−05
MR Test
Inverse variance weighted (fixed effects)
Inverse variance weighted (multiplicative random effects)
MR Egger
MR RAPS
Weighted median
Weighted mode
0.06
0.03
0.00
−0.03
0.09
0.12
0.15
0.18
SNP effect on Protein disulfide−isomerase−like protein of the testis
SNP effect on Gut microbiota abundance (genus Catenibacterium id.2153)

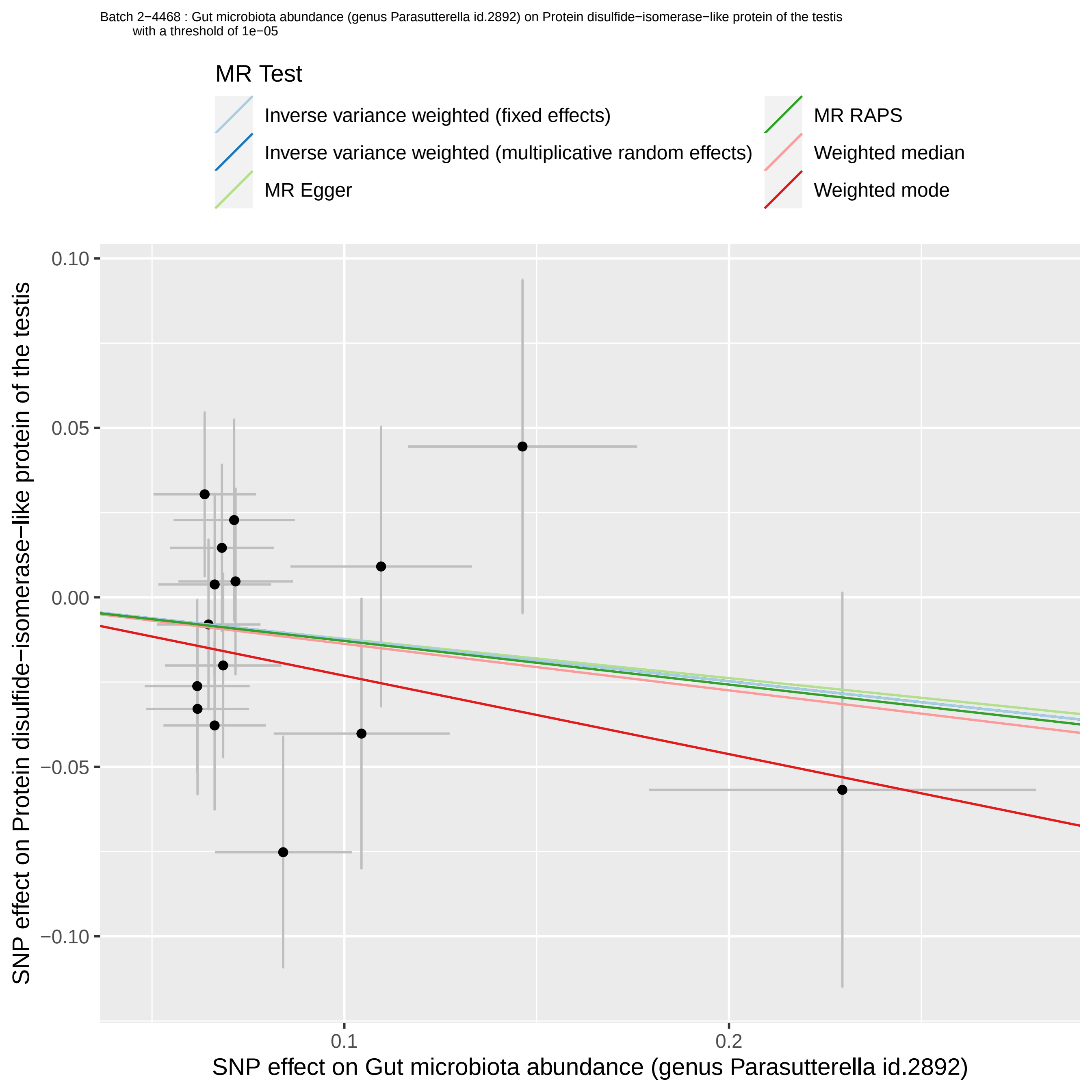

Batch 2−4468 : Gut microbiota abundance (genus Parasutterella id.2892) on Protein disulfide−isomerase−like protein of the testis
with a threshold of 1e−05
MR Test
Inverse variance weighted (fixed effects)
Inverse variance weighted (multiplicative random effects)
MR Egger
MR RAPS
Weighted median
Weighted mode
0.10
0.05
0.00
−0.05
−0.10
0.1
0.2
SNP effect on Protein disulfide−isomerase−like protein of the testis
SNP effect on Gut microbiota abundance (genus Parasutterella id.2892)

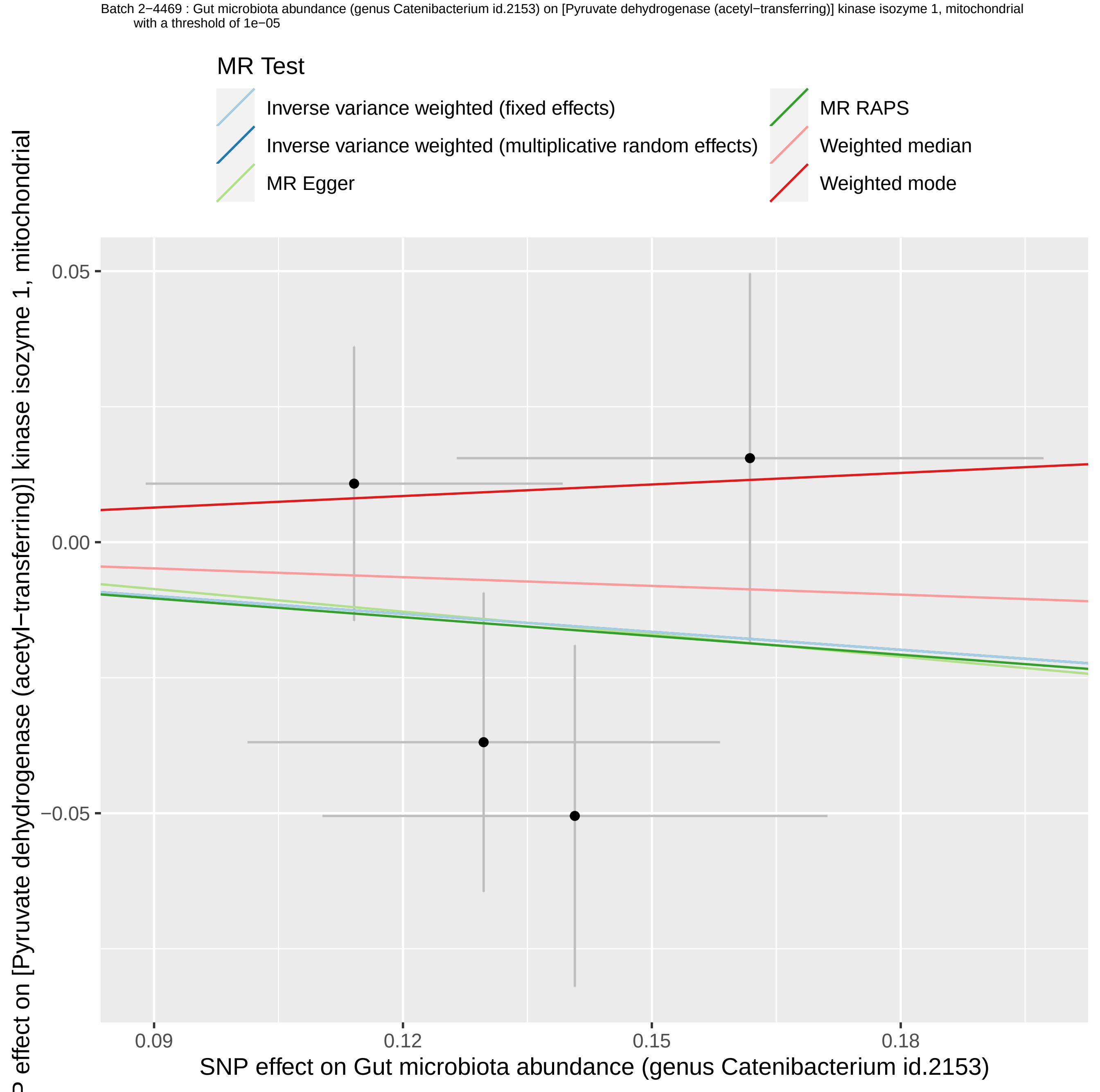

Batch 2−4469 : Gut microbiota abundance (genus Catenibacterium id.2153) on [Pyruvate dehydrogenase (acetyl−transferring)] kinase isozyme 1, mitochondrial
with a threshold of 1e−05
MR Test
Inverse variance weighted (fixed effects)
Inverse variance weighted (multiplicative random effects)
MR Egger
MR RAPS
Weighted median
Weighted mode
0.05
0.00
−0.05
0.09
0.12
0.15
0.18
SNP effect on Gut microbiota abundance (genus Catenibacterium id.2153)
P effect on [Pyruvate dehydrogenase (acetyl−transferring)] kinase isozyme 1, mitochondrial

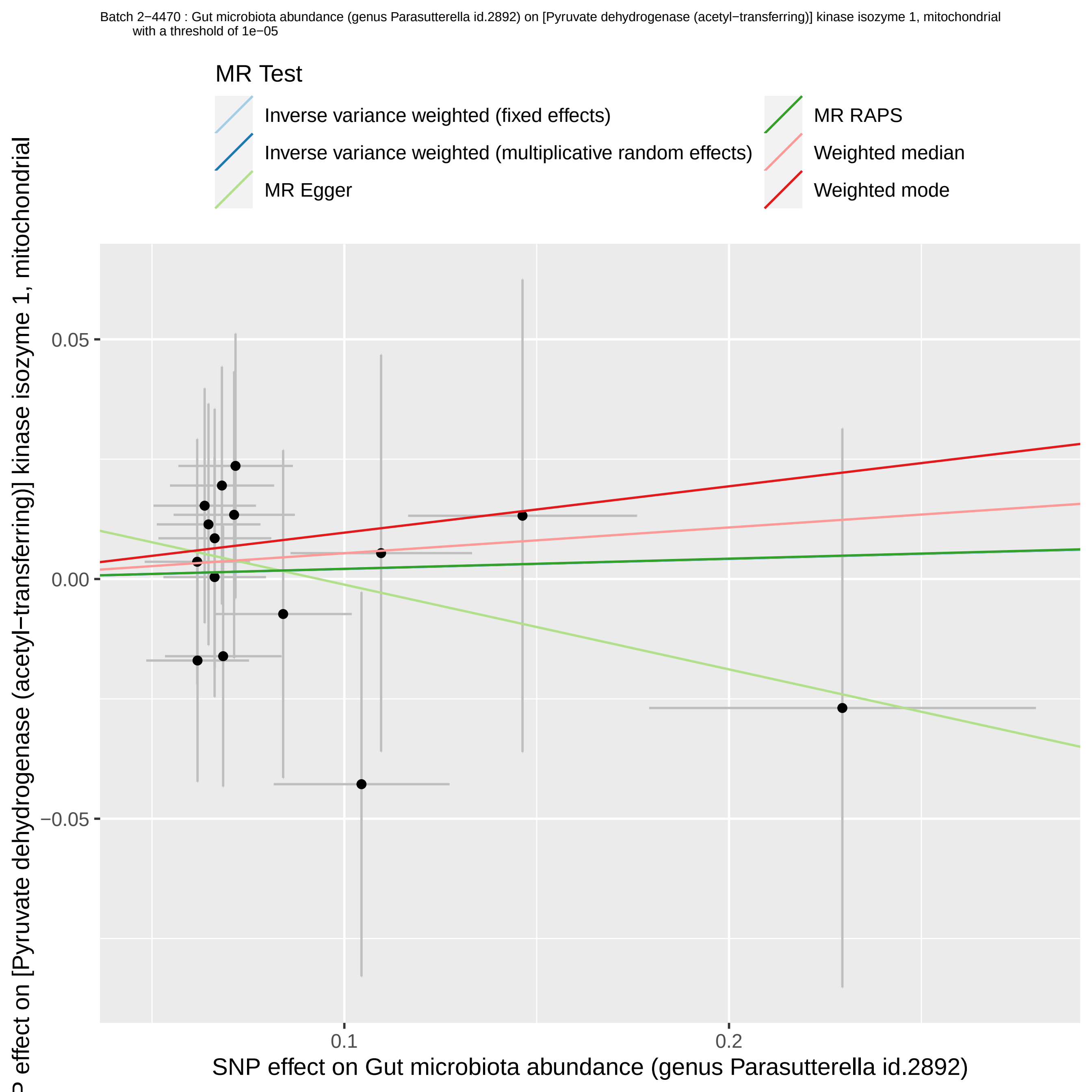
Batch 2−4470 : Gut microbiota abundance (genus Parasutterella id.2892) on [Pyruvate dehydrogenase (acetyl−transferring)] kinase isozyme 1, mitochondrial with a threshold of 1e−05
MR Test
Inverse variance weighted (fixed effects)
Inverse variance weighted (multiplicative random effects)
MR Egger
MR RAPS
Weighted median
Weighted mode
0.05
0.00
−0.05
0.1
0.2
SNP effect on Gut microbiota abundance (genus Parasutterella id.2892)
SNP effect on [Pyruvate dehydrogenase (acetyl−transferring)] kinase isozyme 1, mitochondrial

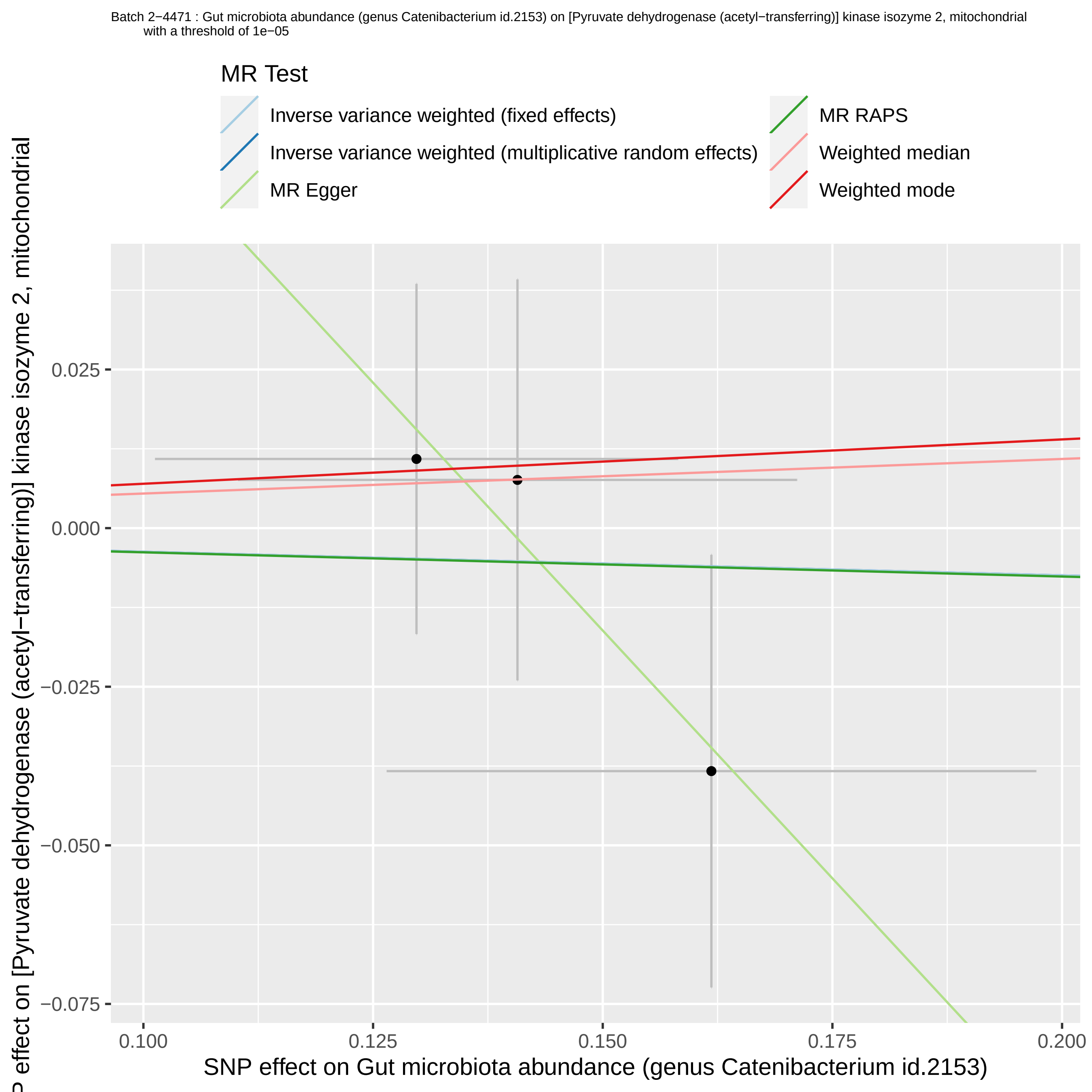

Batch 2−4471 : Gut microbiota abundance (genus Catenibacterium id.2153) on [Pyruvate dehydrogenase (acetyl−transferring)] kinase isozyme 2, mitochondrial with a threshold of 1e−05
MR Test
Inverse variance weighted (fixed effects)
Inverse variance weighted (multiplicative random effects)
MR Egger
MR RAPS
Weighted median
Weighted mode
0.025
0.000
−0.025
−0.050
−0.075
0.100
0.125
0.150
0.175
0.200
SNP effect on Gut microbiota abundance (genus Catenibacterium id.2153)
P effect on [Pyruvate dehydrogenase (acetyl−transferring)] kinase isozyme 2, mitochondrial

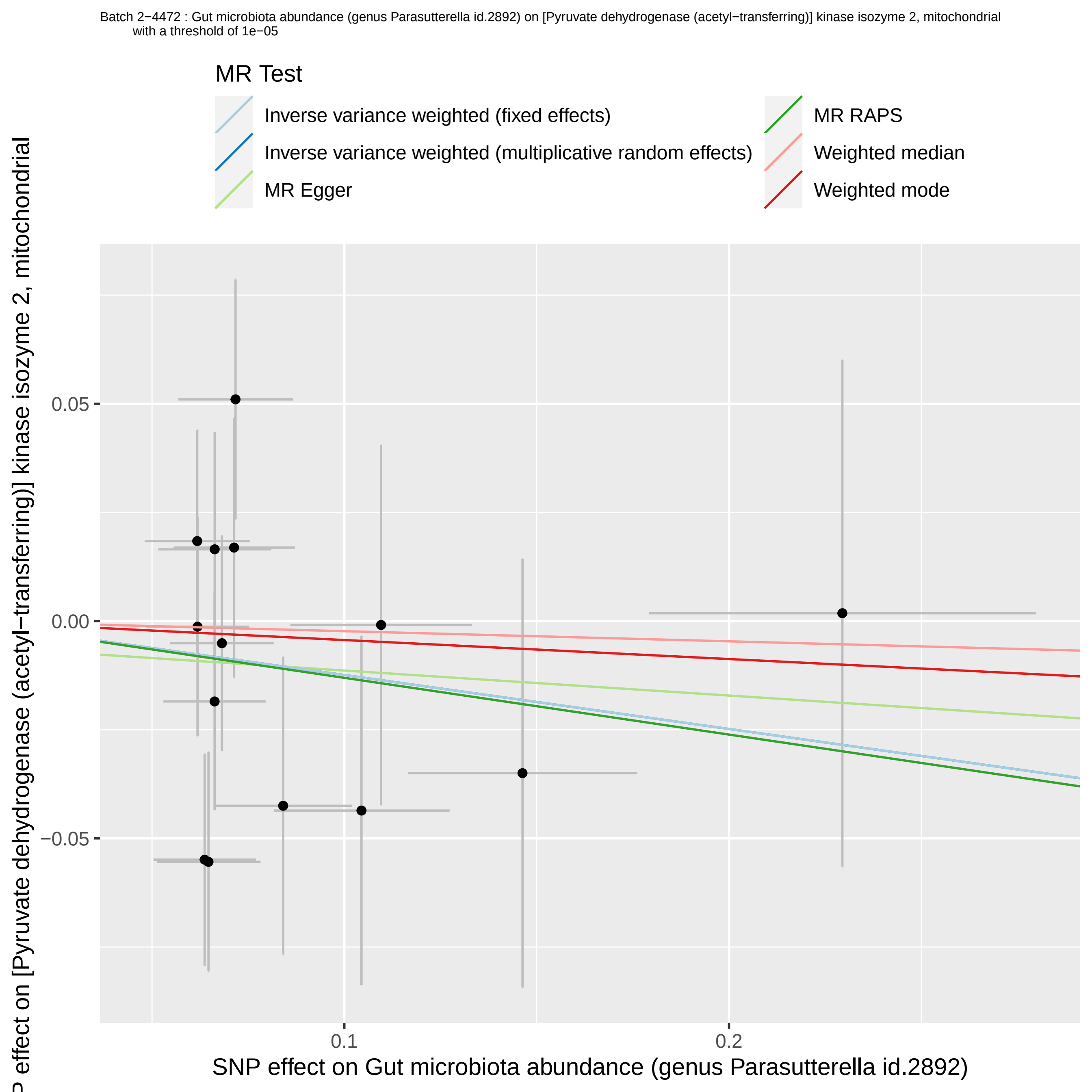
Batch 2−4472 : Gut microbiota abundance (genus Parasutterella id.2892) on [Pyruvate dehydrogenase (acetyl−transferring)] kinase isozyme 2, mitochondrial
with a threshold of 1e−05
MR Test
Inverse variance weighted (fixed effects)
Inverse variance weighted (multiplicative random effects)
MR Egger
MR RAPS
Weighted median
Weighted mode
0.05
0.00
−0.05
0.1
0.2
SNP effect on Gut microbiota abundance (genus Parasutterella id.2892)
P effect on [Pyruvate dehydrogenase (acetyl−transferring)] kinase isozyme 2, mitochondrial

Batch 2−4473 : Gut microbiota abundance (genus Catenibacterium id.2153) on PDZ and LIM domain protein 4
with a threshold of 1e−05

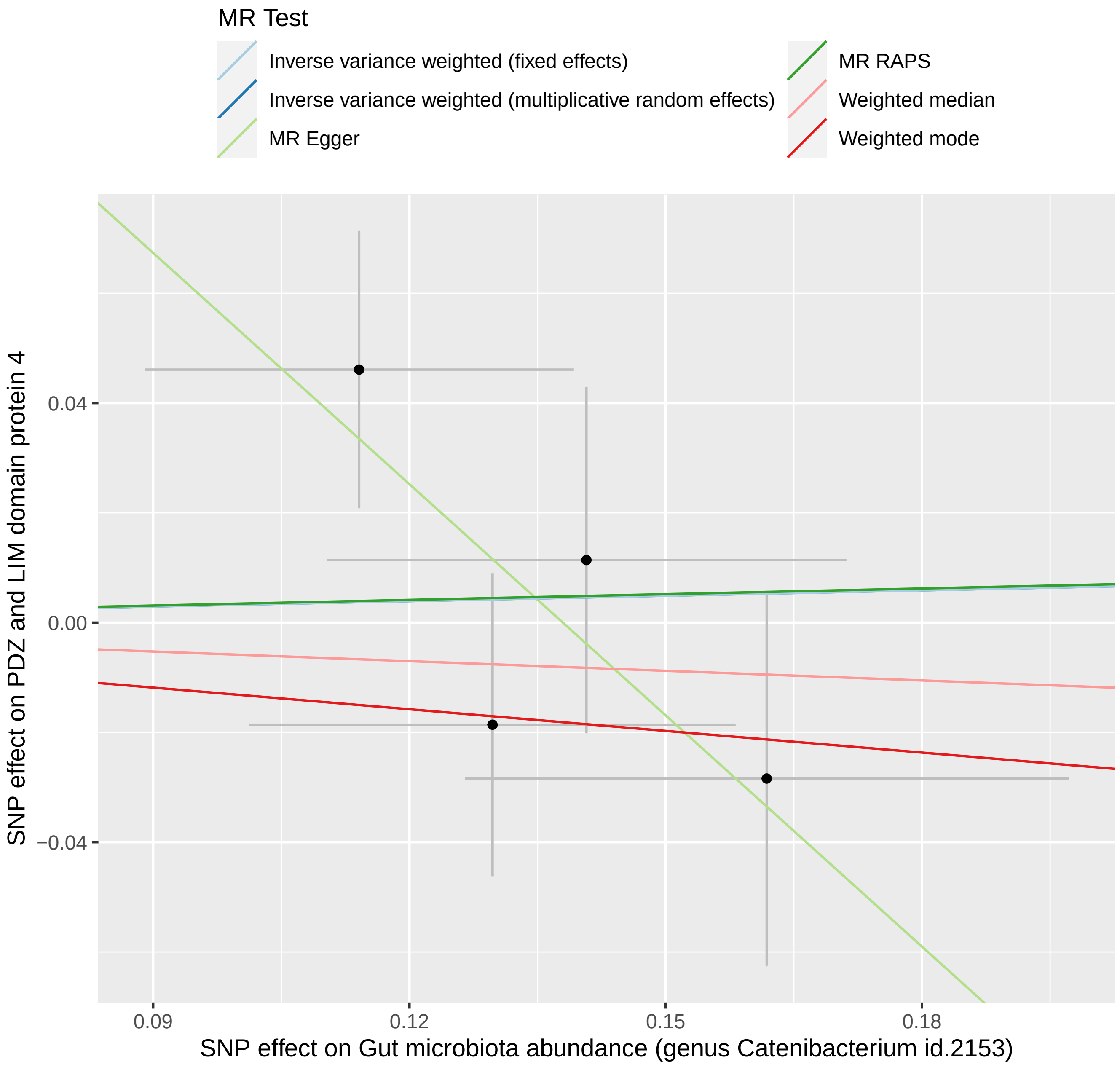

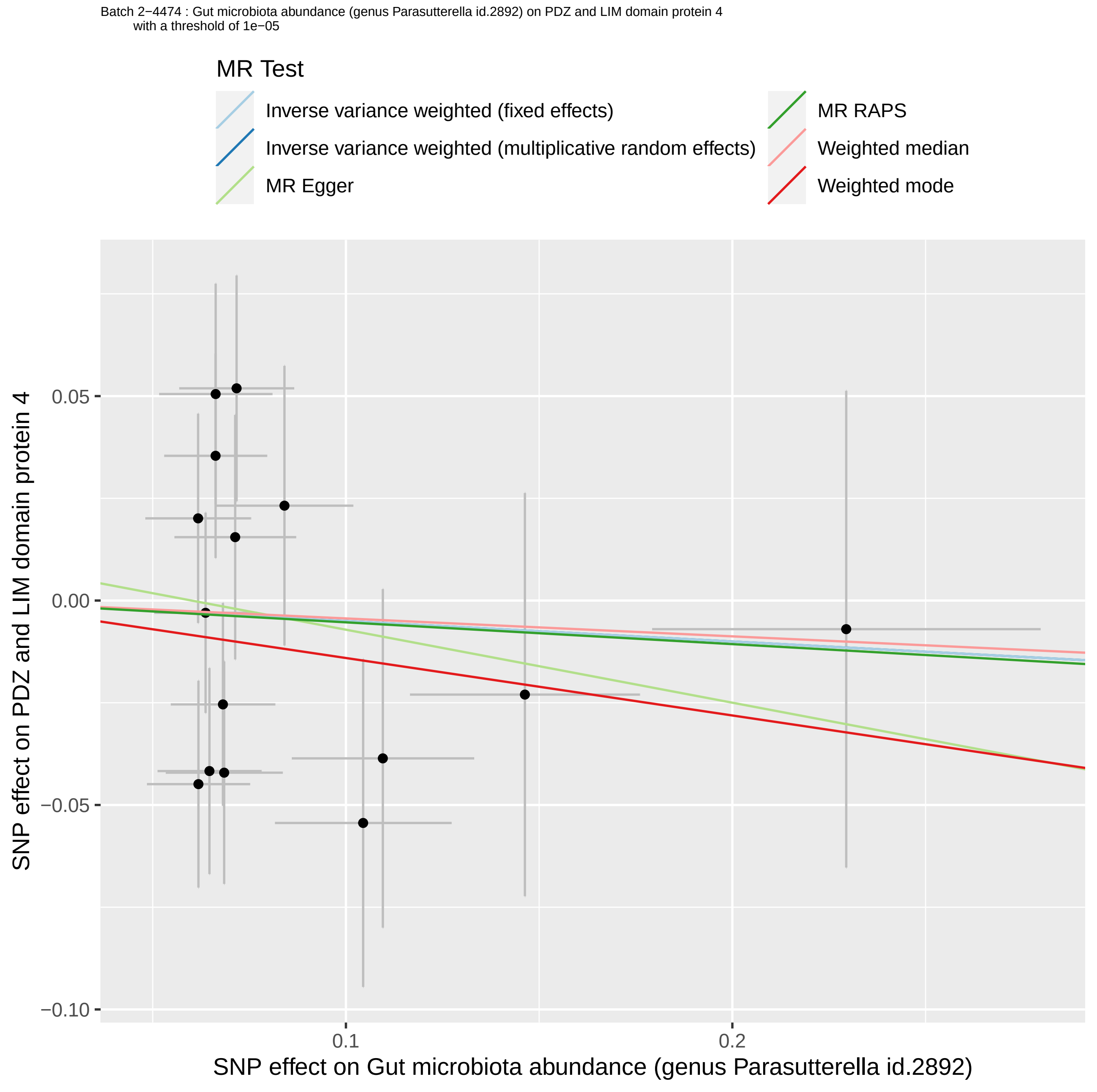
Batch 2−4474 : Gut microbiota abundance (genus Parasutterella id.2892) on PDZ and LIM domain protein 4
with a threshold of 1e−05
MR Test
Inverse variance weighted (fixed effects)
Inverse variance weighted (multiplicative random effects)
MR Egger
MR RAPS
Weighted median
Weighted mode
0.05
0.00
−0.05
−0.10
0.1
0.2
SNP effect on PDZ and LIM domain protein 4
SNP effect on Gut microbiota abundance (genus Parasutterella id.2892)

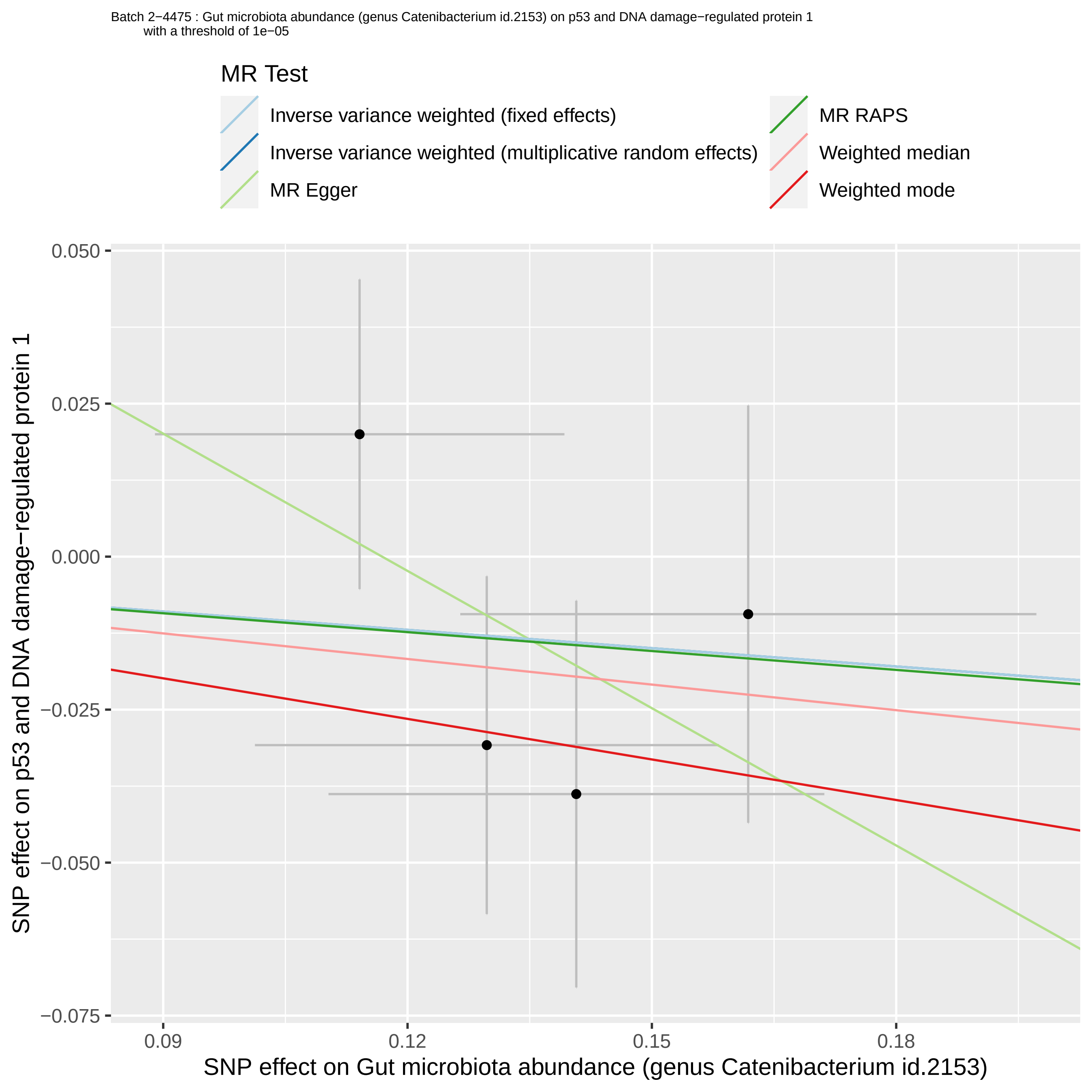
Batch 2−4475 : Gut microbiota abundance (genus Catenibacterium id.2153) on p53 and DNA damage−regulated protein 1 with a threshold of 1e−05
MR Test
Inverse variance weighted (fixed effects)
Inverse variance weighted (multiplicative random effects)
MR Egger
MR RAPS
Weighted median
Weighted mode
0.050
0.025
0.000
−0.025
−0.050
−0.075
0.09
0.12
0.15
0.18
SNP effect on p53 and DNA damage−regulated protein 1
SNP effect on Gut microbiota abundance (genus Catenibacterium id.2153)

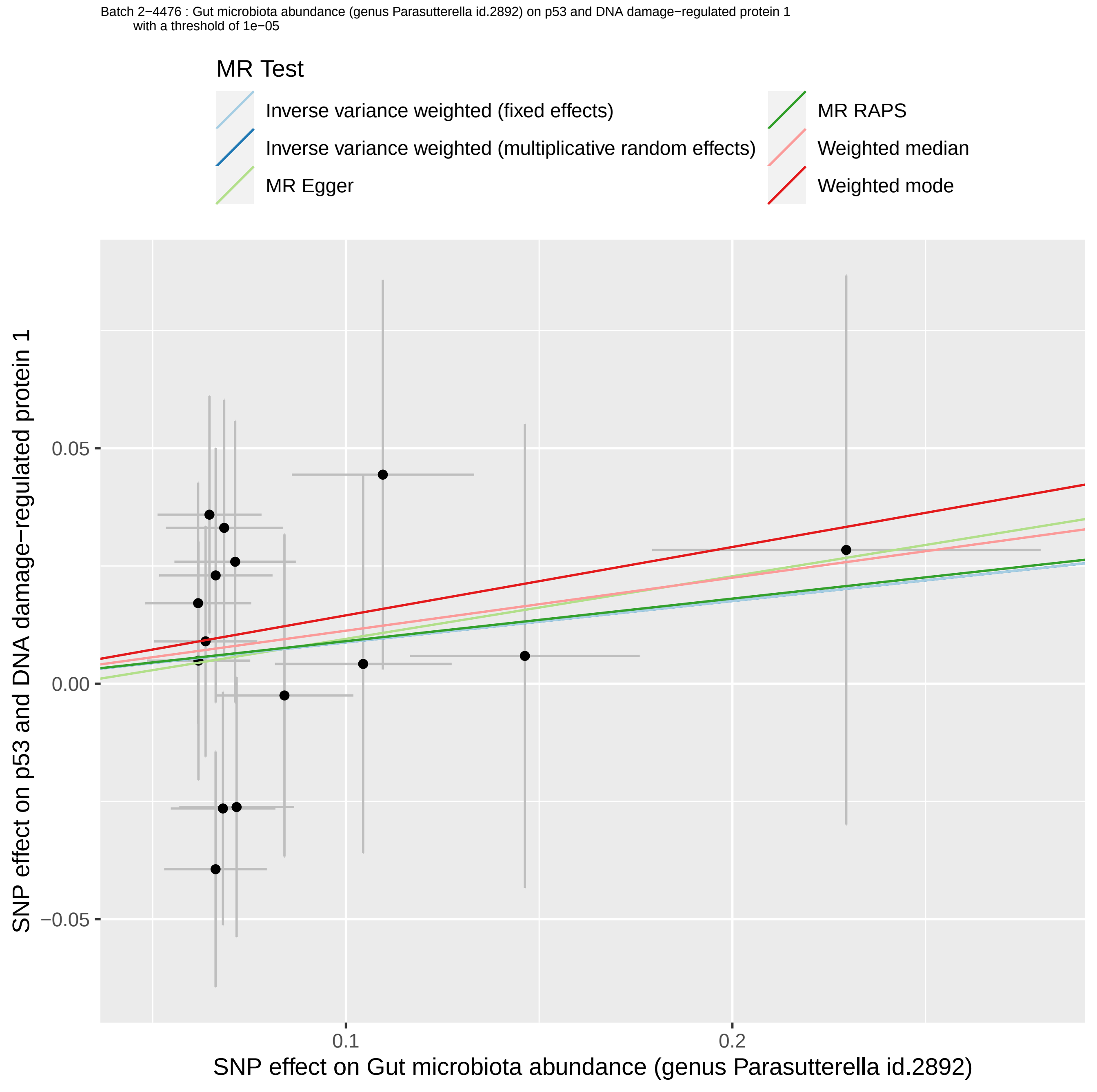
Batch 2−4476 : Gut microbiota abundance (genus Parasutterella id.2892) on p53 and DNA damage−regulated protein 1
with a threshold of 1e−05
MR Test
Inverse variance weighted (fixed effects)
Inverse variance weighted (multiplicative random effects)
MR Egger
MR RAPS
Weighted median
Weighted mode
0.05
0.00
−0.05
0.1
0.2
SNP effect on p53 and DNA damage−regulated protein 1
SNP effect on Gut microbiota abundance (genus Parasutterella id.2892)

Batch 2−4477 : Gut microbiota abundance (genus Catenibacterium id.2153) on Pyridoxal kinase
with a threshold of 1e−05

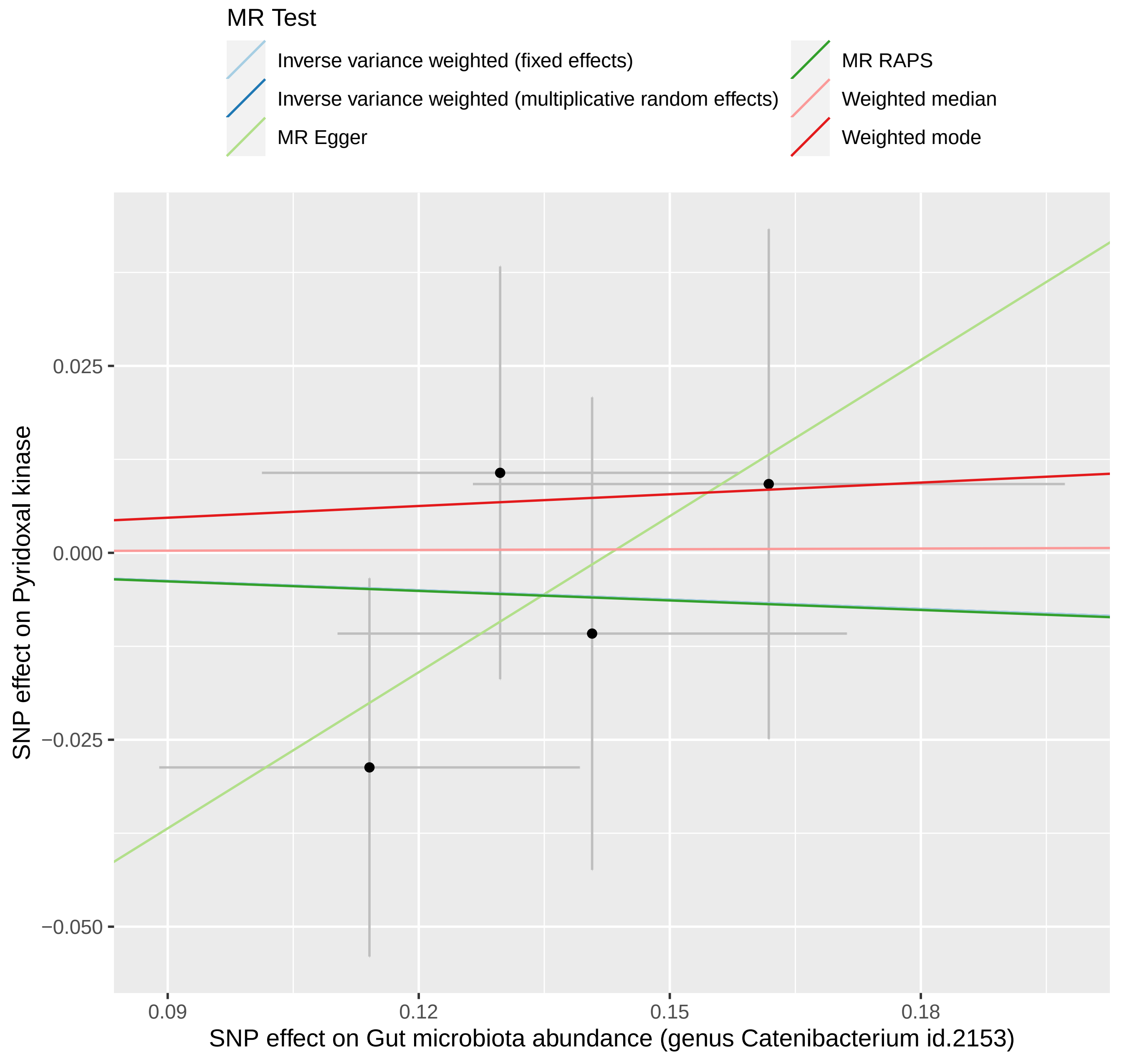

Batch 2−4478 : Gut microbiota abundance (genus Parasutterella id.2892) on Pyridoxal kinase
with a threshold of 1e−05

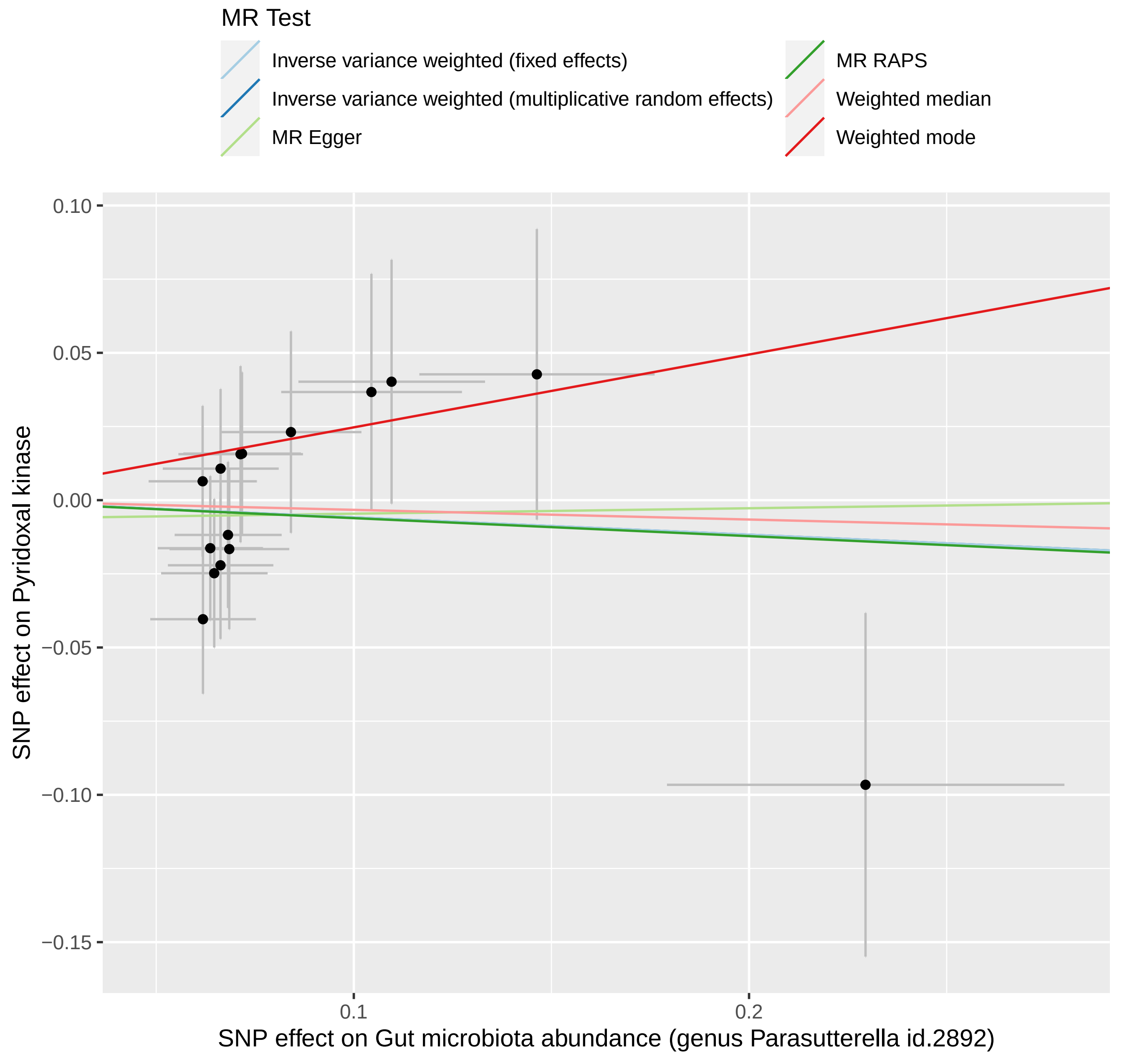

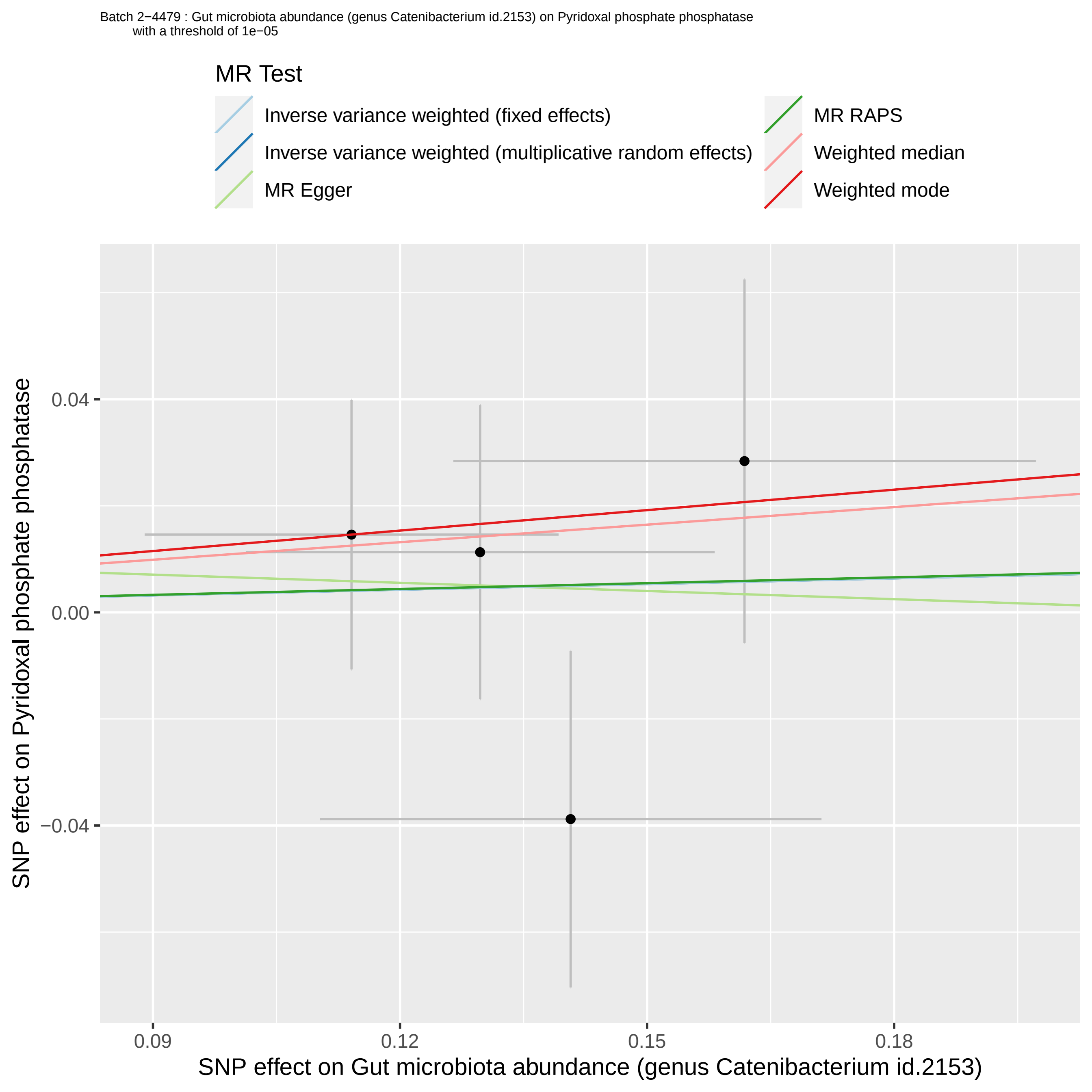

Batch 2−4479 : Gut microbiota abundance (genus Catenibacterium id.2153) on Pyridoxal phosphate phosphatase
with a threshold of 1e−05
MR Test
Inverse variance weighted (fixed effects)
Inverse variance weighted (multiplicative random effects)
MR Egger
MR RAPS
Weighted median
Weighted mode
0.04
0.00
−0.04
0.09
0.12
0.15
0.18
SNP effect on Pyridoxal phosphate phosphatase
SNP effect on Gut microbiota abundance (genus Catenibacterium id.2153)

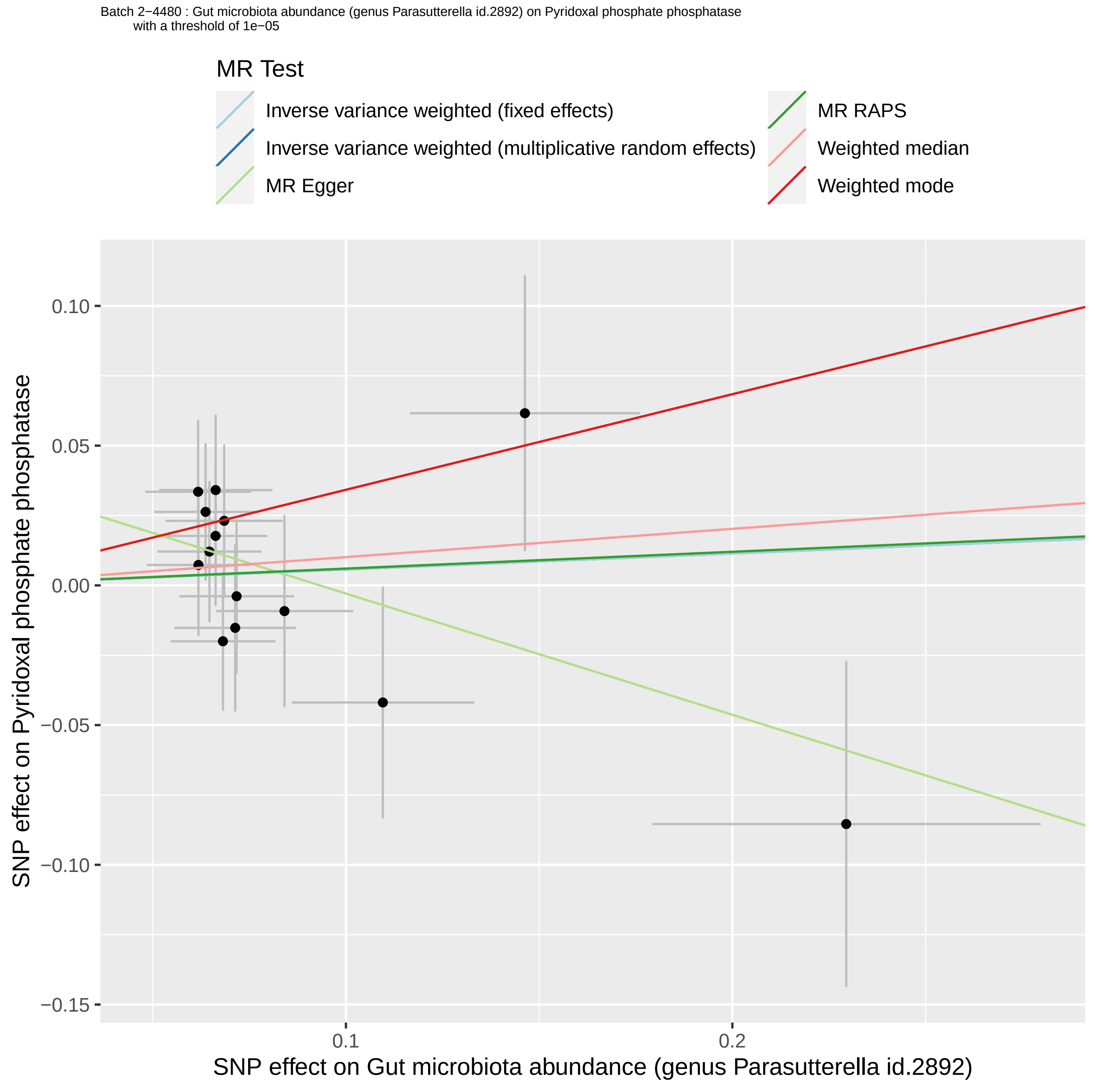

Batch 2−4480 : Gut microbiota abundance (genus Parasutterella id.2892) on Pyridoxal phosphate phosphatase
with a threshold of 1e−05
MR Test
Inverse variance weighted (fixed effects)
Inverse variance weighted (multiplicative random effects)
MR Egger
MR RAPS
Weighted median
Weighted mode
0.10
0.05
0.00
−0.05
−0.10
−0.15
0.1
0.2
SNP effect on Pyridoxal phosphate phosphatase
SNP effect on Gut microbiota abundance (genus Parasutterella id.2892)

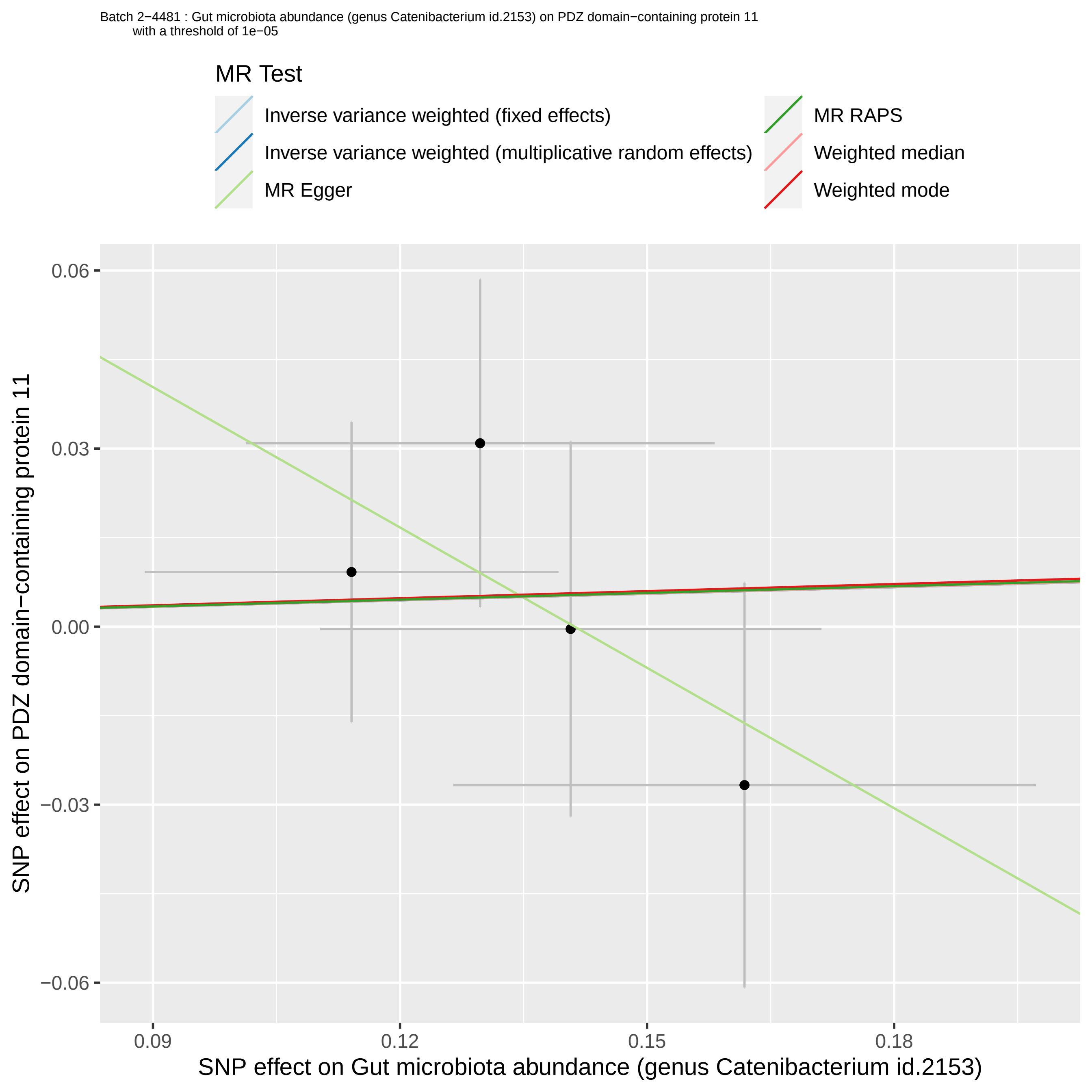
Batch 2−4481 : Gut microbiota abundance (genus Catenibacterium id.2153) on PDZ domain−containing protein 11
with a threshold of 1e−05
MR Test
Inverse variance weighted (fixed effects)
Inverse variance weighted (multiplicative random effects)
MR Egger
MR RAPS
Weighted median
Weighted mode
0.06
0.03
0.00
−0.03
−0.06
0.09
0.12
0.15
0.18
SNP effect on PDZ domain−containing protein 11
SNP effect on Gut microbiota abundance (genus Catenibacterium id.2153)

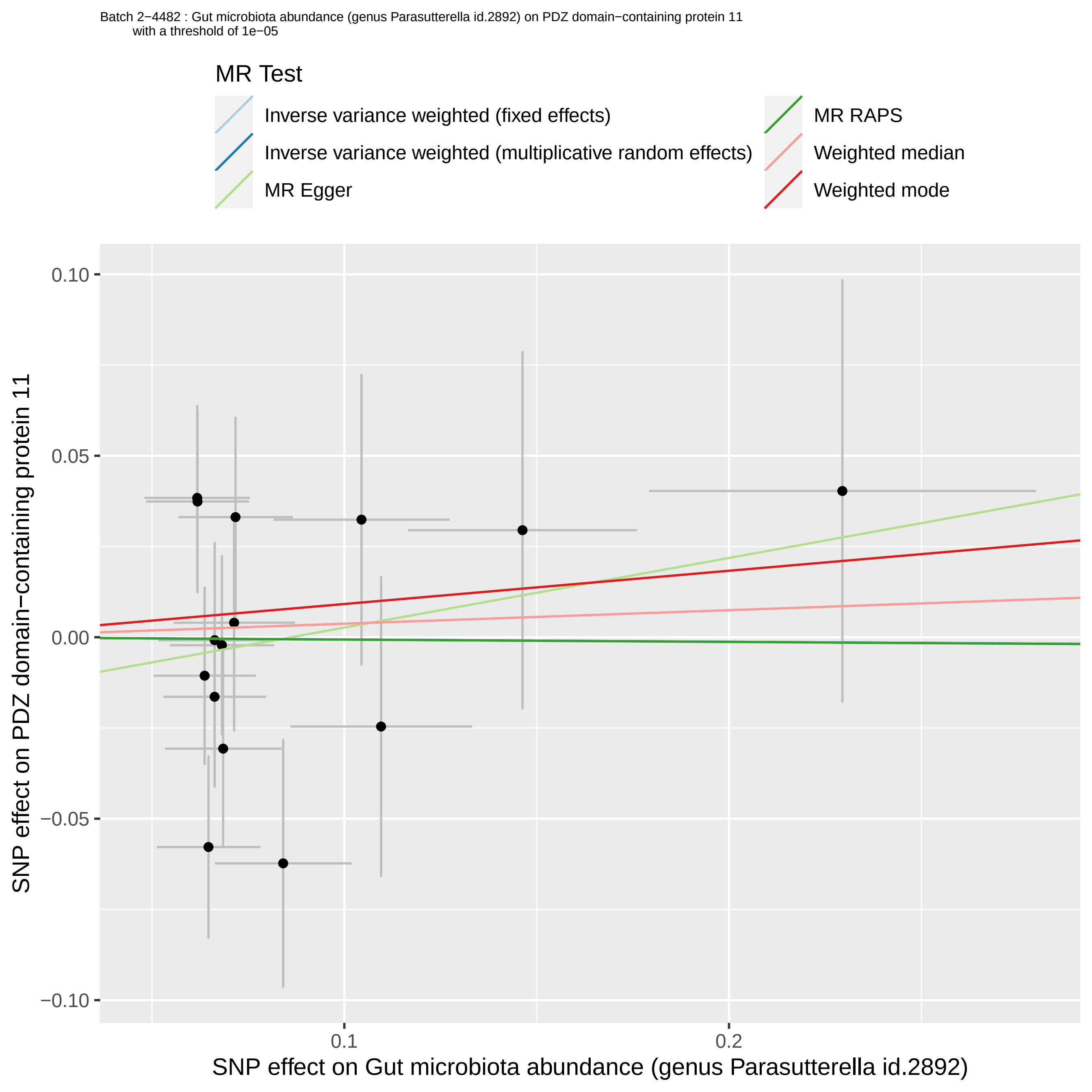
Batch 2−4482 : Gut microbiota abundance (genus Parasutterella id.2892) on PDZ domain−containing protein 11
with a threshold of 1e−05
MR Test
Inverse variance weighted (fixed effects)
Inverse variance weighted (multiplicative random effects)
MR Egger
MR RAPS
Weighted median
Weighted mode
0.10
0.05
0.00
−0.05
−0.10
0.1
0.2
SNP effect on PDZ domain−containing protein 11
SNP effect on Gut microbiota abundance (genus Parasutterella id.2892)

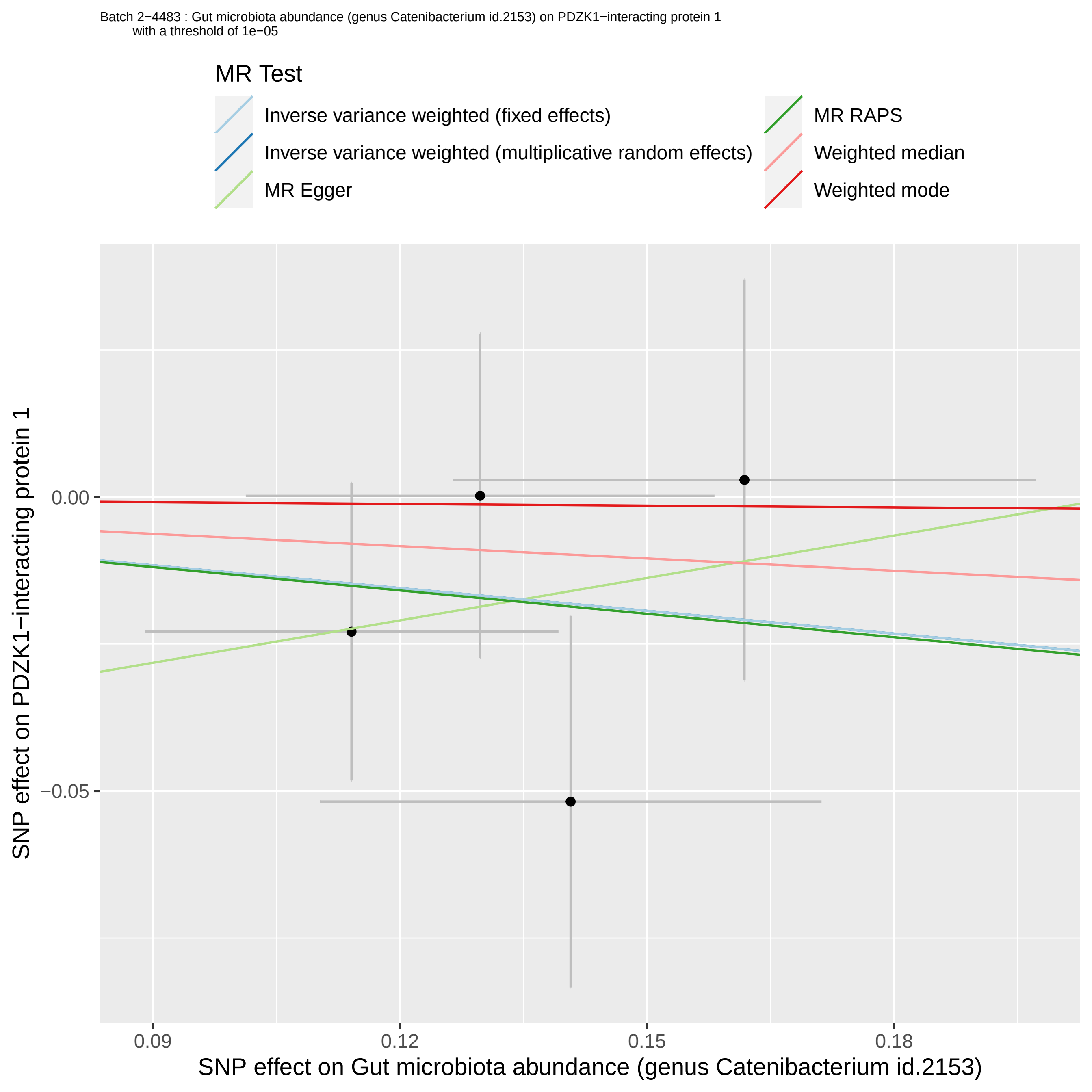
Batch 2−4483 : Gut microbiota abundance (genus Catenibacterium id.2153) on PDZK1−interacting protein 1
with a threshold of 1e−05
MR Test
Inverse variance weighted (fixed effects)
Inverse variance weighted (multiplicative random effects)
MR Egger
MR RAPS
Weighted median
Weighted mode
SNP effect on PDZK1−interacting protein 1
0.00
−0.05
0.09
0.12
0.15
0.18
SNP effect on Gut microbiota abundance (genus Catenibacterium id.2153)

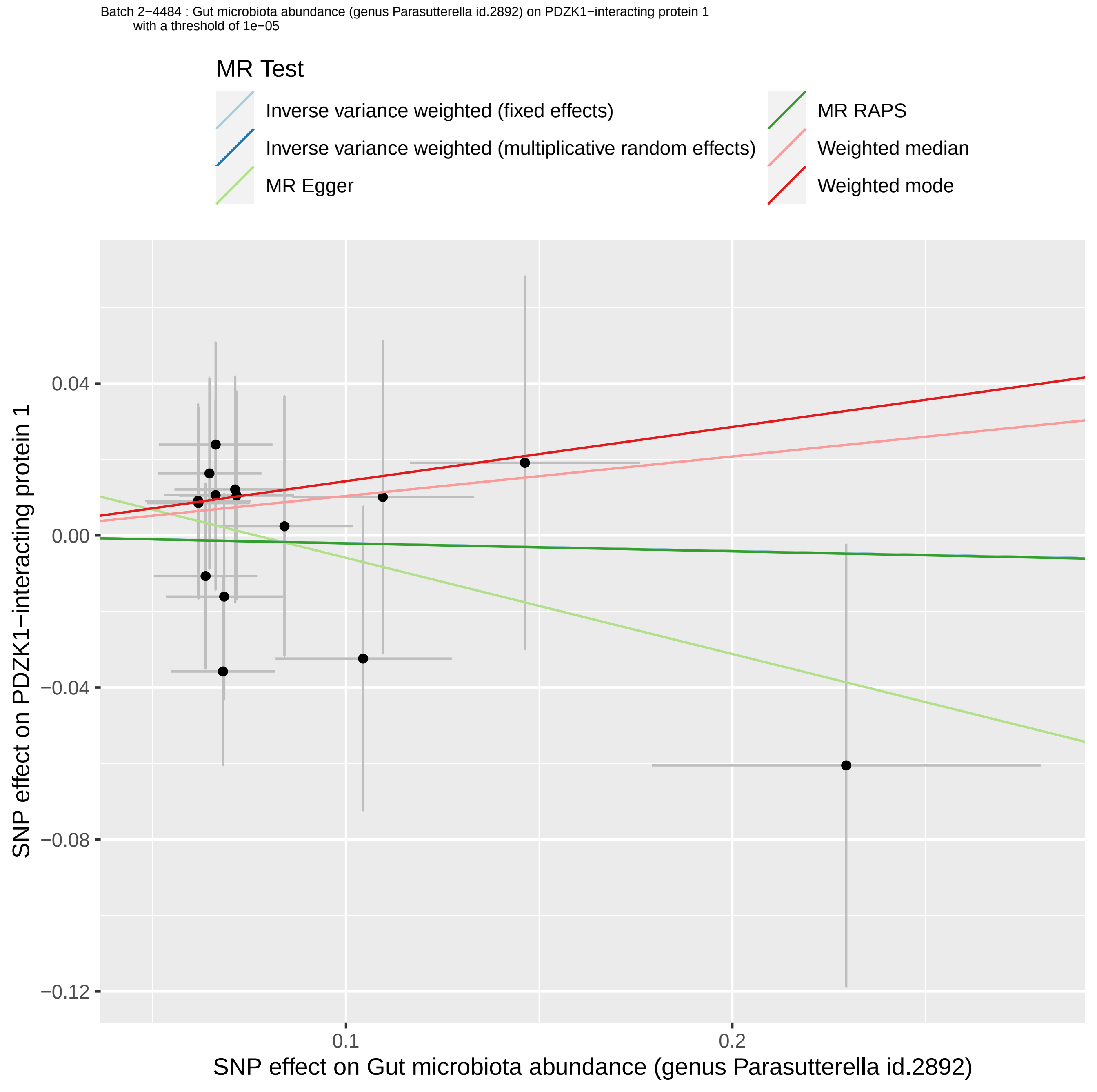

Batch 2−4484 : Gut microbiota abundance (genus Parasutterella id.2892) on PDZK1−interacting protein 1
with a threshold of 1e−05
MR Test
Inverse variance weighted (fixed effects)
Inverse variance weighted (multiplicative random effects)
MR Egger
MR RAPS
Weighted median
Weighted mode
0.04
0.00
−0.04
−0.08
−0.12
0.1
0.2
SNP effect on PDZK1−interacting protein 1
SNP effect on Gut microbiota abundance (genus Parasutterella id.2892)

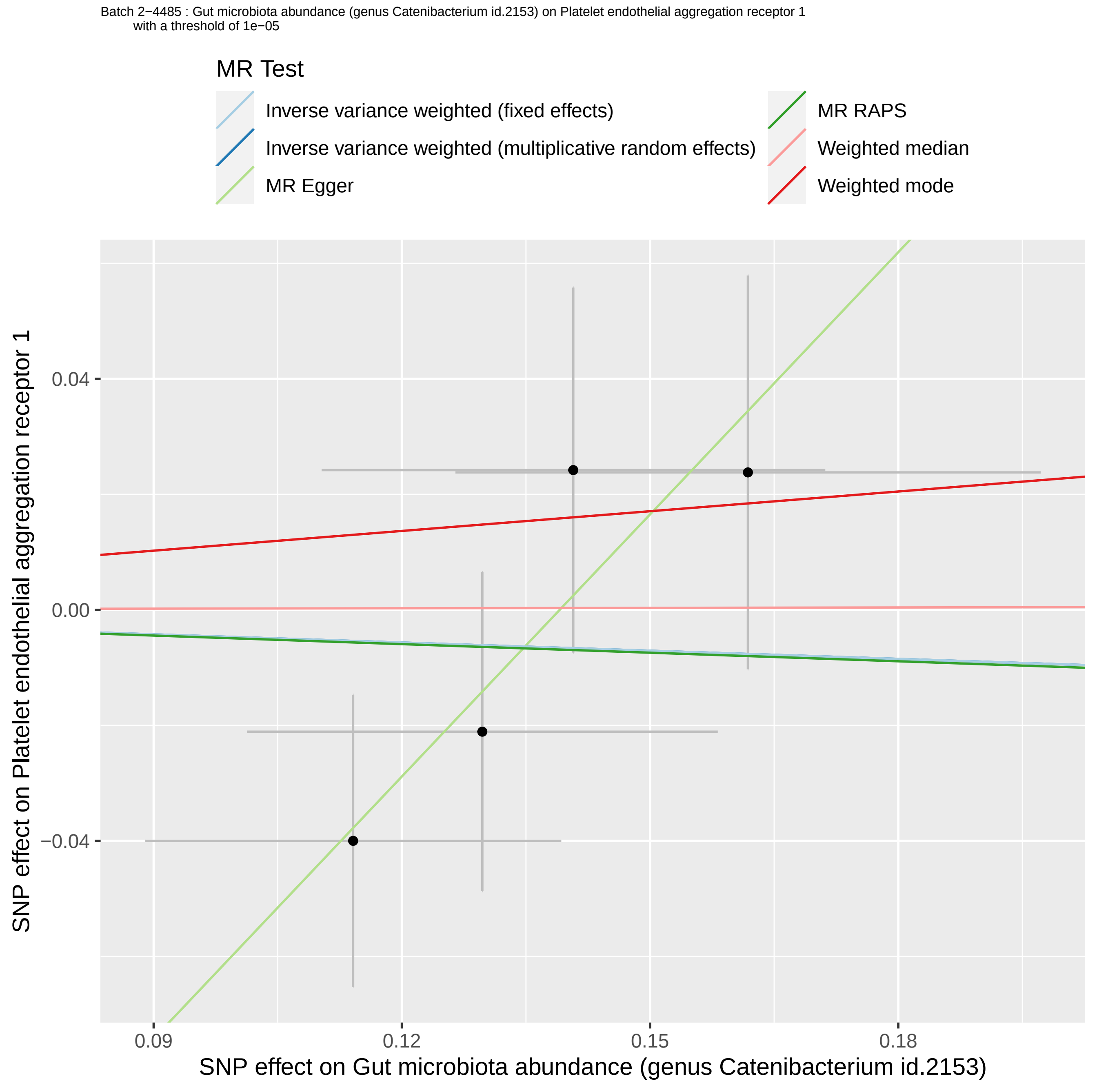
Batch 2−4485 : Gut microbiota abundance (genus Catenibacterium id.2153) on Platelet endothelial aggregation receptor 1
with a threshold of 1e−05
MR Test
Inverse variance weighted (fixed effects)
Inverse variance weighted (multiplicative random effects)
MR Egger
MR RAPS
Weighted median
Weighted mode
0.04
0.00
−0.04
0.09
0.12
0.15
0.18
SNP effect on Platelet endothelial aggregation receptor 1
SNP effect on Gut microbiota abundance (genus Catenibacterium id.2153)

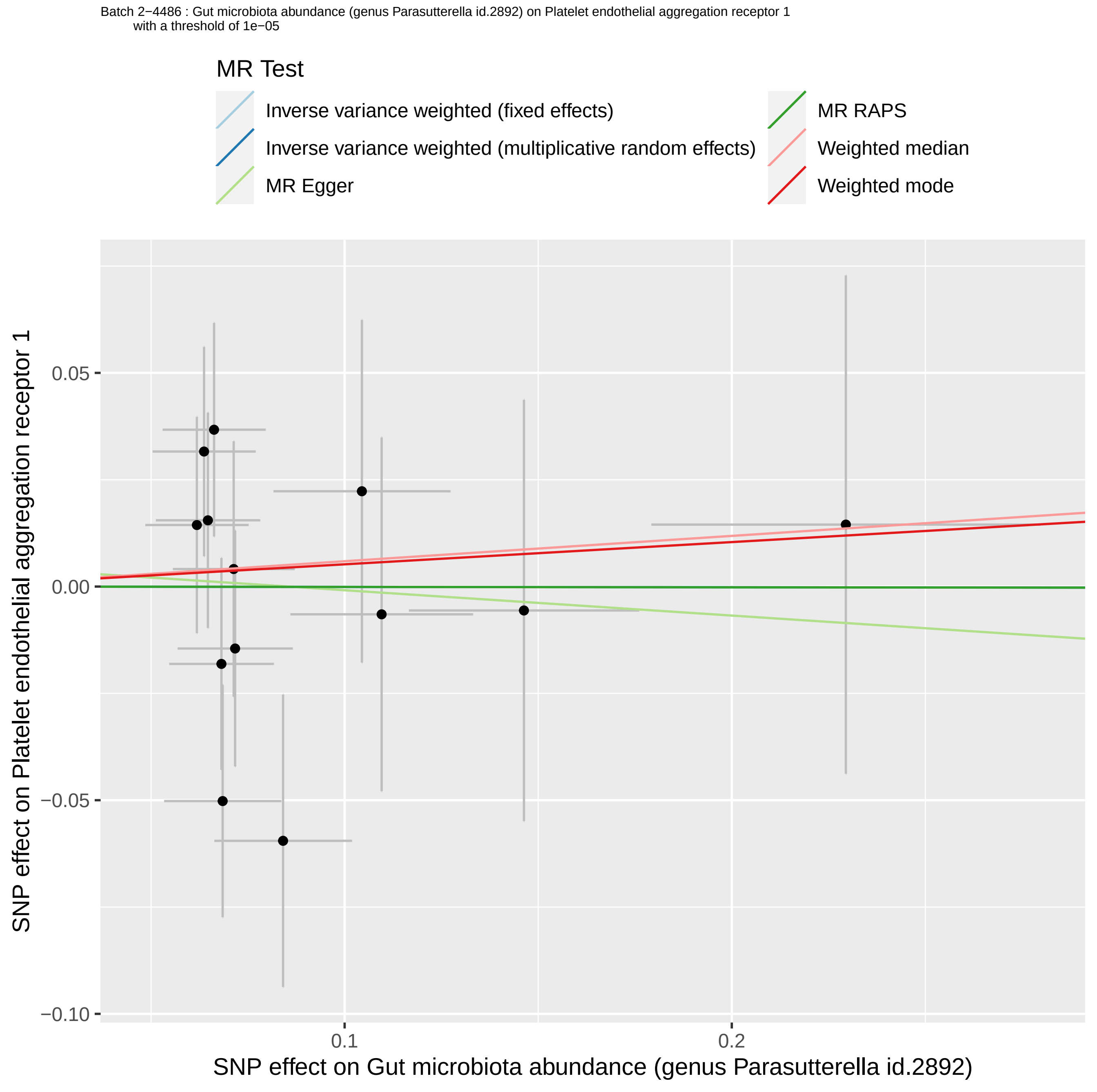

Batch 2−4486 : Gut microbiota abundance (genus Parasutterella id.2892) on Platelet endothelial aggregation receptor 1
with a threshold of 1e−05
MR Test
Inverse variance weighted (fixed effects)
Inverse variance weighted (multiplicative random effects)
MR Egger
MR RAPS
Weighted median
Weighted mode
SNP effect on Platelet endothelial aggregation receptor 1
0.05
0.00
−0.05
−0.10
0.1
0.2
SNP effect on Gut microbiota abundance (genus Parasutterella id.2892)

Batch 2−4487 : Gut microbiota abundance (genus Catenibacterium id.2153) on Platelet endothelial aggregation receptor 1 with a threshold of 1e−05

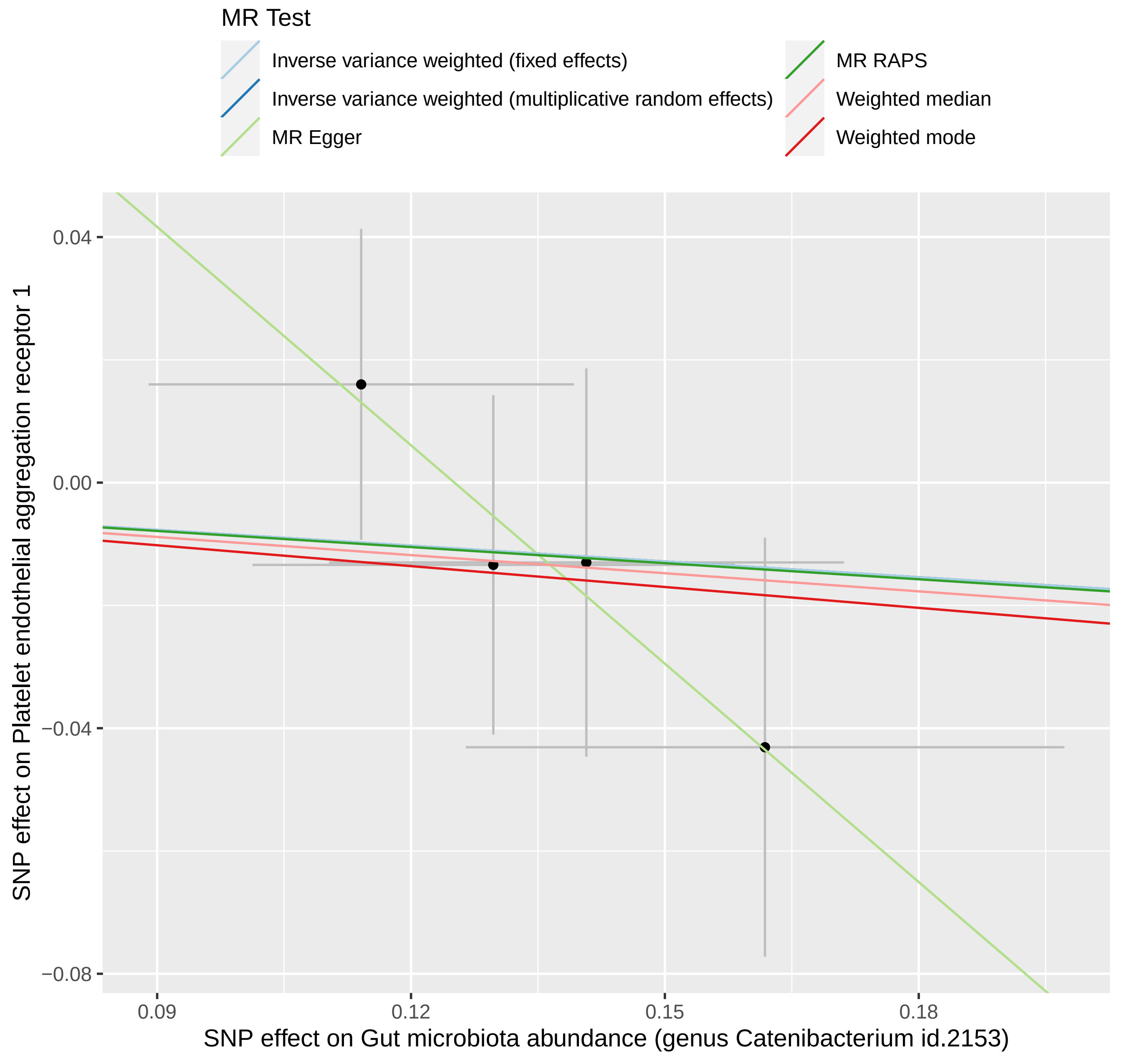

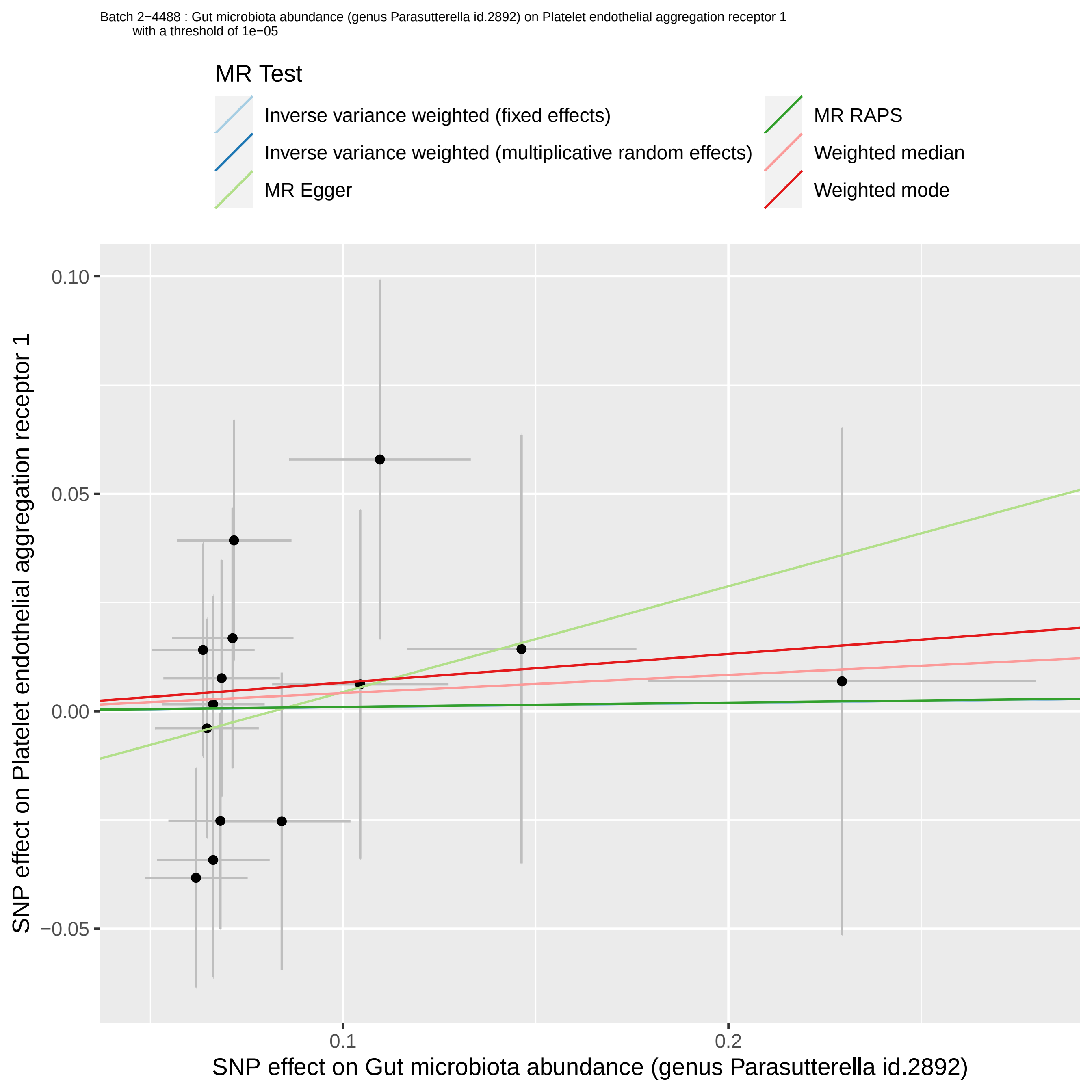
Batch 2−4488 : Gut microbiota abundance (genus Parasutterella id.2892) on Platelet endothelial aggregation receptor 1
with a threshold of 1e−05
MR Test
Inverse variance weighted (fixed effects)
Inverse variance weighted (multiplicative random effects)
MR Egger
MR RAPS
Weighted median
Weighted mode
0.10
0.05
0.00
−0.05
0.1
0.2
SNP effect on Platelet endothelial aggregation receptor 1
SNP effect on Gut microbiota abundance (genus Parasutterella id.2892)

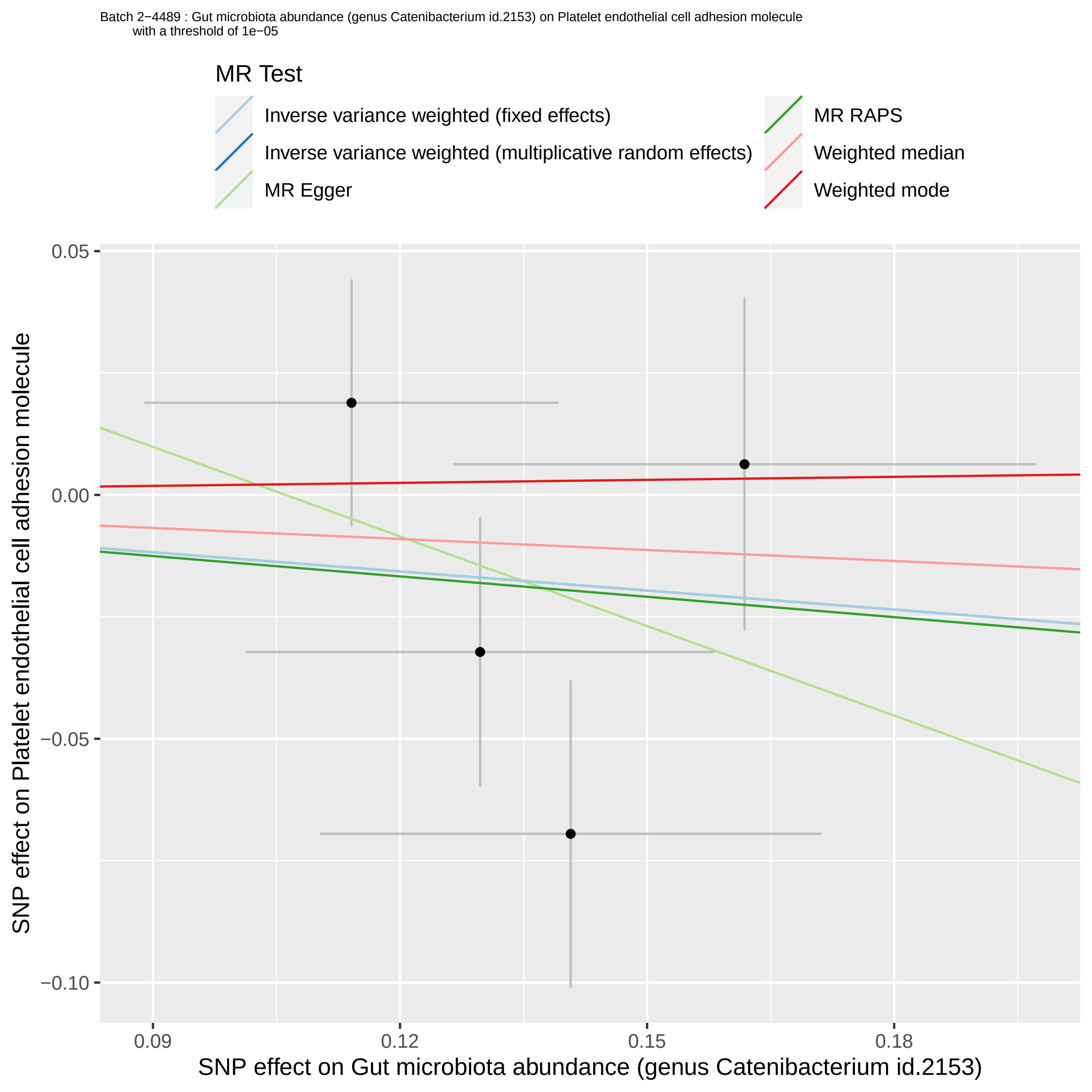
Batch 2−4489 : Gut microbiota abundance (genus Catenibacterium id.2153) on Platelet endothelial cell adhesion molecule
with a threshold of 1e−05
MR Test
Inverse variance weighted (fixed effects)
Inverse variance weighted (multiplicative random effects)
MR Egger
MR RAPS
Weighted median
Weighted mode
0.05
0.00
−0.05
−0.10
0.09
0.12
0.15
0.18
SNP effect on Platelet endothelial cell adhesion molecule
SNP effect on Gut microbiota abundance (genus Catenibacterium id.2153)

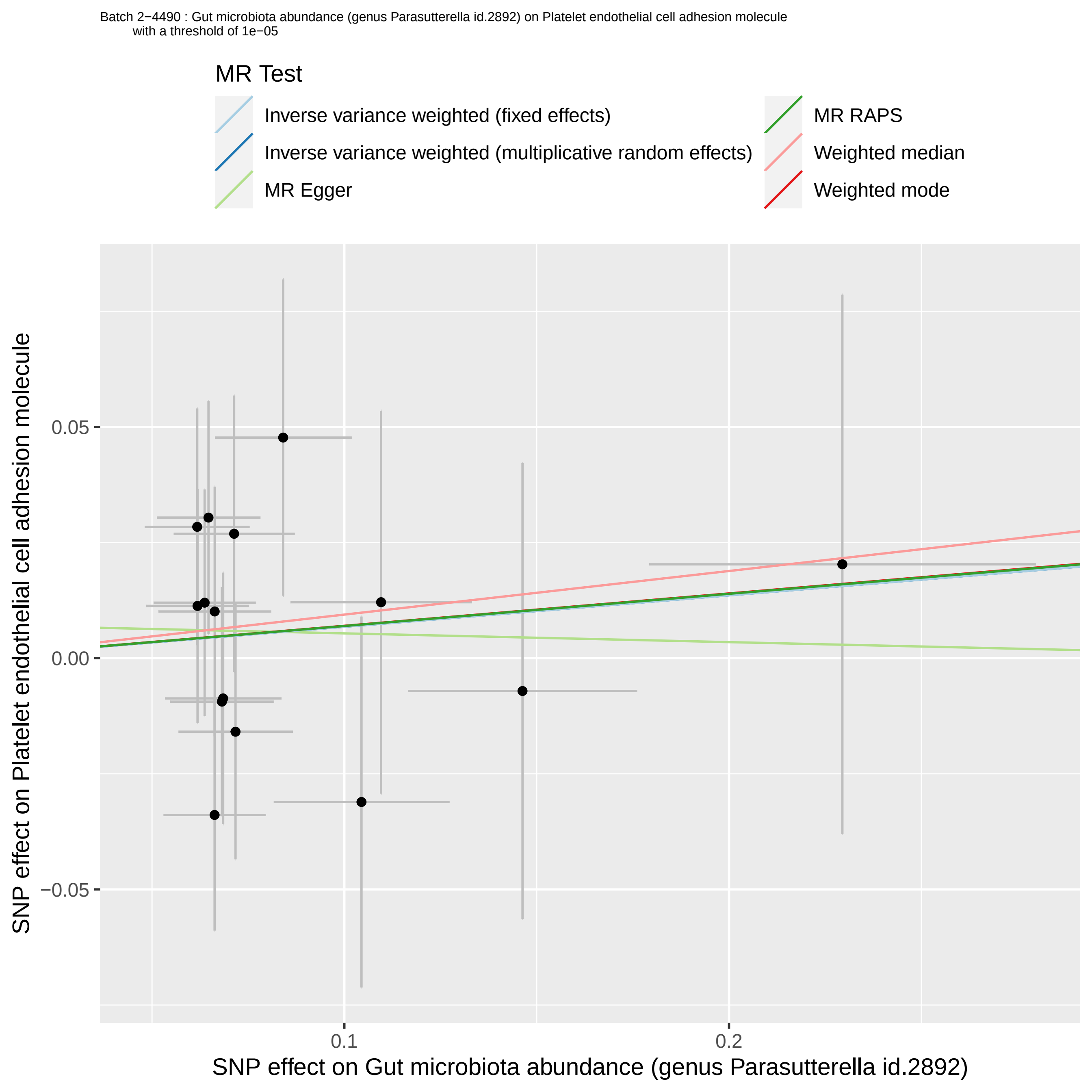

Batch 2−4490 : Gut microbiota abundance (genus Parasutterella id.2892) on Platelet endothelial cell adhesion molecule
with a threshold of 1e−05
MR Test
Inverse variance weighted (fixed effects)
Inverse variance weighted (multiplicative random effects)
MR Egger
MR RAPS
Weighted median
Weighted mode
0.05
0.00
−0.05
0.1
0.2
SNP effect on Platelet endothelial cell adhesion molecule
SNP effect on Gut microbiota abundance (genus Parasutterella id.2892)

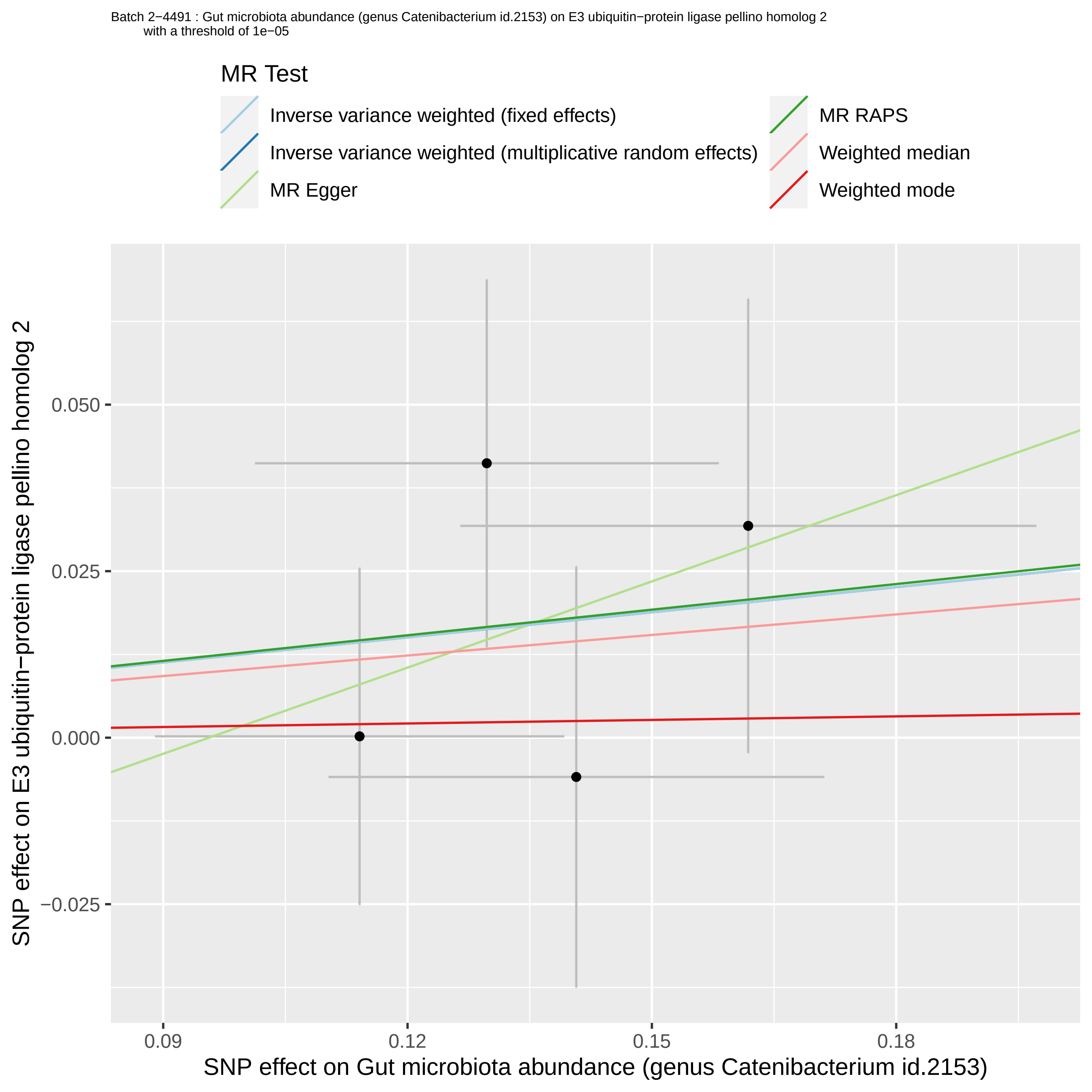
Batch 2−4491 : Gut microbiota abundance (genus Catenibacterium id.2153) on E3 ubiquitin−protein ligase pellino homolog 2
with a threshold of 1e−05
MR Test
Inverse variance weighted (fixed effects)
Inverse variance weighted (multiplicative random effects)
MR Egger
MR RAPS
Weighted median
Weighted mode
SNP effect on E3 ubiquitin−protein ligase pellino homolog 2
0.050
0.025
0.000
−0.025
0.09
0.12
0.15
0.18
SNP effect on Gut microbiota abundance (genus Catenibacterium id.2153)

Batch 2−4492 : Gut microbiota abundance (genus Parasutterella id.2892) on E3 ubiquitin−protein ligase pellino homolog 2 with a threshold of 1e−05

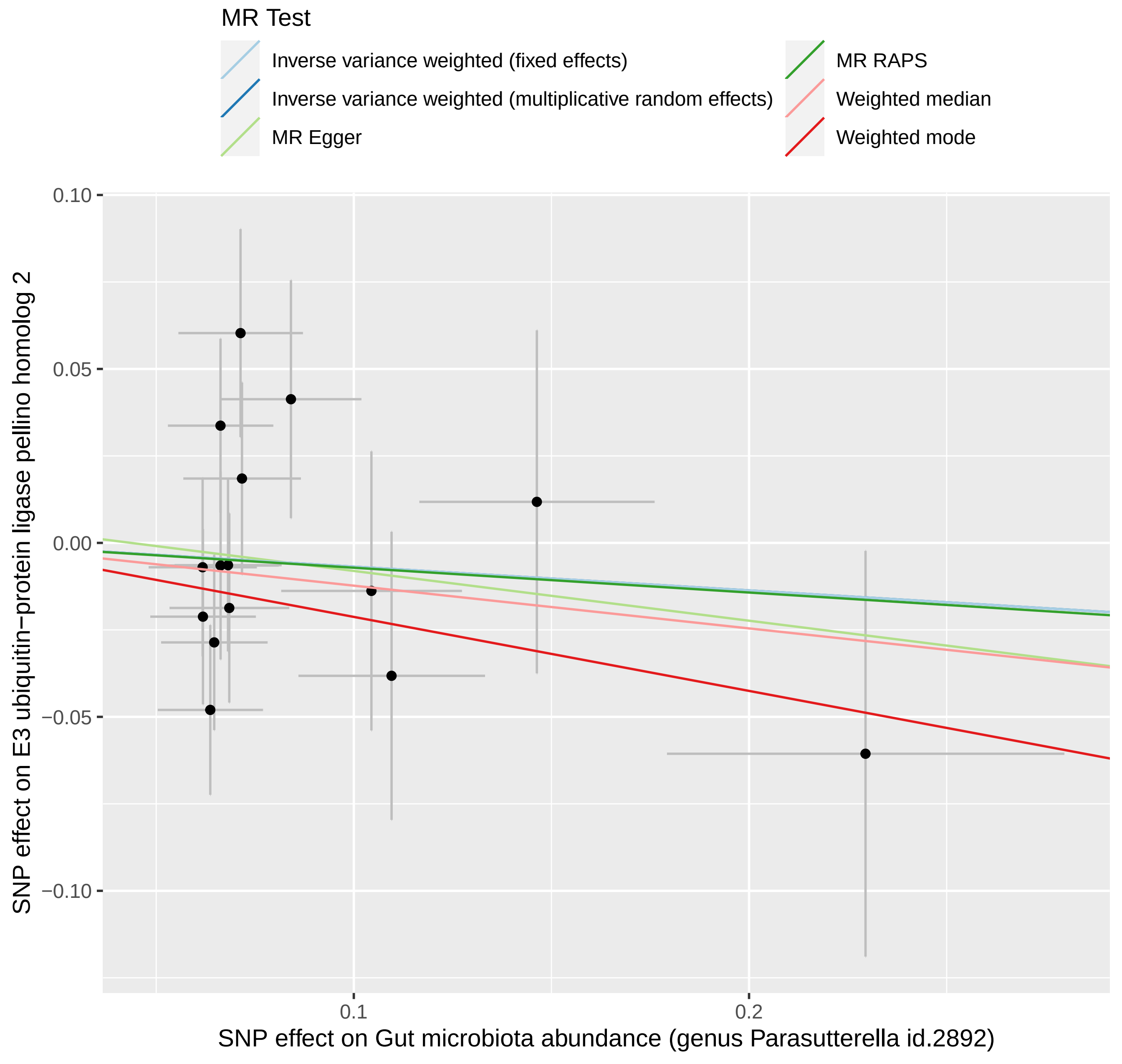

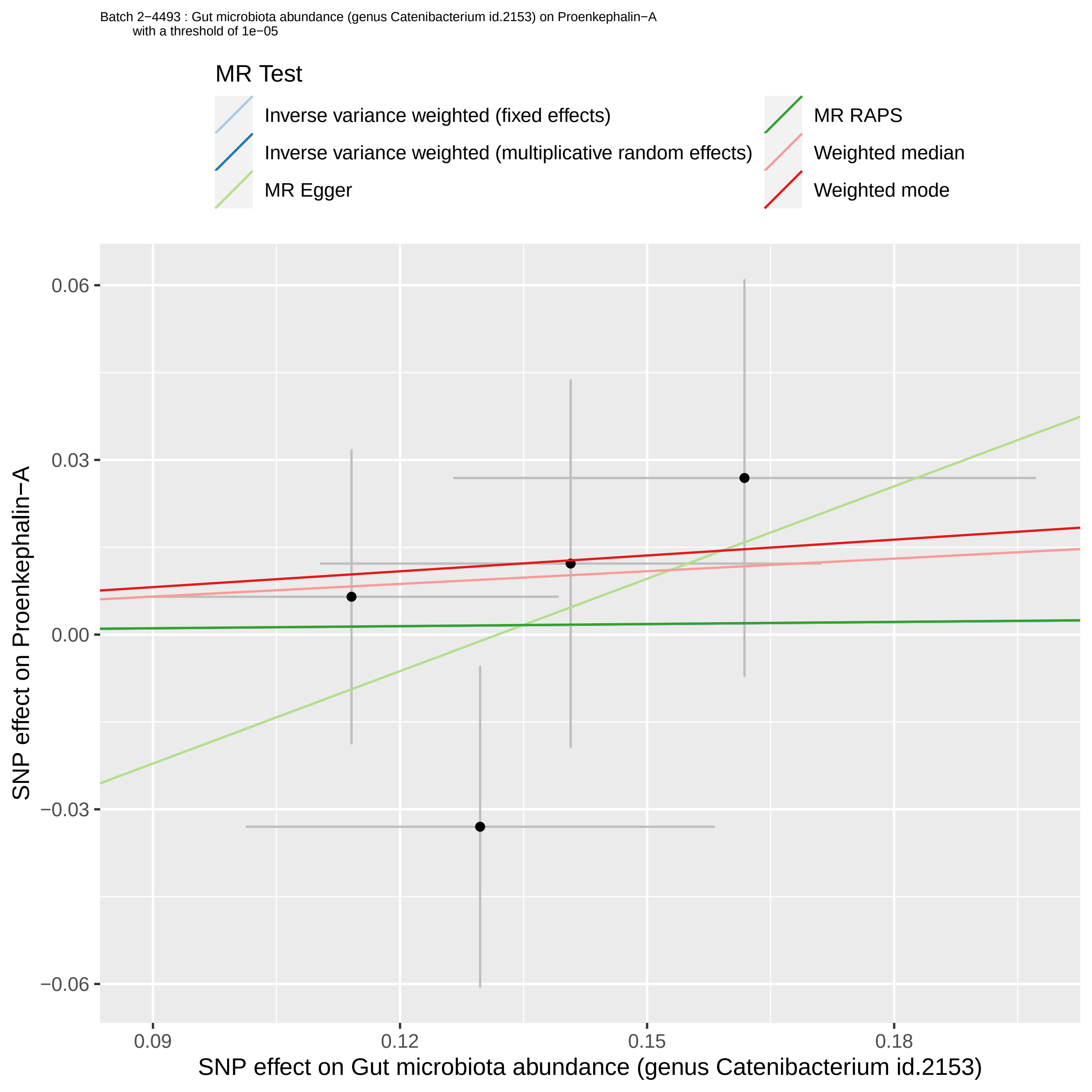
Batch 2−4493 : Gut microbiota abundance (genus Catenibacterium id.2153) on Proenkephalin−A
with a threshold of 1e−05
MR Test
Inverse variance weighted (fixed effects)
Inverse variance weighted (multiplicative random effects)
MR Egger
MR RAPS
Weighted median
Weighted mode
0.06
0.03
0.00
−0.03
−0.06
0.09
0.12
0.15
0.18
SNP effect on Proenkephalin−A
SNP effect on Gut microbiota abundance (genus Catenibacterium id.2153)

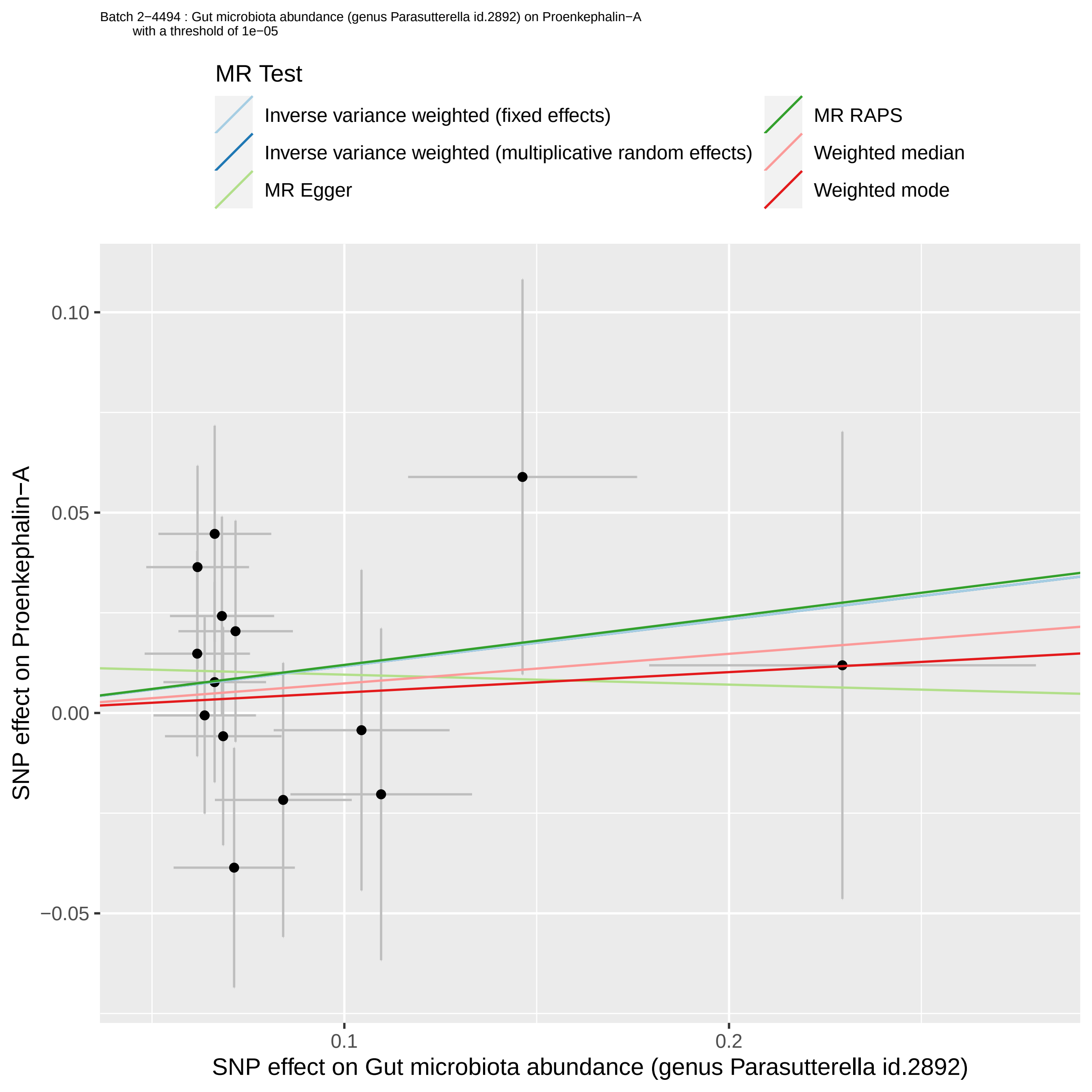

Batch 2−4494 : Gut microbiota abundance (genus Parasutterella id.2892) on Proenkephalin−A
with a threshold of 1e−05
MR Test
Inverse variance weighted (fixed effects)
Inverse variance weighted (multiplicative random effects)
MR Egger
MR RAPS
Weighted median
Weighted mode
0.10
0.05
0.00
−0.05
0.1
0.2
SNP effect on Proenkephalin−A
SNP effect on Gut microbiota abundance (genus Parasutterella id.2892)

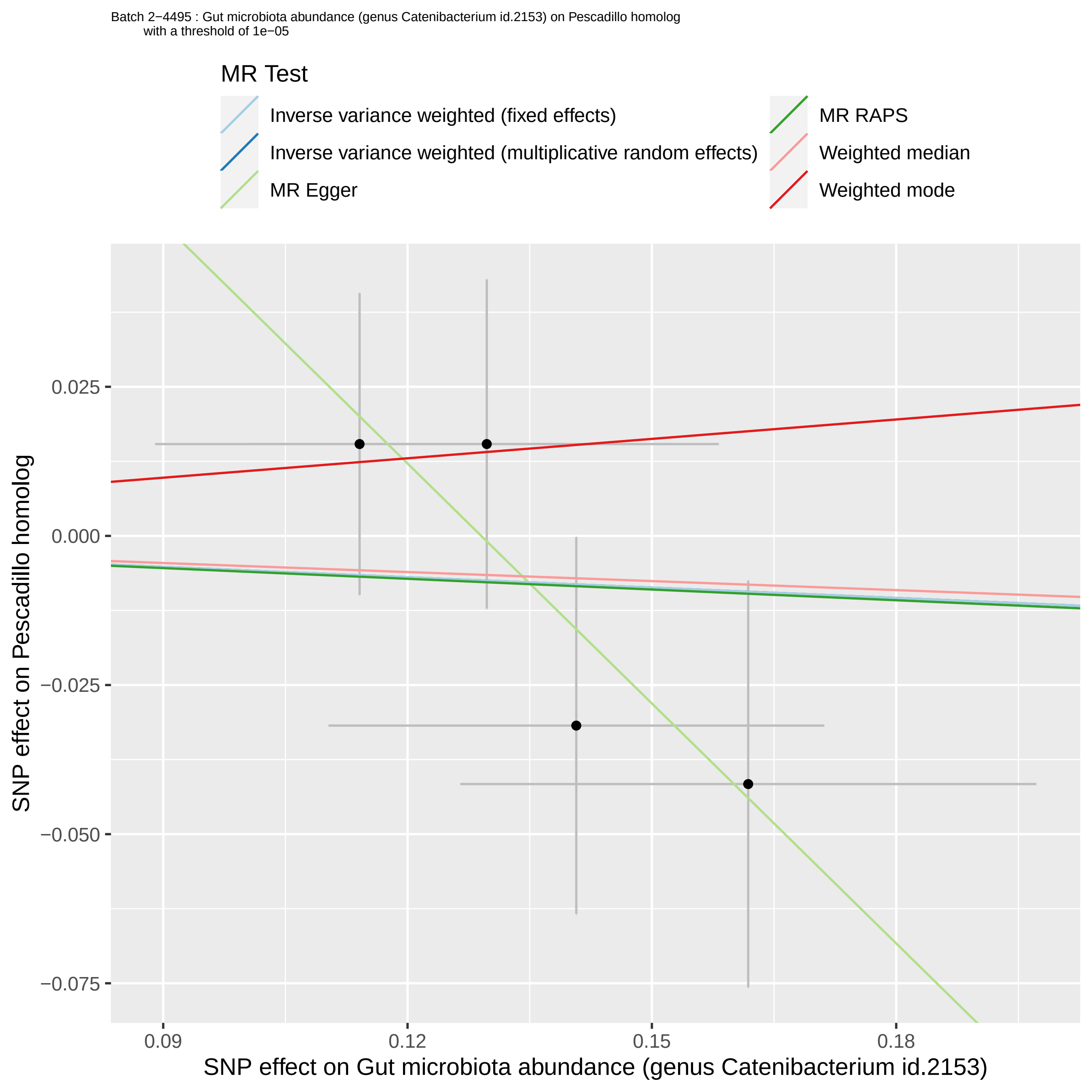

Batch 2−4495 : Gut microbiota abundance (genus Catenibacterium id.2153) on Pescadillo homolog
with a threshold of 1e−05
MR Test
Inverse variance weighted (fixed effects)
Inverse variance weighted (multiplicative random effects)
MR Egger
MR RAPS
Weighted median
Weighted mode
0.025
0.000
−0.025
−0.050
−0.075
0.09
0.12
0.15
0.18
SNP effect on Pescadillo homolog
SNP effect on Gut microbiota abundance (genus Catenibacterium id.2153)

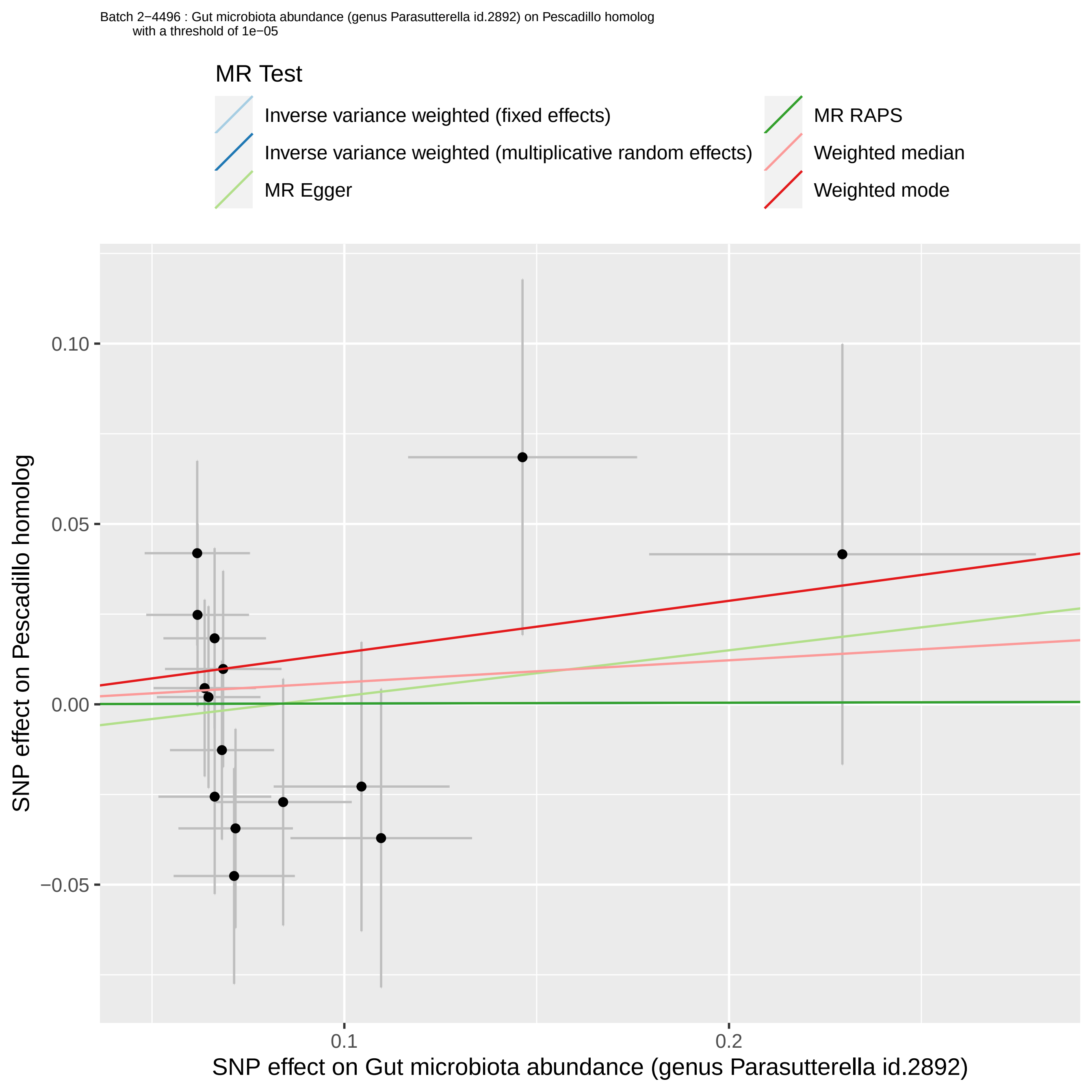
Batch 2−4496 : Gut microbiota abundance (genus Parasutterella id.2892) on Pescadillo homolog
with a threshold of 1e−05
MR Test
Inverse variance weighted (fixed effects)
Inverse variance weighted (multiplicative random effects)
MR Egger
MR RAPS
Weighted median
Weighted mode
0.10
0.05
0.00
−0.05
0.1
0.2
SNP effect on Pescadillo homolog
SNP effect on Gut microbiota abundance (genus Parasutterella id.2892)

Batch 2−4497 : Gut microbiota abundance (genus Catenibacterium id.2153) on Peroxisomal membrane protein PEX14
with a threshold of 1e−05

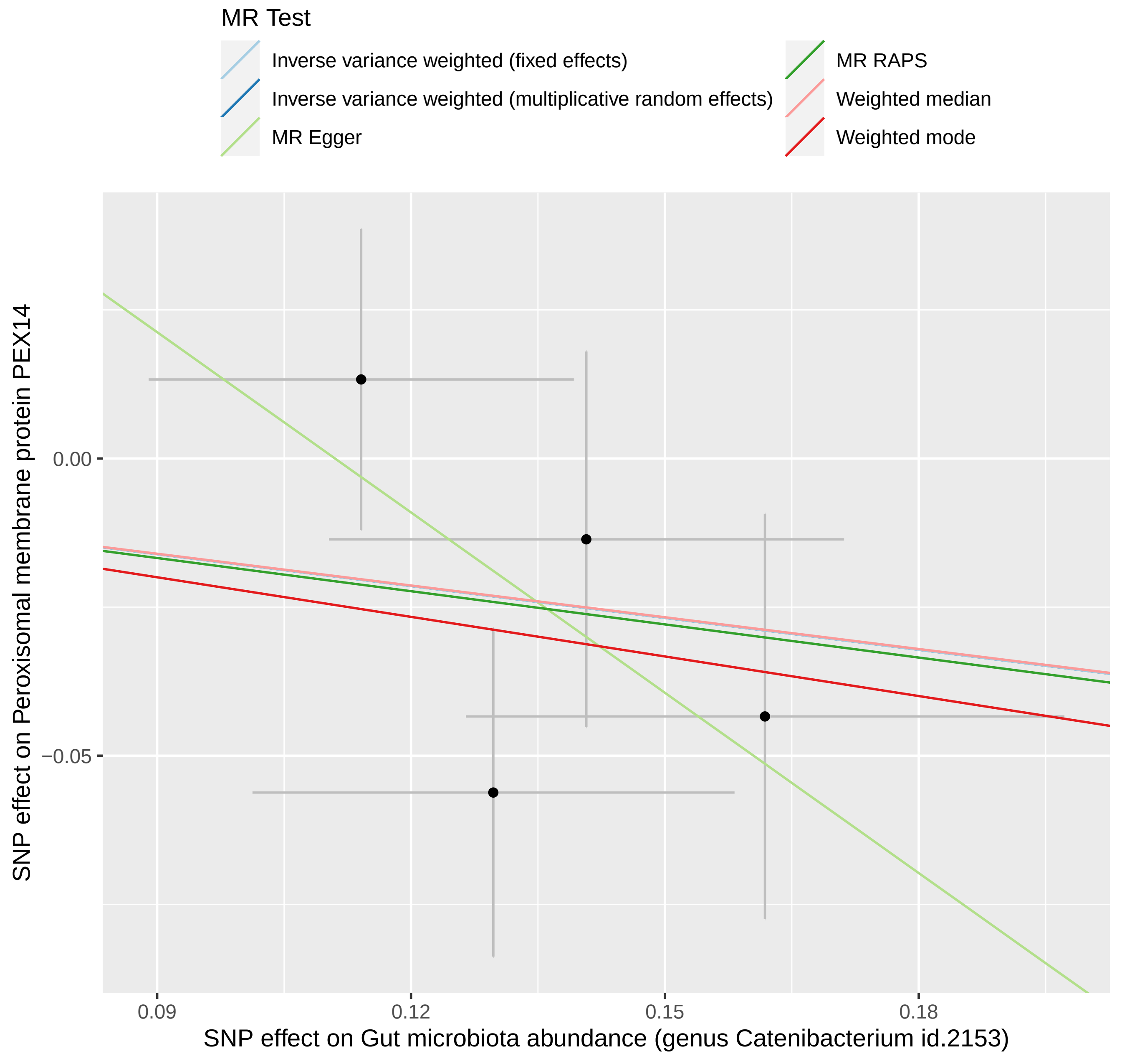

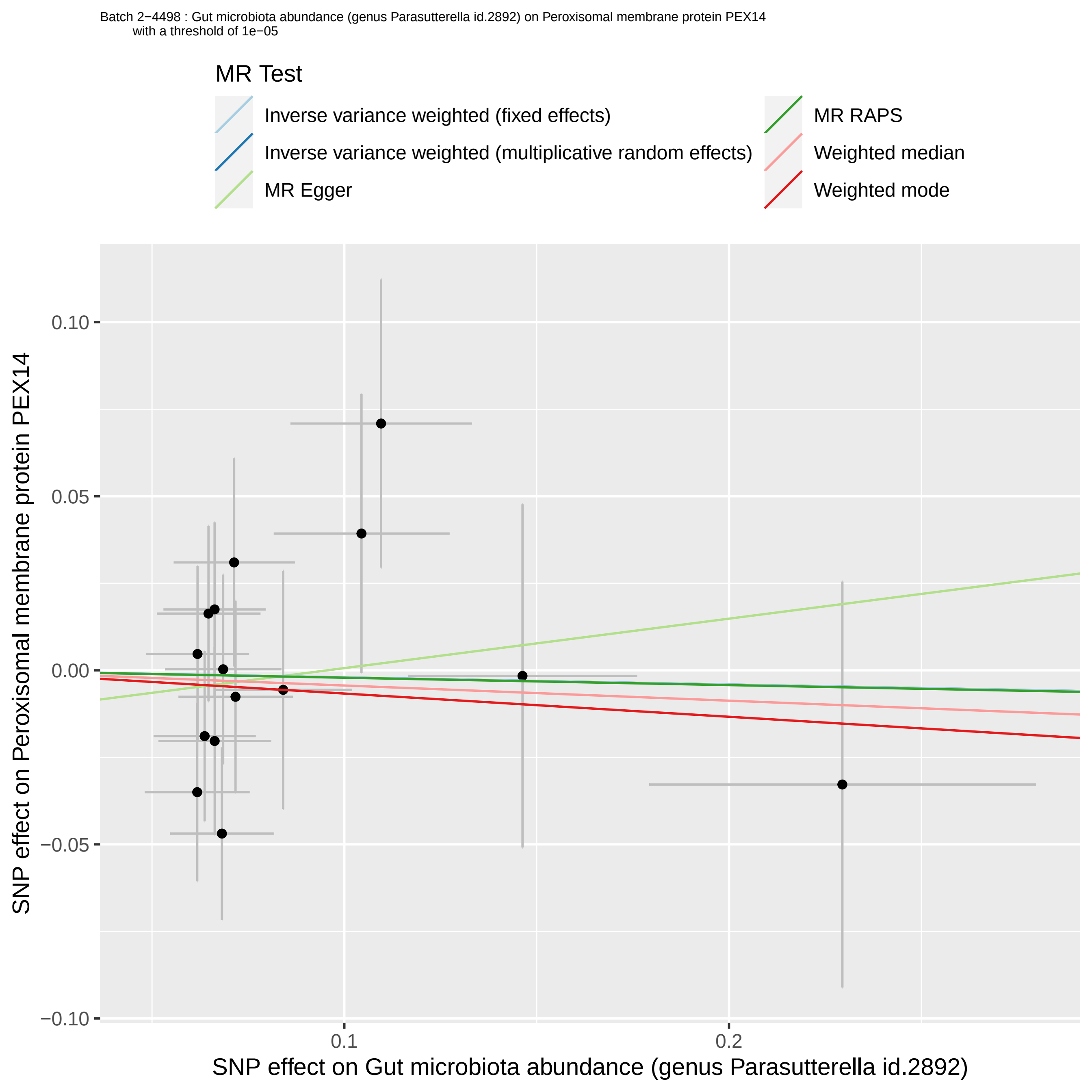

Batch 2−4498 : Gut microbiota abundance (genus Parasutterella id.2892) on Peroxisomal membrane protein PEX14
with a threshold of 1e−05
MR Test
Inverse variance weighted (fixed effects)
Inverse variance weighted (multiplicative random effects)
MR Egger
MR RAPS
Weighted median
Weighted mode
0.10
0.05
0.00
−0.05
−0.10
0.1
0.2
SNP effect on Peroxisomal membrane protein PEX14
SNP effect on Gut microbiota abundance (genus Parasutterella id.2892)

Batch 2−4499 : Gut microbiota abundance (genus Catenibacterium id.2153) on Peroxisomal targeting signal 1 receptor
with a threshold of 1e−05

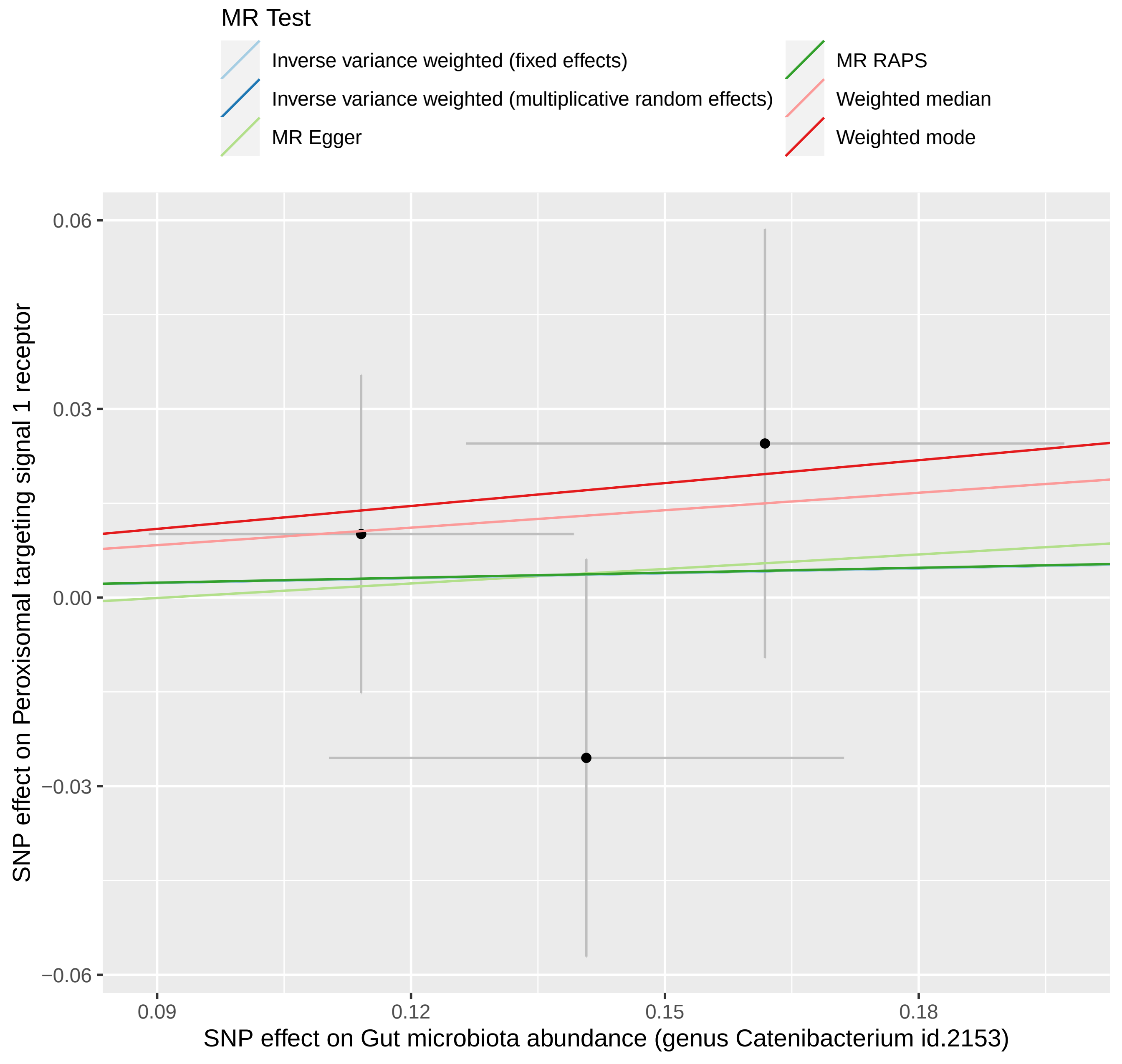

Batch 2−4500 : Gut microbiota abundance (genus Parasutterella id.2892) on Peroxisomal targeting signal 1 receptor
with a threshold of 1e−05

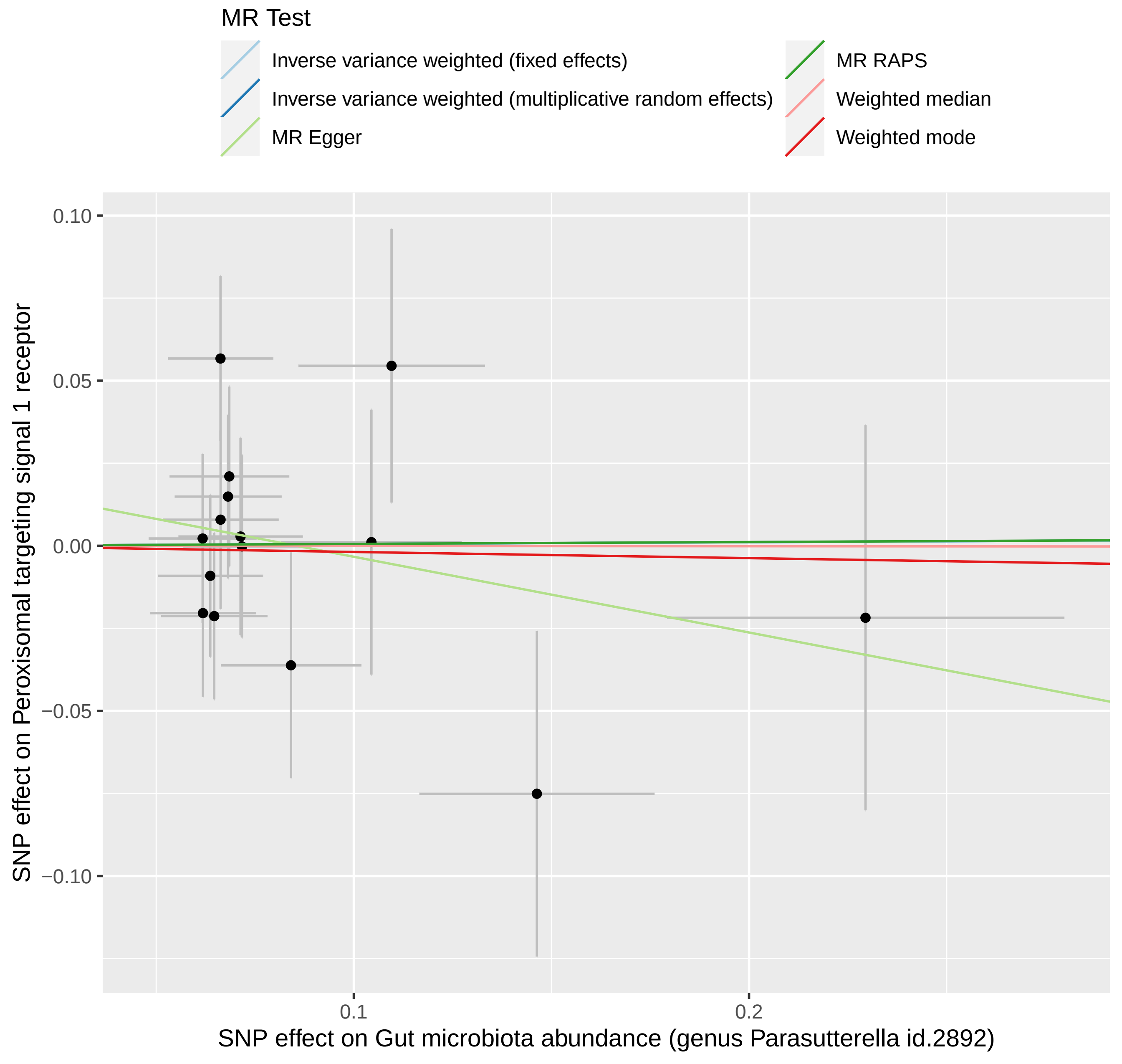

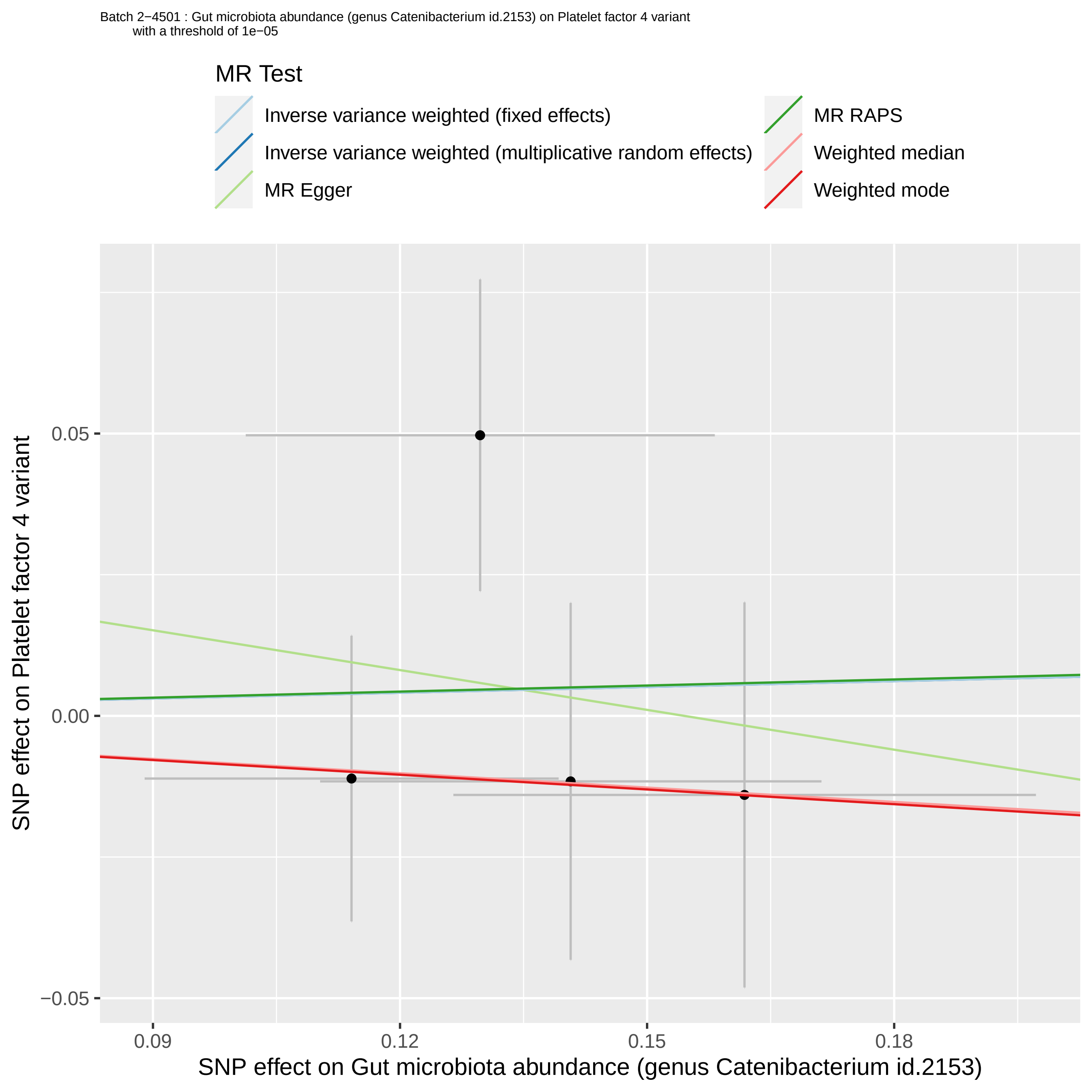

Batch 2−4501 : Gut microbiota abundance (genus Catenibacterium id.2153) on Platelet factor 4 variant
with a threshold of 1e−05
MR Test
Inverse variance weighted (fixed effects)
Inverse variance weighted (multiplicative random effects)
MR Egger
MR RAPS
Weighted median
Weighted mode
0.05
0.00
−0.05
0.09
0.12
0.15
0.18
SNP effect on Platelet factor 4 variant
SNP effect on Gut microbiota abundance (genus Catenibacterium id.2153)

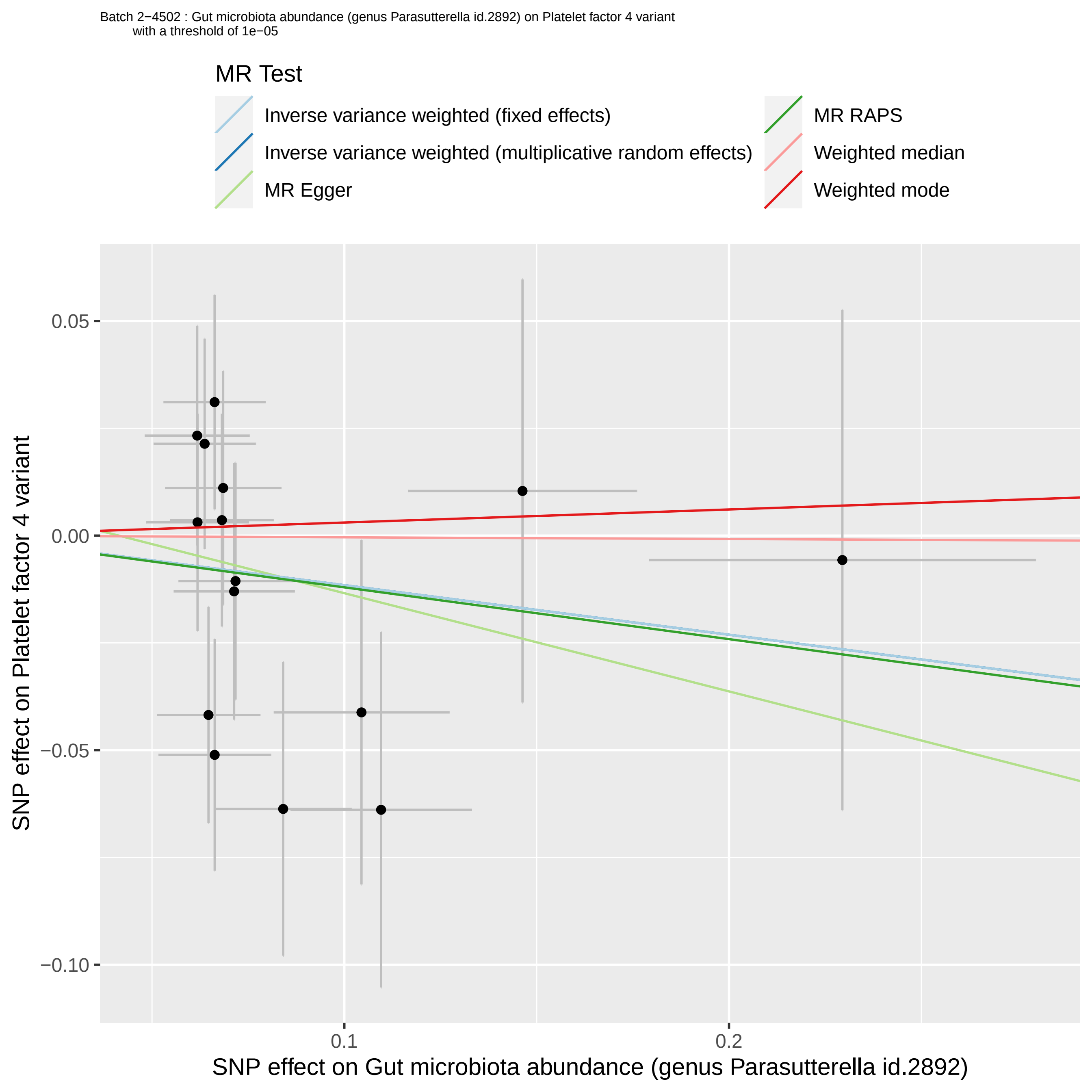

Batch 2−4502 : Gut microbiota abundance (genus Parasutterella id.2892) on Platelet factor 4 variant
with a threshold of 1e−05
MR Test
Inverse variance weighted (fixed effects)
Inverse variance weighted (multiplicative random effects)
MR Egger
MR RAPS
Weighted median
Weighted mode
SNP effect on Platelet factor 4 variant
0.05
0.00
−0.05
−0.10
0.1
0.2
SNP effect on Gut microbiota abundance (genus Parasutterella id.2892)

Batch 2−4503 : Gut microbiota abundance (genus Catenibacterium id.2153) on Prefoldin subunit 5
with a threshold of 1e−05

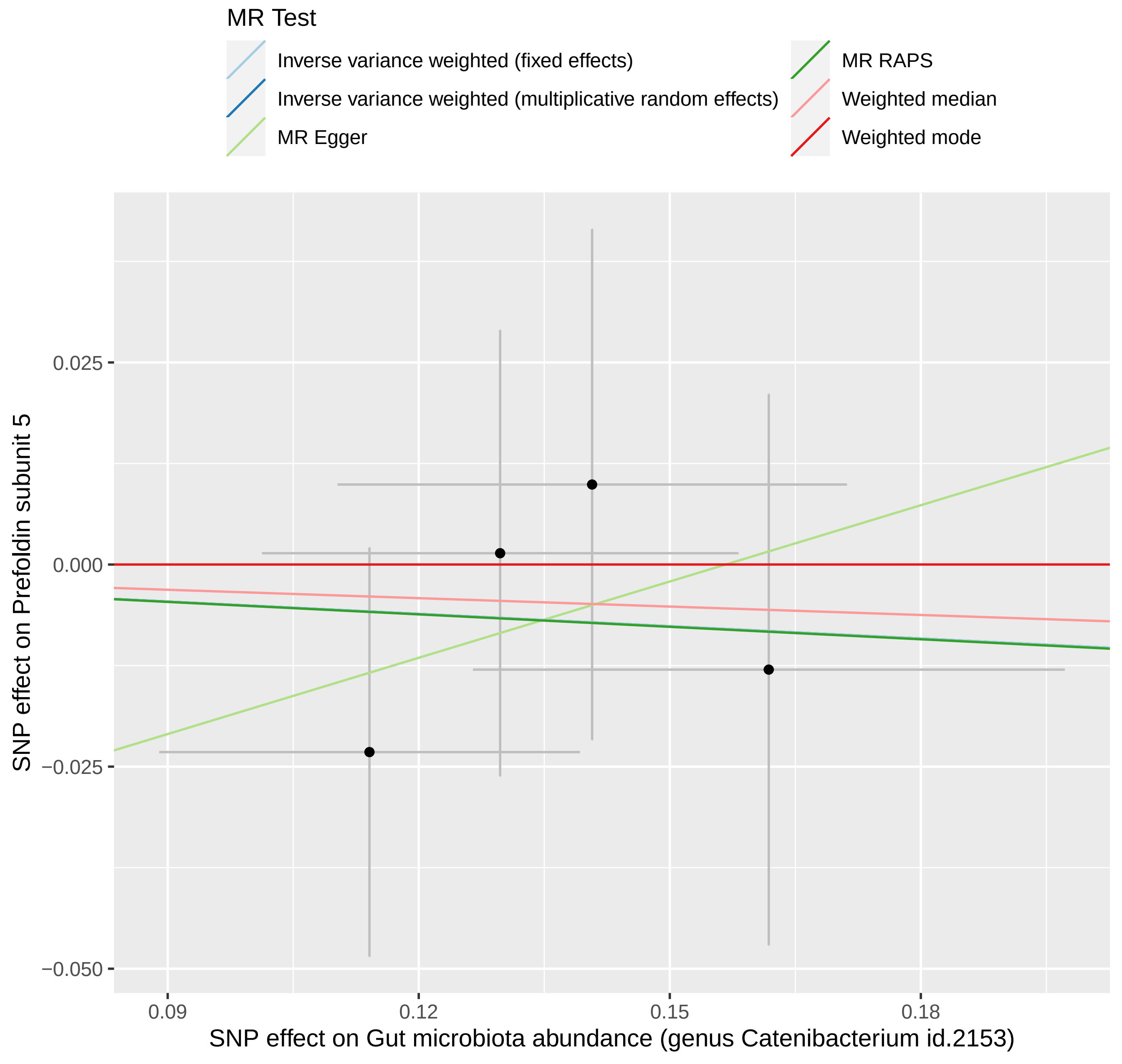

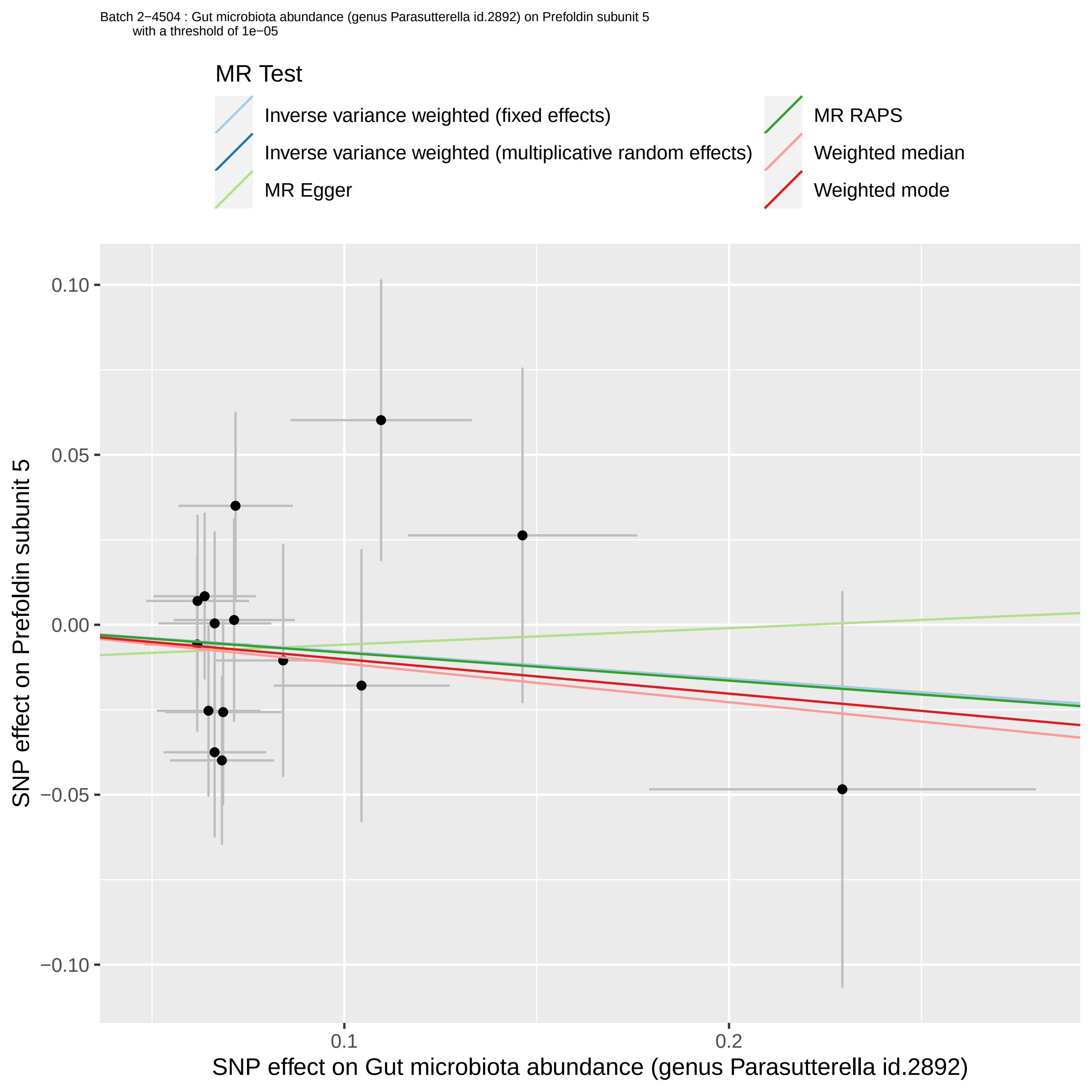

Batch 2−4504 : Gut microbiota abundance (genus Parasutterella id.2892) on Prefoldin subunit 5
with a threshold of 1e−05
MR Test
Inverse variance weighted (fixed effects)
Inverse variance weighted (multiplicative random effects)
MR Egger
MR RAPS
Weighted median
Weighted mode
0.10
0.05
0.00
−0.05
−0.10
0.1
0.2
SNP effect on Prefoldin subunit 5
SNP effect on Gut microbiota abundance (genus Parasutterella id.2892)

Batch 2−4505 : Gut microbiota abundance (genus Catenibacterium id.2153) on Profilin−2
with a threshold of 1e−05

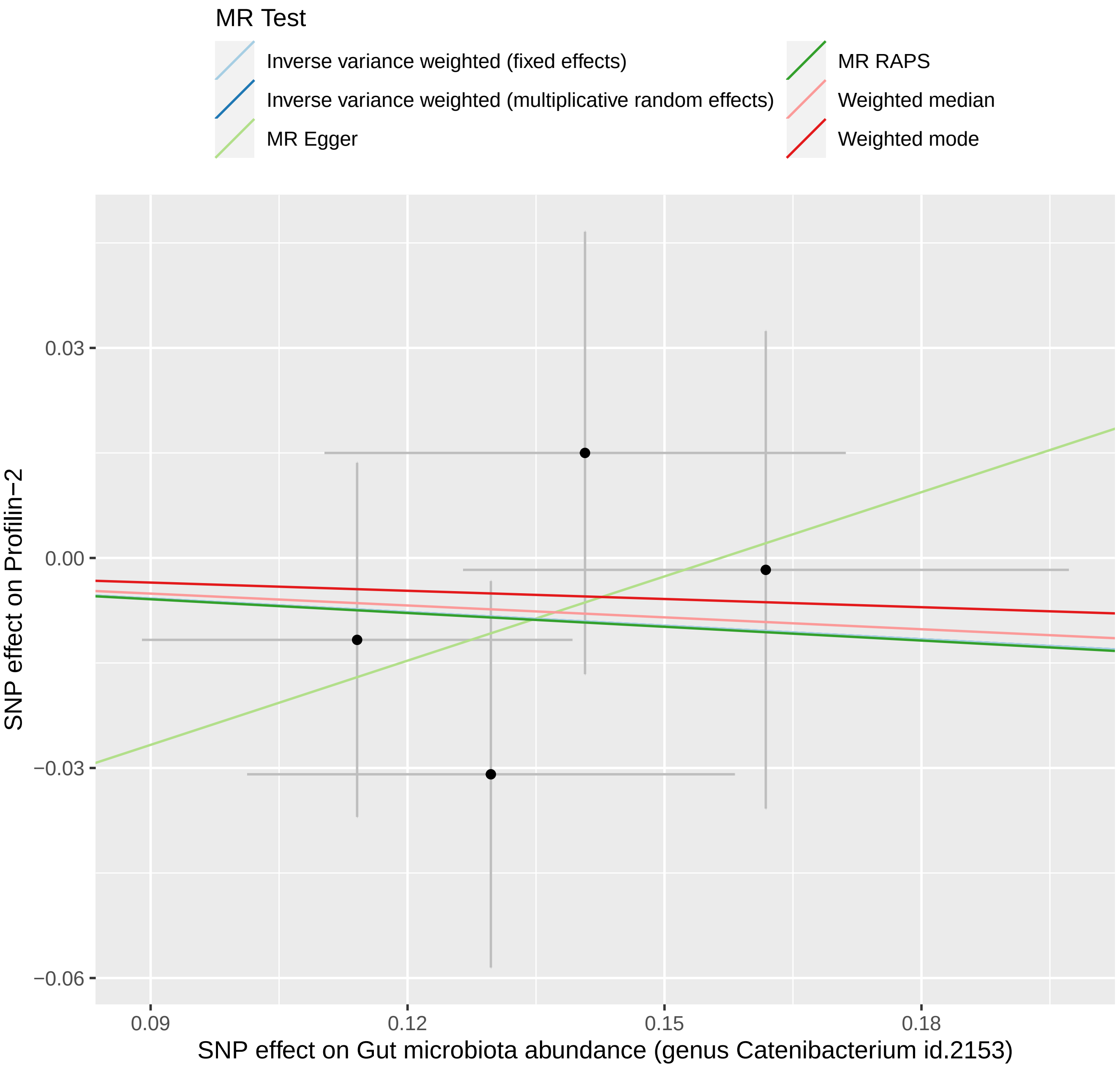

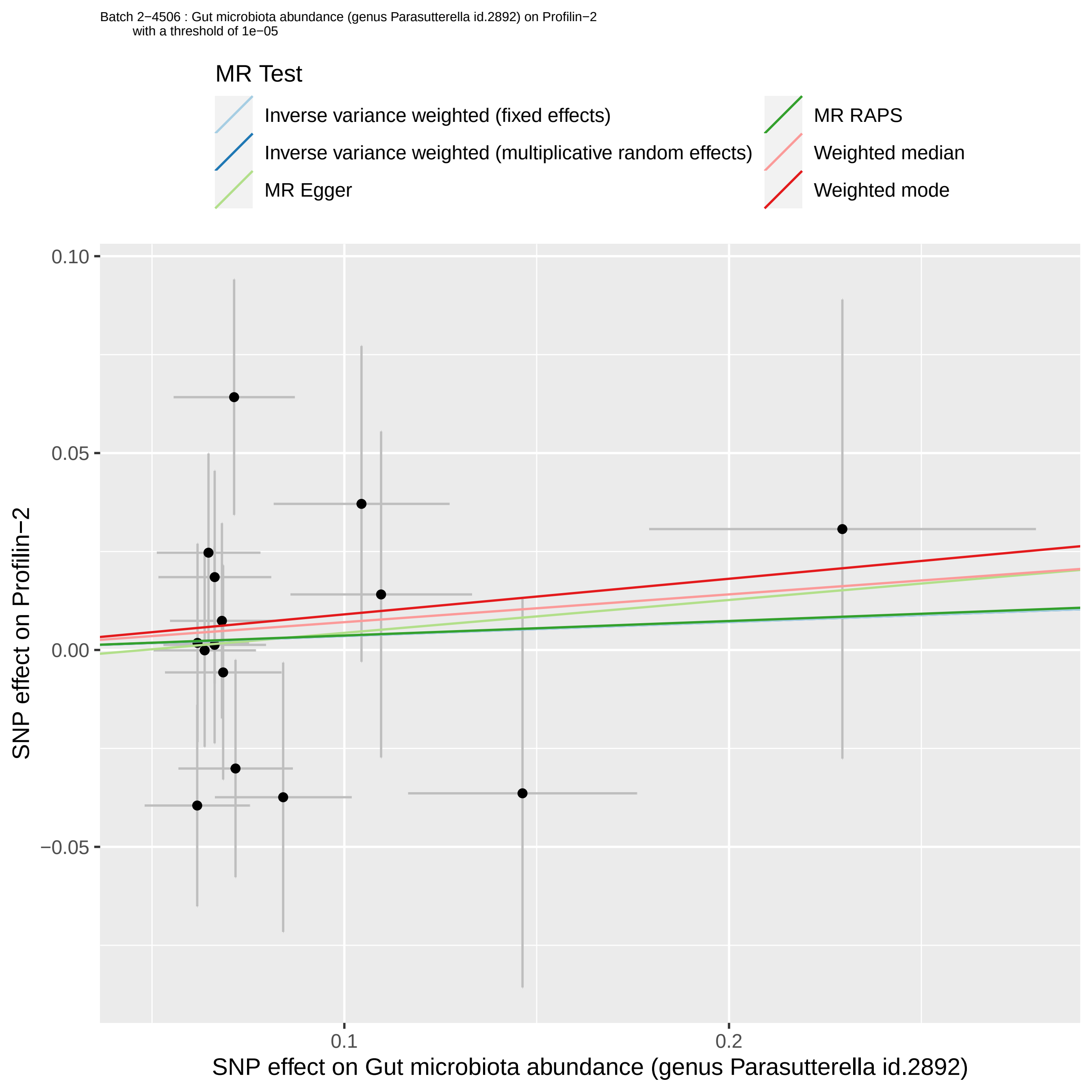

Batch 2−4506 : Gut microbiota abundance (genus Parasutterella id.2892) on Profilin−2
with a threshold of 1e−05
MR Test
Inverse variance weighted (fixed effects)
Inverse variance weighted (multiplicative random effects)
MR Egger
MR RAPS
Weighted median
Weighted mode
0.10
0.05
0.00
−0.05
0.1
0.2
SNP effect on Profilin−2
SNP effect on Gut microbiota abundance (genus Parasutterella id.2892)

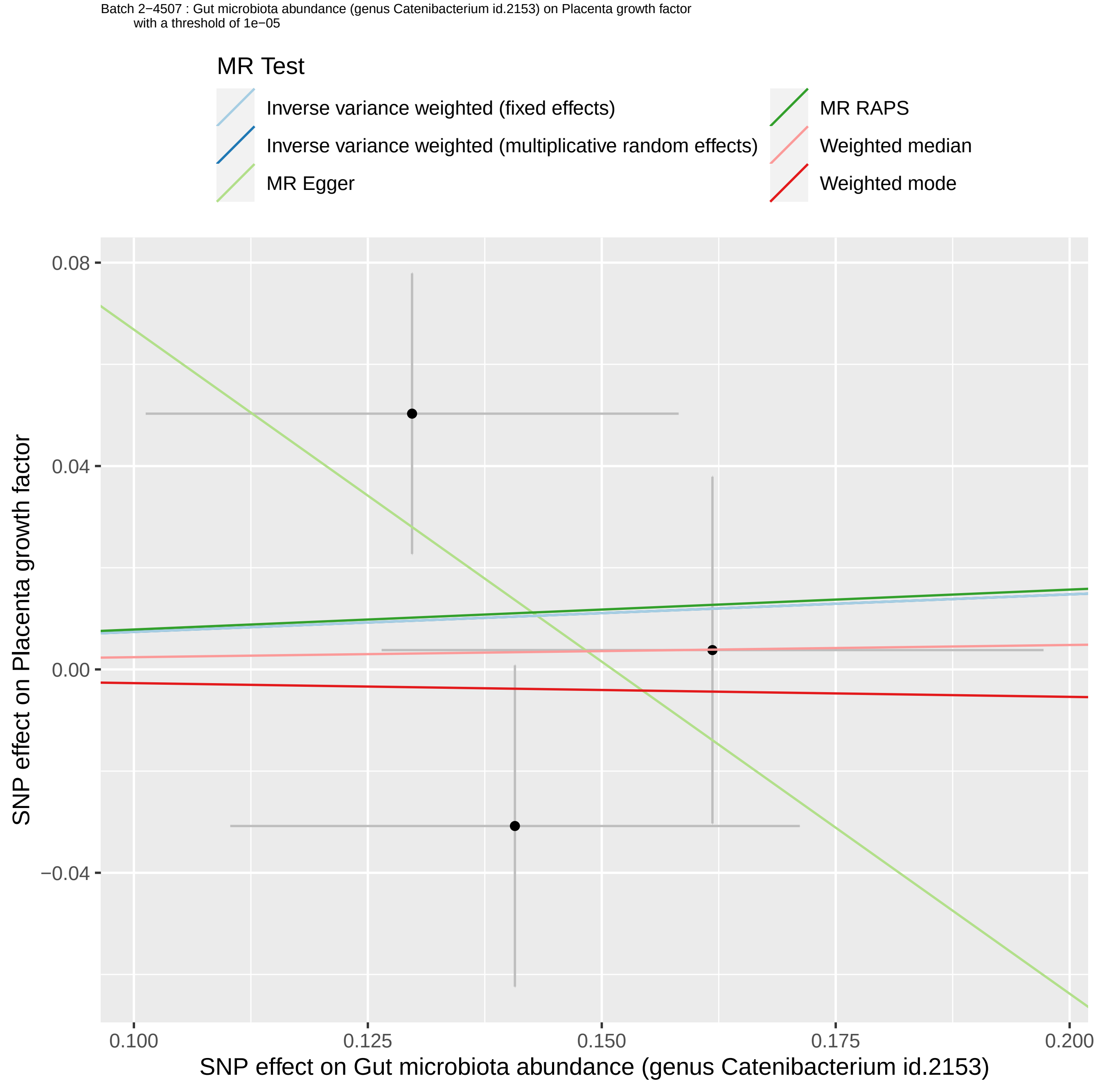

Batch 2−4507 : Gut microbiota abundance (genus Catenibacterium id.2153) on Placenta growth factor
with a threshold of 1e−05
MR Test
Inverse variance weighted (fixed effects)
Inverse variance weighted (multiplicative random effects)
MR Egger
MR RAPS
Weighted median
Weighted mode
0.08
0.04
0.00
−0.04
0.100
0.125
0.150
0.175
0.200
SNP effect on Placenta growth factor
SNP effect on Gut microbiota abundance (genus Catenibacterium id.2153)

Batch 2−4508 : Gut microbiota abundance (genus Parasutterella id.2892) on Placenta growth factor
with a threshold of 1e−05

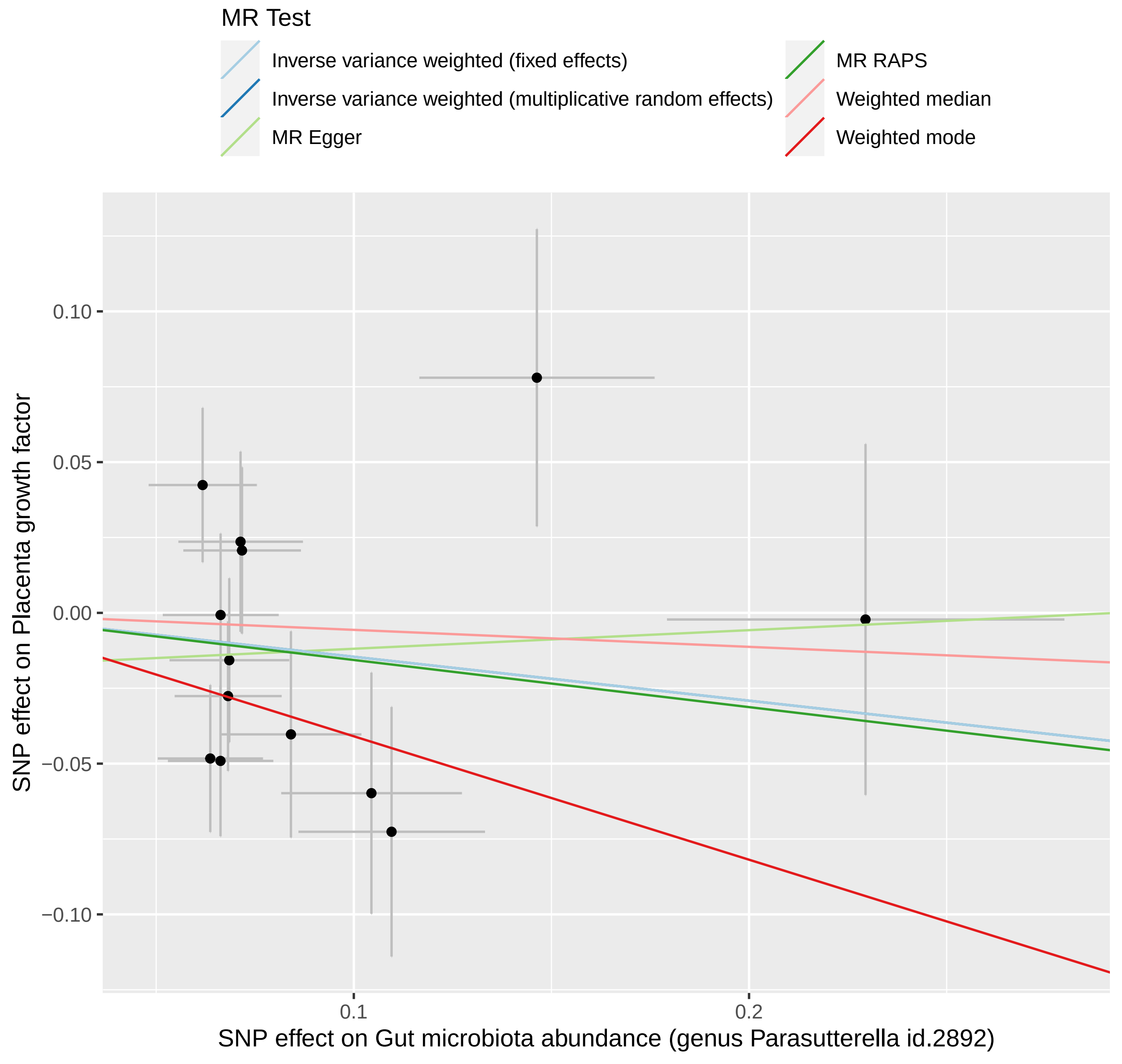

Batch 2−4509 : Gut microbiota abundance (genus Catenibacterium id.2153) on Phosphoglycerate kinase 1
with a threshold of 1e−05

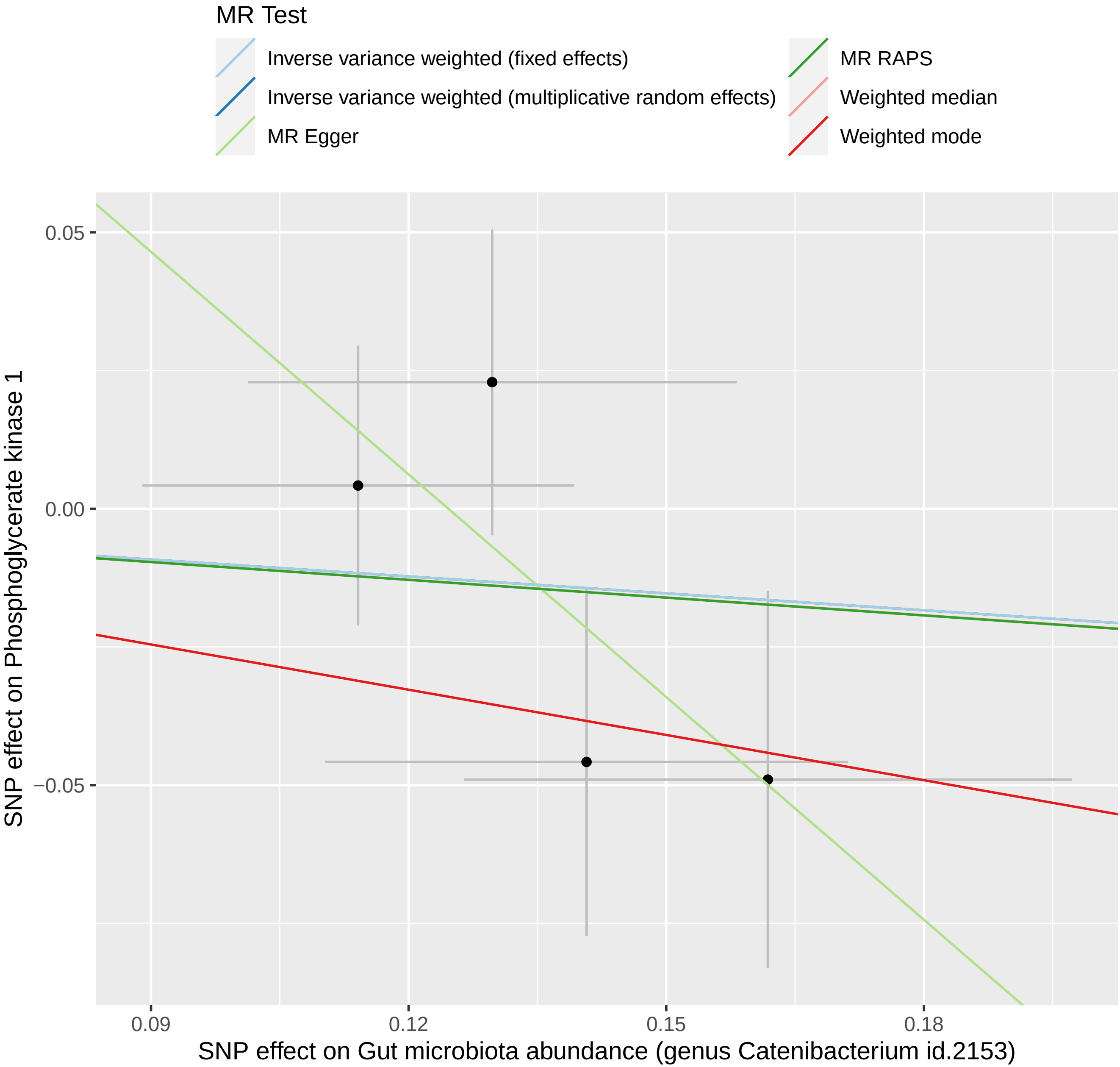

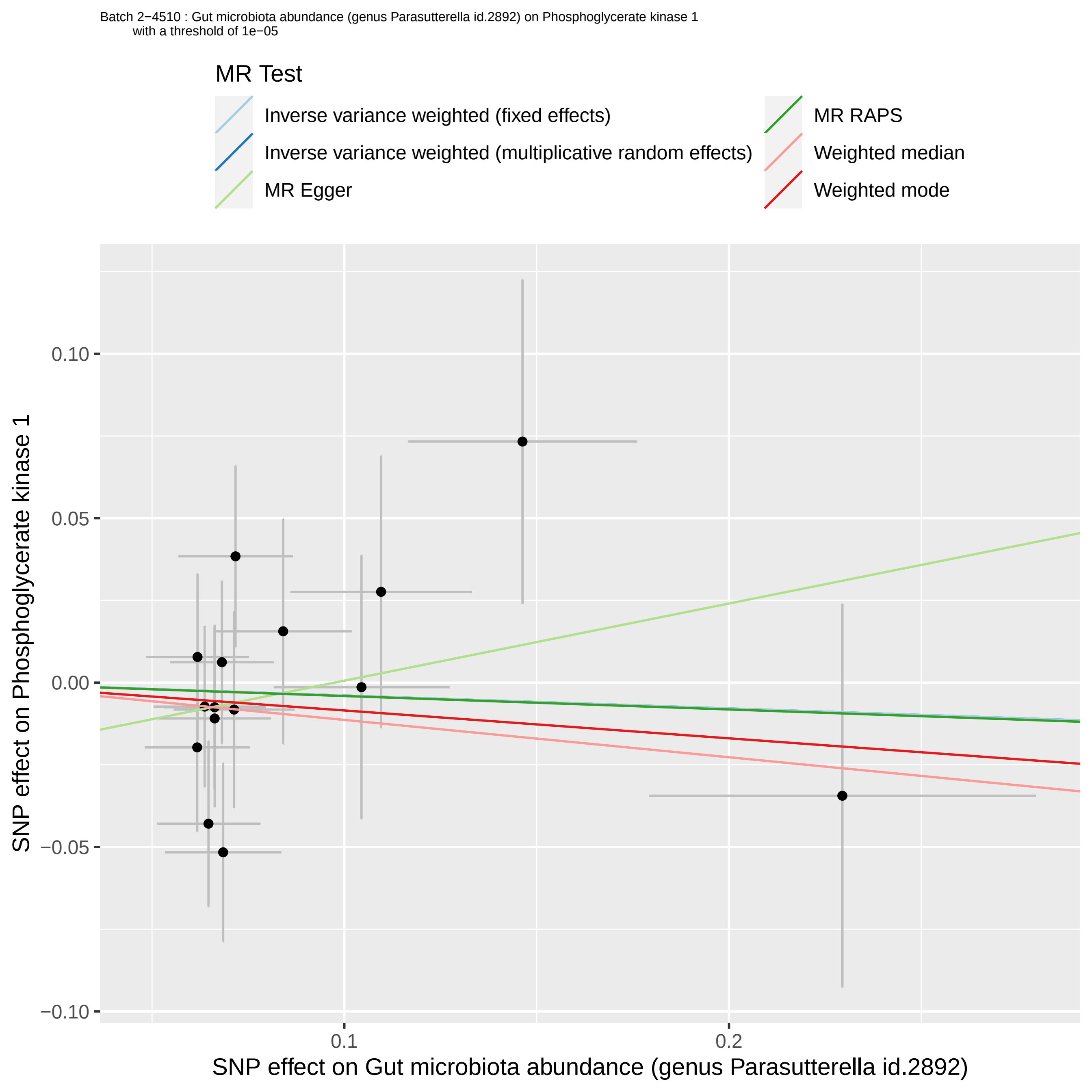
Batch 2−4510 : Gut microbiota abundance (genus Parasutterella id.2892) on Phosphoglycerate kinase 1
with a threshold of 1e−05
MR Test
Inverse variance weighted (fixed effects)
Inverse variance weighted (multiplicative random effects)
MR Egger
MR RAPS
Weighted median
Weighted mode
0.10
0.05
0.00
−0.05
−0.10
0.1
0.2
SNP effect on Phosphoglycerate kinase 1
SNP effect on Gut microbiota abundance (genus Parasutterella id.2892)

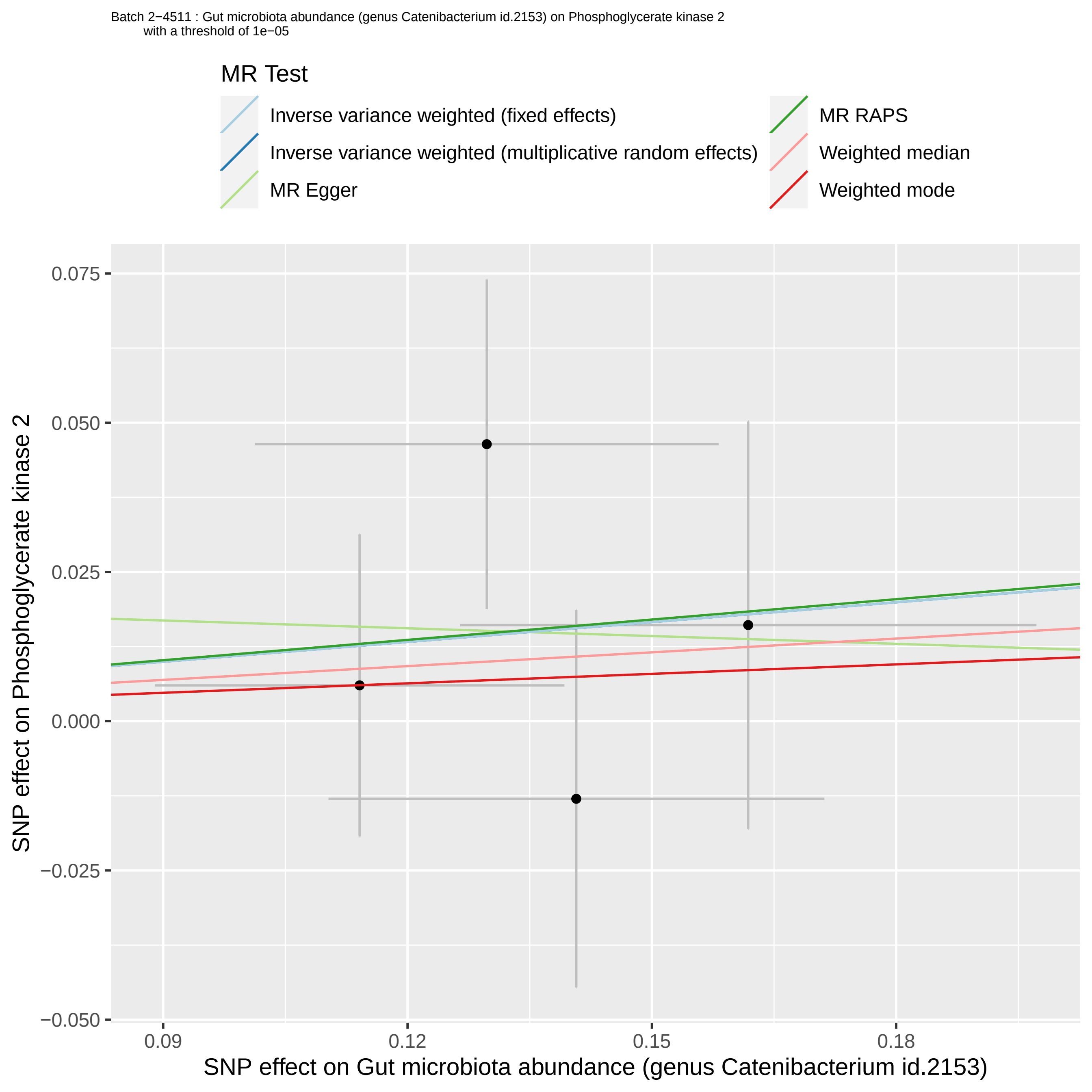
Batch 2−4511 : Gut microbiota abundance (genus Catenibacterium id.2153) on Phosphoglycerate kinase 2
with a threshold of 1e−05
MR Test
Inverse variance weighted (fixed effects)
Inverse variance weighted (multiplicative random effects)
MR Egger
MR RAPS
Weighted median
Weighted mode
0.075
0.050
0.025
0.000
−0.025
−0.050
0.09
0.12
0.15
0.18
SNP effect on Phosphoglycerate kinase 2
SNP effect on Gut microbiota abundance (genus Catenibacterium id.2153)

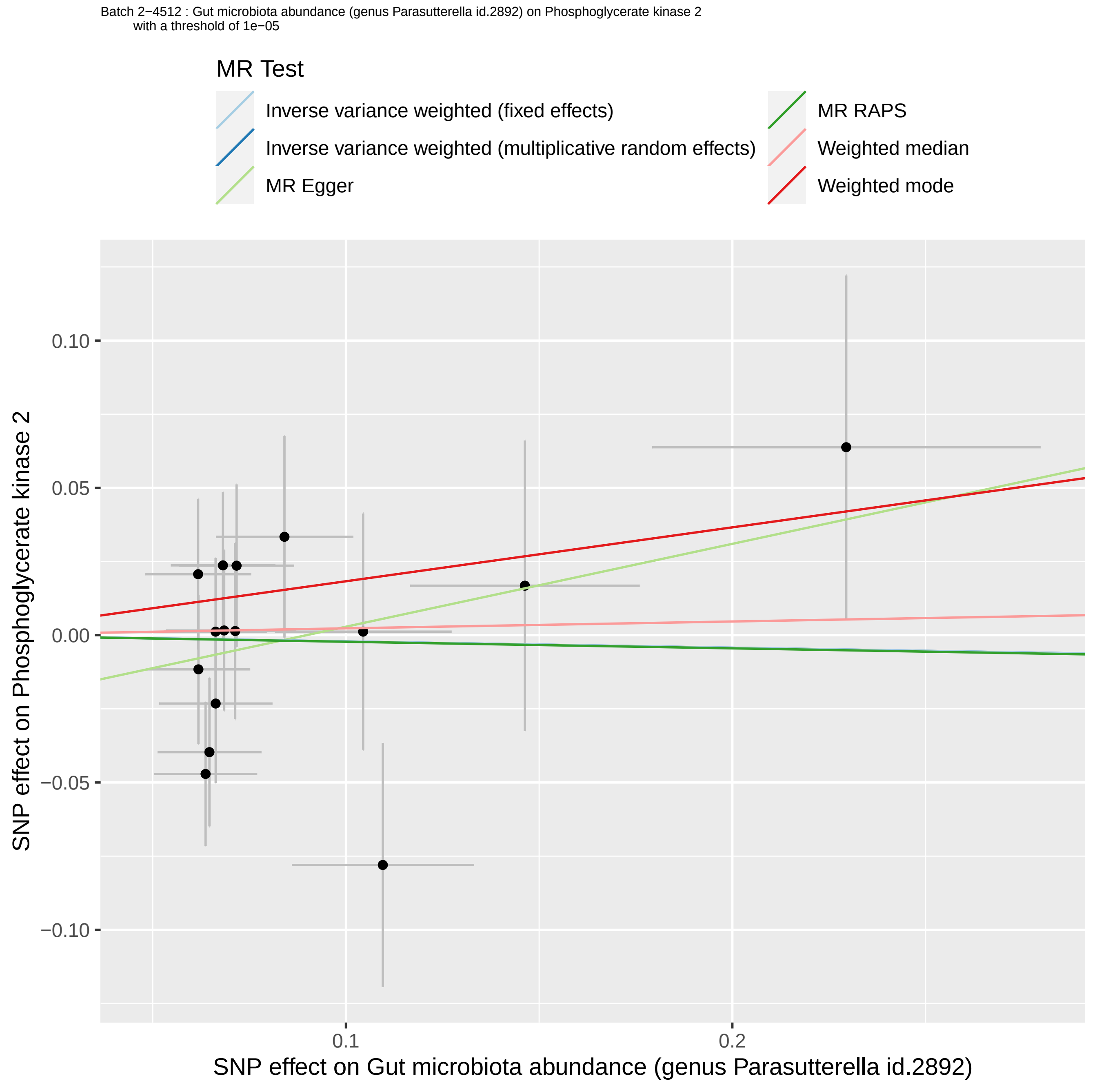

Batch 2−4512 : Gut microbiota abundance (genus Parasutterella id.2892) on Phosphoglycerate kinase 2
with a threshold of 1e−05
MR Test
Inverse variance weighted (fixed effects)
Inverse variance weighted (multiplicative random effects)
MR Egger
MR RAPS
Weighted median
Weighted mode
0.10
0.05
0.00
−0.05
−0.10
0.1
0.2
SNP effect on Phosphoglycerate kinase 2
SNP effect on Gut microbiota abundance (genus Parasutterella id.2892)

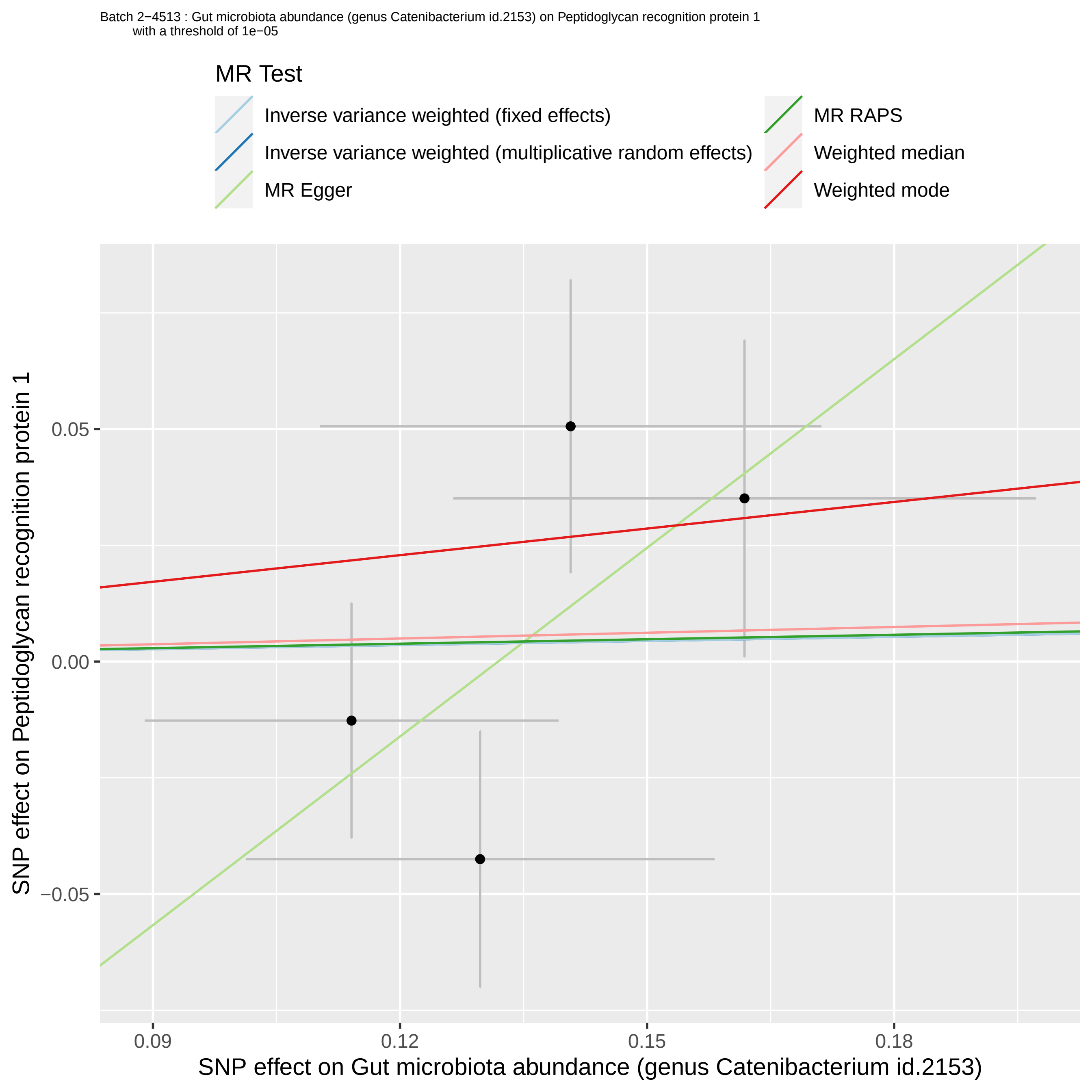
Batch 2−4513 : Gut microbiota abundance (genus Catenibacterium id.2153) on Peptidoglycan recognition protein 1
with a threshold of 1e−05
MR Test
Inverse variance weighted (fixed effects)
Inverse variance weighted (multiplicative random effects)
MR Egger
MR RAPS
Weighted median
Weighted mode
0.05
0.00
−0.05
0.09
0.12
0.15
0.18
SNP effect on Peptidoglycan recognition protein 1
SNP effect on Gut microbiota abundance (genus Catenibacterium id.2153)

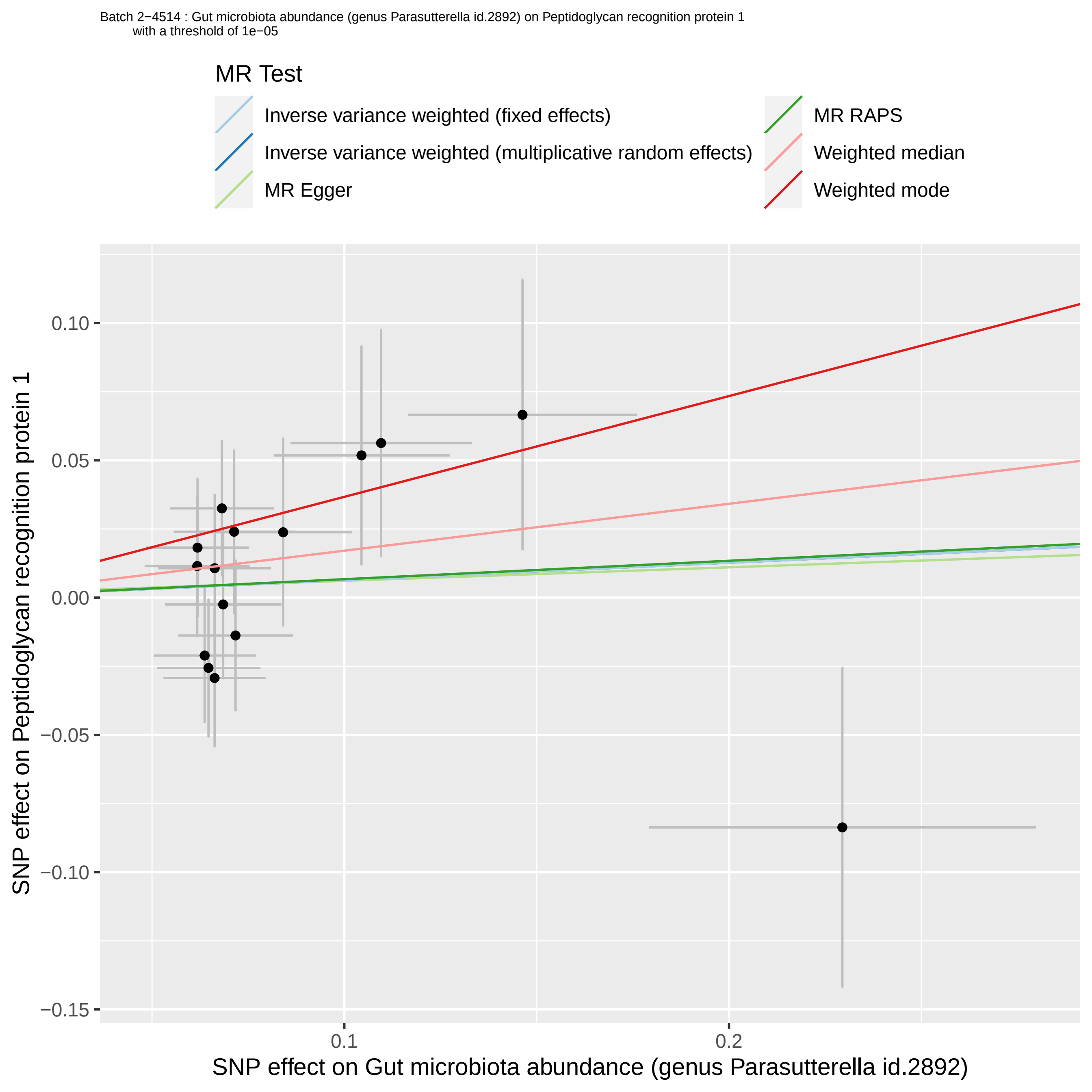
Batch 2−4514 : Gut microbiota abundance (genus Parasutterella id.2892) on Peptidoglycan recognition protein 1
with a threshold of 1e−05
MR Test
Inverse variance weighted (fixed effects)
Inverse variance weighted (multiplicative random effects)
MR Egger
MR RAPS
Weighted median
Weighted mode
0.10
0.05
0.00
−0.05
−0.10
−0.15
0.1
0.2
SNP effect on Peptidoglycan recognition protein 1
SNP effect on Gut microbiota abundance (genus Parasutterella id.2892)

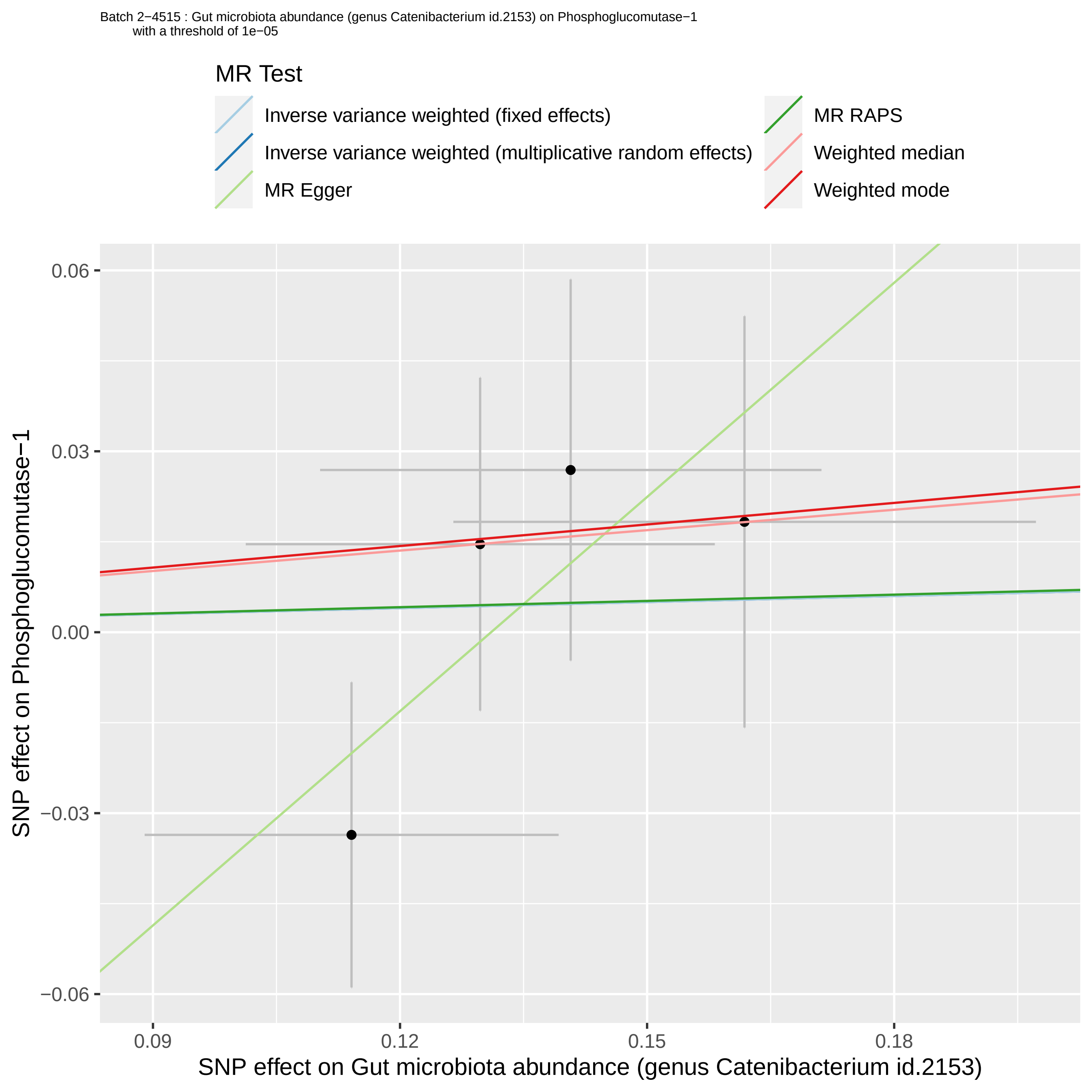
Batch 2−4515 : Gut microbiota abundance (genus Catenibacterium id.2153) on Phosphoglucomutase−1
with a threshold of 1e−05
MR Test
Inverse variance weighted (fixed effects)
Inverse variance weighted (multiplicative random effects)
MR Egger
MR RAPS
Weighted median
Weighted mode
0.06
0.03
0.00
−0.03
−0.06
0.09
0.12
0.15
0.18
SNP effect on Phosphoglucomutase−1
SNP effect on Gut microbiota abundance (genus Catenibacterium id.2153)

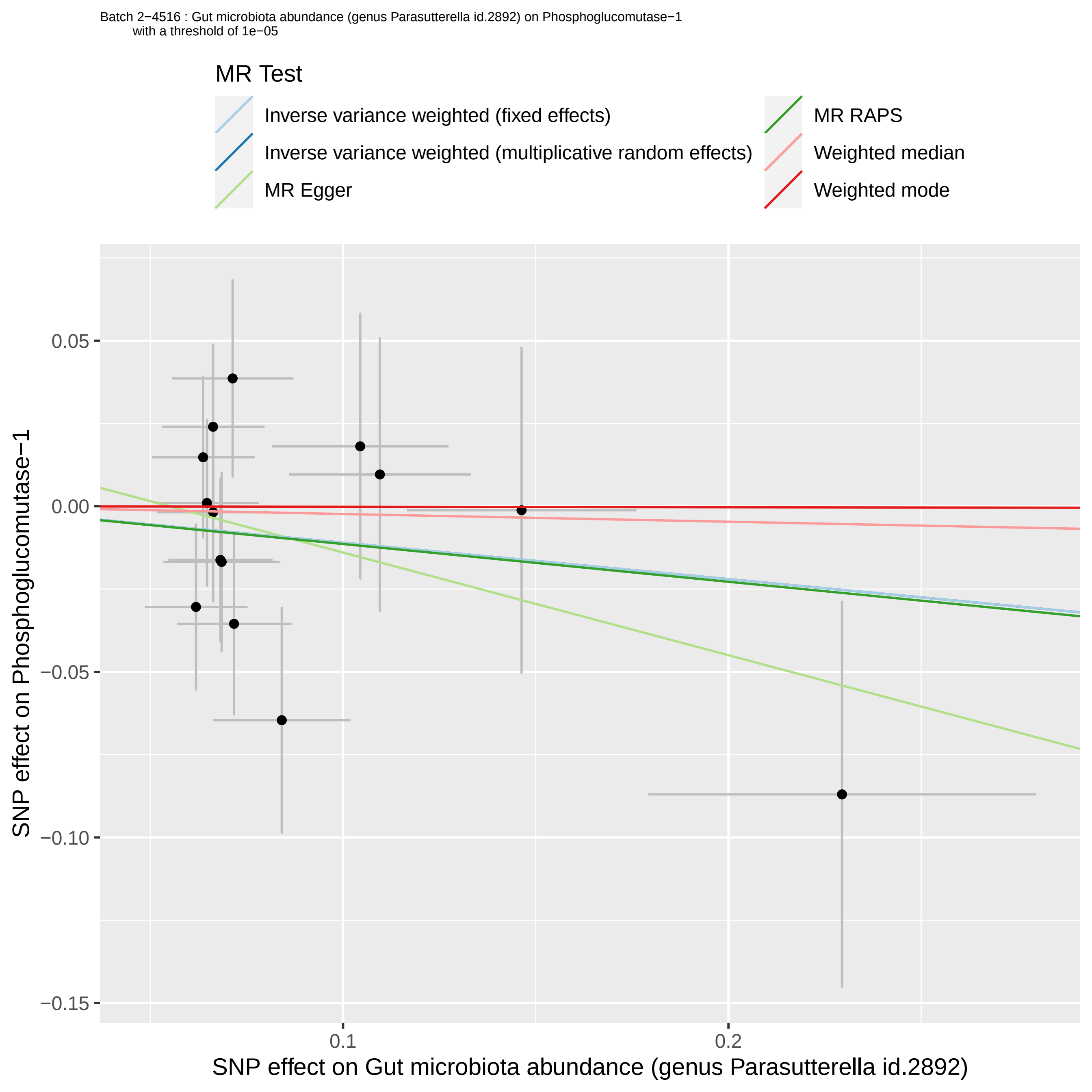
Batch 2−4516 : Gut microbiota abundance (genus Parasutterella id.2892) on Phosphoglucomutase−1
with a threshold of 1e−05
MR Test
Inverse variance weighted (fixed effects)
Inverse variance weighted (multiplicative random effects)
MR Egger
MR RAPS
Weighted median
Weighted mode
0.05
0.00
−0.05
−0.10
−0.15
0.1
0.2
SNP effect on Phosphoglucomutase−1
SNP effect on Gut microbiota abundance (genus Parasutterella id.2892)

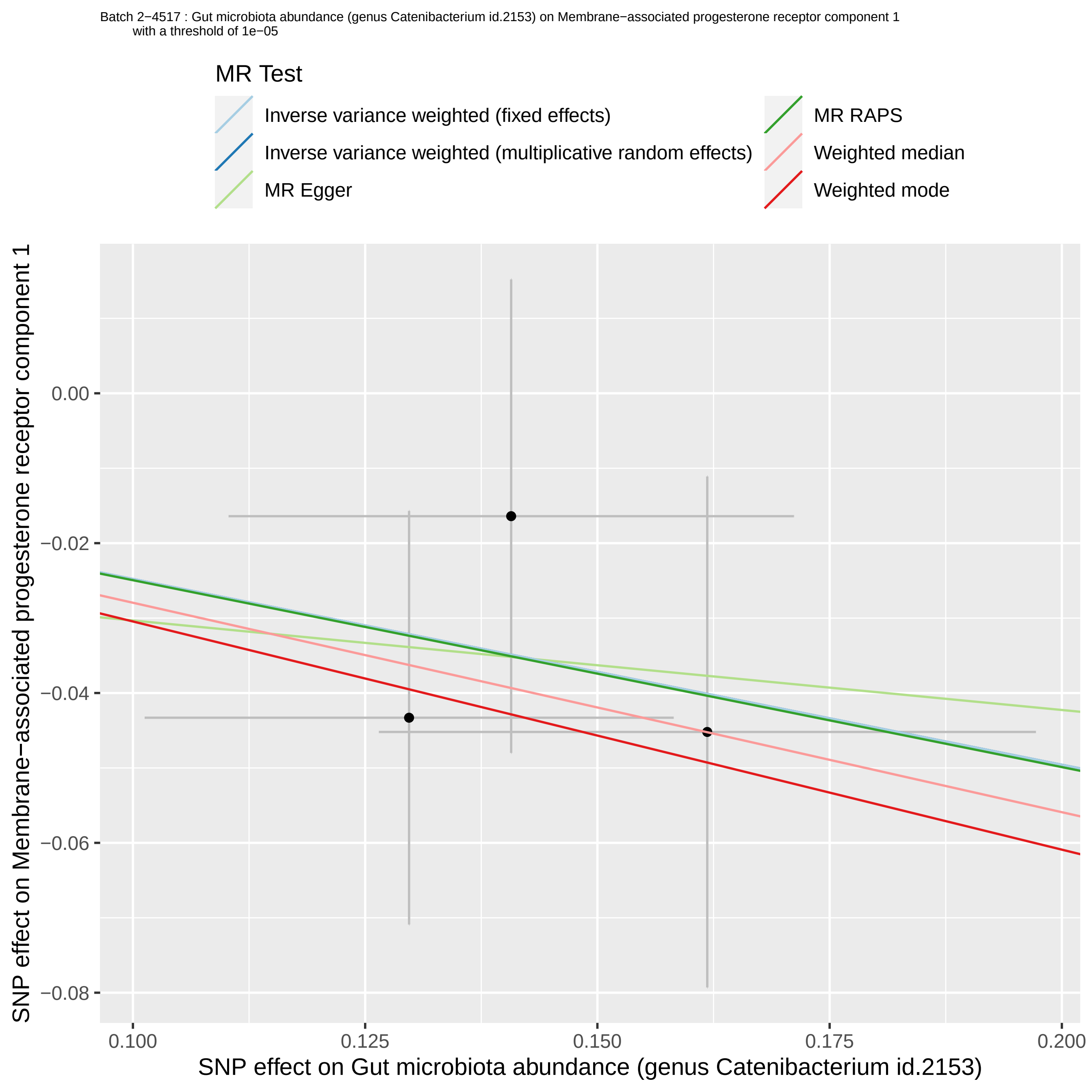
Batch 2−4517 : Gut microbiota abundance (genus Catenibacterium id.2153) on Membrane−associated progesterone receptor component 1
with a threshold of 1e−05
MR Test
Inverse variance weighted (fixed effects)
Inverse variance weighted (multiplicative random effects)
MR Egger
MR RAPS
Weighted median
Weighted mode
SNP effect on Membrane−associated progesterone receptor component 1
0.00
−0.02
−0.04
−0.06
−0.08
0.100
0.125
0.150
0.175
0.200
SNP effect on Gut microbiota abundance (genus Catenibacterium id.2153)

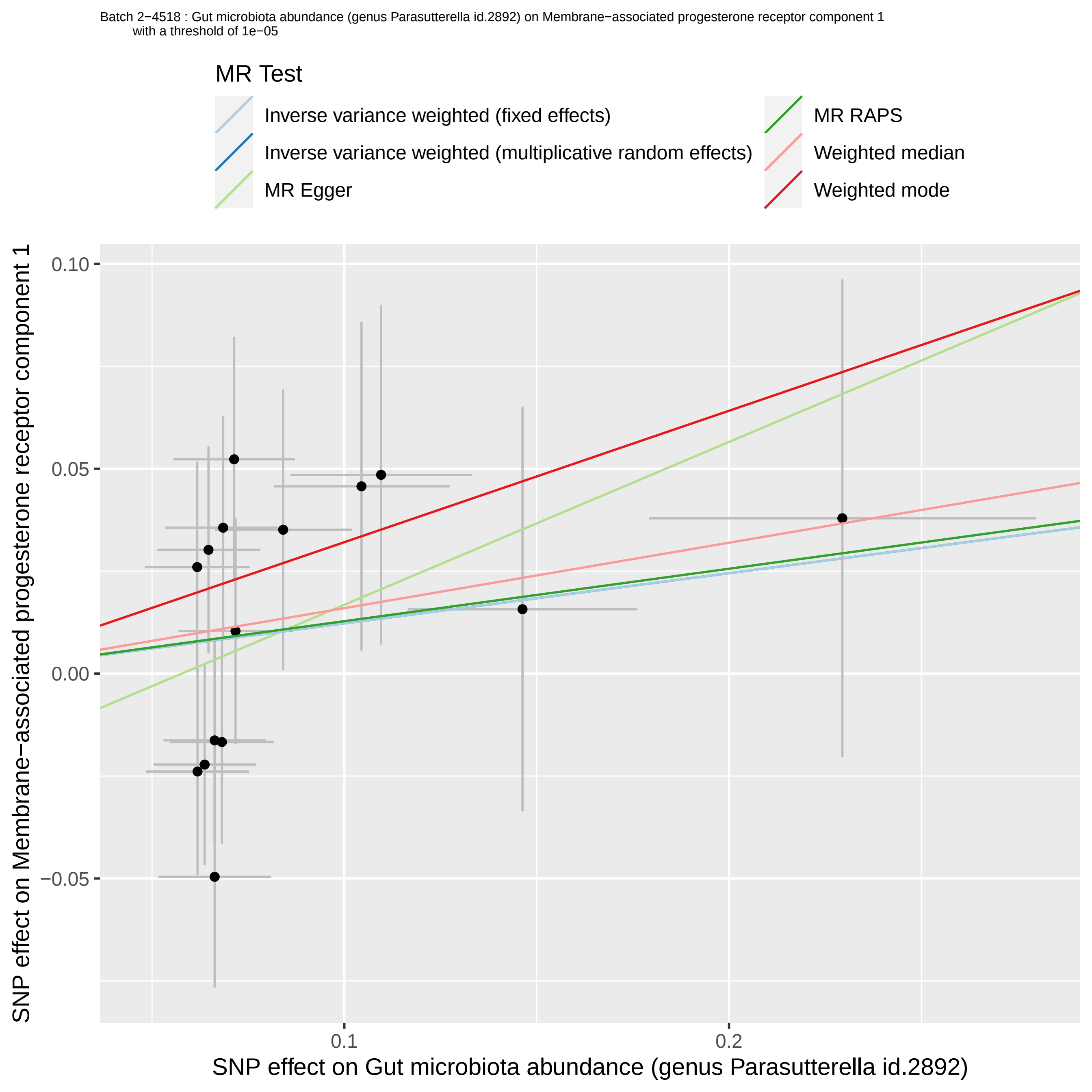
Batch 2−4518 : Gut microbiota abundance (genus Parasutterella id.2892) on Membrane−associated progesterone receptor component 1
with a threshold of 1e−05
MR Test
Inverse variance weighted (fixed effects)
Inverse variance weighted (multiplicative random effects)
MR Egger
MR RAPS
Weighted median
Weighted mode
0.10
0.05
0.00
−0.05
0.1
0.2
SNP effect on Membrane−associated progesterone receptor component 1
SNP effect on Gut microbiota abundance (genus Parasutterella id.2892)

Batch 2−4519 : Gut microbiota abundance (genus Catenibacterium id.2153) on Membrane−associated progesterone receptor component 2 with a threshold of 1e−05

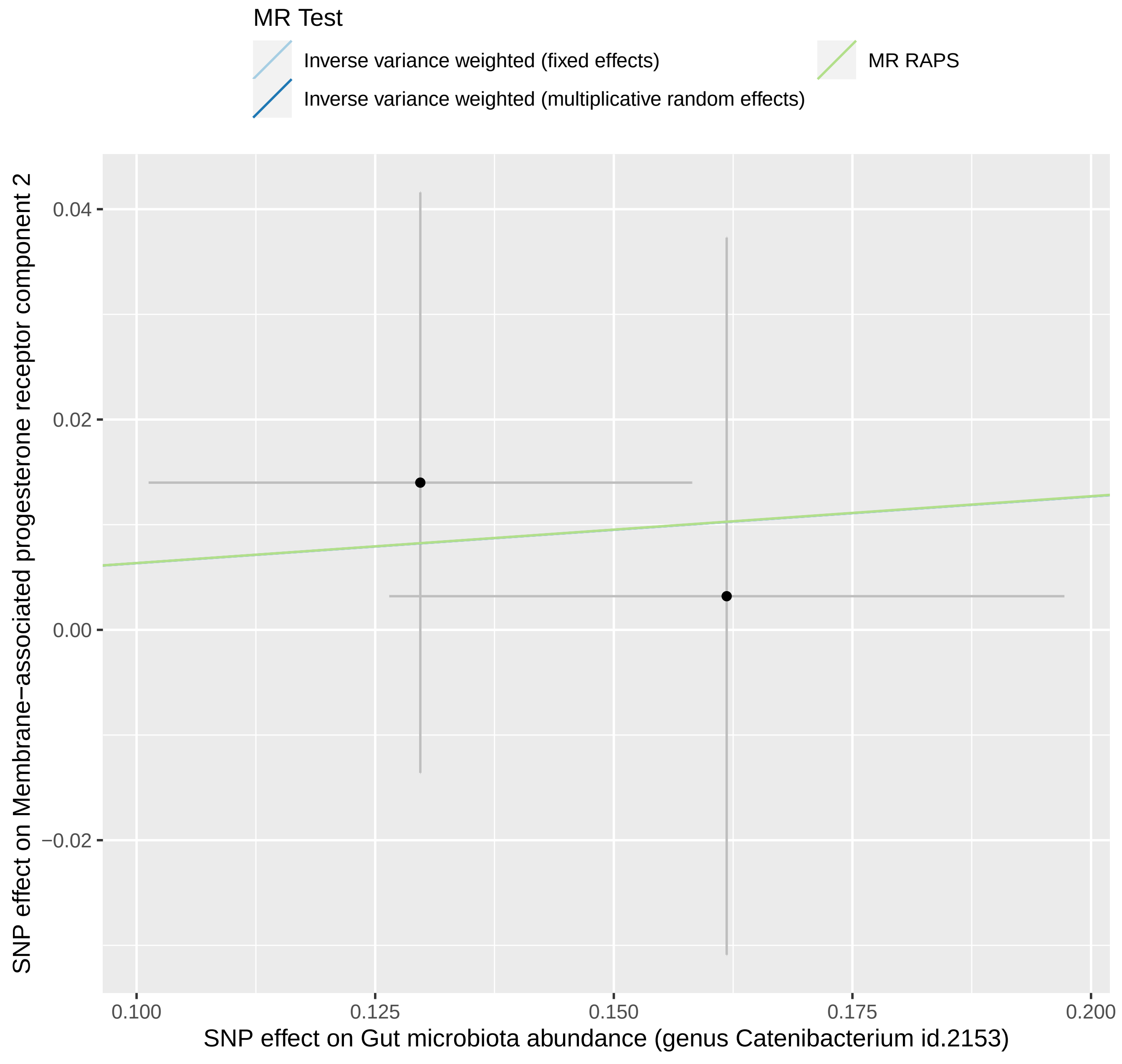

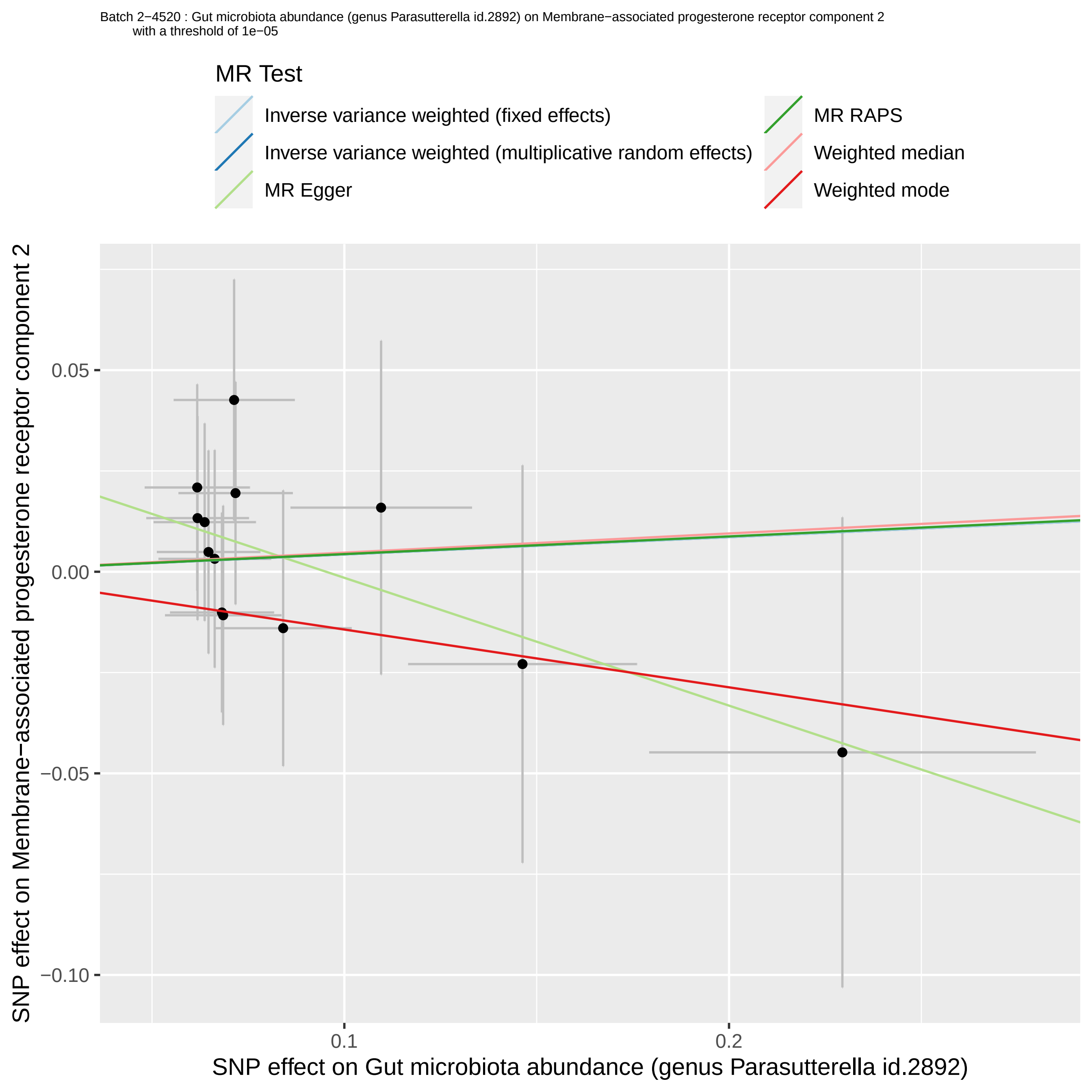

Batch 2−4520 : Gut microbiota abundance (genus Parasutterella id.2892) on Membrane−associated progesterone receptor component 2
with a threshold of 1e−05
MR Test
Inverse variance weighted (fixed effects)
Inverse variance weighted (multiplicative random effects)
MR Egger
MR RAPS
Weighted median
Weighted mode
0.05
0.00
−0.05
−0.10
0.1
0.2
SNP effect on Membrane−associated progesterone receptor component 2
SNP effect on Gut microbiota abundance (genus Parasutterella id.2892)

Batch 2−4521 : Gut microbiota abundance (genus Catenibacterium id.2153) on Membrane−associated progesterone receptor component 2
with a threshold of 1e−05

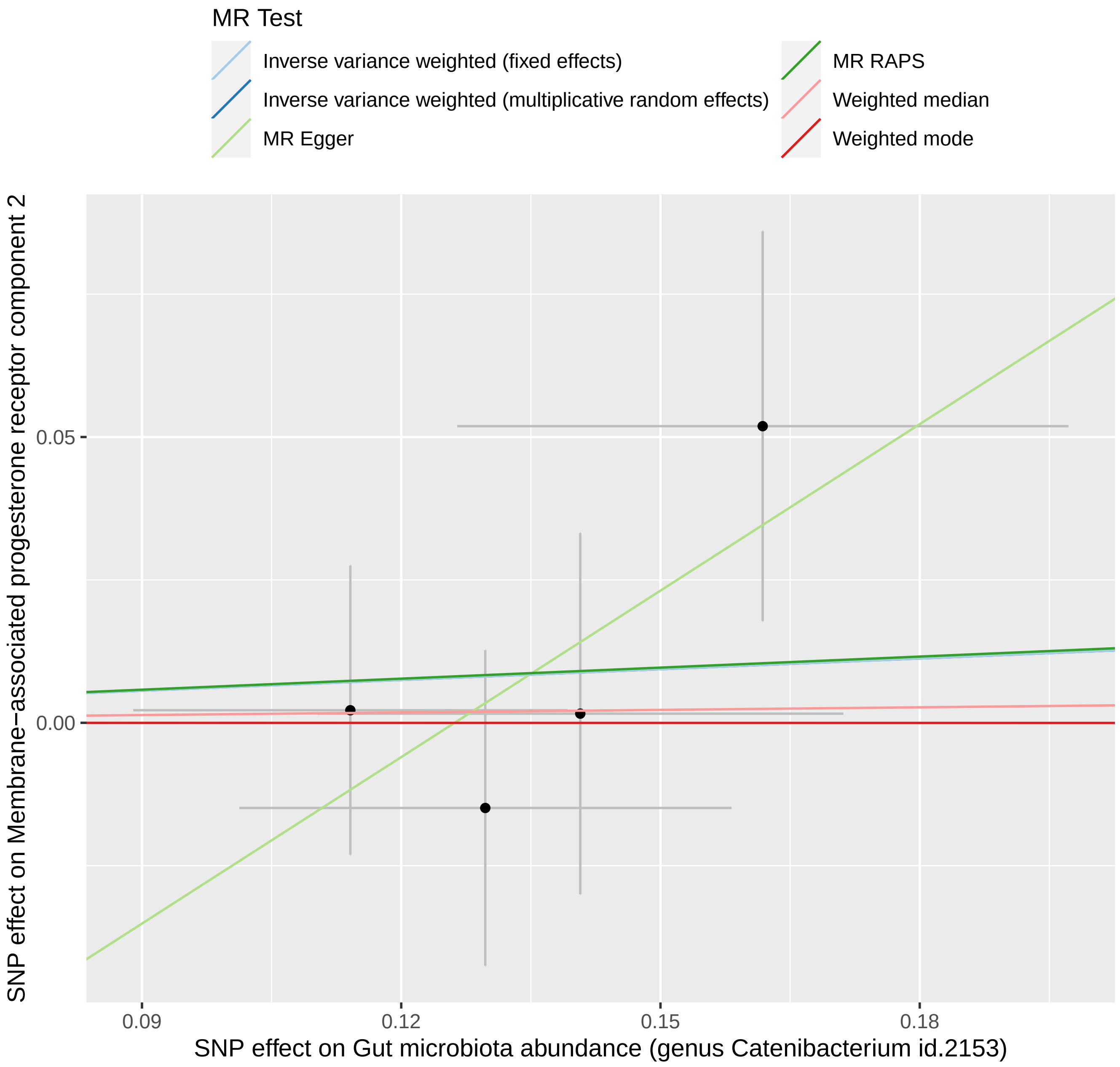

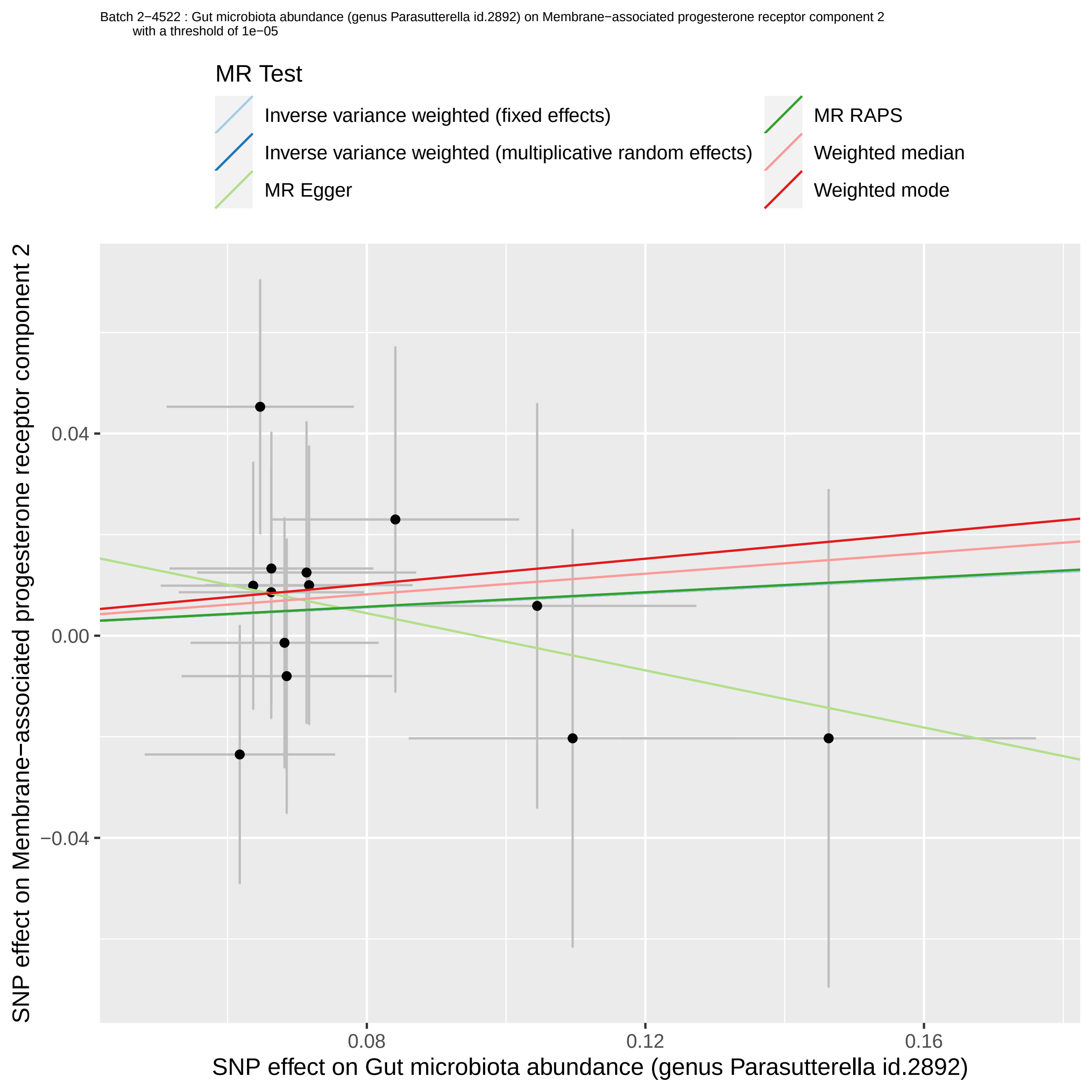

Batch 2−4522 : Gut microbiota abundance (genus Parasutterella id.2892) on Membrane−associated progesterone receptor component 2
with a threshold of 1e−05
MR Test
Inverse variance weighted (fixed effects)
Inverse variance weighted (multiplicative random effects)
MR Egger
MR RAPS
Weighted median
Weighted mode
0.04
0.00
−0.04
0.08
0.12
0.16
SNP effect on Membrane−associated progesterone receptor component 2
SNP effect on Gut microbiota abundance (genus Parasutterella id.2892)

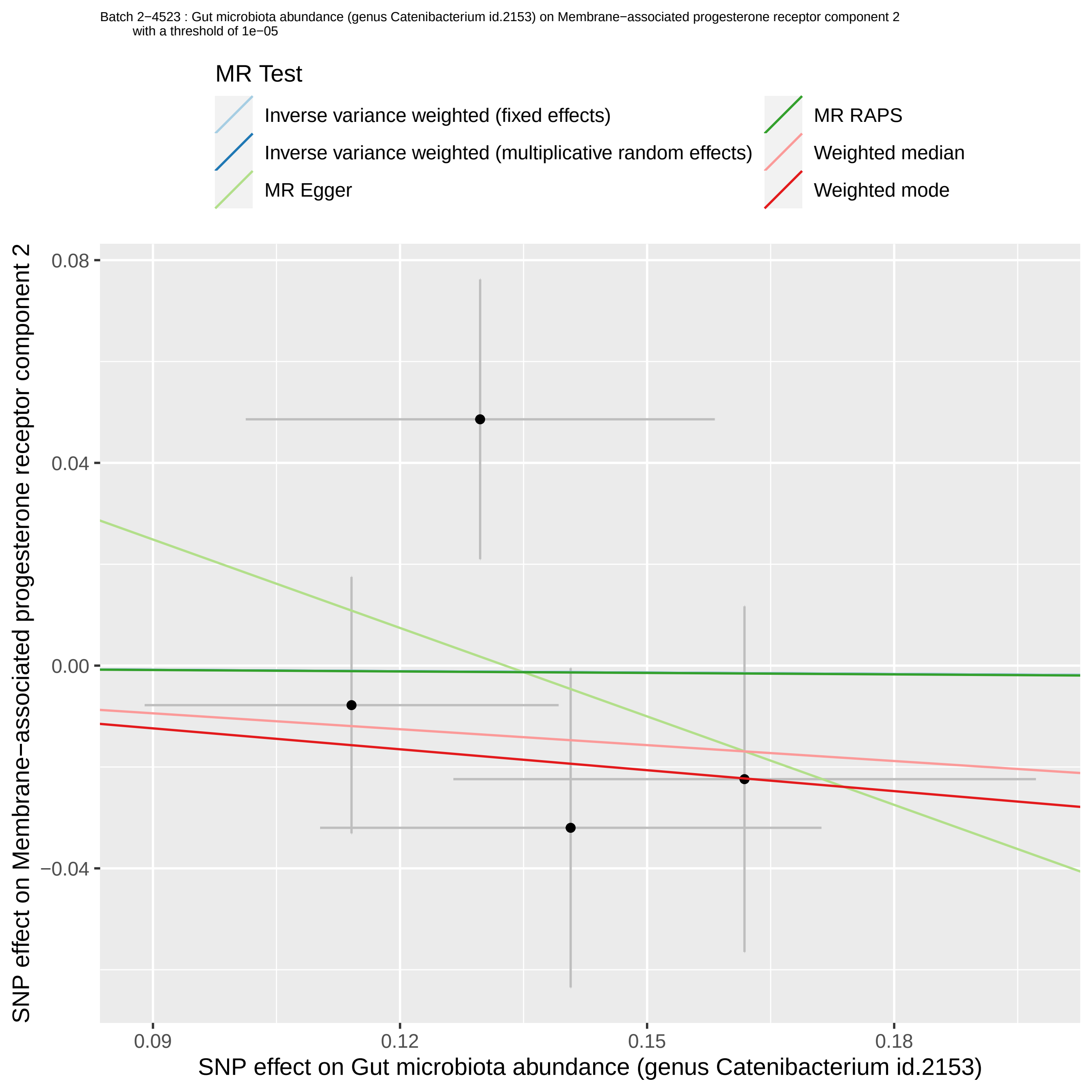
Batch 2−4523 : Gut microbiota abundance (genus Catenibacterium id.2153) on Membrane−associated progesterone receptor component 2
with a threshold of 1e−05
MR Test
Inverse variance weighted (fixed effects)
Inverse variance weighted (multiplicative random effects)
MR Egger
MR RAPS
Weighted median
Weighted mode
0.08
0.04
0.00
−0.04
0.09
0.12
0.15
0.18
SNP effect on Membrane−associated progesterone receptor component 2
SNP effect on Gut microbiota abundance (genus Catenibacterium id.2153)

Batch 2−4524 : Gut microbiota abundance (genus Parasutterella id.2892) on Membrane−associated progesterone receptor component 2
with a threshold of 1e−05

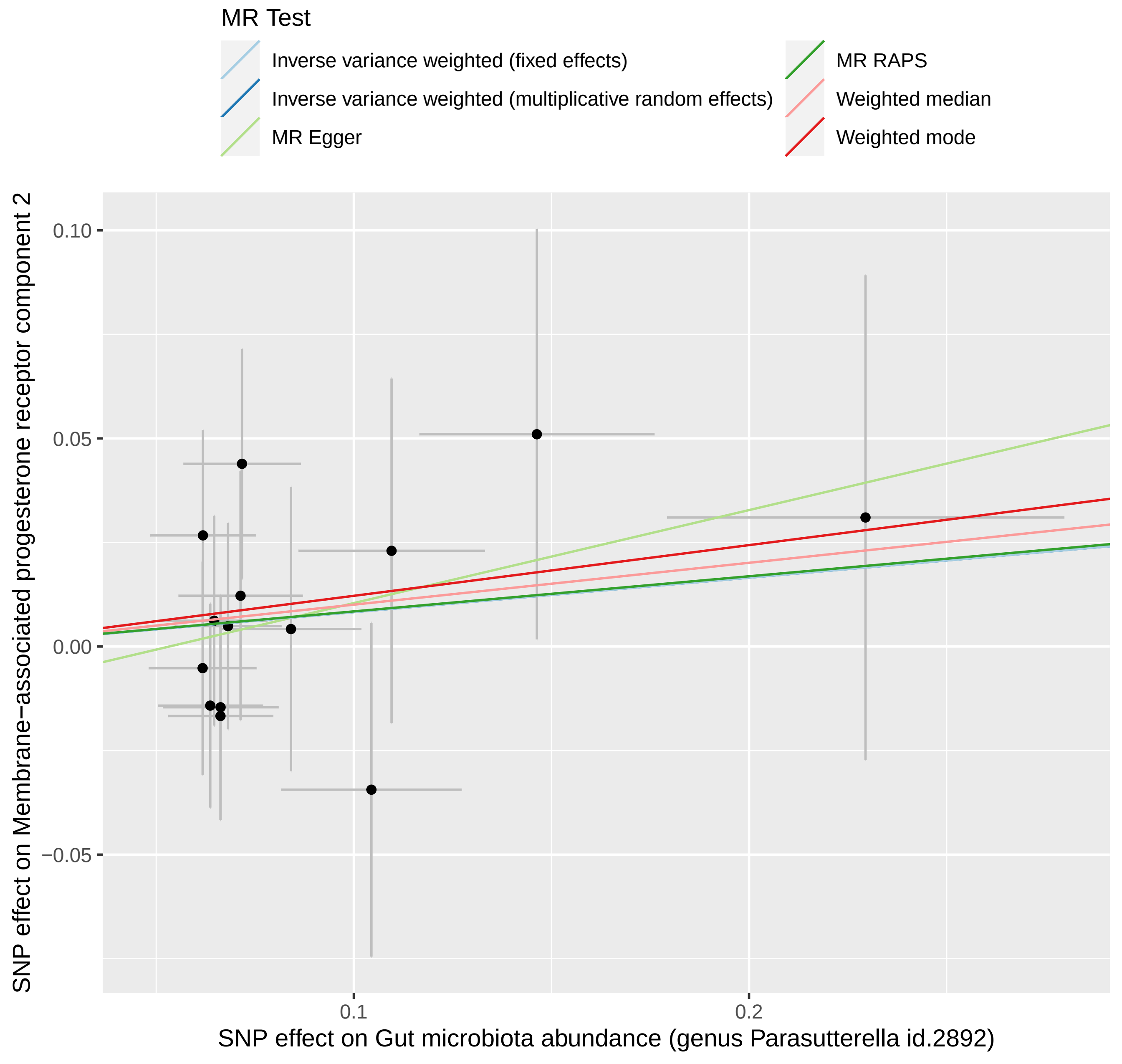

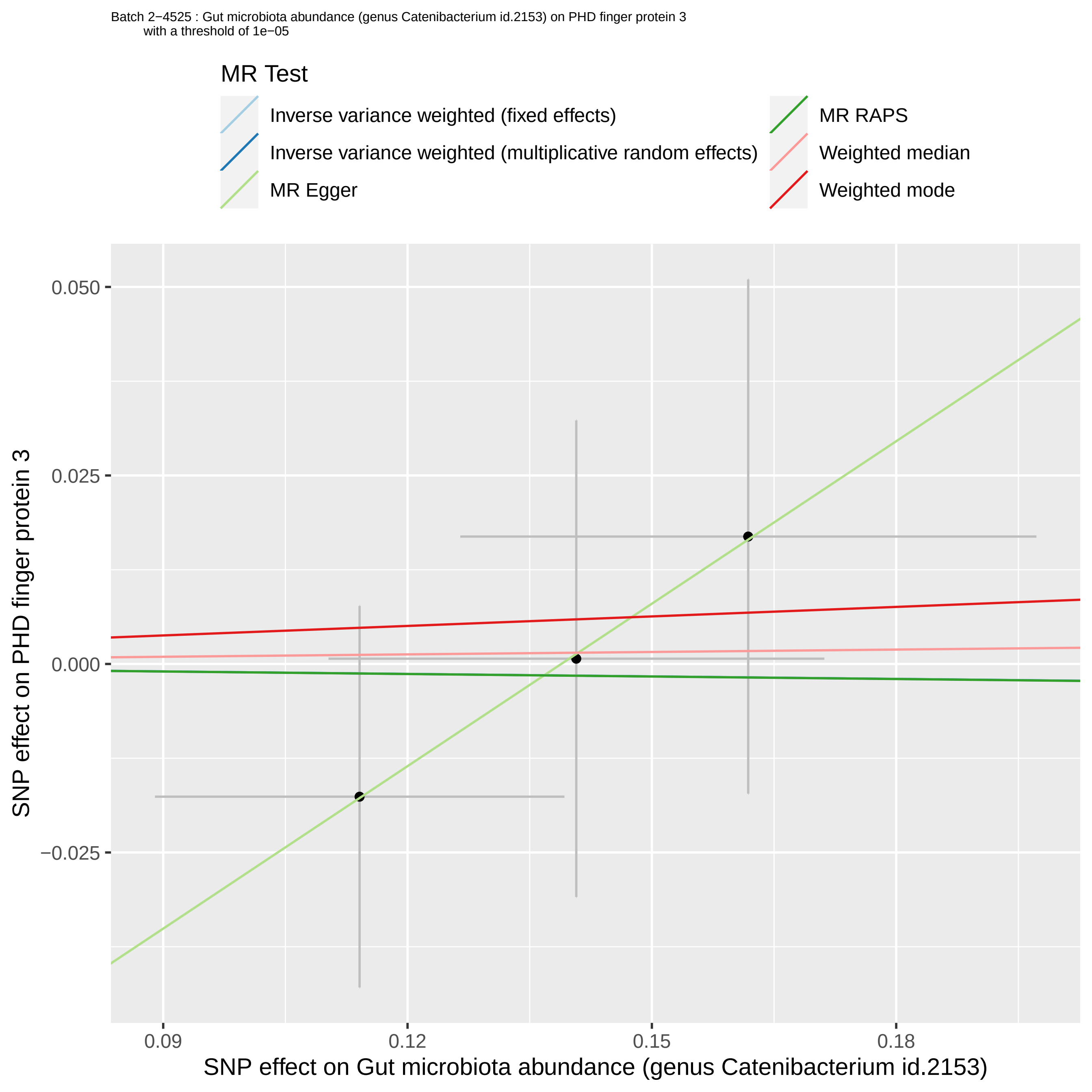

Batch 2−4525 : Gut microbiota abundance (genus Catenibacterium id.2153) on PHD finger protein 3
with a threshold of 1e−05
MR Test
Inverse variance weighted (fixed effects)
Inverse variance weighted (multiplicative random effects)
MR Egger
MR RAPS
Weighted median
Weighted mode
0.050
0.025
0.000
−0.025
0.09
0.12
0.15
0.18
SNP effect on PHD finger protein 3
SNP effect on Gut microbiota abundance (genus Catenibacterium id.2153)

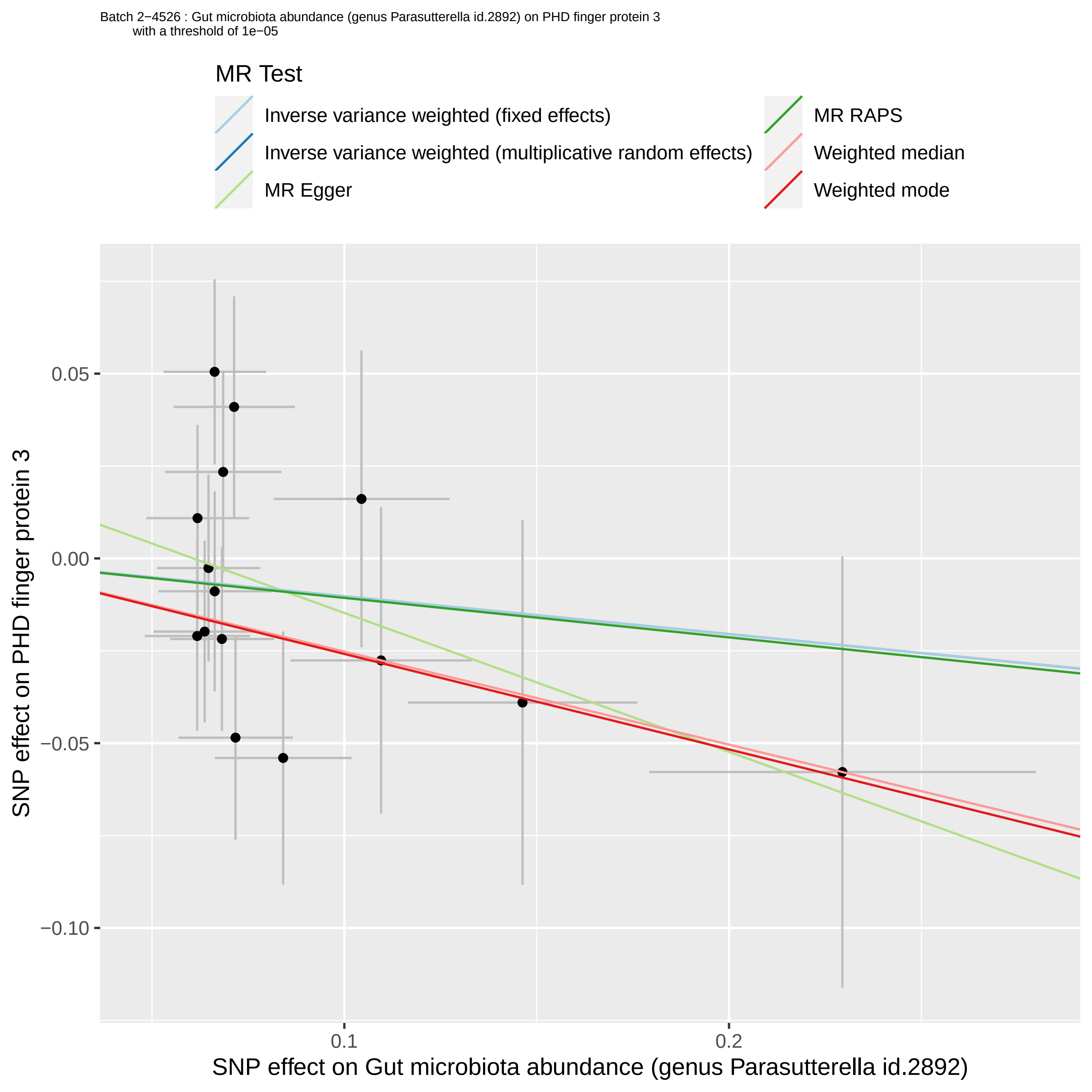

Batch 2−4526 : Gut microbiota abundance (genus Parasutterella id.2892) on PHD finger protein 3
with a threshold of 1e−05
MR Test
Inverse variance weighted (fixed effects)
Inverse variance weighted (multiplicative random effects)
MR Egger
MR RAPS
Weighted median
Weighted mode
0.05
0.00
−0.05
−0.10
0.1
0.2
SNP effect on PHD finger protein 3
SNP effect on Gut microbiota abundance (genus Parasutterella id.2892)

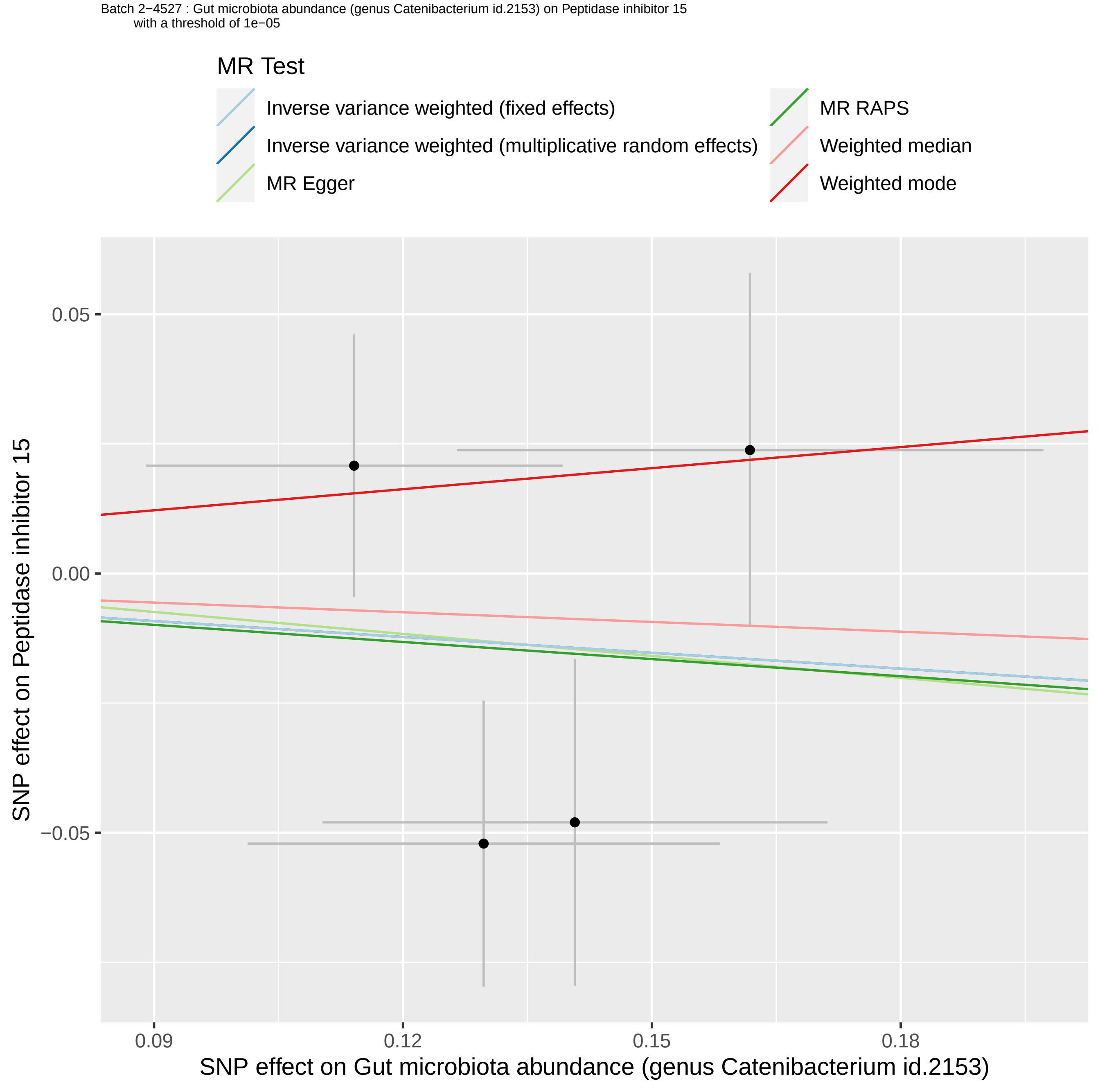
Batch 2−4527 : Gut microbiota abundance (genus Catenibacterium id.2153) on Peptidase inhibitor 15
with a threshold of 1e−05
MR Test
Inverse variance weighted (fixed effects)
Inverse variance weighted (multiplicative random effects)
MR Egger
MR RAPS
Weighted median
Weighted mode
0.05
0.00
−0.05
0.09
0.12
0.15
0.18
SNP effect on Peptidase inhibitor 15
SNP effect on Gut microbiota abundance (genus Catenibacterium id.2153)

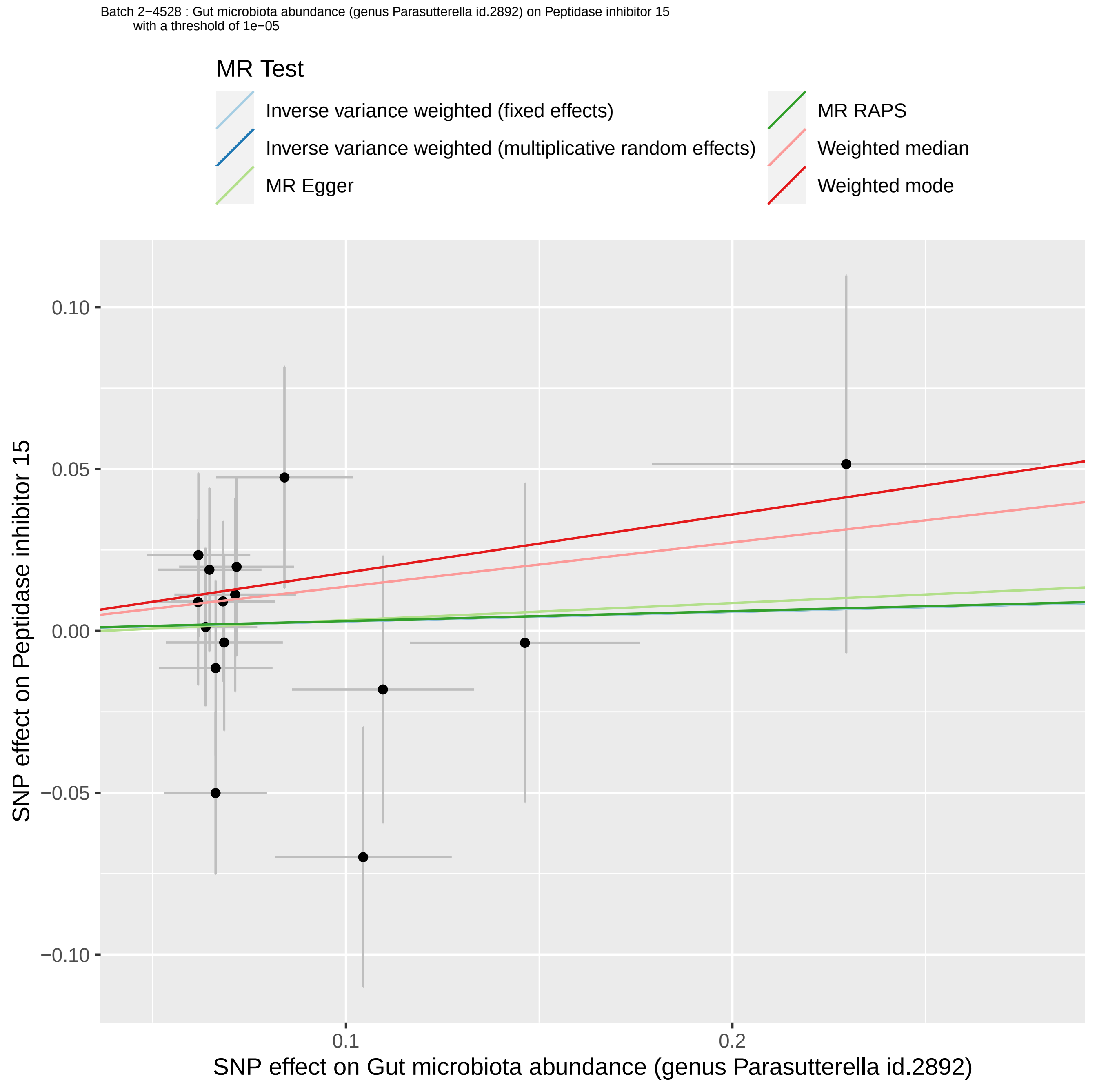
Batch 2−4528 : Gut microbiota abundance (genus Parasutterella id.2892) on Peptidase inhibitor 15
with a threshold of 1e−05
MR Test
Inverse variance weighted (fixed effects)
Inverse variance weighted (multiplicative random effects)
MR Egger
MR RAPS
Weighted median
Weighted mode
0.10
0.05
0.00
−0.05
−0.10
0.1
0.2
SNP effect on Peptidase inhibitor 15
SNP effect on Gut microbiota abundance (genus Parasutterella id.2892)

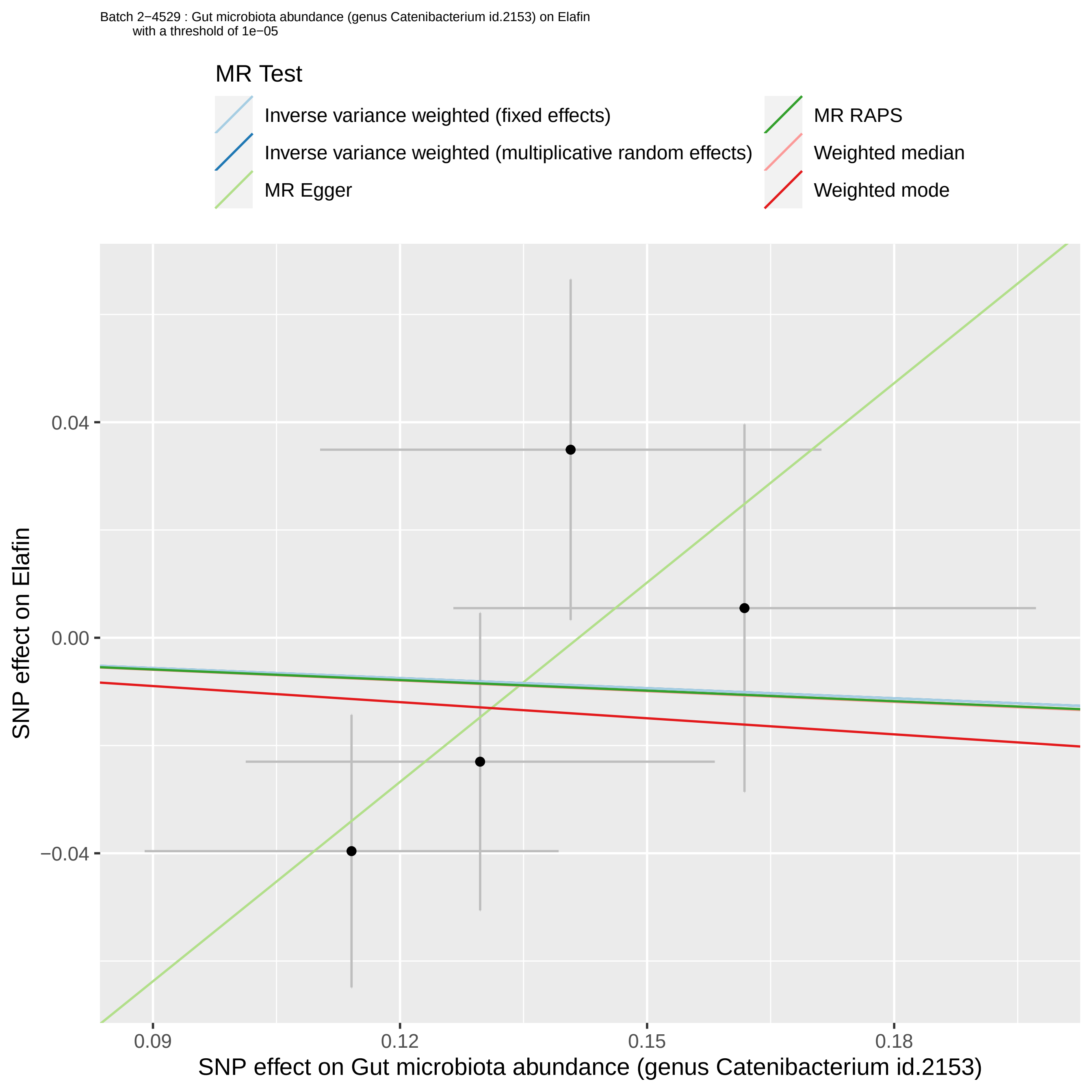

Batch 2−4529 : Gut microbiota abundance (genus Catenibacterium id.2153) on Elafin
with a threshold of 1e−05
MR Test
Inverse variance weighted (fixed effects)
Inverse variance weighted (multiplicative random effects)
MR Egger
MR RAPS
Weighted median
Weighted mode
0.04
0.00
−0.04
0.09
0.12
0.15
0.18
SNP effect on Elafin
SNP effect on Gut microbiota abundance (genus Catenibacterium id.2153)

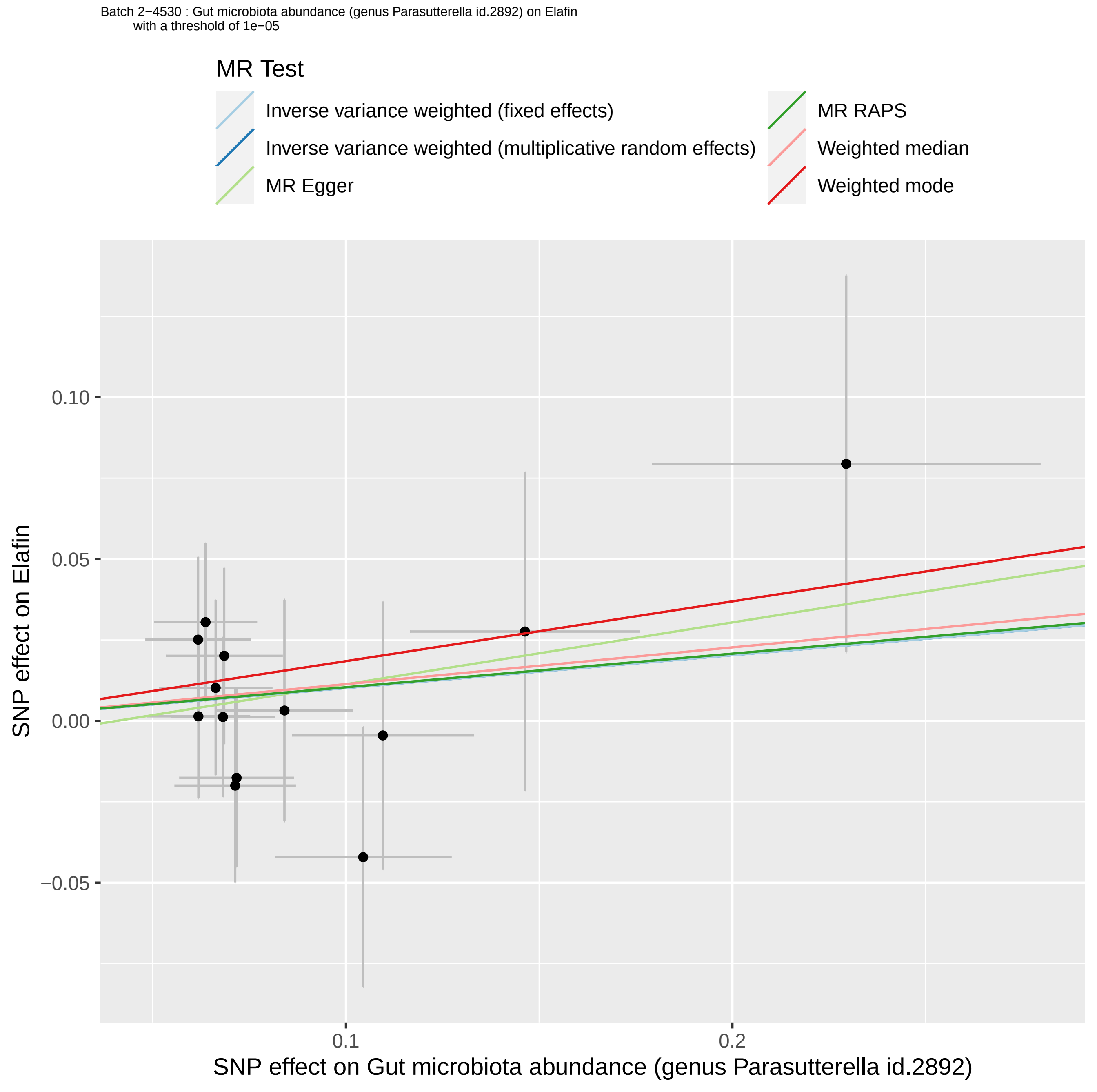

Batch 2−4530 : Gut microbiota abundance (genus Parasutterella id.2892) on Elafin
with a threshold of 1e−05
MR Test
Inverse variance weighted (fixed effects)
Inverse variance weighted (multiplicative random effects)
MR Egger
MR RAPS
Weighted median
Weighted mode
SNP effect on Elafin
0.10
0.05
0.00
−0.05
0.1
0.2
SNP effect on Gut microbiota abundance (genus Parasutterella id.2892)

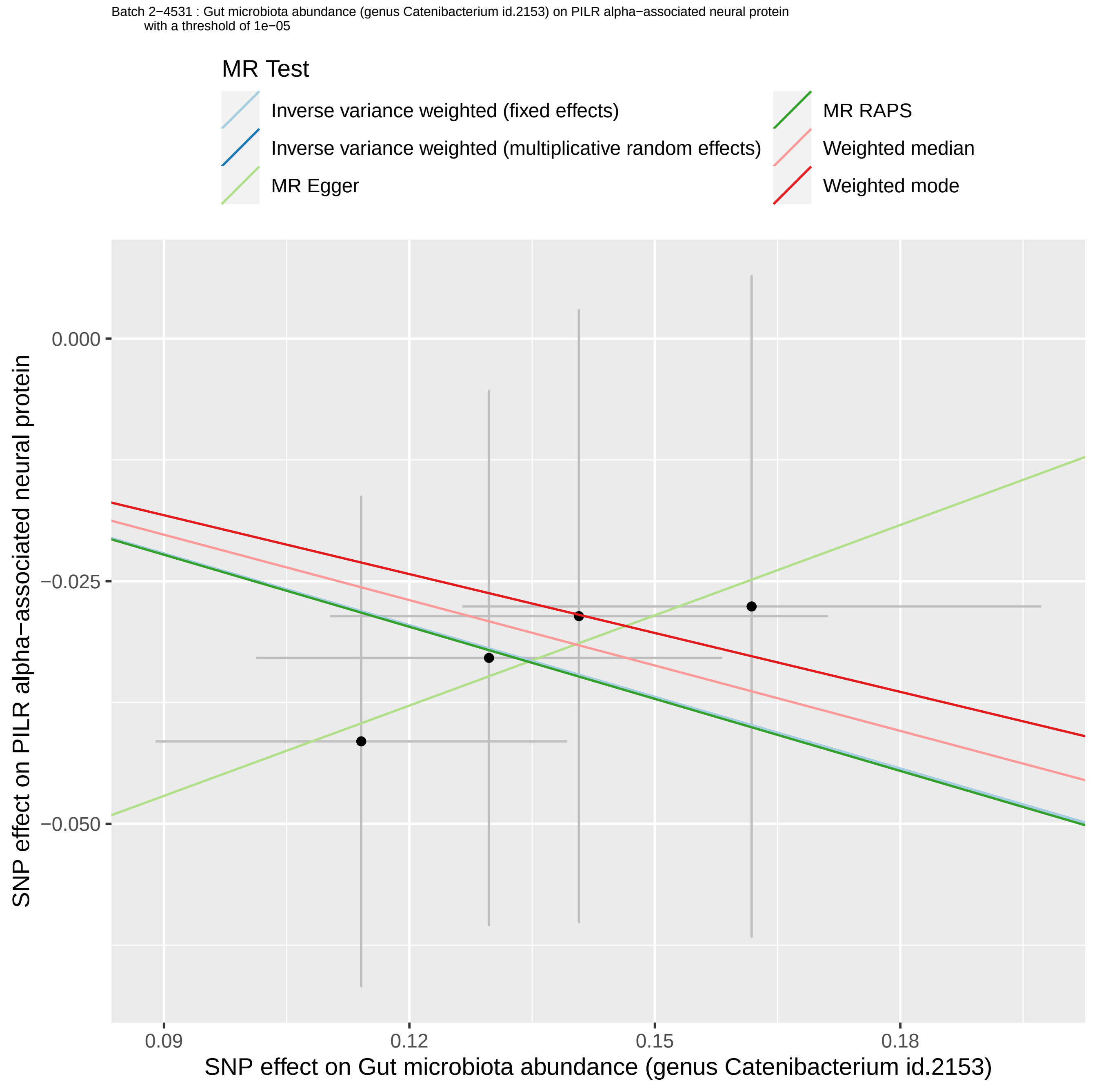
Batch 2−4531 : Gut microbiota abundance (genus Catenibacterium id.2153) on PILR alpha−associated neural protein
with a threshold of 1e−05
MR Test
Inverse variance weighted (fixed effects)
Inverse variance weighted (multiplicative random effects)
MR Egger
MR RAPS
Weighted median
Weighted mode
0.000
−0.025
−0.050
0.09
0.12
0.15
0.18
SNP effect on PILR alpha−associated neural protein
SNP effect on Gut microbiota abundance (genus Catenibacterium id.2153)

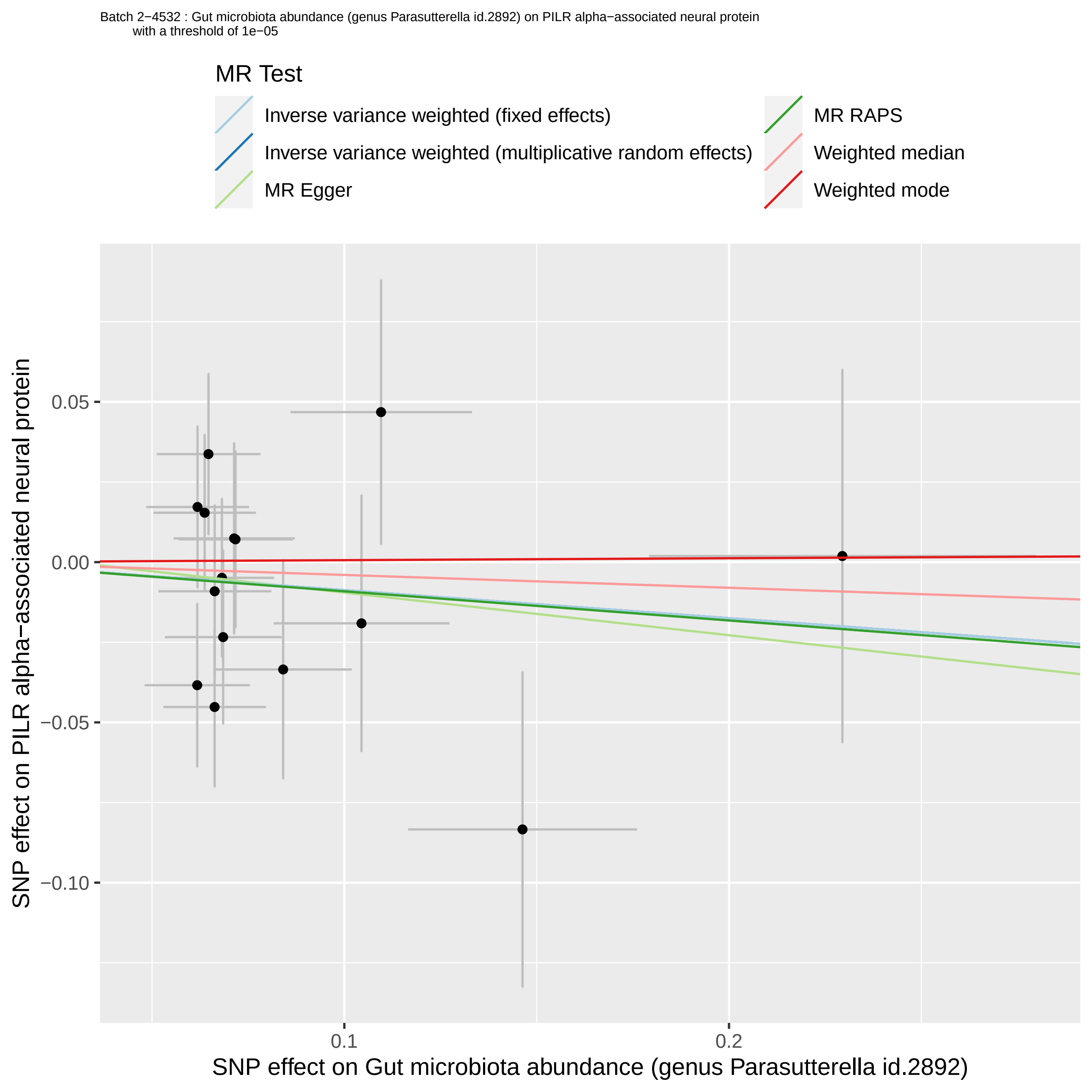
Batch 2−4532 : Gut microbiota abundance (genus Parasutterella id.2892) on PILR alpha−associated neural protein
with a threshold of 1e−05
MR Test
Inverse variance weighted (fixed effects)
Inverse variance weighted (multiplicative random effects)
MR Egger
MR RAPS
Weighted median
Weighted mode
0.05
0.00
−0.05
−0.10
0.1
0.2
SNP effect on PILR alpha−associated neural protein
SNP effect on Gut microbiota abundance (genus Parasutterella id.2892)

Batch 2−4533 : Gut microbiota abundance (genus Catenibacterium id.2153) on PILR alpha−associated neural protein with a threshold of 1e−05

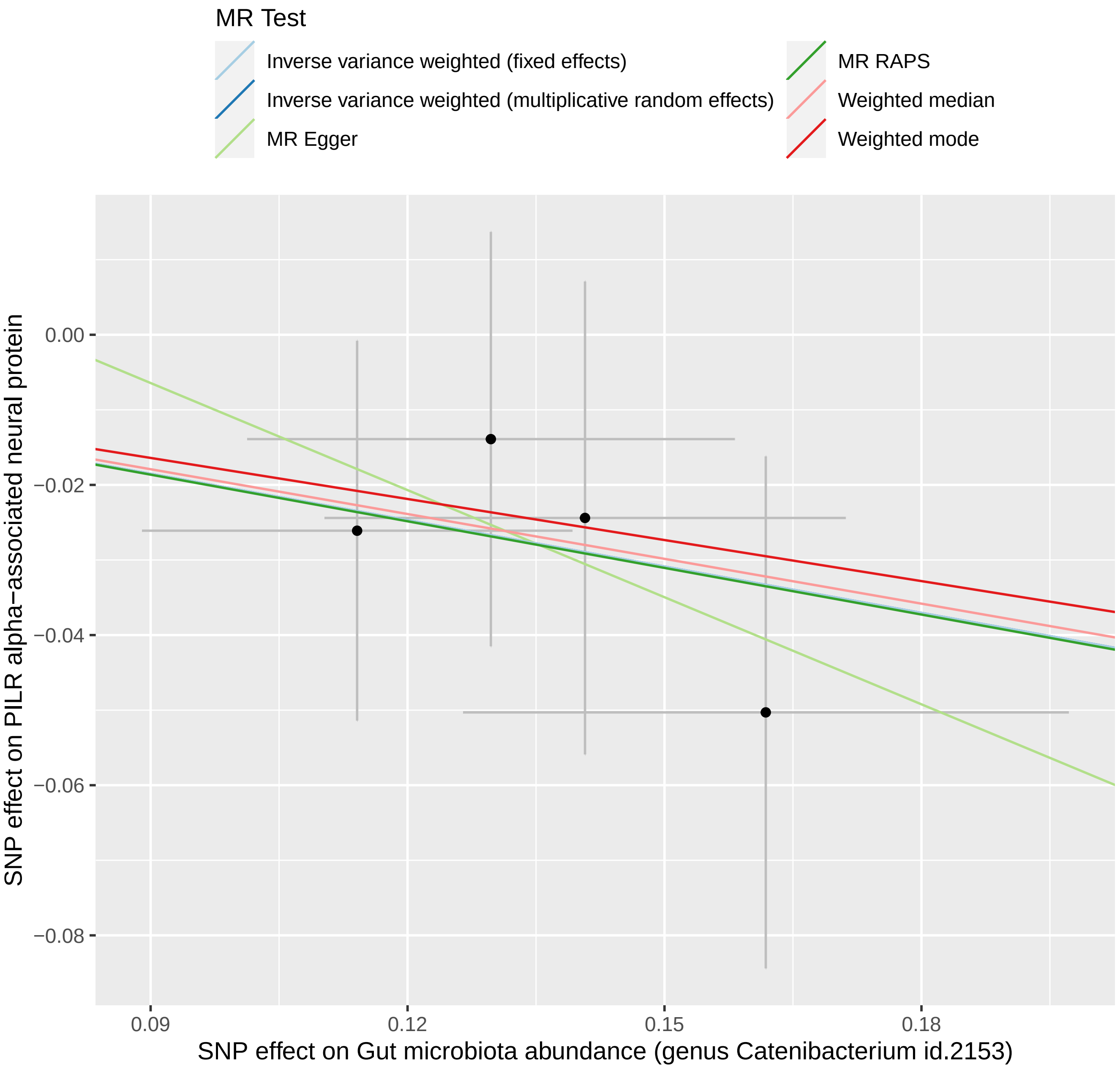

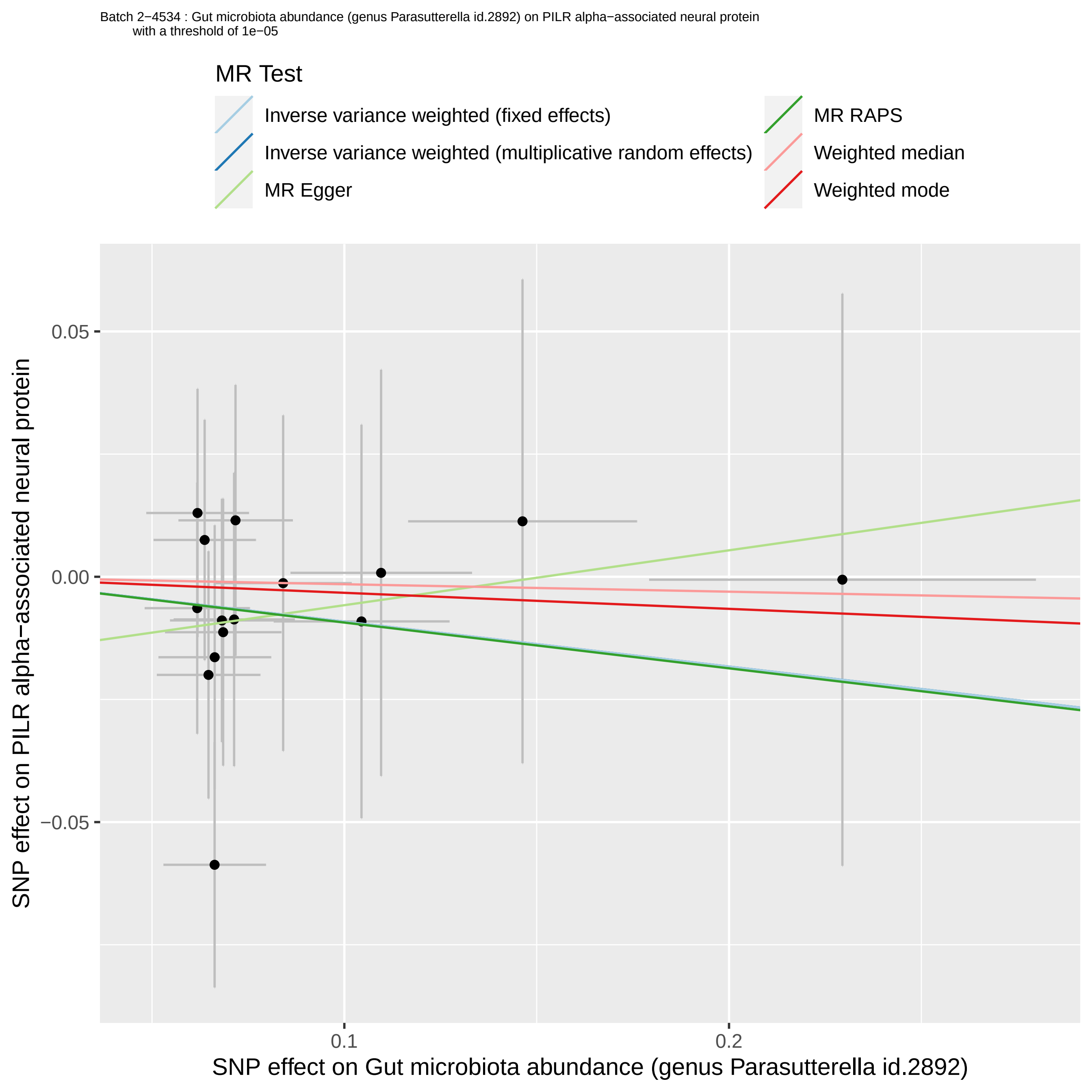
Batch 2−4534 : Gut microbiota abundance (genus Parasutterella id.2892) on PILR alpha−associated neural protein
with a threshold of 1e−05
MR Test
Inverse variance weighted (fixed effects)
Inverse variance weighted (multiplicative random effects)
MR Egger
MR RAPS
Weighted median
Weighted mode
0.05
0.00
−0.05
0.1
0.2
SNP effect on PILR alpha−associated neural protein
SNP effect on Gut microbiota abundance (genus Parasutterella id.2892)

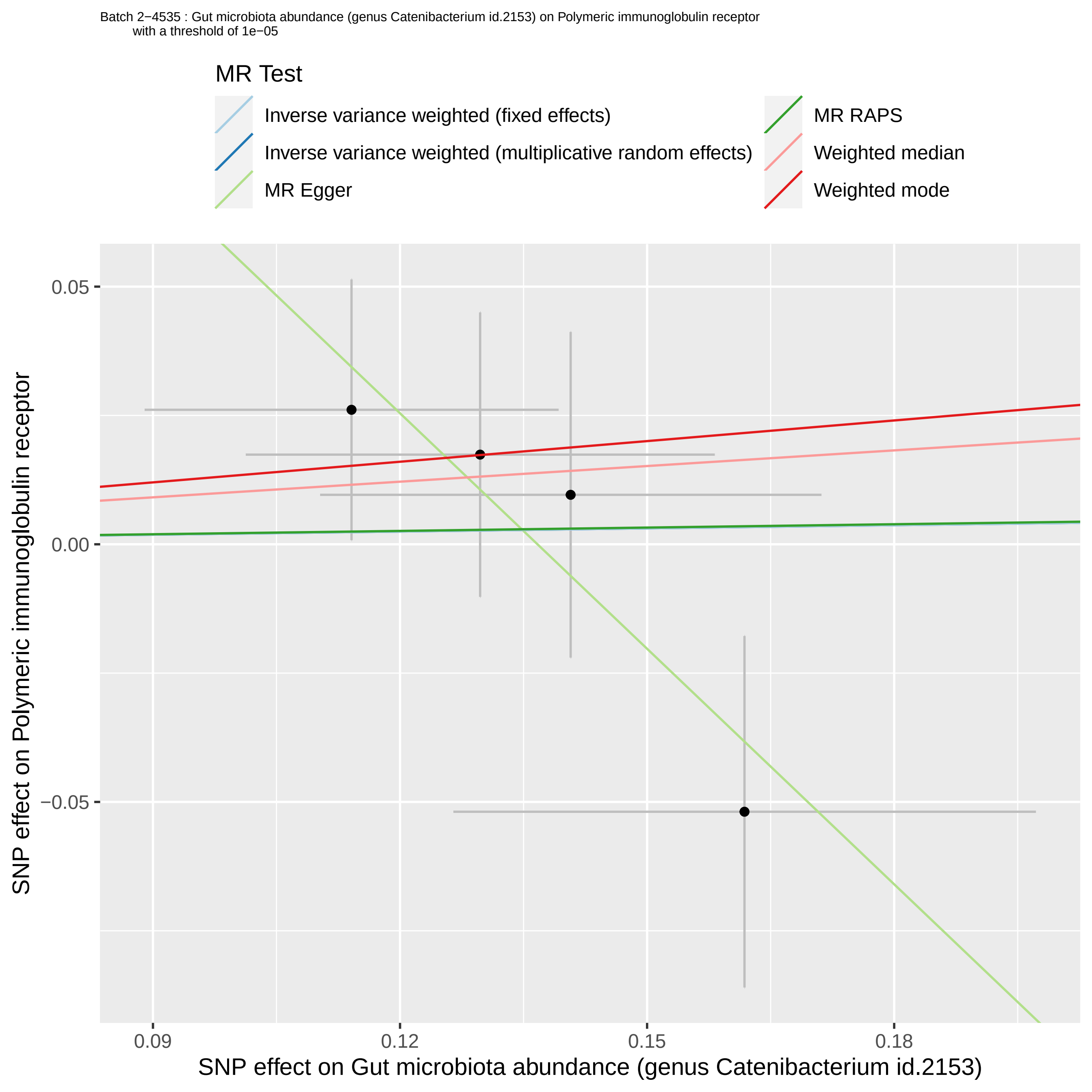
Batch 2−4535 : Gut microbiota abundance (genus Catenibacterium id.2153) on Polymeric immunoglobulin receptor
with a threshold of 1e−05
MR Test
Inverse variance weighted (fixed effects)
Inverse variance weighted (multiplicative random effects)
MR Egger
MR RAPS
Weighted median
Weighted mode
0.05
0.00
−0.05
0.09
0.12
0.15
0.18
SNP effect on Polymeric immunoglobulin receptor
SNP effect on Gut microbiota abundance (genus Catenibacterium id.2153)

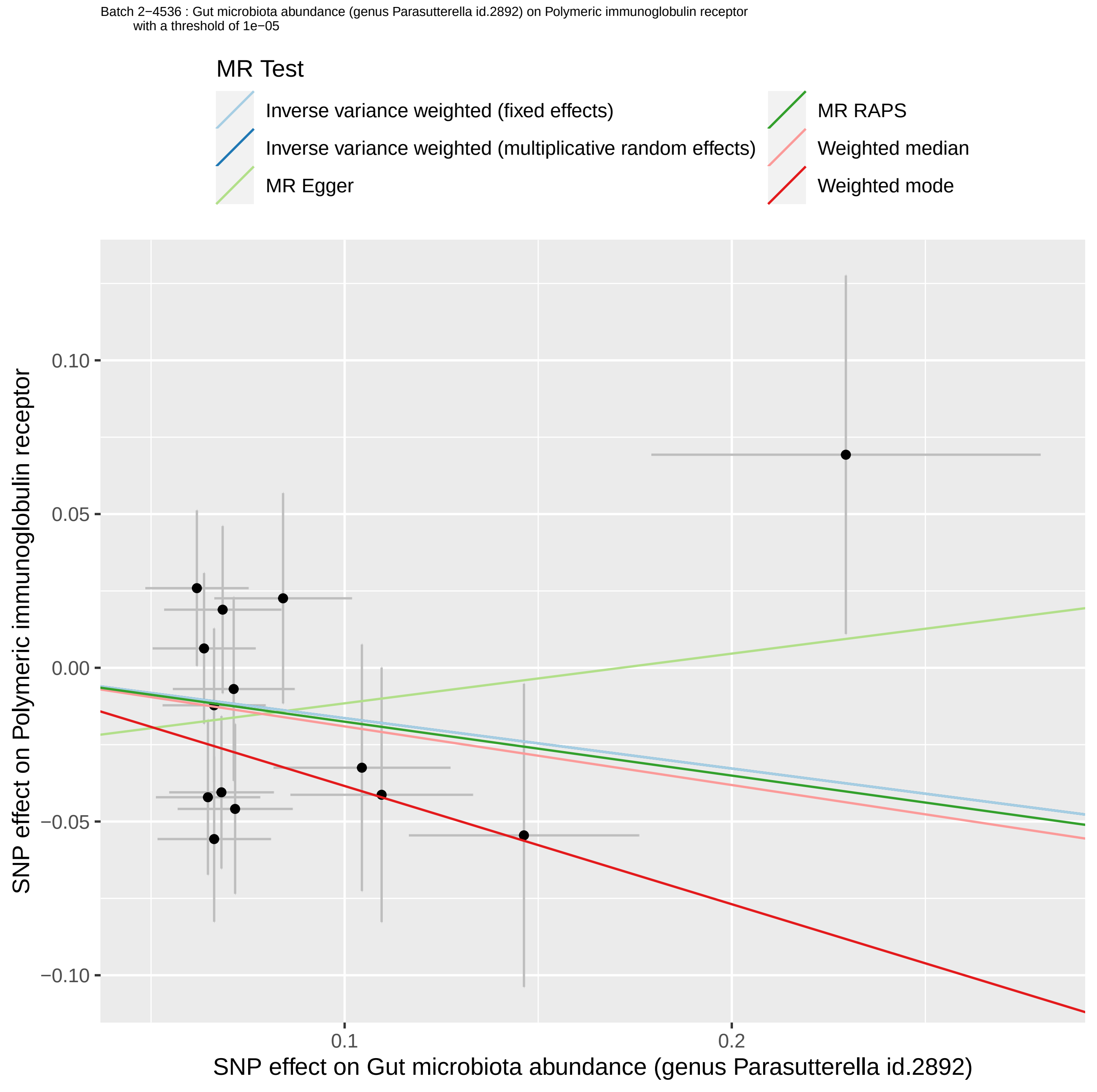

Batch 2−4536 : Gut microbiota abundance (genus Parasutterella id.2892) on Polymeric immunoglobulin receptor
with a threshold of 1e−05
MR Test
Inverse variance weighted (fixed effects)
Inverse variance weighted (multiplicative random effects)
MR Egger
MR RAPS
Weighted median
Weighted mode
0.10
0.05
0.00
−0.05
−0.10
0.1
0.2
SNP effect on Gut microbiota abundance (genus Parasutterella id.2892)
SNP effect on Polymeric immunoglobulin receptor

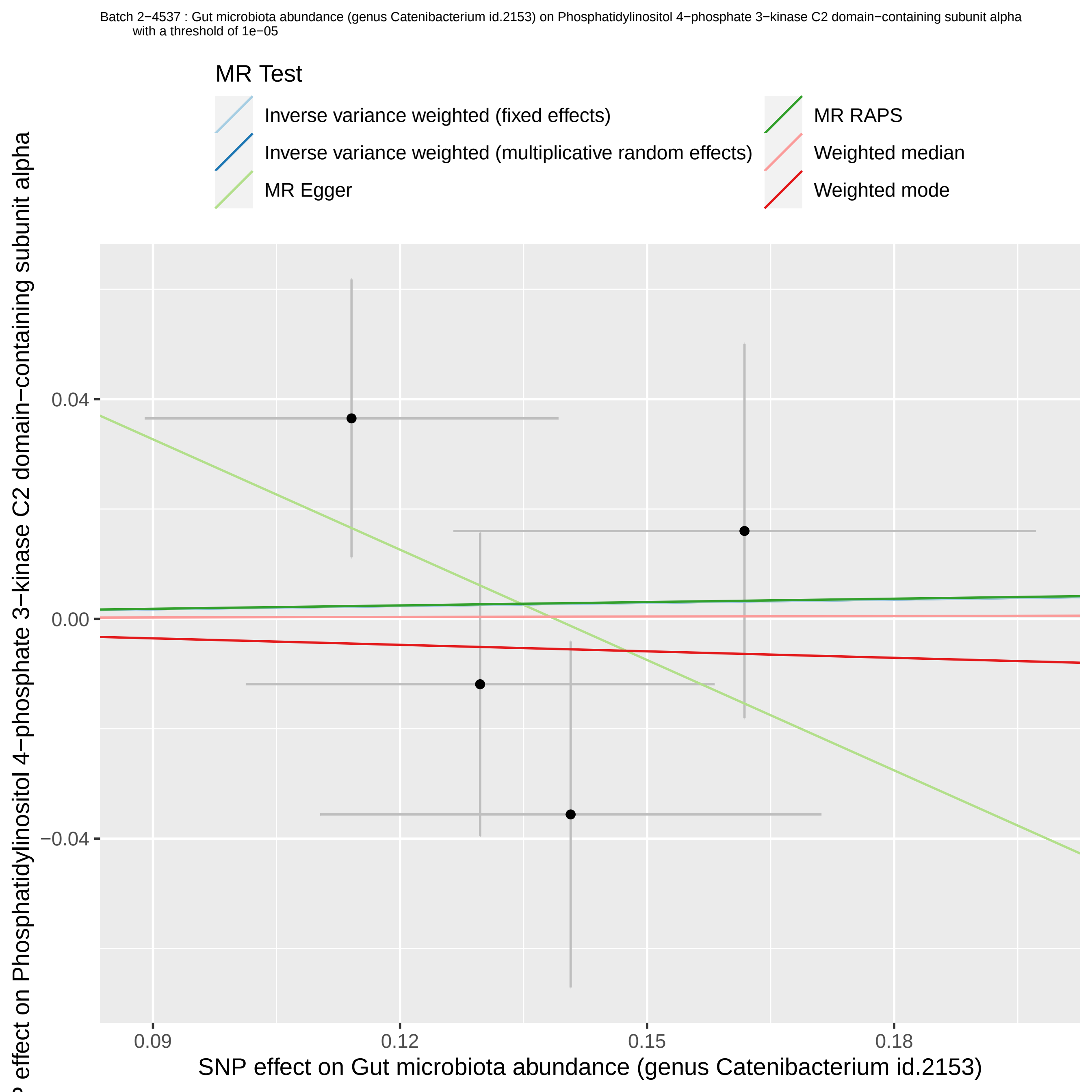

Batch 2−4537 : Gut microbiota abundance (genus Catenibacterium id.2153) on Phosphatidylinositol 4−phosphate 3−kinase C2 domain−containing subunit alpha
with a threshold of 1e−05
MR Test
Inverse variance weighted (fixed effects)
Inverse variance weighted (multiplicative random effects)
MR Egger
MR RAPS
Weighted median
Weighted mode
0.04
0.00
−0.04
0.09
0.12
0.15
0.18
SNP effect on Gut microbiota abundance (genus Catenibacterium id.2153)
SNP effect on Phosphatidylinositol 4−phosphate 3−kinase C2 domain−containing subunit alpha

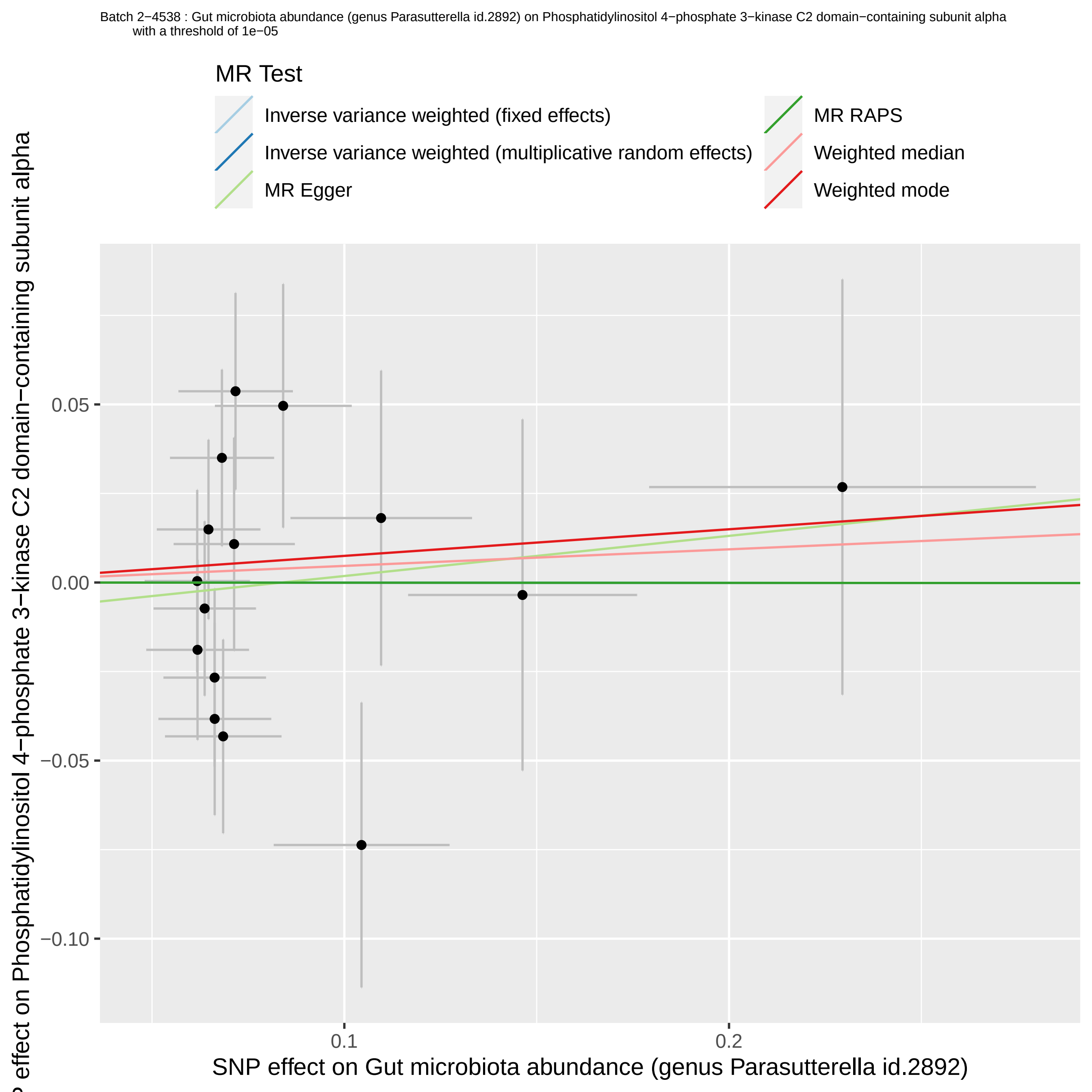

Batch 2−4538 : Gut microbiota abundance (genus Parasutterella id.2892) on Phosphatidylinositol 4−phosphate 3−kinase C2 domain−containing subunit alpha with a threshold of 1e−05
MR Test
Inverse variance weighted (fixed effects)
Inverse variance weighted (multiplicative random effects)
MR Egger
MR RAPS
Weighted median
Weighted mode
0.05
0.00
−0.05
−0.10
0.1
0.2
SNP effect on Gut microbiota abundance (genus Parasutterella id.2892)
effect on Phosphatidylinositol 4−phosphate 3−kinase C2 domain−containing subunit alpha

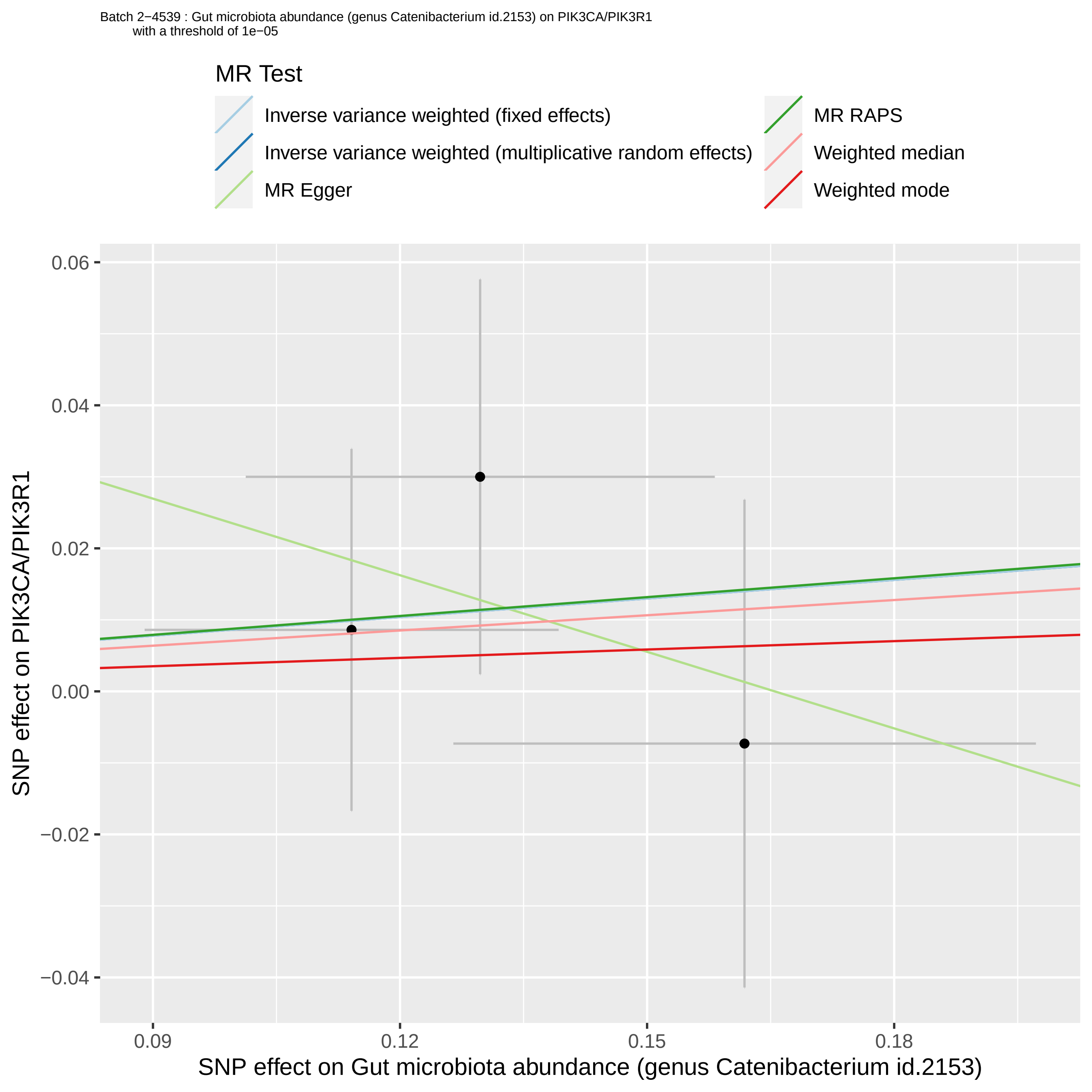
Batch 2−4539 : Gut microbiota abundance (genus Catenibacterium id.2153) on PIK3CA/PIK3R1
with a threshold of 1e−05
MR Test
Inverse variance weighted (fixed effects)
Inverse variance weighted (multiplicative random effects)
MR Egger
MR RAPS
Weighted median
Weighted mode
0.06
0.04
0.02
0.00
−0.02
−0.04
0.09
0.12
0.15
0.18
SNP effect on PIK3CA/PIK3R1
SNP effect on Gut microbiota abundance (genus Catenibacterium id.2153)

Batch 2−4540 : Gut microbiota abundance (genus Parasutterella id.2892) on PIK3CA/PIK3R1
with a threshold of 1e−05

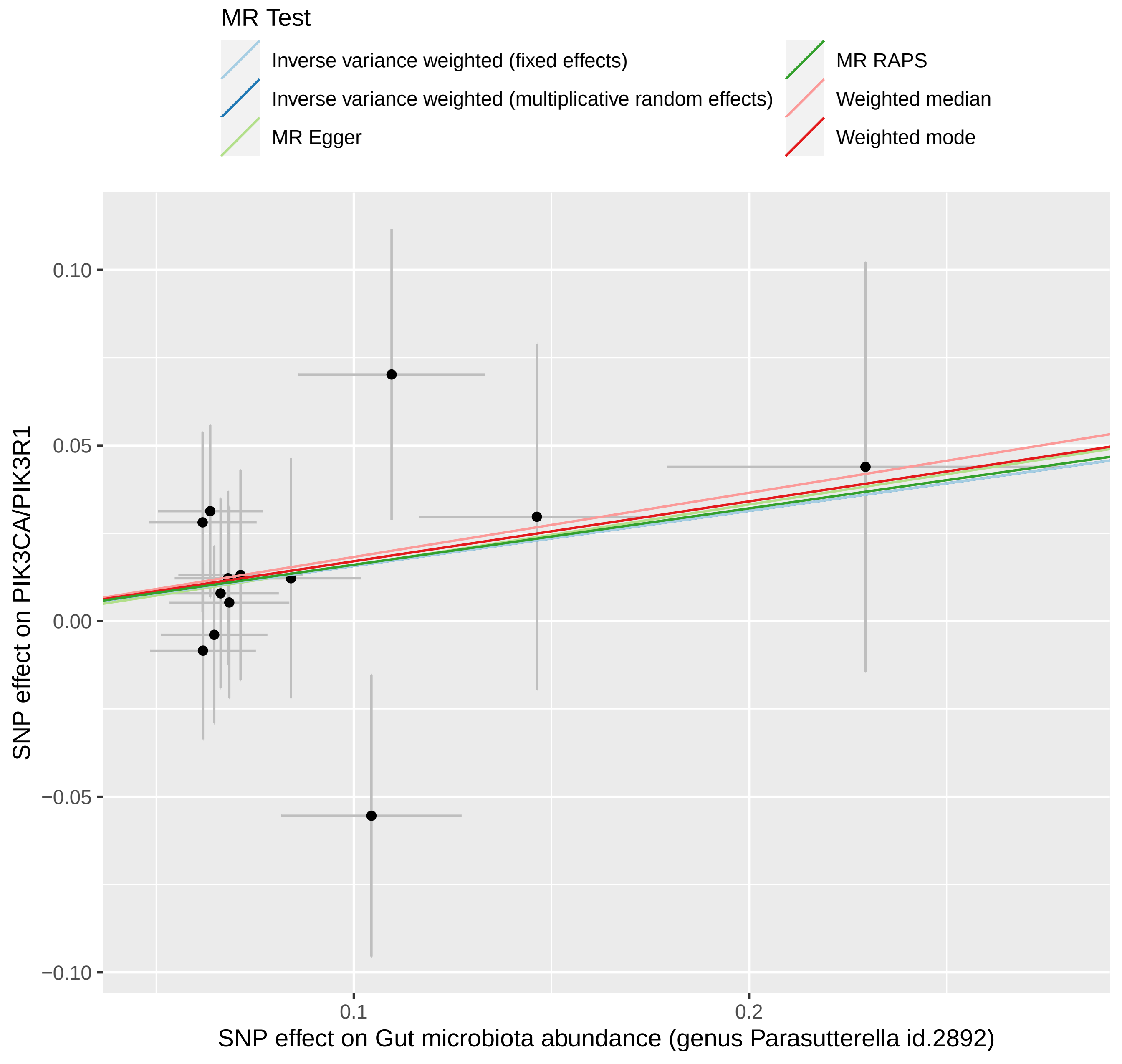

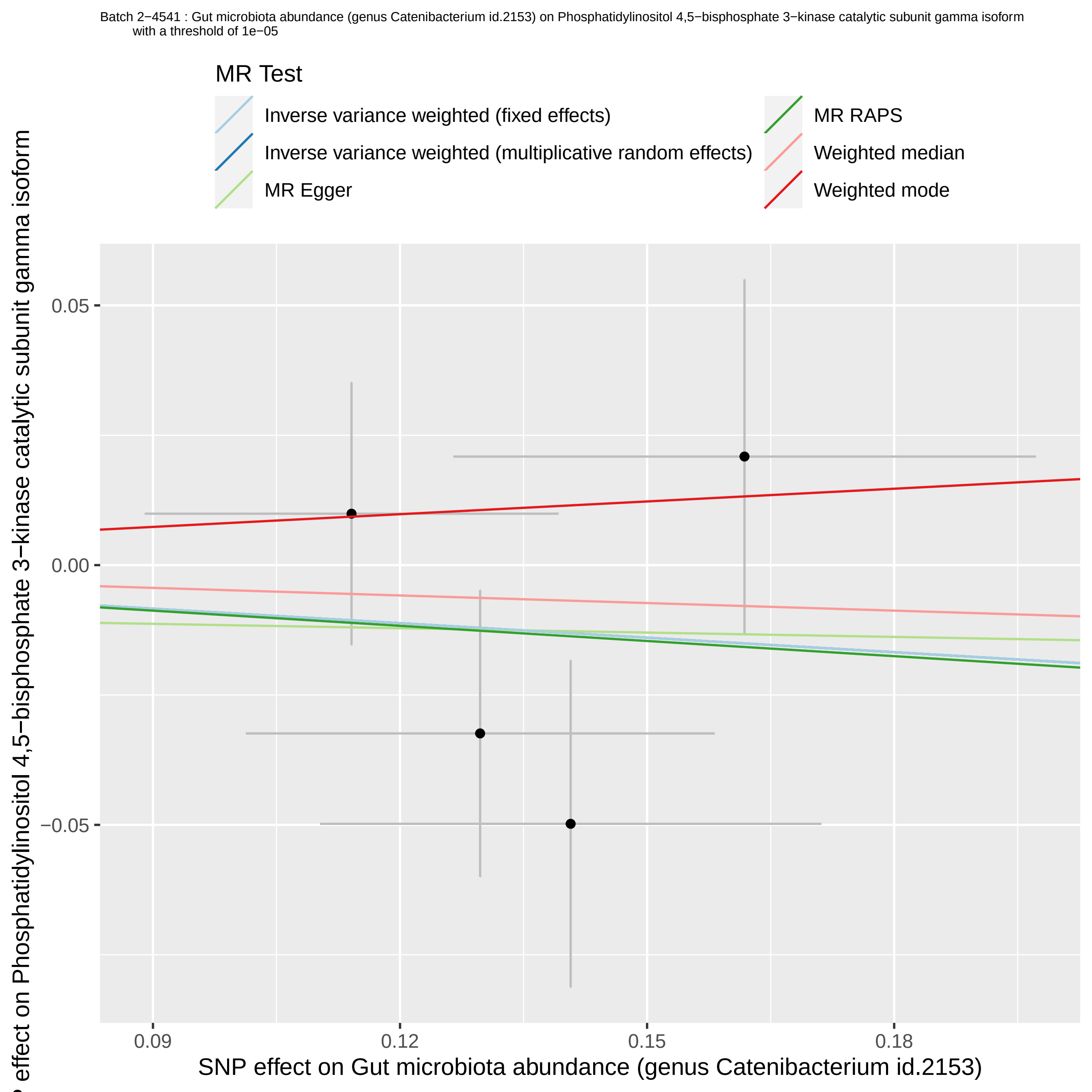
Batch 2−4541 : Gut microbiota abundance (genus Catenibacterium id.2153) on Phosphatidylinositol 4,5−bisphosphate 3−kinase catalytic subunit gamma isoform with a threshold of 1e−05
MR Test
Inverse variance weighted (fixed effects)
Inverse variance weighted (multiplicative random effects)
MR Egger
MR RAPS
Weighted median
Weighted mode
0.05
0.00
−0.05
0.09
0.12
0.15
0.18
SNP effect on Gut microbiota abundance (genus Catenibacterium id.2153)
effect on Phosphatidylinositol 4,5−bisphosphate 3−kinase catalytic subunit gamma isoform

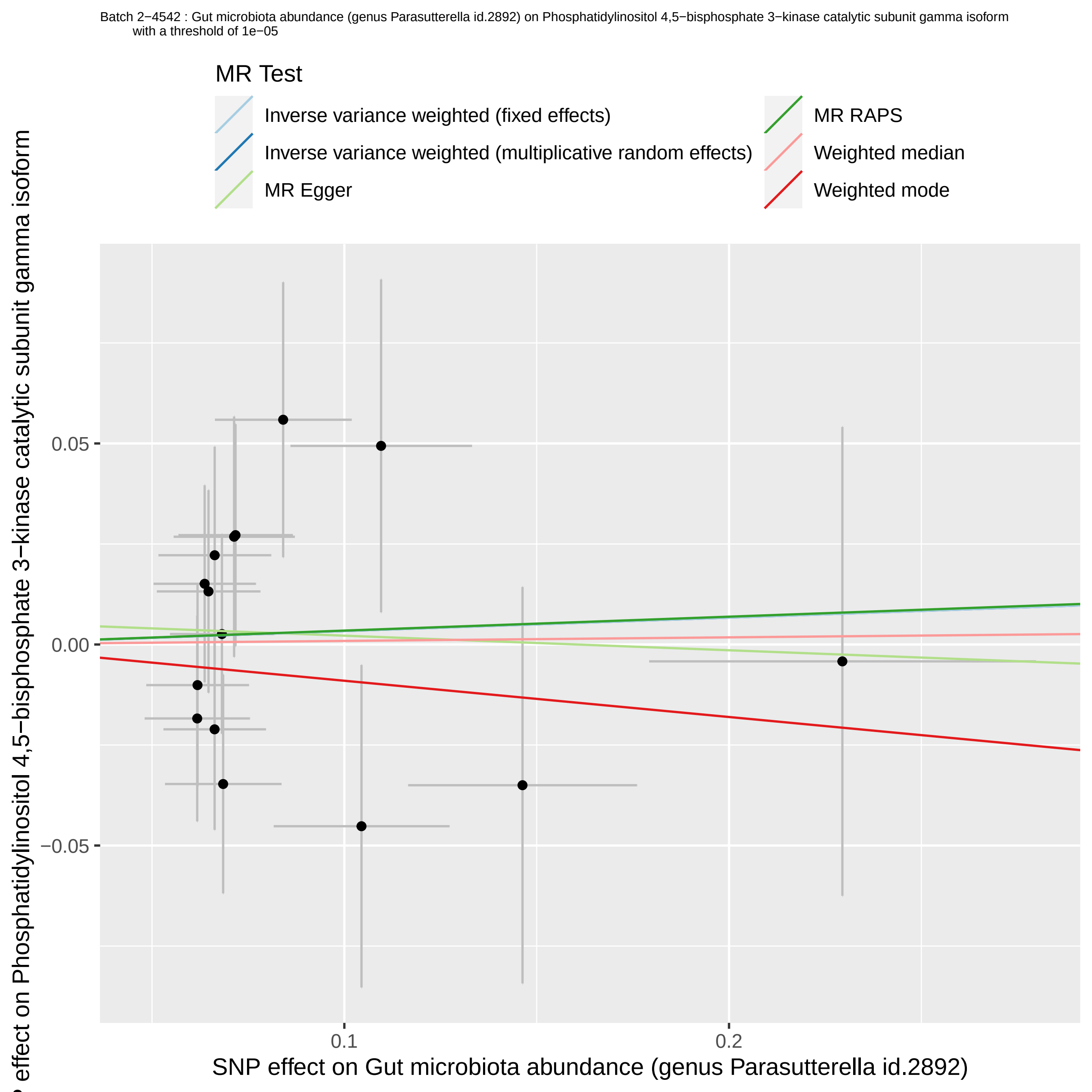
Batch 2−4542 : Gut microbiota abundance (genus Parasutterella id.2892) on Phosphatidylinositol 4,5−bisphosphate 3−kinase catalytic subunit gamma isoform with a threshold of 1e−05
MR Test
Inverse variance weighted (fixed effects)
Inverse variance weighted (multiplicative random effects)
MR Egger
MR RAPS
Weighted median
Weighted mode
0.05
0.00
−0.05
0.1
0.2
SNP effect on Gut microbiota abundance (genus Parasutterella id.2892)
effect on Phosphatidylinositol 4,5−bisphosphate 3−kinase catalytic subunit gamma isoform

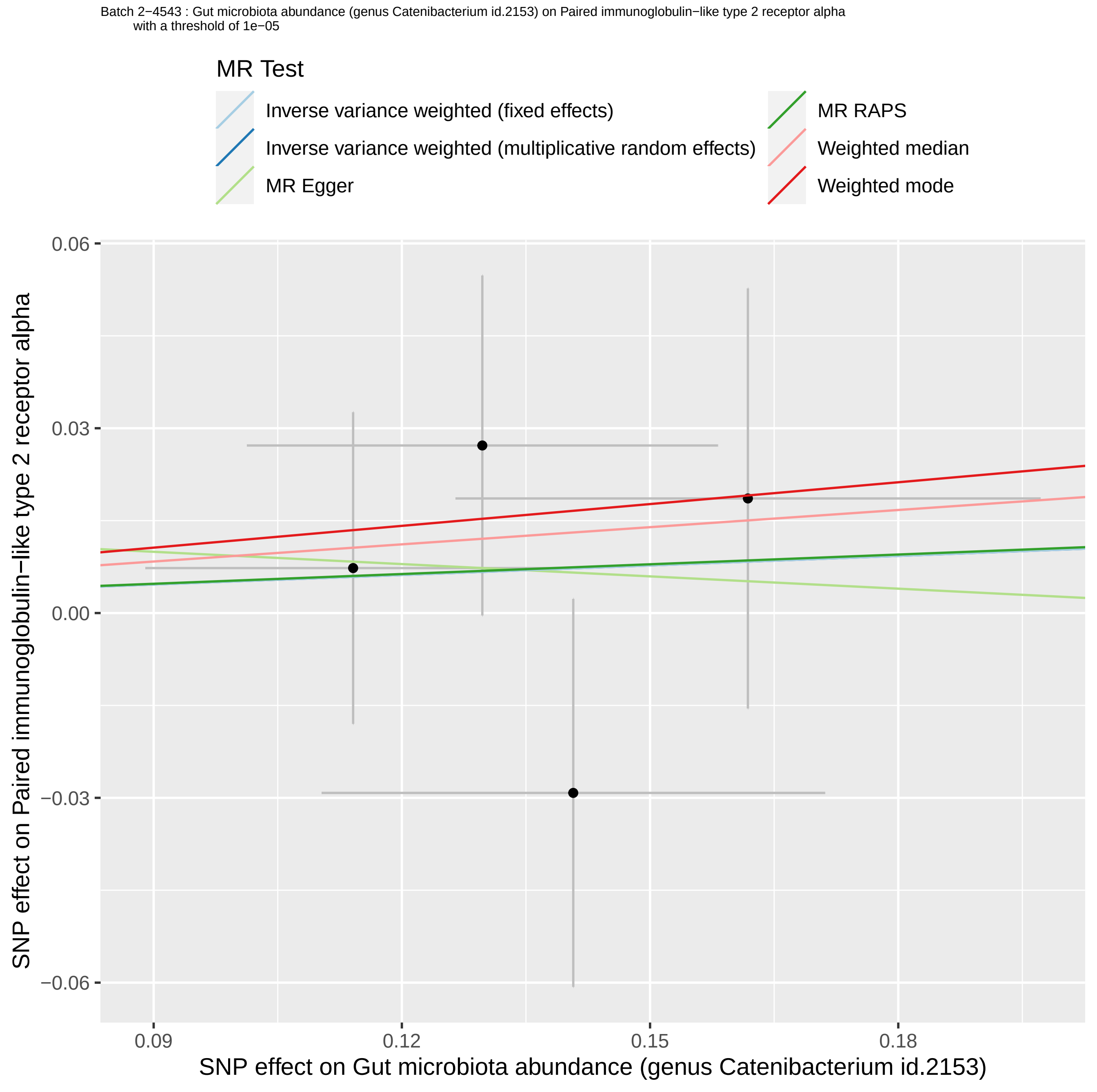

Batch 2−4543 : Gut microbiota abundance (genus Catenibacterium id.2153) on Paired immunoglobulin−like type 2 receptor alpha
with a threshold of 1e−05
MR Test
Inverse variance weighted (fixed effects)
Inverse variance weighted (multiplicative random effects)
MR Egger
MR RAPS
Weighted median
Weighted mode
0.06
0.03
0.00
−0.03
−0.06
SNP effect on Paired immunoglobulin−like type 2 receptor alpha
0.09
0.12
0.15
0.18
SNP effect on Gut microbiota abundance (genus Catenibacterium id.2153)

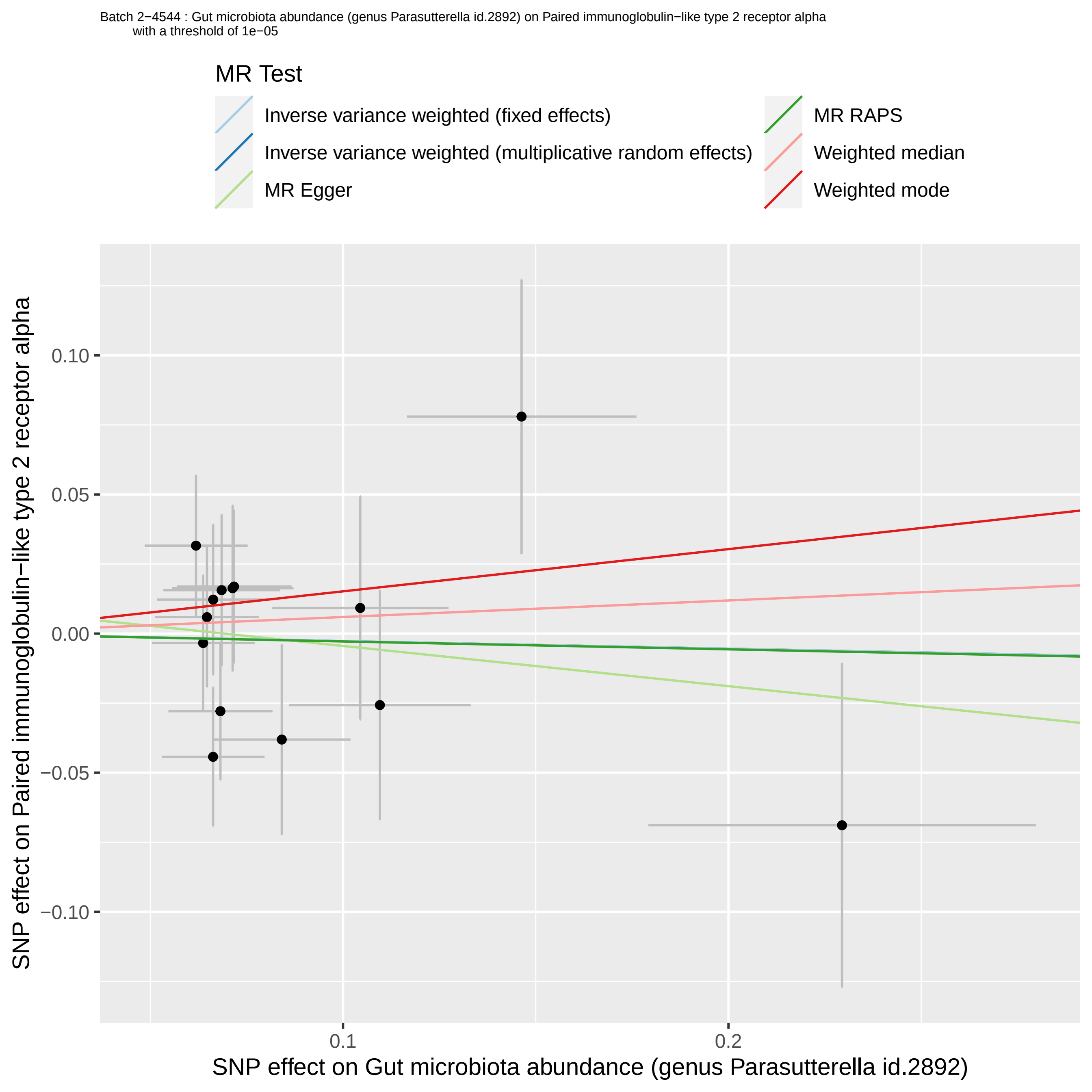
Batch 2−4544 : Gut microbiota abundance (genus Parasutterella id.2892) on Paired immunoglobulin−like type 2 receptor alpha
with a threshold of 1e−05
MR Test
Inverse variance weighted (fixed effects)
Inverse variance weighted (multiplicative random effects)
MR Egger
MR RAPS
Weighted median
Weighted mode
SNP effect on Paired immunoglobulin−like type 2 receptor alpha
0.10
0.05
0.00
−0.05
−0.10
0.1
0.2
SNP effect on Gut microbiota abundance (genus Parasutterella id.2892)

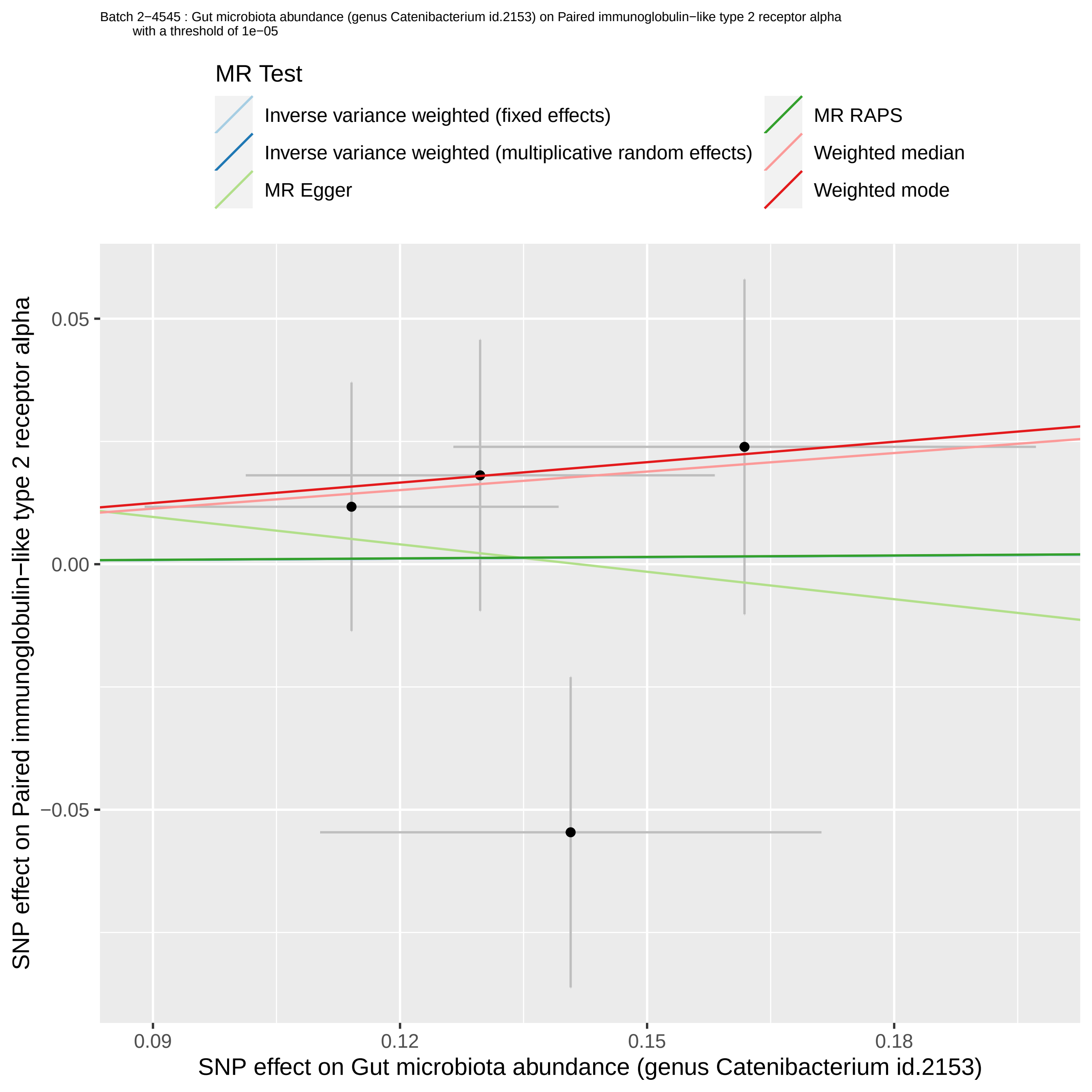
Batch 2−4545 : Gut microbiota abundance (genus Catenibacterium id.2153) on Paired immunoglobulin−like type 2 receptor alpha
with a threshold of 1e−05
MR Test
Inverse variance weighted (fixed effects)
Inverse variance weighted (multiplicative random effects)
MR Egger
MR RAPS
Weighted median
Weighted mode
0.05
0.00
−0.05
0.09
0.12
0.15
0.18
SNP effect on Paired immunoglobulin−like type 2 receptor alpha
SNP effect on Gut microbiota abundance (genus Catenibacterium id.2153)

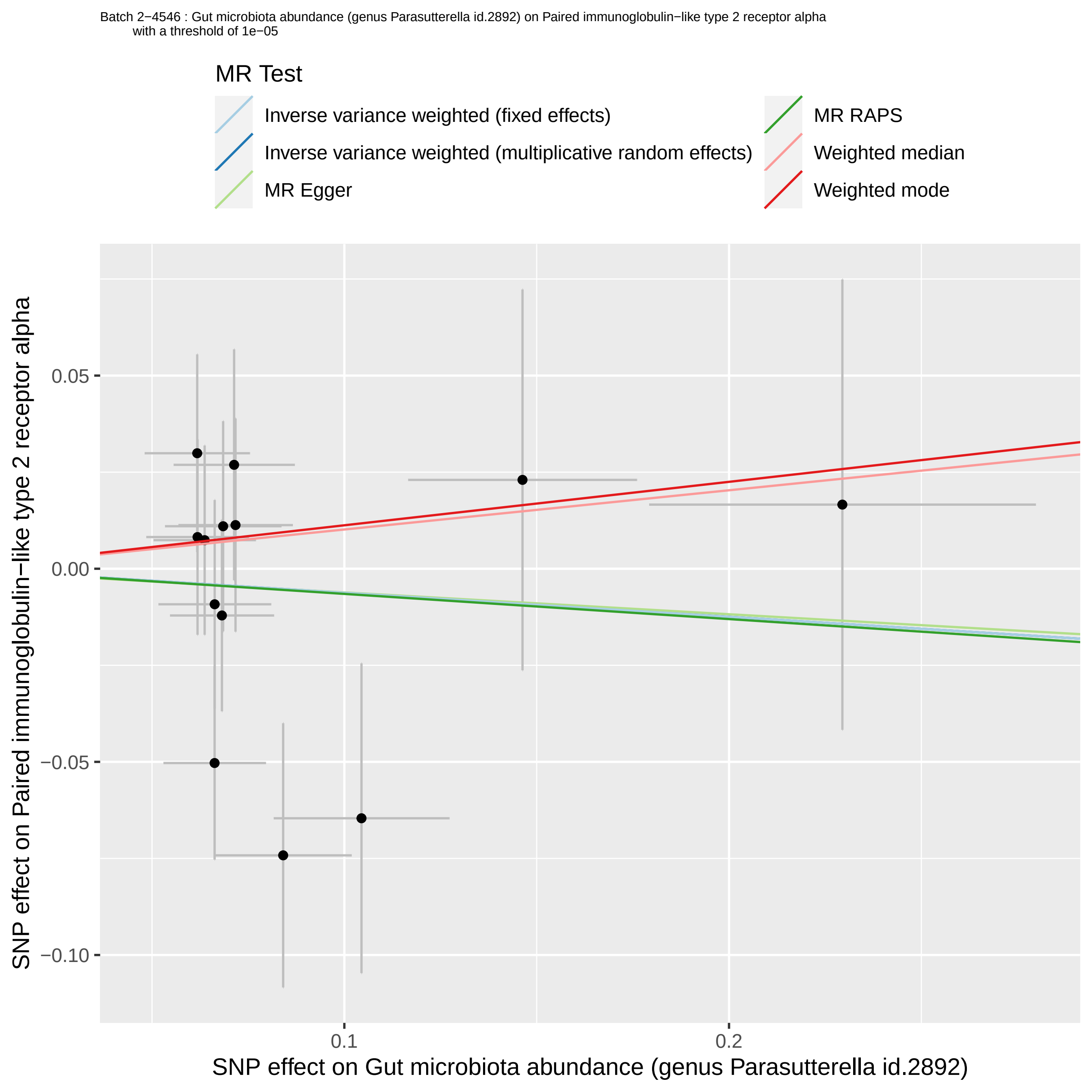

Batch 2−4546 : Gut microbiota abundance (genus Parasutterella id.2892) on Paired immunoglobulin−like type 2 receptor alpha
with a threshold of 1e−05
MR Test
Inverse variance weighted (fixed effects)
Inverse variance weighted (multiplicative random effects)
MR Egger
MR RAPS
Weighted median
Weighted mode
0.05
0.00
−0.05
−0.10
0.1
0.2
SNP effect on Paired immunoglobulin−like type 2 receptor alpha
SNP effect on Gut microbiota abundance (genus Parasutterella id.2892)

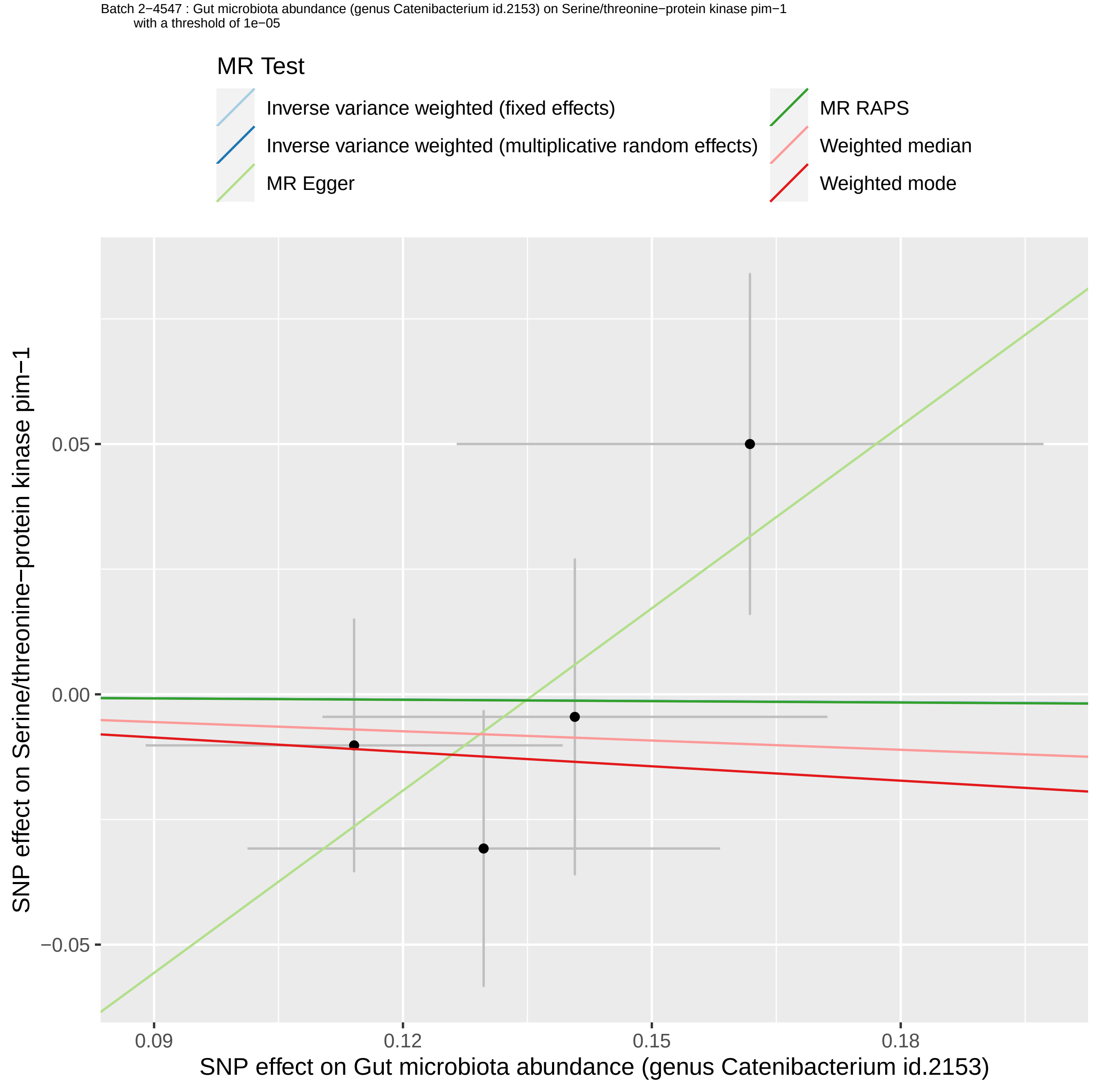
Batch 2−4547 : Gut microbiota abundance (genus Catenibacterium id.2153) on Serine/threonine−protein kinase pim−1
with a threshold of 1e−05
MR Test
Inverse variance weighted (fixed effects)
Inverse variance weighted (multiplicative random effects)
MR Egger
MR RAPS
Weighted median
Weighted mode
0.05
0.00
−0.05
0.09
0.12
0.15
0.18
SNP effect on Serine/threonine−protein kinase pim−1
SNP effect on Gut microbiota abundance (genus Catenibacterium id.2153)

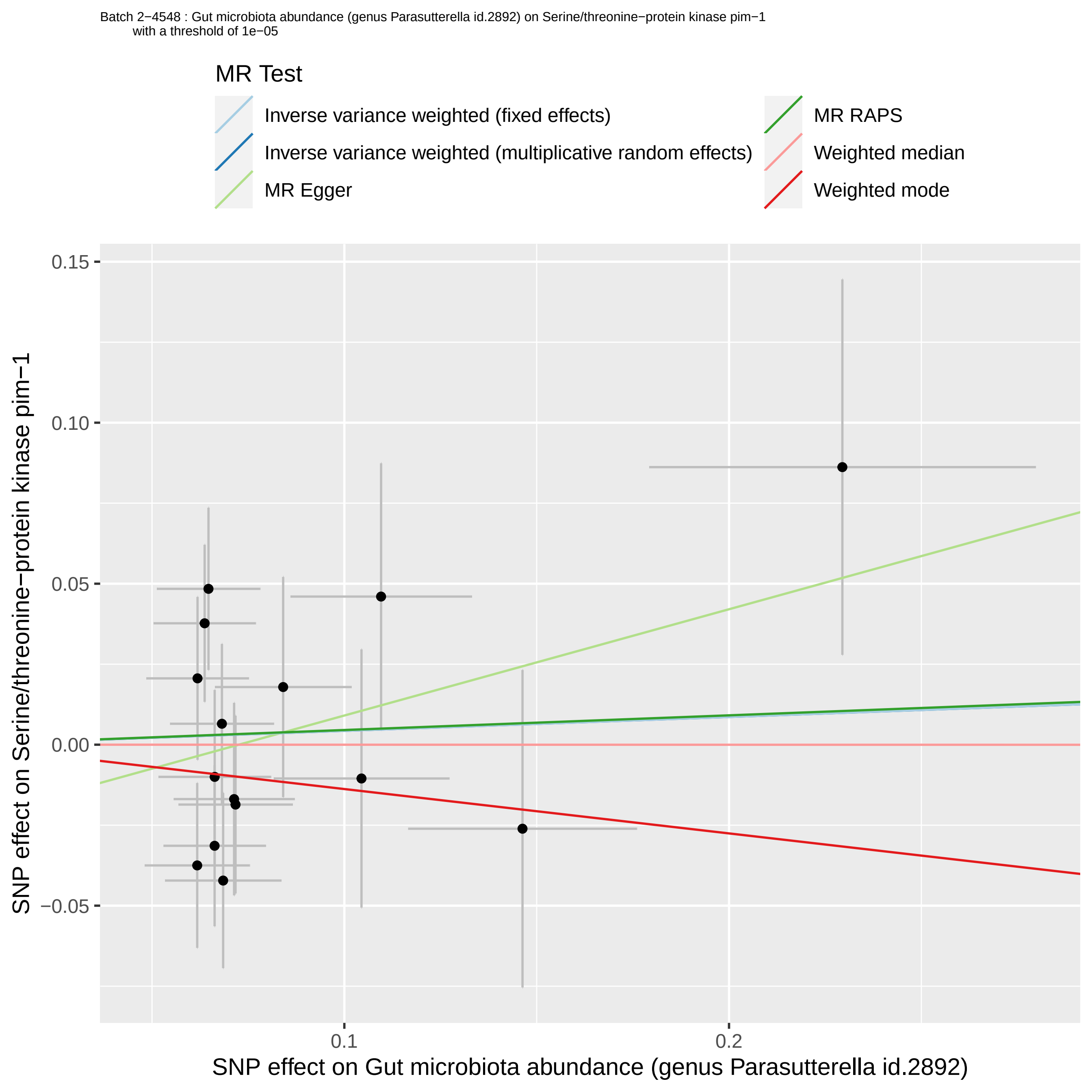

Batch 2−4548 : Gut microbiota abundance (genus Parasutterella id.2892) on Serine/threonine−protein kinase pim−1
with a threshold of 1e−05
MR Test
Inverse variance weighted (fixed effects)
Inverse variance weighted (multiplicative random effects)
MR Egger
MR RAPS
Weighted median
Weighted mode
0.15
0.10
0.05
0.00
−0.05
0.1
0.2
SNP effect on Serine/threonine−protein kinase pim−1
SNP effect on Gut microbiota abundance (genus Parasutterella id.2892)

Batch 2−4549 : Gut microbiota abundance (genus Catenibacterium id.2153) on Peptidyl−prolyl cis−trans isomerase NIMA−interacting 4
with a threshold of 1e−05

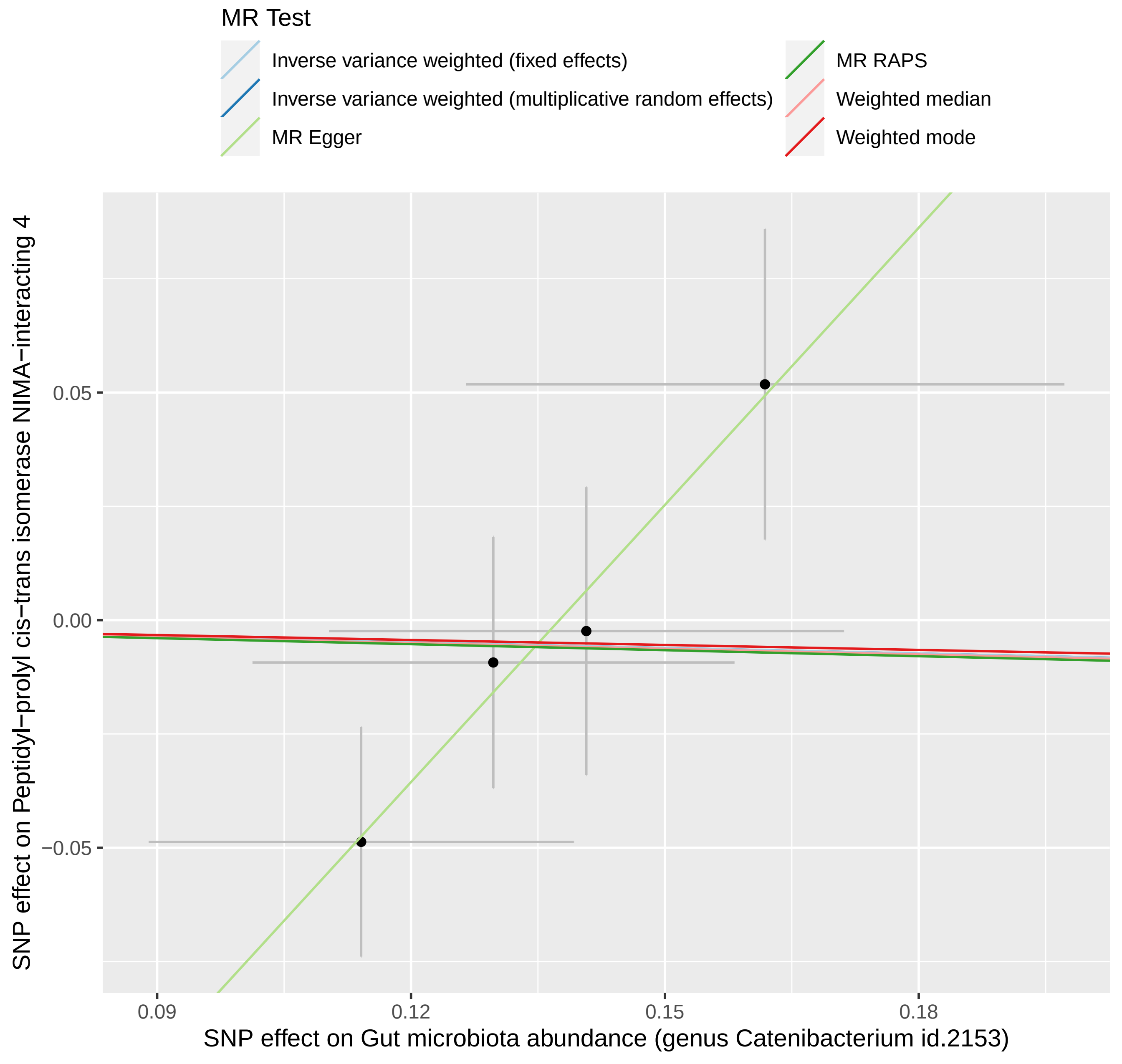

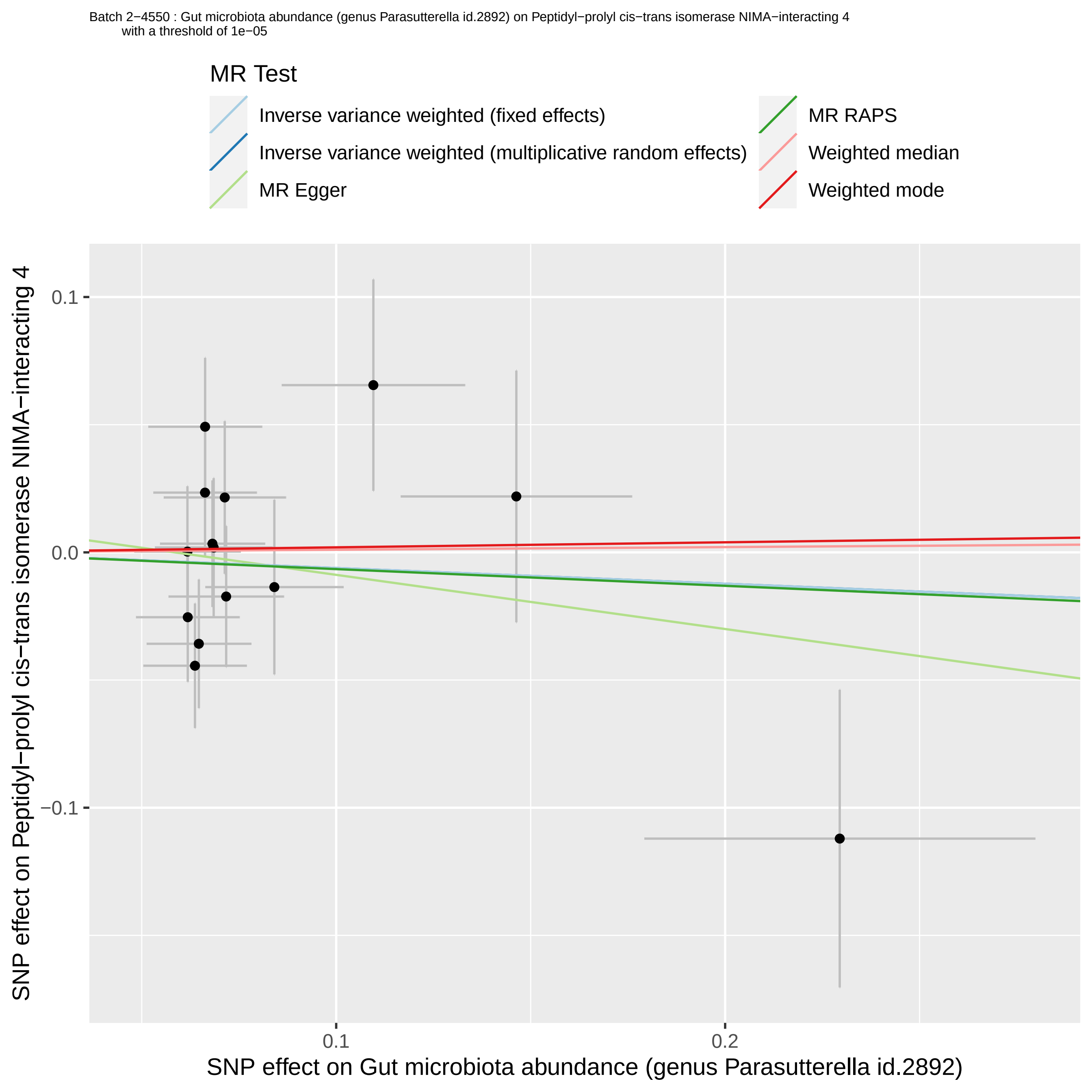
Batch 2−4550 : Gut microbiota abundance (genus Parasutterella id.2892) on Peptidyl−prolyl cis−trans isomerase NIMA−interacting 4
with a threshold of 1e−05
MR Test
Inverse variance weighted (fixed effects)
Inverse variance weighted (multiplicative random effects)
MR Egger
MR RAPS
Weighted median
Weighted mode
0.1
0.0
−0.1
0.1
0.2
SNP effect on Peptidyl−prolyl cis−trans isomerase NIMA−interacting 4
SNP effect on Gut microbiota abundance (genus Parasutterella id.2892)

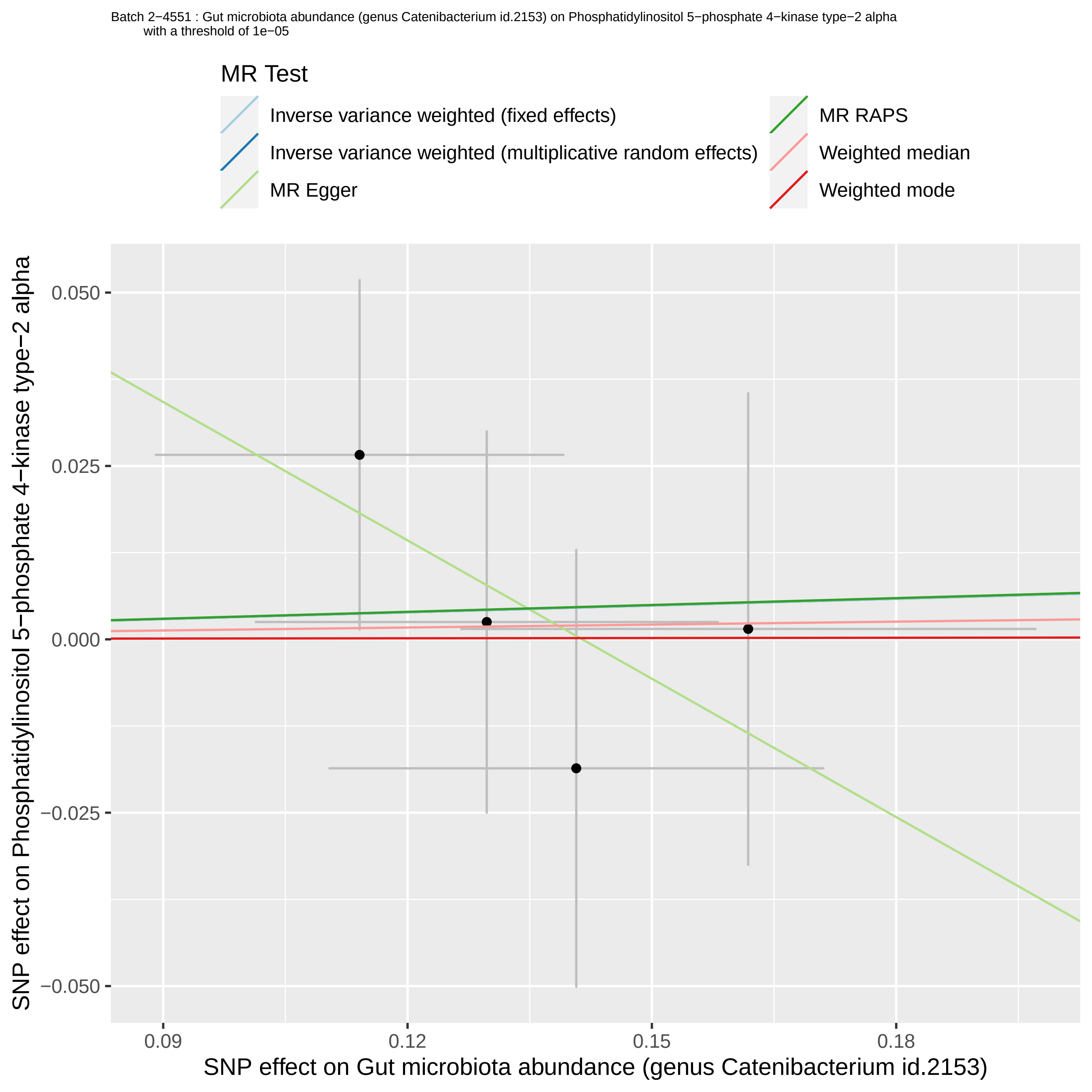
Batch 2−4551 : Gut microbiota abundance (genus Catenibacterium id.2153) on Phosphatidylinositol 5−phosphate 4−kinase type−2 alpha
with a threshold of 1e−05
MR Test
Inverse variance weighted (fixed effects)
Inverse variance weighted (multiplicative random effects)
MR Egger
MR RAPS
Weighted median
Weighted mode
0.050
0.025
0.000
−0.025
−0.050
0.09
0.12
0.15
0.18
SNP effect on Phosphatidylinositol 5−phosphate 4−kinase type−2 alpha
SNP effect on Gut microbiota abundance (genus Catenibacterium id.2153)

Batch 2−4552 : Gut microbiota abundance (genus Parasutterella id.2892) on Phosphatidylinositol 5−phosphate 4−kinase type−2 alpha
with a threshold of 1e−05

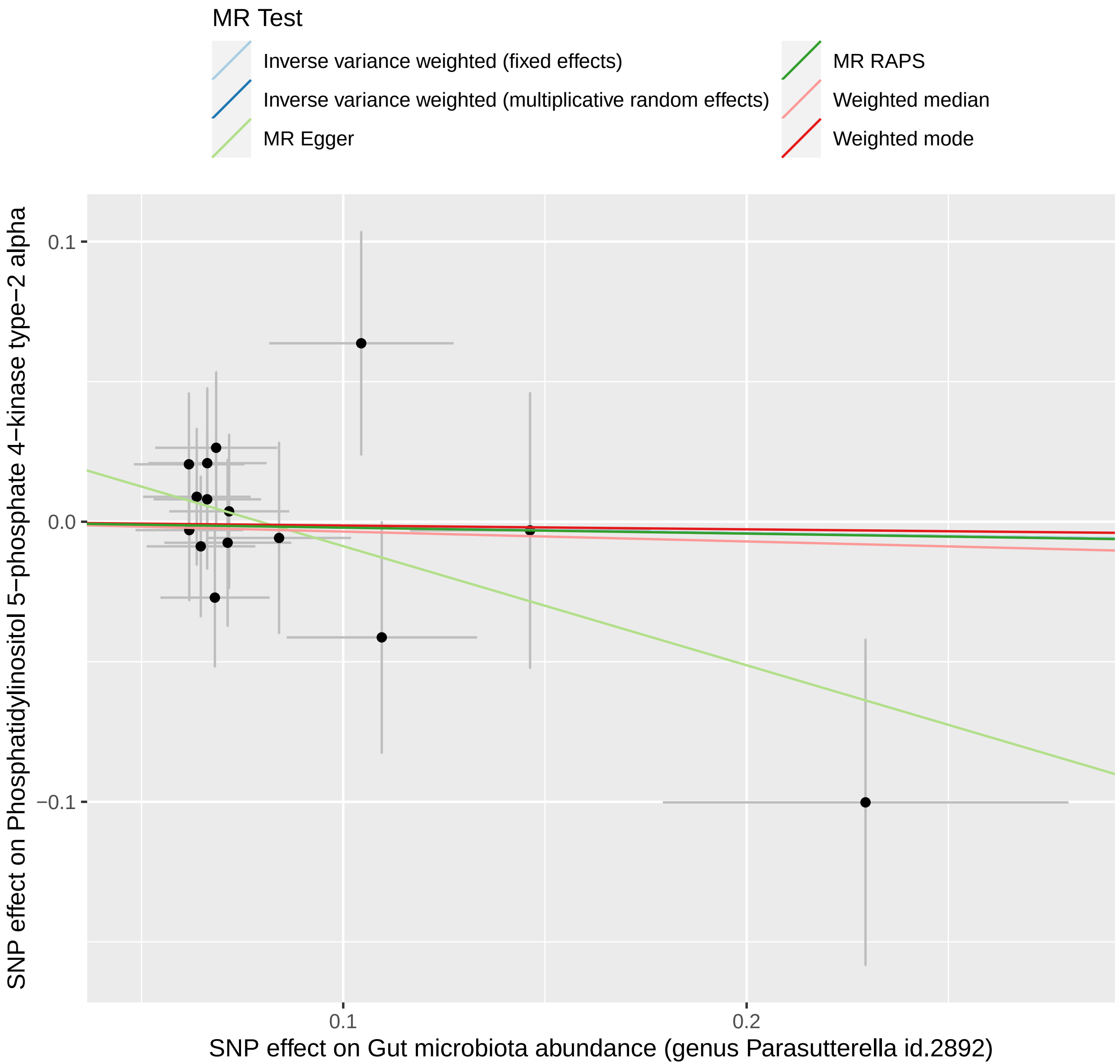

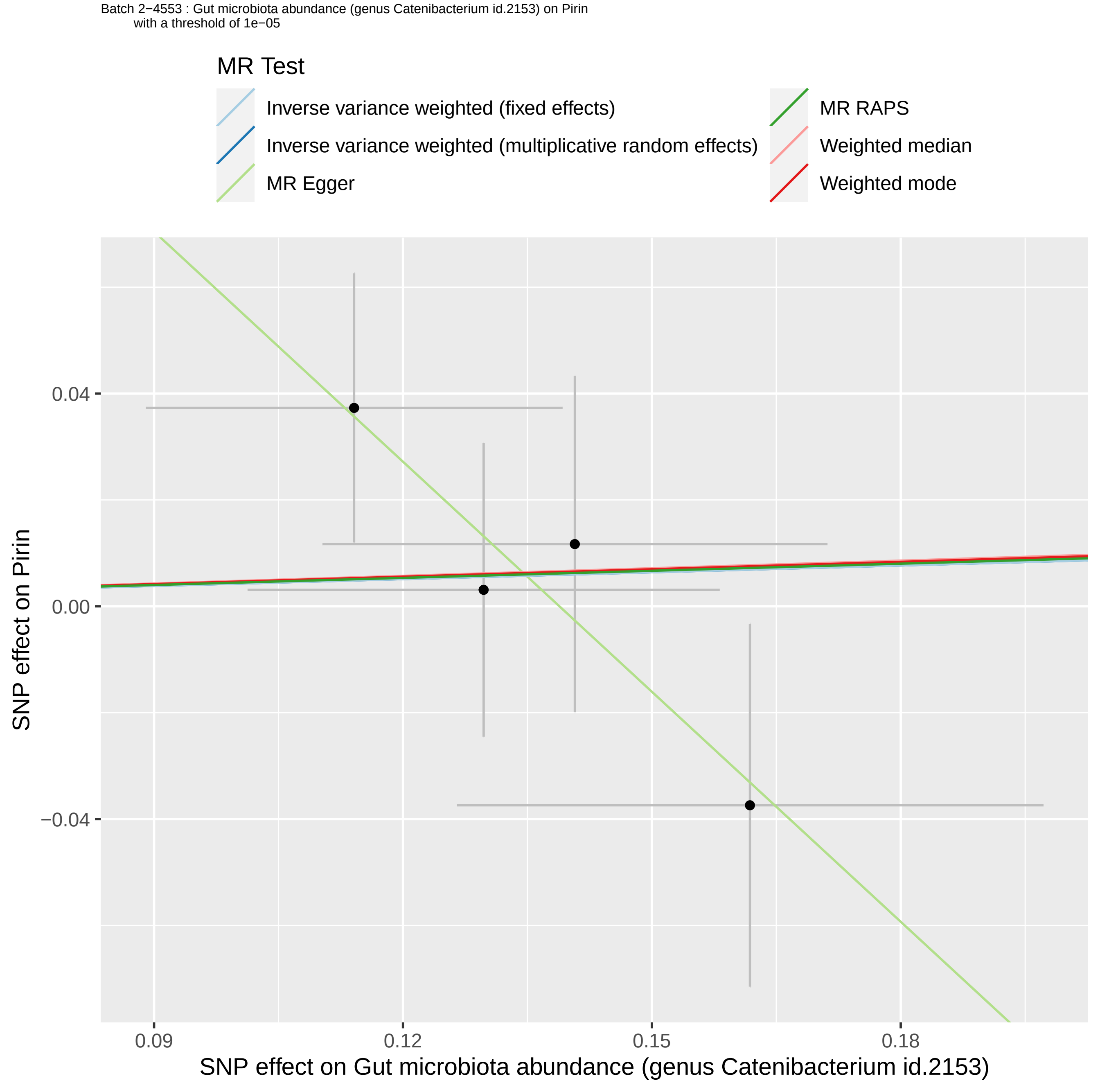

Batch 2−4553 : Gut microbiota abundance (genus Catenibacterium id.2153) on Pirin
with a threshold of 1e−05
MR Test
Inverse variance weighted (fixed effects)
Inverse variance weighted (multiplicative random effects)
MR Egger
MR RAPS
Weighted median
Weighted mode
0.04
0.00
−0.04
0.09
0.12
0.15
0.18
SNP effect on Pirin
SNP effect on Gut microbiota abundance (genus Catenibacterium id.2153)

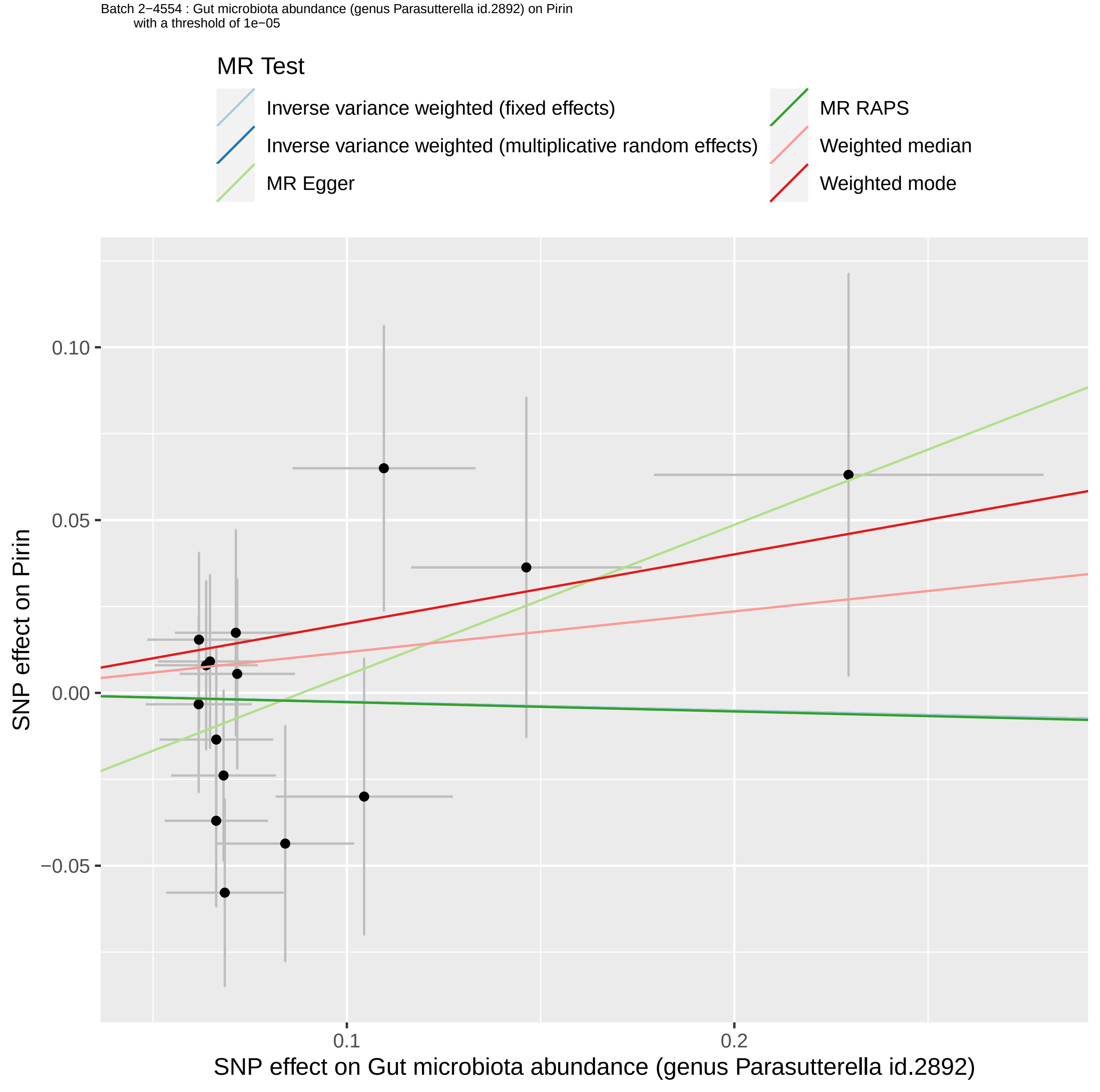

Batch 2−4554 : Gut microbiota abundance (genus Parasutterella id.2892) on Pirin
with a threshold of 1e−05
MR Test
Inverse variance weighted (fixed effects)
Inverse variance weighted (multiplicative random effects)
MR Egger
MR RAPS
Weighted median
Weighted mode
SNP effect on Pirin
0.10
0.05
0.00
−0.05
0.1
0.2
SNP effect on Gut microbiota abundance (genus Parasutterella id.2892)

Batch 2−4555 : Gut microbiota abundance (genus Catenibacterium id.2153) on Phosphatidylinositol transfer protein alpha isoform
with a threshold of 1e−05

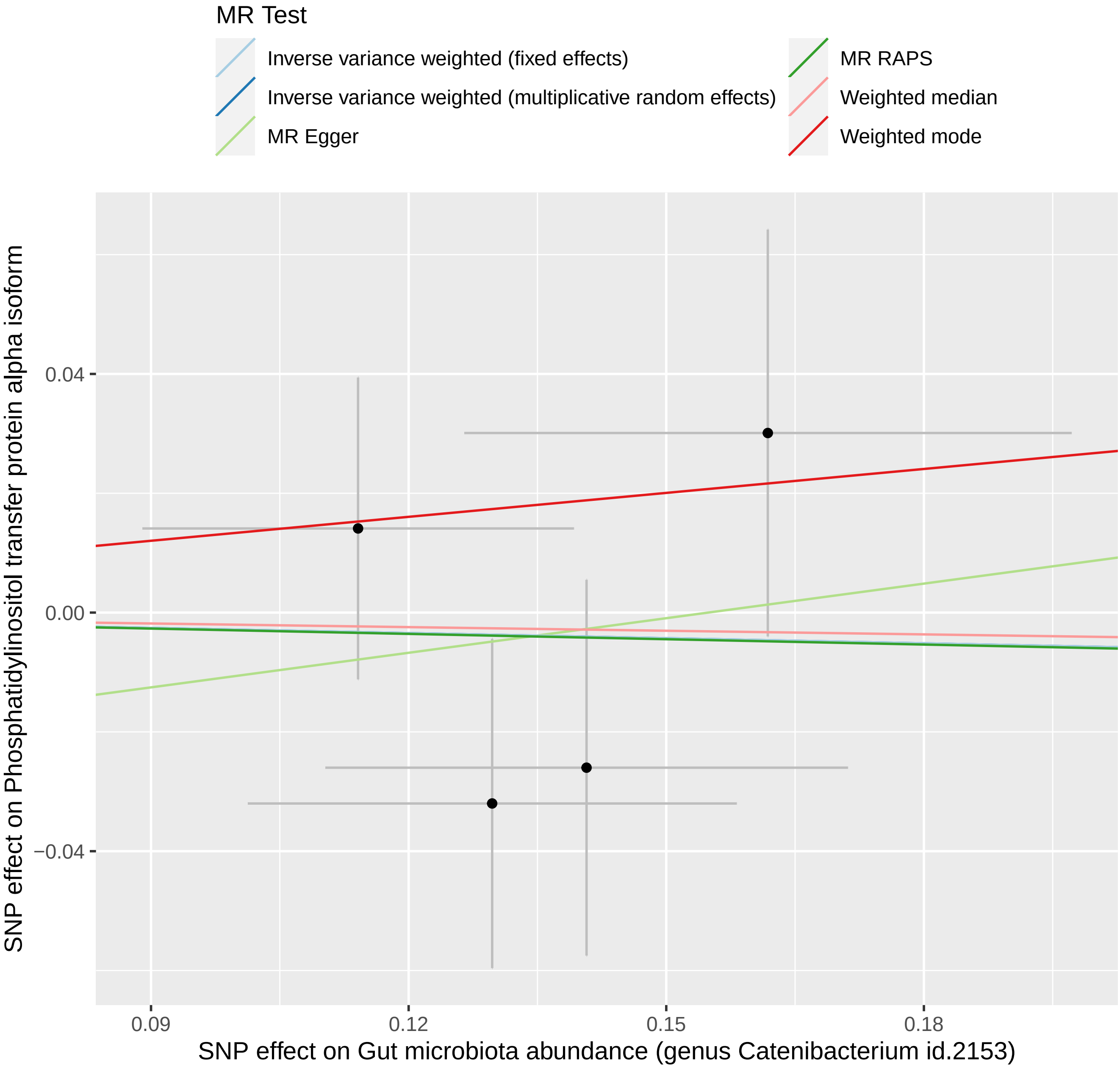

Batch 2−4556 : Gut microbiota abundance (genus Parasutterella id.2892) on Phosphatidylinositol transfer protein alpha isoform with a threshold of 1e−05

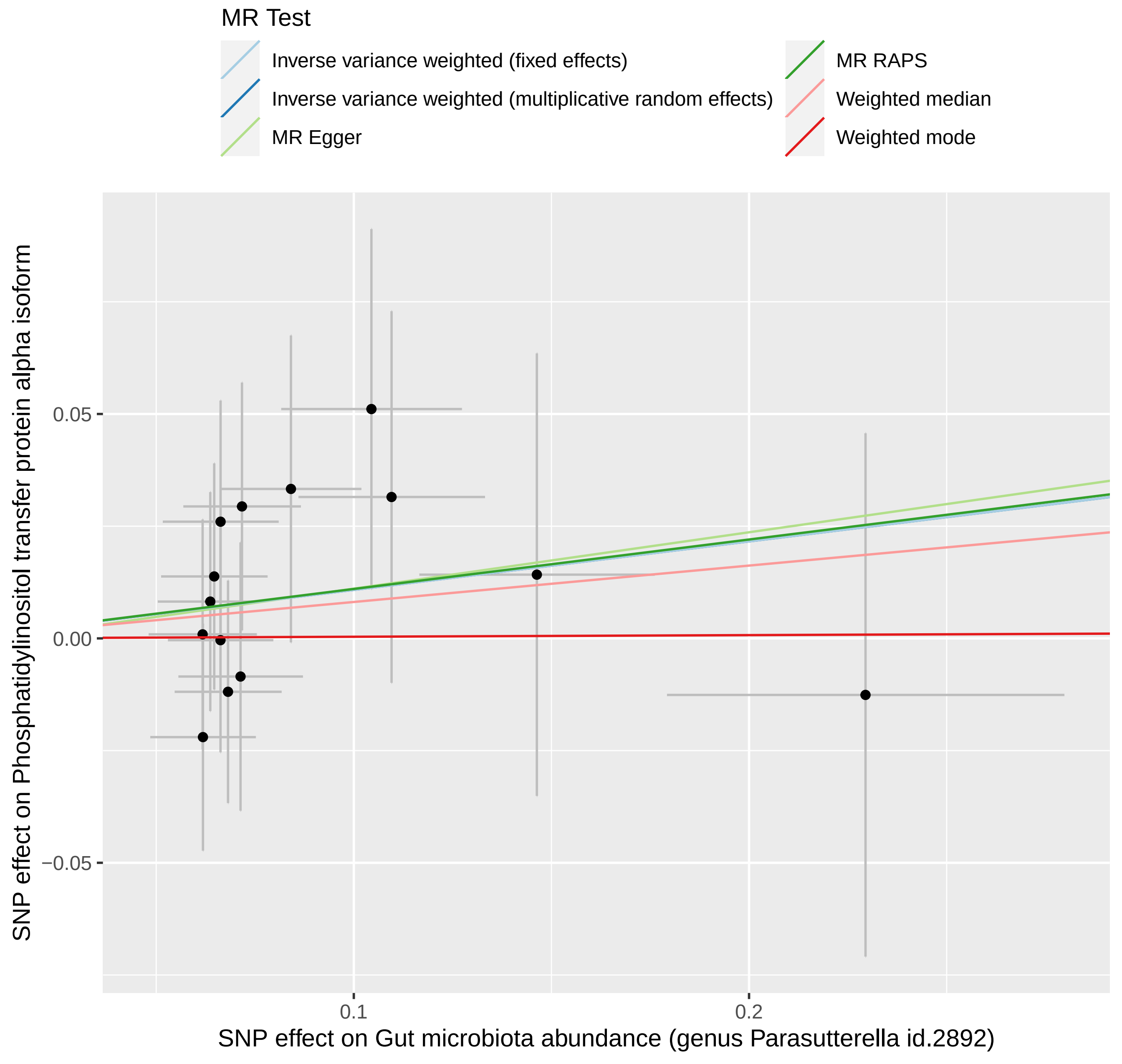

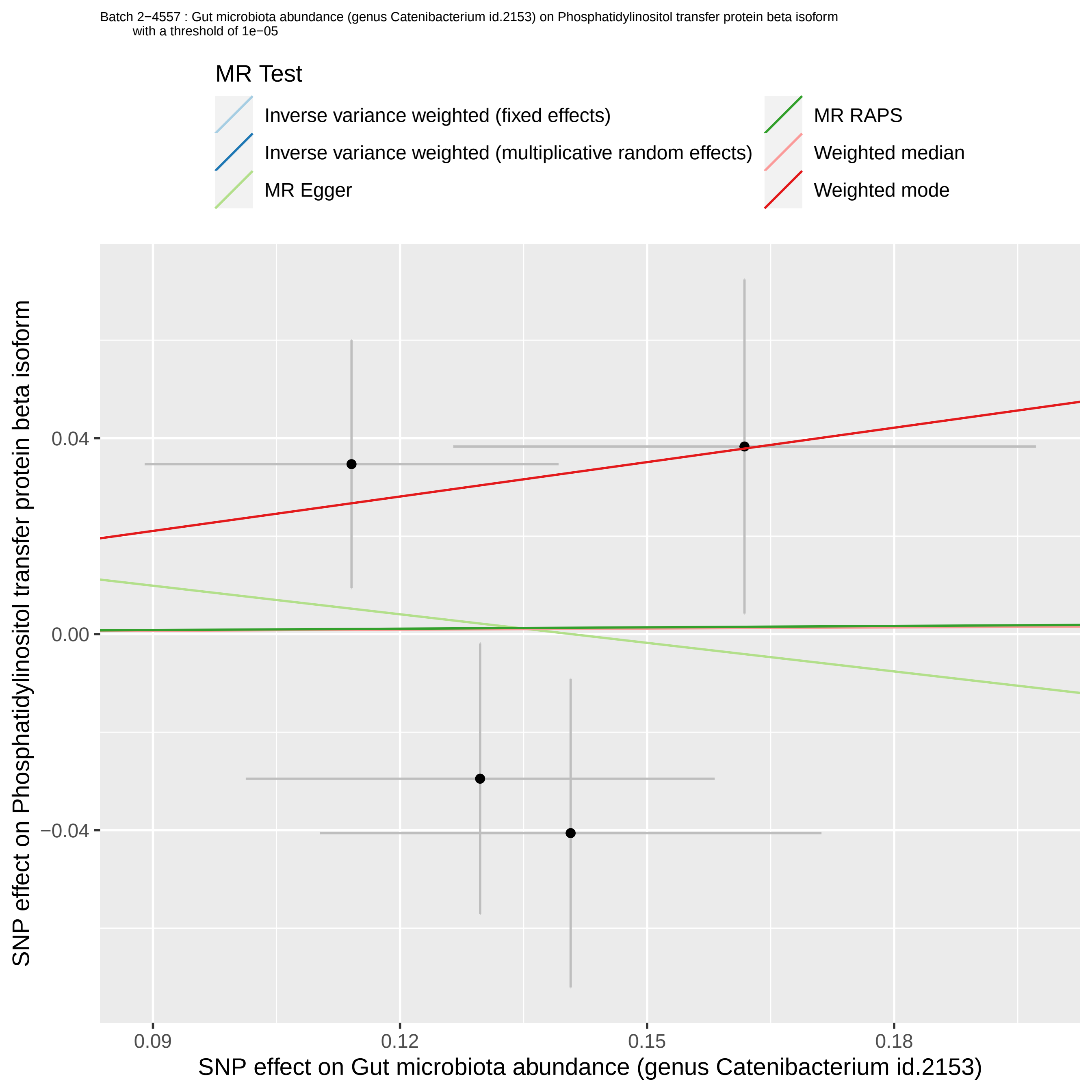
Batch 2−4557 : Gut microbiota abundance (genus Catenibacterium id.2153) on Phosphatidylinositol transfer protein beta isoform
with a threshold of 1e−05
MR Test
Inverse variance weighted (fixed effects)
Inverse variance weighted (multiplicative random effects)
MR Egger
MR RAPS
Weighted median
Weighted mode
0.04
0.00
−0.04
0.09
0.12
0.15
0.18
SNP effect on Phosphatidylinositol transfer protein beta isoform
SNP effect on Gut microbiota abundance (genus Catenibacterium id.2153)

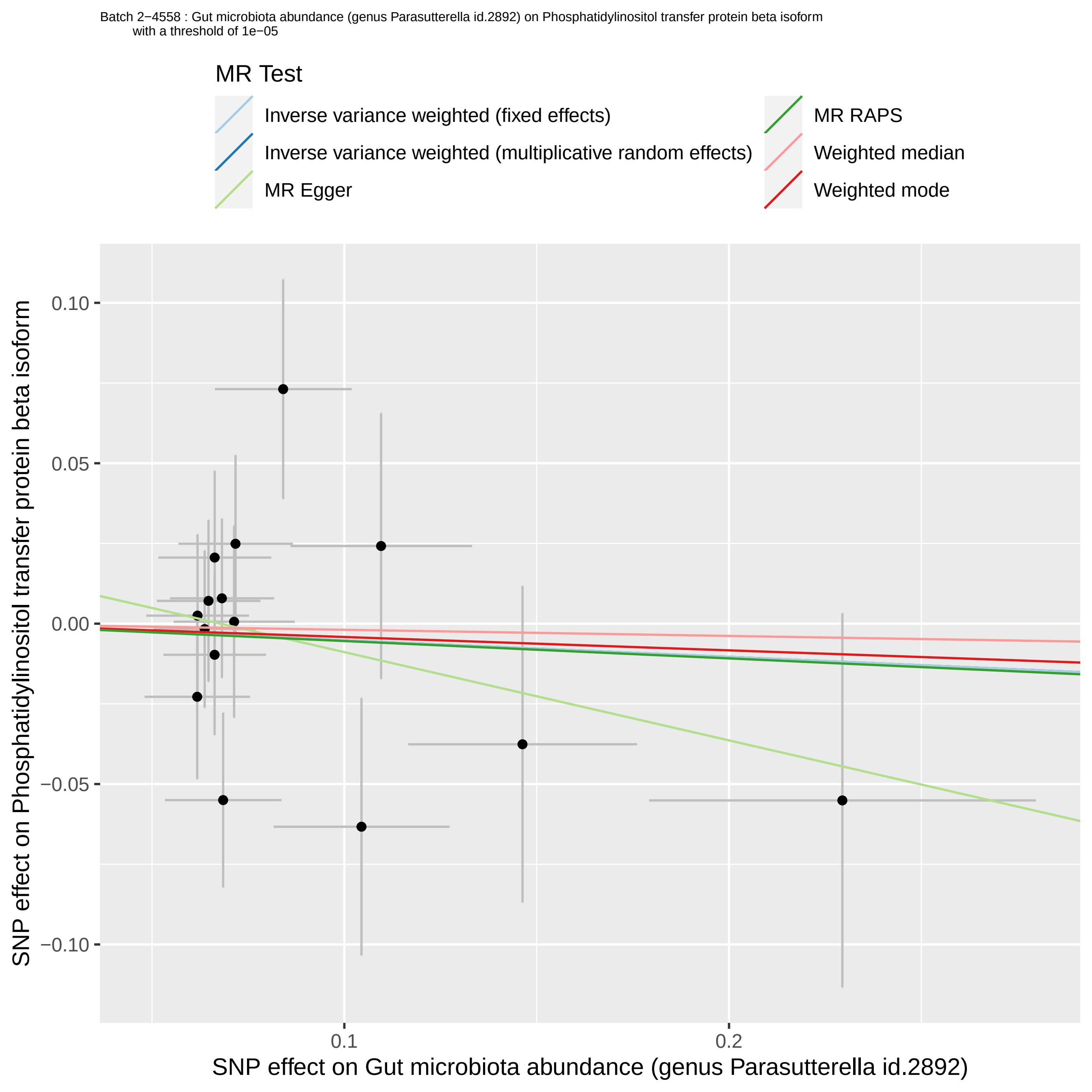
Batch 2−4558 : Gut microbiota abundance (genus Parasutterella id.2892) on Phosphatidylinositol transfer protein beta isoform
with a threshold of 1e−05
MR Test
Inverse variance weighted (fixed effects)
Inverse variance weighted (multiplicative random effects)
MR Egger
MR RAPS
Weighted median
Weighted mode
0.10
0.05
0.00
−0.05
−0.10
0.1
0.2
SNP effect on Phosphatidylinositol transfer protein beta isoform
SNP effect on Gut microbiota abundance (genus Parasutterella id.2892)

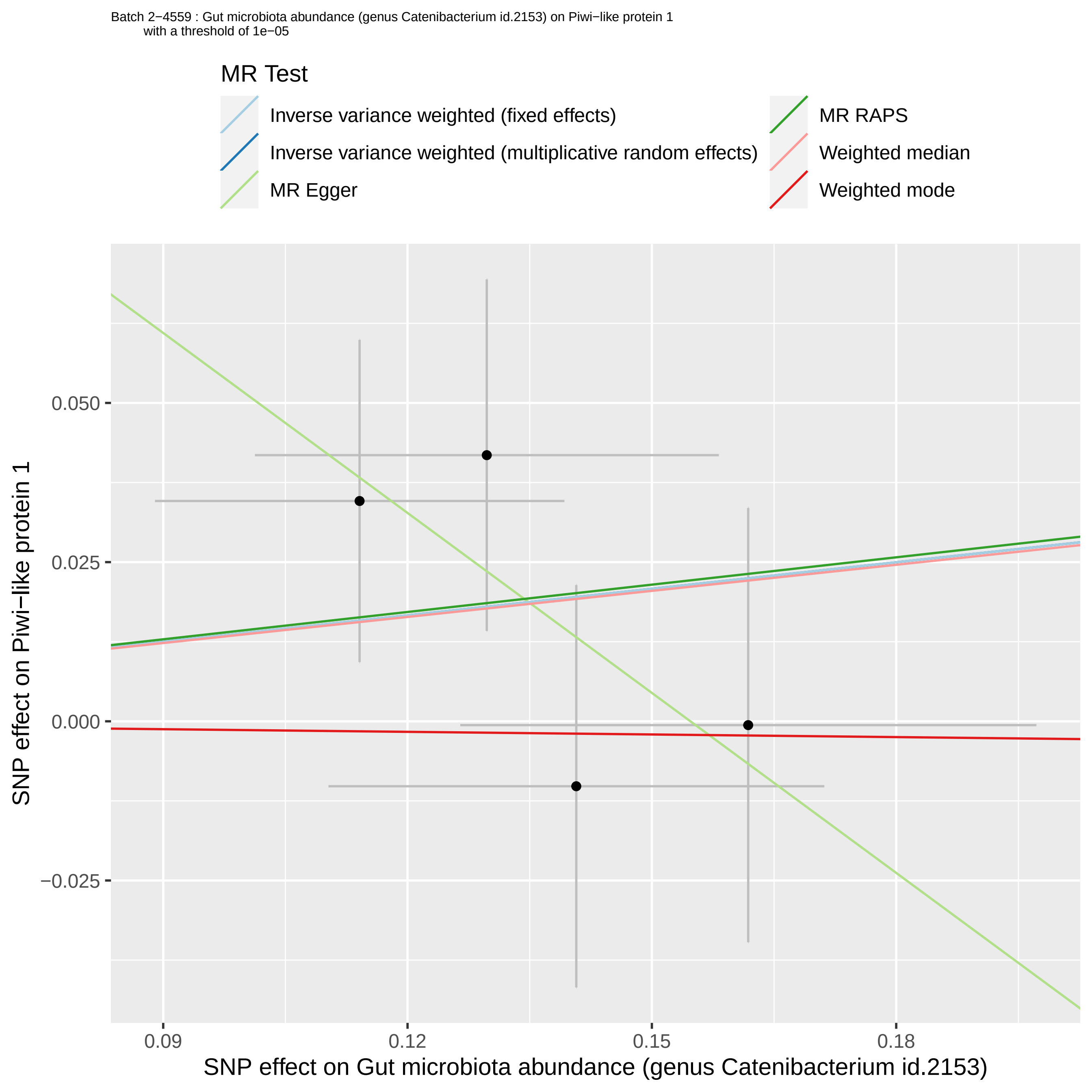

Batch 2−4559 : Gut microbiota abundance (genus Catenibacterium id.2153) on Piwi−like protein 1
with a threshold of 1e−05
MR Test
Inverse variance weighted (fixed effects)
Inverse variance weighted (multiplicative random effects)
MR Egger
MR RAPS
Weighted median
Weighted mode
0.050
0.025
0.000
−0.025
0.09
0.12
0.15
0.18
SNP effect on Gut microbiota abundance (genus Catenibacterium id.2153)
SNP effect on Piwi−like protein 1

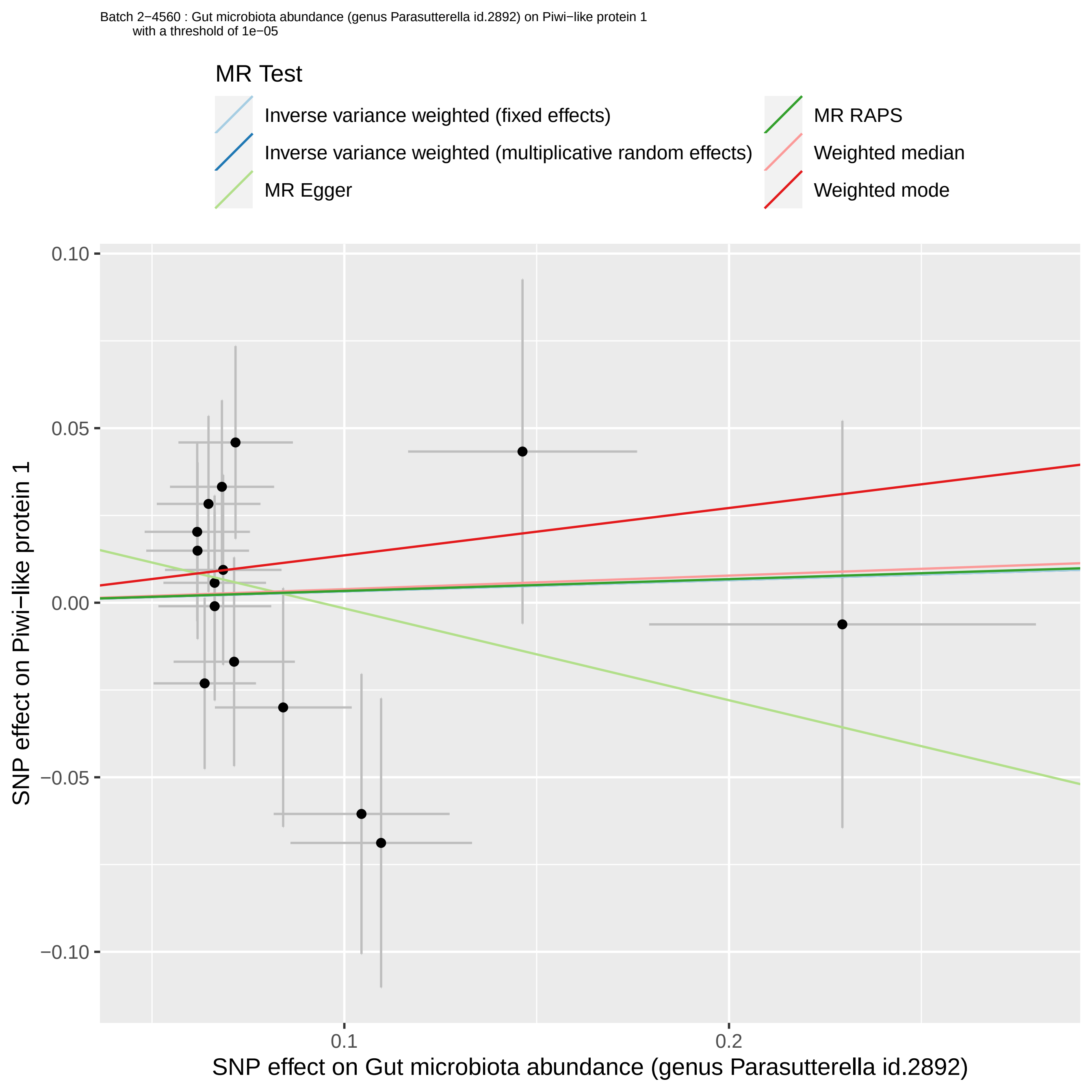
Batch 2−4560 : Gut microbiota abundance (genus Parasutterella id.2892) on Piwi−like protein 1
with a threshold of 1e−05
MR Test
Inverse variance weighted (fixed effects)
Inverse variance weighted (multiplicative random effects)
MR Egger
MR RAPS
Weighted median
Weighted mode
0.10
0.05
0.00
−0.05
−0.10
0.1
0.2
SNP effect on Piwi−like protein 1
SNP effect on Gut microbiota abundance (genus Parasutterella id.2892)

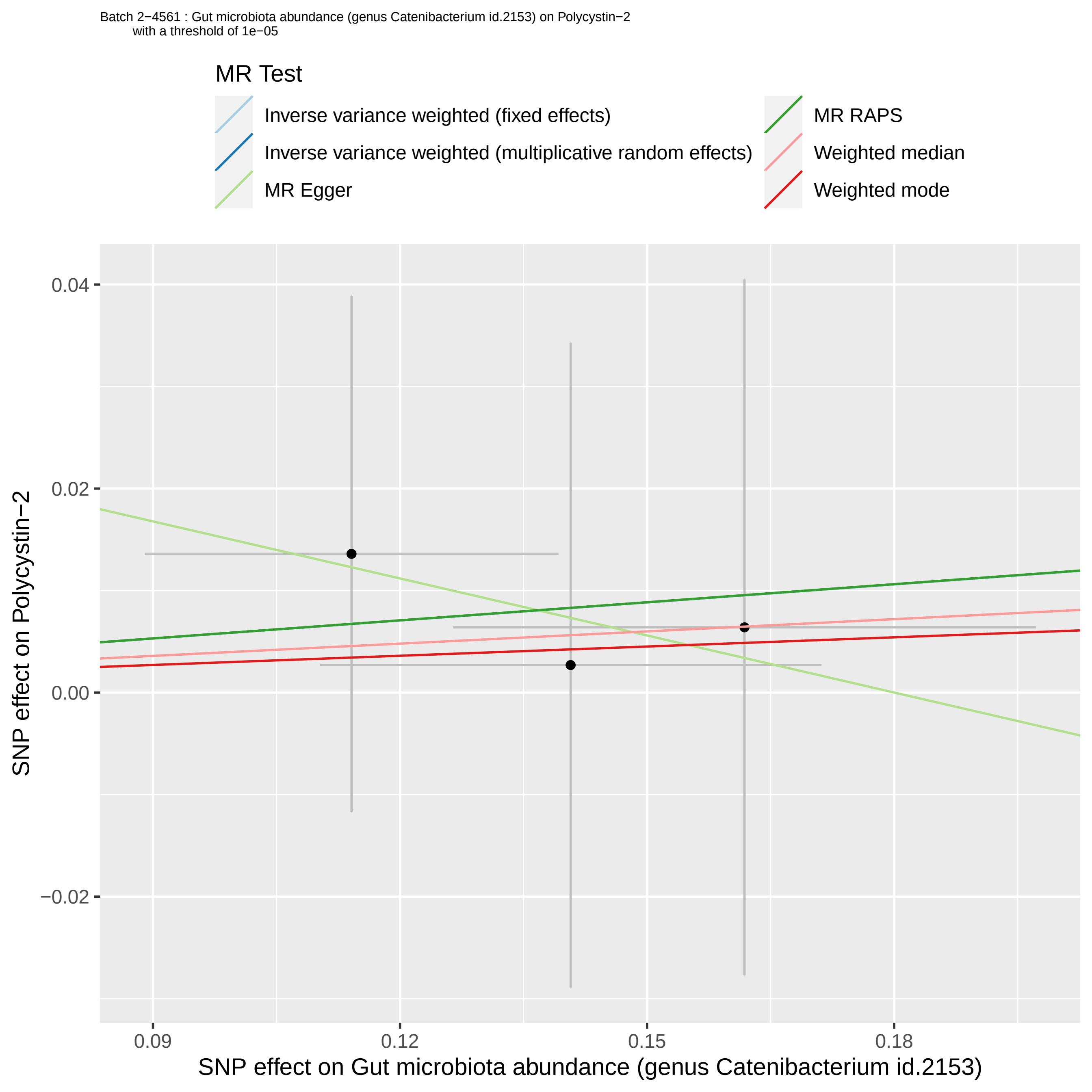
Batch 2−4561 : Gut microbiota abundance (genus Catenibacterium id.2153) on Polycystin−2
with a threshold of 1e−05
MR Test
Inverse variance weighted (fixed effects)
Inverse variance weighted (multiplicative random effects)
MR Egger
MR RAPS
Weighted median
Weighted mode
0.04
0.02
0.00
−0.02
0.09
0.12
0.15
0.18
SNP effect on Polycystin−2
SNP effect on Gut microbiota abundance (genus Catenibacterium id.2153)

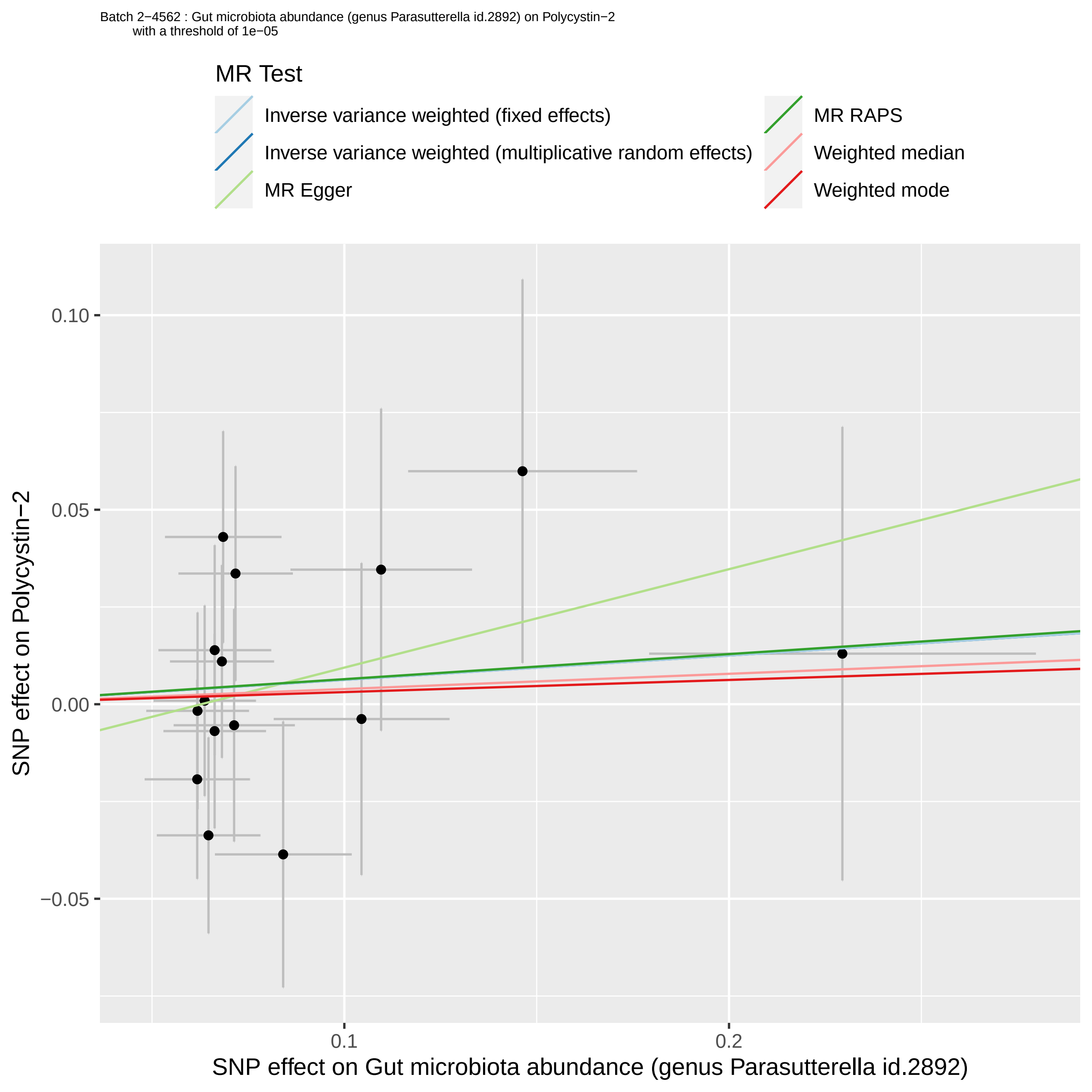

Batch 2−4562 : Gut microbiota abundance (genus Parasutterella id.2892) on Polycystin−2
with a threshold of 1e−05
MR Test
Inverse variance weighted (fixed effects)
Inverse variance weighted (multiplicative random effects)
MR Egger
MR RAPS
Weighted median
Weighted mode
0.10
0.05
0.00
−0.05
0.1
0.2
SNP effect on Polycystin−2
SNP effect on Gut microbiota abundance (genus Parasutterella id.2892)

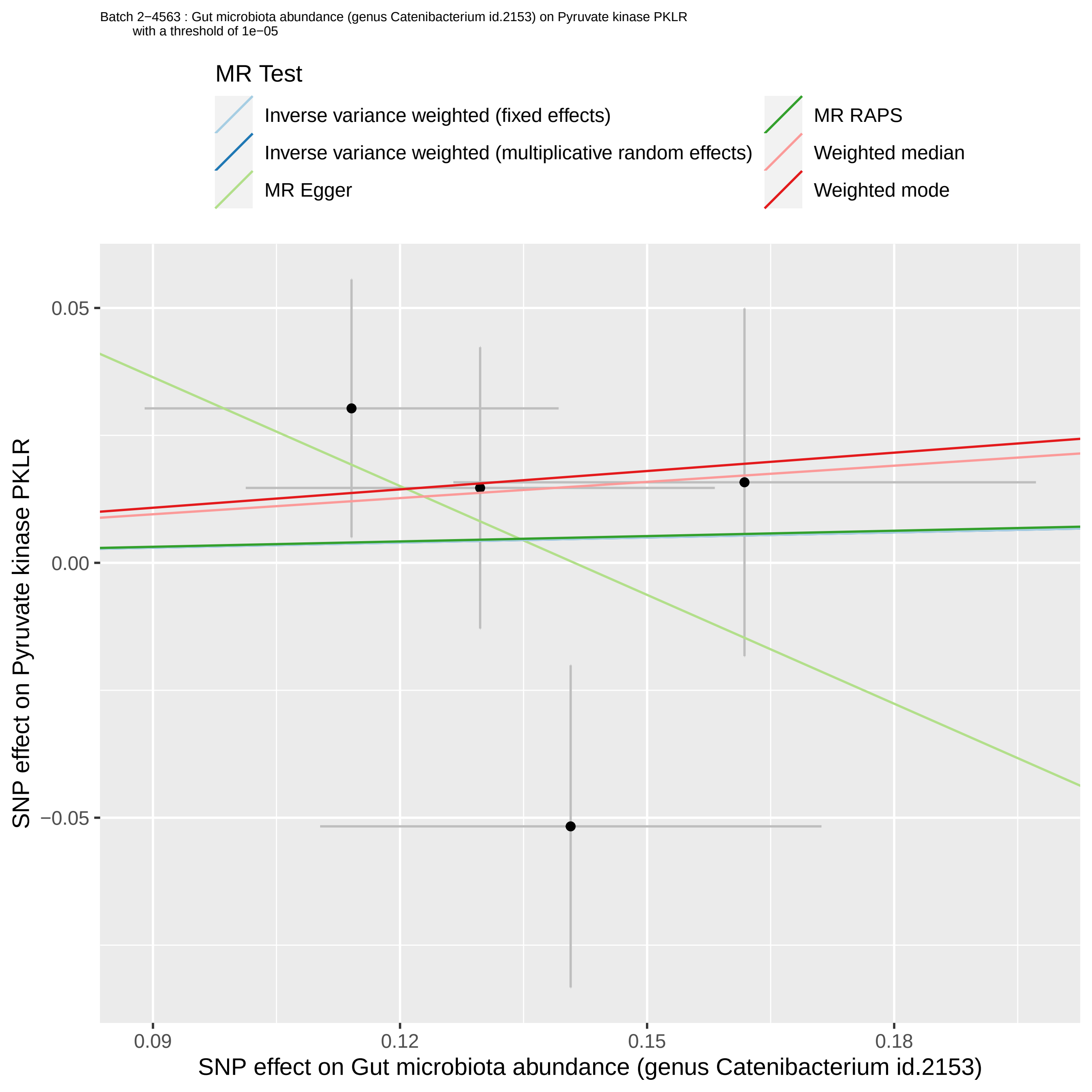

Batch 2−4563 : Gut microbiota abundance (genus Catenibacterium id.2153) on Pyruvate kinase PKLR
with a threshold of 1e−05
MR Test
Inverse variance weighted (fixed effects)
Inverse variance weighted (multiplicative random effects)
MR Egger
MR RAPS
Weighted median
Weighted mode
0.05
0.00
−0.05
0.09
0.12
0.15
0.18
SNP effect on Pyruvate kinase PKLR
SNP effect on Gut microbiota abundance (genus Catenibacterium id.2153)

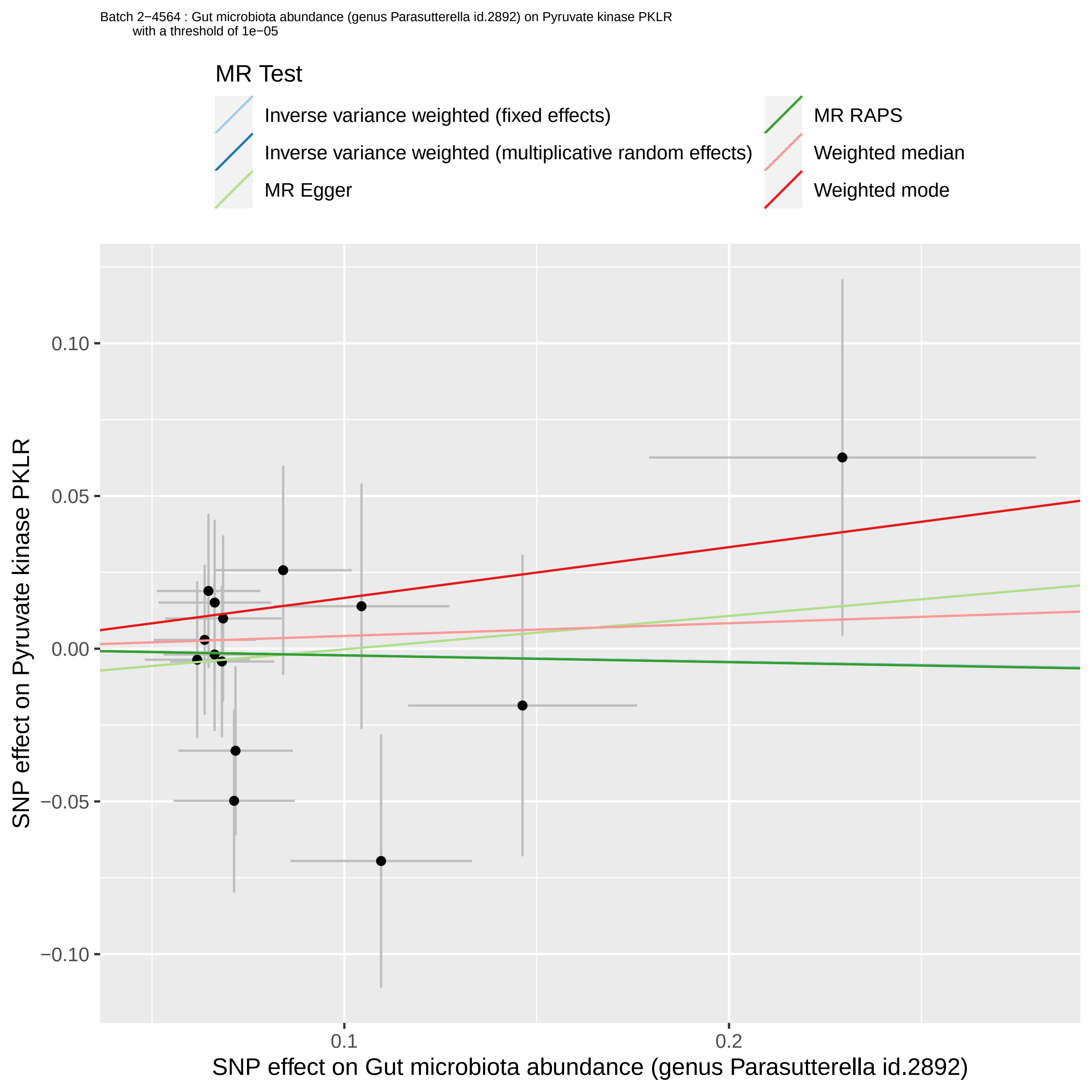
Batch 2−4564 : Gut microbiota abundance (genus Parasutterella id.2892) on Pyruvate kinase PKLR
with a threshold of 1e−05
MR Test
Inverse variance weighted (fixed effects)
Inverse variance weighted (multiplicative random effects)
MR Egger
MR RAPS
Weighted median
Weighted mode
0.10
0.05
0.00
−0.05
−0.10
0.1
0.2
SNP effect on Pyruvate kinase PKLR
SNP effect on Gut microbiota abundance (genus Parasutterella id.2892)

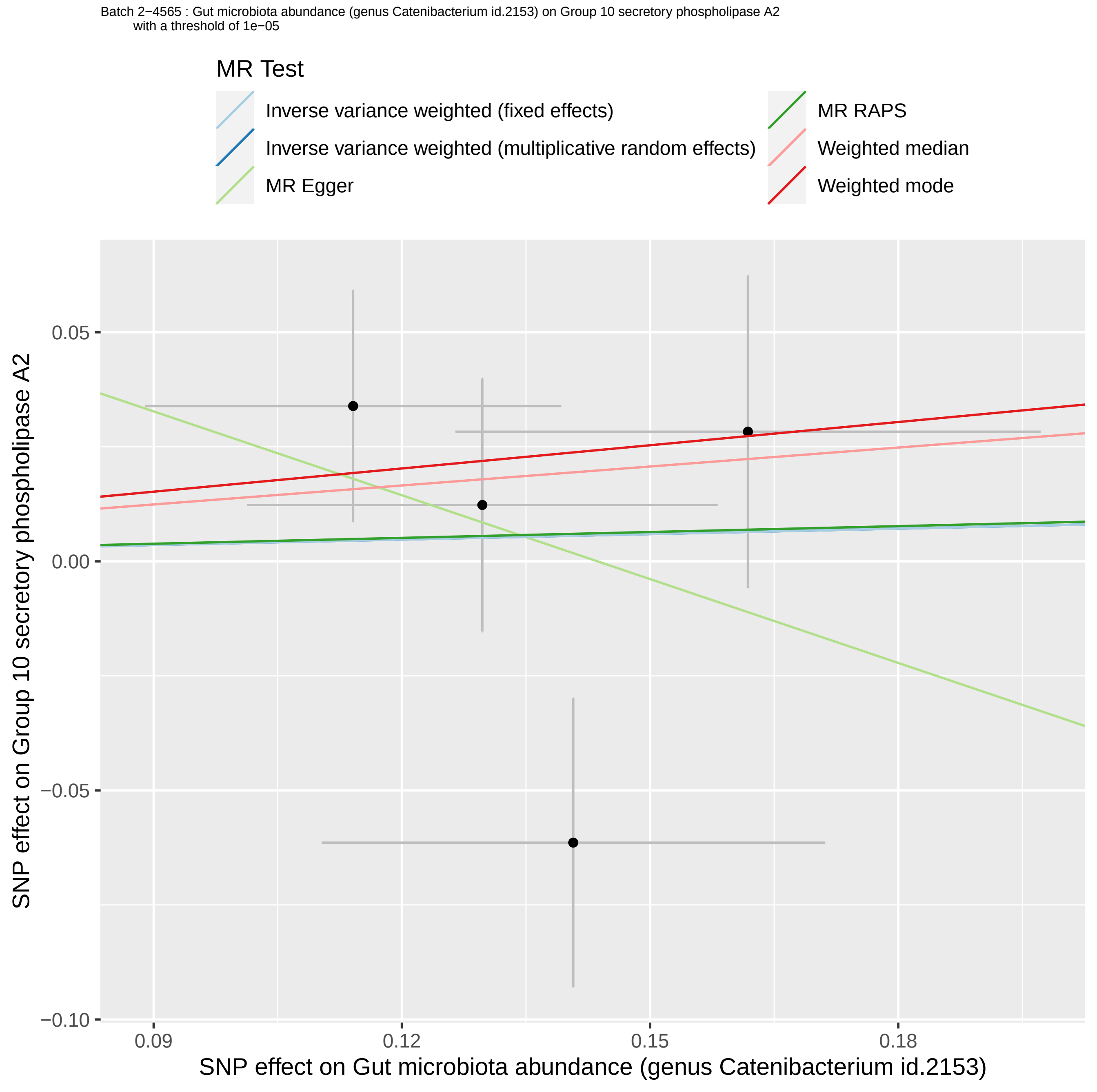
Batch 2−4565 : Gut microbiota abundance (genus Catenibacterium id.2153) on Group 10 secretory phospholipase A2
with a threshold of 1e−05
MR Test
Inverse variance weighted (fixed effects)
Inverse variance weighted (multiplicative random effects)
MR Egger
MR RAPS
Weighted median
Weighted mode
0.05
0.00
−0.05
−0.10
0.09
0.12
0.15
0.18
SNP effect on Group 10 secretory phospholipase A2
SNP effect on Gut microbiota abundance (genus Catenibacterium id.2153)

Batch 2−4566 : Gut microbiota abundance (genus Parasutterella id.2892) on Group 10 secretory phospholipase A2
with a threshold of 1e−05

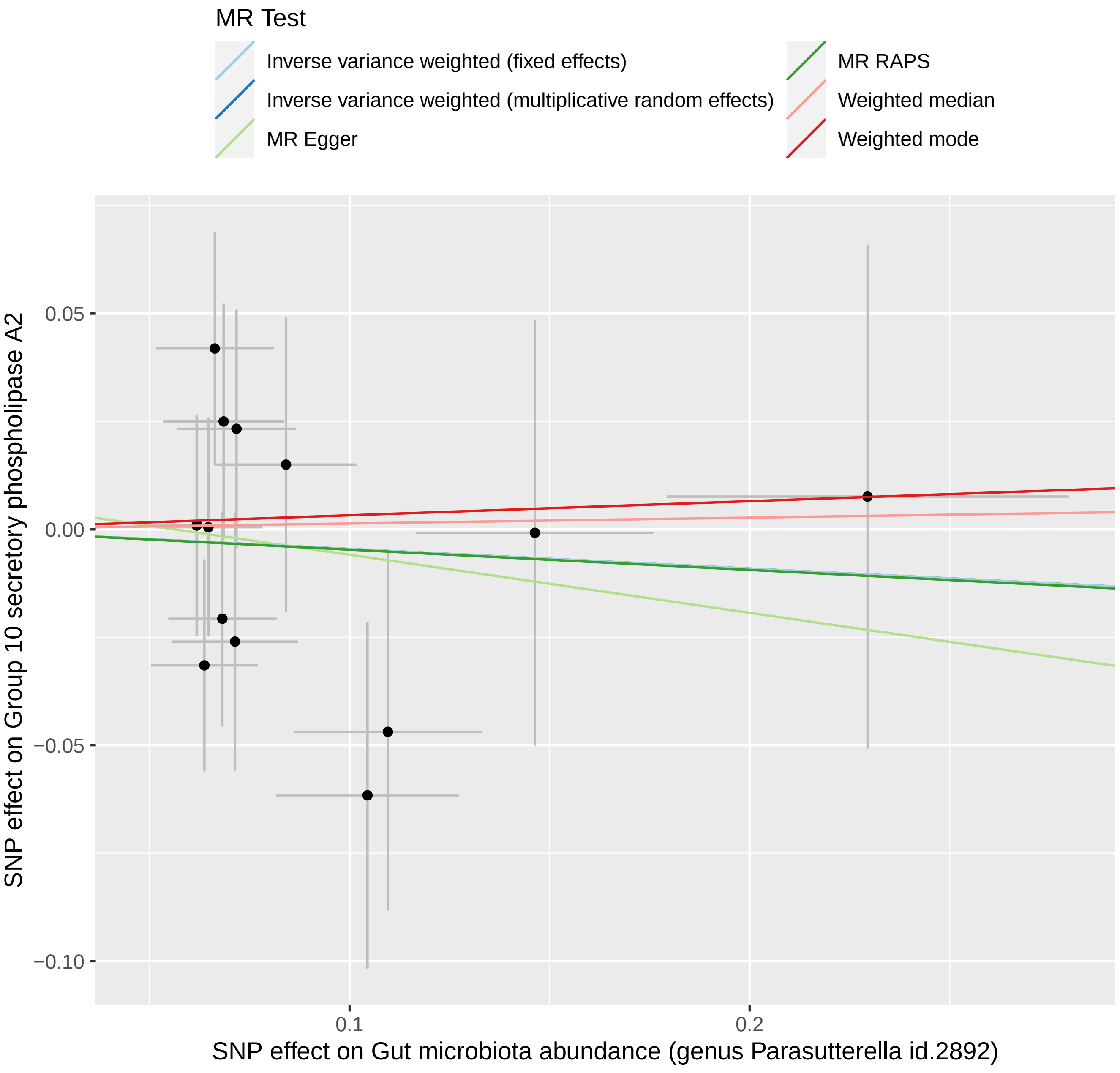

Batch 2−4567 : Gut microbiota abundance (genus Catenibacterium id.2153) on Group XIIB secretory phospholipase A2−like protein
with a threshold of 1e−05

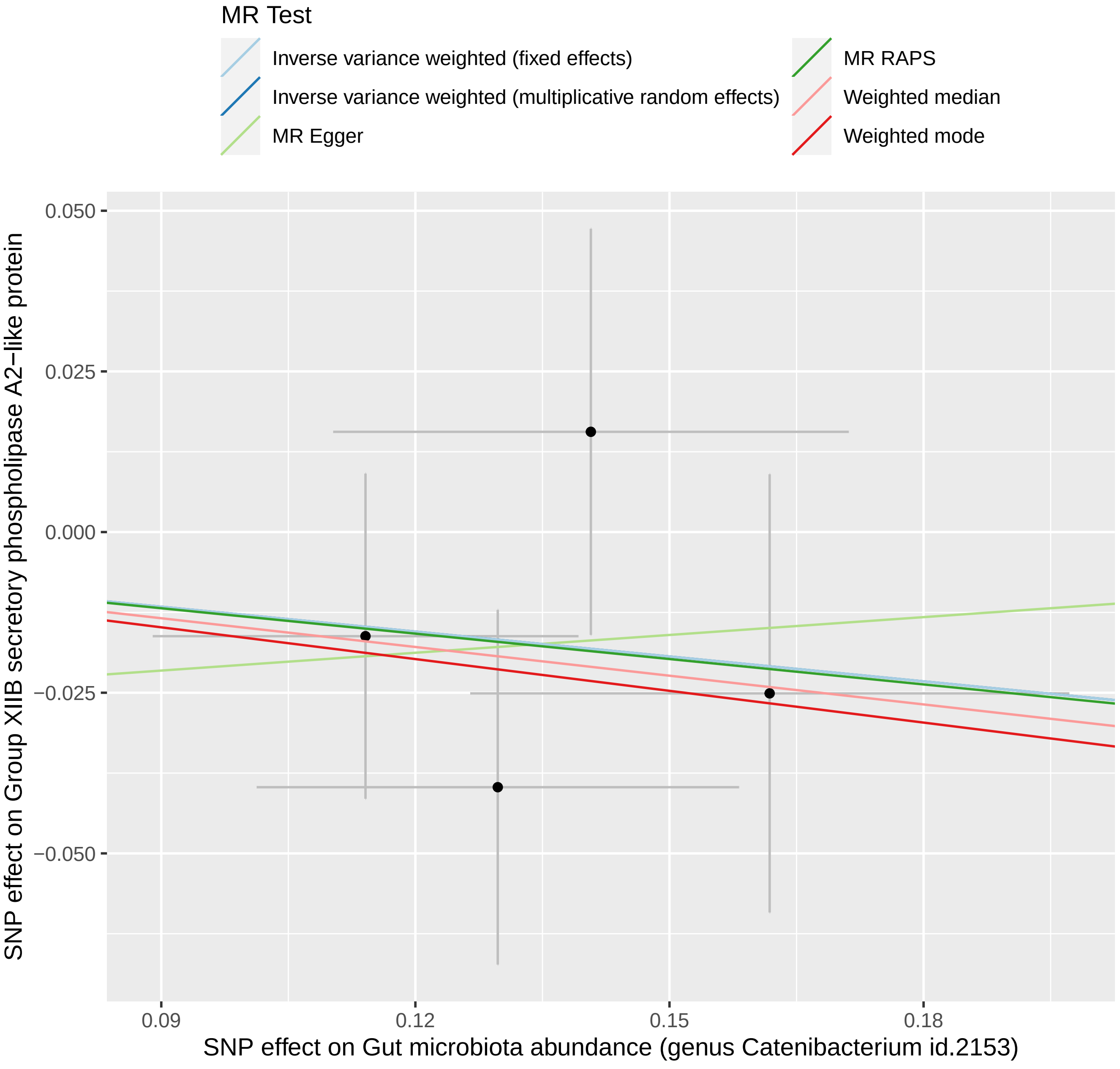

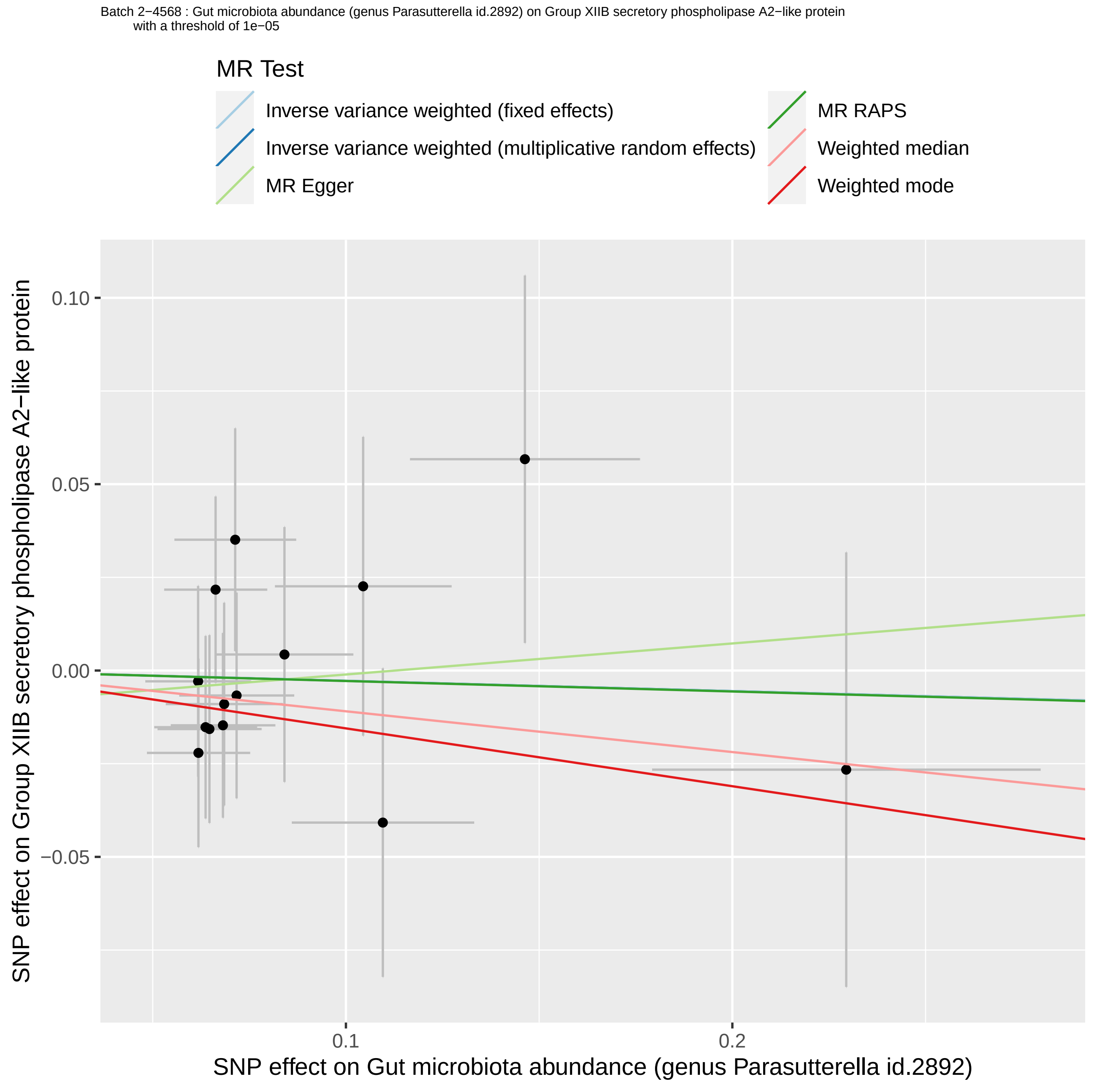

Batch 2−4568 : Gut microbiota abundance (genus Parasutterella id.2892) on Group XIIB secretory phospholipase A2−like protein
with a threshold of 1e−05
MR Test
Inverse variance weighted (fixed effects)
Inverse variance weighted (multiplicative random effects)
MR Egger
MR RAPS
Weighted median
Weighted mode
0.10
0.05
0.00
−0.05
0.1
0.2
SNP effect on Group XIIB secretory phospholipase A2−like protein
SNP effect on Gut microbiota abundance (genus Parasutterella id.2892)

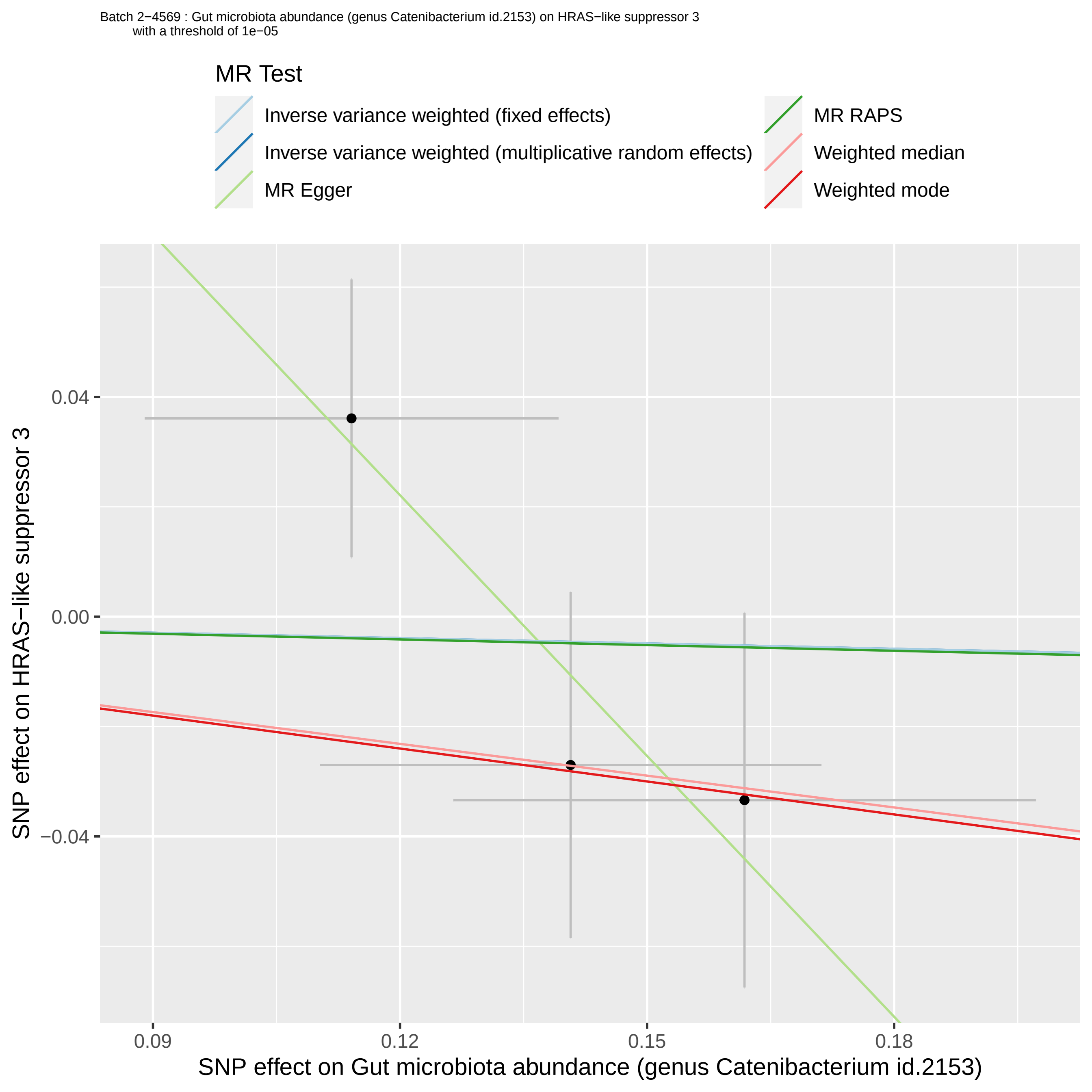

Batch 2−4569 : Gut microbiota abundance (genus Catenibacterium id.2153) on HRAS−like suppressor 3
with a threshold of 1e−05
MR Test
Inverse variance weighted (fixed effects)
Inverse variance weighted (multiplicative random effects)
MR Egger
MR RAPS
Weighted median
Weighted mode
0.04
0.00
−0.04
0.09
0.12
0.15
0.18
SNP effect on HRAS−like suppressor 3
SNP effect on Gut microbiota abundance (genus Catenibacterium id.2153)

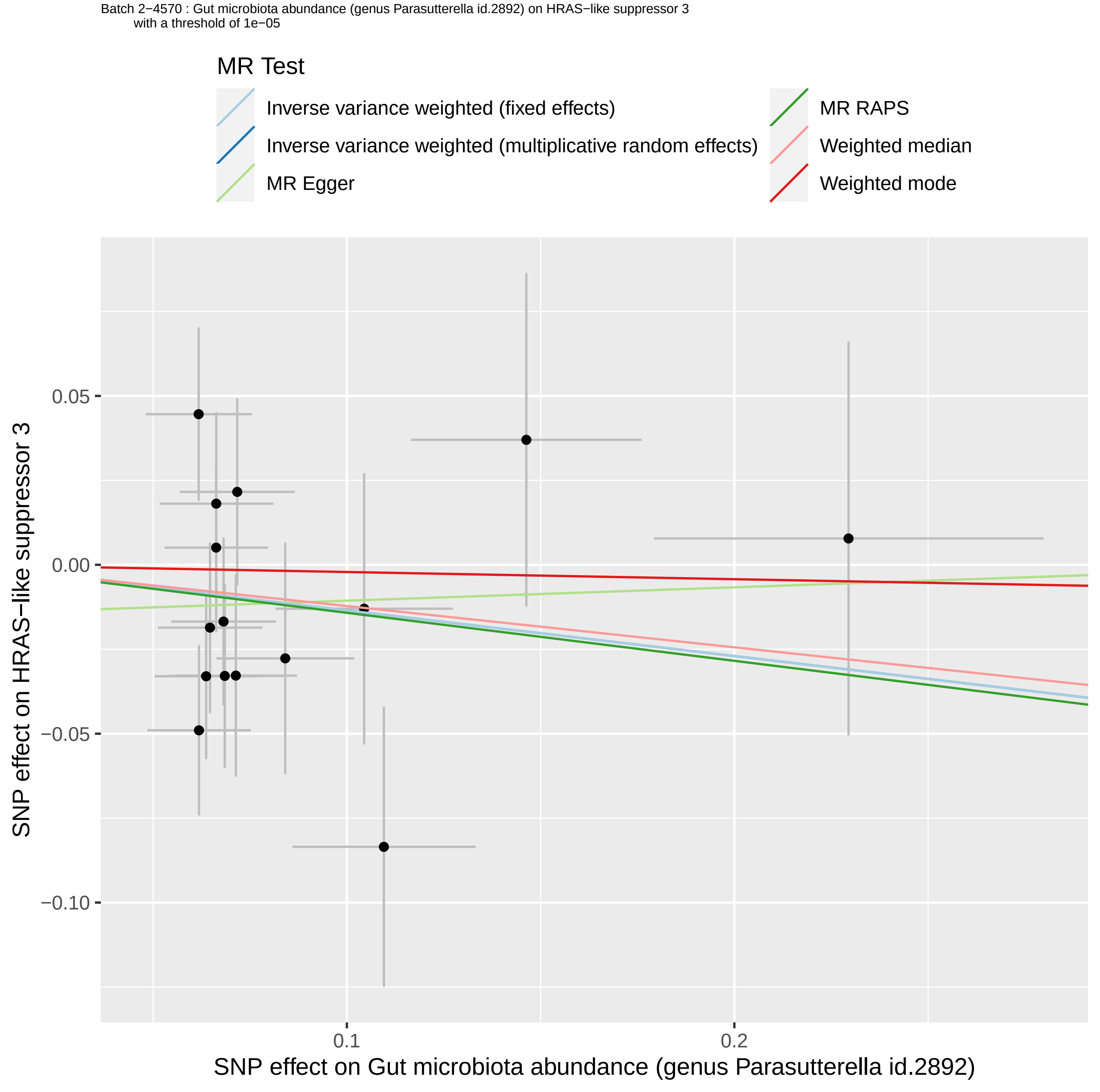

Batch 2−4570 : Gut microbiota abundance (genus Parasutterella id.2892) on HRAS−like suppressor 3
with a threshold of 1e−05
MR Test
Inverse variance weighted (fixed effects)
Inverse variance weighted (multiplicative random effects)
MR Egger
MR RAPS
Weighted median
Weighted mode
0.05
0.00
−0.05
−0.10
0.1
0.2
SNP effect on HRAS−like suppressor 3
SNP effect on Gut microbiota abundance (genus Parasutterella id.2892)

Batch 2−4571 : Gut microbiota abundance (genus Catenibacterium id.2153) on Phospholipase A2, membrane associated
with a threshold of 1e−05

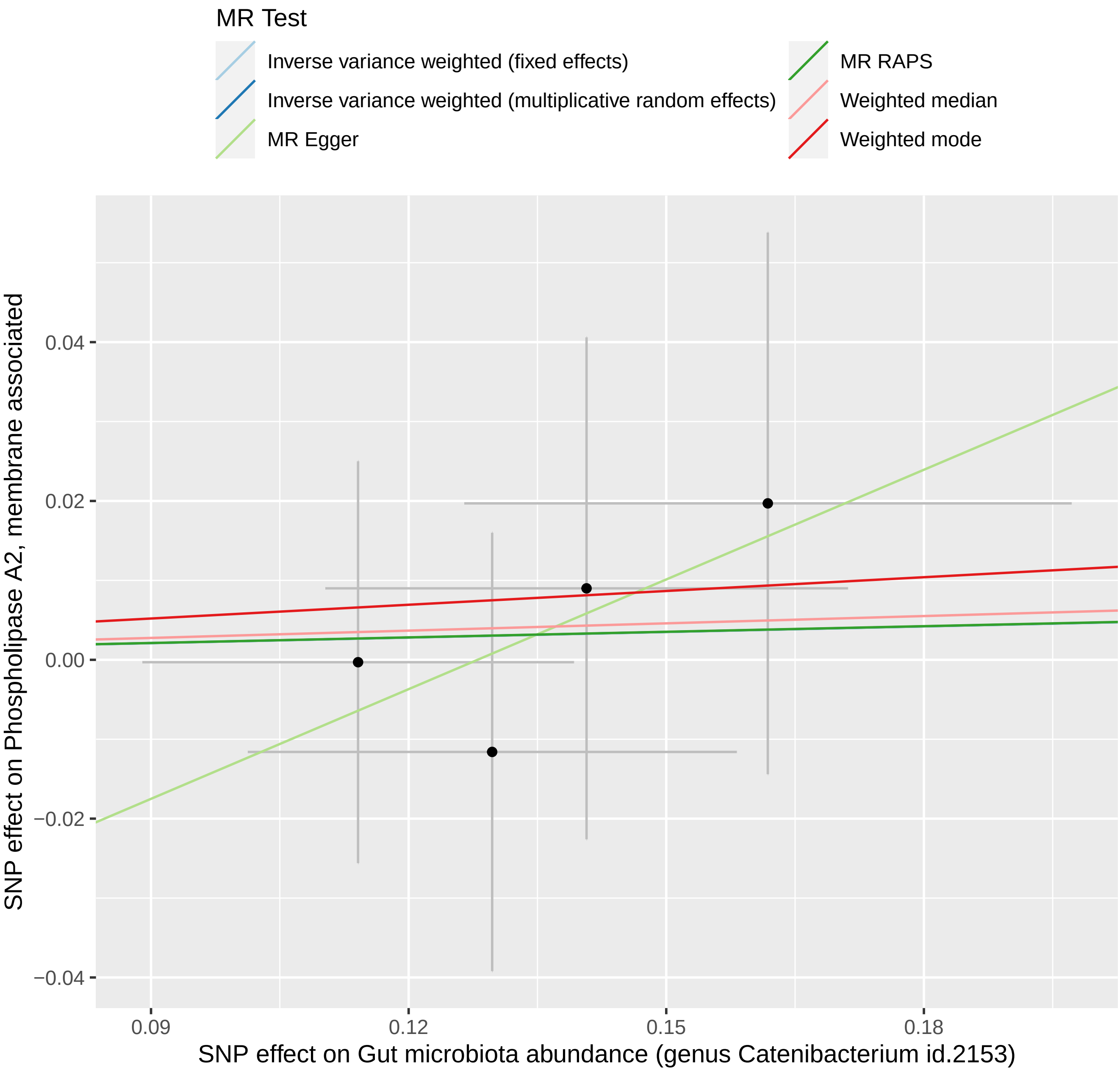

Batch 2−4572 : Gut microbiota abundance (genus Parasutterella id.2892) on Phospholipase A2, membrane associated
with a threshold of 1e−05

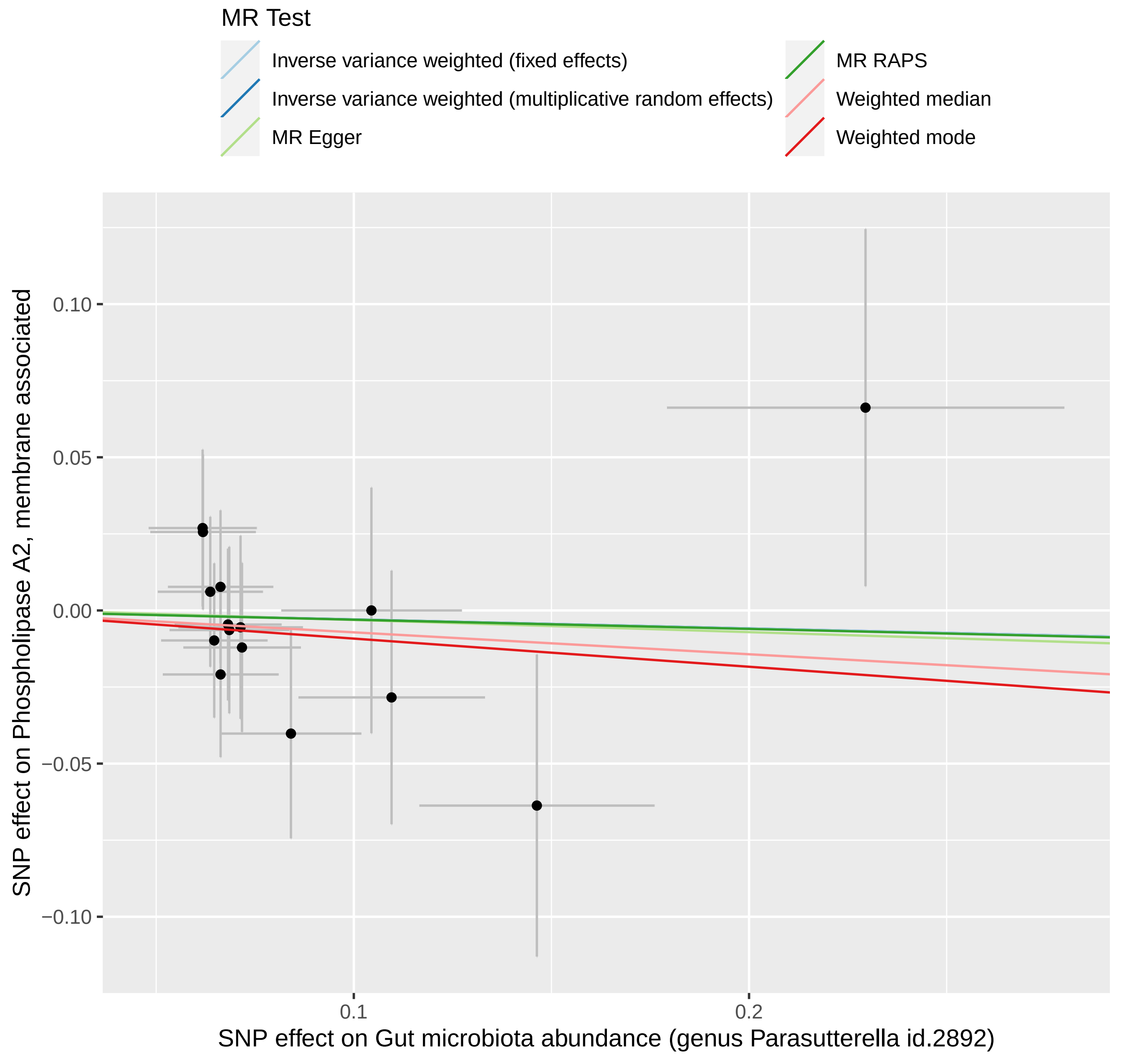

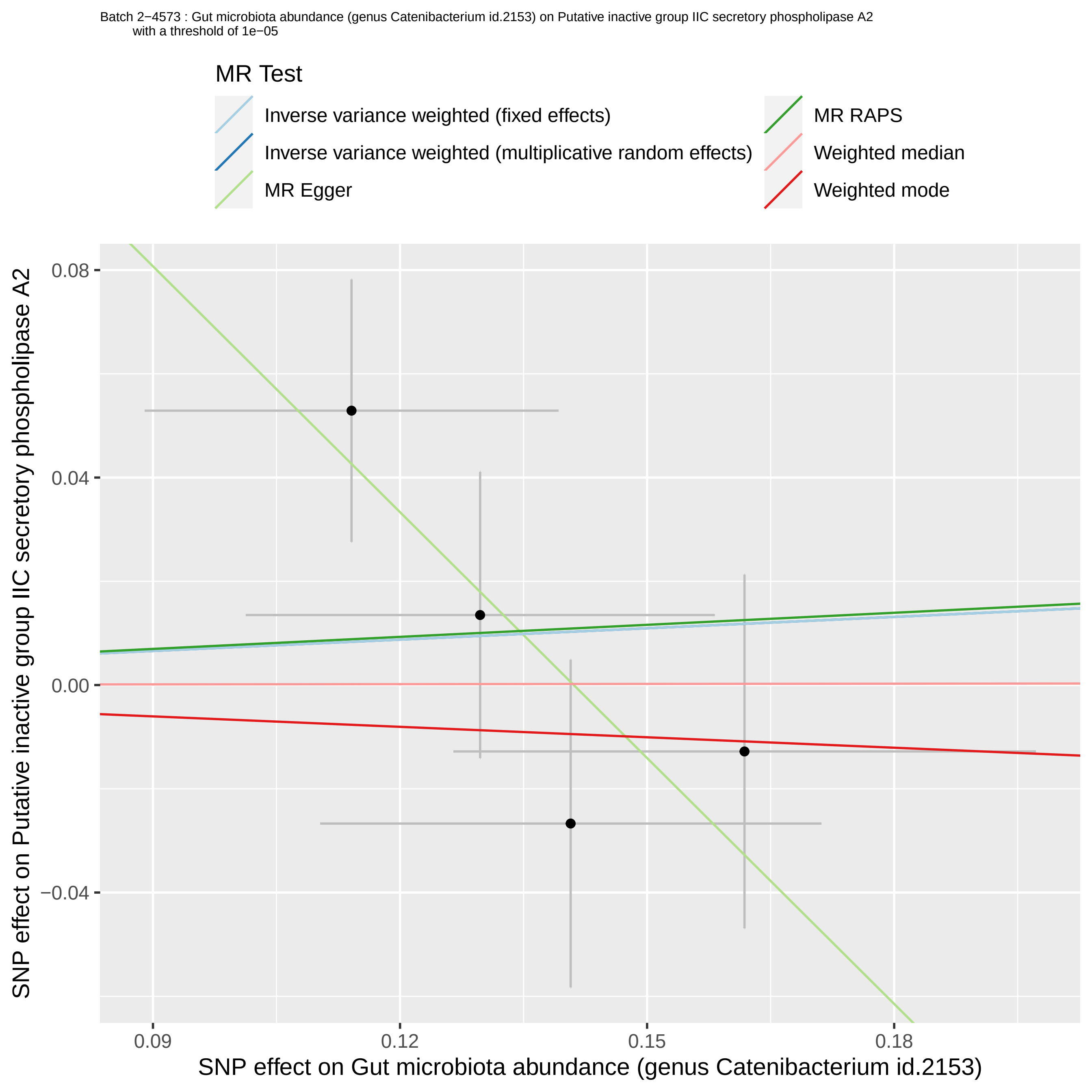

Batch 2−4573 : Gut microbiota abundance (genus Catenibacterium id.2153) on Putative inactive group IIC secretory phospholipase A2
with a threshold of 1e−05
MR Test
Inverse variance weighted (fixed effects)
Inverse variance weighted (multiplicative random effects)
MR Egger
MR RAPS
Weighted median
Weighted mode
0.08
0.04
0.00
−0.04
0.09
0.12
0.15
0.18
SNP effect on Putative inactive group IIC secretory phospholipase A2
SNP effect on Gut microbiota abundance (genus Catenibacterium id.2153)

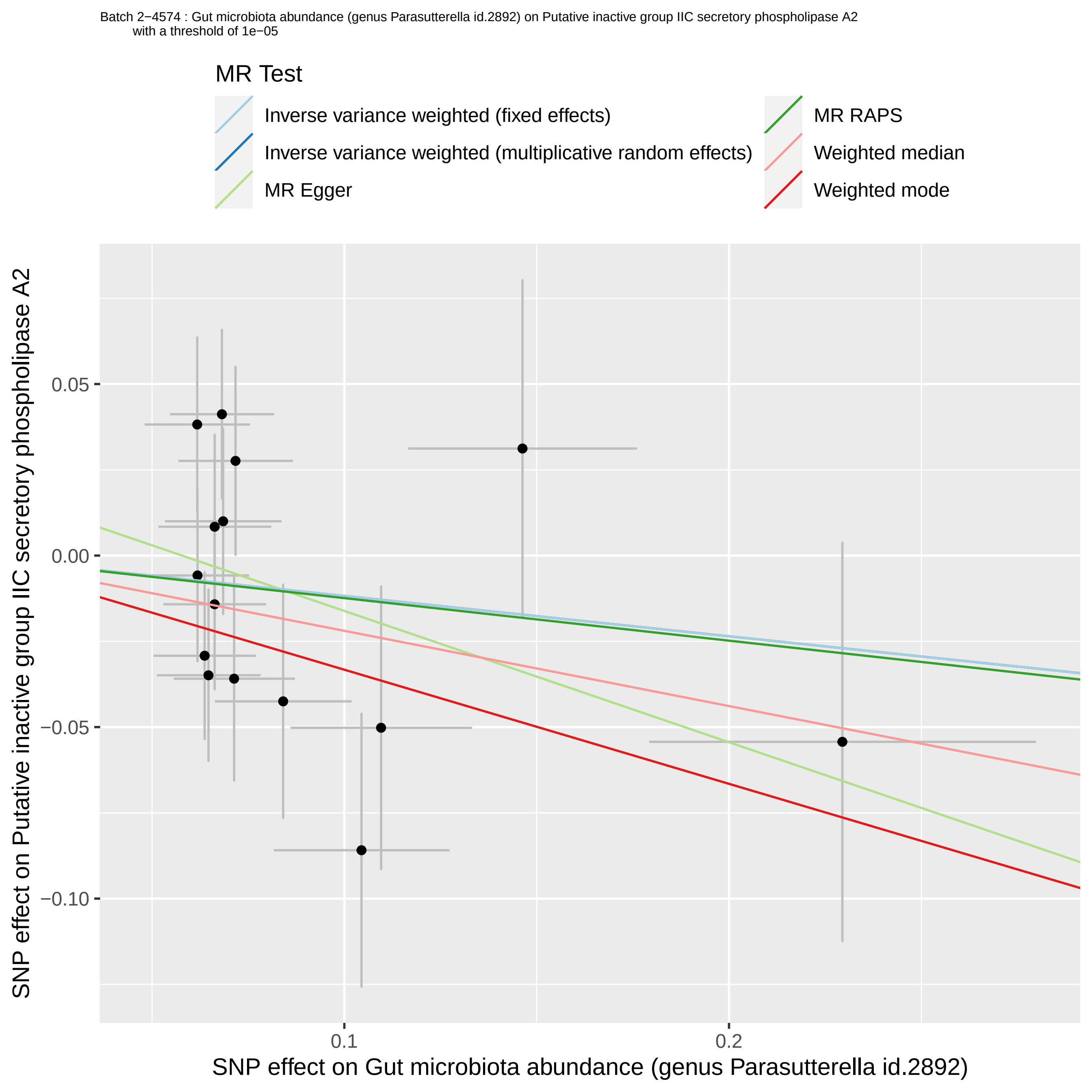
Batch 2−4574 : Gut microbiota abundance (genus Parasutterella id.2892) on Putative inactive group IIC secretory phospholipase A2
with a threshold of 1e−05
MR Test
Inverse variance weighted (fixed effects)
Inverse variance weighted (multiplicative random effects)
MR Egger
MR RAPS
Weighted median
Weighted mode
0.05
0.00
−0.05
−0.10
0.1
0.2
SNP effect on Putative inactive group IIC secretory phospholipase A2
SNP effect on Gut microbiota abundance (genus Parasutterella id.2892)

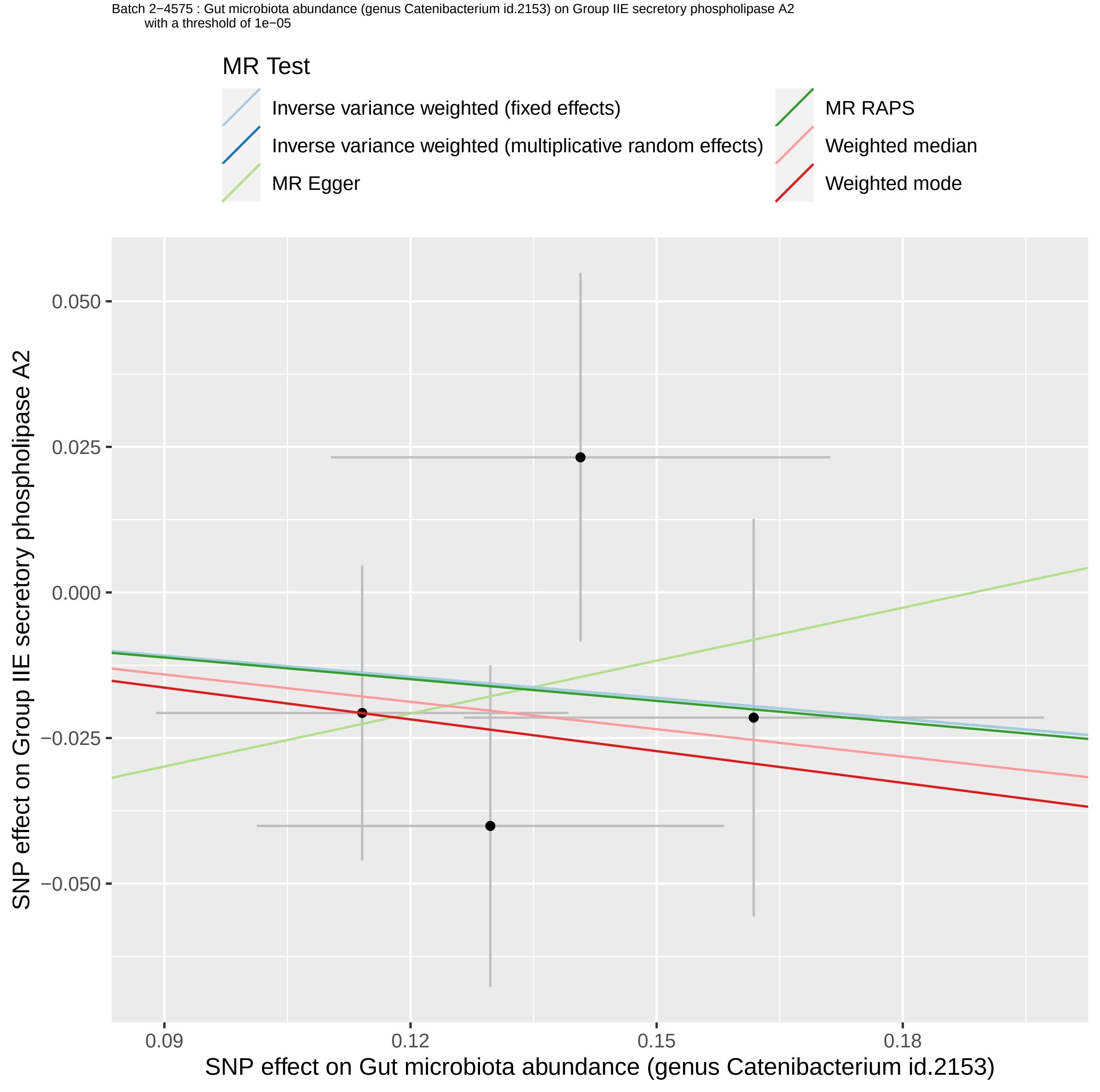

Batch 2−4575 : Gut microbiota abundance (genus Catenibacterium id.2153) on Group IIE secretory phospholipase A2
with a threshold of 1e−05
MR Test
Inverse variance weighted (fixed effects)
Inverse variance weighted (multiplicative random effects)
MR Egger
MR RAPS
Weighted median
Weighted mode
0.050
0.025
0.000
−0.025
−0.050
SNP effect on Group IIE secretory phospholipase A2
0.09
0.12
0.15
0.18
SNP effect on Gut microbiota abundance (genus Catenibacterium id.2153)

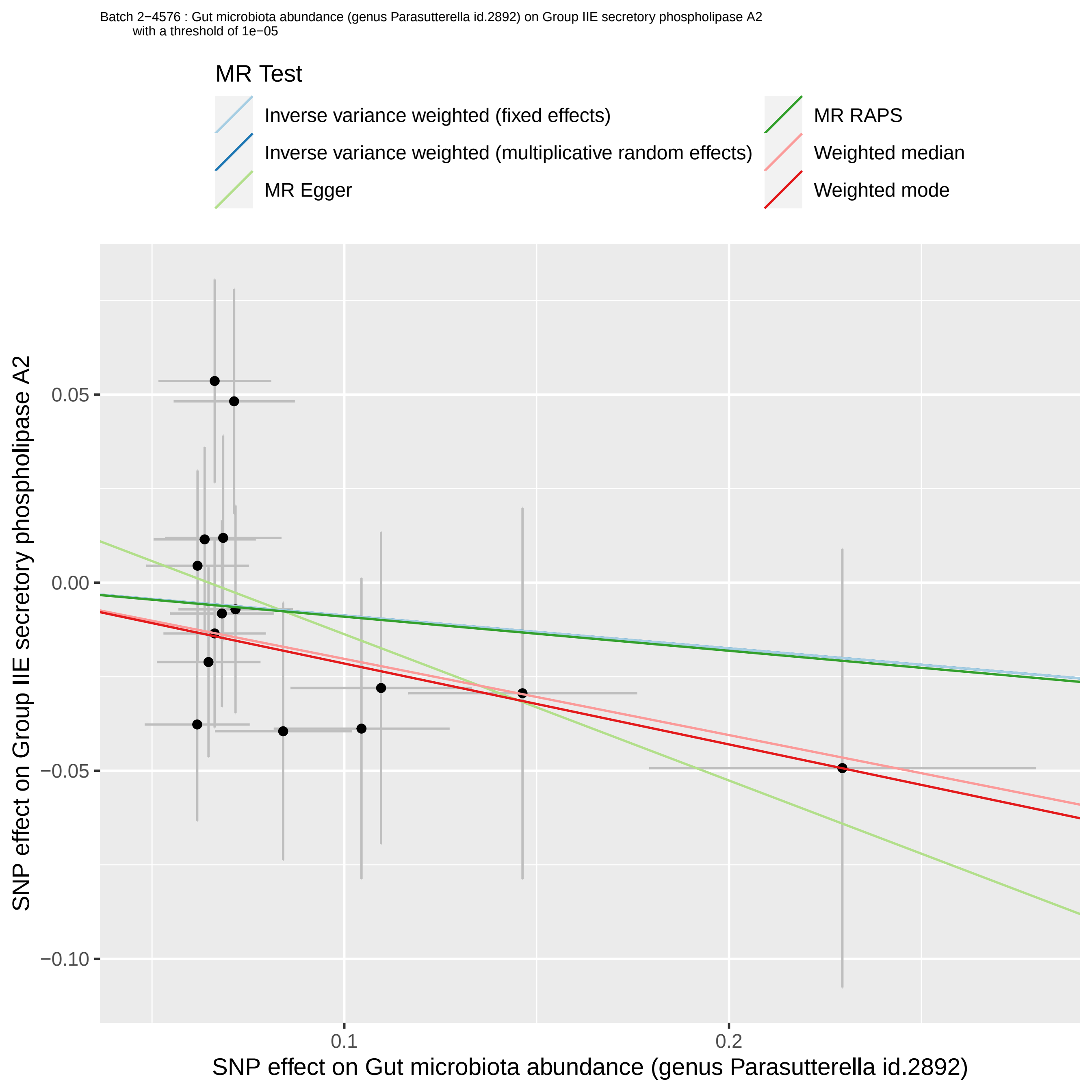
Batch 2−4576 : Gut microbiota abundance (genus Parasutterella id.2892) on Group IIE secretory phospholipase A2
with a threshold of 1e−05
MR Test
Inverse variance weighted (fixed effects)
Inverse variance weighted (multiplicative random effects)
MR Egger
MR RAPS
Weighted median
Weighted mode
SNP effect on Group IIE secretory phospholipase A2
0.05
0.00
−0.05
−0.10
0.1
0.2
SNP effect on Gut microbiota abundance (genus Parasutterella id.2892)

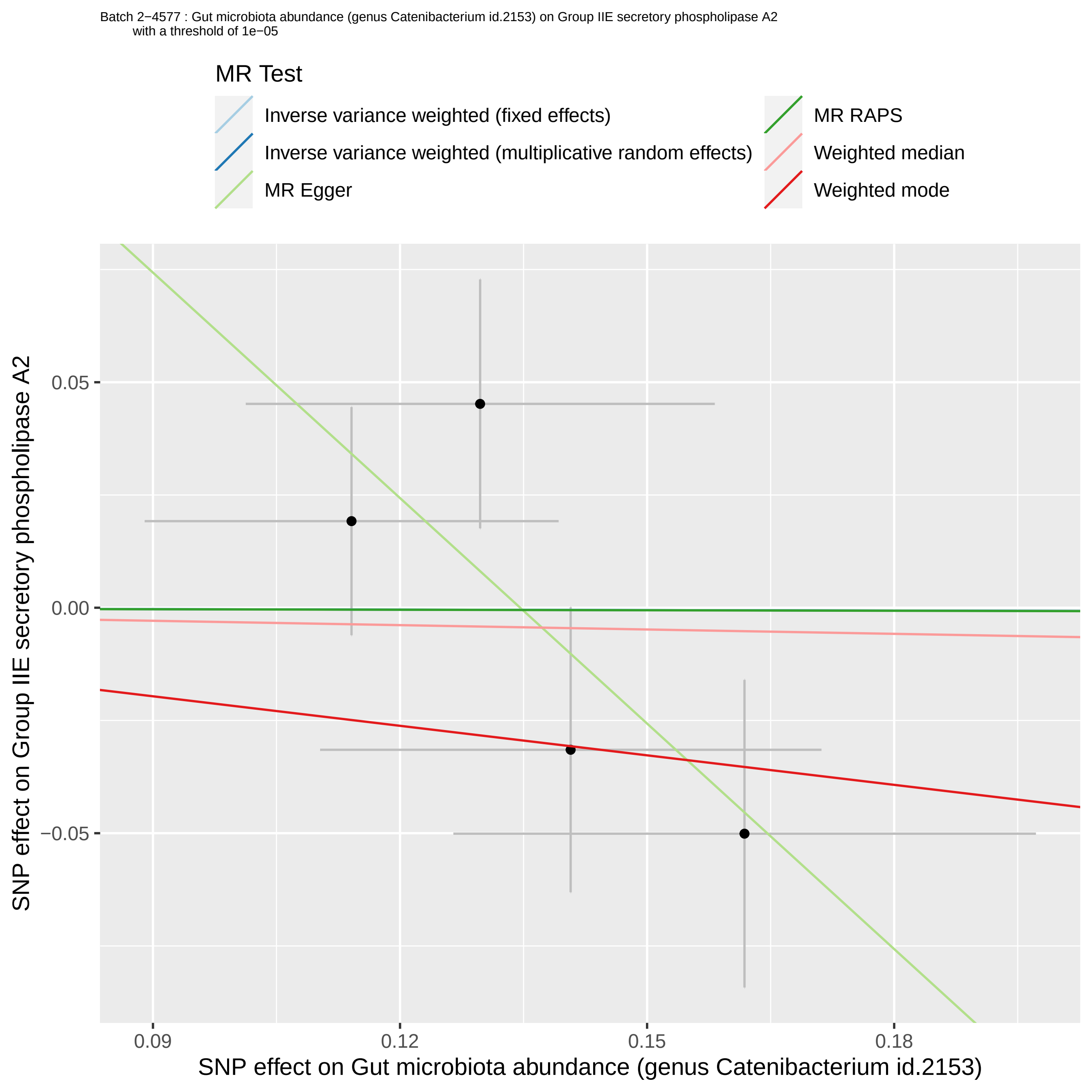
Batch 2−4577 : Gut microbiota abundance (genus Catenibacterium id.2153) on Group IIE secretory phospholipase A2
with a threshold of 1e−05
MR Test
Inverse variance weighted (fixed effects)
Inverse variance weighted (multiplicative random effects)
MR Egger
MR RAPS
Weighted median
Weighted mode
0.05
0.00
−0.05
0.09
0.12
0.15
0.18
SNP effect on Group IIE secretory phospholipase A2
SNP effect on Gut microbiota abundance (genus Catenibacterium id.2153)

Batch 2−4578 : Gut microbiota abundance (genus Parasutterella id.2892) on Group IIE secretory phospholipase A2
with a threshold of 1e−05

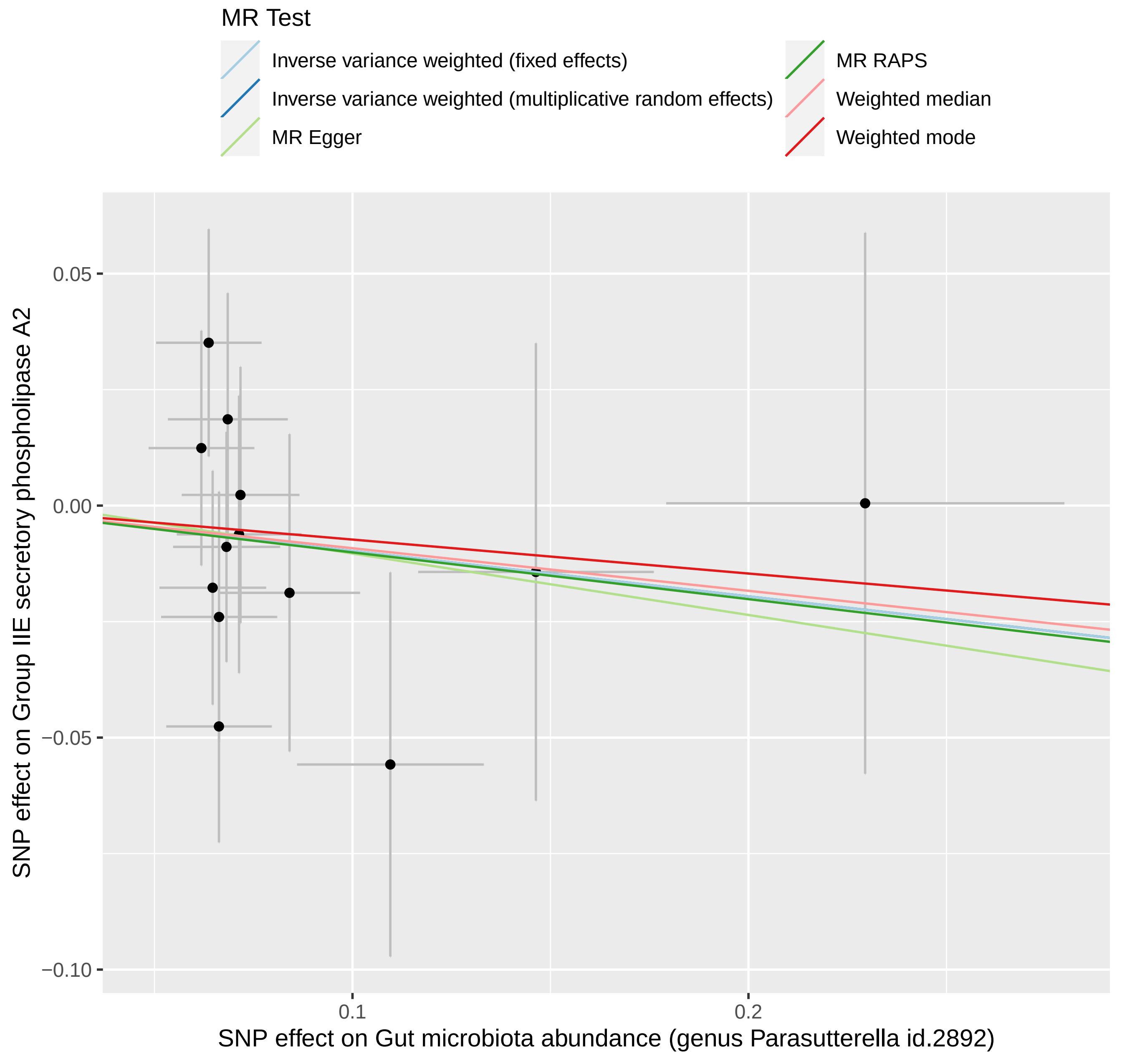

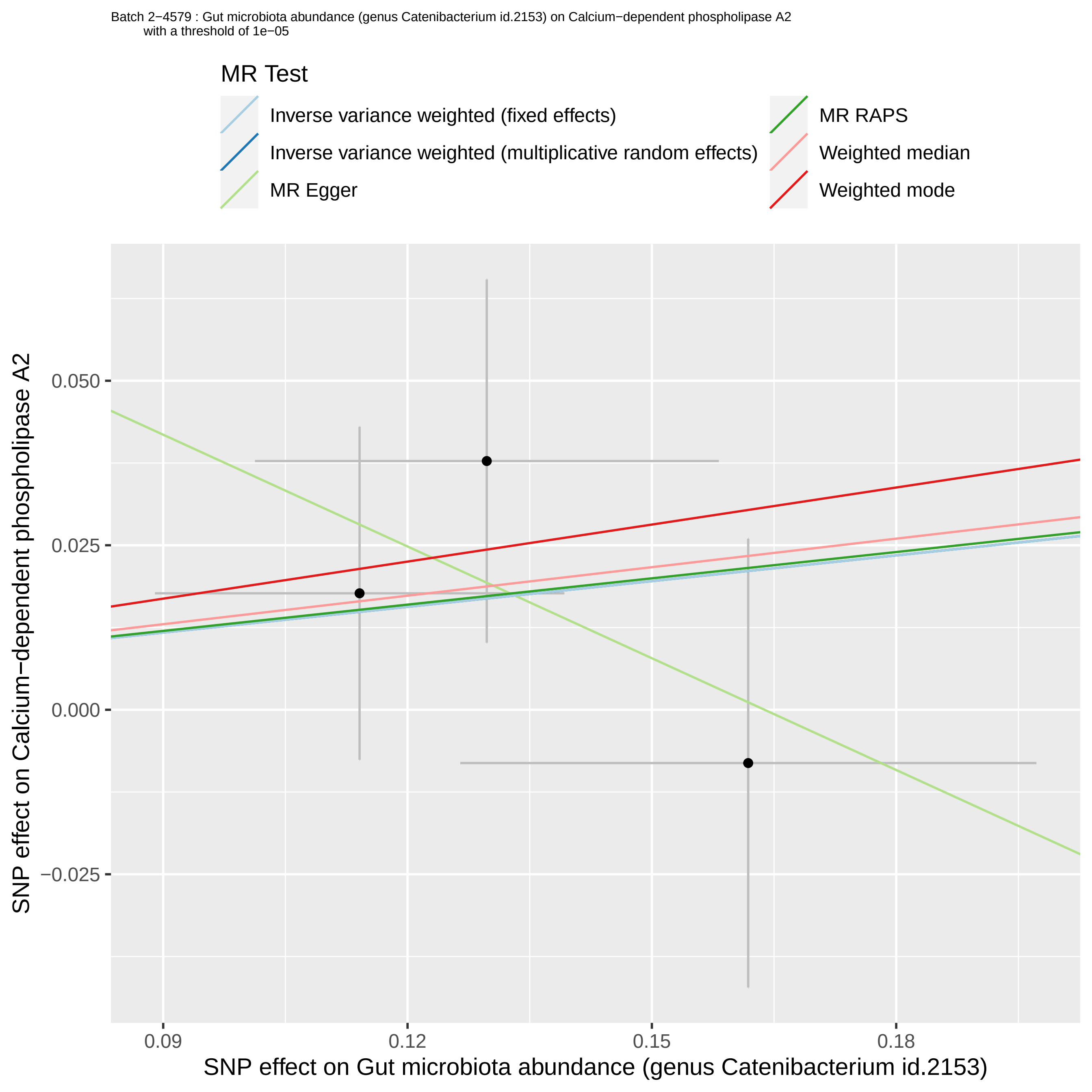

Batch 2−4579 : Gut microbiota abundance (genus Catenibacterium id.2153) on Calcium−dependent phospholipase A2
with a threshold of 1e−05
MR Test
Inverse variance weighted (fixed effects)
Inverse variance weighted (multiplicative random effects)
MR Egger
MR RAPS
Weighted median
Weighted mode
0.050
0.025
0.000
−0.025
0.09
0.12
0.15
0.18
SNP effect on Calcium−dependent phospholipase A2
SNP effect on Gut microbiota abundance (genus Catenibacterium id.2153)

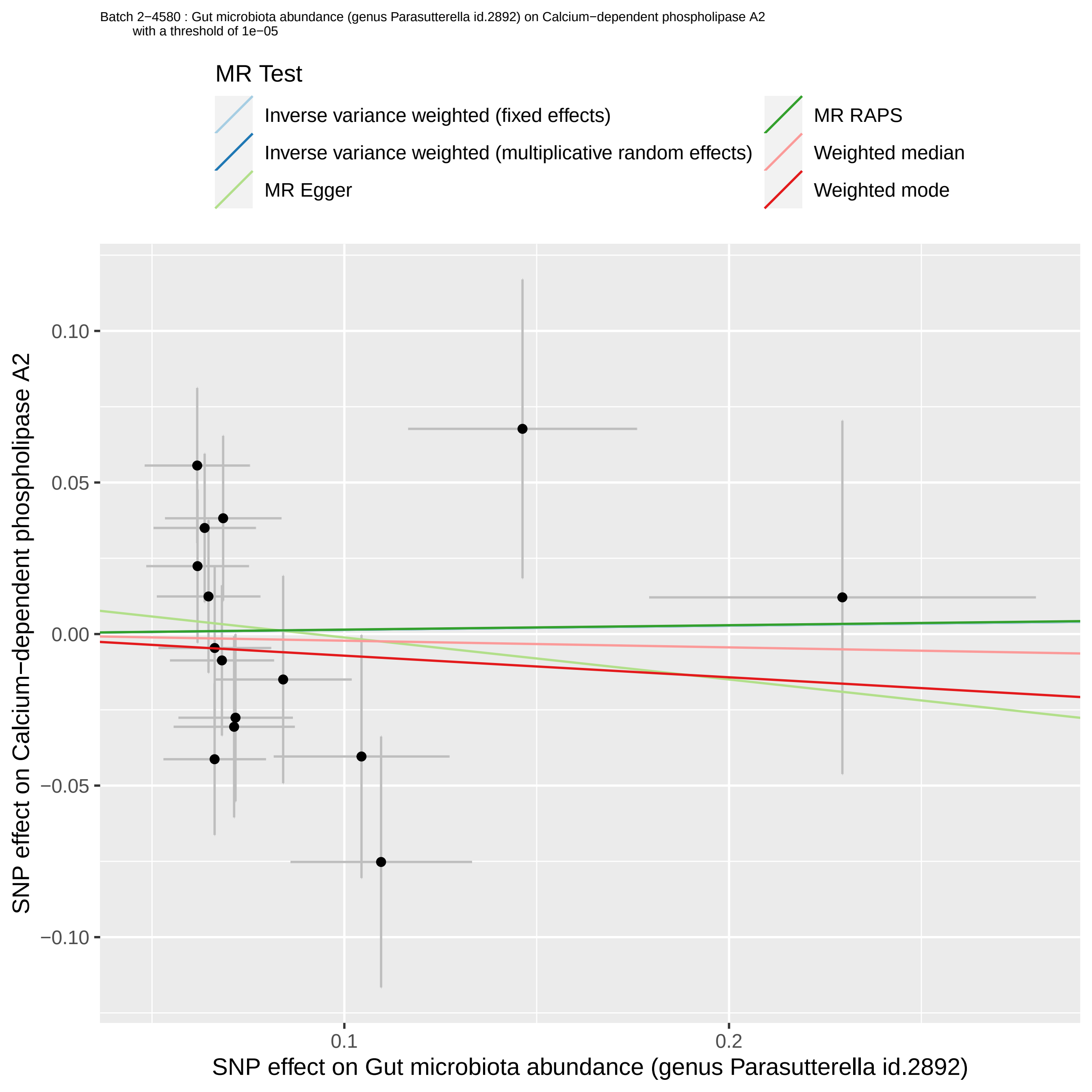

Batch 2−4580 : Gut microbiota abundance (genus Parasutterella id.2892) on Calcium−dependent phospholipase A2
with a threshold of 1e−05
MR Test
Inverse variance weighted (fixed effects)
Inverse variance weighted (multiplicative random effects)
MR Egger
MR RAPS
Weighted median
Weighted mode
0.10
0.05
0.00
−0.05
−0.10
0.1
0.2
SNP effect on Calcium−dependent phospholipase A2
SNP effect on Gut microbiota abundance (genus Parasutterella id.2892)

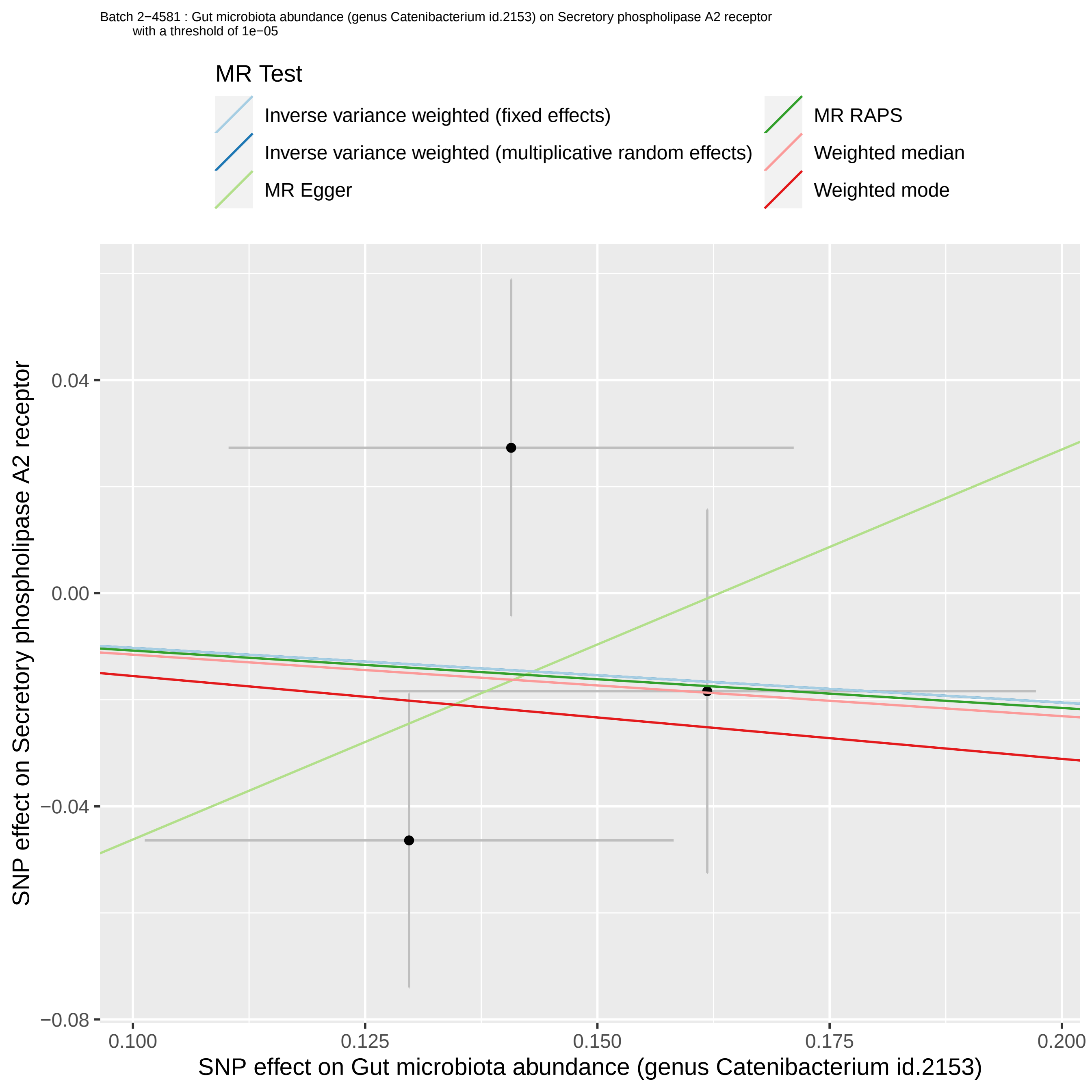
Batch 2−4581 : Gut microbiota abundance (genus Catenibacterium id.2153) on Secretory phospholipase A2 receptor
with a threshold of 1e−05
MR Test
Inverse variance weighted (fixed effects)
Inverse variance weighted (multiplicative random effects)
MR Egger
MR RAPS
Weighted median
Weighted mode
0.04
0.00
−0.04
−0.08
0.100
0.125
0.150
0.175
0.200
SNP effect on Secretory phospholipase A2 receptor
SNP effect on Gut microbiota abundance (genus Catenibacterium id.2153)

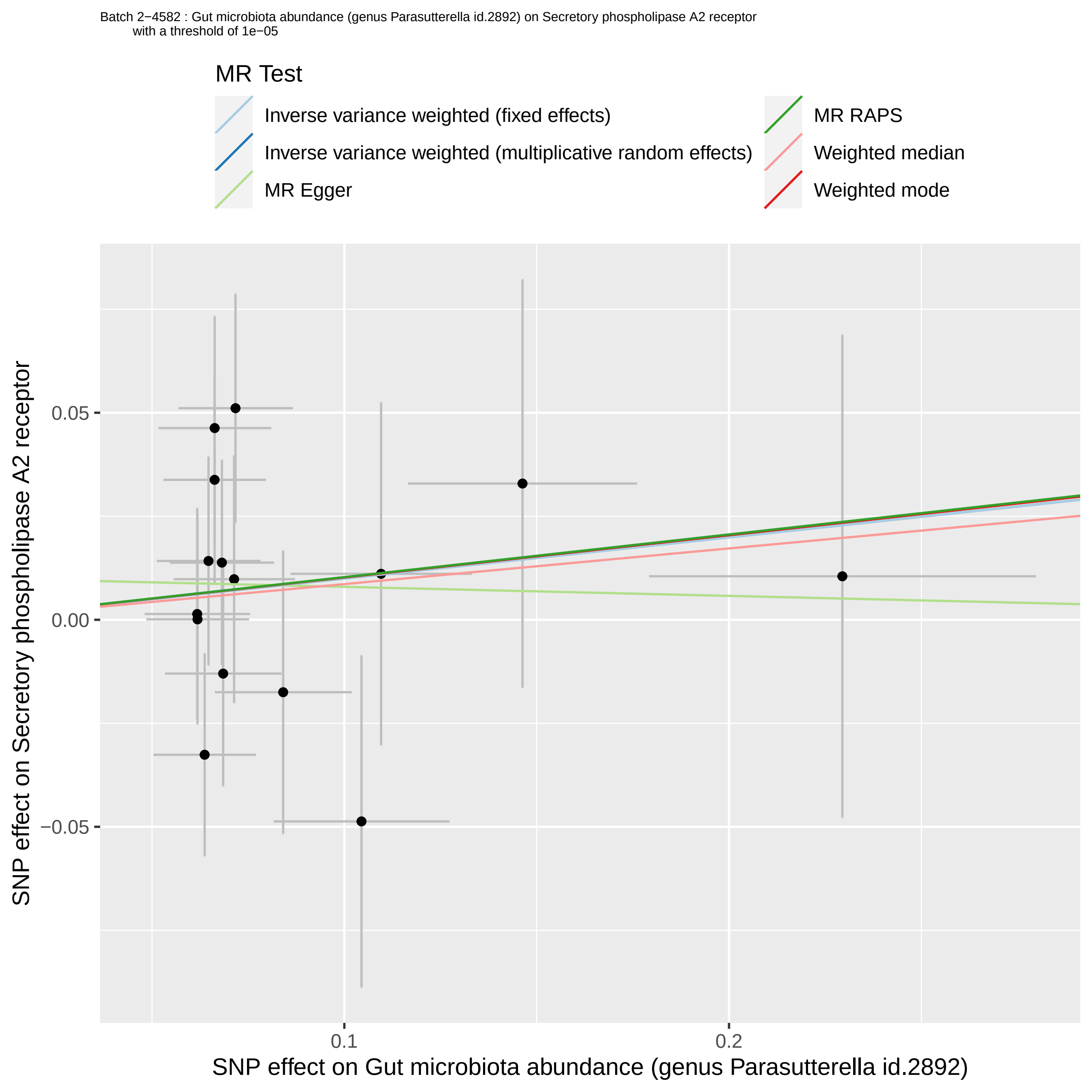
Batch 2−4582 : Gut microbiota abundance (genus Parasutterella id.2892) on Secretory phospholipase A2 receptor
with a threshold of 1e−05
MR Test
Inverse variance weighted (fixed effects)
Inverse variance weighted (multiplicative random effects)
MR Egger
MR RAPS
Weighted median
Weighted mode
SNP effect on Secretory phospholipase A2 receptor
0.05
0.00
−0.05
0.1
0.2
SNP effect on Gut microbiota abundance (genus Parasutterella id.2892)

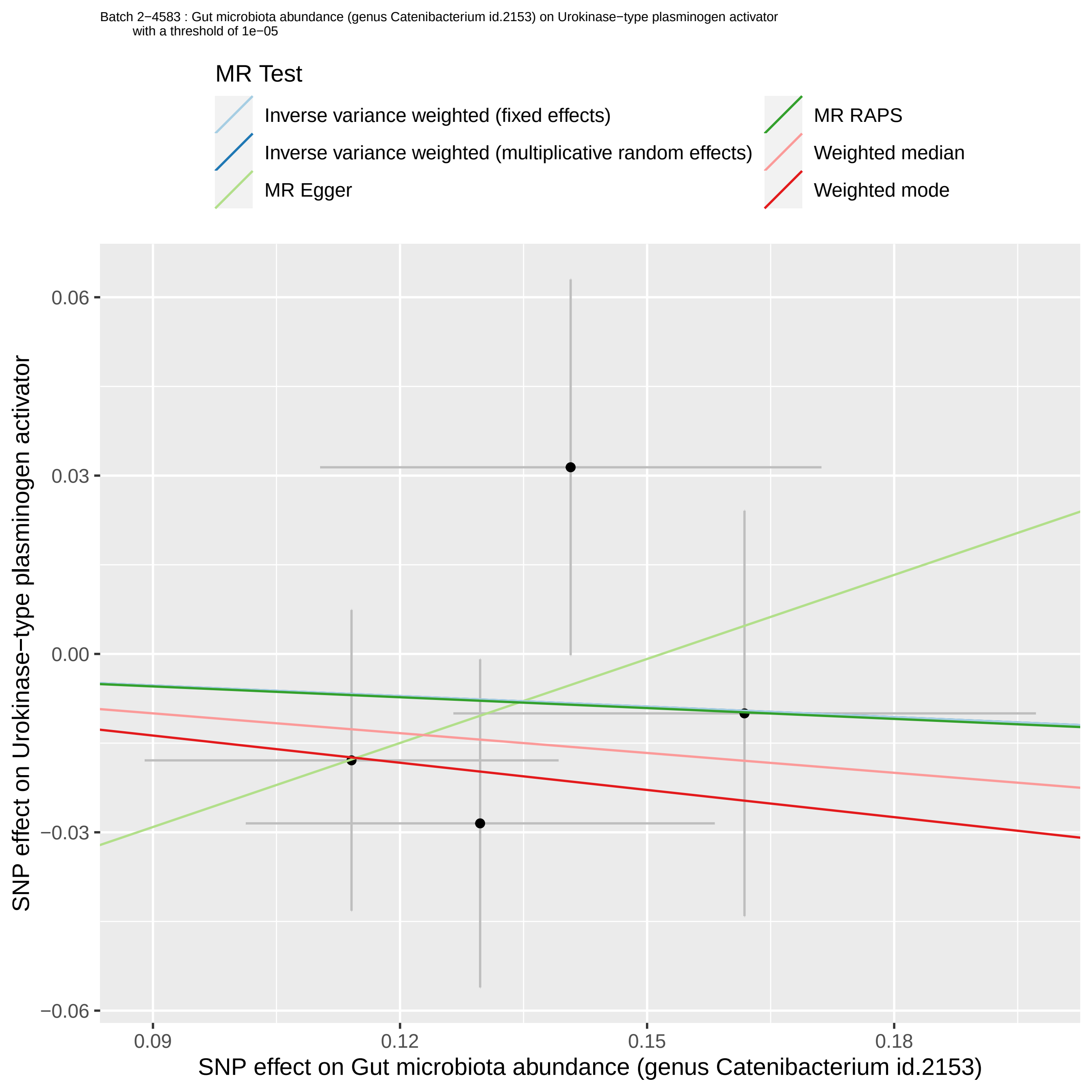
Batch 2−4583 : Gut microbiota abundance (genus Catenibacterium id.2153) on Urokinase−type plasminogen activator
with a threshold of 1e−05
MR Test
Inverse variance weighted (fixed effects)
Inverse variance weighted (multiplicative random effects)
MR Egger
MR RAPS
Weighted median
Weighted mode
0.06
0.03
0.00
−0.03
−0.06
0.09
0.12
0.15
0.18
SNP effect on Urokinase−type plasminogen activator
SNP effect on Gut microbiota abundance (genus Catenibacterium id.2153)

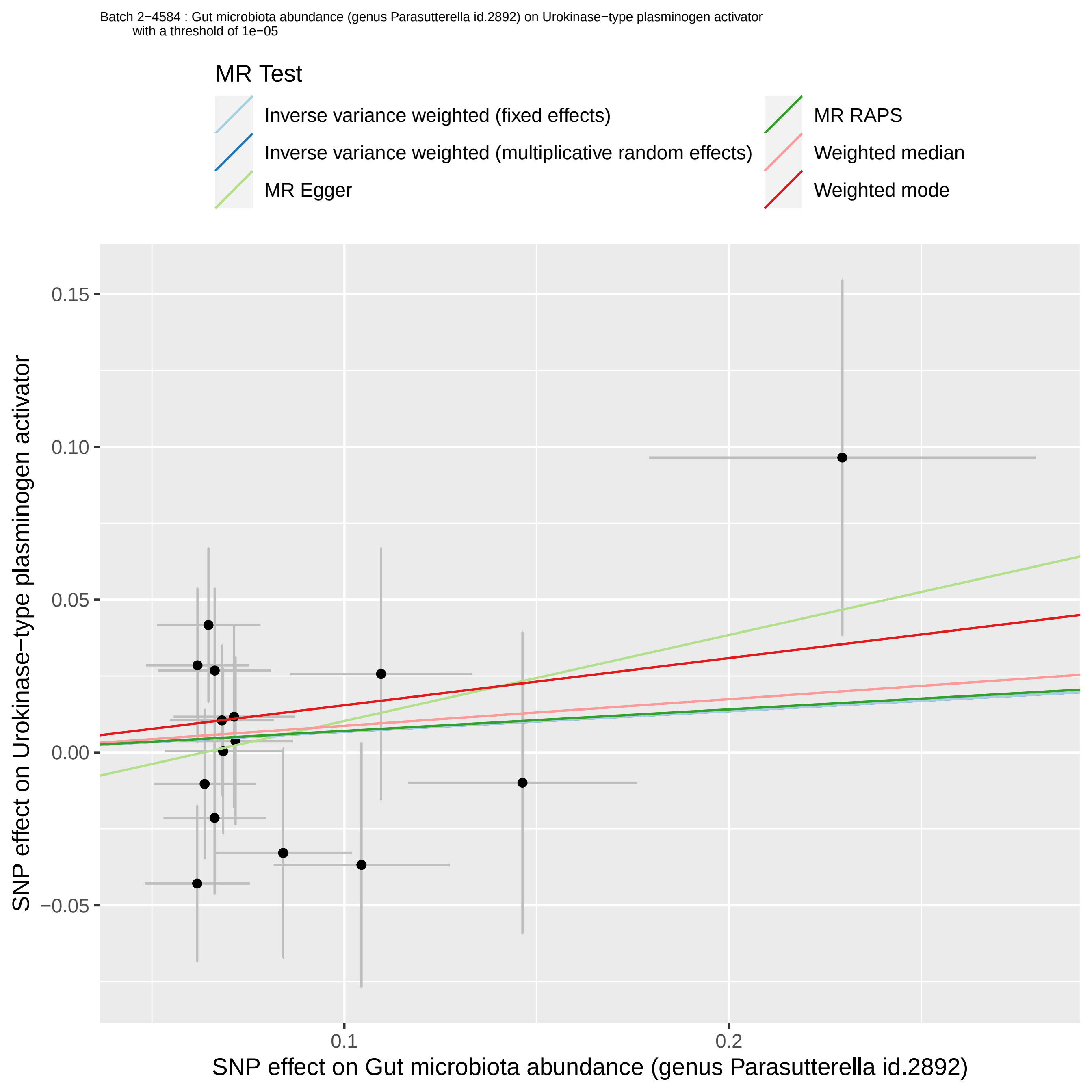

Batch 2−4584 : Gut microbiota abundance (genus Parasutterella id.2892) on Urokinase−type plasminogen activator
with a threshold of 1e−05
MR Test
Inverse variance weighted (fixed effects)
Inverse variance weighted (multiplicative random effects)
MR Egger
MR RAPS
Weighted median
Weighted mode
0.15
0.10
0.05
0.00
−0.05
0.1
0.2
SNP effect on Urokinase−type plasminogen activator
SNP effect on Gut microbiota abundance (genus Parasutterella id.2892)

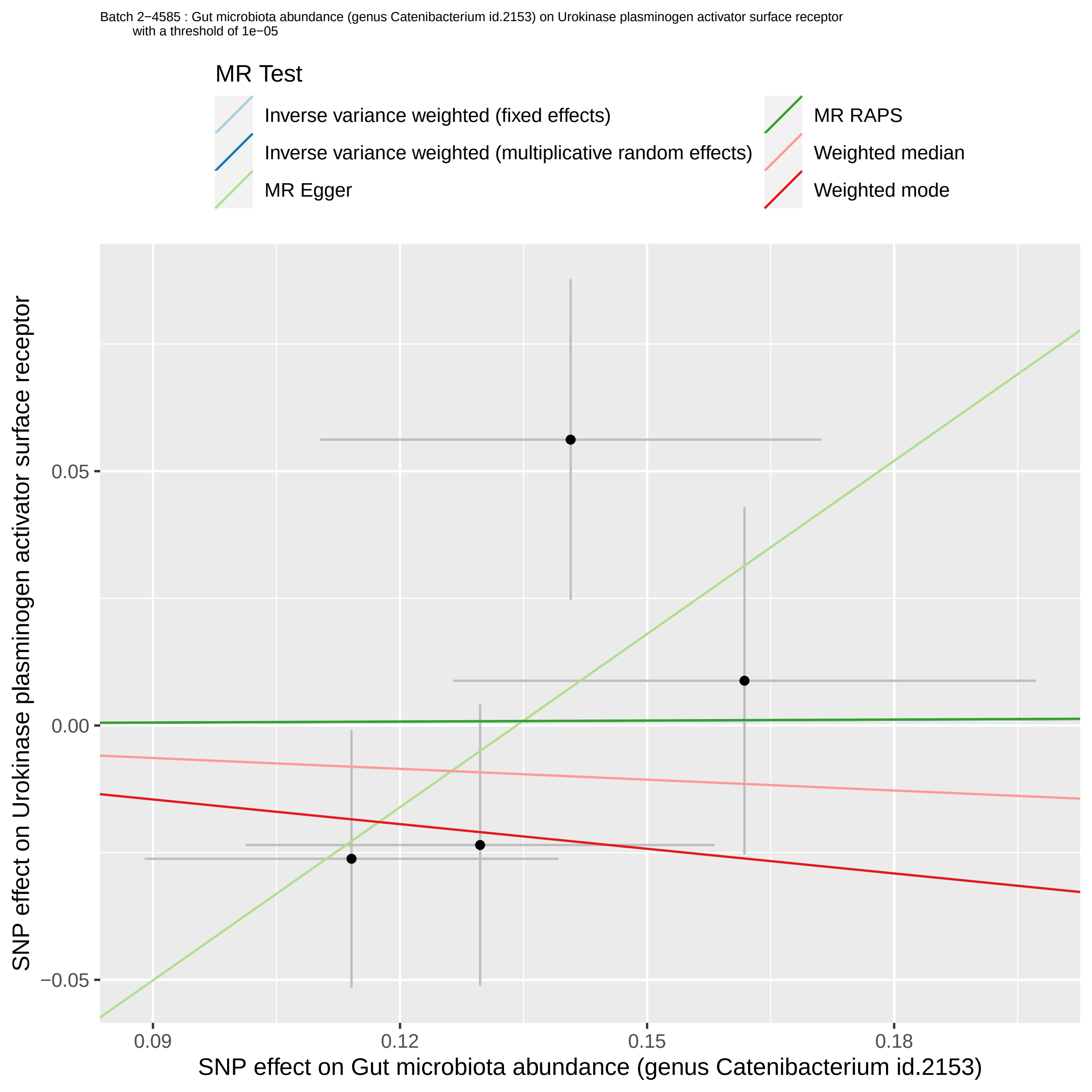

Batch 2−4585 : Gut microbiota abundance (genus Catenibacterium id.2153) on Urokinase plasminogen activator surface receptor with a threshold of 1e−05
MR Test
Inverse variance weighted (fixed effects)
Inverse variance weighted (multiplicative random effects)
MR Egger
MR RAPS
Weighted median
Weighted mode
0.05
0.00
−0.05
0.09
0.12
0.15
0.18
SNP effect on Gut microbiota abundance (genus Catenibacterium id.2153)
SNP effect on Urokinase plasminogen activator surface receptor

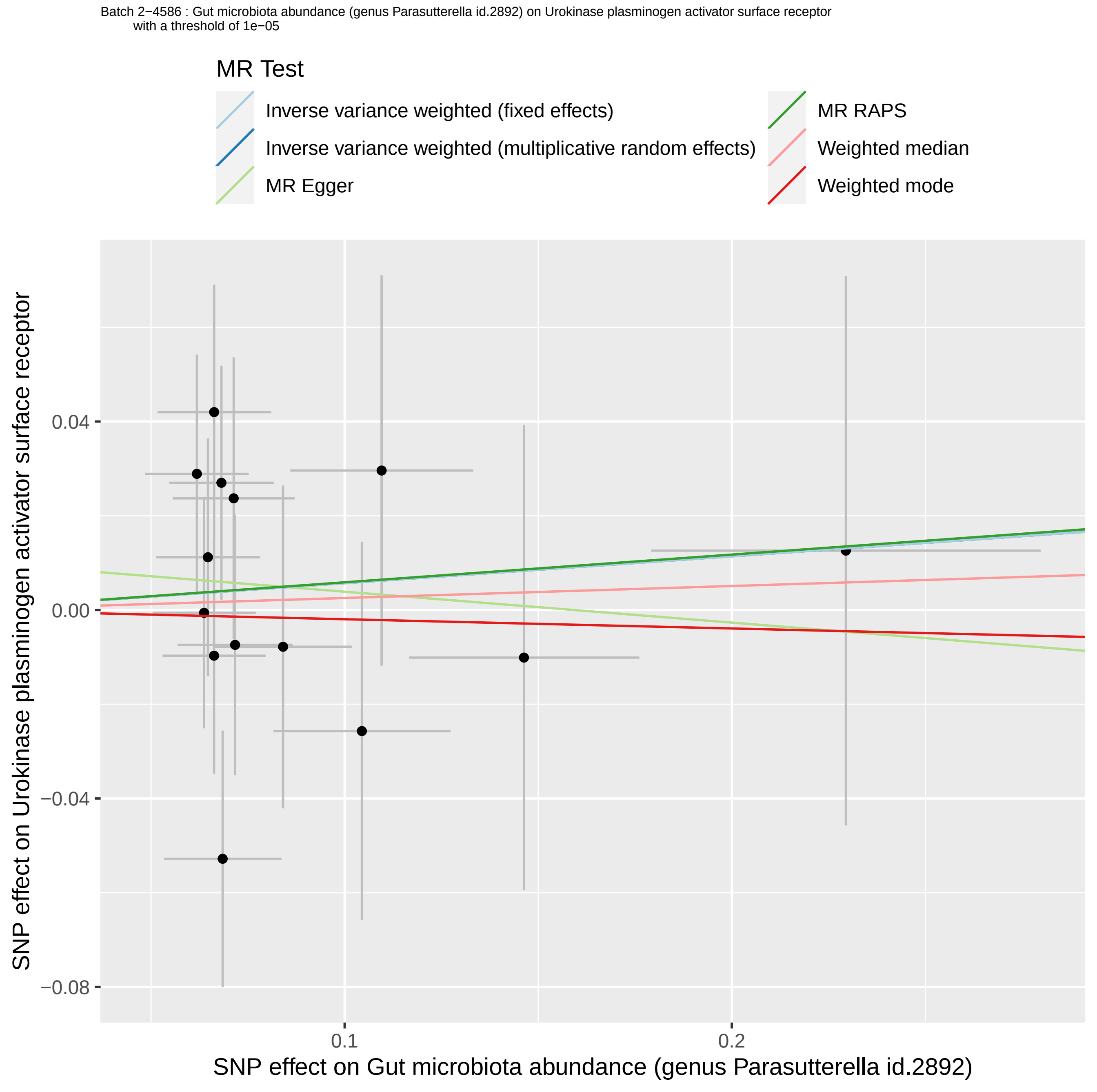
Batch 2−4586 : Gut microbiota abundance (genus Parasutterella id.2892) on Urokinase plasminogen activator surface receptor
with a threshold of 1e−05
MR Test
Inverse variance weighted (fixed effects)
Inverse variance weighted (multiplicative random effects)
MR Egger
MR RAPS
Weighted median
Weighted mode
SNP effect on Urokinase plasminogen activator surface receptor
0.04
0.00
−0.04
−0.08
0.1
0.2
SNP effect on Gut microbiota abundance (genus Parasutterella id.2892)

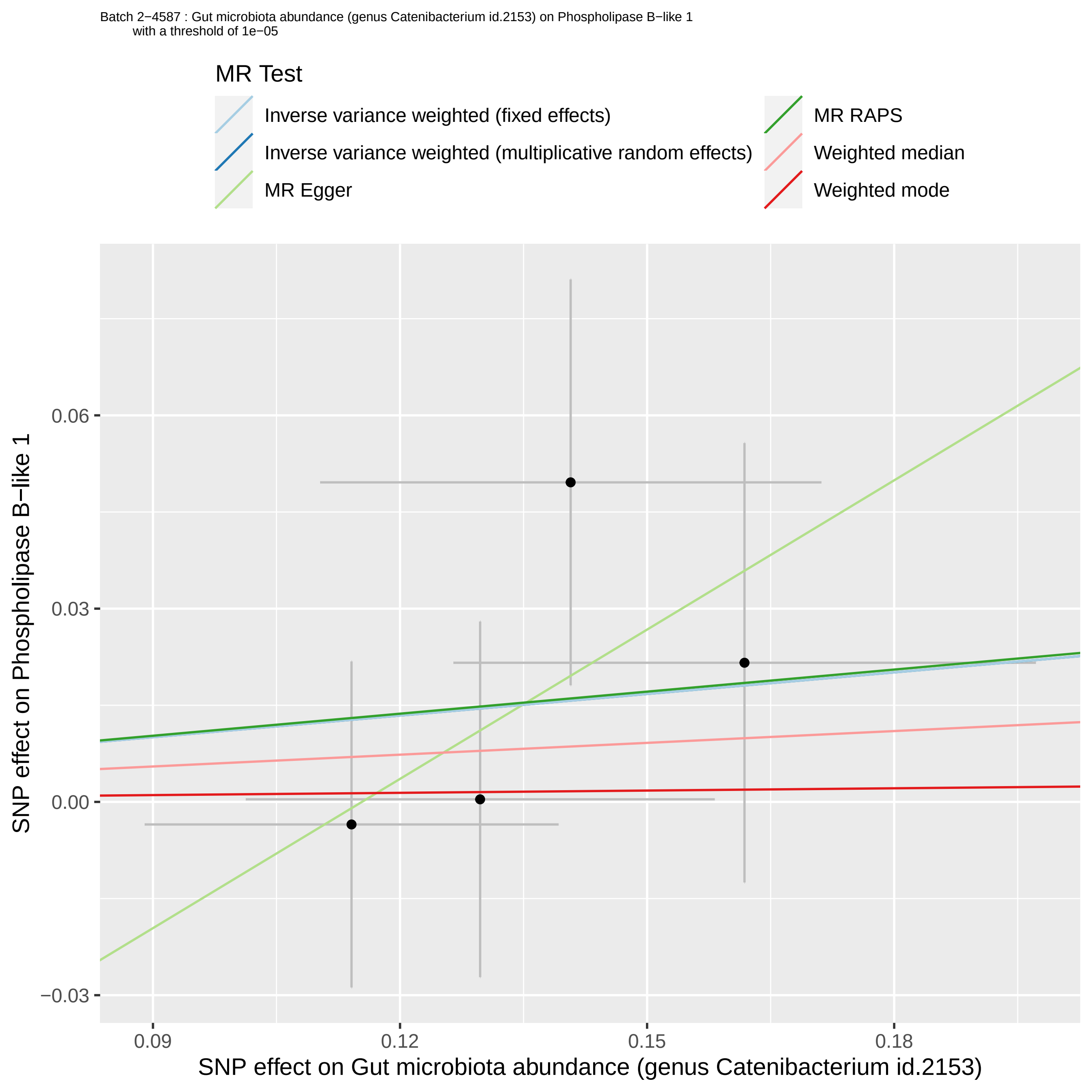

Batch 2−4587 : Gut microbiota abundance (genus Catenibacterium id.2153) on Phospholipase B−like 1
with a threshold of 1e−05
MR Test
Inverse variance weighted (fixed effects)
Inverse variance weighted (multiplicative random effects)
MR Egger
MR RAPS
Weighted median
Weighted mode
0.06
0.03
0.00
−0.03
0.09
0.12
0.15
0.18
SNP effect on Phospholipase B−like 1
SNP effect on Gut microbiota abundance (genus Catenibacterium id.2153)

Batch 2−4588 : Gut microbiota abundance (genus Parasutterella id.2892) on Phospholipase B−like 1
with a threshold of 1e−05

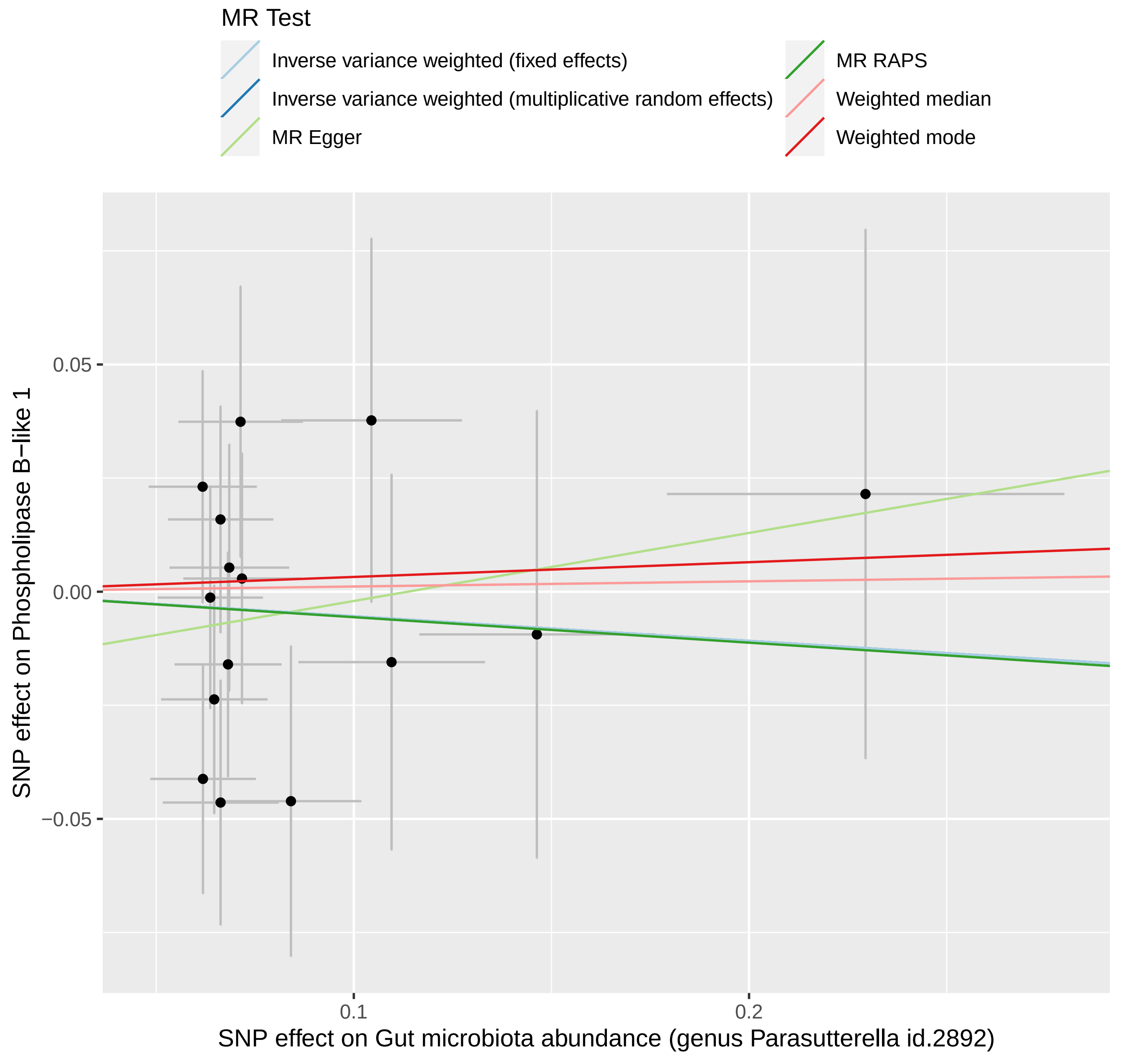

Batch 2−4589 : Gut microbiota abundance (genus Catenibacterium id.2153) on Putative phospholipase B−like 2
with a threshold of 1e−05

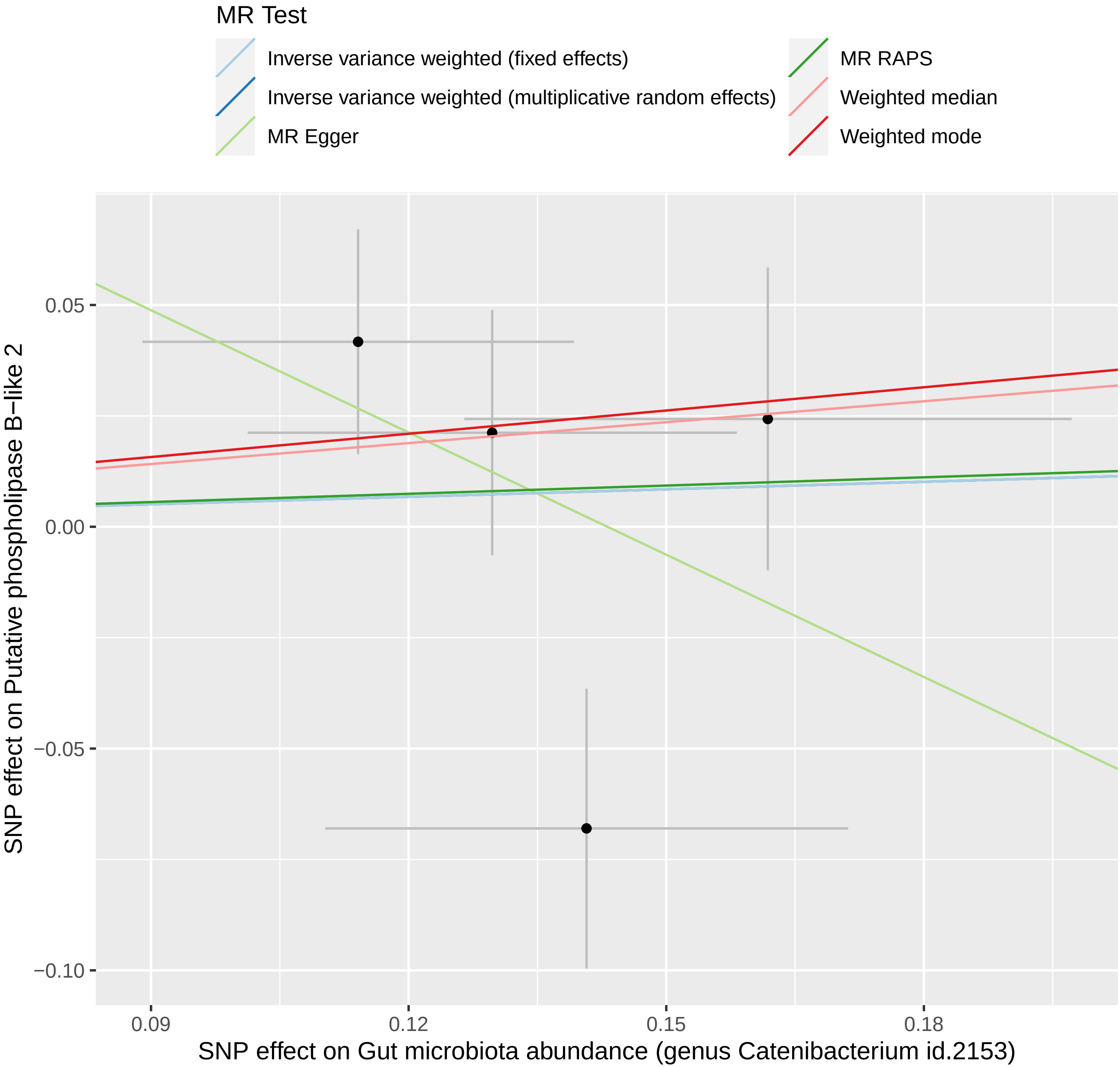

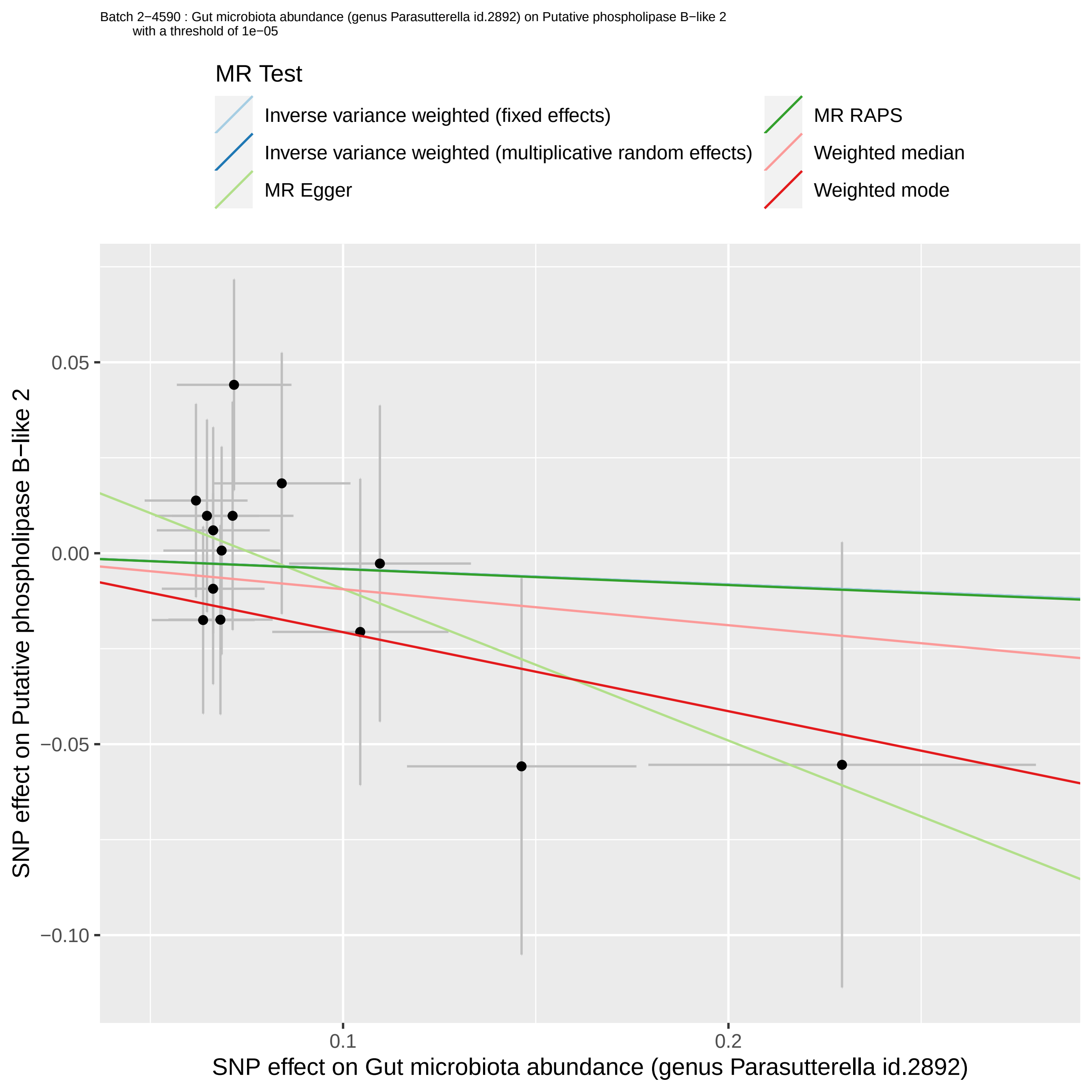
Batch 2−4590 : Gut microbiota abundance (genus Parasutterella id.2892) on Putative phospholipase B−like 2
with a threshold of 1e−05
MR Test
Inverse variance weighted (fixed effects)
Inverse variance weighted (multiplicative random effects)
MR Egger
MR RAPS
Weighted median
Weighted mode
0.05
0.00
−0.05
−0.10
0.1
0.2
SNP effect on Putative phospholipase B−like 2
SNP effect on Gut microbiota abundance (genus Parasutterella id.2892)

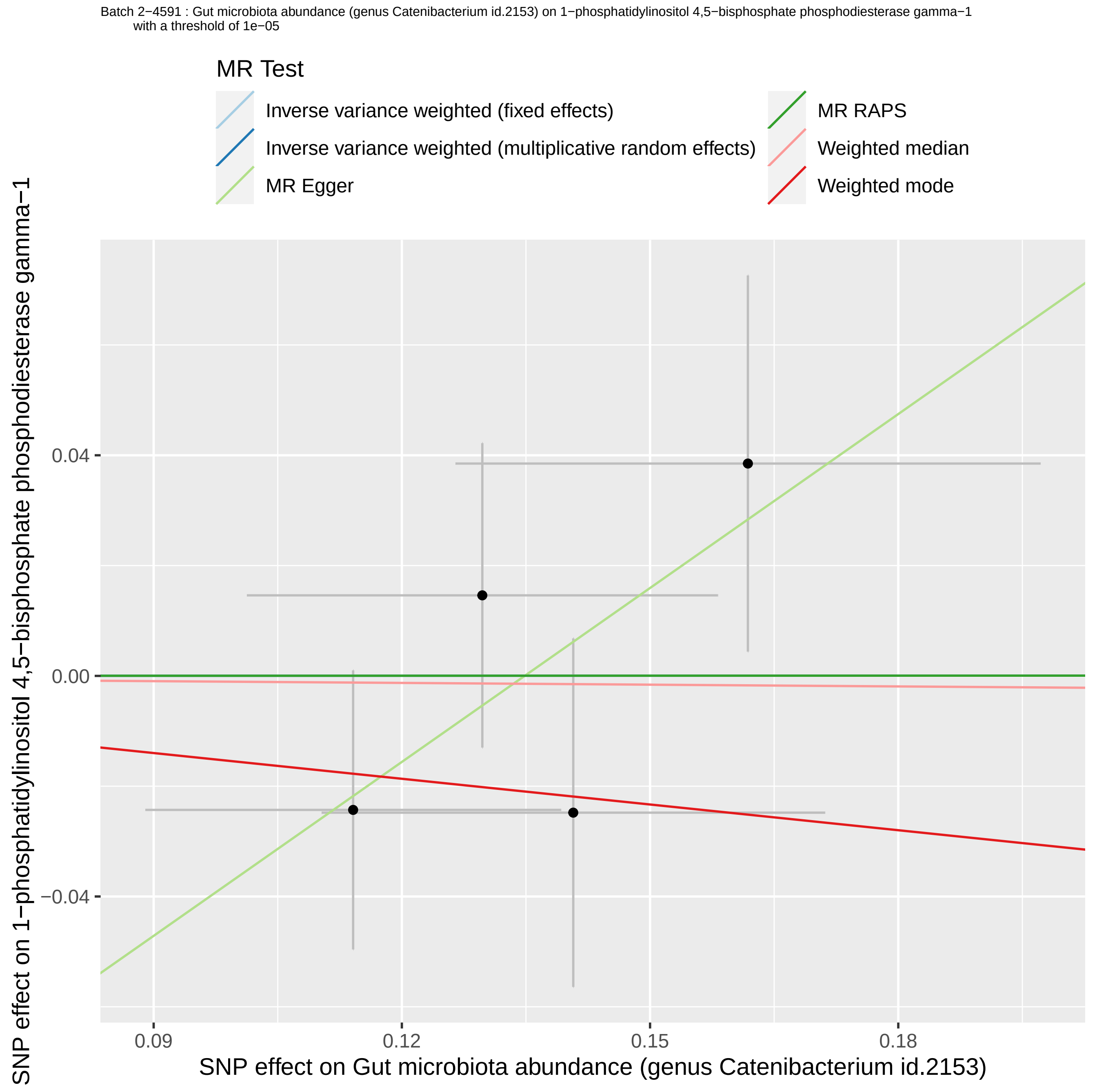
Batch 2−4591 : Gut microbiota abundance (genus Catenibacterium id.2153) on 1−phosphatidylinositol 4,5−bisphosphate phosphodiesterase gamma−1
with a threshold of 1e−05
MR Test
Inverse variance weighted (fixed effects)
Inverse variance weighted (multiplicative random effects)
MR Egger
MR RAPS
Weighted median
Weighted mode
0.04
0.00
−0.04
0.09
0.12
0.15
0.18
SNP effect on Gut microbiota abundance (genus Catenibacterium id.2153)
SNP effect on 1−phosphatidylinositol 4,5−bisphosphate phosphodiesterase gamma−1

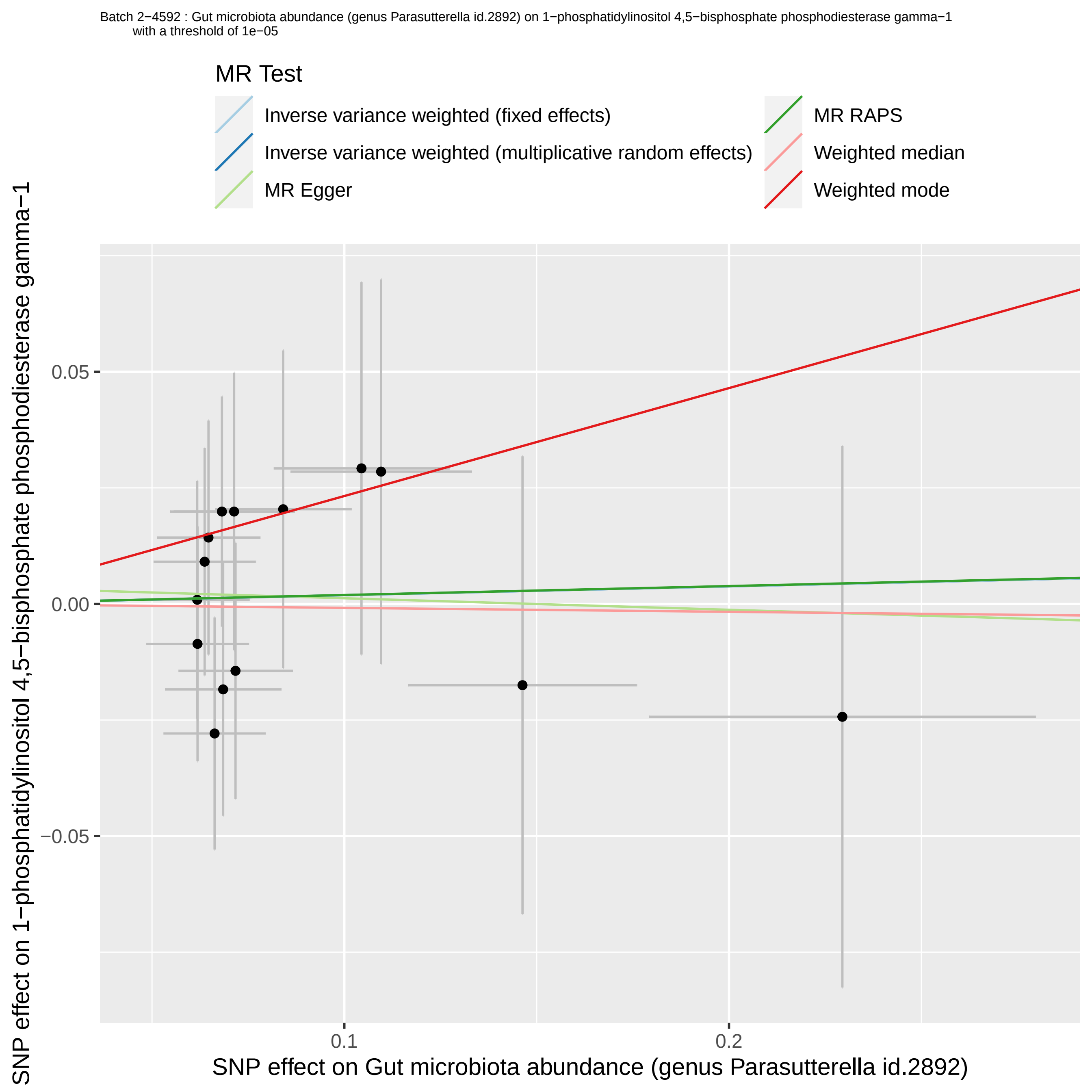

Batch 2−4592 : Gut microbiota abundance (genus Parasutterella id.2892) on 1−phosphatidylinositol 4,5−bisphosphate phosphodiesterase gamma−1
with a threshold of 1e−05
MR Test
Inverse variance weighted (fixed effects)
Inverse variance weighted (multiplicative random effects)
MR Egger
MR RAPS
Weighted median
Weighted mode
0.05
0.00
−0.05
0.1
0.2
SNP effect on 1−phosphatidylinositol 4,5−bisphosphate phosphodiesterase gamma−1
SNP effect on Gut microbiota abundance (genus Parasutterella id.2892)

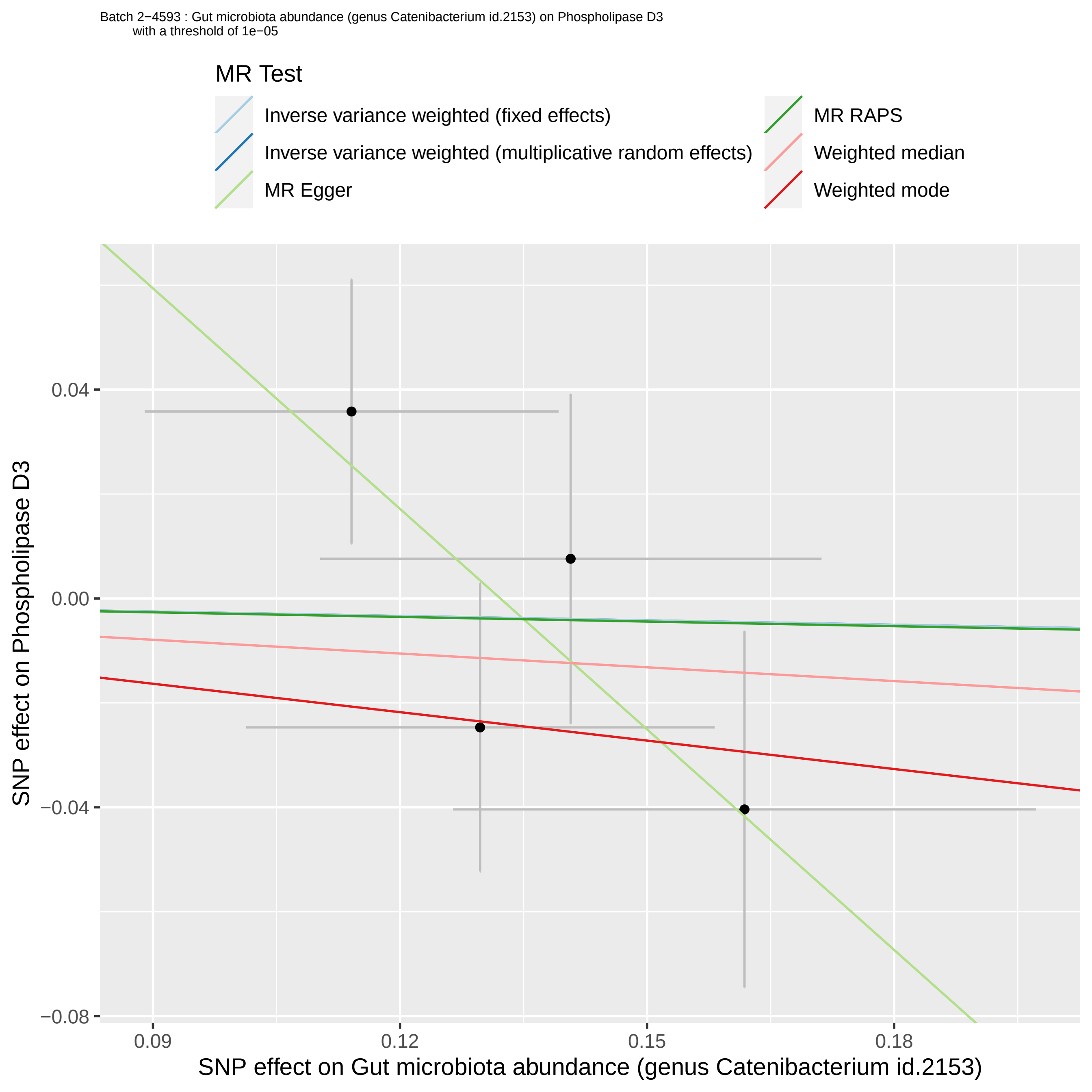

Batch 2−4593 : Gut microbiota abundance (genus Catenibacterium id.2153) on Phospholipase D3
with a threshold of 1e−05
MR Test
Inverse variance weighted (fixed effects)
Inverse variance weighted (multiplicative random effects)
MR Egger
MR RAPS
Weighted median
Weighted mode
0.04
0.00
−0.04
−0.08
0.09
0.12
0.15
0.18
SNP effect on Phospholipase D3
SNP effect on Gut microbiota abundance (genus Catenibacterium id.2153)

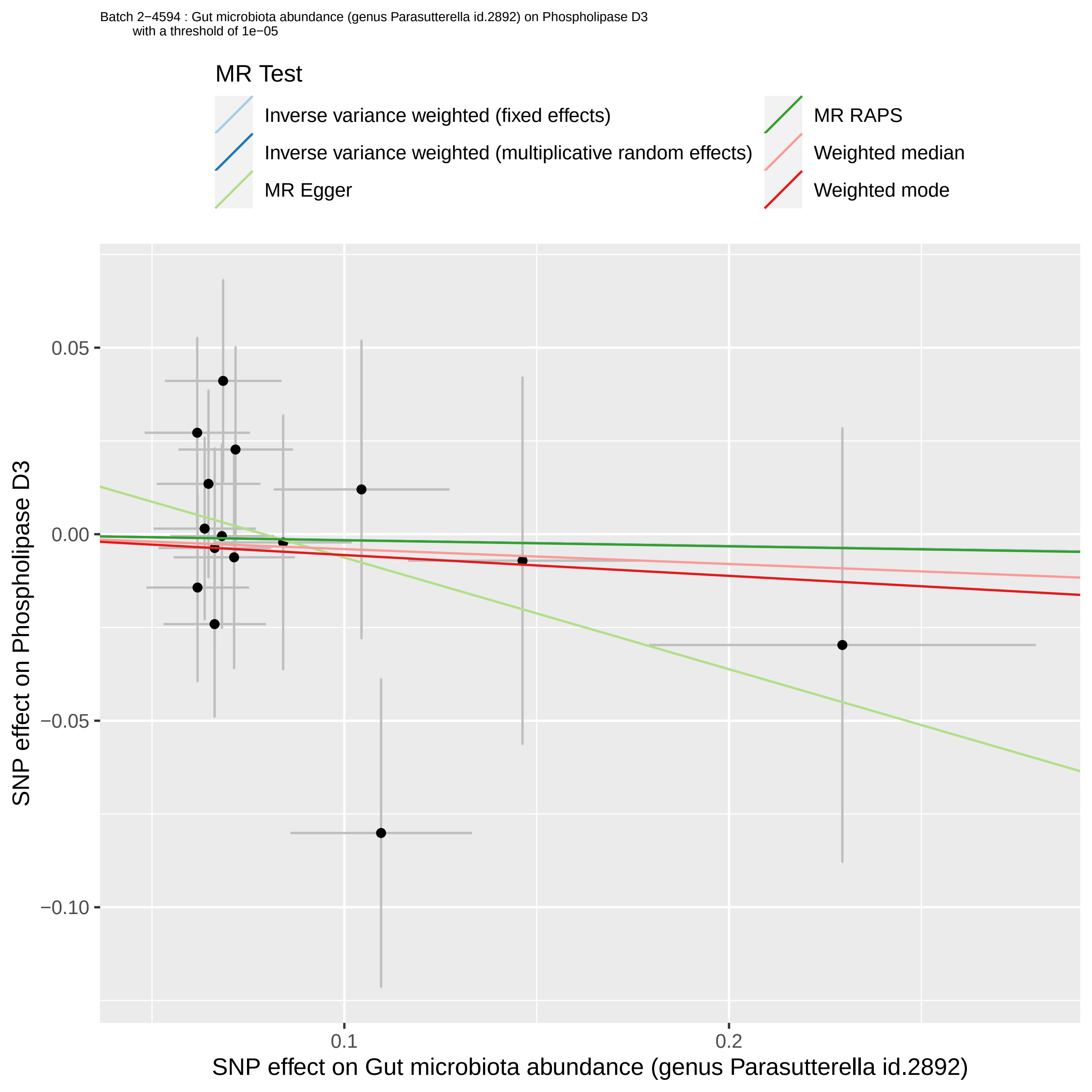

Batch 2−4594 : Gut microbiota abundance (genus Parasutterella id.2892) on Phospholipase D3
with a threshold of 1e−05
MR Test
Inverse variance weighted (fixed effects)
Inverse variance weighted (multiplicative random effects)
MR Egger
MR RAPS
Weighted median
Weighted mode
0.05
0.00
−0.05
−0.10
0.1
0.2
SNP effect on Phospholipase D3
SNP effect on Gut microbiota abundance (genus Parasutterella id.2892)

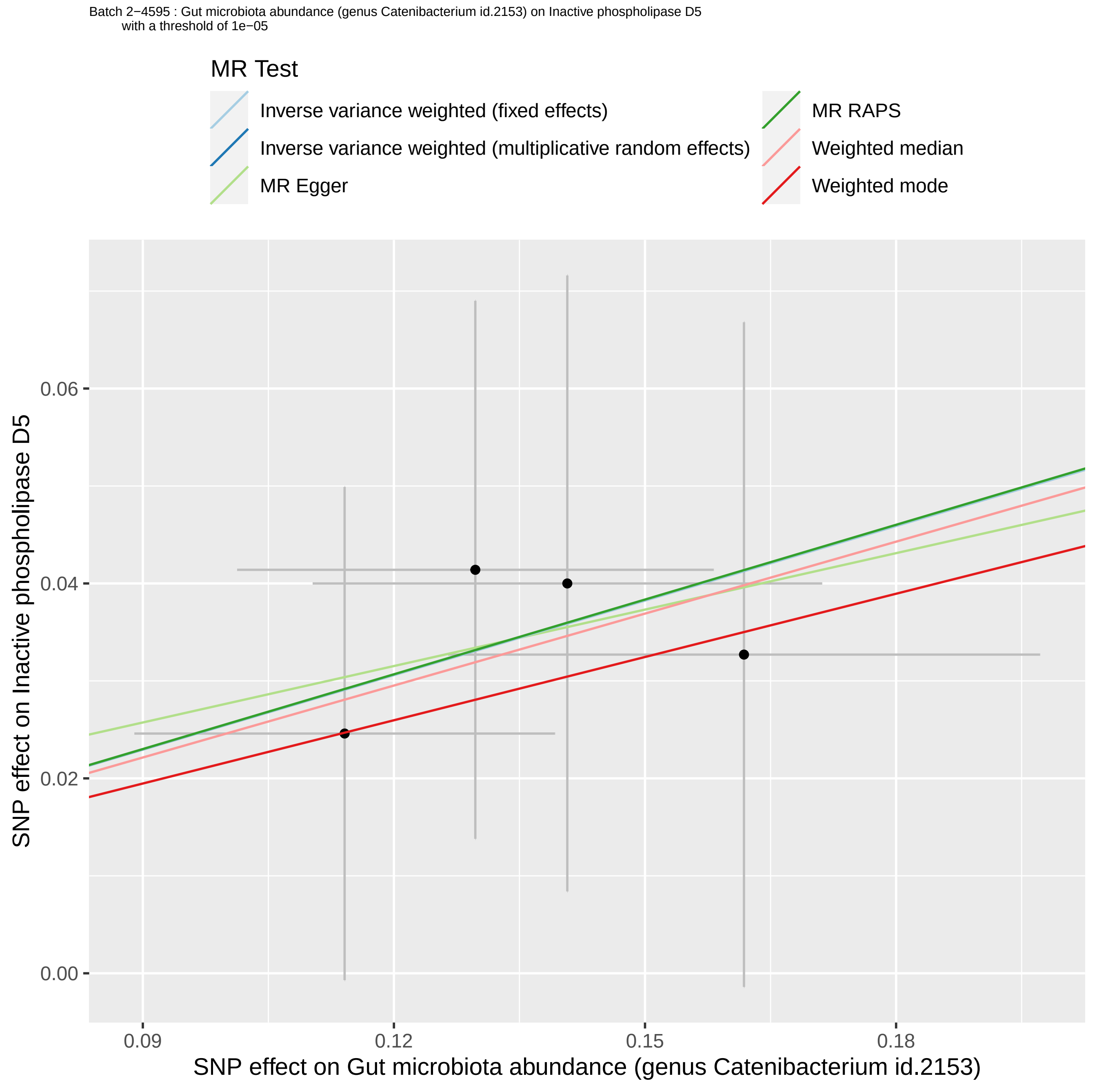

Batch 2−4595 : Gut microbiota abundance (genus Catenibacterium id.2153) on Inactive phospholipase D5
with a threshold of 1e−05
MR Test
Inverse variance weighted (fixed effects)
Inverse variance weighted (multiplicative random effects)
MR Egger
MR RAPS
Weighted median
Weighted mode
0.06
0.04
0.02
0.00
0.09
0.12
0.15
0.18
SNP effect on Inactive phospholipase D5
SNP effect on Gut microbiota abundance (genus Catenibacterium id.2153)

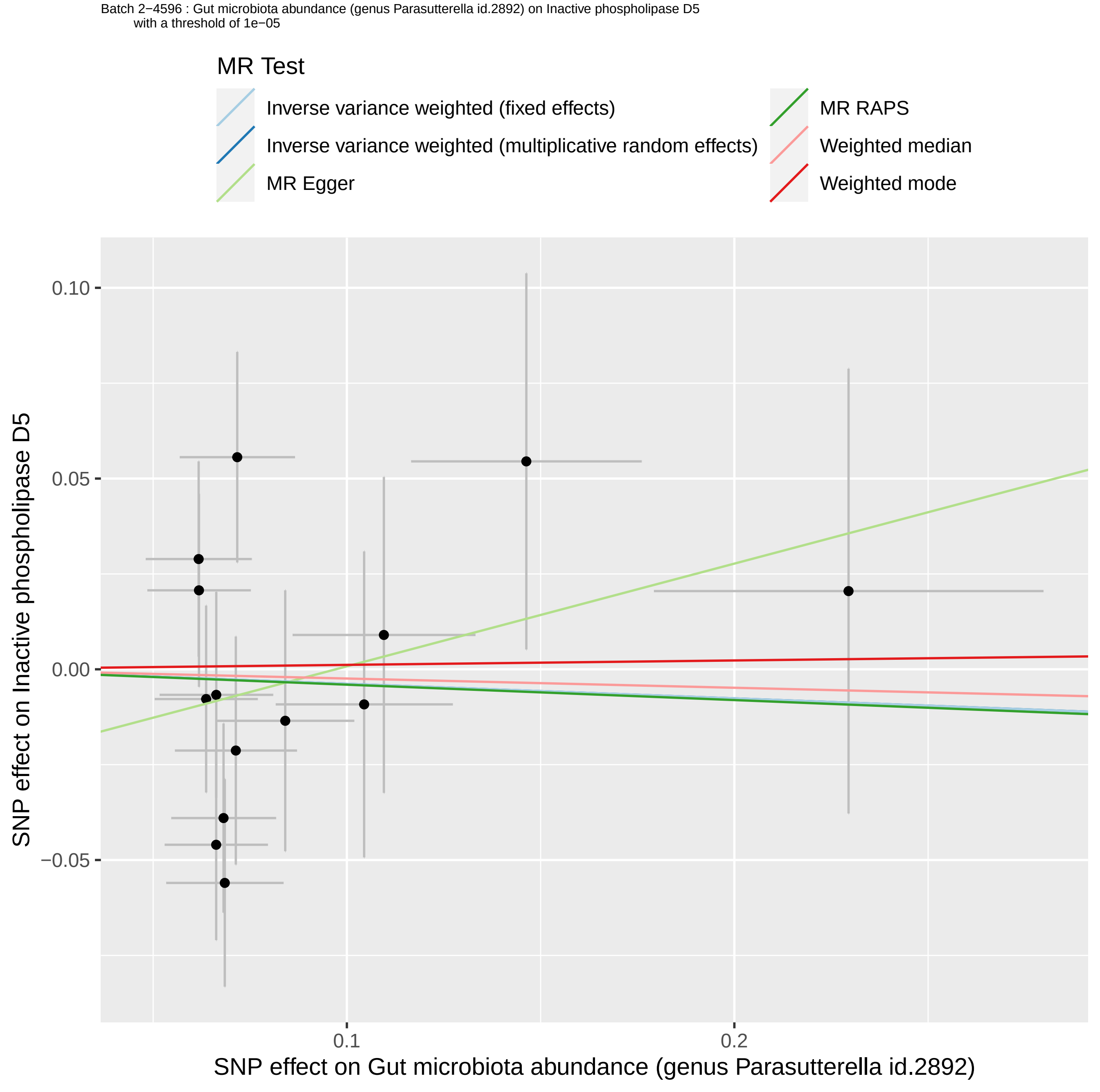
Batch 2−4596 : Gut microbiota abundance (genus Parasutterella id.2892) on Inactive phospholipase D5
with a threshold of 1e−05
MR Test
Inverse variance weighted (fixed effects)
Inverse variance weighted (multiplicative random effects)
MR Egger
MR RAPS
Weighted median
Weighted mode
0.10
0.05
0.00
−0.05
0.1
0.2
SNP effect on Inactive phospholipase D5
SNP effect on Gut microbiota abundance (genus Parasutterella id.2892)

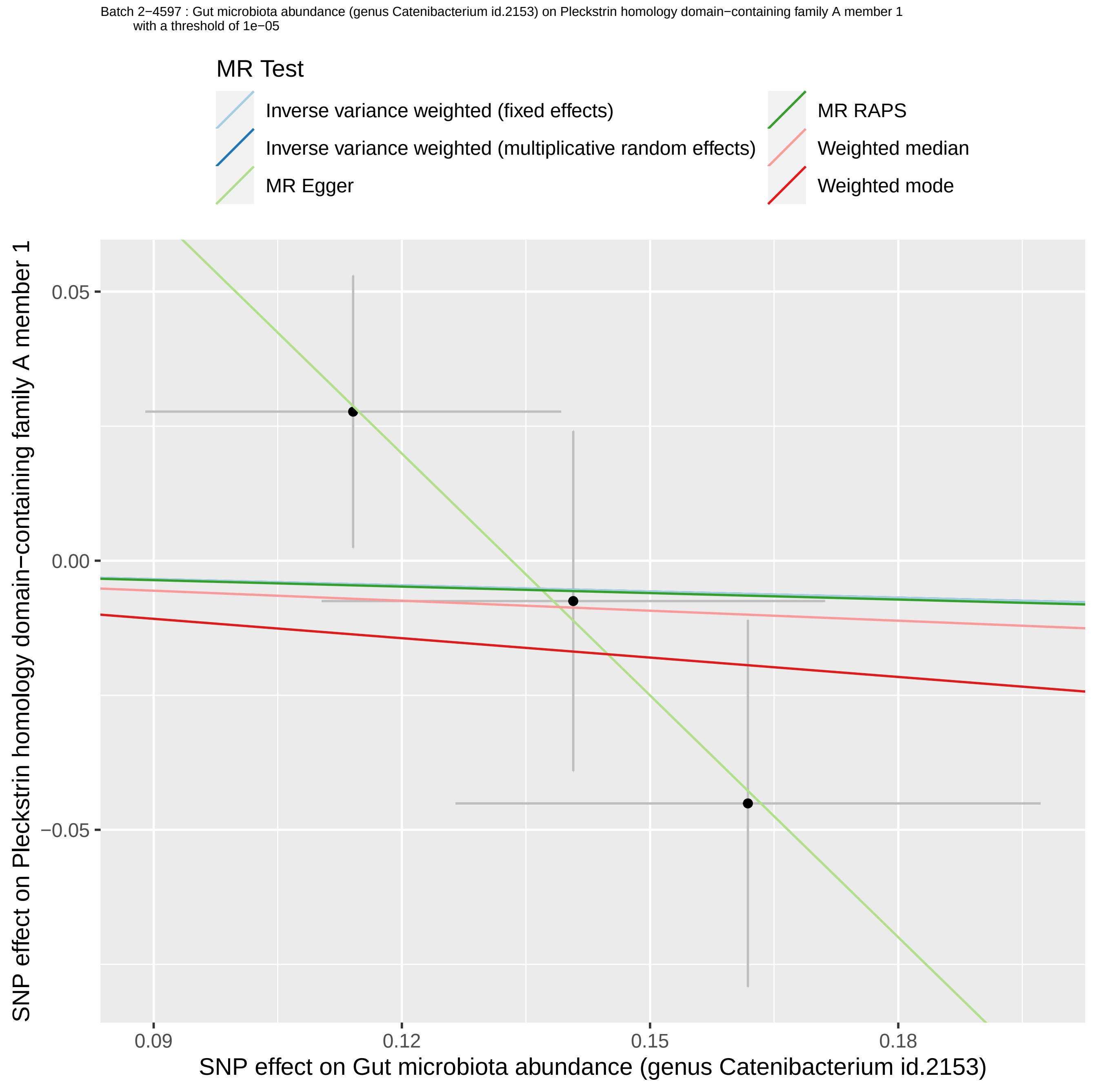

Batch 2−4597 : Gut microbiota abundance (genus Catenibacterium id.2153) on Pleckstrin homology domain−containing family A member 1
with a threshold of 1e−05
MR Test
Inverse variance weighted (fixed effects)
Inverse variance weighted (multiplicative random effects)
MR Egger
MR RAPS
Weighted median
Weighted mode
0.05
0.00
−0.05
0.09
0.12
0.15
0.18
SNP effect on Pleckstrin homology domain−containing family A member 1
SNP effect on Gut microbiota abundance (genus Catenibacterium id.2153)

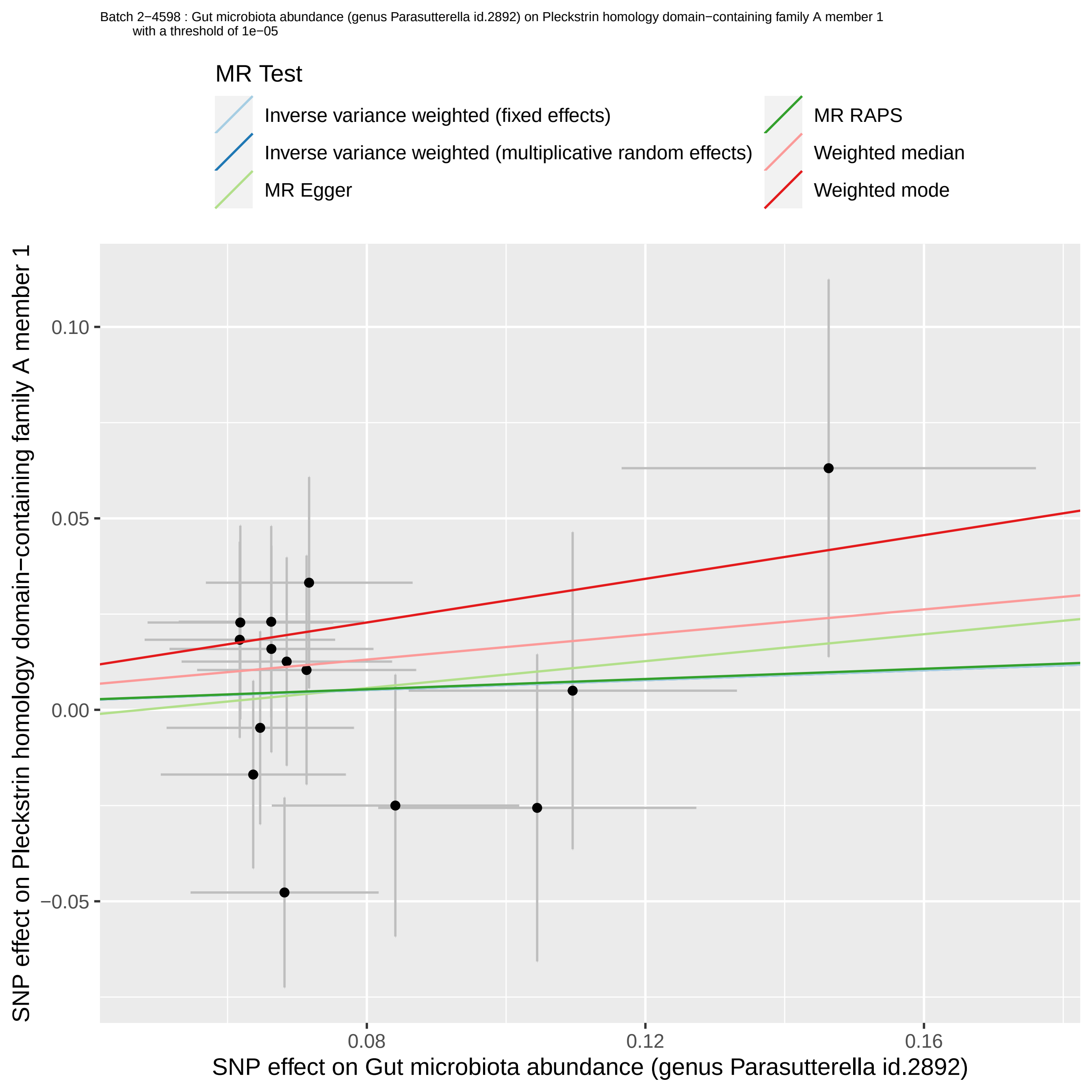
Batch 2−4598 : Gut microbiota abundance (genus Parasutterella id.2892) on Pleckstrin homology domain−containing family A member 1
with a threshold of 1e−05
MR Test
Inverse variance weighted (fixed effects)
Inverse variance weighted (multiplicative random effects)
MR Egger
MR RAPS
Weighted median
Weighted mode
0.10
0.05
0.00
−0.05
0.08
0.12
0.16
SNP effect on Pleckstrin homology domain−containing family A member 1
SNP effect on Gut microbiota abundance (genus Parasutterella id.2892)

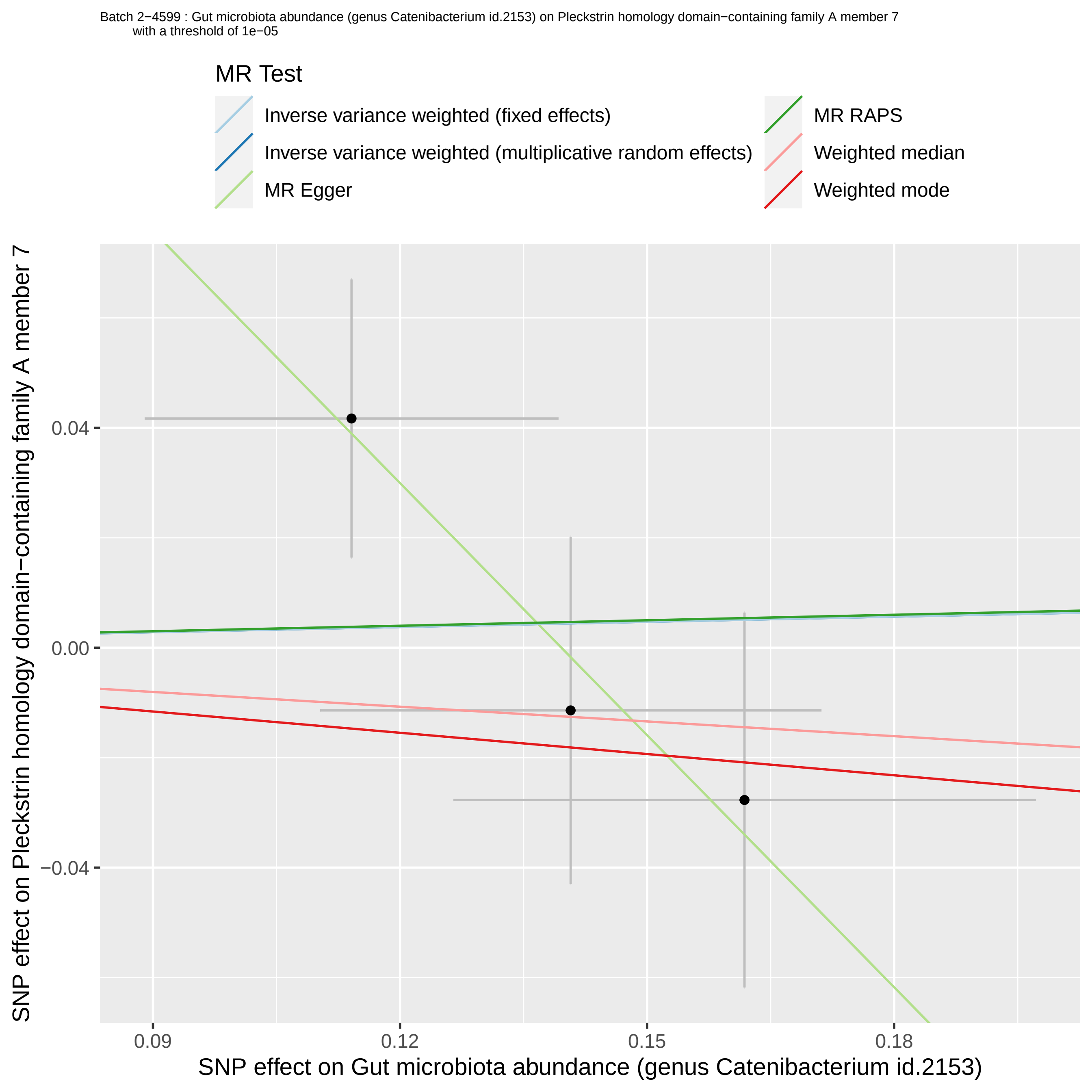
Batch 2−4599 : Gut microbiota abundance (genus Catenibacterium id.2153) on Pleckstrin homology domain−containing family A member 7
with a threshold of 1e−05
MR Test
Inverse variance weighted (fixed effects)
Inverse variance weighted (multiplicative random effects)
MR Egger
MR RAPS
Weighted median
Weighted mode
0.04
0.00
−0.04
0.09
0.12
0.15
0.18
SNP effect on Gut microbiota abundance (genus Catenibacterium id.2153)
SNP effect on Pleckstrin homology domain−containing family A member 7

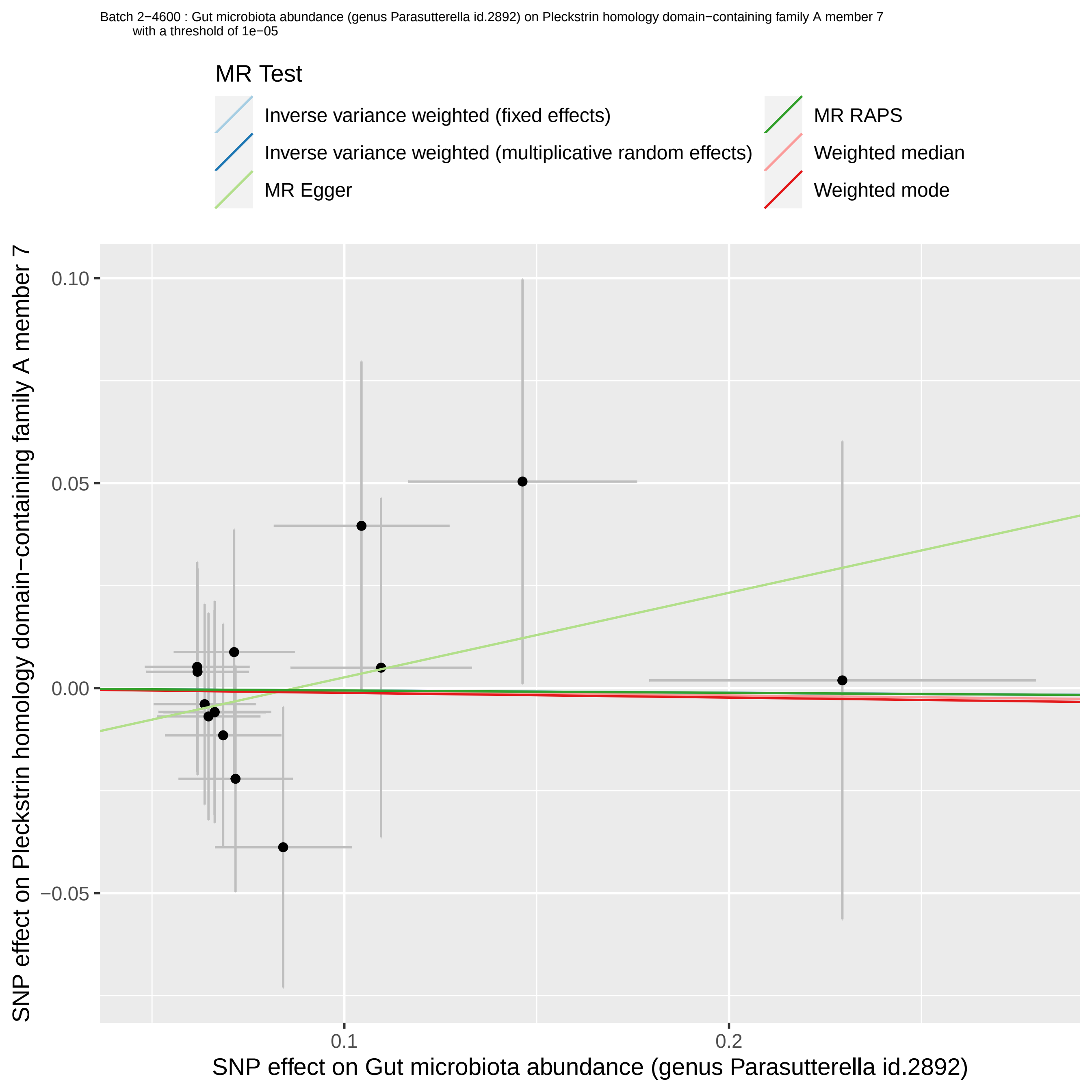
Batch 2−4600 : Gut microbiota abundance (genus Parasutterella id.2892) on Pleckstrin homology domain−containing family A member 7
with a threshold of 1e−05
MR Test
Inverse variance weighted (fixed effects)
Inverse variance weighted (multiplicative random effects)
MR Egger
MR RAPS
Weighted median
Weighted mode
SNP effect on Pleckstrin homology domain−containing family A member 7
0.10
0.05
0.00
−0.05
0.1
0.2
SNP effect on Gut microbiota abundance (genus Parasutterella id.2892)

Batch 2−4601 : Gut microbiota abundance (genus Catenibacterium id.2153) on Angiostatin
with a threshold of 1e−05

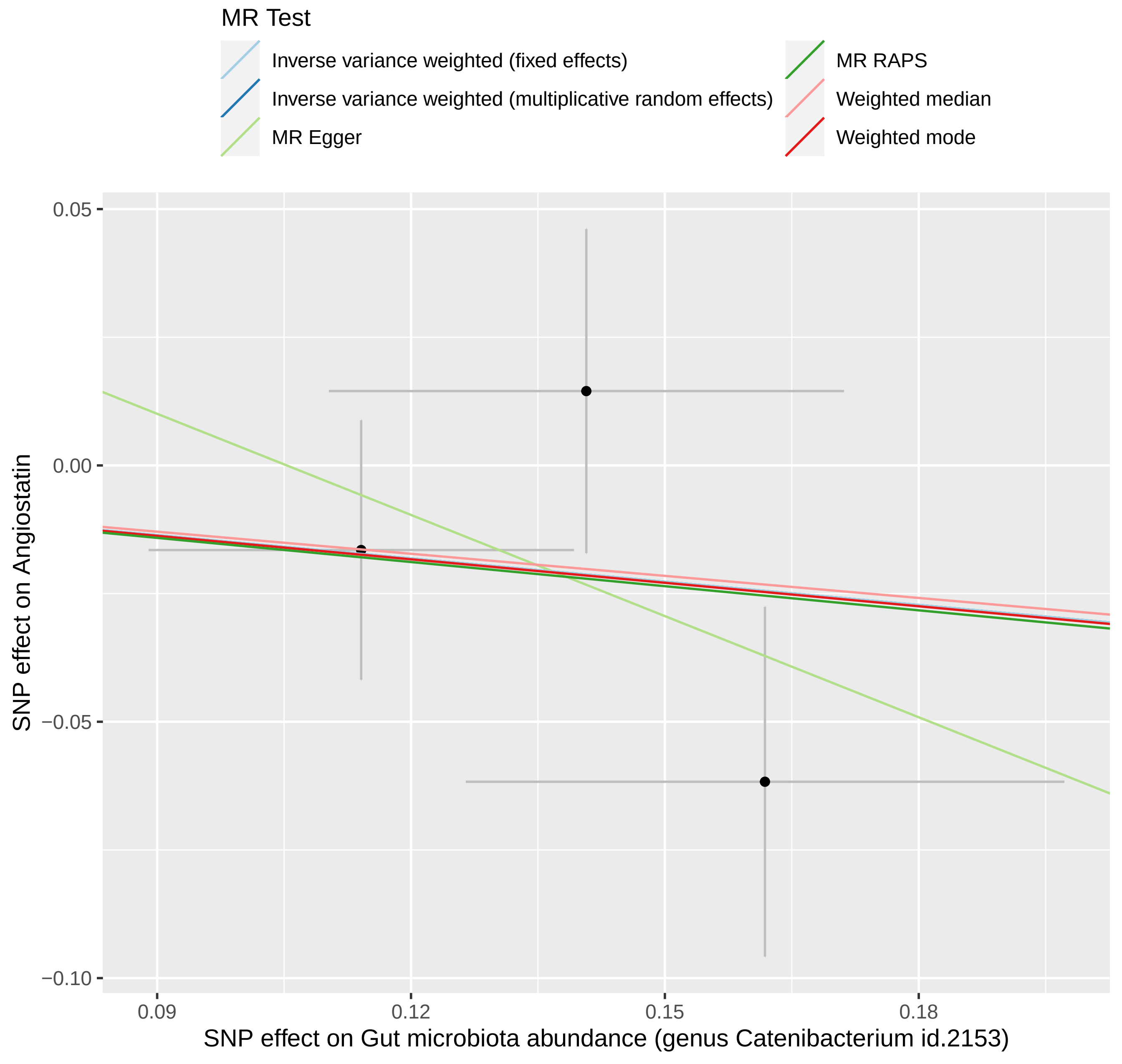

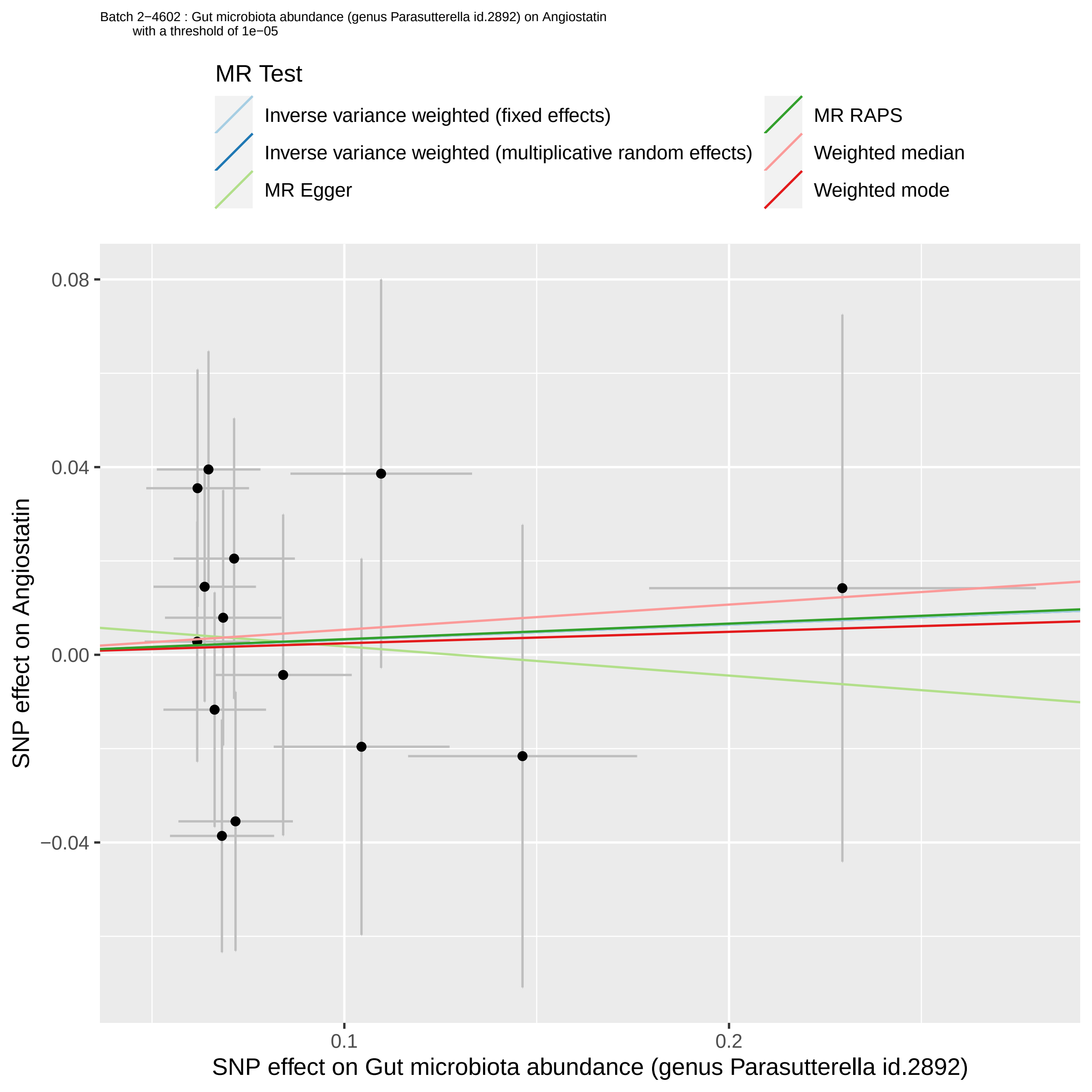

Batch 2−4602 : Gut microbiota abundance (genus Parasutterella id.2892) on Angiostatin
with a threshold of 1e−05
MR Test
Inverse variance weighted (fixed effects)
Inverse variance weighted (multiplicative random effects)
MR Egger
MR RAPS
Weighted median
Weighted mode
0.08
0.04
0.00
−0.04
0.1
0.2
SNP effect on Angiostatin
SNP effect on Gut microbiota abundance (genus Parasutterella id.2892)

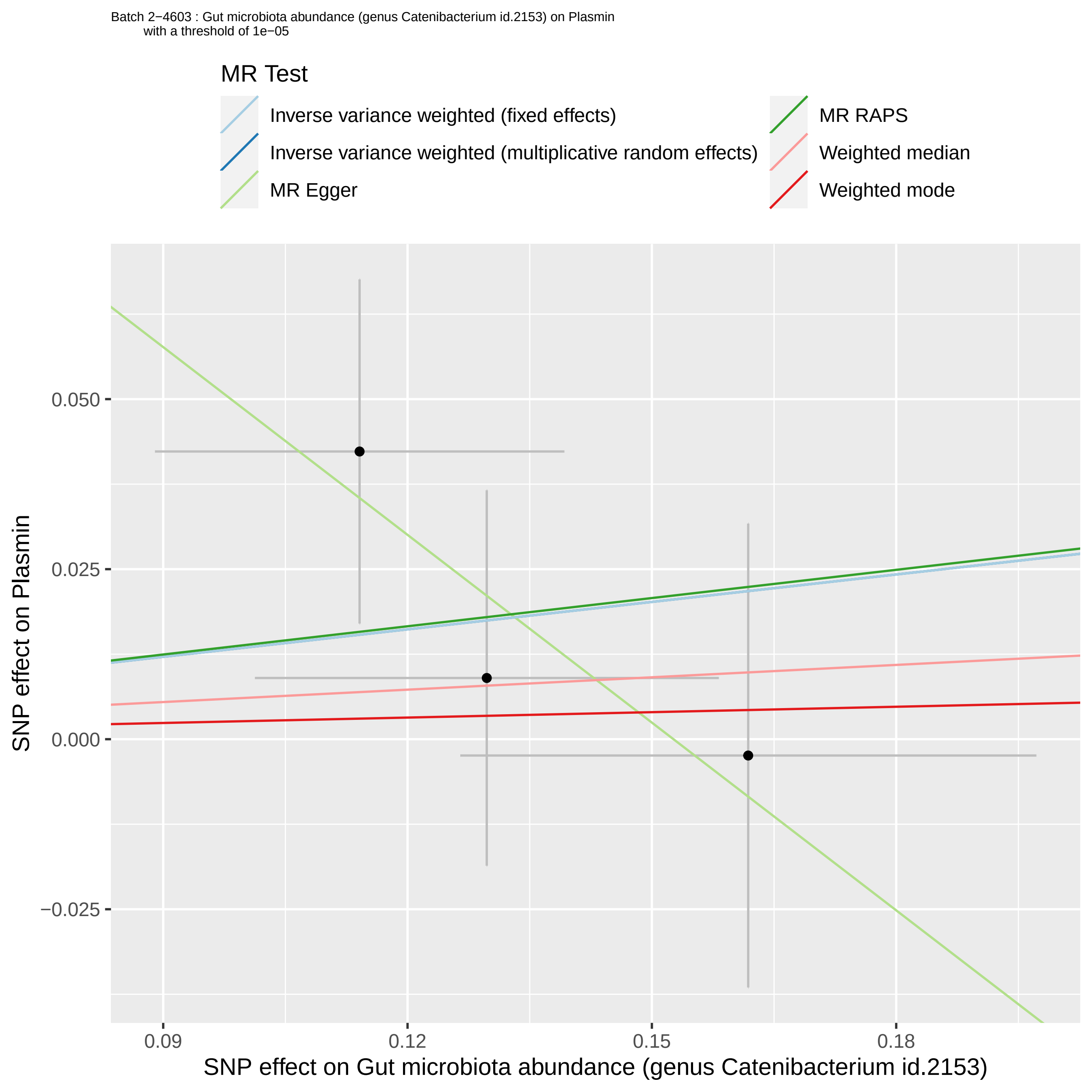

Batch 2−4603 : Gut microbiota abundance (genus Catenibacterium id.2153) on Plasmin
with a threshold of 1e−05
MR Test
Inverse variance weighted (fixed effects)
Inverse variance weighted (multiplicative random effects)
MR Egger
MR RAPS
Weighted median
Weighted mode
SNP effect on Plasmin
0.050
0.025
0.000
−0.025
0.09
0.12
0.15
0.18
SNP effect on Gut microbiota abundance (genus Catenibacterium id.2153)

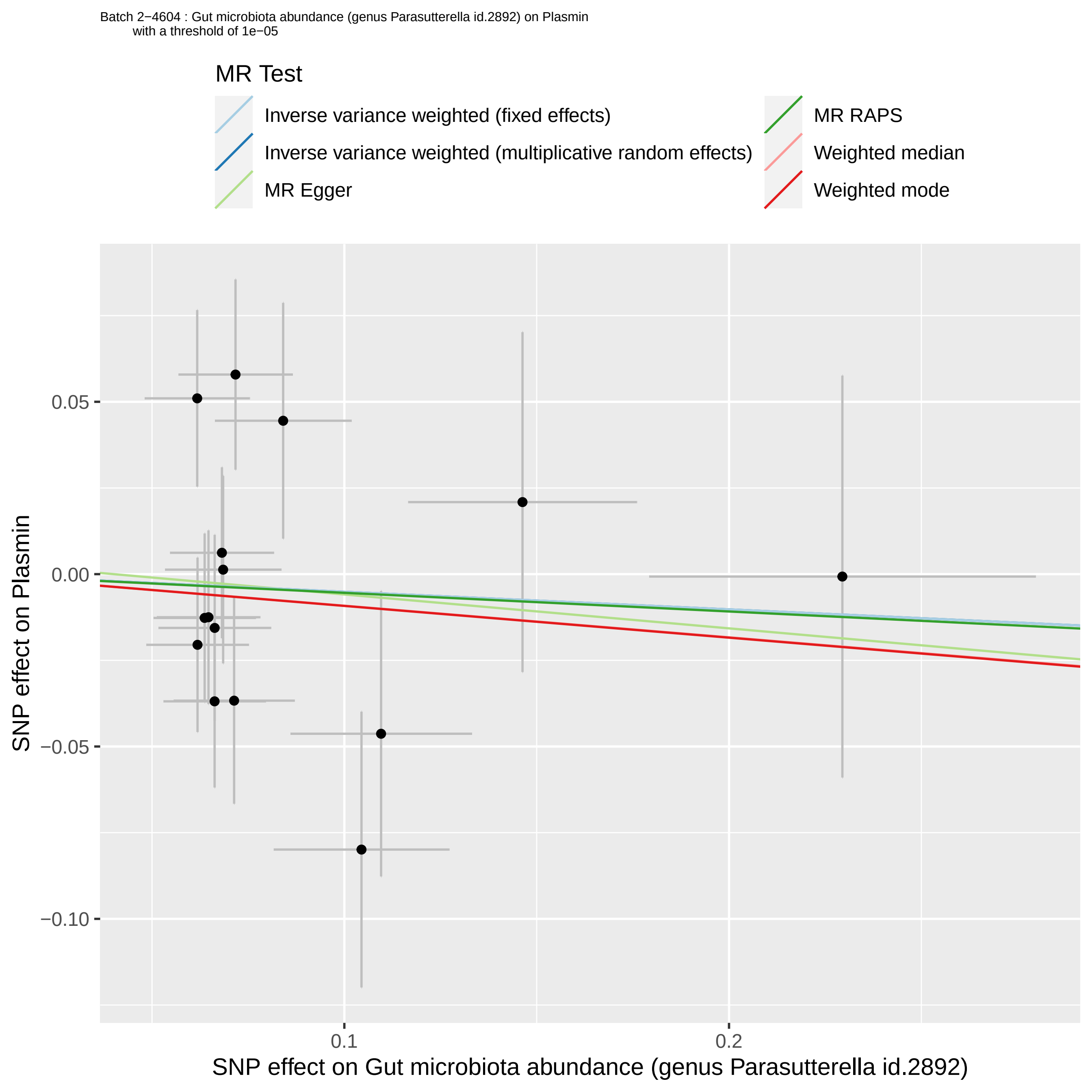

Batch 2−4604 : Gut microbiota abundance (genus Parasutterella id.2892) on Plasmin
with a threshold of 1e−05
MR Test
Inverse variance weighted (fixed effects)
Inverse variance weighted (multiplicative random effects)
MR Egger
MR RAPS
Weighted median
Weighted mode
0.05
0.00
−0.05
−0.10
0.1
0.2
SNP effect on Plasmin
SNP effect on Gut microbiota abundance (genus Parasutterella id.2892)

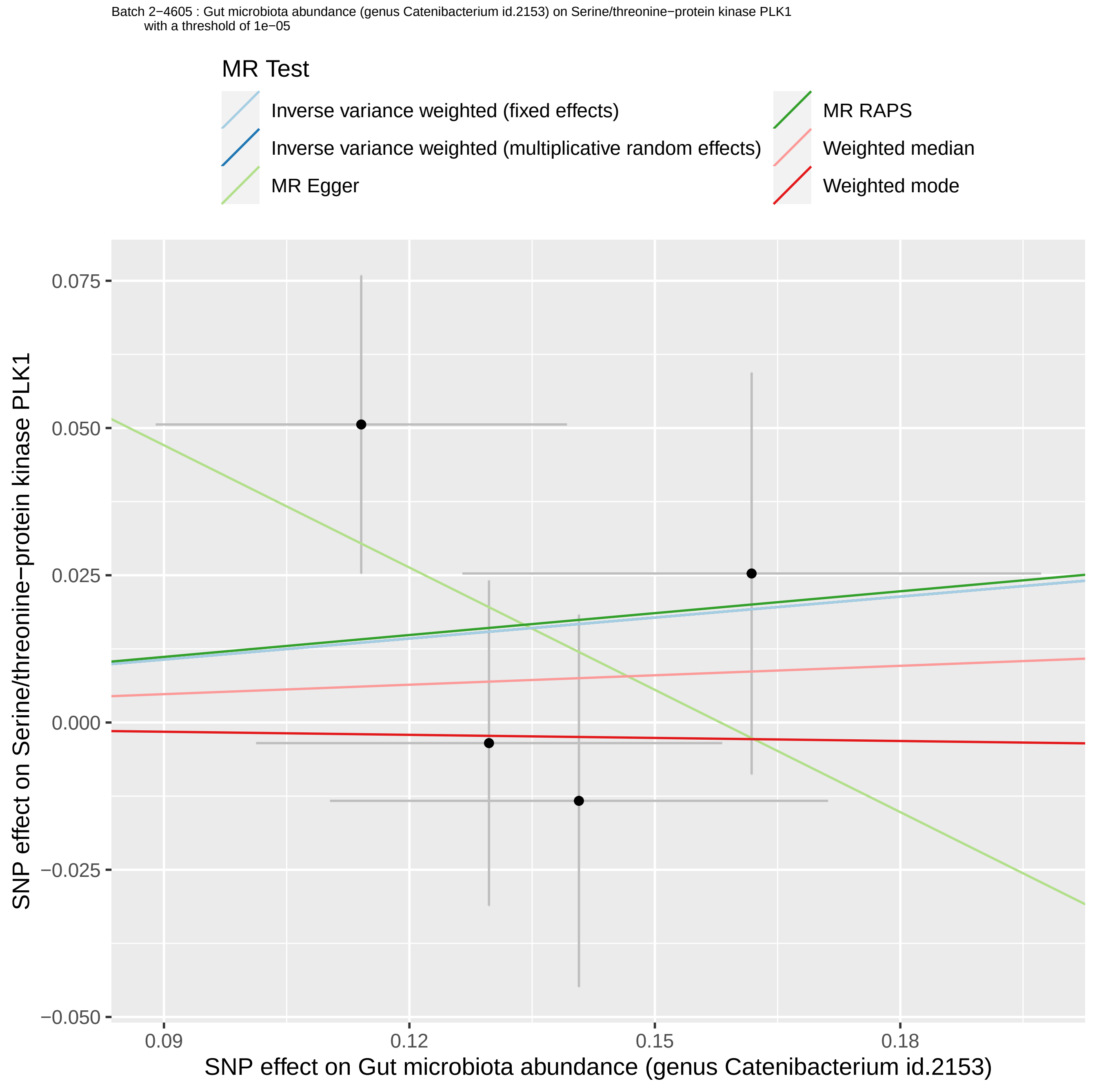
Batch 2−4605 : Gut microbiota abundance (genus Catenibacterium id.2153) on Serine/threonine−protein kinase PLK1
with a threshold of 1e−05
MR Test
Inverse variance weighted (fixed effects)
Inverse variance weighted (multiplicative random effects)
MR Egger
MR RAPS
Weighted median
Weighted mode
SNP effect on Serine/threonine−protein kinase PLK1
0.075
0.050
0.025
0.000
−0.025
−0.050
0.09
0.12
0.15
0.18
SNP effect on Gut microbiota abundance (genus Catenibacterium id.2153)

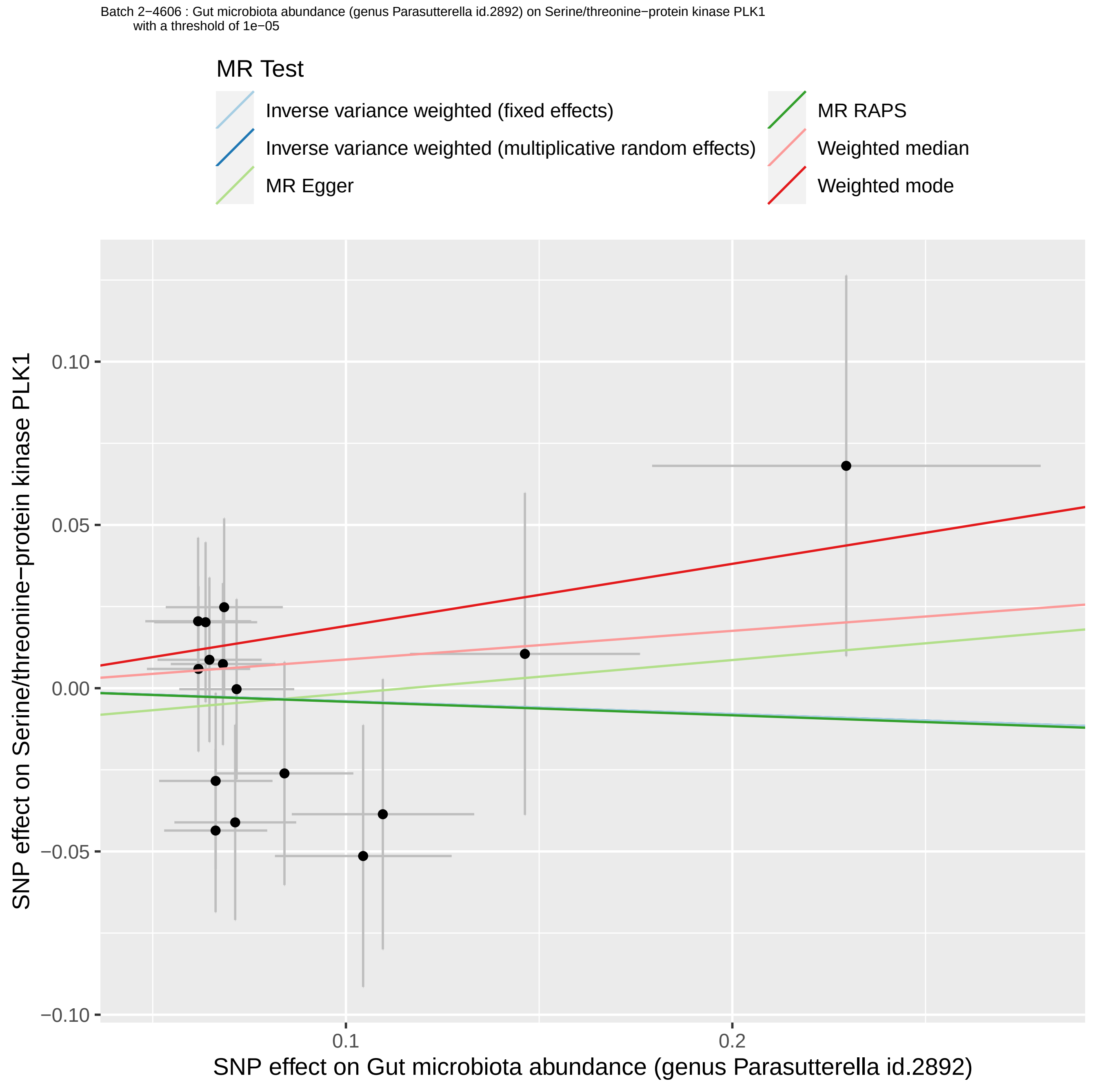

Batch 2−4606 : Gut microbiota abundance (genus Parasutterella id.2892) on Serine/threonine−protein kinase PLK1
with a threshold of 1e−05
MR Test
Inverse variance weighted (fixed effects)
Inverse variance weighted (multiplicative random effects)
MR Egger
MR RAPS
Weighted median
Weighted mode
0.10
0.05
0.00
−0.05
−0.10
0.1
0.2
SNP effect on Serine/threonine−protein kinase PLK1
SNP effect on Gut microbiota abundance (genus Parasutterella id.2892)

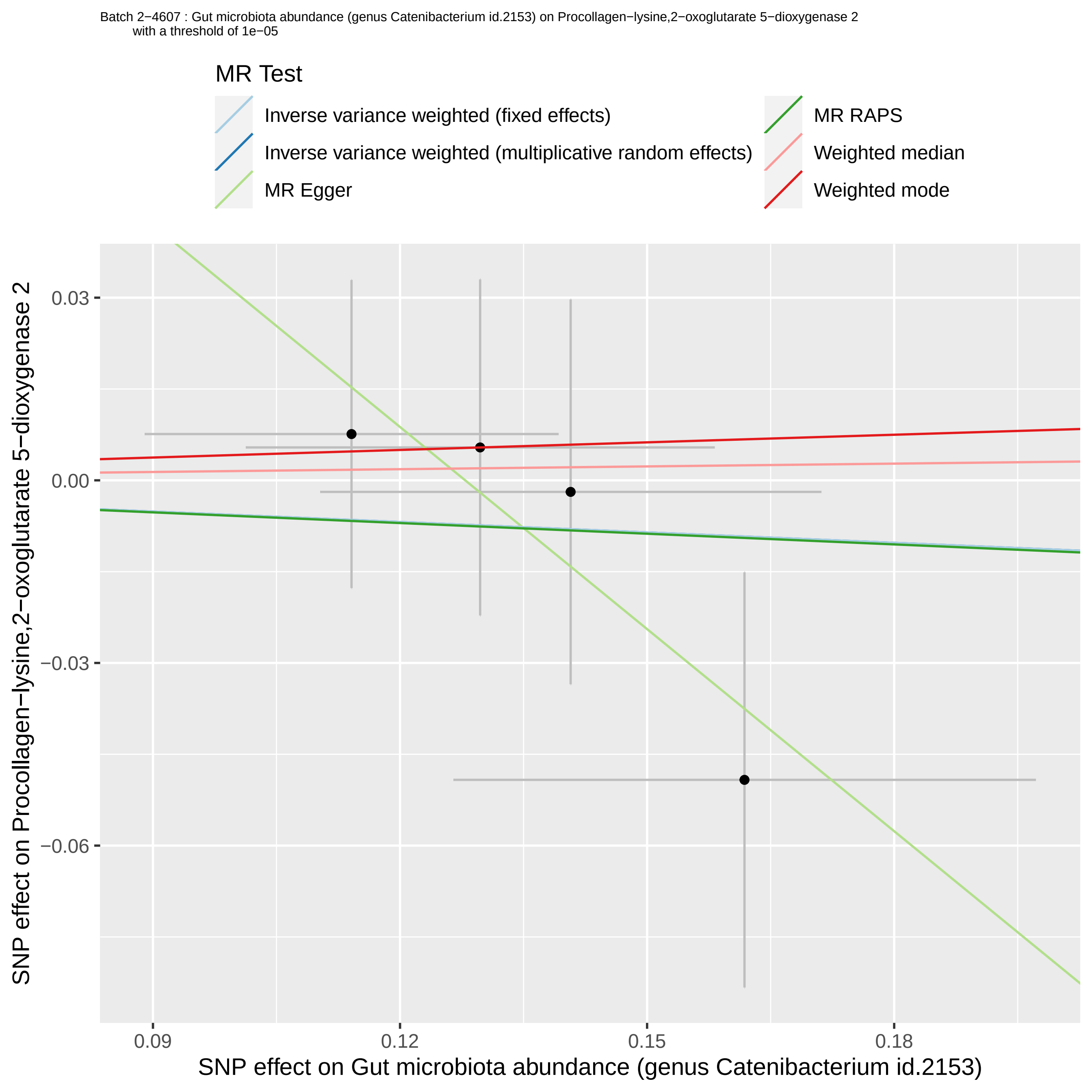

Batch 2−4607 : Gut microbiota abundance (genus Catenibacterium id.2153) on Procollagen−lysine,2−oxoglutarate 5−dioxygenase 2
with a threshold of 1e−05
MR Test
Inverse variance weighted (fixed effects)
Inverse variance weighted (multiplicative random effects)
MR Egger
MR RAPS
Weighted median
Weighted mode
SNP effect on Procollagen−lysine,2−oxoglutarate 5−dioxygenase 2
0.03
0.00
−0.03
−0.06
0.09
0.12
0.15
0.18
SNP effect on Gut microbiota abundance (genus Catenibacterium id.2153)

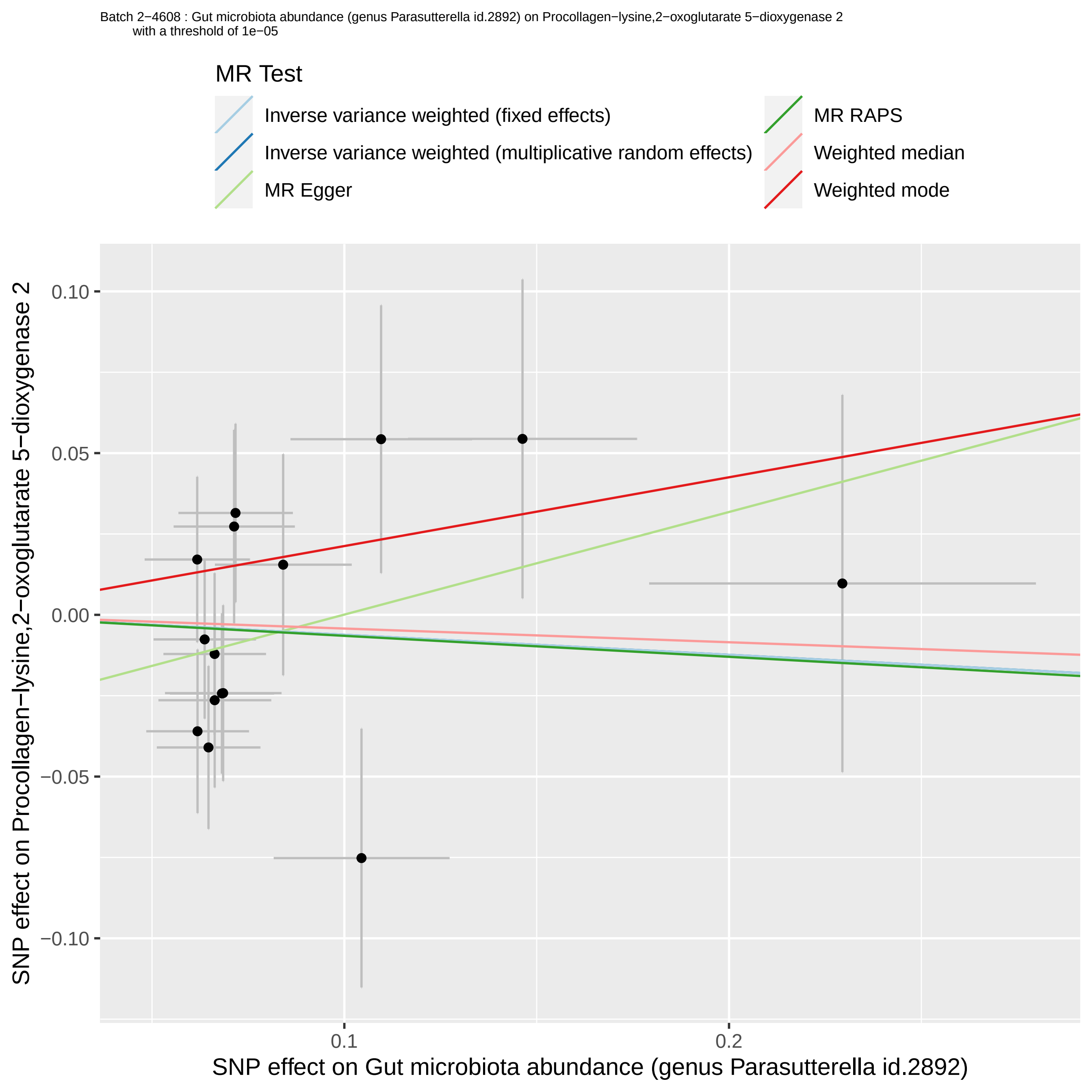
Batch 2−4608 : Gut microbiota abundance (genus Parasutterella id.2892) on Procollagen−lysine,2−oxoglutarate 5−dioxygenase 2
with a threshold of 1e−05
MR Test
Inverse variance weighted (fixed effects)
Inverse variance weighted (multiplicative random effects)
MR Egger
MR RAPS
Weighted median
Weighted mode
SNP effect on Procollagen−lysine,2−oxoglutarate 5−dioxygenase 2
0.10
0.05
0.00
−0.05
−0.10
0.1
0.2
SNP effect on Gut microbiota abundance (genus Parasutterella id.2892)

Batch 2−4609 : Gut microbiota abundance (genus Catenibacterium id.2153) on Procollagen−lysine,2−oxoglutarate 5−dioxygenase 3 with a threshold of 1e−05

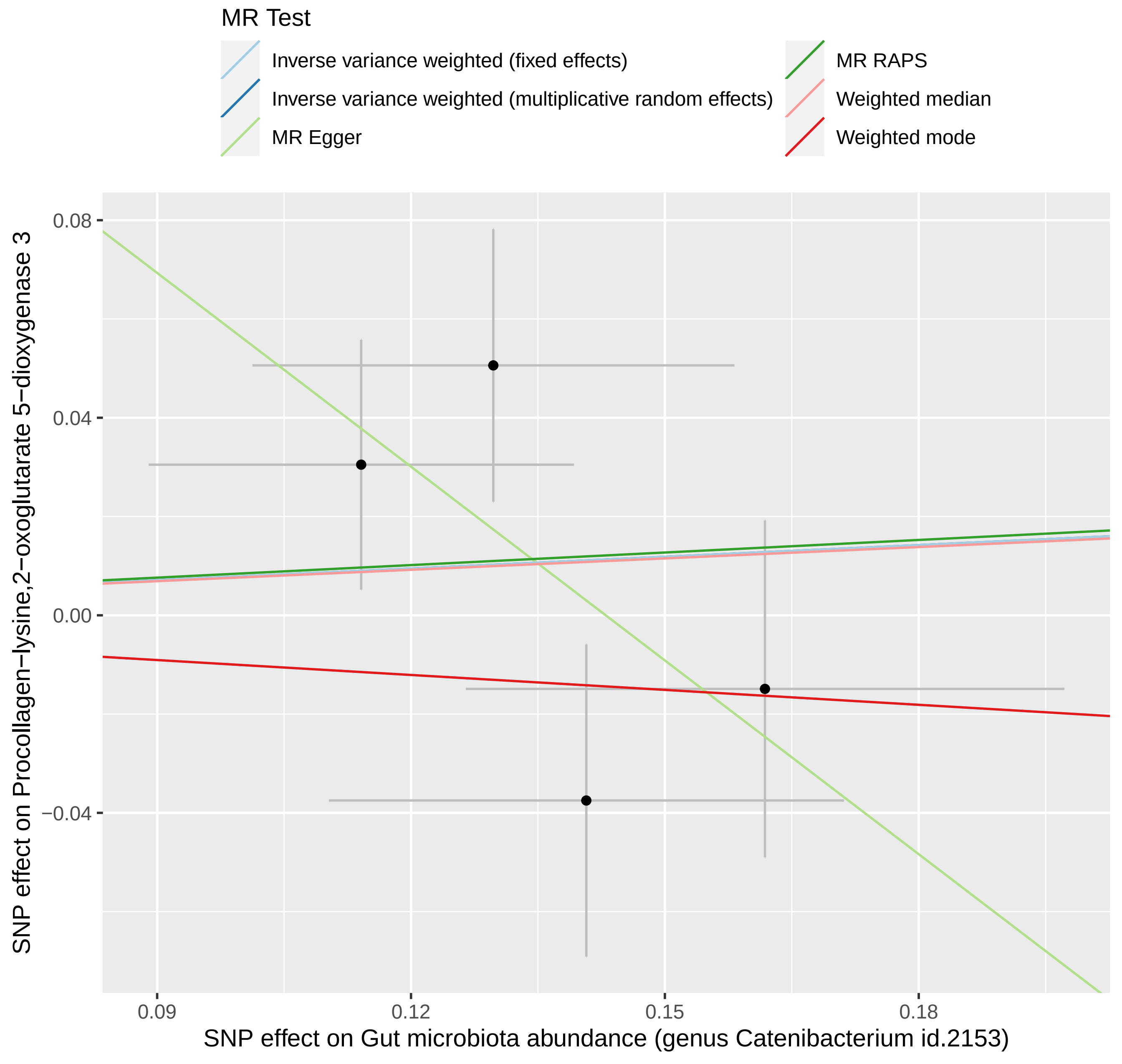

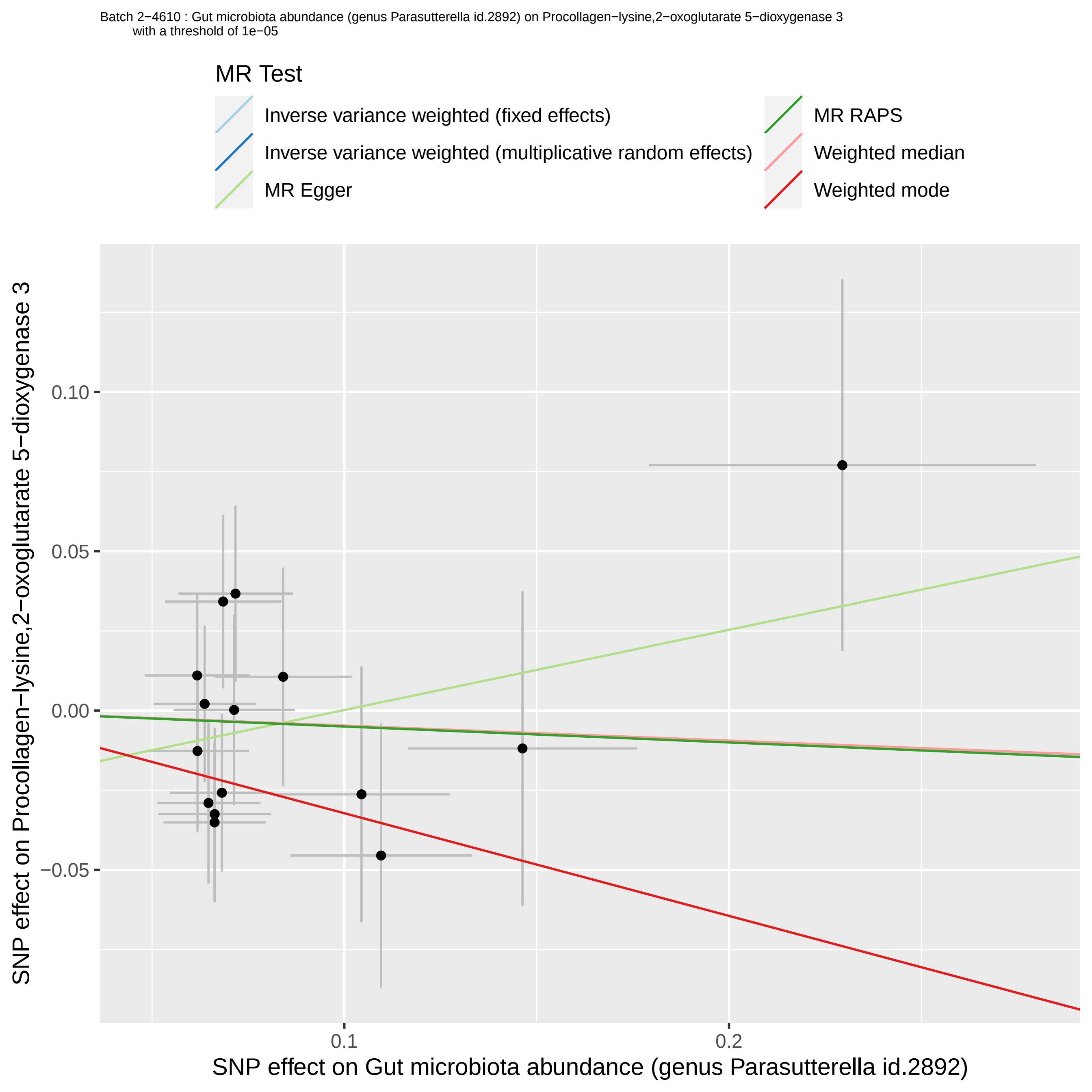
Batch 2−4610 : Gut microbiota abundance (genus Parasutterella id.2892) on Procollagen−lysine,2−oxoglutarate 5−dioxygenase 3
with a threshold of 1e−05
MR Test
Inverse variance weighted (fixed effects)
Inverse variance weighted (multiplicative random effects)
MR Egger
MR RAPS
Weighted median
Weighted mode
0.10
0.05
0.00
−0.05
0.1
0.2
SNP effect on Procollagen−lysine,2−oxoglutarate 5−dioxygenase 3
SNP effect on Gut microbiota abundance (genus Parasutterella id.2892)

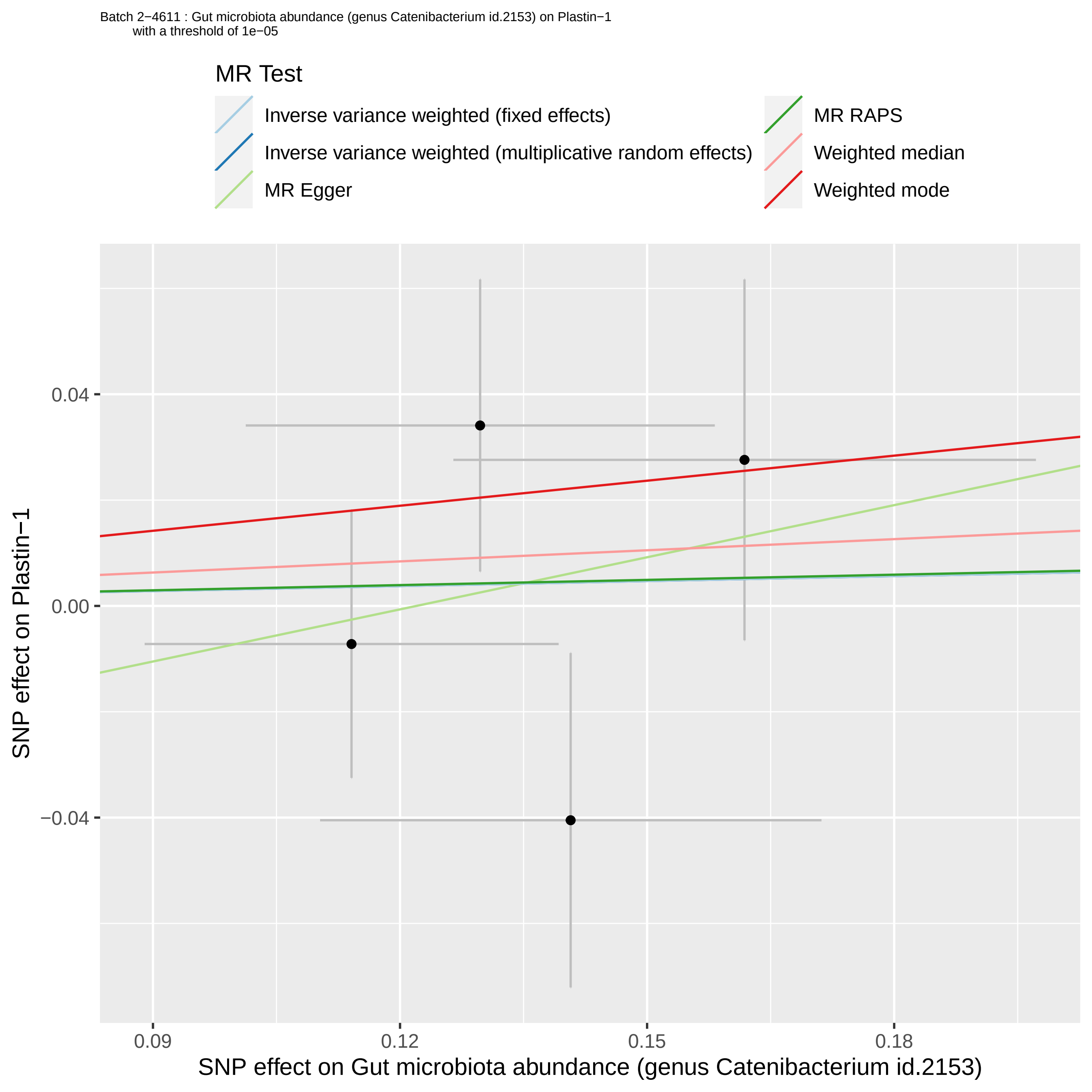
Batch 2−4611 : Gut microbiota abundance (genus Catenibacterium id.2153) on Plastin−1
with a threshold of 1e−05
MR Test
Inverse variance weighted (fixed effects)
Inverse variance weighted (multiplicative random effects)
MR Egger
MR RAPS
Weighted median
Weighted mode
0.04
0.00
−0.04
0.09
0.12
0.15
0.18
SNP effect on Plastin−1
SNP effect on Gut microbiota abundance (genus Catenibacterium id.2153)

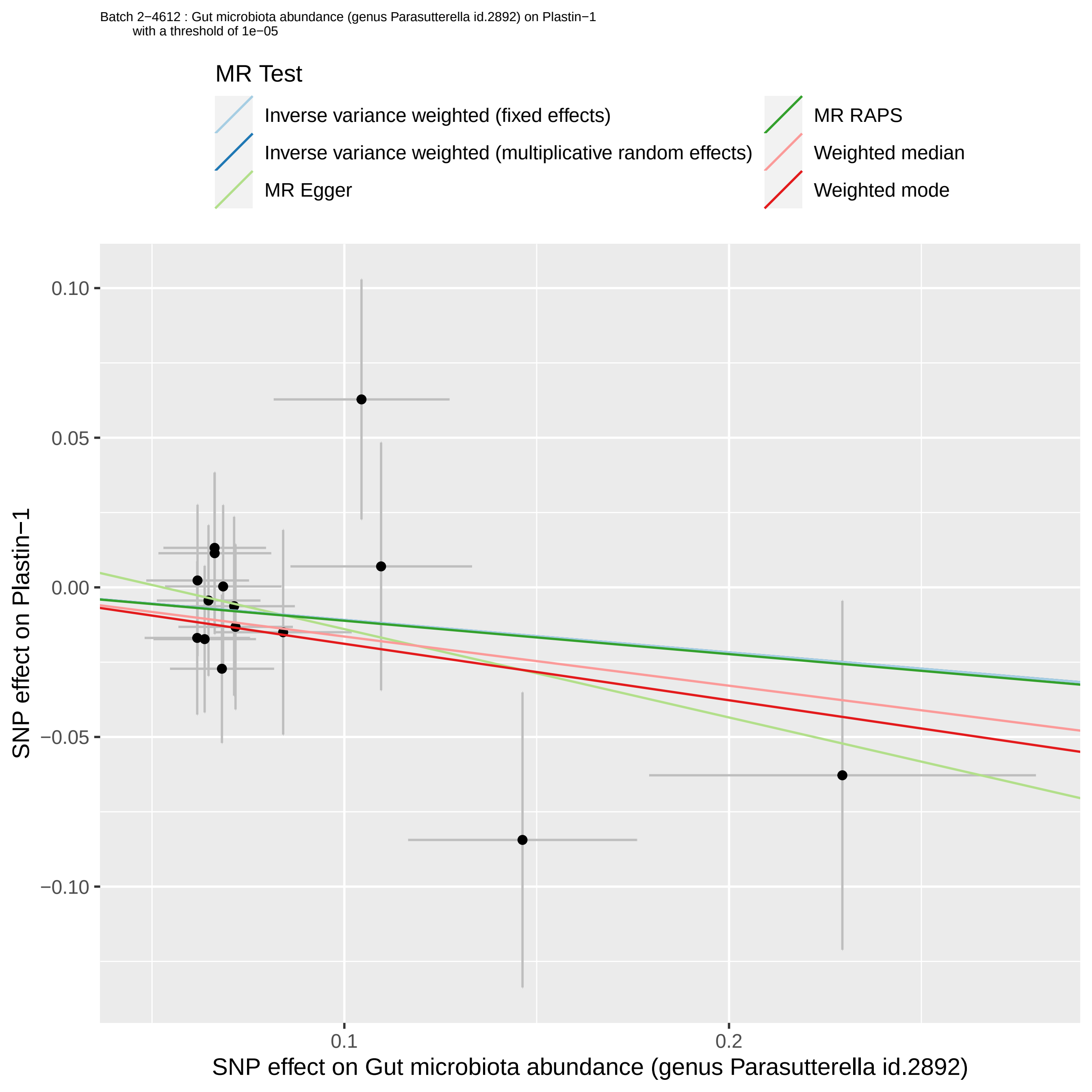

Batch 2−4612 : Gut microbiota abundance (genus Parasutterella id.2892) on Plastin−1
with a threshold of 1e−05
MR Test
Inverse variance weighted (fixed effects)
Inverse variance weighted (multiplicative random effects)
MR Egger
MR RAPS
Weighted median
Weighted mode
0.10
0.05
0.00
−0.05
−0.10
0.1
0.2
SNP effect on Plastin−1
SNP effect on Gut microbiota abundance (genus Parasutterella id.2892)

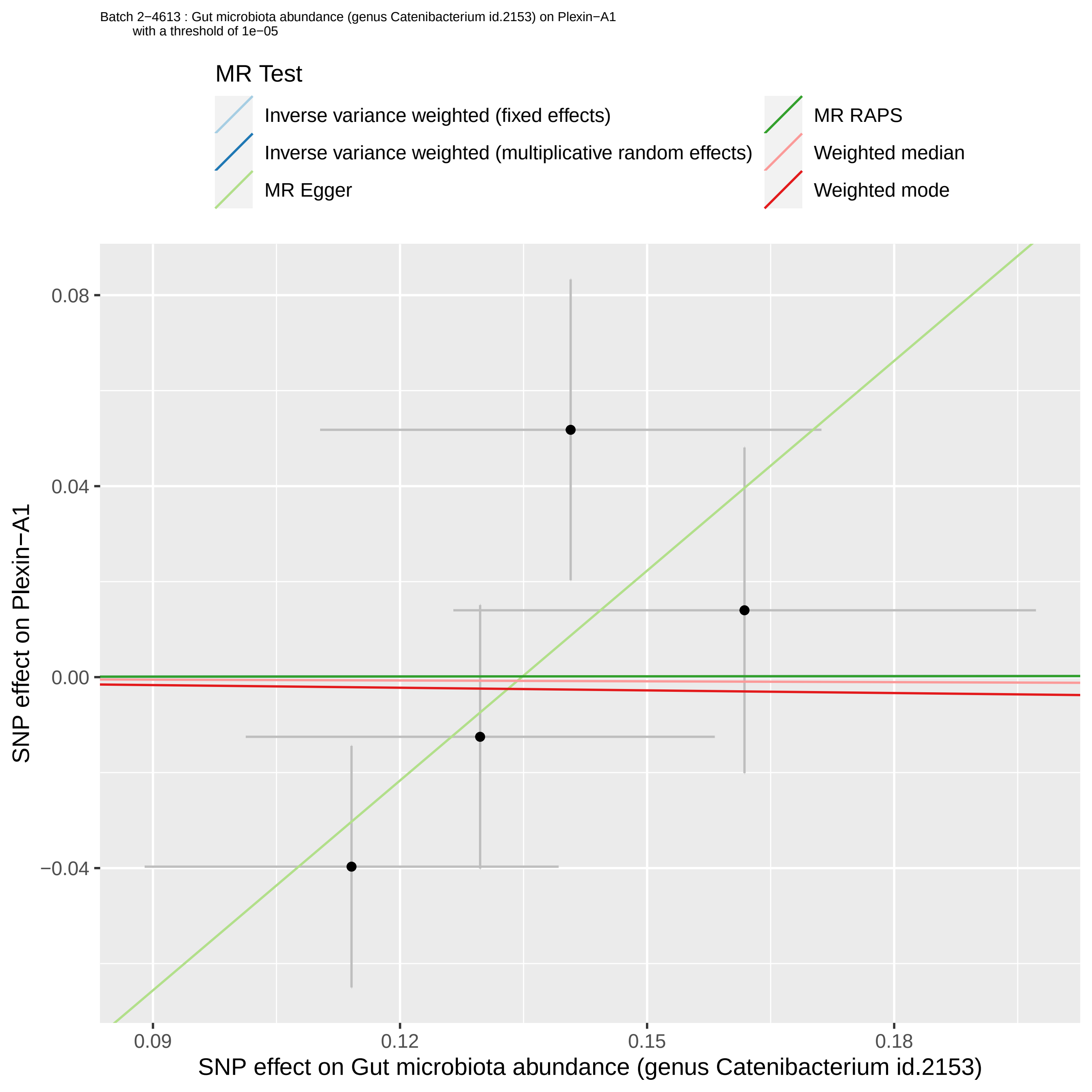

Batch 2−4613 : Gut microbiota abundance (genus Catenibacterium id.2153) on Plexin−A1
with a threshold of 1e−05
MR Test
Inverse variance weighted (fixed effects)
Inverse variance weighted (multiplicative random effects)
MR Egger
MR RAPS
Weighted median
Weighted mode
SNP effect on Plexin−A1
0.08
0.04
0.00
−0.04
0.09
0.12
0.15
0.18
SNP effect on Gut microbiota abundance (genus Catenibacterium id.2153)

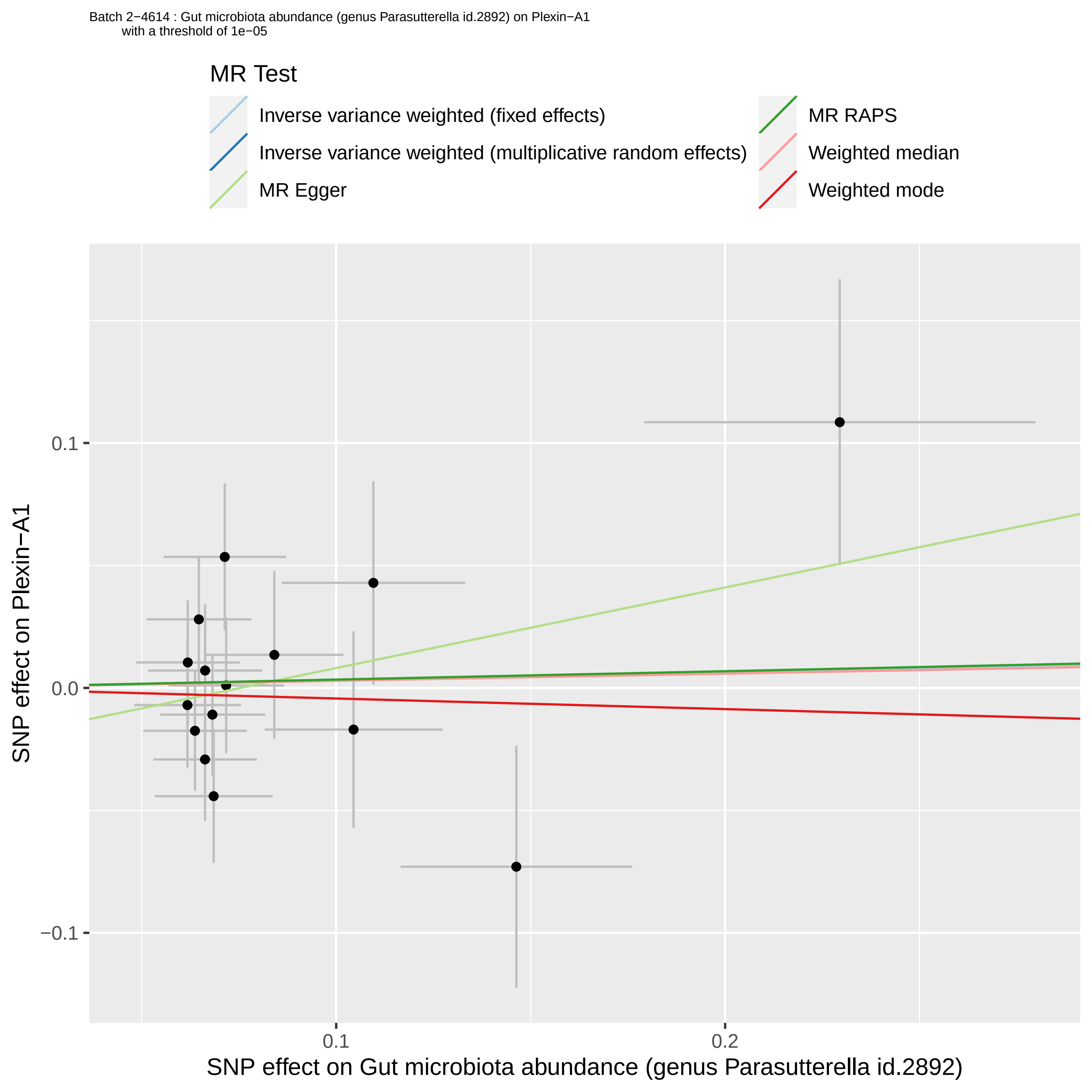

Batch 2−4614 : Gut microbiota abundance (genus Parasutterella id.2892) on Plexin−A1
with a threshold of 1e−05
MR Test
Inverse variance weighted (fixed effects)
Inverse variance weighted (multiplicative random effects)
MR Egger
MR RAPS
Weighted median
Weighted mode
0.1
0.0
−0.1
0.1
0.2
SNP effect on Plexin−A1
SNP effect on Gut microbiota abundance (genus Parasutterella id.2892)

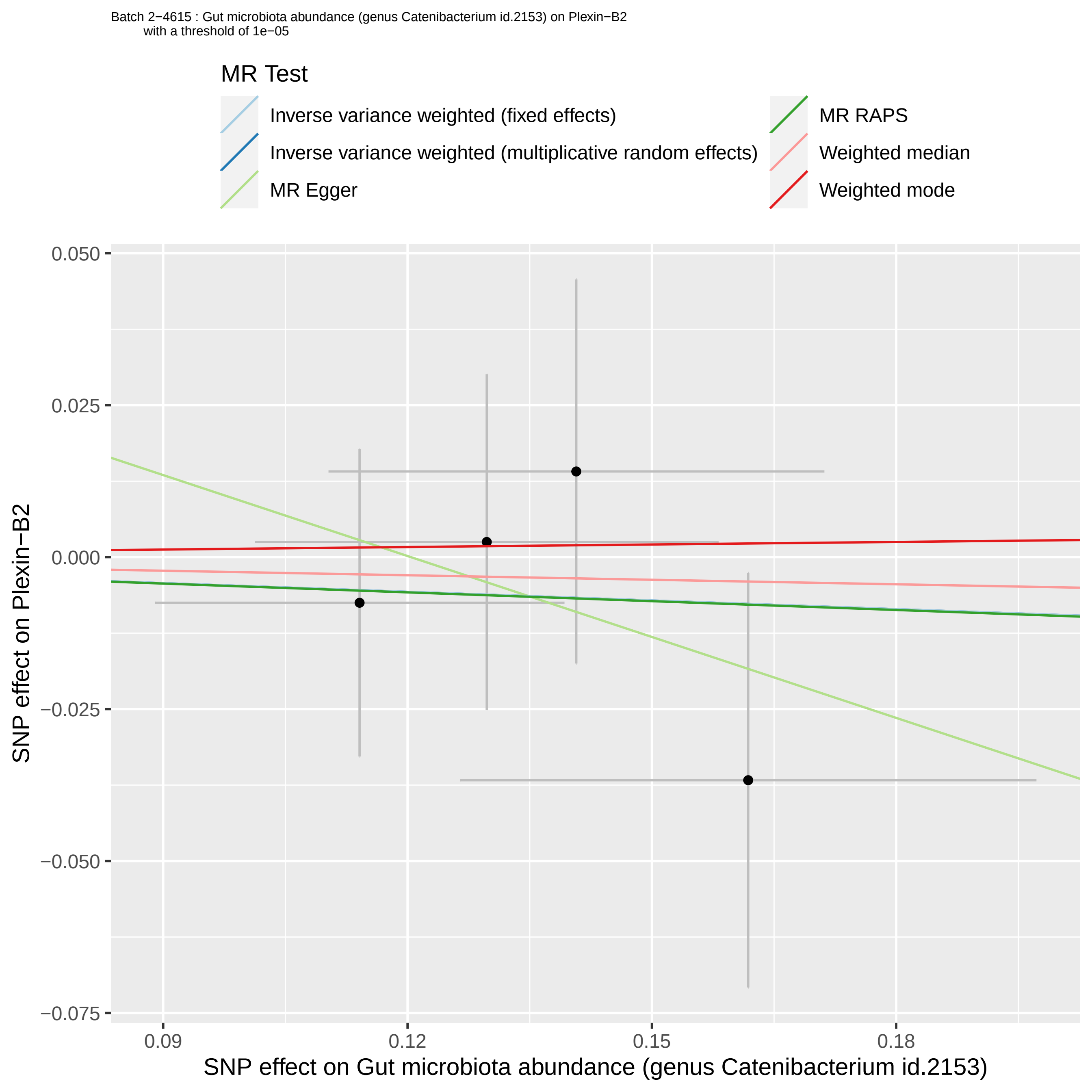
Batch 2−4615 : Gut microbiota abundance (genus Catenibacterium id.2153) on Plexin−B2
with a threshold of 1e−05
MR Test
Inverse variance weighted (fixed effects)
Inverse variance weighted (multiplicative random effects)
MR Egger
MR RAPS
Weighted median
Weighted mode
0.050
0.025
0.000
−0.025
−0.050
−0.075
0.09
0.12
0.15
0.18
SNP effect on Plexin−B2
SNP effect on Gut microbiota abundance (genus Catenibacterium id.2153)

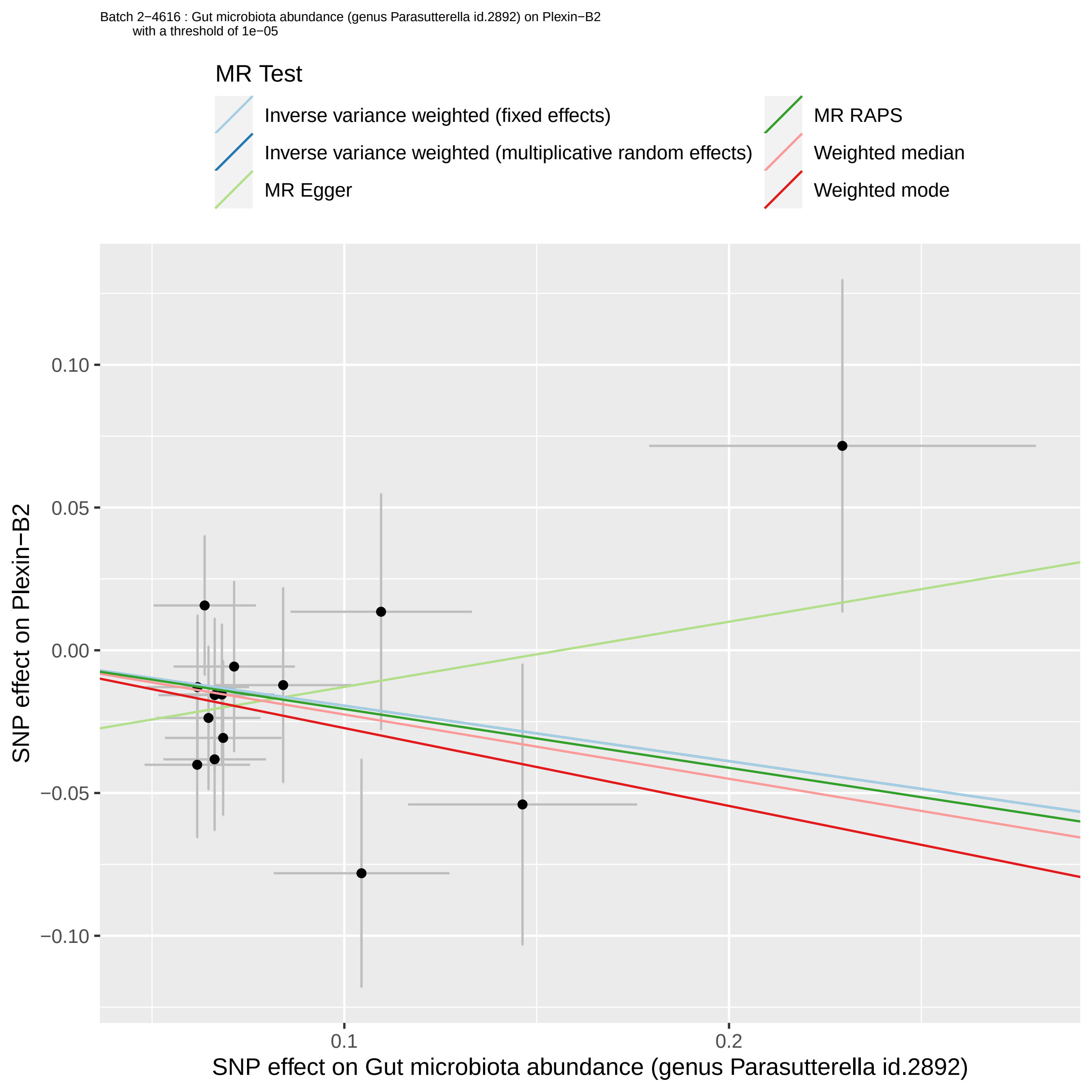
Batch 2−4616 : Gut microbiota abundance (genus Parasutterella id.2892) on Plexin−B2
with a threshold of 1e−05
MR Test
Inverse variance weighted (fixed effects)
Inverse variance weighted (multiplicative random effects)
MR Egger
MR RAPS
Weighted median
Weighted mode
0.10
0.05
0.00
−0.05
−0.10
0.1
0.2
SNP effect on Plexin−B2
SNP effect on Gut microbiota abundance (genus Parasutterella id.2892)

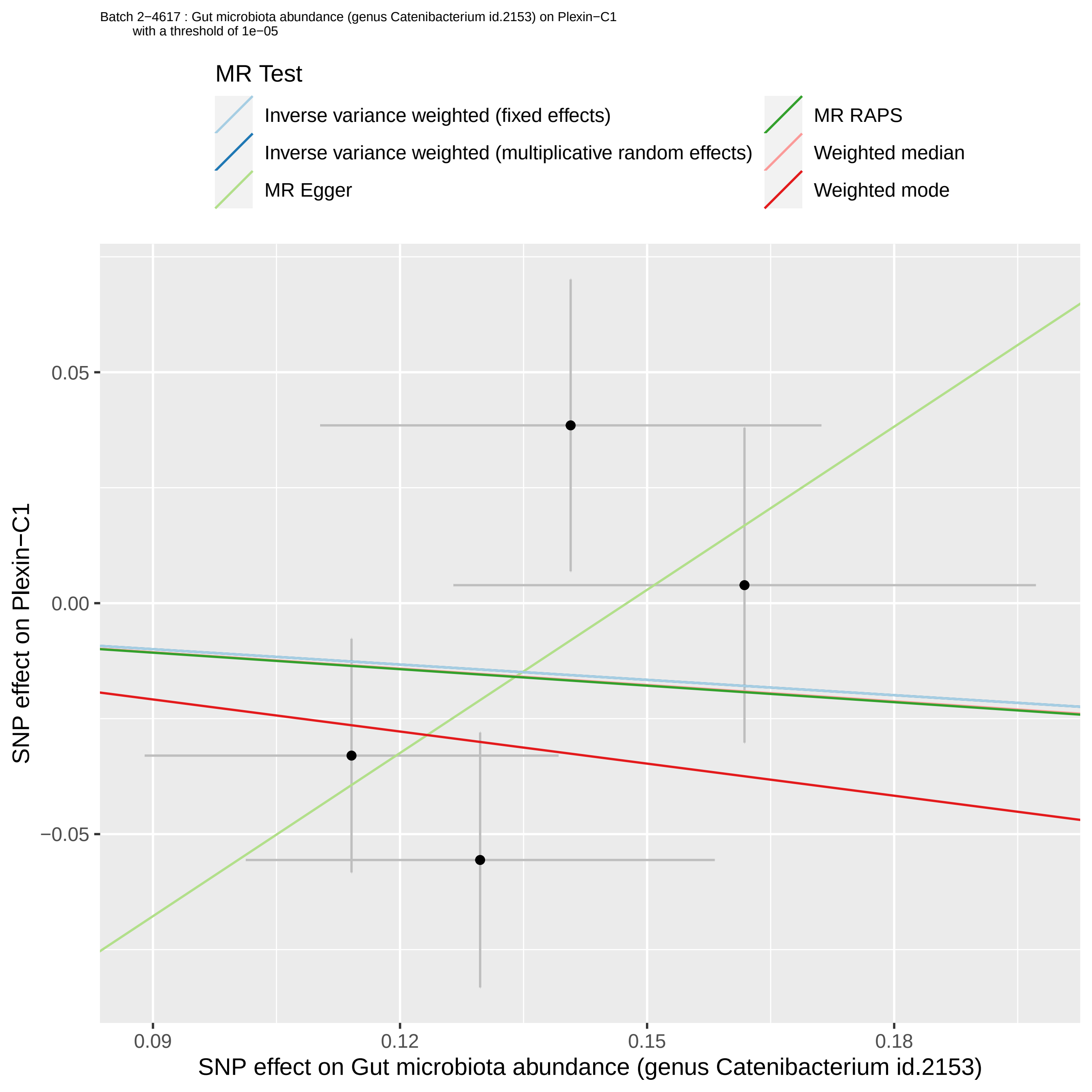
Batch 2−4617 : Gut microbiota abundance (genus Catenibacterium id.2153) on Plexin−C1
with a threshold of 1e−05
MR Test
Inverse variance weighted (fixed effects)
Inverse variance weighted (multiplicative random effects)
MR Egger
MR RAPS
Weighted median
Weighted mode
0.05
0.00
−0.05
0.09
0.12
0.15
0.18
SNP effect on Plexin−C1
SNP effect on Gut microbiota abundance (genus Catenibacterium id.2153)

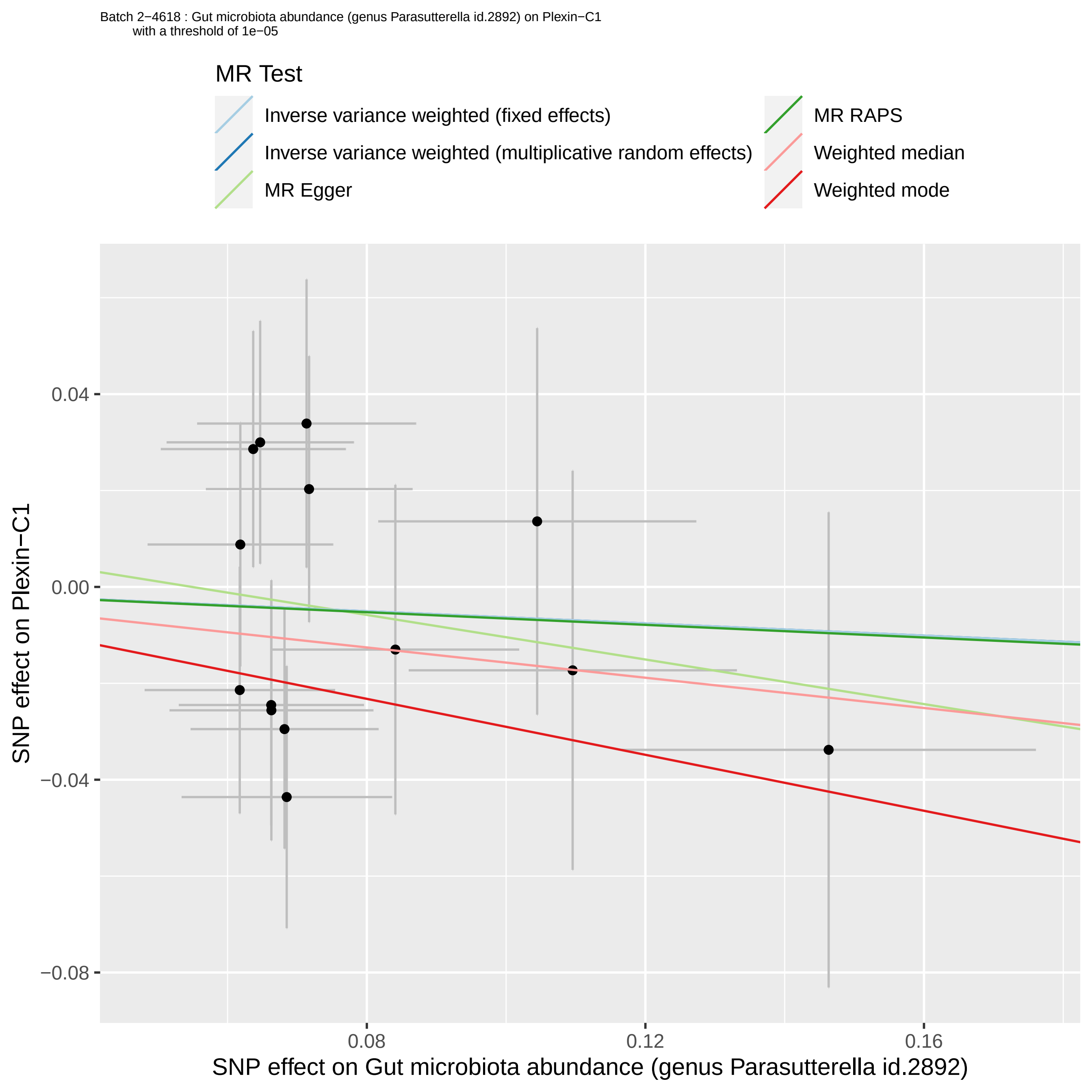

Batch 2−4618 : Gut microbiota abundance (genus Parasutterella id.2892) on Plexin−C1
with a threshold of 1e−05
MR Test
Inverse variance weighted (fixed effects)
Inverse variance weighted (multiplicative random effects)
MR Egger
MR RAPS
Weighted median
Weighted mode
0.04
0.00
−0.04
−0.08
0.08
0.12
0.16
SNP effect on Plexin−C1
SNP effect on Gut microbiota abundance (genus Parasutterella id.2892)

Batch 2−4619 : Gut microbiota abundance (genus Catenibacterium id.2153) on Melanocyte protein PMEL
with a threshold of 1e−05

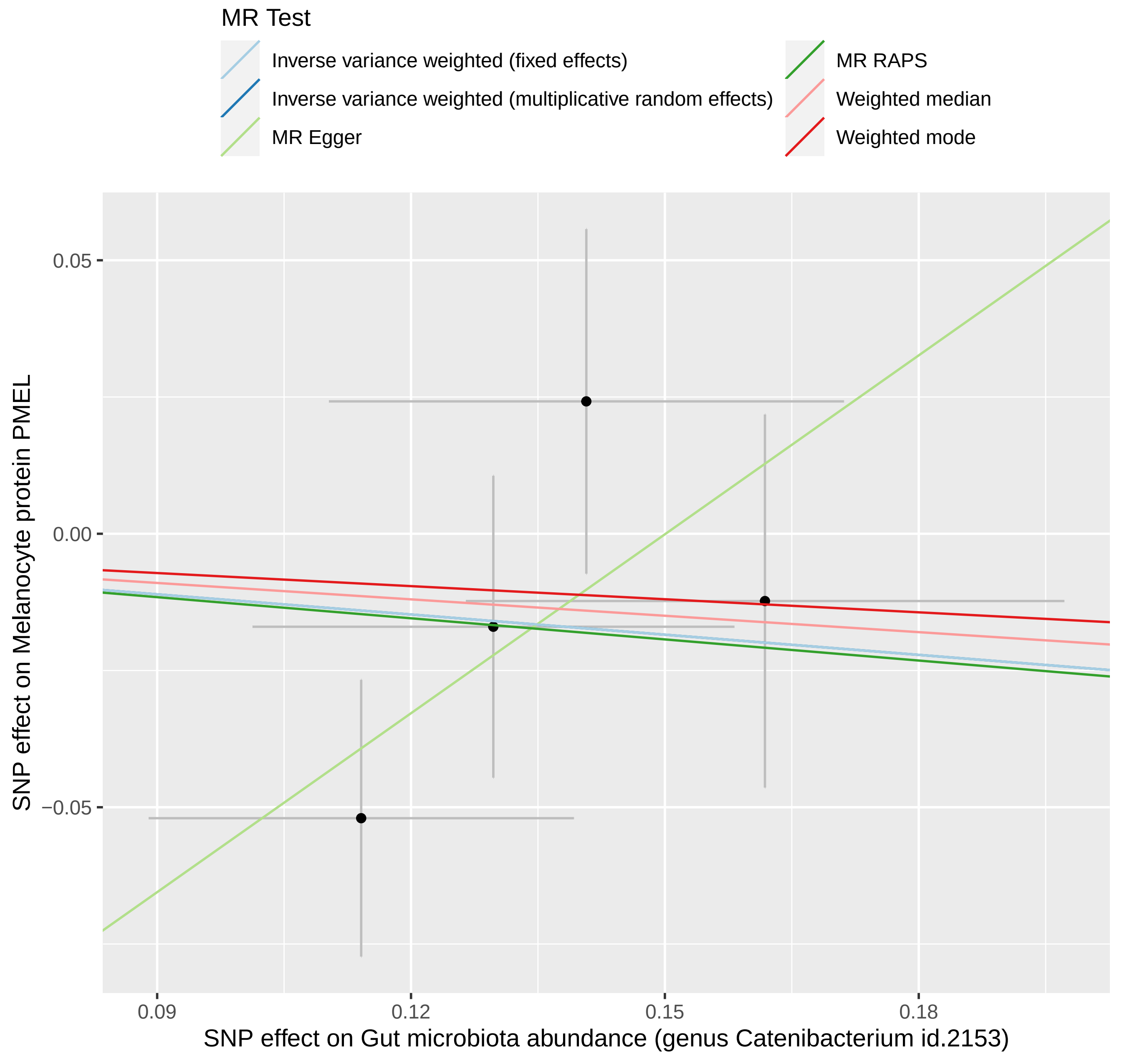

Batch 2−4620 : Gut microbiota abundance (genus Parasutterella id.2892) on Melanocyte protein PMEL
with a threshold of 1e−05

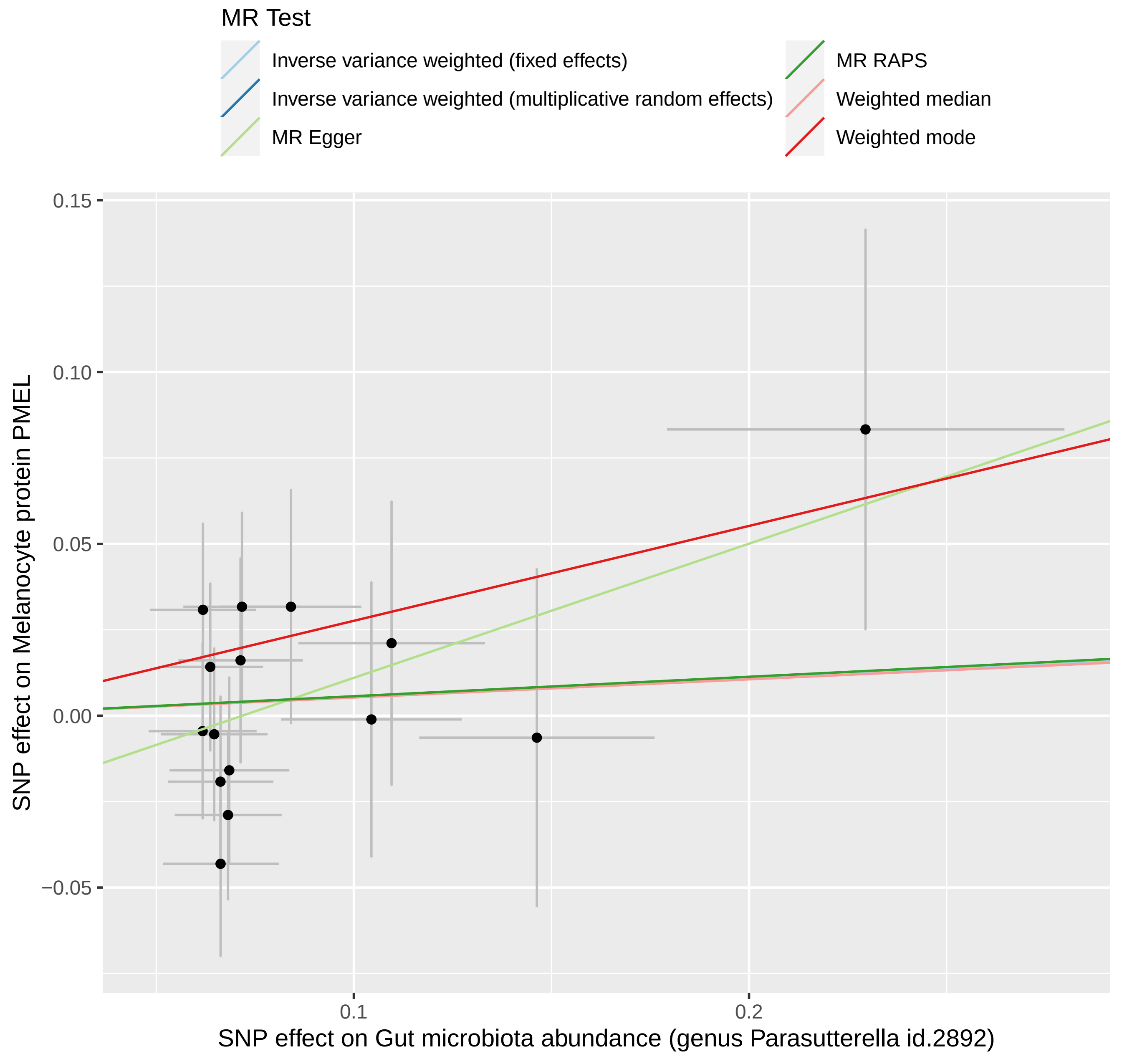

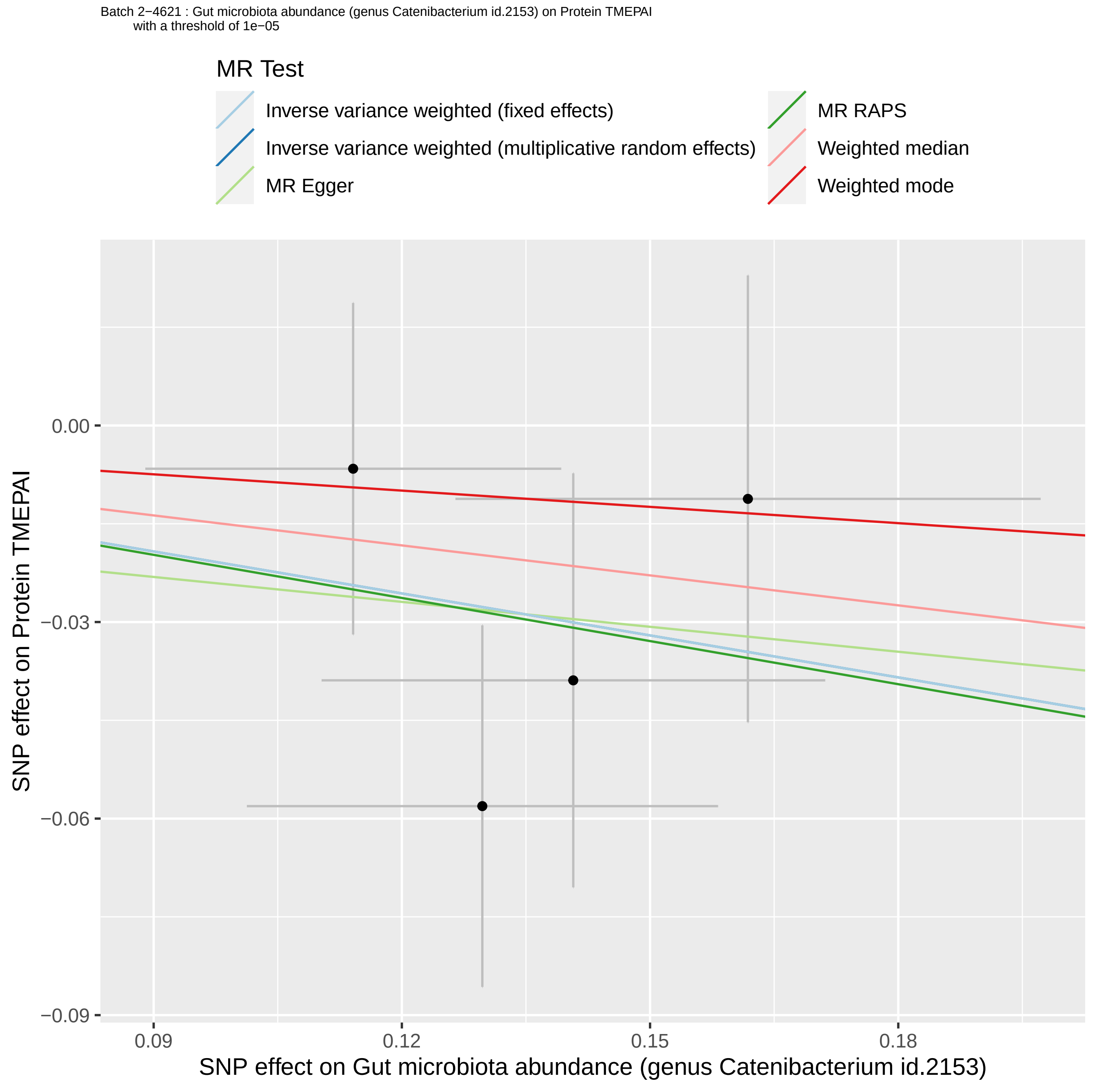
Batch 2−4621 : Gut microbiota abundance (genus Catenibacterium id.2153) on Protein TMEPAI
with a threshold of 1e−05
MR Test
Inverse variance weighted (fixed effects)
Inverse variance weighted (multiplicative random effects)
MR Egger
MR RAPS
Weighted median
Weighted mode
SNP effect on Protein TMEPAI
0.00
−0.03
−0.06
−0.09
0.09
0.12
0.15
0.18
SNP effect on Gut microbiota abundance (genus Catenibacterium id.2153)

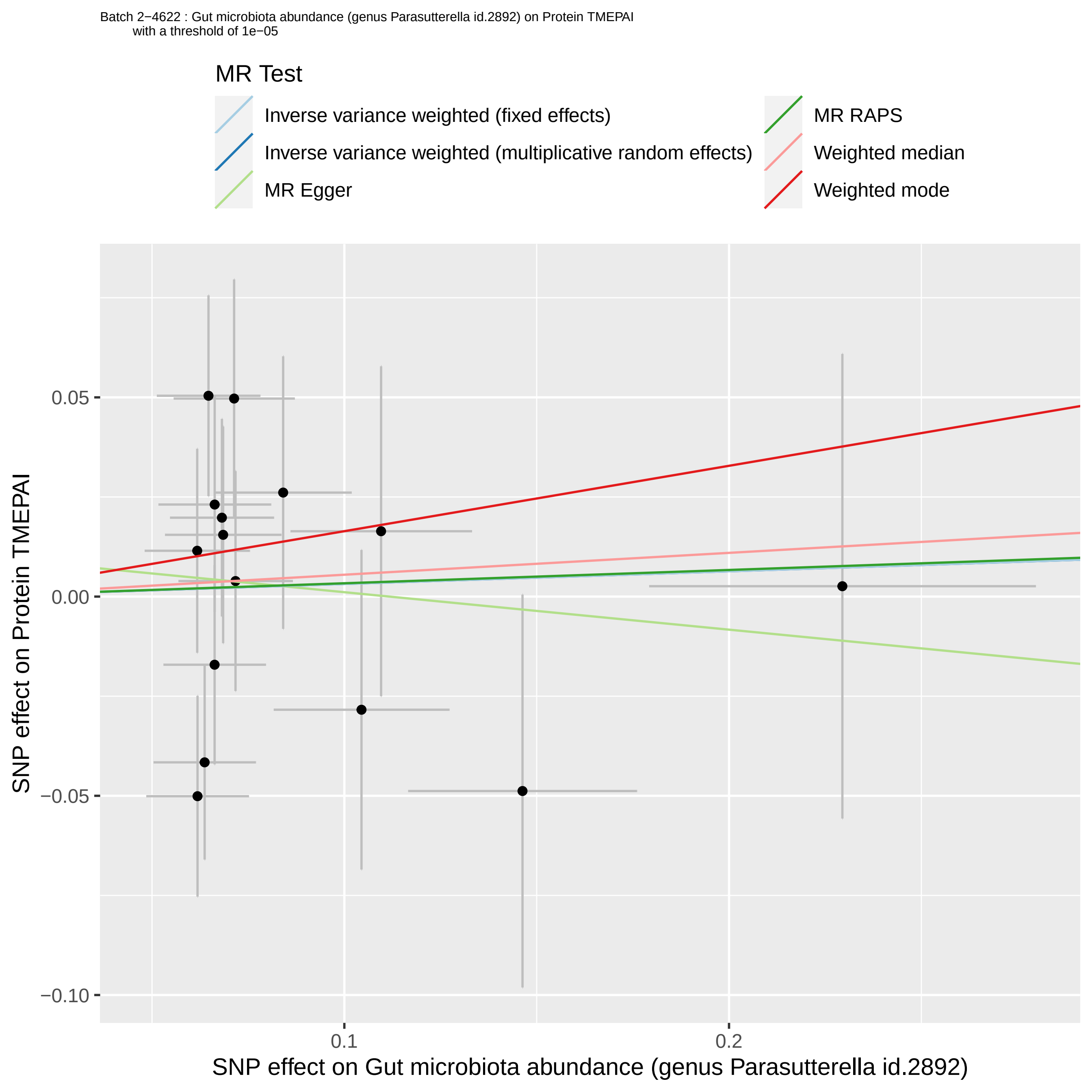

Batch 2−4622 : Gut microbiota abundance (genus Parasutterella id.2892) on Protein TMEPAI
with a threshold of 1e−05
MR Test
Inverse variance weighted (fixed effects)
Inverse variance weighted (multiplicative random effects)
MR Egger
MR RAPS
Weighted median
Weighted mode
SNP effect on Protein TMEPAI
0.05
0.00
−0.05
−0.10
0.1
0.2
SNP effect on Gut microbiota abundance (genus Parasutterella id.2892)

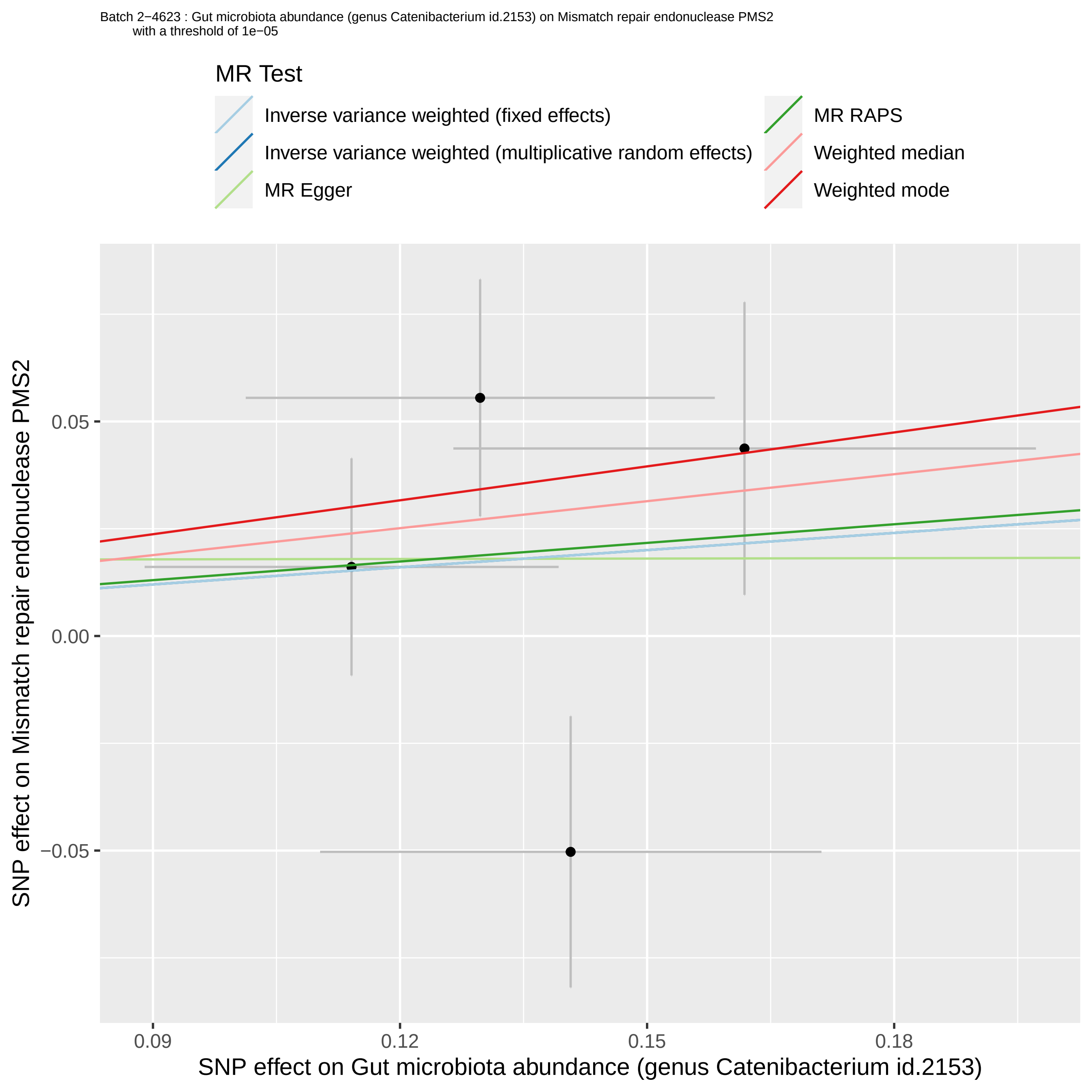
Batch 2−4623 : Gut microbiota abundance (genus Catenibacterium id.2153) on Mismatch repair endonuclease PMS2
with a threshold of 1e−05
MR Test
Inverse variance weighted (fixed effects)
Inverse variance weighted (multiplicative random effects)
MR Egger
MR RAPS
Weighted median
Weighted mode
0.05
0.00
−0.05
0.09
0.12
0.15
0.18
SNP effect on Mismatch repair endonuclease PMS2
SNP effect on Gut microbiota abundance (genus Catenibacterium id.2153)

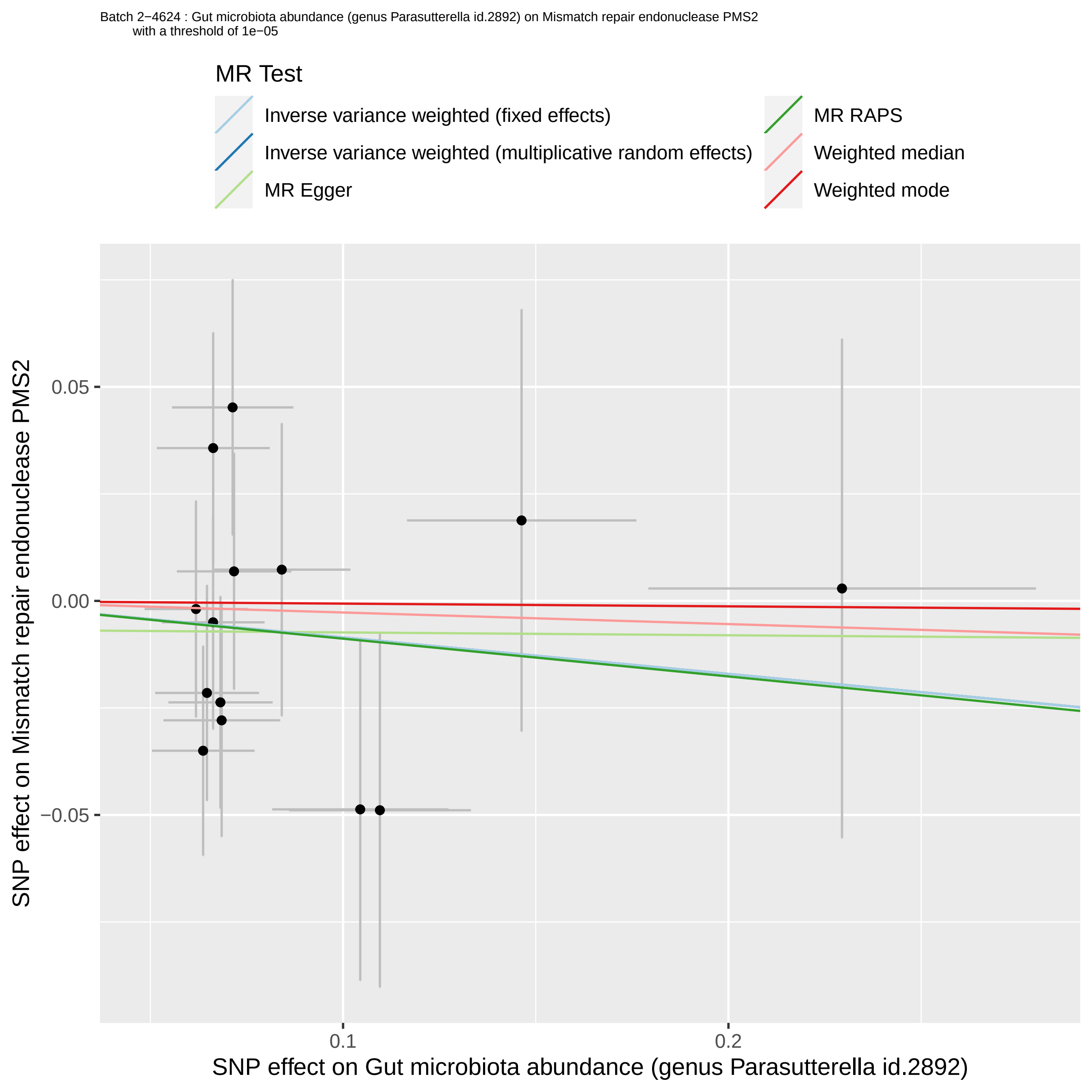
Batch 2−4624 : Gut microbiota abundance (genus Parasutterella id.2892) on Mismatch repair endonuclease PMS2
with a threshold of 1e−05
MR Test
Inverse variance weighted (fixed effects)
Inverse variance weighted (multiplicative random effects)
MR Egger
MR RAPS
Weighted median
Weighted mode
0.05
0.00
−0.05
0.1
0.2
SNP effect on Mismatch repair endonuclease PMS2
SNP effect on Gut microbiota abundance (genus Parasutterella id.2892)

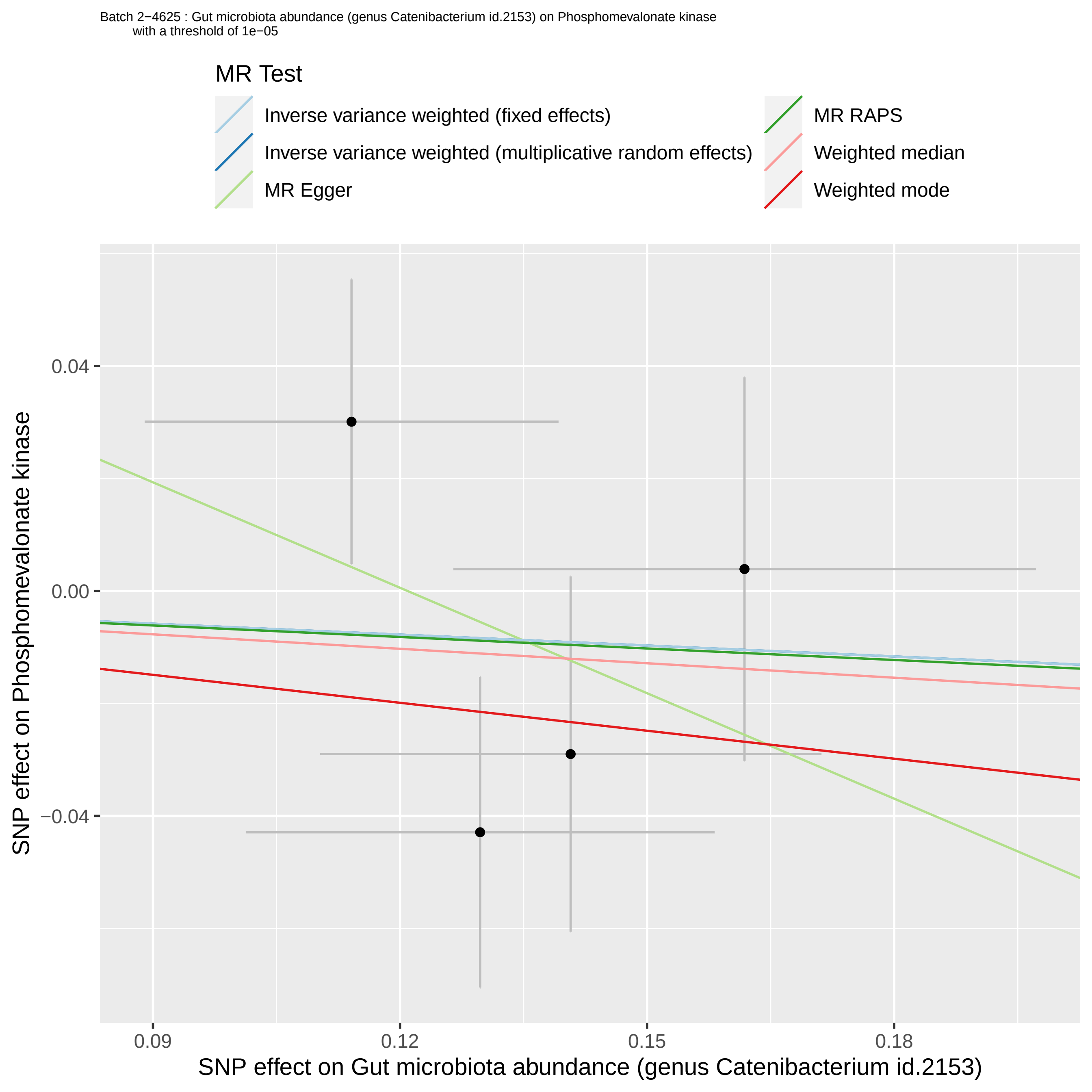

Batch 2−4625 : Gut microbiota abundance (genus Catenibacterium id.2153) on Phosphomevalonate kinase
with a threshold of 1e−05
MR Test
Inverse variance weighted (fixed effects)
Inverse variance weighted (multiplicative random effects)
MR Egger
MR RAPS
Weighted median
Weighted mode
0.04
0.00
−0.04
0.09
0.12
0.15
0.18
SNP effect on Phosphomevalonate kinase
SNP effect on Gut microbiota abundance (genus Catenibacterium id.2153)

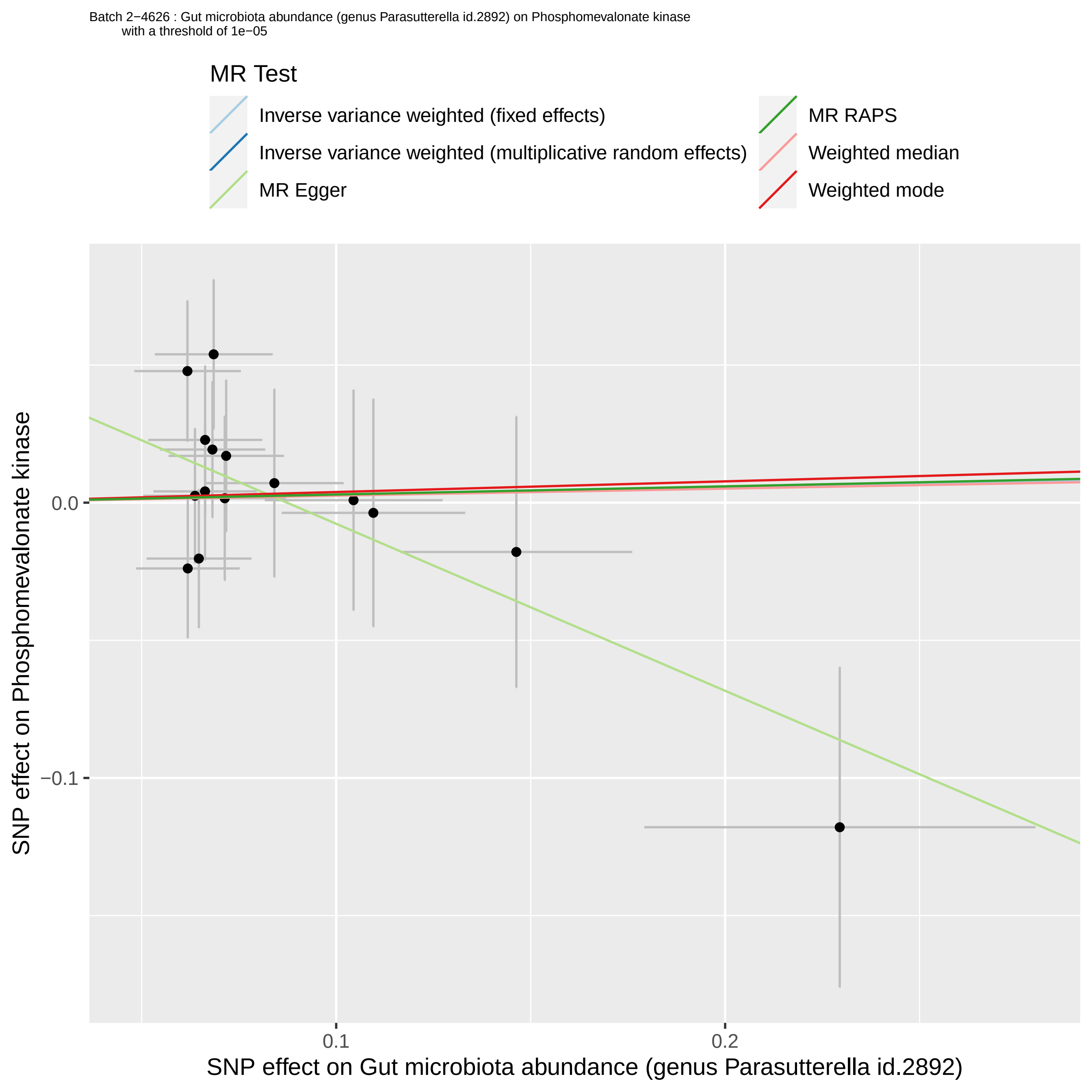
Batch 2−4626 : Gut microbiota abundance (genus Parasutterella id.2892) on Phosphomevalonate kinase
with a threshold of 1e−05
MR Test
Inverse variance weighted (fixed effects)
Inverse variance weighted (multiplicative random effects)
MR Egger
MR RAPS
Weighted median
Weighted mode
SNP effect on Phosphomevalonate kinase
0.0
−0.1
0.1
0.2
SNP effect on Gut microbiota abundance (genus Parasutterella id.2892)

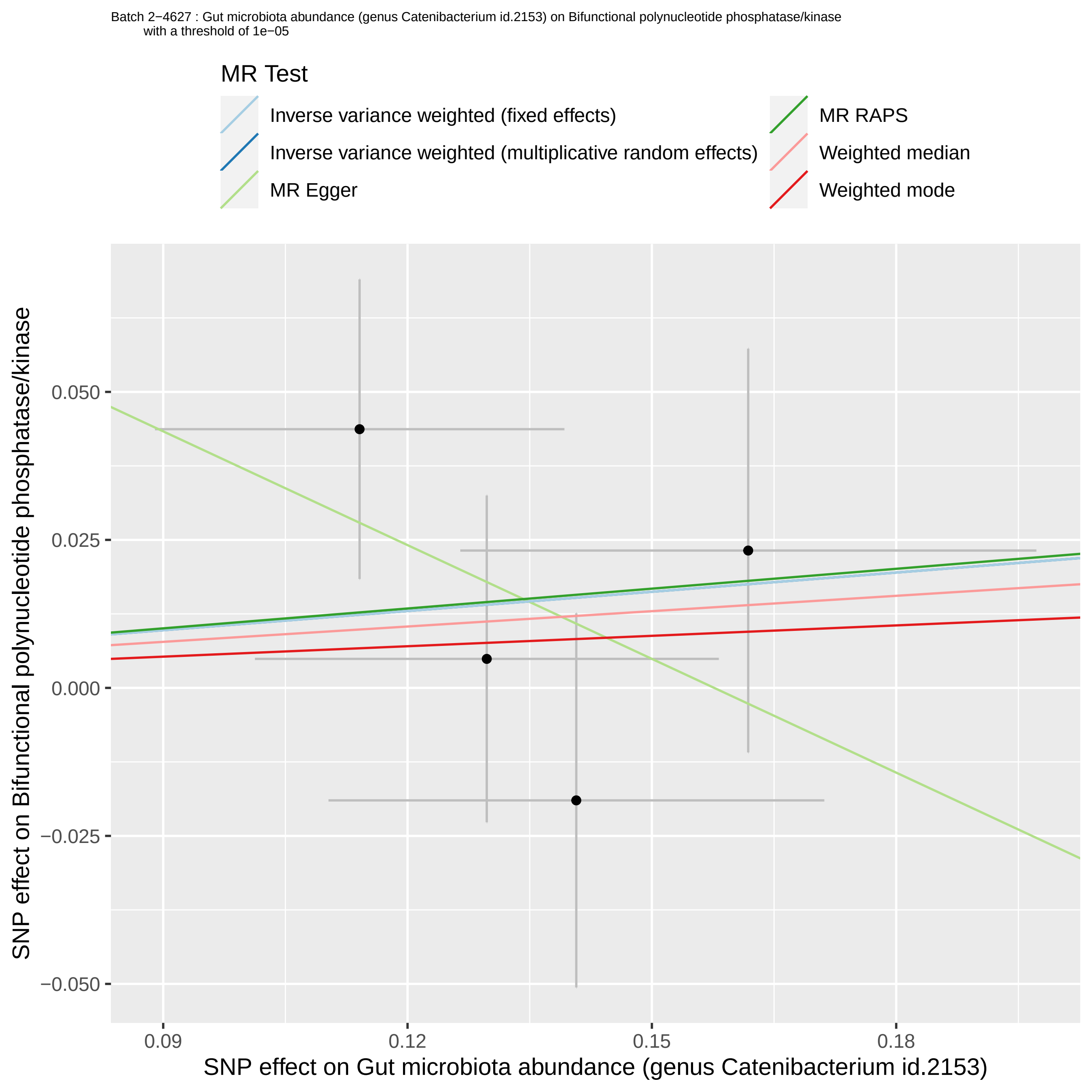
Batch 2−4627 : Gut microbiota abundance (genus Catenibacterium id.2153) on Bifunctional polynucleotide phosphatase/kinase with a threshold of 1e−05
MR Test
Inverse variance weighted (fixed effects)
Inverse variance weighted (multiplicative random effects)
MR Egger
MR RAPS
Weighted median
Weighted mode
0.050
0.025
0.000
−0.025
−0.050
0.09
0.12
0.15
0.18
SNP effect on Bifunctional polynucleotide phosphatase/kinase
SNP effect on Gut microbiota abundance (genus Catenibacterium id.2153)

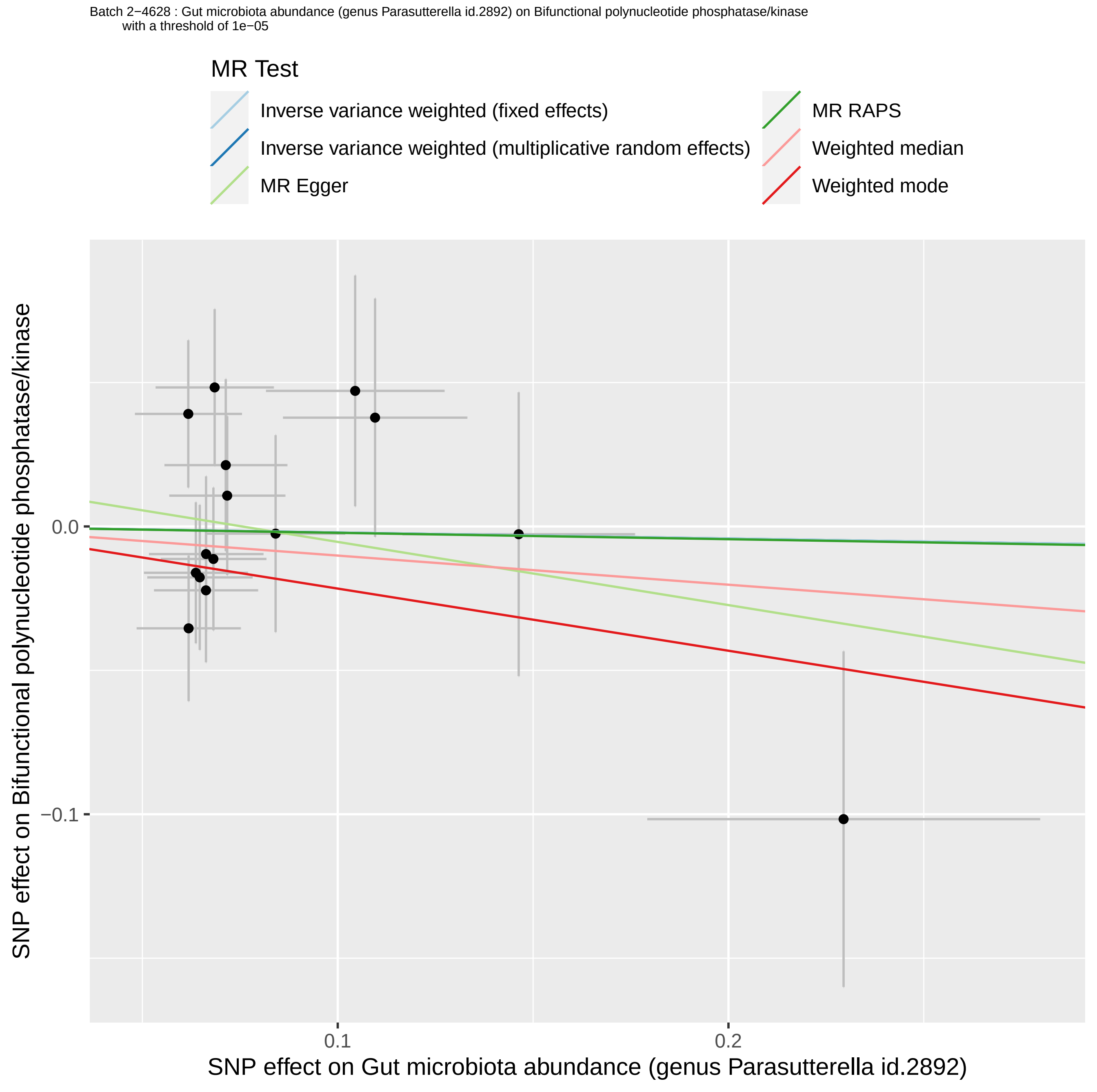
Batch 2−4628 : Gut microbiota abundance (genus Parasutterella id.2892) on Bifunctional polynucleotide phosphatase/kinase with a threshold of 1e−05
MR Test
Inverse variance weighted (fixed effects)
Inverse variance weighted (multiplicative random effects)
MR Egger
MR RAPS
Weighted median
Weighted mode
SNP effect on Bifunctional polynucleotide phosphatase/kinase
0.0
−0.1
0.1
0.2
SNP effect on Gut microbiota abundance (genus Parasutterella id.2892)

Batch 2−4629 : Gut microbiota abundance (genus Catenibacterium id.2153) on Inactive pancreatic lipase−related protein 1
with a threshold of 1e−05

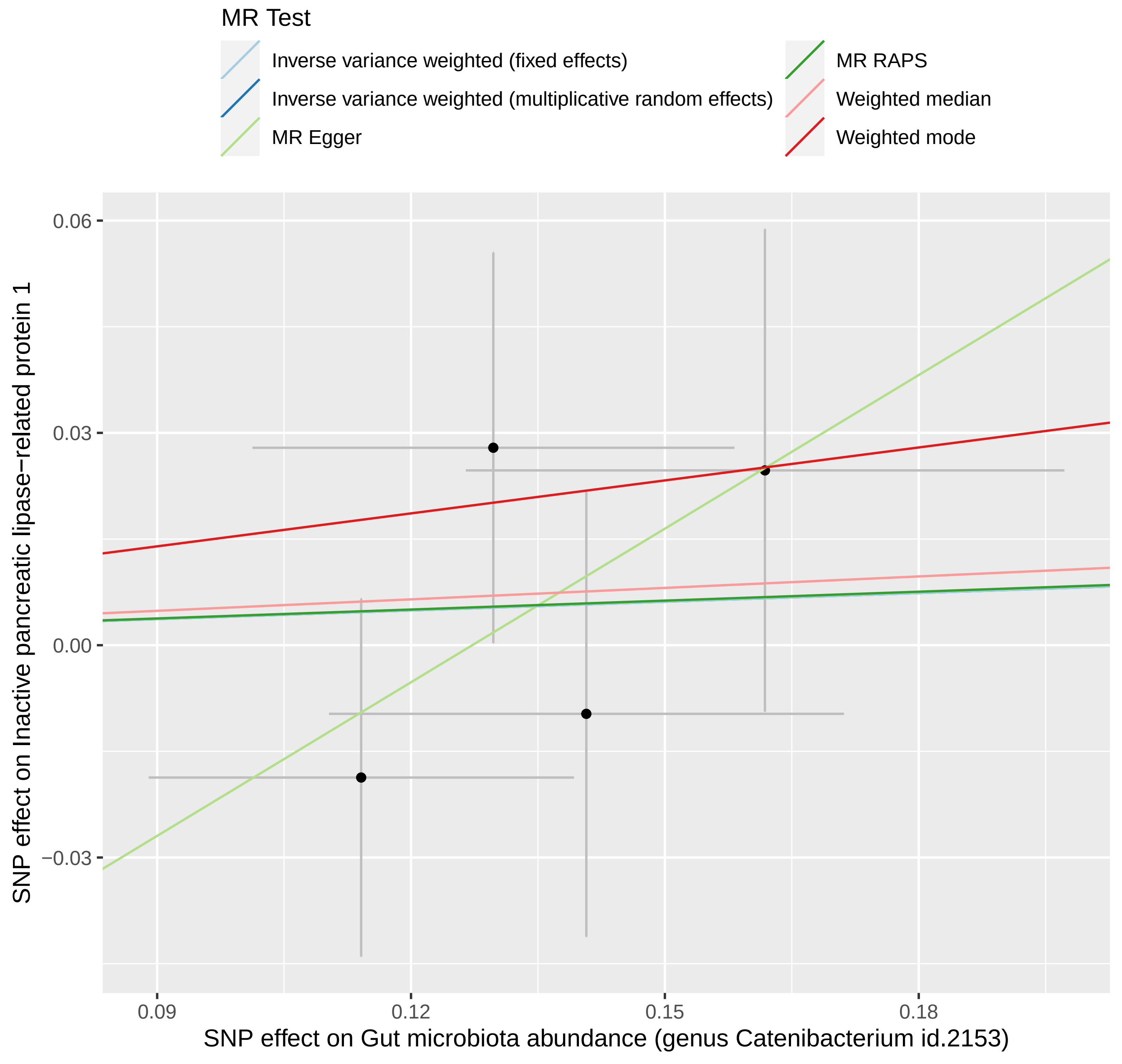

Batch 2−4630 : Gut microbiota abundance (genus Parasutterella id.2892) on Inactive pancreatic lipase−related protein 1
with a threshold of 1e−05

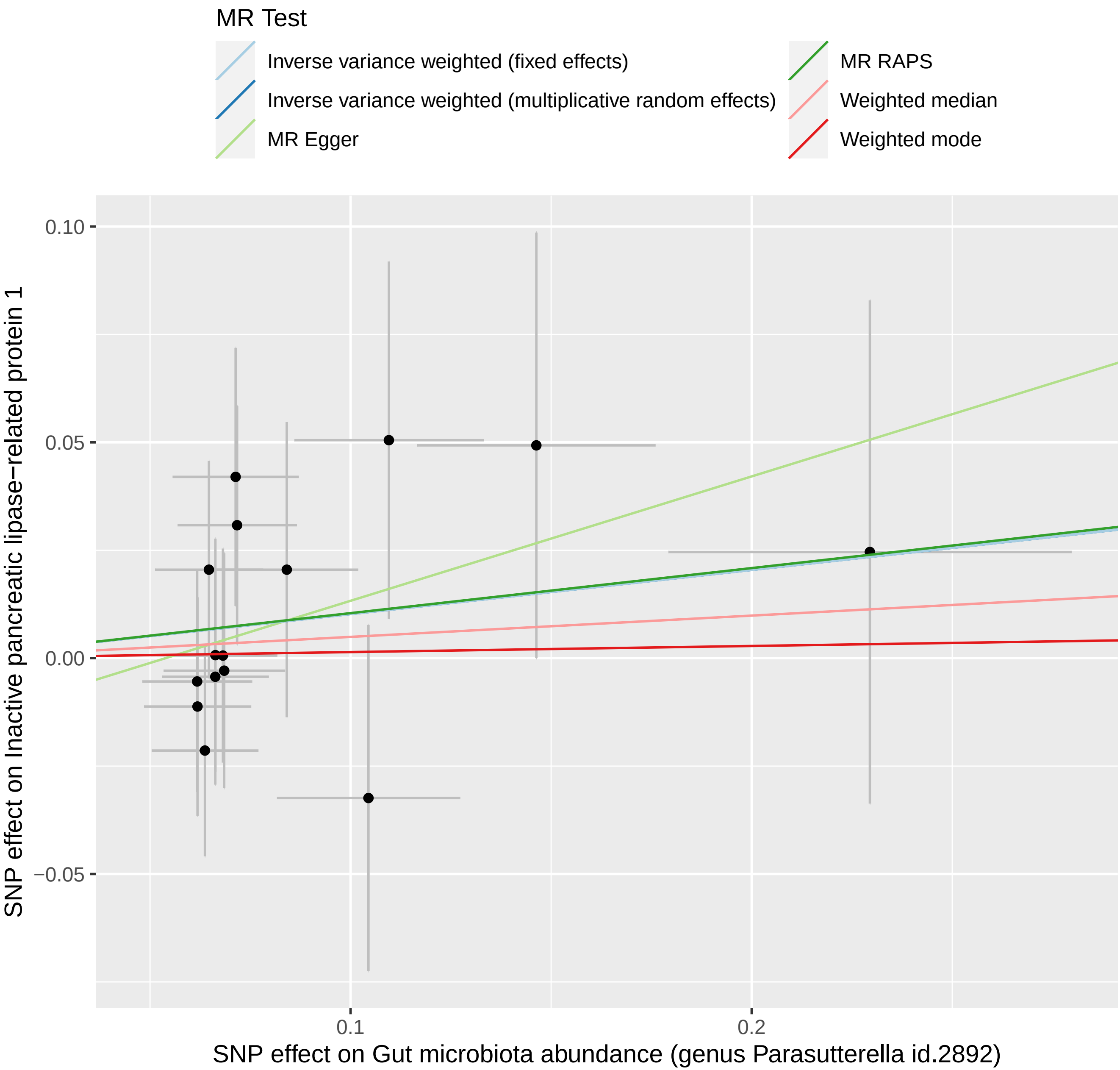

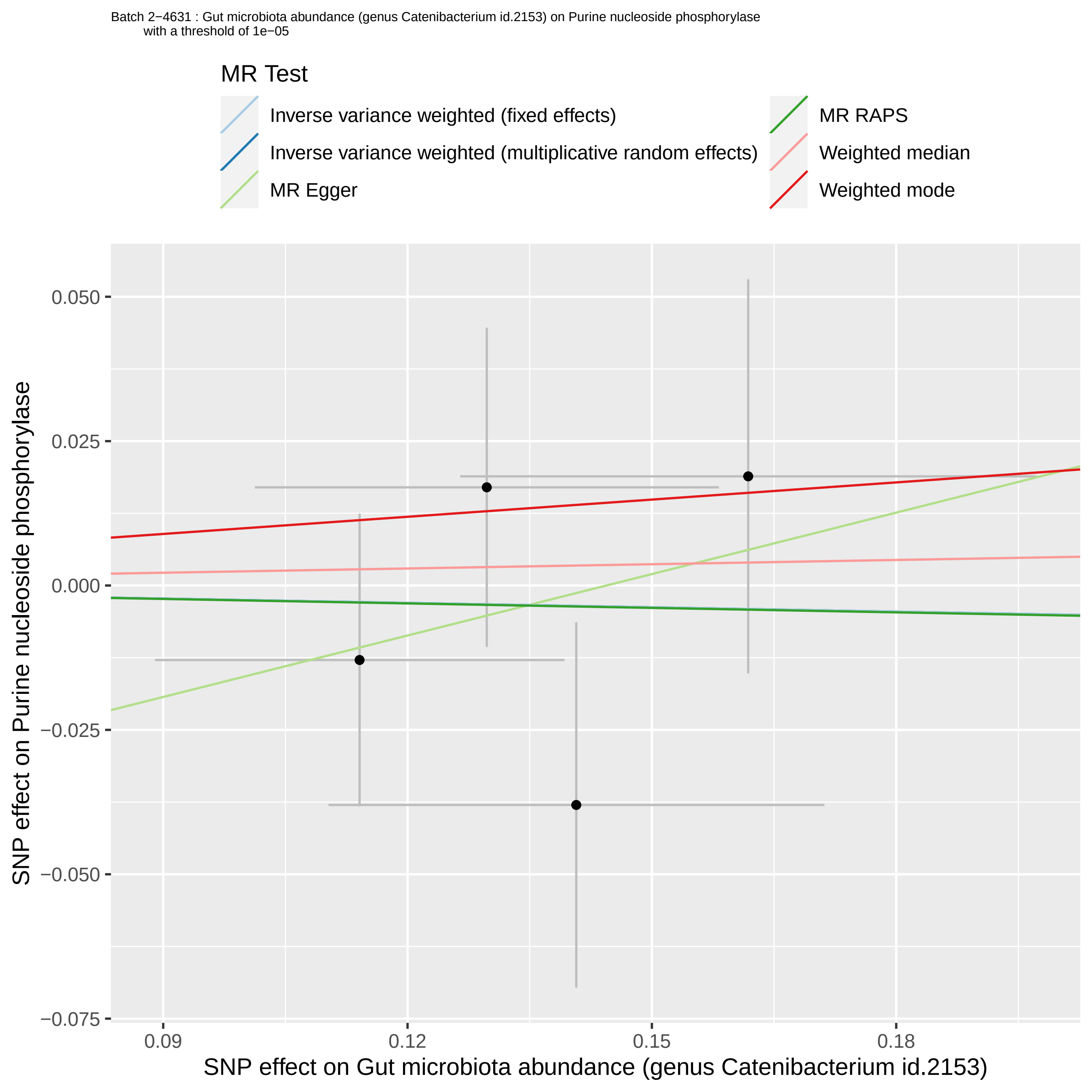
Batch 2−4631 : Gut microbiota abundance (genus Catenibacterium id.2153) on Purine nucleoside phosphorylase
with a threshold of 1e−05
MR Test
Inverse variance weighted (fixed effects)
Inverse variance weighted (multiplicative random effects)
MR Egger
MR RAPS
Weighted median
Weighted mode
SNP effect on Purine nucleoside phosphorylase
0.050
0.025
0.000
−0.025
−0.050
−0.075
0.09
0.12
0.15
0.18
SNP effect on Gut microbiota abundance (genus Catenibacterium id.2153)

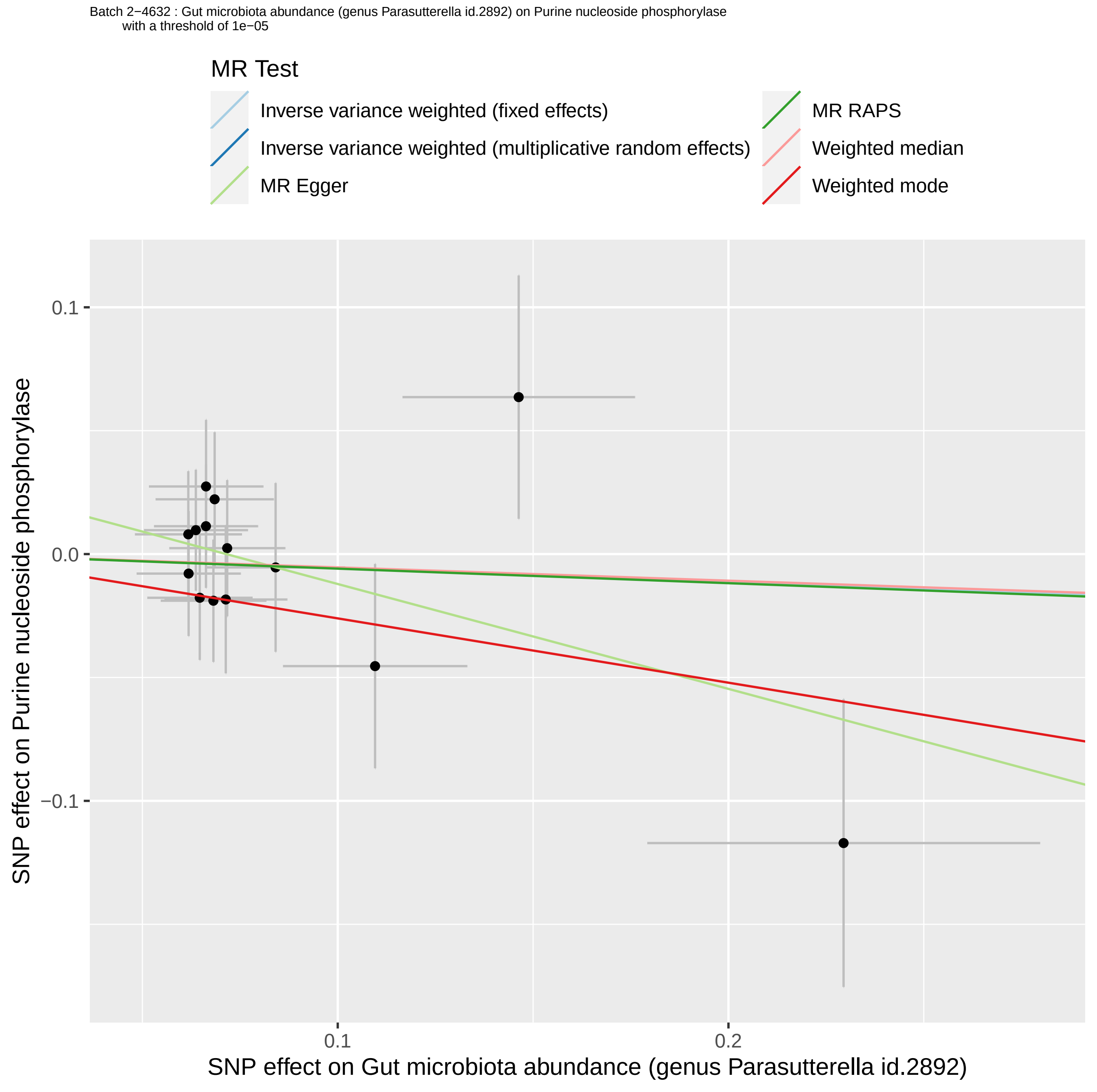

Batch 2−4632 : Gut microbiota abundance (genus Parasutterella id.2892) on Purine nucleoside phosphorylase
with a threshold of 1e−05
MR Test
Inverse variance weighted (fixed effects)
Inverse variance weighted (multiplicative random effects)
MR Egger
MR RAPS
Weighted median
Weighted mode
0.1
0.0
−0.1
0.1
0.2
SNP effect on Purine nucleoside phosphorylase
SNP effect on Gut microbiota abundance (genus Parasutterella id.2892)

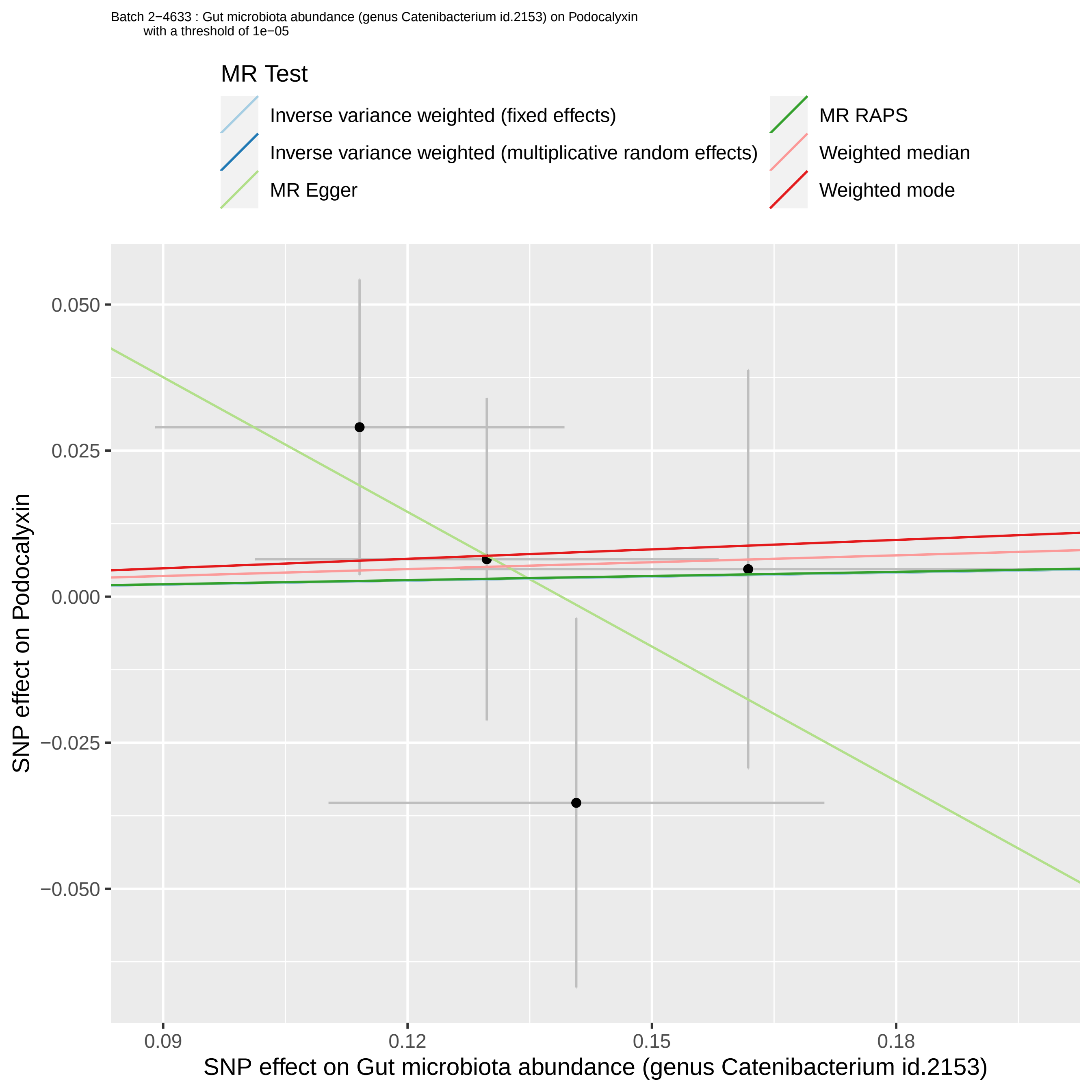
Batch 2−4633 : Gut microbiota abundance (genus Catenibacterium id.2153) on Podocalyxin
with a threshold of 1e−05
MR Test
Inverse variance weighted (fixed effects)
Inverse variance weighted (multiplicative random effects)
MR Egger
MR RAPS
Weighted median
Weighted mode
0.050
0.025
0.000
−0.025
−0.050
0.09
0.12
0.15
0.18
SNP effect on Podocalyxin
SNP effect on Gut microbiota abundance (genus Catenibacterium id.2153)

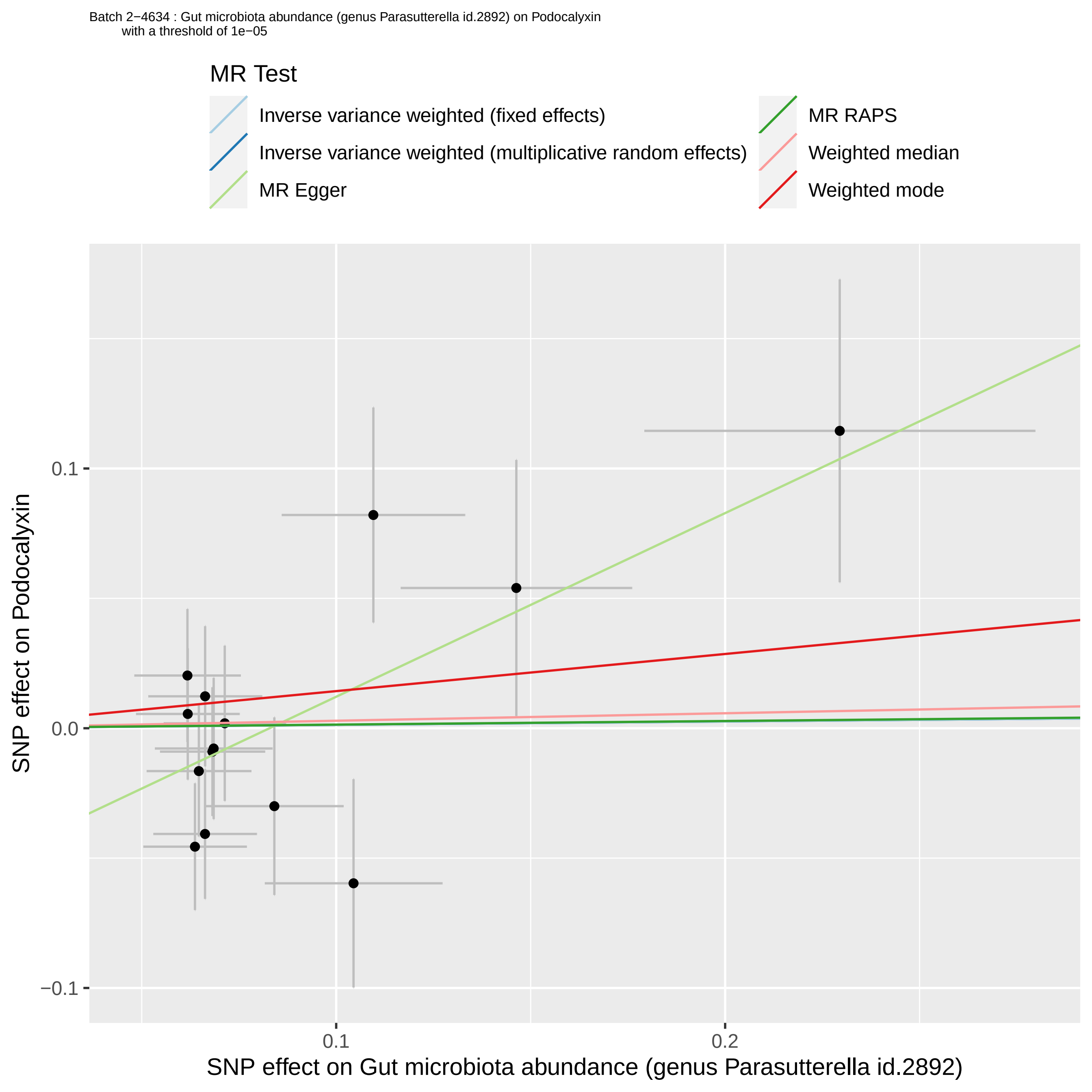
Batch 2−4634 : Gut microbiota abundance (genus Parasutterella id.2892) on Podocalyxin
with a threshold of 1e−05
MR Test
Inverse variance weighted (fixed effects)
Inverse variance weighted (multiplicative random effects)
MR Egger
MR RAPS
Weighted median
Weighted mode
SNP effect on Podocalyxin
0.1
0.0
−0.1
0.1
0.2
SNP effect on Gut microbiota abundance (genus Parasutterella id.2892)

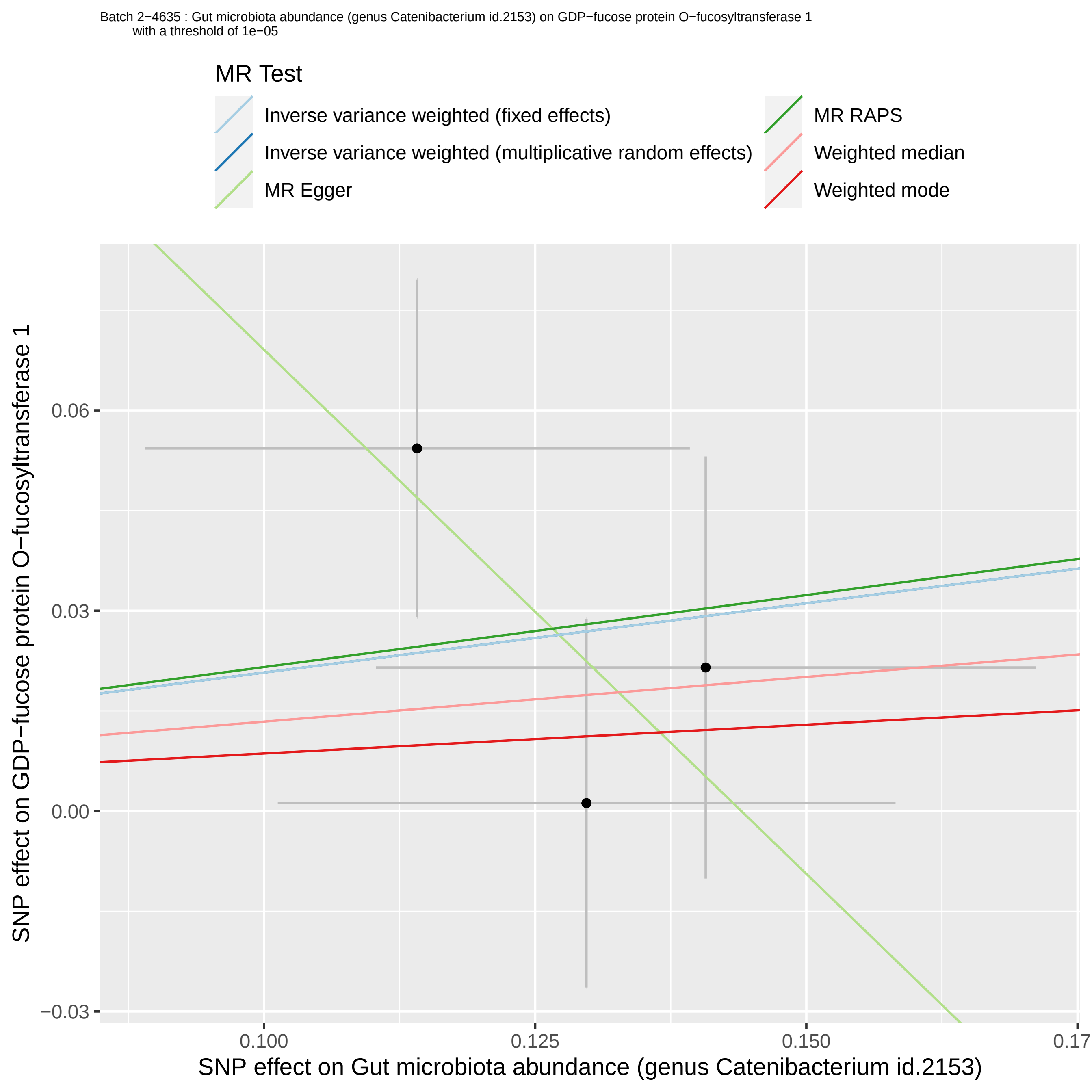
Batch 2−4635 : Gut microbiota abundance (genus Catenibacterium id.2153) on GDP−fucose protein O−fucosyltransferase 1
with a threshold of 1e−05
MR Test
Inverse variance weighted (fixed effects)
Inverse variance weighted (multiplicative random effects)
MR Egger
MR RAPS
Weighted median
Weighted mode
0.06
0.03
0.00
−0.03
0.100
0.125
0.150
0.17
SNP effect on Gut microbiota abundance (genus Catenibacterium id.2153)
SNP effect on GDP−fucose protein O−fucosyltransferase 1

Batch 2−4636 : Gut microbiota abundance (genus Parasutterella id.2892) on GDP−fucose protein O−fucosyltransferase 1
with a threshold of 1e−05

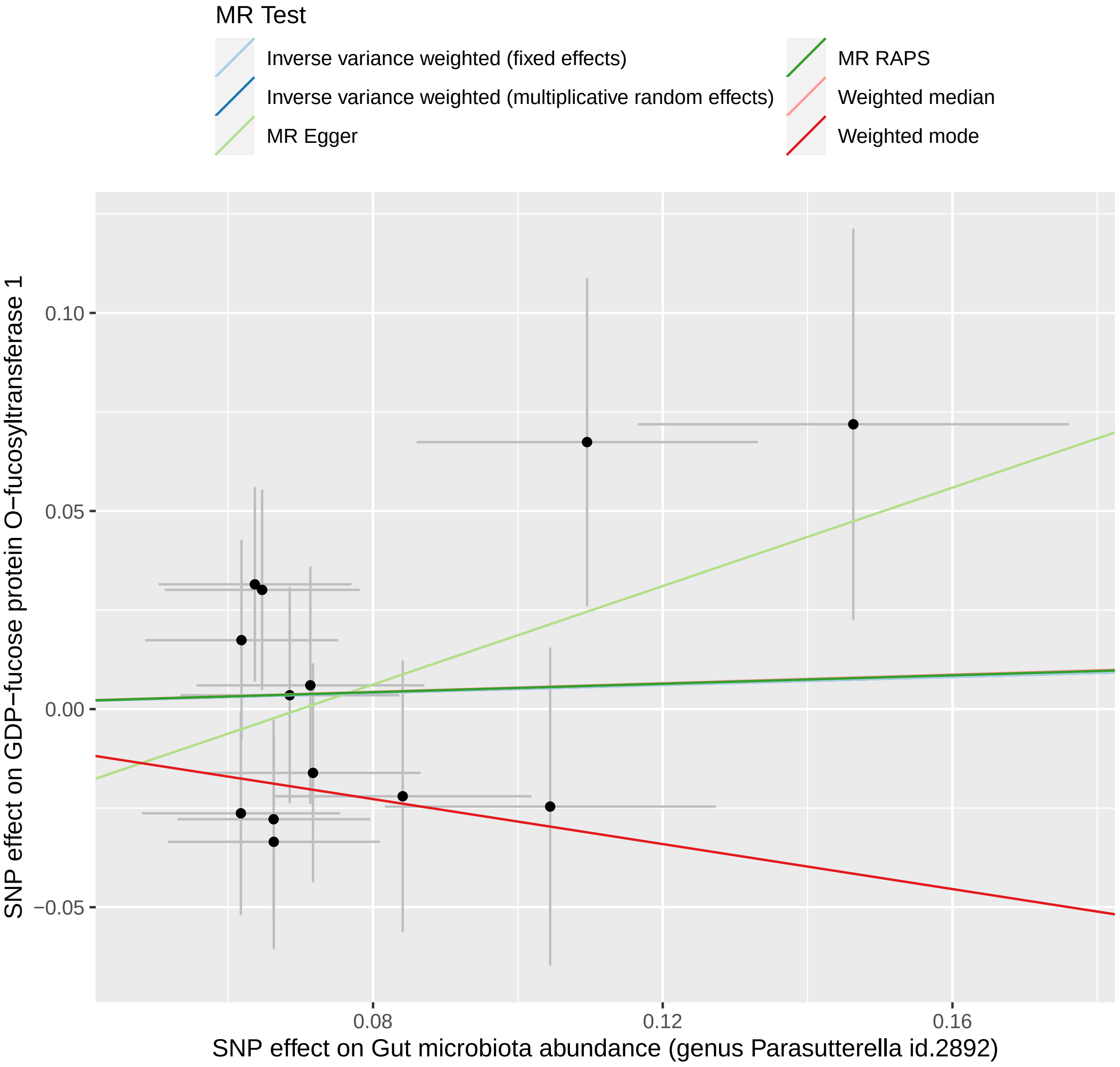

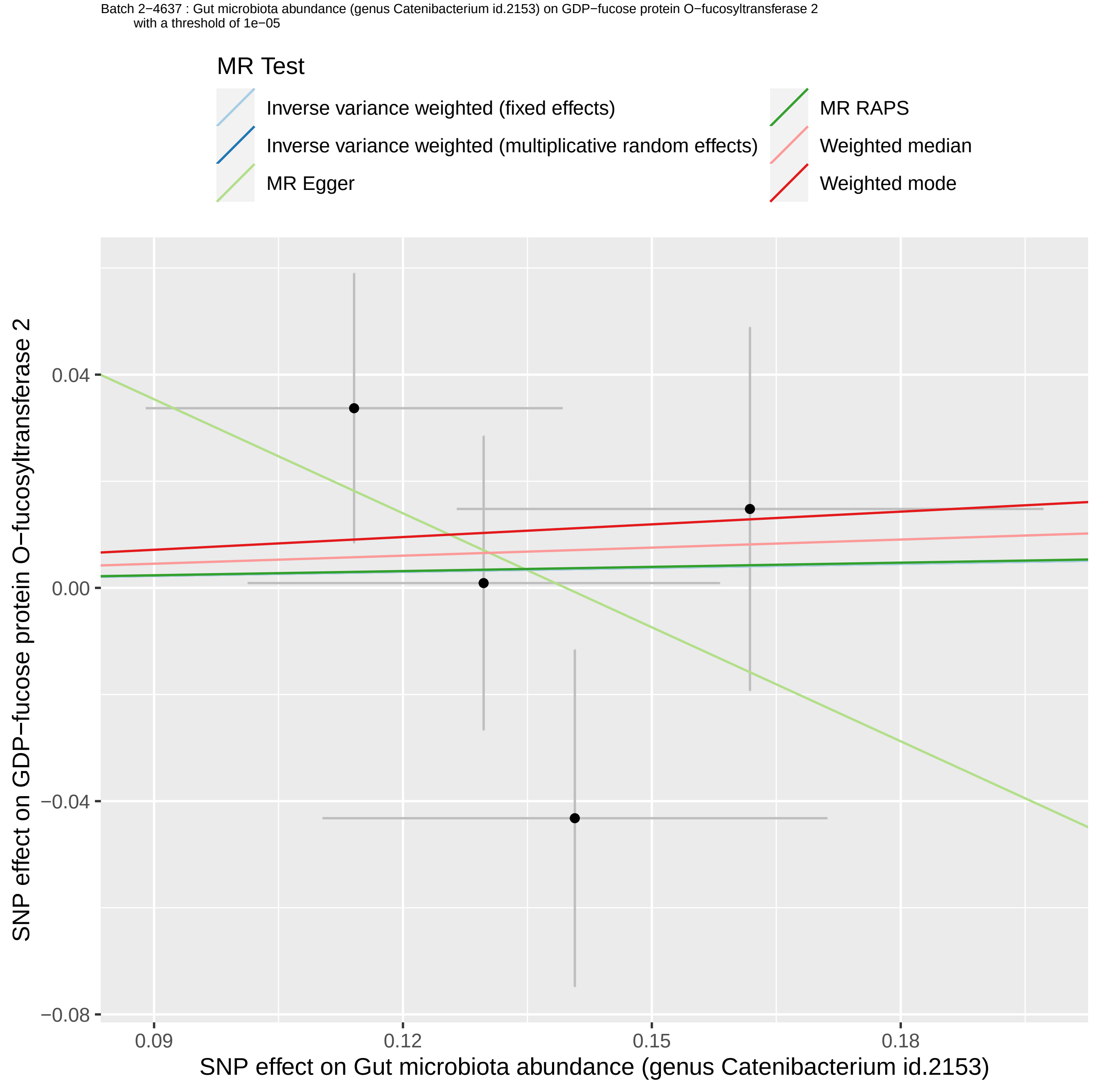

Batch 2−4637 : Gut microbiota abundance (genus Catenibacterium id.2153) on GDP−fucose protein O−fucosyltransferase 2
with a threshold of 1e−05
MR Test
Inverse variance weighted (fixed effects)
Inverse variance weighted (multiplicative random effects)
MR Egger
MR RAPS
Weighted median
Weighted mode
0.04
0.00
−0.04
−0.08
0.09
0.12
0.15
0.18
SNP effect on GDP−fucose protein O−fucosyltransferase 2
SNP effect on Gut microbiota abundance (genus Catenibacterium id.2153)

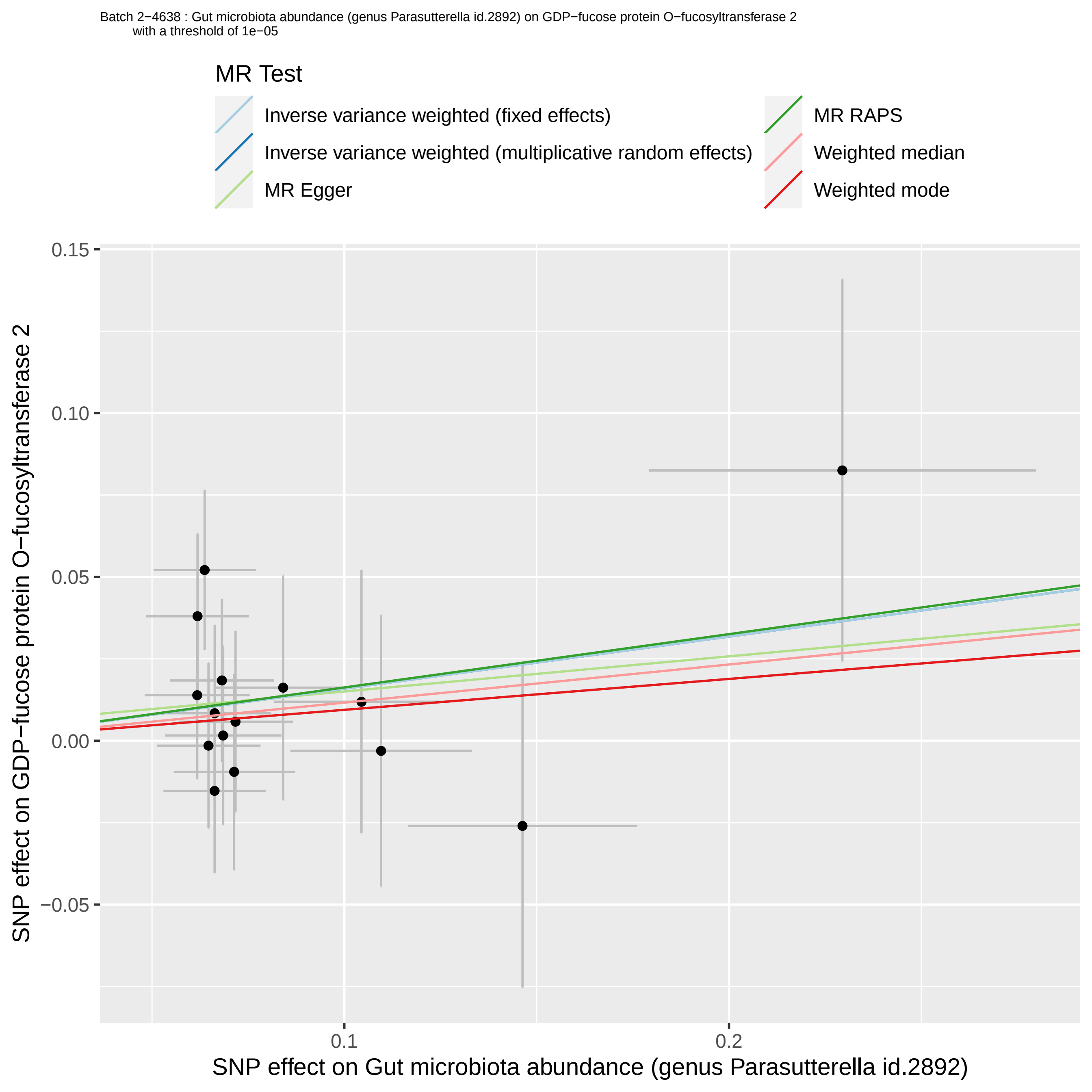

Batch 2−4638 : Gut microbiota abundance (genus Parasutterella id.2892) on GDP−fucose protein O−fucosyltransferase 2
with a threshold of 1e−05
MR Test
Inverse variance weighted (fixed effects)
Inverse variance weighted (multiplicative random effects)
MR Egger
MR RAPS
Weighted median
Weighted mode
0.15
0.10
0.05
0.00
−0.05
0.1
0.2
SNP effect on GDP−fucose protein O−fucosyltransferase 2
SNP effect on Gut microbiota abundance (genus Parasutterella id.2892)

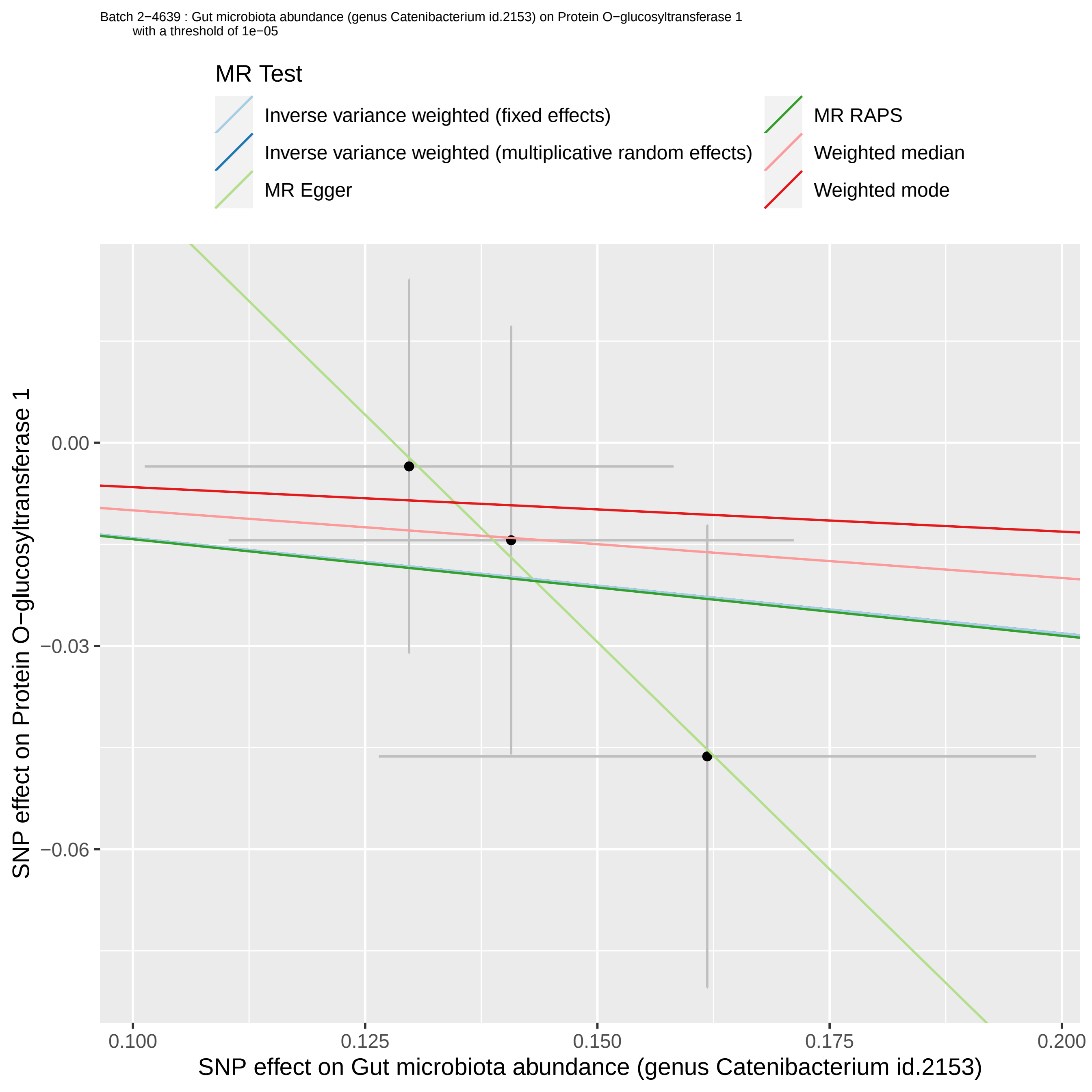

Batch 2−4639 : Gut microbiota abundance (genus Catenibacterium id.2153) on Protein O−glucosyltransferase 1
with a threshold of 1e−05
MR Test
Inverse variance weighted (fixed effects)
Inverse variance weighted (multiplicative random effects)
MR Egger
MR RAPS
Weighted median
Weighted mode
0.00
−0.03
−0.06
0.100
0.125
0.150
0.175
0.200
SNP effect on Protein O−glucosyltransferase 1
SNP effect on Gut microbiota abundance (genus Catenibacterium id.2153)

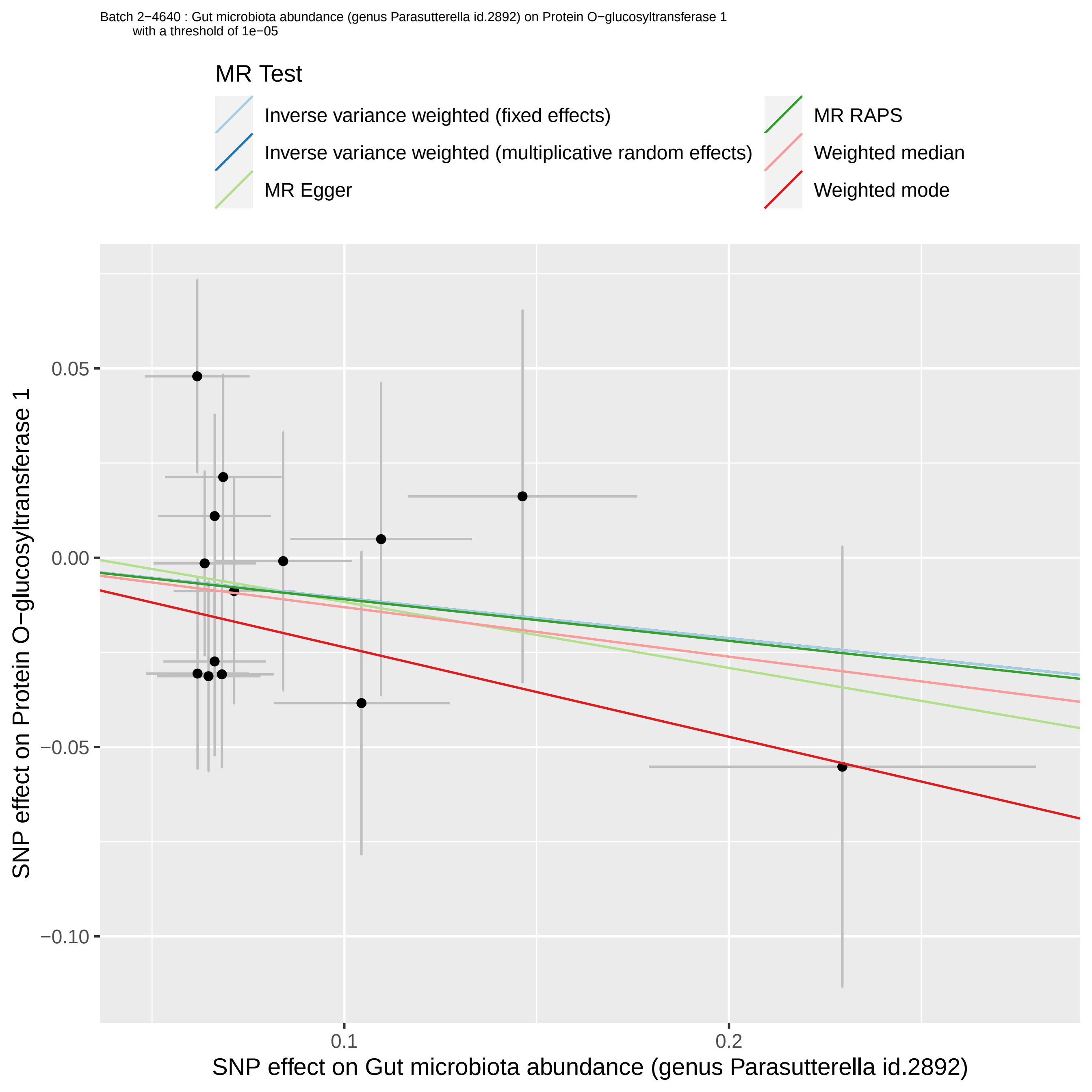
Batch 2−4640 : Gut microbiota abundance (genus Parasutterella id.2892) on Protein O−glucosyltransferase 1
with a threshold of 1e−05
MR Test
Inverse variance weighted (fixed effects)
Inverse variance weighted (multiplicative random effects)
MR Egger
MR RAPS
Weighted median
Weighted mode
0.05
0.00
−0.05
−0.10
0.1
0.2
SNP effect on Protein O−glucosyltransferase 1
SNP effect on Gut microbiota abundance (genus Parasutterella id.2892)

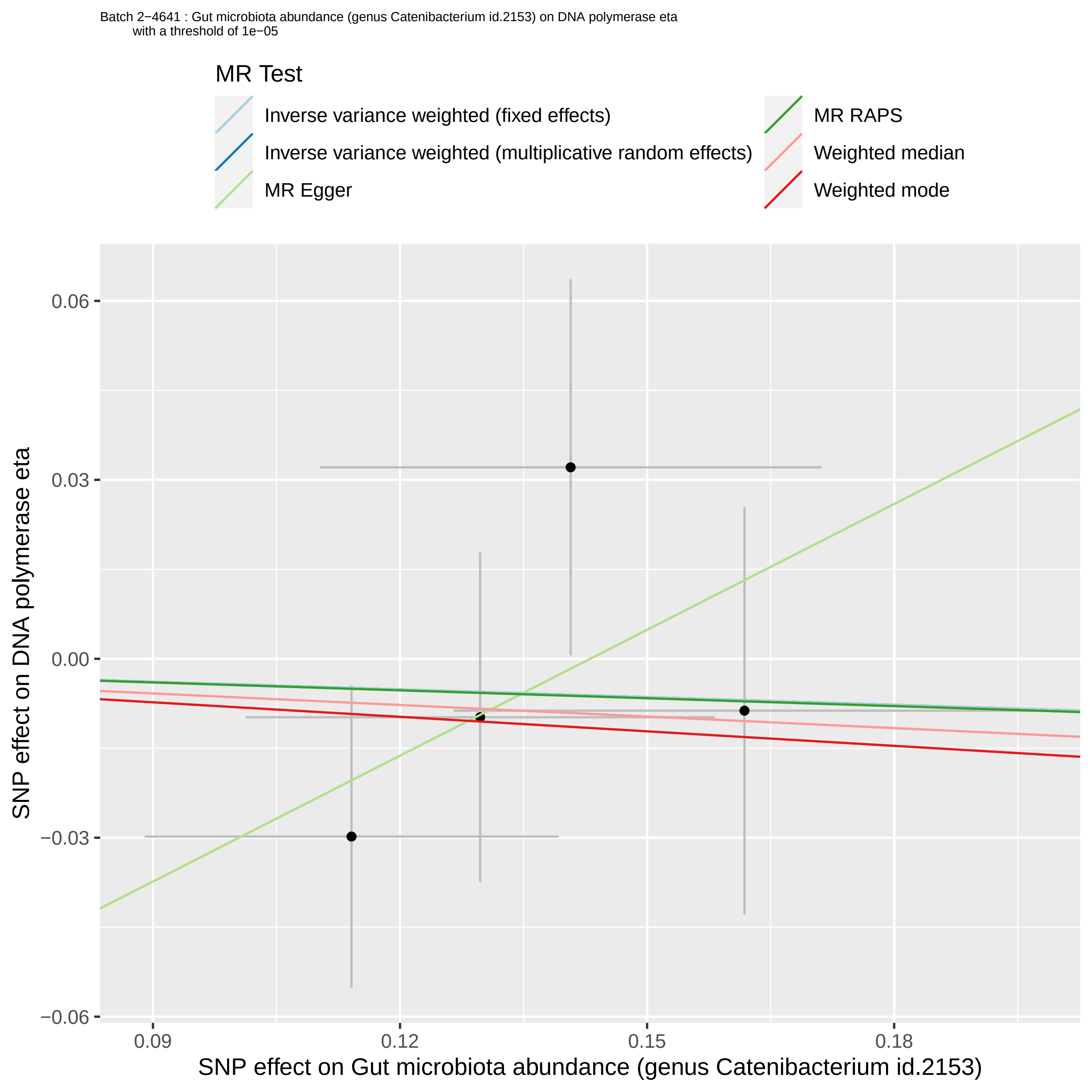
Batch 2−4641 : Gut microbiota abundance (genus Catenibacterium id.2153) on DNA polymerase eta
with a threshold of 1e−05
MR Test
Inverse variance weighted (fixed effects)
Inverse variance weighted (multiplicative random effects)
MR Egger
MR RAPS
Weighted median
Weighted mode
0.06
0.03
0.00
−0.03
−0.06
0.09
0.12
0.15
0.18
SNP effect on DNA polymerase eta
SNP effect on Gut microbiota abundance (genus Catenibacterium id.2153)

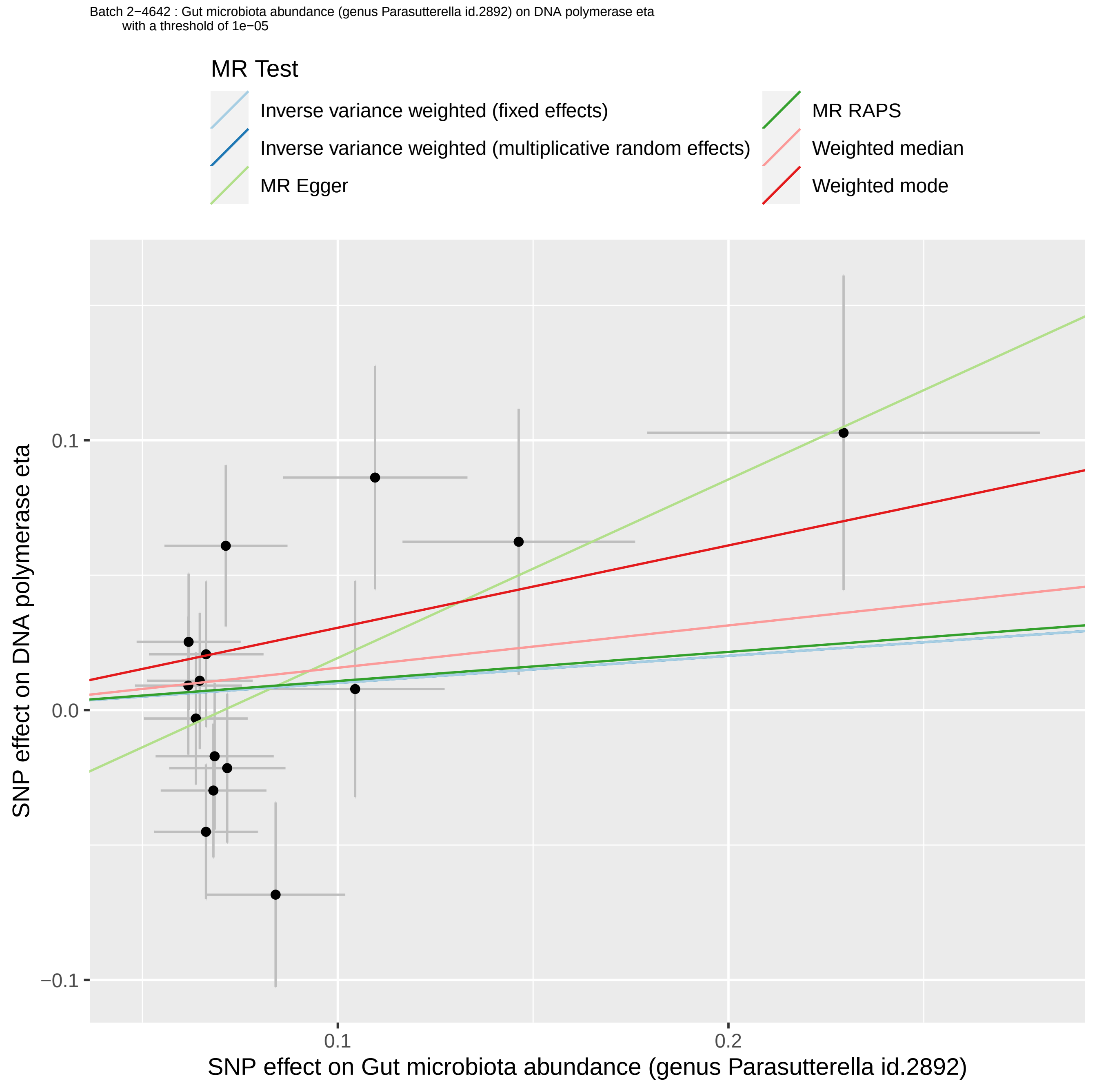
Batch 2−4642 : Gut microbiota abundance (genus Parasutterella id.2892) on DNA polymerase eta
with a threshold of 1e−05
MR Test
Inverse variance weighted (fixed effects)
Inverse variance weighted (multiplicative random effects)
MR Egger
MR RAPS
Weighted median
Weighted mode
SNP effect on DNA polymerase eta
0.1
0.0
−0.1
0.1
0.2
SNP effect on Gut microbiota abundance (genus Parasutterella id.2892)

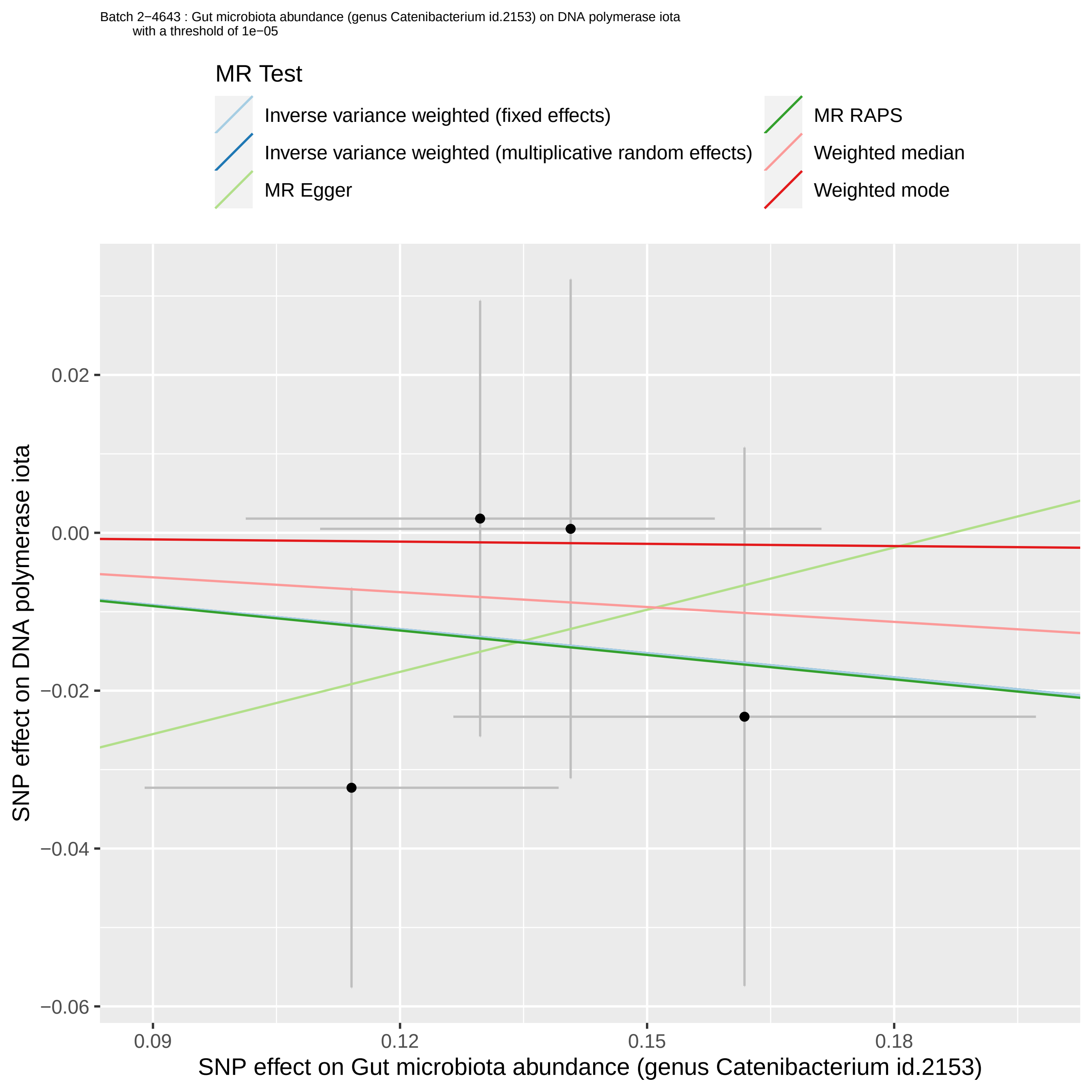
Batch 2−4643 : Gut microbiota abundance (genus Catenibacterium id.2153) on DNA polymerase iota
with a threshold of 1e−05
MR Test
Inverse variance weighted (fixed effects)
Inverse variance weighted (multiplicative random effects)
MR Egger
MR RAPS
Weighted median
Weighted mode
SNP effect on DNA polymerase iota
0.02
0.00
−0.02
−0.04
−0.06
0.09
0.12
0.15
0.18
SNP effect on Gut microbiota abundance (genus Catenibacterium id.2153)

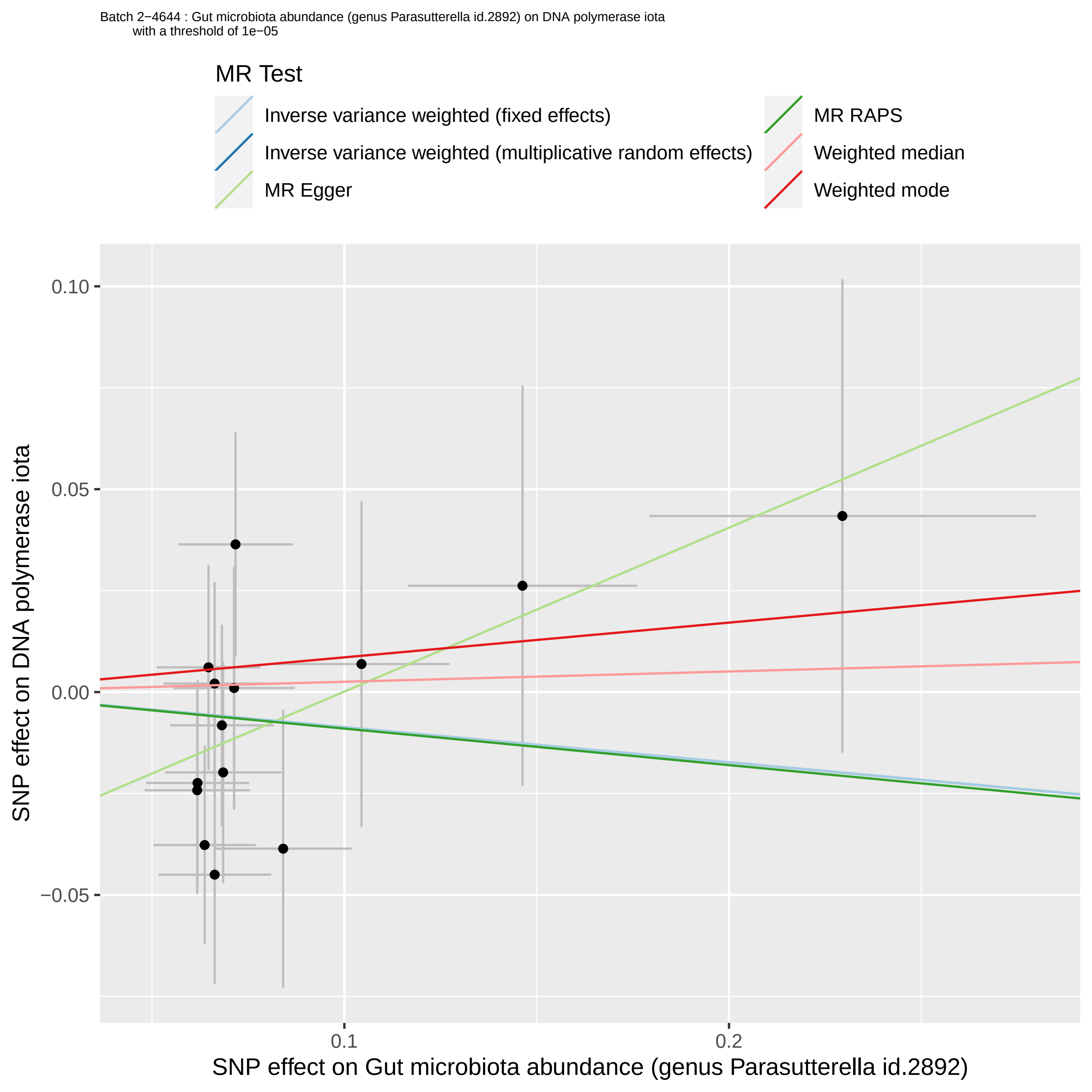

Batch 2−4644 : Gut microbiota abundance (genus Parasutterella id.2892) on DNA polymerase iota
with a threshold of 1e−05
MR Test
Inverse variance weighted (fixed effects)
Inverse variance weighted (multiplicative random effects)
MR Egger
MR RAPS
Weighted median
Weighted mode
0.10
0.05
0.00
−0.05
0.1
0.2
SNP effect on DNA polymerase iota
SNP effect on Gut microbiota abundance (genus Parasutterella id.2892)

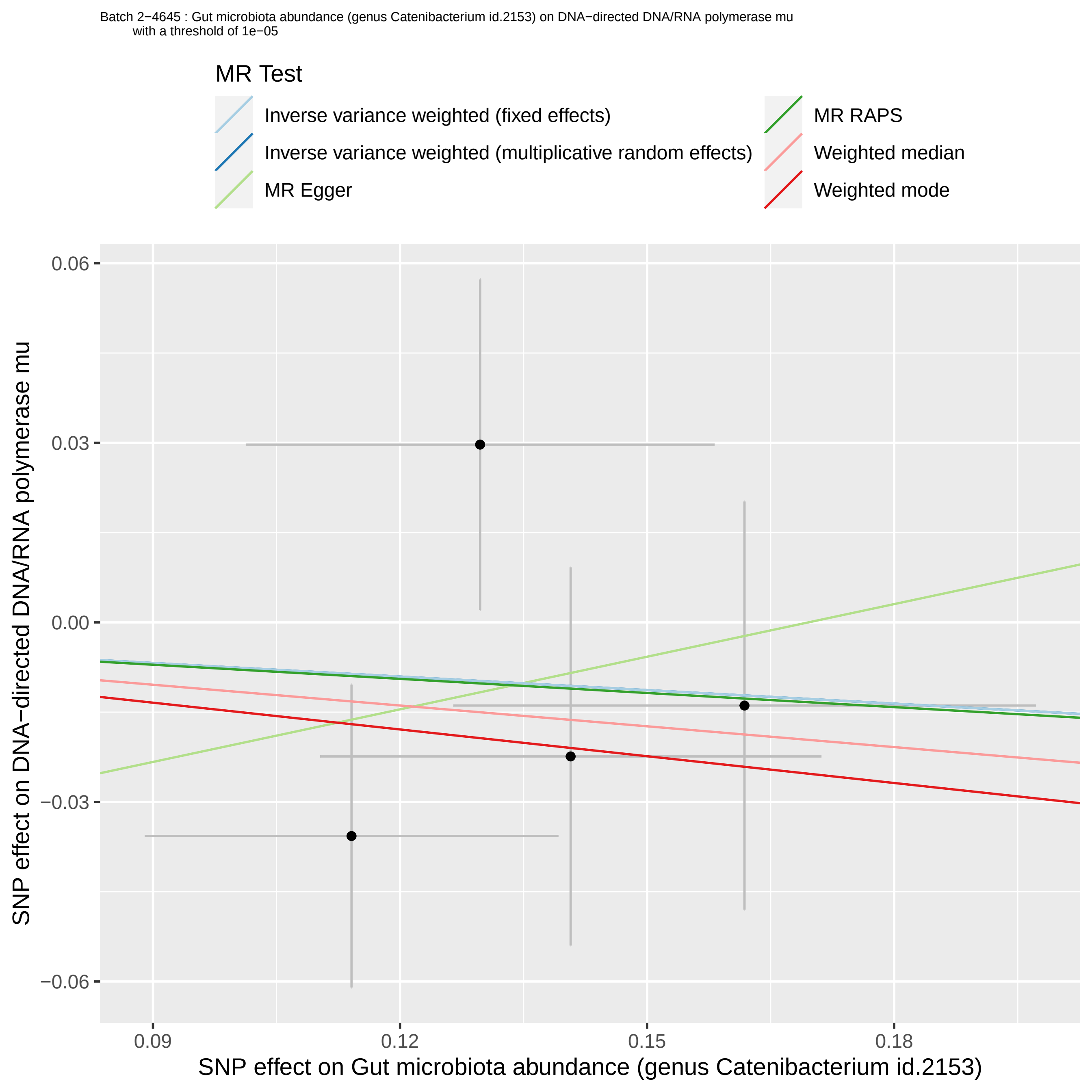

Batch 2−4645 : Gut microbiota abundance (genus Catenibacterium id.2153) on DNA−directed DNA/RNA polymerase mu
with a threshold of 1e−05
MR Test
Inverse variance weighted (fixed effects)
Inverse variance weighted (multiplicative random effects)
MR Egger
MR RAPS
Weighted median
Weighted mode
0.06
0.03
0.00
−0.03
−0.06
0.09
0.12
0.15
0.18
SNP effect on DNA−directed DNA/RNA polymerase mu
SNP effect on Gut microbiota abundance (genus Catenibacterium id.2153)

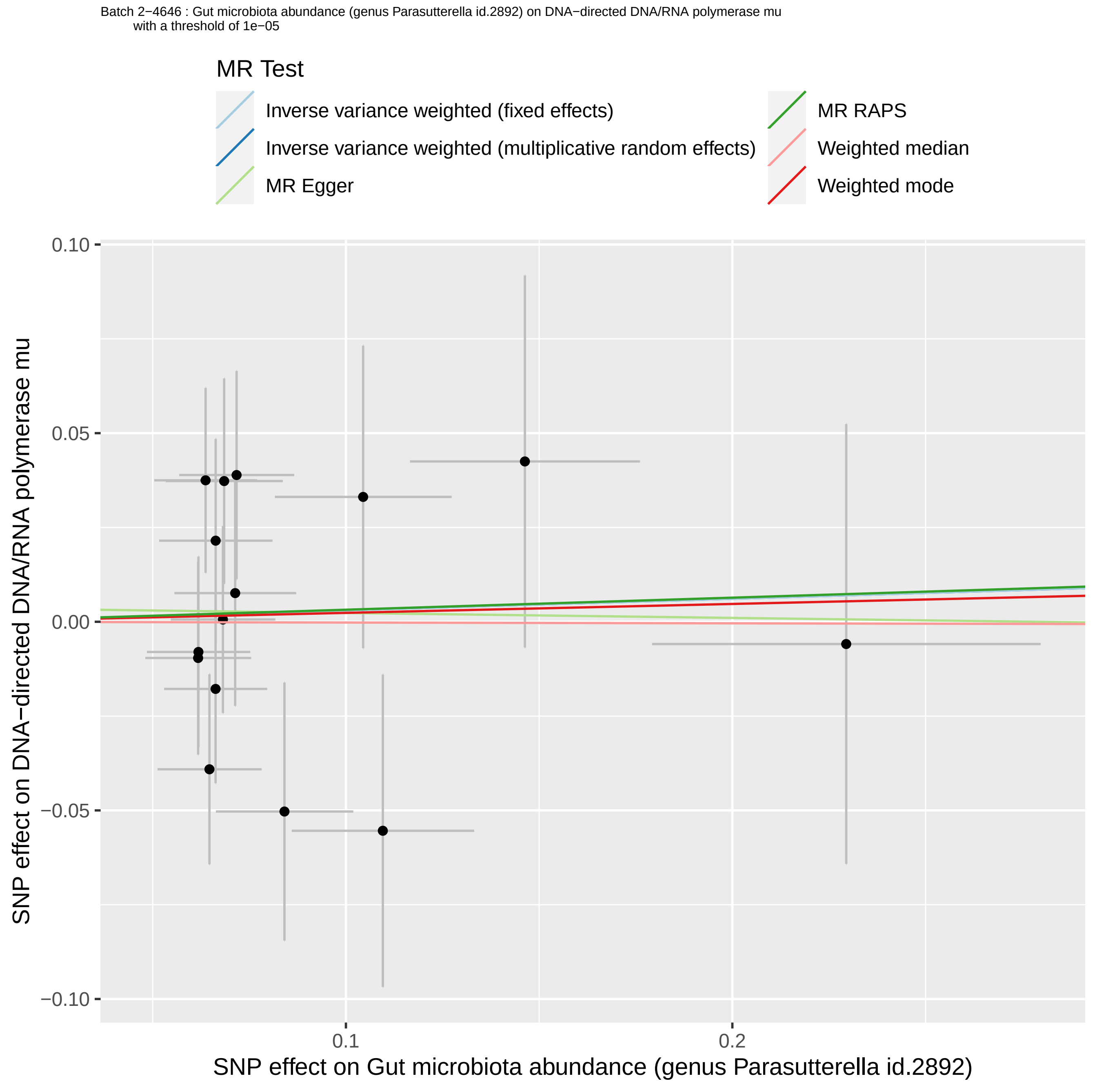

Batch 2−4646 : Gut microbiota abundance (genus Parasutterella id.2892) on DNA−directed DNA/RNA polymerase mu
with a threshold of 1e−05
MR Test
Inverse variance weighted (fixed effects)
Inverse variance weighted (multiplicative random effects)
MR Egger
MR RAPS
Weighted median
Weighted mode
0.10
0.05
0.00
−0.05
−0.10
0.1
0.2
SNP effect on DNA−directed DNA/RNA polymerase mu
SNP effect on Gut microbiota abundance (genus Parasutterella id.2892)

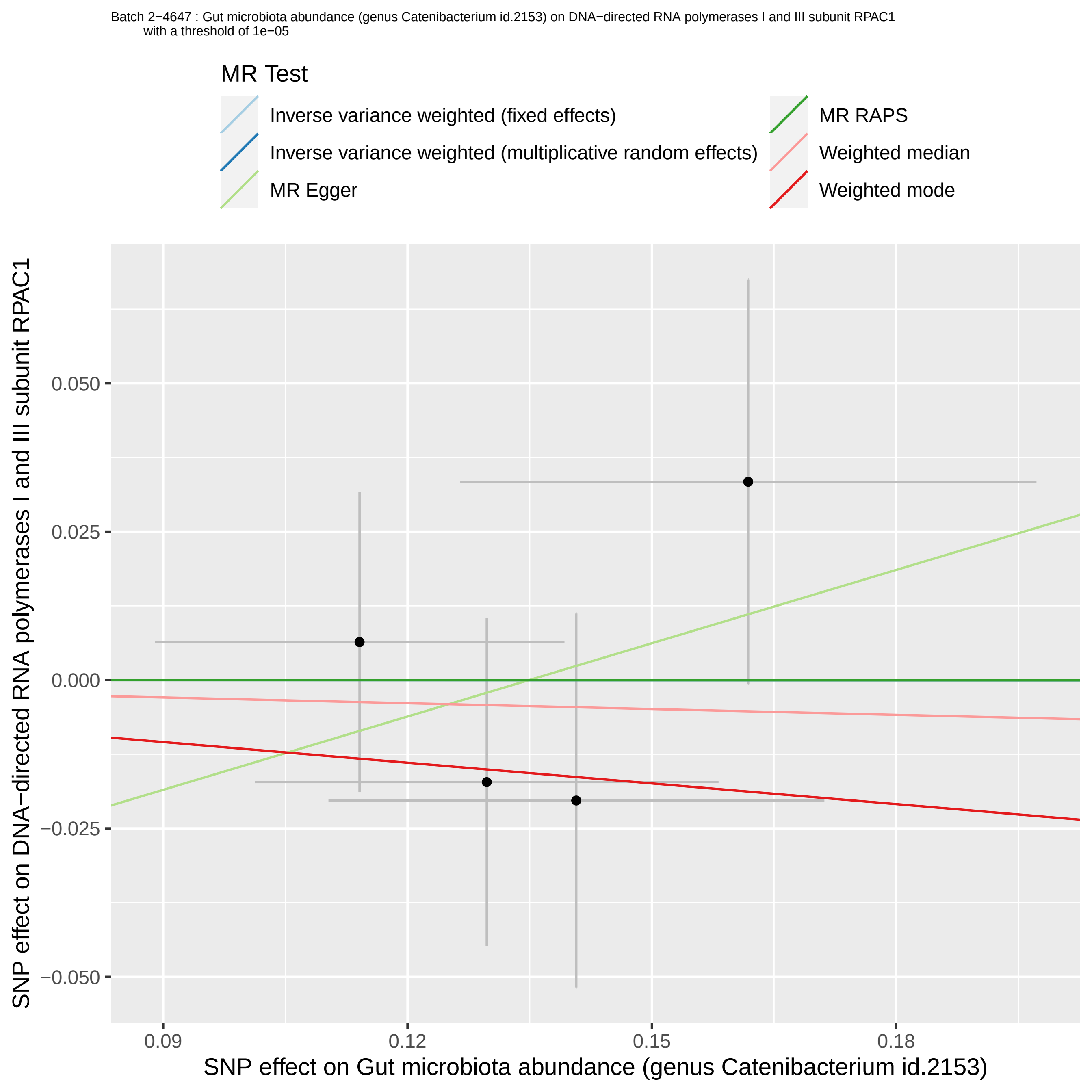
Batch 2−4647 : Gut microbiota abundance (genus Catenibacterium id.2153) on DNA−directed RNA polymerases I and III subunit RPAC1
with a threshold of 1e−05
MR Test
Inverse variance weighted (fixed effects)
Inverse variance weighted (multiplicative random effects)
MR Egger
MR RAPS
Weighted median
Weighted mode
SNP effect on DNA−directed RNA polymerases I and III subunit RPAC1
0.050
0.025
0.000
−0.025
−0.050
0.09
0.12
0.15
0.18
SNP effect on Gut microbiota abundance (genus Catenibacterium id.2153)

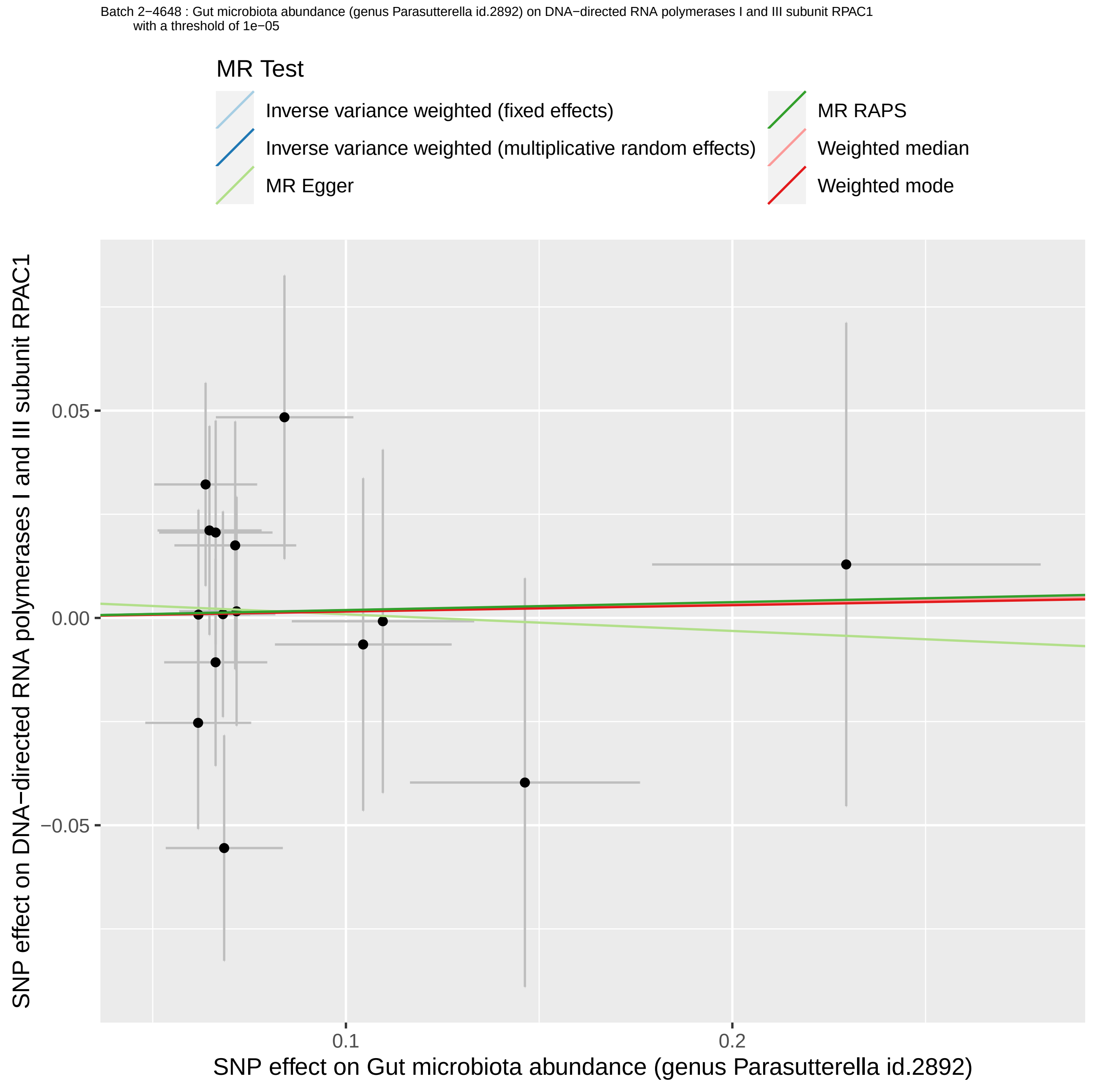
Batch 2−4648 : Gut microbiota abundance (genus Parasutterella id.2892) on DNA−directed RNA polymerases I and III subunit RPAC1
with a threshold of 1e−05
MR Test
Inverse variance weighted (fixed effects)
Inverse variance weighted (multiplicative random effects)
MR Egger
MR RAPS
Weighted median
Weighted mode
0.05
0.00
−0.05
0.1
0.2
SNP effect on DNA−directed RNA polymerases I and III subunit RPAC1
SNP effect on Gut microbiota abundance (genus Parasutterella id.2892)

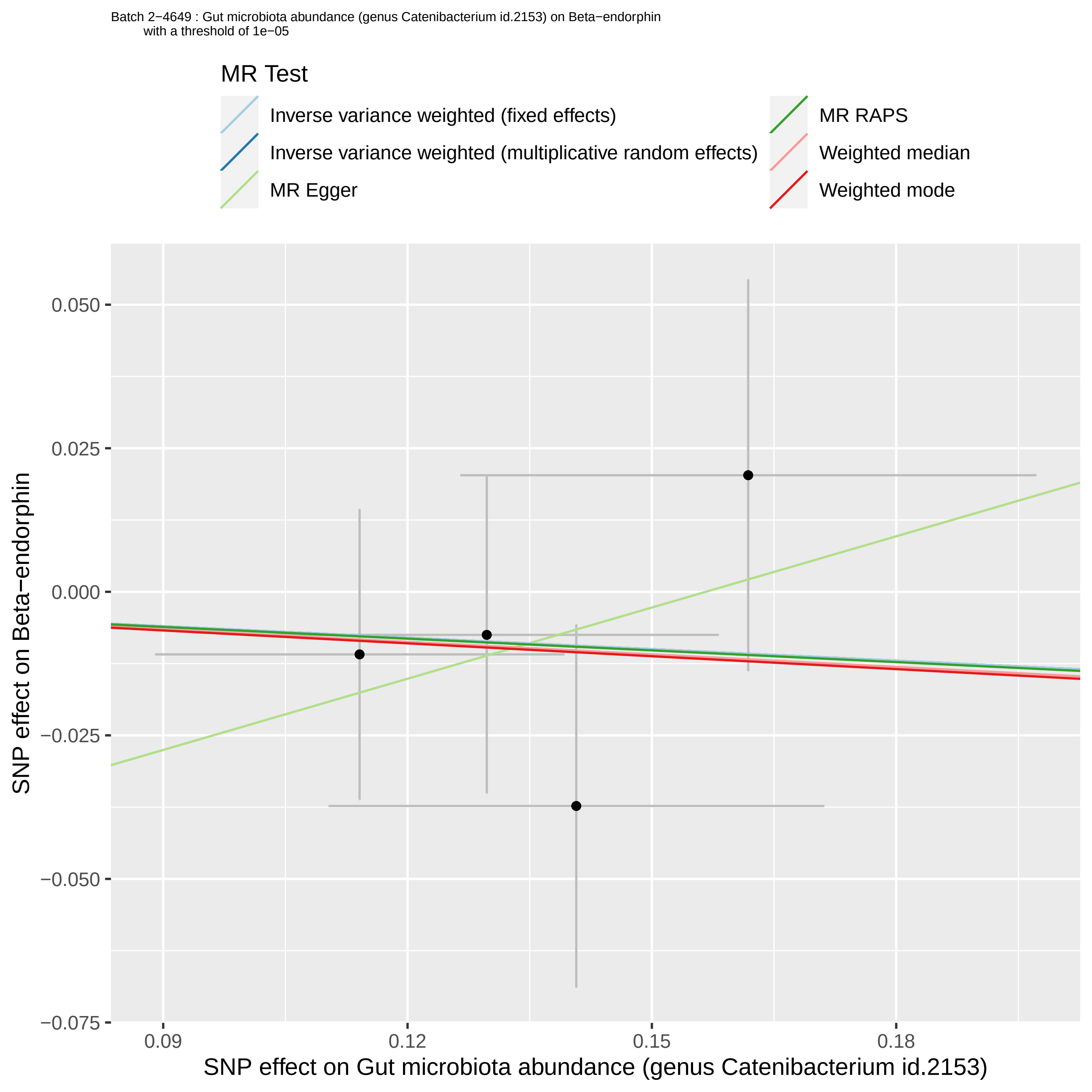
Batch 2−4649 : Gut microbiota abundance (genus Catenibacterium id.2153) on Beta−endorphin
with a threshold of 1e−05
MR Test
Inverse variance weighted (fixed effects)
Inverse variance weighted (multiplicative random effects)
MR Egger
MR RAPS
Weighted median
Weighted mode
0.050
0.025
0.000
−0.025
−0.050
−0.075
0.09
0.12
0.15
0.18
SNP effect on Beta−endorphin
SNP effect on Gut microbiota abundance (genus Catenibacterium id.2153)

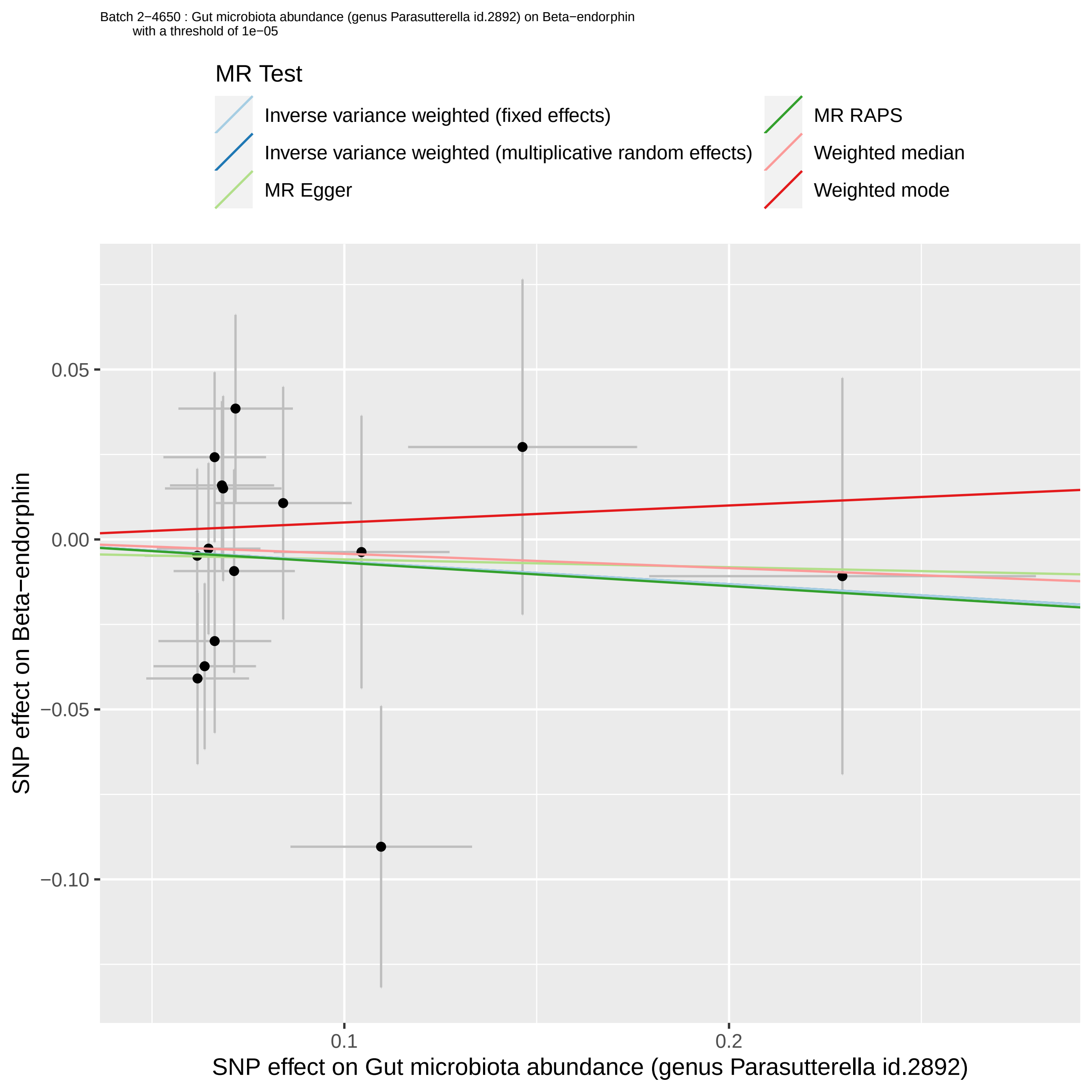

Batch 2−4650 : Gut microbiota abundance (genus Parasutterella id.2892) on Beta−endorphin
with a threshold of 1e−05
MR Test
Inverse variance weighted (fixed effects)
Inverse variance weighted (multiplicative random effects)
MR Egger
MR RAPS
Weighted median
Weighted mode
0.05
0.00
−0.05
−0.10
0.1
0.2
SNP effect on Beta−endorphin
SNP effect on Gut microbiota abundance (genus Parasutterella id.2892)

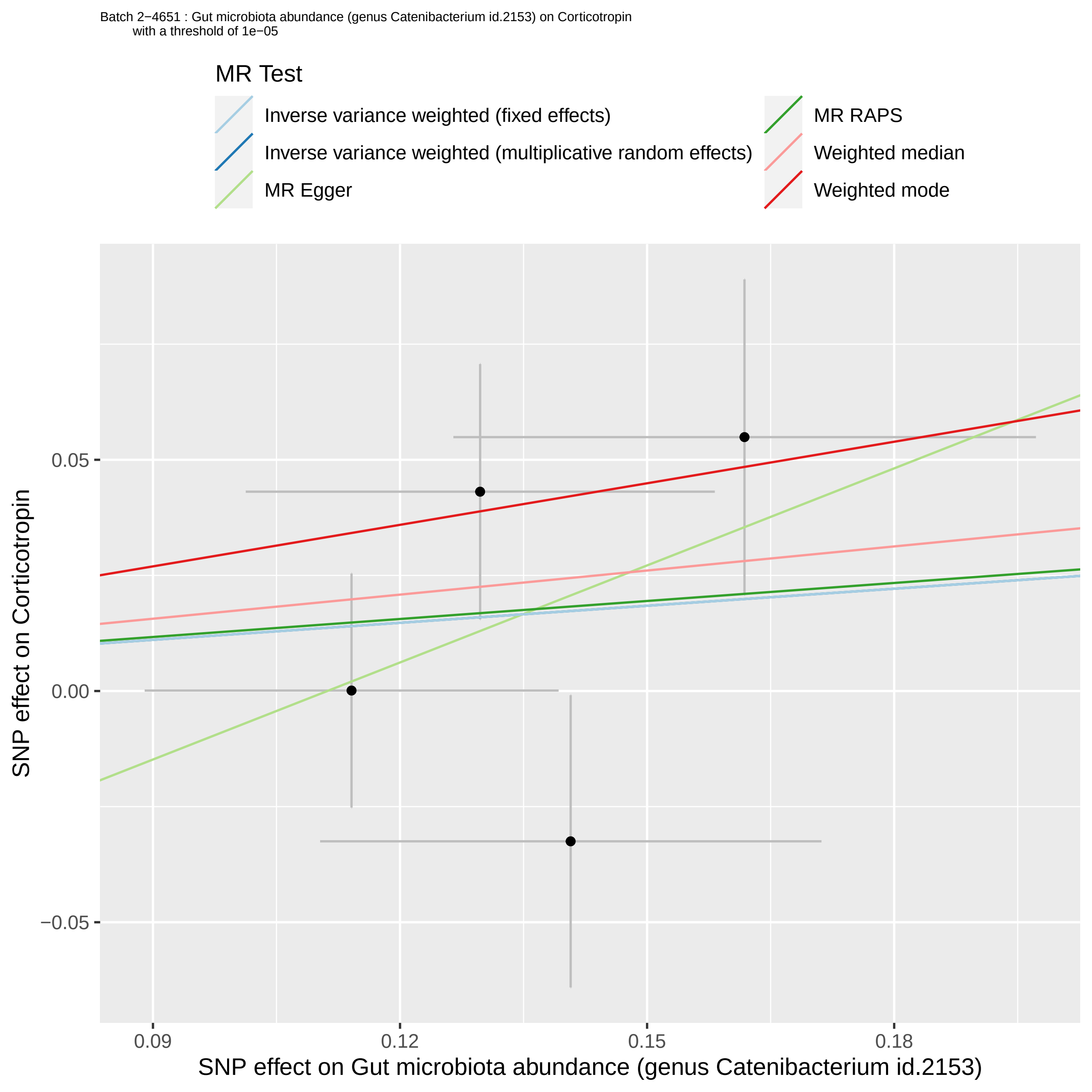

Batch 2−4651 : Gut microbiota abundance (genus Catenibacterium id.2153) on Corticotropin
with a threshold of 1e−05
MR Test
Inverse variance weighted (fixed effects)
Inverse variance weighted (multiplicative random effects)
MR Egger
MR RAPS
Weighted median
Weighted mode
0.05
0.00
−0.05
SNP effect on Corticotropin
0.09
0.12
0.15
0.18
SNP effect on Gut microbiota abundance (genus Catenibacterium id.2153)

Batch 2−4652 : Gut microbiota abundance (genus Parasutterella id.2892) on Corticotropin
with a threshold of 1e−05

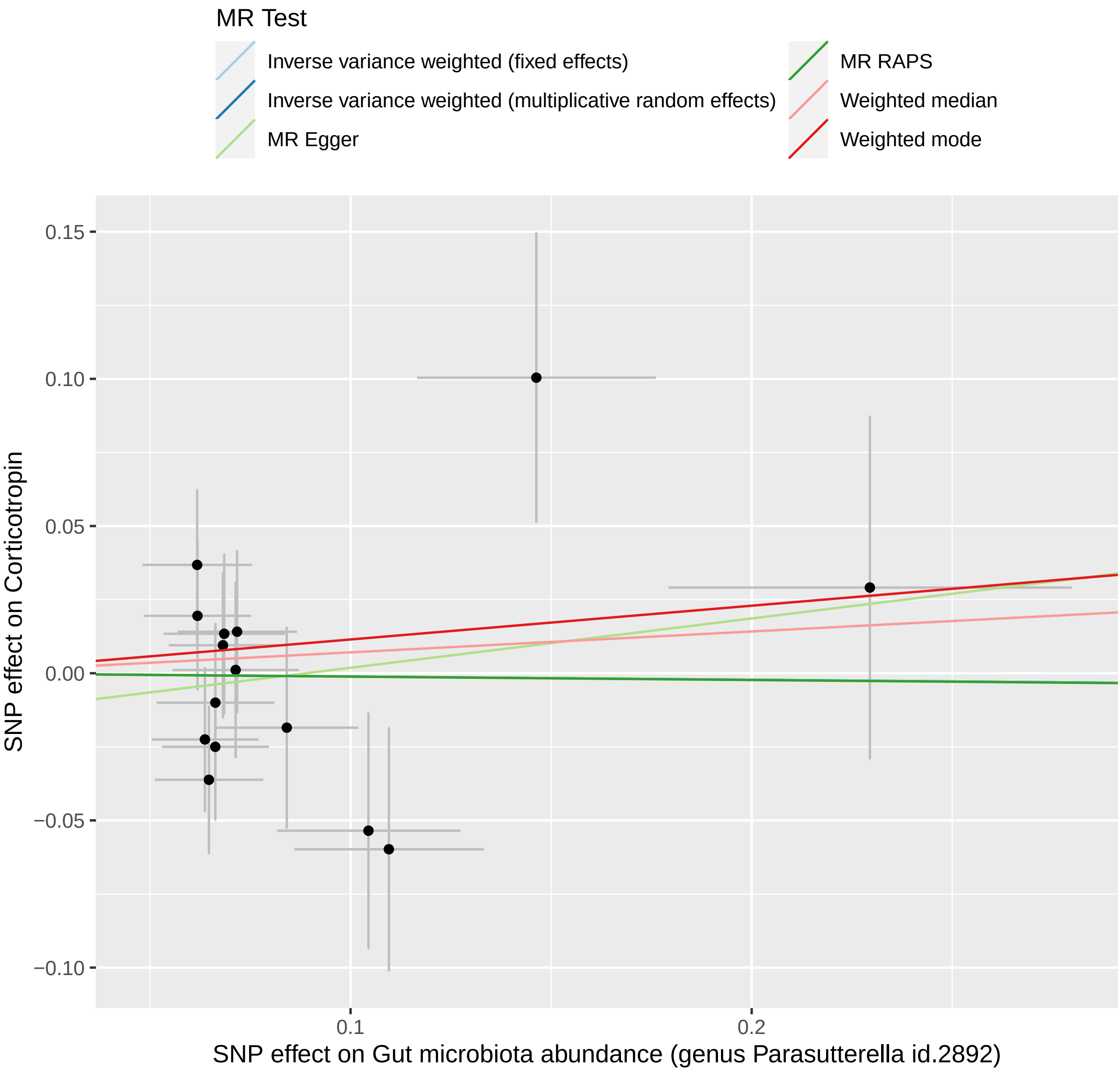

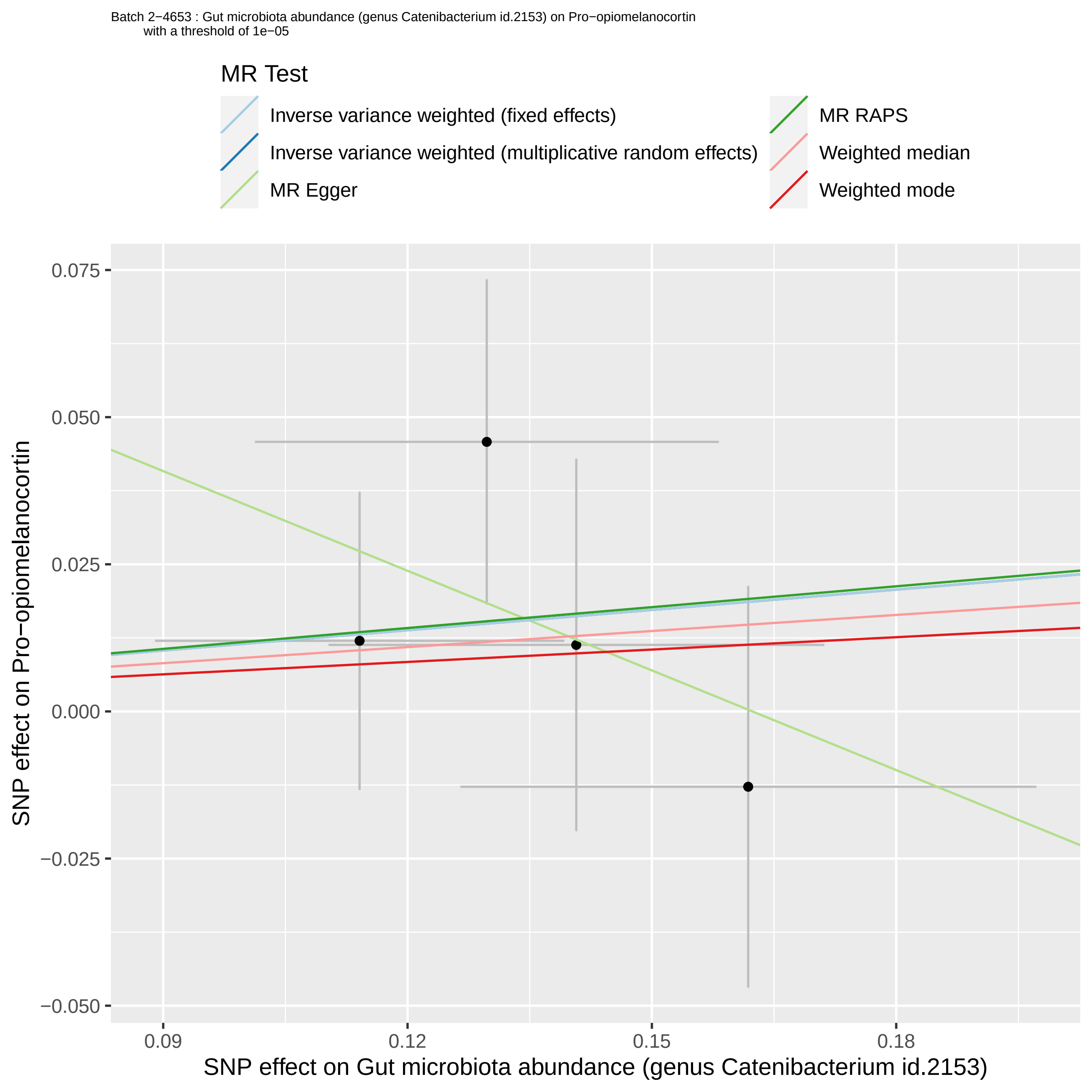
Batch 2−4653 : Gut microbiota abundance (genus Catenibacterium id.2153) on Pro−opiomelanocortin
with a threshold of 1e−05
MR Test
Inverse variance weighted (fixed effects)
Inverse variance weighted (multiplicative random effects)
MR Egger
MR RAPS
Weighted median
Weighted mode
0.075
0.050
0.025
0.000
−0.025
−0.050
SNP effect on Pro−opiomelanocortin
0.09
0.12
0.15
0.18
SNP effect on Gut microbiota abundance (genus Catenibacterium id.2153)

Batch 2−4654 : Gut microbiota abundance (genus Parasutterella id.2892) on Pro−opiomelanocortin
with a threshold of 1e−05

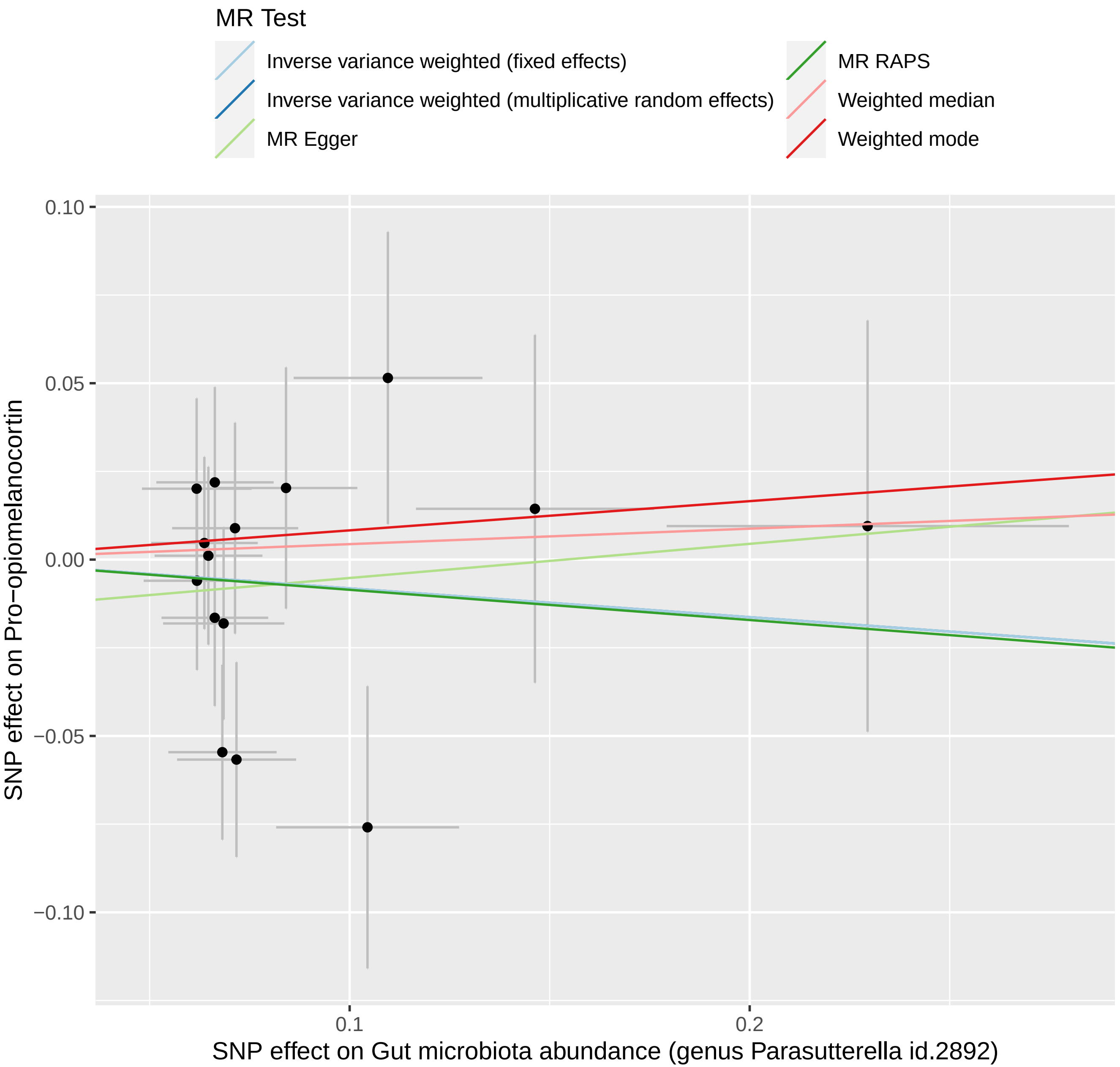

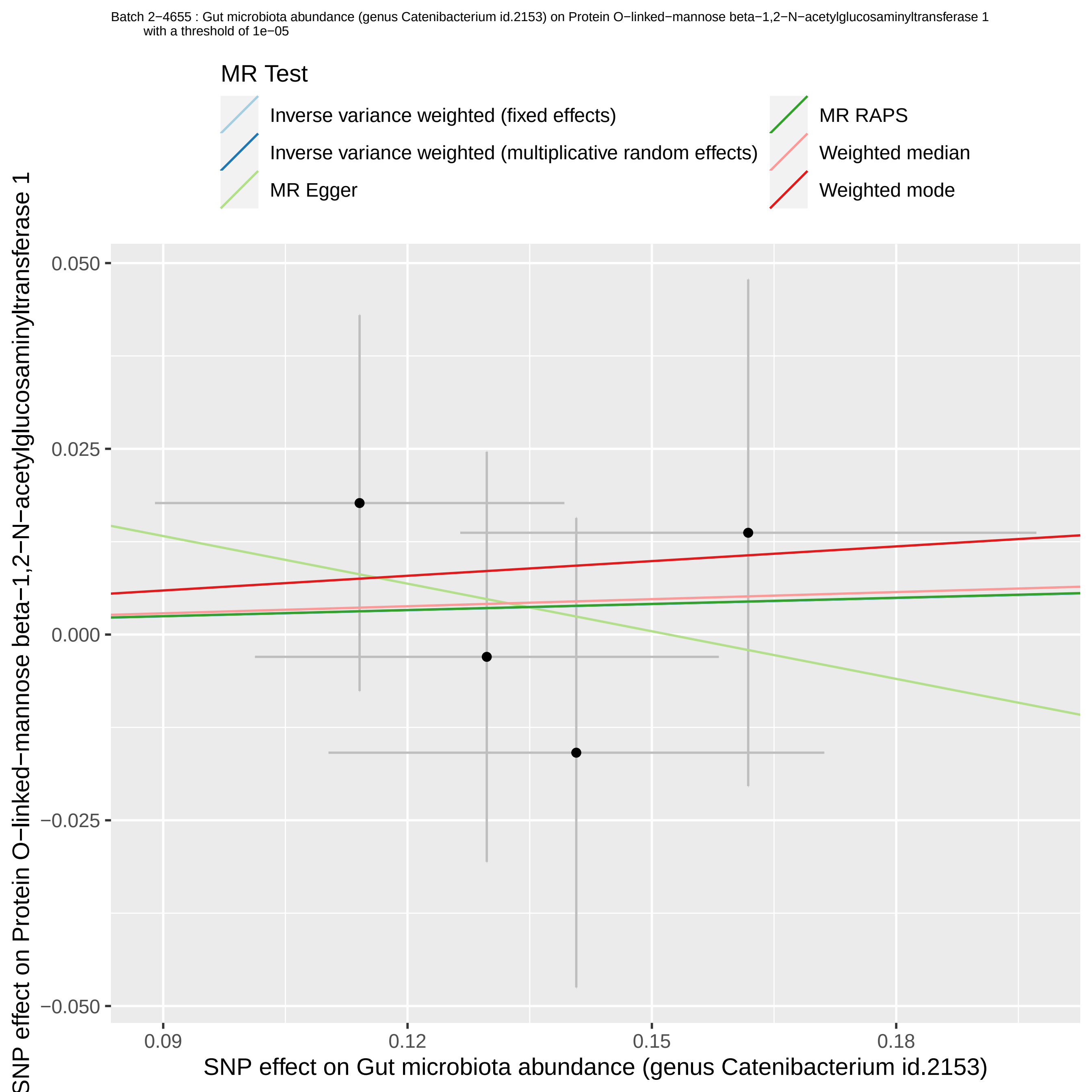

Batch 2−4655 : Gut microbiota abundance (genus Catenibacterium id.2153) on Protein O−linked−mannose beta−1,2−N−acetylglucosaminyltransferase 1
with a threshold of 1e−05
MR Test
Inverse variance weighted (fixed effects)
Inverse variance weighted (multiplicative random effects)
MR Egger
MR RAPS
Weighted median
Weighted mode
0.050
0.025
0.000
−0.025
−0.050
0.09
0.12
0.15
0.18
SNP effect on Gut microbiota abundance (genus Catenibacterium id.2153)
SNP effect on Protein O−linked−mannose beta−1,2−N−acetylglucosaminyltransferase 1

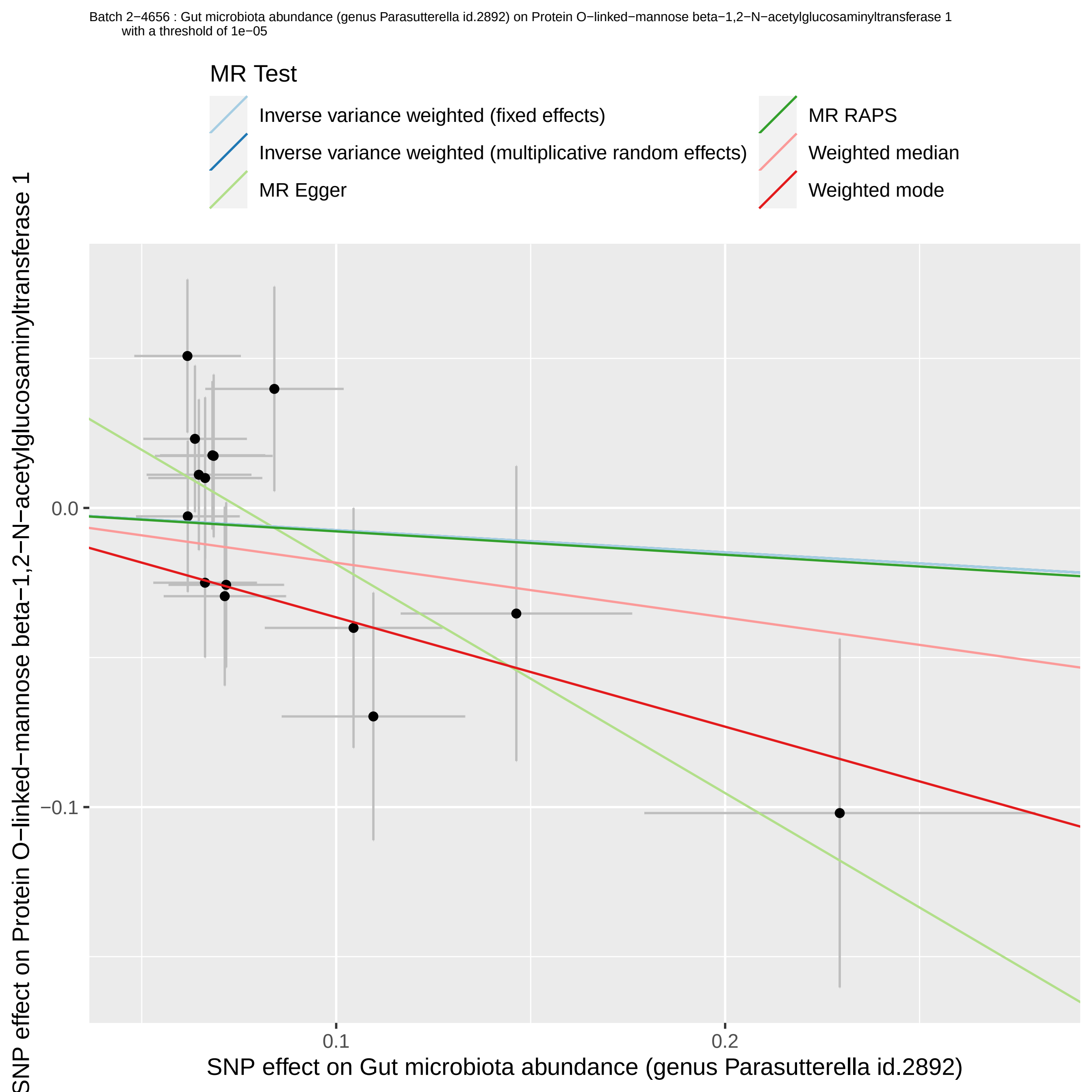
Batch 2−4656 : Gut microbiota abundance (genus Parasutterella id.2892) on Protein O−linked−mannose beta−1,2−N−acetylglucosaminyltransferase 1
with a threshold of 1e−05
MR Test
Inverse variance weighted (fixed effects)
Inverse variance weighted (multiplicative random effects)
MR Egger
MR RAPS
Weighted median
Weighted mode
0.0
−0.1
0.1
0.2
SNP effect on Gut microbiota abundance (genus Parasutterella id.2892)
SNP effect on Protein O−linked−mannose beta−1,2−N−acetylglucosaminyltransferase 1

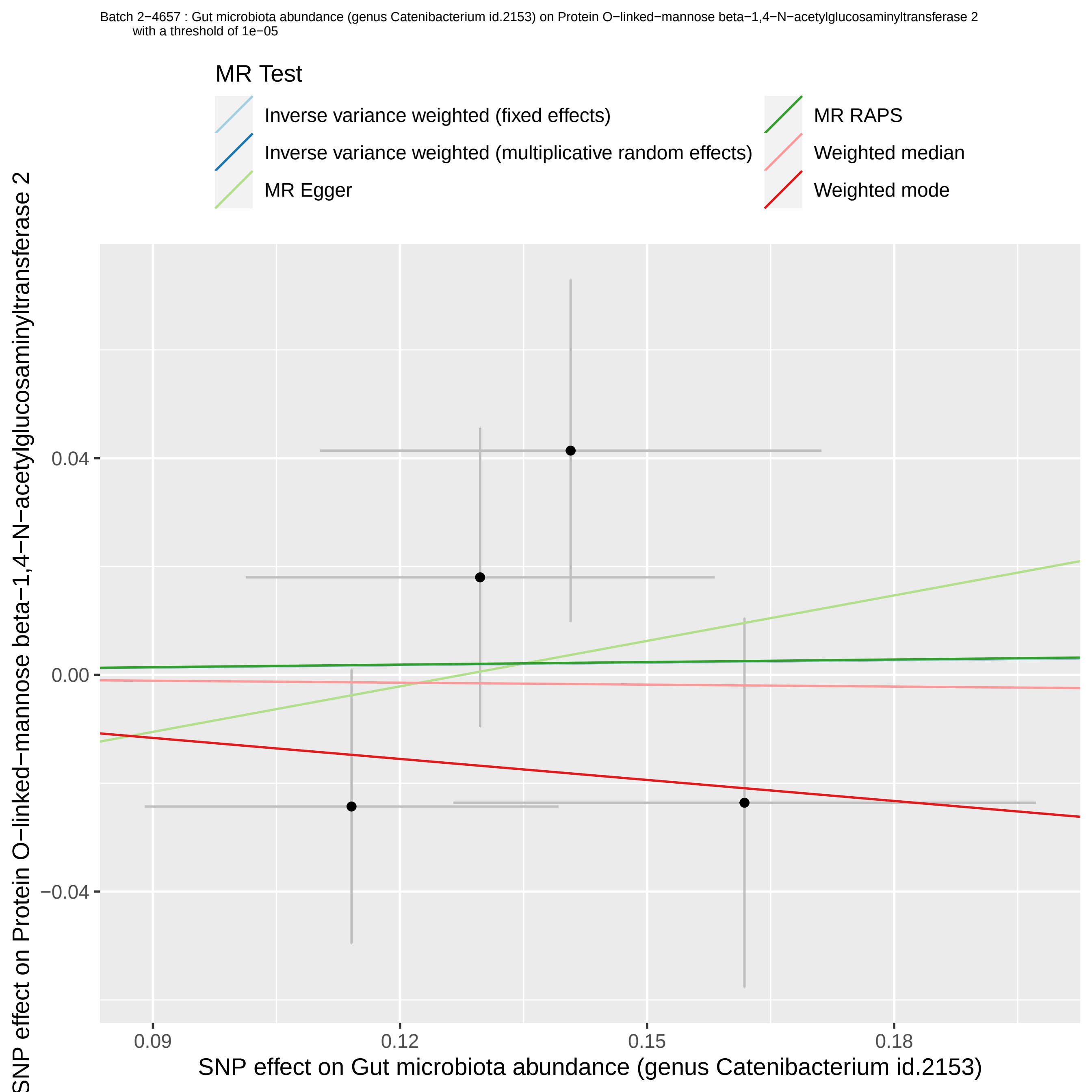
Batch 2−4657 : Gut microbiota abundance (genus Catenibacterium id.2153) on Protein O−linked−mannose beta−1,4−N−acetylglucosaminyltransferase 2
with a threshold of 1e−05
MR Test
Inverse variance weighted (fixed effects)
Inverse variance weighted (multiplicative random effects)
MR Egger
MR RAPS
Weighted median
Weighted mode
SNP effect on Protein O−linked−mannose beta−1,4−N−acetylglucosaminyltransferase 2
0.04
0.00
−0.04
0.09
0.12
0.15
0.18
SNP effect on Gut microbiota abundance (genus Catenibacterium id.2153)

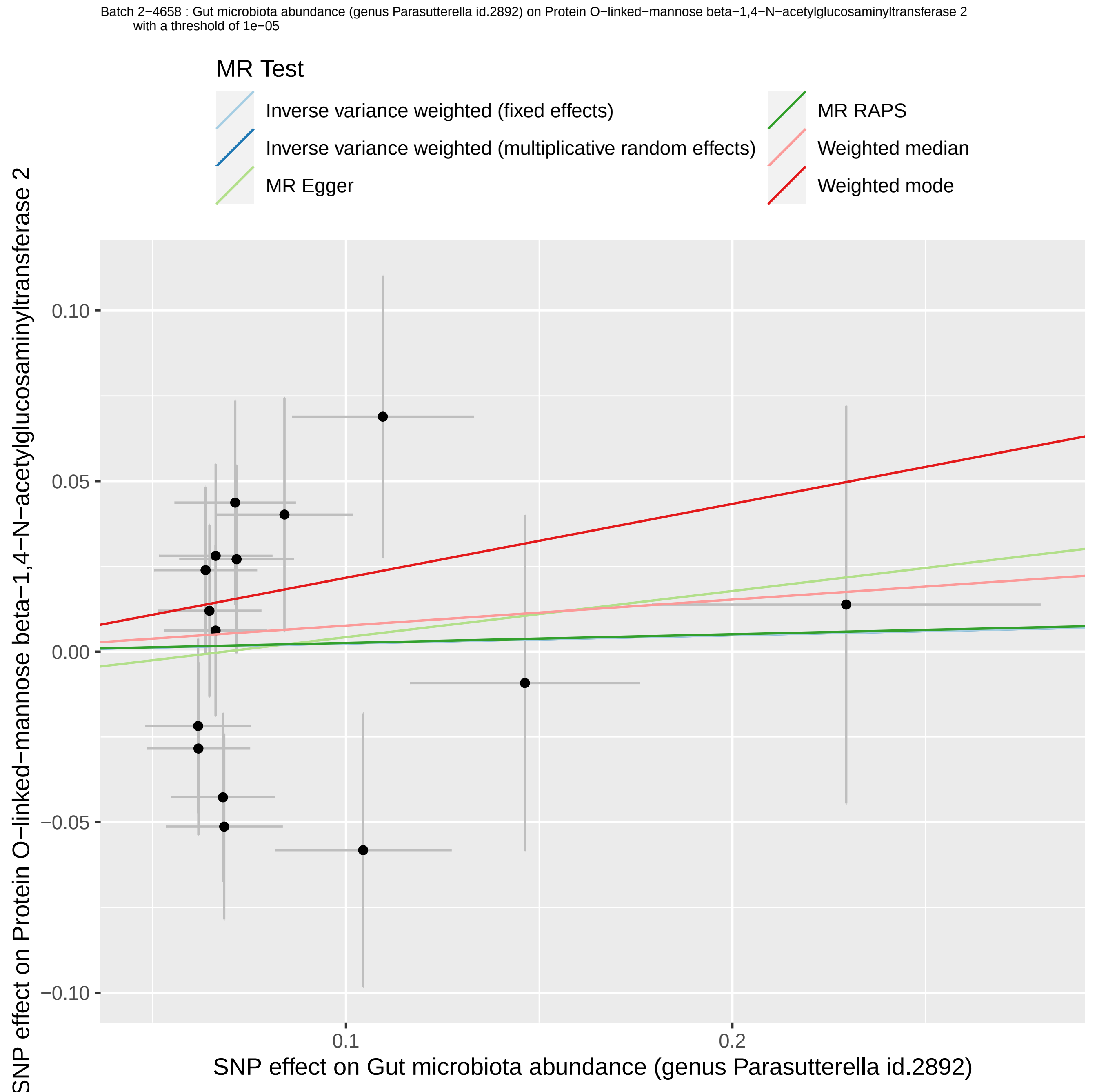

Batch 2−4658 : Gut microbiota abundance (genus Parasutterella id.2892) on Protein O−linked−mannose beta−1,4−N−acetylglucosaminyltransferase 2 with a threshold of 1e−05
MR Test
Inverse variance weighted (fixed effects)
Inverse variance weighted (multiplicative random effects)
MR Egger
MR RAPS
Weighted median
Weighted mode
0.10
0.05
0.00
−0.05
−0.10
0.1
0.2
SNP effect on Protein O−linked−mannose beta−1,4−N−acetylglucosaminyltransferase 2
SNP effect on Gut microbiota abundance (genus Parasutterella id.2892)

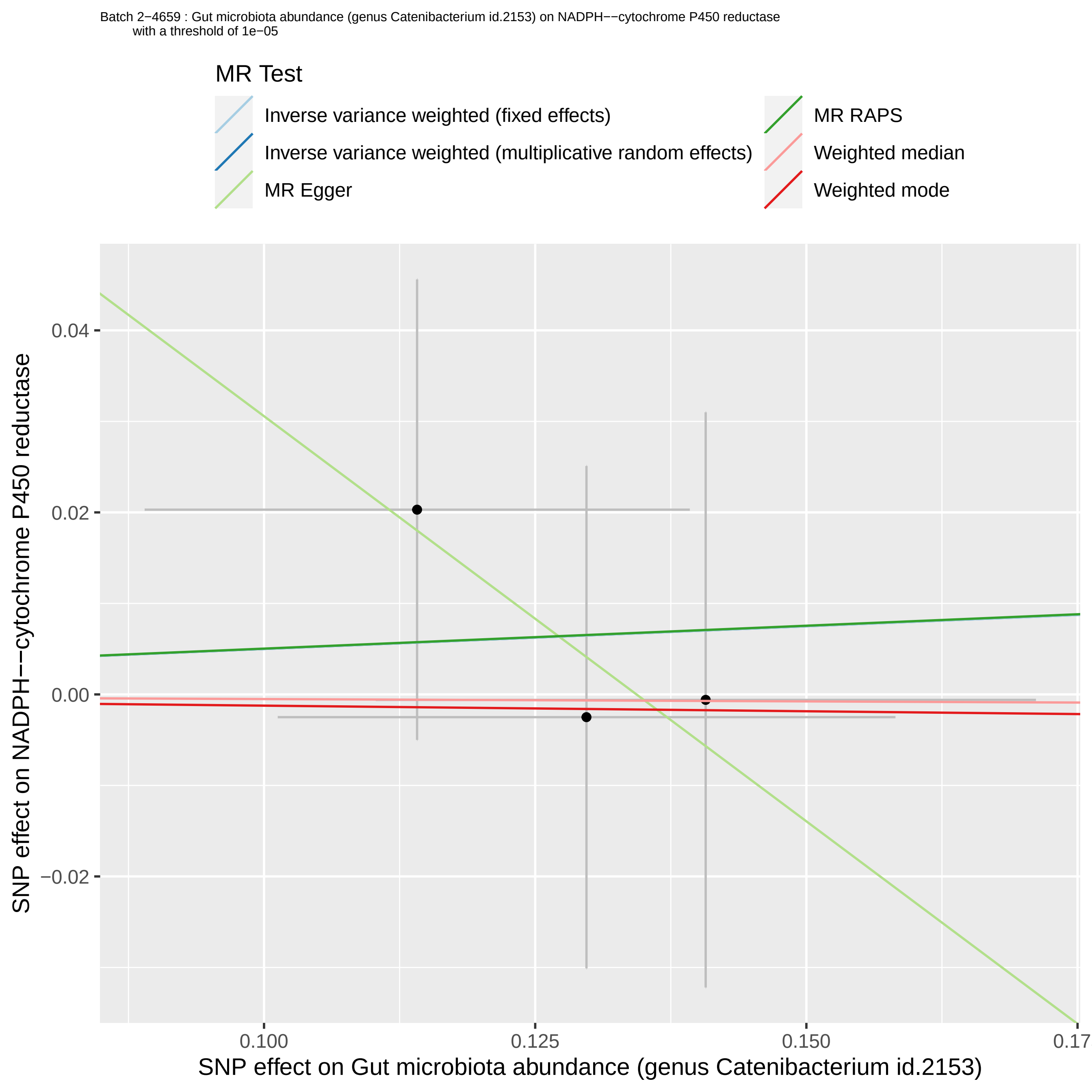

Batch 2−4659 : Gut microbiota abundance (genus Catenibacterium id.2153) on NADPH−−cytochrome P450 reductase
with a threshold of 1e−05
MR Test
Inverse variance weighted (fixed effects)
Inverse variance weighted (multiplicative random effects)
MR Egger
MR RAPS
Weighted median
Weighted mode
SNP effect on NADPH−−cytochrome P450 reductase
0.04
0.02
0.00
−0.02
0.100
0.125
0.150
0.17
SNP effect on Gut microbiota abundance (genus Catenibacterium id.2153)

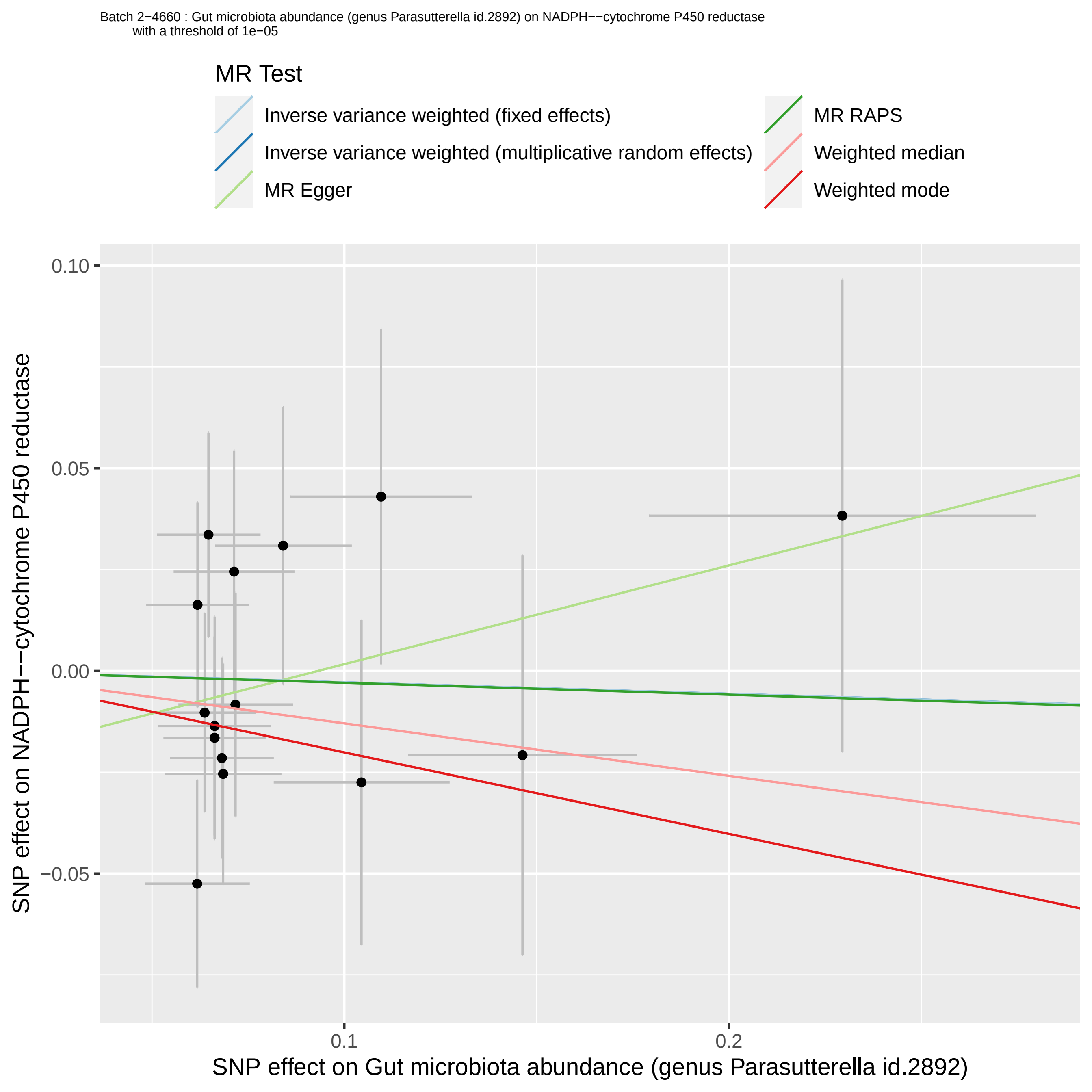

Batch 2−4660 : Gut microbiota abundance (genus Parasutterella id.2892) on NADPH−−cytochrome P450 reductase
with a threshold of 1e−05
MR Test
Inverse variance weighted (fixed effects)
Inverse variance weighted (multiplicative random effects)
MR Egger
MR RAPS
Weighted median
Weighted mode
0.10
0.05
0.00
−0.05
0.1
0.2
SNP effect on NADPH−−cytochrome P450 reductase
SNP effect on Gut microbiota abundance (genus Parasutterella id.2892)

Batch 2−4661 : Gut microbiota abundance (genus Catenibacterium id.2153) on Periostin
with a threshold of 1e−05

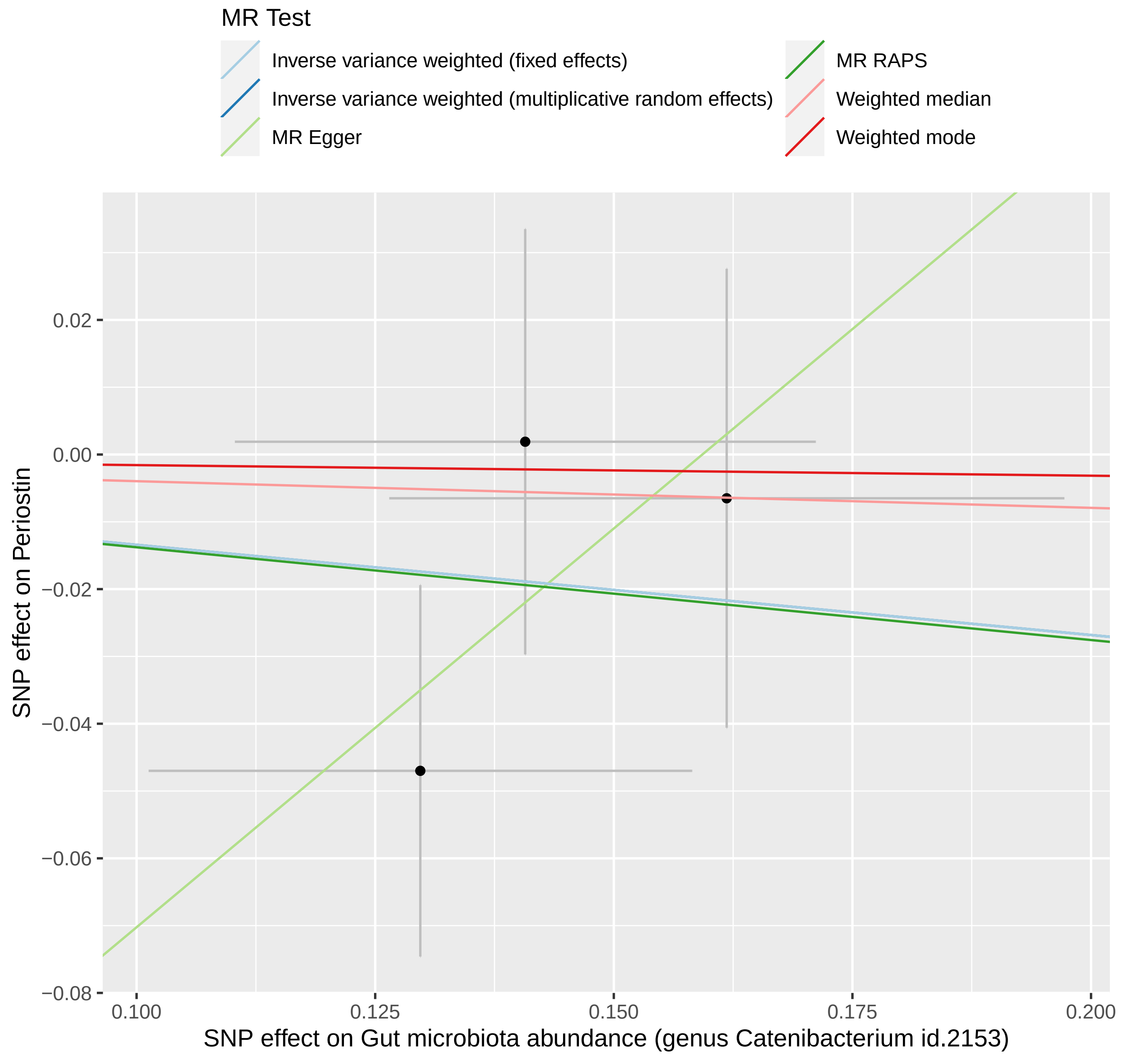

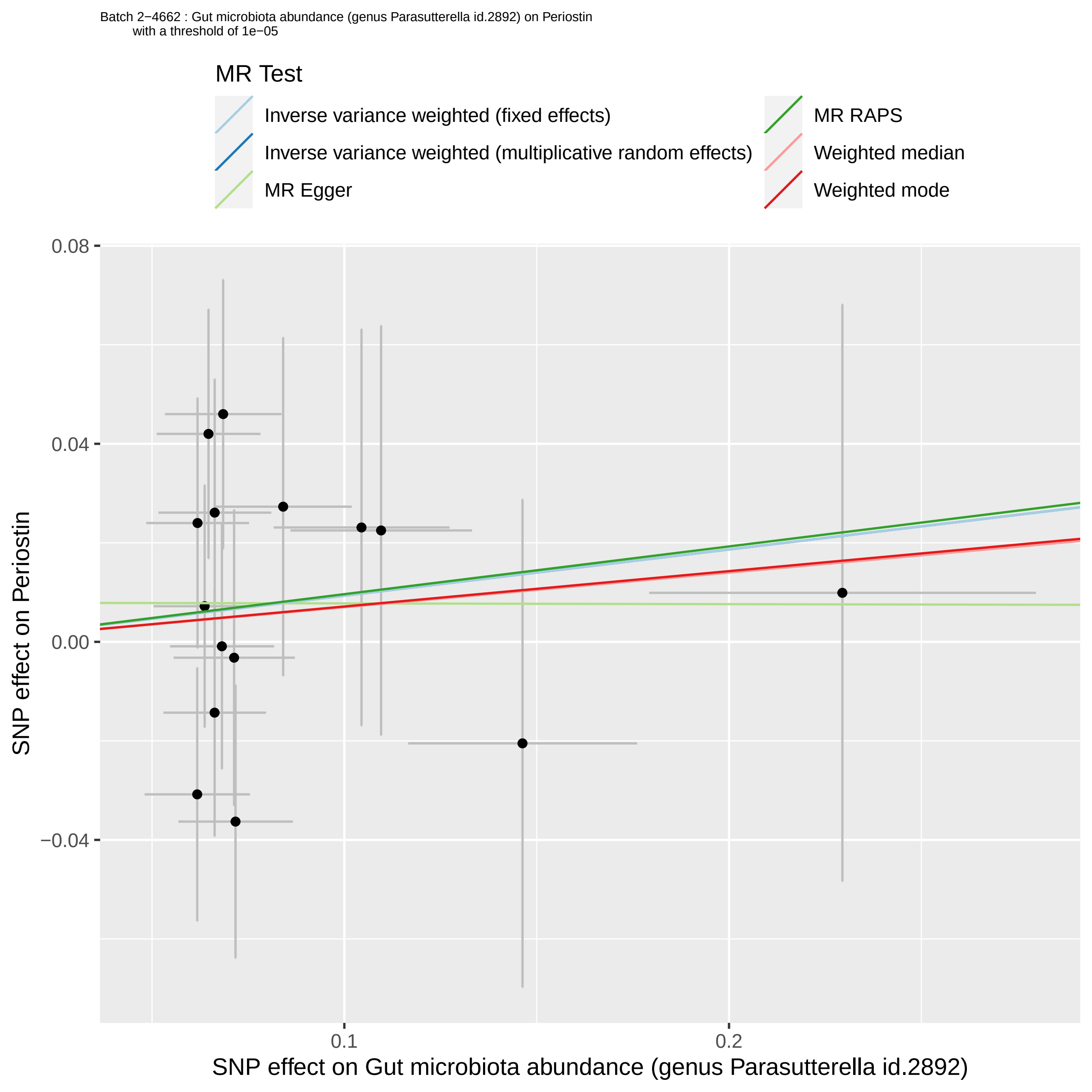

Batch 2−4662 : Gut microbiota abundance (genus Parasutterella id.2892) on Periostin
with a threshold of 1e−05
MR Test
Inverse variance weighted (fixed effects)
Inverse variance weighted (multiplicative random effects)
MR Egger
MR RAPS
Weighted median
Weighted mode
0.08
0.04
0.00
−0.04
0.1
0.2
SNP effect on Periostin
SNP effect on Gut microbiota abundance (genus Parasutterella id.2892)

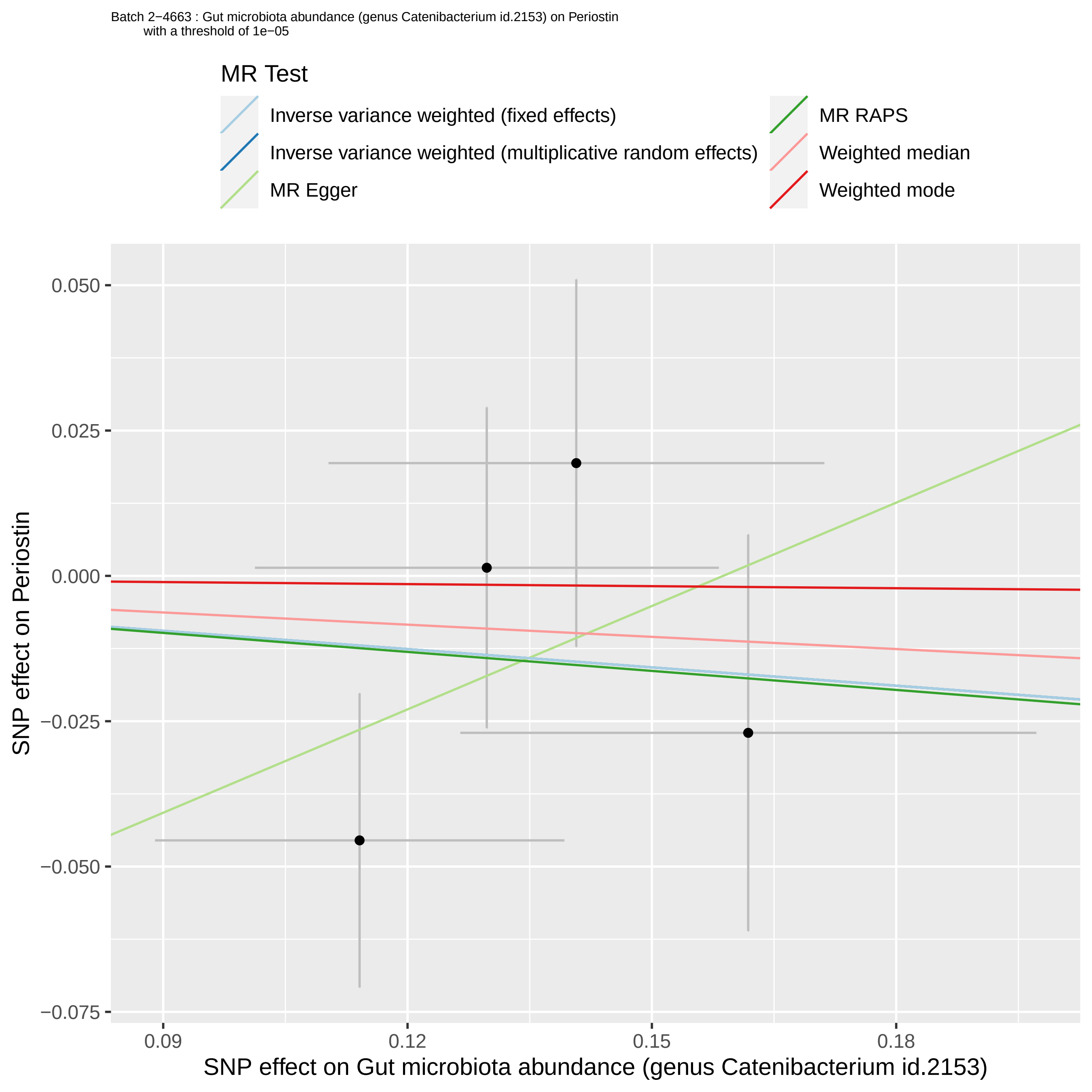
Batch 2−4663 : Gut microbiota abundance (genus Catenibacterium id.2153) on Periostin
with a threshold of 1e−05
MR Test
Inverse variance weighted (fixed effects)
Inverse variance weighted (multiplicative random effects)
MR Egger
MR RAPS
Weighted median
Weighted mode
0.050
0.025
0.000
−0.025
−0.050
−0.075
0.09
0.12
0.15
0.18
SNP effect on Periostin
SNP effect on Gut microbiota abundance (genus Catenibacterium id.2153)

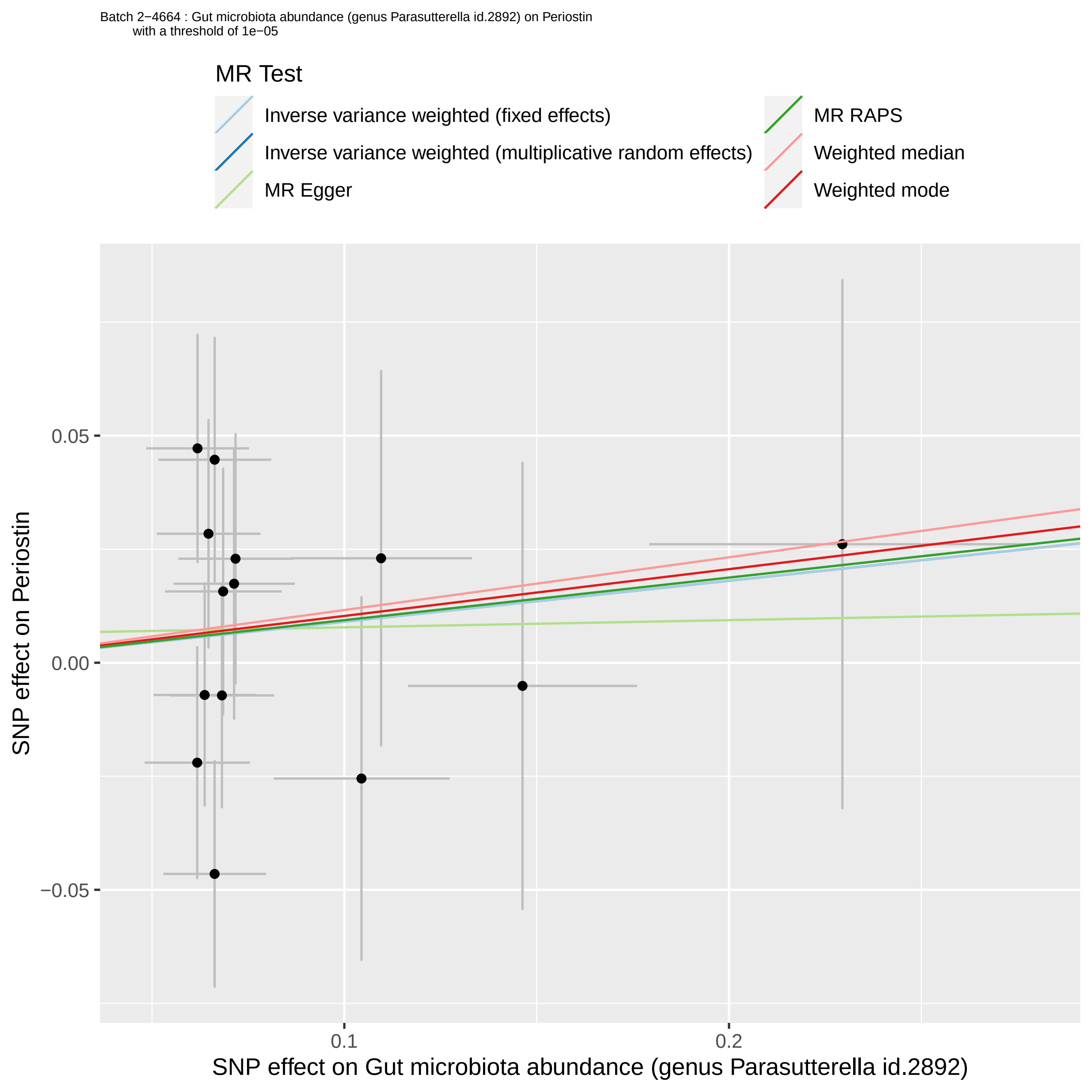
Batch 2−4664 : Gut microbiota abundance (genus Parasutterella id.2892) on Periostin
with a threshold of 1e−05
MR Test
Inverse variance weighted (fixed effects)
Inverse variance weighted (multiplicative random effects)
MR Egger
MR RAPS
Weighted median
Weighted mode
0.05
0.00
−0.05
0.1
0.2
SNP effect on Periostin
SNP effect on Gut microbiota abundance (genus Parasutterella id.2892)

Batch 2−4665 : Gut microbiota abundance (genus Catenibacterium id.2153) on Periostin
with a threshold of 1e−05

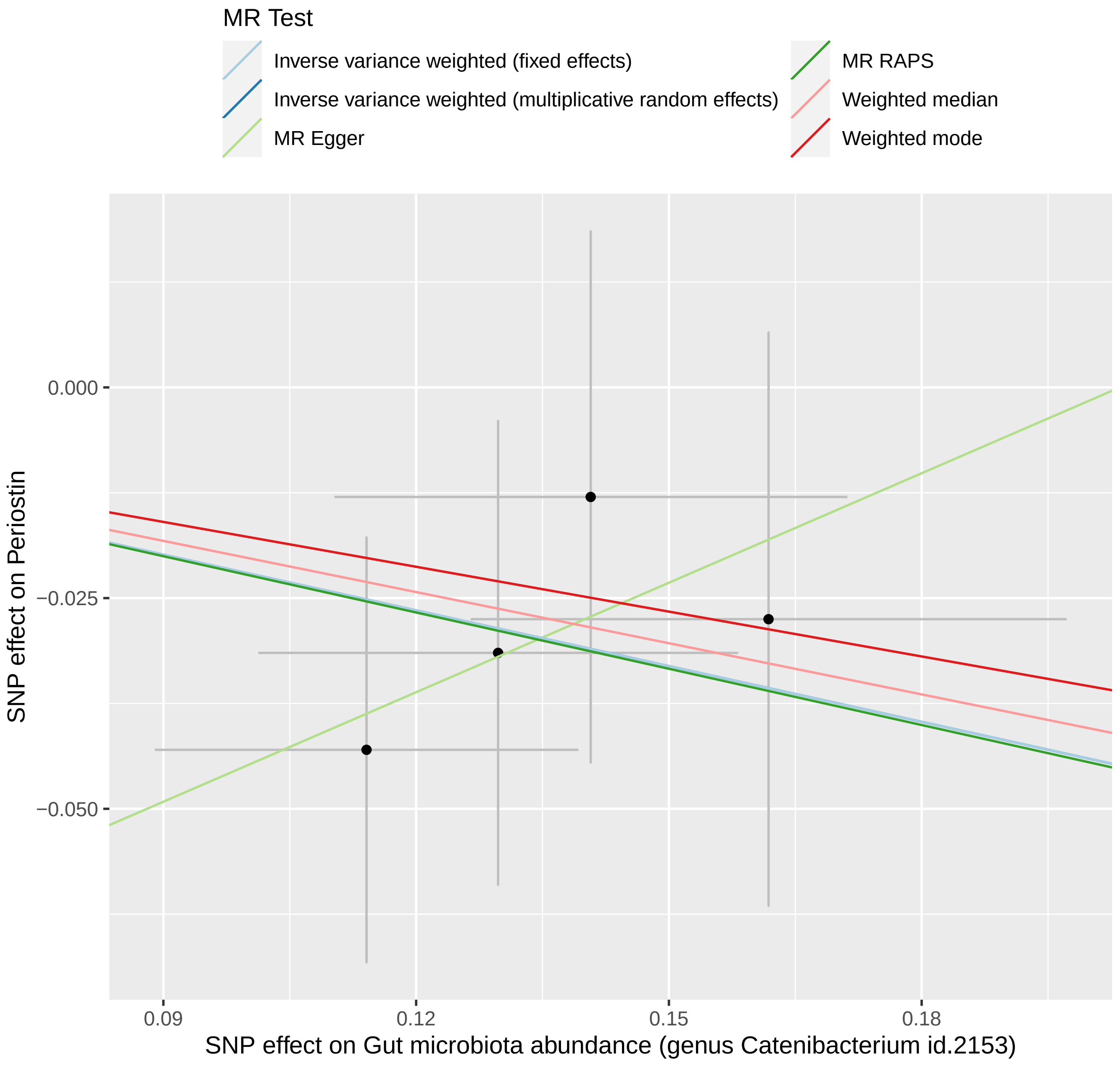

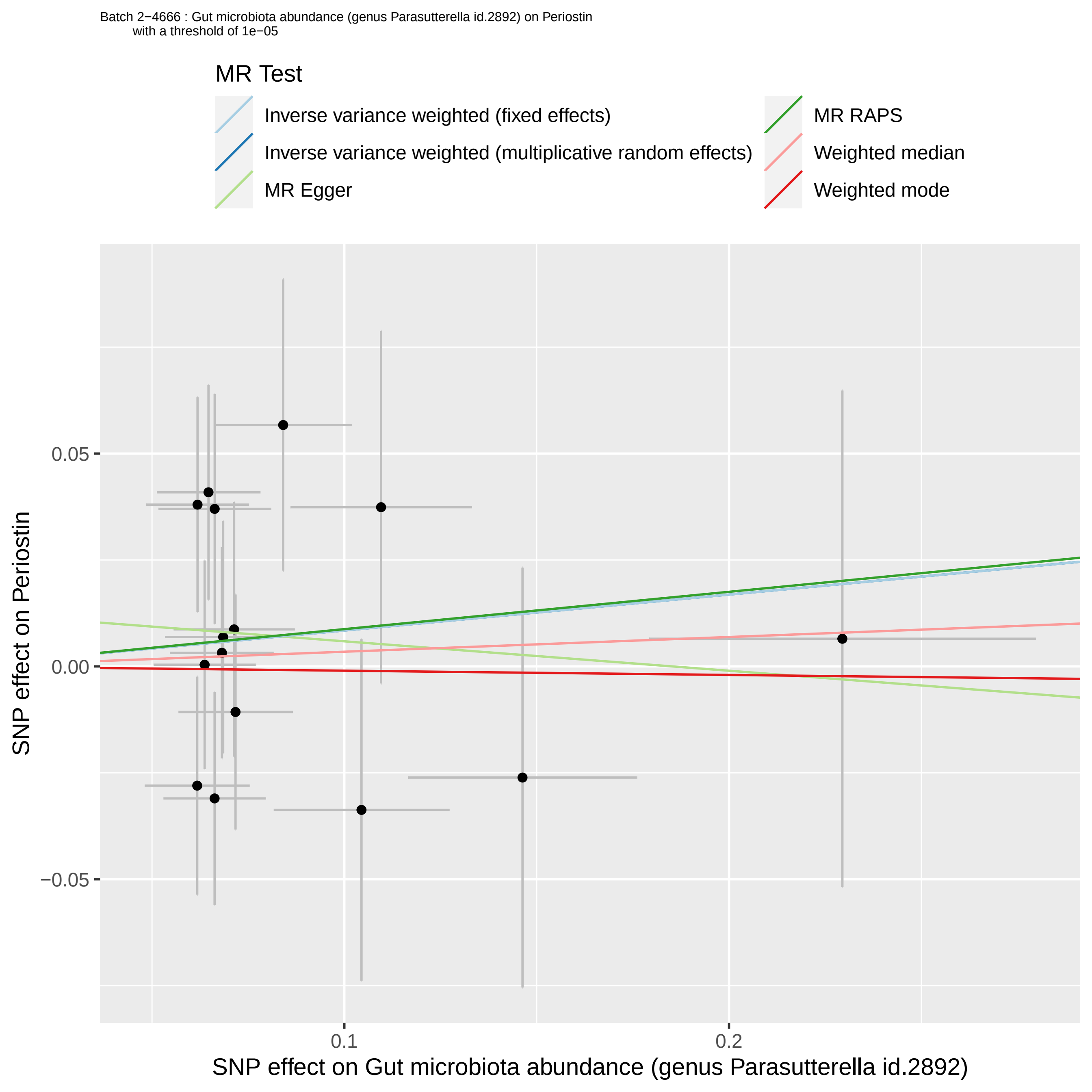
Batch 2−4666 : Gut microbiota abundance (genus Parasutterella id.2892) on Periostin
with a threshold of 1e−05
MR Test
Inverse variance weighted (fixed effects)
Inverse variance weighted (multiplicative random effects)
MR Egger
MR RAPS
Weighted median
Weighted mode
0.05
0.00
−0.05
0.1
0.2
SNP effect on Periostin
SNP effect on Gut microbiota abundance (genus Parasutterella id.2892)

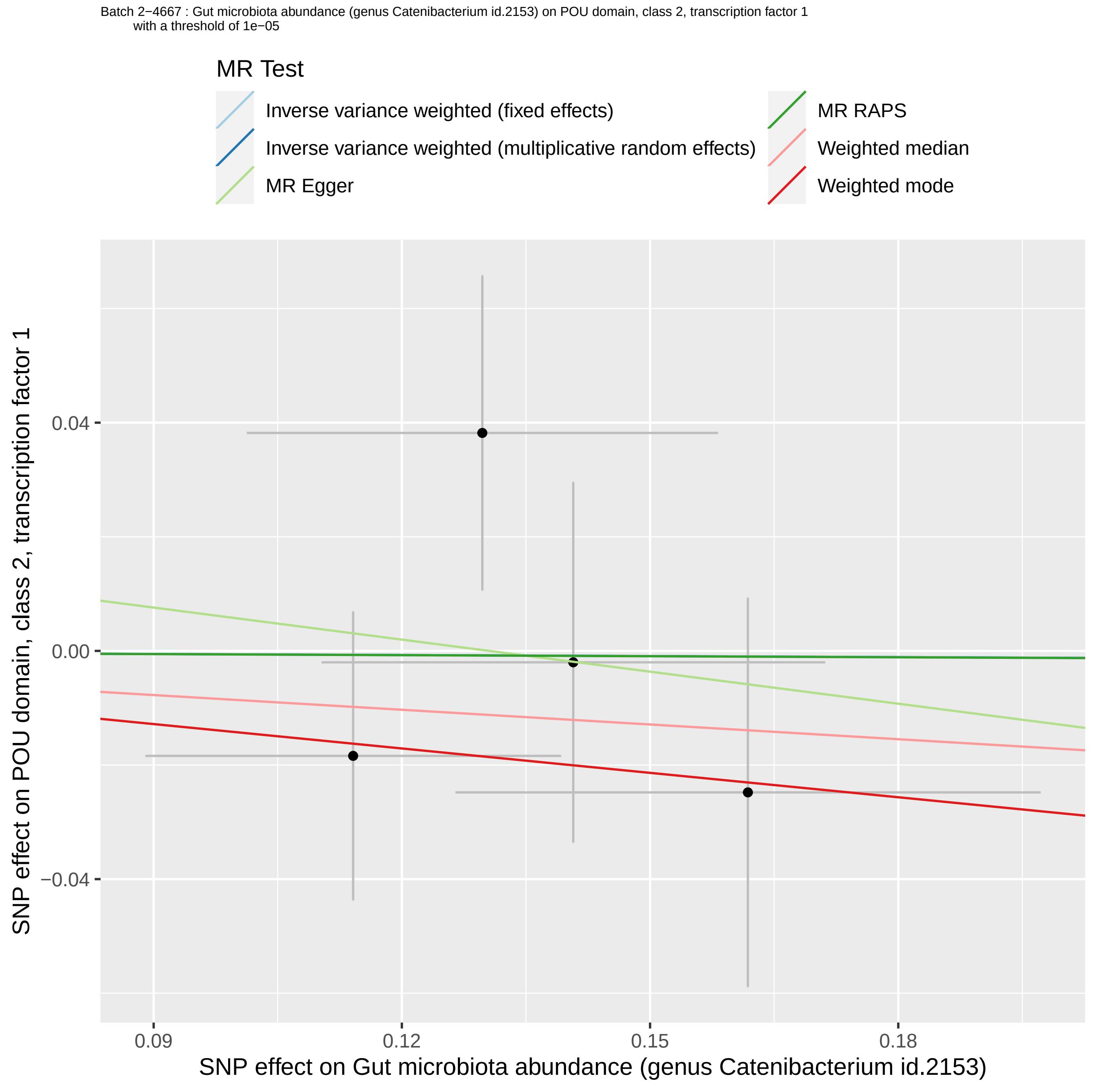

Batch 2−4667 : Gut microbiota abundance (genus Catenibacterium id.2153) on POU domain, class 2, transcription factor 1
with a threshold of 1e−05
MR Test
Inverse variance weighted (fixed effects)
Inverse variance weighted (multiplicative random effects)
MR Egger
MR RAPS
Weighted median
Weighted mode
0.04
0.00
−0.04
0.09
0.12
0.15
0.18
SNP effect on POU domain, class 2, transcription factor 1
SNP effect on Gut microbiota abundance (genus Catenibacterium id.2153)

Batch 2−4668 : Gut microbiota abundance (genus Parasutterella id.2892) on POU domain, class 2, transcription factor 1
with a threshold of 1e−05

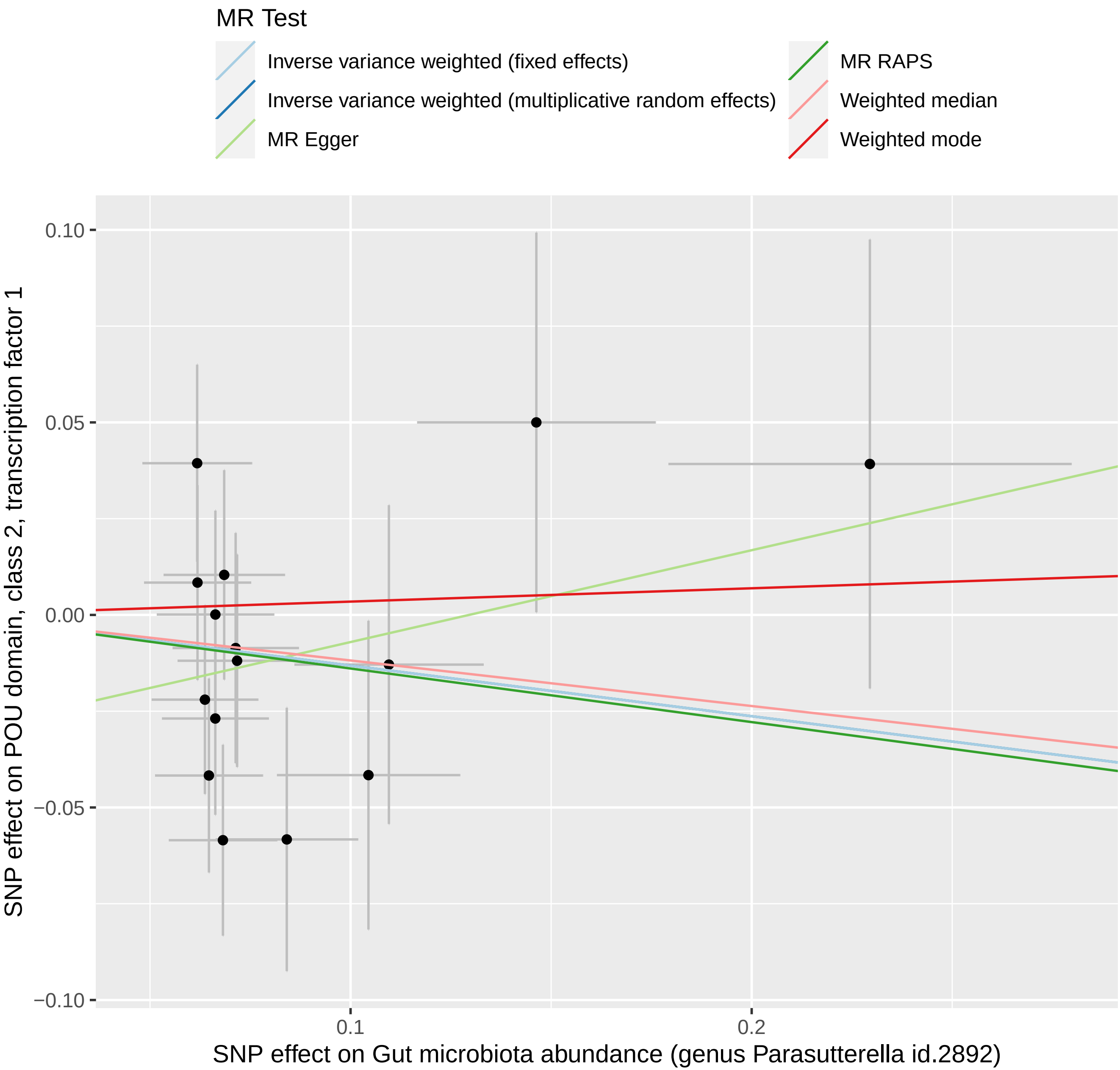

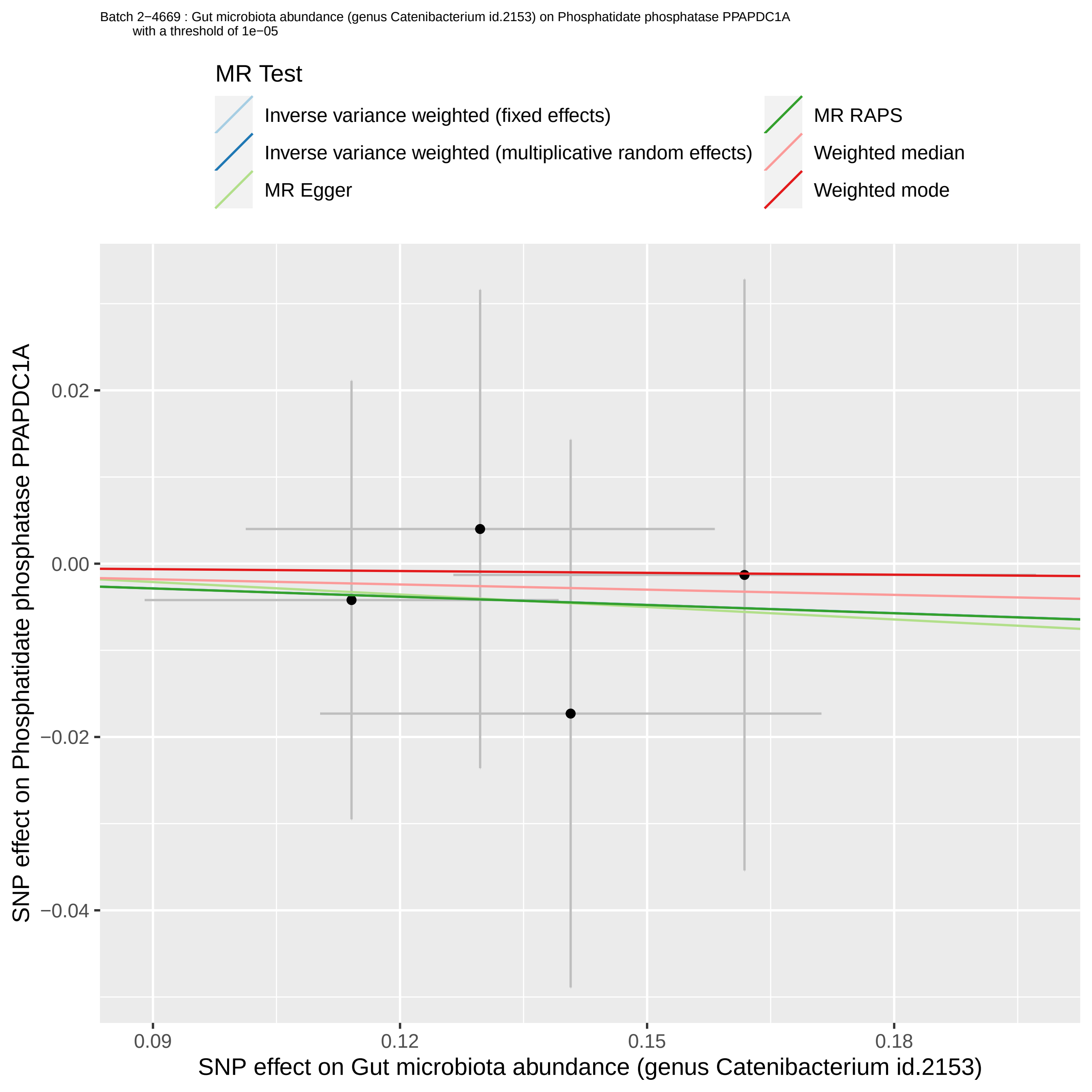
Batch 2−4669 : Gut microbiota abundance (genus Catenibacterium id.2153) on Phosphatidate phosphatase PPAPDC1A
with a threshold of 1e−05
MR Test
Inverse variance weighted (fixed effects)
Inverse variance weighted (multiplicative random effects)
MR Egger
MR RAPS
Weighted median
Weighted mode
0.02
0.00
−0.02
−0.04
0.09
0.12
0.15
0.18
SNP effect on Phosphatidate phosphatase PPAPDC1A
SNP effect on Gut microbiota abundance (genus Catenibacterium id.2153)

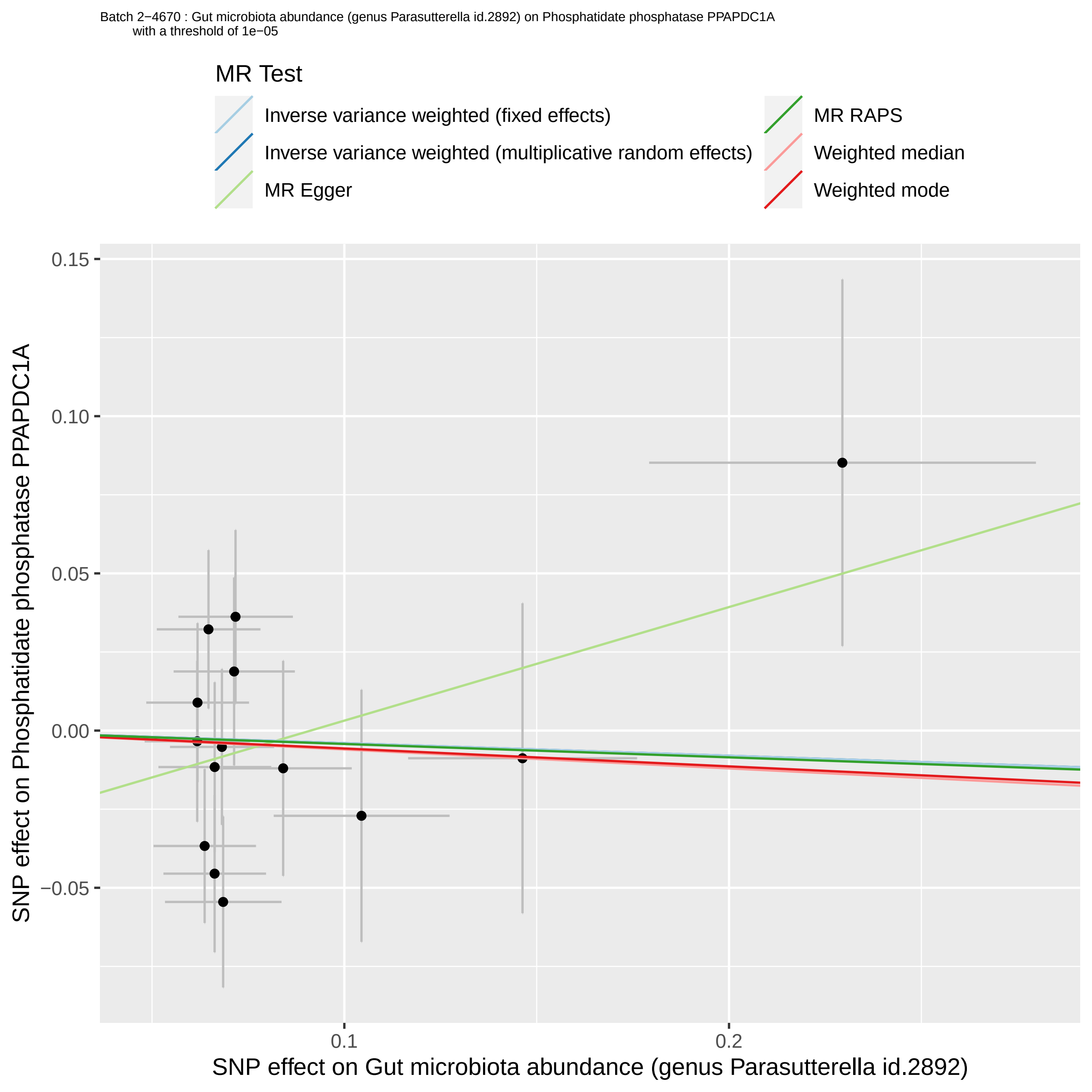
Batch 2−4670 : Gut microbiota abundance (genus Parasutterella id.2892) on Phosphatidate phosphatase PPAPDC1A
with a threshold of 1e−05
MR Test
Inverse variance weighted (fixed effects)
Inverse variance weighted (multiplicative random effects)
MR Egger
MR RAPS
Weighted median
Weighted mode
0.15
0.10
0.05
0.00
−0.05
0.1
0.2
SNP effect on Phosphatidate phosphatase PPAPDC1A
SNP effect on Gut microbiota abundance (genus Parasutterella id.2892)

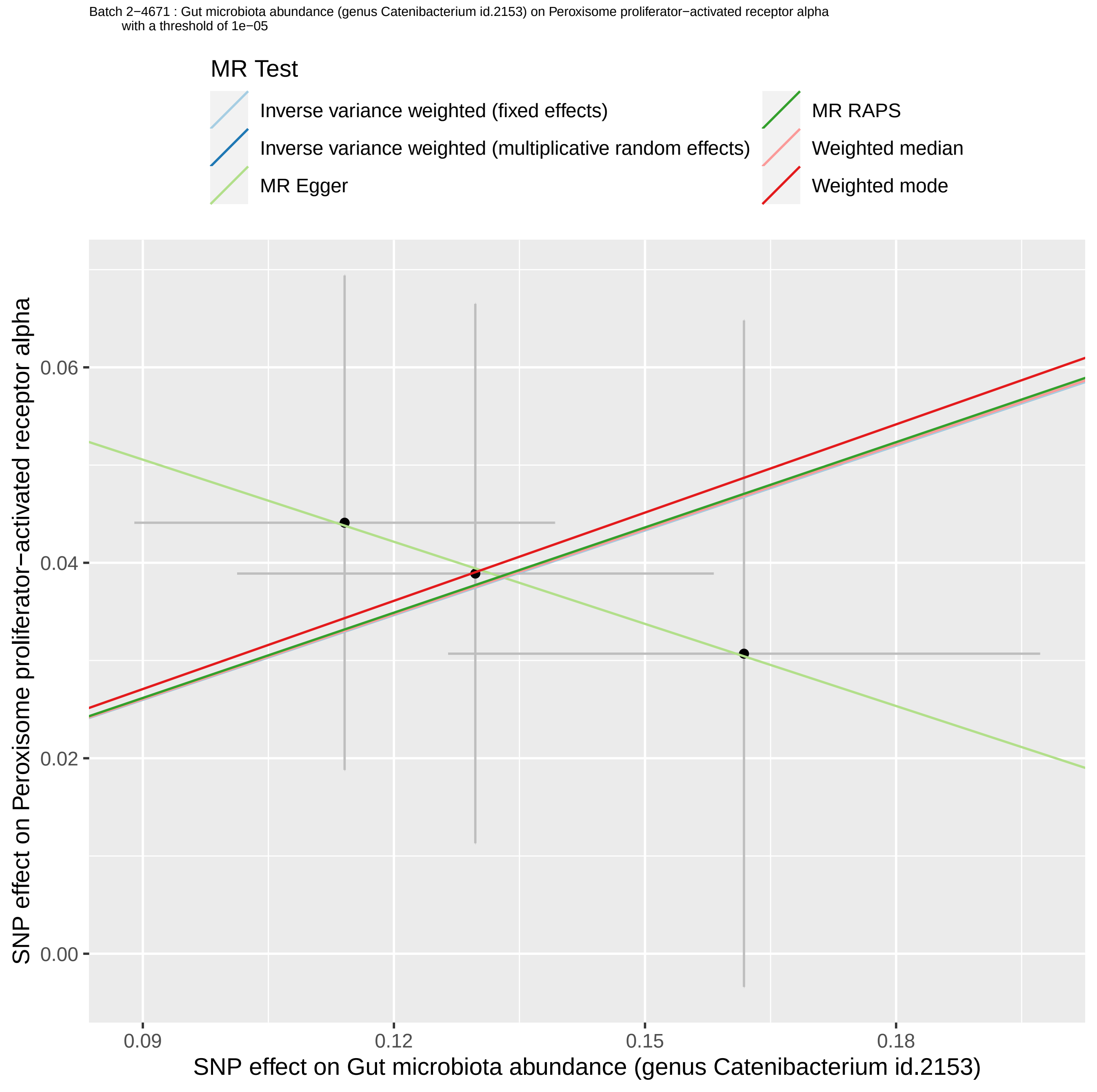
Batch 2−4671 : Gut microbiota abundance (genus Catenibacterium id.2153) on Peroxisome proliferator−activated receptor alpha
with a threshold of 1e−05
MR Test
Inverse variance weighted (fixed effects)
Inverse variance weighted (multiplicative random effects)
MR Egger
MR RAPS
Weighted median
Weighted mode
0.06
0.04
0.02
0.00
0.09
0.12
0.15
0.18
SNP effect on Peroxisome proliferator−activated receptor alpha
SNP effect on Gut microbiota abundance (genus Catenibacterium id.2153)

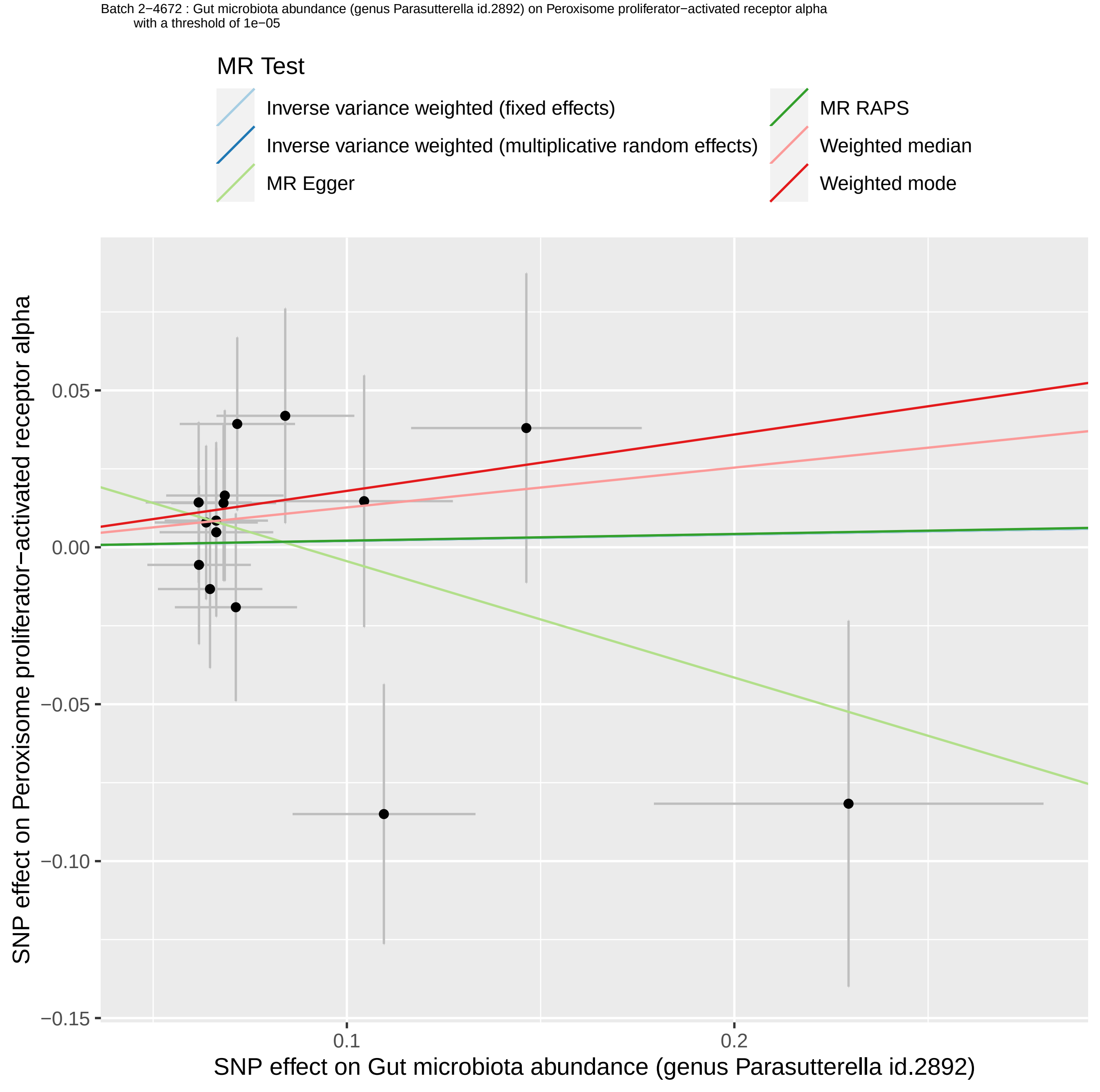

Batch 2−4672 : Gut microbiota abundance (genus Parasutterella id.2892) on Peroxisome proliferator−activated receptor alpha
with a threshold of 1e−05
MR Test
Inverse variance weighted (fixed effects)
Inverse variance weighted (multiplicative random effects)
MR Egger
MR RAPS
Weighted median
Weighted mode
0.05
0.00
−0.05
−0.10
−0.15
0.1
0.2
SNP effect on Peroxisome proliferator−activated receptor alpha
SNP effect on Gut microbiota abundance (genus Parasutterella id.2892)

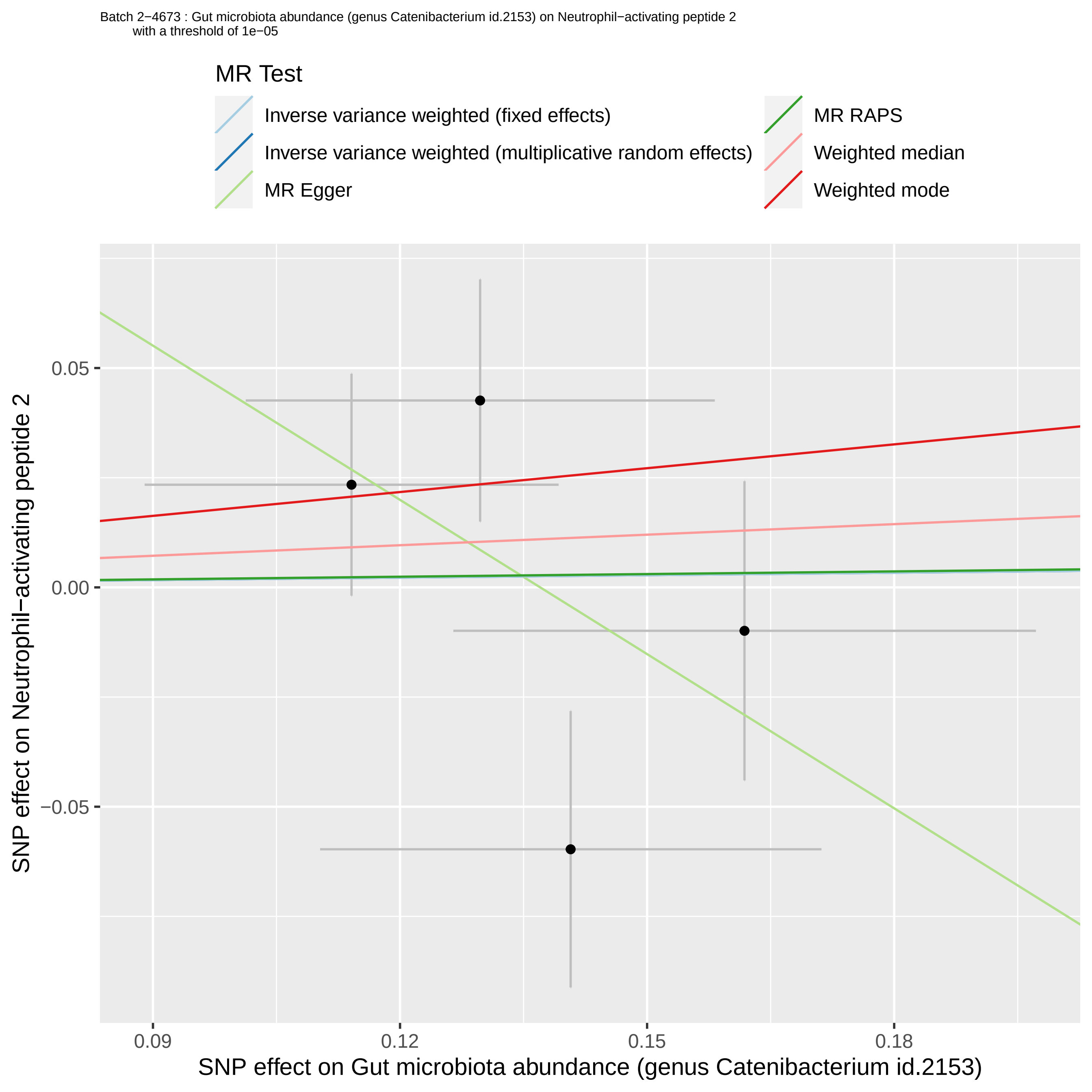
Batch 2−4673 : Gut microbiota abundance (genus Catenibacterium id.2153) on Neutrophil−activating peptide 2
with a threshold of 1e−05
MR Test
Inverse variance weighted (fixed effects)
Inverse variance weighted (multiplicative random effects)
MR Egger
MR RAPS
Weighted median
Weighted mode
0.05
0.00
−0.05
0.09
0.12
0.15
0.18
SNP effect on Neutrophil−activating peptide 2
SNP effect on Gut microbiota abundance (genus Catenibacterium id.2153)

Batch 2−4674 : Gut microbiota abundance (genus Parasutterella id.2892) on Neutrophil−activating peptide 2
with a threshold of 1e−05

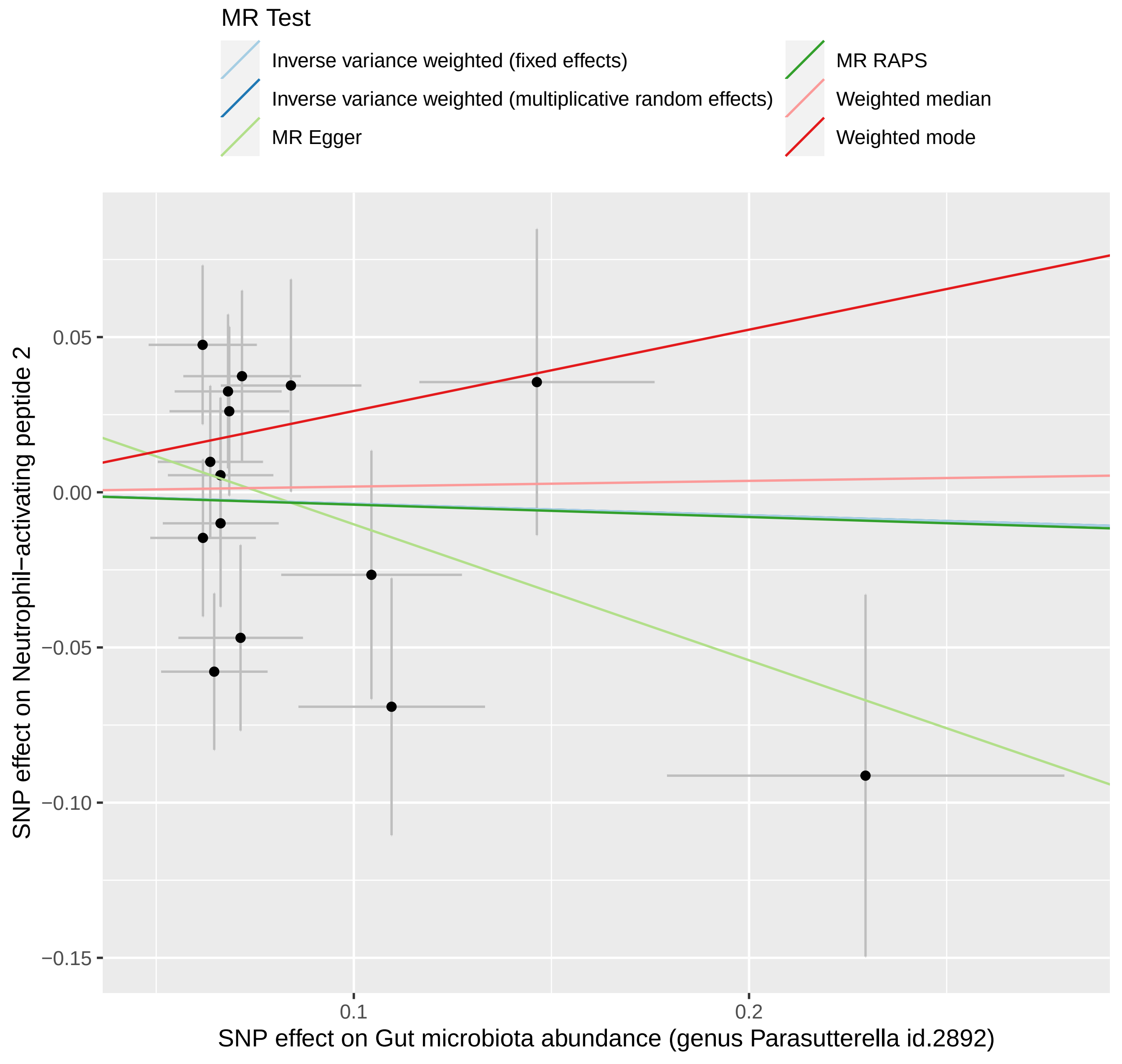

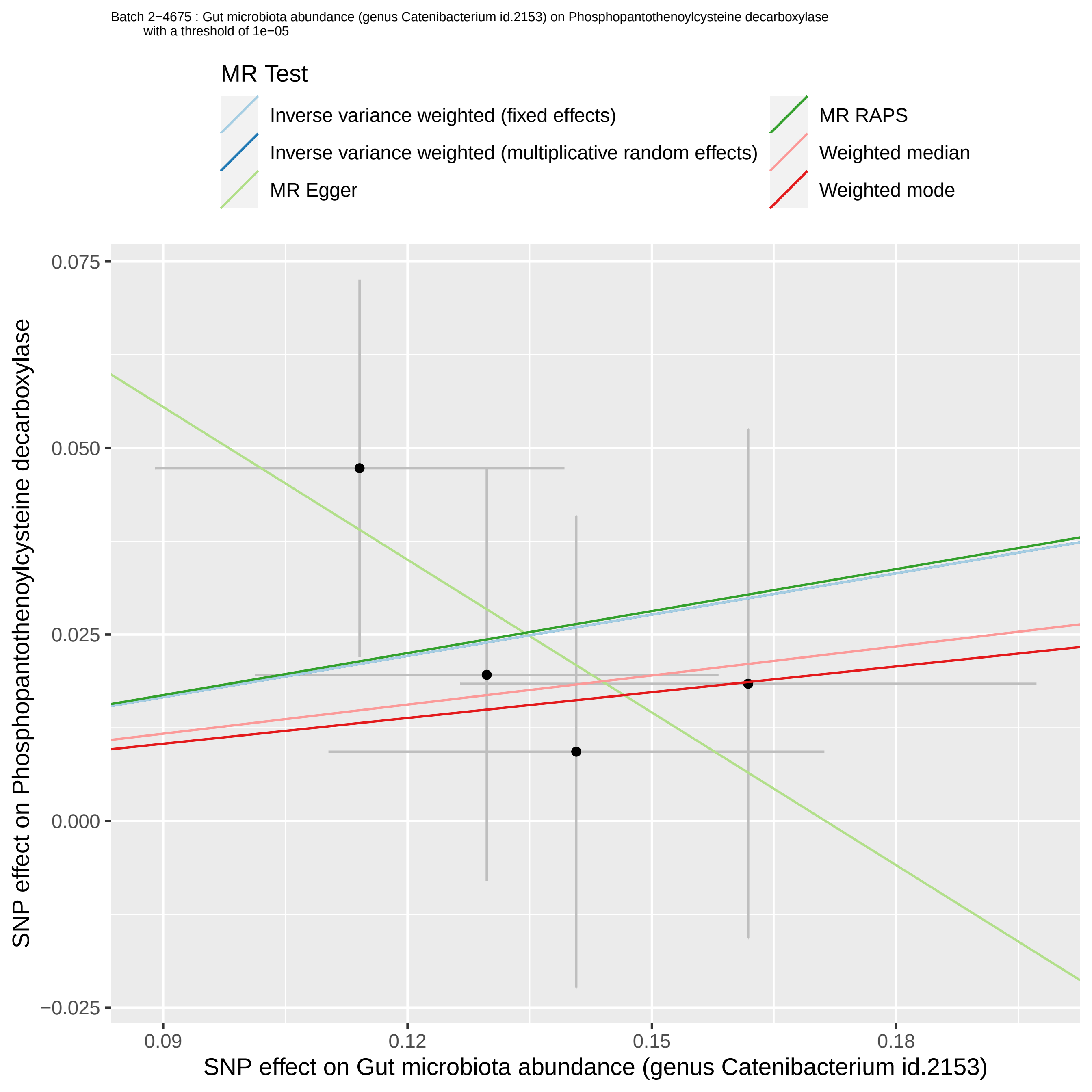
Batch 2−4675 : Gut microbiota abundance (genus Catenibacterium id.2153) on Phosphopantothenoylcysteine decarboxylase
with a threshold of 1e−05
MR Test
Inverse variance weighted (fixed effects)
Inverse variance weighted (multiplicative random effects)
MR Egger
MR RAPS
Weighted median
Weighted mode
SNP effect on Phosphopantothenoylcysteine decarboxylase
0.075
0.050
0.025
0.000
−0.025
0.09
0.12
0.15
0.18
SNP effect on Gut microbiota abundance (genus Catenibacterium id.2153)

Batch 2−4676 : Gut microbiota abundance (genus Parasutterella id.2892) on Phosphopantothenoylcysteine decarboxylase with a threshold of 1e−05

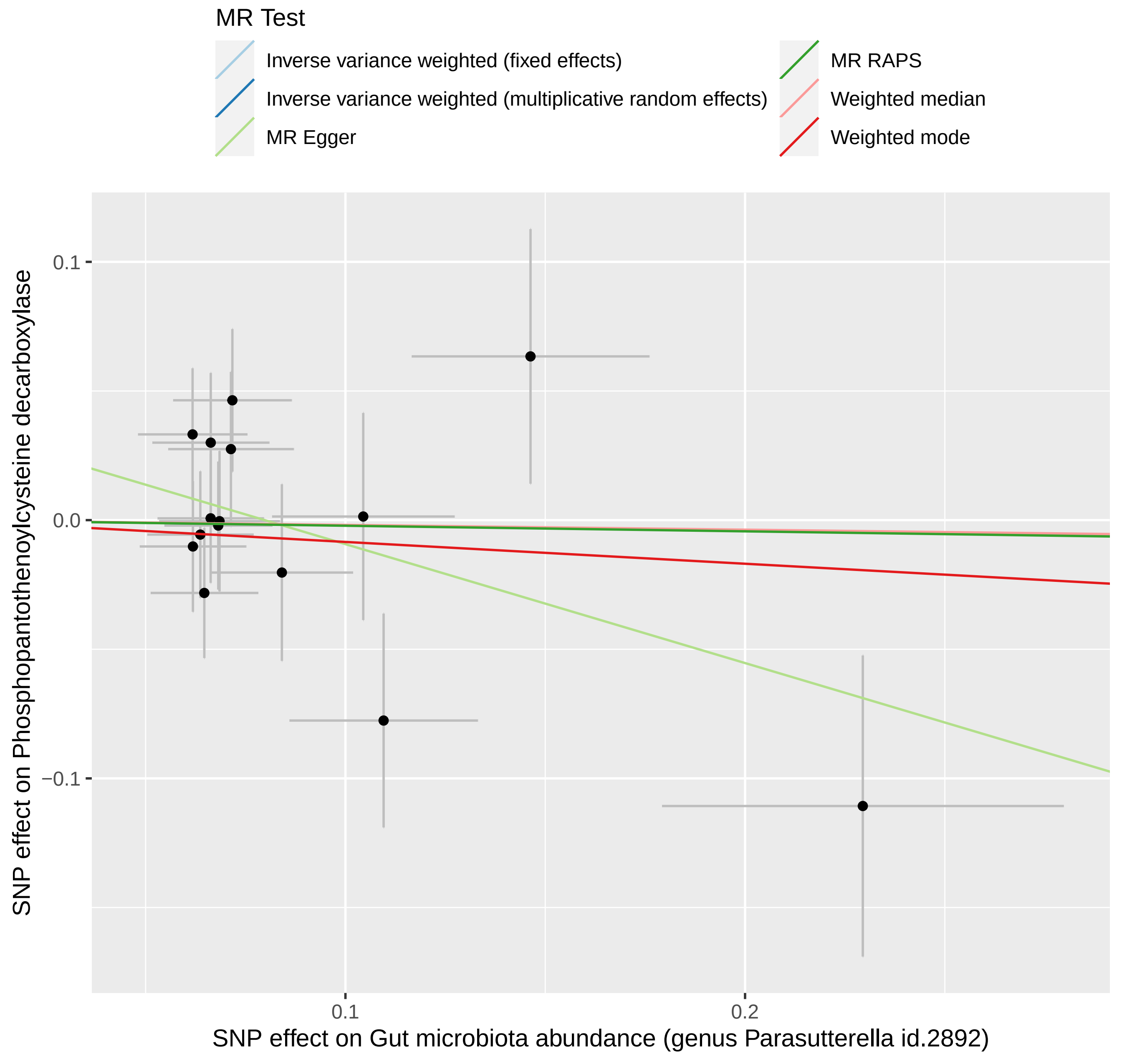

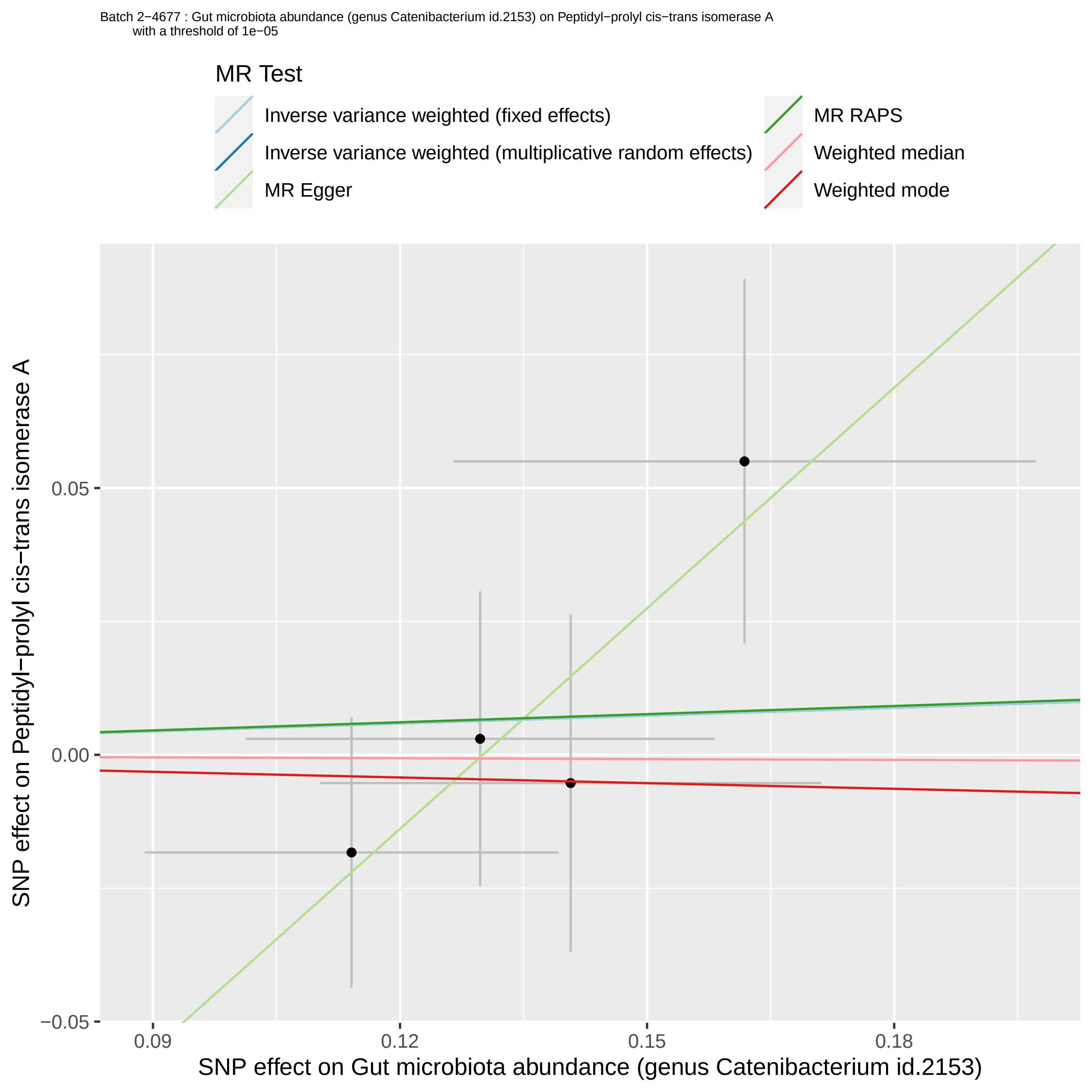

Batch 2−4677 : Gut microbiota abundance (genus Catenibacterium id.2153) on Peptidyl−prolyl cis−trans isomerase A
with a threshold of 1e−05
MR Test
Inverse variance weighted (fixed effects)
Inverse variance weighted (multiplicative random effects)
MR Egger
MR RAPS
Weighted median
Weighted mode
0.05
0.00
−0.05
0.09
0.12
0.15
0.18
SNP effect on Peptidyl−prolyl cis−trans isomerase A
SNP effect on Gut microbiota abundance (genus Catenibacterium id.2153)

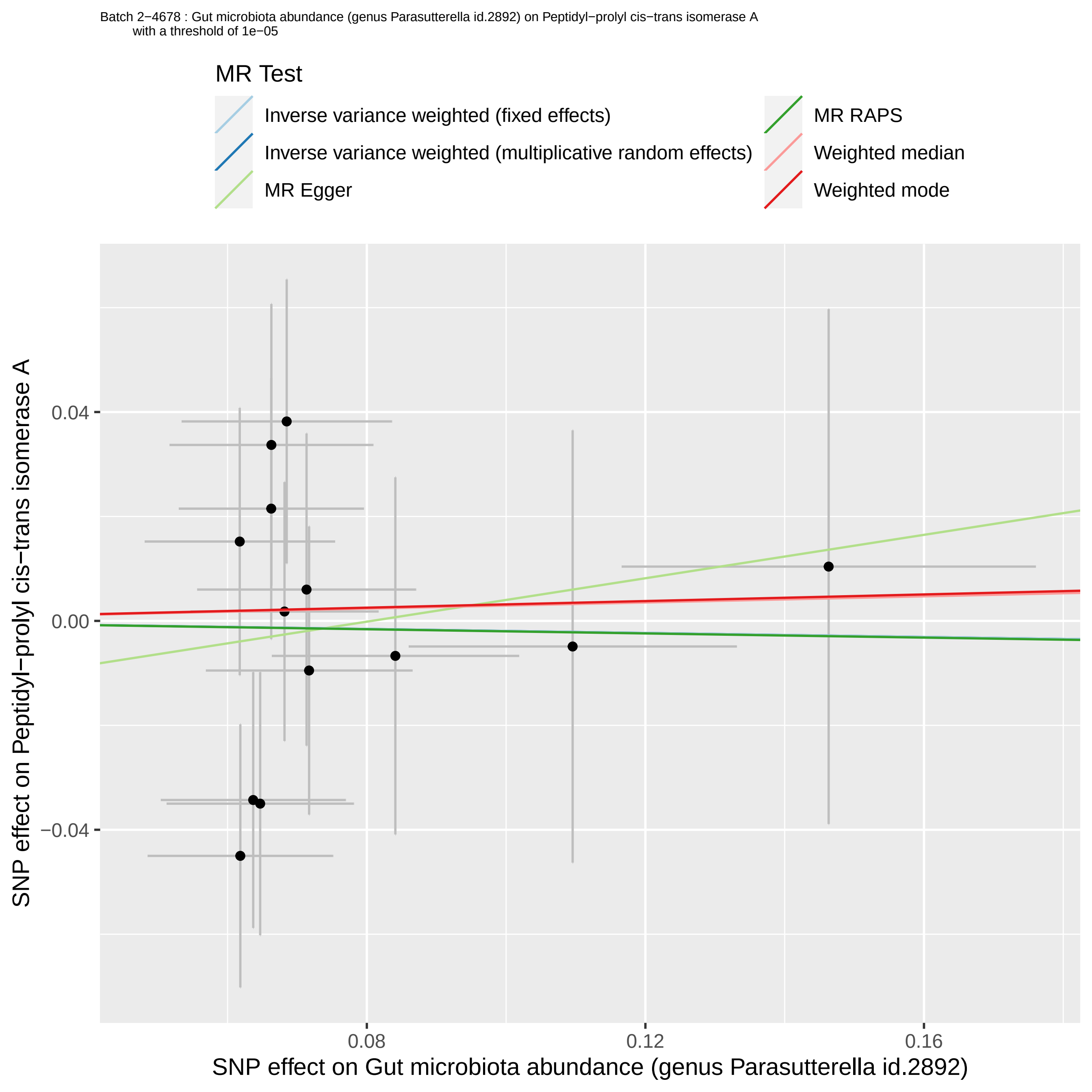

Batch 2−4678 : Gut microbiota abundance (genus Parasutterella id.2892) on Peptidyl−prolyl cis−trans isomerase A
with a threshold of 1e−05
MR Test
Inverse variance weighted (fixed effects)
Inverse variance weighted (multiplicative random effects)
MR Egger
MR RAPS
Weighted median
Weighted mode
0.04
0.00
−0.04
0.08
0.12
0.16
SNP effect on Peptidyl−prolyl cis−trans isomerase A
SNP effect on Gut microbiota abundance (genus Parasutterella id.2892)

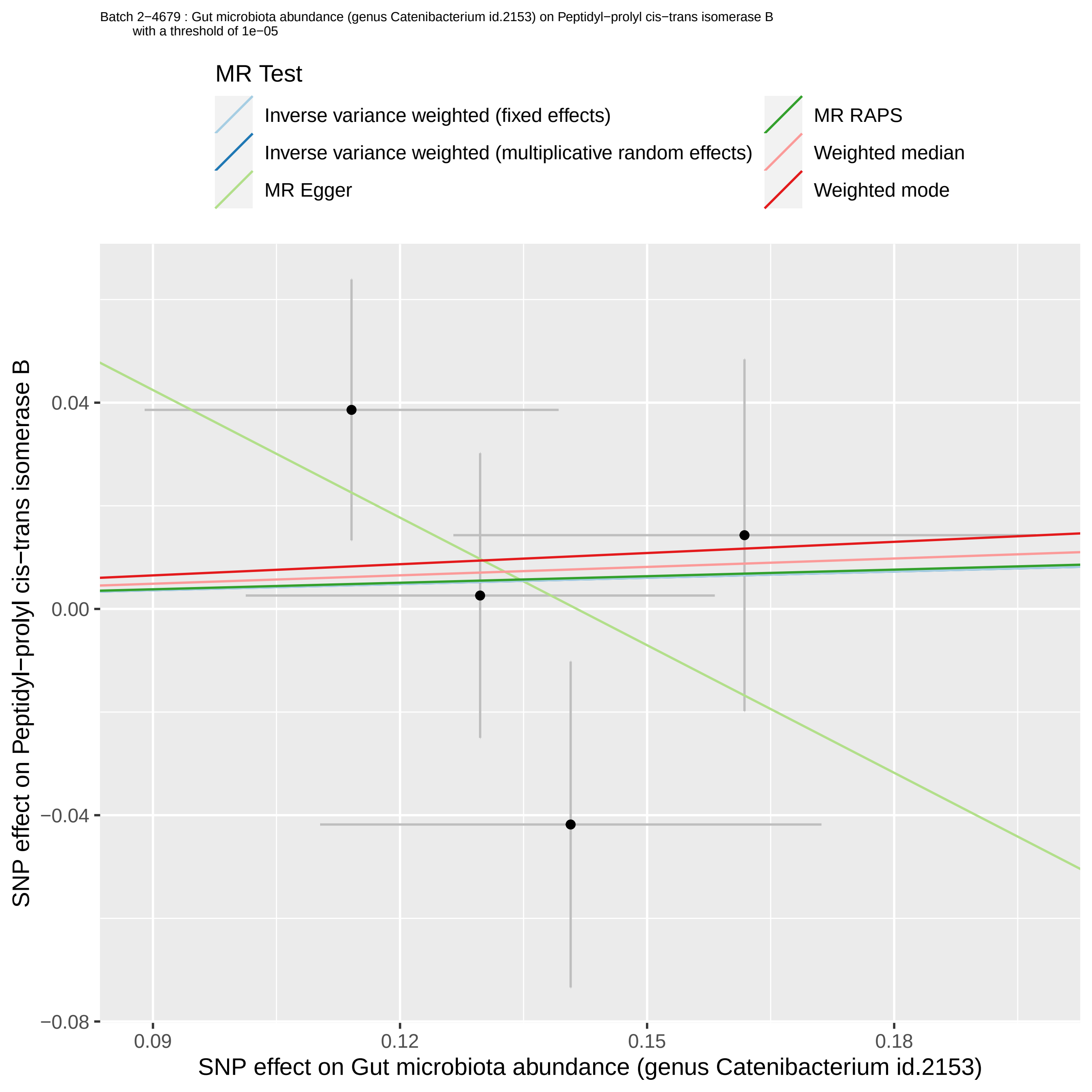
Batch 2−4679 : Gut microbiota abundance (genus Catenibacterium id.2153) on Peptidyl−prolyl cis−trans isomerase B
with a threshold of 1e−05
MR Test
Inverse variance weighted (fixed effects)
Inverse variance weighted (multiplicative random effects)
MR Egger
MR RAPS
Weighted median
Weighted mode
SNP effect on Peptidyl−prolyl cis−trans isomerase B
0.04
0.00
−0.04
−0.08
0.09
0.12
0.15
0.18
SNP effect on Gut microbiota abundance (genus Catenibacterium id.2153)

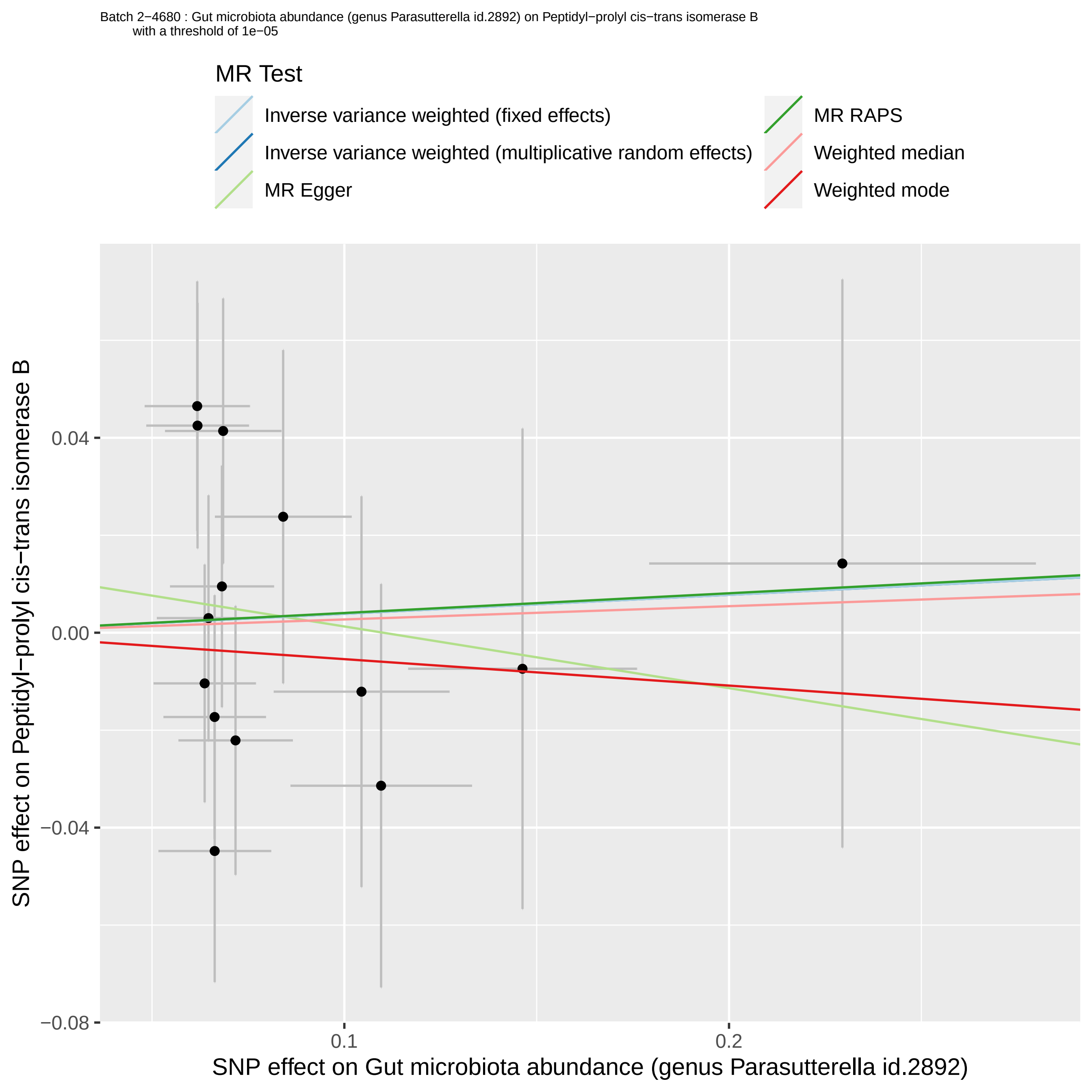

Batch 2−4680 : Gut microbiota abundance (genus Parasutterella id.2892) on Peptidyl−prolyl cis−trans isomerase B
with a threshold of 1e−05
MR Test
Inverse variance weighted (fixed effects)
Inverse variance weighted (multiplicative random effects)
MR Egger
MR RAPS
Weighted median
Weighted mode
0.04
0.00
−0.04
−0.08
0.1
0.2
SNP effect on Peptidyl−prolyl cis−trans isomerase B
SNP effect on Gut microbiota abundance (genus Parasutterella id.2892)

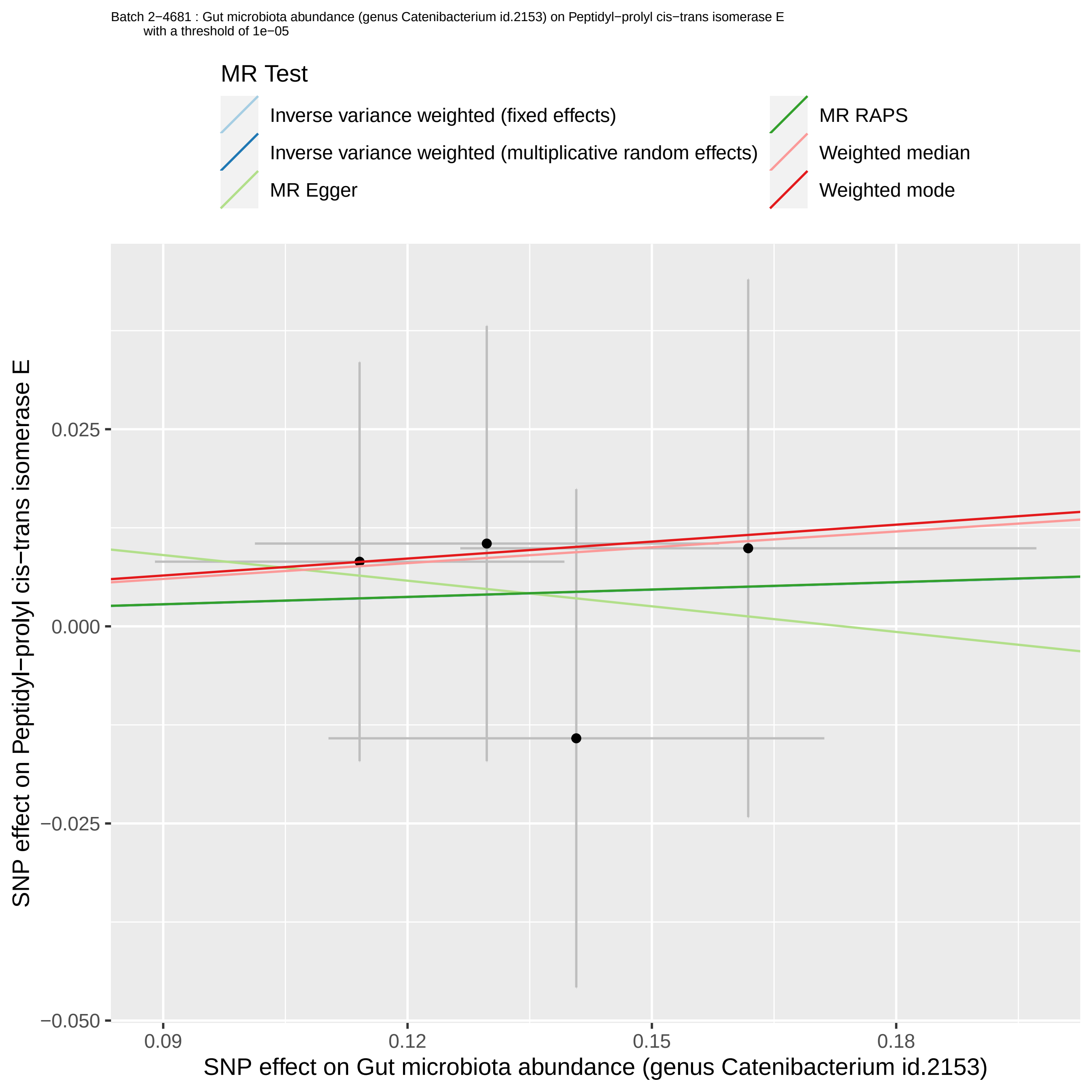

Batch 2−4681 : Gut microbiota abundance (genus Catenibacterium id.2153) on Peptidyl−prolyl cis−trans isomerase E
with a threshold of 1e−05
MR Test
Inverse variance weighted (fixed effects)
Inverse variance weighted (multiplicative random effects)
MR Egger
MR RAPS
Weighted median
Weighted mode
0.025
0.000
−0.025
−0.050
0.09
0.12
0.15
0.18
SNP effect on Peptidyl−prolyl cis−trans isomerase E
SNP effect on Gut microbiota abundance (genus Catenibacterium id.2153)

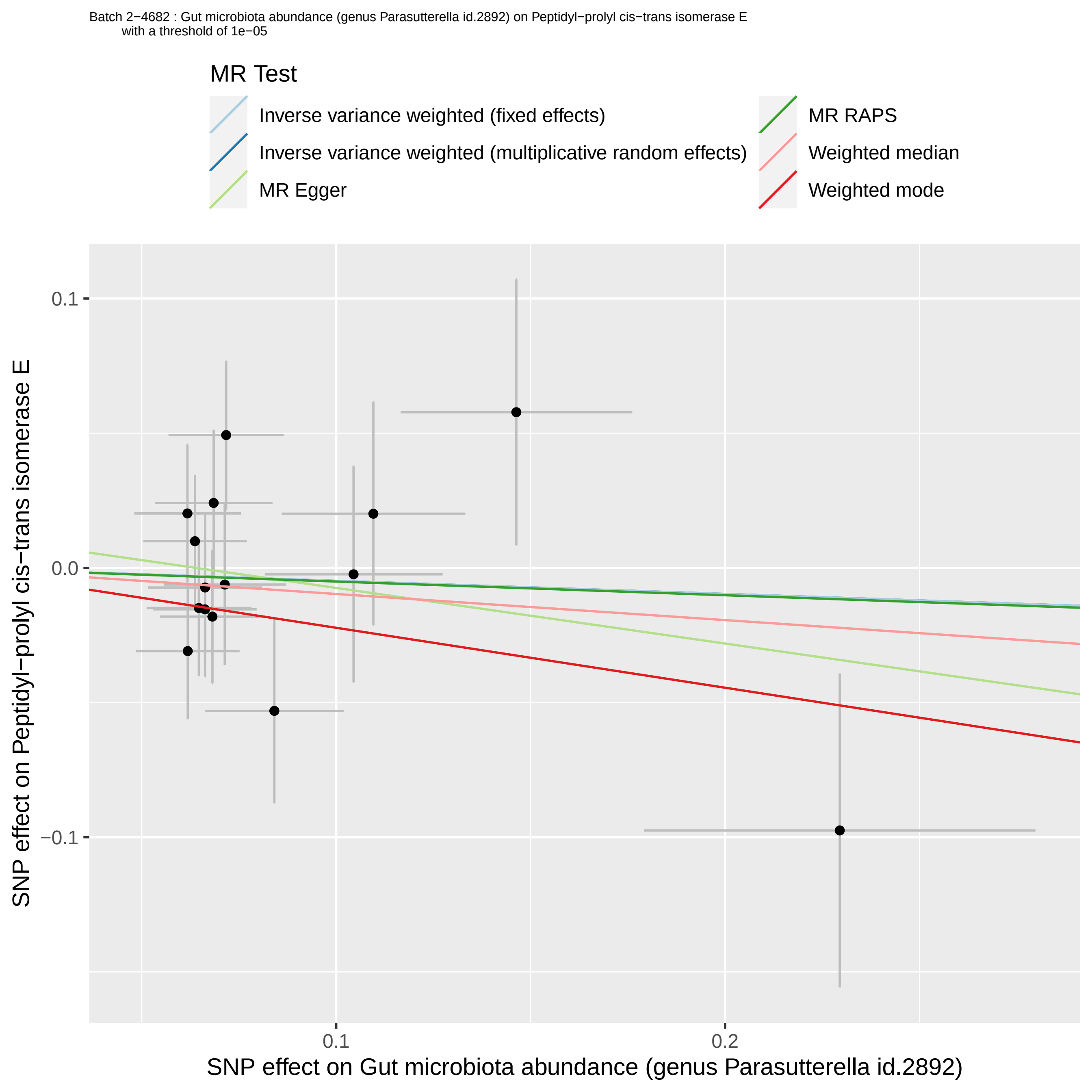
Batch 2−4682 : Gut microbiota abundance (genus Parasutterella id.2892) on Peptidyl−prolyl cis−trans isomerase E
with a threshold of 1e−05
MR Test
Inverse variance weighted (fixed effects)
Inverse variance weighted (multiplicative random effects)
MR Egger
MR RAPS
Weighted median
Weighted mode
0.1
0.0
−0.1
0.1
0.2
SNP effect on Peptidyl−prolyl cis−trans isomerase E
SNP effect on Gut microbiota abundance (genus Parasutterella id.2892)

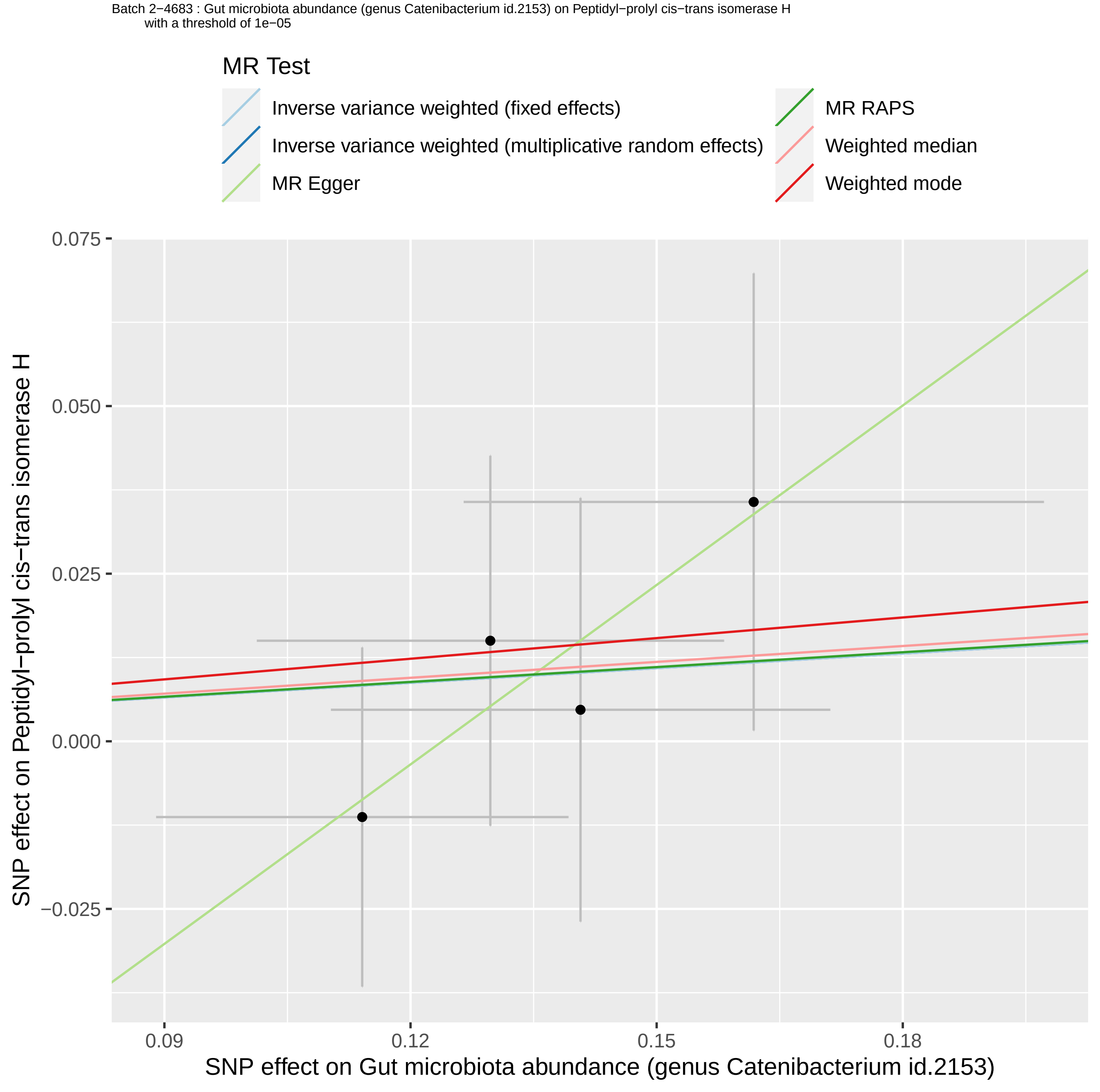
Batch 2−4683 : Gut microbiota abundance (genus Catenibacterium id.2153) on Peptidyl−prolyl cis−trans isomerase H
with a threshold of 1e−05
MR Test
Inverse variance weighted (fixed effects)
Inverse variance weighted (multiplicative random effects)
MR Egger
MR RAPS
Weighted median
Weighted mode
0.075
0.050
0.025
0.000
−0.025
0.09
0.12
0.15
0.18
SNP effect on Peptidyl−prolyl cis−trans isomerase H
SNP effect on Gut microbiota abundance (genus Catenibacterium id.2153)

Batch 2−4684 : Gut microbiota abundance (genus Parasutterella id.2892) on Peptidyl−prolyl cis−trans isomerase H
with a threshold of 1e−05

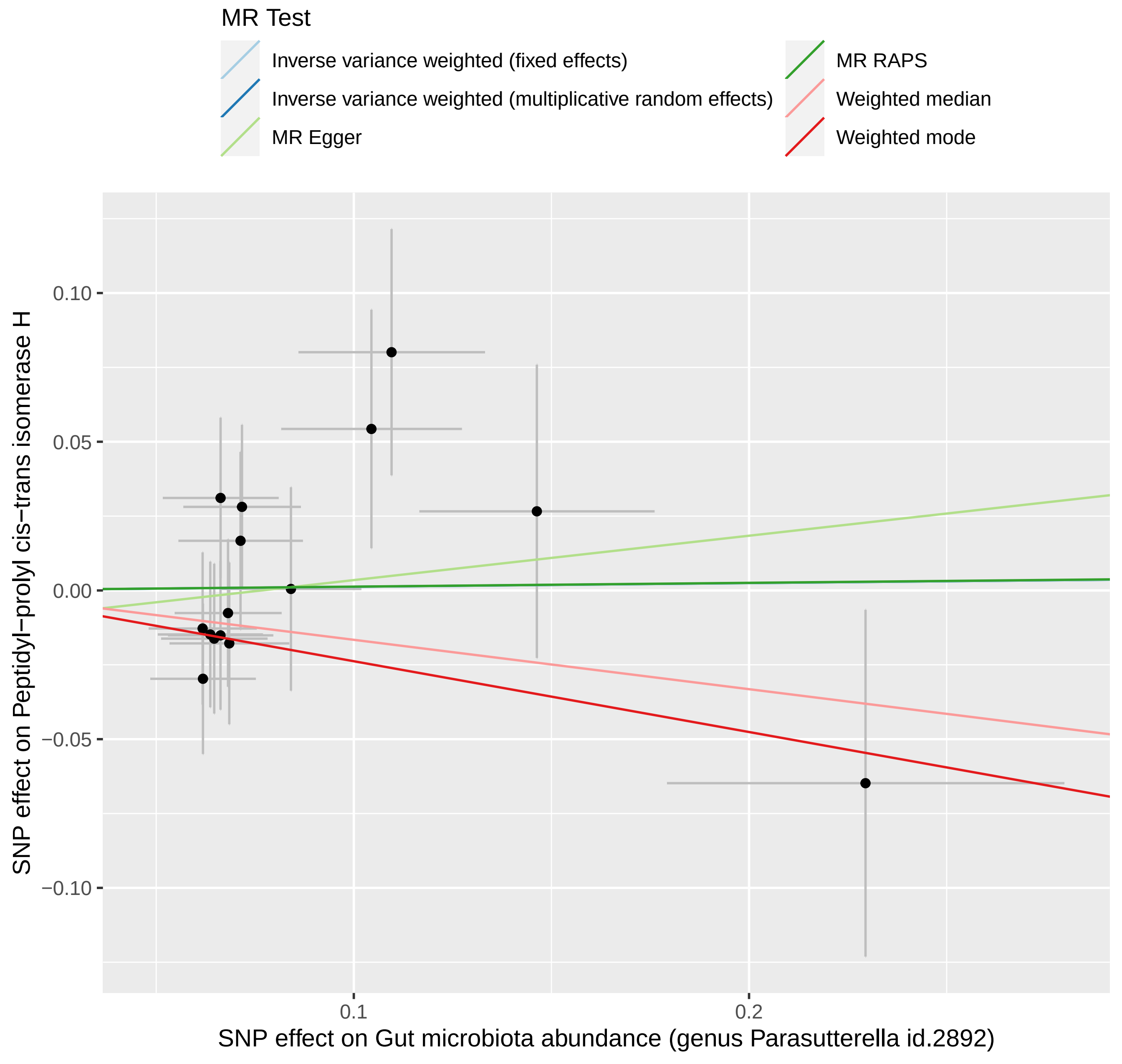

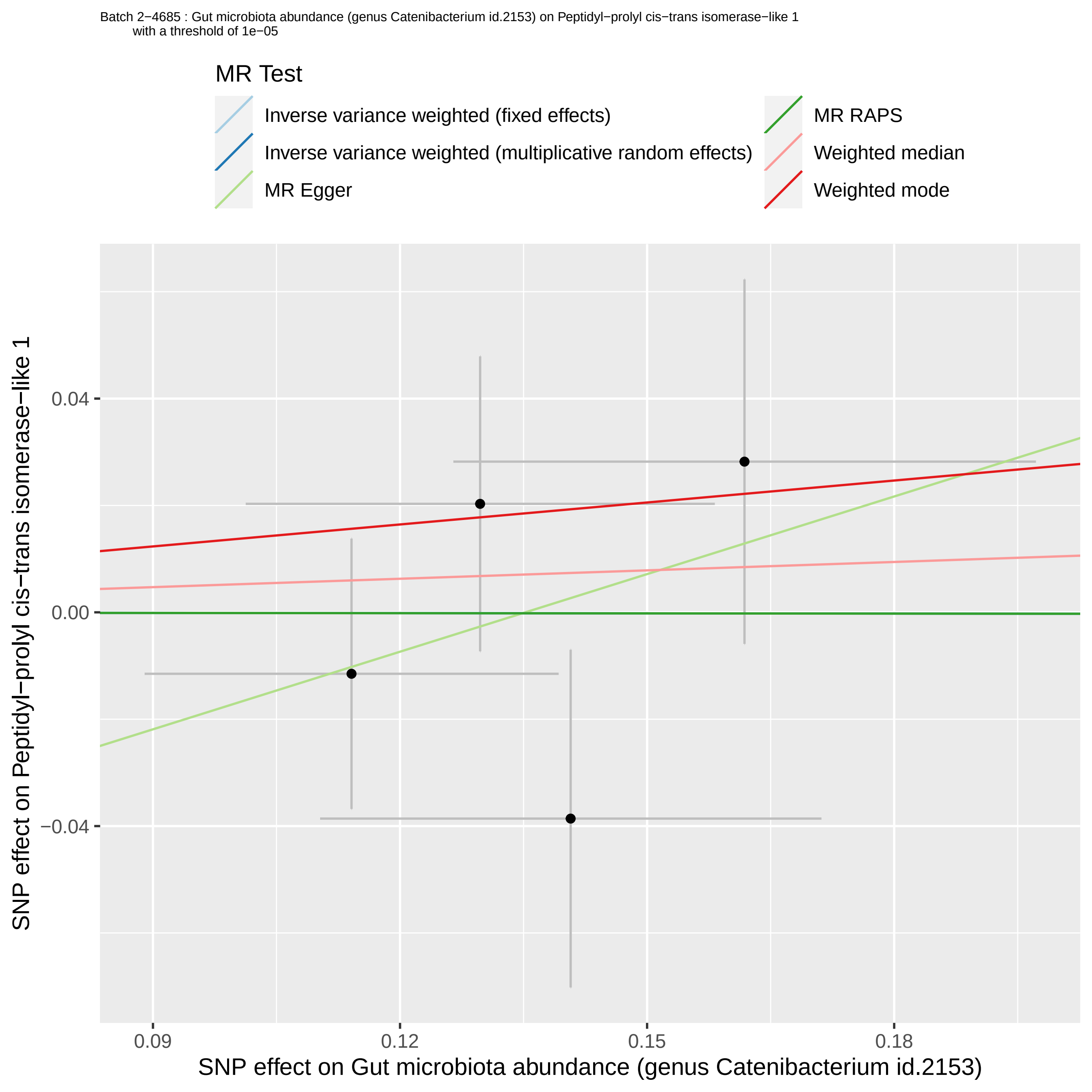
Batch 2−4685 : Gut microbiota abundance (genus Catenibacterium id.2153) on Peptidyl−prolyl cis−trans isomerase−like 1
with a threshold of 1e−05
MR Test
Inverse variance weighted (fixed effects)
Inverse variance weighted (multiplicative random effects)
MR Egger
MR RAPS
Weighted median
Weighted mode
0.04
0.00
−0.04
0.09
0.12
0.15
0.18
SNP effect on Peptidyl−prolyl cis−trans isomerase−like 1
SNP effect on Gut microbiota abundance (genus Catenibacterium id.2153)

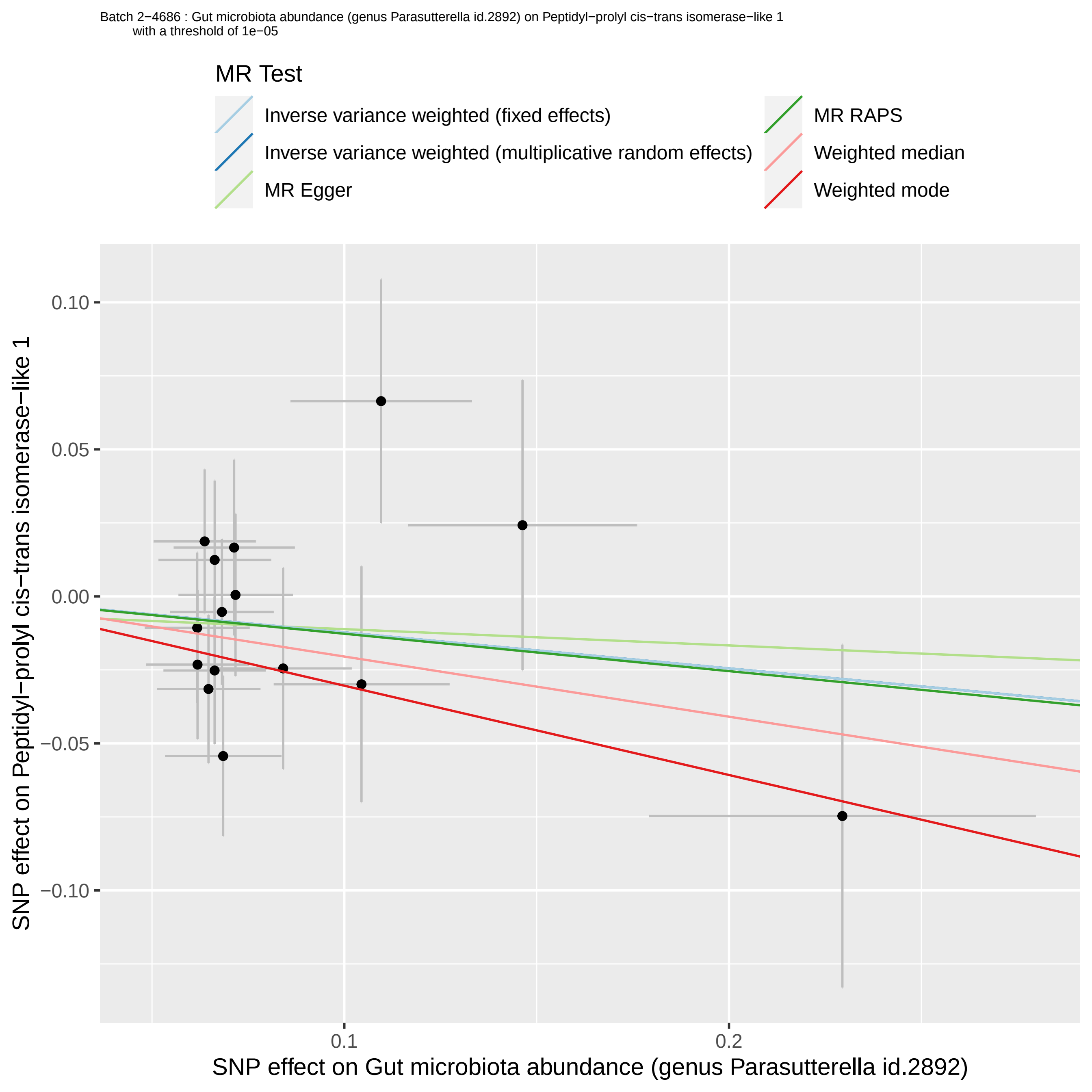

Batch 2−4686 : Gut microbiota abundance (genus Parasutterella id.2892) on Peptidyl−prolyl cis−trans isomerase−like 1
with a threshold of 1e−05
MR Test
Inverse variance weighted (fixed effects)
Inverse variance weighted (multiplicative random effects)
MR Egger
MR RAPS
Weighted median
Weighted mode
0.10
0.05
0.00
−0.05
−0.10
0.1
0.2
SNP effect on Peptidyl−prolyl cis−trans isomerase−like 1
SNP effect on Gut microbiota abundance (genus Parasutterella id.2892)

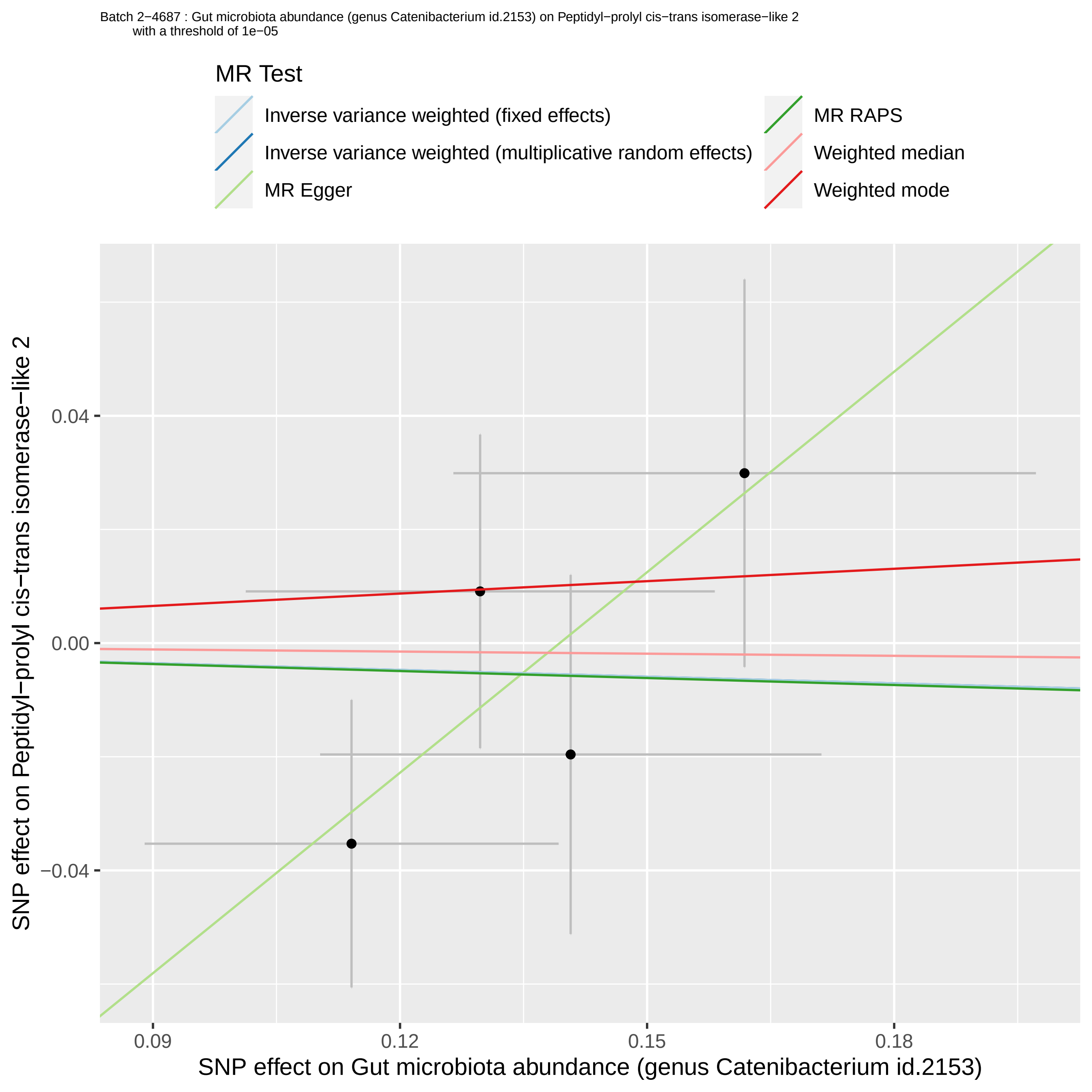

Batch 2−4687 : Gut microbiota abundance (genus Catenibacterium id.2153) on Peptidyl−prolyl cis−trans isomerase−like 2
with a threshold of 1e−05
MR Test
Inverse variance weighted (fixed effects)
Inverse variance weighted (multiplicative random effects)
MR Egger
MR RAPS
Weighted median
Weighted mode
0.04
0.00
−0.04
0.09
0.12
0.15
0.18
SNP effect on Peptidyl−prolyl cis−trans isomerase−like 2
SNP effect on Gut microbiota abundance (genus Catenibacterium id.2153)

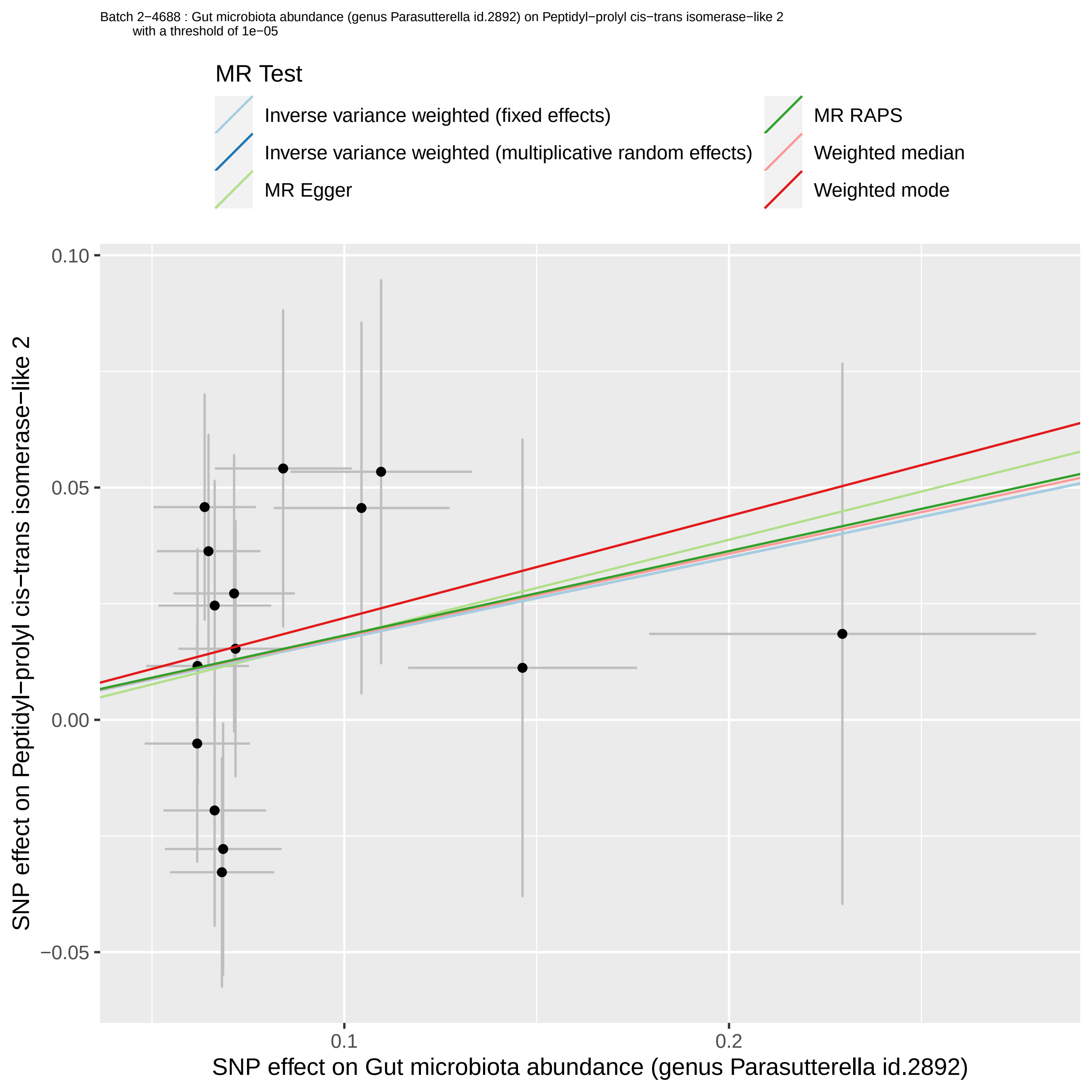
Batch 2−4688 : Gut microbiota abundance (genus Parasutterella id.2892) on Peptidyl−prolyl cis−trans isomerase−like 2
with a threshold of 1e−05
MR Test
Inverse variance weighted (fixed effects)
Inverse variance weighted (multiplicative random effects)
MR Egger
MR RAPS
Weighted median
Weighted mode
0.10
0.05
0.00
−0.05
0.1
0.2
SNP effect on Peptidyl−prolyl cis−trans isomerase−like 2
SNP effect on Gut microbiota abundance (genus Parasutterella id.2892)

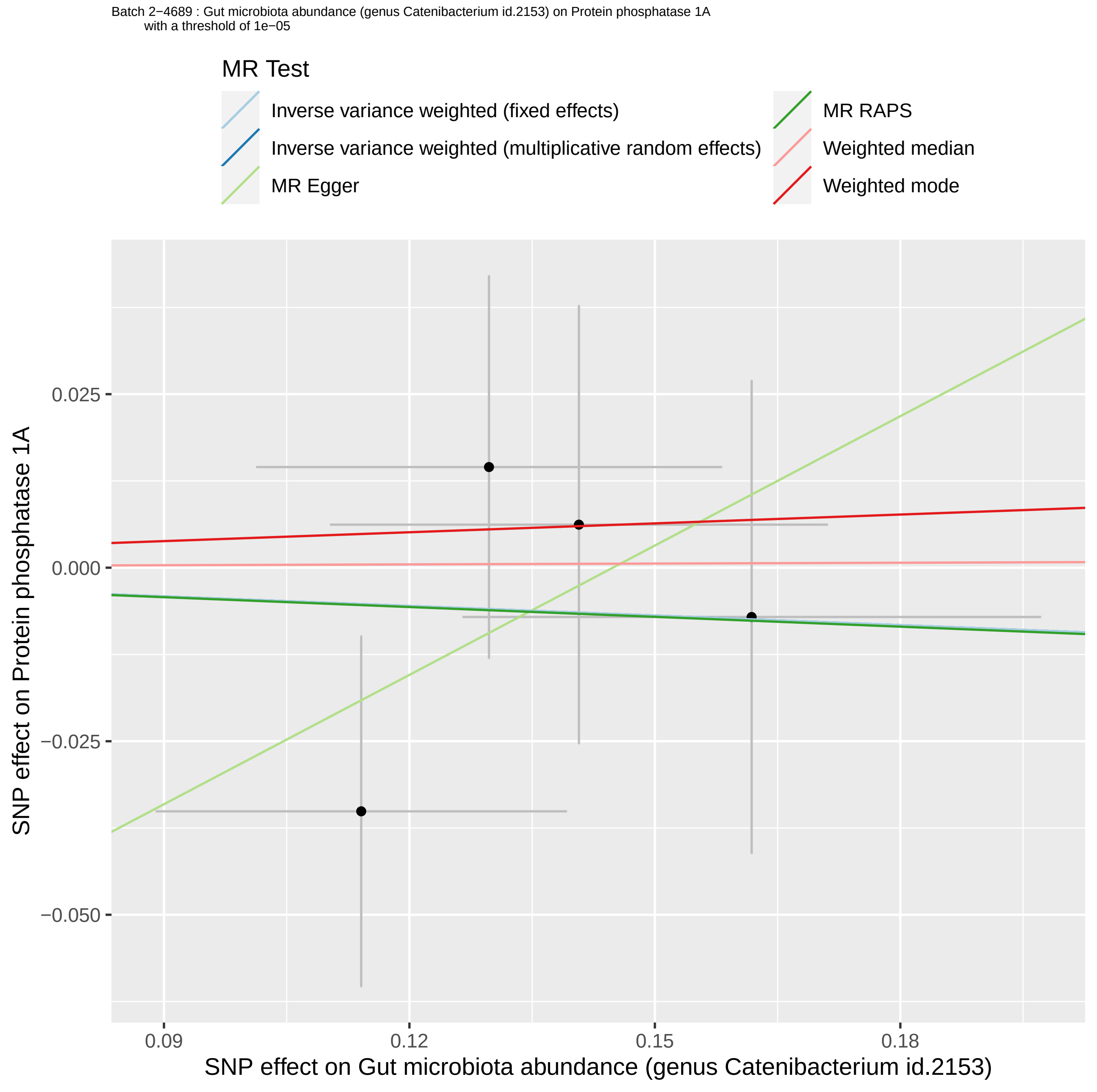
Batch 2−4689 : Gut microbiota abundance (genus Catenibacterium id.2153) on Protein phosphatase 1A
with a threshold of 1e−05
MR Test
Inverse variance weighted (fixed effects)
Inverse variance weighted (multiplicative random effects)
MR Egger
MR RAPS
Weighted median
Weighted mode
0.025
0.000
−0.025
−0.050
0.09
0.12
0.15
0.18
SNP effect on Protein phosphatase 1A
SNP effect on Gut microbiota abundance (genus Catenibacterium id.2153)

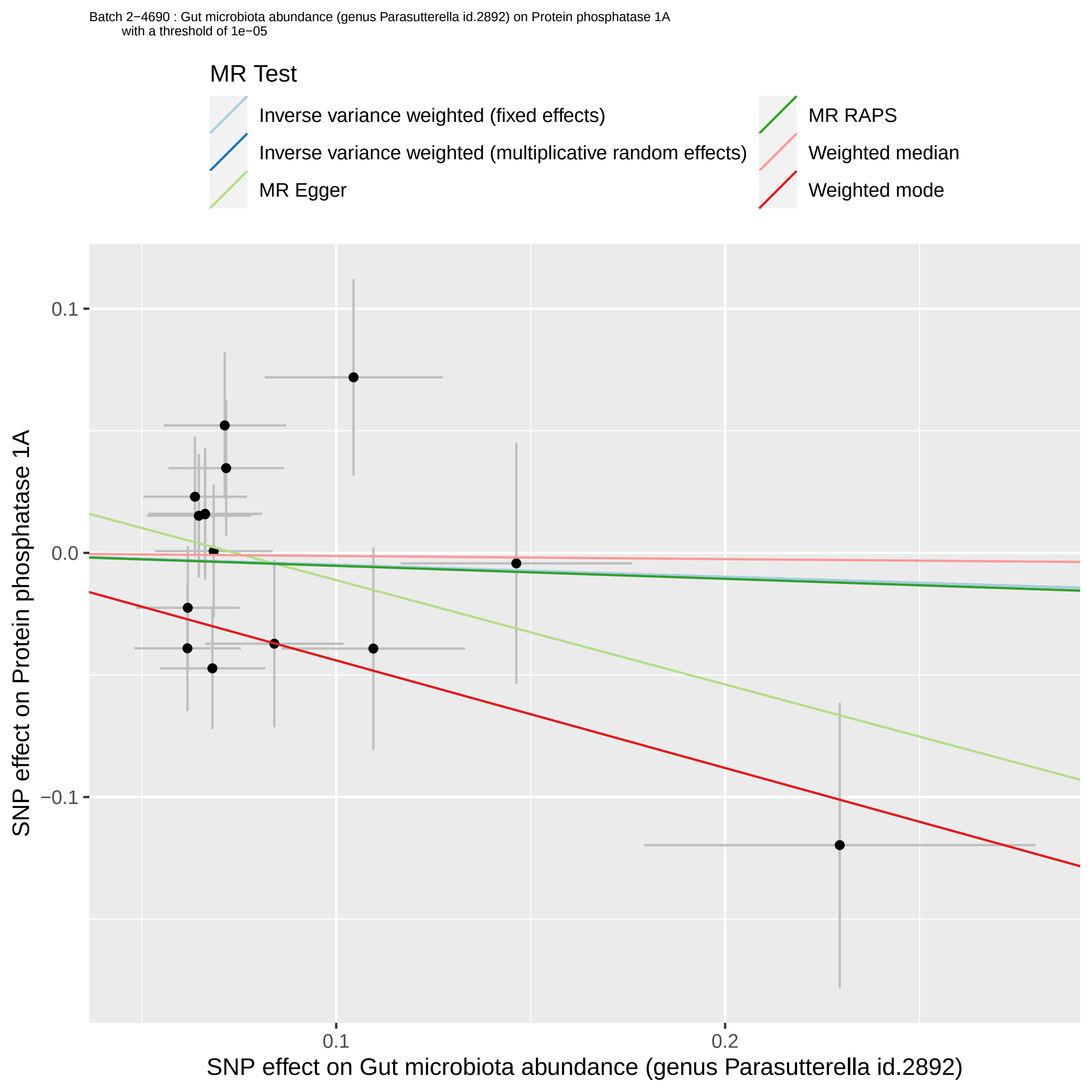

Batch 2−4690 : Gut microbiota abundance (genus Parasutterella id.2892) on Protein phosphatase 1A
with a threshold of 1e−05
MR Test
Inverse variance weighted (fixed effects)
Inverse variance weighted (multiplicative random effects)
MR Egger
MR RAPS
Weighted median
Weighted mode
0.1
0.0
−0.1
0.1
0.2
SNP effect on Protein phosphatase 1A
SNP effect on Gut microbiota abundance (genus Parasutterella id.2892)

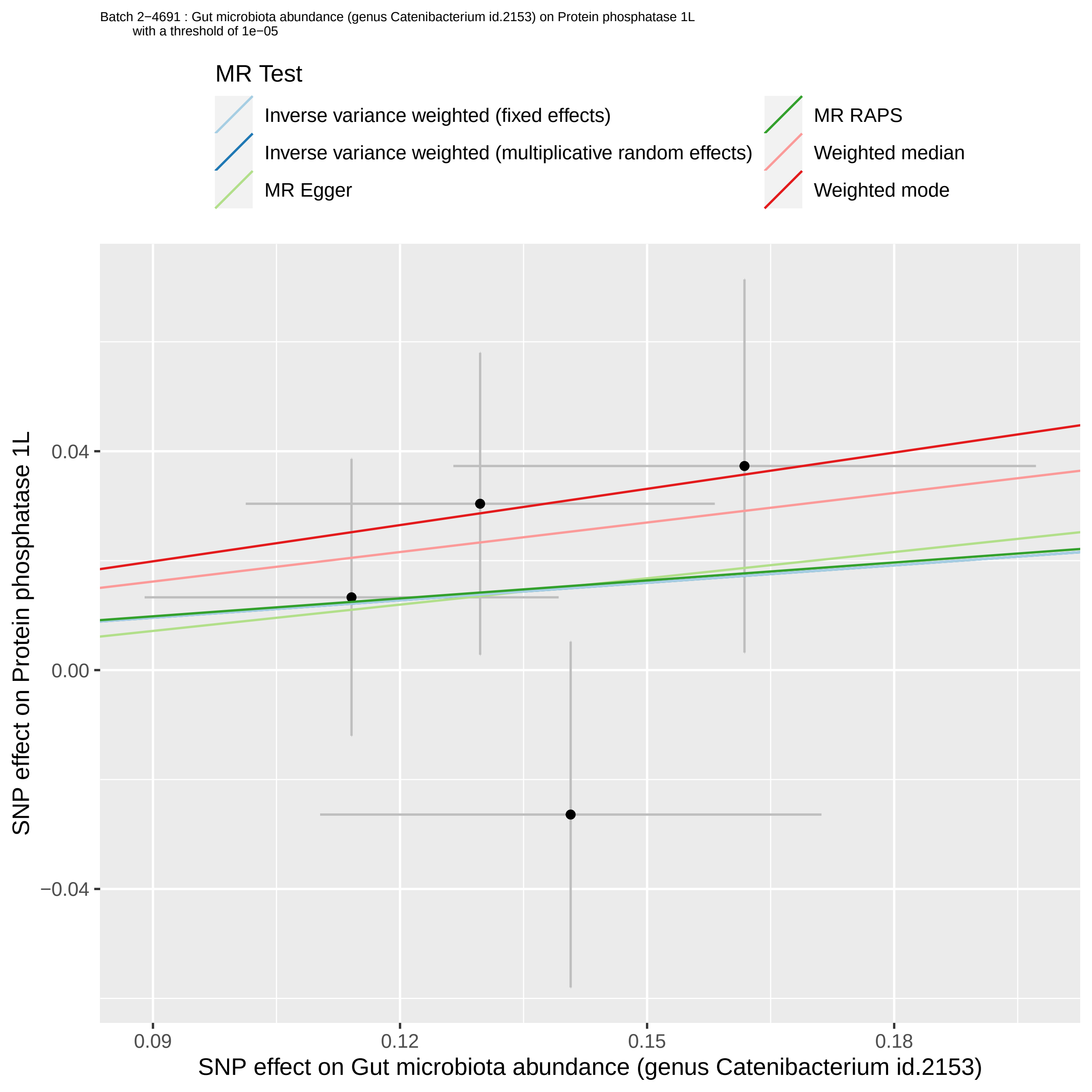

Batch 2−4691 : Gut microbiota abundance (genus Catenibacterium id.2153) on Protein phosphatase 1L
with a threshold of 1e−05
MR Test
Inverse variance weighted (fixed effects)
Inverse variance weighted (multiplicative random effects)
MR Egger
MR RAPS
Weighted median
Weighted mode
SNP effect on Protein phosphatase 1L
0.04
0.00
−0.04
0.09
0.12
0.15
0.18
SNP effect on Gut microbiota abundance (genus Catenibacterium id.2153)

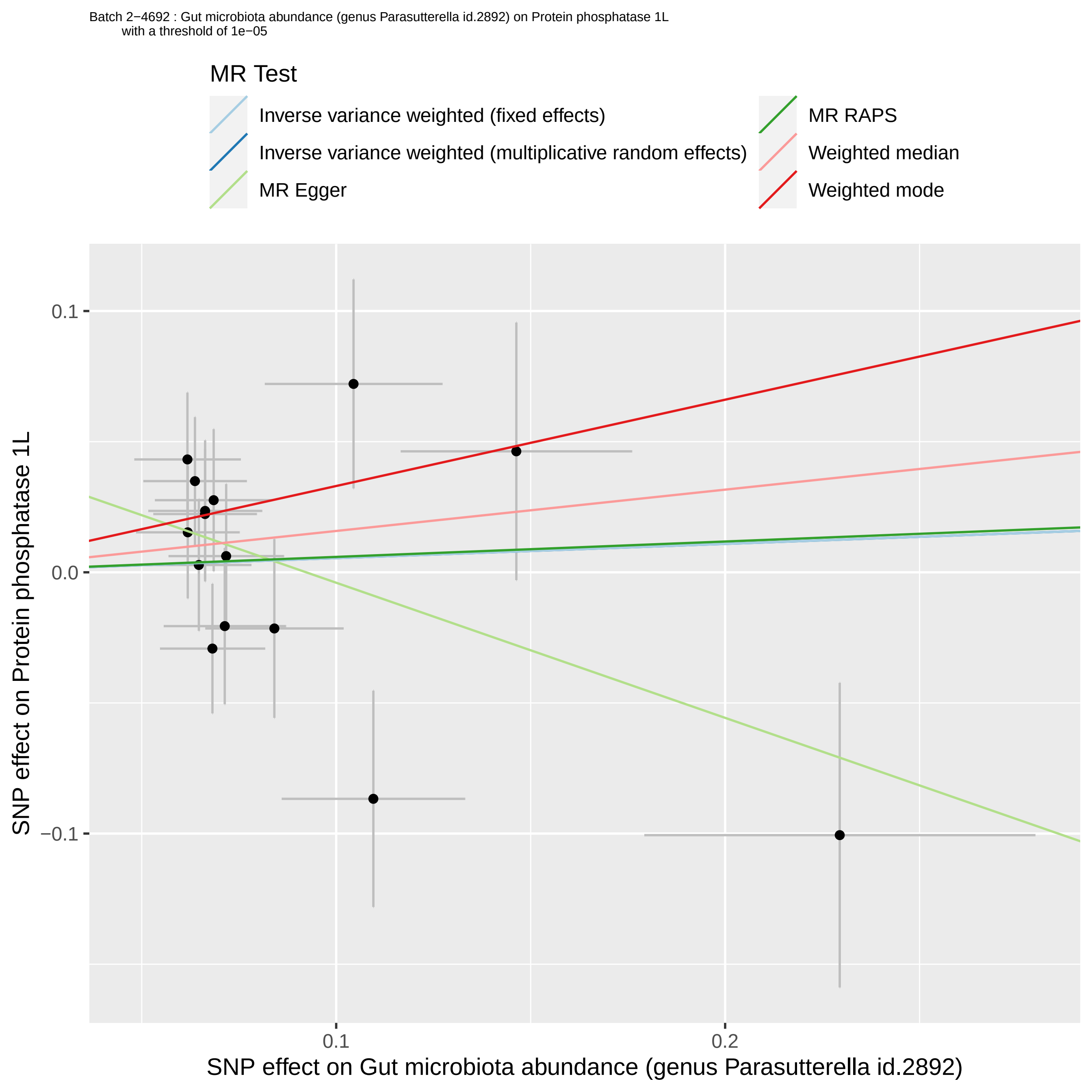
Batch 2−4692 : Gut microbiota abundance (genus Parasutterella id.2892) on Protein phosphatase 1L
with a threshold of 1e−05
MR Test
Inverse variance weighted (fixed effects)
Inverse variance weighted (multiplicative random effects)
MR Egger
MR RAPS
Weighted median
Weighted mode
0.1
0.0
−0.1
0.1
0.2
SNP effect on Protein phosphatase 1L
SNP effect on Gut microbiota abundance (genus Parasutterella id.2892)

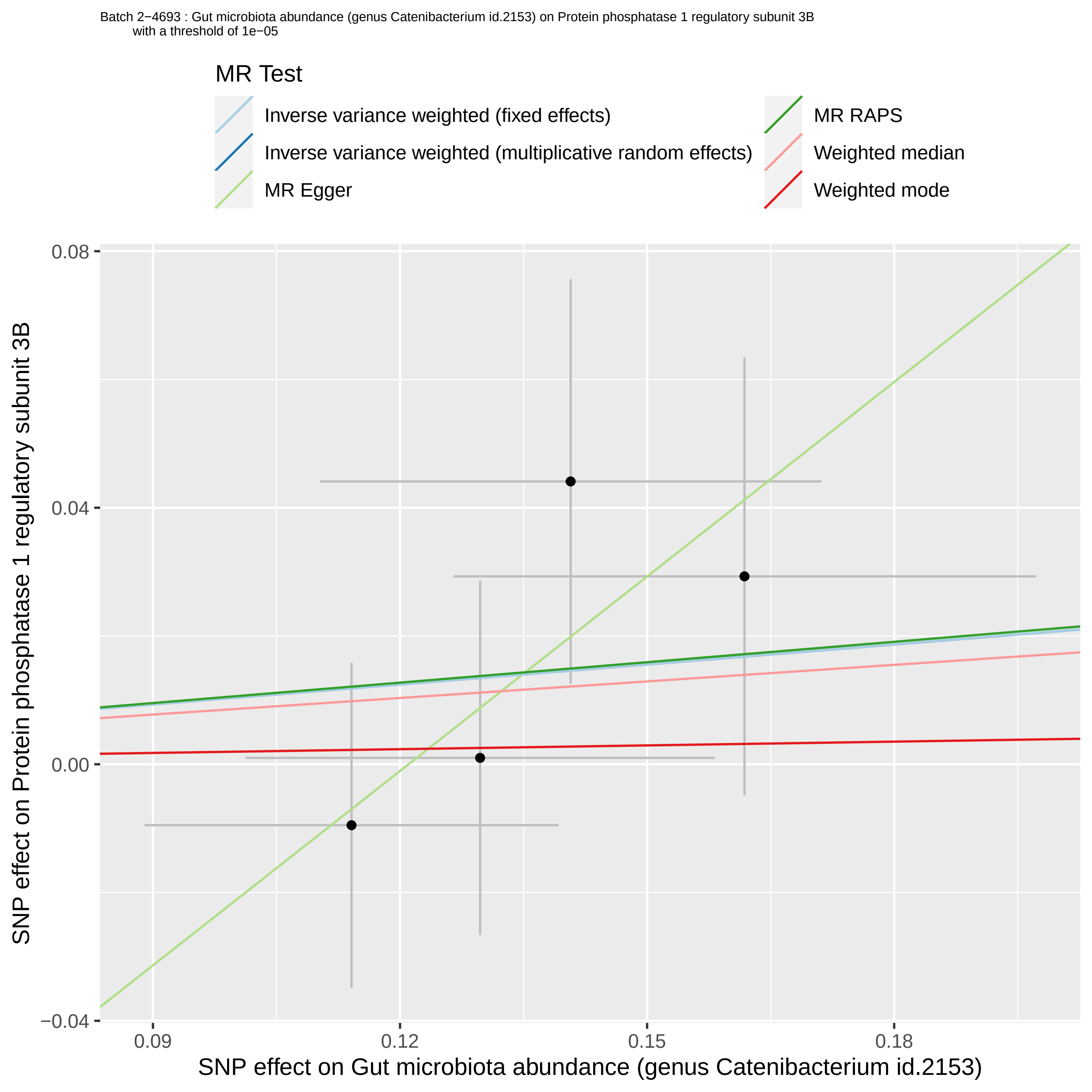
Batch 2−4693 : Gut microbiota abundance (genus Catenibacterium id.2153) on Protein phosphatase 1 regulatory subunit 3B
with a threshold of 1e−05
MR Test
Inverse variance weighted (fixed effects)
Inverse variance weighted (multiplicative random effects)
MR Egger
MR RAPS
Weighted median
Weighted mode
0.08
0.04
0.00
−0.04
0.09
0.12
0.15
0.18
SNP effect on Protein phosphatase 1 regulatory subunit 3B
SNP effect on Gut microbiota abundance (genus Catenibacterium id.2153)

Batch 2−4694 : Gut microbiota abundance (genus Parasutterella id.2892) on Protein phosphatase 1 regulatory subunit 3B
with a threshold of 1e−05

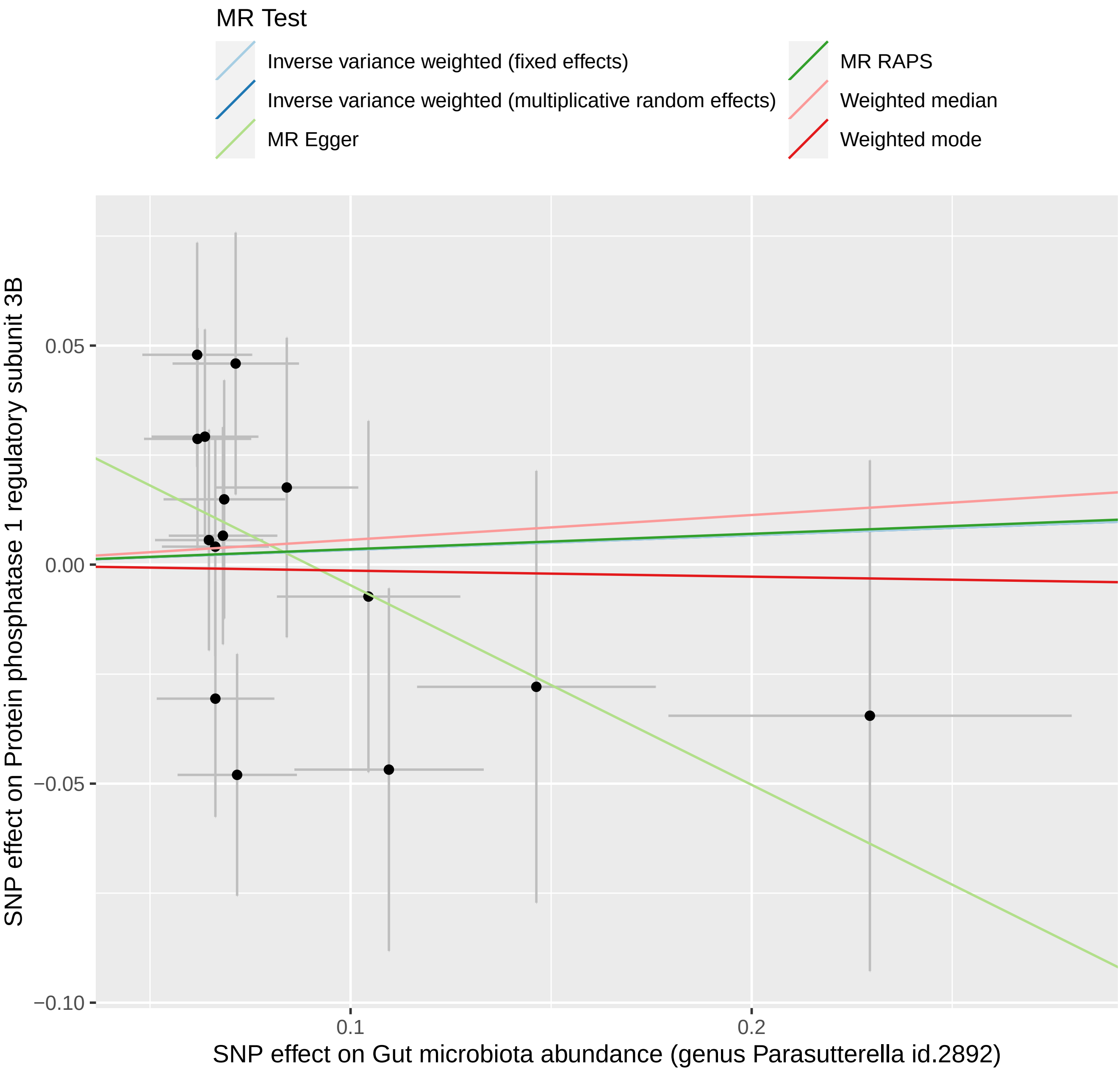

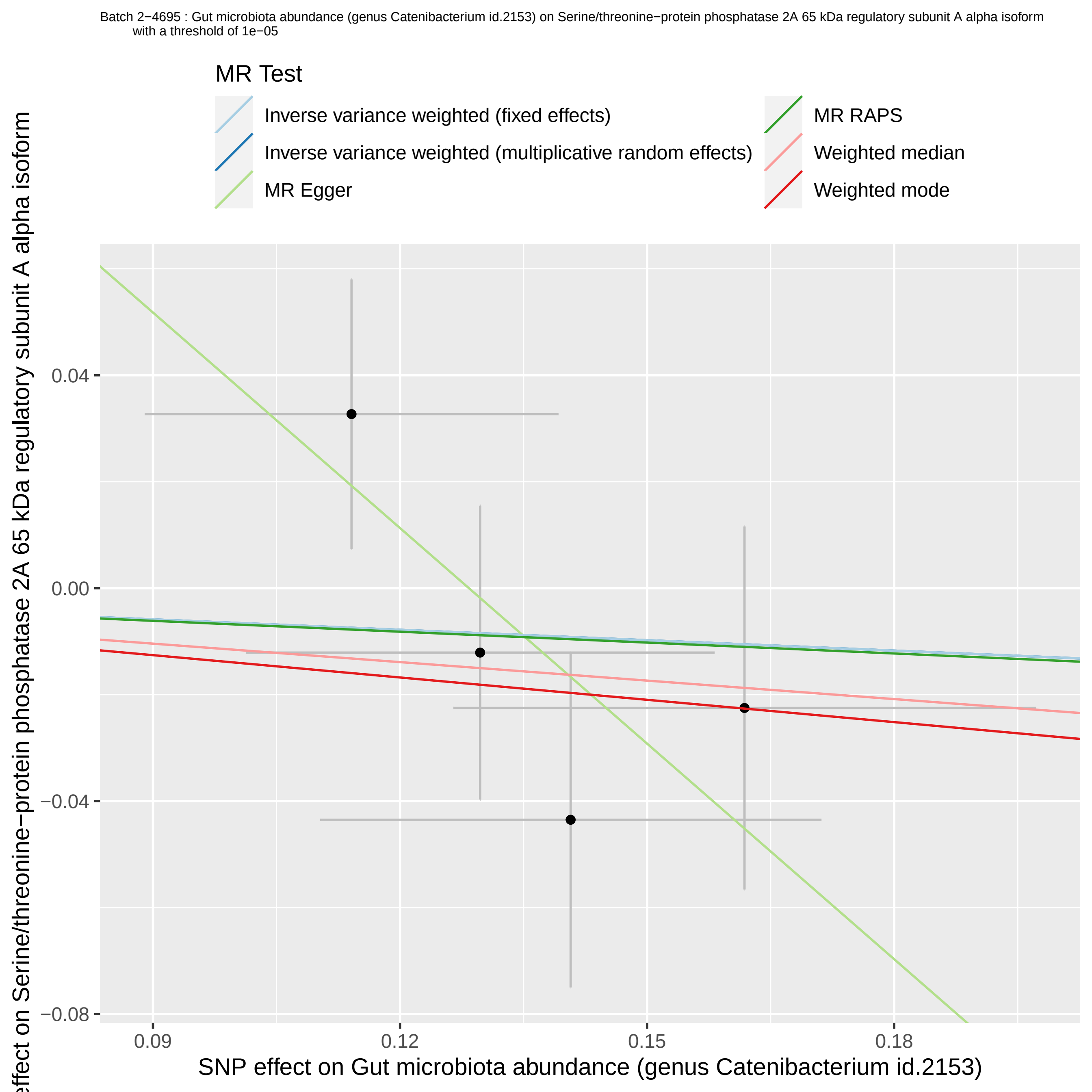
Batch 2−4695 : Gut microbiota abundance (genus Catenibacterium id.2153) on Serine/threonine−protein phosphatase 2A 65 kDa regulatory subunit A alpha isoform with a threshold of 1e−05
MR Test
Inverse variance weighted (fixed effects)
Inverse variance weighted (multiplicative random effects)
MR Egger
MR RAPS
Weighted median
Weighted mode
0.04
0.00
−0.04
−0.08
0.09
0.12
0.15
0.18
SNP effect on Gut microbiota abundance (genus Catenibacterium id.2153)
effect on Serine/threonine−protein phosphatase 2A 65 kDa regulatory subunit A alpha isoform

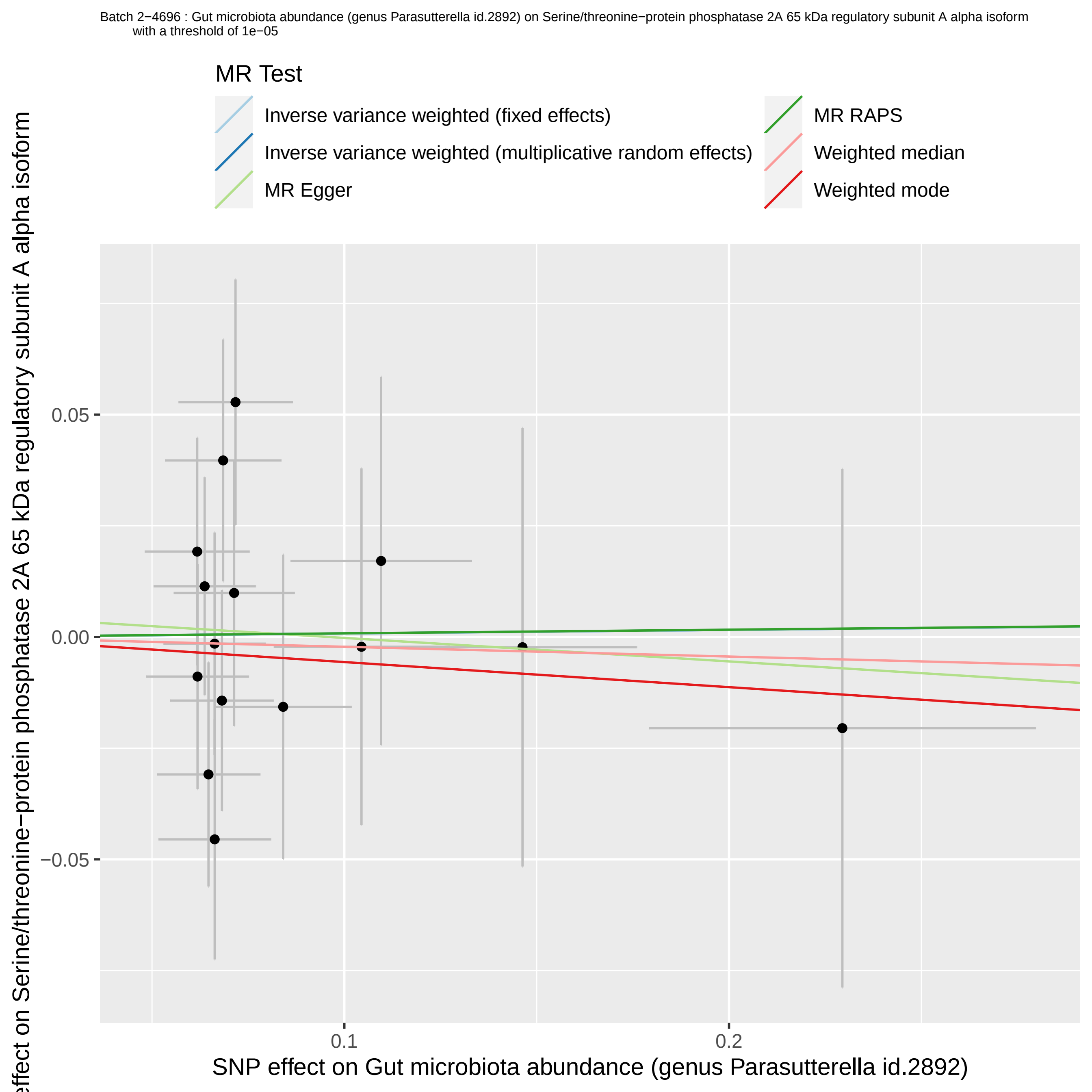
Batch 2−4696 : Gut microbiota abundance (genus Parasutterella id.2892) on Serine/threonine−protein phosphatase 2A 65 kDa regulatory subunit A alpha isoform with a threshold of 1e−05
MR Test
Inverse variance weighted (fixed effects)
Inverse variance weighted (multiplicative random effects)
MR Egger
MR RAPS
Weighted median
Weighted mode
0.05
0.00
−0.05
0.1
0.2
SNP effect on Gut microbiota abundance (genus Parasutterella id.2892)
effect on Serine/threonine−protein phosphatase 2A 65 kDa regulatory subunit A alpha isoform

Batch 2−4697 : Gut microbiota abundance (genus Catenibacterium id.2153) on Serine/threonine−protein phosphatase 2A regulatory subunit B" subunit alpha
with a threshold of 1e−05

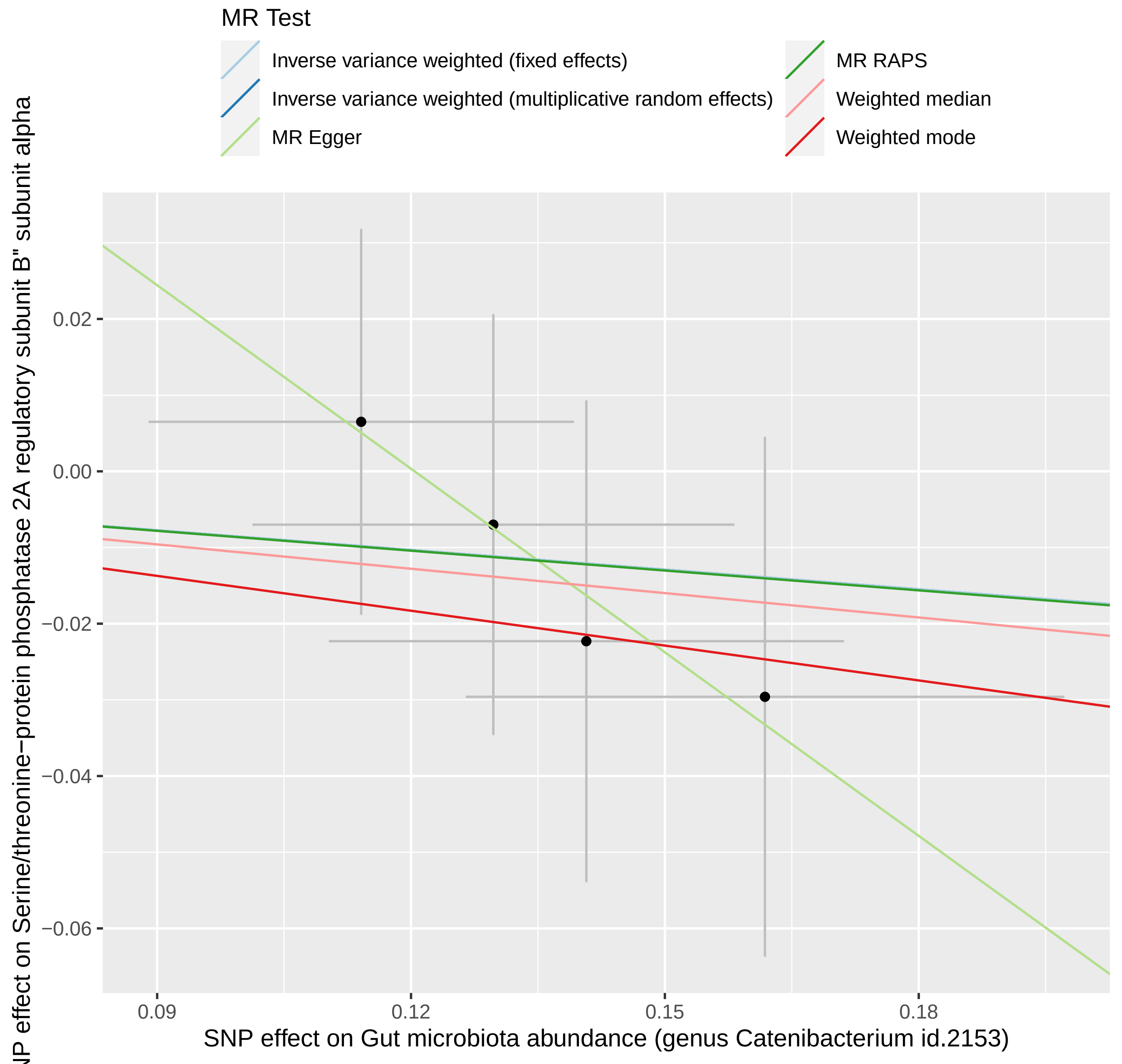

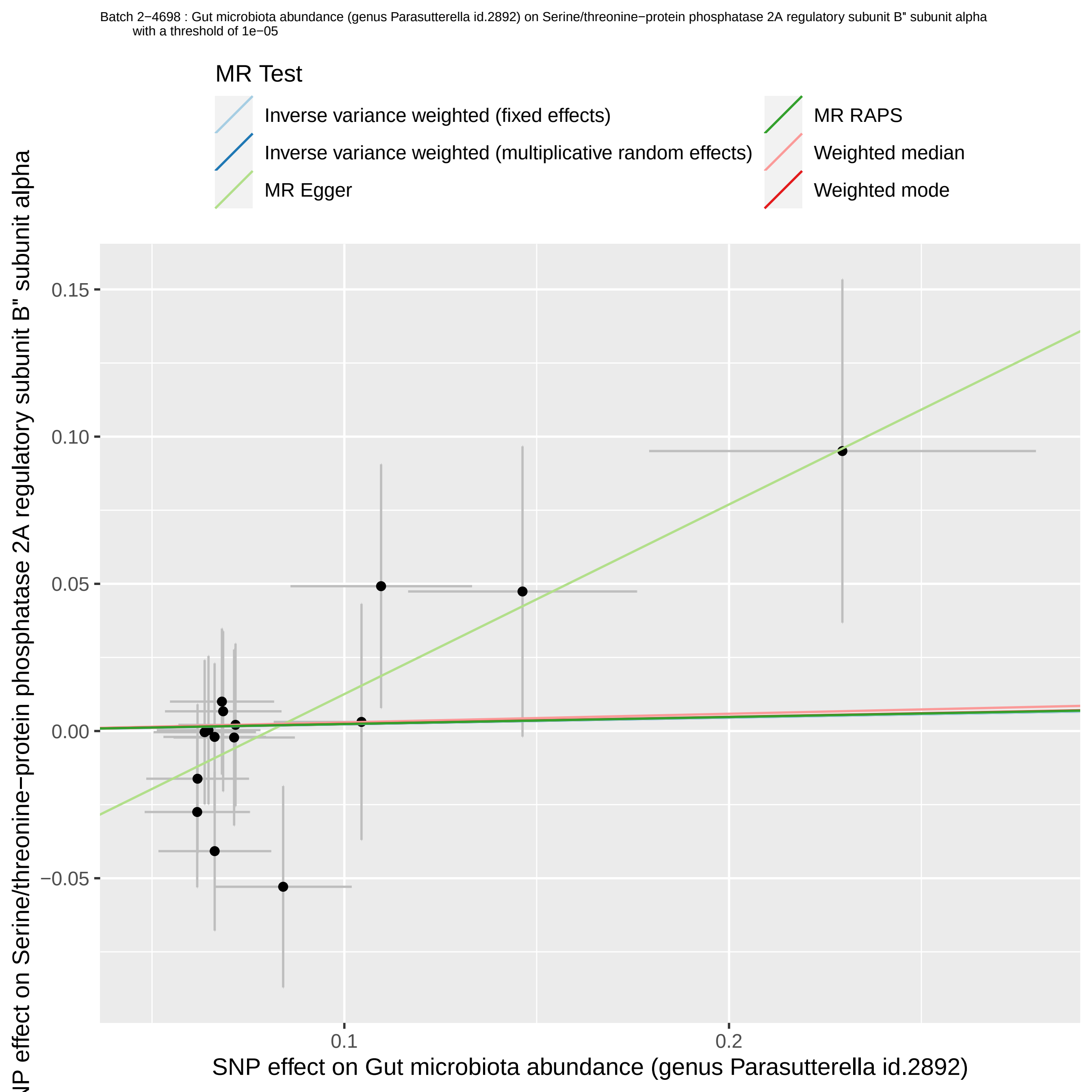
Batch 2−4698 : Gut microbiota abundance (genus Parasutterella id.2892) on Serine/threonine−protein phosphatase 2A regulatory subunit B" subunit alpha
with a threshold of 1e−05
MR Test
Inverse variance weighted (fixed effects)
Inverse variance weighted (multiplicative random effects)
MR Egger
MR RAPS
Weighted median
Weighted mode
0.15
0.10
0.05
0.00
−0.05
0.1
0.2
SNP effect on Gut microbiota abundance (genus Parasutterella id.2892)
SNP effect on Serine/threonine−protein phosphatase 2A regulatory subunit B" subunit alpha

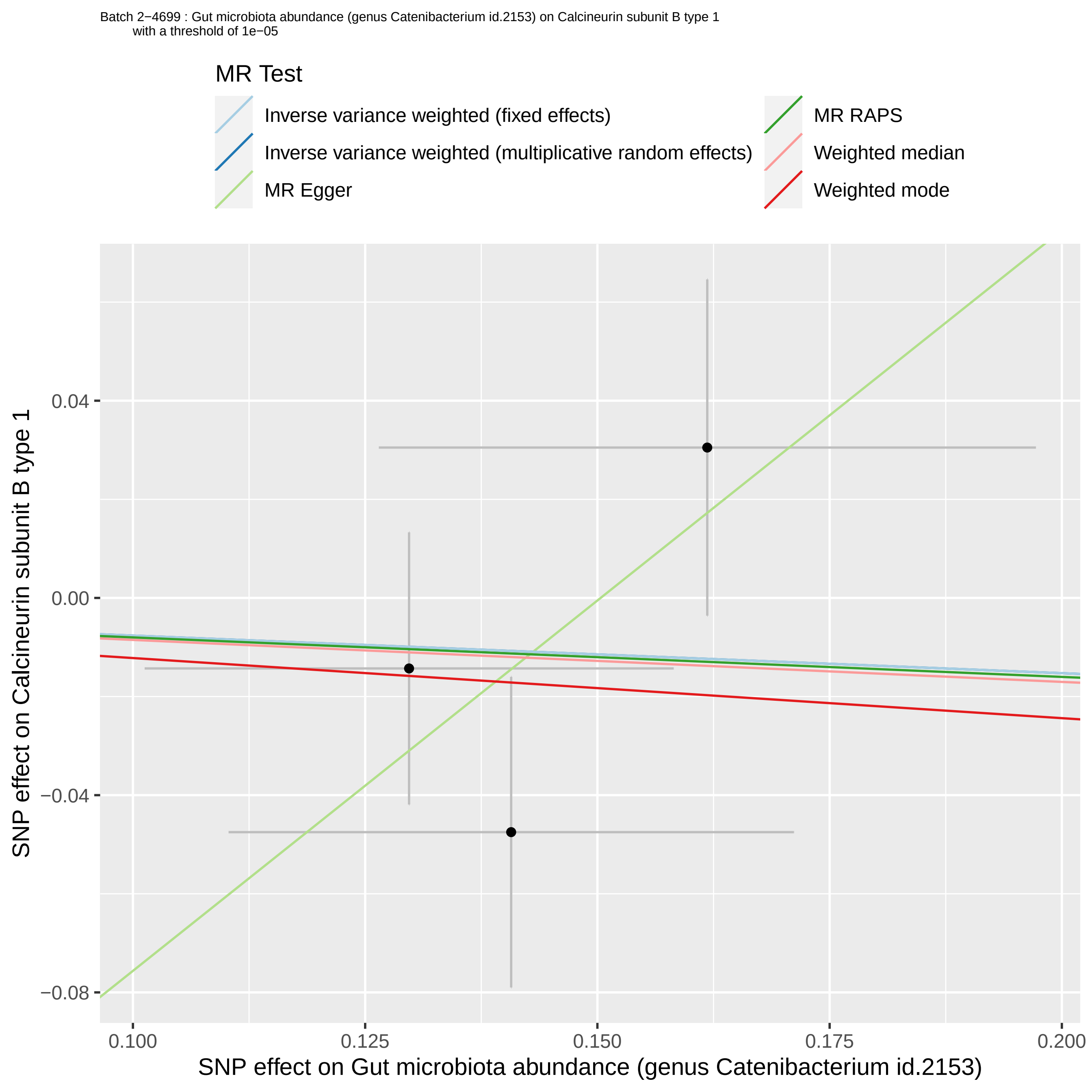

Batch 2−4699 : Gut microbiota abundance (genus Catenibacterium id.2153) on Calcineurin subunit B type 1
with a threshold of 1e−05
MR Test
Inverse variance weighted (fixed effects)
Inverse variance weighted (multiplicative random effects)
MR Egger
MR RAPS
Weighted median
Weighted mode
0.04
0.00
−0.04
−0.08
0.100
0.125
0.150
0.175
0.200
SNP effect on Calcineurin subunit B type 1
SNP effect on Gut microbiota abundance (genus Catenibacterium id.2153)

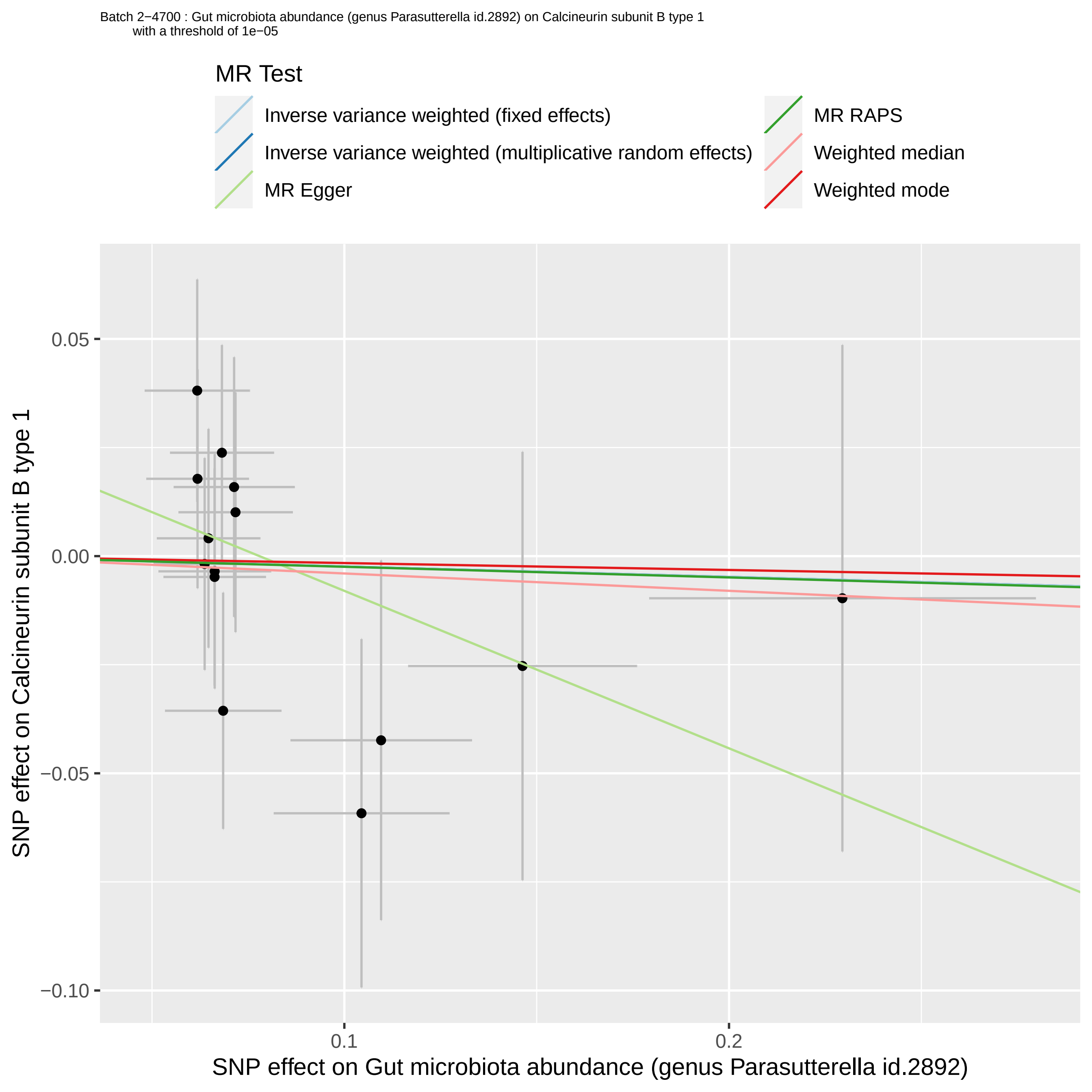

Batch 2−4700 : Gut microbiota abundance (genus Parasutterella id.2892) on Calcineurin subunit B type 1
with a threshold of 1e−05
MR Test
Inverse variance weighted (fixed effects)
Inverse variance weighted (multiplicative random effects)
MR Egger
MR RAPS
Weighted median
Weighted mode
0.05
0.00
−0.05
−0.10
0.1
0.2
SNP effect on Calcineurin subunit B type 1
SNP effect on Gut microbiota abundance (genus Parasutterella id.2892)

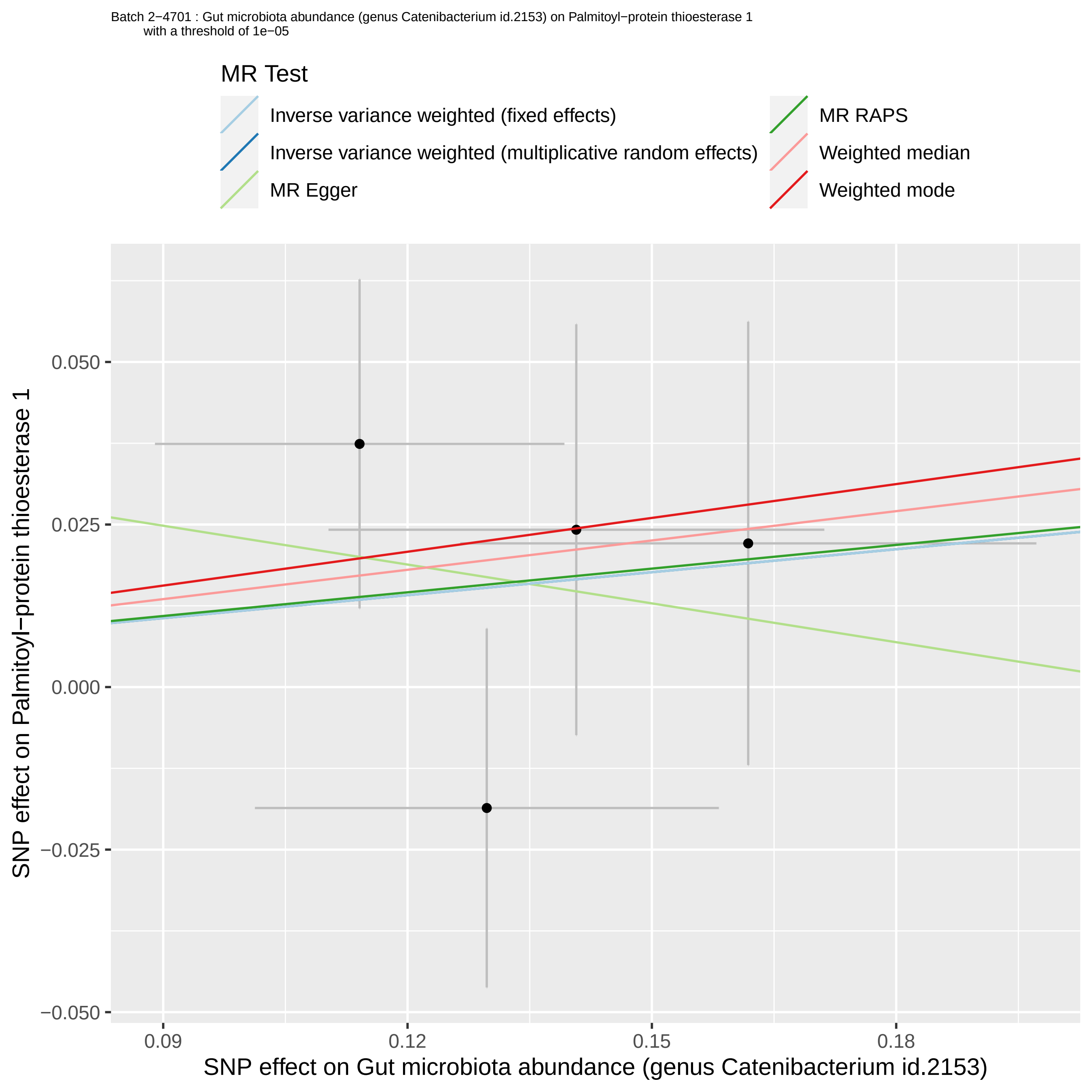

Batch 2−4701 : Gut microbiota abundance (genus Catenibacterium id.2153) on Palmitoyl−protein thioesterase 1
with a threshold of 1e−05
MR Test
Inverse variance weighted (fixed effects)
Inverse variance weighted (multiplicative random effects)
MR Egger
MR RAPS
Weighted median
Weighted mode
SNP effect on Palmitoyl−protein thioesterase 1
0.050
0.025
0.000
−0.025
−0.050
0.09
0.12
0.15
0.18
SNP effect on Gut microbiota abundance (genus Catenibacterium id.2153)

Batch 2−4702 : Gut microbiota abundance (genus Parasutterella id.2892) on Palmitoyl−protein thioesterase 1
with a threshold of 1e−05

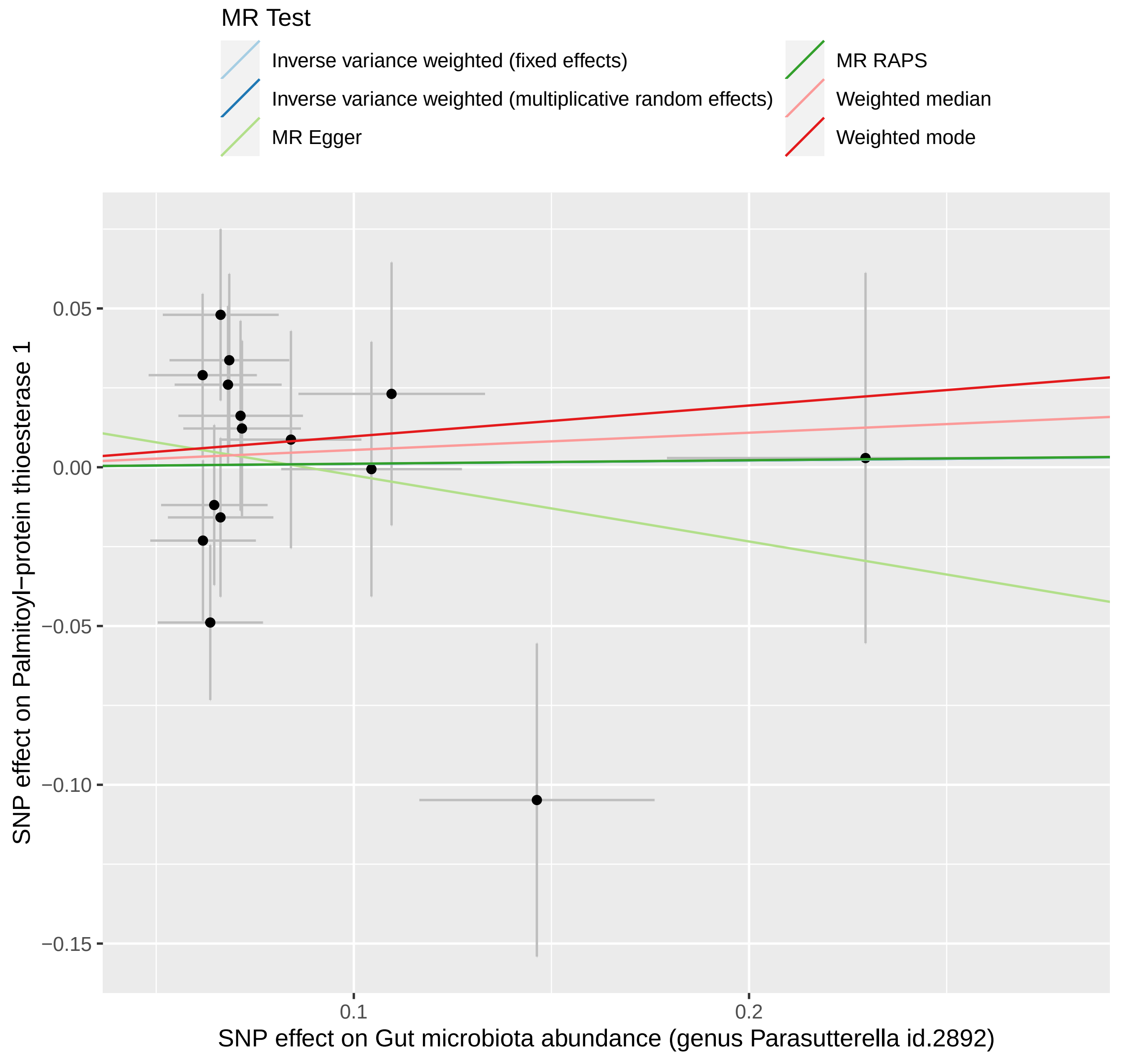

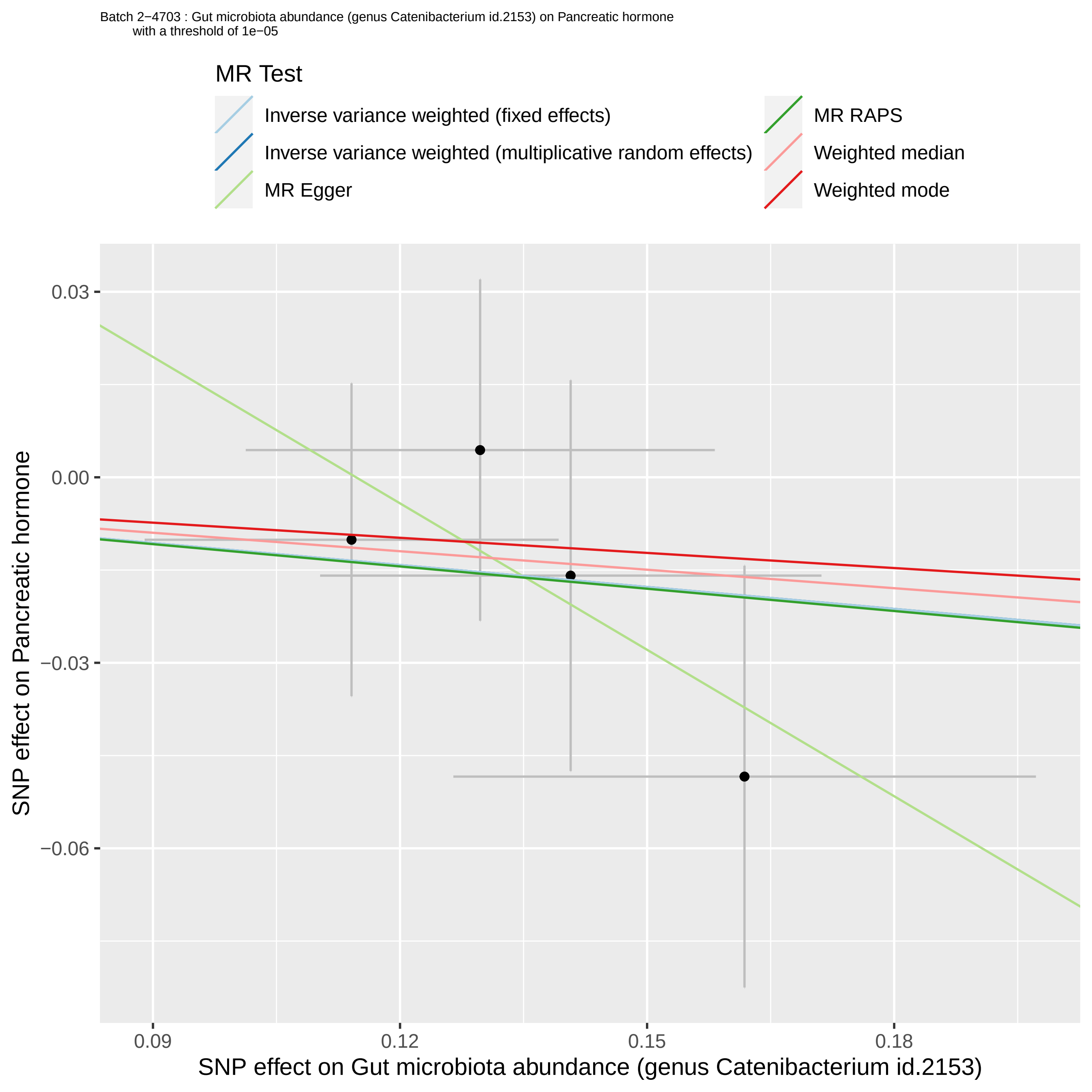

Batch 2−4703 : Gut microbiota abundance (genus Catenibacterium id.2153) on Pancreatic hormone
with a threshold of 1e−05
MR Test
Inverse variance weighted (fixed effects)
Inverse variance weighted (multiplicative random effects)
MR Egger
MR RAPS
Weighted median
Weighted mode
0.03
0.00
−0.03
−0.06
SNP effect on Pancreatic hormone
0.09
0.12
0.15
0.18
SNP effect on Gut microbiota abundance (genus Catenibacterium id.2153)

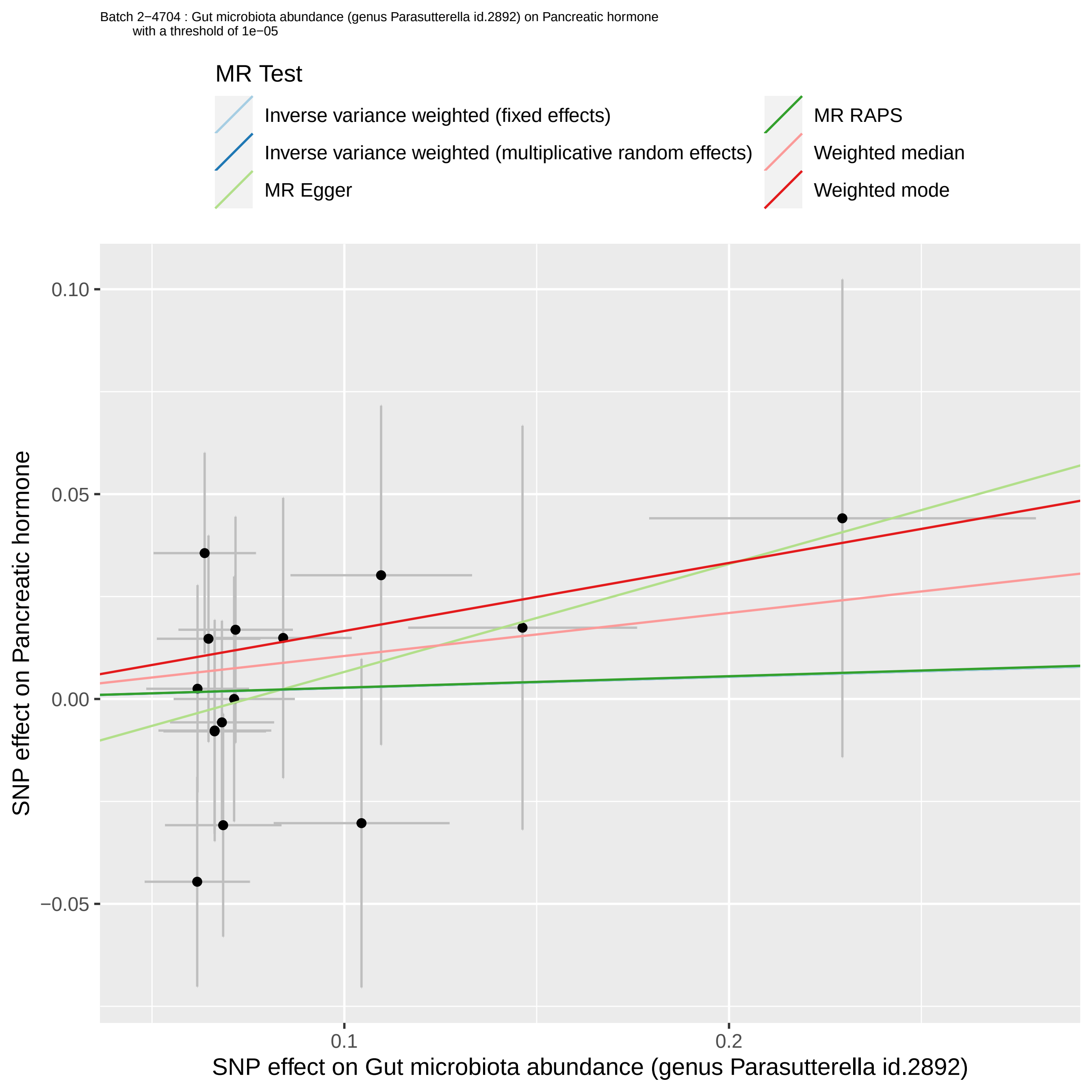
Batch 2−4704 : Gut microbiota abundance (genus Parasutterella id.2892) on Pancreatic hormone
with a threshold of 1e−05
MR Test
Inverse variance weighted (fixed effects)
Inverse variance weighted (multiplicative random effects)
MR Egger
MR RAPS
Weighted median
Weighted mode
0.10
0.05
0.00
−0.05
0.1
0.2
SNP effect on Pancreatic hormone
SNP effect on Gut microbiota abundance (genus Parasutterella id.2892)

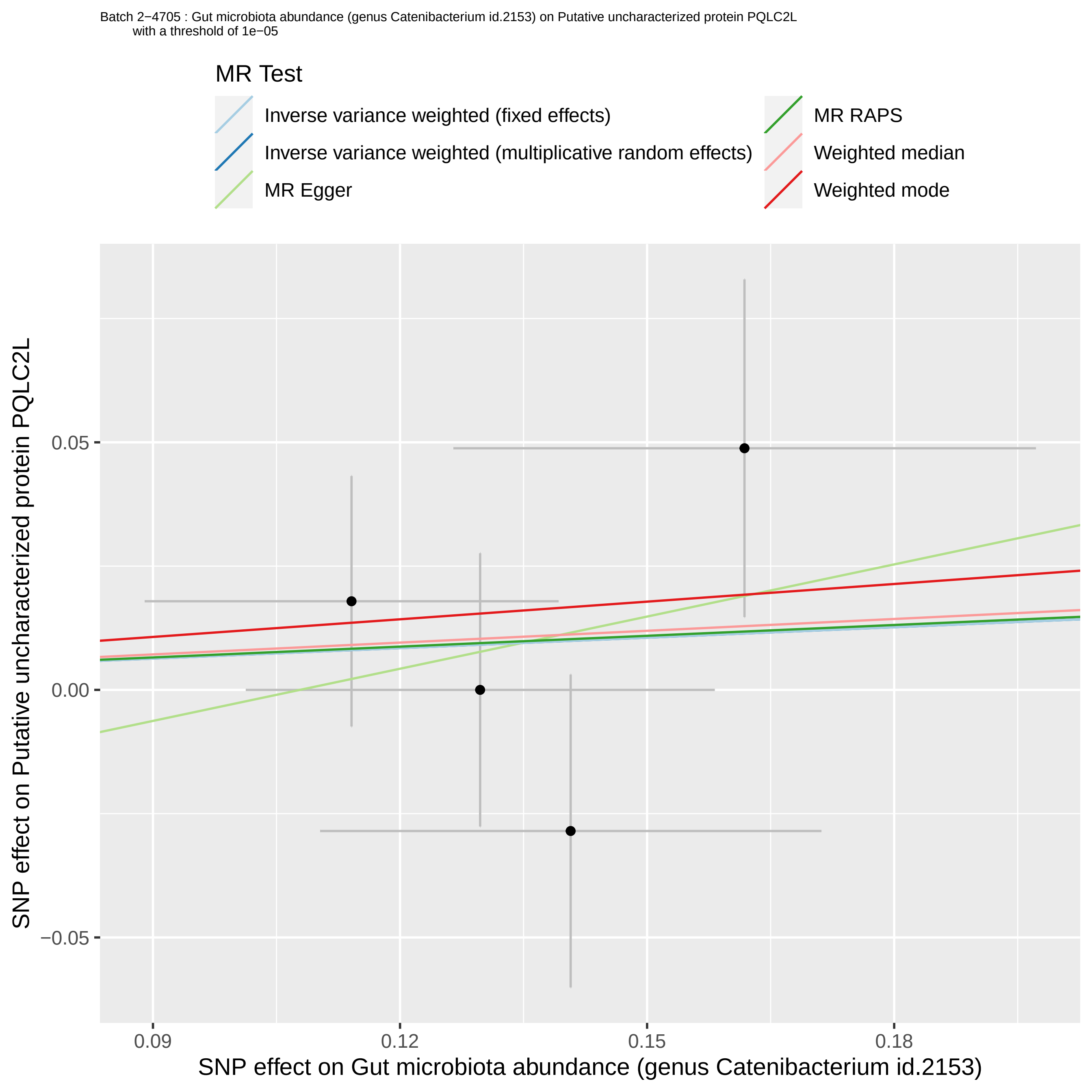
Batch 2−4705 : Gut microbiota abundance (genus Catenibacterium id.2153) on Putative uncharacterized protein PQLC2L
with a threshold of 1e−05
MR Test
Inverse variance weighted (fixed effects)
Inverse variance weighted (multiplicative random effects)
MR Egger
MR RAPS
Weighted median
Weighted mode
0.05
0.00
−0.05
0.09
0.12
0.15
0.18
SNP effect on Putative uncharacterized protein PQLC2L
SNP effect on Gut microbiota abundance (genus Catenibacterium id.2153)

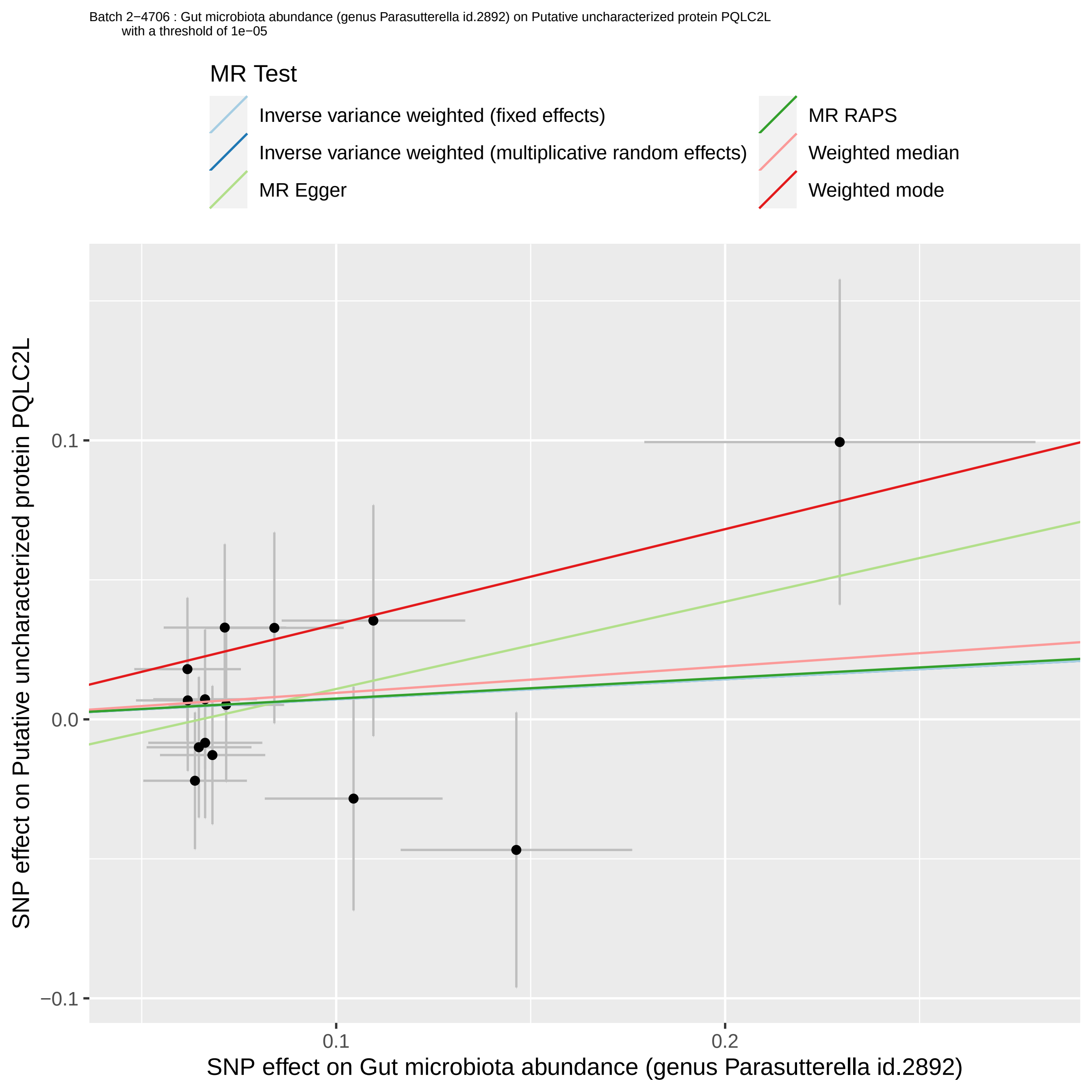
Batch 2−4706 : Gut microbiota abundance (genus Parasutterella id.2892) on Putative uncharacterized protein PQLC2L
with a threshold of 1e−05
MR Test
Inverse variance weighted (fixed effects)
Inverse variance weighted (multiplicative random effects)
MR Egger
MR RAPS
Weighted median
Weighted mode
SNP effect on Putative uncharacterized protein PQLC2L
0.1
0.0
−0.1
0.1
0.2
SNP effect on Gut microbiota abundance (genus Parasutterella id.2892)

Batch 2−4707 : Gut microbiota abundance (genus Catenibacterium id.2153) on Basic salivary proline−rich protein 4
with a threshold of 1e−05

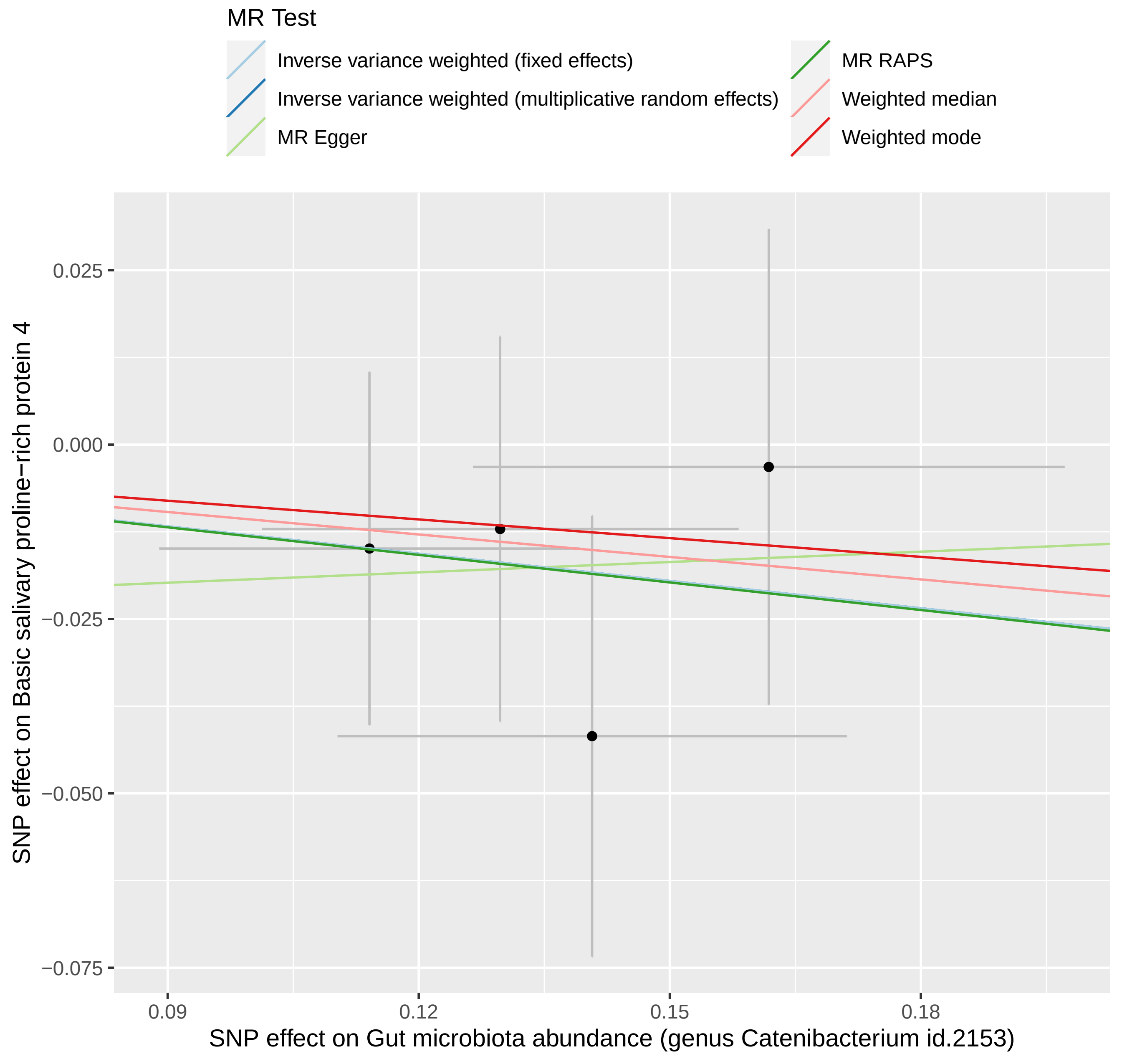

Batch 2−4708 : Gut microbiota abundance (genus Parasutterella id.2892) on Basic salivary proline−rich protein 4
with a threshold of 1e−05

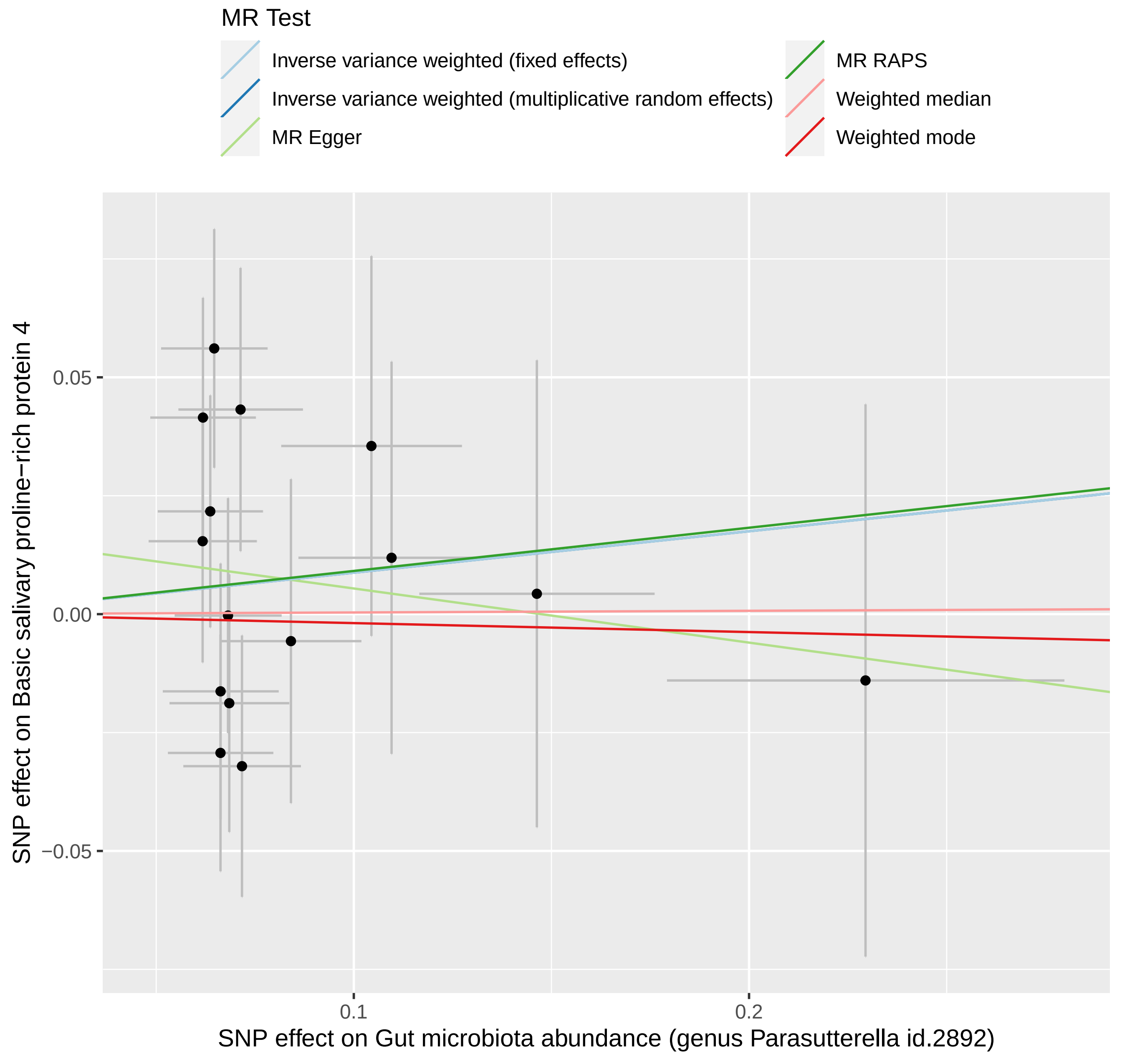

Batch 2−4709 : Gut microbiota abundance (genus Catenibacterium id.2153) on Protein regulator of cytokinesis 1
with a threshold of 1e−05

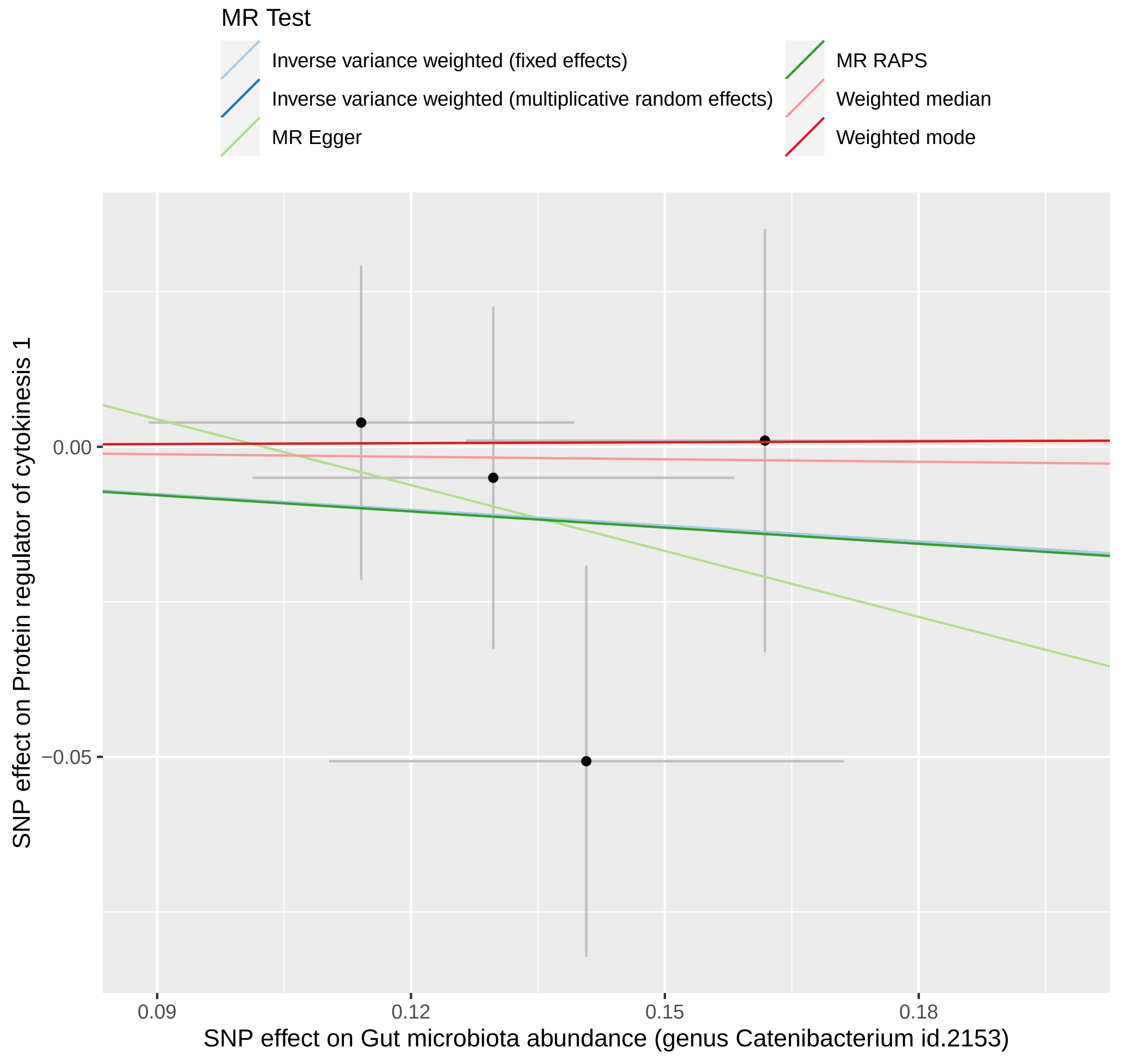

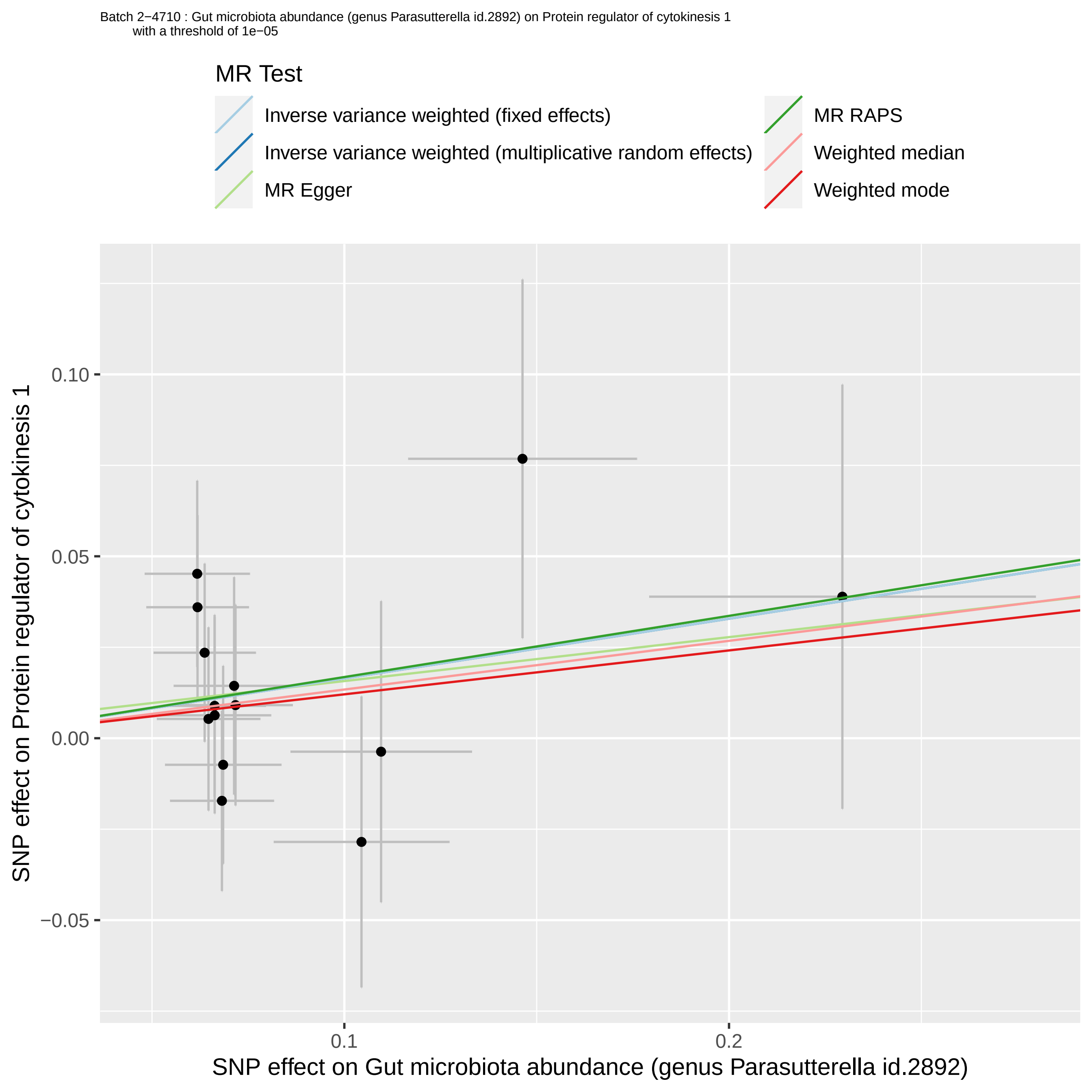
Batch 2−4710 : Gut microbiota abundance (genus Parasutterella id.2892) on Protein regulator of cytokinesis 1
with a threshold of 1e−05
MR Test
Inverse variance weighted (fixed effects)
Inverse variance weighted (multiplicative random effects)
MR Egger
MR RAPS
Weighted median
Weighted mode
0.10
0.05
0.00
−0.05
0.1
0.2
SNP effect on Protein regulator of cytokinesis 1
SNP effect on Gut microbiota abundance (genus Parasutterella id.2892)

Batch 2−4711 : Gut microbiota abundance (genus Catenibacterium id.2153) on Lysosomal Pro−X carboxypeptidase
with a threshold of 1e−05

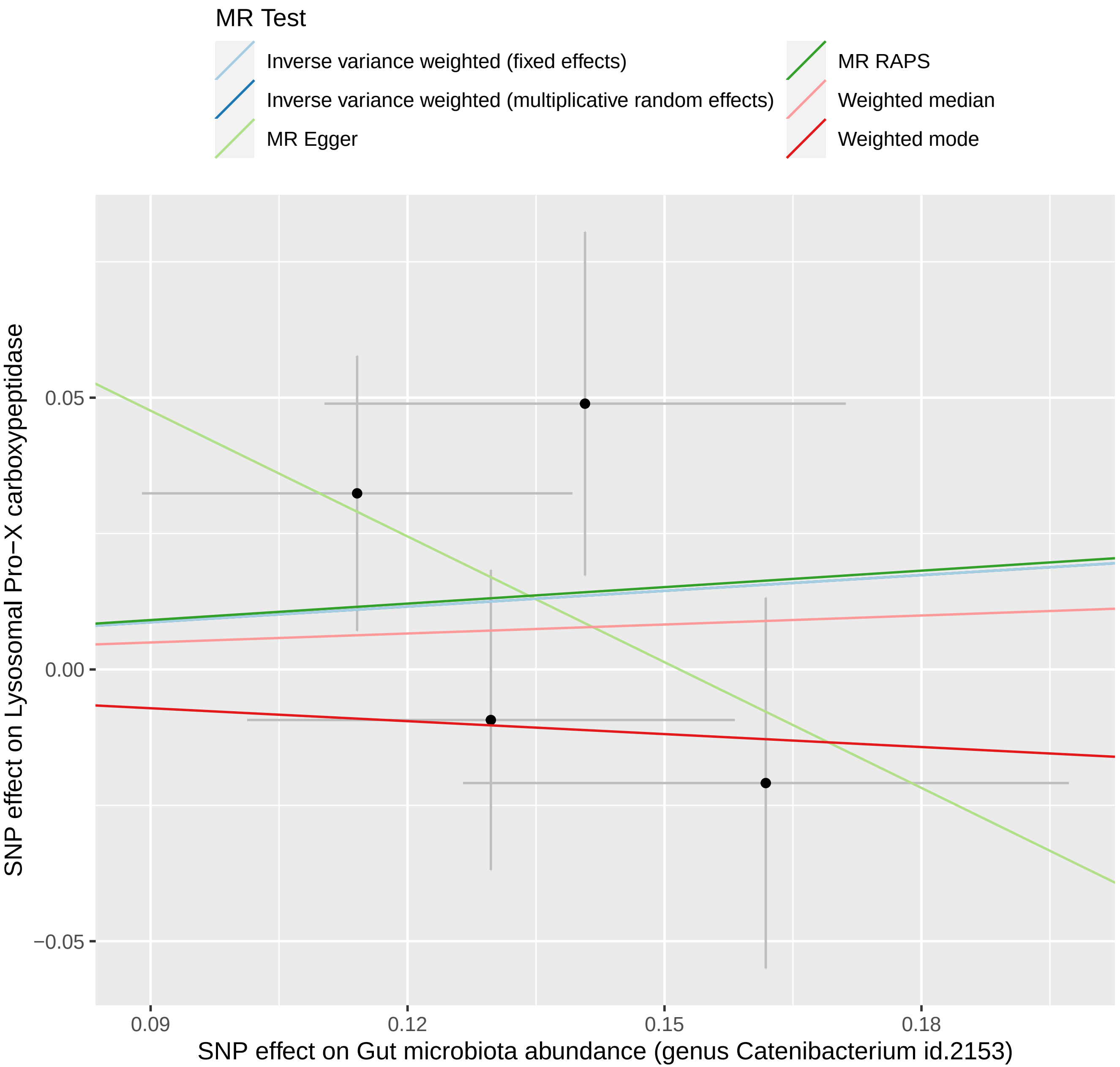

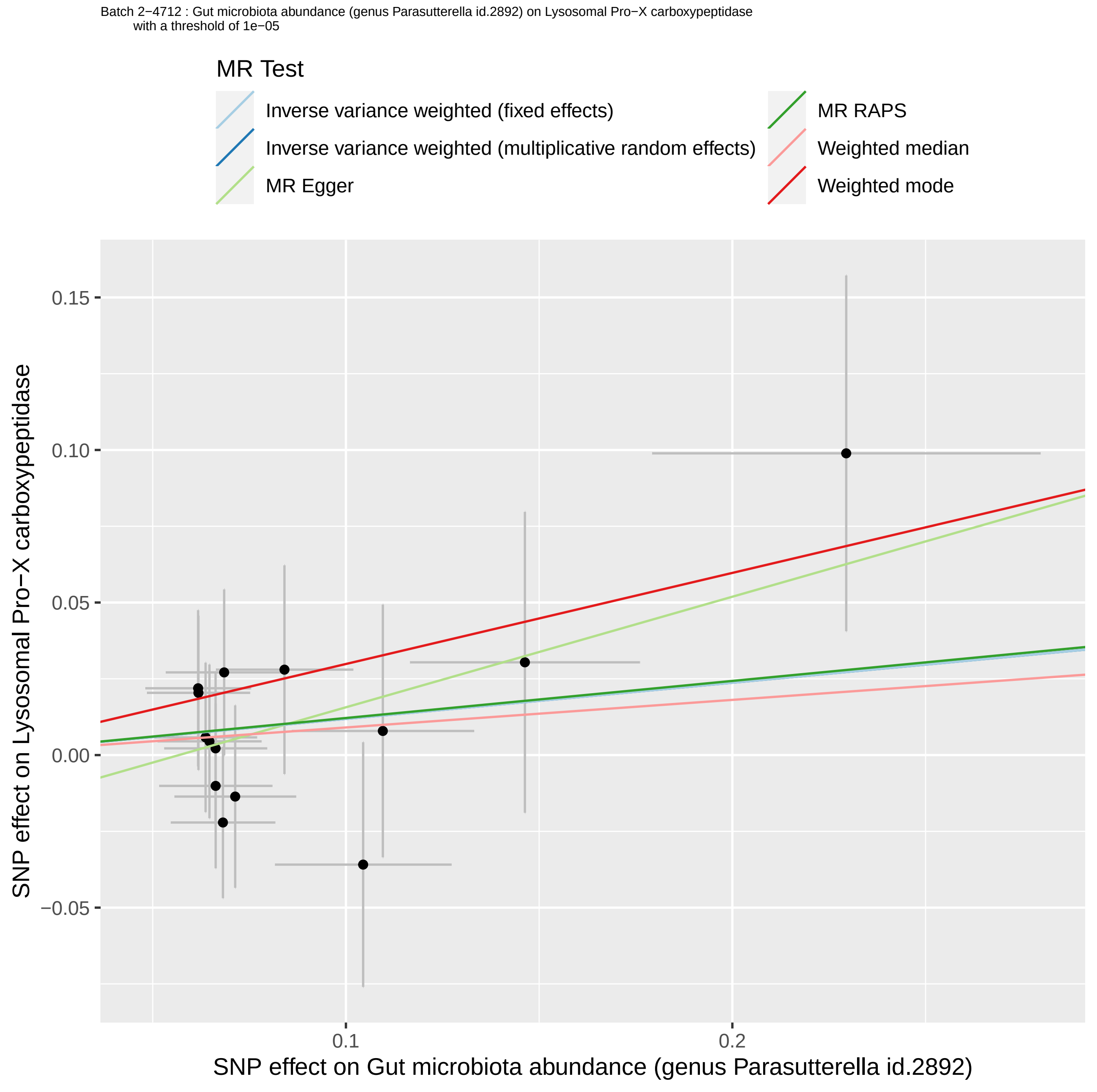
Batch 2−4712 : Gut microbiota abundance (genus Parasutterella id.2892) on Lysosomal Pro−X carboxypeptidase
with a threshold of 1e−05
MR Test
Inverse variance weighted (fixed effects)
Inverse variance weighted (multiplicative random effects)
MR Egger
MR RAPS
Weighted median
Weighted mode
0.15
0.10
0.05
0.00
−0.05
0.1
0.2
SNP effect on Lysosomal Pro−X carboxypeptidase
SNP effect on Gut microbiota abundance (genus Parasutterella id.2892)

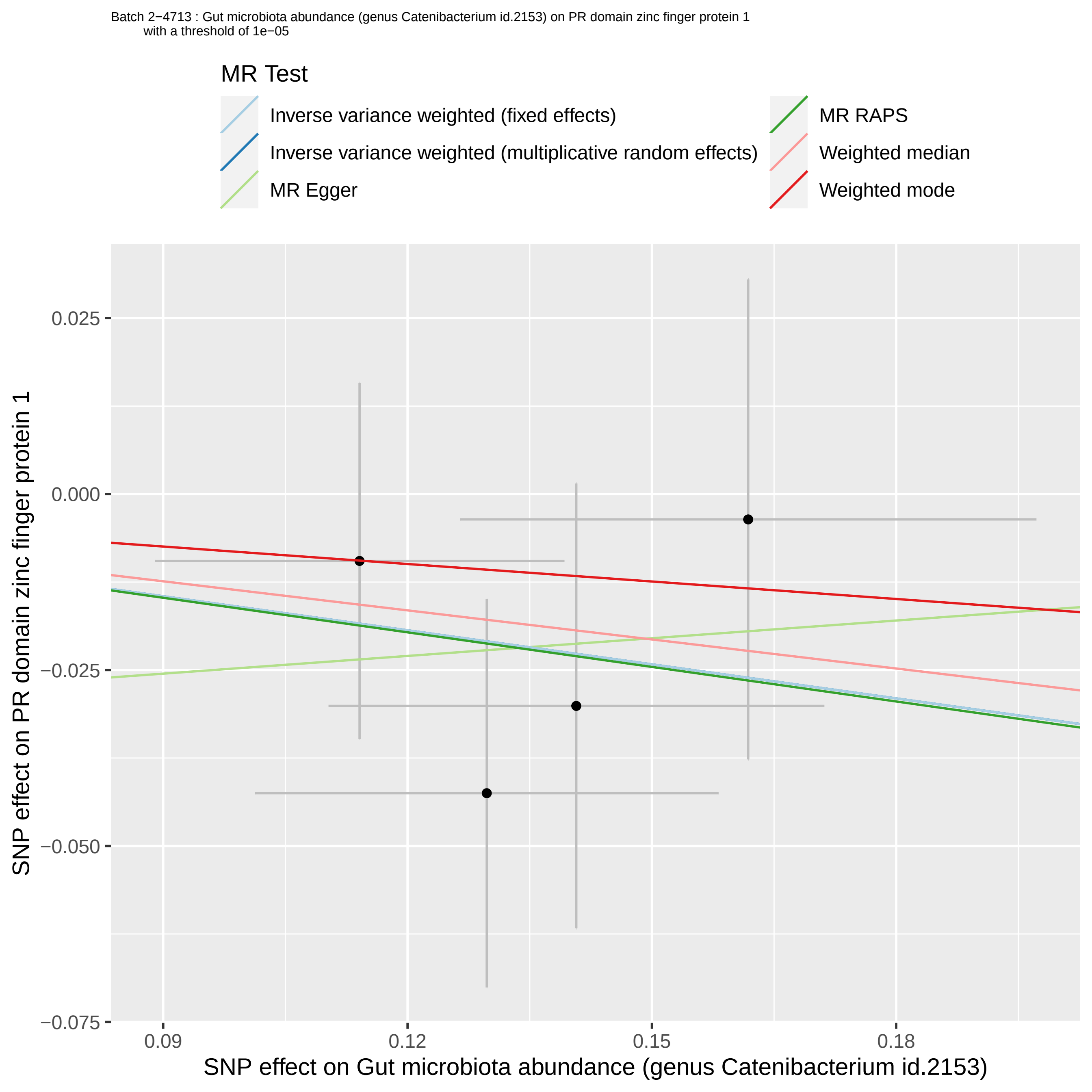

Batch 2−4713 : Gut microbiota abundance (genus Catenibacterium id.2153) on PR domain zinc finger protein 1
with a threshold of 1e−05
MR Test
Inverse variance weighted (fixed effects)
Inverse variance weighted (multiplicative random effects)
MR Egger
MR RAPS
Weighted median
Weighted mode
0.025
0.000
−0.025
−0.050
−0.075
0.09
0.12
0.15
0.18
SNP effect on PR domain zinc finger protein 1
SNP effect on Gut microbiota abundance (genus Catenibacterium id.2153)

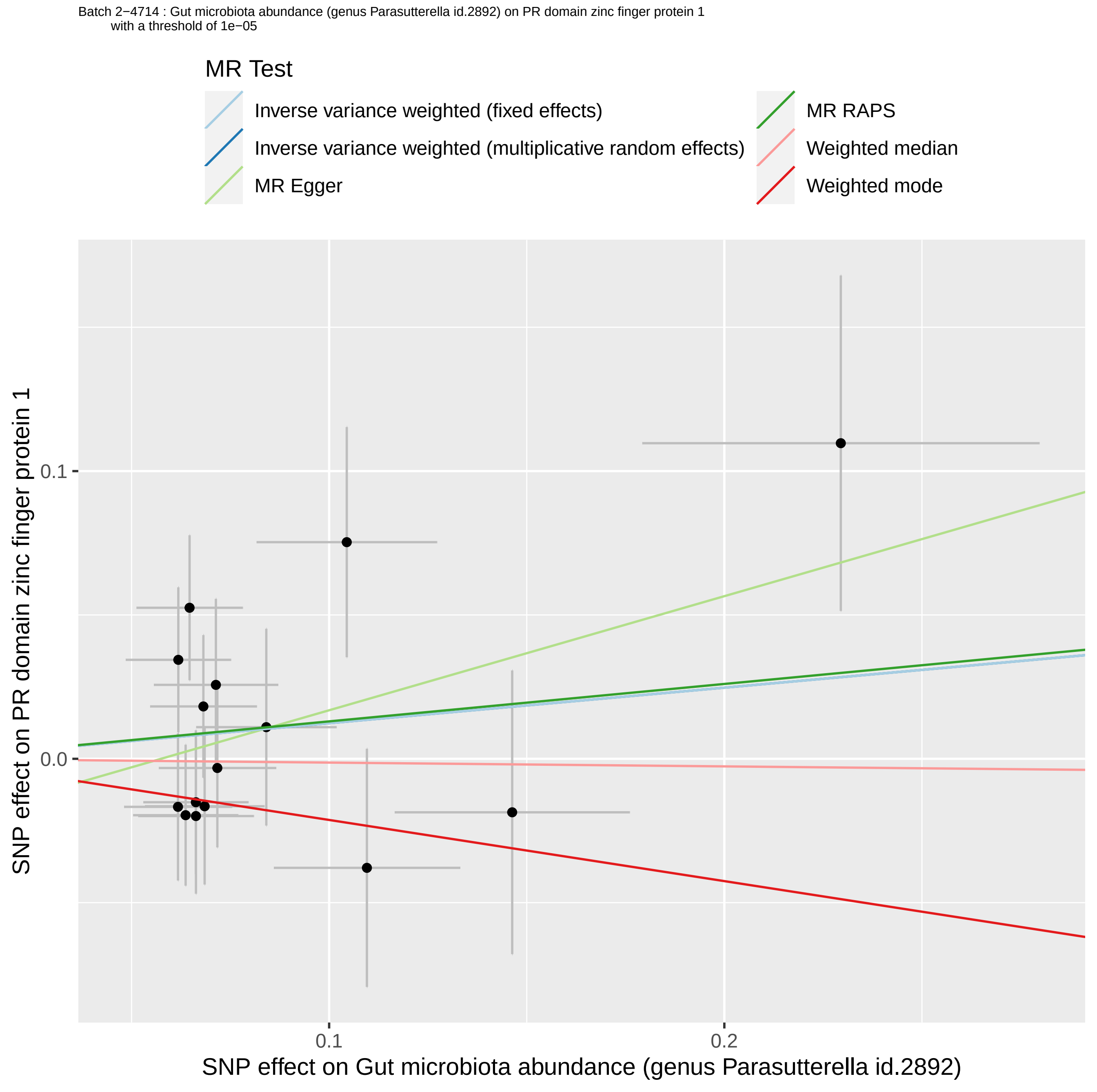
Batch 2−4714 : Gut microbiota abundance (genus Parasutterella id.2892) on PR domain zinc finger protein 1
with a threshold of 1e−05
MR Test
Inverse variance weighted (fixed effects)
Inverse variance weighted (multiplicative random effects)
MR Egger
MR RAPS
Weighted median
Weighted mode
0.1
0.0
0.1
0.2
SNP effect on PR domain zinc finger protein 1
SNP effect on Gut microbiota abundance (genus Parasutterella id.2892)

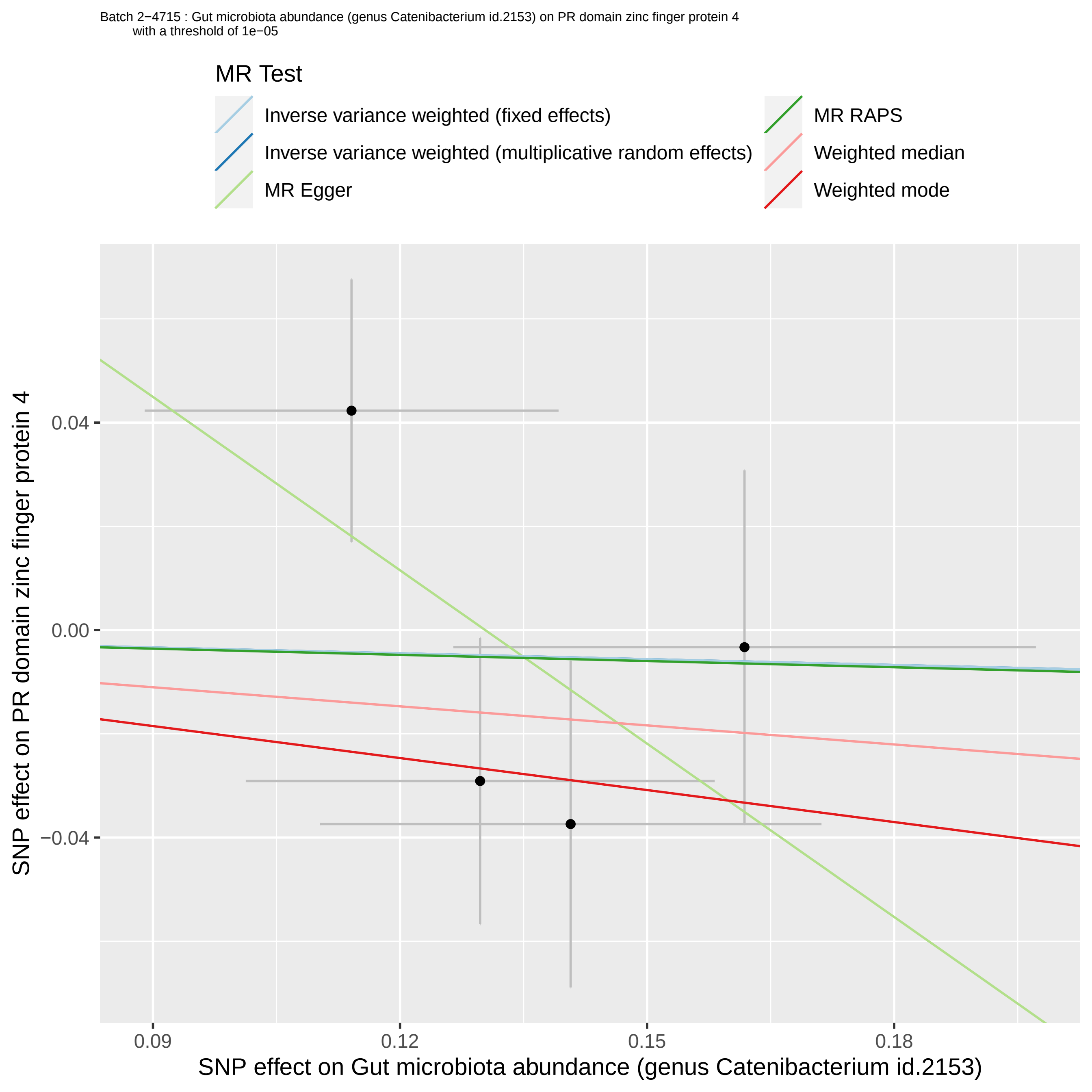

Batch 2−4715 : Gut microbiota abundance (genus Catenibacterium id.2153) on PR domain zinc finger protein 4
with a threshold of 1e−05
MR Test
Inverse variance weighted (fixed effects)
Inverse variance weighted (multiplicative random effects)
MR Egger
MR RAPS
Weighted median
Weighted mode
0.04
0.00
−0.04
0.09
0.12
0.15
0.18
SNP effect on PR domain zinc finger protein 4
SNP effect on Gut microbiota abundance (genus Catenibacterium id.2153)

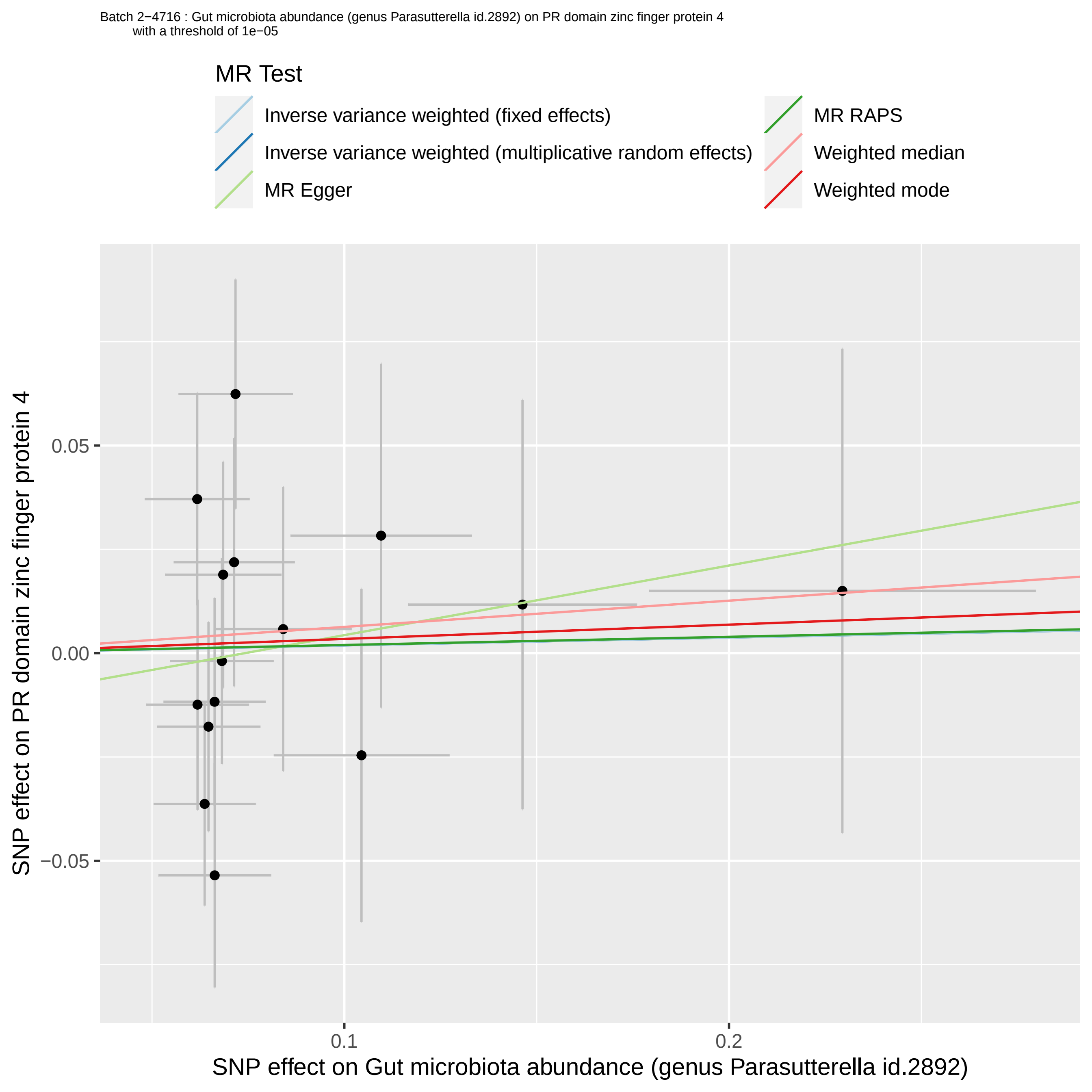

Batch 2−4716 : Gut microbiota abundance (genus Parasutterella id.2892) on PR domain zinc finger protein 4
with a threshold of 1e−05
MR Test
Inverse variance weighted (fixed effects)
Inverse variance weighted (multiplicative random effects)
MR Egger
MR RAPS
Weighted median
Weighted mode
0.05
0.00
−0.05
0.1
0.2
SNP effect on PR domain zinc finger protein 4
SNP effect on Gut microbiota abundance (genus Parasutterella id.2892)

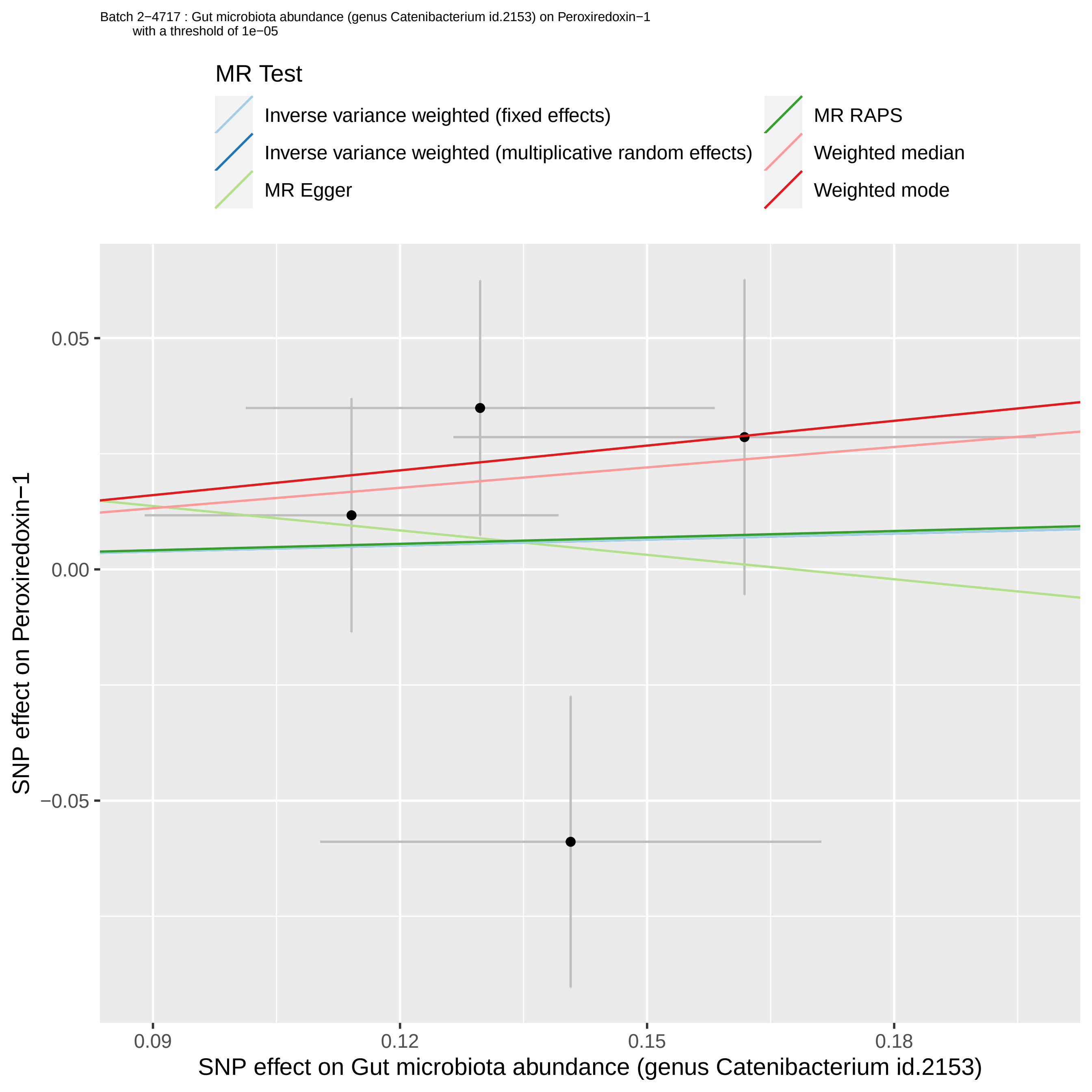
Batch 2−4717 : Gut microbiota abundance (genus Catenibacterium id.2153) on Peroxiredoxin−1
with a threshold of 1e−05
MR Test
Inverse variance weighted (fixed effects)
Inverse variance weighted (multiplicative random effects)
MR Egger
MR RAPS
Weighted median
Weighted mode
0.05
0.00
−0.05
0.09
0.12
0.15
0.18
SNP effect on Peroxiredoxin−1
SNP effect on Gut microbiota abundance (genus Catenibacterium id.2153)

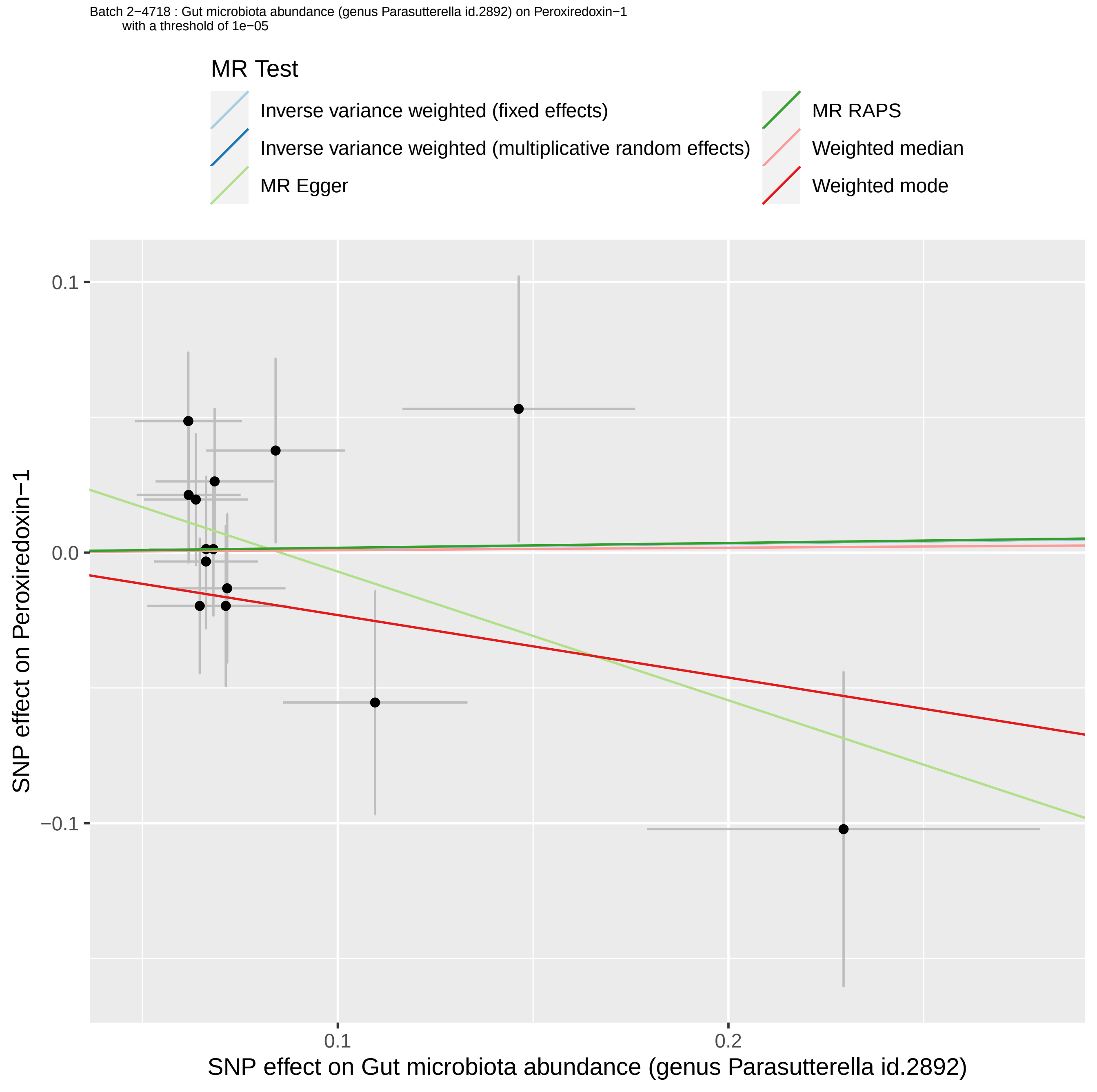

Batch 2−4718 : Gut microbiota abundance (genus Parasutterella id.2892) on Peroxiredoxin−1
with a threshold of 1e−05
MR Test
Inverse variance weighted (fixed effects)
Inverse variance weighted (multiplicative random effects)
MR Egger
MR RAPS
Weighted median
Weighted mode
0.1
0.0
−0.1
0.1
0.2
SNP effect on Peroxiredoxin−1
SNP effect on Gut microbiota abundance (genus Parasutterella id.2892)

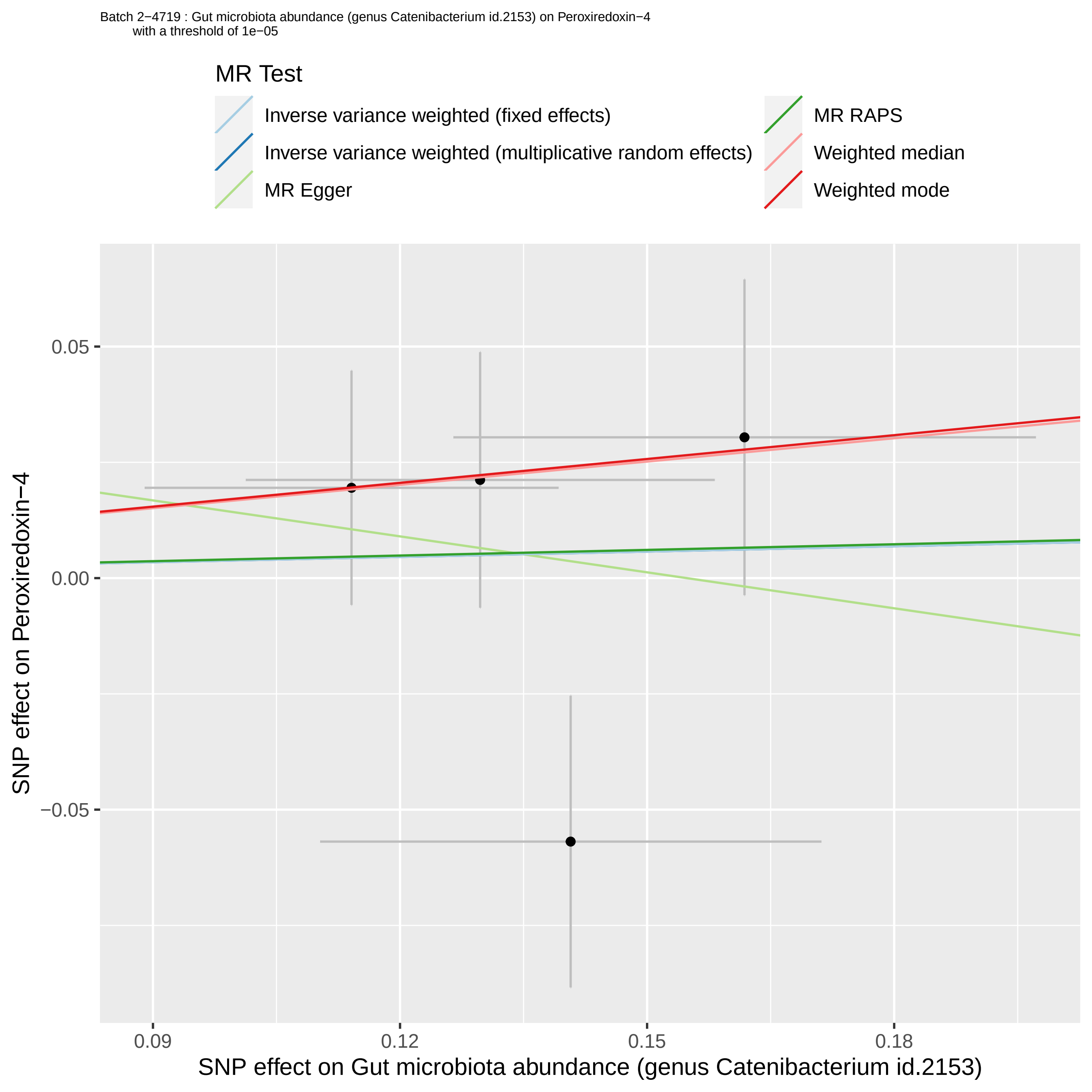

Batch 2−4719 : Gut microbiota abundance (genus Catenibacterium id.2153) on Peroxiredoxin−4
with a threshold of 1e−05
MR Test
Inverse variance weighted (fixed effects)
Inverse variance weighted (multiplicative random effects)
MR Egger
MR RAPS
Weighted median
Weighted mode
0.05
0.00
−0.05
0.09
0.12
0.15
0.18
SNP effect on Peroxiredoxin−4
SNP effect on Gut microbiota abundance (genus Catenibacterium id.2153)

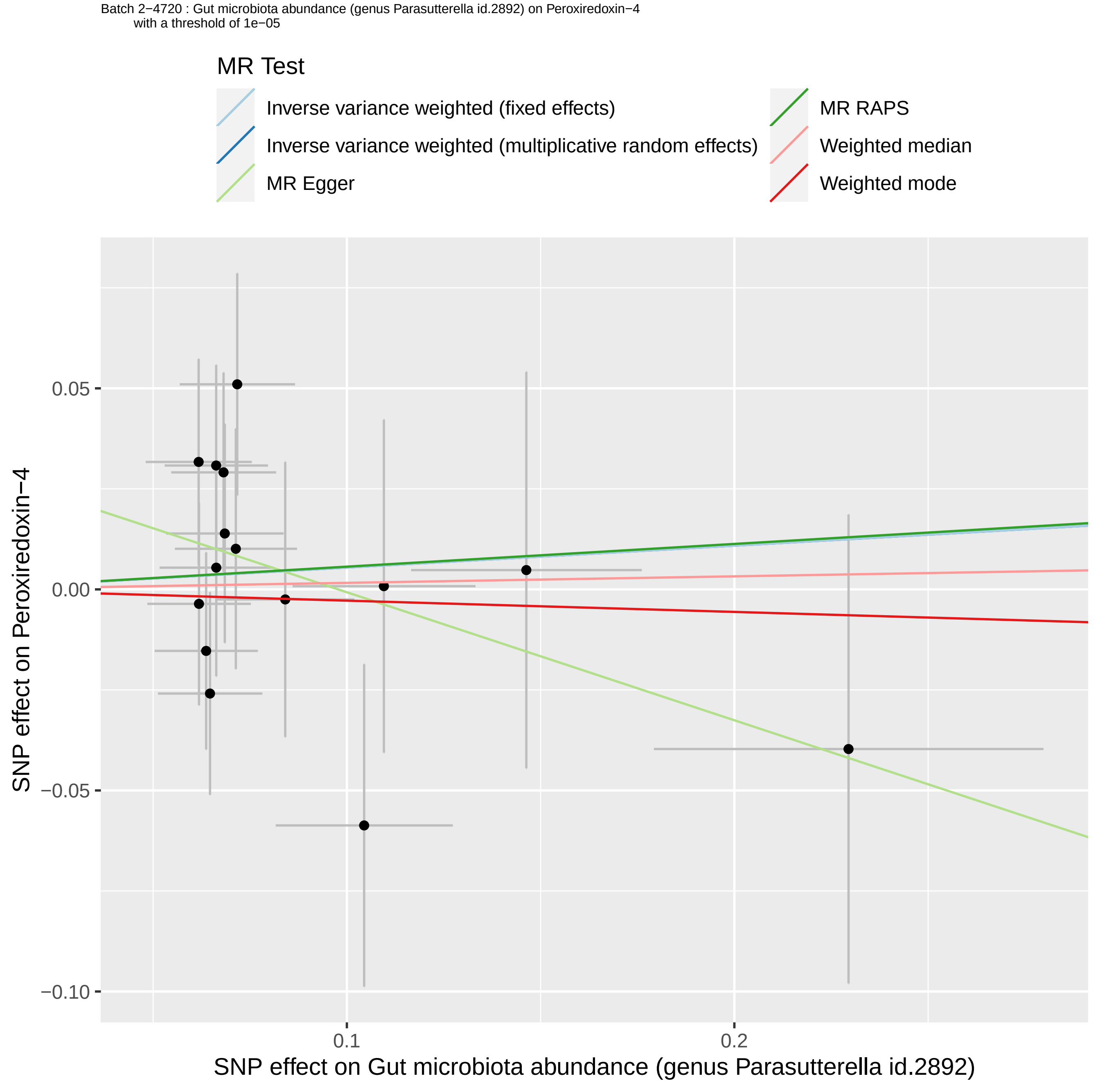

Batch 2−4720 : Gut microbiota abundance (genus Parasutterella id.2892) on Peroxiredoxin−4
with a threshold of 1e−05
MR Test
Inverse variance weighted (fixed effects)
Inverse variance weighted (multiplicative random effects)
MR Egger
MR RAPS
Weighted median
Weighted mode
0.05
0.00
−0.05
−0.10
0.1
0.2
SNP effect on Peroxiredoxin−4
SNP effect on Gut microbiota abundance (genus Parasutterella id.2892)

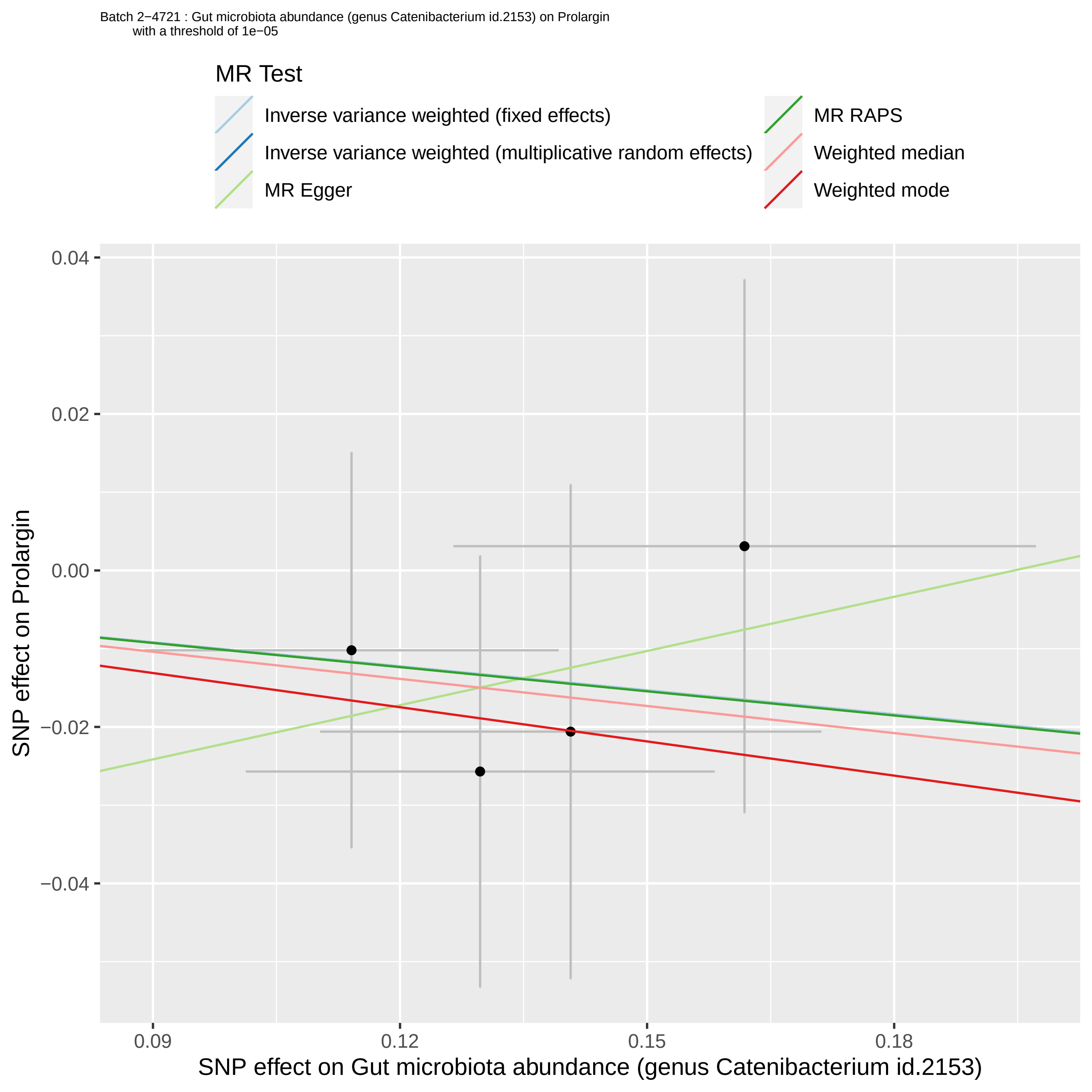
Batch 2−4721 : Gut microbiota abundance (genus Catenibacterium id.2153) on Prolargin
with a threshold of 1e−05
MR Test
Inverse variance weighted (fixed effects)
Inverse variance weighted (multiplicative random effects)
MR Egger
MR RAPS
Weighted median
Weighted mode
0.04
0.02
0.00
−0.02
−0.04
0.09
0.12
0.15
0.18
SNP effect on Prolargin
SNP effect on Gut microbiota abundance (genus Catenibacterium id.2153)

Batch 2−4722 : Gut microbiota abundance (genus Parasutterella id.2892) on Prolargin
with a threshold of 1e−05

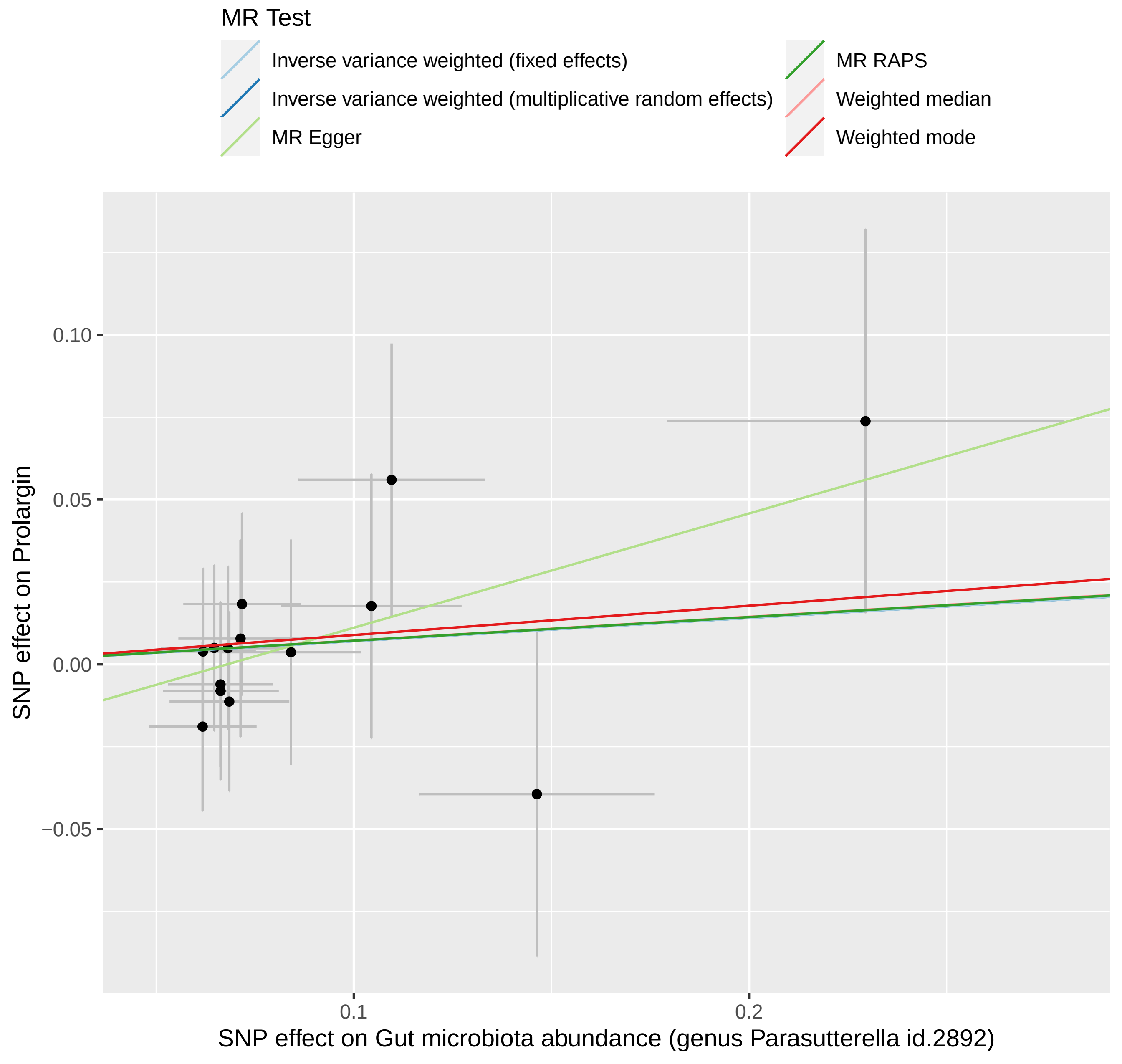

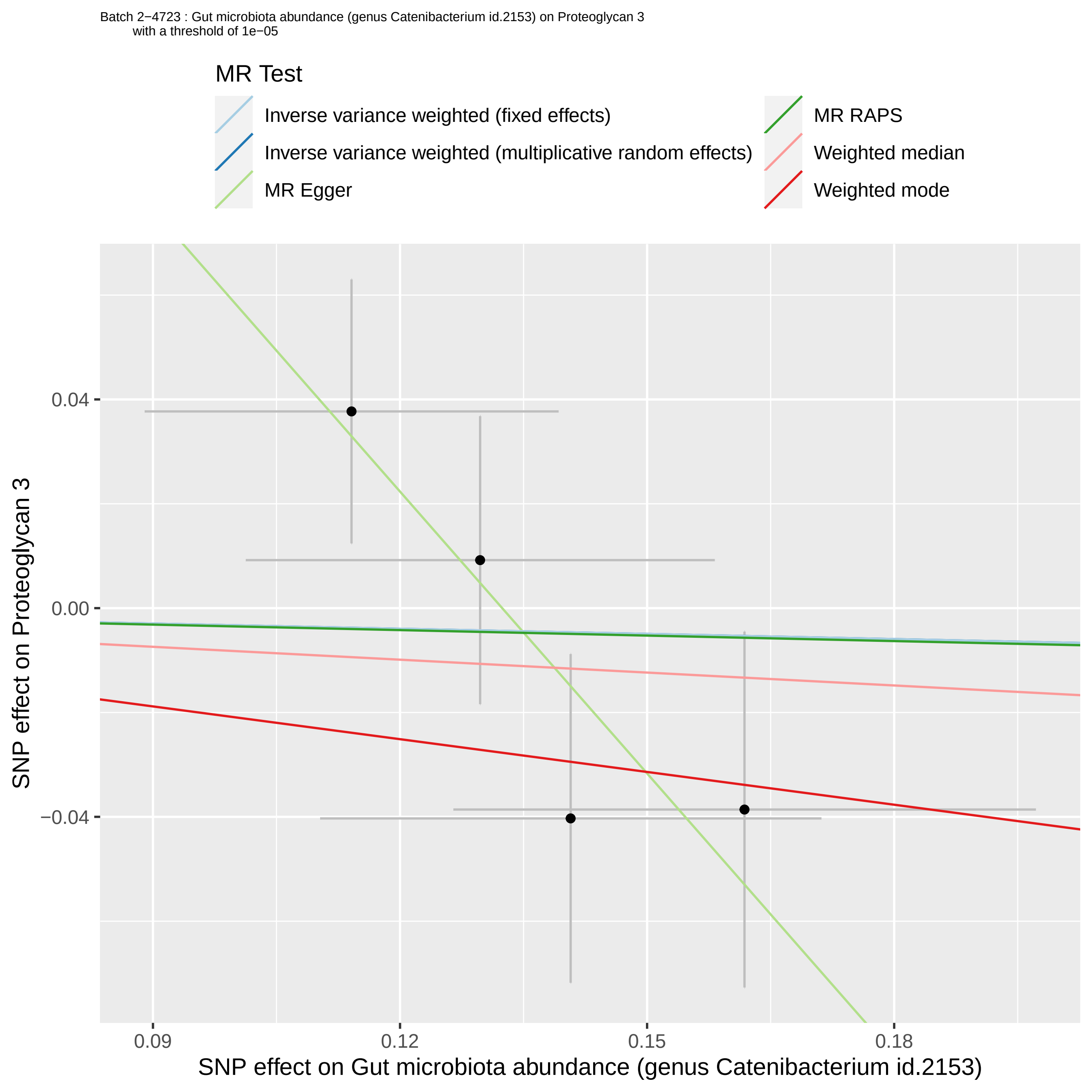
Batch 2−4723 : Gut microbiota abundance (genus Catenibacterium id.2153) on Proteoglycan 3
with a threshold of 1e−05
MR Test
Inverse variance weighted (fixed effects)
Inverse variance weighted (multiplicative random effects)
MR Egger
MR RAPS
Weighted median
Weighted mode
0.04
0.00
−0.04
0.09
0.12
0.15
0.18
SNP effect on Proteoglycan 3
SNP effect on Gut microbiota abundance (genus Catenibacterium id.2153)

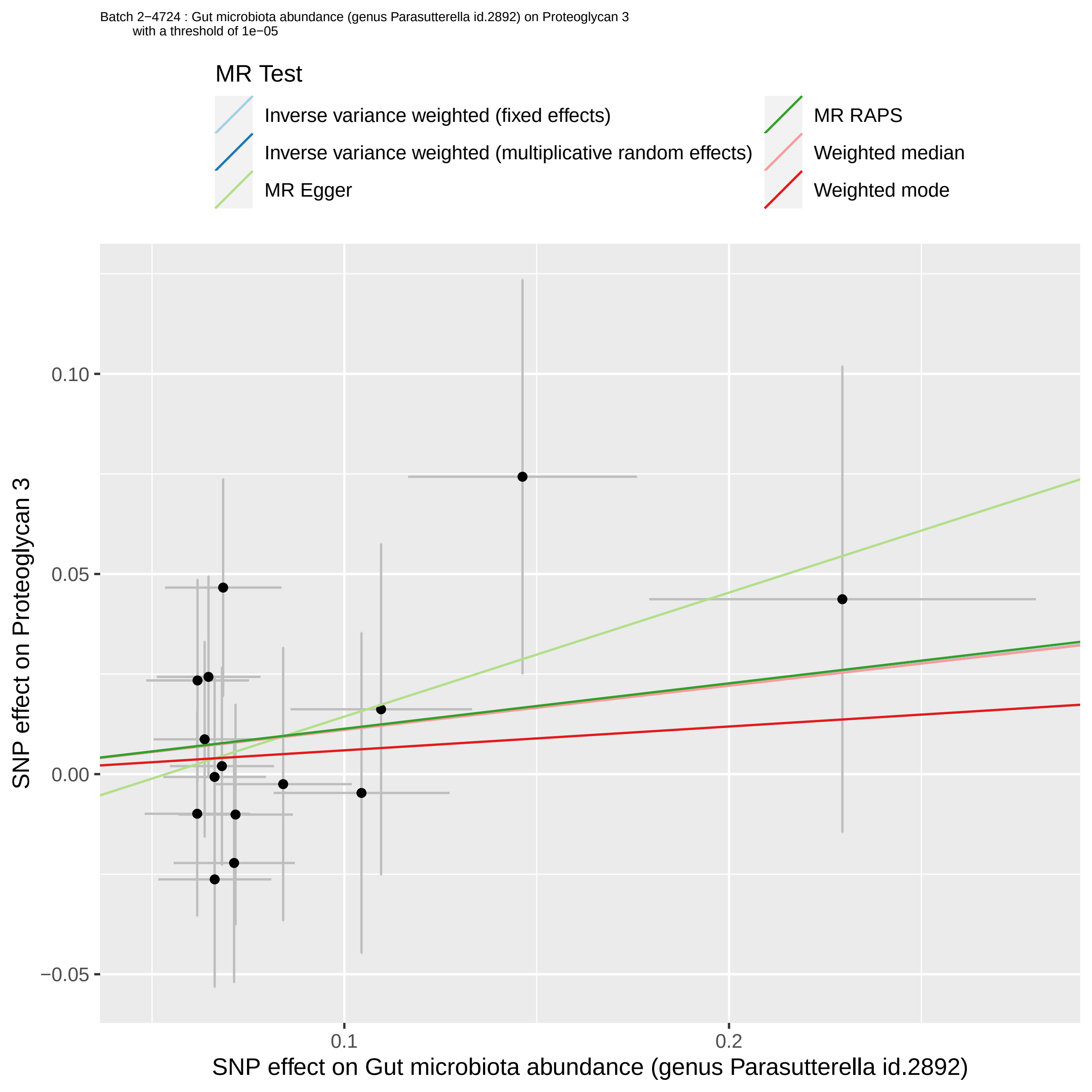

Batch 2−4724 : Gut microbiota abundance (genus Parasutterella id.2892) on Proteoglycan 3
with a threshold of 1e−05
MR Test
Inverse variance weighted (fixed effects)
Inverse variance weighted (multiplicative random effects)
MR Egger
MR RAPS
Weighted median
Weighted mode
SNP effect on Proteoglycan 3
0.10
0.05
0.00
−0.05
0.1
0.2
SNP effect on Gut microbiota abundance (genus Parasutterella id.2892)

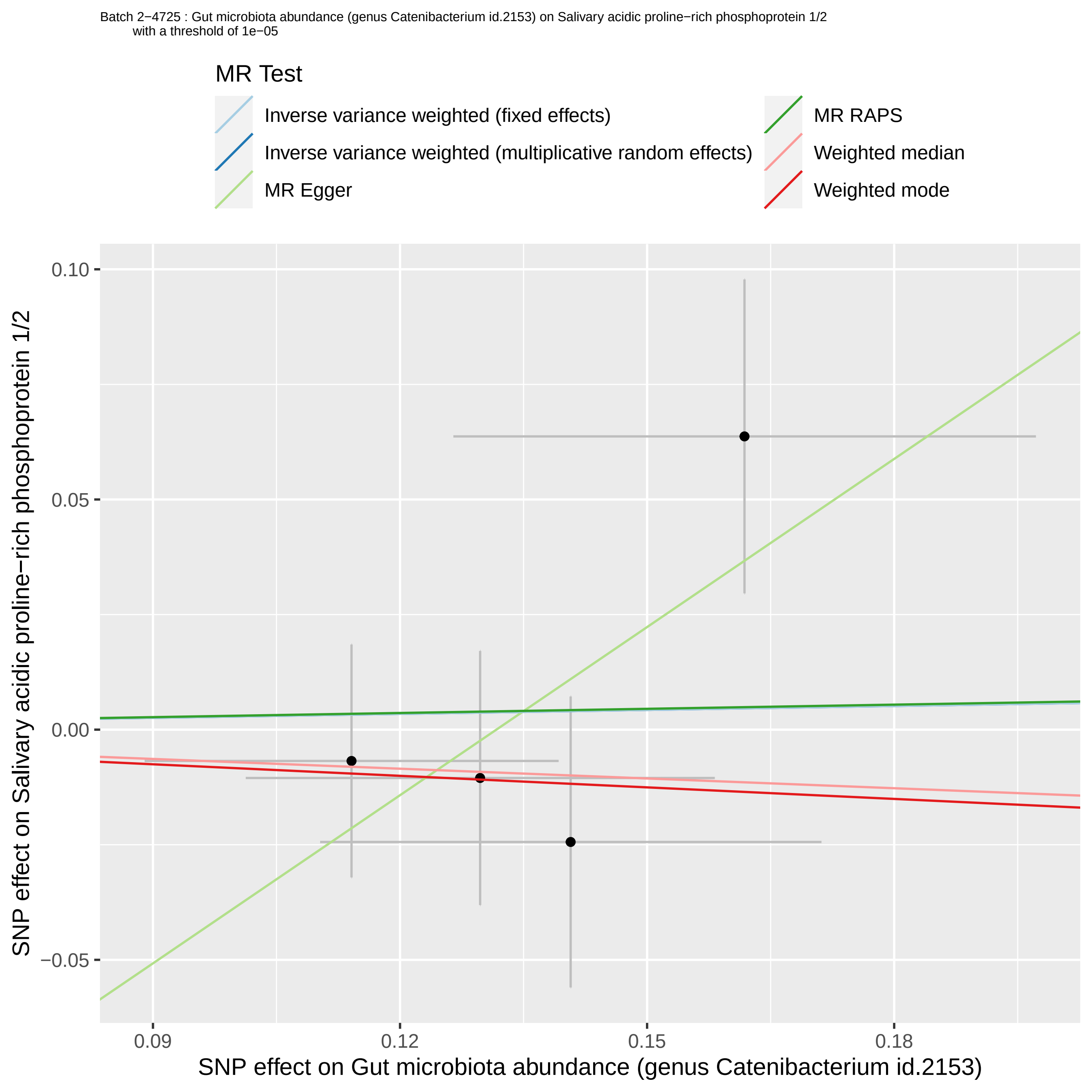

Batch 2−4725 : Gut microbiota abundance (genus Catenibacterium id.2153) on Salivary acidic proline−rich phosphoprotein 1/2
with a threshold of 1e−05
MR Test
Inverse variance weighted (fixed effects)
Inverse variance weighted (multiplicative random effects)
MR Egger
MR RAPS
Weighted median
Weighted mode
0.10
0.05
0.00
−0.05
0.09
0.12
0.15
0.18
SNP effect on Salivary acidic proline−rich phosphoprotein 1/2
SNP effect on Gut microbiota abundance (genus Catenibacterium id.2153)

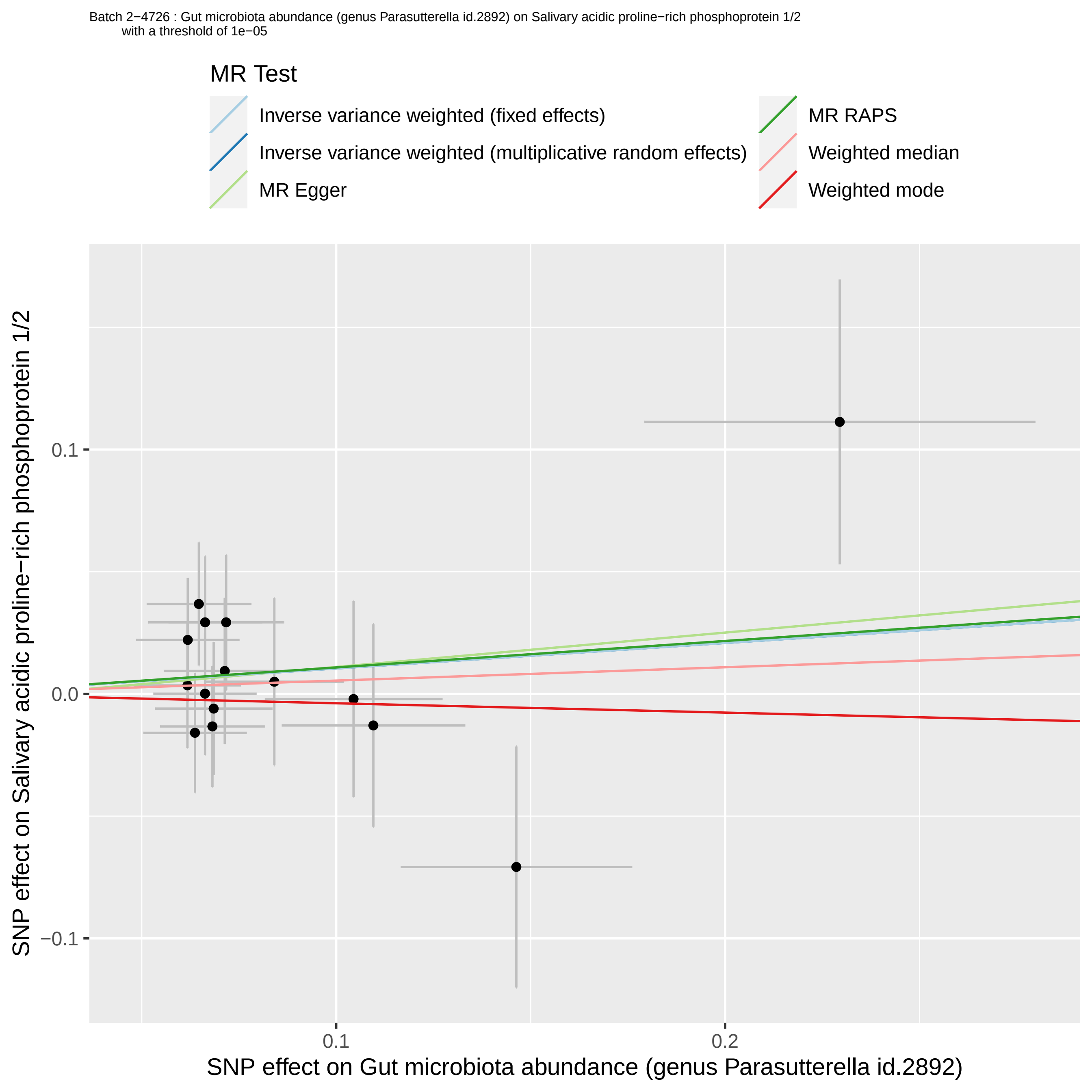

Batch 2−4726 : Gut microbiota abundance (genus Parasutterella id.2892) on Salivary acidic proline−rich phosphoprotein 1/2
with a threshold of 1e−05
MR Test
Inverse variance weighted (fixed effects)
Inverse variance weighted (multiplicative random effects)
MR Egger
MR RAPS
Weighted median
Weighted mode
0.1
0.0
−0.1
0.1
0.2
SNP effect on Salivary acidic proline−rich phosphoprotein 1/2
SNP effect on Gut microbiota abundance (genus Parasutterella id.2892)

Batch 2−4727 : Gut microbiota abundance (genus Catenibacterium id.2153) on DNA primase small subunit
with a threshold of 1e−05

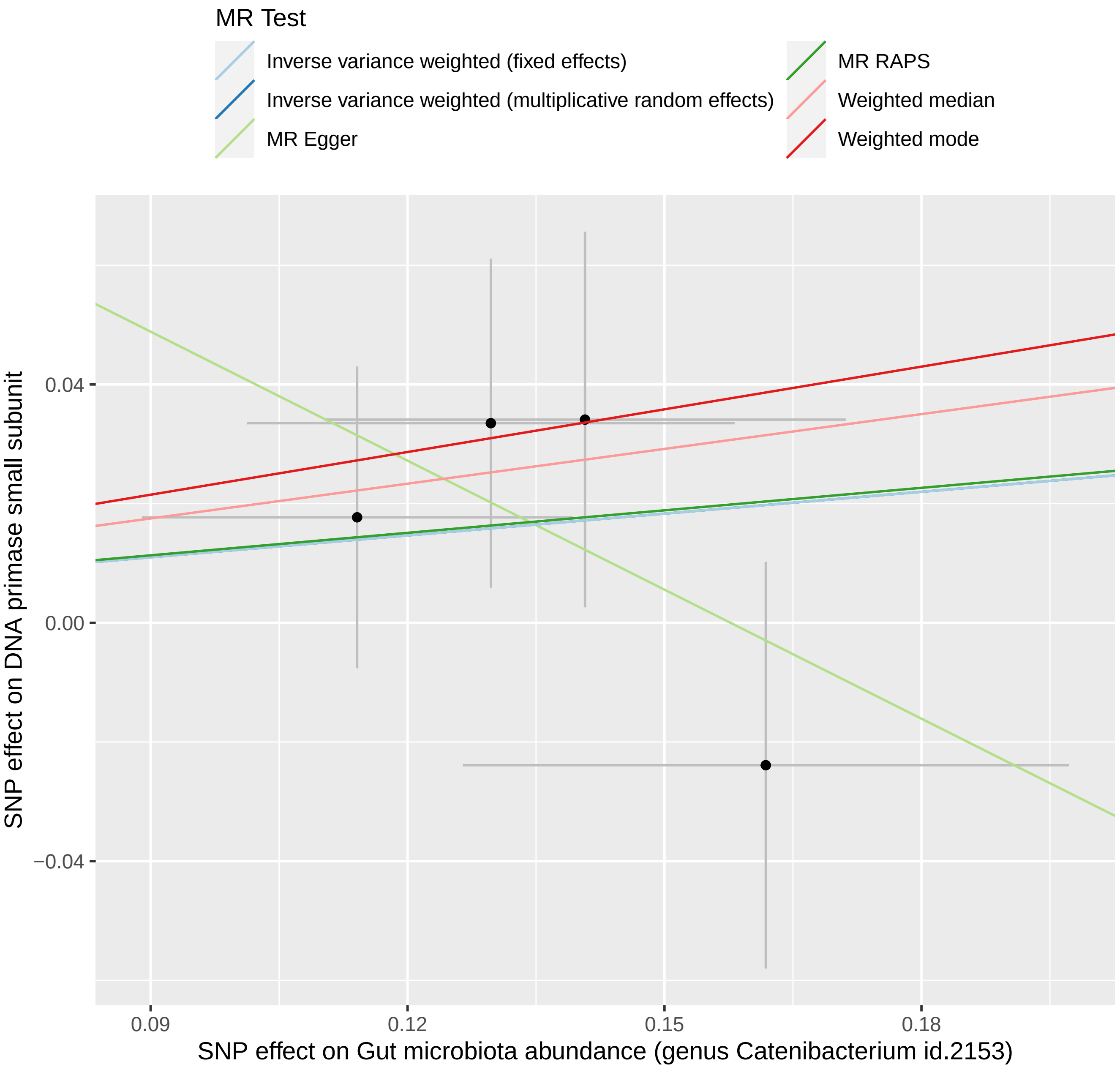

Batch 2−4728 : Gut microbiota abundance (genus Parasutterella id.2892) on DNA primase small subunit
with a threshold of 1e−05

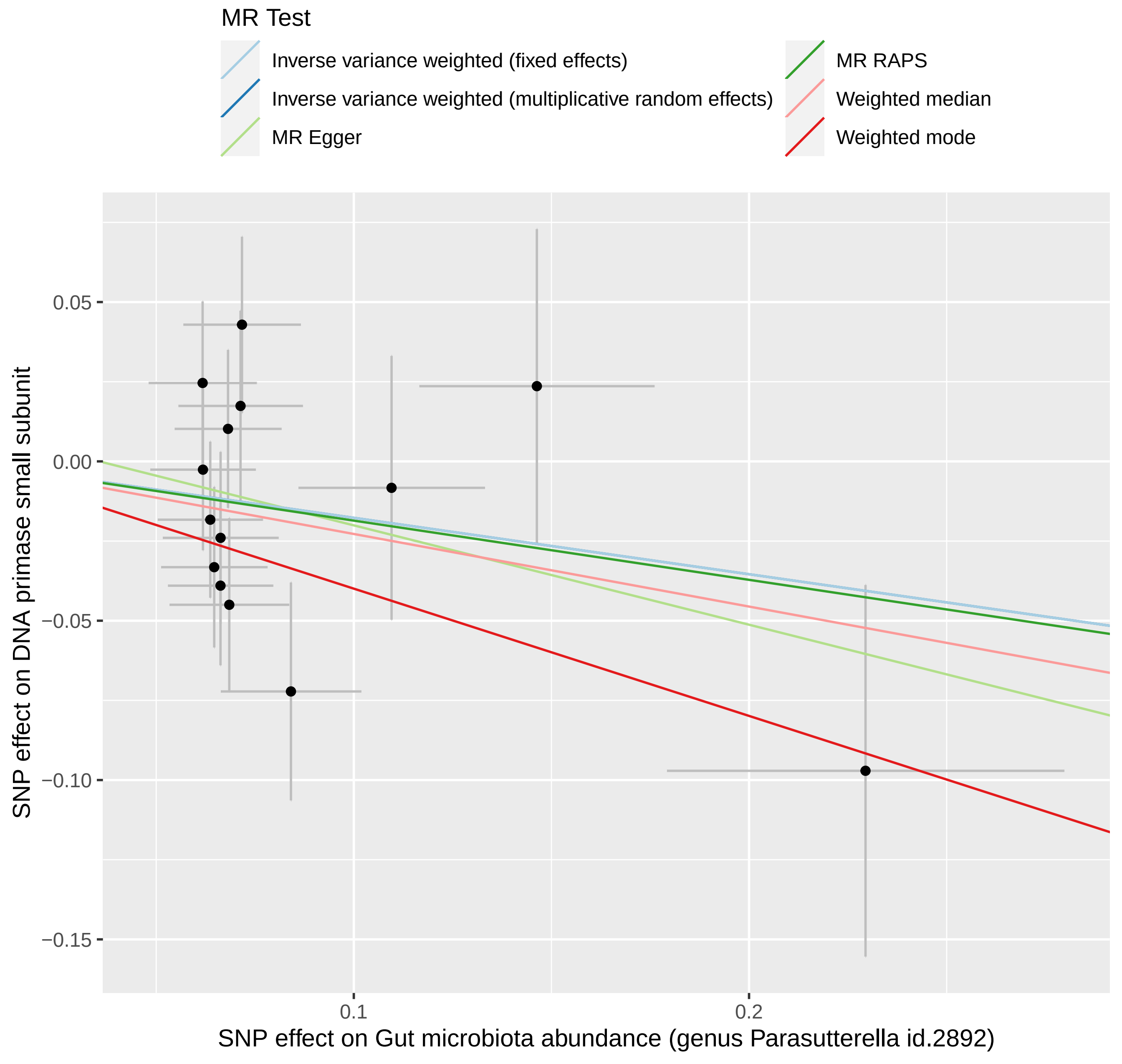

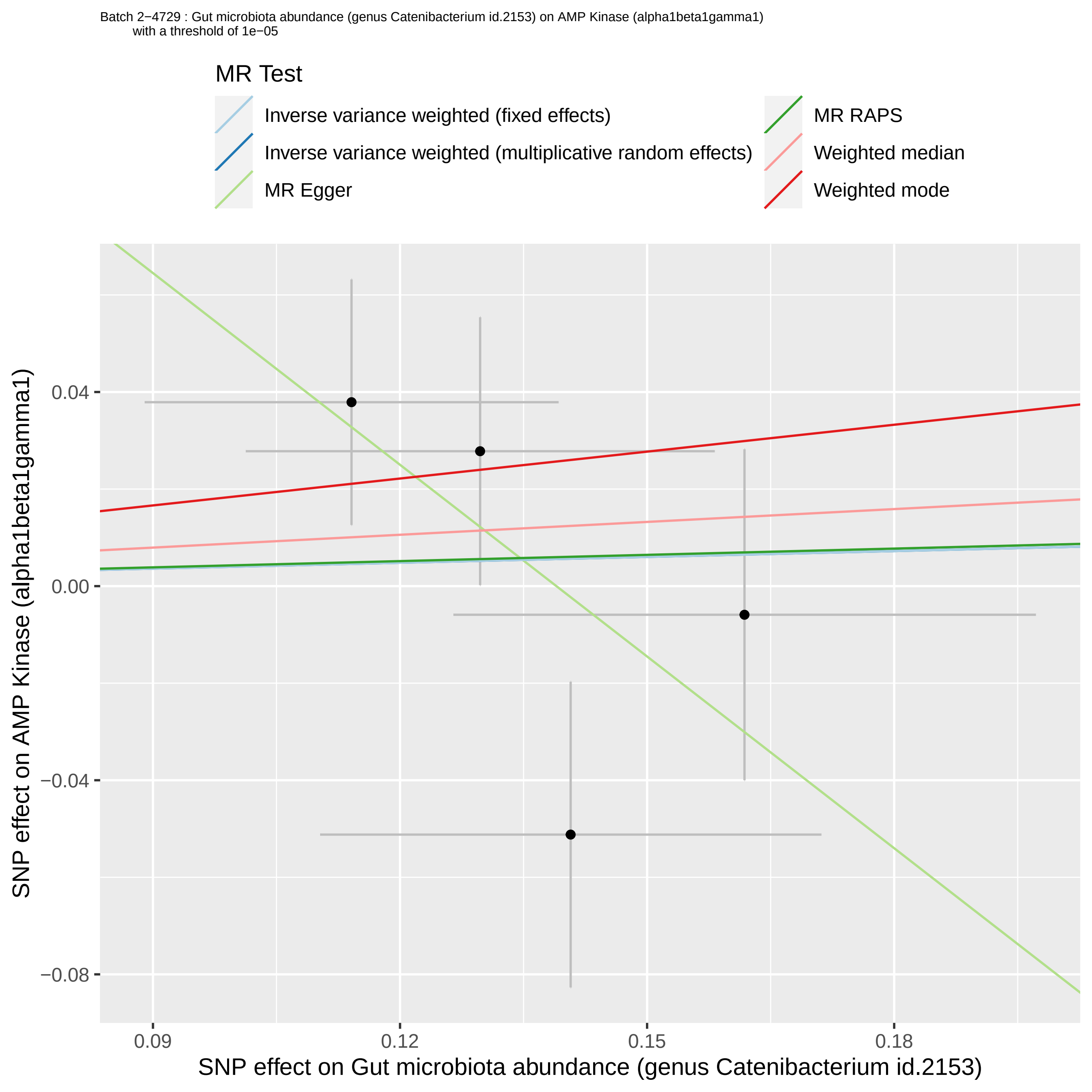
Batch 2−4729 : Gut microbiota abundance (genus Catenibacterium id.2153) on AMP Kinase (alpha1beta1gamma1)
with a threshold of 1e−05
MR Test
Inverse variance weighted (fixed effects)
Inverse variance weighted (multiplicative random effects)
MR Egger
MR RAPS
Weighted median
Weighted mode
0.04
0.00
−0.04
−0.08
0.09
0.12
0.15
0.18
SNP effect on AMP Kinase (alpha1beta1gamma1)
SNP effect on Gut microbiota abundance (genus Catenibacterium id.2153)

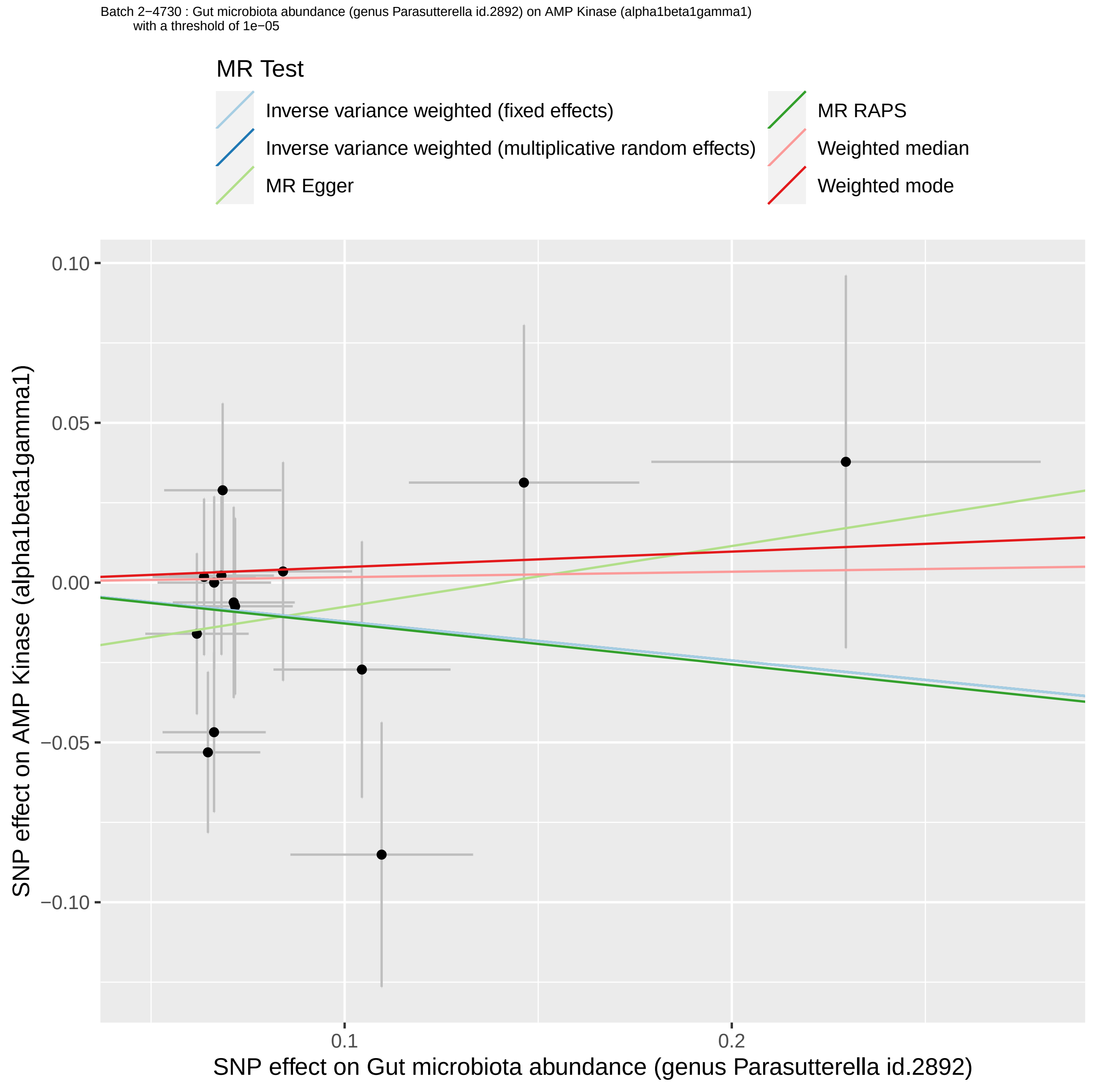

Batch 2−4730 : Gut microbiota abundance (genus Parasutterella id.2892) on AMP Kinase (alpha1beta1gamma1)
with a threshold of 1e−05
MR Test
Inverse variance weighted (fixed effects)
Inverse variance weighted (multiplicative random effects)
MR Egger
MR RAPS
Weighted median
Weighted mode
0.10
0.05
0.00
−0.05
−0.10
0.1
0.2
SNP effect on AMP Kinase (alpha1beta1gamma1)
SNP effect on Gut microbiota abundance (genus Parasutterella id.2892)

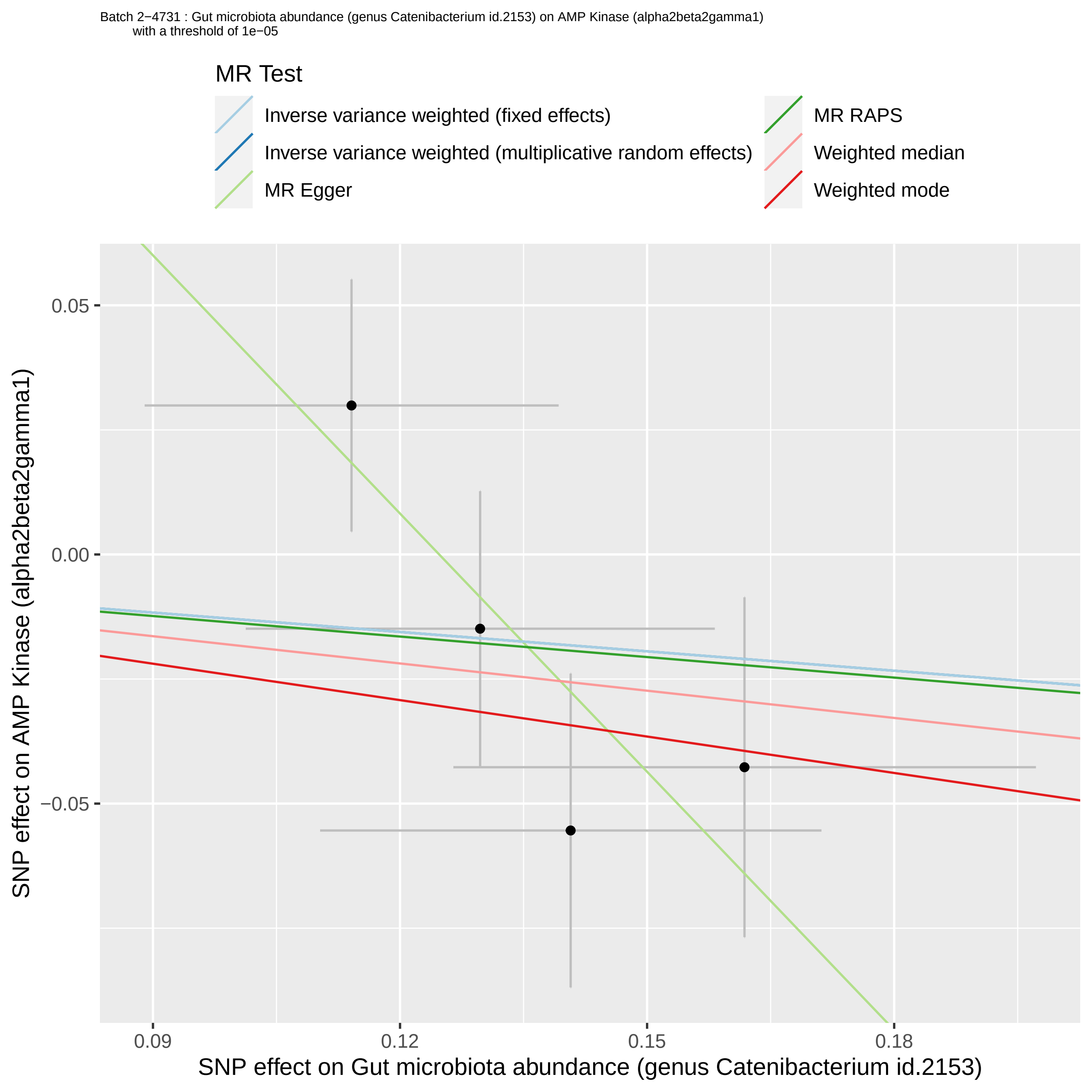
Batch 2−4731 : Gut microbiota abundance (genus Catenibacterium id.2153) on AMP Kinase (alpha2beta2gamma1)
with a threshold of 1e−05
MR Test
Inverse variance weighted (fixed effects)
Inverse variance weighted (multiplicative random effects)
MR Egger
MR RAPS
Weighted median
Weighted mode
0.05
0.00
−0.05
0.09
0.12
0.15
0.18
SNP effect on AMP Kinase (alpha2beta2gamma1)
SNP effect on Gut microbiota abundance (genus Catenibacterium id.2153)

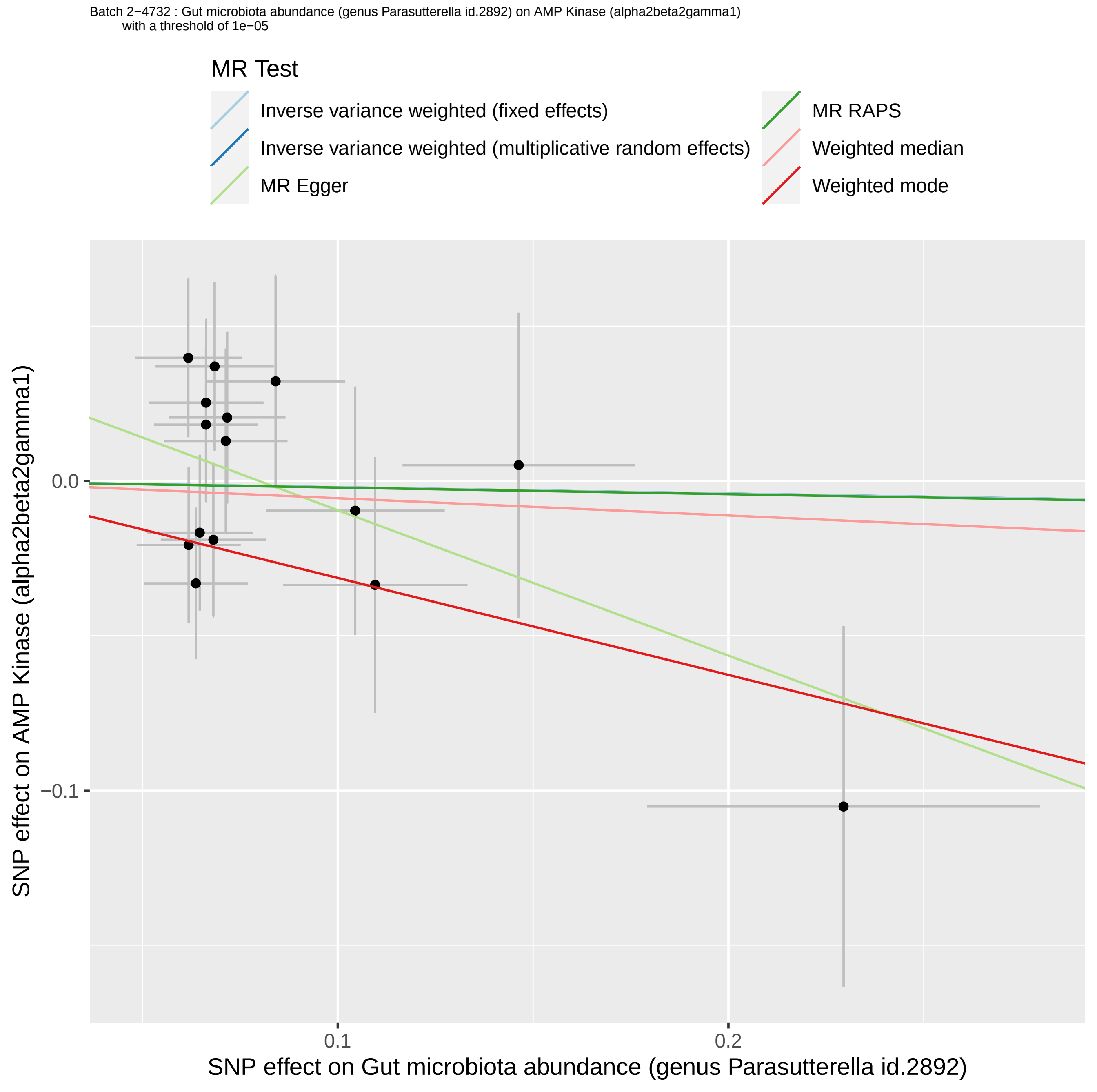

Batch 2−4732 : Gut microbiota abundance (genus Parasutterella id.2892) on AMP Kinase (alpha2beta2gamma1)
with a threshold of 1e−05
MR Test
Inverse variance weighted (fixed effects)
Inverse variance weighted (multiplicative random effects)
MR Egger
MR RAPS
Weighted median
Weighted mode
0.0
−0.1
0.1
0.2
SNP effect on AMP Kinase (alpha2beta2gamma1)
SNP effect on Gut microbiota abundance (genus Parasutterella id.2892)

Batch 2−4733 : Gut microbiota abundance (genus Catenibacterium id.2153) on cAMP−dependent protein kinase catalytic subunit alpha
with a threshold of 1e−05

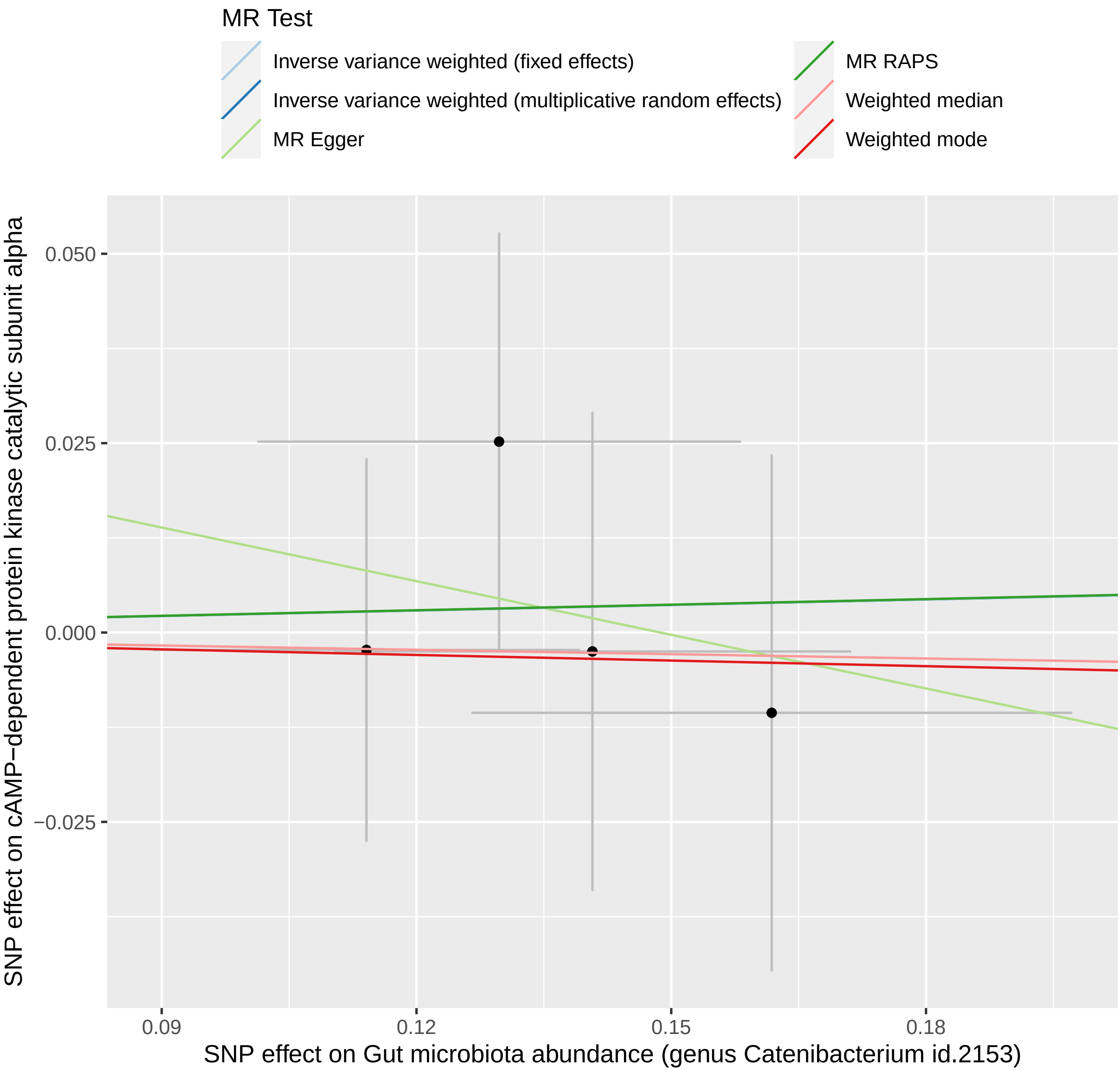

Batch 2−4734 : Gut microbiota abundance (genus Parasutterella id.2892) on cAMP−dependent protein kinase catalytic subunit alpha
with a threshold of 1e−05

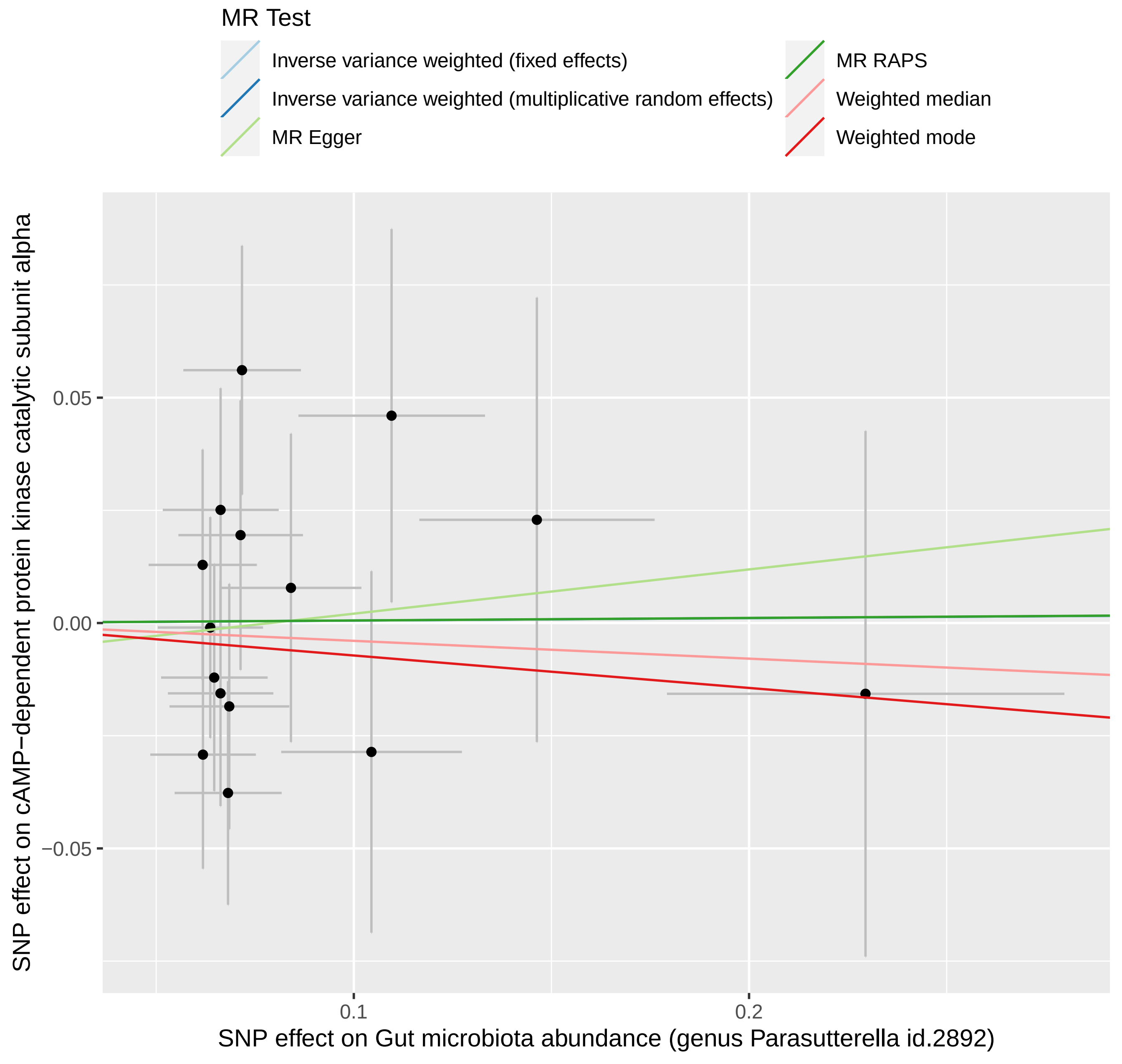

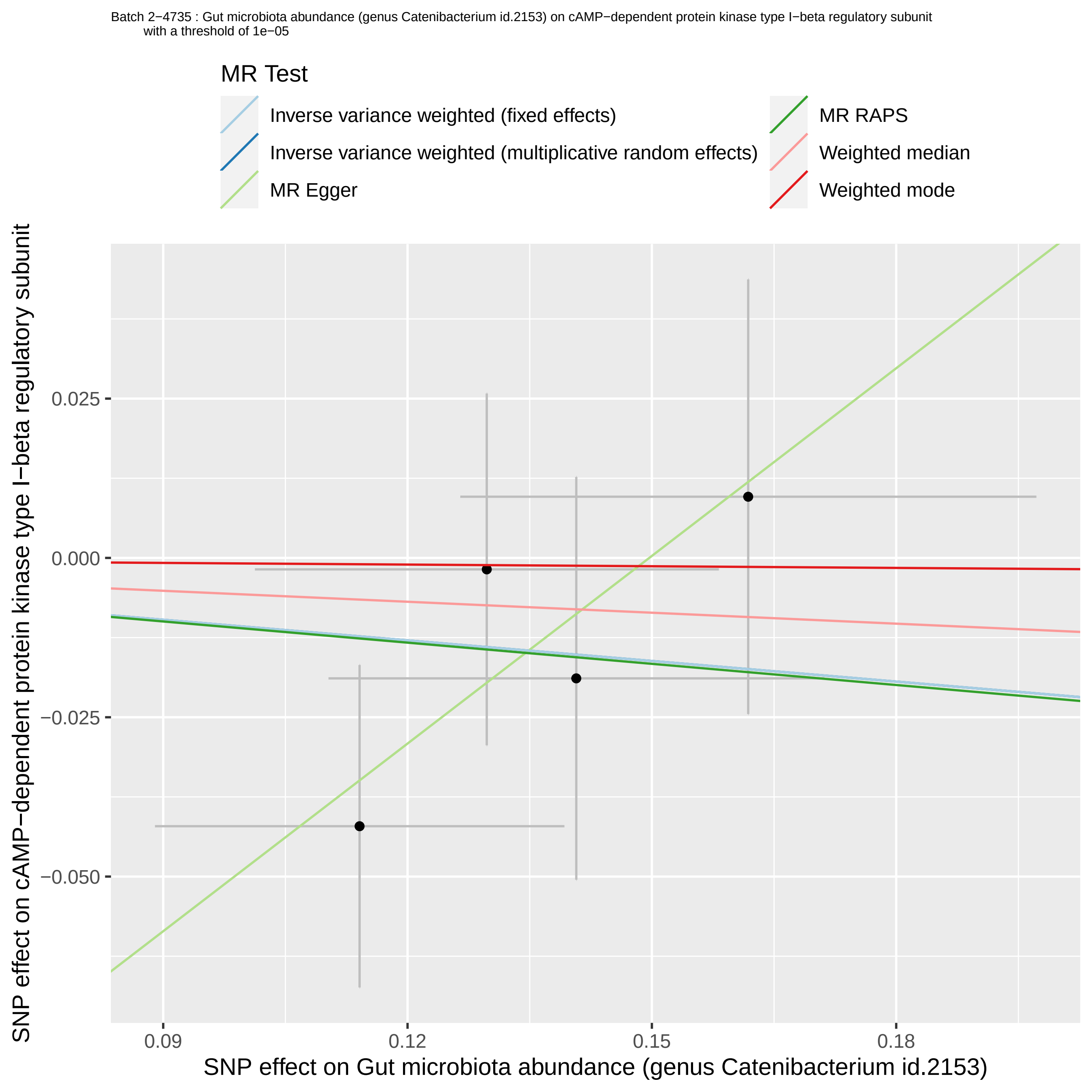
Batch 2−4735 : Gut microbiota abundance (genus Catenibacterium id.2153) on cAMP−dependent protein kinase type I−beta regulatory subunit
with a threshold of 1e−05
MR Test
Inverse variance weighted (fixed effects)
Inverse variance weighted (multiplicative random effects)
MR Egger
MR RAPS
Weighted median
Weighted mode
0.025
0.000
−0.025
−0.050
0.09
0.12
0.15
0.18
SNP effect on cAMP−dependent protein kinase type I−beta regulatory subunit
SNP effect on Gut microbiota abundance (genus Catenibacterium id.2153)

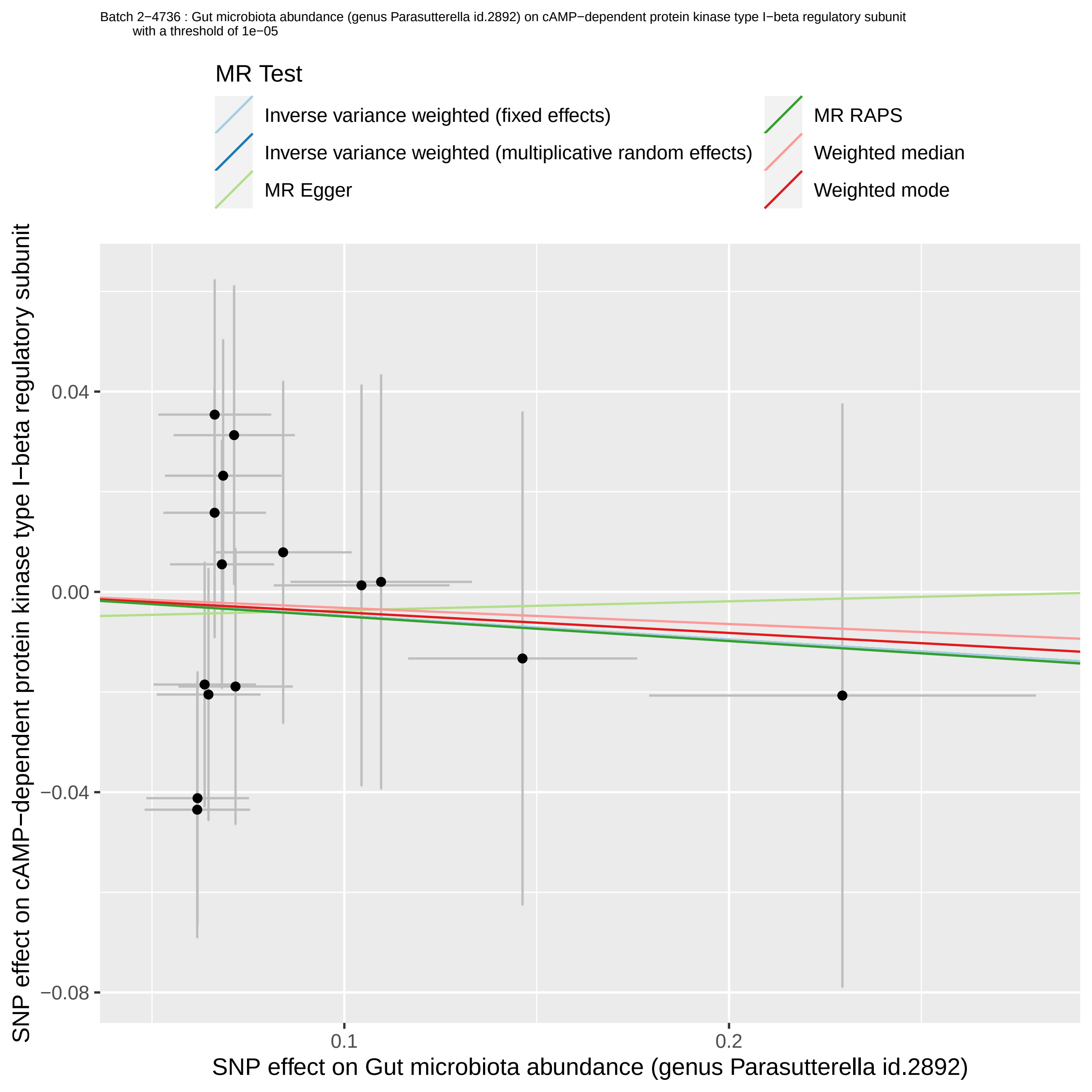

Batch 2−4736 : Gut microbiota abundance (genus Parasutterella id.2892) on cAMP−dependent protein kinase type I−beta regulatory subunit
with a threshold of 1e−05
MR Test
Inverse variance weighted (fixed effects)
Inverse variance weighted (multiplicative random effects)
MR Egger
MR RAPS
Weighted median
Weighted mode
0.04
0.00
−0.04
−0.08
0.1
0.2
SNP effect on Gut microbiota abundance (genus Parasutterella id.2892)
SNP effect on cAMP−dependent protein kinase type I−beta regulatory subunit

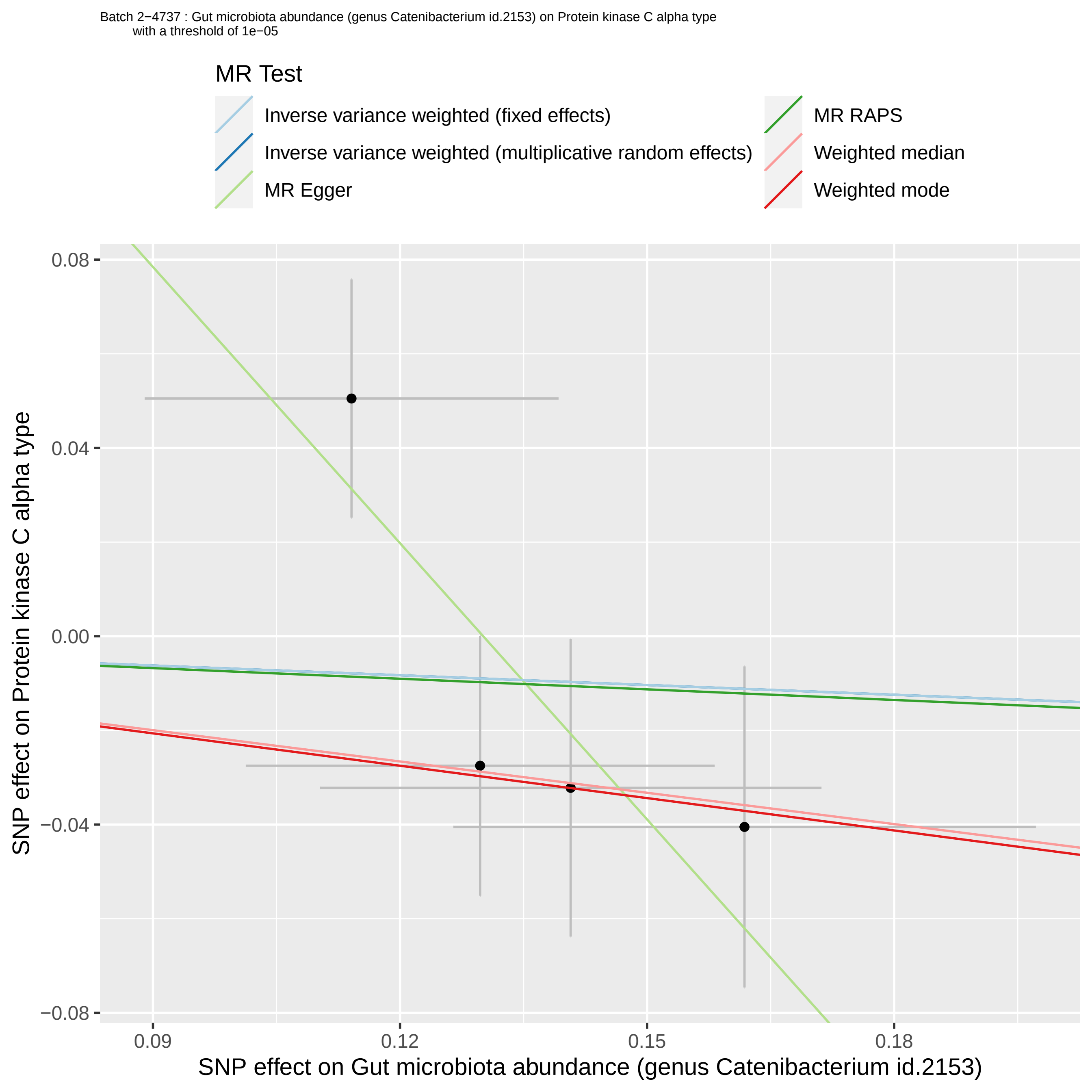

Batch 2−4737 : Gut microbiota abundance (genus Catenibacterium id.2153) on Protein kinase C alpha type
with a threshold of 1e−05
MR Test
Inverse variance weighted (fixed effects)
Inverse variance weighted (multiplicative random effects)
MR Egger
MR RAPS
Weighted median
Weighted mode
0.08
0.04
0.00
−0.04
−0.08
0.09
0.12
0.15
0.18
SNP effect on Protein kinase C alpha type
SNP effect on Gut microbiota abundance (genus Catenibacterium id.2153)

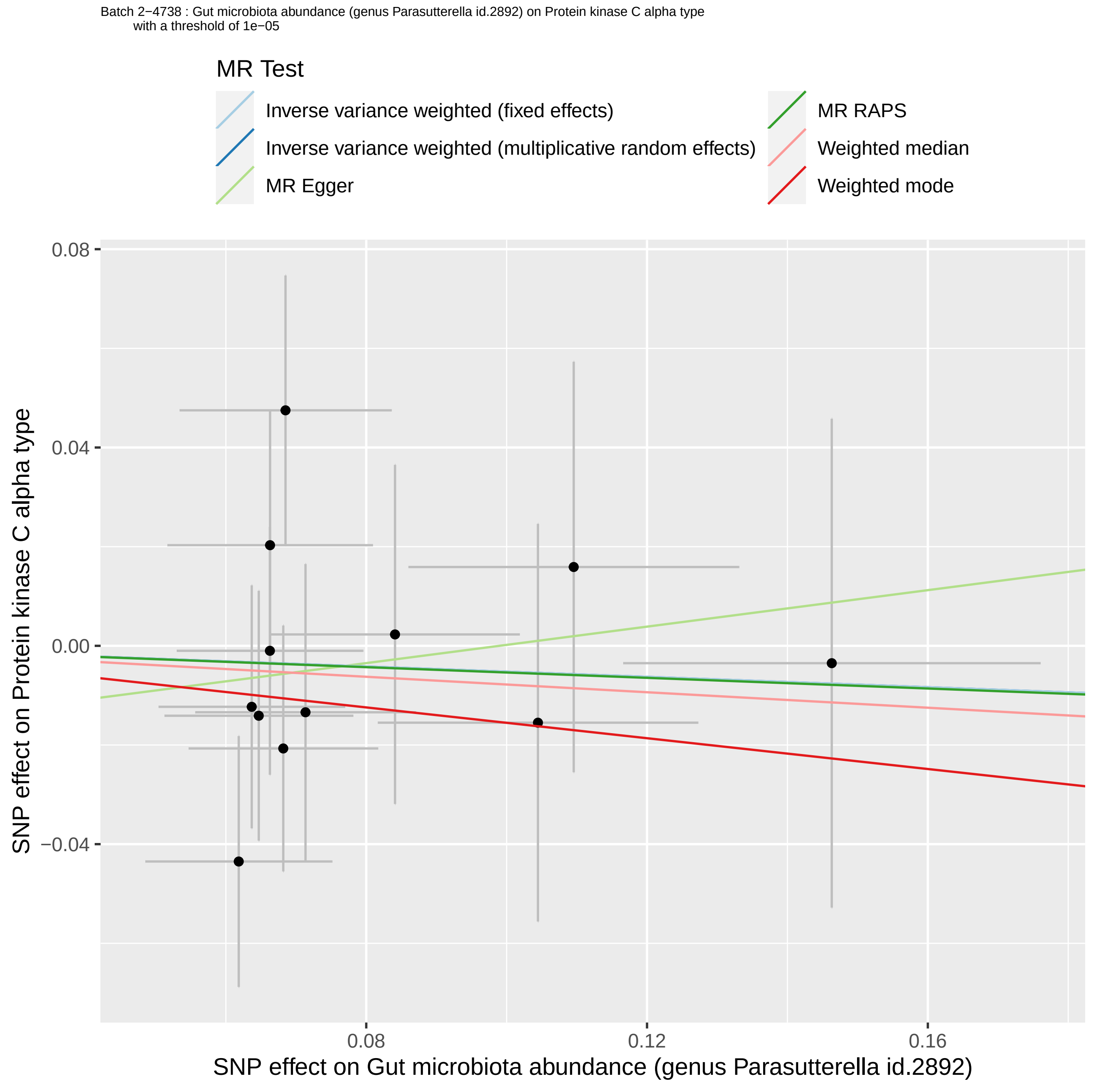

Batch 2−4738 : Gut microbiota abundance (genus Parasutterella id.2892) on Protein kinase C alpha type
with a threshold of 1e−05
MR Test
Inverse variance weighted (fixed effects)
Inverse variance weighted (multiplicative random effects)
MR Egger
MR RAPS
Weighted median
Weighted mode
0.08
0.04
0.00
−0.04
0.08
0.12
0.16
SNP effect on Protein kinase C alpha type
SNP effect on Gut microbiota abundance (genus Parasutterella id.2892)

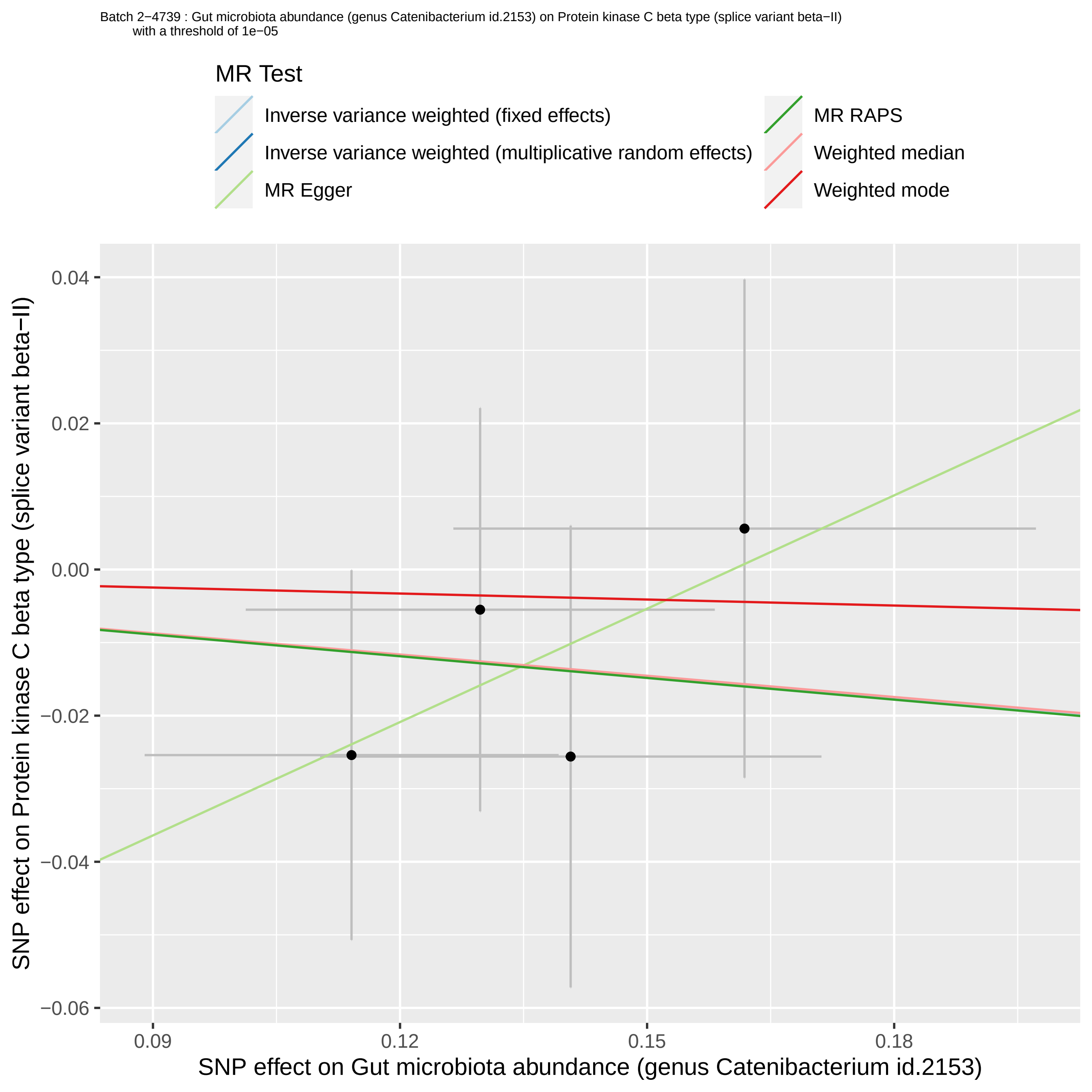
Batch 2−4739 : Gut microbiota abundance (genus Catenibacterium id.2153) on Protein kinase C beta type (splice variant beta−II)
with a threshold of 1e−05
MR Test
Inverse variance weighted (fixed effects)
Inverse variance weighted (multiplicative random effects)
MR Egger
MR RAPS
Weighted median
Weighted mode
0.04
0.02
0.00
−0.02
−0.04
−0.06
0.09
0.12
0.15
0.18
SNP effect on Protein kinase C beta type (splice variant beta−II)
SNP effect on Gut microbiota abundance (genus Catenibacterium id.2153)

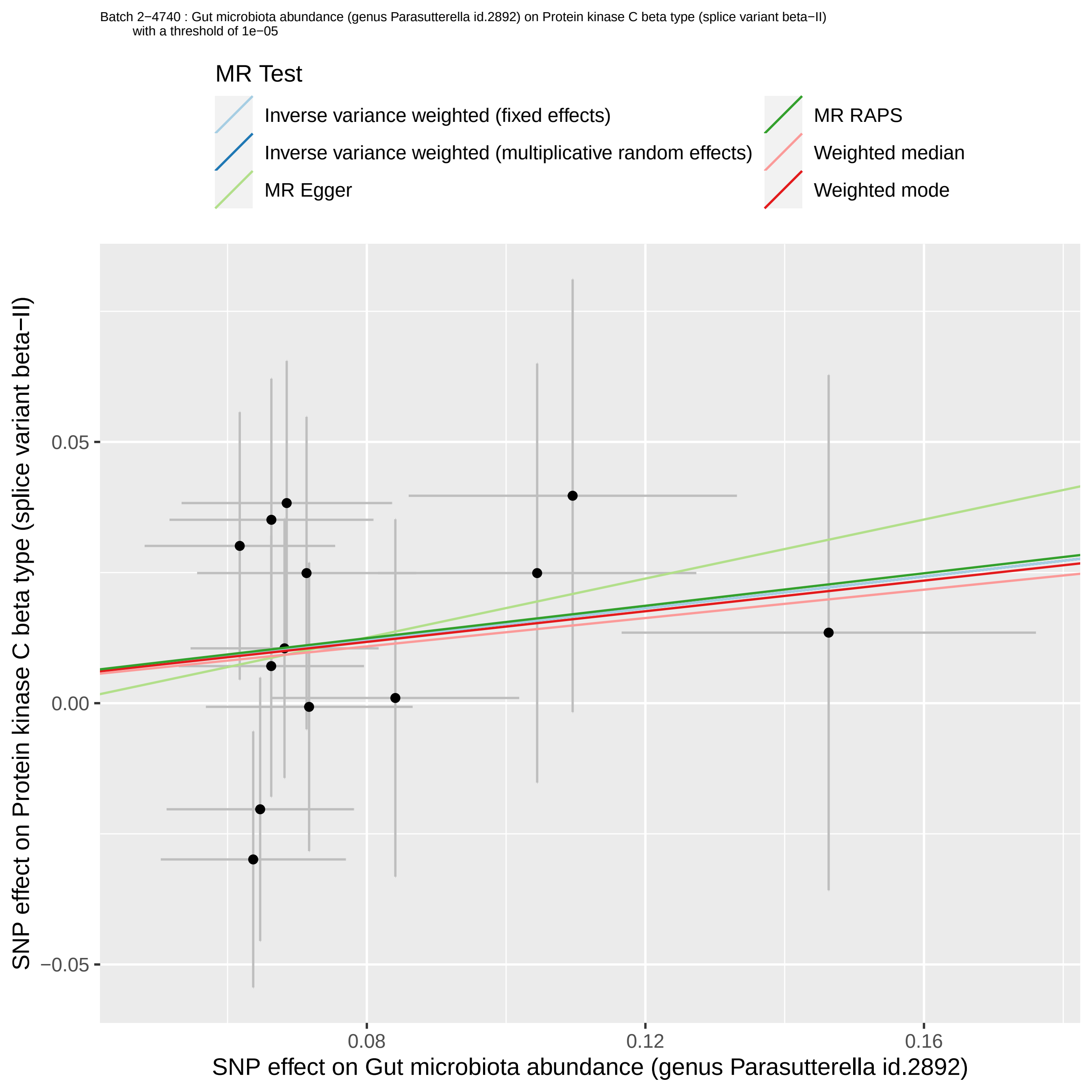
Batch 2−4740 : Gut microbiota abundance (genus Parasutterella id.2892) on Protein kinase C beta type (splice variant beta−II)
with a threshold of 1e−05
MR Test
Inverse variance weighted (fixed effects)
Inverse variance weighted (multiplicative random effects)
MR Egger
MR RAPS
Weighted median
Weighted mode
0.05
0.00
−0.05
0.08
0.12
0.16
SNP effect on Protein kinase C beta type (splice variant beta−II)
SNP effect on Gut microbiota abundance (genus Parasutterella id.2892)

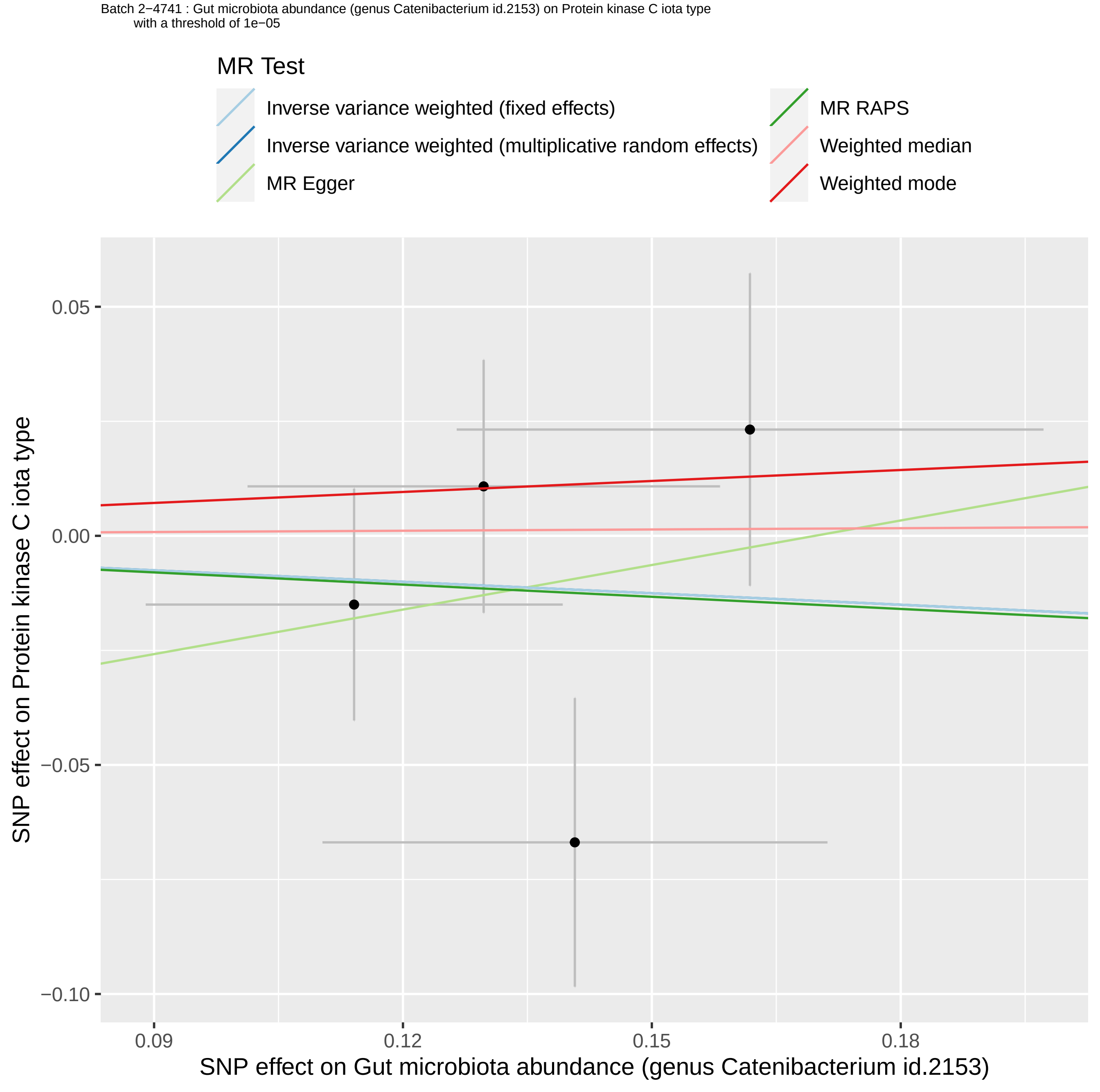

Batch 2−4741 : Gut microbiota abundance (genus Catenibacterium id.2153) on Protein kinase C iota type
with a threshold of 1e−05
MR Test
Inverse variance weighted (fixed effects)
Inverse variance weighted (multiplicative random effects)
MR Egger
MR RAPS
Weighted median
Weighted mode
0.05
0.00
−0.05
−0.10
0.09
0.12
0.15
0.18
SNP effect on Protein kinase C iota type
SNP effect on Gut microbiota abundance (genus Catenibacterium id.2153)

Batch 2−4742 : Gut microbiota abundance (genus Parasutterella id.2892) on Protein kinase C iota type
with a threshold of 1e−05

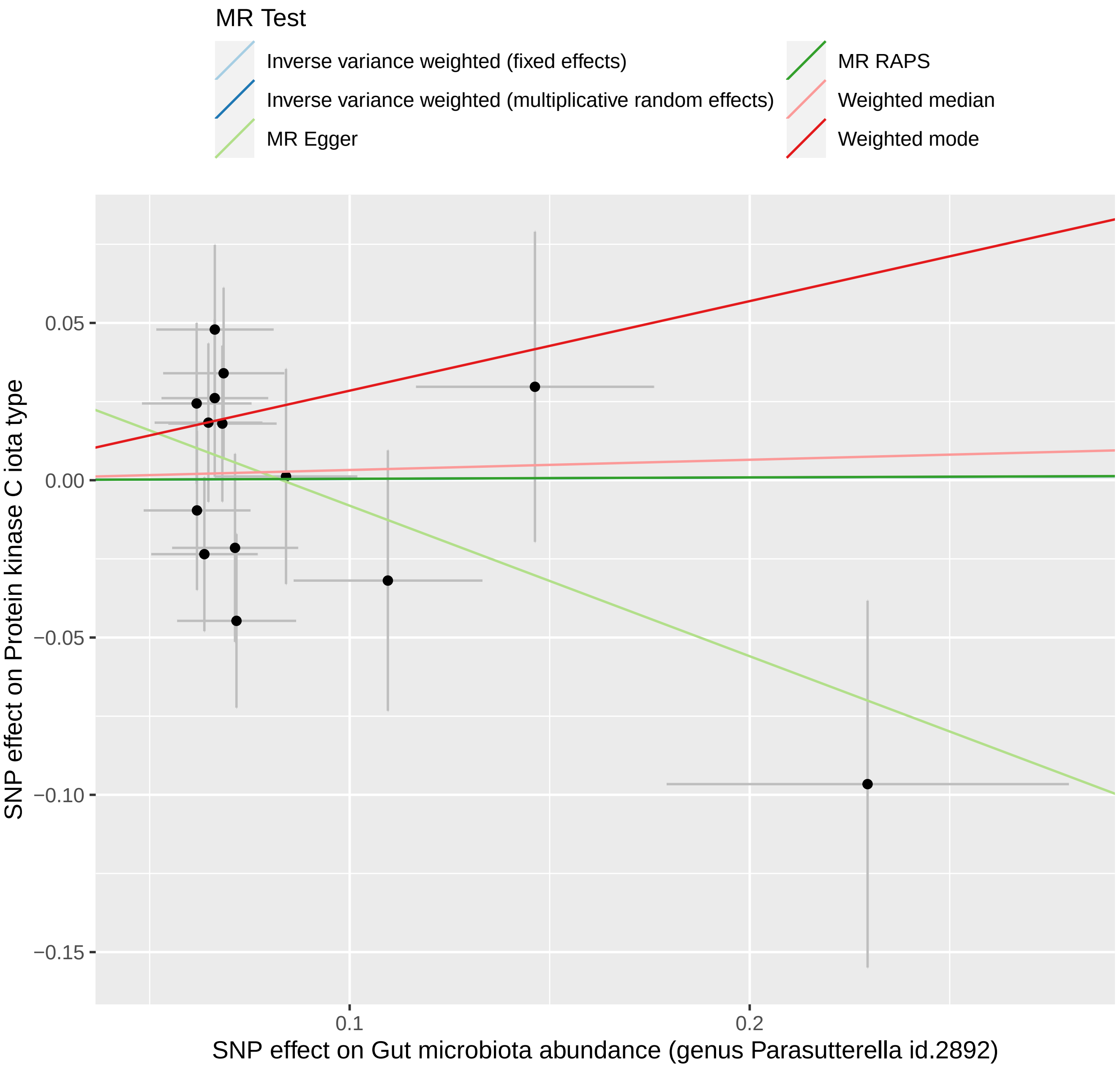

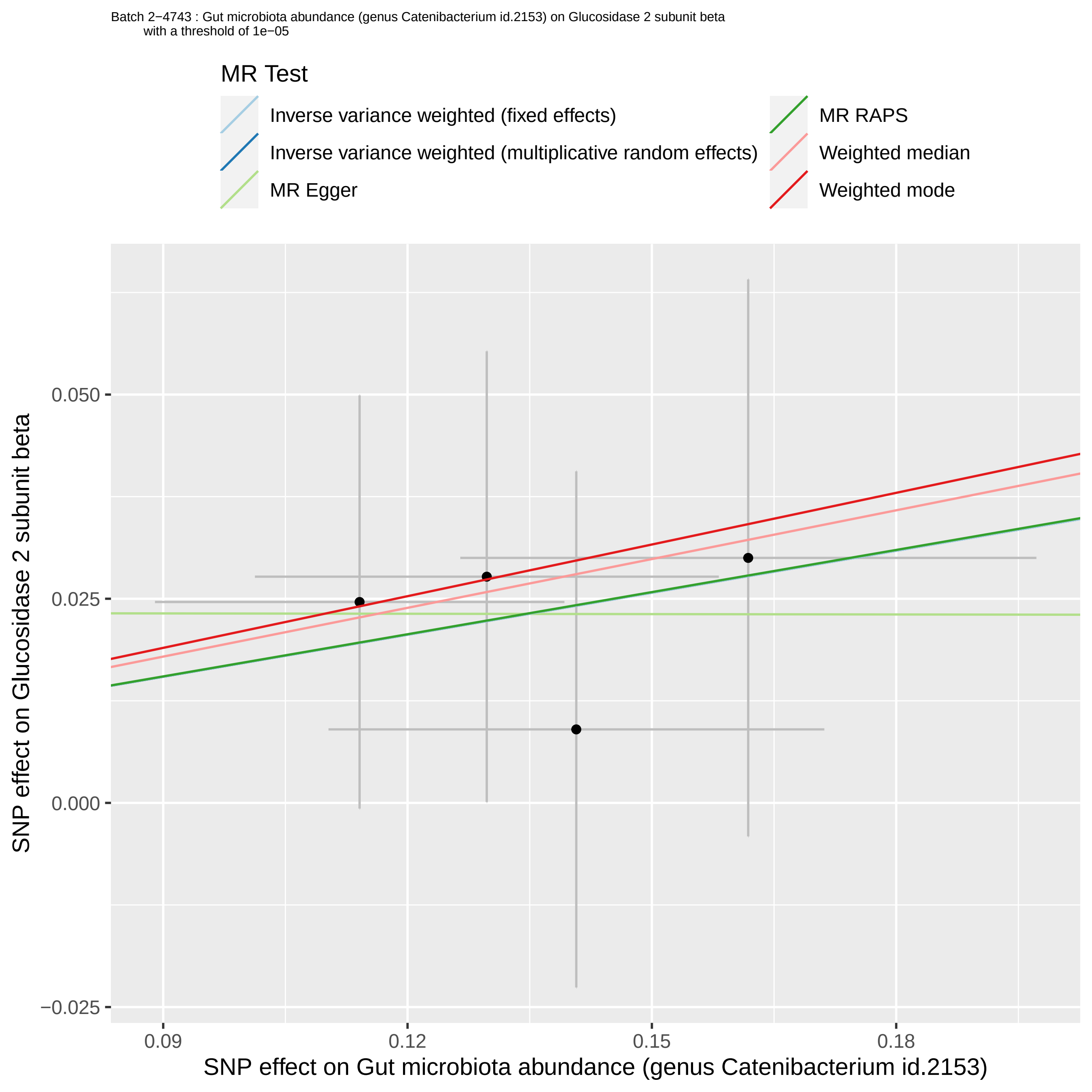

Batch 2−4743 : Gut microbiota abundance (genus Catenibacterium id.2153) on Glucosidase 2 subunit beta
with a threshold of 1e−05
MR Test
Inverse variance weighted (fixed effects)
Inverse variance weighted (multiplicative random effects)
MR Egger
MR RAPS
Weighted median
Weighted mode
0.050
0.025
0.000
−0.025
0.09
0.12
0.15
0.18
SNP effect on Glucosidase 2 subunit beta
SNP effect on Gut microbiota abundance (genus Catenibacterium id.2153)

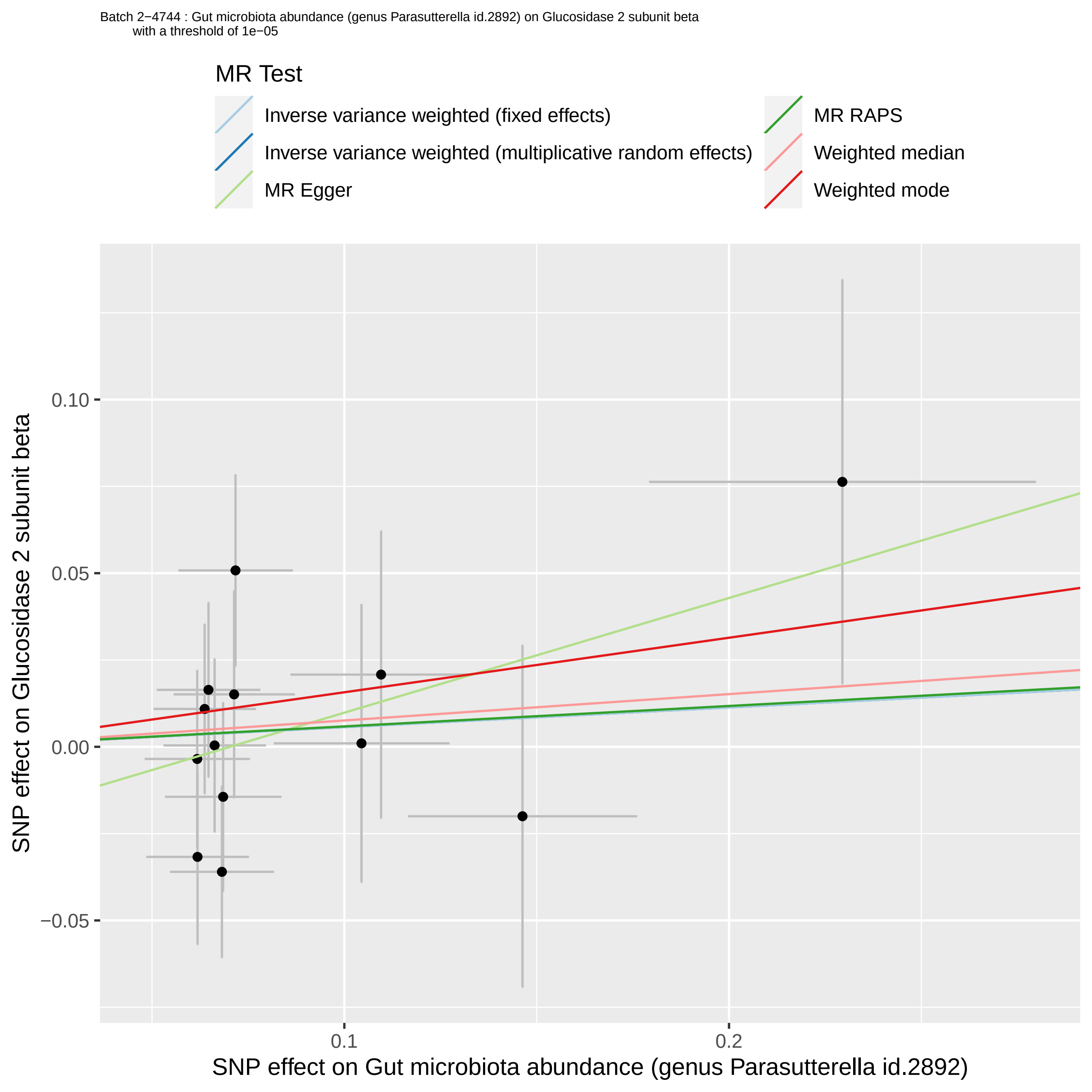
Batch 2−4744 : Gut microbiota abundance (genus Parasutterella id.2892) on Glucosidase 2 subunit beta
with a threshold of 1e−05
MR Test
Inverse variance weighted (fixed effects)
Inverse variance weighted (multiplicative random effects)
MR Egger
MR RAPS
Weighted median
Weighted mode
0.10
0.05
0.00
−0.05
0.1
0.2
SNP effect on Glucosidase 2 subunit beta
SNP effect on Gut microbiota abundance (genus Parasutterella id.2892)

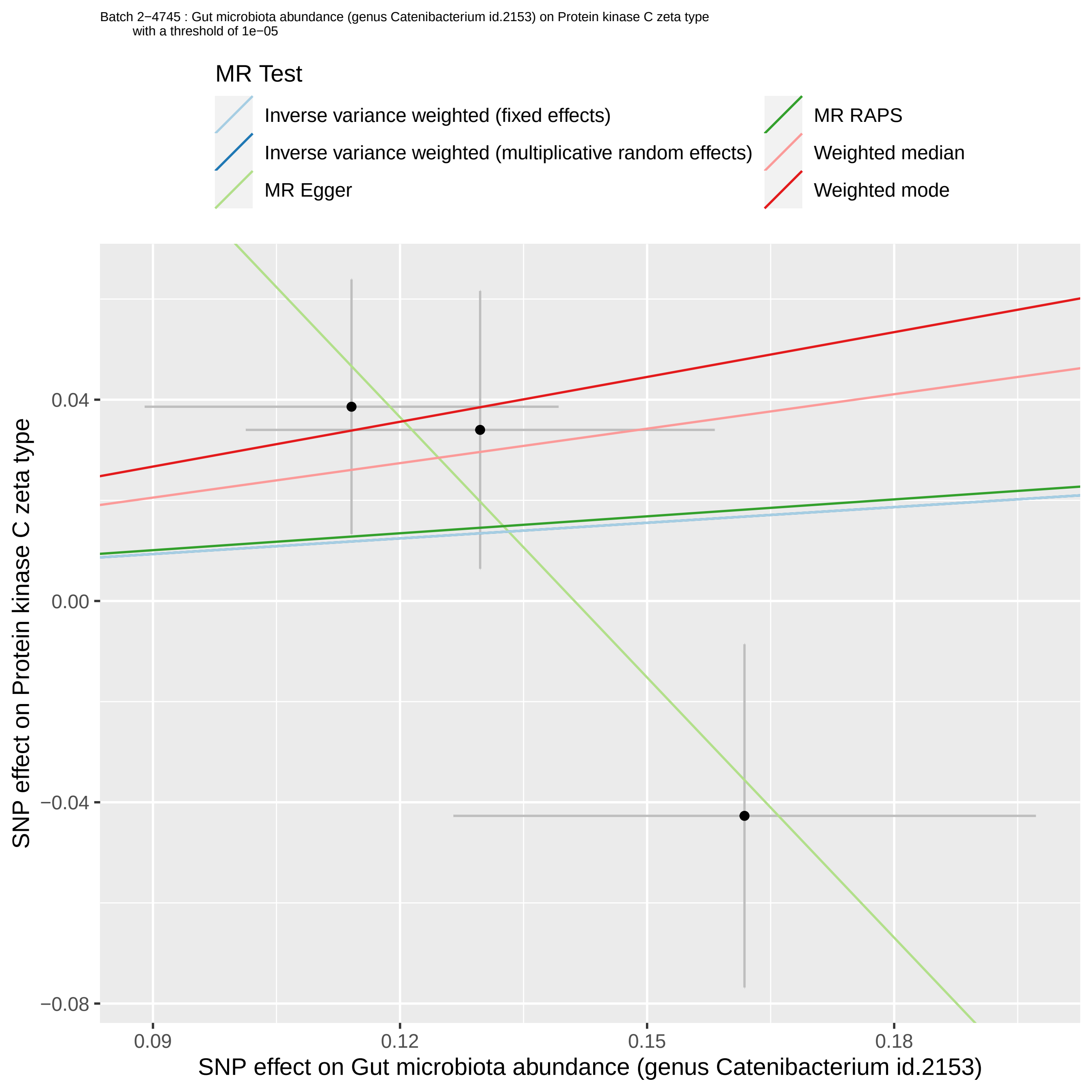
Batch 2−4745 : Gut microbiota abundance (genus Catenibacterium id.2153) on Protein kinase C zeta type
with a threshold of 1e−05
MR Test
Inverse variance weighted (fixed effects)
Inverse variance weighted (multiplicative random effects)
MR Egger
MR RAPS
Weighted median
Weighted mode
0.04
0.00
−0.04
−0.08
0.09
0.12
0.15
0.18
SNP effect on Protein kinase C zeta type
SNP effect on Gut microbiota abundance (genus Catenibacterium id.2153)

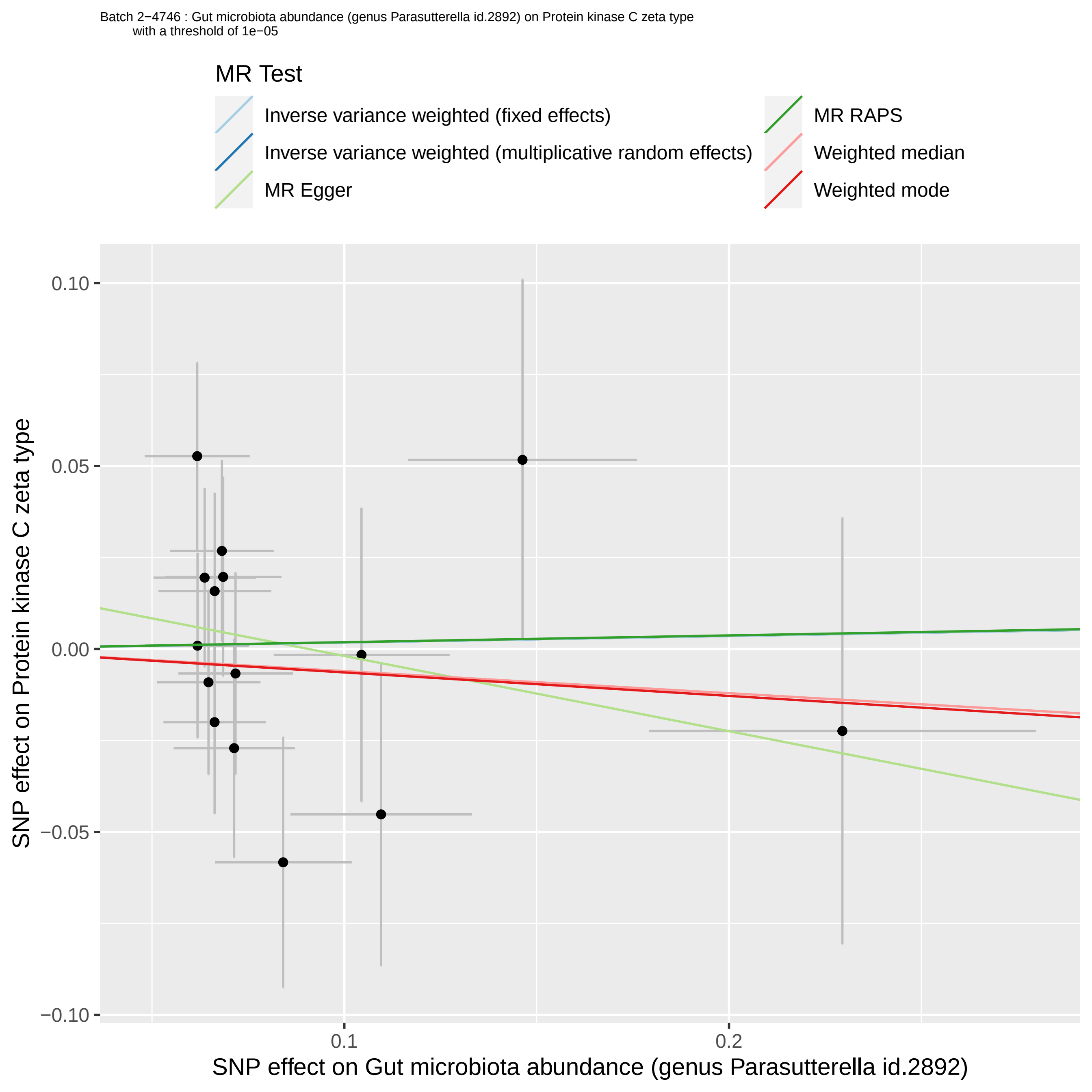

Batch 2−4746 : Gut microbiota abundance (genus Parasutterella id.2892) on Protein kinase C zeta type
with a threshold of 1e−05
MR Test
Inverse variance weighted (fixed effects)
Inverse variance weighted (multiplicative random effects)
MR Egger
MR RAPS
Weighted median
Weighted mode
0.10
0.05
0.00
−0.05
−0.10
0.1
0.2
SNP effect on Protein kinase C zeta type
SNP effect on Gut microbiota abundance (genus Parasutterella id.2892)

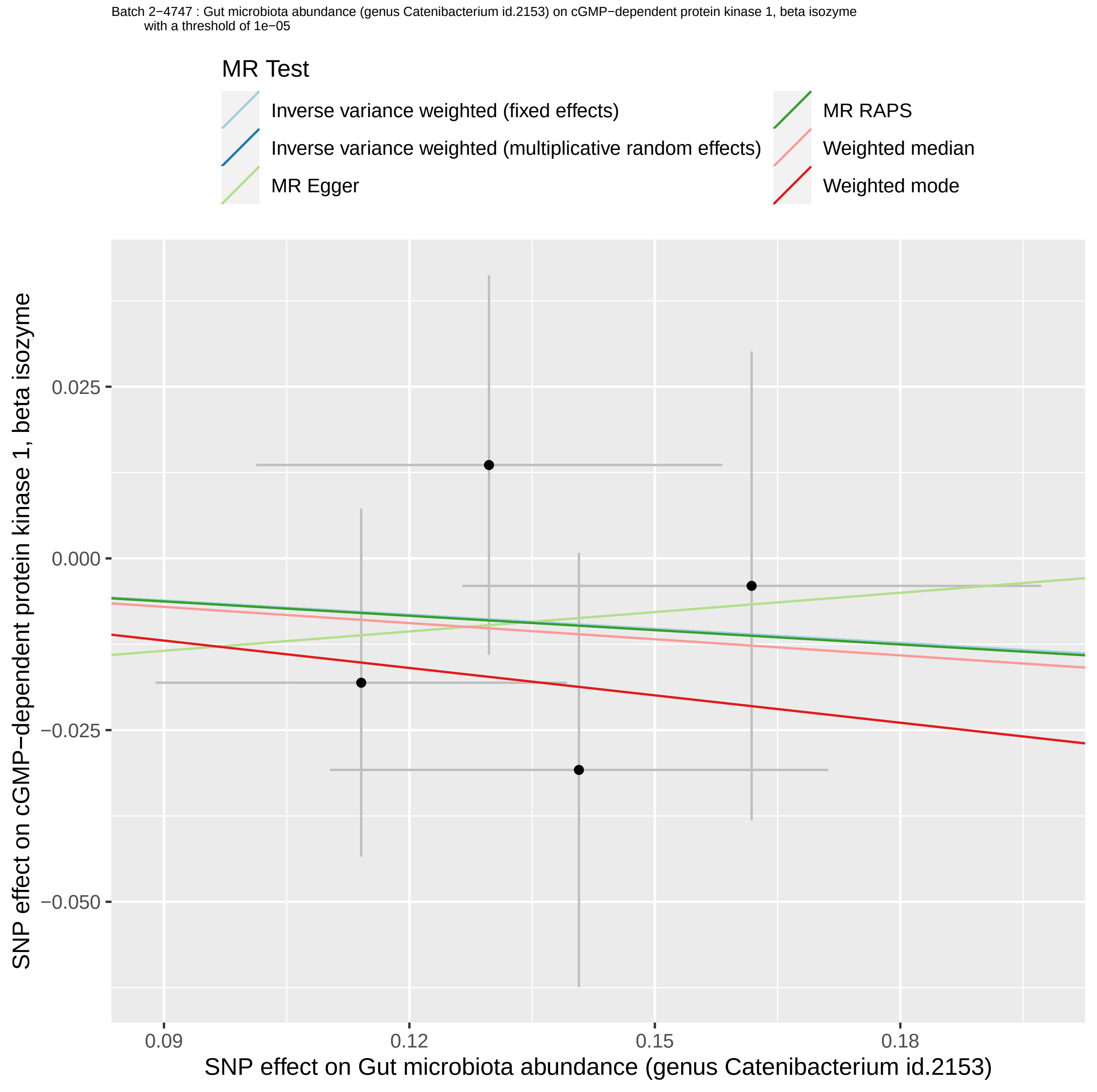
Batch 2−4747 : Gut microbiota abundance (genus Catenibacterium id.2153) on cGMP−dependent protein kinase 1, beta isozyme
with a threshold of 1e−05
MR Test
Inverse variance weighted (fixed effects)
Inverse variance weighted (multiplicative random effects)
MR Egger
MR RAPS
Weighted median
Weighted mode
0.025
0.000
−0.025
−0.050
0.09
0.12
0.15
0.18
SNP effect on cGMP−dependent protein kinase 1, beta isozyme
SNP effect on Gut microbiota abundance (genus Catenibacterium id.2153)

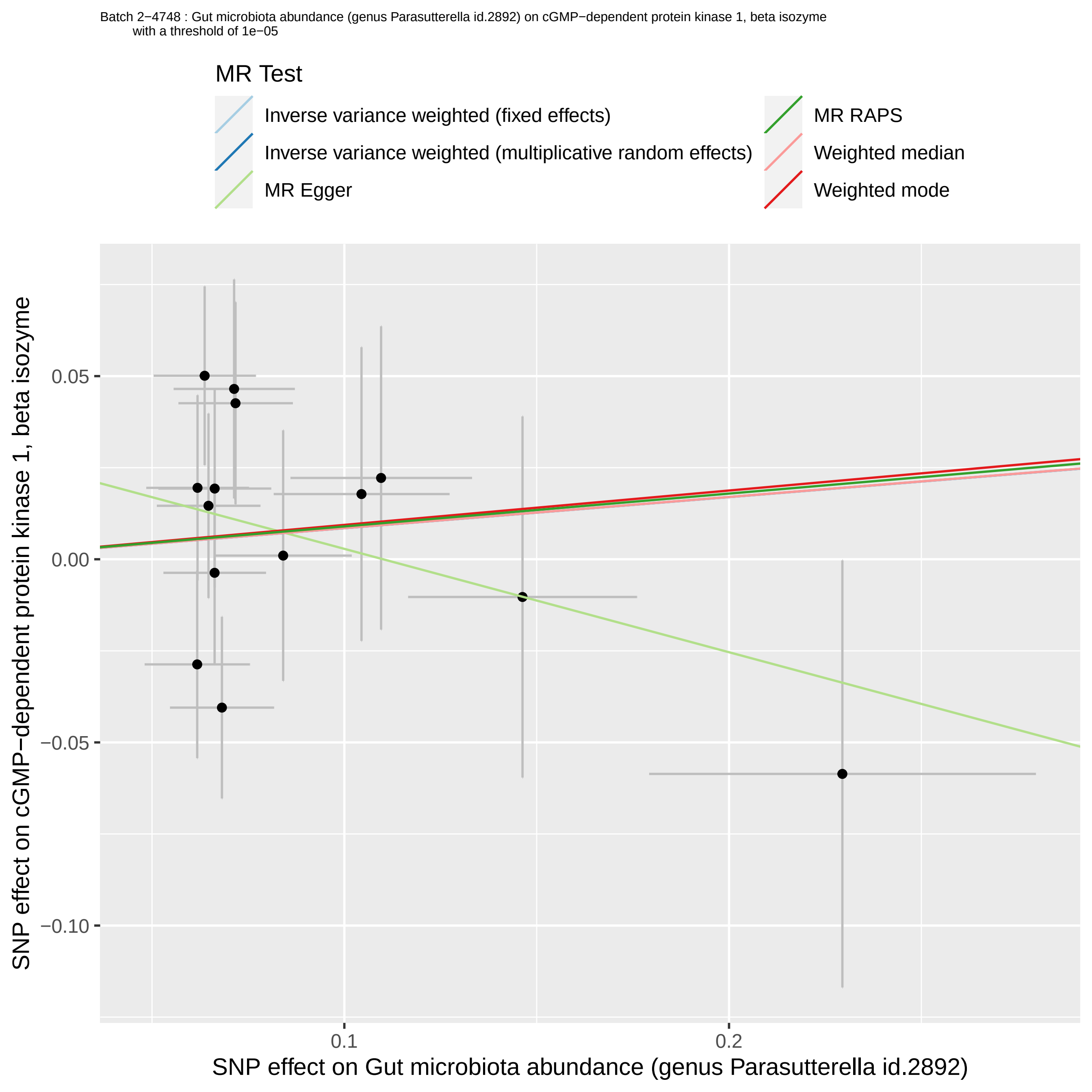
Batch 2−4748 : Gut microbiota abundance (genus Parasutterella id.2892) on cGMP−dependent protein kinase 1, beta isozyme
with a threshold of 1e−05
MR Test
Inverse variance weighted (fixed effects)
Inverse variance weighted (multiplicative random effects)
MR Egger
MR RAPS
Weighted median
Weighted mode
SNP effect on cGMP−dependent protein kinase 1, beta isozyme
0.05
0.00
−0.05
−0.10
0.1
0.2
SNP effect on Gut microbiota abundance (genus Parasutterella id.2892)

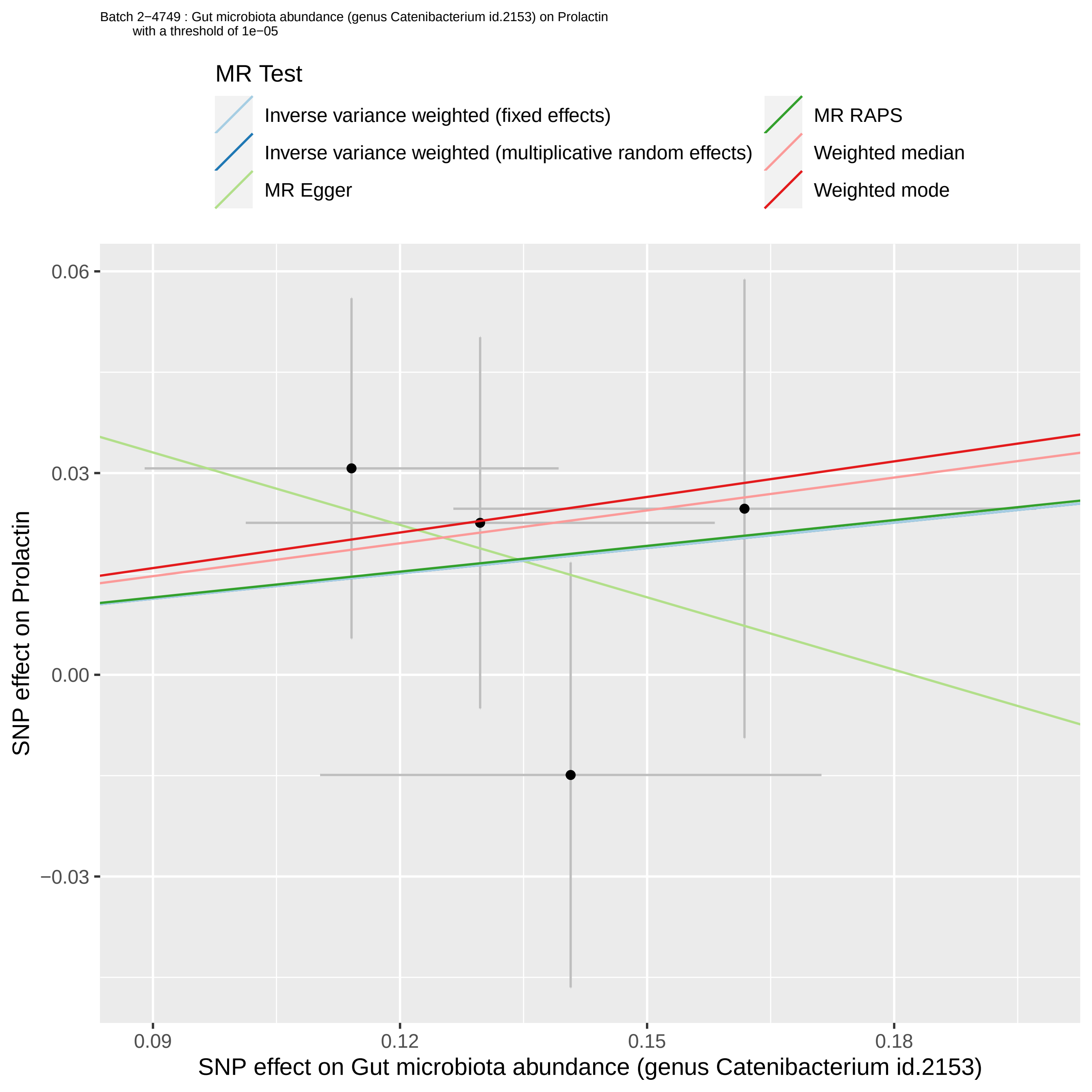
Batch 2−4749 : Gut microbiota abundance (genus Catenibacterium id.2153) on Prolactin
with a threshold of 1e−05
MR Test
Inverse variance weighted (fixed effects)
Inverse variance weighted (multiplicative random effects)
MR Egger
MR RAPS
Weighted median
Weighted mode
0.06
0.03
0.00
−0.03
0.09
0.12
0.15
0.18
SNP effect on Prolactin
SNP effect on Gut microbiota abundance (genus Catenibacterium id.2153)

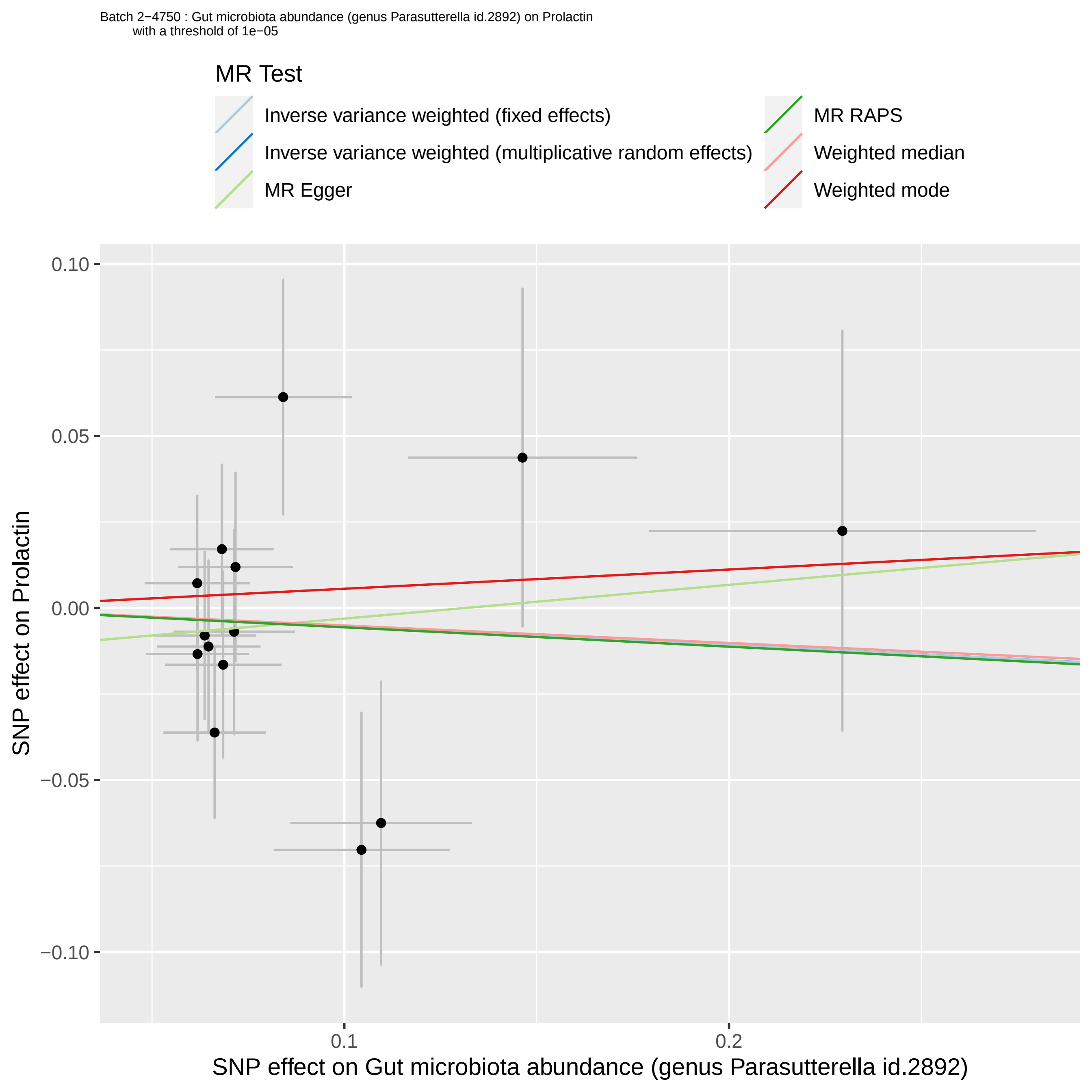

Batch 2−4750 : Gut microbiota abundance (genus Parasutterella id.2892) on Prolactin
with a threshold of 1e−05
MR Test
Inverse variance weighted (fixed effects)
Inverse variance weighted (multiplicative random effects)
MR Egger
MR RAPS
Weighted median
Weighted mode
SNP effect on Prolactin
0.10
0.05
0.00
−0.05
−0.10
0.1
0.2
SNP effect on Gut microbiota abundance (genus Parasutterella id.2892)

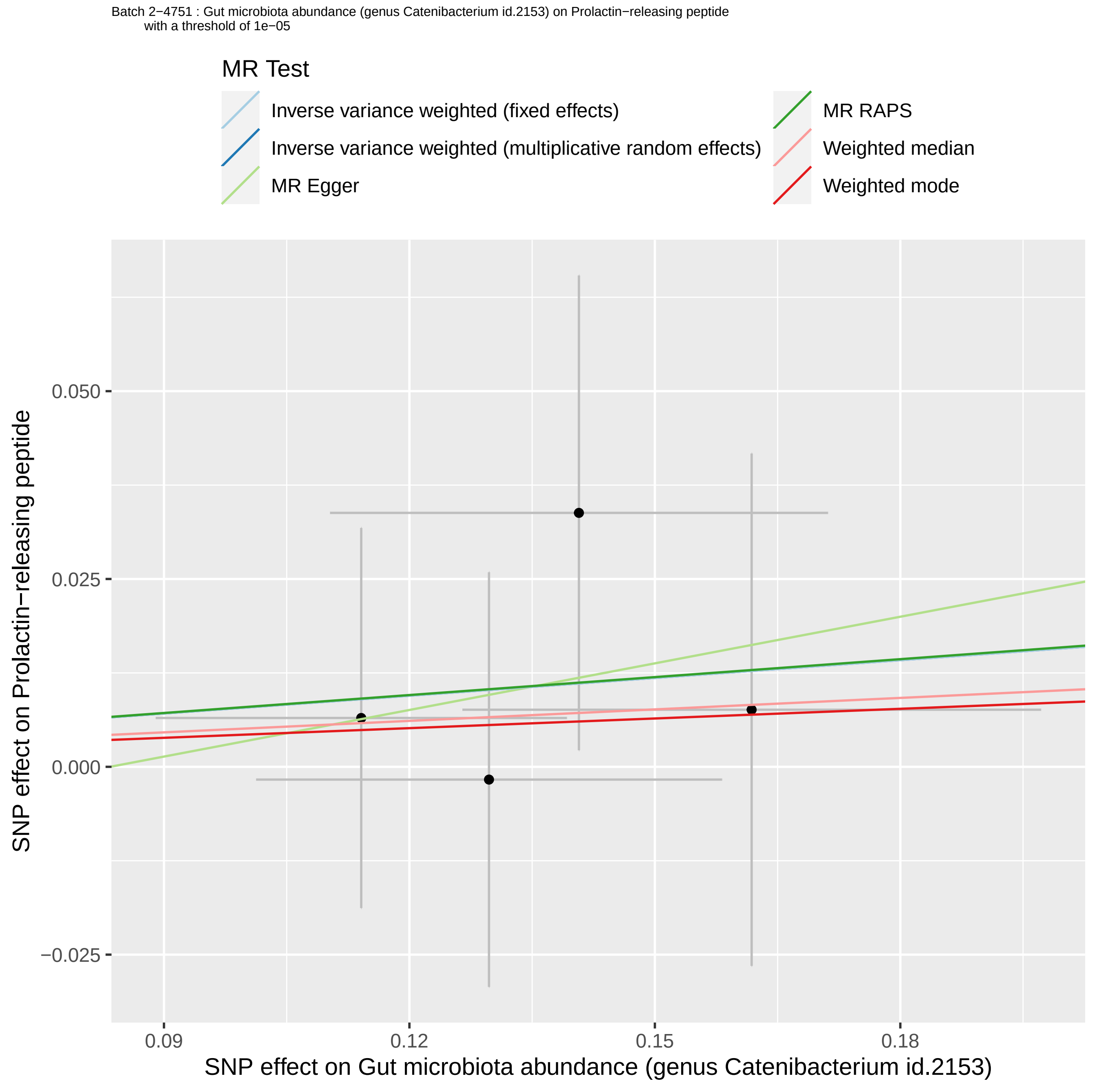

Batch 2−4751 : Gut microbiota abundance (genus Catenibacterium id.2153) on Prolactin−releasing peptide with a threshold of 1e−05
MR Test
Inverse variance weighted (fixed effects)
Inverse variance weighted (multiplicative random effects)
MR Egger
MR RAPS
Weighted median
Weighted mode
0.050
0.025
0.000
−0.025
SNP effect on Prolactin−releasing peptide
0.09
0.12
0.15
0.18
SNP effect on Gut microbiota abundance (genus Catenibacterium id.2153)

Batch 2−4752 : Gut microbiota abundance (genus Parasutterella id.2892) on Prolactin−releasing peptide
with a threshold of 1e−05

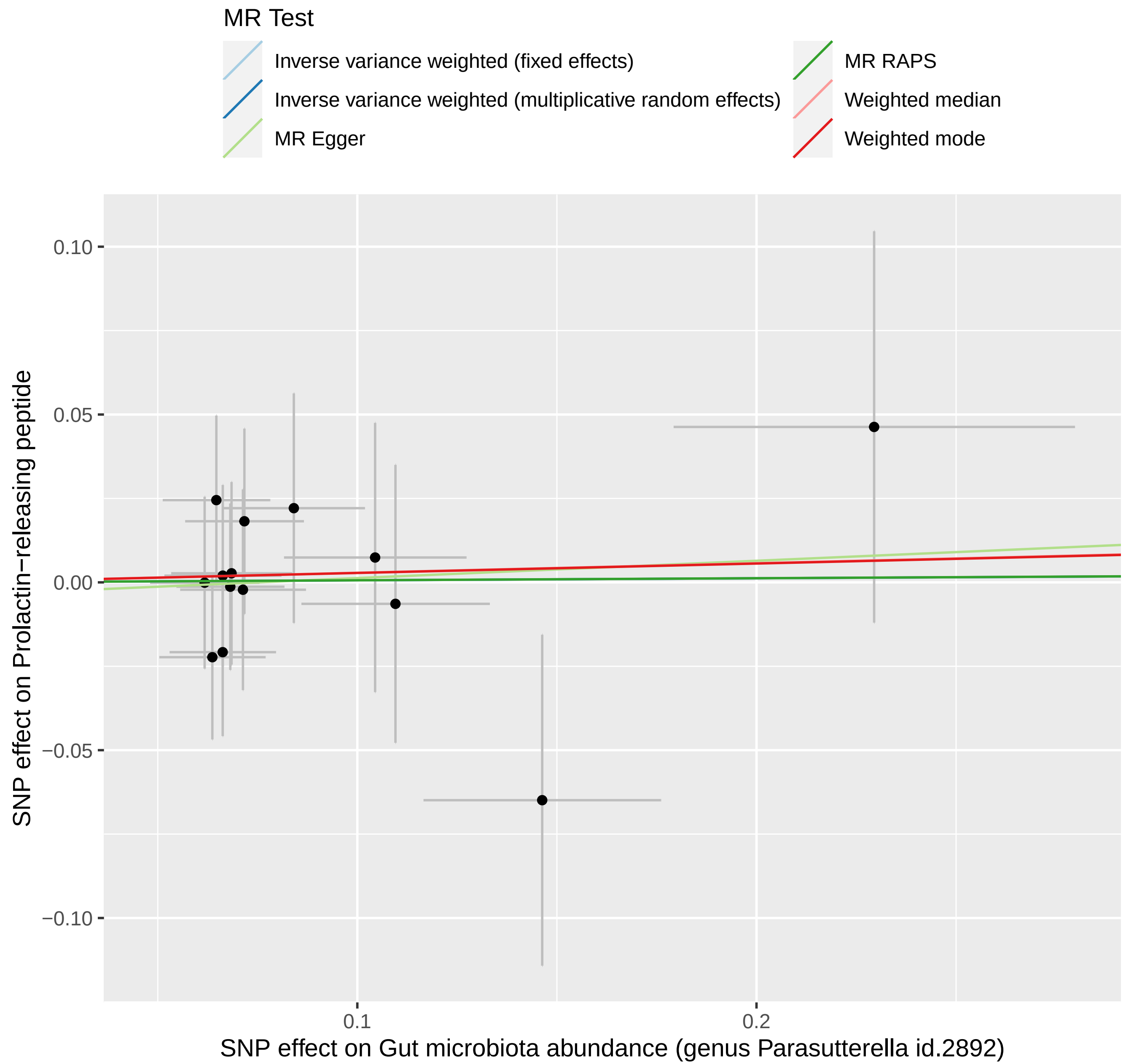

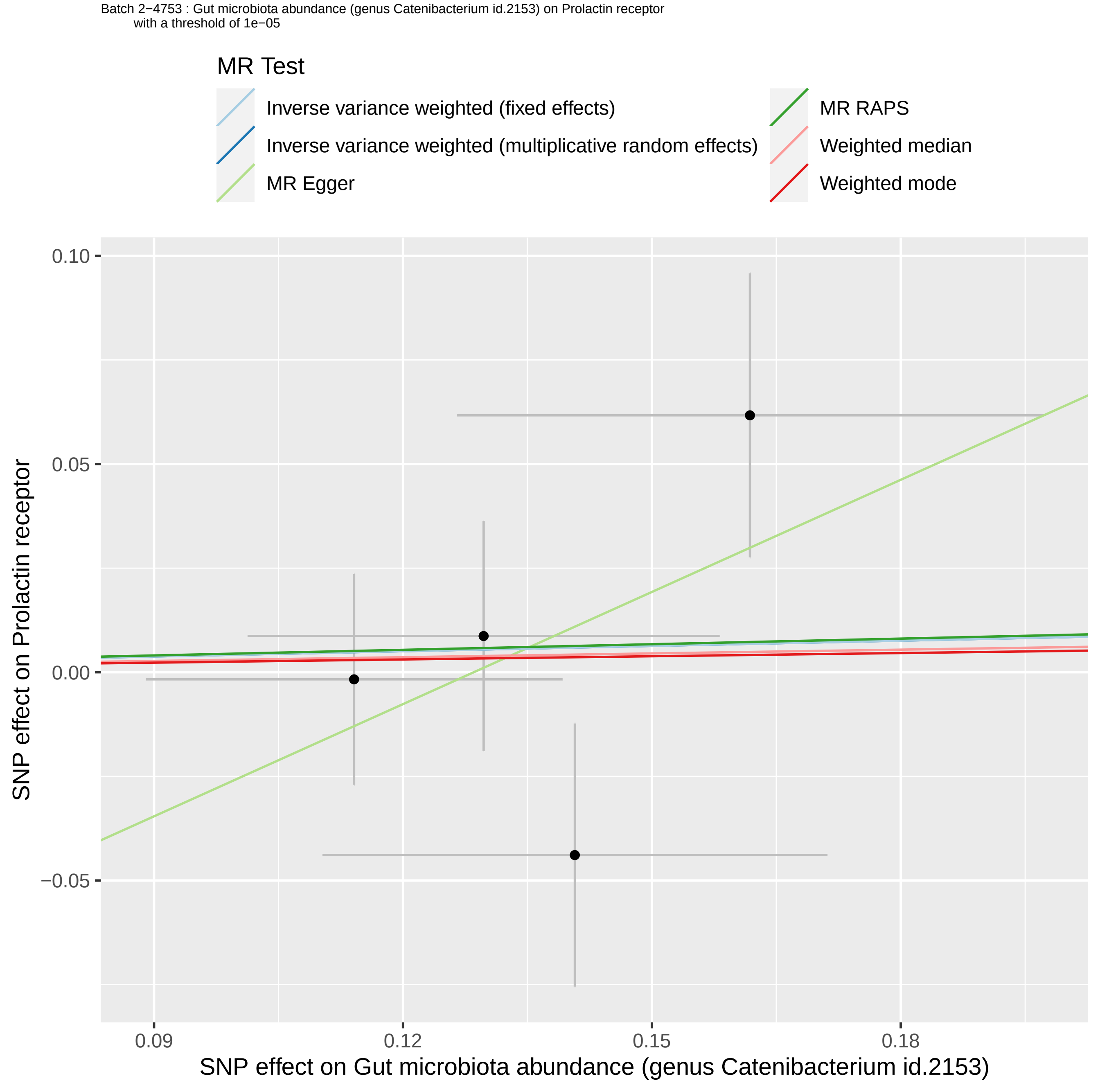
Batch 2−4753 : Gut microbiota abundance (genus Catenibacterium id.2153) on Prolactin receptor
with a threshold of 1e−05
MR Test
Inverse variance weighted (fixed effects)
Inverse variance weighted (multiplicative random effects)
MR Egger
MR RAPS
Weighted median
Weighted mode
0.10
0.05
0.00
−0.05
0.09
0.12
0.15
0.18
SNP effect on Prolactin receptor
SNP effect on Gut microbiota abundance (genus Catenibacterium id.2153)

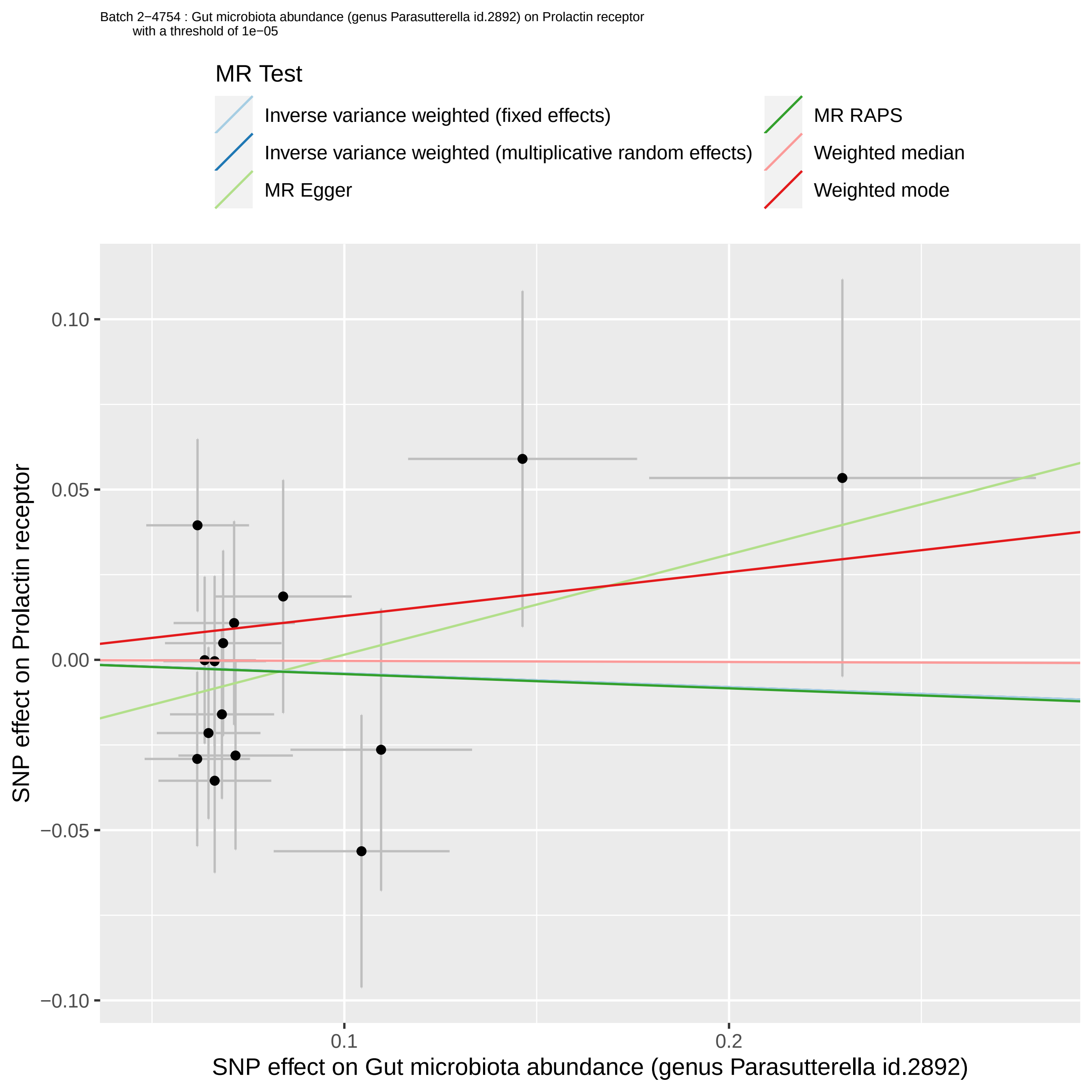
Batch 2−4754 : Gut microbiota abundance (genus Parasutterella id.2892) on Prolactin receptor
with a threshold of 1e−05
MR Test
Inverse variance weighted (fixed effects)
Inverse variance weighted (multiplicative random effects)
MR Egger
MR RAPS
Weighted median
Weighted mode
0.10
0.05
0.00
−0.05
−0.10
0.1
0.2
SNP effect on Prolactin receptor
SNP effect on Gut microbiota abundance (genus Parasutterella id.2892)

Batch 2−4755 : Gut microbiota abundance (genus Catenibacterium id.2153) on Protein arginine N−methyltransferase 3
with a threshold of 1e−05

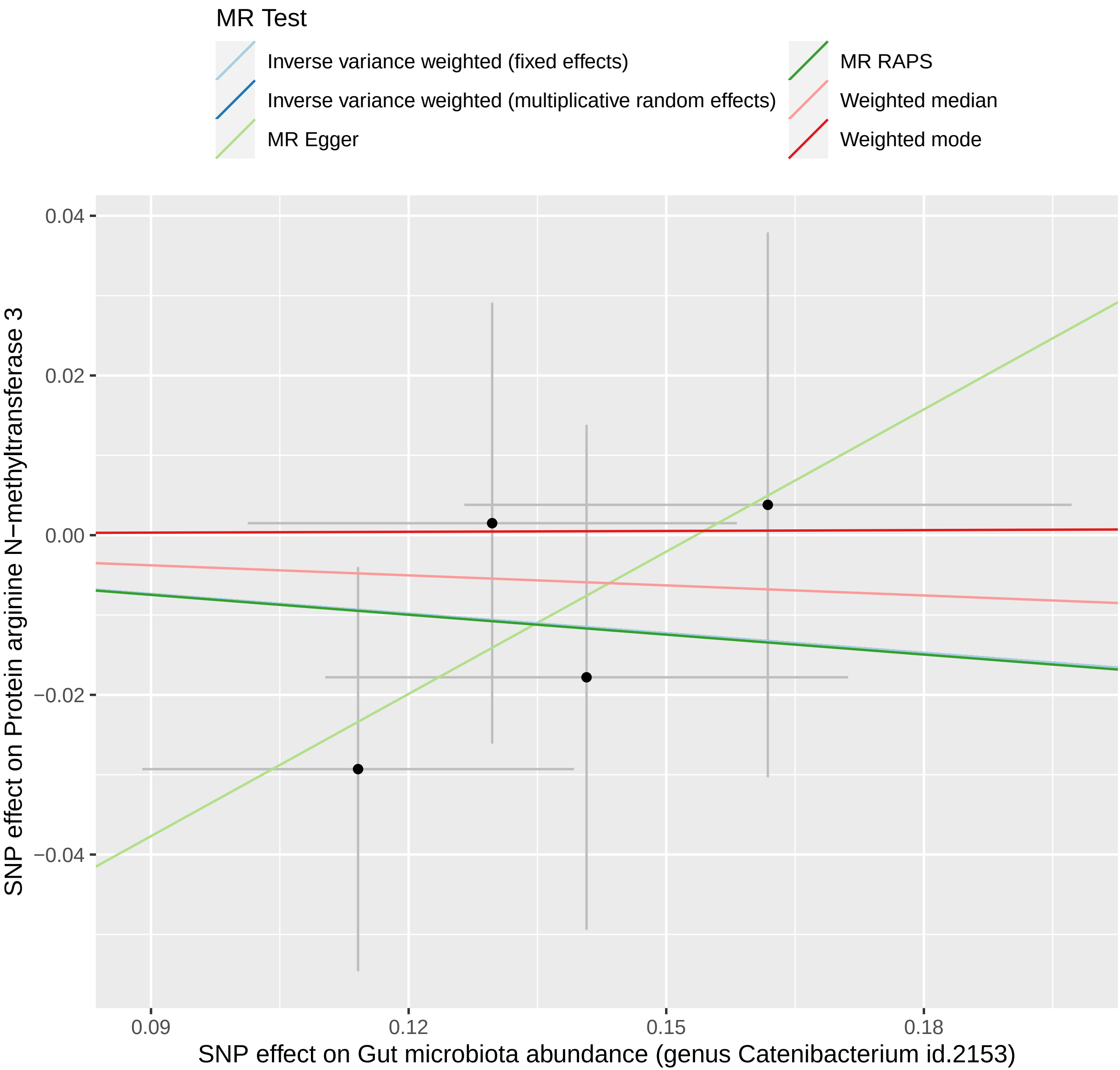

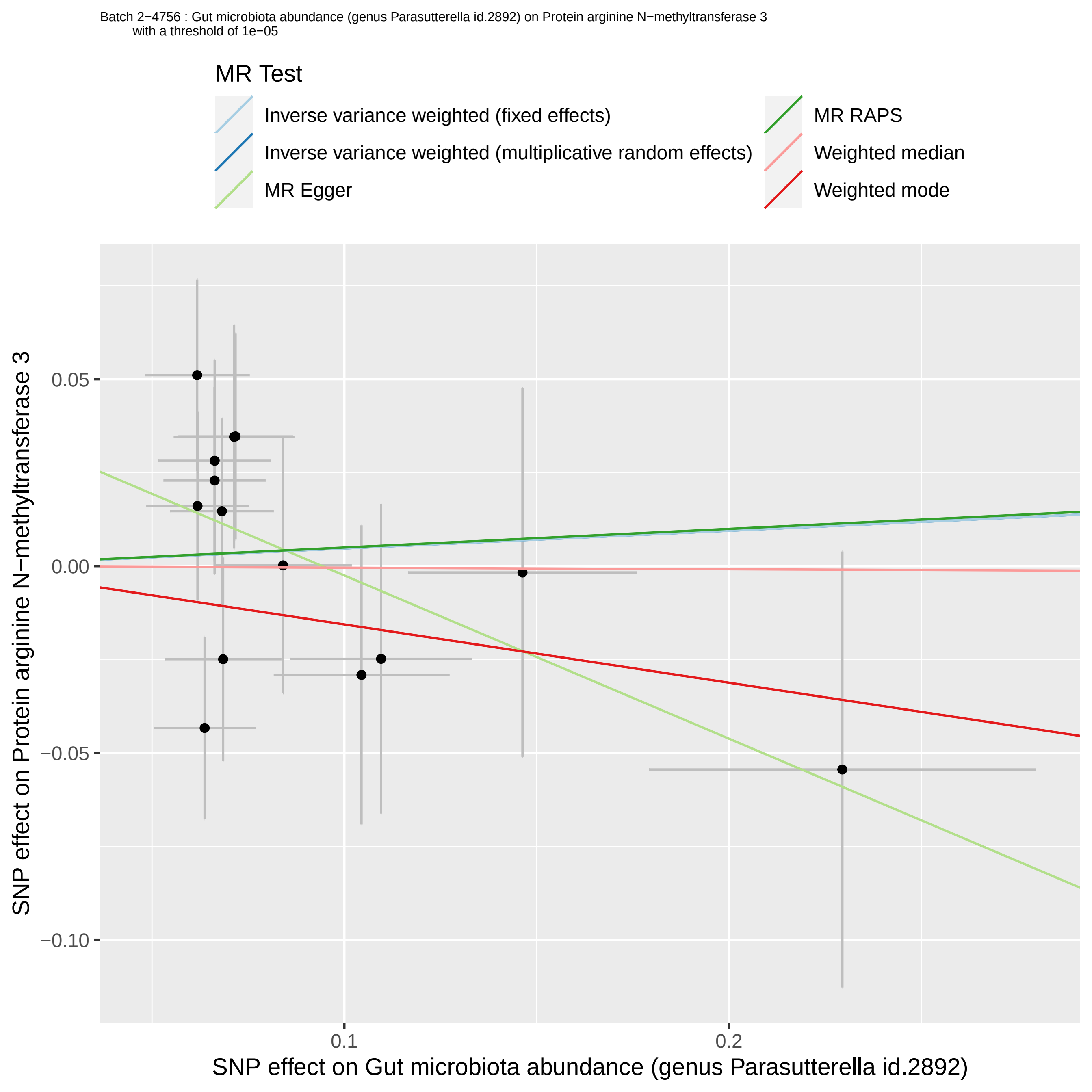
Batch 2−4756 : Gut microbiota abundance (genus Parasutterella id.2892) on Protein arginine N−methyltransferase 3
with a threshold of 1e−05
MR Test
Inverse variance weighted (fixed effects)
Inverse variance weighted (multiplicative random effects)
MR Egger
MR RAPS
Weighted median
Weighted mode
0.05
0.00
−0.05
−0.10
0.1
0.2
SNP effect on Protein arginine N−methyltransferase 3
SNP effect on Gut microbiota abundance (genus Parasutterella id.2892)

Batch 2−4757 : Gut microbiota abundance (genus Catenibacterium id.2153) on Major prion protein
with a threshold of 1e−05

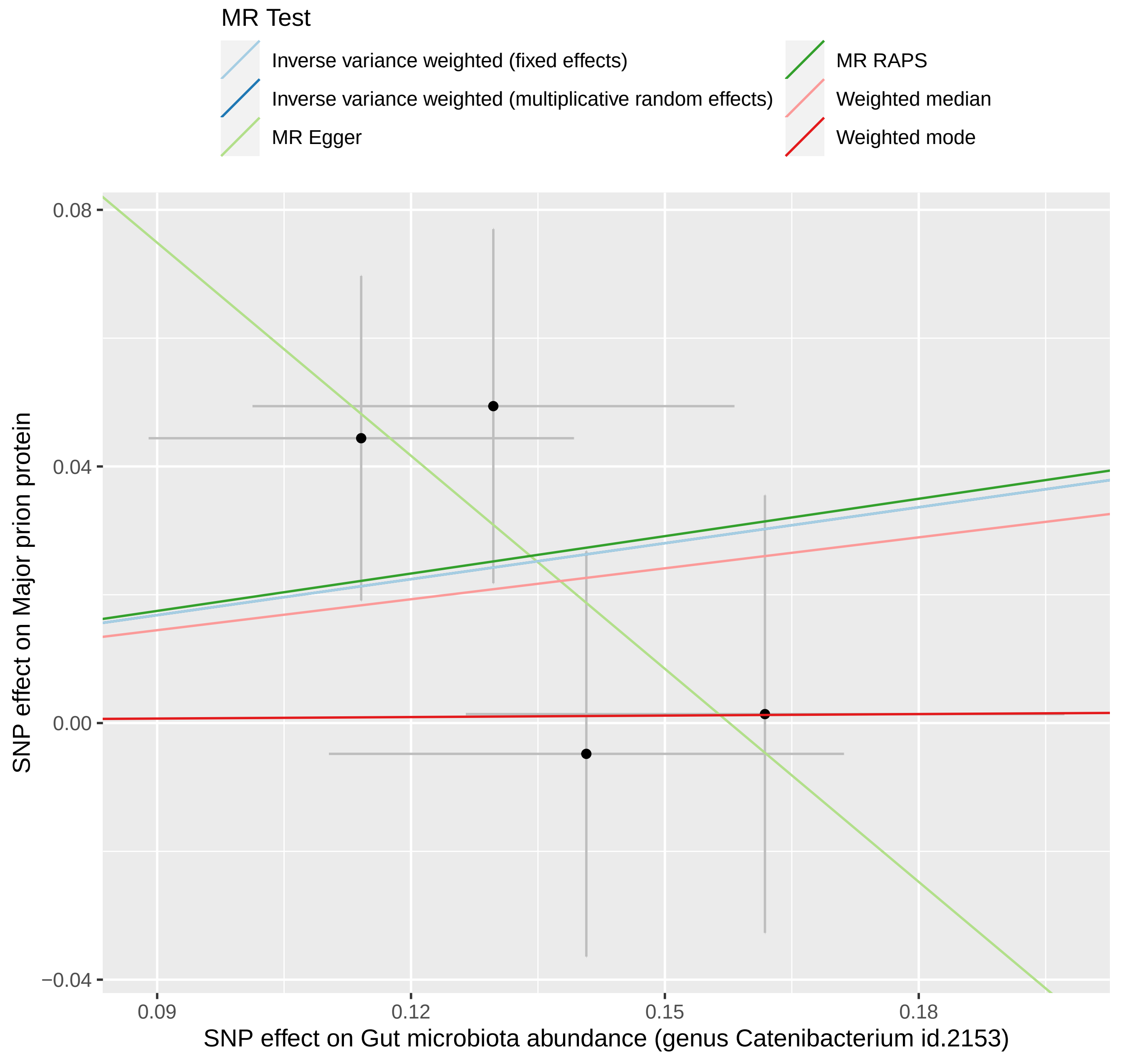

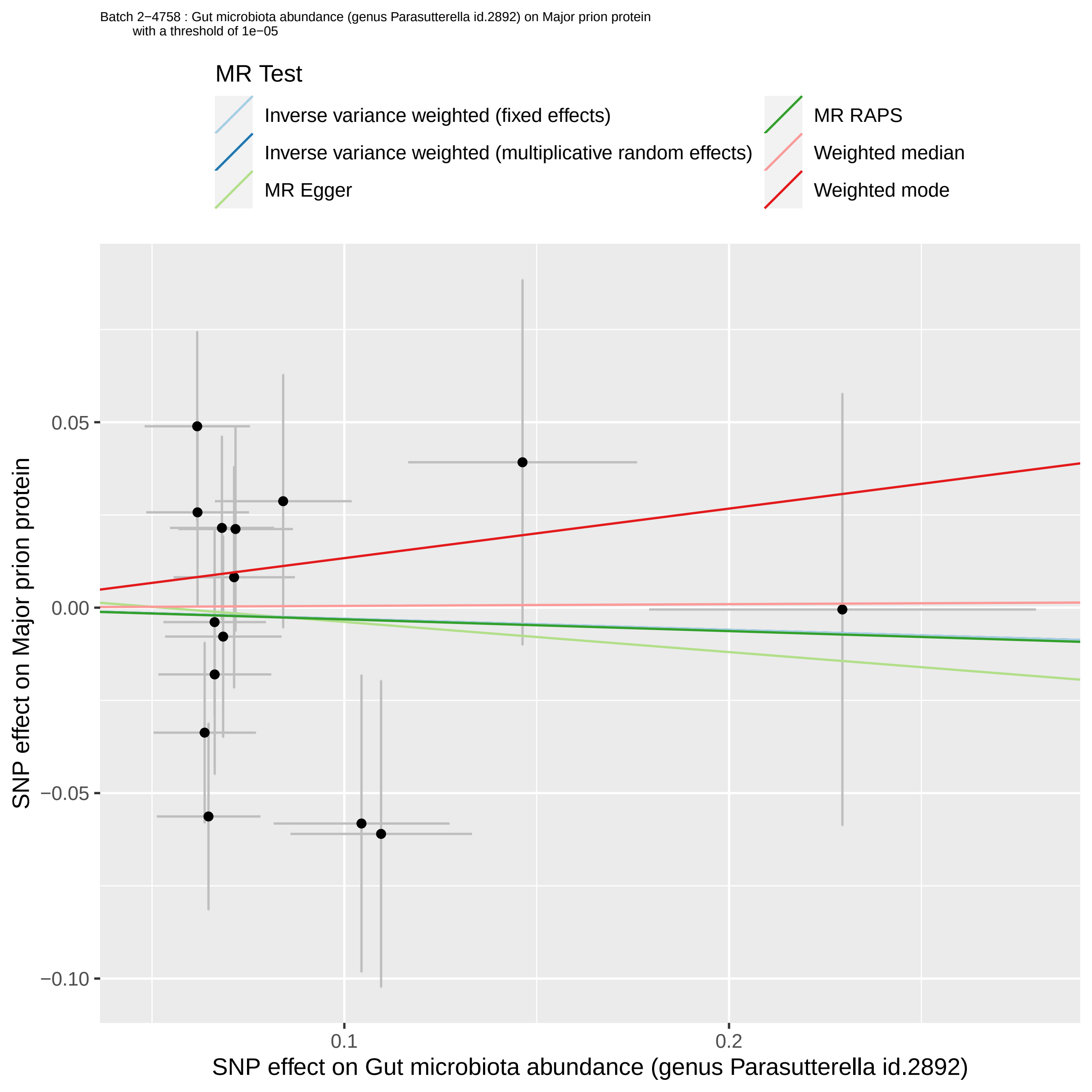
Batch 2−4758 : Gut microbiota abundance (genus Parasutterella id.2892) on Major prion protein
with a threshold of 1e−05
MR Test
Inverse variance weighted (fixed effects)
Inverse variance weighted (multiplicative random effects)
MR Egger
MR RAPS
Weighted median
Weighted mode
SNP effect on Major prion protein
0.05
0.00
−0.05
−0.10
0.1
0.2
SNP effect on Gut microbiota abundance (genus Parasutterella id.2892)

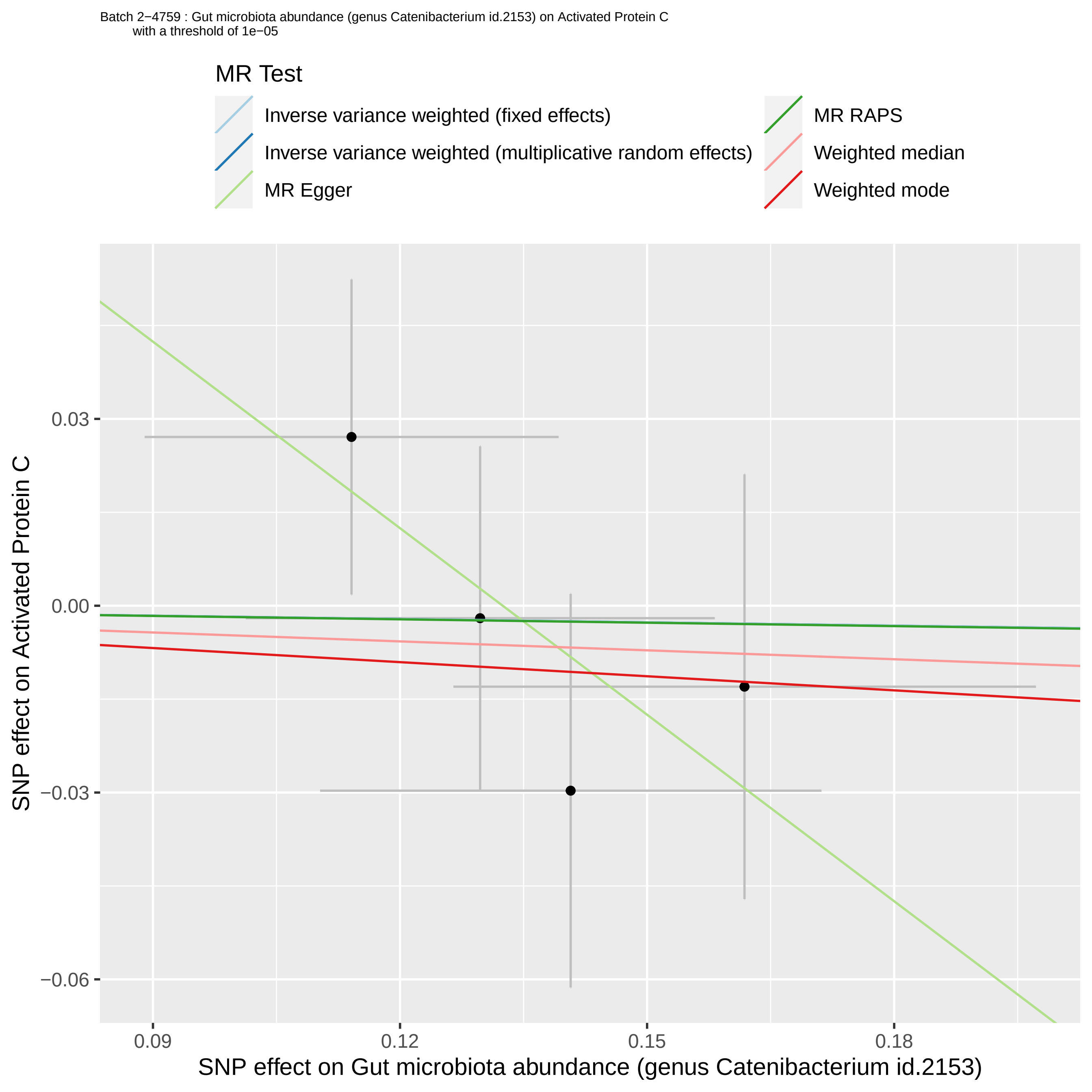
Batch 2−4759 : Gut microbiota abundance (genus Catenibacterium id.2153) on Activated Protein C
with a threshold of 1e−05
MR Test
Inverse variance weighted (fixed effects)
Inverse variance weighted (multiplicative random effects)
MR Egger
MR RAPS
Weighted median
Weighted mode
0.03
0.00
−0.03
−0.06
0.09
0.12
0.15
0.18
SNP effect on Activated Protein C
SNP effect on Gut microbiota abundance (genus Catenibacterium id.2153)

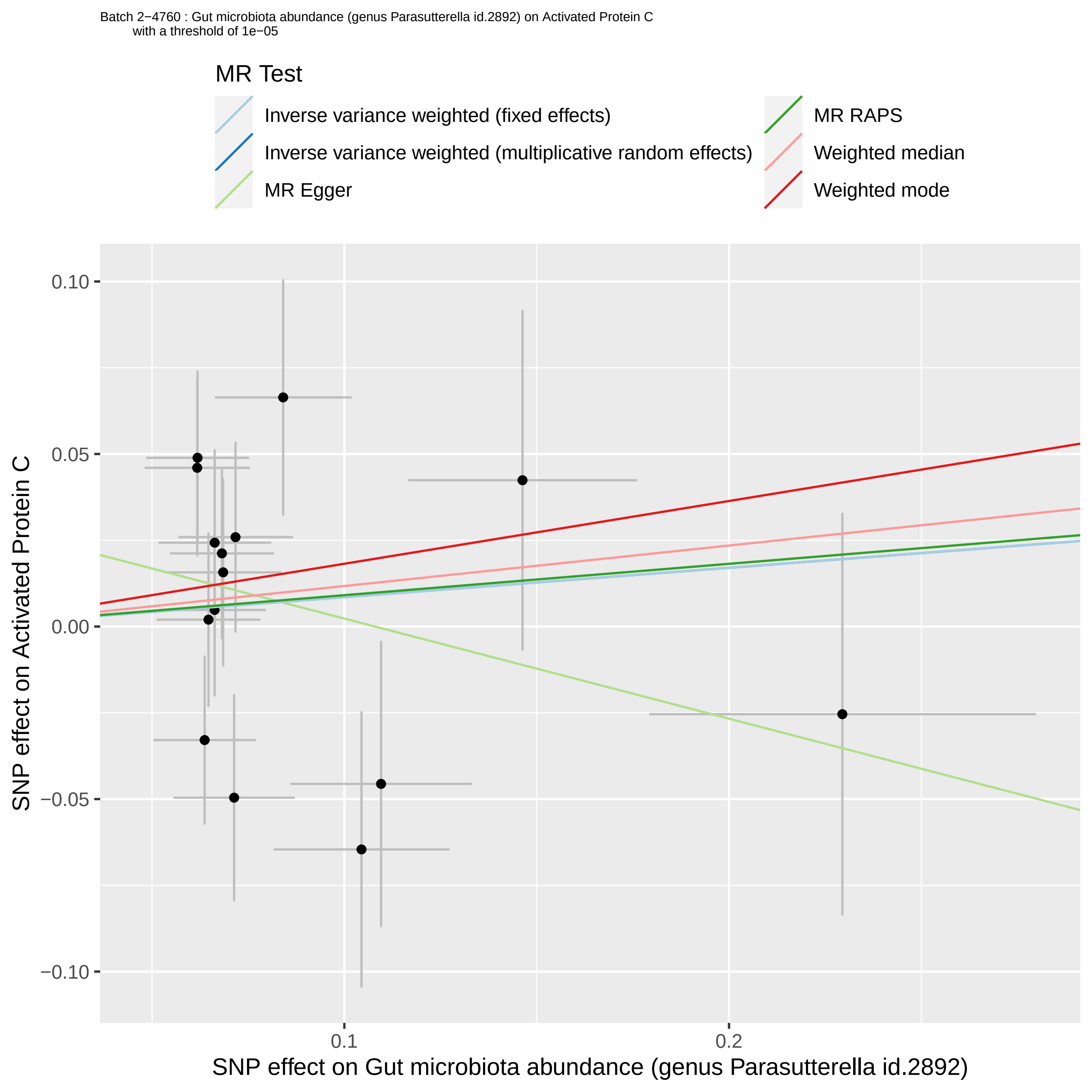
Batch 2−4760 : Gut microbiota abundance (genus Parasutterella id.2892) on Activated Protein C
with a threshold of 1e−05
MR Test
Inverse variance weighted (fixed effects)
Inverse variance weighted (multiplicative random effects)
MR Egger
MR RAPS
Weighted median
Weighted mode
0.10
0.05
0.00
−0.05
−0.10
0.1
0.2
SNP effect on Activated Protein C
SNP effect on Gut microbiota abundance (genus Parasutterella id.2892)

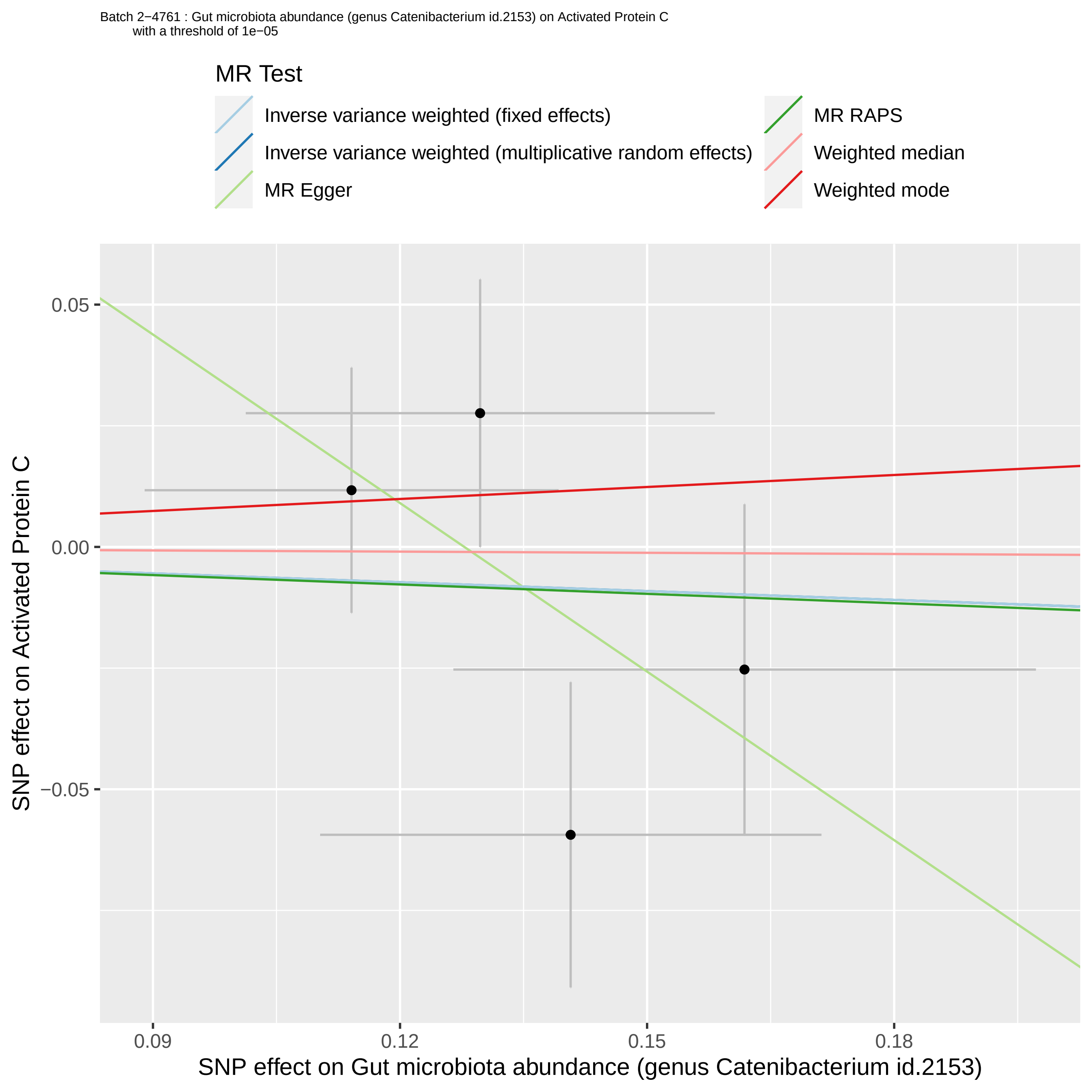
Batch 2−4761 : Gut microbiota abundance (genus Catenibacterium id.2153) on Activated Protein C
with a threshold of 1e−05
MR Test
Inverse variance weighted (fixed effects)
Inverse variance weighted (multiplicative random effects)
MR Egger
MR RAPS
Weighted median
Weighted mode
0.05
0.00
−0.05
0.09
0.12
0.15
0.18
SNP effect on Activated Protein C
SNP effect on Gut microbiota abundance (genus Catenibacterium id.2153)

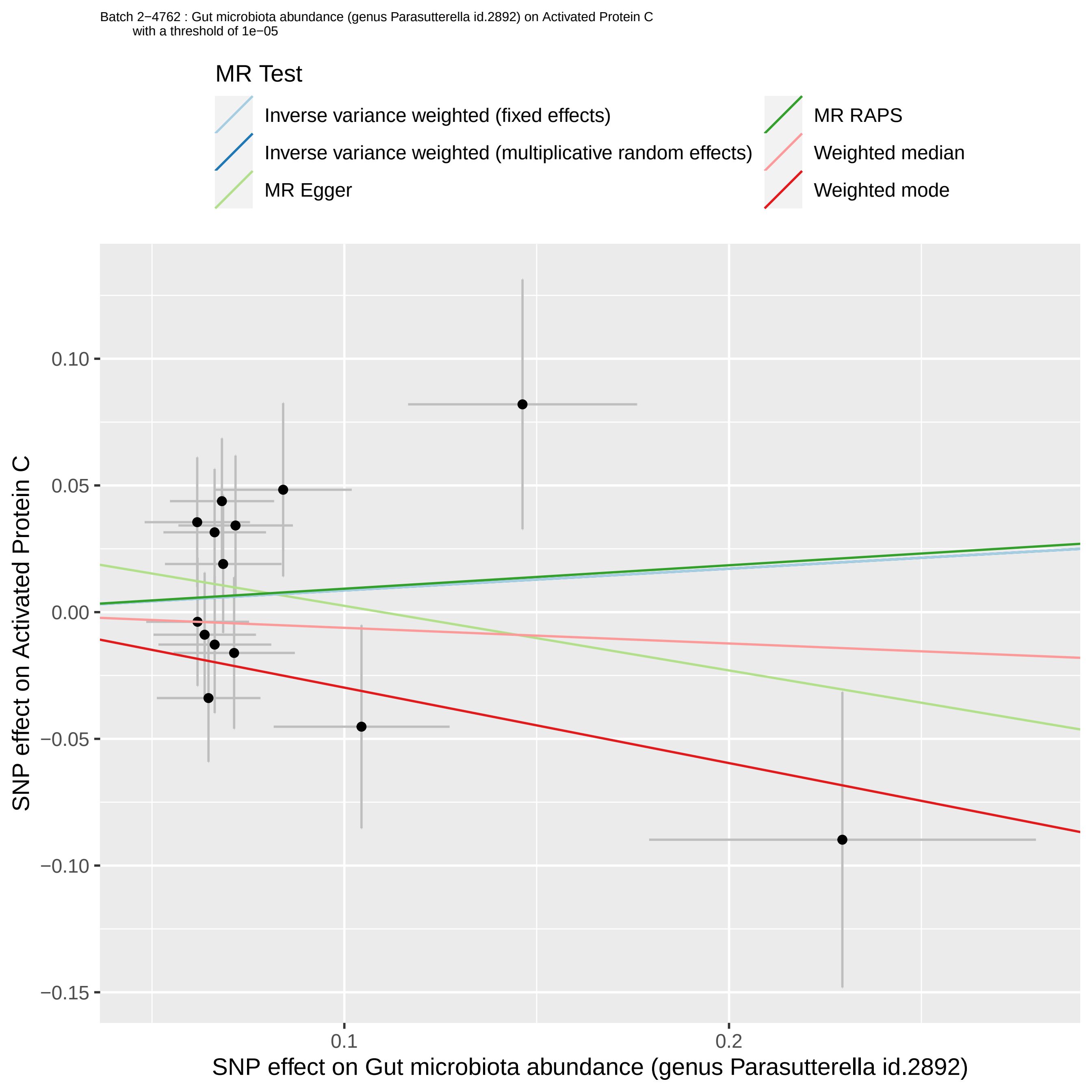

Batch 2−4762 : Gut microbiota abundance (genus Parasutterella id.2892) on Activated Protein C
with a threshold of 1e−05
MR Test
Inverse variance weighted (fixed effects)
Inverse variance weighted (multiplicative random effects)
MR Egger
MR RAPS
Weighted median
Weighted mode
0.10
0.05
0.00
−0.05
−0.10
−0.15
SNP effect on Activated Protein C
0.1
0.2
SNP effect on Gut microbiota abundance (genus Parasutterella id.2892)

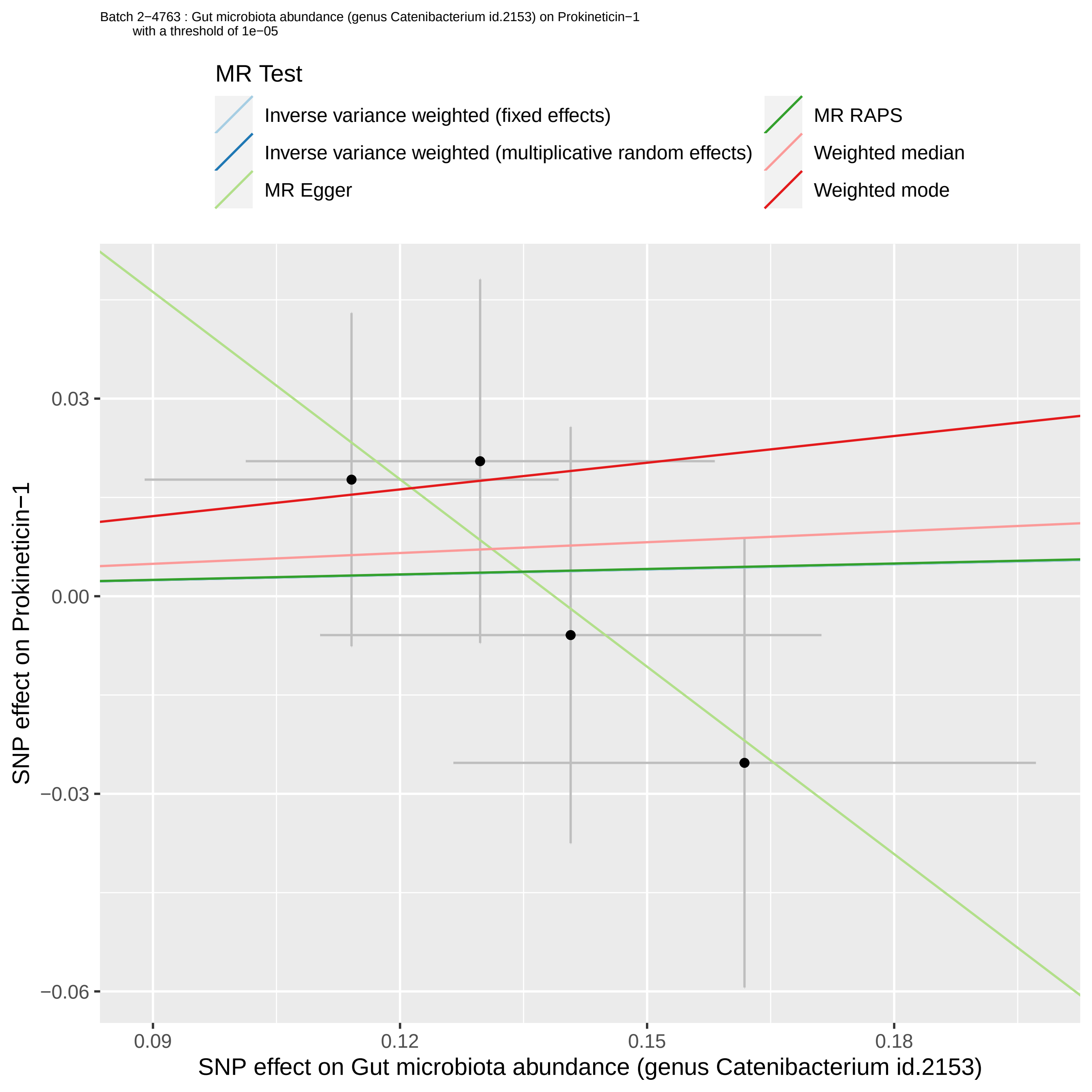
Batch 2−4763 : Gut microbiota abundance (genus Catenibacterium id.2153) on Prokineticin−1
with a threshold of 1e−05
MR Test
Inverse variance weighted (fixed effects)
Inverse variance weighted (multiplicative random effects)
MR Egger
MR RAPS
Weighted median
Weighted mode
0.03
0.00
−0.03
−0.06
0.09
0.12
0.15
0.18
SNP effect on Prokineticin−1
SNP effect on Gut microbiota abundance (genus Catenibacterium id.2153)

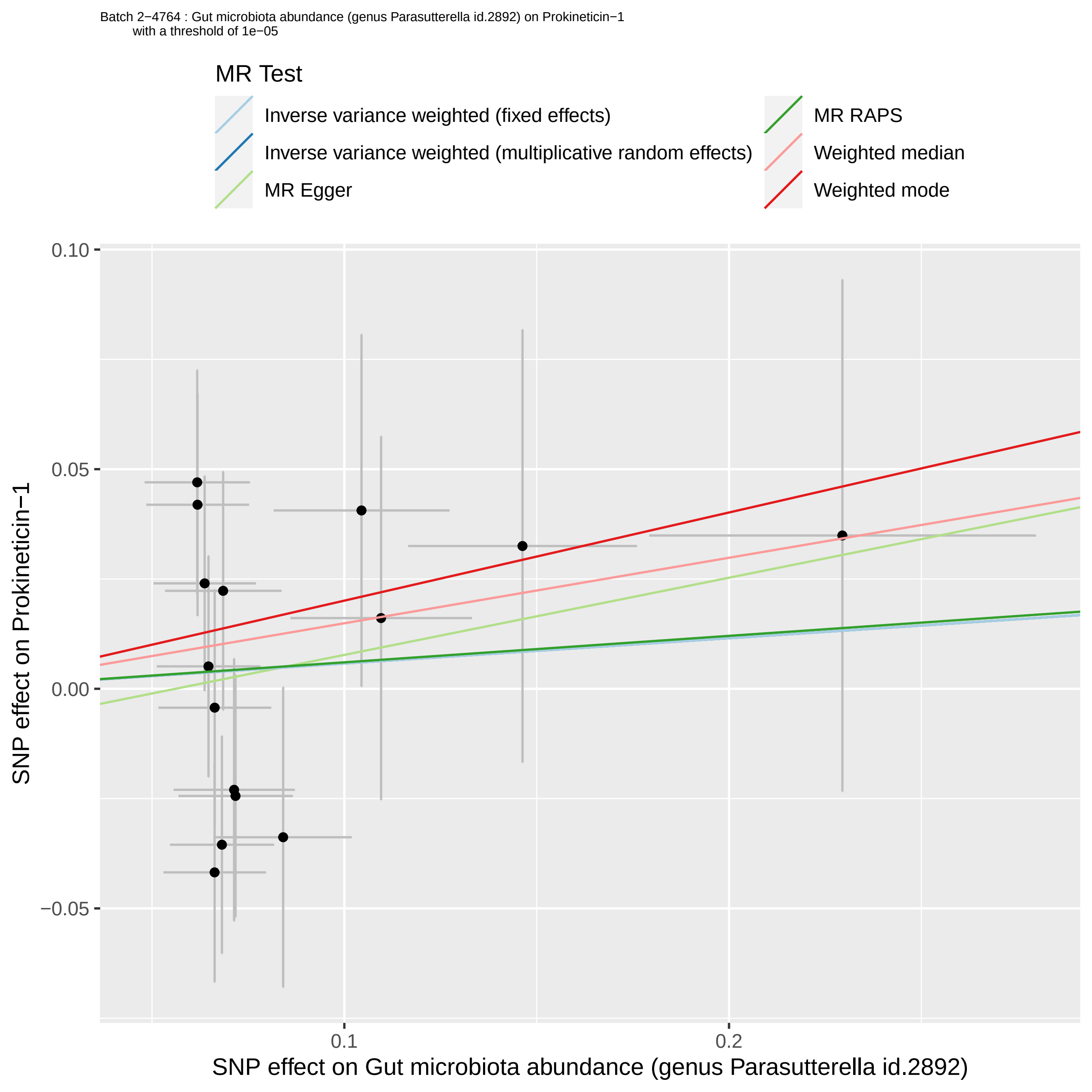
Batch 2−4764 : Gut microbiota abundance (genus Parasutterella id.2892) on Prokineticin−1
with a threshold of 1e−05
MR Test
Inverse variance weighted (fixed effects)
Inverse variance weighted (multiplicative random effects)
MR Egger
MR RAPS
Weighted median
Weighted mode
0.10
0.05
0.00
−0.05
0.1
0.2
SNP effect on Prokineticin−1
SNP effect on Gut microbiota abundance (genus Parasutterella id.2892)

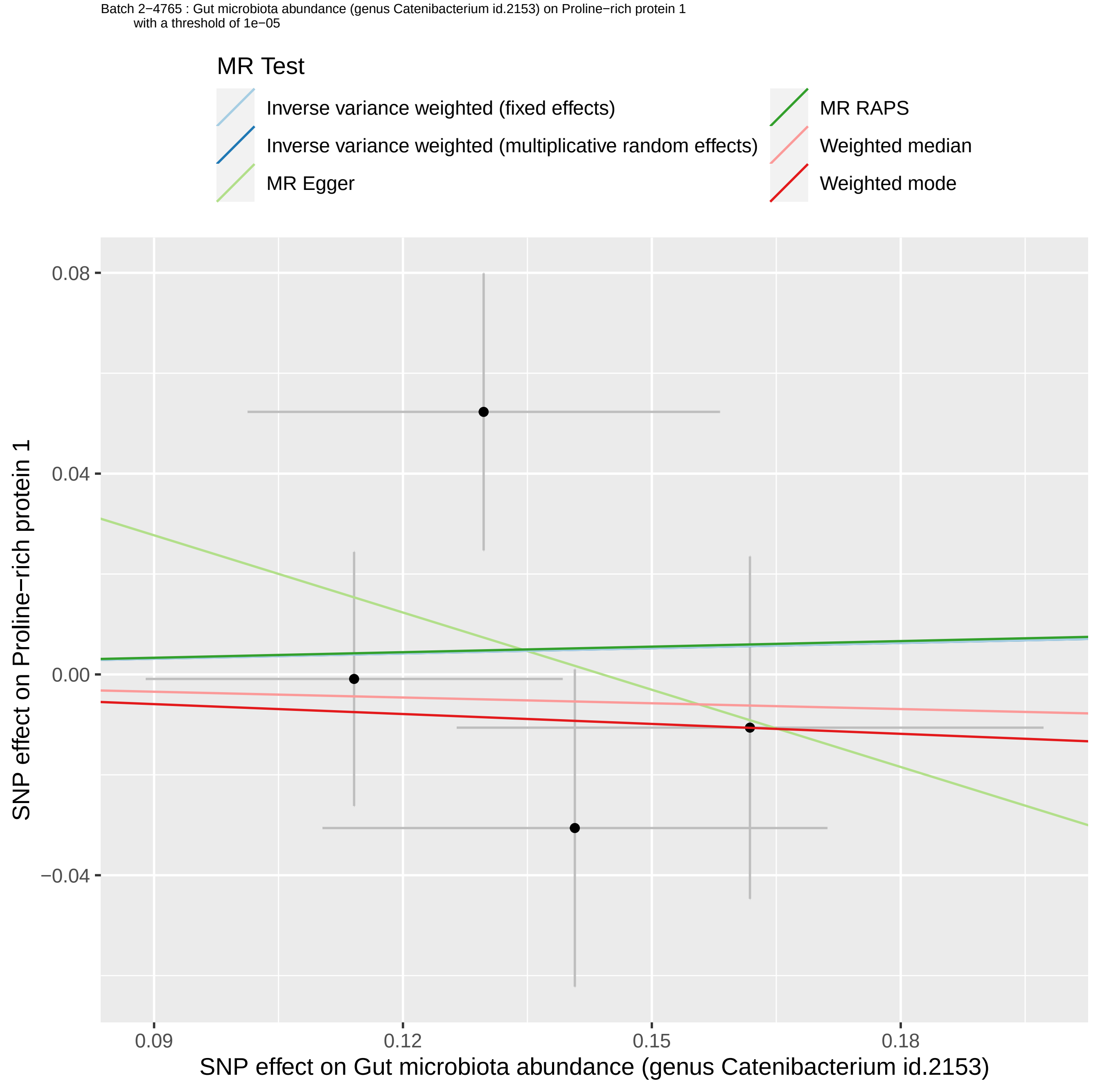

Batch 2−4765 : Gut microbiota abundance (genus Catenibacterium id.2153) on Proline−rich protein 1
with a threshold of 1e−05
MR Test
Inverse variance weighted (fixed effects)
Inverse variance weighted (multiplicative random effects)
MR Egger
MR RAPS
Weighted median
Weighted mode
0.08
0.04
0.00
−0.04
0.09
0.12
0.15
0.18
SNP effect on Proline−rich protein 1
SNP effect on Gut microbiota abundance (genus Catenibacterium id.2153)

Batch 2−4766 : Gut microbiota abundance (genus Parasutterella id.2892) on Proline−rich protein 1
with a threshold of 1e−05

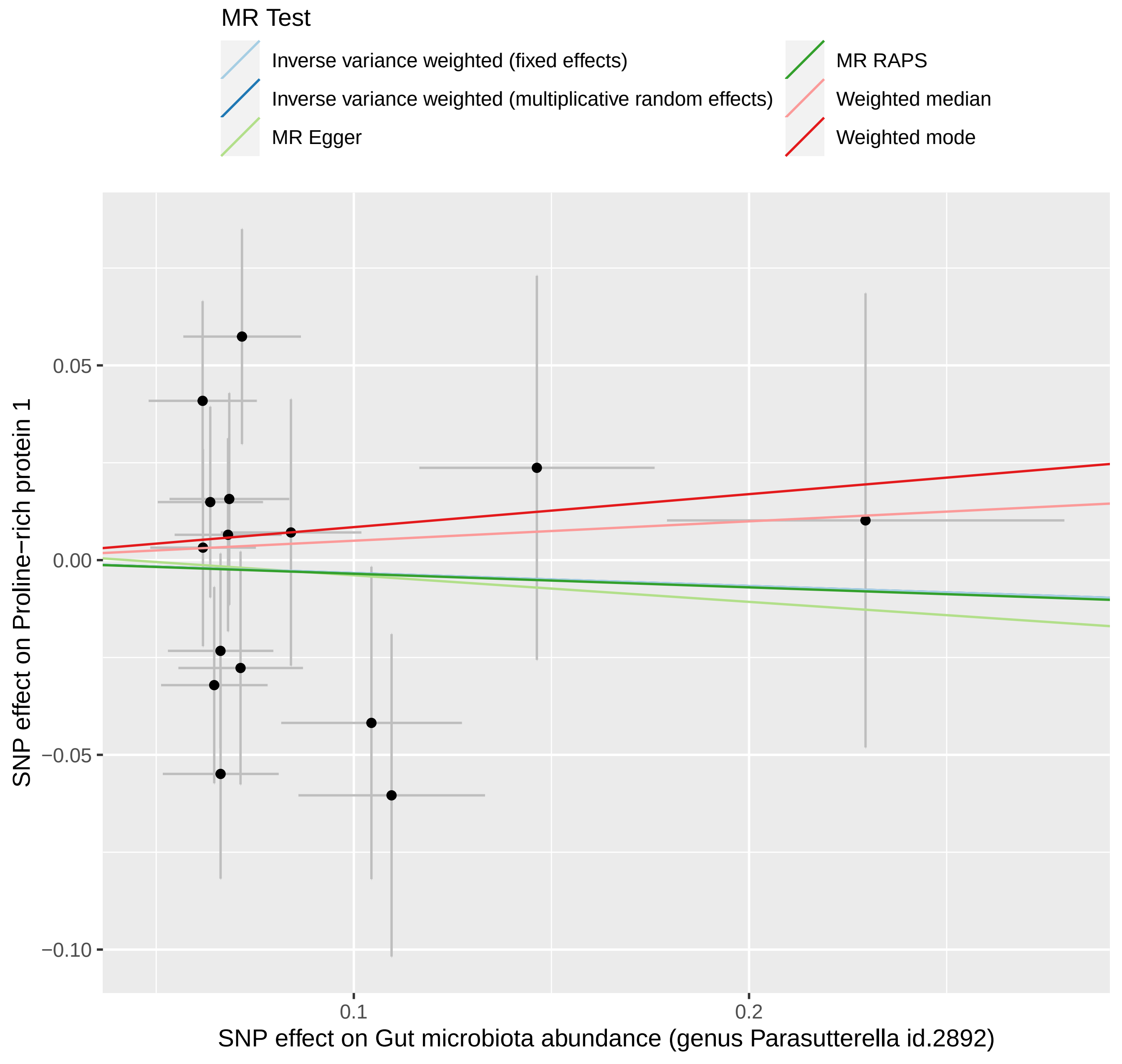

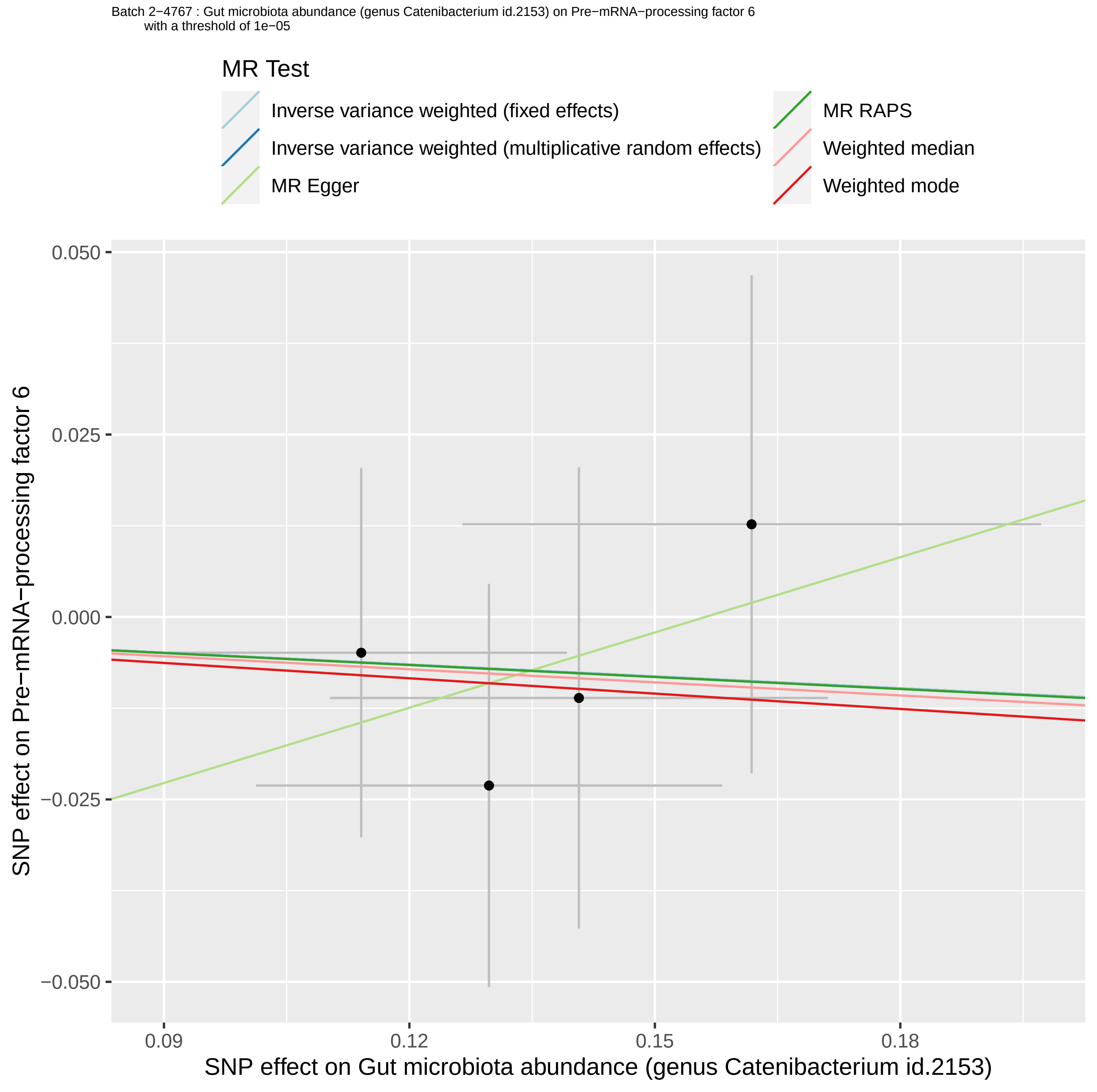
Batch 2−4767 : Gut microbiota abundance (genus Catenibacterium id.2153) on Pre−mRNA−processing factor 6
with a threshold of 1e−05
MR Test
Inverse variance weighted (fixed effects)
Inverse variance weighted (multiplicative random effects)
MR Egger
MR RAPS
Weighted median
Weighted mode
0.050
0.025
0.000
−0.025
−0.050
0.09
0.12
0.15
0.18
SNP effect on Pre−mRNA−processing factor 6
SNP effect on Gut microbiota abundance (genus Catenibacterium id.2153)

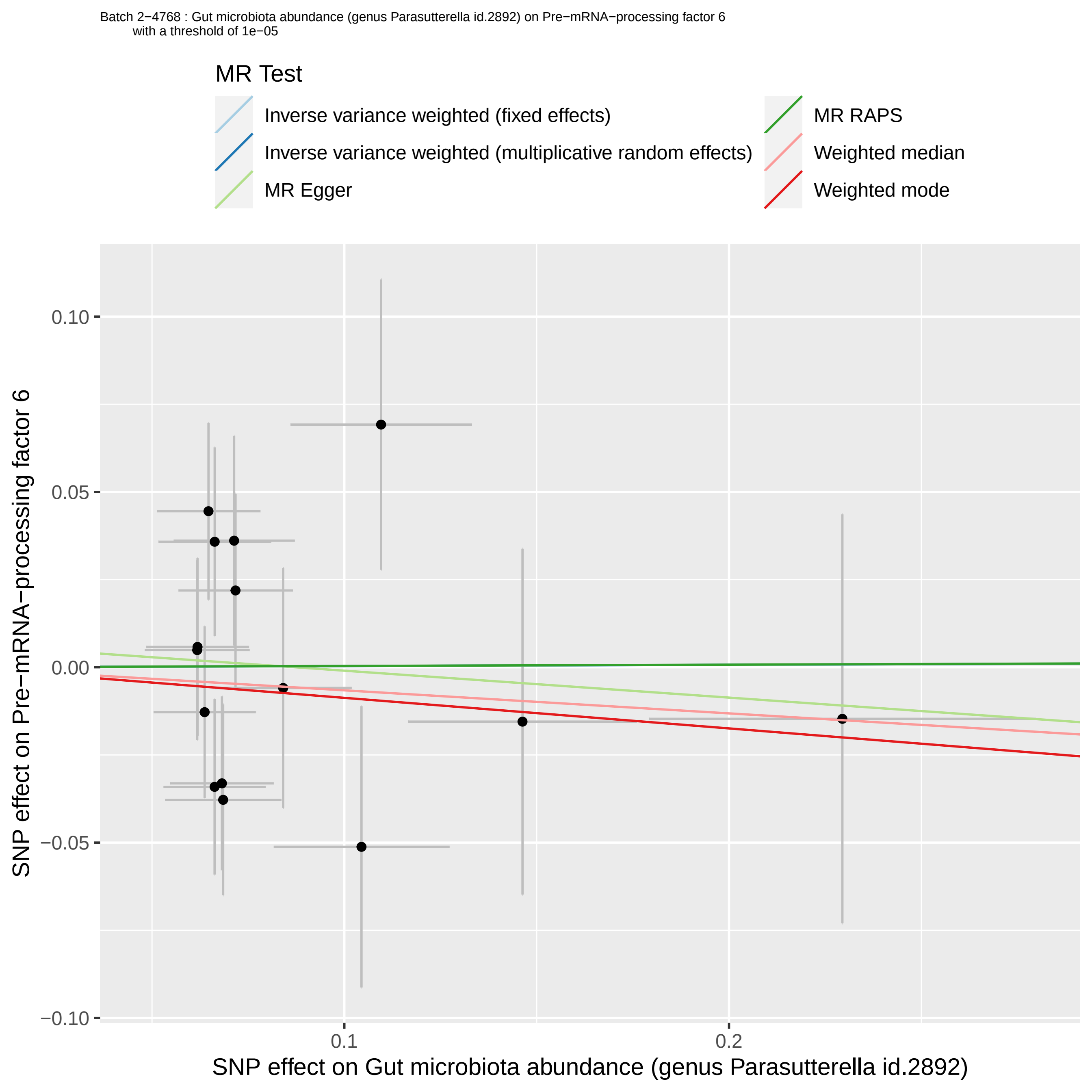
Batch 2−4768 : Gut microbiota abundance (genus Parasutterella id.2892) on Pre−mRNA−processing factor 6
with a threshold of 1e−05
MR Test
Inverse variance weighted (fixed effects)
Inverse variance weighted (multiplicative random effects)
MR Egger
MR RAPS
Weighted median
Weighted mode
0.10
0.05
0.00
−0.05
−0.10
0.1
0.2
SNP effect on Pre−mRNA−processing factor 6
SNP effect on Gut microbiota abundance (genus Parasutterella id.2892)

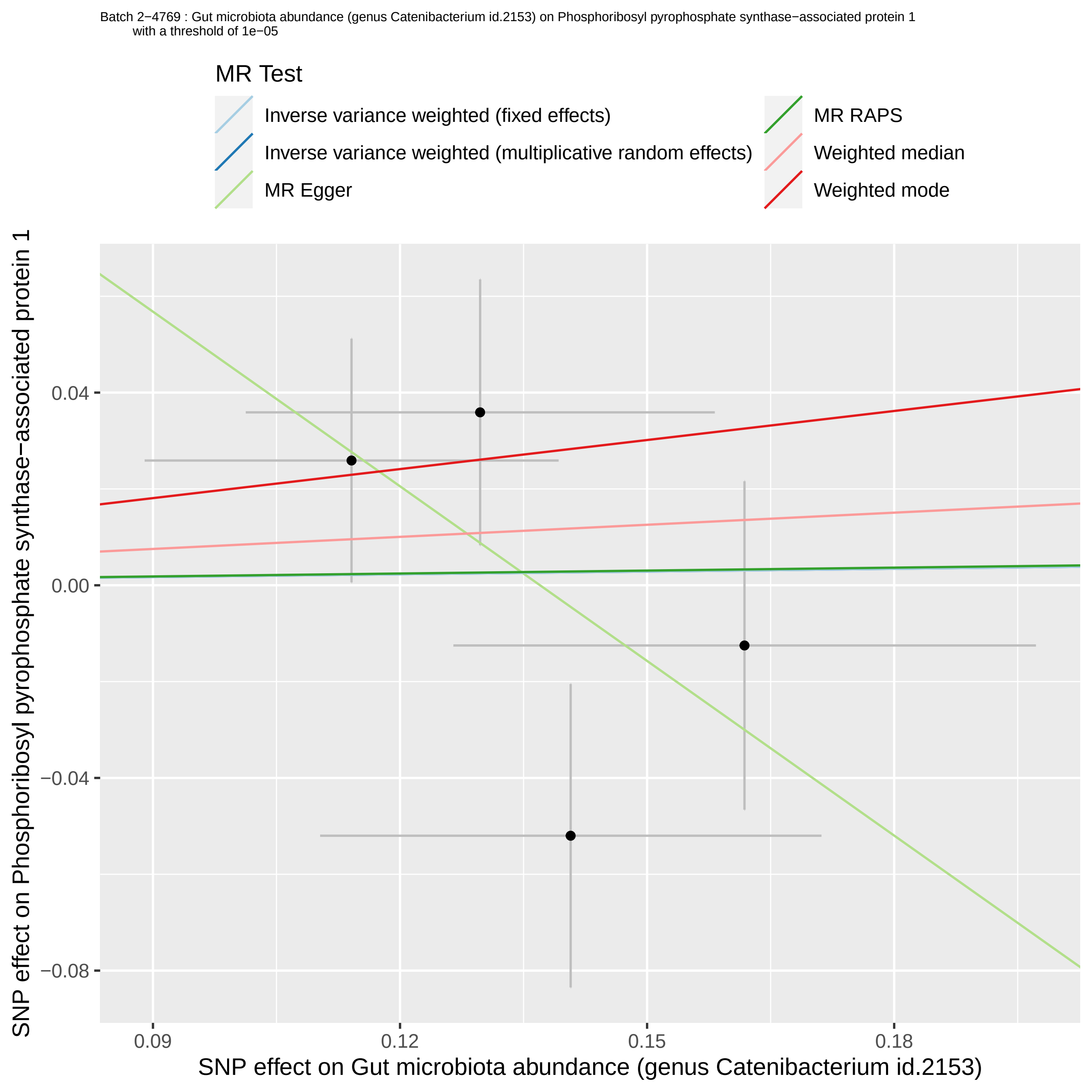
Batch 2−4769 : Gut microbiota abundance (genus Catenibacterium id.2153) on Phosphoribosyl pyrophosphate synthase−associated protein 1
with a threshold of 1e−05
MR Test
Inverse variance weighted (fixed effects)
Inverse variance weighted (multiplicative random effects)
MR Egger
MR RAPS
Weighted median
Weighted mode
SNP effect on Phosphoribosyl pyrophosphate synthase−associated protein 1
0.04
0.00
−0.04
−0.08
0.09
0.12
0.15
0.18
SNP effect on Gut microbiota abundance (genus Catenibacterium id.2153)

Batch 2−4770 : Gut microbiota abundance (genus Parasutterella id.2892) on Phosphoribosyl pyrophosphate synthase−associated protein 1
with a threshold of 1e−05

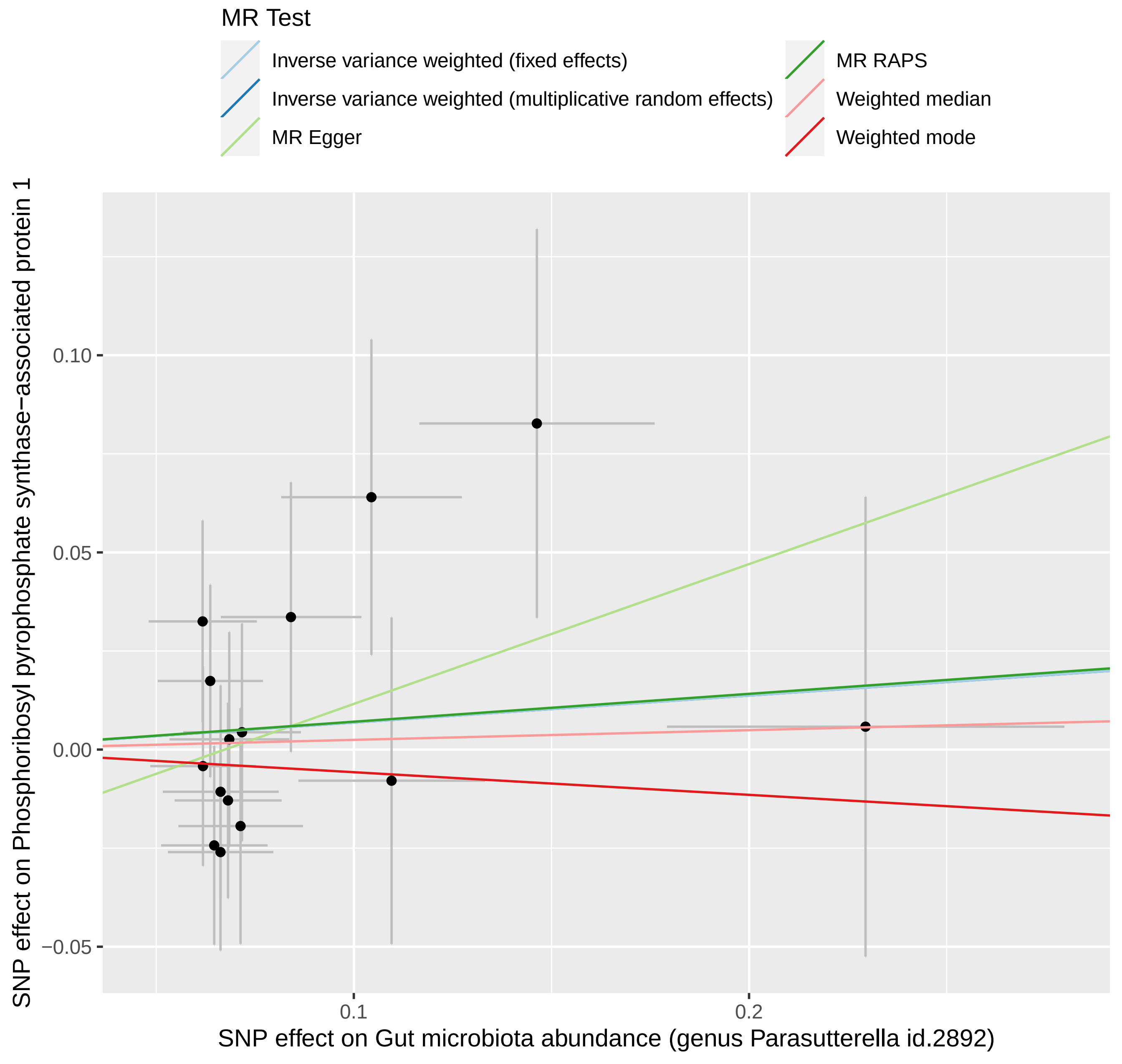

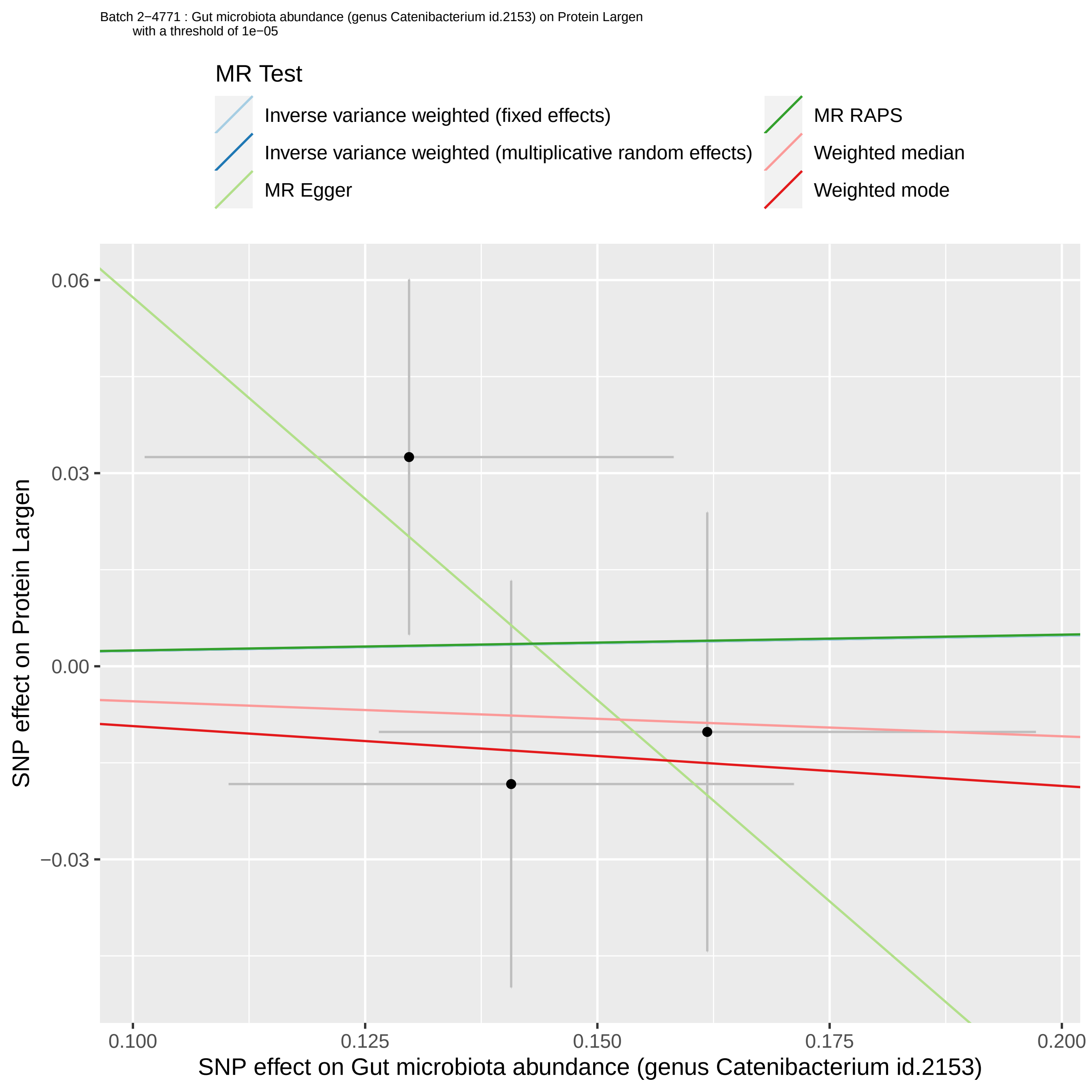

Batch 2−4771 : Gut microbiota abundance (genus Catenibacterium id.2153) on Protein Largen
with a threshold of 1e−05
MR Test
Inverse variance weighted (fixed effects)
Inverse variance weighted (multiplicative random effects)
MR Egger
MR RAPS
Weighted median
Weighted mode
0.06
0.03
0.00
−0.03
0.100
0.125
0.150
0.175
0.200
SNP effect on Protein Largen
SNP effect on Gut microbiota abundance (genus Catenibacterium id.2153)

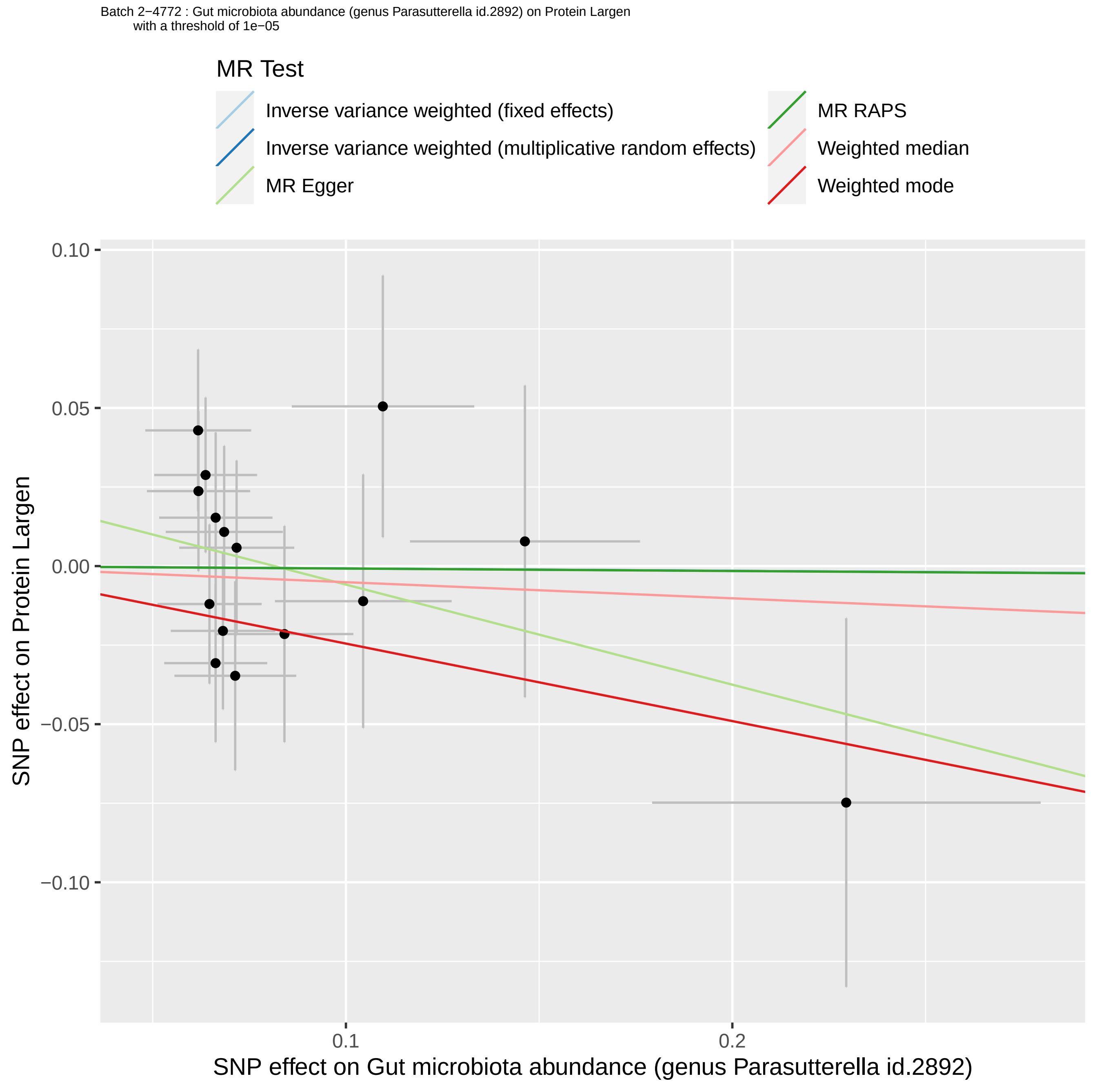

Batch 2−4772 : Gut microbiota abundance (genus Parasutterella id.2892) on Protein Largen
with a threshold of 1e−05
MR Test
Inverse variance weighted (fixed effects)
Inverse variance weighted (multiplicative random effects)
MR Egger
MR RAPS
Weighted median
Weighted mode
0.10
0.05
0.00
−0.05
−0.10
0.1
0.2
SNP effect on Protein Largen
SNP effect on Gut microbiota abundance (genus Parasutterella id.2892)

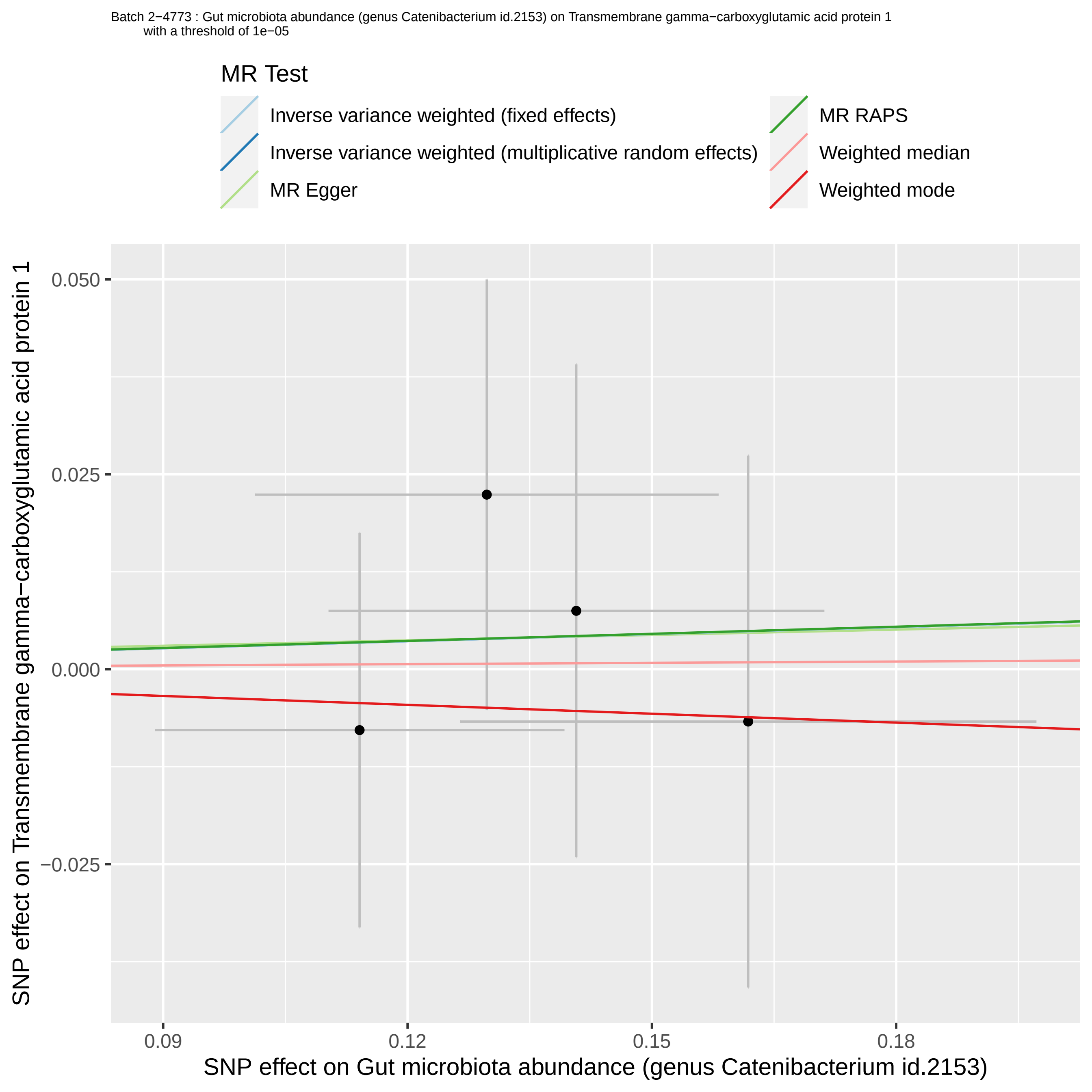
Batch 2−4773 : Gut microbiota abundance (genus Catenibacterium id.2153) on Transmembrane gamma−carboxyglutamic acid protein 1 with a threshold of 1e−05
MR Test
Inverse variance weighted (fixed effects)
Inverse variance weighted (multiplicative random effects)
MR Egger
MR RAPS
Weighted median
Weighted mode
0.050
0.025
0.000
−0.025
0.09
0.12
0.15
0.18
SNP effect on Transmembrane gamma−carboxyglutamic acid protein 1
SNP effect on Gut microbiota abundance (genus Catenibacterium id.2153)

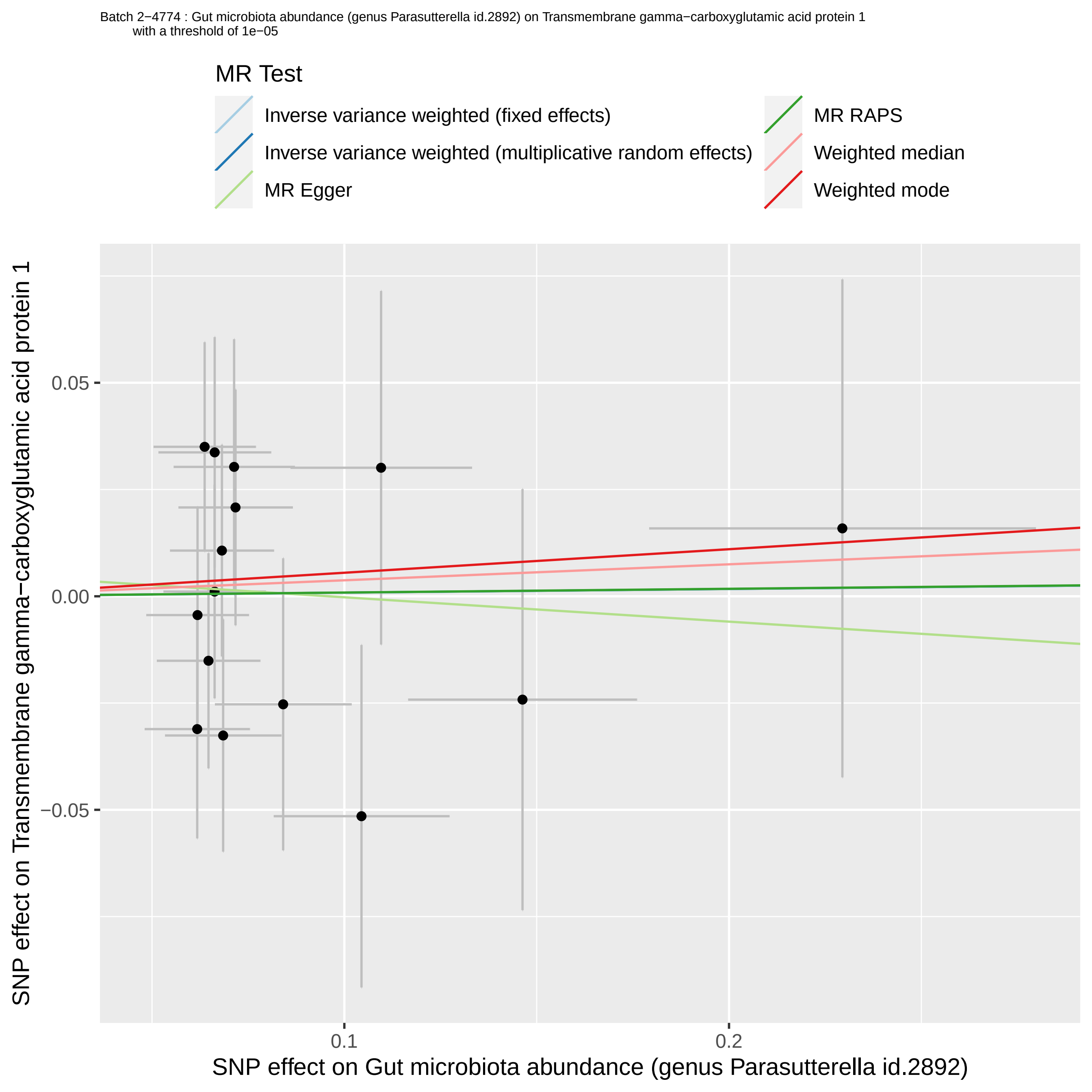
Batch 2−4774 : Gut microbiota abundance (genus Parasutterella id.2892) on Transmembrane gamma−carboxyglutamic acid protein 1
with a threshold of 1e−05
MR Test
Inverse variance weighted (fixed effects)
Inverse variance weighted (multiplicative random effects)
MR Egger
MR RAPS
Weighted median
Weighted mode
SNP effect on Transmembrane gamma−carboxyglutamic acid protein 1
0.05
0.00
−0.05
0.1
0.2
SNP effect on Gut microbiota abundance (genus Parasutterella id.2892)

Batch 2−4775 : Gut microbiota abundance (genus Catenibacterium id.2153) on Transmembrane gamma−carboxyglutamic acid protein 1 with a threshold of 1e−05

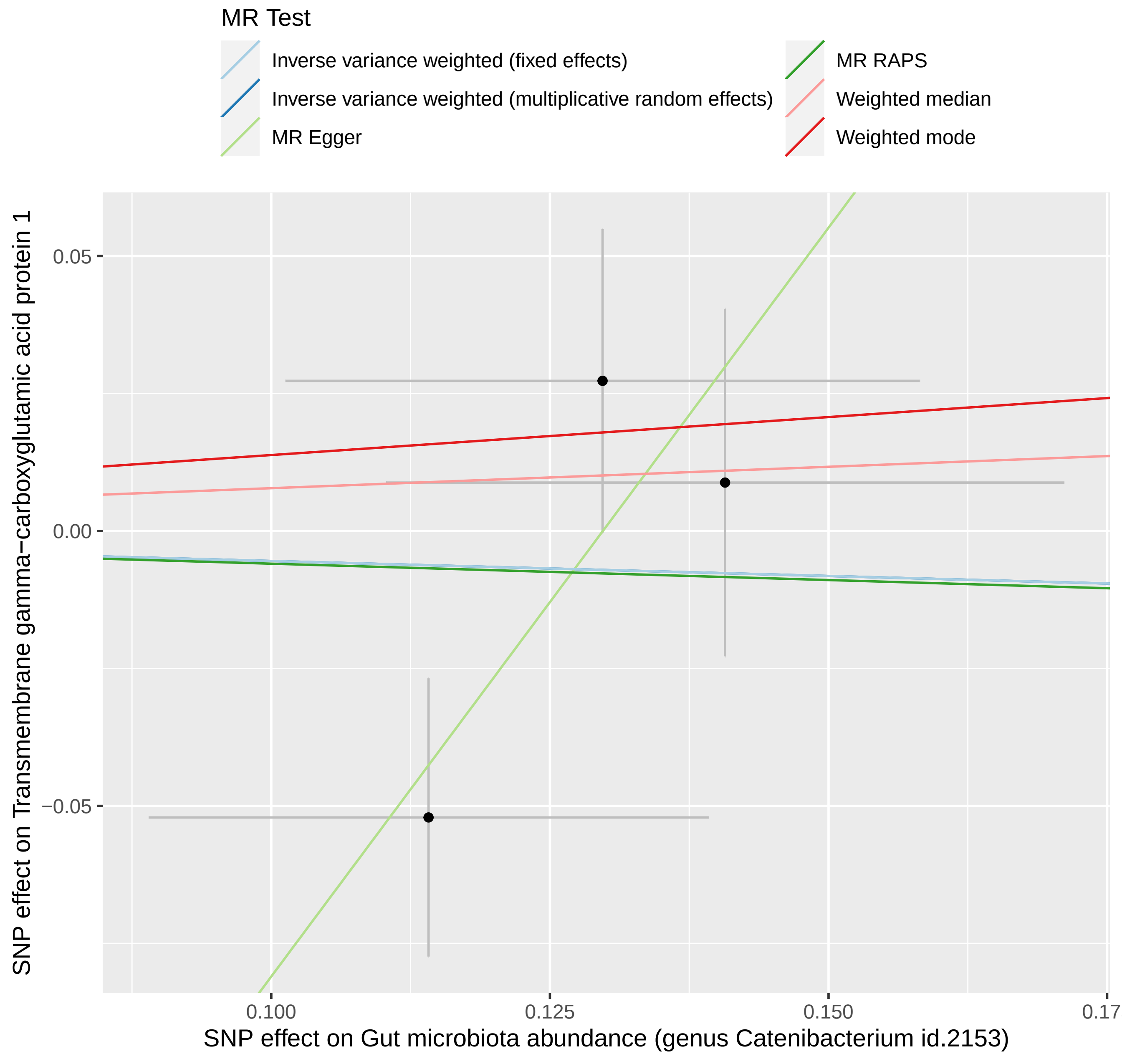

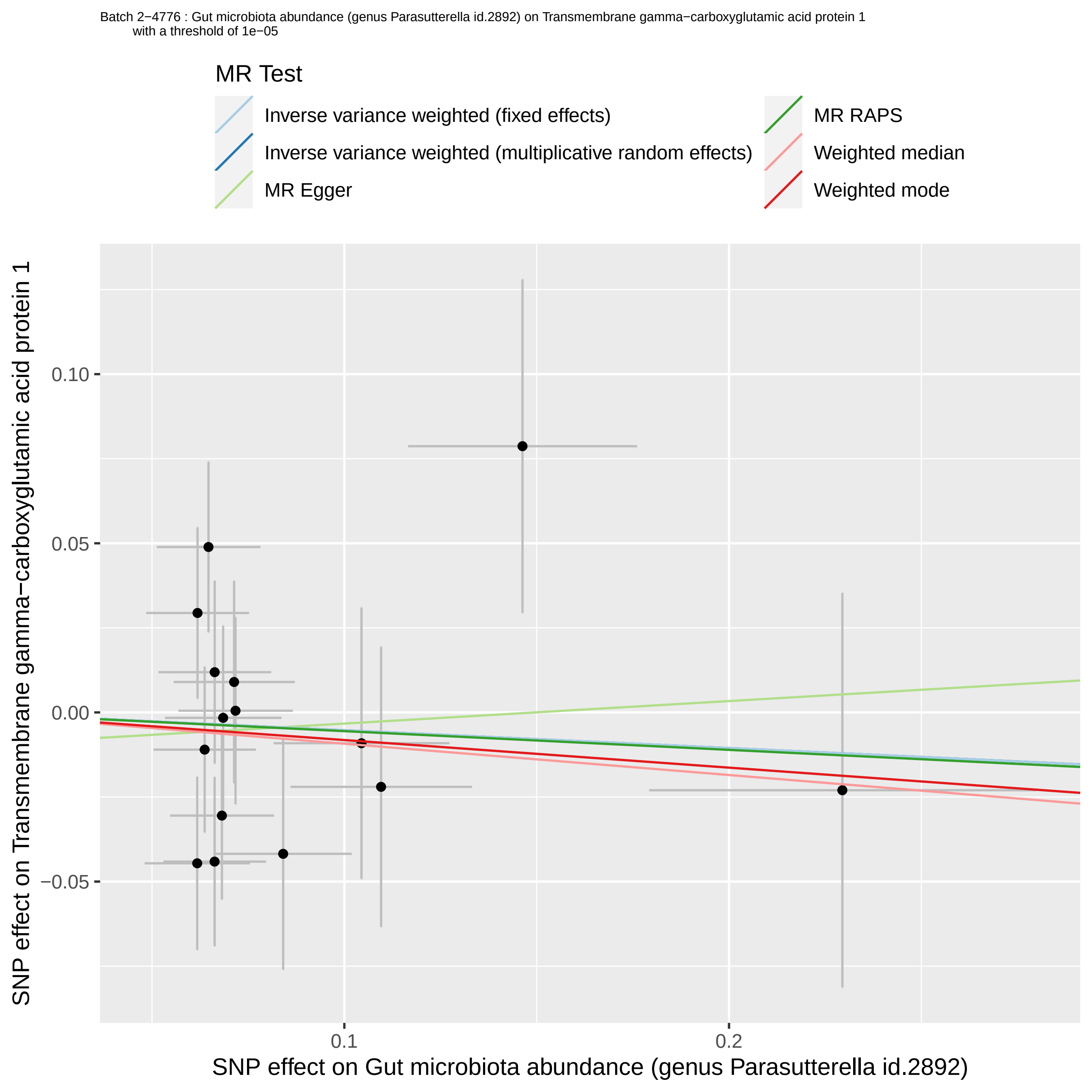
Batch 2−4776 : Gut microbiota abundance (genus Parasutterella id.2892) on Transmembrane gamma−carboxyglutamic acid protein 1
with a threshold of 1e−05
MR Test
Inverse variance weighted (fixed effects)
Inverse variance weighted (multiplicative random effects)
MR Egger
MR RAPS
Weighted median
Weighted mode
0.10
0.05
0.00
−0.05
0.1
0.2
SNP effect on Transmembrane gamma−carboxyglutamic acid protein 1
SNP effect on Gut microbiota abundance (genus Parasutterella id.2892)

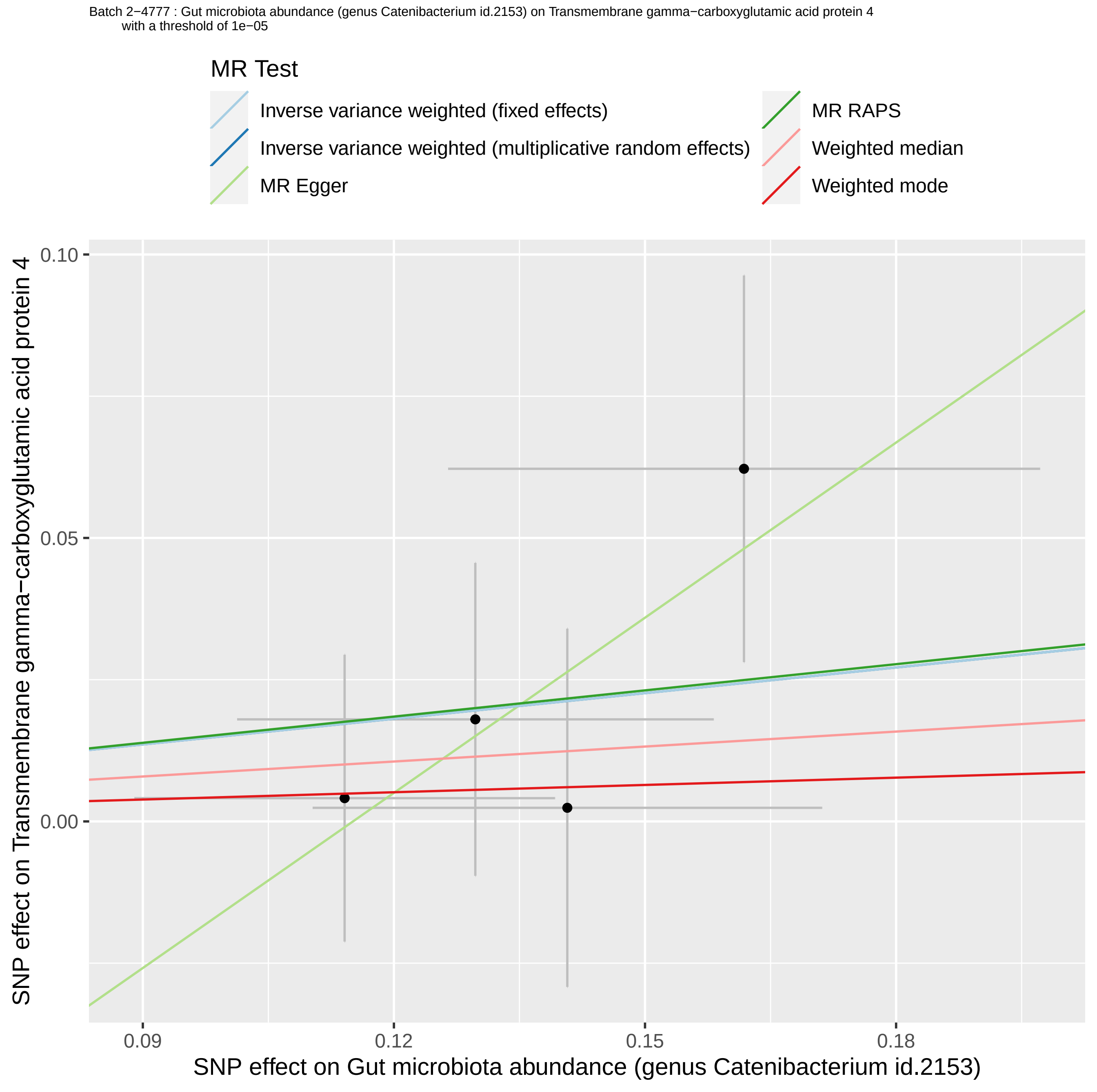

Batch 2−4777 : Gut microbiota abundance (genus Catenibacterium id.2153) on Transmembrane gamma−carboxyglutamic acid protein 4
with a threshold of 1e−05
MR Test
Inverse variance weighted (fixed effects)
Inverse variance weighted (multiplicative random effects)
MR Egger
MR RAPS
Weighted median
Weighted mode
0.10
0.05
0.00
0.09
0.12
0.15
0.18
SNP effect on Transmembrane gamma−carboxyglutamic acid protein 4
SNP effect on Gut microbiota abundance (genus Catenibacterium id.2153)

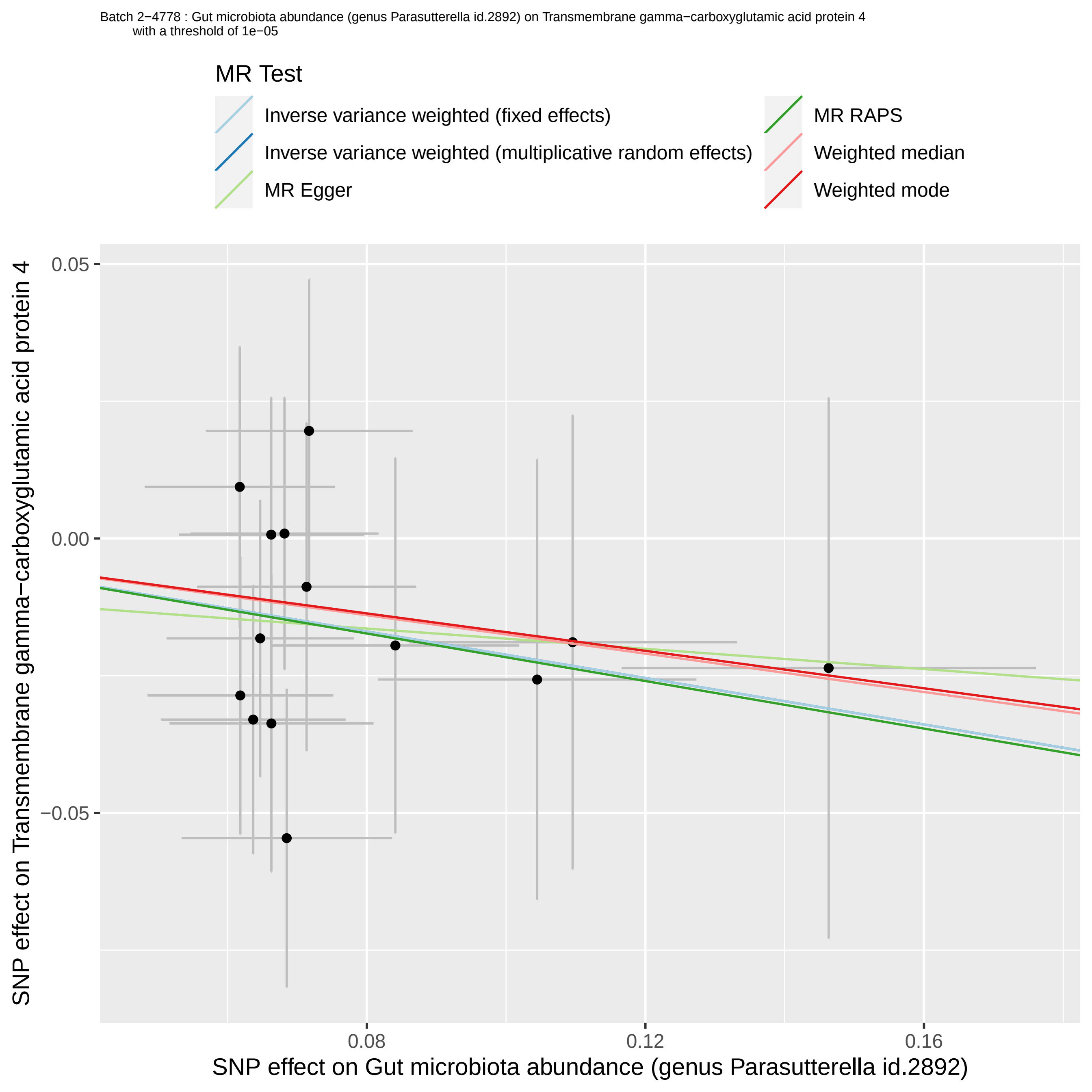
Batch 2−4778 : Gut microbiota abundance (genus Parasutterella id.2892) on Transmembrane gamma−carboxyglutamic acid protein 4
with a threshold of 1e−05
MR Test
Inverse variance weighted (fixed effects)
Inverse variance weighted (multiplicative random effects)
MR Egger
MR RAPS
Weighted median
Weighted mode
0.05
0.00
−0.05
0.08
0.12
0.16
SNP effect on Transmembrane gamma−carboxyglutamic acid protein 4
SNP effect on Gut microbiota abundance (genus Parasutterella id.2892)

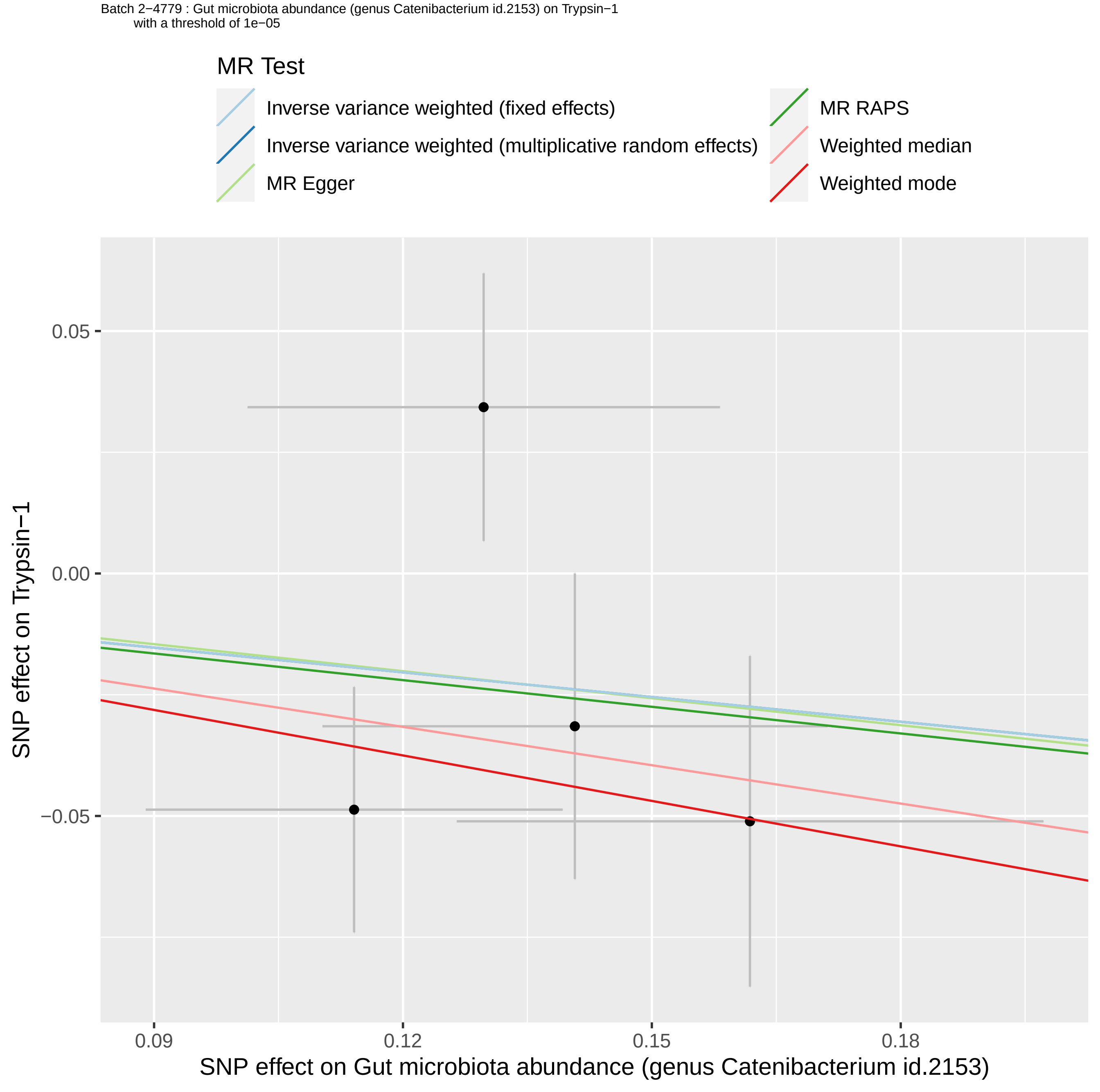
Batch 2−4779 : Gut microbiota abundance (genus Catenibacterium id.2153) on Trypsin−1
with a threshold of 1e−05
MR Test
Inverse variance weighted (fixed effects)
Inverse variance weighted (multiplicative random effects)
MR Egger
MR RAPS
Weighted median
Weighted mode
0.05
0.00
−0.05
0.09
0.12
0.15
0.18
SNP effect on Trypsin−1
SNP effect on Gut microbiota abundance (genus Catenibacterium id.2153)

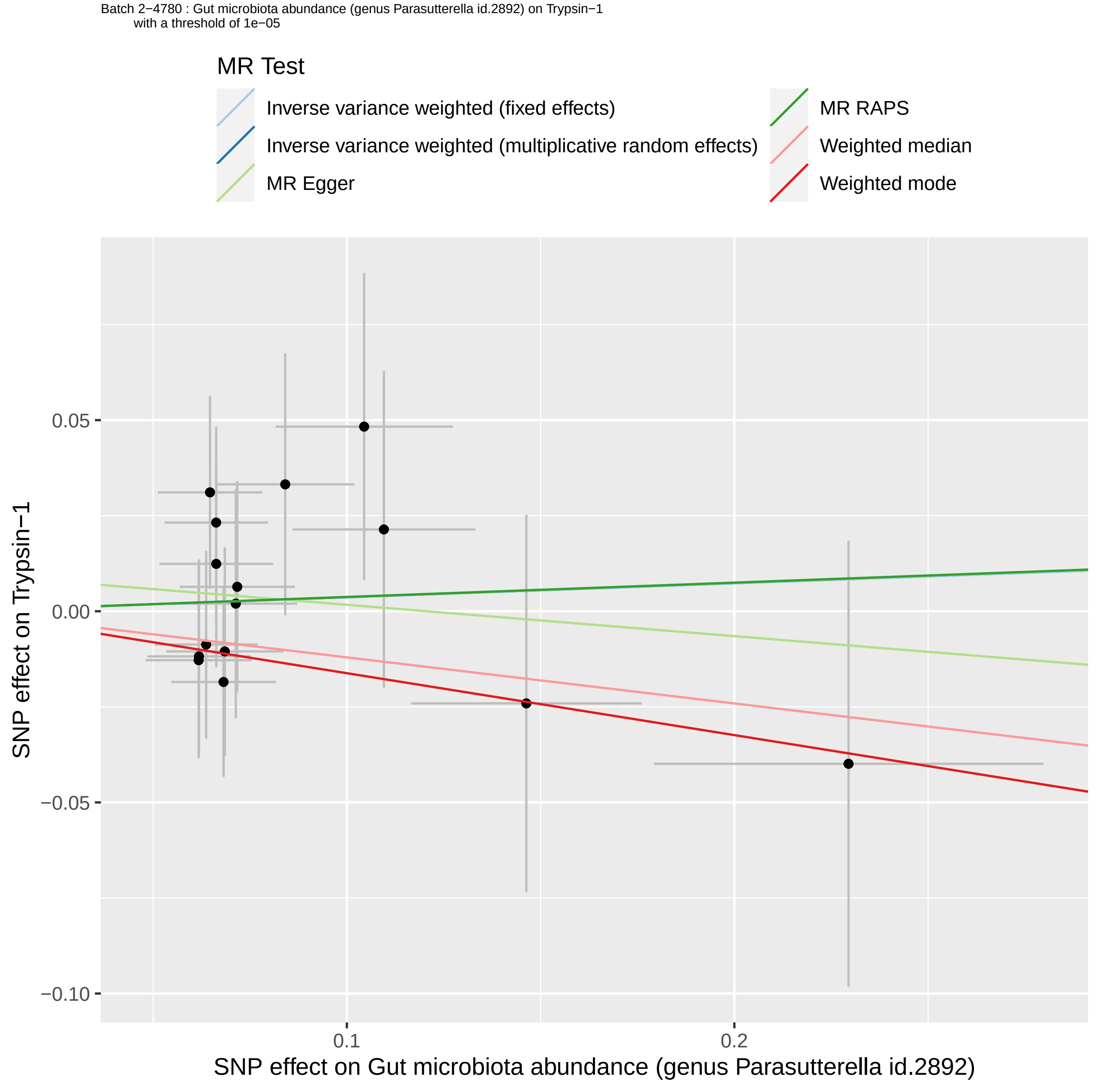
Batch 2−4780 : Gut microbiota abundance (genus Parasutterella id.2892) on Trypsin−1
with a threshold of 1e−05
MR Test
Inverse variance weighted (fixed effects)
Inverse variance weighted (multiplicative random effects)
MR Egger
MR RAPS
Weighted median
Weighted mode
SNP effect on Trypsin−1
0.05
0.00
−0.05
−0.10
0.1
0.2
SNP effect on Gut microbiota abundance (genus Parasutterella id.2892)

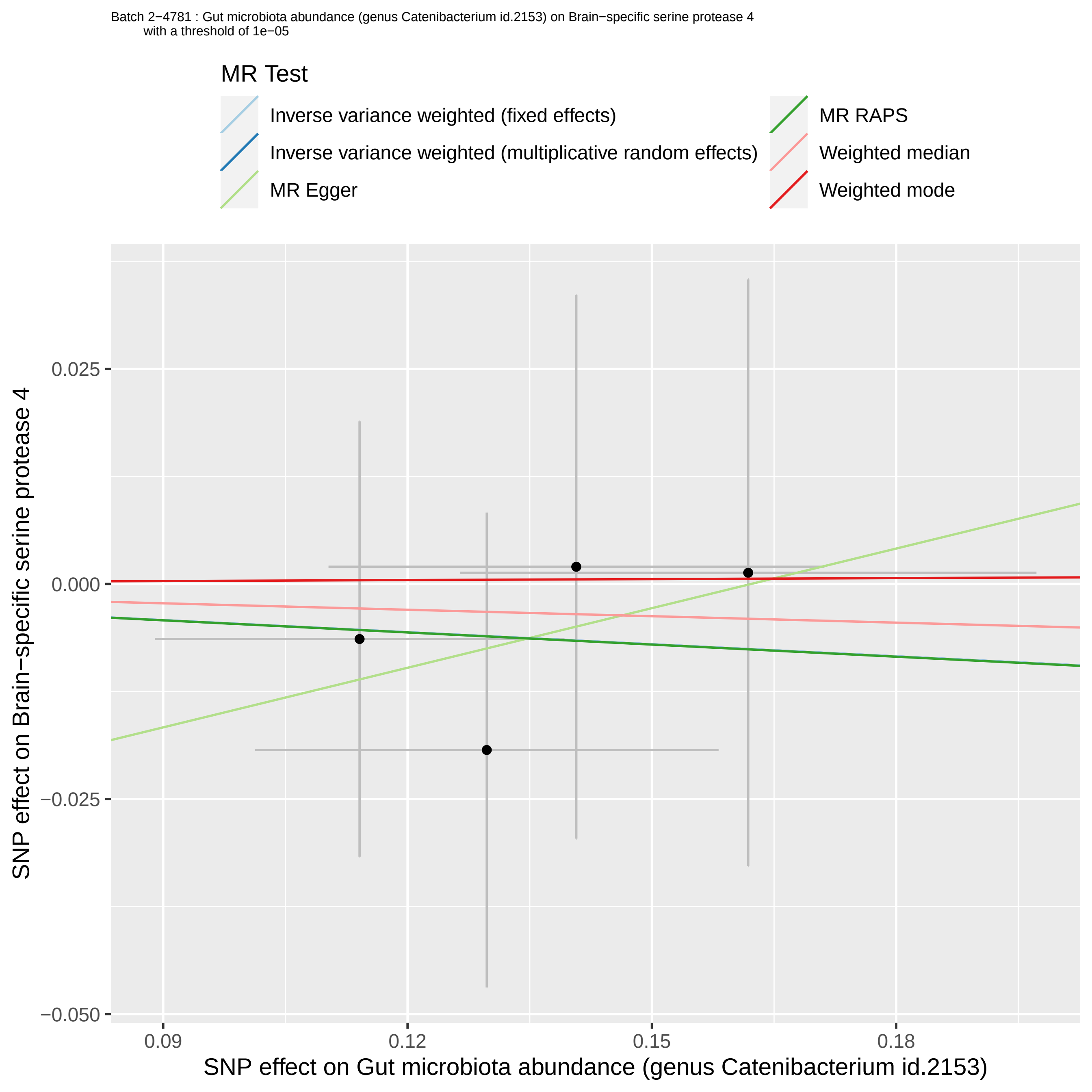
Batch 2−4781 : Gut microbiota abundance (genus Catenibacterium id.2153) on Brain−specific serine protease 4
with a threshold of 1e−05
MR Test
Inverse variance weighted (fixed effects)
Inverse variance weighted (multiplicative random effects)
MR Egger
MR RAPS
Weighted median
Weighted mode
0.025
0.000
−0.025
−0.050
0.09
0.12
0.15
0.18
SNP effect on Brain−specific serine protease 4
SNP effect on Gut microbiota abundance (genus Catenibacterium id.2153)

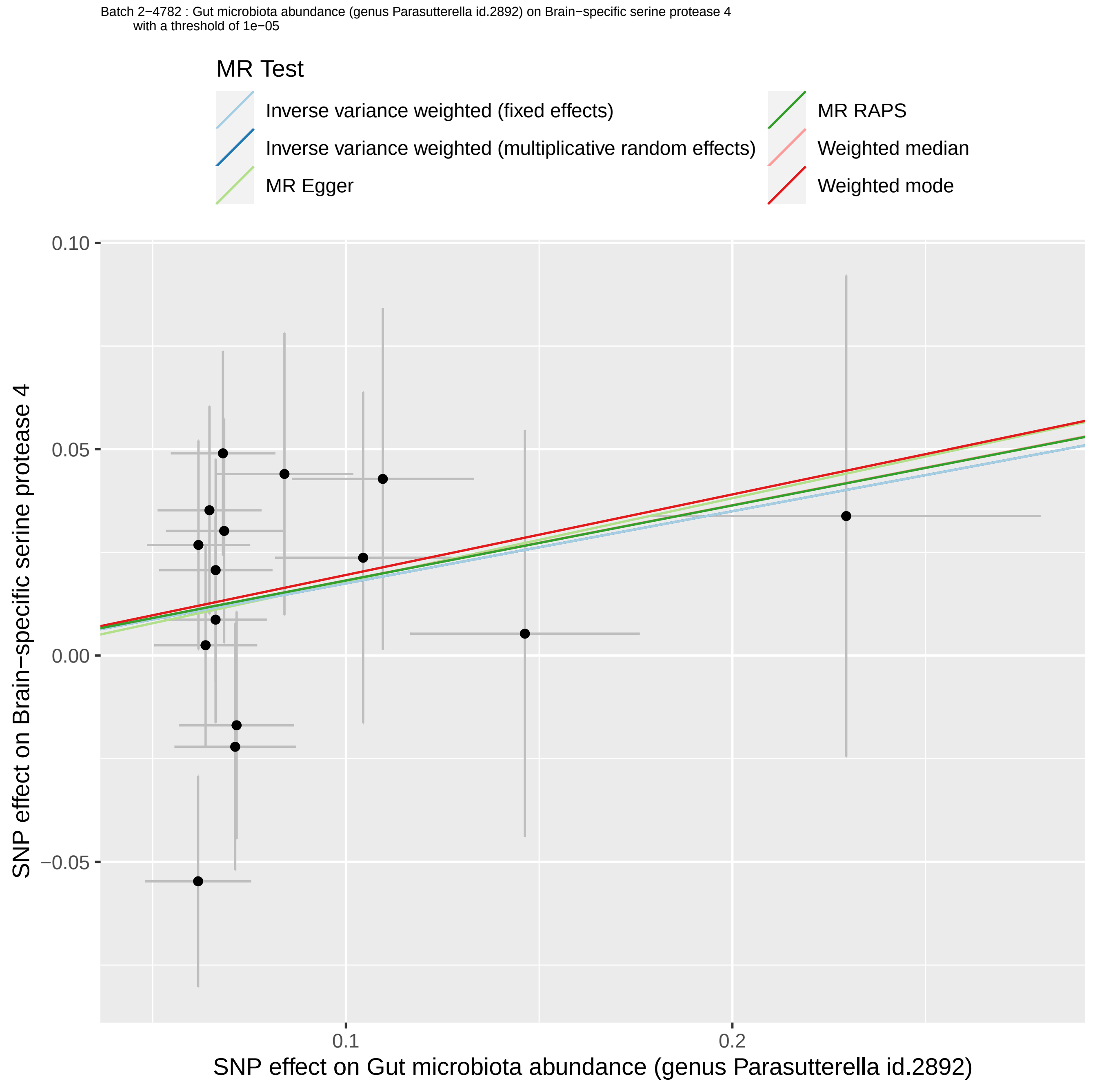

Batch 2−4782 : Gut microbiota abundance (genus Parasutterella id.2892) on Brain−specific serine protease 4
with a threshold of 1e−05
MR Test
Inverse variance weighted (fixed effects)
Inverse variance weighted (multiplicative random effects)
MR Egger
MR RAPS
Weighted median
Weighted mode
0.10
0.05
0.00
−0.05
0.1
0.2
SNP effect on Brain−specific serine protease 4
SNP effect on Gut microbiota abundance (genus Parasutterella id.2892)

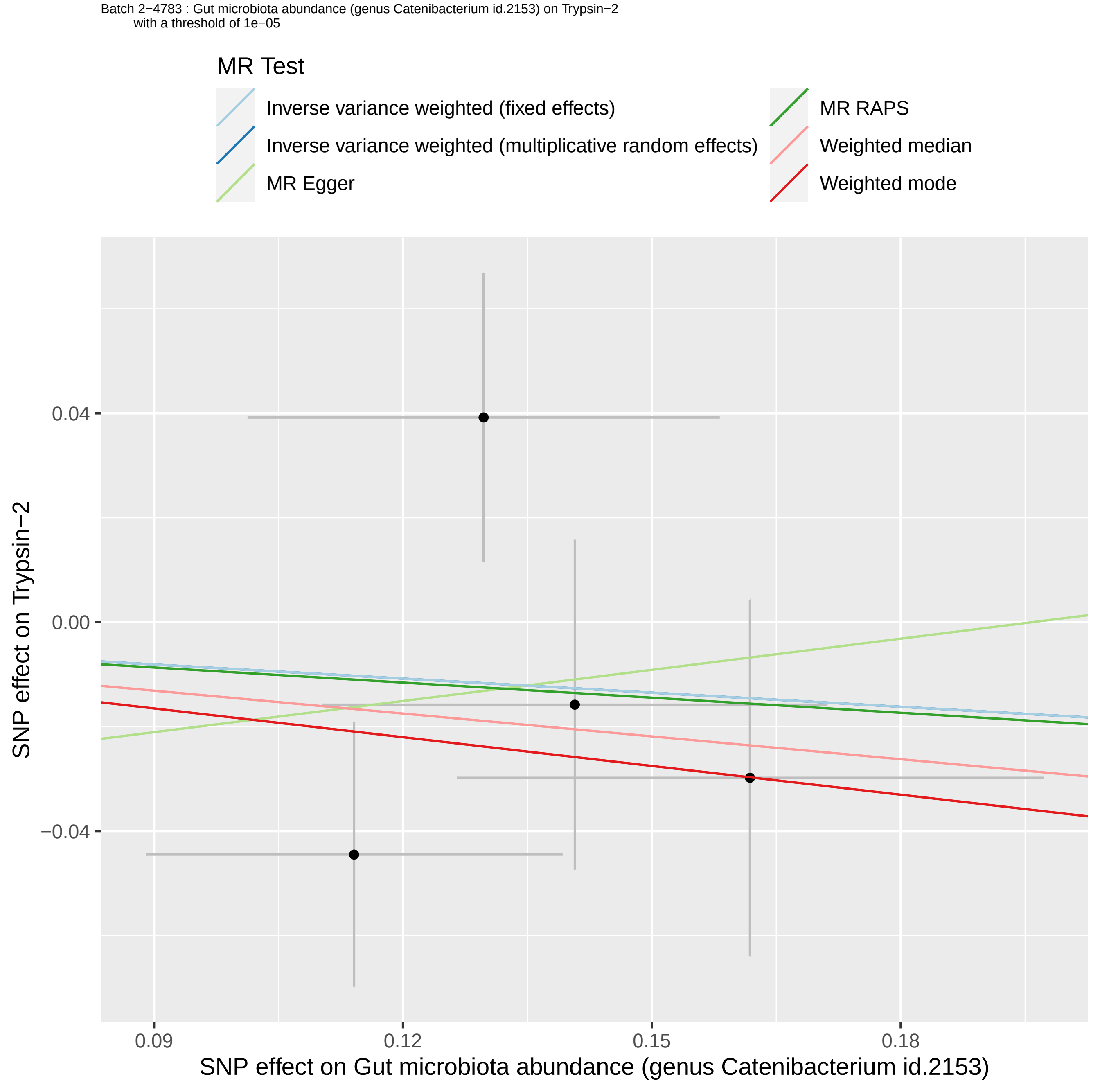
Batch 2−4783 : Gut microbiota abundance (genus Catenibacterium id.2153) on Trypsin−2
with a threshold of 1e−05
MR Test
Inverse variance weighted (fixed effects)
Inverse variance weighted (multiplicative random effects)
MR Egger
MR RAPS
Weighted median
Weighted mode
0.04
0.00
−0.04
0.09
0.12
0.15
0.18
SNP effect on Trypsin−2
SNP effect on Gut microbiota abundance (genus Catenibacterium id.2153)

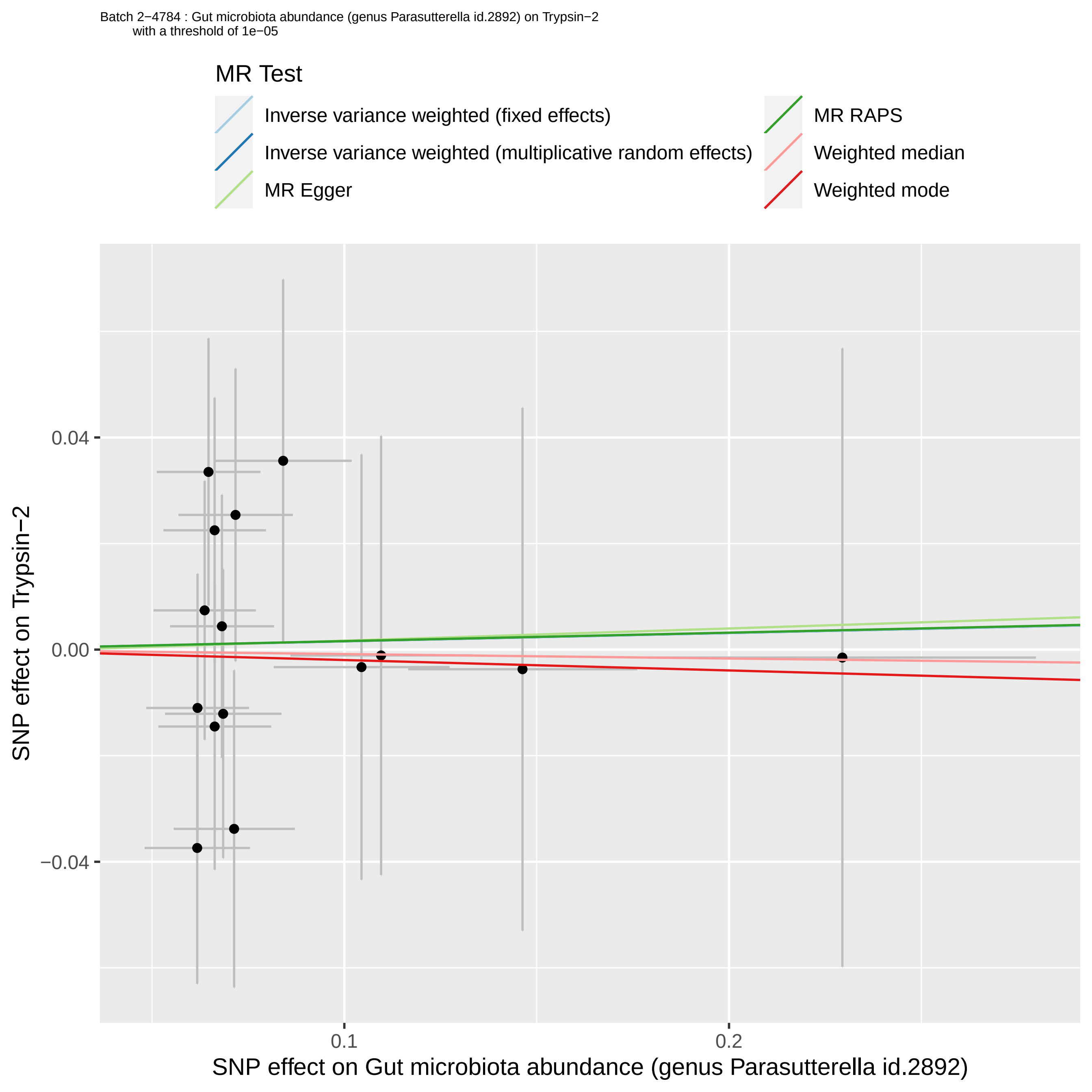
Batch 2−4784 : Gut microbiota abundance (genus Parasutterella id.2892) on Trypsin−2
with a threshold of 1e−05
MR Test
Inverse variance weighted (fixed effects)
Inverse variance weighted (multiplicative random effects)
MR Egger
MR RAPS
Weighted median
Weighted mode
0.04
0.00
−0.04
0.1
0.2
SNP effect on Trypsin−2
SNP effect on Gut microbiota abundance (genus Parasutterella id.2892)

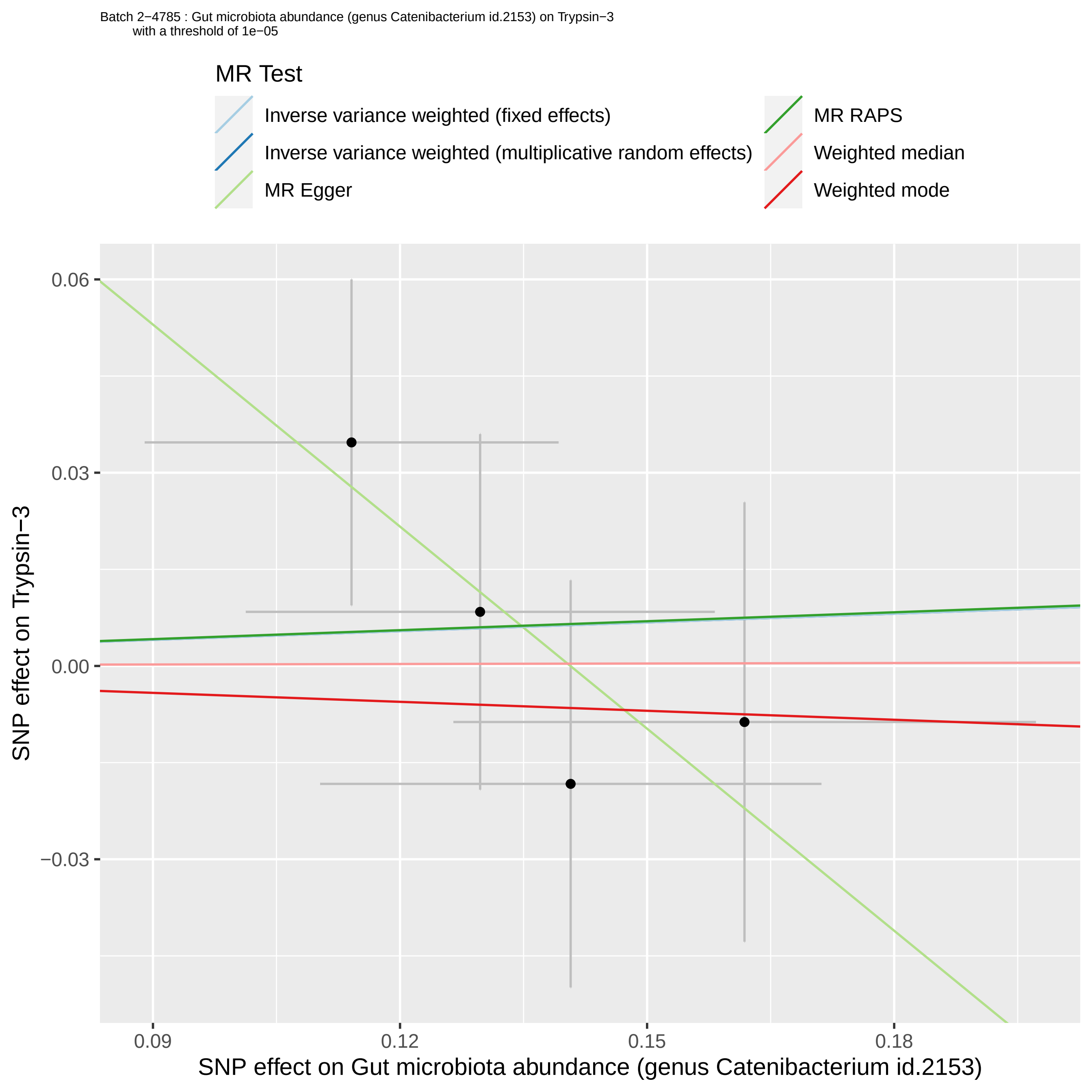

Batch 2−4785 : Gut microbiota abundance (genus Catenibacterium id.2153) on Trypsin−3
with a threshold of 1e−05
MR Test
Inverse variance weighted (fixed effects)
Inverse variance weighted (multiplicative random effects)
MR Egger
MR RAPS
Weighted median
Weighted mode
0.06
0.03
0.00
−0.03
0.09
0.12
0.15
0.18
SNP effect on Trypsin−3
SNP effect on Gut microbiota abundance (genus Catenibacterium id.2153)

Batch 2−4786 : Gut microbiota abundance (genus Parasutterella id.2892) on Trypsin−3
with a threshold of 1e−05

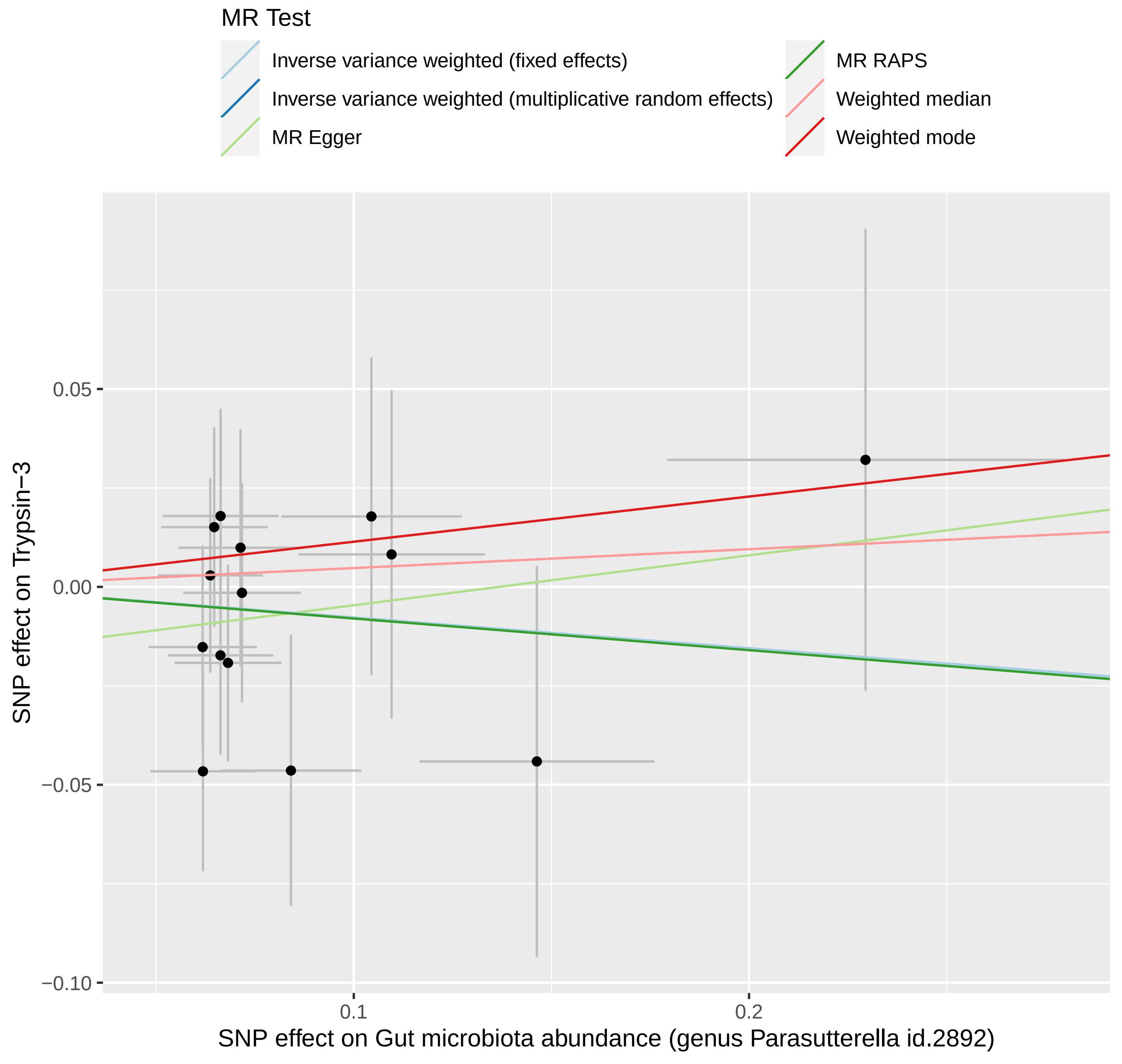

Batch 2−4787 : Gut microbiota abundance (genus Catenibacterium id.2153) on Probable inactive serine protease 37 with a threshold of 1e−05

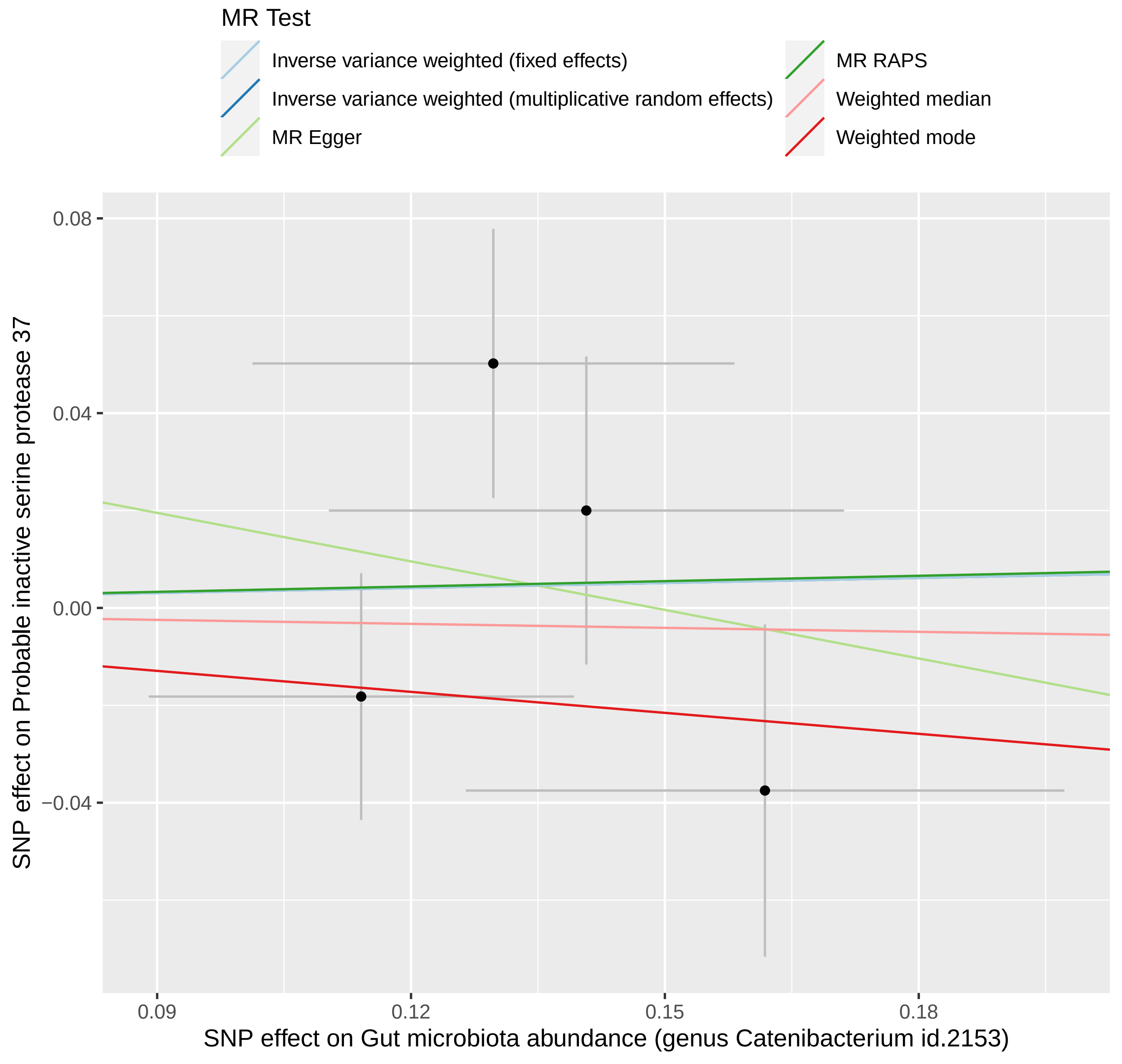

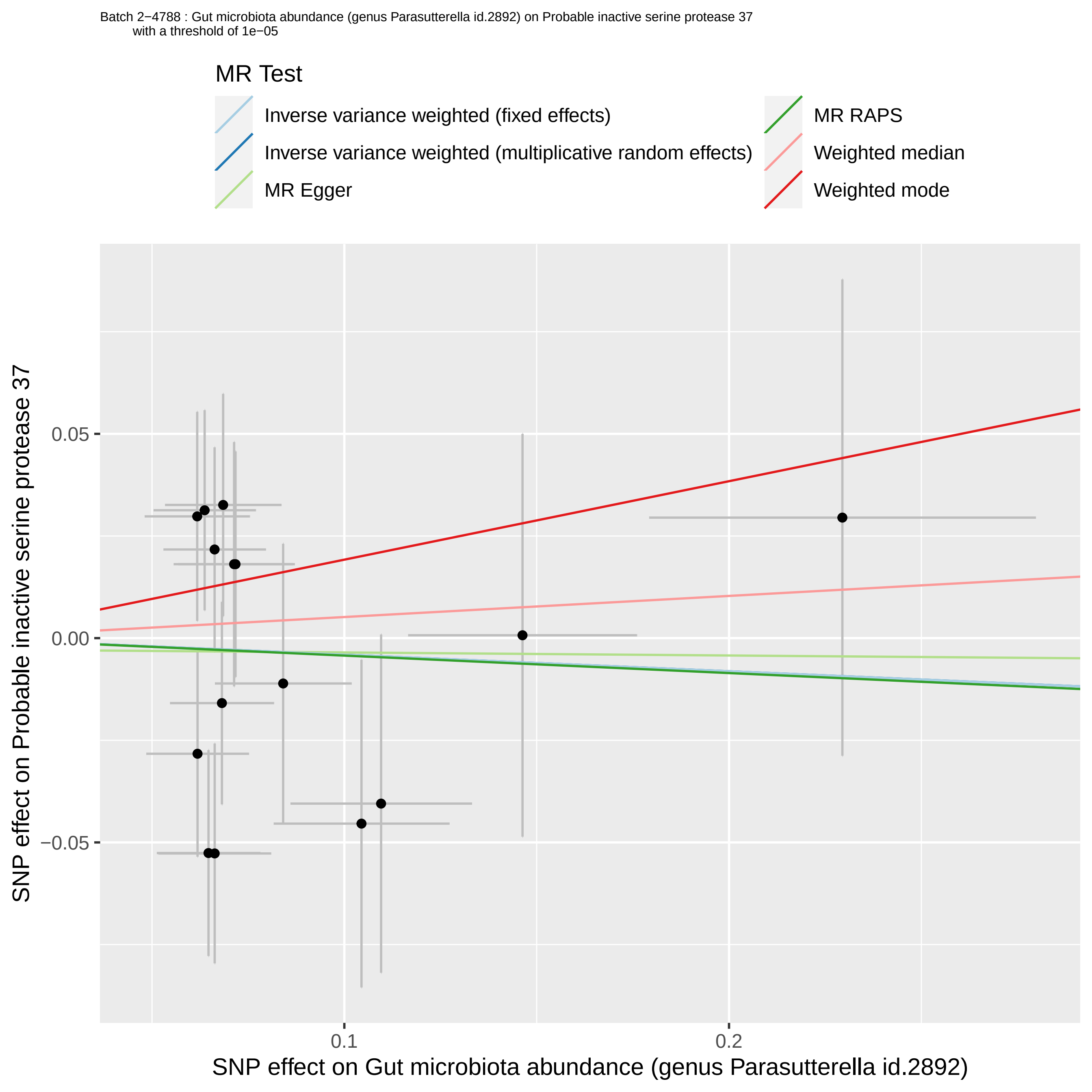
Batch 2−4788 : Gut microbiota abundance (genus Parasutterella id.2892) on Probable inactive serine protease 37
with a threshold of 1e−05
MR Test
Inverse variance weighted (fixed effects)
Inverse variance weighted (multiplicative random effects)
MR Egger
MR RAPS
Weighted median
Weighted mode
SNP effect on Probable inactive serine protease 37
0.05
0.00
−0.05
0.1
0.2
SNP effect on Gut microbiota abundance (genus Parasutterella id.2892)

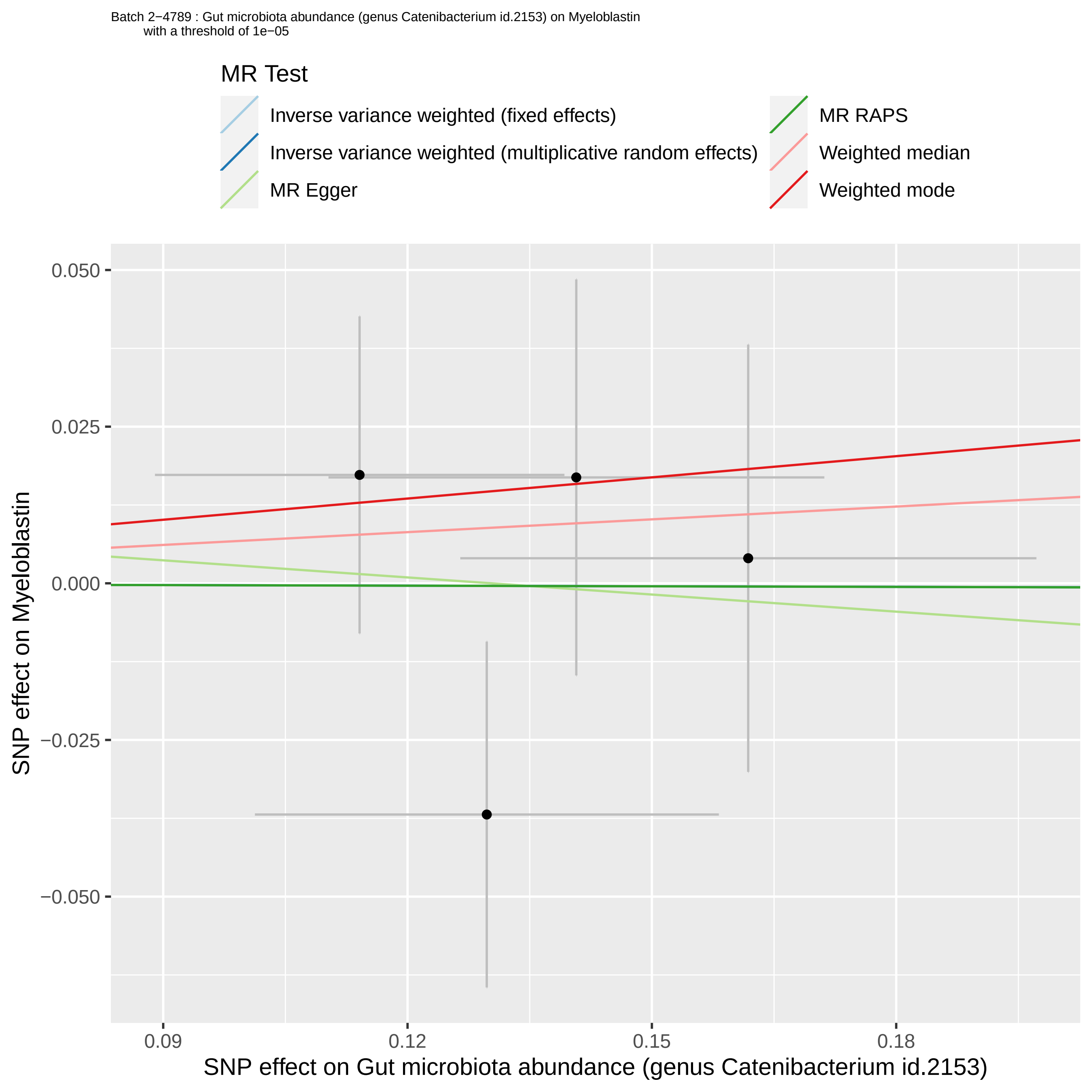

Batch 2−4789 : Gut microbiota abundance (genus Catenibacterium id.2153) on Myeloblastin
with a threshold of 1e−05
MR Test
Inverse variance weighted (fixed effects)
Inverse variance weighted (multiplicative random effects)
MR Egger
MR RAPS
Weighted median
Weighted mode
0.050
0.025
0.000
−0.025
−0.050
0.09
0.12
0.15
0.18
SNP effect on Myeloblastin
SNP effect on Gut microbiota abundance (genus Catenibacterium id.2153)

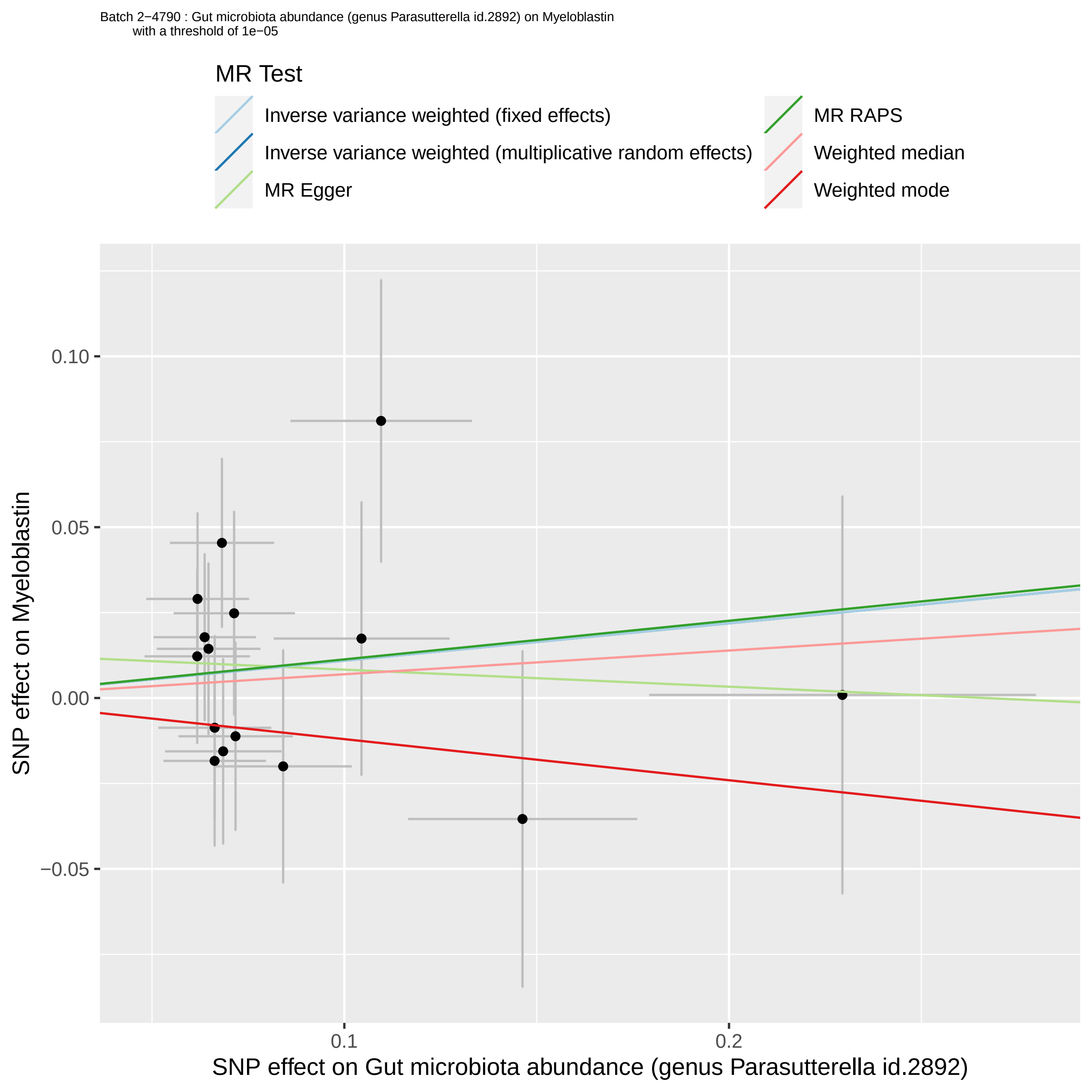
Batch 2−4790 : Gut microbiota abundance (genus Parasutterella id.2892) on Myeloblastin
with a threshold of 1e−05
MR Test
Inverse variance weighted (fixed effects)
Inverse variance weighted (multiplicative random effects)
MR Egger
MR RAPS
Weighted median
Weighted mode
0.10
0.05
0.00
−0.05
0.1
0.2
SNP effect on Myeloblastin
SNP effect on Gut microbiota abundance (genus Parasutterella id.2892)

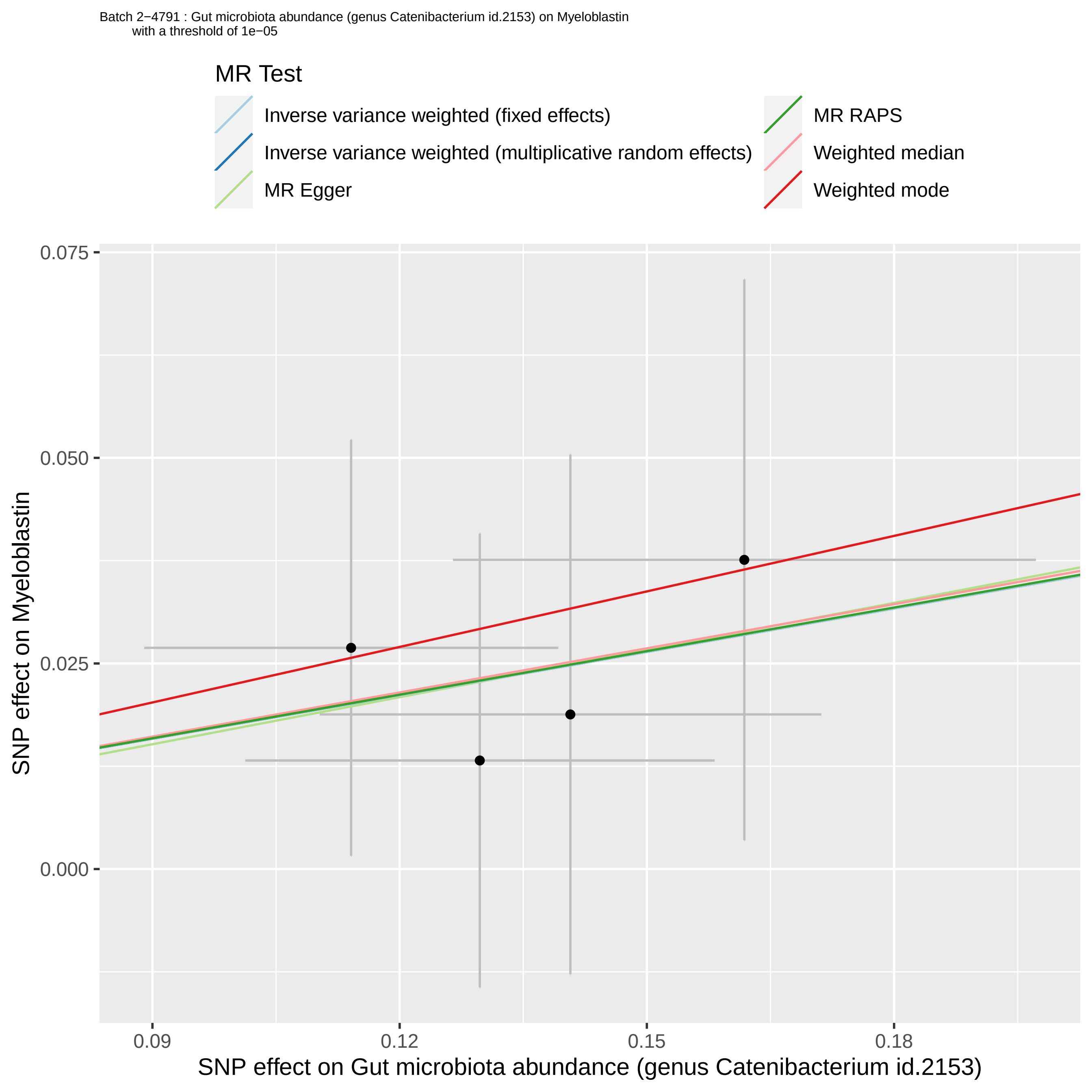

Batch 2−4791 : Gut microbiota abundance (genus Catenibacterium id.2153) on Myeloblastin
with a threshold of 1e−05
MR Test
Inverse variance weighted (fixed effects)
Inverse variance weighted (multiplicative random effects)
MR Egger
MR RAPS
Weighted median
Weighted mode
0.075
0.050
0.025
0.000
SNP effect on Myeloblastin
0.09
0.12
0.15
0.18
SNP effect on Gut microbiota abundance (genus Catenibacterium id.2153)

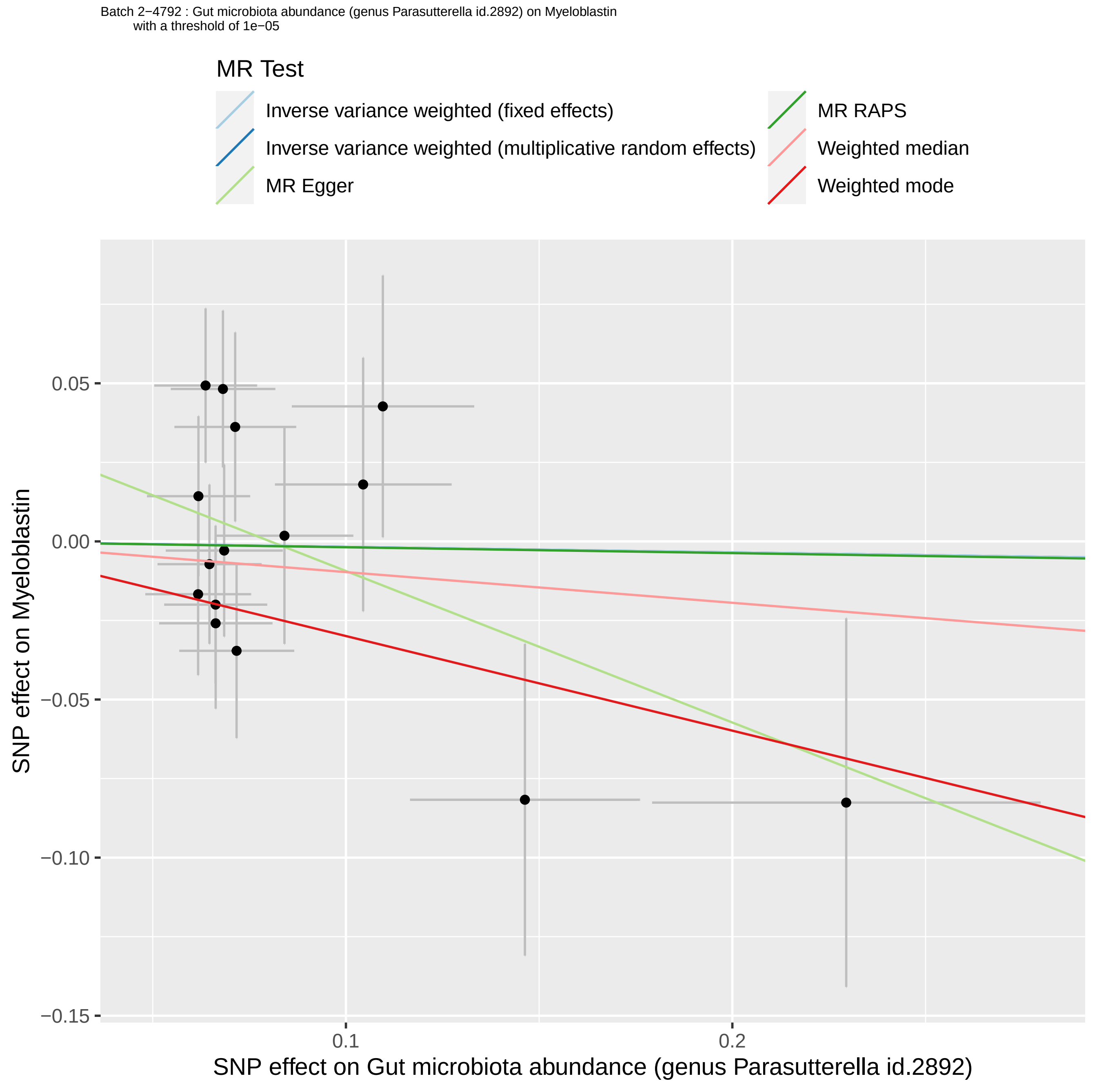

Batch 2−4792 : Gut microbiota abundance (genus Parasutterella id.2892) on Myeloblastin
with a threshold of 1e−05
MR Test
Inverse variance weighted (fixed effects)
Inverse variance weighted (multiplicative random effects)
MR Egger
MR RAPS
Weighted median
Weighted mode
0.05
0.00
−0.05
−0.10
−0.15
0.1
0.2
SNP effect on Myeloblastin
SNP effect on Gut microbiota abundance (genus Parasutterella id.2892)

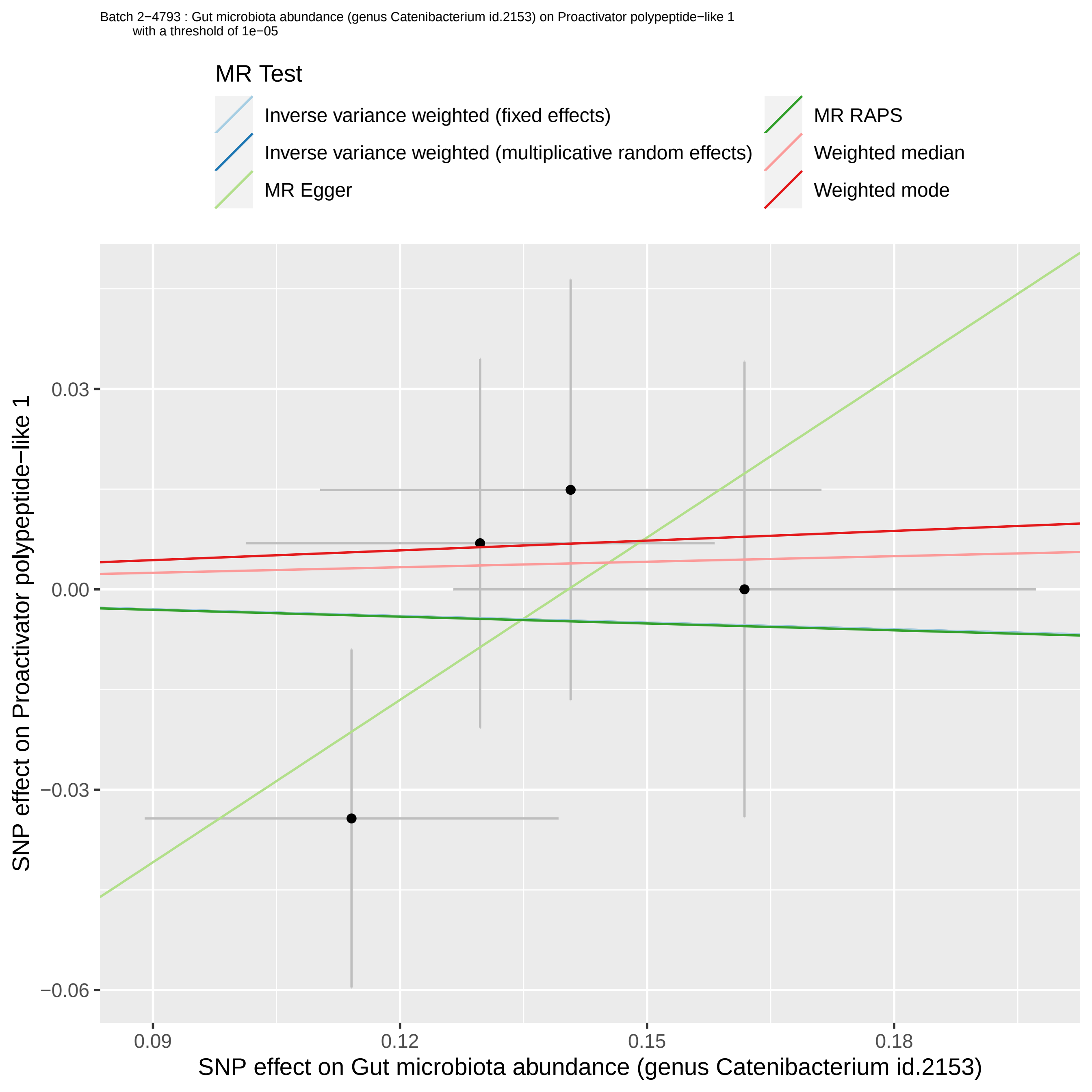

Batch 2−4793 : Gut microbiota abundance (genus Catenibacterium id.2153) on Proactivator polypeptide−like 1
with a threshold of 1e−05
MR Test
Inverse variance weighted (fixed effects)
Inverse variance weighted (multiplicative random effects)
MR Egger
MR RAPS
Weighted median
Weighted mode
0.03
0.00
−0.03
−0.06
0.09
0.12
0.15
0.18
SNP effect on Proactivator polypeptide−like 1
SNP effect on Gut microbiota abundance (genus Catenibacterium id.2153)

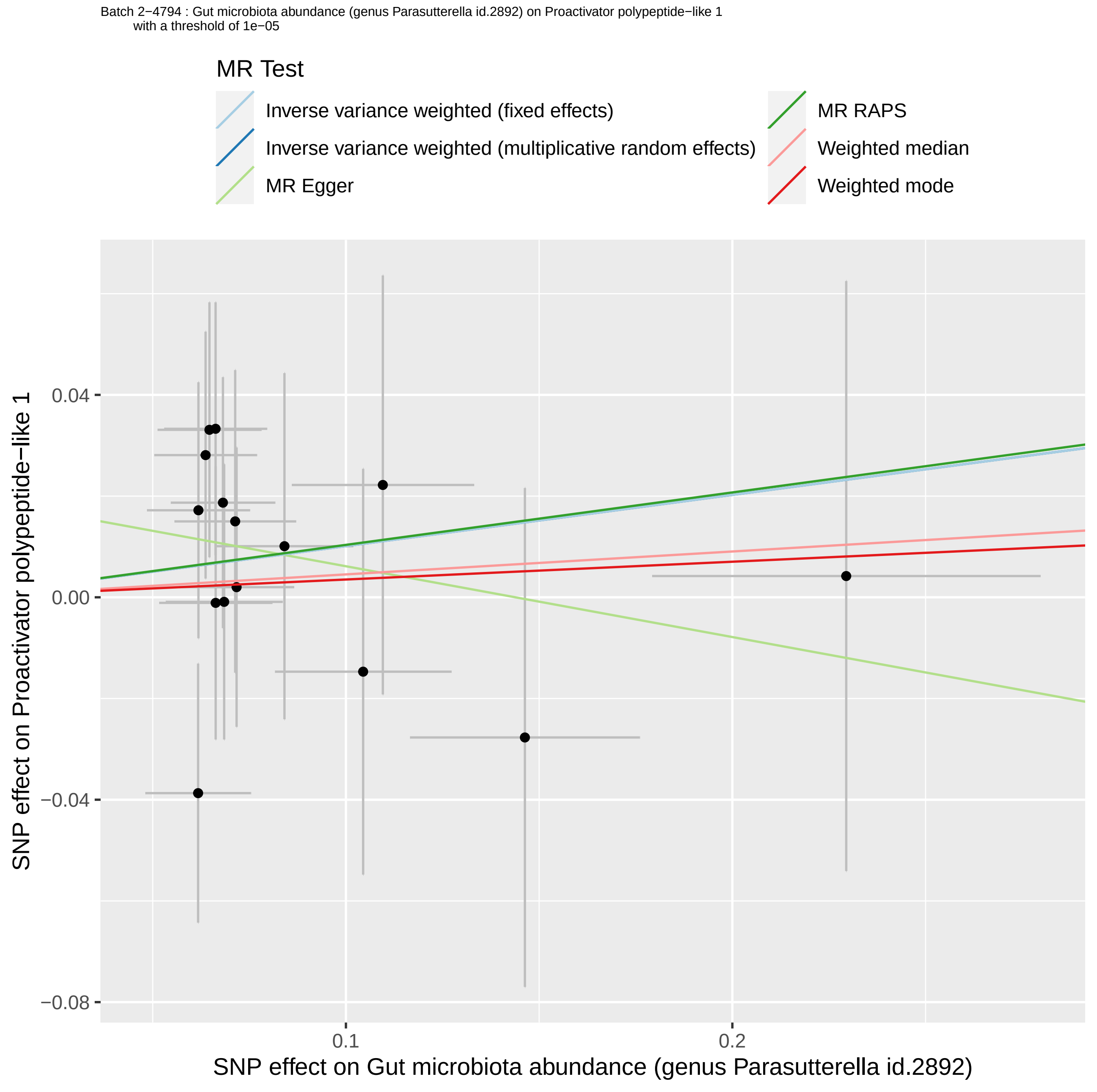

Batch 2−4794 : Gut microbiota abundance (genus Parasutterella id.2892) on Proactivator polypeptide−like 1
with a threshold of 1e−05
MR Test
Inverse variance weighted (fixed effects)
Inverse variance weighted (multiplicative random effects)
MR Egger
MR RAPS
Weighted median
Weighted mode
0.04
0.00
−0.04
−0.08
0.1
0.2
SNP effect on Proactivator polypeptide−like 1
SNP effect on Gut microbiota abundance (genus Parasutterella id.2892)

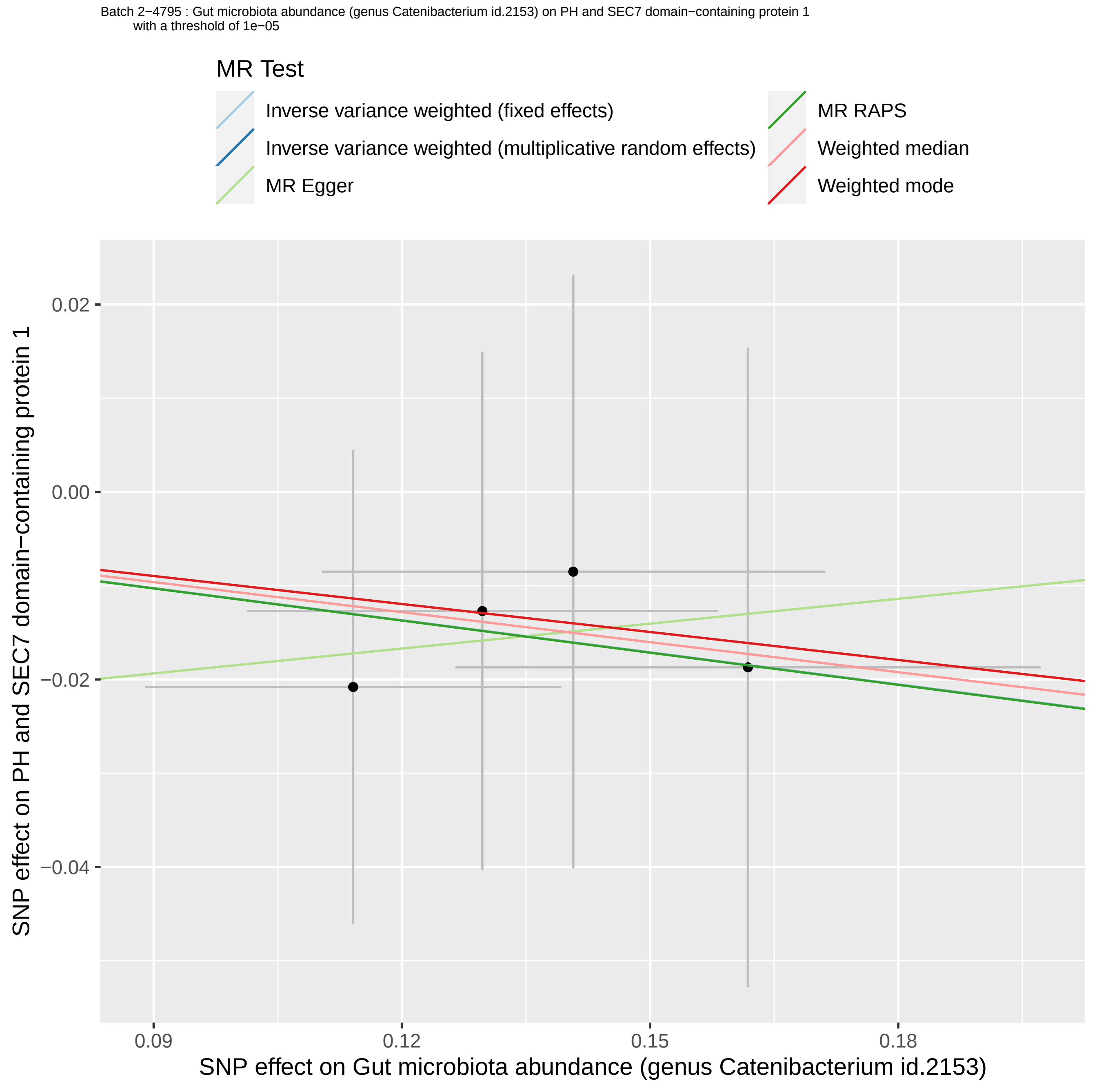
Batch 2−4795 : Gut microbiota abundance (genus Catenibacterium id.2153) on PH and SEC7 domain−containing protein 1
with a threshold of 1e−05
MR Test
Inverse variance weighted (fixed effects)
Inverse variance weighted (multiplicative random effects)
MR Egger
MR RAPS
Weighted median
Weighted mode
0.02
0.00
−0.02
−0.04
0.09
0.12
0.15
0.18
SNP effect on PH and SEC7 domain−containing protein 1
SNP effect on Gut microbiota abundance (genus Catenibacterium id.2153)

Batch 2−4796 : Gut microbiota abundance (genus Parasutterella id.2892) on PH and SEC7 domain−containing protein 1
with a threshold of 1e−05

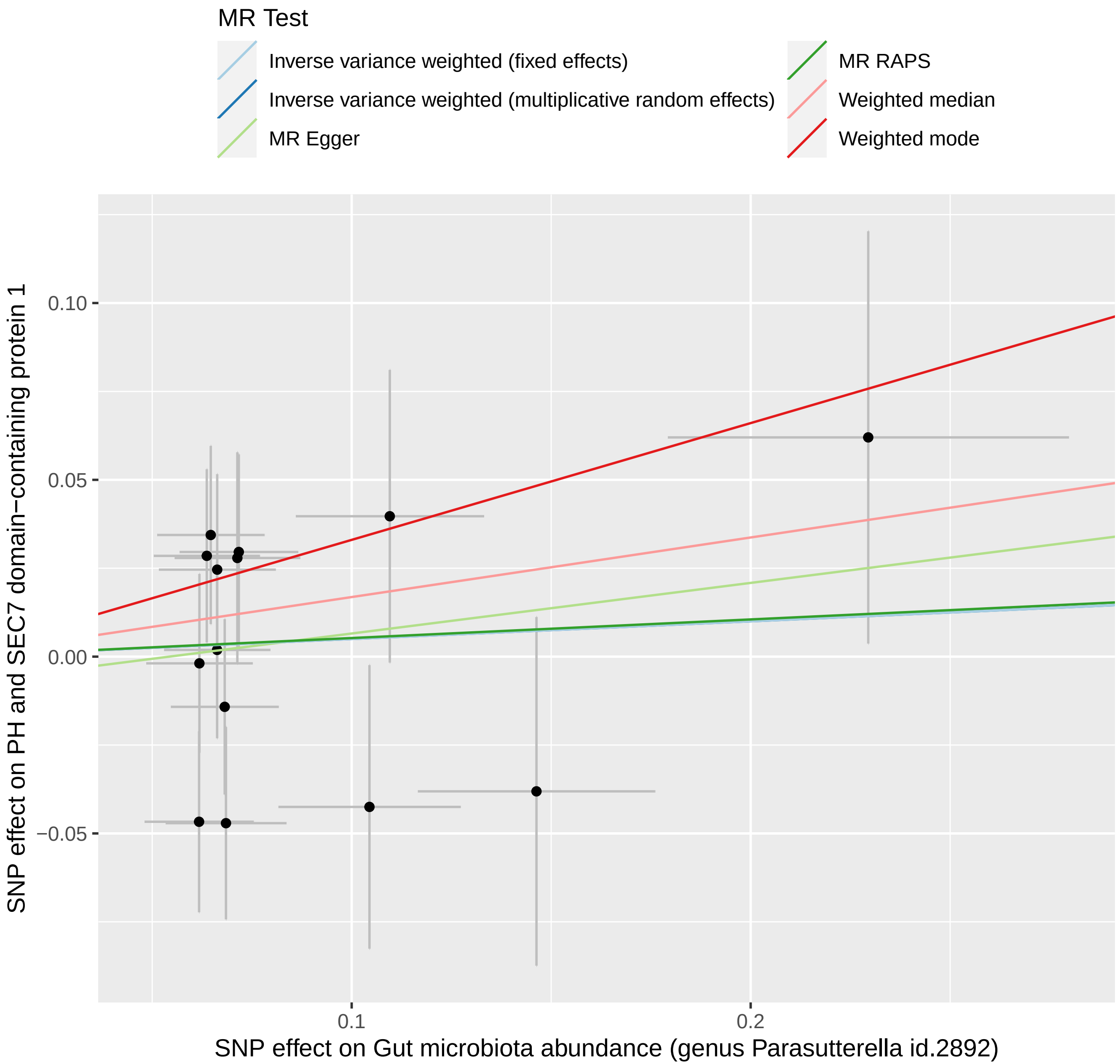

Batch 2−4797 : Gut microbiota abundance (genus Catenibacterium id.2153) on PH and SEC7 domain−containing protein 2
with a threshold of 1e−05

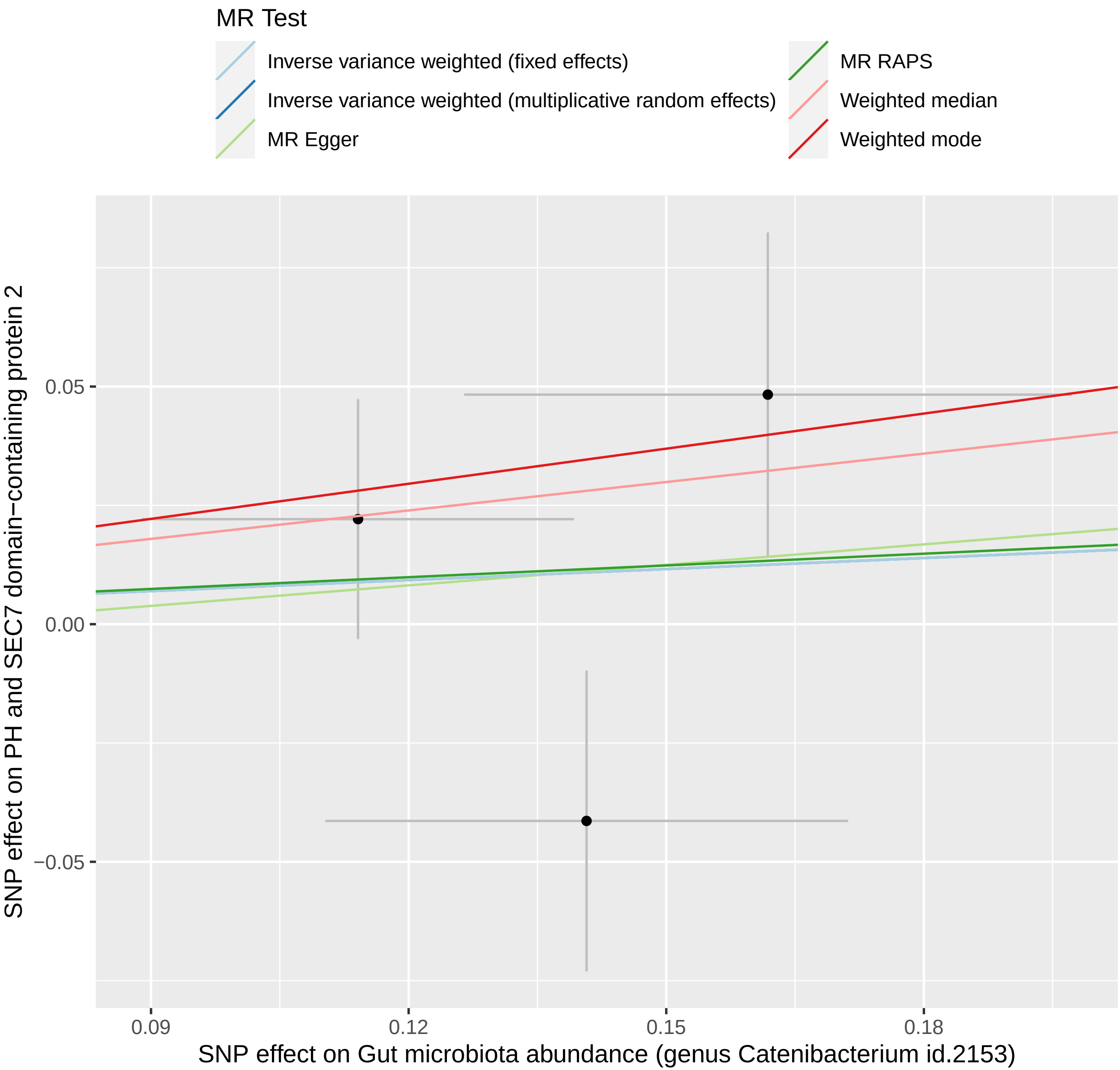

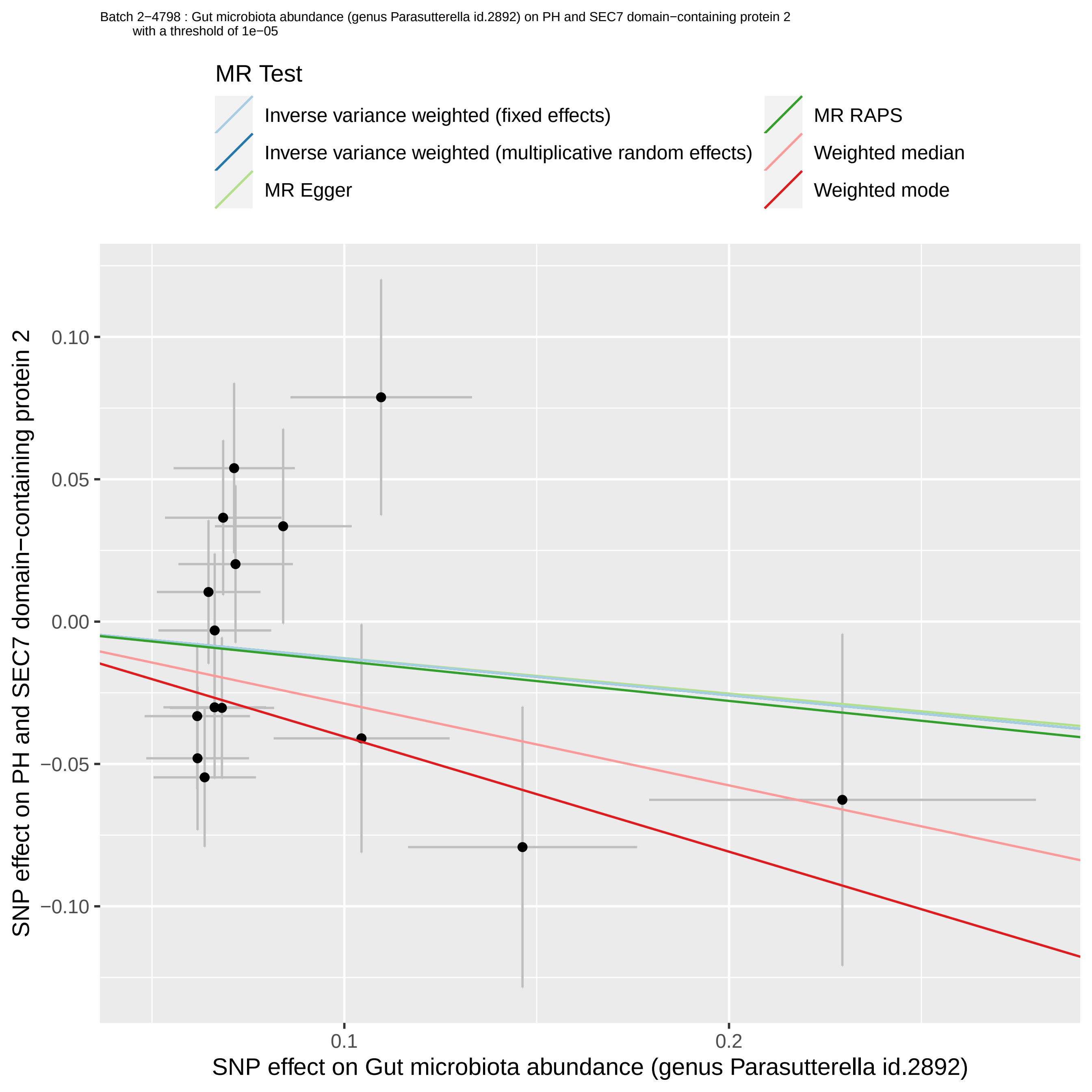

Batch 2−4798 : Gut microbiota abundance (genus Parasutterella id.2892) on PH and SEC7 domain−containing protein 2
with a threshold of 1e−05
MR Test
Inverse variance weighted (fixed effects)
Inverse variance weighted (multiplicative random effects)
MR Egger
MR RAPS
Weighted median
Weighted mode
0.10
0.05
0.00
−0.05
−0.10
0.1
0.2
SNP effect on PH and SEC7 domain−containing protein 2
SNP effect on Gut microbiota abundance (genus Parasutterella id.2892)

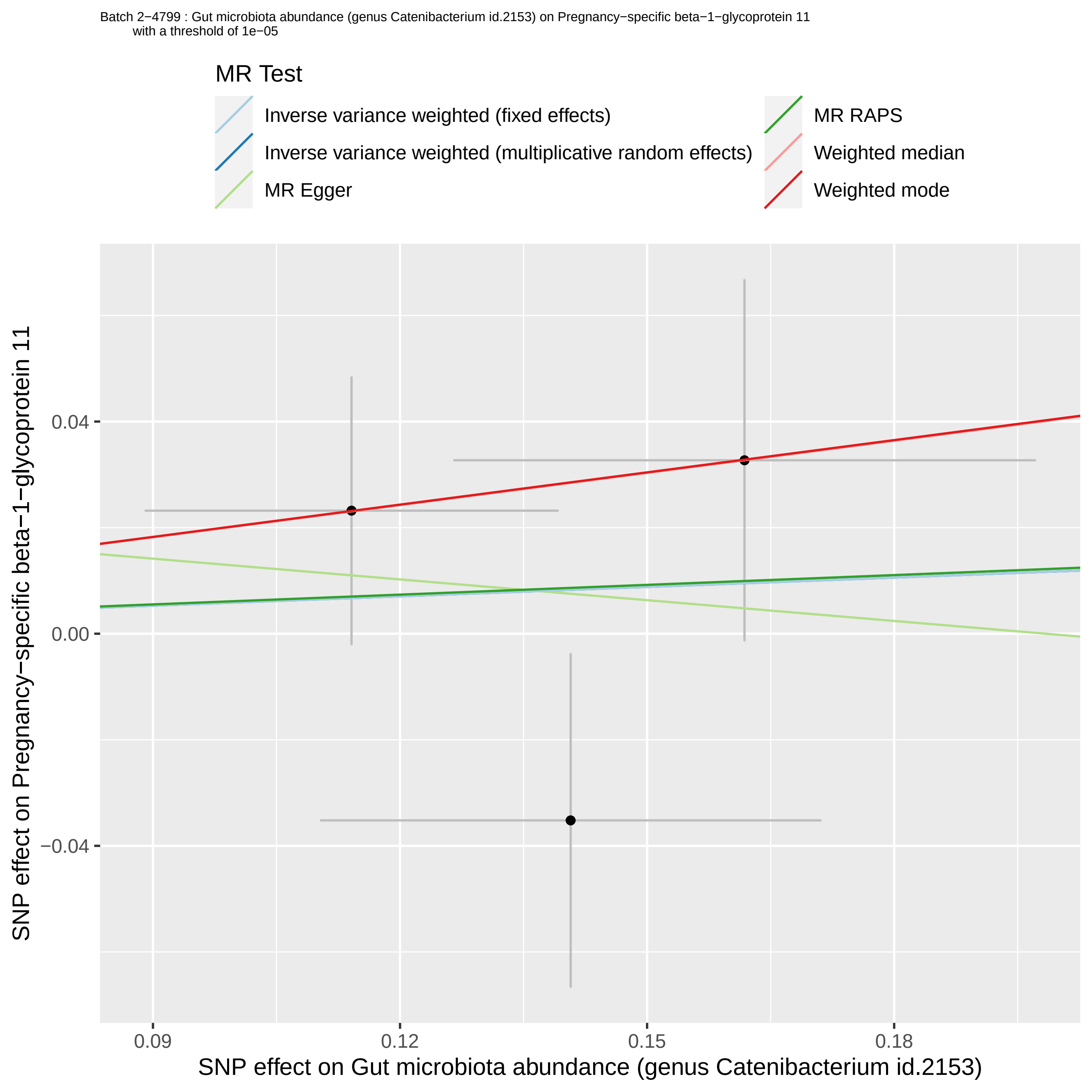

Batch 2−4799 : Gut microbiota abundance (genus Catenibacterium id.2153) on Pregnancy−specific beta−1−glycoprotein 11
with a threshold of 1e−05
MR Test
Inverse variance weighted (fixed effects)
Inverse variance weighted (multiplicative random effects)
MR Egger
MR RAPS
Weighted median
Weighted mode
0.04
0.00
−0.04
0.09
0.12
0.15
0.18
SNP effect on Pregnancy−specific beta−1−glycoprotein 11
SNP effect on Gut microbiota abundance (genus Catenibacterium id.2153)

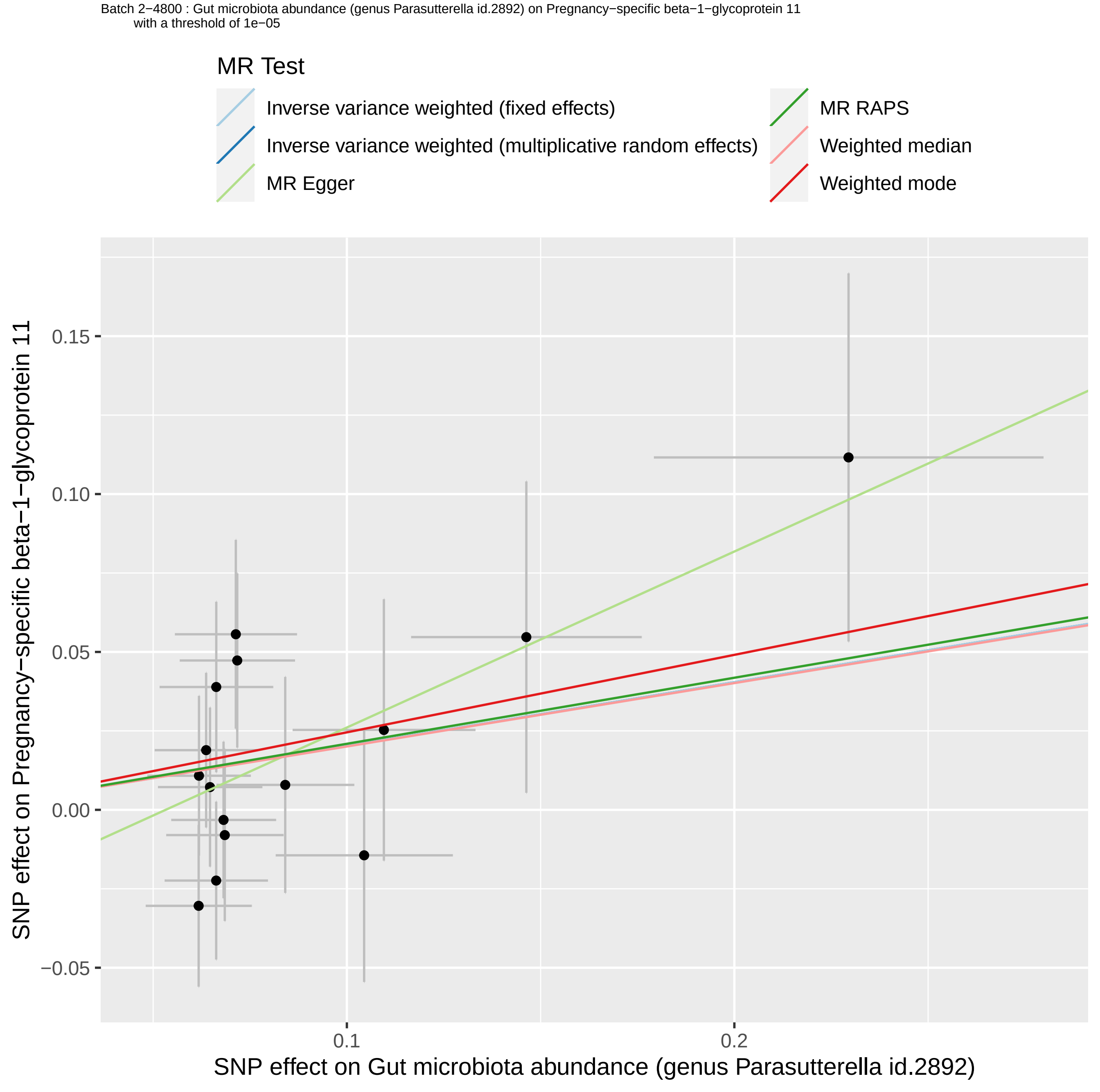
Batch 2−4800 : Gut microbiota abundance (genus Parasutterella id.2892) on Pregnancy−specific beta−1−glycoprotein 11
with a threshold of 1e−05
MR Test
Inverse variance weighted (fixed effects)
Inverse variance weighted (multiplicative random effects)
MR Egger
MR RAPS
Weighted median
Weighted mode
0.15
0.10
0.05
0.00
−0.05
0.1
0.2
SNP effect on Pregnancy−specific beta−1−glycoprotein 11
SNP effect on Gut microbiota abundance (genus Parasutterella id.2892)

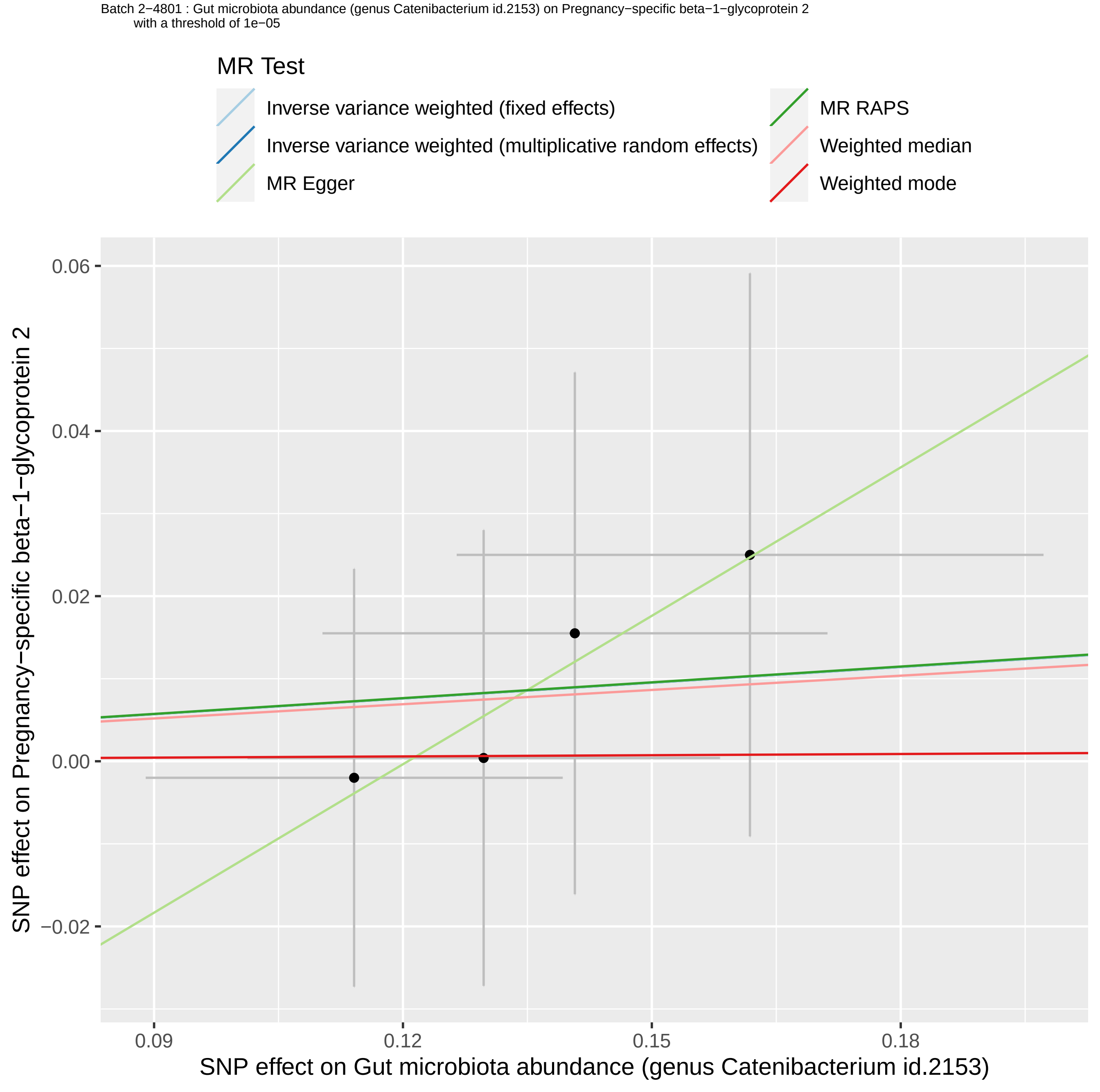

Batch 2−4801 : Gut microbiota abundance (genus Catenibacterium id.2153) on Pregnancy−specific beta−1−glycoprotein 2
with a threshold of 1e−05
MR Test
Inverse variance weighted (fixed effects)
Inverse variance weighted (multiplicative random effects)
MR Egger
MR RAPS
Weighted median
Weighted mode
0.06
0.04
0.02
0.00
−0.02
0.09
0.12
0.15
0.18
SNP effect on Pregnancy−specific beta−1−glycoprotein 2
SNP effect on Gut microbiota abundance (genus Catenibacterium id.2153)

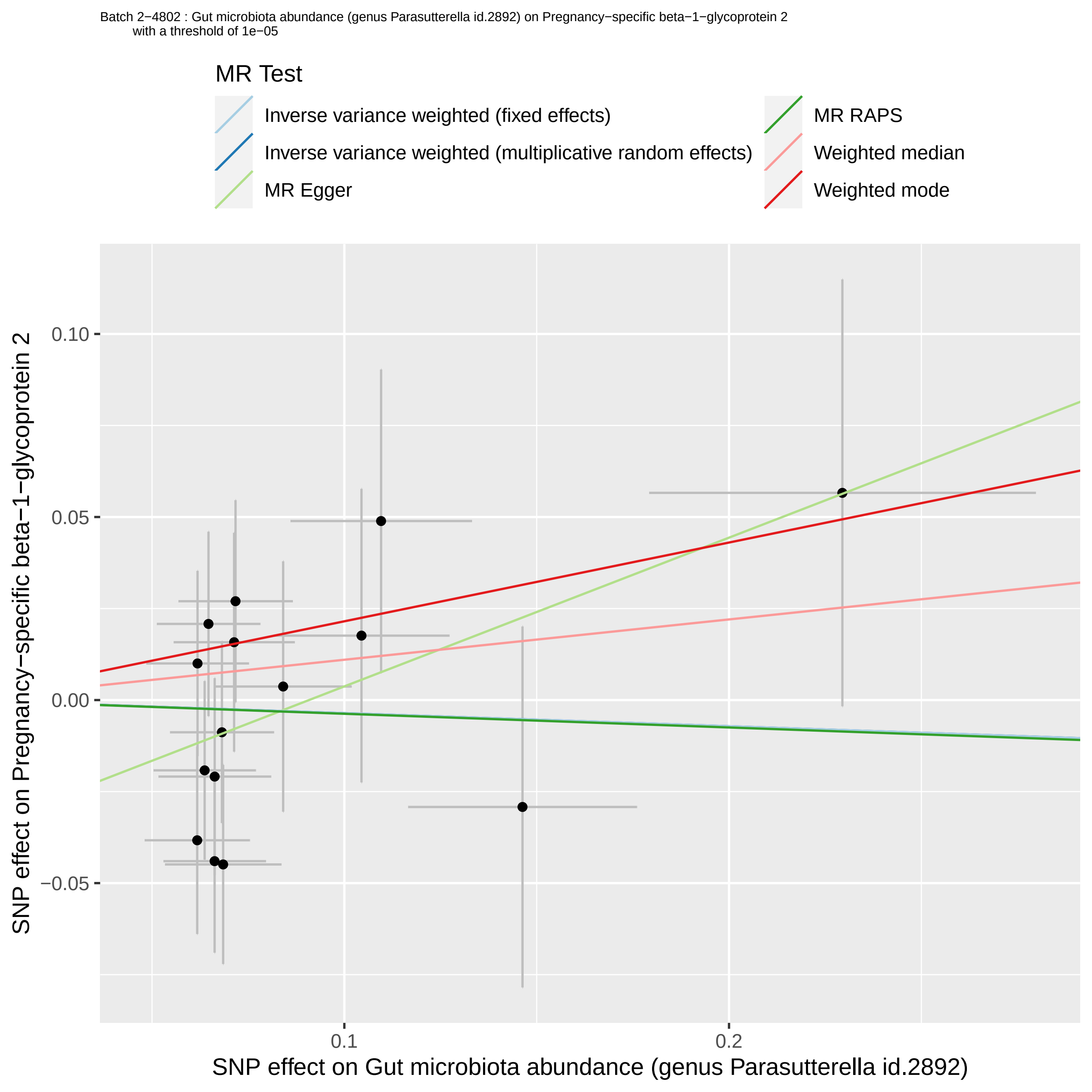
Batch 2−4802 : Gut microbiota abundance (genus Parasutterella id.2892) on Pregnancy−specific beta−1−glycoprotein 2
with a threshold of 1e−05
MR Test
Inverse variance weighted (fixed effects)
Inverse variance weighted (multiplicative random effects)
MR Egger
MR RAPS
Weighted median
Weighted mode
0.10
0.05
0.00
−0.05
0.1
0.2
SNP effect on Pregnancy−specific beta−1−glycoprotein 2
SNP effect on Gut microbiota abundance (genus Parasutterella id.2892)

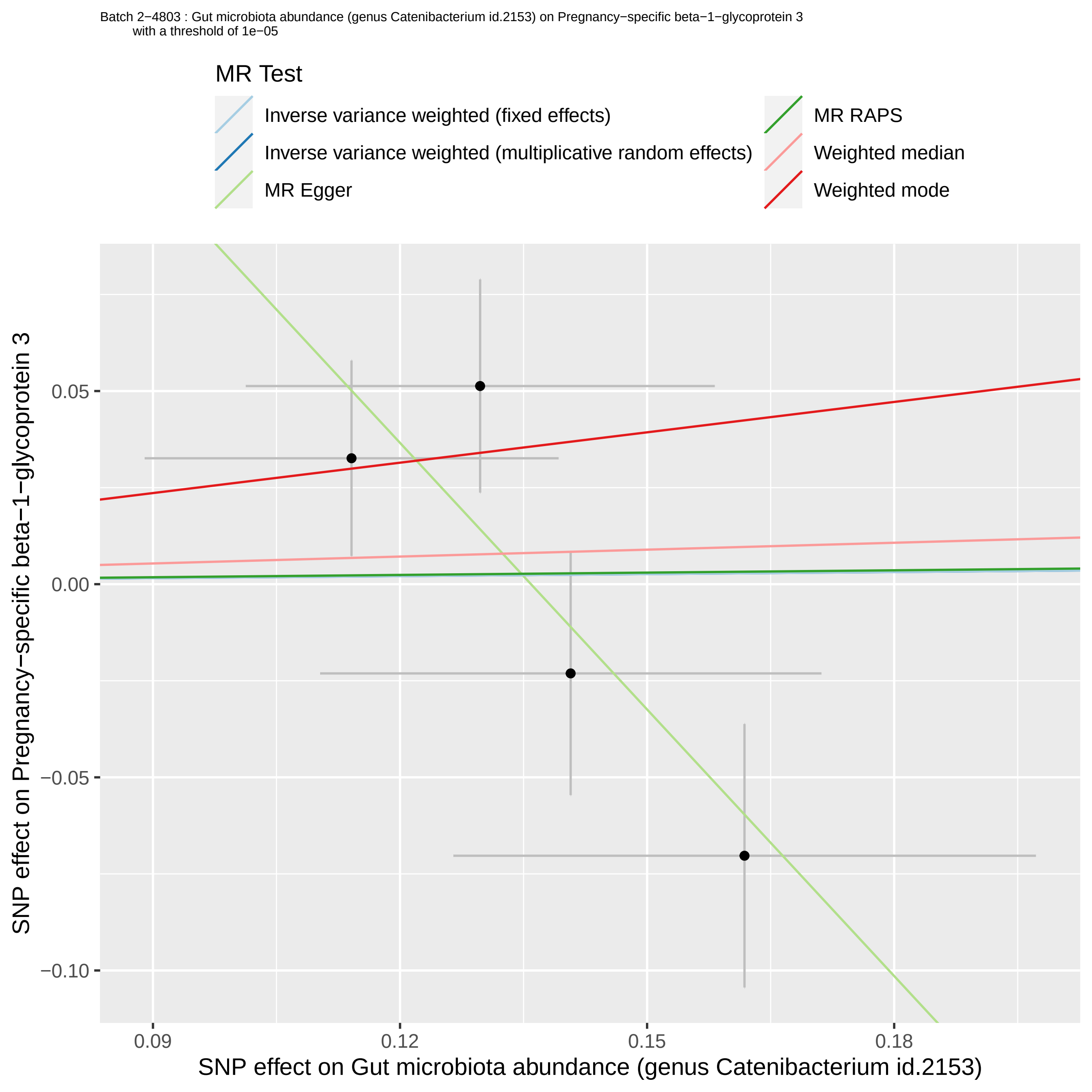
Batch 2−4803 : Gut microbiota abundance (genus Catenibacterium id.2153) on Pregnancy−specific beta−1−glycoprotein 3
with a threshold of 1e−05
MR Test
Inverse variance weighted (fixed effects)
Inverse variance weighted (multiplicative random effects)
MR Egger
MR RAPS
Weighted median
Weighted mode
0.05
0.00
−0.05
−0.10
0.09
0.12
0.15
0.18
SNP effect on Pregnancy−specific beta−1−glycoprotein 3
SNP effect on Gut microbiota abundance (genus Catenibacterium id.2153)

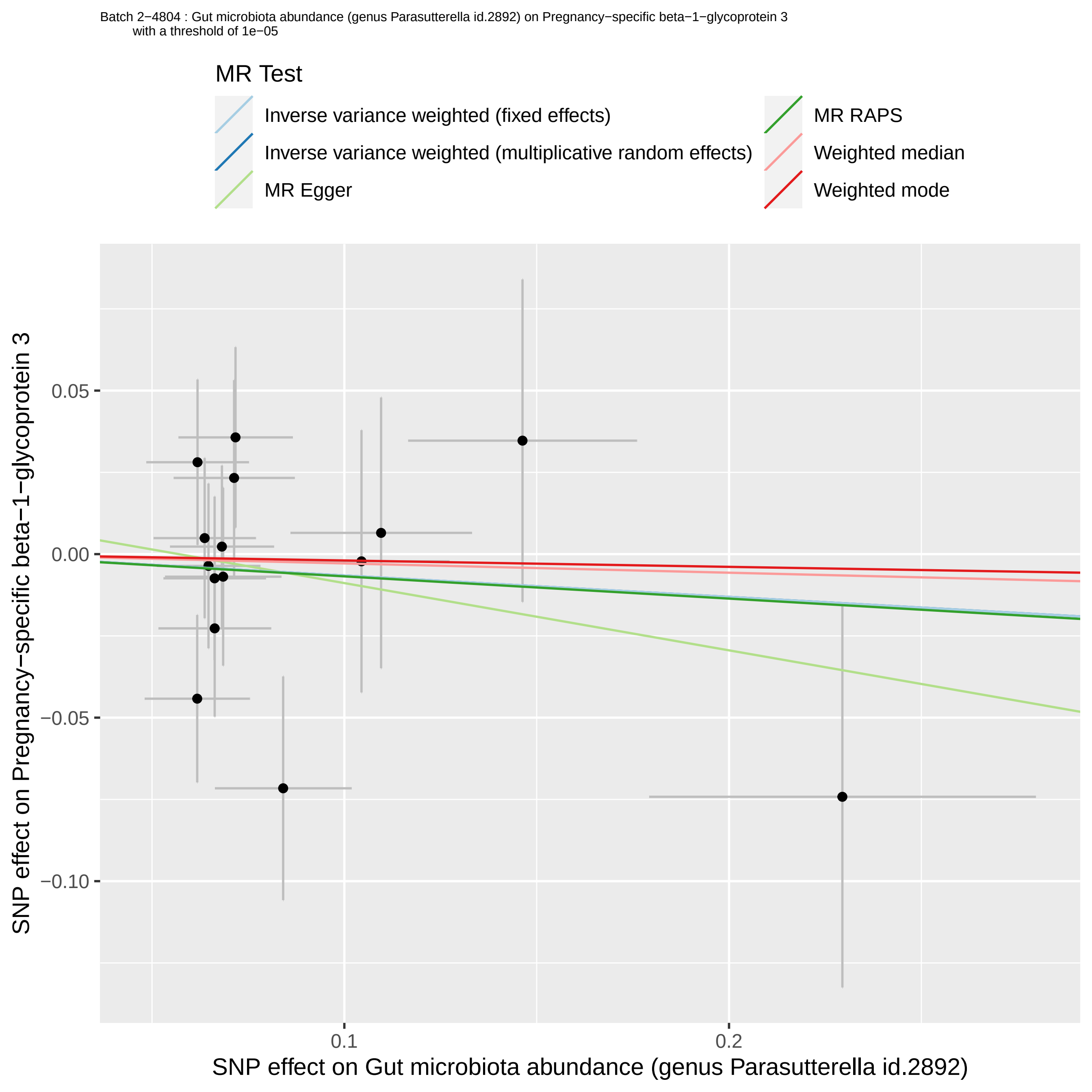
Batch 2−4804 : Gut microbiota abundance (genus Parasutterella id.2892) on Pregnancy−specific beta−1−glycoprotein 3
with a threshold of 1e−05
MR Test
Inverse variance weighted (fixed effects)
Inverse variance weighted (multiplicative random effects)
MR Egger
MR RAPS
Weighted median
Weighted mode
0.05
0.00
−0.05
−0.10
0.1
0.2
SNP effect on Pregnancy−specific beta−1−glycoprotein 3
SNP effect on Gut microbiota abundance (genus Parasutterella id.2892)

Batch 2−4805 : Gut microbiota abundance (genus Catenibacterium id.2153) on Pregnancy−specific beta−1−glycoprotein 4
with a threshold of 1e−05

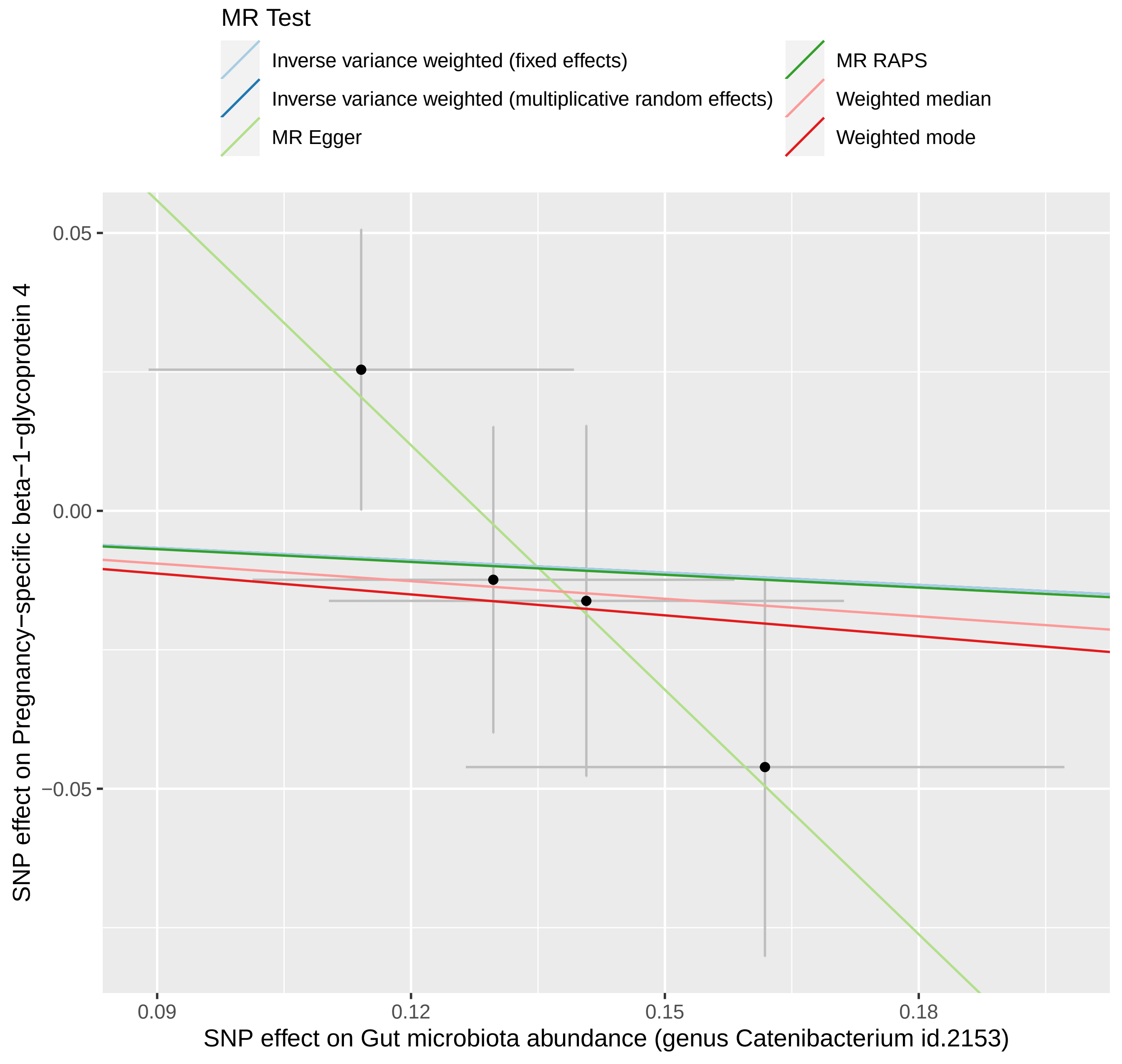

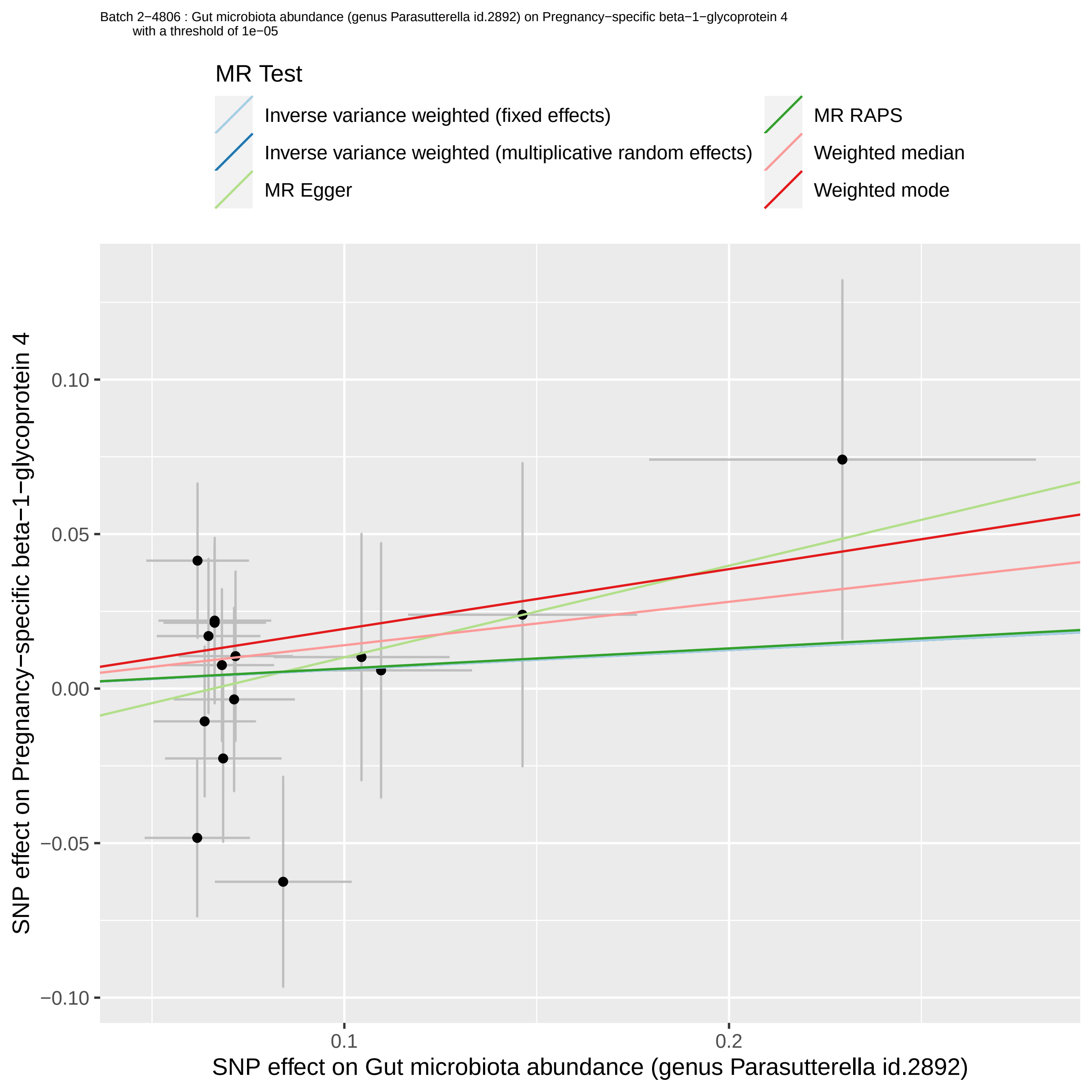

Batch 2−4806 : Gut microbiota abundance (genus Parasutterella id.2892) on Pregnancy−specific beta−1−glycoprotein 4
with a threshold of 1e−05
MR Test
Inverse variance weighted (fixed effects)
Inverse variance weighted (multiplicative random effects)
MR Egger
MR RAPS
Weighted median
Weighted mode
SNP effect on Pregnancy−specific beta−1−glycoprotein 4
0.10
0.05
0.00
−0.05
−0.10
0.1
0.2
SNP effect on Gut microbiota abundance (genus Parasutterella id.2892)

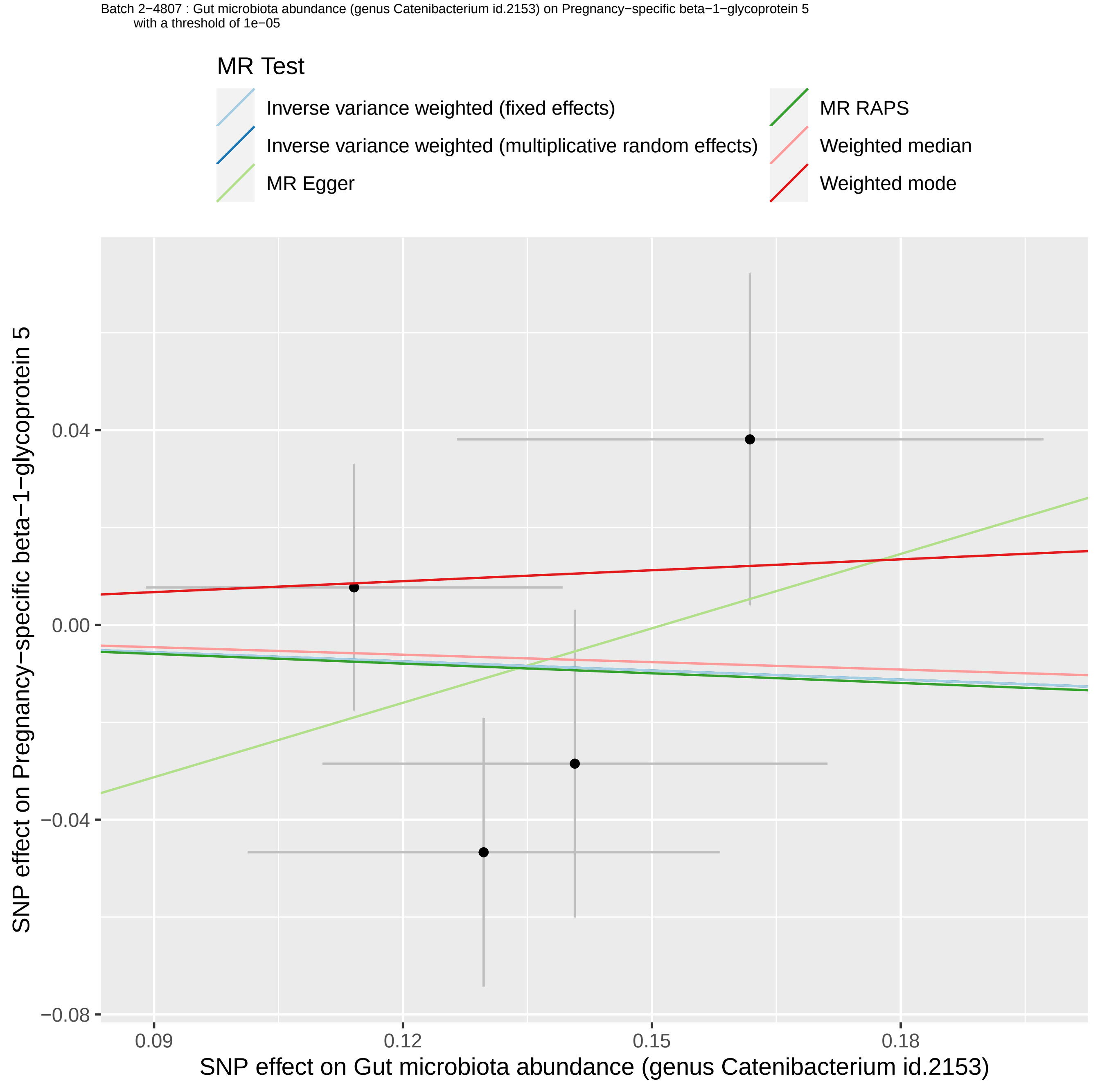

Batch 2−4807 : Gut microbiota abundance (genus Catenibacterium id.2153) on Pregnancy−specific beta−1−glycoprotein 5
with a threshold of 1e−05
MR Test
Inverse variance weighted (fixed effects)
Inverse variance weighted (multiplicative random effects)
MR Egger
MR RAPS
Weighted median
Weighted mode
SNP effect on Pregnancy−specific beta−1−glycoprotein 5
0.04
0.00
−0.04
−0.08
0.09
0.12
0.15
0.18
SNP effect on Gut microbiota abundance (genus Catenibacterium id.2153)

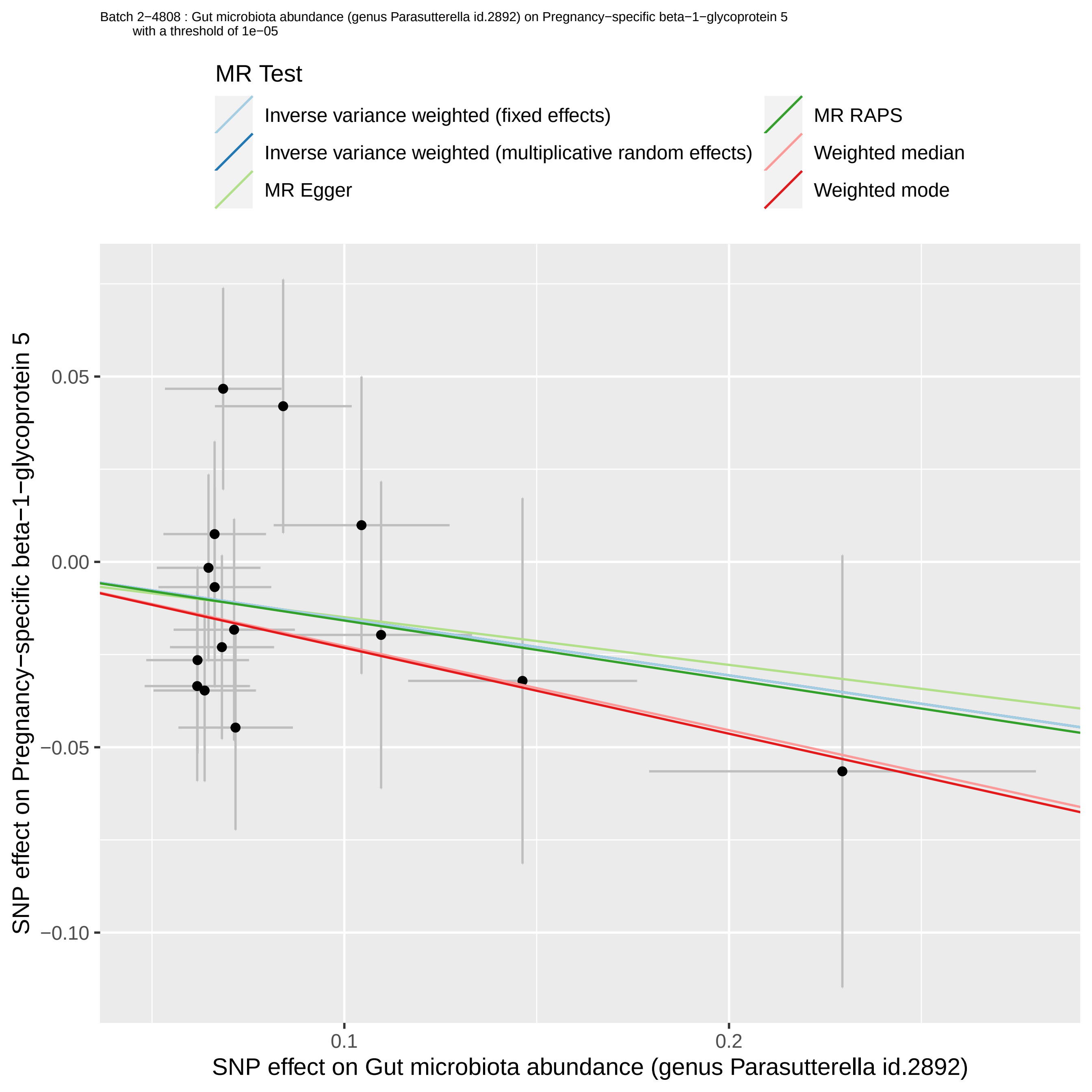
Batch 2−4808 : Gut microbiota abundance (genus Parasutterella id.2892) on Pregnancy−specific beta−1−glycoprotein 5
with a threshold of 1e−05
MR Test
Inverse variance weighted (fixed effects)
Inverse variance weighted (multiplicative random effects)
MR Egger
MR RAPS
Weighted median
Weighted mode
0.05
0.00
−0.05
−0.10
0.1
0.2
SNP effect on Pregnancy−specific beta−1−glycoprotein 5
SNP effect on Gut microbiota abundance (genus Parasutterella id.2892)

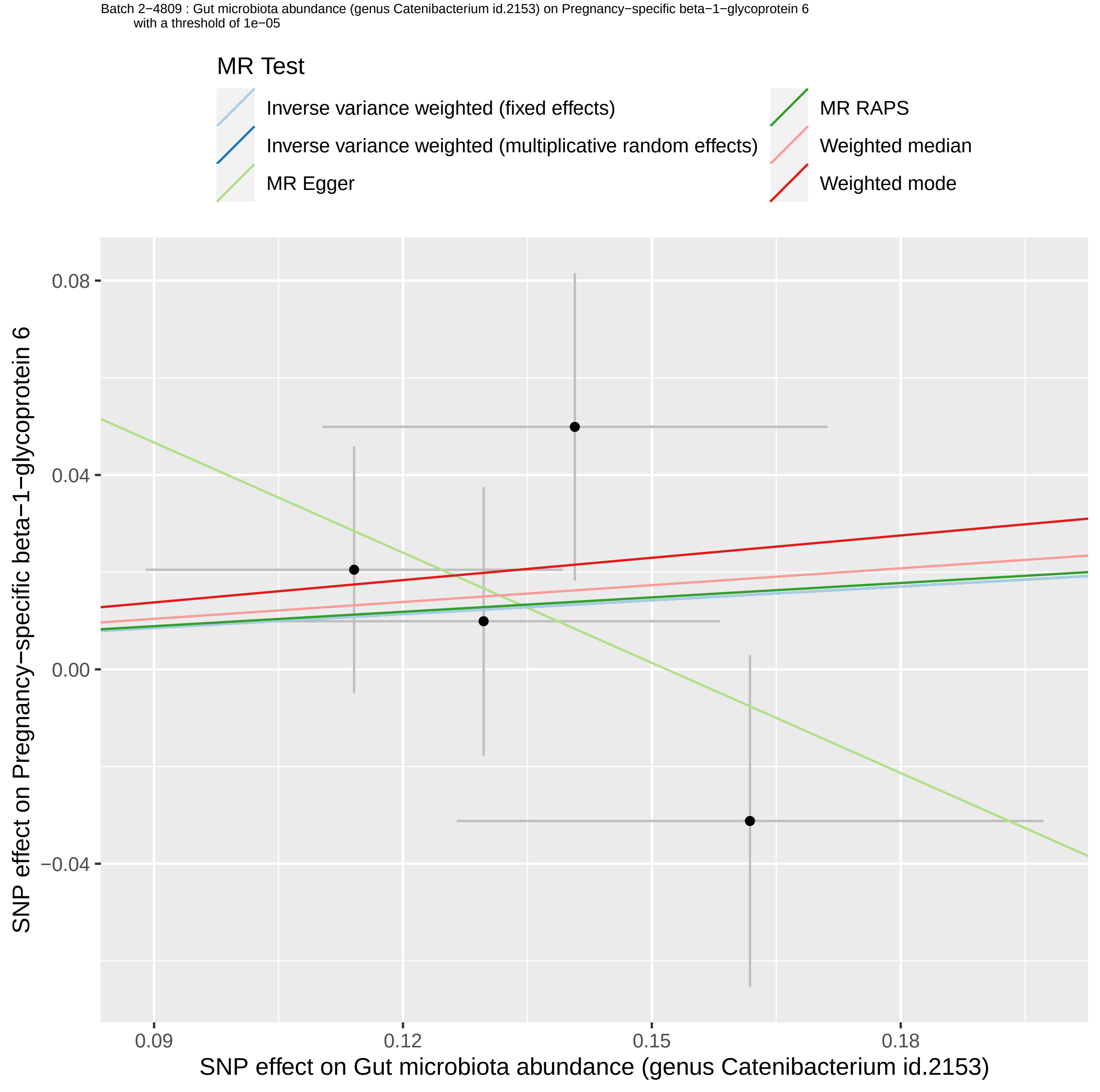

Batch 2−4809 : Gut microbiota abundance (genus Catenibacterium id.2153) on Pregnancy−specific beta−1−glycoprotein 6
with a threshold of 1e−05
MR Test
Inverse variance weighted (fixed effects)
Inverse variance weighted (multiplicative random effects)
MR Egger
MR RAPS
Weighted median
Weighted mode
0.08
0.04
0.00
−0.04
0.09
0.12
0.15
0.18
SNP effect on Pregnancy−specific beta−1−glycoprotein 6
SNP effect on Gut microbiota abundance (genus Catenibacterium id.2153)

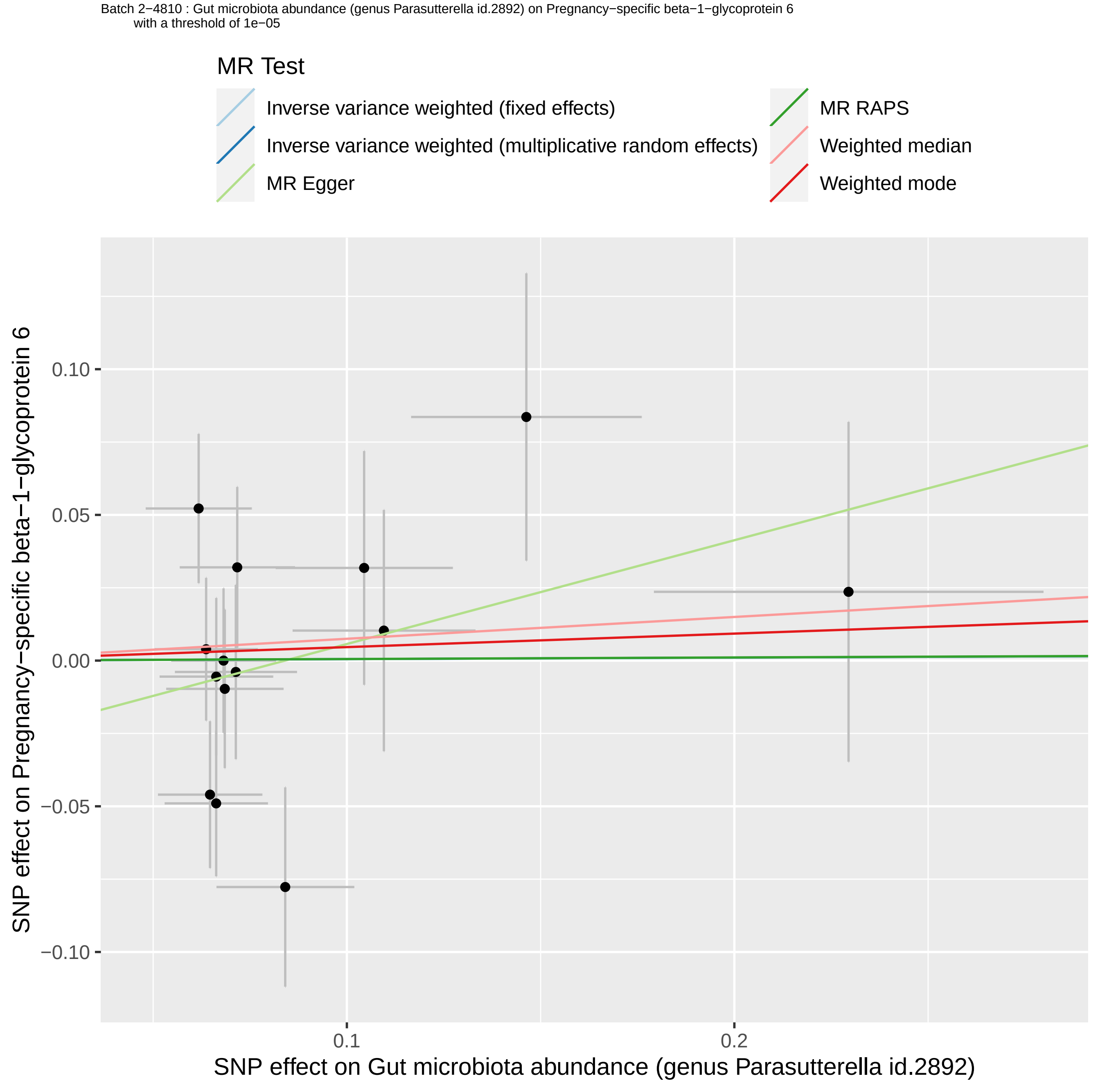

Batch 2−4810 : Gut microbiota abundance (genus Parasutterella id.2892) on Pregnancy−specific beta−1−glycoprotein 6
with a threshold of 1e−05
MR Test
Inverse variance weighted (fixed effects)
Inverse variance weighted (multiplicative random effects)
MR Egger
MR RAPS
Weighted median
Weighted mode
SNP effect on Pregnancy−specific beta−1−glycoprotein 6
0.10
0.05
0.00
−0.05
−0.10
0.1
0.2
SNP effect on Gut microbiota abundance (genus Parasutterella id.2892)

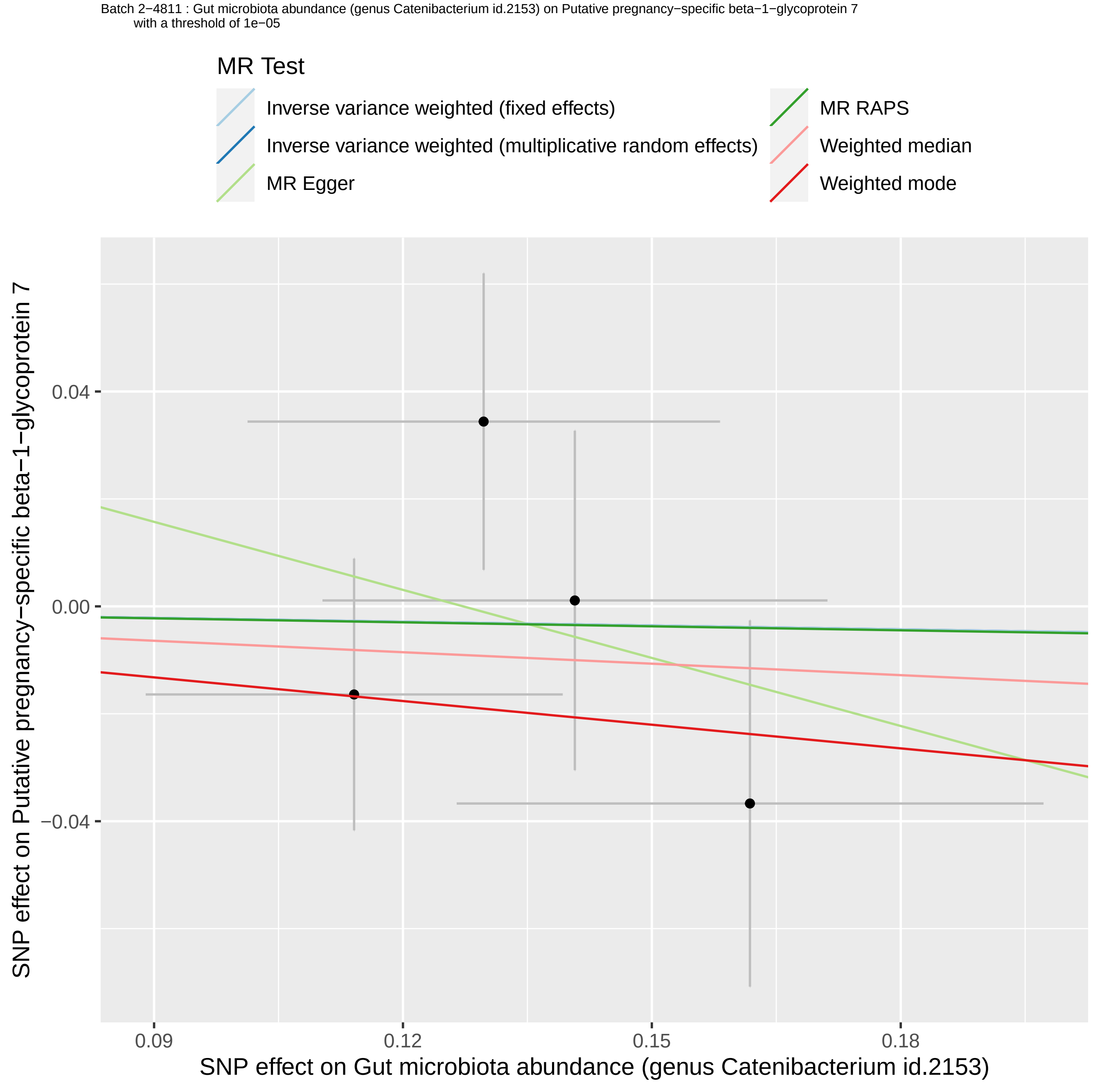

Batch 2−4811 : Gut microbiota abundance (genus Catenibacterium id.2153) on Putative pregnancy−specific beta−1−glycoprotein 7
with a threshold of 1e−05
MR Test
Inverse variance weighted (fixed effects)
Inverse variance weighted (multiplicative random effects)
MR Egger
MR RAPS
Weighted median
Weighted mode
0.04
0.00
−0.04
0.09
0.12
0.15
0.18
SNP effect on Putative pregnancy−specific beta−1−glycoprotein 7
SNP effect on Gut microbiota abundance (genus Catenibacterium id.2153)

Batch 2−4812 : Gut microbiota abundance (genus Parasutterella id.2892) on Putative pregnancy−specific beta−1−glycoprotein 7
with a threshold of 1e−05

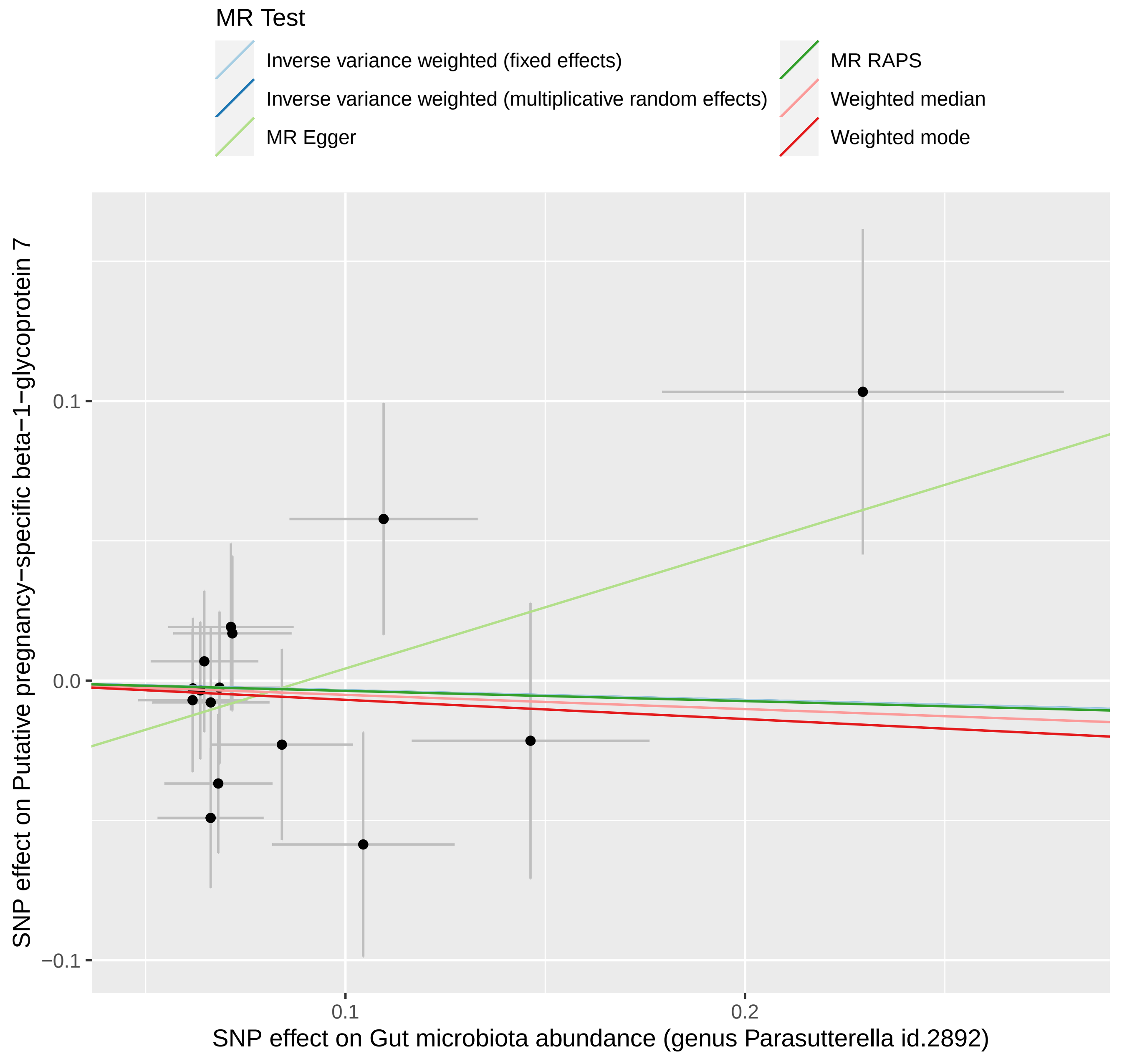

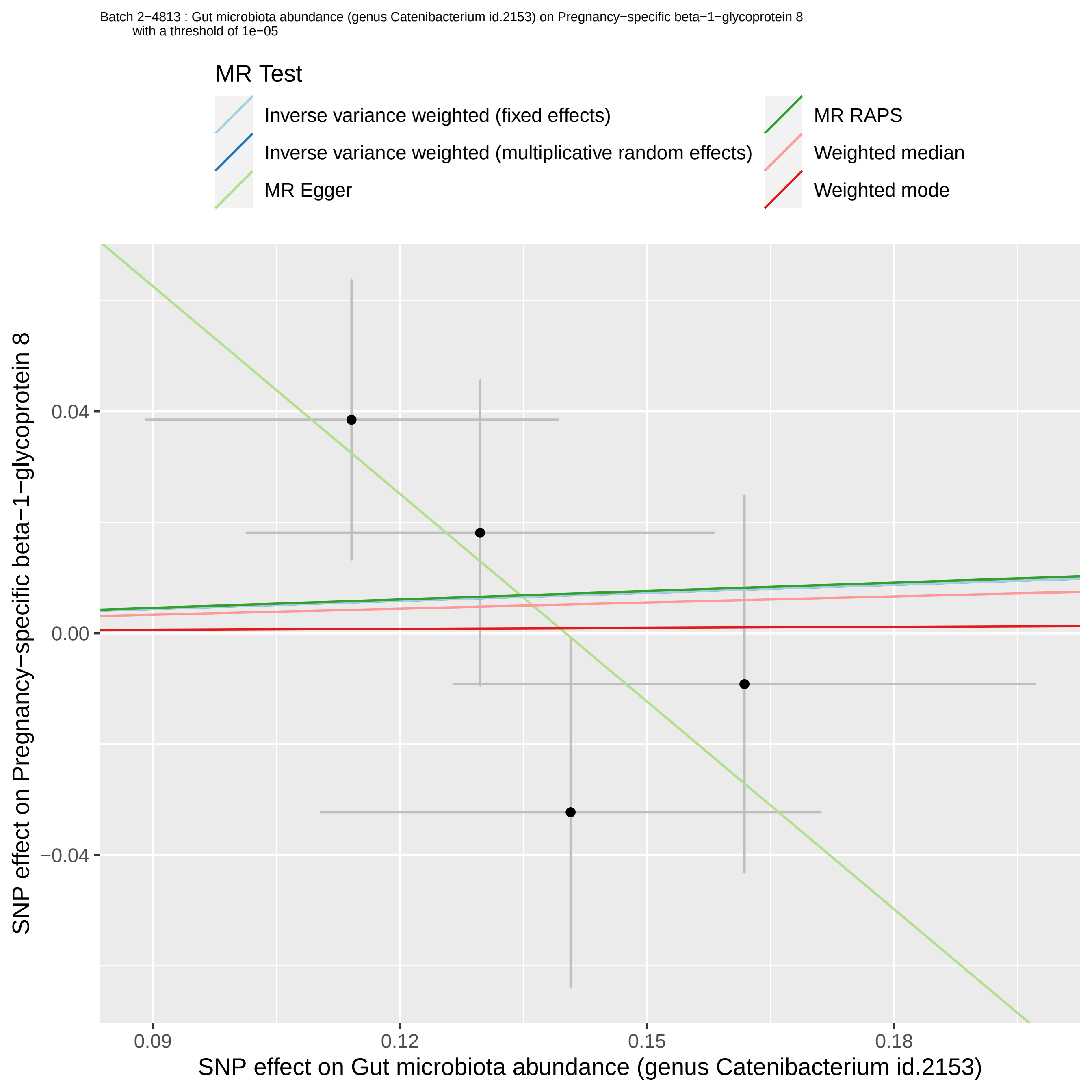

Batch 2−4813 : Gut microbiota abundance (genus Catenibacterium id.2153) on Pregnancy−specific beta−1−glycoprotein 8
with a threshold of 1e−05
MR Test
Inverse variance weighted (fixed effects)
Inverse variance weighted (multiplicative random effects)
MR Egger
MR RAPS
Weighted median
Weighted mode
0.04
0.00
−0.04
0.09
0.12
0.15
0.18
SNP effect on Pregnancy−specific beta−1−glycoprotein 8
SNP effect on Gut microbiota abundance (genus Catenibacterium id.2153)

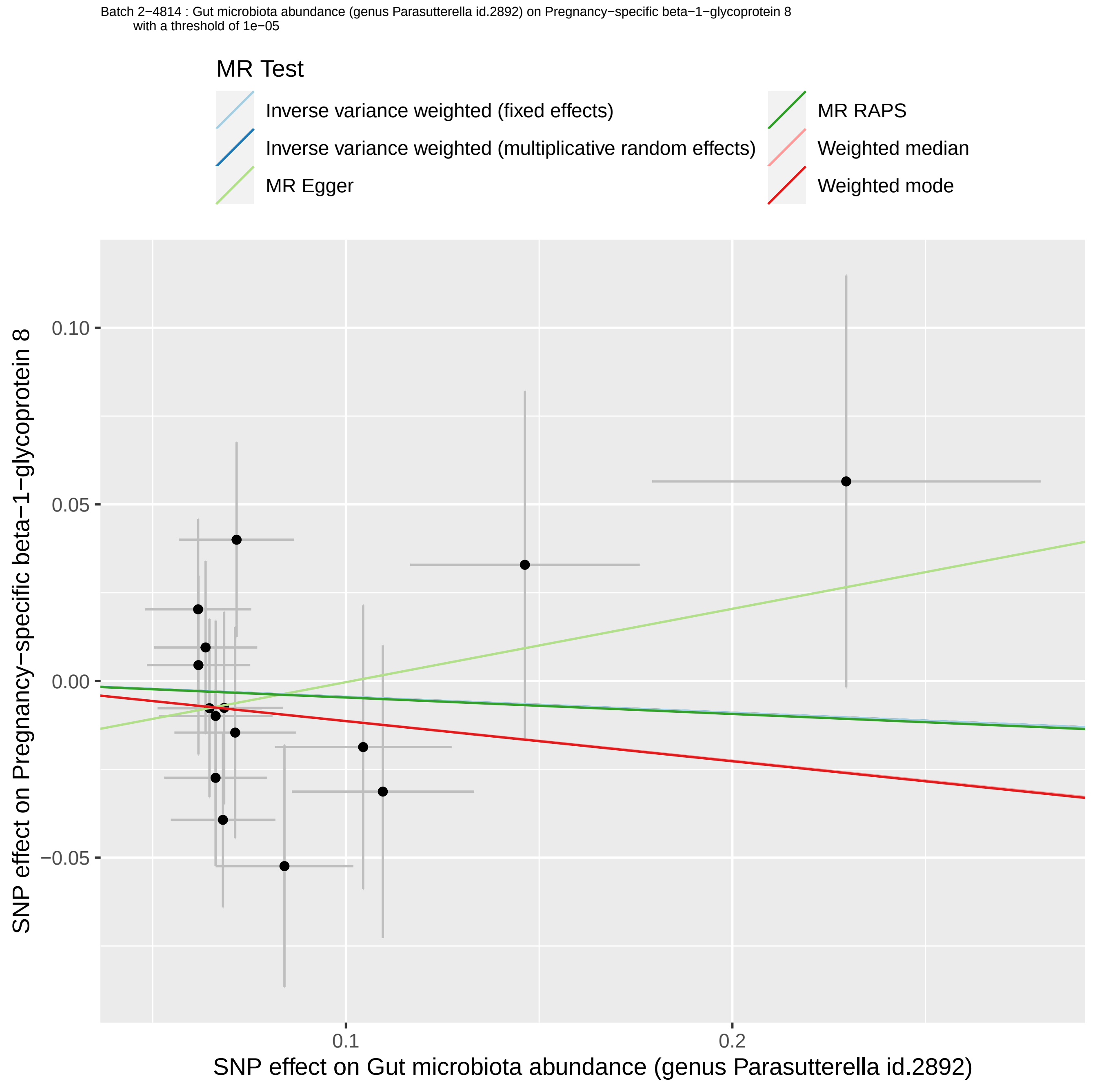

Batch 2−4814 : Gut microbiota abundance (genus Parasutterella id.2892) on Pregnancy−specific beta−1−glycoprotein 8
with a threshold of 1e−05
MR Test
Inverse variance weighted (fixed effects)
Inverse variance weighted (multiplicative random effects)
MR Egger
MR RAPS
Weighted median
Weighted mode
0.10
0.05
0.00
−0.05
0.1
0.2
SNP effect on Pregnancy−specific beta−1−glycoprotein 8
SNP effect on Gut microbiota abundance (genus Parasutterella id.2892)

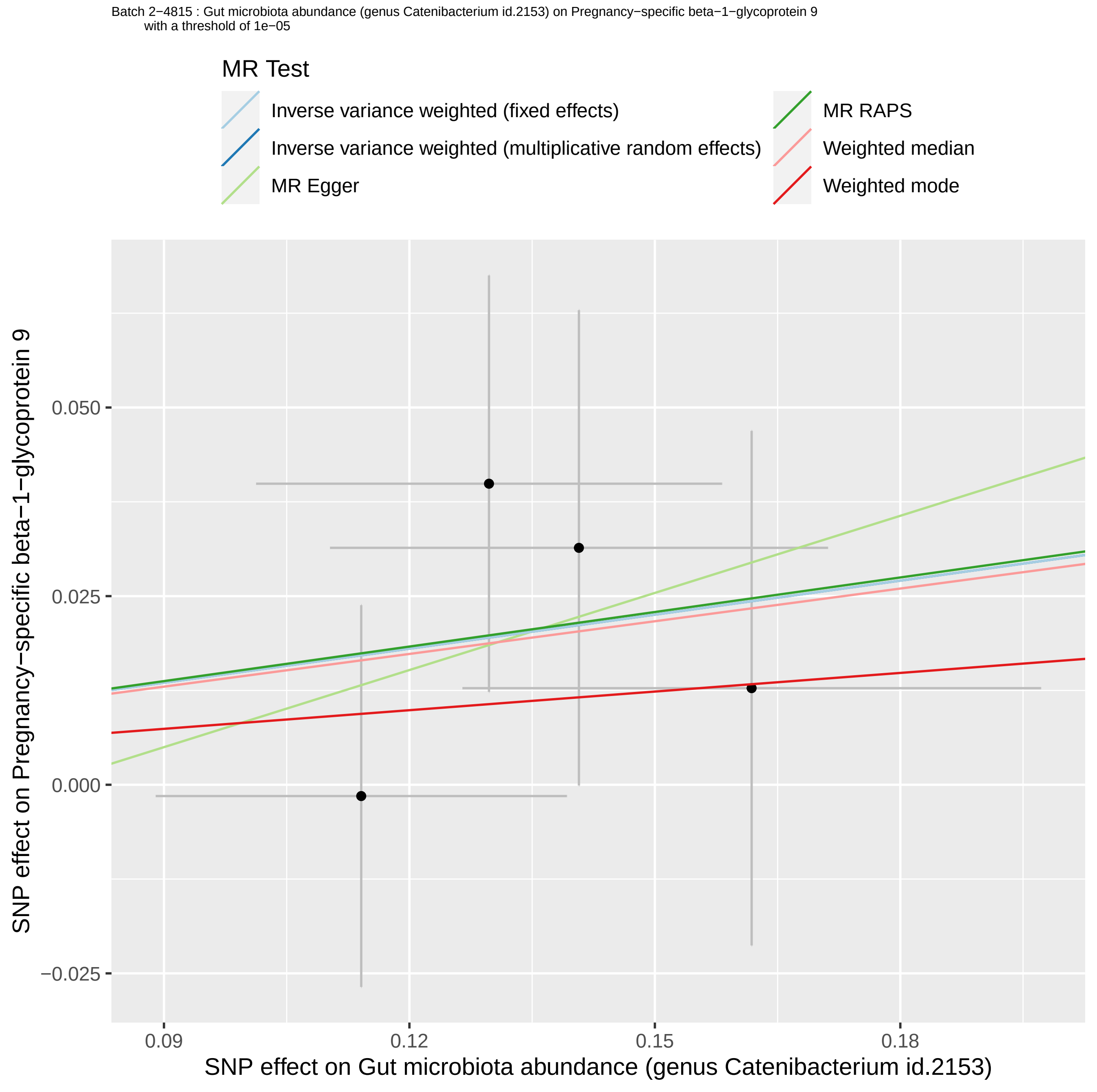

Batch 2−4815 : Gut microbiota abundance (genus Catenibacterium id.2153) on Pregnancy−specific beta−1−glycoprotein 9
with a threshold of 1e−05
MR Test
Inverse variance weighted (fixed effects)
Inverse variance weighted (multiplicative random effects)
MR Egger
MR RAPS
Weighted median
Weighted mode
0.050
0.025
0.000
−0.025
0.09
0.12
0.15
0.18
SNP effect on Pregnancy−specific beta−1−glycoprotein 9
SNP effect on Gut microbiota abundance (genus Catenibacterium id.2153)

Batch 2−4816 : Gut microbiota abundance (genus Parasutterella id.2892) on Pregnancy−specific beta−1−glycoprotein 9
with a threshold of 1e−05

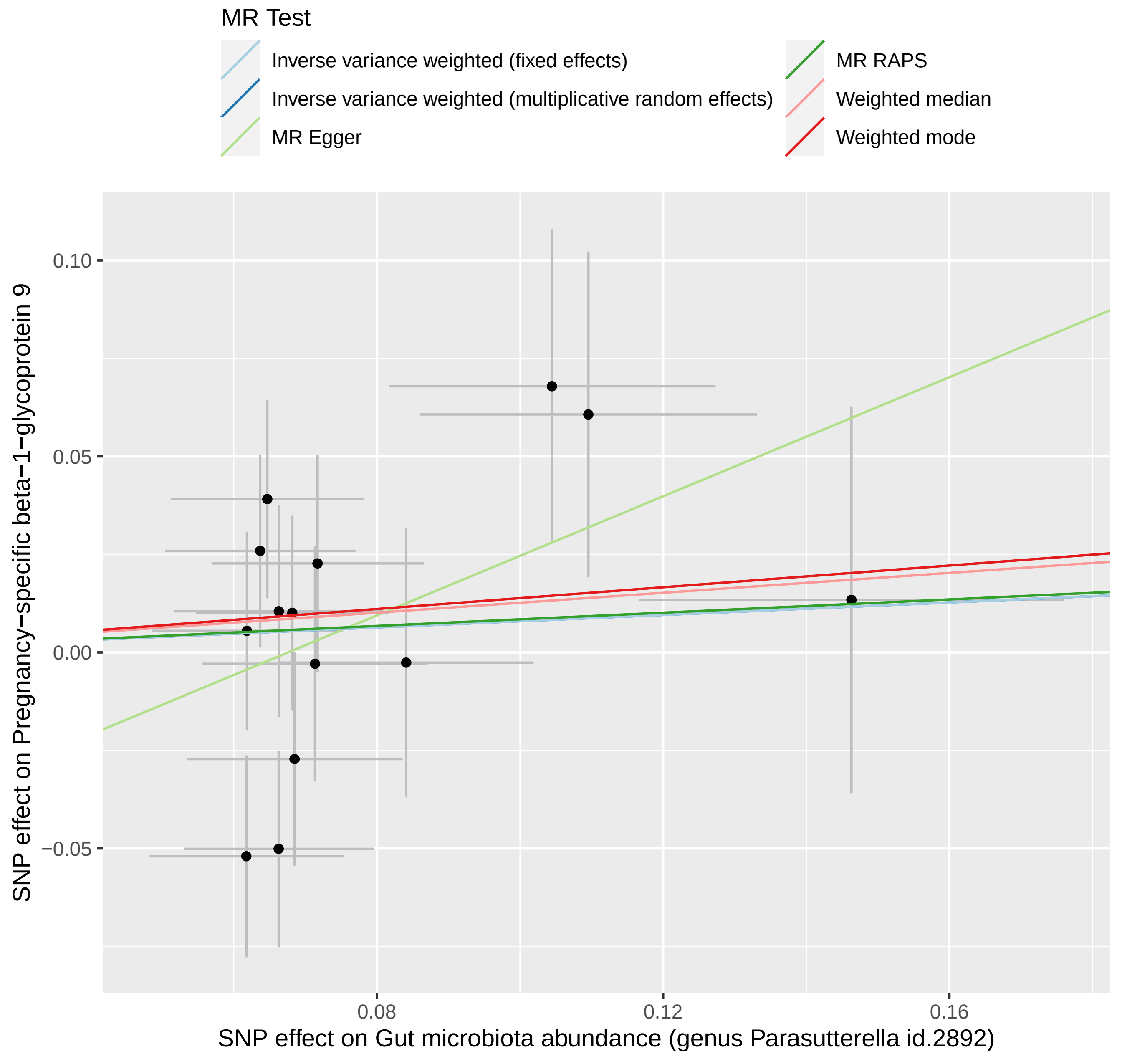

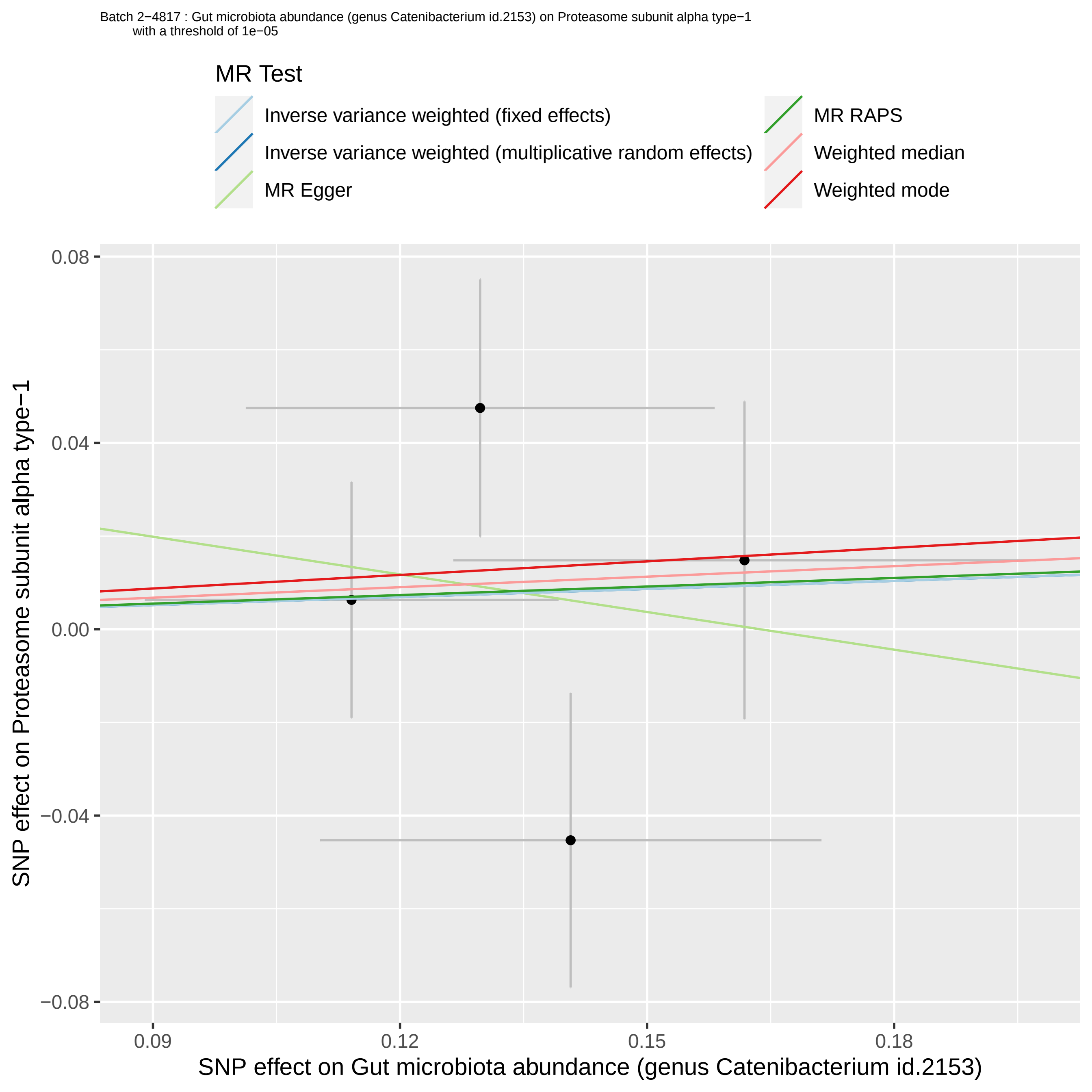

Batch 2−4817 : Gut microbiota abundance (genus Catenibacterium id.2153) on Proteasome subunit alpha type−1
with a threshold of 1e−05
MR Test
Inverse variance weighted (fixed effects)
Inverse variance weighted (multiplicative random effects)
MR Egger
MR RAPS
Weighted median
Weighted mode
0.08
0.04
0.00
−0.04
−0.08
0.09
0.12
0.15
0.18
SNP effect on Proteasome subunit alpha type−1
SNP effect on Gut microbiota abundance (genus Catenibacterium id.2153)

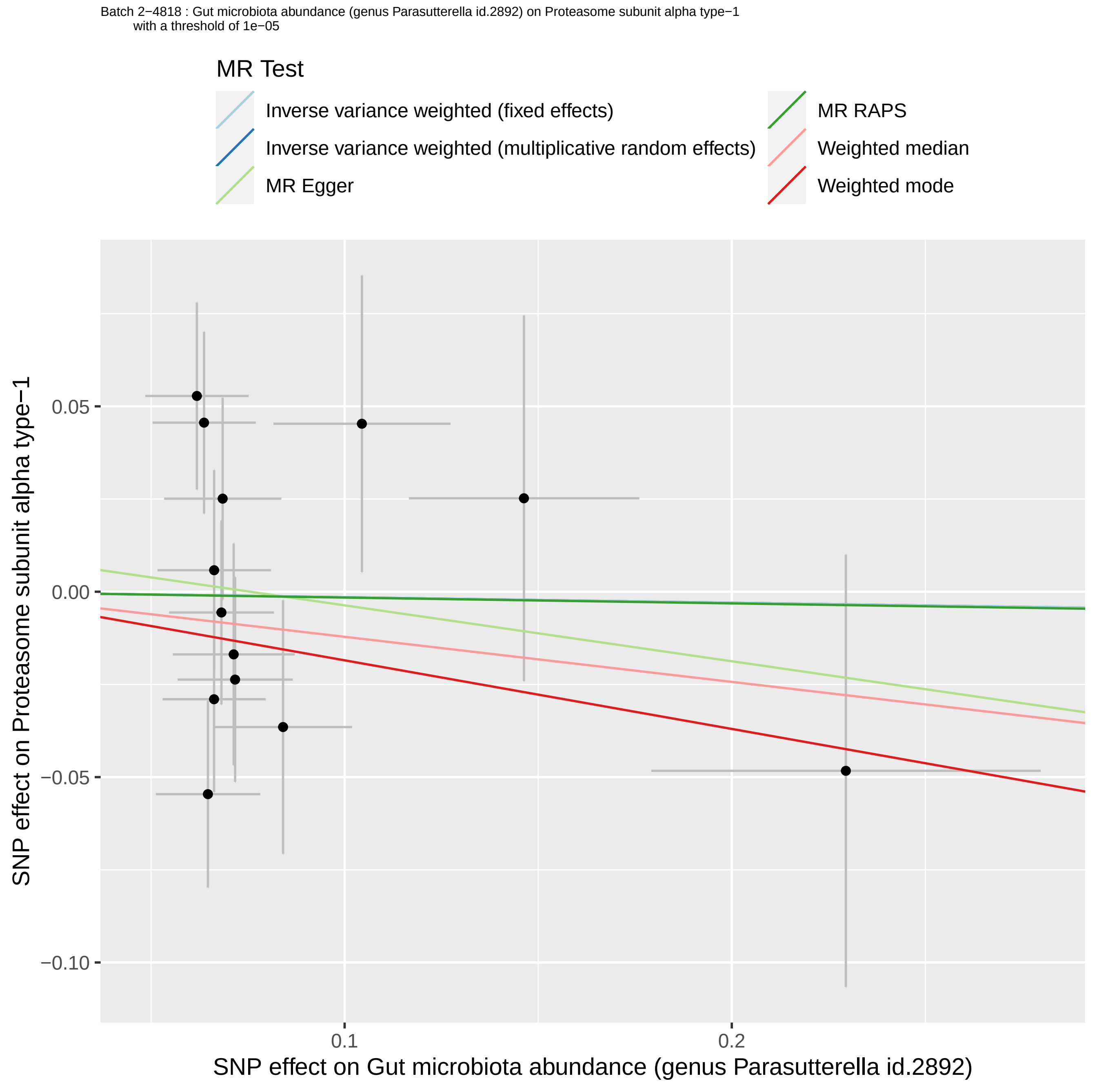
Batch 2−4818 : Gut microbiota abundance (genus Parasutterella id.2892) on Proteasome subunit alpha type−1
with a threshold of 1e−05
MR Test
Inverse variance weighted (fixed effects)
Inverse variance weighted (multiplicative random effects)
MR Egger
MR RAPS
Weighted median
Weighted mode
0.05
0.00
−0.05
−0.10
0.1
0.2
SNP effect on Proteasome subunit alpha type−1
SNP effect on Gut microbiota abundance (genus Parasutterella id.2892)

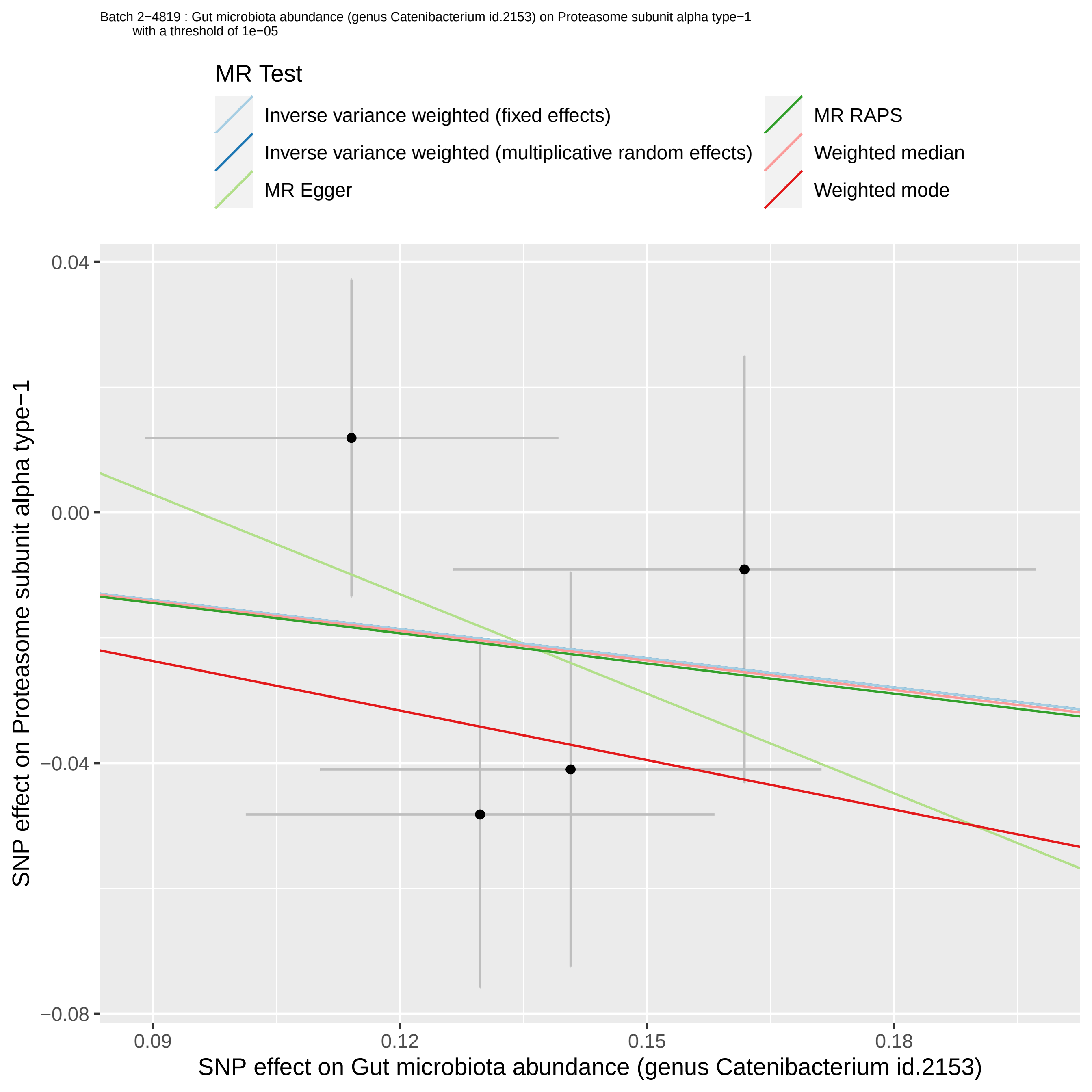

Batch 2−4819 : Gut microbiota abundance (genus Catenibacterium id.2153) on Proteasome subunit alpha type−1
with a threshold of 1e−05
MR Test
Inverse variance weighted (fixed effects)
Inverse variance weighted (multiplicative random effects)
MR Egger
MR RAPS
Weighted median
Weighted mode
0.04
0.00
−0.04
−0.08
0.09
0.12
0.15
0.18
SNP effect on Proteasome subunit alpha type−1
SNP effect on Gut microbiota abundance (genus Catenibacterium id.2153)

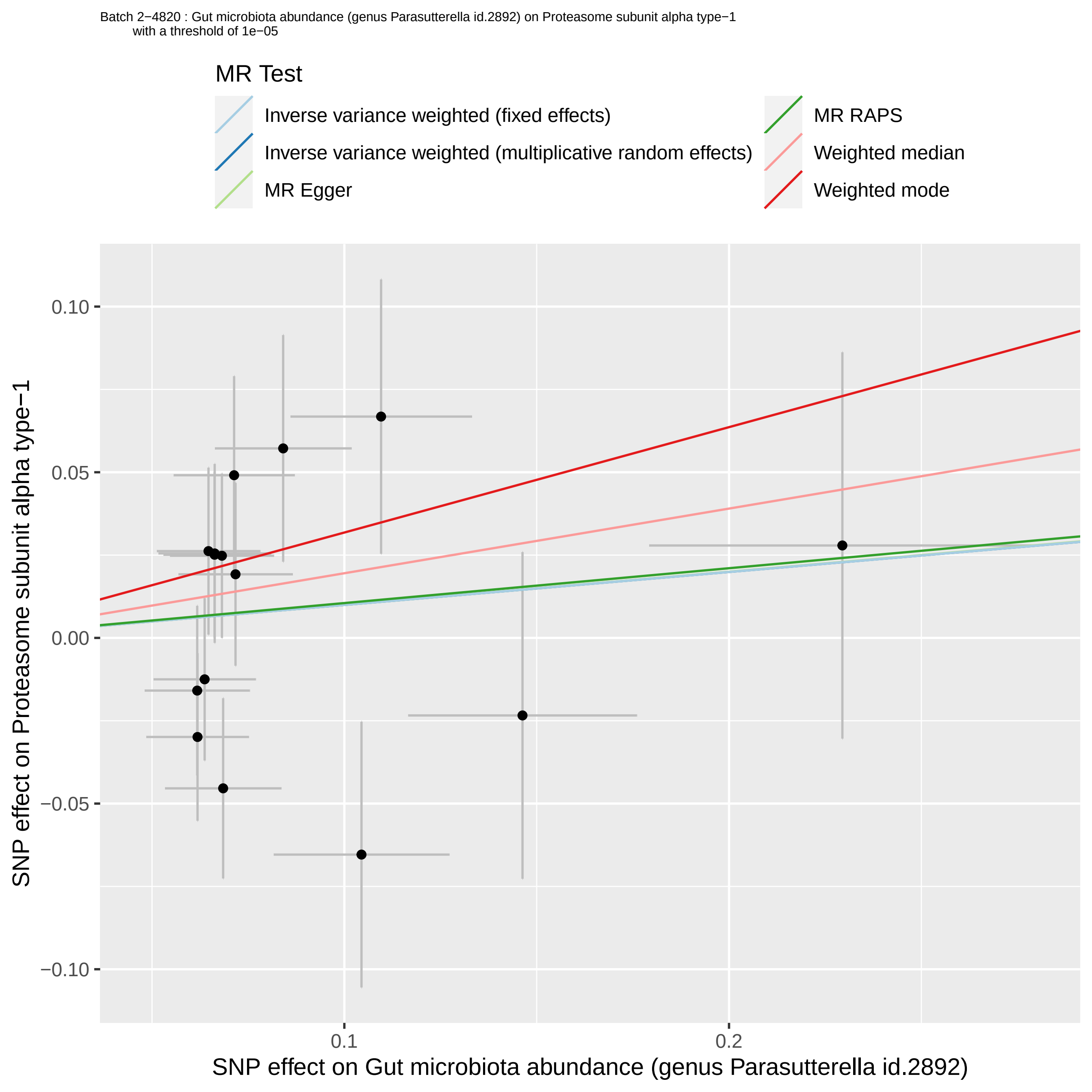

Batch 2−4820 : Gut microbiota abundance (genus Parasutterella id.2892) on Proteasome subunit alpha type−1
with a threshold of 1e−05
MR Test
Inverse variance weighted (fixed effects)
Inverse variance weighted (multiplicative random effects)
MR Egger
MR RAPS
Weighted median
Weighted mode
0.10
0.05
0.00
−0.05
−0.10
0.1
0.2
SNP effect on Proteasome subunit alpha type−1
SNP effect on Gut microbiota abundance (genus Parasutterella id.2892)

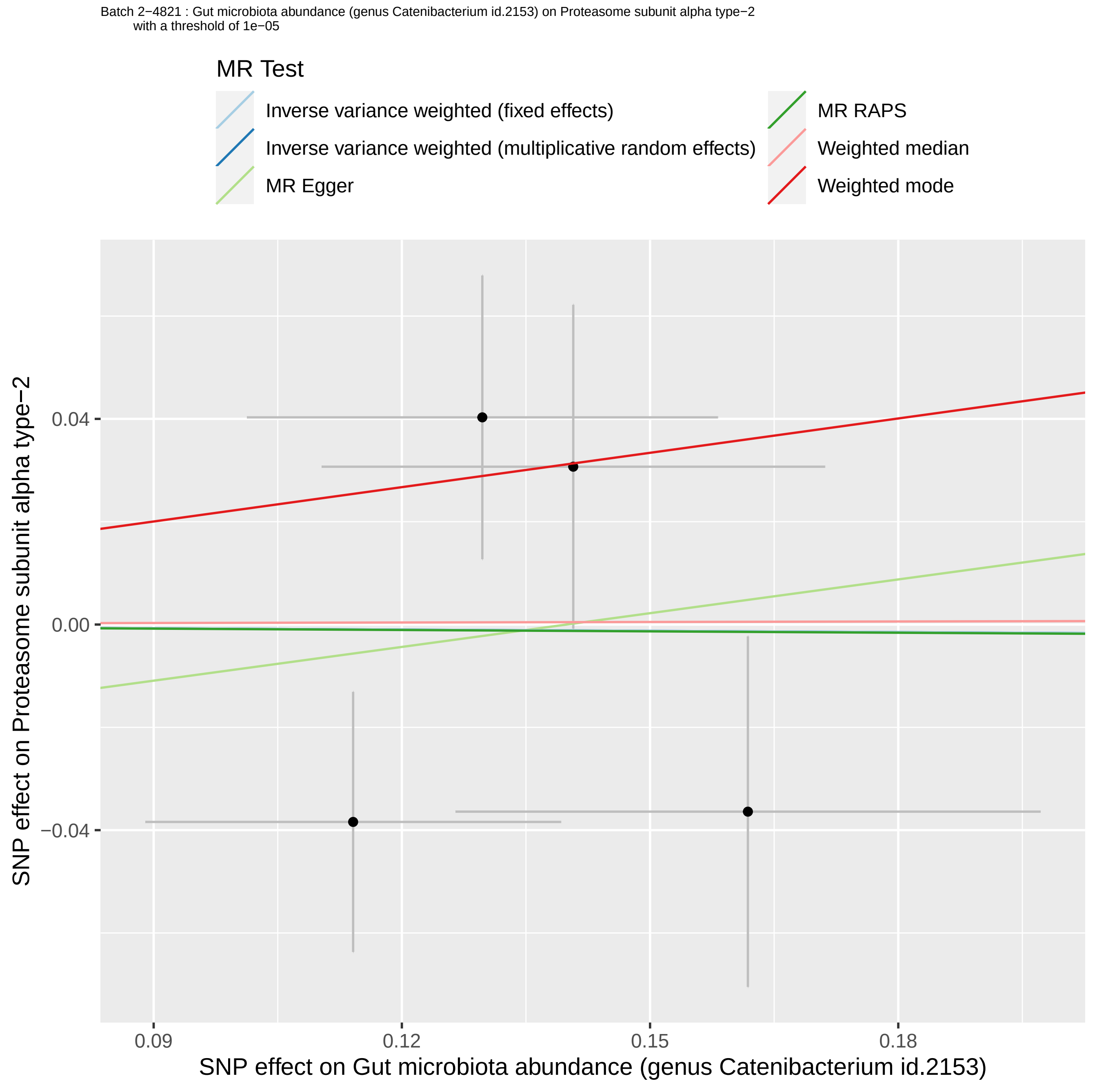
Batch 2−4821 : Gut microbiota abundance (genus Catenibacterium id.2153) on Proteasome subunit alpha type−2
with a threshold of 1e−05
MR Test
Inverse variance weighted (fixed effects)
Inverse variance weighted (multiplicative random effects)
MR Egger
MR RAPS
Weighted median
Weighted mode
0.04
0.00
−0.04
0.09
0.12
0.15
0.18
SNP effect on Proteasome subunit alpha type−2
SNP effect on Gut microbiota abundance (genus Catenibacterium id.2153)

Batch 2−4822 : Gut microbiota abundance (genus Parasutterella id.2892) on Proteasome subunit alpha type−2
with a threshold of 1e−05

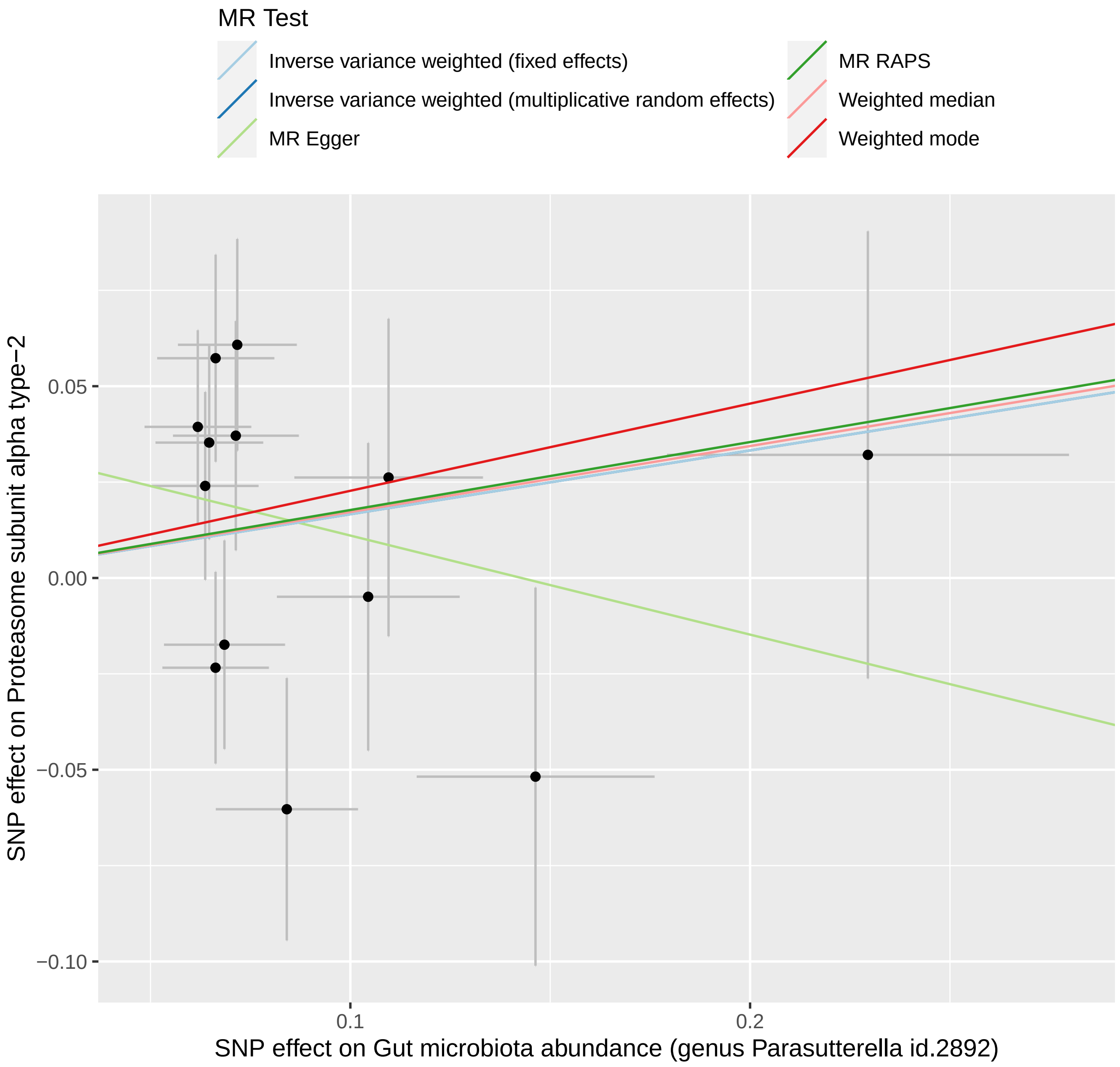

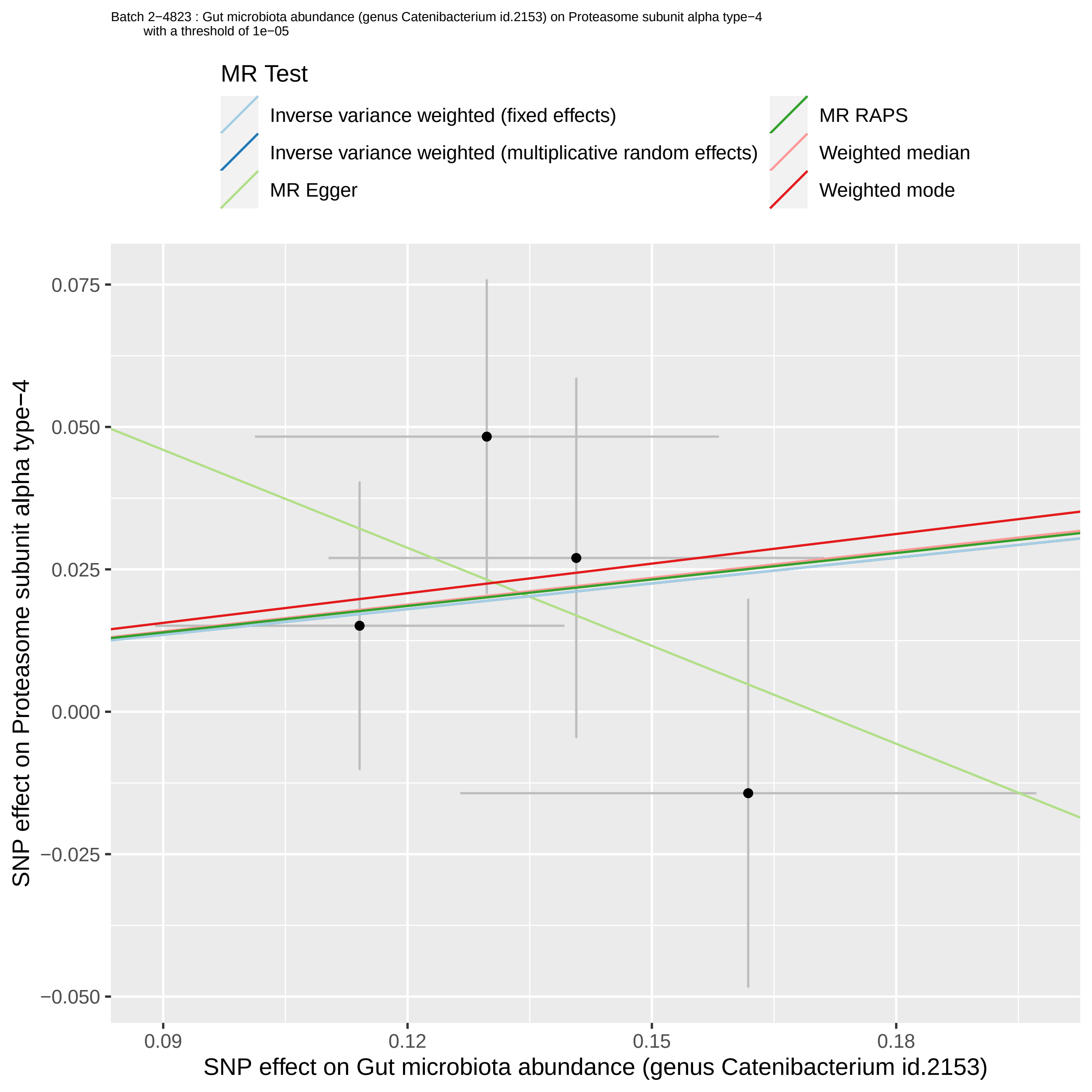
Batch 2−4823 : Gut microbiota abundance (genus Catenibacterium id.2153) on Proteasome subunit alpha type−4
with a threshold of 1e−05
MR Test
Inverse variance weighted (fixed effects)
Inverse variance weighted (multiplicative random effects)
MR Egger
MR RAPS
Weighted median
Weighted mode
0.075
0.050
0.025
0.000
−0.025
−0.050
SNP effect on Proteasome subunit alpha type−4
0.09
0.12
0.15
0.18
SNP effect on Gut microbiota abundance (genus Catenibacterium id.2153)

Batch 2−4824 : Gut microbiota abundance (genus Parasutterella id.2892) on Proteasome subunit alpha type−4
with a threshold of 1e−05

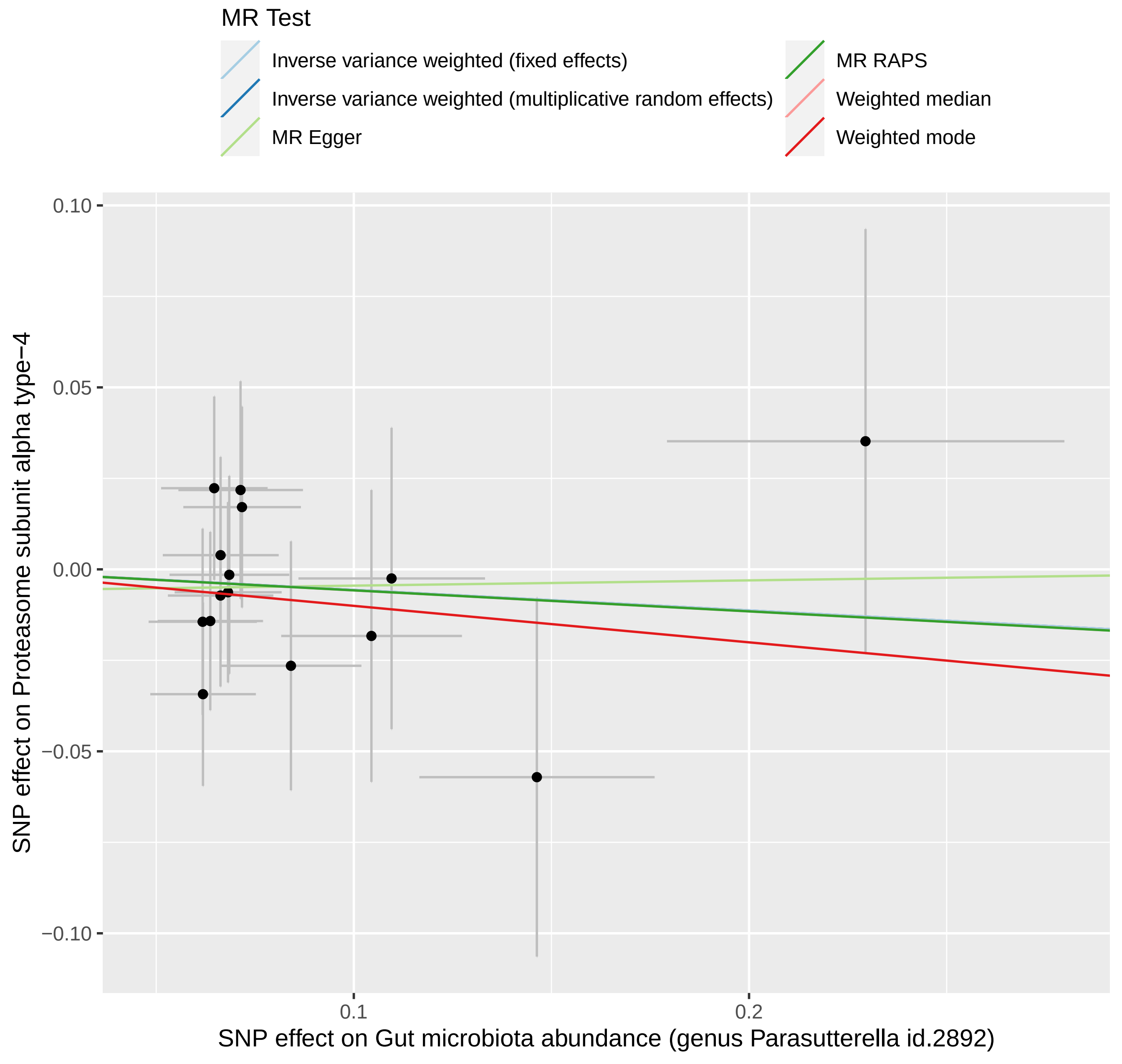

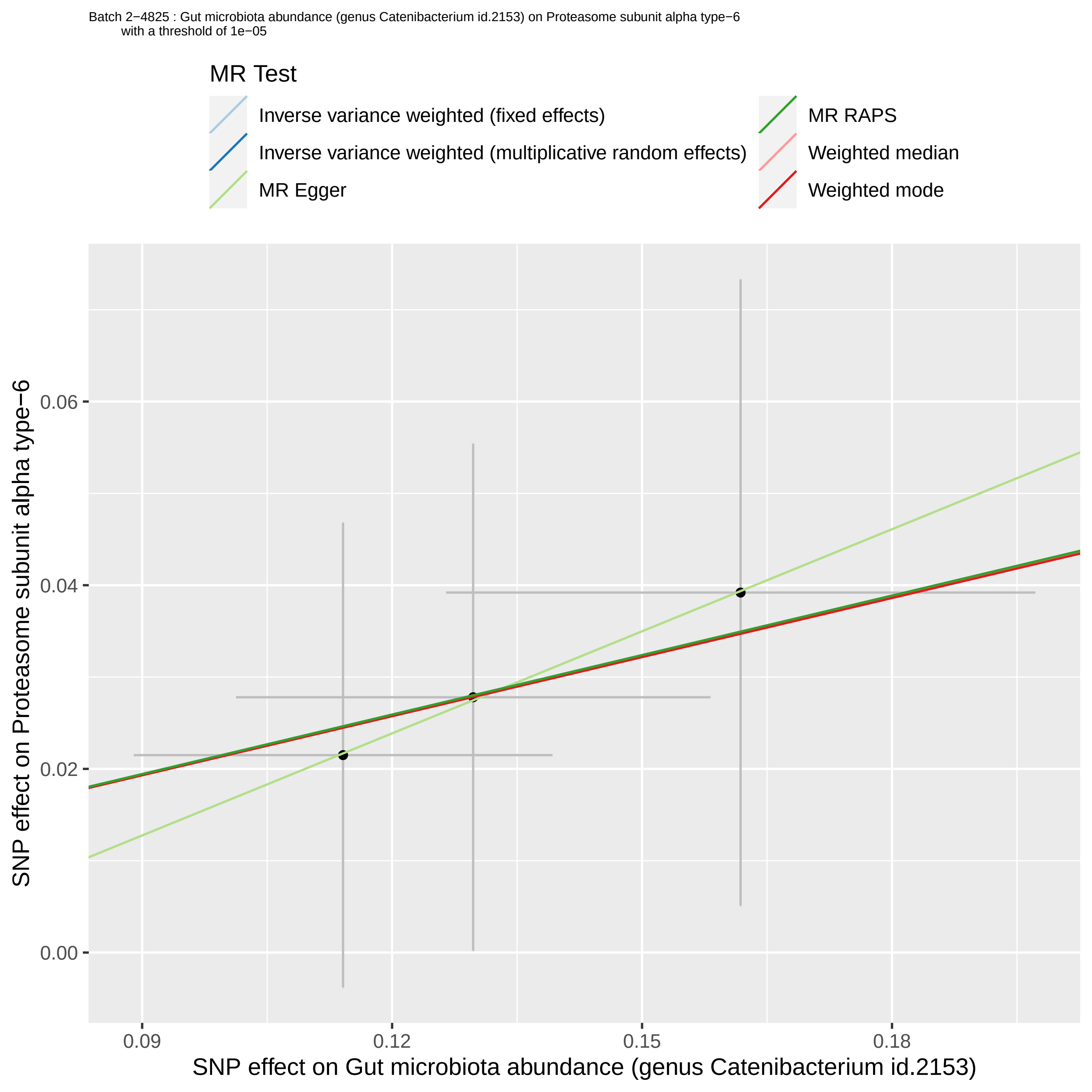
Batch 2−4825 : Gut microbiota abundance (genus Catenibacterium id.2153) on Proteasome subunit alpha type−6
with a threshold of 1e−05
MR Test
Inverse variance weighted (fixed effects)
Inverse variance weighted (multiplicative random effects)
MR Egger
MR RAPS
Weighted median
Weighted mode
0.06
0.04
0.02
0.00
0.09
0.12
0.15
0.18
SNP effect on Proteasome subunit alpha type−6
SNP effect on Gut microbiota abundance (genus Catenibacterium id.2153)

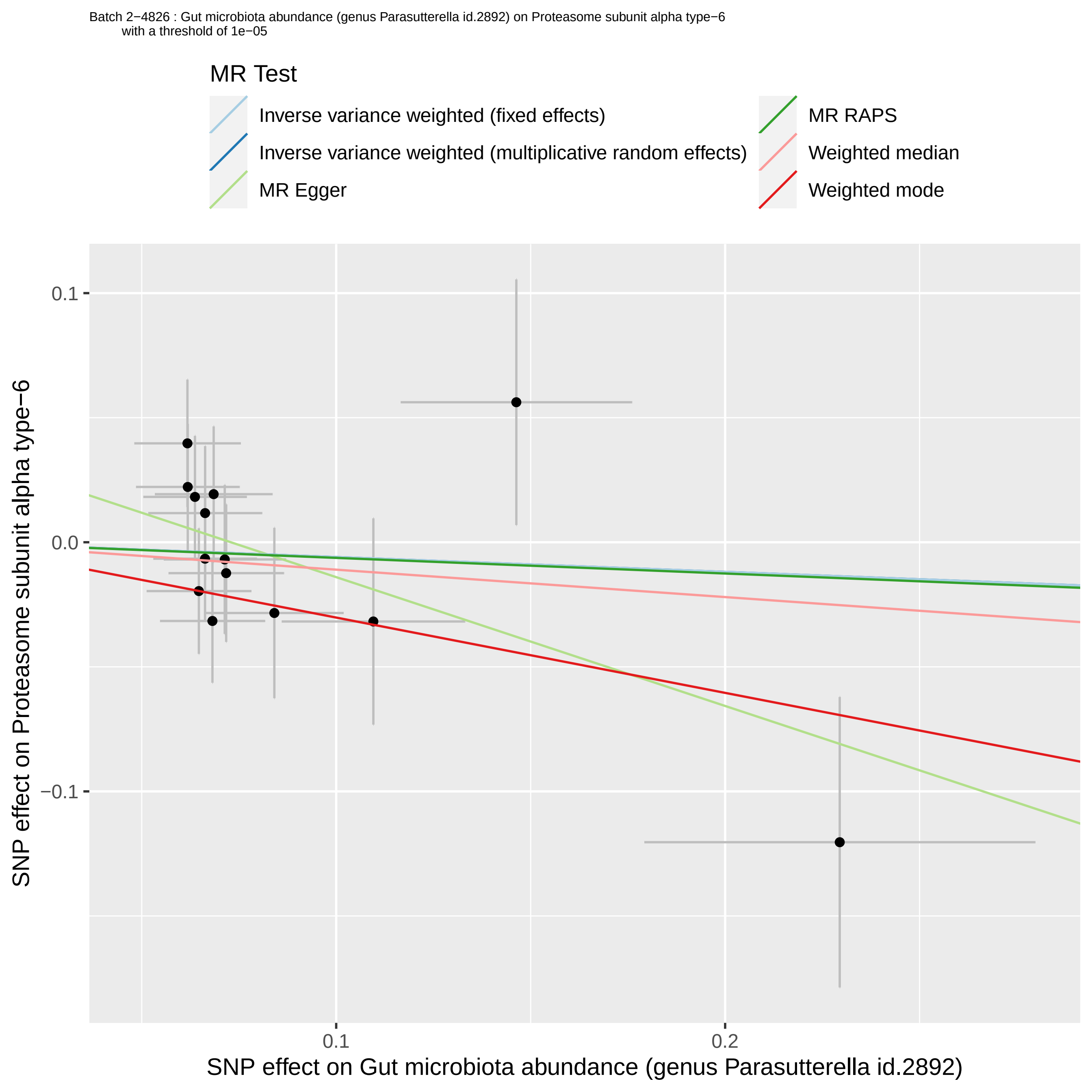

Batch 2−4826 : Gut microbiota abundance (genus Parasutterella id.2892) on Proteasome subunit alpha type−6
with a threshold of 1e−05
MR Test
Inverse variance weighted (fixed effects)
Inverse variance weighted (multiplicative random effects)
MR Egger
MR RAPS
Weighted median
Weighted mode
0.1
0.0
−0.1
0.1
0.2
SNP effect on Proteasome subunit alpha type−6
SNP effect on Gut microbiota abundance (genus Parasutterella id.2892)

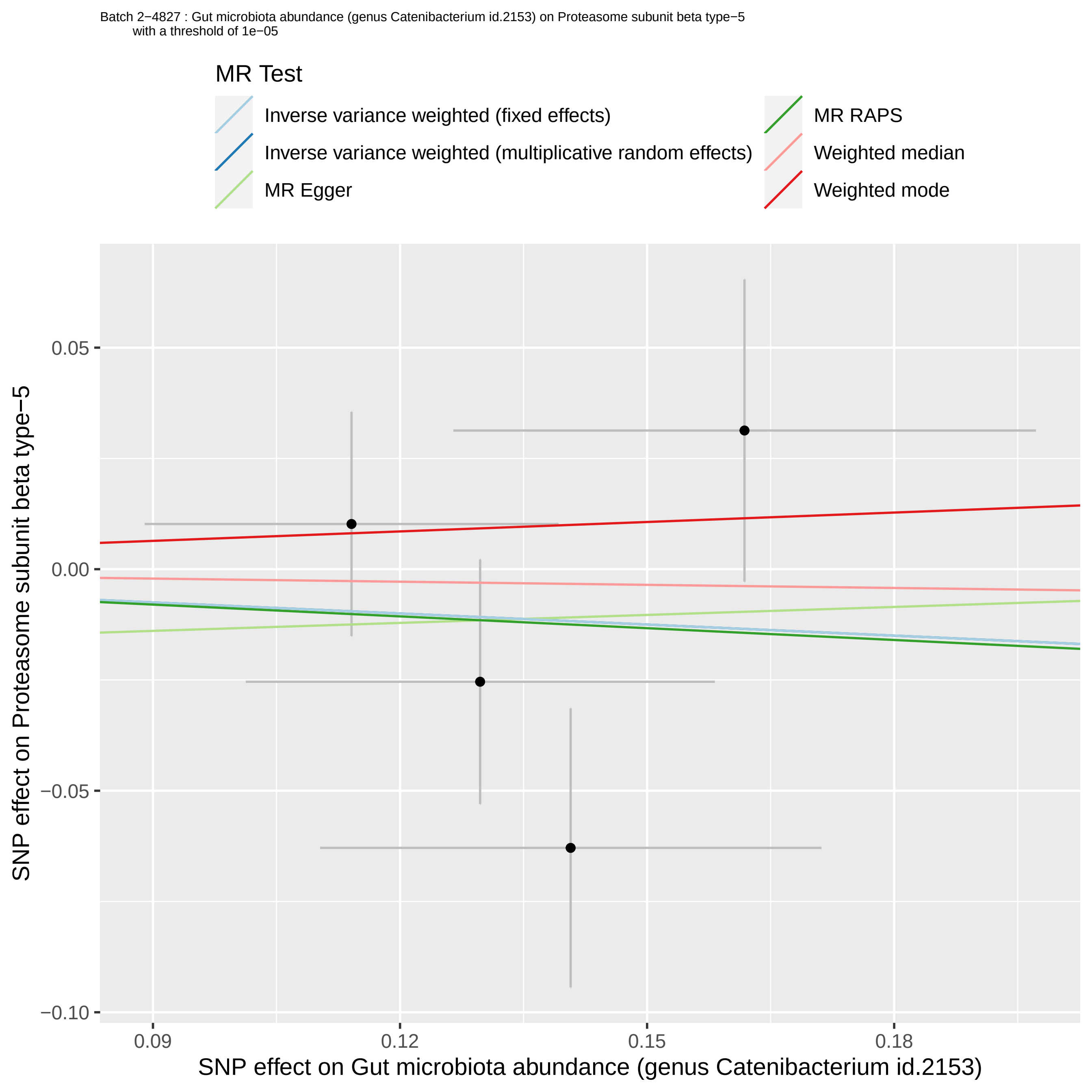

Batch 2−4827 : Gut microbiota abundance (genus Catenibacterium id.2153) on Proteasome subunit beta type−5
with a threshold of 1e−05
MR Test
Inverse variance weighted (fixed effects)
Inverse variance weighted (multiplicative random effects)
MR Egger
MR RAPS
Weighted median
Weighted mode
0.05
0.00
−0.05
−0.10
0.09
0.12
0.15
0.18
SNP effect on Proteasome subunit beta type−5
SNP effect on Gut microbiota abundance (genus Catenibacterium id.2153)

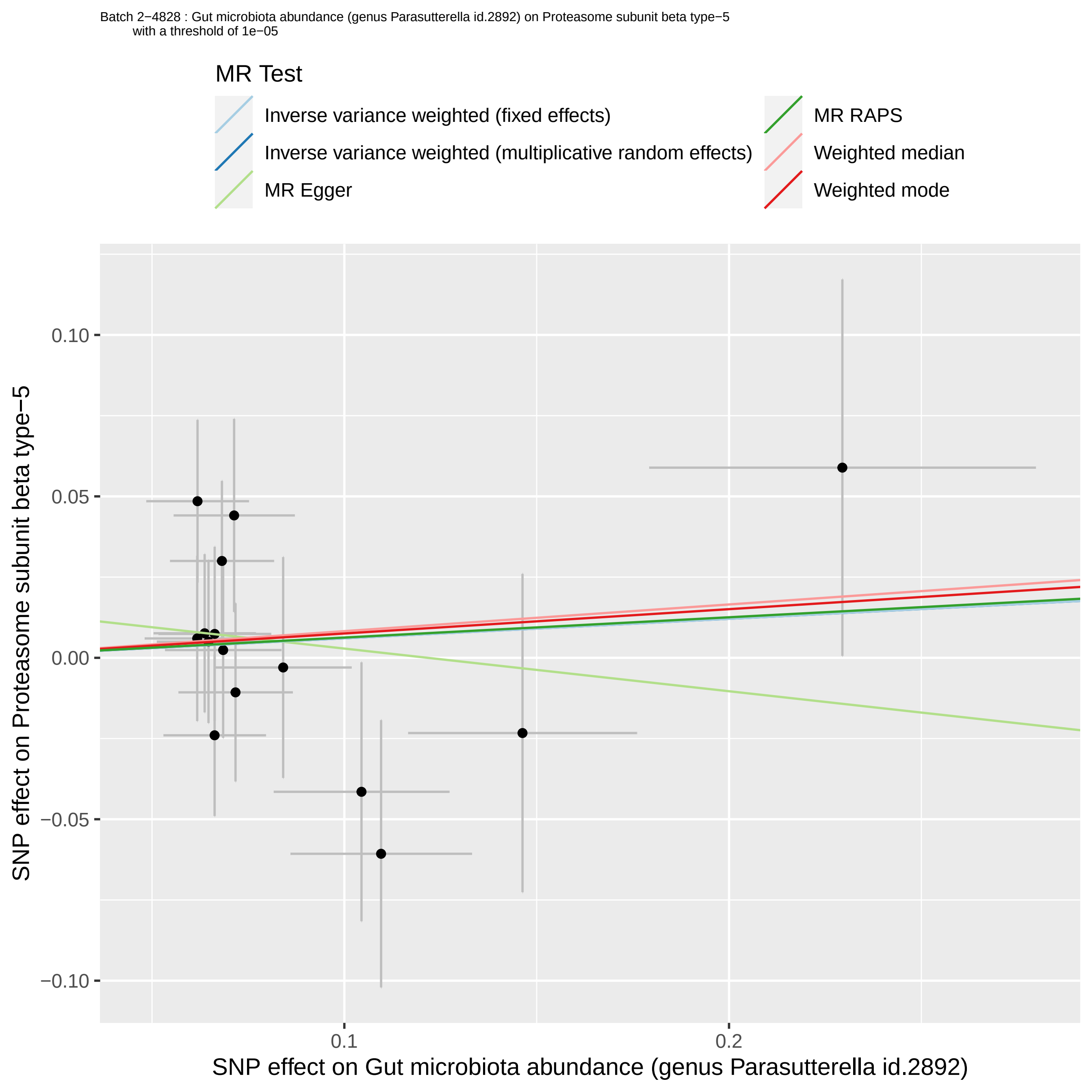

Batch 2−4828 : Gut microbiota abundance (genus Parasutterella id.2892) on Proteasome subunit beta type−5
with a threshold of 1e−05
MR Test
Inverse variance weighted (fixed effects)
Inverse variance weighted (multiplicative random effects)
MR Egger
MR RAPS
Weighted median
Weighted mode
0.10
0.05
0.00
−0.05
−0.10
0.1
0.2
SNP effect on Proteasome subunit beta type−5
SNP effect on Gut microbiota abundance (genus Parasutterella id.2892)

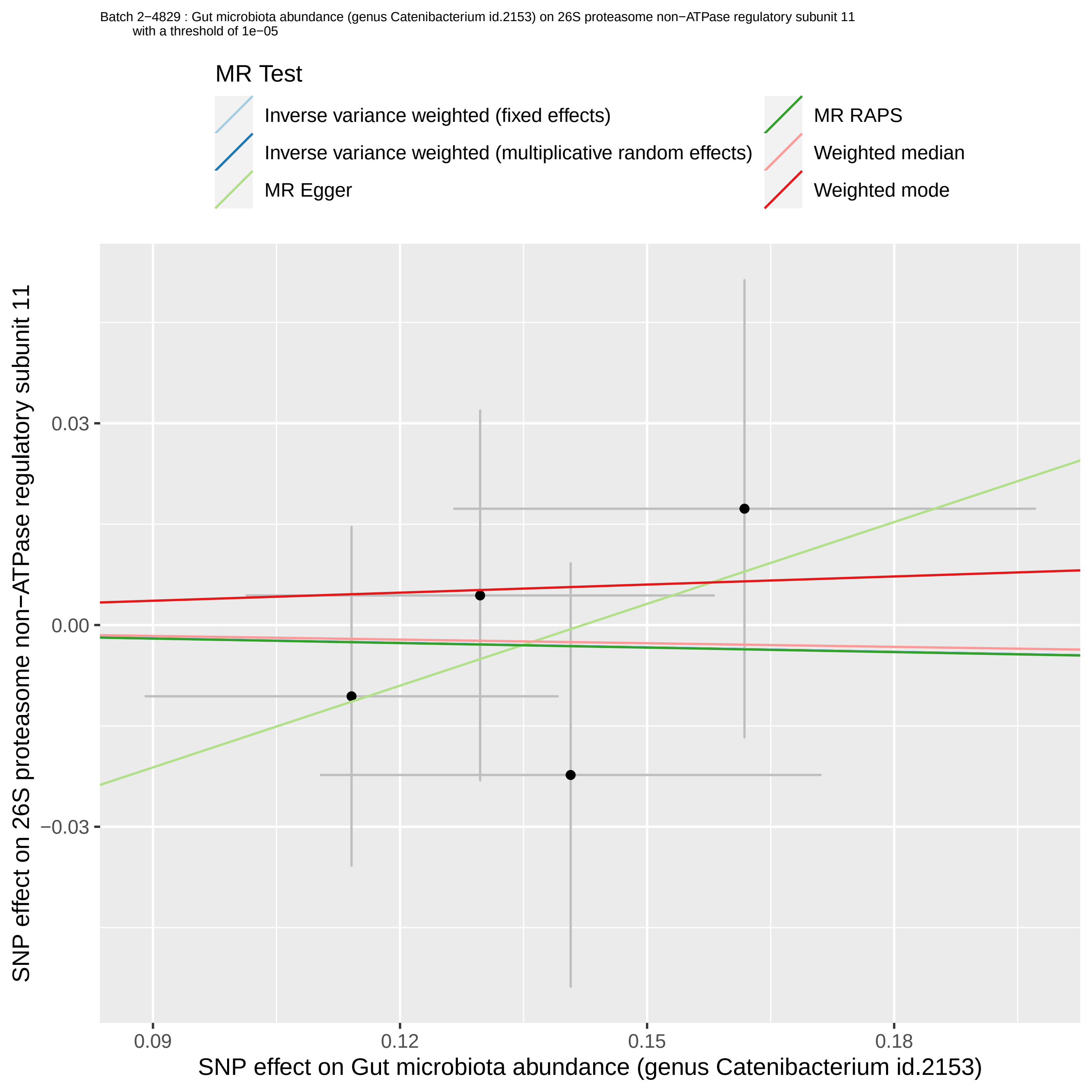
Batch 2−4829 : Gut microbiota abundance (genus Catenibacterium id.2153) on 26S proteasome non−ATPase regulatory subunit 11
with a threshold of 1e−05
MR Test
Inverse variance weighted (fixed effects)
Inverse variance weighted (multiplicative random effects)
MR Egger
MR RAPS
Weighted median
Weighted mode
0.03
0.00
−0.03
0.09
0.12
0.15
0.18
SNP effect on 26S proteasome non−ATPase regulatory subunit 11
SNP effect on Gut microbiota abundance (genus Catenibacterium id.2153)

Batch 2−4830 : Gut microbiota abundance (genus Parasutterella id.2892) on 26S proteasome non−ATPase regulatory subunit 11
with a threshold of 1e−05

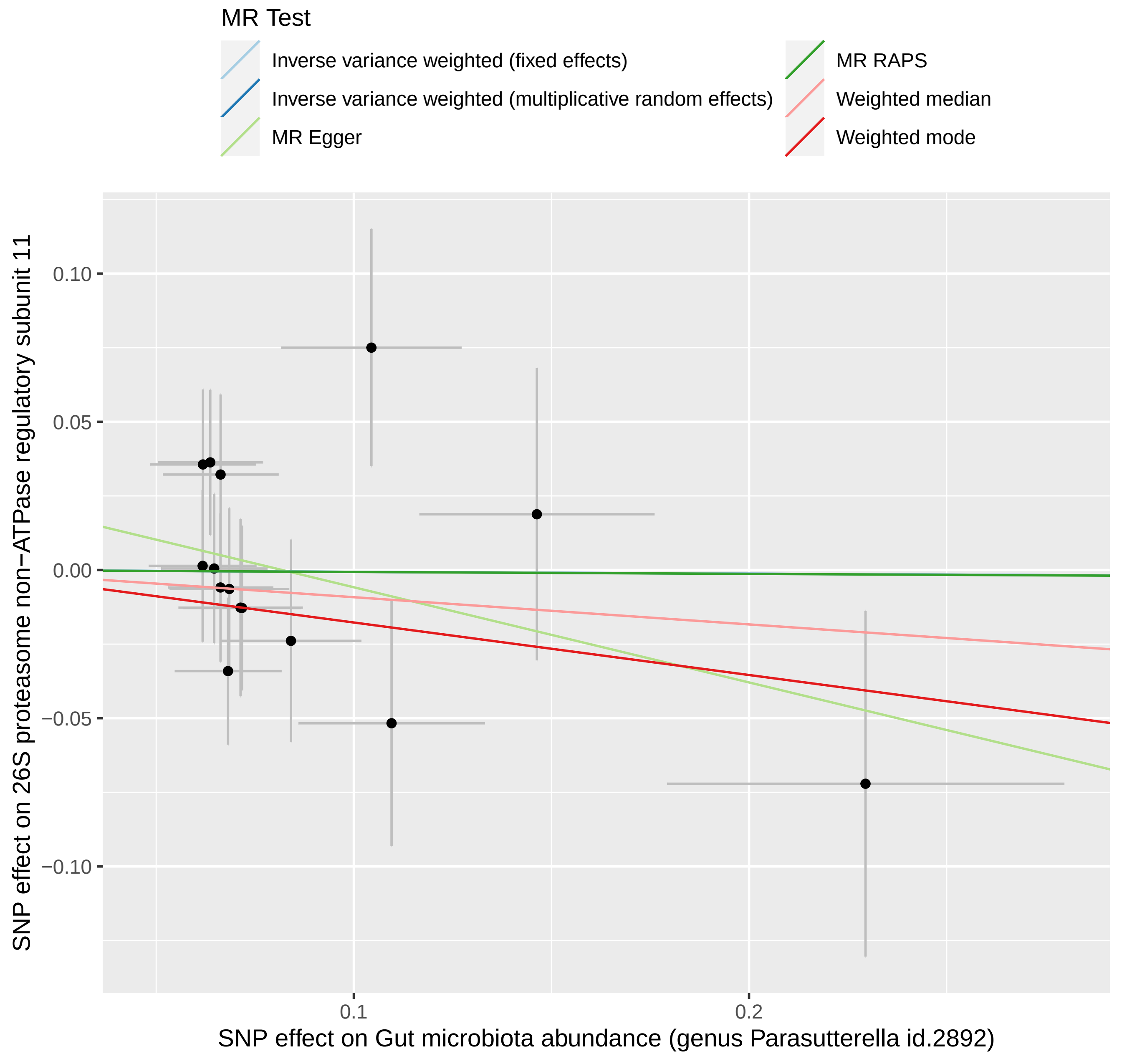

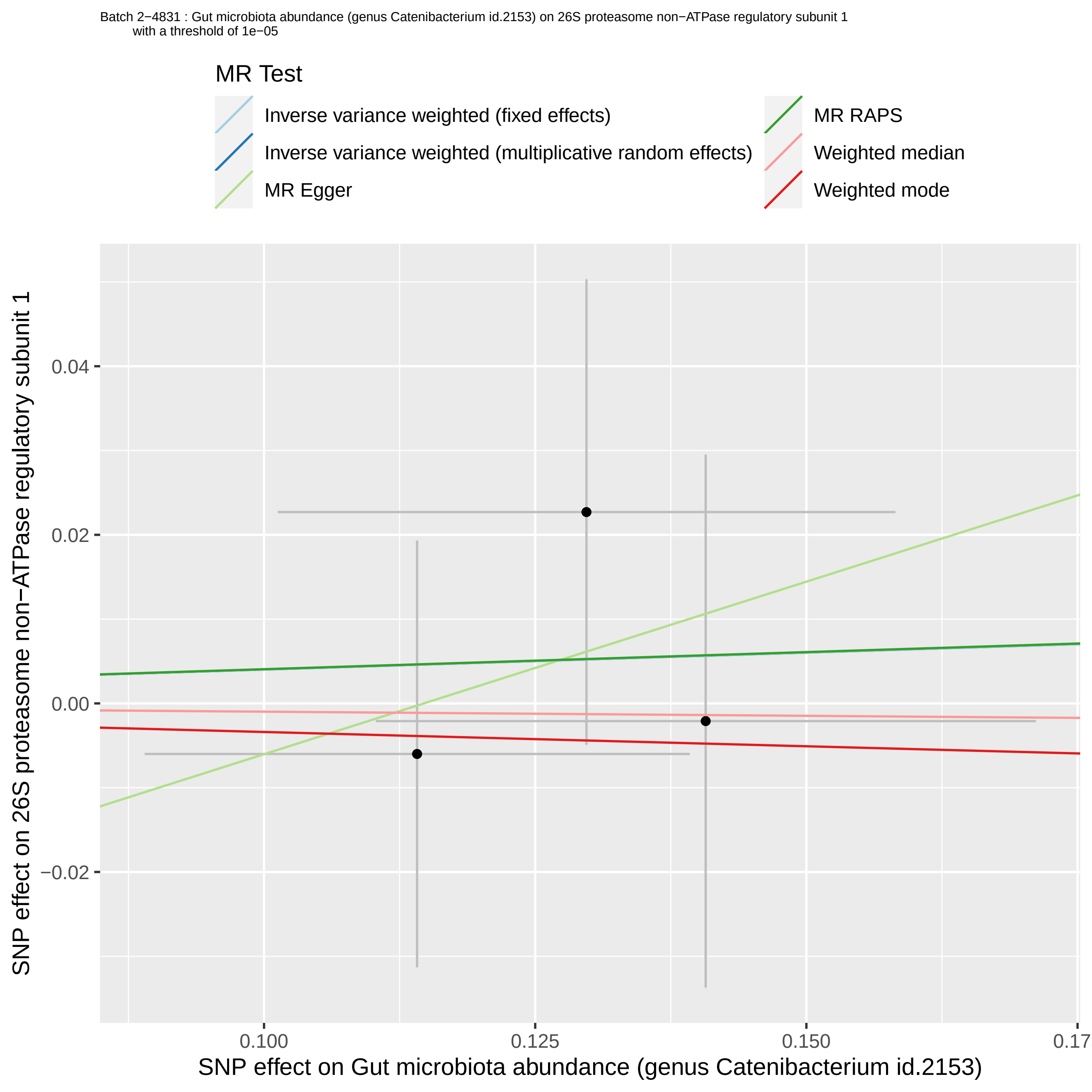

Batch 2−4831 : Gut microbiota abundance (genus Catenibacterium id.2153) on 26S proteasome non−ATPase regulatory subunit 1
with a threshold of 1e−05
MR Test
Inverse variance weighted (fixed effects)
Inverse variance weighted (multiplicative random effects)
MR Egger
MR RAPS
Weighted median
Weighted mode
SNP effect on 26S proteasome non−ATPase regulatory subunit 1
0.04
0.02
0.00
−0.02
0.100
0.125
0.150
0.17
SNP effect on Gut microbiota abundance (genus Catenibacterium id.2153)

Batch 2−4832 : Gut microbiota abundance (genus Parasutterella id.2892) on 26S proteasome non−ATPase regulatory subunit 1
with a threshold of 1e−05

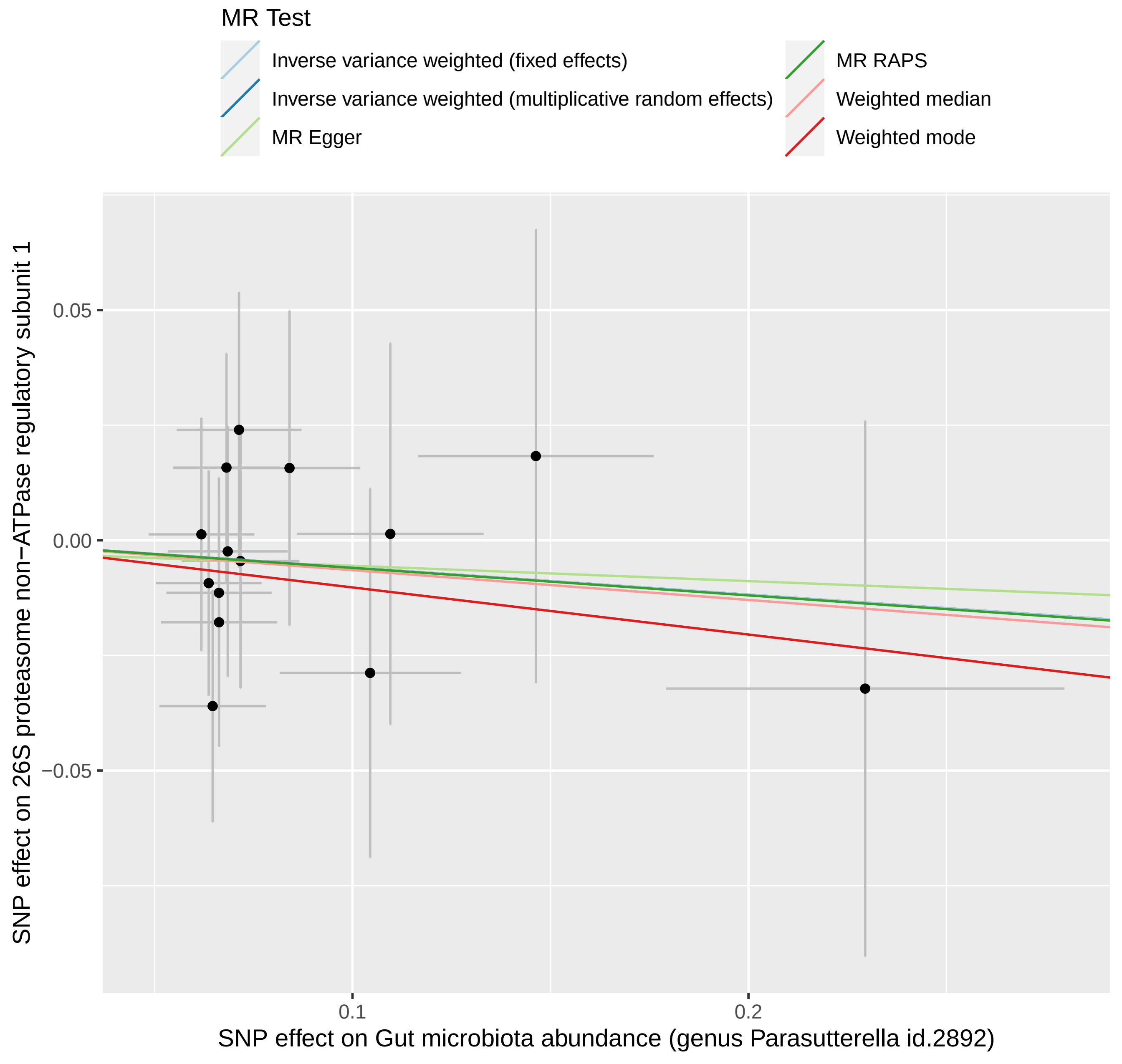

Batch 2−4833 : Gut microbiota abundance (genus Catenibacterium id.2153) on 26S proteasome non−ATPase regulatory subunit 4
with a threshold of 1e−05

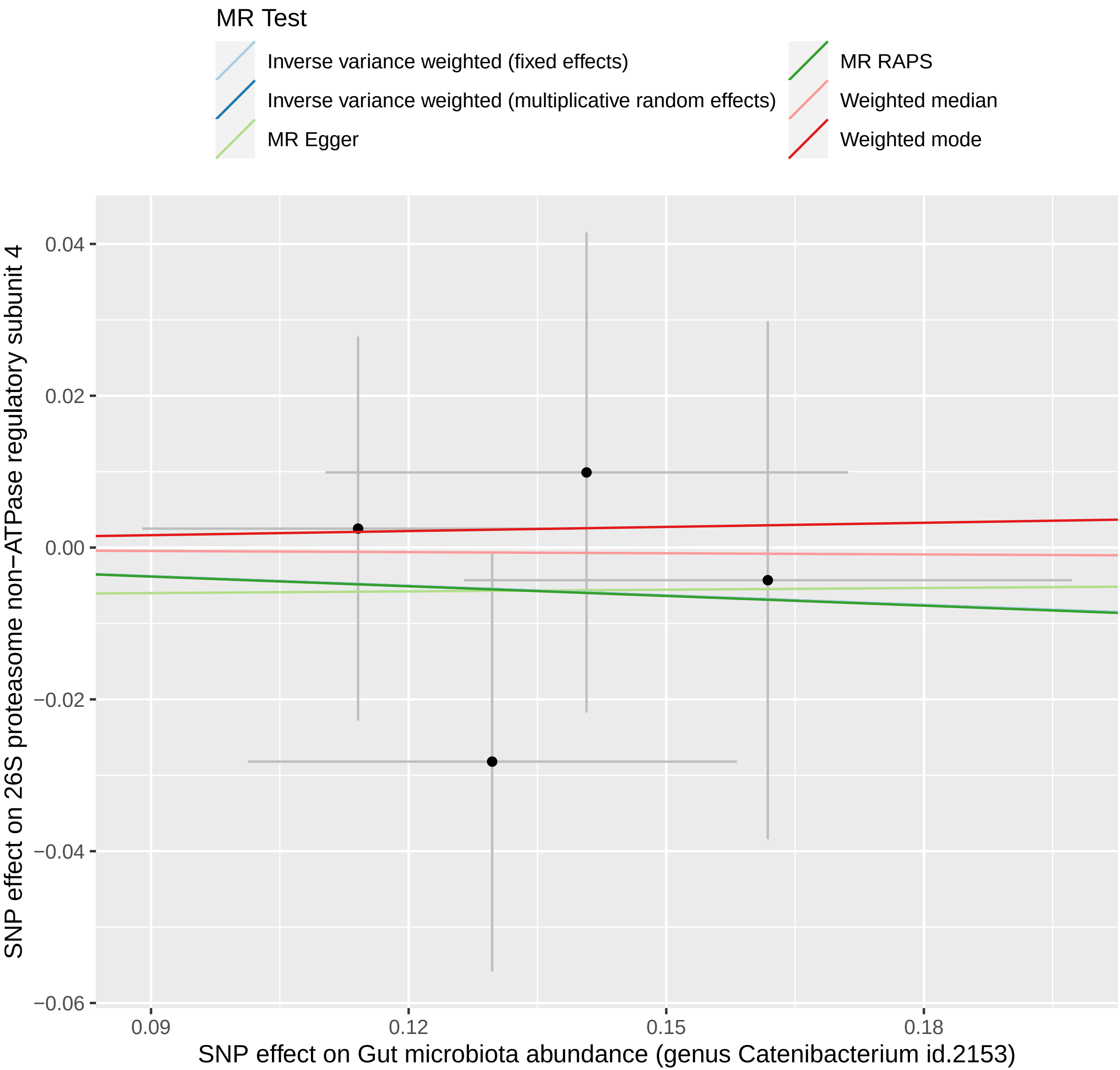

Batch 2−4834 : Gut microbiota abundance (genus Parasutterella id.2892) on 26S proteasome non−ATPase regulatory subunit 4
with a threshold of 1e−05

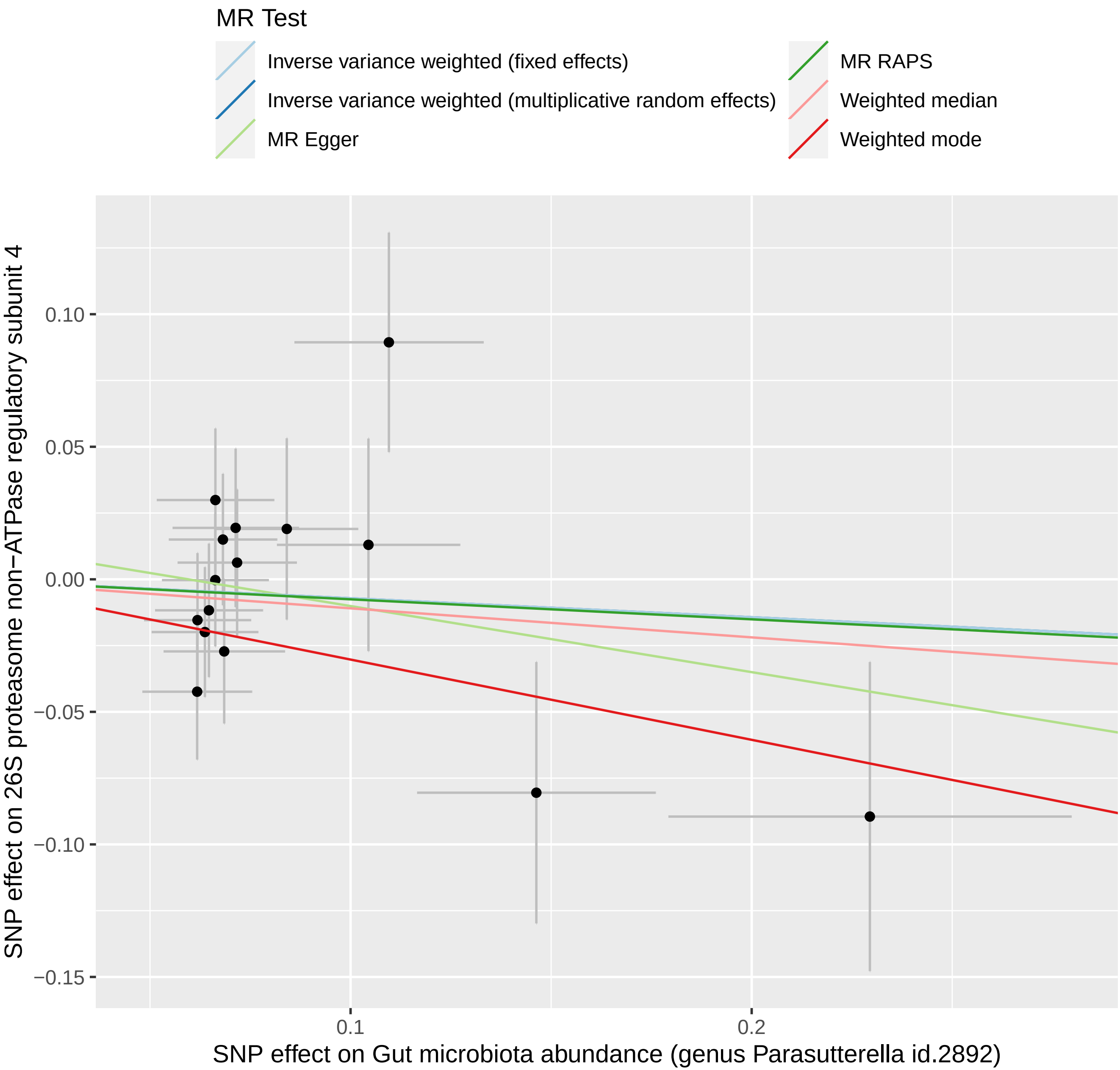

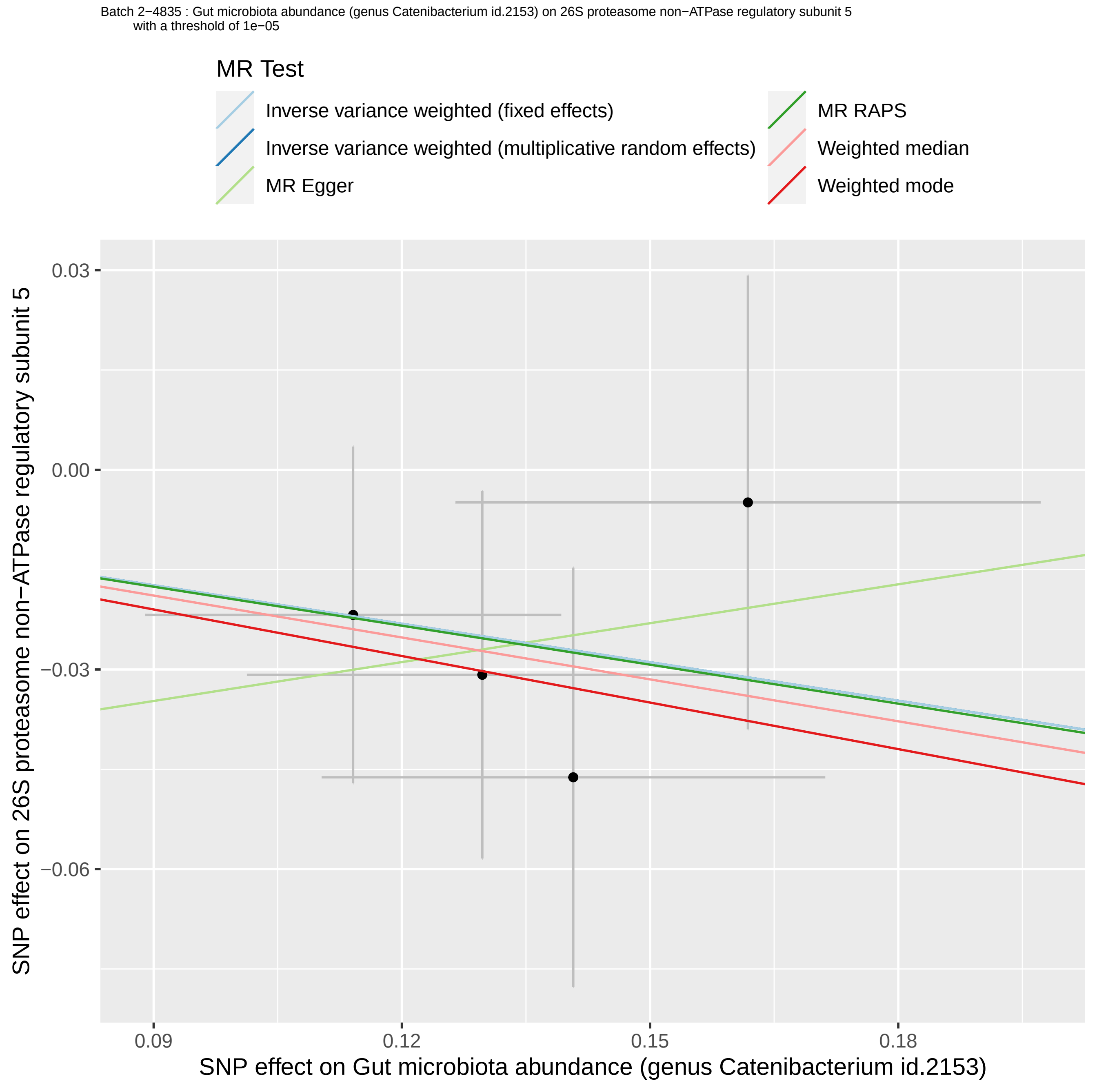
Batch 2−4835 : Gut microbiota abundance (genus Catenibacterium id.2153) on 26S proteasome non−ATPase regulatory subunit 5
with a threshold of 1e−05
MR Test
Inverse variance weighted (fixed effects)
Inverse variance weighted (multiplicative random effects)
MR Egger
MR RAPS
Weighted median
Weighted mode
0.03
0.00
−0.03
−0.06
0.09
0.12
0.15
0.18
SNP effect on 26S proteasome non−ATPase regulatory subunit 5
SNP effect on Gut microbiota abundance (genus Catenibacterium id.2153)

Batch 2−4836 : Gut microbiota abundance (genus Parasutterella id.2892) on 26S proteasome non−ATPase regulatory subunit 5
with a threshold of 1e−05

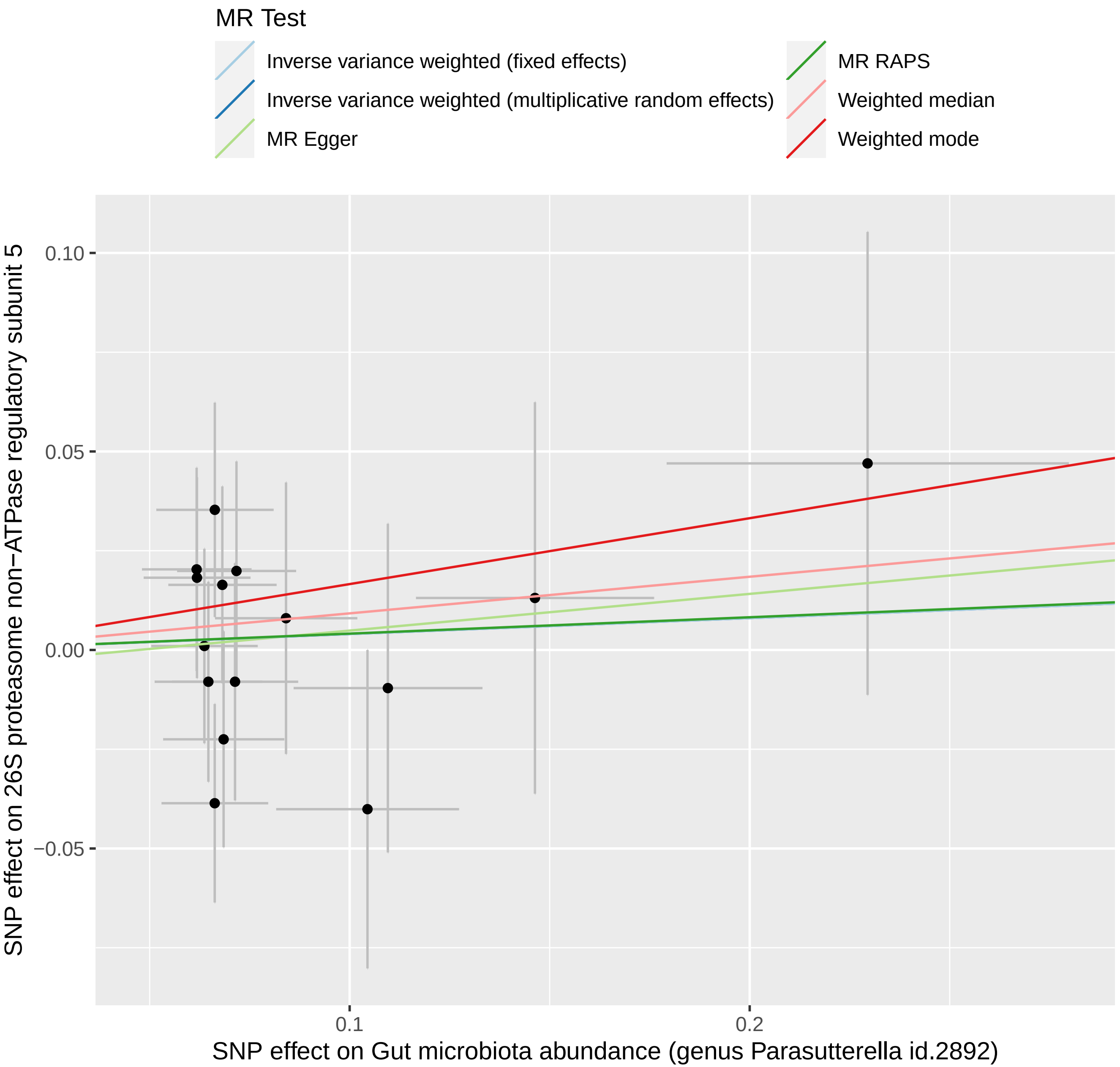

Batch 2−4837 : Gut microbiota abundance (genus Catenibacterium id.2153) on 26S proteasome non−ATPase regulatory subunit 7
with a threshold of 1e−05

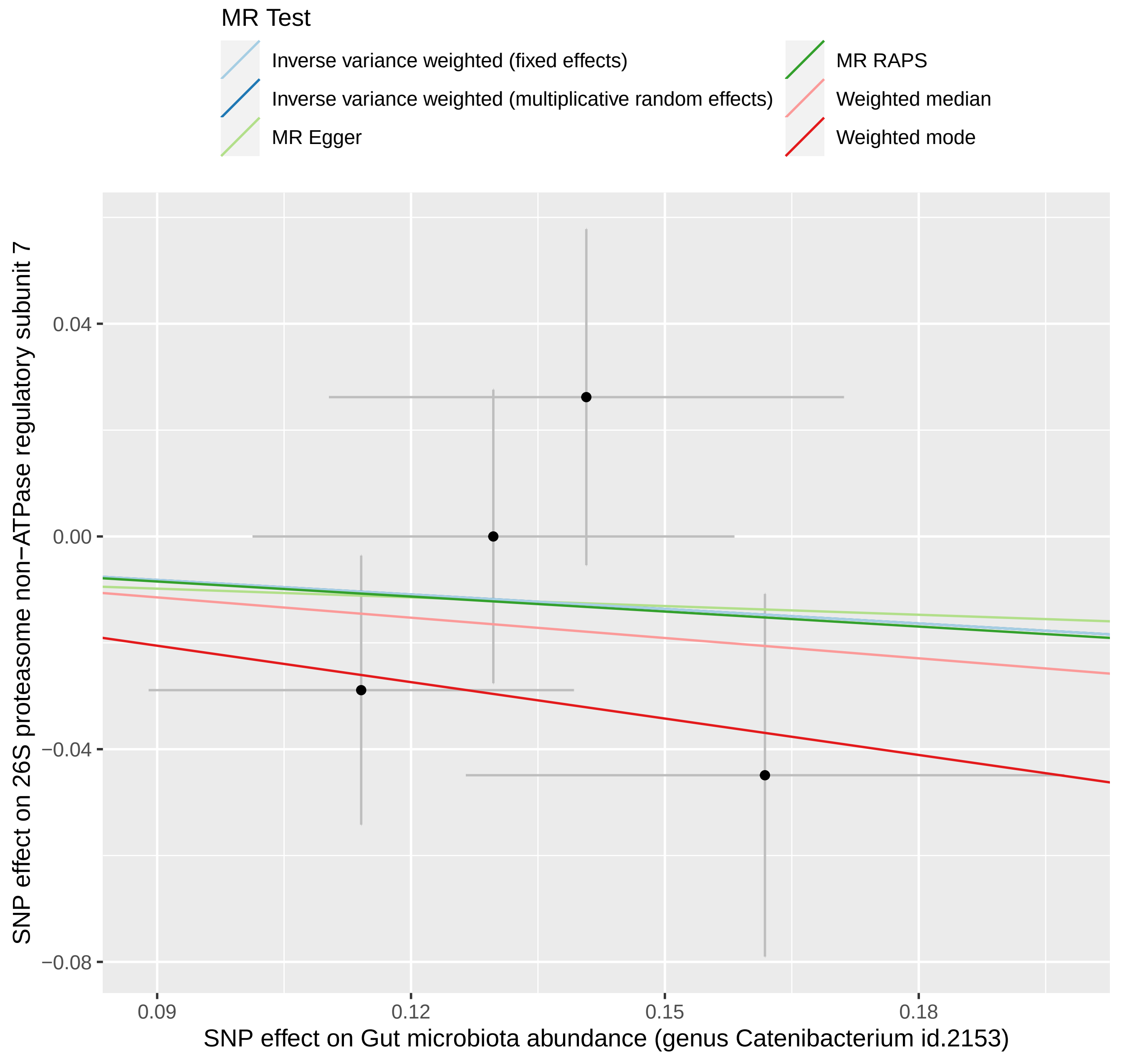

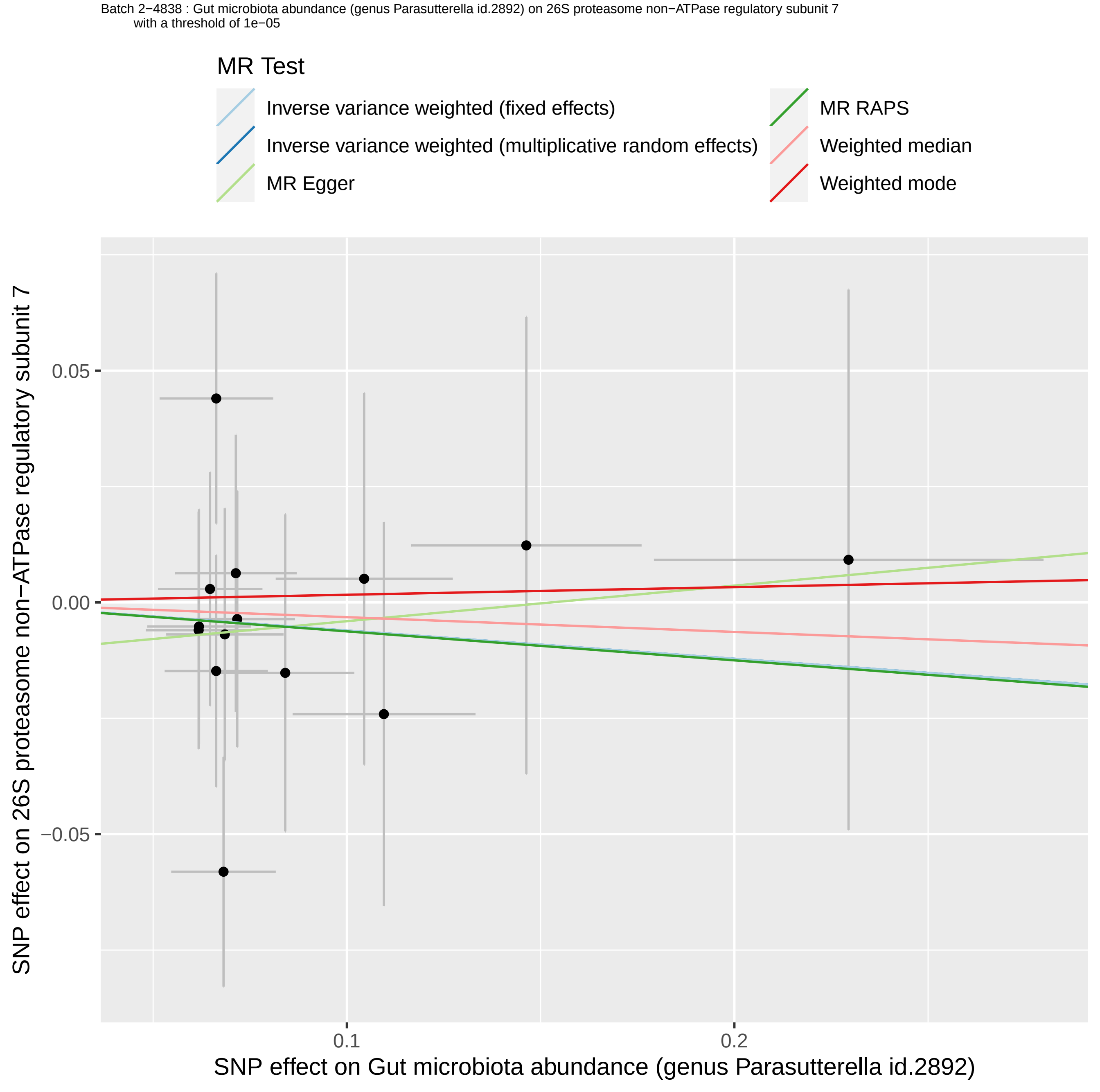

Batch 2−4838 : Gut microbiota abundance (genus Parasutterella id.2892) on 26S proteasome non−ATPase regulatory subunit 7
with a threshold of 1e−05
MR Test
Inverse variance weighted (fixed effects)
Inverse variance weighted (multiplicative random effects)
MR Egger
MR RAPS
Weighted median
Weighted mode
0.05
0.00
−0.05
0.1
0.2
SNP effect on 26S proteasome non−ATPase regulatory subunit 7
SNP effect on Gut microbiota abundance (genus Parasutterella id.2892)

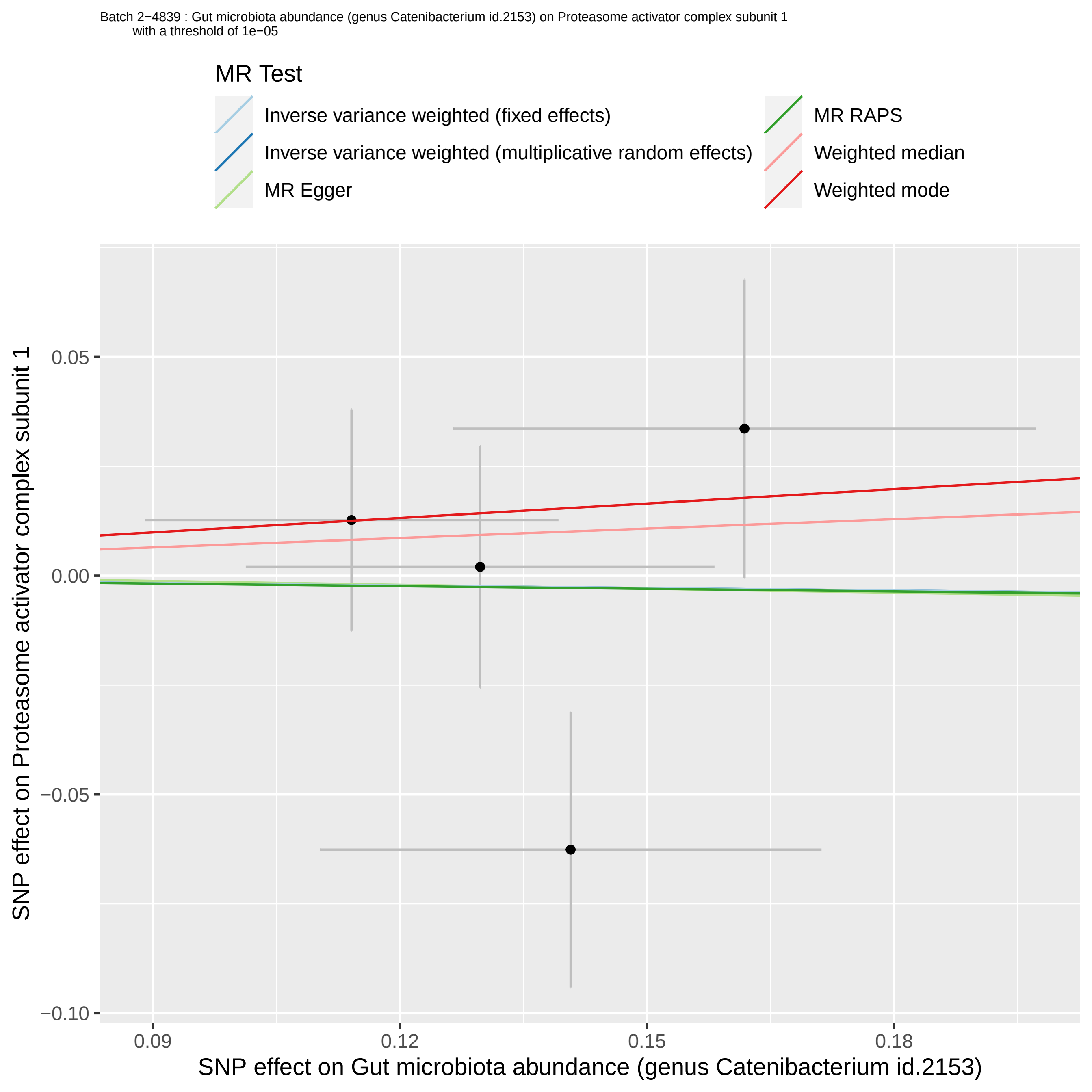
Batch 2−4839 : Gut microbiota abundance (genus Catenibacterium id.2153) on Proteasome activator complex subunit 1
with a threshold of 1e−05
MR Test
Inverse variance weighted (fixed effects)
Inverse variance weighted (multiplicative random effects)
MR Egger
MR RAPS
Weighted median
Weighted mode
SNP effect on Proteasome activator complex subunit 1
0.05
0.00
−0.05
−0.10
0.09
0.12
0.15
0.18
SNP effect on Gut microbiota abundance (genus Catenibacterium id.2153)

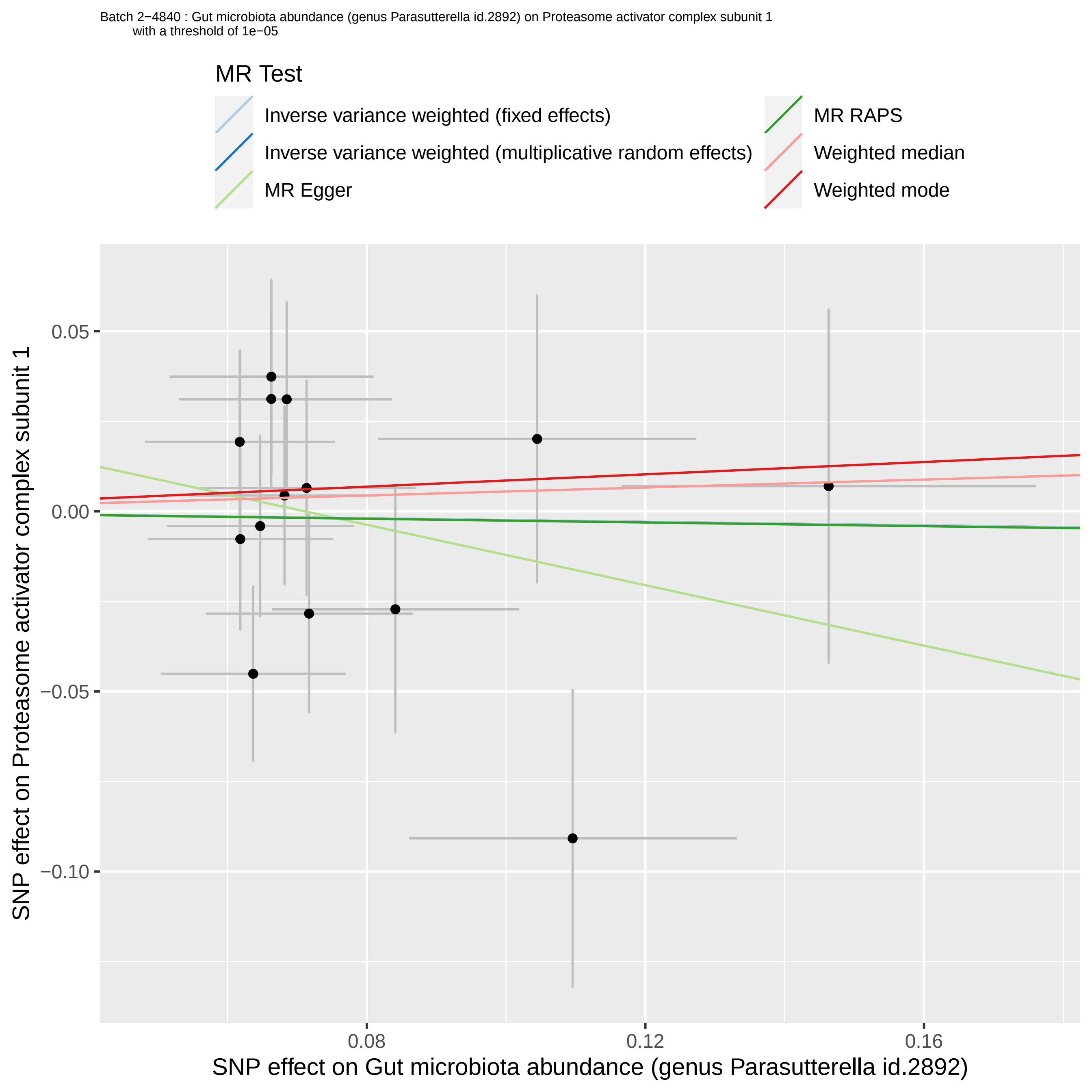

Batch 2−4840 : Gut microbiota abundance (genus Parasutterella id.2892) on Proteasome activator complex subunit 1
with a threshold of 1e−05
MR Test
Inverse variance weighted (fixed effects)
Inverse variance weighted (multiplicative random effects)
MR Egger
MR RAPS
Weighted median
Weighted mode
0.05
0.00
−0.05
−0.10
0.08
0.12
0.16
SNP effect on Proteasome activator complex subunit 1
SNP effect on Gut microbiota abundance (genus Parasutterella id.2892)

Batch 2−4841 : Gut microbiota abundance (genus Catenibacterium id.2153) on Proteasome activator complex subunit 3
with a threshold of 1e−05

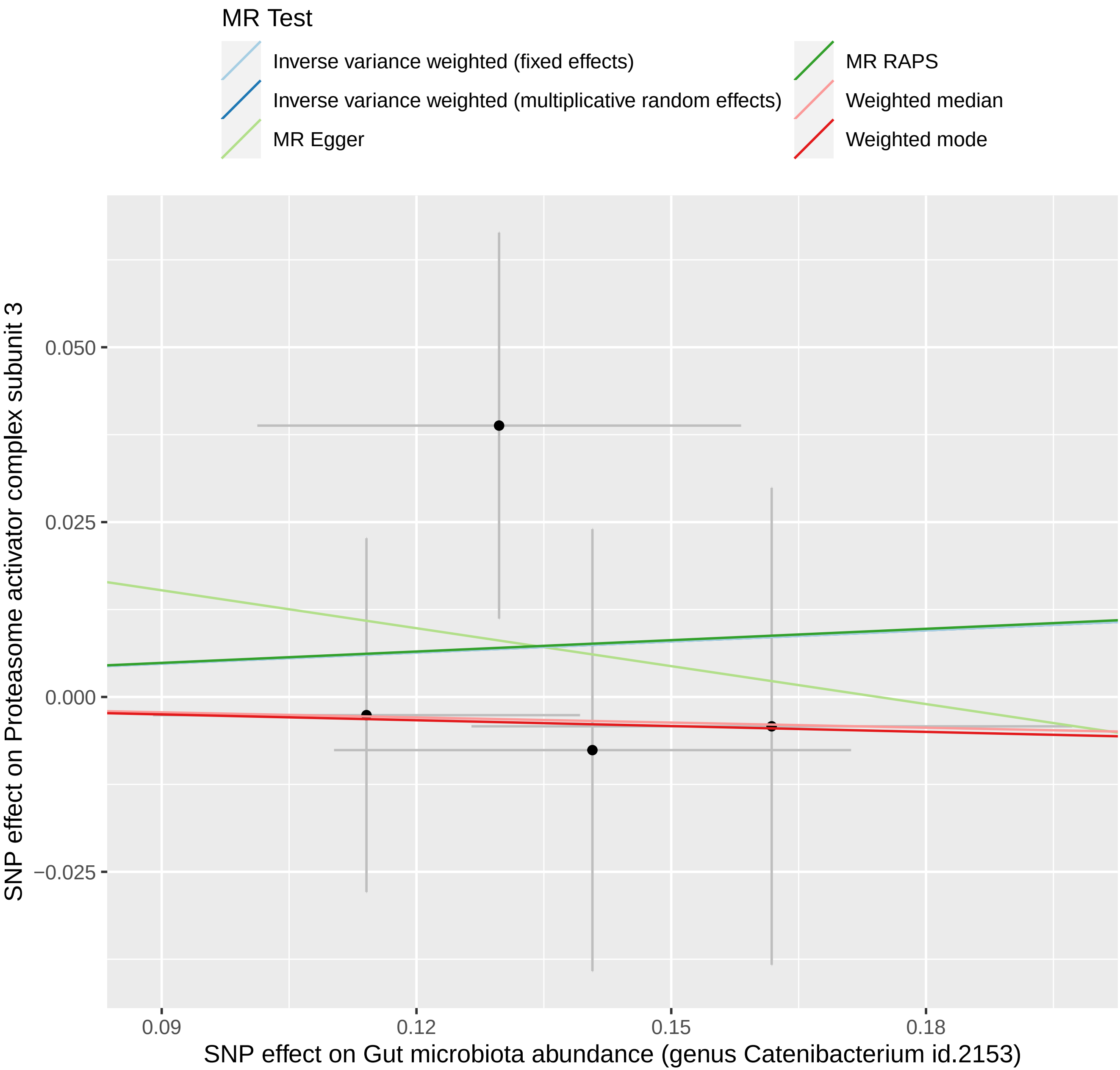

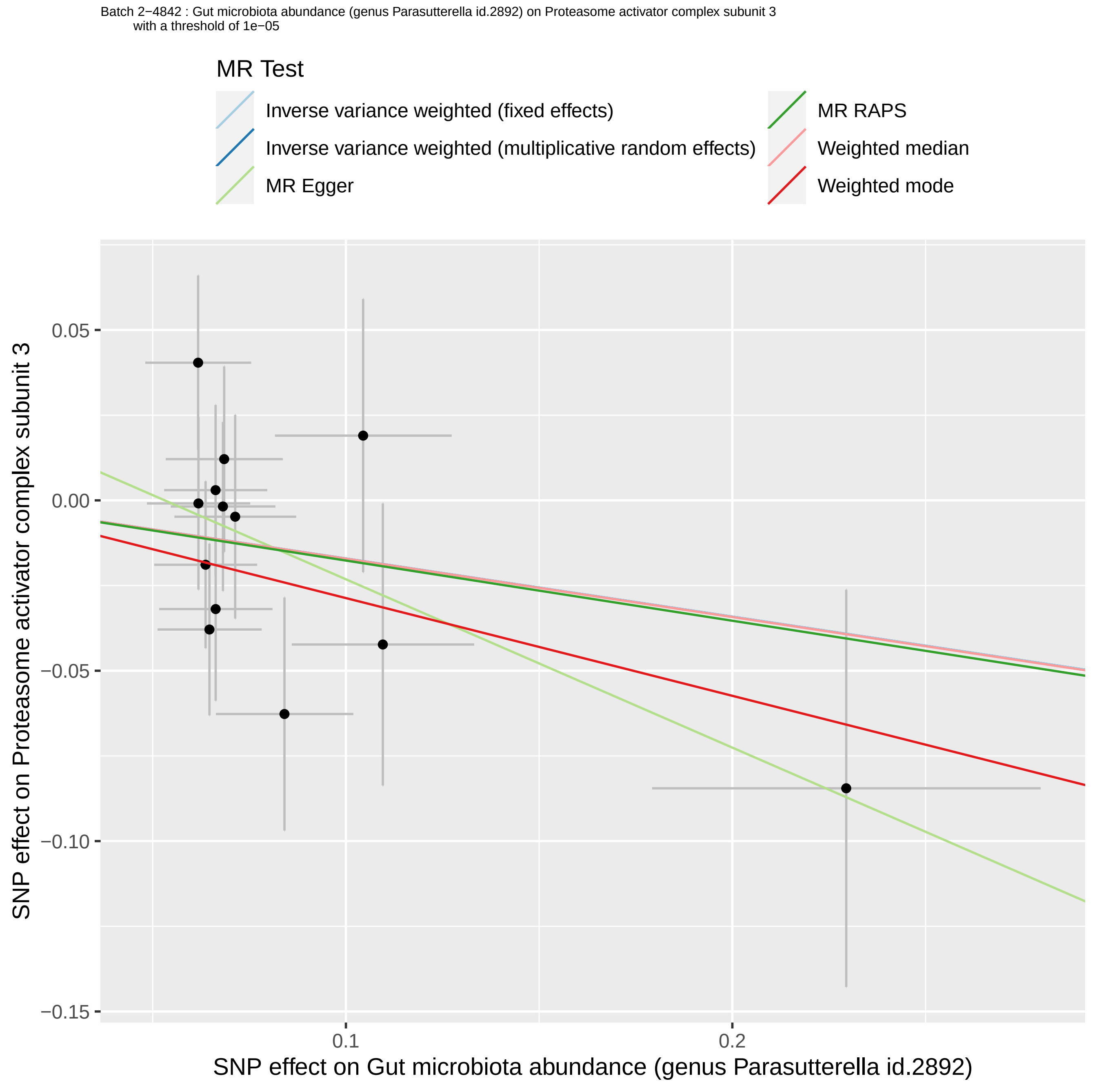
Batch 2−4842 : Gut microbiota abundance (genus Parasutterella id.2892) on Proteasome activator complex subunit 3
with a threshold of 1e−05
MR Test
Inverse variance weighted (fixed effects)
Inverse variance weighted (multiplicative random effects)
MR Egger
MR RAPS
Weighted median
Weighted mode
0.05
0.00
−0.05
−0.10
−0.15
0.1
0.2
SNP effect on Proteasome activator complex subunit 3
SNP effect on Gut microbiota abundance (genus Parasutterella id.2892)

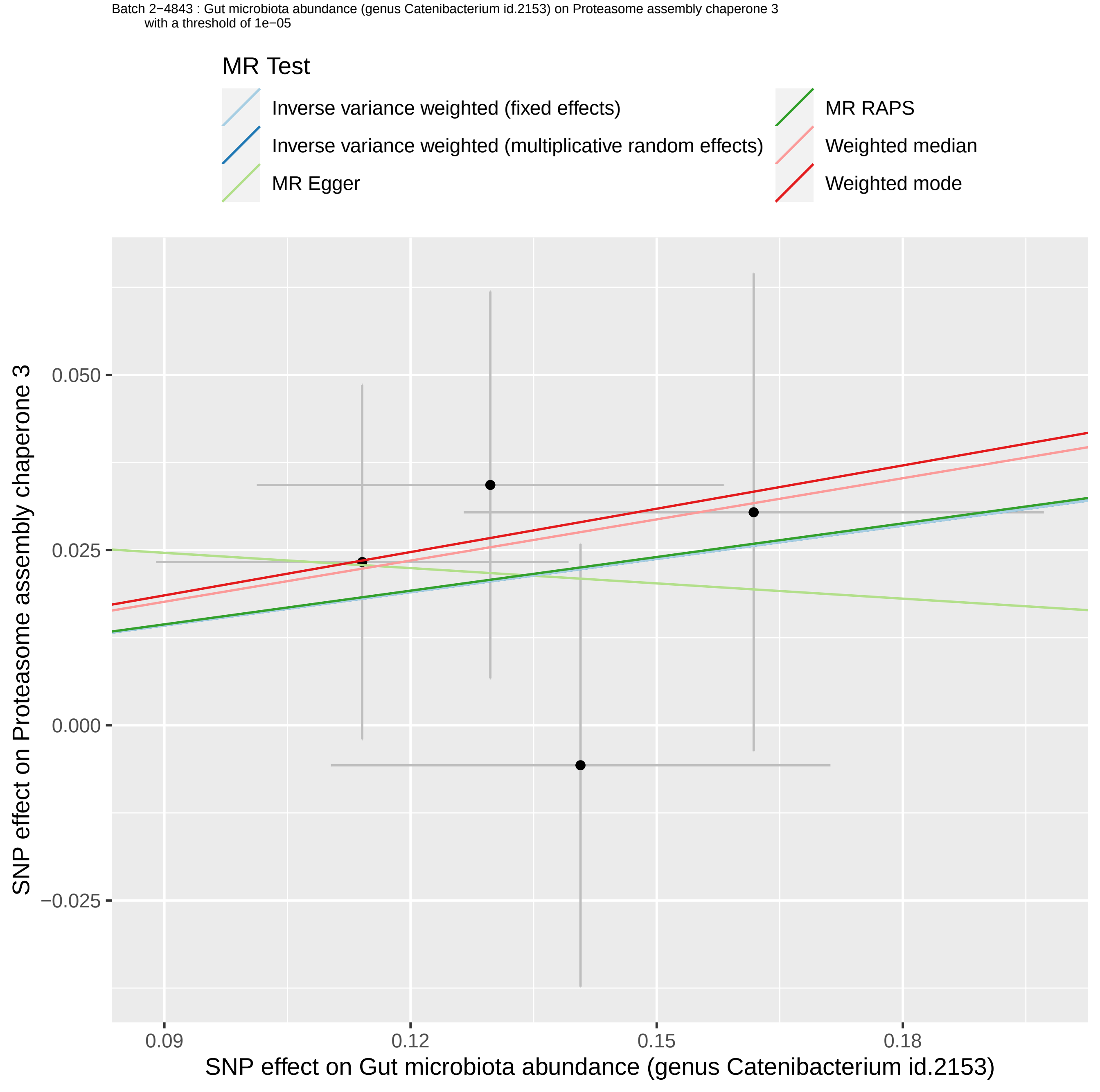

Batch 2−4843 : Gut microbiota abundance (genus Catenibacterium id.2153) on Proteasome assembly chaperone 3
with a threshold of 1e−05
MR Test
Inverse variance weighted (fixed effects)
Inverse variance weighted (multiplicative random effects)
MR Egger
MR RAPS
Weighted median
Weighted mode
0.050
0.025
0.000
−0.025
0.09
0.12
0.15
0.18
SNP effect on Proteasome assembly chaperone 3
SNP effect on Gut microbiota abundance (genus Catenibacterium id.2153)

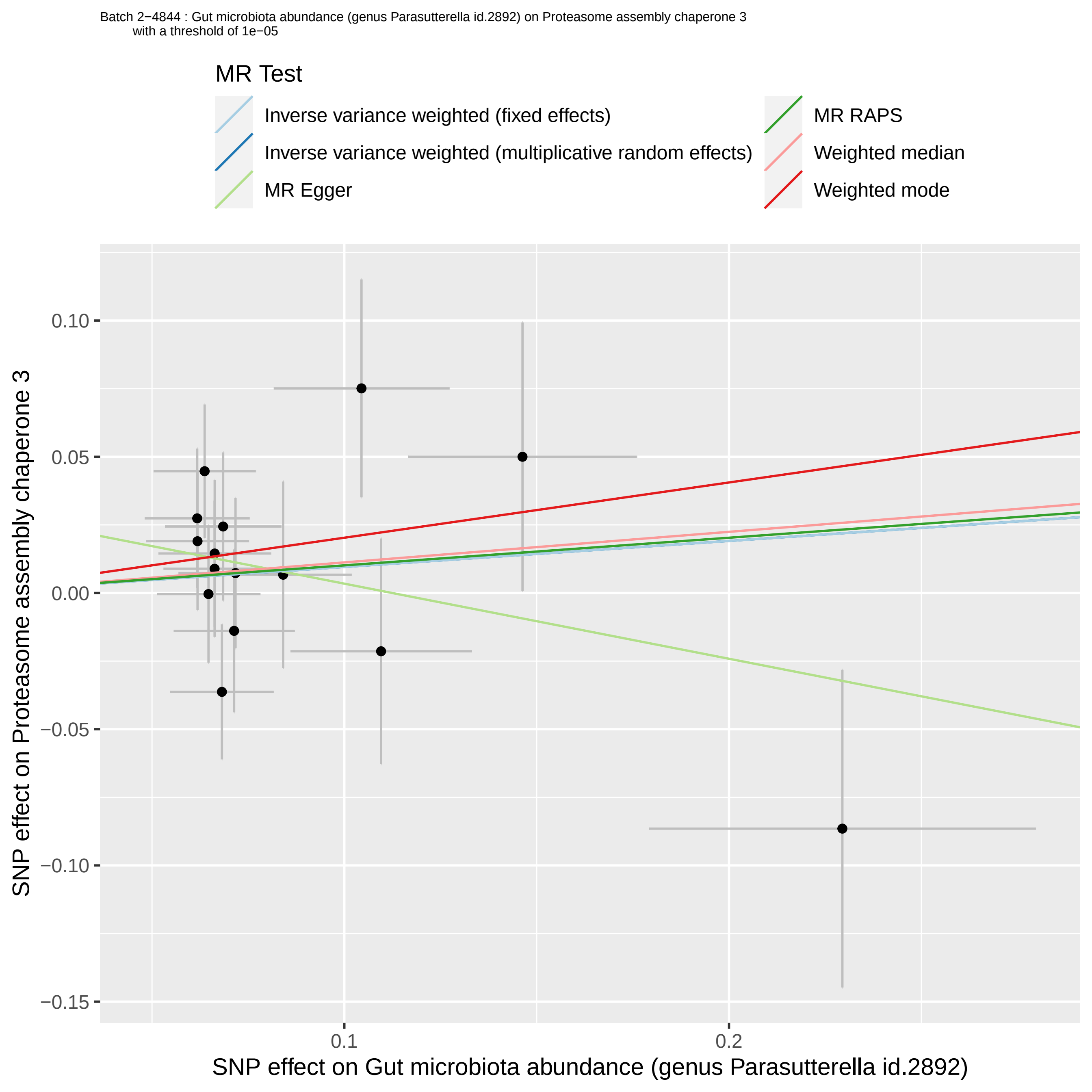
Batch 2−4844 : Gut microbiota abundance (genus Parasutterella id.2892) on Proteasome assembly chaperone 3
with a threshold of 1e−05
MR Test
Inverse variance weighted (fixed effects)
Inverse variance weighted (multiplicative random effects)
MR Egger
MR RAPS
Weighted median
Weighted mode
0.10
0.05
0.00
−0.05
−0.10
−0.15
0.1
0.2
SNP effect on Proteasome assembly chaperone 3
SNP effect on Gut microbiota abundance (genus Parasutterella id.2892)

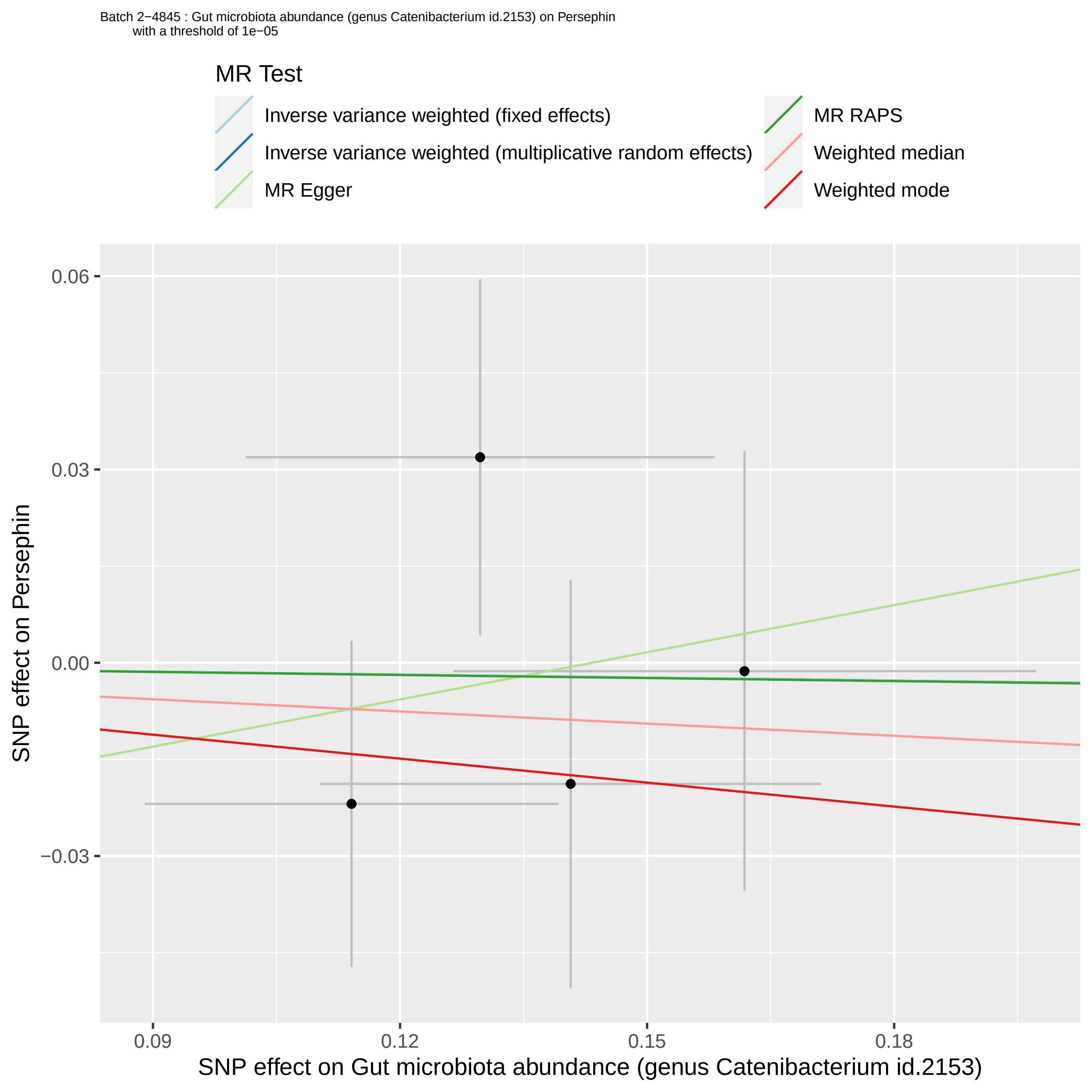
Batch 2−4845 : Gut microbiota abundance (genus Catenibacterium id.2153) on Persephin
with a threshold of 1e−05
MR Test
Inverse variance weighted (fixed effects)
Inverse variance weighted (multiplicative random effects)
MR Egger
MR RAPS
Weighted median
Weighted mode
0.06
0.03
0.00
−0.03
0.09
0.12
0.15
0.18
SNP effect on Persephin
SNP effect on Gut microbiota abundance (genus Catenibacterium id.2153)

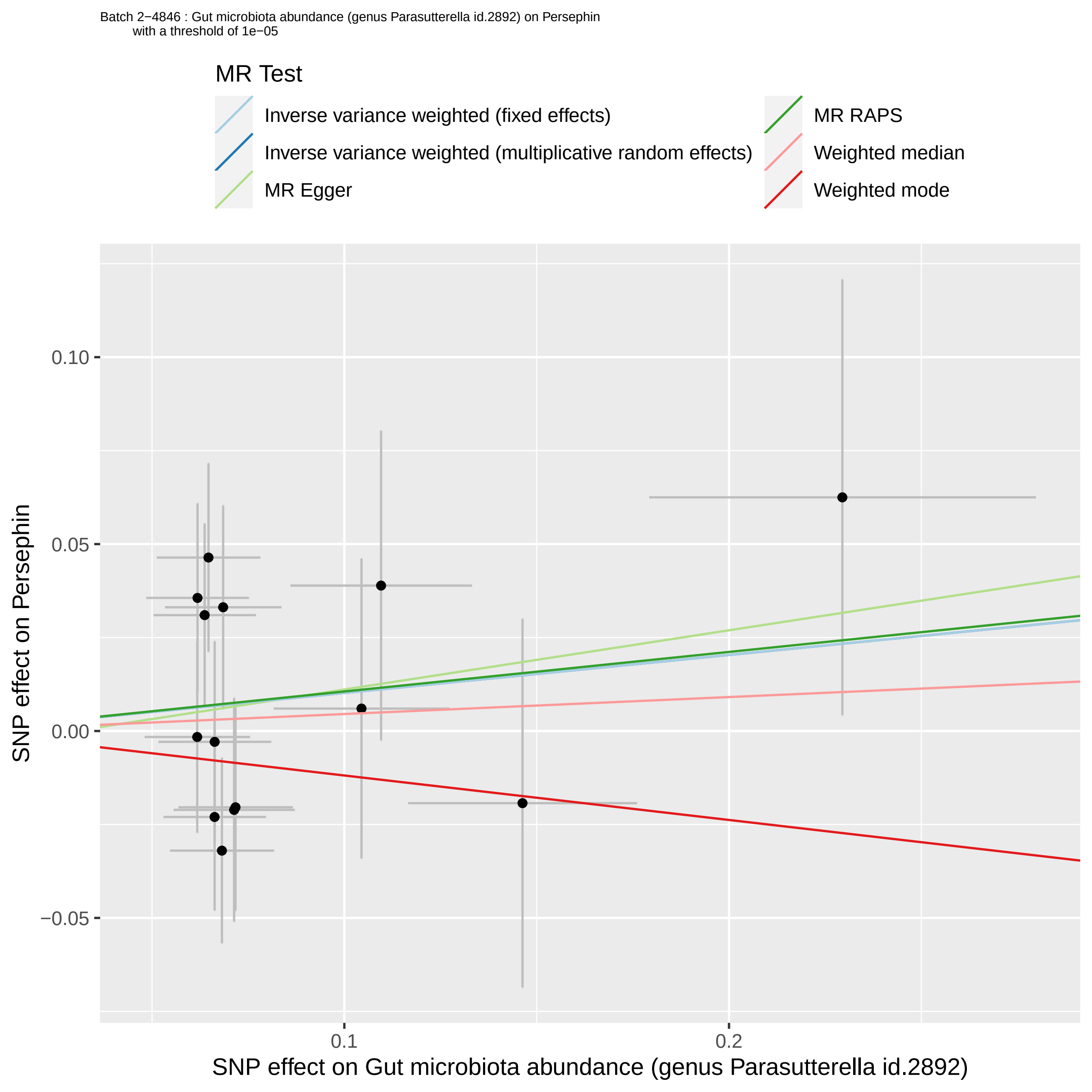

Batch 2−4846 : Gut microbiota abundance (genus Parasutterella id.2892) on Persephin
with a threshold of 1e−05
MR Test
Inverse variance weighted (fixed effects)
Inverse variance weighted (multiplicative random effects)
MR Egger
MR RAPS
Weighted median
Weighted mode
0.10
0.05
0.00
−0.05
0.1
0.2
SNP effect on Persephin
SNP effect on Gut microbiota abundance (genus Parasutterella id.2892)

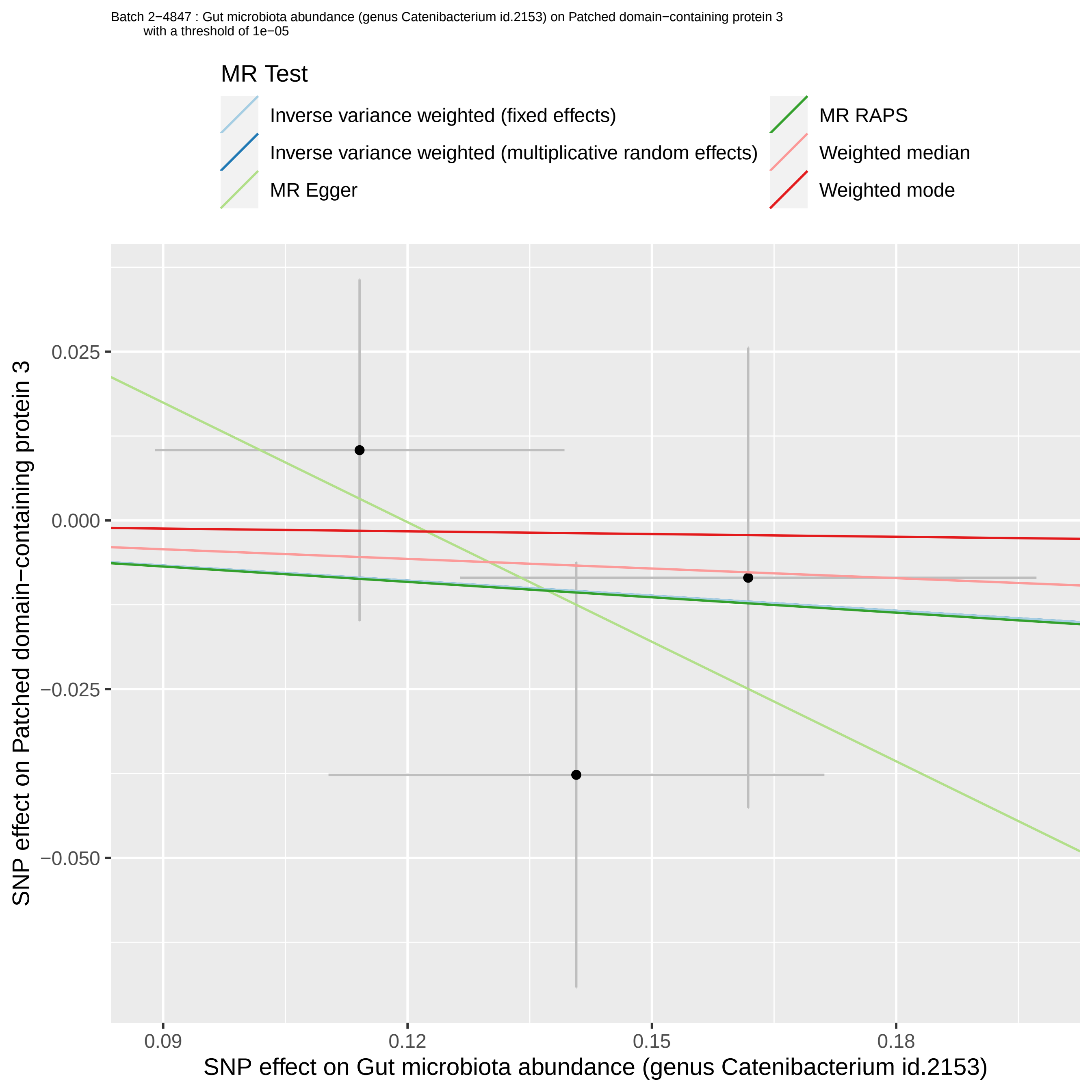

Batch 2−4847 : Gut microbiota abundance (genus Catenibacterium id.2153) on Patched domain−containing protein 3
with a threshold of 1e−05
MR Test
Inverse variance weighted (fixed effects)
Inverse variance weighted (multiplicative random effects)
MR Egger
MR RAPS
Weighted median
Weighted mode
0.025
0.000
−0.025
−0.050
0.09
0.12
0.15
0.18
SNP effect on Patched domain−containing protein 3
SNP effect on Gut microbiota abundance (genus Catenibacterium id.2153)

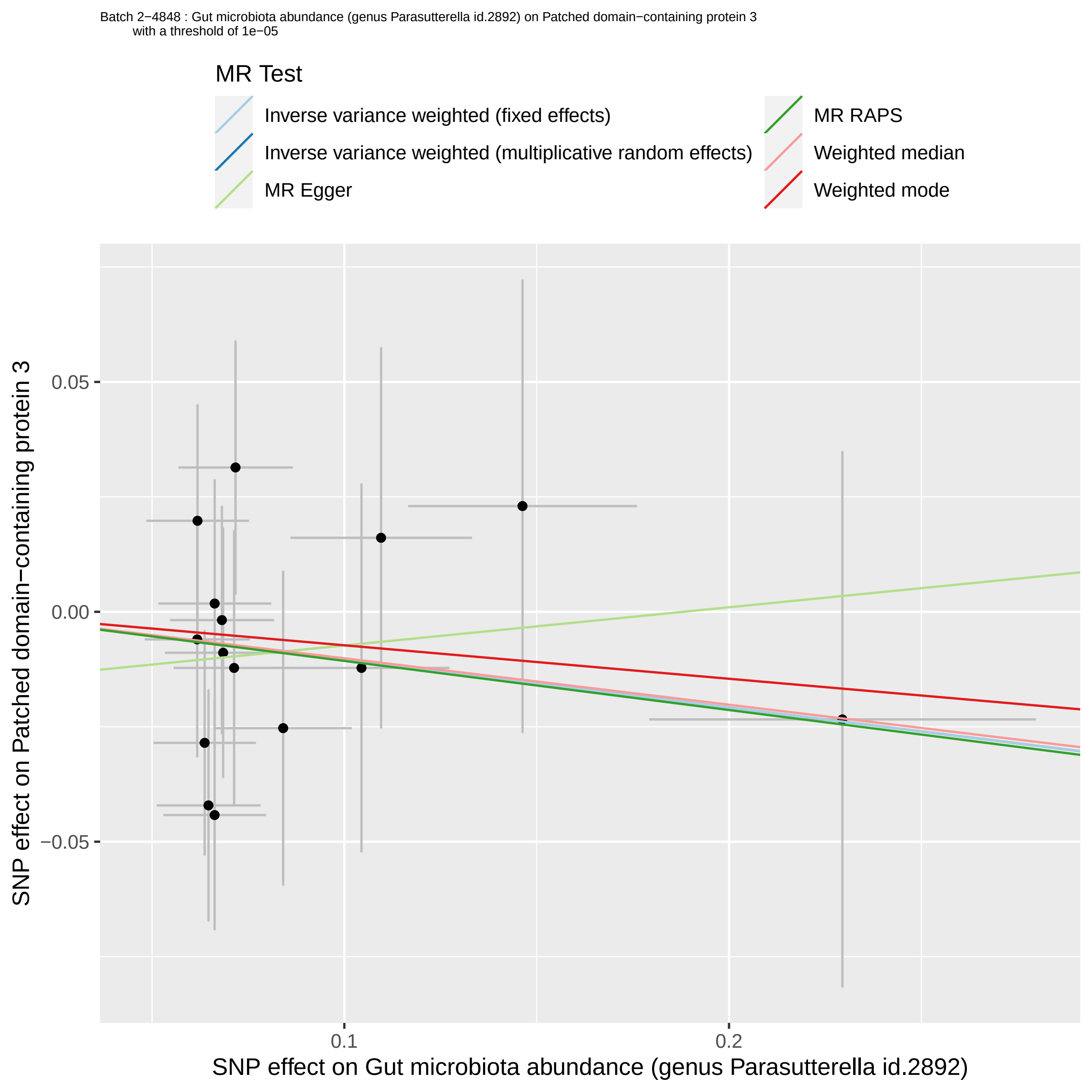
Batch 2−4848 : Gut microbiota abundance (genus Parasutterella id.2892) on Patched domain−containing protein 3
with a threshold of 1e−05
MR Test
Inverse variance weighted (fixed effects)
Inverse variance weighted (multiplicative random effects)
MR Egger
MR RAPS
Weighted median
Weighted mode
0.05
0.00
−0.05
0.1
0.2
SNP effect on Patched domain−containing protein 3
SNP effect on Gut microbiota abundance (genus Parasutterella id.2892)

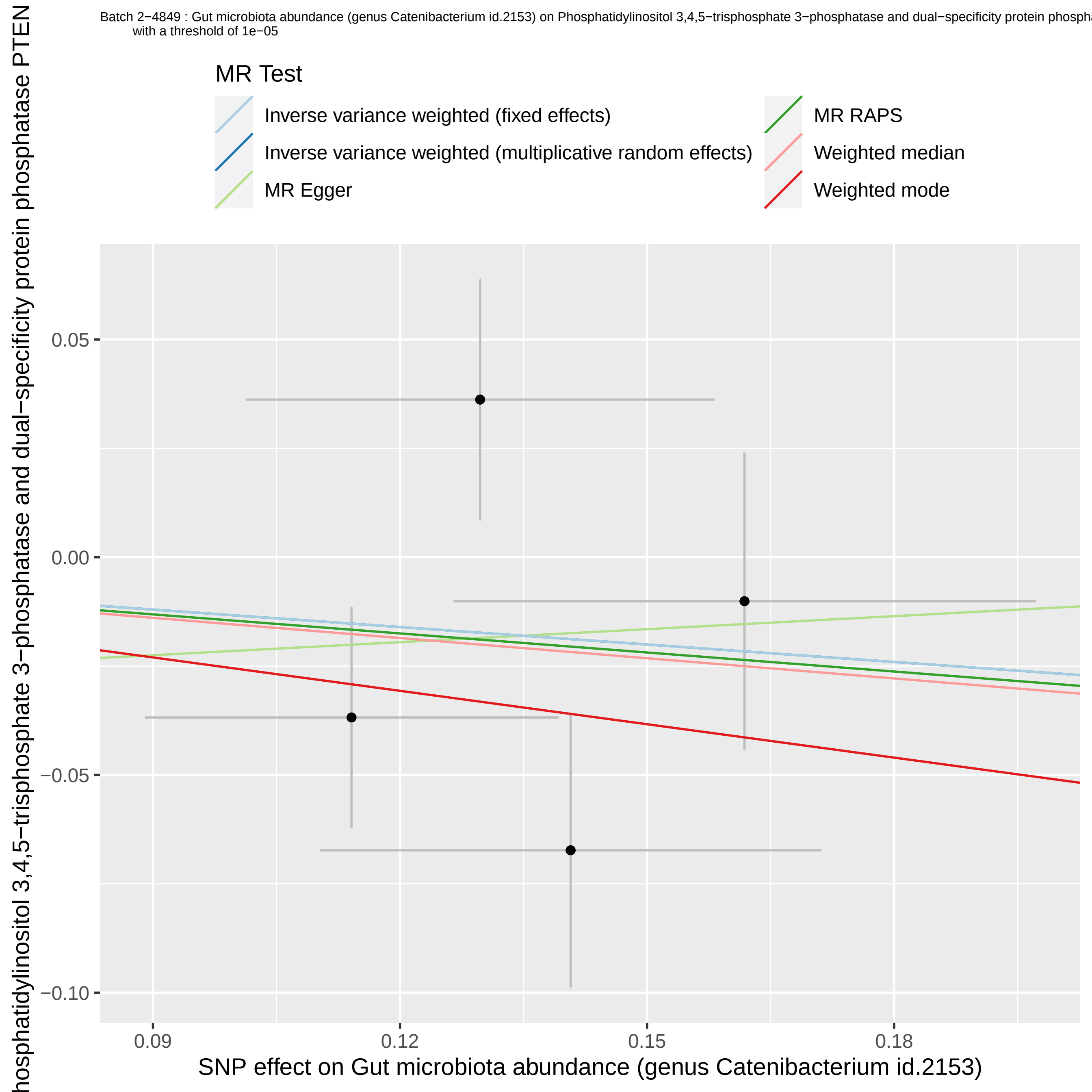
Batch 2−4849 : Gut microbiota abundance (genus Catenibacterium id.2153) on Phosphatidylinositol 3,4,5−trisphosphate 3−phosphatase and dual−specificity protein phospha
with a threshold of 1e−05
MR Test
Inverse variance weighted (fixed effects)
Inverse variance weighted (multiplicative random effects)
MR Egger
MR RAPS
Weighted median
Weighted mode
0.05
0.00
−0.05
−0.10
0.09
0.12
0.15
0.18
SNP effect on Gut microbiota abundance (genus Catenibacterium id.2153)
hosphatidylinositol 3,4,5−trisphosphate 3−phosphatase and dual−specificity protein phosphatase PTEN

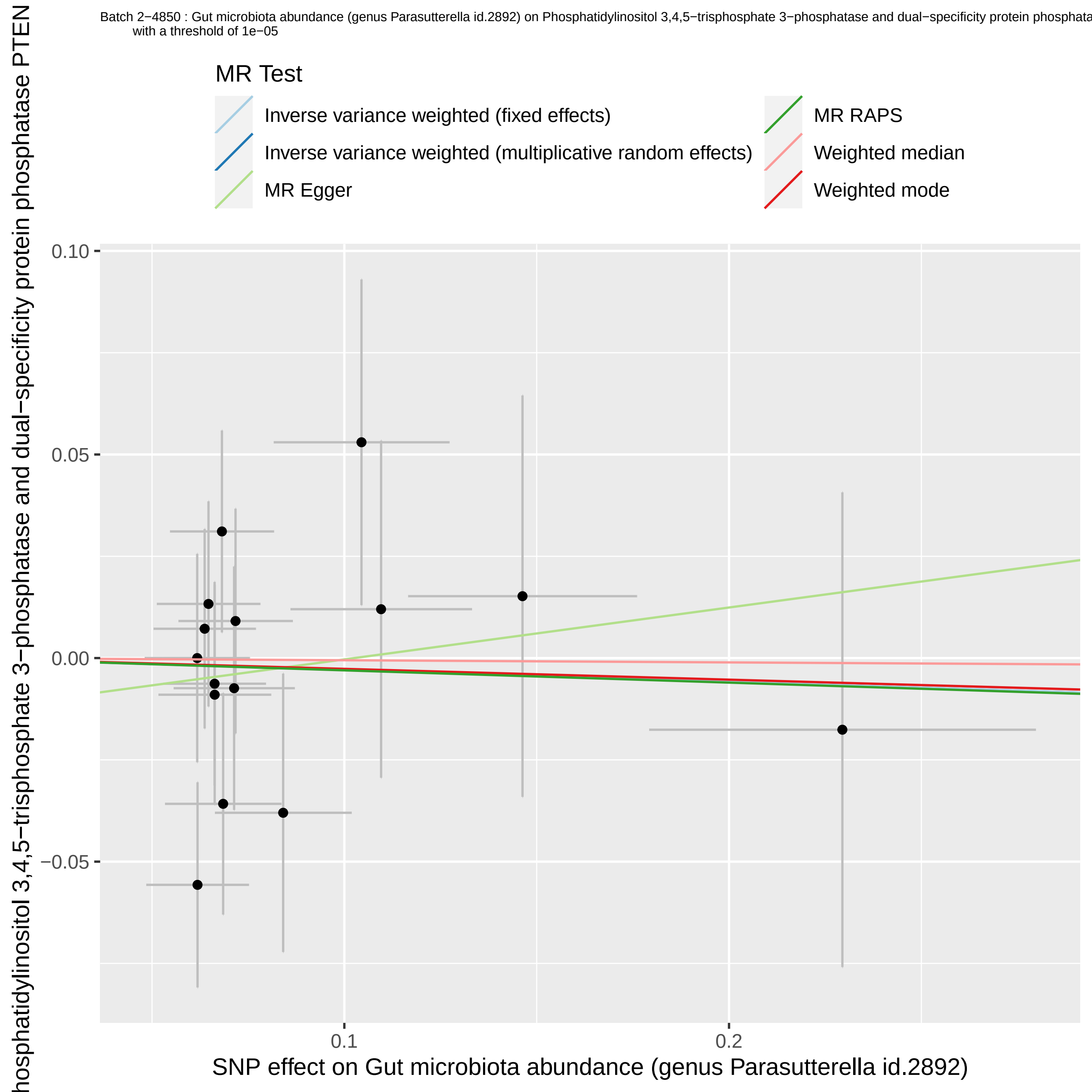

Batch 2−4850 : Gut microbiota abundance (genus Parasutterella id.2892) on Phosphatidylinositol 3,4,5−trisphosphate 3−phosphatase and dual−specificity protein phosphata
with a threshold of 1e−05
MR Test
Inverse variance weighted (fixed effects)
Inverse variance weighted (multiplicative random effects)
MR Egger
MR RAPS
Weighted median
Weighted mode
0.10
0.05
0.00
−0.05
0.1
0.2
SNP effect on Gut microbiota abundance (genus Parasutterella id.2892)
hosphatidylinositol 3,4,5−trisphosphate 3−phosphatase and dual−specificity protein phosphatase PTEN

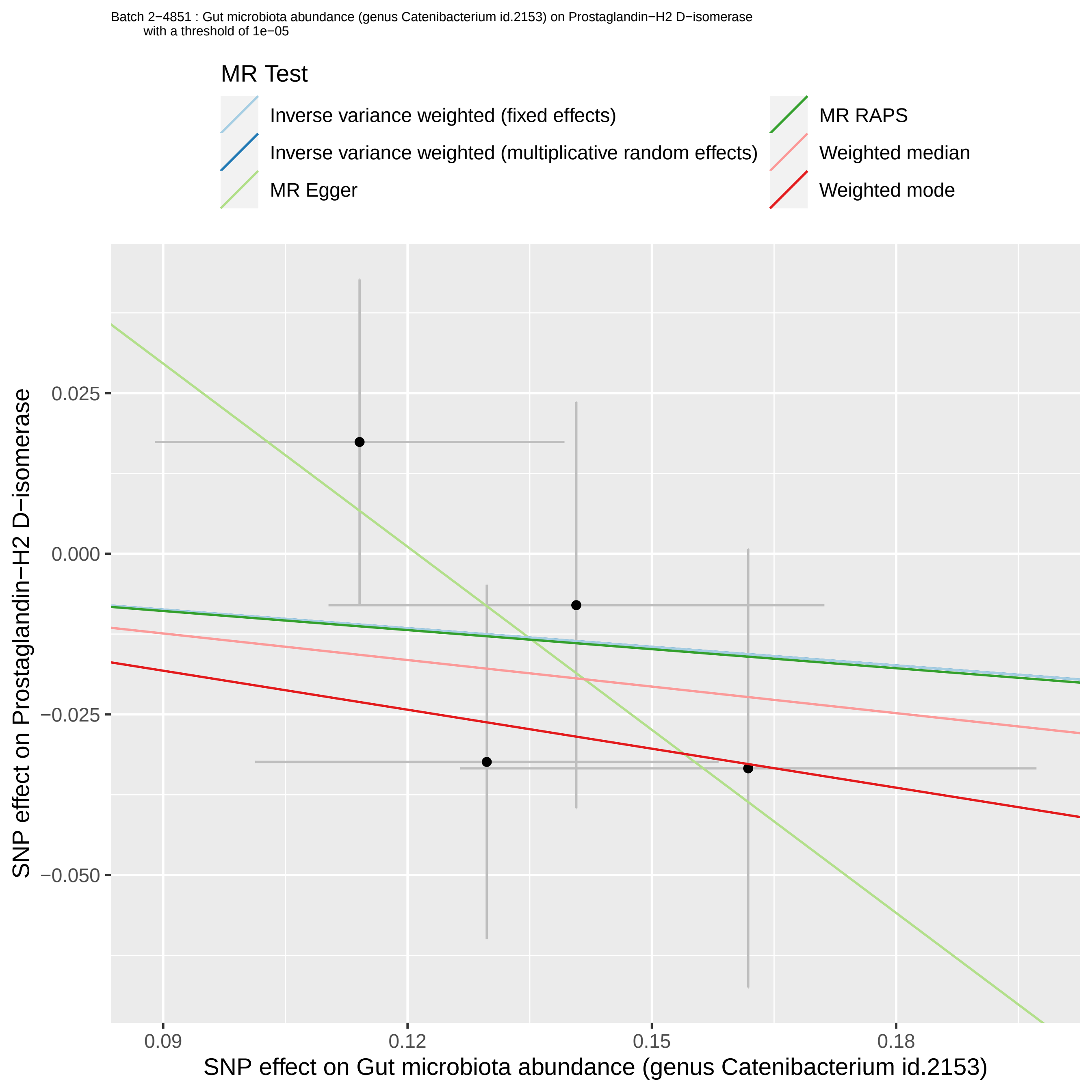
Batch 2−4851 : Gut microbiota abundance (genus Catenibacterium id.2153) on Prostaglandin−H2 D−isomerase
with a threshold of 1e−05
MR Test
Inverse variance weighted (fixed effects)
Inverse variance weighted (multiplicative random effects)
MR Egger
MR RAPS
Weighted median
Weighted mode
0.025
0.000
−0.025
−0.050
SNP effect on Prostaglandin−H2 D−isomerase
0.09
0.12
0.15
0.18
SNP effect on Gut microbiota abundance (genus Catenibacterium id.2153)

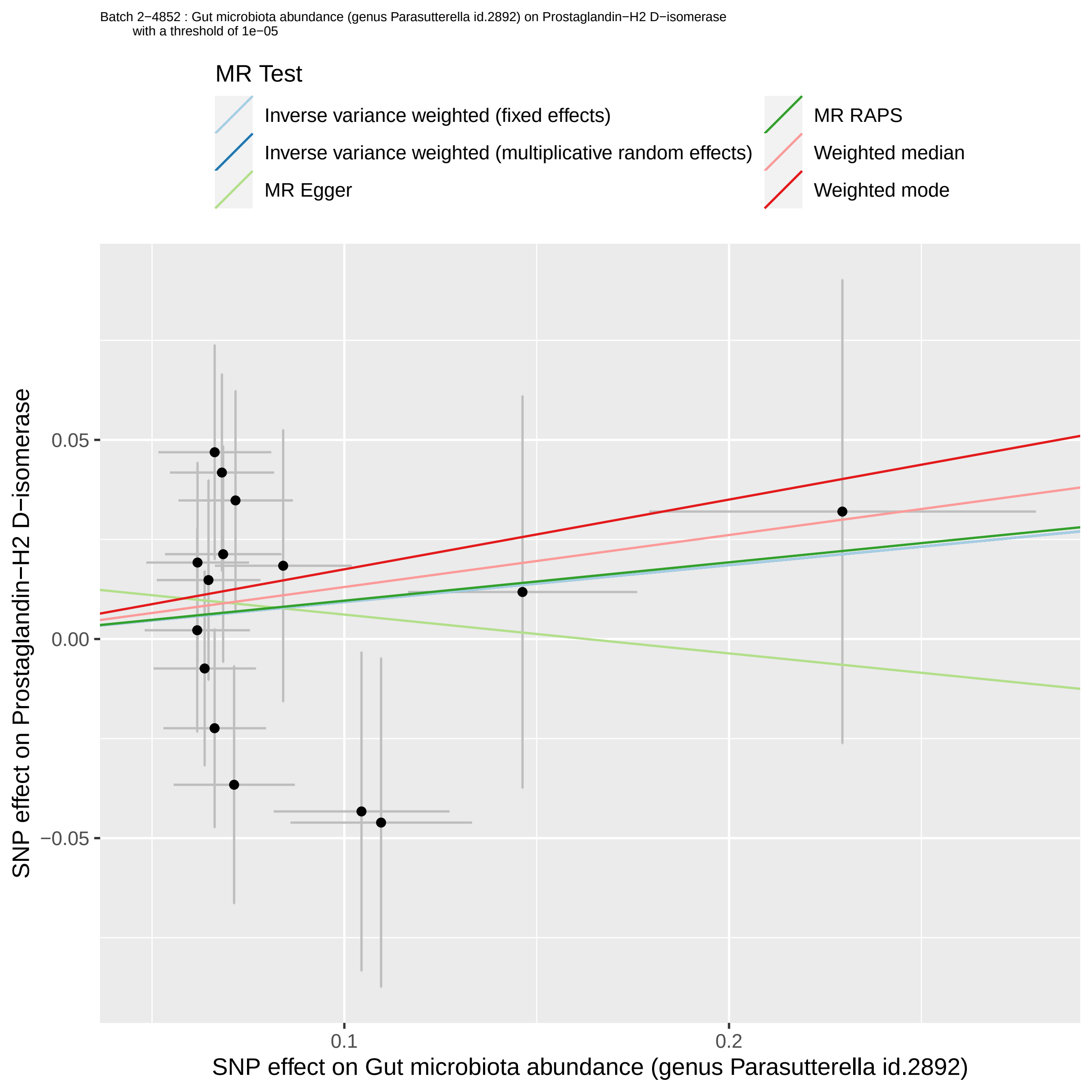
Batch 2−4852 : Gut microbiota abundance (genus Parasutterella id.2892) on Prostaglandin−H2 D−isomerase
with a threshold of 1e−05
MR Test
Inverse variance weighted (fixed effects)
Inverse variance weighted (multiplicative random effects)
MR Egger
MR RAPS
Weighted median
Weighted mode
SNP effect on Prostaglandin−H2 D−isomerase
0.05
0.00
−0.05
0.1
0.2
SNP effect on Gut microbiota abundance (genus Parasutterella id.2892)

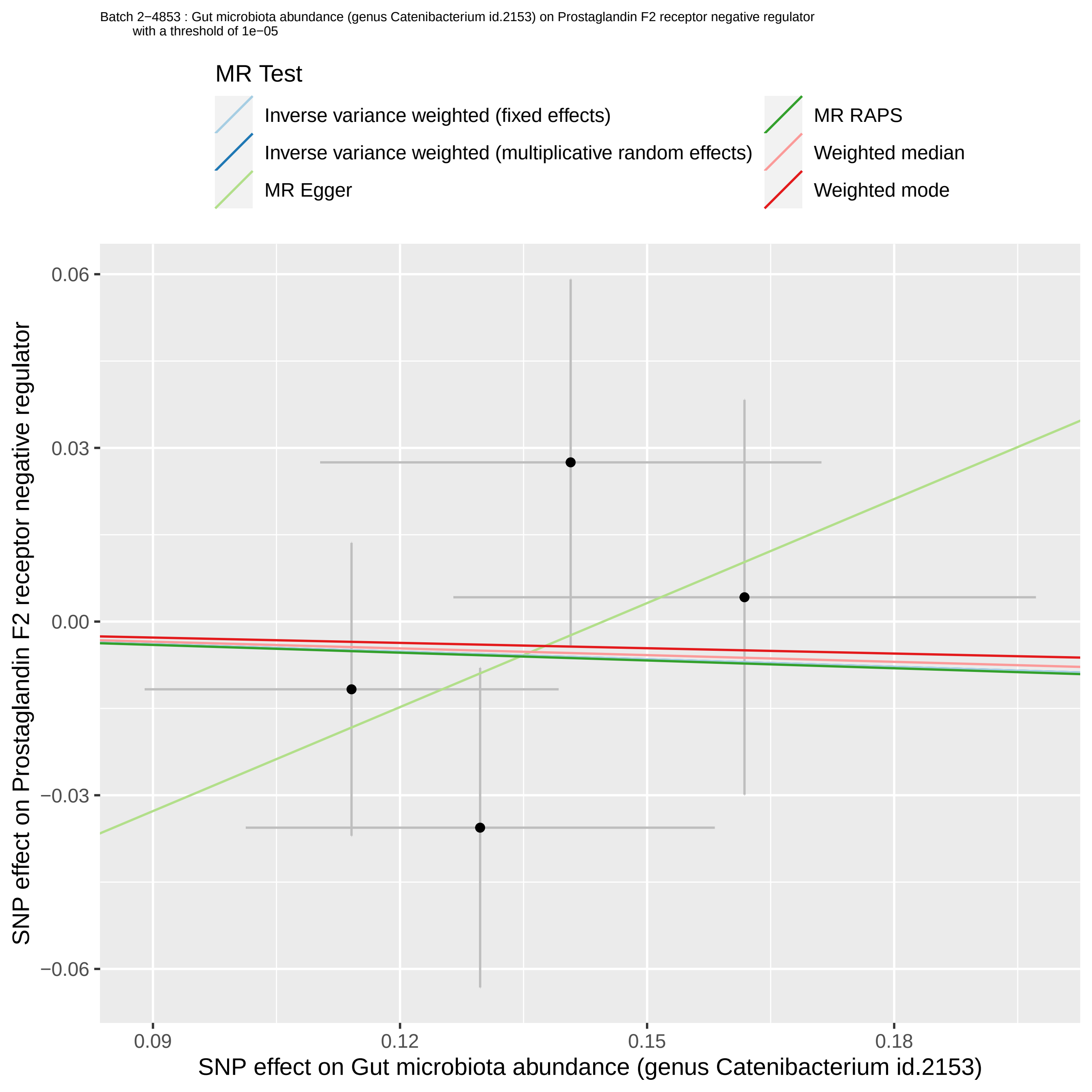

Batch 2−4853 : Gut microbiota abundance (genus Catenibacterium id.2153) on Prostaglandin F2 receptor negative regulator
with a threshold of 1e−05
MR Test
Inverse variance weighted (fixed effects)
Inverse variance weighted (multiplicative random effects)
MR Egger
MR RAPS
Weighted median
Weighted mode
0.06
0.03
0.00
−0.03
−0.06
0.09
0.12
0.15
0.18
SNP effect on Prostaglandin F2 receptor negative regulator
SNP effect on Gut microbiota abundance (genus Catenibacterium id.2153)

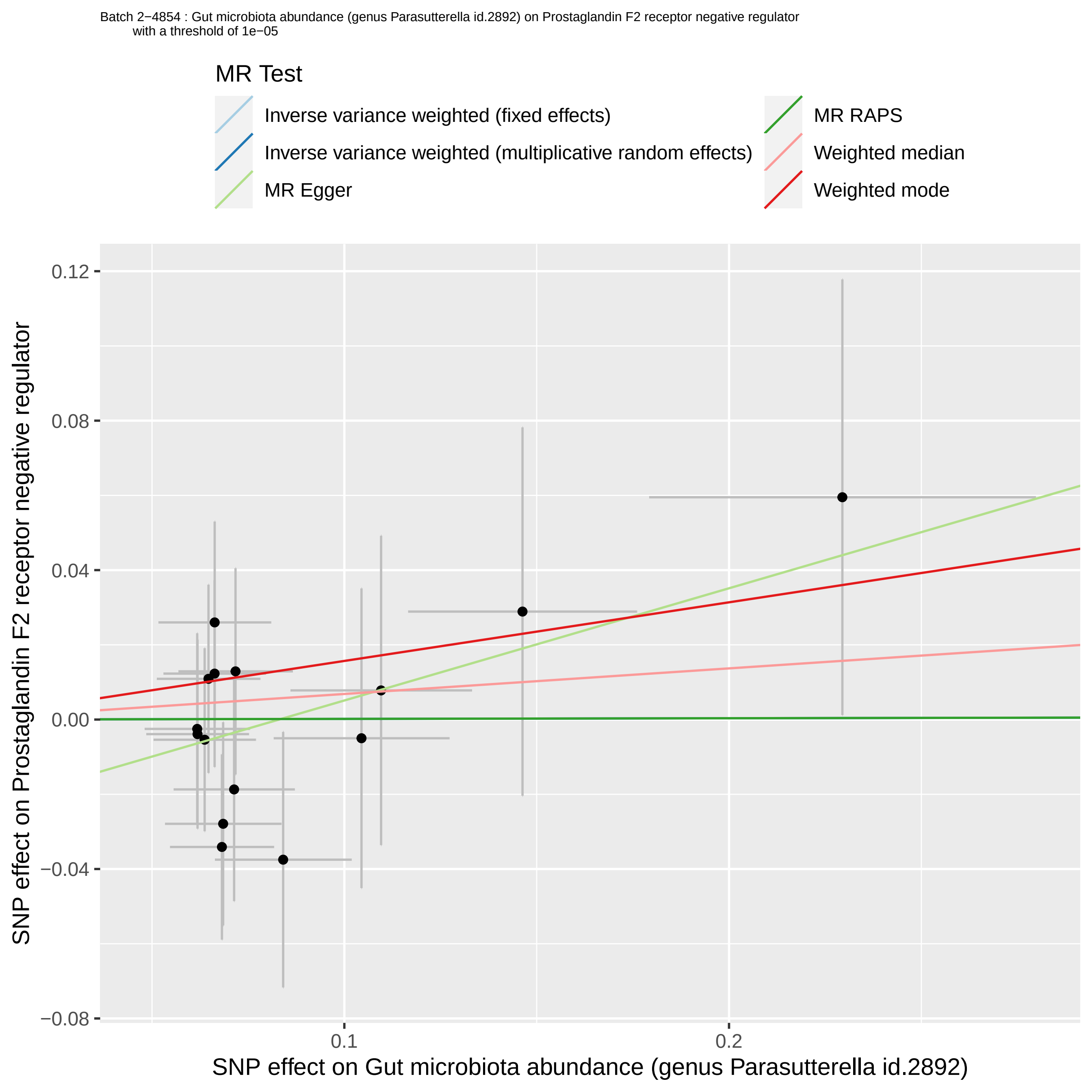
Batch 2−4854 : Gut microbiota abundance (genus Parasutterella id.2892) on Prostaglandin F2 receptor negative regulator
with a threshold of 1e−05
MR Test
Inverse variance weighted (fixed effects)
Inverse variance weighted (multiplicative random effects)
MR Egger
MR RAPS
Weighted median
Weighted mode
0.12
0.08
0.04
0.00
−0.04
−0.08
0.1
0.2
SNP effect on Prostaglandin F2 receptor negative regulator
SNP effect on Gut microbiota abundance (genus Parasutterella id.2892)

Batch 2−4855 : Gut microbiota abundance (genus Catenibacterium id.2153) on Prostaglandin reductase 1
with a threshold of 1e−05

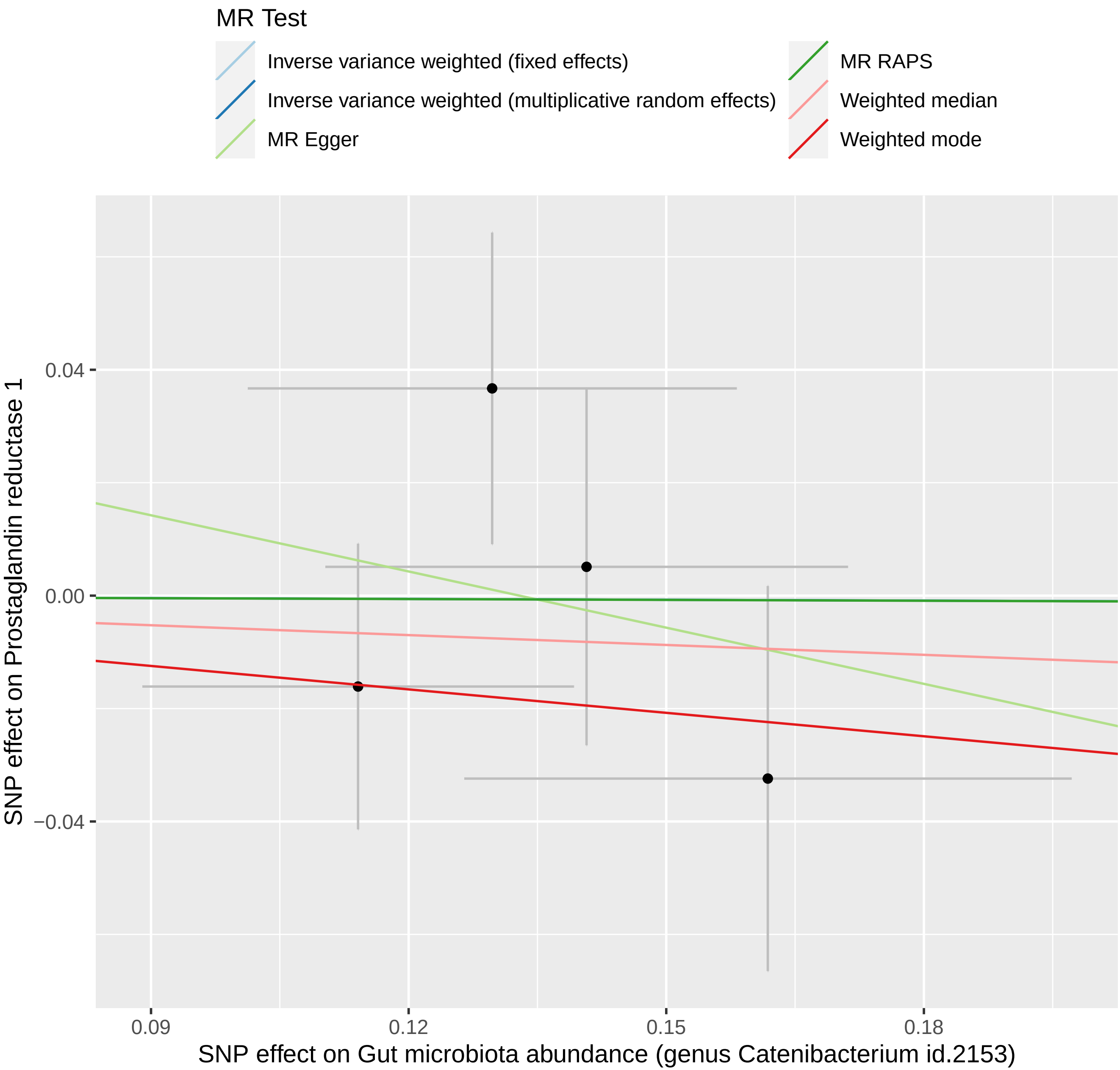

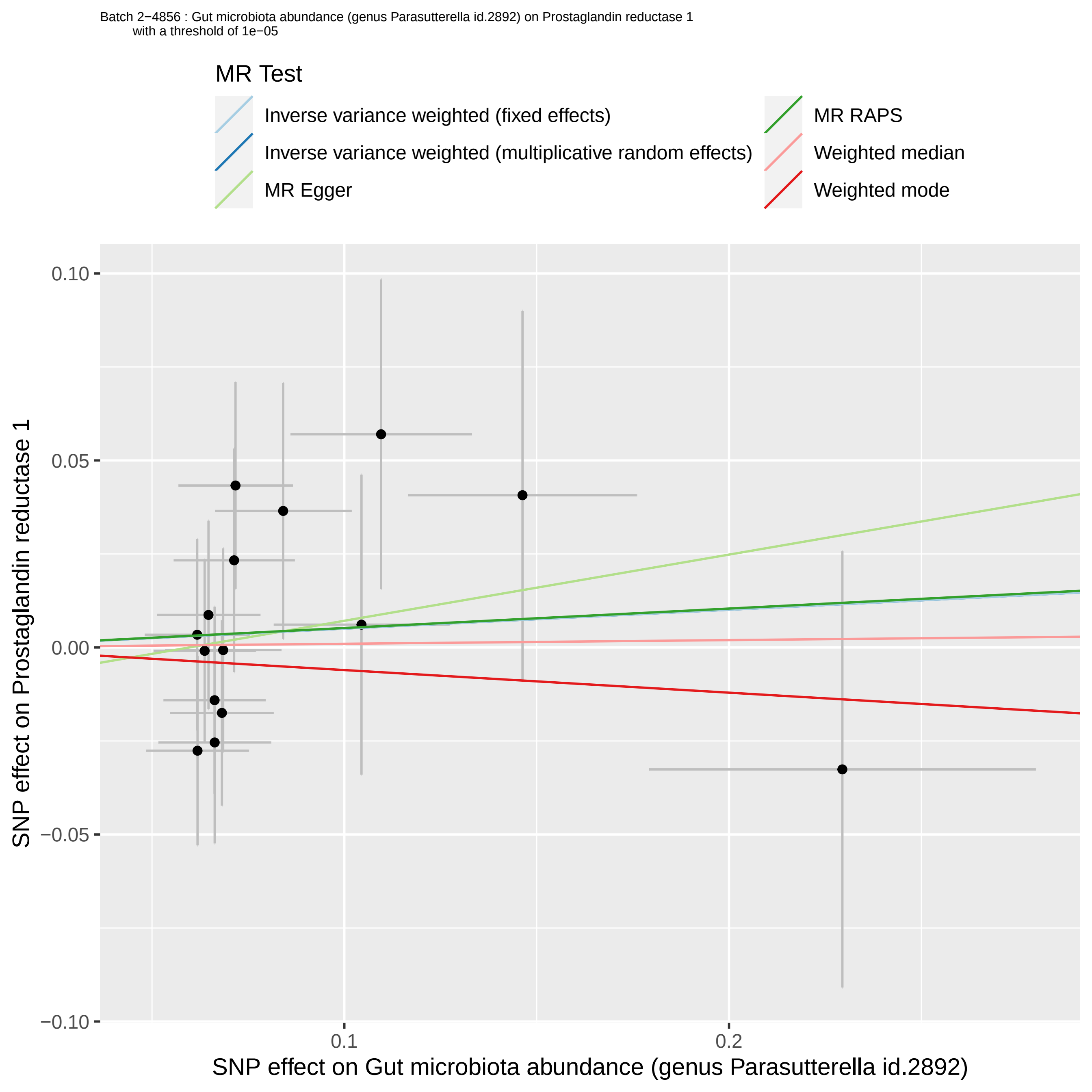
Batch 2−4856 : Gut microbiota abundance (genus Parasutterella id.2892) on Prostaglandin reductase 1
with a threshold of 1e−05
MR Test
Inverse variance weighted (fixed effects)
Inverse variance weighted (multiplicative random effects)
MR Egger
MR RAPS
Weighted median
Weighted mode
0.10
0.05
0.00
−0.05
−0.10
0.1
0.2
SNP effect on Prostaglandin reductase 1
SNP effect on Gut microbiota abundance (genus Parasutterella id.2892)

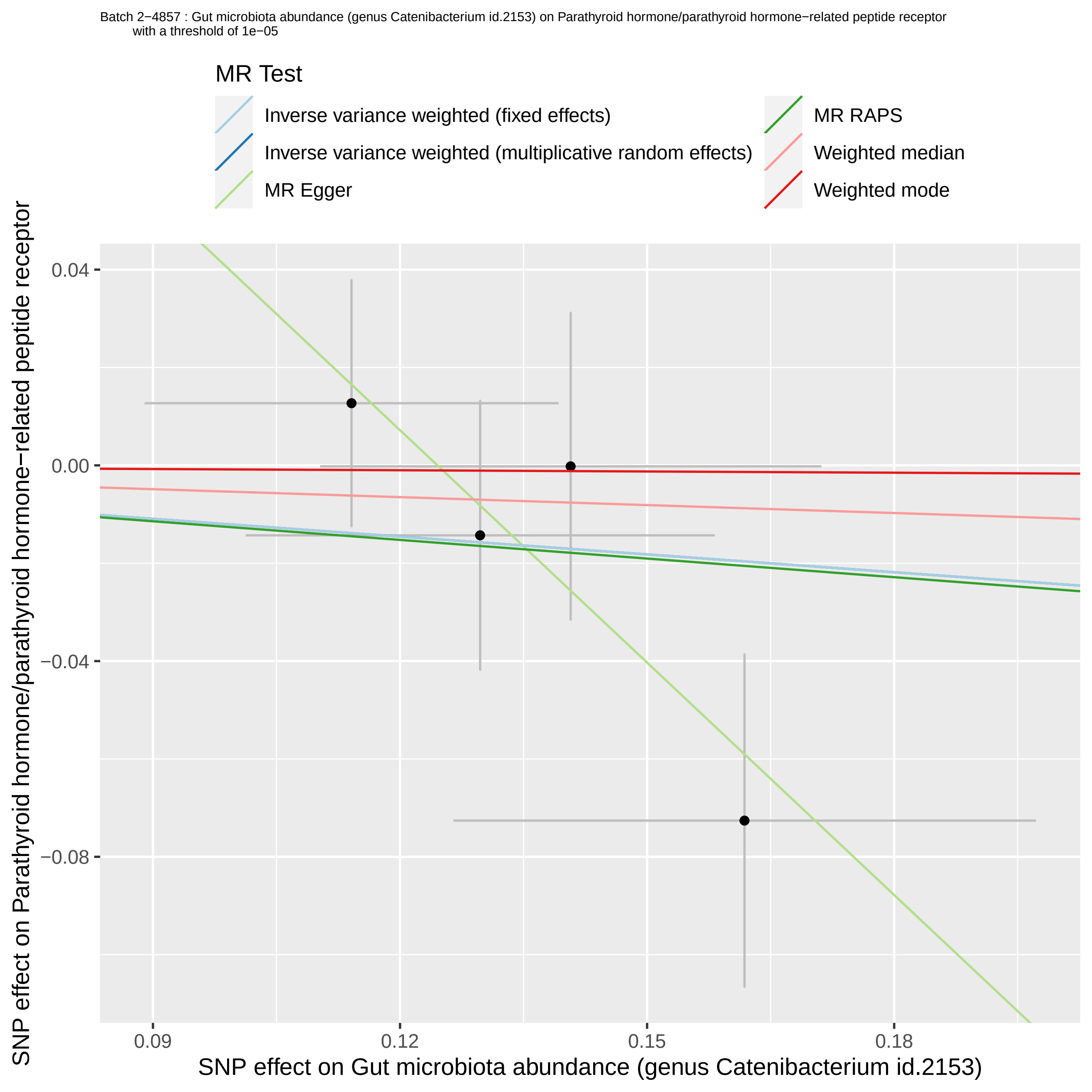

Batch 2−4857 : Gut microbiota abundance (genus Catenibacterium id.2153) on Parathyroid hormone/parathyroid hormone−related peptide receptor
with a threshold of 1e−05
MR Test
Inverse variance weighted (fixed effects)
Inverse variance weighted (multiplicative random effects)
MR Egger
MR RAPS
Weighted median
Weighted mode
0.04
0.00
−0.04
−0.08
0.09
0.12
0.15
0.18
SNP effect on Parathyroid hormone/parathyroid hormone−related peptide receptor
SNP effect on Gut microbiota abundance (genus Catenibacterium id.2153)

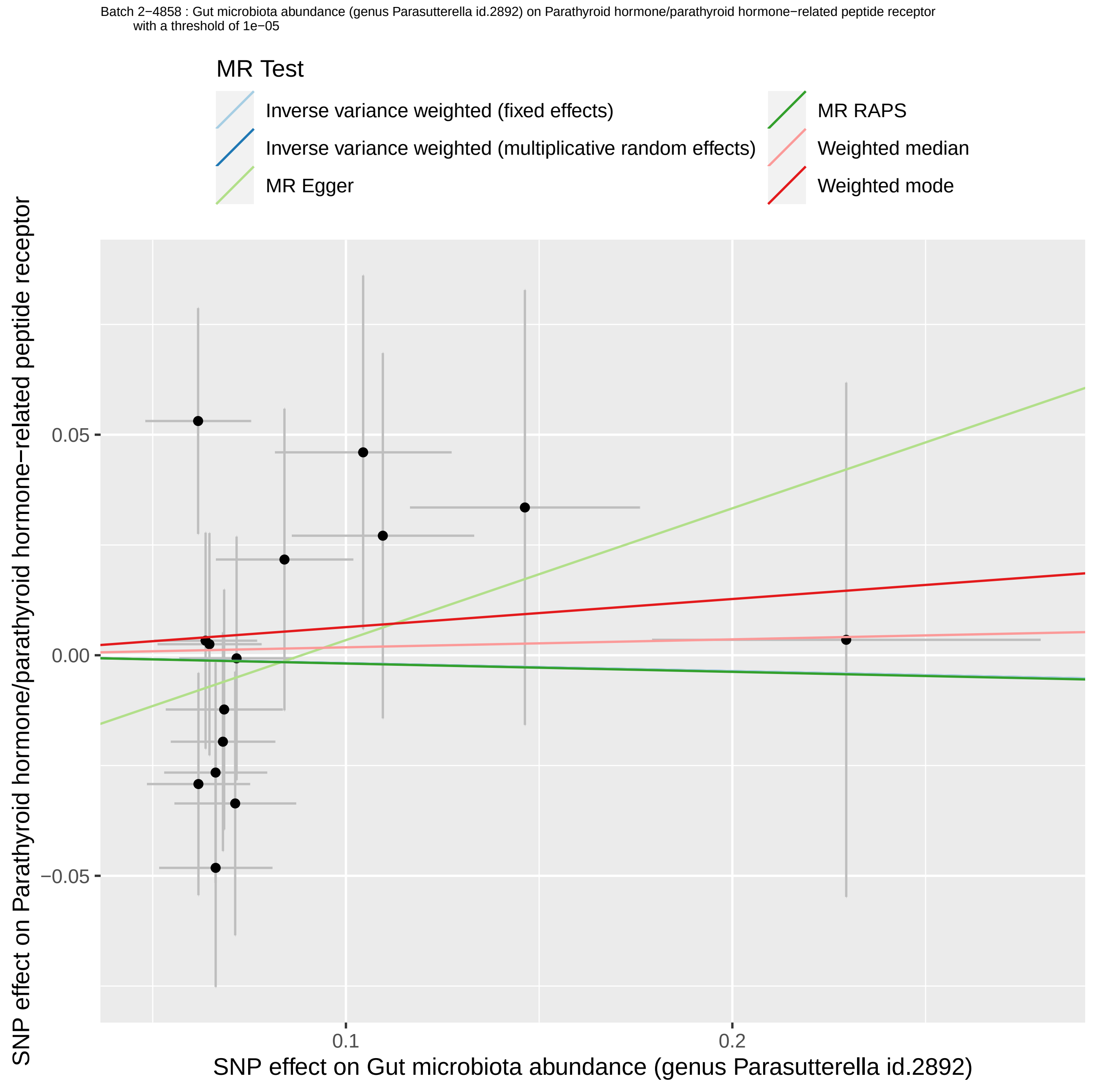
Batch 2−4858 : Gut microbiota abundance (genus Parasutterella id.2892) on Parathyroid hormone/parathyroid hormone−related peptide receptor
with a threshold of 1e−05
MR Test
Inverse variance weighted (fixed effects)
Inverse variance weighted (multiplicative random effects)
MR Egger
MR RAPS
Weighted median
Weighted mode
0.05
0.00
−0.05
0.1
0.2
SNP effect on Gut microbiota abundance (genus Parasutterella id.2892)
SNP effect on Parathyroid hormone/parathyroid hormone−related peptide receptor

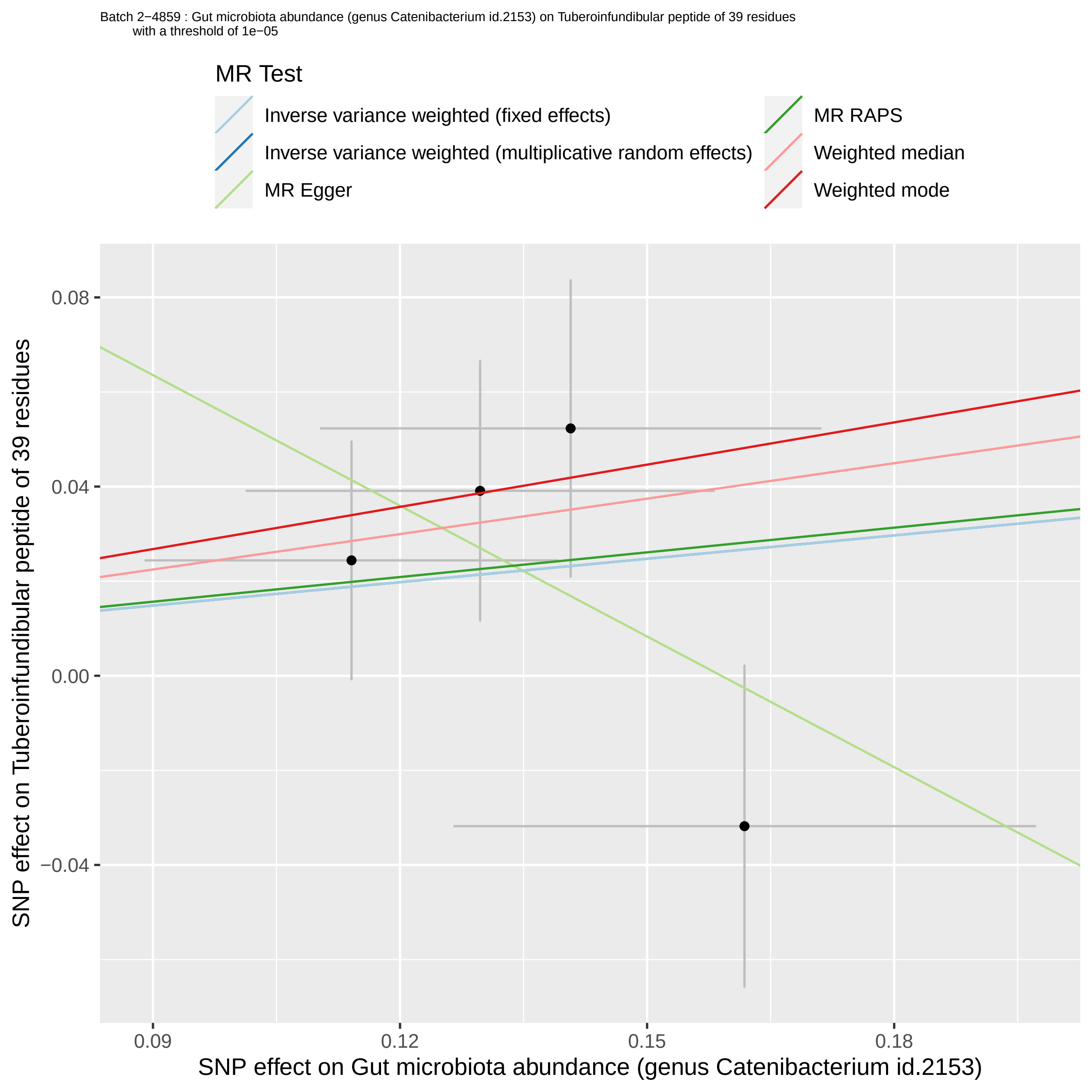

Batch 2−4859 : Gut microbiota abundance (genus Catenibacterium id.2153) on Tuberoinfundibular peptide of 39 residues
with a threshold of 1e−05
MR Test
Inverse variance weighted (fixed effects)
Inverse variance weighted (multiplicative random effects)
MR Egger
MR RAPS
Weighted median
Weighted mode
0.08
0.04
0.00
−0.04
0.09
0.12
0.15
0.18
SNP effect on Tuberoinfundibular peptide of 39 residues
SNP effect on Gut microbiota abundance (genus Catenibacterium id.2153)

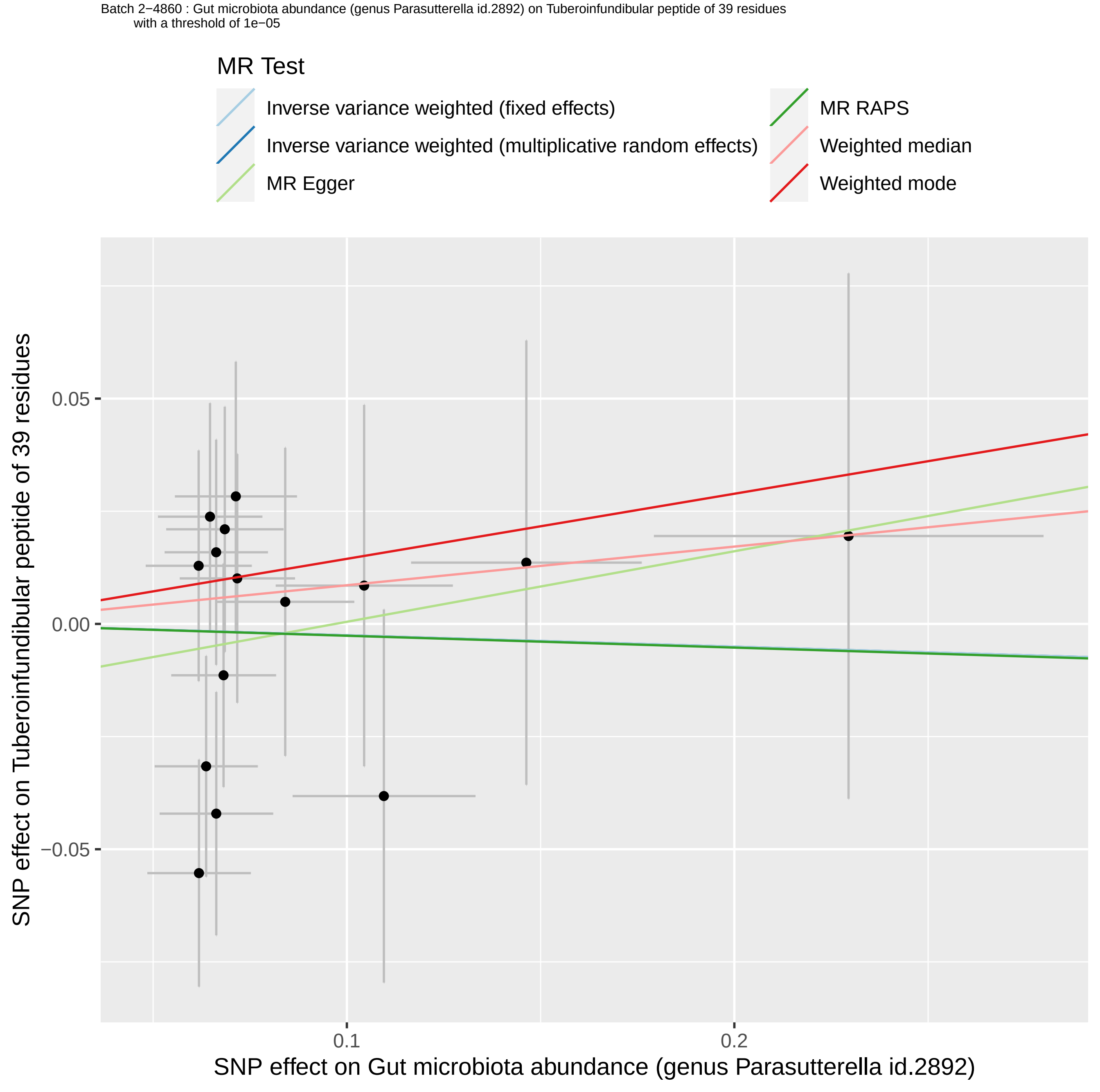
Batch 2−4860 : Gut microbiota abundance (genus Parasutterella id.2892) on Tuberoinfundibular peptide of 39 residues
with a threshold of 1e−05
MR Test
Inverse variance weighted (fixed effects)
Inverse variance weighted (multiplicative random effects)
MR Egger
MR RAPS
Weighted median
Weighted mode
0.05
0.00
−0.05
0.1
0.2
SNP effect on Tuberoinfundibular peptide of 39 residues
SNP effect on Gut microbiota abundance (genus Parasutterella id.2892)

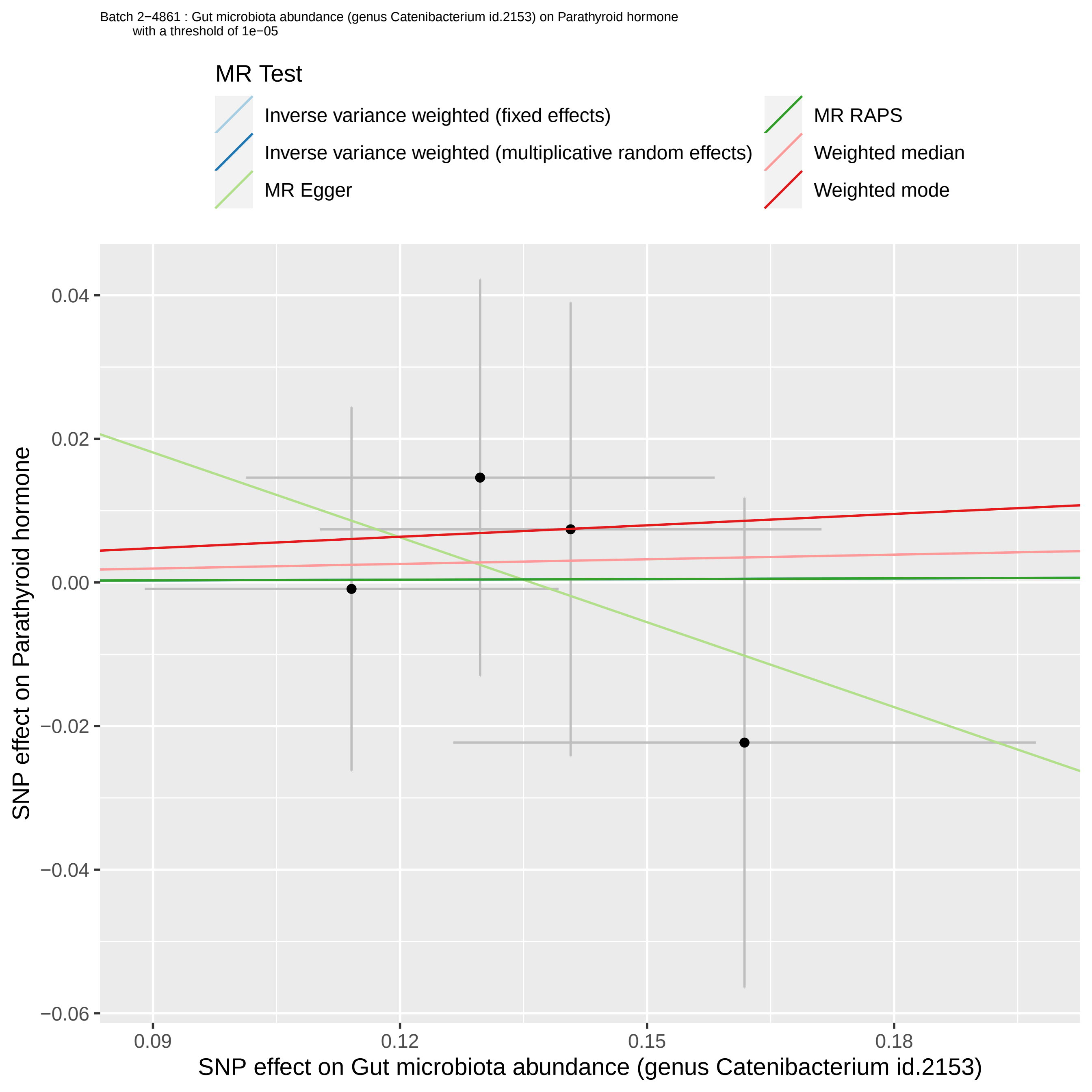
Batch 2−4861 : Gut microbiota abundance (genus Catenibacterium id.2153) on Parathyroid hormone
with a threshold of 1e−05
MR Test
Inverse variance weighted (fixed effects)
Inverse variance weighted (multiplicative random effects)
MR Egger
MR RAPS
Weighted median
Weighted mode
0.04
0.02
0.00
−0.02
−0.04
−0.06
0.09
0.12
0.15
0.18
SNP effect on Parathyroid hormone
SNP effect on Gut microbiota abundance (genus Catenibacterium id.2153)

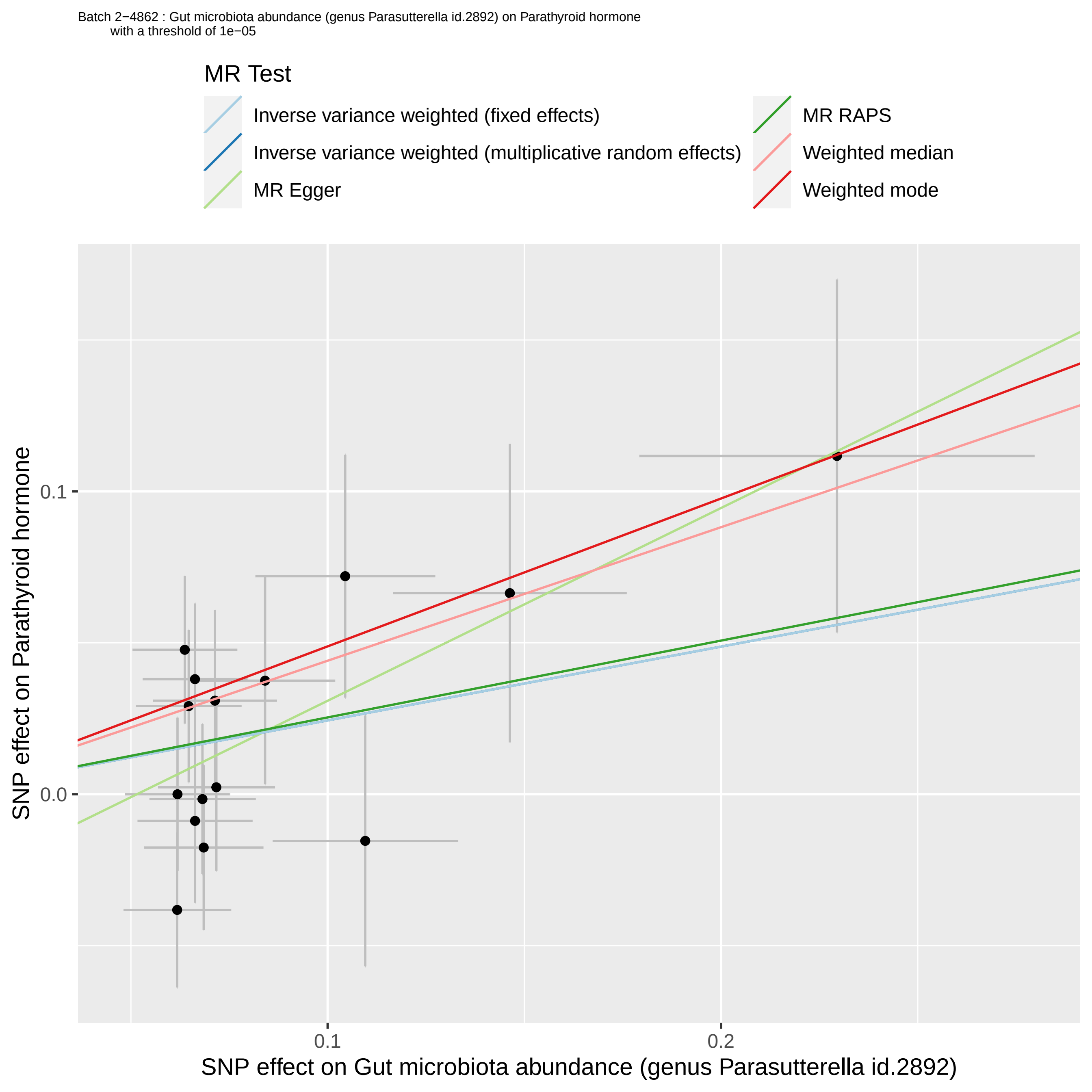
Batch 2−4862 : Gut microbiota abundance (genus Parasutterella id.2892) on Parathyroid hormone
with a threshold of 1e−05
MR Test
Inverse variance weighted (fixed effects)
Inverse variance weighted (multiplicative random effects)
MR Egger
MR RAPS
Weighted median
Weighted mode
SNP effect on Parathyroid hormone
0.1
0.0
0.1
0.2
SNP effect on Gut microbiota abundance (genus Parasutterella id.2892)

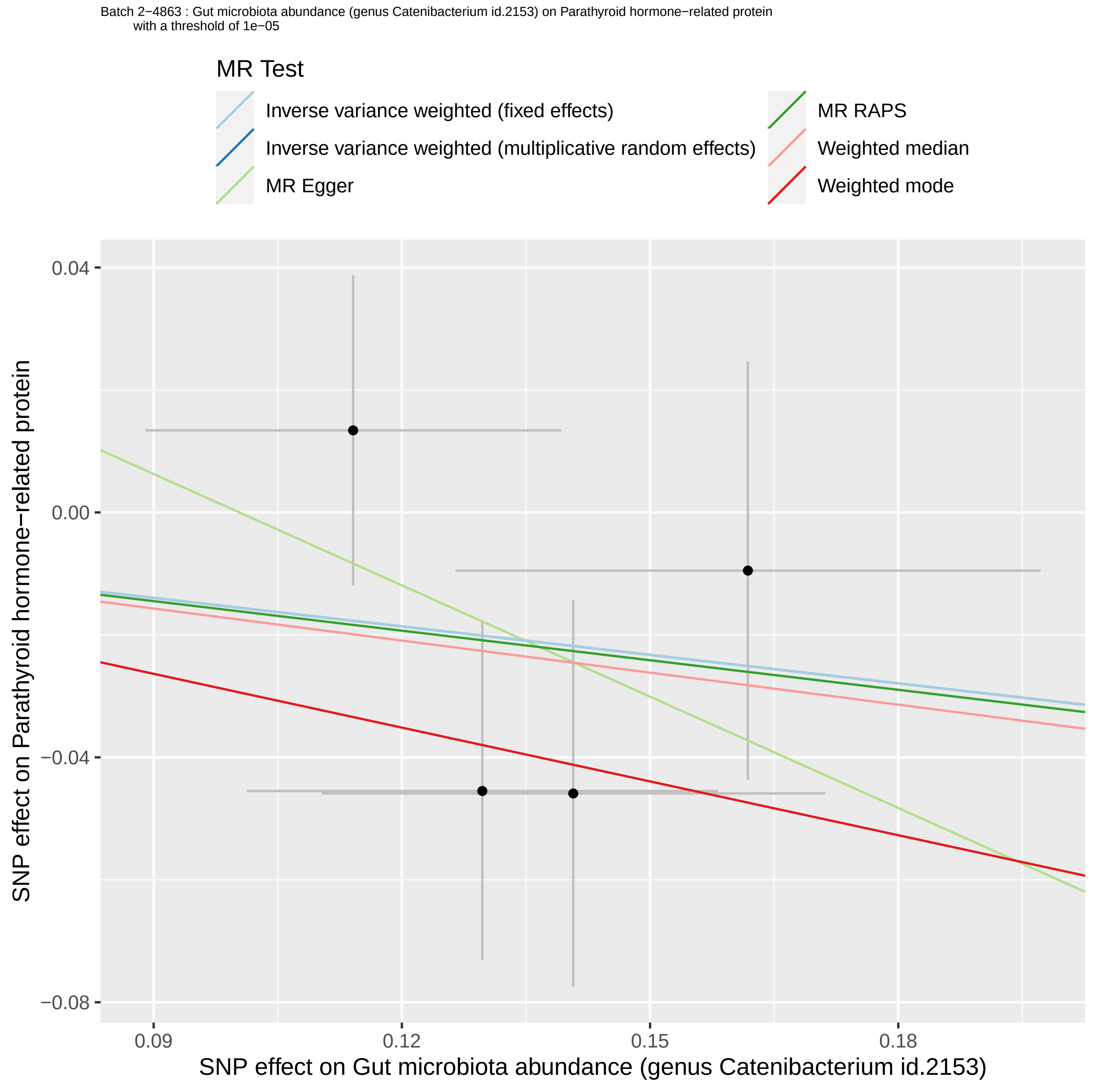

Batch 2−4863 : Gut microbiota abundance (genus Catenibacterium id.2153) on Parathyroid hormone−related protein
with a threshold of 1e−05
MR Test
Inverse variance weighted (fixed effects)
Inverse variance weighted (multiplicative random effects)
MR Egger
MR RAPS
Weighted median
Weighted mode
0.04
0.00
−0.04
−0.08
0.09
0.12
0.15
0.18
SNP effect on Parathyroid hormone−related protein
SNP effect on Gut microbiota abundance (genus Catenibacterium id.2153)

Batch 2−4864 : Gut microbiota abundance (genus Parasutterella id.2892) on Parathyroid hormone−related protein with a threshold of 1e−05

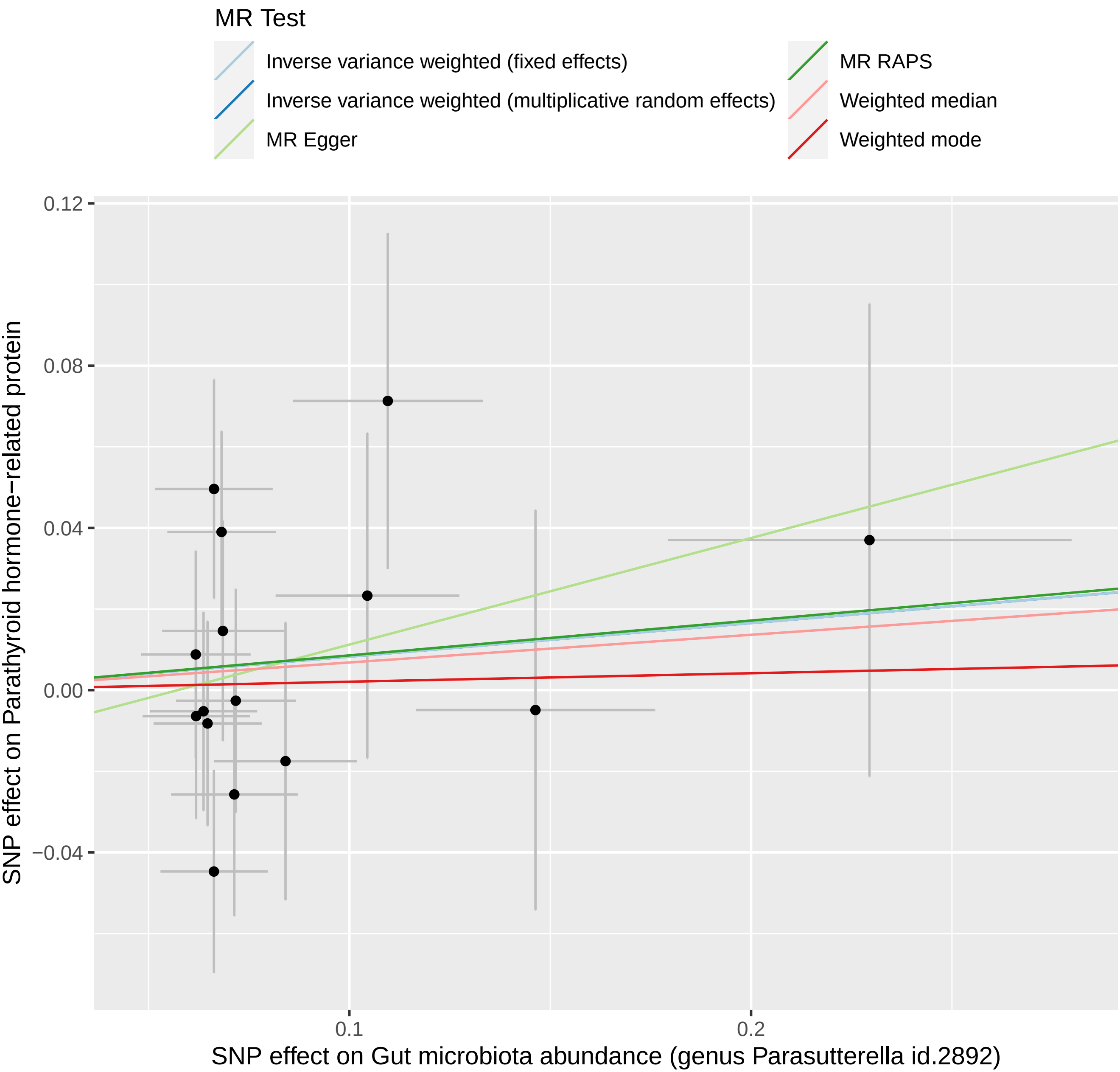

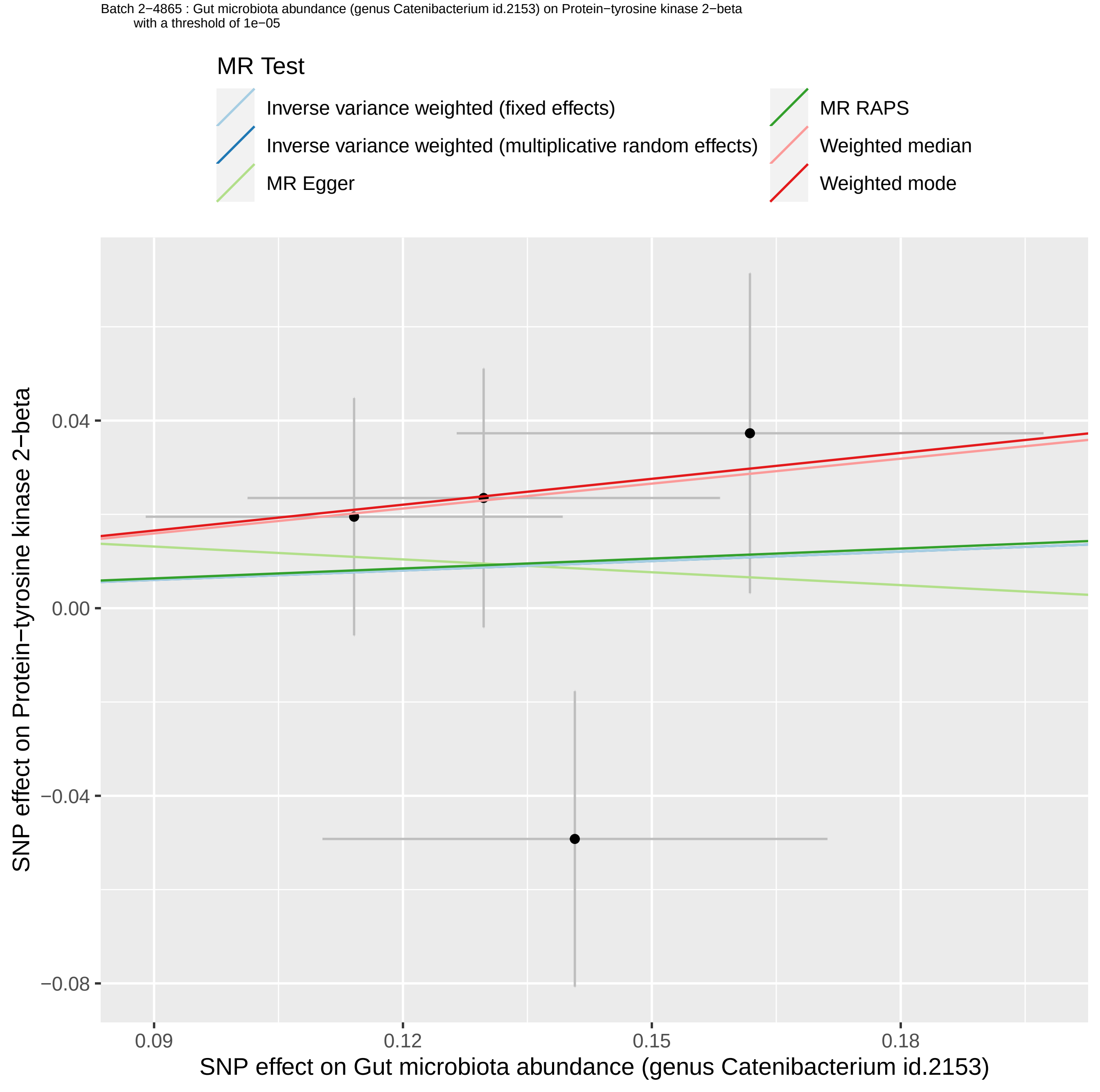

Batch 2−4865 : Gut microbiota abundance (genus Catenibacterium id.2153) on Protein−tyrosine kinase 2−beta
with a threshold of 1e−05
MR Test
Inverse variance weighted (fixed effects)
Inverse variance weighted (multiplicative random effects)
MR Egger
MR RAPS
Weighted median
Weighted mode
0.04
0.00
−0.04
−0.08
0.09
0.12
0.15
0.18
SNP effect on Protein−tyrosine kinase 2−beta
SNP effect on Gut microbiota abundance (genus Catenibacterium id.2153)

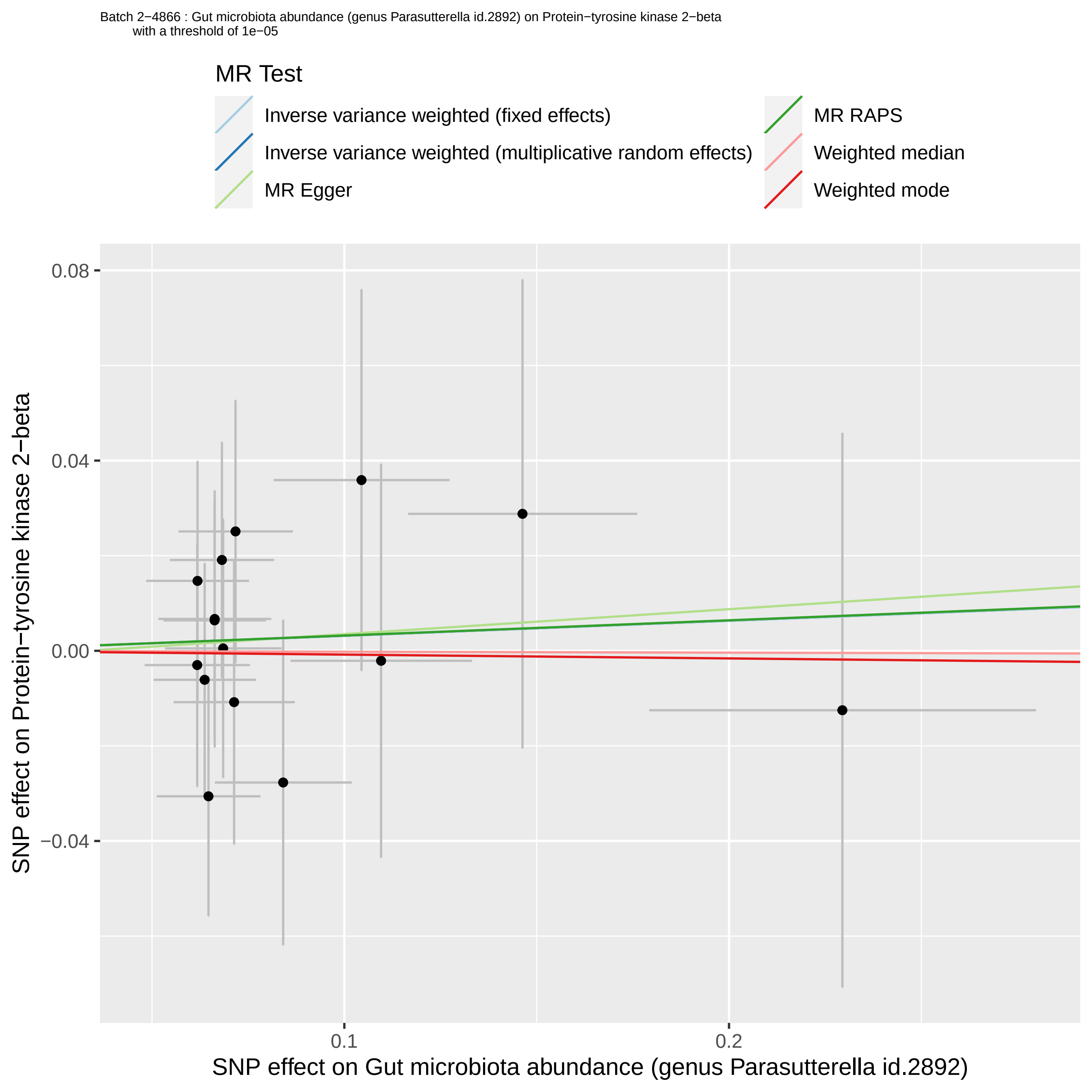
Batch 2−4866 : Gut microbiota abundance (genus Parasutterella id.2892) on Protein−tyrosine kinase 2−beta
with a threshold of 1e−05
MR Test
Inverse variance weighted (fixed effects)
Inverse variance weighted (multiplicative random effects)
MR Egger
MR RAPS
Weighted median
Weighted mode
0.08
0.04
0.00
−0.04
0.1
0.2
SNP effect on Protein−tyrosine kinase 2−beta
SNP effect on Gut microbiota abundance (genus Parasutterella id.2892)

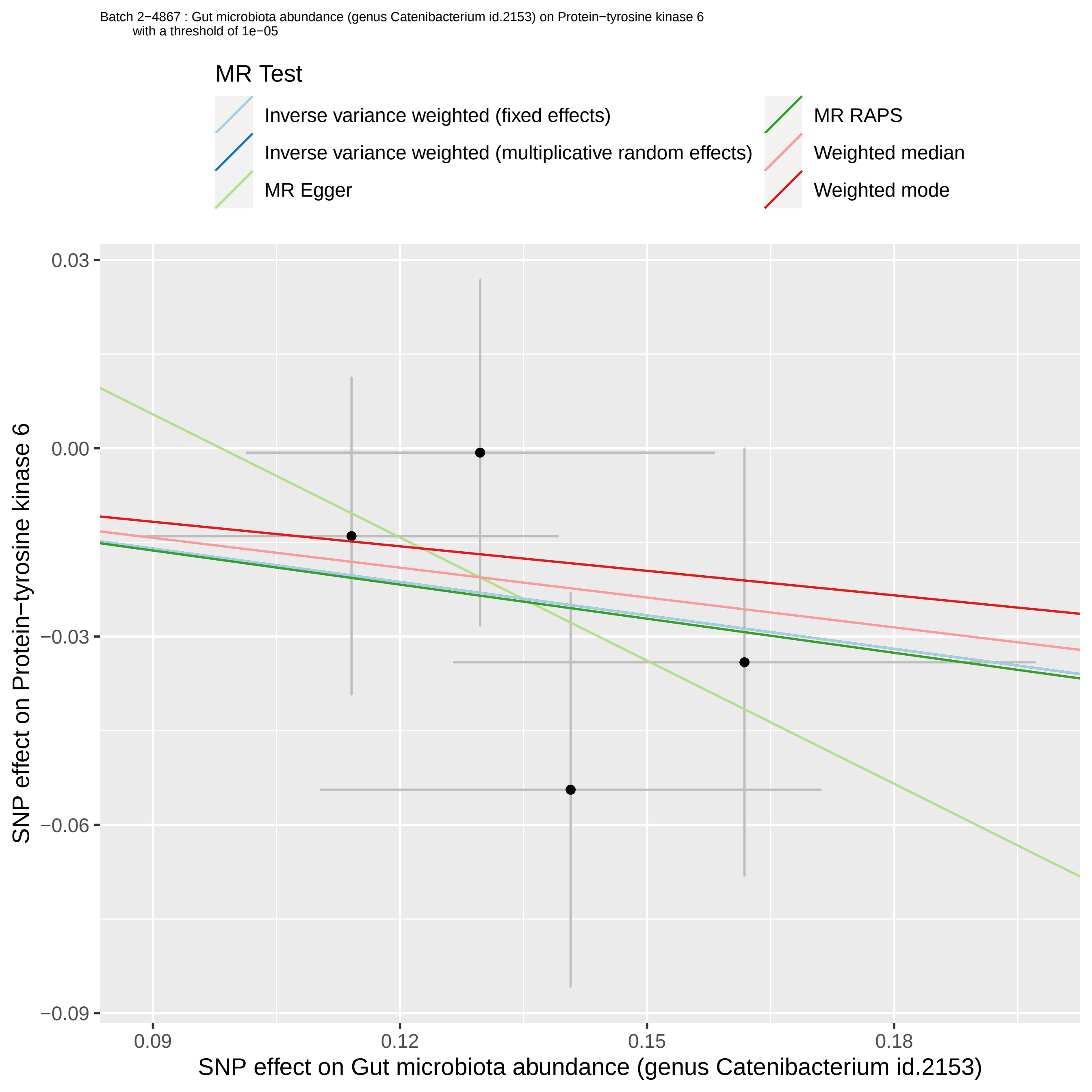
Batch 2−4867 : Gut microbiota abundance (genus Catenibacterium id.2153) on Protein−tyrosine kinase 6
with a threshold of 1e−05
MR Test
Inverse variance weighted (fixed effects)
Inverse variance weighted (multiplicative random effects)
MR Egger
MR RAPS
Weighted median
Weighted mode
0.03
0.00
−0.03
−0.06
−0.09
0.09
0.12
0.15
0.18
SNP effect on Protein−tyrosine kinase 6
SNP effect on Gut microbiota abundance (genus Catenibacterium id.2153)

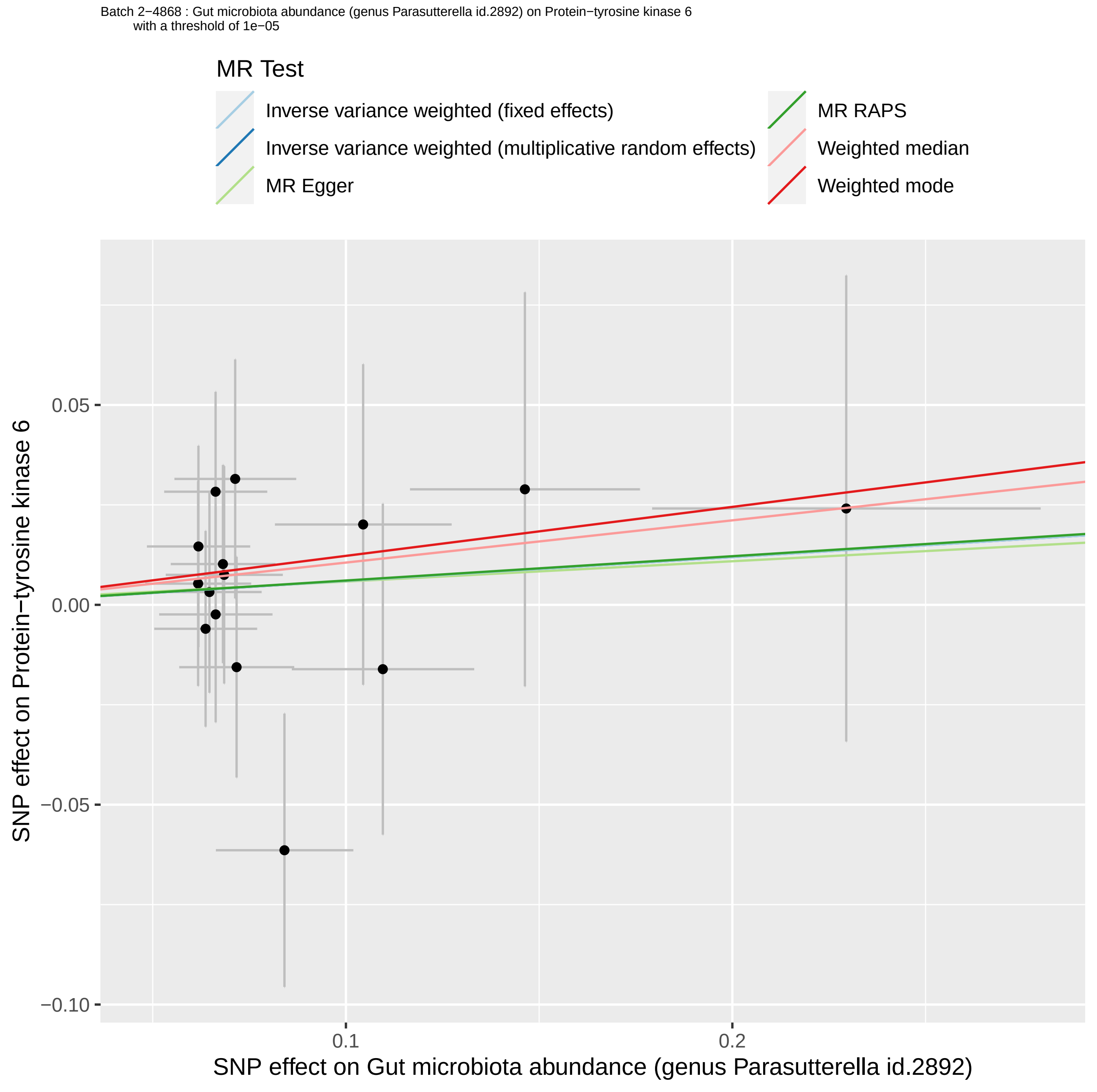
Batch 2−4868 : Gut microbiota abundance (genus Parasutterella id.2892) on Protein−tyrosine kinase 6
with a threshold of 1e−05
MR Test
Inverse variance weighted (fixed effects)
Inverse variance weighted (multiplicative random effects)
MR Egger
MR RAPS
Weighted median
Weighted mode
0.05
0.00
−0.05
−0.10
0.1
0.2
SNP effect on Protein−tyrosine kinase 6
SNP effect on Gut microbiota abundance (genus Parasutterella id.2892)

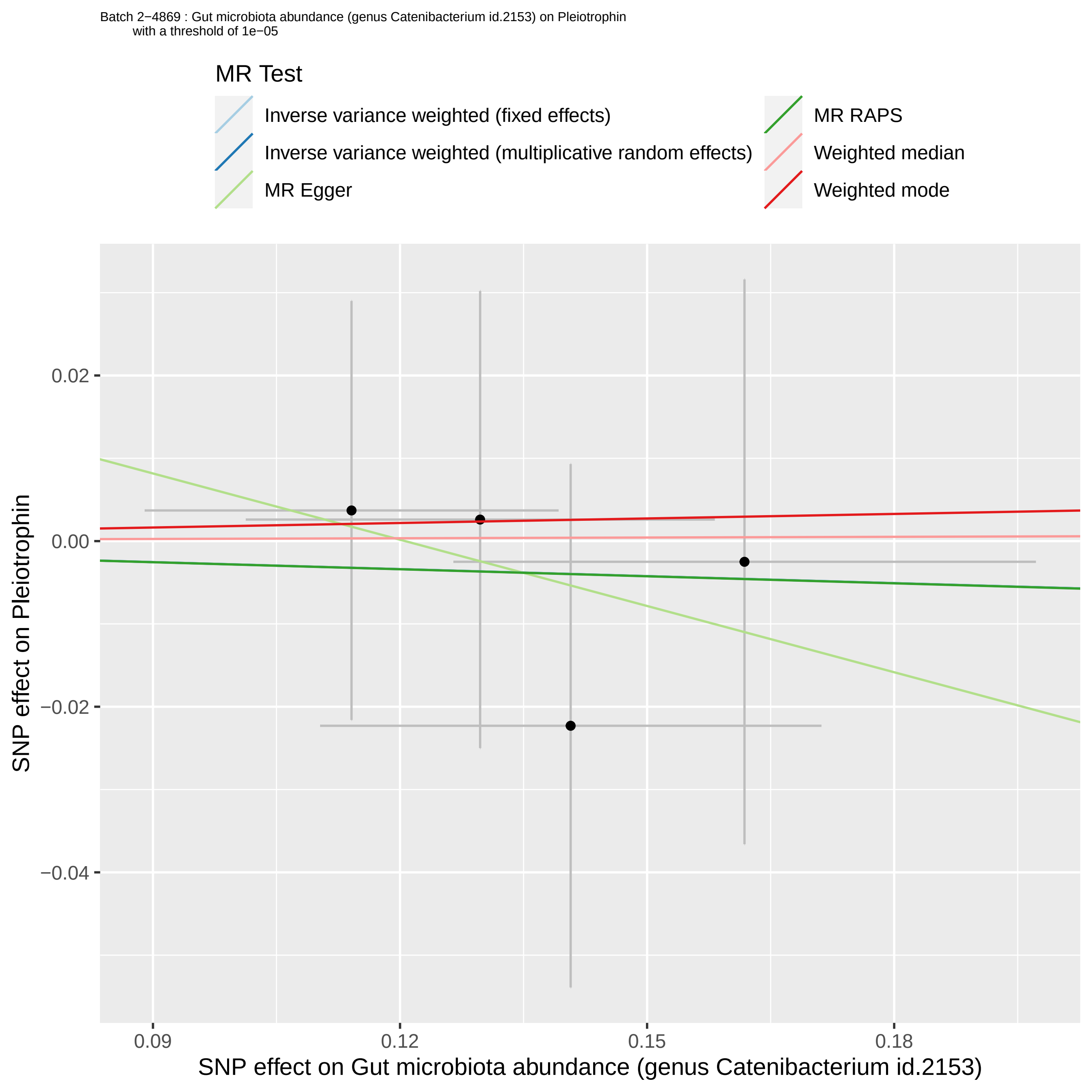
Batch 2−4869 : Gut microbiota abundance (genus Catenibacterium id.2153) on Pleiotrophin
with a threshold of 1e−05
MR Test
Inverse variance weighted (fixed effects)
Inverse variance weighted (multiplicative random effects)
MR Egger
MR RAPS
Weighted median
Weighted mode
0.02
0.00
−0.02
−0.04
0.09
0.12
0.15
0.18
SNP effect on Pleiotrophin
SNP effect on Gut microbiota abundance (genus Catenibacterium id.2153)

Batch 2−4870 : Gut microbiota abundance (genus Parasutterella id.2892) on Pleiotrophin
with a threshold of 1e−05

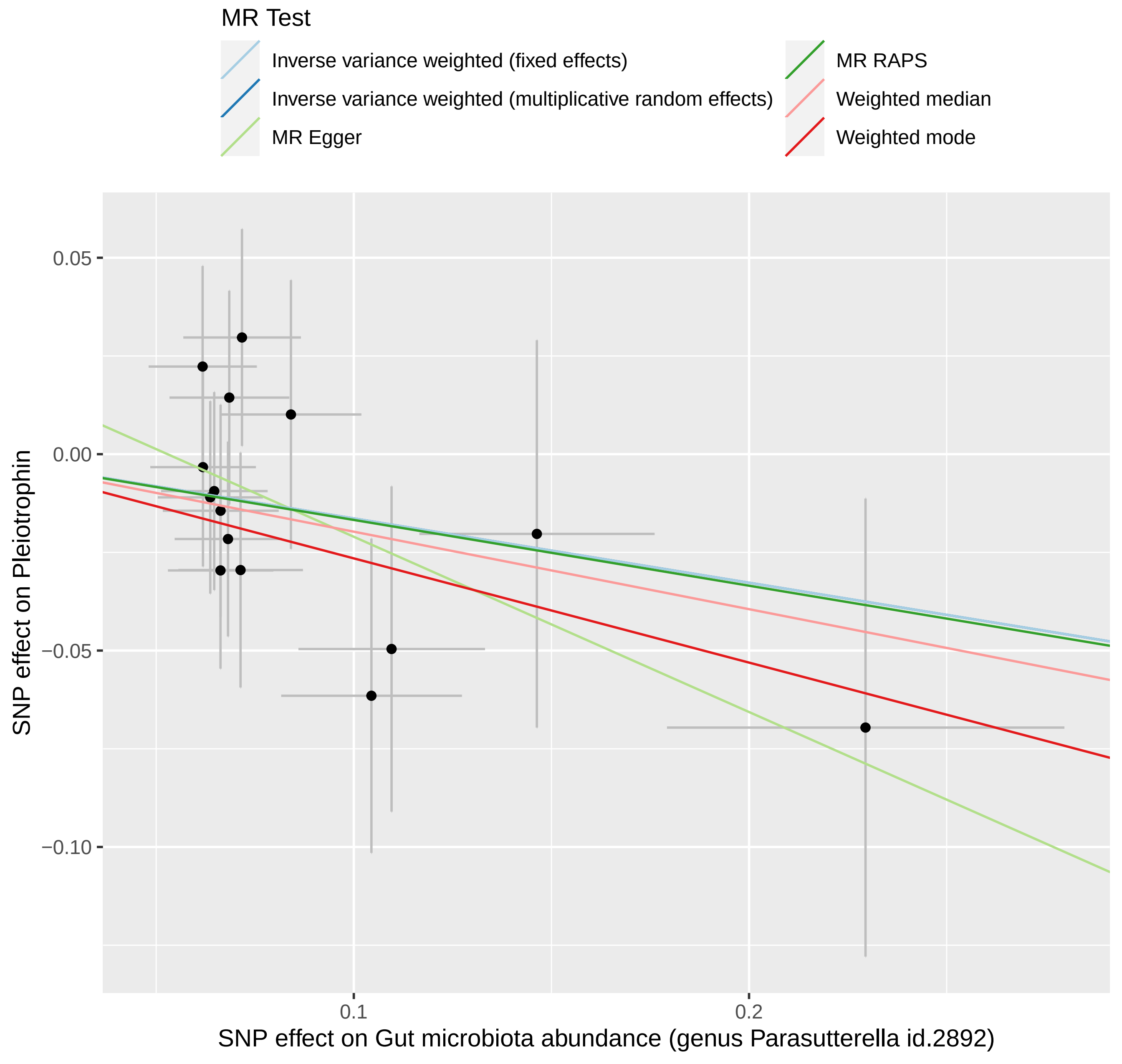

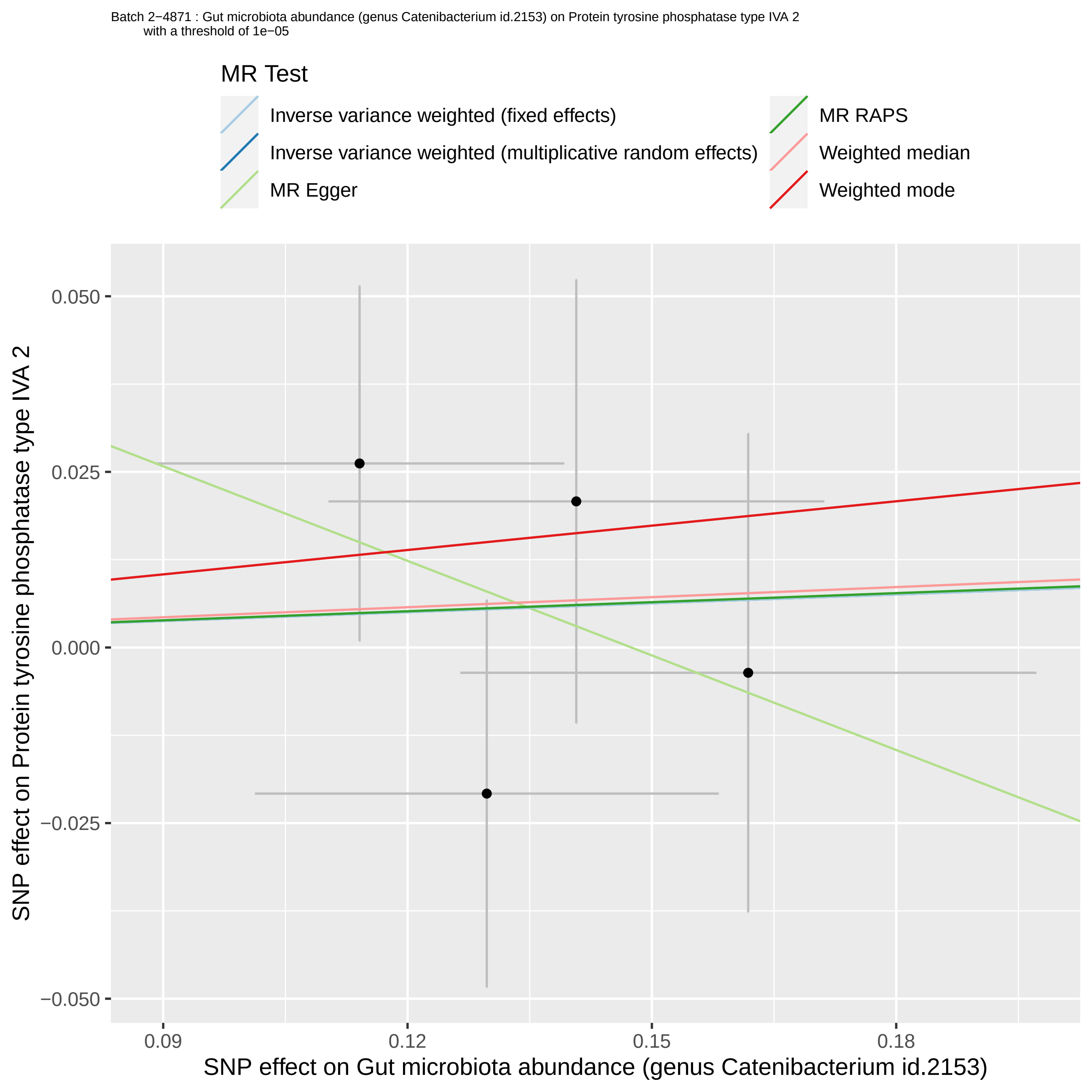
Batch 2−4871 : Gut microbiota abundance (genus Catenibacterium id.2153) on Protein tyrosine phosphatase type IVA 2
with a threshold of 1e−05
MR Test
Inverse variance weighted (fixed effects)
Inverse variance weighted (multiplicative random effects)
MR Egger
MR RAPS
Weighted median
Weighted mode
0.050
0.025
0.000
−0.025
−0.050
0.09
0.12
0.15
0.18
SNP effect on Protein tyrosine phosphatase type IVA 2
SNP effect on Gut microbiota abundance (genus Catenibacterium id.2153)

Batch 2−4872 : Gut microbiota abundance (genus Parasutterella id.2892) on Protein tyrosine phosphatase type IVA 2
with a threshold of 1e−05

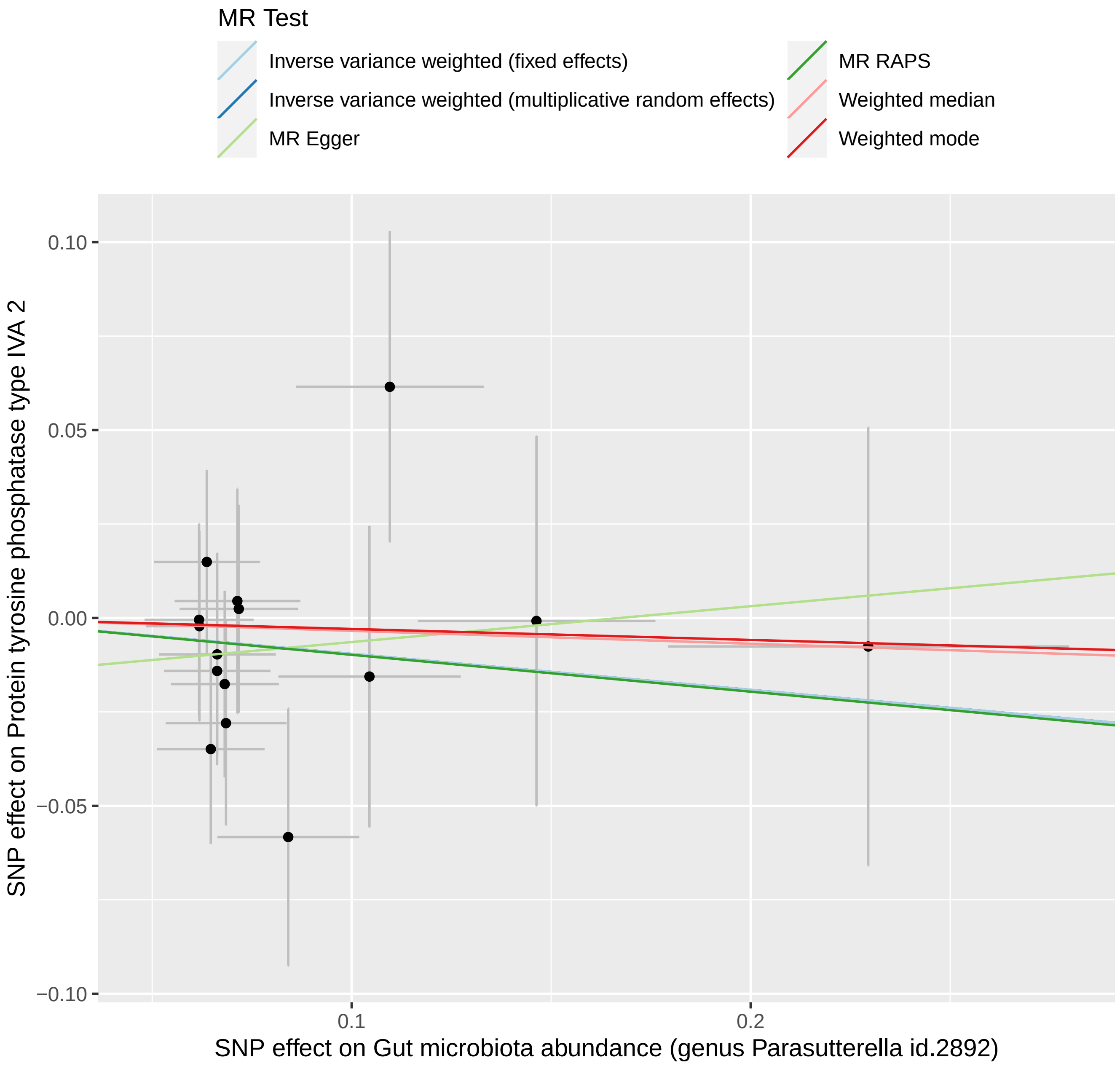

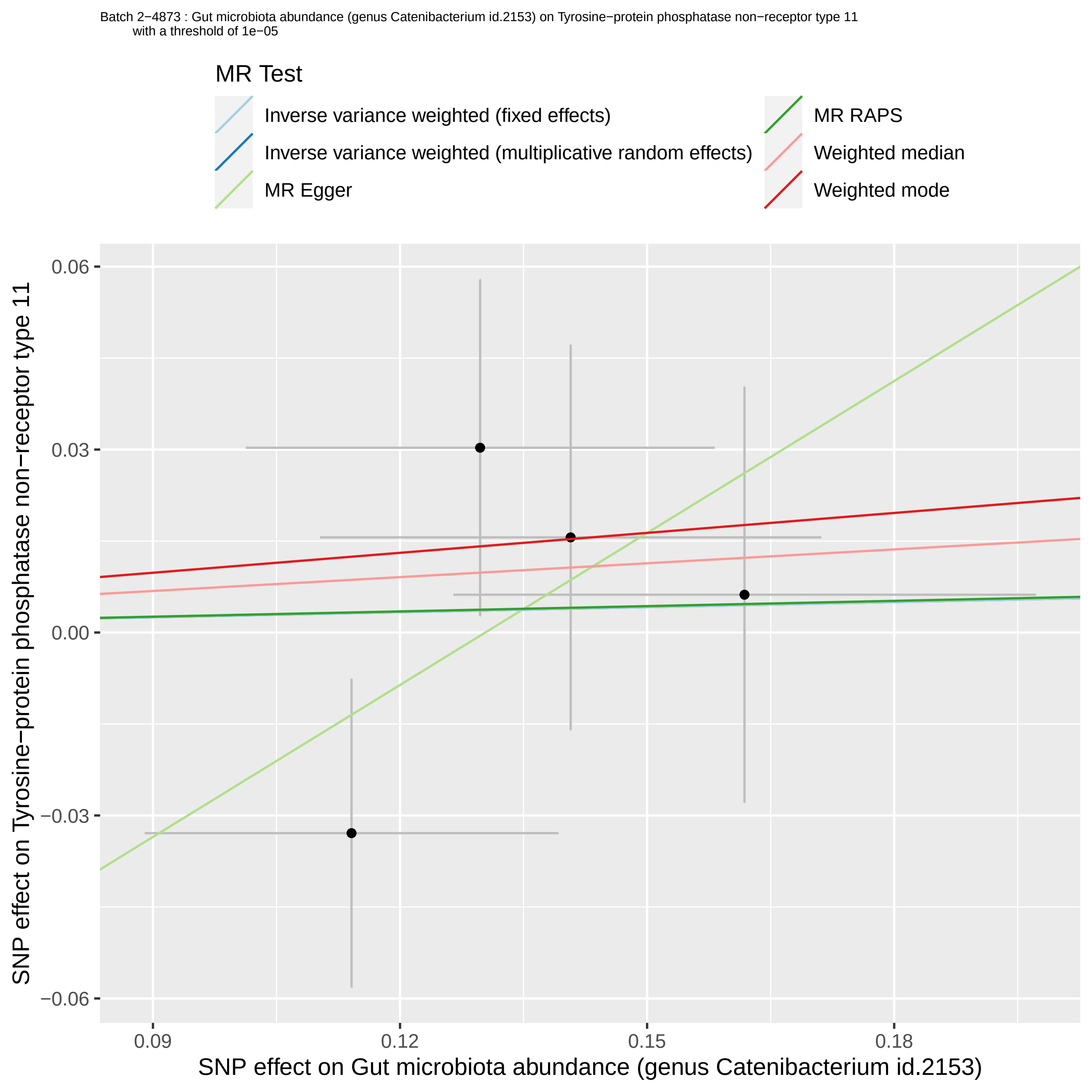
Batch 2−4873 : Gut microbiota abundance (genus Catenibacterium id.2153) on Tyrosine−protein phosphatase non−receptor type 11
with a threshold of 1e−05
MR Test
Inverse variance weighted (fixed effects)
Inverse variance weighted (multiplicative random effects)
MR Egger
MR RAPS
Weighted median
Weighted mode
0.06
0.03
0.00
−0.03
−0.06
0.09
0.12
0.15
0.18
SNP effect on Tyrosine−protein phosphatase non−receptor type 11
SNP effect on Gut microbiota abundance (genus Catenibacterium id.2153)

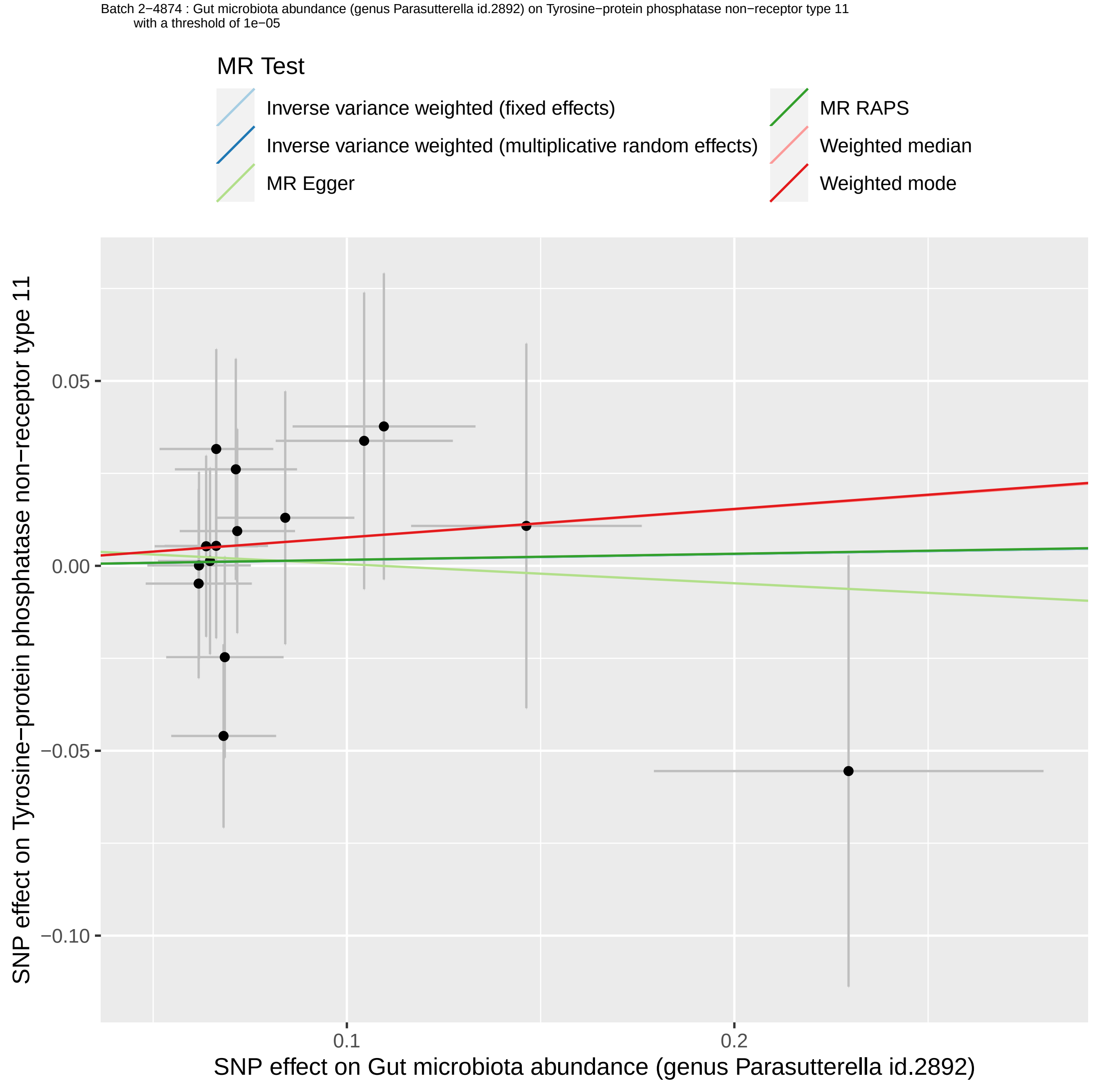
Batch 2−4874 : Gut microbiota abundance (genus Parasutterella id.2892) on Tyrosine−protein phosphatase non−receptor type 11
with a threshold of 1e−05
MR Test
Inverse variance weighted (fixed effects)
Inverse variance weighted (multiplicative random effects)
MR Egger
MR RAPS
Weighted median
Weighted mode
0.05
0.00
−0.05
−0.10
0.1
0.2
SNP effect on Tyrosine−protein phosphatase non−receptor type 11
SNP effect on Gut microbiota abundance (genus Parasutterella id.2892)

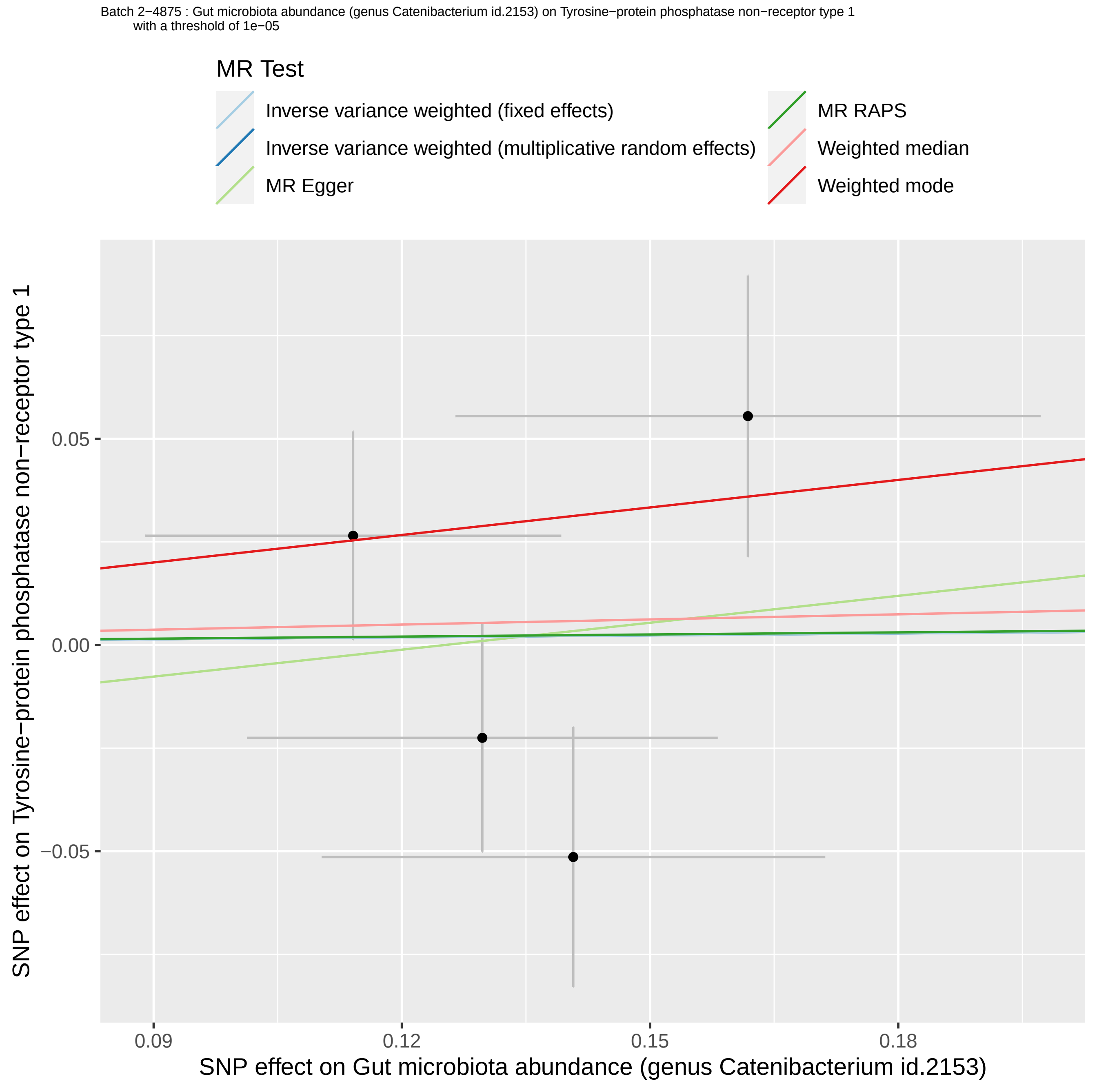

Batch 2−4875 : Gut microbiota abundance (genus Catenibacterium id.2153) on Tyrosine−protein phosphatase non−receptor type 1
with a threshold of 1e−05
MR Test
Inverse variance weighted (fixed effects)
Inverse variance weighted (multiplicative random effects)
MR Egger
MR RAPS
Weighted median
Weighted mode
0.05
0.00
−0.05
0.09
0.12
0.15
0.18
SNP effect on Tyrosine−protein phosphatase non−receptor type 1
SNP effect on Gut microbiota abundance (genus Catenibacterium id.2153)

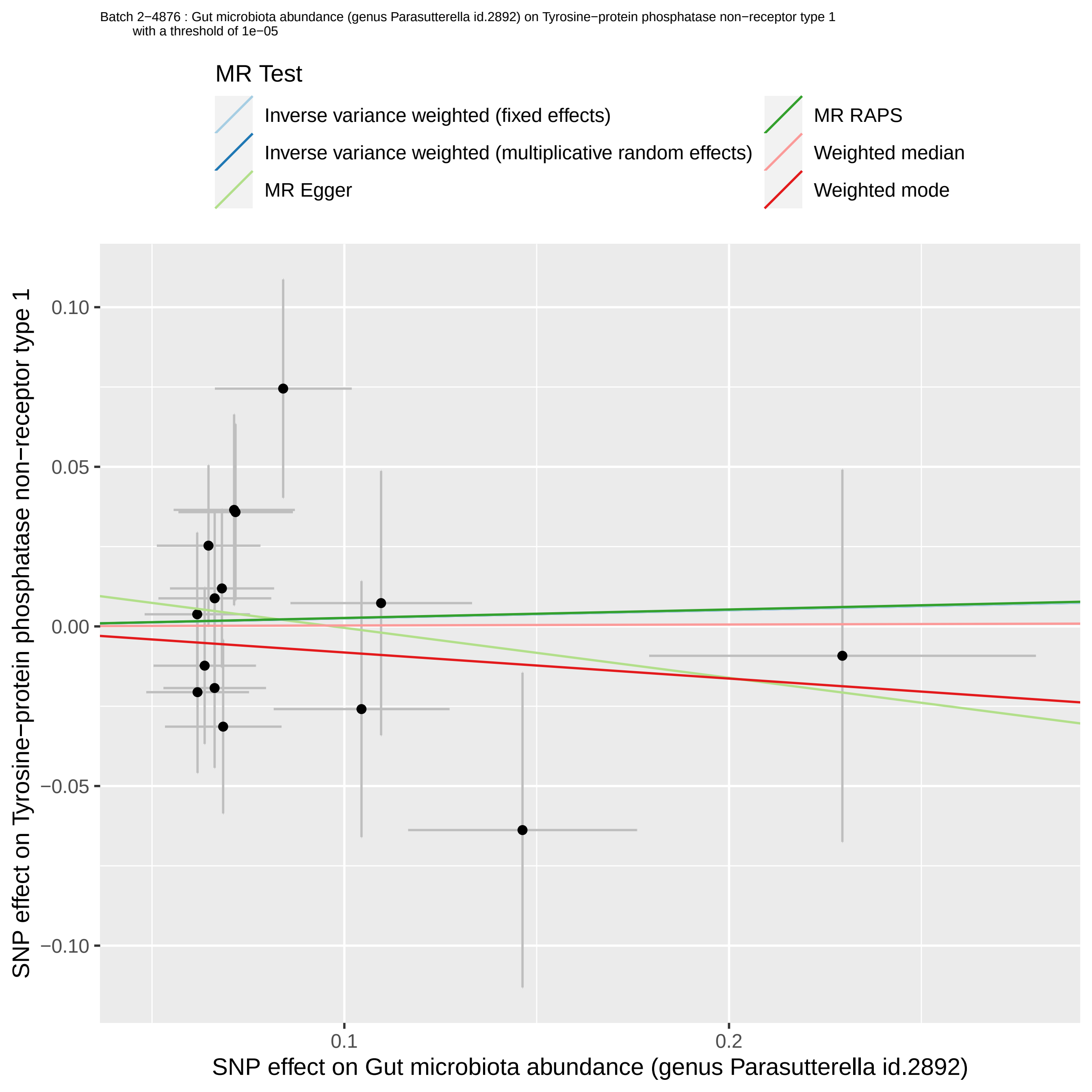
Batch 2−4876 : Gut microbiota abundance (genus Parasutterella id.2892) on Tyrosine−protein phosphatase non−receptor type 1
with a threshold of 1e−05
MR Test
Inverse variance weighted (fixed effects)
Inverse variance weighted (multiplicative random effects)
MR Egger
MR RAPS
Weighted median
Weighted mode
0.10
0.05
0.00
−0.05
−0.10
0.1
0.2
SNP effect on Tyrosine−protein phosphatase non−receptor type 1
SNP effect on Gut microbiota abundance (genus Parasutterella id.2892)

Batch 2−4877 : Gut microbiota abundance (genus Catenibacterium id.2153) on Tyrosine−protein phosphatase non−receptor type 2
with a threshold of 1e−05

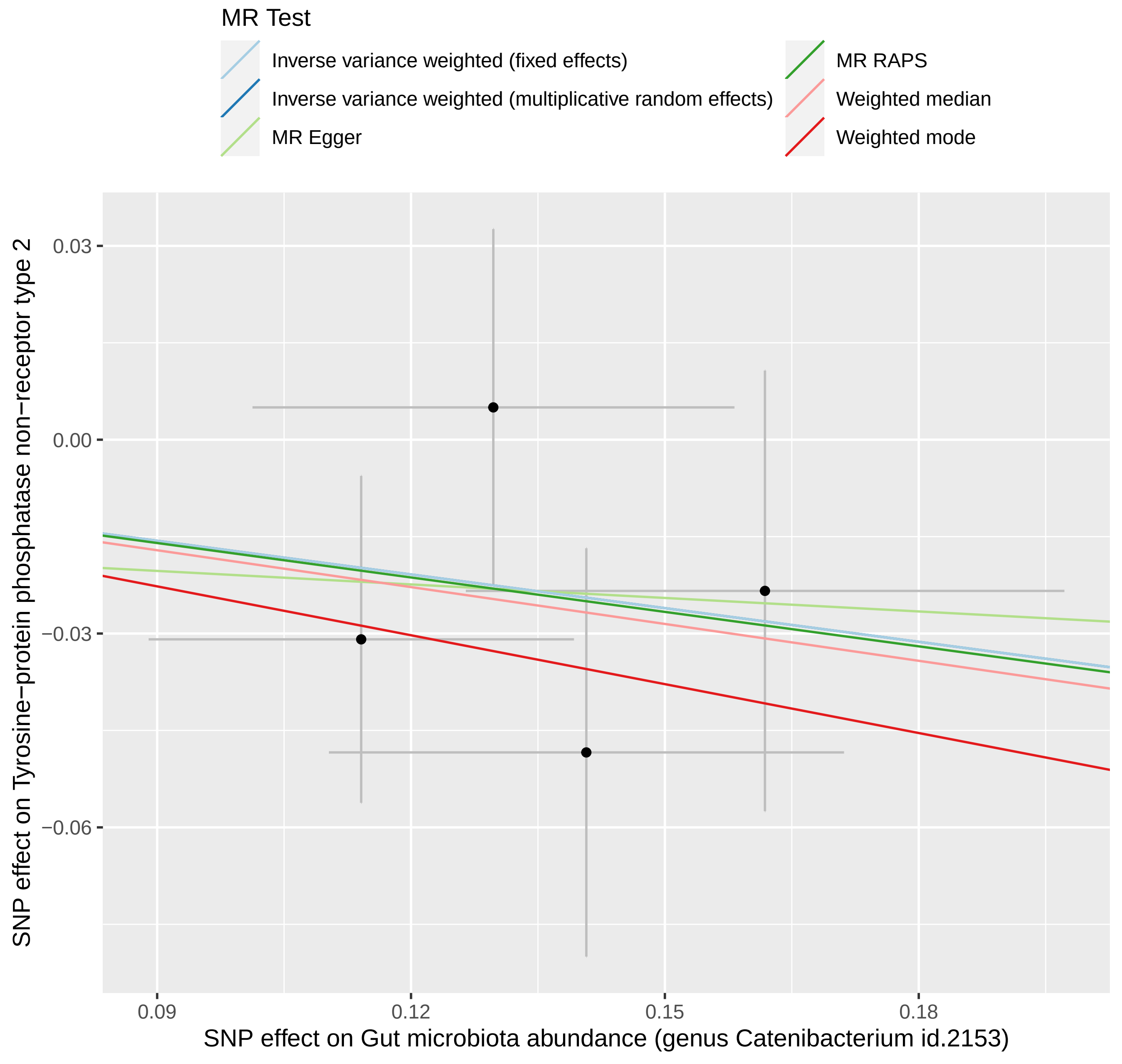

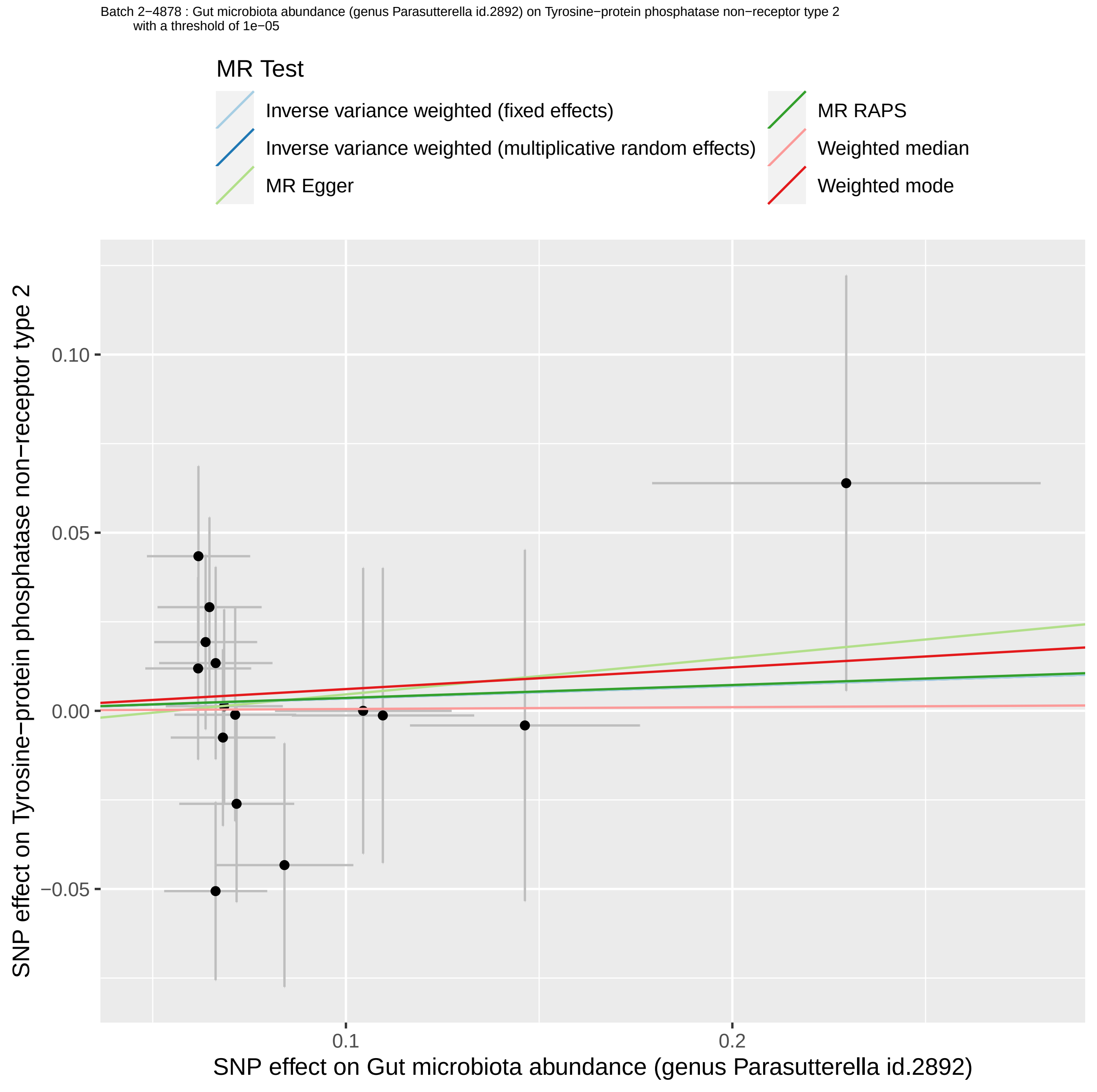
Batch 2−4878 : Gut microbiota abundance (genus Parasutterella id.2892) on Tyrosine−protein phosphatase non−receptor type 2
with a threshold of 1e−05
MR Test
Inverse variance weighted (fixed effects)
Inverse variance weighted (multiplicative random effects)
MR Egger
MR RAPS
Weighted median
Weighted mode
SNP effect on Tyrosine−protein phosphatase non−receptor type 2
0.10
0.05
0.00
−0.05
0.1
0.2
SNP effect on Gut microbiota abundance (genus Parasutterella id.2892)

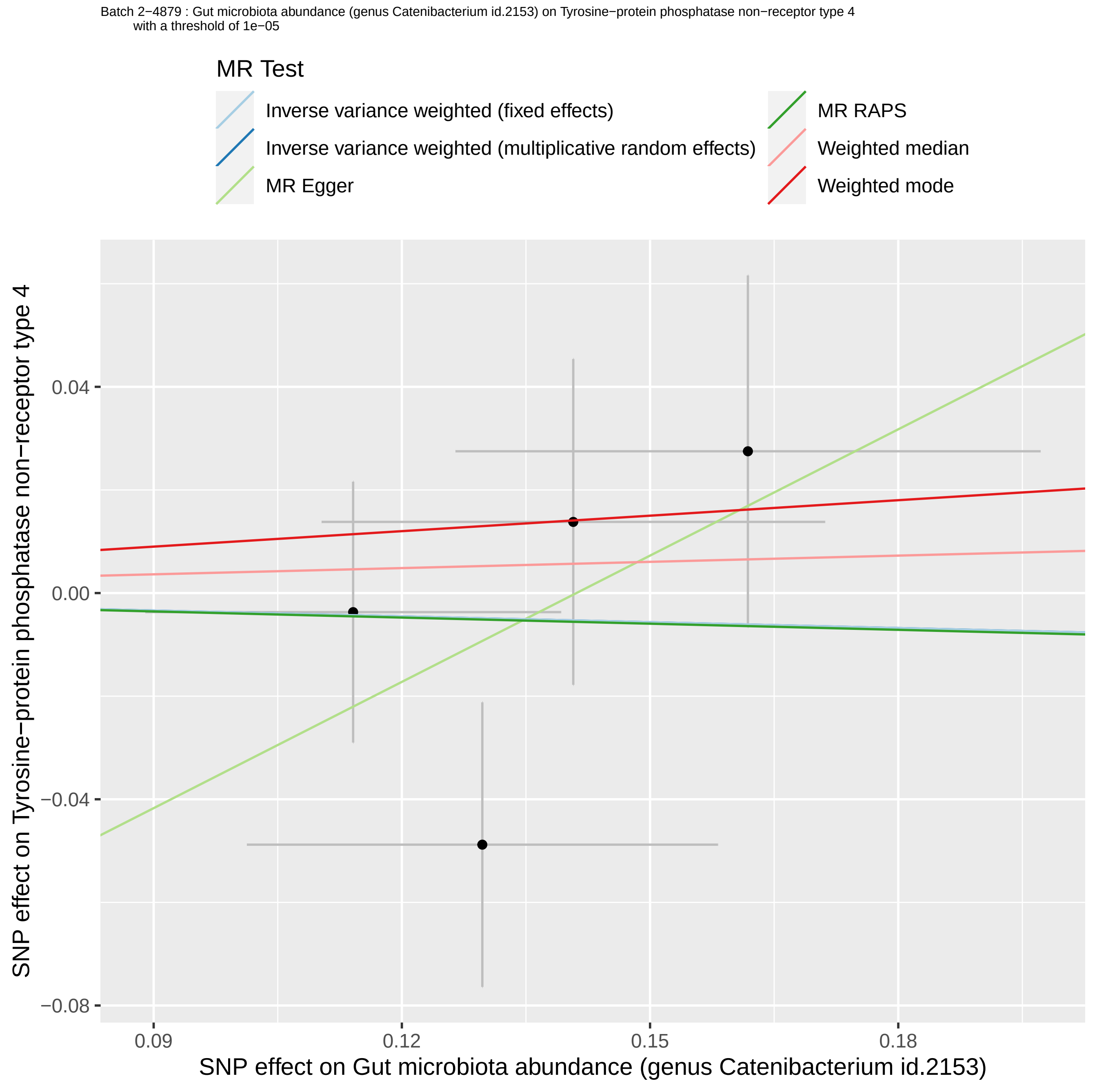

Batch 2−4879 : Gut microbiota abundance (genus Catenibacterium id.2153) on Tyrosine−protein phosphatase non−receptor type 4
with a threshold of 1e−05
MR Test
Inverse variance weighted (fixed effects)
Inverse variance weighted (multiplicative random effects)
MR Egger
MR RAPS
Weighted median
Weighted mode
0.04
0.00
−0.04
−0.08
0.09
0.12
0.15
0.18
SNP effect on Tyrosine−protein phosphatase non−receptor type 4
SNP effect on Gut microbiota abundance (genus Catenibacterium id.2153)

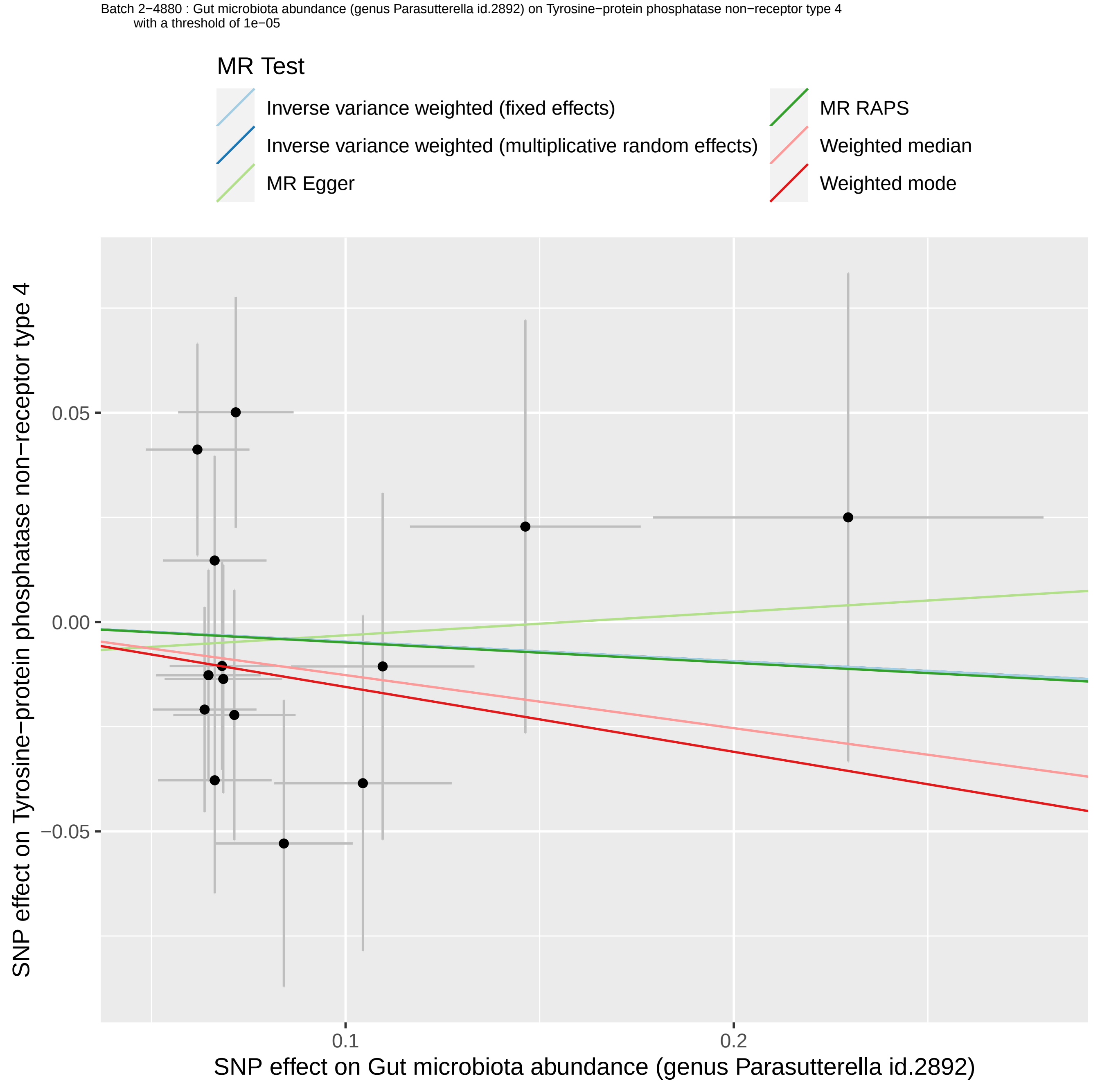
Batch 2−4880 : Gut microbiota abundance (genus Parasutterella id.2892) on Tyrosine−protein phosphatase non−receptor type 4
with a threshold of 1e−05
MR Test
Inverse variance weighted (fixed effects)
Inverse variance weighted (multiplicative random effects)
MR Egger
MR RAPS
Weighted median
Weighted mode
SNP effect on Tyrosine−protein phosphatase non−receptor type 4
0.05
0.00
−0.05
0.1
0.2
SNP effect on Gut microbiota abundance (genus Parasutterella id.2892)

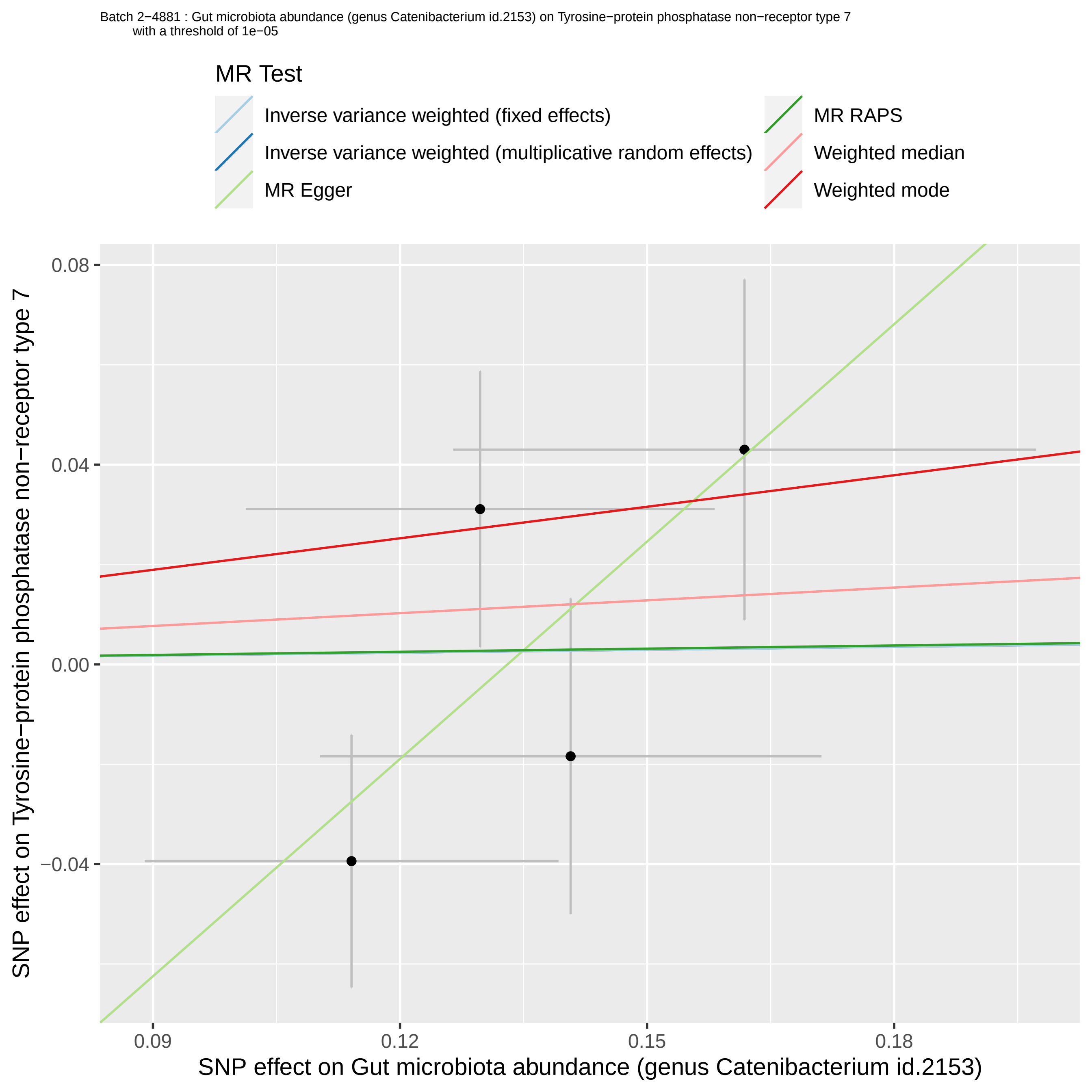

Batch 2−4881 : Gut microbiota abundance (genus Catenibacterium id.2153) on Tyrosine−protein phosphatase non−receptor type 7
with a threshold of 1e−05
MR Test
Inverse variance weighted (fixed effects)
Inverse variance weighted (multiplicative random effects)
MR Egger
MR RAPS
Weighted median
Weighted mode
0.08
0.04
0.00
−0.04
0.09
0.12
0.15
0.18
SNP effect on Tyrosine−protein phosphatase non−receptor type 7
SNP effect on Gut microbiota abundance (genus Catenibacterium id.2153)

Batch 2−4882 : Gut microbiota abundance (genus Parasutterella id.2892) on Tyrosine−protein phosphatase non−receptor type 7
with a threshold of 1e−05

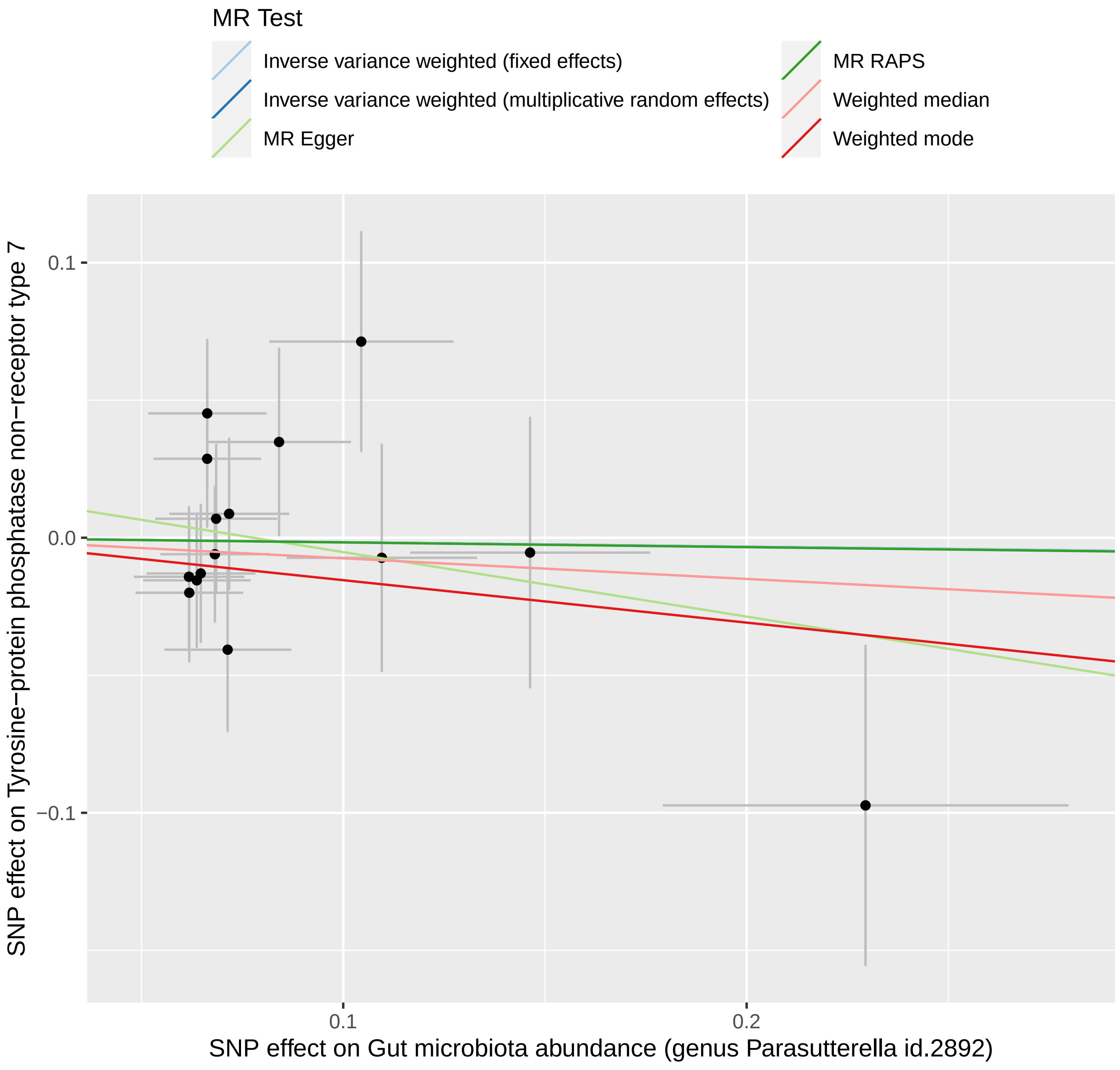

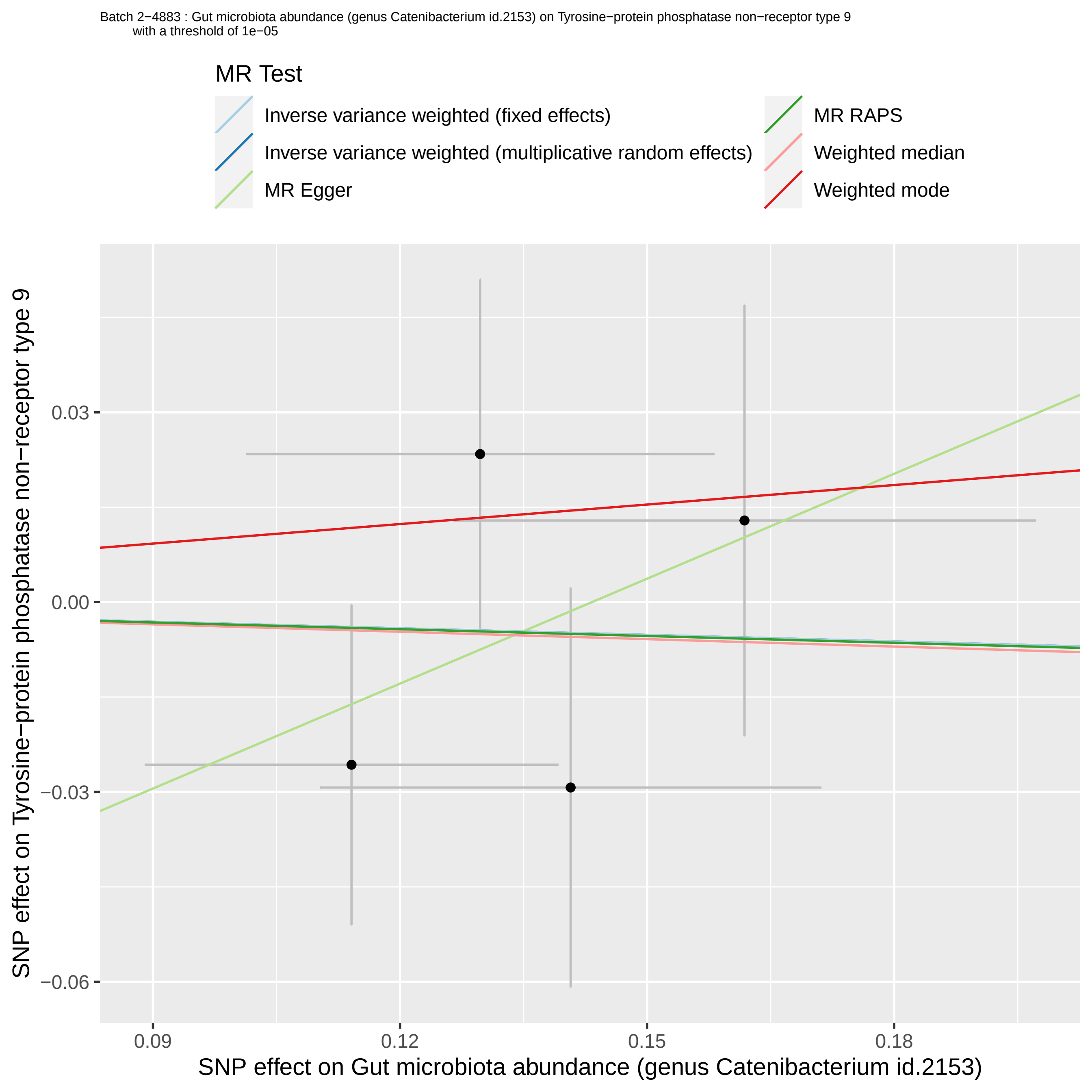
Batch 2−4883 : Gut microbiota abundance (genus Catenibacterium id.2153) on Tyrosine−protein phosphatase non−receptor type 9
with a threshold of 1e−05
MR Test
Inverse variance weighted (fixed effects)
Inverse variance weighted (multiplicative random effects)
MR Egger
MR RAPS
Weighted median
Weighted mode
0.03
0.00
−0.03
−0.06
0.09
0.12
0.15
0.18
SNP effect on Tyrosine−protein phosphatase non−receptor type 9
SNP effect on Gut microbiota abundance (genus Catenibacterium id.2153)

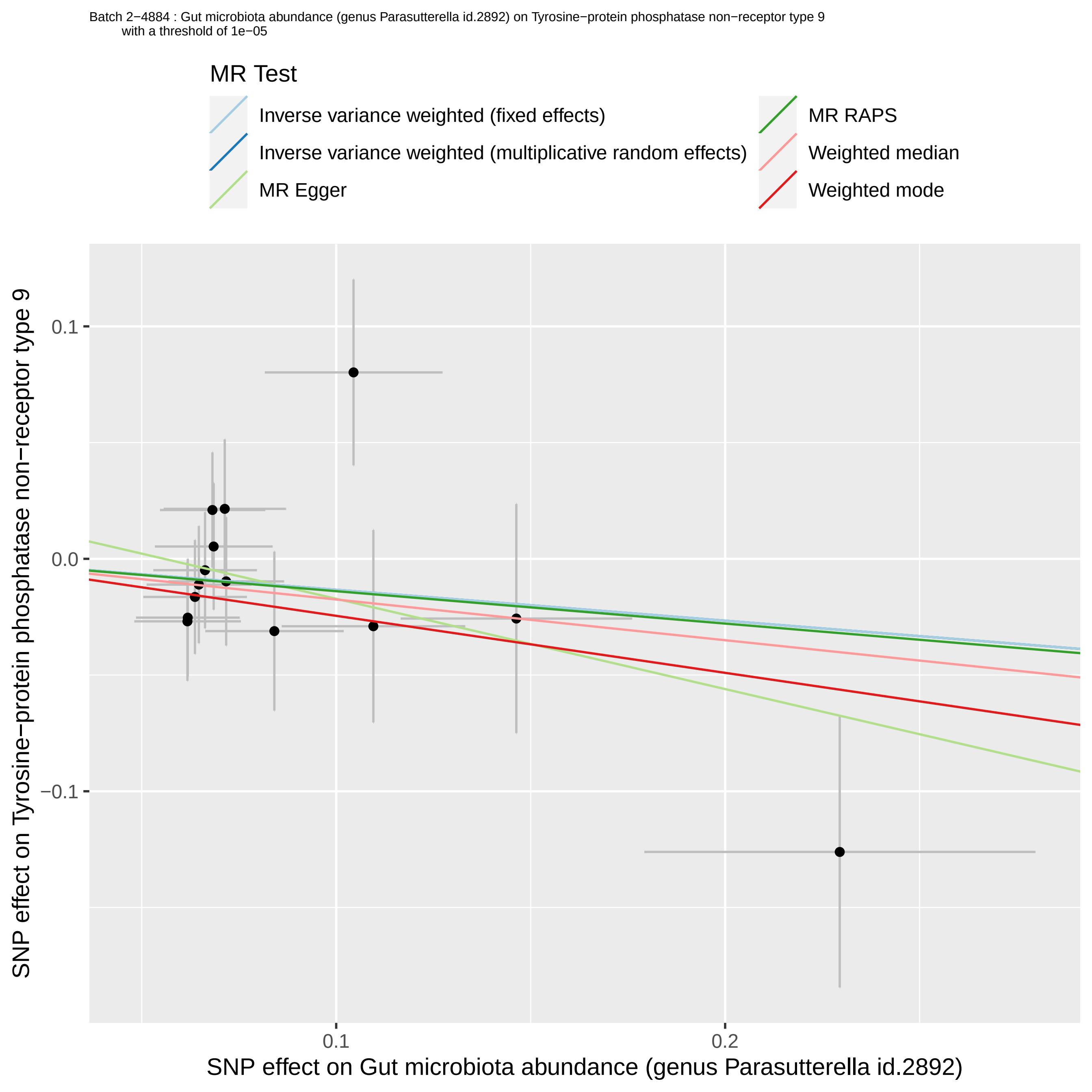

Batch 2−4884 : Gut microbiota abundance (genus Parasutterella id.2892) on Tyrosine−protein phosphatase non−receptor type 9
with a threshold of 1e−05
MR Test
Inverse variance weighted (fixed effects)
Inverse variance weighted (multiplicative random effects)
MR Egger
MR RAPS
Weighted median
Weighted mode
0.1
0.0
−0.1
0.1
0.2
SNP effect on Tyrosine−protein phosphatase non−receptor type 9
SNP effect on Gut microbiota abundance (genus Parasutterella id.2892)

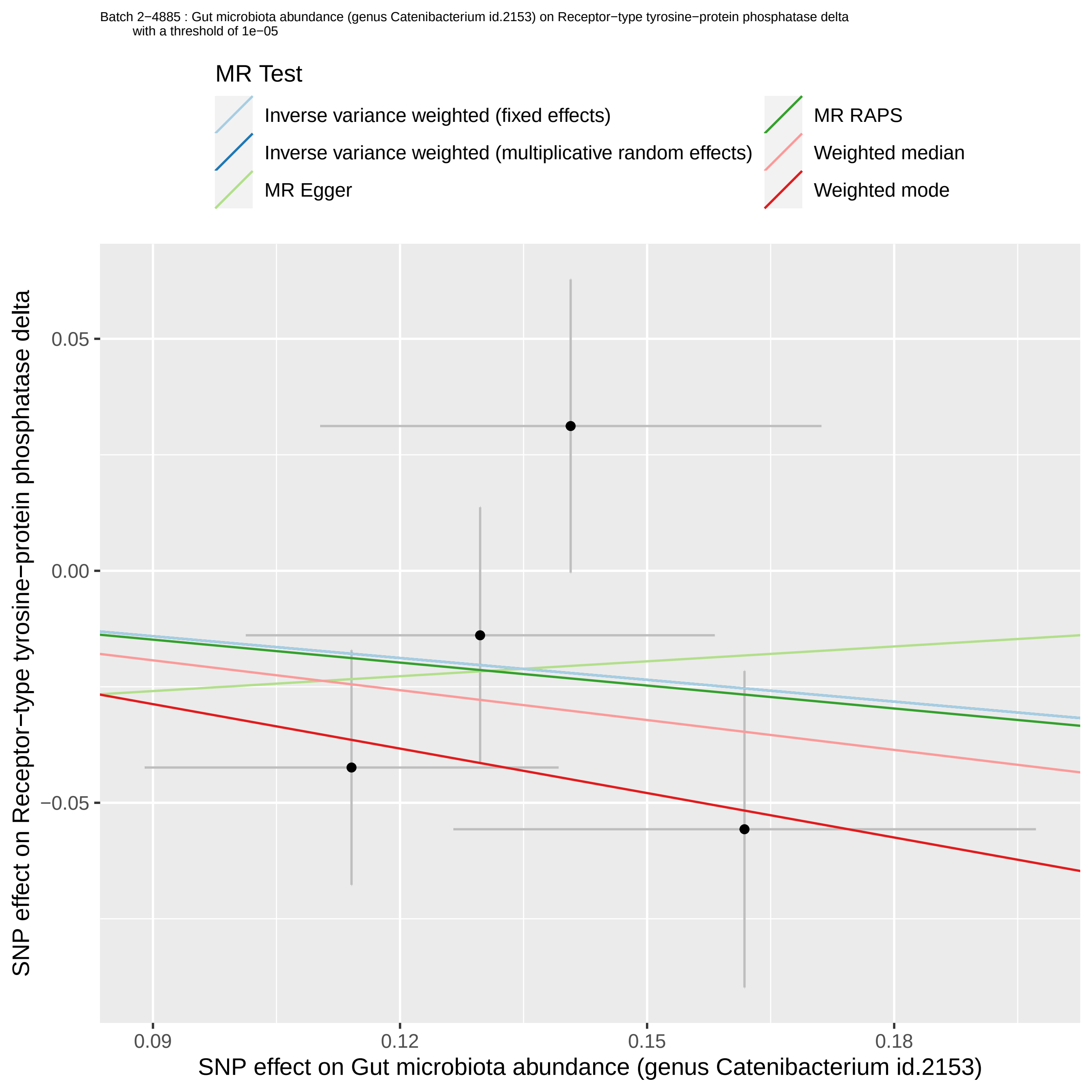

Batch 2−4885 : Gut microbiota abundance (genus Catenibacterium id.2153) on Receptor−type tyrosine−protein phosphatase delta
with a threshold of 1e−05
MR Test
Inverse variance weighted (fixed effects)
Inverse variance weighted (multiplicative random effects)
MR Egger
MR RAPS
Weighted median
Weighted mode
SNP effect on Receptor−type tyrosine−protein phosphatase delta
0.05
0.00
−0.05
0.09
0.12
0.15
0.18
SNP effect on Gut microbiota abundance (genus Catenibacterium id.2153)

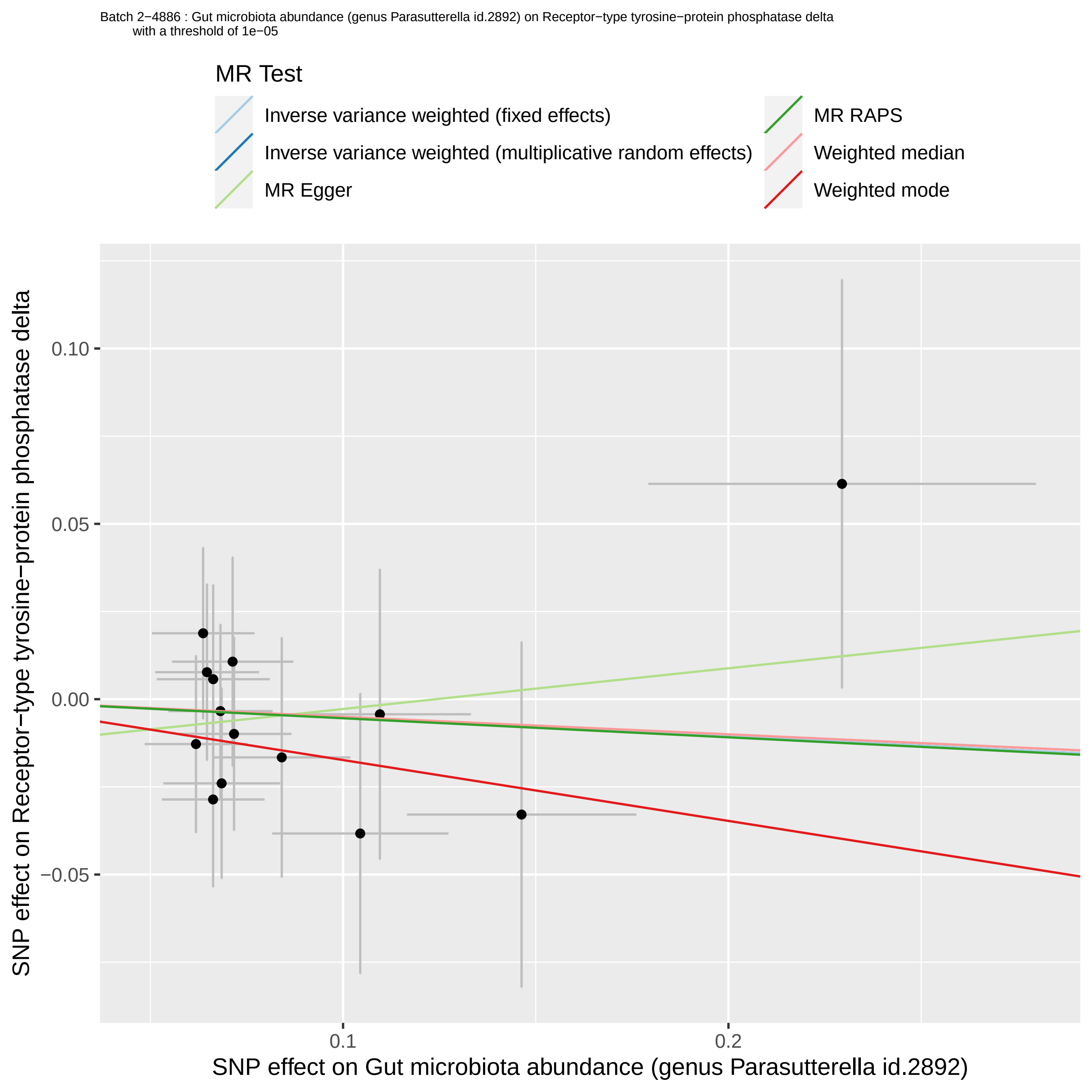

Batch 2−4886 : Gut microbiota abundance (genus Parasutterella id.2892) on Receptor−type tyrosine−protein phosphatase delta
with a threshold of 1e−05
MR Test
Inverse variance weighted (fixed effects)
Inverse variance weighted (multiplicative random effects)
MR Egger
MR RAPS
Weighted median
Weighted mode
0.10
0.05
0.00
−0.05
0.1
0.2
SNP effect on Receptor−type tyrosine−protein phosphatase delta
SNP effect on Gut microbiota abundance (genus Parasutterella id.2892)

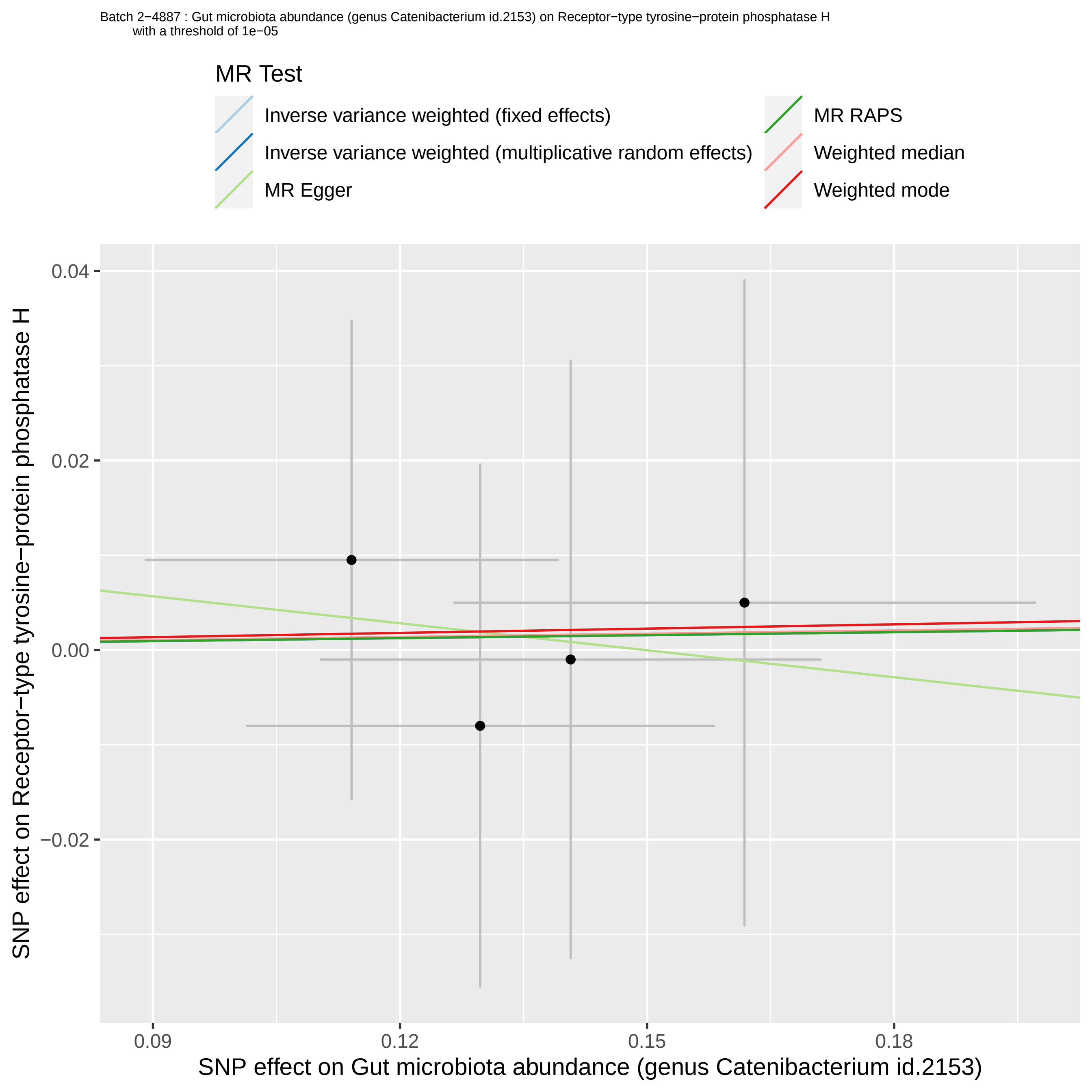
Batch 2−4887 : Gut microbiota abundance (genus Catenibacterium id.2153) on Receptor−type tyrosine−protein phosphatase H
with a threshold of 1e−05
MR Test
Inverse variance weighted (fixed effects)
Inverse variance weighted (multiplicative random effects)
MR Egger
MR RAPS
Weighted median
Weighted mode
SNP effect on Receptor−type tyrosine−protein phosphatase H
0.04
0.02
0.00
−0.02
0.09
0.12
0.15
0.18
SNP effect on Gut microbiota abundance (genus Catenibacterium id.2153)

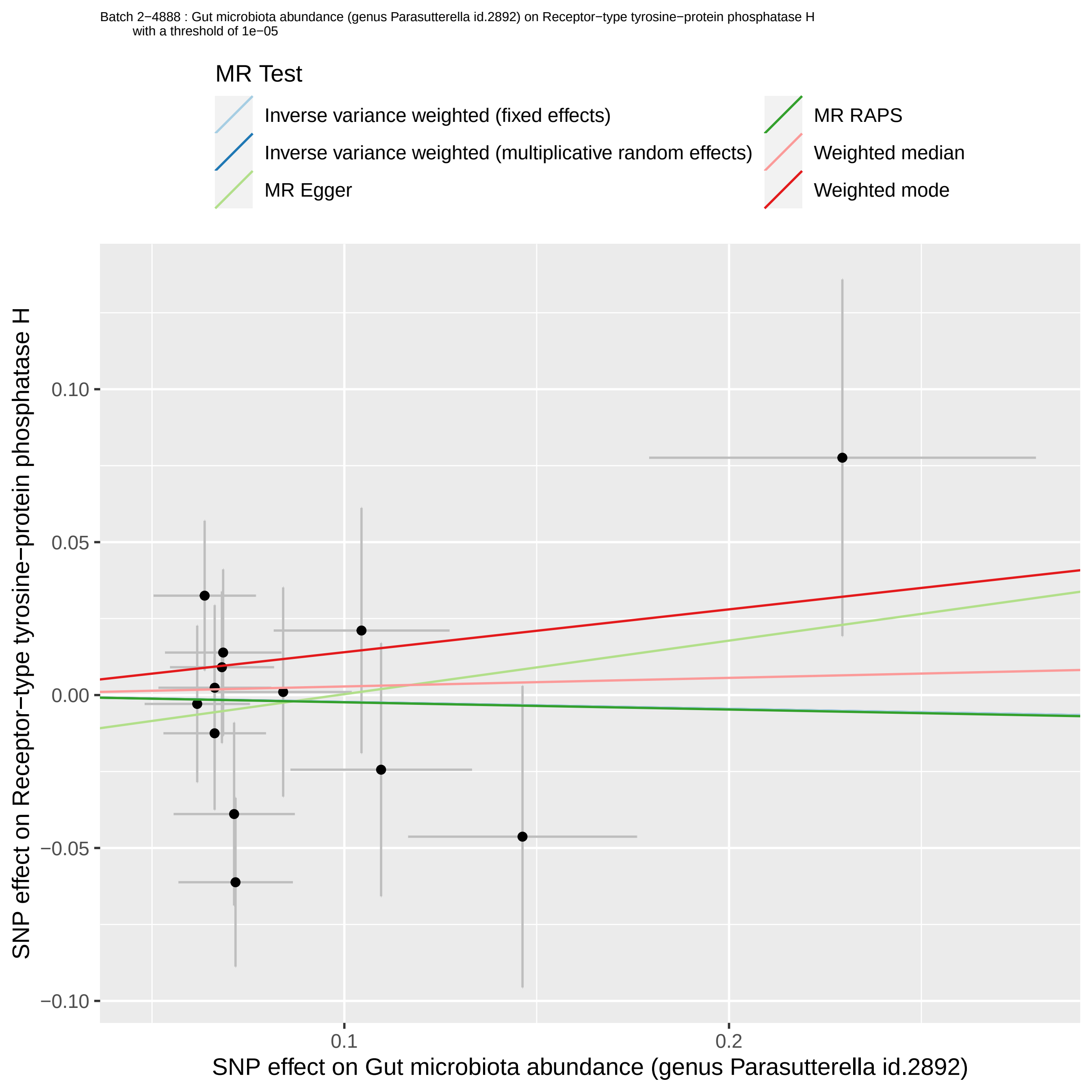
Batch 2−4888 : Gut microbiota abundance (genus Parasutterella id.2892) on Receptor−type tyrosine−protein phosphatase H
with a threshold of 1e−05
MR Test
Inverse variance weighted (fixed effects)
Inverse variance weighted (multiplicative random effects)
MR Egger
MR RAPS
Weighted median
Weighted mode
SNP effect on Receptor−type tyrosine−protein phosphatase H
0.10
0.05
0.00
−0.05
−0.10
0.1
0.2
SNP effect on Gut microbiota abundance (genus Parasutterella id.2892)

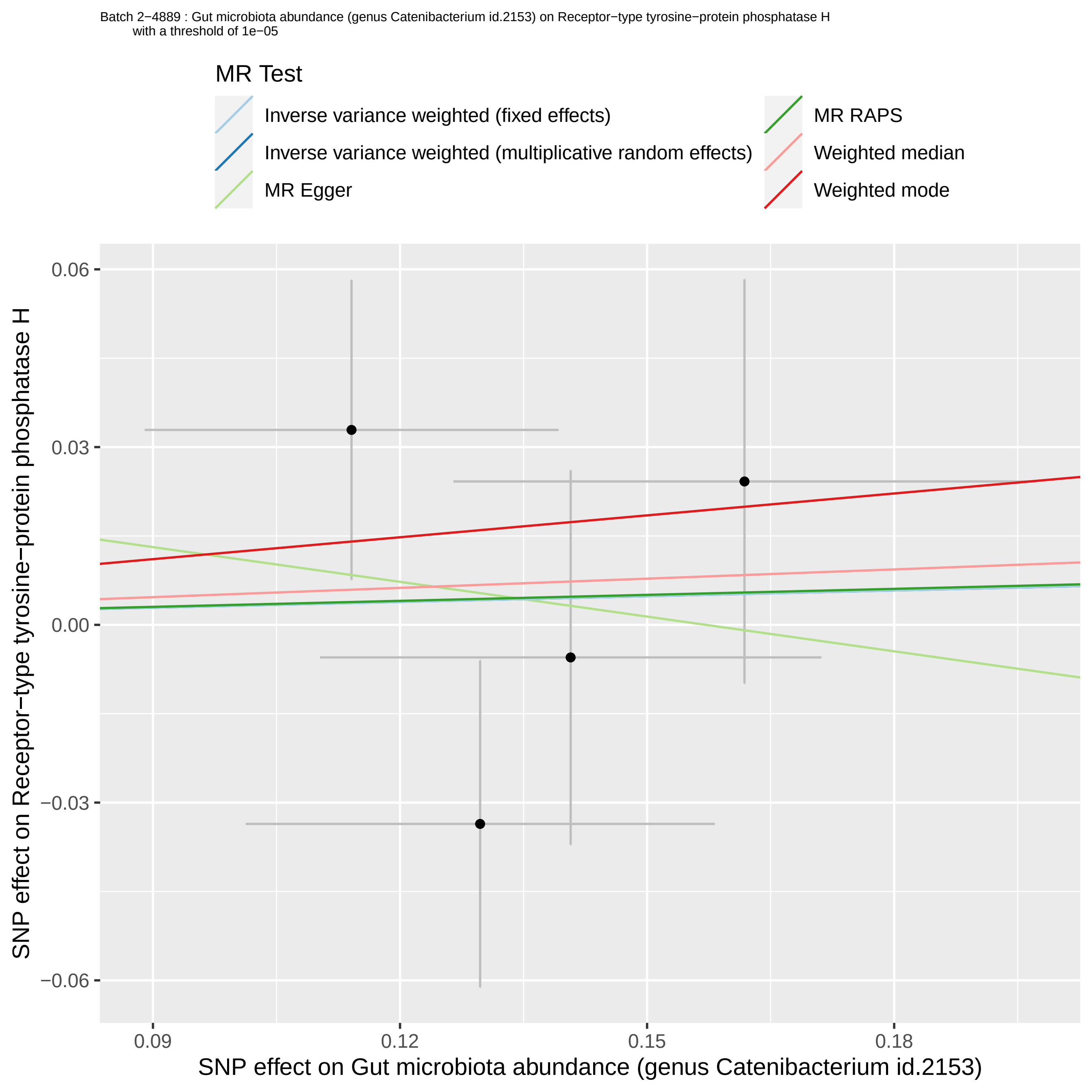

Batch 2−4889 : Gut microbiota abundance (genus Catenibacterium id.2153) on Receptor−type tyrosine−protein phosphatase H
with a threshold of 1e−05
MR Test
Inverse variance weighted (fixed effects)
Inverse variance weighted (multiplicative random effects)
MR Egger
MR RAPS
Weighted median
Weighted mode
0.06
0.03
0.00
−0.03
−0.06
0.09
0.12
0.15
0.18
SNP effect on Receptor−type tyrosine−protein phosphatase H
SNP effect on Gut microbiota abundance (genus Catenibacterium id.2153)

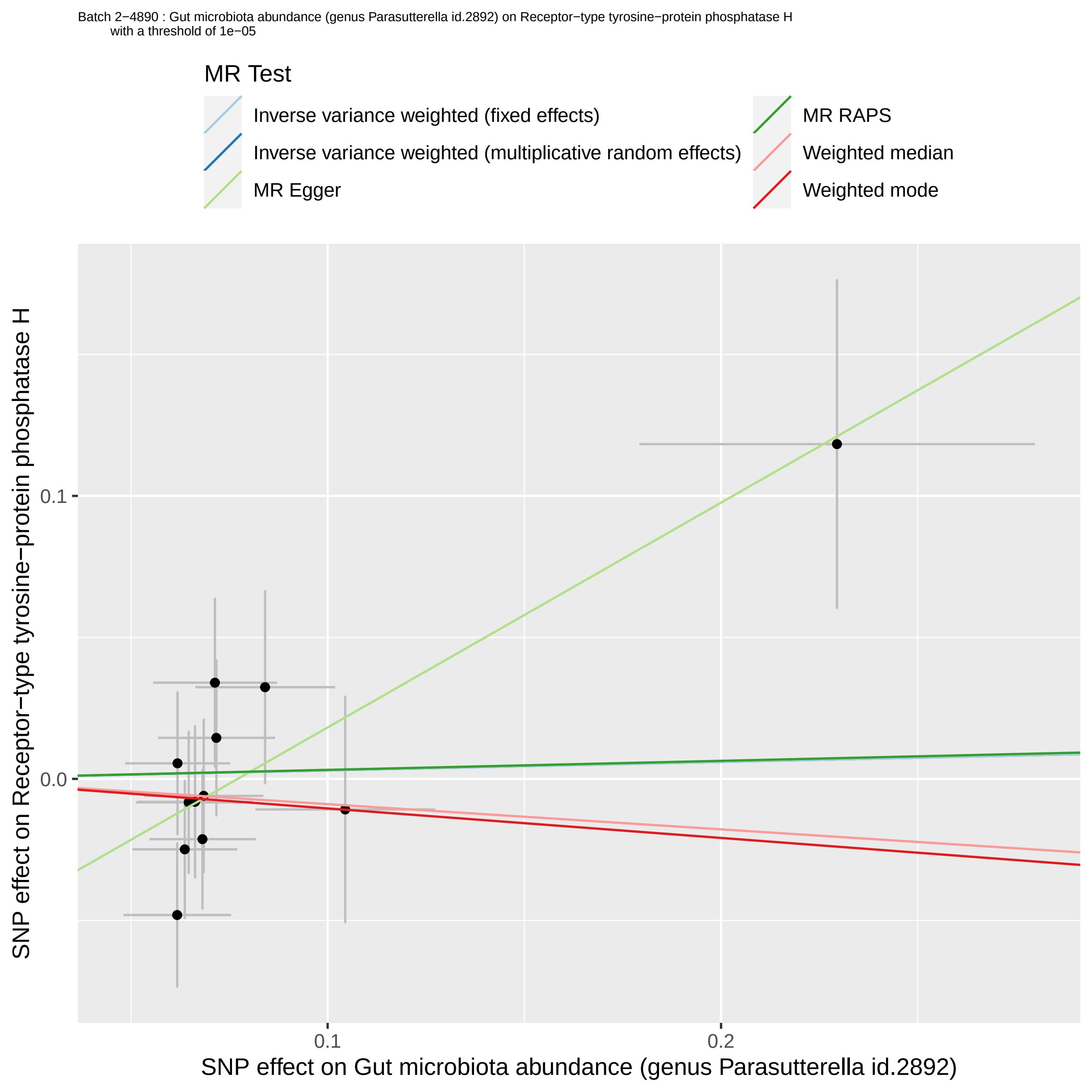
Batch 2−4890 : Gut microbiota abundance (genus Parasutterella id.2892) on Receptor−type tyrosine−protein phosphatase H
with a threshold of 1e−05
MR Test
Inverse variance weighted (fixed effects)
Inverse variance weighted (multiplicative random effects)
MR Egger
MR RAPS
Weighted median
Weighted mode
0.1
0.0
0.1
0.2
SNP effect on Receptor−type tyrosine−protein phosphatase H
SNP effect on Gut microbiota abundance (genus Parasutterella id.2892)

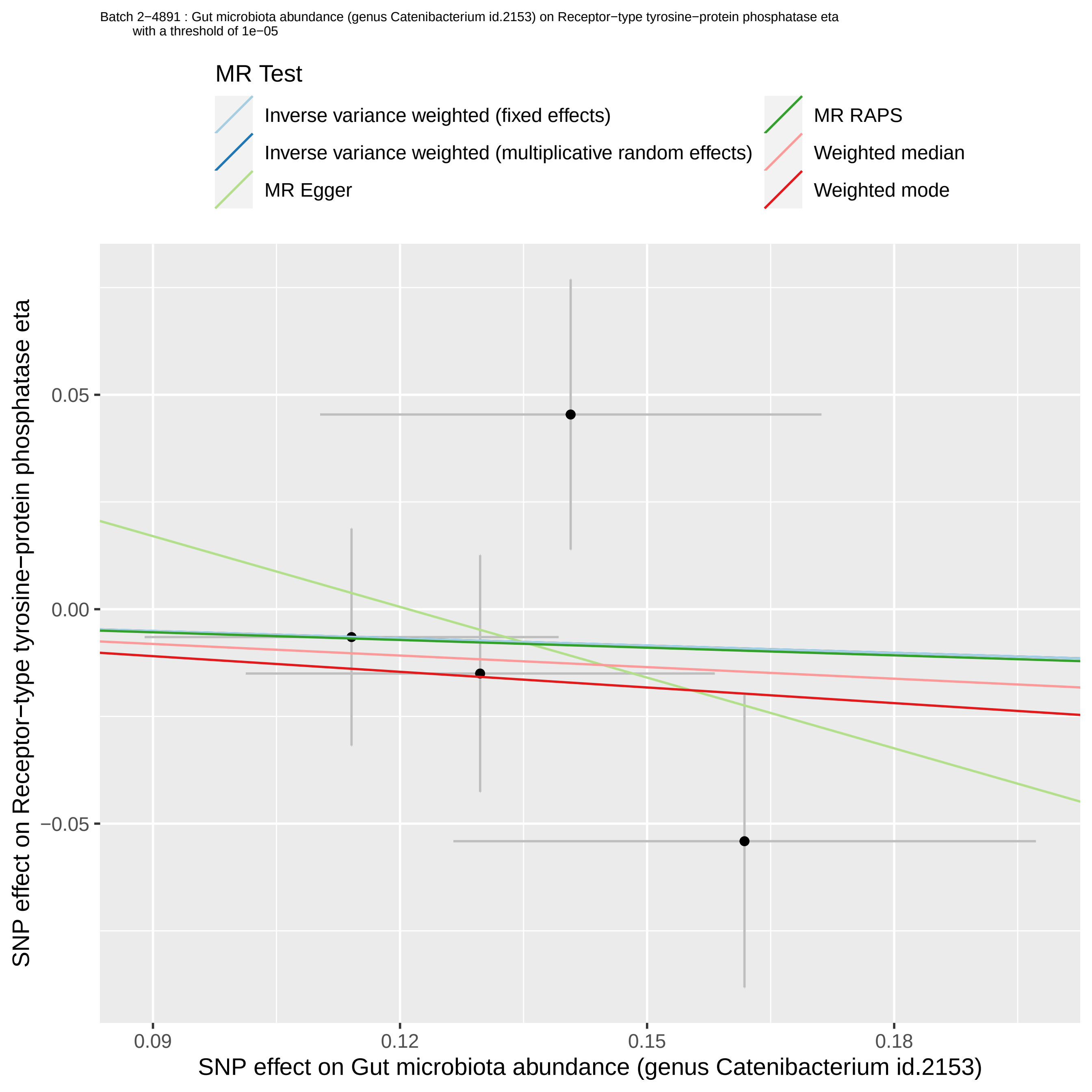

Batch 2−4891 : Gut microbiota abundance (genus Catenibacterium id.2153) on Receptor−type tyrosine−protein phosphatase eta
with a threshold of 1e−05
MR Test
Inverse variance weighted (fixed effects)
Inverse variance weighted (multiplicative random effects)
MR Egger
MR RAPS
Weighted median
Weighted mode
SNP effect on Receptor−type tyrosine−protein phosphatase eta
0.05
0.00
−0.05
0.09
0.12
0.15
0.18
SNP effect on Gut microbiota abundance (genus Catenibacterium id.2153)

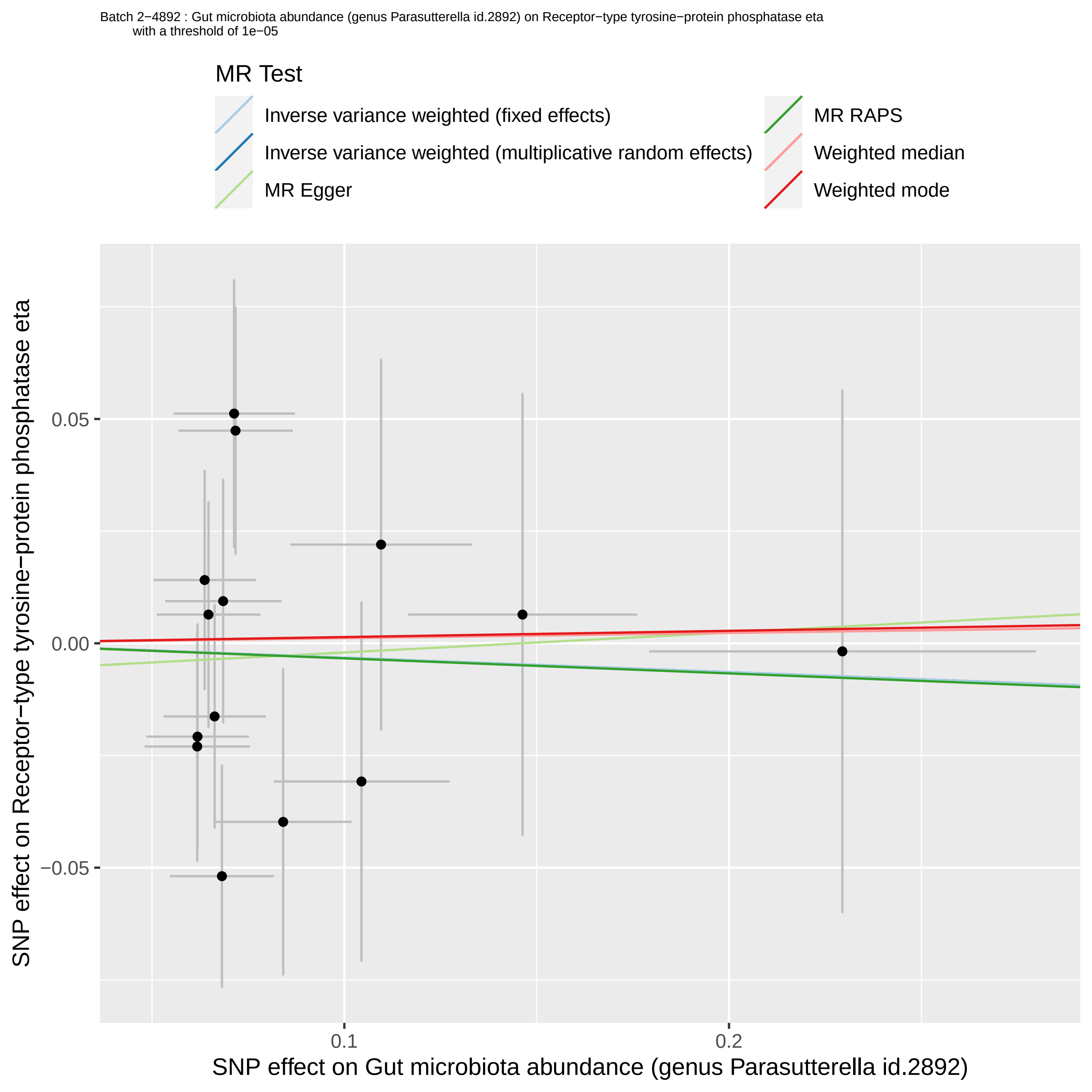
Batch 2−4892 : Gut microbiota abundance (genus Parasutterella id.2892) on Receptor−type tyrosine−protein phosphatase eta
with a threshold of 1e−05
MR Test
Inverse variance weighted (fixed effects)
Inverse variance weighted (multiplicative random effects)
MR Egger
MR RAPS
Weighted median
Weighted mode
0.05
0.00
−0.05
0.1
0.2
SNP effect on Receptor−type tyrosine−protein phosphatase eta
SNP effect on Gut microbiota abundance (genus Parasutterella id.2892)

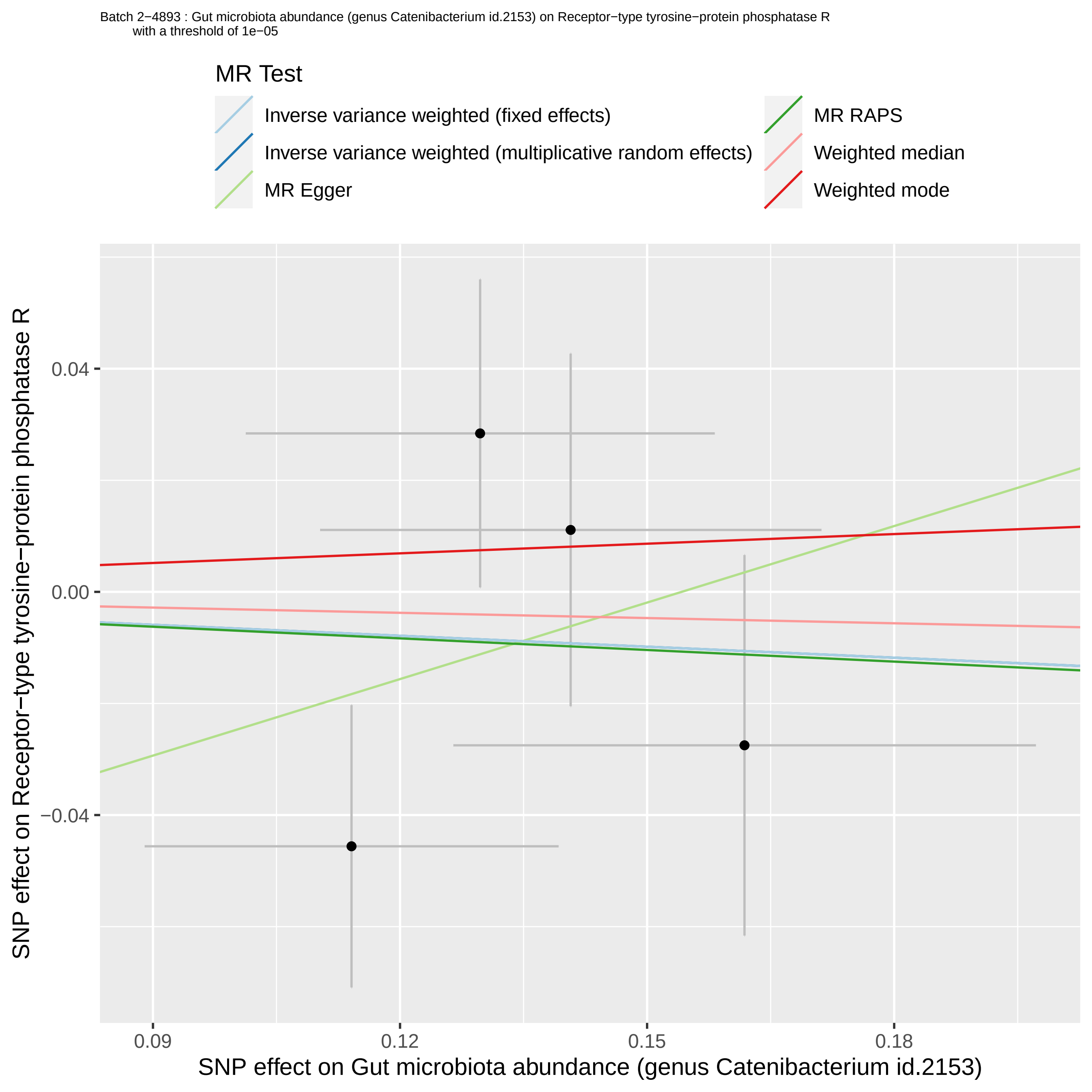
Batch 2−4893 : Gut microbiota abundance (genus Catenibacterium id.2153) on Receptor−type tyrosine−protein phosphatase R
with a threshold of 1e−05
MR Test
Inverse variance weighted (fixed effects)
Inverse variance weighted (multiplicative random effects)
MR Egger
MR RAPS
Weighted median
Weighted mode
0.04
0.00
−0.04
0.09
0.12
0.15
0.18
SNP effect on Receptor−type tyrosine−protein phosphatase R
SNP effect on Gut microbiota abundance (genus Catenibacterium id.2153)

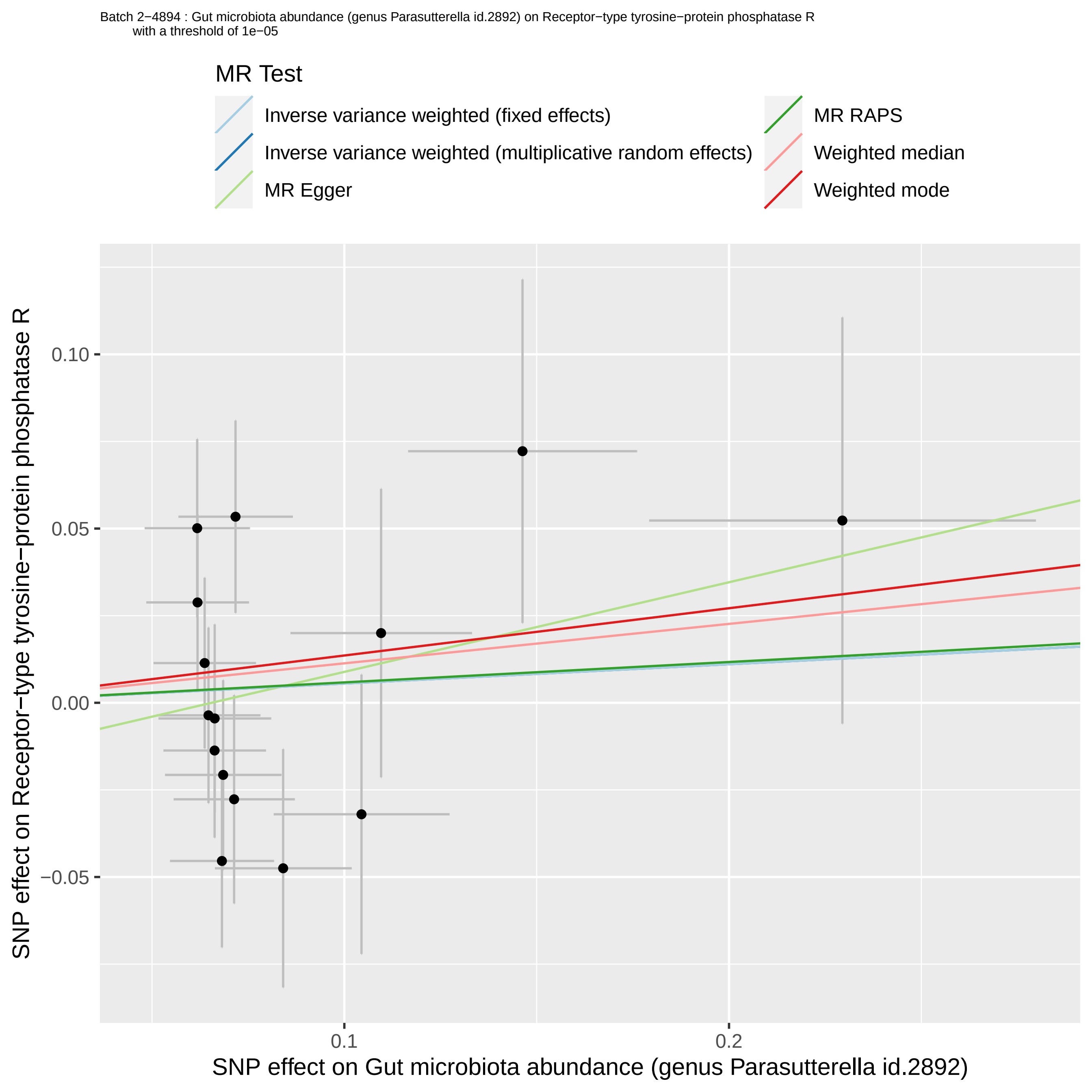
Batch 2−4894 : Gut microbiota abundance (genus Parasutterella id.2892) on Receptor−type tyrosine−protein phosphatase R
with a threshold of 1e−05
MR Test
Inverse variance weighted (fixed effects)
Inverse variance weighted (multiplicative random effects)
MR Egger
MR RAPS
Weighted median
Weighted mode
0.10
0.05
0.00
−0.05
0.1
0.2
SNP effect on Receptor−type tyrosine−protein phosphatase R
SNP effect on Gut microbiota abundance (genus Parasutterella id.2892)

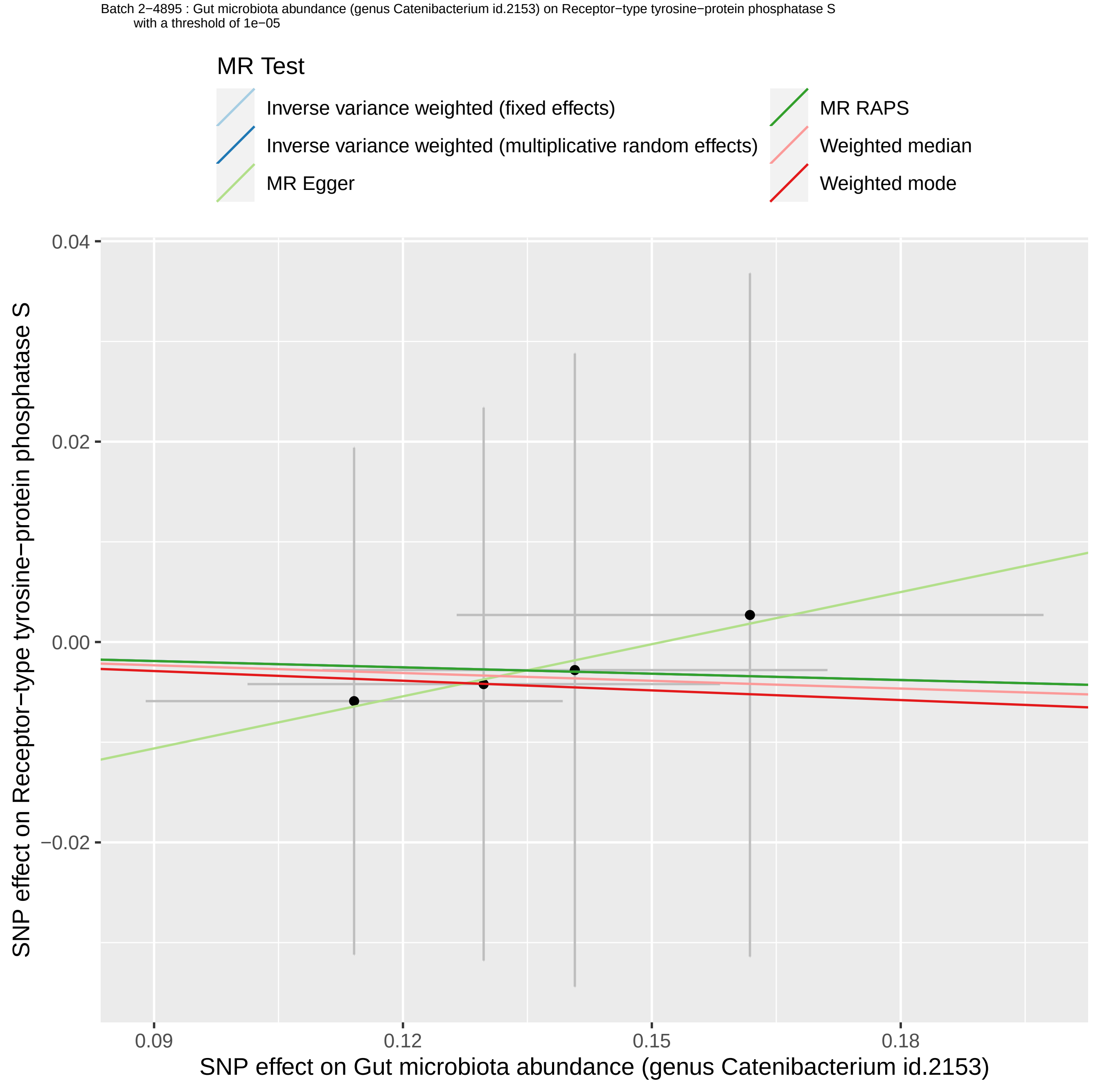

Batch 2−4895 : Gut microbiota abundance (genus Catenibacterium id.2153) on Receptor−type tyrosine−protein phosphatase S with a threshold of 1e−05
MR Test
Inverse variance weighted (fixed effects)
Inverse variance weighted (multiplicative random effects)
MR Egger
MR RAPS
Weighted median
Weighted mode
0.04
0.02
0.00
−0.02
0.09
0.12
0.15
0.18
SNP effect on Receptor−type tyrosine−protein phosphatase S
SNP effect on Gut microbiota abundance (genus Catenibacterium id.2153)

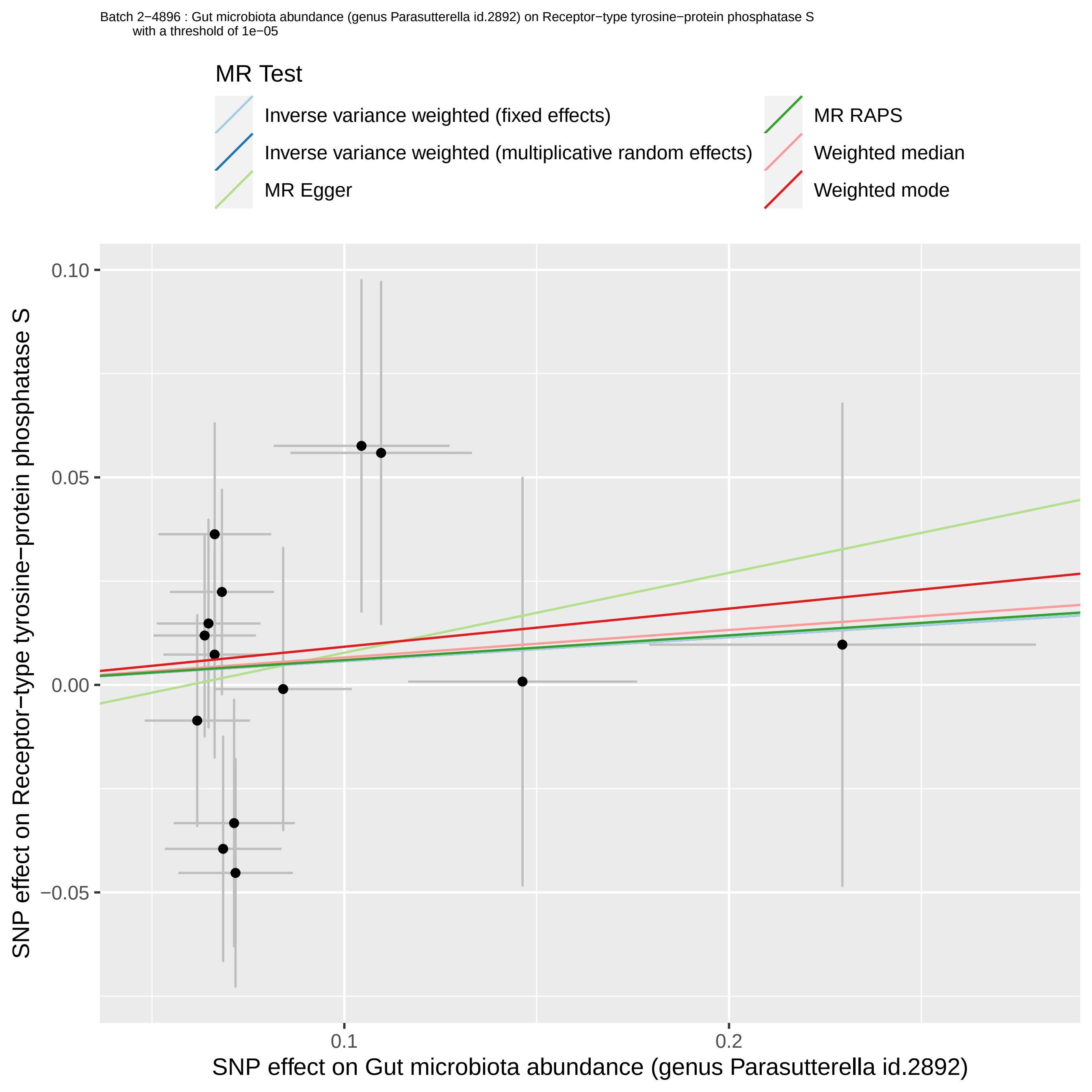
Batch 2−4896 : Gut microbiota abundance (genus Parasutterella id.2892) on Receptor−type tyrosine−protein phosphatase S
with a threshold of 1e−05
MR Test
Inverse variance weighted (fixed effects)
Inverse variance weighted (multiplicative random effects)
MR Egger
MR RAPS
Weighted median
Weighted mode
0.10
0.05
0.00
−0.05
0.1
0.2
SNP effect on Receptor−type tyrosine−protein phosphatase S
SNP effect on Gut microbiota abundance (genus Parasutterella id.2892)

Batch 2−4897 : Gut microbiota abundance (genus Catenibacterium id.2153) on Receptor−type tyrosine−protein phosphatase U
with a threshold of 1e−05

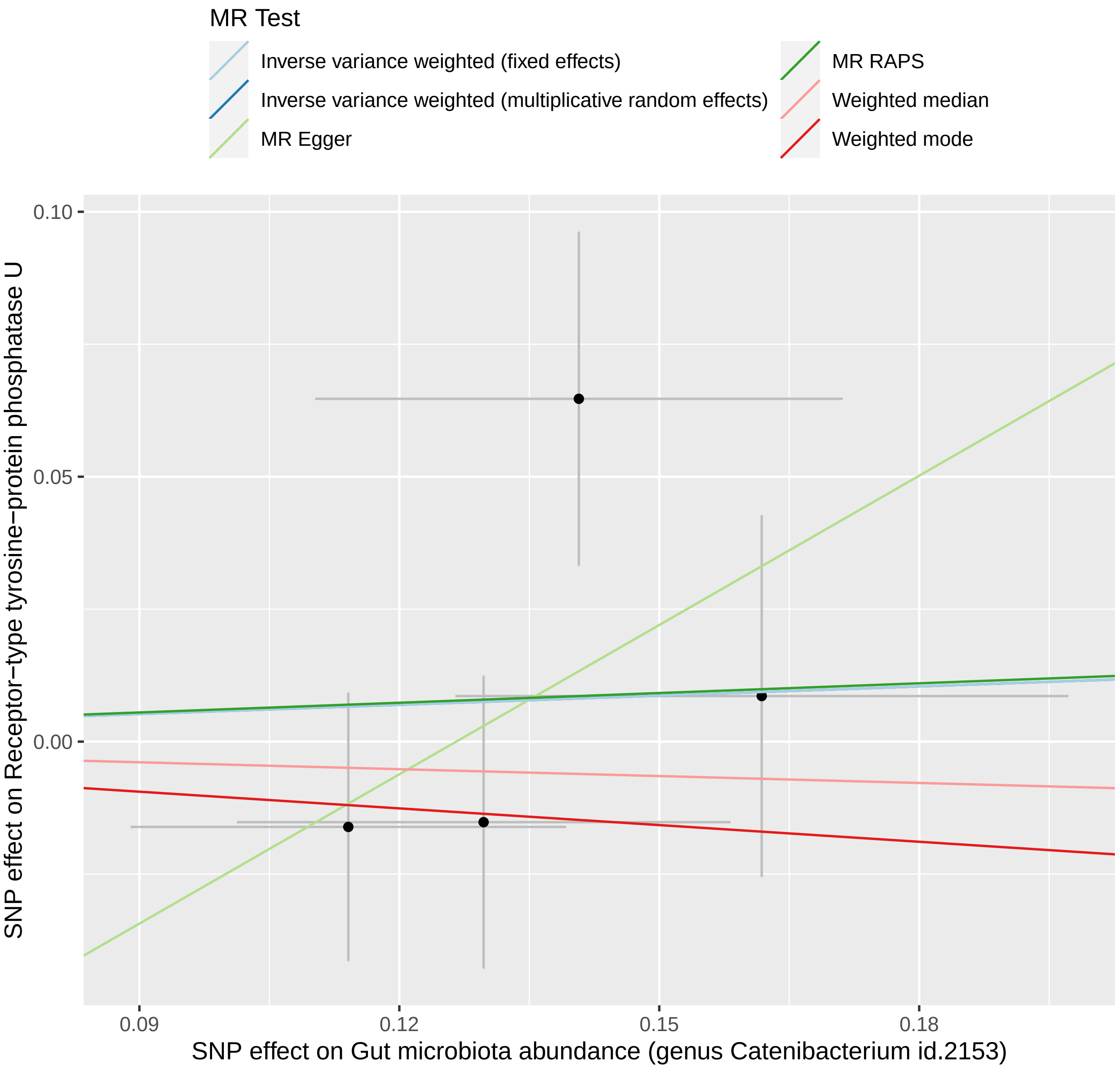

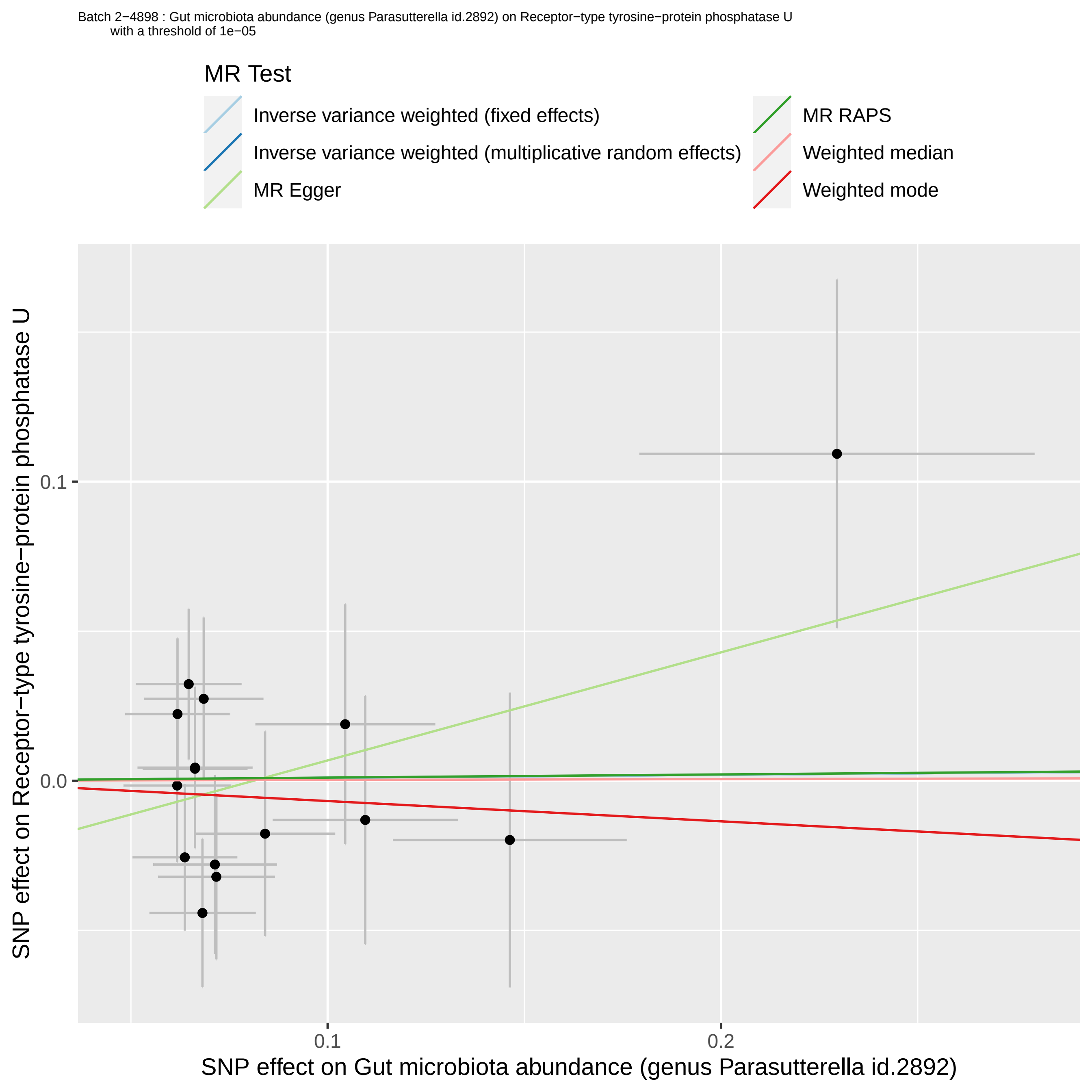

Batch 2−4898 : Gut microbiota abundance (genus Parasutterella id.2892) on Receptor−type tyrosine−protein phosphatase U
with a threshold of 1e−05
MR Test
Inverse variance weighted (fixed effects)
Inverse variance weighted (multiplicative random effects)
MR Egger
MR RAPS
Weighted median
Weighted mode
0.1
0.0
0.1
0.2
SNP effect on Receptor−type tyrosine−protein phosphatase U
SNP effect on Gut microbiota abundance (genus Parasutterella id.2892)

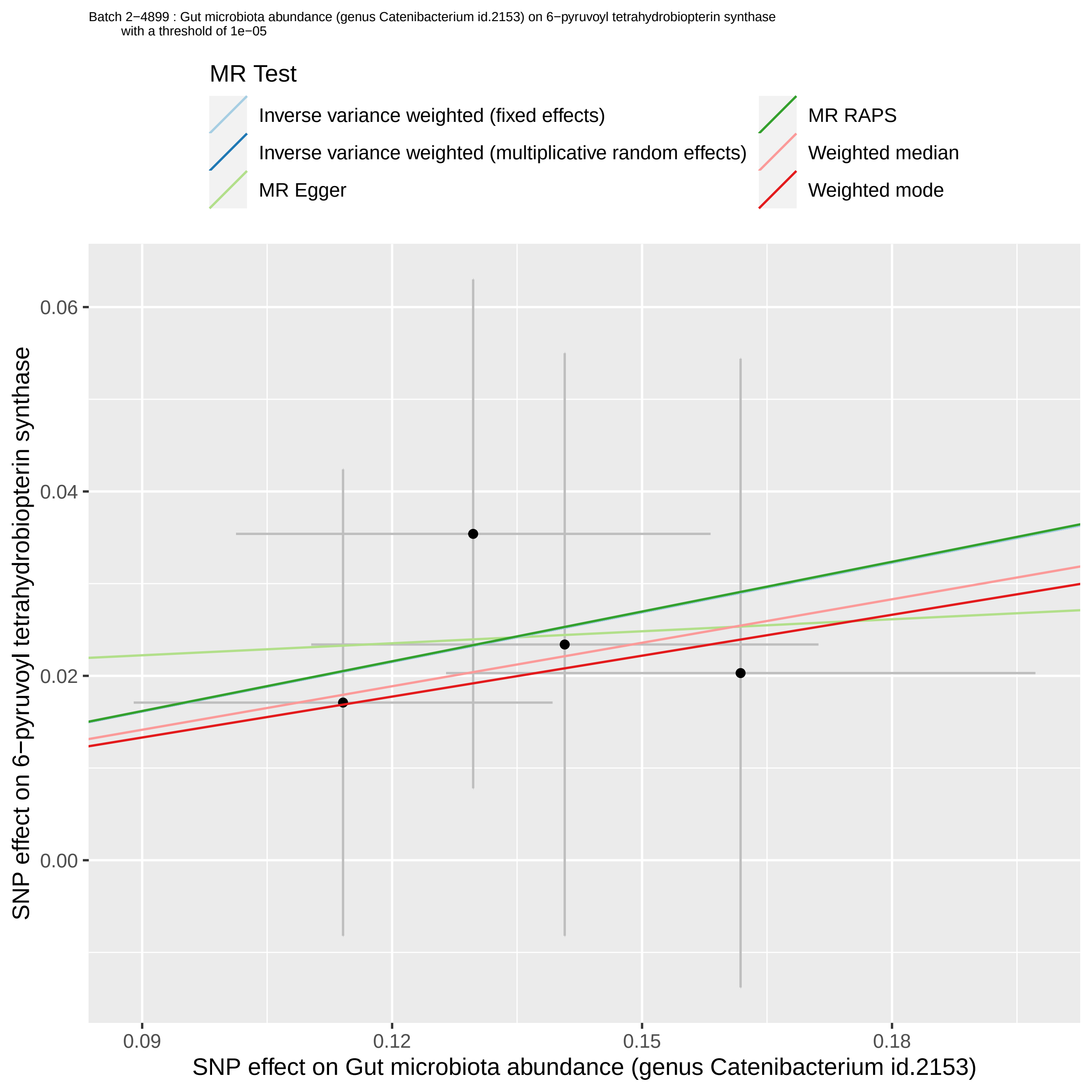
Batch 2−4899 : Gut microbiota abundance (genus Catenibacterium id.2153) on 6−pyruvoyl tetrahydrobiopterin synthase
with a threshold of 1e−05
MR Test
Inverse variance weighted (fixed effects)
Inverse variance weighted (multiplicative random effects)
MR Egger
MR RAPS
Weighted median
Weighted mode
0.06
0.04
0.02
0.00
0.09
0.12
0.15
0.18
SNP effect on 6−pyruvoyl tetrahydrobiopterin synthase
SNP effect on Gut microbiota abundance (genus Catenibacterium id.2153)

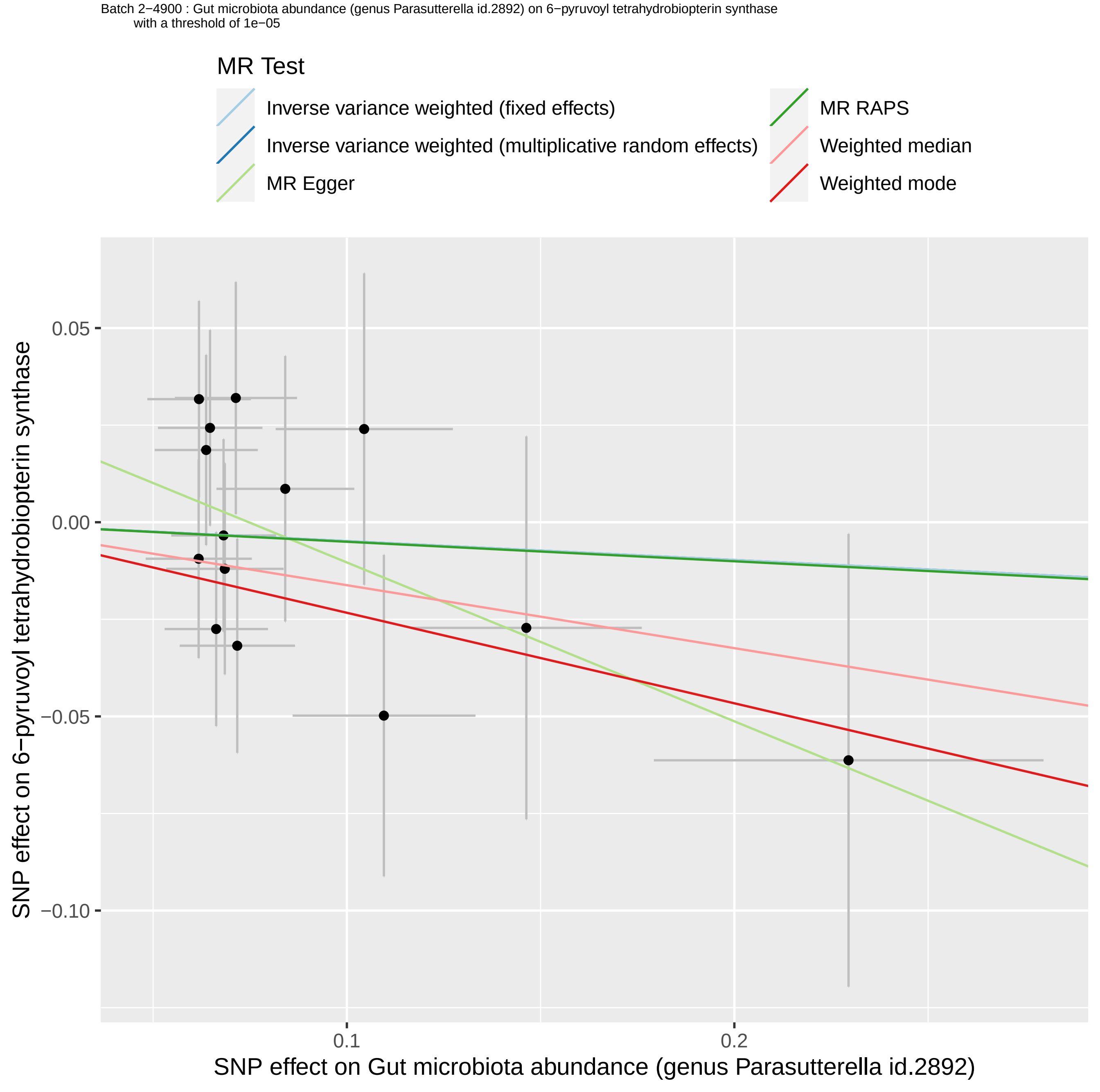
Batch 2−4900 : Gut microbiota abundance (genus Parasutterella id.2892) on 6−pyruvoyl tetrahydrobiopterin synthase
with a threshold of 1e−05
MR Test
Inverse variance weighted (fixed effects)
Inverse variance weighted (multiplicative random effects)
MR Egger
MR RAPS
Weighted median
Weighted mode
0.05
0.00
−0.05
−0.10
0.1
0.2
SNP effect on 6−pyruvoyl tetrahydrobiopterin synthase
SNP effect on Gut microbiota abundance (genus Parasutterella id.2892)

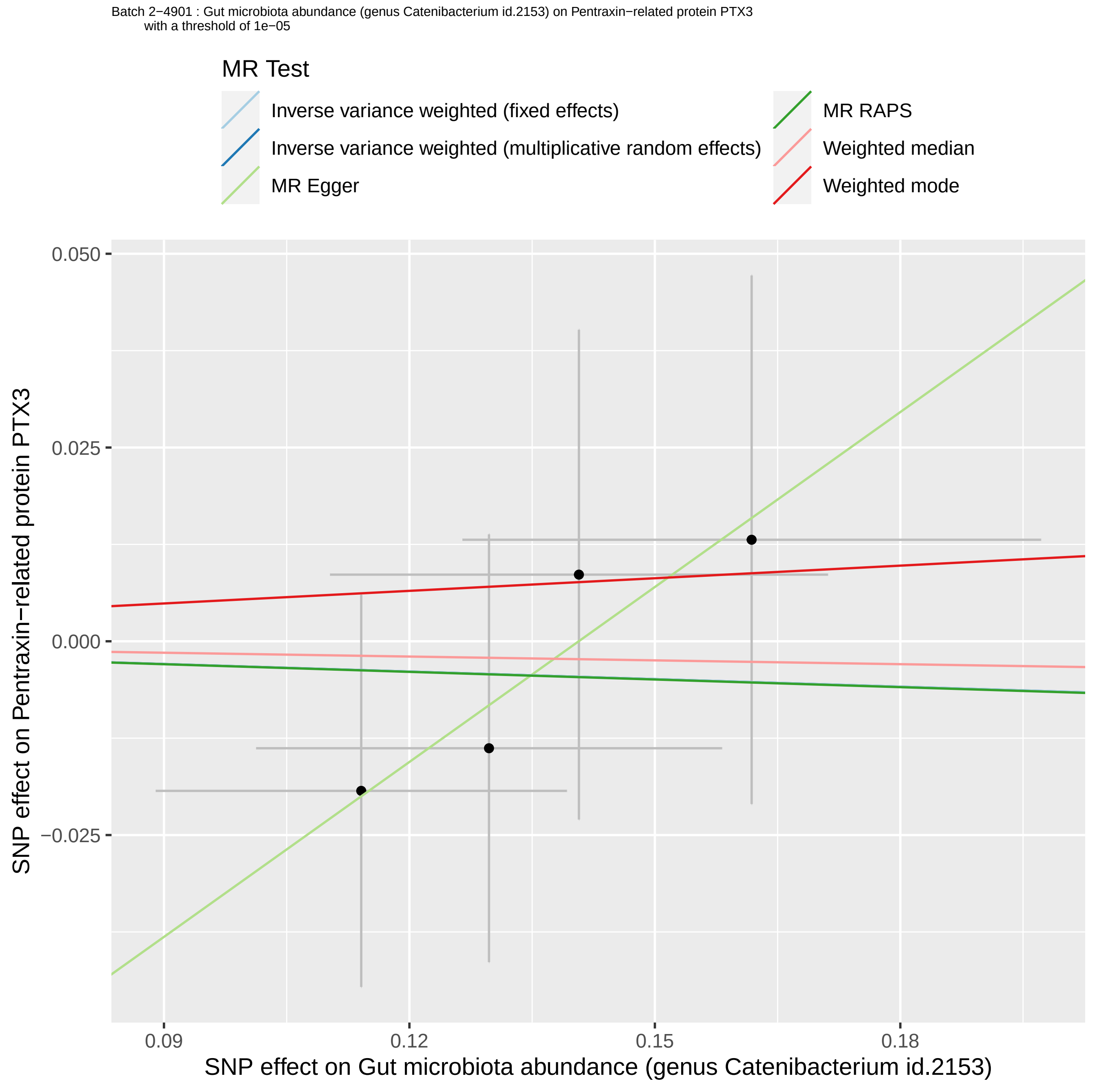

Batch 2−4901 : Gut microbiota abundance (genus Catenibacterium id.2153) on Pentraxin−related protein PTX3
with a threshold of 1e−05
MR Test
Inverse variance weighted (fixed effects)
Inverse variance weighted (multiplicative random effects)
MR Egger
MR RAPS
Weighted median
Weighted mode
0.050
0.025
0.000
−0.025
0.09
0.12
0.15
0.18
SNP effect on Pentraxin−related protein PTX3
SNP effect on Gut microbiota abundance (genus Catenibacterium id.2153)

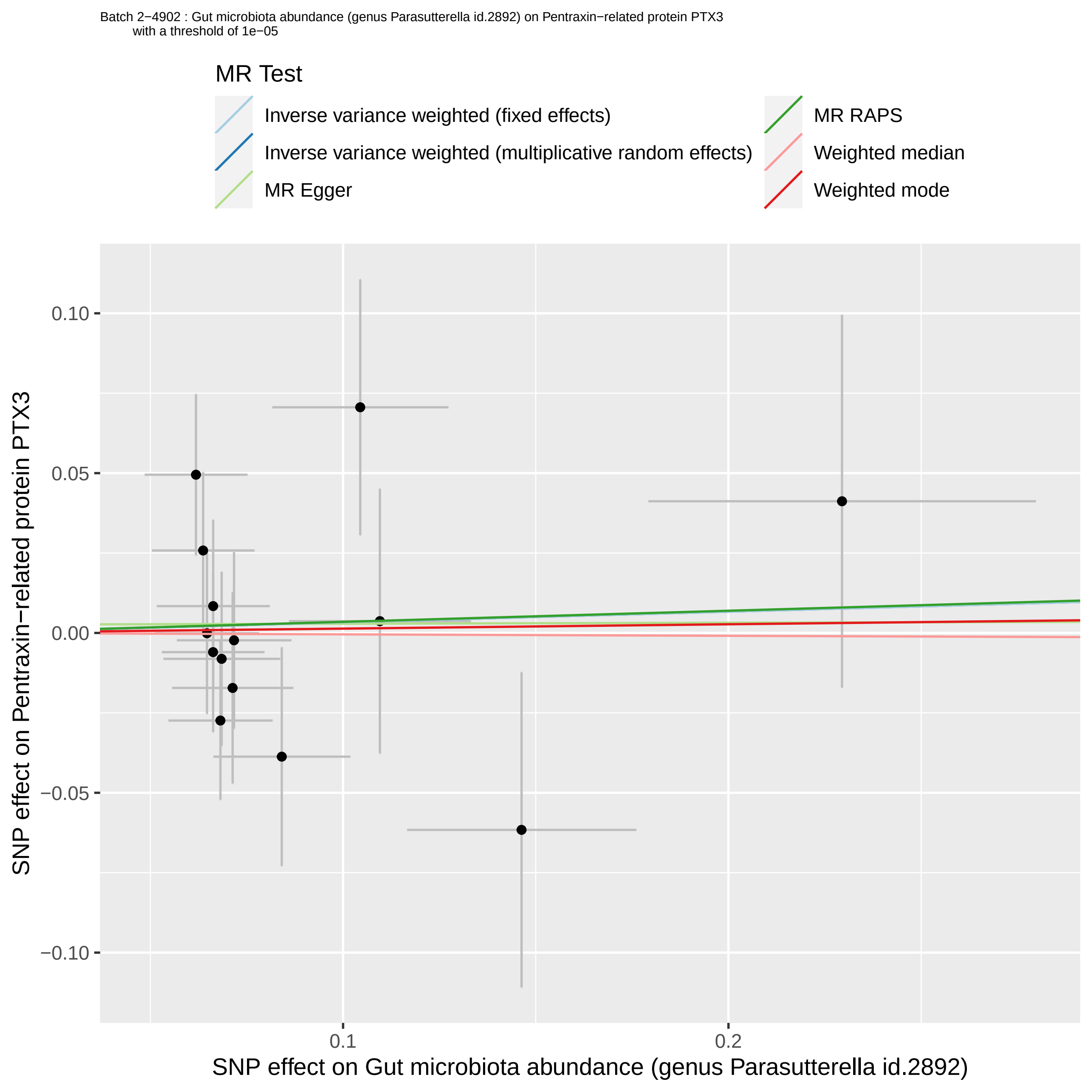
Batch 2−4902 : Gut microbiota abundance (genus Parasutterella id.2892) on Pentraxin−related protein PTX3
with a threshold of 1e−05
MR Test
Inverse variance weighted (fixed effects)
Inverse variance weighted (multiplicative random effects)
MR Egger
MR RAPS
Weighted median
Weighted mode
0.10
0.05
0.00
−0.05
−0.10
0.1
0.2
SNP effect on Pentraxin−related protein PTX3
SNP effect on Gut microbiota abundance (genus Parasutterella id.2892)

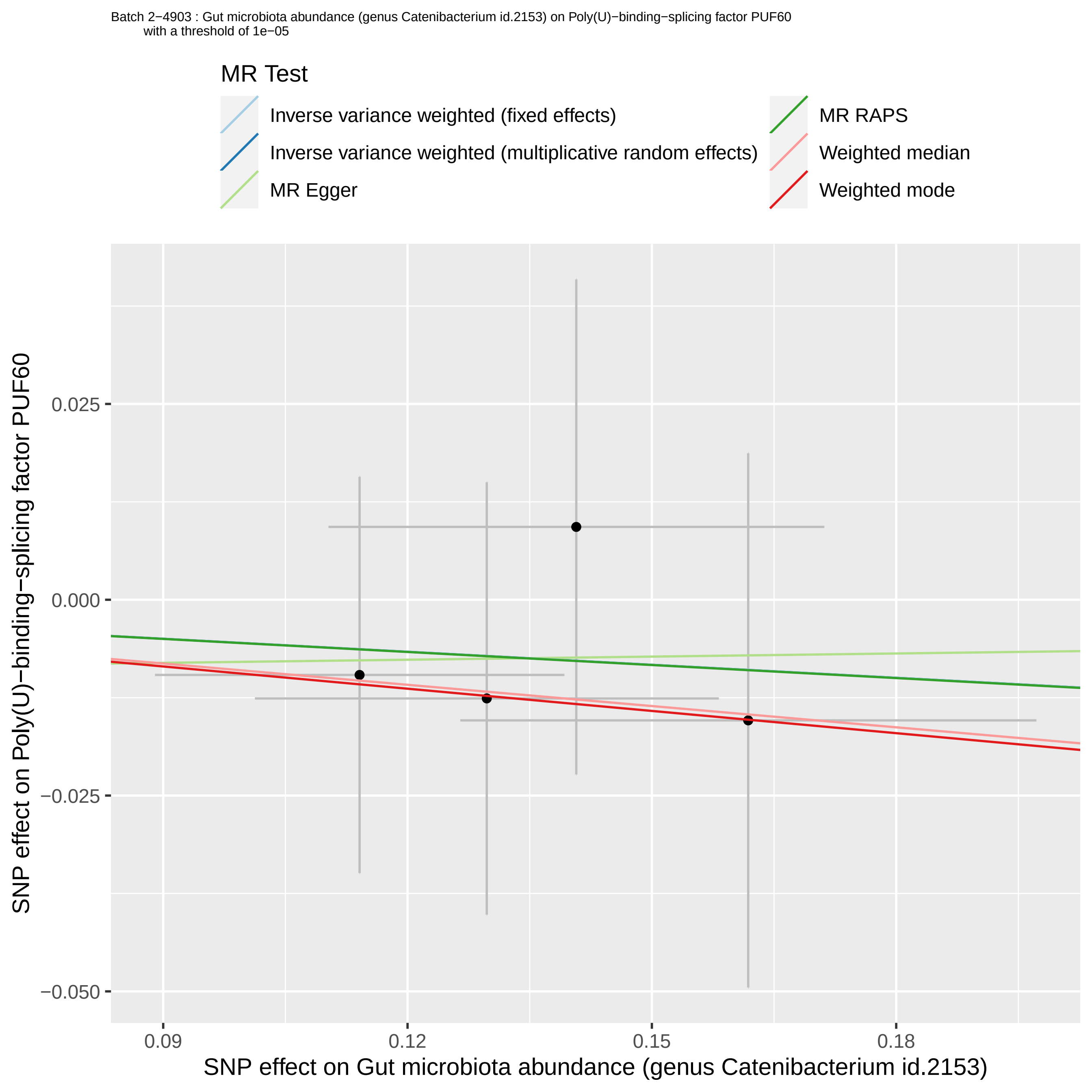

Batch 2−4903 : Gut microbiota abundance (genus Catenibacterium id.2153) on Poly(U)−binding−splicing factor PUF60
with a threshold of 1e−05
MR Test
Inverse variance weighted (fixed effects)
Inverse variance weighted (multiplicative random effects)
MR Egger
MR RAPS
Weighted median
Weighted mode
0.025
0.000
−0.025
−0.050
0.09
0.12
0.15
0.18
SNP effect on Gut microbiota abundance (genus Catenibacterium id.2153)
SNP effect on Poly(U)−binding−splicing factor PUF60

Batch 2−4904 : Gut microbiota abundance (genus Parasutterella id.2892) on Poly(U)−binding−splicing factor PUF60
with a threshold of 1e−05

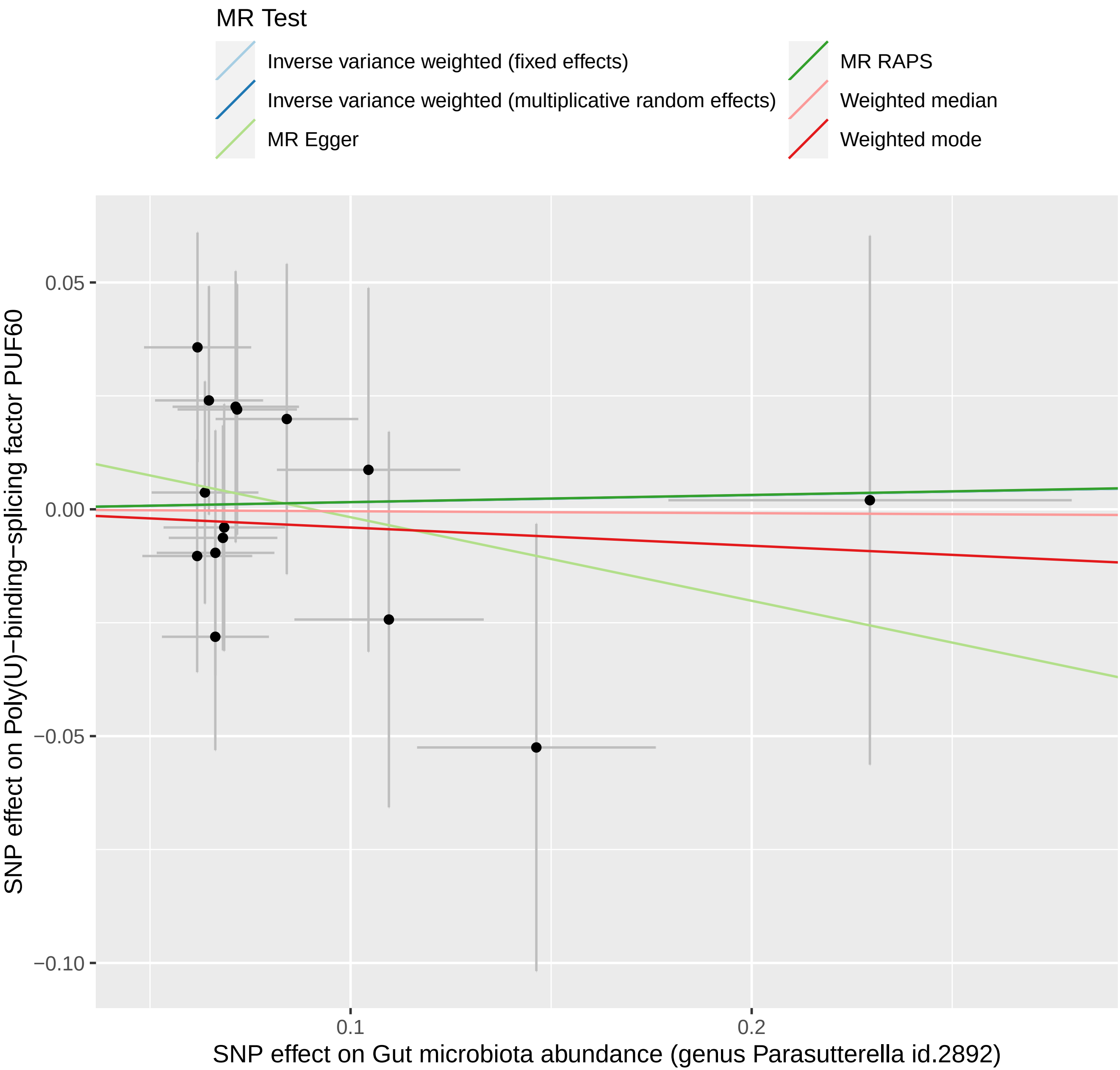

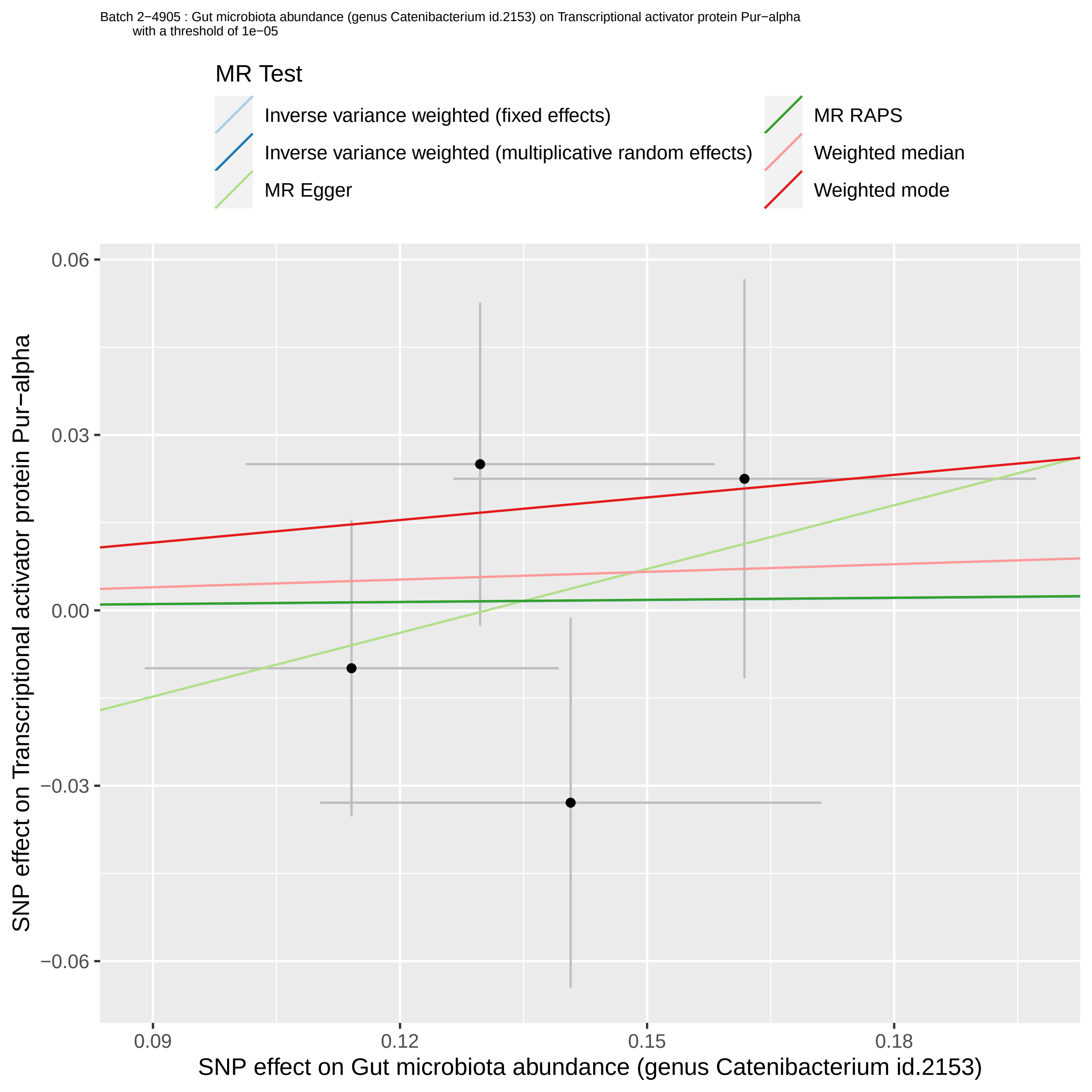

Batch 2−4905 : Gut microbiota abundance (genus Catenibacterium id.2153) on Transcriptional activator protein Pur−alpha
with a threshold of 1e−05
MR Test
Inverse variance weighted (fixed effects)
Inverse variance weighted (multiplicative random effects)
MR Egger
MR RAPS
Weighted median
Weighted mode
0.06
0.03
0.00
−0.03
−0.06
0.09
0.12
0.15
0.18
SNP effect on Transcriptional activator protein Pur−alpha
SNP effect on Gut microbiota abundance (genus Catenibacterium id.2153)

Batch 2−4906 : Gut microbiota abundance (genus Parasutterella id.2892) on Transcriptional activator protein Pur−alpha
with a threshold of 1e−05

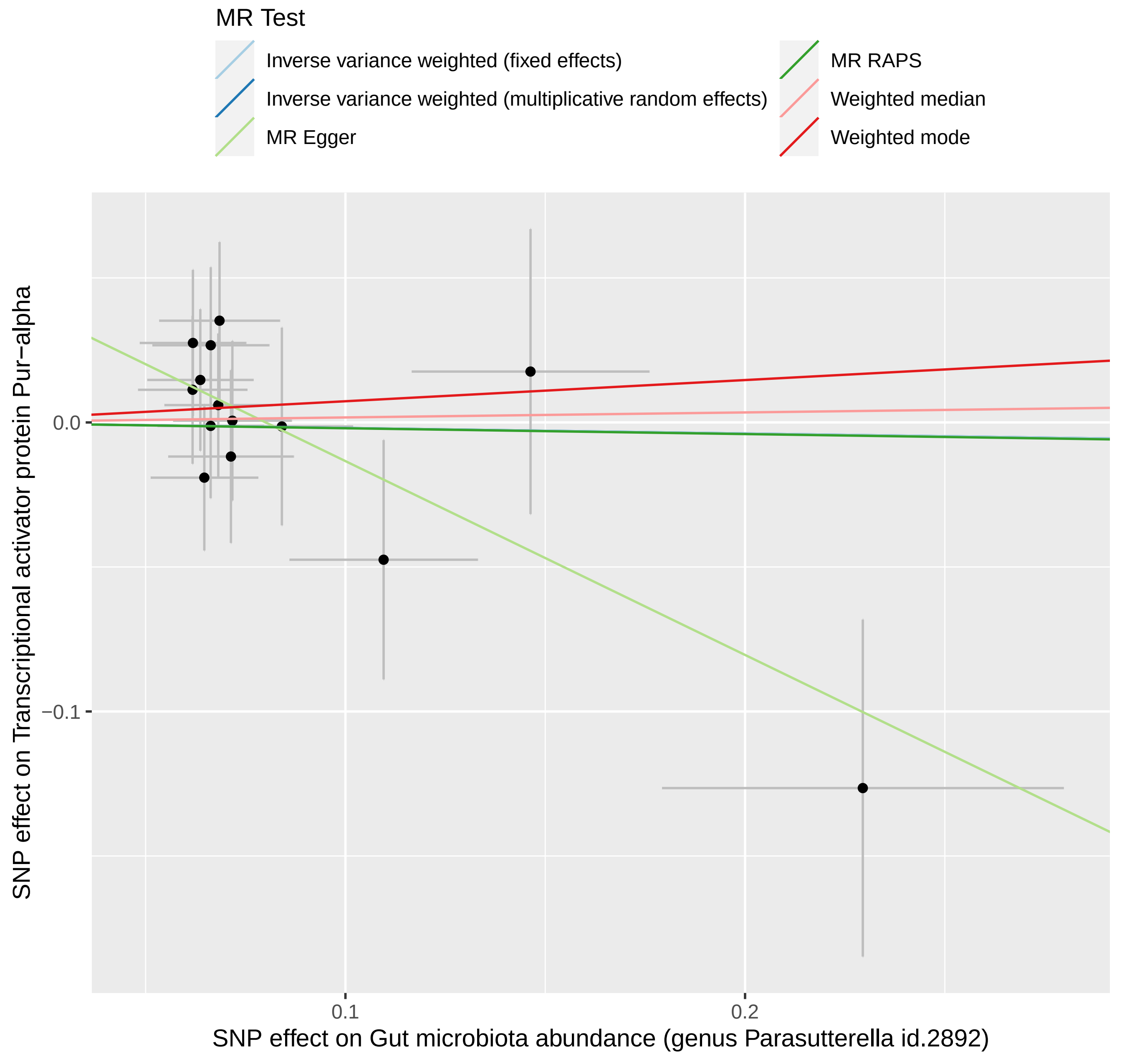

Batch 2−4907 : Gut microbiota abundance (genus Catenibacterium id.2153) on tRNA pseudouridine synthase A, mitochondrial
with a threshold of 1e−05

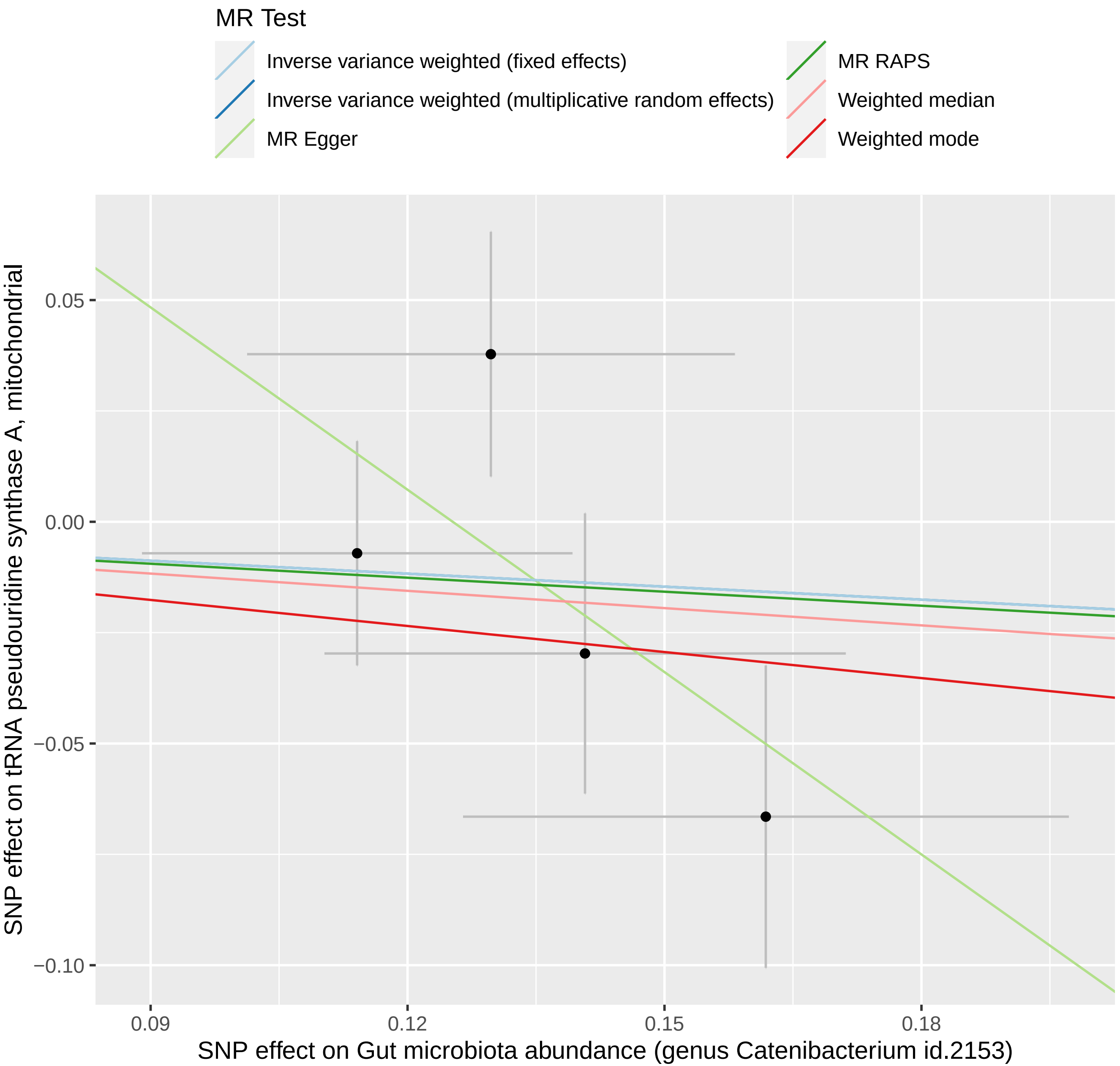

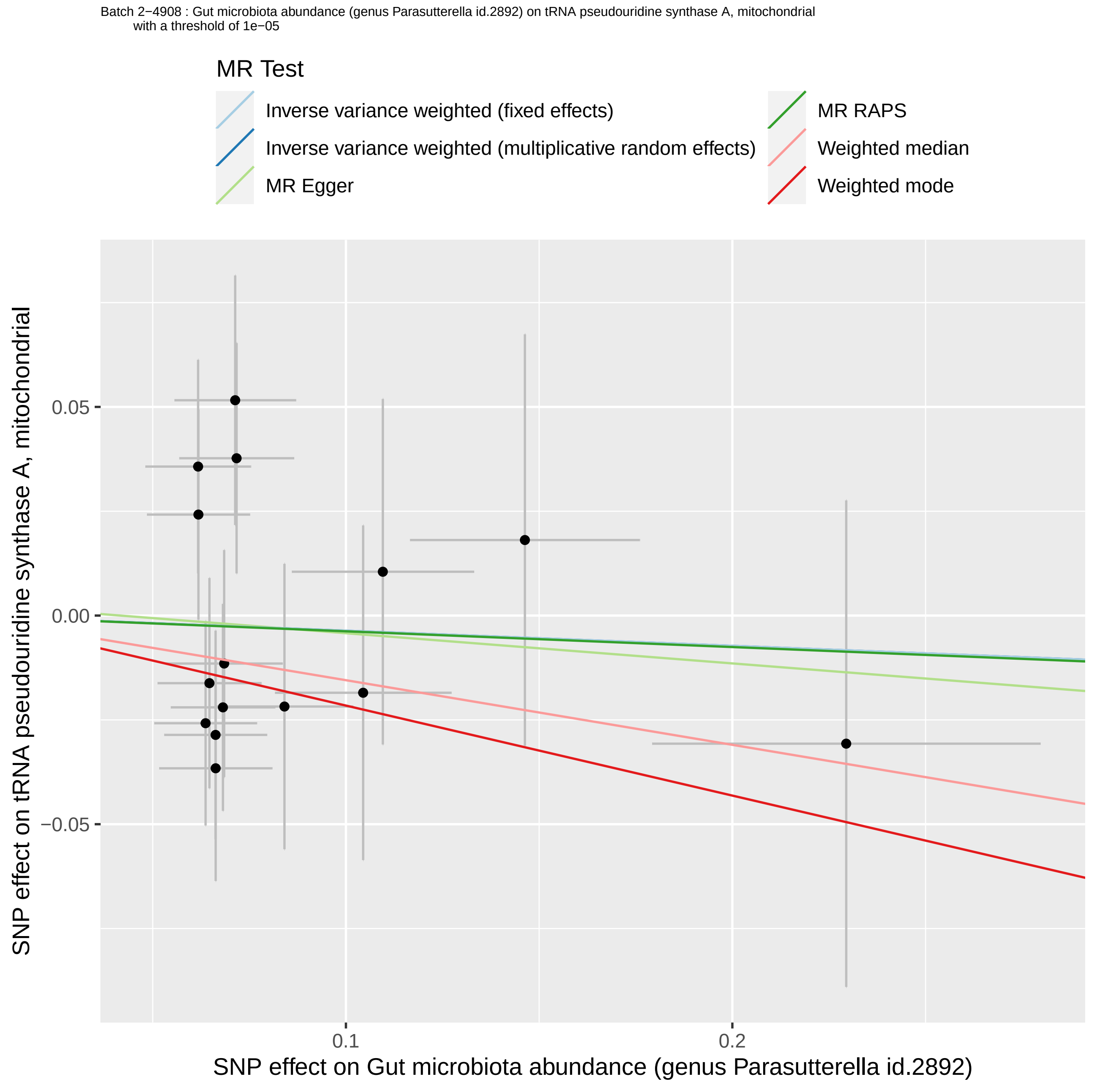
Batch 2−4908 : Gut microbiota abundance (genus Parasutterella id.2892) on tRNA pseudouridine synthase A, mitochondrial
with a threshold of 1e−05
MR Test
Inverse variance weighted (fixed effects)
Inverse variance weighted (multiplicative random effects)
MR Egger
MR RAPS
Weighted median
Weighted mode
0.05
0.00
−0.05
0.1
0.2
SNP effect on tRNA pseudouridine synthase A, mitochondrial
SNP effect on Gut microbiota abundance (genus Parasutterella id.2892)

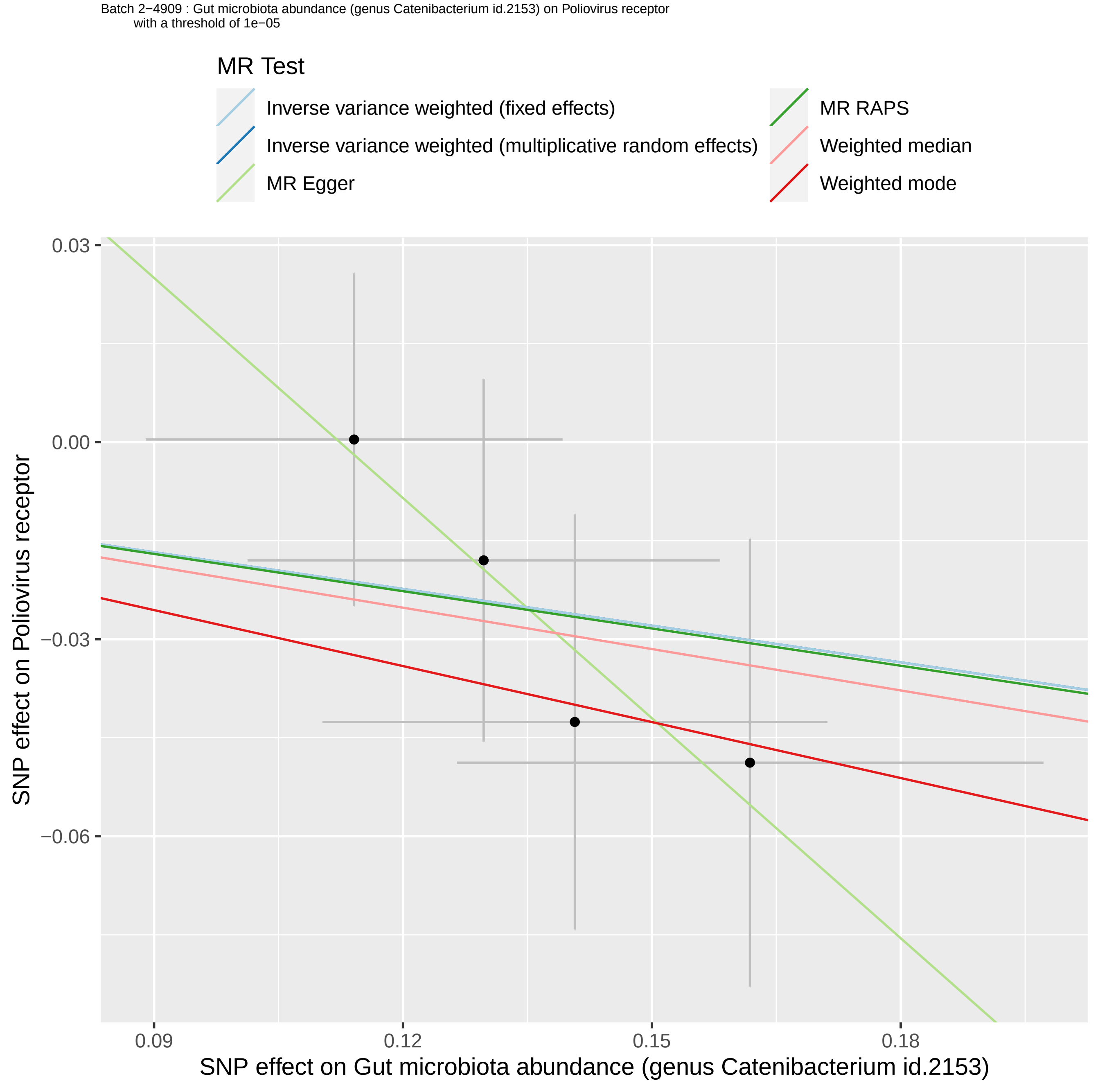
Batch 2−4909 : Gut microbiota abundance (genus Catenibacterium id.2153) on Poliovirus receptor
with a threshold of 1e−05
MR Test
Inverse variance weighted (fixed effects)
Inverse variance weighted (multiplicative random effects)
MR Egger
MR RAPS
Weighted median
Weighted mode
0.03
0.00
−0.03
−0.06
0.09
0.12
0.15
0.18
SNP effect on Poliovirus receptor
SNP effect on Gut microbiota abundance (genus Catenibacterium id.2153)

Batch 2−4910 : Gut microbiota abundance (genus Parasutterella id.2892) on Poliovirus receptor
with a threshold of 1e−05

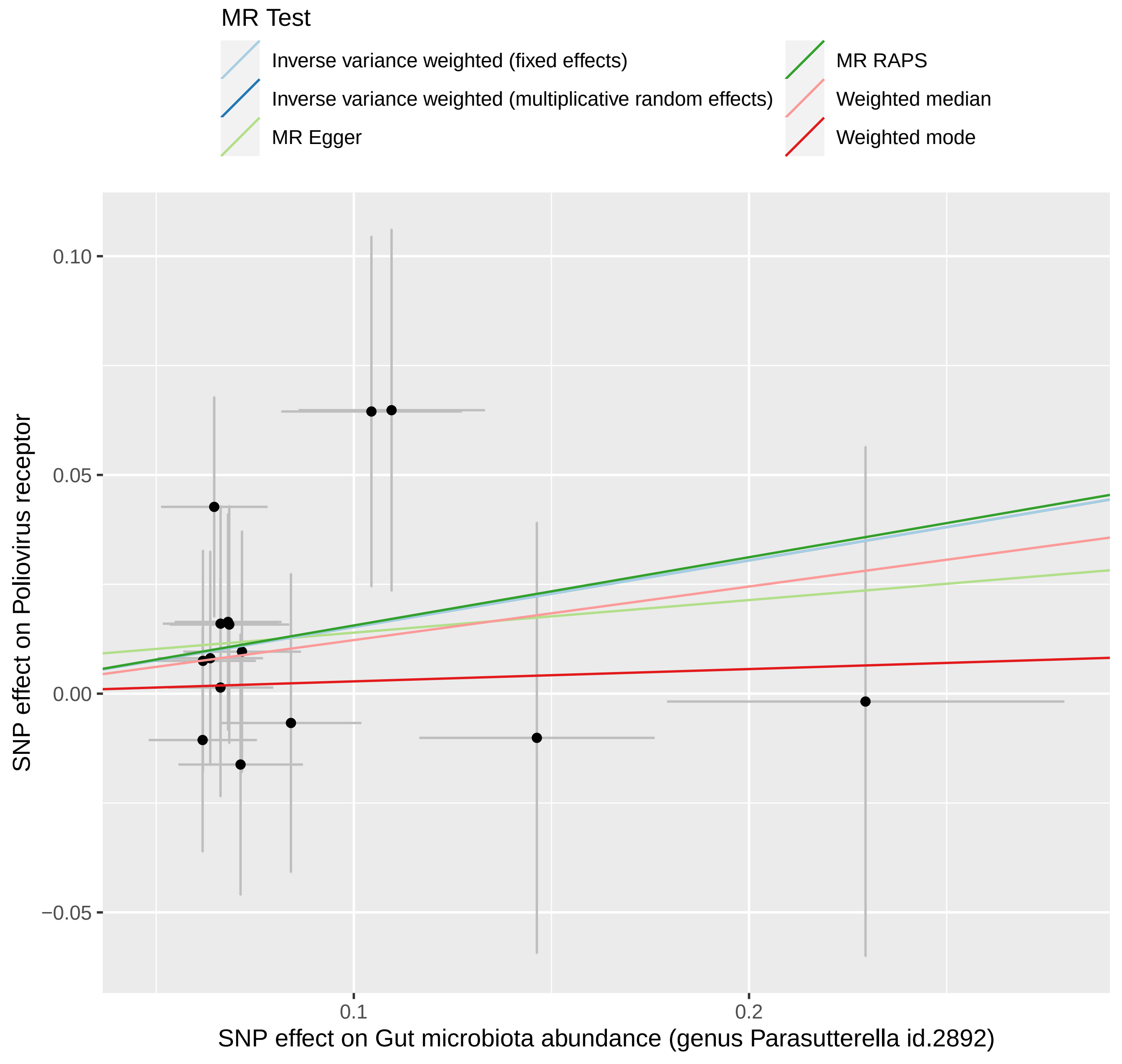

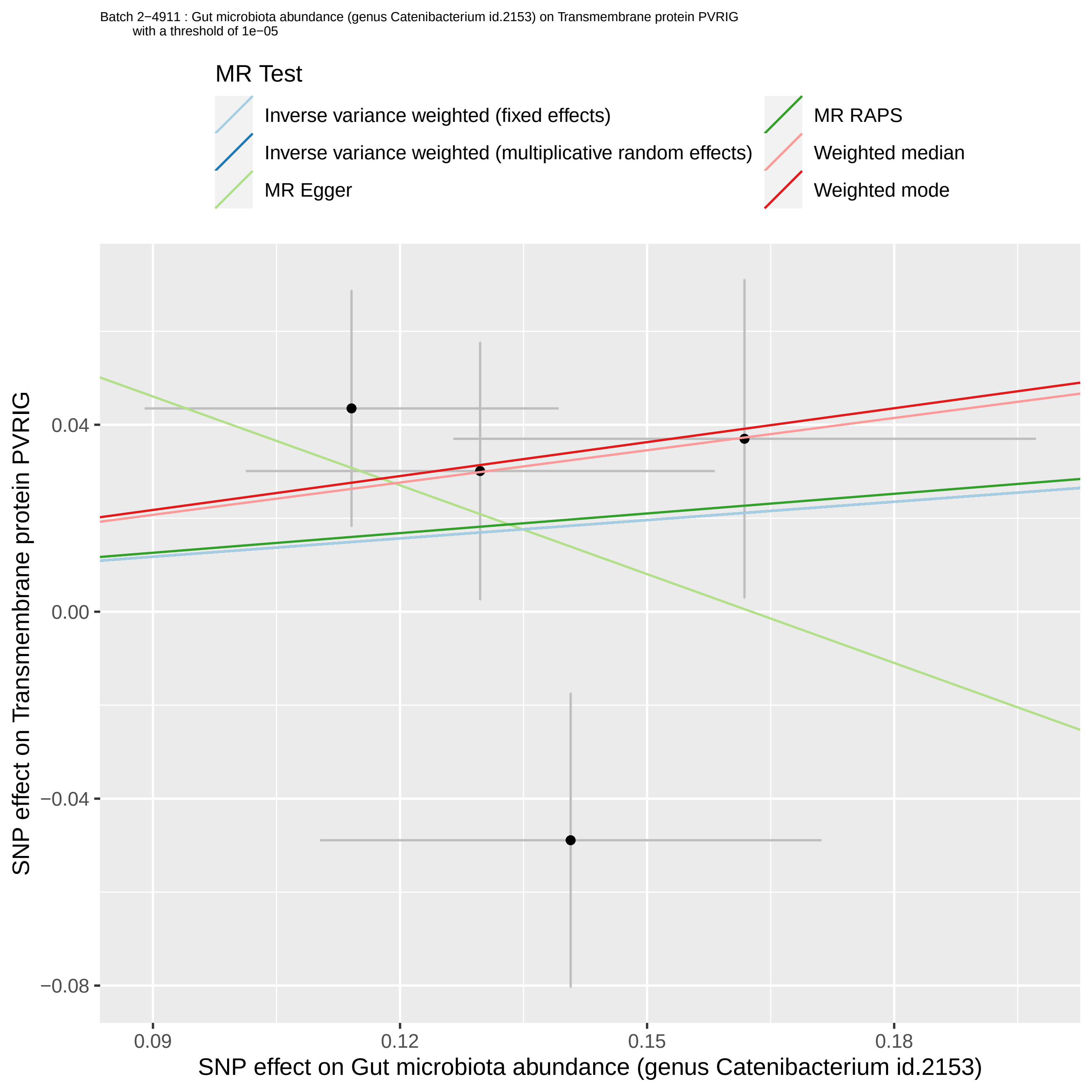

Batch 2−4911 : Gut microbiota abundance (genus Catenibacterium id.2153) on Transmembrane protein PVRIG
with a threshold of 1e−05
MR Test
Inverse variance weighted (fixed effects)
Inverse variance weighted (multiplicative random effects)
MR Egger
MR RAPS
Weighted median
Weighted mode
0.04
0.00
−0.04
−0.08
0.09
0.12
0.15
0.18
SNP effect on Transmembrane protein PVRIG
SNP effect on Gut microbiota abundance (genus Catenibacterium id.2153)

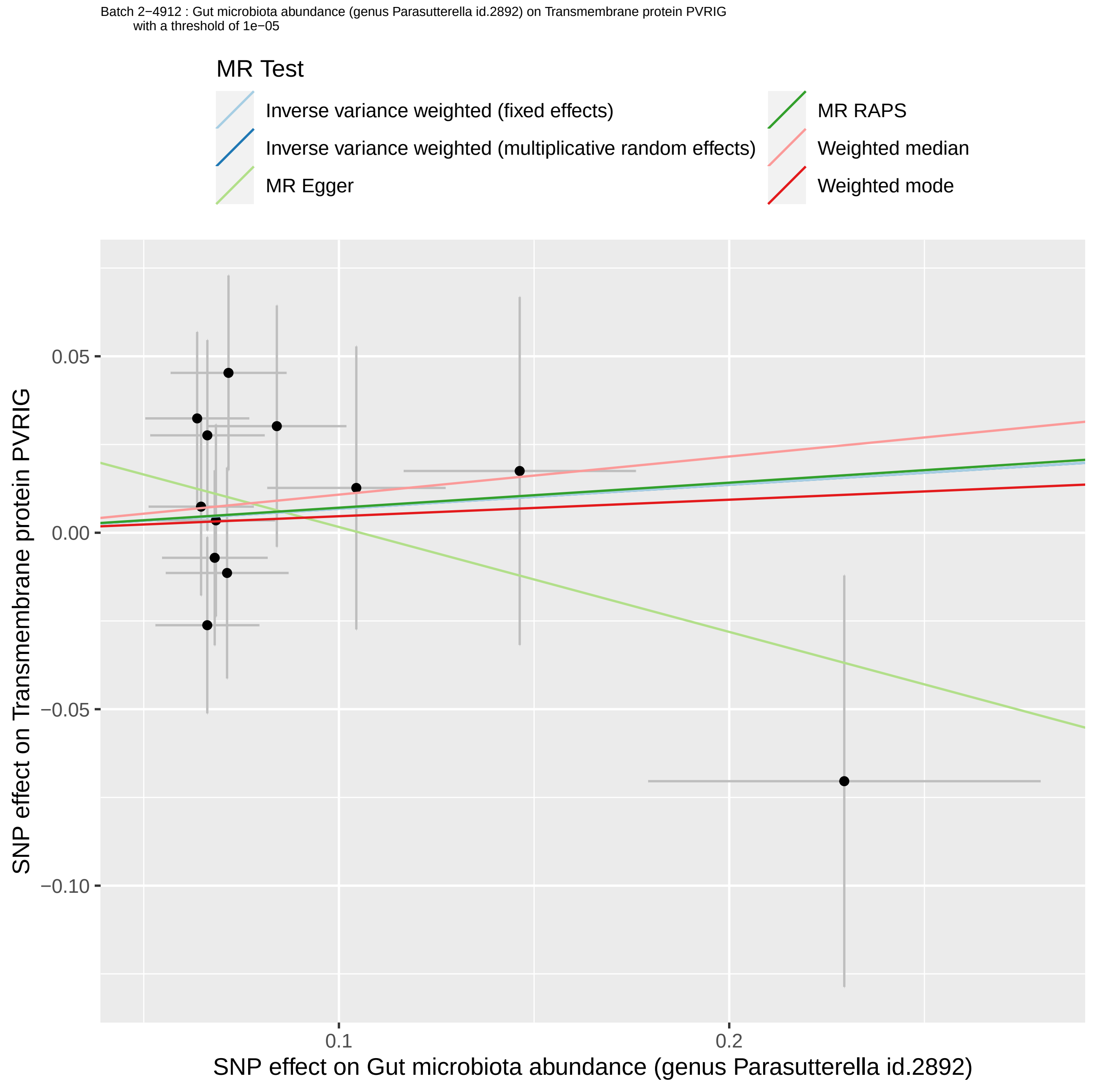
Batch 2−4912 : Gut microbiota abundance (genus Parasutterella id.2892) on Transmembrane protein PVRIG
with a threshold of 1e−05
MR Test
Inverse variance weighted (fixed effects)
Inverse variance weighted (multiplicative random effects)
MR Egger
MR RAPS
Weighted median
Weighted mode
SNP effect on Transmembrane protein PVRIG
0.05
0.00
−0.05
−0.10
0.1
0.2
SNP effect on Gut microbiota abundance (genus Parasutterella id.2892)

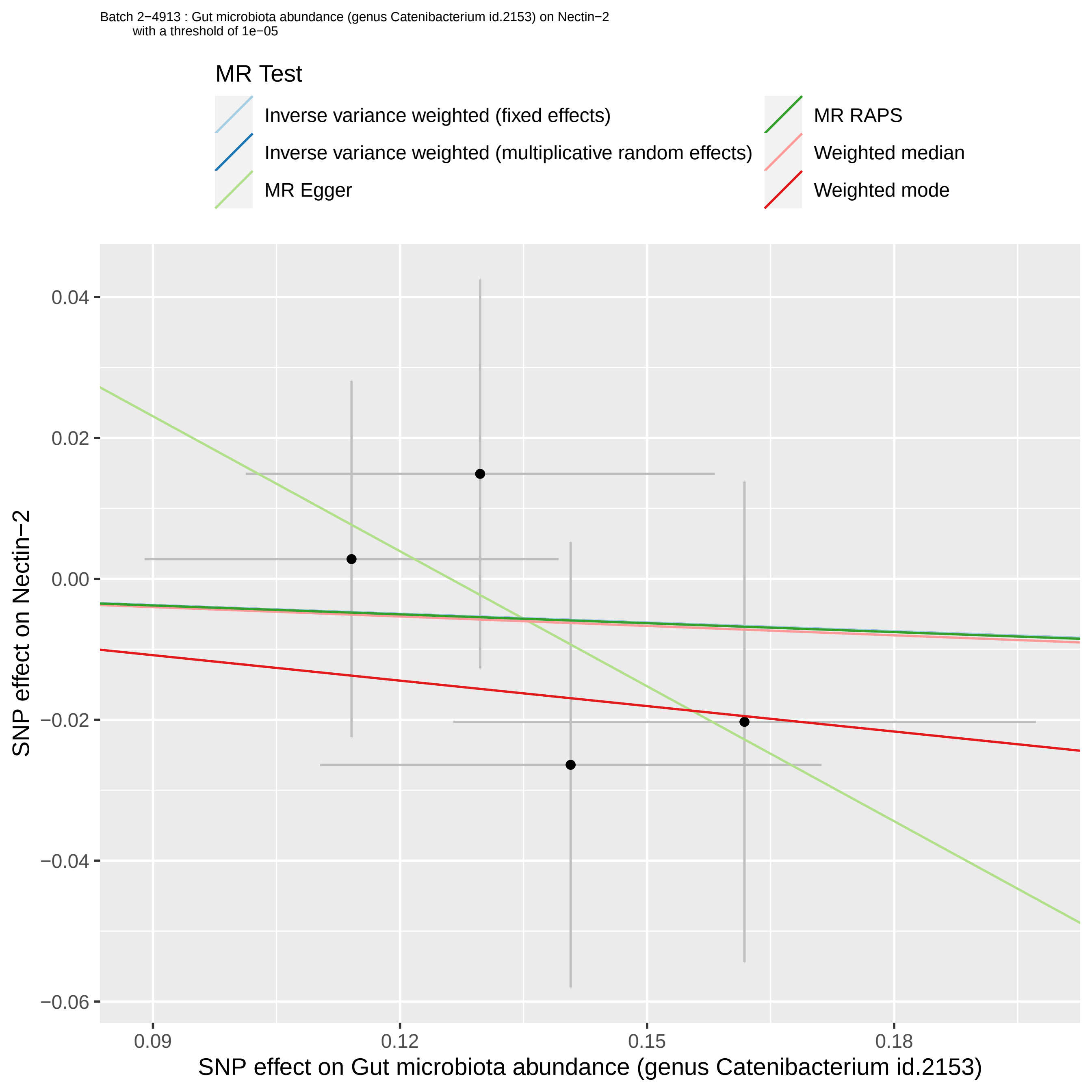

Batch 2−4913 : Gut microbiota abundance (genus Catenibacterium id.2153) on Nectin−2
with a threshold of 1e−05
MR Test
Inverse variance weighted (fixed effects)
Inverse variance weighted (multiplicative random effects)
MR Egger
MR RAPS
Weighted median
Weighted mode
0.04
0.02
0.00
−0.02
−0.04
−0.06
0.09
0.12
0.15
0.18
SNP effect on Nectin−2
SNP effect on Gut microbiota abundance (genus Catenibacterium id.2153)

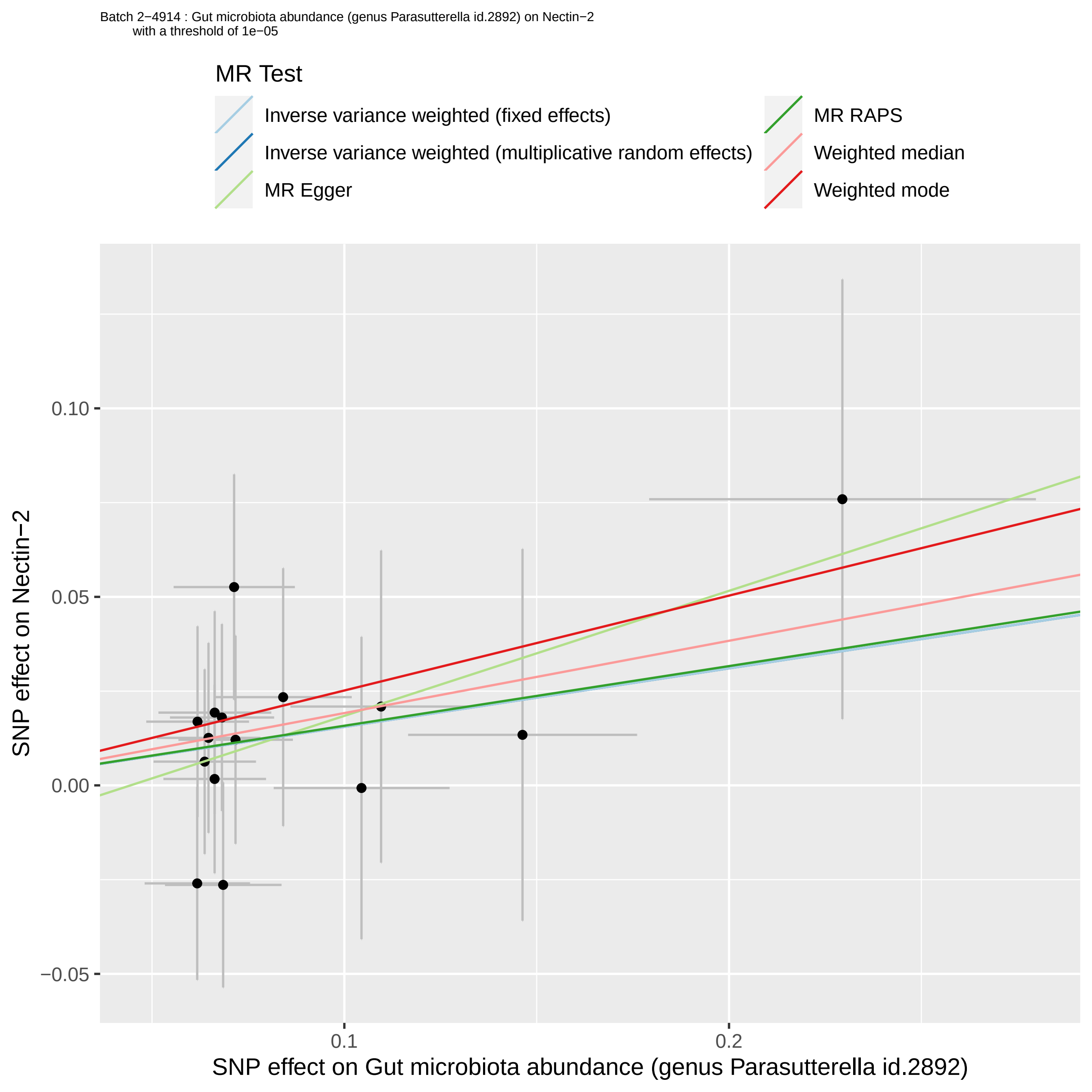
Batch 2−4914 : Gut microbiota abundance (genus Parasutterella id.2892) on Nectin−2
with a threshold of 1e−05
MR Test
Inverse variance weighted (fixed effects)
Inverse variance weighted (multiplicative random effects)
MR Egger
MR RAPS
Weighted median
Weighted mode
0.10
0.05
0.00
−0.05
0.1
0.2
SNP effect on Nectin−2
SNP effect on Gut microbiota abundance (genus Parasutterella id.2892)

Batch 2−4915 : Gut microbiota abundance (genus Catenibacterium id.2153) on Nectin−3
with a threshold of 1e−05

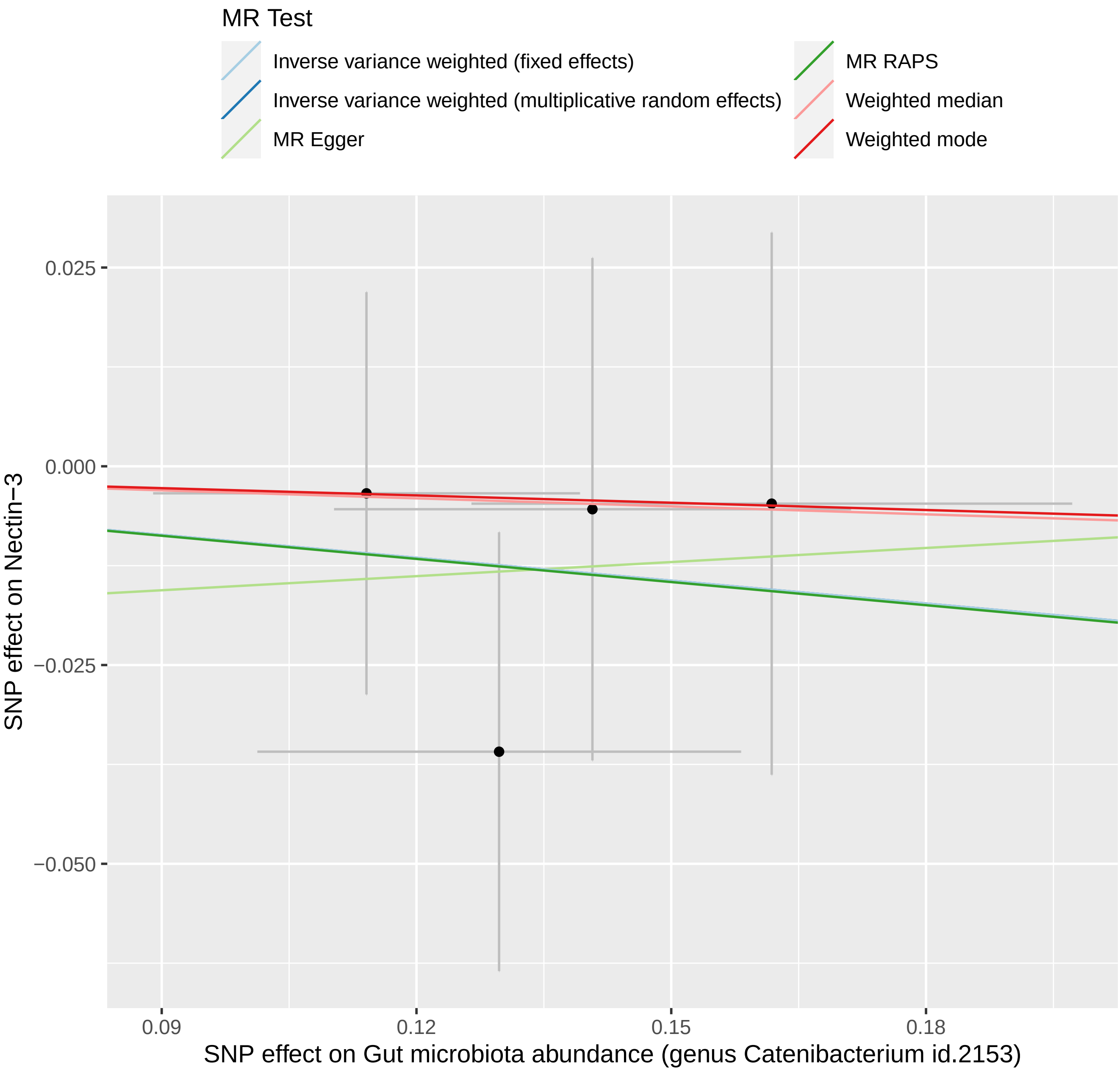

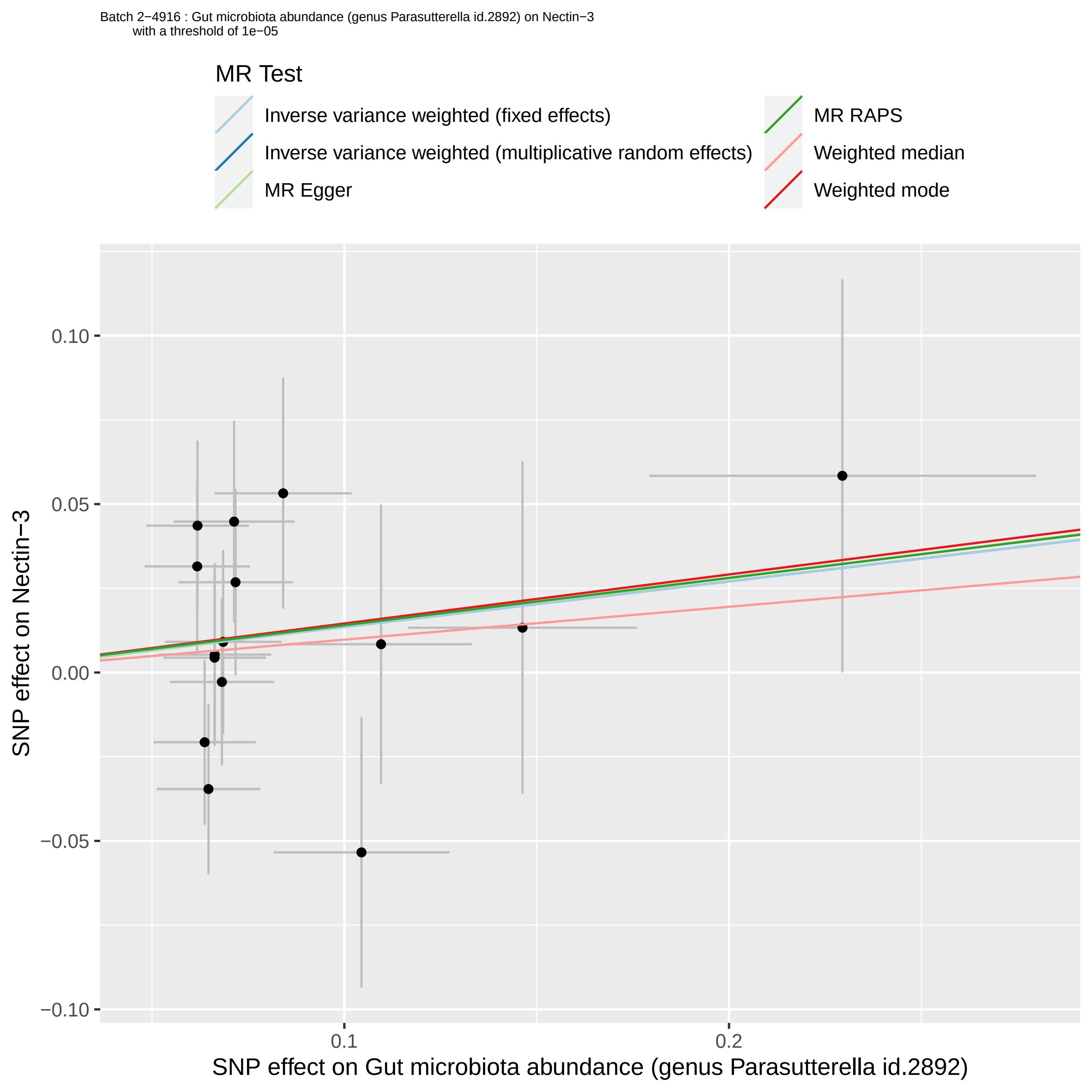
Batch 2−4916 : Gut microbiota abundance (genus Parasutterella id.2892) on Nectin−3
with a threshold of 1e−05
MR Test
Inverse variance weighted (fixed effects)
Inverse variance weighted (multiplicative random effects)
MR Egger
MR RAPS
Weighted median
Weighted mode
0.10
0.05
0.00
−0.05
−0.10
SNP effect on Nectin−3
0.1
0.2
SNP effect on Gut microbiota abundance (genus Parasutterella id.2892)

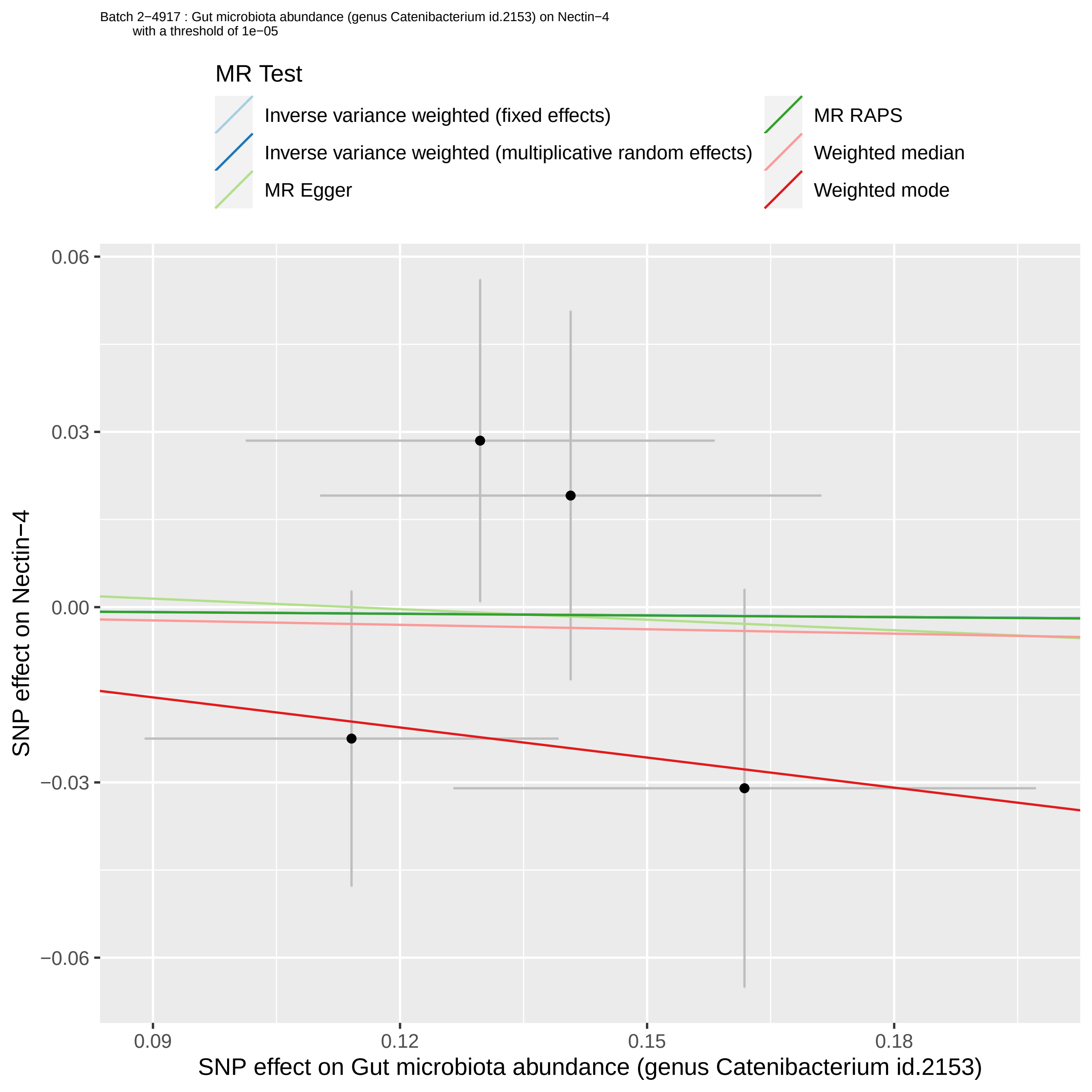

Batch 2−4917 : Gut microbiota abundance (genus Catenibacterium id.2153) on Nectin−4
with a threshold of 1e−05
MR Test
Inverse variance weighted (fixed effects)
Inverse variance weighted (multiplicative random effects)
MR Egger
MR RAPS
Weighted median
Weighted mode
0.06
0.03
0.00
−0.03
−0.06
SNP effect on Nectin−4
0.09
0.12
0.15
0.18
SNP effect on Gut microbiota abundance (genus Catenibacterium id.2153)

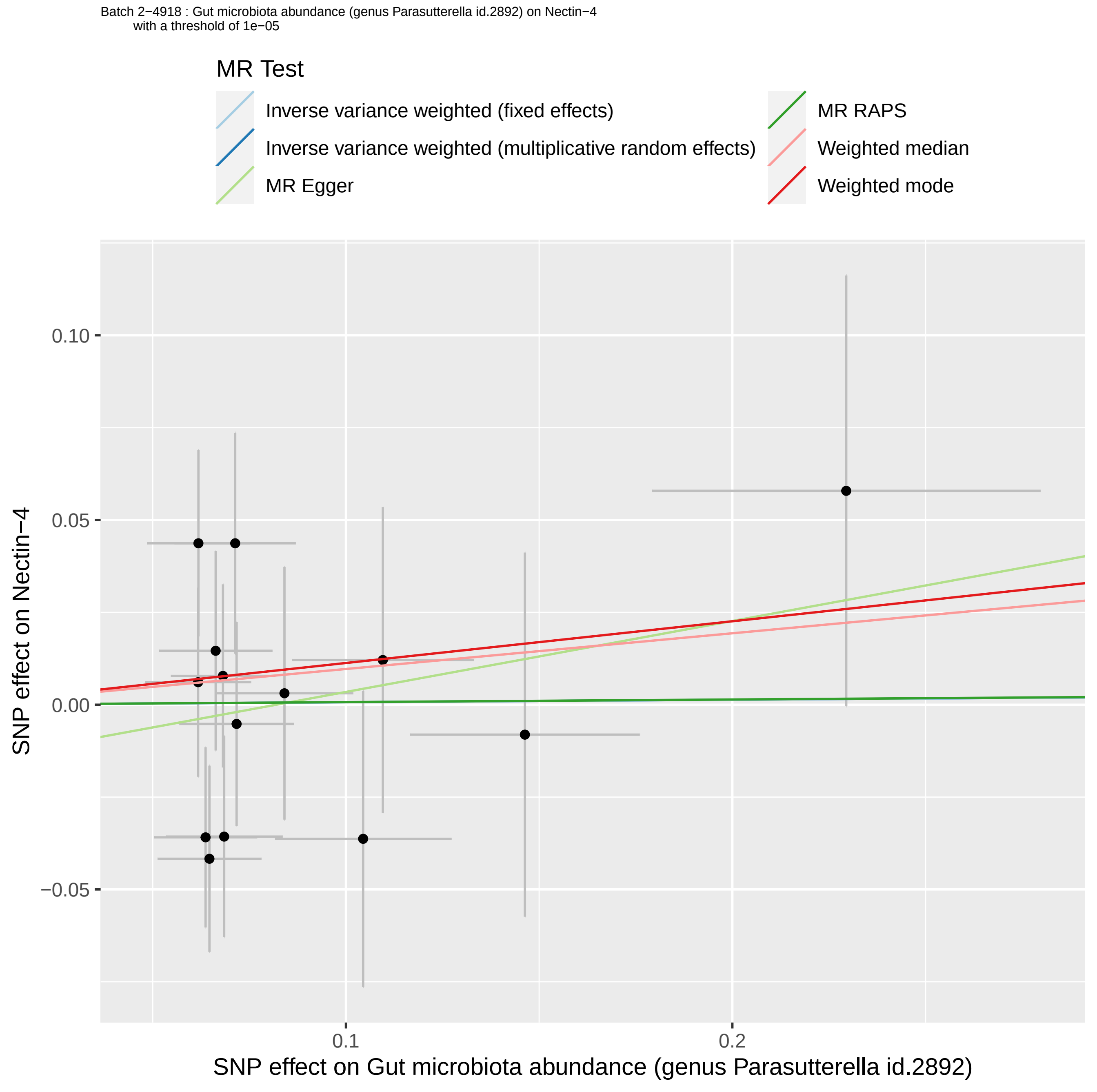
Batch 2−4918 : Gut microbiota abundance (genus Parasutterella id.2892) on Nectin−4
with a threshold of 1e−05
MR Test
Inverse variance weighted (fixed effects)
Inverse variance weighted (multiplicative random effects)
MR Egger
MR RAPS
Weighted median
Weighted mode
0.10
0.05
0.00
−0.05
SNP effect on Nectin−4
0.1
0.2
SNP effect on Gut microbiota abundance (genus Parasutterella id.2892)

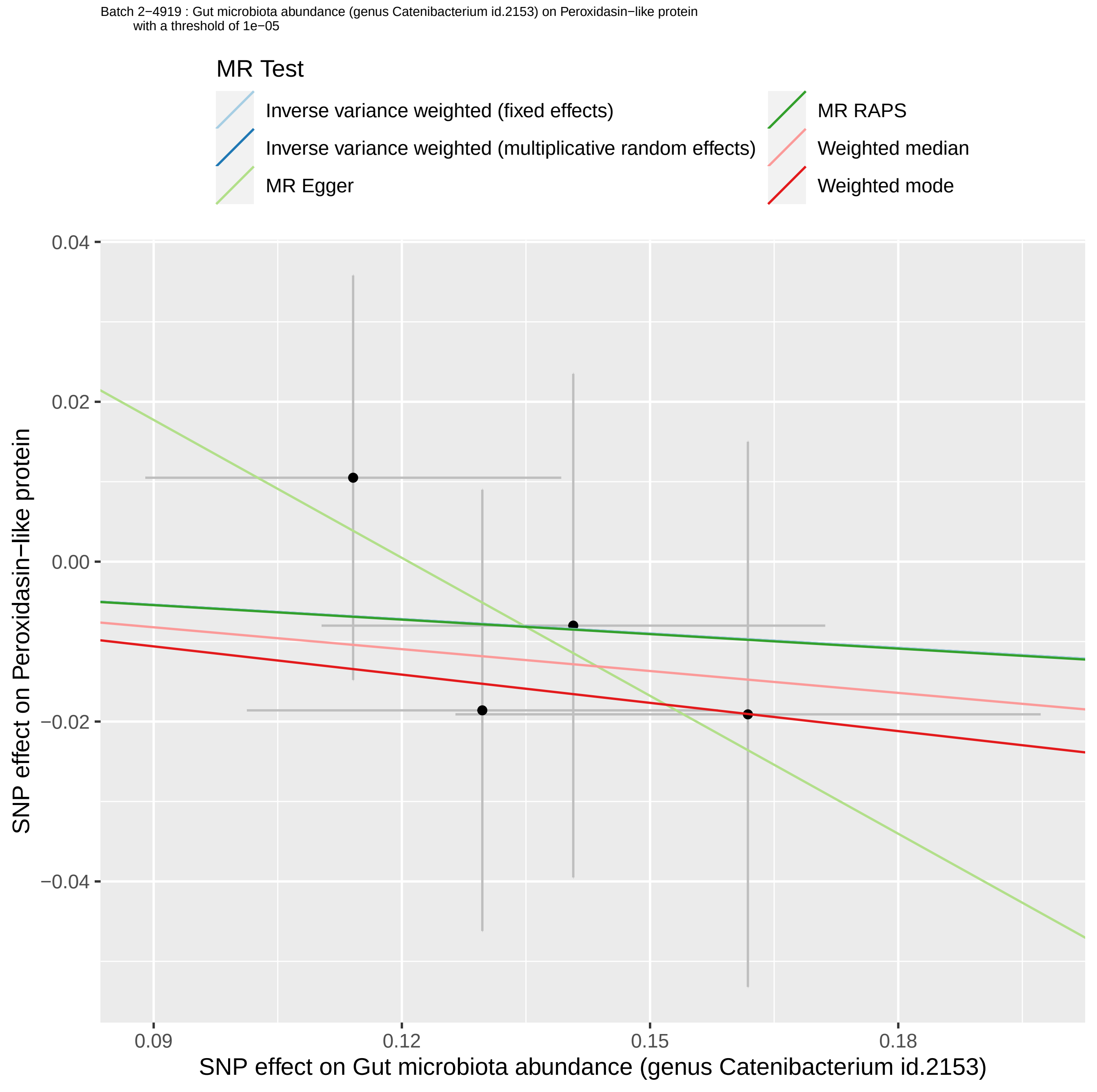

Batch 2−4919 : Gut microbiota abundance (genus Catenibacterium id.2153) on Peroxidasin−like protein
with a threshold of 1e−05
MR Test
Inverse variance weighted (fixed effects)
Inverse variance weighted (multiplicative random effects)
MR Egger
MR RAPS
Weighted median
Weighted mode
0.04
0.02
0.00
−0.02
−0.04
SNP effect on Peroxidasin−like protein
0.09
0.12
0.15
0.18
SNP effect on Gut microbiota abundance (genus Catenibacterium id.2153)

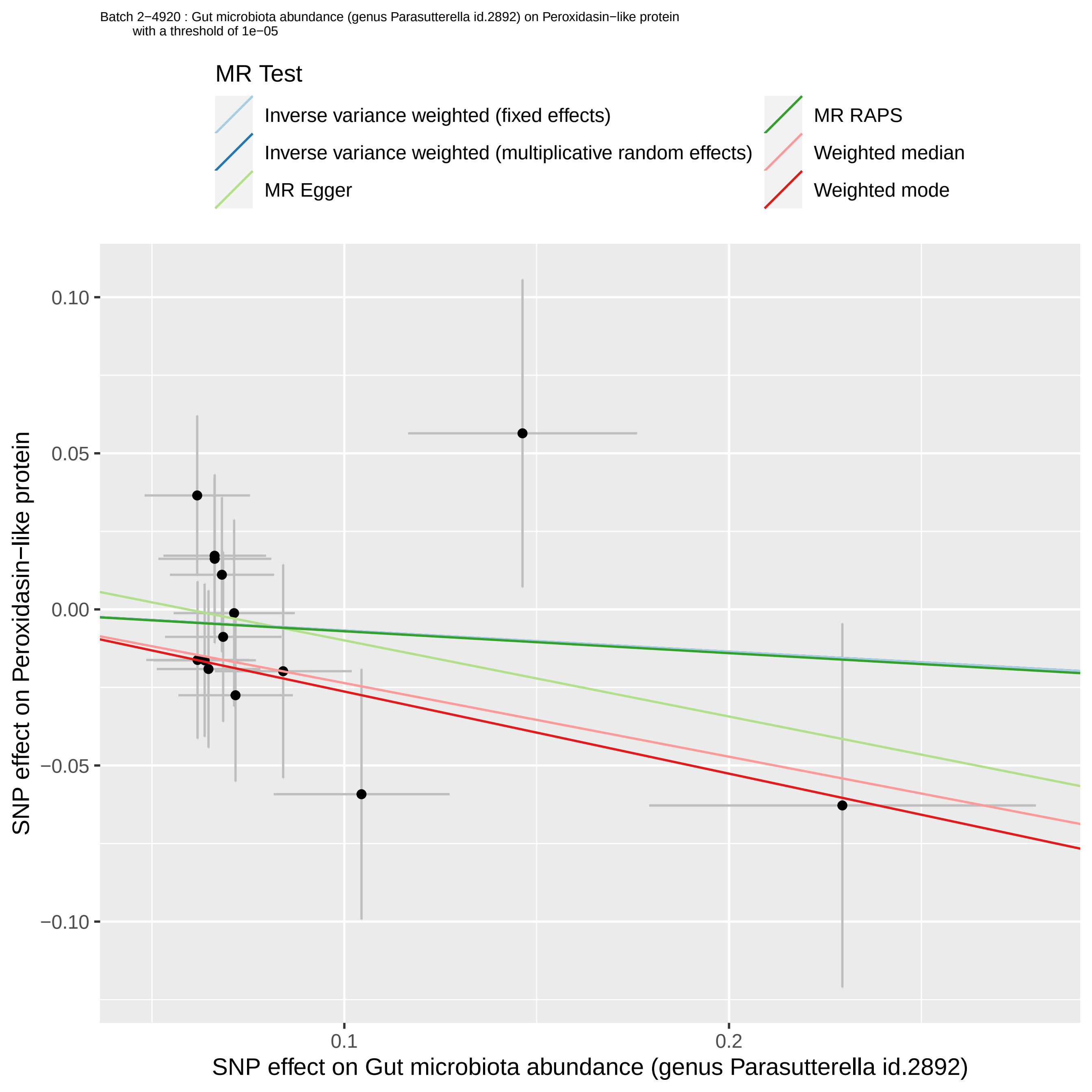

Batch 2−4920 : Gut microbiota abundance (genus Parasutterella id.2892) on Peroxidasin−like protein
with a threshold of 1e−05
MR Test
Inverse variance weighted (fixed effects)
Inverse variance weighted (multiplicative random effects)
MR Egger
MR RAPS
Weighted median
Weighted mode
0.10
0.05
0.00
−0.05
−0.10
0.1
0.2
SNP effect on Peroxidasin−like protein
SNP effect on Gut microbiota abundance (genus Parasutterella id.2892)

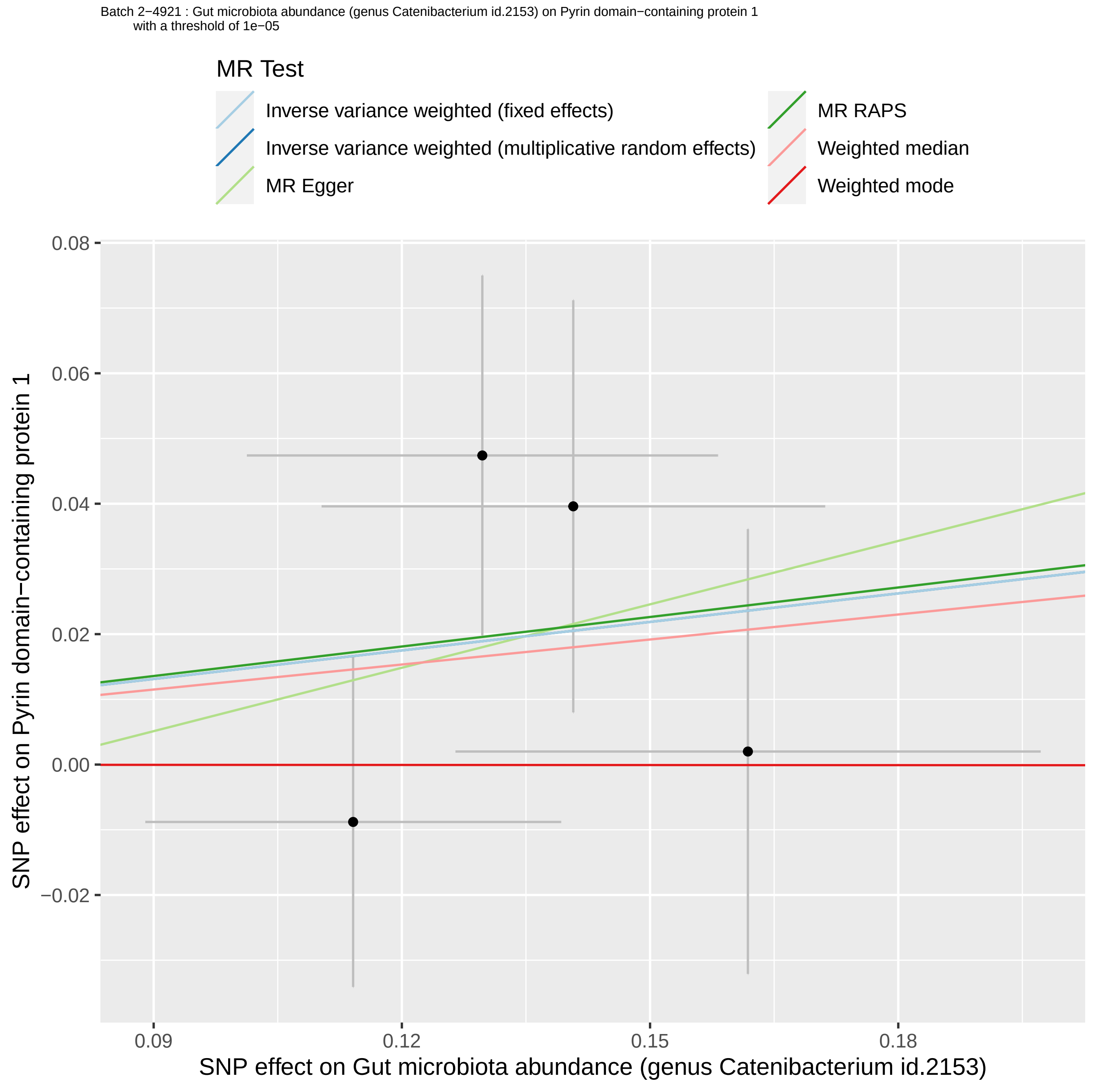
Batch 2−4921 : Gut microbiota abundance (genus Catenibacterium id.2153) on Pyrin domain−containing protein 1
with a threshold of 1e−05
MR Test
Inverse variance weighted (fixed effects)
Inverse variance weighted (multiplicative random effects)
MR Egger
MR RAPS
Weighted median
Weighted mode
0.08
0.06
0.04
0.02
0.00
−0.02
0.09
0.12
0.15
0.18
SNP effect on Pyrin domain−containing protein 1
SNP effect on Gut microbiota abundance (genus Catenibacterium id.2153)

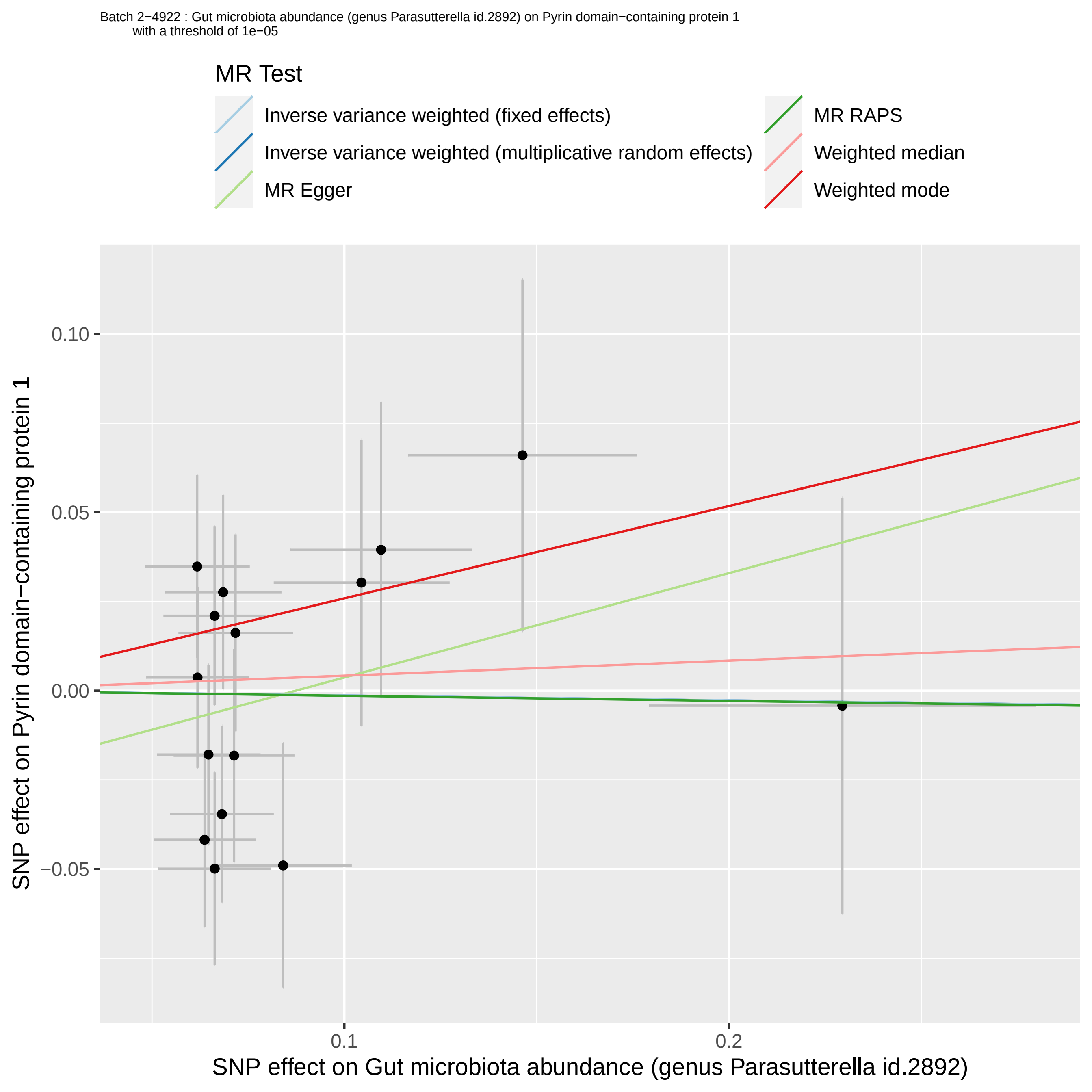

Batch 2−4922 : Gut microbiota abundance (genus Parasutterella id.2892) on Pyrin domain−containing protein 1
with a threshold of 1e−05
MR Test
Inverse variance weighted (fixed effects)
Inverse variance weighted (multiplicative random effects)
MR Egger
MR RAPS
Weighted median
Weighted mode
0.10
0.05
0.00
−0.05
0.1
0.2
SNP effect on Pyrin domain−containing protein 1
SNP effect on Gut microbiota abundance (genus Parasutterella id.2892)

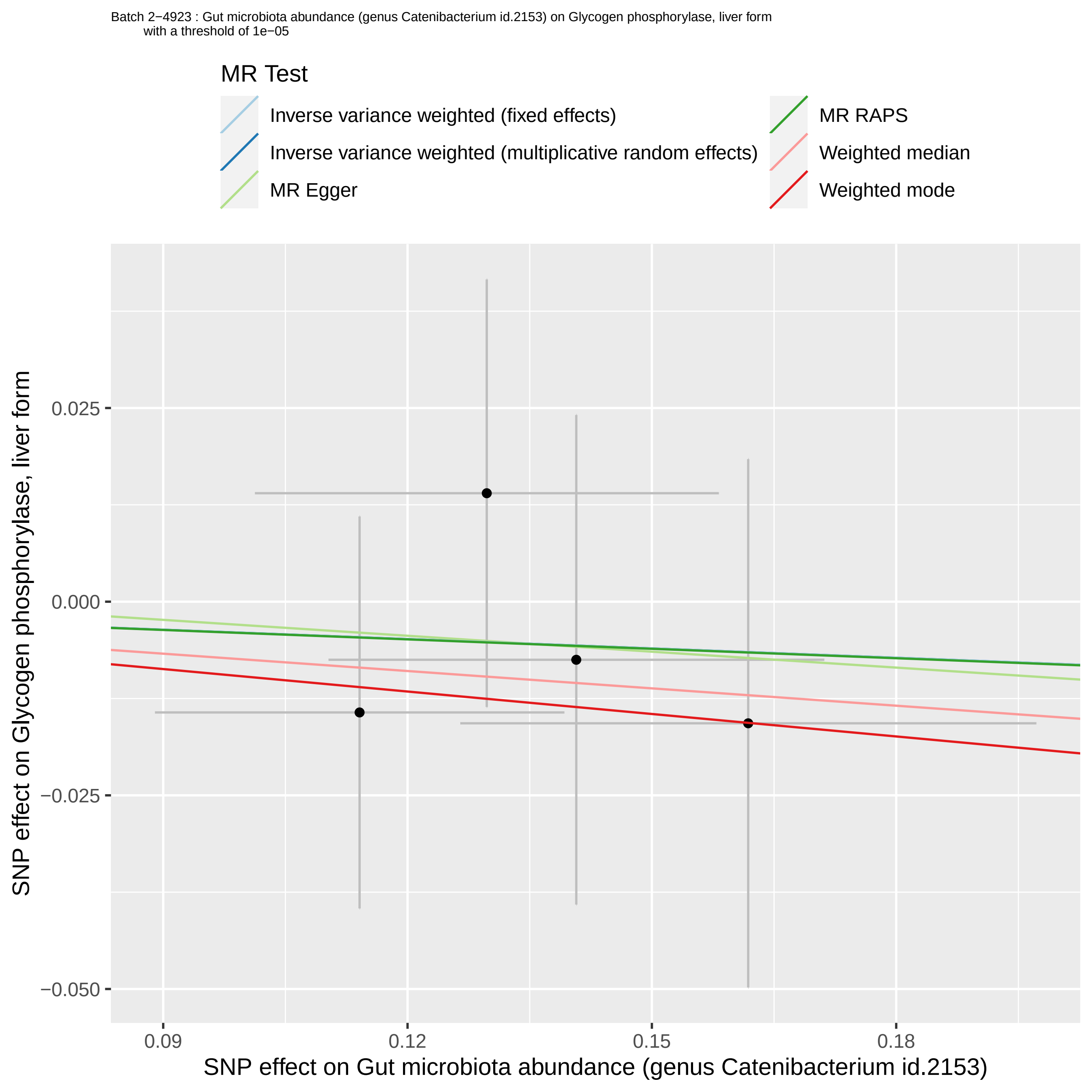

Batch 2−4923 : Gut microbiota abundance (genus Catenibacterium id.2153) on Glycogen phosphorylase, liver form
with a threshold of 1e−05
MR Test
Inverse variance weighted (fixed effects)
Inverse variance weighted (multiplicative random effects)
MR Egger
MR RAPS
Weighted median
Weighted mode
0.025
0.000
−0.025
−0.050
0.09
0.12
0.15
0.18
SNP effect on Glycogen phosphorylase, liver form
SNP effect on Gut microbiota abundance (genus Catenibacterium id.2153)

Batch 2−4924 : Gut microbiota abundance (genus Parasutterella id.2892) on Glycogen phosphorylase, liver form
with a threshold of 1e−05

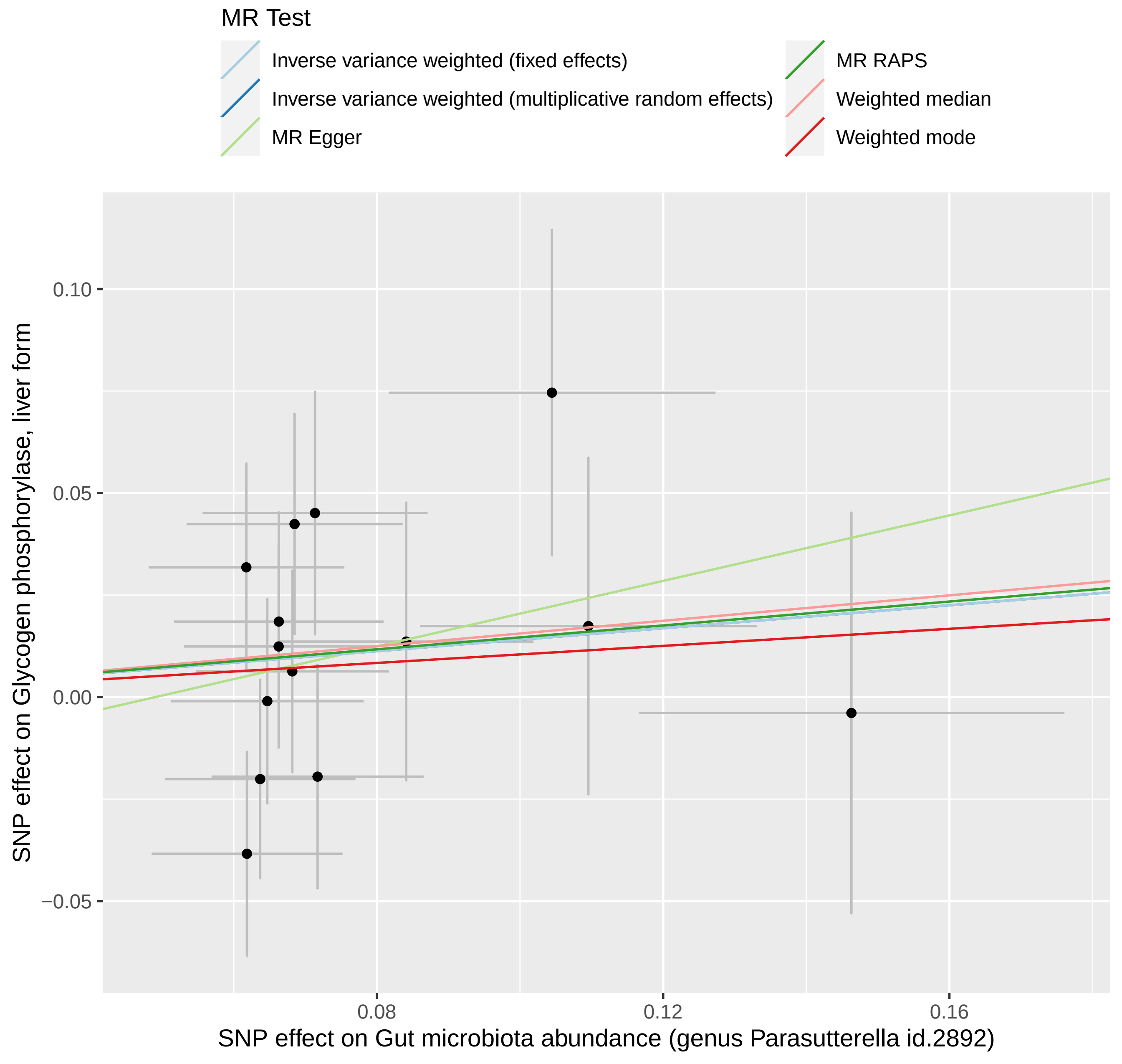

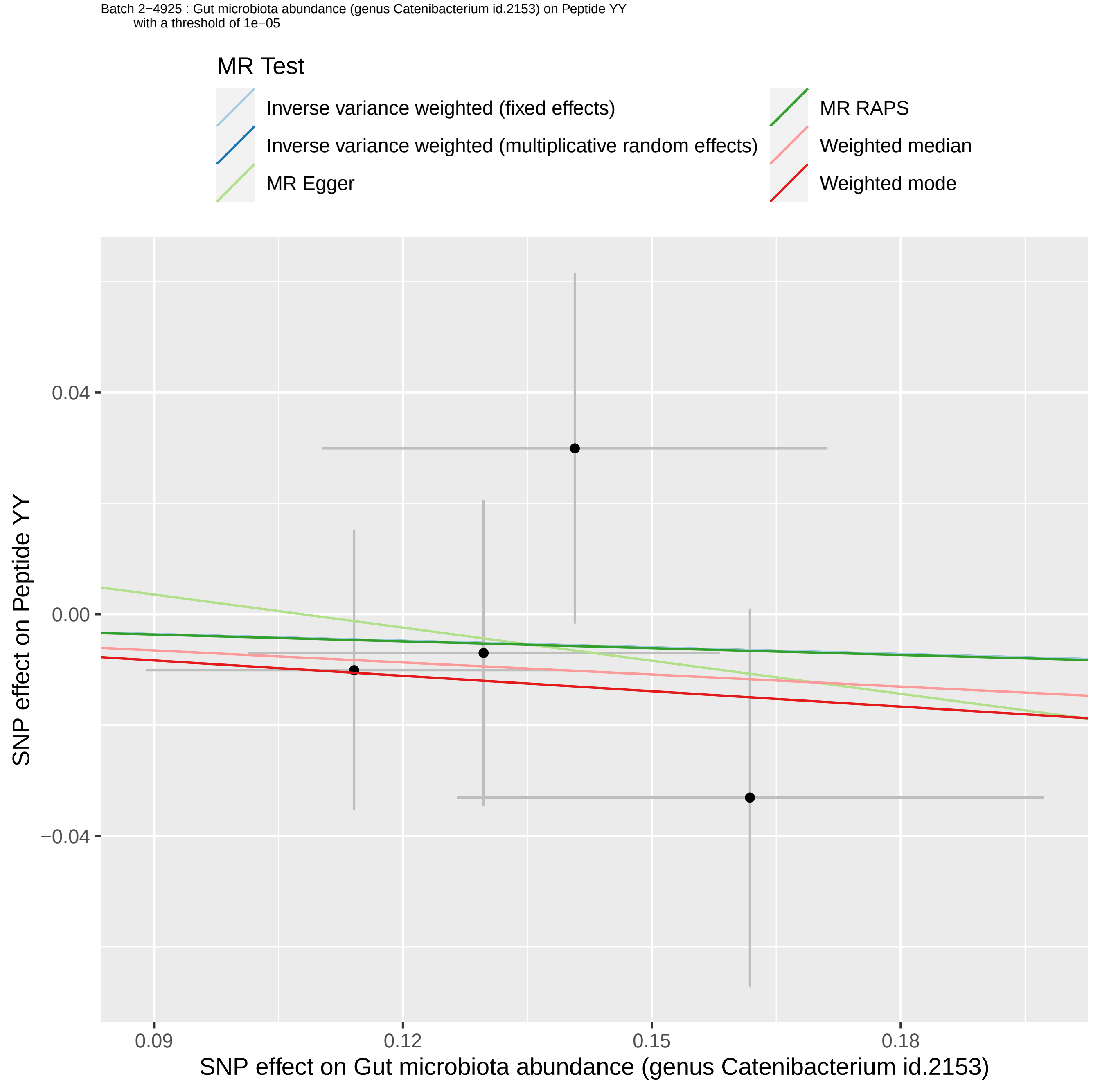
Batch 2−4925 : Gut microbiota abundance (genus Catenibacterium id.2153) on Peptide YY
with a threshold of 1e−05
MR Test
Inverse variance weighted (fixed effects)
Inverse variance weighted (multiplicative random effects)
MR Egger
MR RAPS
Weighted median
Weighted mode
0.04
0.00
−0.04
0.09
0.12
0.15
0.18
SNP effect on Peptide YY
SNP effect on Gut microbiota abundance (genus Catenibacterium id.2153)

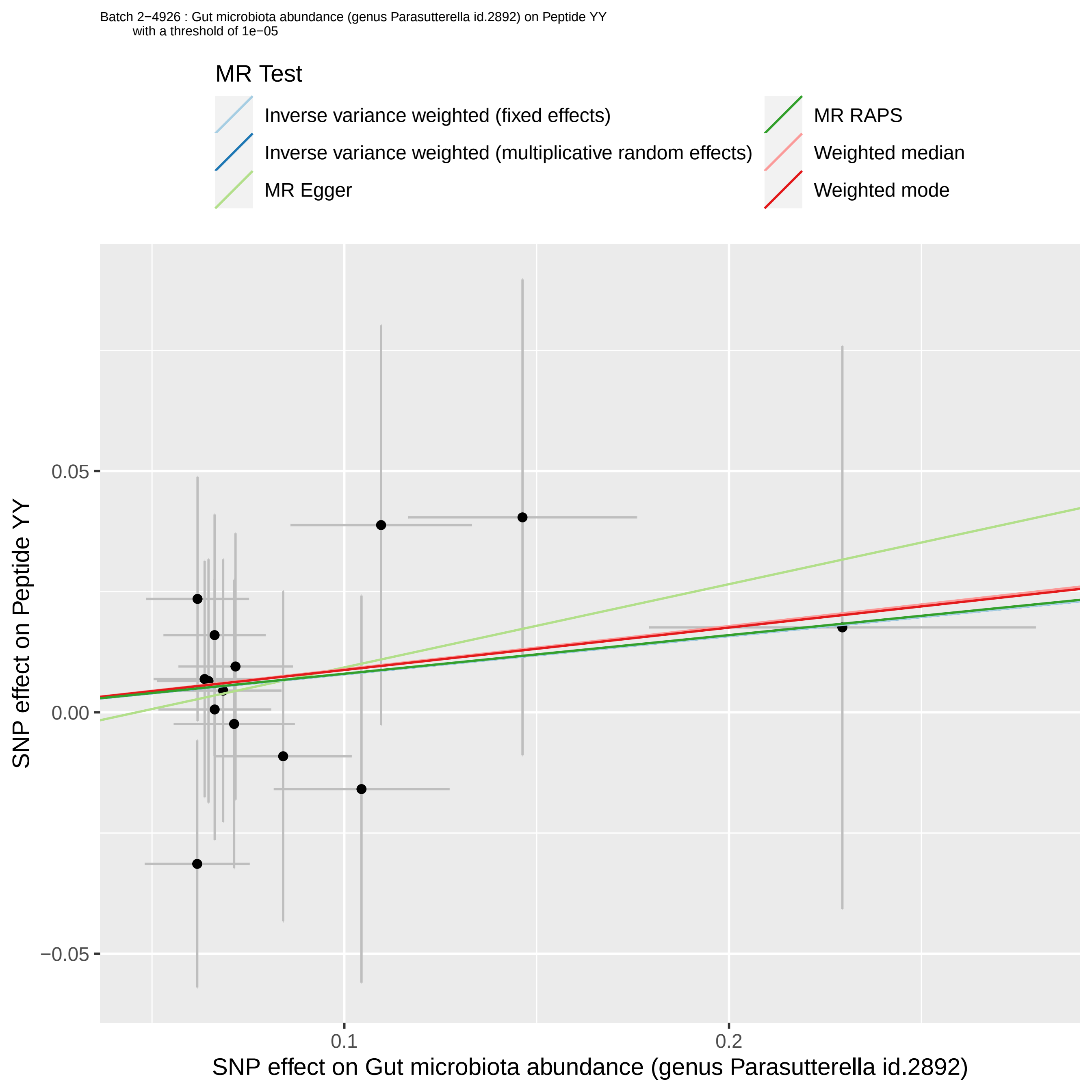

Batch 2−4926 : Gut microbiota abundance (genus Parasutterella id.2892) on Peptide YY
with a threshold of 1e−05
MR Test
Inverse variance weighted (fixed effects)
Inverse variance weighted (multiplicative random effects)
MR Egger
MR RAPS
Weighted median
Weighted mode
0.05
0.00
−0.05
0.1
0.2
SNP effect on Peptide YY
SNP effect on Gut microbiota abundance (genus Parasutterella id.2892)

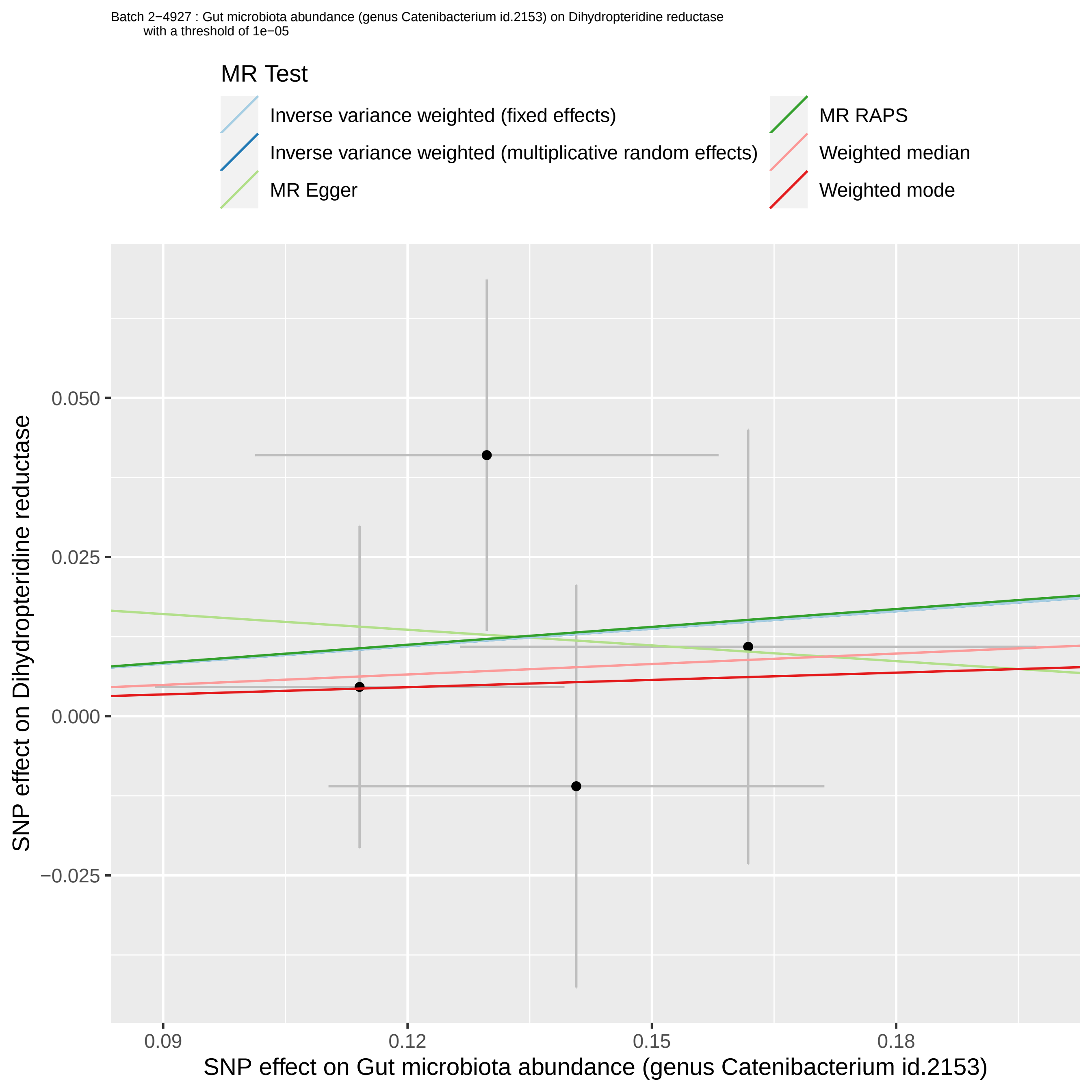

Batch 2−4927 : Gut microbiota abundance (genus Catenibacterium id.2153) on Dihydropteridine reductase
with a threshold of 1e−05
MR Test
Inverse variance weighted (fixed effects)
Inverse variance weighted (multiplicative random effects)
MR Egger
MR RAPS
Weighted median
Weighted mode
0.050
0.025
0.000
−0.025
0.09
0.12
0.15
0.18
SNP effect on Dihydropteridine reductase
SNP effect on Gut microbiota abundance (genus Catenibacterium id.2153)

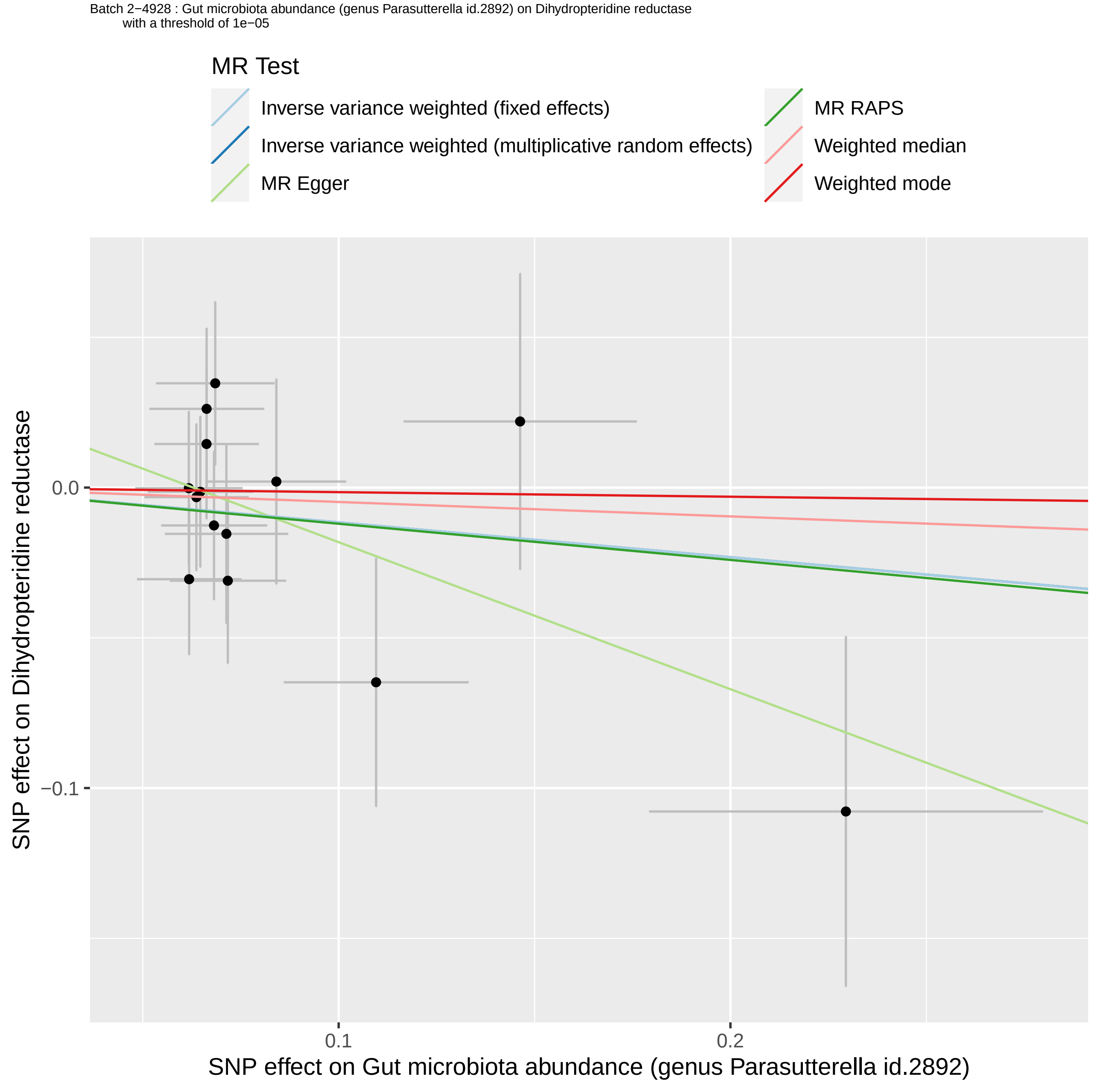

Batch 2−4928 : Gut microbiota abundance (genus Parasutterella id.2892) on Dihydropteridine reductase
with a threshold of 1e−05
MR Test
Inverse variance weighted (fixed effects)
Inverse variance weighted (multiplicative random effects)
MR Egger
MR RAPS
Weighted median
Weighted mode
0.0
−0.1
0.1
0.2
SNP effect on Dihydropteridine reductase
SNP effect on Gut microbiota abundance (genus Parasutterella id.2892)

Batch 2−4929 : Gut microbiota abundance (genus Catenibacterium id.2153) on Glutaminyl−peptide cyclotransferase
with a threshold of 1e−05

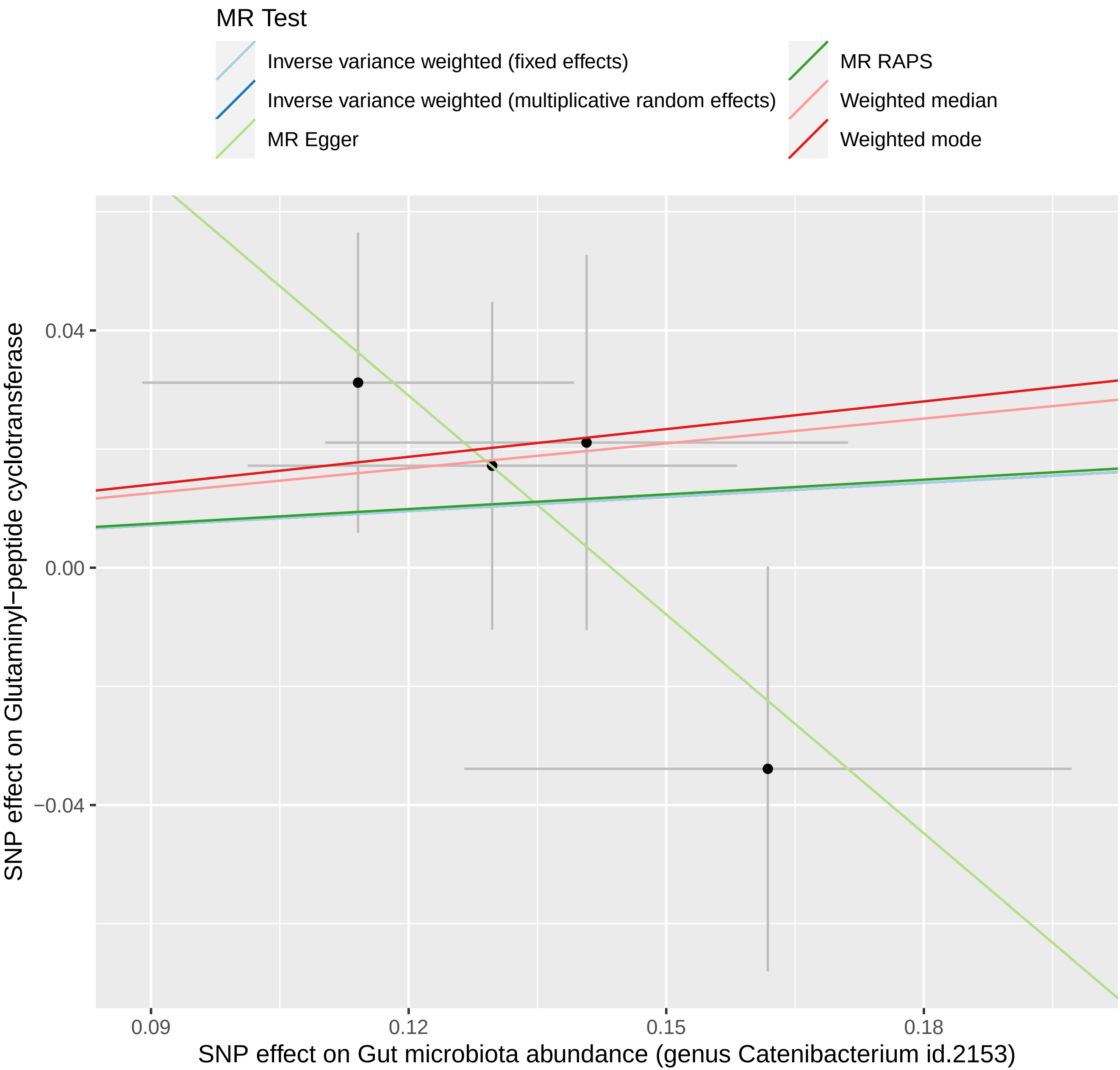

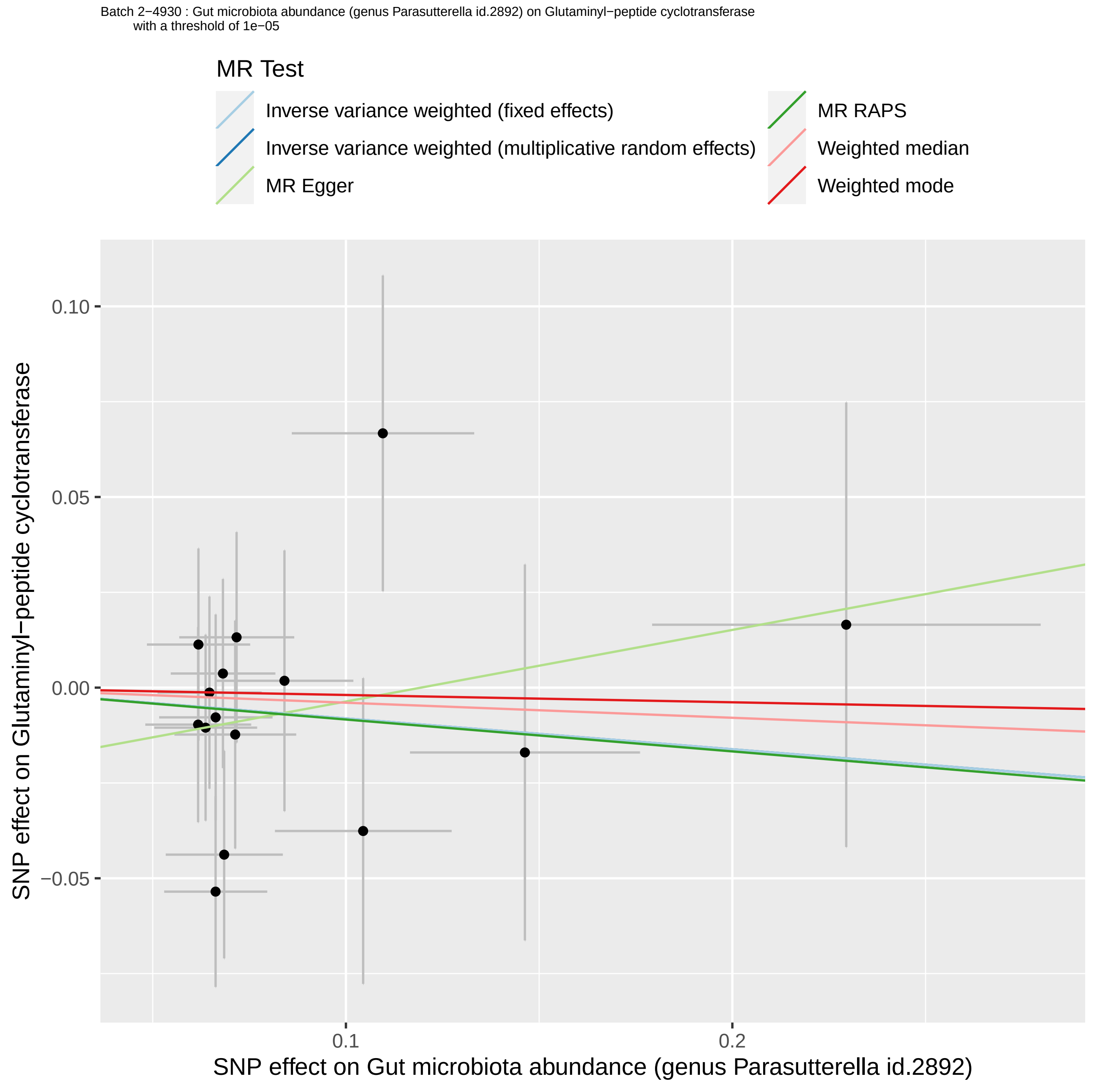

Batch 2−4930 : Gut microbiota abundance (genus Parasutterella id.2892) on Glutaminyl−peptide cyclotransferase
with a threshold of 1e−05
MR Test
Inverse variance weighted (fixed effects)
Inverse variance weighted (multiplicative random effects)
MR Egger
MR RAPS
Weighted median
Weighted mode
0.10
0.05
0.00
−0.05
0.1
0.2
SNP effect on Glutaminyl−peptide cyclotransferase
SNP effect on Gut microbiota abundance (genus Parasutterella id.2892)

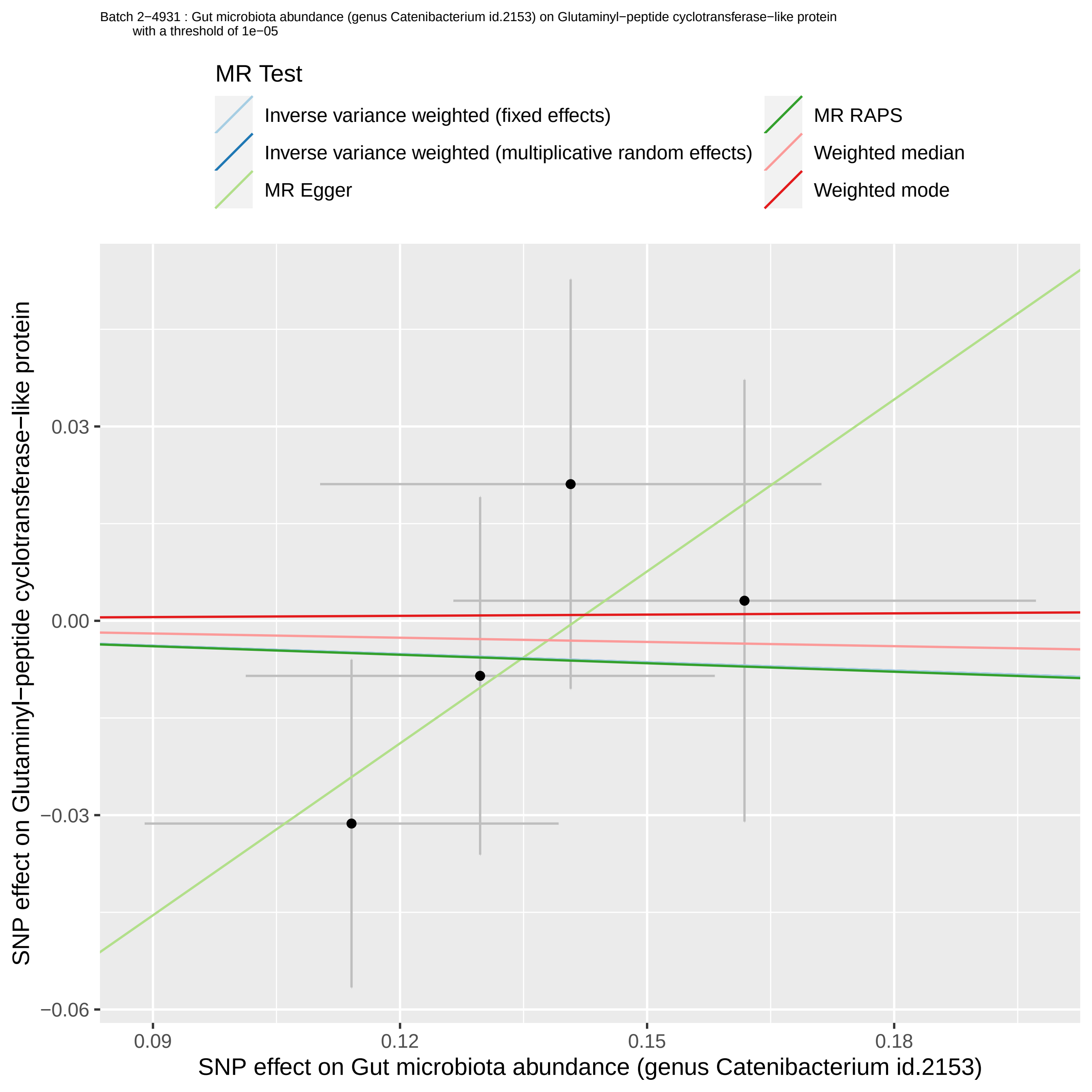
Batch 2−4931 : Gut microbiota abundance (genus Catenibacterium id.2153) on Glutaminyl−peptide cyclotransferase−like protein
with a threshold of 1e−05
MR Test
Inverse variance weighted (fixed effects)
Inverse variance weighted (multiplicative random effects)
MR Egger
MR RAPS
Weighted median
Weighted mode
SNP effect on Glutaminyl−peptide cyclotransferase−like protein
0.03
0.00
−0.03
−0.06
0.09
0.12
0.15
0.18
SNP effect on Gut microbiota abundance (genus Catenibacterium id.2153)

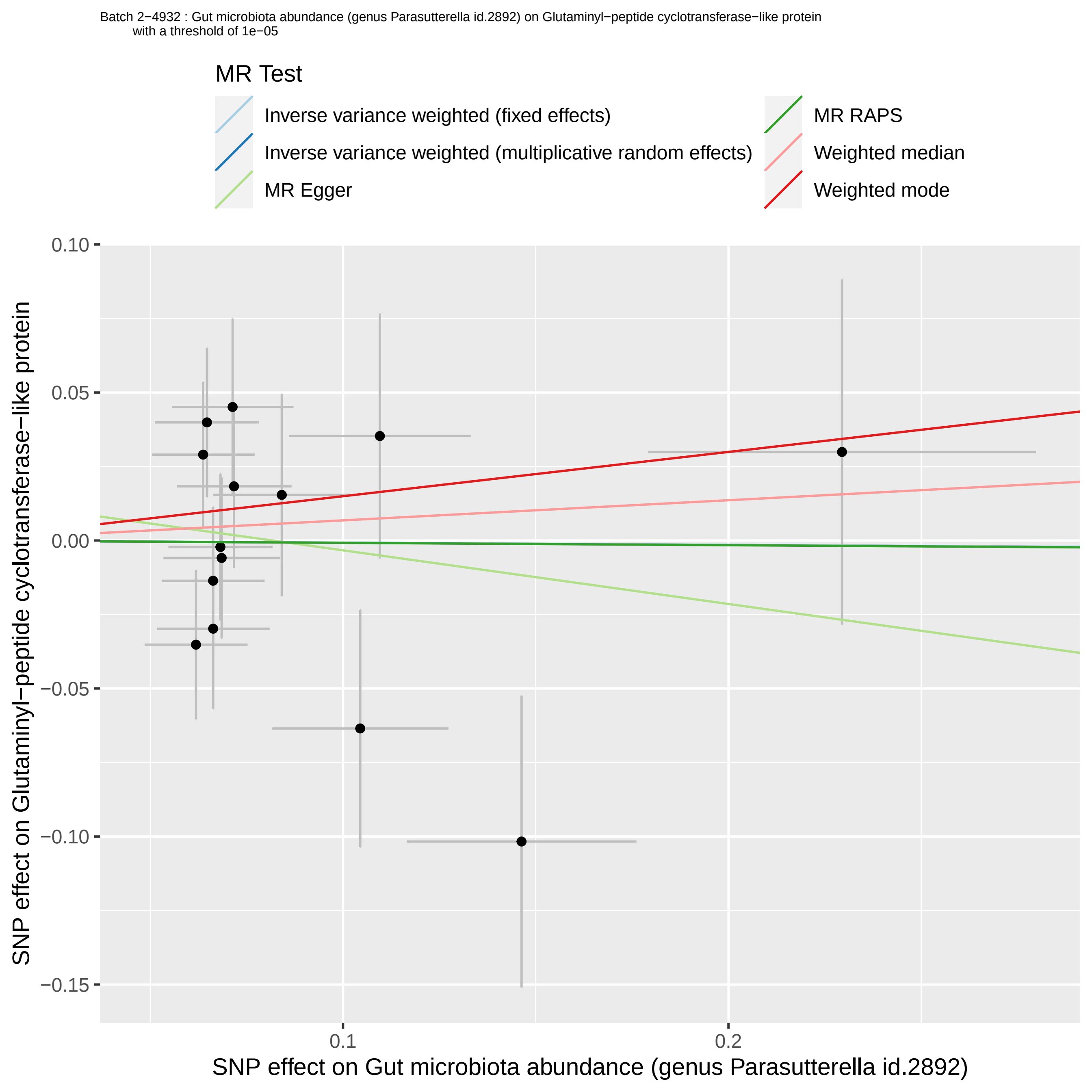
Batch 2−4932 : Gut microbiota abundance (genus Parasutterella id.2892) on Glutaminyl−peptide cyclotransferase−like protein
with a threshold of 1e−05
MR Test
Inverse variance weighted (fixed effects)
Inverse variance weighted (multiplicative random effects)
MR Egger
MR RAPS
Weighted median
Weighted mode
0.10
0.05
0.00
−0.05
−0.10
−0.15
0.1
0.2
SNP effect on Glutaminyl−peptide cyclotransferase−like protein
SNP effect on Gut microbiota abundance (genus Parasutterella id.2892)

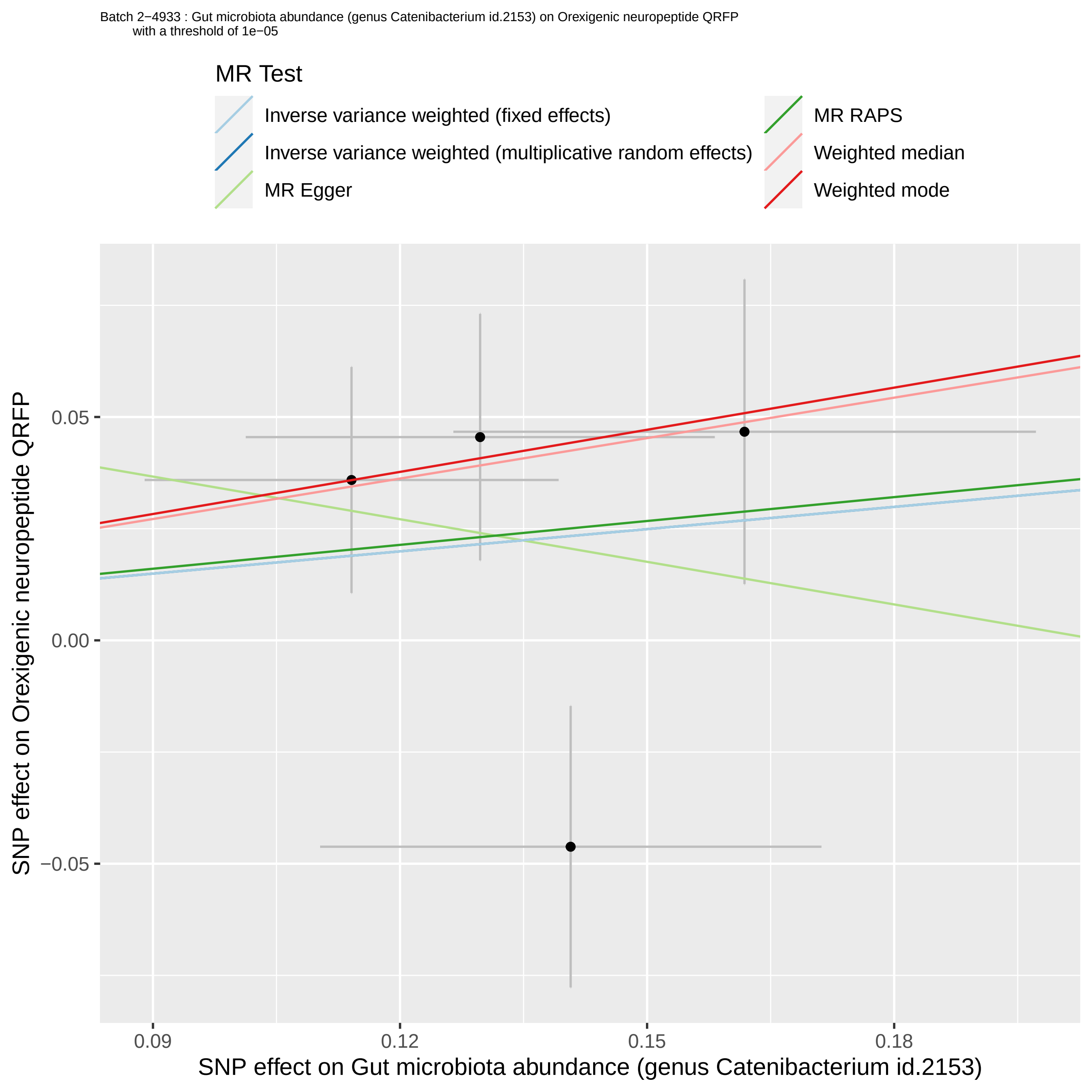
Batch 2−4933 : Gut microbiota abundance (genus Catenibacterium id.2153) on Orexigenic neuropeptide QRFP
with a threshold of 1e−05
MR Test
Inverse variance weighted (fixed effects)
Inverse variance weighted (multiplicative random effects)
MR Egger
MR RAPS
Weighted median
Weighted mode
0.05
0.00
−0.05
0.09
0.12
0.15
0.18
SNP effect on Orexigenic neuropeptide QRFP
SNP effect on Gut microbiota abundance (genus Catenibacterium id.2153)

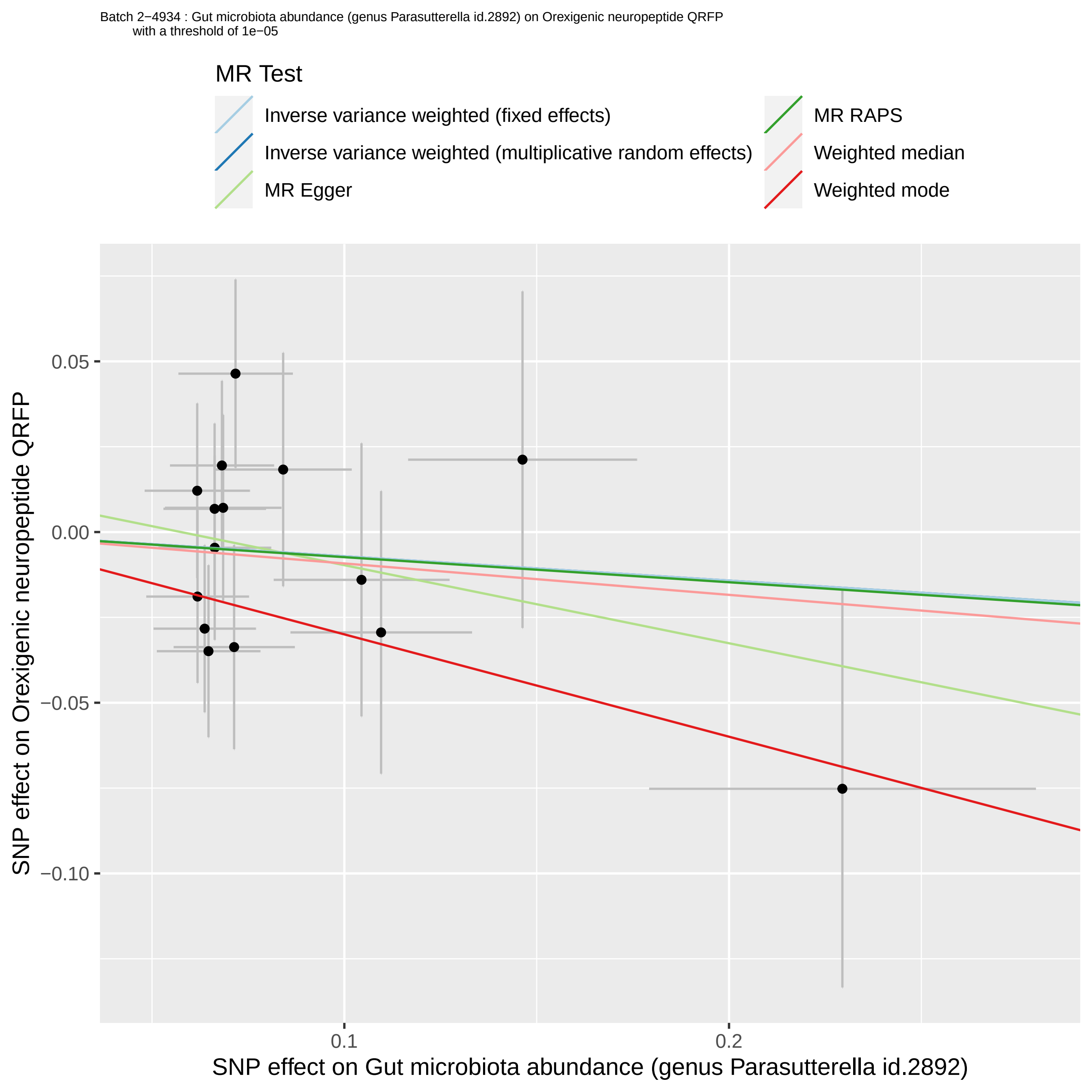
Batch 2−4934 : Gut microbiota abundance (genus Parasutterella id.2892) on Orexigenic neuropeptide QRFP
with a threshold of 1e−05
MR Test
Inverse variance weighted (fixed effects)
Inverse variance weighted (multiplicative random effects)
MR Egger
MR RAPS
Weighted median
Weighted mode
0.05
0.00
−0.05
−0.10
0.1
0.2
SNP effect on Orexigenic neuropeptide QRFP
SNP effect on Gut microbiota abundance (genus Parasutterella id.2892)

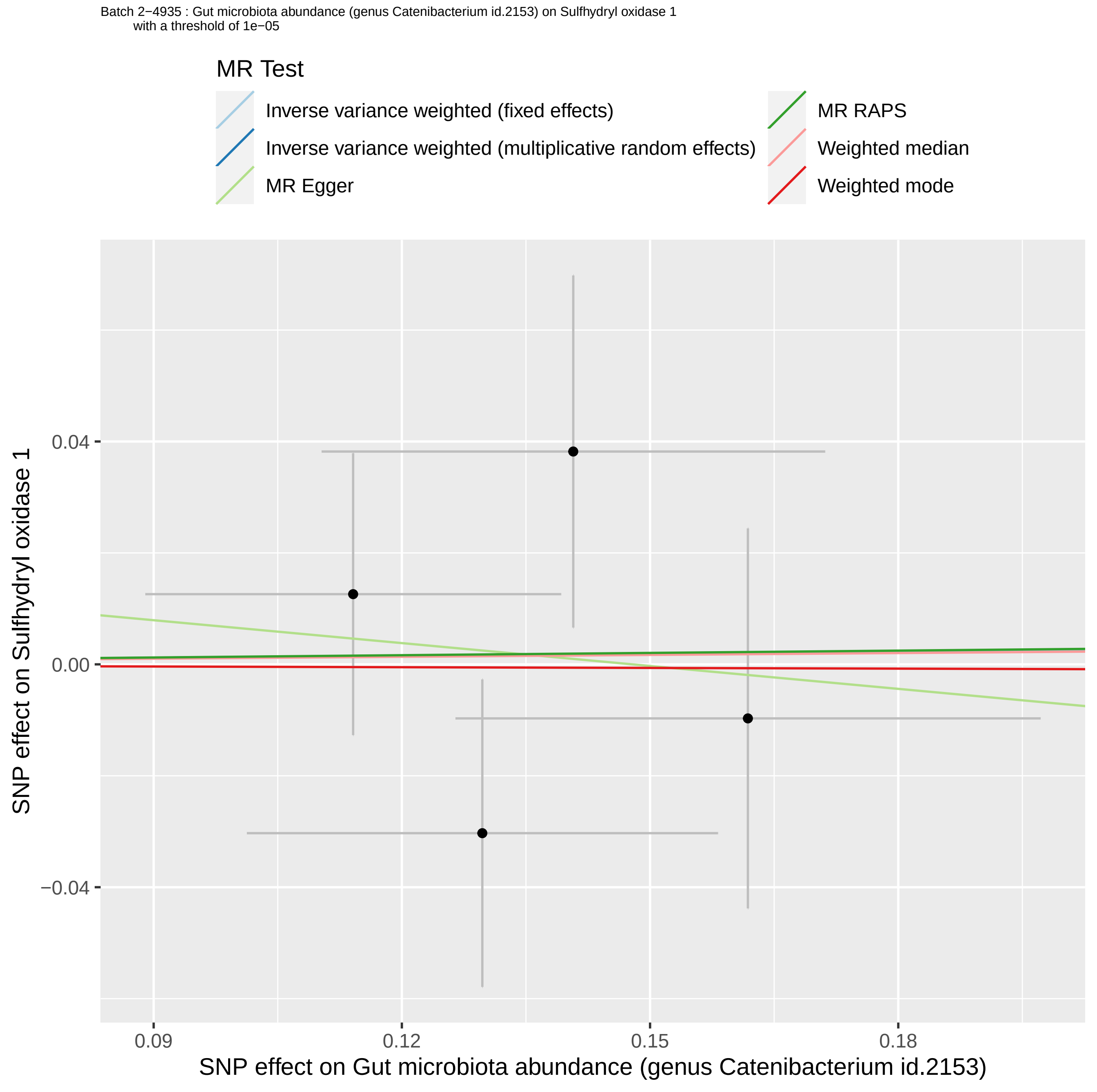
Batch 2−4935 : Gut microbiota abundance (genus Catenibacterium id.2153) on Sulfhydryl oxidase 1
with a threshold of 1e−05
MR Test
Inverse variance weighted (fixed effects)
Inverse variance weighted (multiplicative random effects)
MR Egger
MR RAPS
Weighted median
Weighted mode
0.04
0.00
−0.04
0.09
0.12
0.15
0.18
SNP effect on Sulfhydryl oxidase 1
SNP effect on Gut microbiota abundance (genus Catenibacterium id.2153)

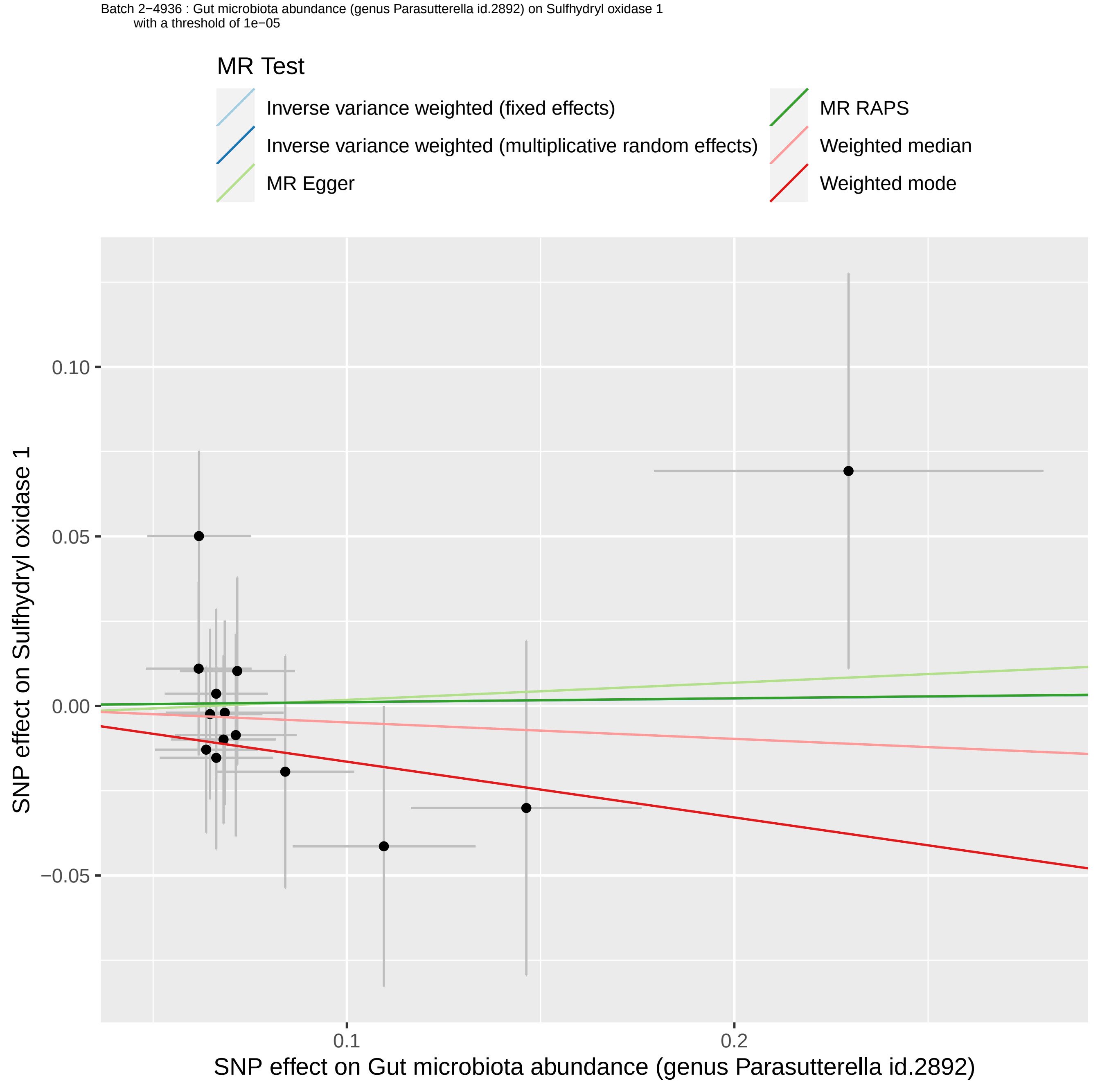

Batch 2−4936 : Gut microbiota abundance (genus Parasutterella id.2892) on Sulfhydryl oxidase 1
with a threshold of 1e−05
MR Test
Inverse variance weighted (fixed effects)
Inverse variance weighted (multiplicative random effects)
MR Egger
MR RAPS
Weighted median
Weighted mode
0.10
0.05
0.00
−0.05
0.1
0.2
SNP effect on Sulfhydryl oxidase 1
SNP effect on Gut microbiota abundance (genus Parasutterella id.2892)

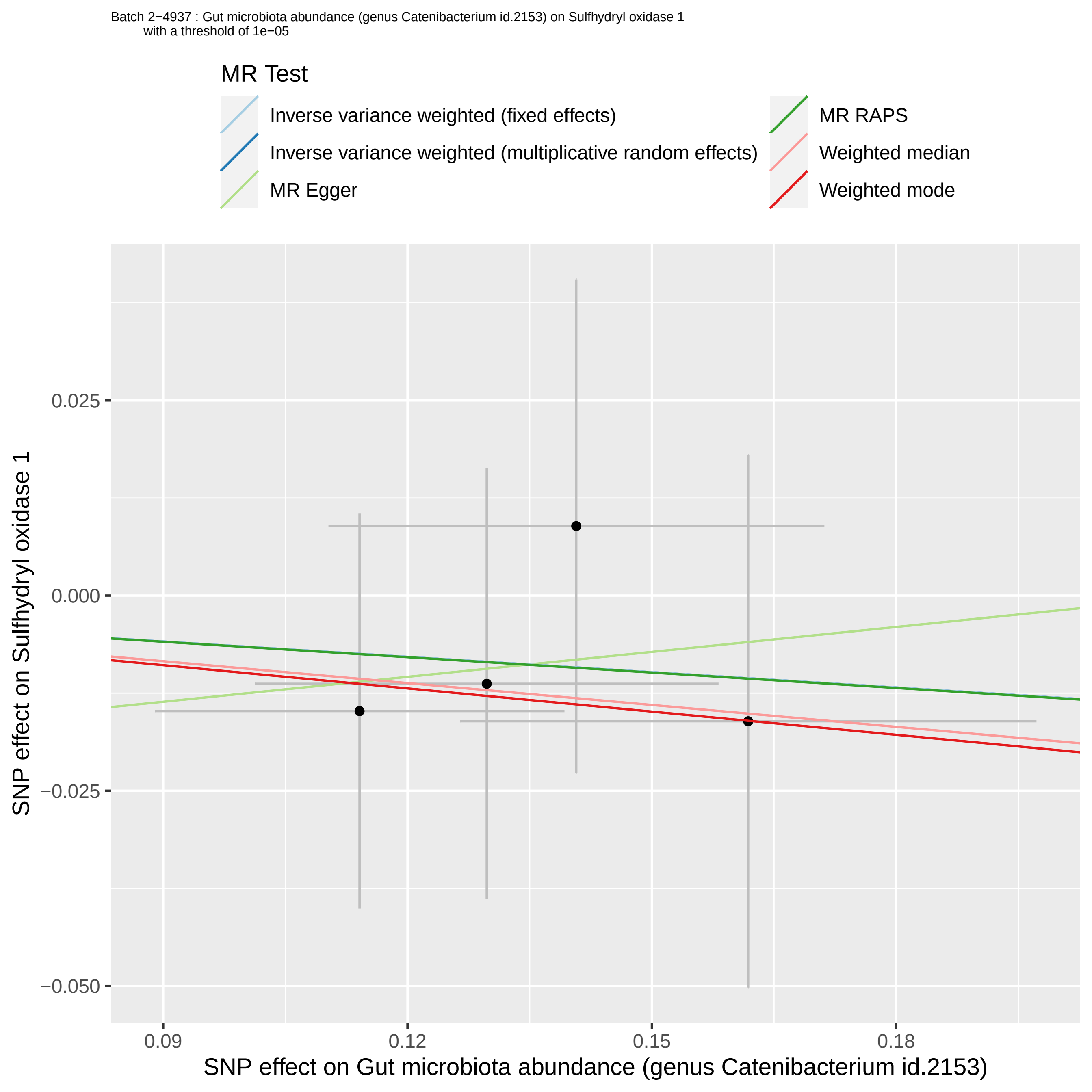

Batch 2−4937 : Gut microbiota abundance (genus Catenibacterium id.2153) on Sulfhydryl oxidase 1
with a threshold of 1e−05
MR Test
Inverse variance weighted (fixed effects)
Inverse variance weighted (multiplicative random effects)
MR Egger
MR RAPS
Weighted median
Weighted mode
0.025
0.000
−0.025
−0.050
0.09
0.12
0.15
0.18
SNP effect on Sulfhydryl oxidase 1
SNP effect on Gut microbiota abundance (genus Catenibacterium id.2153)

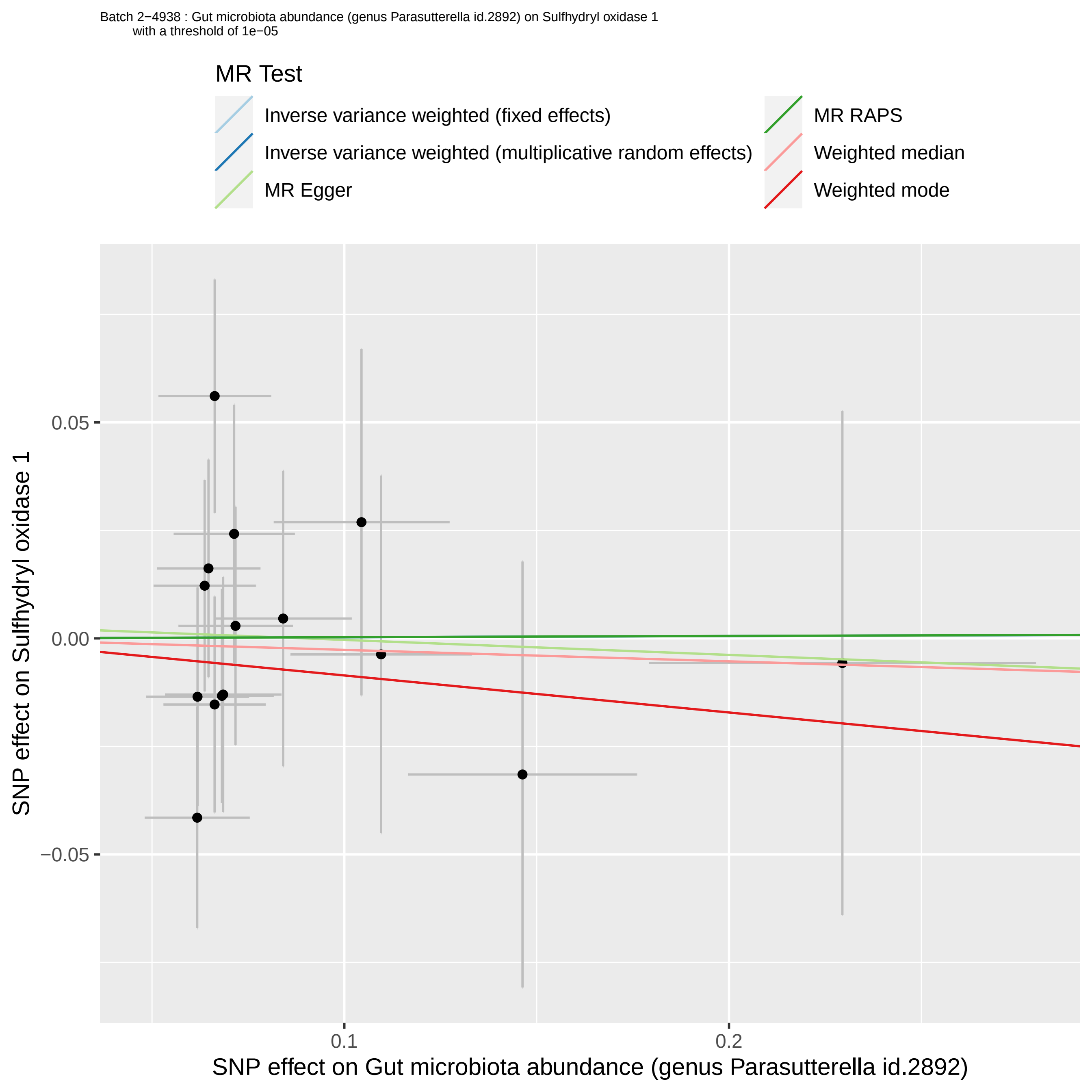
Batch 2−4938 : Gut microbiota abundance (genus Parasutterella id.2892) on Sulfhydryl oxidase 1
with a threshold of 1e−05
MR Test
Inverse variance weighted (fixed effects)
Inverse variance weighted (multiplicative random effects)
MR Egger
MR RAPS
Weighted median
Weighted mode
0.05
0.00
−0.05
0.1
0.2
SNP effect on Sulfhydryl oxidase 1
SNP effect on Gut microbiota abundance (genus Parasutterella id.2892)

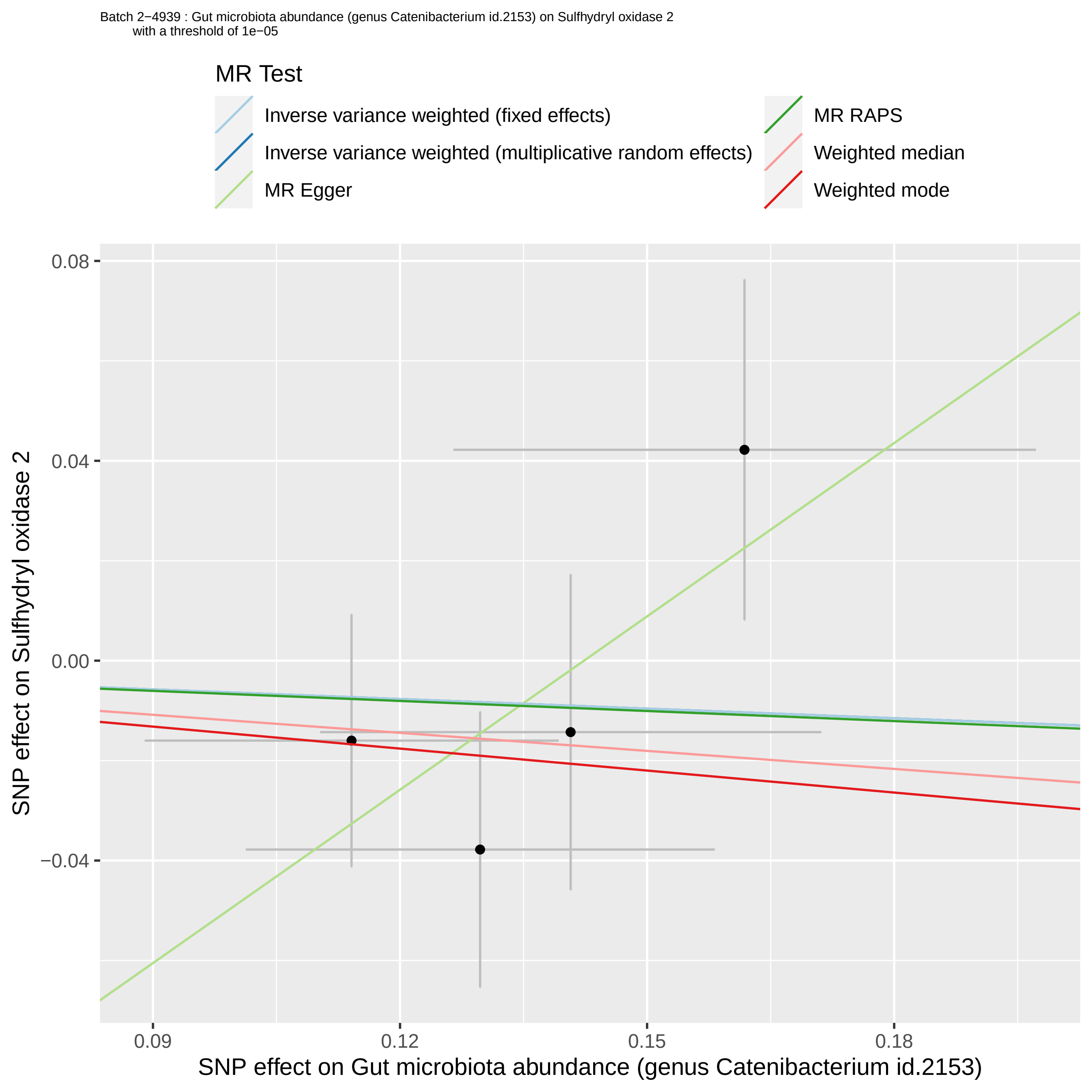

Batch 2−4939 : Gut microbiota abundance (genus Catenibacterium id.2153) on Sulfhydryl oxidase 2
with a threshold of 1e−05
MR Test
Inverse variance weighted (fixed effects)
Inverse variance weighted (multiplicative random effects)
MR Egger
MR RAPS
Weighted median
Weighted mode
0.08
0.04
0.00
−0.04
0.09
0.12
0.15
0.18
SNP effect on Sulfhydryl oxidase 2
SNP effect on Gut microbiota abundance (genus Catenibacterium id.2153)

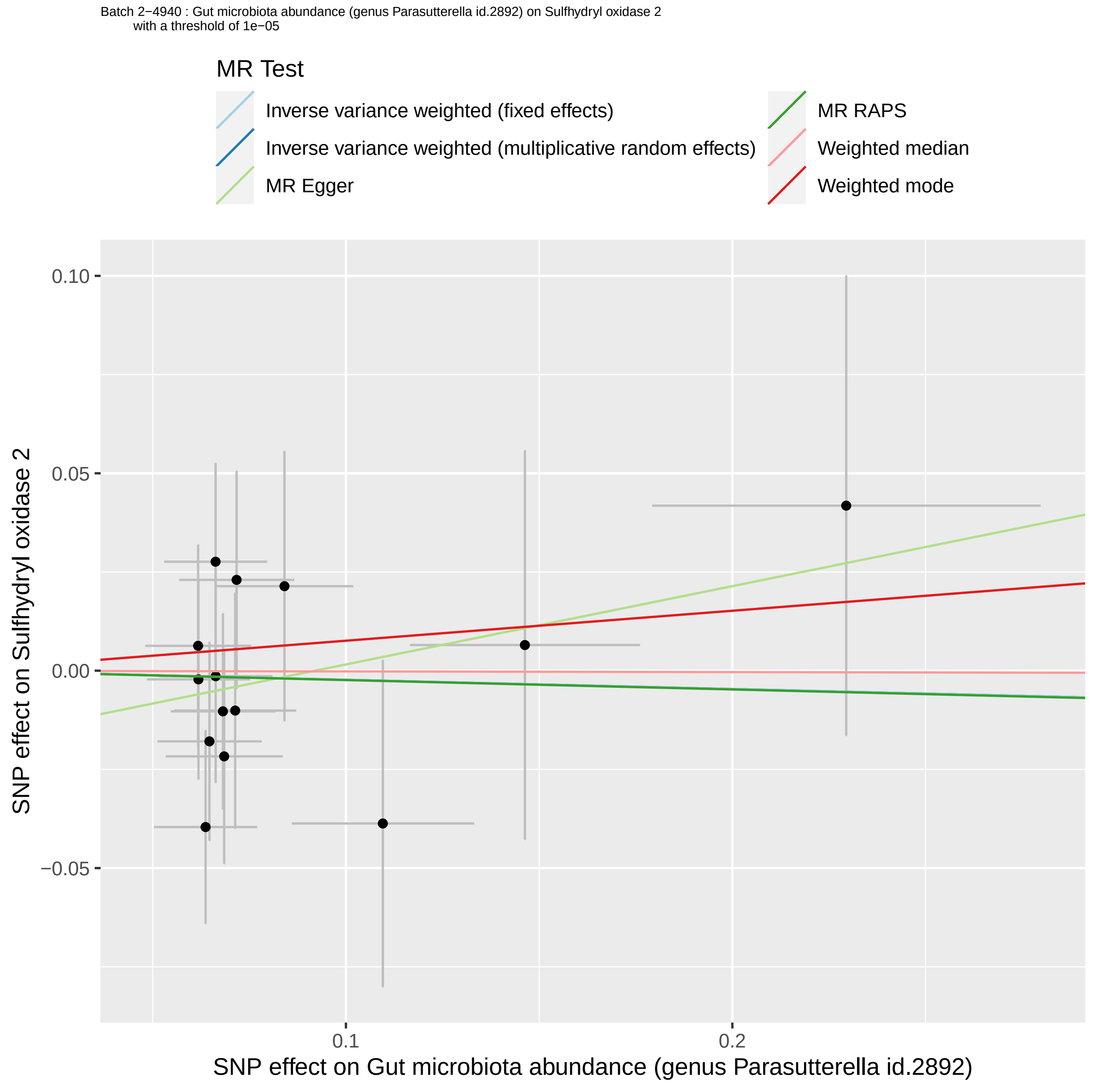
Batch 2−4940 : Gut microbiota abundance (genus Parasutterella id.2892) on Sulfhydryl oxidase 2
with a threshold of 1e−05
MR Test
Inverse variance weighted (fixed effects)
Inverse variance weighted (multiplicative random effects)
MR Egger
MR RAPS
Weighted median
Weighted mode
0.10
0.05
0.00
−0.05
0.1
0.2
SNP effect on Sulfhydryl oxidase 2
SNP effect on Gut microbiota abundance (genus Parasutterella id.2892)

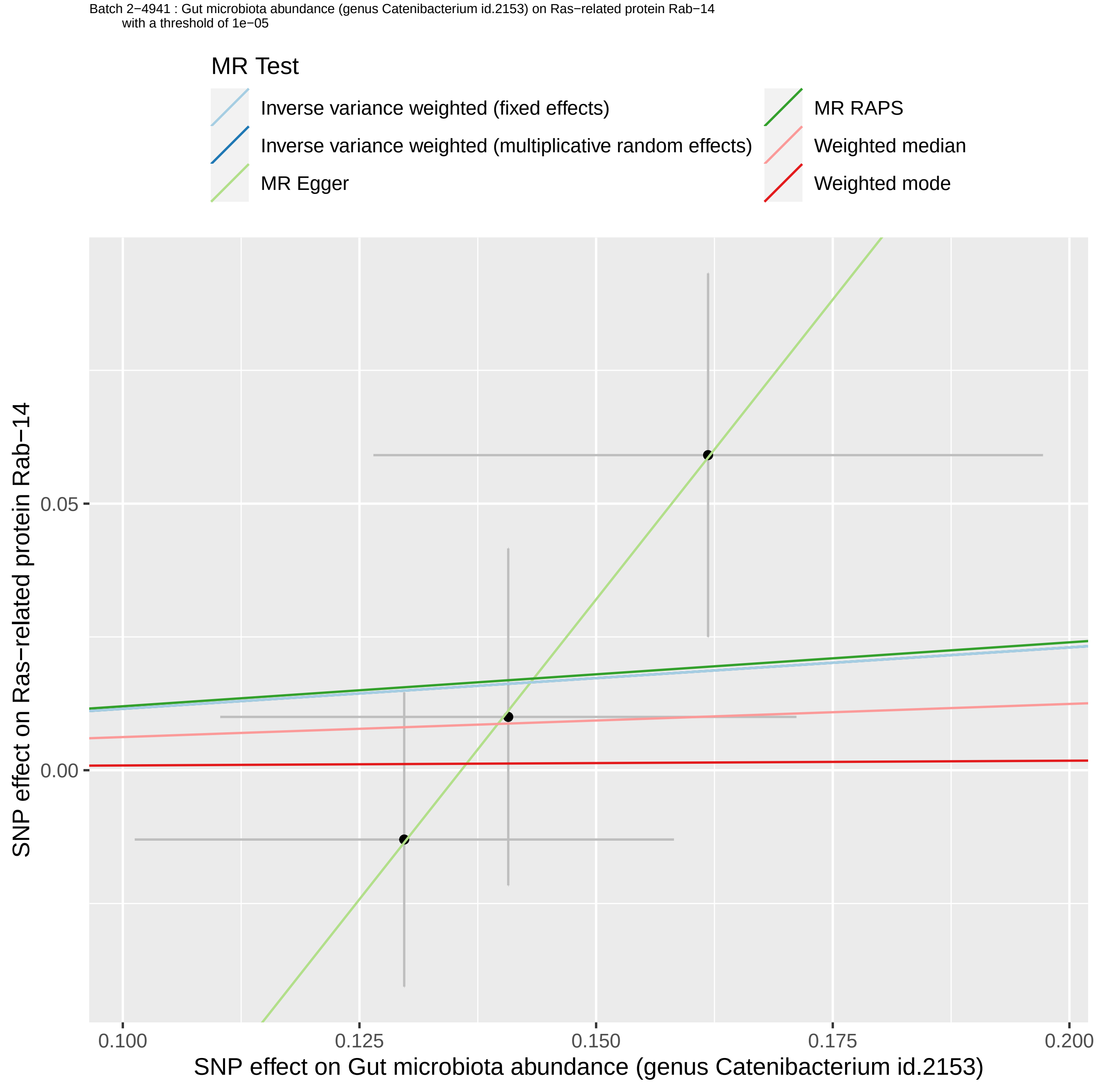

Batch 2−4941 : Gut microbiota abundance (genus Catenibacterium id.2153) on Ras−related protein Rab−14
with a threshold of 1e−05
MR Test
Inverse variance weighted (fixed effects)
Inverse variance weighted (multiplicative random effects)
MR Egger
MR RAPS
Weighted median
Weighted mode
SNP effect on Ras−related protein Rab−14
0.05
0.00
0.100
0.125
0.150
0.175
0.200
SNP effect on Gut microbiota abundance (genus Catenibacterium id.2153)

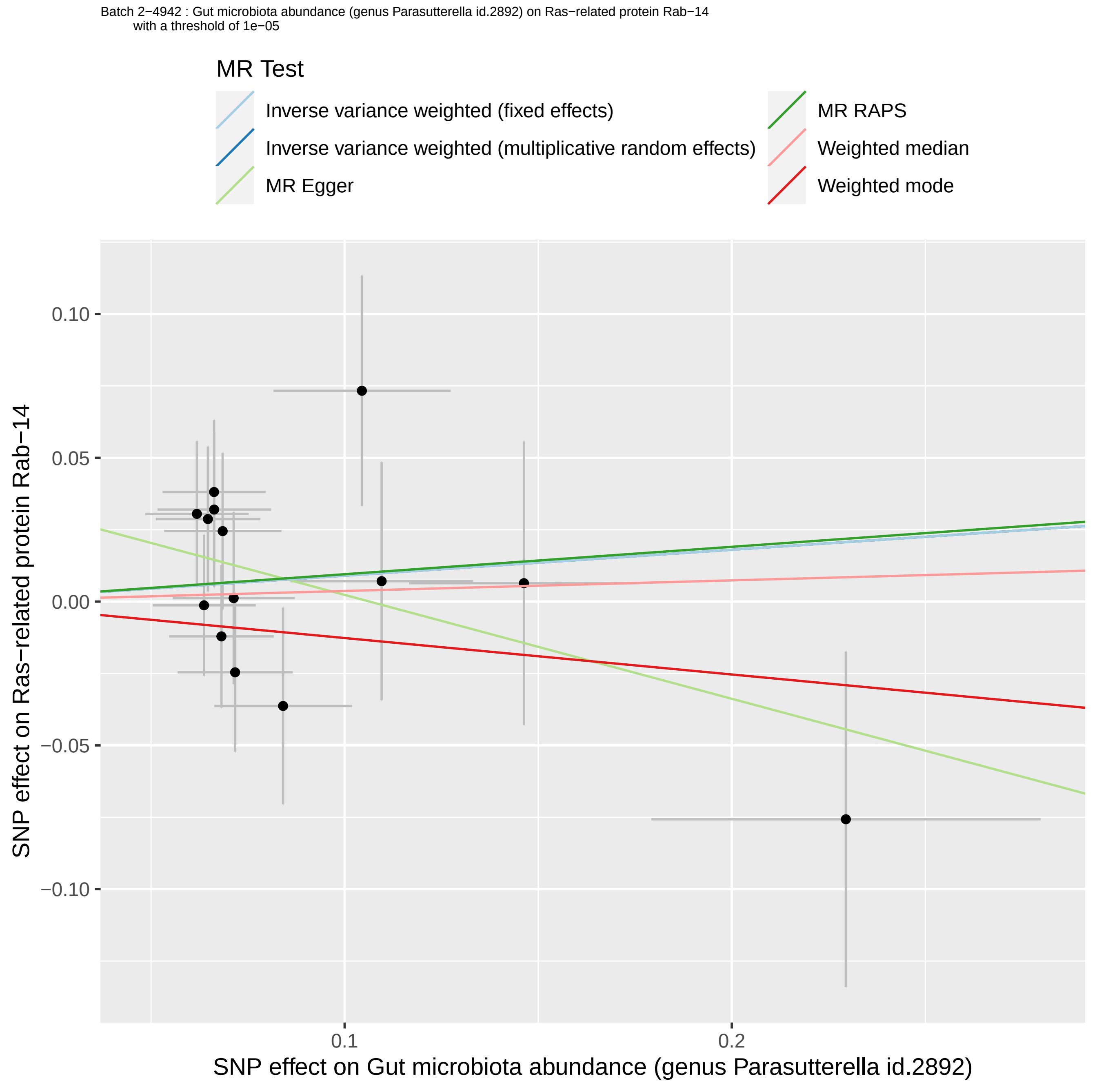

Batch 2−4942 : Gut microbiota abundance (genus Parasutterella id.2892) on Ras−related protein Rab−14
with a threshold of 1e−05
MR Test
Inverse variance weighted (fixed effects)
Inverse variance weighted (multiplicative random effects)
MR Egger
MR RAPS
Weighted median
Weighted mode
0.10
0.05
0.00
−0.05
−0.10
0.1
0.2
SNP effect on Ras−related protein Rab−14
SNP effect on Gut microbiota abundance (genus Parasutterella id.2892)

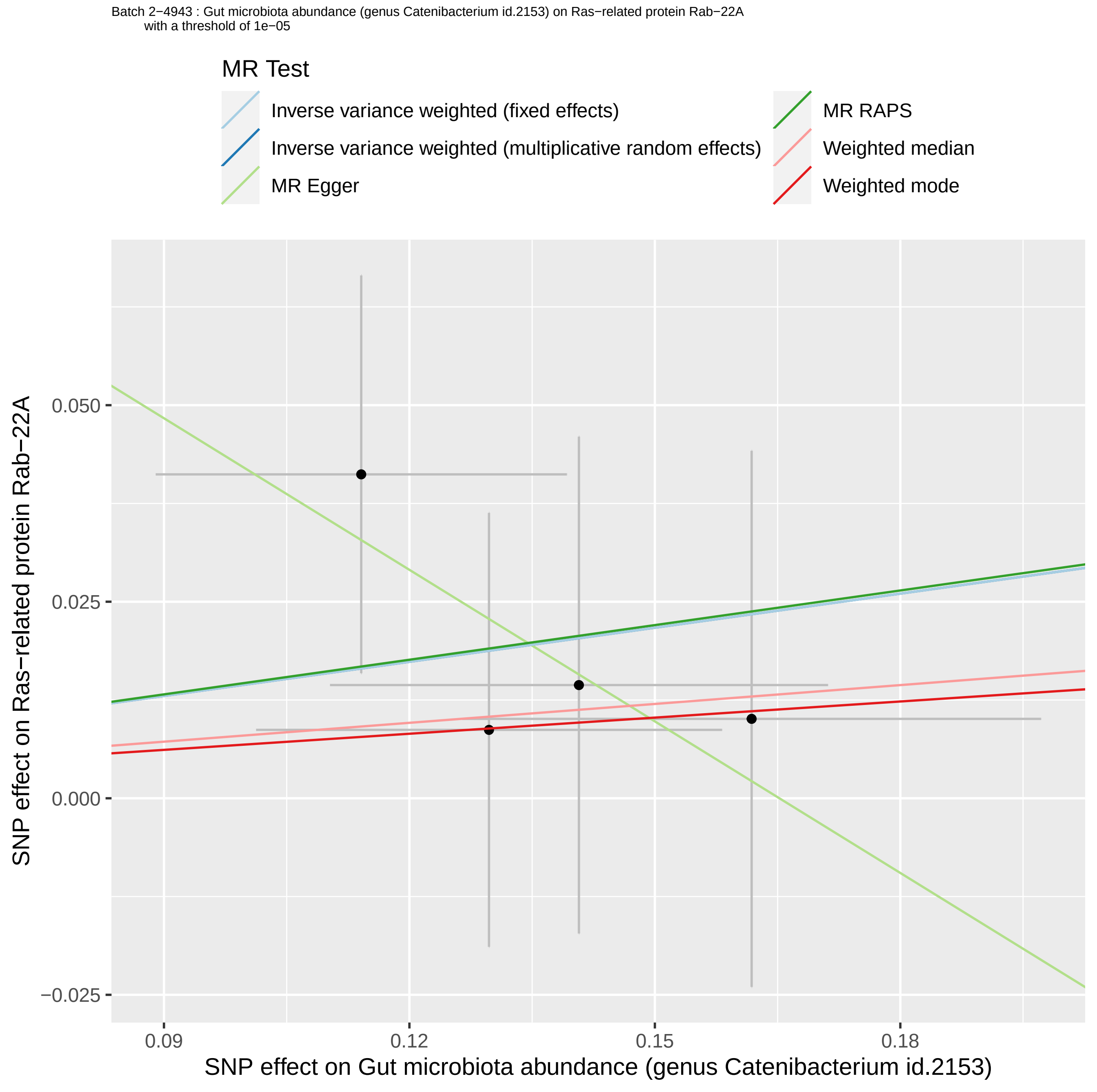
Batch 2−4943 : Gut microbiota abundance (genus Catenibacterium id.2153) on Ras−related protein Rab−22A
with a threshold of 1e−05
MR Test
Inverse variance weighted (fixed effects)
Inverse variance weighted (multiplicative random effects)
MR Egger
MR RAPS
Weighted median
Weighted mode
0.050
0.025
0.000
−0.025
0.09
0.12
0.15
0.18
SNP effect on Ras−related protein Rab−22A
SNP effect on Gut microbiota abundance (genus Catenibacterium id.2153)

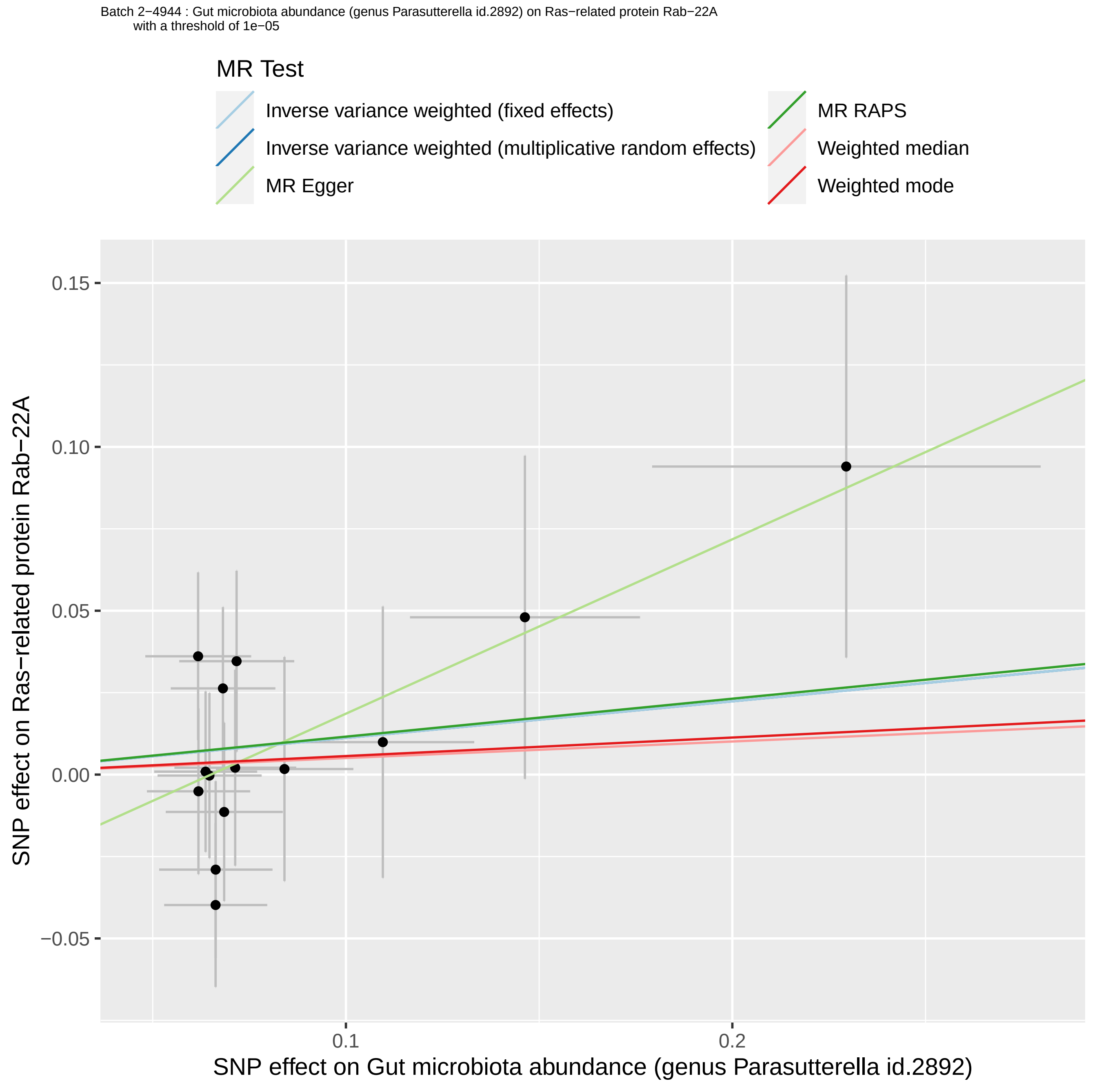

Batch 2−4944 : Gut microbiota abundance (genus Parasutterella id.2892) on Ras−related protein Rab−22A
with a threshold of 1e−05
MR Test
Inverse variance weighted (fixed effects)
Inverse variance weighted (multiplicative random effects)
MR Egger
MR RAPS
Weighted median
Weighted mode
0.15
0.10
0.05
0.00
−0.05
0.1
0.2
SNP effect on Ras−related protein Rab−22A
SNP effect on Gut microbiota abundance (genus Parasutterella id.2892)

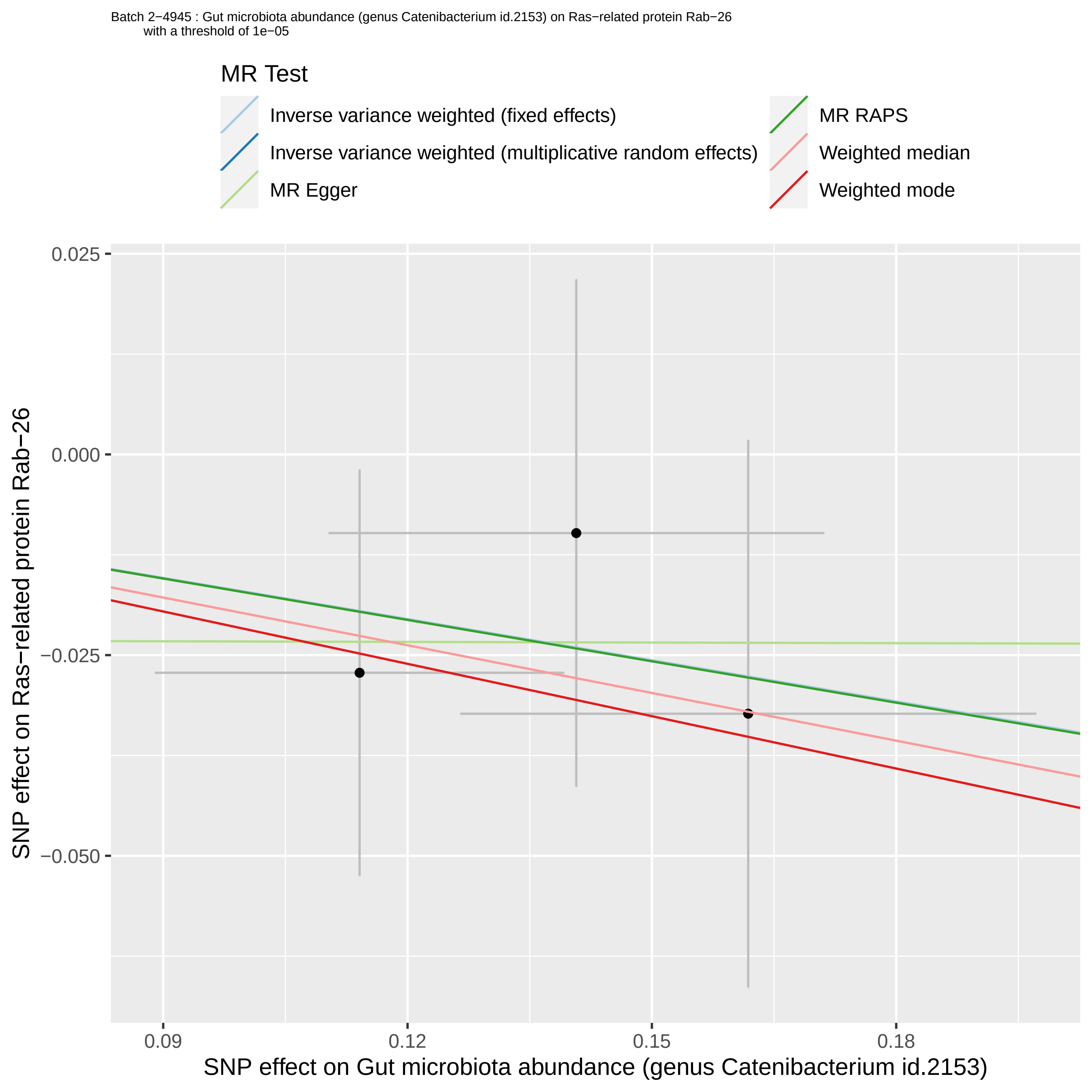

Batch 2−4945 : Gut microbiota abundance (genus Catenibacterium id.2153) on Ras−related protein Rab−26
with a threshold of 1e−05
MR Test
Inverse variance weighted (fixed effects)
Inverse variance weighted (multiplicative random effects)
MR Egger
MR RAPS
Weighted median
Weighted mode
0.025
0.000
−0.025
−0.050
SNP effect on Ras−related protein Rab−26
0.09
0.12
0.15
0.18
SNP effect on Gut microbiota abundance (genus Catenibacterium id.2153)

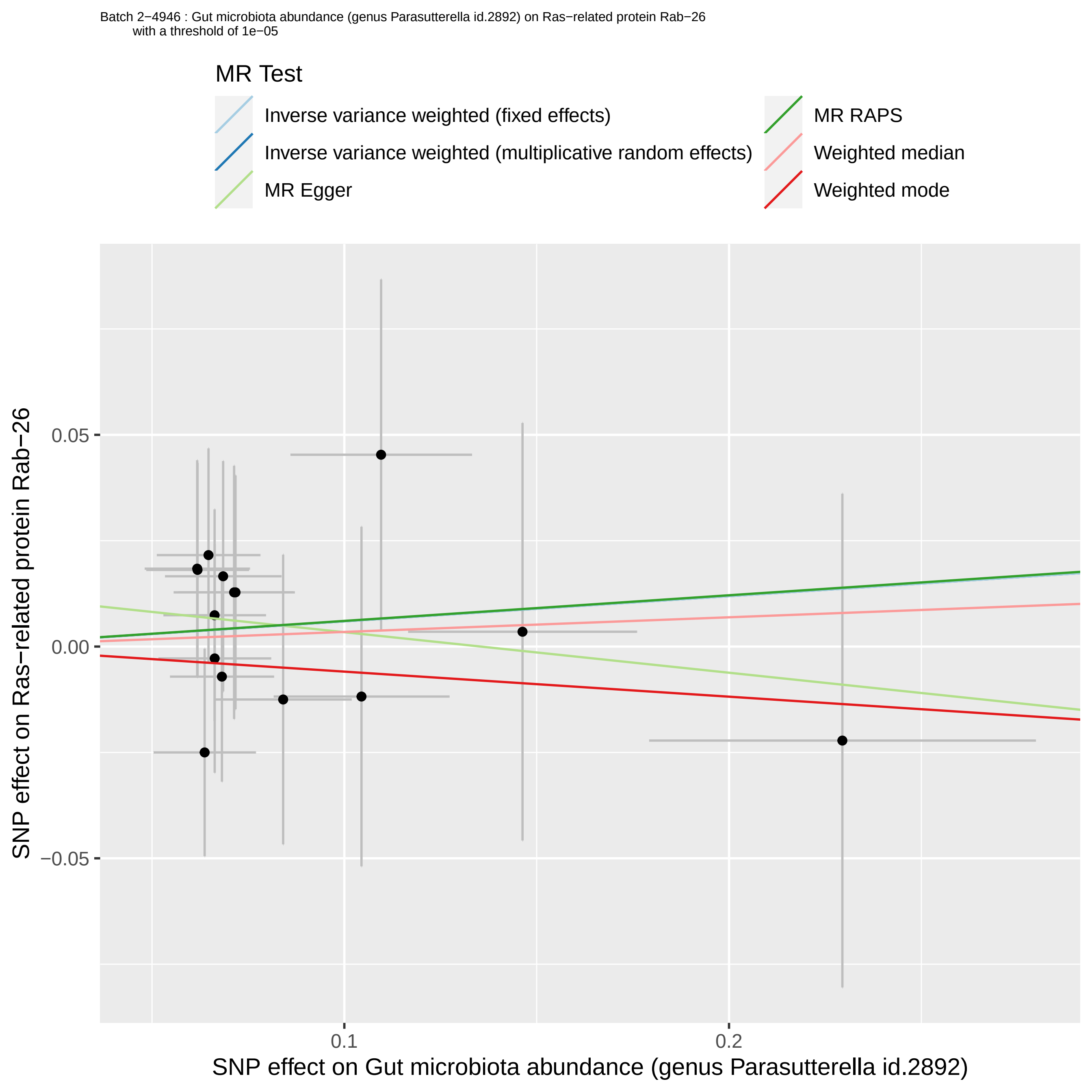

Batch 2−4946 : Gut microbiota abundance (genus Parasutterella id.2892) on Ras−related protein Rab−26
with a threshold of 1e−05
MR Test
Inverse variance weighted (fixed effects)
Inverse variance weighted (multiplicative random effects)
MR Egger
MR RAPS
Weighted median
Weighted mode
0.05
0.00
−0.05
0.1
0.2
SNP effect on Ras−related protein Rab−26
SNP effect on Gut microbiota abundance (genus Parasutterella id.2892)

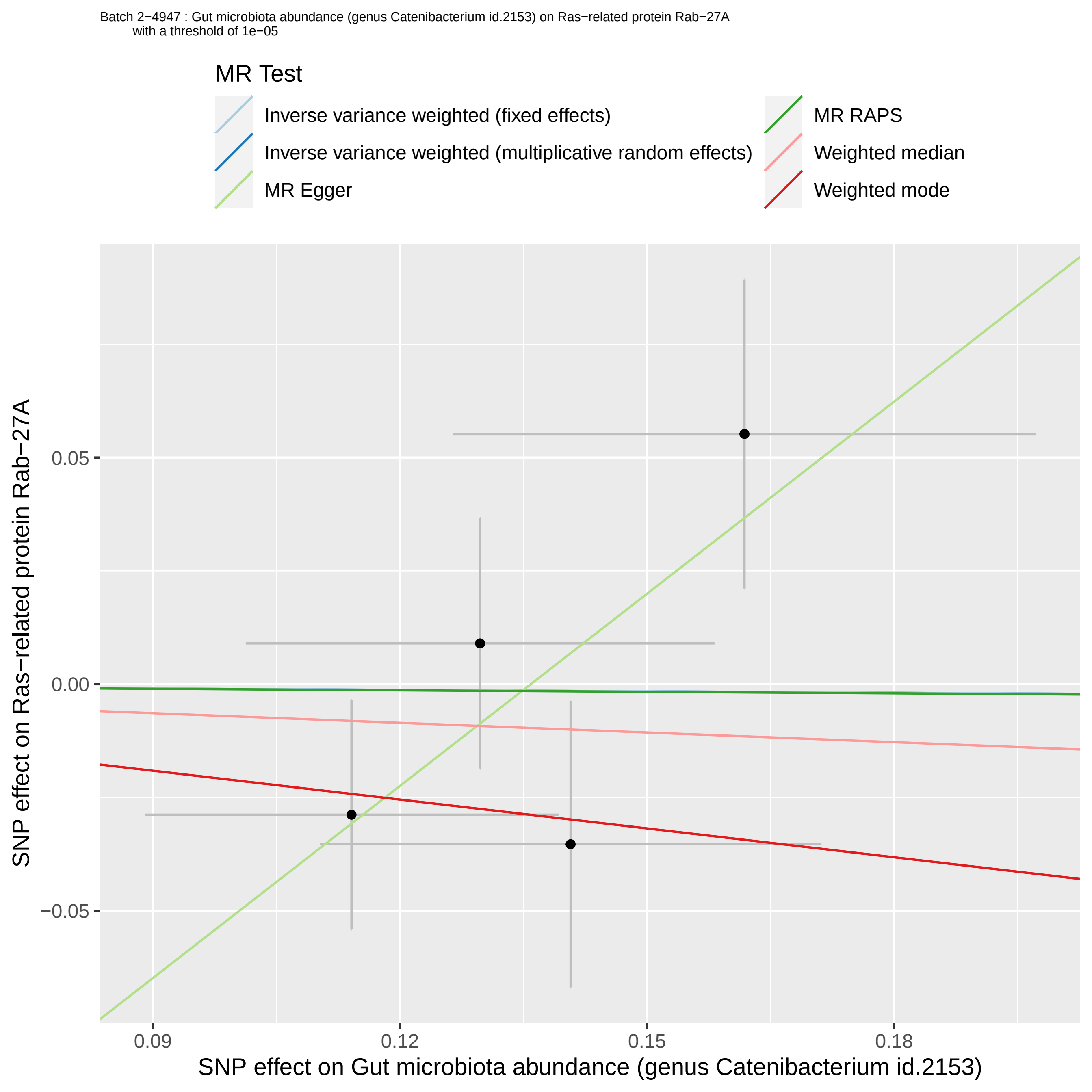

Batch 2−4947 : Gut microbiota abundance (genus Catenibacterium id.2153) on Ras−related protein Rab−27A
with a threshold of 1e−05
MR Test
Inverse variance weighted (fixed effects)
Inverse variance weighted (multiplicative random effects)
MR Egger
MR RAPS
Weighted median
Weighted mode
SNP effect on Ras−related protein Rab−27A
0.05
0.00
−0.05
0.09
0.12
0.15
0.18
SNP effect on Gut microbiota abundance (genus Catenibacterium id.2153)

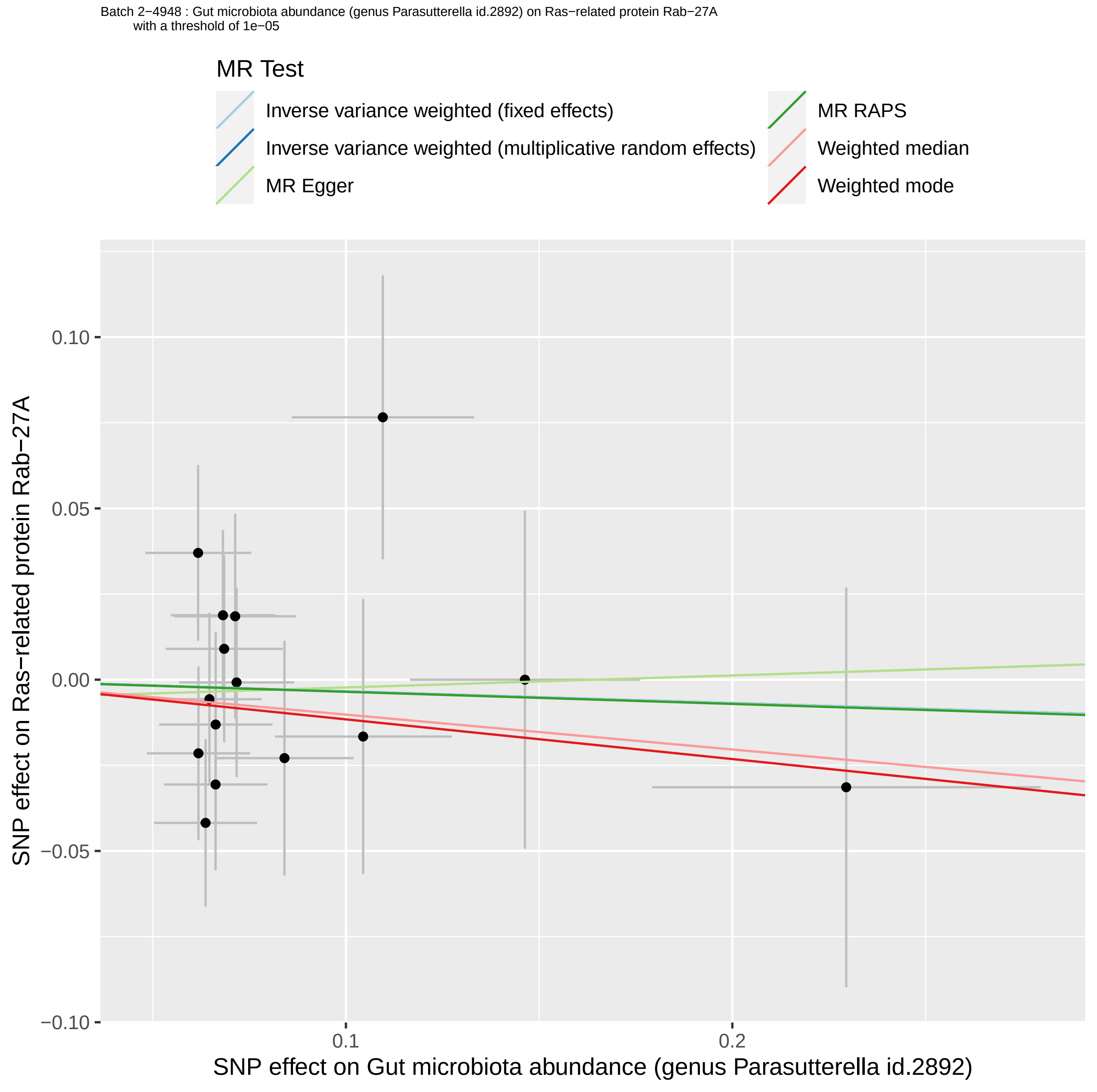
Batch 2−4948 : Gut microbiota abundance (genus Parasutterella id.2892) on Ras−related protein Rab−27A
with a threshold of 1e−05
MR Test
Inverse variance weighted (fixed effects)
Inverse variance weighted (multiplicative random effects)
MR Egger
MR RAPS
Weighted median
Weighted mode
SNP effect on Ras−related protein Rab−27A
0.10
0.05
0.00
−0.05
−0.10
0.1
0.2
SNP effect on Gut microbiota abundance (genus Parasutterella id.2892)

Batch 2−4949 : Gut microbiota abundance (genus Catenibacterium id.2153) on Ras−related protein Rab−27B
with a threshold of 1e−05

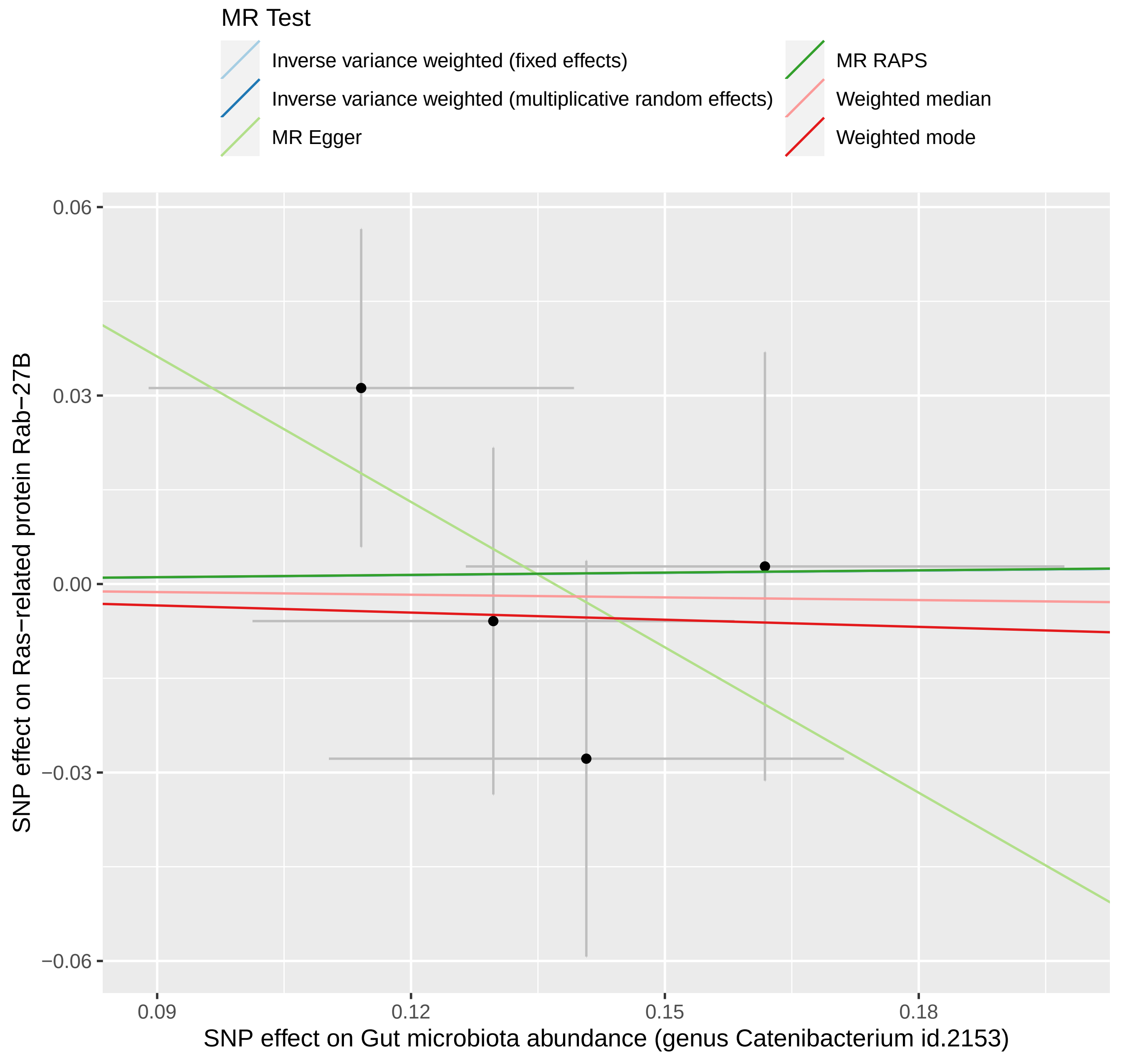

Batch 2−4950 : Gut microbiota abundance (genus Parasutterella id.2892) on Ras−related protein Rab−27B
with a threshold of 1e−05

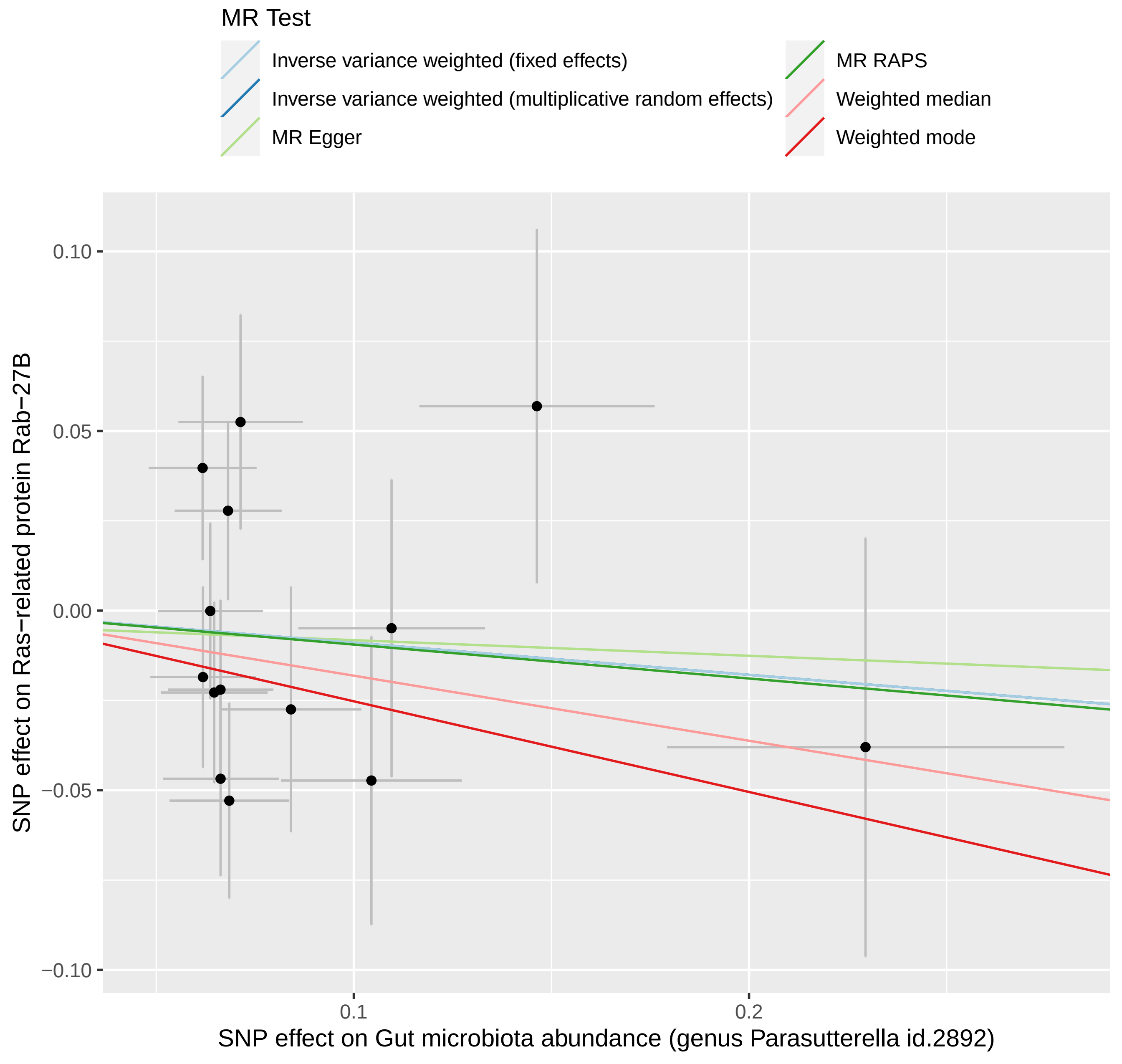

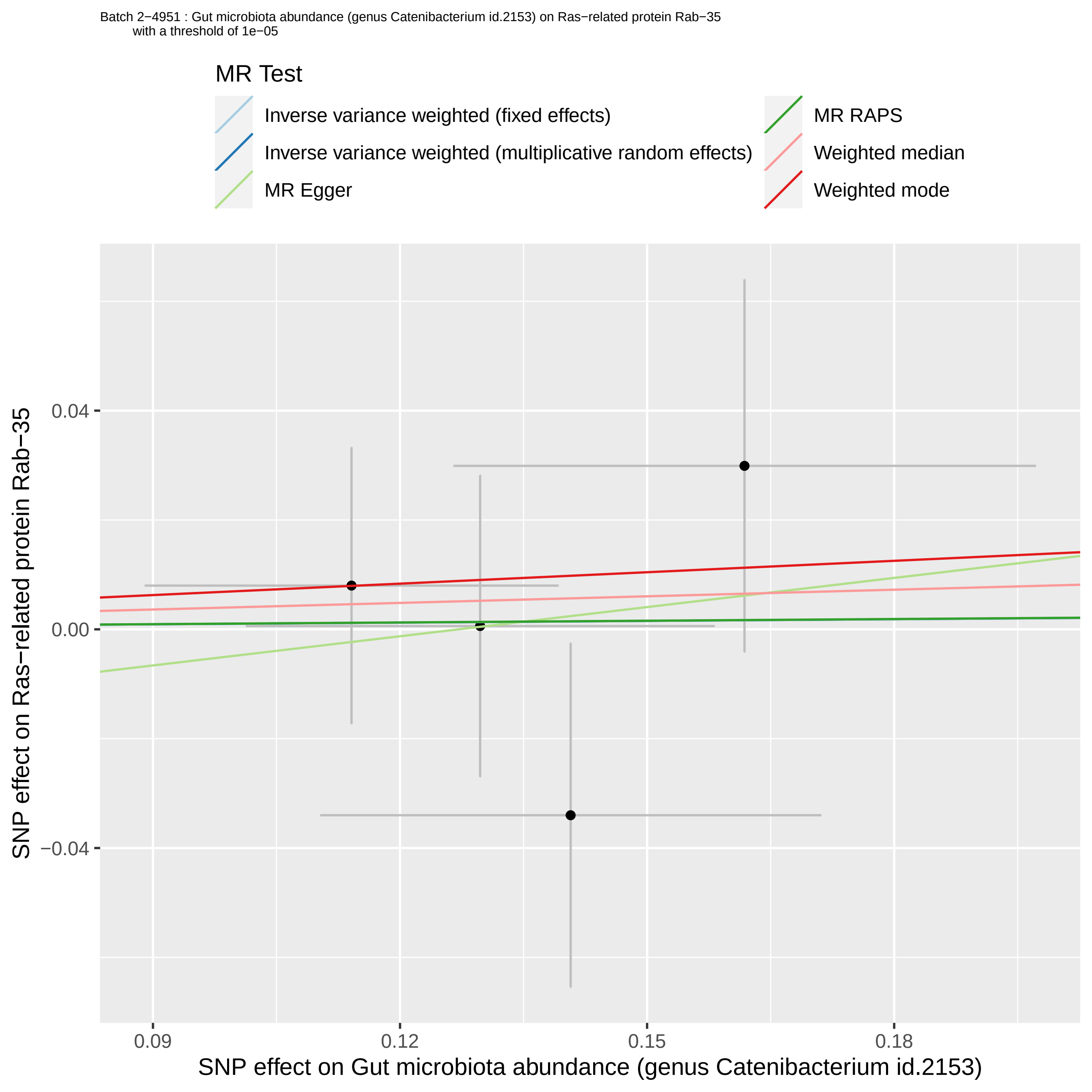

Batch 2−4951 : Gut microbiota abundance (genus Catenibacterium id.2153) on Ras−related protein Rab−35
with a threshold of 1e−05
MR Test
Inverse variance weighted (fixed effects)
Inverse variance weighted (multiplicative random effects)
MR Egger
MR RAPS
Weighted median
Weighted mode
0.04
0.00
−0.04
0.09
0.12
0.15
0.18
SNP effect on Ras−related protein Rab−35
SNP effect on Gut microbiota abundance (genus Catenibacterium id.2153)

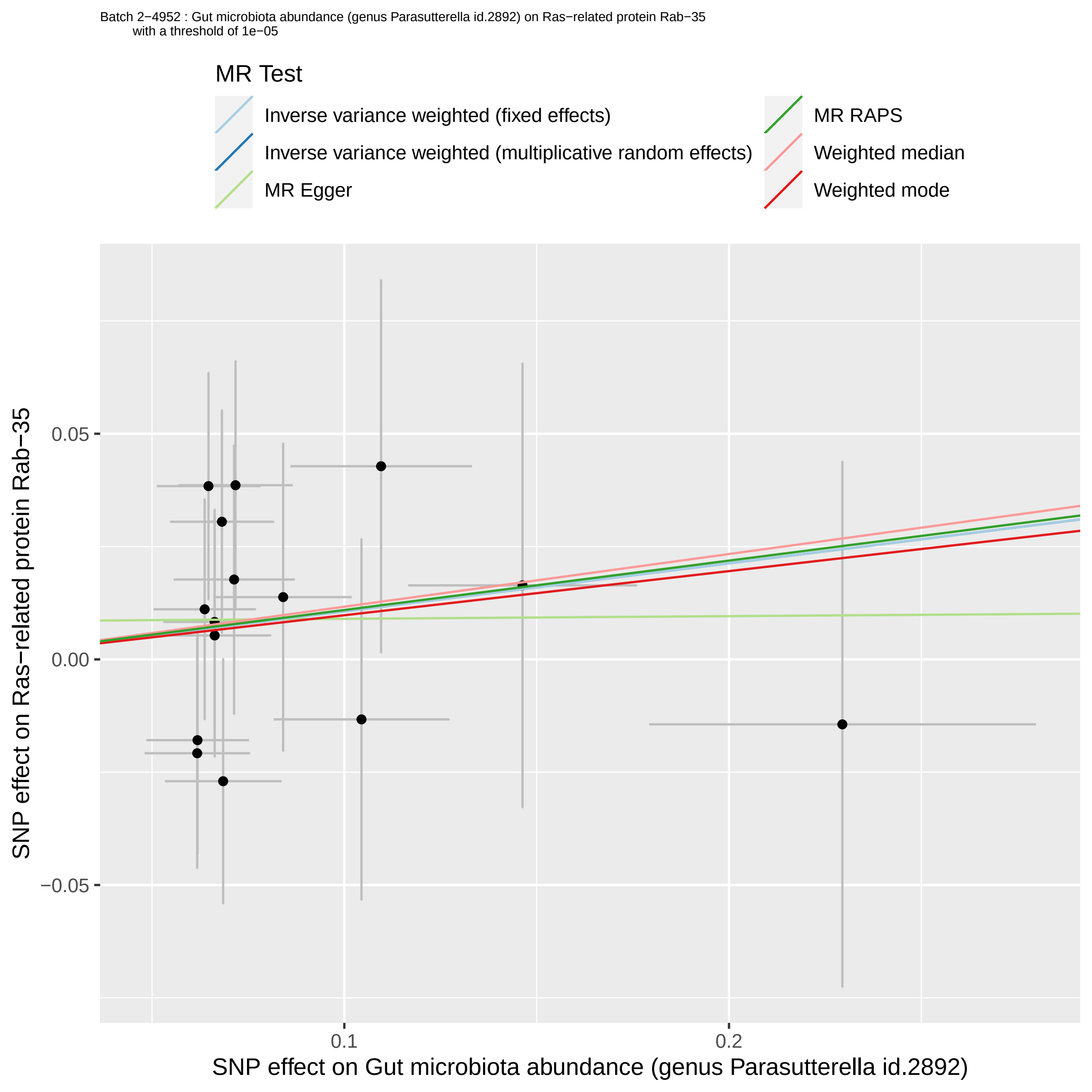

Batch 2−4952 : Gut microbiota abundance (genus Parasutterella id.2892) on Ras−related protein Rab−35
with a threshold of 1e−05
MR Test
Inverse variance weighted (fixed effects)
Inverse variance weighted (multiplicative random effects)
MR Egger
MR RAPS
Weighted median
Weighted mode
SNP effect on Ras−related protein Rab−35
0.05
0.00
−0.05
0.1
0.2
SNP effect on Gut microbiota abundance (genus Parasutterella id.2892)

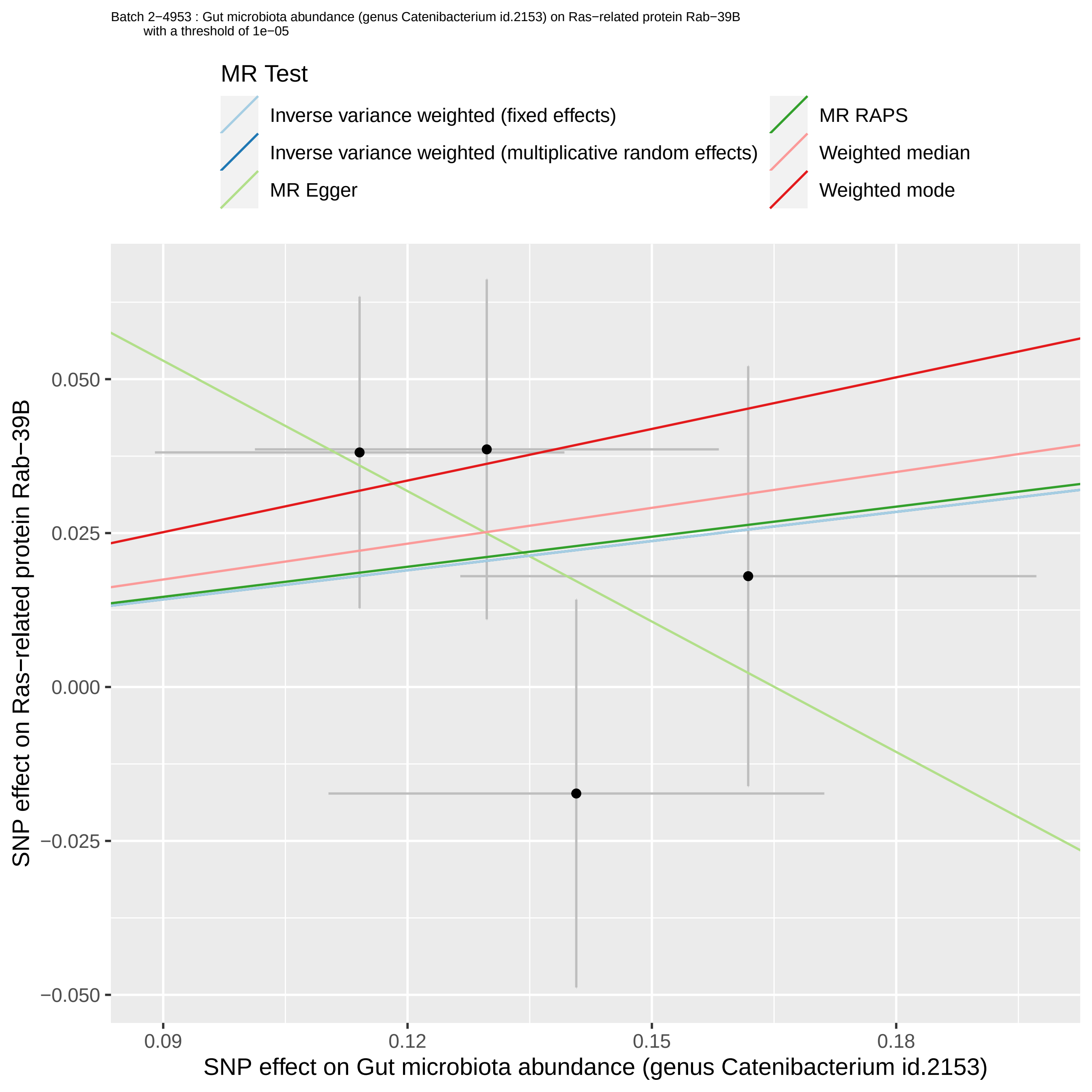

Batch 2−4953 : Gut microbiota abundance (genus Catenibacterium id.2153) on Ras−related protein Rab−39B
with a threshold of 1e−05
MR Test
Inverse variance weighted (fixed effects)
Inverse variance weighted (multiplicative random effects)
MR Egger
MR RAPS
Weighted median
Weighted mode
0.050
0.025
0.000
−0.025
−0.050
0.09
0.12
0.15
0.18
SNP effect on Ras−related protein Rab−39B
SNP effect on Gut microbiota abundance (genus Catenibacterium id.2153)

Batch 2−4954 : Gut microbiota abundance (genus Parasutterella id.2892) on Ras−related protein Rab−39B
with a threshold of 1e−05

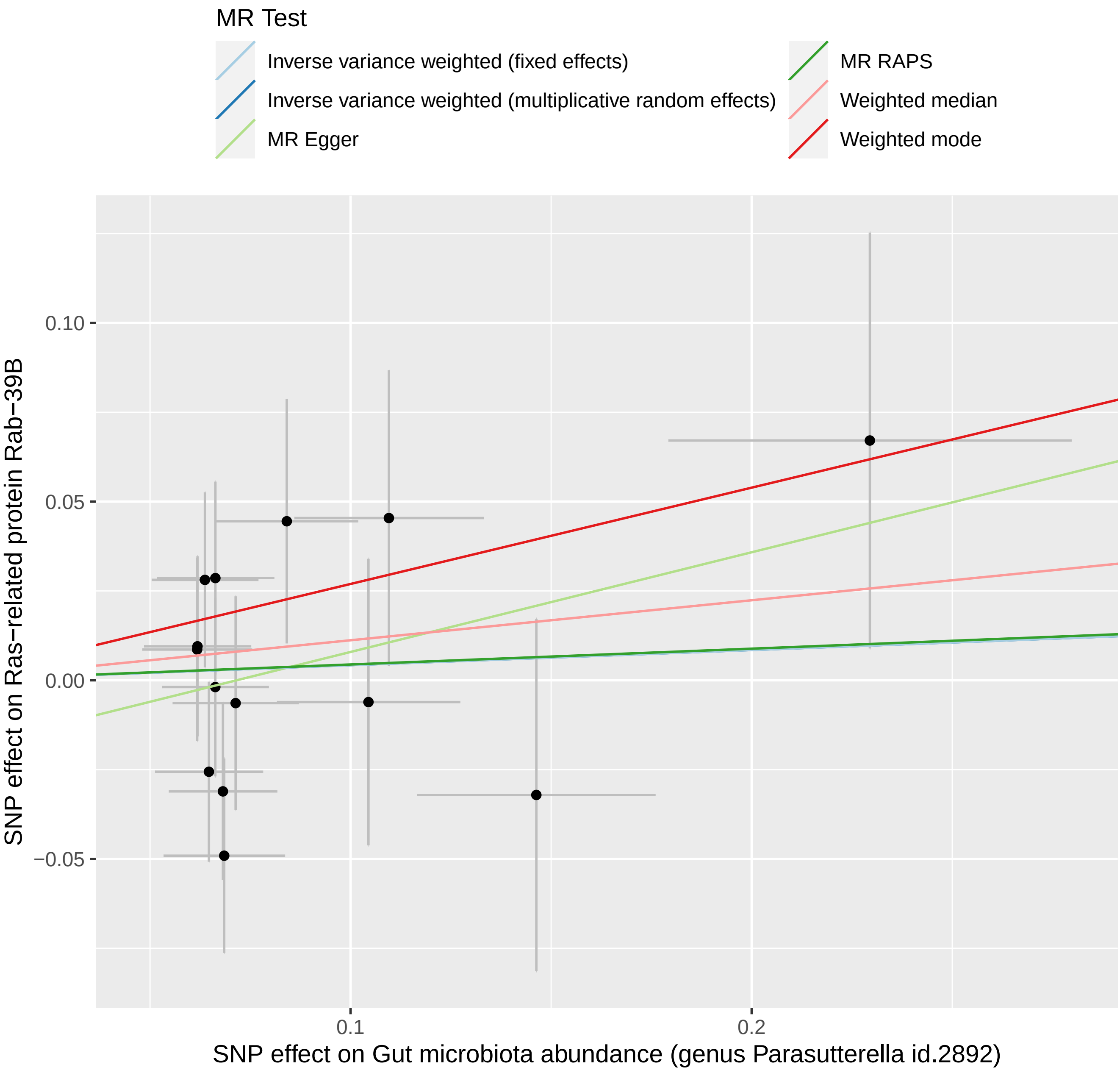

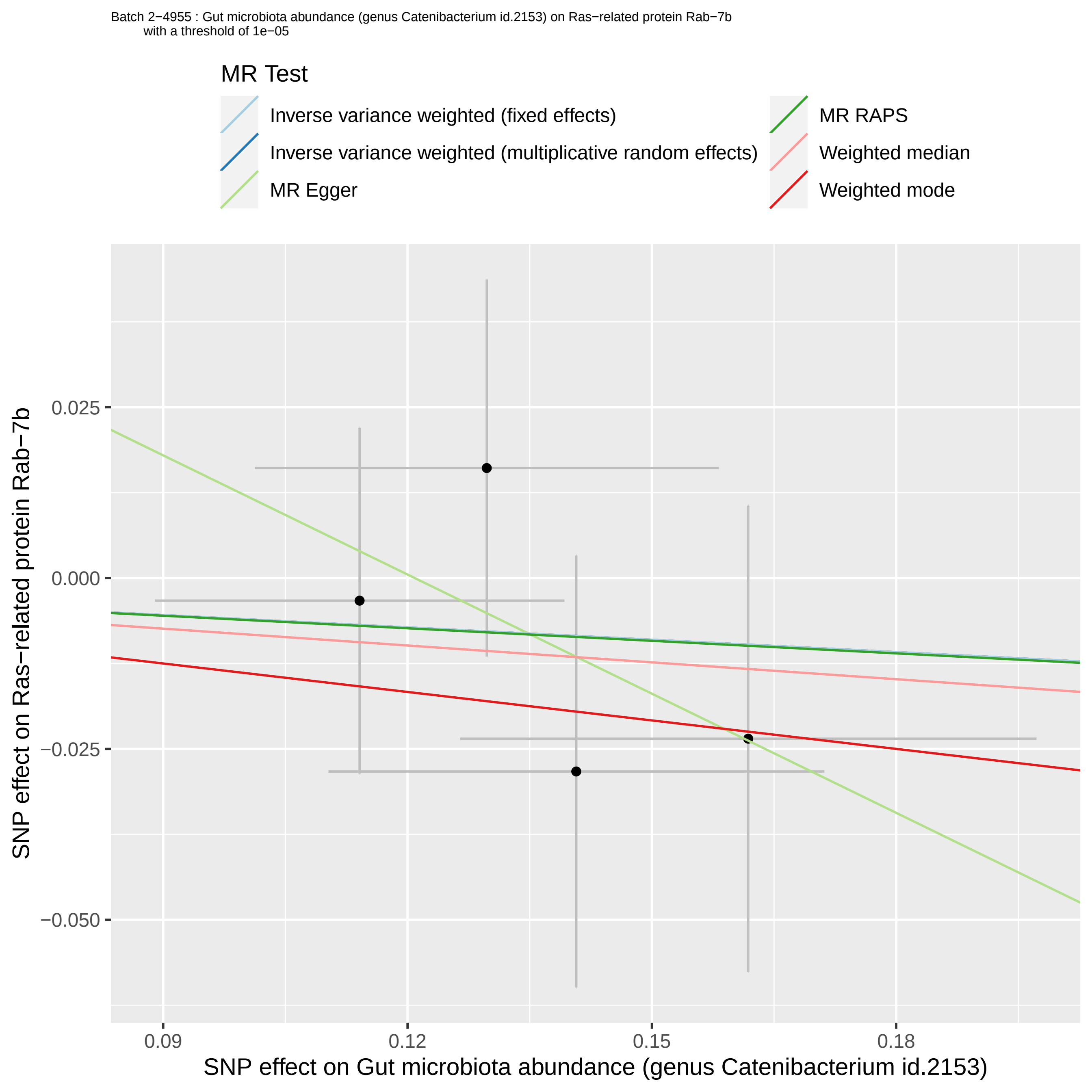

Batch 2−4955 : Gut microbiota abundance (genus Catenibacterium id.2153) on Ras−related protein Rab−7b
with a threshold of 1e−05
MR Test
Inverse variance weighted (fixed effects)
Inverse variance weighted (multiplicative random effects)
MR Egger
MR RAPS
Weighted median
Weighted mode
0.025
0.000
−0.025
−0.050
SNP effect on Ras−related protein Rab−7b
0.09
0.12
0.15
0.18
SNP effect on Gut microbiota abundance (genus Catenibacterium id.2153)

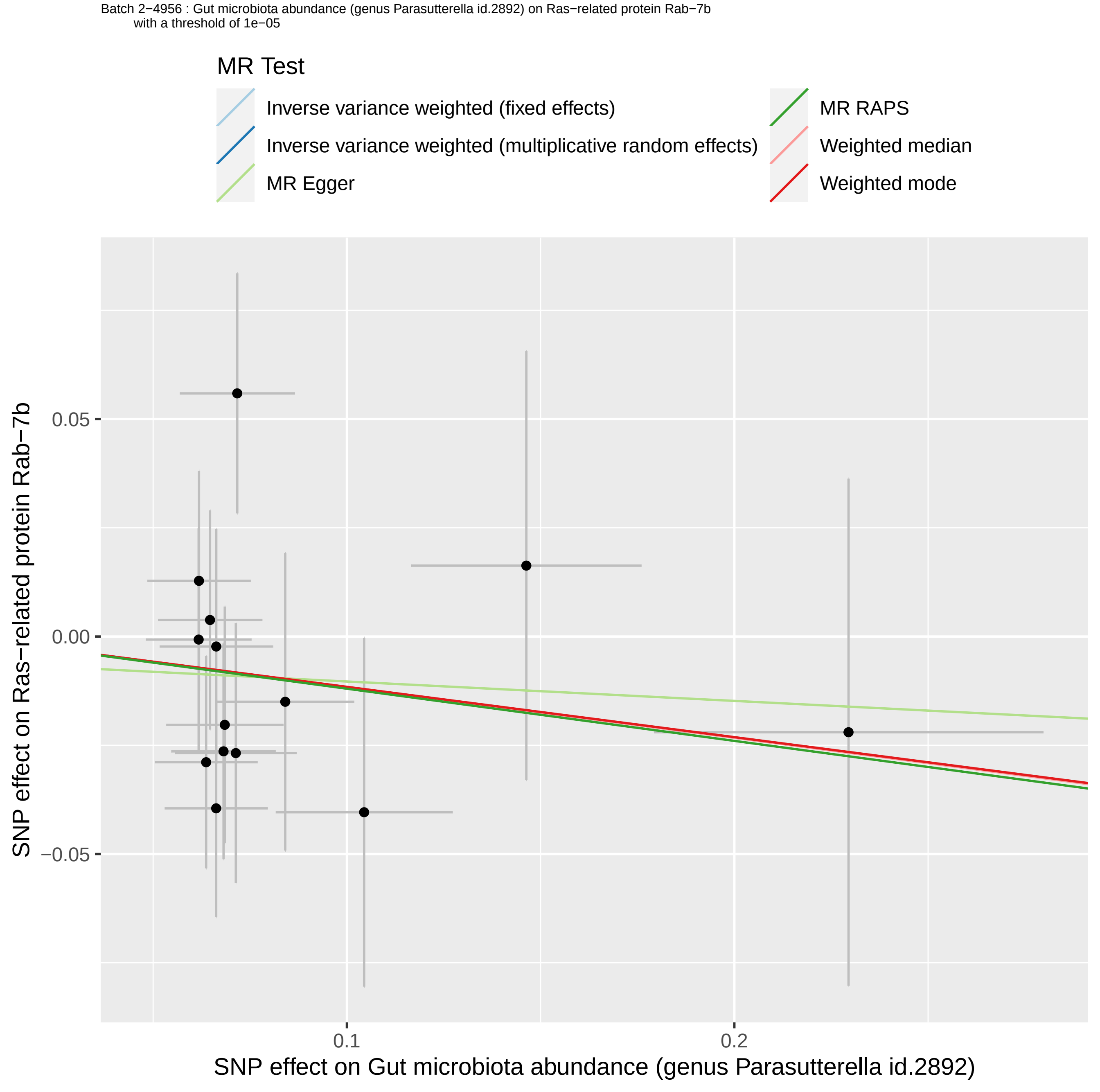
Batch 2−4956 : Gut microbiota abundance (genus Parasutterella id.2892) on Ras−related protein Rab−7b
with a threshold of 1e−05
MR Test
Inverse variance weighted (fixed effects)
Inverse variance weighted (multiplicative random effects)
MR Egger
MR RAPS
Weighted median
Weighted mode
0.05
0.00
−0.05
0.1
0.2
SNP effect on Ras−related protein Rab−7b
SNP effect on Gut microbiota abundance (genus Parasutterella id.2892)

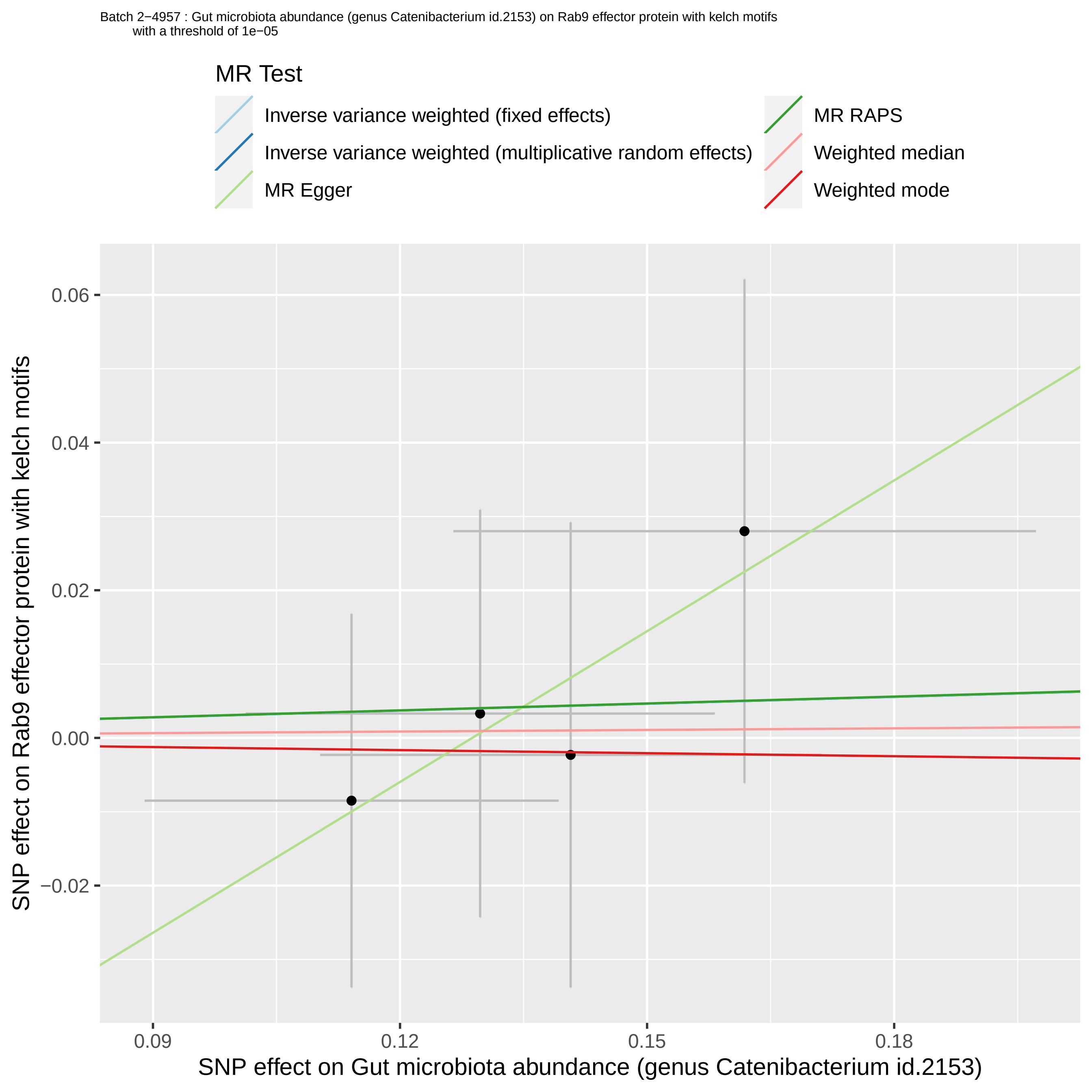
Batch 2−4957 : Gut microbiota abundance (genus Catenibacterium id.2153) on Rab9 effector protein with kelch motifs
with a threshold of 1e−05
MR Test
Inverse variance weighted (fixed effects)
Inverse variance weighted (multiplicative random effects)
MR Egger
MR RAPS
Weighted median
Weighted mode
0.06
0.04
0.02
0.00
−0.02
0.09
0.12
0.15
0.18
SNP effect on Rab9 effector protein with kelch motifs
SNP effect on Gut microbiota abundance (genus Catenibacterium id.2153)

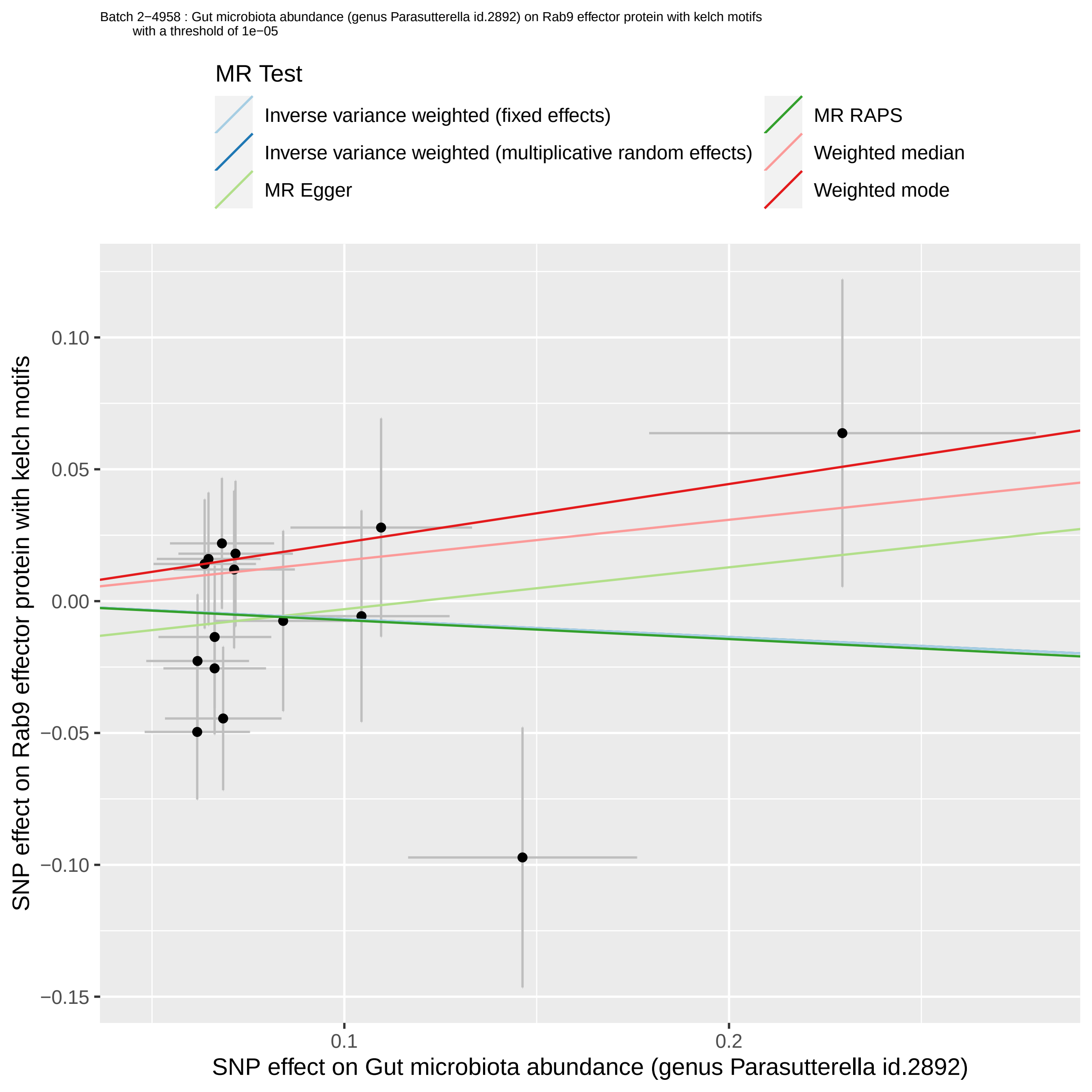
Batch 2−4958 : Gut microbiota abundance (genus Parasutterella id.2892) on Rab9 effector protein with kelch motifs
with a threshold of 1e−05
MR Test
Inverse variance weighted (fixed effects)
Inverse variance weighted (multiplicative random effects)
MR Egger
MR RAPS
Weighted median
Weighted mode
SNP effect on Rab9 effector protein with kelch motifs
0.10
0.05
0.00
−0.05
−0.10
−0.15
0.1
0.2
SNP effect on Gut microbiota abundance (genus Parasutterella id.2892)

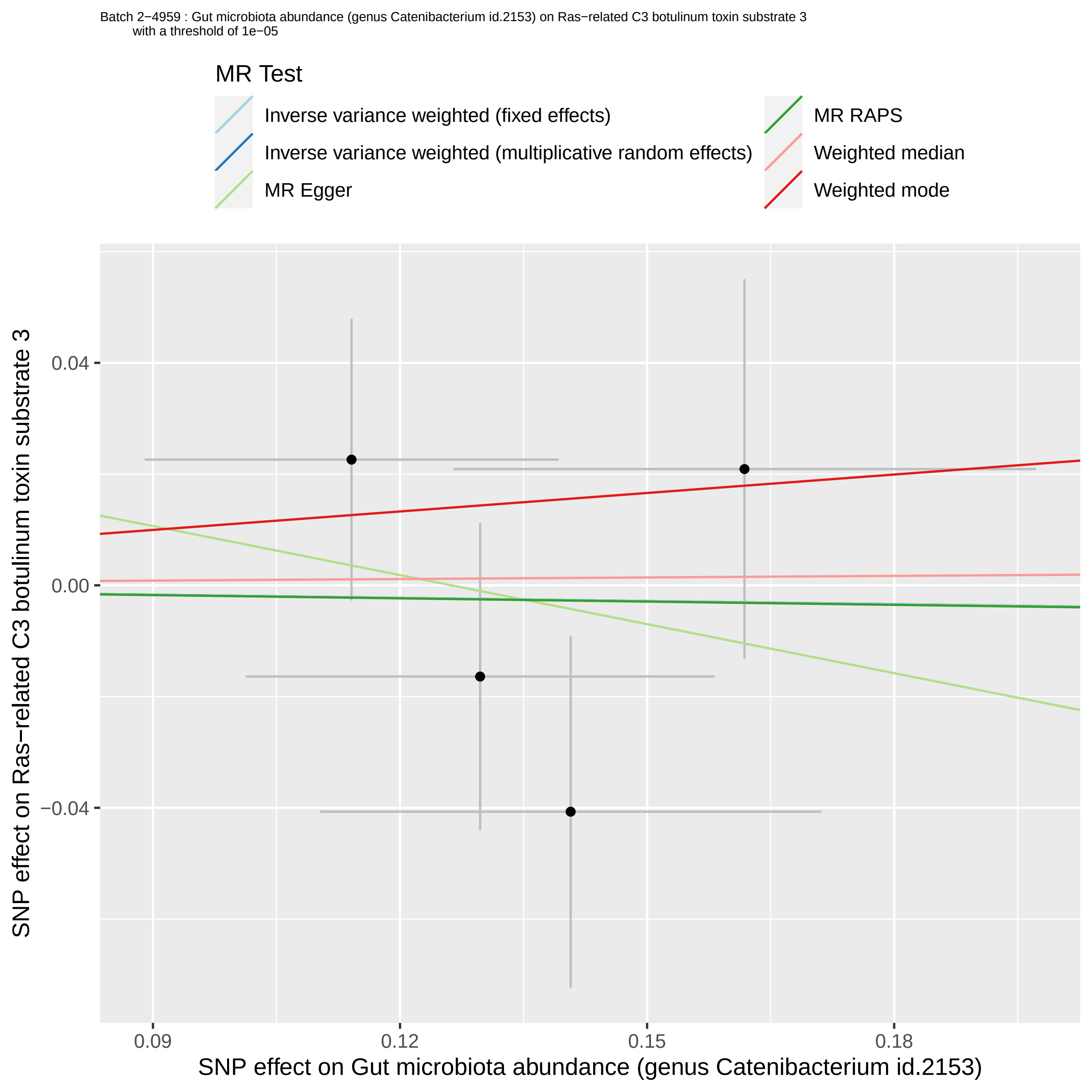
Batch 2−4959 : Gut microbiota abundance (genus Catenibacterium id.2153) on Ras−related C3 botulinum toxin substrate 3
with a threshold of 1e−05
MR Test
Inverse variance weighted (fixed effects)
Inverse variance weighted (multiplicative random effects)
MR Egger
MR RAPS
Weighted median
Weighted mode
SNP effect on Ras−related C3 botulinum toxin substrate 3
0.04
0.00
−0.04
0.09
0.12
0.15
0.18
SNP effect on Gut microbiota abundance (genus Catenibacterium id.2153)

Batch 2−4960 : Gut microbiota abundance (genus Parasutterella id.2892) on Ras−related C3 botulinum toxin substrate 3
with a threshold of 1e−05

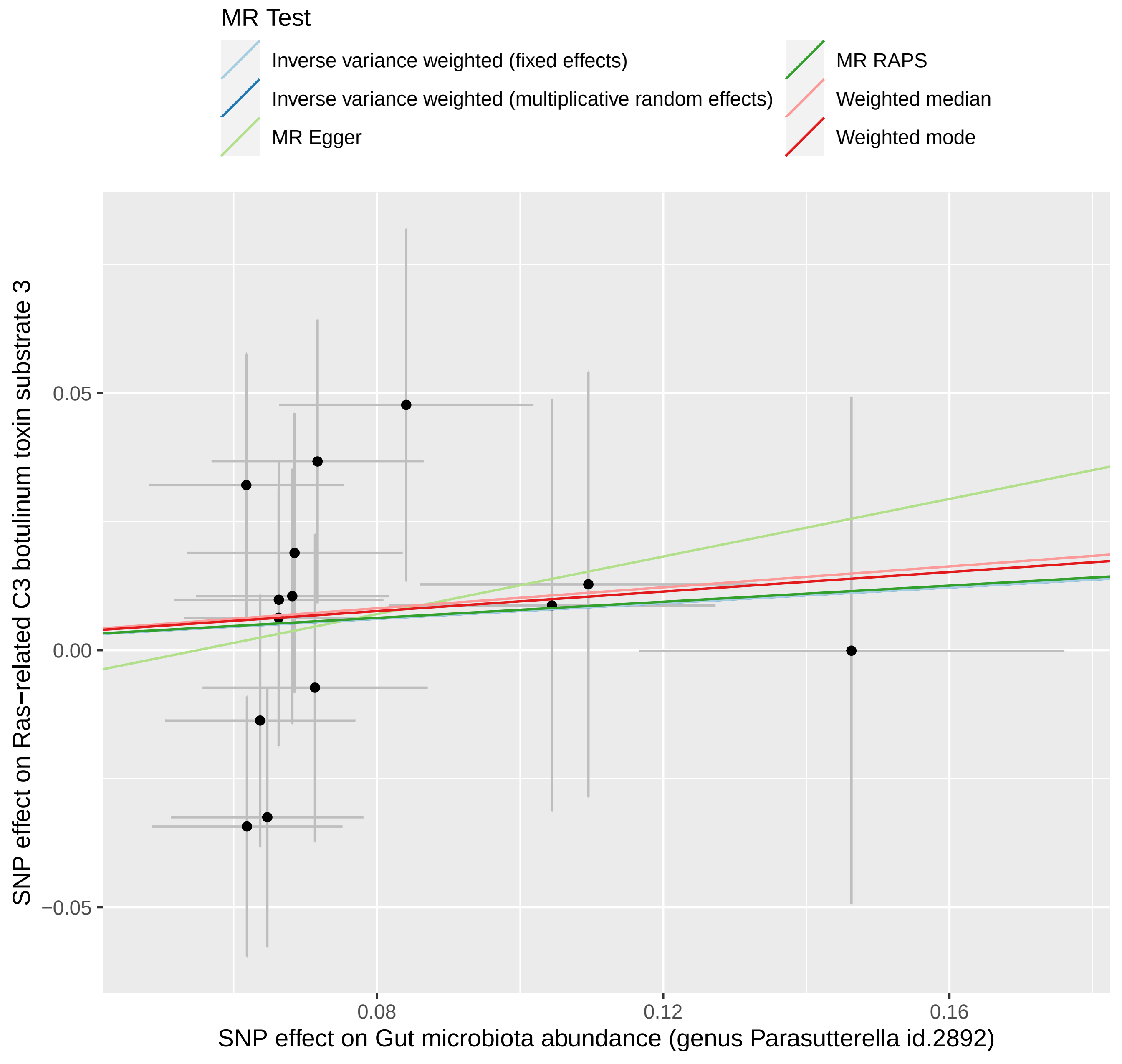

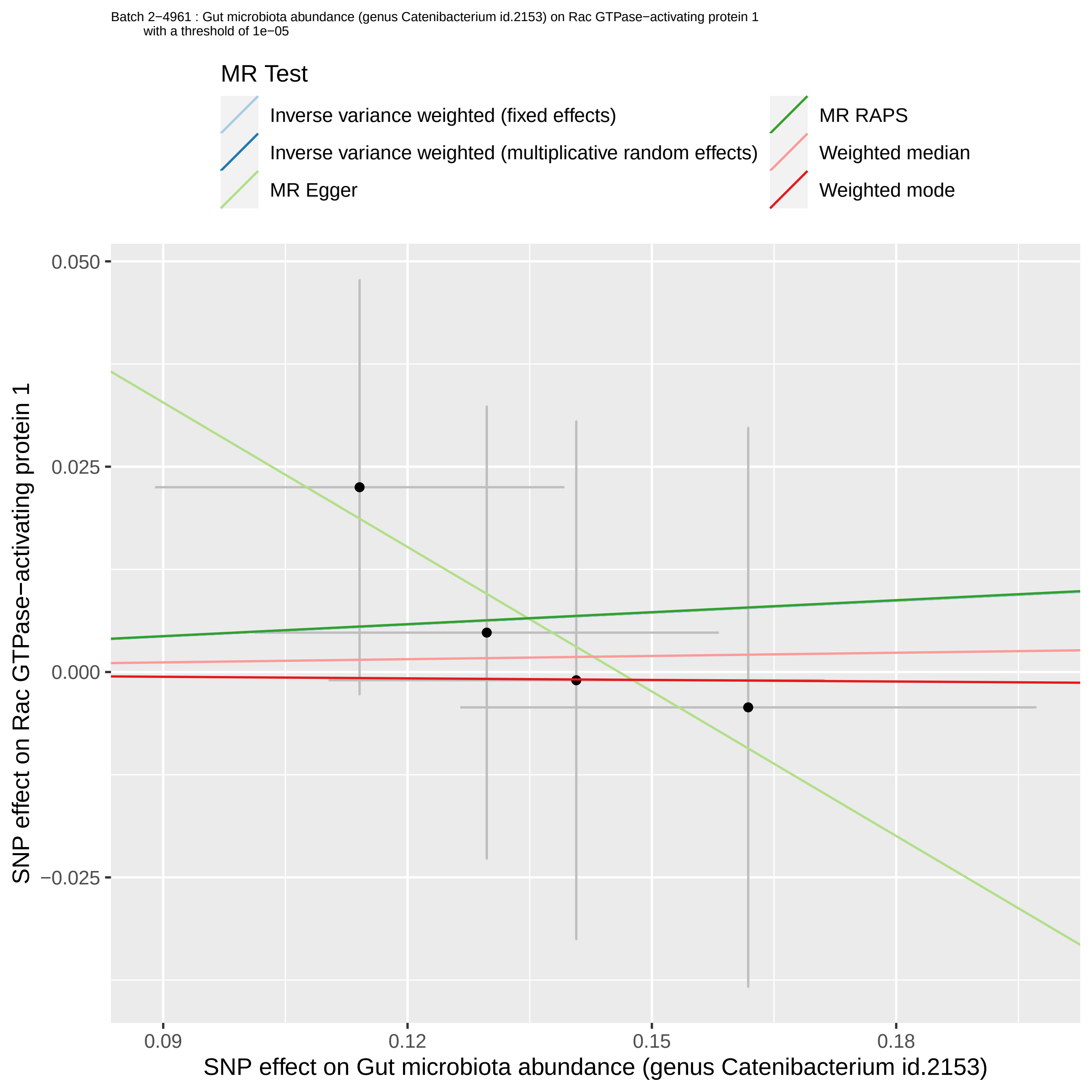
Batch 2−4961 : Gut microbiota abundance (genus Catenibacterium id.2153) on Rac GTPase−activating protein 1
with a threshold of 1e−05
MR Test
Inverse variance weighted (fixed effects)
Inverse variance weighted (multiplicative random effects)
MR Egger
MR RAPS
Weighted median
Weighted mode
0.050
0.025
0.000
−0.025
0.09
0.12
0.15
0.18
SNP effect on Rac GTPase−activating protein 1
SNP effect on Gut microbiota abundance (genus Catenibacterium id.2153)

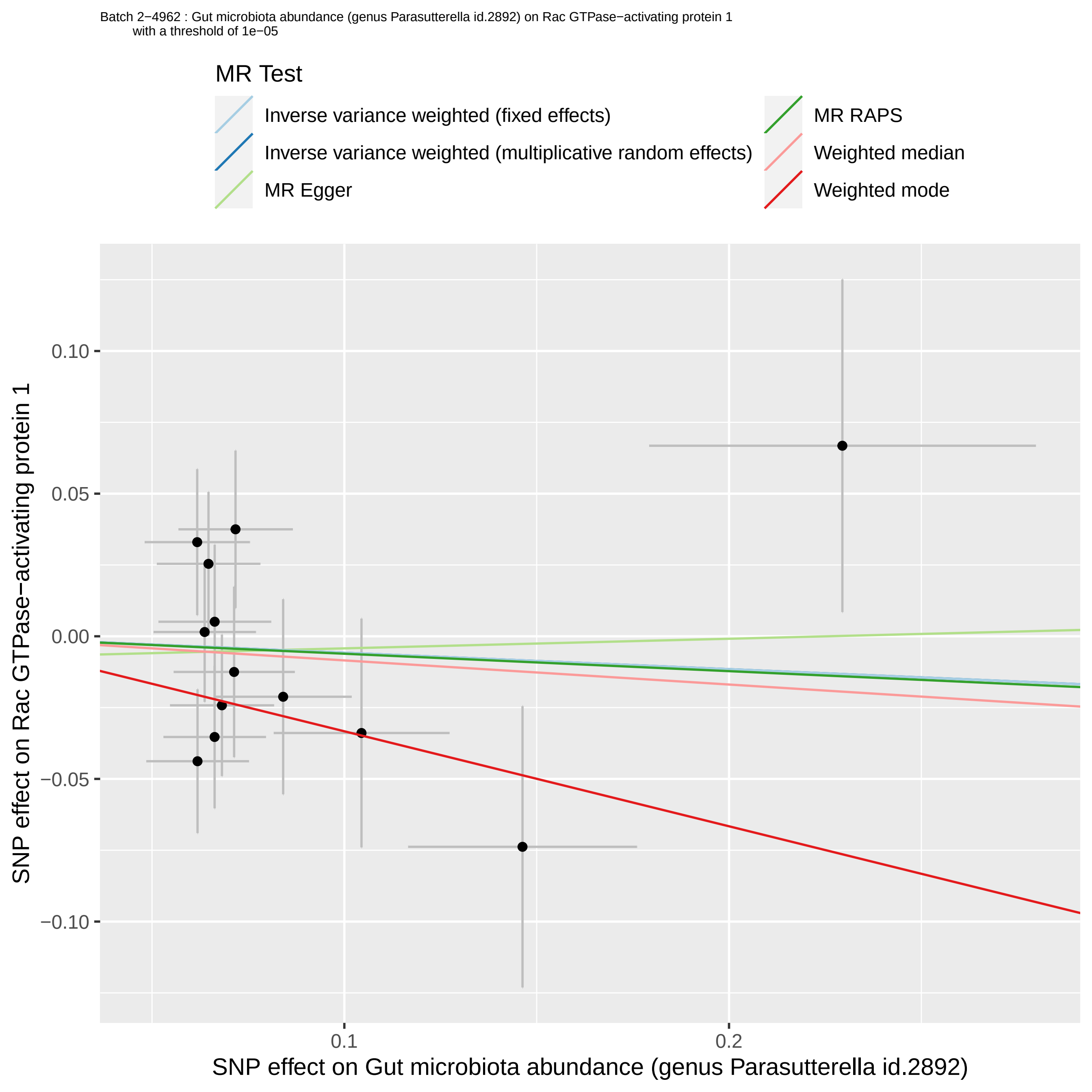

Batch 2−4962 : Gut microbiota abundance (genus Parasutterella id.2892) on Rac GTPase−activating protein 1
with a threshold of 1e−05
MR Test
Inverse variance weighted (fixed effects)
Inverse variance weighted (multiplicative random effects)
MR Egger
MR RAPS
Weighted median
Weighted mode
0.10
0.05
0.00
−0.05
−0.10
0.1
0.2
SNP effect on Rac GTPase−activating protein 1
SNP effect on Gut microbiota abundance (genus Parasutterella id.2892)

Batch 2−4963 : Gut microbiota abundance (genus Catenibacterium id.2153) on Cell cycle checkpoint protein RAD1
with a threshold of 1e−05

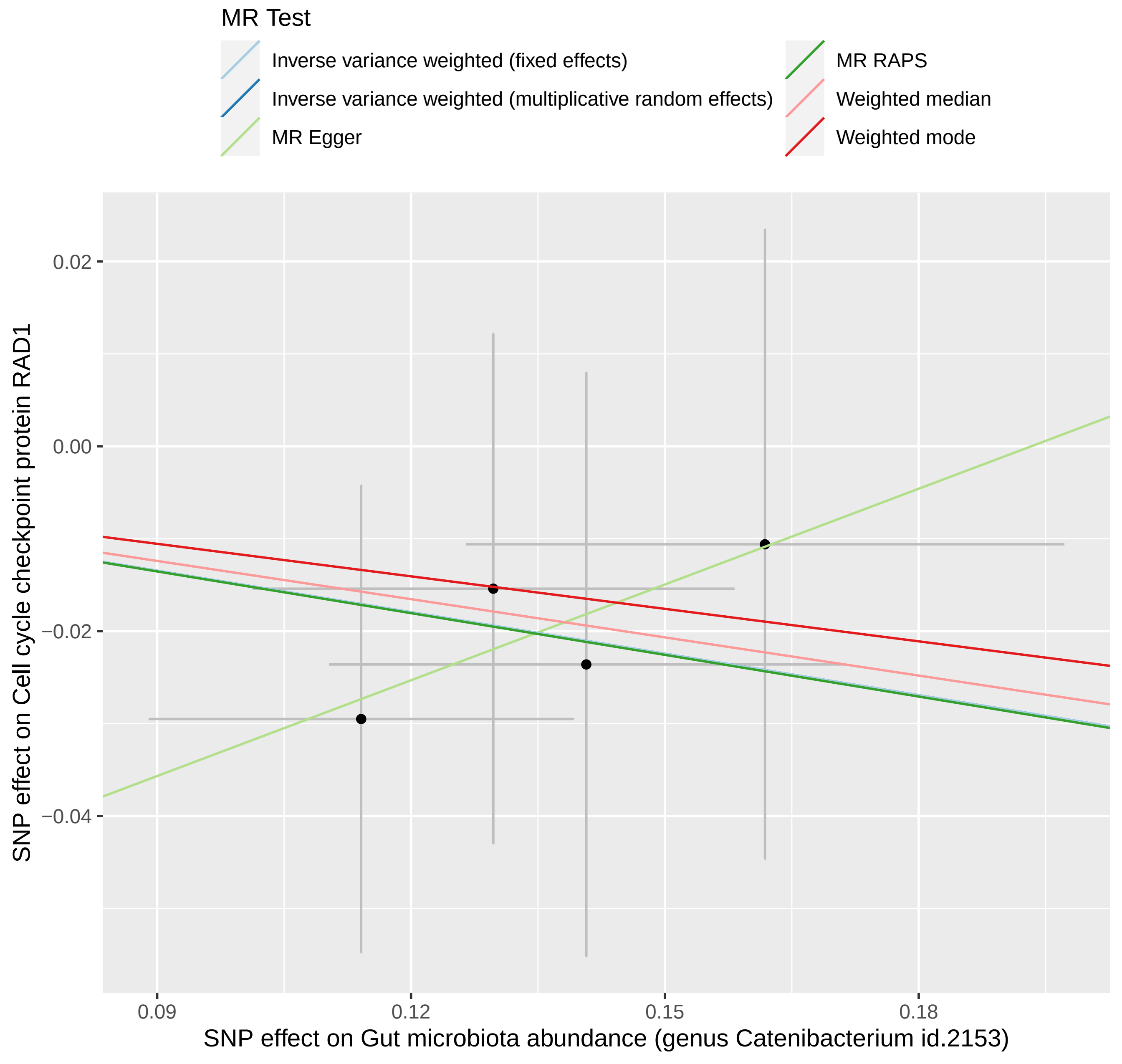

Batch 2−4964 : Gut microbiota abundance (genus Parasutterella id.2892) on Cell cycle checkpoint protein RAD1
with a threshold of 1e−05

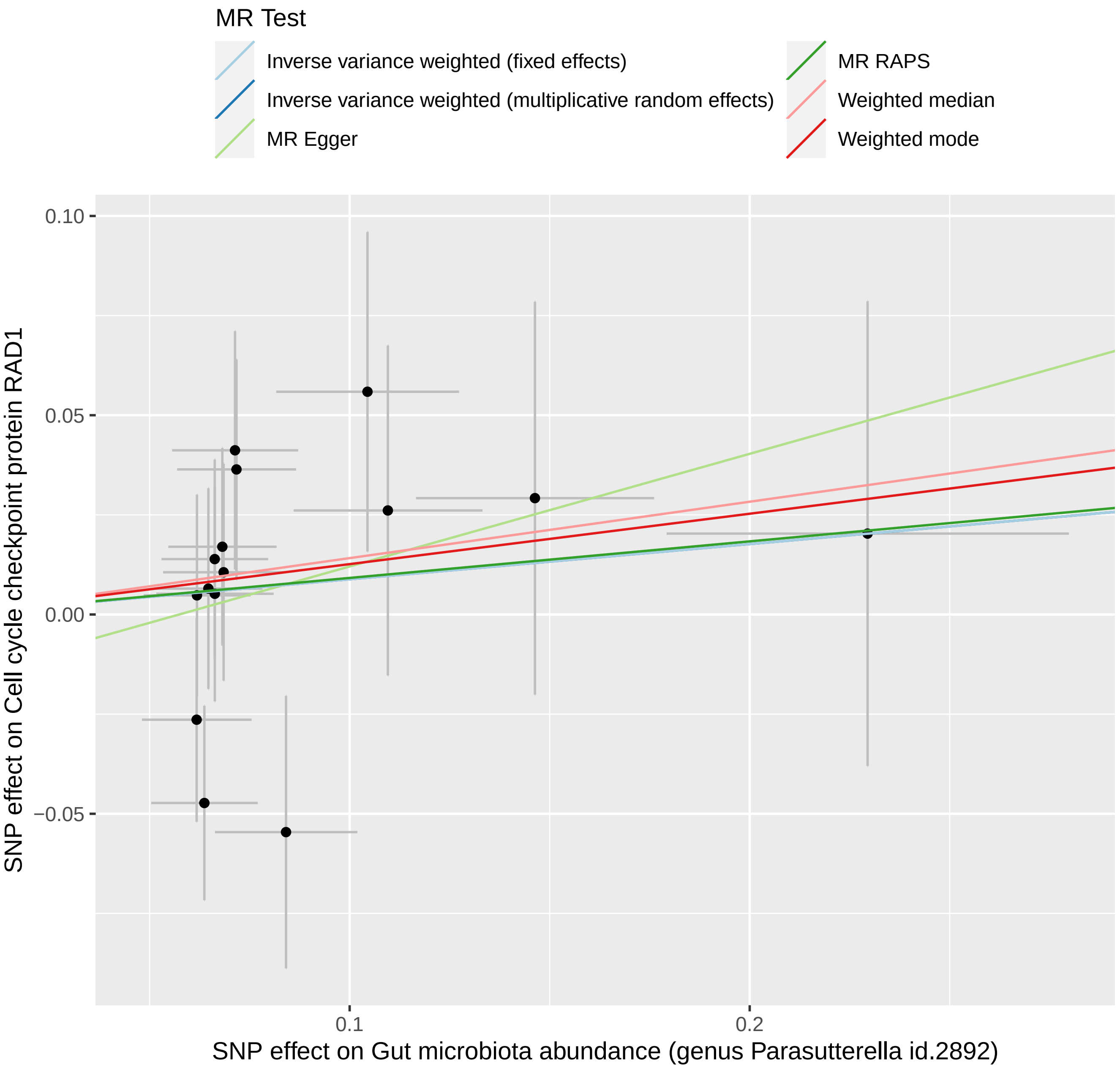

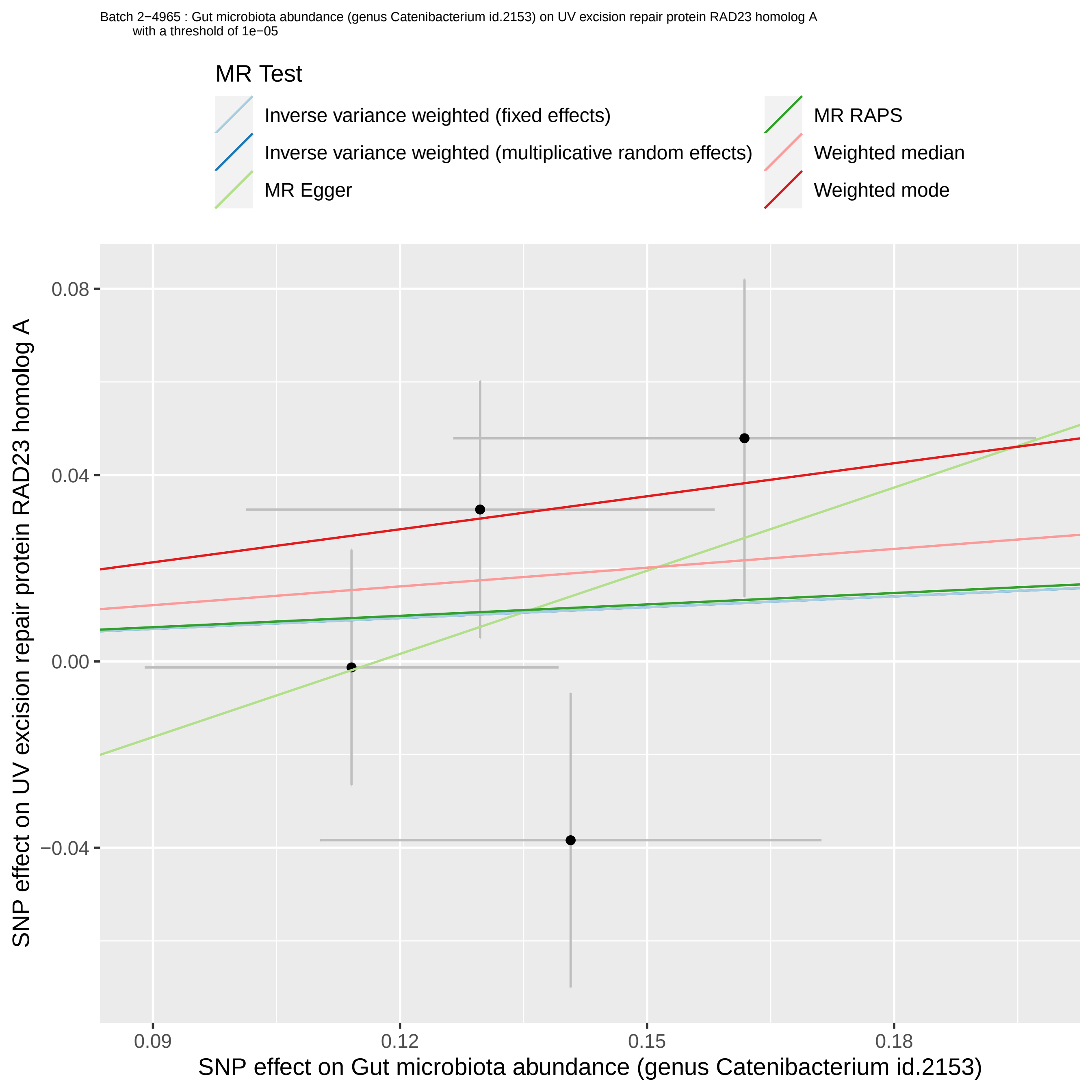
Batch 2−4965 : Gut microbiota abundance (genus Catenibacterium id.2153) on UV excision repair protein RAD23 homolog A with a threshold of 1e−05
MR Test
Inverse variance weighted (fixed effects)
Inverse variance weighted (multiplicative random effects)
MR Egger
MR RAPS
Weighted median
Weighted mode
0.08
0.04
0.00
−0.04
0.09
0.12
0.15
0.18
SNP effect on UV excision repair protein RAD23 homolog A
SNP effect on Gut microbiota abundance (genus Catenibacterium id.2153)

Batch 2−4966 : Gut microbiota abundance (genus Parasutterella id.2892) on UV excision repair protein RAD23 homolog A
with a threshold of 1e−05

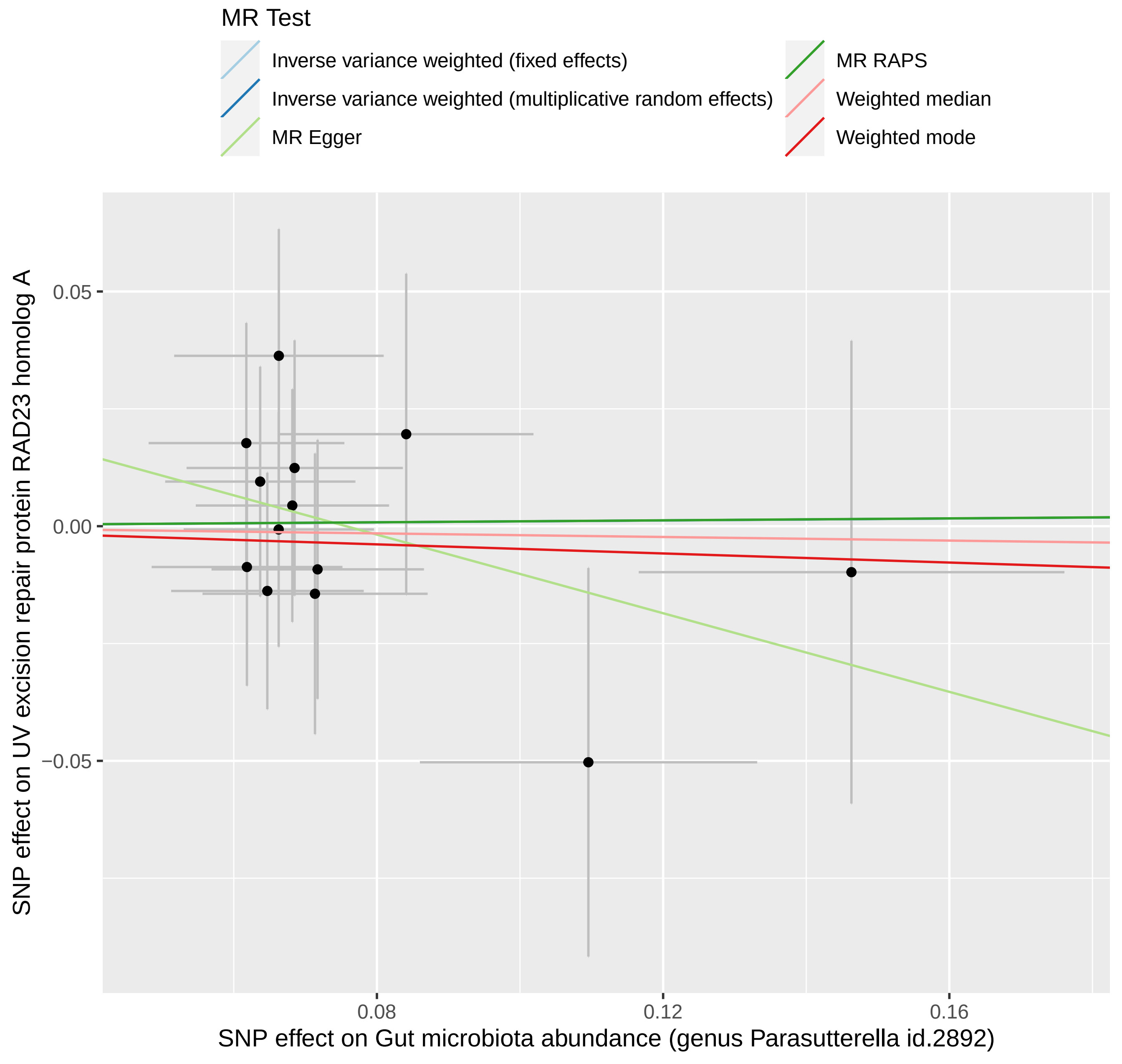

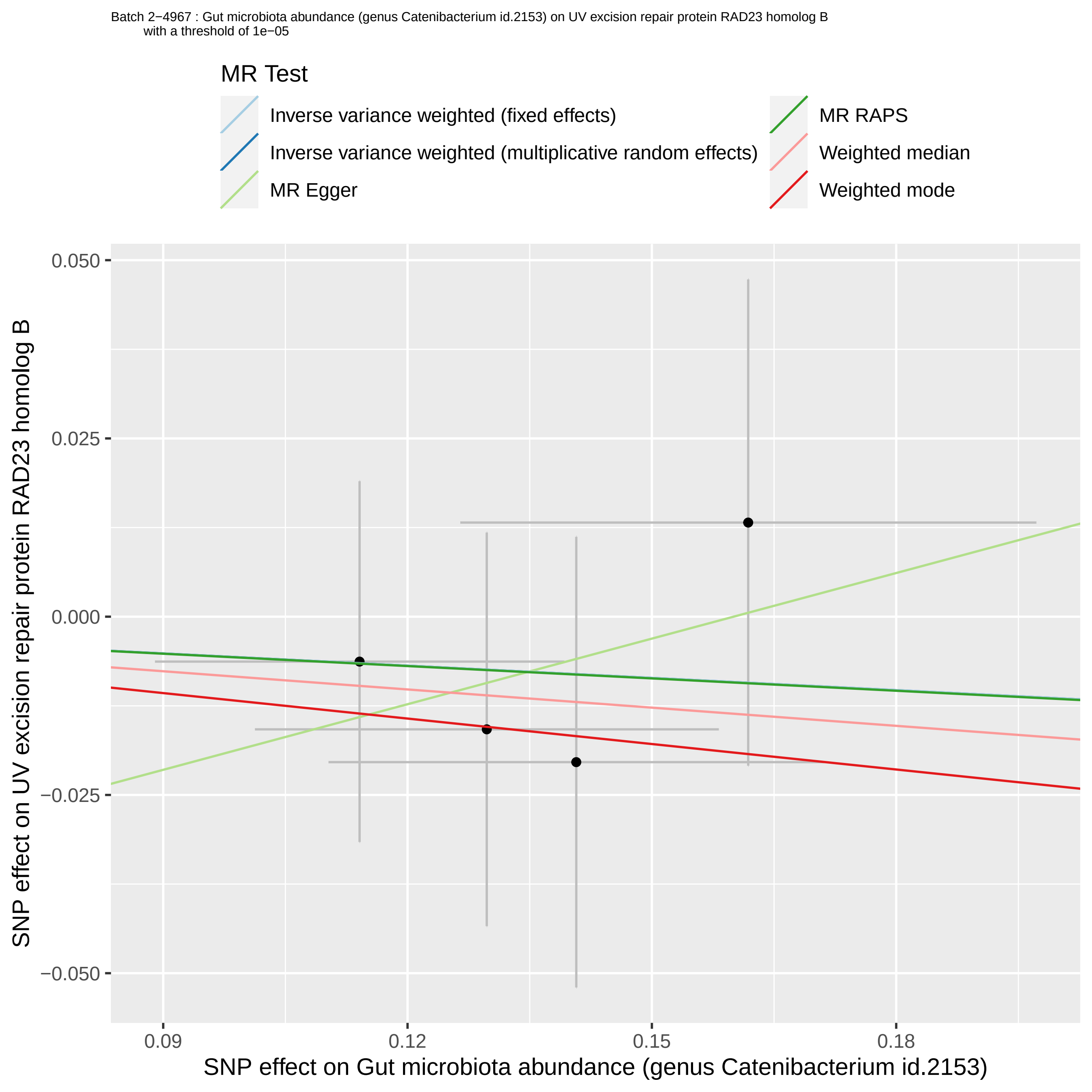

Batch 2−4967 : Gut microbiota abundance (genus Catenibacterium id.2153) on UV excision repair protein RAD23 homolog B
with a threshold of 1e−05
MR Test
Inverse variance weighted (fixed effects)
Inverse variance weighted (multiplicative random effects)
MR Egger
MR RAPS
Weighted median
Weighted mode
0.050
0.025
0.000
−0.025
−0.050
0.09
0.12
0.15
0.18
SNP effect on UV excision repair protein RAD23 homolog B
SNP effect on Gut microbiota abundance (genus Catenibacterium id.2153)

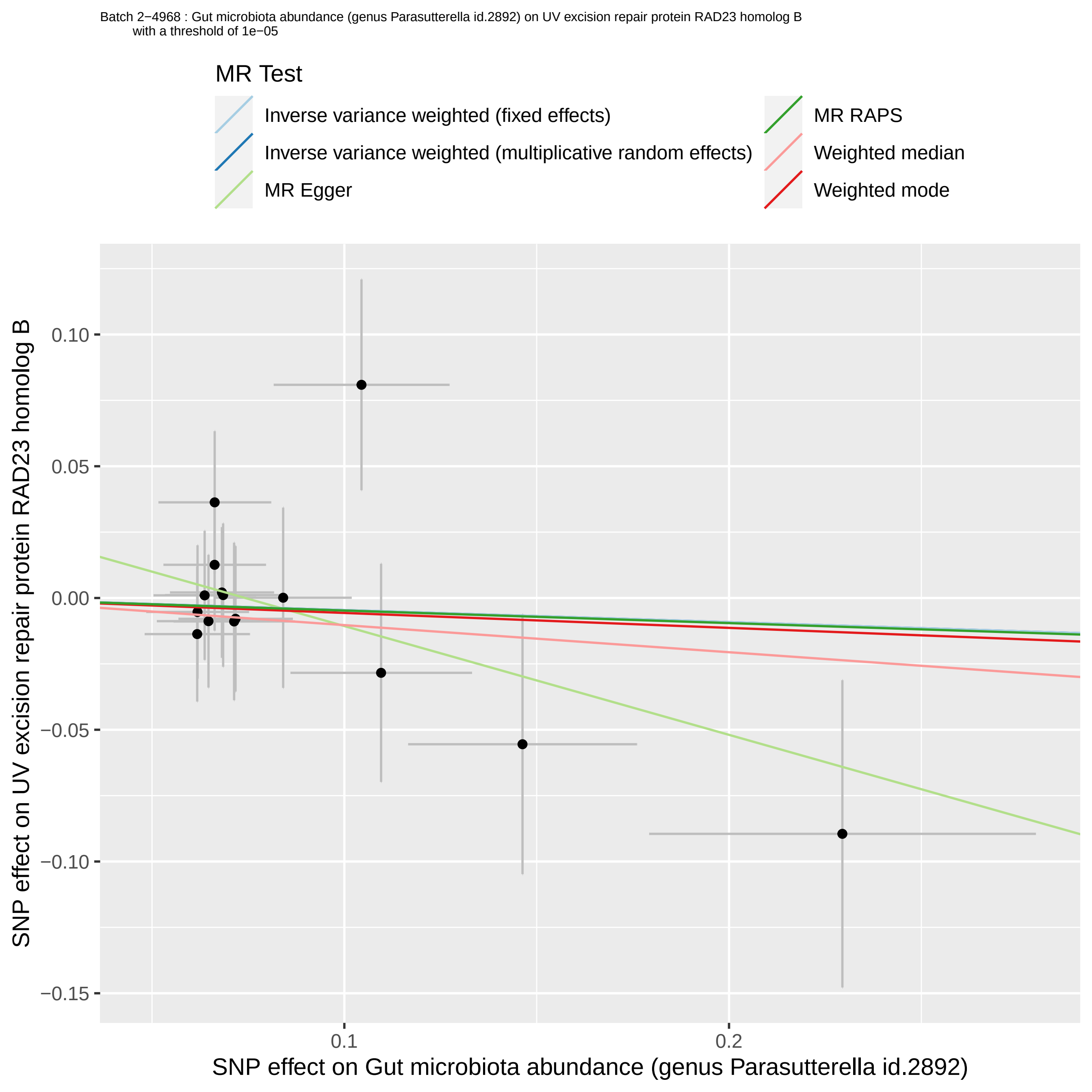

Batch 2−4968 : Gut microbiota abundance (genus Parasutterella id.2892) on UV excision repair protein RAD23 homolog B
with a threshold of 1e−05
MR Test
Inverse variance weighted (fixed effects)
Inverse variance weighted (multiplicative random effects)
MR Egger
MR RAPS
Weighted median
Weighted mode
0.10
0.05
0.00
−0.05
−0.10
−0.15
0.1
0.2
SNP effect on UV excision repair protein RAD23 homolog B
SNP effect on Gut microbiota abundance (genus Parasutterella id.2892)

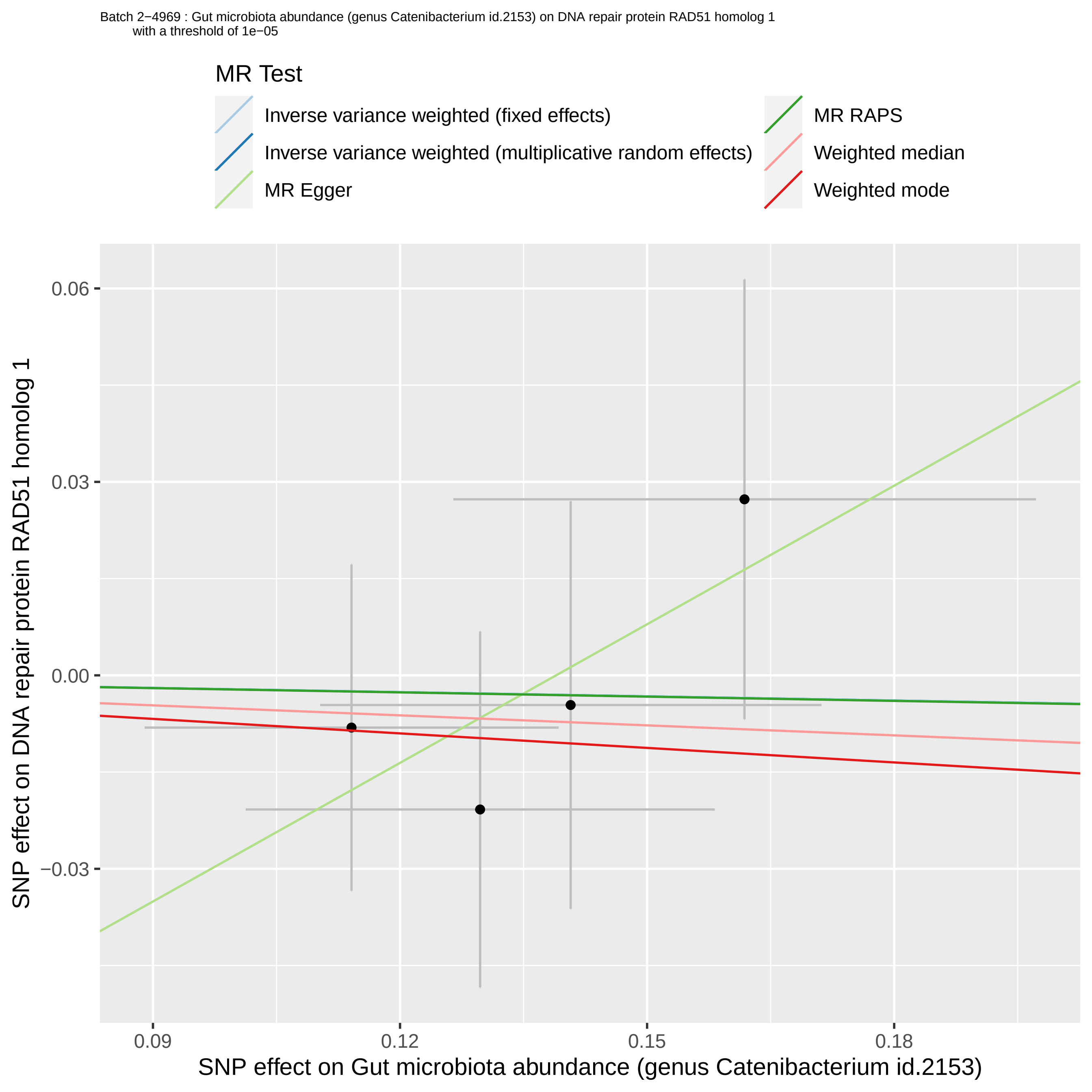
Batch 2−4969 : Gut microbiota abundance (genus Catenibacterium id.2153) on DNA repair protein RAD51 homolog 1
with a threshold of 1e−05
MR Test
Inverse variance weighted (fixed effects)
Inverse variance weighted (multiplicative random effects)
MR Egger
MR RAPS
Weighted median
Weighted mode
0.06
0.03
0.00
−0.03
0.09
0.12
0.15
0.18
SNP effect on DNA repair protein RAD51 homolog 1
SNP effect on Gut microbiota abundance (genus Catenibacterium id.2153)

Batch 2−4970 : Gut microbiota abundance (genus Parasutterella id.2892) on DNA repair protein RAD51 homolog 1
with a threshold of 1e−05

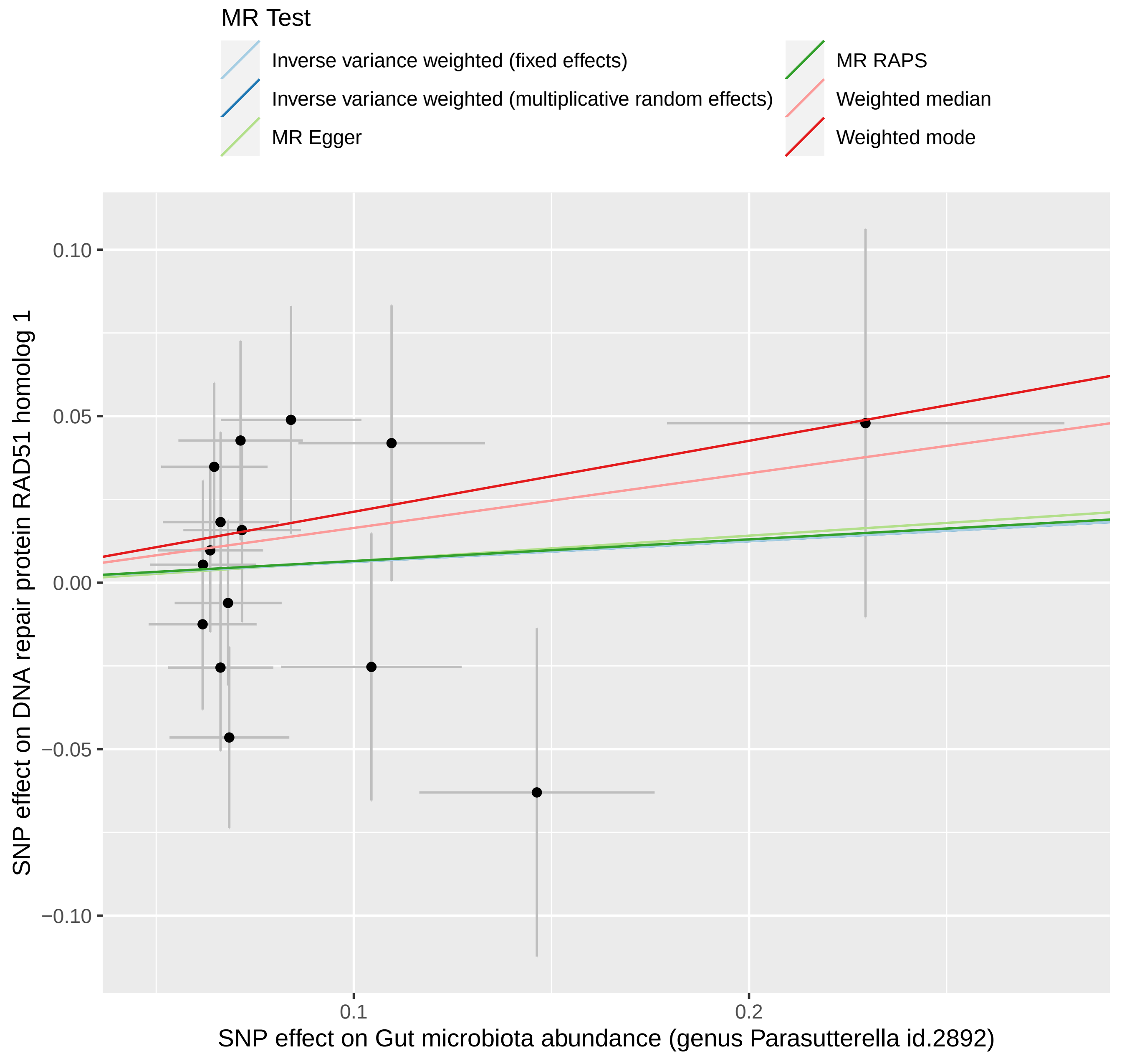

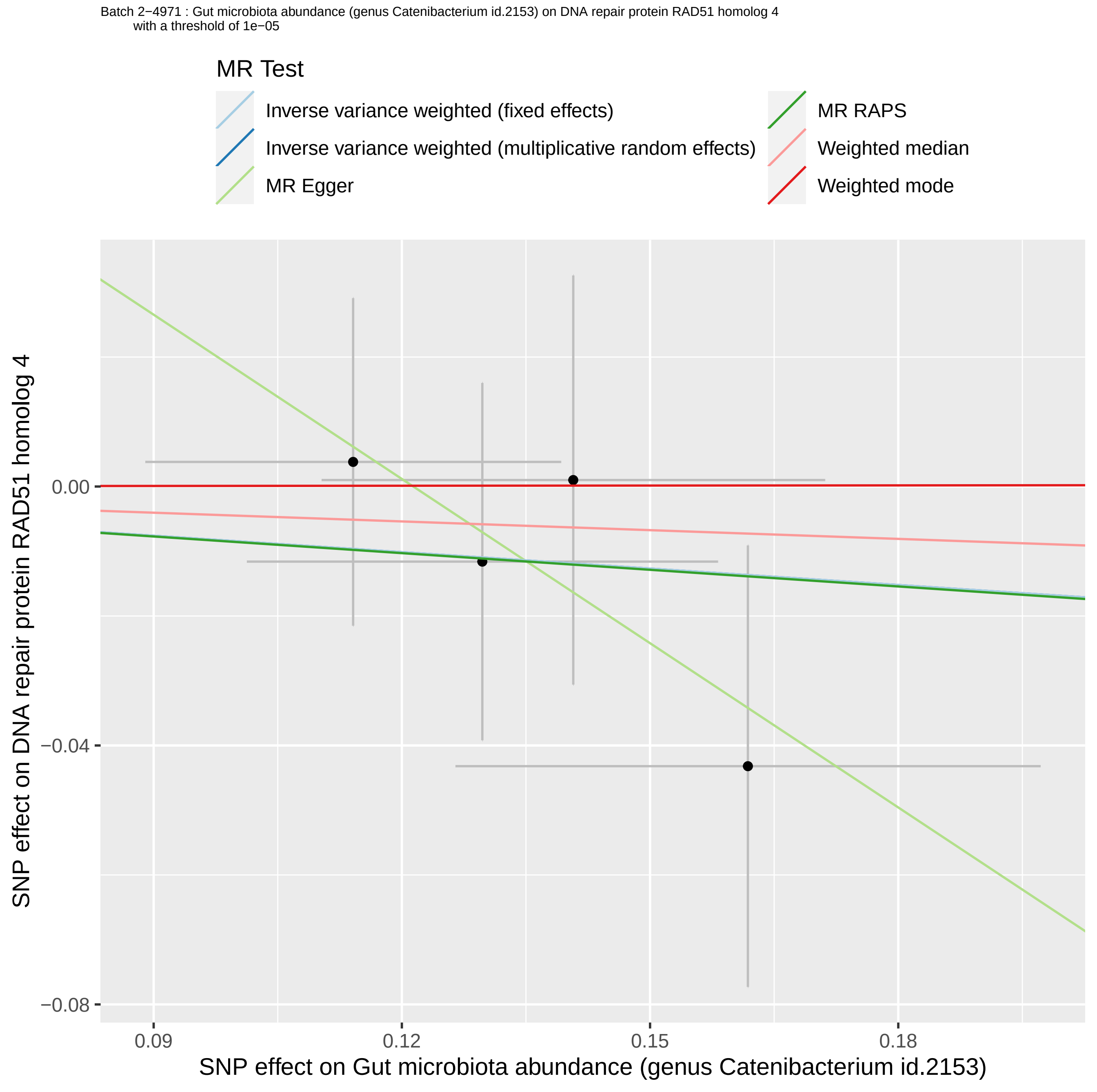

Batch 2−4971 : Gut microbiota abundance (genus Catenibacterium id.2153) on DNA repair protein RAD51 homolog 4
with a threshold of 1e−05
MR Test
Inverse variance weighted (fixed effects)
Inverse variance weighted (multiplicative random effects)
MR Egger
MR RAPS
Weighted median
Weighted mode
SNP effect on DNA repair protein RAD51 homolog 4
0.00
−0.04
−0.08
0.09
0.12
0.15
0.18
SNP effect on Gut microbiota abundance (genus Catenibacterium id.2153)

Batch 2−4972 : Gut microbiota abundance (genus Parasutterella id.2892) on DNA repair protein RAD51 homolog 4
with a threshold of 1e−05

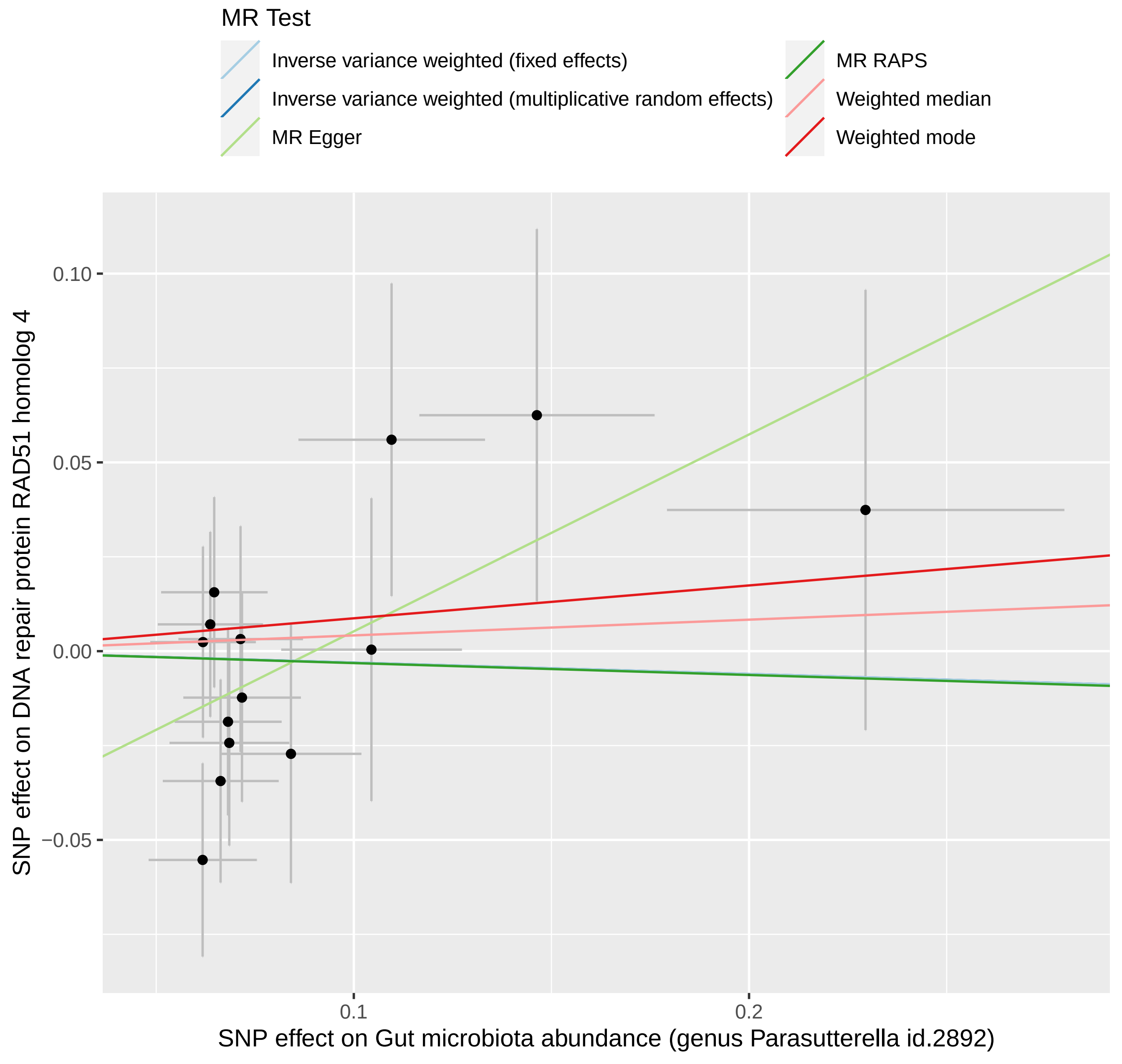

Batch 2−4973 : Gut microbiota abundance (genus Catenibacterium id.2153) on NKG2D ligand 4
with a threshold of 1e−05

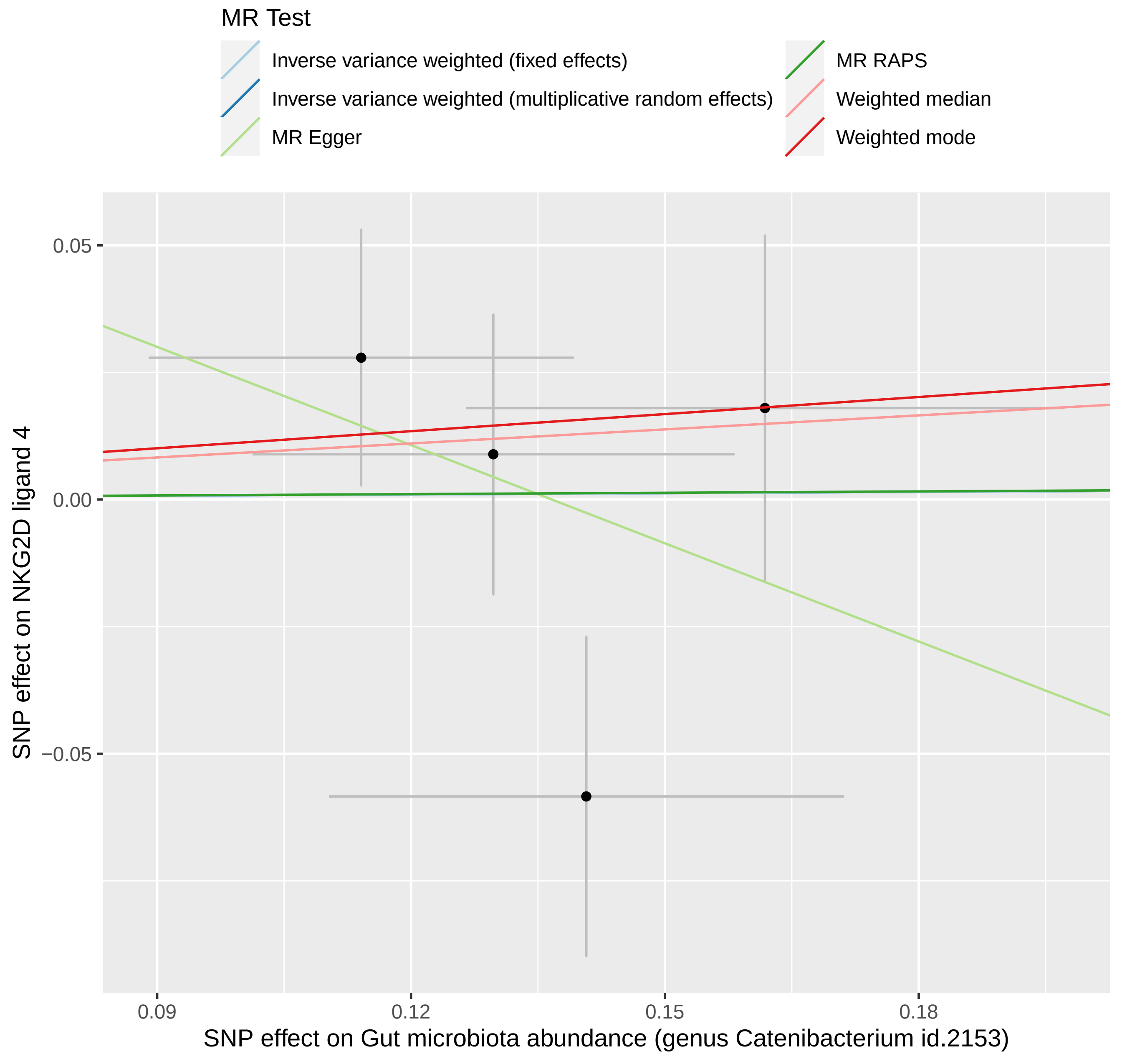

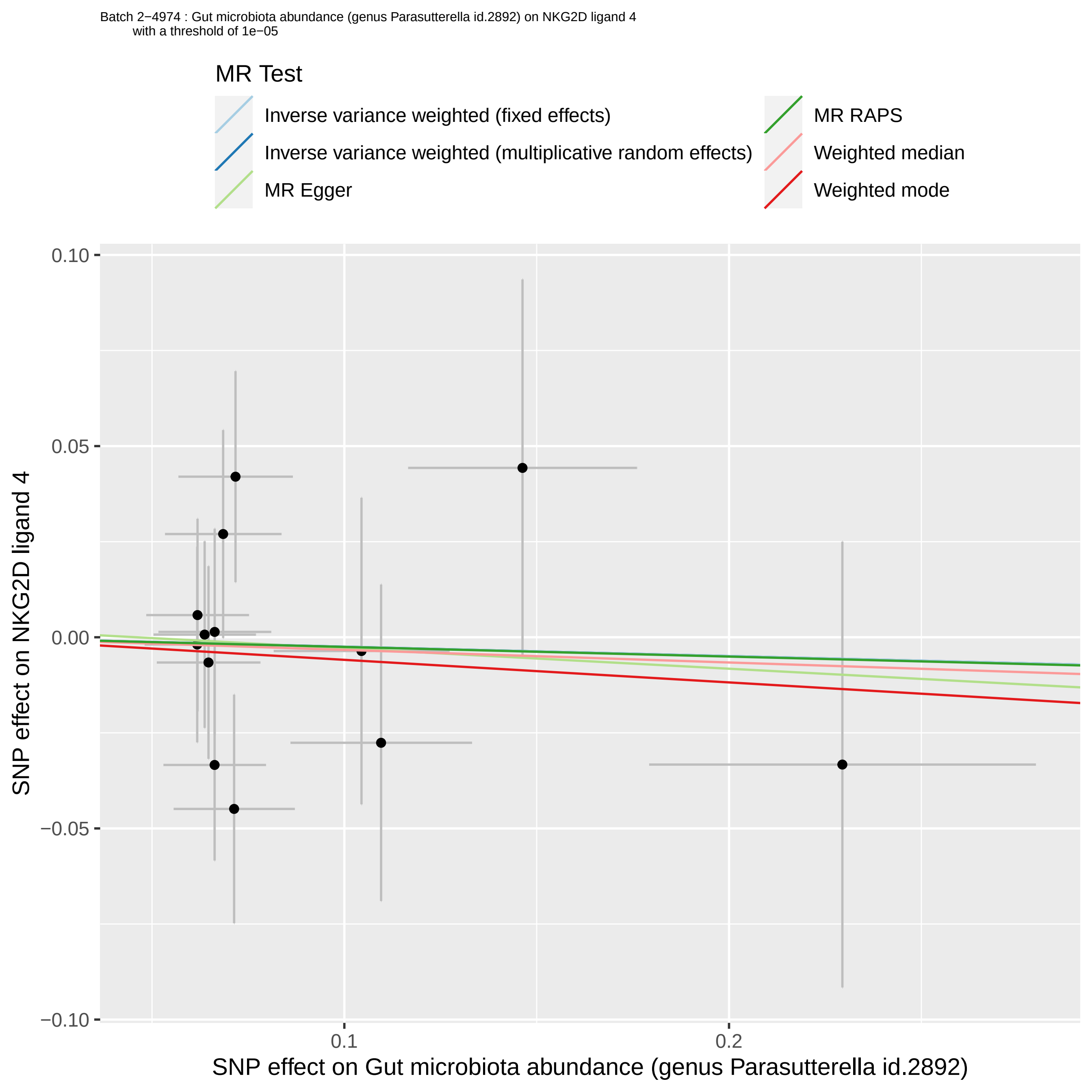
Batch 2−4974 : Gut microbiota abundance (genus Parasutterella id.2892) on NKG2D ligand 4
with a threshold of 1e−05
MR Test
Inverse variance weighted (fixed effects)
Inverse variance weighted (multiplicative random effects)
MR Egger
MR RAPS
Weighted median
Weighted mode
0.10
0.05
0.00
−0.05
−0.10
0.1
0.2
SNP effect on NKG2D ligand 4
SNP effect on Gut microbiota abundance (genus Parasutterella id.2892)

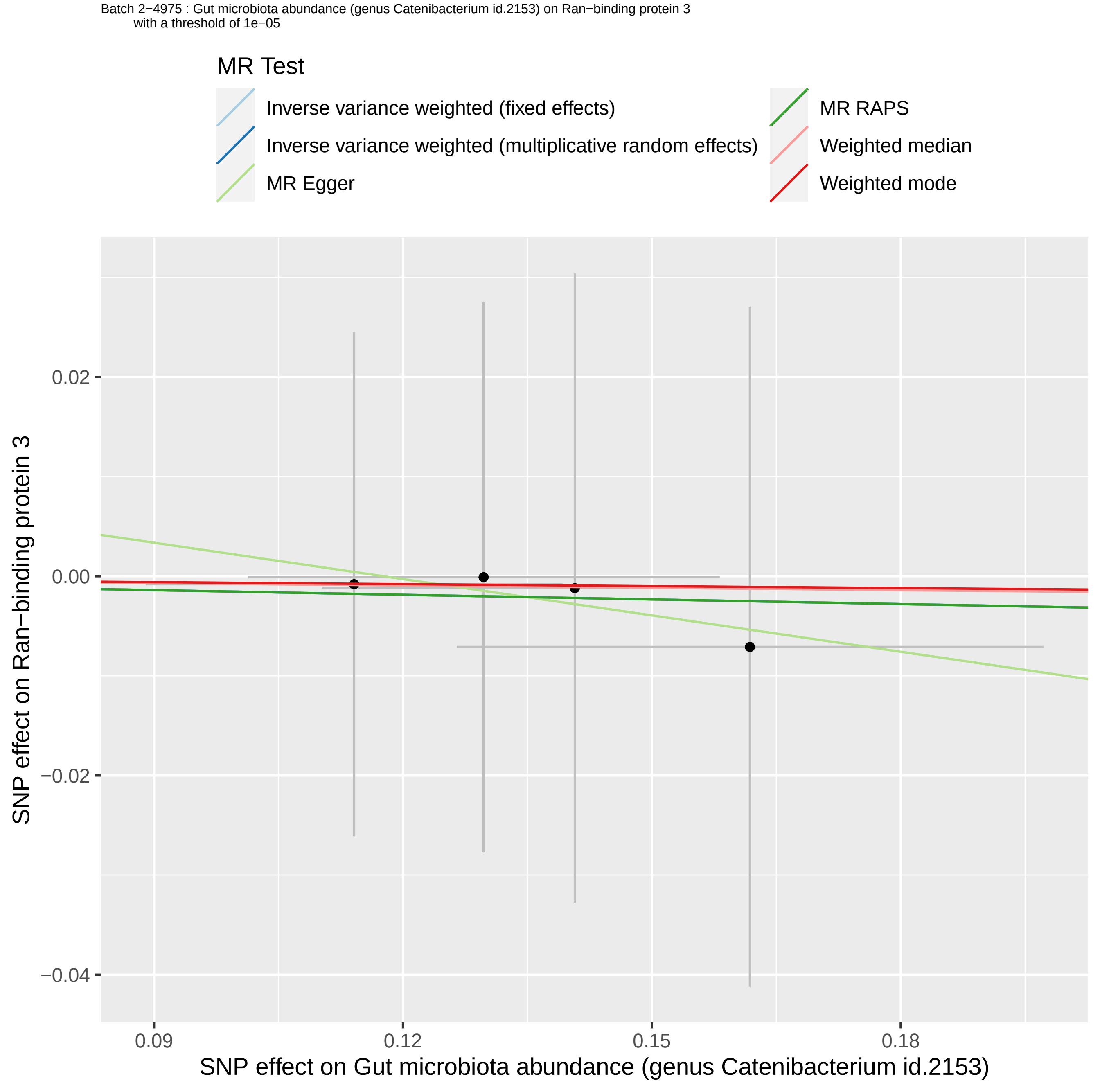

Batch 2−4975 : Gut microbiota abundance (genus Catenibacterium id.2153) on Ran−binding protein 3
with a threshold of 1e−05
MR Test
Inverse variance weighted (fixed effects)
Inverse variance weighted (multiplicative random effects)
MR Egger
MR RAPS
Weighted median
Weighted mode
0.02
0.00
−0.02
−0.04
0.09
0.12
0.15
0.18
SNP effect on Ran−binding protein 3
SNP effect on Gut microbiota abundance (genus Catenibacterium id.2153)

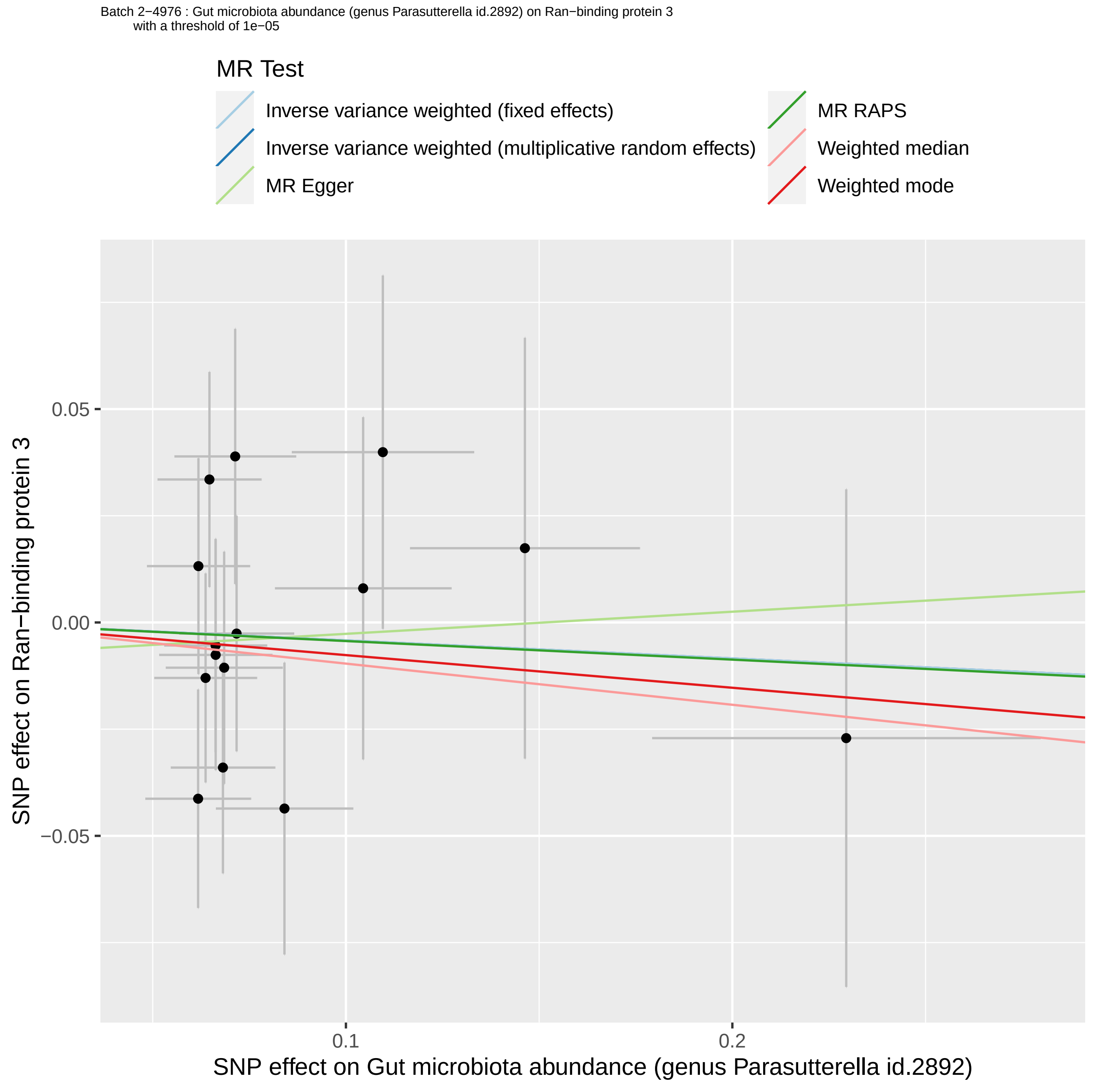
Batch 2−4976 : Gut microbiota abundance (genus Parasutterella id.2892) on Ran−binding protein 3
with a threshold of 1e−05
MR Test
Inverse variance weighted (fixed effects)
Inverse variance weighted (multiplicative random effects)
MR Egger
MR RAPS
Weighted median
Weighted mode
0.05
0.00
−0.05
0.1
0.2
SNP effect on Ran−binding protein 3
SNP effect on Gut microbiota abundance (genus Parasutterella id.2892)

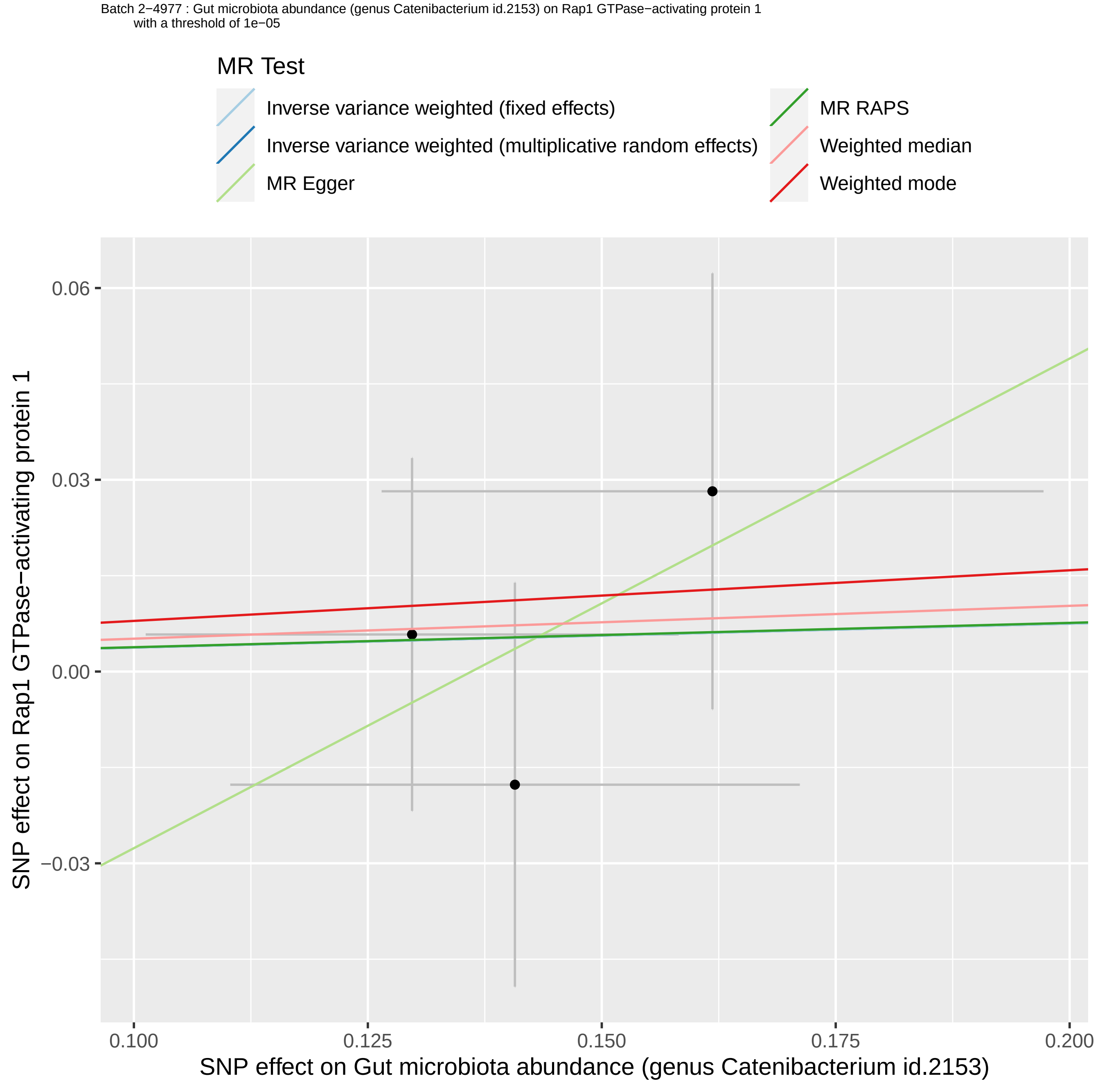
Batch 2−4977 : Gut microbiota abundance (genus Catenibacterium id.2153) on Rap1 GTPase−activating protein 1
with a threshold of 1e−05
MR Test
Inverse variance weighted (fixed effects)
Inverse variance weighted (multiplicative random effects)
MR Egger
MR RAPS
Weighted median
Weighted mode
SNP effect on Rap1 GTPase−activating protein 1
0.06
0.03
0.00
−0.03
0.100
0.125
0.150
0.175
0.200
SNP effect on Gut microbiota abundance (genus Catenibacterium id.2153)

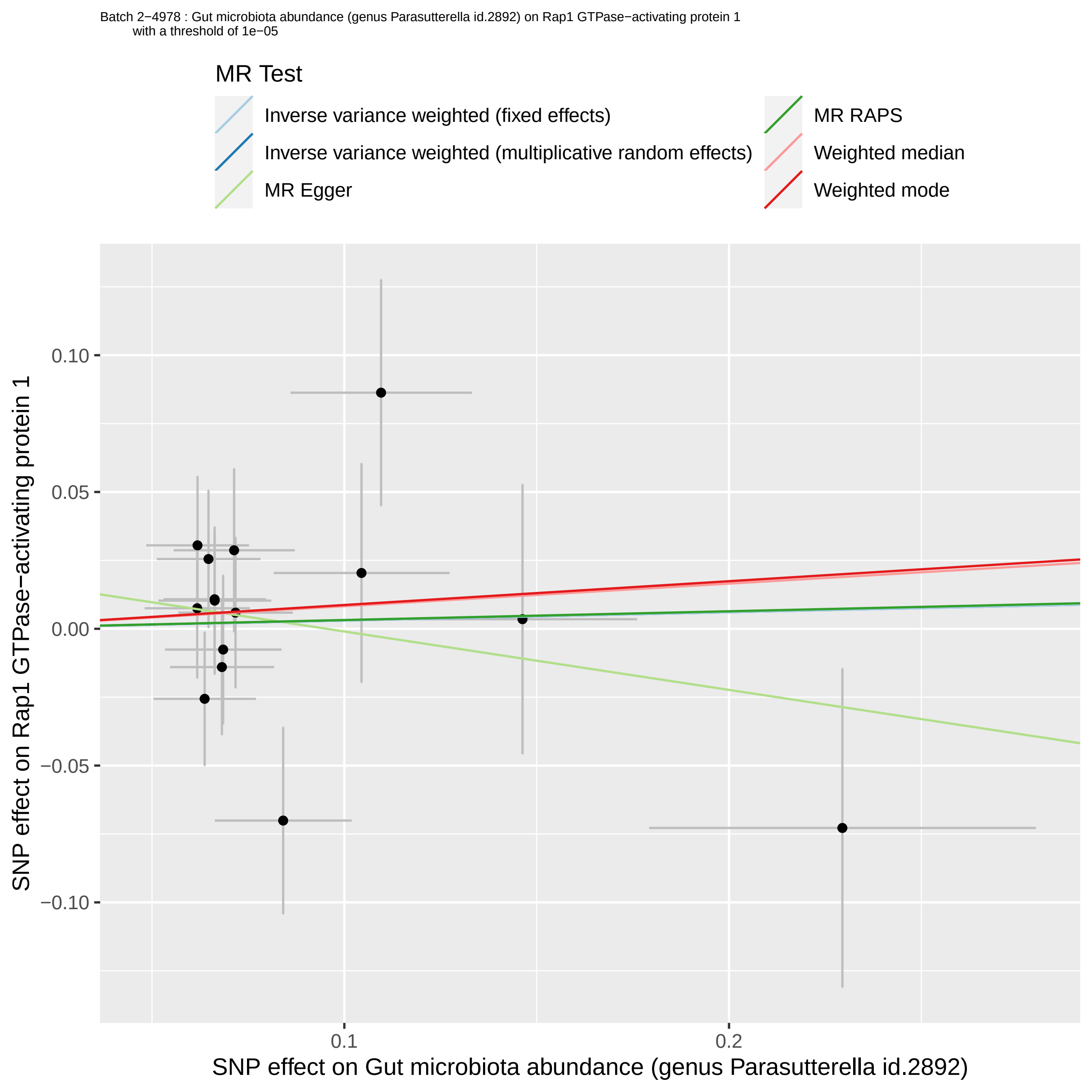
Batch 2−4978 : Gut microbiota abundance (genus Parasutterella id.2892) on Rap1 GTPase−activating protein 1
with a threshold of 1e−05
MR Test
Inverse variance weighted (fixed effects)
Inverse variance weighted (multiplicative random effects)
MR Egger
MR RAPS
Weighted median
Weighted mode
0.10
0.05
0.00
−0.05
−0.10
0.1
0.2
SNP effect on Rap1 GTPase−activating protein 1
SNP effect on Gut microbiota abundance (genus Parasutterella id.2892)

Batch 2−4979 : Gut microbiota abundance (genus Catenibacterium id.2153) on Rap1 GTPase−GDP dissociation stimulator 1
with a threshold of 1e−05

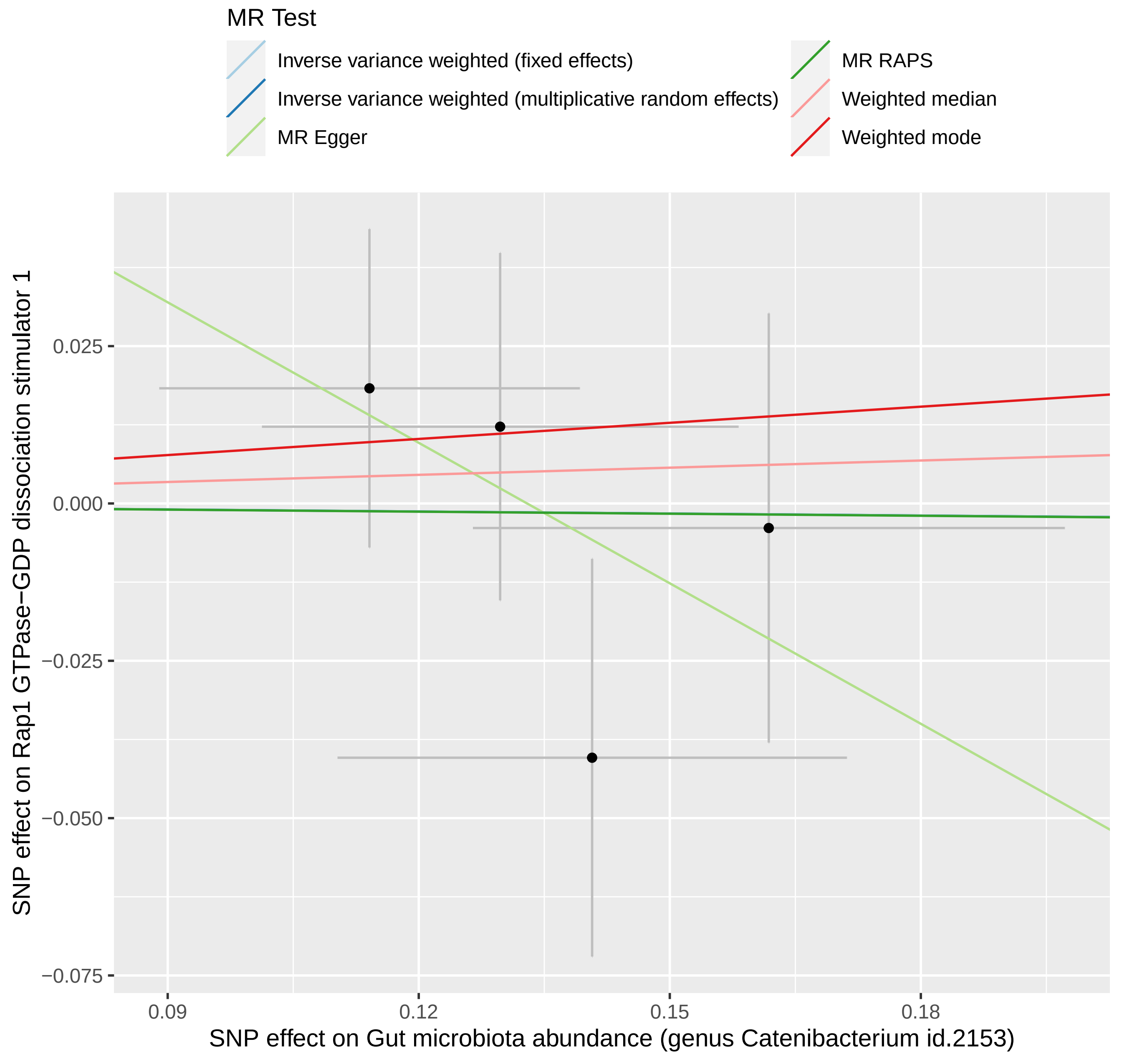

Batch 2−4980 : Gut microbiota abundance (genus Parasutterella id.2892) on Rap1 GTPase−GDP dissociation stimulator 1 with a threshold of 1e−05

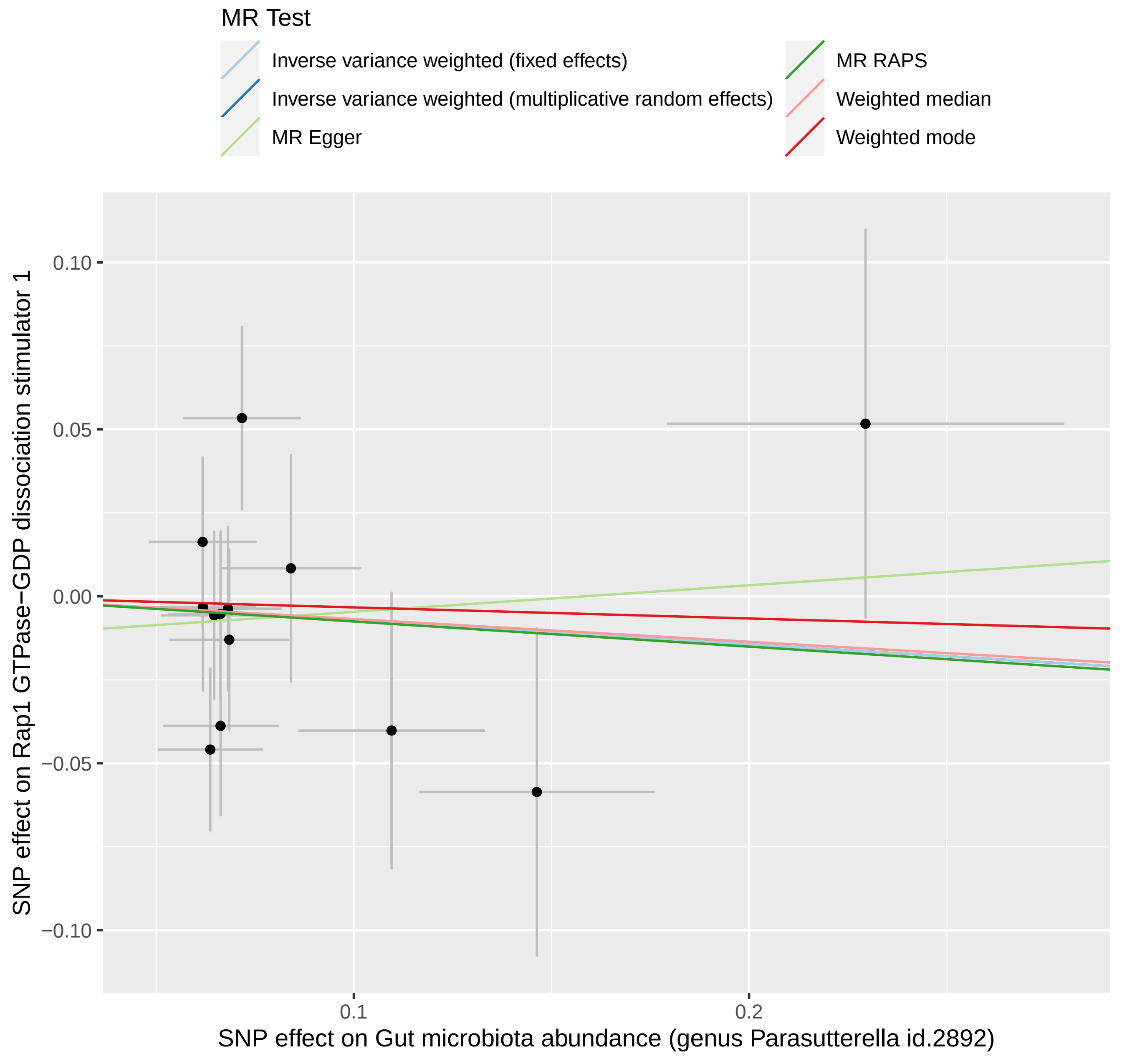

Batch 2−4981 : Gut microbiota abundance (genus Catenibacterium id.2153) on Ras−related protein Rap−2a
with a threshold of 1e−05

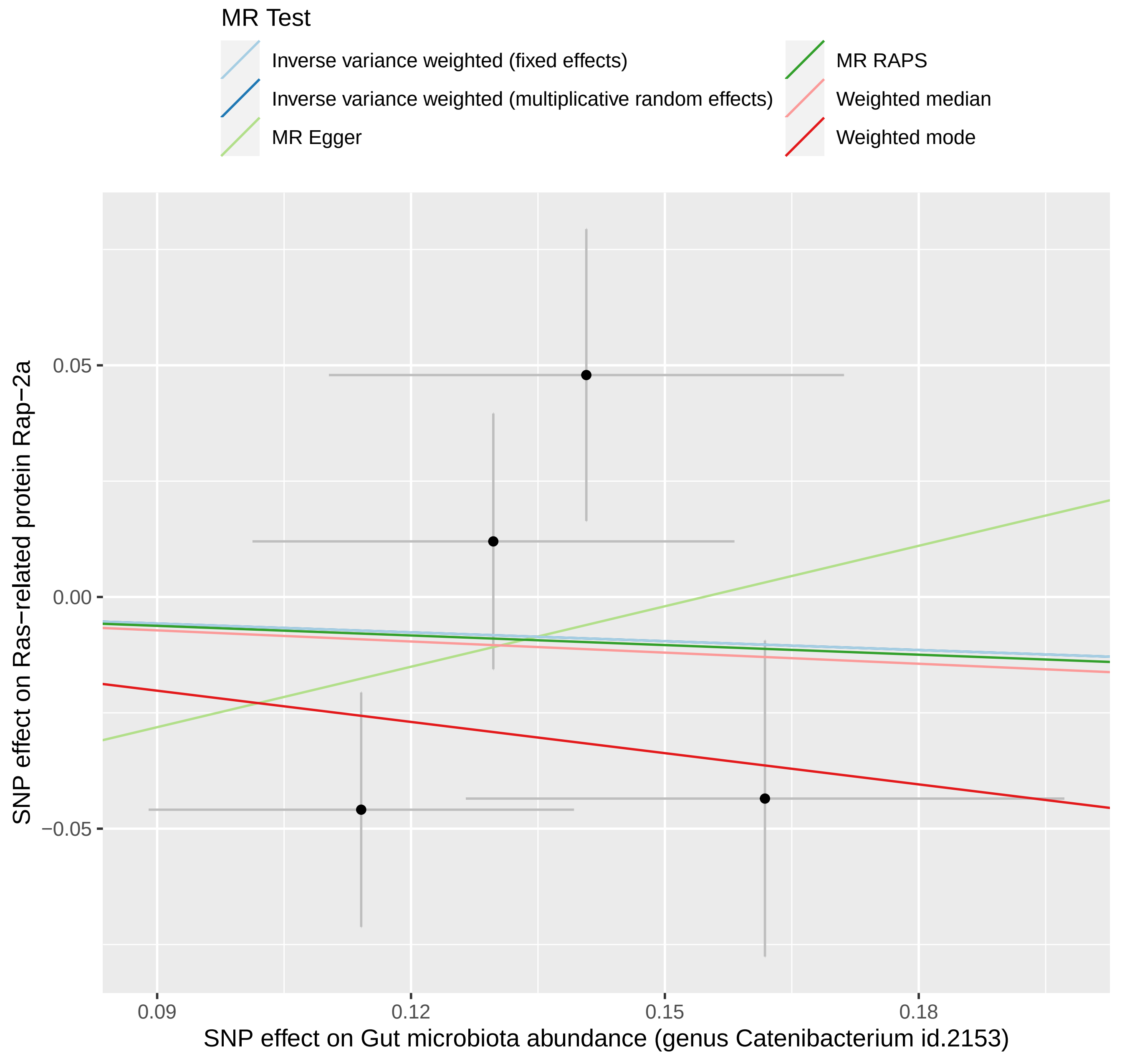

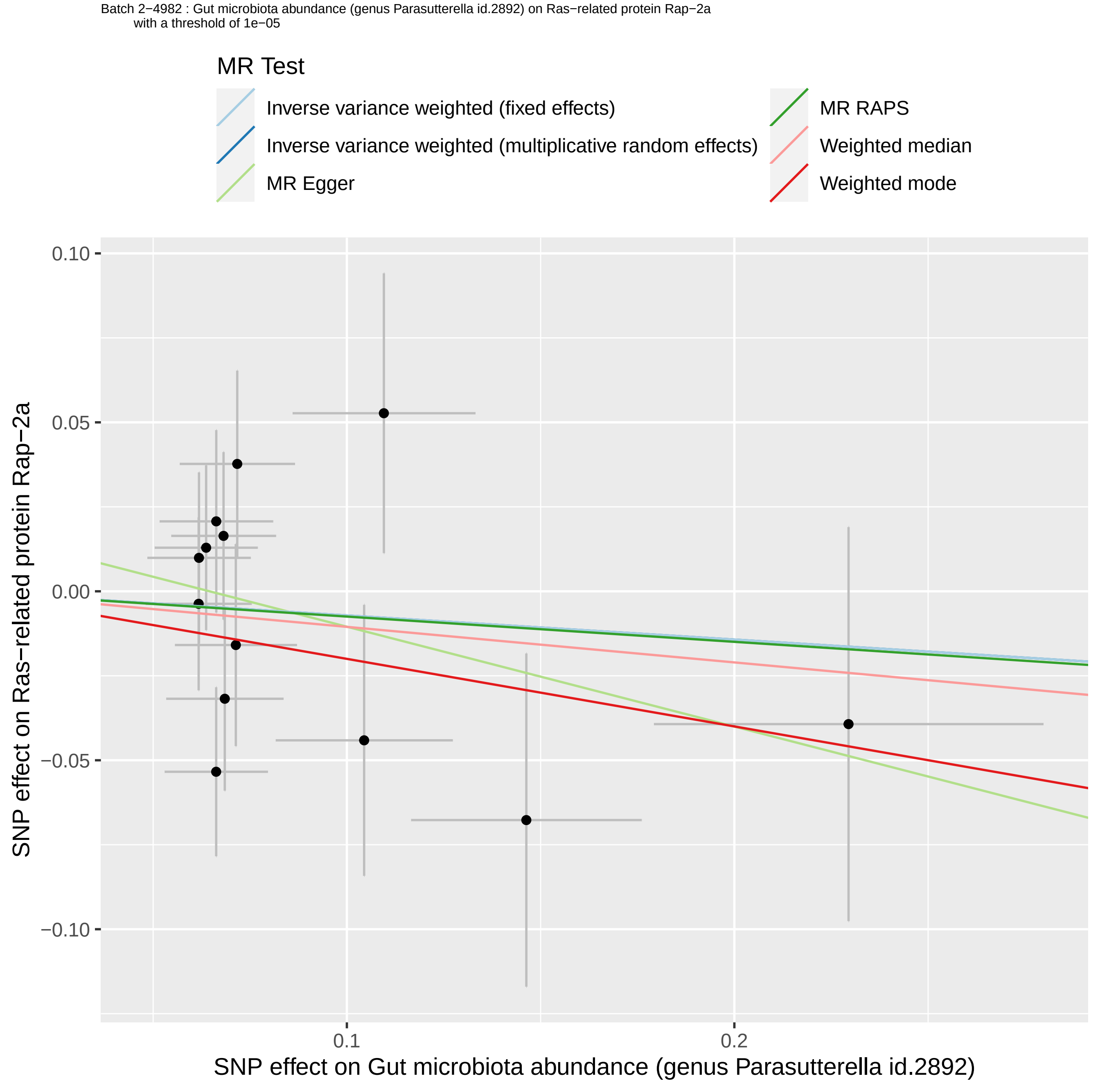

Batch 2−4982 : Gut microbiota abundance (genus Parasutterella id.2892) on Ras−related protein Rap−2a
with a threshold of 1e−05
MR Test
Inverse variance weighted (fixed effects)
Inverse variance weighted (multiplicative random effects)
MR Egger
MR RAPS
Weighted median
Weighted mode
0.10
0.05
0.00
−0.05
−0.10
0.1
0.2
SNP effect on Ras−related protein Rap−2a
SNP effect on Gut microbiota abundance (genus Parasutterella id.2892)

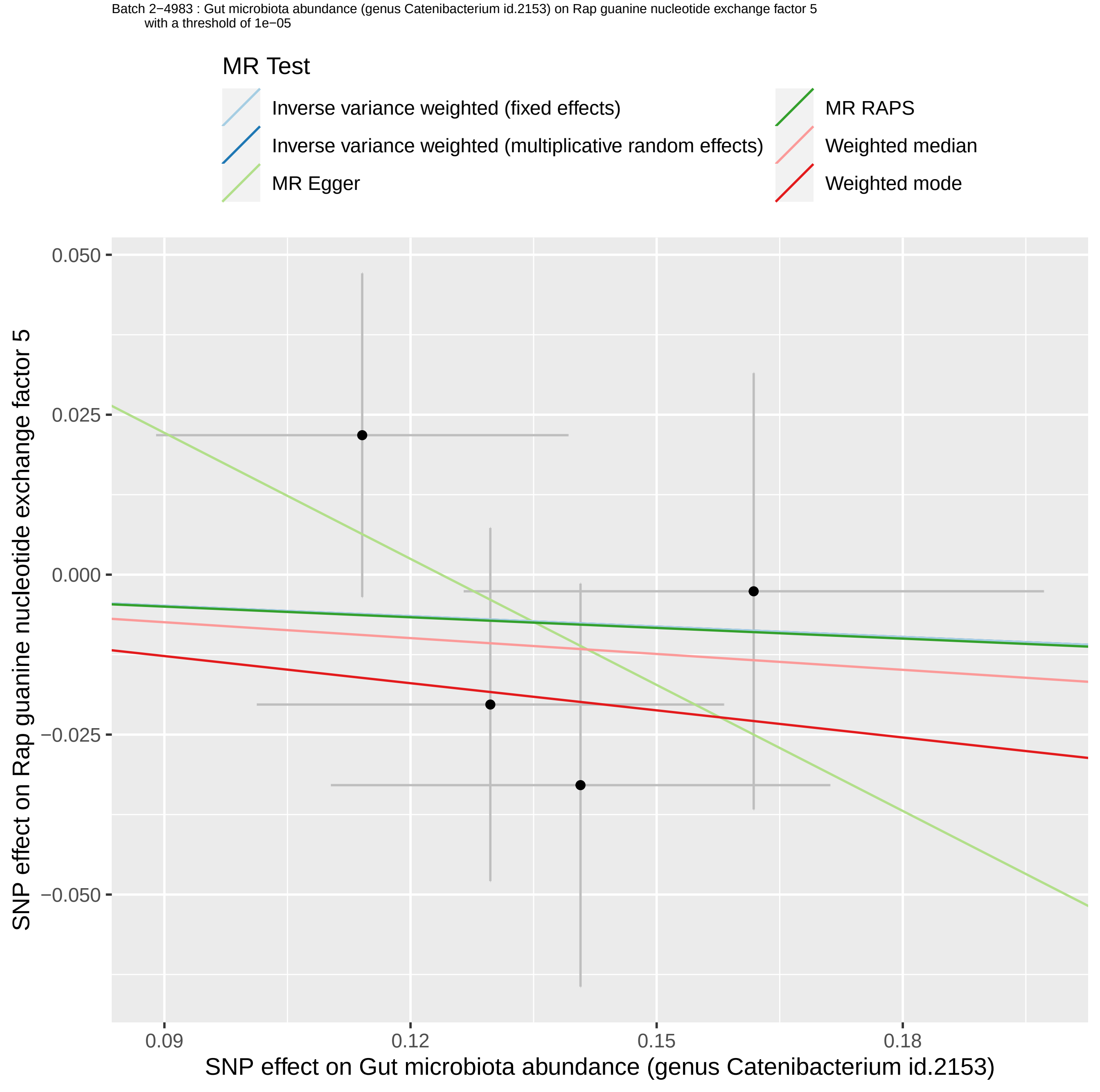
Batch 2−4983 : Gut microbiota abundance (genus Catenibacterium id.2153) on Rap guanine nucleotide exchange factor 5
with a threshold of 1e−05
MR Test
Inverse variance weighted (fixed effects)
Inverse variance weighted (multiplicative random effects)
MR Egger
MR RAPS
Weighted median
Weighted mode
0.050
0.025
0.000
−0.025
−0.050
0.09
0.12
0.15
0.18
SNP effect on Rap guanine nucleotide exchange factor 5
SNP effect on Gut microbiota abundance (genus Catenibacterium id.2153)

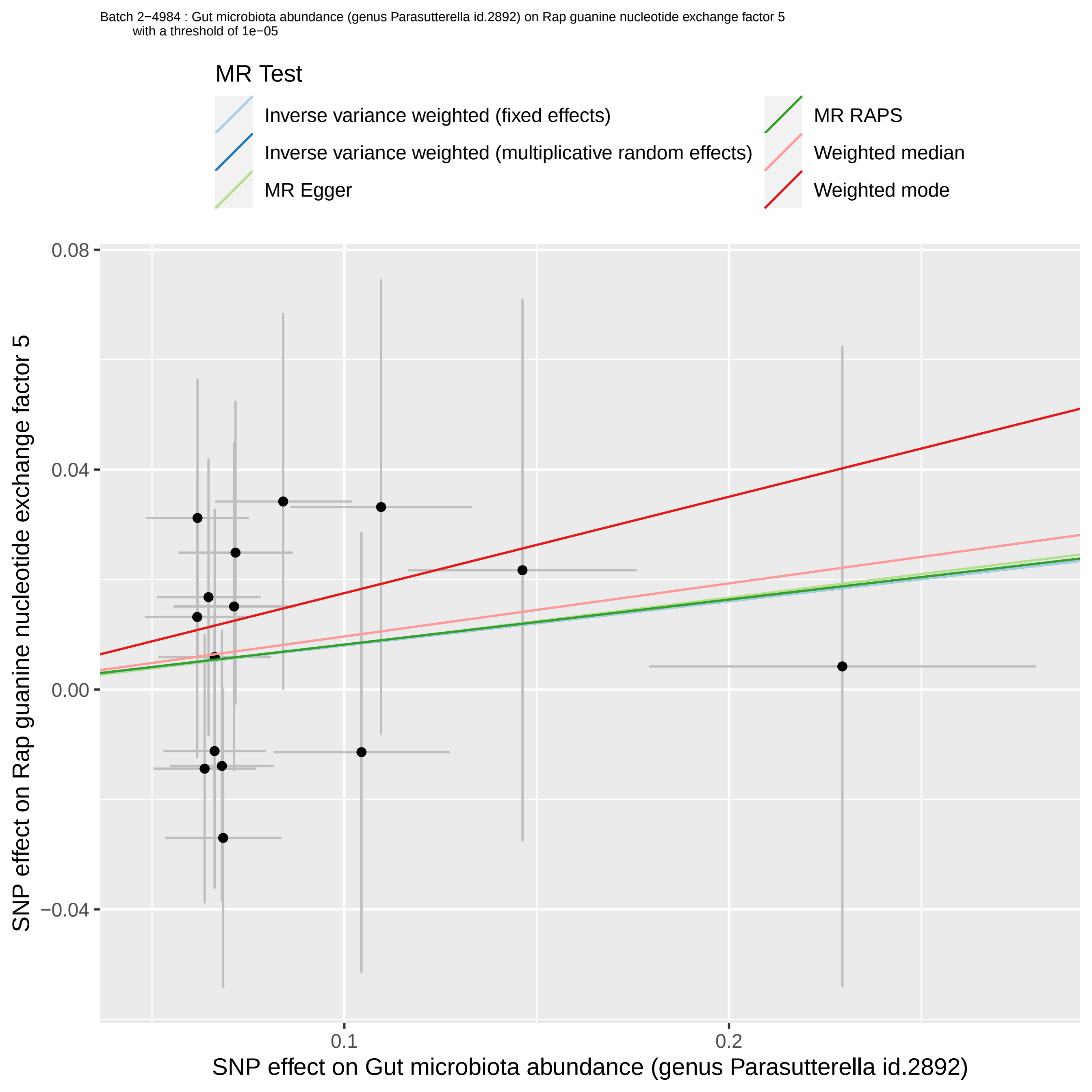
Batch 2−4984 : Gut microbiota abundance (genus Parasutterella id.2892) on Rap guanine nucleotide exchange factor 5
with a threshold of 1e−05
MR Test
Inverse variance weighted (fixed effects)
Inverse variance weighted (multiplicative random effects)
MR Egger
MR RAPS
Weighted median
Weighted mode
0.08
0.04
0.00
−0.04
0.1
0.2
SNP effect on Rap guanine nucleotide exchange factor 5
SNP effect on Gut microbiota abundance (genus Parasutterella id.2892)

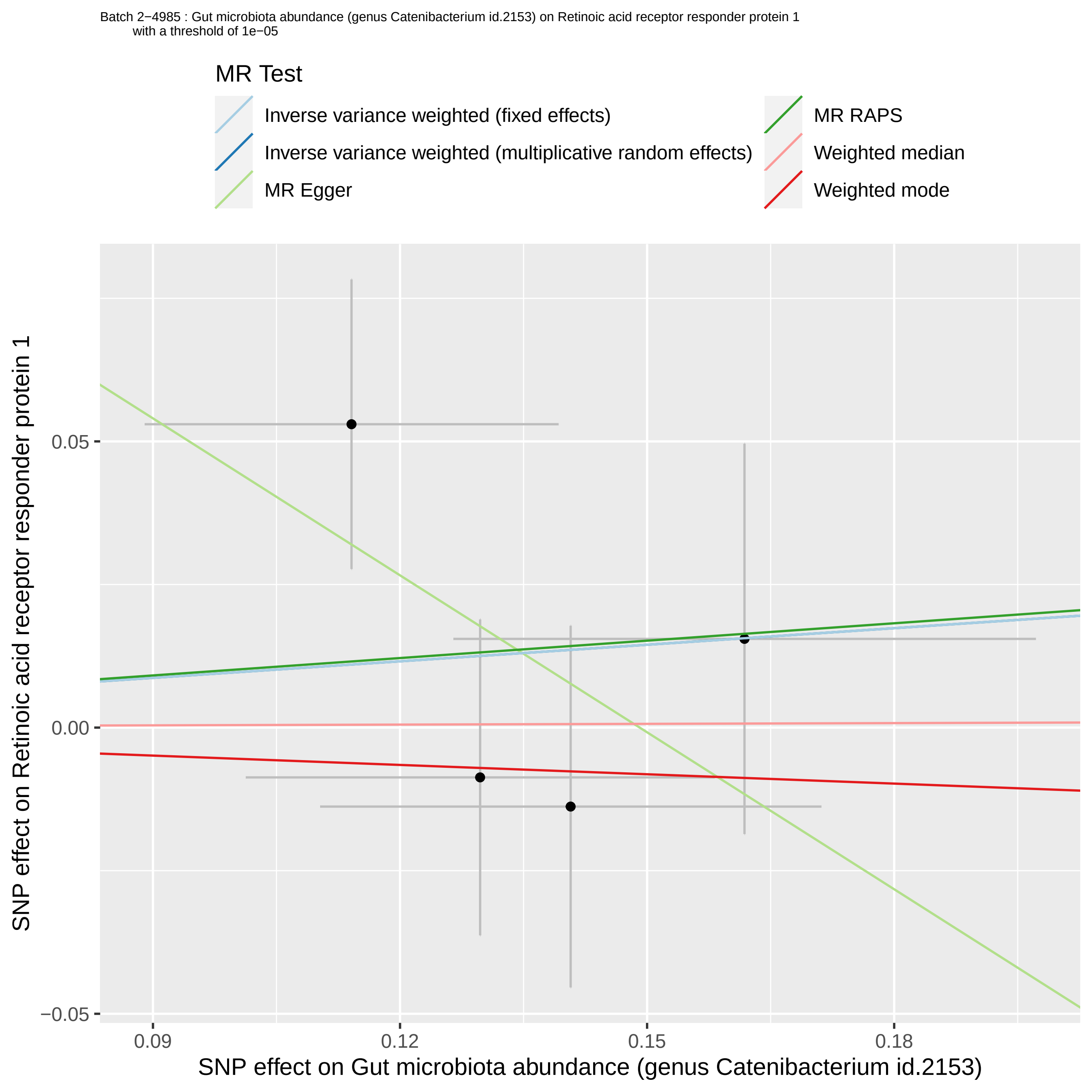

Batch 2−4985 : Gut microbiota abundance (genus Catenibacterium id.2153) on Retinoic acid receptor responder protein 1
with a threshold of 1e−05
MR Test
Inverse variance weighted (fixed effects)
Inverse variance weighted (multiplicative random effects)
MR Egger
MR RAPS
Weighted median
Weighted mode
0.05
0.00
−0.05
0.09
0.12
0.15
0.18
SNP effect on Retinoic acid receptor responder protein 1
SNP effect on Gut microbiota abundance (genus Catenibacterium id.2153)

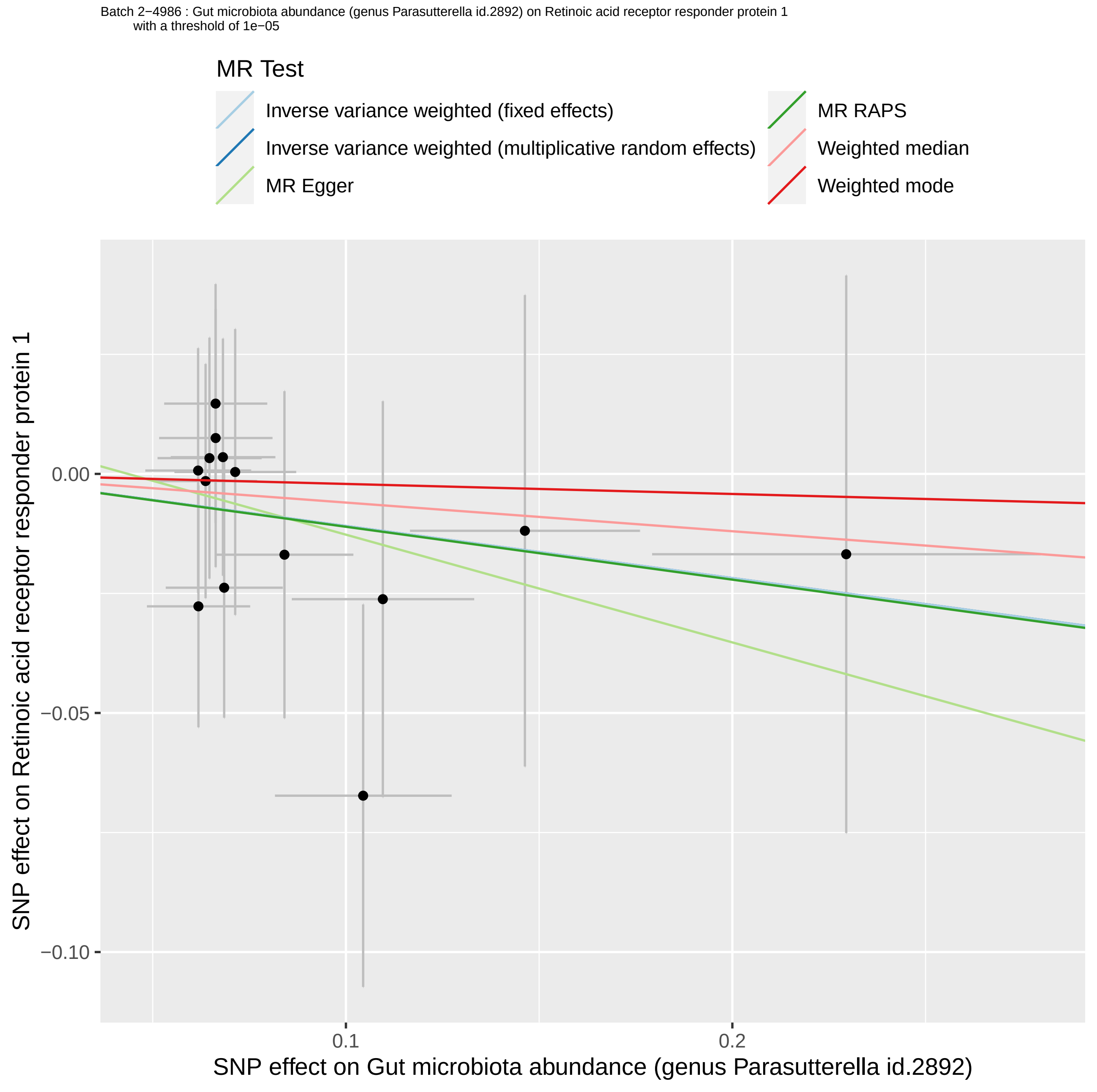

Batch 2−4986 : Gut microbiota abundance (genus Parasutterella id.2892) on Retinoic acid receptor responder protein 1
with a threshold of 1e−05
MR Test
Inverse variance weighted (fixed effects)
Inverse variance weighted (multiplicative random effects)
MR Egger
MR RAPS
Weighted median
Weighted mode
SNP effect on Retinoic acid receptor responder protein 1
0.00
−0.05
−0.10
0.1
0.2
SNP effect on Gut microbiota abundance (genus Parasutterella id.2892)

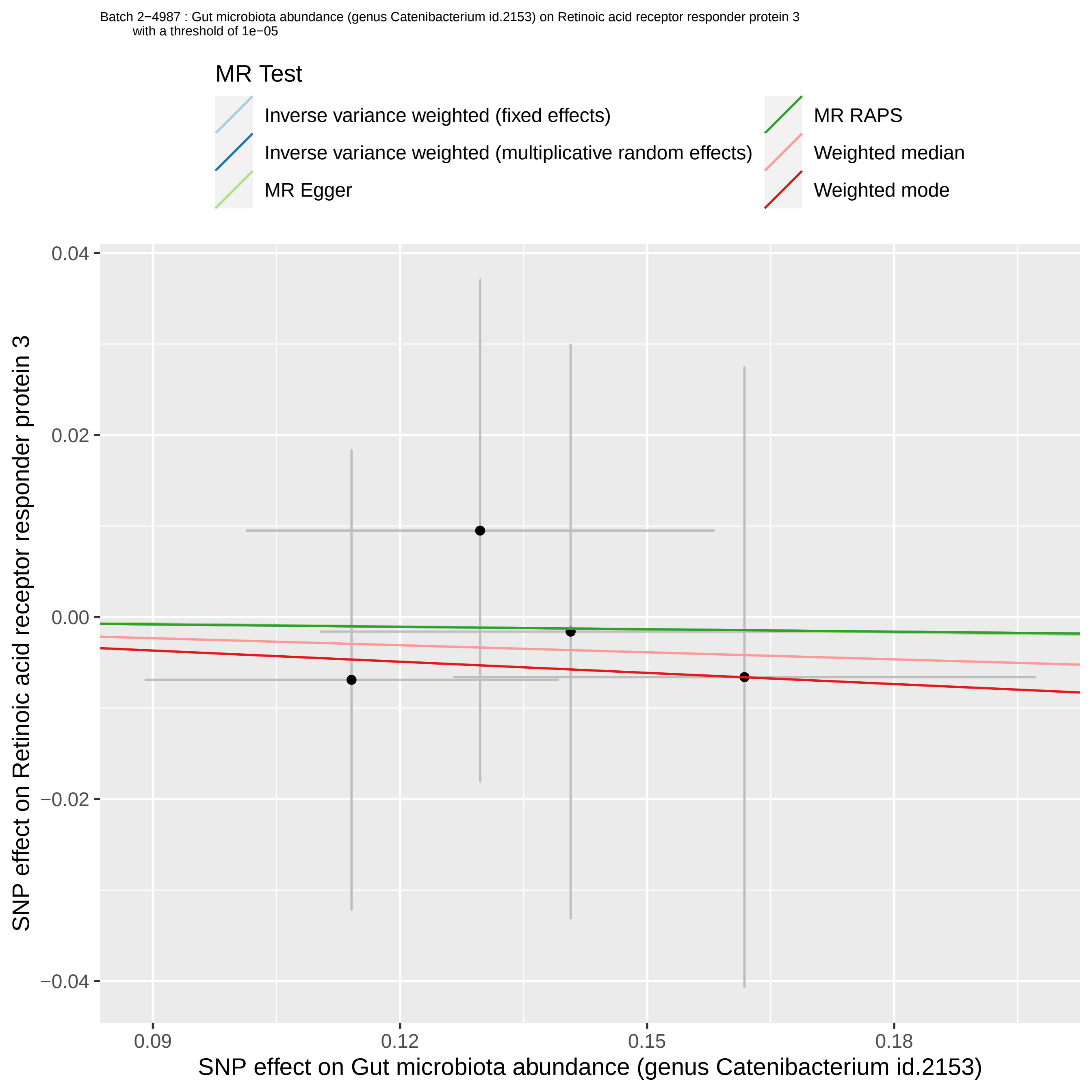
Batch 2−4987 : Gut microbiota abundance (genus Catenibacterium id.2153) on Retinoic acid receptor responder protein 3
with a threshold of 1e−05
MR Test
Inverse variance weighted (fixed effects)
Inverse variance weighted (multiplicative random effects)
MR Egger
MR RAPS
Weighted median
Weighted mode
0.04
0.02
0.00
−0.02
−0.04
0.09
0.12
0.15
0.18
SNP effect on Retinoic acid receptor responder protein 3
SNP effect on Gut microbiota abundance (genus Catenibacterium id.2153)

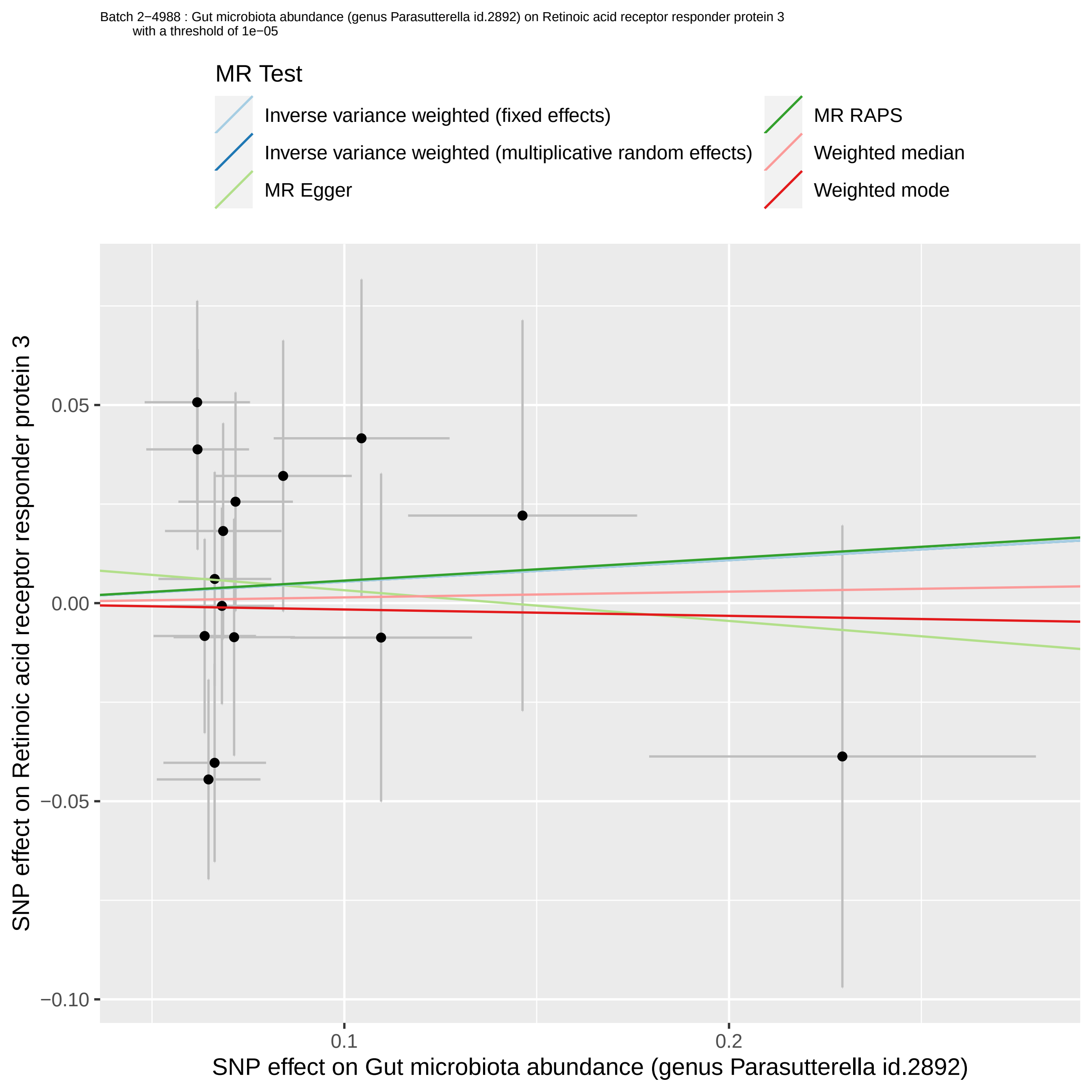
Batch 2−4988 : Gut microbiota abundance (genus Parasutterella id.2892) on Retinoic acid receptor responder protein 3
with a threshold of 1e−05
MR Test
Inverse variance weighted (fixed effects)
Inverse variance weighted (multiplicative random effects)
MR Egger
MR RAPS
Weighted median
Weighted mode
0.05
0.00
−0.05
−0.10
0.1
0.2
SNP effect on Retinoic acid receptor responder protein 3
SNP effect on Gut microbiota abundance (genus Parasutterella id.2892)

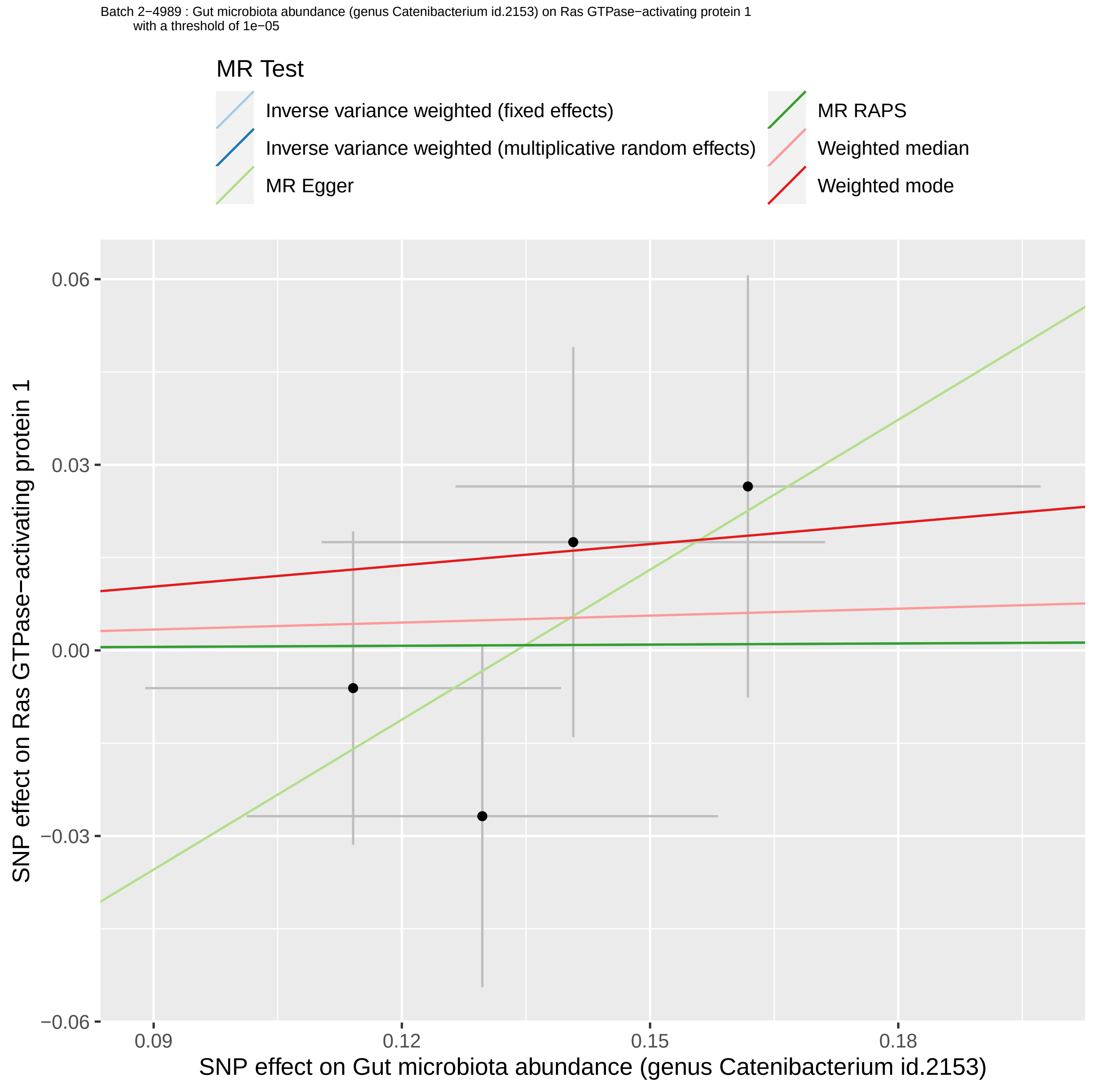

Batch 2−4989 : Gut microbiota abundance (genus Catenibacterium id.2153) on Ras GTPase−activating protein 1
with a threshold of 1e−05
MR Test
Inverse variance weighted (fixed effects)
Inverse variance weighted (multiplicative random effects)
MR Egger
MR RAPS
Weighted median
Weighted mode
0.06
0.03
0.00
−0.03
−0.06
0.09
0.12
0.15
0.18
SNP effect on Ras GTPase−activating protein 1
SNP effect on Gut microbiota abundance (genus Catenibacterium id.2153)

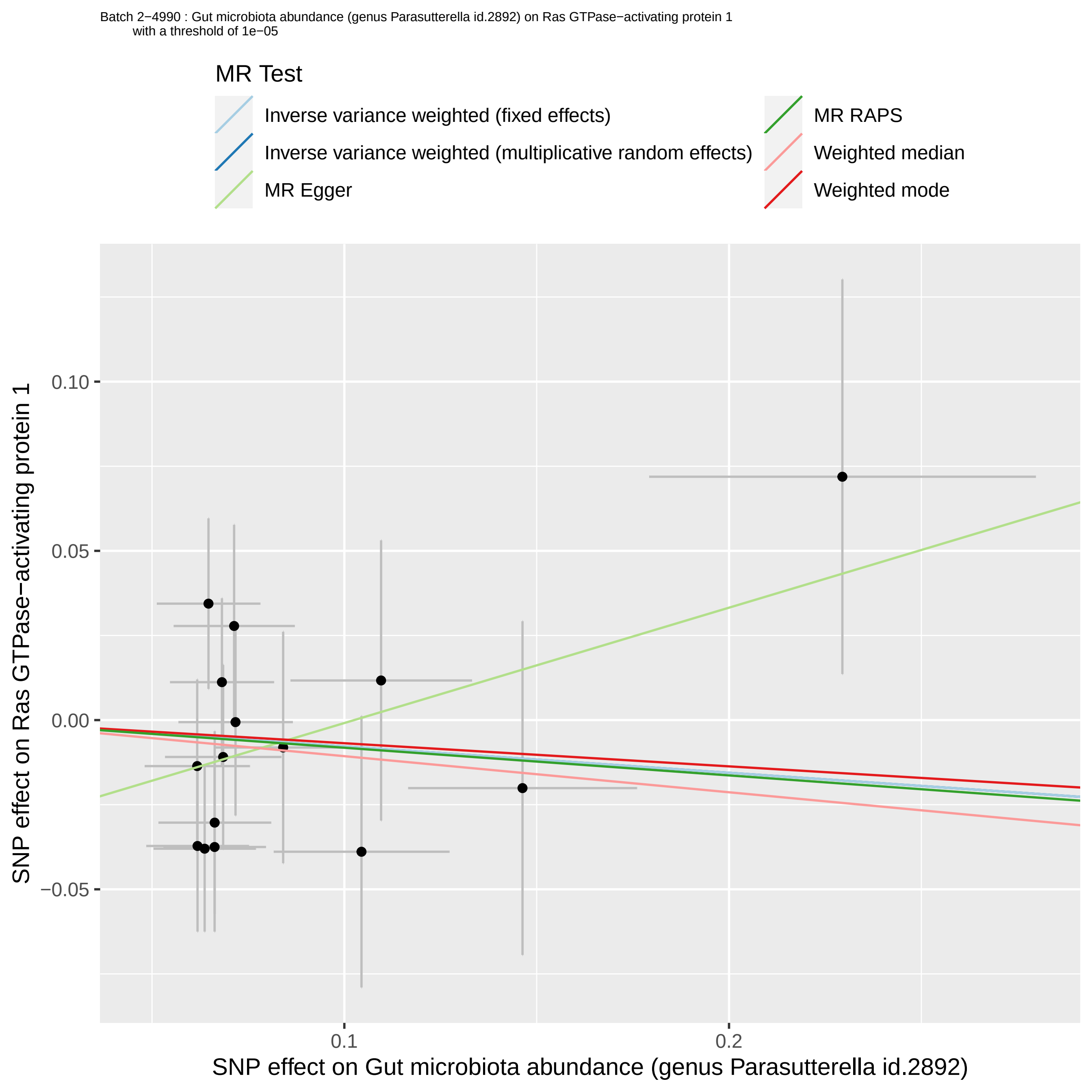

Batch 2−4990 : Gut microbiota abundance (genus Parasutterella id.2892) on Ras GTPase−activating protein 1
with a threshold of 1e−05
MR Test
Inverse variance weighted (fixed effects)
Inverse variance weighted (multiplicative random effects)
MR Egger
MR RAPS
Weighted median
Weighted mode
0.10
0.05
0.00
−0.05
0.1
0.2
SNP effect on Ras GTPase−activating protein 1
SNP effect on Gut microbiota abundance (genus Parasutterella id.2892)

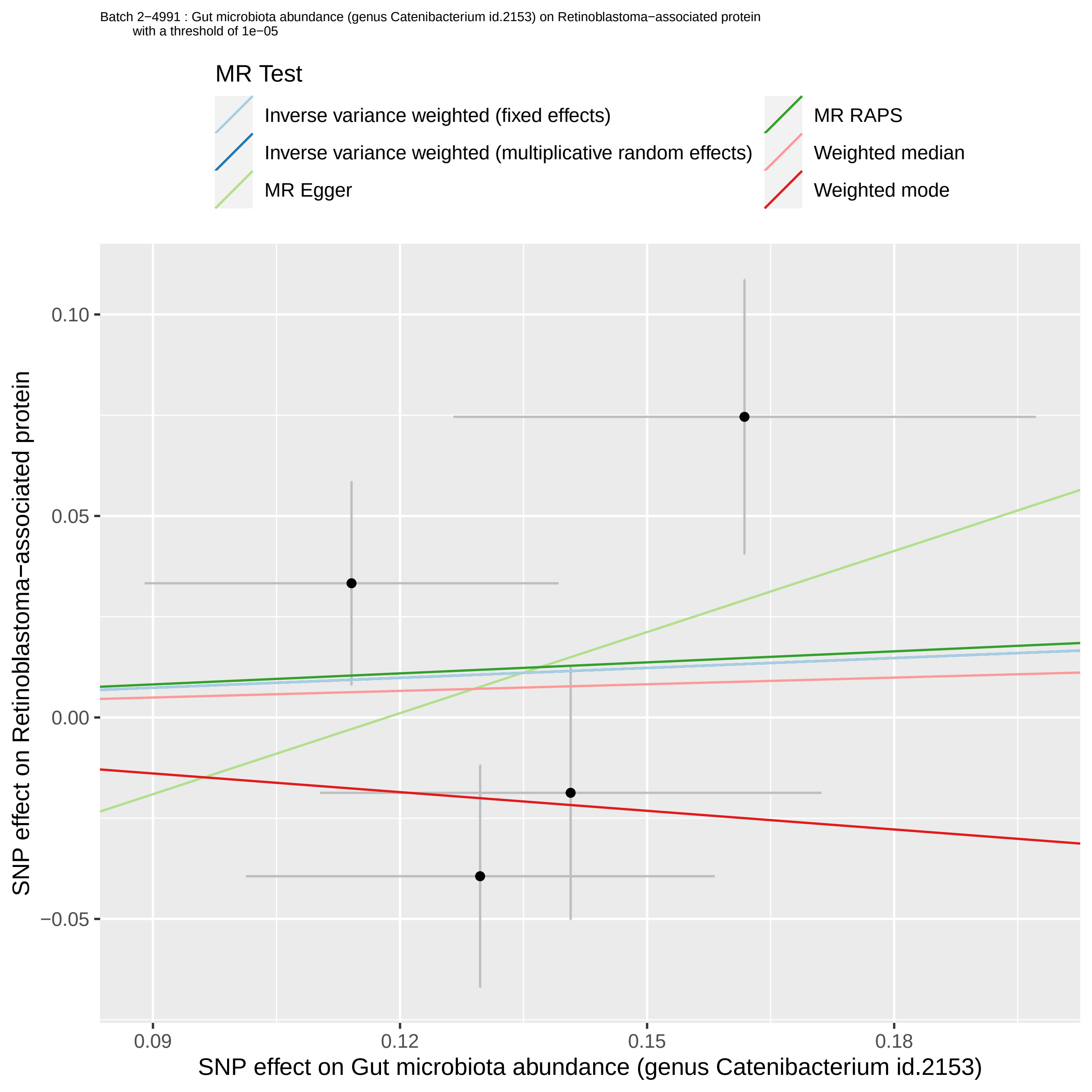

Batch 2−4991 : Gut microbiota abundance (genus Catenibacterium id.2153) on Retinoblastoma−associated protein
with a threshold of 1e−05
MR Test
Inverse variance weighted (fixed effects)
Inverse variance weighted (multiplicative random effects)
MR Egger
MR RAPS
Weighted median
Weighted mode
0.10
0.05
0.00
−0.05
0.09
0.12
0.15
0.18
SNP effect on Retinoblastoma−associated protein
SNP effect on Gut microbiota abundance (genus Catenibacterium id.2153)

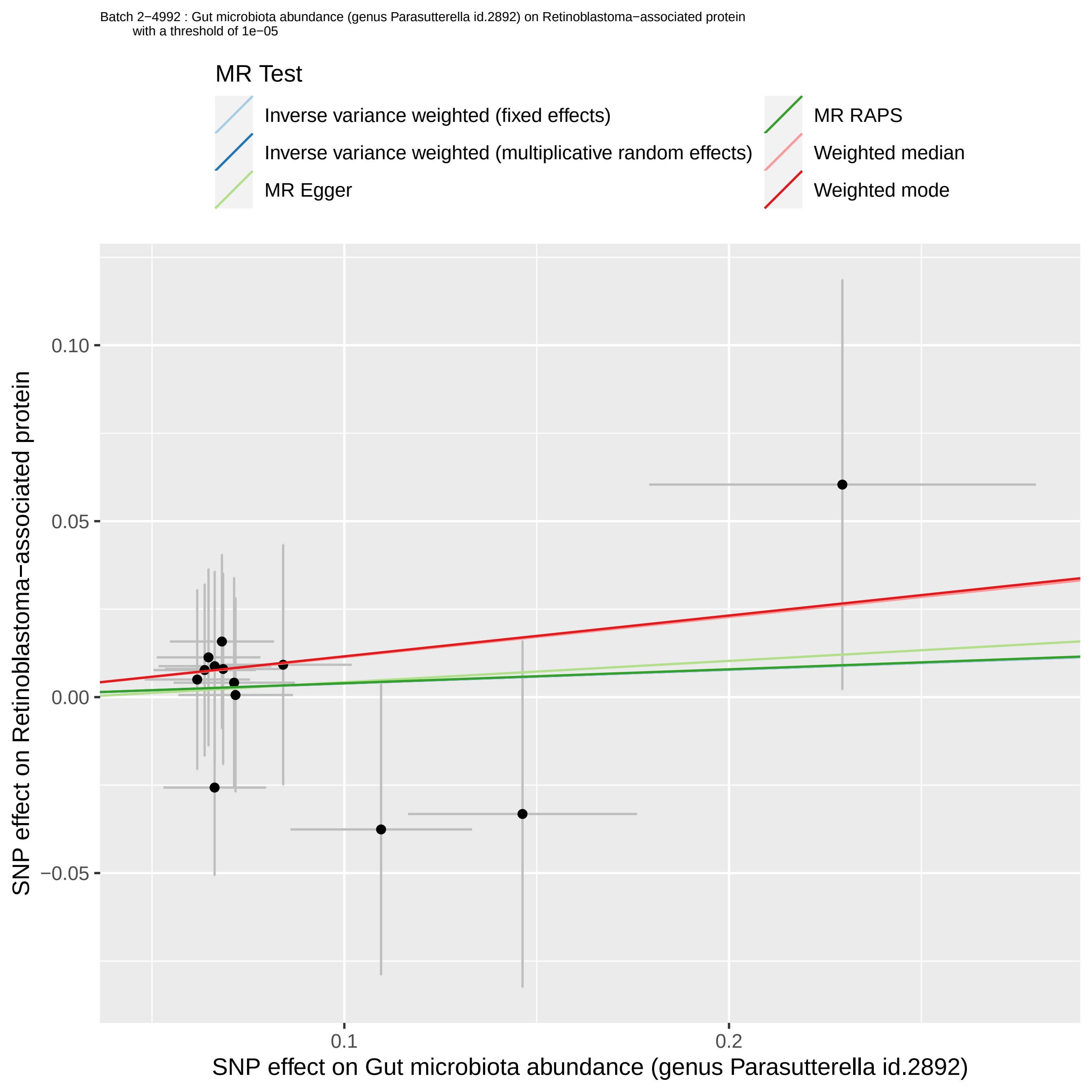
Batch 2−4992 : Gut microbiota abundance (genus Parasutterella id.2892) on Retinoblastoma−associated protein
with a threshold of 1e−05
MR Test
Inverse variance weighted (fixed effects)
Inverse variance weighted (multiplicative random effects)
MR Egger
MR RAPS
Weighted median
Weighted mode
0.10
0.05
0.00
−0.05
0.1
0.2
SNP effect on Retinoblastoma−associated protein
SNP effect on Gut microbiota abundance (genus Parasutterella id.2892)

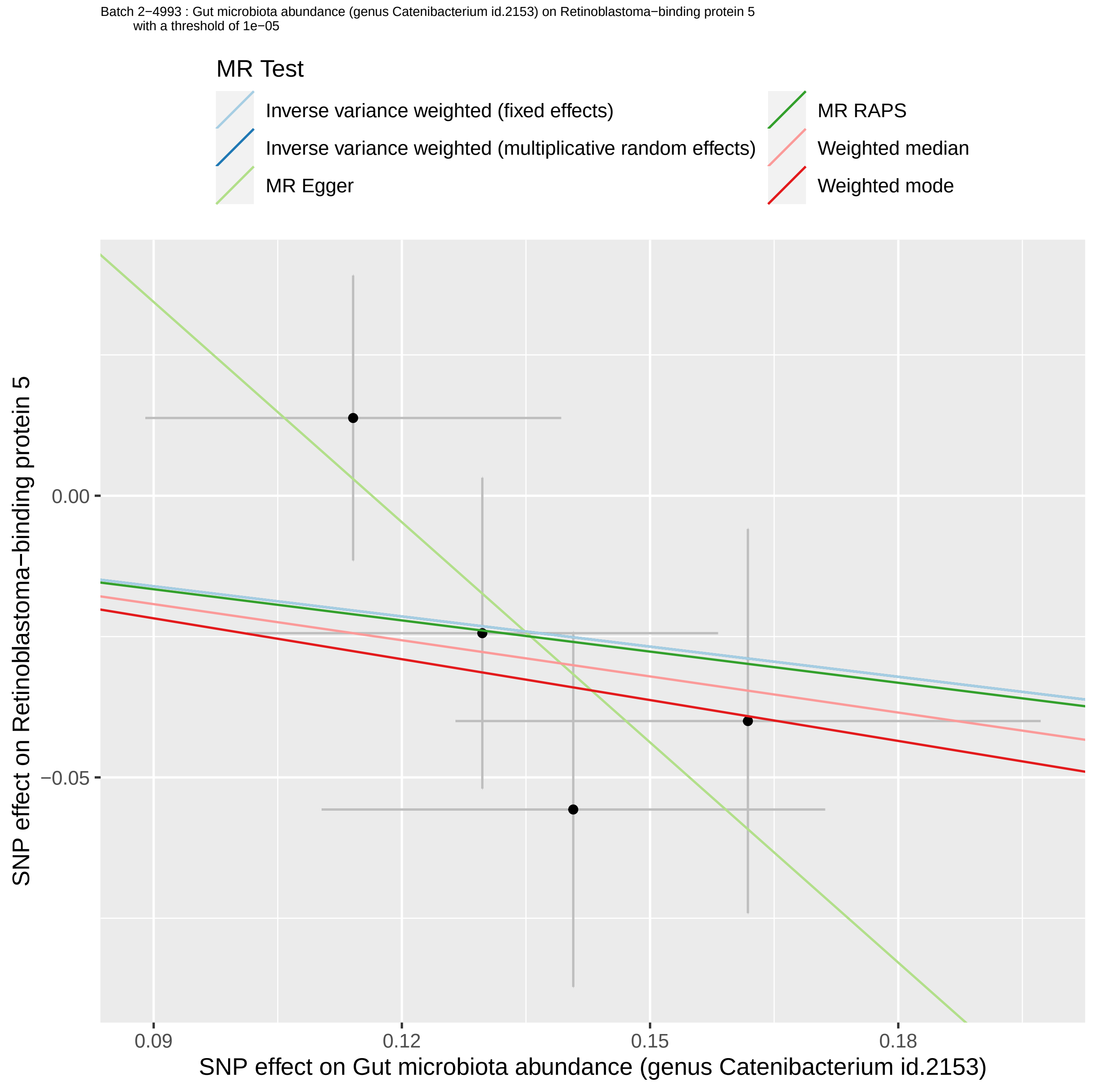
Batch 2−4993 : Gut microbiota abundance (genus Catenibacterium id.2153) on Retinoblastoma−binding protein 5
with a threshold of 1e−05
MR Test
Inverse variance weighted (fixed effects)
Inverse variance weighted (multiplicative random effects)
MR Egger
MR RAPS
Weighted median
Weighted mode
0.00
−0.05
0.09
0.12
0.15
0.18
SNP effect on Retinoblastoma−binding protein 5
SNP effect on Gut microbiota abundance (genus Catenibacterium id.2153)

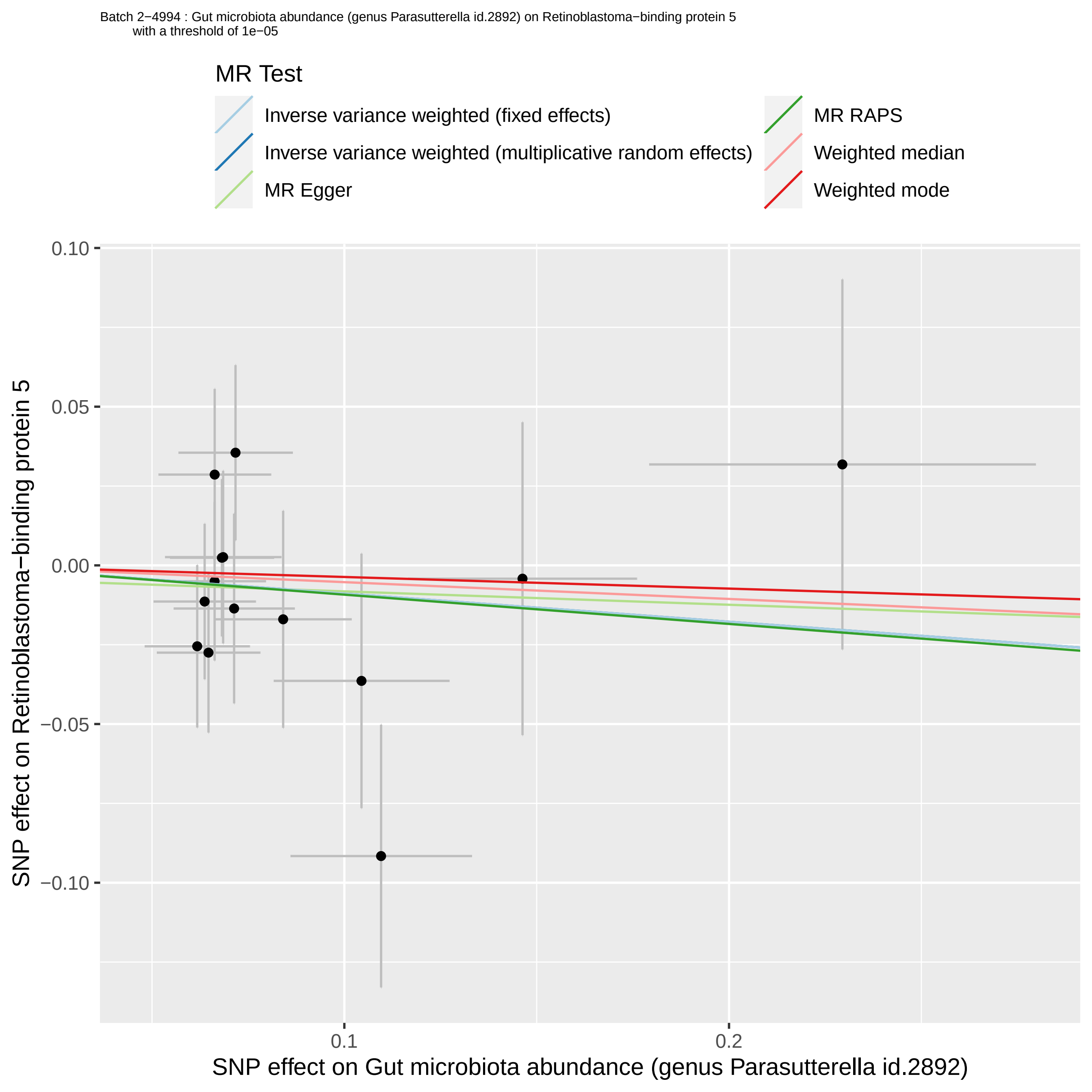
Batch 2−4994 : Gut microbiota abundance (genus Parasutterella id.2892) on Retinoblastoma−binding protein 5
with a threshold of 1e−05
MR Test
Inverse variance weighted (fixed effects)
Inverse variance weighted (multiplicative random effects)
MR Egger
MR RAPS
Weighted median
Weighted mode
0.10
0.05
0.00
−0.05
−0.10
0.1
0.2
SNP effect on Retinoblastoma−binding protein 5
SNP effect on Gut microbiota abundance (genus Parasutterella id.2892)

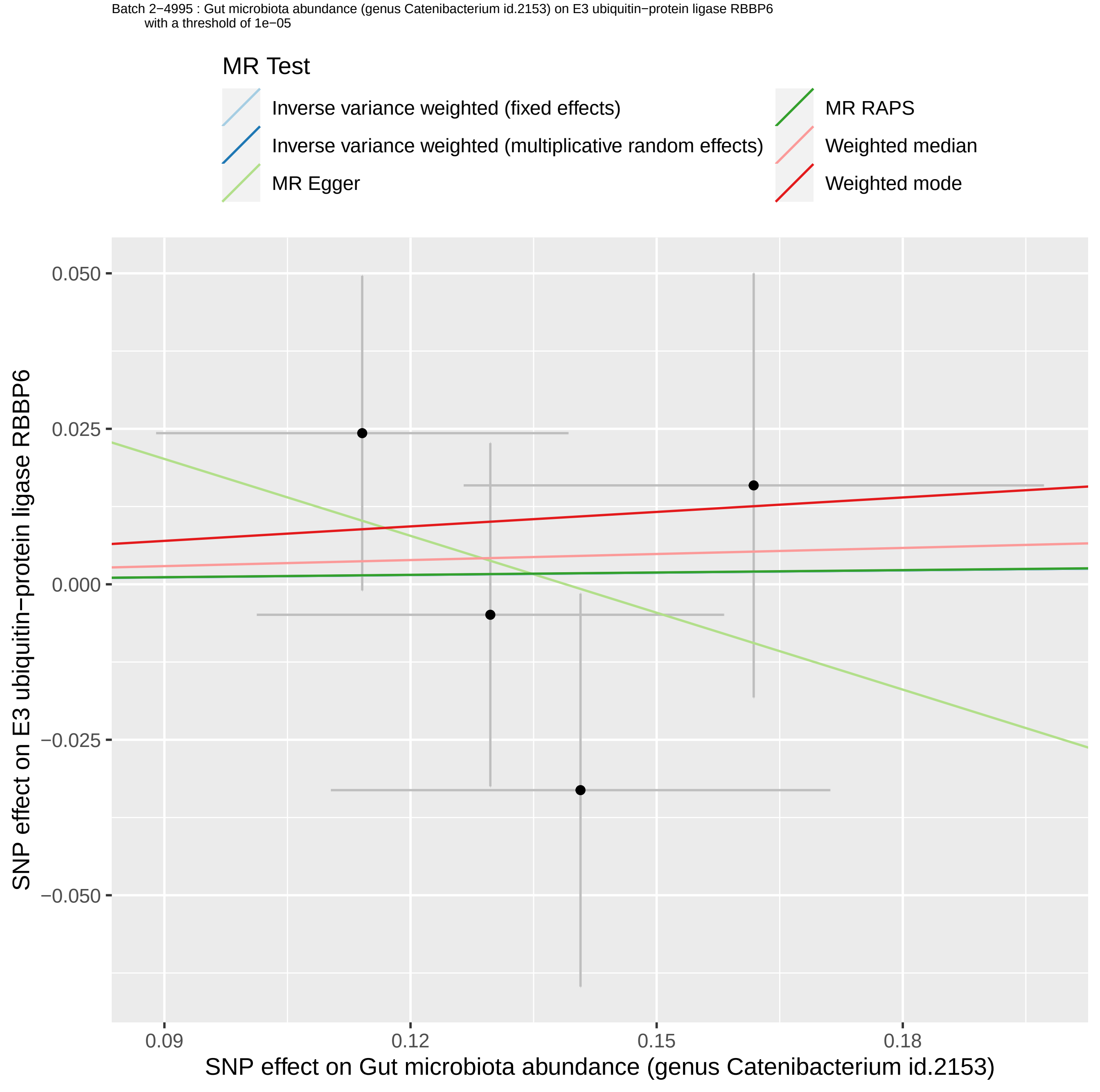
Batch 2−4995 : Gut microbiota abundance (genus Catenibacterium id.2153) on E3 ubiquitin−protein ligase RBBP6
with a threshold of 1e−05
MR Test
Inverse variance weighted (fixed effects)
Inverse variance weighted (multiplicative random effects)
MR Egger
MR RAPS
Weighted median
Weighted mode
0.050
0.025
0.000
−0.025
−0.050
0.09
0.12
0.15
0.18
SNP effect on E3 ubiquitin−protein ligase RBBP6
SNP effect on Gut microbiota abundance (genus Catenibacterium id.2153)

Batch 2−4996 : Gut microbiota abundance (genus Parasutterella id.2892) on E3 ubiquitin−protein ligase RBBP6
with a threshold of 1e−05

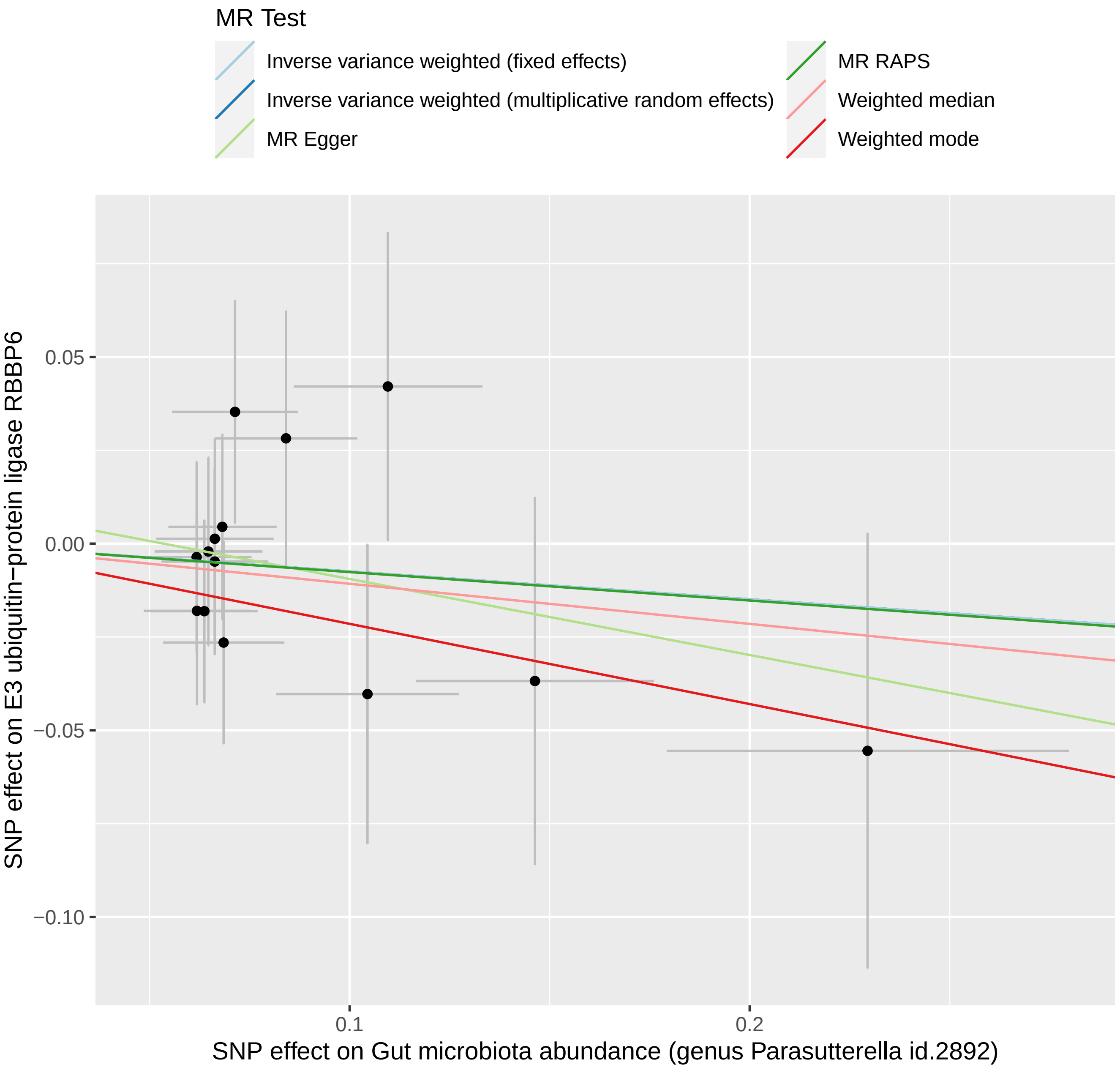

Batch 2−4997 : Gut microbiota abundance (genus Catenibacterium id.2153) on RNA binding protein fox−1 homolog 2
with a threshold of 1e−05

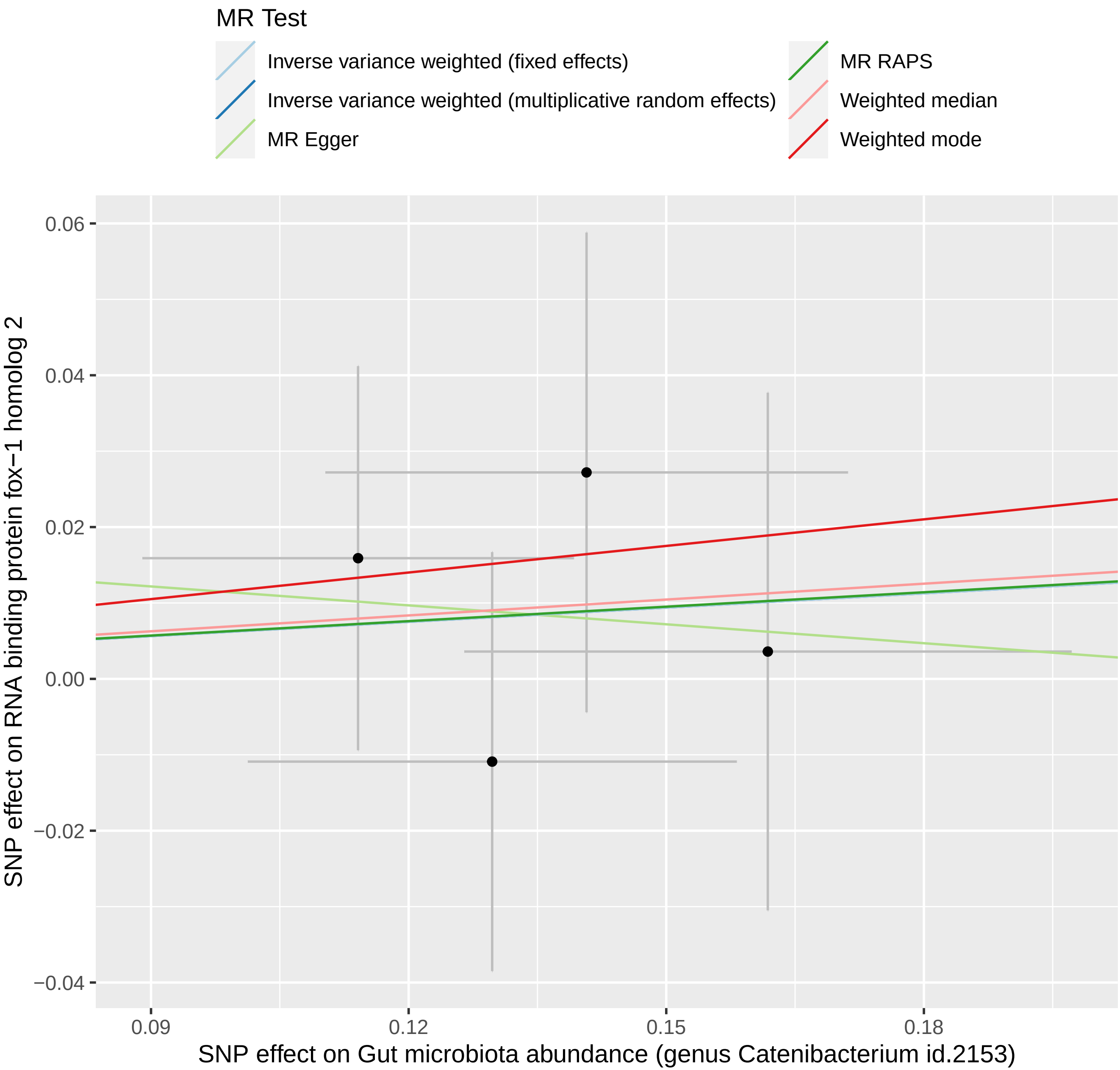

Batch 2−4998 : Gut microbiota abundance (genus Parasutterella id.2892) on RNA binding protein fox−1 homolog 2
with a threshold of 1e−05

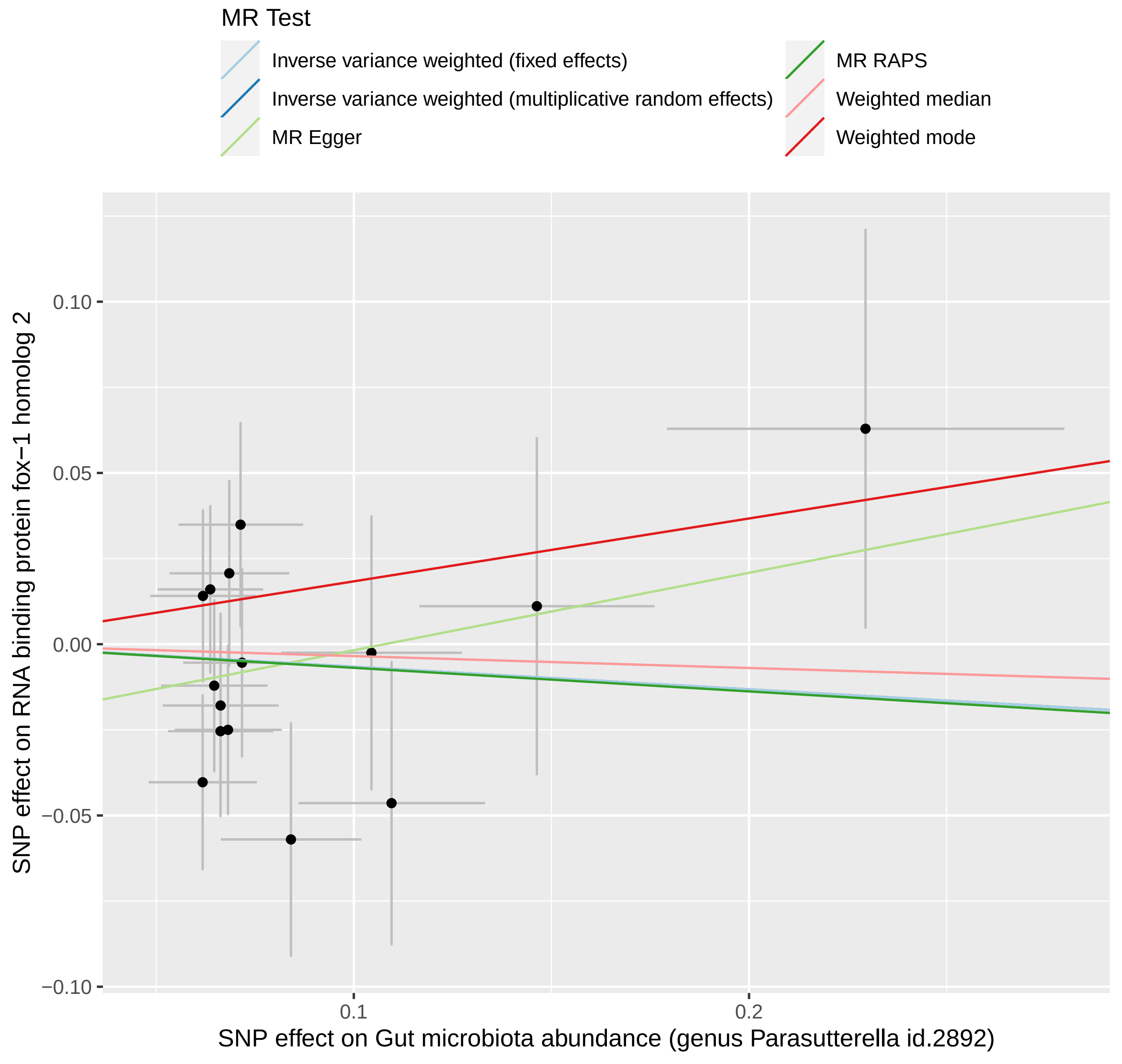

Batch 2−4999 : Gut microbiota abundance (genus Catenibacterium id.2153) on Retinoblastoma−like protein 1
with a threshold of 1e−05

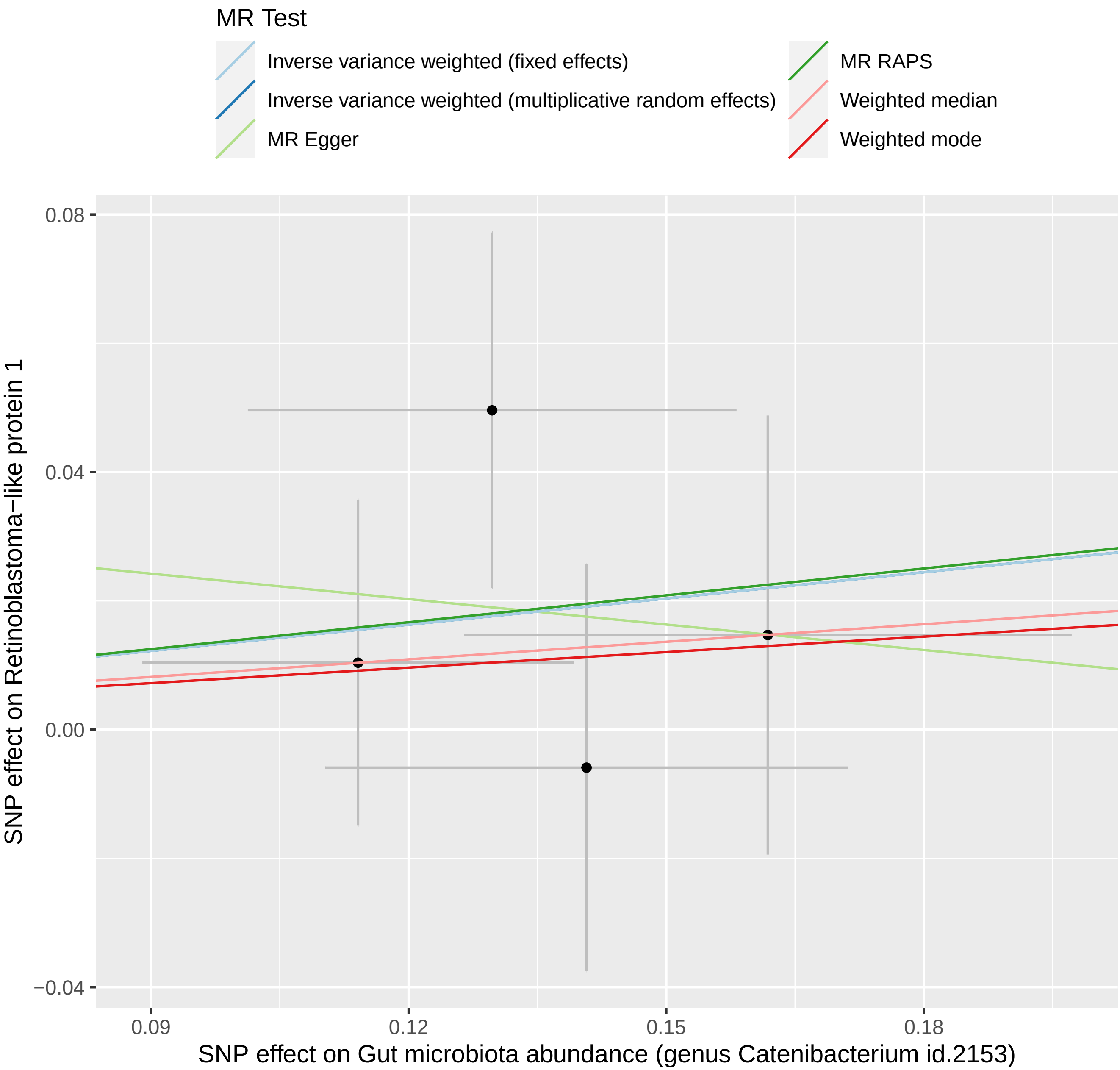

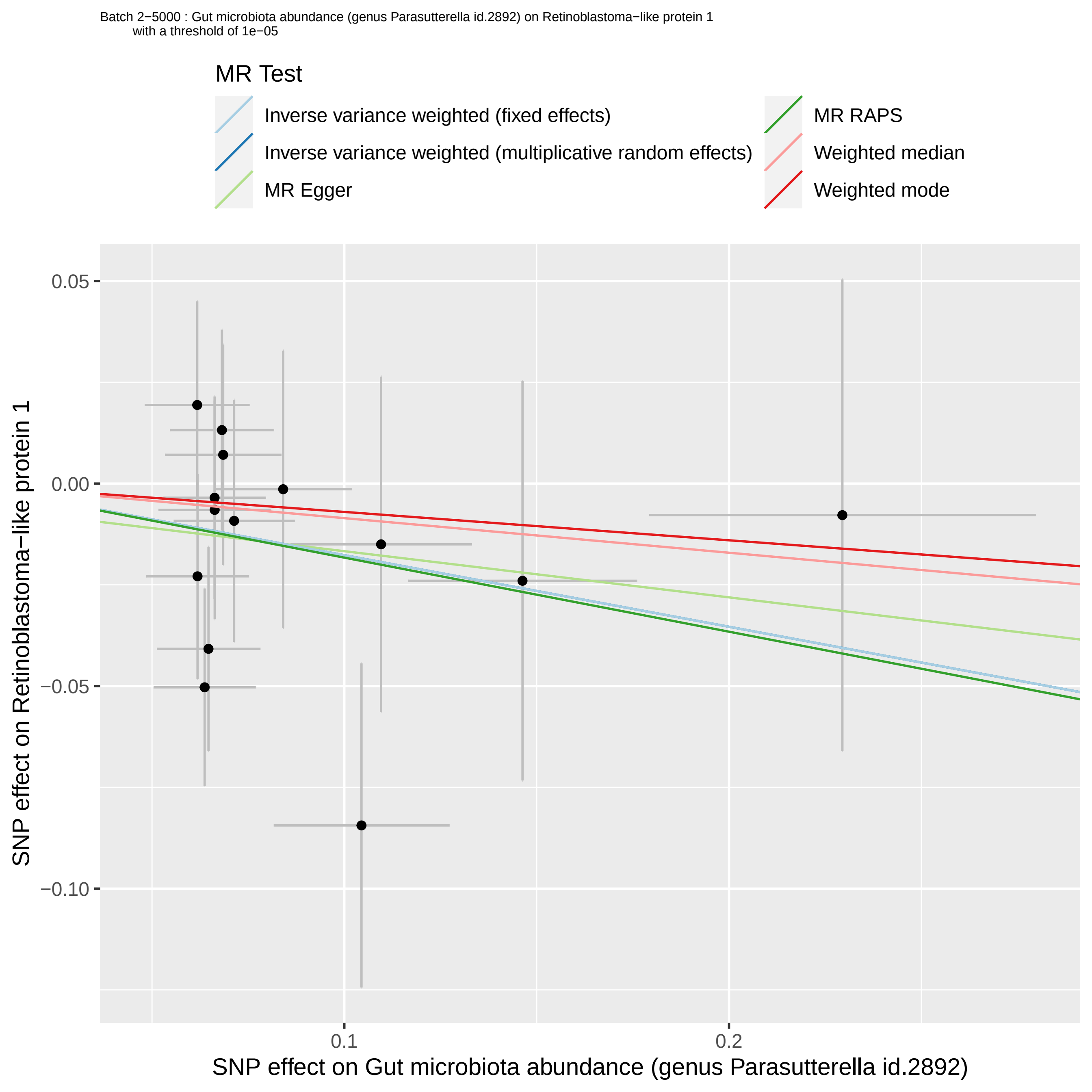

Batch 2−5000 : Gut microbiota abundance (genus Parasutterella id.2892) on Retinoblastoma−like protein 1
with a threshold of 1e−05
MR Test
Inverse variance weighted (fixed effects)
Inverse variance weighted (multiplicative random effects)
MR Egger
MR RAPS
Weighted median
Weighted mode
0.05
0.00
−0.05
−0.10
0.1
0.2
SNP effect on Retinoblastoma−like protein 1
SNP effect on Gut microbiota abundance (genus Parasutterella id.2892)

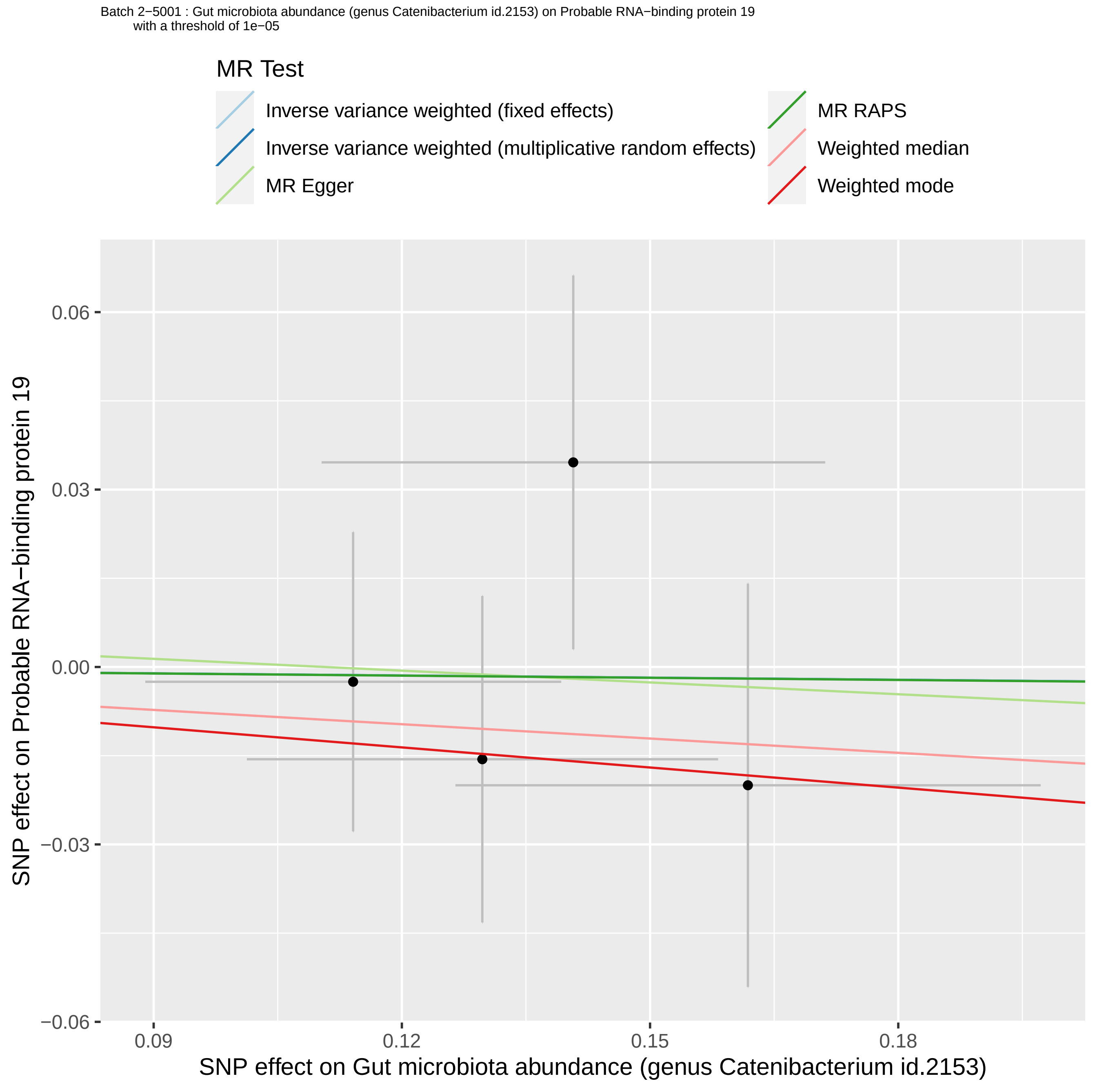
Batch 2−5001 : Gut microbiota abundance (genus Catenibacterium id.2153) on Probable RNA−binding protein 19
with a threshold of 1e−05
MR Test
Inverse variance weighted (fixed effects)
Inverse variance weighted (multiplicative random effects)
MR Egger
MR RAPS
Weighted median
Weighted mode
0.06
0.03
0.00
−0.03
−0.06
0.09
0.12
0.15
0.18
SNP effect on Probable RNA−binding protein 19
SNP effect on Gut microbiota abundance (genus Catenibacterium id.2153)

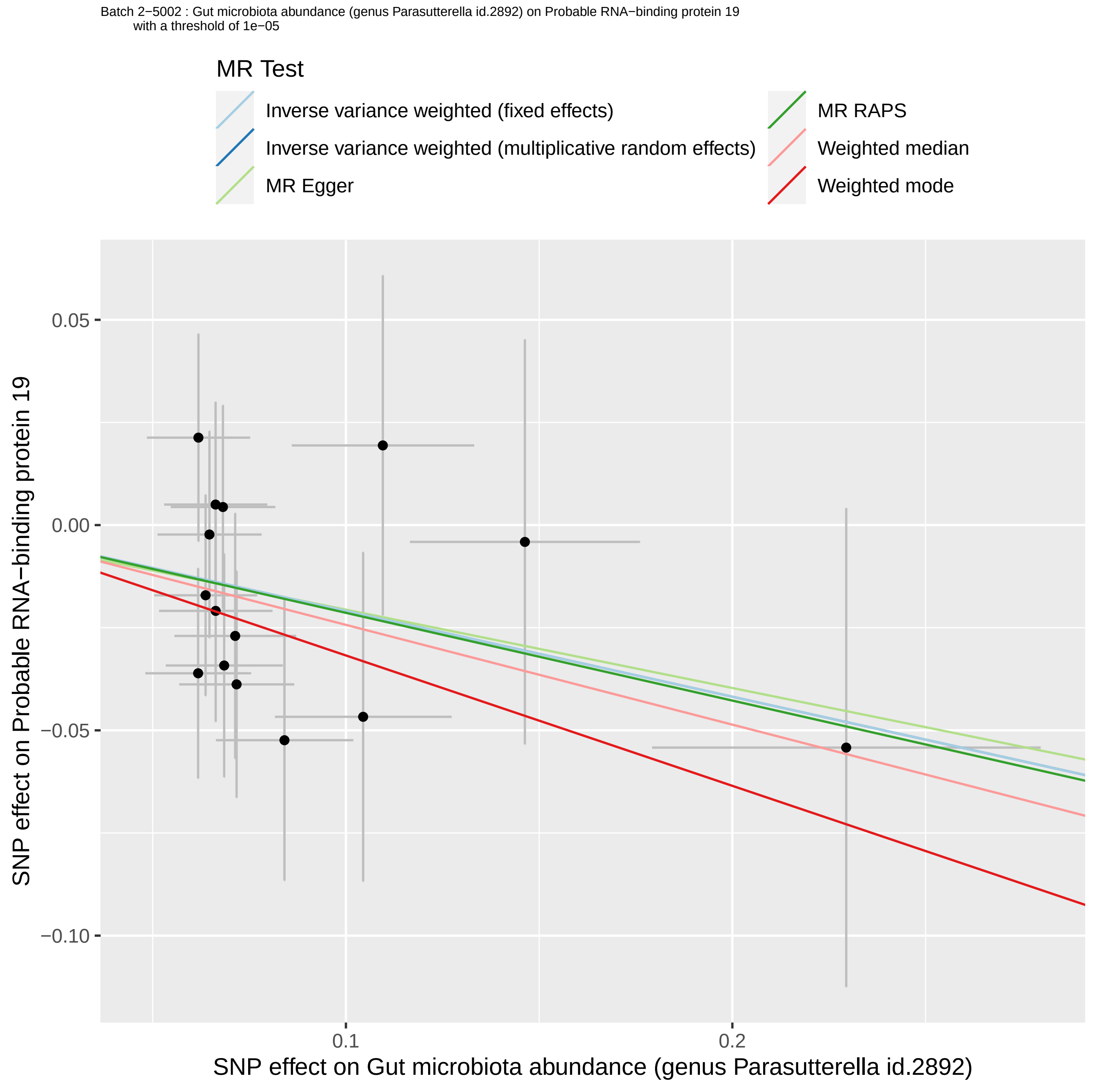
Batch 2−5002 : Gut microbiota abundance (genus Parasutterella id.2892) on Probable RNA−binding protein 19
with a threshold of 1e−05
MR Test
Inverse variance weighted (fixed effects)
Inverse variance weighted (multiplicative random effects)
MR Egger
MR RAPS
Weighted median
Weighted mode
0.05
0.00
−0.05
−0.10
SNP effect on Probable RNA−binding protein 19
0.1
0.2
SNP effect on Gut microbiota abundance (genus Parasutterella id.2892)

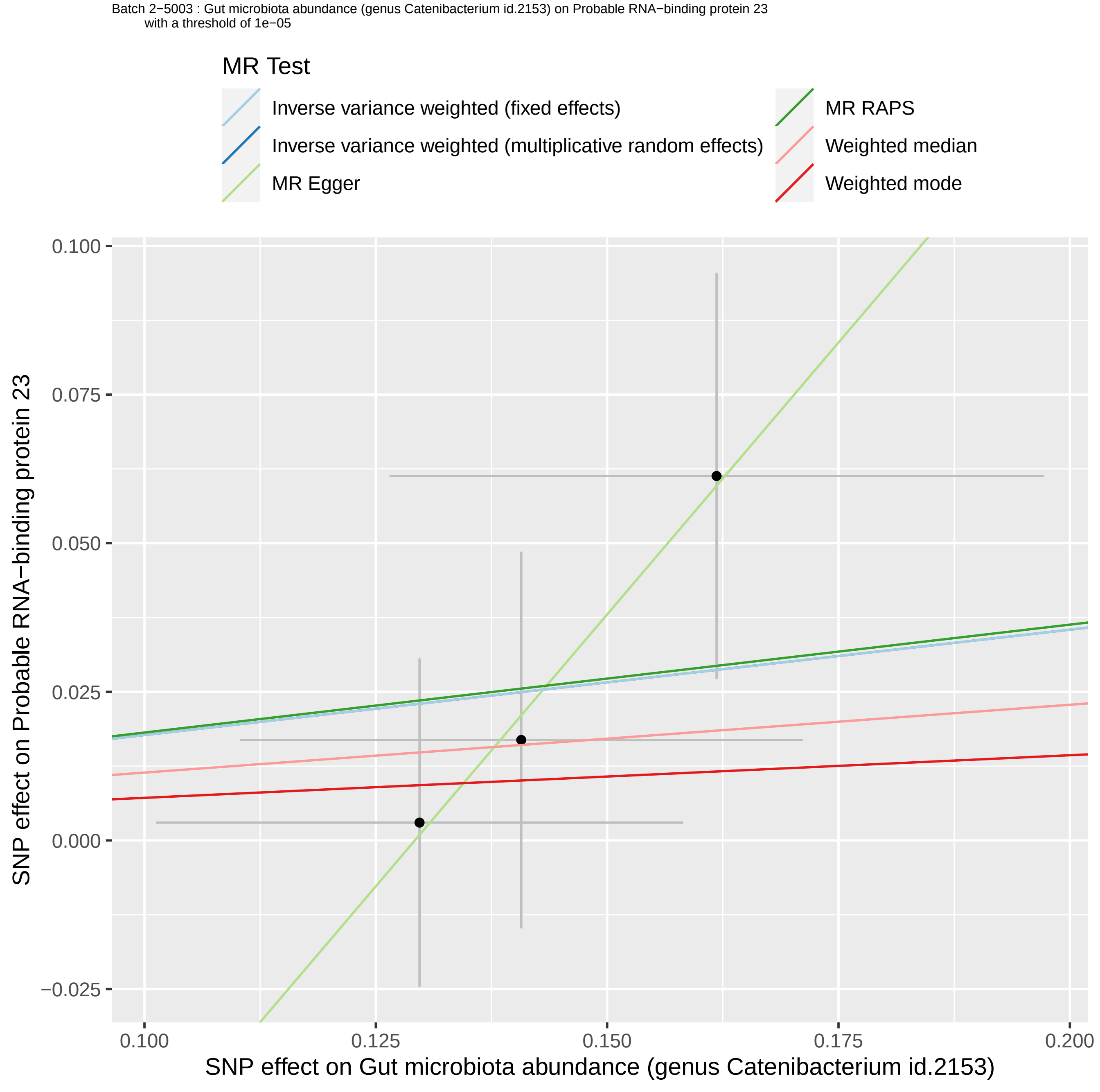
Batch 2−5003 : Gut microbiota abundance (genus Catenibacterium id.2153) on Probable RNA−binding protein 23
with a threshold of 1e−05
MR Test
Inverse variance weighted (fixed effects)
Inverse variance weighted (multiplicative random effects)
MR Egger
MR RAPS
Weighted median
Weighted mode
0.100
0.075
0.050
0.025
0.000
−0.025
0.100
0.125
0.150
0.175
0.200
SNP effect on Probable RNA−binding protein 23
SNP effect on Gut microbiota abundance (genus Catenibacterium id.2153)

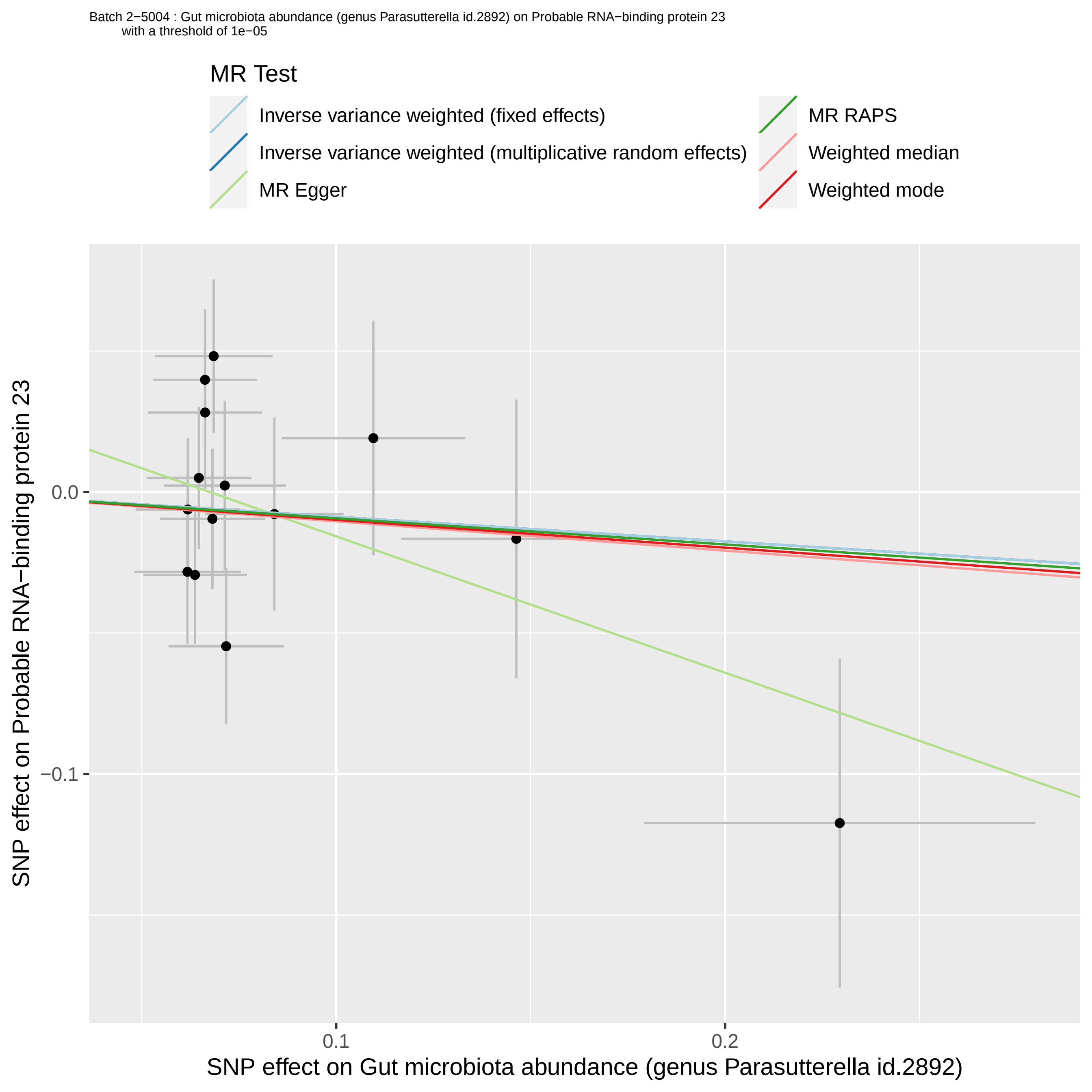

Batch 2−5004 : Gut microbiota abundance (genus Parasutterella id.2892) on Probable RNA−binding protein 23
with a threshold of 1e−05
MR Test
Inverse variance weighted (fixed effects)
Inverse variance weighted (multiplicative random effects)
MR Egger
MR RAPS
Weighted median
Weighted mode
0.0
−0.1
0.1
0.2
SNP effect on Probable RNA−binding protein 23
SNP effect on Gut microbiota abundance (genus Parasutterella id.2892)

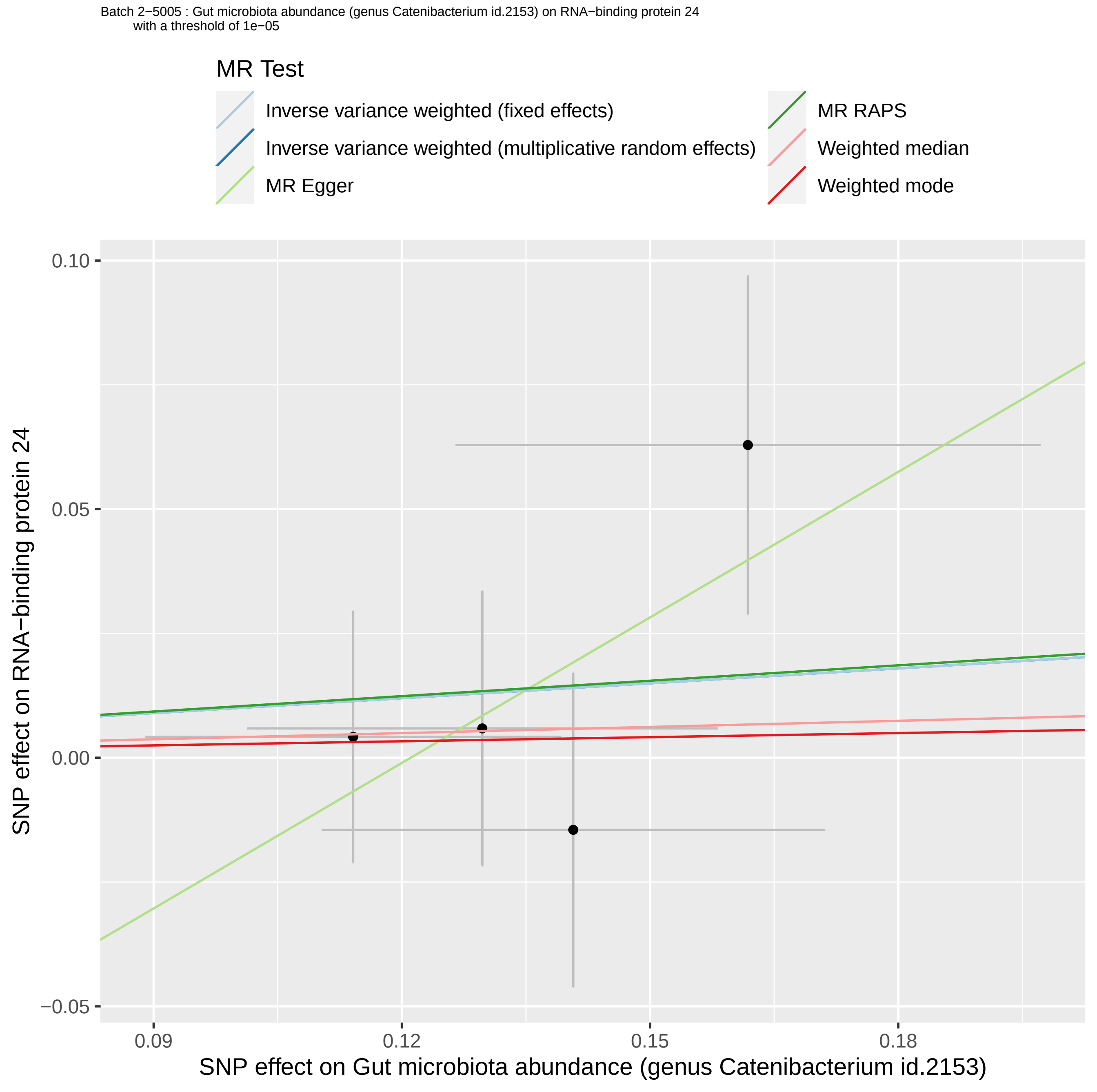
Batch 2−5005 : Gut microbiota abundance (genus Catenibacterium id.2153) on RNA−binding protein 24
with a threshold of 1e−05
MR Test
Inverse variance weighted (fixed effects)
Inverse variance weighted (multiplicative random effects)
MR Egger
MR RAPS
Weighted median
Weighted mode
0.10
0.05
0.00
−0.05
0.09
0.12
0.15
0.18
SNP effect on RNA−binding protein 24
SNP effect on Gut microbiota abundance (genus Catenibacterium id.2153)

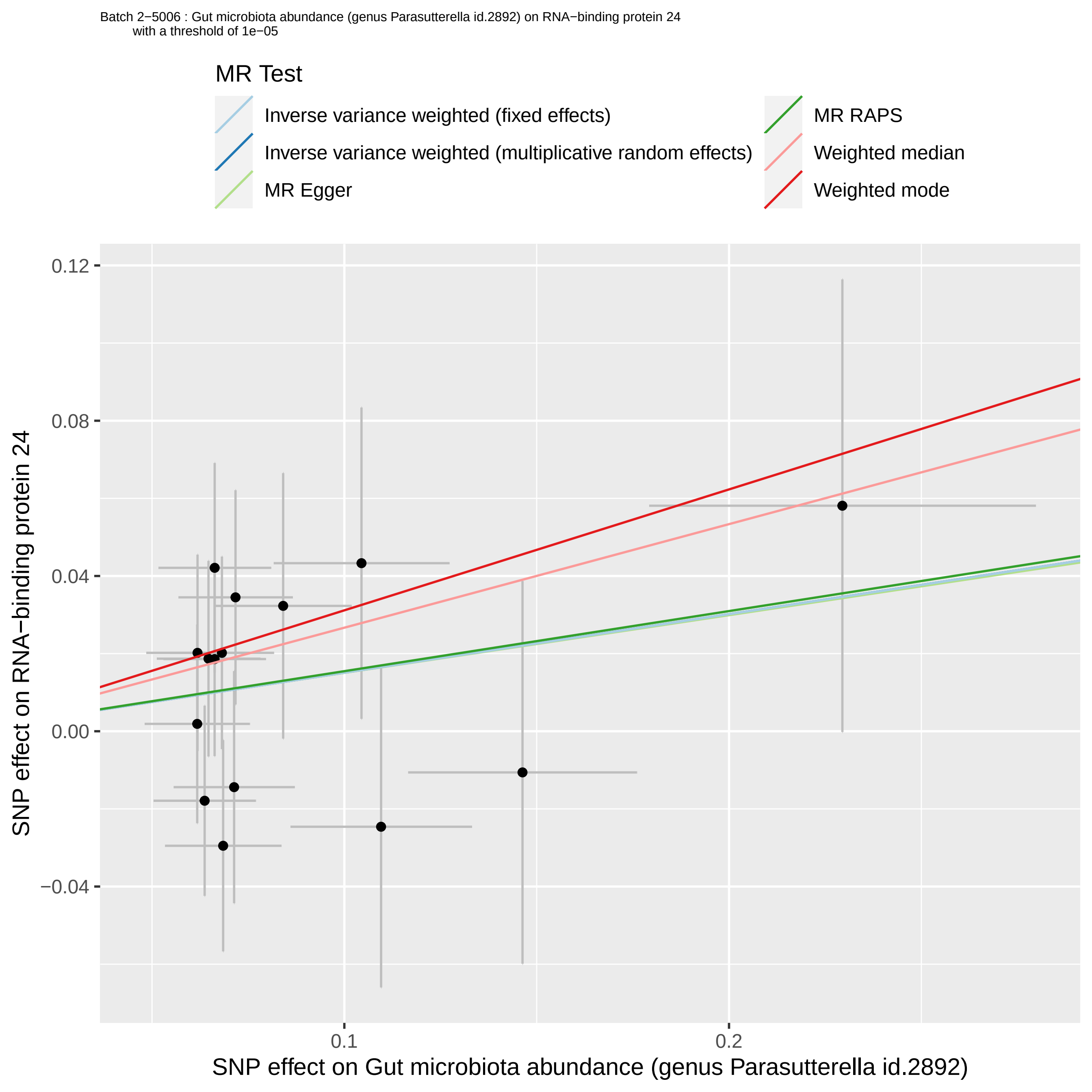
Batch 2−5006 : Gut microbiota abundance (genus Parasutterella id.2892) on RNA−binding protein 24
with a threshold of 1e−05
MR Test
Inverse variance weighted (fixed effects)
Inverse variance weighted (multiplicative random effects)
MR Egger
MR RAPS
Weighted median
Weighted mode
0.12
0.08
0.04
0.00
−0.04
0.1
0.2
SNP effect on RNA−binding protein 24
SNP effect on Gut microbiota abundance (genus Parasutterella id.2892)

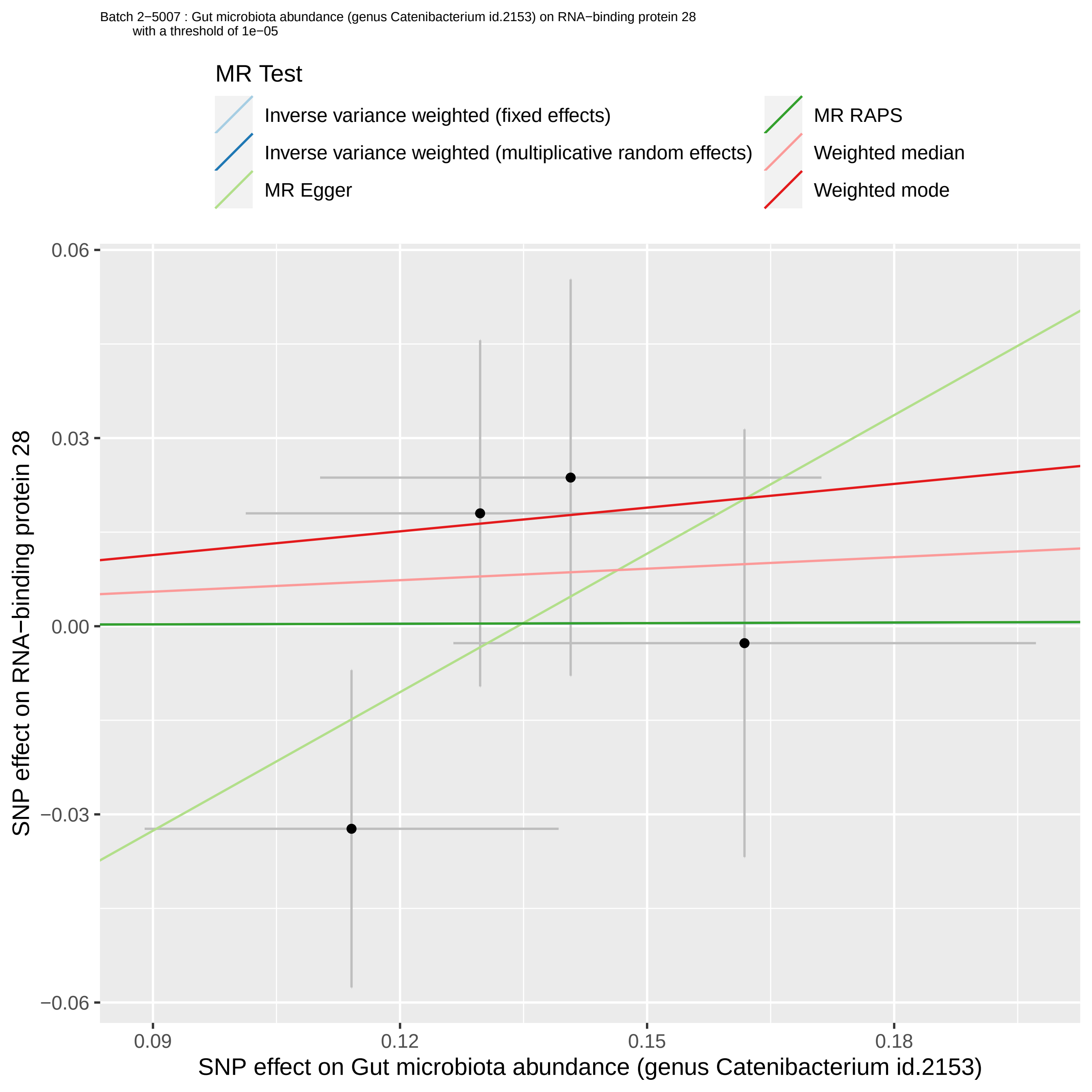

Batch 2−5007 : Gut microbiota abundance (genus Catenibacterium id.2153) on RNA−binding protein 28
with a threshold of 1e−05
MR Test
Inverse variance weighted (fixed effects)
Inverse variance weighted (multiplicative random effects)
MR Egger
MR RAPS
Weighted median
Weighted mode
0.06
0.03
0.00
−0.03
−0.06
0.09
0.12
0.15
0.18
SNP effect on RNA−binding protein 28
SNP effect on Gut microbiota abundance (genus Catenibacterium id.2153)

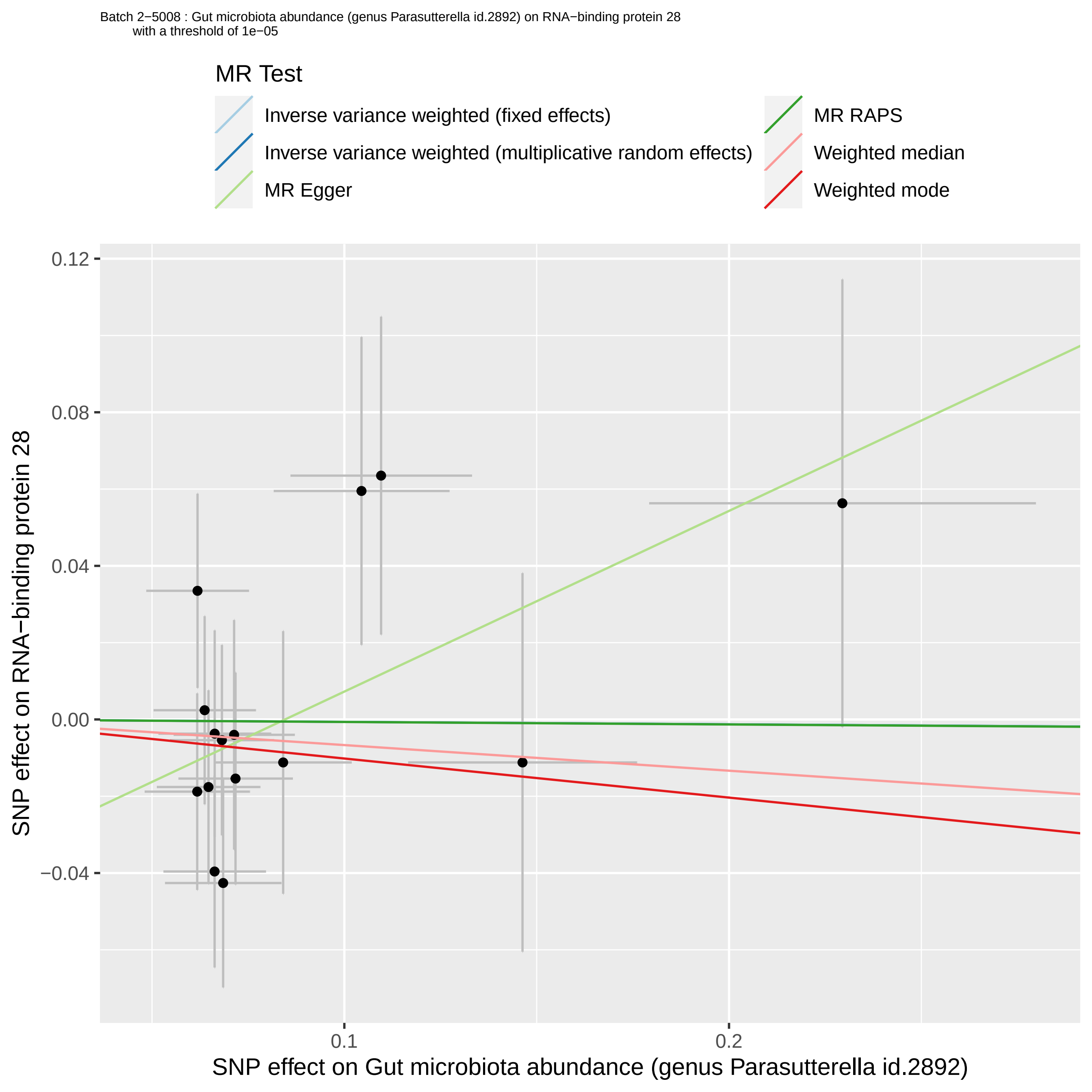

Batch 2−5008 : Gut microbiota abundance (genus Parasutterella id.2892) on RNA−binding protein 28
with a threshold of 1e−05
MR Test
Inverse variance weighted (fixed effects)
Inverse variance weighted (multiplicative random effects)
MR Egger
MR RAPS
Weighted median
Weighted mode
0.12
0.08
0.04
0.00
−0.04
0.1
0.2
SNP effect on RNA−binding protein 28
SNP effect on Gut microbiota abundance (genus Parasutterella id.2892)

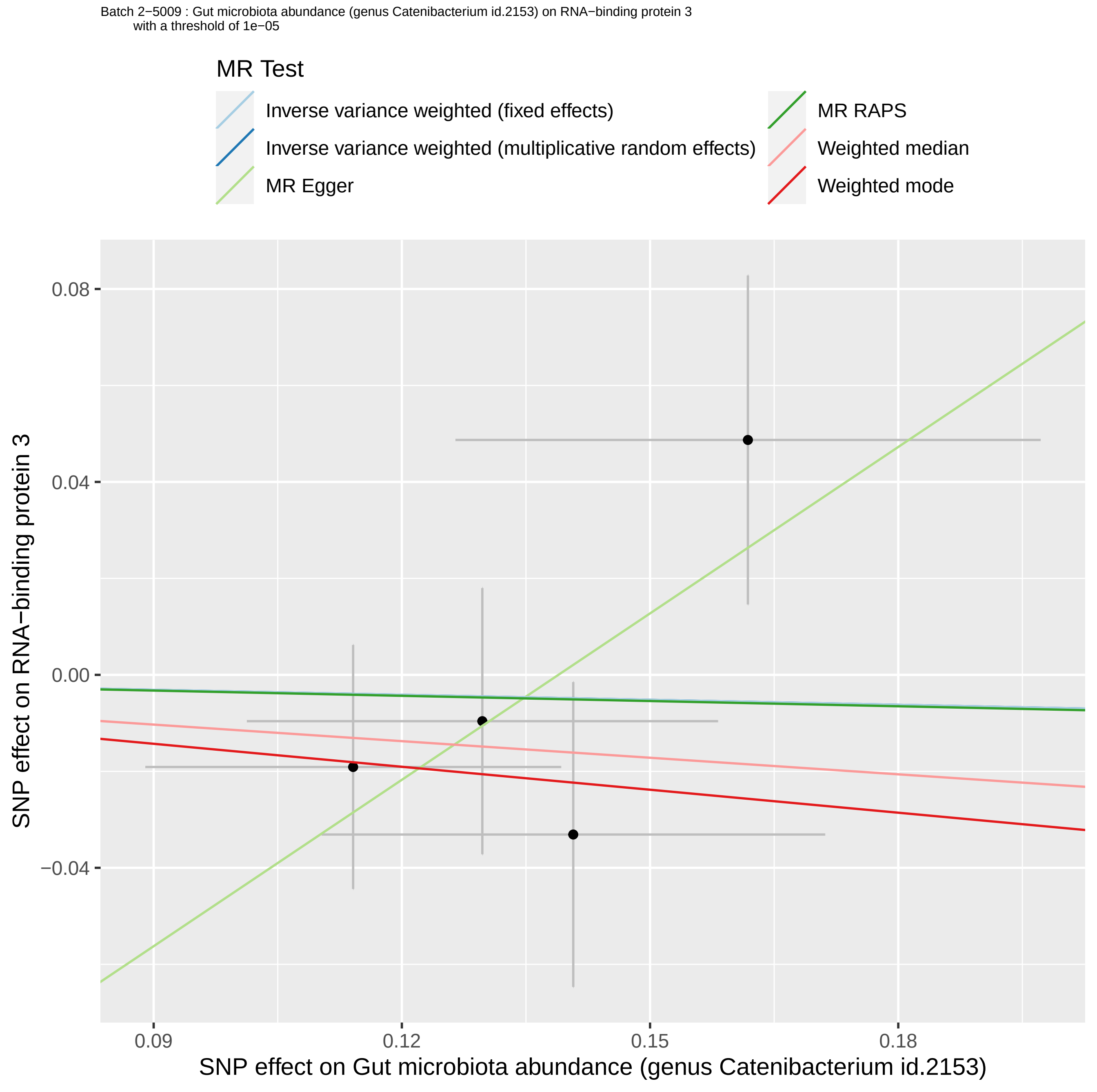
Batch 2−5009 : Gut microbiota abundance (genus Catenibacterium id.2153) on RNA−binding protein 3
with a threshold of 1e−05
MR Test
Inverse variance weighted (fixed effects)
Inverse variance weighted (multiplicative random effects)
MR Egger
MR RAPS
Weighted median
Weighted mode
0.08
0.04
0.00
−0.04
0.09
0.12
0.15
0.18
SNP effect on RNA−binding protein 3
SNP effect on Gut microbiota abundance (genus Catenibacterium id.2153)

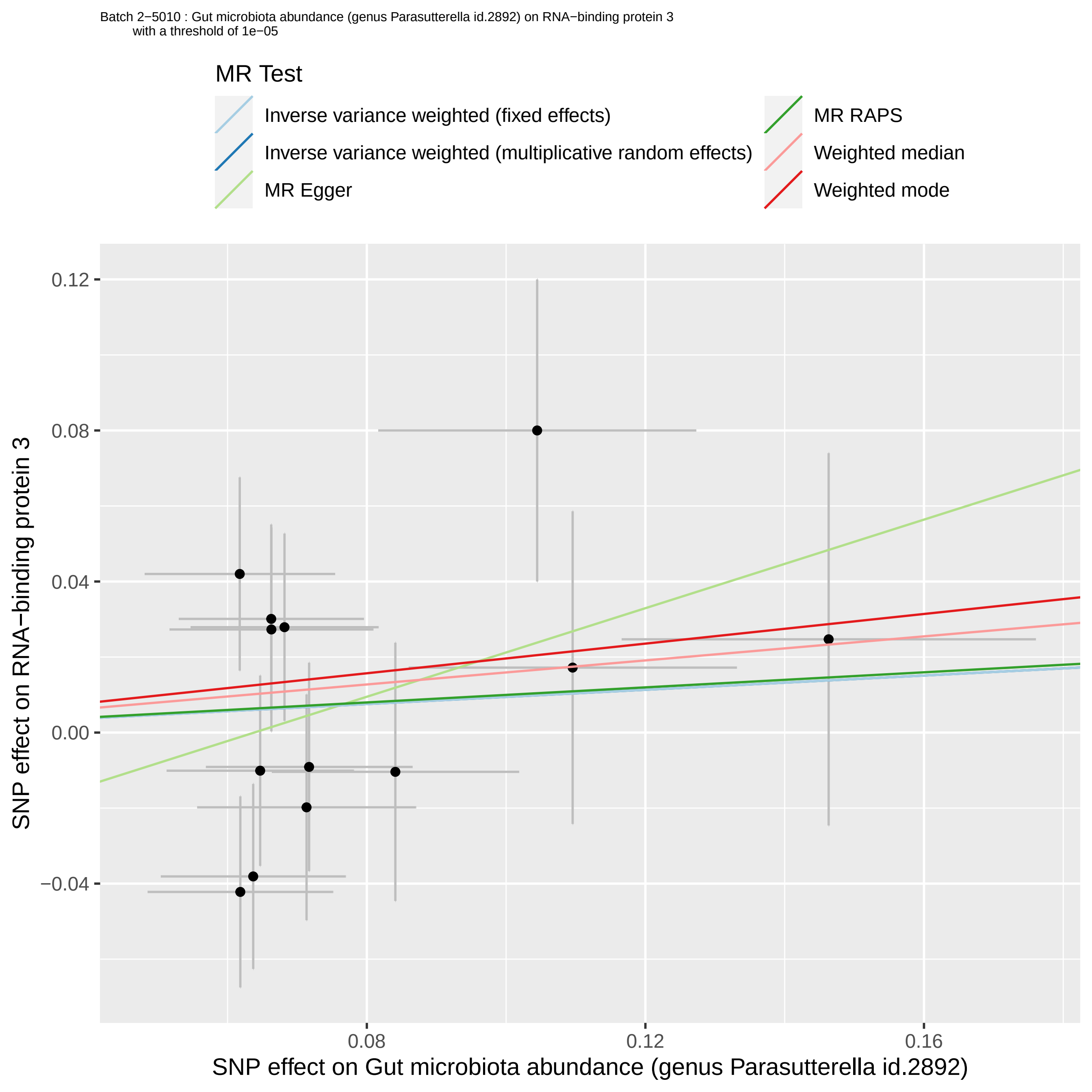
Batch 2−5010 : Gut microbiota abundance (genus Parasutterella id.2892) on RNA−binding protein 3
with a threshold of 1e−05
MR Test
Inverse variance weighted (fixed effects)
Inverse variance weighted (multiplicative random effects)
MR Egger
MR RAPS
Weighted median
Weighted mode
SNP effect on RNA−binding protein 3
0.12
0.08
0.04
0.00
−0.04
0.08
0.12
0.16
SNP effect on Gut microbiota abundance (genus Parasutterella id.2892)

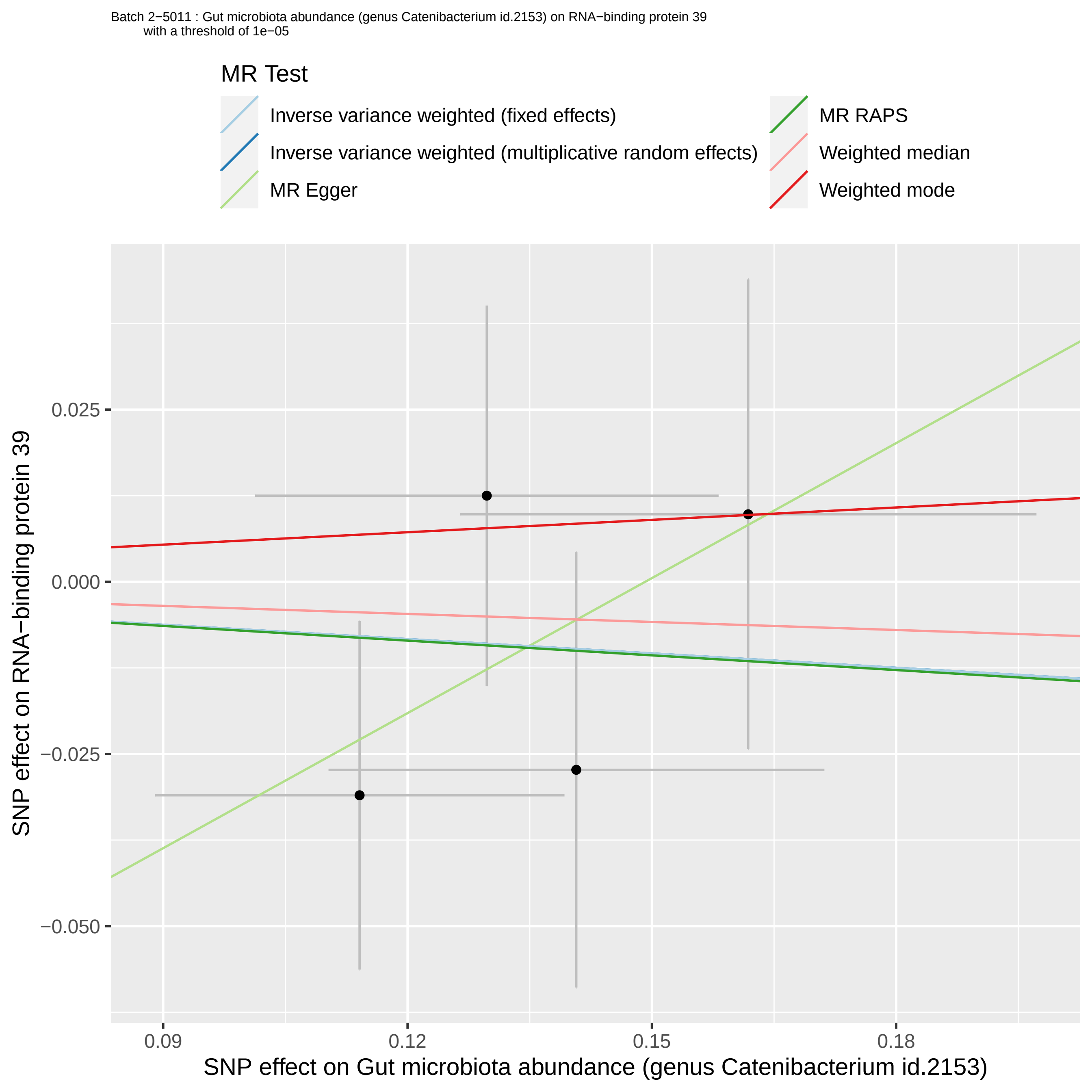
Batch 2−5011 : Gut microbiota abundance (genus Catenibacterium id.2153) on RNA−binding protein 39
with a threshold of 1e−05
MR Test
Inverse variance weighted (fixed effects)
Inverse variance weighted (multiplicative random effects)
MR Egger
MR RAPS
Weighted median
Weighted mode
0.025
0.000
−0.025
−0.050
0.09
0.12
0.15
0.18
SNP effect on RNA−binding protein 39
SNP effect on Gut microbiota abundance (genus Catenibacterium id.2153)

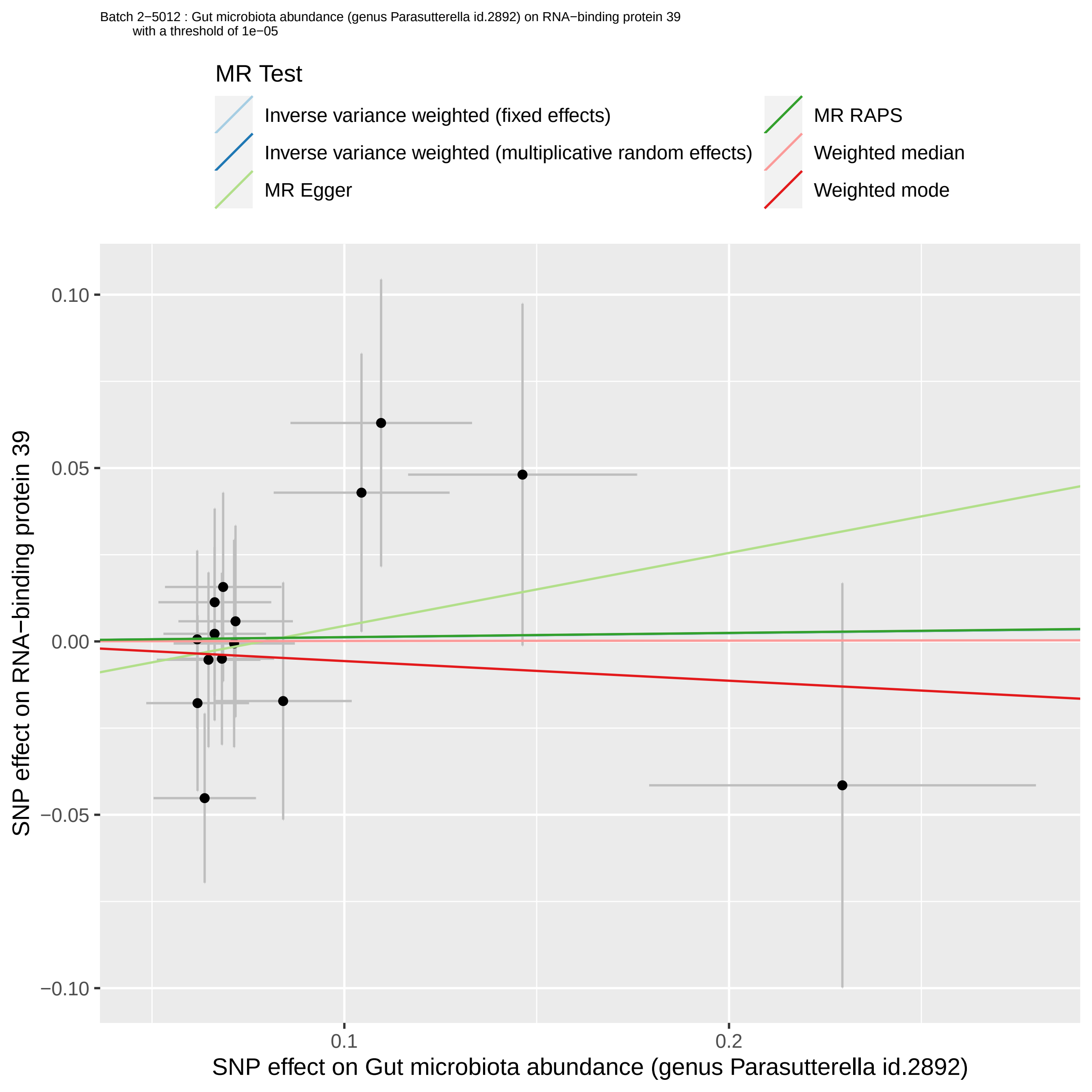
Batch 2−5012 : Gut microbiota abundance (genus Parasutterella id.2892) on RNA−binding protein 39
with a threshold of 1e−05
MR Test
Inverse variance weighted (fixed effects)
Inverse variance weighted (multiplicative random effects)
MR Egger
MR RAPS
Weighted median
Weighted mode
0.10
0.05
0.00
−0.05
−0.10
0.1
0.2
SNP effect on RNA−binding protein 39
SNP effect on Gut microbiota abundance (genus Parasutterella id.2892)

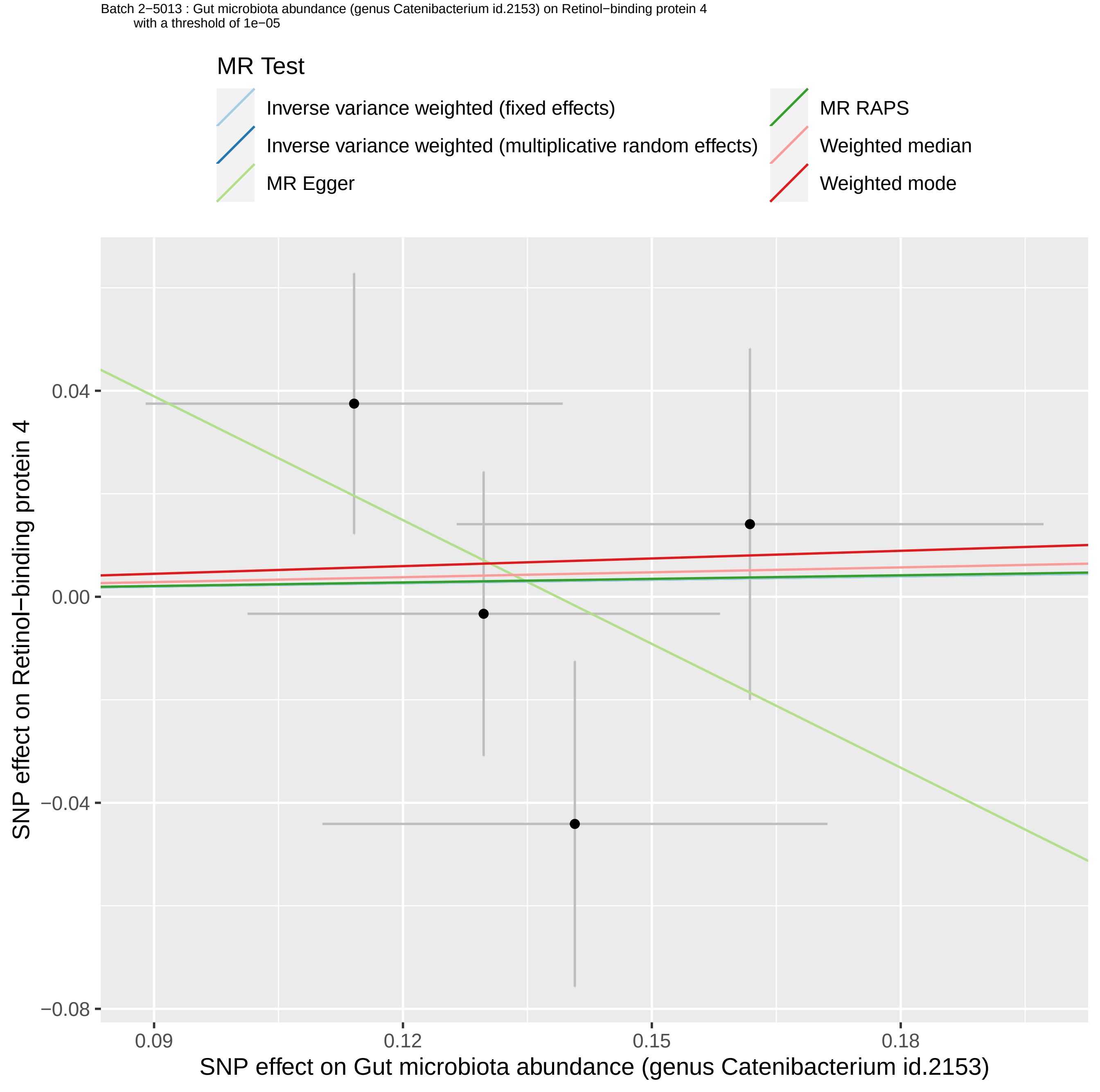
Batch 2−5013 : Gut microbiota abundance (genus Catenibacterium id.2153) on Retinol−binding protein 4
with a threshold of 1e−05
MR Test
Inverse variance weighted (fixed effects)
Inverse variance weighted (multiplicative random effects)
MR Egger
MR RAPS
Weighted median
Weighted mode
0.04
0.00
−0.04
−0.08
0.09
0.12
0.15
0.18
SNP effect on Gut microbiota abundance (genus Catenibacterium id.2153)
SNP effect on Retinol−binding protein 4

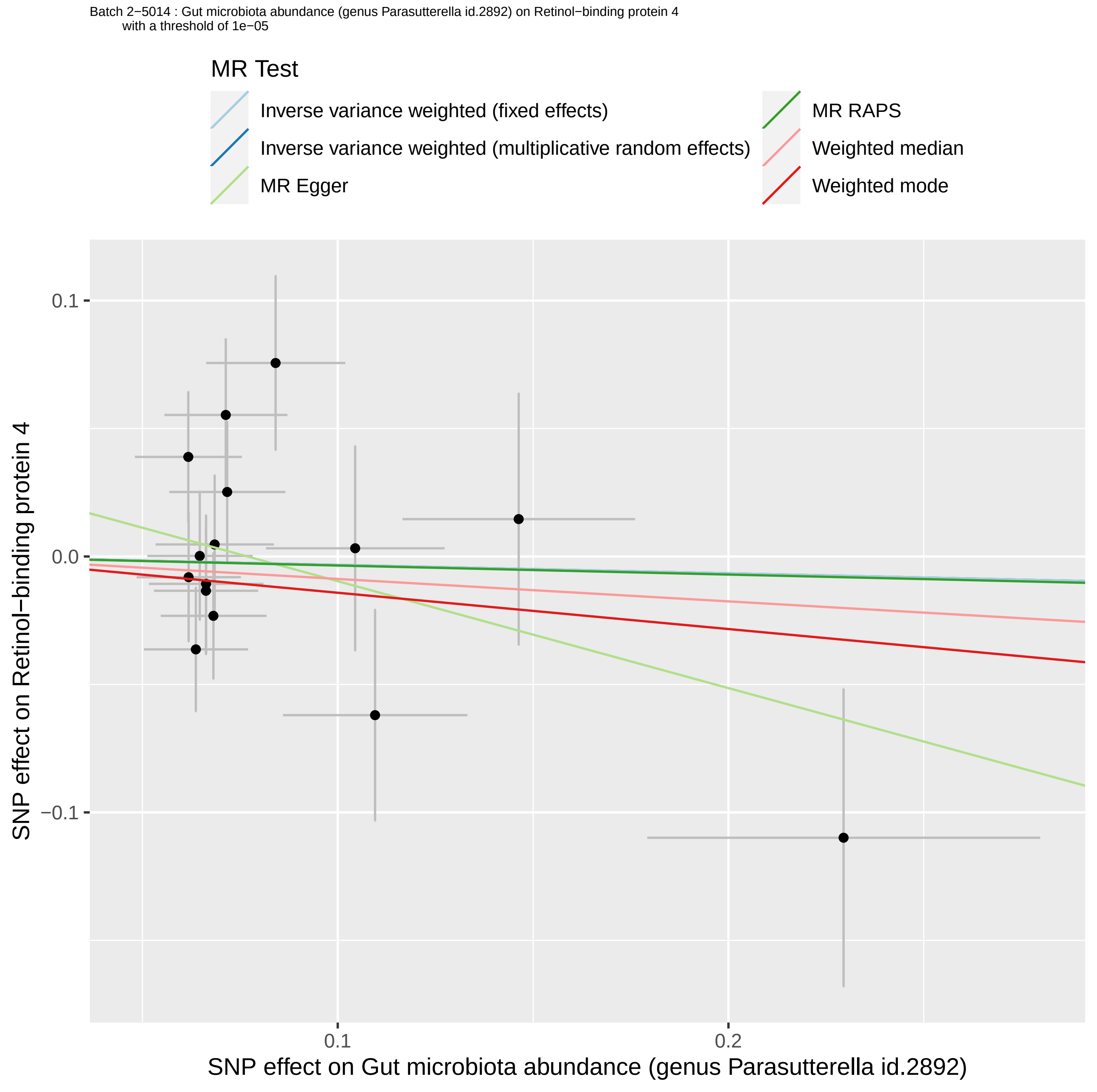

Batch 2−5014 : Gut microbiota abundance (genus Parasutterella id.2892) on Retinol−binding protein 4
with a threshold of 1e−05
MR Test
Inverse variance weighted (fixed effects)
Inverse variance weighted (multiplicative random effects)
MR Egger
MR RAPS
Weighted median
Weighted mode
0.1
0.0
−0.1
0.1
0.2
SNP effect on Retinol−binding protein 4
SNP effect on Gut microbiota abundance (genus Parasutterella id.2892)

Batch 2−5015 : Gut microbiota abundance (genus Catenibacterium id.2153) on Retinoid−binding protein 7
with a threshold of 1e−05

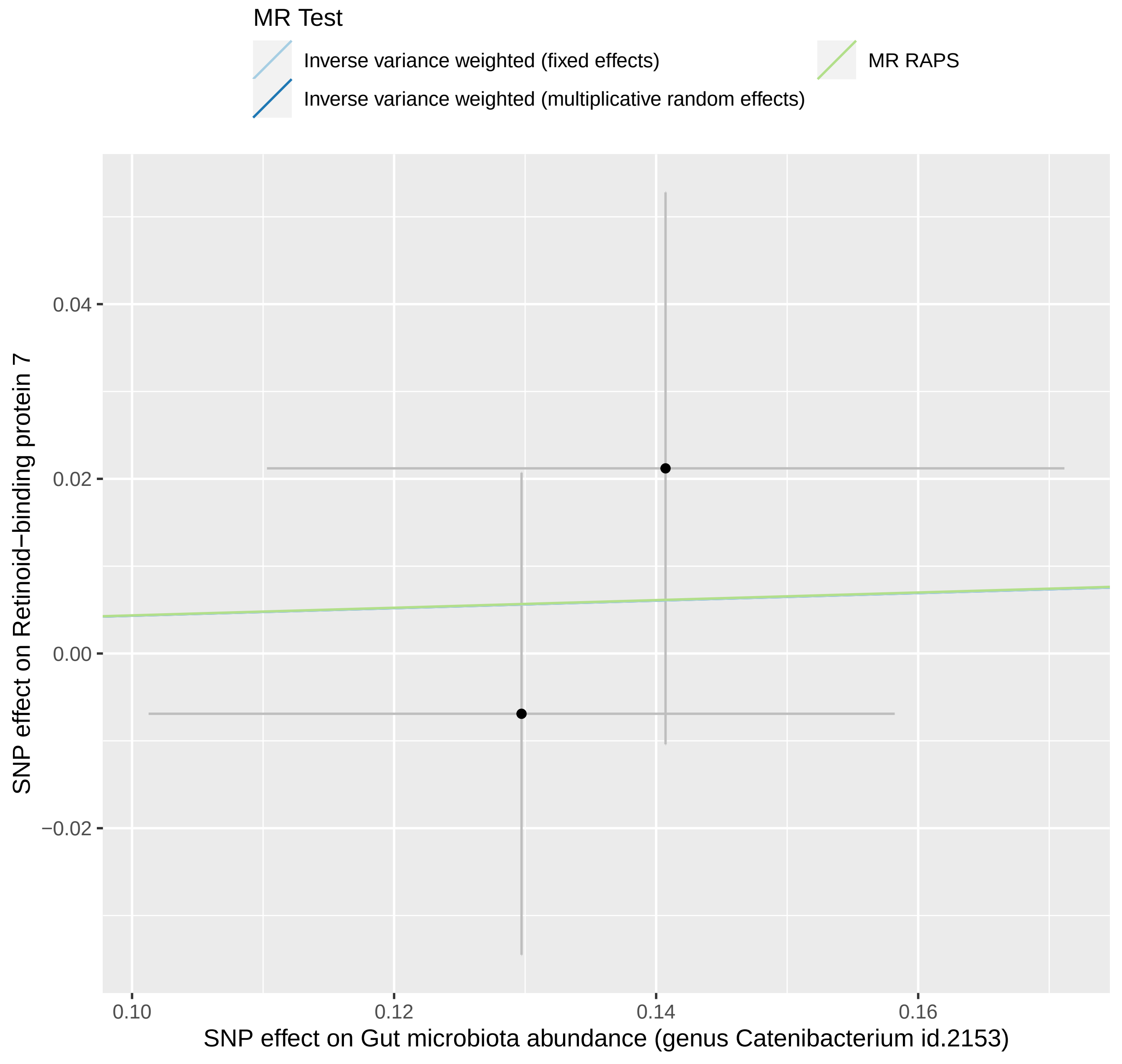

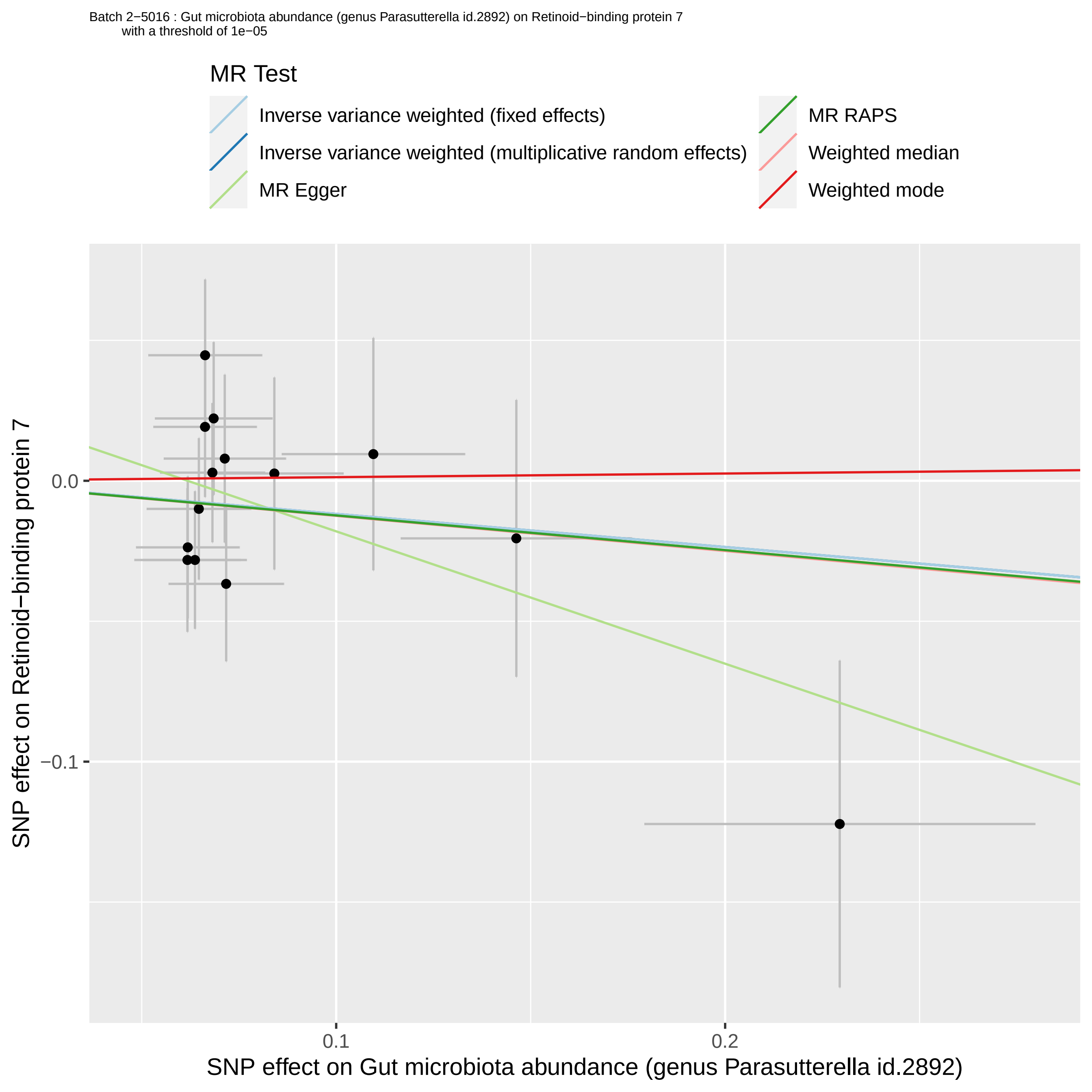
Batch 2−5016 : Gut microbiota abundance (genus Parasutterella id.2892) on Retinoid−binding protein 7
with a threshold of 1e−05
MR Test
Inverse variance weighted (fixed effects)
Inverse variance weighted (multiplicative random effects)
MR Egger
MR RAPS
Weighted median
Weighted mode
0.0
−0.1
0.1
0.2
SNP effect on Retinoid−binding protein 7
SNP effect on Gut microbiota abundance (genus Parasutterella id.2892)

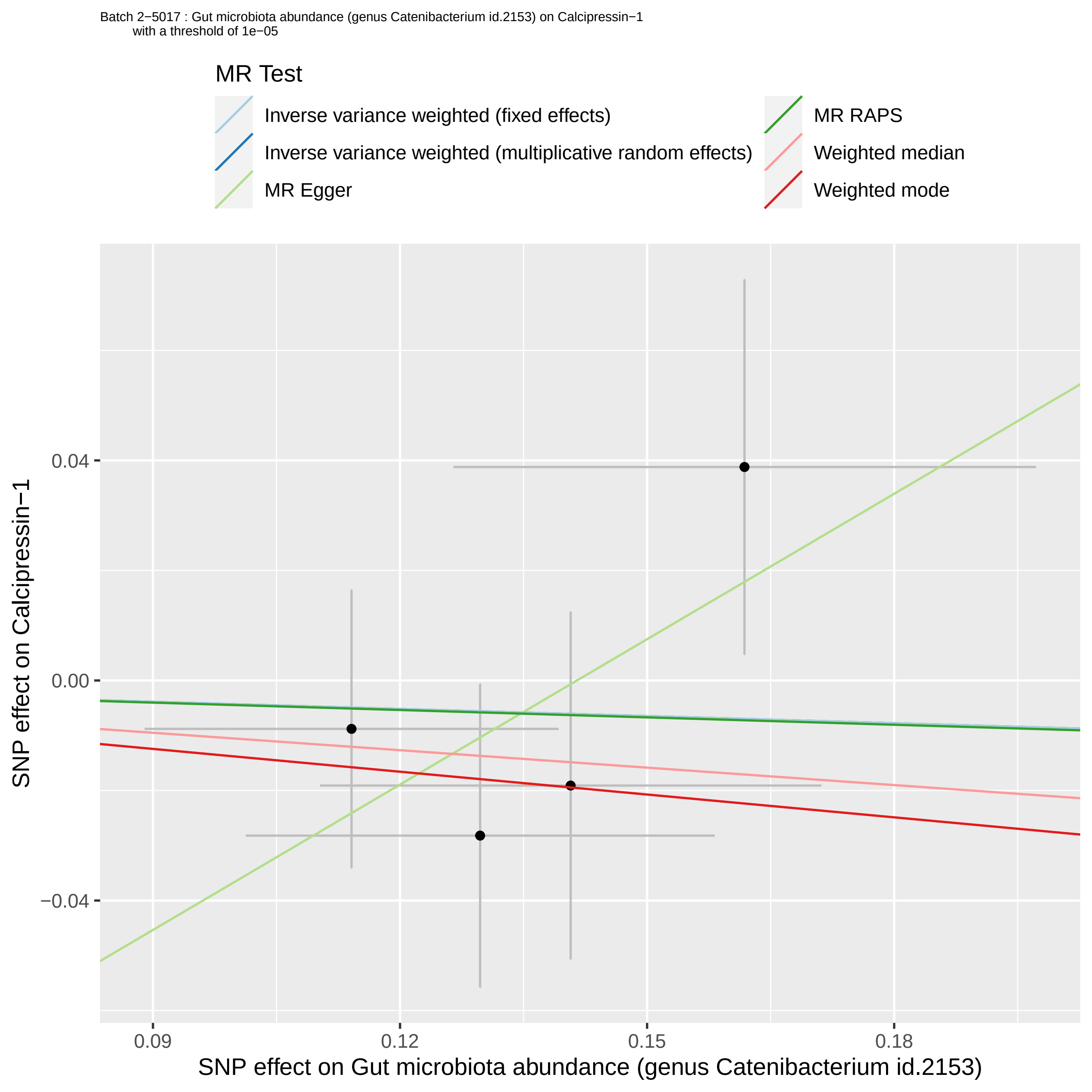

Batch 2−5017 : Gut microbiota abundance (genus Catenibacterium id.2153) on Calcipressin−1
with a threshold of 1e−05
MR Test
Inverse variance weighted (fixed effects)
Inverse variance weighted (multiplicative random effects)
MR Egger
MR RAPS
Weighted median
Weighted mode
0.04
0.00
−0.04
SNP effect on Calcipressin−1
0.09
0.12
0.15
0.18
SNP effect on Gut microbiota abundance (genus Catenibacterium id.2153)

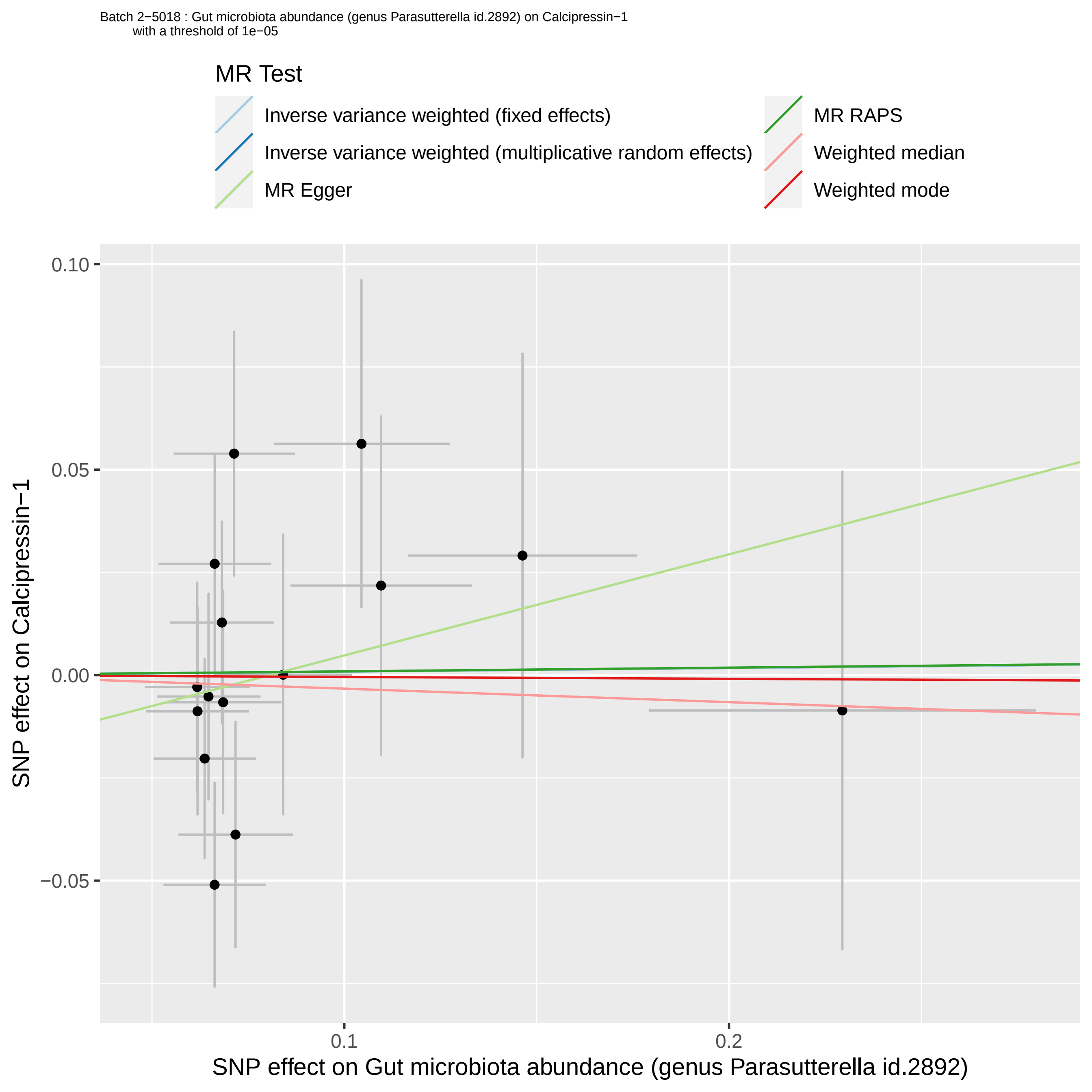

Batch 2−5018 : Gut microbiota abundance (genus Parasutterella id.2892) on Calcipressin−1
with a threshold of 1e−05
MR Test
Inverse variance weighted (fixed effects)
Inverse variance weighted (multiplicative random effects)
MR Egger
MR RAPS
Weighted median
Weighted mode
0.10
0.05
0.00
−0.05
0.1
0.2
SNP effect on Calcipressin−1
SNP effect on Gut microbiota abundance (genus Parasutterella id.2892)

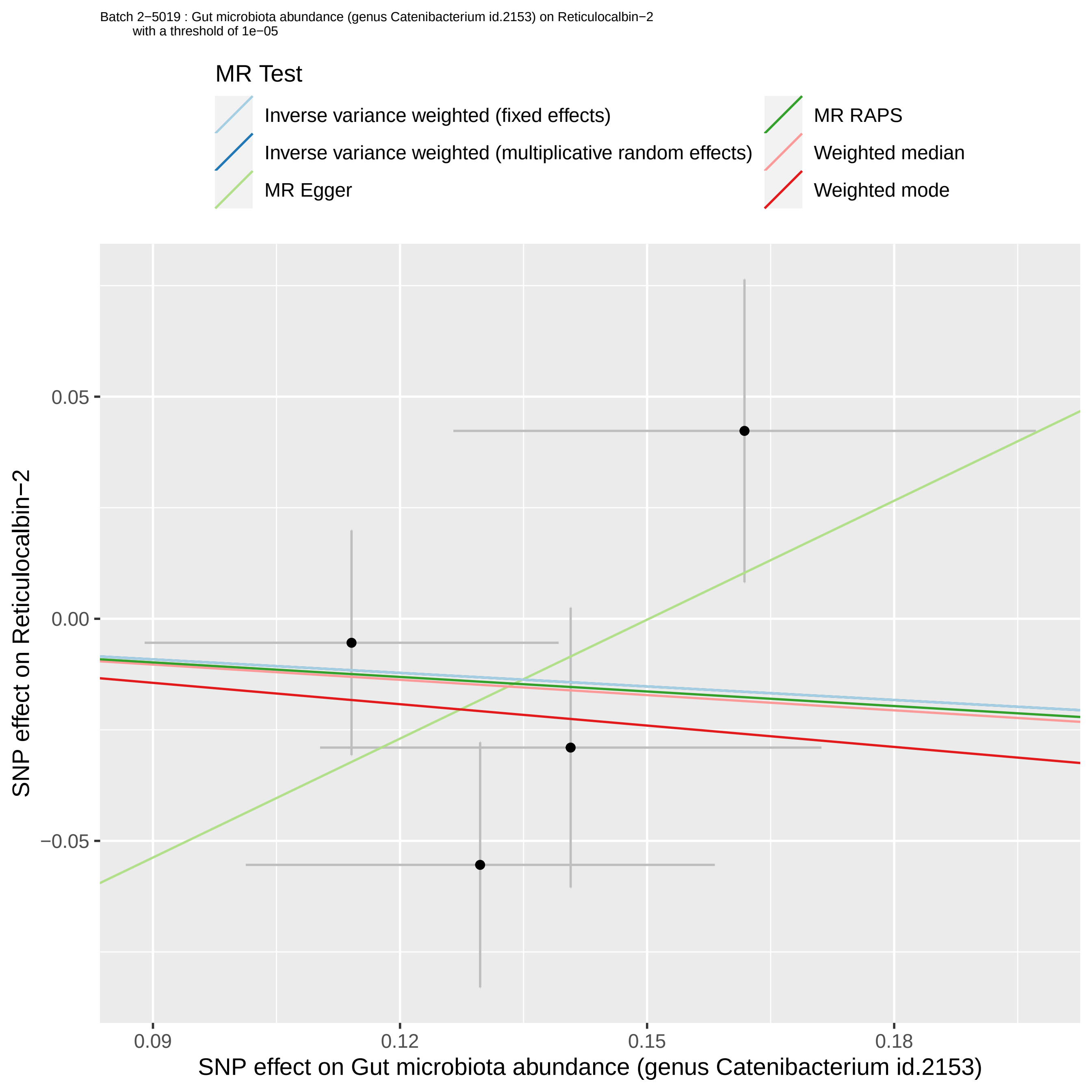
Batch 2−5019 : Gut microbiota abundance (genus Catenibacterium id.2153) on Reticulocalbin−2
with a threshold of 1e−05
MR Test
Inverse variance weighted (fixed effects)
Inverse variance weighted (multiplicative random effects)
MR Egger
MR RAPS
Weighted median
Weighted mode
0.05
0.00
−0.05
0.09
0.12
0.15
0.18
SNP effect on Reticulocalbin−2
SNP effect on Gut microbiota abundance (genus Catenibacterium id.2153)

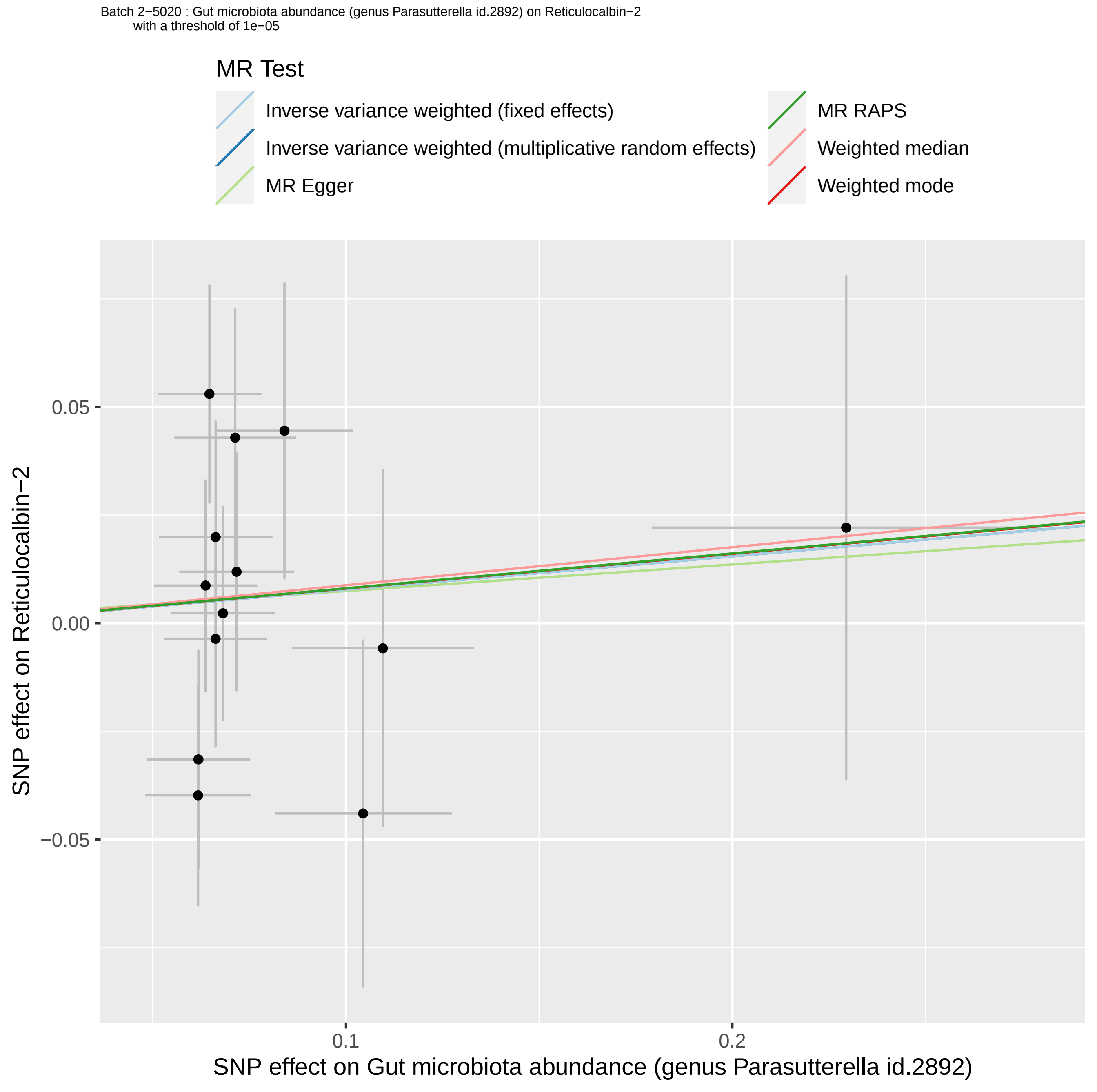
Batch 2−5020 : Gut microbiota abundance (genus Parasutterella id.2892) on Reticulocalbin−2
with a threshold of 1e−05
MR Test
Inverse variance weighted (fixed effects)
Inverse variance weighted (multiplicative random effects)
MR Egger
MR RAPS
Weighted median
Weighted mode
SNP effect on Reticulocalbin−2
0.05
0.00
−0.05
0.1
0.2
SNP effect on Gut microbiota abundance (genus Parasutterella id.2892)

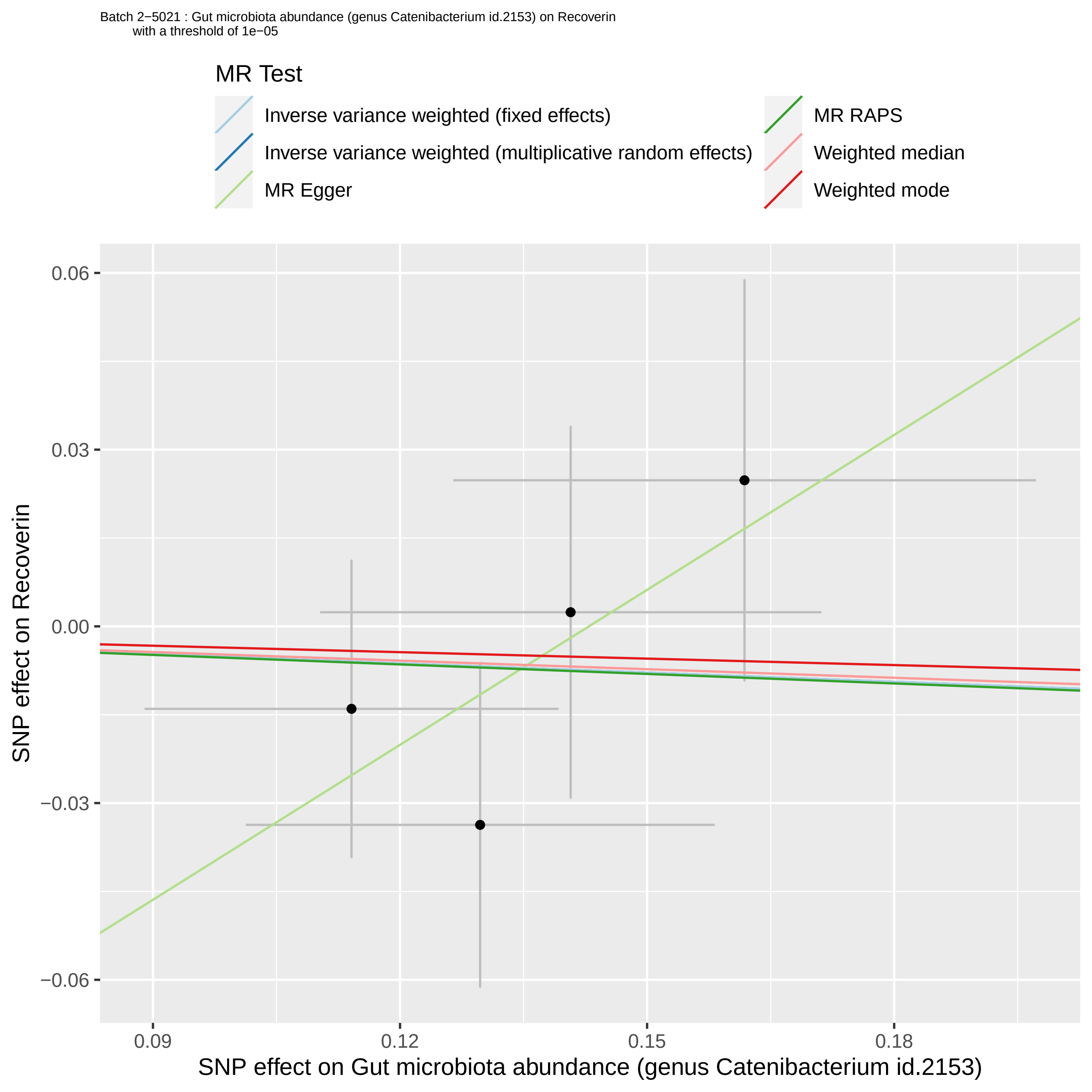
Batch 2−5021 : Gut microbiota abundance (genus Catenibacterium id.2153) on Recoverin
with a threshold of 1e−05
MR Test
Inverse variance weighted (fixed effects)
Inverse variance weighted (multiplicative random effects)
MR Egger
MR RAPS
Weighted median
Weighted mode
0.06
0.03
0.00
−0.03
−0.06
SNP effect on Recoverin
0.09
0.12
0.15
0.18
SNP effect on Gut microbiota abundance (genus Catenibacterium id.2153)

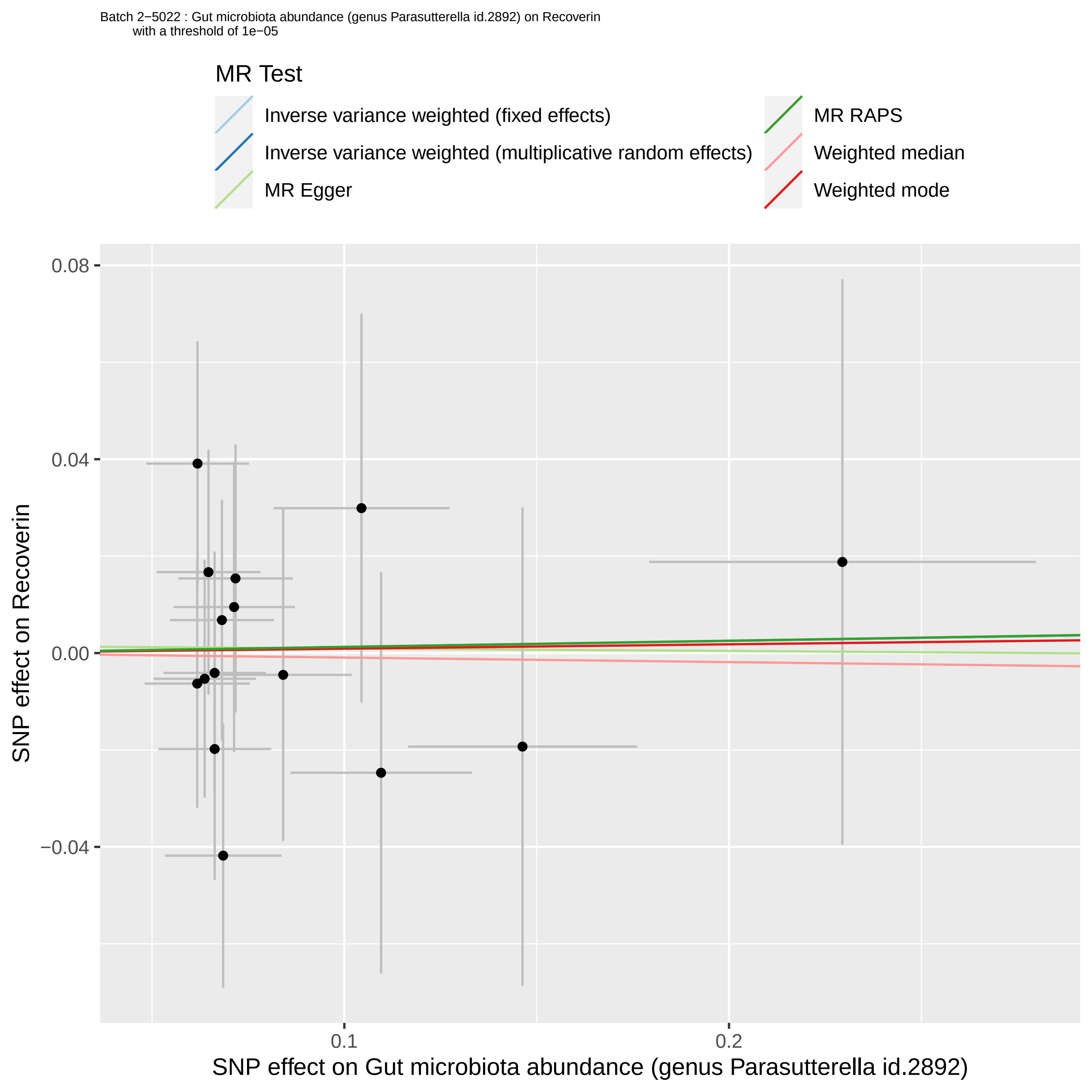

Batch 2−5022 : Gut microbiota abundance (genus Parasutterella id.2892) on Recoverin
with a threshold of 1e−05
MR Test
Inverse variance weighted (fixed effects)
Inverse variance weighted (multiplicative random effects)
MR Egger
MR RAPS
Weighted median
Weighted mode
0.08
0.04
0.00
−0.04
0.1
0.2
SNP effect on Recoverin
SNP effect on Gut microbiota abundance (genus Parasutterella id.2892)

Batch 2−5023 : Gut microbiota abundance (genus Catenibacterium id.2153) on Retinol dehydrogenase 16
with a threshold of 1e−05

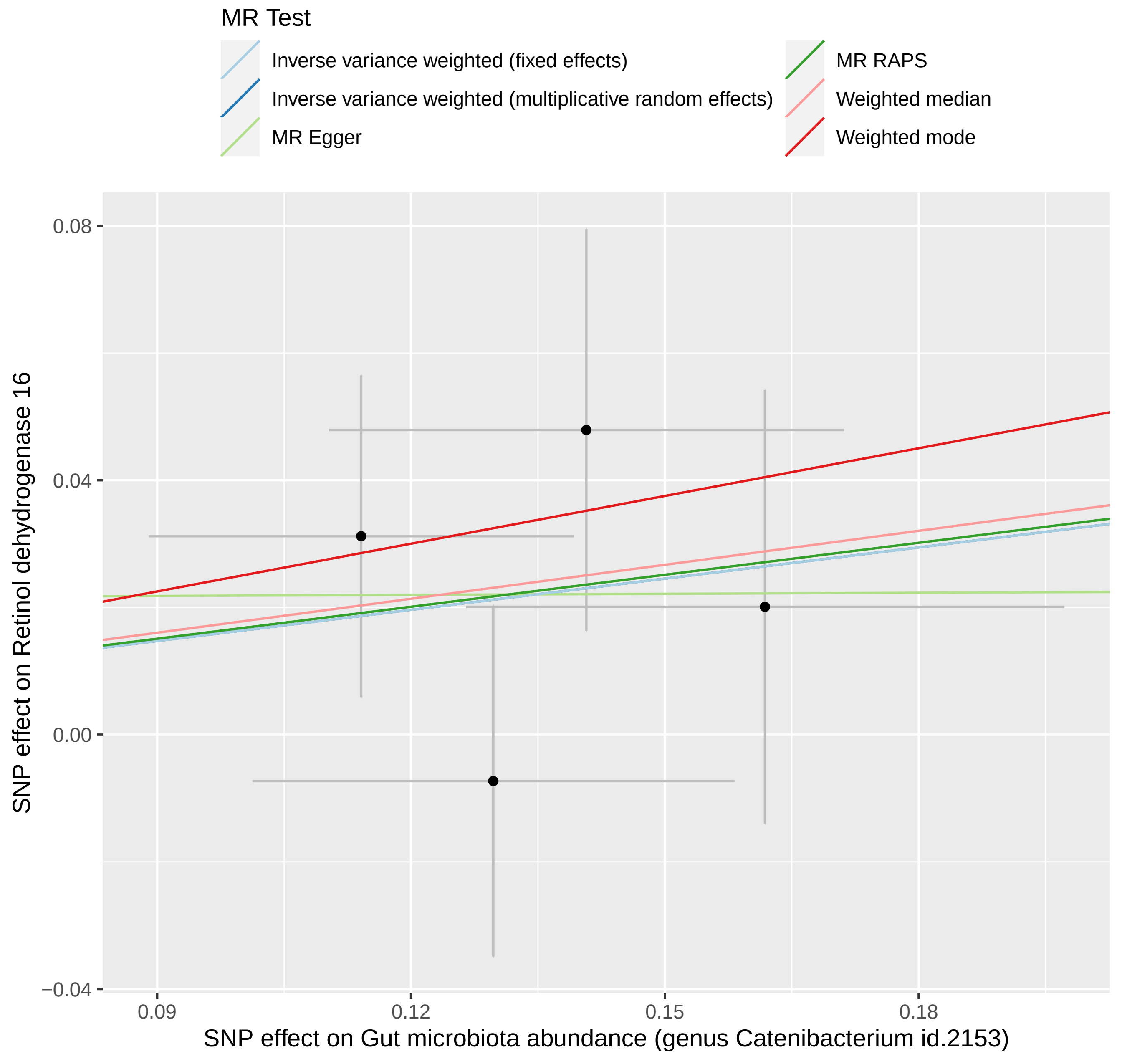

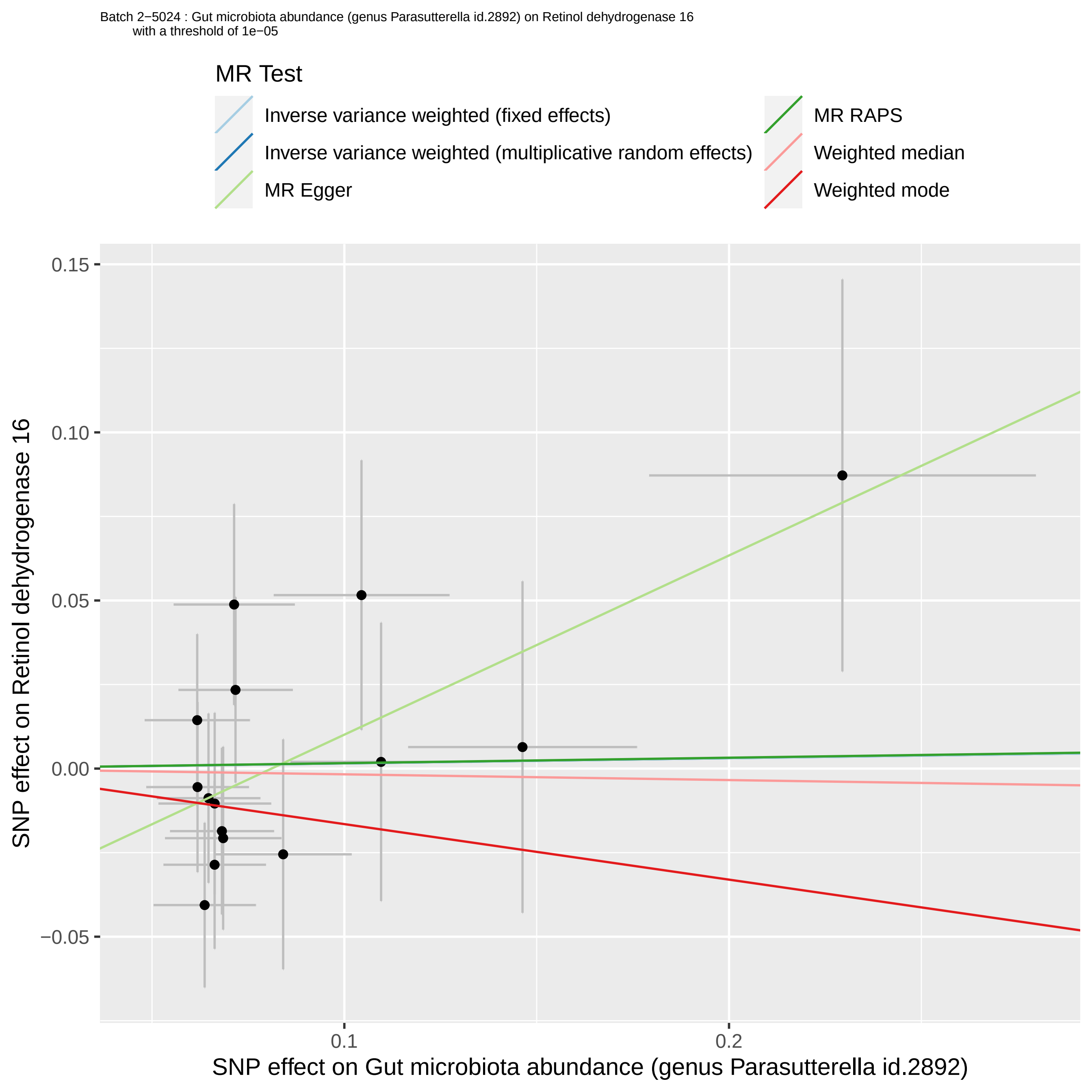

Batch 2−5024 : Gut microbiota abundance (genus Parasutterella id.2892) on Retinol dehydrogenase 16
with a threshold of 1e−05
MR Test
Inverse variance weighted (fixed effects)
Inverse variance weighted (multiplicative random effects)
MR Egger
MR RAPS
Weighted median
Weighted mode
0.15
0.10
0.05
0.00
−0.05
0.1
0.2
SNP effect on Retinol dehydrogenase 16
SNP effect on Gut microbiota abundance (genus Parasutterella id.2892)

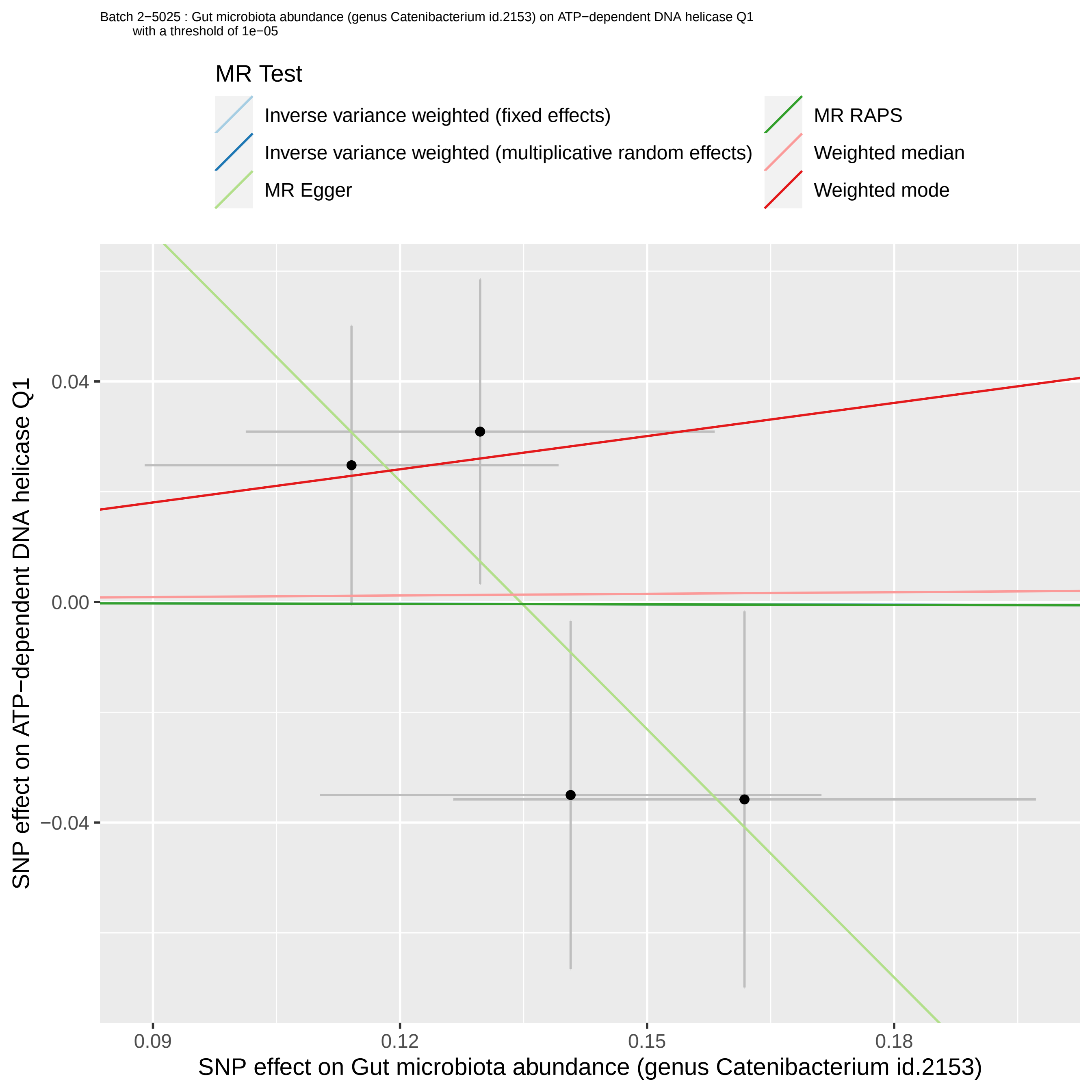
Batch 2−5025 : Gut microbiota abundance (genus Catenibacterium id.2153) on ATP−dependent DNA helicase Q1
with a threshold of 1e−05
MR Test
Inverse variance weighted (fixed effects)
Inverse variance weighted (multiplicative random effects)
MR Egger
MR RAPS
Weighted median
Weighted mode
0.04
0.00
−0.04
0.09
0.12
0.15
0.18
SNP effect on ATP−dependent DNA helicase Q1
SNP effect on Gut microbiota abundance (genus Catenibacterium id.2153)

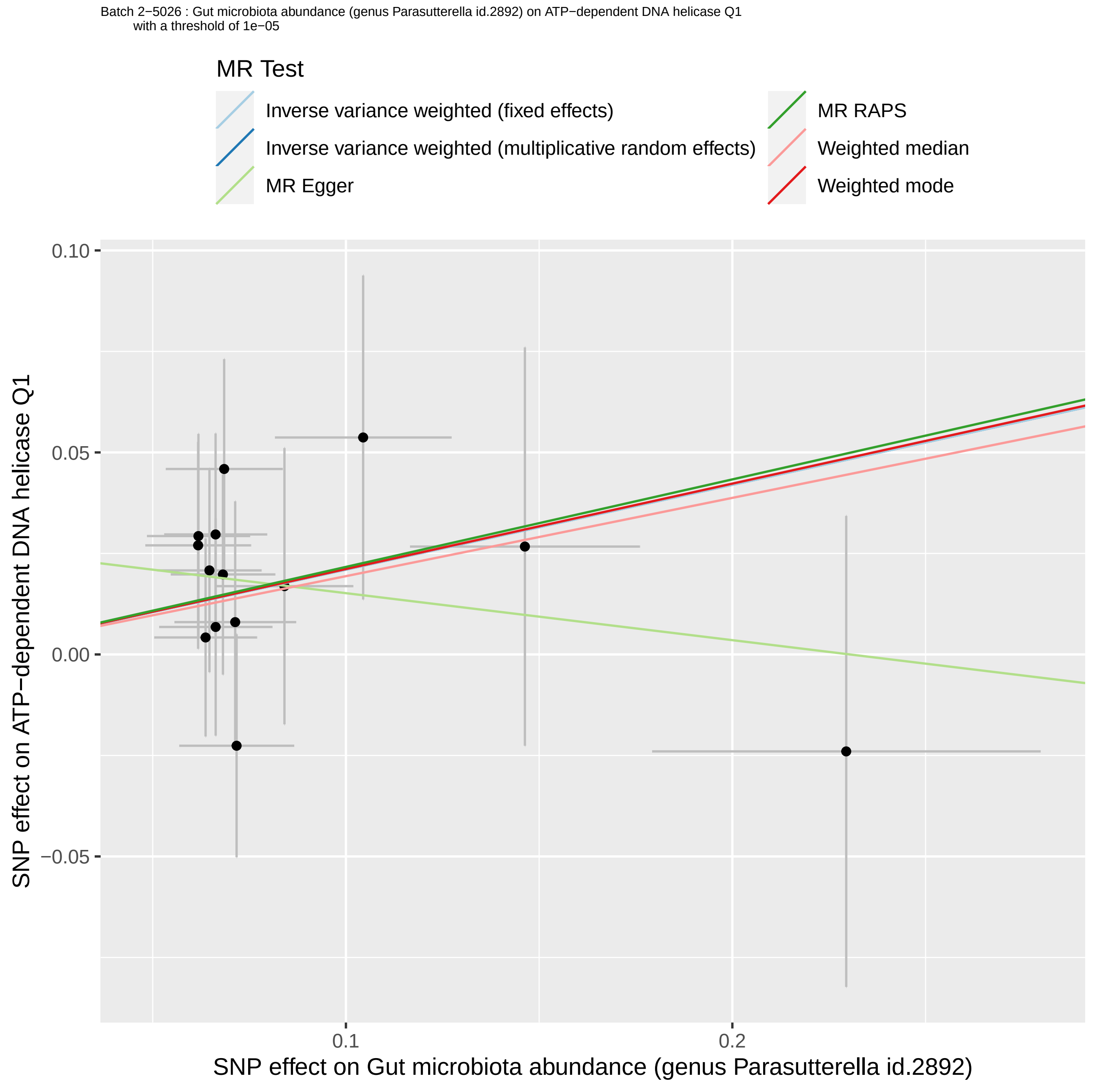

Batch 2−5026 : Gut microbiota abundance (genus Parasutterella id.2892) on ATP−dependent DNA helicase Q1
with a threshold of 1e−05
MR Test
Inverse variance weighted (fixed effects)
Inverse variance weighted (multiplicative random effects)
MR Egger
MR RAPS
Weighted median
Weighted mode
0.10
0.05
0.00
−0.05
0.1
0.2
SNP effect on ATP−dependent DNA helicase Q1
SNP effect on Gut microbiota abundance (genus Parasutterella id.2892)

Batch 2−5027 : Gut microbiota abundance (genus Catenibacterium id.2153) on Regenerating islet−derived protein 3−alpha
with a threshold of 1e−05

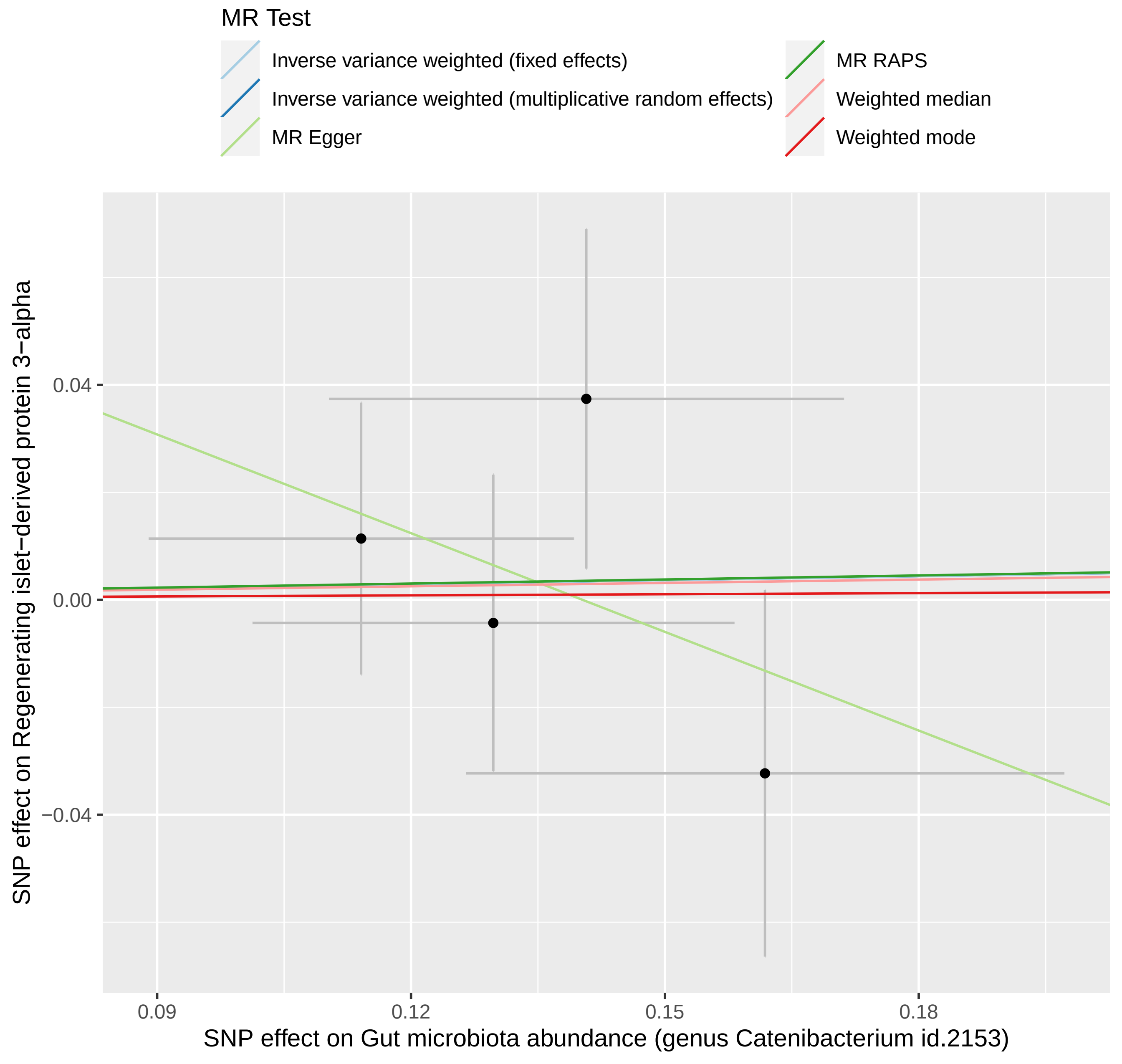

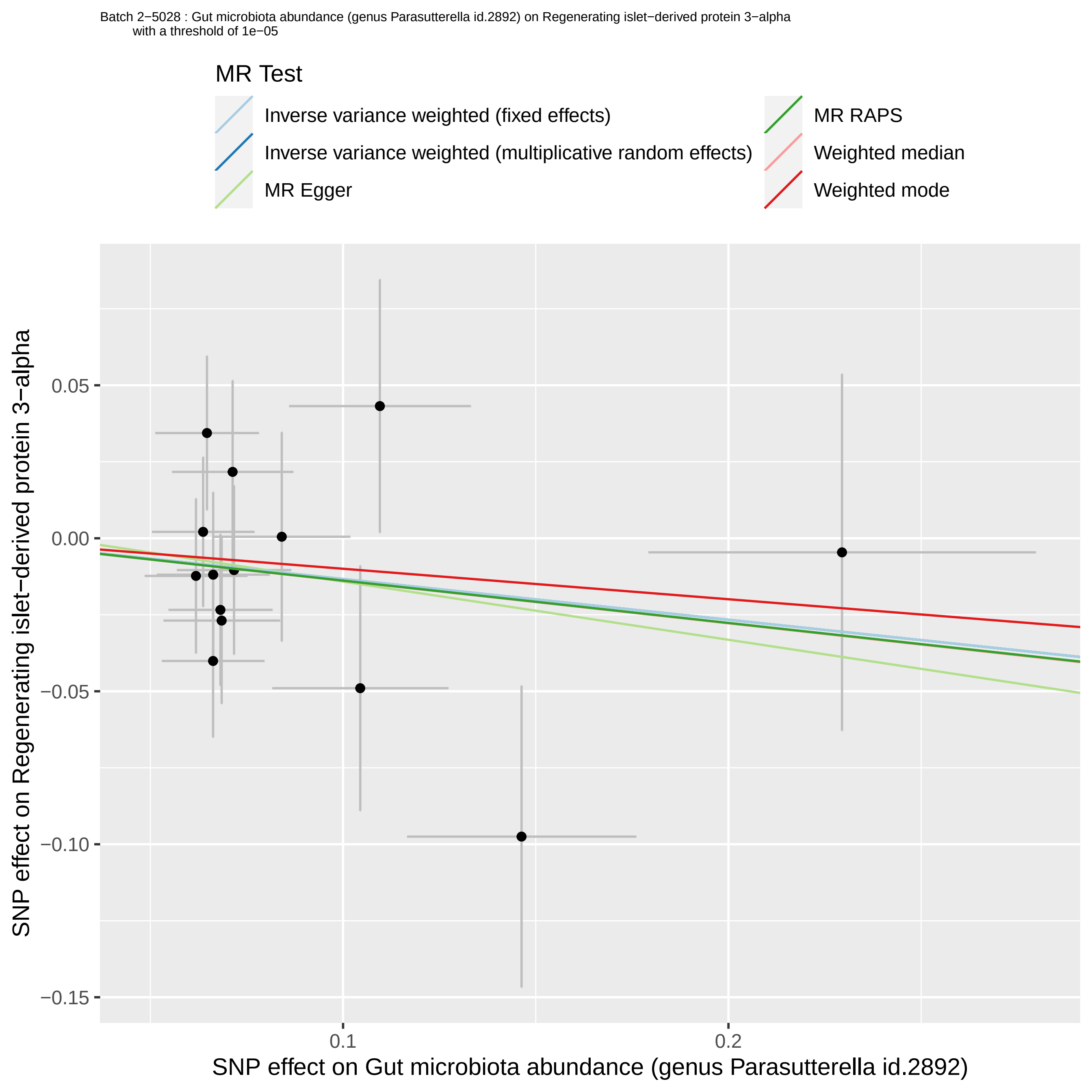

Batch 2−5028 : Gut microbiota abundance (genus Parasutterella id.2892) on Regenerating islet−derived protein 3−alpha
with a threshold of 1e−05
MR Test
Inverse variance weighted (fixed effects)
Inverse variance weighted (multiplicative random effects)
MR Egger
MR RAPS
Weighted median
Weighted mode
0.05
0.00
−0.05
−0.10
−0.15
0.1
0.2
SNP effect on Regenerating islet−derived protein 3−alpha
SNP effect on Gut microbiota abundance (genus Parasutterella id.2892)

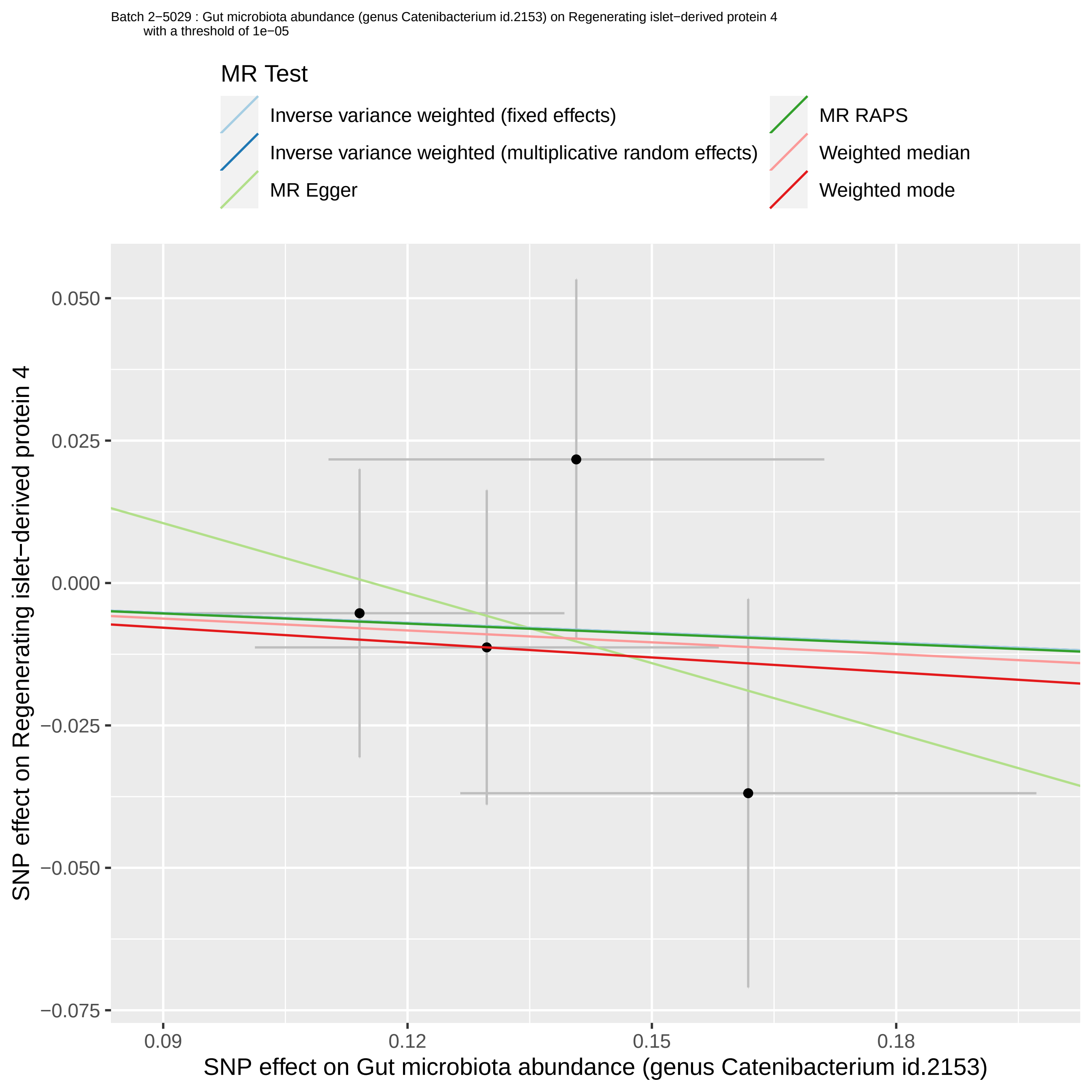
Batch 2−5029 : Gut microbiota abundance (genus Catenibacterium id.2153) on Regenerating islet−derived protein 4
with a threshold of 1e−05
MR Test
Inverse variance weighted (fixed effects)
Inverse variance weighted (multiplicative random effects)
MR Egger
MR RAPS
Weighted median
Weighted mode
SNP effect on Regenerating islet−derived protein 4
0.050
0.025
0.000
−0.025
−0.050
−0.075
0.09
0.12
0.15
0.18
SNP effect on Gut microbiota abundance (genus Catenibacterium id.2153)

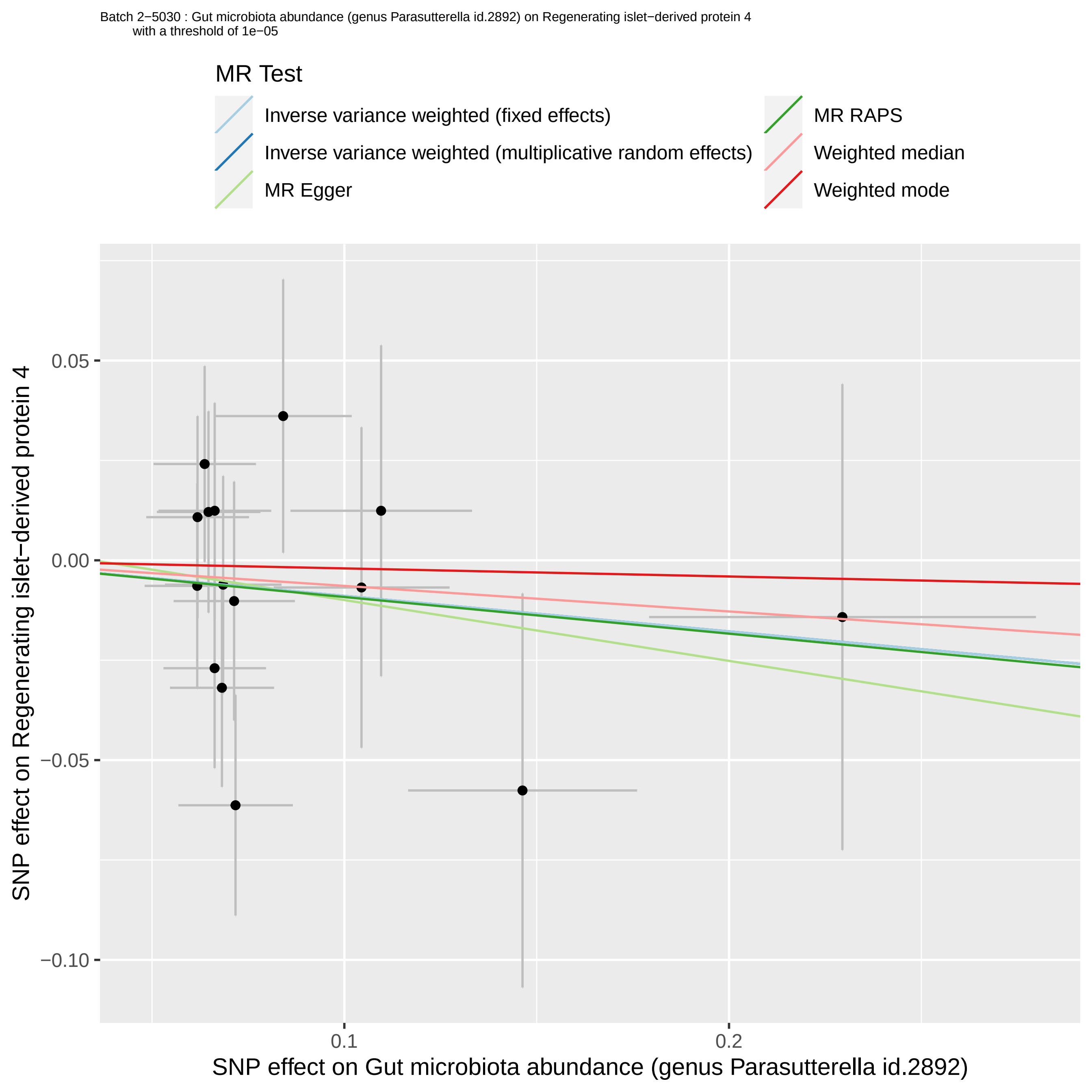
Batch 2−5030 : Gut microbiota abundance (genus Parasutterella id.2892) on Regenerating islet−derived protein 4
with a threshold of 1e−05
MR Test
Inverse variance weighted (fixed effects)
Inverse variance weighted (multiplicative random effects)
MR Egger
MR RAPS
Weighted median
Weighted mode
SNP effect on Regenerating islet−derived protein 4
0.05
0.00
−0.05
−0.10
0.1
0.2
SNP effect on Gut microbiota abundance (genus Parasutterella id.2892)

Batch 2−5031 : Gut microbiota abundance (genus Catenibacterium id.2153) on Transcription factor RelB
with a threshold of 1e−05

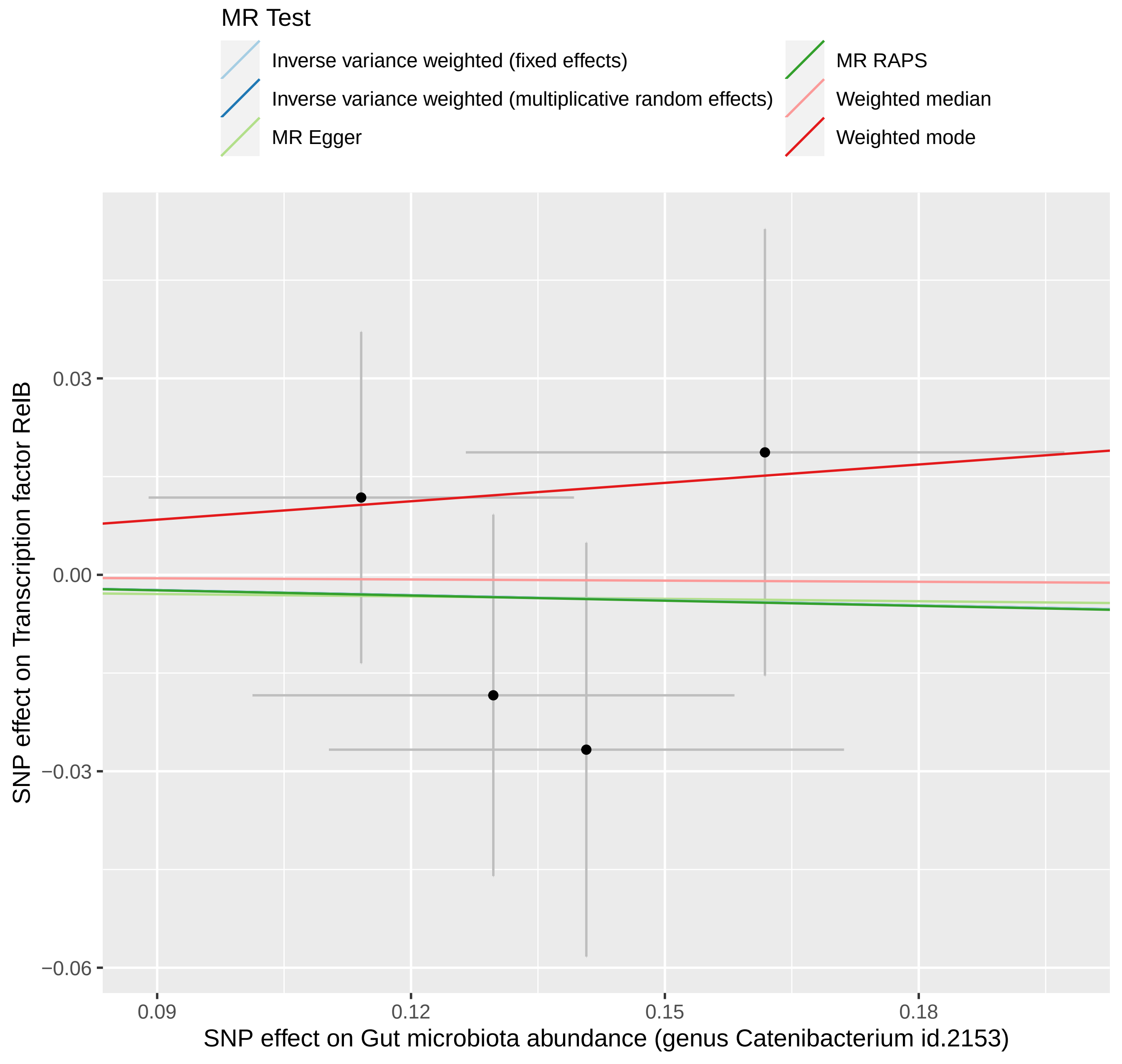

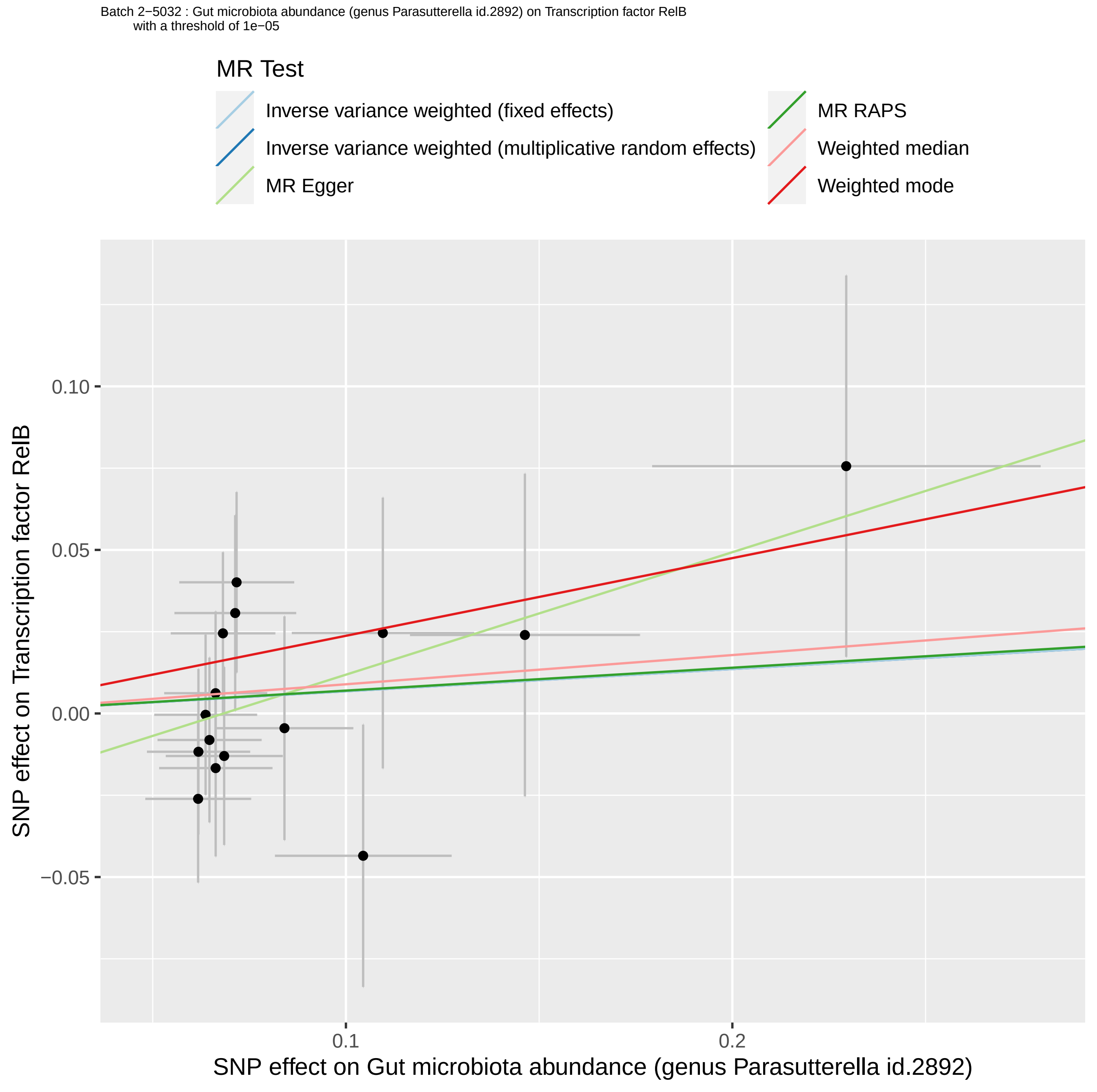

Batch 2−5032 : Gut microbiota abundance (genus Parasutterella id.2892) on Transcription factor RelB
with a threshold of 1e−05
MR Test
Inverse variance weighted (fixed effects)
Inverse variance weighted (multiplicative random effects)
MR Egger
MR RAPS
Weighted median
Weighted mode
0.10
0.05
0.00
−0.05
0.1
0.2
SNP effect on Transcription factor RelB
SNP effect on Gut microbiota abundance (genus Parasutterella id.2892)

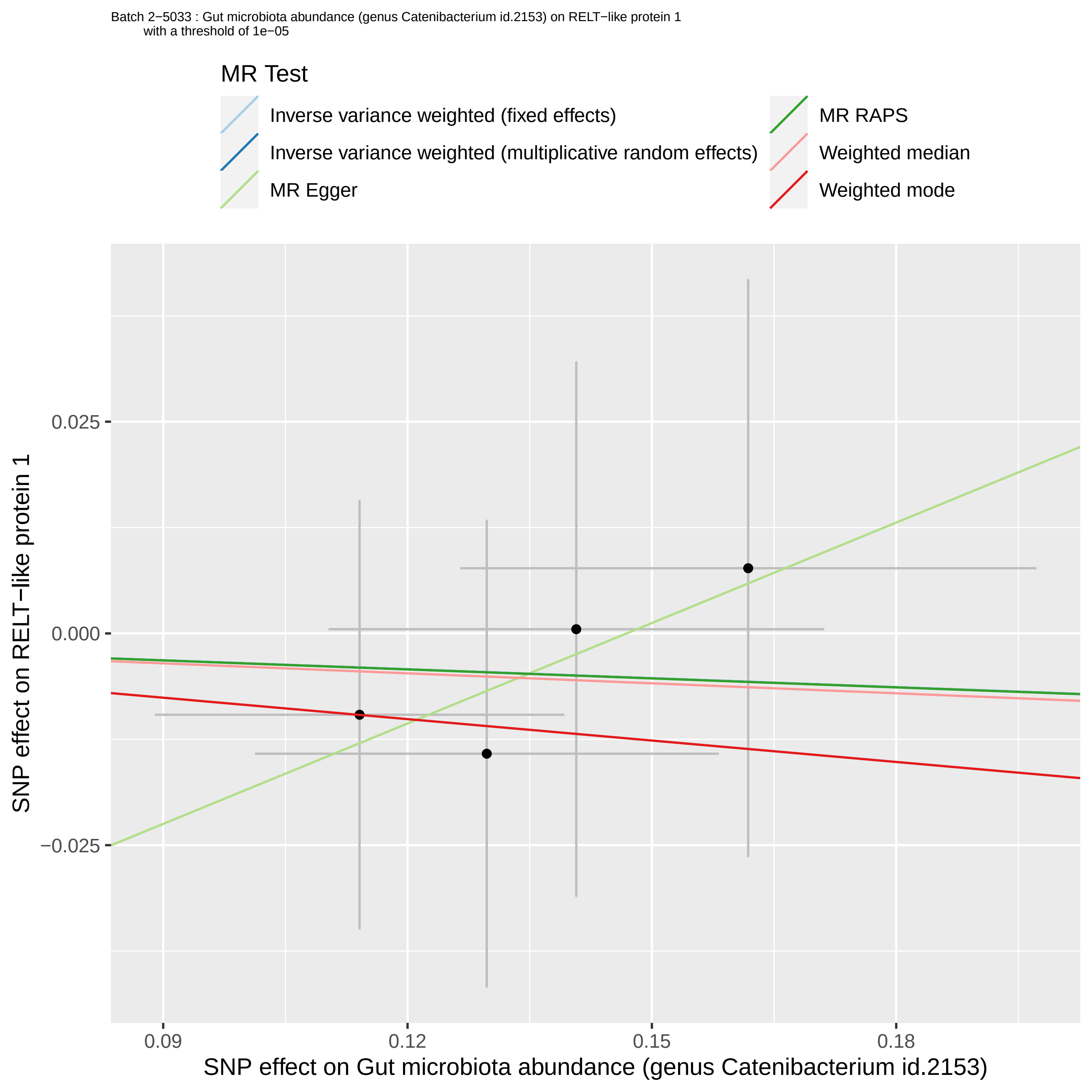
Batch 2−5033 : Gut microbiota abundance (genus Catenibacterium id.2153) on RELT−like protein 1
with a threshold of 1e−05
MR Test
Inverse variance weighted (fixed effects)
Inverse variance weighted (multiplicative random effects)
MR Egger
MR RAPS
Weighted median
Weighted mode
SNP effect on RELT−like protein 1
0.025
0.000
−0.025
0.09
0.12
0.15
0.18
SNP effect on Gut microbiota abundance (genus Catenibacterium id.2153)

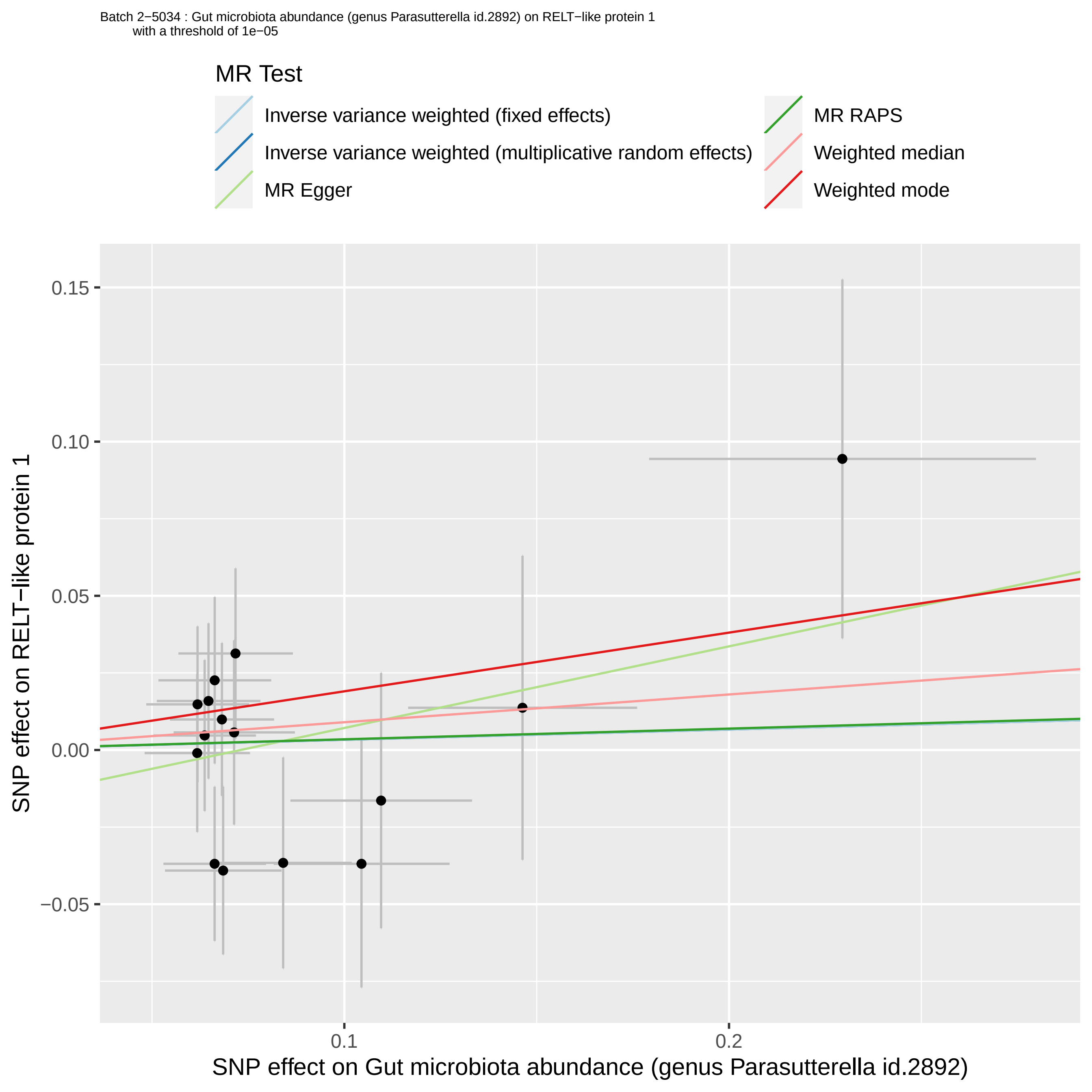

Batch 2−5034 : Gut microbiota abundance (genus Parasutterella id.2892) on RELT−like protein 1
with a threshold of 1e−05
MR Test
Inverse variance weighted (fixed effects)
Inverse variance weighted (multiplicative random effects)
MR Egger
MR RAPS
Weighted median
Weighted mode
0.15
0.10
0.05
0.00
−0.05
0.1
0.2
SNP effect on RELT−like protein 1
SNP effect on Gut microbiota abundance (genus Parasutterella id.2892)

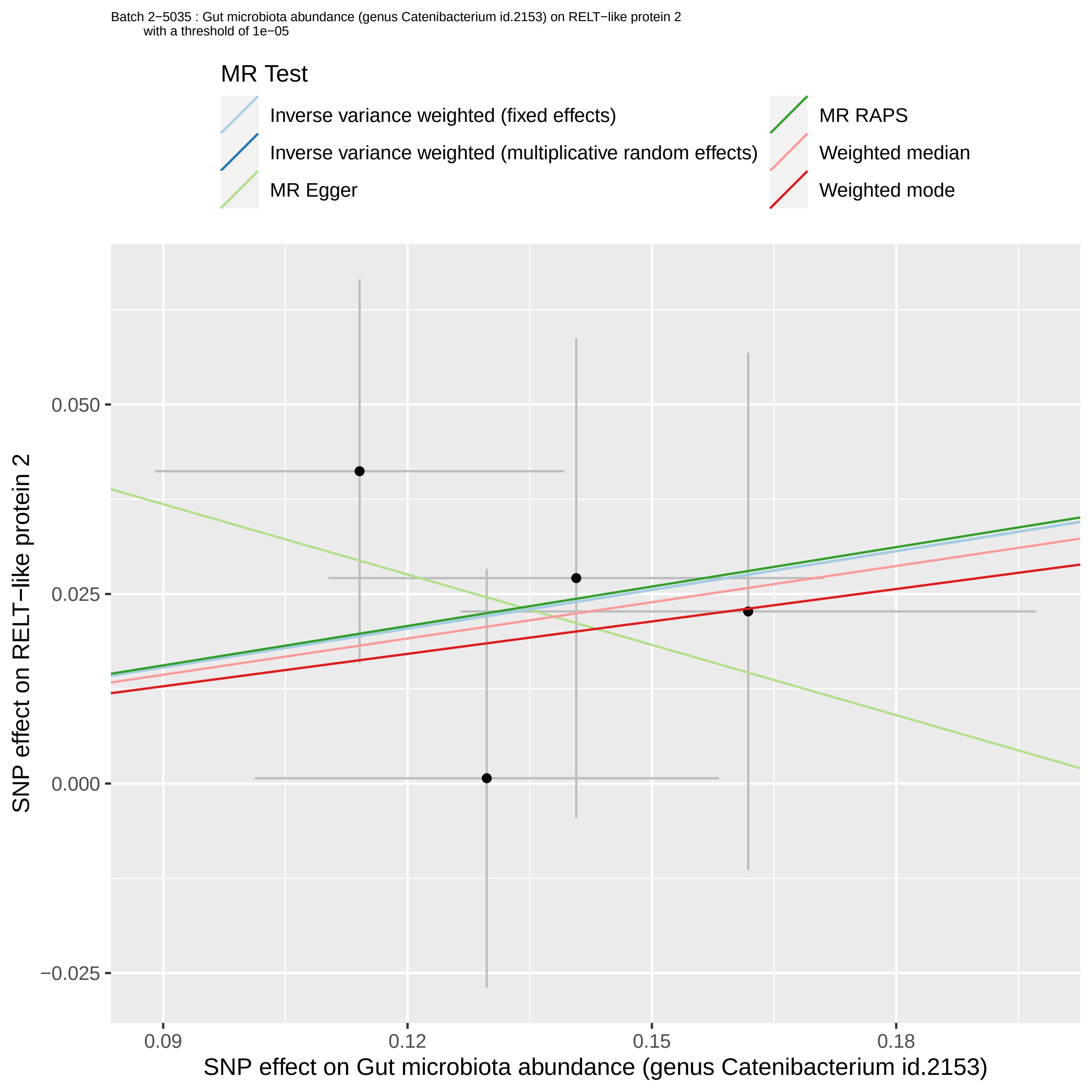
Batch 2−5035 : Gut microbiota abundance (genus Catenibacterium id.2153) on RELT−like protein 2
with a threshold of 1e−05
MR Test
Inverse variance weighted (fixed effects)
Inverse variance weighted (multiplicative random effects)
MR Egger
MR RAPS
Weighted median
Weighted mode
SNP effect on RELT−like protein 2
0.050
0.025
0.000
−0.025
0.09
0.12
0.15
0.18
SNP effect on Gut microbiota abundance (genus Catenibacterium id.2153)

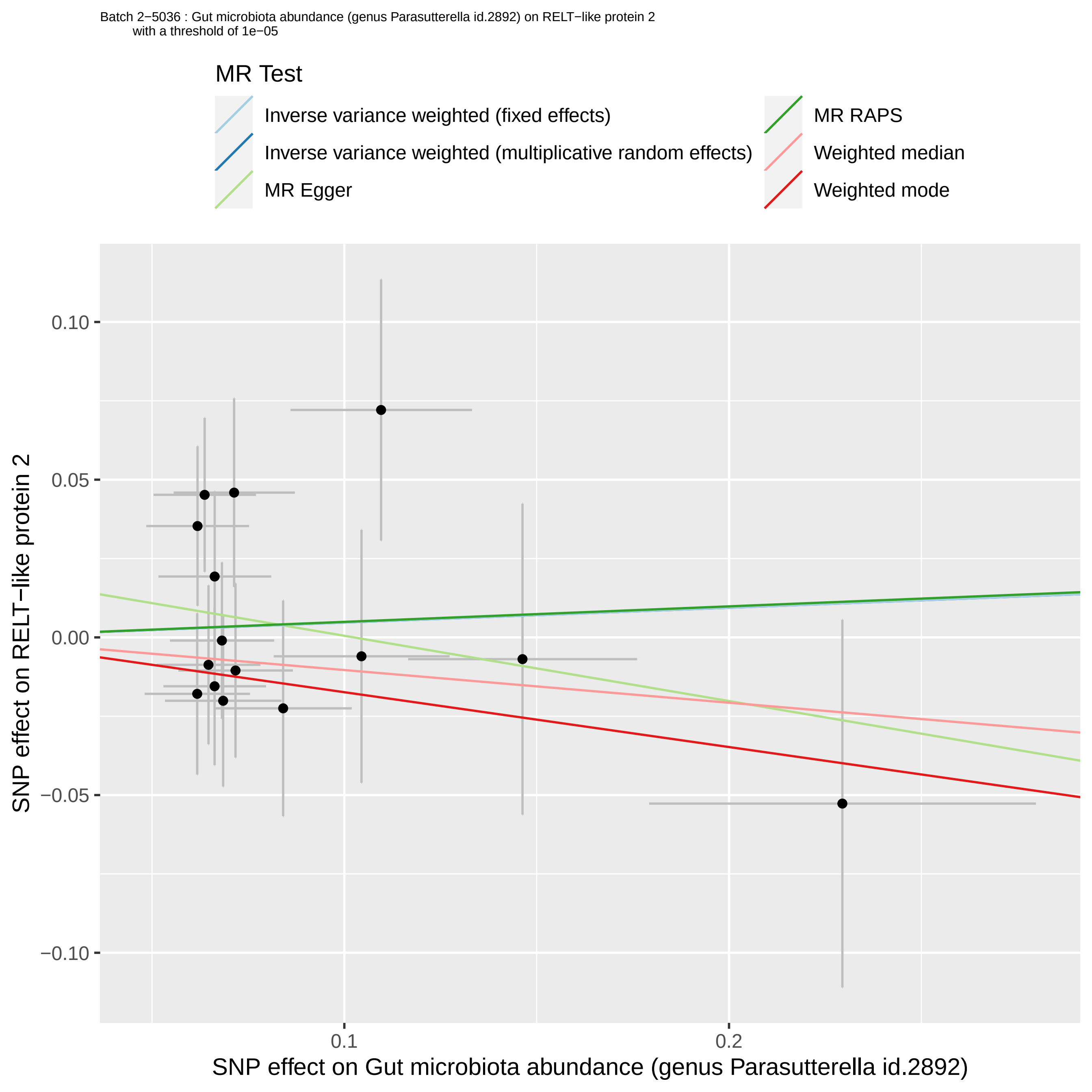

Batch 2−5036 : Gut microbiota abundance (genus Parasutterella id.2892) on RELT−like protein 2
with a threshold of 1e−05
MR Test
Inverse variance weighted (fixed effects)
Inverse variance weighted (multiplicative random effects)
MR Egger
MR RAPS
Weighted median
Weighted mode
0.10
0.05
0.00
−0.05
−0.10
0.1
0.2
SNP effect on RELT−like protein 2
SNP effect on Gut microbiota abundance (genus Parasutterella id.2892)

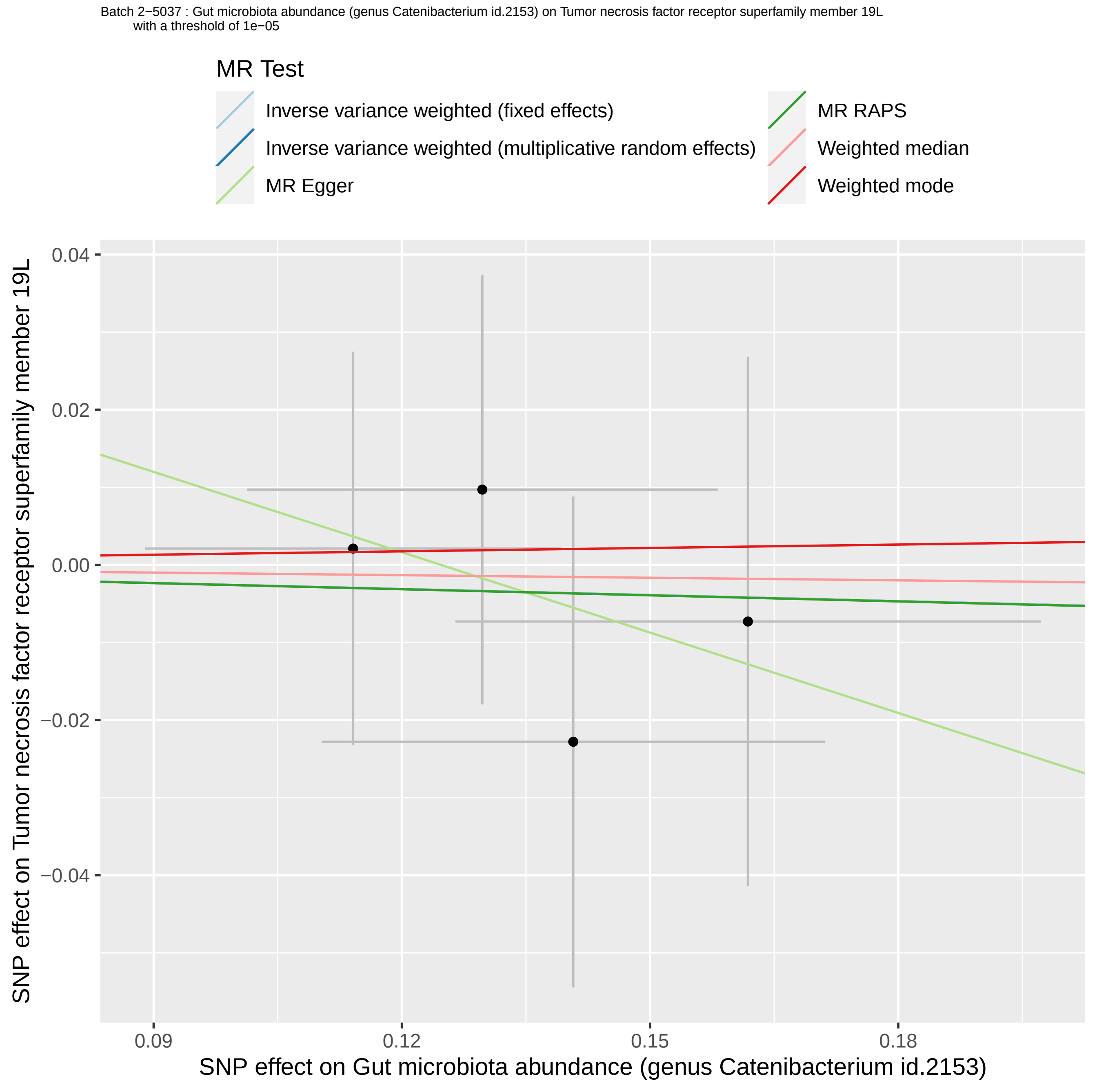
Batch 2−5037 : Gut microbiota abundance (genus Catenibacterium id.2153) on Tumor necrosis factor receptor superfamily member 19L
with a threshold of 1e−05
MR Test
Inverse variance weighted (fixed effects)
Inverse variance weighted (multiplicative random effects)
MR Egger
MR RAPS
Weighted median
Weighted mode
0.04
0.02
0.00
−0.02
−0.04
0.09
0.12
0.15
0.18
SNP effect on Tumor necrosis factor receptor superfamily member 19L
SNP effect on Gut microbiota abundance (genus Catenibacterium id.2153)

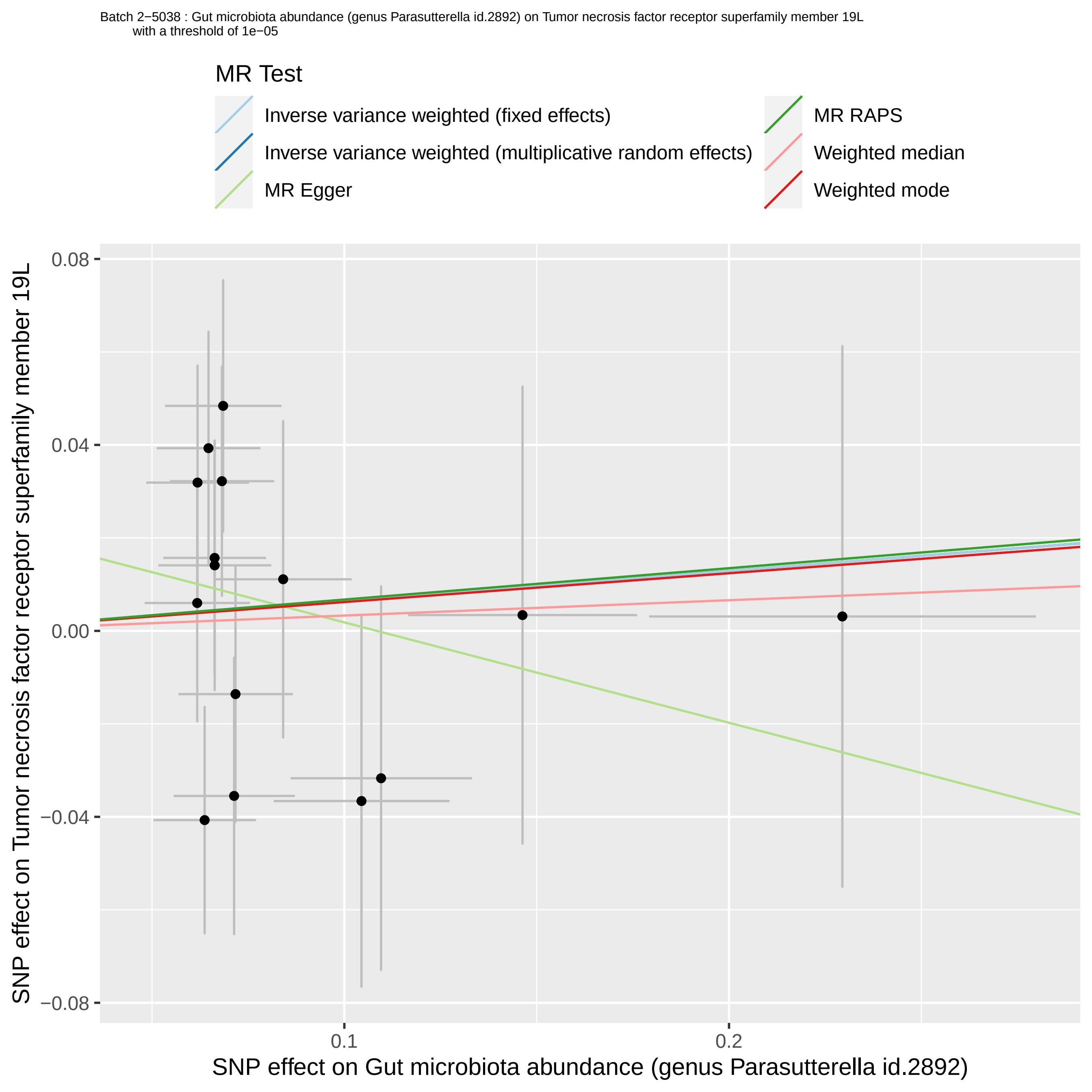
Batch 2−5038 : Gut microbiota abundance (genus Parasutterella id.2892) on Tumor necrosis factor receptor superfamily member 19L
with a threshold of 1e−05
MR Test
Inverse variance weighted (fixed effects)
Inverse variance weighted (multiplicative random effects)
MR Egger
MR RAPS
Weighted median
Weighted mode
0.08
0.04
0.00
−0.04
−0.08
0.1
0.2
SNP effect on Tumor necrosis factor receptor superfamily member 19L
SNP effect on Gut microbiota abundance (genus Parasutterella id.2892)

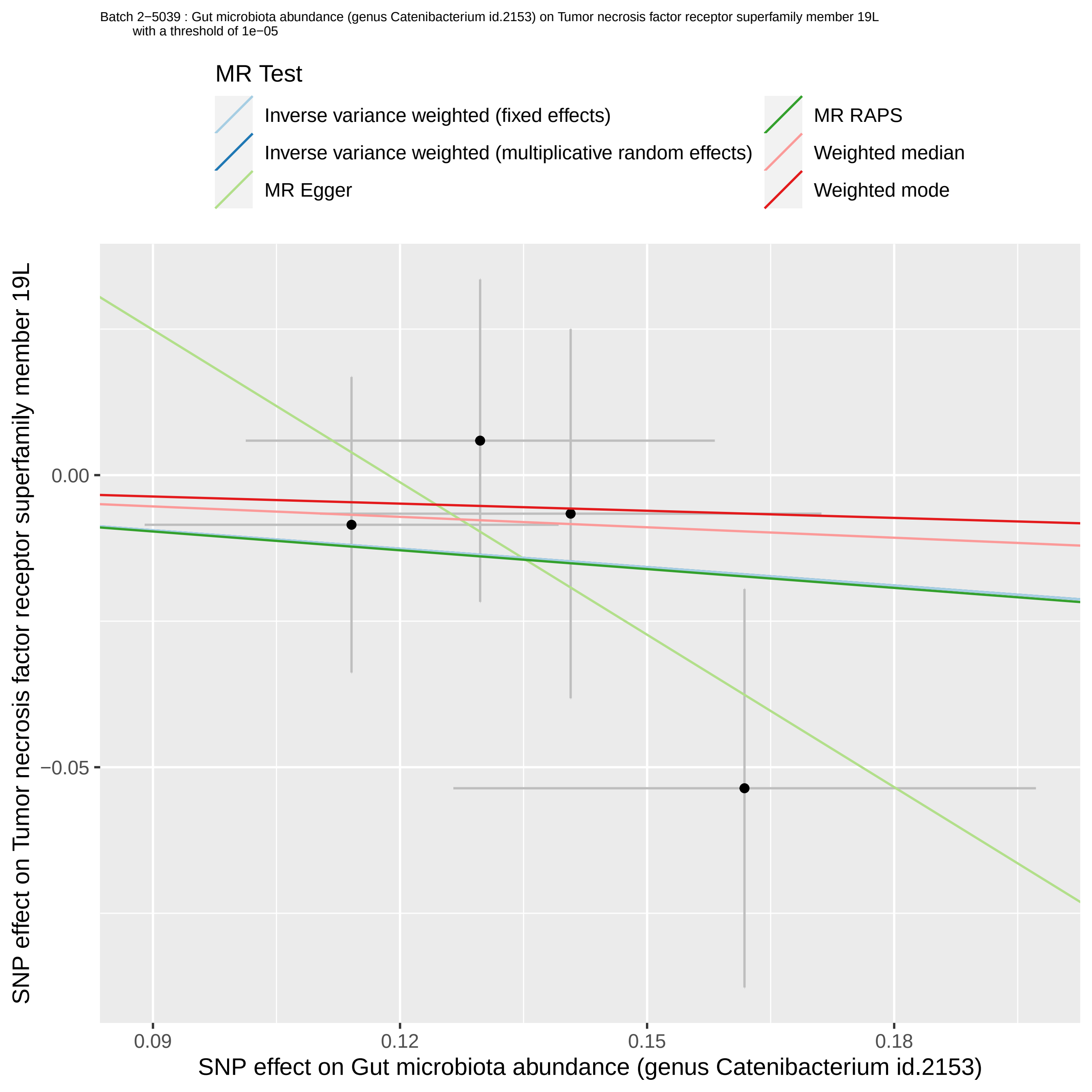

Batch 2−5039 : Gut microbiota abundance (genus Catenibacterium id.2153) on Tumor necrosis factor receptor superfamily member 19L
with a threshold of 1e−05
MR Test
Inverse variance weighted (fixed effects)
Inverse variance weighted (multiplicative random effects)
MR Egger
MR RAPS
Weighted median
Weighted mode
SNP effect on Tumor necrosis factor receptor superfamily member 19L
0.00
−0.05
0.09
0.12
0.15
0.18
SNP effect on Gut microbiota abundance (genus Catenibacterium id.2153)

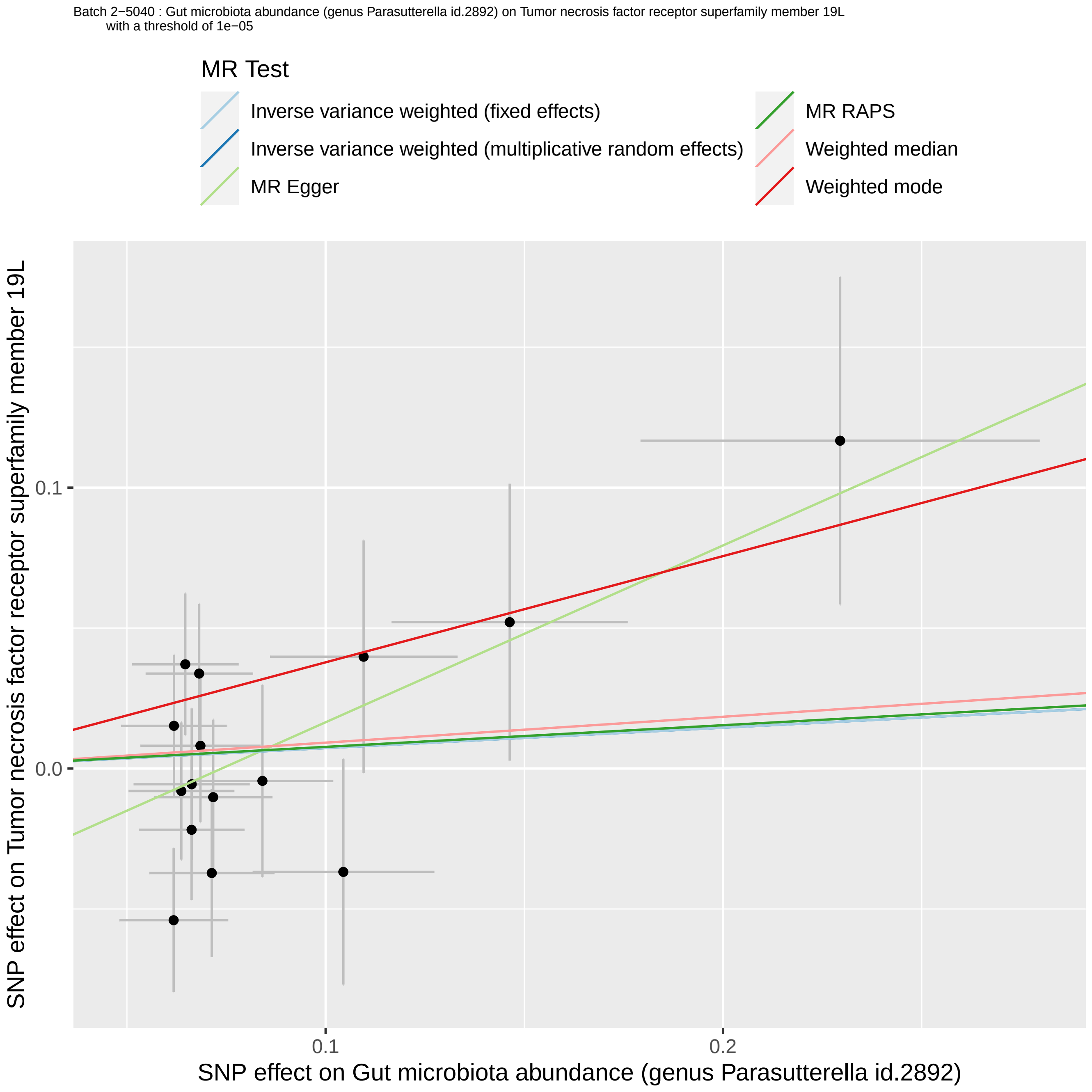
Batch 2−5040 : Gut microbiota abundance (genus Parasutterella id.2892) on Tumor necrosis factor receptor superfamily member 19L
with a threshold of 1e−05
MR Test
Inverse variance weighted (fixed effects)
Inverse variance weighted (multiplicative random effects)
MR Egger
MR RAPS
Weighted median
Weighted mode
0.1
0.0
0.1
0.2
SNP effect on Tumor necrosis factor receptor superfamily member 19L
SNP effect on Gut microbiota abundance (genus Parasutterella id.2892)

Batch 2−5041 : Gut microbiota abundance (genus Catenibacterium id.2153) on Renin
with a threshold of 1e−05

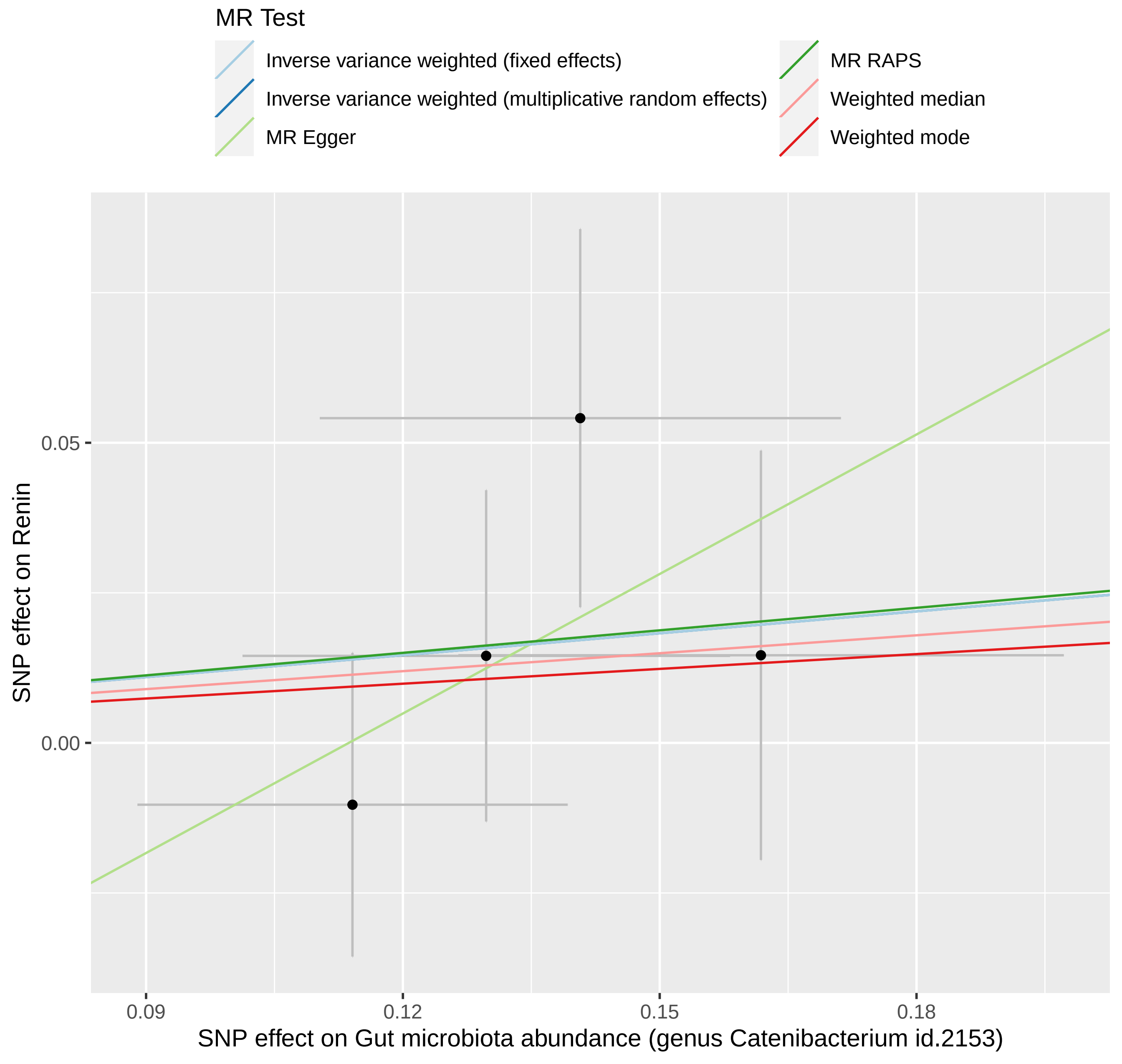

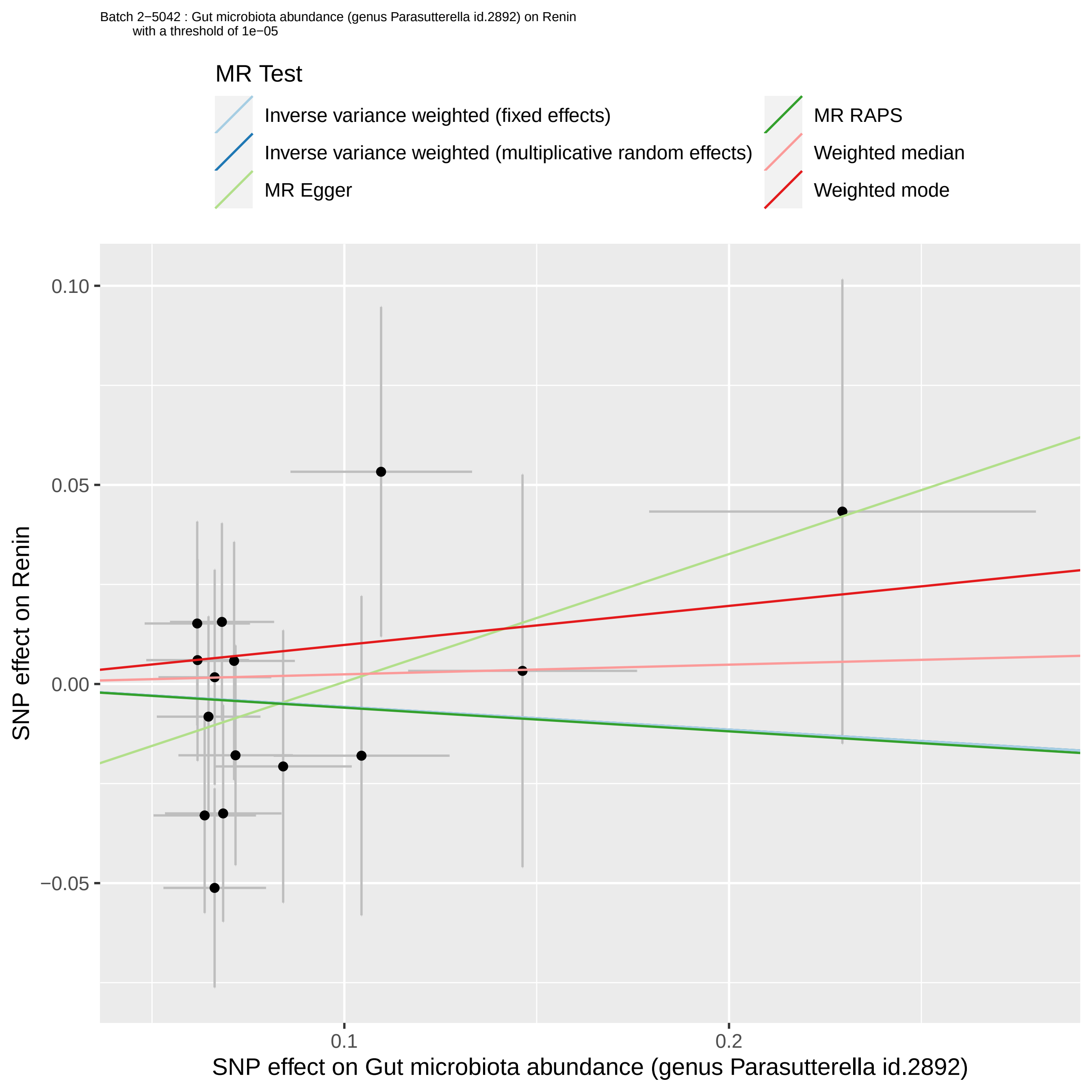
Batch 2−5042 : Gut microbiota abundance (genus Parasutterella id.2892) on Renin
with a threshold of 1e−05
MR Test
Inverse variance weighted (fixed effects)
Inverse variance weighted (multiplicative random effects)
MR Egger
MR RAPS
Weighted median
Weighted mode
0.10
0.05
0.00
−0.05
0.1
0.2
SNP effect on Renin
SNP effect on Gut microbiota abundance (genus Parasutterella id.2892)

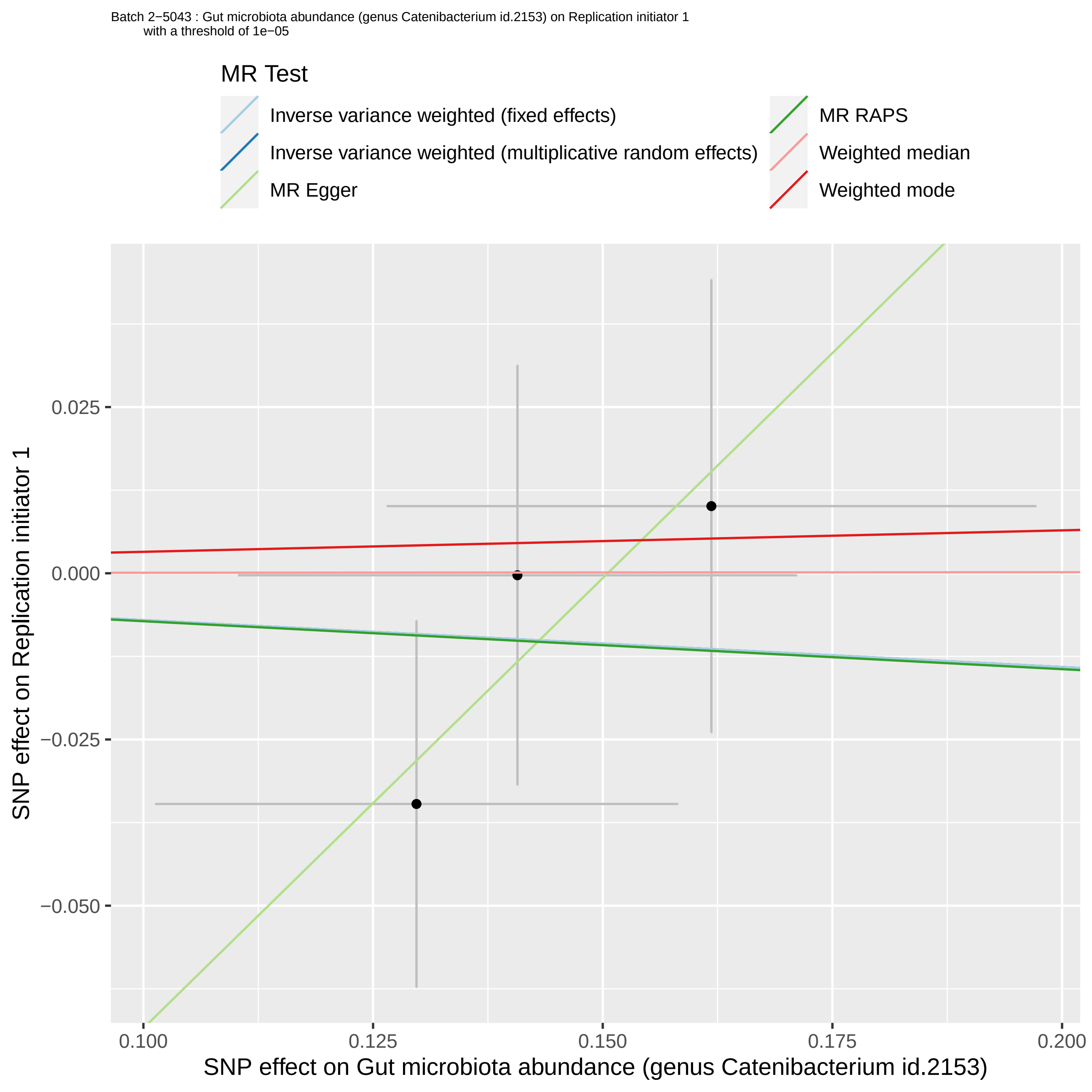
Batch 2−5043 : Gut microbiota abundance (genus Catenibacterium id.2153) on Replication initiator 1
with a threshold of 1e−05
MR Test
Inverse variance weighted (fixed effects)
Inverse variance weighted (multiplicative random effects)
MR Egger
MR RAPS
Weighted median
Weighted mode
0.025
0.000
−0.025
−0.050
0.100
0.125
0.150
0.175
0.200
SNP effect on Replication initiator 1
SNP effect on Gut microbiota abundance (genus Catenibacterium id.2153)

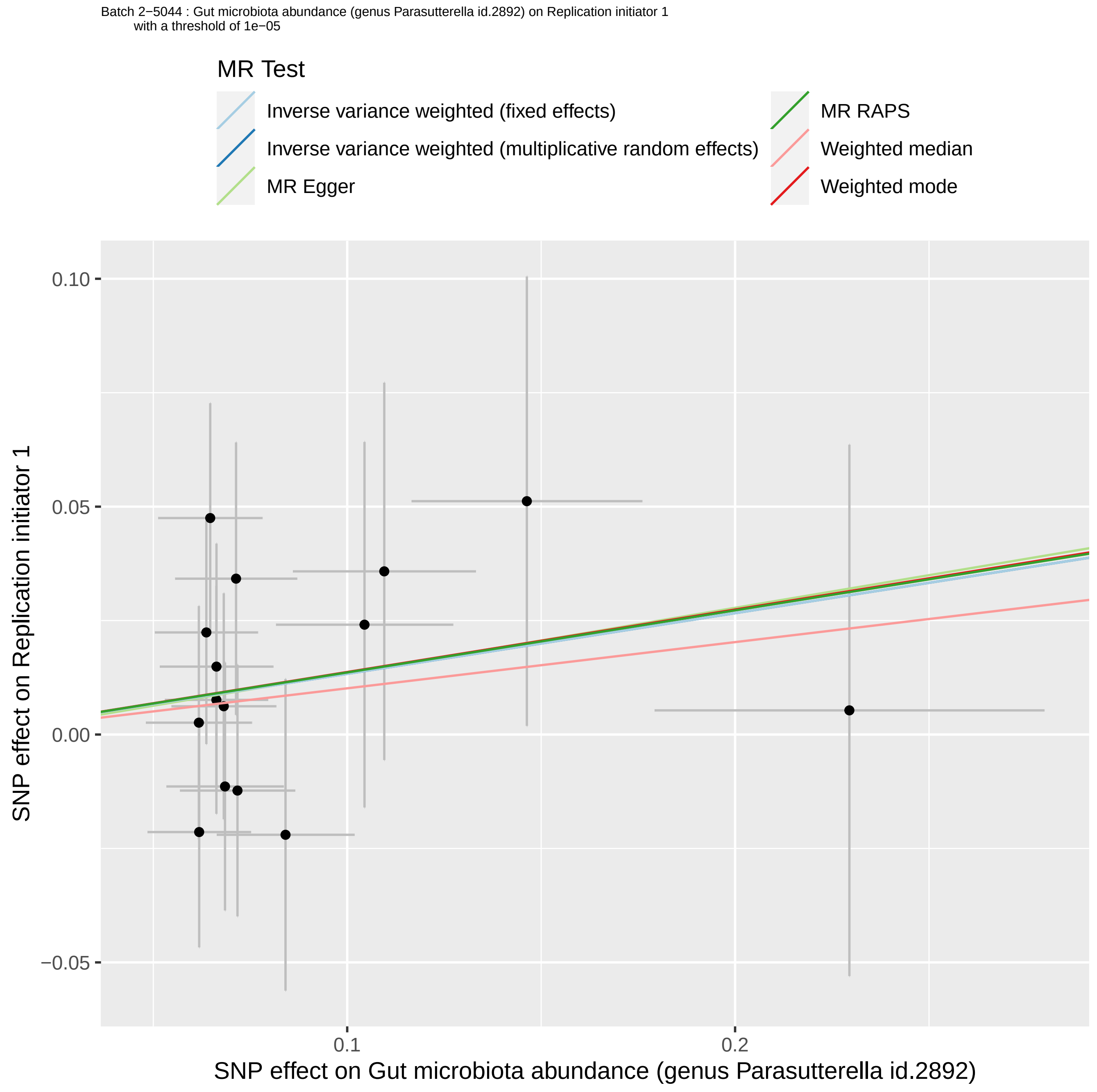

Batch 2−5044 : Gut microbiota abundance (genus Parasutterella id.2892) on Replication initiator 1
with a threshold of 1e−05
MR Test
Inverse variance weighted (fixed effects)
Inverse variance weighted (multiplicative random effects)
MR Egger
MR RAPS
Weighted median
Weighted mode
0.10
0.05
0.00
−0.05
0.1
0.2
SNP effect on Replication initiator 1
SNP effect on Gut microbiota abundance (genus Parasutterella id.2892)

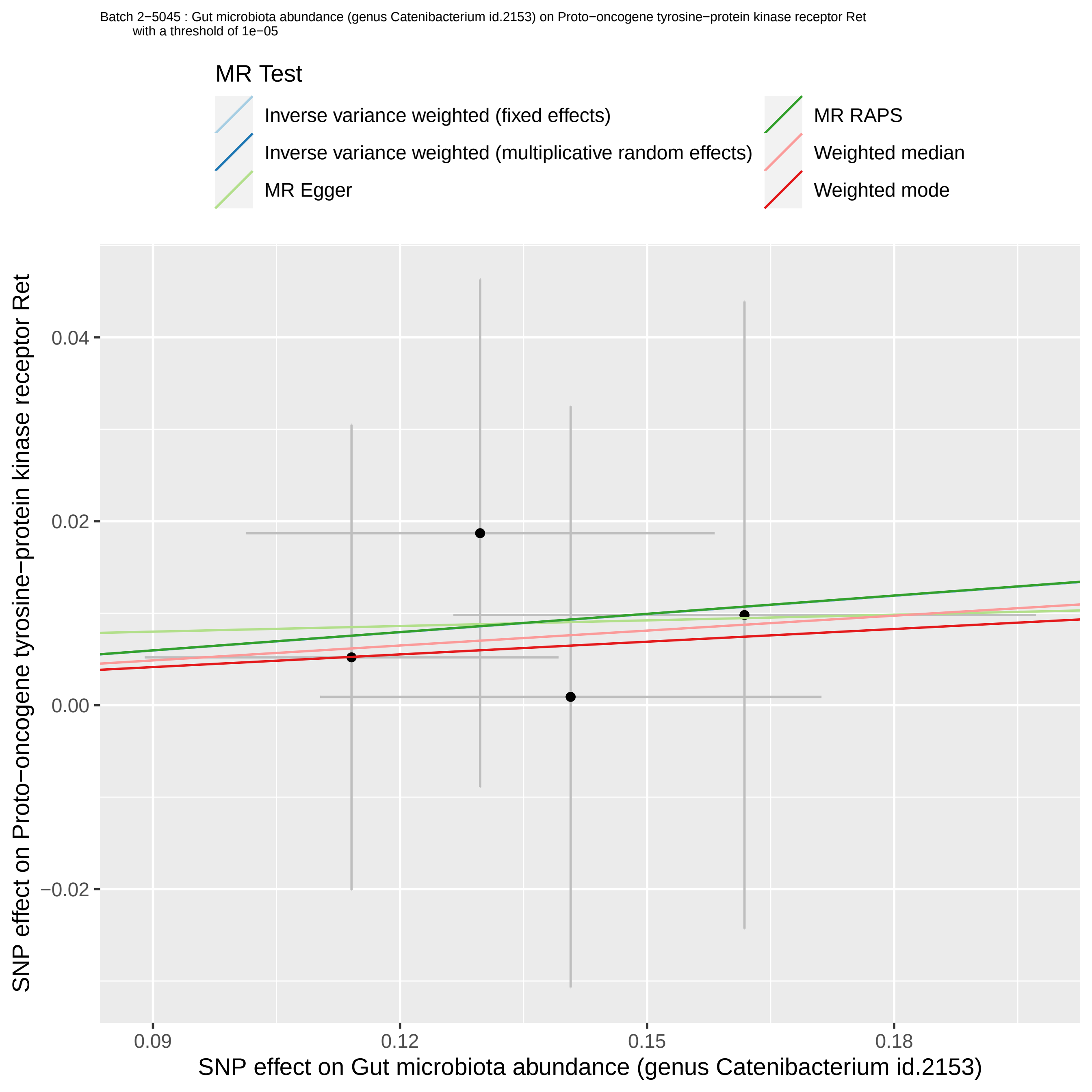

Batch 2−5045 : Gut microbiota abundance (genus Catenibacterium id.2153) on Proto−oncogene tyrosine−protein kinase receptor Ret
with a threshold of 1e−05
MR Test
Inverse variance weighted (fixed effects)
Inverse variance weighted (multiplicative random effects)
MR Egger
MR RAPS
Weighted median
Weighted mode
SNP effect on Proto−oncogene tyrosine−protein kinase receptor Ret
0.04
0.02
0.00
−0.02
0.09
0.12
0.15
0.18
SNP effect on Gut microbiota abundance (genus Catenibacterium id.2153)

Batch 2−5046 : Gut microbiota abundance (genus Parasutterella id.2892) on Proto−oncogene tyrosine−protein kinase receptor Ret
with a threshold of 1e−05

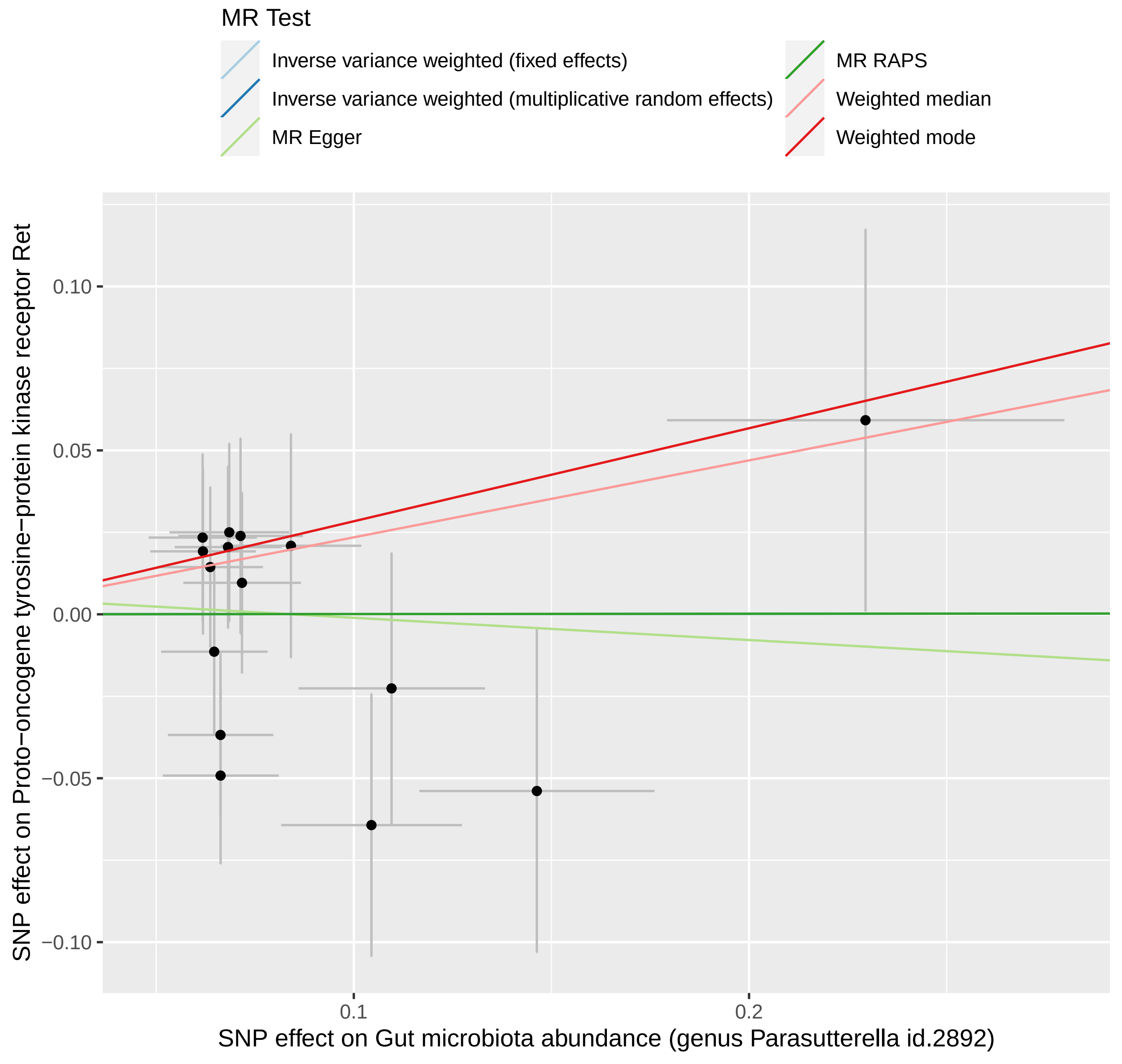

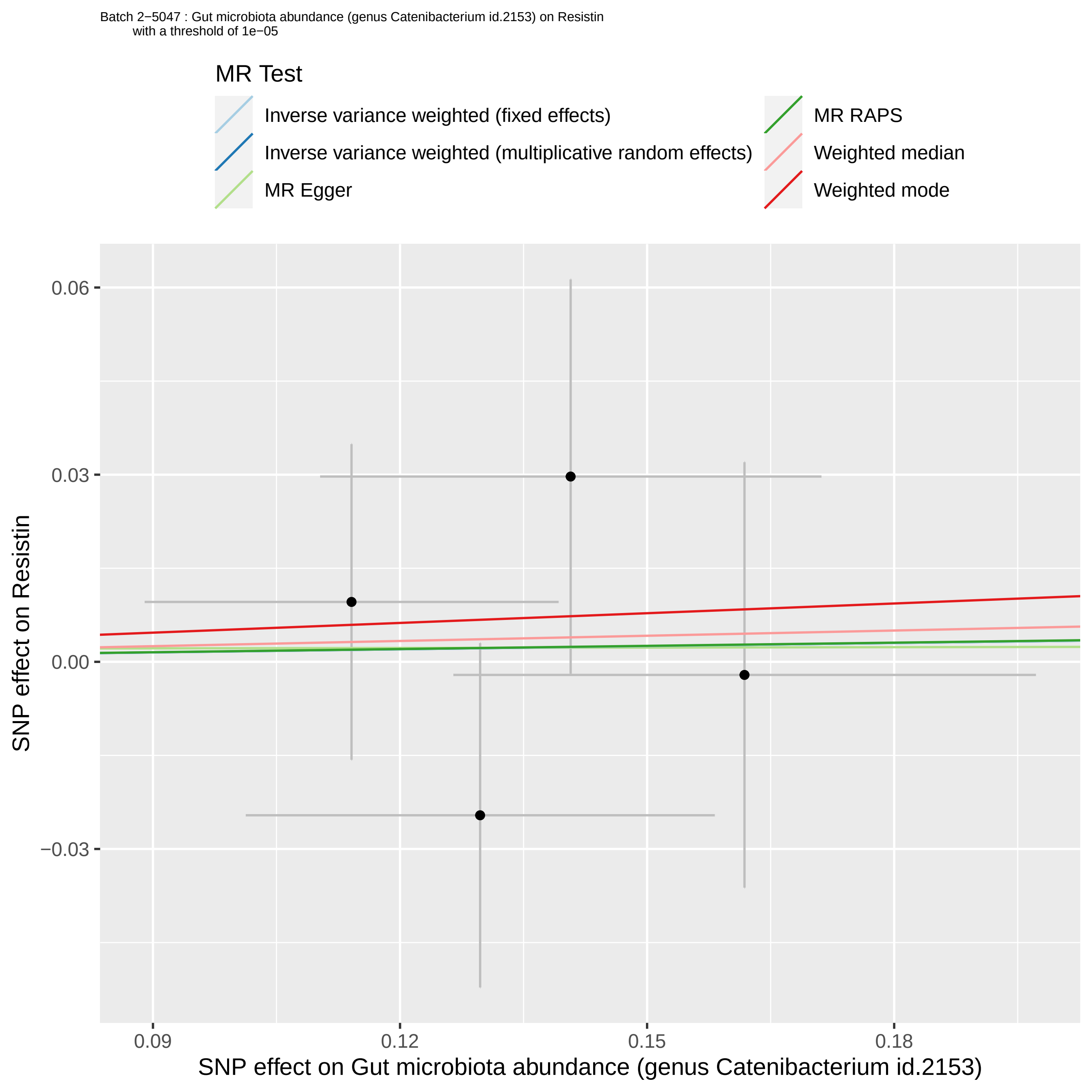

Batch 2−5047 : Gut microbiota abundance (genus Catenibacterium id.2153) on Resistin
with a threshold of 1e−05
MR Test
Inverse variance weighted (fixed effects)
Inverse variance weighted (multiplicative random effects)
MR Egger
MR RAPS
Weighted median
Weighted mode
0.06
0.03
0.00
−0.03
0.09
0.12
0.15
0.18
SNP effect on Resistin
SNP effect on Gut microbiota abundance (genus Catenibacterium id.2153)

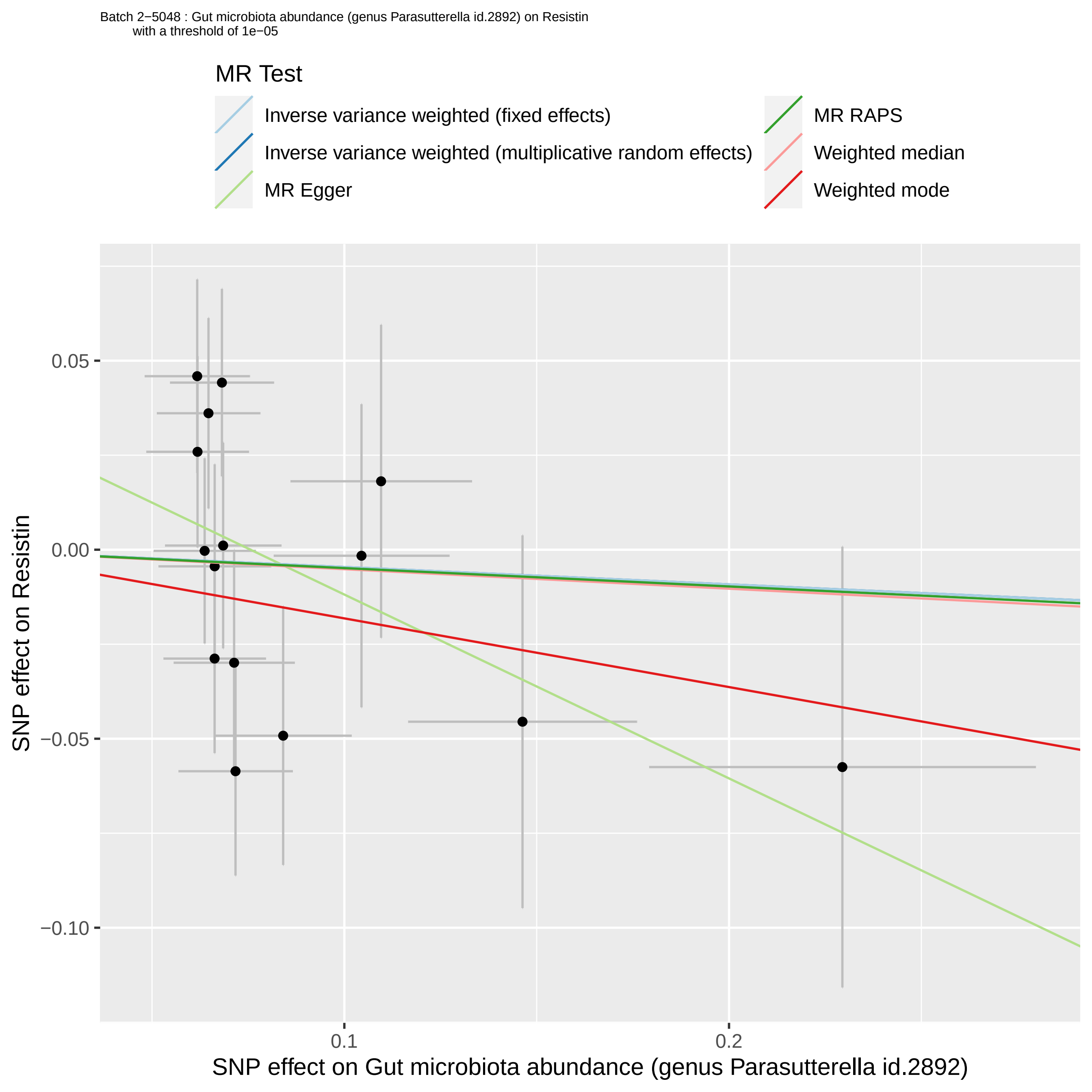

Batch 2−5048 : Gut microbiota abundance (genus Parasutterella id.2892) on Resistin
with a threshold of 1e−05
MR Test
Inverse variance weighted (fixed effects)
Inverse variance weighted (multiplicative random effects)
MR Egger
MR RAPS
Weighted median
Weighted mode
0.05
0.00
−0.05
−0.10
0.1
0.2
SNP effect on Resistin
SNP effect on Gut microbiota abundance (genus Parasutterella id.2892)

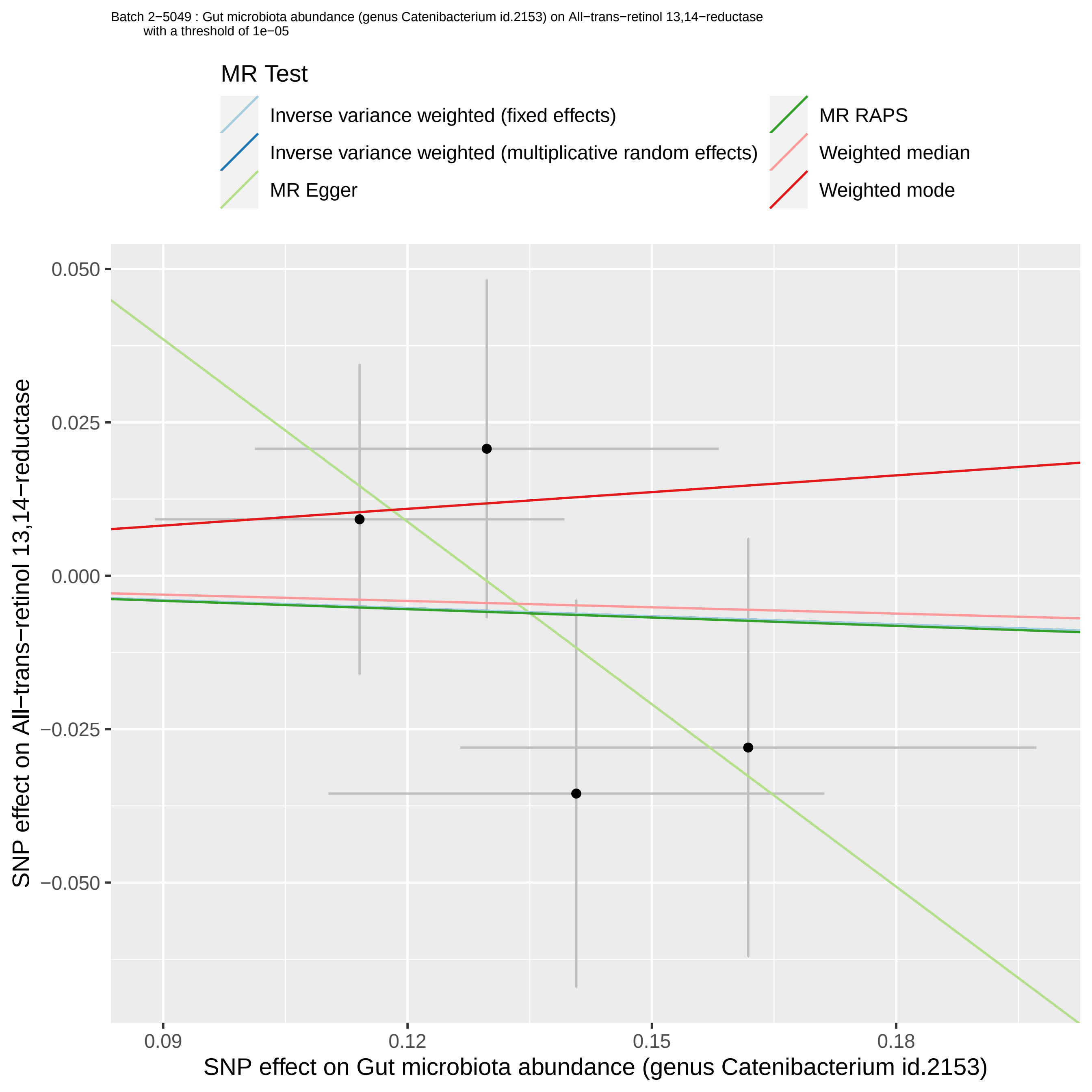
Batch 2−5049 : Gut microbiota abundance (genus Catenibacterium id.2153) on All−trans−retinol 13,14−reductase
with a threshold of 1e−05
MR Test
Inverse variance weighted (fixed effects)
Inverse variance weighted (multiplicative random effects)
MR Egger
MR RAPS
Weighted median
Weighted mode
0.050
0.025
0.000
−0.025
−0.050
0.09
0.12
0.15
0.18
SNP effect on All−trans−retinol 13,14−reductase
SNP effect on Gut microbiota abundance (genus Catenibacterium id.2153)

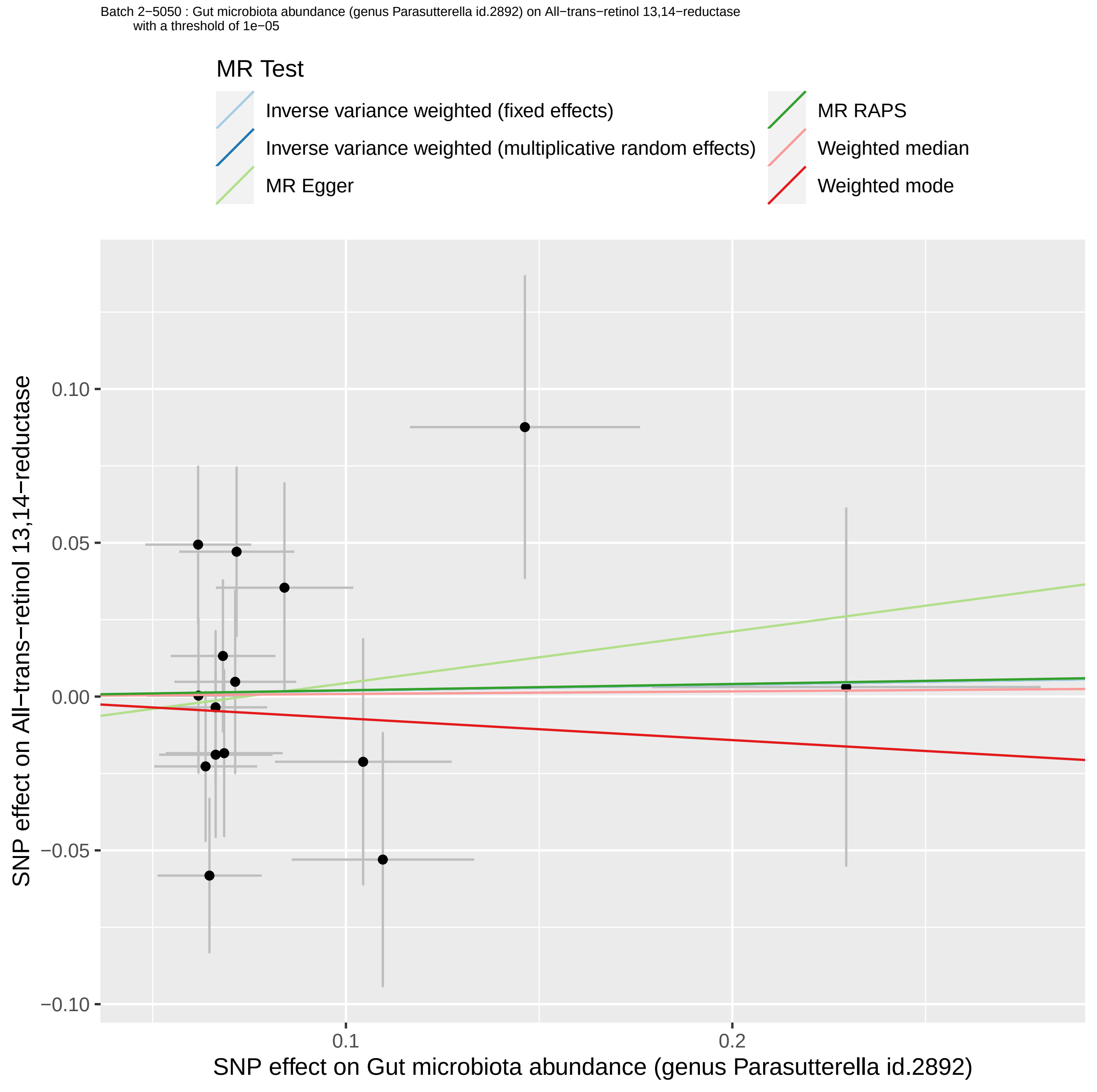
Batch 2−5050 : Gut microbiota abundance (genus Parasutterella id.2892) on All−trans−retinol 13,14−reductase
with a threshold of 1e−05
MR Test
Inverse variance weighted (fixed effects)
Inverse variance weighted (multiplicative random effects)
MR Egger
MR RAPS
Weighted median
Weighted mode
0.10
0.05
0.00
−0.05
−0.10
0.1
0.2
SNP effect on All−trans−retinol 13,14−reductase
SNP effect on Gut microbiota abundance (genus Parasutterella id.2892)

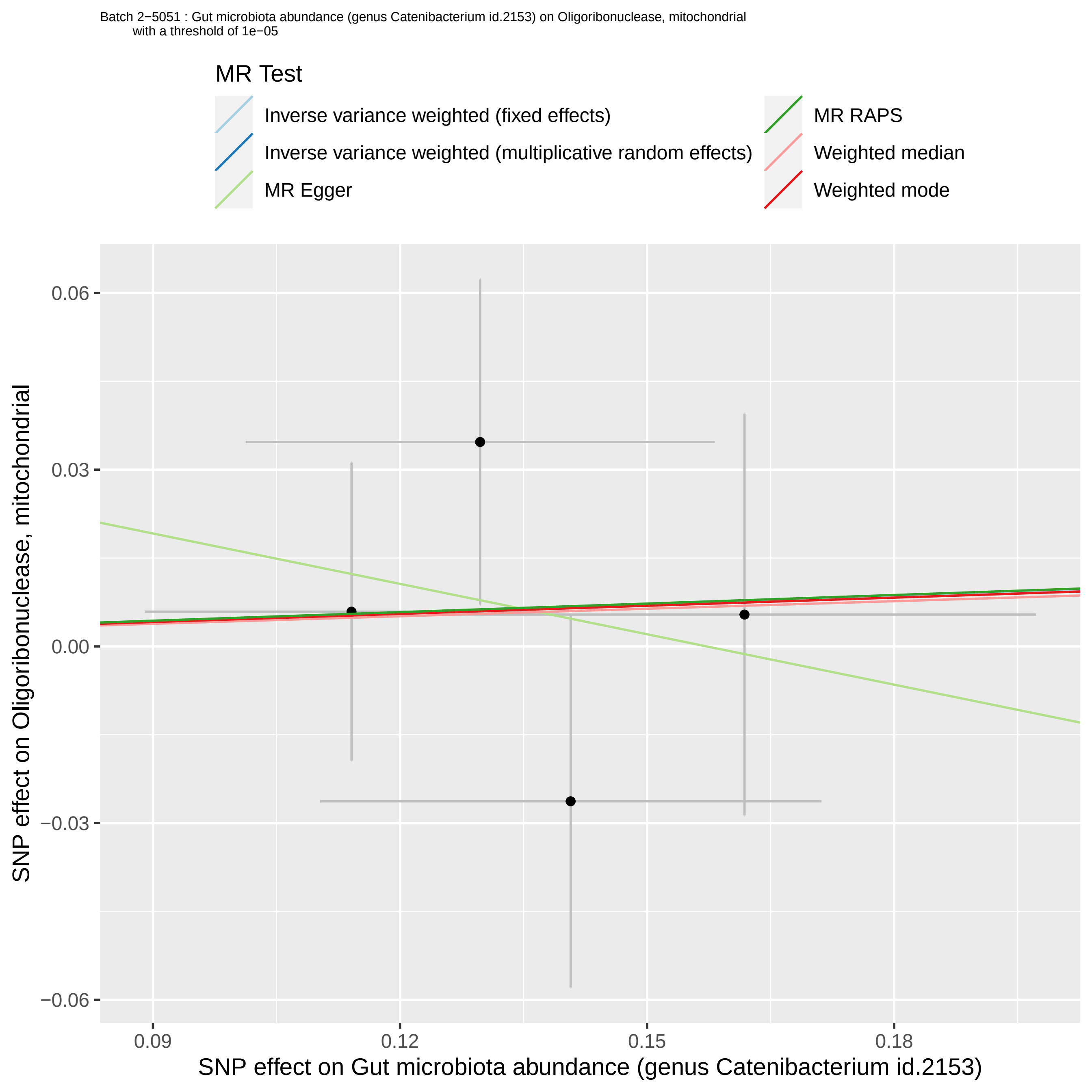

Batch 2−5051 : Gut microbiota abundance (genus Catenibacterium id.2153) on Oligoribonuclease, mitochondrial
with a threshold of 1e−05
MR Test
Inverse variance weighted (fixed effects)
Inverse variance weighted (multiplicative random effects)
MR Egger
MR RAPS
Weighted median
Weighted mode
0.06
0.03
0.00
−0.03
−0.06
0.09
0.12
0.15
0.18
SNP effect on Oligoribonuclease, mitochondrial
SNP effect on Gut microbiota abundance (genus Catenibacterium id.2153)

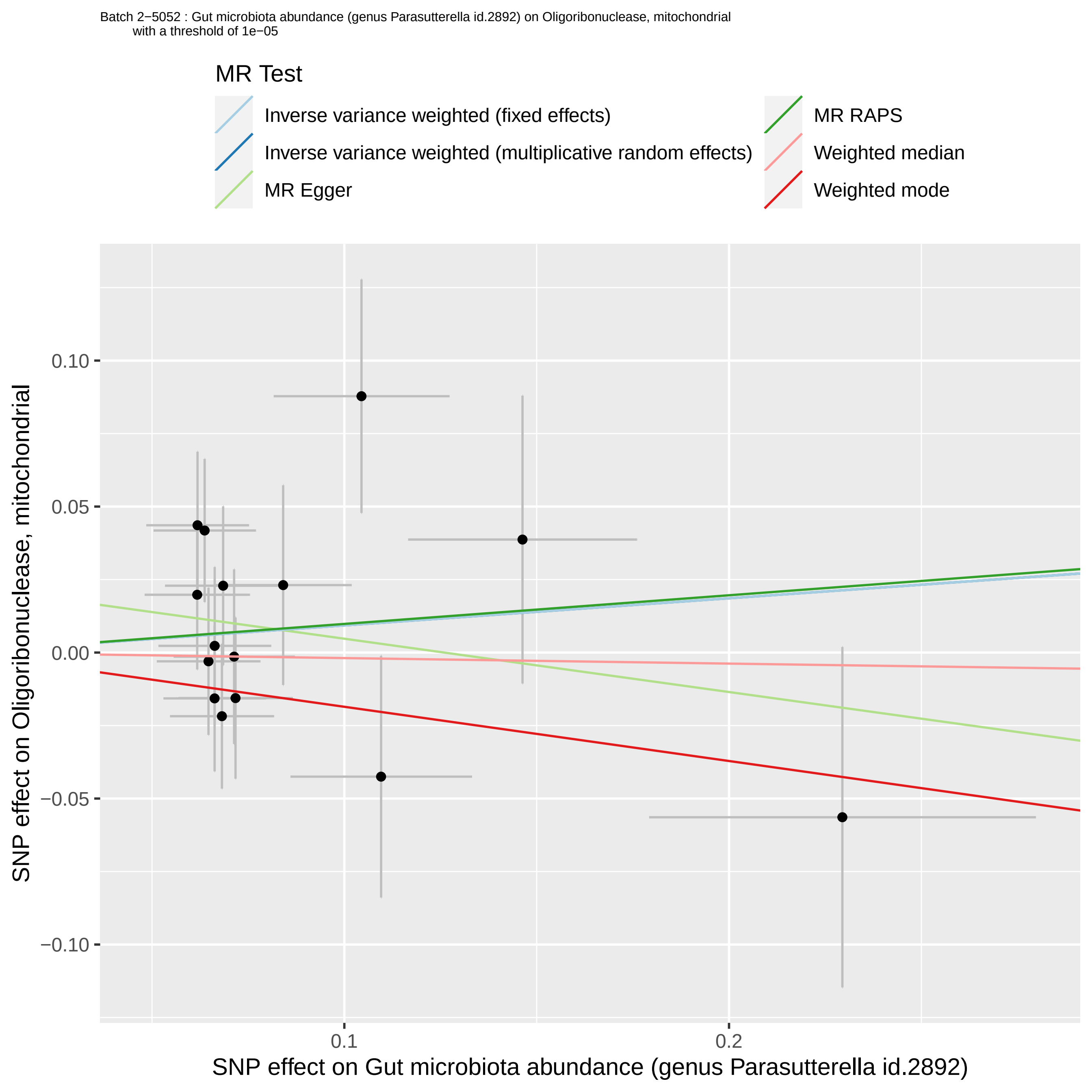
Batch 2−5052 : Gut microbiota abundance (genus Parasutterella id.2892) on Oligoribonuclease, mitochondrial
with a threshold of 1e−05
MR Test
Inverse variance weighted (fixed effects)
Inverse variance weighted (multiplicative random effects)
MR Egger
MR RAPS
Weighted median
Weighted mode
0.10
0.05
0.00
−0.05
−0.10
0.1
0.2
SNP effect on Oligoribonuclease, mitochondrial
SNP effect on Gut microbiota abundance (genus Parasutterella id.2892)

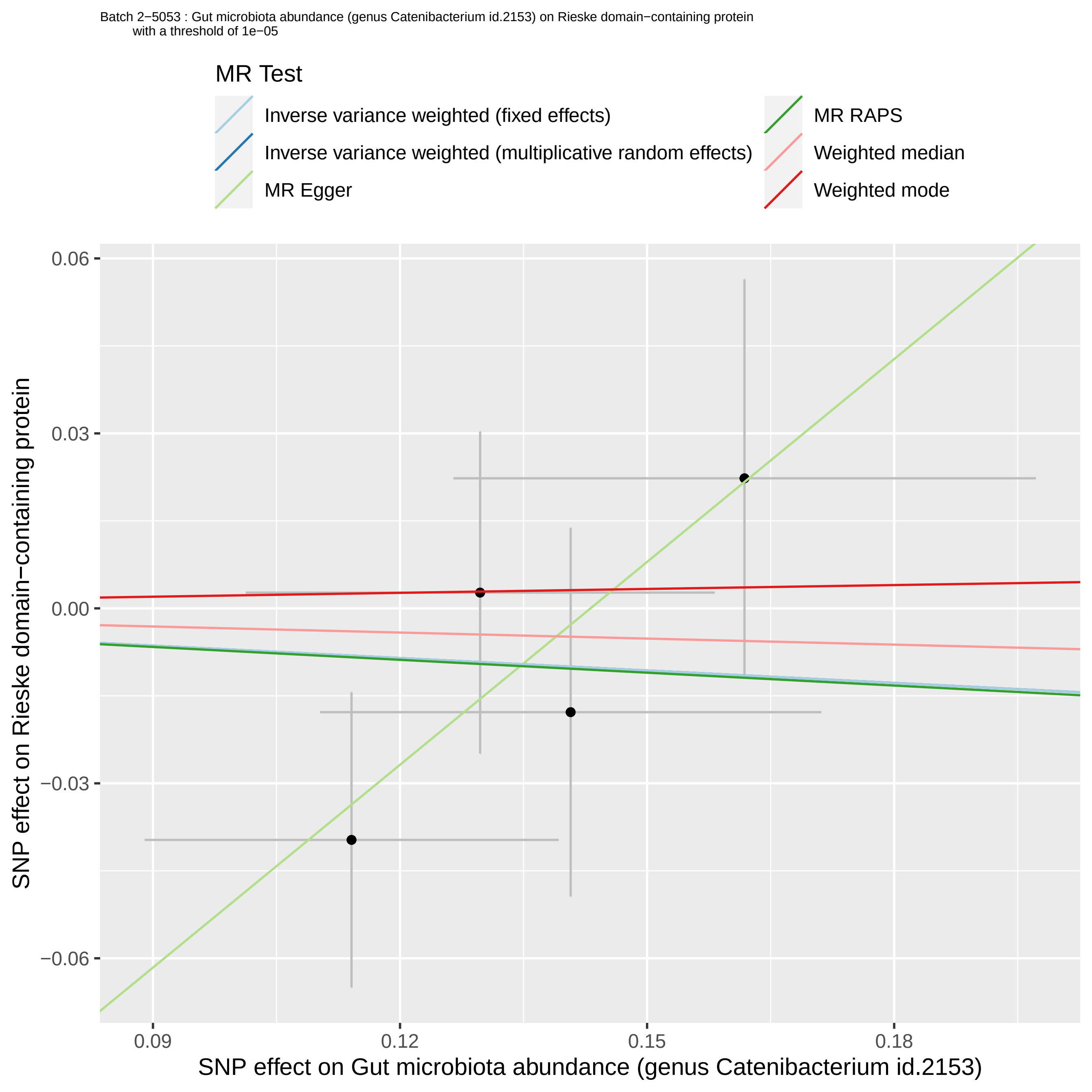
Batch 2−5053 : Gut microbiota abundance (genus Catenibacterium id.2153) on Rieske domain−containing protein
with a threshold of 1e−05
MR Test
Inverse variance weighted (fixed effects)
Inverse variance weighted (multiplicative random effects)
MR Egger
MR RAPS
Weighted median
Weighted mode
0.06
0.03
0.00
−0.03
−0.06
0.09
0.12
0.15
0.18
SNP effect on Rieske domain−containing protein
SNP effect on Gut microbiota abundance (genus Catenibacterium id.2153)

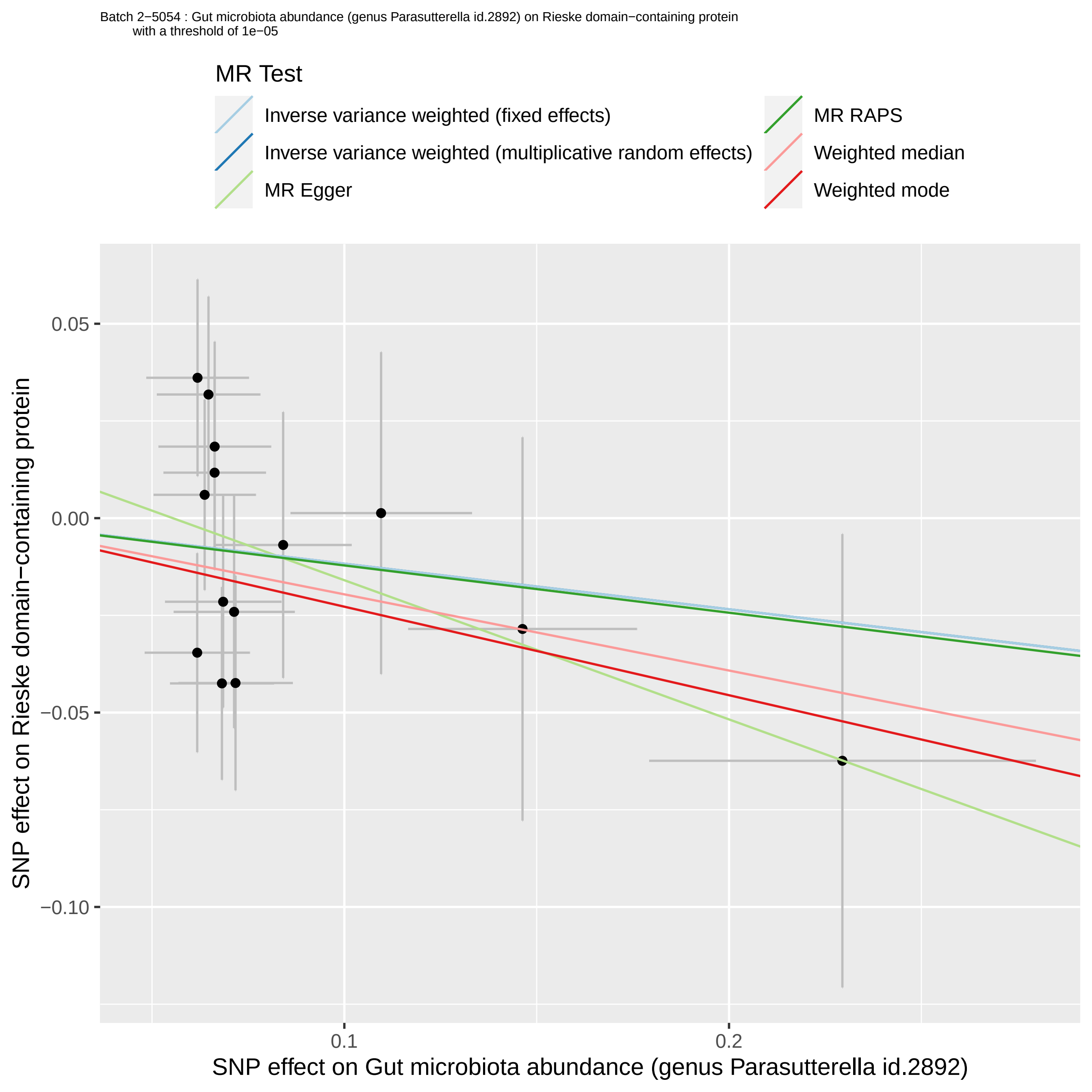

Batch 2−5054 : Gut microbiota abundance (genus Parasutterella id.2892) on Rieske domain−containing protein
with a threshold of 1e−05
MR Test
Inverse variance weighted (fixed effects)
Inverse variance weighted (multiplicative random effects)
MR Egger
MR RAPS
Weighted median
Weighted mode
0.05
0.00
−0.05
−0.10
0.1
0.2
SNP effect on Rieske domain−containing protein
SNP effect on Gut microbiota abundance (genus Parasutterella id.2892)

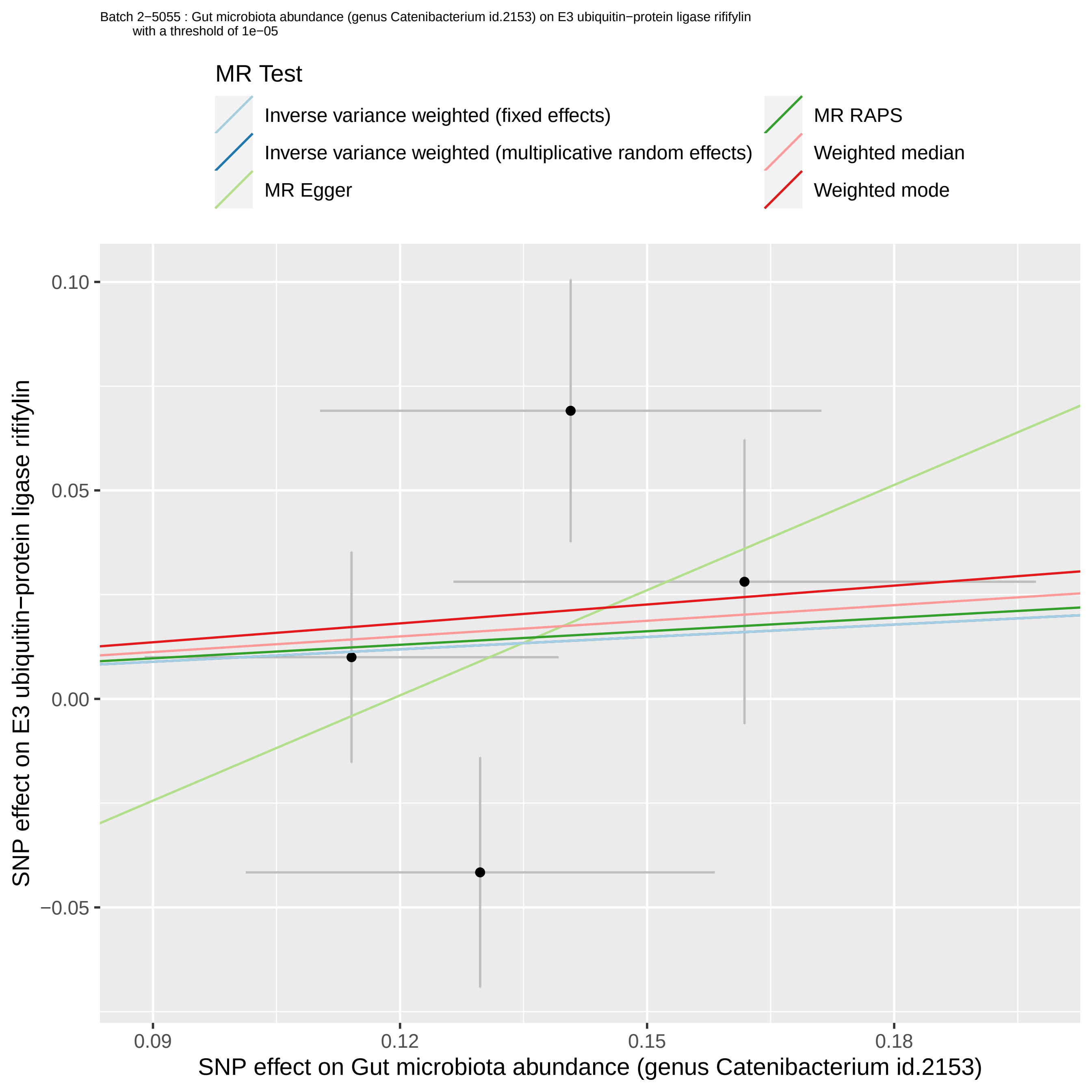
Batch 2−5055 : Gut microbiota abundance (genus Catenibacterium id.2153) on E3 ubiquitin−protein ligase rififylin
with a threshold of 1e−05
MR Test
Inverse variance weighted (fixed effects)
Inverse variance weighted (multiplicative random effects)
MR Egger
MR RAPS
Weighted median
Weighted mode
0.10
0.05
0.00
−0.05
0.09
0.12
0.15
0.18
SNP effect on E3 ubiquitin−protein ligase rififylin
SNP effect on Gut microbiota abundance (genus Catenibacterium id.2153)

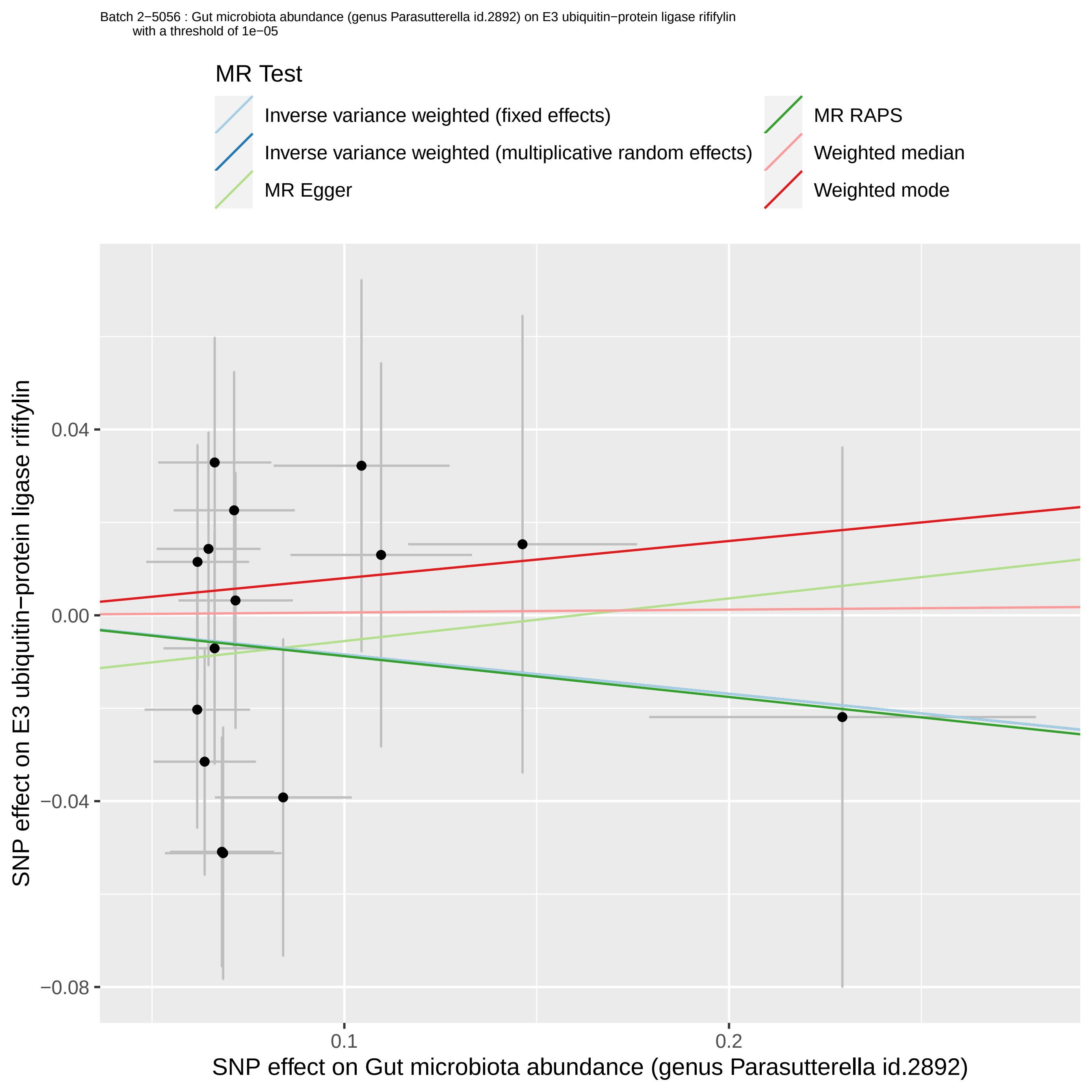
Batch 2−5056 : Gut microbiota abundance (genus Parasutterella id.2892) on E3 ubiquitin−protein ligase rififylin
with a threshold of 1e−05
MR Test
Inverse variance weighted (fixed effects)
Inverse variance weighted (multiplicative random effects)
MR Egger
MR RAPS
Weighted median
Weighted mode
0.04
0.00
−0.04
−0.08
0.1
0.2
SNP effect on E3 ubiquitin−protein ligase rififylin
SNP effect on Gut microbiota abundance (genus Parasutterella id.2892)

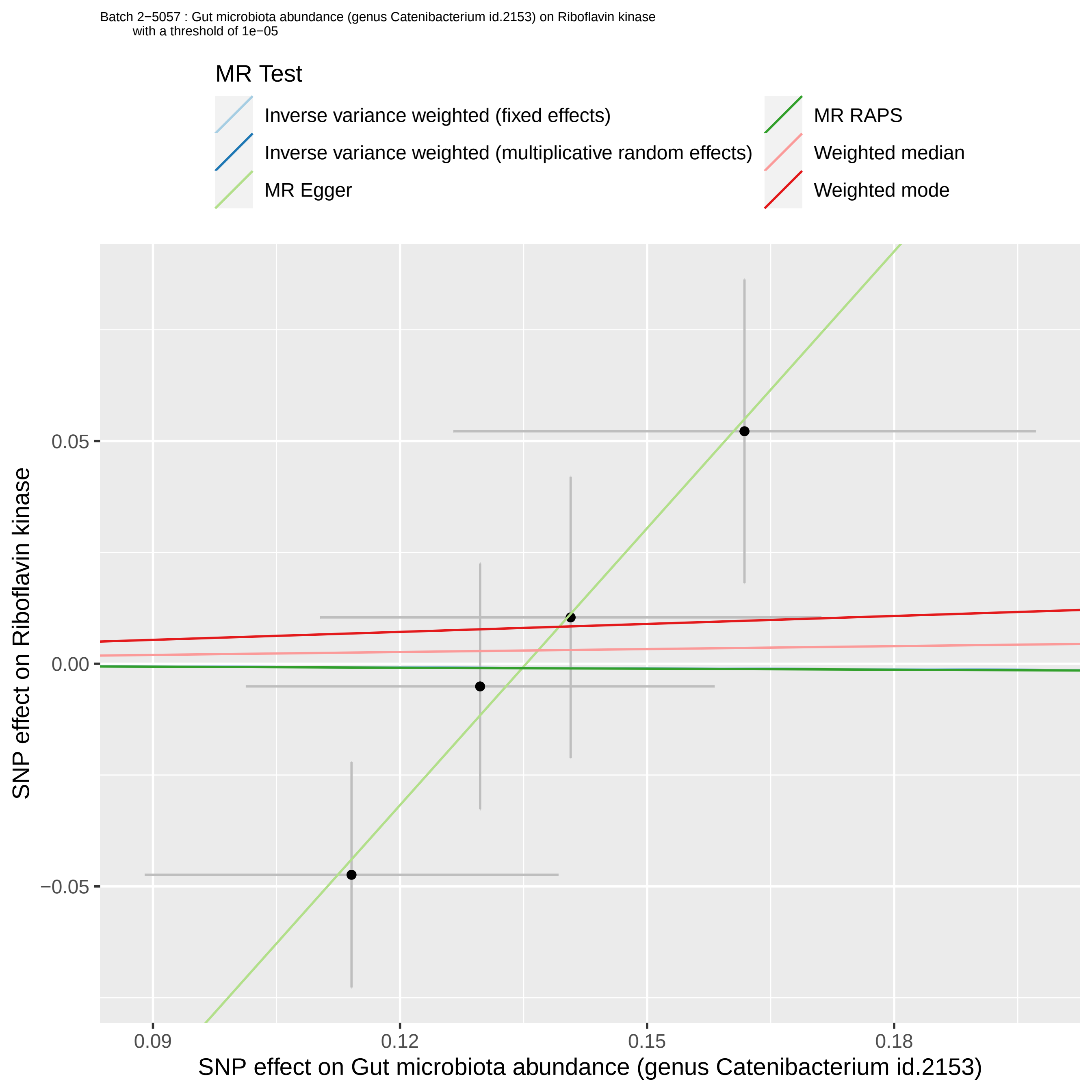
Batch 2−5057 : Gut microbiota abundance (genus Catenibacterium id.2153) on Riboflavin kinase
with a threshold of 1e−05
MR Test
Inverse variance weighted (fixed effects)
Inverse variance weighted (multiplicative random effects)
MR Egger
MR RAPS
Weighted median
Weighted mode
0.05
0.00
−0.05
0.09
0.12
0.15
0.18
SNP effect on Riboflavin kinase
SNP effect on Gut microbiota abundance (genus Catenibacterium id.2153)

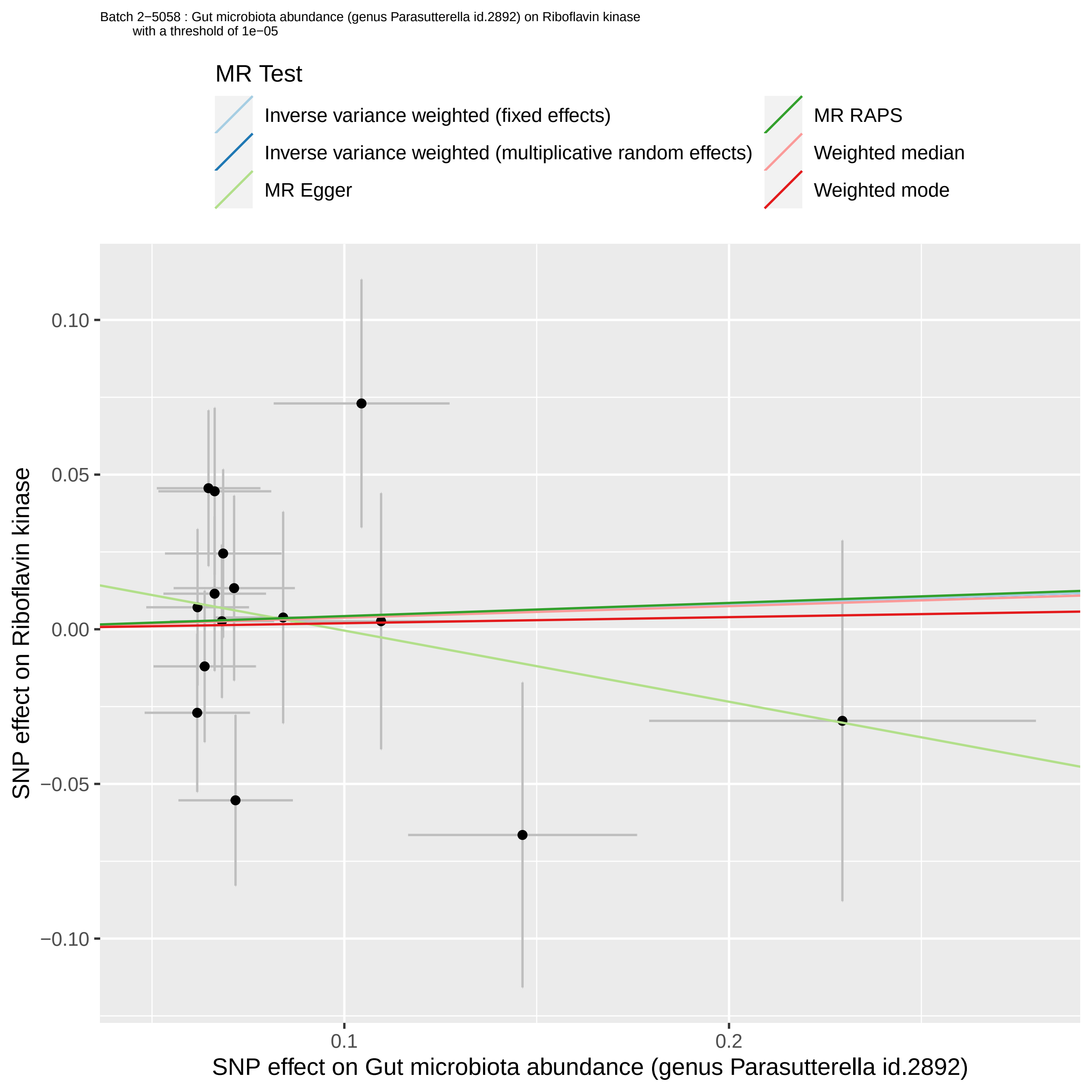
Batch 2−5058 : Gut microbiota abundance (genus Parasutterella id.2892) on Riboflavin kinase
with a threshold of 1e−05
MR Test
Inverse variance weighted (fixed effects)
Inverse variance weighted (multiplicative random effects)
MR Egger
MR RAPS
Weighted median
Weighted mode
0.10
0.05
0.00
−0.05
−0.10
0.1
0.2
SNP effect on Riboflavin kinase
SNP effect on Gut microbiota abundance (genus Parasutterella id.2892)

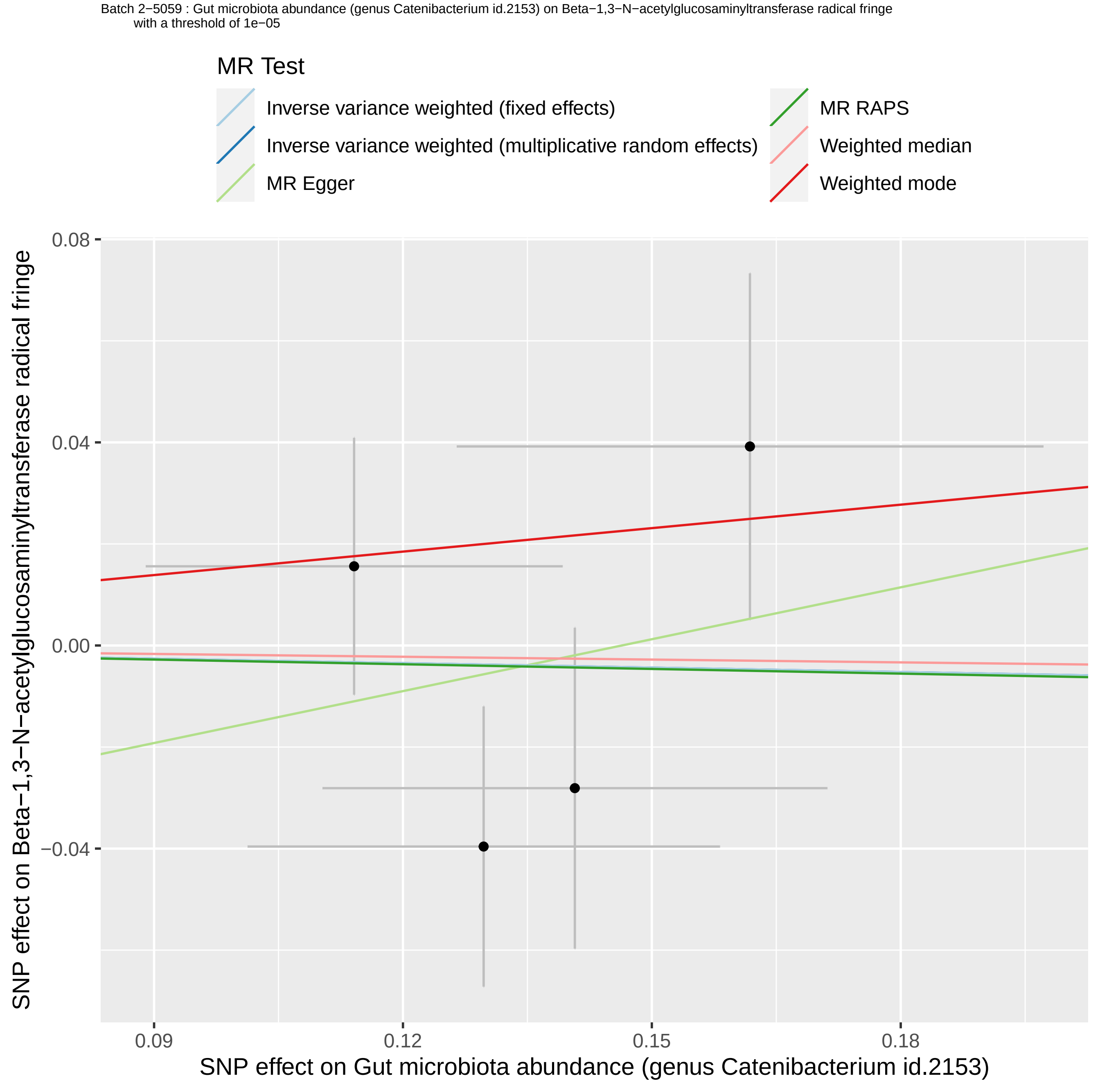

Batch 2−5059 : Gut microbiota abundance (genus Catenibacterium id.2153) on Beta−1,3−N−acetylglucosaminyltransferase radical fringe
with a threshold of 1e−05
MR Test
Inverse variance weighted (fixed effects)
Inverse variance weighted (multiplicative random effects)
MR Egger
MR RAPS
Weighted median
Weighted mode
0.08
0.04
0.00
−0.04
0.09
0.12
0.15
0.18
SNP effect on Beta−1,3−N−acetylglucosaminyltransferase radical fringe
SNP effect on Gut microbiota abundance (genus Catenibacterium id.2153)

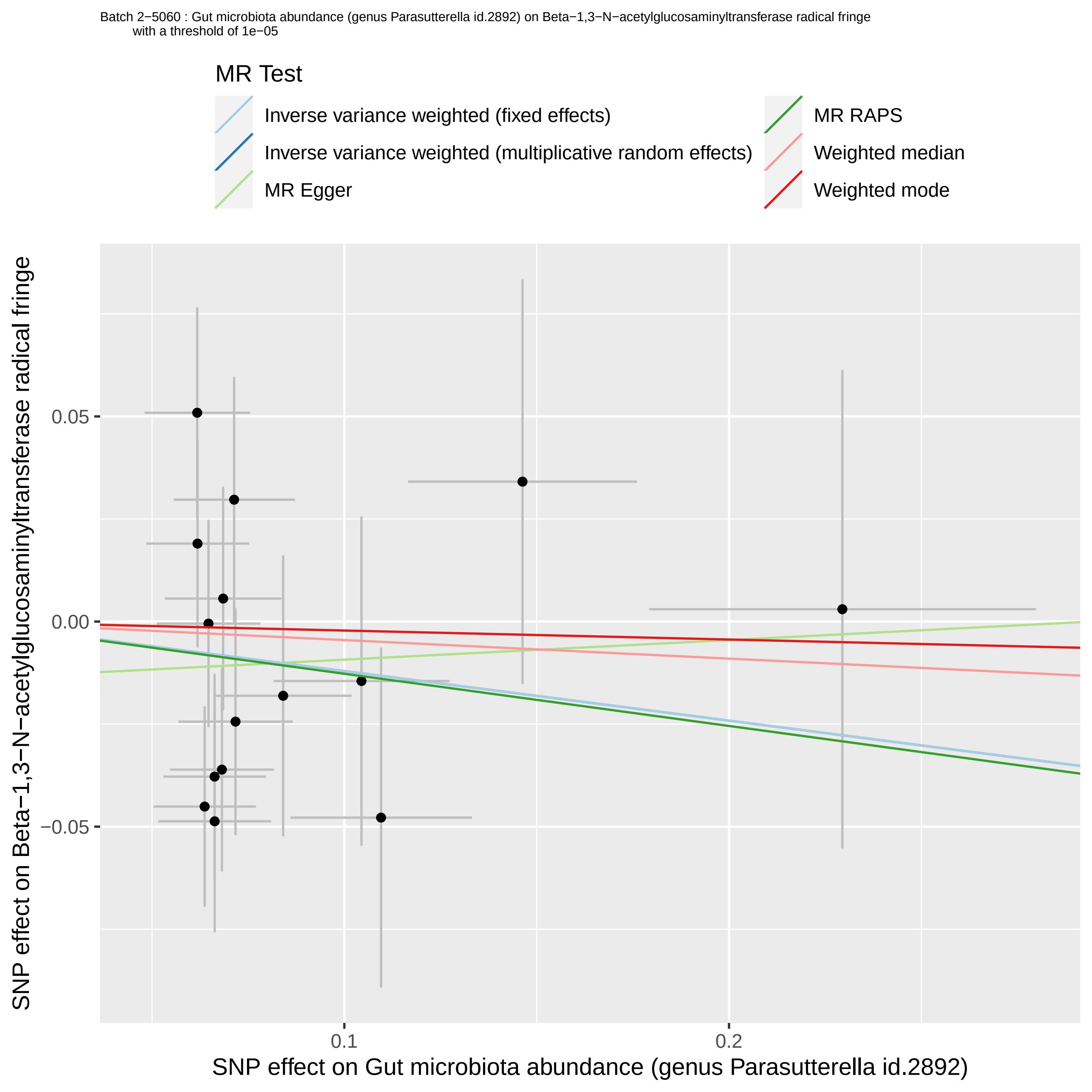

Batch 2−5060 : Gut microbiota abundance (genus Parasutterella id.2892) on Beta−1,3−N−acetylglucosaminyltransferase radical fringe
with a threshold of 1e−05
MR Test
Inverse variance weighted (fixed effects)
Inverse variance weighted (multiplicative random effects)
MR Egger
MR RAPS
Weighted median
Weighted mode
0.05
0.00
−0.05
0.1
0.2
SNP effect on Beta−1,3−N−acetylglucosaminyltransferase radical fringe
SNP effect on Gut microbiota abundance (genus Parasutterella id.2892)

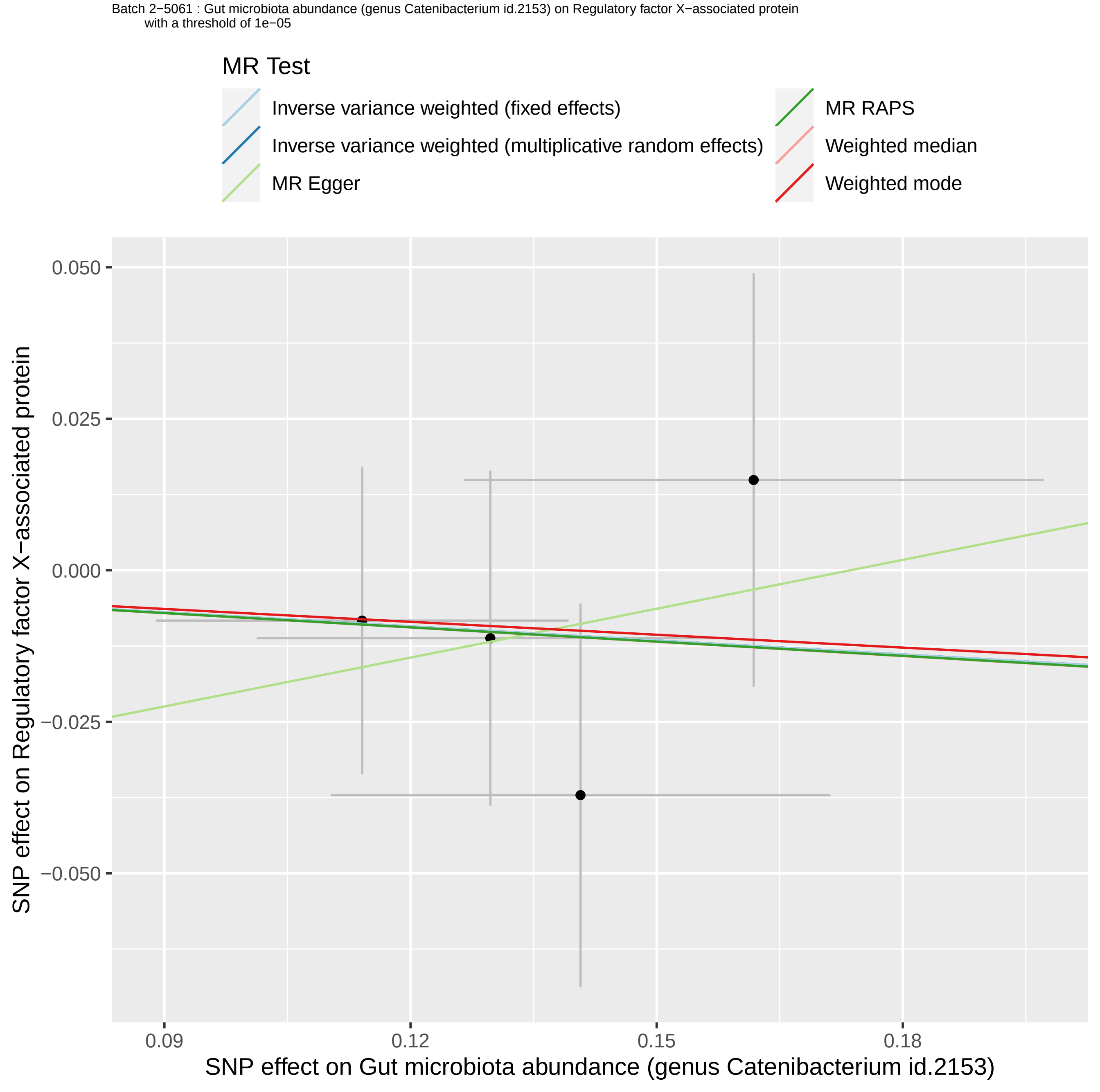

Batch 2−5061 : Gut microbiota abundance (genus Catenibacterium id.2153) on Regulatory factor X−associated protein
with a threshold of 1e−05
MR Test
Inverse variance weighted (fixed effects)
Inverse variance weighted (multiplicative random effects)
MR Egger
MR RAPS
Weighted median
Weighted mode
0.050
0.025
0.000
−0.025
−0.050
0.09
0.12
0.15
0.18
SNP effect on Regulatory factor X−associated protein
SNP effect on Gut microbiota abundance (genus Catenibacterium id.2153)

Batch 2−5062 : Gut microbiota abundance (genus Parasutterella id.2892) on Regulatory factor X−associated protein
with a threshold of 1e−05

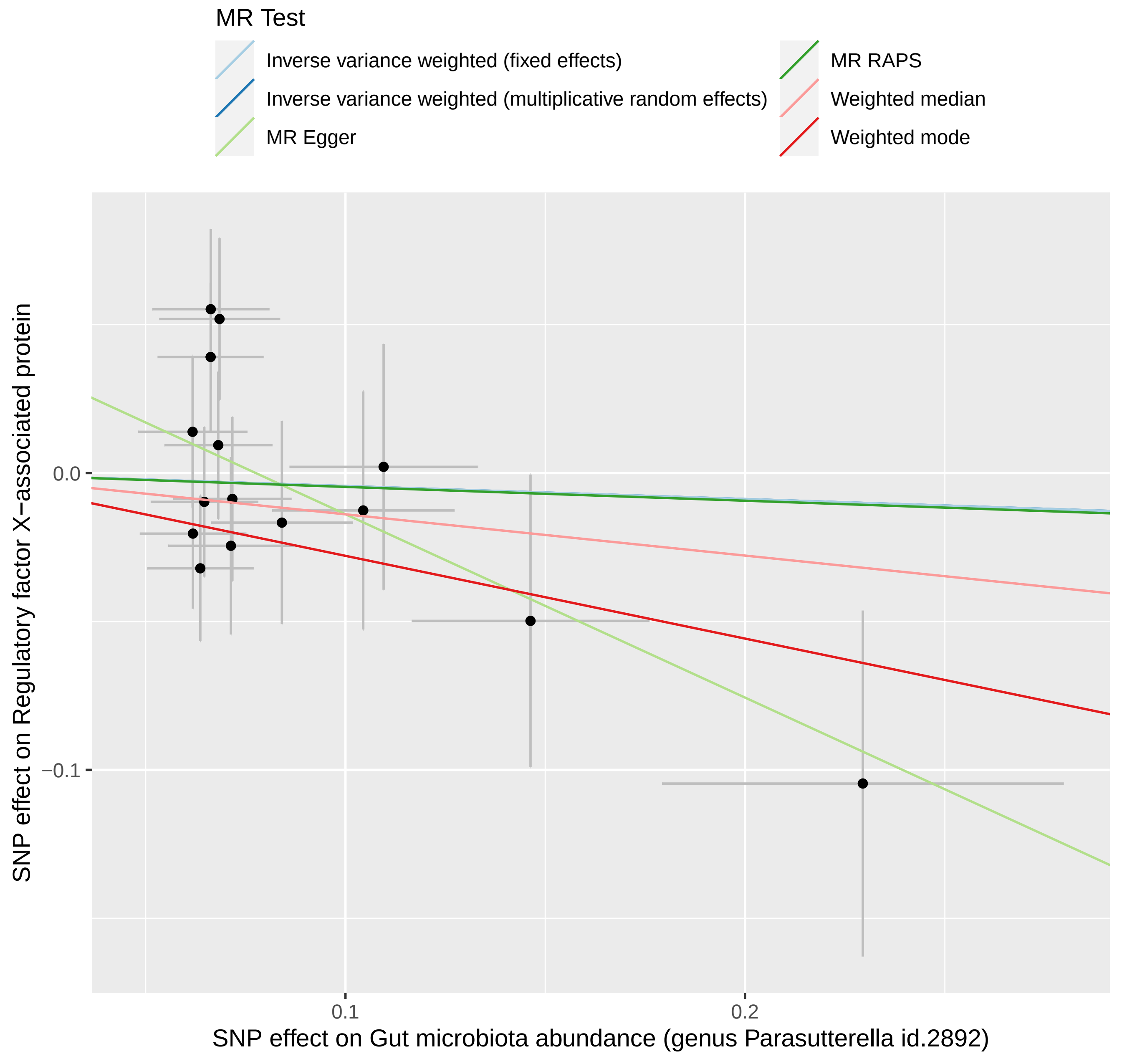

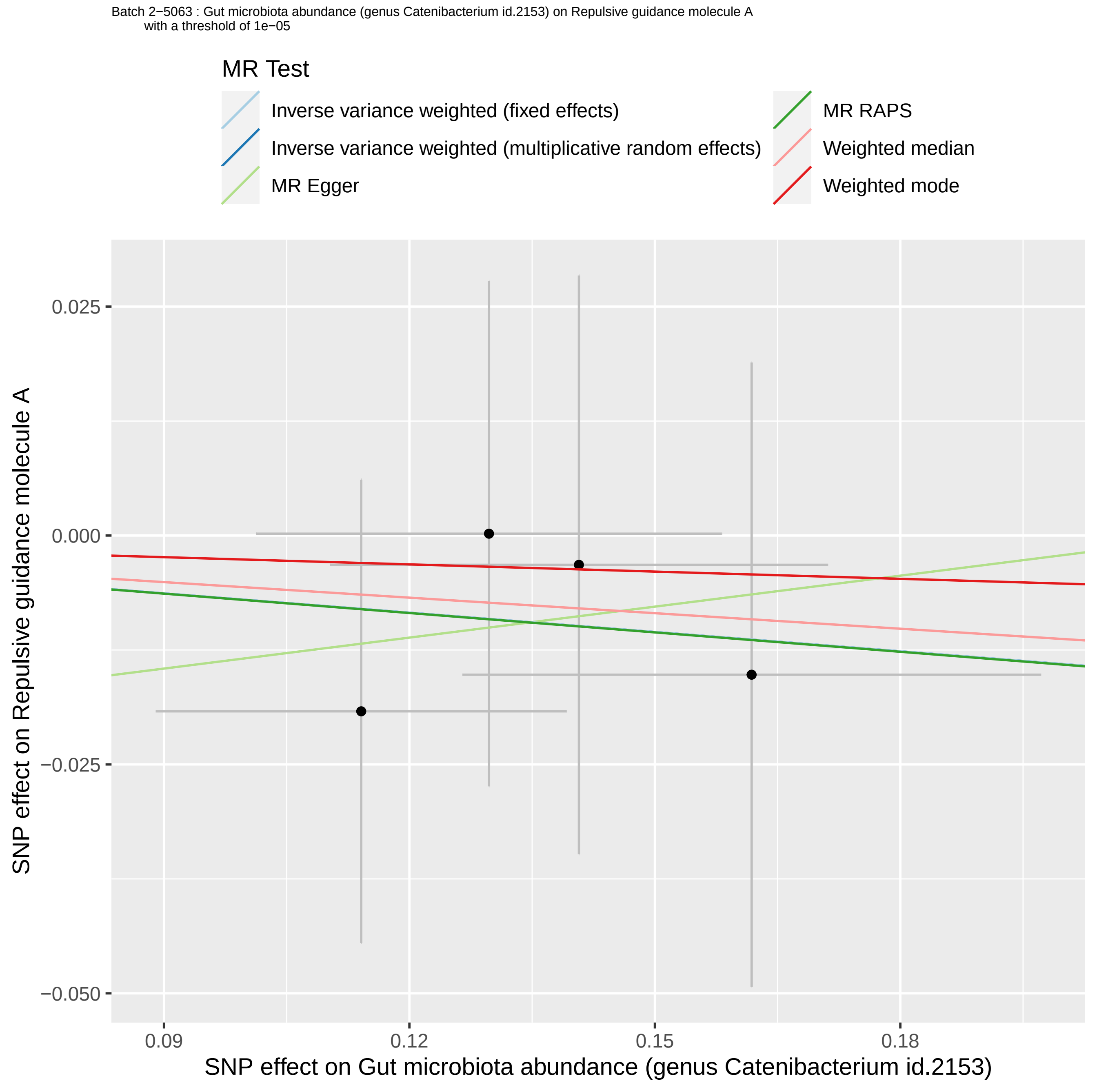

Batch 2−5063 : Gut microbiota abundance (genus Catenibacterium id.2153) on Repulsive guidance molecule A
with a threshold of 1e−05
MR Test
Inverse variance weighted (fixed effects)
Inverse variance weighted (multiplicative random effects)
MR Egger
MR RAPS
Weighted median
Weighted mode
0.025
0.000
−0.025
−0.050
0.09
0.12
0.15
0.18
SNP effect on Repulsive guidance molecule A
SNP effect on Gut microbiota abundance (genus Catenibacterium id.2153)

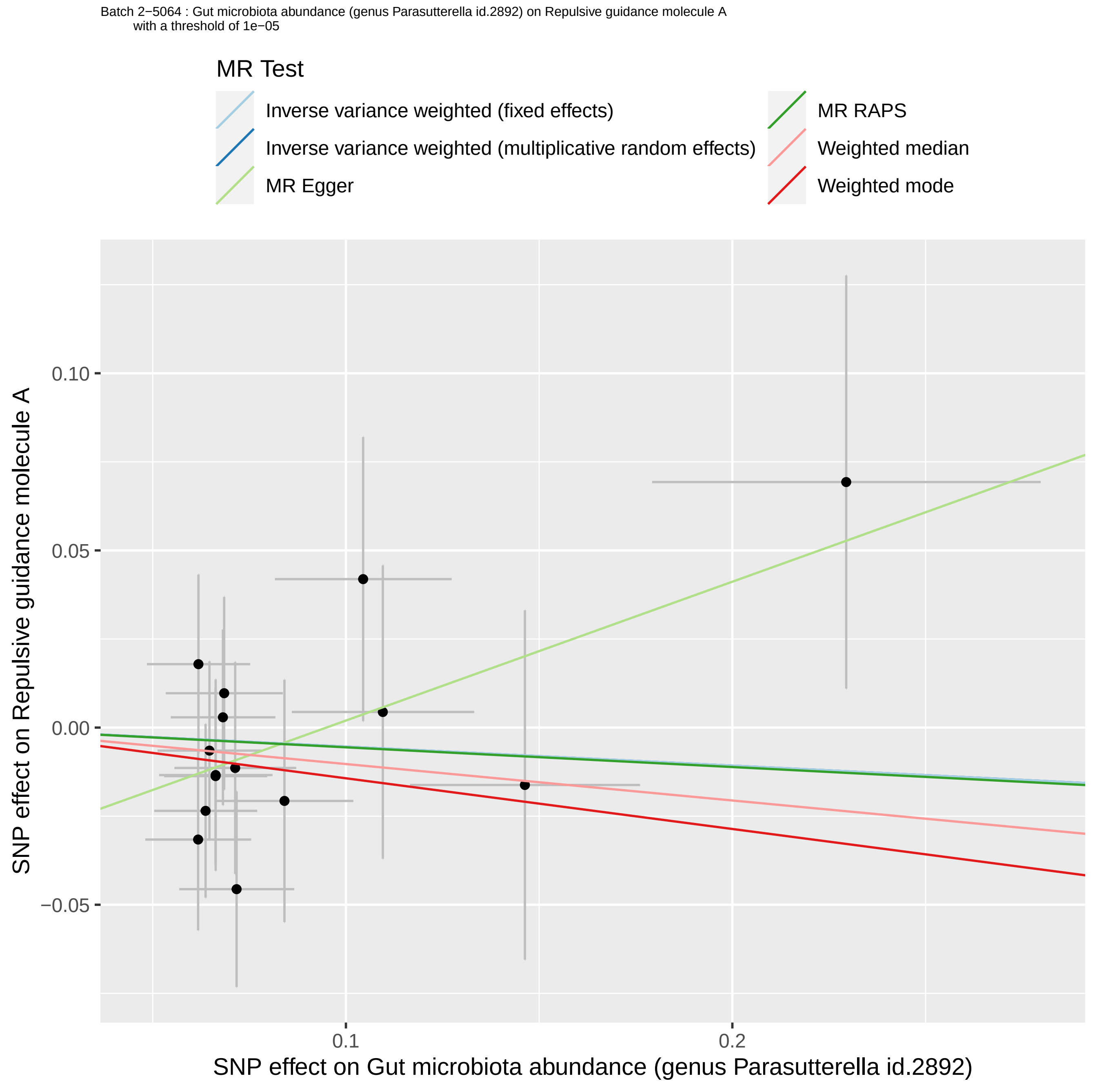

Batch 2−5064 : Gut microbiota abundance (genus Parasutterella id.2892) on Repulsive guidance molecule A
with a threshold of 1e−05
MR Test
Inverse variance weighted (fixed effects)
Inverse variance weighted (multiplicative random effects)
MR Egger
MR RAPS
Weighted median
Weighted mode
0.10
0.05
0.00
−0.05
0.1
0.2
SNP effect on Repulsive guidance molecule A
SNP effect on Gut microbiota abundance (genus Parasutterella id.2892)

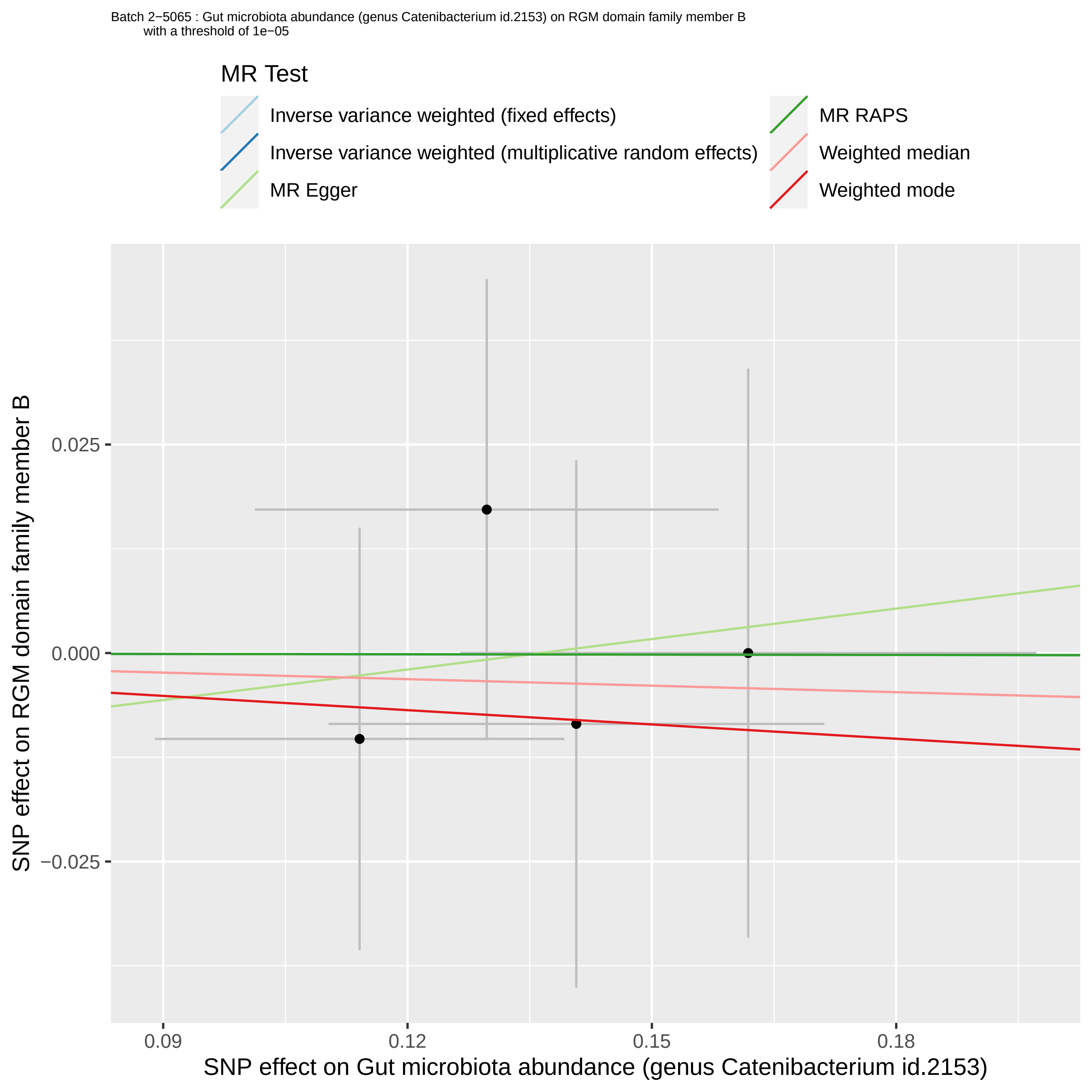

Batch 2−5065 : Gut microbiota abundance (genus Catenibacterium id.2153) on RGM domain family member B
with a threshold of 1e−05
MR Test
Inverse variance weighted (fixed effects)
Inverse variance weighted (multiplicative random effects)
MR Egger
MR RAPS
Weighted median
Weighted mode
0.025
0.000
−0.025
0.09
0.12
0.15
0.18
SNP effect on RGM domain family member B
SNP effect on Gut microbiota abundance (genus Catenibacterium id.2153)

Batch 2−5066 : Gut microbiota abundance (genus Parasutterella id.2892) on RGM domain family member B
with a threshold of 1e−05

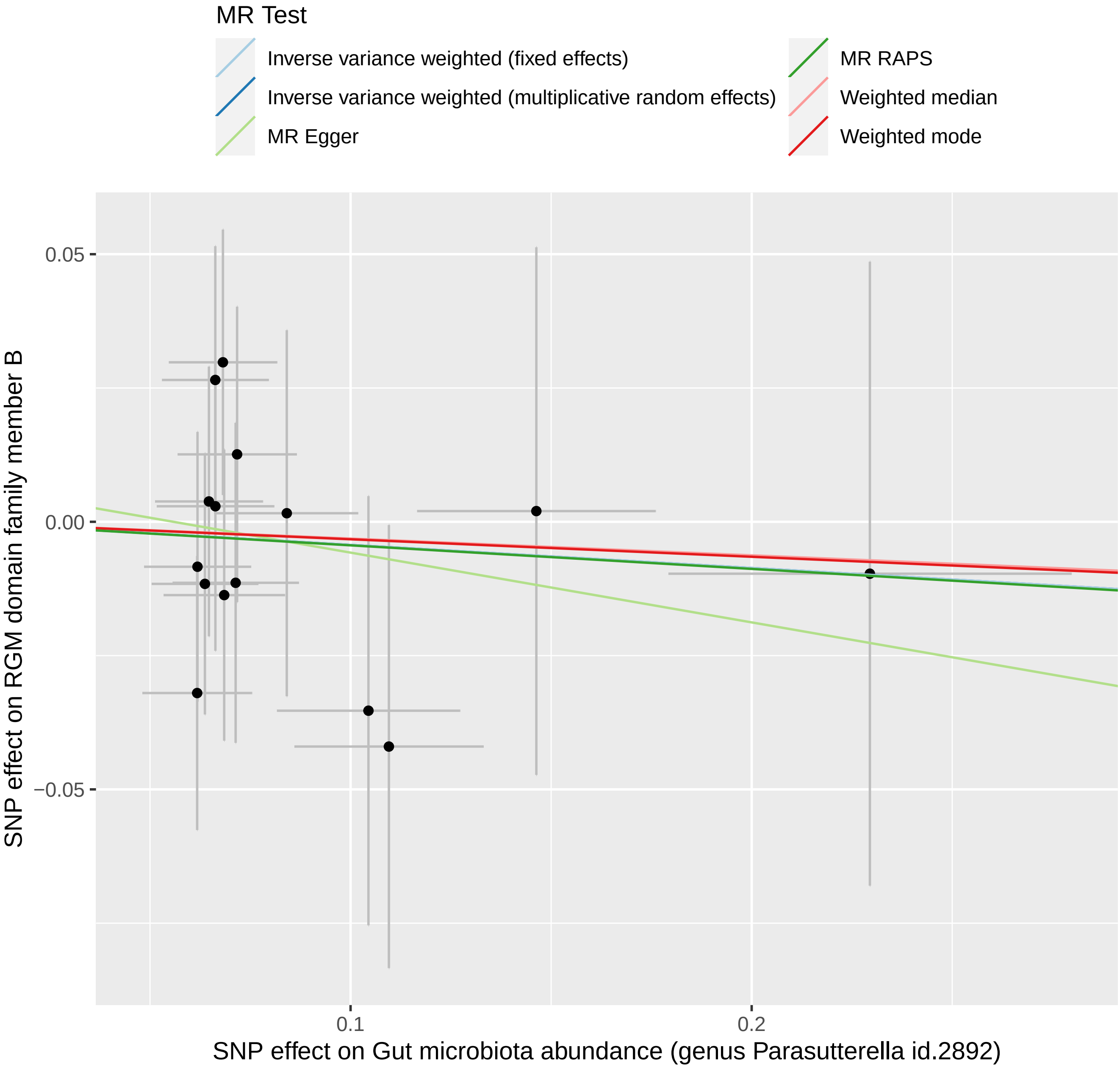

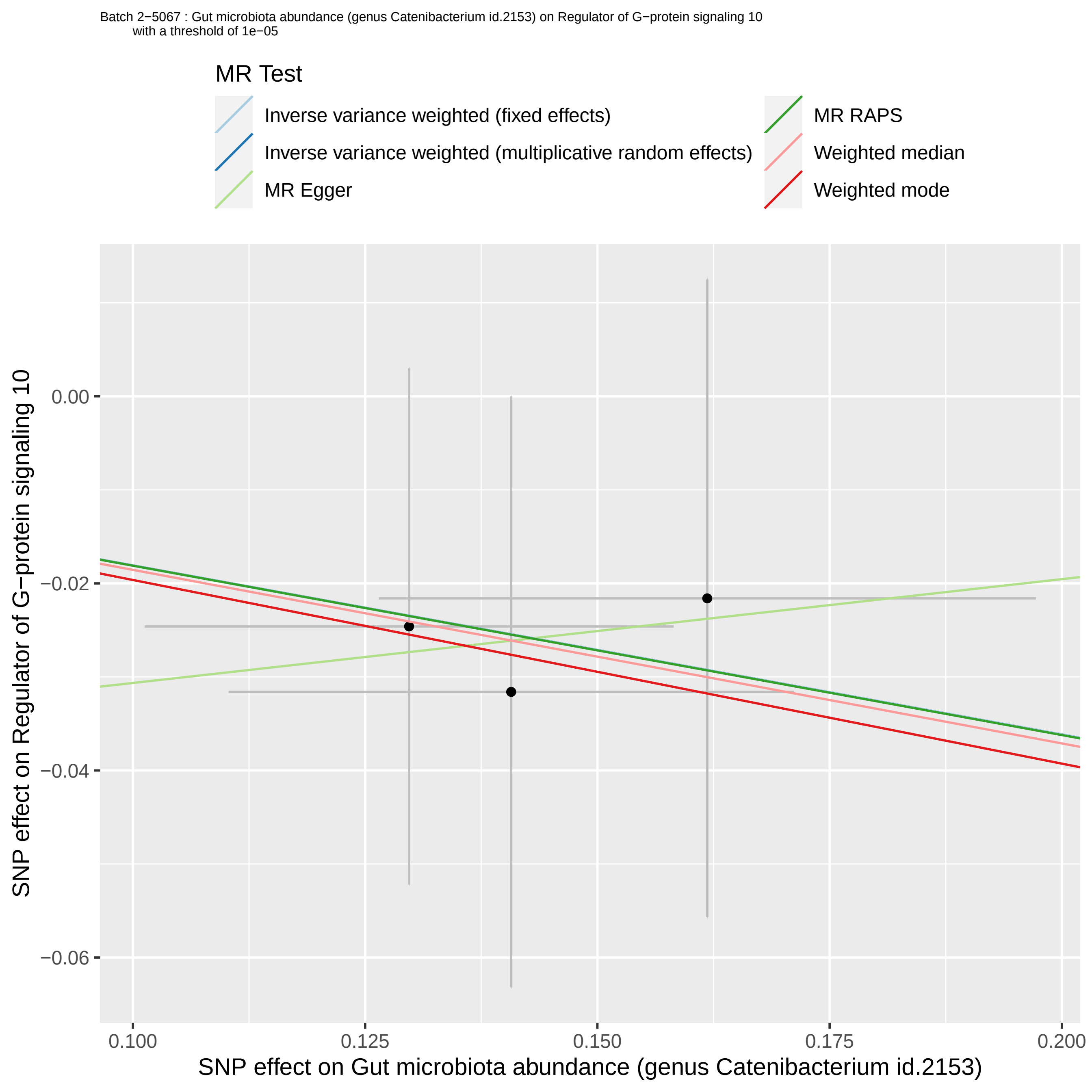

Batch 2−5067 : Gut microbiota abundance (genus Catenibacterium id.2153) on Regulator of G−protein signaling 10
with a threshold of 1e−05
MR Test
Inverse variance weighted (fixed effects)
Inverse variance weighted (multiplicative random effects)
MR Egger
MR RAPS
Weighted median
Weighted mode
SNP effect on Regulator of G−protein signaling 10
0.00
−0.02
−0.04
−0.06
0.100
0.125
0.150
0.175
0.200
SNP effect on Gut microbiota abundance (genus Catenibacterium id.2153)

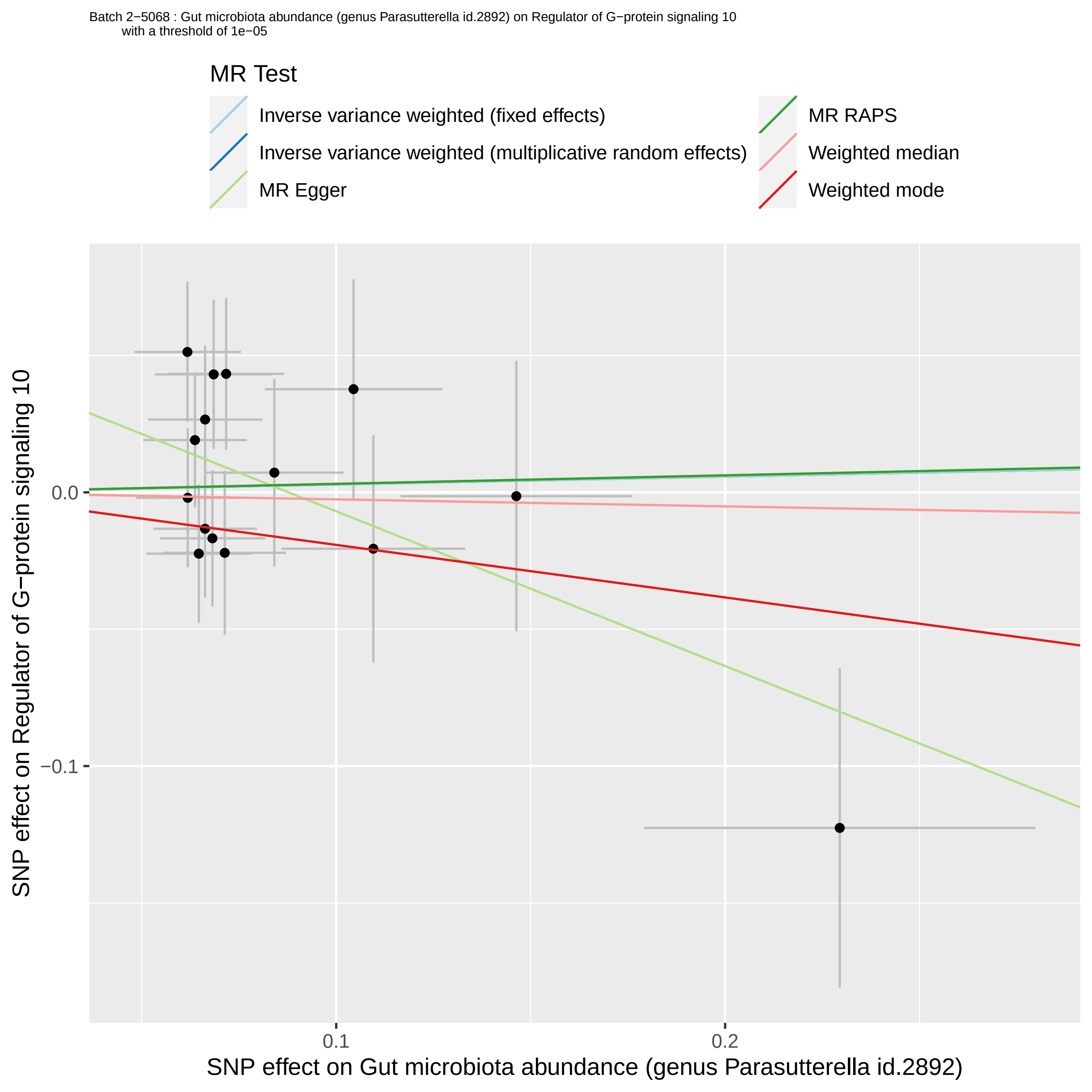

Batch 2−5068 : Gut microbiota abundance (genus Parasutterella id.2892) on Regulator of G−protein signaling 10
with a threshold of 1e−05
MR Test
Inverse variance weighted (fixed effects)
Inverse variance weighted (multiplicative random effects)
MR Egger
MR RAPS
Weighted median
Weighted mode
SNP effect on Regulator of G−protein signaling 10
0.0
−0.1
0.1
0.2
SNP effect on Gut microbiota abundance (genus Parasutterella id.2892)

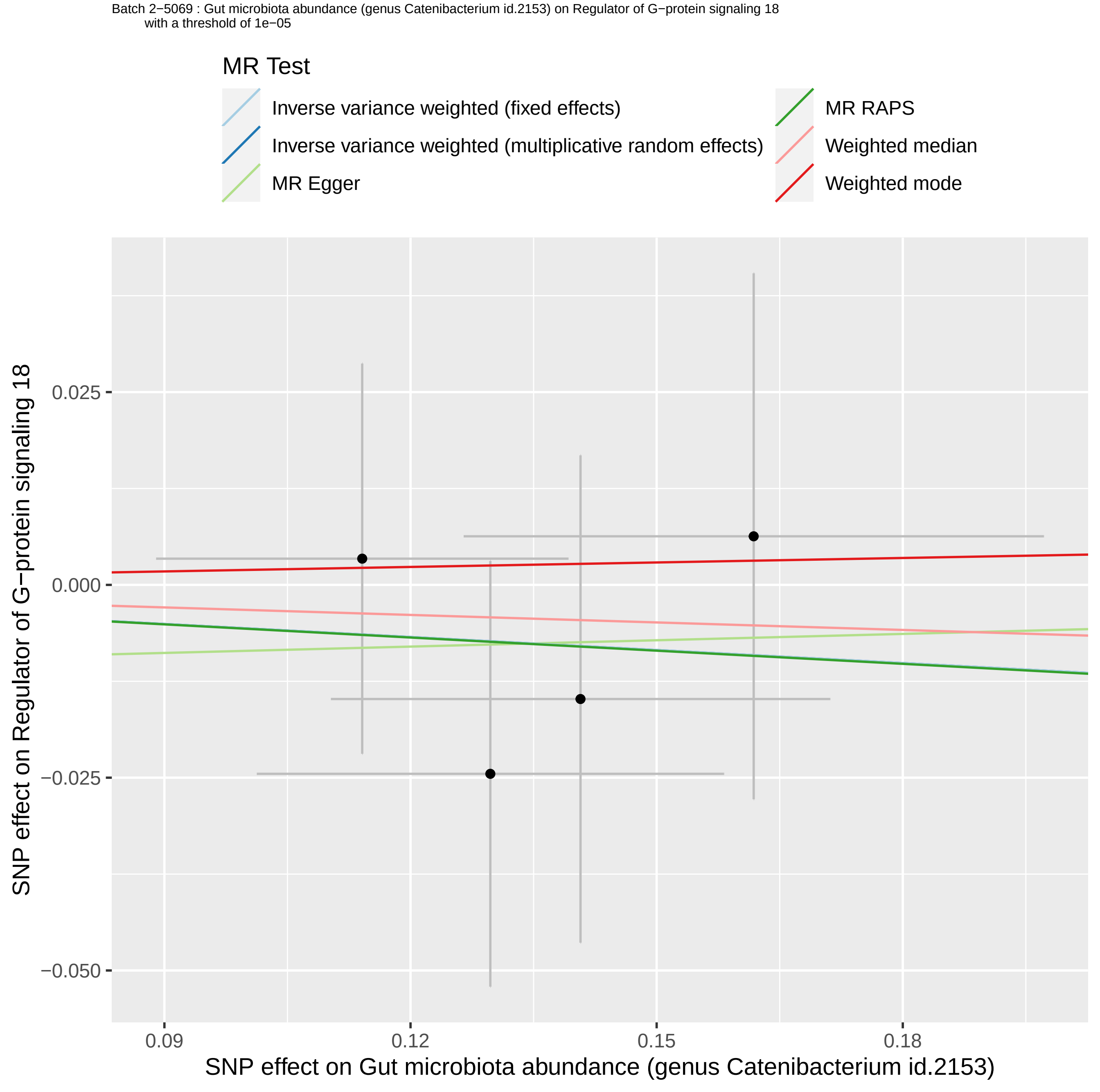

Batch 2−5069 : Gut microbiota abundance (genus Catenibacterium id.2153) on Regulator of G−protein signaling 18
with a threshold of 1e−05
MR Test
Inverse variance weighted (fixed effects)
Inverse variance weighted (multiplicative random effects)
MR Egger
MR RAPS
Weighted median
Weighted mode
0.025
0.000
−0.025
−0.050
0.09
0.12
0.15
0.18
SNP effect on Regulator of G−protein signaling 18
SNP effect on Gut microbiota abundance (genus Catenibacterium id.2153)

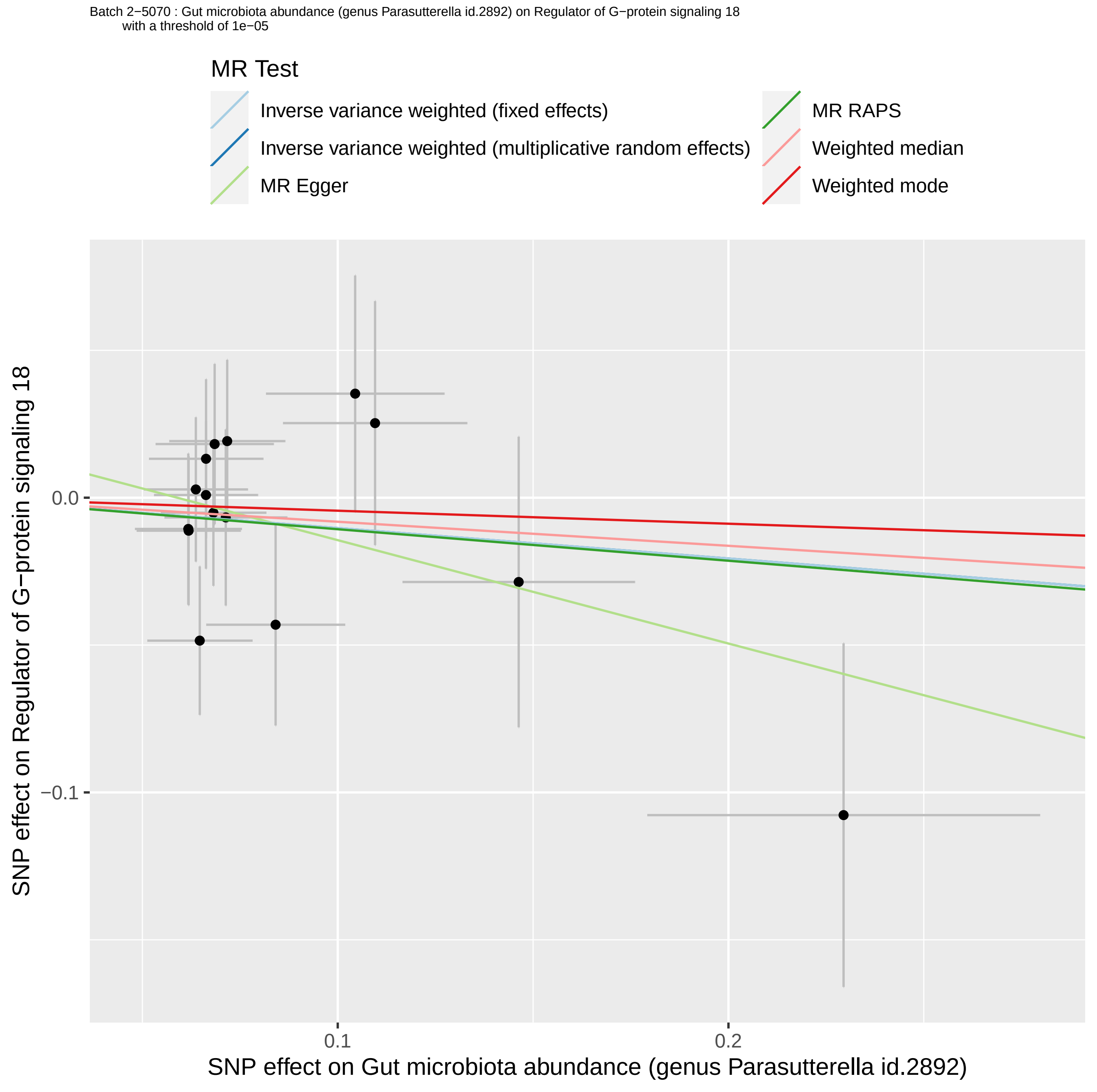

Batch 2−5070 : Gut microbiota abundance (genus Parasutterella id.2892) on Regulator of G−protein signaling 18
with a threshold of 1e−05
MR Test
Inverse variance weighted (fixed effects)
Inverse variance weighted (multiplicative random effects)
MR Egger
MR RAPS
Weighted median
Weighted mode
0.0
−0.1
0.1
0.2
SNP effect on Regulator of G−protein signaling 18
SNP effect on Gut microbiota abundance (genus Parasutterella id.2892)

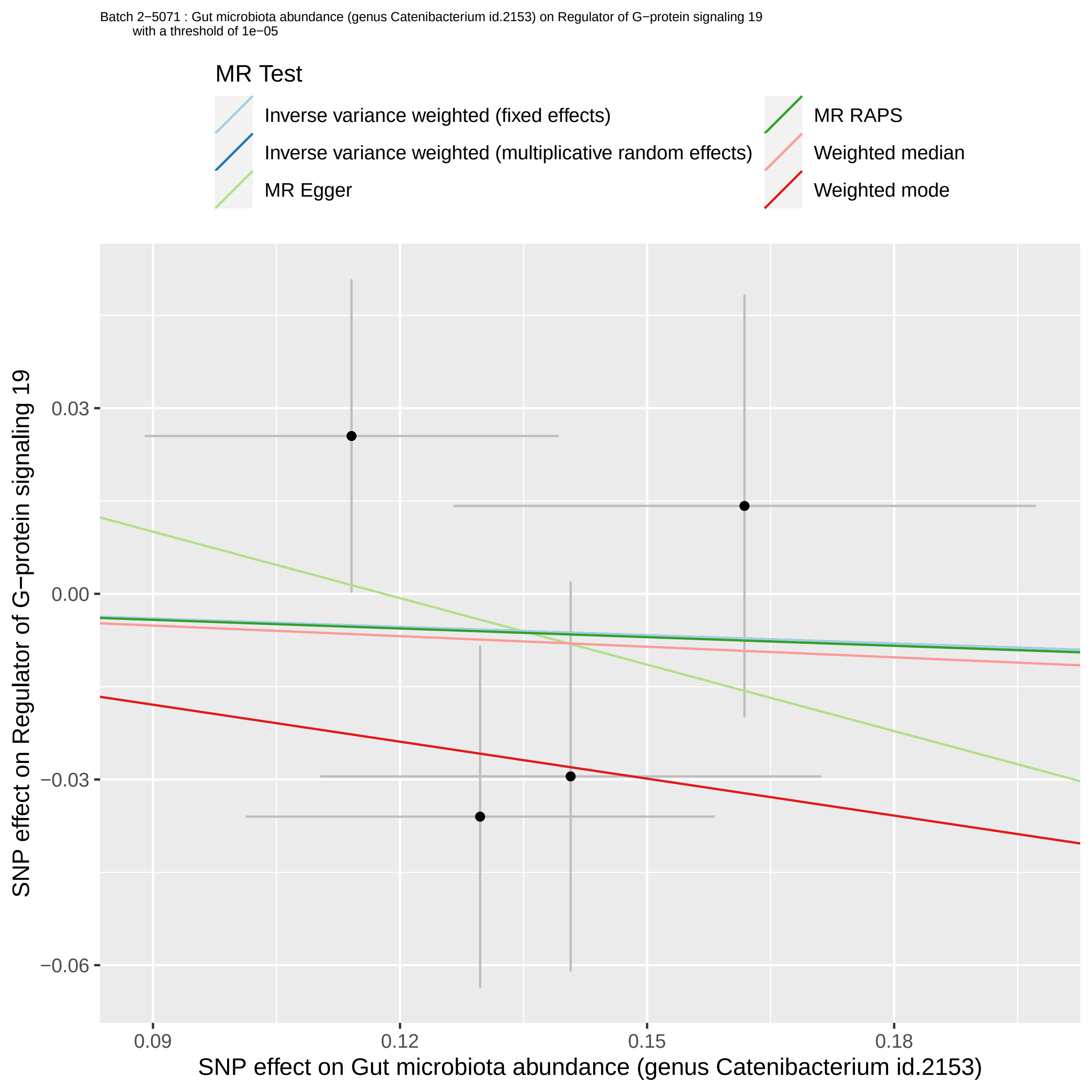
Batch 2−5071 : Gut microbiota abundance (genus Catenibacterium id.2153) on Regulator of G−protein signaling 19
with a threshold of 1e−05
MR Test
Inverse variance weighted (fixed effects)
Inverse variance weighted (multiplicative random effects)
MR Egger
MR RAPS
Weighted median
Weighted mode
SNP effect on Regulator of G−protein signaling 19
0.03
0.00
−0.03
−0.06
0.09
0.12
0.15
0.18
SNP effect on Gut microbiota abundance (genus Catenibacterium id.2153)

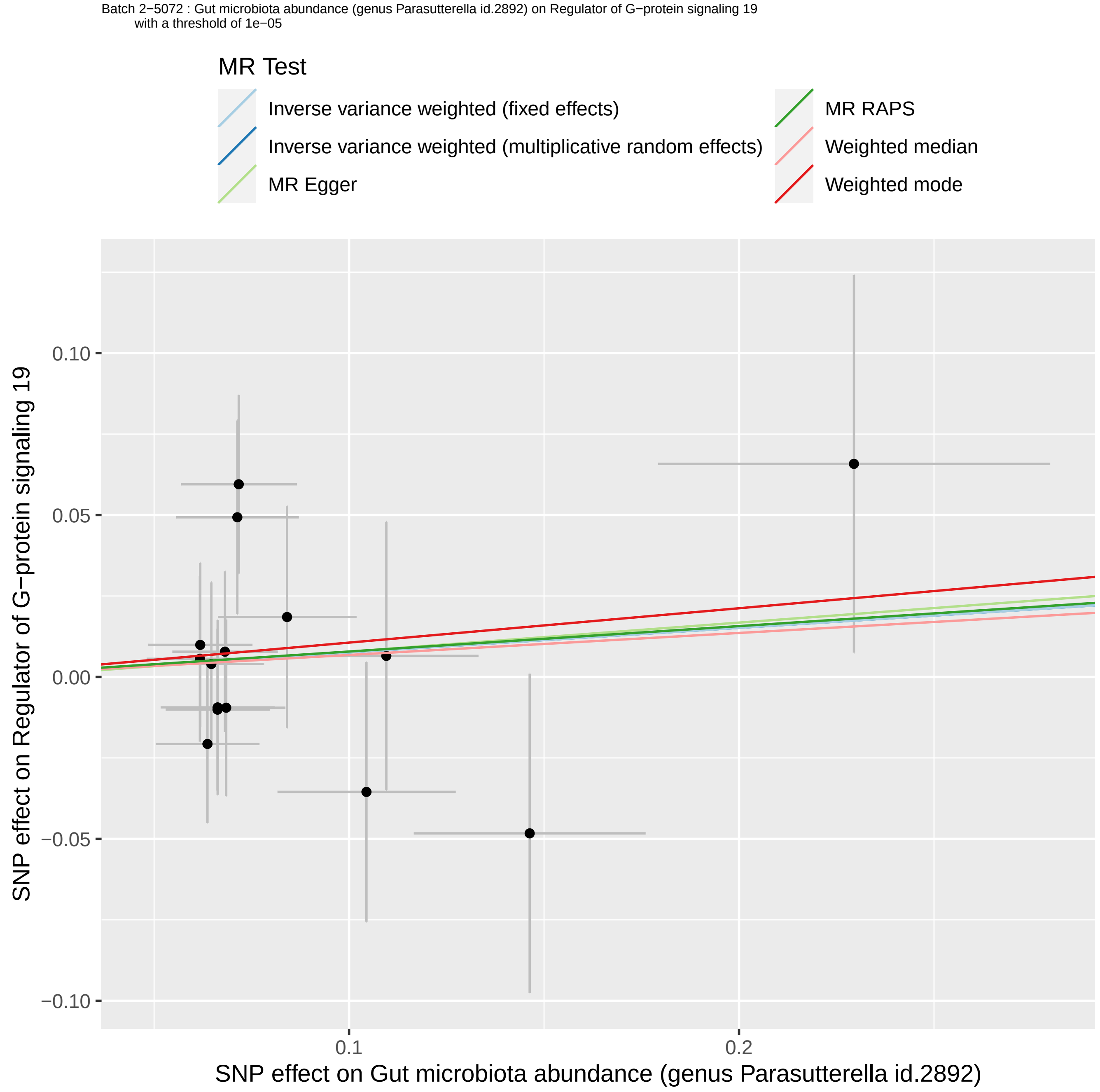
Batch 2−5072 : Gut microbiota abundance (genus Parasutterella id.2892) on Regulator of G−protein signaling 19
with a threshold of 1e−05
MR Test
Inverse variance weighted (fixed effects)
Inverse variance weighted (multiplicative random effects)
MR Egger
MR RAPS
Weighted median
Weighted mode
0.10
0.05
0.00
−0.05
−0.10
0.1
0.2
SNP effect on Regulator of G−protein signaling 19
SNP effect on Gut microbiota abundance (genus Parasutterella id.2892)

Batch 2−5073 : Gut microbiota abundance (genus Catenibacterium id.2153) on Regulator of G−protein signaling 3
with a threshold of 1e−05

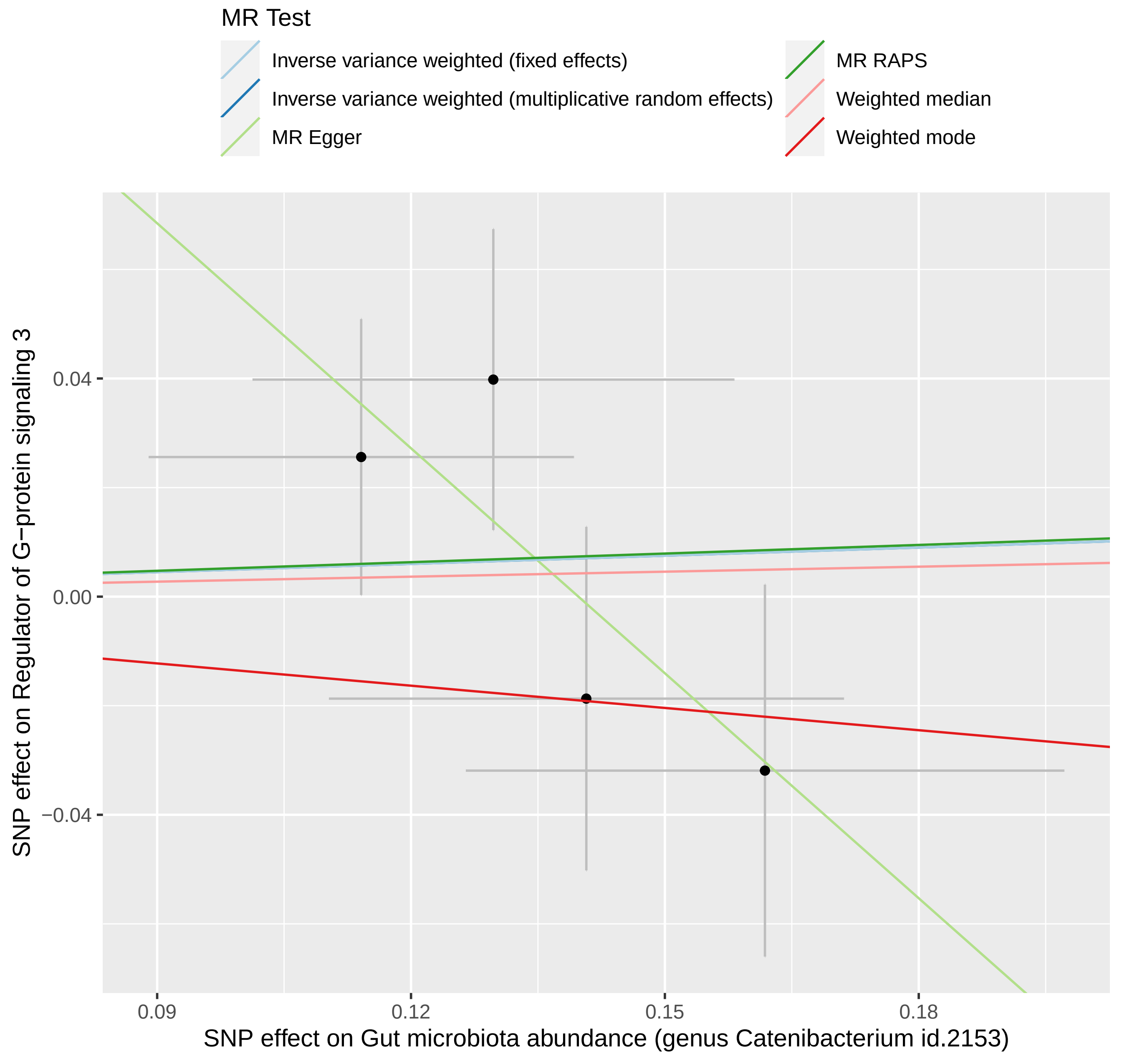

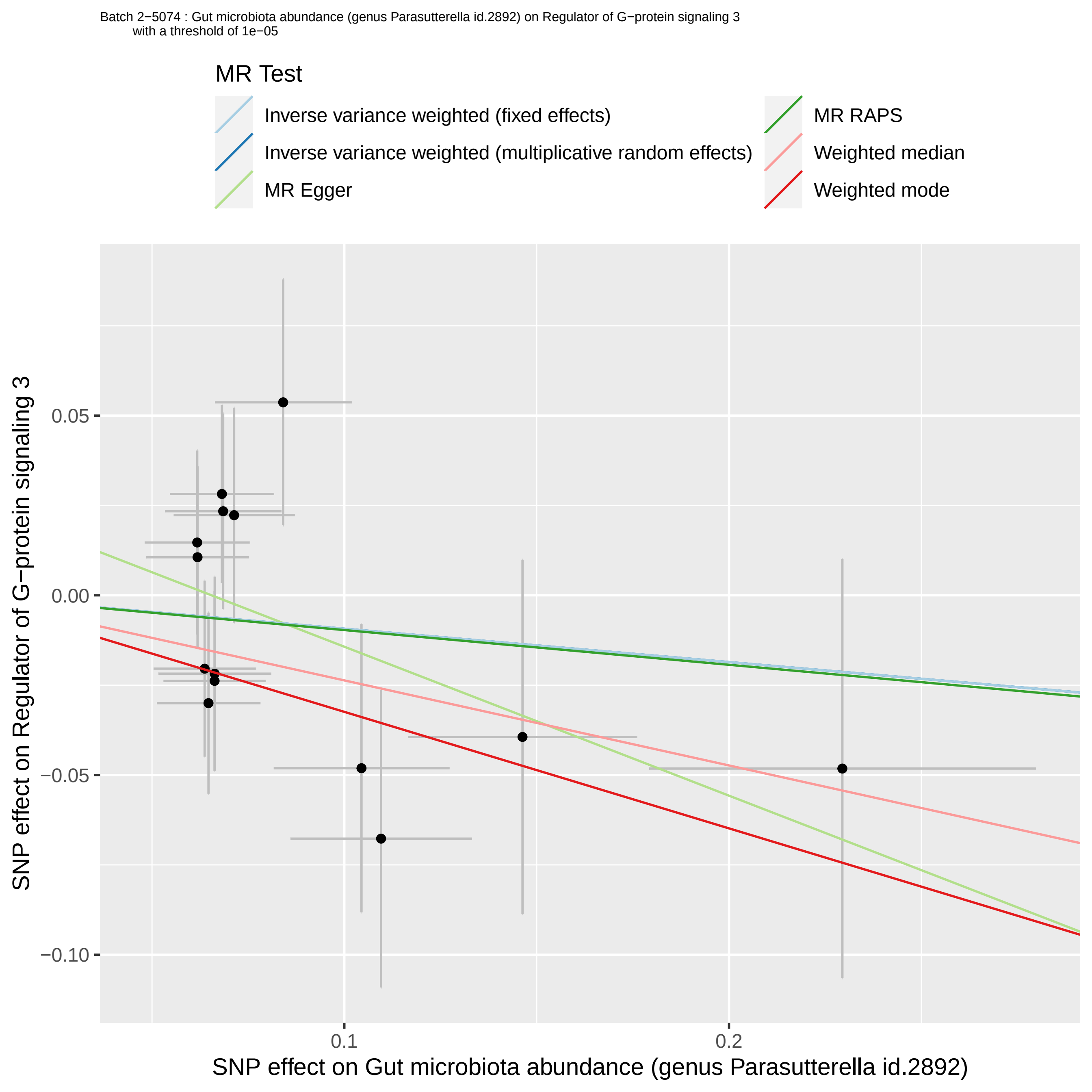

Batch 2−5074 : Gut microbiota abundance (genus Parasutterella id.2892) on Regulator of G−protein signaling 3
with a threshold of 1e−05
MR Test
Inverse variance weighted (fixed effects)
Inverse variance weighted (multiplicative random effects)
MR Egger
MR RAPS
Weighted median
Weighted mode
SNP effect on Regulator of G−protein signaling 3
0.05
0.00
−0.05
−0.10
0.1
0.2
SNP effect on Gut microbiota abundance (genus Parasutterella id.2892)

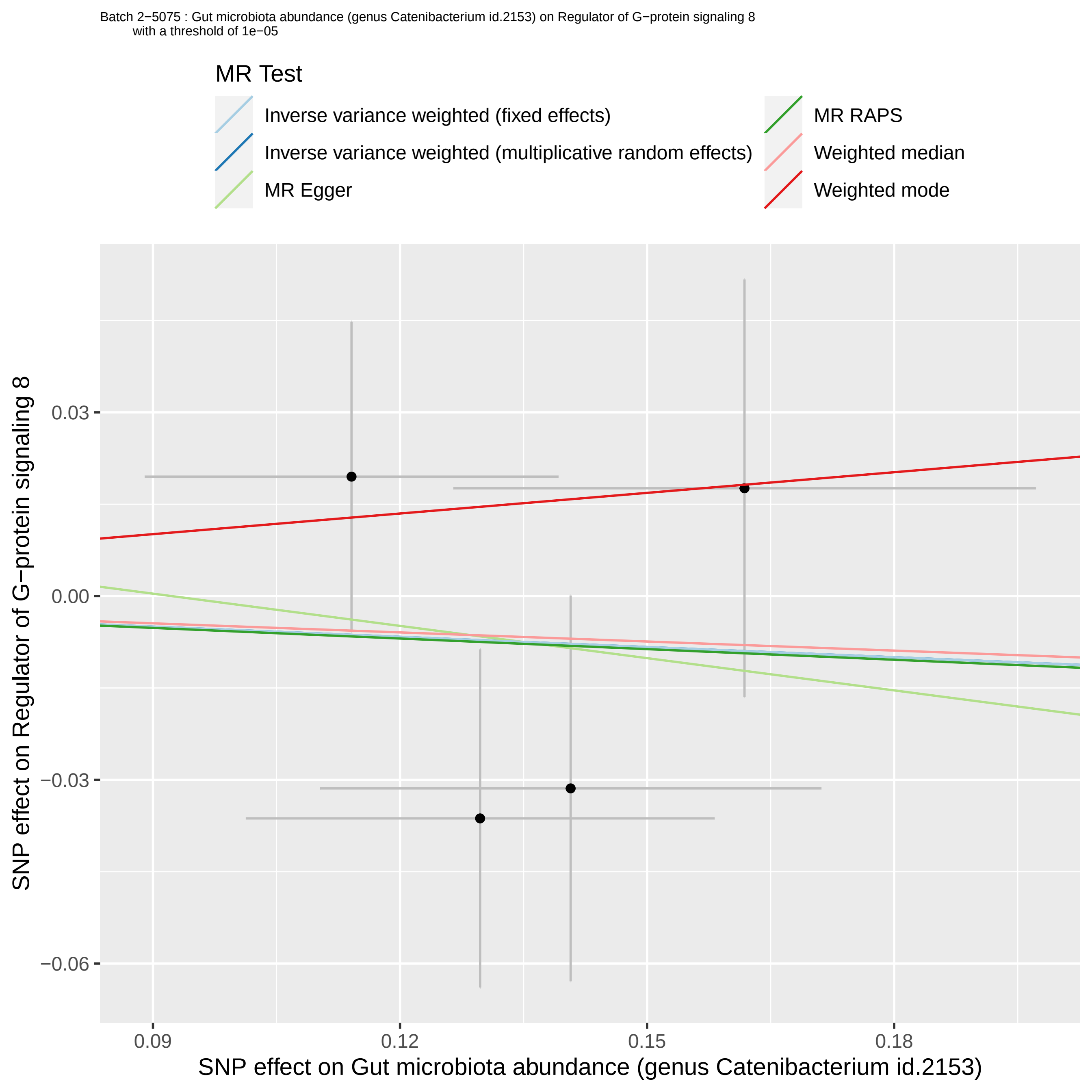
Batch 2−5075 : Gut microbiota abundance (genus Catenibacterium id.2153) on Regulator of G−protein signaling 8
with a threshold of 1e−05
MR Test
Inverse variance weighted (fixed effects)
Inverse variance weighted (multiplicative random effects)
MR Egger
MR RAPS
Weighted median
Weighted mode
0.03
0.00
−0.03
−0.06
0.09
0.12
0.15
0.18
SNP effect on Regulator of G−protein signaling 8
SNP effect on Gut microbiota abundance (genus Catenibacterium id.2153)

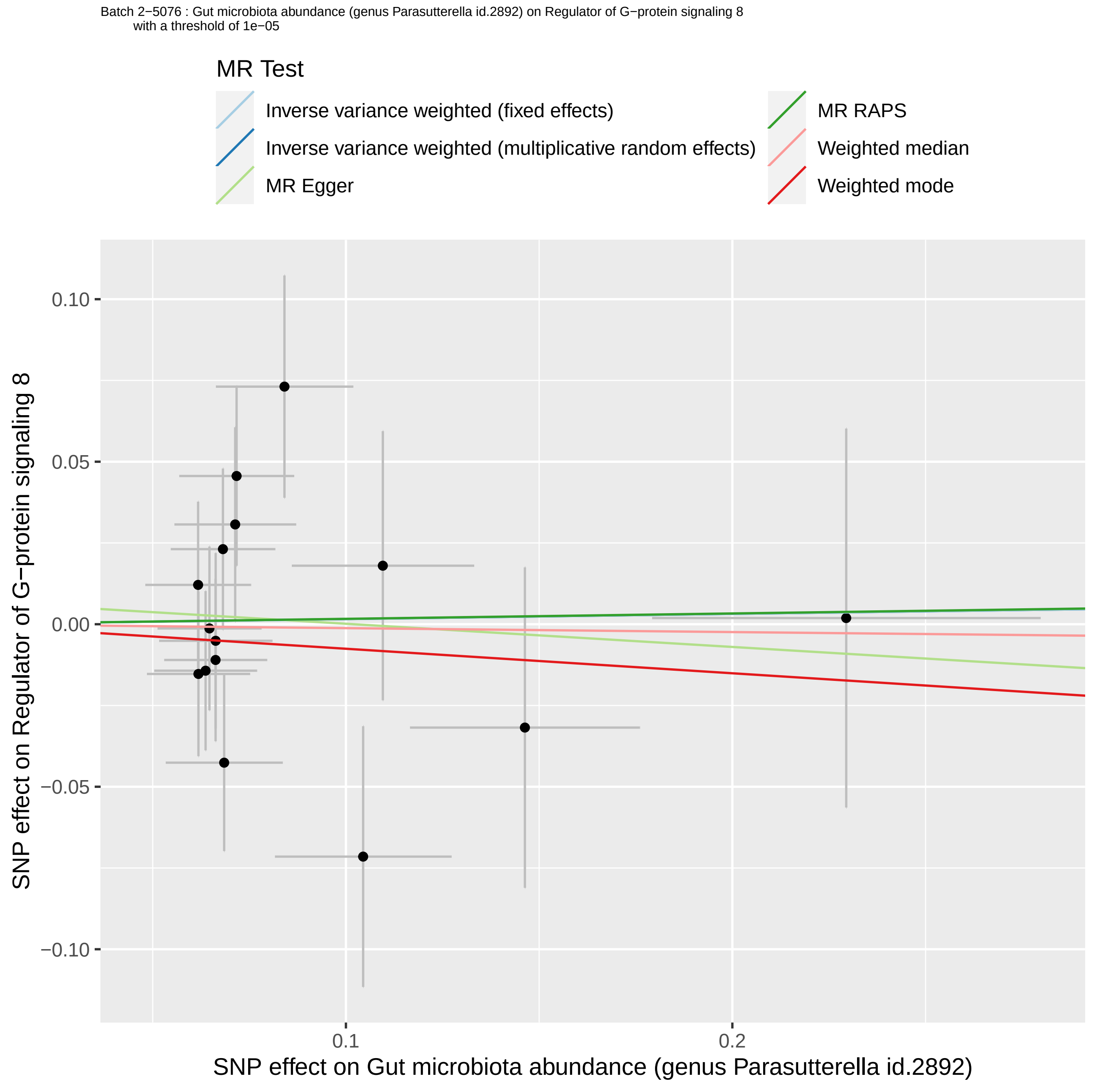
Batch 2−5076 : Gut microbiota abundance (genus Parasutterella id.2892) on Regulator of G−protein signaling 8
with a threshold of 1e−05
MR Test
Inverse variance weighted (fixed effects)
Inverse variance weighted (multiplicative random effects)
MR Egger
MR RAPS
Weighted median
Weighted mode
0.10
0.05
0.00
−0.05
−0.10
0.1
0.2
SNP effect on Regulator of G−protein signaling 8
SNP effect on Gut microbiota abundance (genus Parasutterella id.2892)

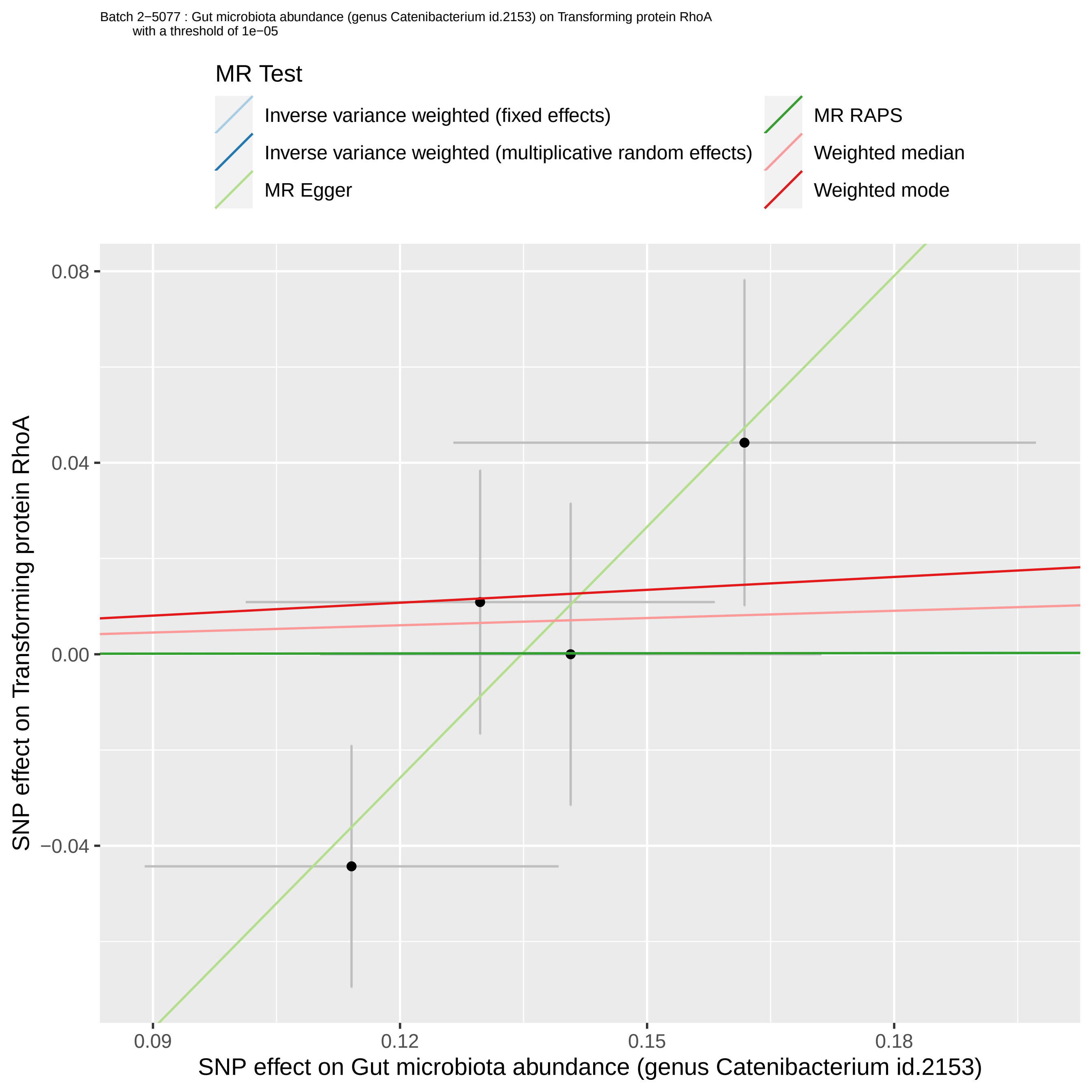
Batch 2−5077 : Gut microbiota abundance (genus Catenibacterium id.2153) on Transforming protein RhoA
with a threshold of 1e−05
MR Test
Inverse variance weighted (fixed effects)
Inverse variance weighted (multiplicative random effects)
MR Egger
MR RAPS
Weighted median
Weighted mode
0.08
0.04
0.00
−0.04
0.09
0.12
0.15
0.18
SNP effect on Transforming protein RhoA
SNP effect on Gut microbiota abundance (genus Catenibacterium id.2153)

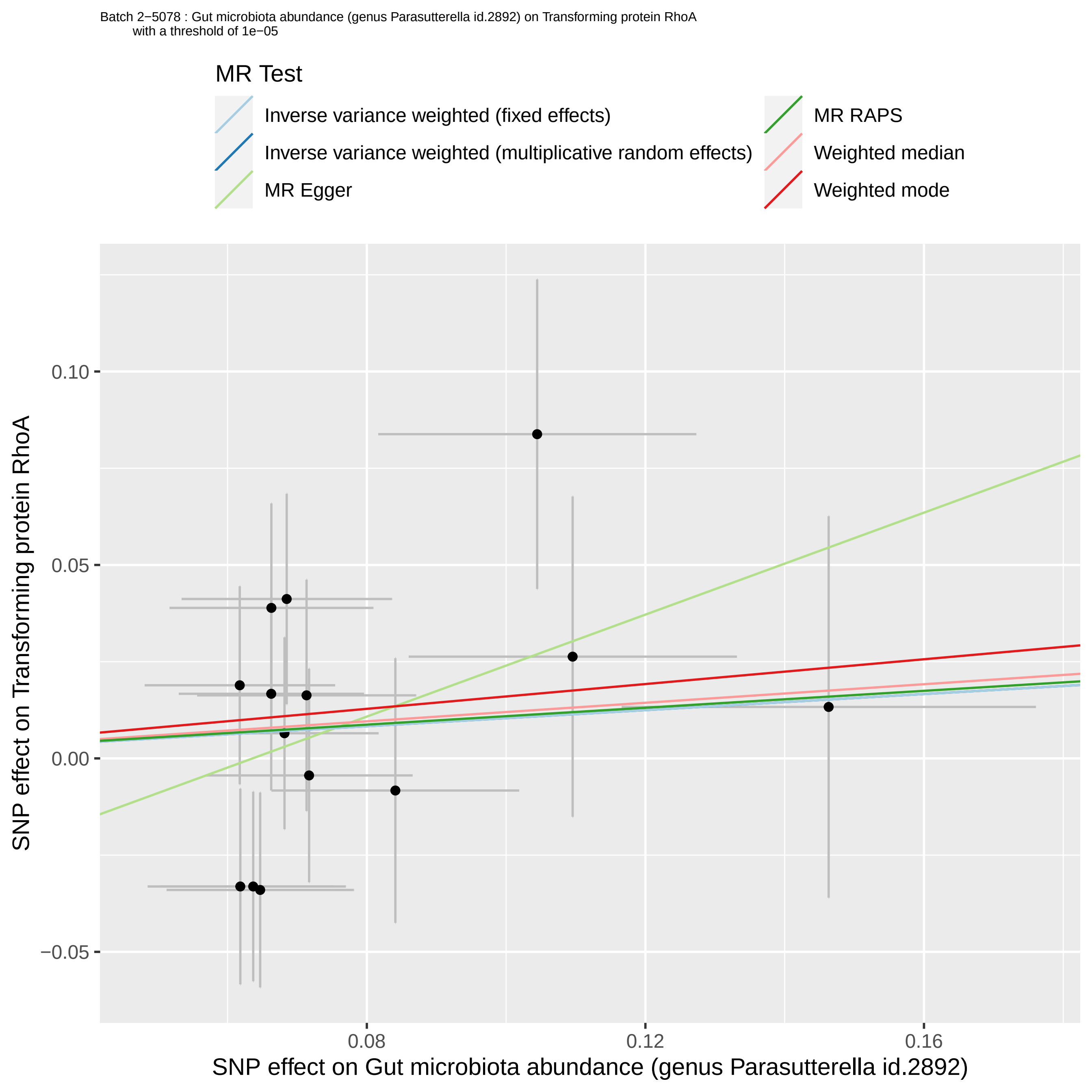

Batch 2−5078 : Gut microbiota abundance (genus Parasutterella id.2892) on Transforming protein RhoA
with a threshold of 1e−05
MR Test
Inverse variance weighted (fixed effects)
Inverse variance weighted (multiplicative random effects)
MR Egger
MR RAPS
Weighted median
Weighted mode
0.10
0.05
0.00
−0.05
SNP effect on Transforming protein RhoA
0.08
0.12
0.16
SNP effect on Gut microbiota abundance (genus Parasutterella id.2892)

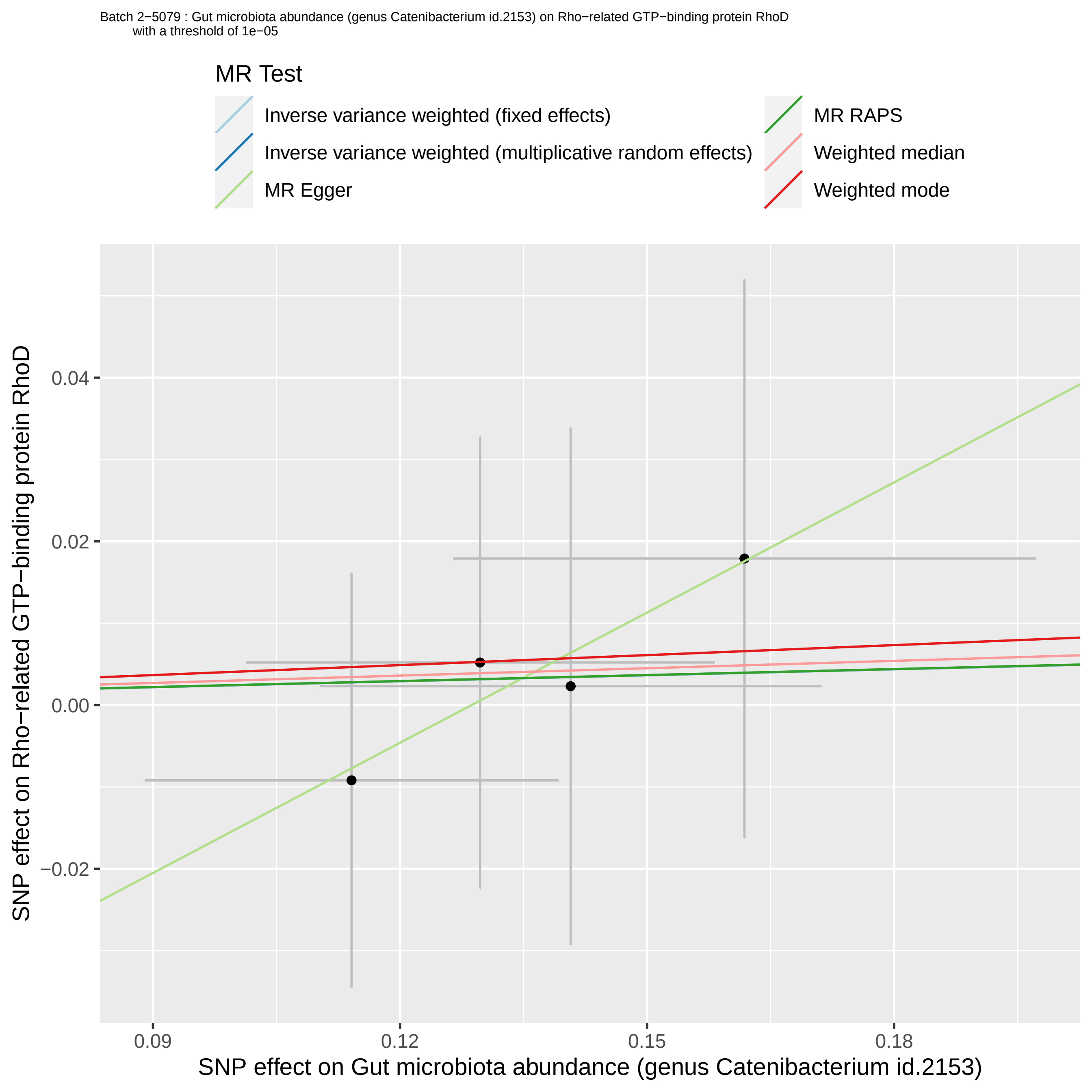
Batch 2−5079 : Gut microbiota abundance (genus Catenibacterium id.2153) on Rho−related GTP−binding protein RhoD
with a threshold of 1e−05
MR Test
Inverse variance weighted (fixed effects)
Inverse variance weighted (multiplicative random effects)
MR Egger
MR RAPS
Weighted median
Weighted mode
0.04
0.02
0.00
−0.02
0.09
0.12
0.15
0.18
SNP effect on Rho−related GTP−binding protein RhoD
SNP effect on Gut microbiota abundance (genus Catenibacterium id.2153)

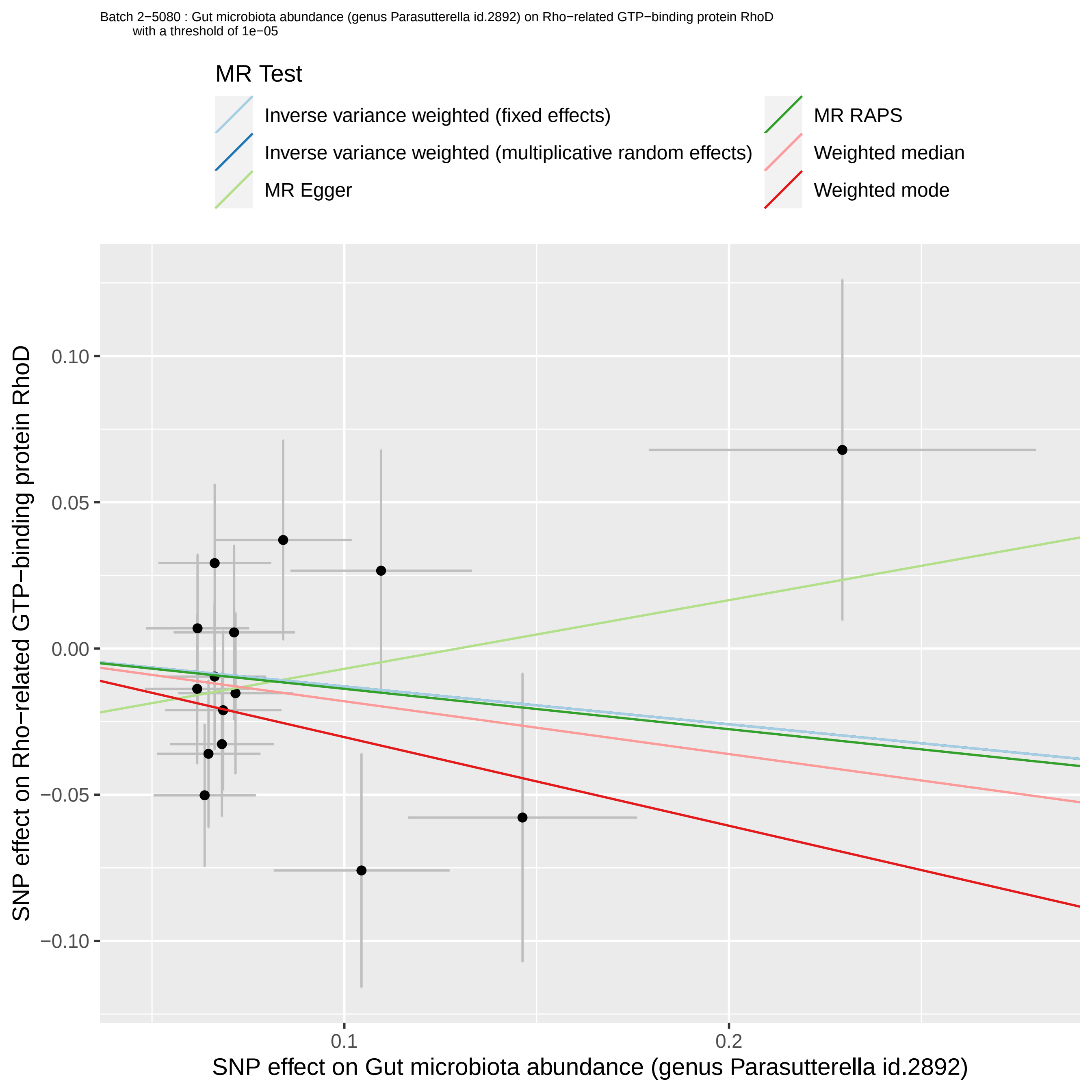

Batch 2−5080 : Gut microbiota abundance (genus Parasutterella id.2892) on Rho−related GTP−binding protein RhoD
with a threshold of 1e−05
MR Test
Inverse variance weighted (fixed effects)
Inverse variance weighted (multiplicative random effects)
MR Egger
MR RAPS
Weighted median
Weighted mode
0.10
0.05
0.00
−0.05
−0.10
0.1
0.2
SNP effect on Rho−related GTP−binding protein RhoD
SNP effect on Gut microbiota abundance (genus Parasutterella id.2892)

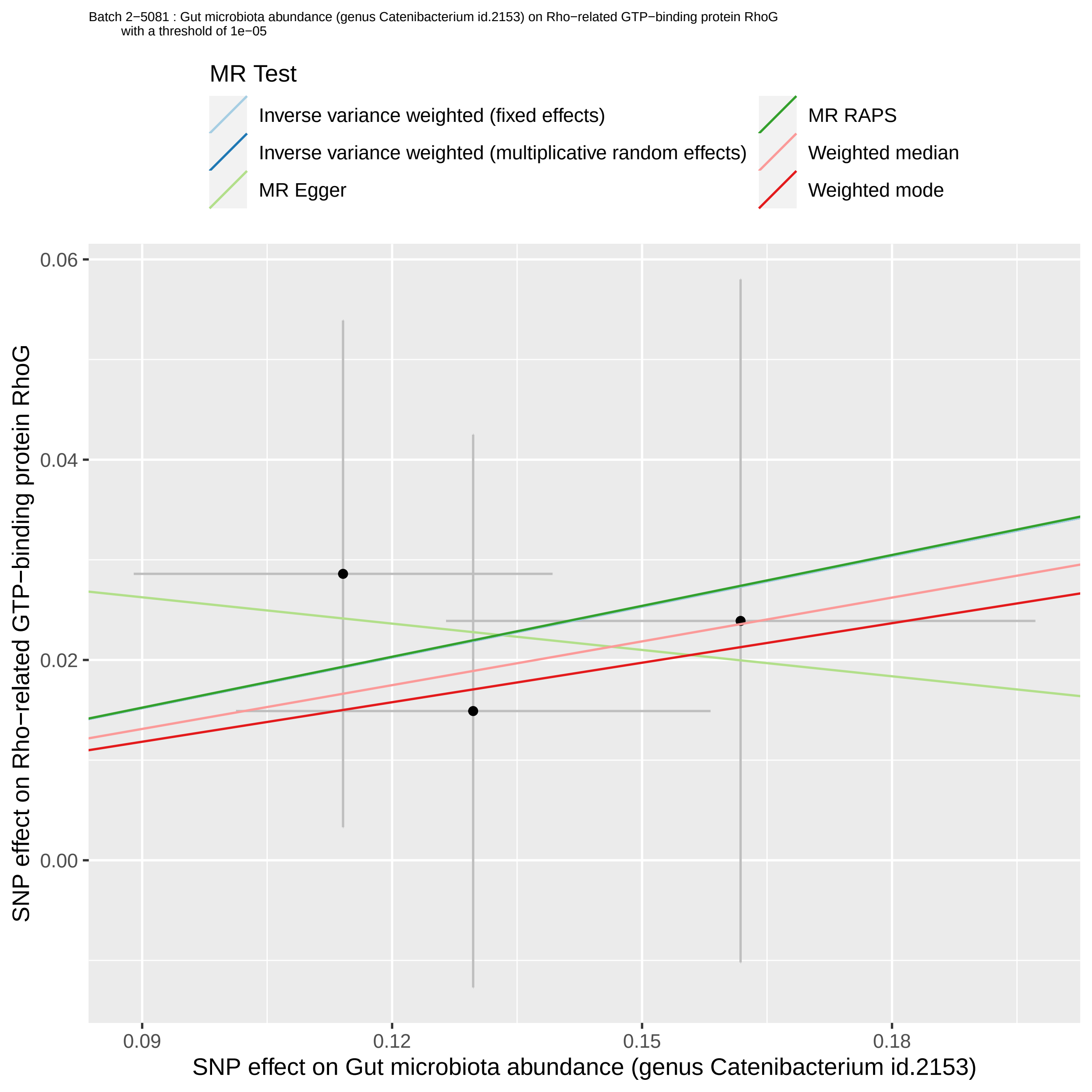
Batch 2−5081 : Gut microbiota abundance (genus Catenibacterium id.2153) on Rho−related GTP−binding protein RhoG
with a threshold of 1e−05
MR Test
Inverse variance weighted (fixed effects)
Inverse variance weighted (multiplicative random effects)
MR Egger
MR RAPS
Weighted median
Weighted mode
0.06
0.04
0.02
0.00
0.09
0.12
0.15
0.18
SNP effect on Rho−related GTP−binding protein RhoG
SNP effect on Gut microbiota abundance (genus Catenibacterium id.2153)

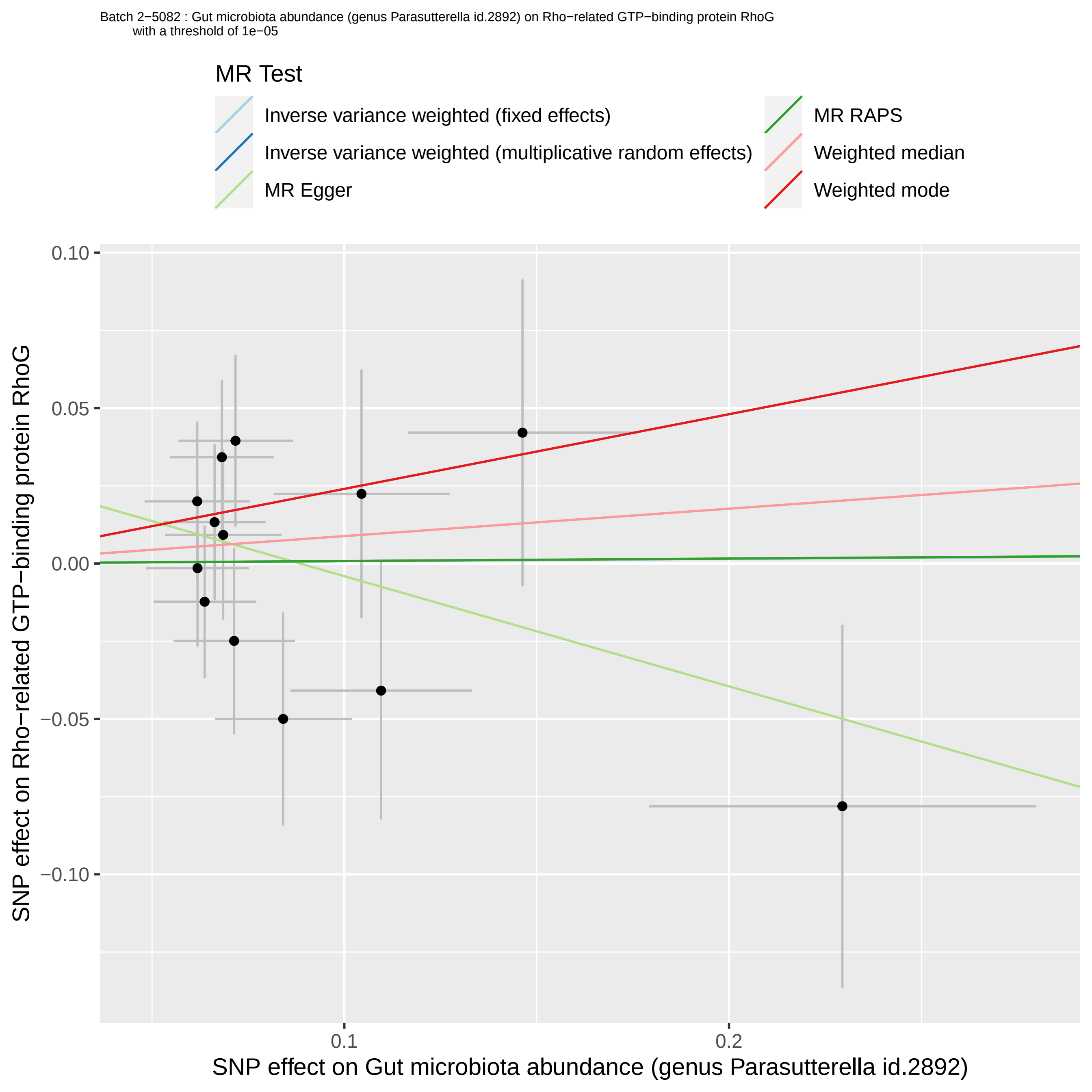

Batch 2−5082 : Gut microbiota abundance (genus Parasutterella id.2892) on Rho−related GTP−binding protein RhoG
with a threshold of 1e−05
MR Test
Inverse variance weighted (fixed effects)
Inverse variance weighted (multiplicative random effects)
MR Egger
MR RAPS
Weighted median
Weighted mode
0.10
0.05
0.00
−0.05
−0.10
0.1
0.2
SNP effect on Rho−related GTP−binding protein RhoG
SNP effect on Gut microbiota abundance (genus Parasutterella id.2892)

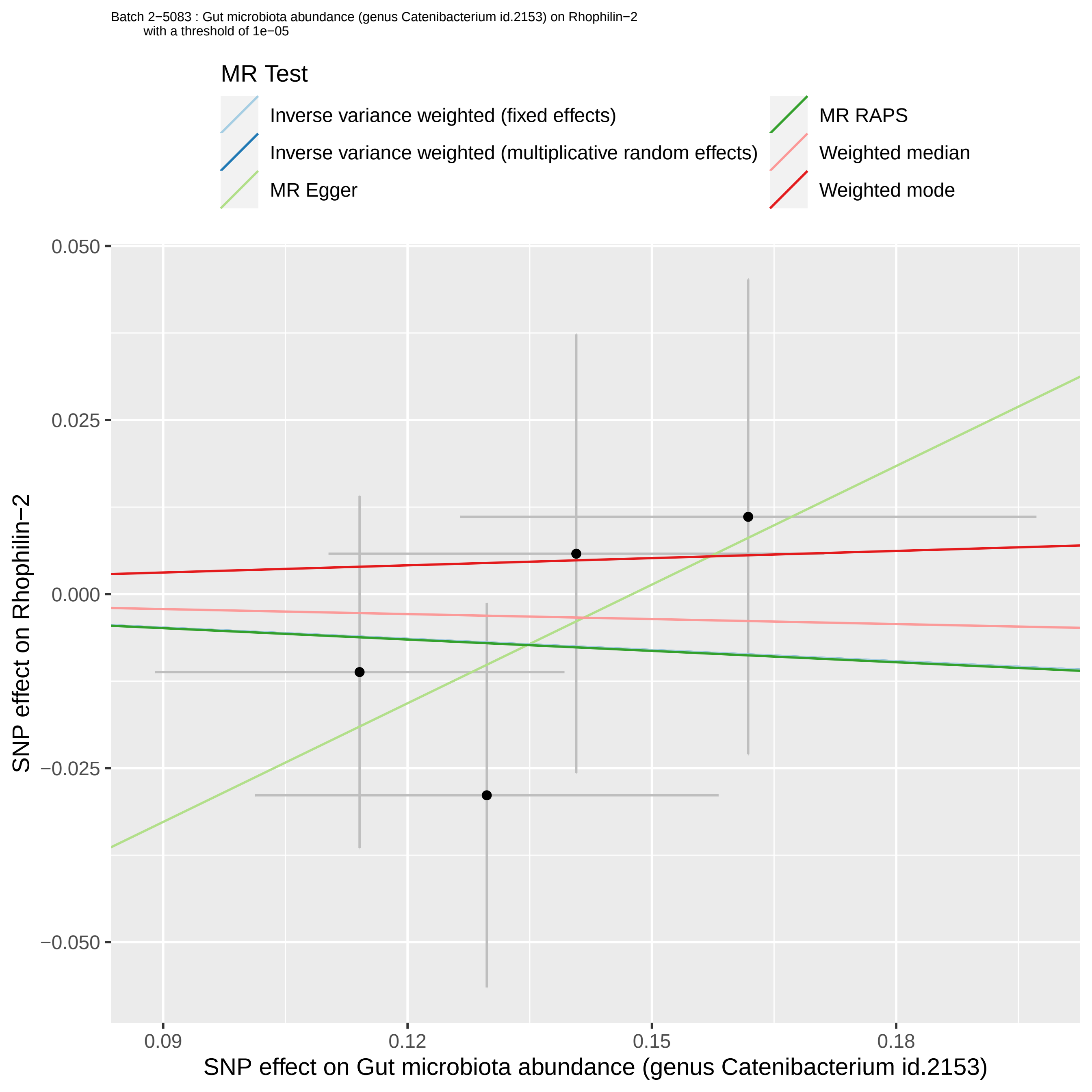

Batch 2−5083 : Gut microbiota abundance (genus Catenibacterium id.2153) on Rhophilin−2
with a threshold of 1e−05
MR Test
Inverse variance weighted (fixed effects)
Inverse variance weighted (multiplicative random effects)
MR Egger
MR RAPS
Weighted median
Weighted mode
0.050
0.025
0.000
−0.025
−0.050
SNP effect on Rhophilin−2
0.09
0.12
0.15
0.18
SNP effect on Gut microbiota abundance (genus Catenibacterium id.2153)

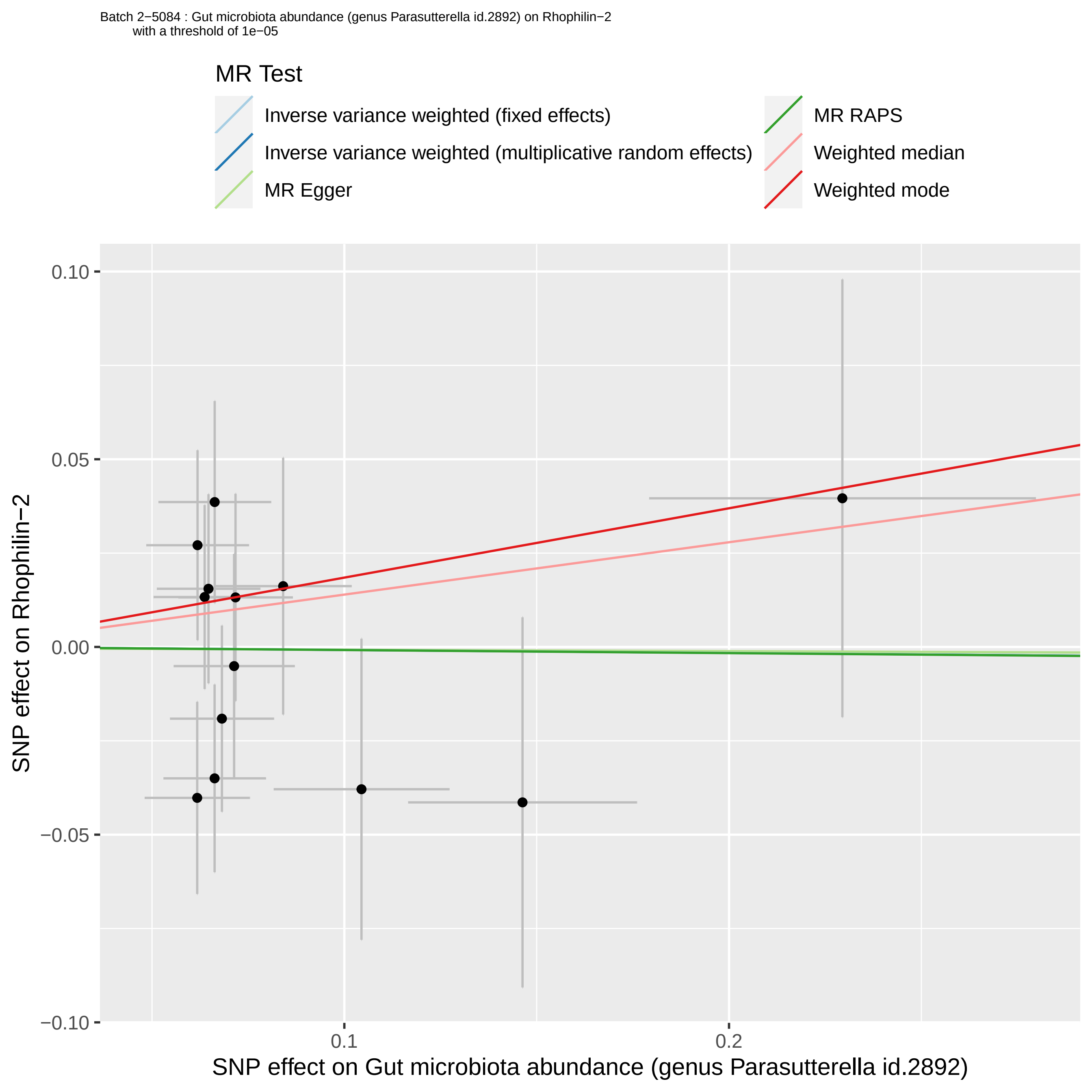

Batch 2−5084 : Gut microbiota abundance (genus Parasutterella id.2892) on Rhophilin−2
with a threshold of 1e−05
MR Test
Inverse variance weighted (fixed effects)
Inverse variance weighted (multiplicative random effects)
MR Egger
MR RAPS
Weighted median
Weighted mode
0.10
0.05
0.00
−0.05
−0.10
0.1
0.2
SNP effect on Rhophilin−2
SNP effect on Gut microbiota abundance (genus Parasutterella id.2892)

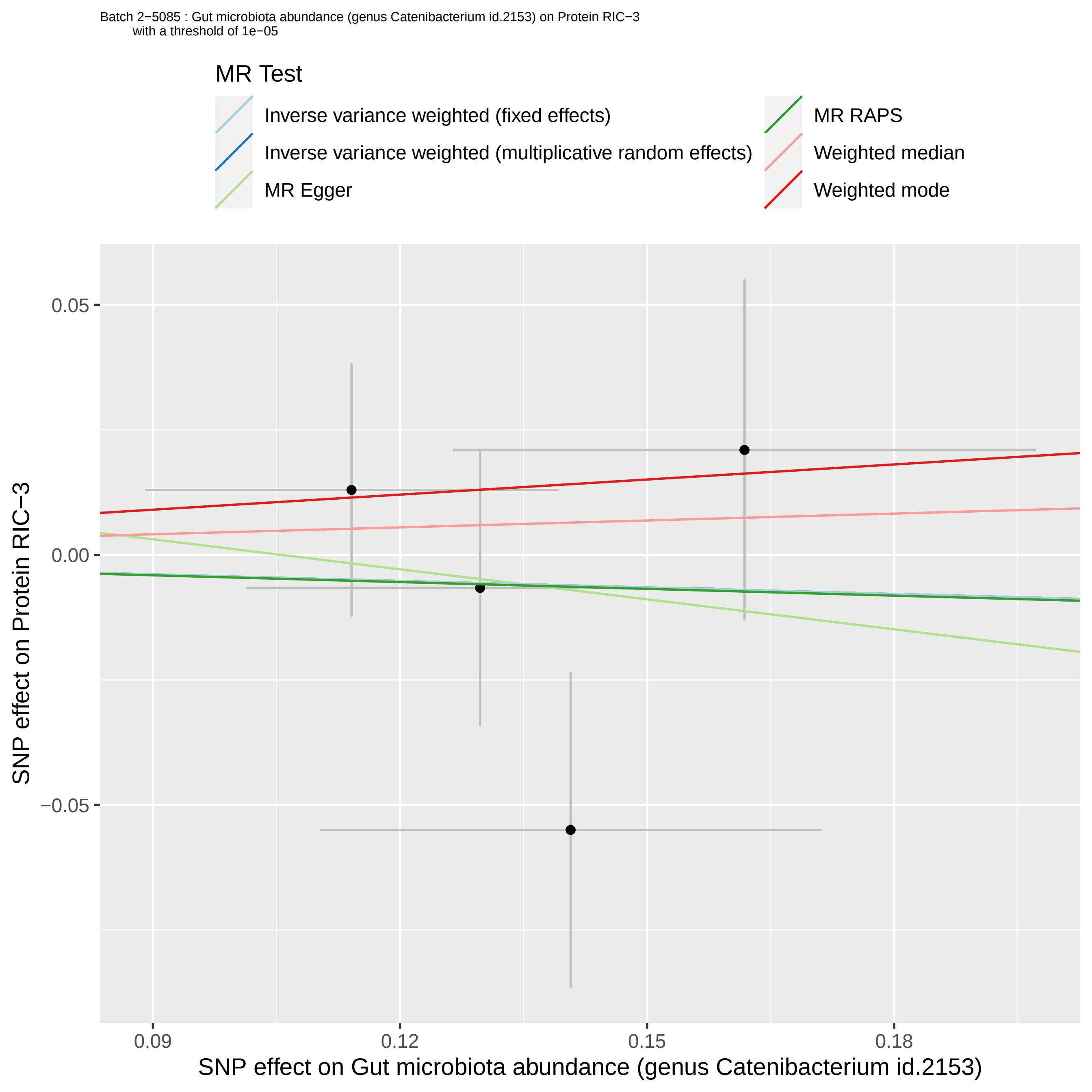
Batch 2−5085 : Gut microbiota abundance (genus Catenibacterium id.2153) on Protein RIC−3
with a threshold of 1e−05
MR Test
Inverse variance weighted (fixed effects)
Inverse variance weighted (multiplicative random effects)
MR Egger
MR RAPS
Weighted median
Weighted mode
0.05
0.00
−0.05
0.09
0.12
0.15
0.18
SNP effect on Protein RIC−3
SNP effect on Gut microbiota abundance (genus Catenibacterium id.2153)

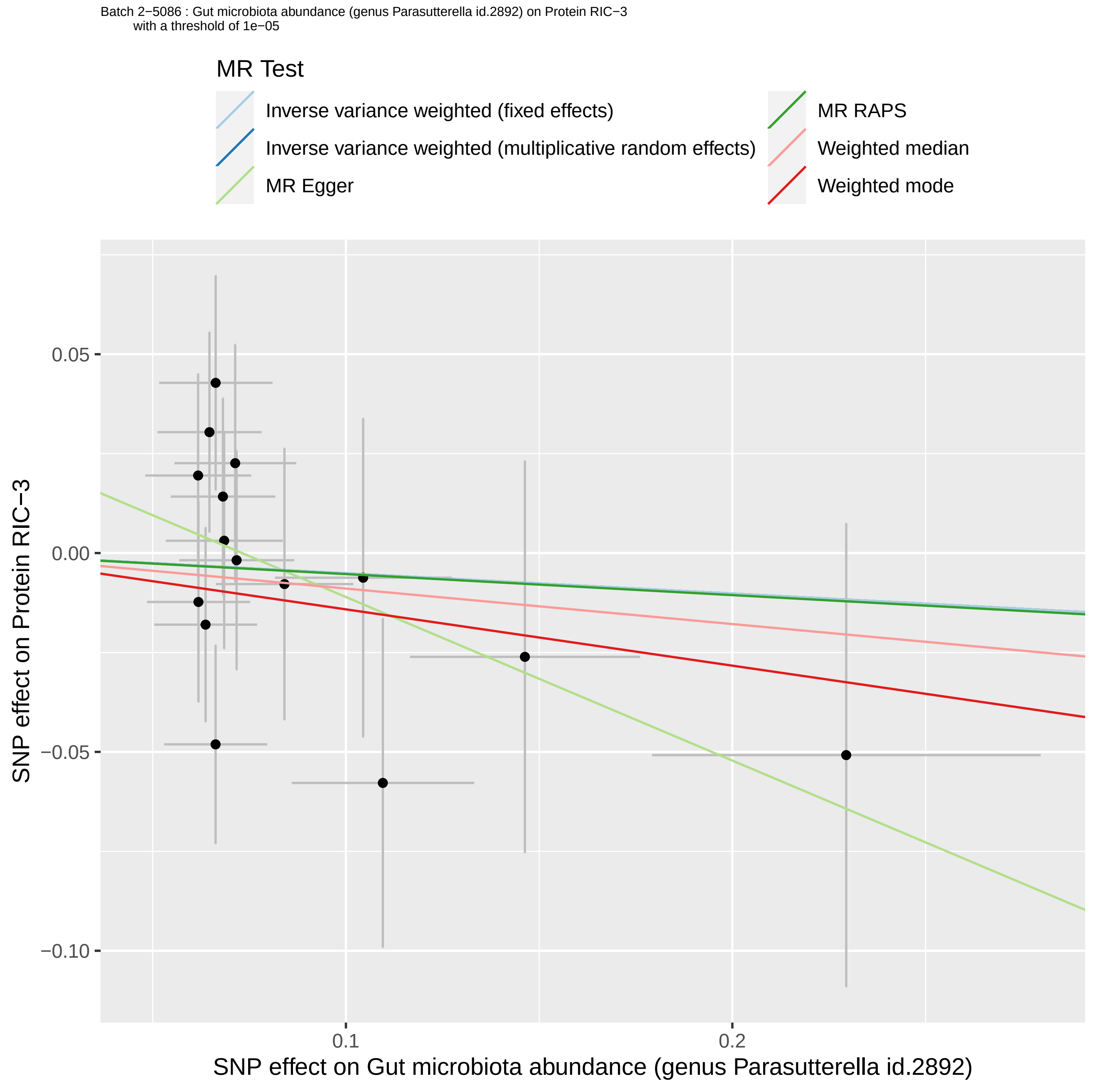

Batch 2−5086 : Gut microbiota abundance (genus Parasutterella id.2892) on Protein RIC−3
with a threshold of 1e−05
MR Test
Inverse variance weighted (fixed effects)
Inverse variance weighted (multiplicative random effects)
MR Egger
MR RAPS
Weighted median
Weighted mode
0.05
0.00
−0.05
−0.10
0.1
0.2
SNP effect on Protein RIC−3
SNP effect on Gut microbiota abundance (genus Parasutterella id.2892)

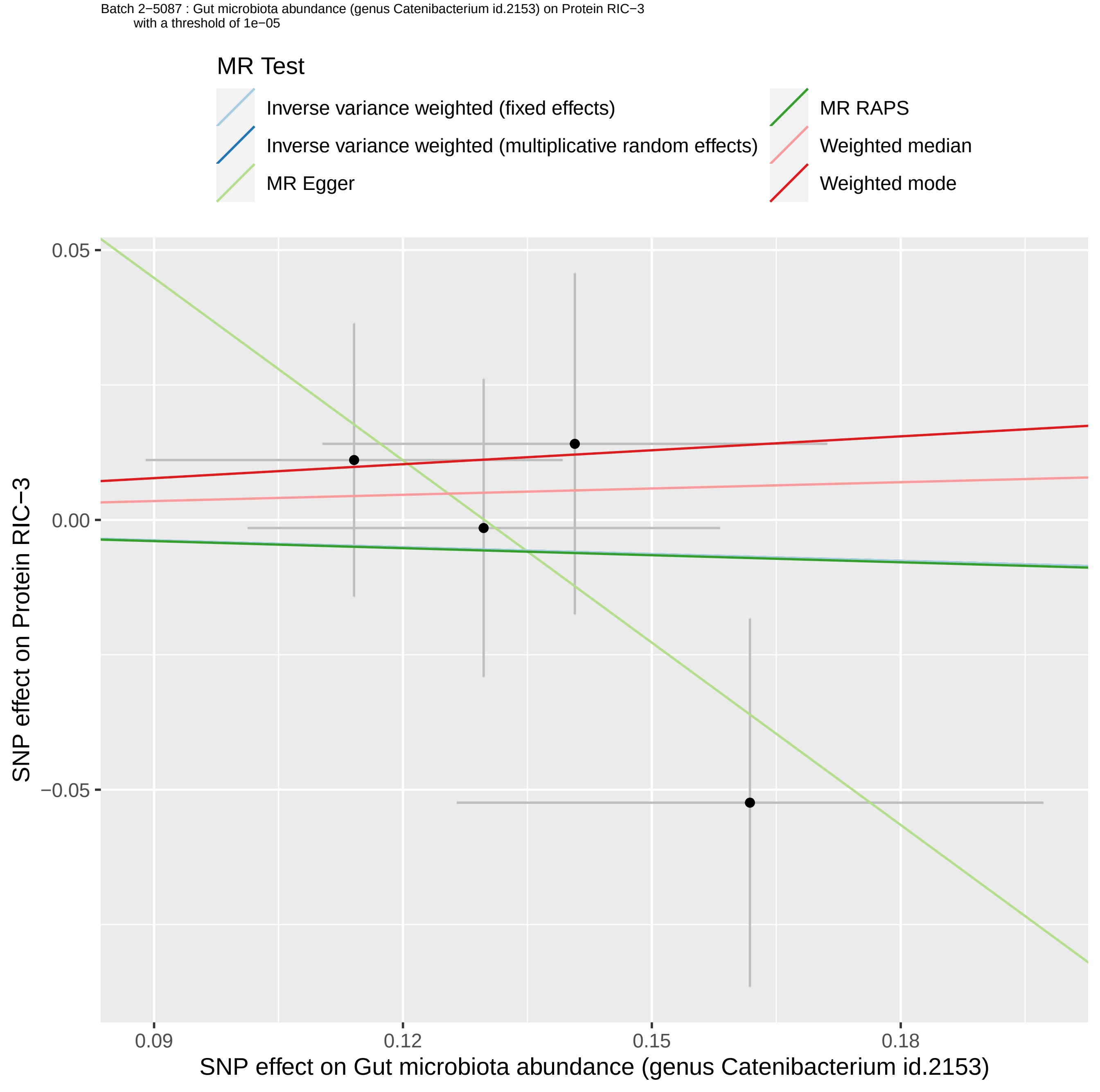
Batch 2−5087 : Gut microbiota abundance (genus Catenibacterium id.2153) on Protein RIC−3
with a threshold of 1e−05
MR Test
Inverse variance weighted (fixed effects)
Inverse variance weighted (multiplicative random effects)
MR Egger
MR RAPS
Weighted median
Weighted mode
0.05
0.00
−0.05
0.09
0.12
0.15
0.18
SNP effect on Protein RIC−3
SNP effect on Gut microbiota abundance (genus Catenibacterium id.2153)

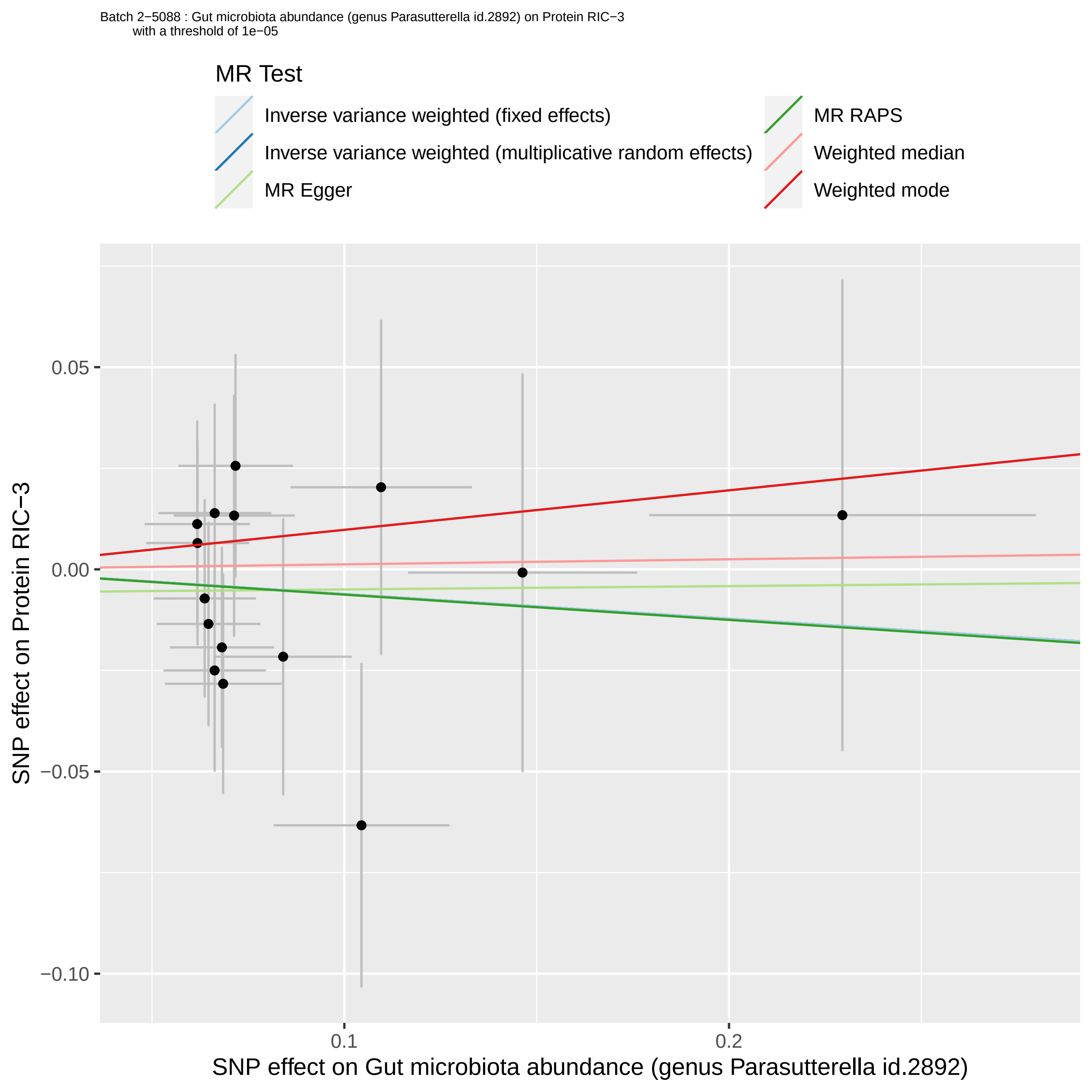
Batch 2−5088 : Gut microbiota abundance (genus Parasutterella id.2892) on Protein RIC−3
with a threshold of 1e−05
MR Test
Inverse variance weighted (fixed effects)
Inverse variance weighted (multiplicative random effects)
MR Egger
MR RAPS
Weighted median
Weighted mode
0.05
0.00
−0.05
−0.10
0.1
0.2
SNP effect on Protein RIC−3
SNP effect on Gut microbiota abundance (genus Parasutterella id.2892)

Batch 2−5089 : Gut microbiota abundance (genus Catenibacterium id.2153) on Receptor−interacting serine/threonine−protein kinase 2
with a threshold of 1e−05

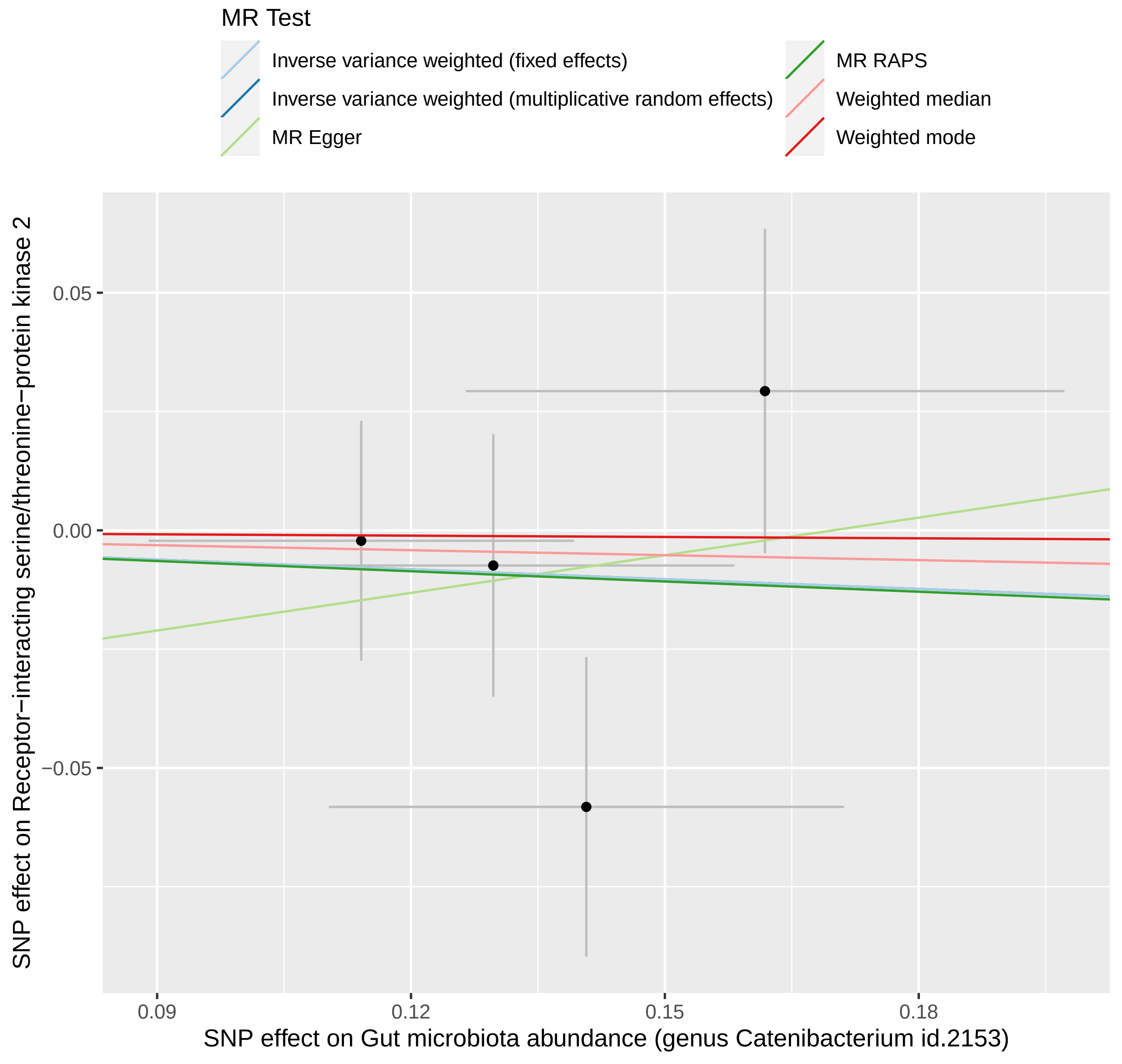

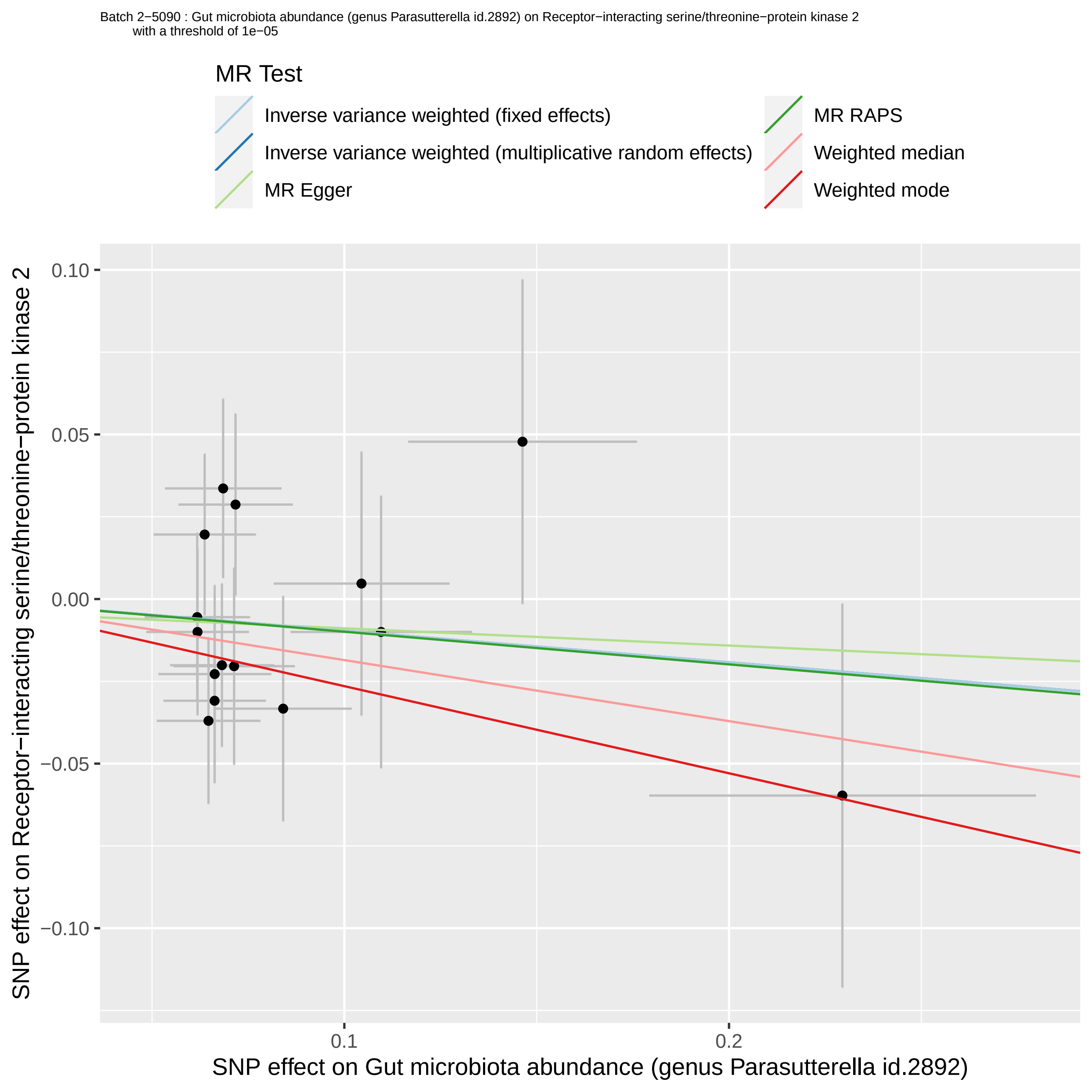
Batch 2−5090 : Gut microbiota abundance (genus Parasutterella id.2892) on Receptor−interacting serine/threonine−protein kinase 2
with a threshold of 1e−05
MR Test
Inverse variance weighted (fixed effects)
Inverse variance weighted (multiplicative random effects)
MR Egger
MR RAPS
Weighted median
Weighted mode
0.10
0.05
0.00
−0.05
−0.10
SNP effect on Receptor−interacting serine/threonine−protein kinase 2
0.1
0.2
SNP effect on Gut microbiota abundance (genus Parasutterella id.2892)

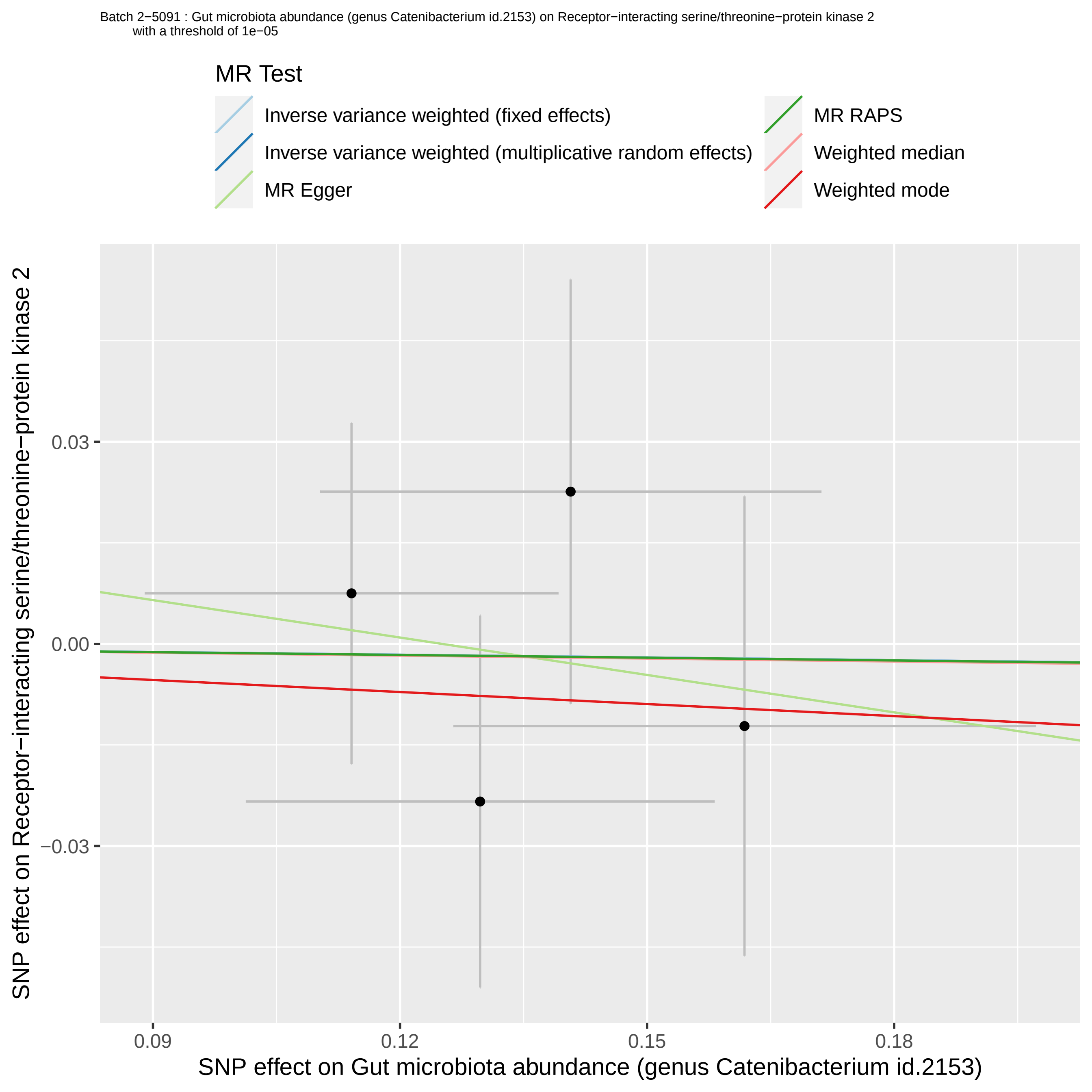

Batch 2−5091 : Gut microbiota abundance (genus Catenibacterium id.2153) on Receptor−interacting serine/threonine−protein kinase 2
with a threshold of 1e−05
MR Test
Inverse variance weighted (fixed effects)
Inverse variance weighted (multiplicative random effects)
MR Egger
MR RAPS
Weighted median
Weighted mode
0.03
0.00
−0.03
0.09
0.12
0.15
0.18
SNP effect on Receptor−interacting serine/threonine−protein kinase 2
SNP effect on Gut microbiota abundance (genus Catenibacterium id.2153)

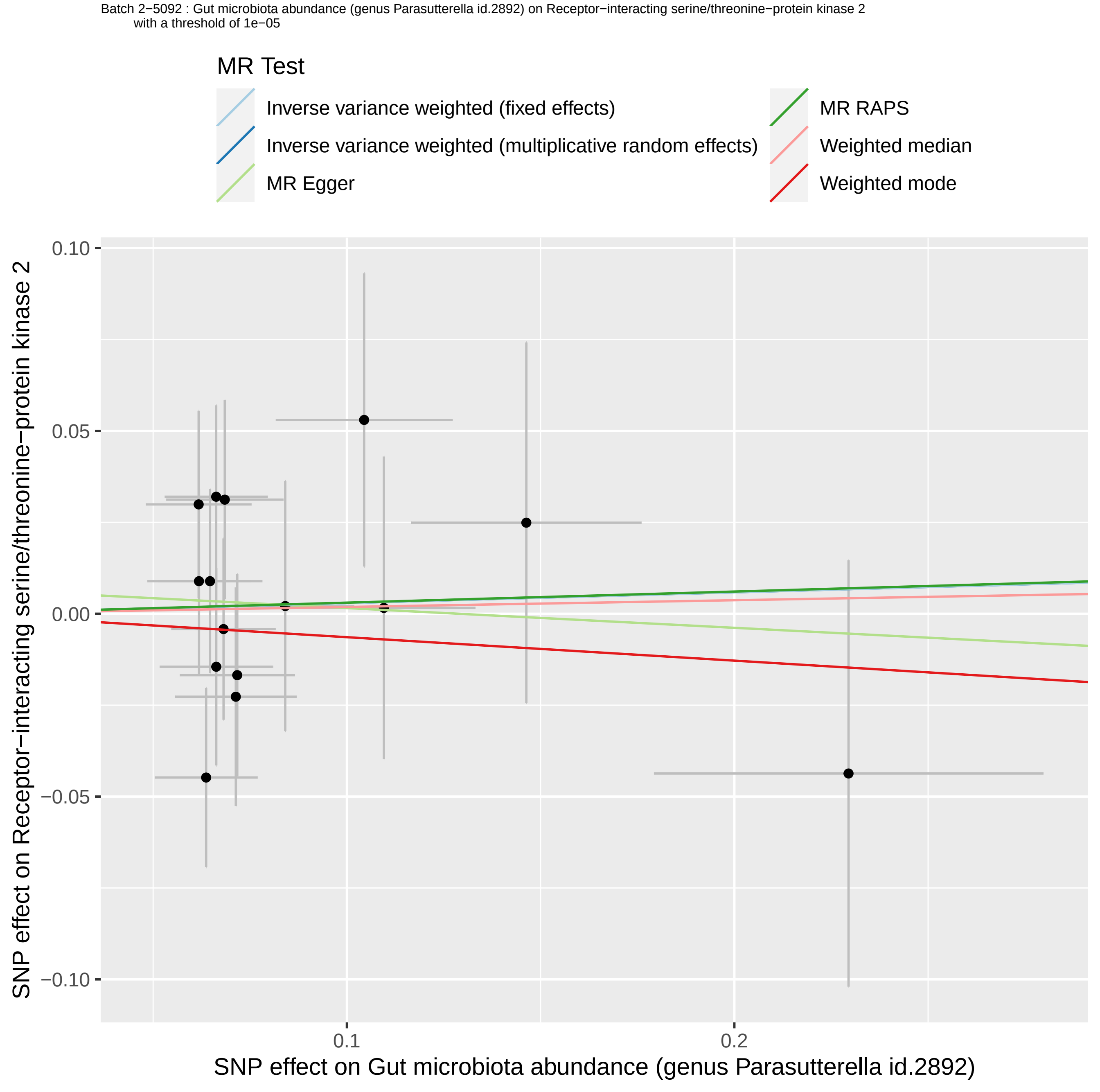

Batch 2−5092 : Gut microbiota abundance (genus Parasutterella id.2892) on Receptor−interacting serine/threonine−protein kinase 2
with a threshold of 1e−05
MR Test
Inverse variance weighted (fixed effects)
Inverse variance weighted (multiplicative random effects)
MR Egger
MR RAPS
Weighted median
Weighted mode
0.10
0.05
0.00
−0.05
−0.10
0.1
0.2
SNP effect on Receptor−interacting serine/threonine−protein kinase 2
SNP effect on Gut microbiota abundance (genus Parasutterella id.2892)

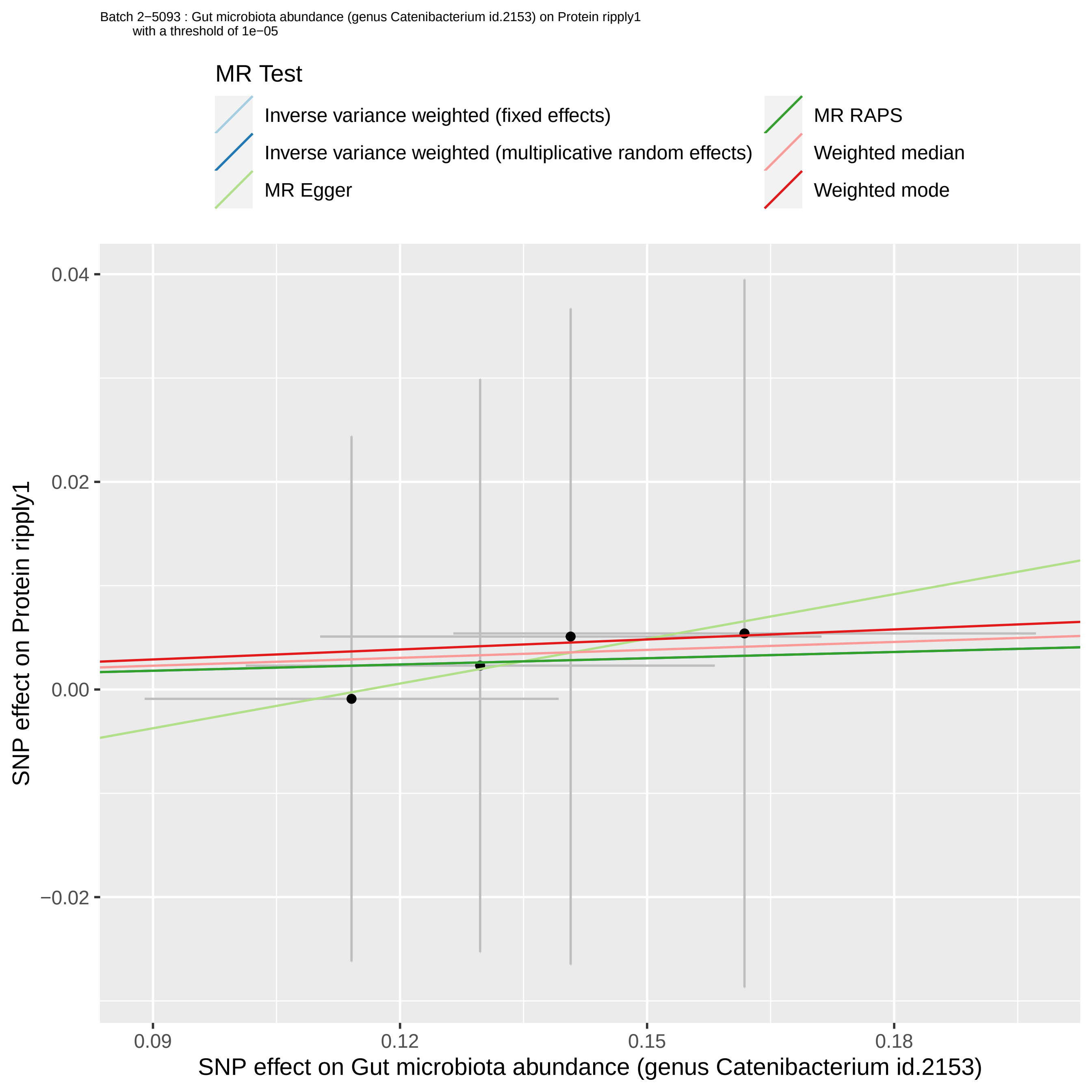
Batch 2−5093 : Gut microbiota abundance (genus Catenibacterium id.2153) on Protein ripply1
with a threshold of 1e−05
MR Test
Inverse variance weighted (fixed effects)
Inverse variance weighted (multiplicative random effects)
MR Egger
MR RAPS
Weighted median
Weighted mode
0.04
0.02
0.00
−0.02
0.09
0.12
0.15
0.18
SNP effect on Protein ripply1
SNP effect on Gut microbiota abundance (genus Catenibacterium id.2153)

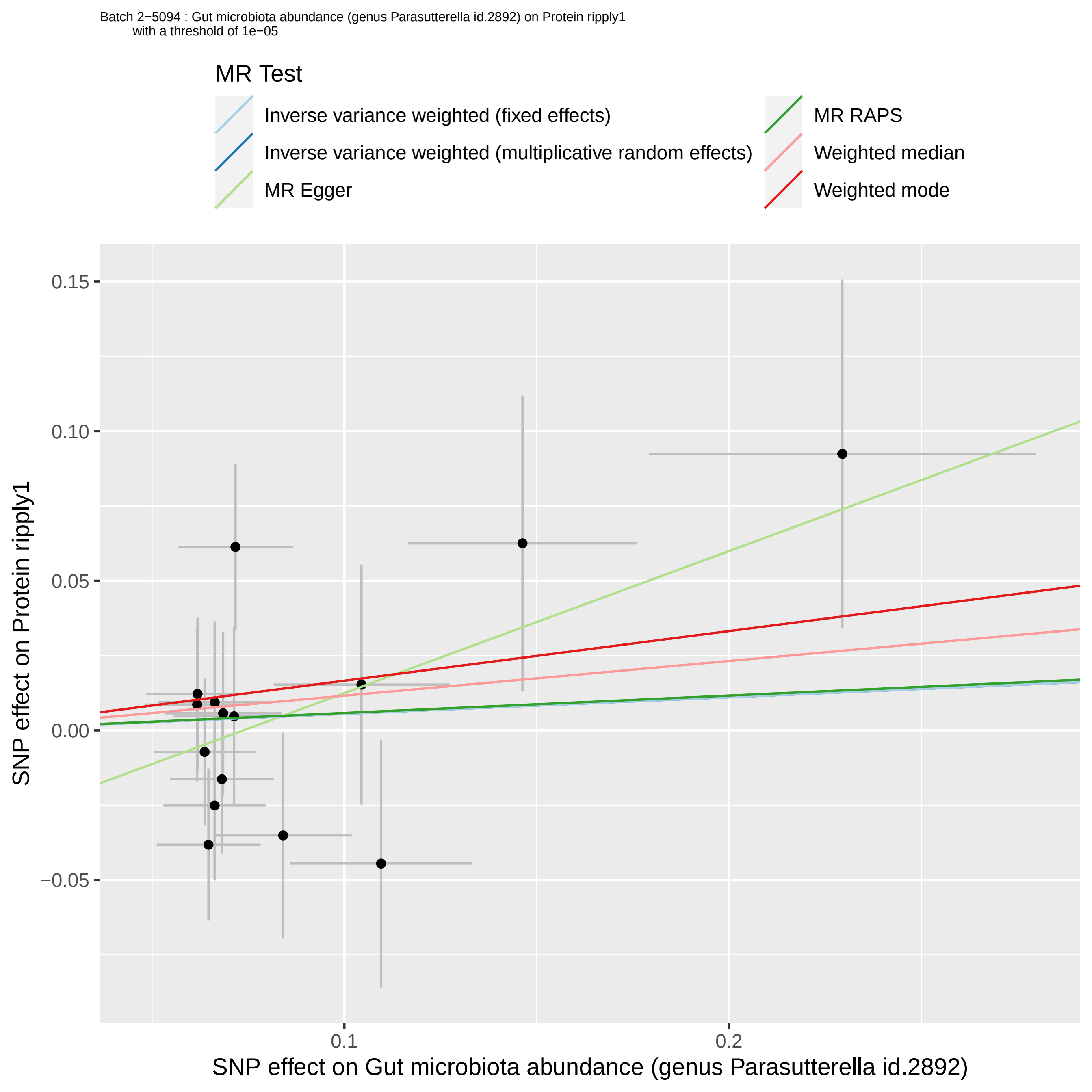

Batch 2−5094 : Gut microbiota abundance (genus Parasutterella id.2892) on Protein ripply1
with a threshold of 1e−05
MR Test
Inverse variance weighted (fixed effects)
Inverse variance weighted (multiplicative random effects)
MR Egger
MR RAPS
Weighted median
Weighted mode
SNP effect on Protein ripply1
0.15
0.10
0.05
0.00
−0.05
0.1
0.2
SNP effect on Gut microbiota abundance (genus Parasutterella id.2892)

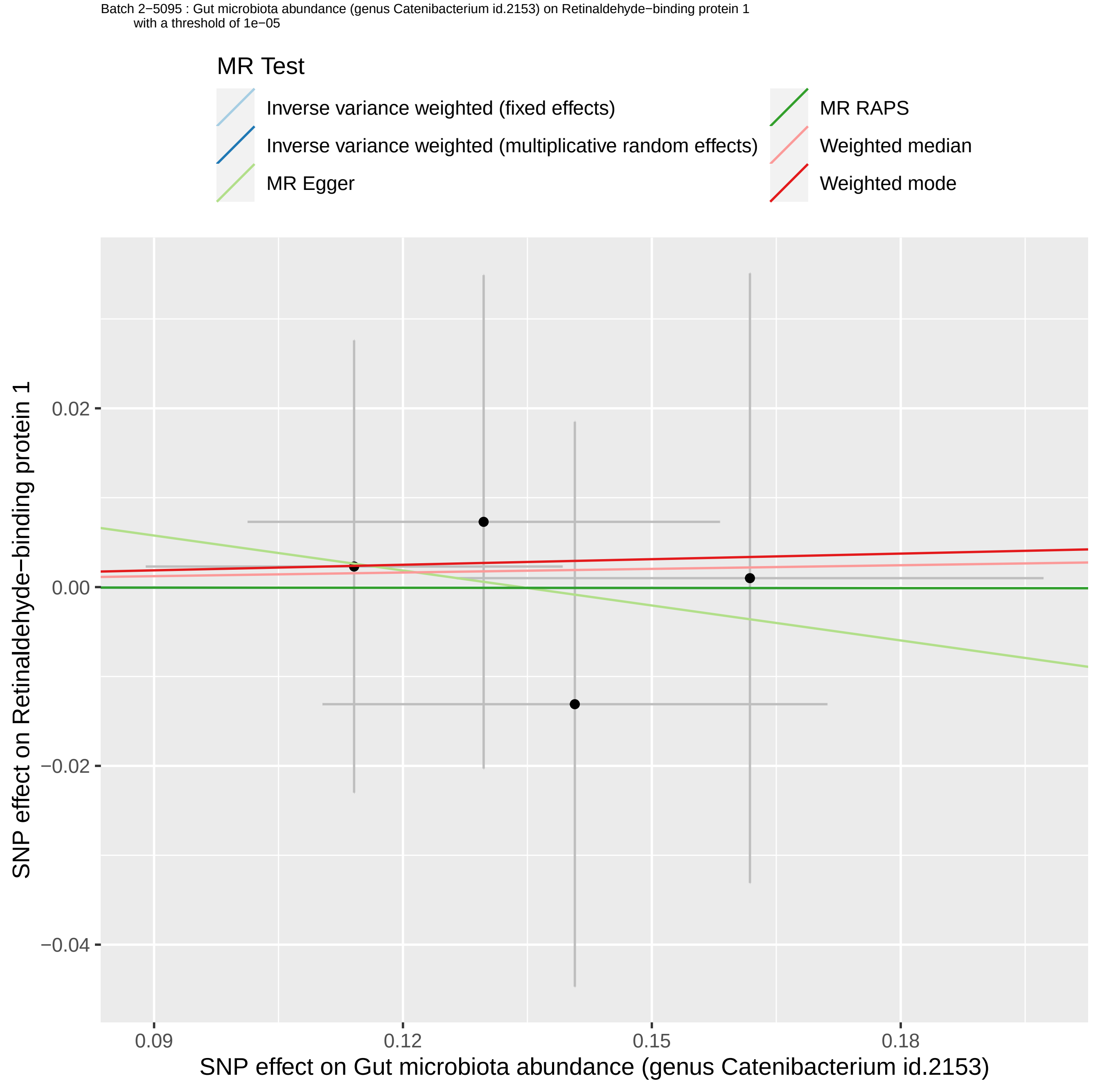
Batch 2−5095 : Gut microbiota abundance (genus Catenibacterium id.2153) on Retinaldehyde−binding protein 1
with a threshold of 1e−05
MR Test
Inverse variance weighted (fixed effects)
Inverse variance weighted (multiplicative random effects)
MR Egger
MR RAPS
Weighted median
Weighted mode
0.02
0.00
−0.02
−0.04
0.09
0.12
0.15
0.18
SNP effect on Retinaldehyde−binding protein 1
SNP effect on Gut microbiota abundance (genus Catenibacterium id.2153)

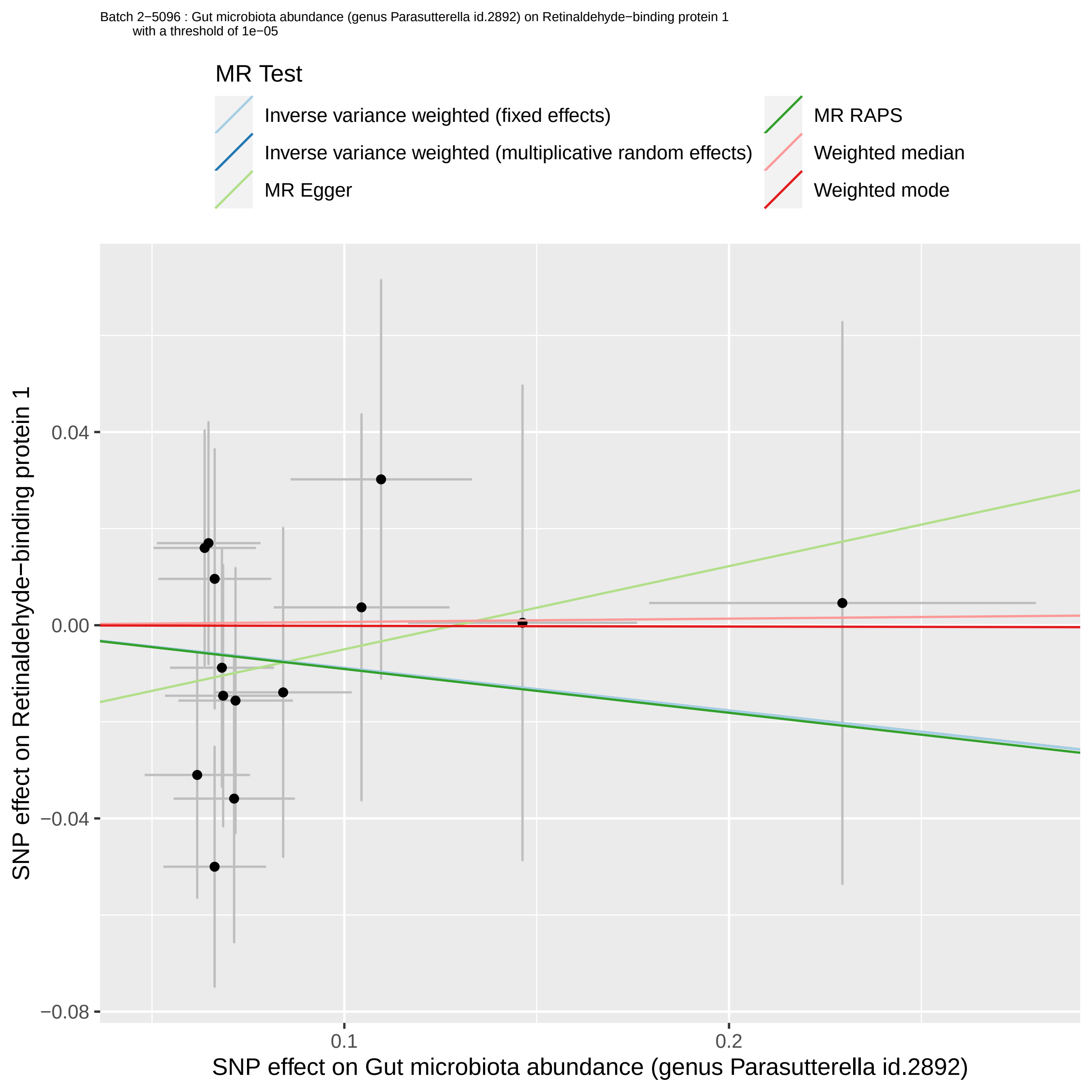

Batch 2−5096 : Gut microbiota abundance (genus Parasutterella id.2892) on Retinaldehyde−binding protein 1
with a threshold of 1e−05
MR Test
Inverse variance weighted (fixed effects)
Inverse variance weighted (multiplicative random effects)
MR Egger
MR RAPS
Weighted median
Weighted mode
0.04
0.00
−0.04
−0.08
0.1
0.2
SNP effect on Retinaldehyde−binding protein 1
SNP effect on Gut microbiota abundance (genus Parasutterella id.2892)

Batch 2−5097 : Gut microbiota abundance (genus Catenibacterium id.2153) on Regulator of microtubule dynamics protein 1
with a threshold of 1e−05

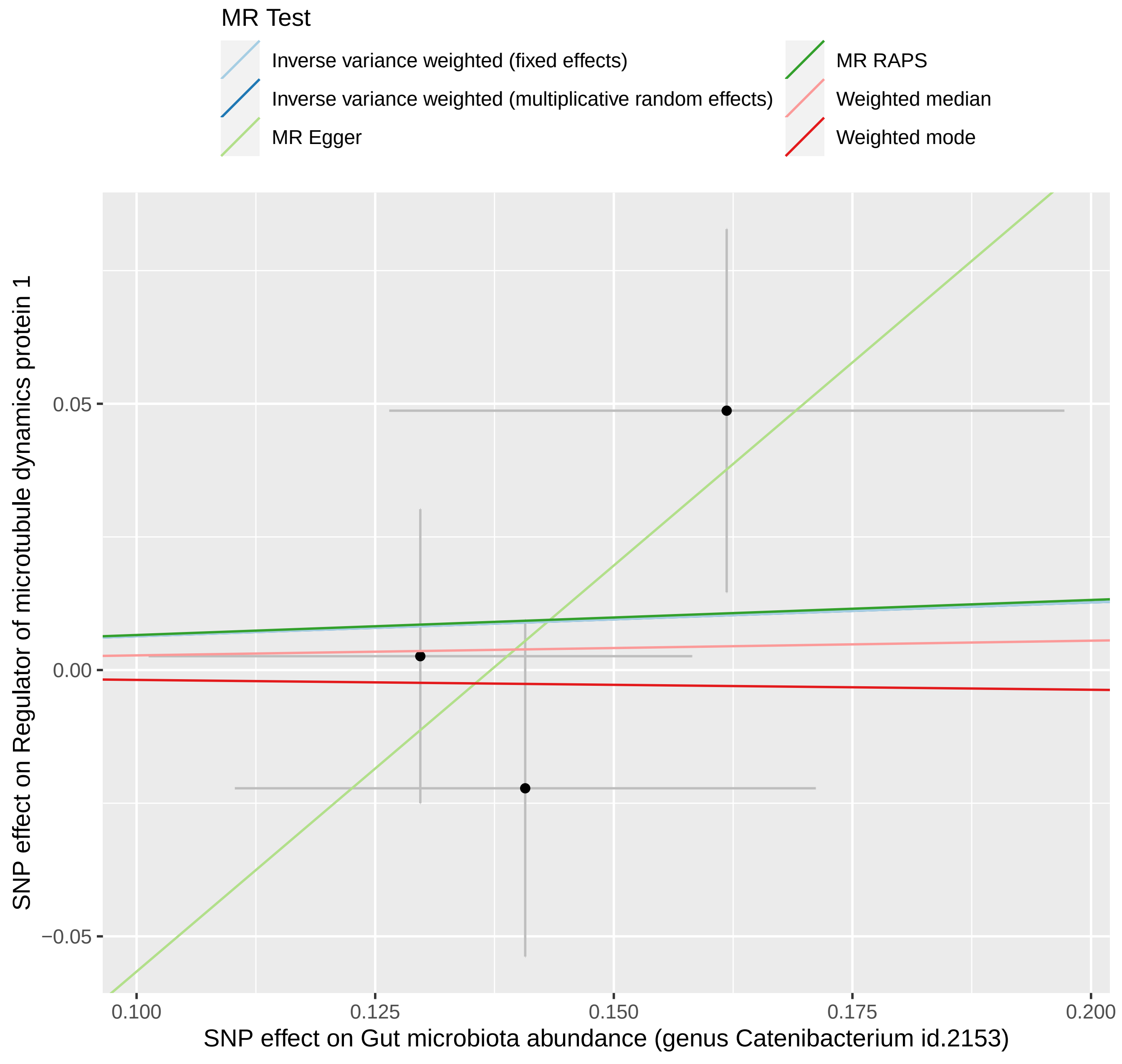

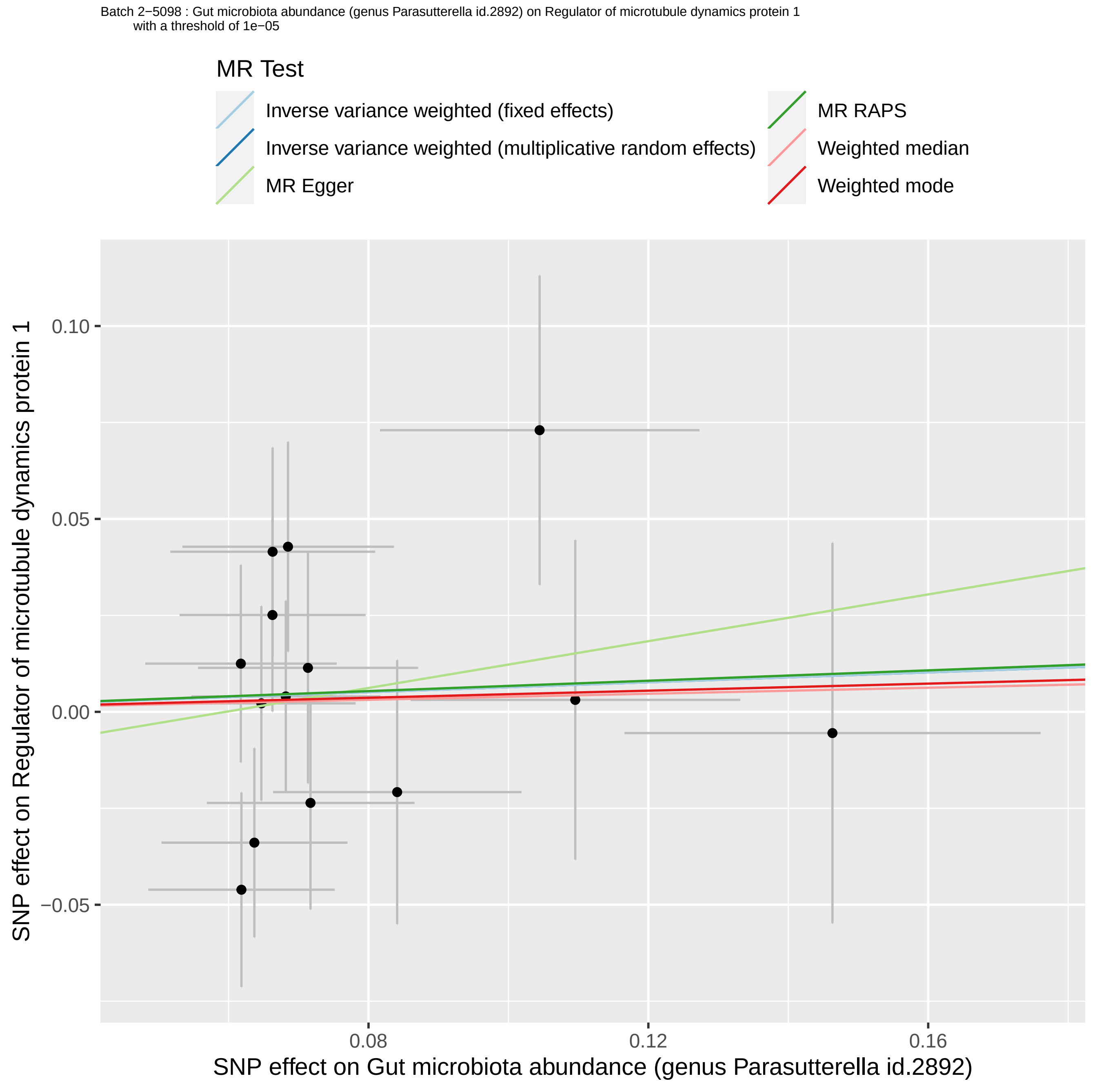

Batch 2−5098 : Gut microbiota abundance (genus Parasutterella id.2892) on Regulator of microtubule dynamics protein 1
with a threshold of 1e−05
MR Test
Inverse variance weighted (fixed effects)
Inverse variance weighted (multiplicative random effects)
MR Egger
MR RAPS
Weighted median
Weighted mode
0.10
0.05
0.00
−0.05
0.08
0.12
0.16
SNP effect on Regulator of microtubule dynamics protein 1
SNP effect on Gut microbiota abundance (genus Parasutterella id.2892)

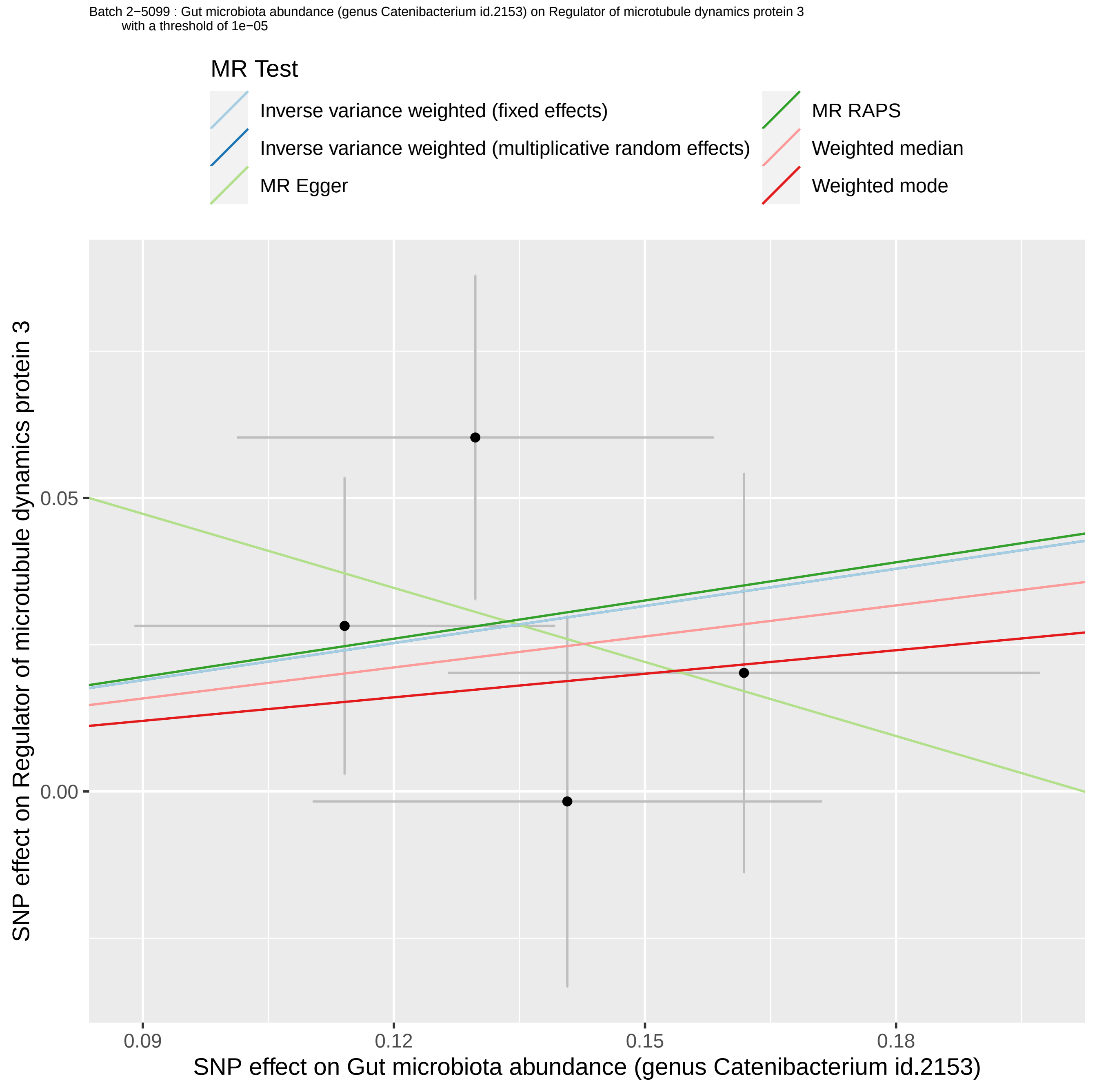
Batch 2−5099 : Gut microbiota abundance (genus Catenibacterium id.2153) on Regulator of microtubule dynamics protein 3
with a threshold of 1e−05
MR Test
Inverse variance weighted (fixed effects)
Inverse variance weighted (multiplicative random effects)
MR Egger
MR RAPS
Weighted median
Weighted mode
0.05
0.00
0.09
0.12
0.15
0.18
SNP effect on Regulator of microtubule dynamics protein 3
SNP effect on Gut microbiota abundance (genus Catenibacterium id.2153)

Batch 2−5100 : Gut microbiota abundance (genus Parasutterella id.2892) on Regulator of microtubule dynamics protein 3
with a threshold of 1e−05

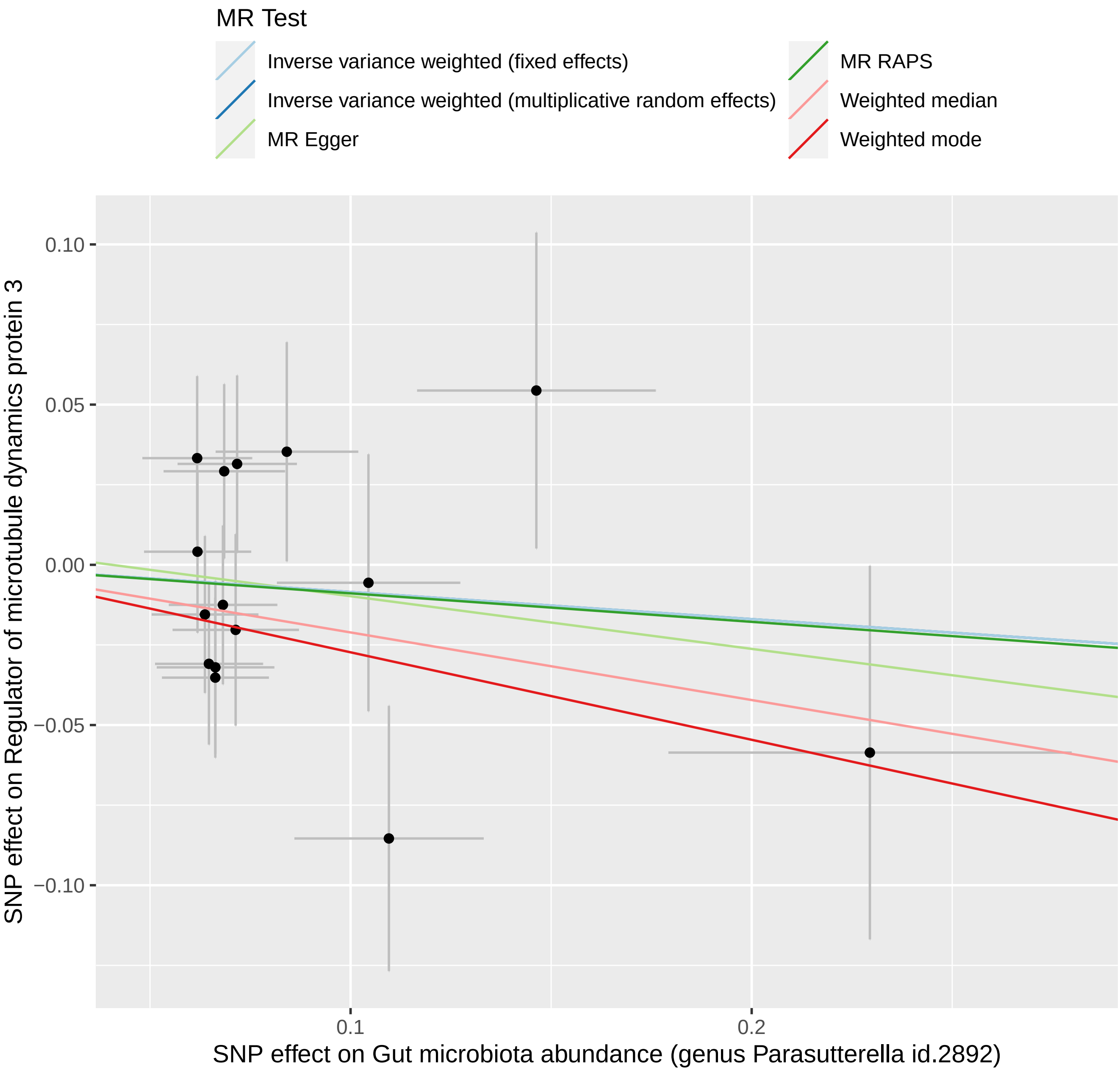

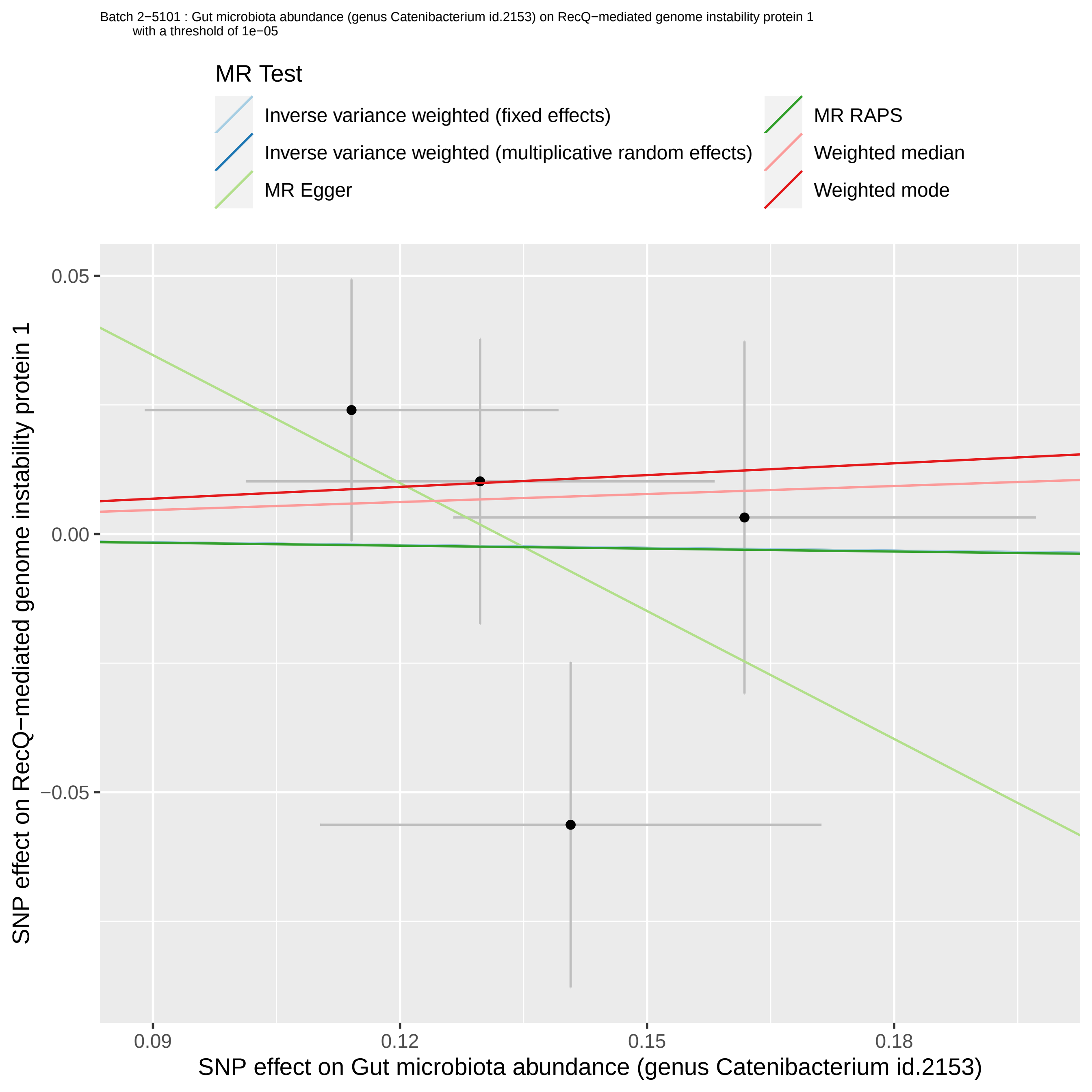
Batch 2−5101 : Gut microbiota abundance (genus Catenibacterium id.2153) on RecQ−mediated genome instability protein 1
with a threshold of 1e−05
MR Test
Inverse variance weighted (fixed effects)
Inverse variance weighted (multiplicative random effects)
MR Egger
MR RAPS
Weighted median
Weighted mode
0.05
0.00
−0.05
0.09
0.12
0.15
0.18
SNP effect on RecQ−mediated genome instability protein 1
SNP effect on Gut microbiota abundance (genus Catenibacterium id.2153)

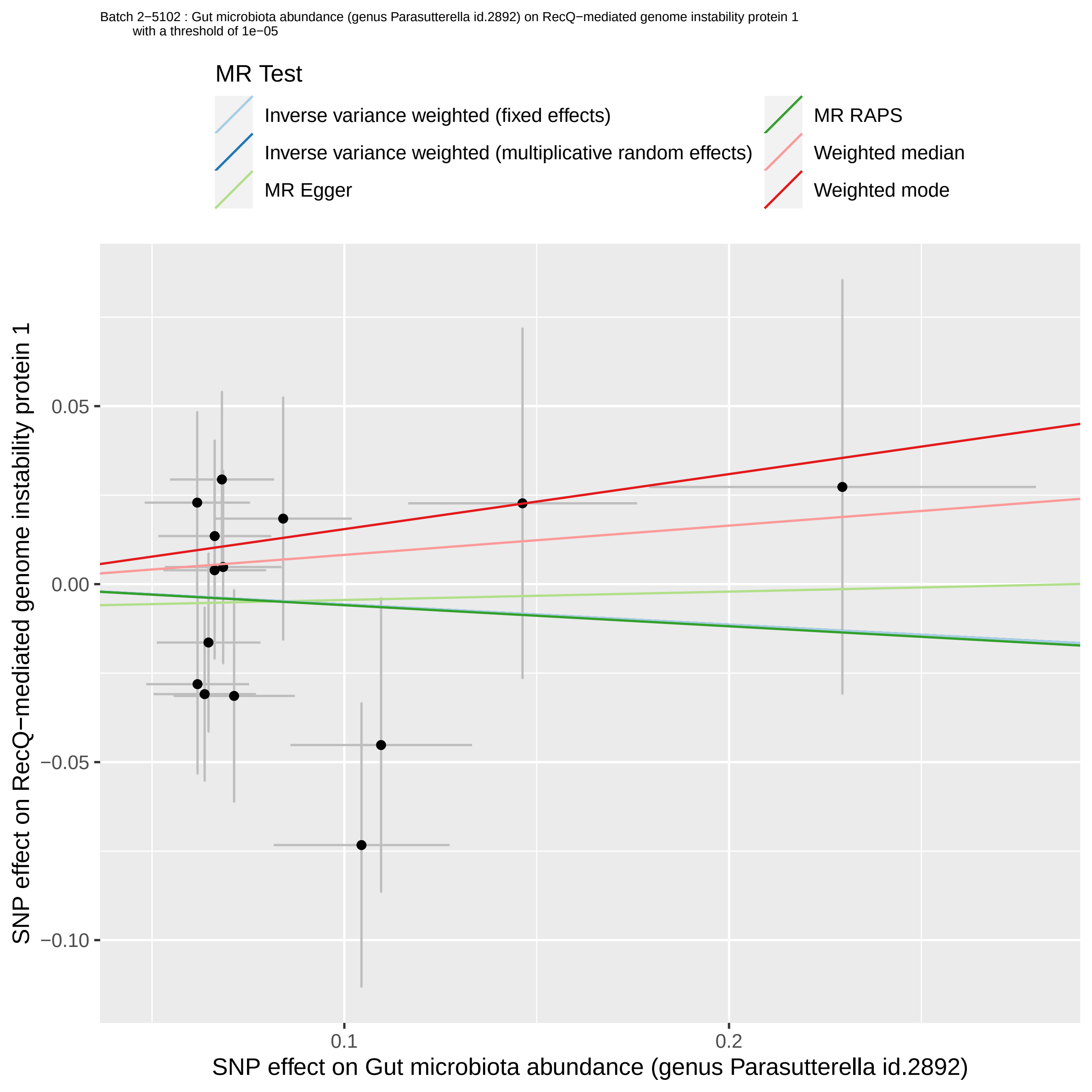

Batch 2−5102 : Gut microbiota abundance (genus Parasutterella id.2892) on RecQ−mediated genome instability protein 1
with a threshold of 1e−05
MR Test
Inverse variance weighted (fixed effects)
Inverse variance weighted (multiplicative random effects)
MR Egger
MR RAPS
Weighted median
Weighted mode
SNP effect on RecQ−mediated genome instability protein 1
0.05
0.00
−0.05
−0.10
0.1
0.2
SNP effect on Gut microbiota abundance (genus Parasutterella id.2892)

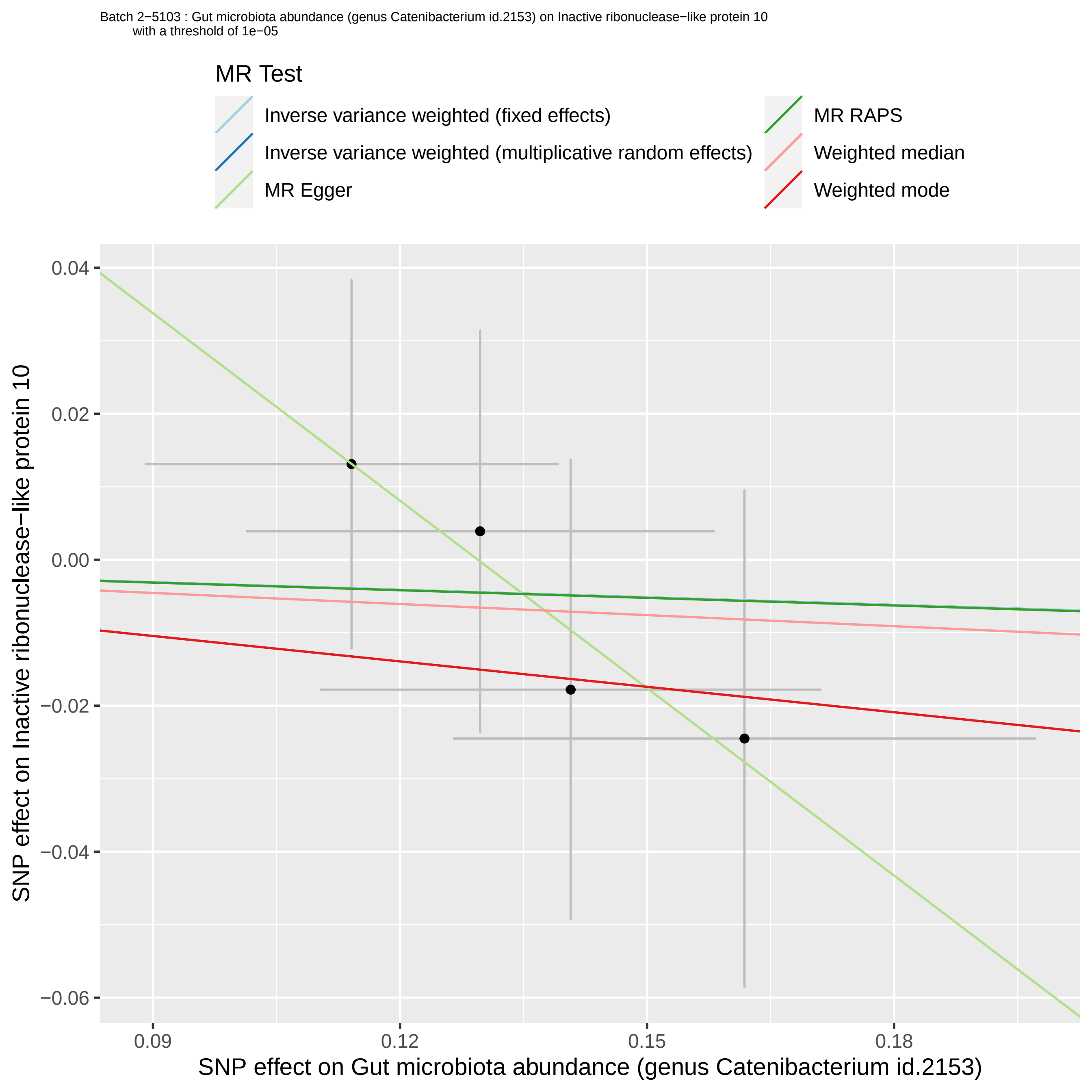

Batch 2−5103 : Gut microbiota abundance (genus Catenibacterium id.2153) on Inactive ribonuclease−like protein 10
with a threshold of 1e−05
MR Test
Inverse variance weighted (fixed effects)
Inverse variance weighted (multiplicative random effects)
MR Egger
MR RAPS
Weighted median
Weighted mode
0.04
0.02
0.00
−0.02
−0.04
−0.06
0.09
0.12
0.15
0.18
SNP effect on Inactive ribonuclease−like protein 10
SNP effect on Gut microbiota abundance (genus Catenibacterium id.2153)

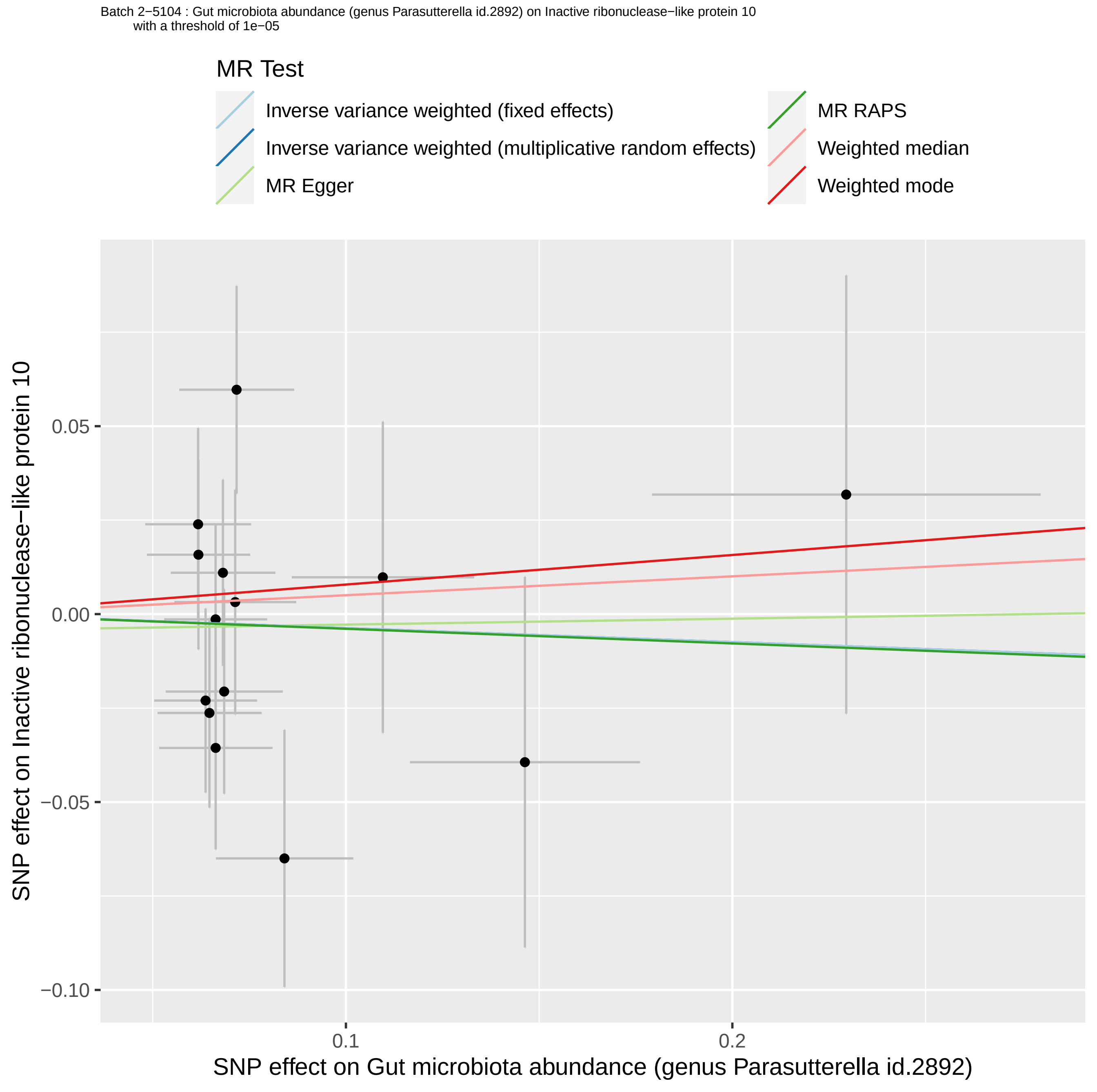

Batch 2−5104 : Gut microbiota abundance (genus Parasutterella id.2892) on Inactive ribonuclease−like protein 10
with a threshold of 1e−05
MR Test
Inverse variance weighted (fixed effects)
Inverse variance weighted (multiplicative random effects)
MR Egger
MR RAPS
Weighted median
Weighted mode
SNP effect on Inactive ribonuclease−like protein 10
0.05
0.00
−0.05
−0.10
0.1
0.2
SNP effect on Gut microbiota abundance (genus Parasutterella id.2892)

Batch 2−5105 : Gut microbiota abundance (genus Catenibacterium id.2153) on Probable inactive ribonuclease−like protein 13
with a threshold of 1e−05

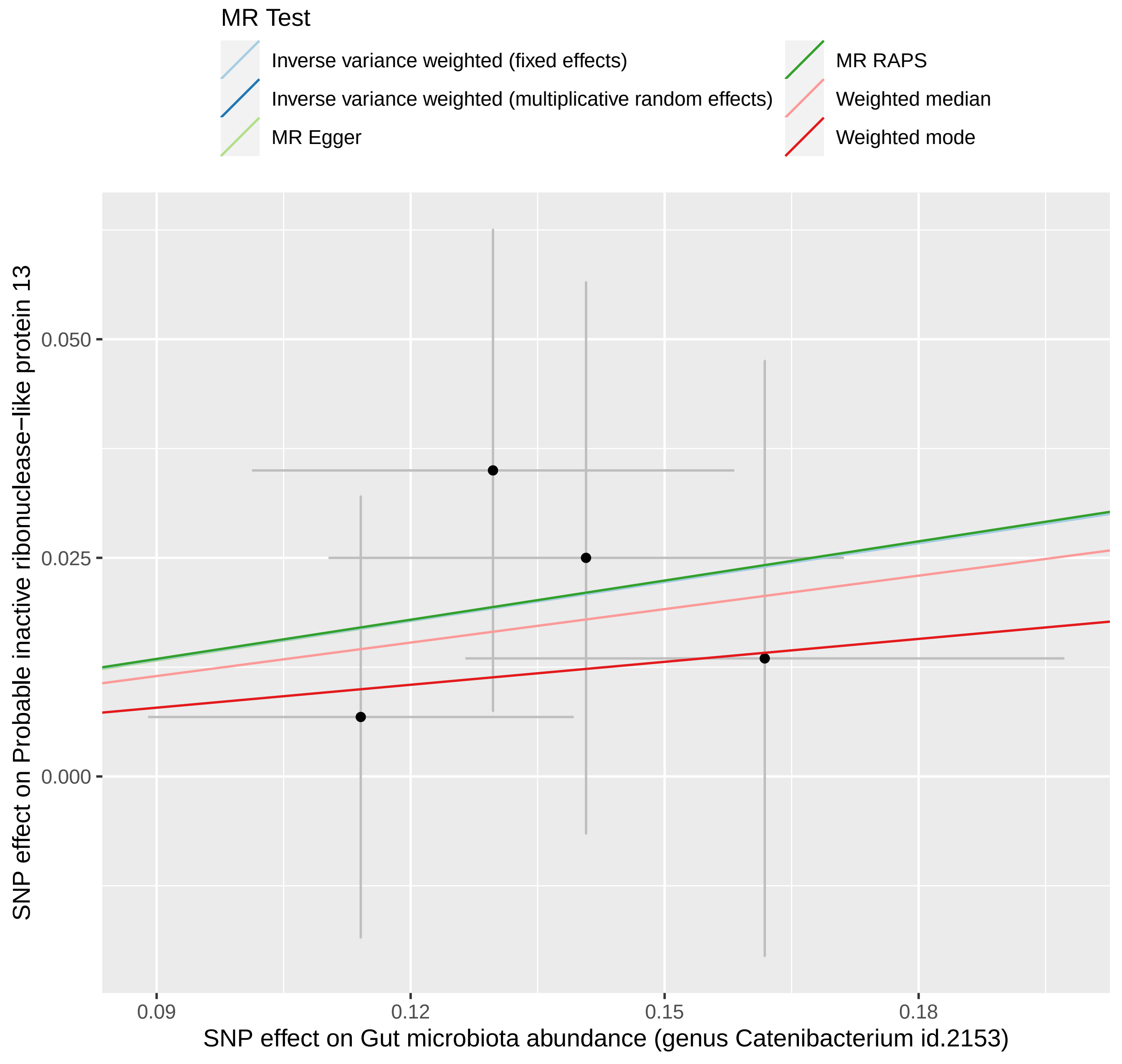

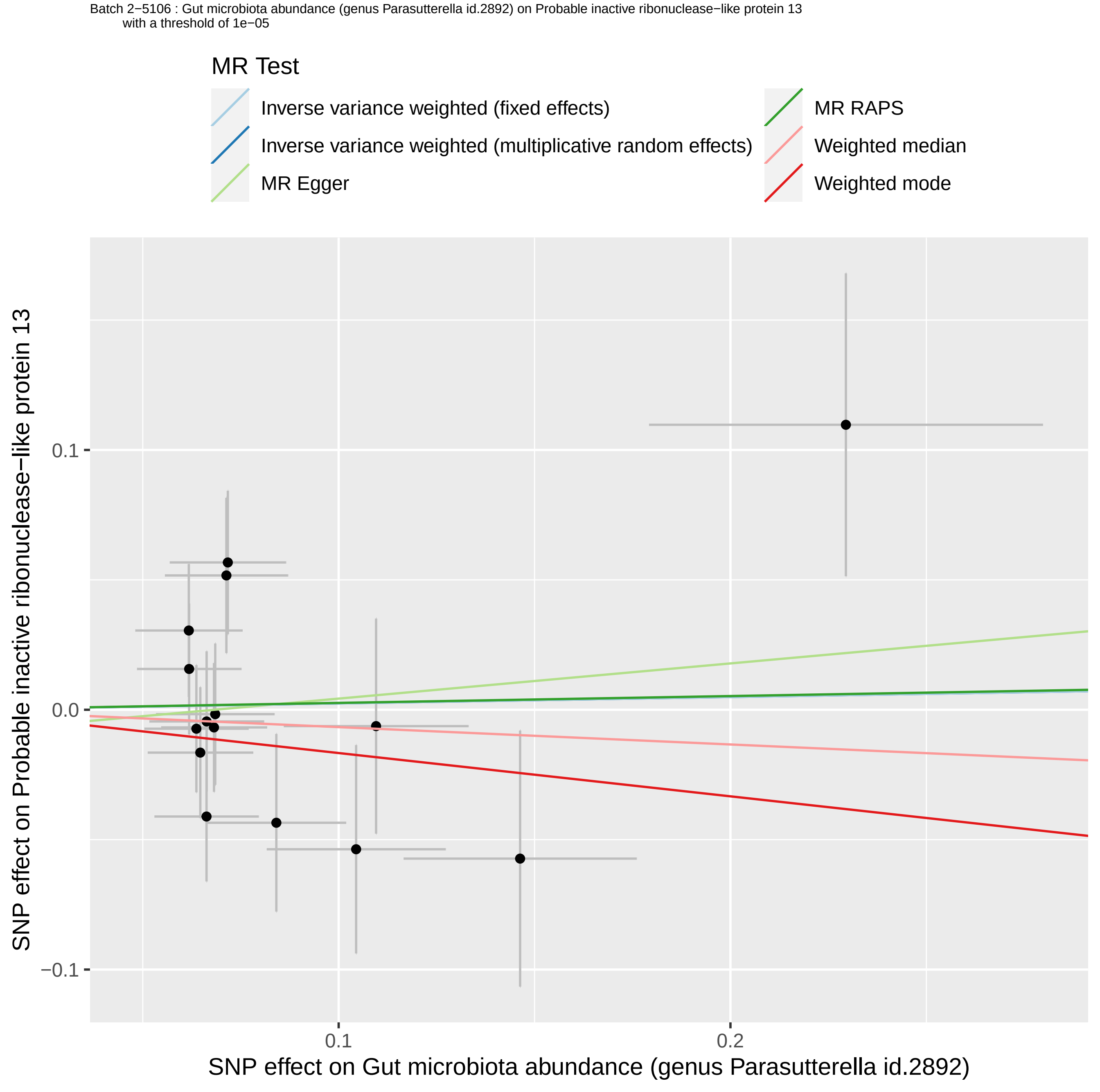

Batch 2−5106 : Gut microbiota abundance (genus Parasutterella id.2892) on Probable inactive ribonuclease−like protein 13
with a threshold of 1e−05
MR Test
Inverse variance weighted (fixed effects)
Inverse variance weighted (multiplicative random effects)
MR Egger
MR RAPS
Weighted median
Weighted mode
0.1
0.0
−0.1
0.1
0.2
SNP effect on Probable inactive ribonuclease−like protein 13
SNP effect on Gut microbiota abundance (genus Parasutterella id.2892)

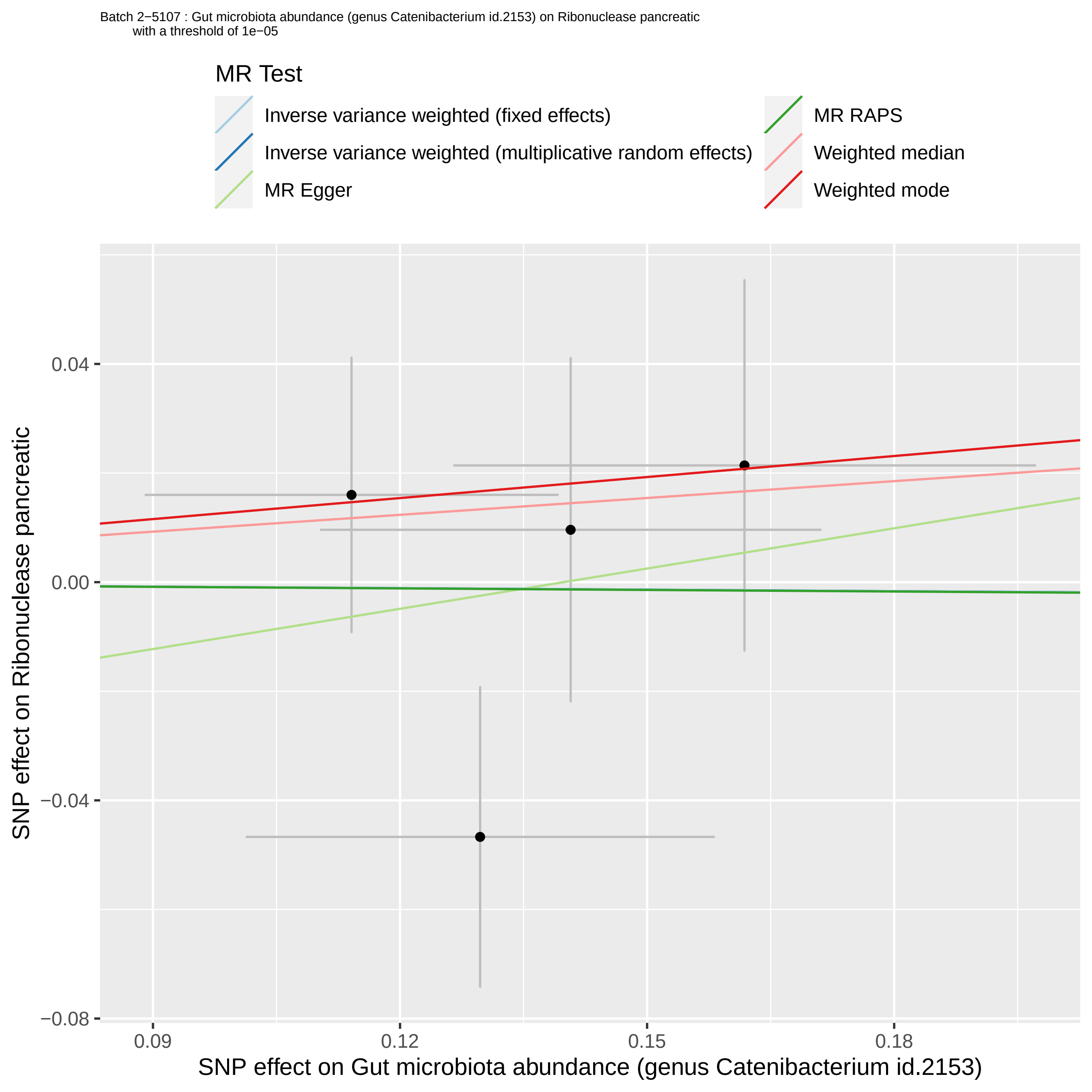
Batch 2−5107 : Gut microbiota abundance (genus Catenibacterium id.2153) on Ribonuclease pancreatic
with a threshold of 1e−05
MR Test
Inverse variance weighted (fixed effects)
Inverse variance weighted (multiplicative random effects)
MR Egger
MR RAPS
Weighted median
Weighted mode
0.04
0.00
−0.04
−0.08
0.09
0.12
0.15
0.18
SNP effect on Ribonuclease pancreatic
SNP effect on Gut microbiota abundance (genus Catenibacterium id.2153)

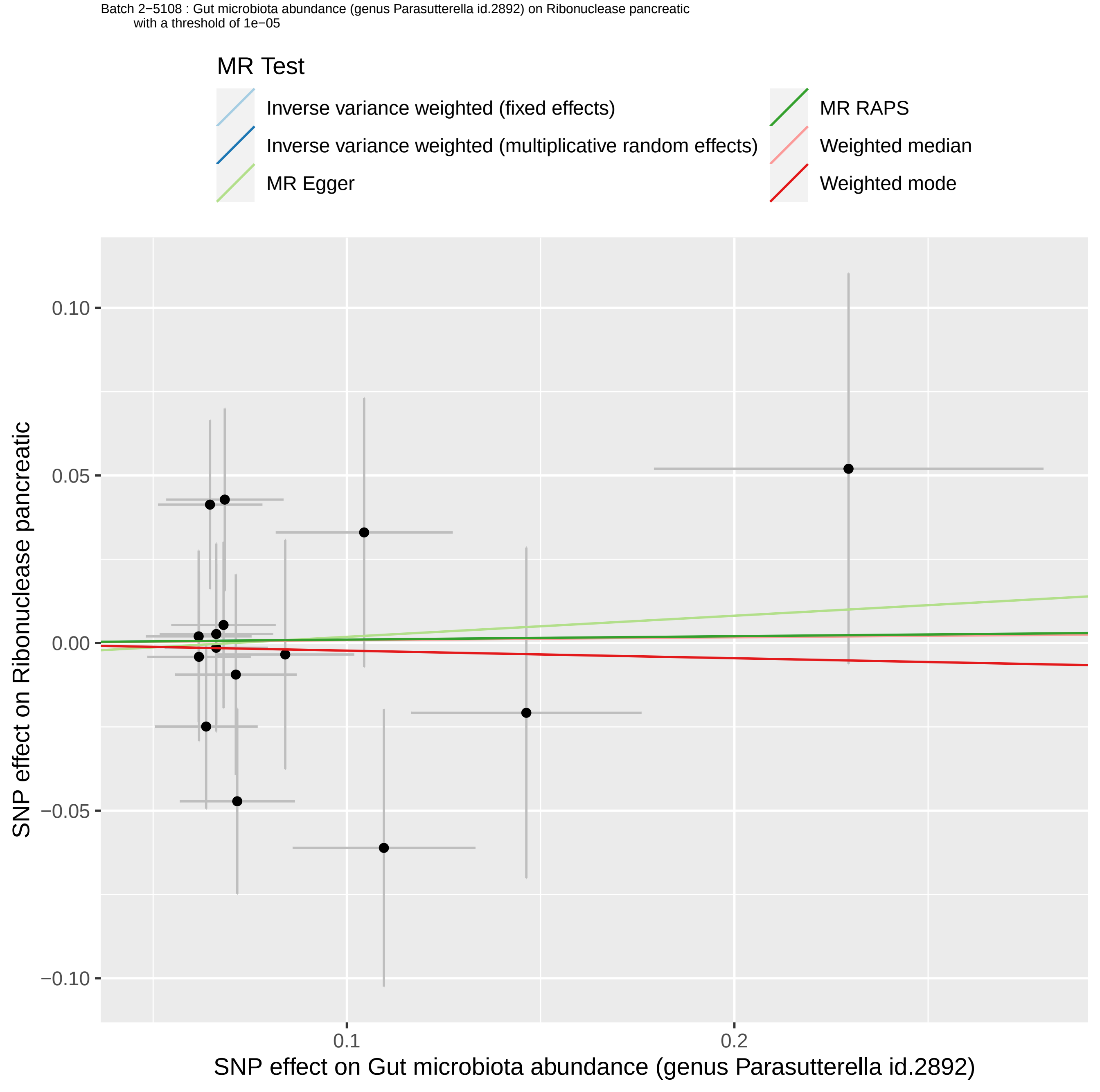

Batch 2−5108 : Gut microbiota abundance (genus Parasutterella id.2892) on Ribonuclease pancreatic
with a threshold of 1e−05
MR Test
Inverse variance weighted (fixed effects)
Inverse variance weighted (multiplicative random effects)
MR Egger
MR RAPS
Weighted median
Weighted mode
SNP effect on Ribonuclease pancreatic
0.10
0.05
0.00
−0.05
−0.10
0.1
0.2
SNP effect on Gut microbiota abundance (genus Parasutterella id.2892)

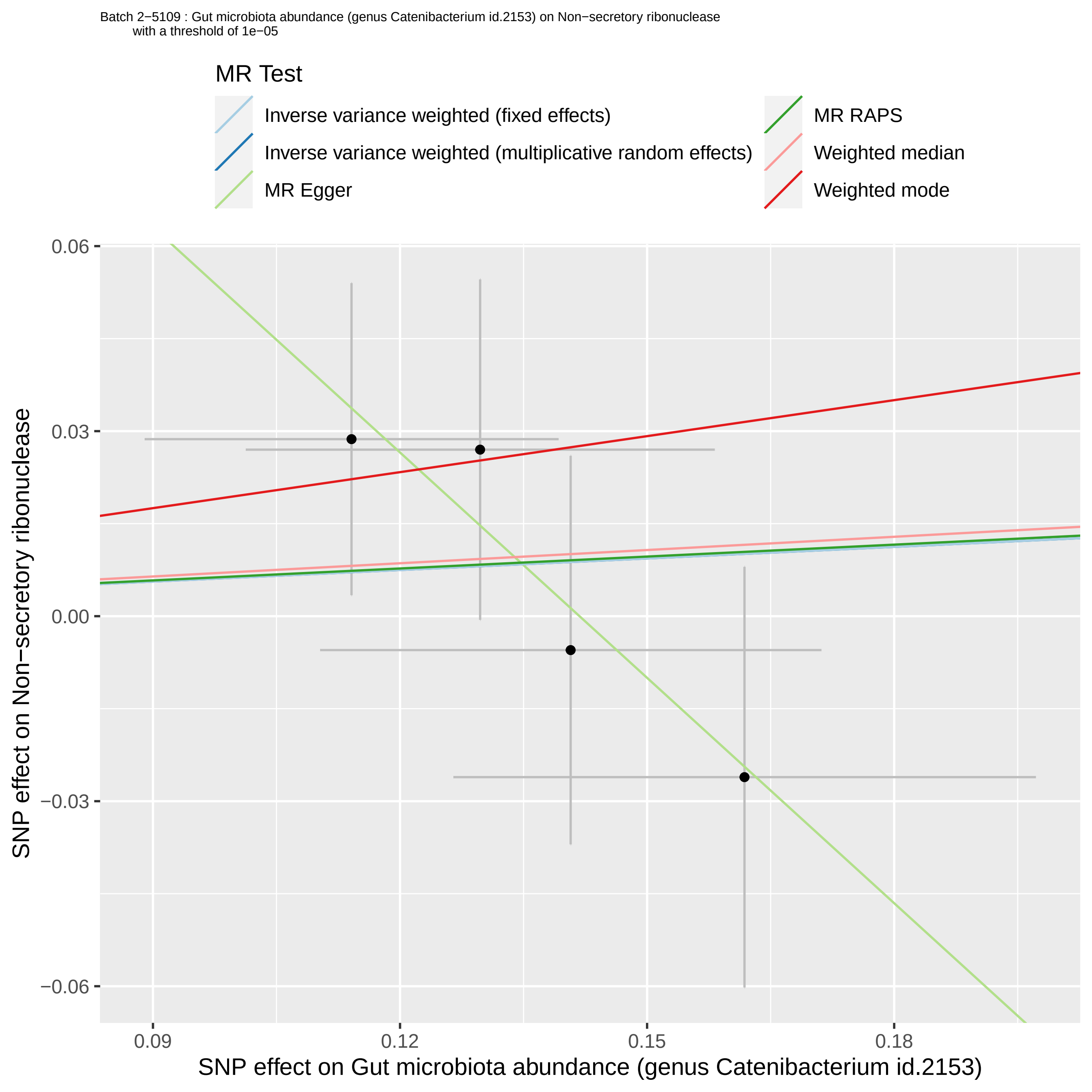
Batch 2−5109 : Gut microbiota abundance (genus Catenibacterium id.2153) on Non−secretory ribonuclease
with a threshold of 1e−05
MR Test
Inverse variance weighted (fixed effects)
Inverse variance weighted (multiplicative random effects)
MR Egger
MR RAPS
Weighted median
Weighted mode
0.06
0.03
0.00
−0.03
−0.06
0.09
0.12
0.15
0.18
SNP effect on Gut microbiota abundance (genus Catenibacterium id.2153)
SNP effect on Non−secretory ribonuclease

Batch 2−5110 : Gut microbiota abundance (genus Parasutterella id.2892) on Non−secretory ribonuclease with a threshold of 1e−05

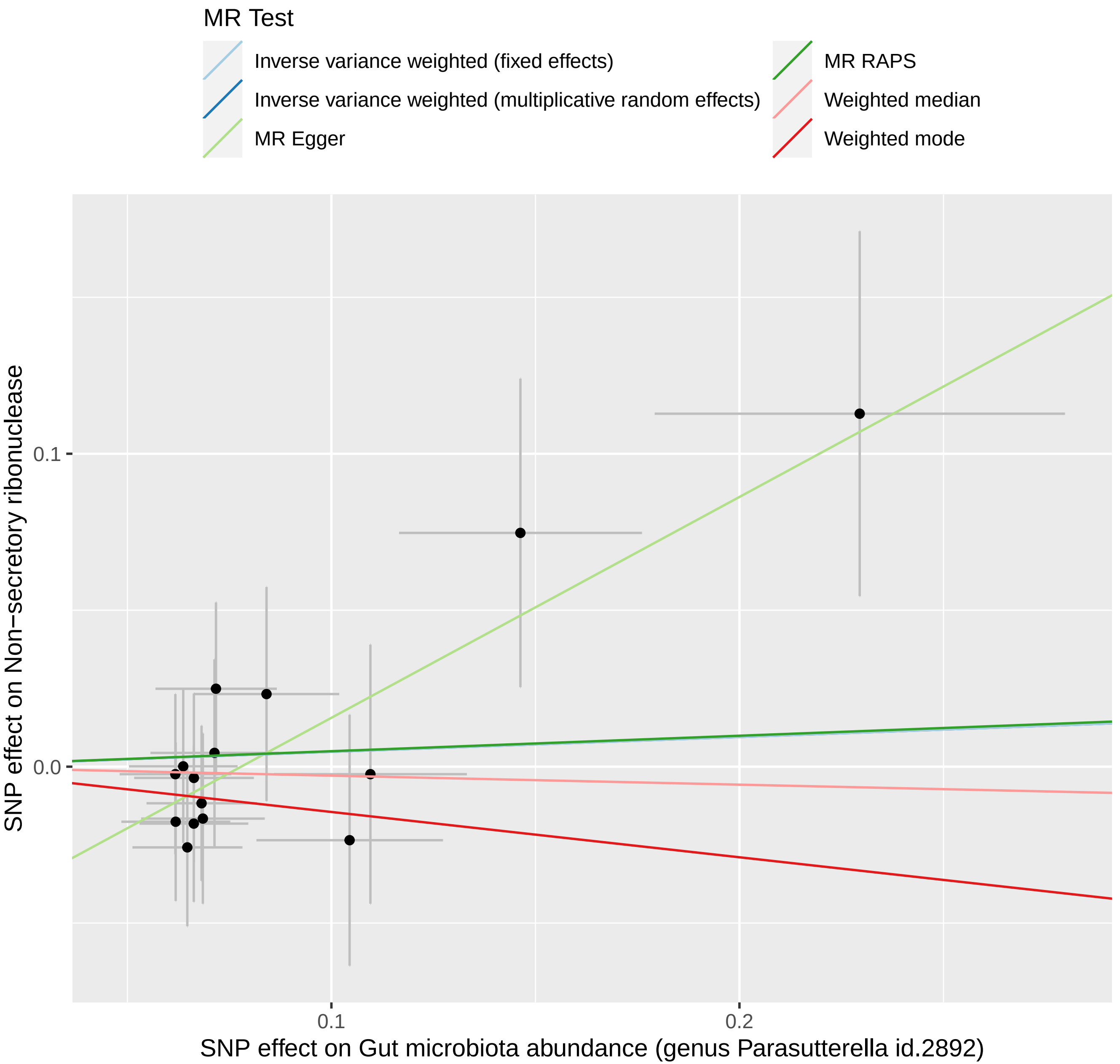

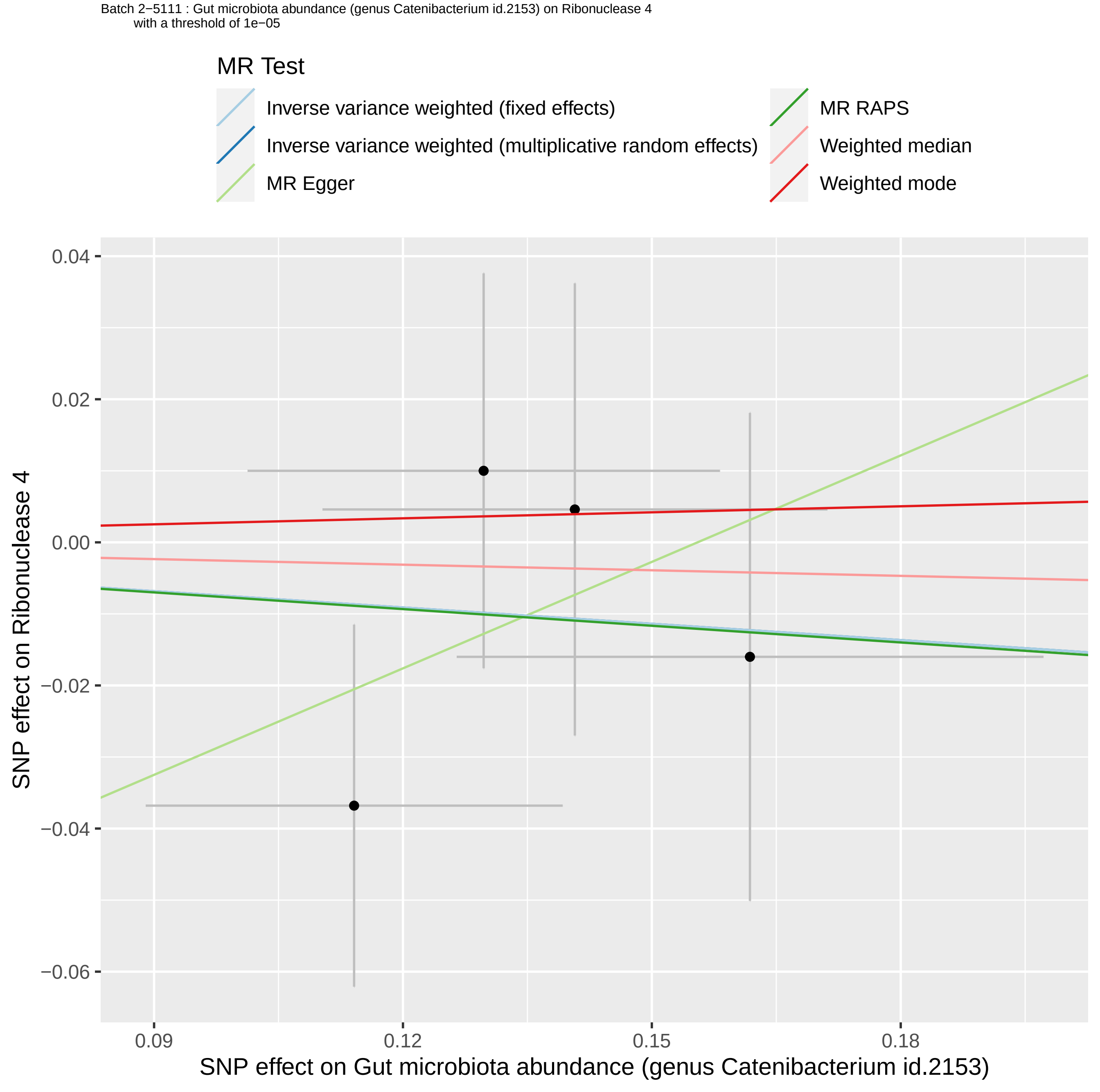
Batch 2−5111 : Gut microbiota abundance (genus Catenibacterium id.2153) on Ribonuclease 4
with a threshold of 1e−05
MR Test
Inverse variance weighted (fixed effects)
Inverse variance weighted (multiplicative random effects)
MR Egger
MR RAPS
Weighted median
Weighted mode
0.04
0.02
0.00
−0.02
−0.04
−0.06
0.09
0.12
0.15
0.18
SNP effect on Ribonuclease 4
SNP effect on Gut microbiota abundance (genus Catenibacterium id.2153)

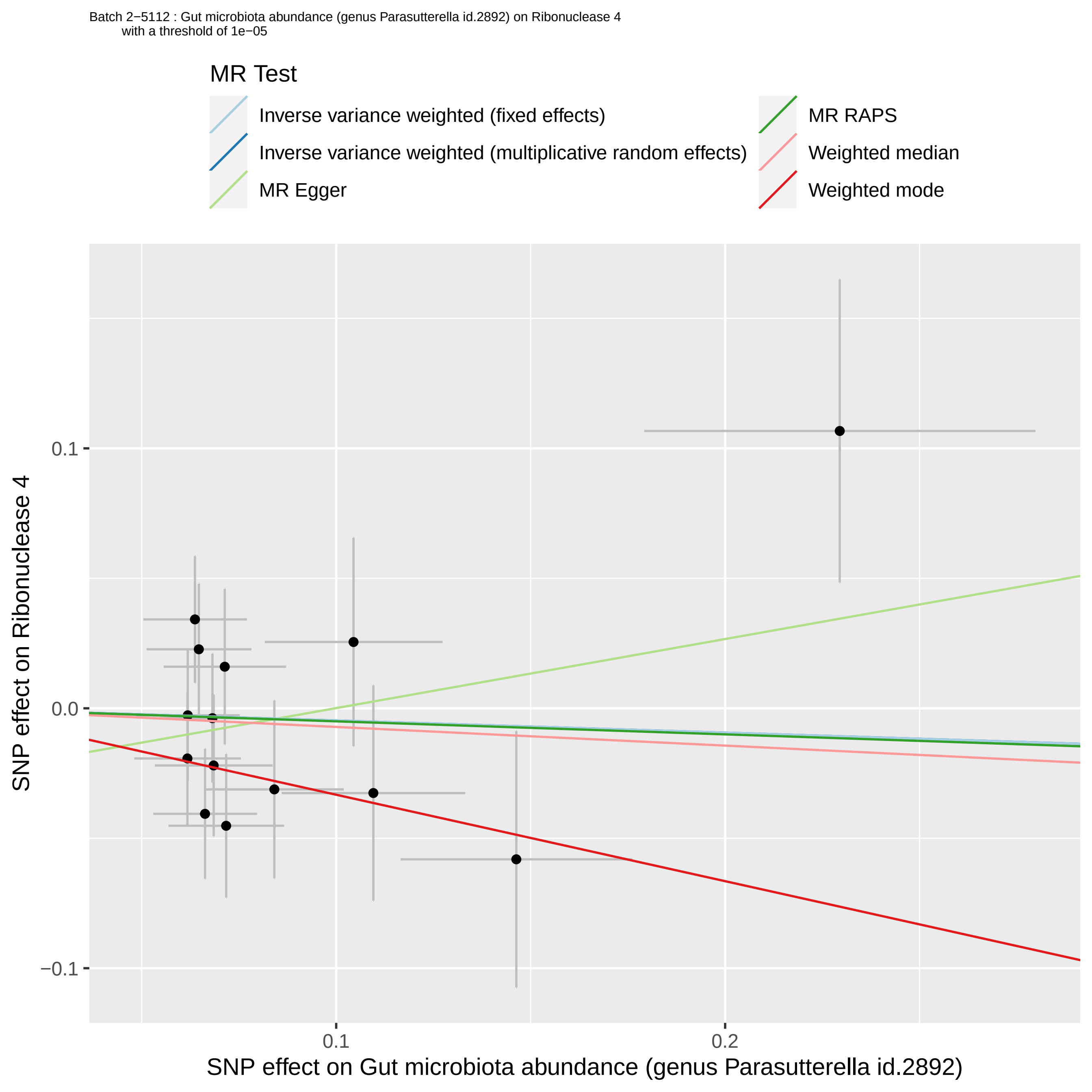

Batch 2−5112 : Gut microbiota abundance (genus Parasutterella id.2892) on Ribonuclease 4
with a threshold of 1e−05
MR Test
Inverse variance weighted (fixed effects)
Inverse variance weighted (multiplicative random effects)
MR Egger
MR RAPS
Weighted median
Weighted mode
0.1
0.0
−0.1
0.1
0.2
SNP effect on Ribonuclease 4
SNP effect on Gut microbiota abundance (genus Parasutterella id.2892)

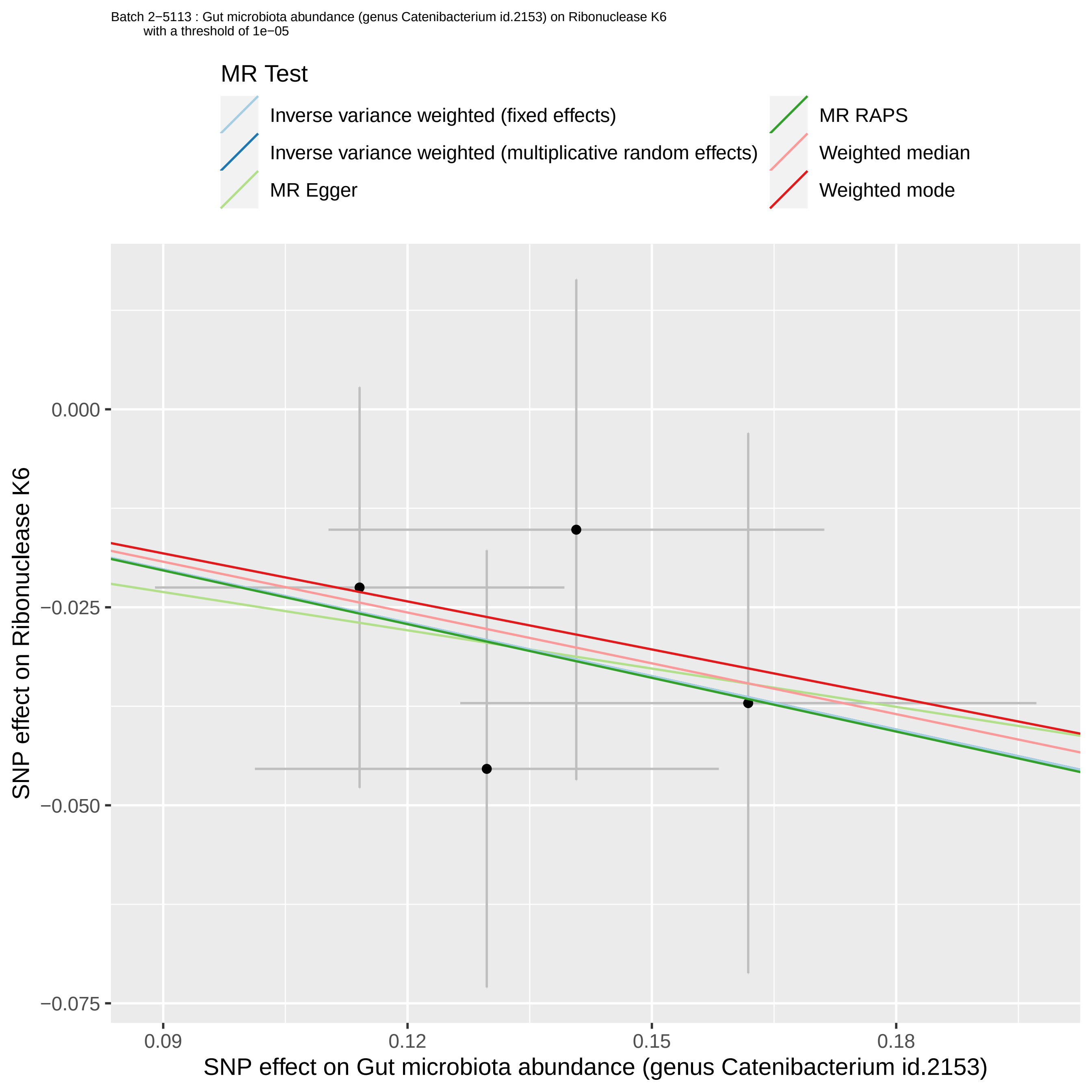
Batch 2−5113 : Gut microbiota abundance (genus Catenibacterium id.2153) on Ribonuclease K6
with a threshold of 1e−05
MR Test
Inverse variance weighted (fixed effects)
Inverse variance weighted (multiplicative random effects)
MR Egger
MR RAPS
Weighted median
Weighted mode
0.000
−0.025
−0.050
−0.075
0.09
0.12
0.15
0.18
SNP effect on Ribonuclease K6
SNP effect on Gut microbiota abundance (genus Catenibacterium id.2153)

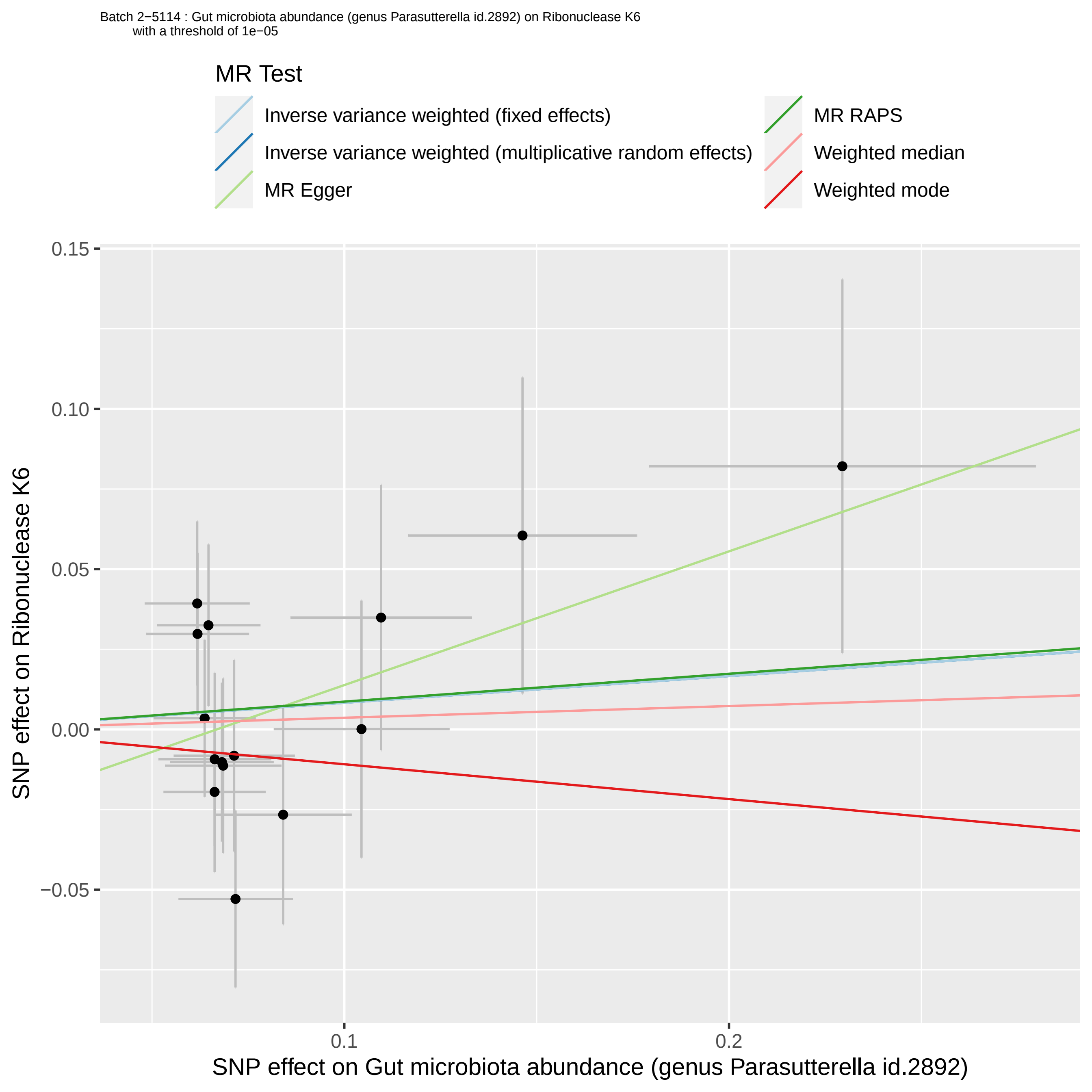
Batch 2−5114 : Gut microbiota abundance (genus Parasutterella id.2892) on Ribonuclease K6
with a threshold of 1e−05
MR Test
Inverse variance weighted (fixed effects)
Inverse variance weighted (multiplicative random effects)
MR Egger
MR RAPS
Weighted median
Weighted mode
0.15
0.10
0.05
0.00
−0.05
SNP effect on Ribonuclease K6
0.1
0.2
SNP effect on Gut microbiota abundance (genus Parasutterella id.2892)

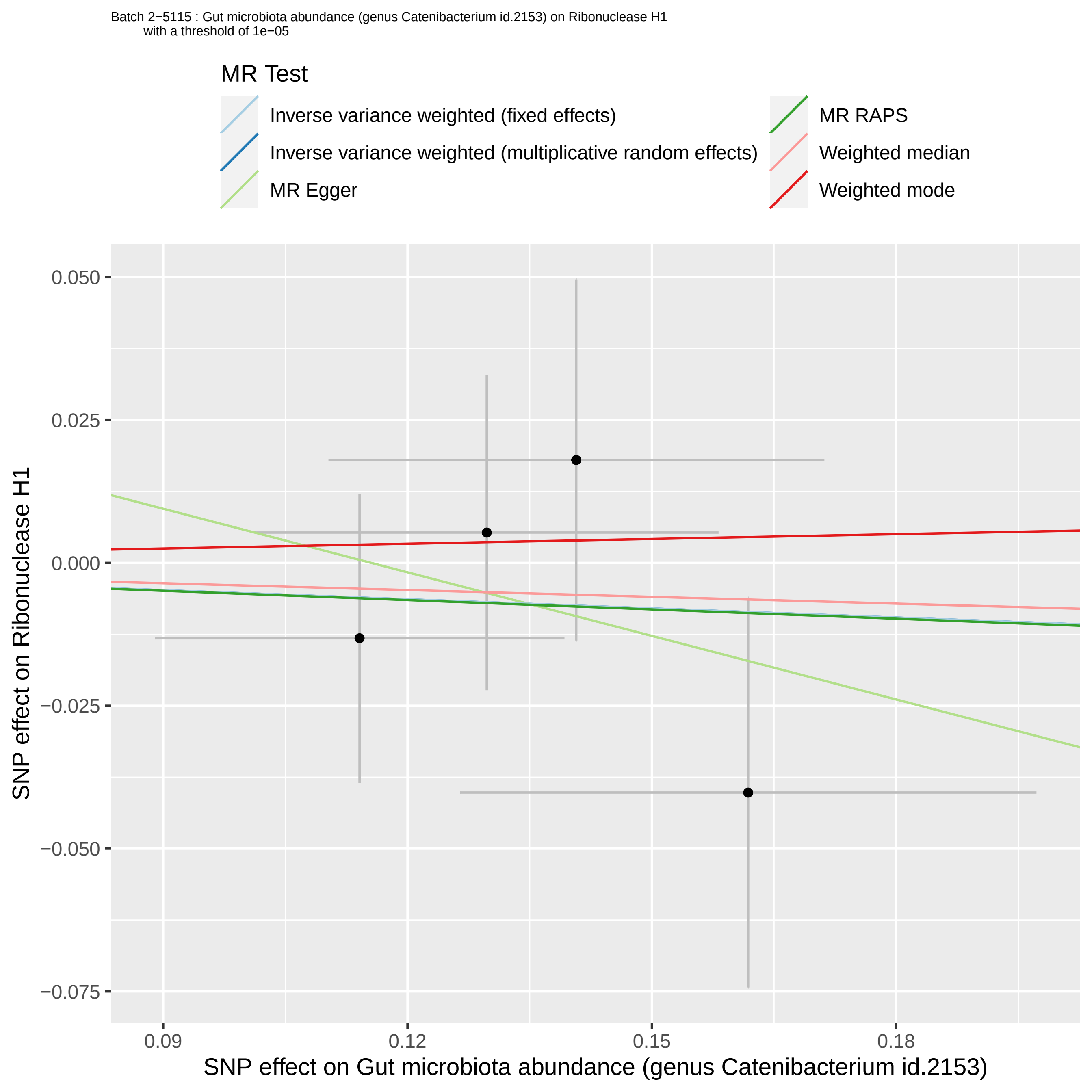
Batch 2−5115 : Gut microbiota abundance (genus Catenibacterium id.2153) on Ribonuclease H1
with a threshold of 1e−05
MR Test
Inverse variance weighted (fixed effects)
Inverse variance weighted (multiplicative random effects)
MR Egger
MR RAPS
Weighted median
Weighted mode
0.050
0.025
0.000
−0.025
−0.050
−0.075
0.09
0.12
0.15
0.18
SNP effect on Ribonuclease H1
SNP effect on Gut microbiota abundance (genus Catenibacterium id.2153)

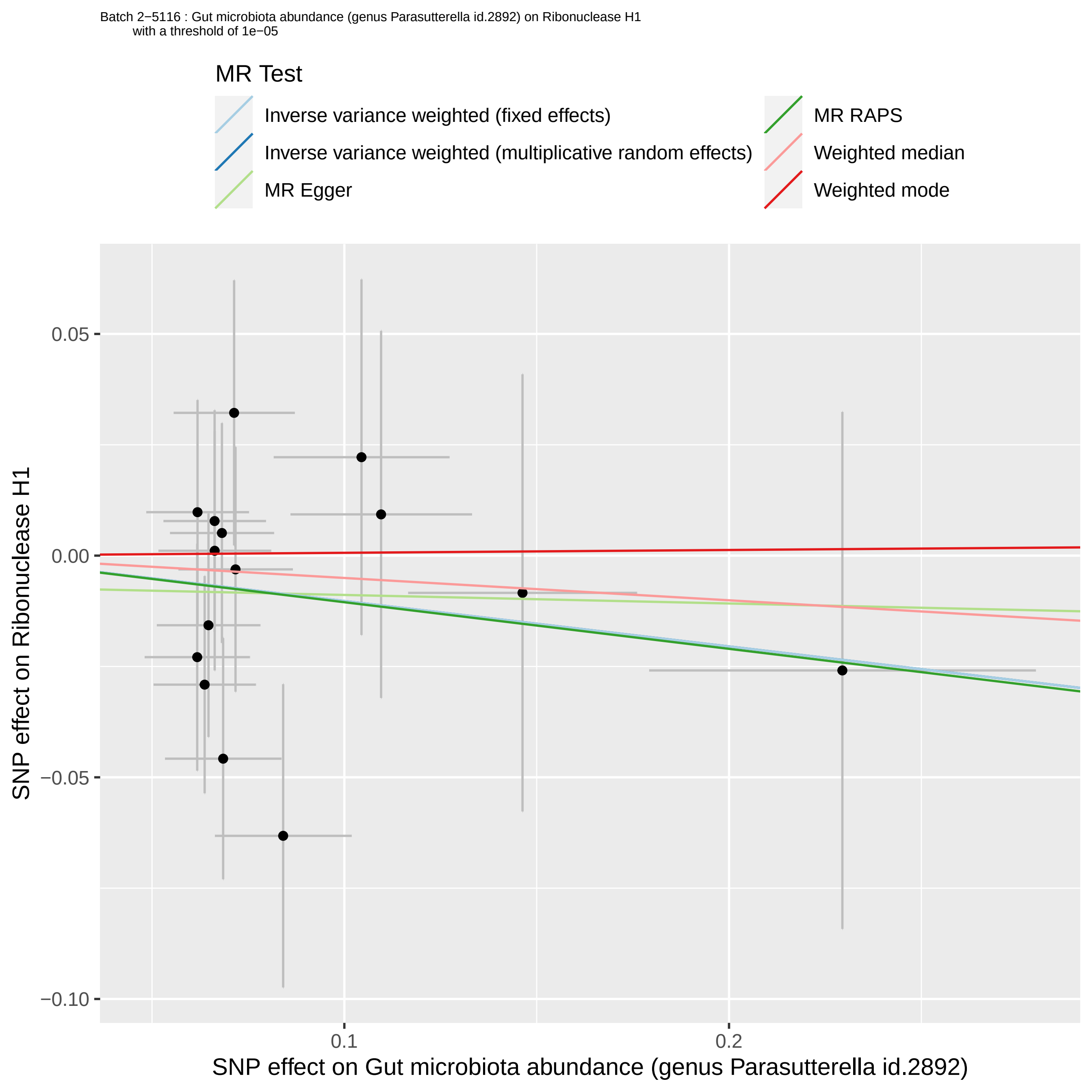

Batch 2−5116 : Gut microbiota abundance (genus Parasutterella id.2892) on Ribonuclease H1
with a threshold of 1e−05
MR Test
Inverse variance weighted (fixed effects)
Inverse variance weighted (multiplicative random effects)
MR Egger
MR RAPS
Weighted median
Weighted mode
SNP effect on Ribonuclease H1
0.05
0.00
−0.05
−0.10
0.1
0.2
SNP effect on Gut microbiota abundance (genus Parasutterella id.2892)

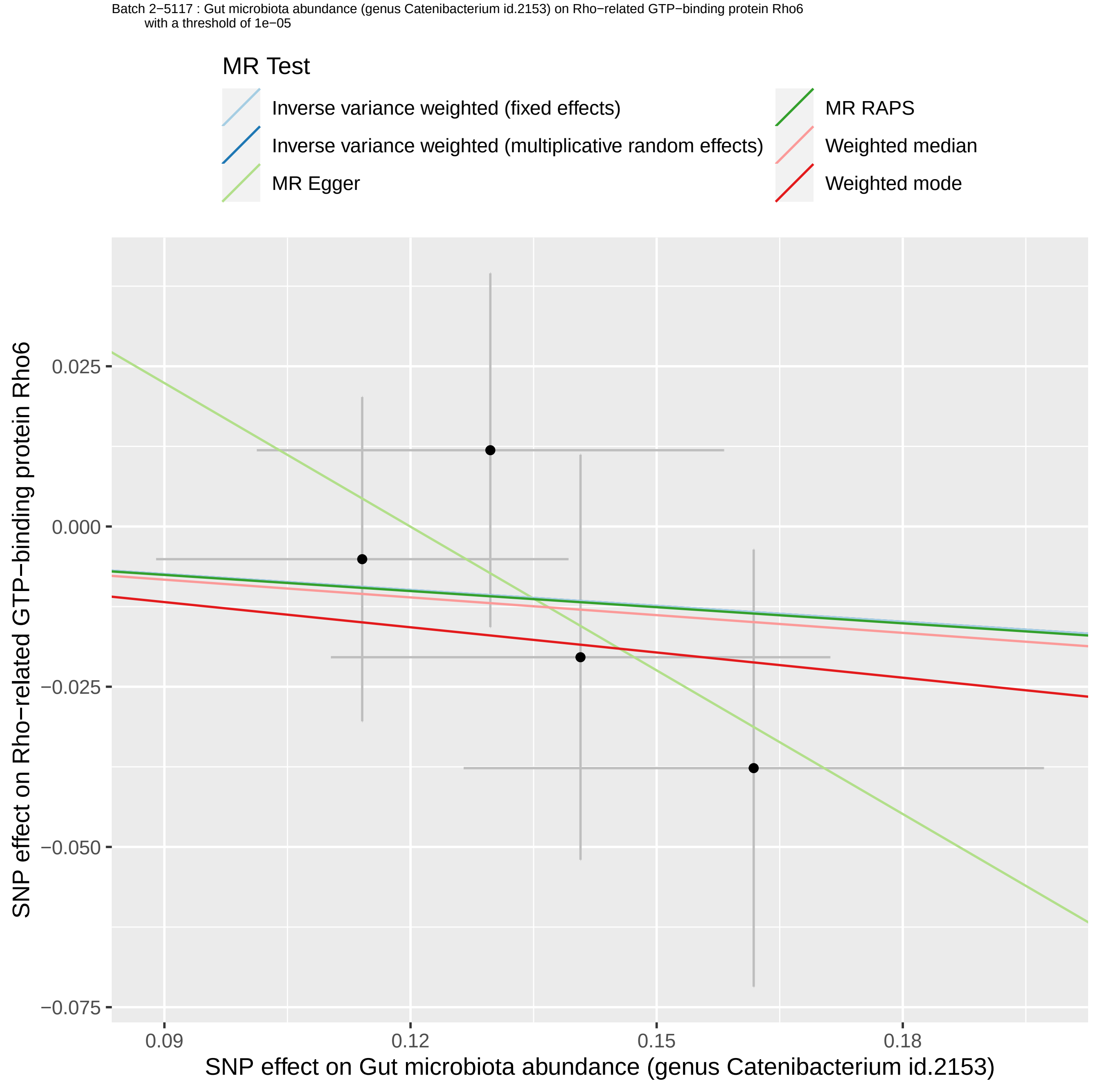

Batch 2−5117 : Gut microbiota abundance (genus Catenibacterium id.2153) on Rho−related GTP−binding protein Rho6
with a threshold of 1e−05
MR Test
Inverse variance weighted (fixed effects)
Inverse variance weighted (multiplicative random effects)
MR Egger
MR RAPS
Weighted median
Weighted mode
0.025
0.000
−0.025
−0.050
−0.075
0.09
0.12
0.15
0.18
SNP effect on Rho−related GTP−binding protein Rho6
SNP effect on Gut microbiota abundance (genus Catenibacterium id.2153)

Batch 2−5118 : Gut microbiota abundance (genus Parasutterella id.2892) on Rho−related GTP−binding protein Rho6
with a threshold of 1e−05

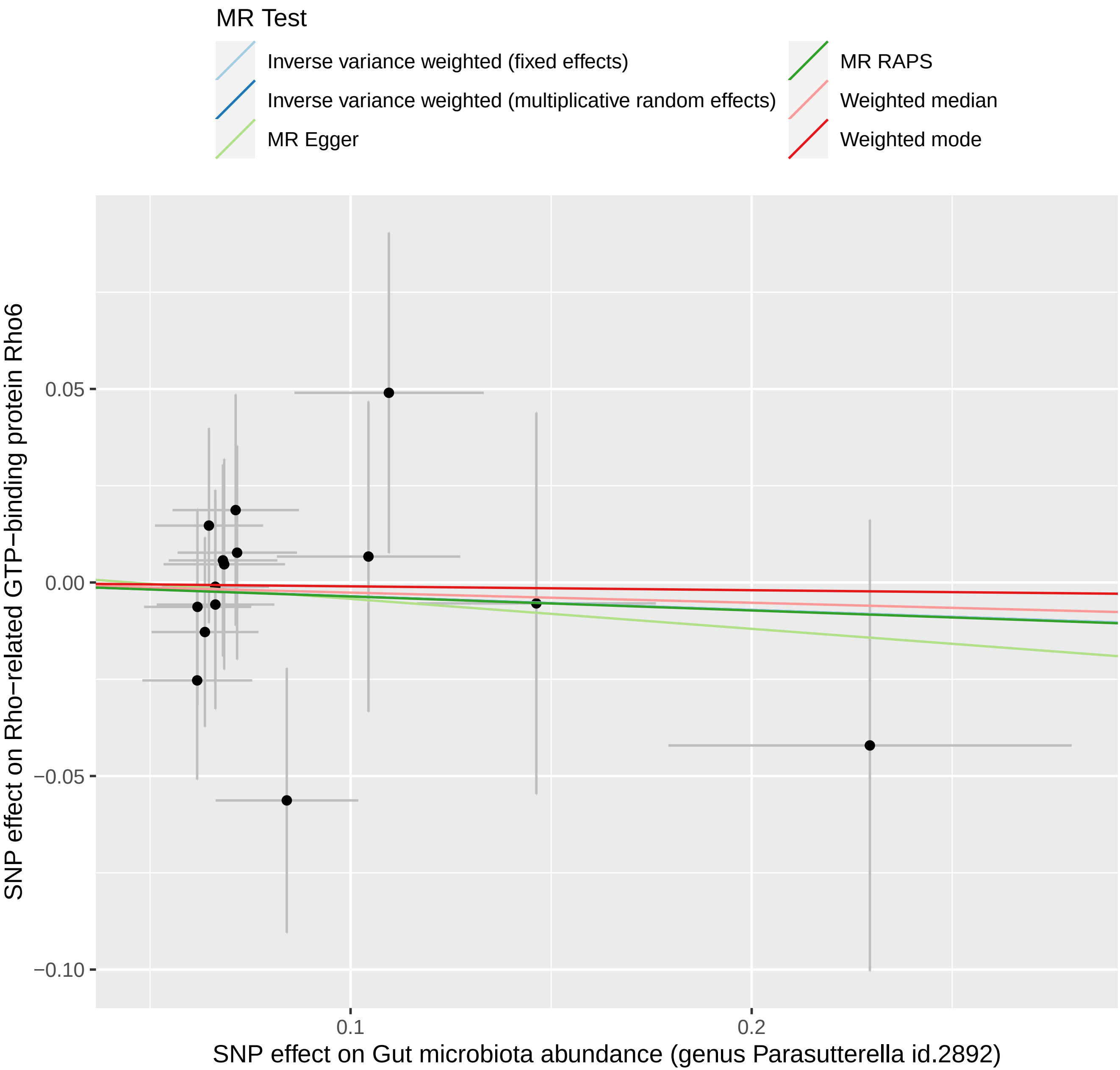

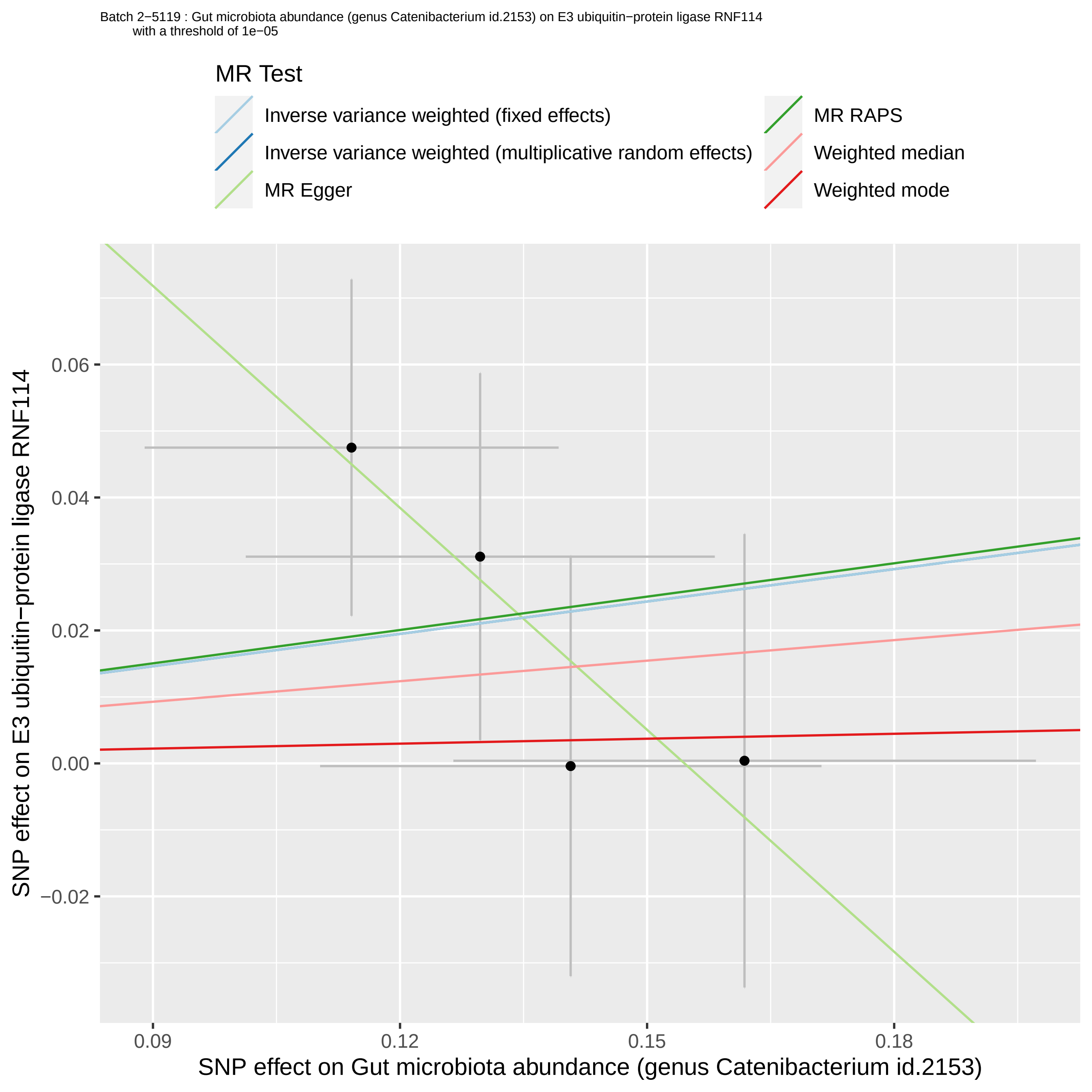

Batch 2−5119 : Gut microbiota abundance (genus Catenibacterium id.2153) on E3 ubiquitin−protein ligase RNF114
with a threshold of 1e−05
MR Test
Inverse variance weighted (fixed effects)
Inverse variance weighted (multiplicative random effects)
MR Egger
MR RAPS
Weighted median
Weighted mode
0.06
0.04
0.02
0.00
−0.02
0.09
0.12
0.15
0.18
SNP effect on E3 ubiquitin−protein ligase RNF114
SNP effect on Gut microbiota abundance (genus Catenibacterium id.2153)

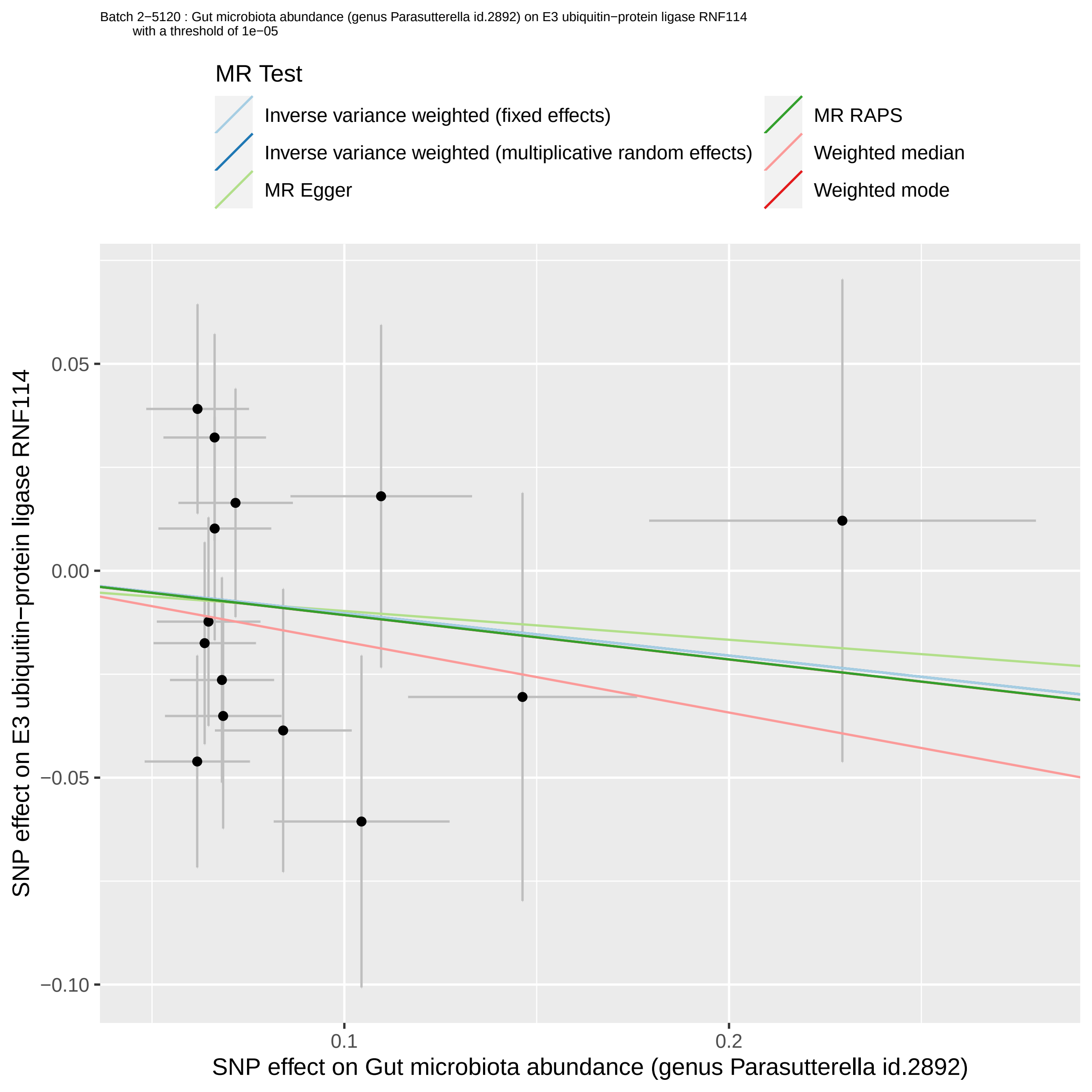

Batch 2−5120 : Gut microbiota abundance (genus Parasutterella id.2892) on E3 ubiquitin−protein ligase RNF114
with a threshold of 1e−05
MR Test
Inverse variance weighted (fixed effects)
Inverse variance weighted (multiplicative random effects)
MR Egger
MR RAPS
Weighted median
Weighted mode
SNP effect on E3 ubiquitin−protein ligase RNF114
0.05
0.00
−0.05
−0.10
0.1
0.2
SNP effect on Gut microbiota abundance (genus Parasutterella id.2892)

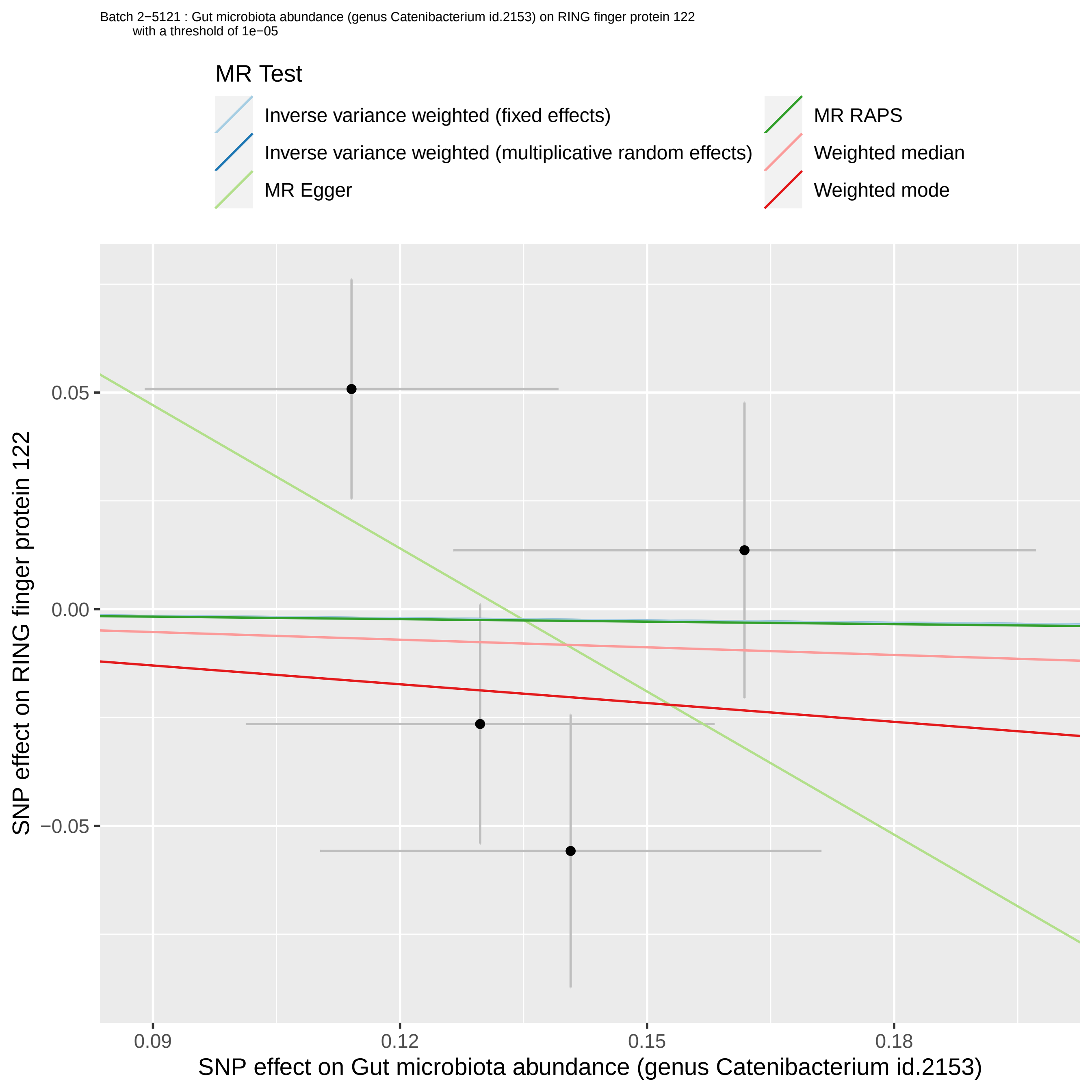

Batch 2−5121 : Gut microbiota abundance (genus Catenibacterium id.2153) on RING finger protein 122
with a threshold of 1e−05
MR Test
Inverse variance weighted (fixed effects)
Inverse variance weighted (multiplicative random effects)
MR Egger
MR RAPS
Weighted median
Weighted mode
SNP effect on RING finger protein 122
0.05
0.00
−0.05
0.09
0.12
0.15
0.18
SNP effect on Gut microbiota abundance (genus Catenibacterium id.2153)

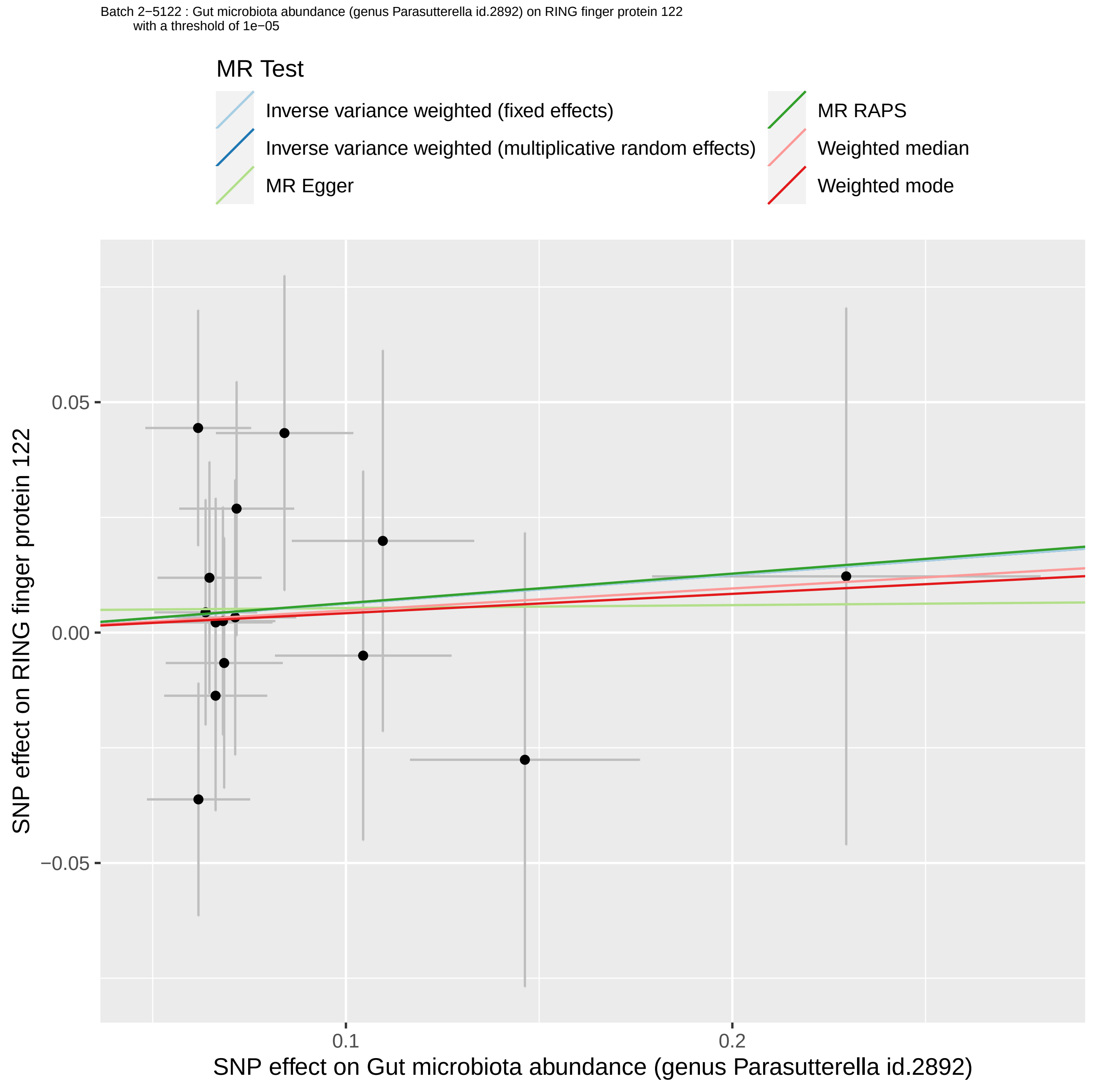
Batch 2−5122 : Gut microbiota abundance (genus Parasutterella id.2892) on RING finger protein 122
with a threshold of 1e−05
MR Test
Inverse variance weighted (fixed effects)
Inverse variance weighted (multiplicative random effects)
MR Egger
MR RAPS
Weighted median
Weighted mode
0.05
0.00
−0.05
0.1
0.2
SNP effect on RING finger protein 122
SNP effect on Gut microbiota abundance (genus Parasutterella id.2892)

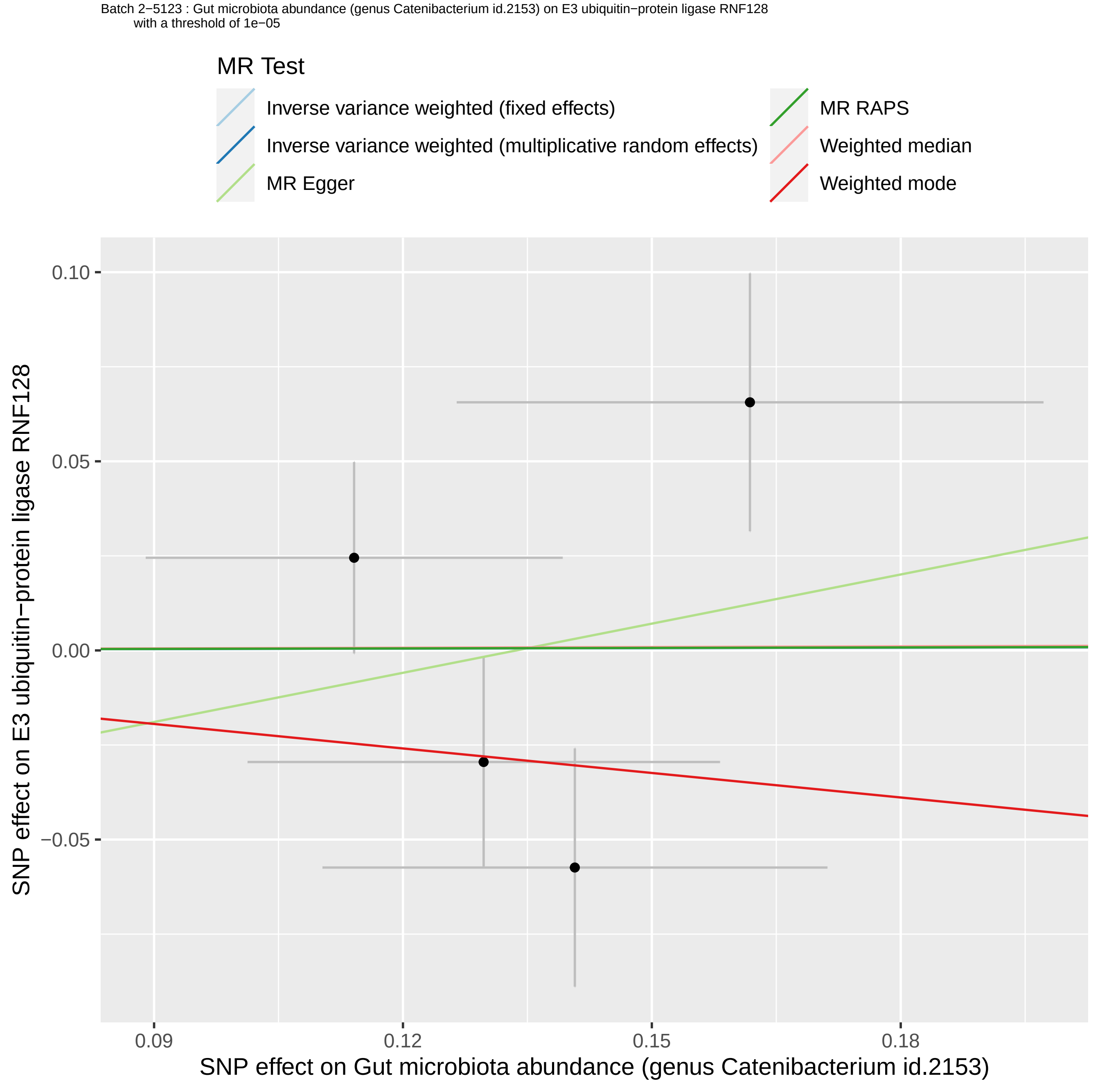
Batch 2−5123 : Gut microbiota abundance (genus Catenibacterium id.2153) on E3 ubiquitin−protein ligase RNF128
with a threshold of 1e−05
MR Test
Inverse variance weighted (fixed effects)
Inverse variance weighted (multiplicative random effects)
MR Egger
MR RAPS
Weighted median
Weighted mode
0.10
0.05
0.00
−0.05
0.09
0.12
0.15
0.18
SNP effect on E3 ubiquitin−protein ligase RNF128
SNP effect on Gut microbiota abundance (genus Catenibacterium id.2153)

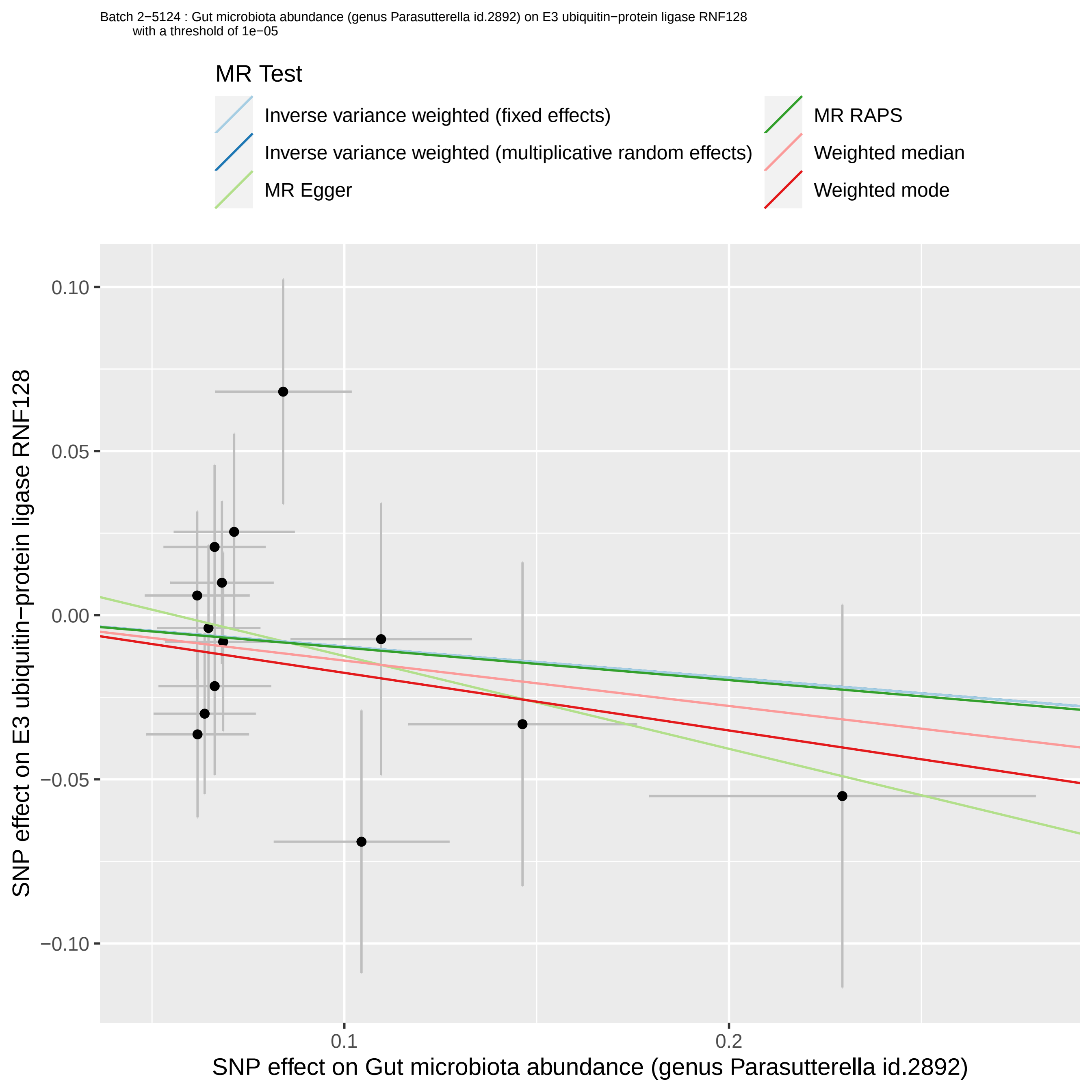

Batch 2−5124 : Gut microbiota abundance (genus Parasutterella id.2892) on E3 ubiquitin−protein ligase RNF128
with a threshold of 1e−05
MR Test
Inverse variance weighted (fixed effects)
Inverse variance weighted (multiplicative random effects)
MR Egger
MR RAPS
Weighted median
Weighted mode
SNP effect on E3 ubiquitin−protein ligase RNF128
0.10
0.05
0.00
−0.05
−0.10
0.1
0.2
SNP effect on Gut microbiota abundance (genus Parasutterella id.2892)

Batch 2−5125 : Gut microbiota abundance (genus Catenibacterium id.2153) on E3 ubiquitin−protein ligase RNF13
with a threshold of 1e−05

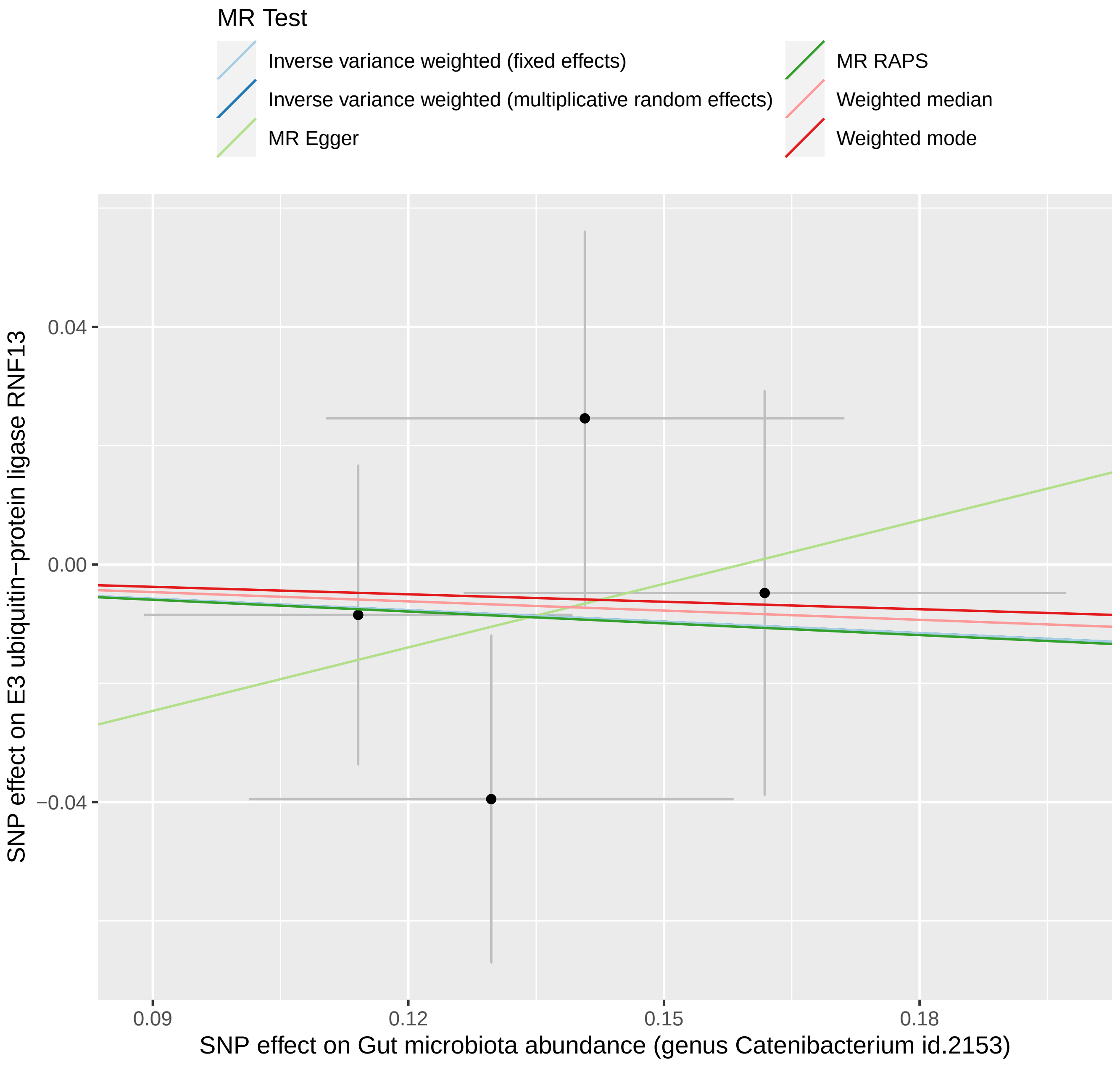

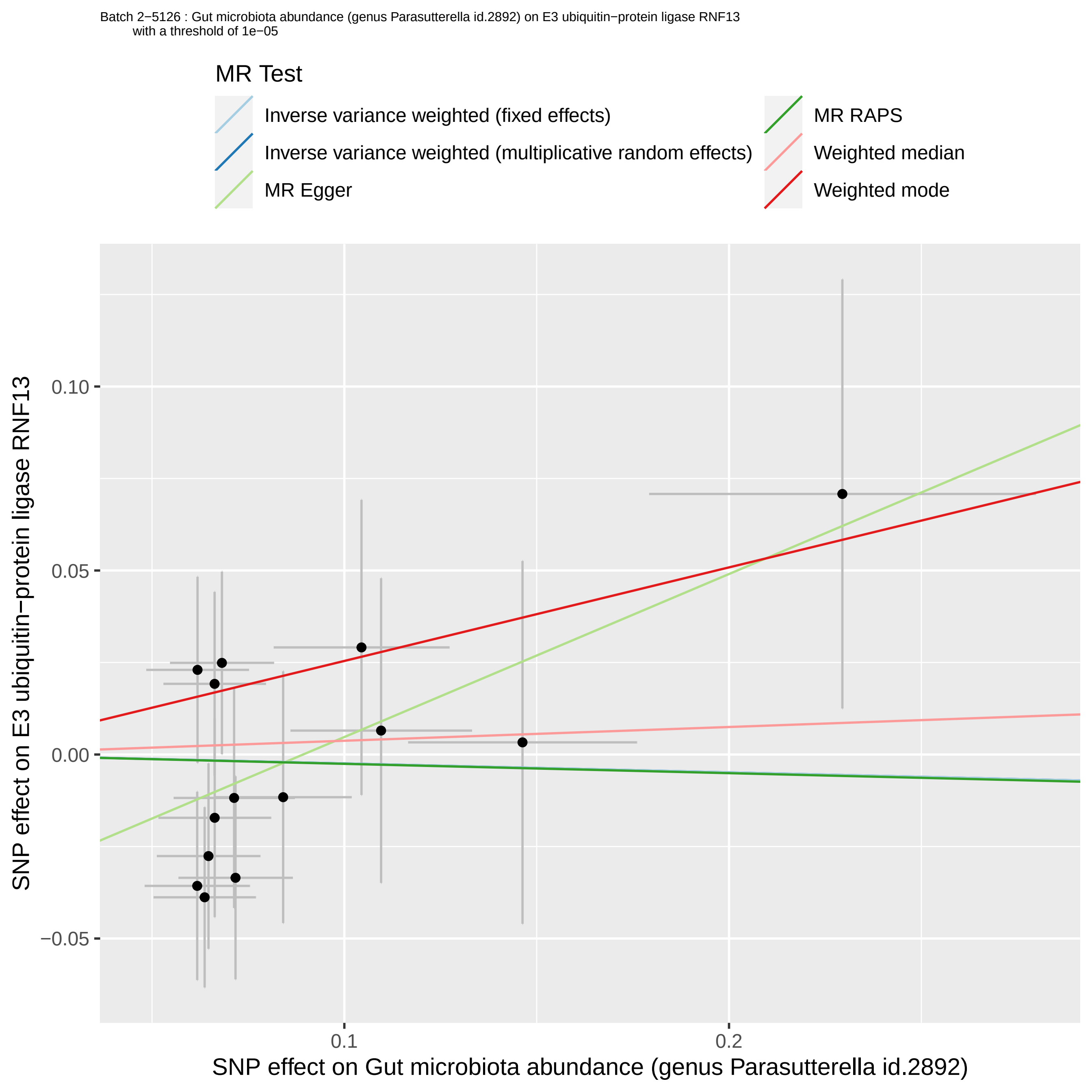
Batch 2−5126 : Gut microbiota abundance (genus Parasutterella id.2892) on E3 ubiquitin−protein ligase RNF13
with a threshold of 1e−05
MR Test
Inverse variance weighted (fixed effects)
Inverse variance weighted (multiplicative random effects)
MR Egger
MR RAPS
Weighted median
Weighted mode
0.10
0.05
0.00
−0.05
0.1
0.2
SNP effect on E3 ubiquitin−protein ligase RNF13
SNP effect on Gut microbiota abundance (genus Parasutterella id.2892)

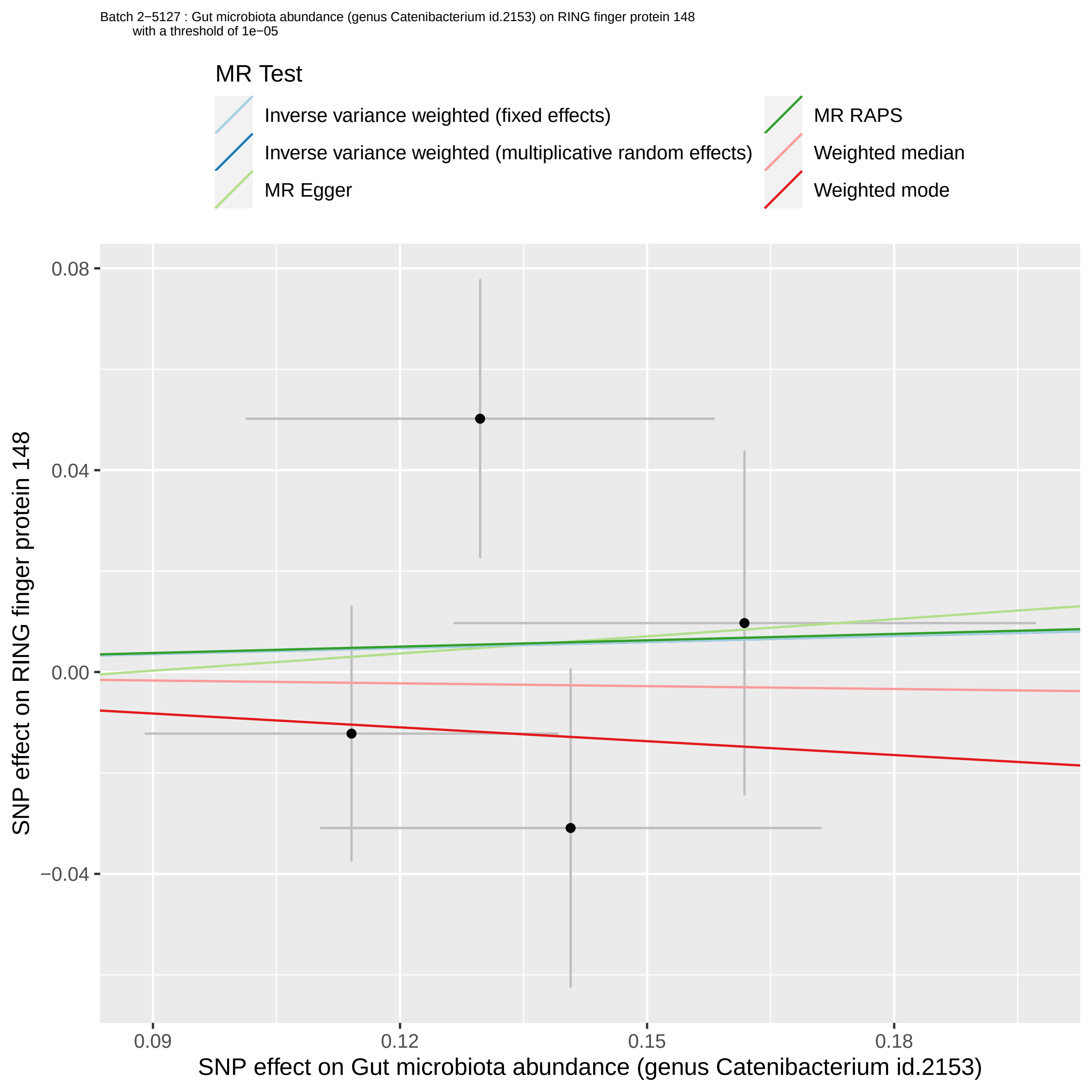

Batch 2−5127 : Gut microbiota abundance (genus Catenibacterium id.2153) on RING finger protein 148
with a threshold of 1e−05
MR Test
Inverse variance weighted (fixed effects)
Inverse variance weighted (multiplicative random effects)
MR Egger
MR RAPS
Weighted median
Weighted mode
0.08
0.04
0.00
−0.04
0.09
0.12
0.15
0.18
SNP effect on RING finger protein 148
SNP effect on Gut microbiota abundance (genus Catenibacterium id.2153)

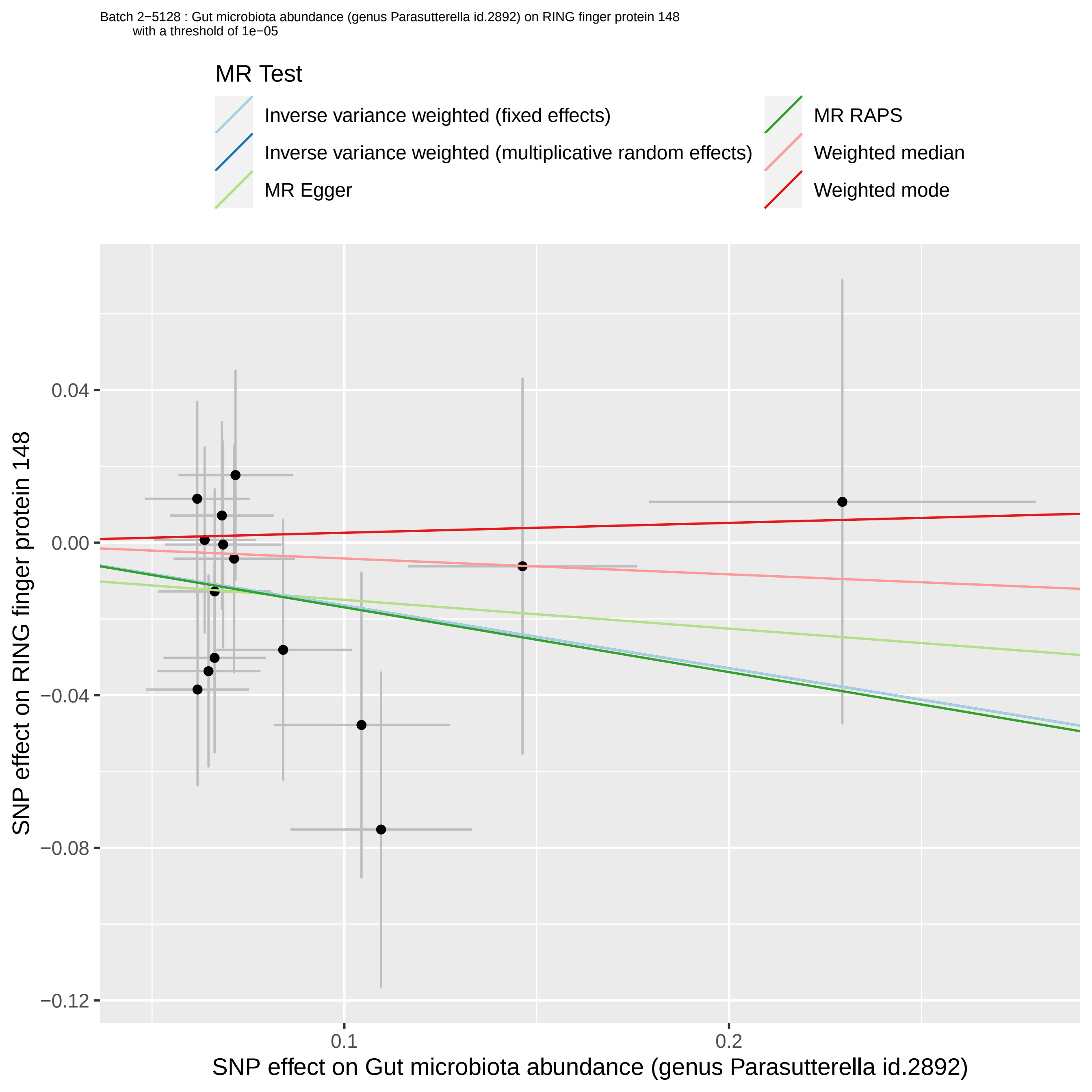

Batch 2−5128 : Gut microbiota abundance (genus Parasutterella id.2892) on RING finger protein 148
with a threshold of 1e−05
MR Test
Inverse variance weighted (fixed effects)
Inverse variance weighted (multiplicative random effects)
MR Egger
MR RAPS
Weighted median
Weighted mode
0.04
0.00
−0.04
−0.08
−0.12
0.1
0.2
SNP effect on RING finger protein 148
SNP effect on Gut microbiota abundance (genus Parasutterella id.2892)

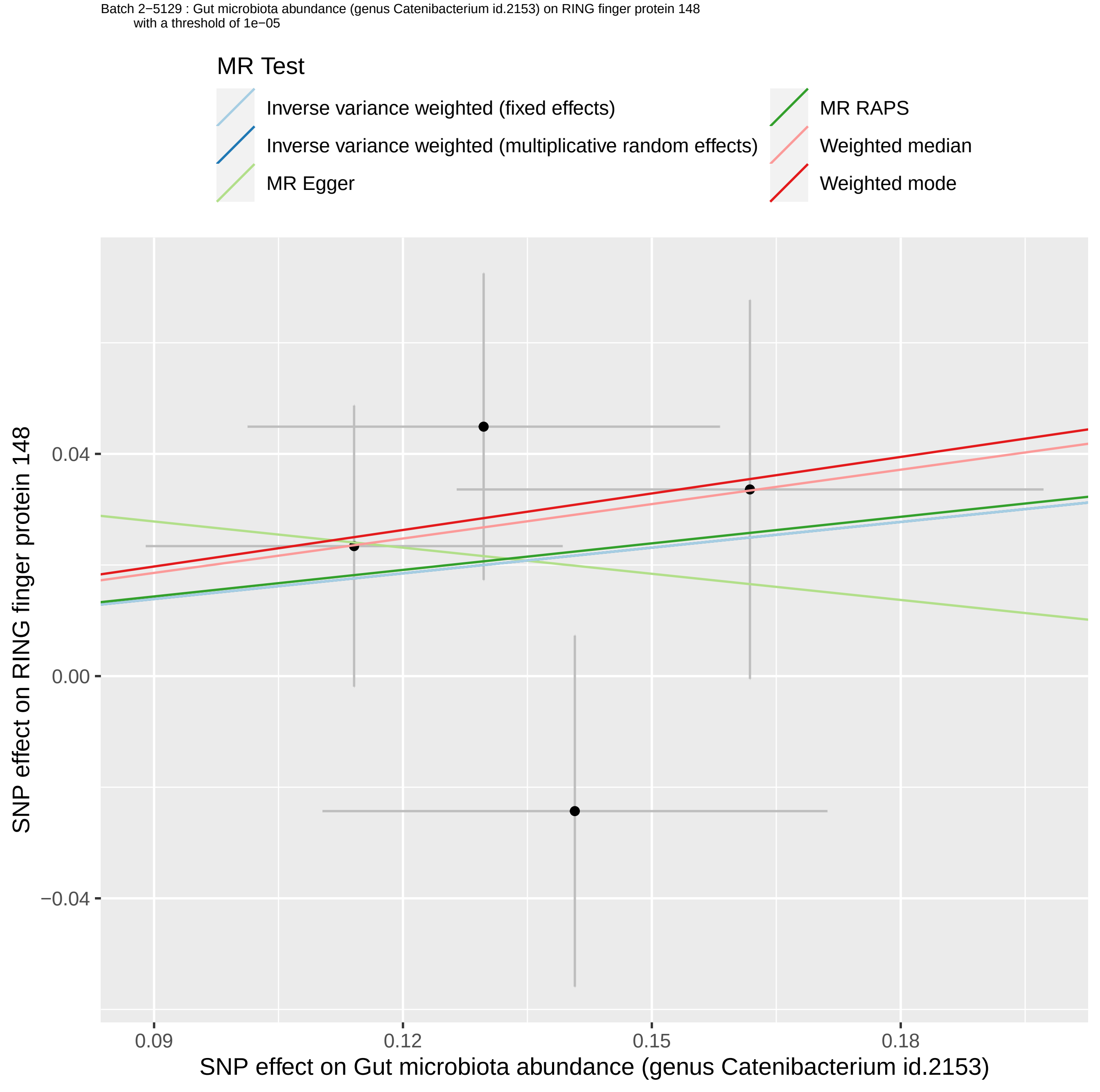

Batch 2−5129 : Gut microbiota abundance (genus Catenibacterium id.2153) on RING finger protein 148
with a threshold of 1e−05
MR Test
Inverse variance weighted (fixed effects)
Inverse variance weighted (multiplicative random effects)
MR Egger
MR RAPS
Weighted median
Weighted mode
SNP effect on RING finger protein 148
0.04
0.00
−0.04
0.09
0.12
0.15
0.18
SNP effect on Gut microbiota abundance (genus Catenibacterium id.2153)

Batch 2−5130 : Gut microbiota abundance (genus Parasutterella id.2892) on RING finger protein 148
with a threshold of 1e−05

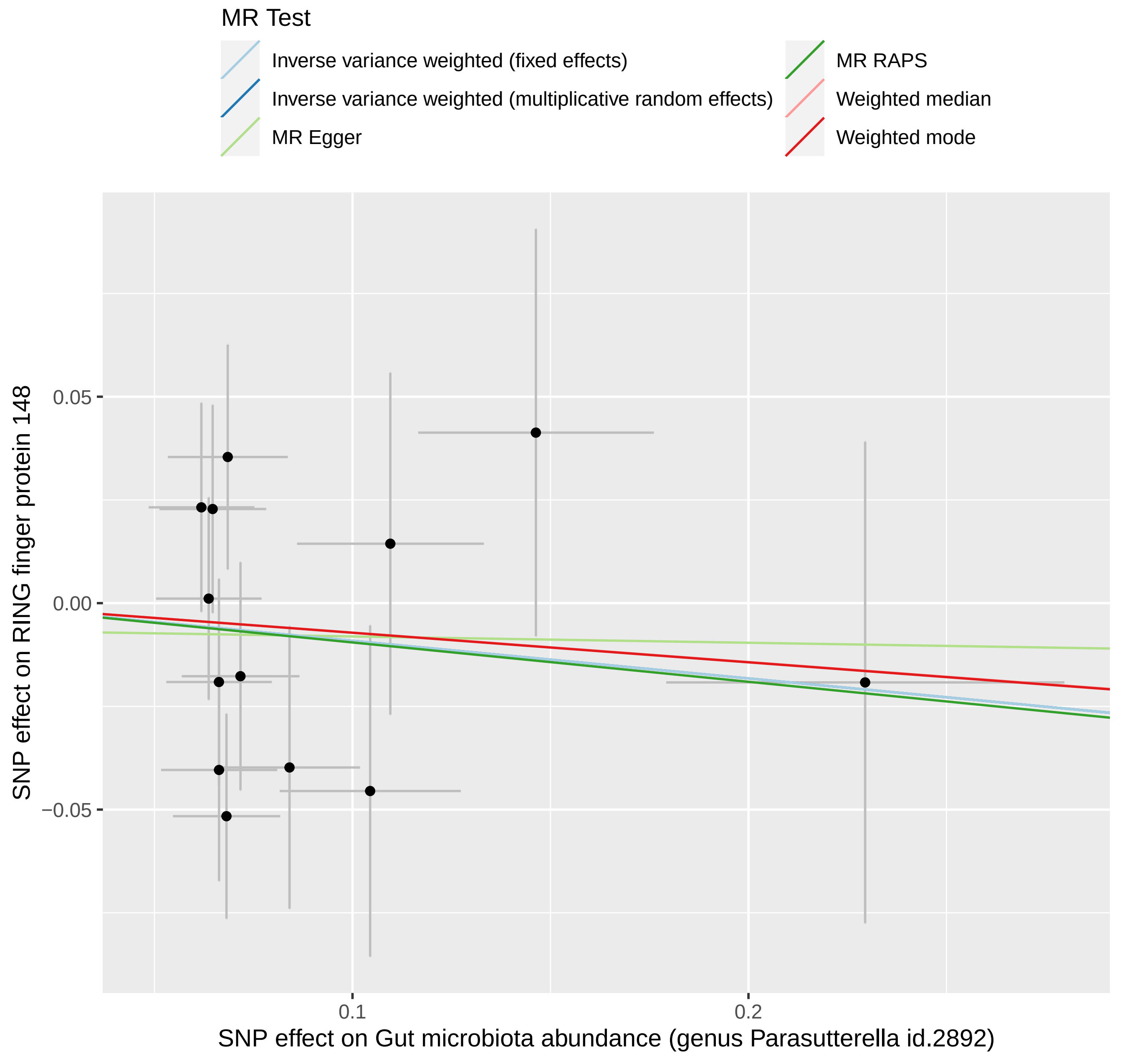

Batch 2−5131 : Gut microbiota abundance (genus Catenibacterium id.2153) on E3 ubiquitin−protein ligase RNF149
with a threshold of 1e−05

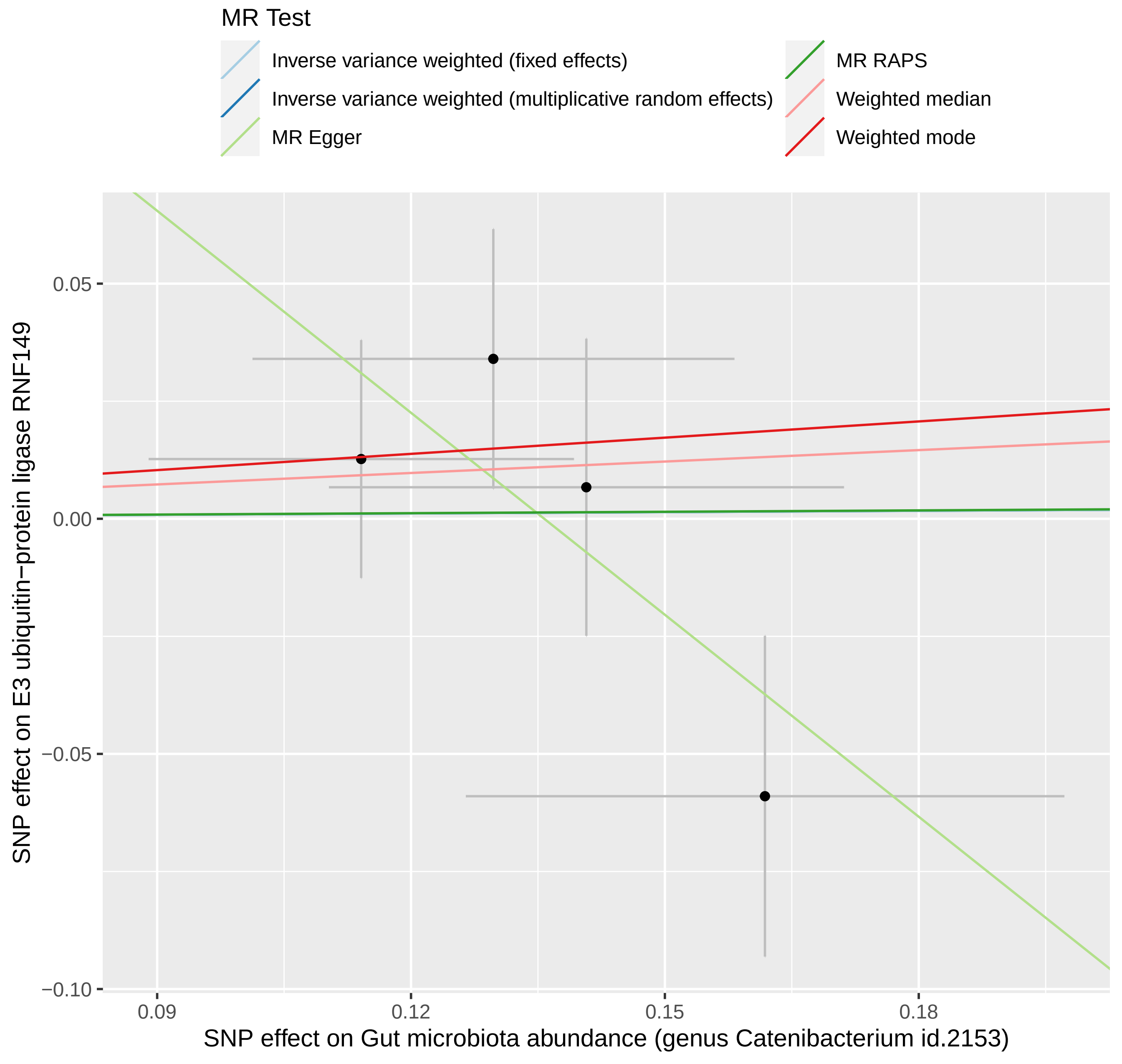

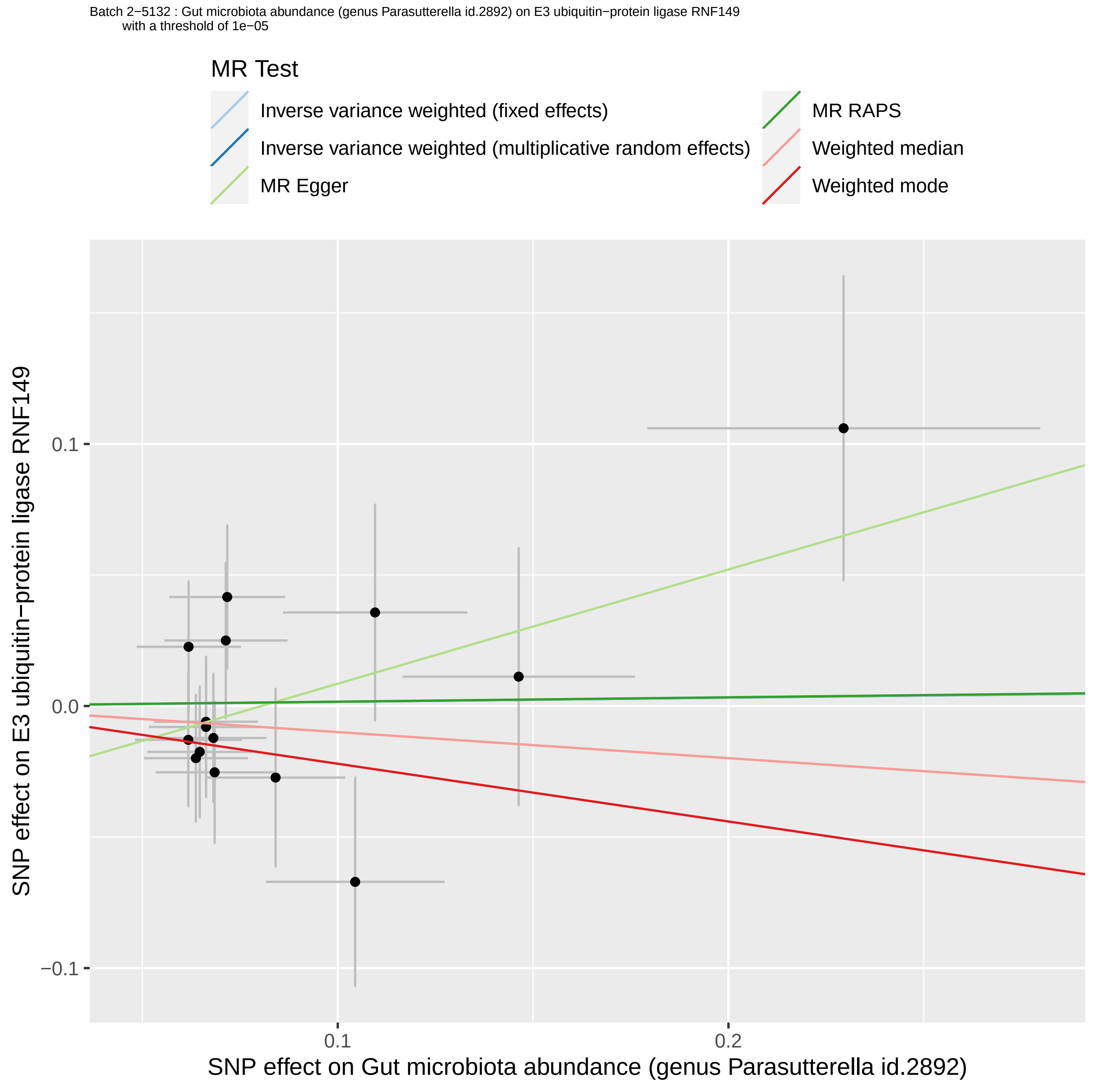
Batch 2−5132 : Gut microbiota abundance (genus Parasutterella id.2892) on E3 ubiquitin−protein ligase RNF149
with a threshold of 1e−05
MR Test
Inverse variance weighted (fixed effects)
Inverse variance weighted (multiplicative random effects)
MR Egger
MR RAPS
Weighted median
Weighted mode
0.1
0.0
−0.1
0.1
0.2
SNP effect on E3 ubiquitin−protein ligase RNF149
SNP effect on Gut microbiota abundance (genus Parasutterella id.2892)

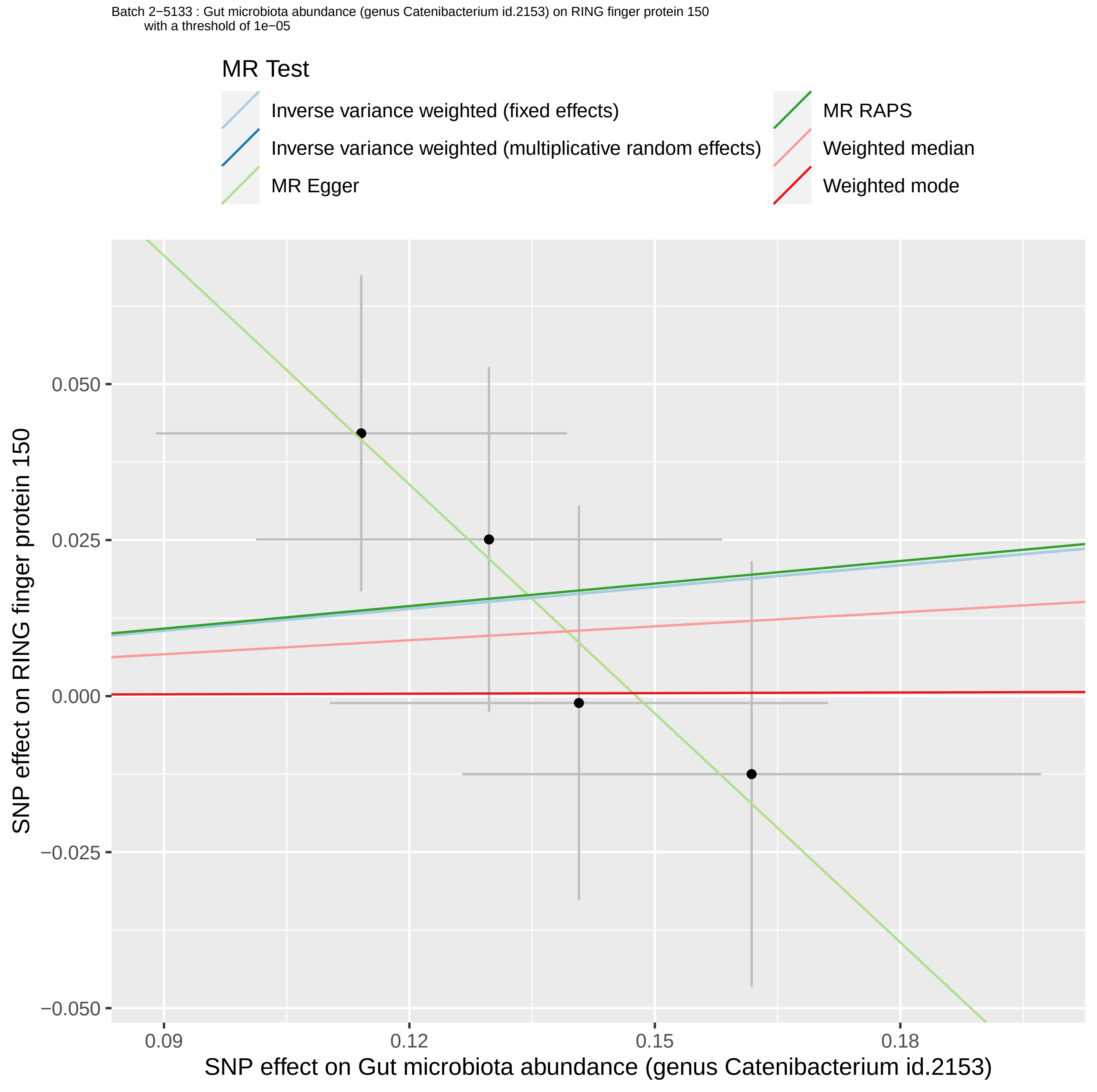

Batch 2−5133 : Gut microbiota abundance (genus Catenibacterium id.2153) on RING finger protein 150
with a threshold of 1e−05
MR Test
Inverse variance weighted (fixed effects)
Inverse variance weighted (multiplicative random effects)
MR Egger
MR RAPS
Weighted median
Weighted mode
0.050
0.025
0.000
−0.025
−0.050
0.09
0.12
0.15
0.18
SNP effect on RING finger protein 150
SNP effect on Gut microbiota abundance (genus Catenibacterium id.2153)

Batch 2−5134 : Gut microbiota abundance (genus Parasutterella id.2892) on RING finger protein 150
with a threshold of 1e−05

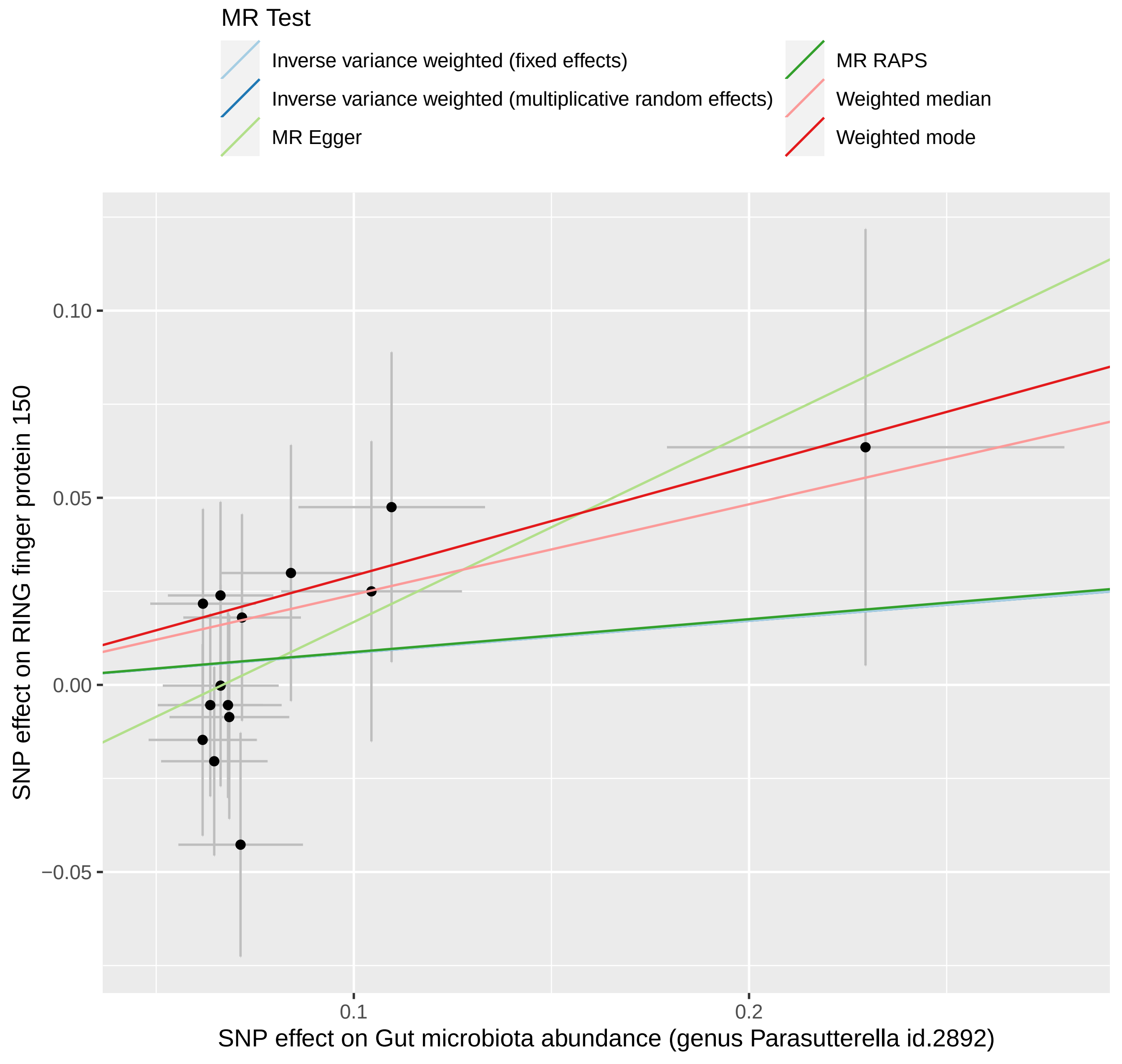

Batch 2−5135 : Gut microbiota abundance (genus Catenibacterium id.2153) on RING finger protein 165
with a threshold of 1e−05

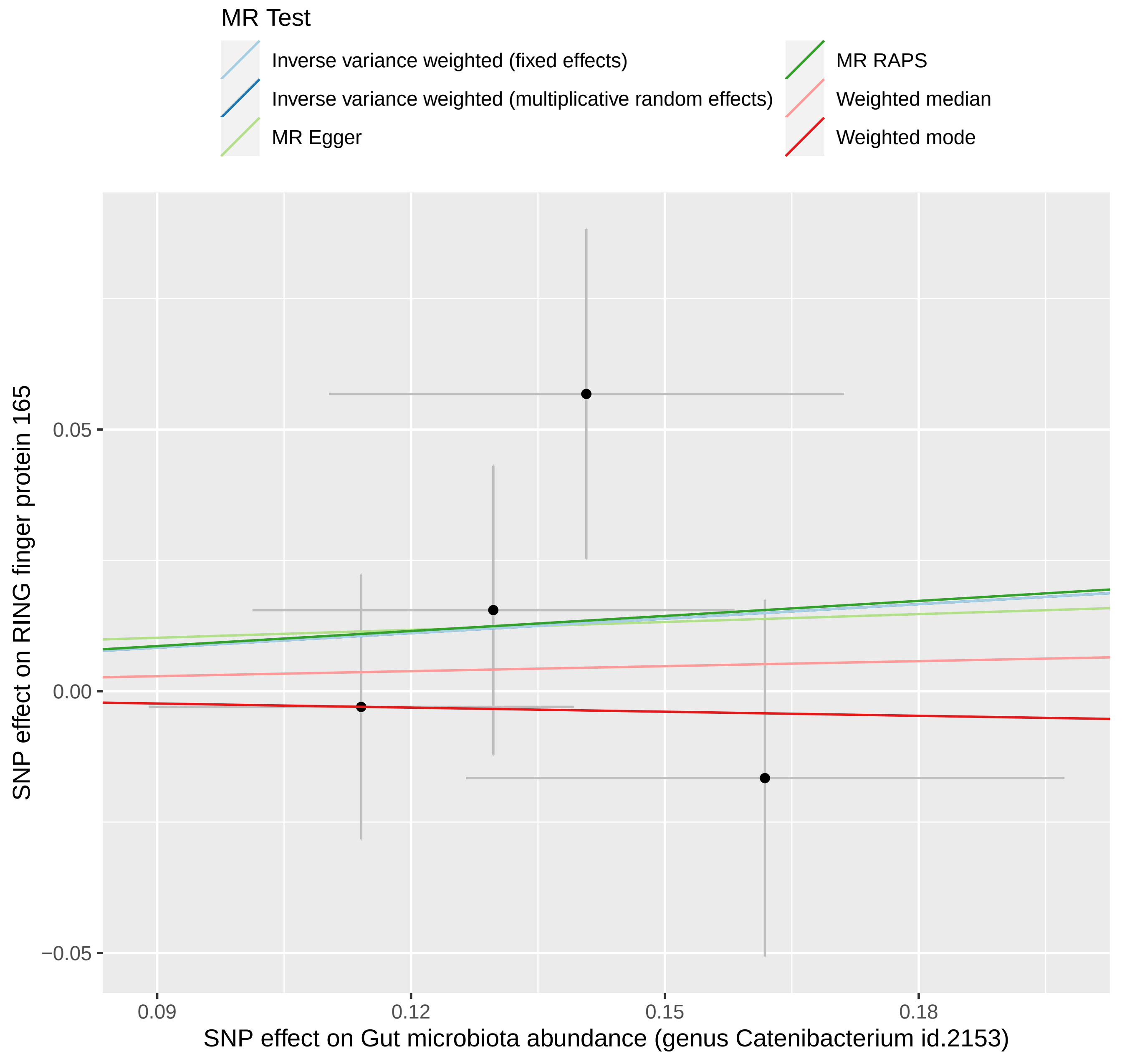

Batch 2−5136 : Gut microbiota abundance (genus Parasutterella id.2892) on RING finger protein 165
with a threshold of 1e−05

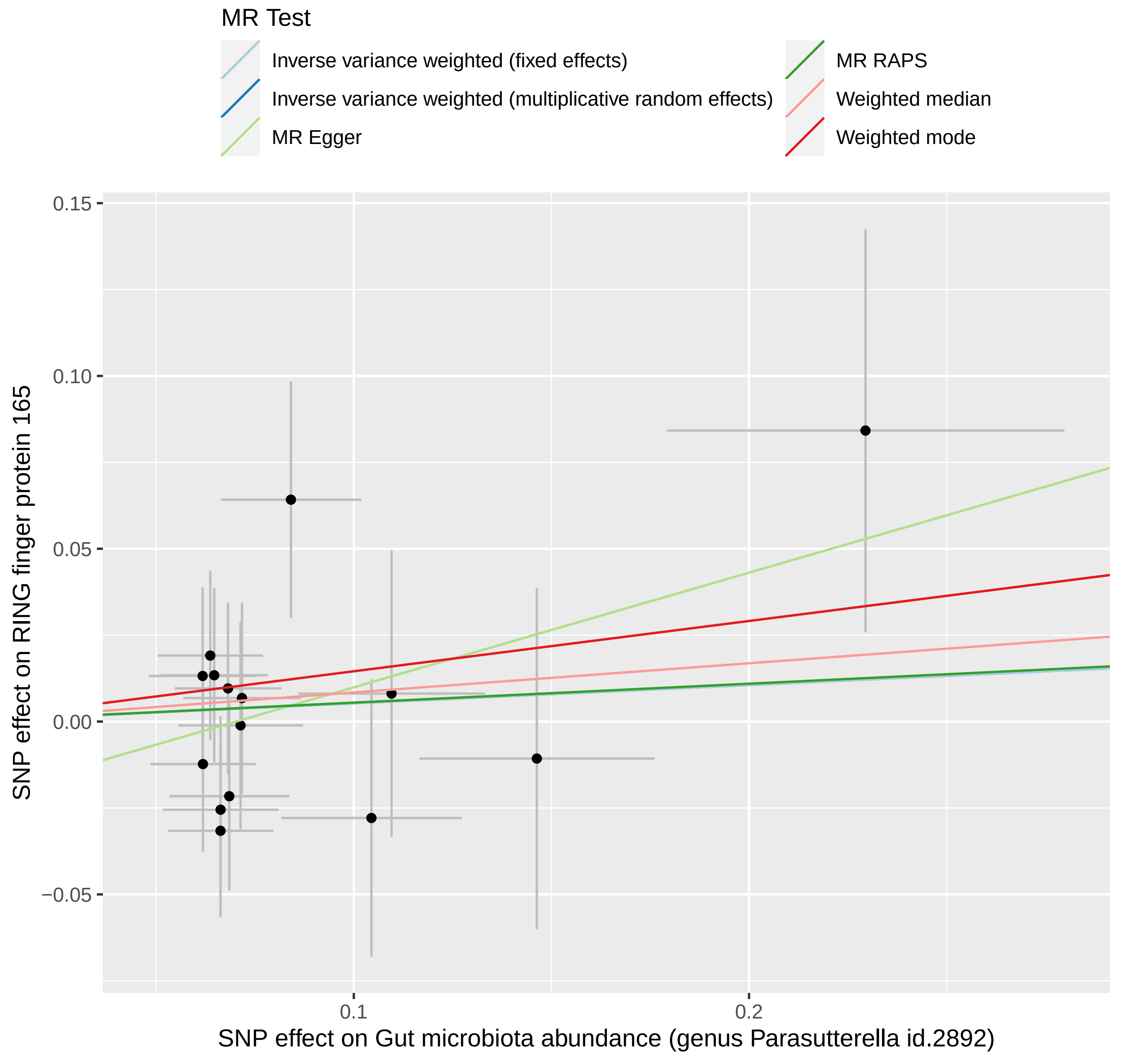

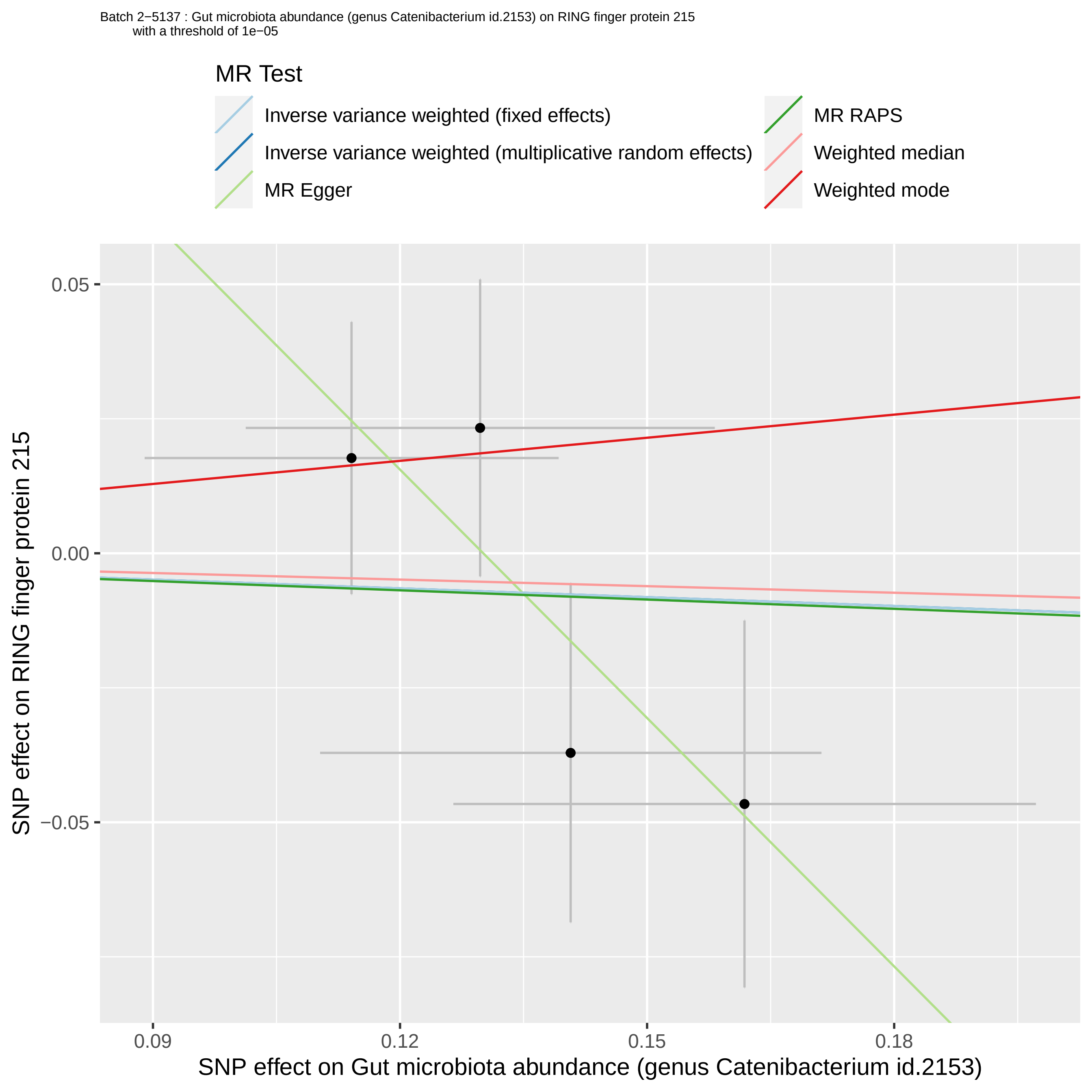
Batch 2−5137 : Gut microbiota abundance (genus Catenibacterium id.2153) on RING finger protein 215
with a threshold of 1e−05
MR Test
Inverse variance weighted (fixed effects)
Inverse variance weighted (multiplicative random effects)
MR Egger
MR RAPS
Weighted median
Weighted mode
0.05
0.00
−0.05
0.09
0.12
0.15
0.18
SNP effect on RING finger protein 215
SNP effect on Gut microbiota abundance (genus Catenibacterium id.2153)

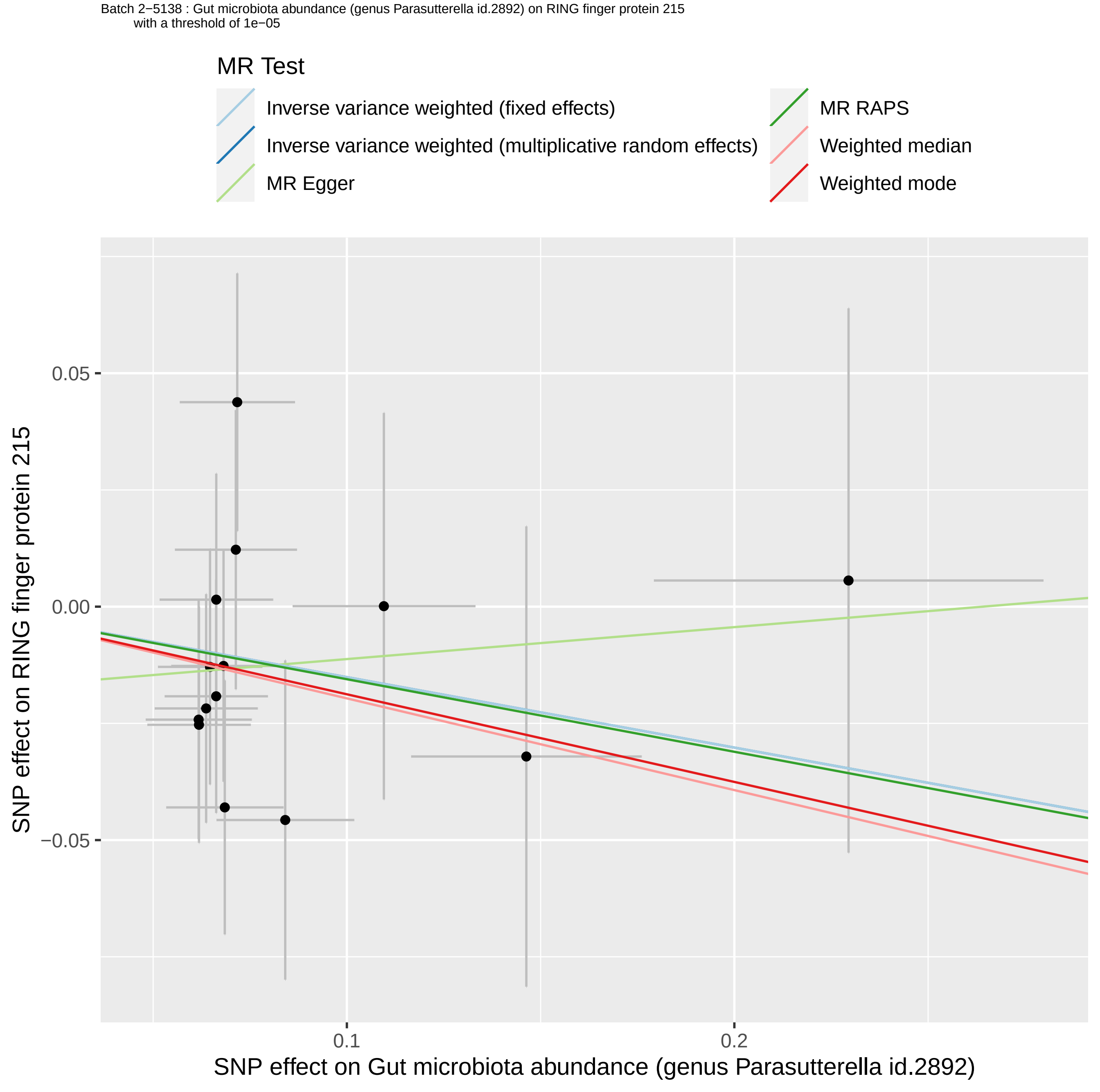
Batch 2−5138 : Gut microbiota abundance (genus Parasutterella id.2892) on RING finger protein 215
with a threshold of 1e−05
MR Test
Inverse variance weighted (fixed effects)
Inverse variance weighted (multiplicative random effects)
MR Egger
MR RAPS
Weighted median
Weighted mode
0.05
0.00
−0.05
0.1
0.2
SNP effect on RING finger protein 215
SNP effect on Gut microbiota abundance (genus Parasutterella id.2892)

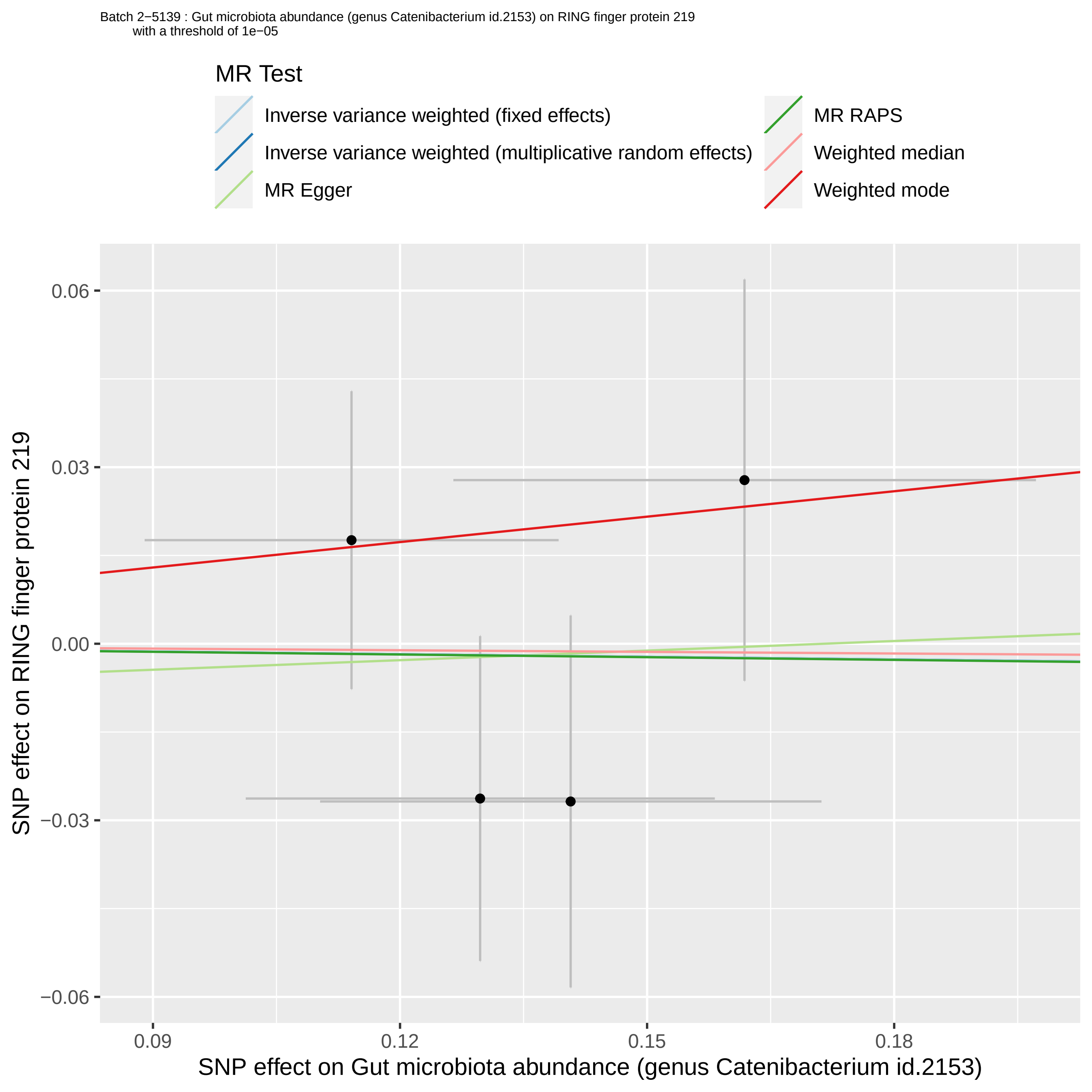

Batch 2−5139 : Gut microbiota abundance (genus Catenibacterium id.2153) on RING finger protein 219
with a threshold of 1e−05
MR Test
Inverse variance weighted (fixed effects)
Inverse variance weighted (multiplicative random effects)
MR Egger
MR RAPS
Weighted median
Weighted mode
0.06
0.03
0.00
−0.03
−0.06
0.09
0.12
0.15
0.18
SNP effect on RING finger protein 219
SNP effect on Gut microbiota abundance (genus Catenibacterium id.2153)

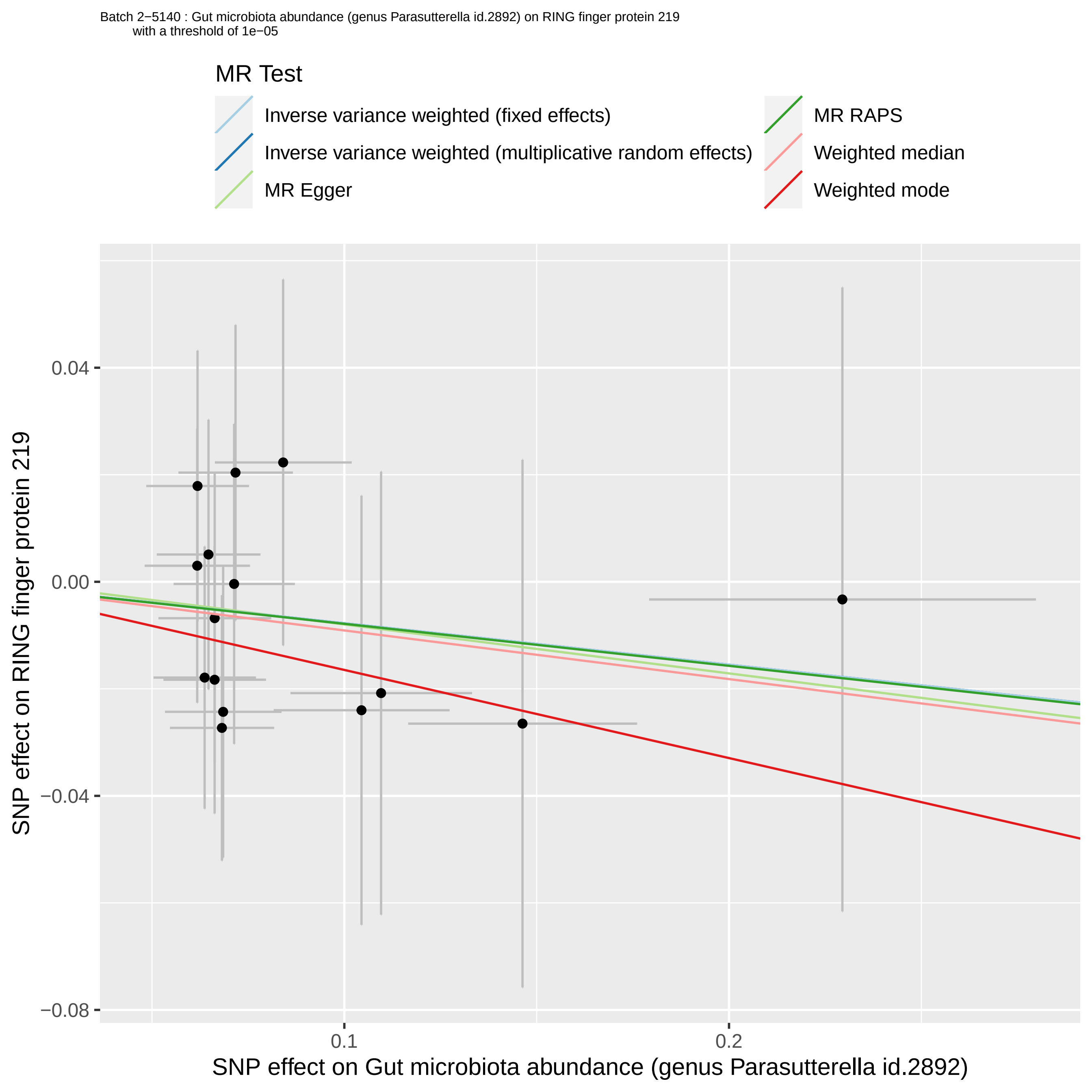

Batch 2−5140 : Gut microbiota abundance (genus Parasutterella id.2892) on RING finger protein 219
with a threshold of 1e−05
MR Test
Inverse variance weighted (fixed effects)
Inverse variance weighted (multiplicative random effects)
MR Egger
MR RAPS
Weighted median
Weighted mode
0.04
0.00
−0.04
−0.08
0.1
0.2
SNP effect on RING finger protein 219
SNP effect on Gut microbiota abundance (genus Parasutterella id.2892)

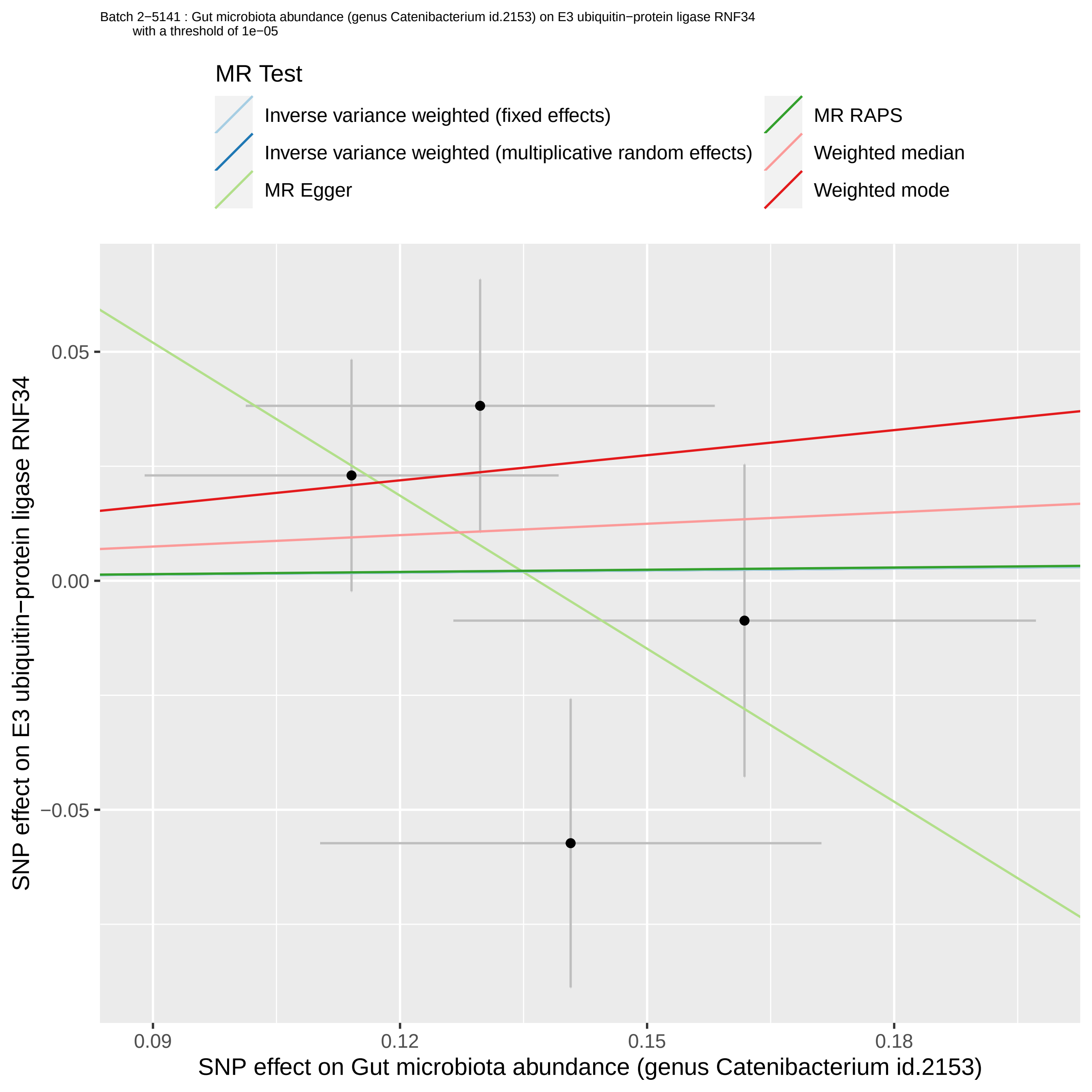
Batch 2−5141 : Gut microbiota abundance (genus Catenibacterium id.2153) on E3 ubiquitin−protein ligase RNF34
with a threshold of 1e−05
MR Test
Inverse variance weighted (fixed effects)
Inverse variance weighted (multiplicative random effects)
MR Egger
MR RAPS
Weighted median
Weighted mode
0.05
0.00
−0.05
0.09
0.12
0.15
0.18
SNP effect on E3 ubiquitin−protein ligase RNF34
SNP effect on Gut microbiota abundance (genus Catenibacterium id.2153)

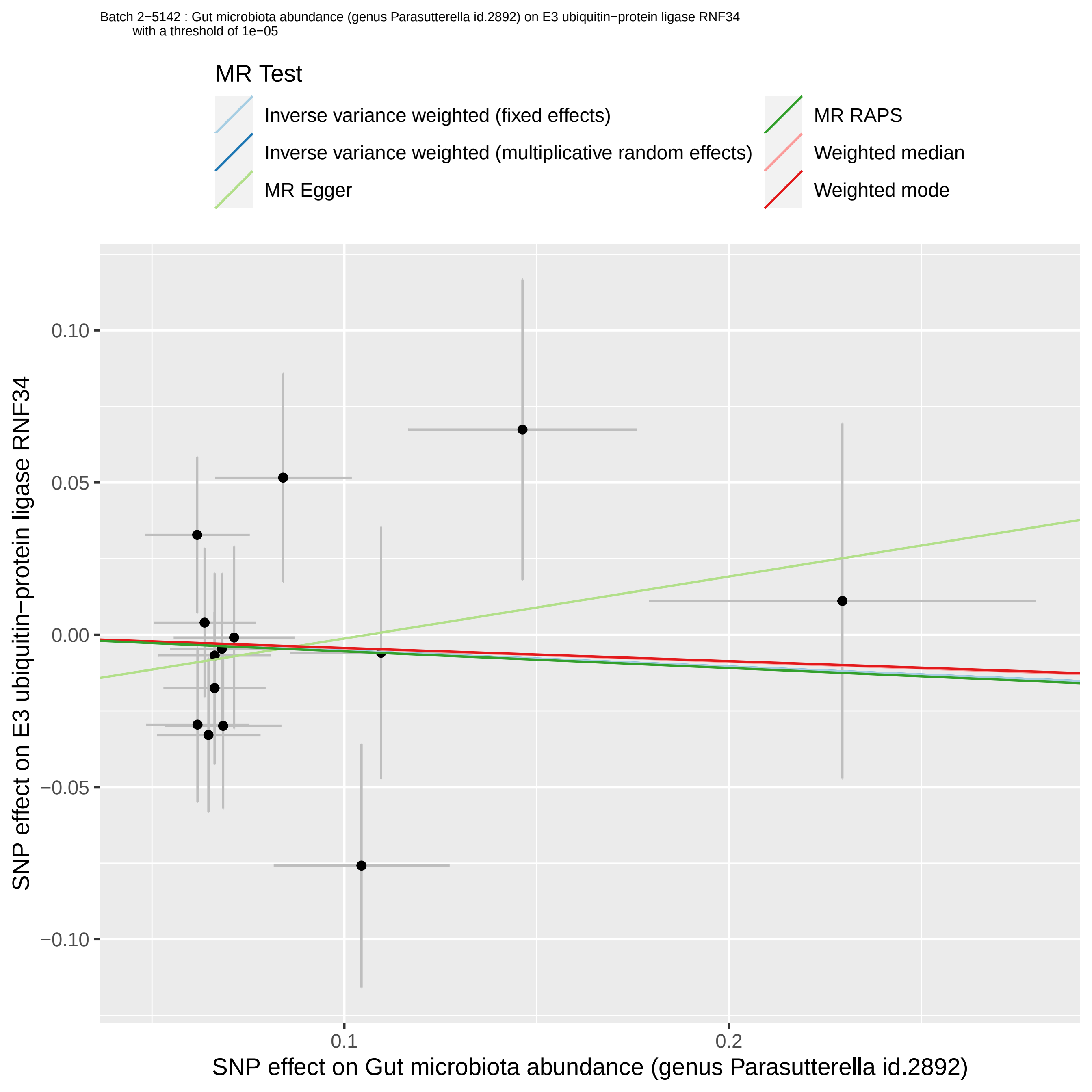

Batch 2−5142 : Gut microbiota abundance (genus Parasutterella id.2892) on E3 ubiquitin−protein ligase RNF34
with a threshold of 1e−05
MR Test
Inverse variance weighted (fixed effects)
Inverse variance weighted (multiplicative random effects)
MR Egger
MR RAPS
Weighted median
Weighted mode
0.10
0.05
0.00
−0.05
−0.10
0.1
0.2
SNP effect on Gut microbiota abundance (genus Parasutterella id.2892)
SNP effect on E3 ubiquitin−protein ligase RNF34

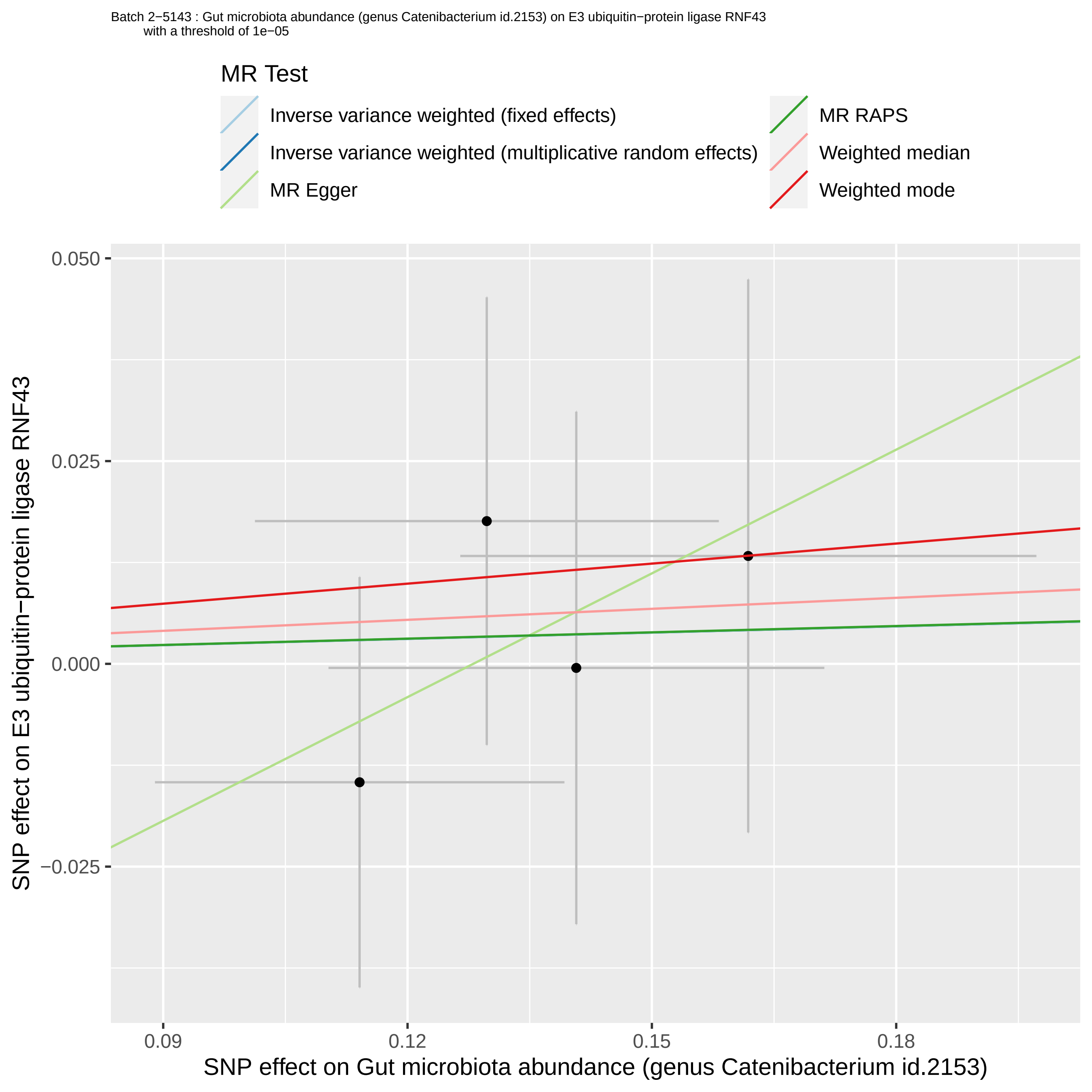

Batch 2−5143 : Gut microbiota abundance (genus Catenibacterium id.2153) on E3 ubiquitin−protein ligase RNF43
with a threshold of 1e−05
MR Test
Inverse variance weighted (fixed effects)
Inverse variance weighted (multiplicative random effects)
MR Egger
MR RAPS
Weighted median
Weighted mode
0.050
0.025
0.000
−0.025
0.09
0.12
0.15
0.18
SNP effect on E3 ubiquitin−protein ligase RNF43
SNP effect on Gut microbiota abundance (genus Catenibacterium id.2153)

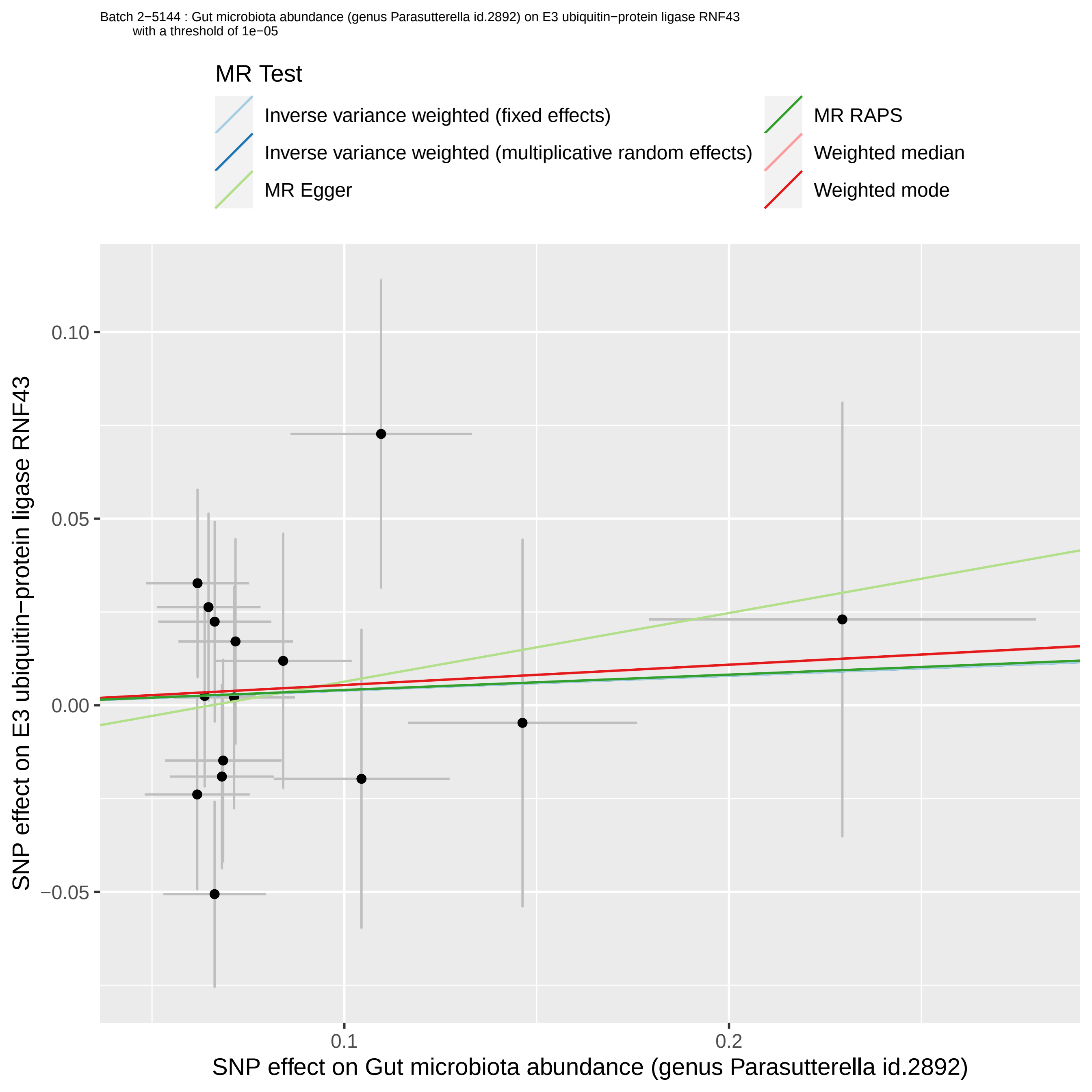

Batch 2−5144 : Gut microbiota abundance (genus Parasutterella id.2892) on E3 ubiquitin−protein ligase RNF43
with a threshold of 1e−05
MR Test
Inverse variance weighted (fixed effects)
Inverse variance weighted (multiplicative random effects)
MR Egger
MR RAPS
Weighted median
Weighted mode
0.10
0.05
0.00
−0.05
0.1
0.2
SNP effect on E3 ubiquitin−protein ligase RNF43
SNP effect on Gut microbiota abundance (genus Parasutterella id.2892)

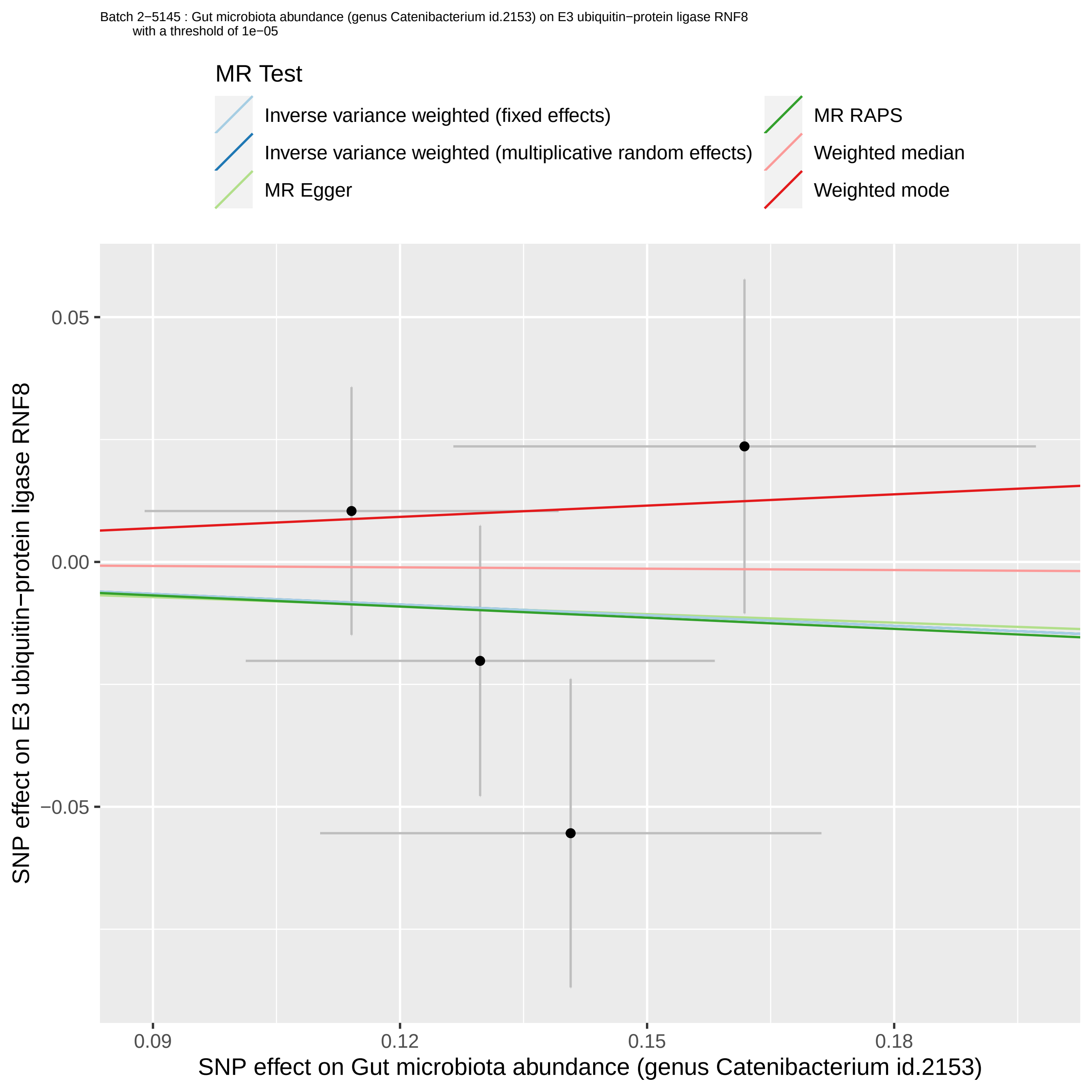

Batch 2−5145 : Gut microbiota abundance (genus Catenibacterium id.2153) on E3 ubiquitin−protein ligase RNF8
with a threshold of 1e−05
MR Test
Inverse variance weighted (fixed effects)
Inverse variance weighted (multiplicative random effects)
MR Egger
MR RAPS
Weighted median
Weighted mode
0.05
0.00
−0.05
0.09
0.12
0.15
0.18
SNP effect on E3 ubiquitin−protein ligase RNF8
SNP effect on Gut microbiota abundance (genus Catenibacterium id.2153)

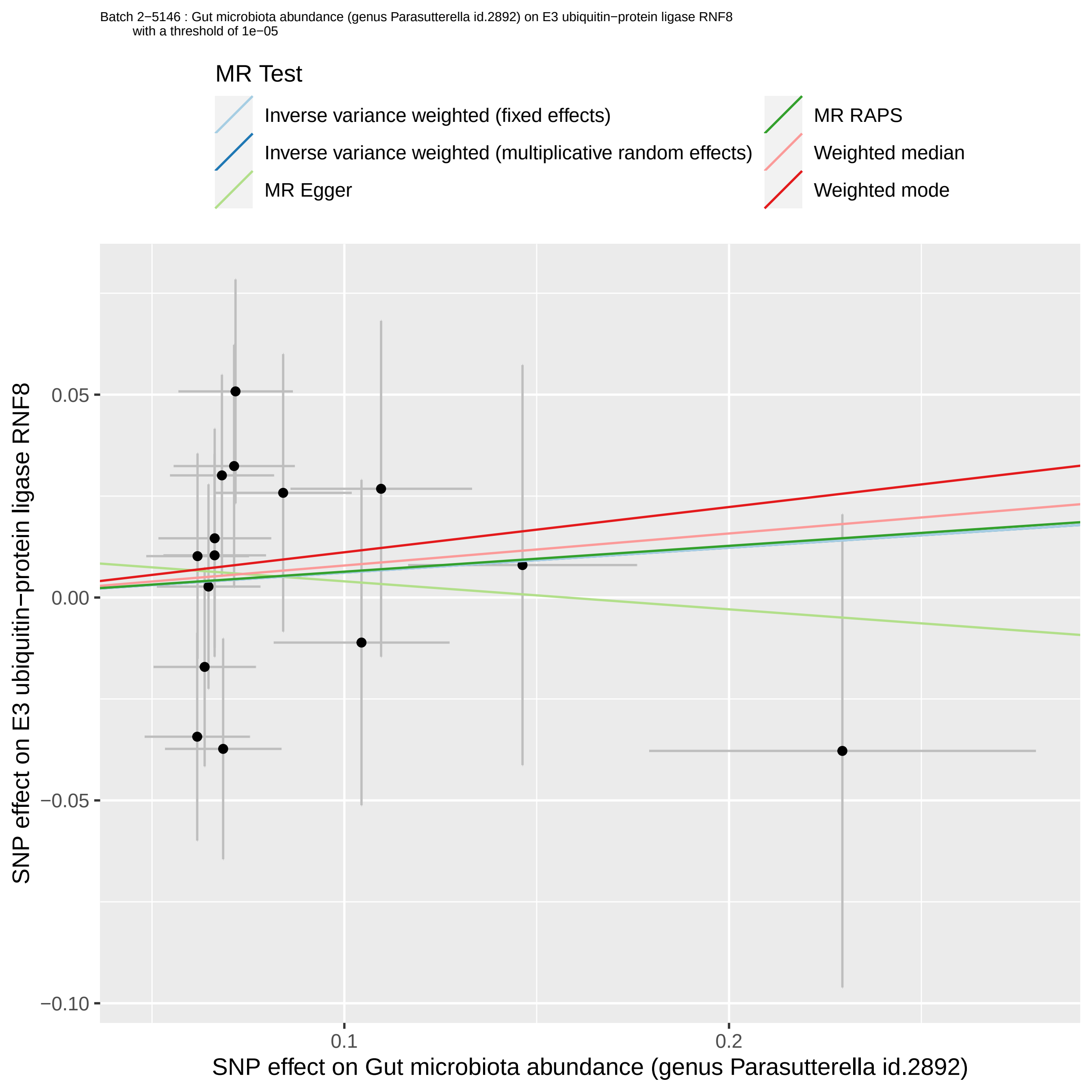

Batch 2−5146 : Gut microbiota abundance (genus Parasutterella id.2892) on E3 ubiquitin−protein ligase RNF8
with a threshold of 1e−05
MR Test
Inverse variance weighted (fixed effects)
Inverse variance weighted (multiplicative random effects)
MR Egger
MR RAPS
Weighted median
Weighted mode
0.05
0.00
−0.05
−0.10
0.1
0.2
SNP effect on E3 ubiquitin−protein ligase RNF8
SNP effect on Gut microbiota abundance (genus Parasutterella id.2892)

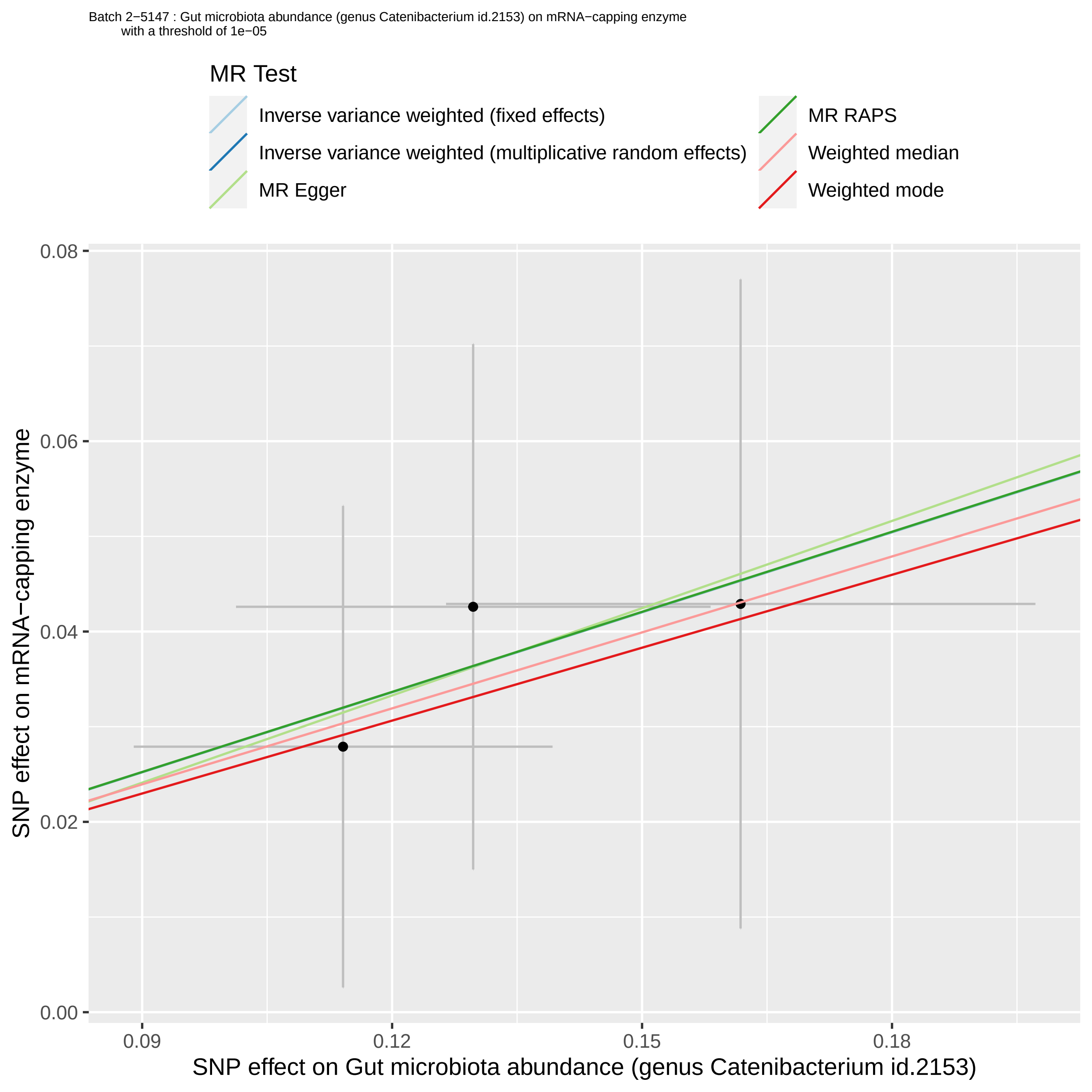
Batch 2−5147 : Gut microbiota abundance (genus Catenibacterium id.2153) on mRNA−capping enzyme
with a threshold of 1e−05
MR Test
Inverse variance weighted (fixed effects)
Inverse variance weighted (multiplicative random effects)
MR Egger
MR RAPS
Weighted median
Weighted mode
0.08
0.06
0.04
0.02
0.00
0.09
0.12
0.15
0.18
SNP effect on mRNA−capping enzyme
SNP effect on Gut microbiota abundance (genus Catenibacterium id.2153)

Batch 2−5148 : Gut microbiota abundance (genus Parasutterella id.2892) on mRNA−capping enzyme
with a threshold of 1e−05

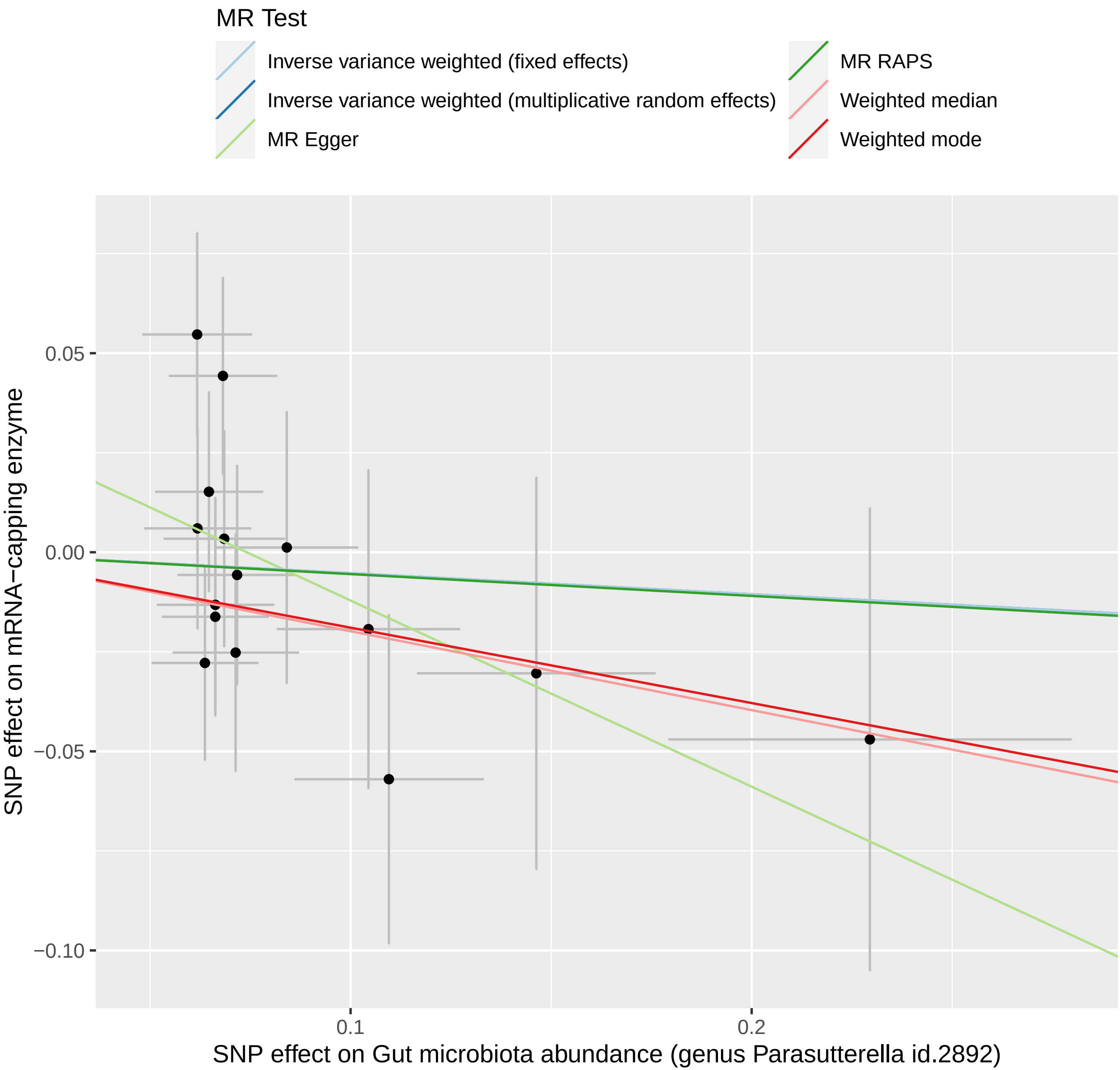

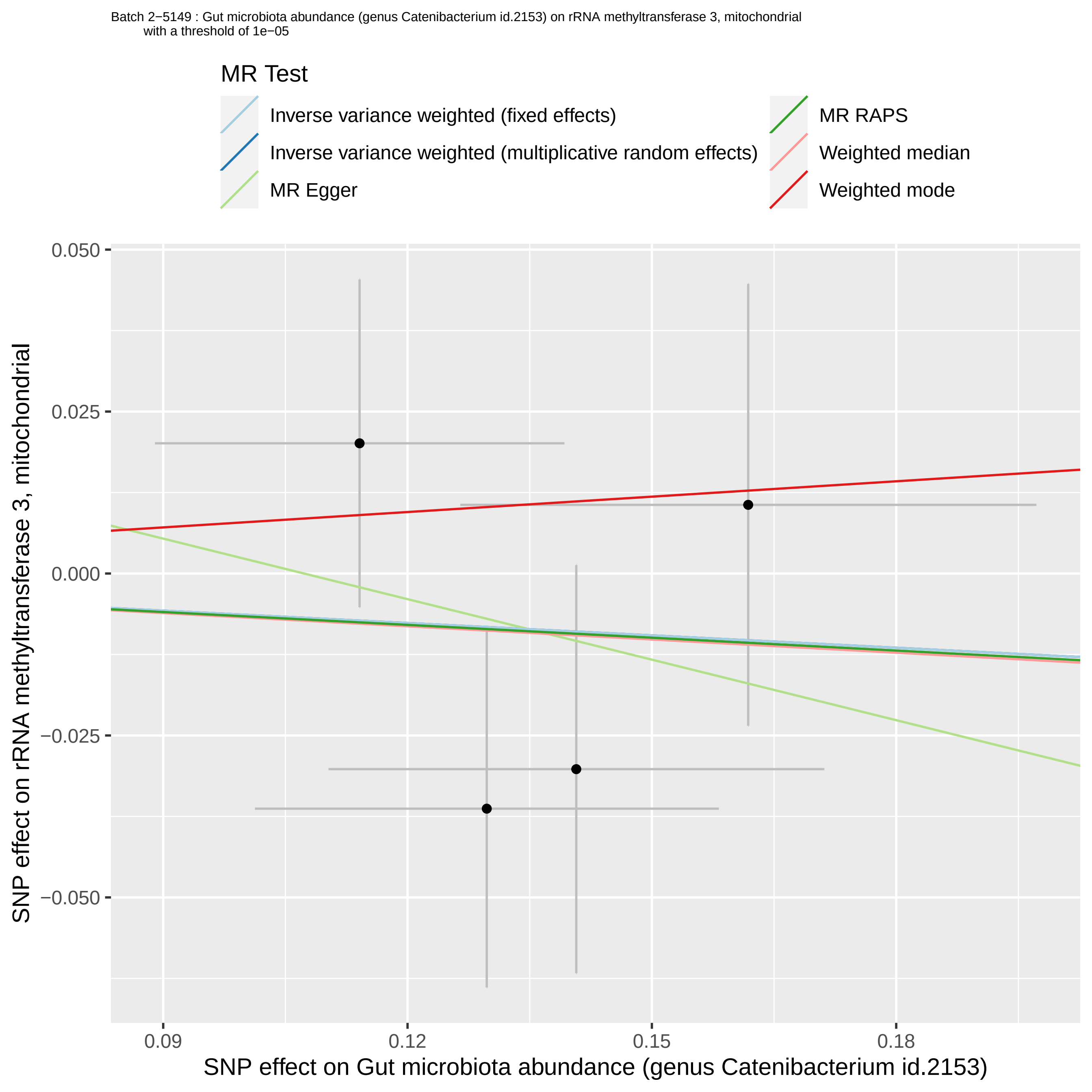

Batch 2−5149 : Gut microbiota abundance (genus Catenibacterium id.2153) on rRNA methyltransferase 3, mitochondrial
with a threshold of 1e−05
MR Test
Inverse variance weighted (fixed effects)
Inverse variance weighted (multiplicative random effects)
MR Egger
MR RAPS
Weighted median
Weighted mode
0.050
0.025
0.000
−0.025
−0.050
0.09
0.12
0.15
0.18
SNP effect on rRNA methyltransferase 3, mitochondrial
SNP effect on Gut microbiota abundance (genus Catenibacterium id.2153)

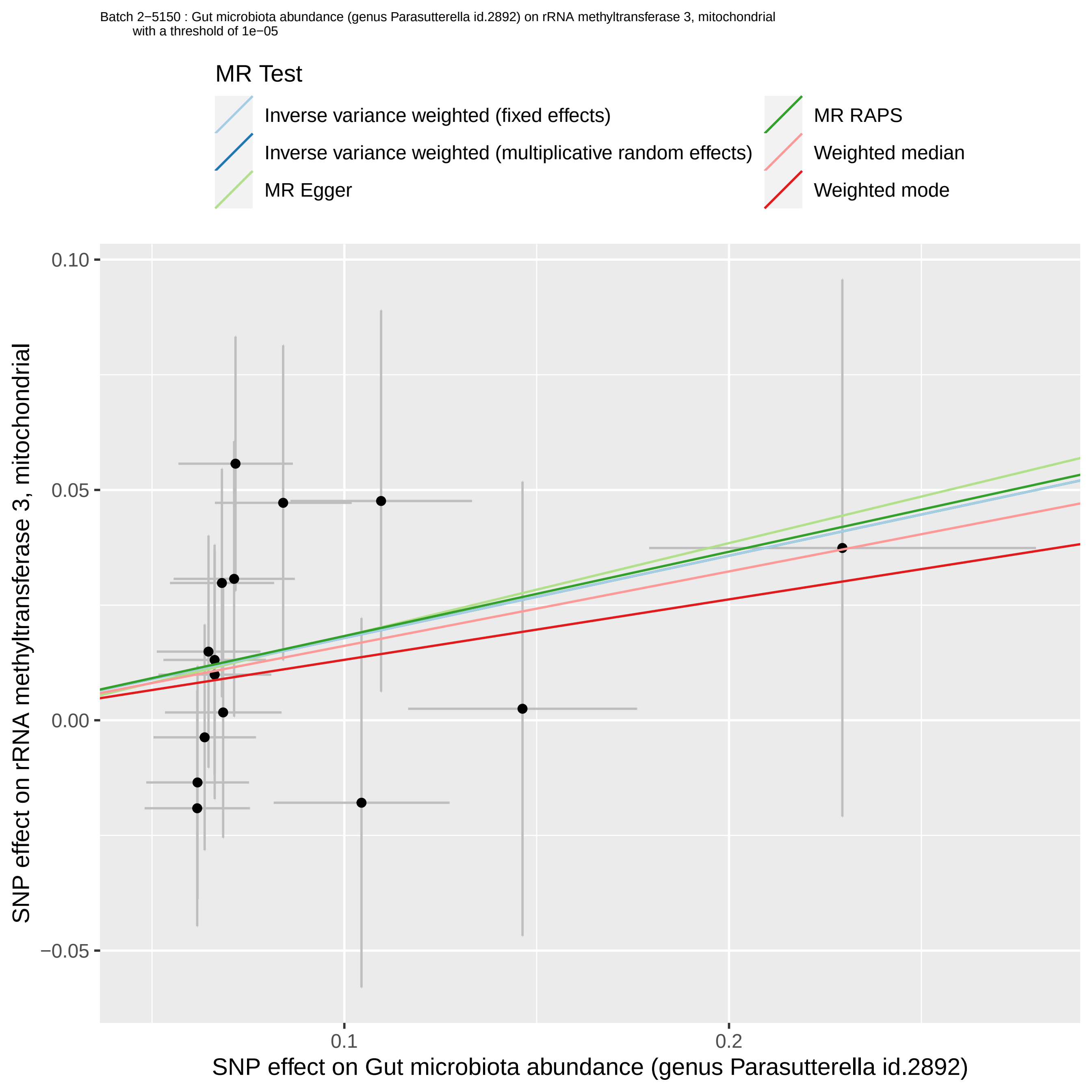
Batch 2−5150 : Gut microbiota abundance (genus Parasutterella id.2892) on rRNA methyltransferase 3, mitochondrial
with a threshold of 1e−05
MR Test
Inverse variance weighted (fixed effects)
Inverse variance weighted (multiplicative random effects)
MR Egger
MR RAPS
Weighted median
Weighted mode
0.10
0.05
0.00
−0.05
0.1
0.2
SNP effect on rRNA methyltransferase 3, mitochondrial
SNP effect on Gut microbiota abundance (genus Parasutterella id.2892)

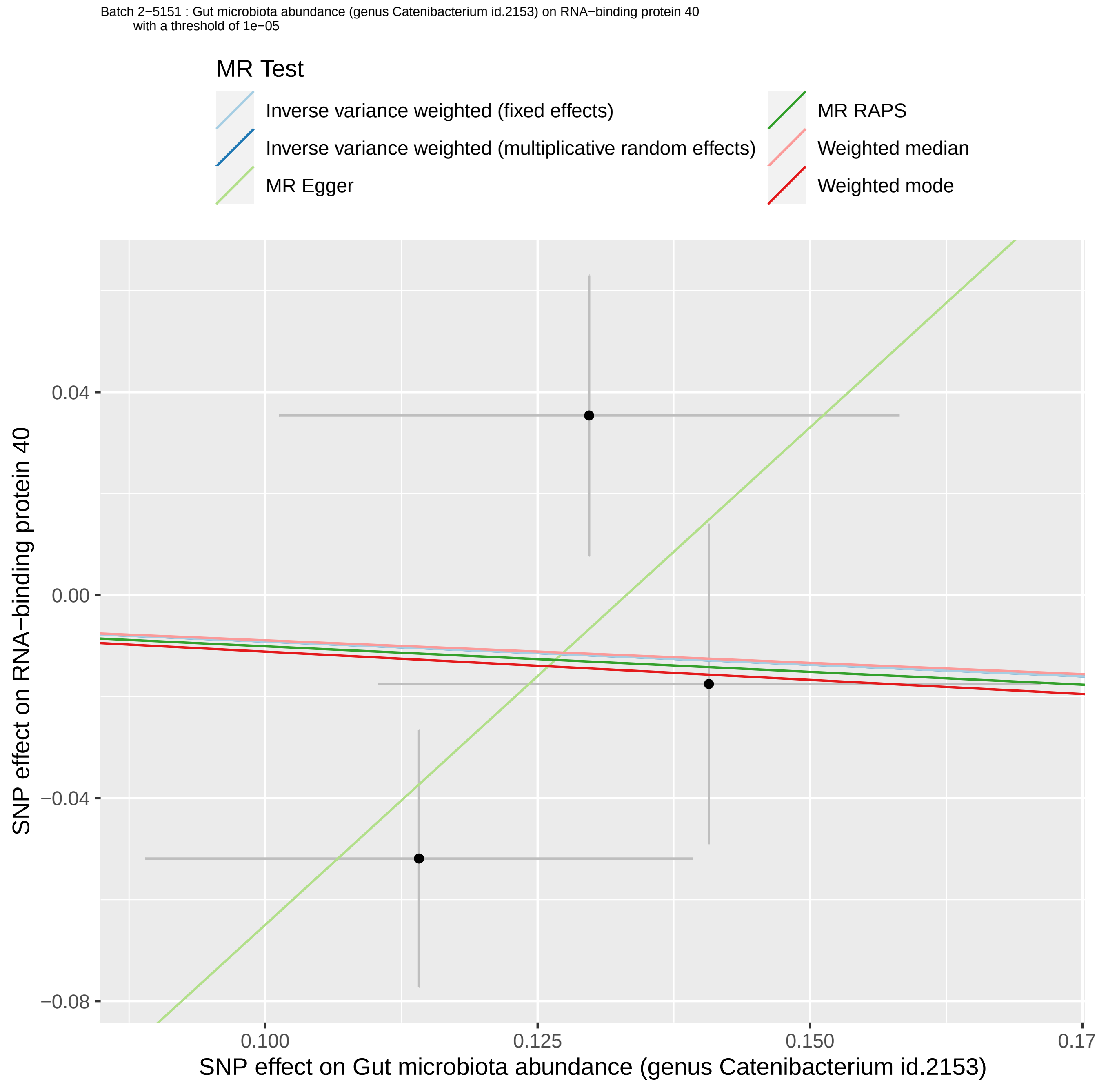
Batch 2−5151 : Gut microbiota abundance (genus Catenibacterium id.2153) on RNA−binding protein 40
with a threshold of 1e−05
MR Test
Inverse variance weighted (fixed effects)
Inverse variance weighted (multiplicative random effects)
MR Egger
MR RAPS
Weighted median
Weighted mode
0.04
0.00
−0.04
−0.08
0.100
0.125
0.150
0.17
SNP effect on RNA−binding protein 40
SNP effect on Gut microbiota abundance (genus Catenibacterium id.2153)

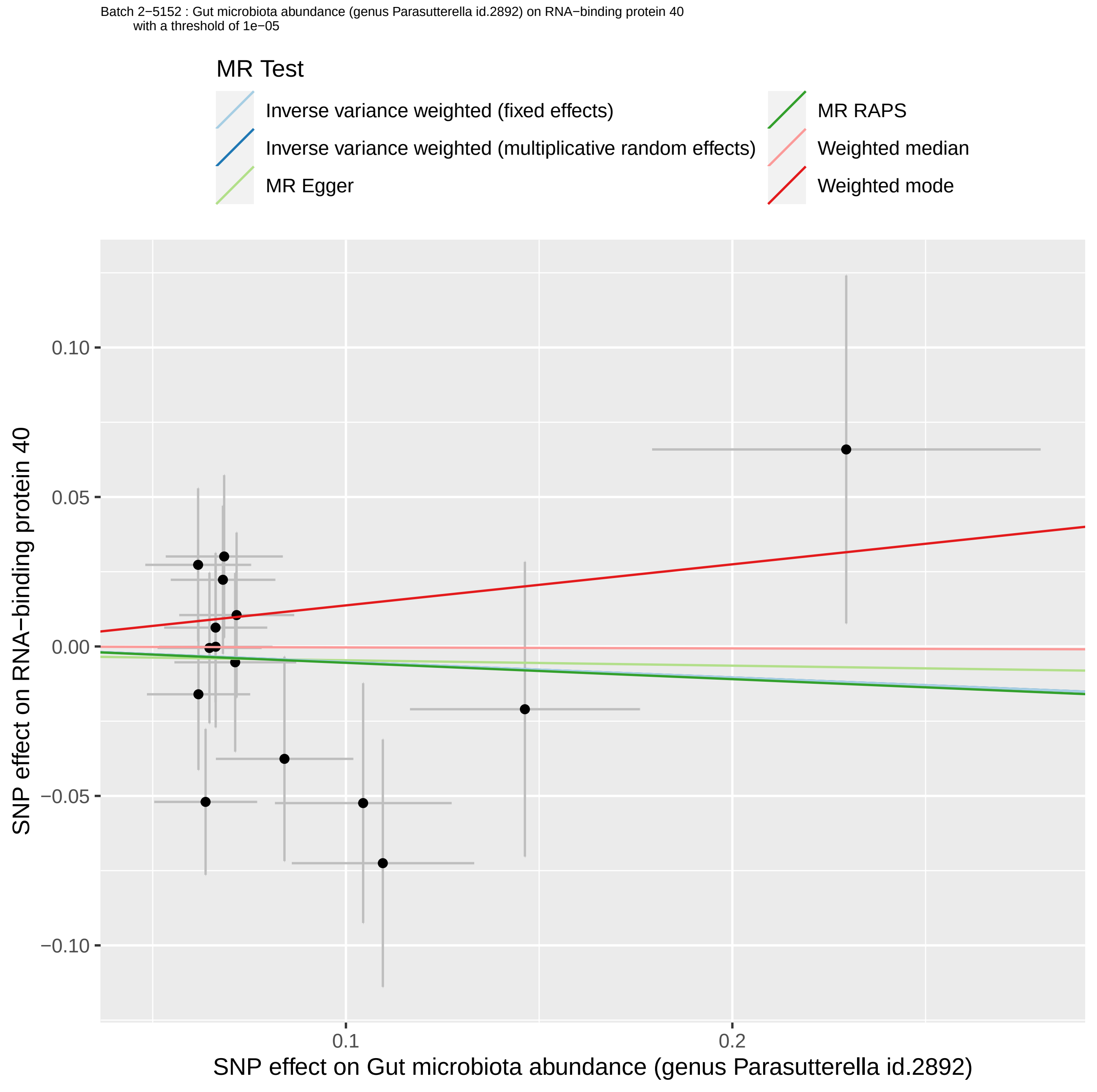

Batch 2−5152 : Gut microbiota abundance (genus Parasutterella id.2892) on RNA−binding protein 40
with a threshold of 1e−05
MR Test
Inverse variance weighted (fixed effects)
Inverse variance weighted (multiplicative random effects)
MR Egger
MR RAPS
Weighted median
Weighted mode
0.10
0.05
0.00
−0.05
−0.10
0.1
0.2
SNP effect on RNA−binding protein 40
SNP effect on Gut microbiota abundance (genus Parasutterella id.2892)

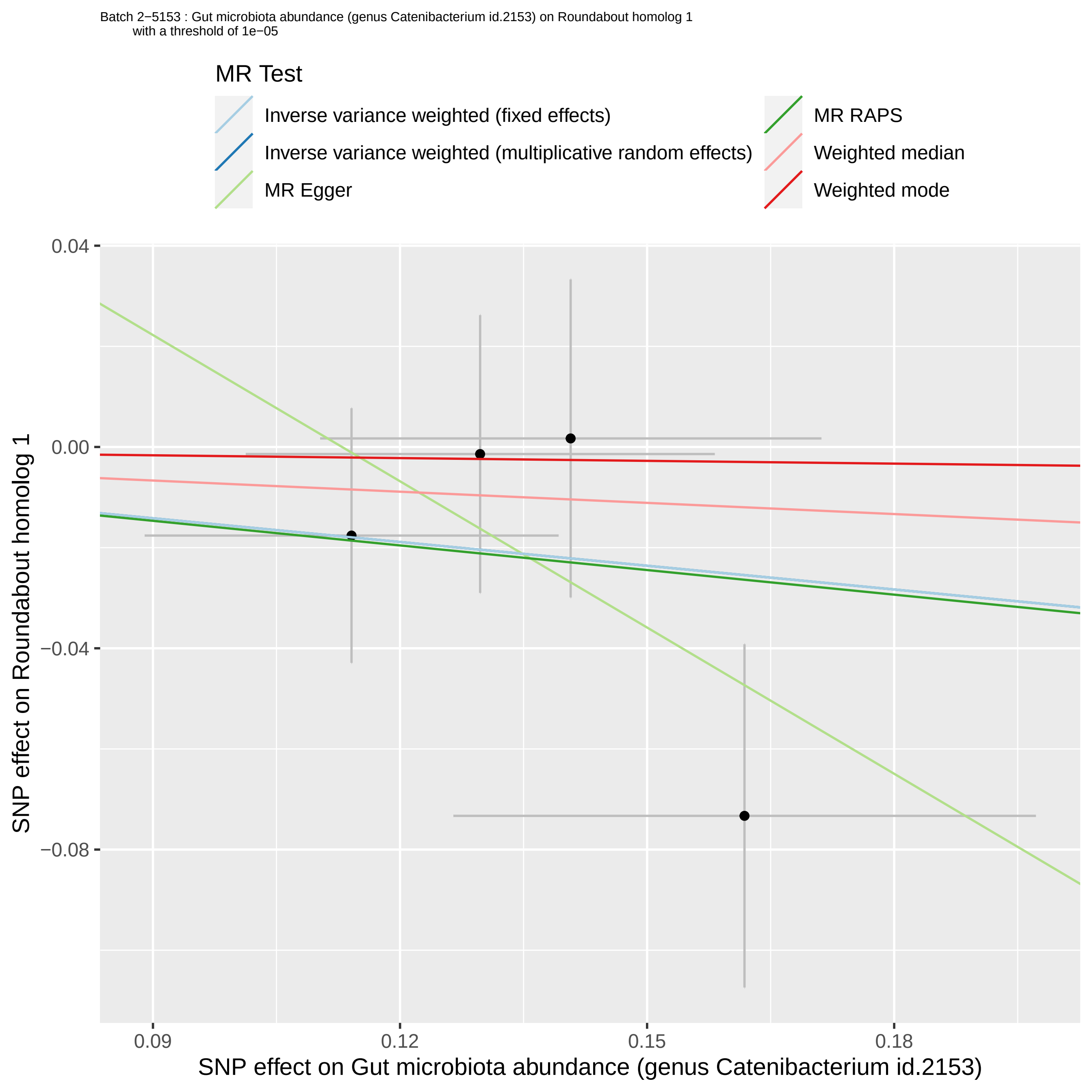

Batch 2−5153 : Gut microbiota abundance (genus Catenibacterium id.2153) on Roundabout homolog 1
with a threshold of 1e−05
MR Test
Inverse variance weighted (fixed effects)
Inverse variance weighted (multiplicative random effects)
MR Egger
MR RAPS
Weighted median
Weighted mode
0.04
0.00
−0.04
−0.08
0.09
0.12
0.15
0.18
SNP effect on Roundabout homolog 1
SNP effect on Gut microbiota abundance (genus Catenibacterium id.2153)

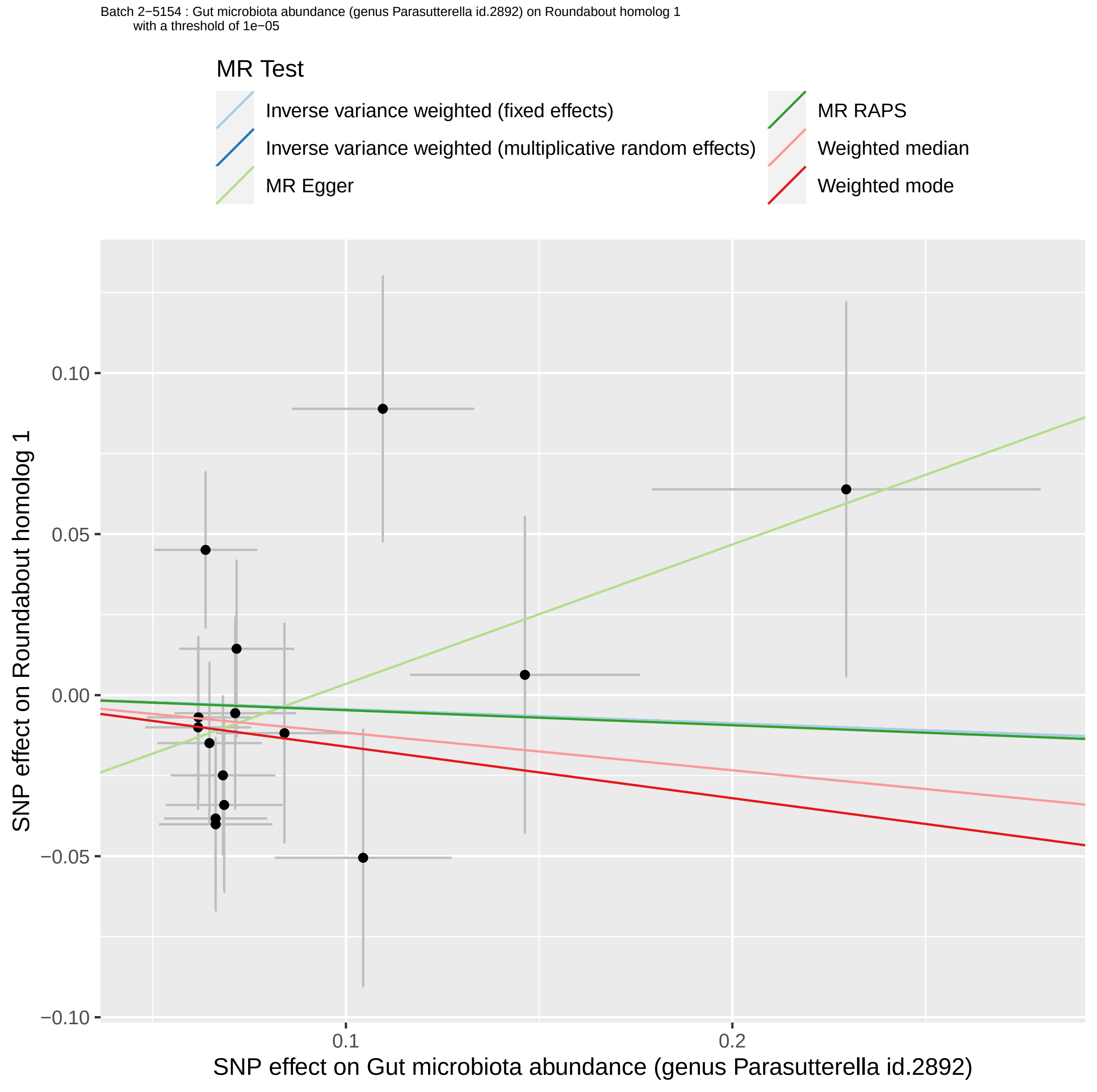

Batch 2−5154 : Gut microbiota abundance (genus Parasutterella id.2892) on Roundabout homolog 1
with a threshold of 1e−05
MR Test
Inverse variance weighted (fixed effects)
Inverse variance weighted (multiplicative random effects)
MR Egger
MR RAPS
Weighted median
Weighted mode
0.10
0.05
0.00
−0.05
−0.10
0.1
0.2
SNP effect on Roundabout homolog 1
SNP effect on Gut microbiota abundance (genus Parasutterella id.2892)

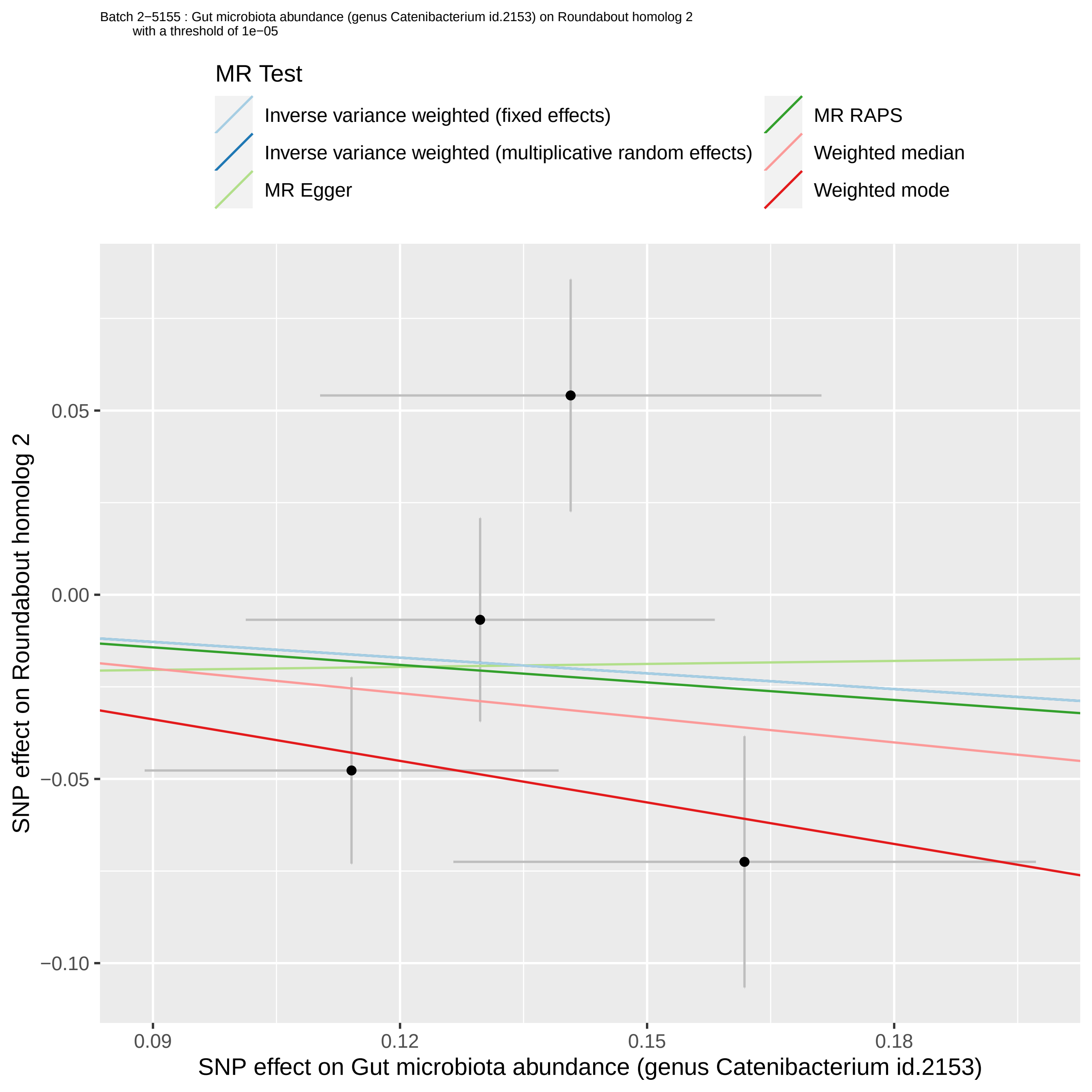
Batch 2−5155 : Gut microbiota abundance (genus Catenibacterium id.2153) on Roundabout homolog 2
with a threshold of 1e−05
MR Test
Inverse variance weighted (fixed effects)
Inverse variance weighted (multiplicative random effects)
MR Egger
MR RAPS
Weighted median
Weighted mode
0.05
0.00
−0.05
−0.10
0.09
0.12
0.15
0.18
SNP effect on Roundabout homolog 2
SNP effect on Gut microbiota abundance (genus Catenibacterium id.2153)

Batch 2−5156 : Gut microbiota abundance (genus Parasutterella id.2892) on Roundabout homolog 2
with a threshold of 1e−05

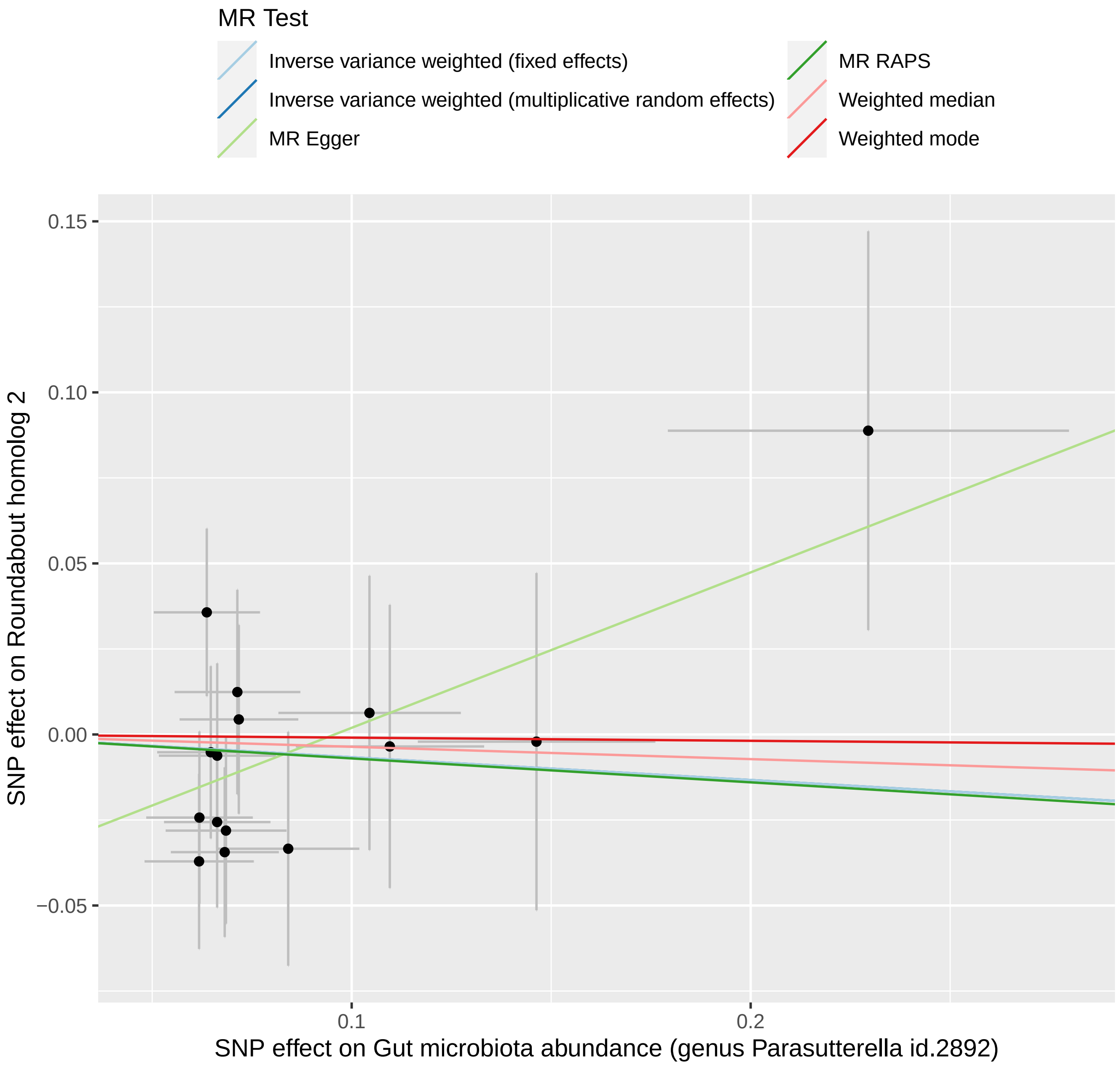

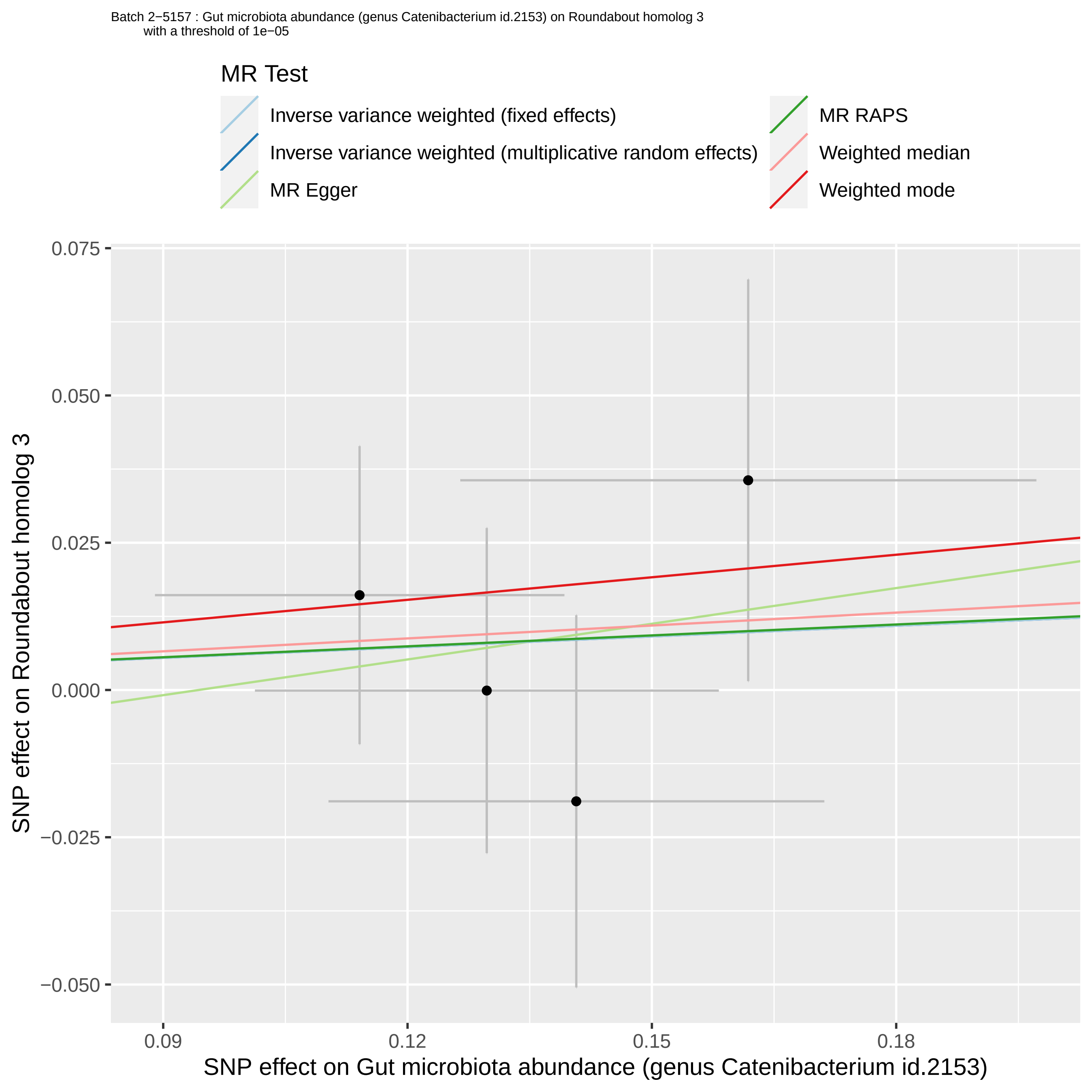

Batch 2−5157 : Gut microbiota abundance (genus Catenibacterium id.2153) on Roundabout homolog 3
with a threshold of 1e−05
MR Test
Inverse variance weighted (fixed effects)
Inverse variance weighted (multiplicative random effects)
MR Egger
MR RAPS
Weighted median
Weighted mode
0.075
0.050
0.025
0.000
−0.025
−0.050
0.09
0.12
0.15
0.18
SNP effect on Roundabout homolog 3
SNP effect on Gut microbiota abundance (genus Catenibacterium id.2153)

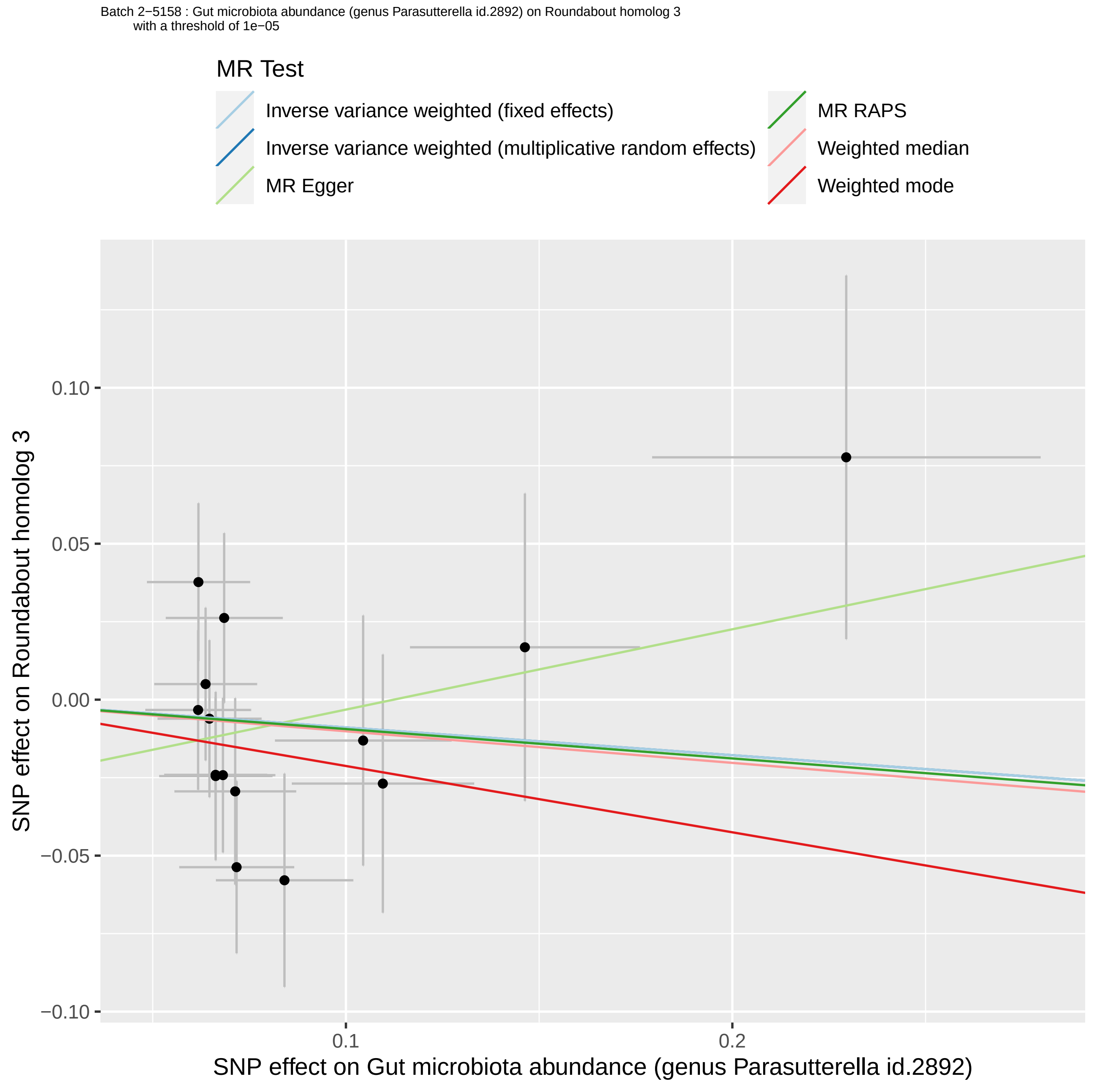

Batch 2−5158 : Gut microbiota abundance (genus Parasutterella id.2892) on Roundabout homolog 3
with a threshold of 1e−05
MR Test
Inverse variance weighted (fixed effects)
Inverse variance weighted (multiplicative random effects)
MR Egger
MR RAPS
Weighted median
Weighted mode
SNP effect on Roundabout homolog 3
0.10
0.05
0.00
−0.05
−0.10
0.1
0.2
SNP effect on Gut microbiota abundance (genus Parasutterella id.2892)

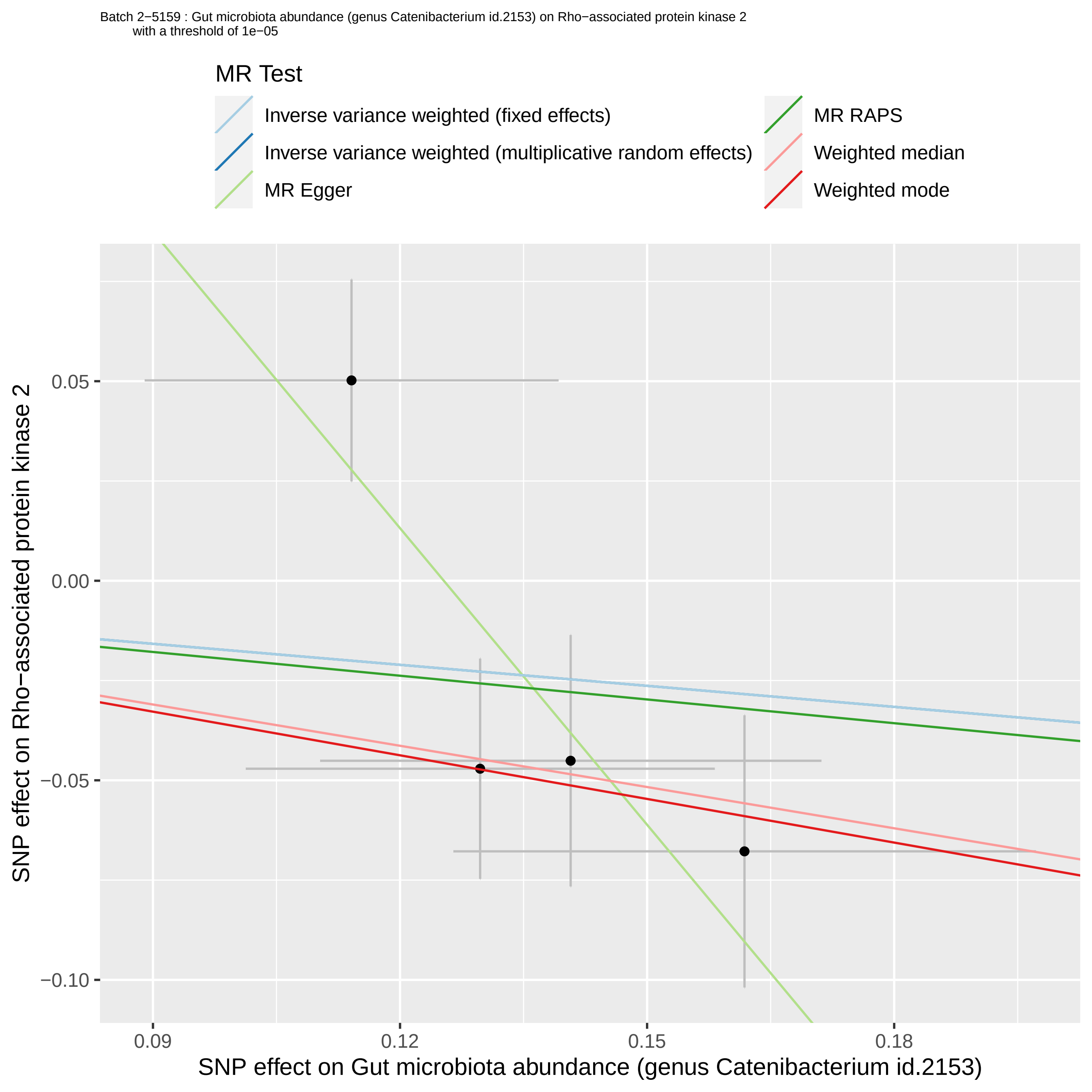

Batch 2−5159 : Gut microbiota abundance (genus Catenibacterium id.2153) on Rho−associated protein kinase 2
with a threshold of 1e−05
MR Test
Inverse variance weighted (fixed effects)
Inverse variance weighted (multiplicative random effects)
MR Egger
MR RAPS
Weighted median
Weighted mode
SNP effect on Rho−associated protein kinase 2
0.05
0.00
−0.05
−0.10
0.09
0.12
0.15
0.18
SNP effect on Gut microbiota abundance (genus Catenibacterium id.2153)

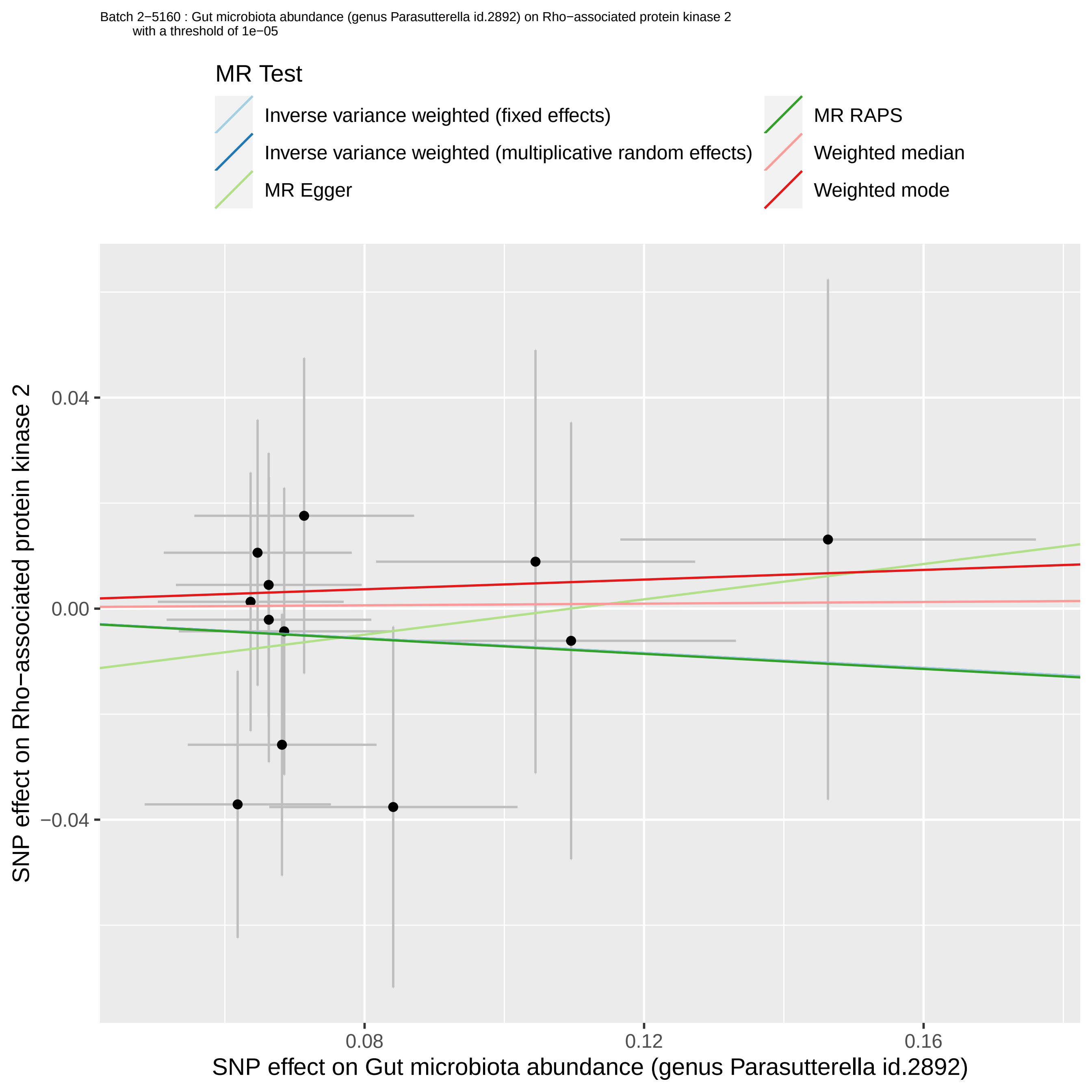

Batch 2−5160 : Gut microbiota abundance (genus Parasutterella id.2892) on Rho−associated protein kinase 2
with a threshold of 1e−05
MR Test
Inverse variance weighted (fixed effects)
Inverse variance weighted (multiplicative random effects)
MR Egger
MR RAPS
Weighted median
Weighted mode
0.04
0.00
−0.04
0.08
0.12
0.16
SNP effect on Rho−associated protein kinase 2
SNP effect on Gut microbiota abundance (genus Parasutterella id.2892)

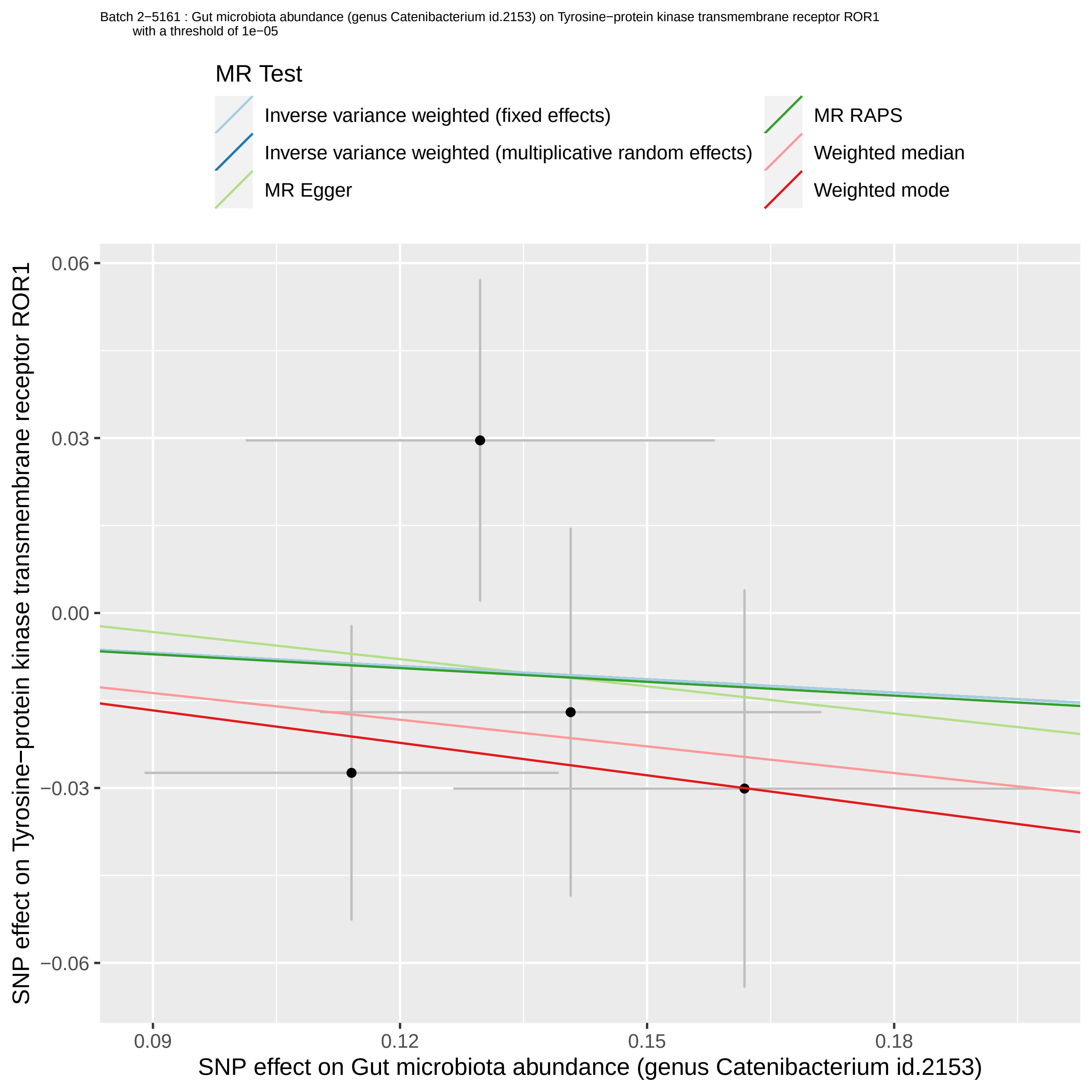

Batch 2−5161 : Gut microbiota abundance (genus Catenibacterium id.2153) on Tyrosine−protein kinase transmembrane receptor ROR1
with a threshold of 1e−05
MR Test
Inverse variance weighted (fixed effects)
Inverse variance weighted (multiplicative random effects)
MR Egger
MR RAPS
Weighted median
Weighted mode
0.06
0.03
0.00
−0.03
−0.06
0.09
0.12
0.15
0.18
SNP effect on Tyrosine−protein kinase transmembrane receptor ROR1
SNP effect on Gut microbiota abundance (genus Catenibacterium id.2153)

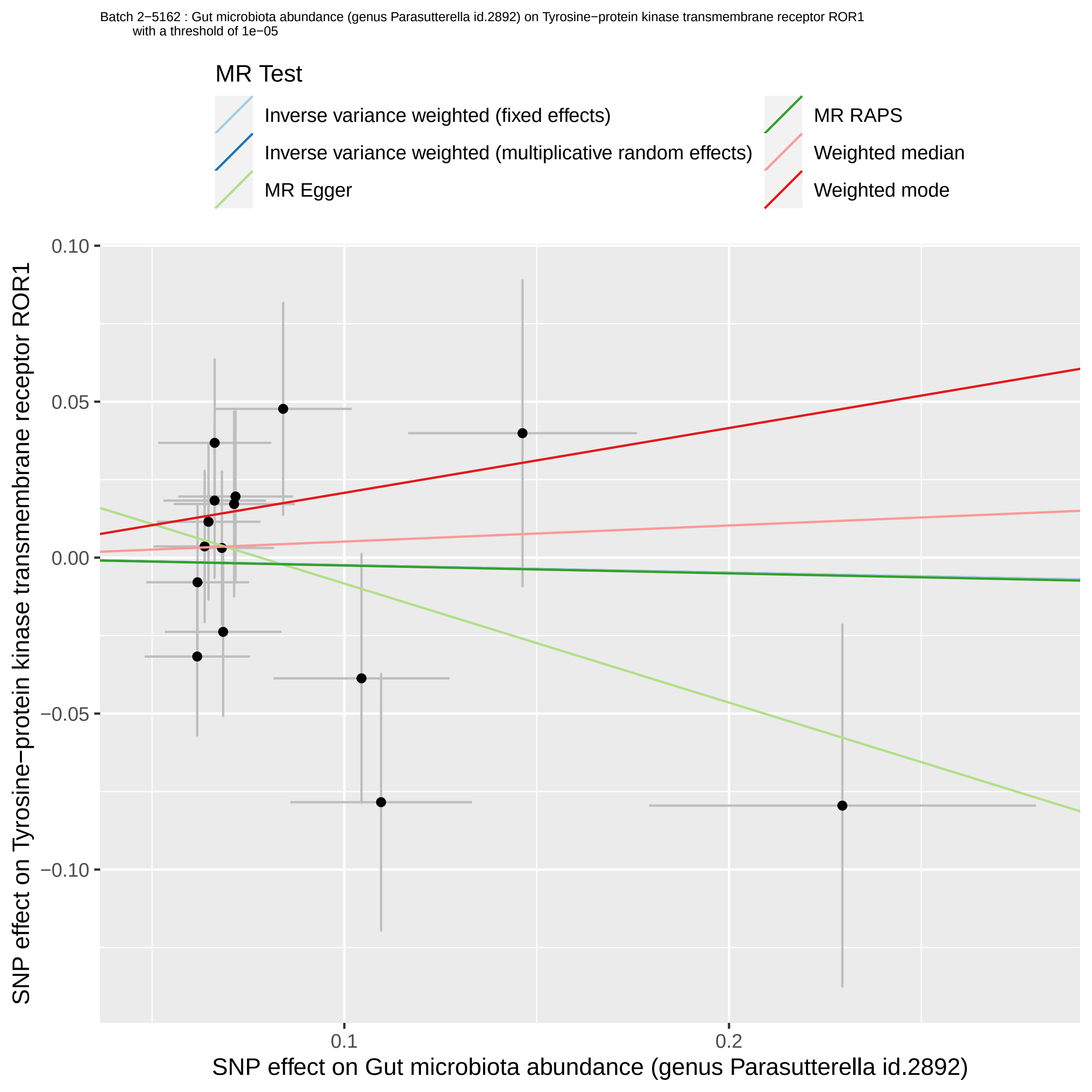
Batch 2−5162 : Gut microbiota abundance (genus Parasutterella id.2892) on Tyrosine−protein kinase transmembrane receptor ROR1
with a threshold of 1e−05
MR Test
Inverse variance weighted (fixed effects)
Inverse variance weighted (multiplicative random effects)
MR Egger
MR RAPS
Weighted median
Weighted mode
0.10
0.05
0.00
−0.05
−0.10
0.1
0.2
SNP effect on Gut microbiota abundance (genus Parasutterella id.2892)
SNP effect on Tyrosine−protein kinase transmembrane receptor ROR1

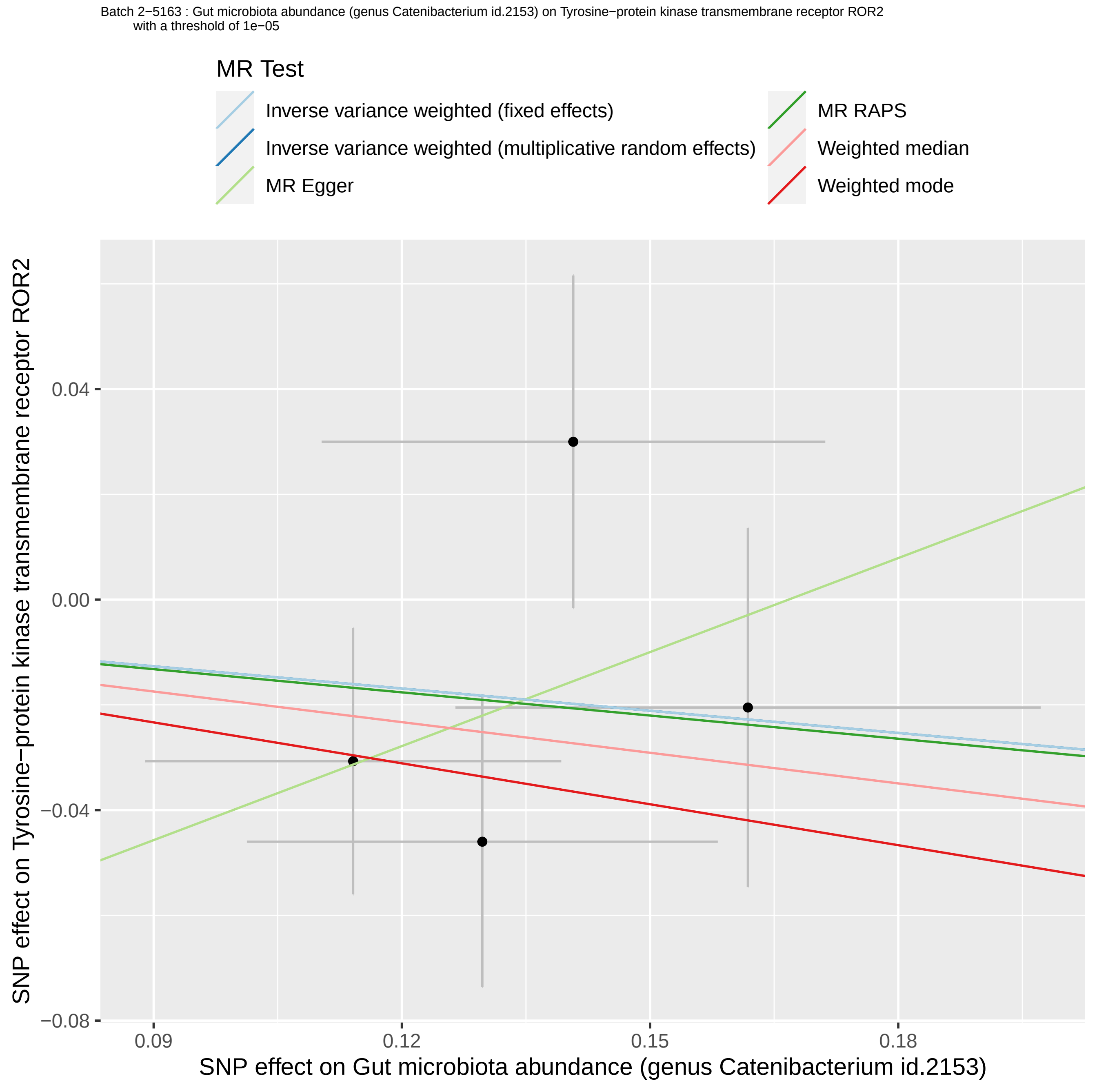
Batch 2−5163 : Gut microbiota abundance (genus Catenibacterium id.2153) on Tyrosine−protein kinase transmembrane receptor ROR2
with a threshold of 1e−05
MR Test
Inverse variance weighted (fixed effects)
Inverse variance weighted (multiplicative random effects)
MR Egger
MR RAPS
Weighted median
Weighted mode
SNP effect on Tyrosine−protein kinase transmembrane receptor ROR2
0.04
0.00
−0.04
−0.08
0.09
0.12
0.15
0.18
SNP effect on Gut microbiota abundance (genus Catenibacterium id.2153)

Batch 2−5164 : Gut microbiota abundance (genus Parasutterella id.2892) on Tyrosine−protein kinase transmembrane receptor ROR2
with a threshold of 1e−05

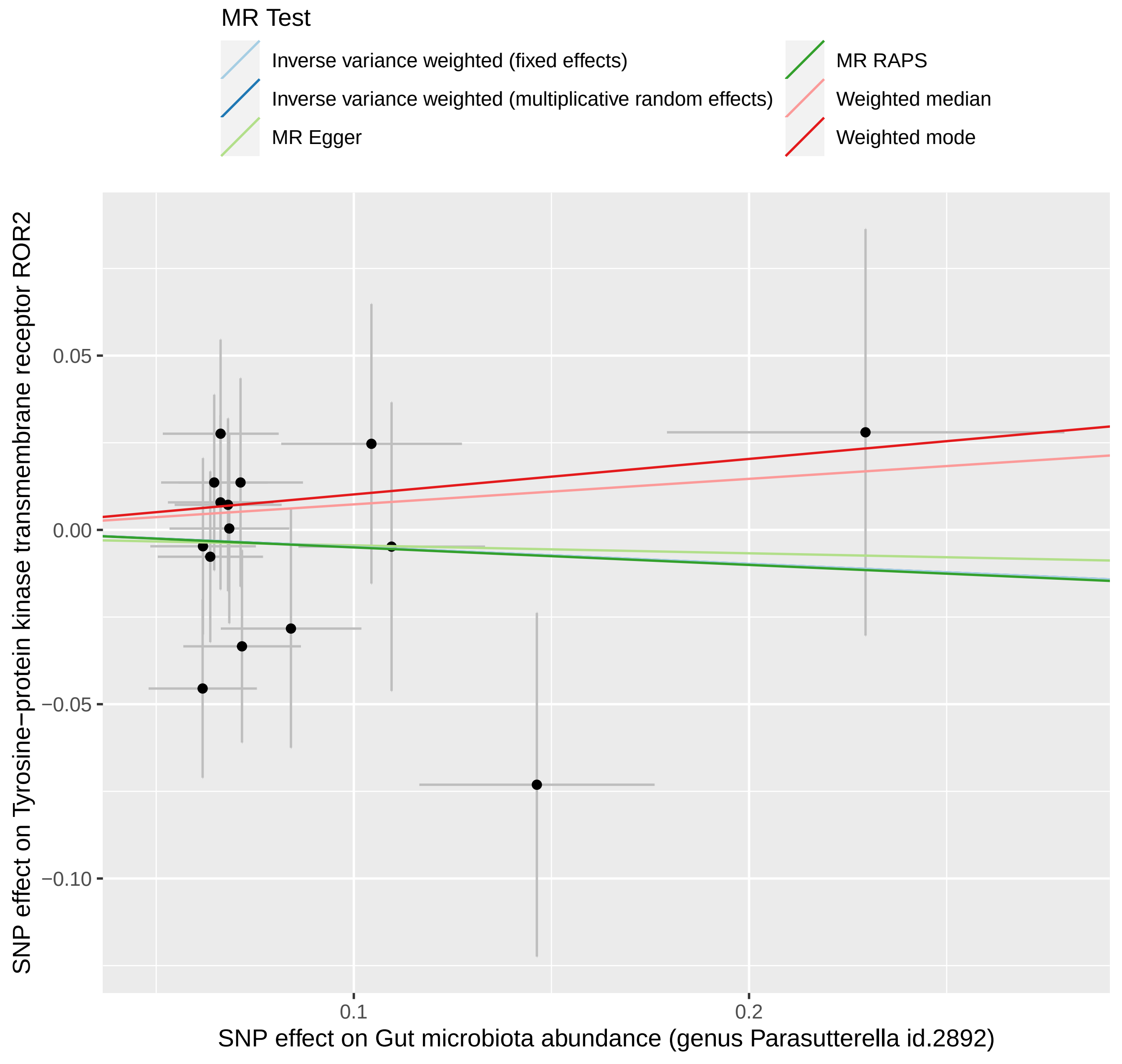

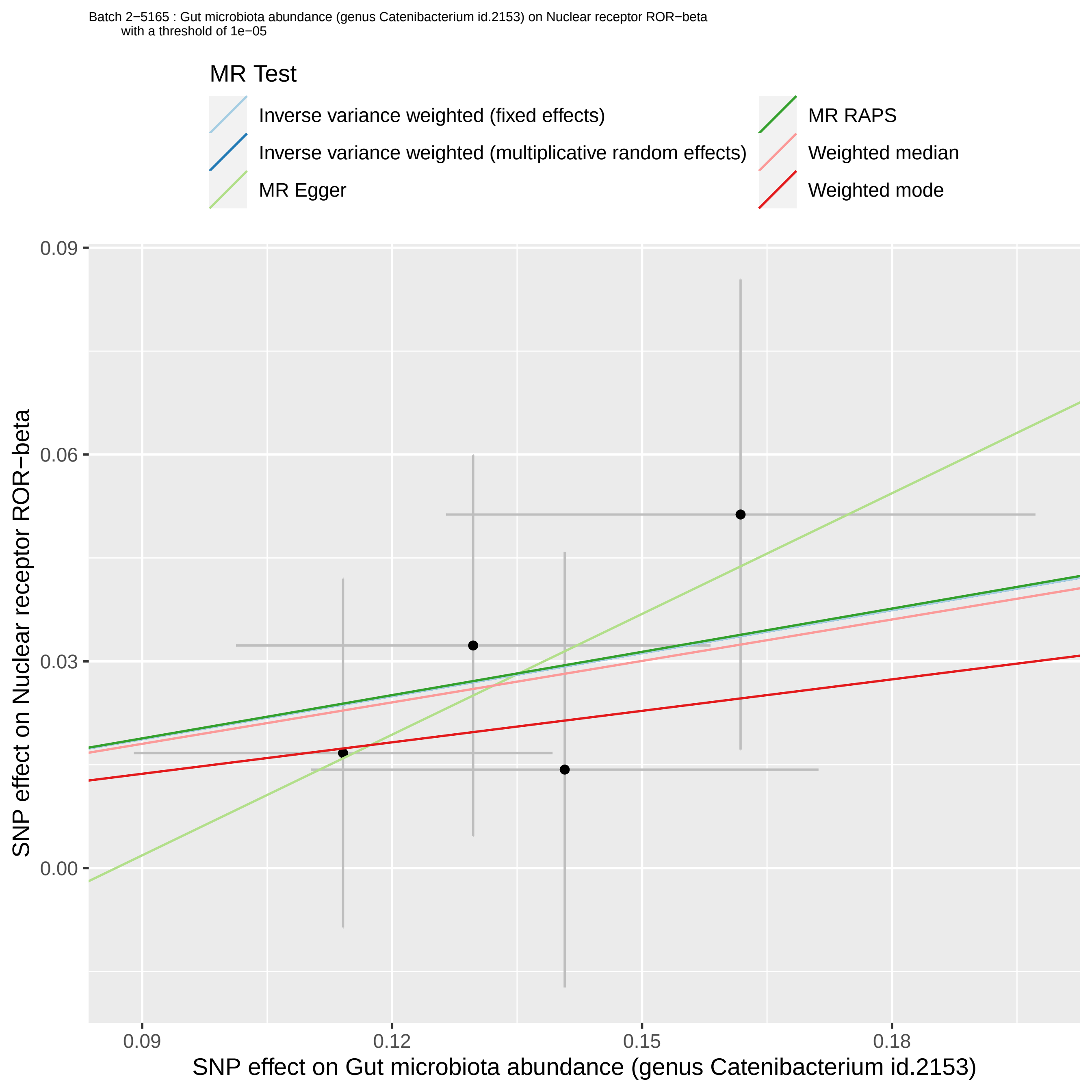

Batch 2−5165 : Gut microbiota abundance (genus Catenibacterium id.2153) on Nuclear receptor ROR−beta
with a threshold of 1e−05
MR Test
Inverse variance weighted (fixed effects)
Inverse variance weighted (multiplicative random effects)
MR Egger
MR RAPS
Weighted median
Weighted mode
0.09
0.06
0.03
0.00
0.09
0.12
0.15
0.18
SNP effect on Nuclear receptor ROR−beta
SNP effect on Gut microbiota abundance (genus Catenibacterium id.2153)

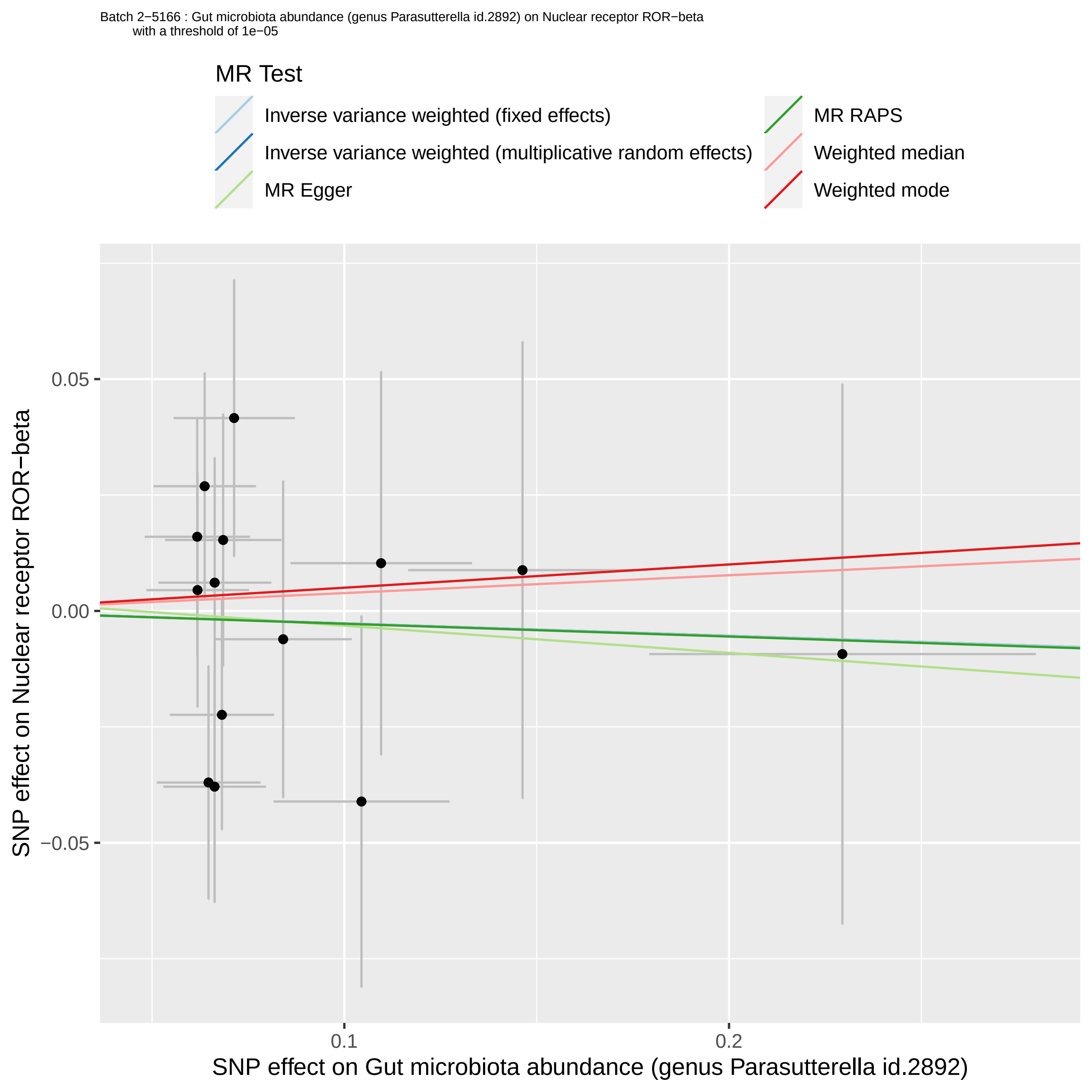

Batch 2−5166 : Gut microbiota abundance (genus Parasutterella id.2892) on Nuclear receptor ROR−beta
with a threshold of 1e−05
MR Test
Inverse variance weighted (fixed effects)
Inverse variance weighted (multiplicative random effects)
MR Egger
MR RAPS
Weighted median
Weighted mode
0.05
0.00
−0.05
0.1
0.2
SNP effect on Nuclear receptor ROR−beta
SNP effect on Gut microbiota abundance (genus Parasutterella id.2892)

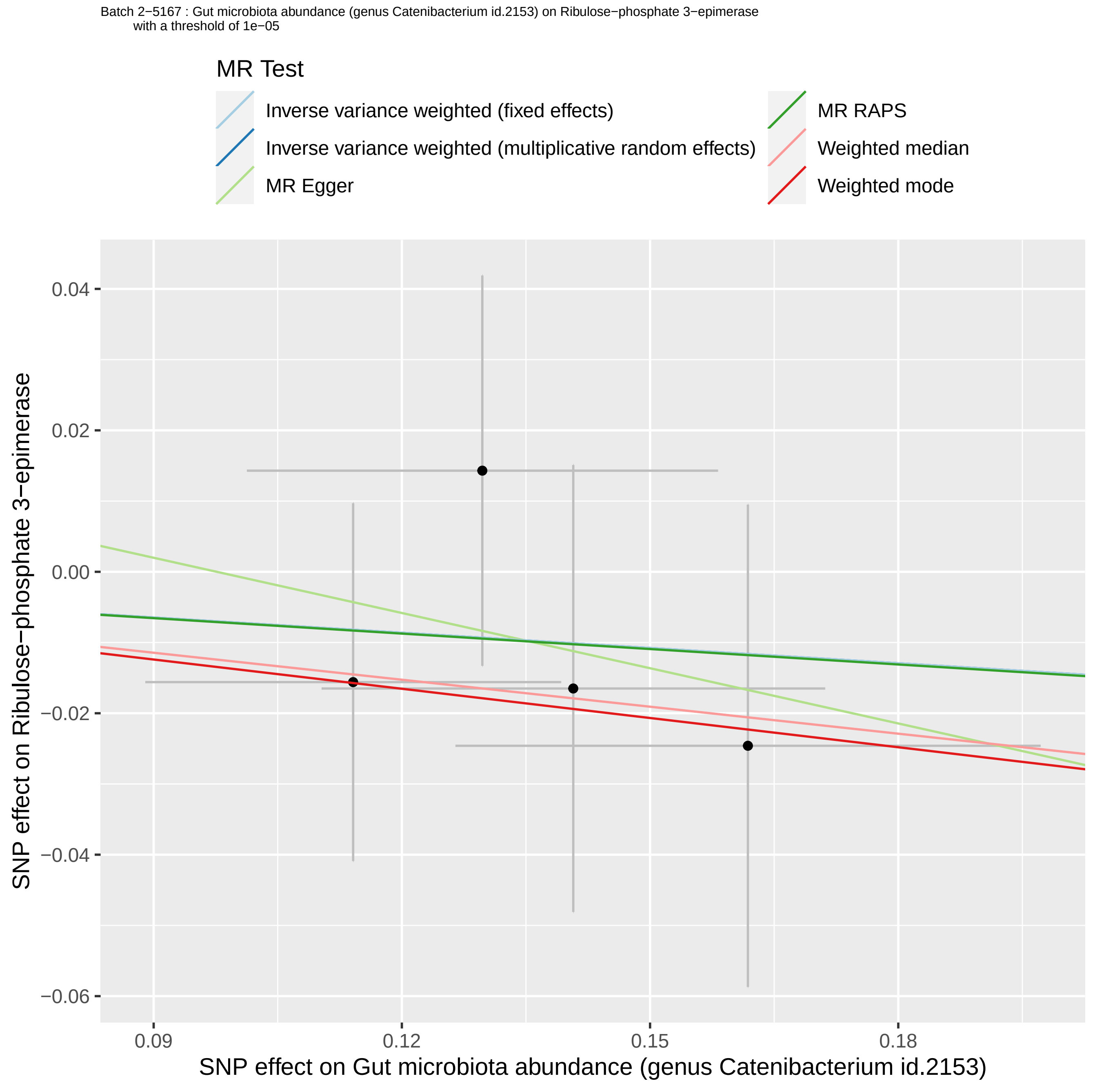

Batch 2−5167 : Gut microbiota abundance (genus Catenibacterium id.2153) on Ribulose−phosphate 3−epimerase
with a threshold of 1e−05
MR Test
Inverse variance weighted (fixed effects)
Inverse variance weighted (multiplicative random effects)
MR Egger
MR RAPS
Weighted median
Weighted mode
SNP effect on Ribulose−phosphate 3−epimerase
0.04
0.02
0.00
−0.02
−0.04
−0.06
0.09
0.12
0.15
0.18
SNP effect on Gut microbiota abundance (genus Catenibacterium id.2153)

Batch 2−5168 : Gut microbiota abundance (genus Parasutterella id.2892) on Ribulose−phosphate 3−epimerase
with a threshold of 1e−05

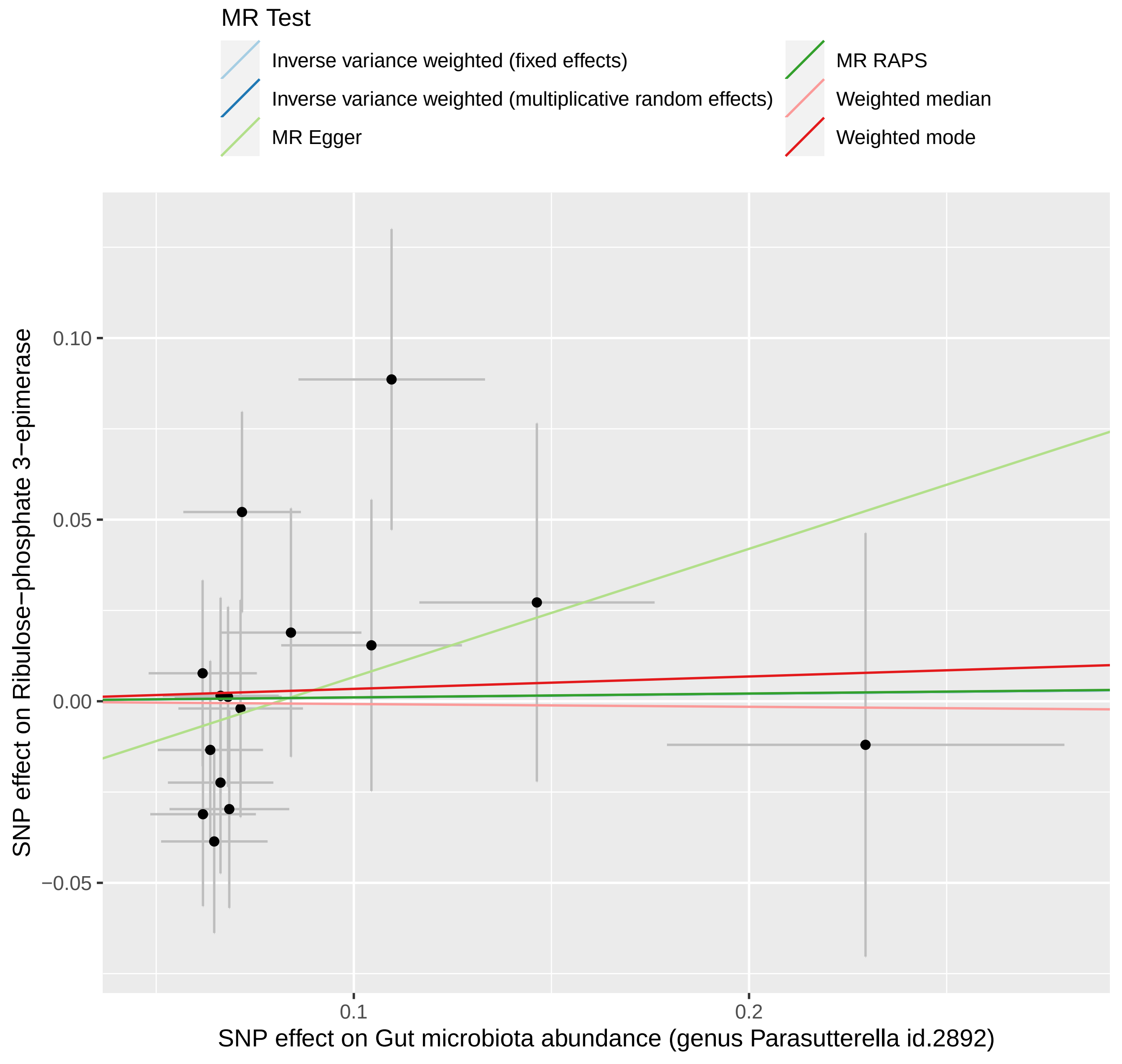

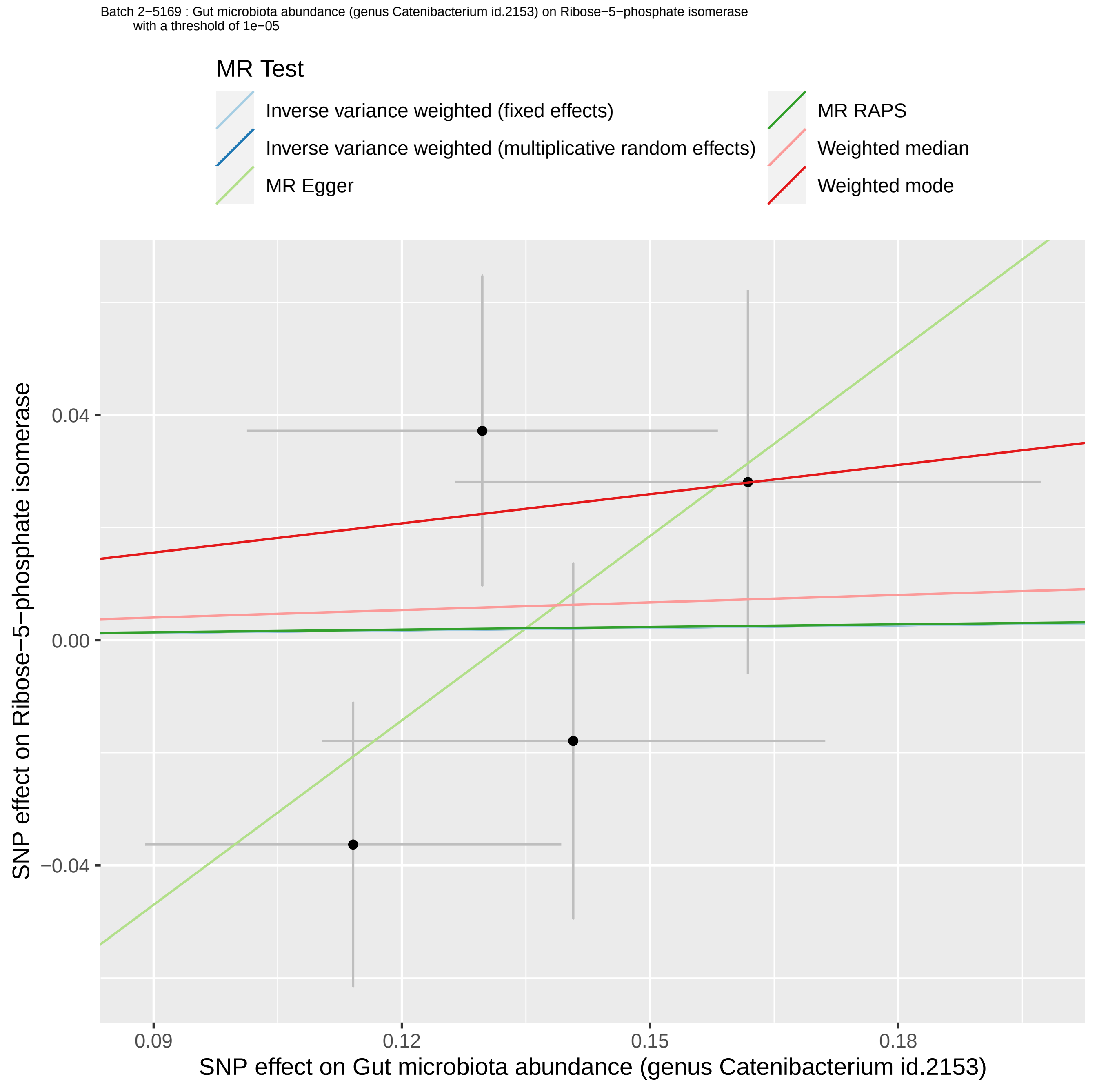

Batch 2−5169 : Gut microbiota abundance (genus Catenibacterium id.2153) on Ribose−5−phosphate isomerase
with a threshold of 1e−05
MR Test
Inverse variance weighted (fixed effects)
Inverse variance weighted (multiplicative random effects)
MR Egger
MR RAPS
Weighted median
Weighted mode
0.04
0.00
−0.04
0.09
0.12
0.15
0.18
SNP effect on Ribose−5−phosphate isomerase
SNP effect on Gut microbiota abundance (genus Catenibacterium id.2153)

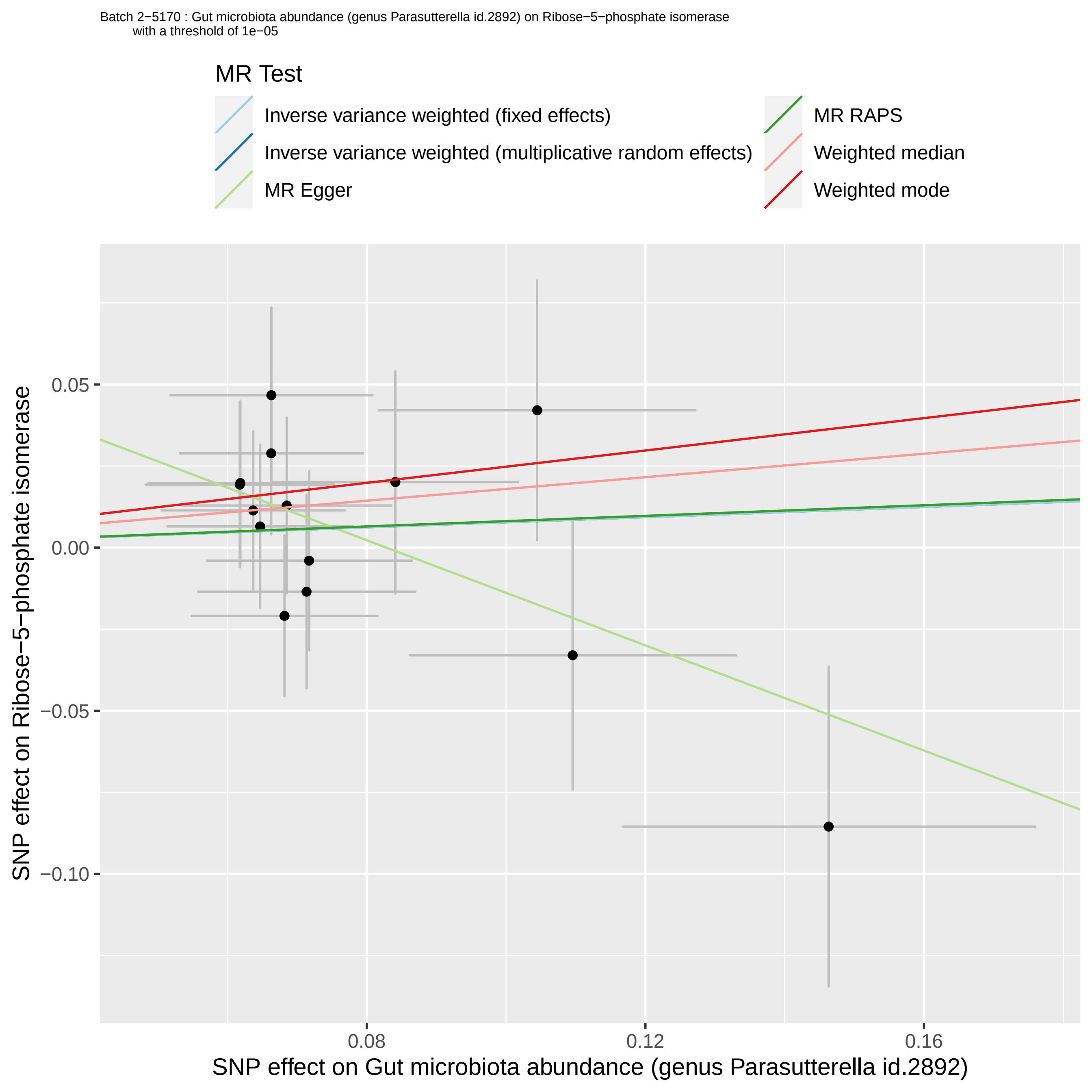

Batch 2−5170 : Gut microbiota abundance (genus Parasutterella id.2892) on Ribose−5−phosphate isomerase
with a threshold of 1e−05
MR Test
Inverse variance weighted (fixed effects)
Inverse variance weighted (multiplicative random effects)
MR Egger
MR RAPS
Weighted median
Weighted mode
0.05
0.00
−0.05
−0.10
0.08
0.12
0.16
SNP effect on Ribose−5−phosphate isomerase
SNP effect on Gut microbiota abundance (genus Parasutterella id.2892)

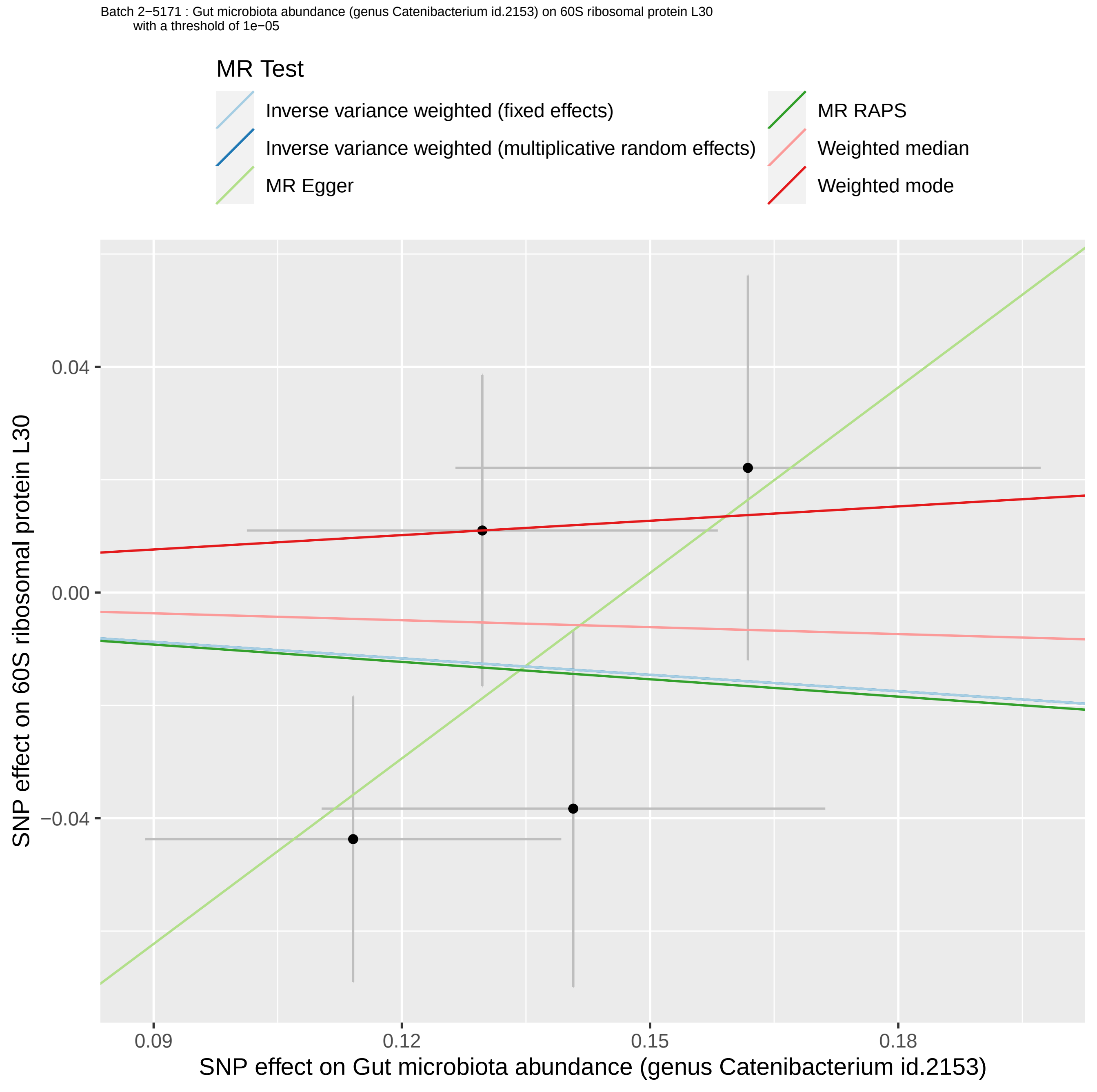
Batch 2−5171 : Gut microbiota abundance (genus Catenibacterium id.2153) on 60S ribosomal protein L30
with a threshold of 1e−05
MR Test
Inverse variance weighted (fixed effects)
Inverse variance weighted (multiplicative random effects)
MR Egger
MR RAPS
Weighted median
Weighted mode
0.04
0.00
−0.04
0.09
0.12
0.15
0.18
SNP effect on 60S ribosomal protein L30
SNP effect on Gut microbiota abundance (genus Catenibacterium id.2153)

Batch 2−5172 : Gut microbiota abundance (genus Parasutterella id.2892) on 60S ribosomal protein L30
with a threshold of 1e−05

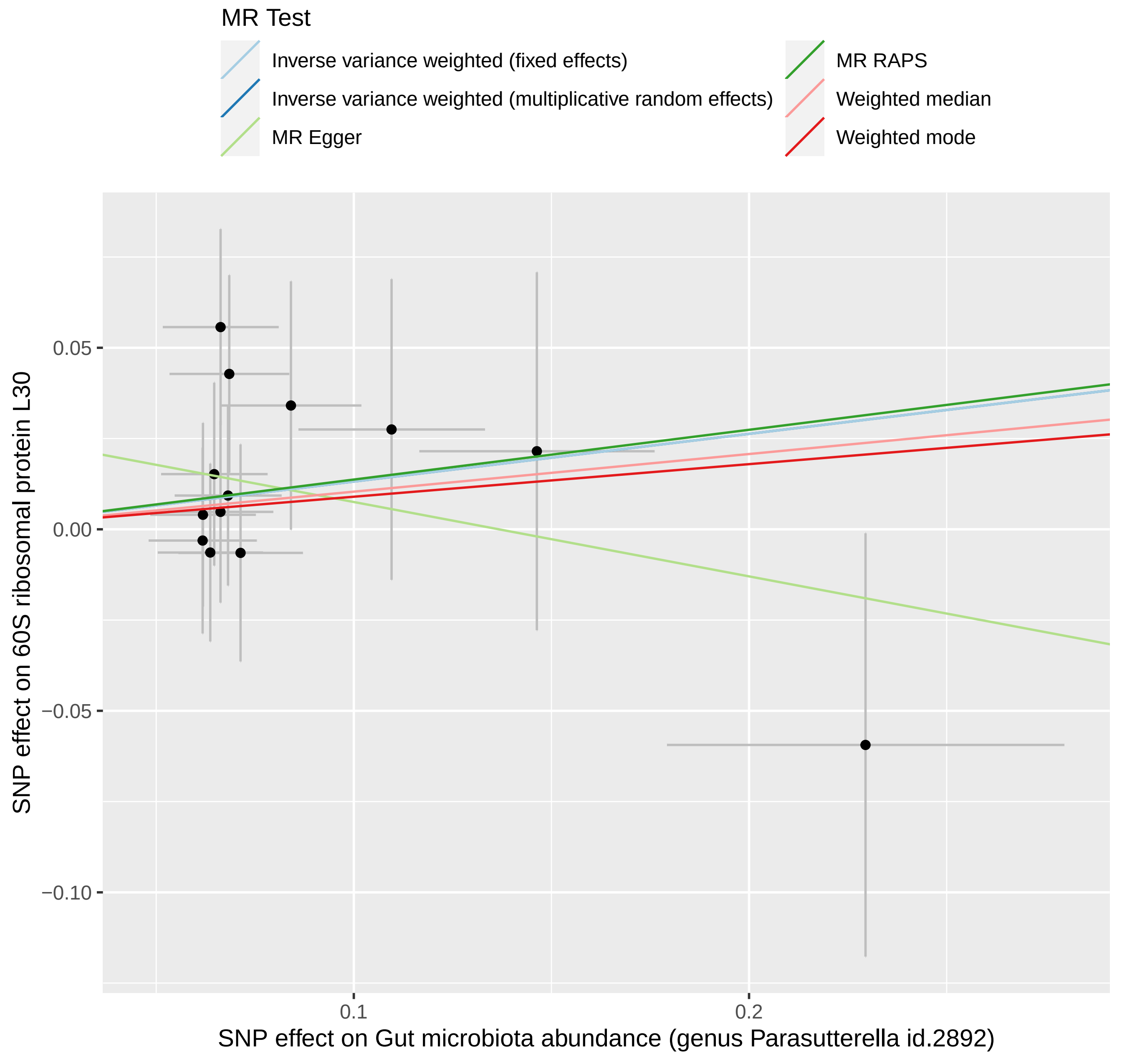

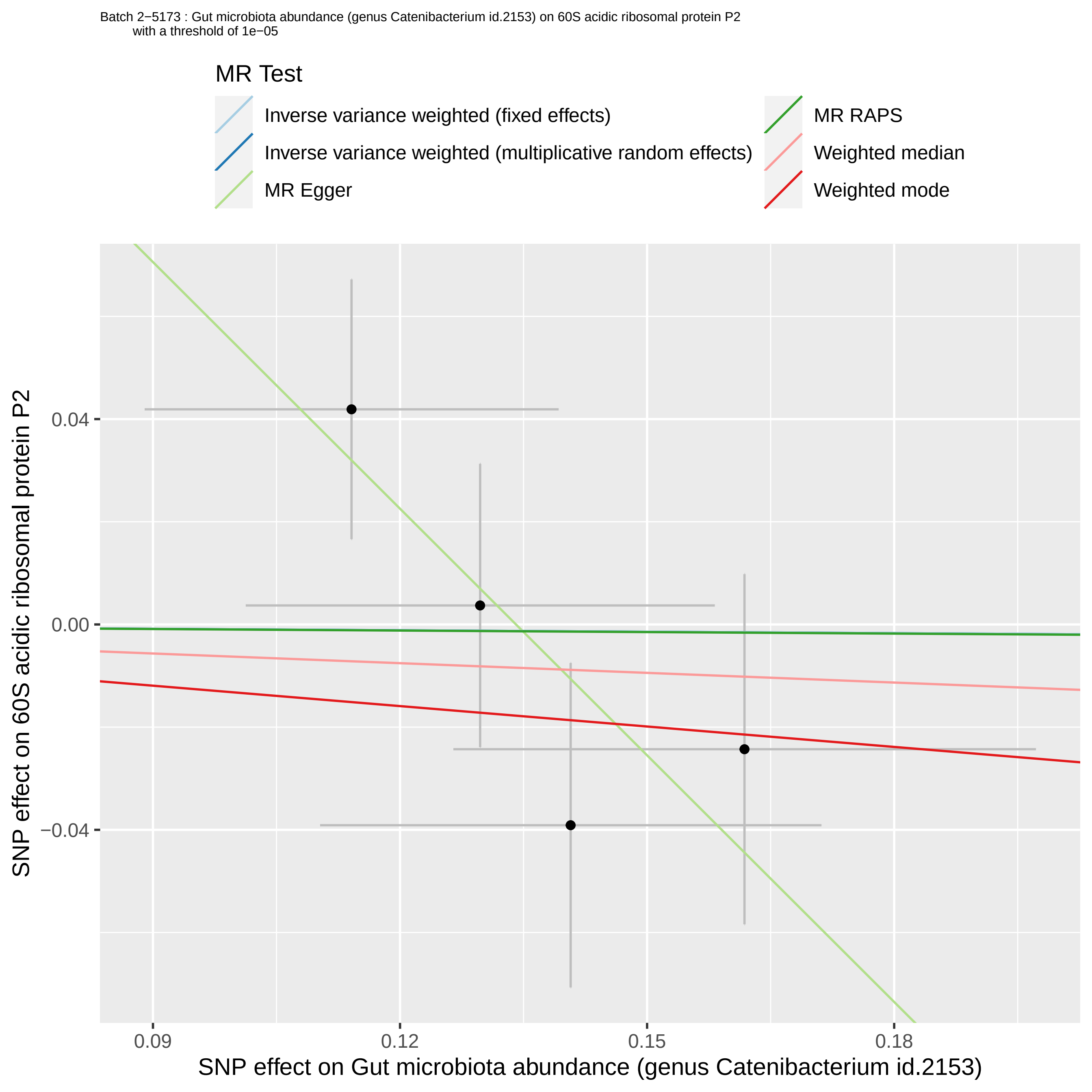
Batch 2−5173 : Gut microbiota abundance (genus Catenibacterium id.2153) on 60S acidic ribosomal protein P2
with a threshold of 1e−05
MR Test
Inverse variance weighted (fixed effects)
Inverse variance weighted (multiplicative random effects)
MR Egger
MR RAPS
Weighted median
Weighted mode
SNP effect on 60S acidic ribosomal protein P2
0.04
0.00
−0.04
0.09
0.12
0.15
0.18
SNP effect on Gut microbiota abundance (genus Catenibacterium id.2153)

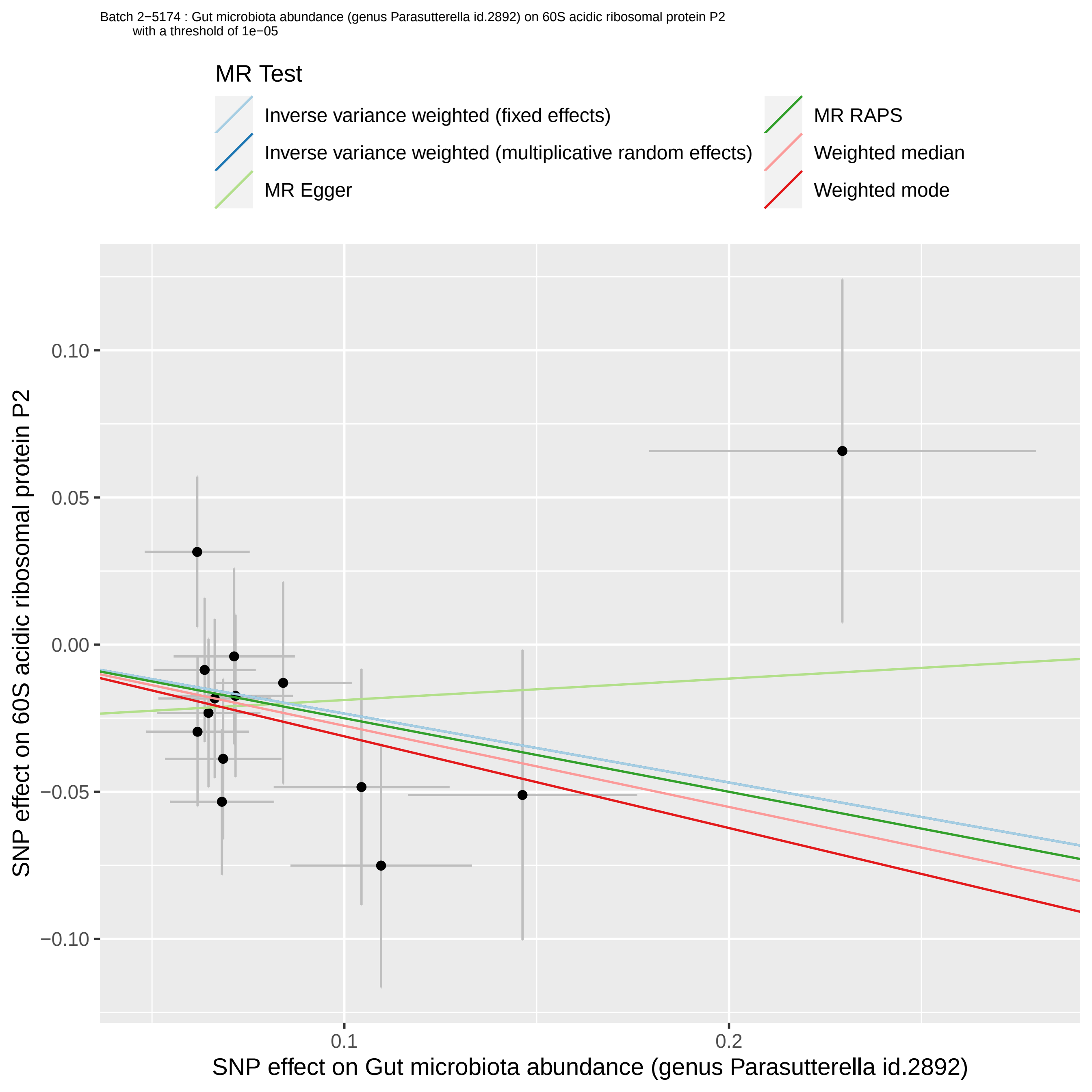

Batch 2−5174 : Gut microbiota abundance (genus Parasutterella id.2892) on 60S acidic ribosomal protein P2
with a threshold of 1e−05
MR Test
Inverse variance weighted (fixed effects)
Inverse variance weighted (multiplicative random effects)
MR Egger
MR RAPS
Weighted median
Weighted mode
0.10
0.05
0.00
−0.05
−0.10
0.1
0.2
SNP effect on 60S acidic ribosomal protein P2
SNP effect on Gut microbiota abundance (genus Parasutterella id.2892)

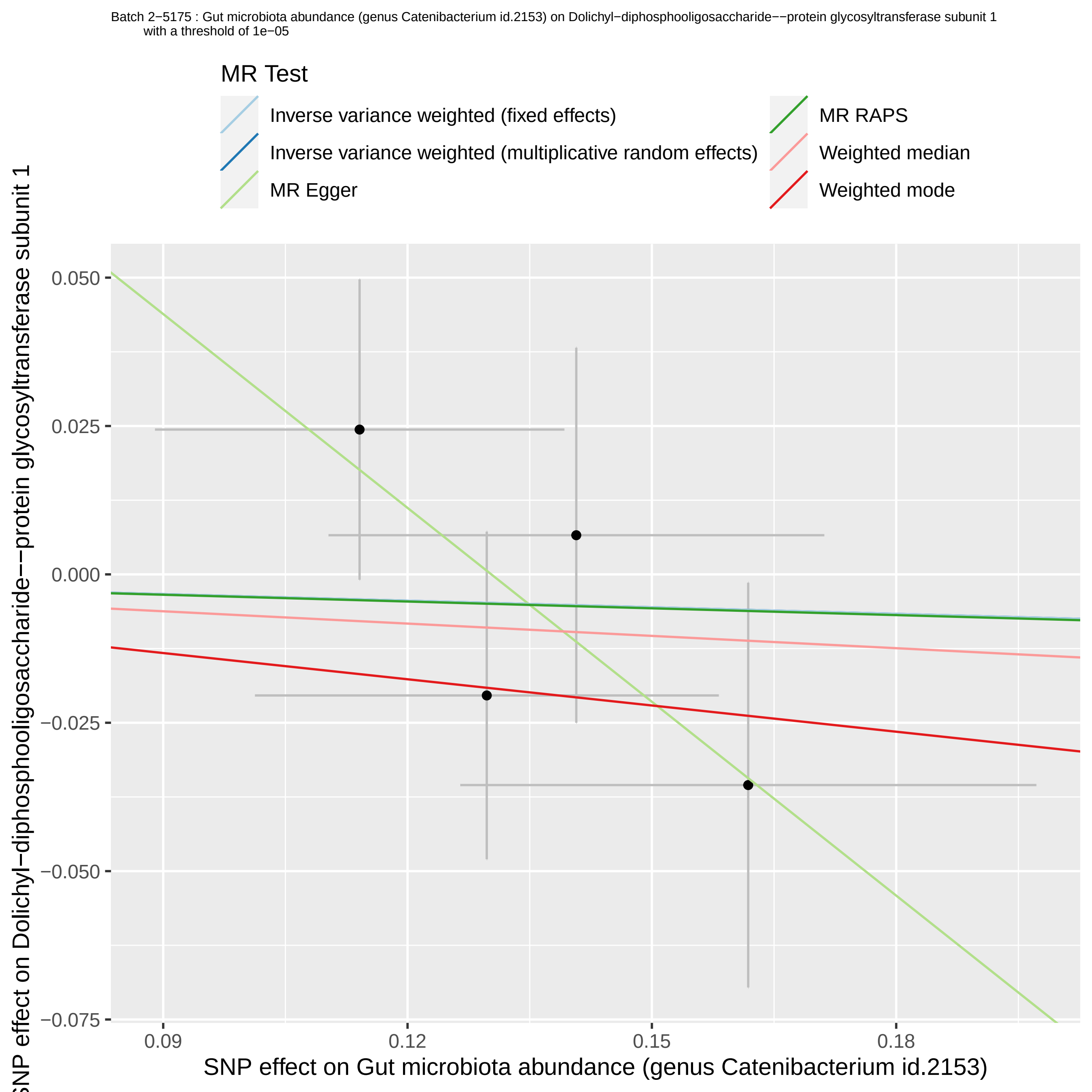
Batch 2−5175 : Gut microbiota abundance (genus Catenibacterium id.2153) on Dolichyl−diphosphooligosaccharide−−protein glycosyltransferase subunit 1 with a threshold of 1e−05
MR Test
Inverse variance weighted (fixed effects)
Inverse variance weighted (multiplicative random effects)
MR Egger
MR RAPS
Weighted median
Weighted mode
0.050
0.025
0.000
−0.025
−0.050
−0.075
0.09
0.12
0.15
0.18
SNP effect on Gut microbiota abundance (genus Catenibacterium id.2153)
SNP effect on Dolichyl−diphosphooligosaccharide−−protein glycosyltransferase subunit 1

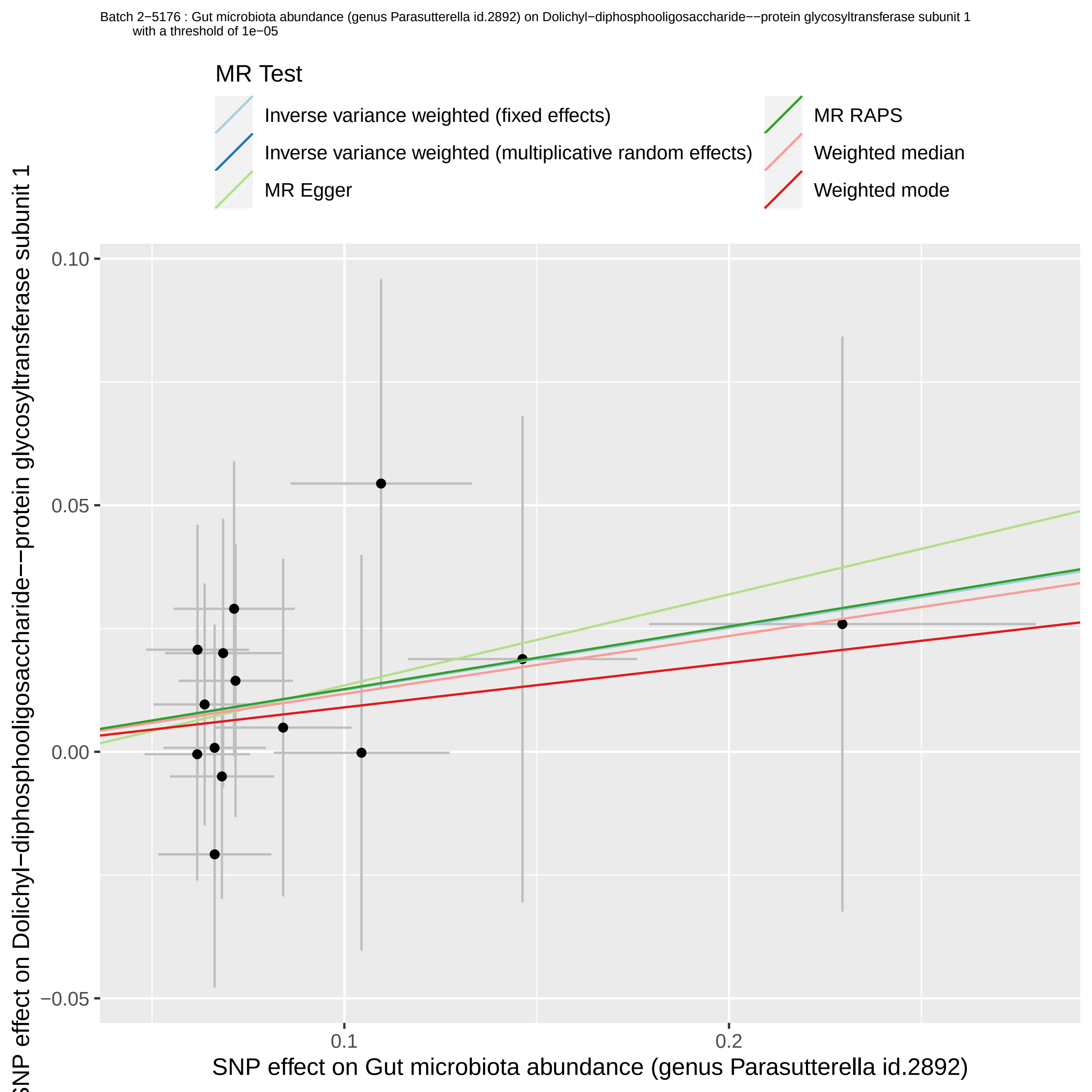
Batch 2−5176 : Gut microbiota abundance (genus Parasutterella id.2892) on Dolichyl−diphosphooligosaccharide−−protein glycosyltransferase subunit 1
with a threshold of 1e−05
MR Test
Inverse variance weighted (fixed effects)
Inverse variance weighted (multiplicative random effects)
MR Egger
MR RAPS
Weighted median
Weighted mode
0.10
0.05
0.00
−0.05
0.1
0.2
SNP effect on Gut microbiota abundance (genus Parasutterella id.2892)
SNP effect on Dolichyl−diphosphooligosaccharide−−protein glycosyltransferase subunit 1

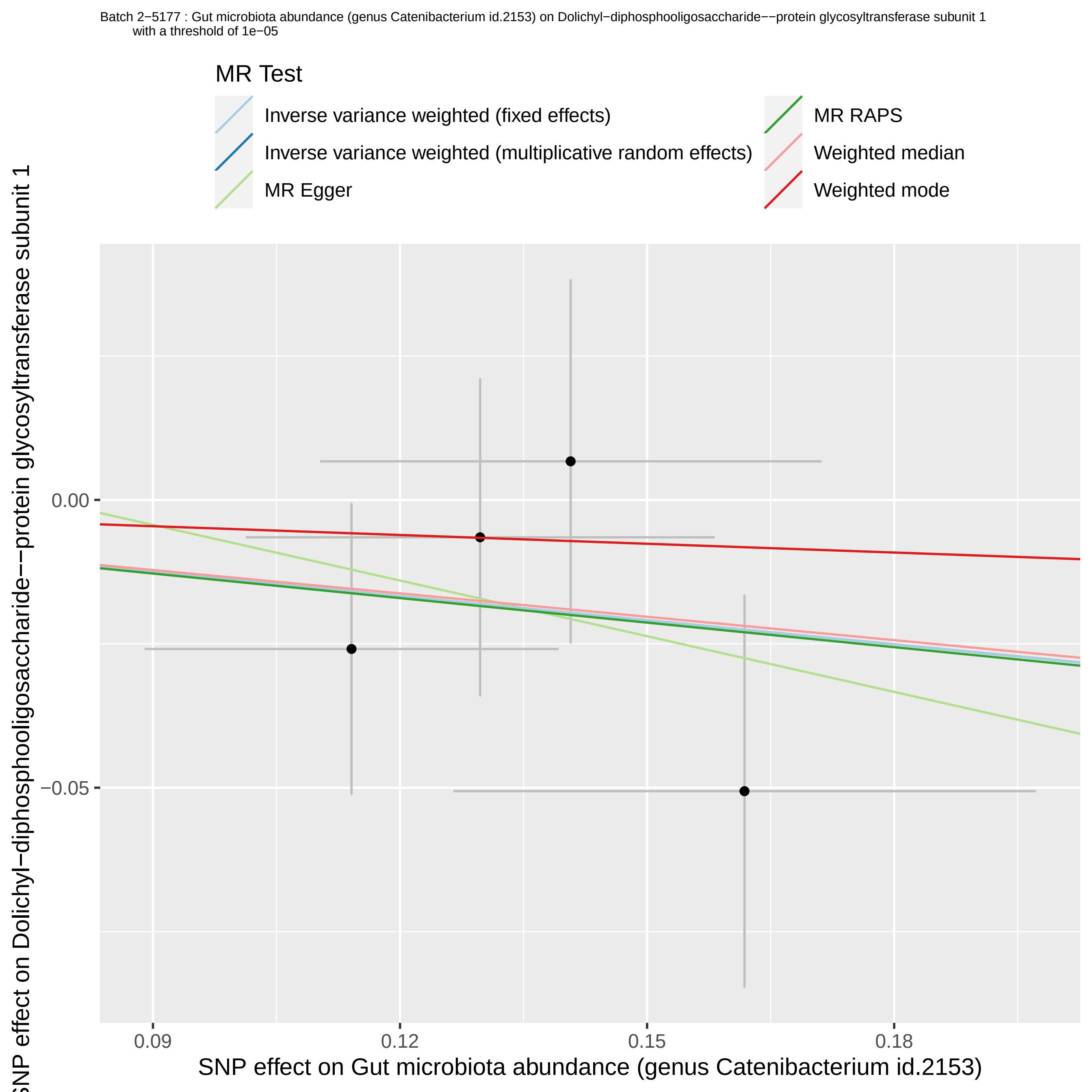
Batch 2−5177 : Gut microbiota abundance (genus Catenibacterium id.2153) on Dolichyl−diphosphooligosaccharide−−protein glycosyltransferase subunit 1
with a threshold of 1e−05
MR Test
Inverse variance weighted (fixed effects)
Inverse variance weighted (multiplicative random effects)
MR Egger
MR RAPS
Weighted median
Weighted mode
0.00
−0.05
0.09
0.12
0.15
0.18
SNP effect on Gut microbiota abundance (genus Catenibacterium id.2153)
SNP effect on Dolichyl−diphosphooligosaccharide−−protein glycosyltransferase subunit 1

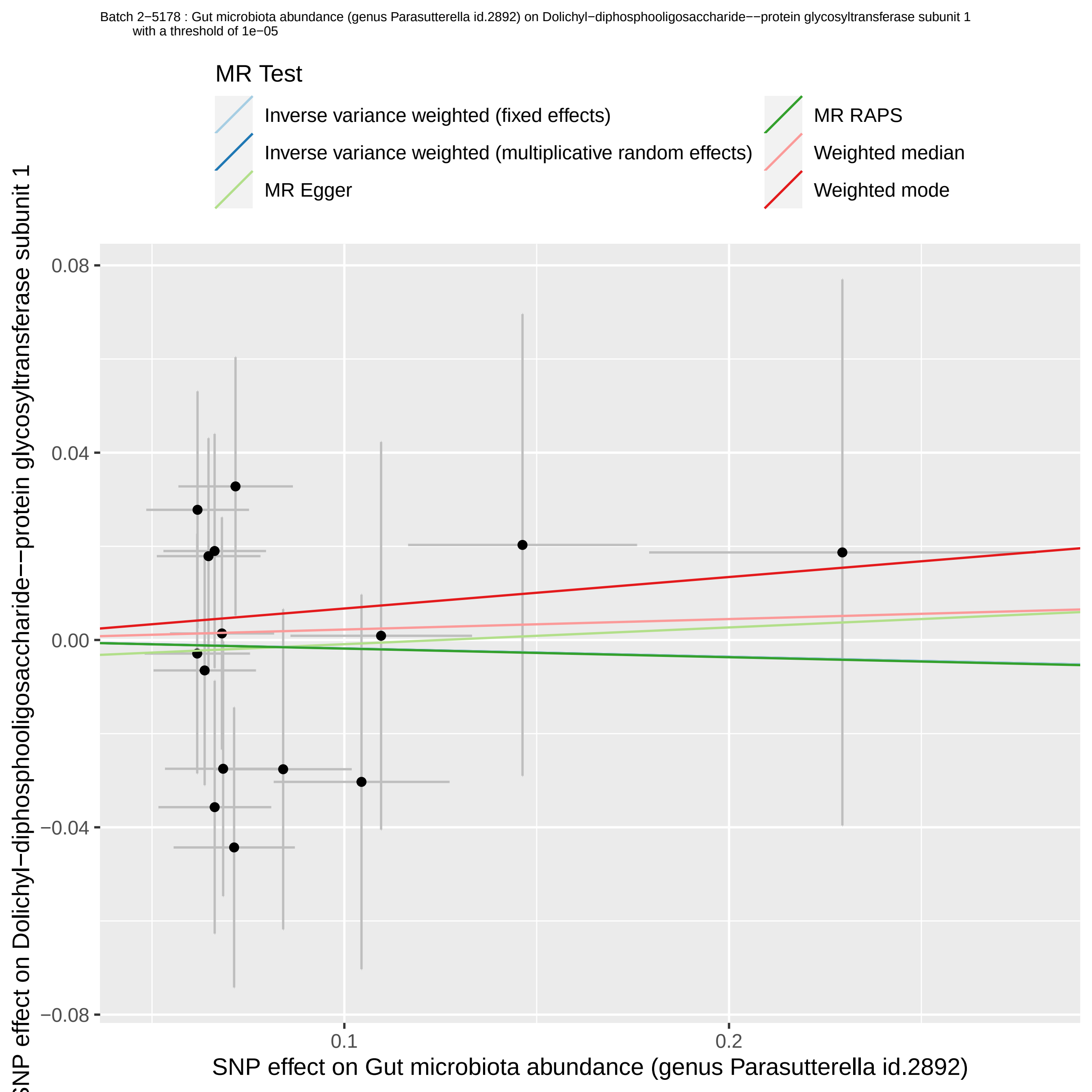

Batch 2−5178 : Gut microbiota abundance (genus Parasutterella id.2892) on Dolichyl−diphosphooligosaccharide−−protein glycosyltransferase subunit 1
with a threshold of 1e−05
MR Test
Inverse variance weighted (fixed effects)
Inverse variance weighted (multiplicative random effects)
MR Egger
MR RAPS
Weighted median
Weighted mode
0.08
0.04
0.00
−0.04
−0.08
0.1
0.2
SNP effect on Gut microbiota abundance (genus Parasutterella id.2892)
SNP effect on Dolichyl−diphosphooligosaccharide−−protein glycosyltransferase subunit 1

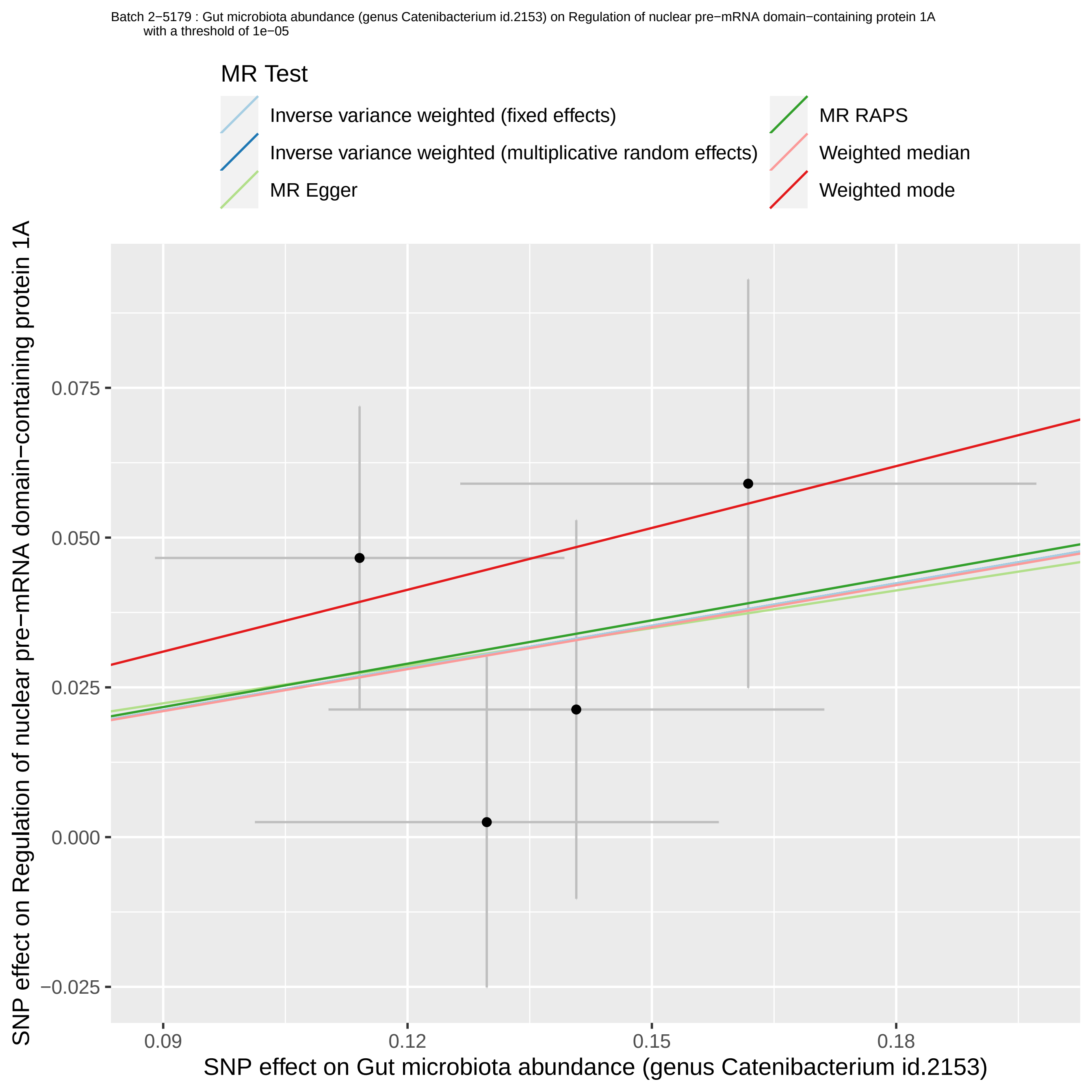

Batch 2−5179 : Gut microbiota abundance (genus Catenibacterium id.2153) on Regulation of nuclear pre−mRNA domain−containing protein 1A
with a threshold of 1e−05
MR Test
Inverse variance weighted (fixed effects)
Inverse variance weighted (multiplicative random effects)
MR Egger
MR RAPS
Weighted median
Weighted mode
0.075
0.050
0.025
0.000
−0.025
0.09
0.12
0.15
0.18
SNP effect on Regulation of nuclear pre−mRNA domain−containing protein 1A
SNP effect on Gut microbiota abundance (genus Catenibacterium id.2153)

Batch 2−5180 : Gut microbiota abundance (genus Parasutterella id.2892) on Regulation of nuclear pre−mRNA domain−containing protein 1A
with a threshold of 1e−05

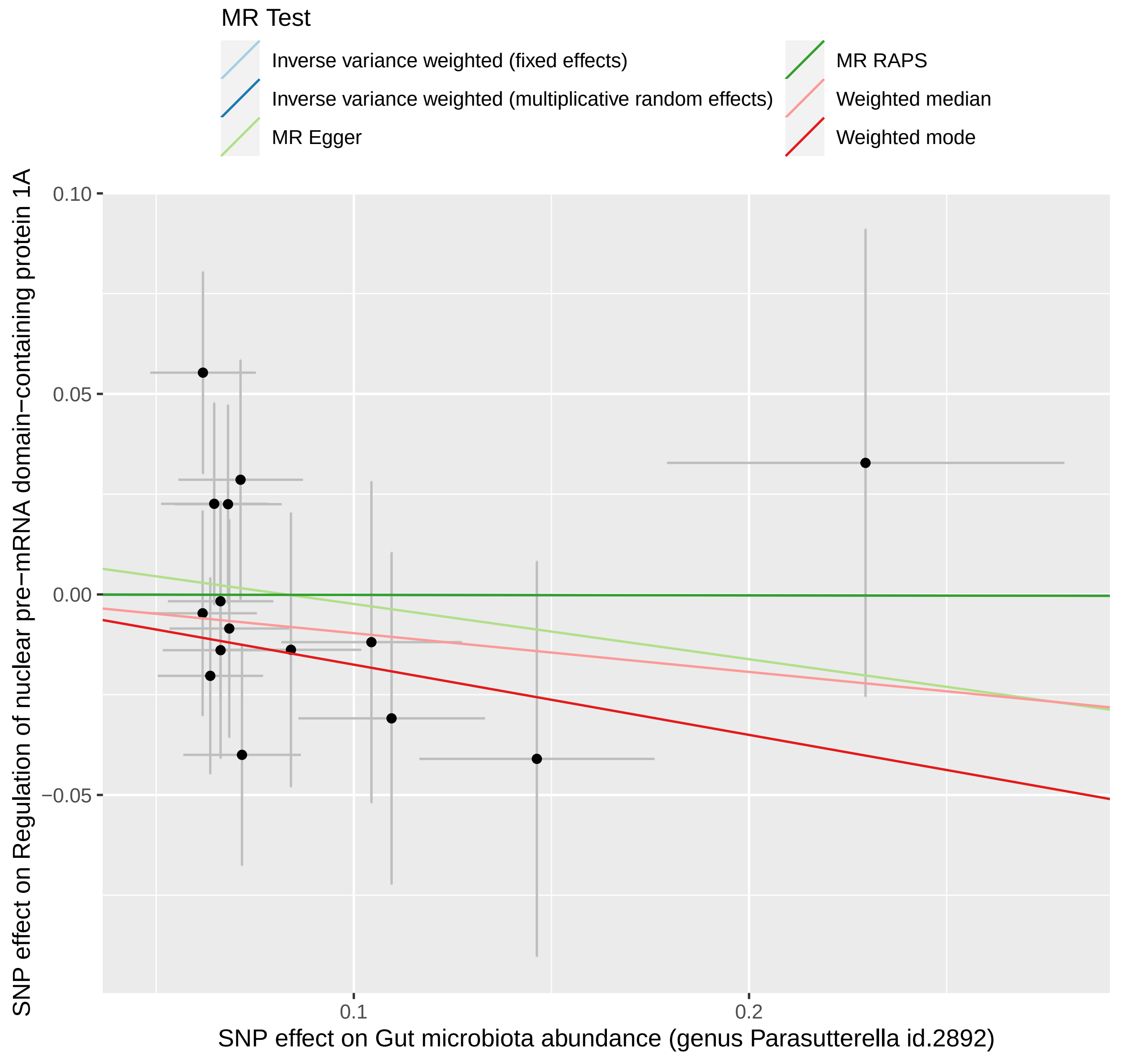

Batch 2−5181 : Gut microbiota abundance (genus Catenibacterium id.2153) on Ubiquitin+1, truncated mutation for UbB
with a threshold of 1e−05

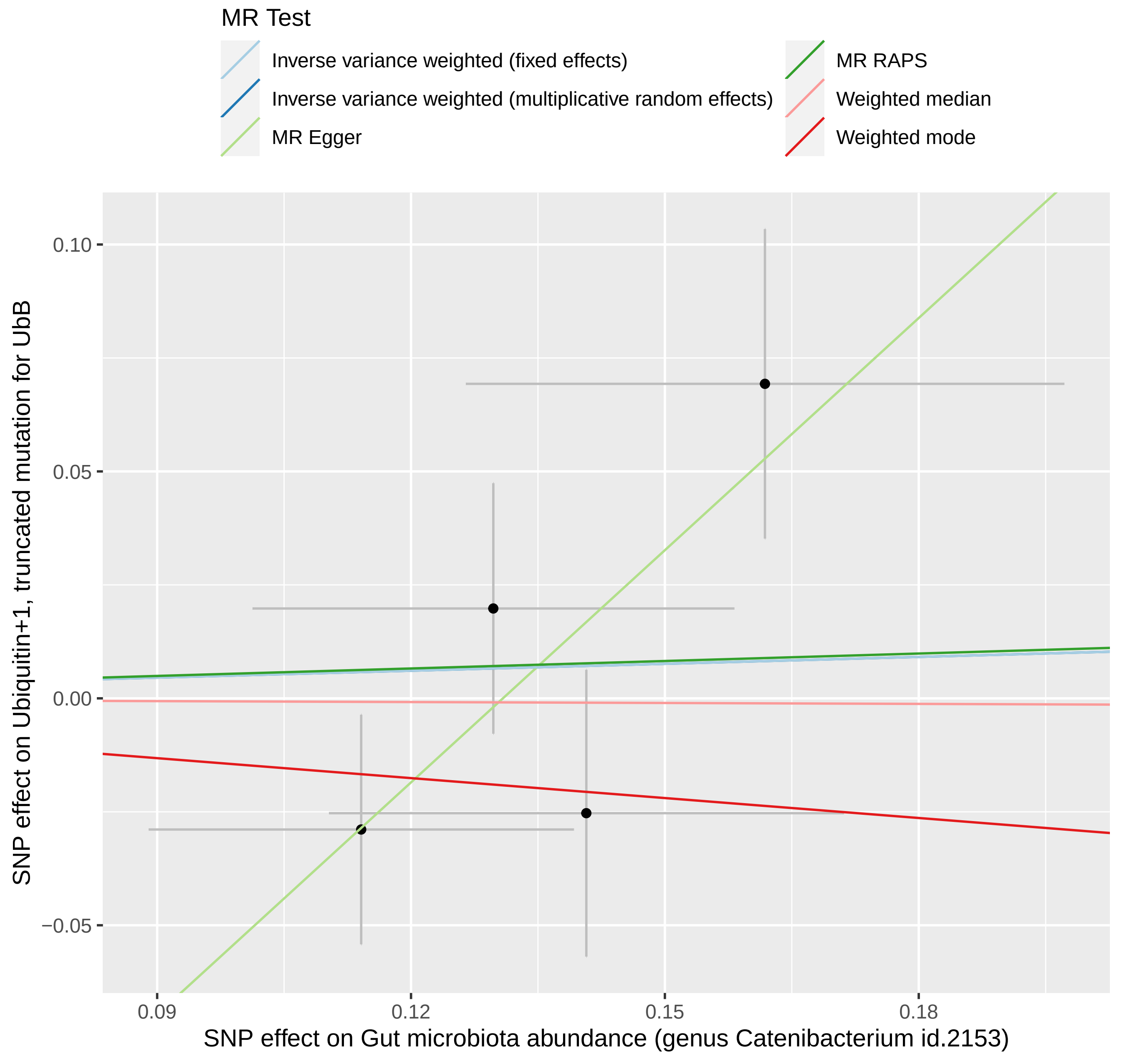

Batch 2−5182 : Gut microbiota abundance (genus Parasutterella id.2892) on Ubiquitin+1, truncated mutation for UbB with a threshold of 1e−05

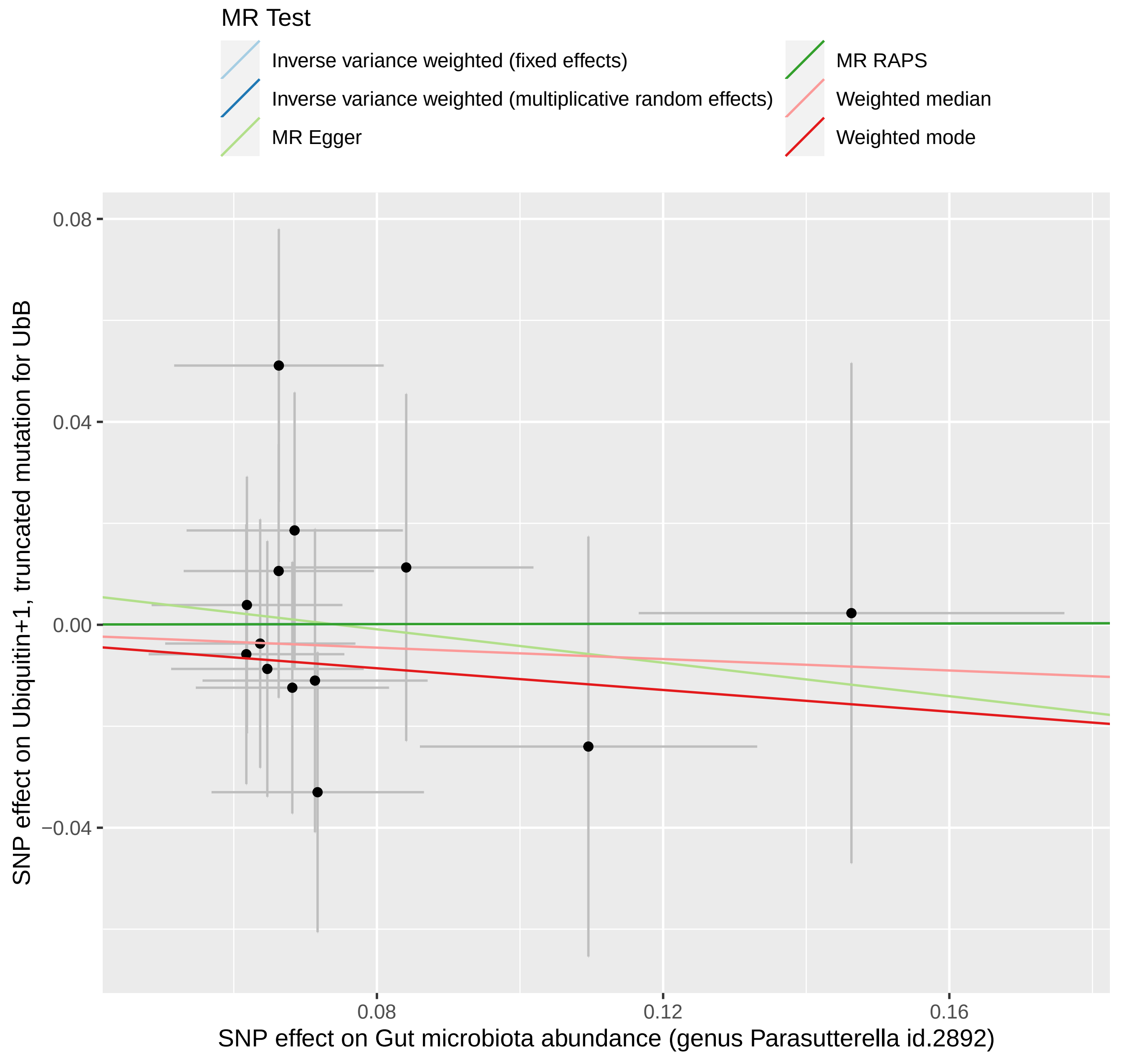

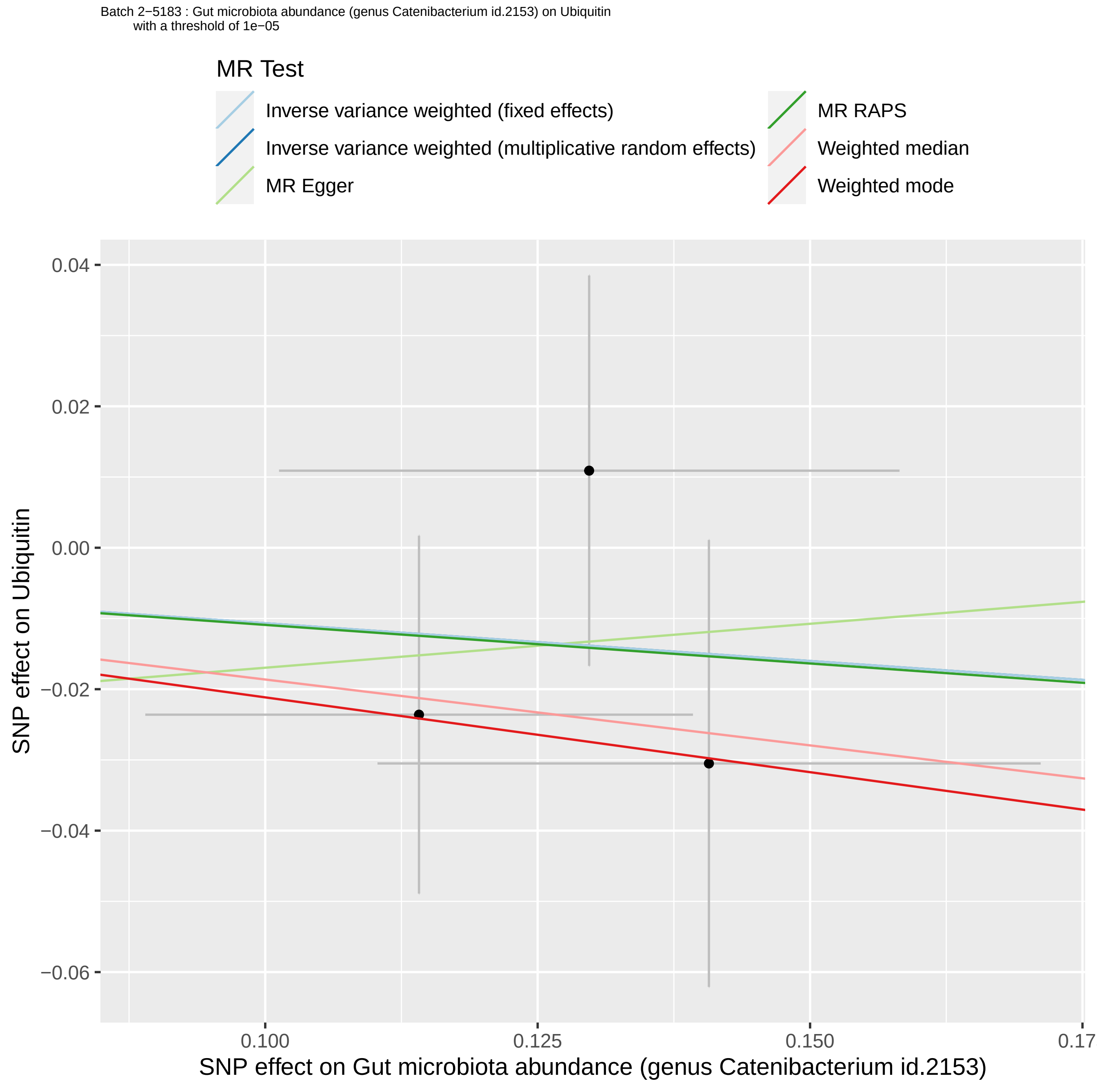
Batch 2−5183 : Gut microbiota abundance (genus Catenibacterium id.2153) on Ubiquitin
with a threshold of 1e−05
MR Test
Inverse variance weighted (fixed effects)
Inverse variance weighted (multiplicative random effects)
MR Egger
MR RAPS
Weighted median
Weighted mode
0.04
0.02
0.00
−0.02
−0.04
−0.06
SNP effect on Ubiquitin
0.100
0.125
0.150
0.17
SNP effect on Gut microbiota abundance (genus Catenibacterium id.2153)

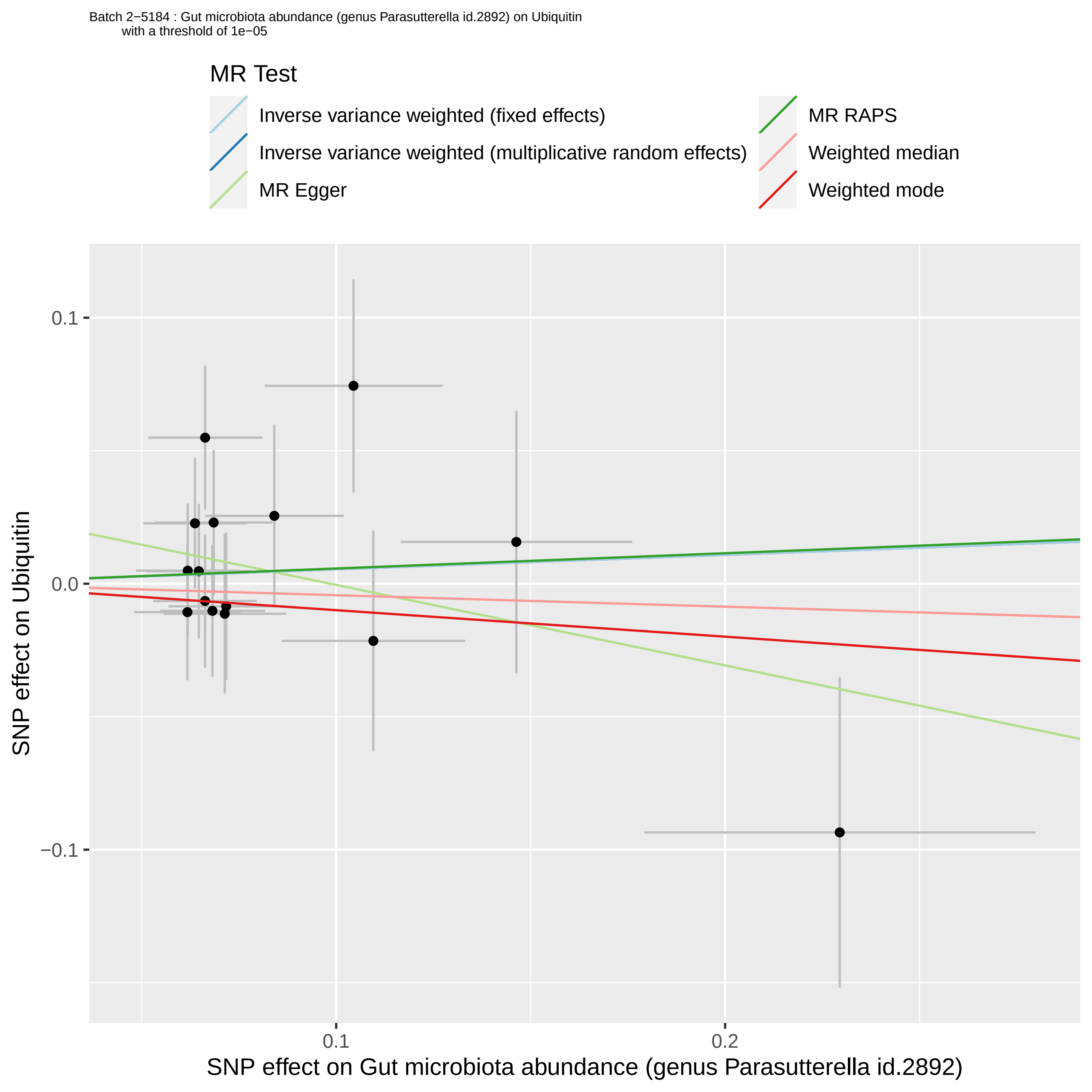

Batch 2−5184 : Gut microbiota abundance (genus Parasutterella id.2892) on Ubiquitin
with a threshold of 1e−05
MR Test
Inverse variance weighted (fixed effects)
Inverse variance weighted (multiplicative random effects)
MR Egger
MR RAPS
Weighted median
Weighted mode
0.1
0.0
−0.1
0.1
0.2
SNP effect on Ubiquitin
SNP effect on Gut microbiota abundance (genus Parasutterella id.2892)

Batch 2−5185 : Gut microbiota abundance (genus Catenibacterium id.2153) on 40S ribosomal protein S3
with a threshold of 1e−05

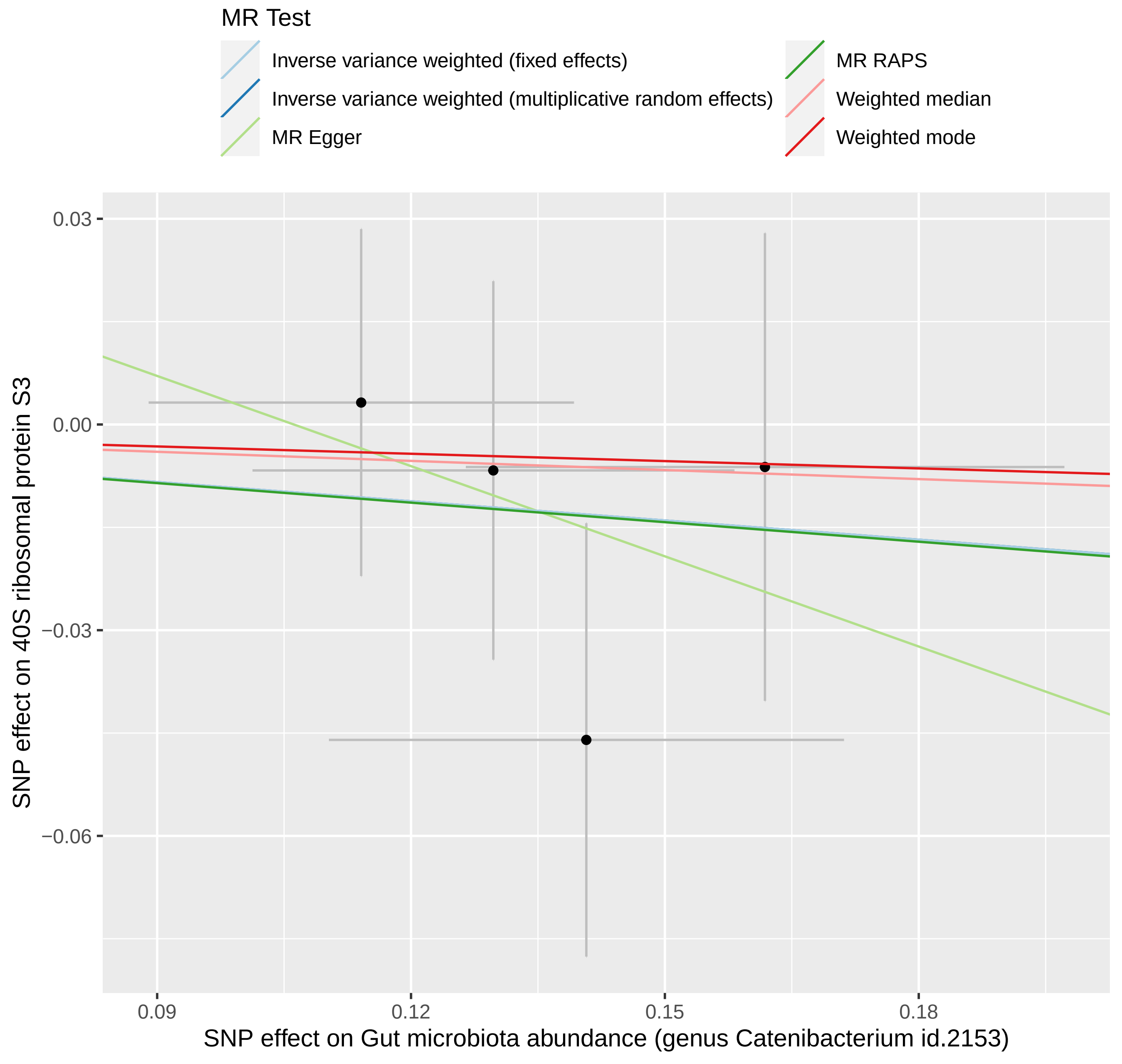

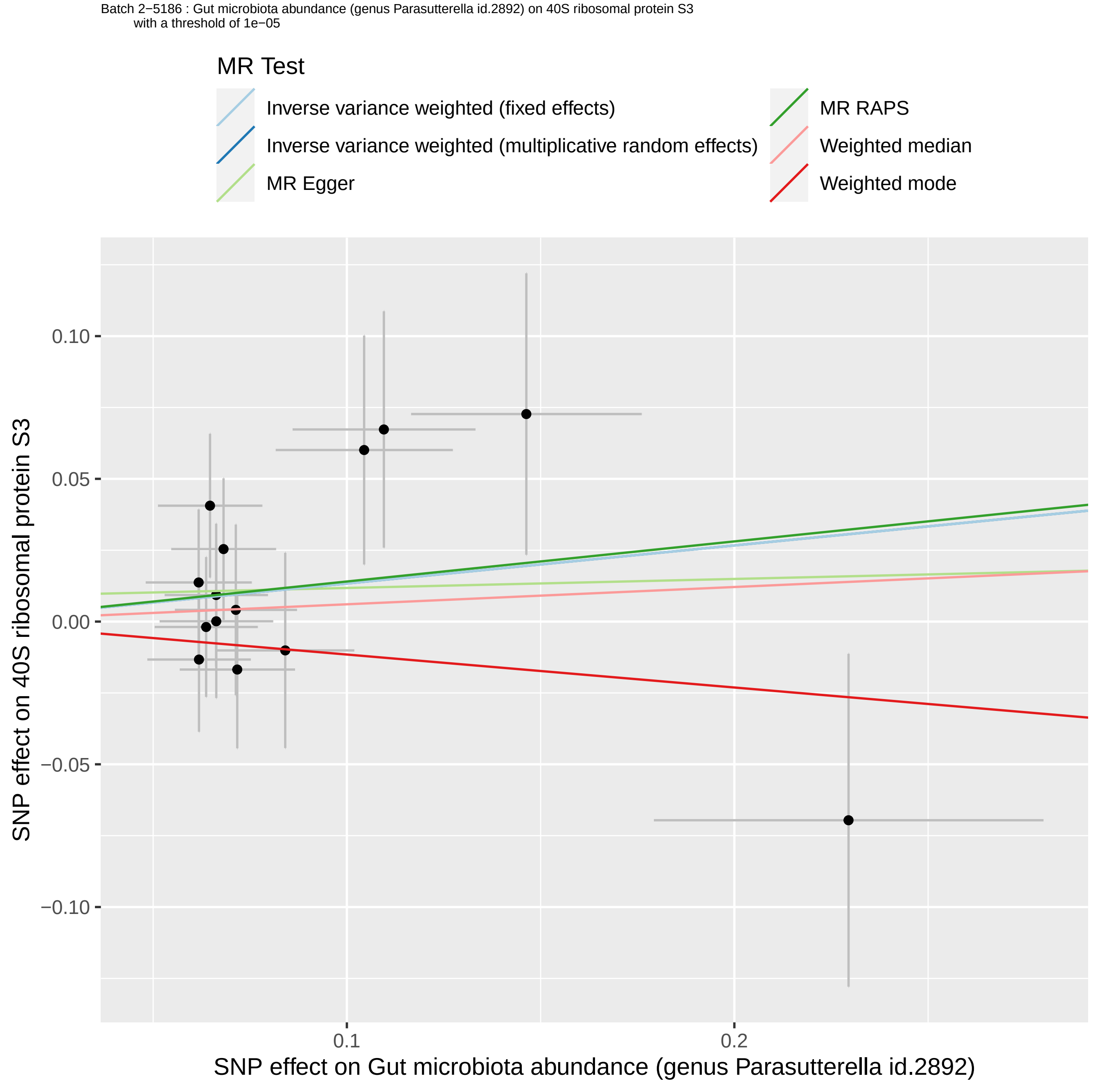
Batch 2−5186 : Gut microbiota abundance (genus Parasutterella id.2892) on 40S ribosomal protein S3
with a threshold of 1e−05
MR Test
Inverse variance weighted (fixed effects)
Inverse variance weighted (multiplicative random effects)
MR Egger
MR RAPS
Weighted median
Weighted mode
0.10
0.05
0.00
−0.05
−0.10
0.1
0.2
SNP effect on 40S ribosomal protein S3
SNP effect on Gut microbiota abundance (genus Parasutterella id.2892)

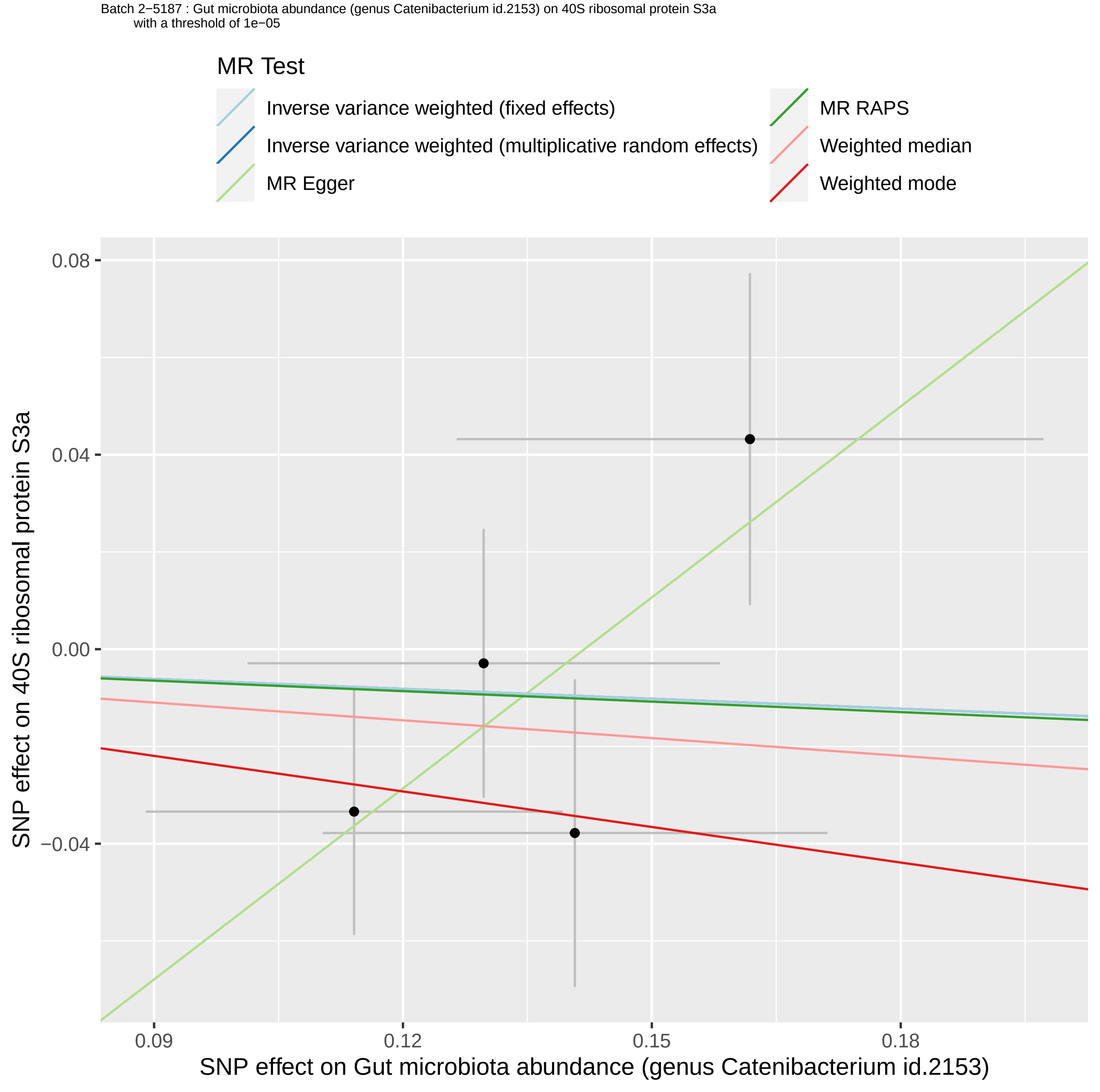

Batch 2−5187 : Gut microbiota abundance (genus Catenibacterium id.2153) on 40S ribosomal protein S3a
with a threshold of 1e−05
MR Test
Inverse variance weighted (fixed effects)
Inverse variance weighted (multiplicative random effects)
MR Egger
MR RAPS
Weighted median
Weighted mode
0.08
0.04
0.00
−0.04
0.09
0.12
0.15
0.18
SNP effect on 40S ribosomal protein S3a
SNP effect on Gut microbiota abundance (genus Catenibacterium id.2153)

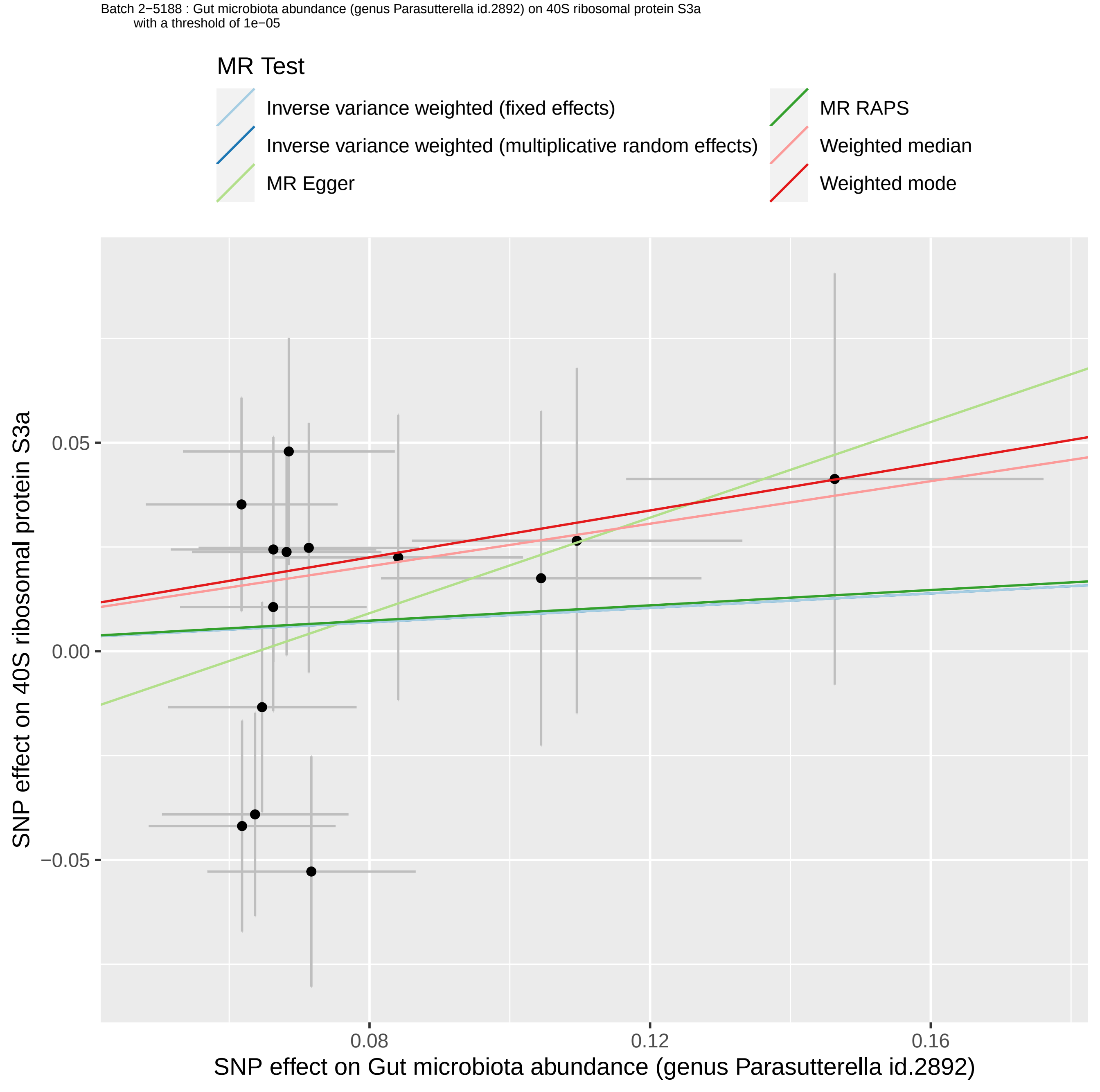
Batch 2−5188 : Gut microbiota abundance (genus Parasutterella id.2892) on 40S ribosomal protein S3a
with a threshold of 1e−05
MR Test
Inverse variance weighted (fixed effects)
Inverse variance weighted (multiplicative random effects)
MR Egger
MR RAPS
Weighted median
Weighted mode
SNP effect on 40S ribosomal protein S3a
0.05
0.00
−0.05
0.08
0.12
0.16
SNP effect on Gut microbiota abundance (genus Parasutterella id.2892)

Batch 2−5189 : Gut microbiota abundance (genus Catenibacterium id.2153) on 40S ribosomal protein S4, X isoform
with a threshold of 1e−05

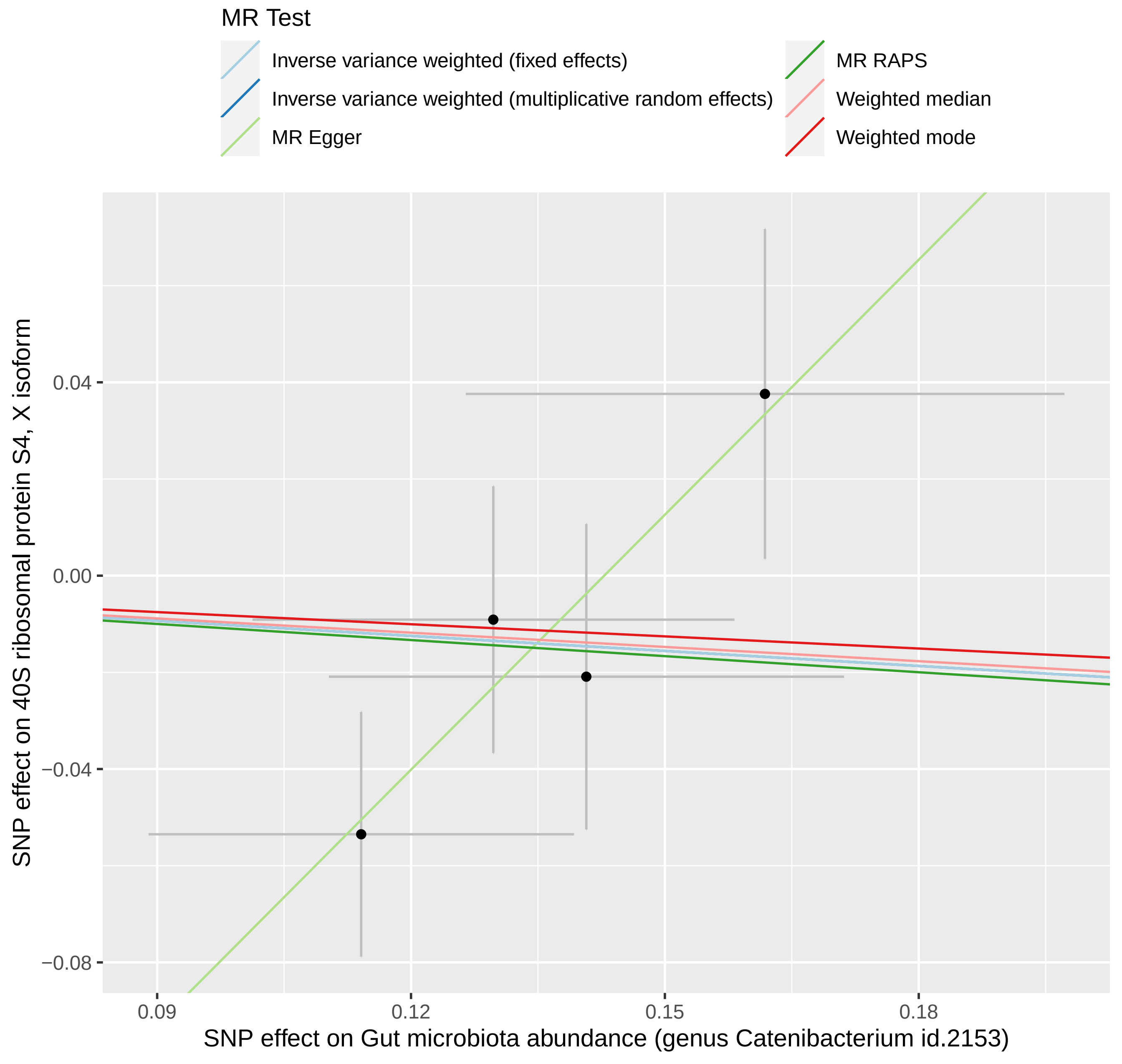

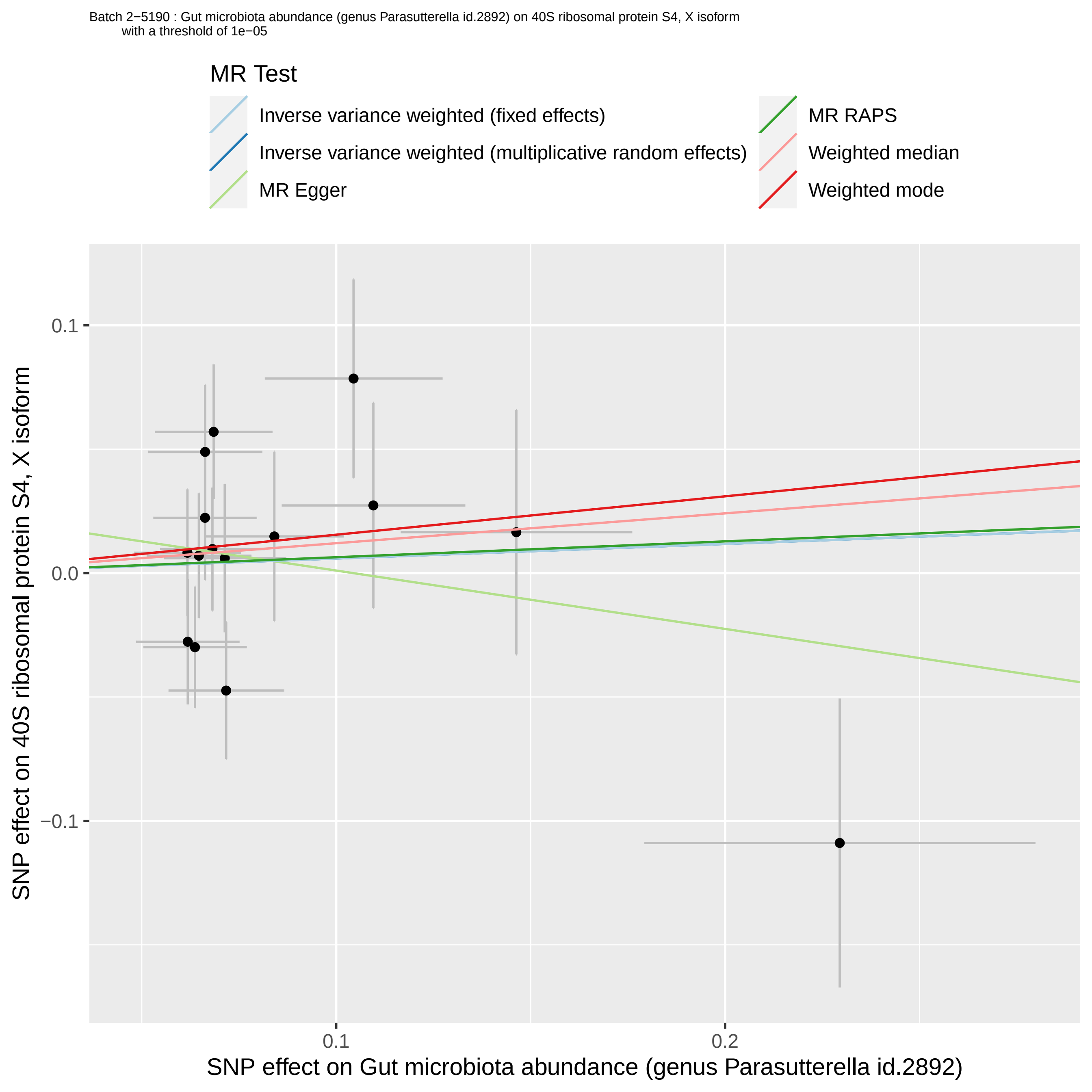

Batch 2−5190 : Gut microbiota abundance (genus Parasutterella id.2892) on 40S ribosomal protein S4, X isoform
with a threshold of 1e−05
MR Test
Inverse variance weighted (fixed effects)
Inverse variance weighted (multiplicative random effects)
MR Egger
MR RAPS
Weighted median
Weighted mode
0.1
0.0
−0.1
0.1
0.2
SNP effect on 40S ribosomal protein S4, X isoform
SNP effect on Gut microbiota abundance (genus Parasutterella id.2892)

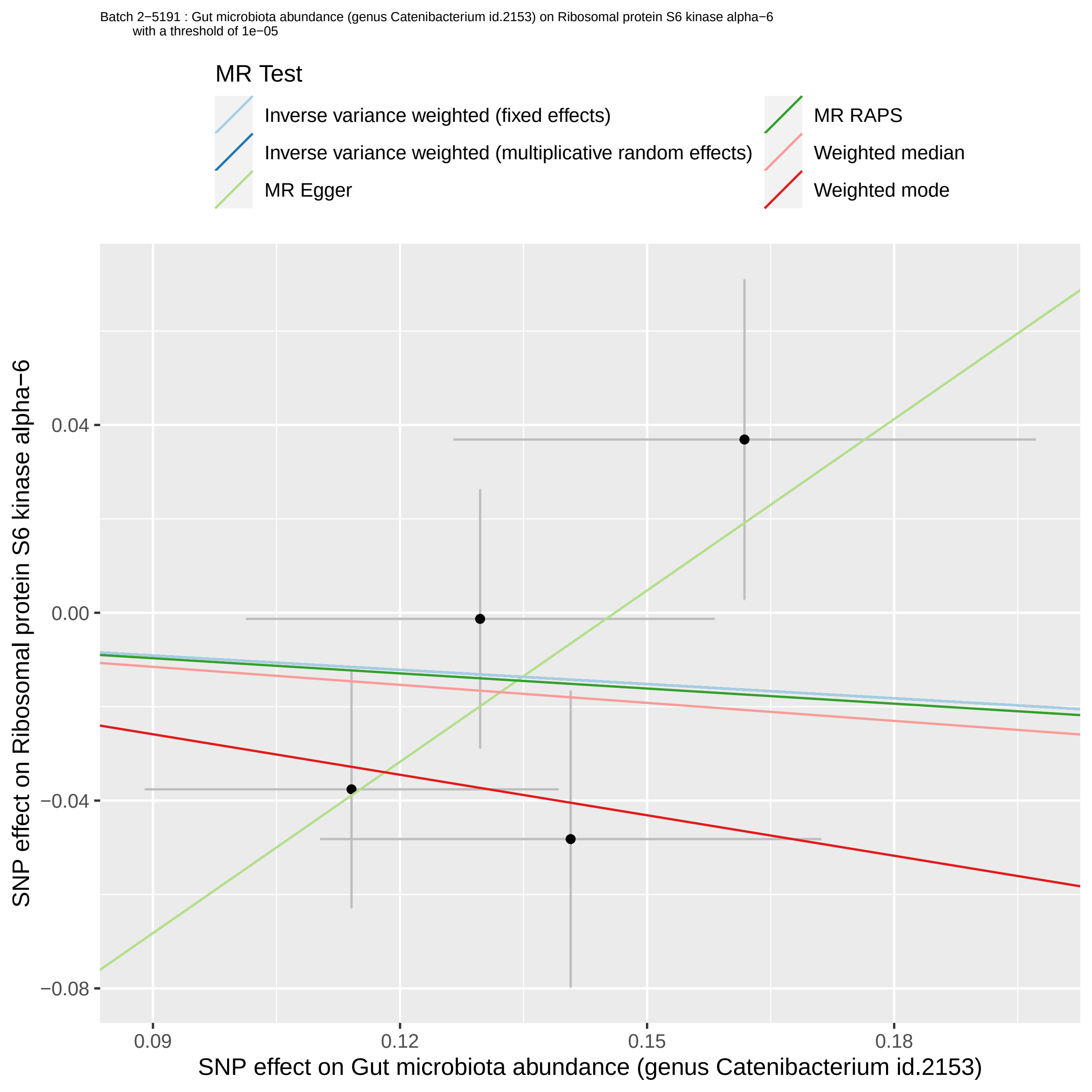
Batch 2−5191 : Gut microbiota abundance (genus Catenibacterium id.2153) on Ribosomal protein S6 kinase alpha−6
with a threshold of 1e−05
MR Test
Inverse variance weighted (fixed effects)
Inverse variance weighted (multiplicative random effects)
MR Egger
MR RAPS
Weighted median
Weighted mode
0.04
0.00
−0.04
−0.08
0.09
0.12
0.15
0.18
SNP effect on Ribosomal protein S6 kinase alpha−6
SNP effect on Gut microbiota abundance (genus Catenibacterium id.2153)

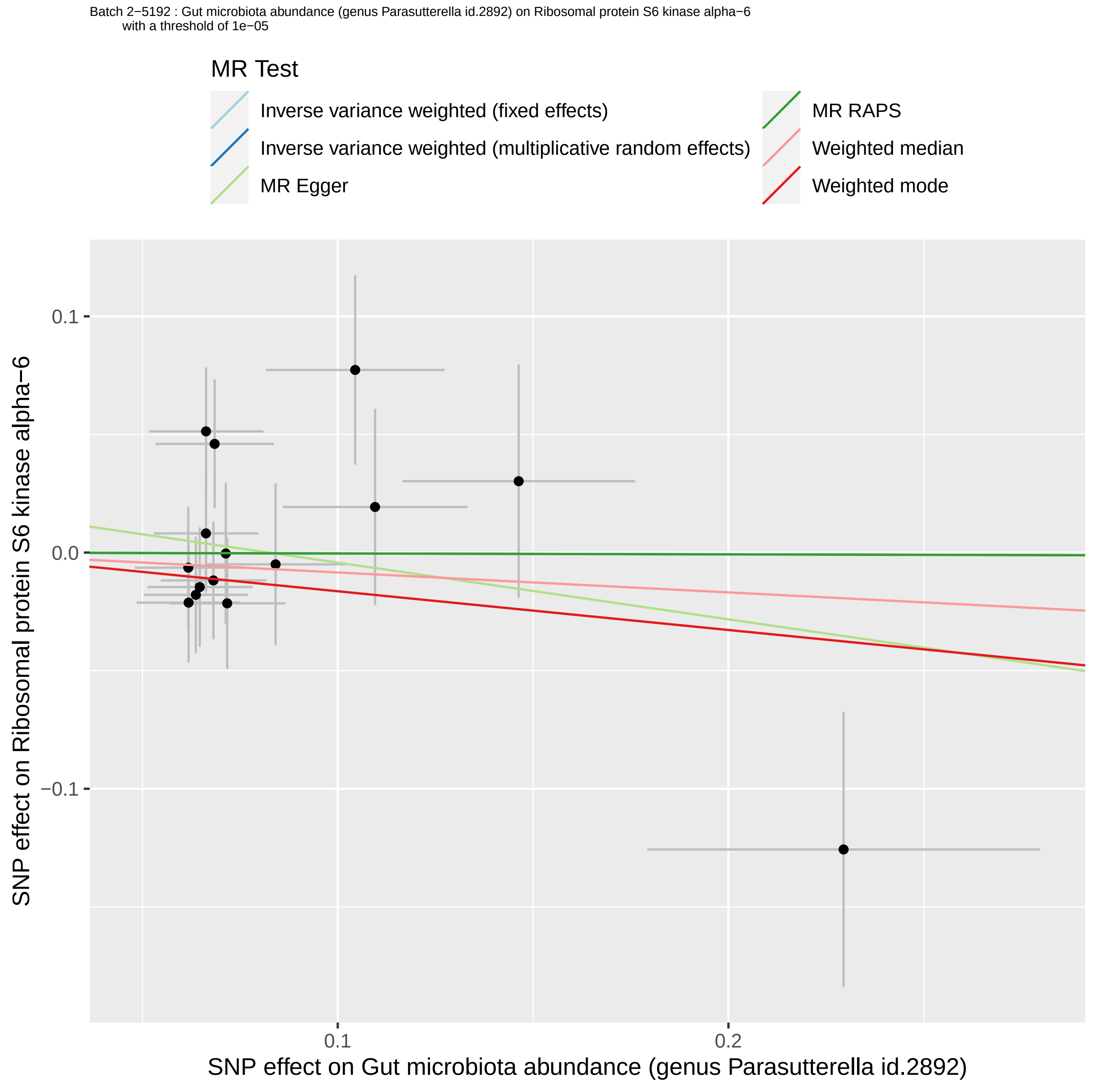

Batch 2−5192 : Gut microbiota abundance (genus Parasutterella id.2892) on Ribosomal protein S6 kinase alpha−6
with a threshold of 1e−05
MR Test
Inverse variance weighted (fixed effects)
Inverse variance weighted (multiplicative random effects)
MR Egger
MR RAPS
Weighted median
Weighted mode
0.1
0.0
−0.1
0.1
0.2
SNP effect on Ribosomal protein S6 kinase alpha−6
SNP effect on Gut microbiota abundance (genus Parasutterella id.2892)

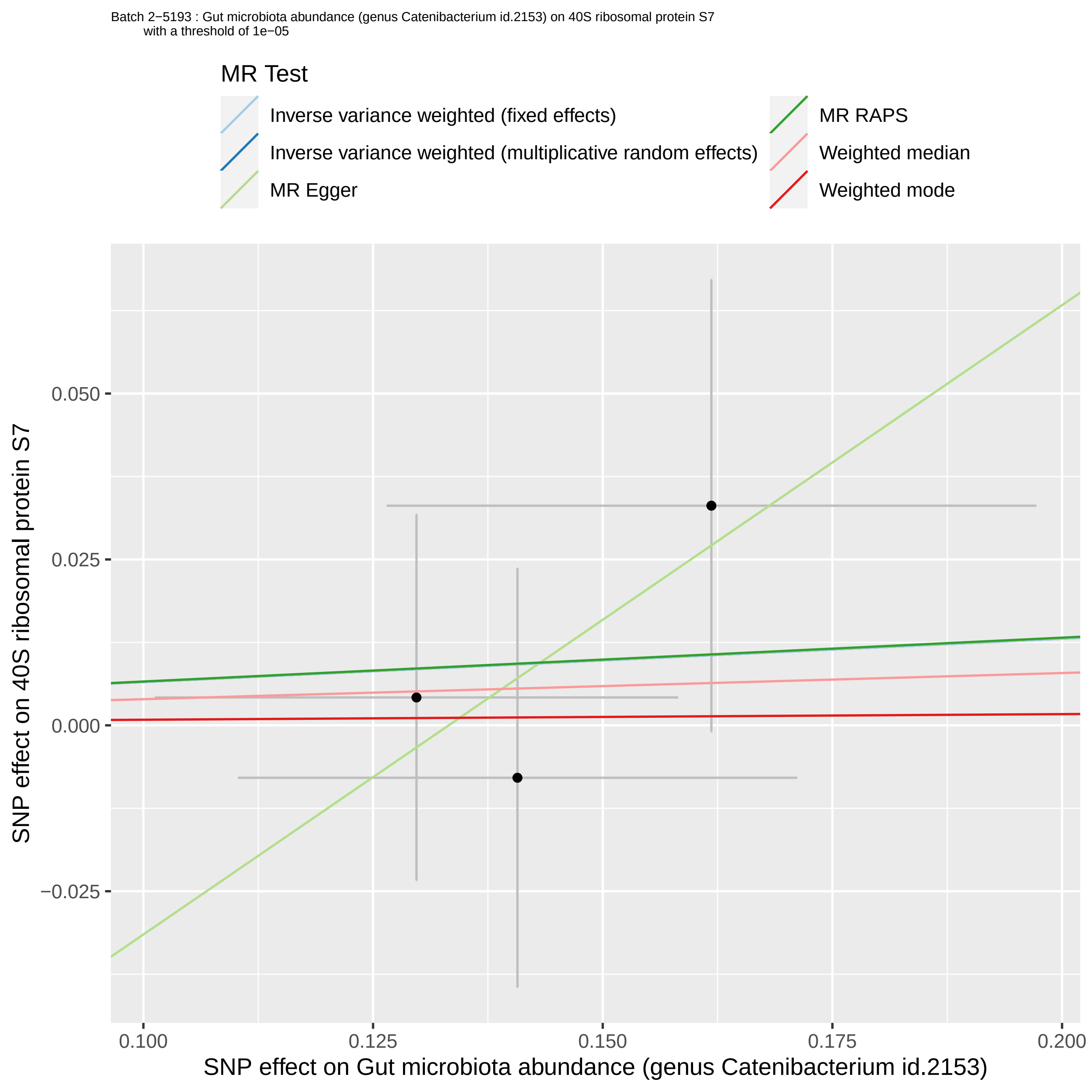
Batch 2−5193 : Gut microbiota abundance (genus Catenibacterium id.2153) on 40S ribosomal protein S7
with a threshold of 1e−05
MR Test
Inverse variance weighted (fixed effects)
Inverse variance weighted (multiplicative random effects)
MR Egger
MR RAPS
Weighted median
Weighted mode
SNP effect on 40S ribosomal protein S7
0.050
0.025
0.000
−0.025
0.100
0.125
0.150
0.175
0.200
SNP effect on Gut microbiota abundance (genus Catenibacterium id.2153)

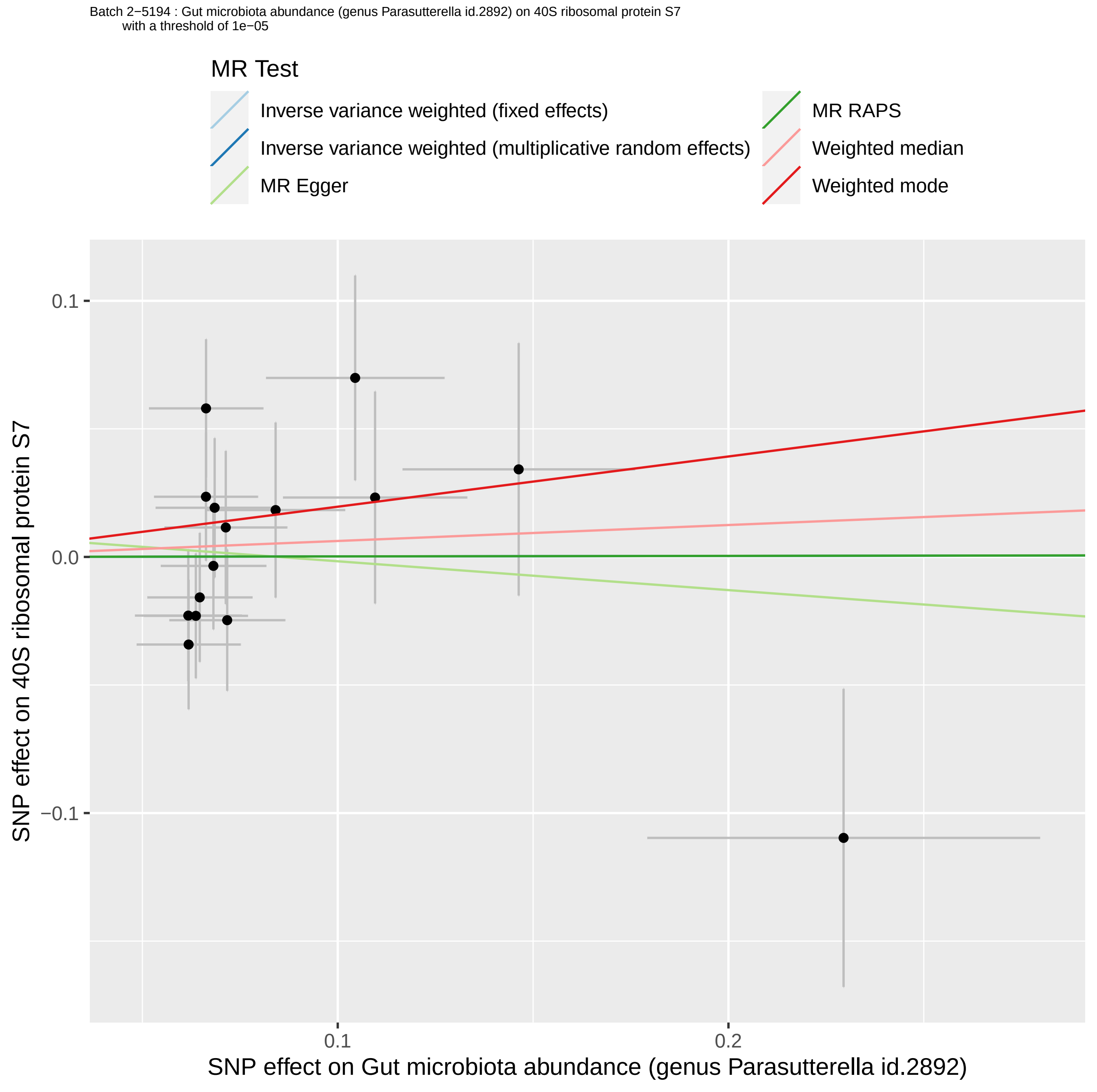

Batch 2−5194 : Gut microbiota abundance (genus Parasutterella id.2892) on 40S ribosomal protein S7
with a threshold of 1e−05
MR Test
Inverse variance weighted (fixed effects)
Inverse variance weighted (multiplicative random effects)
MR Egger
MR RAPS
Weighted median
Weighted mode
0.1
0.0
−0.1
0.1
0.2
SNP effect on 40S ribosomal protein S7
SNP effect on Gut microbiota abundance (genus Parasutterella id.2892)

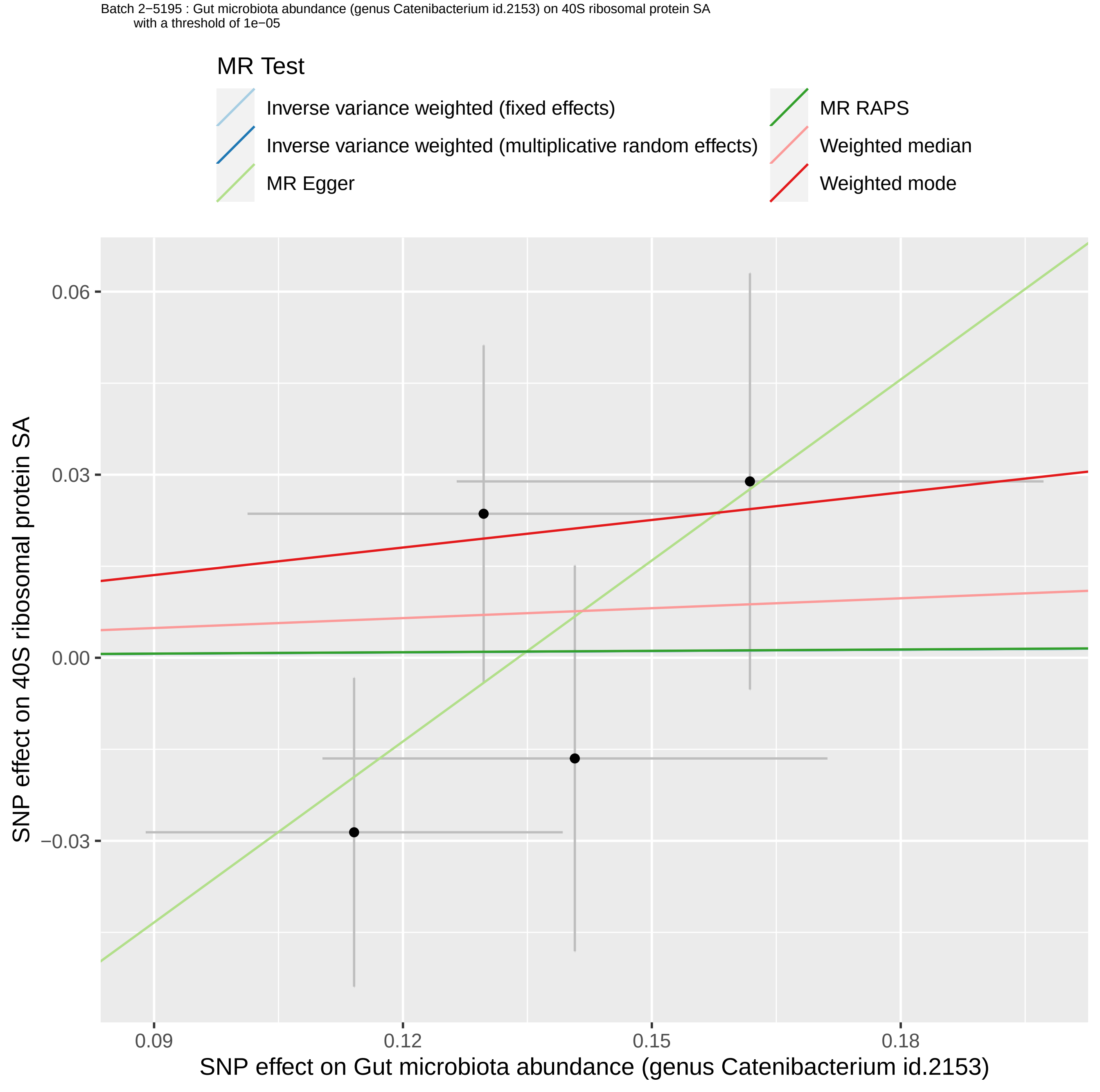
Batch 2−5195 : Gut microbiota abundance (genus Catenibacterium id.2153) on 40S ribosomal protein SA
with a threshold of 1e−05
MR Test
Inverse variance weighted (fixed effects)
Inverse variance weighted (multiplicative random effects)
MR Egger
MR RAPS
Weighted median
Weighted mode
SNP effect on 40S ribosomal protein SA
0.06
0.03
0.00
−0.03
0.09
0.12
0.15
0.18
SNP effect on Gut microbiota abundance (genus Catenibacterium id.2153)

Batch 2−5196 : Gut microbiota abundance (genus Parasutterella id.2892) on 40S ribosomal protein SA
with a threshold of 1e−05

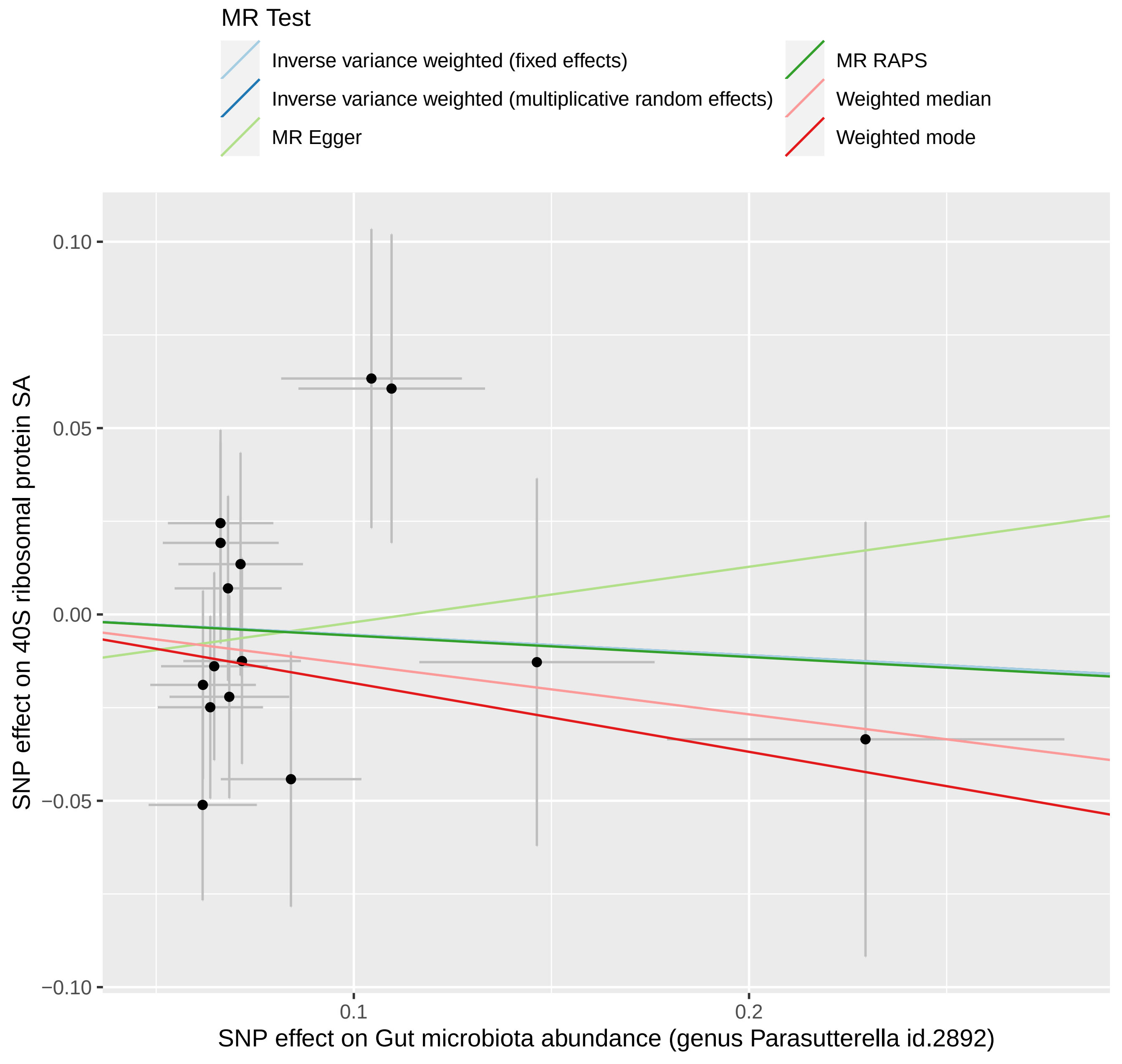

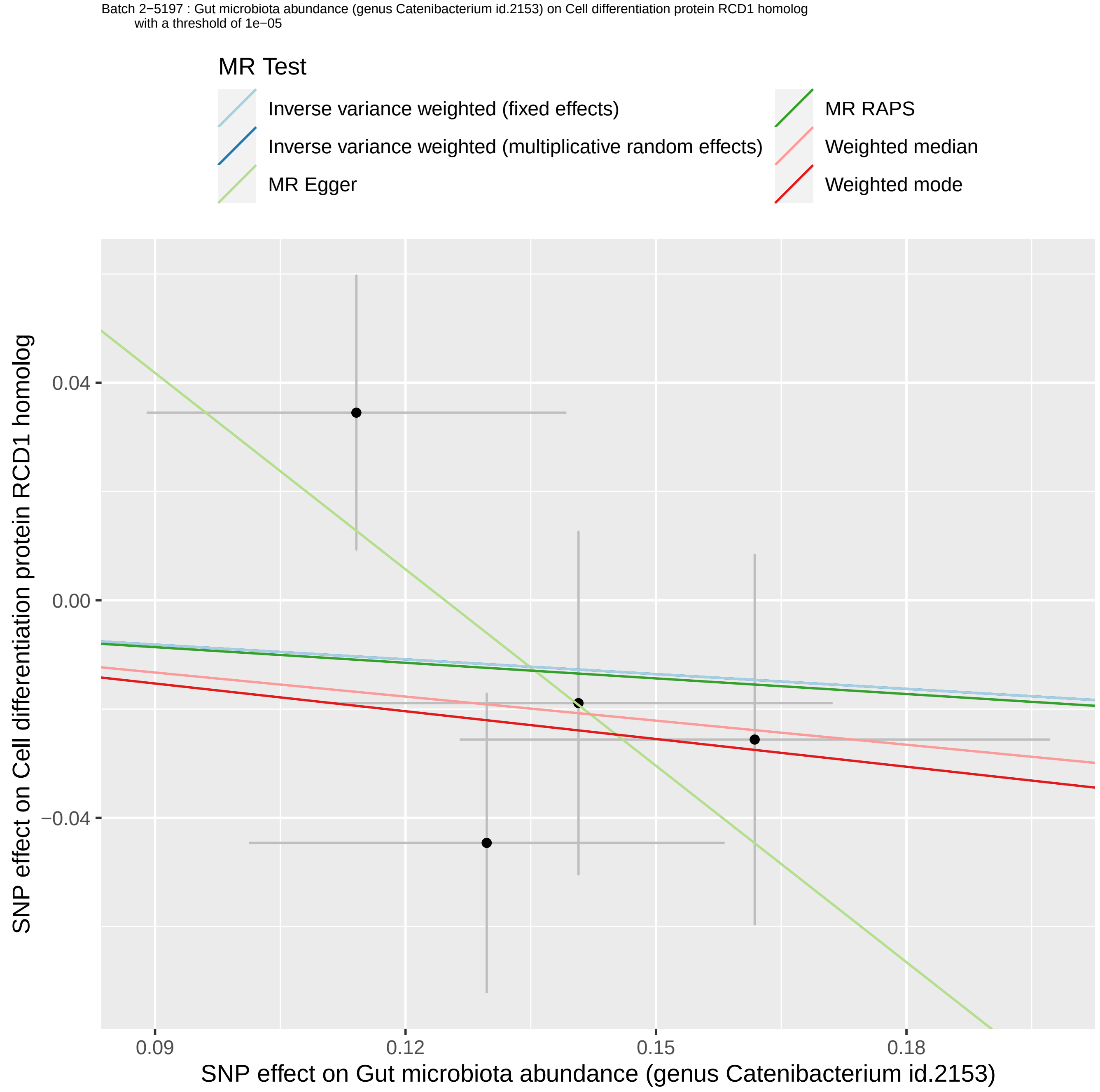

Batch 2−5197 : Gut microbiota abundance (genus Catenibacterium id.2153) on Cell differentiation protein RCD1 homolog
with a threshold of 1e−05
MR Test
Inverse variance weighted (fixed effects)
Inverse variance weighted (multiplicative random effects)
MR Egger
MR RAPS
Weighted median
Weighted mode
SNP effect on Cell differentiation protein RCD1 homolog
0.04
0.00
−0.04
0.09
0.12
0.15
0.18
SNP effect on Gut microbiota abundance (genus Catenibacterium id.2153)

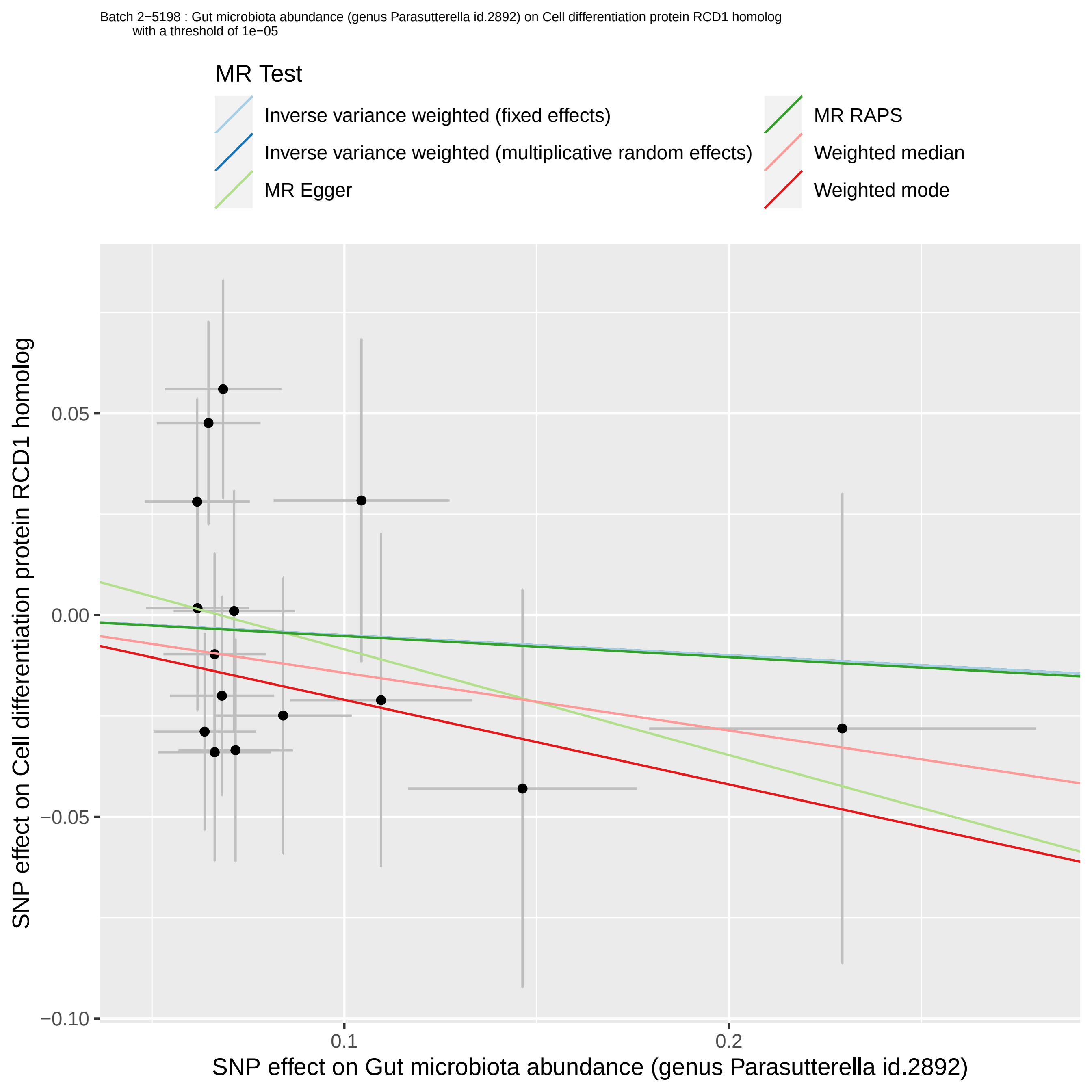
Batch 2−5198 : Gut microbiota abundance (genus Parasutterella id.2892) on Cell differentiation protein RCD1 homolog
with a threshold of 1e−05
MR Test
Inverse variance weighted (fixed effects)
Inverse variance weighted (multiplicative random effects)
MR Egger
MR RAPS
Weighted median
Weighted mode
0.05
0.00
−0.05
−0.10
0.1
0.2
SNP effect on Cell differentiation protein RCD1 homolog
SNP effect on Gut microbiota abundance (genus Parasutterella id.2892)

Batch 2−5199 : Gut microbiota abundance (genus Catenibacterium id.2153) on Ribosome−binding protein 1
with a threshold of 1e−05

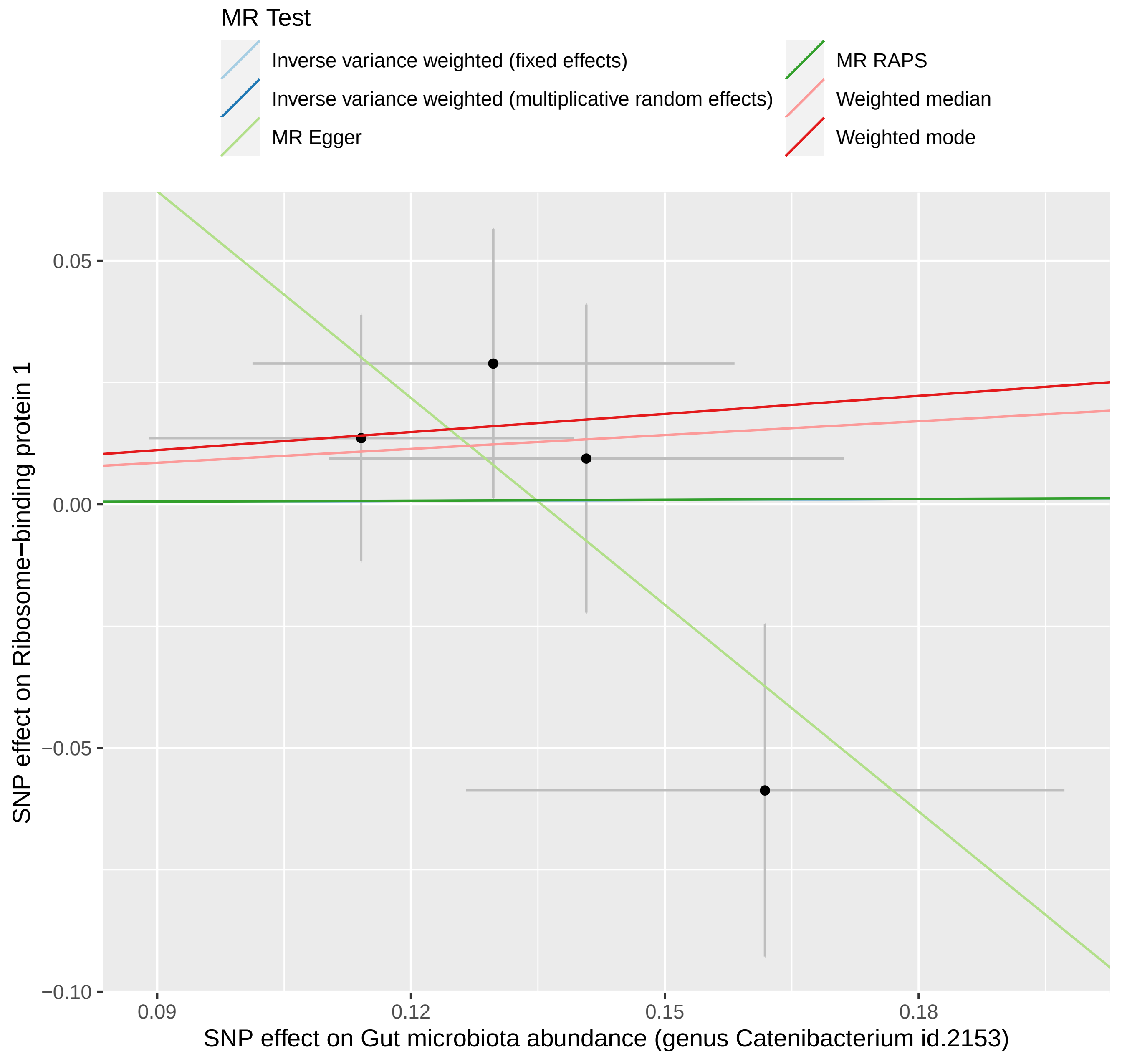

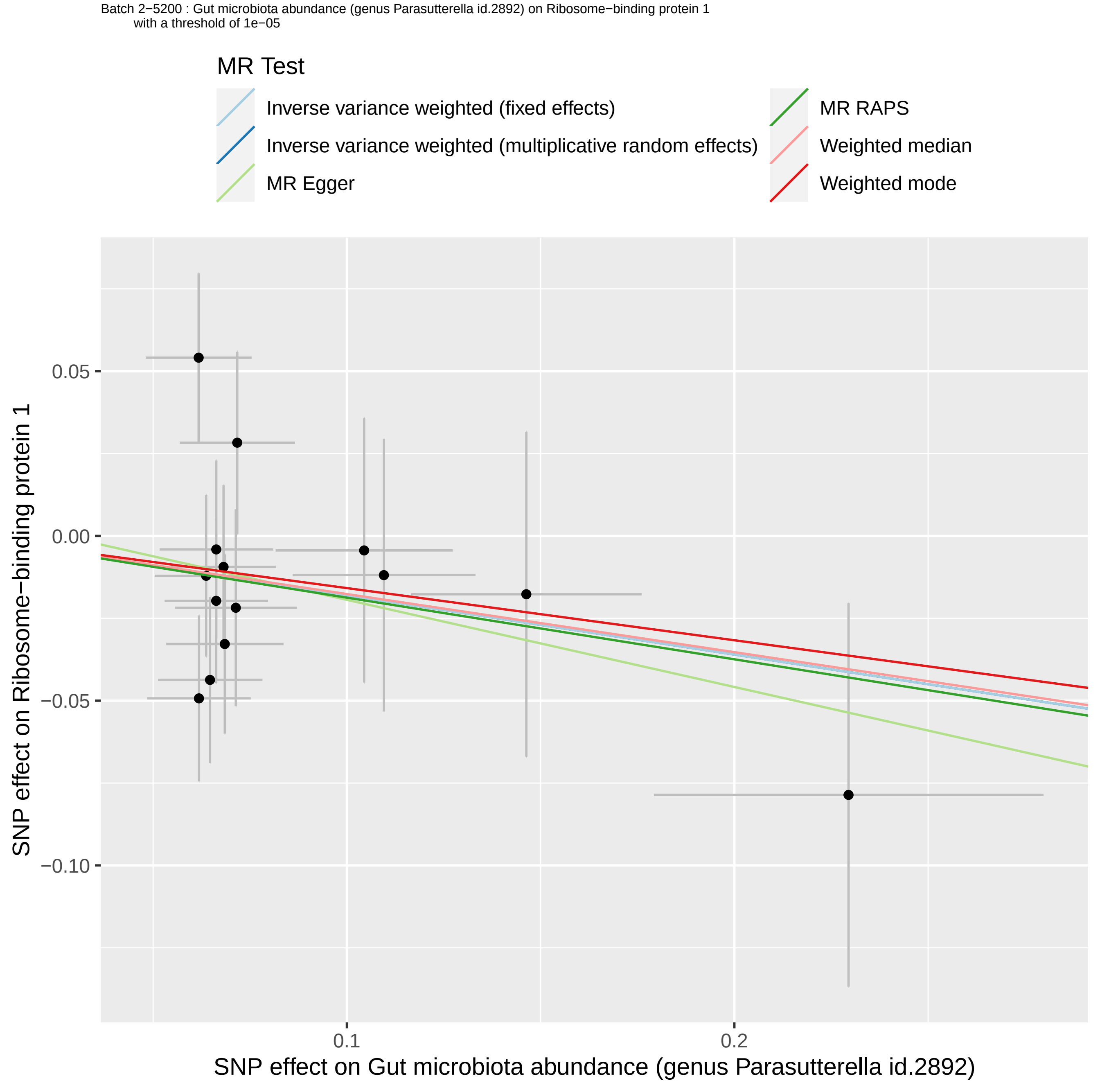

Batch 2−5200 : Gut microbiota abundance (genus Parasutterella id.2892) on Ribosome−binding protein 1
with a threshold of 1e−05
MR Test
Inverse variance weighted (fixed effects)
Inverse variance weighted (multiplicative random effects)
MR Egger
MR RAPS
Weighted median
Weighted mode
0.05
0.00
−0.05
−0.10
0.1
0.2
SNP effect on Ribosome−binding protein 1
SNP effect on Gut microbiota abundance (genus Parasutterella id.2892)

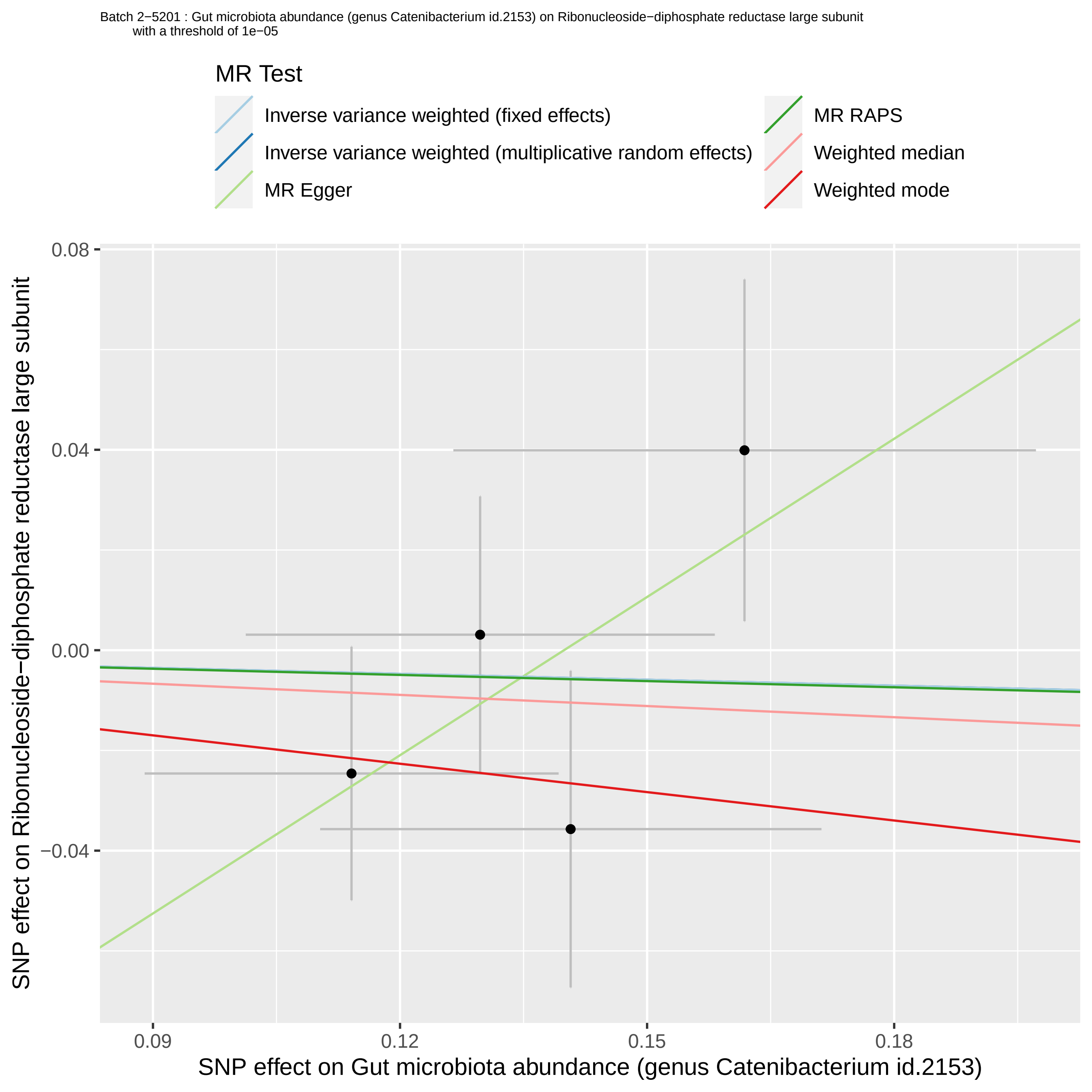

Batch 2−5201 : Gut microbiota abundance (genus Catenibacterium id.2153) on Ribonucleoside−diphosphate reductase large subunit with a threshold of 1e−05
MR Test
Inverse variance weighted (fixed effects)
Inverse variance weighted (multiplicative random effects)
MR Egger
MR RAPS
Weighted median
Weighted mode
0.08
0.04
0.00
−0.04
0.09
0.12
0.15
0.18
SNP effect on Ribonucleoside−diphosphate reductase large subunit
SNP effect on Gut microbiota abundance (genus Catenibacterium id.2153)

Batch 2−5202 : Gut microbiota abundance (genus Parasutterella id.2892) on Ribonucleoside−diphosphate reductase large subunit
with a threshold of 1e−05

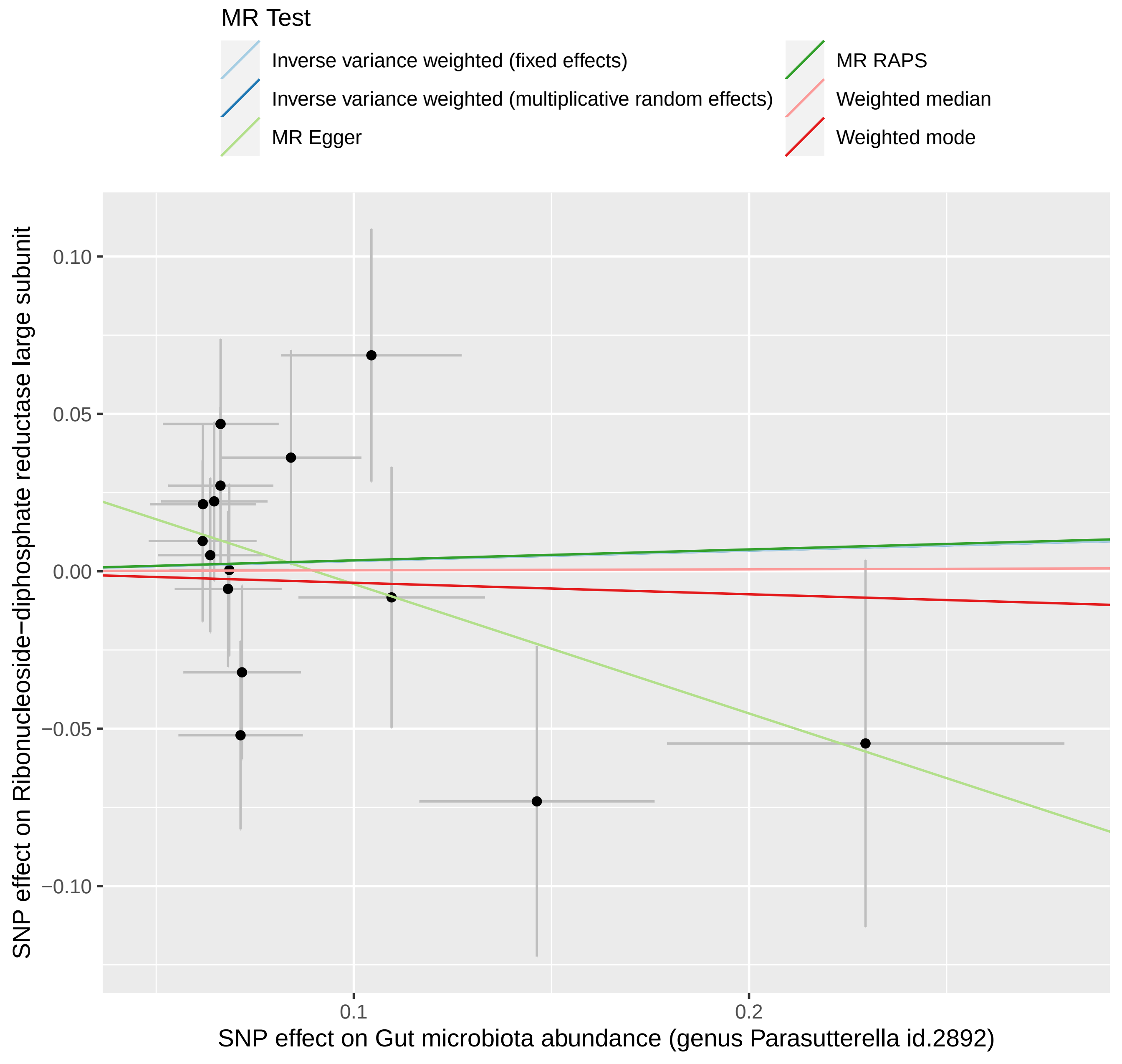

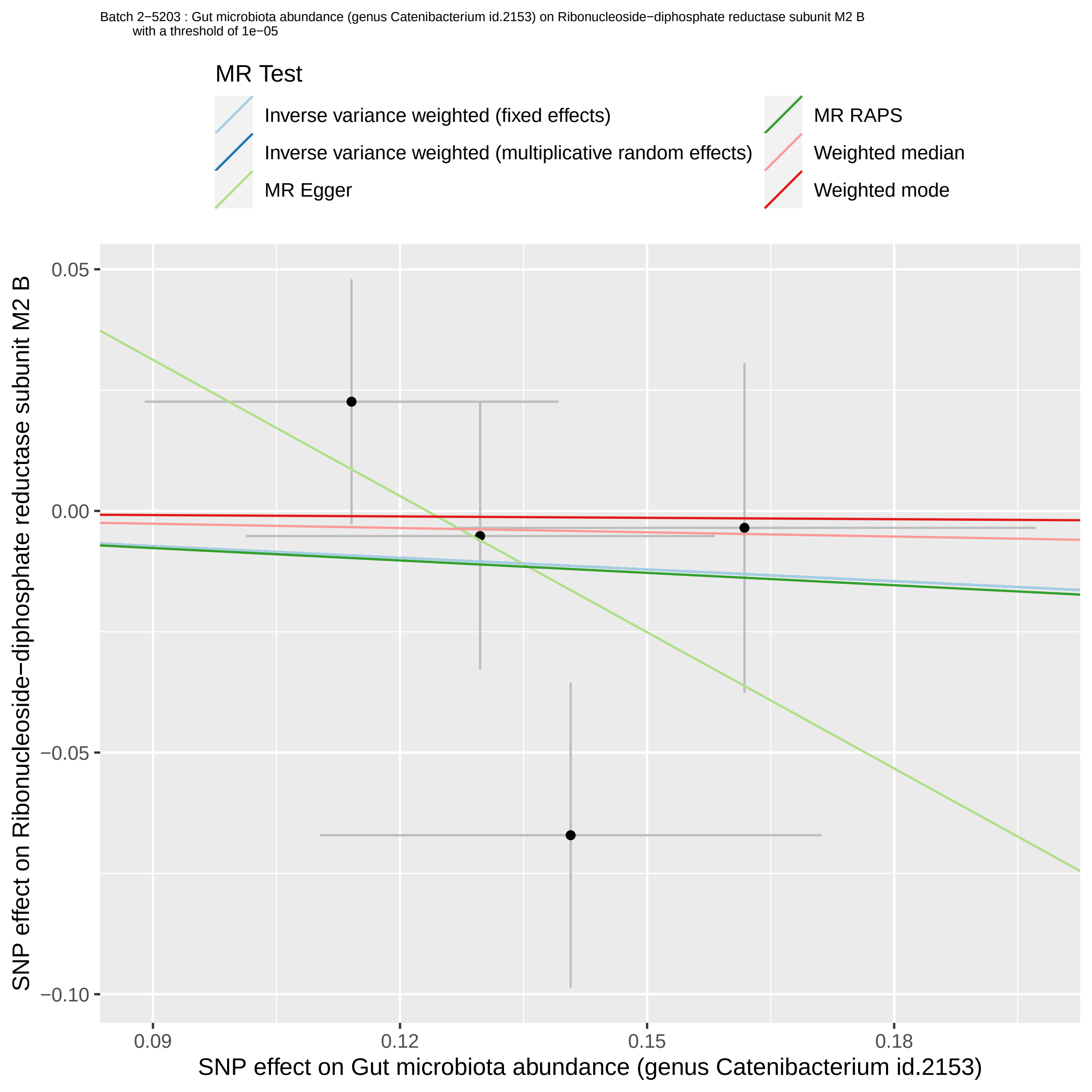
Batch 2−5203 : Gut microbiota abundance (genus Catenibacterium id.2153) on Ribonucleoside−diphosphate reductase subunit M2 B
with a threshold of 1e−05
MR Test
Inverse variance weighted (fixed effects)
Inverse variance weighted (multiplicative random effects)
MR Egger
MR RAPS
Weighted median
Weighted mode
SNP effect on Ribonucleoside−diphosphate reductase subunit M2 B
SNP effect on Gut microbiota abundance (genus Catenibacterium id.2153)
0.05
0.00
−0.05
−0.10
0.09
0.12
0.15
0.18

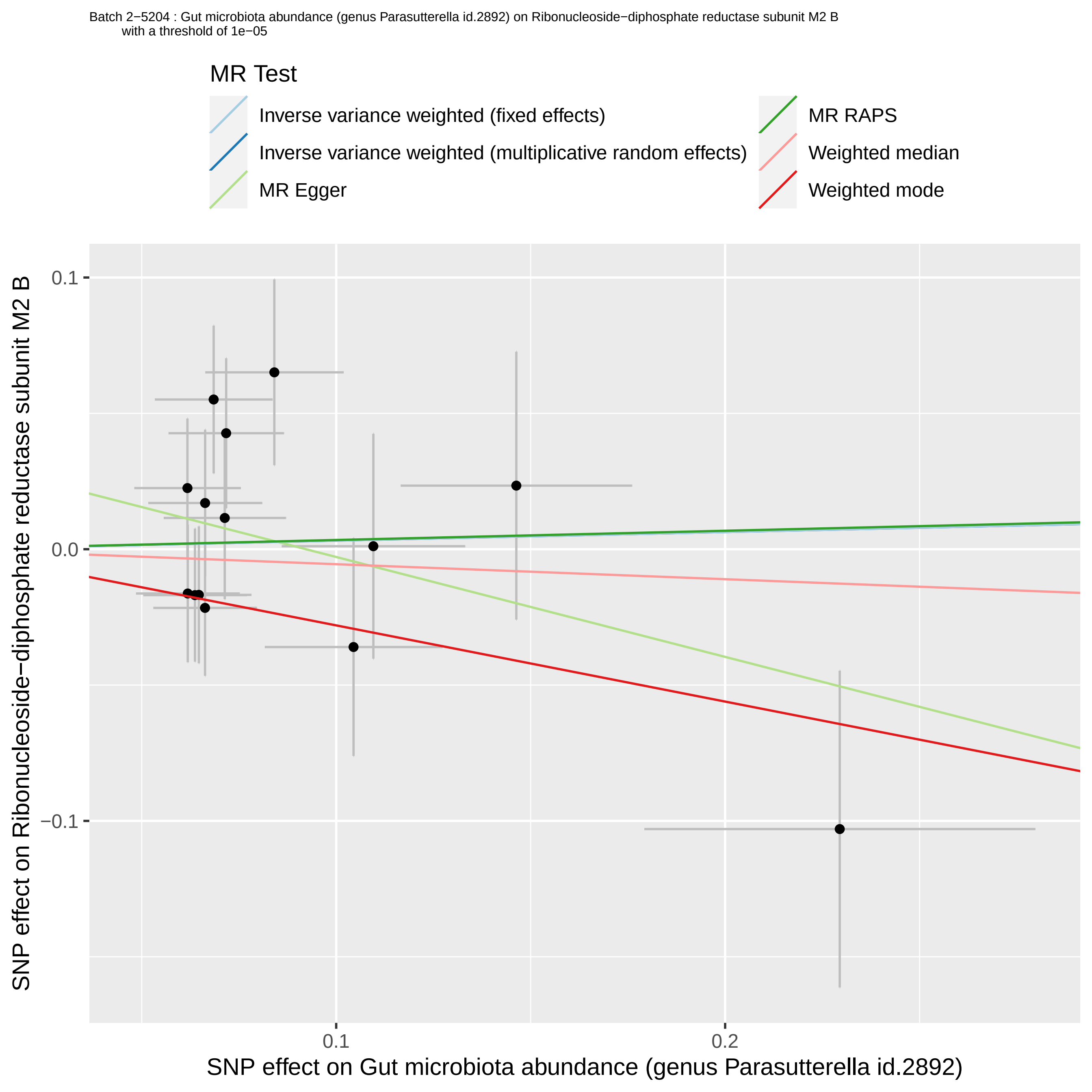

Batch 2−5204 : Gut microbiota abundance (genus Parasutterella id.2892) on Ribonucleoside−diphosphate reductase subunit M2 B
with a threshold of 1e−05
MR Test
Inverse variance weighted (fixed effects)
Inverse variance weighted (multiplicative random effects)
MR Egger
MR RAPS
Weighted median
Weighted mode
0.1
0.0
−0.1
0.1
0.2
SNP effect on Ribonucleoside−diphosphate reductase subunit M2 B
SNP effect on Gut microbiota abundance (genus Parasutterella id.2892)

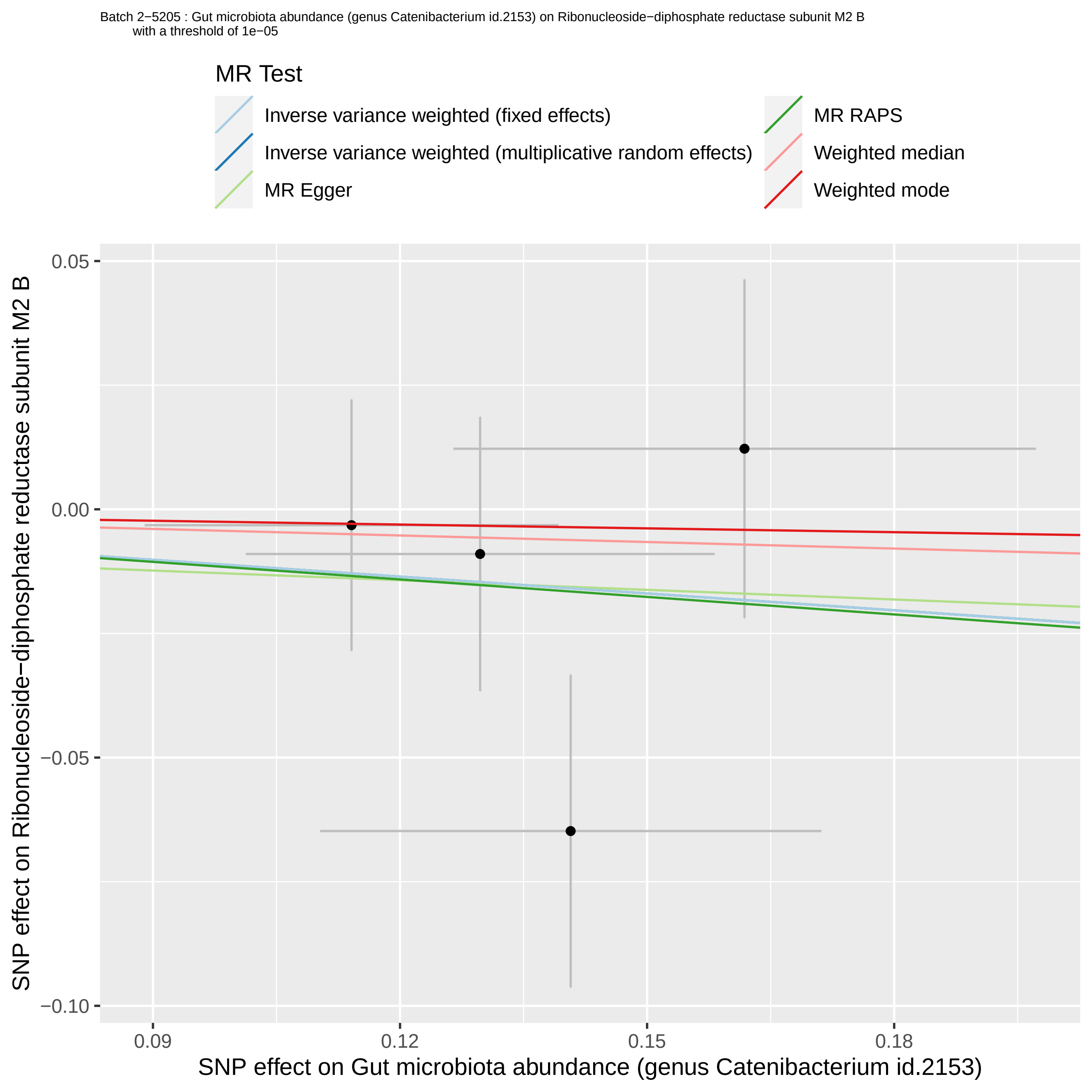

Batch 2−5205 : Gut microbiota abundance (genus Catenibacterium id.2153) on Ribonucleoside−diphosphate reductase subunit M2 B
with a threshold of 1e−05
MR Test
Inverse variance weighted (fixed effects)
Inverse variance weighted (multiplicative random effects)
MR Egger
MR RAPS
Weighted median
Weighted mode
0.05
0.00
−0.05
−0.10
0.09
0.12
0.15
0.18
SNP effect on Ribonucleoside−diphosphate reductase subunit M2 B
SNP effect on Gut microbiota abundance (genus Catenibacterium id.2153)

Batch 2−5206 : Gut microbiota abundance (genus Parasutterella id.2892) on Ribonucleoside−diphosphate reductase subunit M2 B
with a threshold of 1e−05

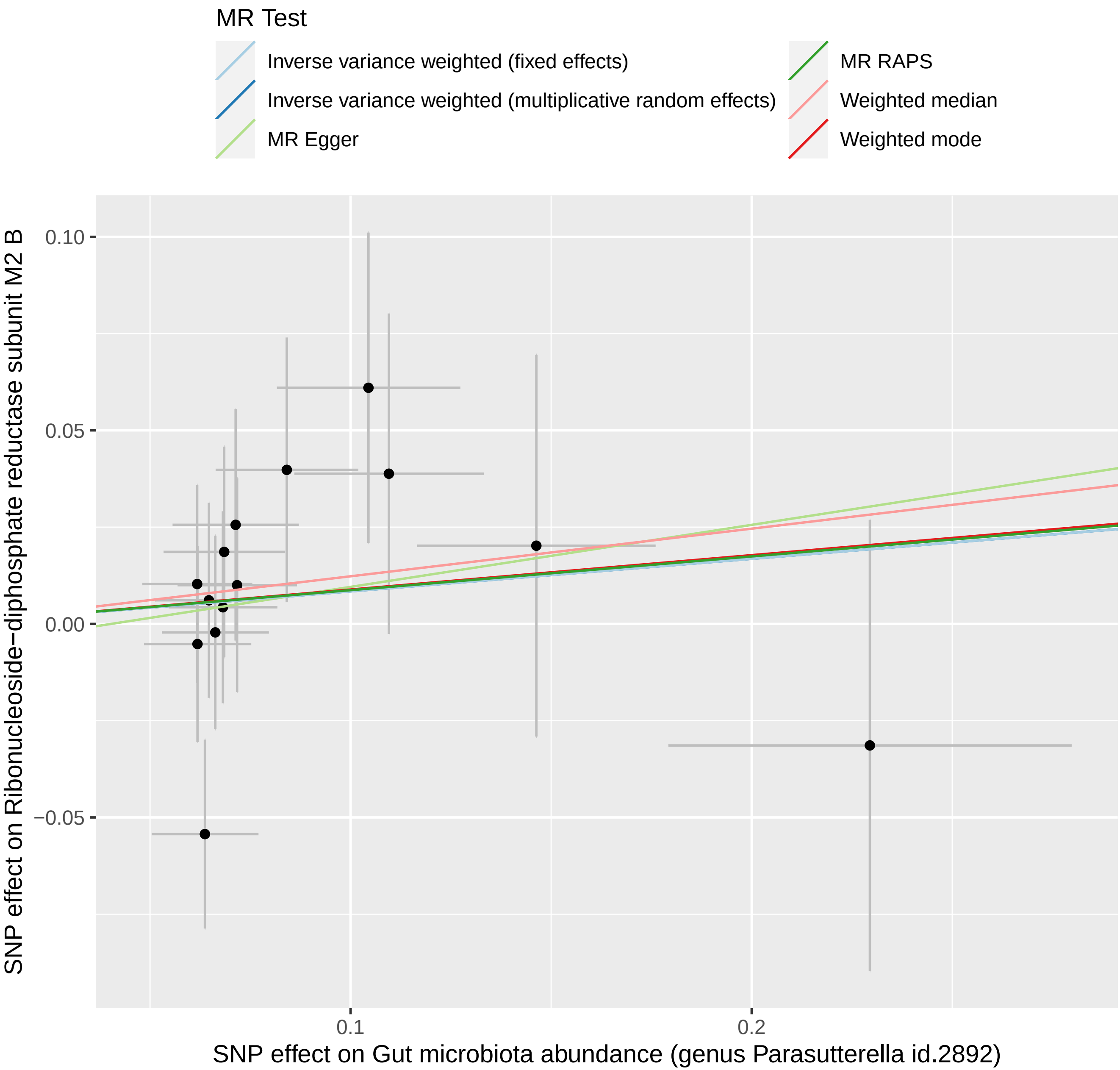

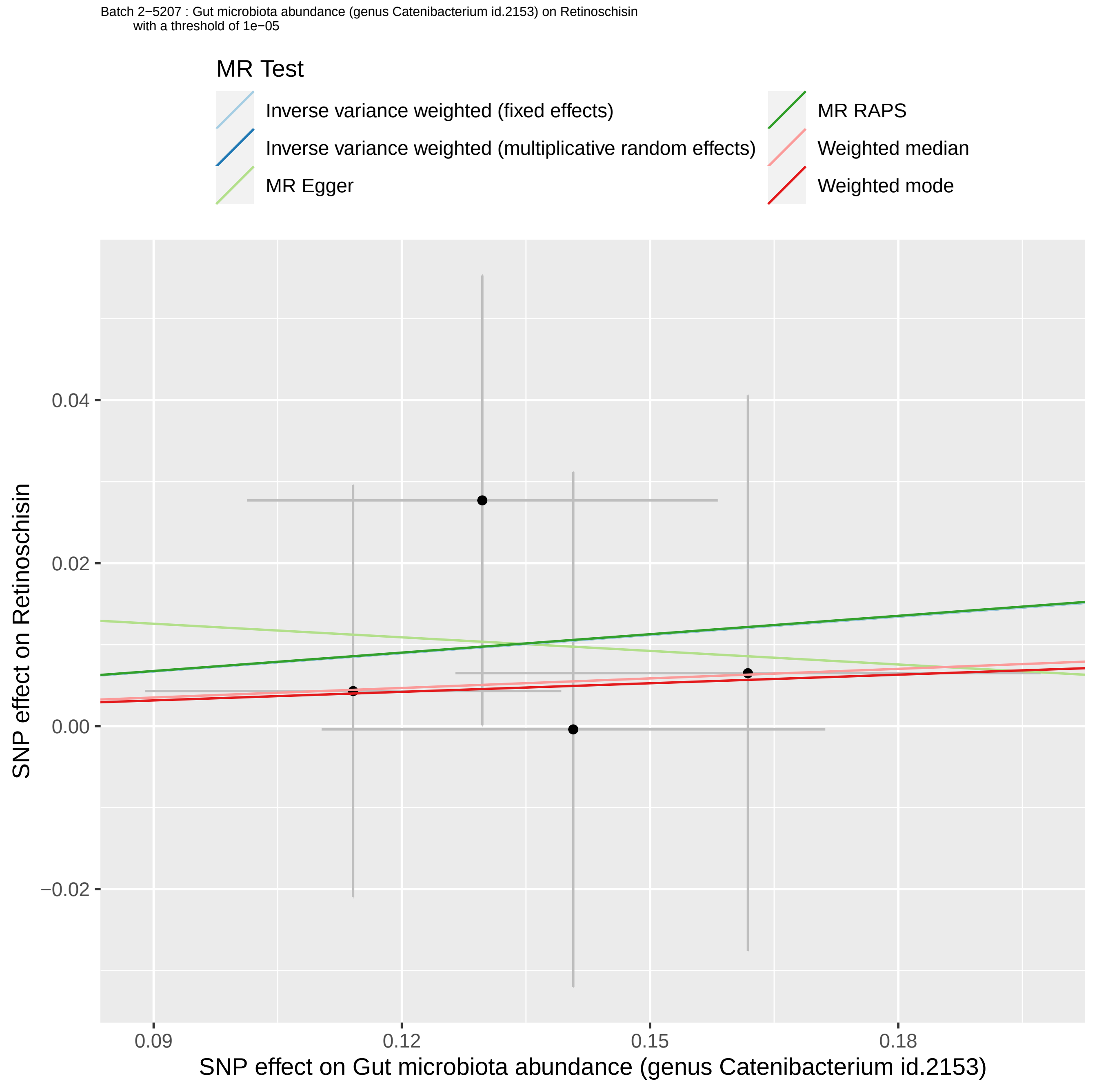
Batch 2−5207 : Gut microbiota abundance (genus Catenibacterium id.2153) on Retinoschisin
with a threshold of 1e−05
MR Test
Inverse variance weighted (fixed effects)
Inverse variance weighted (multiplicative random effects)
MR Egger
MR RAPS
Weighted median
Weighted mode
0.04
0.02
0.00
−0.02
0.09
0.12
0.15
0.18
SNP effect on Retinoschisin
SNP effect on Gut microbiota abundance (genus Catenibacterium id.2153)

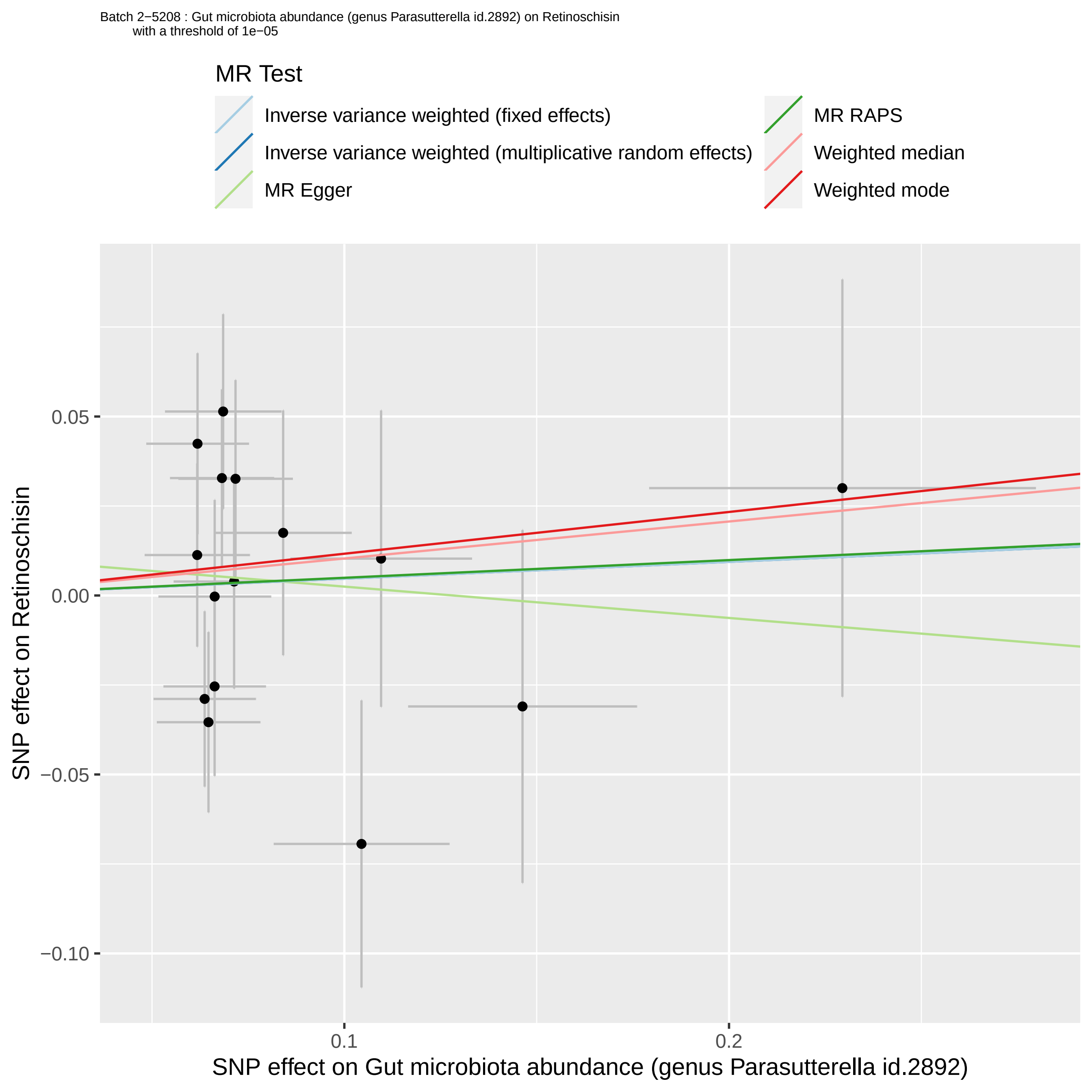
Batch 2−5208 : Gut microbiota abundance (genus Parasutterella id.2892) on Retinoschisin
with a threshold of 1e−05
MR Test
Inverse variance weighted (fixed effects)
Inverse variance weighted (multiplicative random effects)
MR Egger
MR RAPS
Weighted median
Weighted mode
0.05
0.00
−0.05
−0.10
0.1
0.2
SNP effect on Retinoschisin
SNP effect on Gut microbiota abundance (genus Parasutterella id.2892)

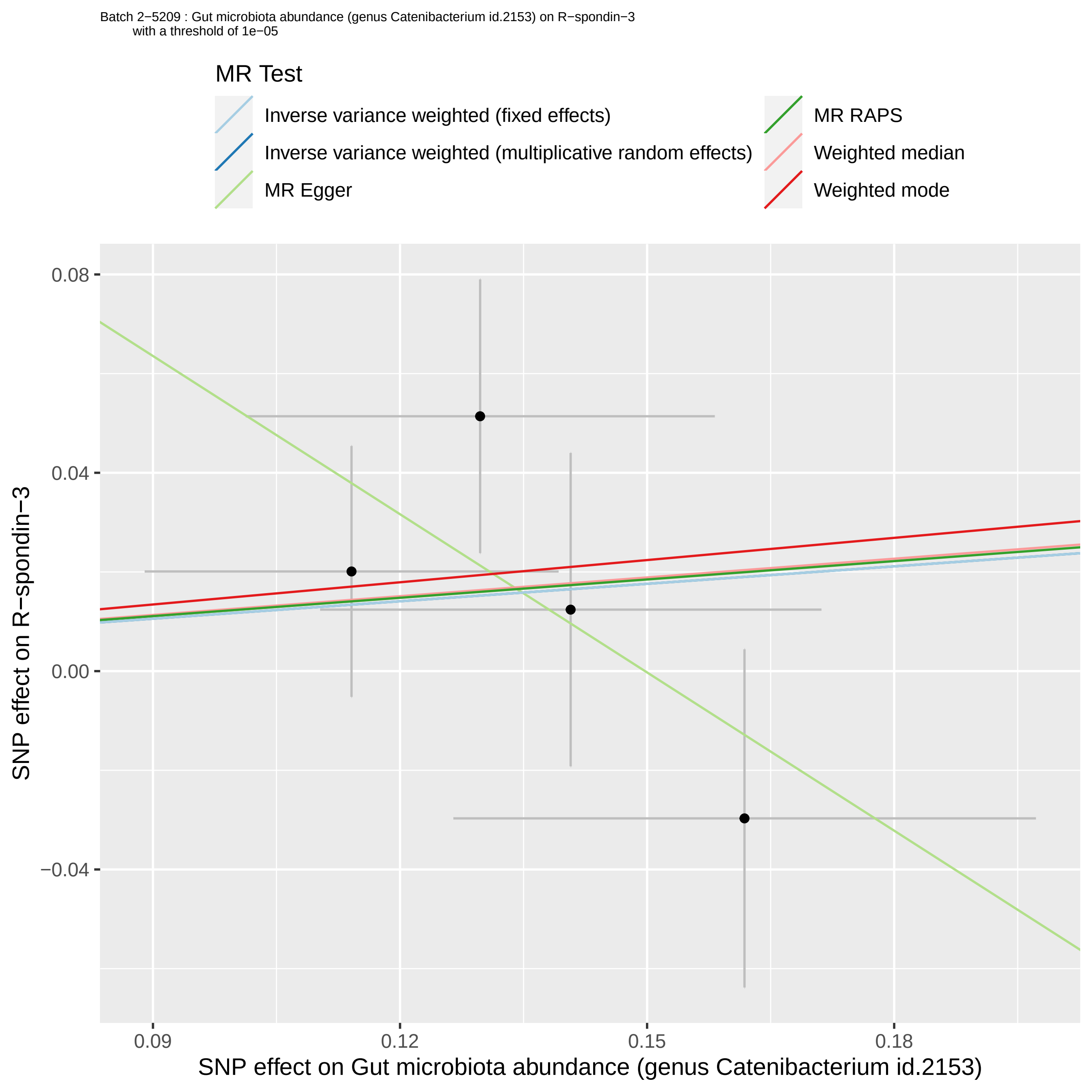
Batch 2−5209 : Gut microbiota abundance (genus Catenibacterium id.2153) on R−spondin−3
with a threshold of 1e−05
MR Test
Inverse variance weighted (fixed effects)
Inverse variance weighted (multiplicative random effects)
MR Egger
MR RAPS
Weighted median
Weighted mode
0.08
0.04
0.00
−0.04
0.09
0.12
0.15
0.18
SNP effect on R−spondin−3
SNP effect on Gut microbiota abundance (genus Catenibacterium id.2153)

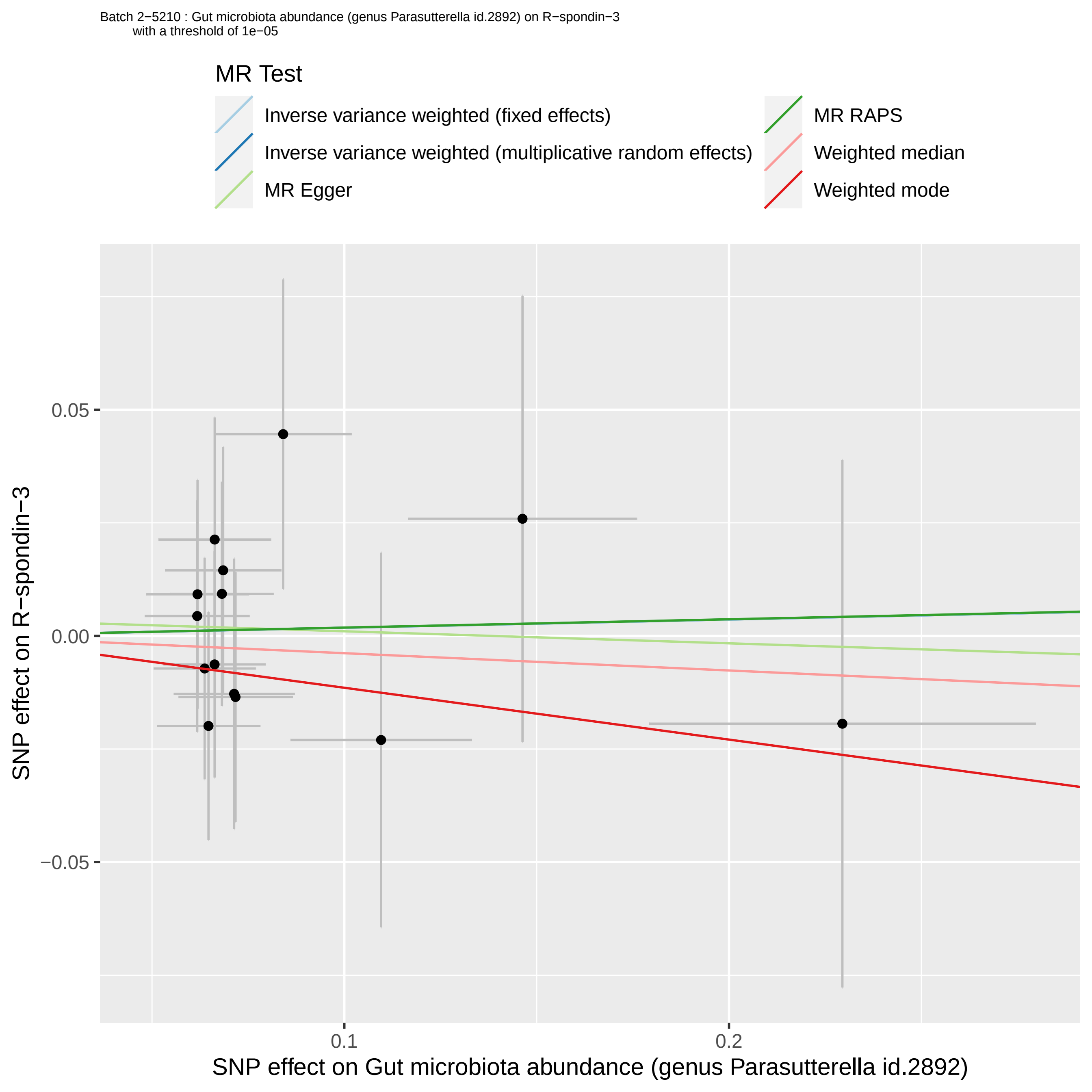
Batch 2−5210 : Gut microbiota abundance (genus Parasutterella id.2892) on R−spondin−3
with a threshold of 1e−05
MR Test
Inverse variance weighted (fixed effects)
Inverse variance weighted (multiplicative random effects)
MR Egger
MR RAPS
Weighted median
Weighted mode
0.05
0.00
−0.05
0.1
0.2
SNP effect on R−spondin−3
SNP effect on Gut microbiota abundance (genus Parasutterella id.2892)

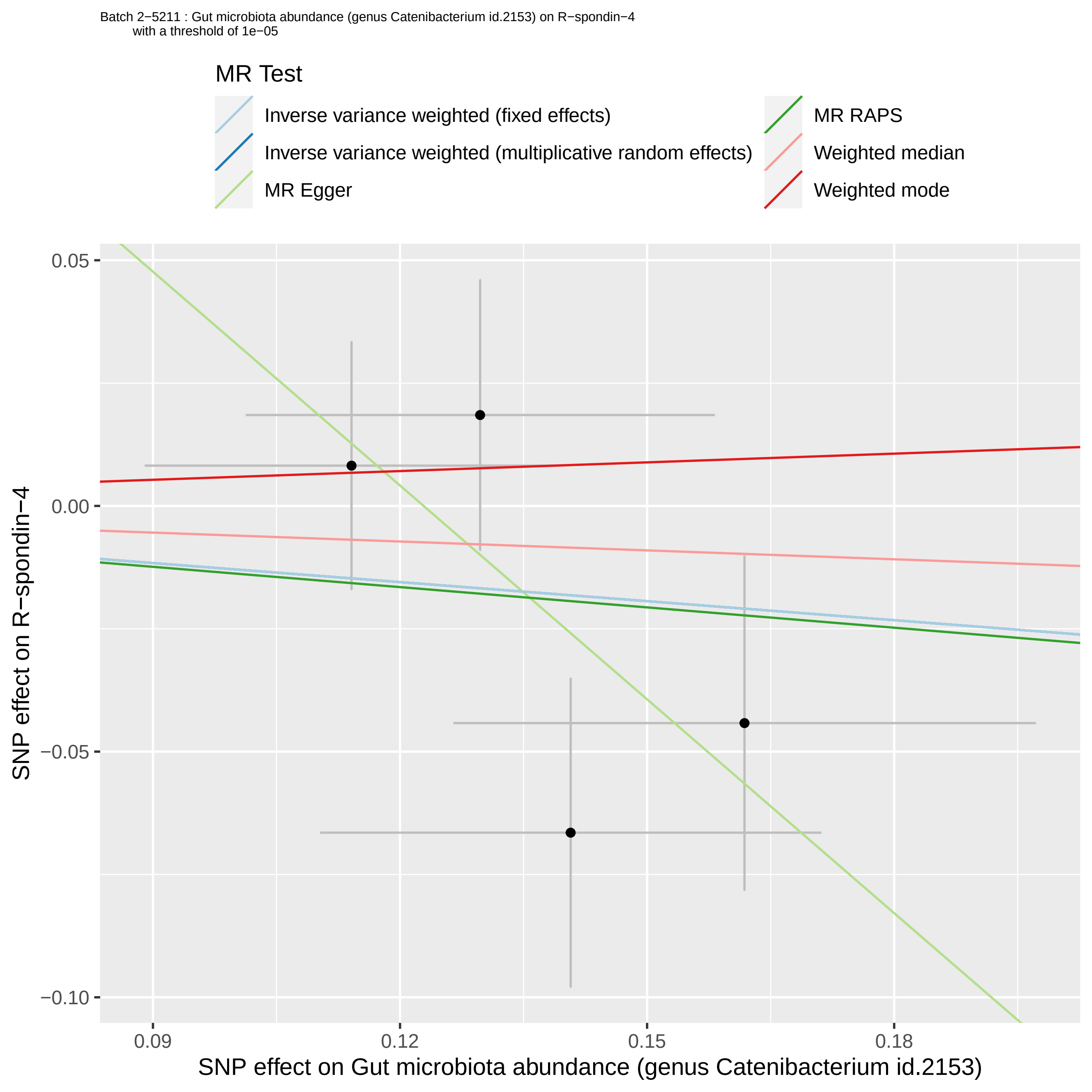

Batch 2−5211 : Gut microbiota abundance (genus Catenibacterium id.2153) on R−spondin−4
with a threshold of 1e−05
MR Test
Inverse variance weighted (fixed effects)
Inverse variance weighted (multiplicative random effects)
MR Egger
MR RAPS
Weighted median
Weighted mode
0.05
0.00
−0.05
−0.10
0.09
0.12
0.15
0.18
SNP effect on R−spondin−4
SNP effect on Gut microbiota abundance (genus Catenibacterium id.2153)

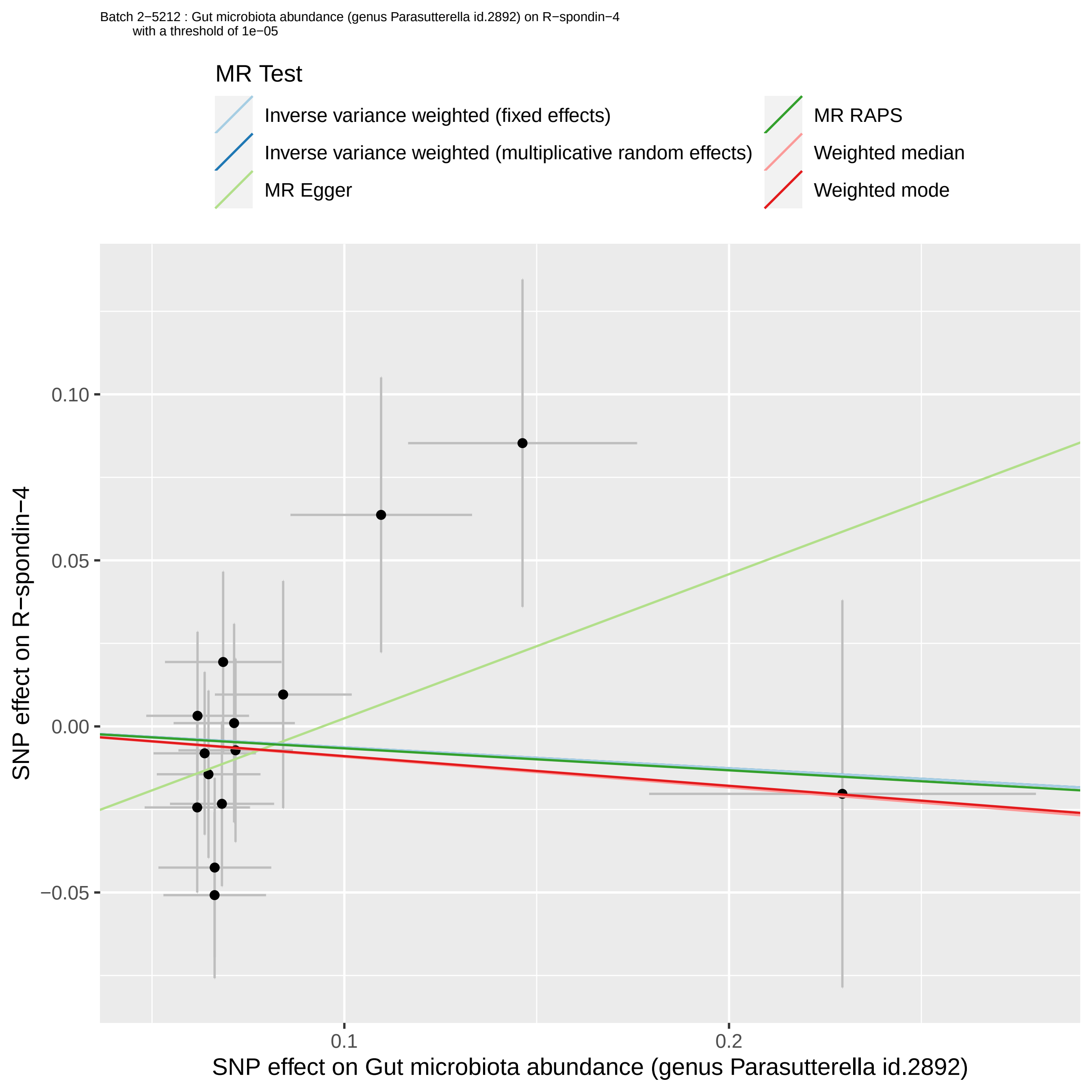
Batch 2−5212 : Gut microbiota abundance (genus Parasutterella id.2892) on R−spondin−4
with a threshold of 1e−05
MR Test
Inverse variance weighted (fixed effects)
Inverse variance weighted (multiplicative random effects)
MR Egger
MR RAPS
Weighted median
Weighted mode
SNP effect on R−spondin−4
0.10
0.05
0.00
−0.05
0.1
0.2
SNP effect on Gut microbiota abundance (genus Parasutterella id.2892)

Batch 2−5213 : Gut microbiota abundance (genus Catenibacterium id.2153) on Arginine/serine−rich protein 1
with a threshold of 1e−05

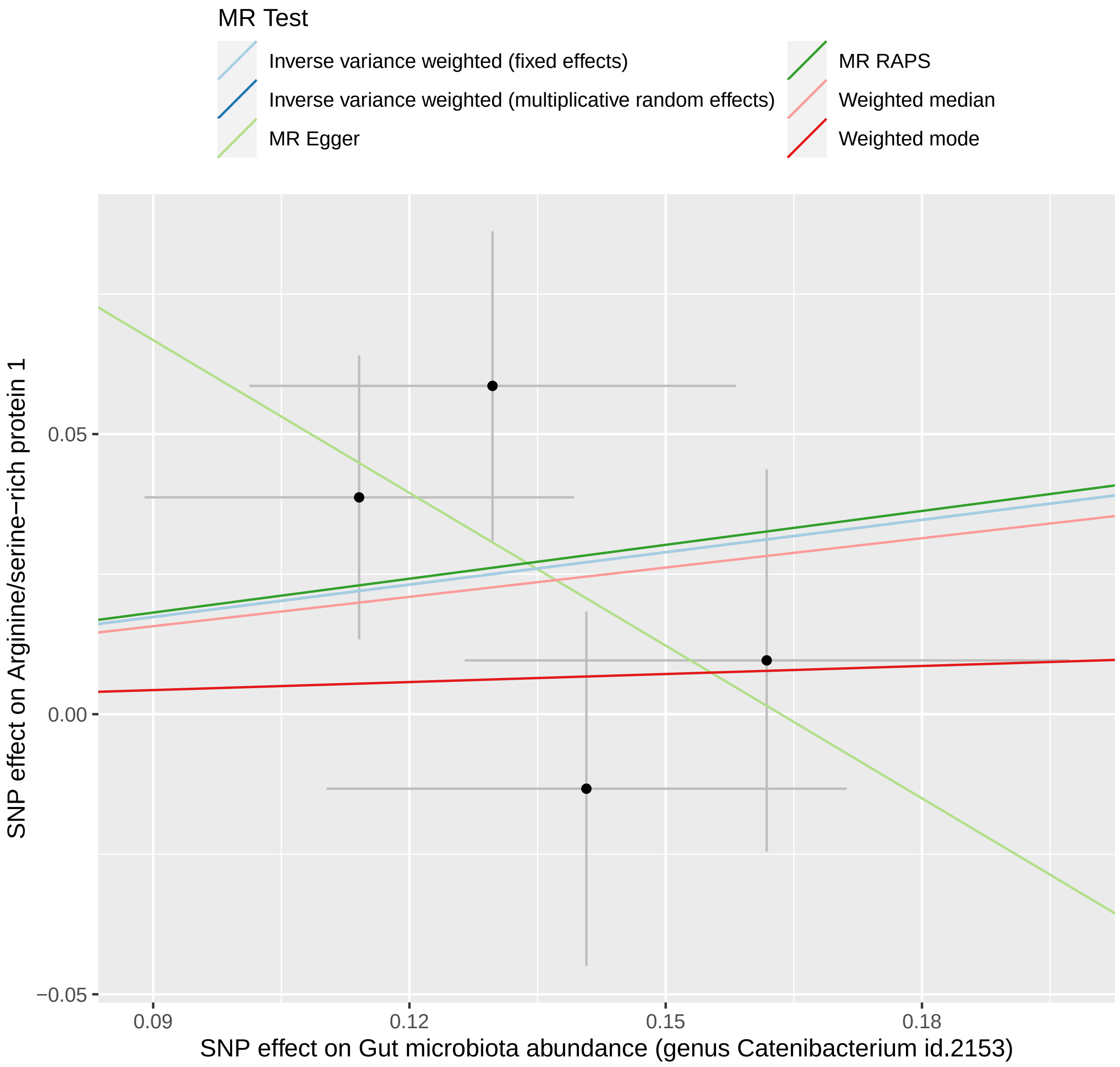

Batch 2−5214 : Gut microbiota abundance (genus Parasutterella id.2892) on Arginine/serine−rich protein 1
with a threshold of 1e−05

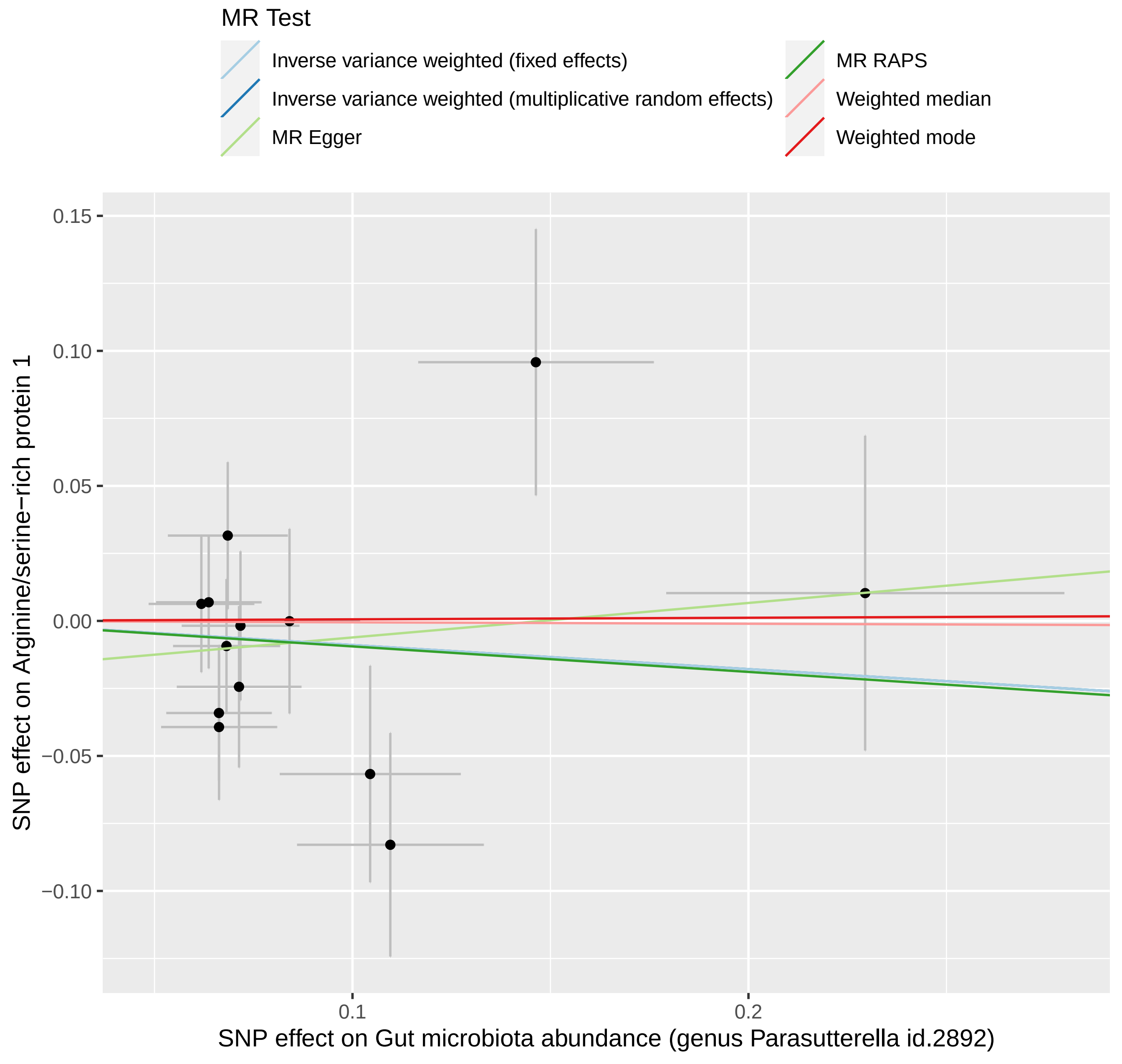

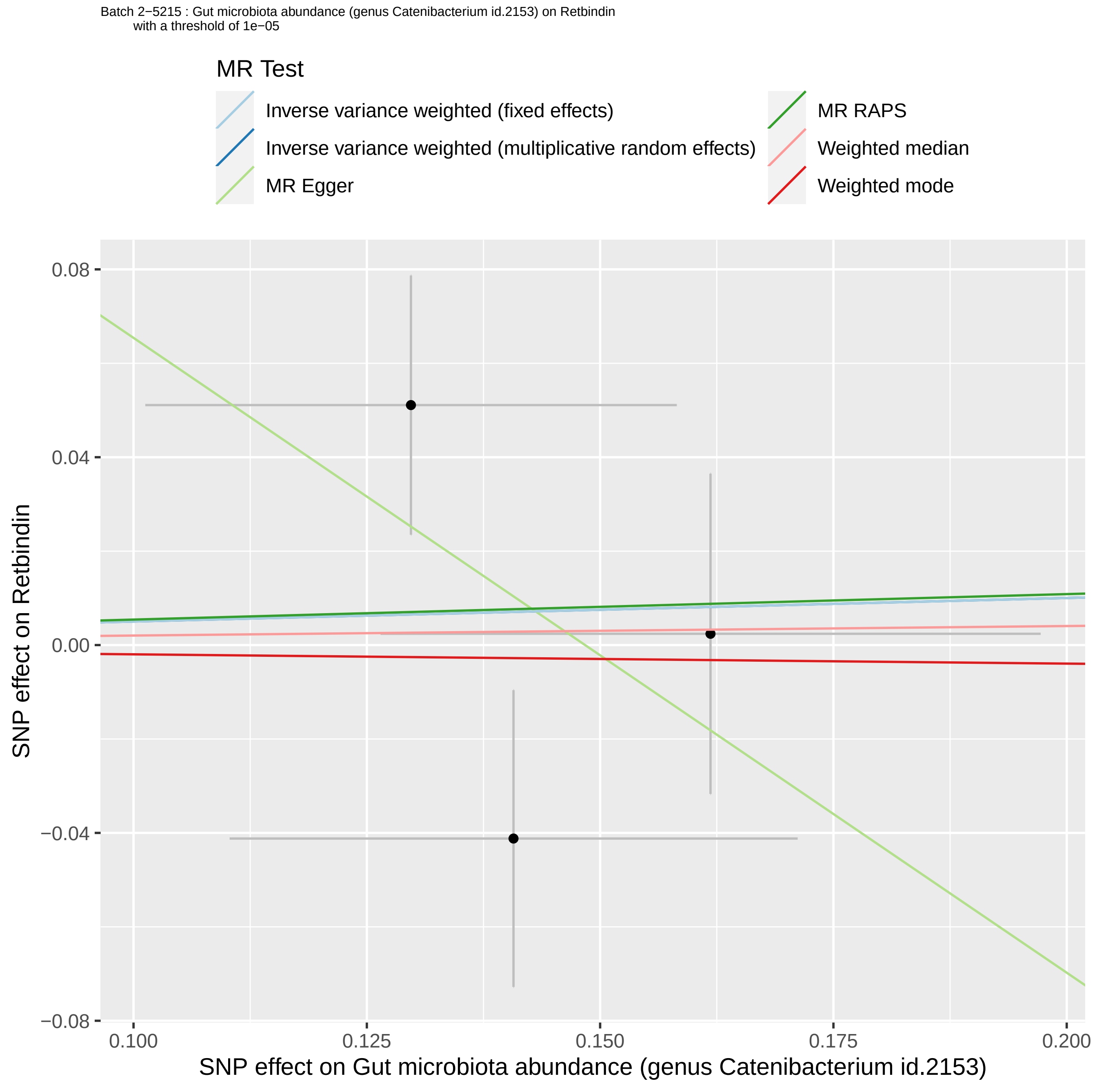
Batch 2−5215 : Gut microbiota abundance (genus Catenibacterium id.2153) on Retbindin
with a threshold of 1e−05
MR Test
Inverse variance weighted (fixed effects)
Inverse variance weighted (multiplicative random effects)
MR Egger
MR RAPS
Weighted median
Weighted mode
0.08
0.04
0.00
−0.04
−0.08
0.100
0.125
0.150
0.175
0.200
SNP effect on Retbindin
SNP effect on Gut microbiota abundance (genus Catenibacterium id.2153)

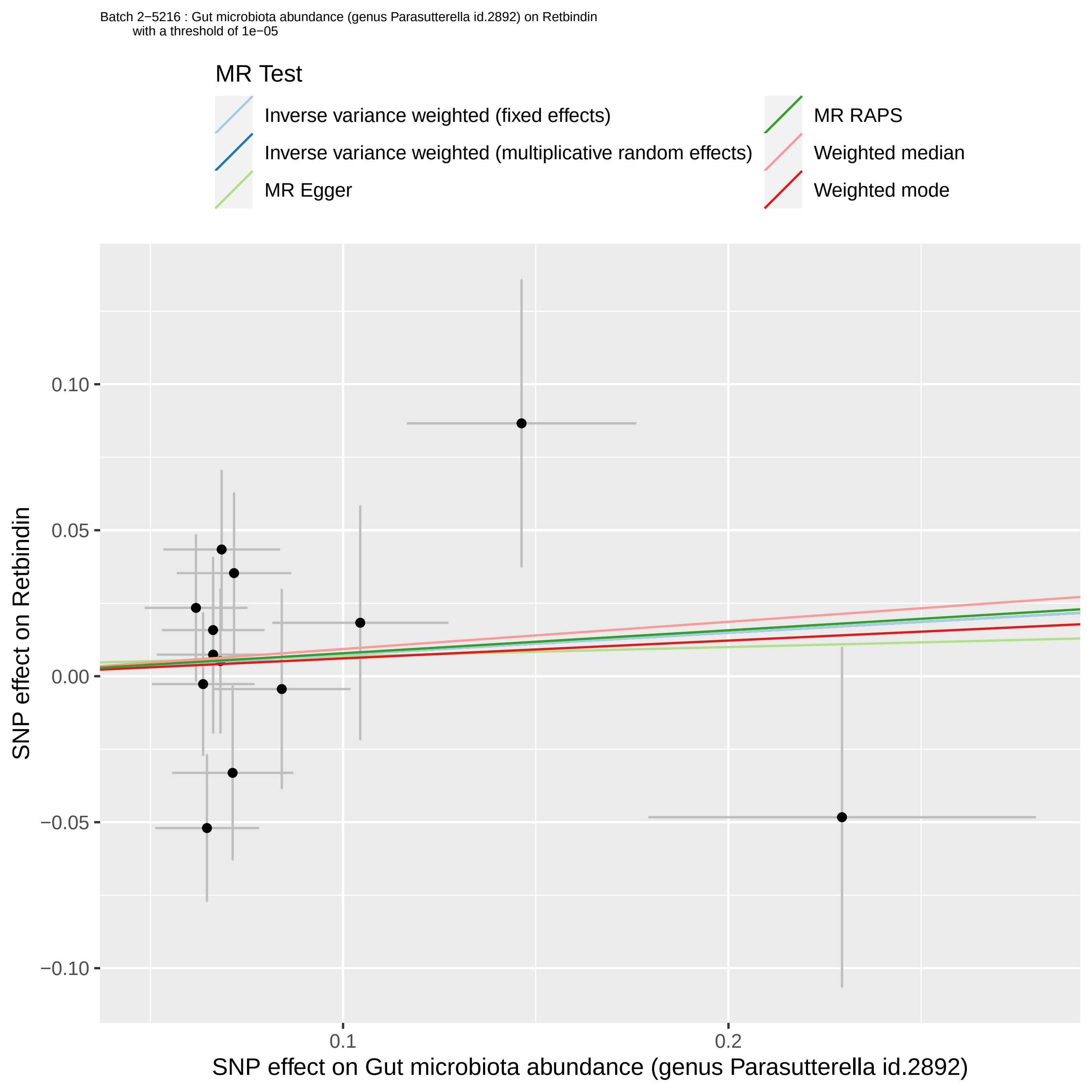

Batch 2−5216 : Gut microbiota abundance (genus Parasutterella id.2892) on Retbindin
with a threshold of 1e−05
MR Test
Inverse variance weighted (fixed effects)
Inverse variance weighted (multiplicative random effects)
MR Egger
MR RAPS
Weighted median
Weighted mode
0.10
0.05
0.00
−0.05
−0.10
SNP effect on Retbindin
0.1
0.2
SNP effect on Gut microbiota abundance (genus Parasutterella id.2892)

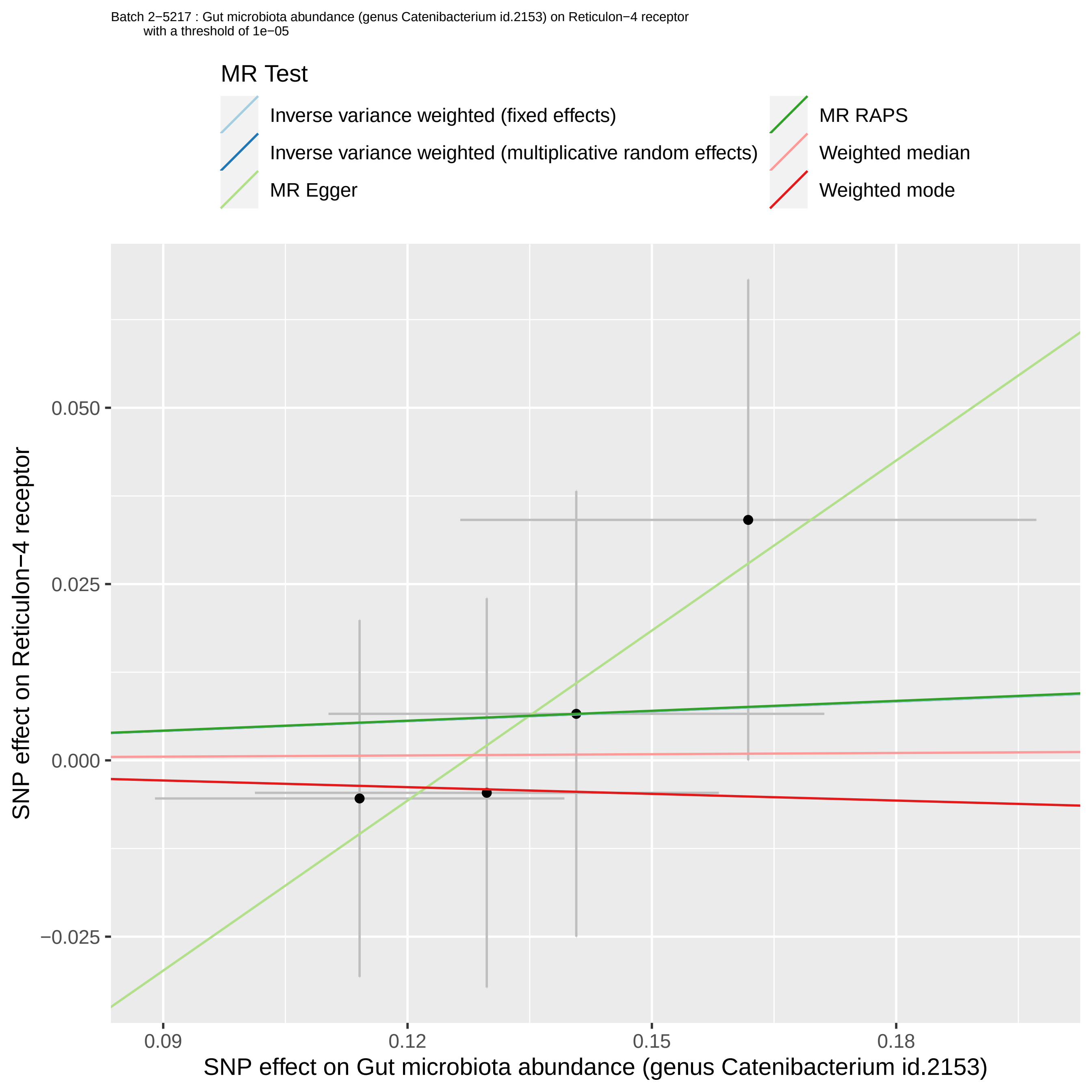
Batch 2−5217 : Gut microbiota abundance (genus Catenibacterium id.2153) on Reticulon−4 receptor
with a threshold of 1e−05
MR Test
Inverse variance weighted (fixed effects)
Inverse variance weighted (multiplicative random effects)
MR Egger
MR RAPS
Weighted median
Weighted mode
0.050
0.025
0.000
−0.025
0.09
0.12
0.15
0.18
SNP effect on Reticulon−4 receptor
SNP effect on Gut microbiota abundance (genus Catenibacterium id.2153)

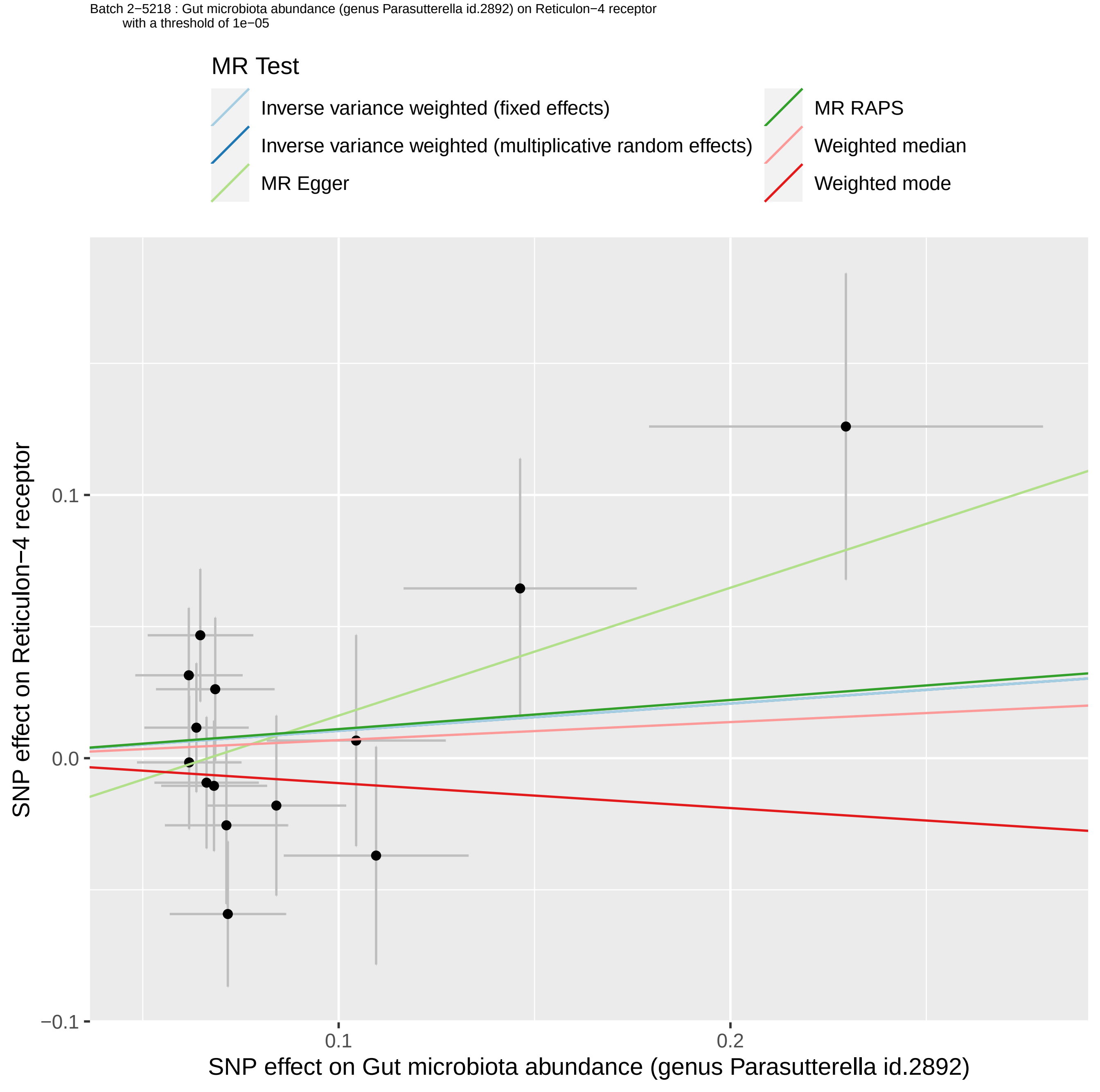
Batch 2−5218 : Gut microbiota abundance (genus Parasutterella id.2892) on Reticulon−4 receptor
with a threshold of 1e−05
MR Test
Inverse variance weighted (fixed effects)
Inverse variance weighted (multiplicative random effects)
MR Egger
MR RAPS
Weighted median
Weighted mode
0.1
0.0
−0.1
0.1
0.2
SNP effect on Reticulon−4 receptor
SNP effect on Gut microbiota abundance (genus Parasutterella id.2892)

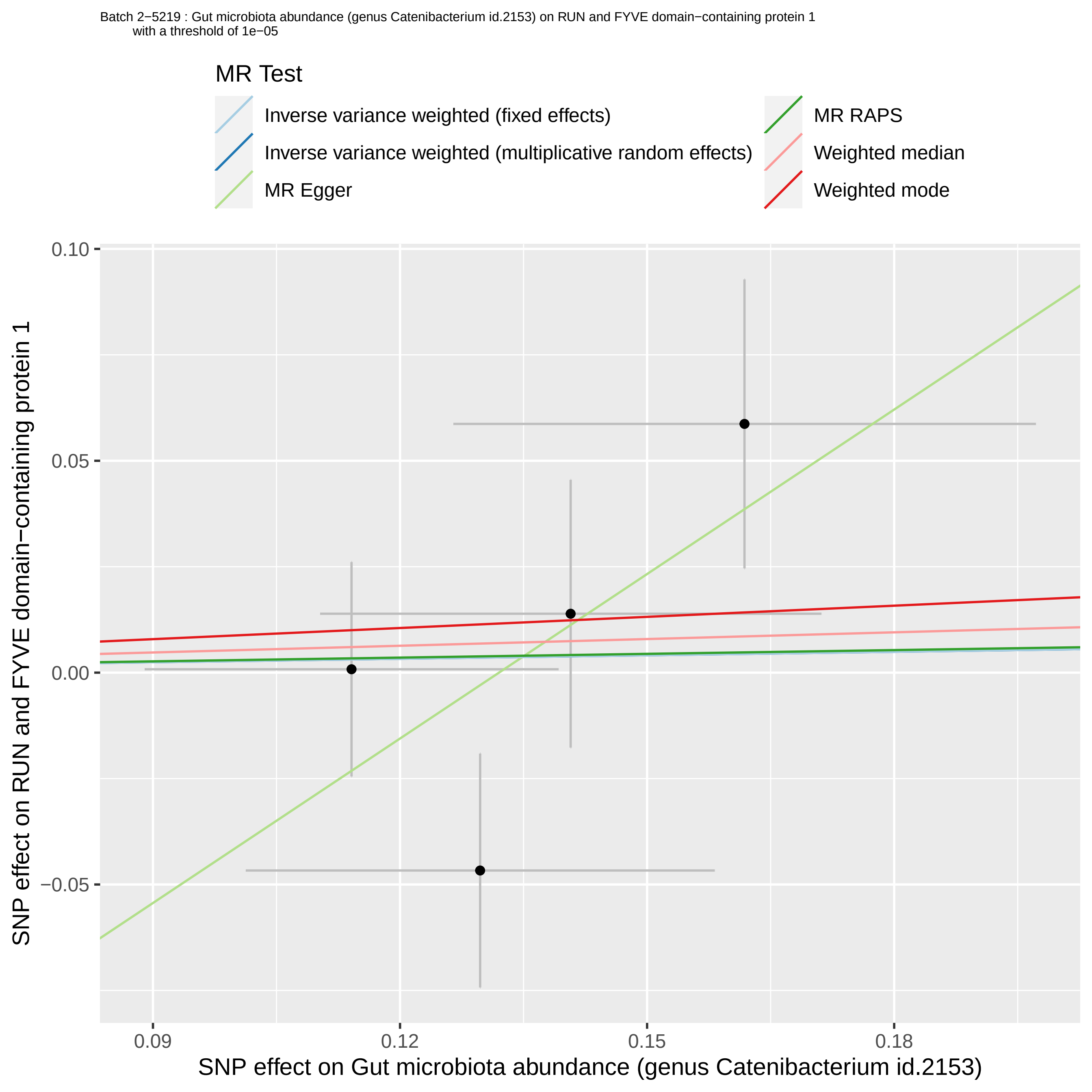

Batch 2−5219 : Gut microbiota abundance (genus Catenibacterium id.2153) on RUN and FYVE domain−containing protein 1 with a threshold of 1e−05
MR Test
Inverse variance weighted (fixed effects)
Inverse variance weighted (multiplicative random effects)
MR Egger
MR RAPS
Weighted median
Weighted mode
0.10
0.05
0.00
−0.05
0.09
0.12
0.15
0.18
SNP effect on RUN and FYVE domain−containing protein 1
SNP effect on Gut microbiota abundance (genus Catenibacterium id.2153)

Batch 2−5220 : Gut microbiota abundance (genus Parasutterella id.2892) on RUN and FYVE domain−containing protein 1
with a threshold of 1e−05

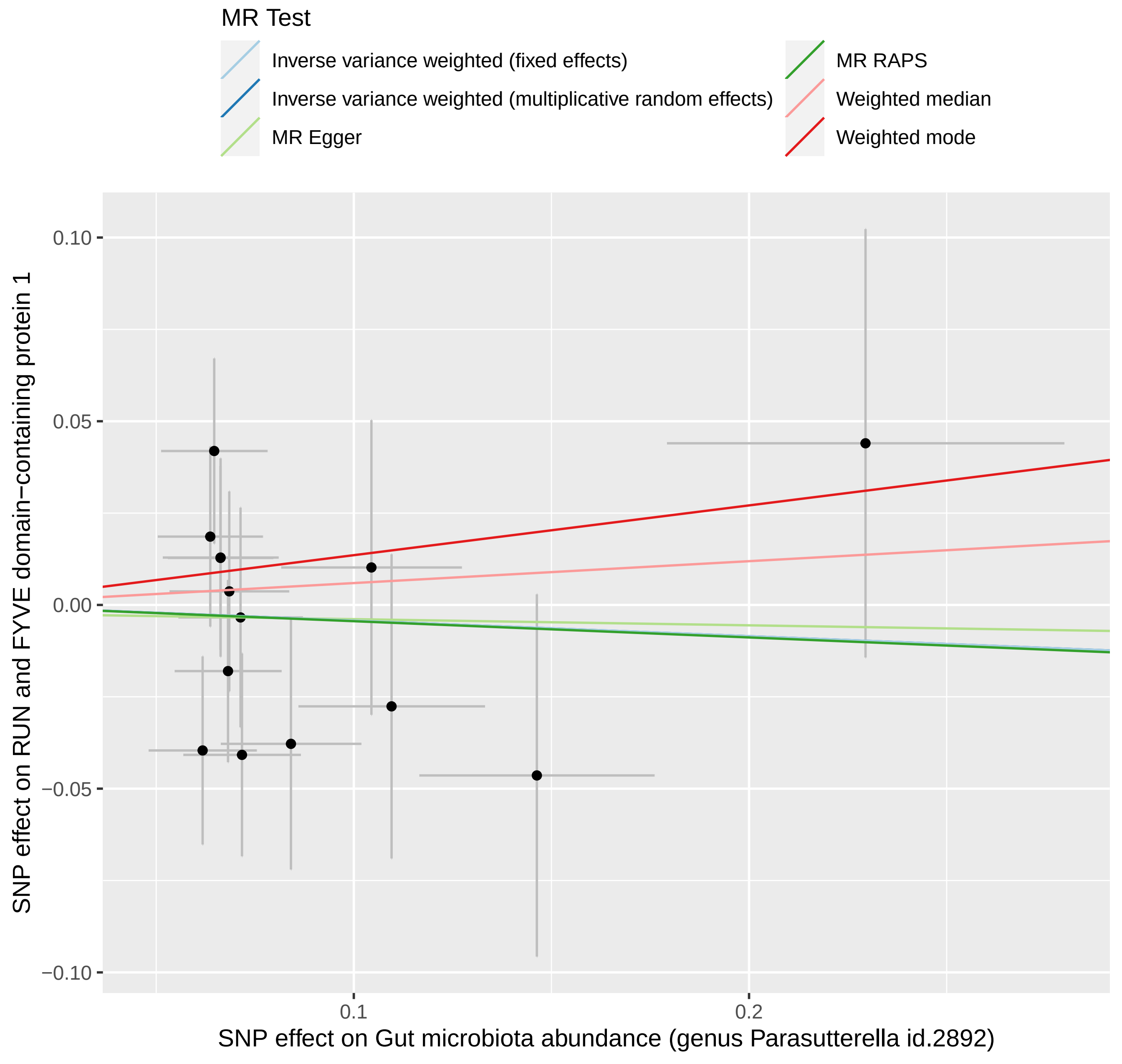

Batch 2−5221 : Gut microbiota abundance (genus Catenibacterium id.2153) on Runt−related transcription factor 3
with a threshold of 1e−05

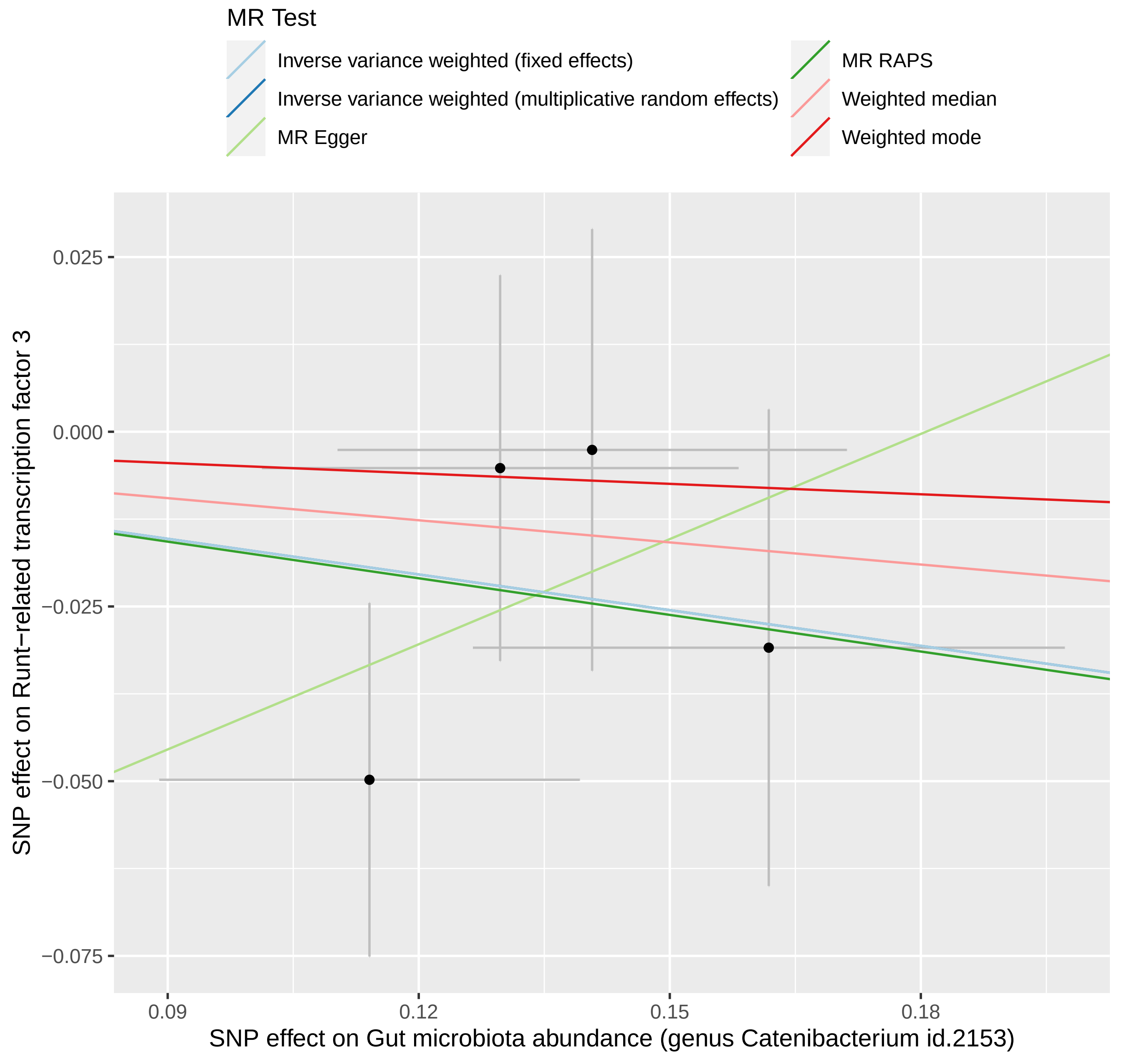

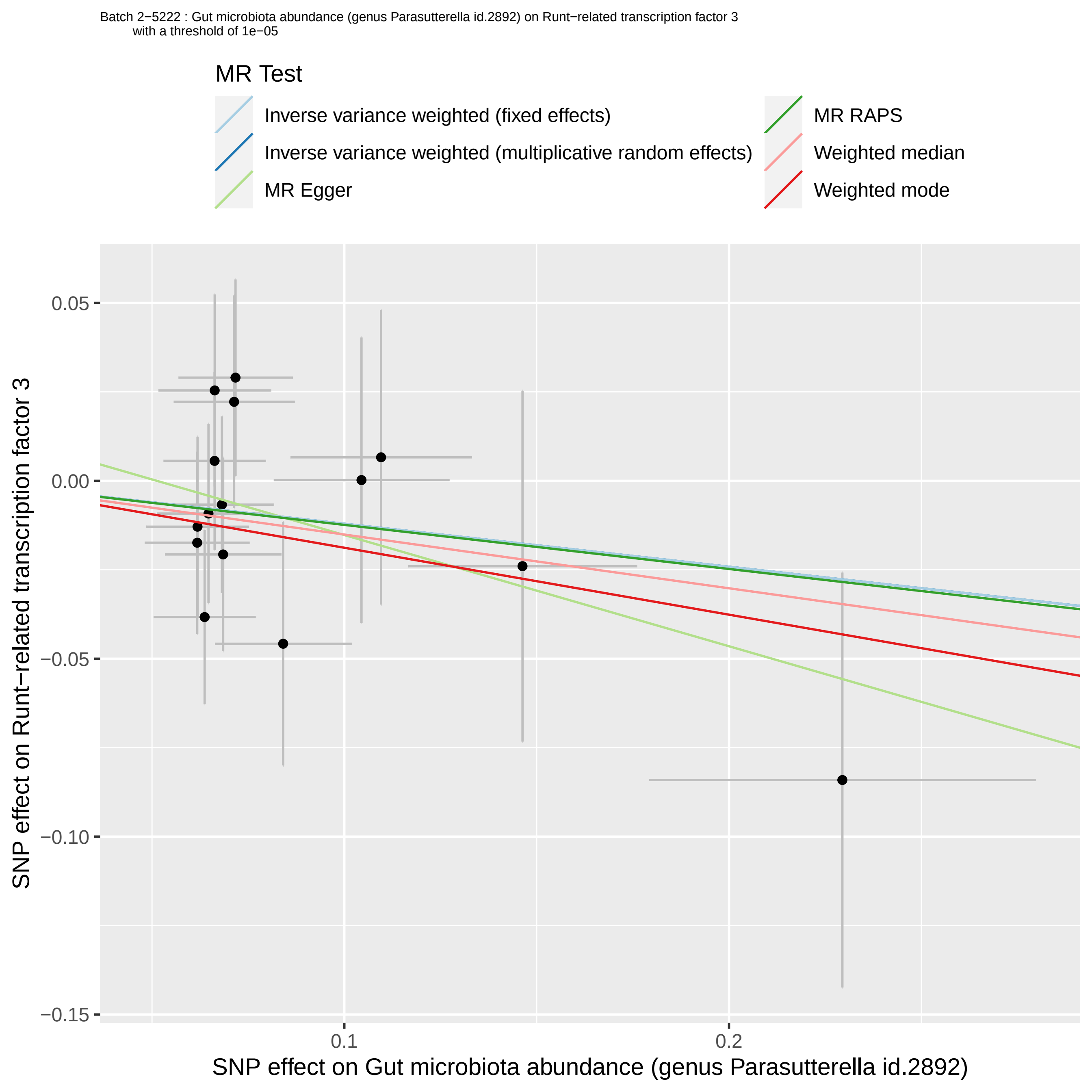
Batch 2−5222 : Gut microbiota abundance (genus Parasutterella id.2892) on Runt−related transcription factor 3
with a threshold of 1e−05
MR Test
Inverse variance weighted (fixed effects)
Inverse variance weighted (multiplicative random effects)
MR Egger
MR RAPS
Weighted median
Weighted mode
0.05
0.00
−0.05
−0.10
−0.15
0.1
0.2
SNP effect on Runt−related transcription factor 3
SNP effect on Gut microbiota abundance (genus Parasutterella id.2892)

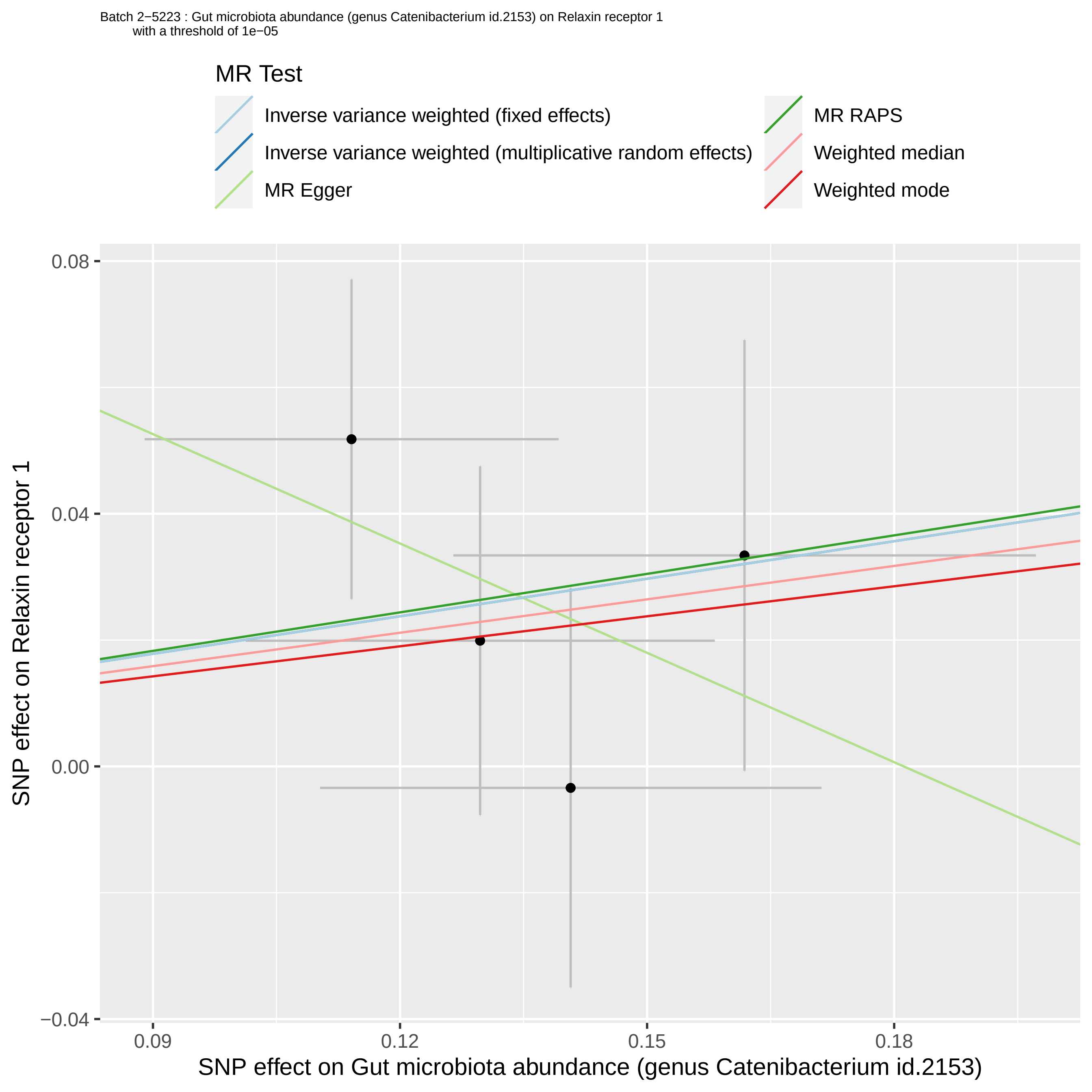
Batch 2−5223 : Gut microbiota abundance (genus Catenibacterium id.2153) on Relaxin receptor 1
with a threshold of 1e−05
MR Test
Inverse variance weighted (fixed effects)
Inverse variance weighted (multiplicative random effects)
MR Egger
MR RAPS
Weighted median
Weighted mode
0.08
0.04
0.00
−0.04
0.09
0.12
0.15
0.18
SNP effect on Relaxin receptor 1
SNP effect on Gut microbiota abundance (genus Catenibacterium id.2153)

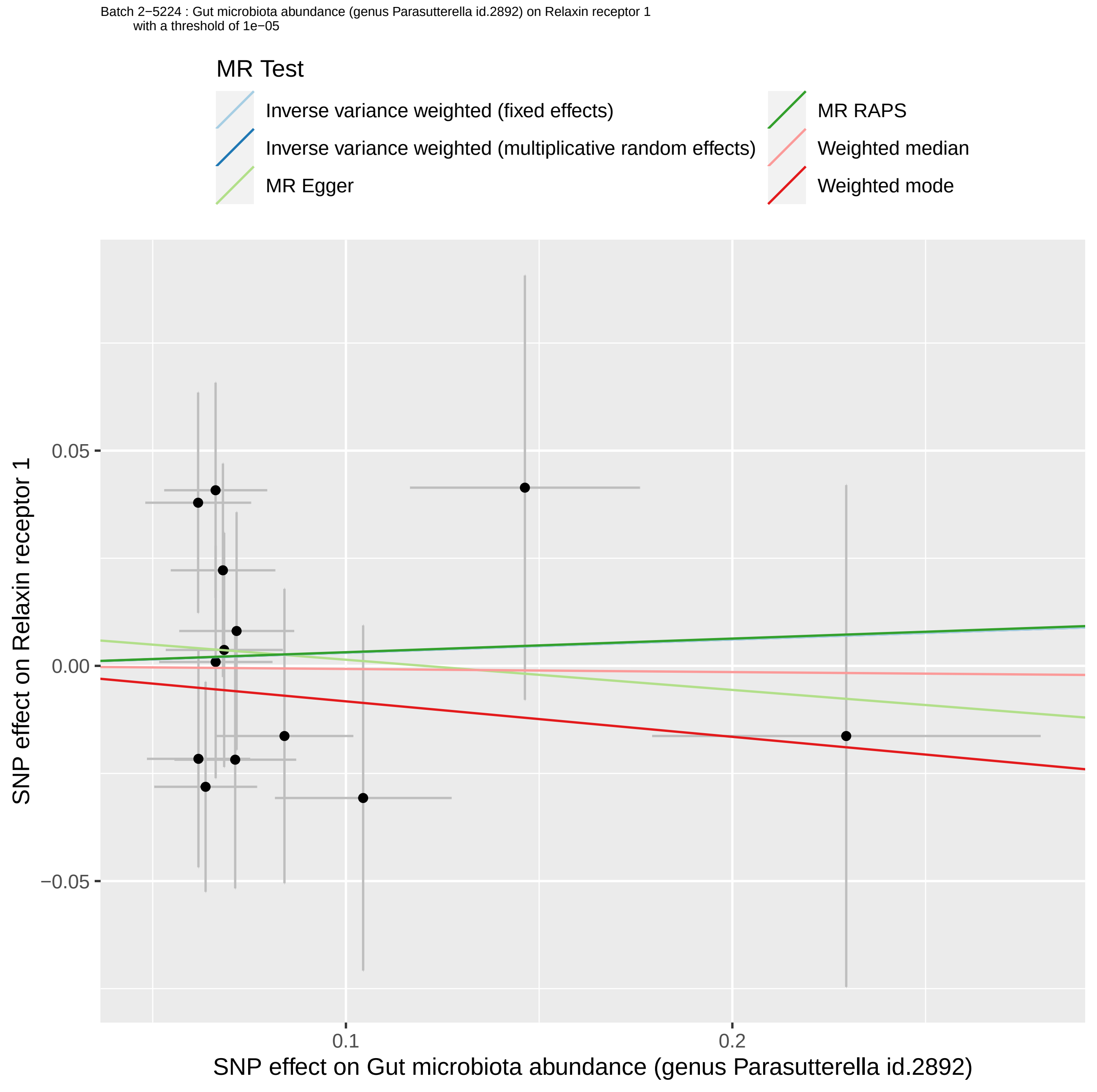

Batch 2−5224 : Gut microbiota abundance (genus Parasutterella id.2892) on Relaxin receptor 1
with a threshold of 1e−05
MR Test
Inverse variance weighted (fixed effects)
Inverse variance weighted (multiplicative random effects)
MR Egger
MR RAPS
Weighted median
Weighted mode
0.05
0.00
−0.05
0.1
0.2
SNP effect on Relaxin receptor 1
SNP effect on Gut microbiota abundance (genus Parasutterella id.2892)

Batch 2−5225 : Gut microbiota abundance (genus Catenibacterium id.2153) on Tyrosine−protein kinase RYK
with a threshold of 1e−05

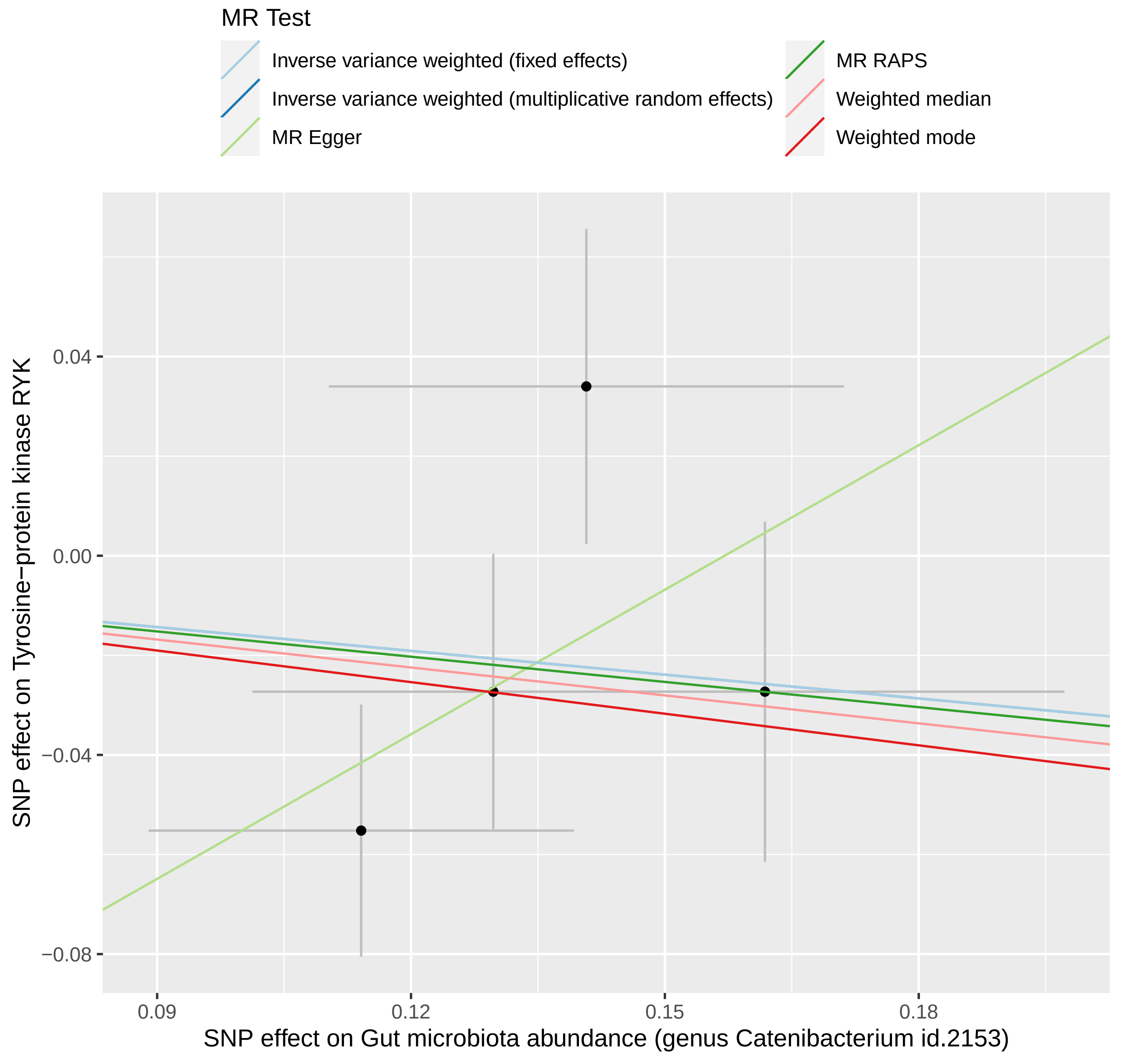

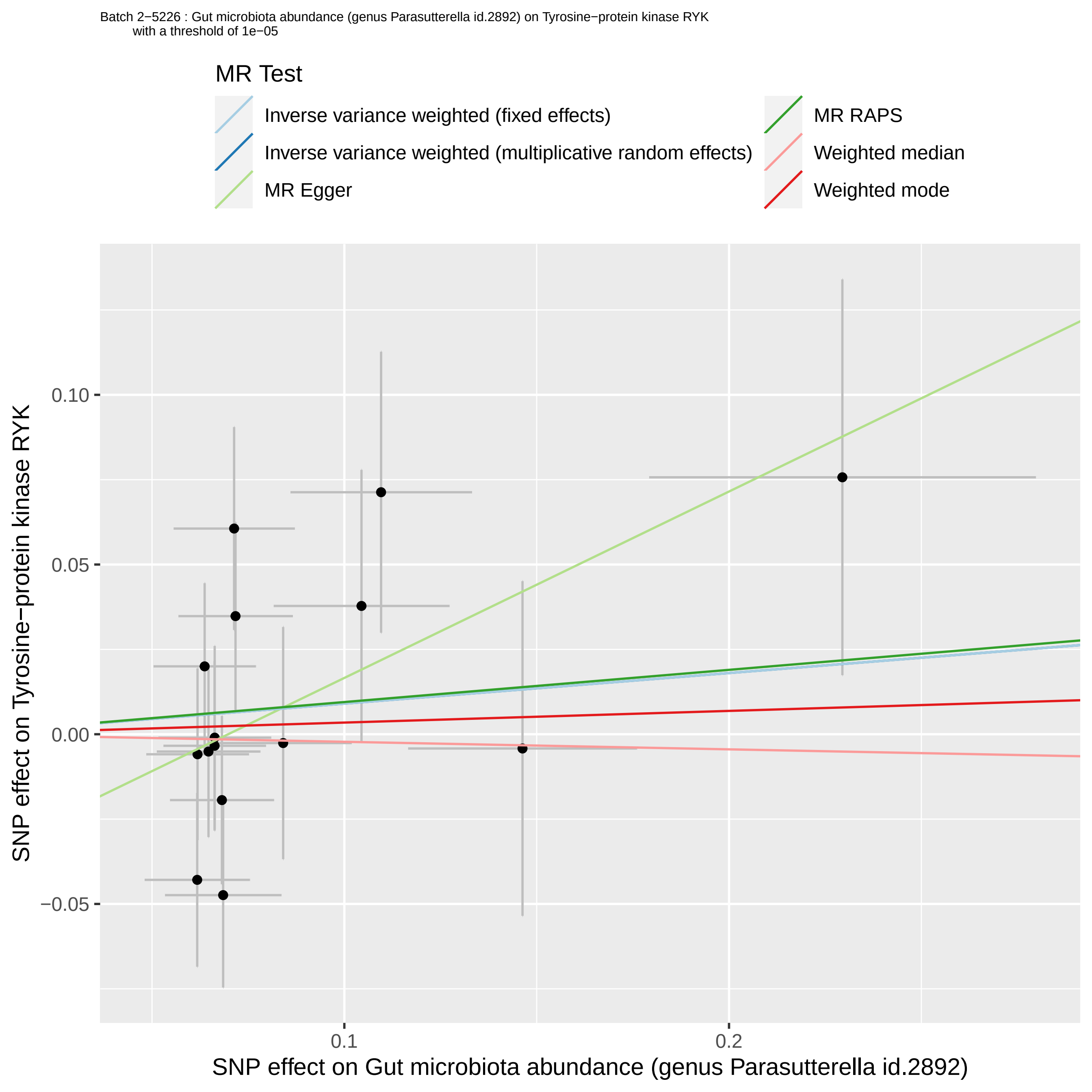

Batch 2−5226 : Gut microbiota abundance (genus Parasutterella id.2892) on Tyrosine−protein kinase RYK
with a threshold of 1e−05
MR Test
Inverse variance weighted (fixed effects)
Inverse variance weighted (multiplicative random effects)
MR Egger
MR RAPS
Weighted median
Weighted mode
0.10
0.05
0.00
−0.05
0.1
0.2
SNP effect on Tyrosine−protein kinase RYK
SNP effect on Gut microbiota abundance (genus Parasutterella id.2892)

Batch 2−5227 : Gut microbiota abundance (genus Catenibacterium id.2153) on Protein S100−A11
with a threshold of 1e−05

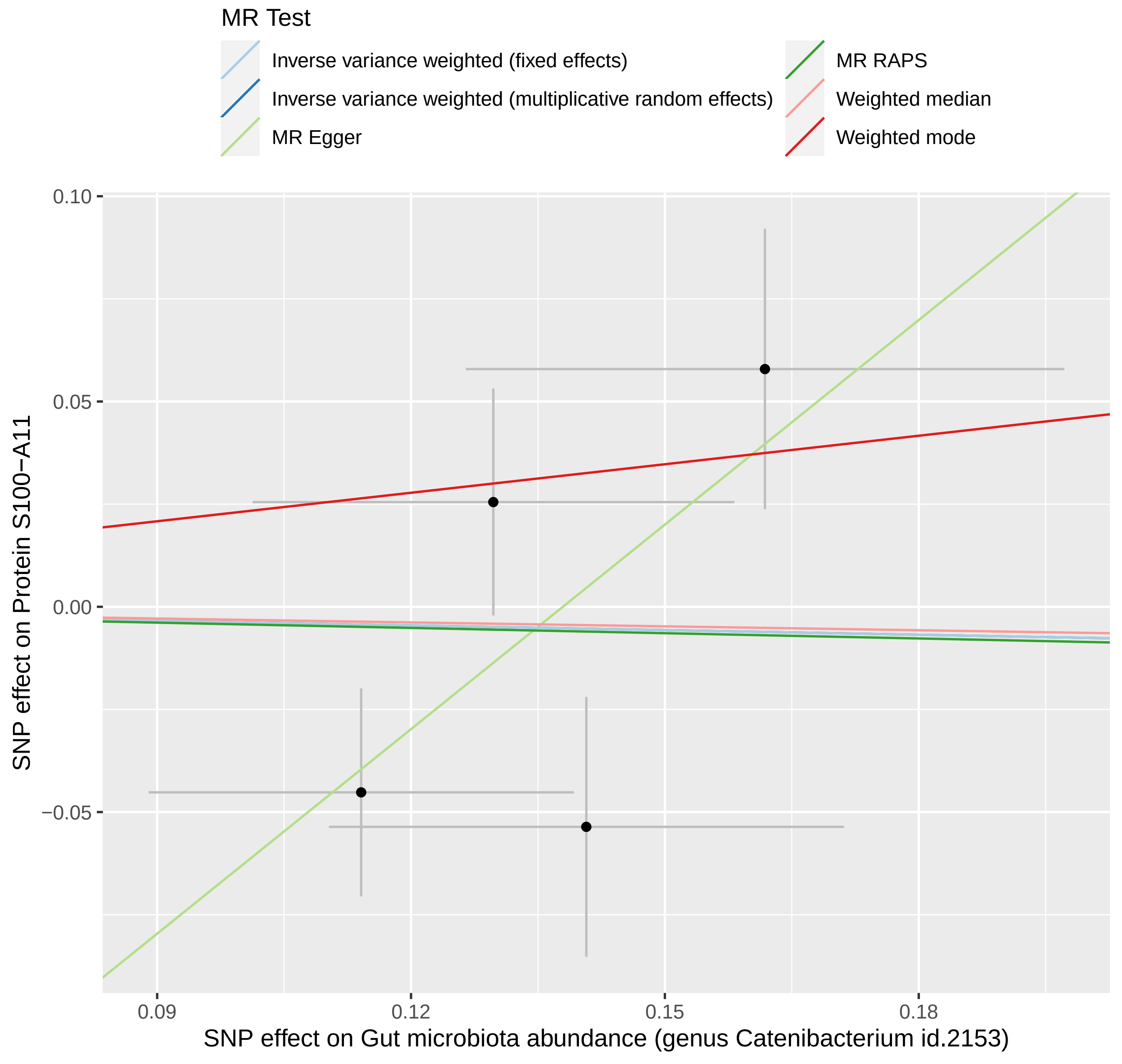

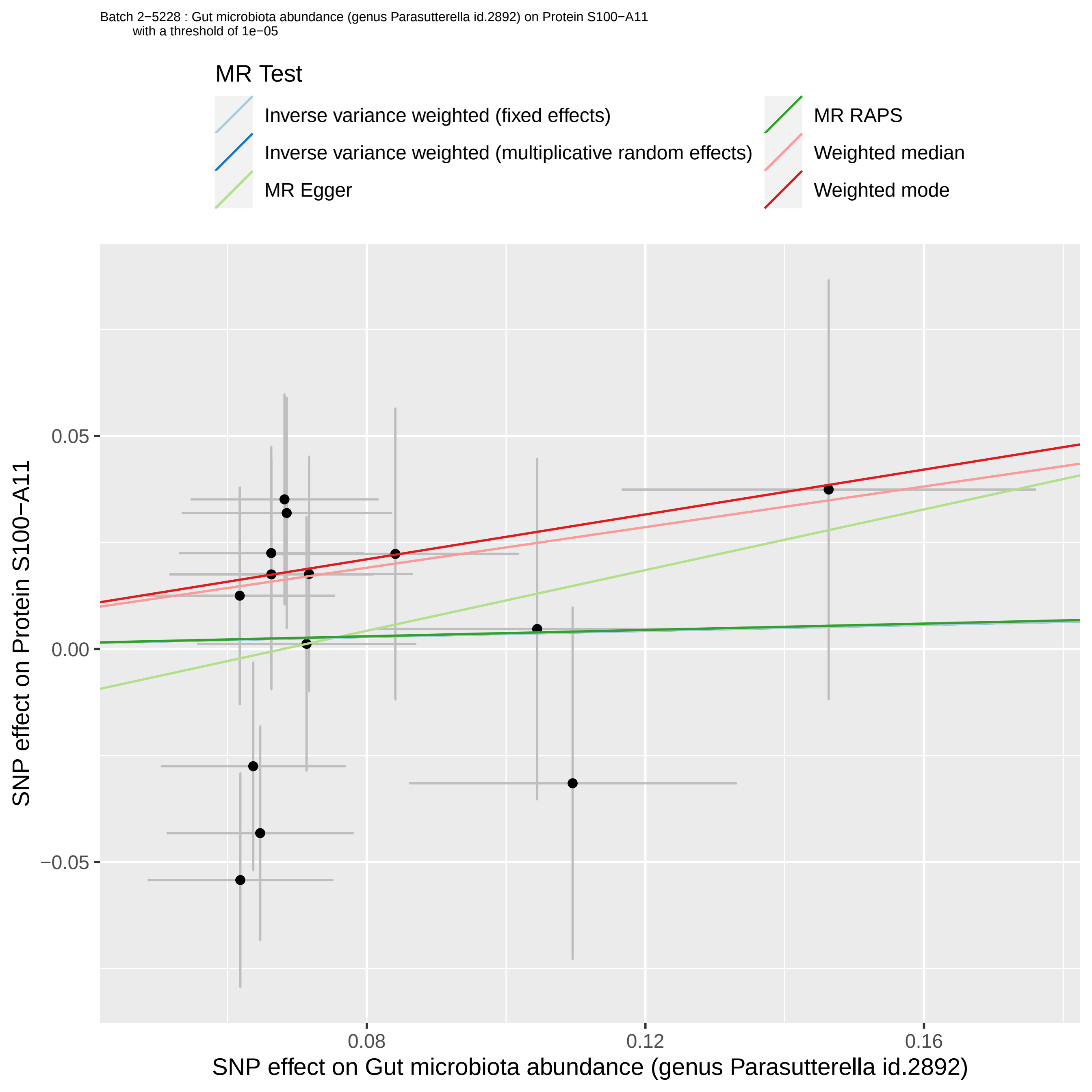

Batch 2−5228 : Gut microbiota abundance (genus Parasutterella id.2892) on Protein S100−A11
with a threshold of 1e−05
MR Test
Inverse variance weighted (fixed effects)
Inverse variance weighted (multiplicative random effects)
MR Egger
MR RAPS
Weighted median
Weighted mode
0.05
0.00
−0.05
0.08
0.12
0.16
SNP effect on Protein S100−A11
SNP effect on Gut microbiota abundance (genus Parasutterella id.2892)

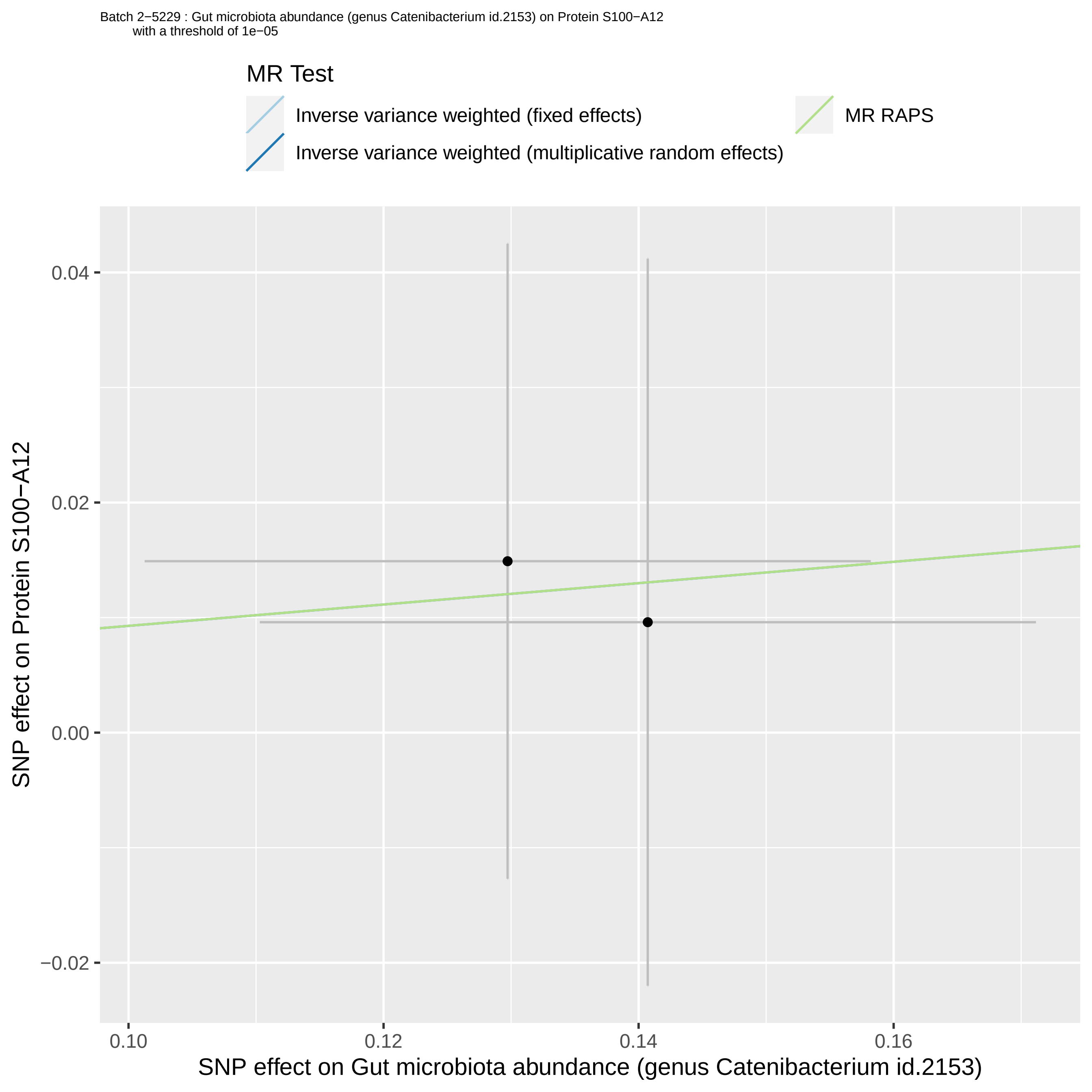

Batch 2−5229 : Gut microbiota abundance (genus Catenibacterium id.2153) on Protein S100−A12
with a threshold of 1e−05
MR Test
Inverse variance weighted (fixed effects)
Inverse variance weighted (multiplicative random effects)
MR RAPS
0.04
0.02
0.00
−0.02
SNP effect on Protein S100−A12
0.10
0.12
0.14
0.16
SNP effect on Gut microbiota abundance (genus Catenibacterium id.2153)

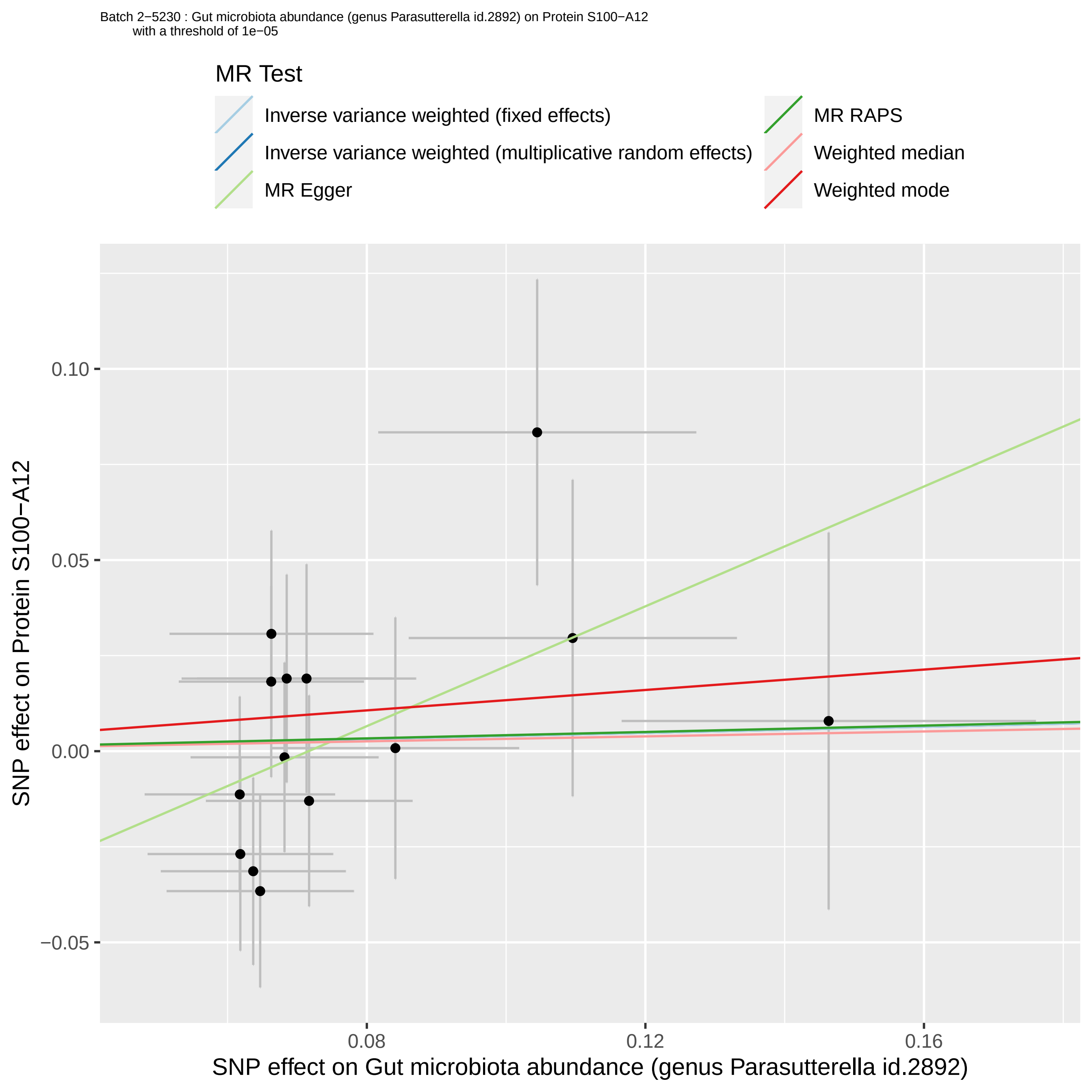

Batch 2−5230 : Gut microbiota abundance (genus Parasutterella id.2892) on Protein S100−A12
with a threshold of 1e−05
MR Test
Inverse variance weighted (fixed effects)
Inverse variance weighted (multiplicative random effects)
MR Egger
MR RAPS
Weighted median
Weighted mode
0.10
0.05
0.00
−0.05
0.08
0.12
0.16
SNP effect on Protein S100−A12
SNP effect on Gut microbiota abundance (genus Parasutterella id.2892)

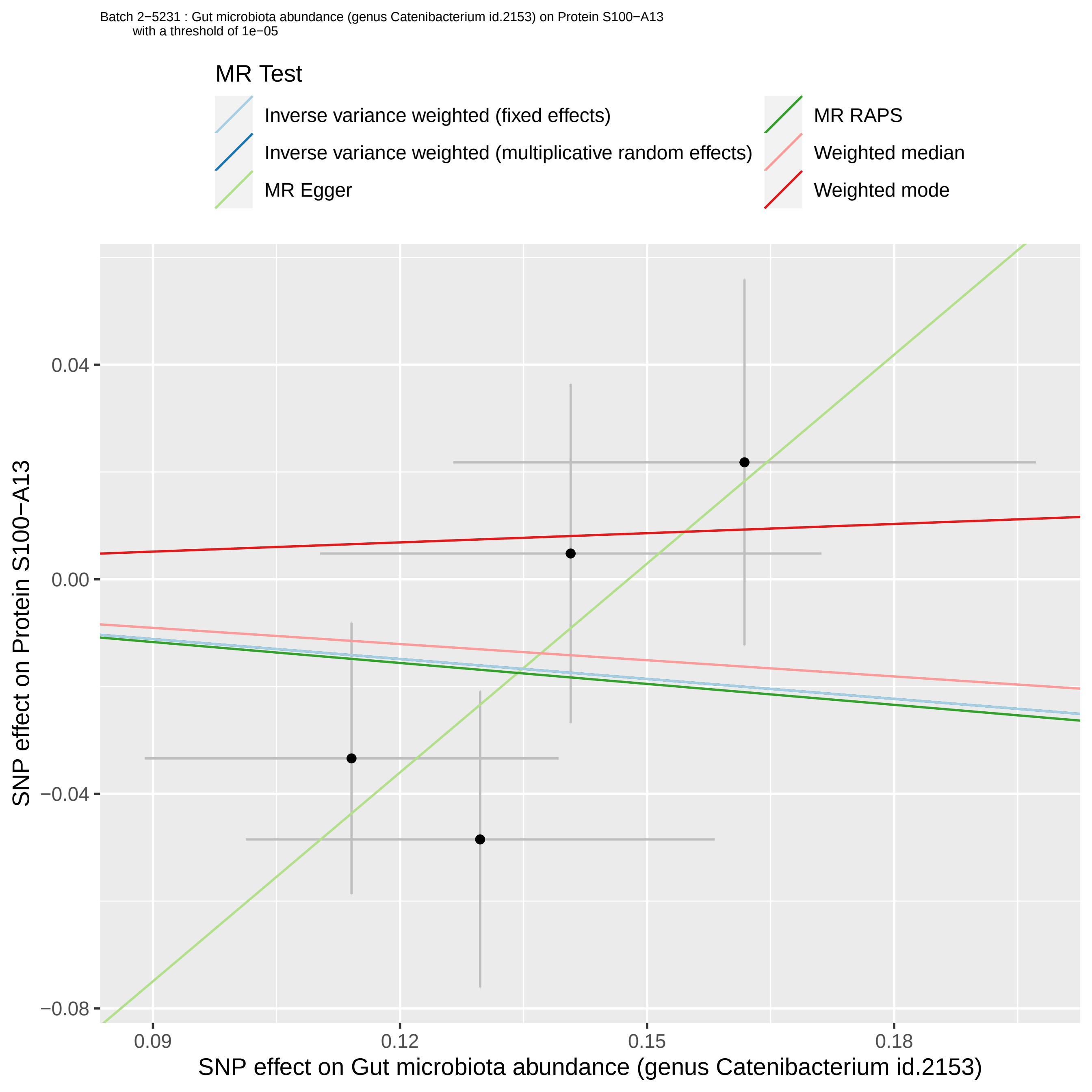
Batch 2−5231 : Gut microbiota abundance (genus Catenibacterium id.2153) on Protein S100−A13
with a threshold of 1e−05
MR Test
Inverse variance weighted (fixed effects)
Inverse variance weighted (multiplicative random effects)
MR Egger
MR RAPS
Weighted median
Weighted mode
SNP effect on Protein S100−A13
0.04
0.00
−0.04
−0.08
0.09
0.12
0.15
0.18
SNP effect on Gut microbiota abundance (genus Catenibacterium id.2153)

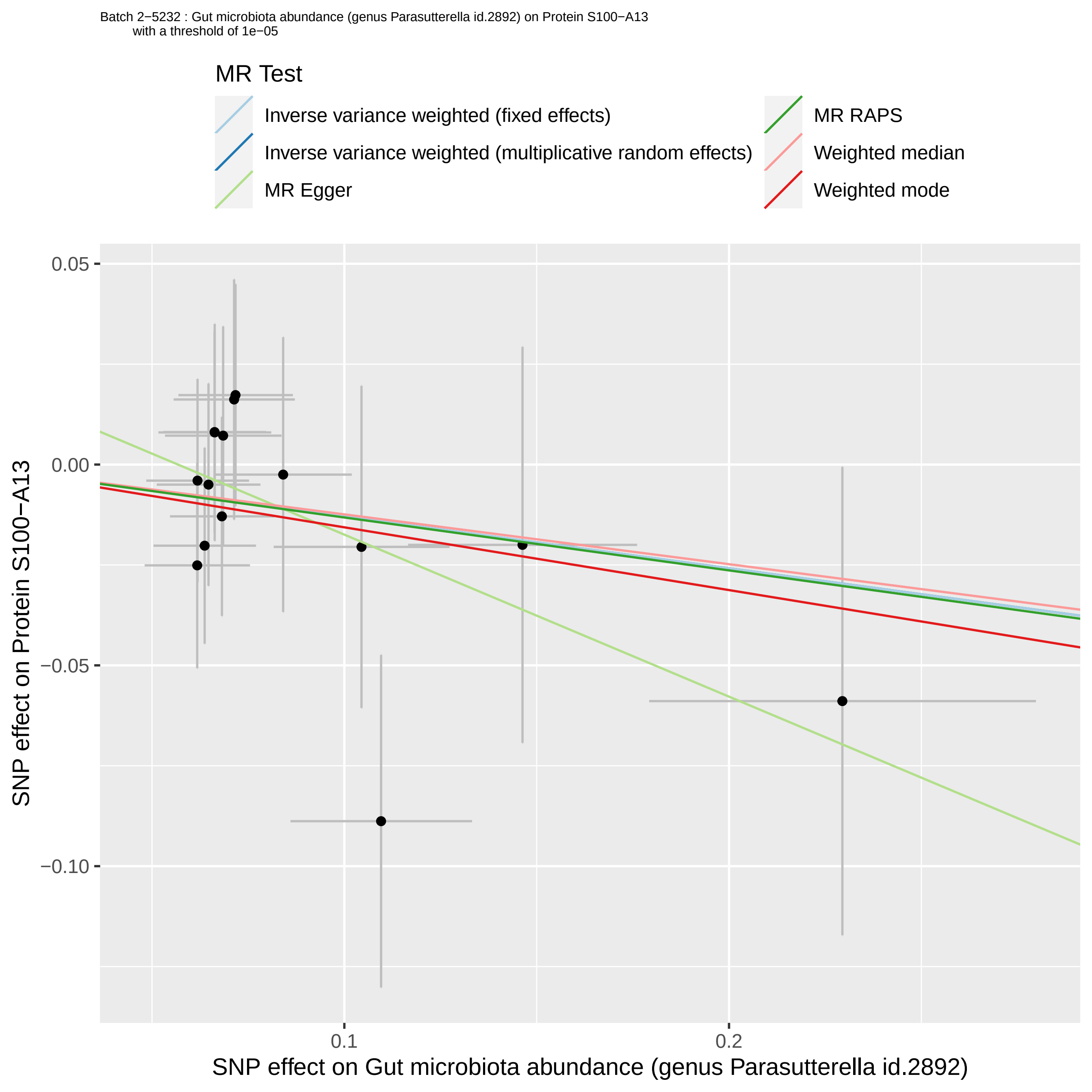
Batch 2−5232 : Gut microbiota abundance (genus Parasutterella id.2892) on Protein S100−A13
with a threshold of 1e−05
MR Test
Inverse variance weighted (fixed effects)
Inverse variance weighted (multiplicative random effects)
MR Egger
MR RAPS
Weighted median
Weighted mode
0.05
0.00
−0.05
−0.10
0.1
0.2
SNP effect on Gut microbiota abundance (genus Parasutterella id.2892)
SNP effect on Protein S100−A13

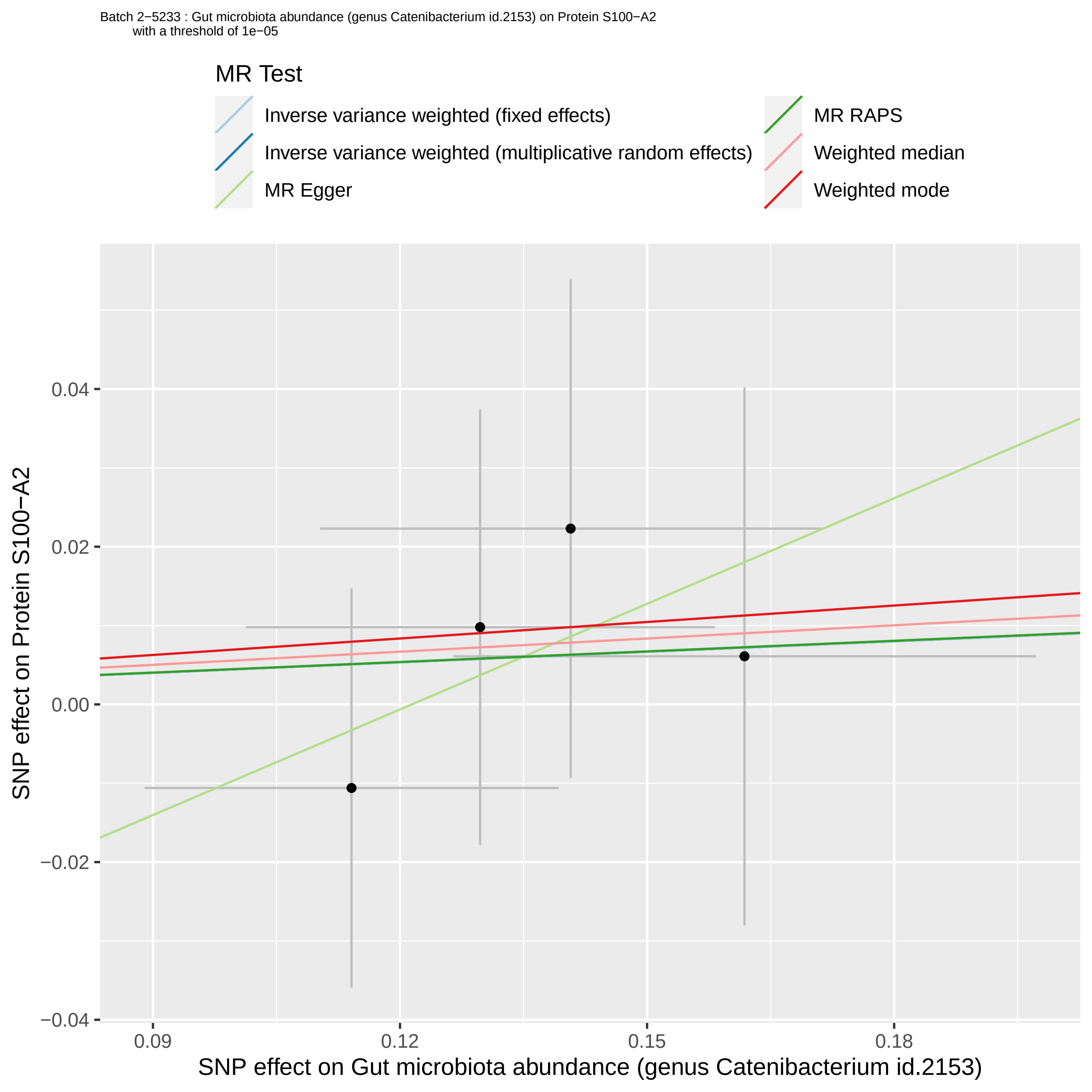
Batch 2−5233 : Gut microbiota abundance (genus Catenibacterium id.2153) on Protein S100−A2
with a threshold of 1e−05
MR Test
Inverse variance weighted (fixed effects)
Inverse variance weighted (multiplicative random effects)
MR Egger
MR RAPS
Weighted median
Weighted mode
SNP effect on Protein S100−A2
0.04
0.02
0.00
−0.02
−0.04
0.09
0.12
0.15
0.18
SNP effect on Gut microbiota abundance (genus Catenibacterium id.2153)

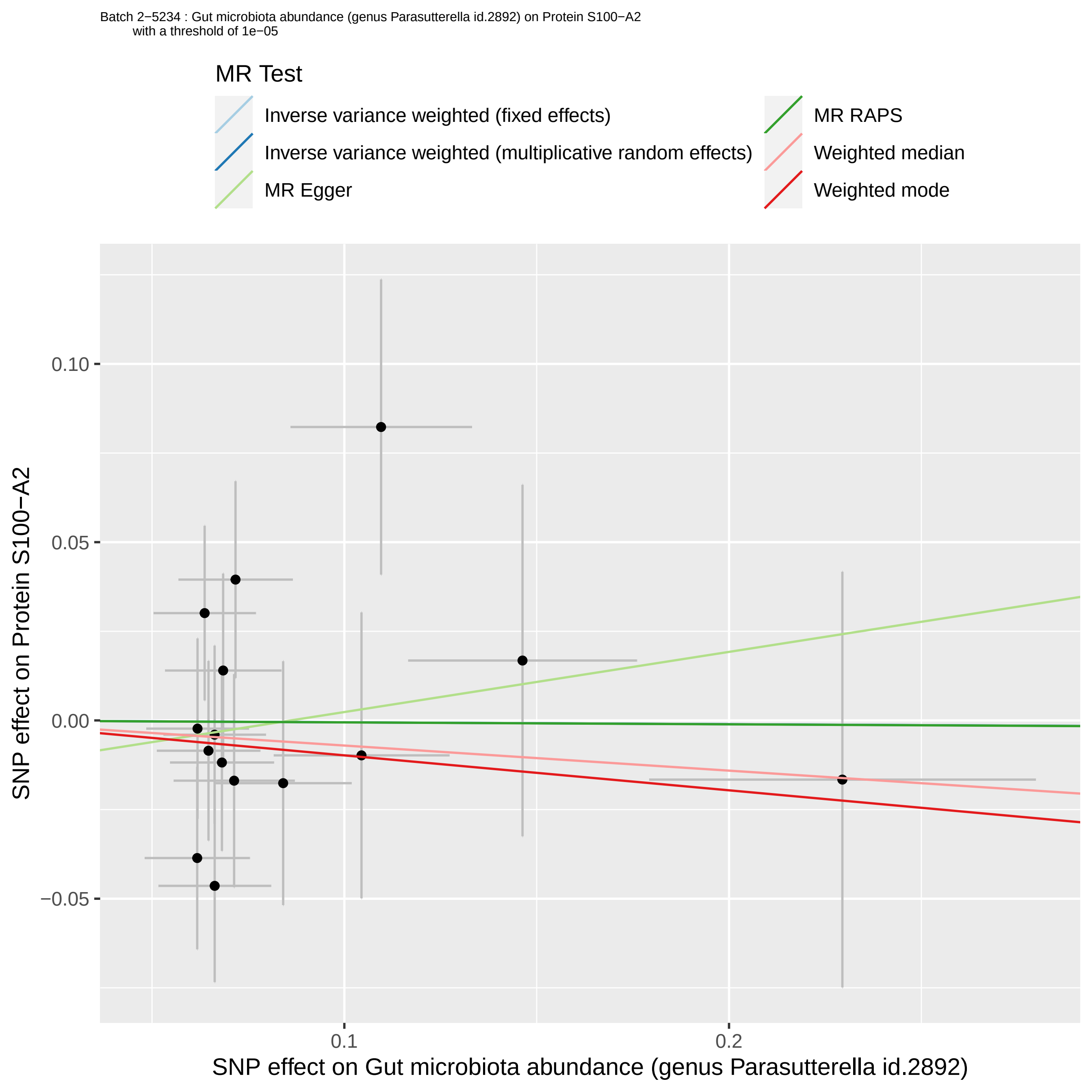
Batch 2−5234 : Gut microbiota abundance (genus Parasutterella id.2892) on Protein S100−A2
with a threshold of 1e−05
MR Test
Inverse variance weighted (fixed effects)
Inverse variance weighted (multiplicative random effects)
MR Egger
MR RAPS
Weighted median
Weighted mode
0.10
0.05
0.00
−0.05
0.1
0.2
SNP effect on Protein S100−A2
SNP effect on Gut microbiota abundance (genus Parasutterella id.2892)

Batch 2−5235 : Gut microbiota abundance (genus Catenibacterium id.2153) on Protein S100−A4
with a threshold of 1e−05

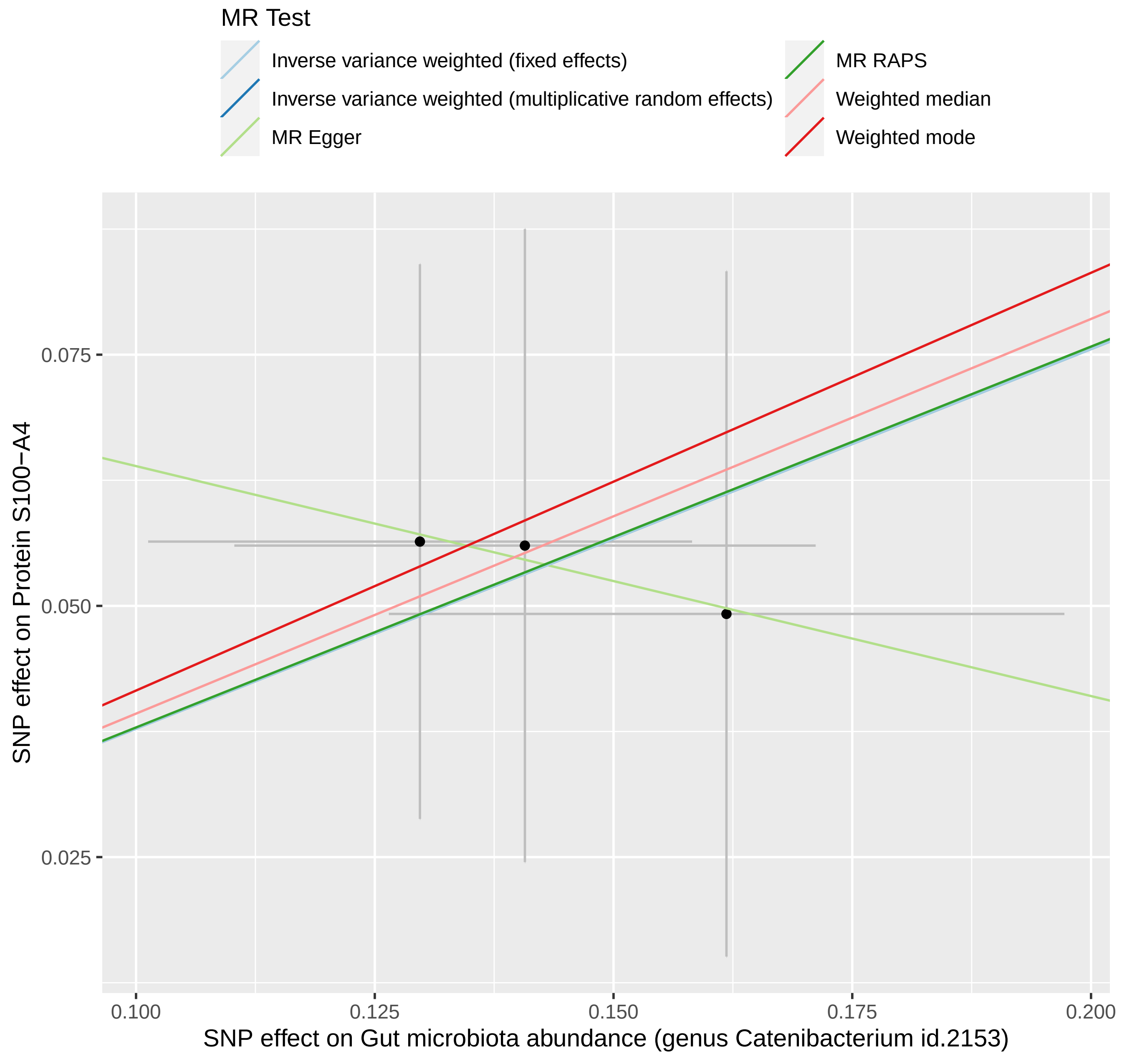

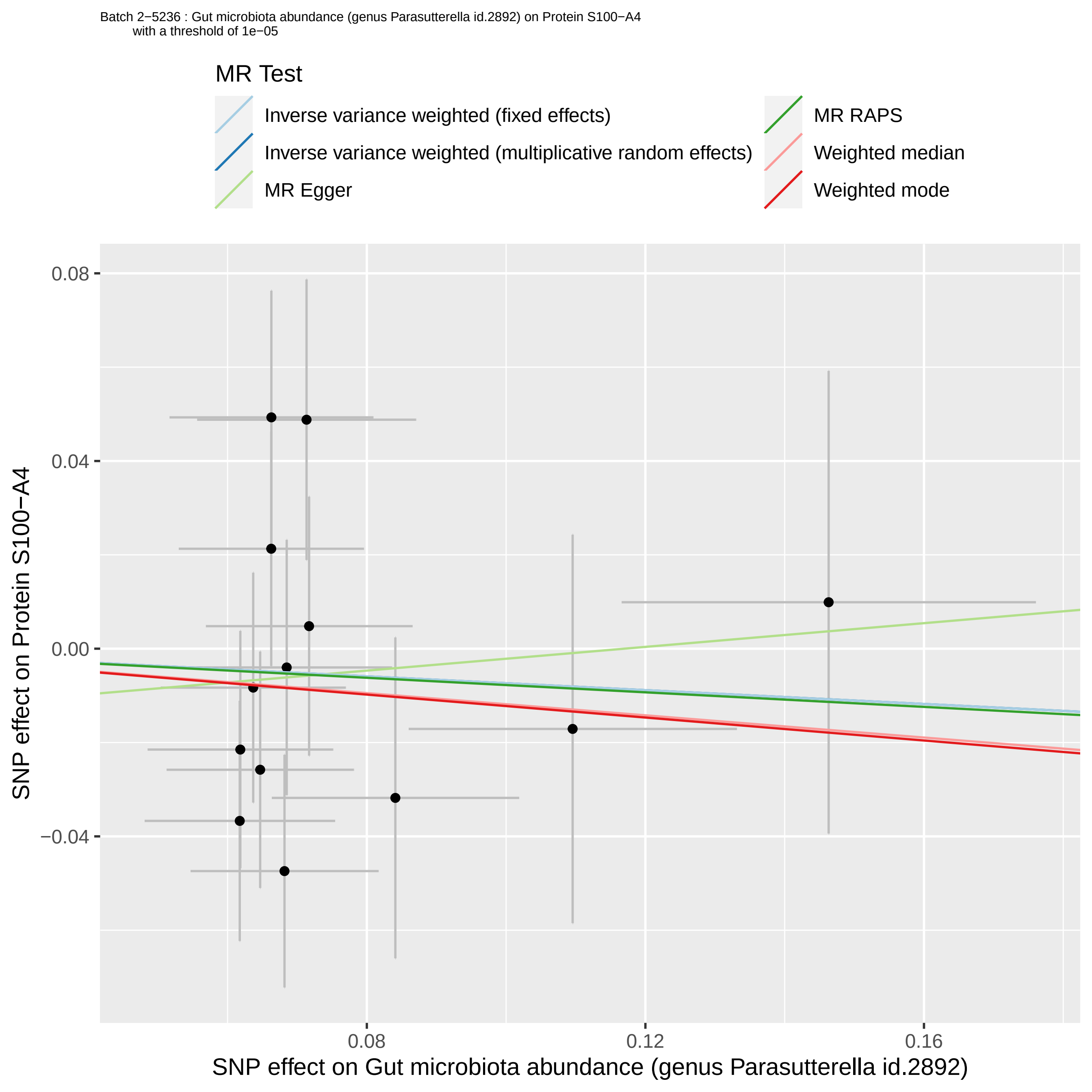

Batch 2−5236 : Gut microbiota abundance (genus Parasutterella id.2892) on Protein S100−A4
with a threshold of 1e−05
MR Test
Inverse variance weighted (fixed effects)
Inverse variance weighted (multiplicative random effects)
MR Egger
MR RAPS
Weighted median
Weighted mode
0.08
0.04
0.00
−0.04
0.08
0.12
0.16
SNP effect on Protein S100−A4
SNP effect on Gut microbiota abundance (genus Parasutterella id.2892)

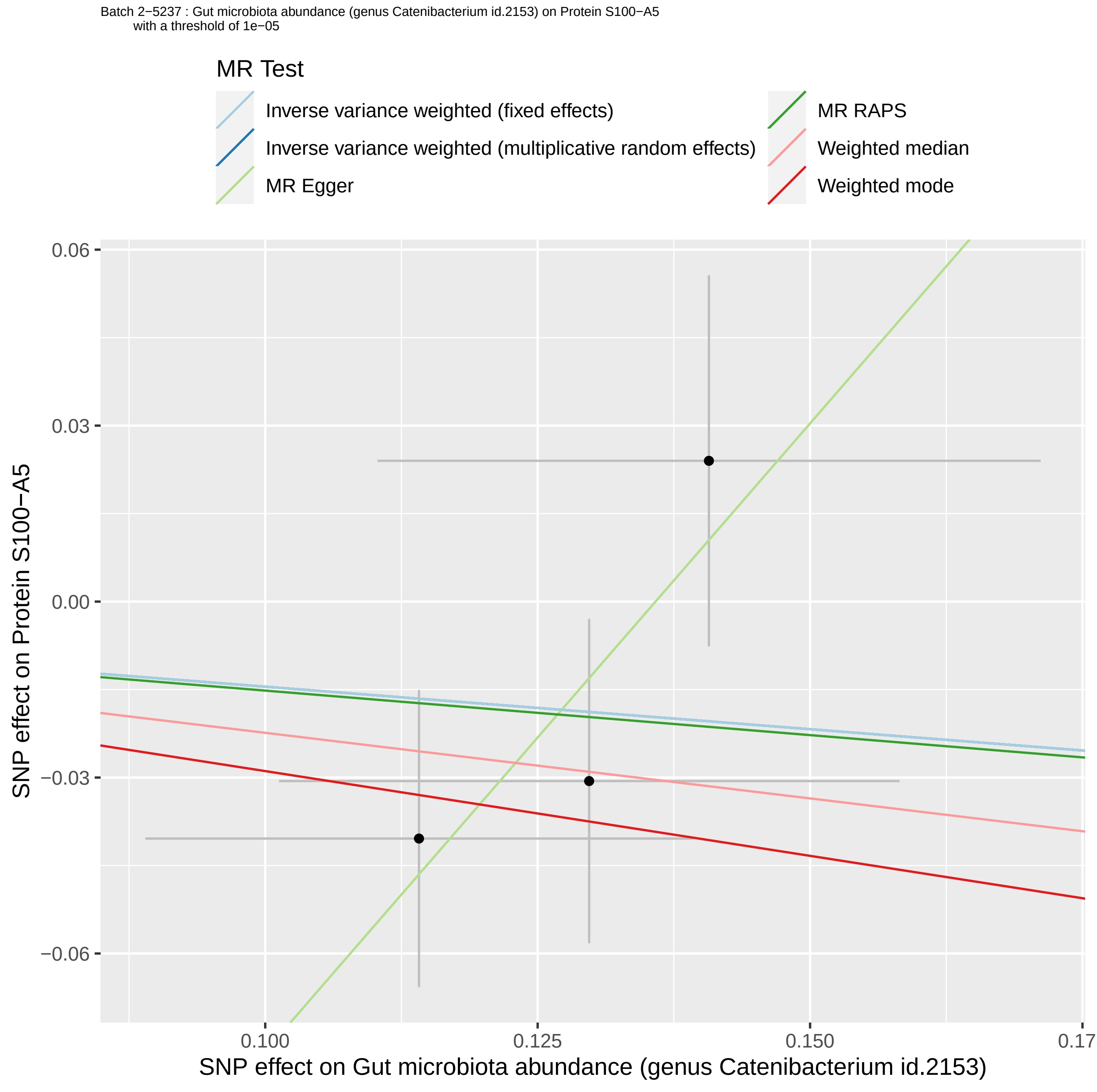

Batch 2−5237 : Gut microbiota abundance (genus Catenibacterium id.2153) on Protein S100−A5
with a threshold of 1e−05
MR Test
Inverse variance weighted (fixed effects)
Inverse variance weighted (multiplicative random effects)
[truncated: 371,440 more chars]
