# Supplementary material for: Investigating the role of gut microbiota in diabetic nephropathy through plasma proteome mediated analysis
Source: Sci Rep. 2025 Feb 14;15:5457. doi: 10.1038/s41598-025-90306-7 (PMC11828962; doi:10.1038/s41598-025-90306-7)
Supplement: Supplementary file 2 — Supplementary Material 2 [file 41598_2025_90306_MOESM2_ESM.pdf]

Supplementary Figure 2: Funnel plots of all MR analyses in this study

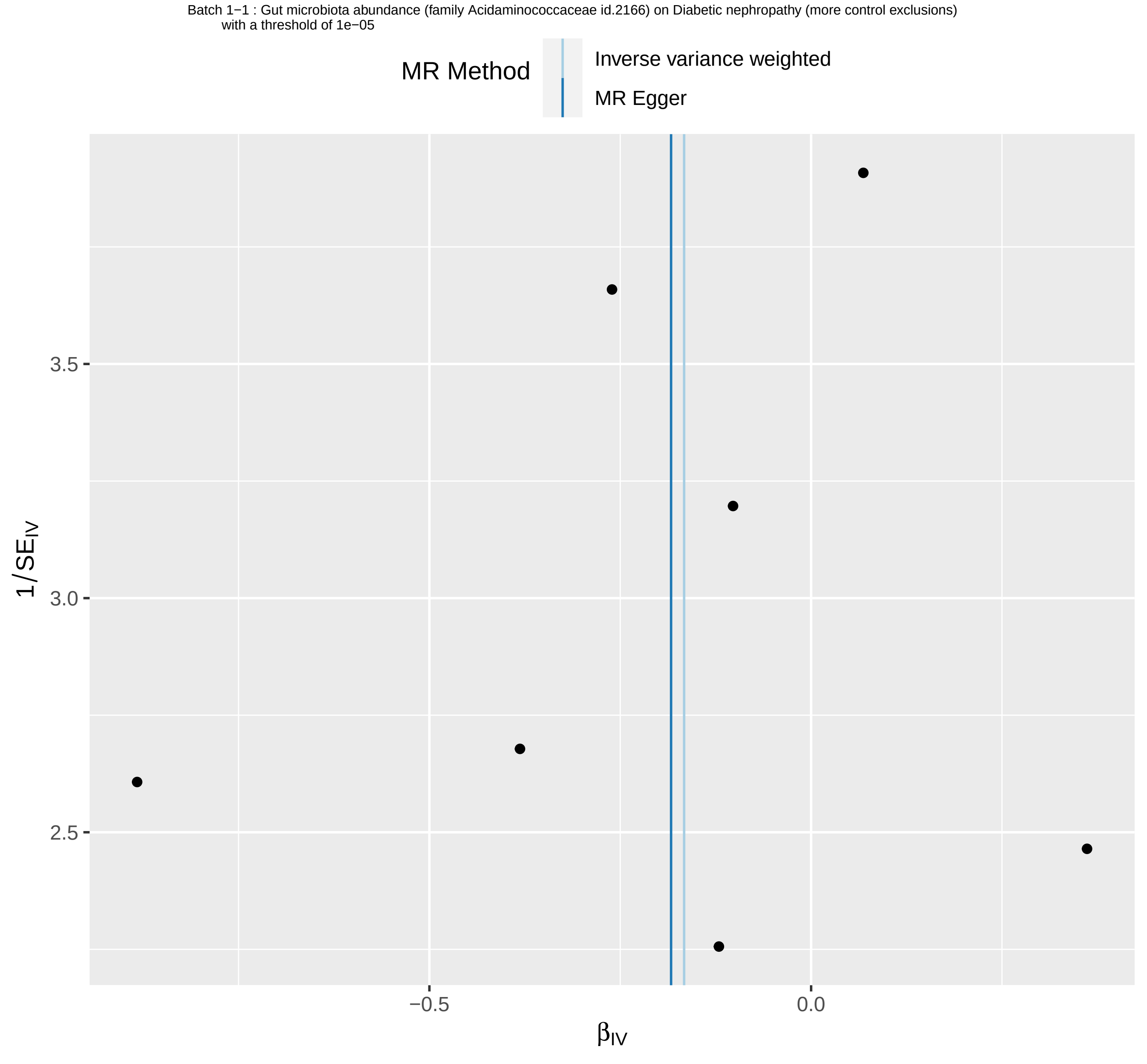

Batch 1−2 : Gut microbiota abundance (family Actinomycetaceae id.421) on Diabetic nephropathy (more control exclusions) with a threshold of 1e−05

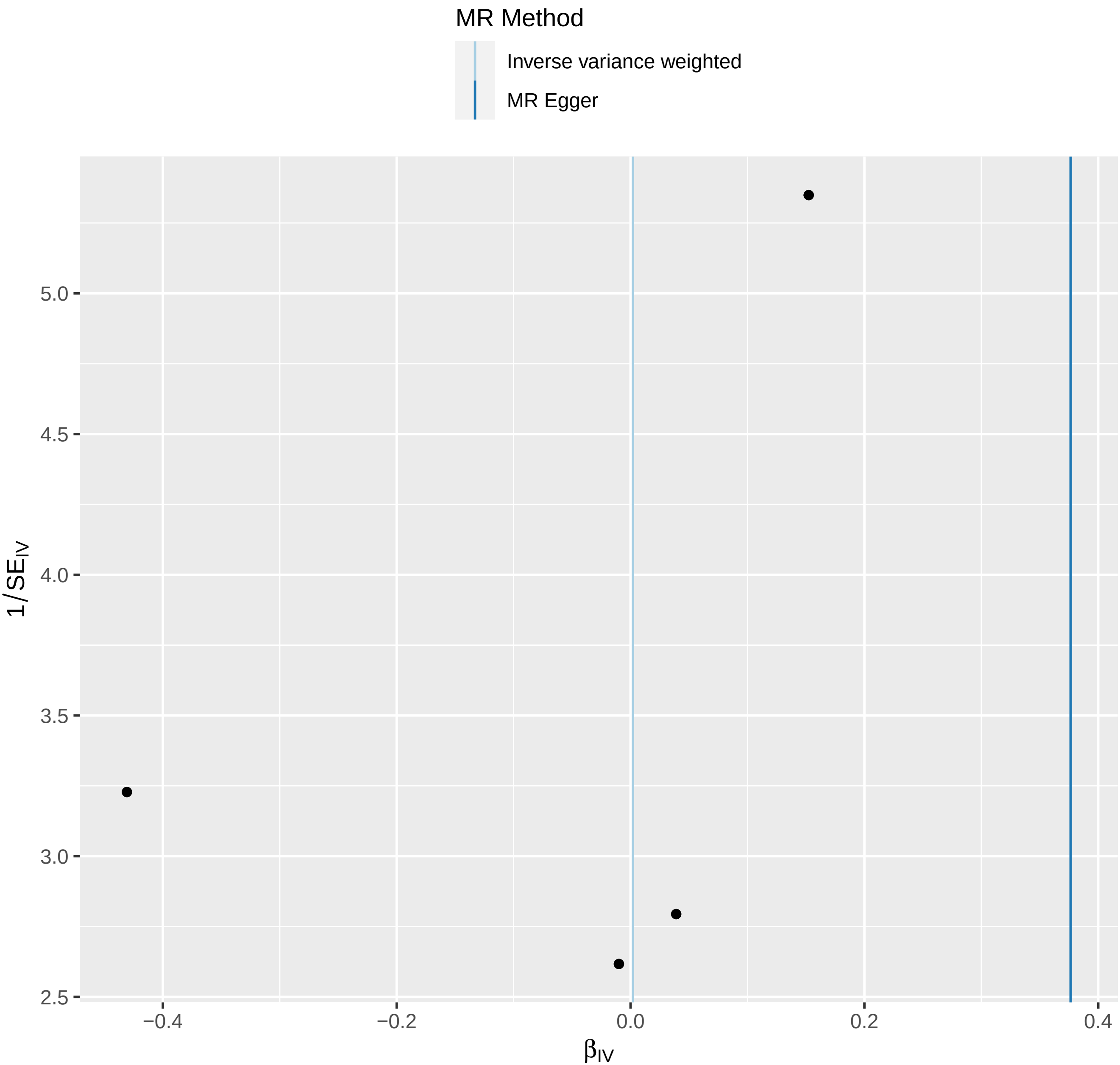

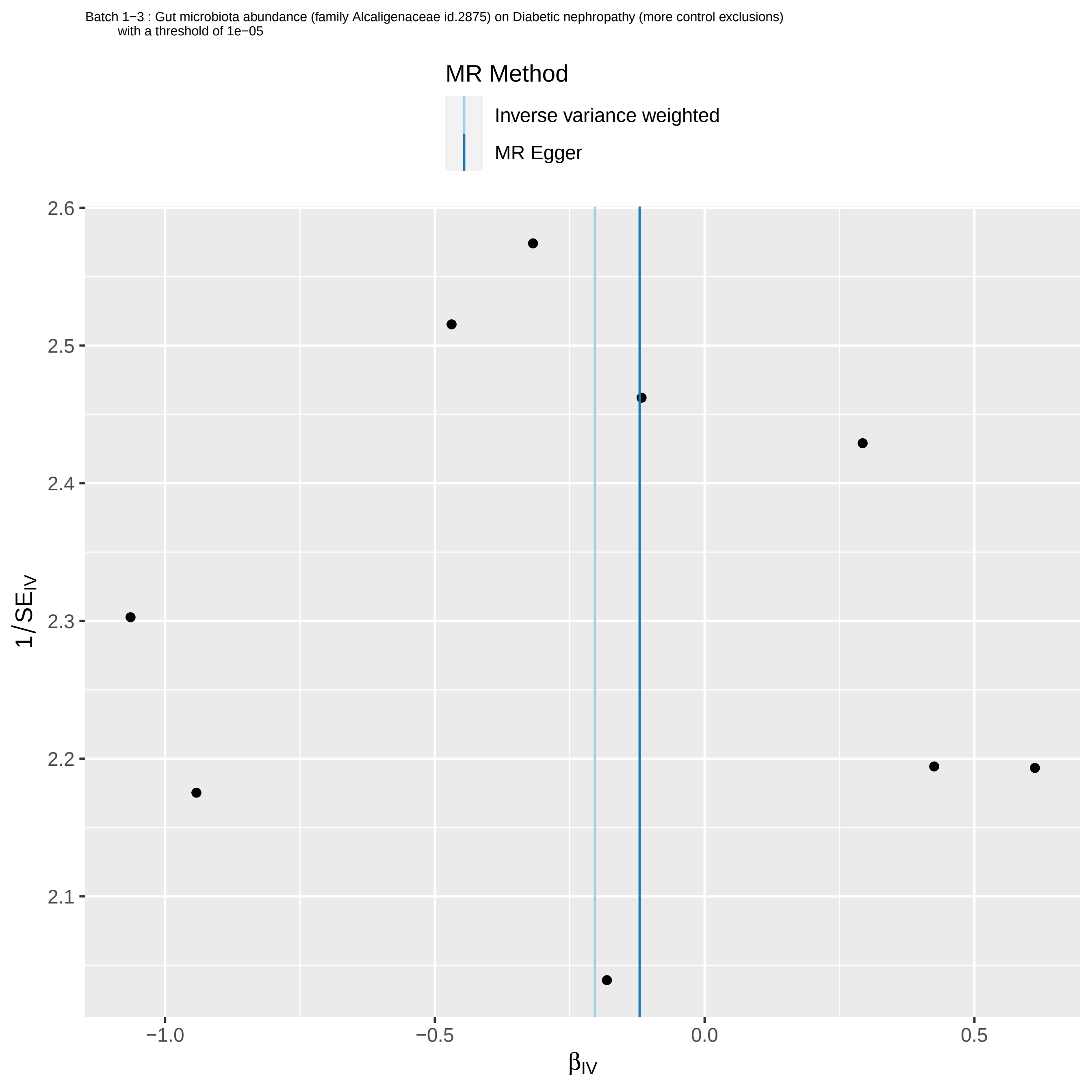
Batch 1−3 : Gut microbiota abundance (family Alcaligenaceae id.2875) on Diabetic nephropathy (more control exclusions)
with a threshold of 1e−05
MR Method
Inverse variance weighted
MR Egger
2.6
2.5
2.4
2.3
2.2
2.1
1/SE_IV
−1.0
−0.5
0.0
0.5
β_IV

Batch 1−4 : Gut microbiota abundance (family Bacteroidaceae id.917) on Diabetic nephropathy (more control exclusions) with a threshold of 1e−05

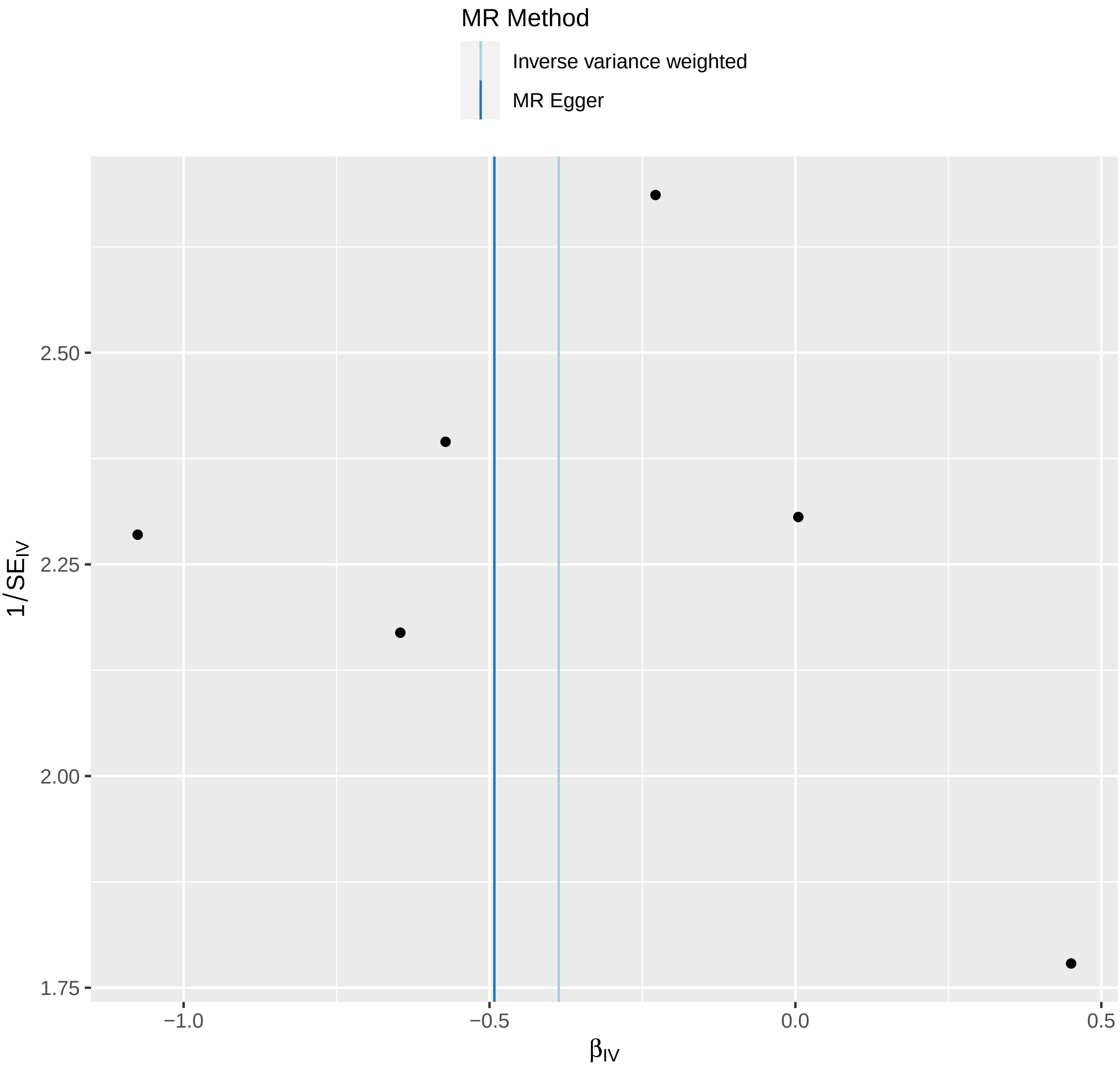

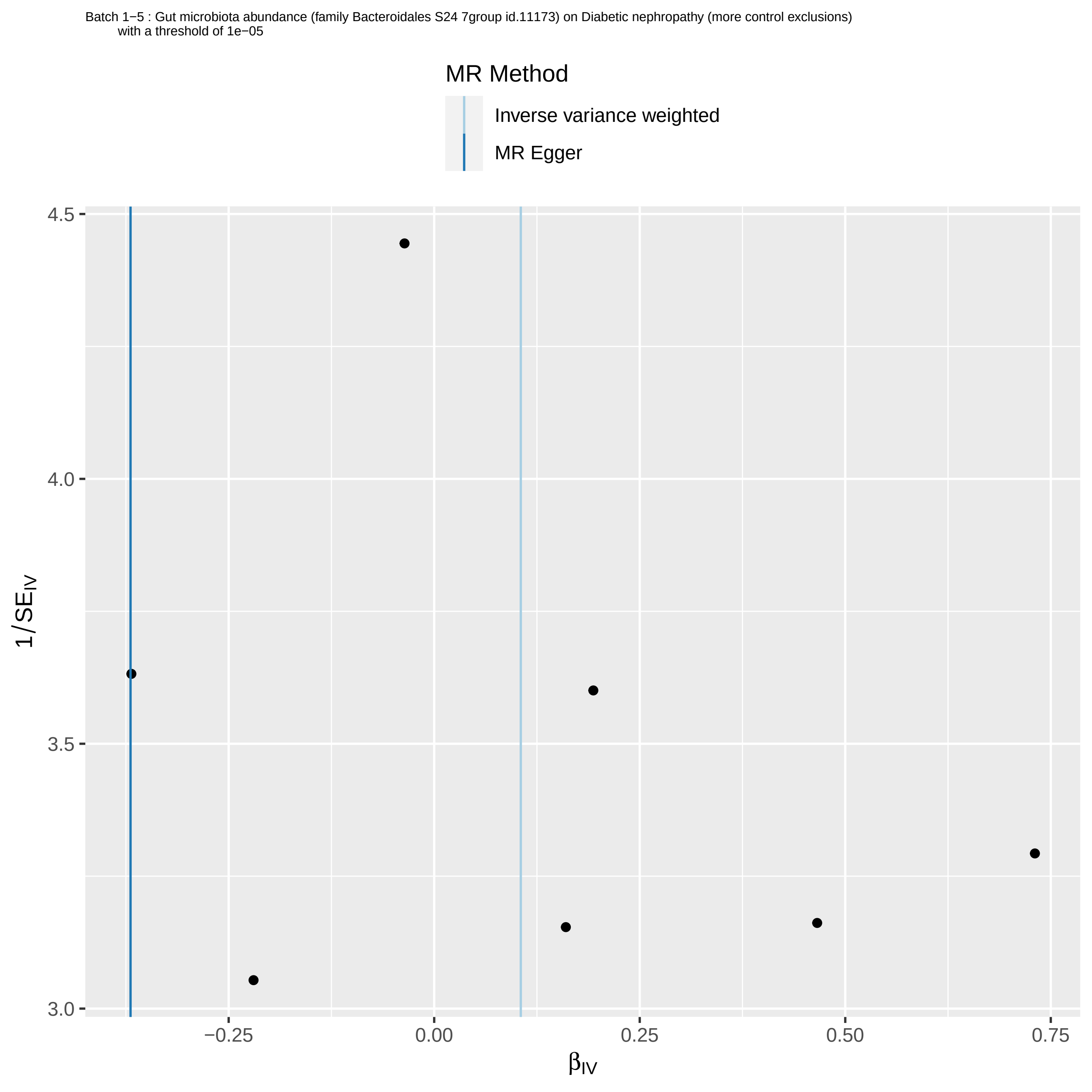
Batch 1−5 : Gut microbiota abundance (family Bacteroidales S24 7group id.11173) on Diabetic nephropathy (more control exclusions)
with a threshold of 1e−05
MR Method
Inverse variance weighted
MR Egger
4.5
4.0
3.5
3.0
1/SE_IV
−0.25
0.00
0.25
0.50
0.75
β_IV

Batch 1−6 : Gut microbiota abundance (family Bifidobacteriaceae id.433) on Diabetic nephropathy (more control exclusions)
with a threshold of 1e−05

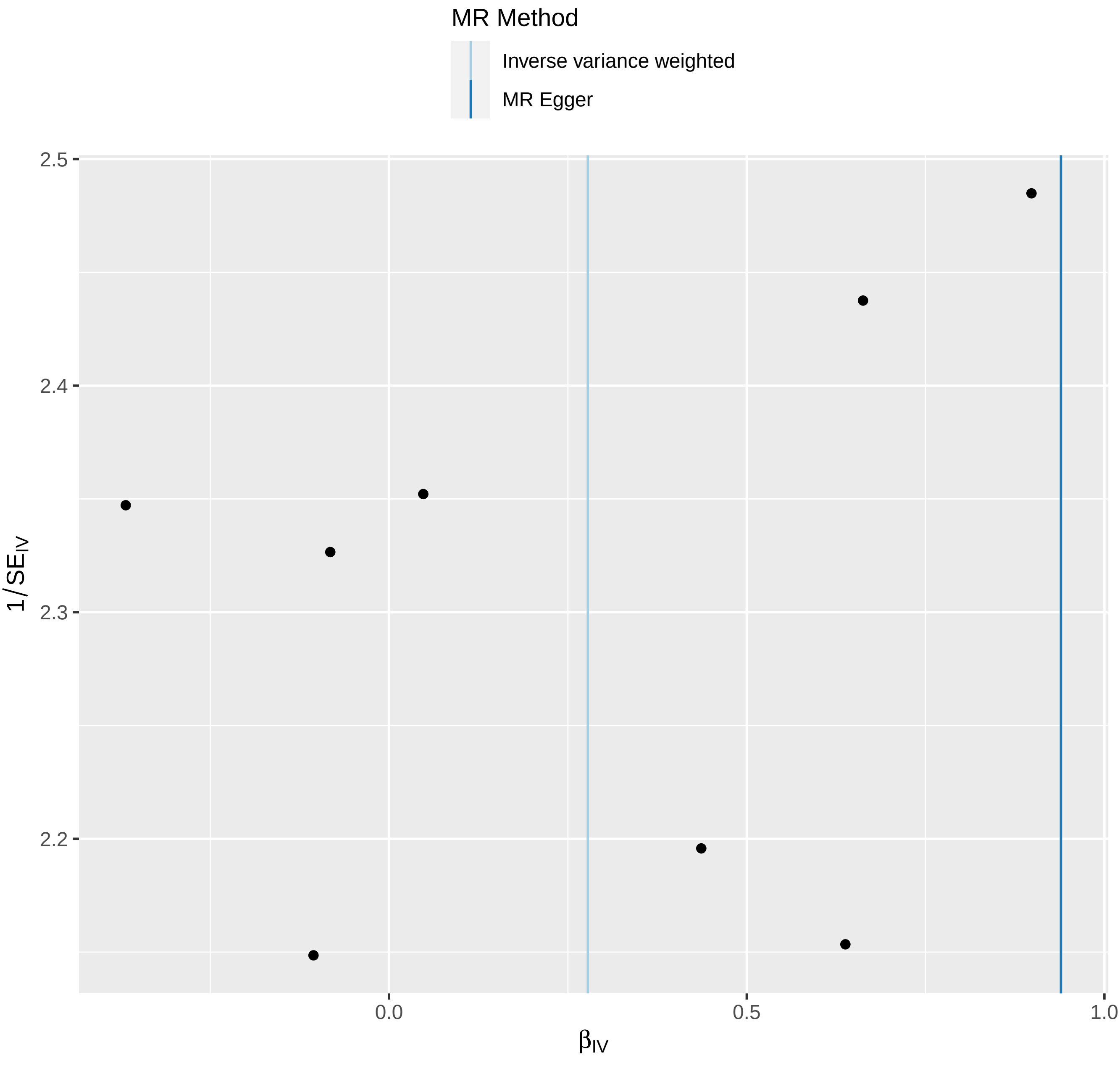

Batch 1−8 : Gut microbiota abundance (family Clostridiaceae1 id.1869) on Diabetic nephropathy (more control exclusions) with a threshold of 1e−05

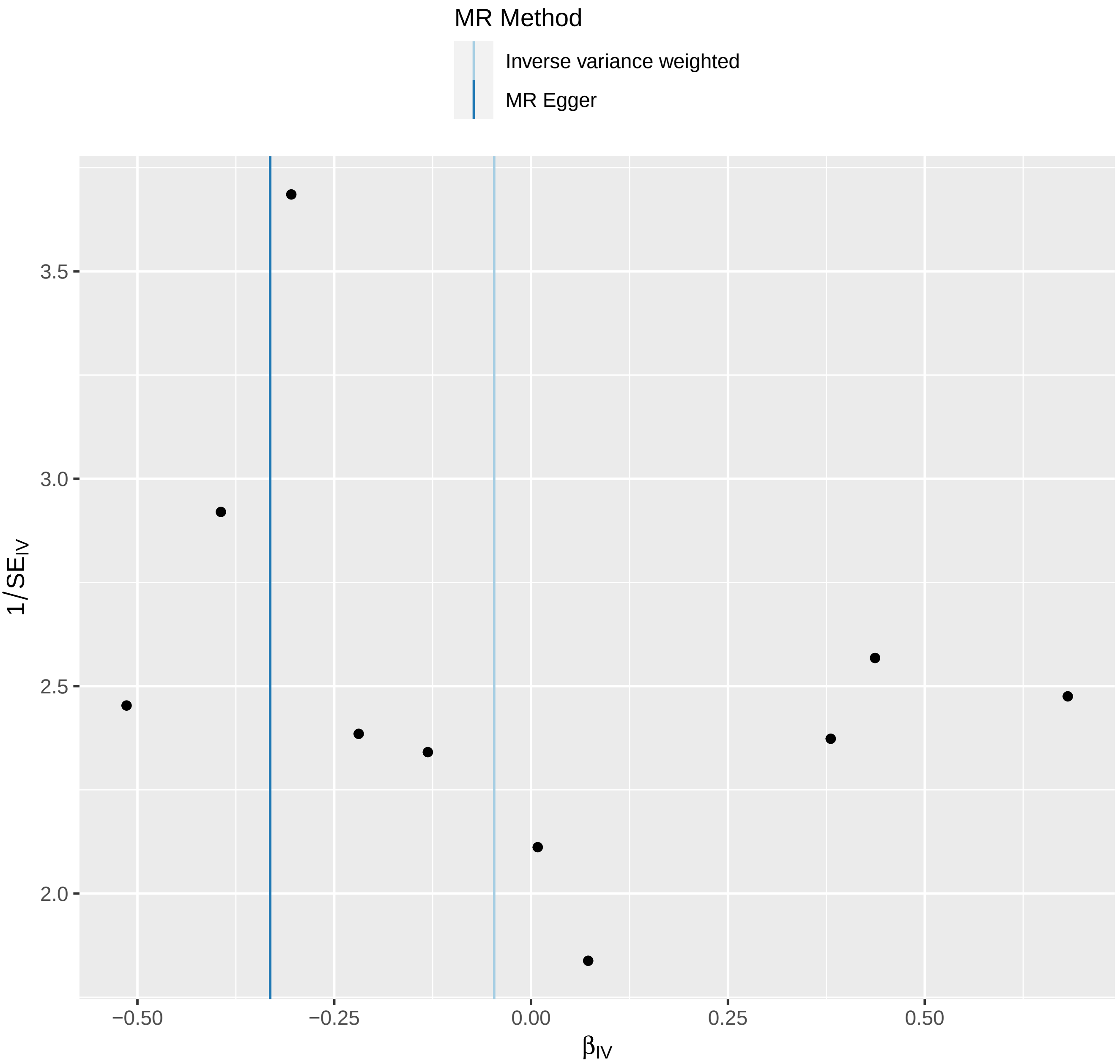

Batch 1−9 : Gut microbiota abundance (family Clostridiales vadin BB60 group id.11286) on Diabetic nephropathy (more control exclusions) with a threshold of 1e−05

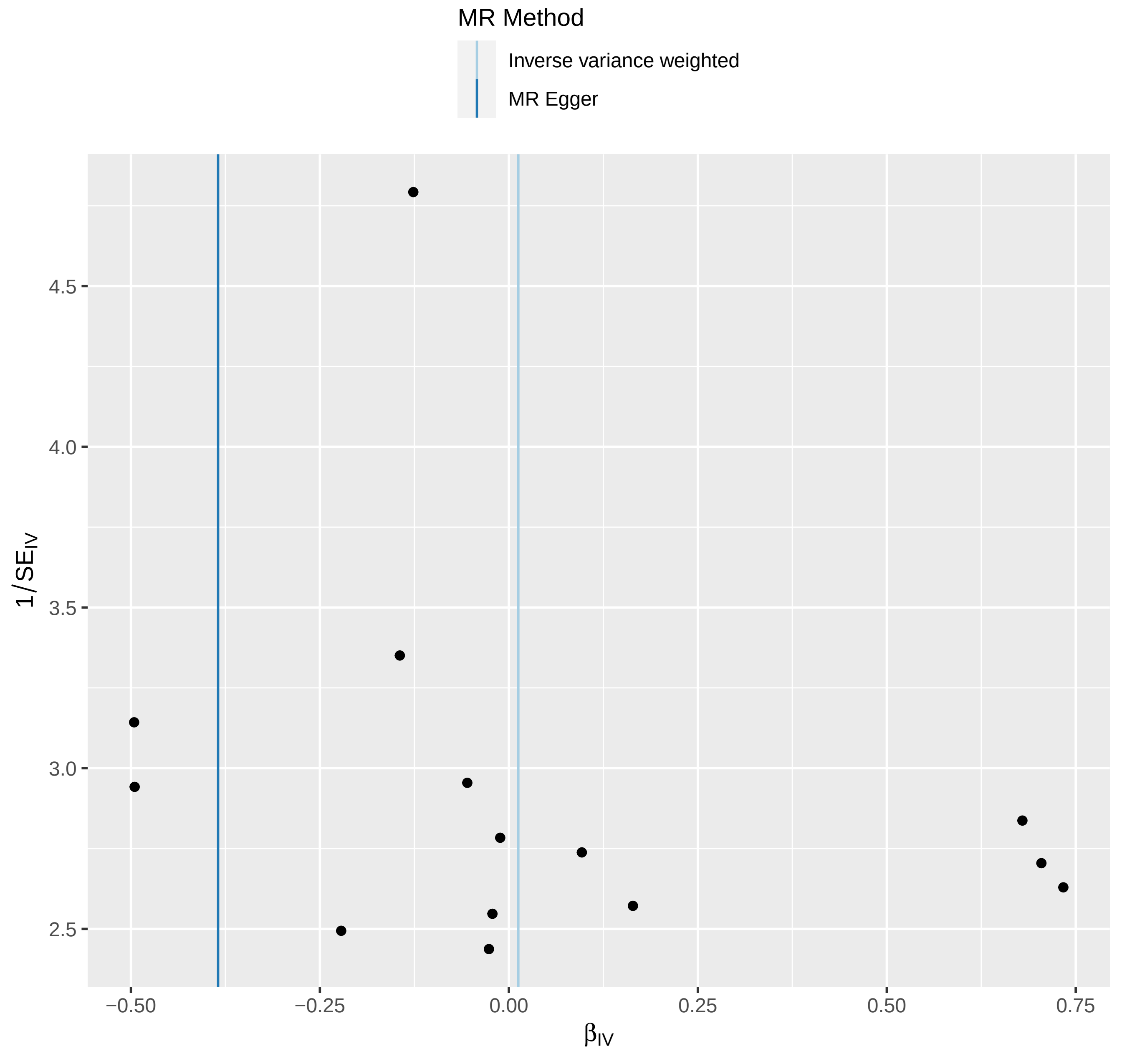

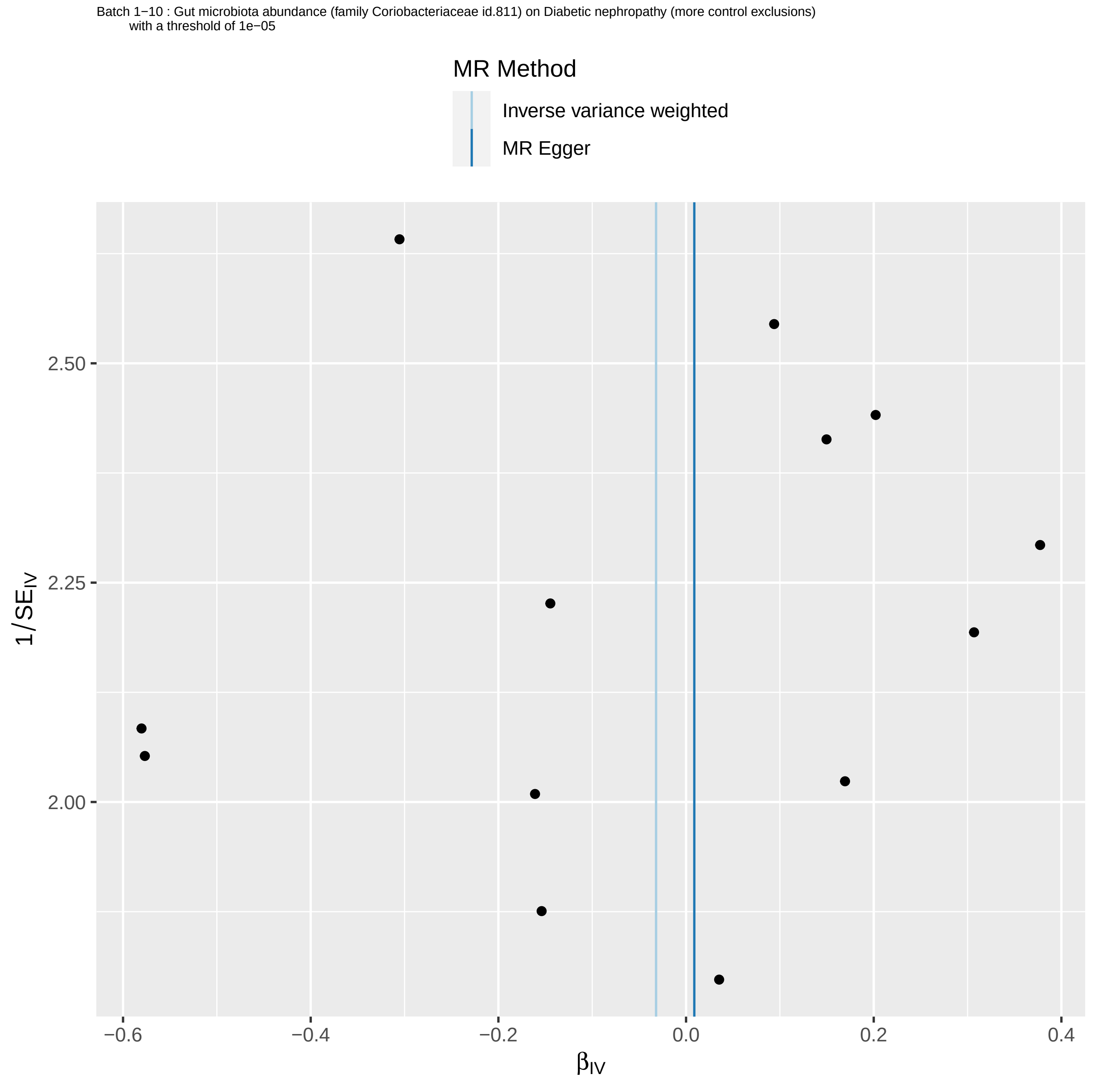
Batch 1−10 : Gut microbiota abundance (family Coriobacteriaceae id.811) on Diabetic nephropathy (more control exclusions) with a threshold of 1e−05
MR Method
Inverse variance weighted
MR Egger
2.50
2.25
2.00
1/SE_IV
−0.6
−0.4
−0.2
0.0
0.2
0.4
β_IV

Batch 1−11 : Gut microbiota abundance (family Defluviitaleaceae id.1924) on Diabetic nephropathy (more control exclusions) with a threshold of 1e−05

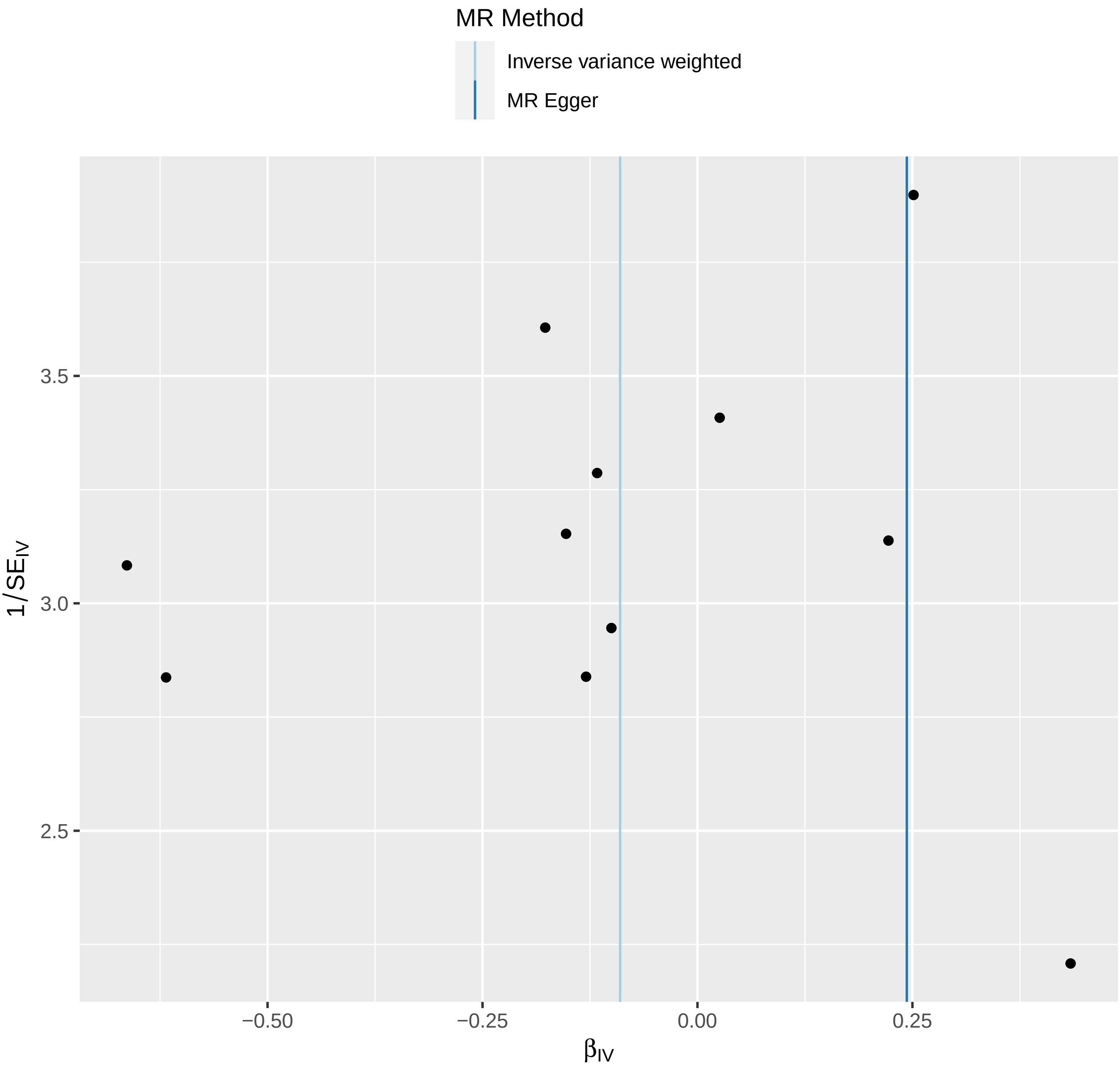

Batch 1−12 : Gut microbiota abundance (family Desulfovibrionaceae id.3169) on Diabetic nephropathy (more control exclusions) with a threshold of 1e−05

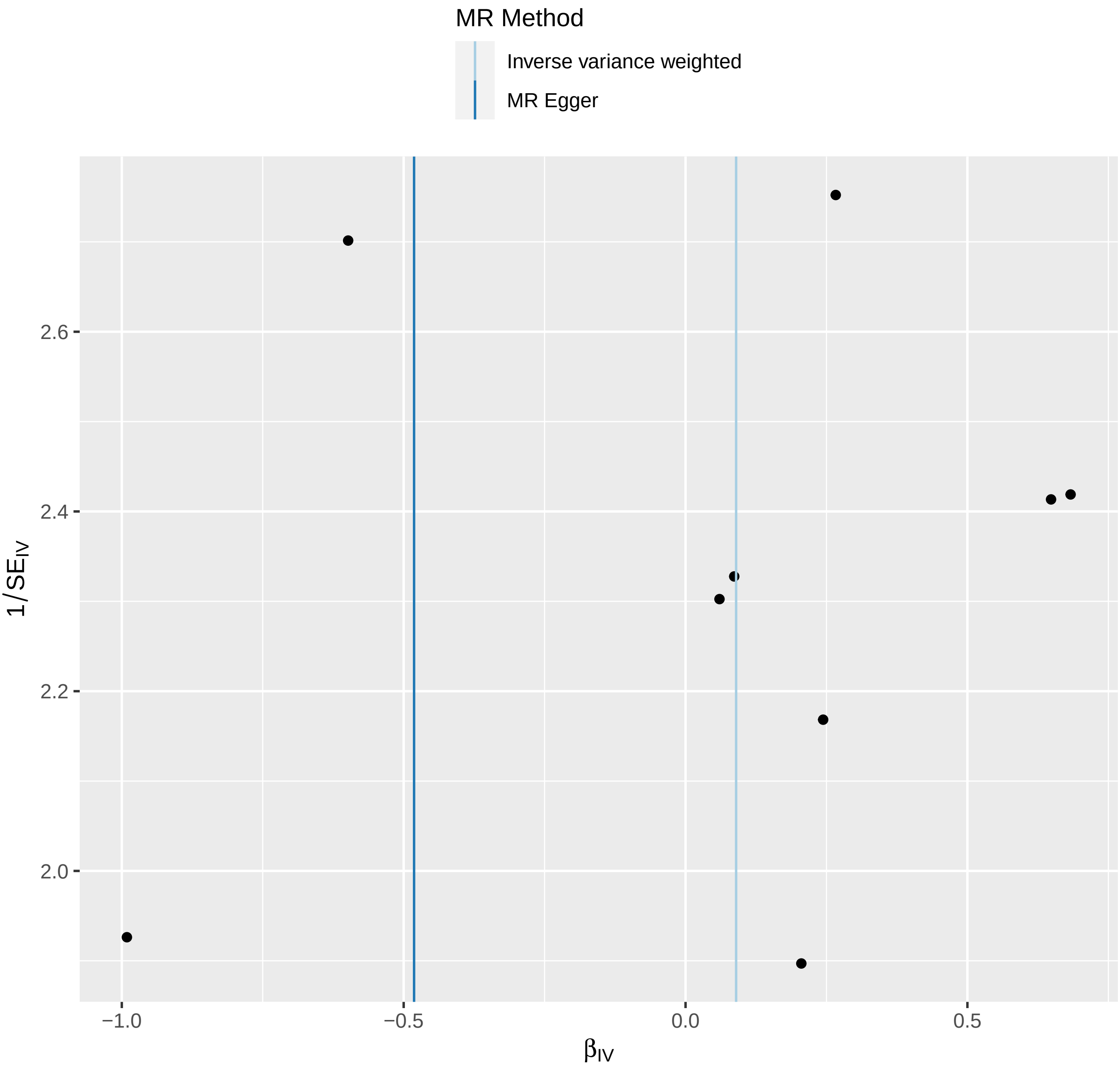

Batch 1−13 : Gut microbiota abundance (family Enterobacteriaceae id.3469) on Diabetic nephropathy (more control exclusions) with a threshold of 1e−05

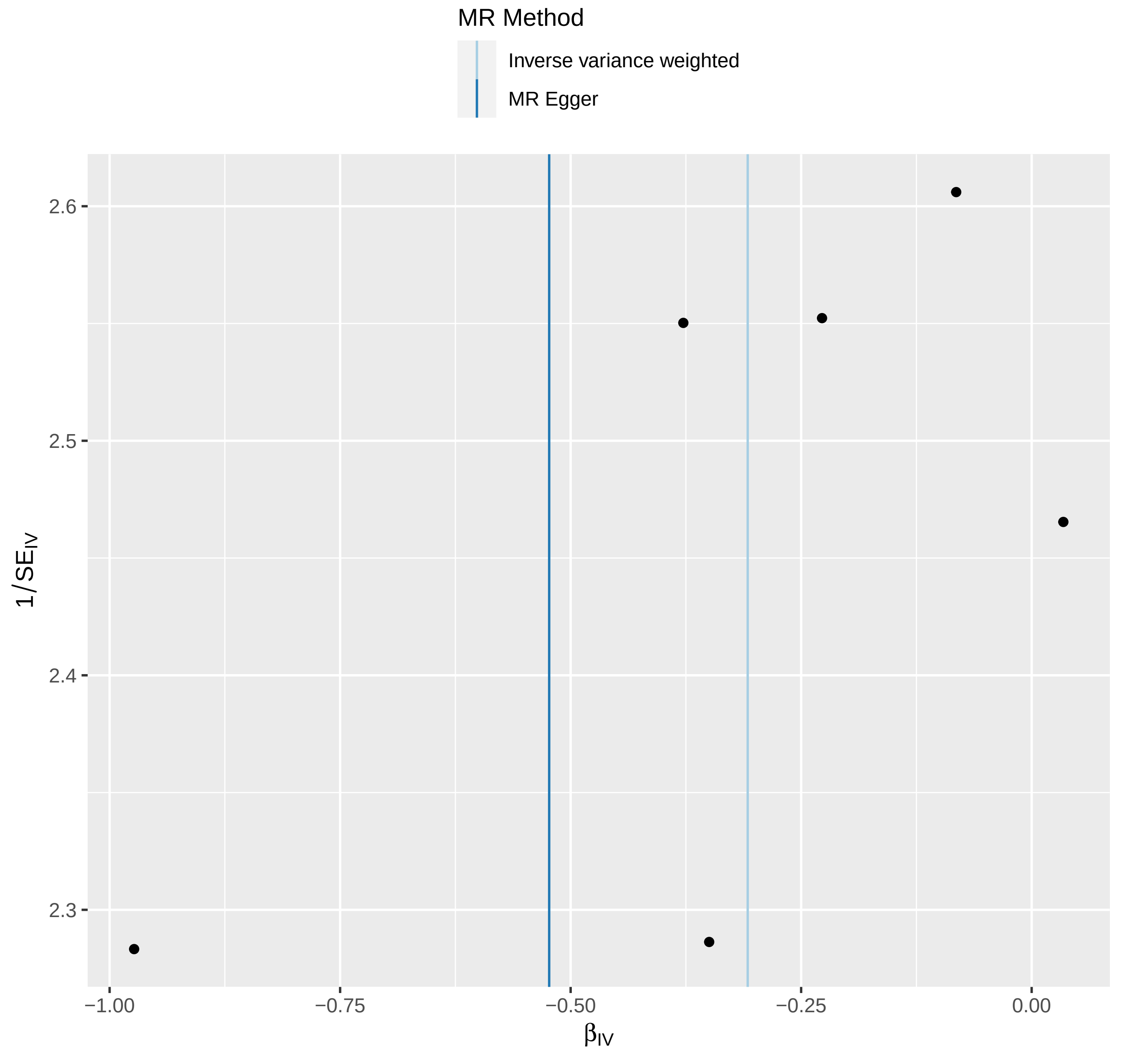

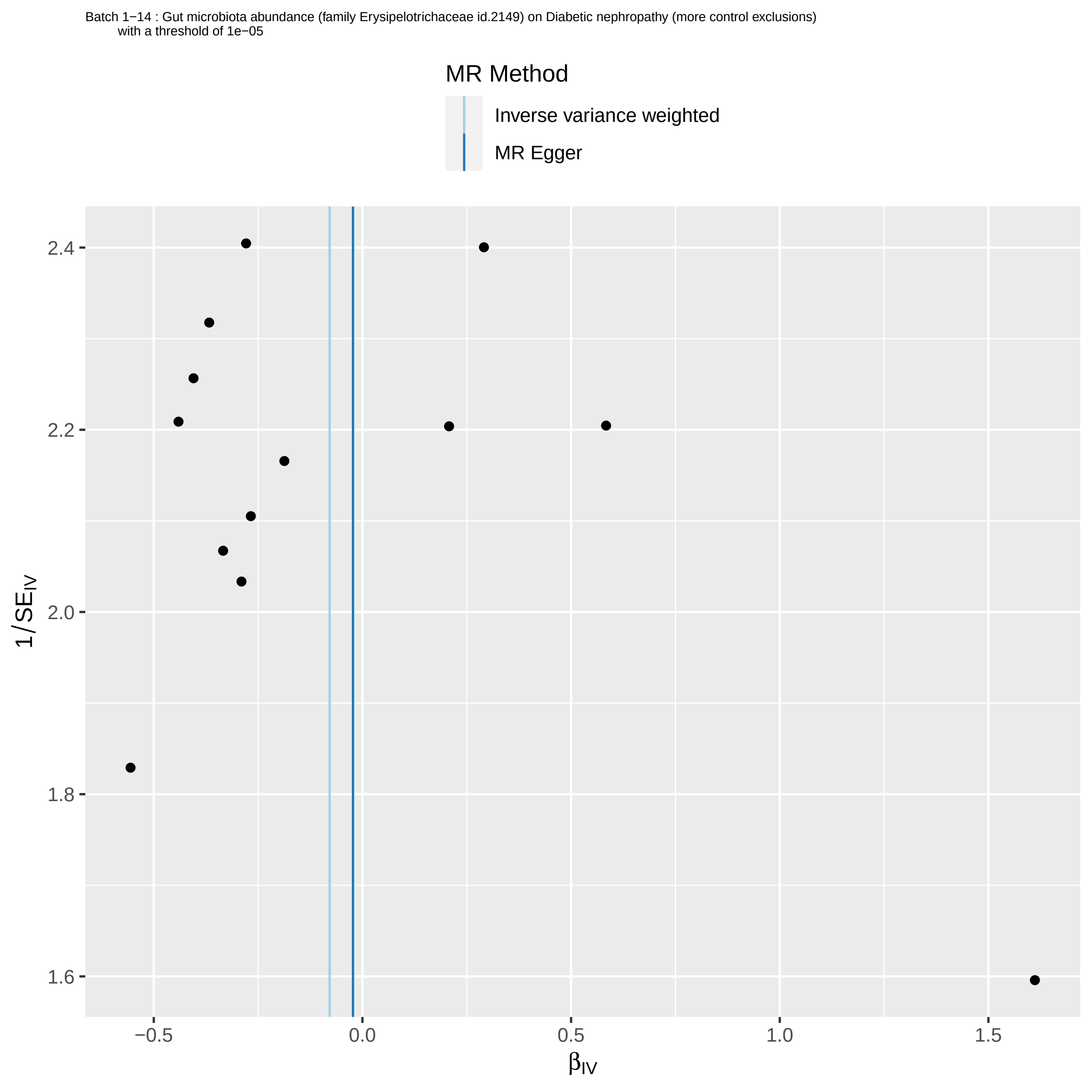
Batch 1−14 : Gut microbiota abundance (family Erysipelotrichaceae id.2149) on Diabetic nephropathy (more control exclusions)
with a threshold of 1e−05
MR Method
Inverse variance weighted
MR Egger
2.4
2.2
2.0
1.8
1.6
1/SE_IV
−0.5
0.0
0.5
1.0
1.5
β_IV

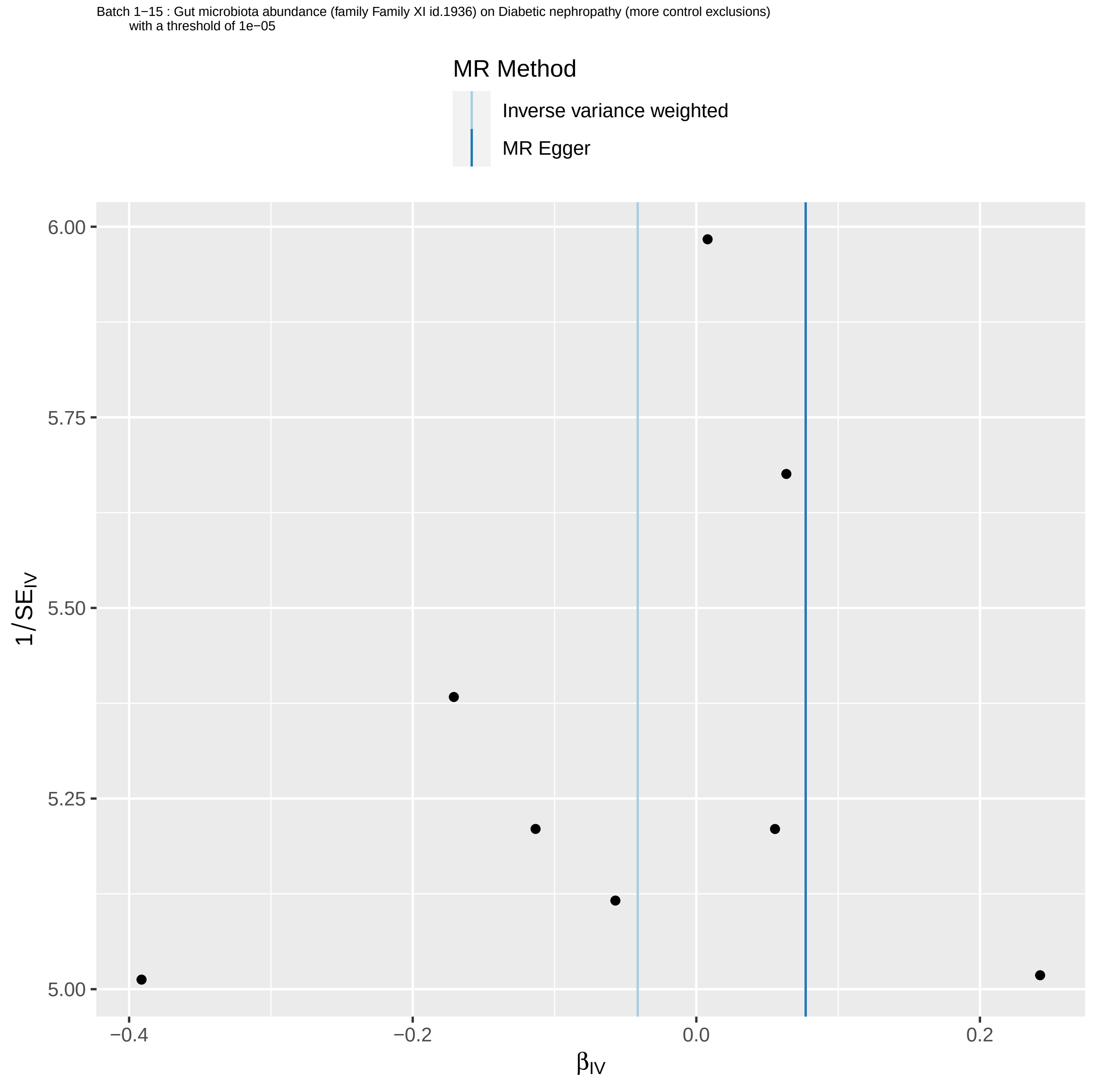
Batch 1−15 : Gut microbiota abundance (family Family XI id.1936) on Diabetic nephropathy (more control exclusions)
with a threshold of 1e−05
MR Method
Inverse variance weighted
MR Egger
6.00
5.75
5.50
5.25
5.00
1/SE_IV
−0.4
−0.2
0.0
0.2
β_IV

Batch 1−16 : Gut microbiota abundance (family Family XIII id.1957) on Diabetic nephropathy (more control exclusions) with a threshold of 1e−05

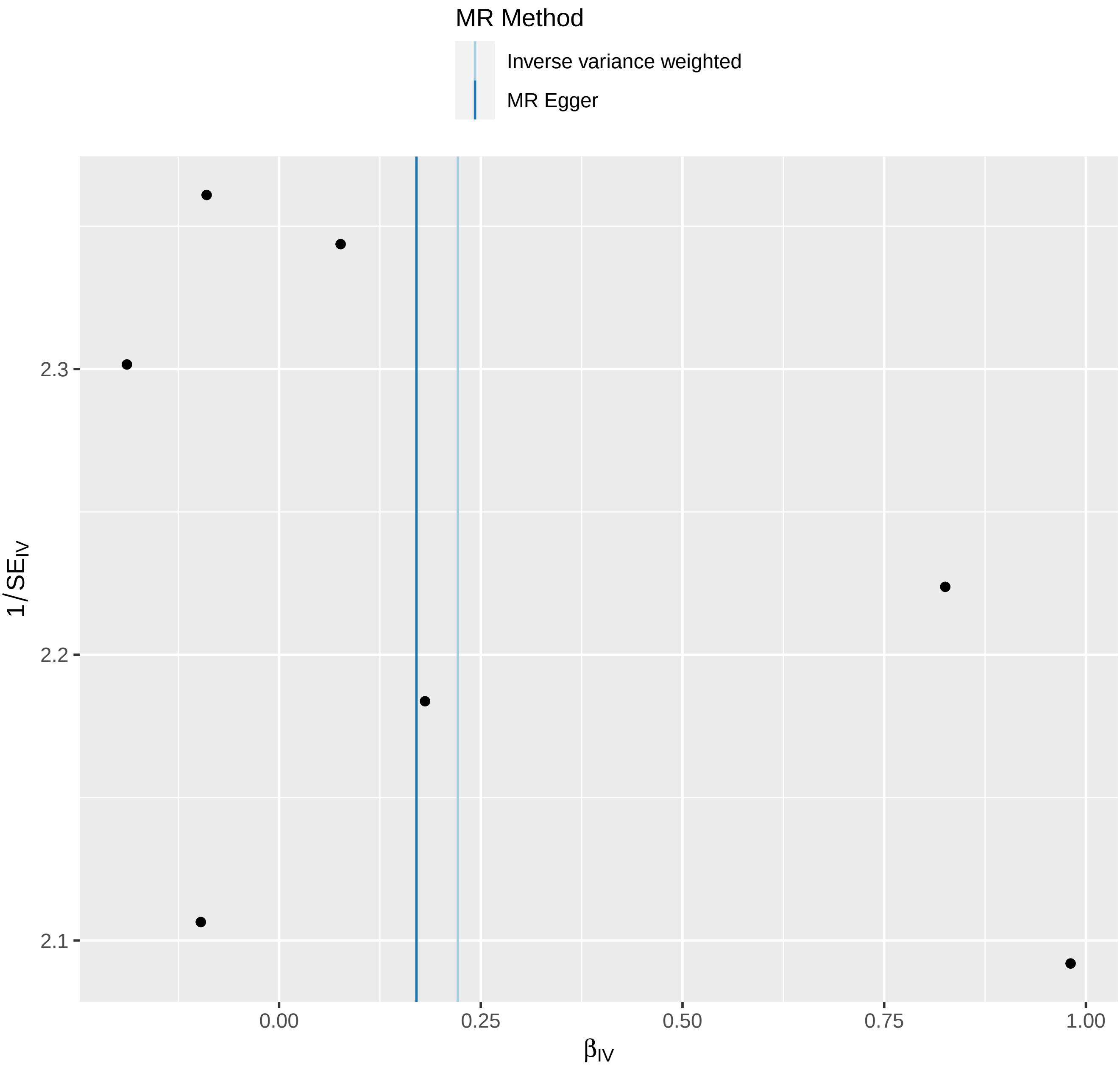

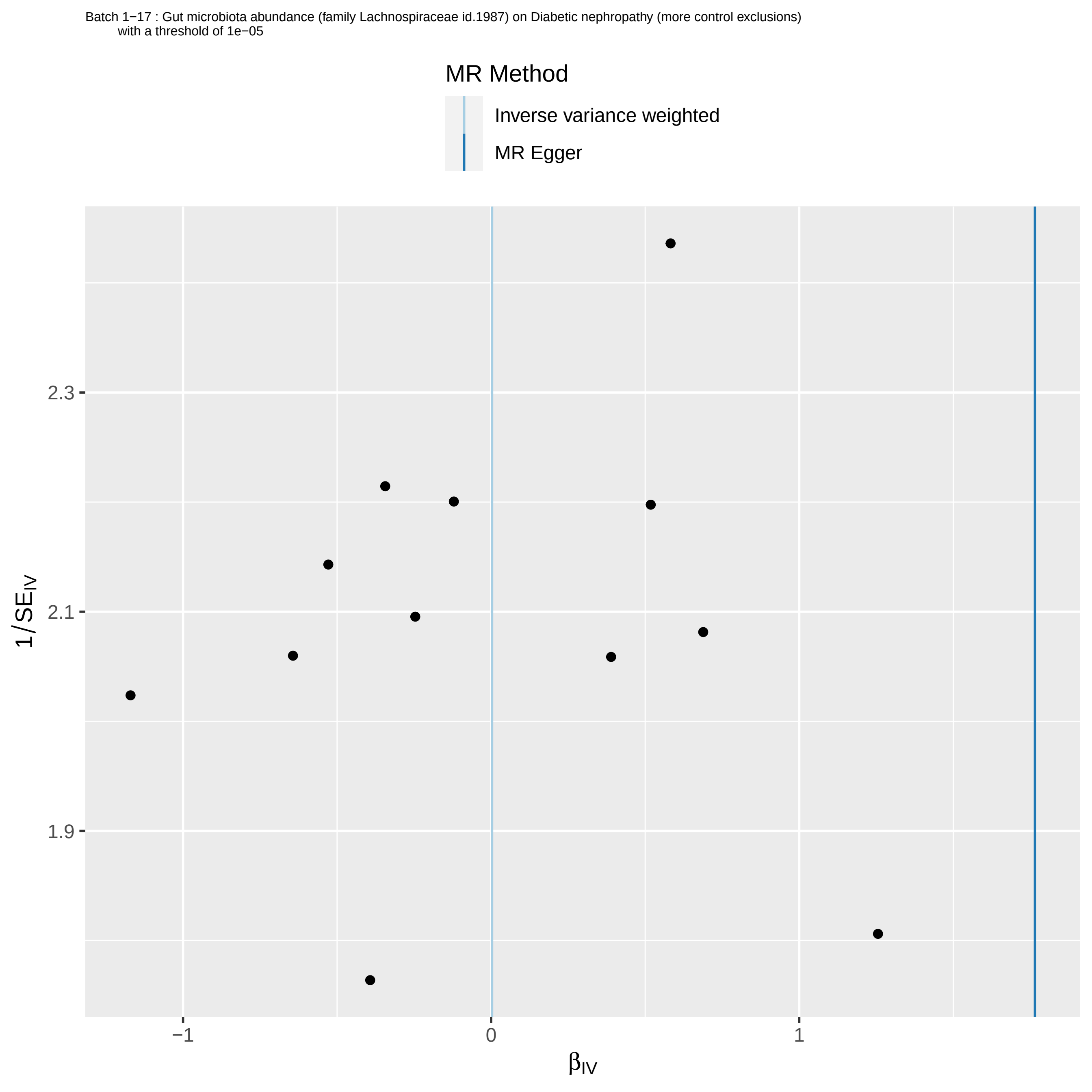
Batch 1−17 : Gut microbiota abundance (family Lachnospiraceae id.1987) on Diabetic nephropathy (more control exclusions)
with a threshold of 1e−05
MR Method
Inverse variance weighted
MR Egger
2.3
2.1
1.9
1/SE_IV
−1
0
1
β_IV

Batch 1−18 : Gut microbiota abundance (family Lactobacillaceae id.1836) on Diabetic nephropathy (more control exclusions) with a threshold of 1e−05

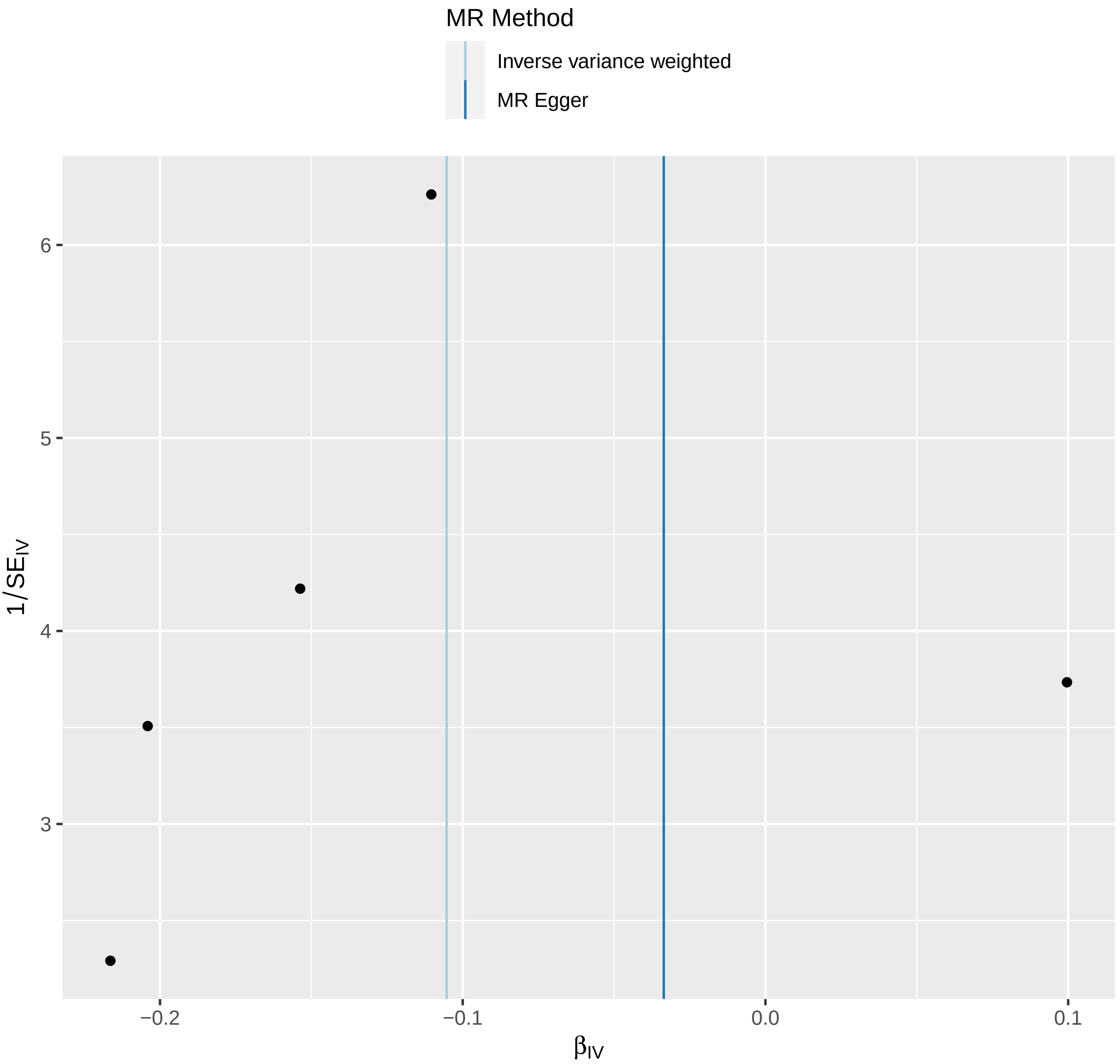

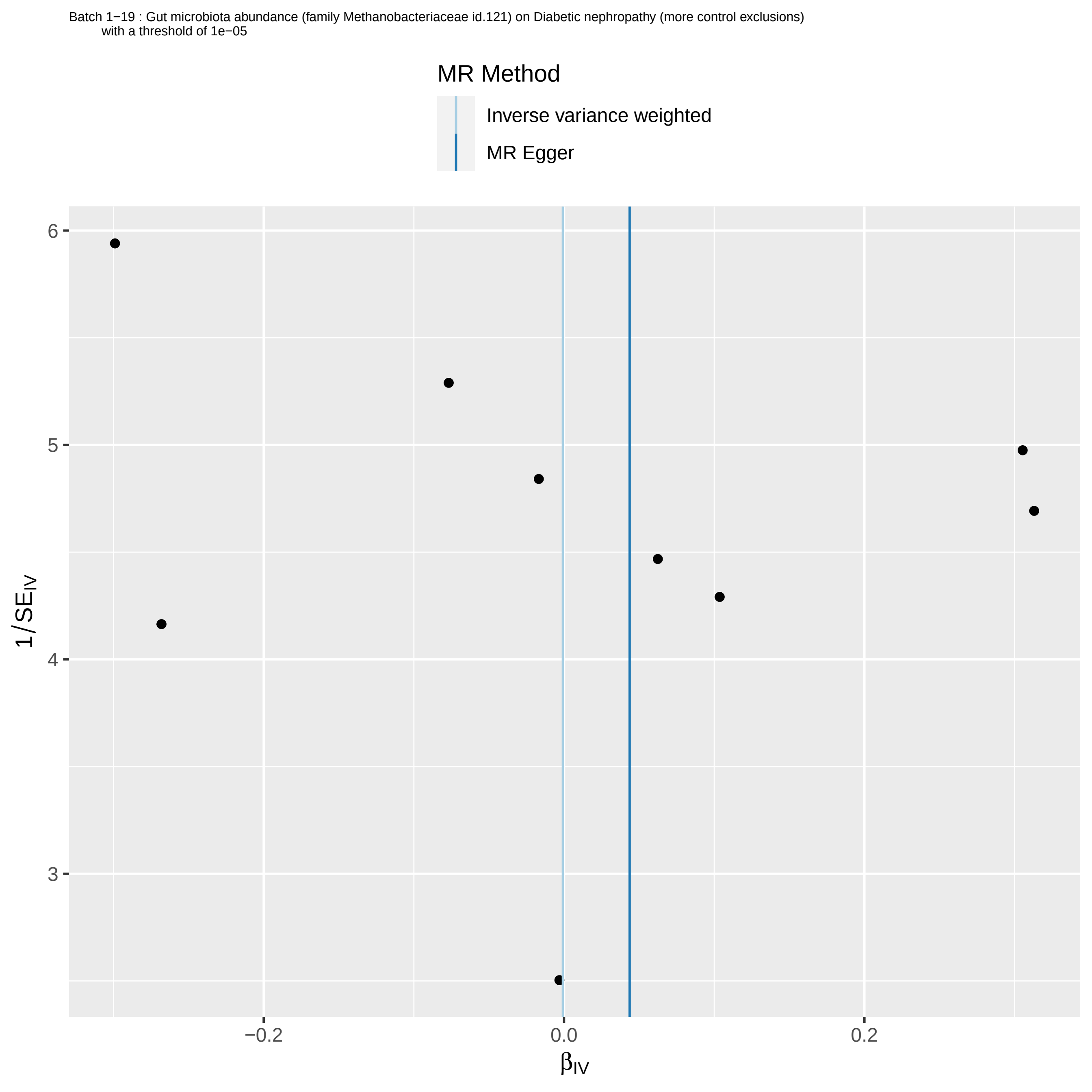
Batch 1−19 : Gut microbiota abundance (family Methanobacteriaceae id.121) on Diabetic nephropathy (more control exclusions)
with a threshold of 1e−05
MR Method
Inverse variance weighted
MR Egger
6
5
4
3
1/SE_IV
−0.2
0.0
0.2
β_IV

Batch 1−20 : Gut microbiota abundance (family Oxalobacteraceae id.2966) on Diabetic nephropathy (more control exclusions)
with a threshold of 1e−05

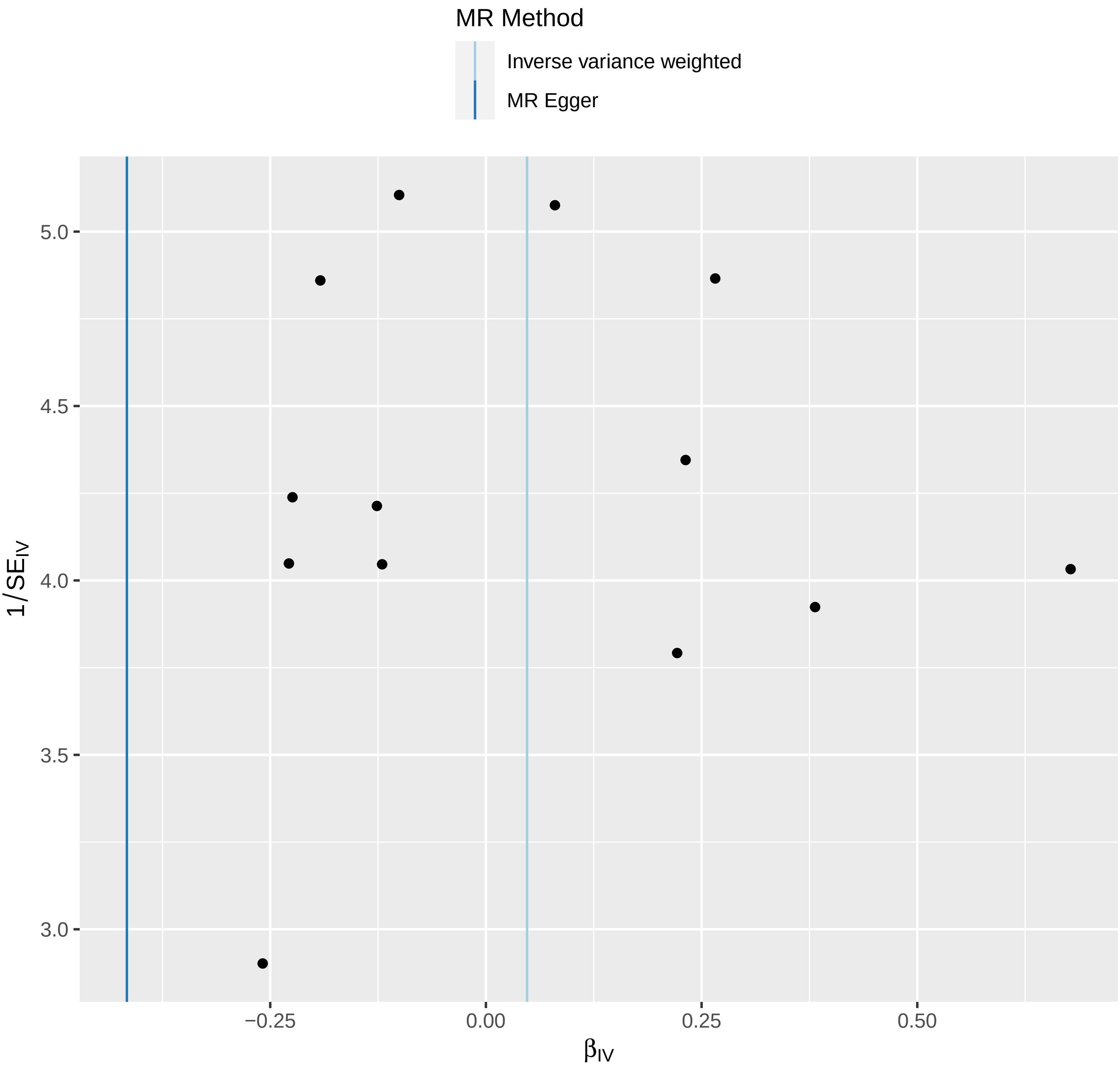

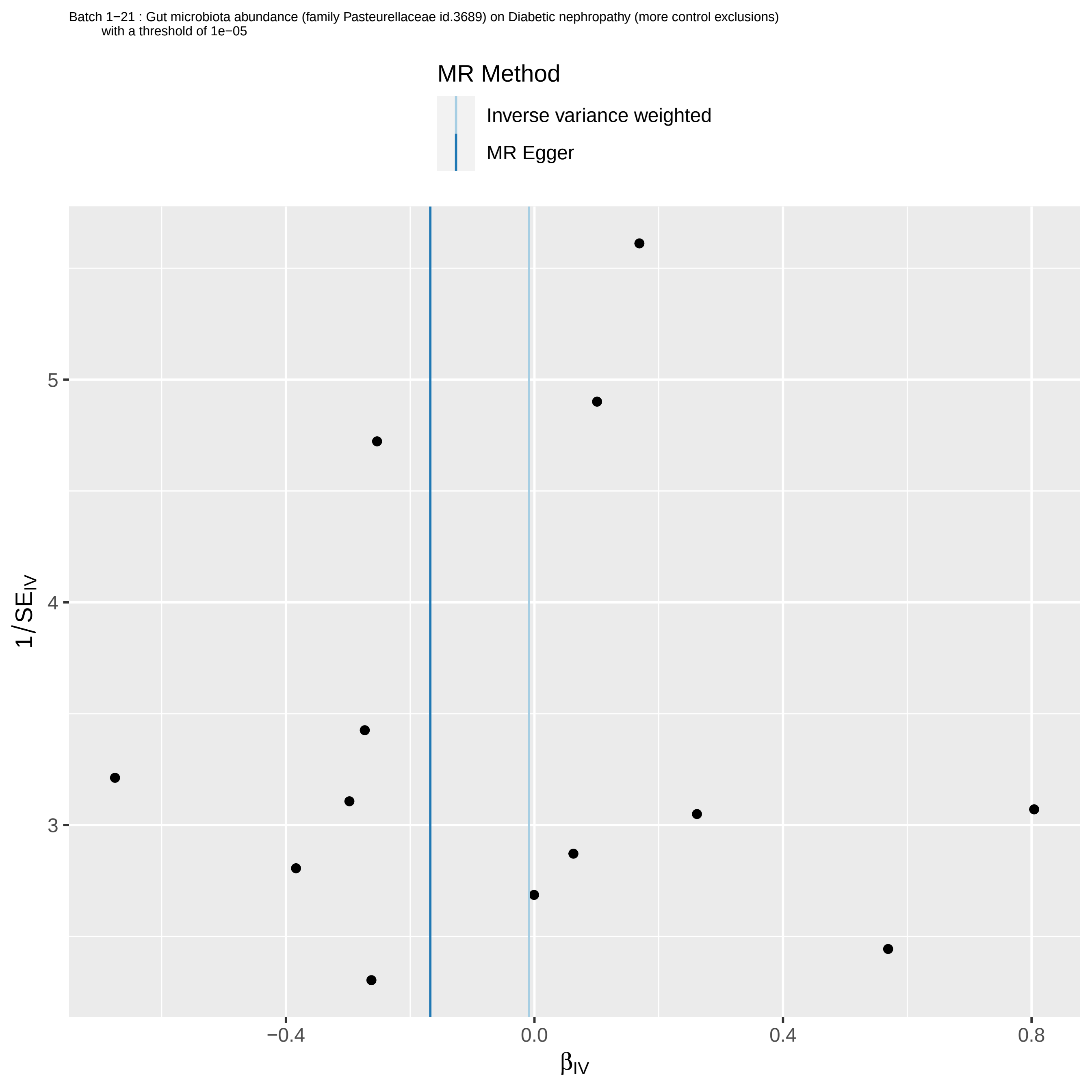

Batch 1−21 : Gut microbiota abundance (family Pasteurellaceae id.3689) on Diabetic nephropathy (more control exclusions)
with a threshold of 1e−05
MR Method
Inverse variance weighted
MR Egger
5
4
3
1/SE_IV
−0.4
0.0
0.4
0.8
β_IV

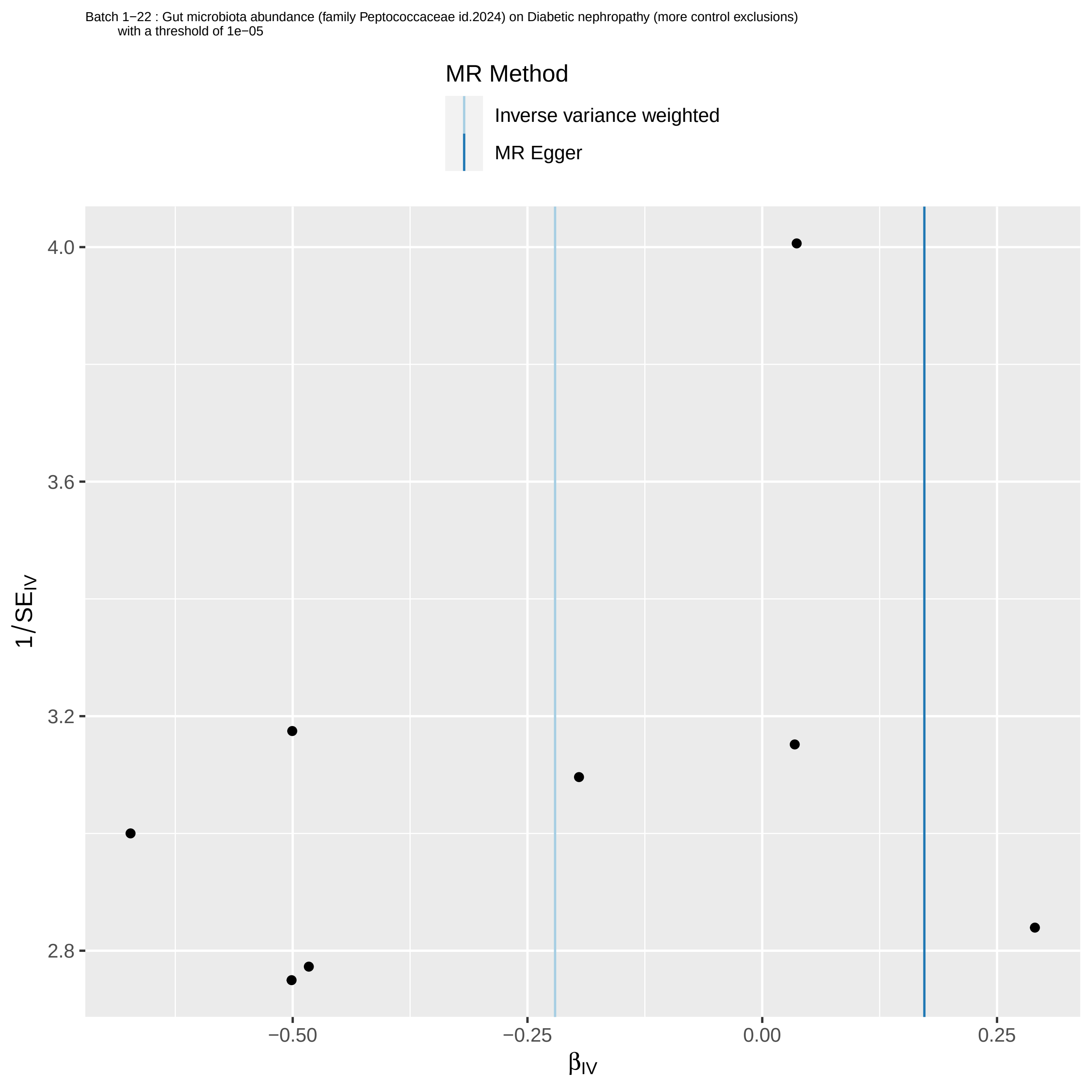
Batch 1−22 : Gut microbiota abundance (family Peptococcaceae id.2024) on Diabetic nephropathy (more control exclusions)
with a threshold of 1e−05
MR Method
Inverse variance weighted
MR Egger
4.0
3.6
3.2
2.8
1/SE_IV
−0.50
−0.25
0.00
0.25
β_IV

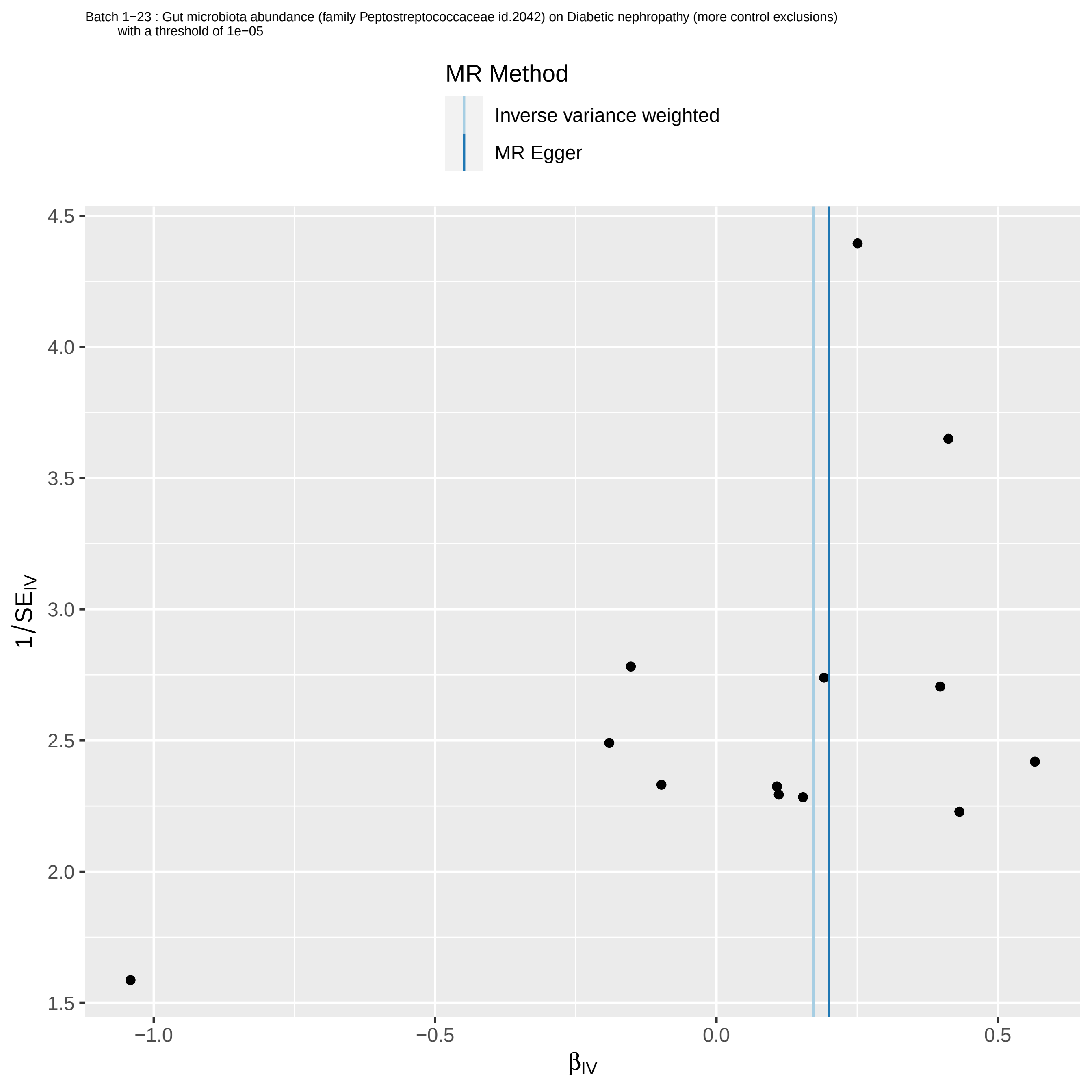

Batch 1−23 : Gut microbiota abundance (family Peptostreptococcaceae id.2042) on Diabetic nephropathy (more control exclusions) with a threshold of 1e−05
MR Method
Inverse variance weighted
MR Egger
4.5
4.0
3.5
3.0
2.5
2.0
1.5
1/SE_IV
−1.0
−0.5
0.0
0.5
β_IV

Batch 1−24 : Gut microbiota abundance (family Porphyromonadaceae id.943) on Diabetic nephropathy (more control exclusions) with a threshold of 1e−05

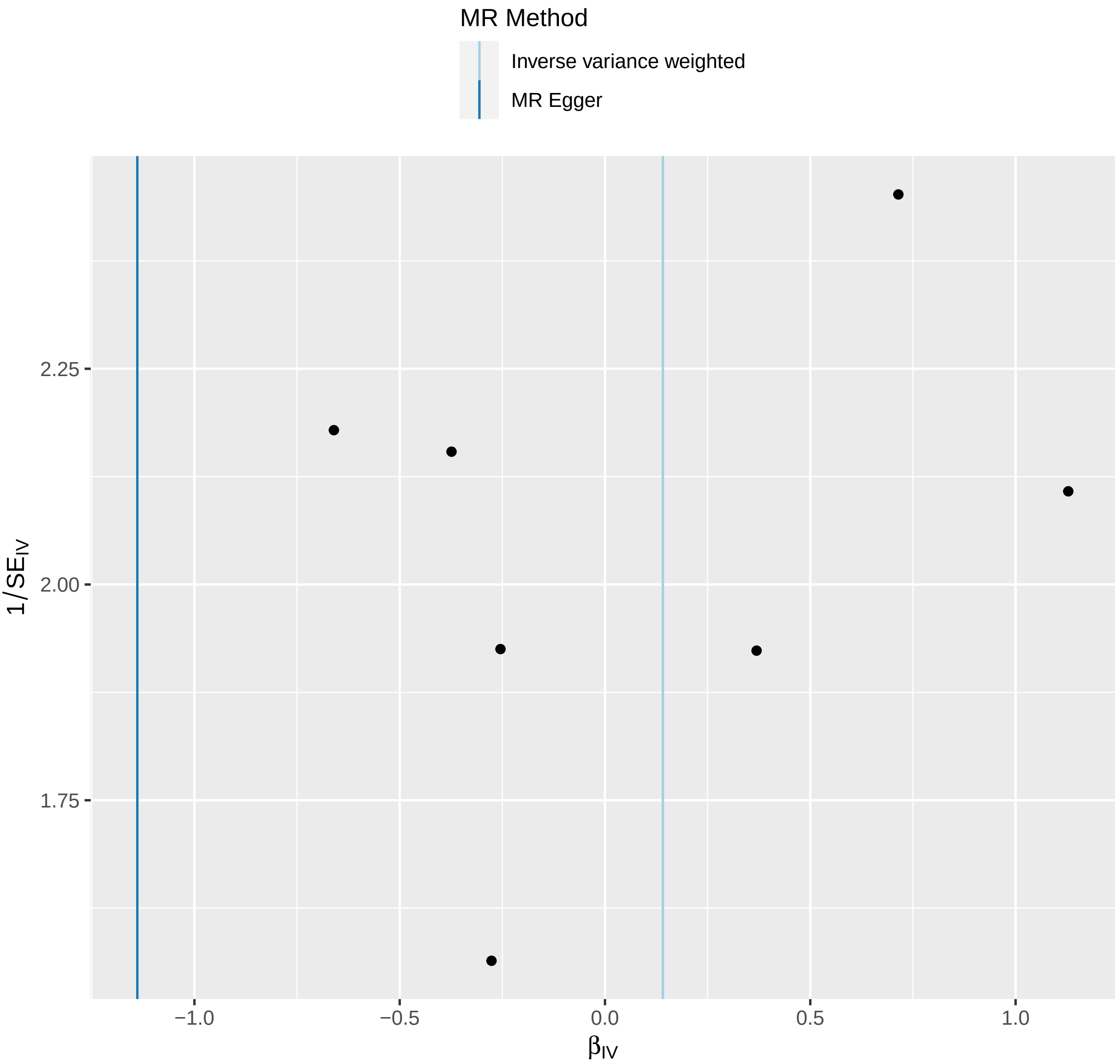

Batch 1−25 : Gut microbiota abundance (family Prevotellaceae id.960) on Diabetic nephropathy (more control exclusions) with a threshold of 1e−05

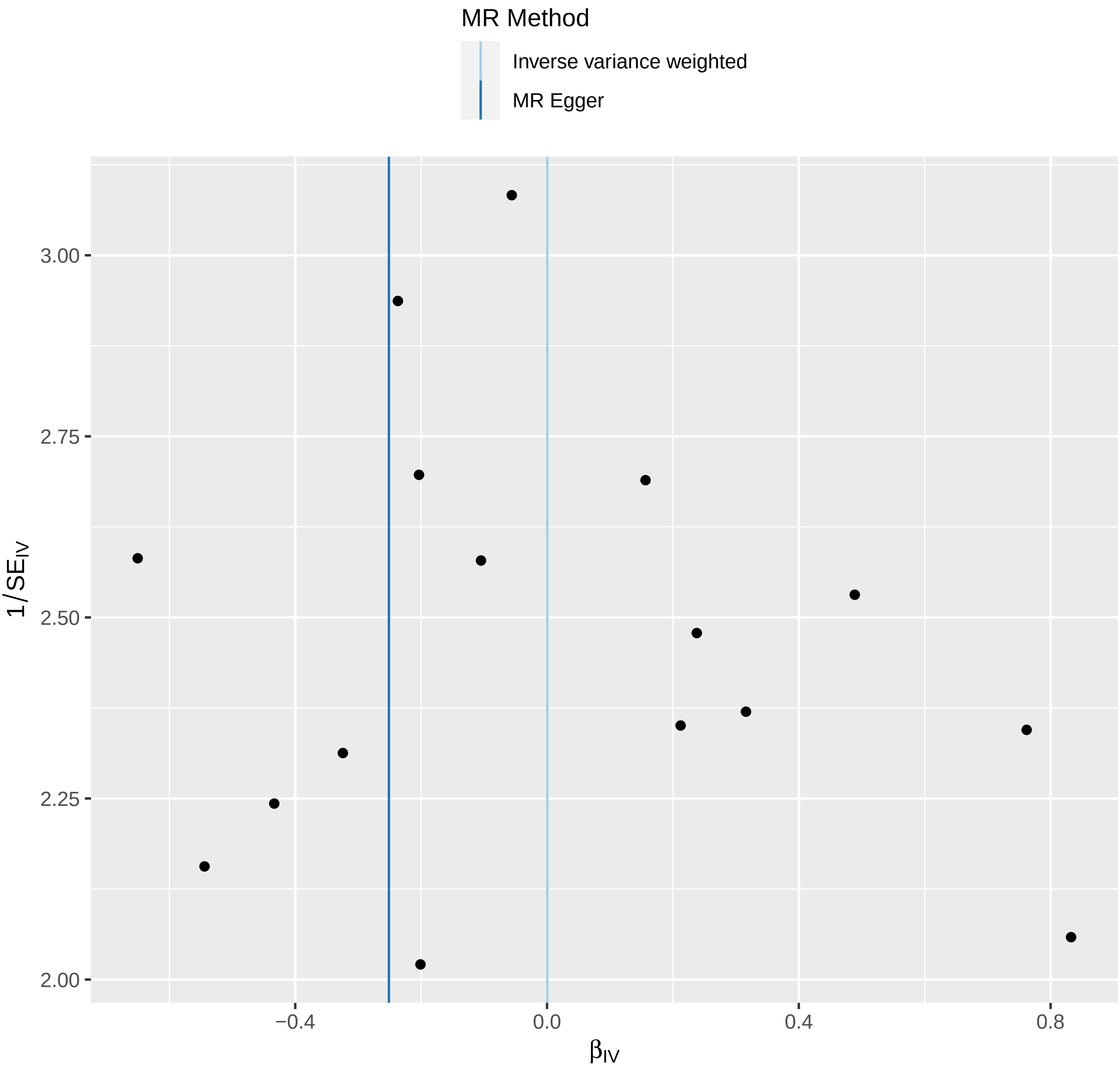

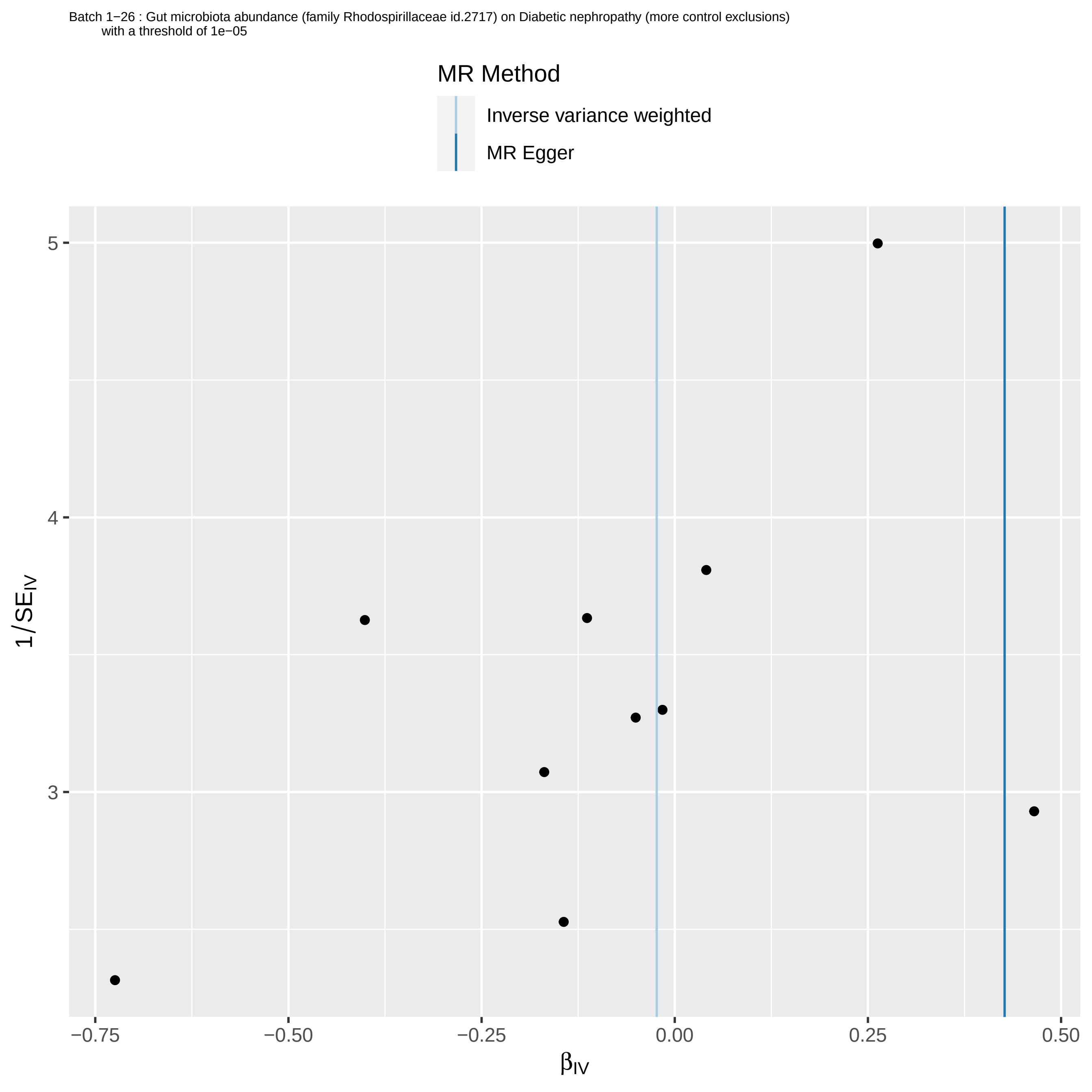

Batch 1−26 : Gut microbiota abundance (family Rhodospirillaceae id.2717) on Diabetic nephropathy (more control exclusions) with a threshold of 1e−05
MR Method
Inverse variance weighted
MR Egger
5
4
3
1/SE_IV
−0.75
−0.50
−0.25
0.00
0.25
0.50
β_IV

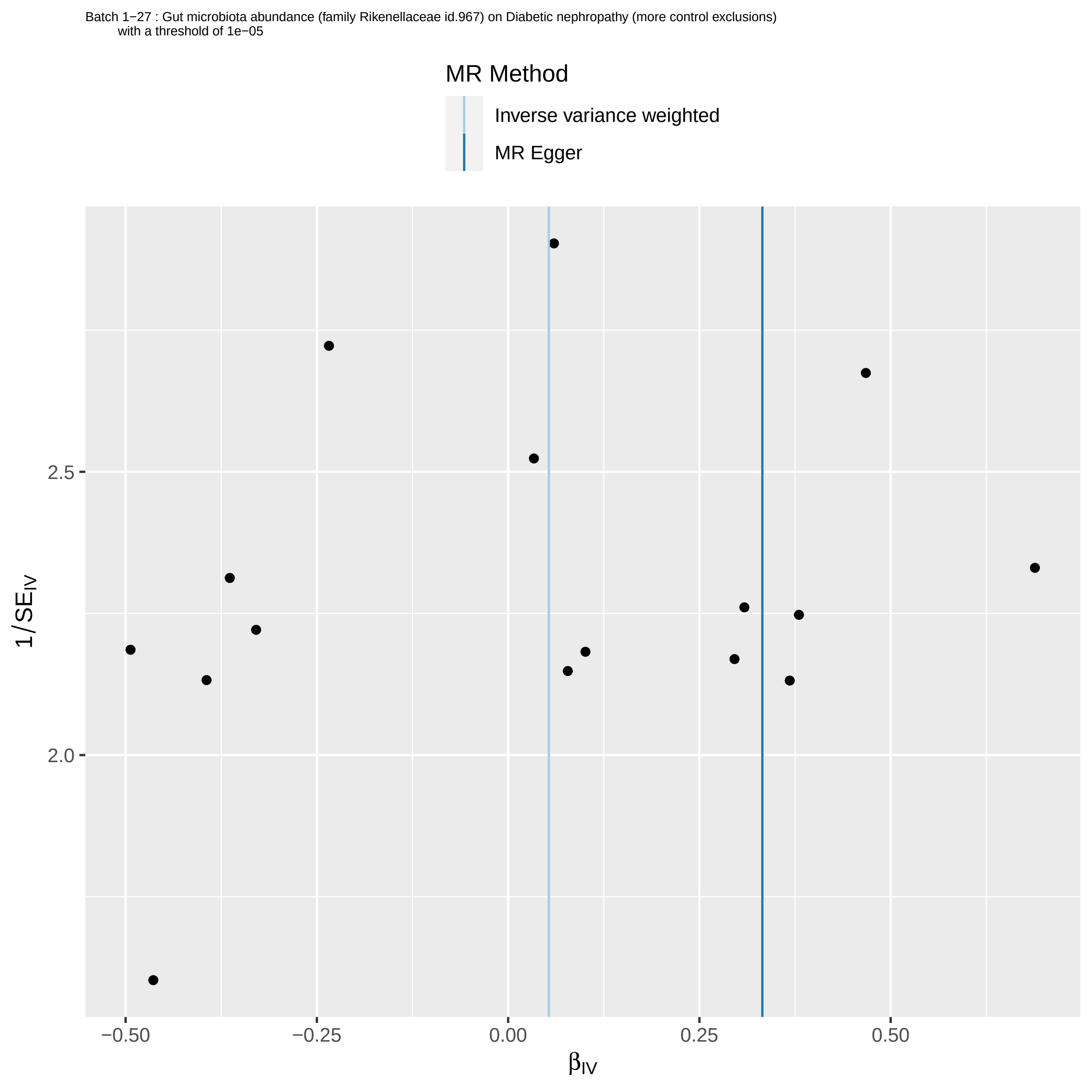

Batch 1−27 : Gut microbiota abundance (family Rikenellaceae id.967) on Diabetic nephropathy (more control exclusions)
with a threshold of 1e−05
MR Method
Inverse variance weighted
MR Egger
2.5
2.0
1/SE_IV
−0.50
−0.25
0.00
0.25
0.50
β_IV

Batch 1−28 : Gut microbiota abundance (family Ruminococcaceae id.2050) on Diabetic nephropathy (more control exclusions)
with a threshold of 1e−05

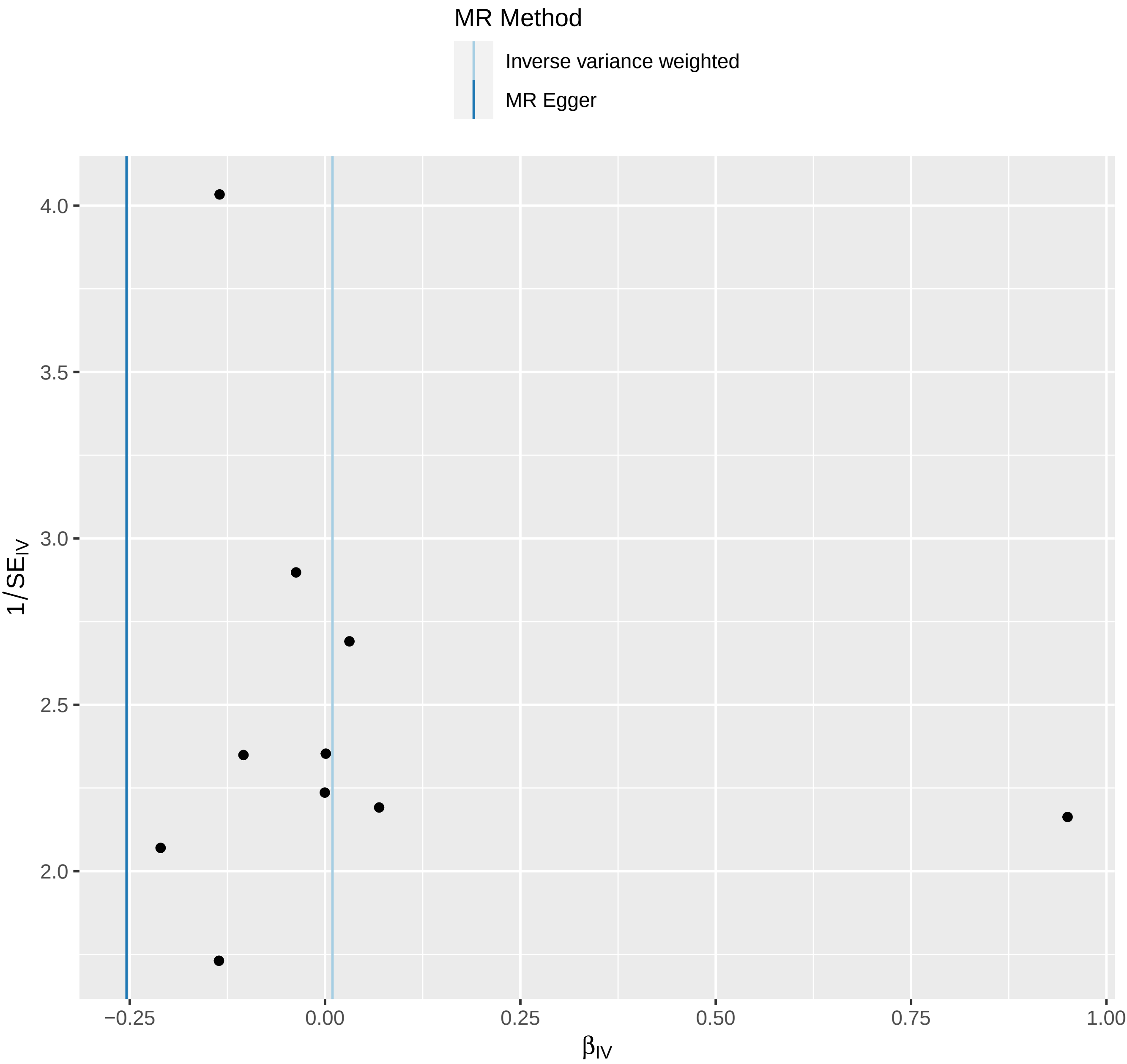

Batch 1−29 : Gut microbiota abundance (family Streptococcaceae id.1850) on Diabetic nephropathy (more control exclusions) with a threshold of 1e−05

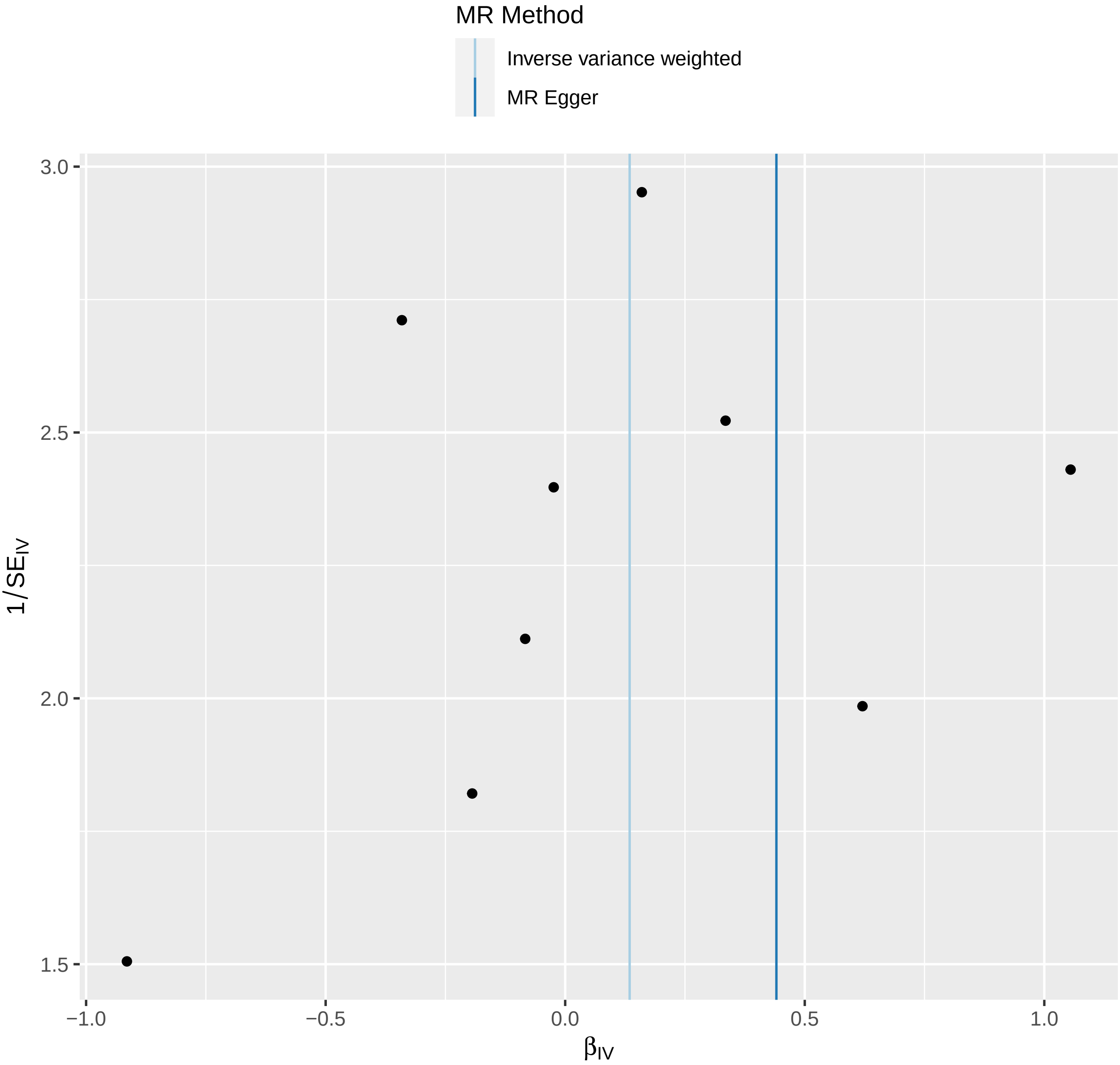

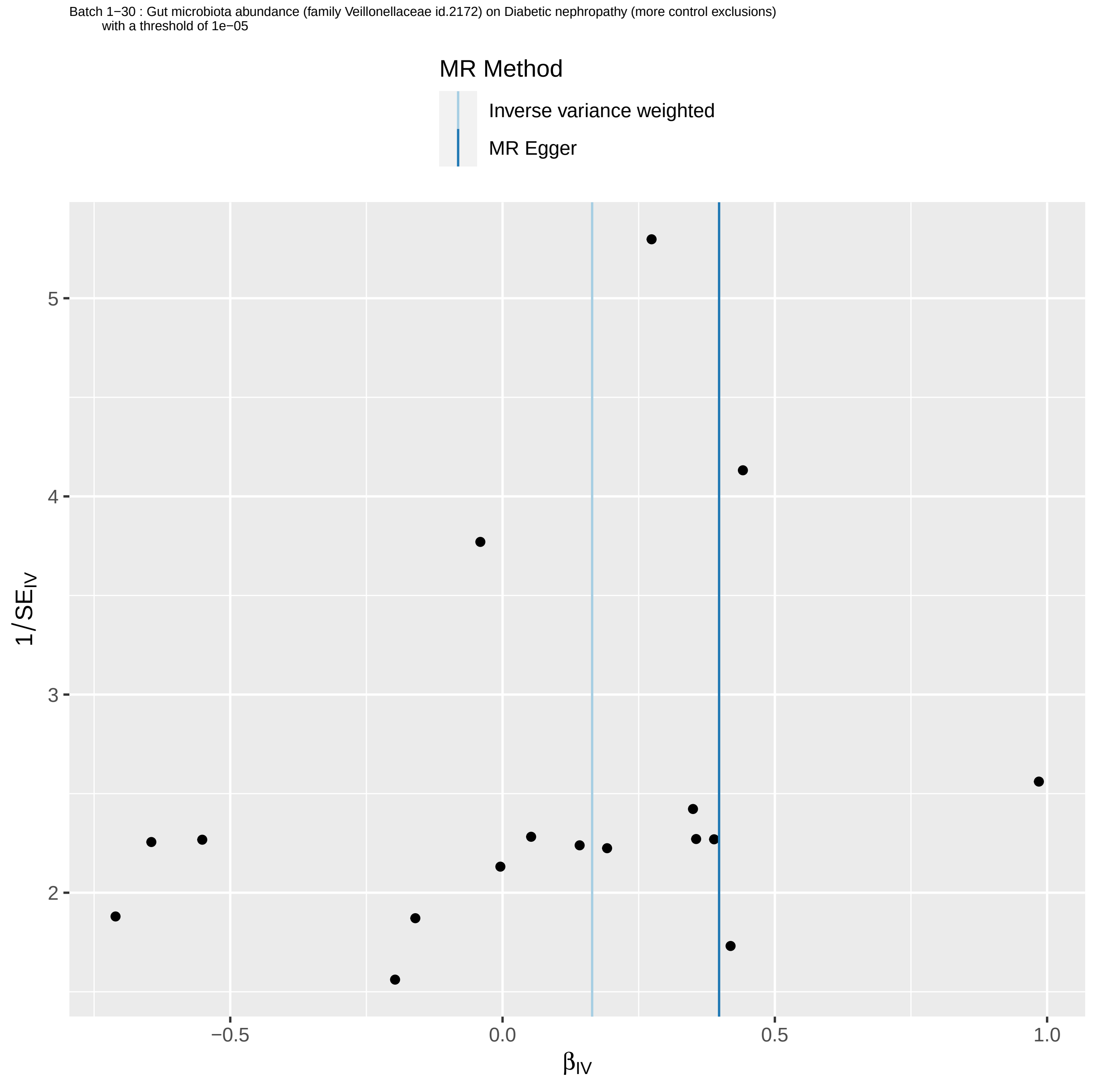

Batch 1−30 : Gut microbiota abundance (family Veillonellaceae id.2172) on Diabetic nephropathy (more control exclusions)
with a threshold of 1e−05
MR Method
Inverse variance weighted
MR Egger
1/SE_IV
5
4
3
2
−0.5
0.0
0.5
1.0
β_IV

Batch 1−31 : Gut microbiota abundance (family Verrucomicrobiaceae id.4036) on Diabetic nephropathy (more control exclusions) with a threshold of 1e−05

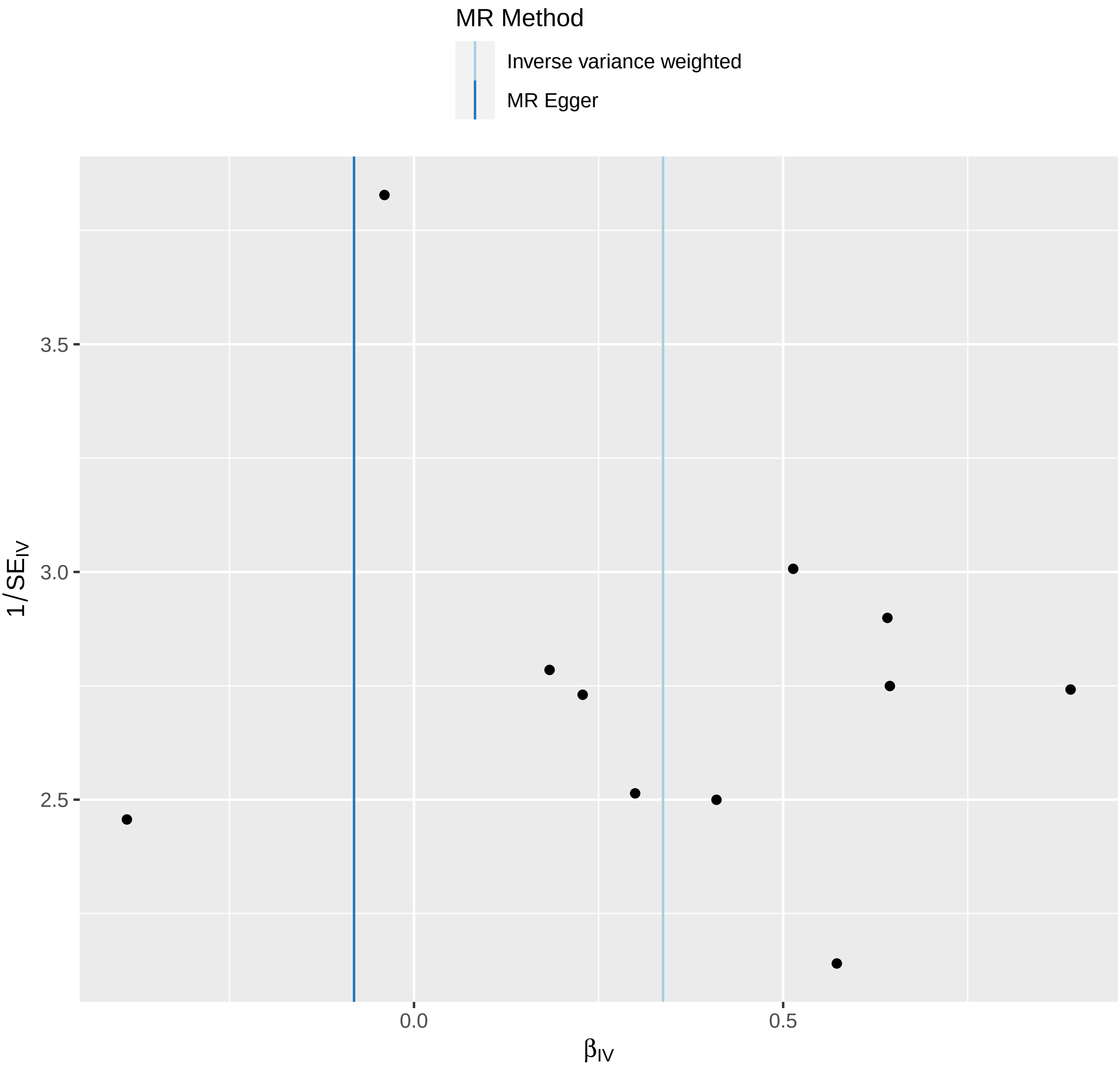

Batch 1−32 : Gut microbiota abundance (family Victivallaceae id.2255) on Diabetic nephropathy (more control exclusions)
with a threshold of 1e−05

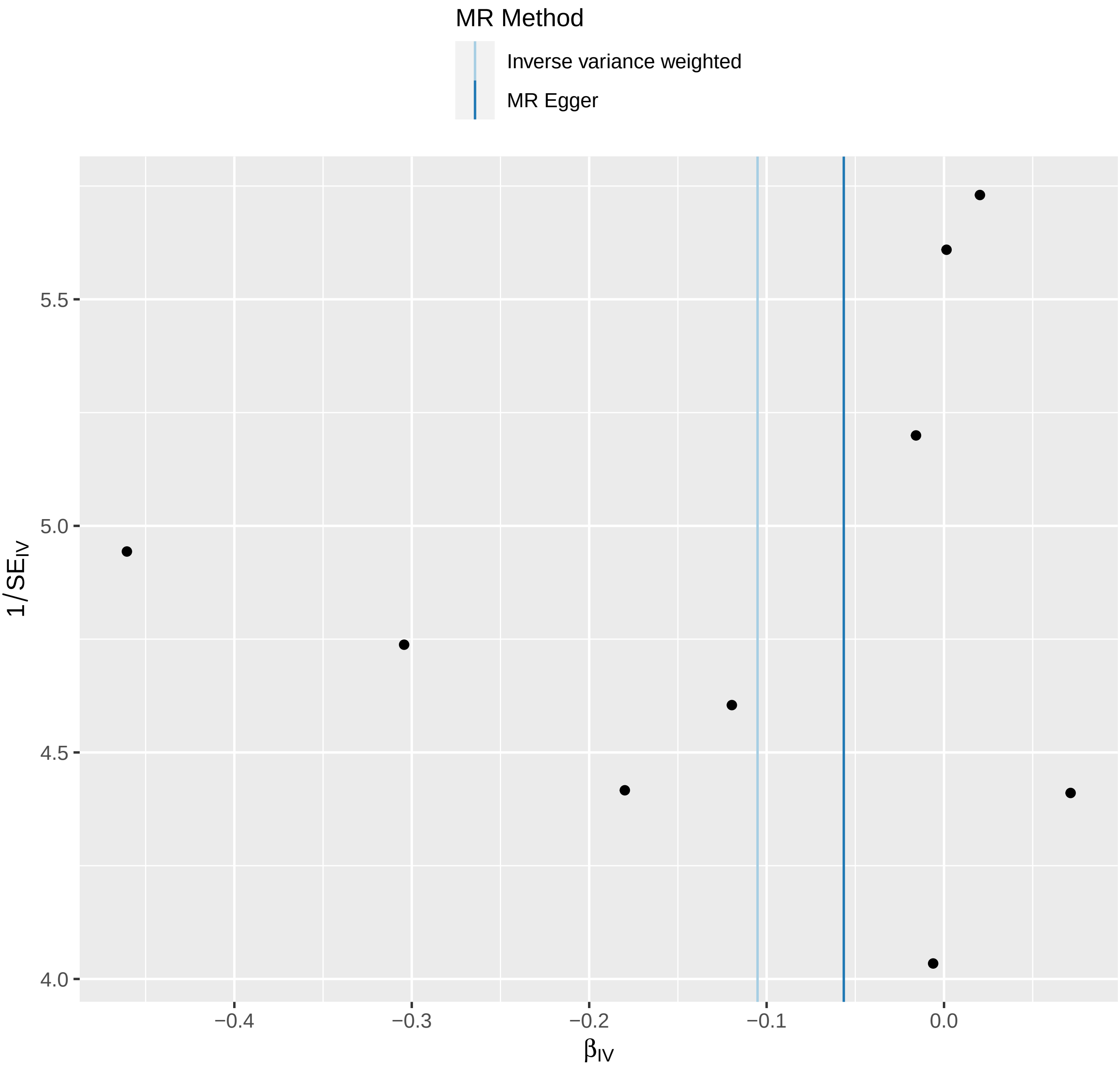

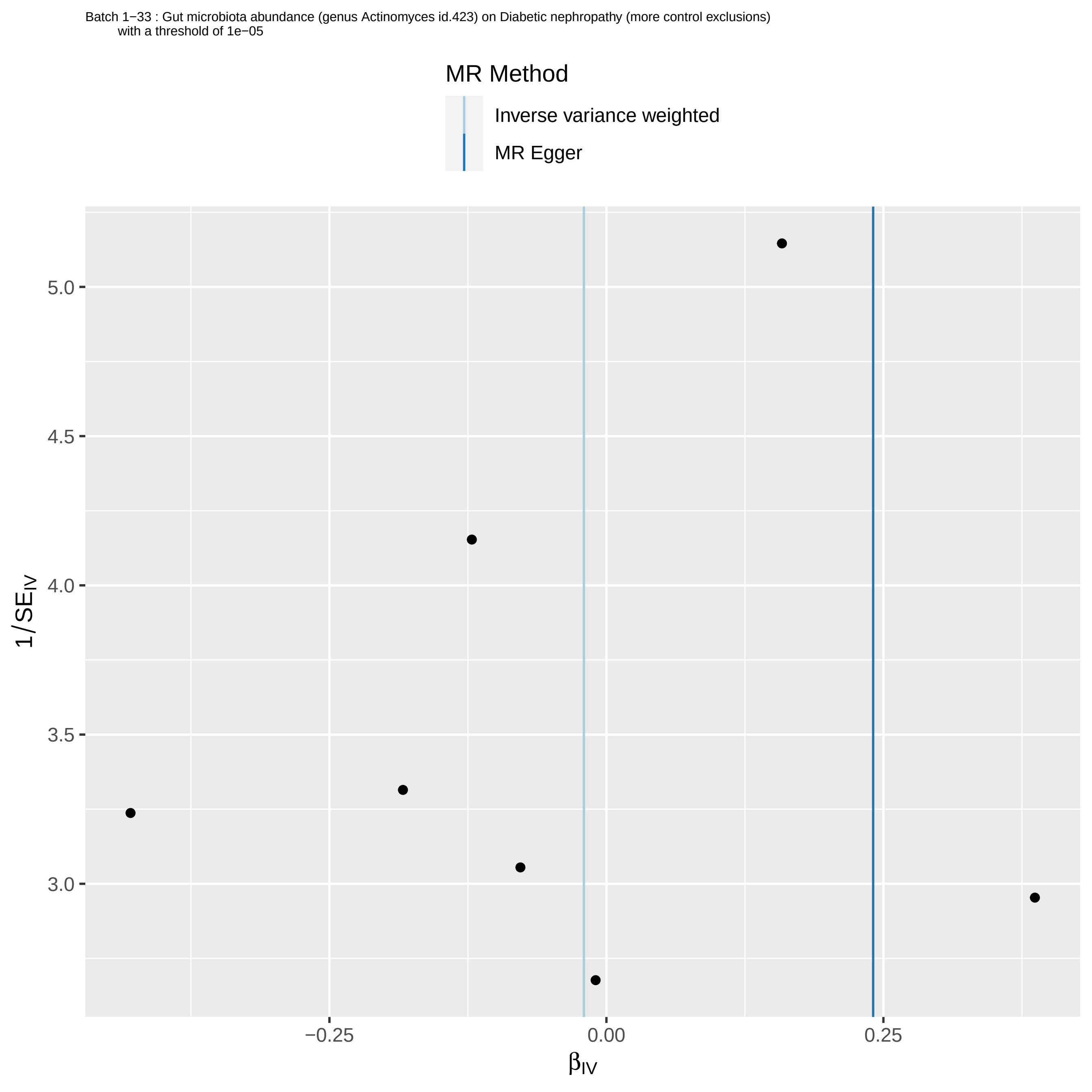
Batch 1−33 : Gut microbiota abundance (genus Actinomyces id.423) on Diabetic nephropathy (more control exclusions) with a threshold of 1e−05
MR Method
Inverse variance weighted
MR Egger
5.0
4.5
4.0
3.5
3.0
1/SE$_{IV}$
−0.25
0.00
0.25
β_{IV}

Batch 1−34 : Gut microbiota abundance (genus Adlercreutzia id.812) on Diabetic nephropathy (more control exclusions) with a threshold of 1e−05

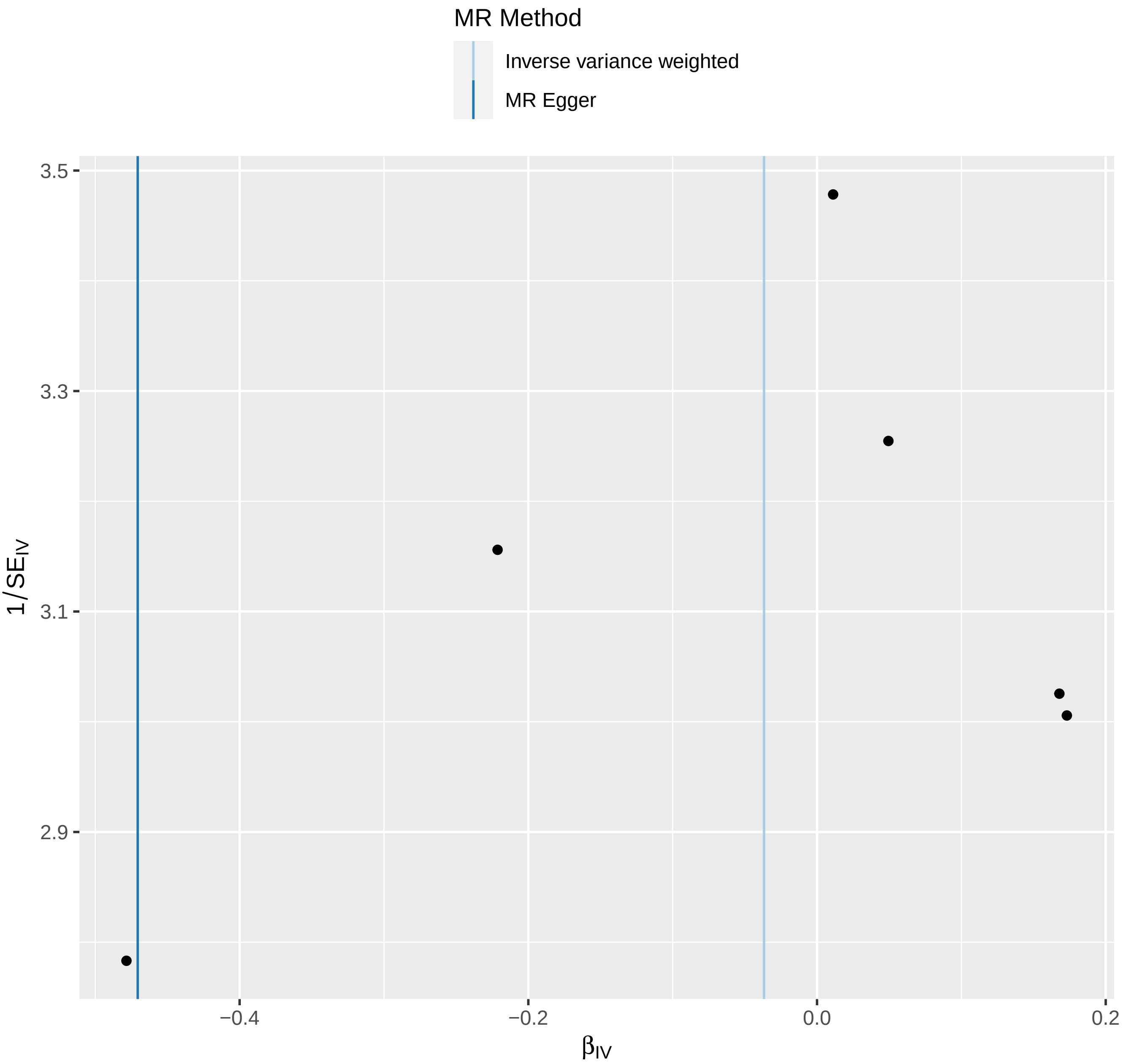

Batch 1−35 : Gut microbiota abundance (genus Akkermansia id.4037) on Diabetic nephropathy (more control exclusions) with a threshold of 1e−05

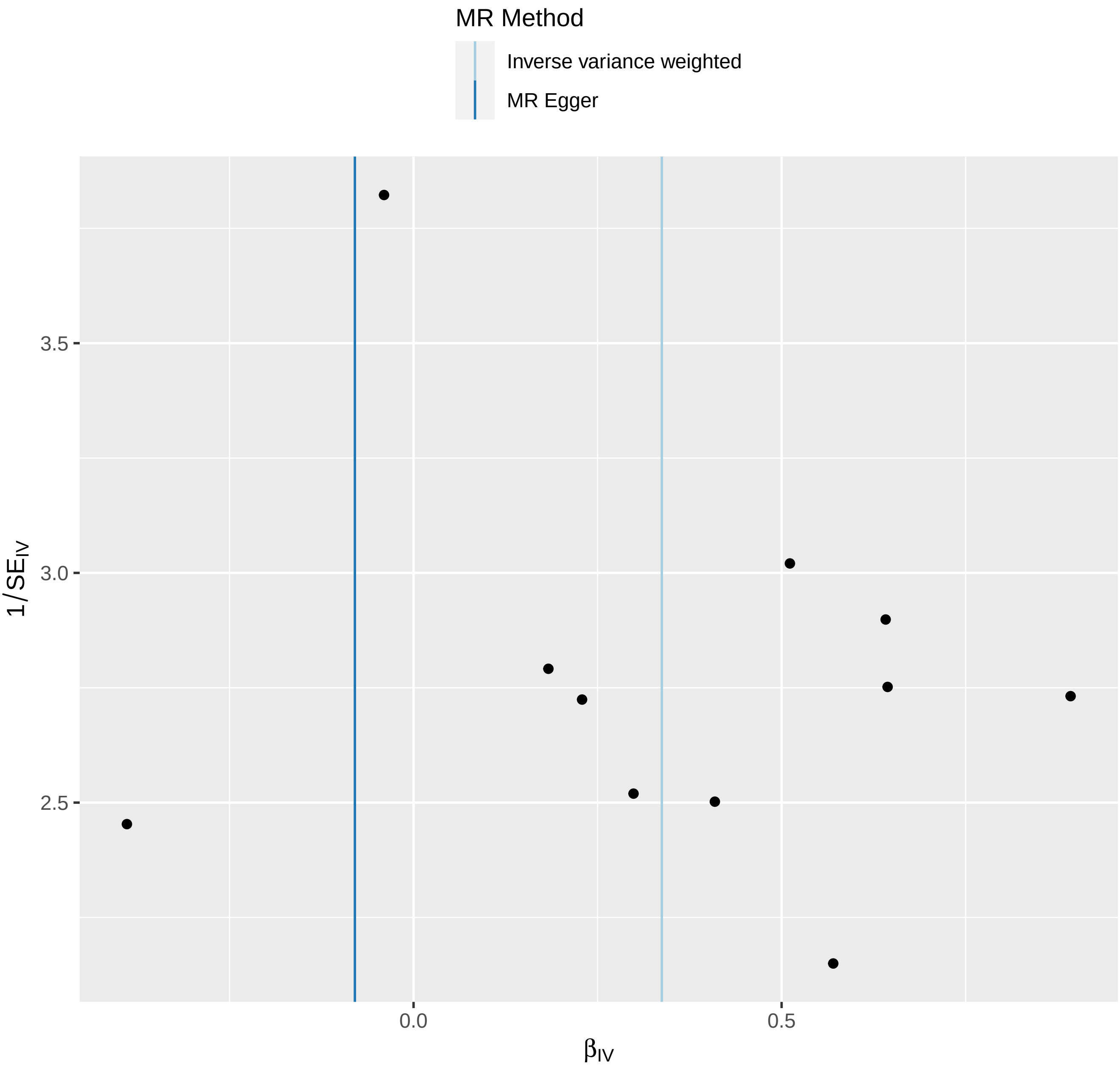

Batch 1−36 : Gut microbiota abundance (genus Alistipes id.968) on Diabetic nephropathy (more control exclusions) with a threshold of 1e−05

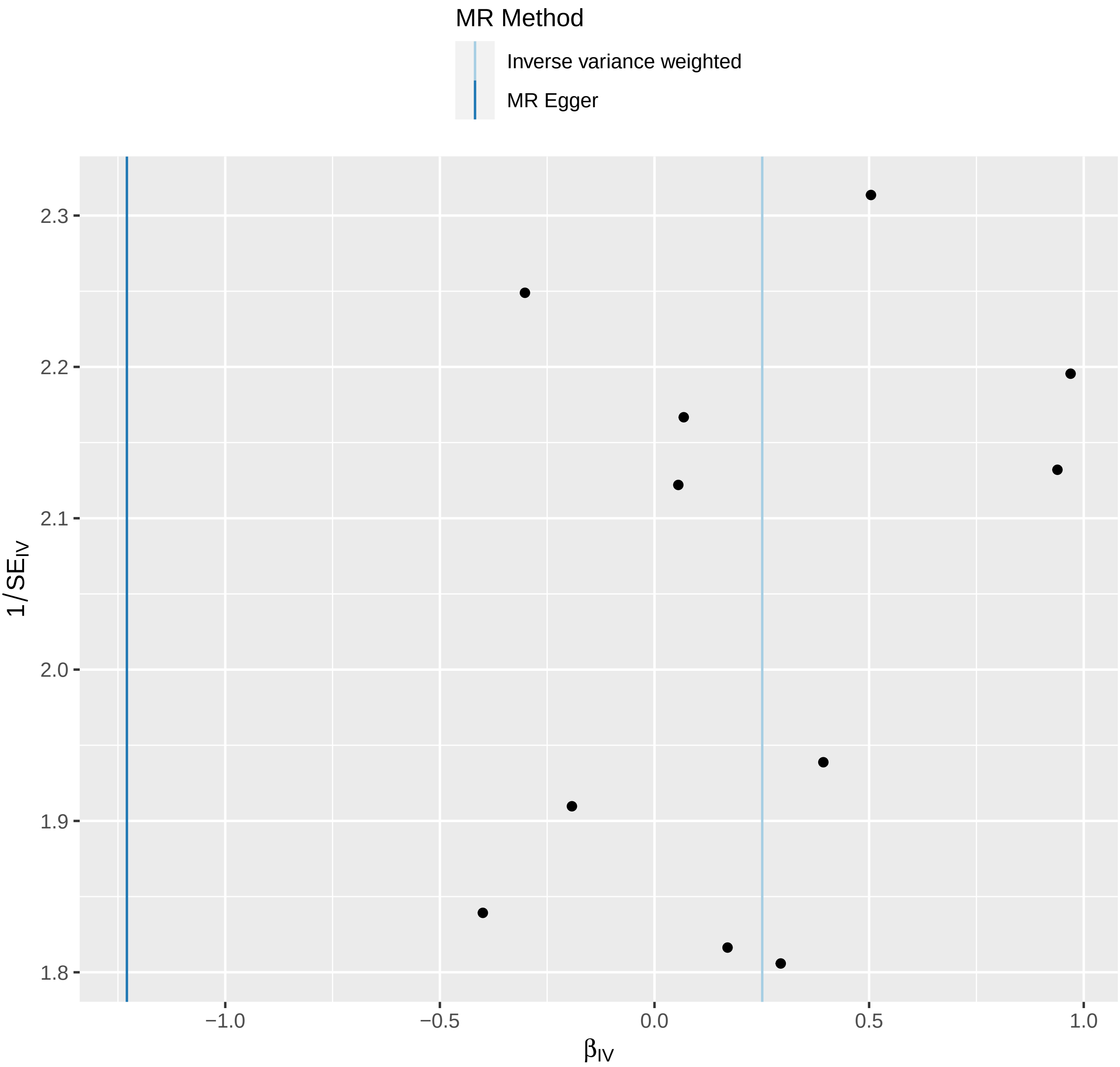

Batch 1−37 : Gut microbiota abundance (genus Allisonella id.2174) on Diabetic nephropathy (more control exclusions) with a threshold of 1e−05

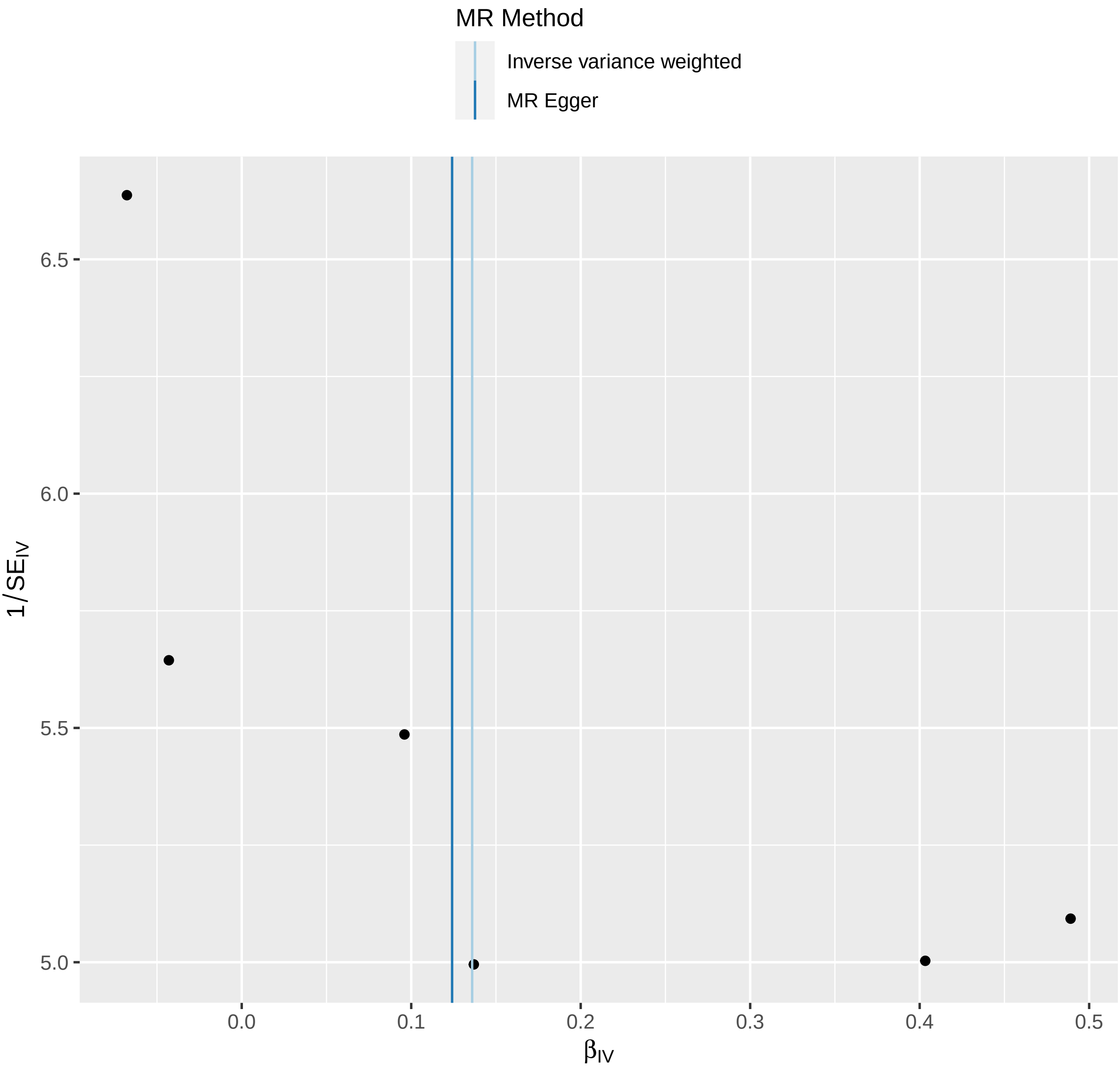

Batch 1−38 : Gut microbiota abundance (genus Alloprevotella id.961) on Diabetic nephropathy (more control exclusions) with a threshold of 1e−05

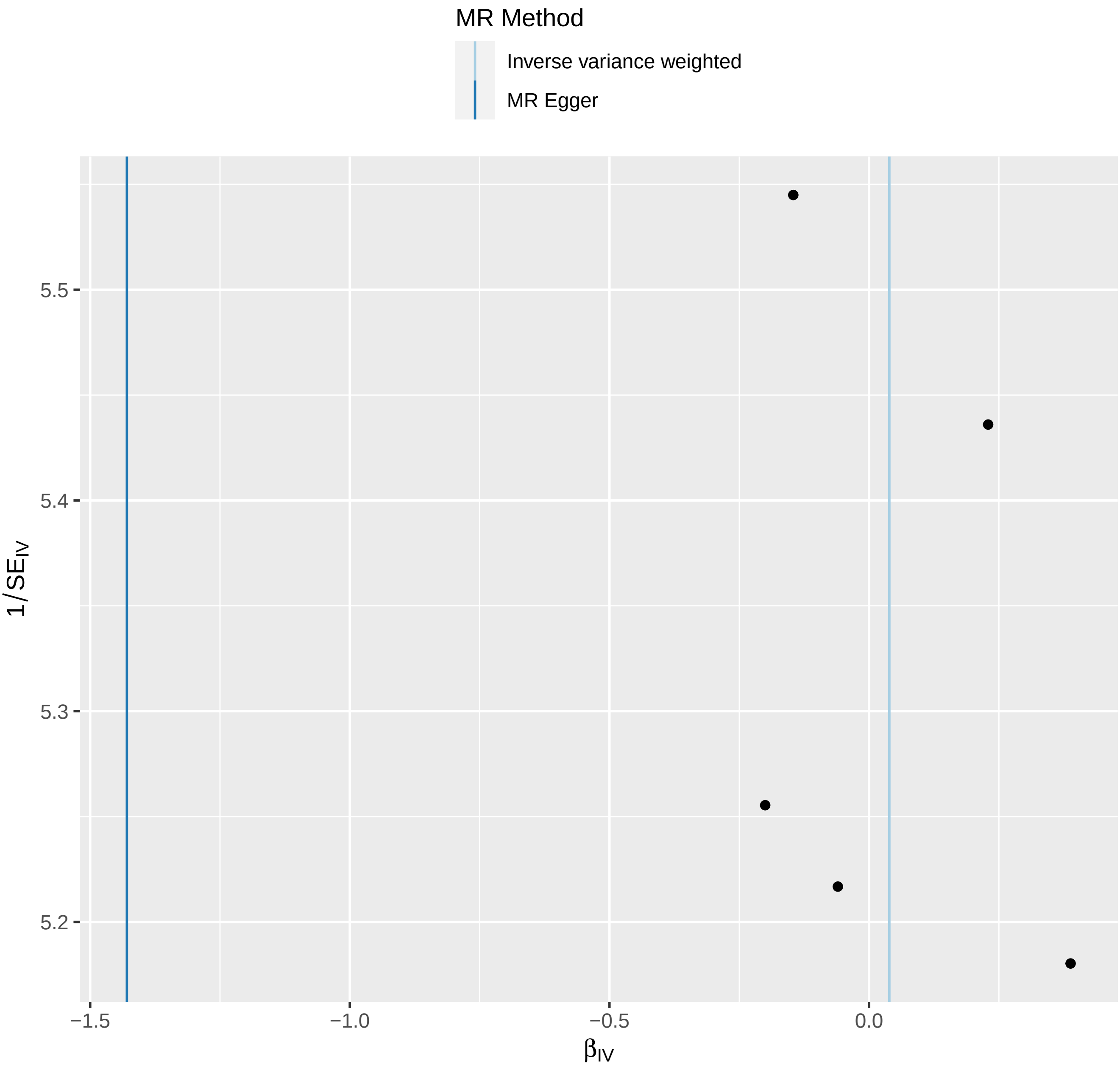

Batch 1−39 : Gut microbiota abundance (genus Anaerofilum id.2053) on Diabetic nephropathy (more control exclusions) with a threshold of 1e−05

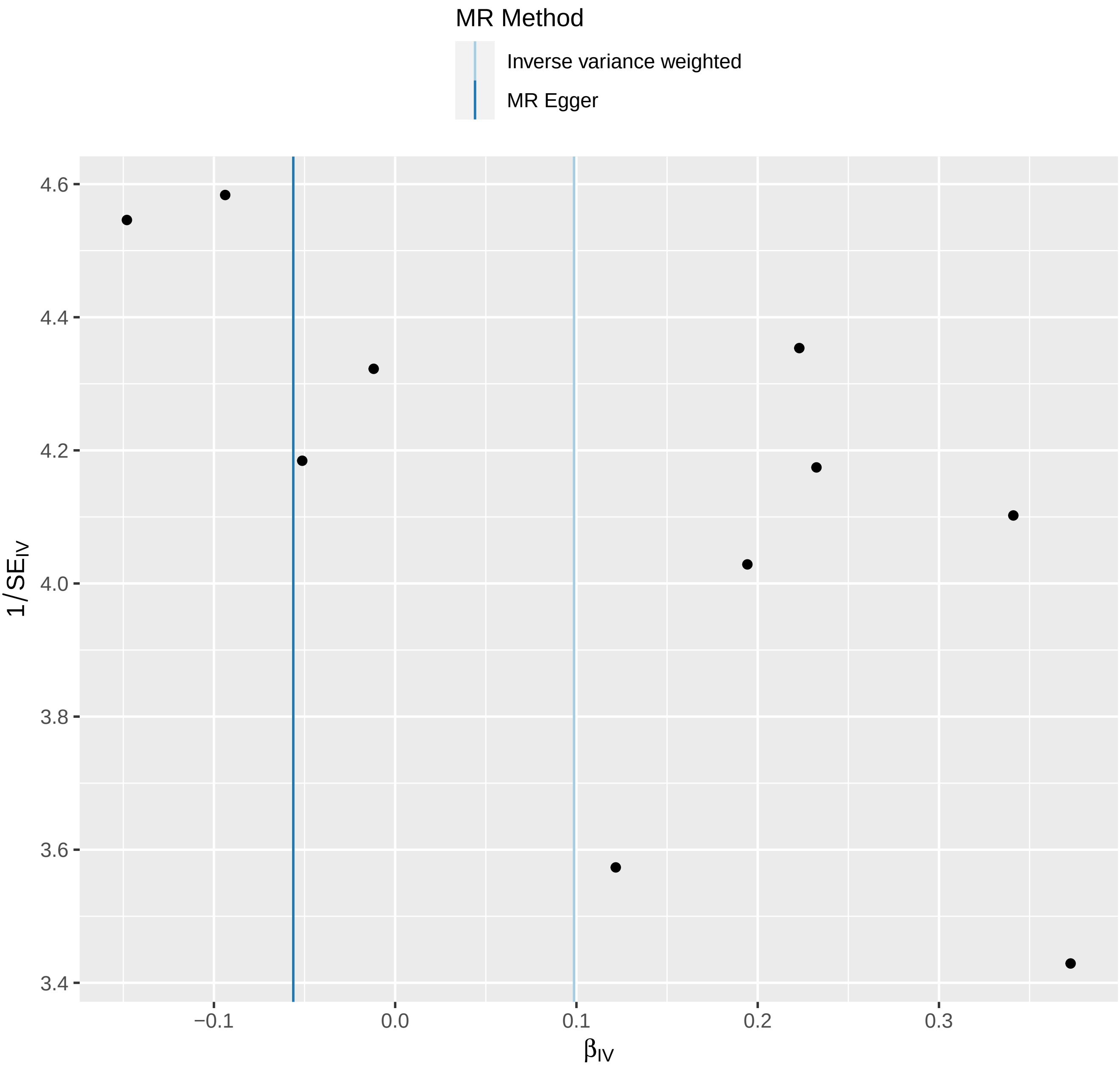

Batch 1−40 : Gut microbiota abundance (genus Anaerostipes id.1991) on Diabetic nephropathy (more control exclusions)
with a threshold of 1e−05

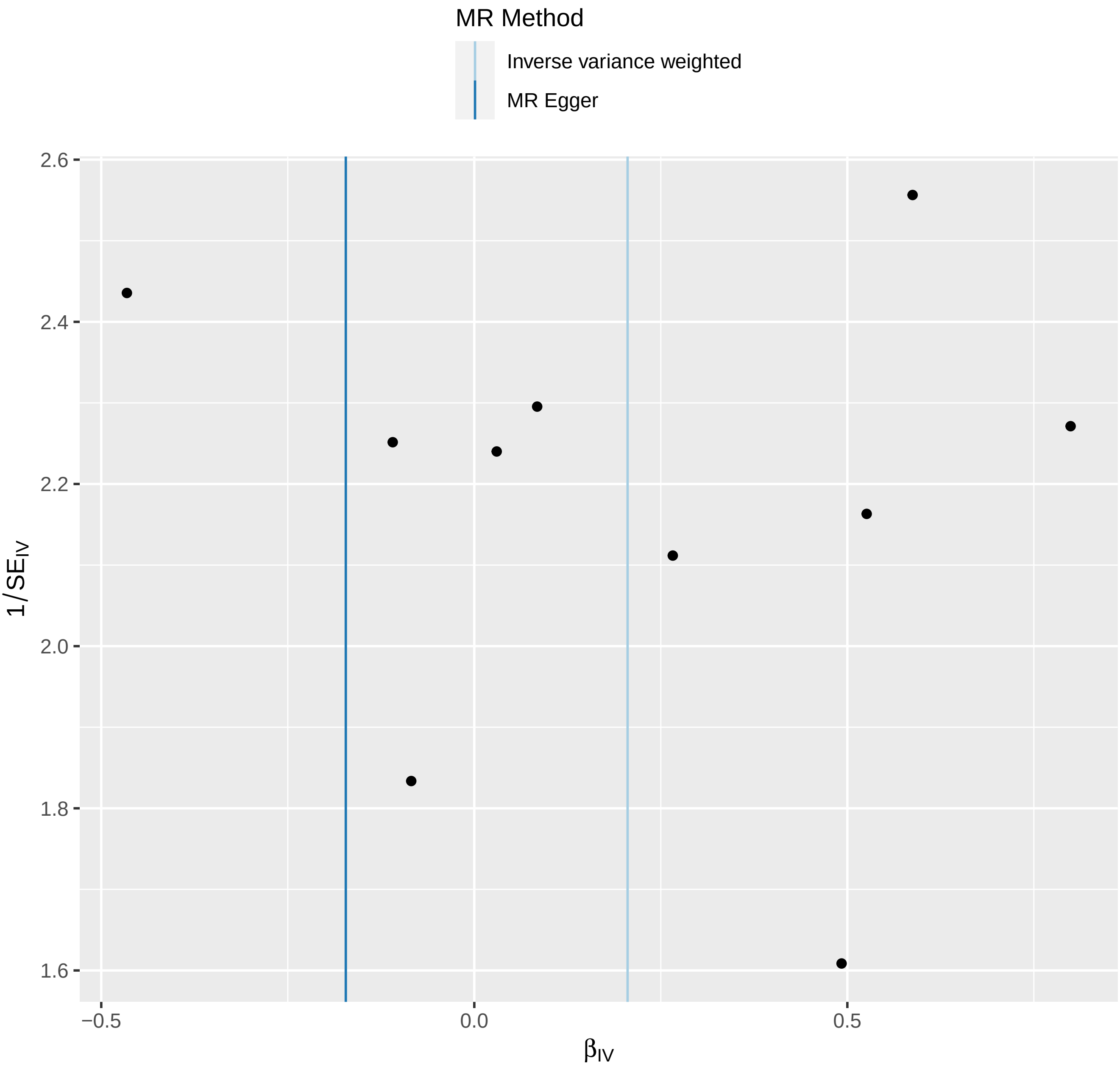

Batch 1−41 : Gut microbiota abundance (genus Anaerotruncus id.2054) on Diabetic nephropathy (more control exclusions) with a threshold of 1e−05

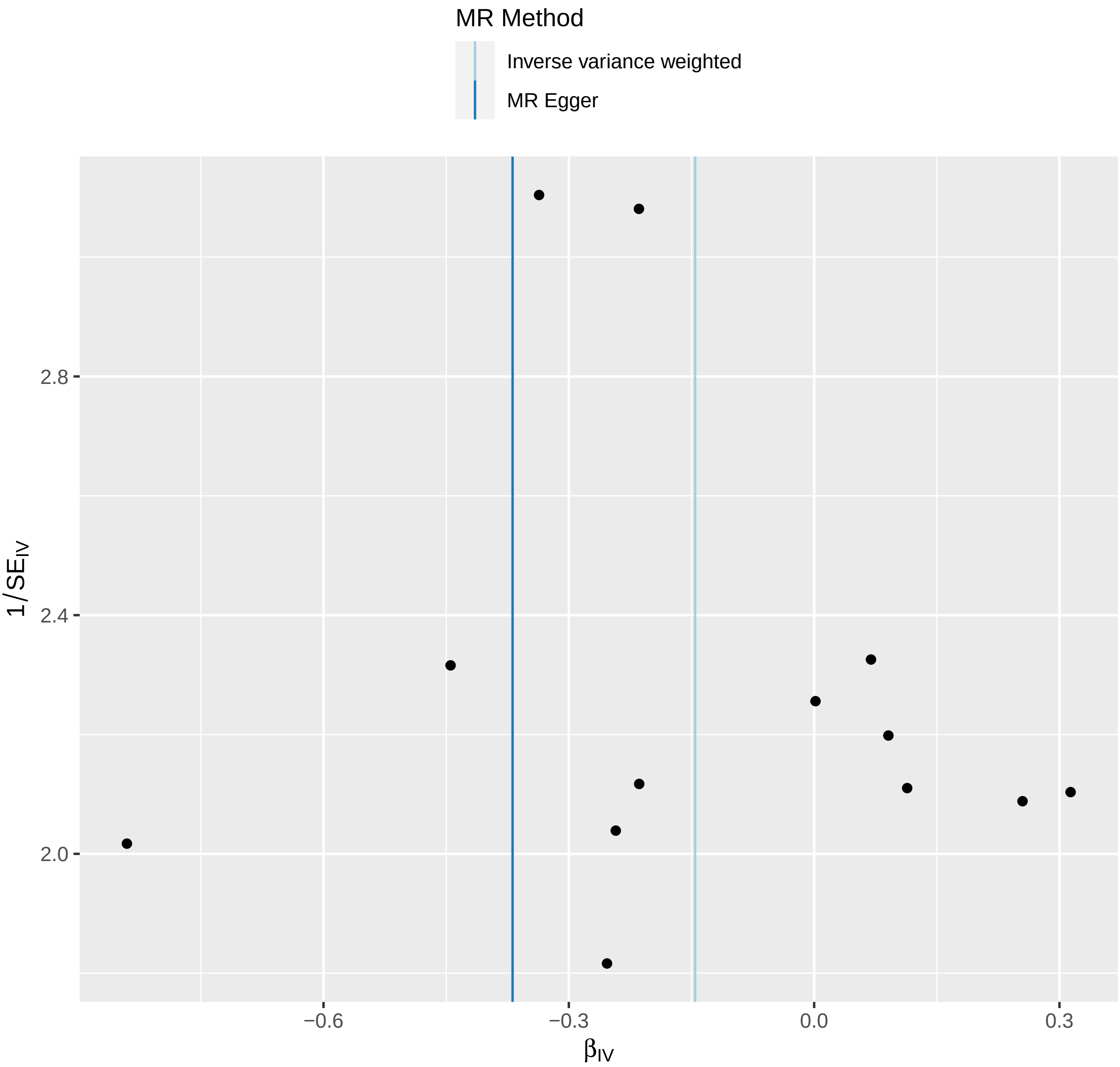

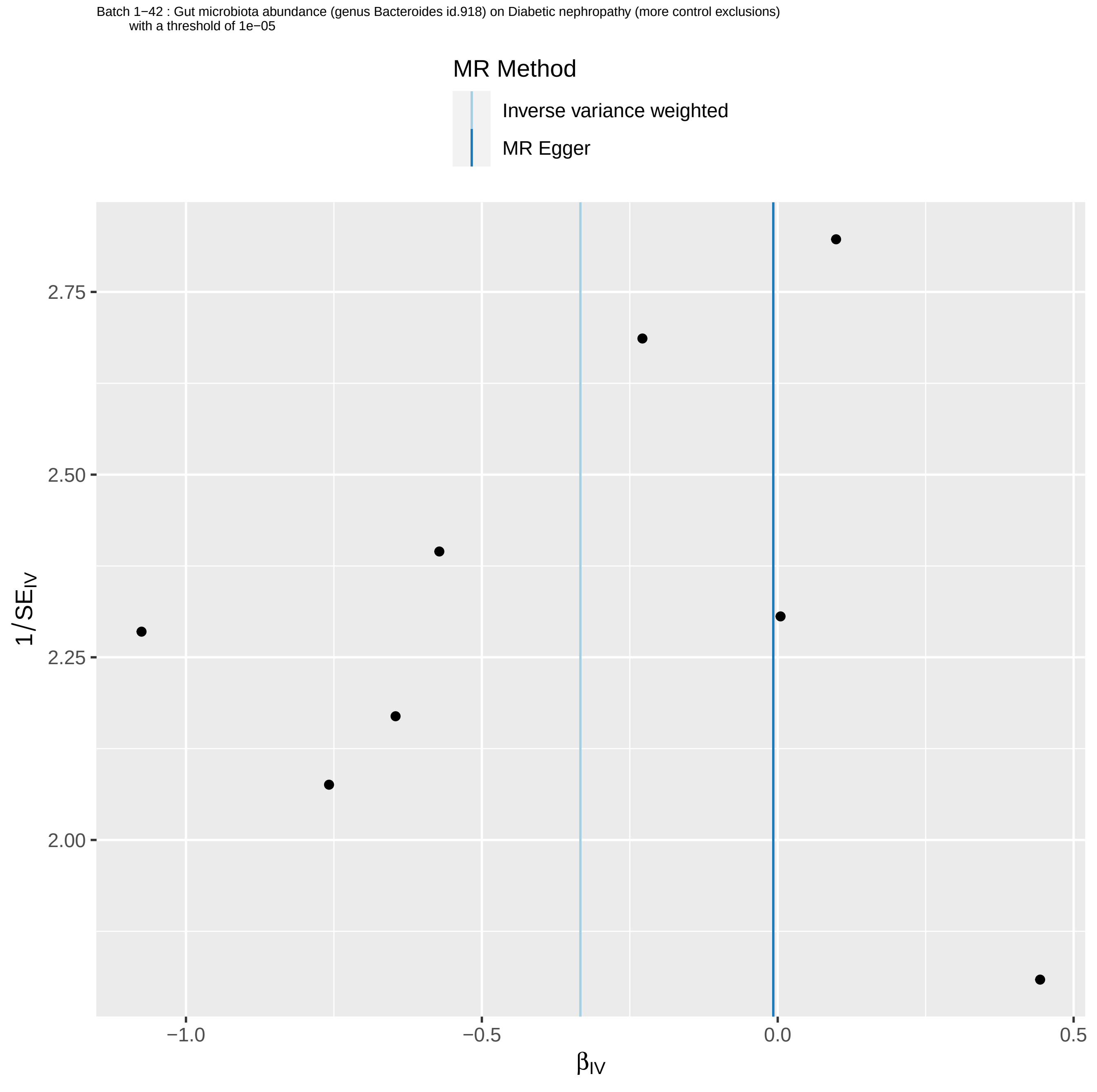
Batch 1−42 : Gut microbiota abundance (genus Bacteroides id.918) on Diabetic nephropathy (more control exclusions)
with a threshold of 1e−05
MR Method
Inverse variance weighted
MR Egger
2.75
2.50
2.25
2.00
1/SE_IV
−1.0
−0.5
0.0
0.5
β_IV

Batch 1−43 : Gut microbiota abundance (genus Barnesiella id.944) on Diabetic nephropathy (more control exclusions) with a threshold of 1e−05

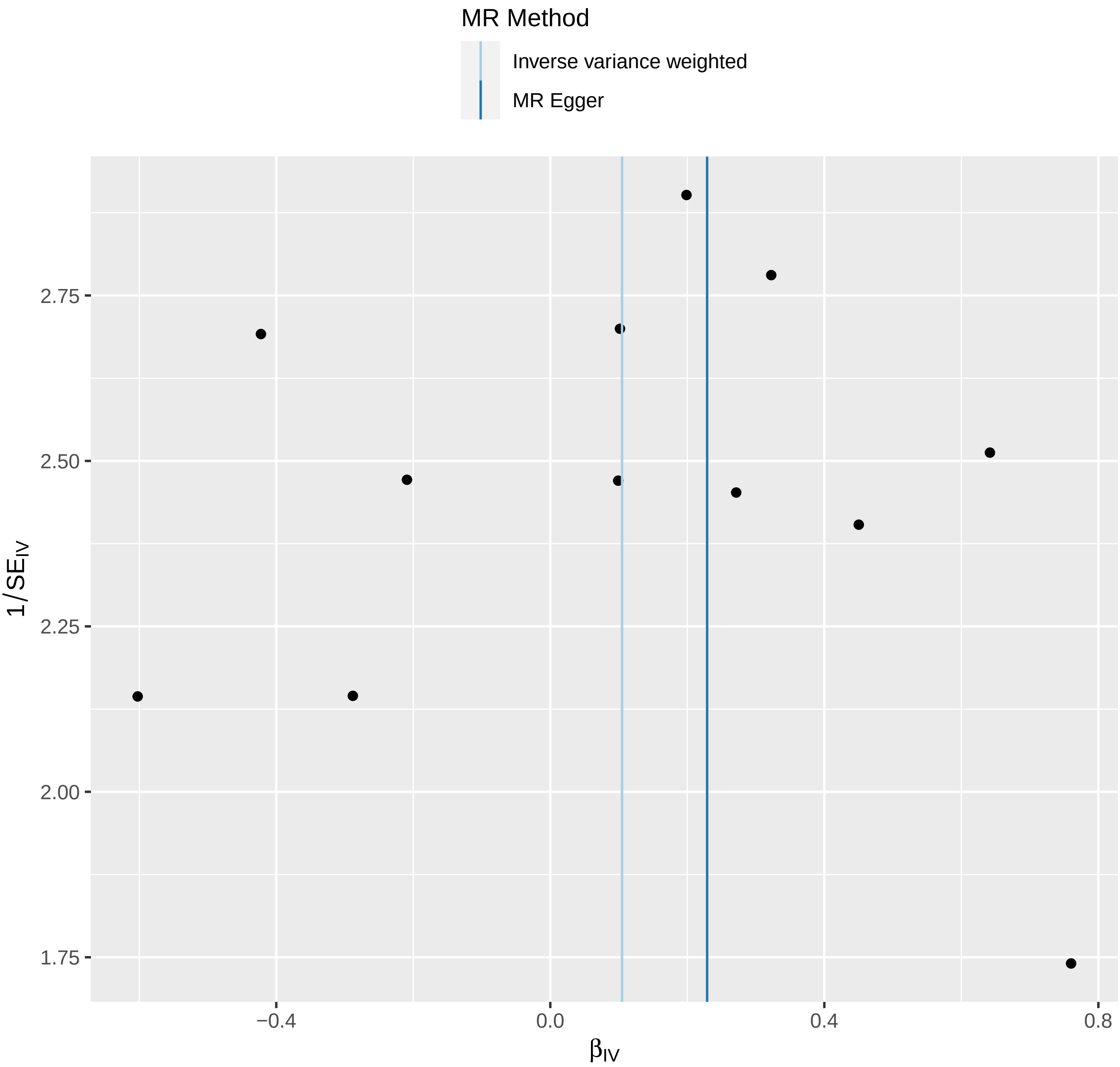

Batch 1−44 : Gut microbiota abundance (genus Bifidobacterium id.436) on Diabetic nephropathy (more control exclusions) with a threshold of 1e−05

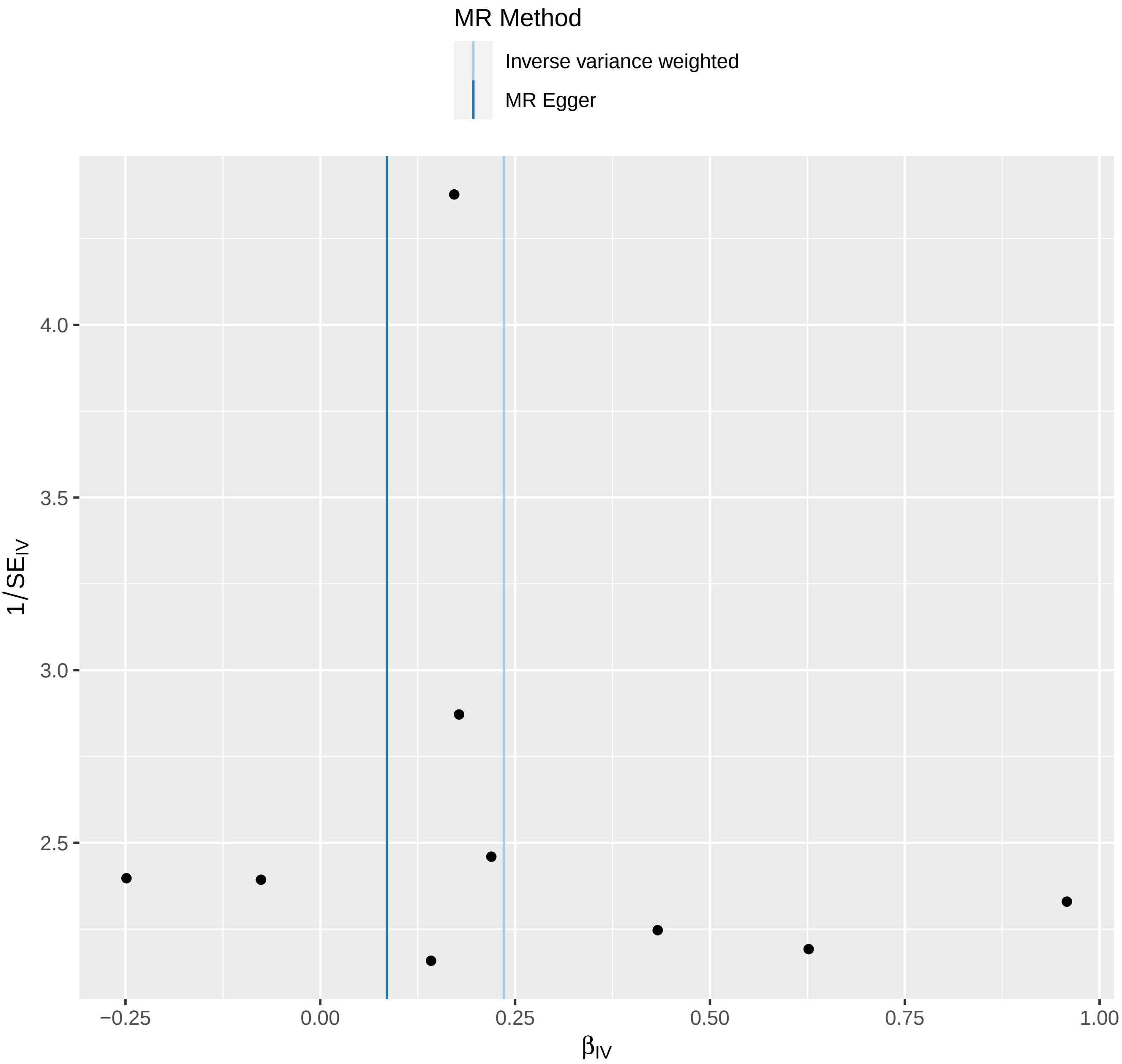

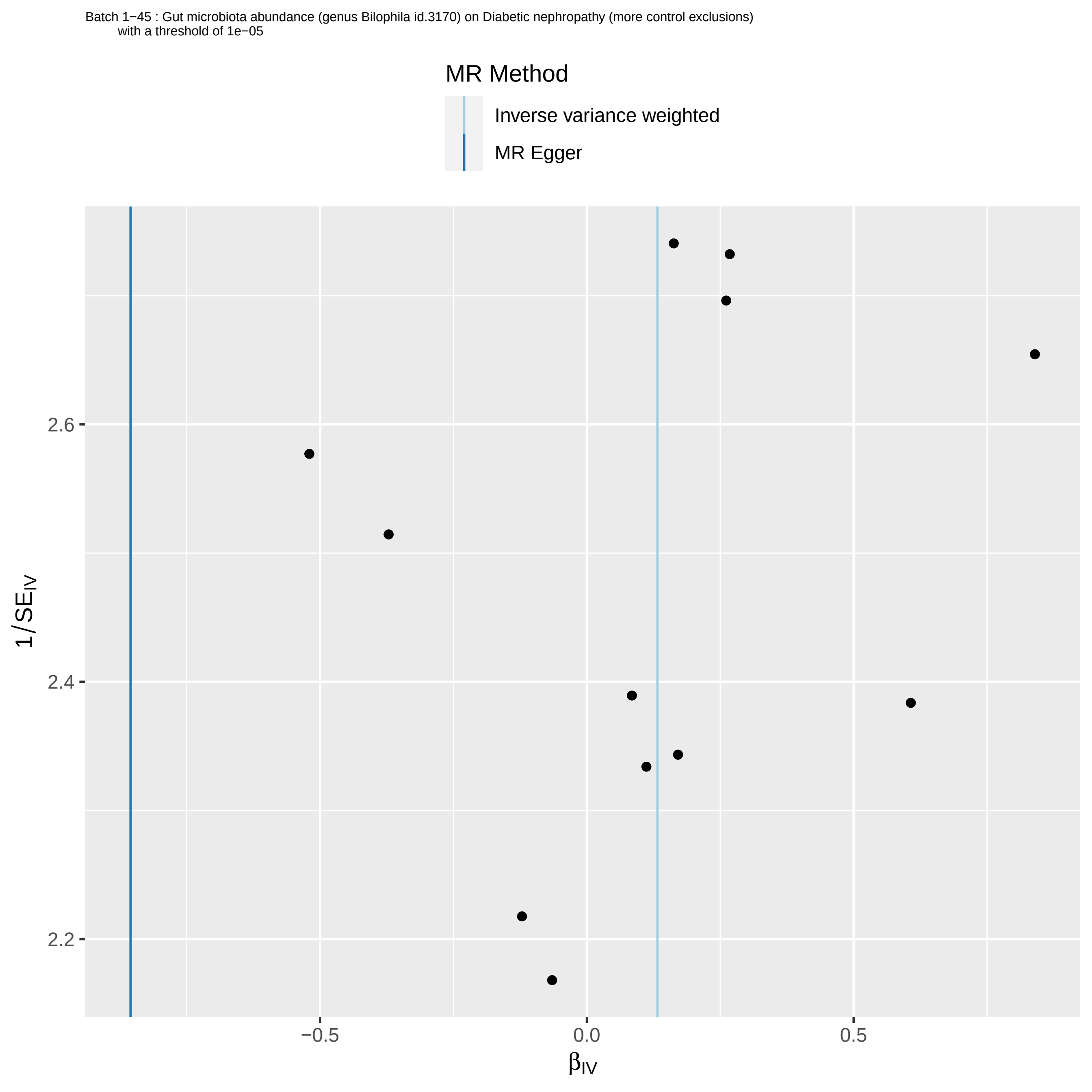

Batch 1−45 : Gut microbiota abundance (genus Bilophila id.3170) on Diabetic nephropathy (more control exclusions)
with a threshold of 1e−05
MR Method
Inverse variance weighted
MR Egger
2.6
2.4
2.2
1/SE_IV
−0.5
0.0
0.5
β_IV

Batch 1−46 : Gut microbiota abundance (genus Blautia id.1992) on Diabetic nephropathy (more control exclusions) with a threshold of 1e−05

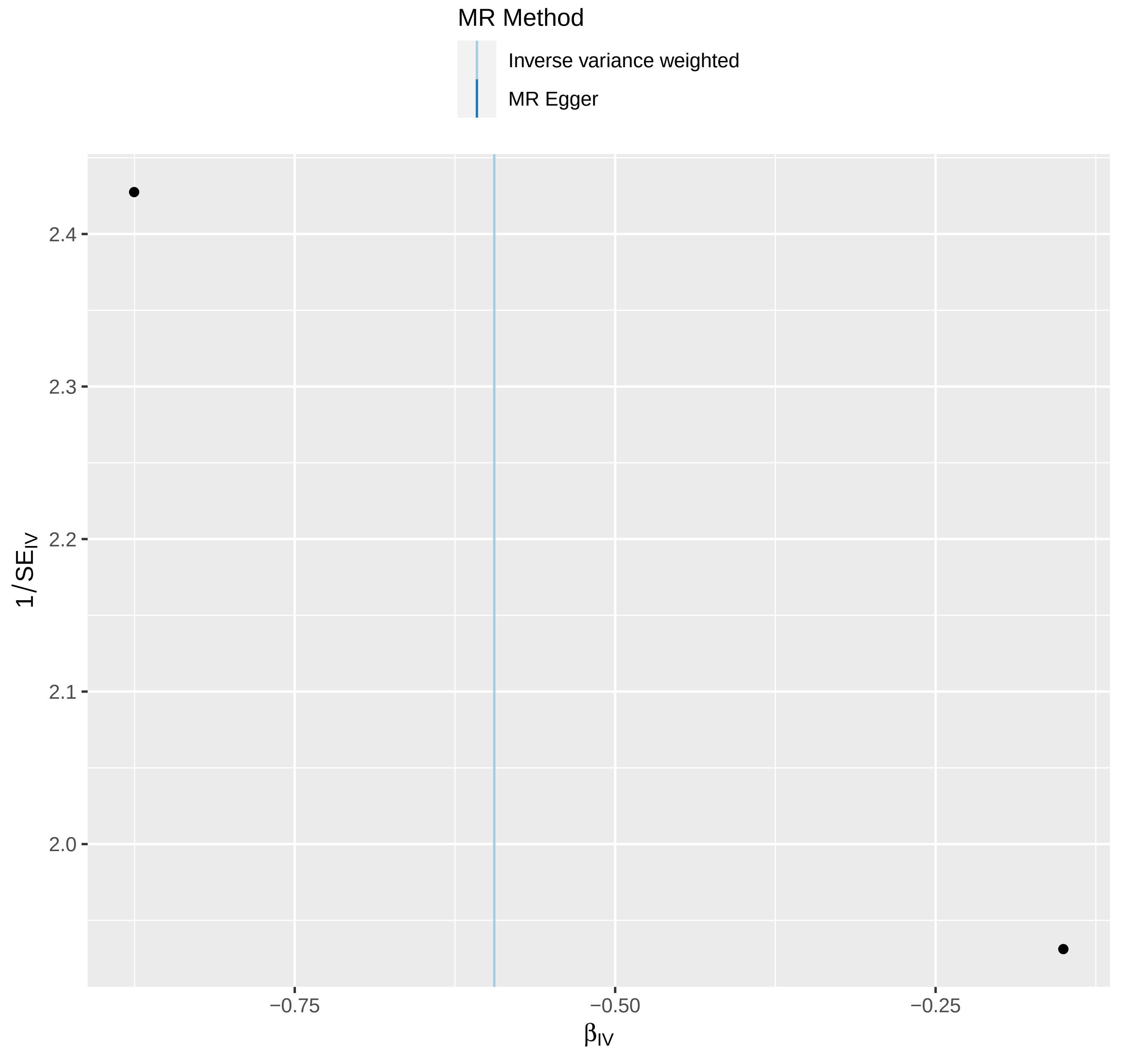

Batch 1−47 : Gut microbiota abundance (genus Butyricicoccus id.2055) on Diabetic nephropathy (more control exclusions) with a threshold of 1e−05

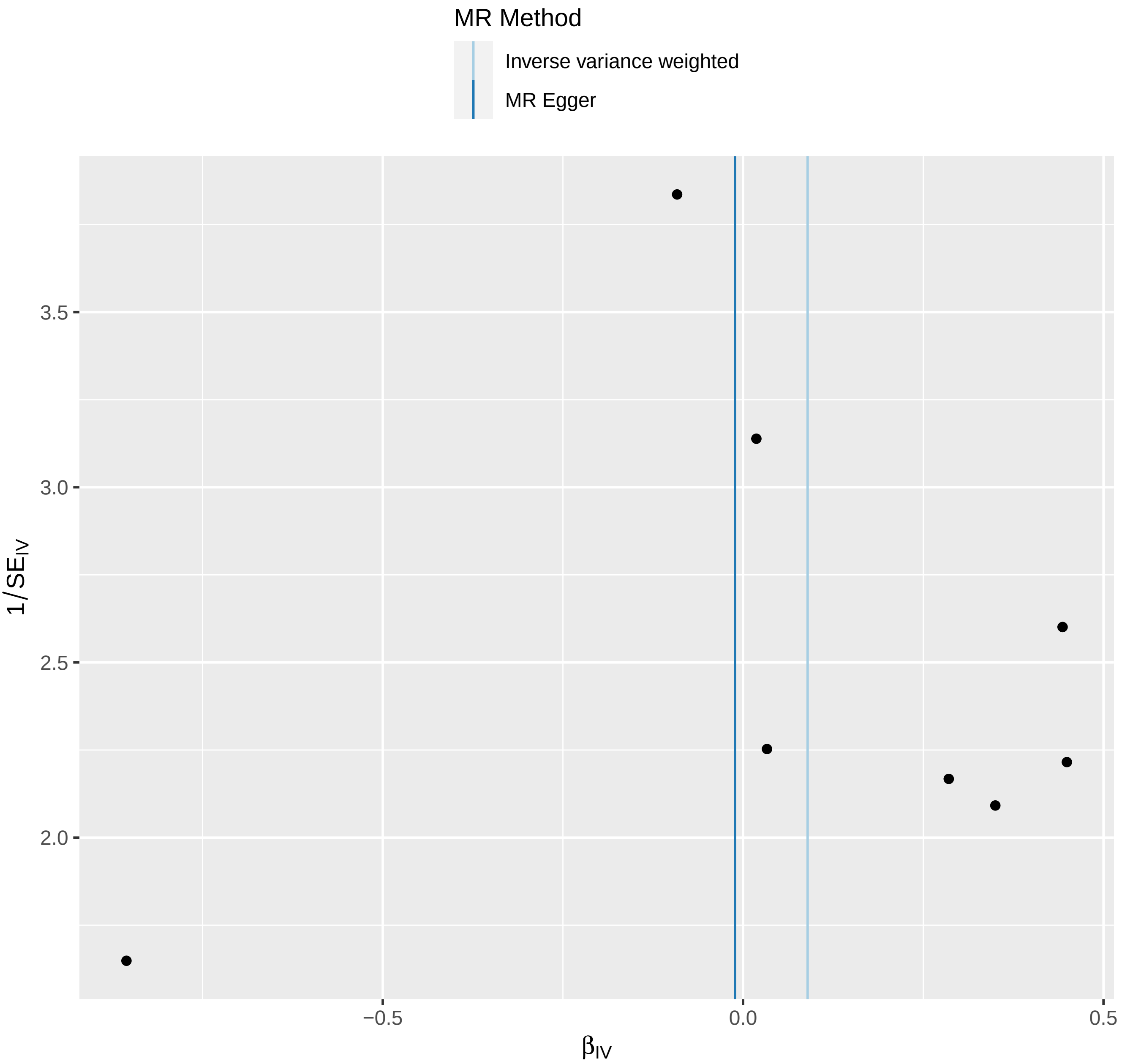

Batch 1−48 : Gut microbiota abundance (genus Butyricimonas id.945) on Diabetic nephropathy (more control exclusions)
with a threshold of 1e−05

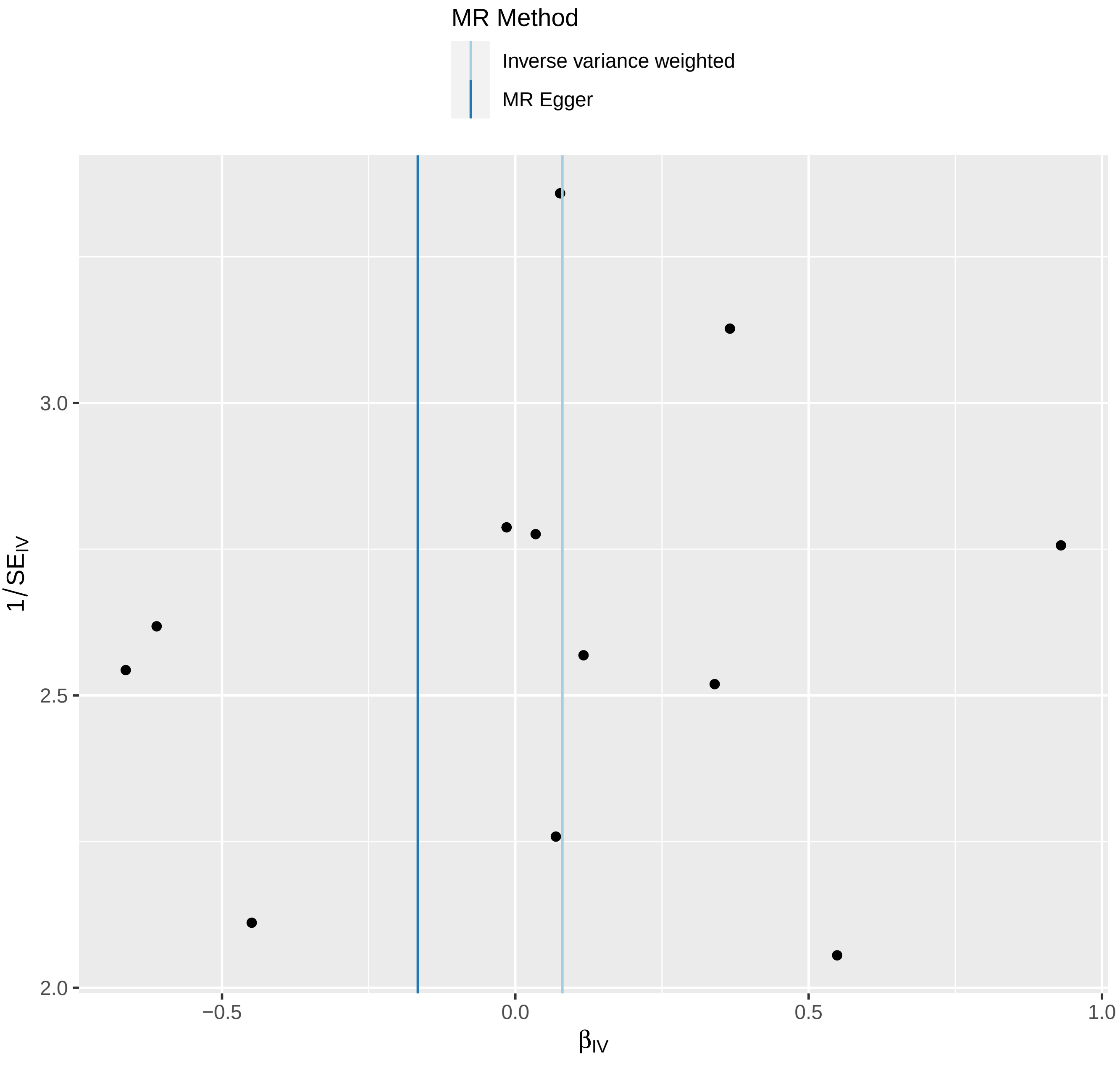

Batch 1−49 : Gut microbiota abundance (genus Butyrivibrio id.1993) on Diabetic nephropathy (more control exclusions)
with a threshold of 1e−05

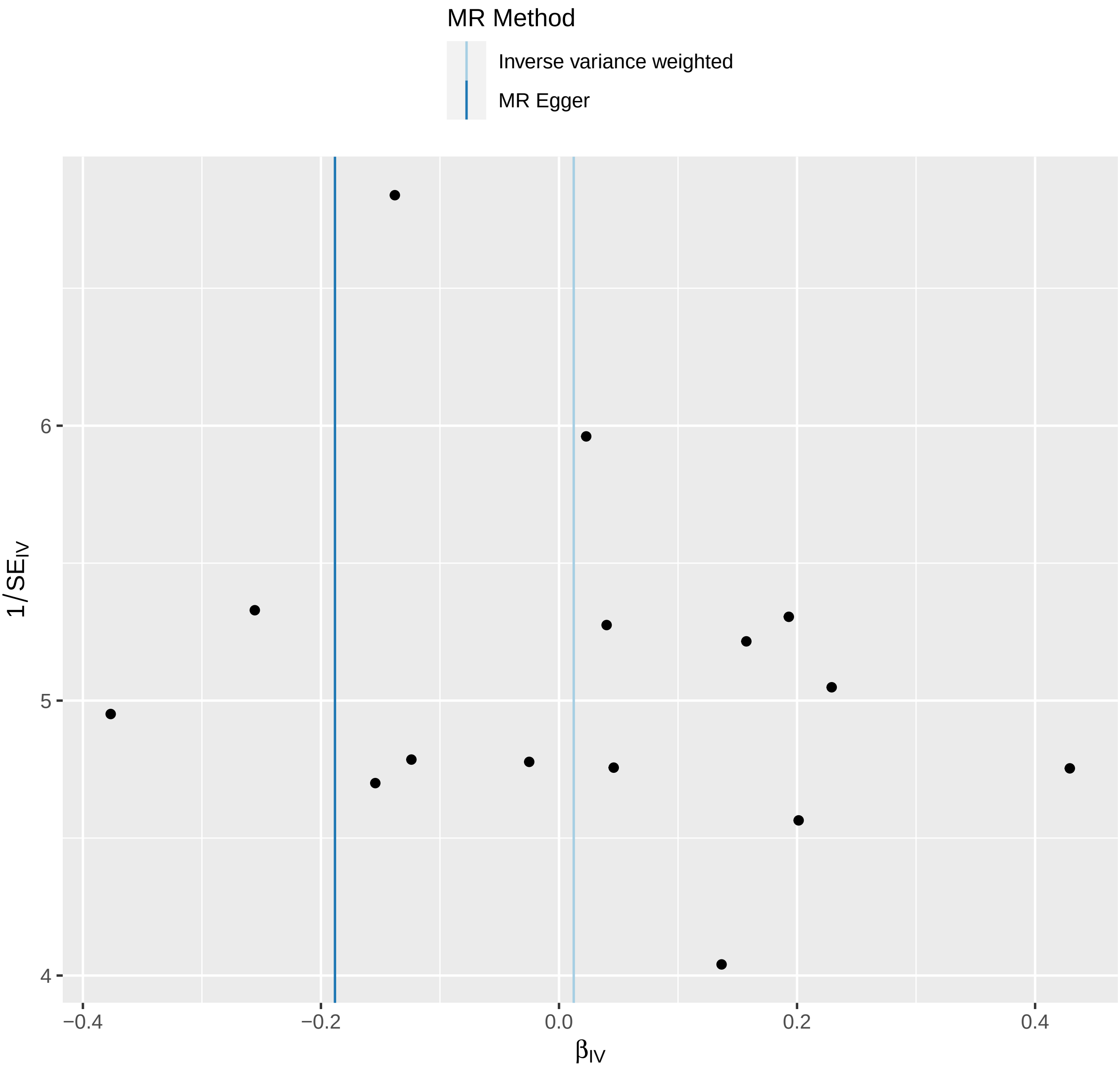

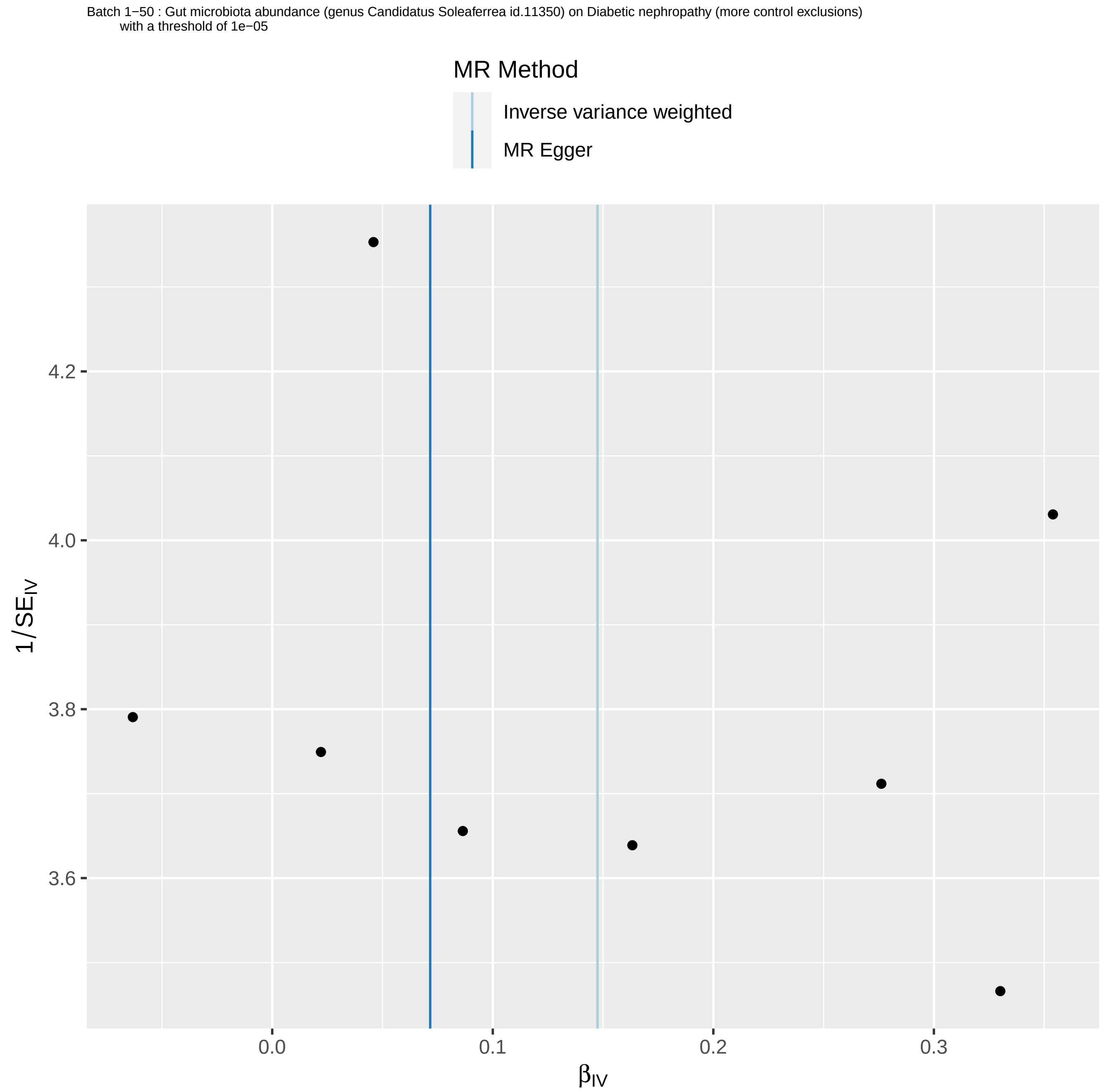

Batch 1−50 : Gut microbiota abundance (genus Candidatus Soleaferrea id.11350) on Diabetic nephropathy (more control exclusions)
with a threshold of 1e−05
MR Method
Inverse variance weighted
MR Egger
4.2
4.0
3.8
3.6
1/SE_IV
0.0
0.1
0.2
0.3
β_IV

Batch 1−51 : Gut microbiota abundance (genus Catenibacterium id.2153) on Diabetic nephropathy (more control exclusions) with a threshold of 1e−05

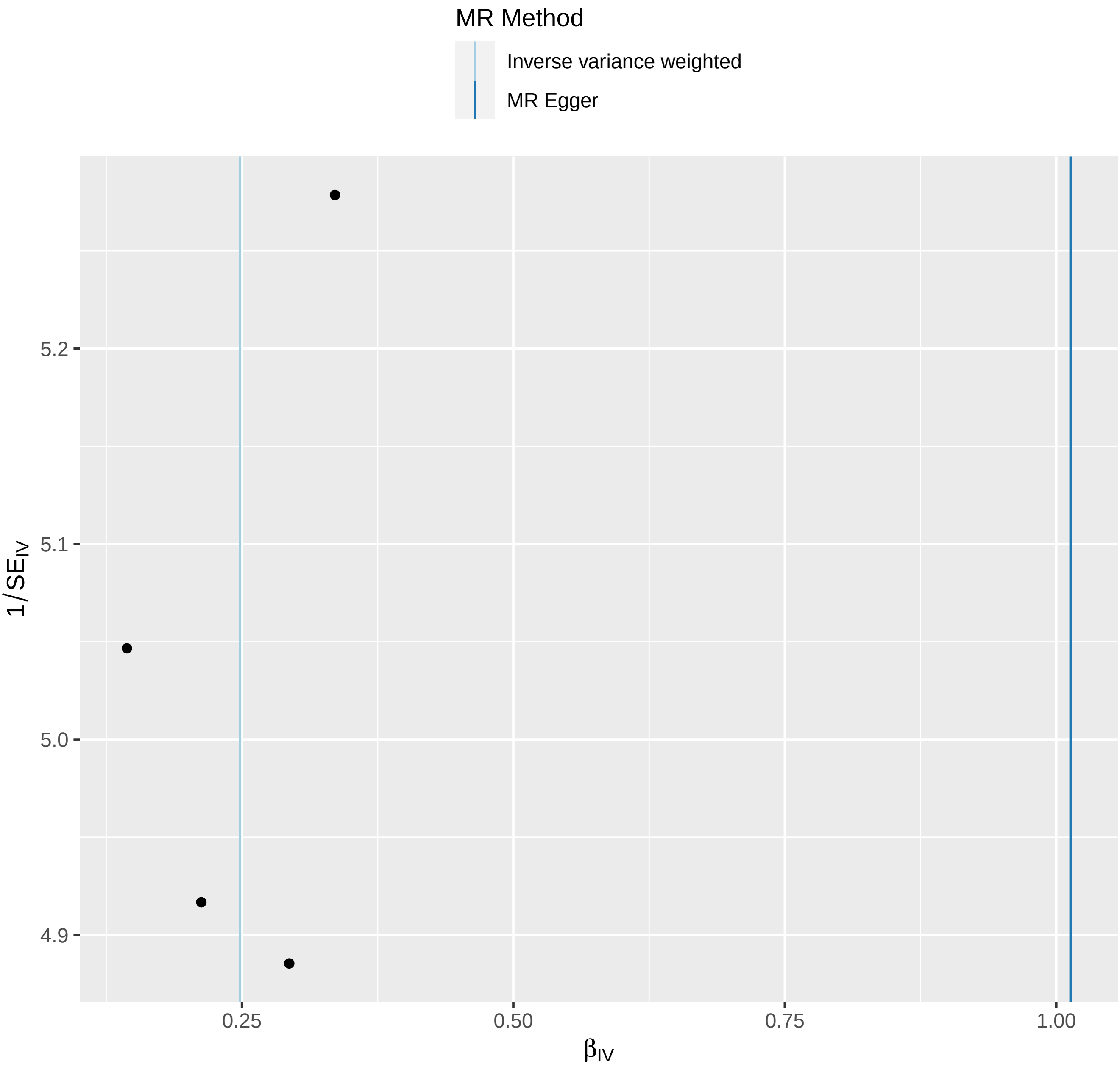

Batch 1−52 : Gut microbiota abundance (genus Christensenellaceae R 7group id.11283) on Diabetic nephropathy (more control exclusions) with a threshold of 1e−05

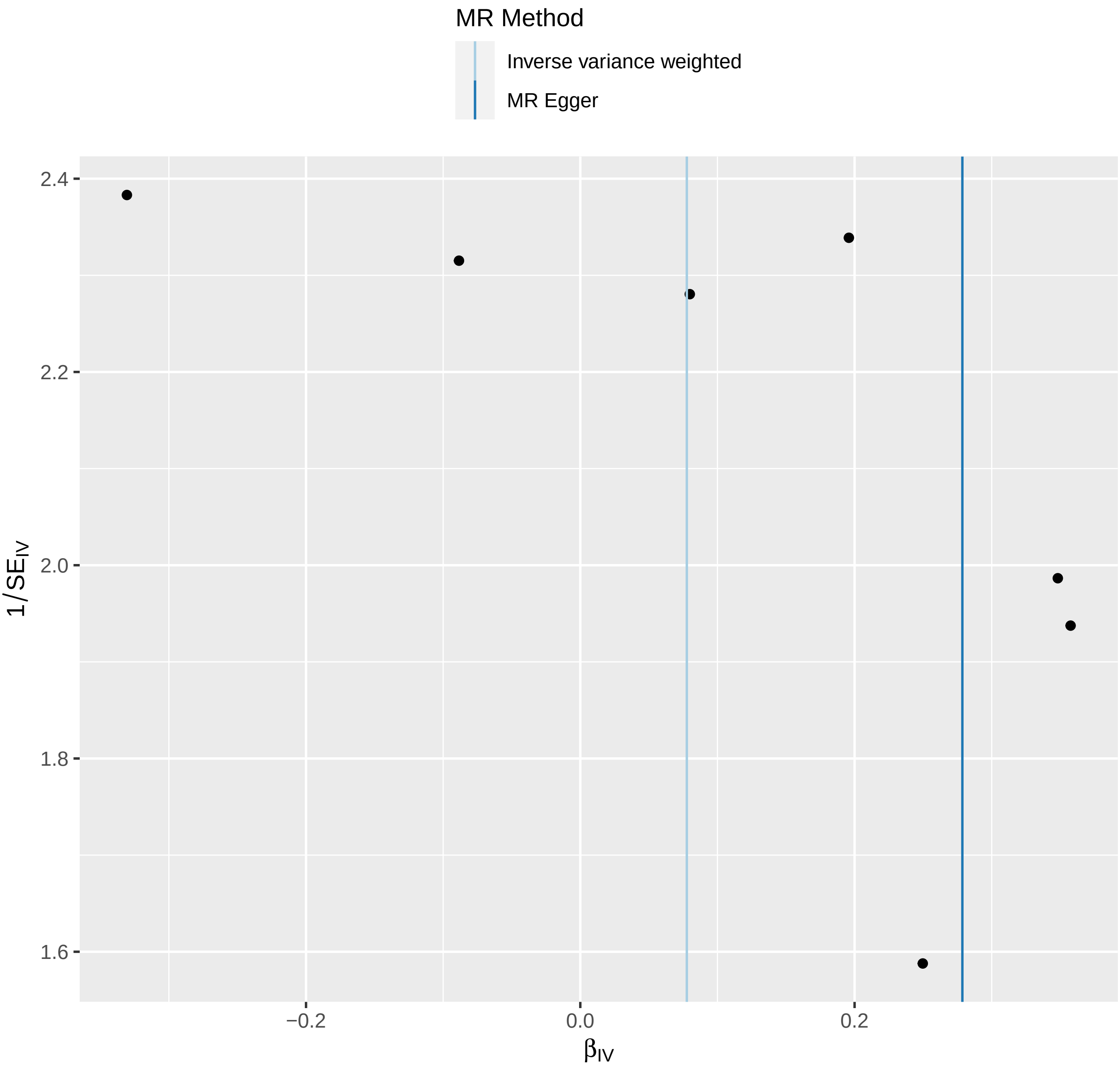

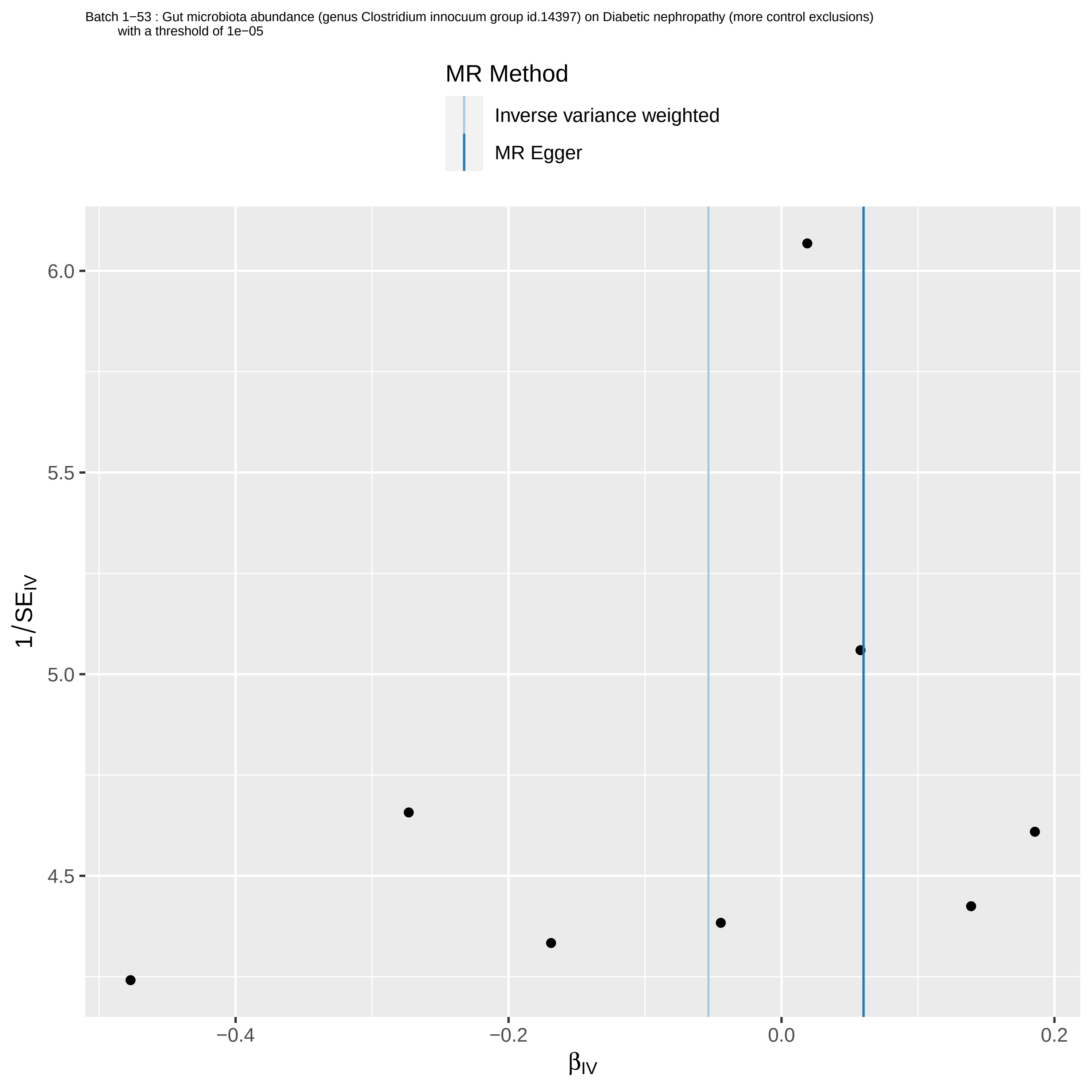
Batch 1−53 : Gut microbiota abundance (genus Clostridium innocuum group id.14397) on Diabetic nephropathy (more control exclusions) with a threshold of 1e−05
MR Method
Inverse variance weighted
MR Egger
6.0
5.5
5.0
4.5
$1/SE_{IV}$
−0.4
−0.2
0.0
0.2
β_{IV}

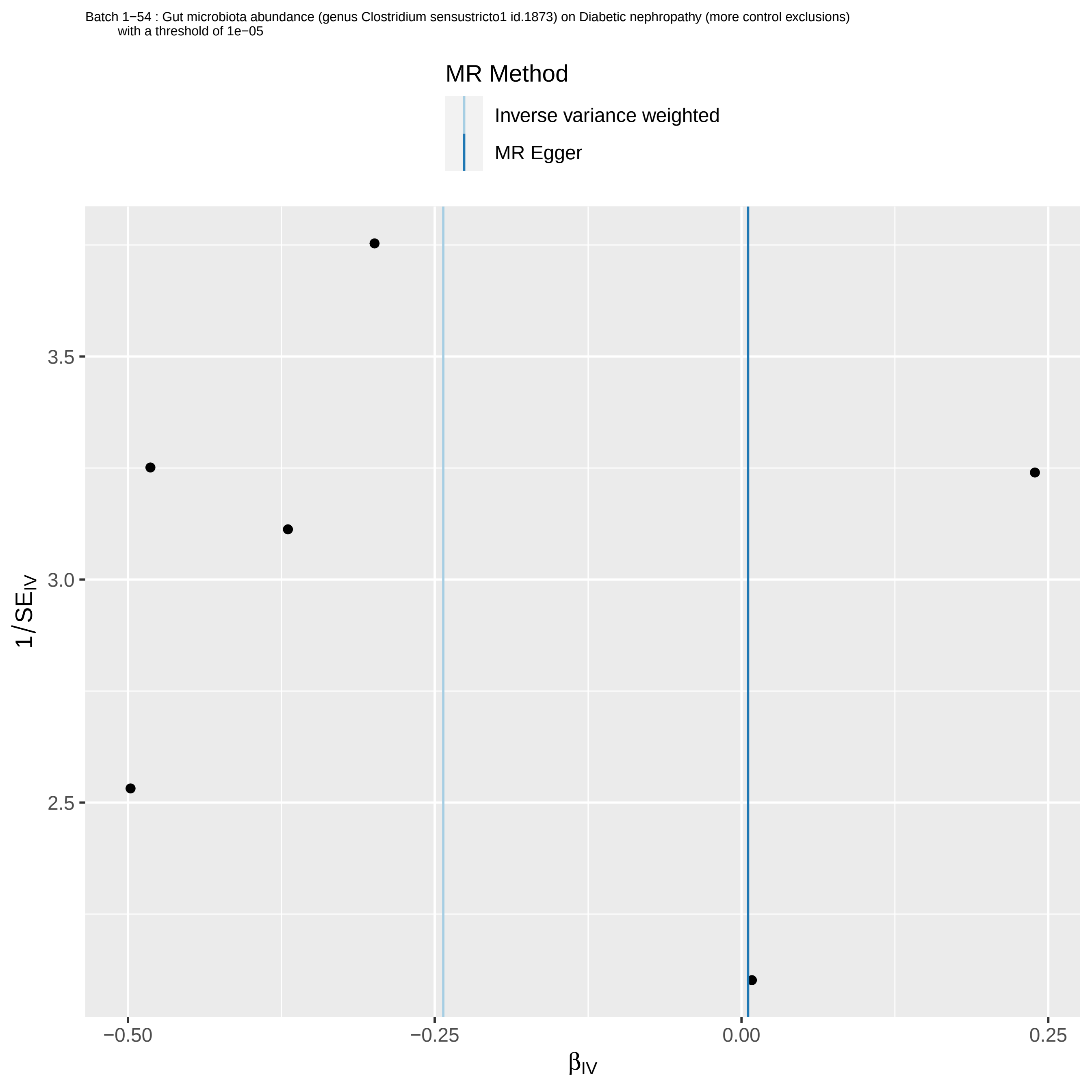
Batch 1−54 : Gut microbiota abundance (genus Clostridium sensustricto1 id.1873) on Diabetic nephropathy (more control exclusions)
with a threshold of 1e−05
MR Method
Inverse variance weighted
MR Egger
3.5
3.0
2.5
1/SE_IV
−0.50
−0.25
0.00
0.25
β_IV

Batch 1−55 : Gut microbiota abundance (genus Collinsella id.815) on Diabetic nephropathy (more control exclusions) with a threshold of 1e−05

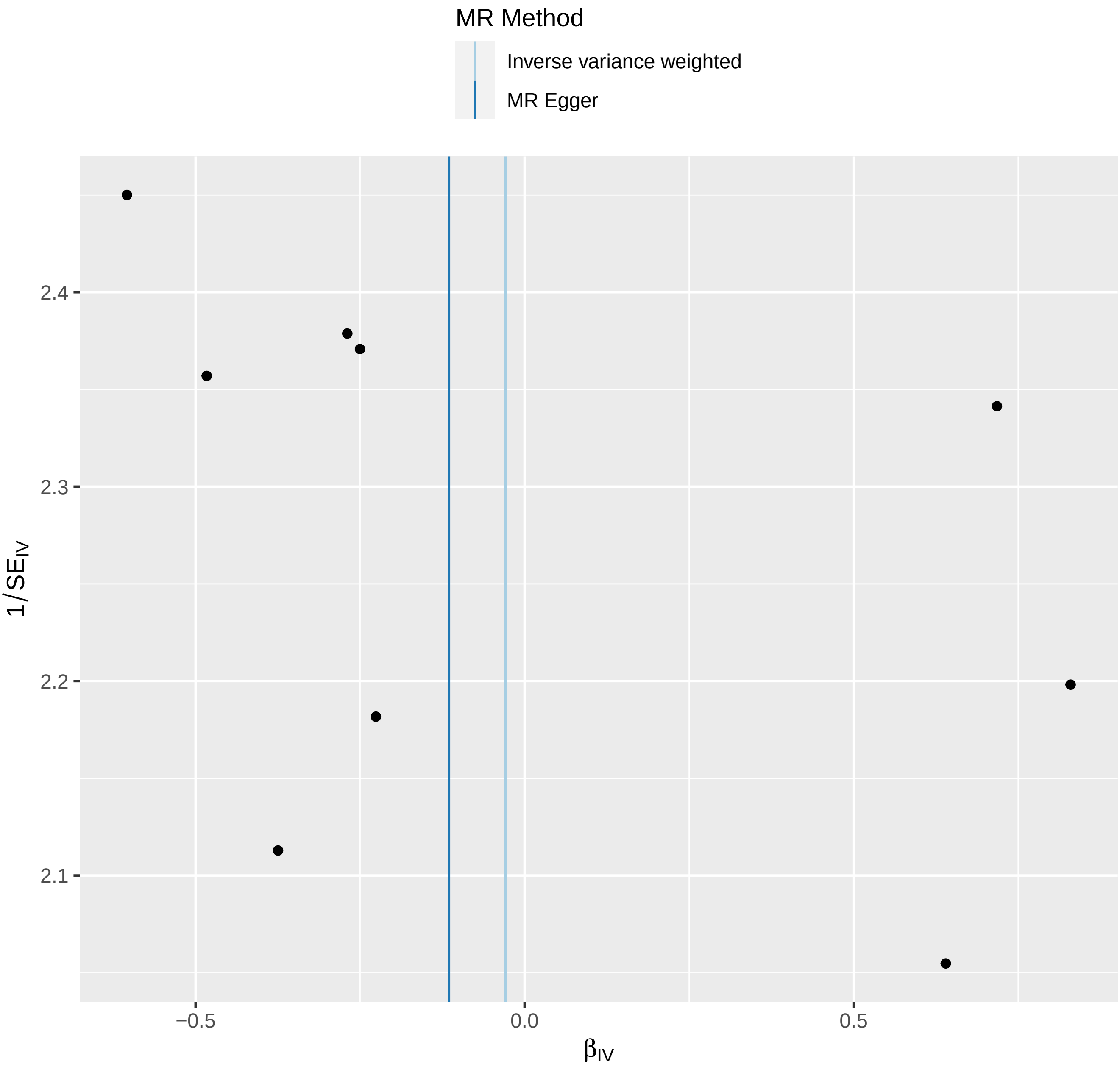

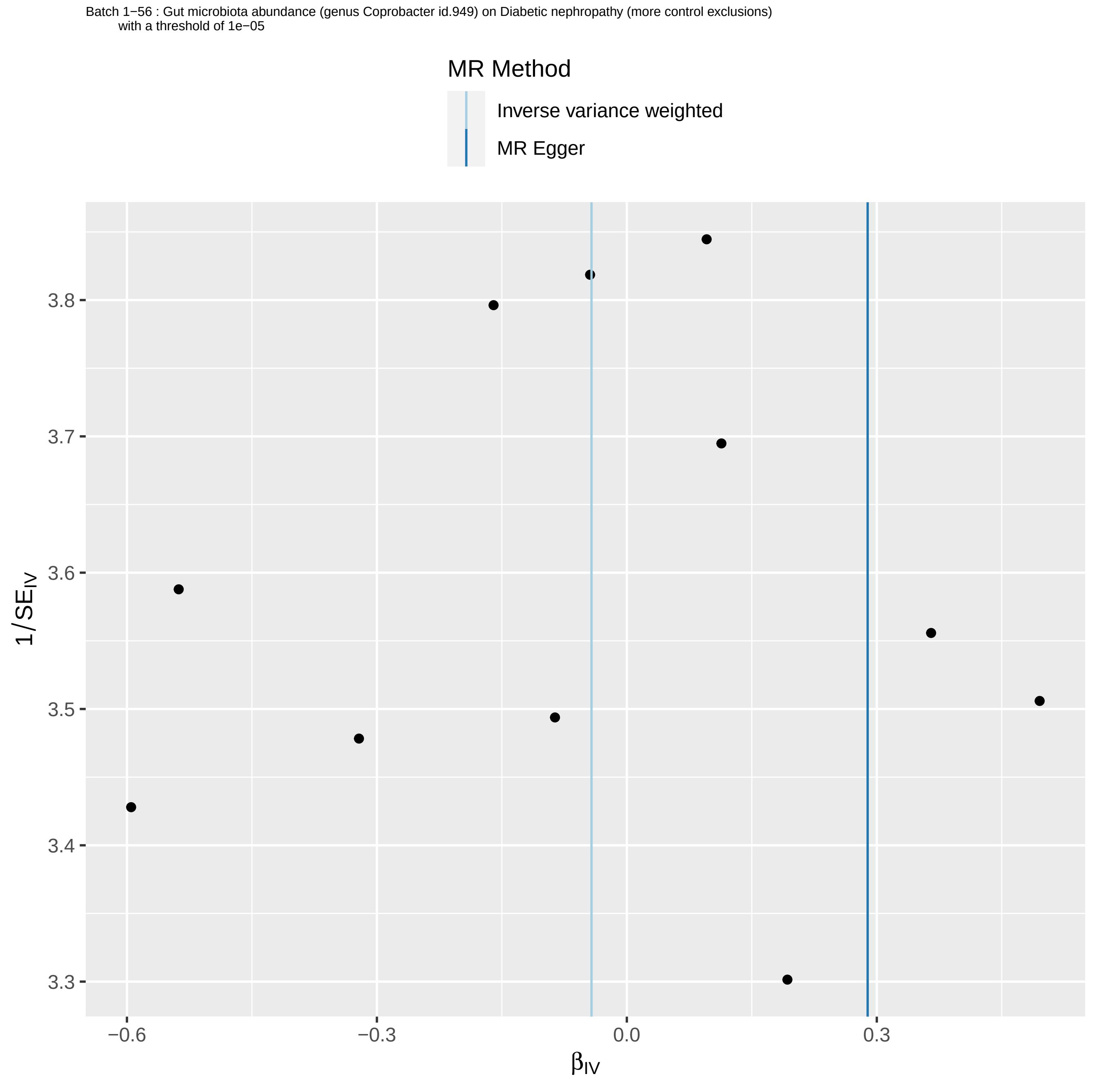
Batch 1−56 : Gut microbiota abundance (genus Coprobacter id.949) on Diabetic nephropathy (more control exclusions)
with a threshold of 1e−05
MR Method
Inverse variance weighted
MR Egger
3.8
3.7
3.6
3.5
3.4
3.3
1/SE_IV
−0.6
−0.3
0.0
0.3
β_IV

Batch 1−57 : Gut microbiota abundance (genus Coprococcus1 id.11301) on Diabetic nephropathy (more control exclusions) with a threshold of 1e−05

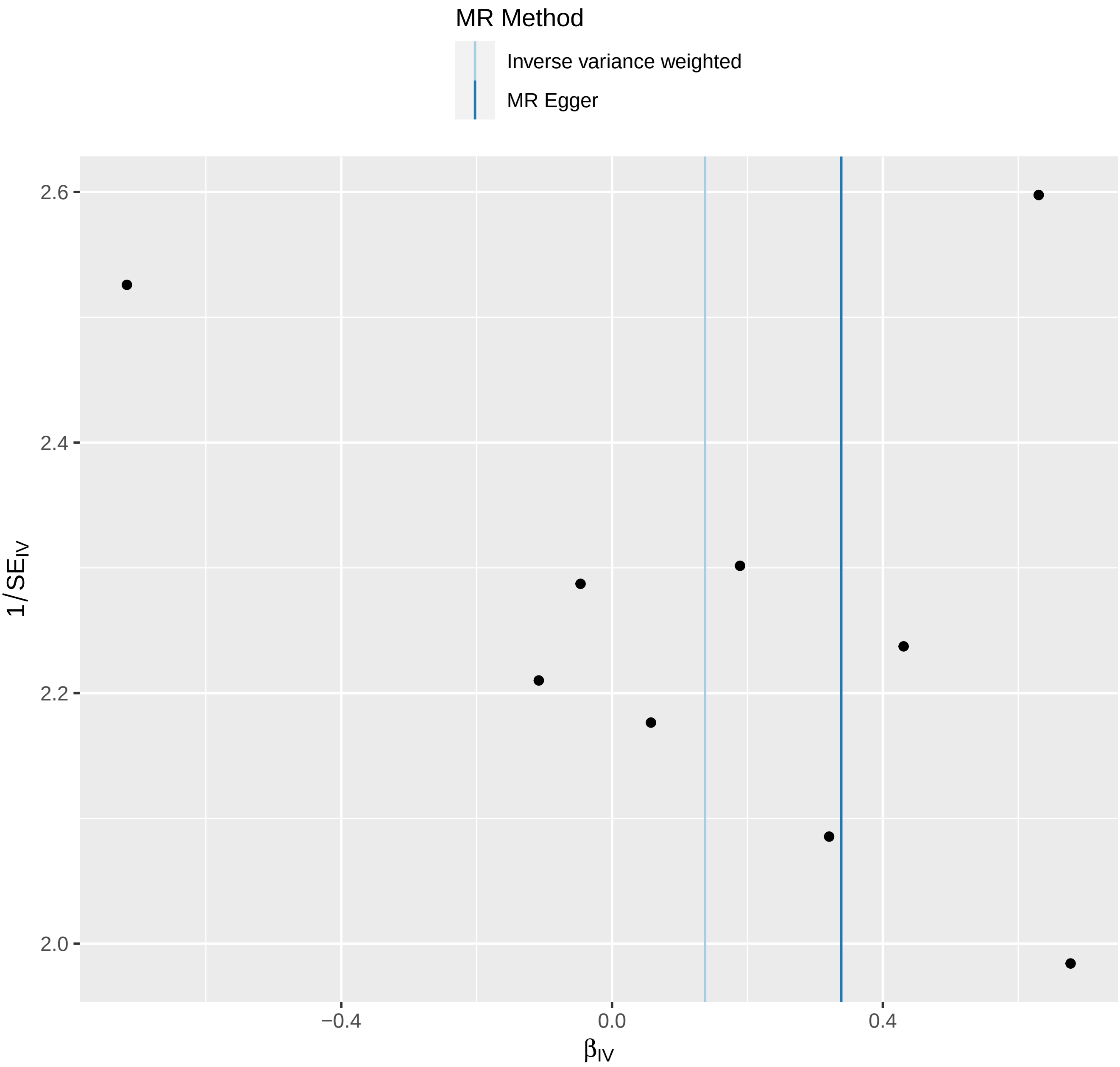

Batch 1−58 : Gut microbiota abundance (genus Coprococcus2 id.11302) on Diabetic nephropathy (more control exclusions) with a threshold of 1e−05

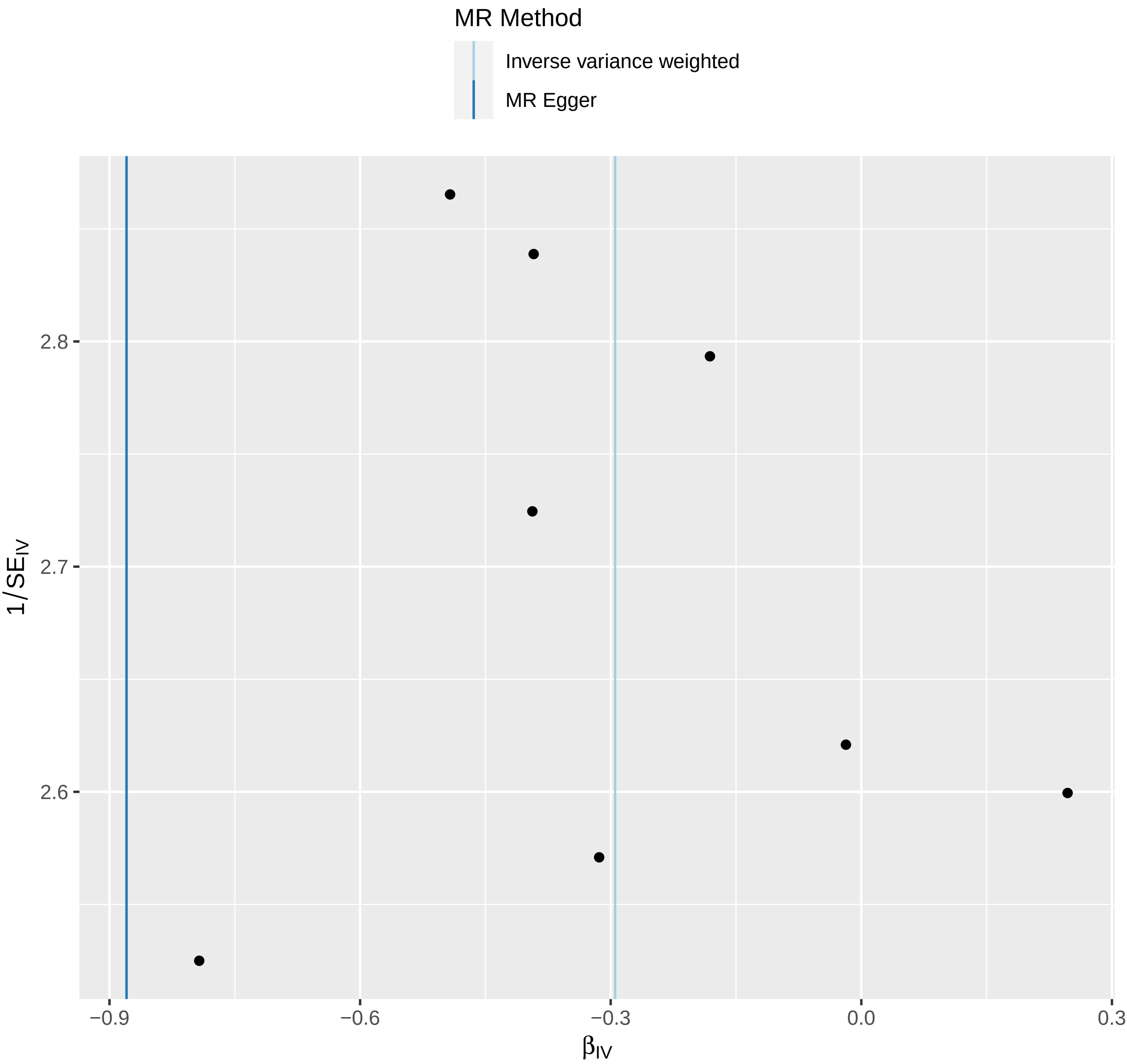

Batch 1−59 : Gut microbiota abundance (genus Coprococcus3 id.11303) on Diabetic nephropathy (more control exclusions) with a threshold of 1e−05

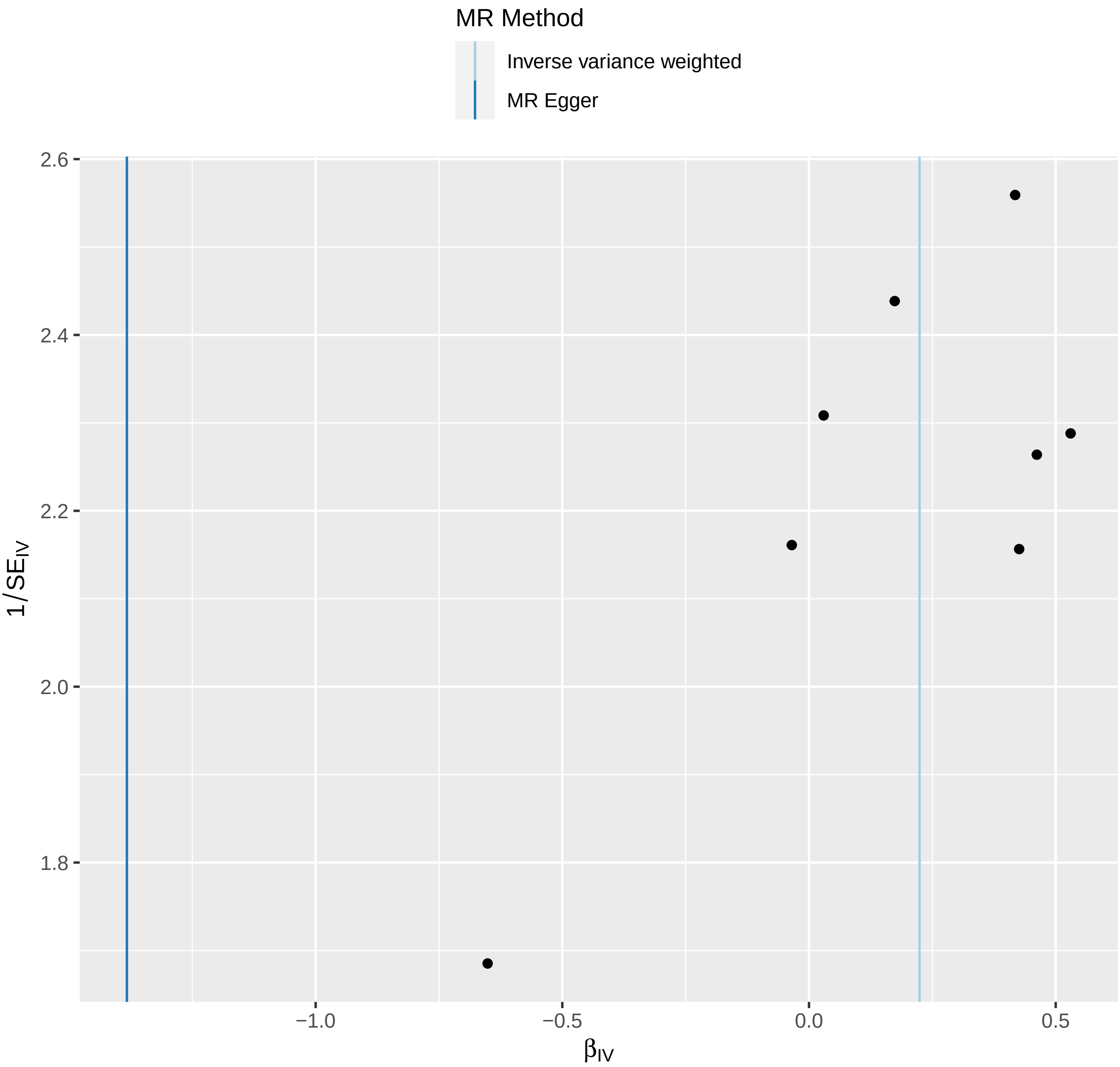

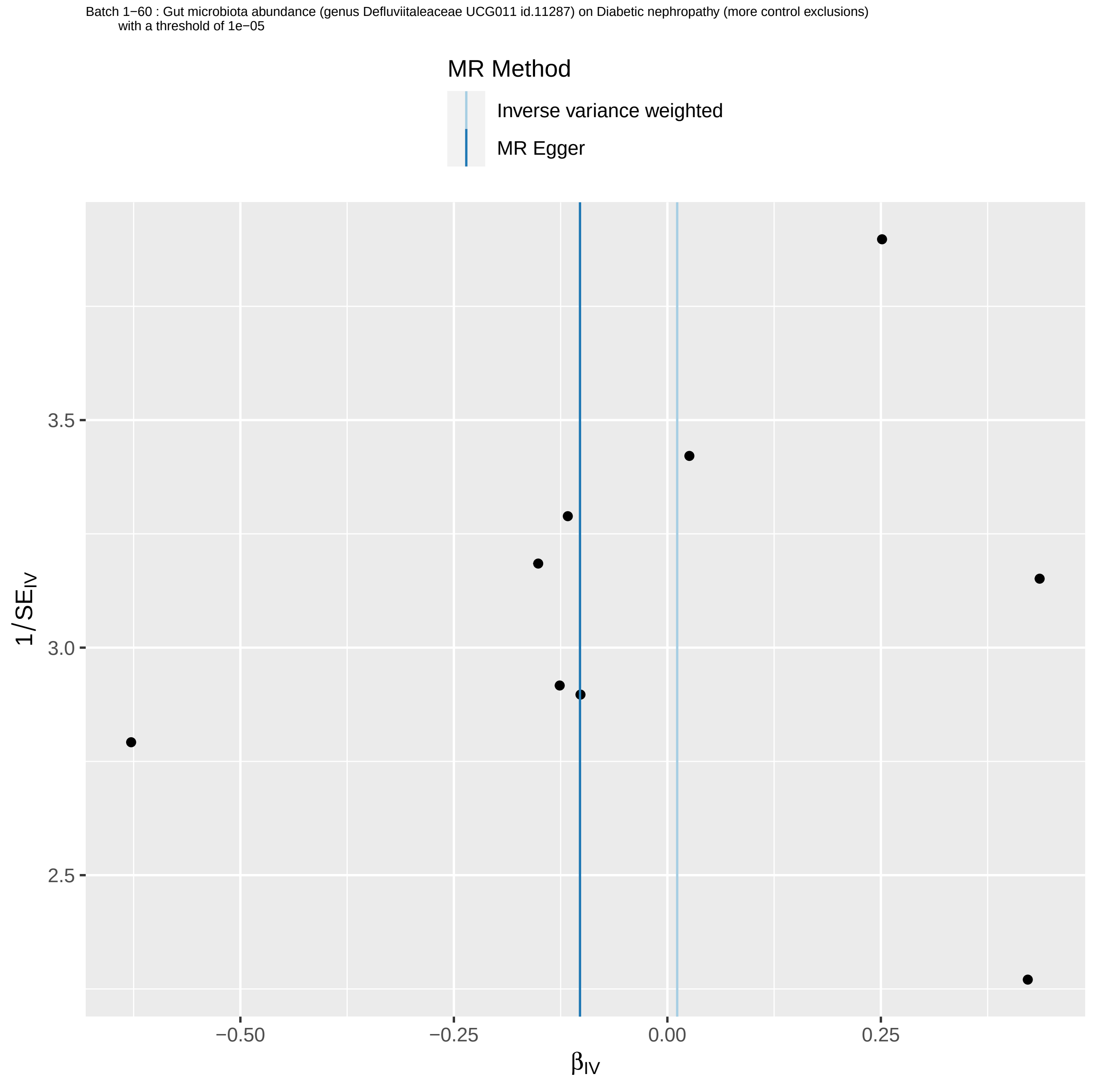

Batch 1−60 : Gut microbiota abundance (genus Defluviitaleaceae UCG011 id.11287) on Diabetic nephropathy (more control exclusions)
with a threshold of 1e−05
MR Method
Inverse variance weighted
MR Egger
3.5
3.0
2.5
1/SE_IV
−0.50
−0.25
0.00
0.25
β_IV

Batch 1−61 : Gut microbiota abundance (genus Desulfovibrio id.3173) on Diabetic nephropathy (more control exclusions) with a threshold of 1e−05

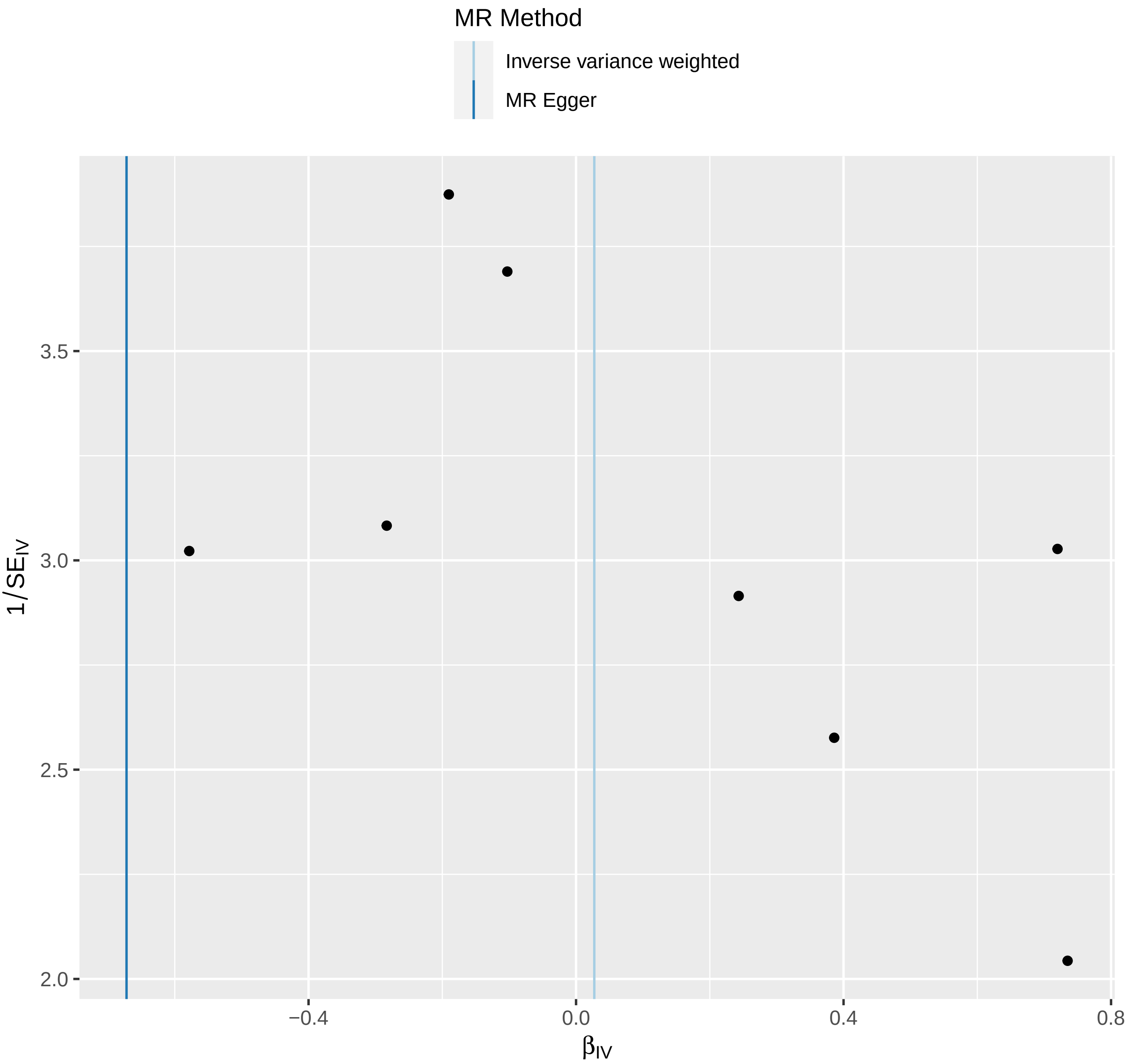

Batch 1−62 : Gut microbiota abundance (genus Dialister id.2183) on Diabetic nephropathy (more control exclusions) with a threshold of 1e−05

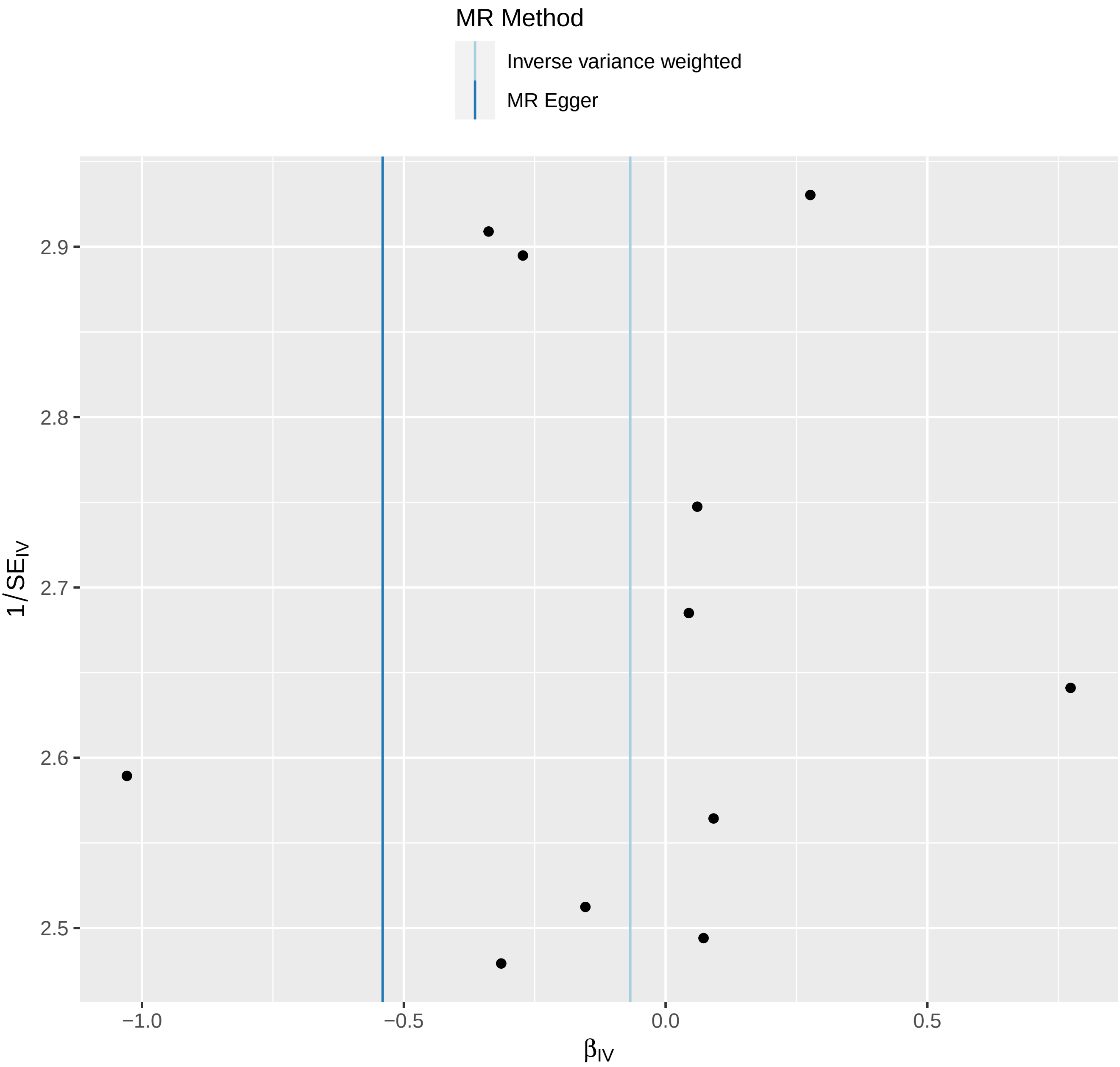

Batch 1−63 : Gut microbiota abundance (genus Dorea id.1997) on Diabetic nephropathy (more control exclusions)
with a threshold of 1e−05

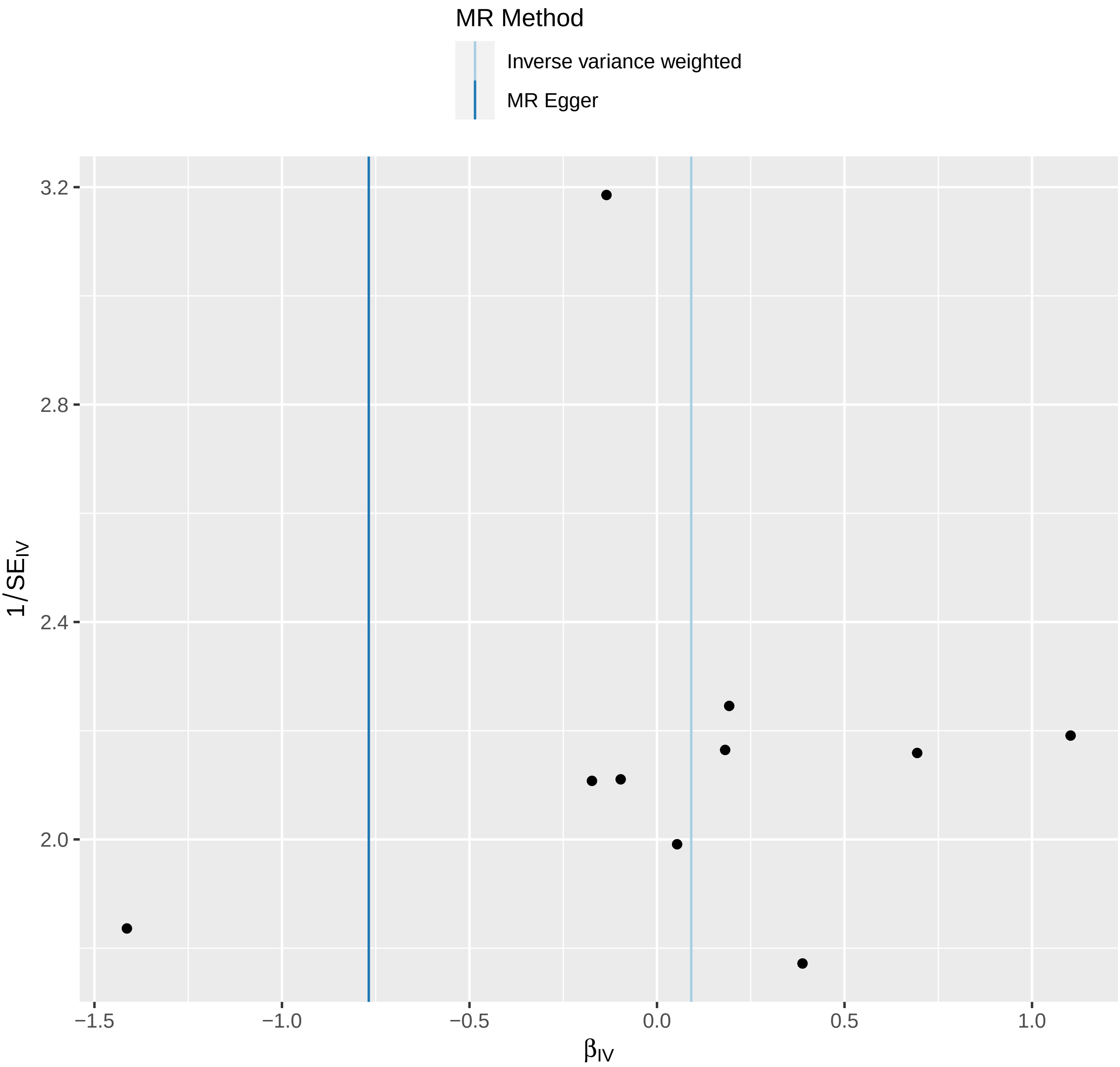

Batch 1−64 : Gut microbiota abundance (genus Eggerthella id.819) on Diabetic nephropathy (more control exclusions) with a threshold of 1e−05

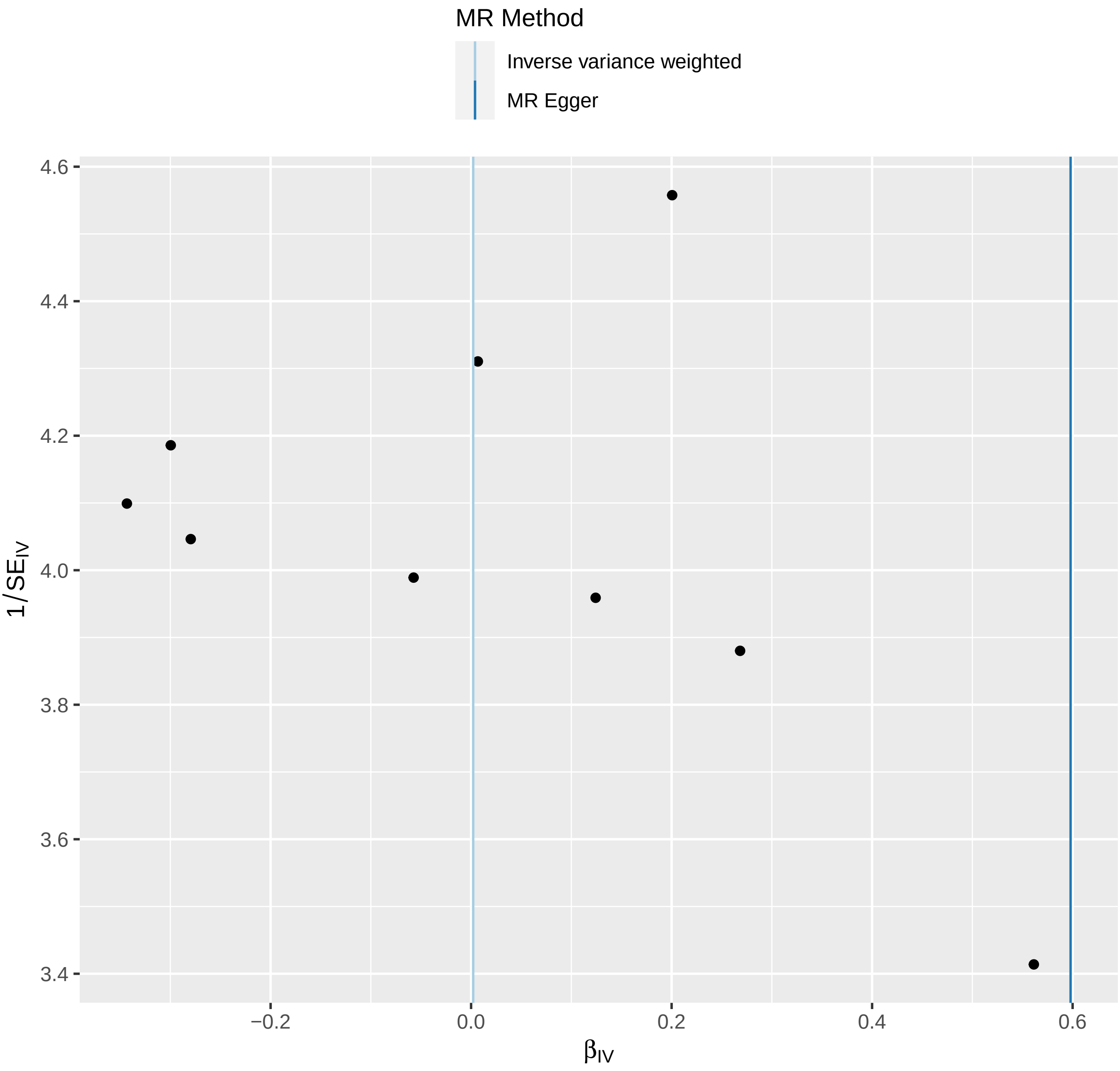

Batch 1−65 : Gut microbiota abundance (genus Eisenbergiella id.11304) on Diabetic nephropathy (more control exclusions) with a threshold of 1e−05

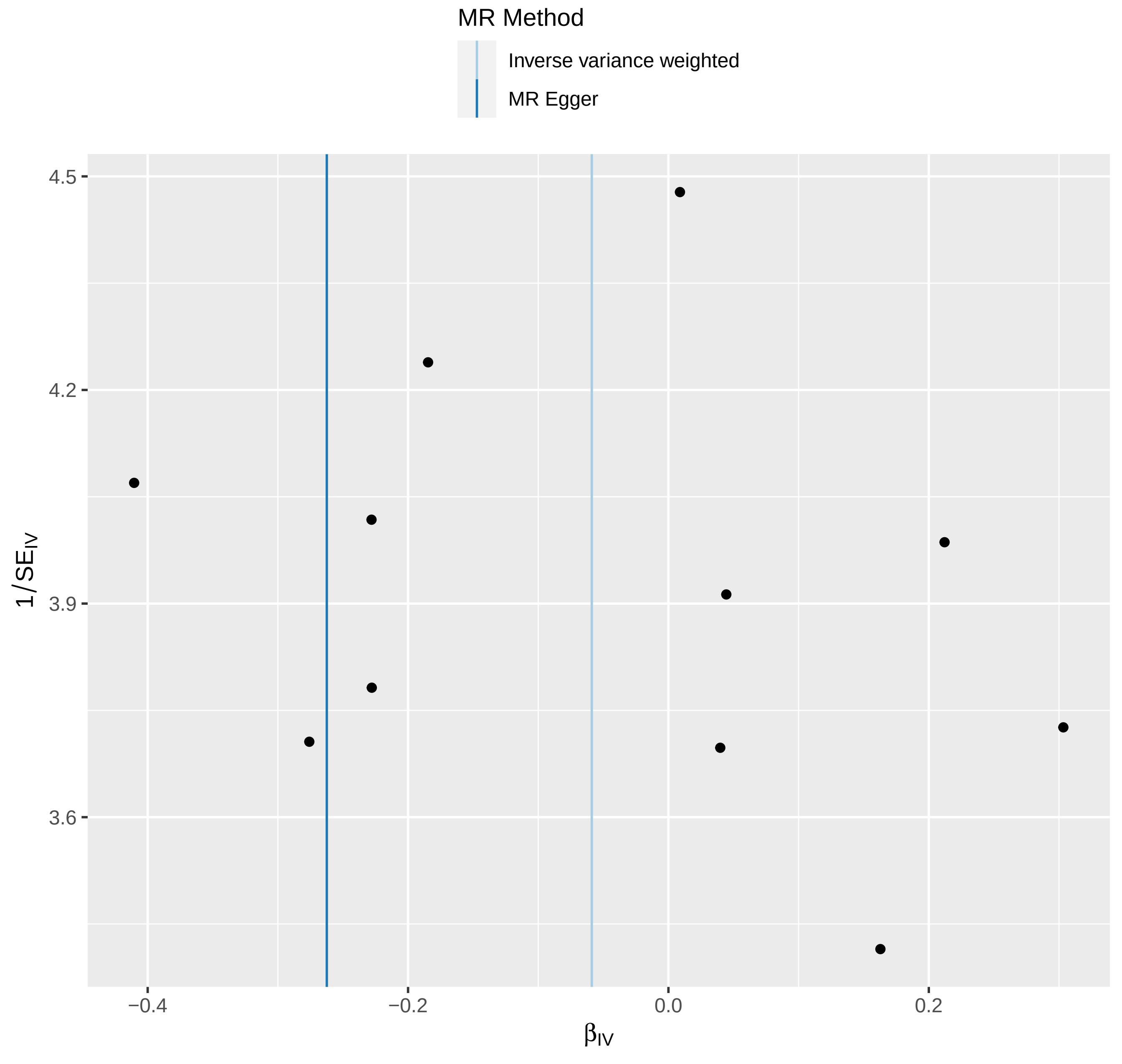

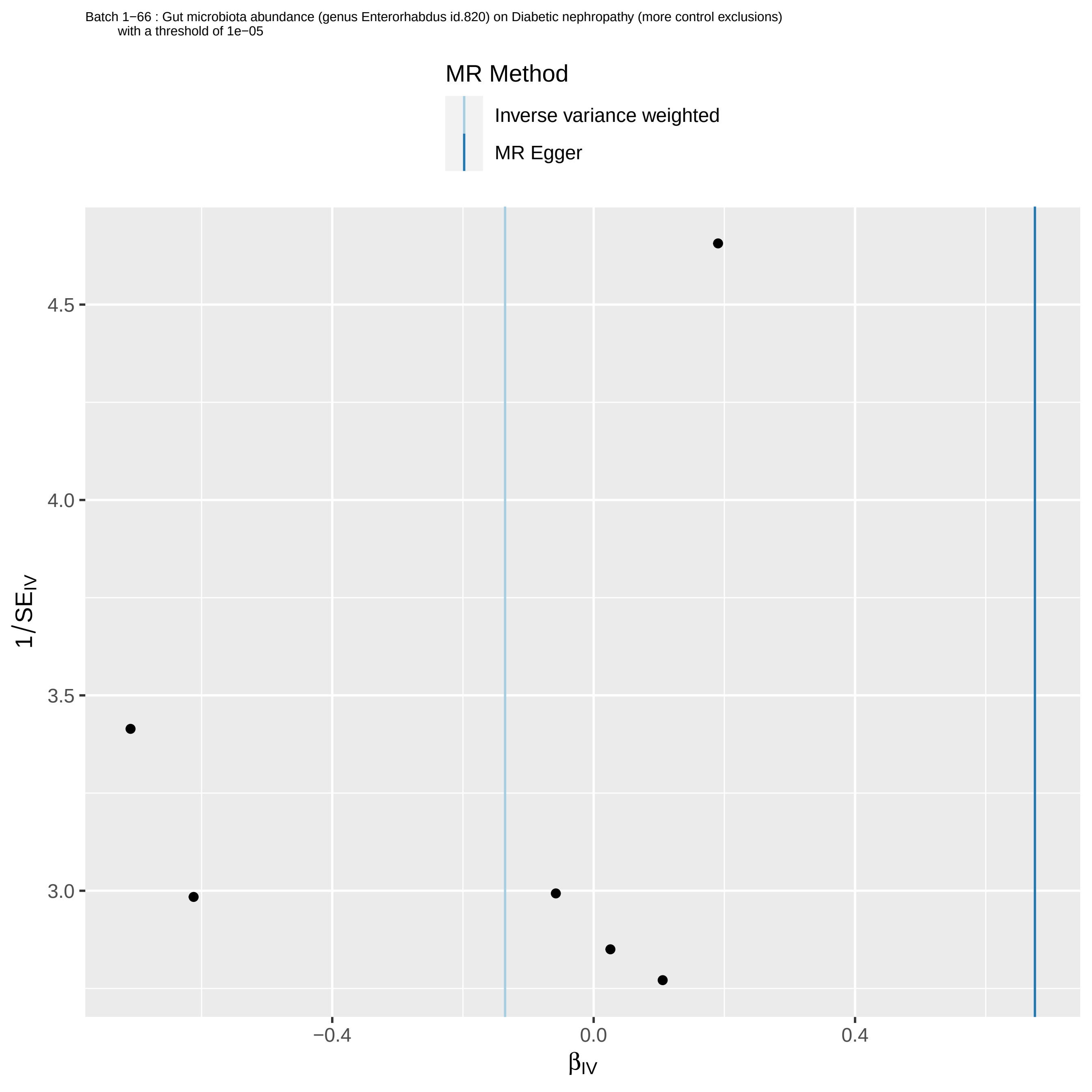

Batch 1−66 : Gut microbiota abundance (genus Enterorhabdus id.820) on Diabetic nephropathy (more control exclusions)
with a threshold of 1e−05
MR Method
Inverse variance weighted
MR Egger
4.5
4.0
3.5
3.0
1/SE_IV
−0.4
0.0
0.4
β_IV

Batch 1−67 : Gut microbiota abundance (genus Erysipelatoclostridium id.11381) on Diabetic nephropathy (more control exclusions) with a threshold of 1e−05

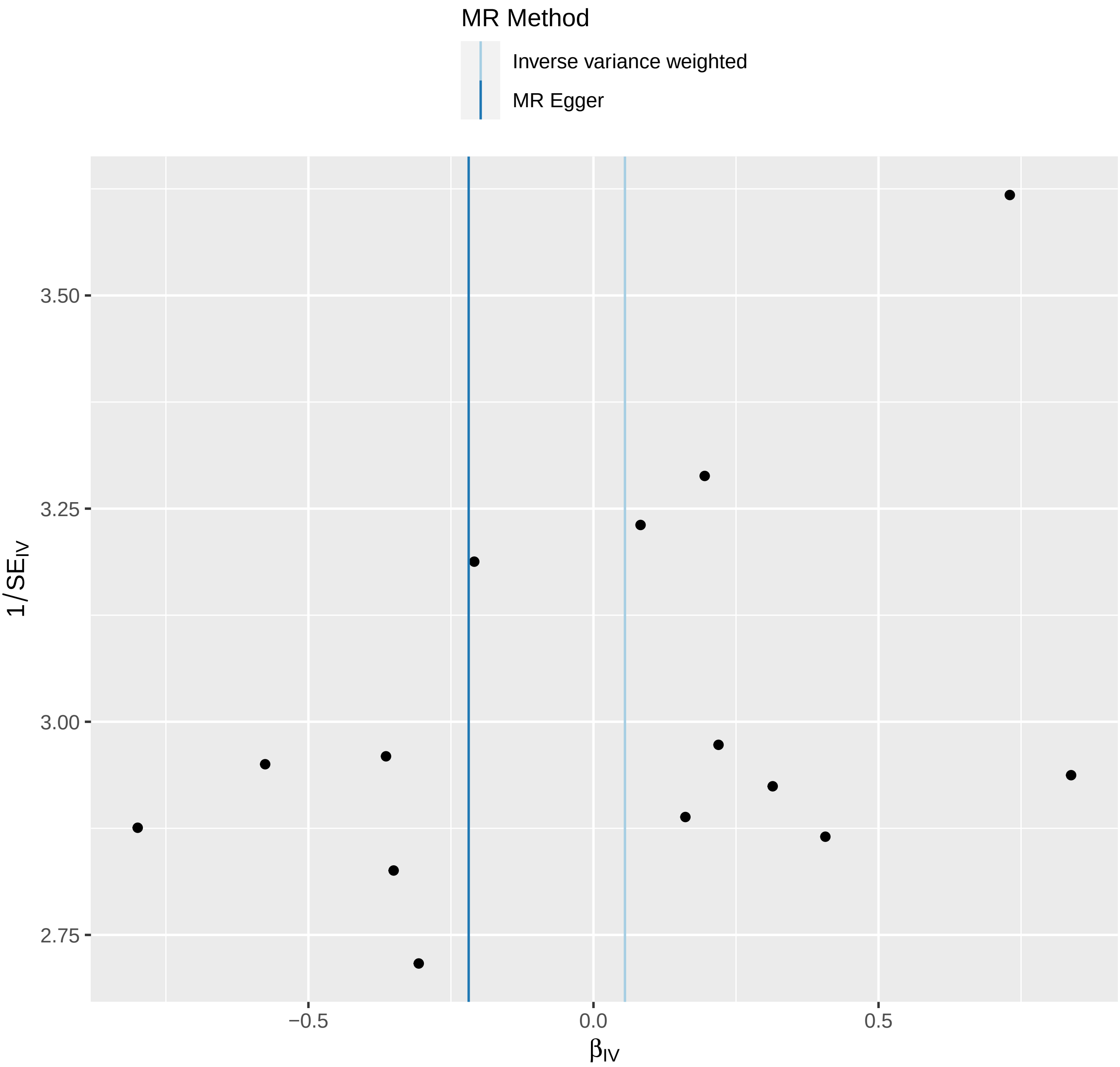

Batch 1−69 : Gut microbiota abundance (genus Escherichia Shigella id.3504) on Diabetic nephropathy (more control exclusions) with a threshold of 1e−05

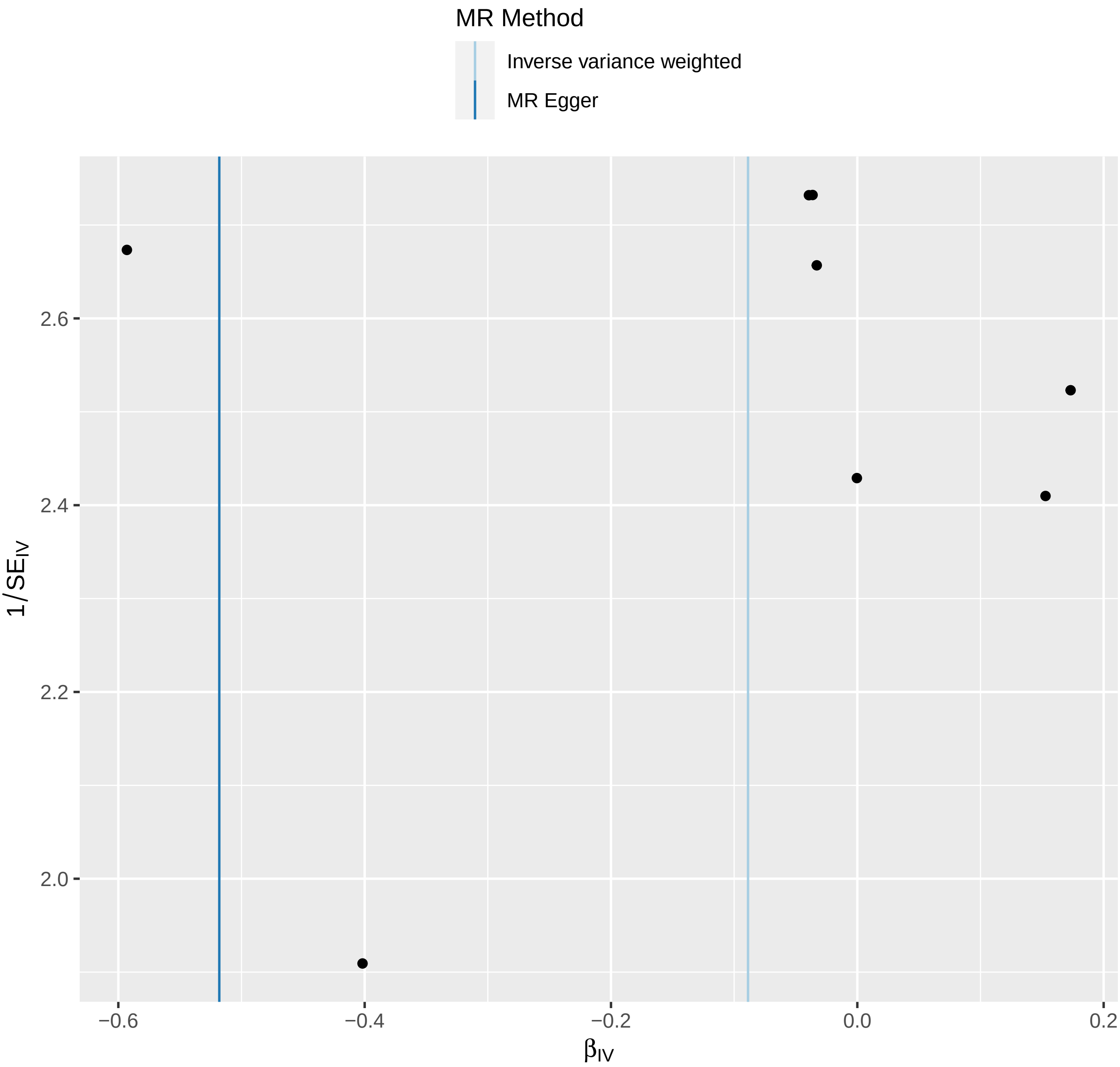

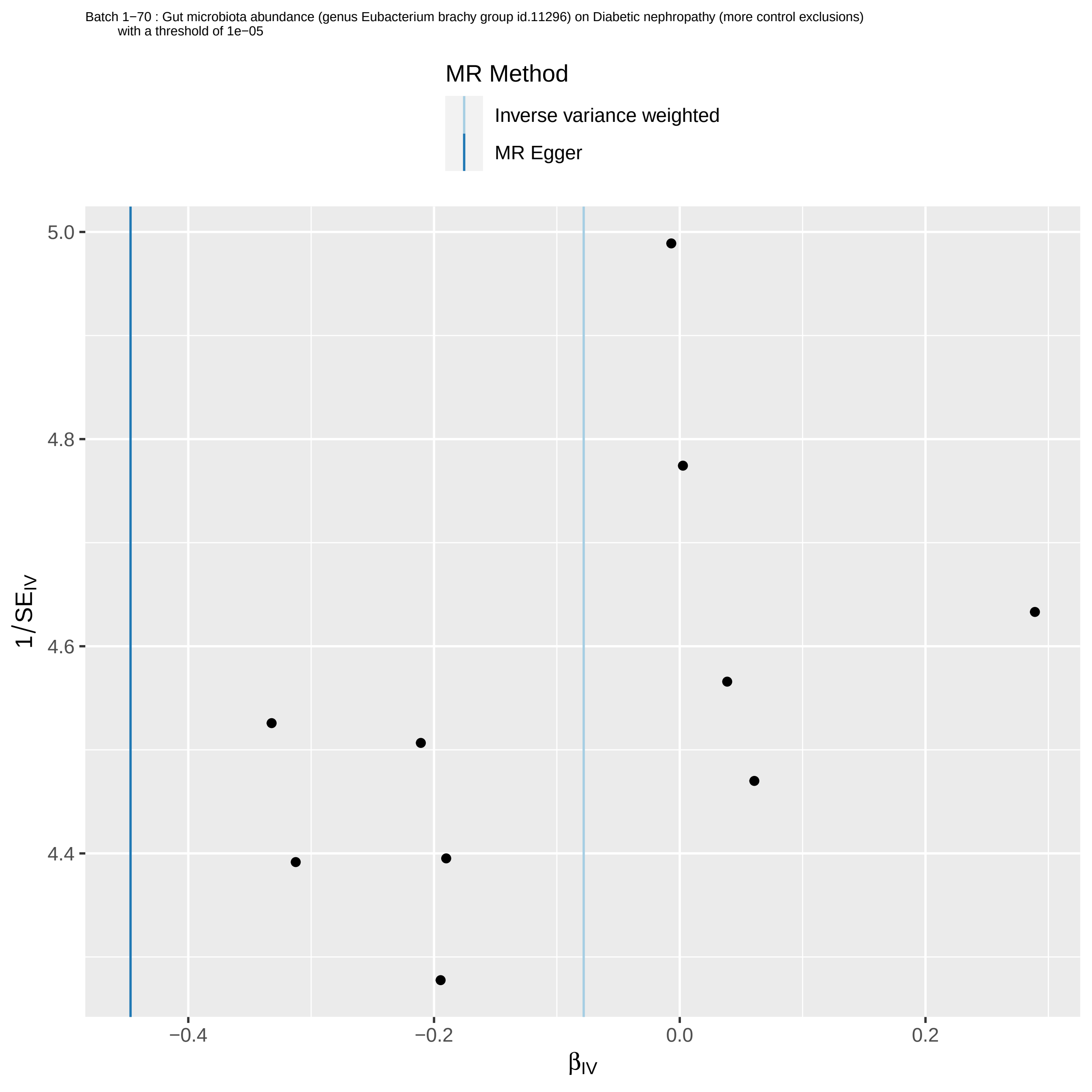
Batch 1−70 : Gut microbiota abundance (genus Eubacterium brachy group id.11296) on Diabetic nephropathy (more control exclusions)
with a threshold of 1e−05
MR Method
Inverse variance weighted
MR Egger
5.0
4.8
4.6
4.4
1/SE_IV
−0.4
−0.2
0.0
0.2
β_IV

Batch 1−71 : Gut microbiota abundance (genus Eubacterium coprostanoligenes group id.11375) on Diabetic nephropathy (more control exclusions) with a threshold of 1e−05

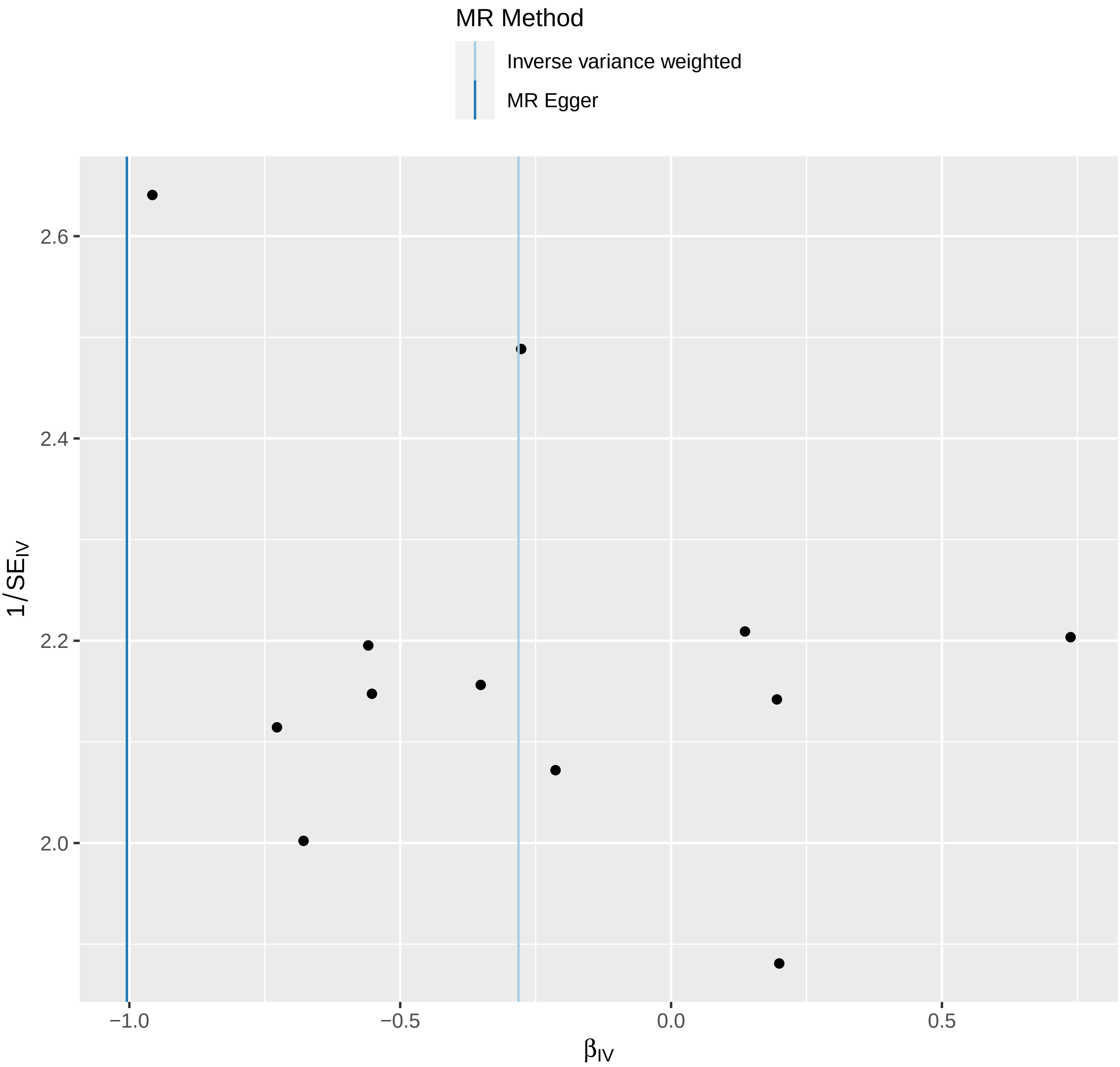

Batch 1−72 : Gut microbiota abundance (genus Eubacterium eligens group id.14372) on Diabetic nephropathy (more control exclusions) with a threshold of 1e−05

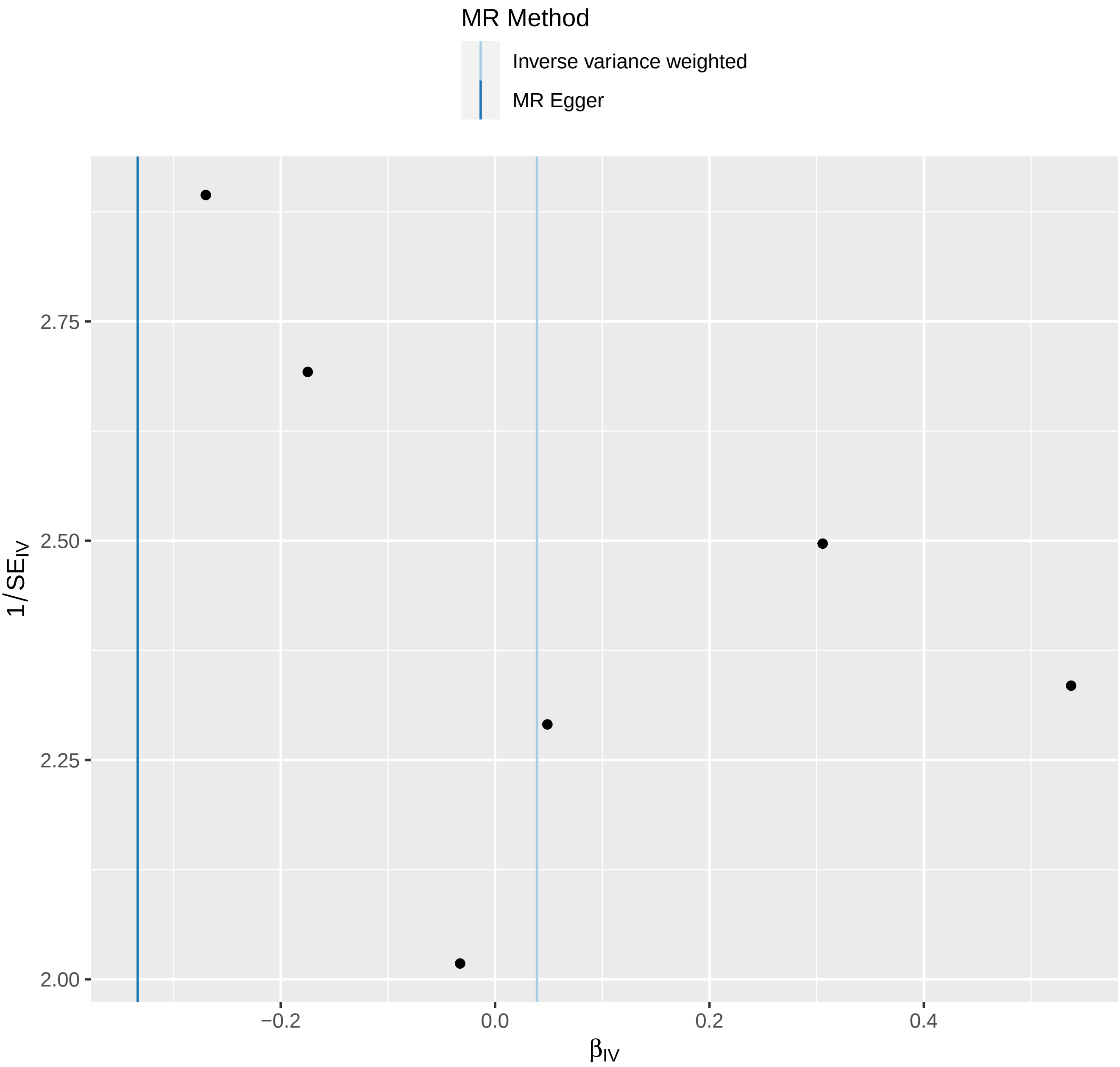

Batch 1−73 : Gut microbiota abundance (genus Eubacterium fissicatena group id.14373) on Diabetic nephropathy (more control exclusions) with a threshold of 1e−05

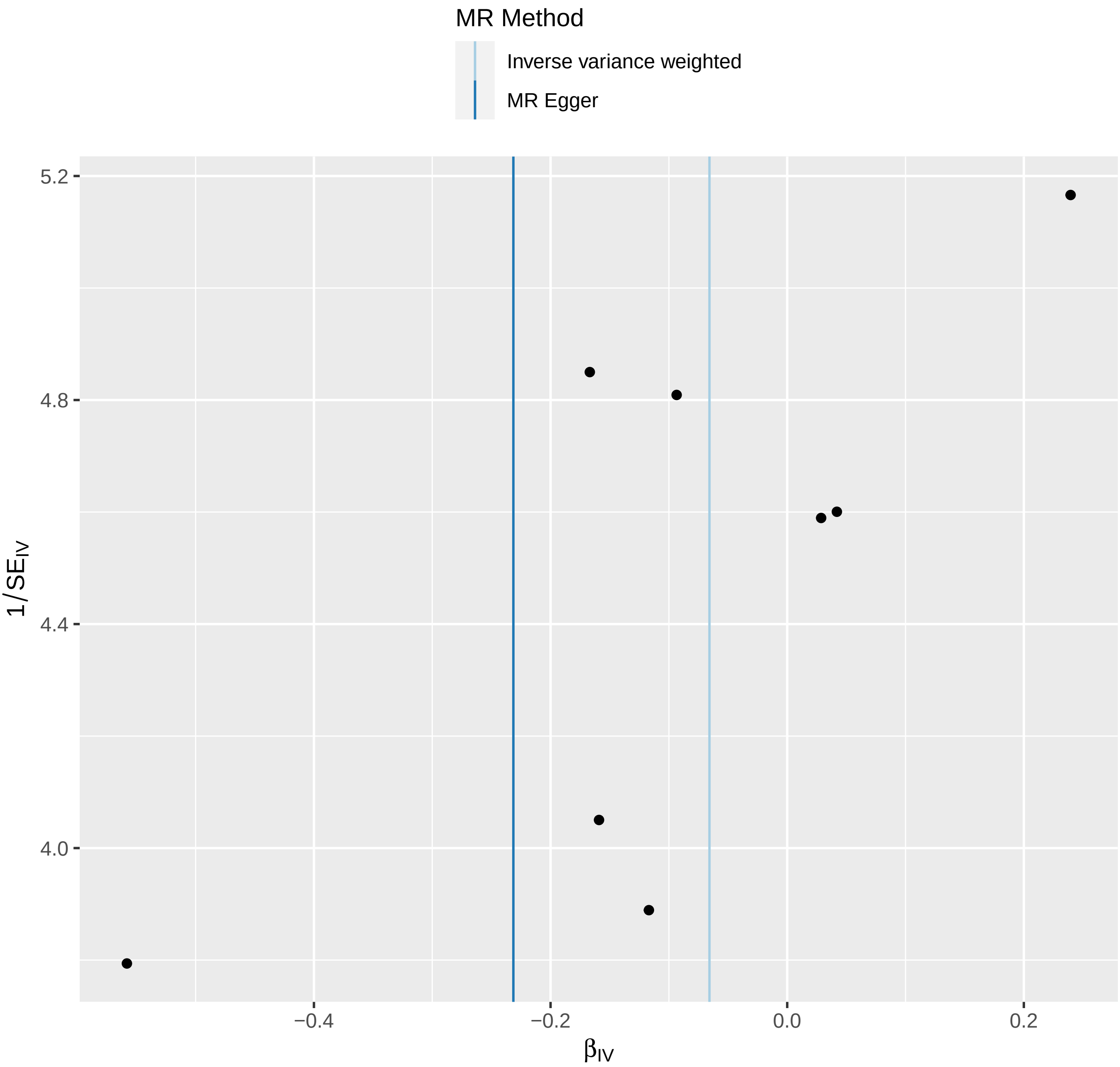

Batch 1−74 : Gut microbiota abundance (genus Eubacterium hallii group id.11338) on Diabetic nephropathy (more control exclusions) with a threshold of 1e−05

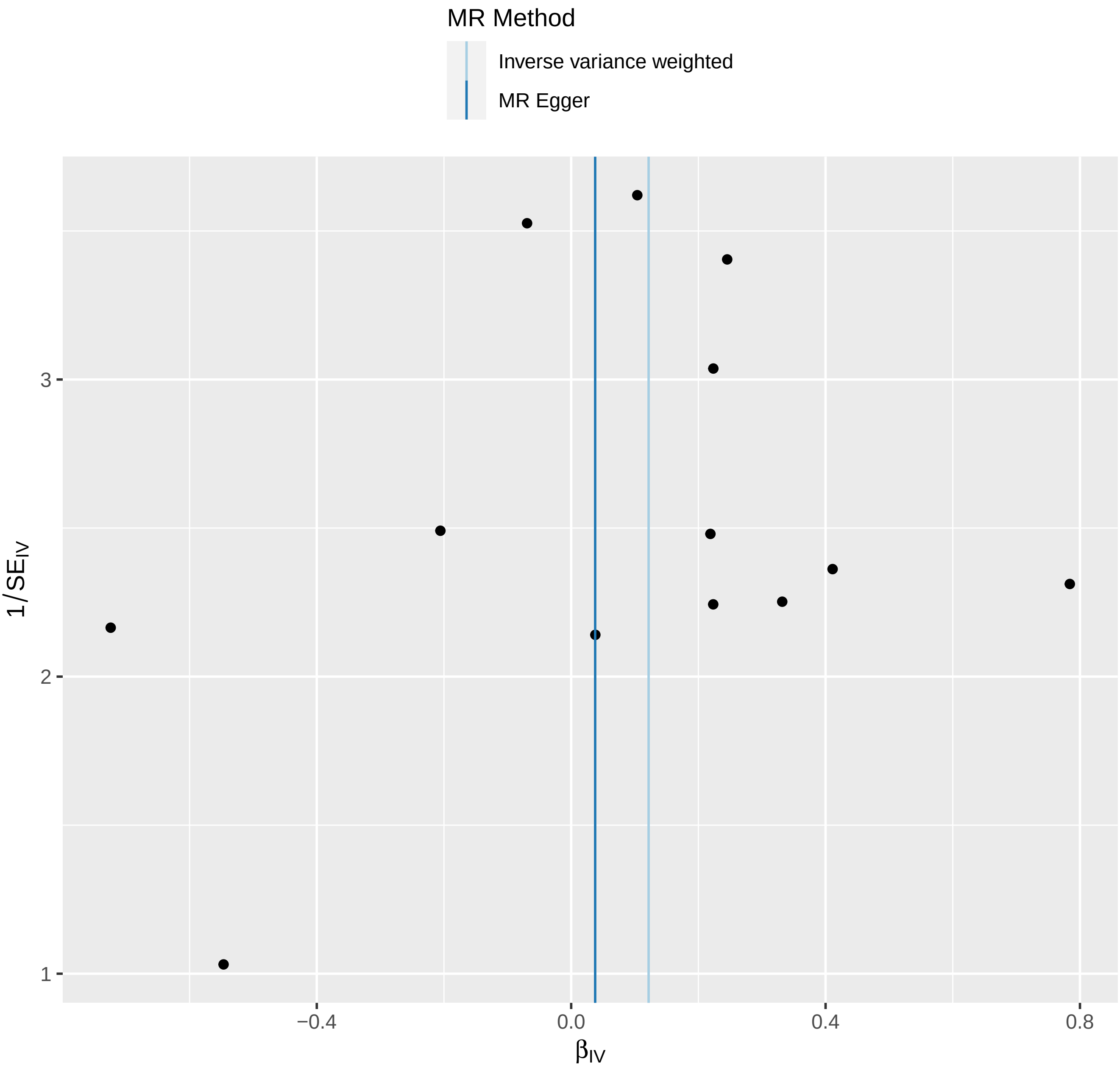

Batch 1−75 : Gut microbiota abundance (genus Eubacterium nodatum group id.11297) on Diabetic nephropathy (more control exclusions) with a threshold of 1e−05

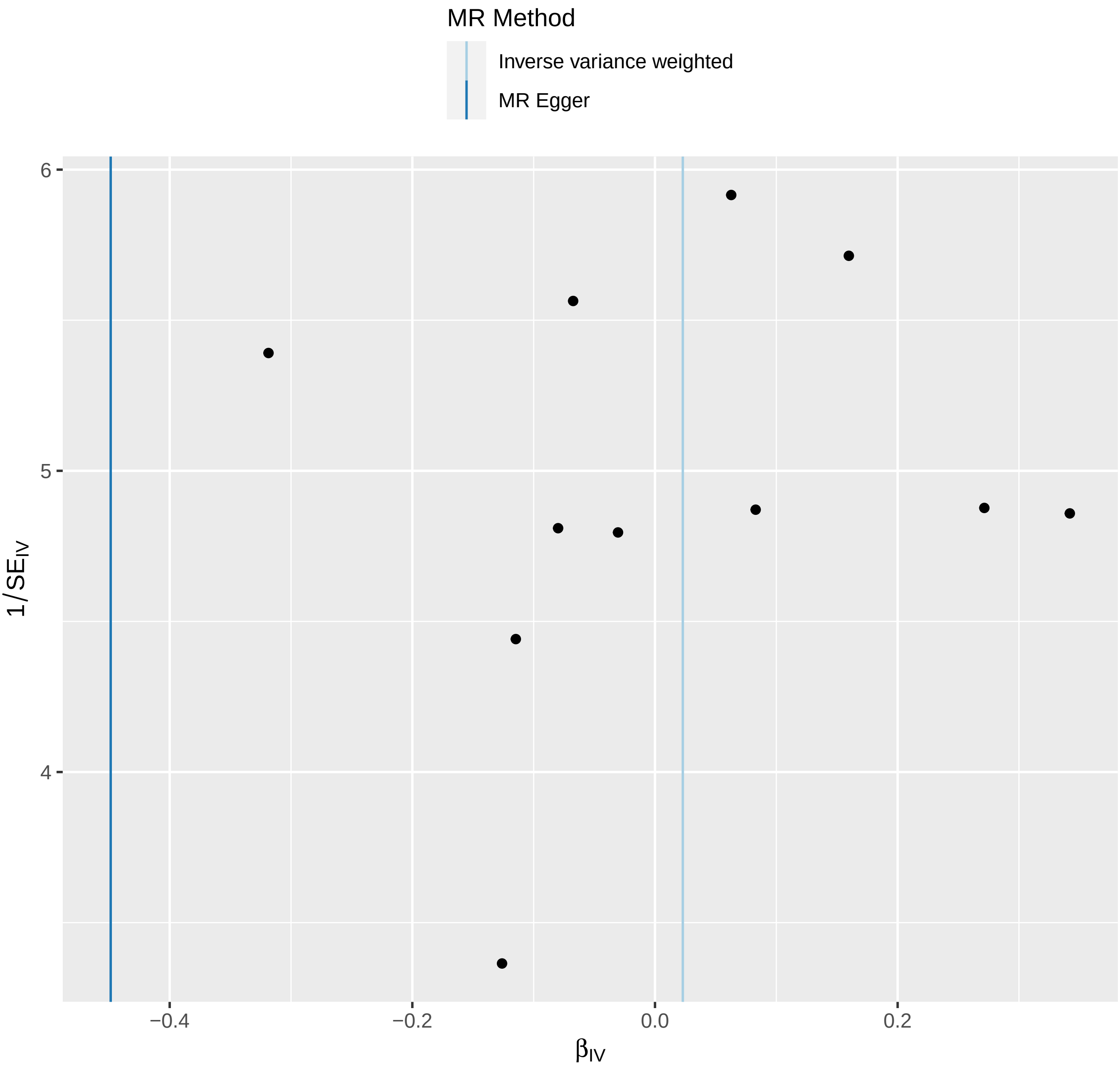

Batch 1−76 : Gut microbiota abundance (genus Eubacterium oxidoreducens group id.11339) on Diabetic nephropathy (more control exclusions) with a threshold of 1e−05

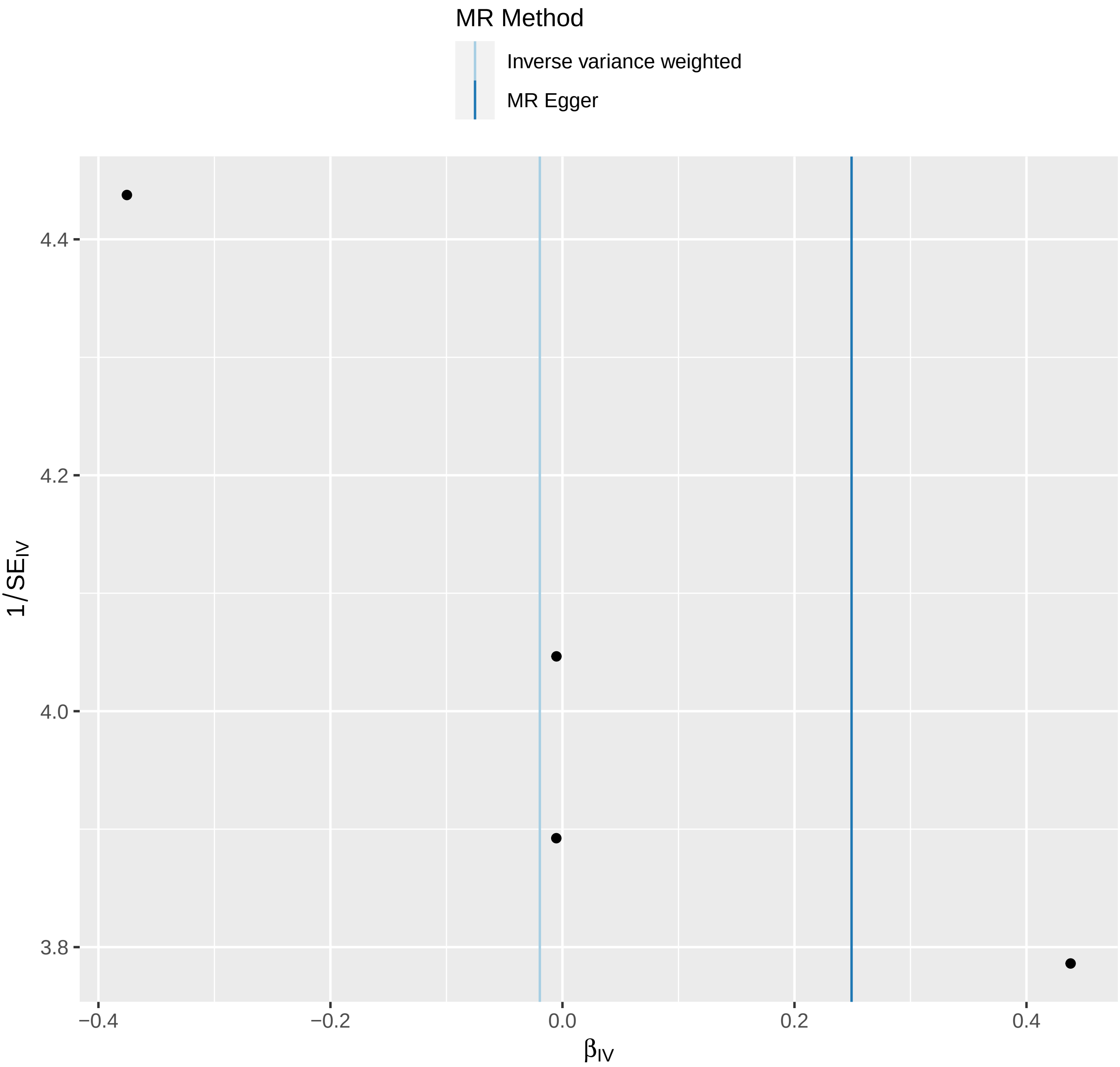

Batch 1−77 : Gut microbiota abundance (genus Eubacterium rectale group id.14374) on Diabetic nephropathy (more control exclusions) with a threshold of 1e−05

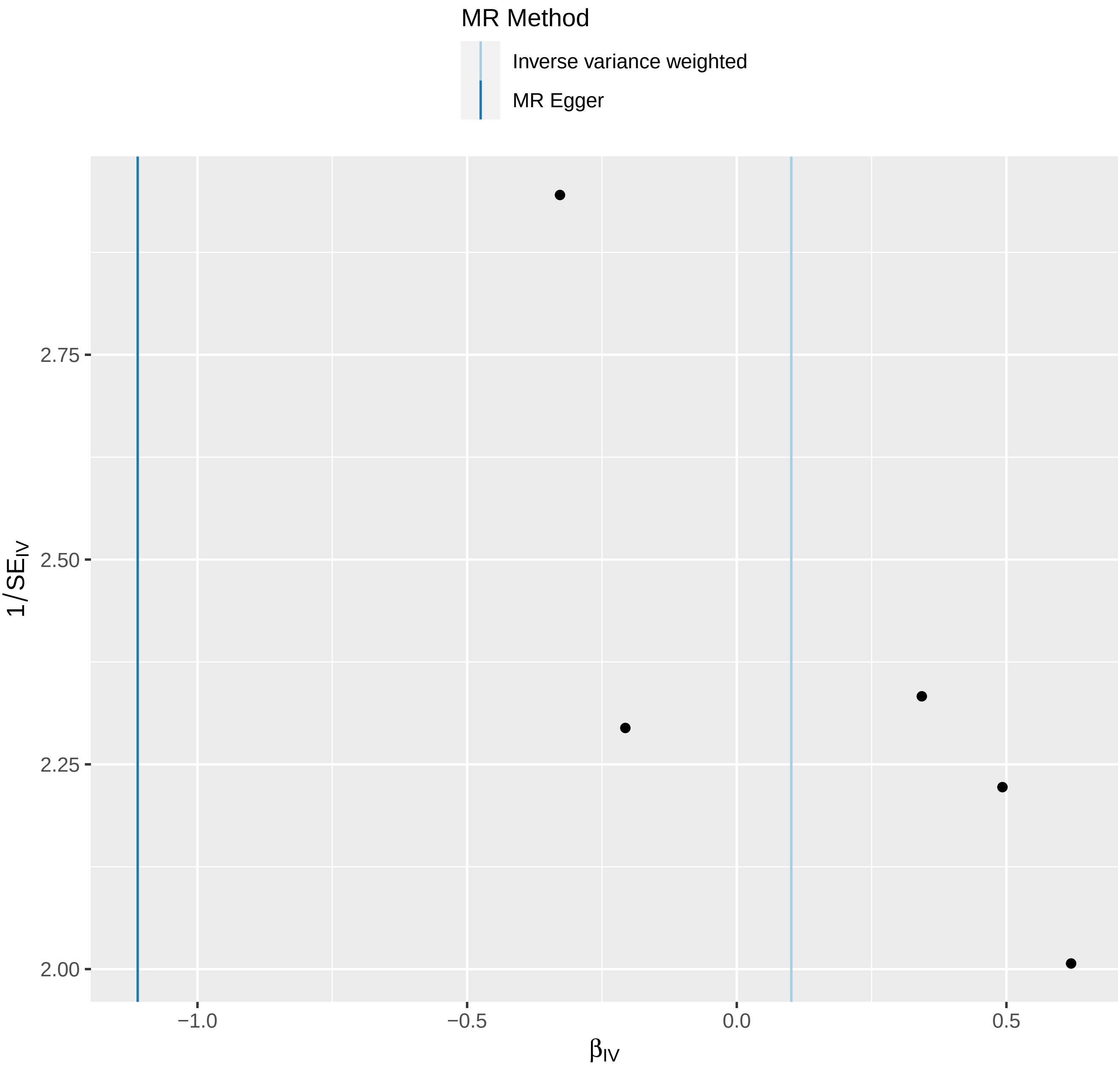

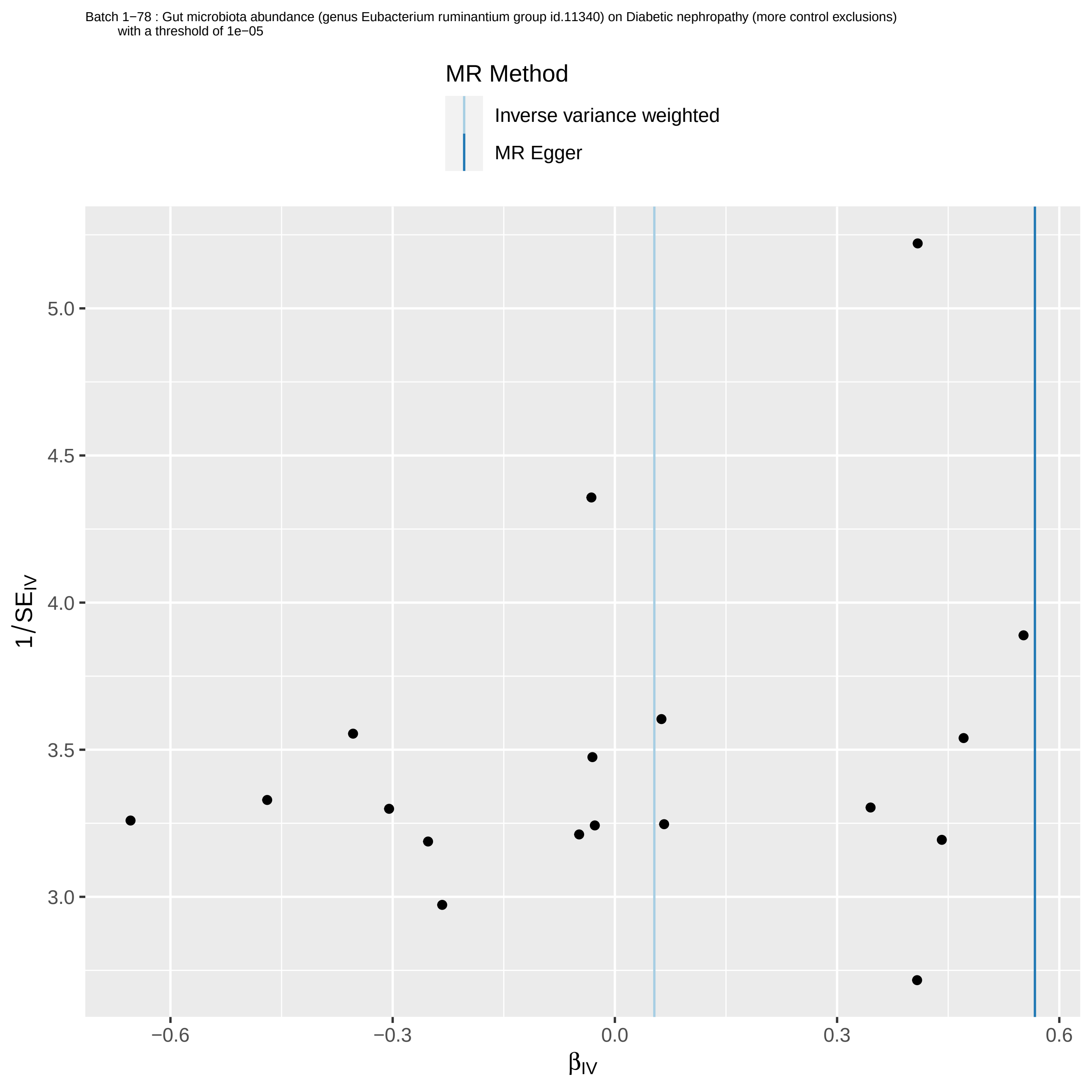
Batch 1−78 : Gut microbiota abundance (genus Eubacterium ruminantium group id.11340) on Diabetic nephropathy (more control exclusions)
with a threshold of 1e−05
MR Method
Inverse variance weighted
MR Egger
5.0
4.5
4.0
3.5
3.0
1/SE_IV
−0.6
−0.3
0.0
0.3
0.6
β_IV

Batch 1−79 : Gut microbiota abundance (genus Eubacterium ventriosum group id.11341) on Diabetic nephropathy (more control exclusions) with a threshold of 1e−05

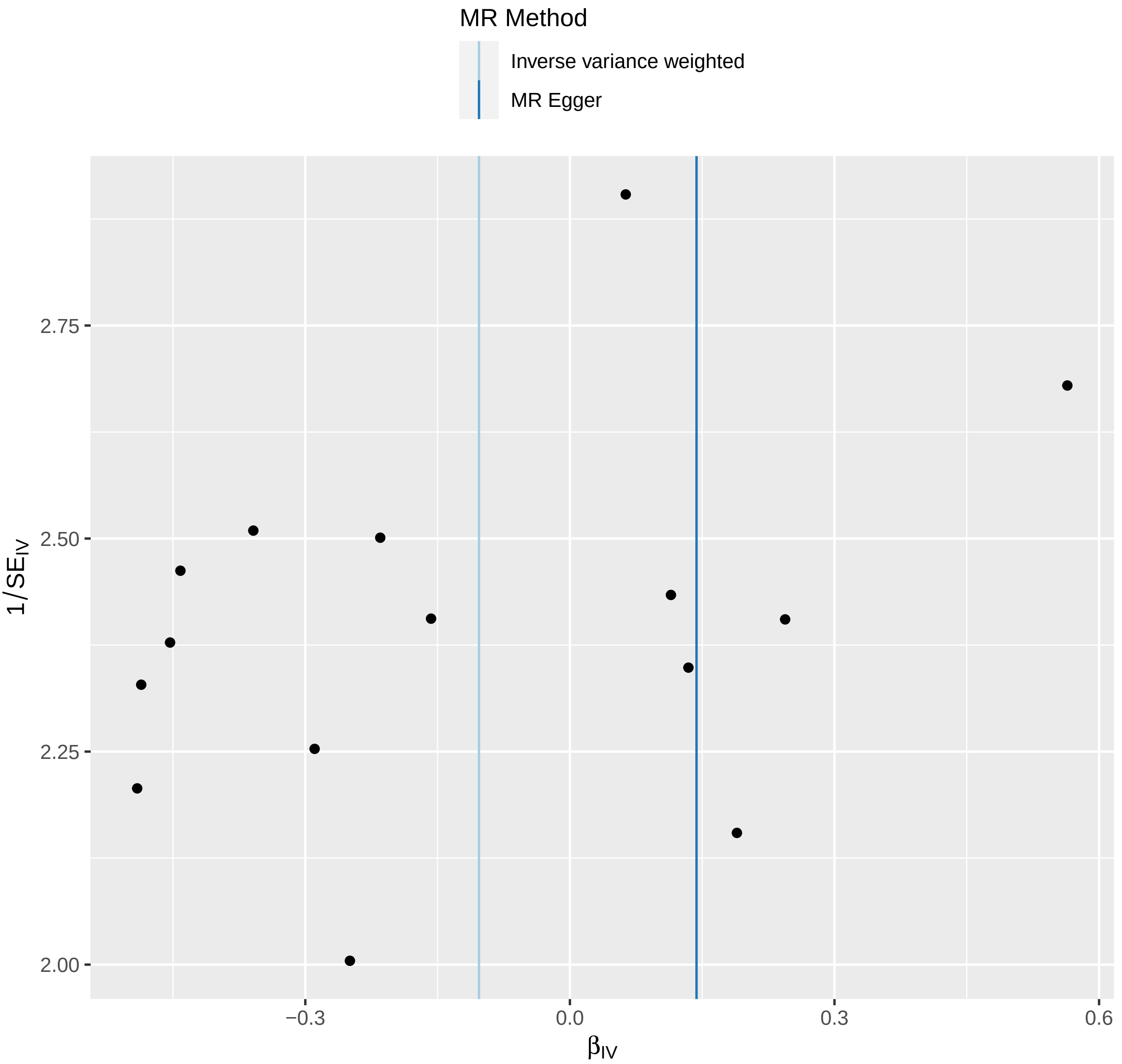

Batch 1−80 : Gut microbiota abundance (genus Eubacterium xylanophilum group id.14375) on Diabetic nephropathy (more control exclusions) with a threshold of 1e−05

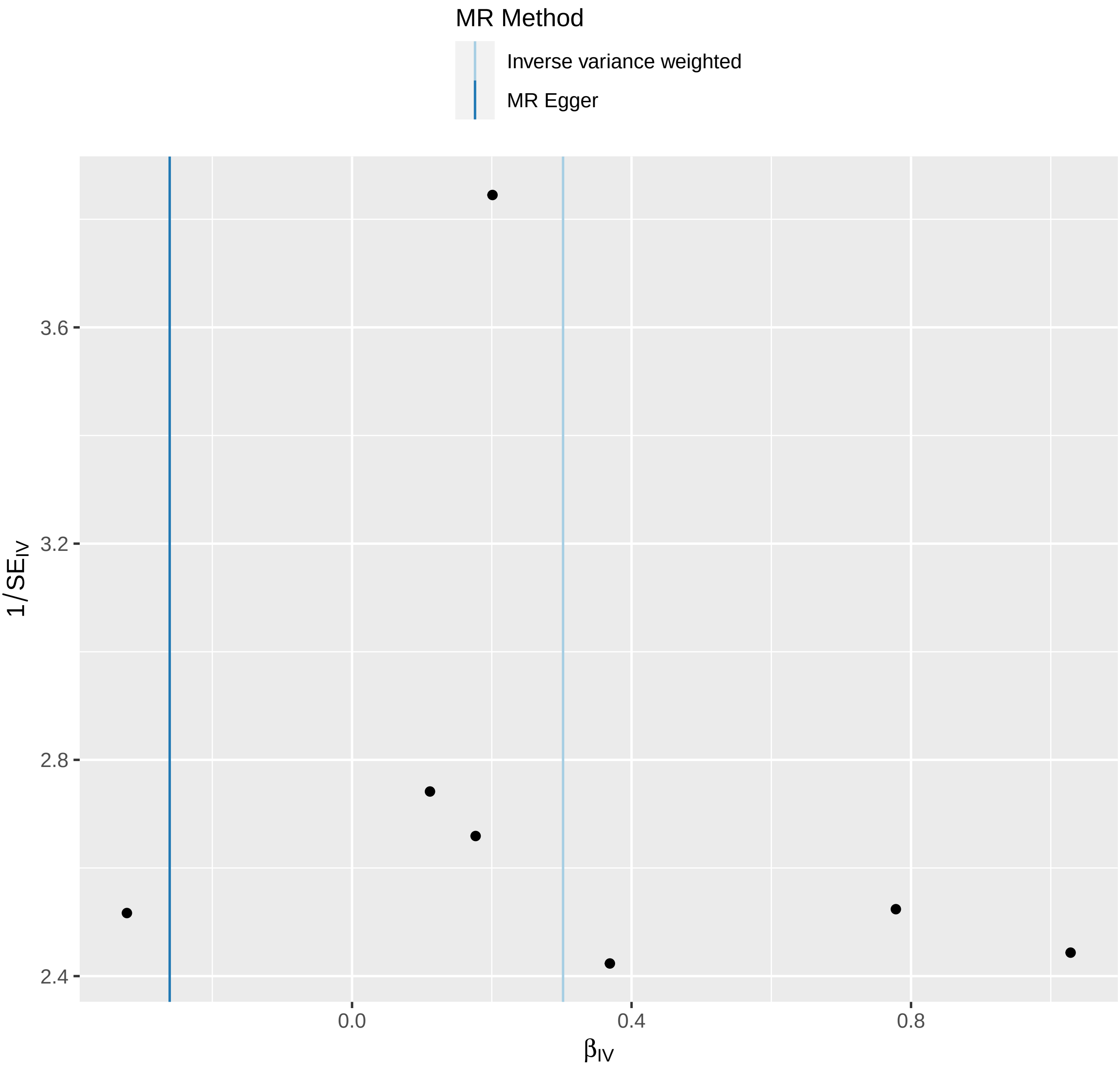

Batch 1−81 : Gut microbiota abundance (genus Faecalibacterium id.2057) on Diabetic nephropathy (more control exclusions) with a threshold of 1e−05

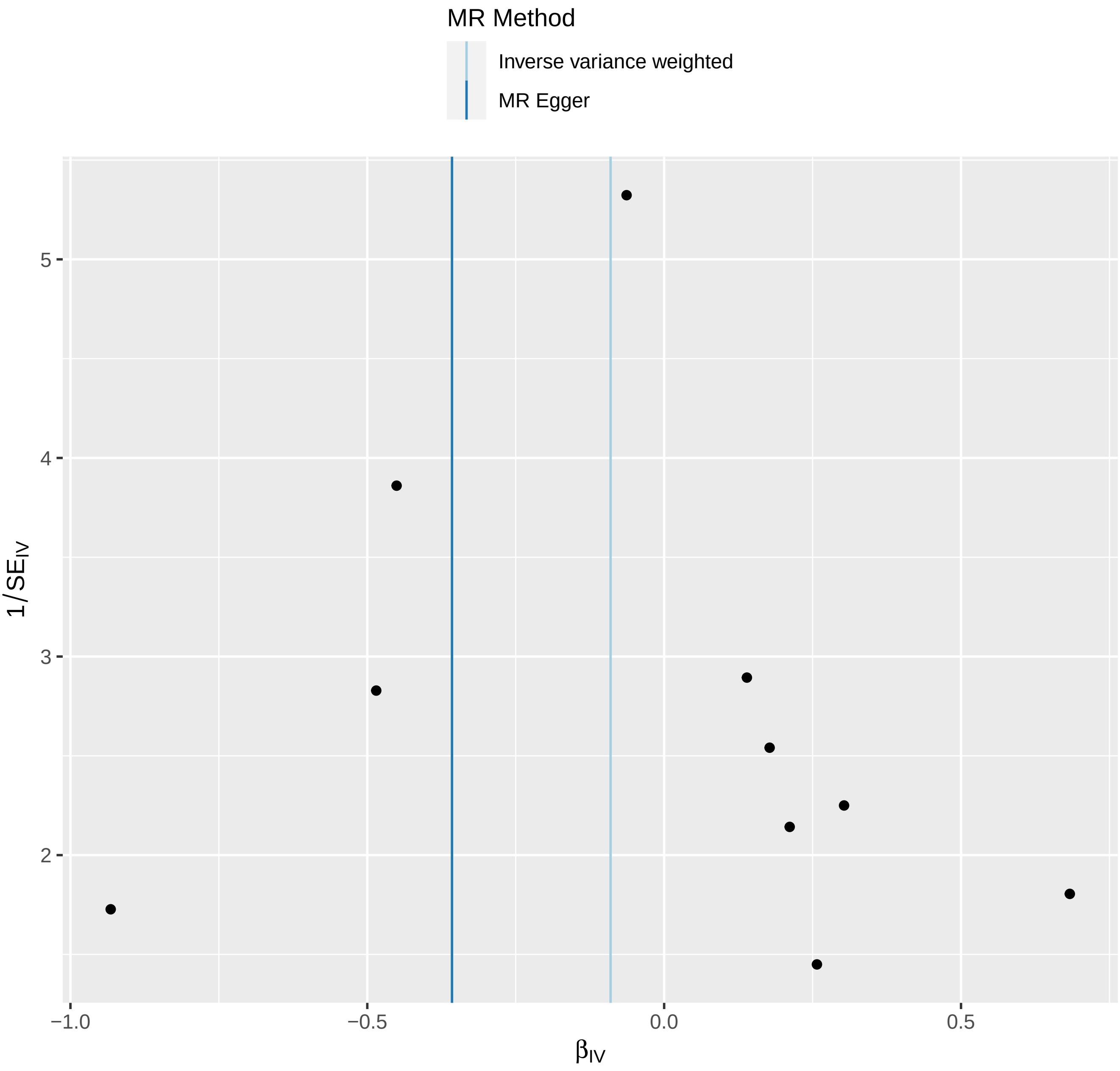

Batch 1−82 : Gut microbiota abundance (genus Family XIII AD3011 group id.11293) on Diabetic nephropathy (more control exclusions) with a threshold of 1e−05

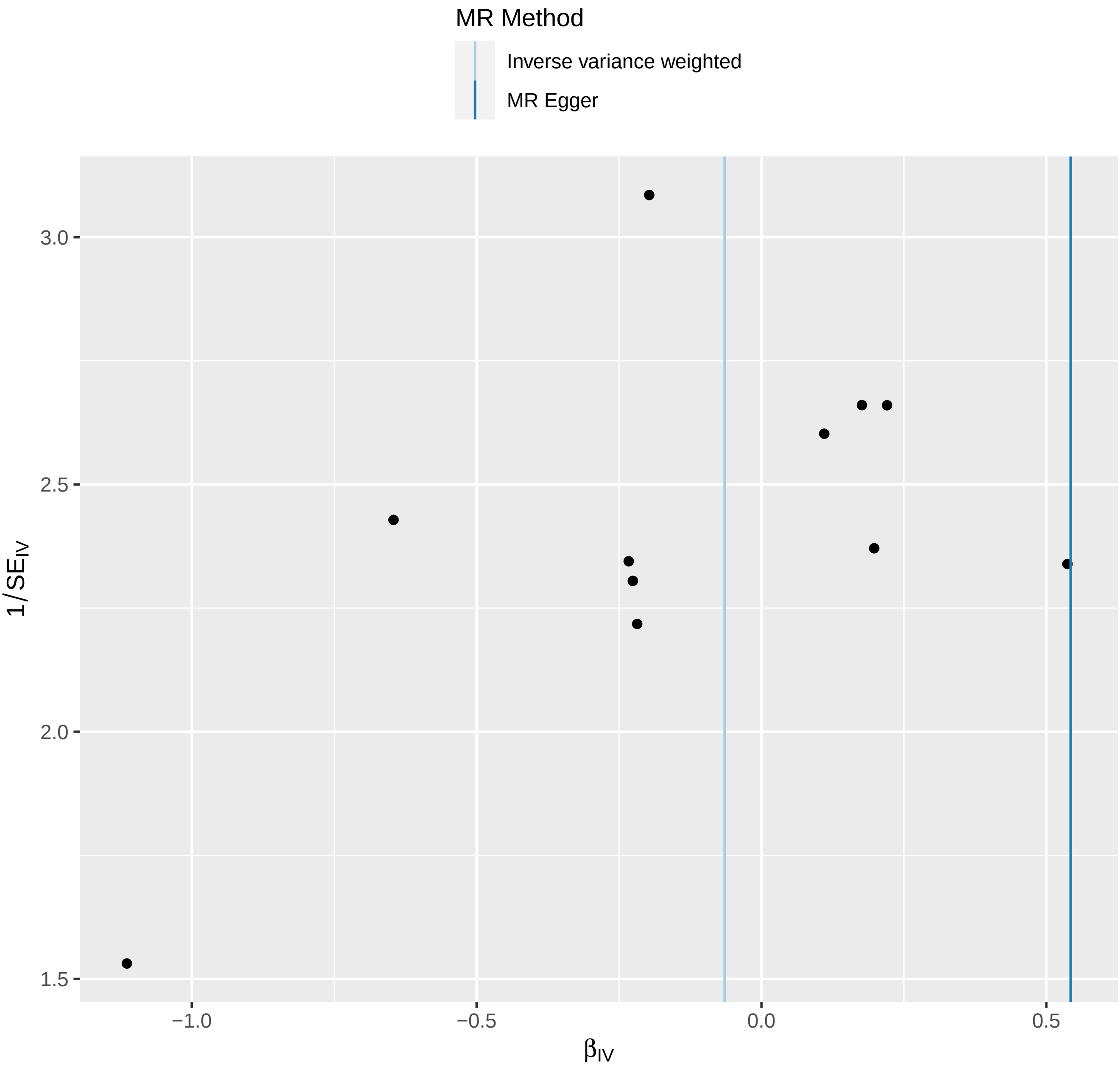

Batch 1−83 : Gut microbiota abundance (genus Family XIII UCG001 id.11294) on Diabetic nephropathy (more control exclusions) with a threshold of 1e−05

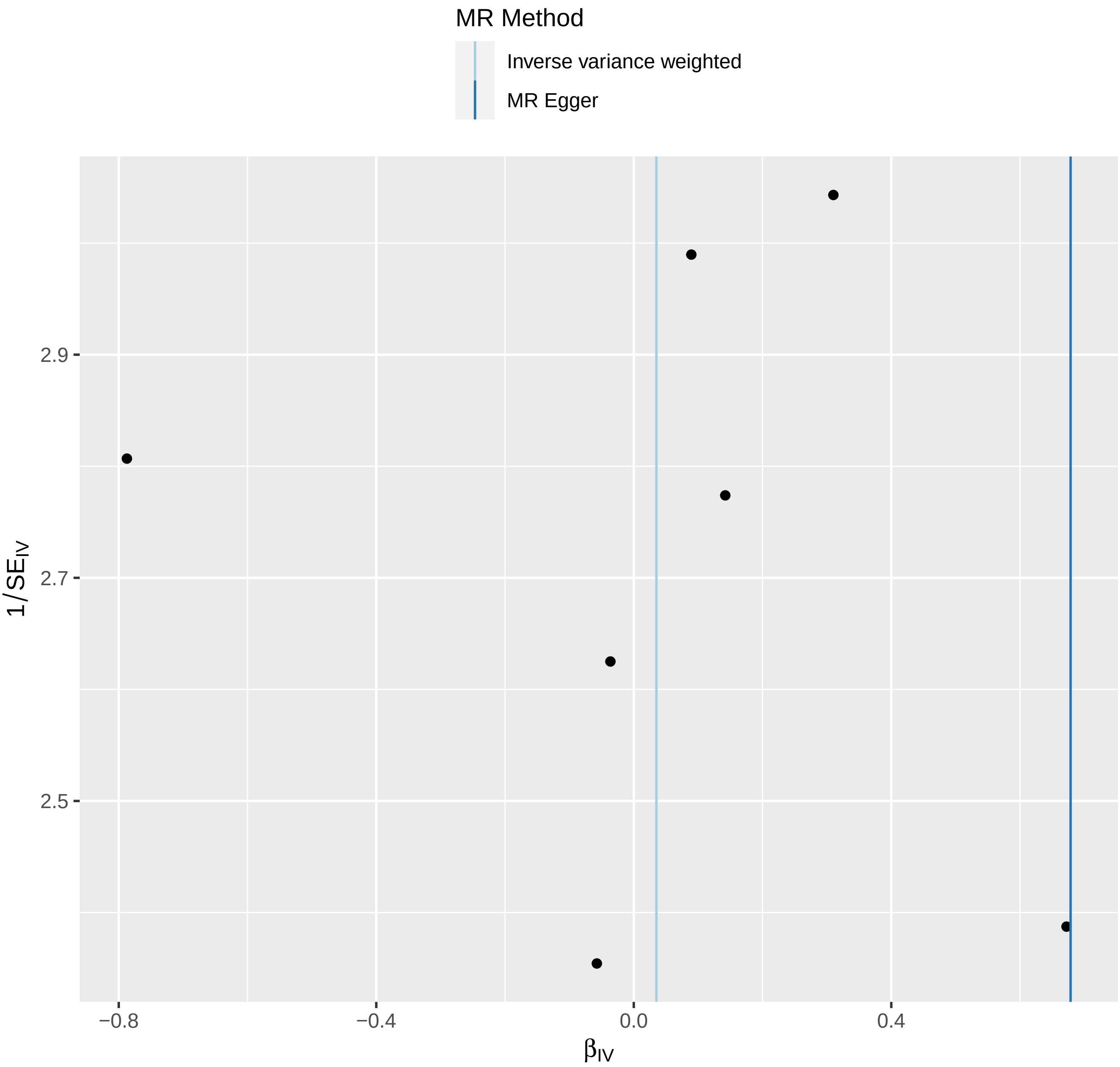

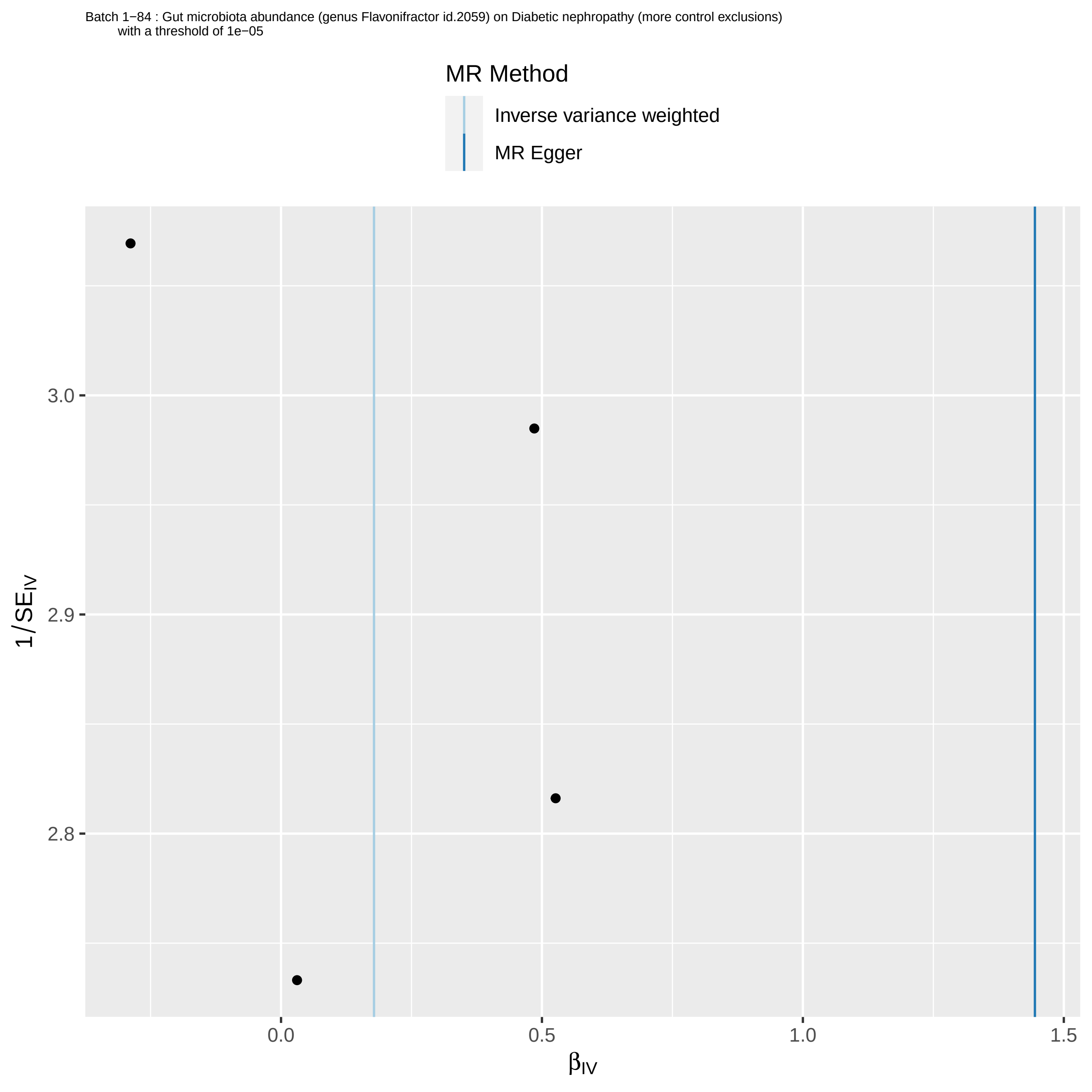

Batch 1−84 : Gut microbiota abundance (genus Flavonifractor id.2059) on Diabetic nephropathy (more control exclusions)
with a threshold of 1e−05
MR Method
Inverse variance weighted
MR Egger
3.0
2.9
2.8
1/SE_IV
0.0
0.5
1.0
1.5
β_IV

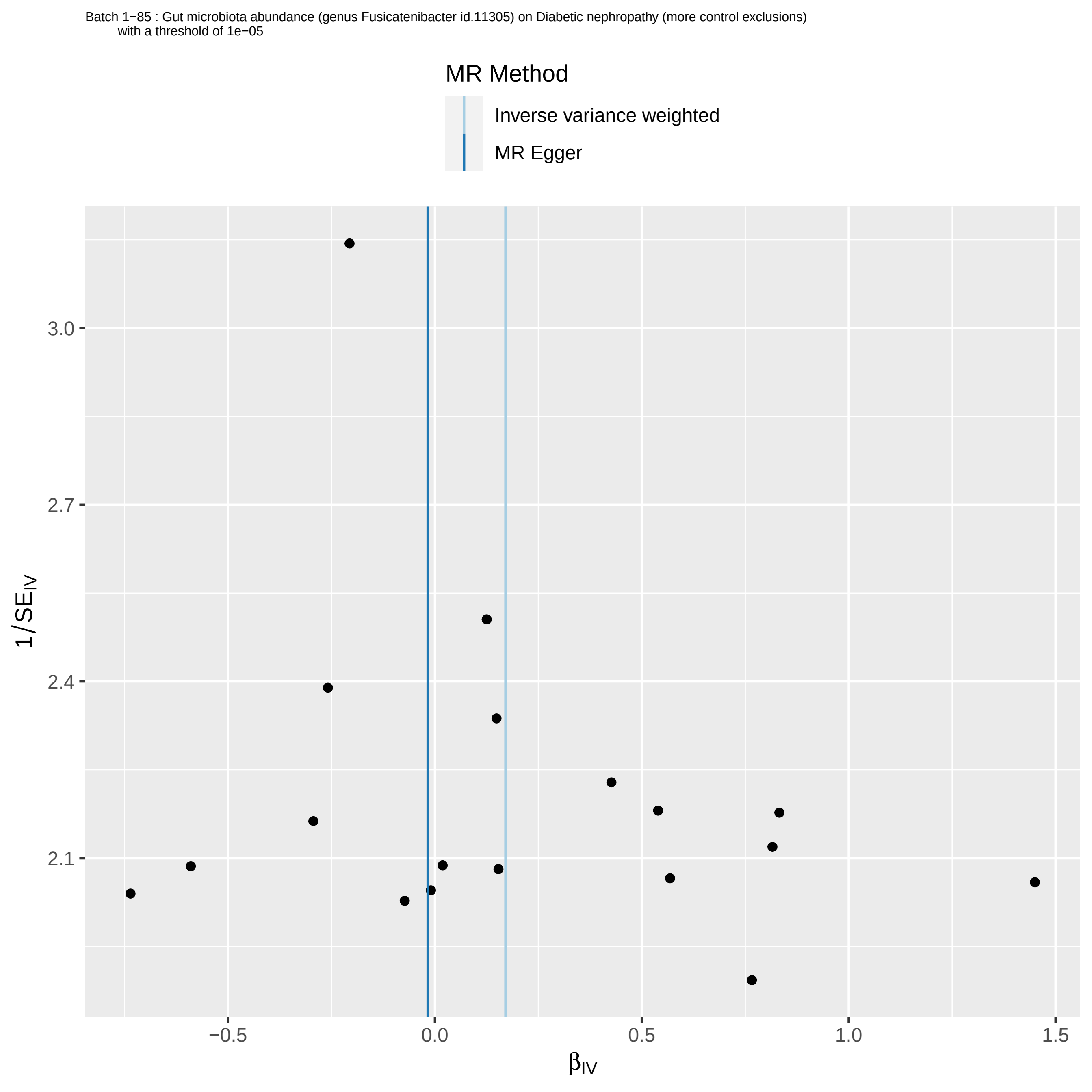
Batch 1−85 : Gut microbiota abundance (genus Fusicatenibacter id.11305) on Diabetic nephropathy (more control exclusions)
with a threshold of 1e−05
MR Method
Inverse variance weighted
MR Egger
3.0
2.7
2.4
2.1
1/SE_IV
−0.5
0.0
0.5
1.0
1.5
β_IV

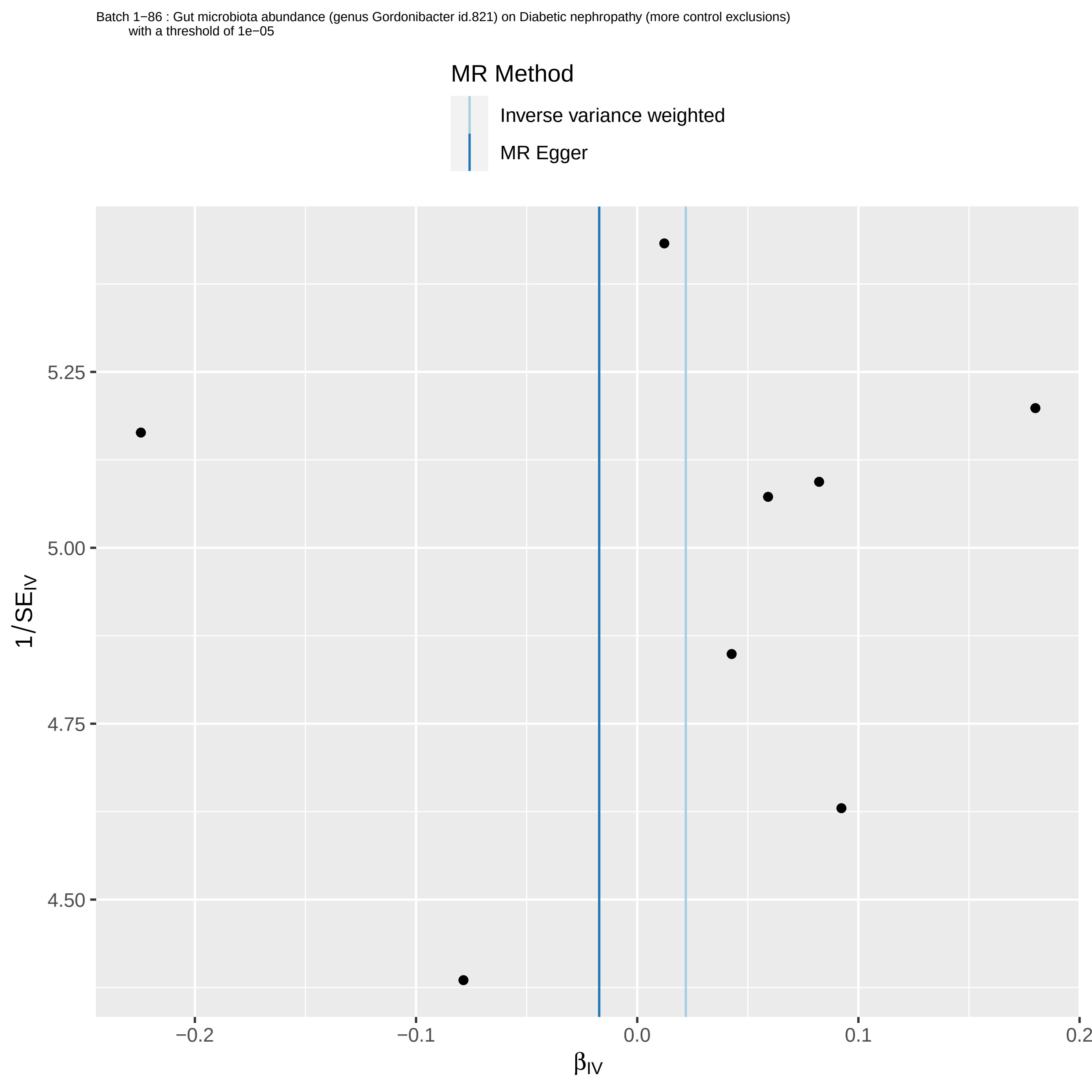
Batch 1−86 : Gut microbiota abundance (genus Gordonibacter id.821) on Diabetic nephropathy (more control exclusions)
with a threshold of 1e−05
MR Method
Inverse variance weighted
MR Egger
5.25
5.00
4.75
4.50
1/SE_IV
−0.2
−0.1
0.0
0.1
0.2
β_IV

Batch 1−87 : Gut microbiota abundance (genus Haemophilus id.3698) on Diabetic nephropathy (more control exclusions) with a threshold of 1e−05

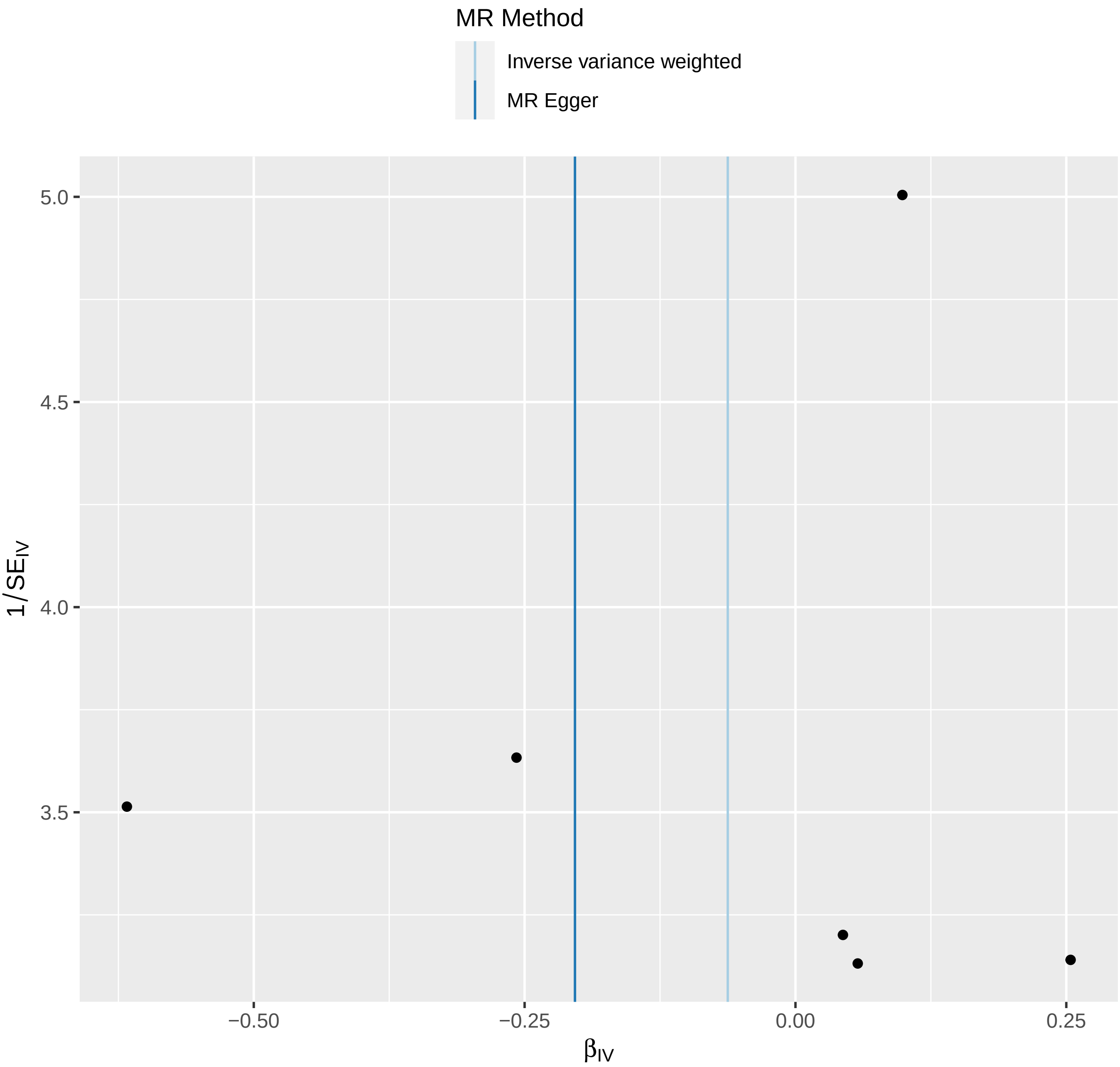

Batch 1−88 : Gut microbiota abundance (genus Holdemanella id.11393) on Diabetic nephropathy (more control exclusions)
with a threshold of 1e−05

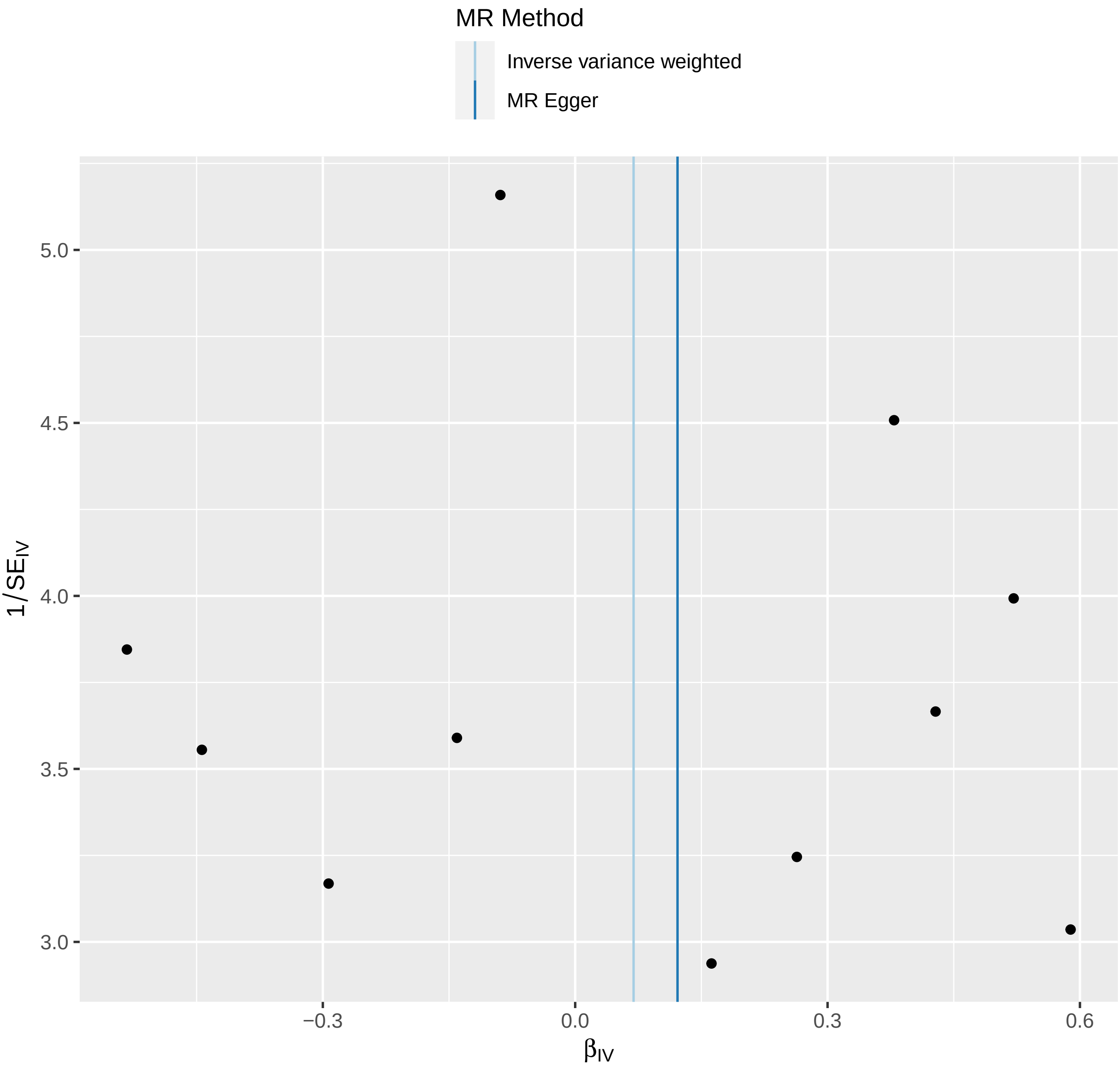

Batch 1−89 : Gut microbiota abundance (genus Holdemania id.2157) on Diabetic nephropathy (more control exclusions) with a threshold of 1e−05

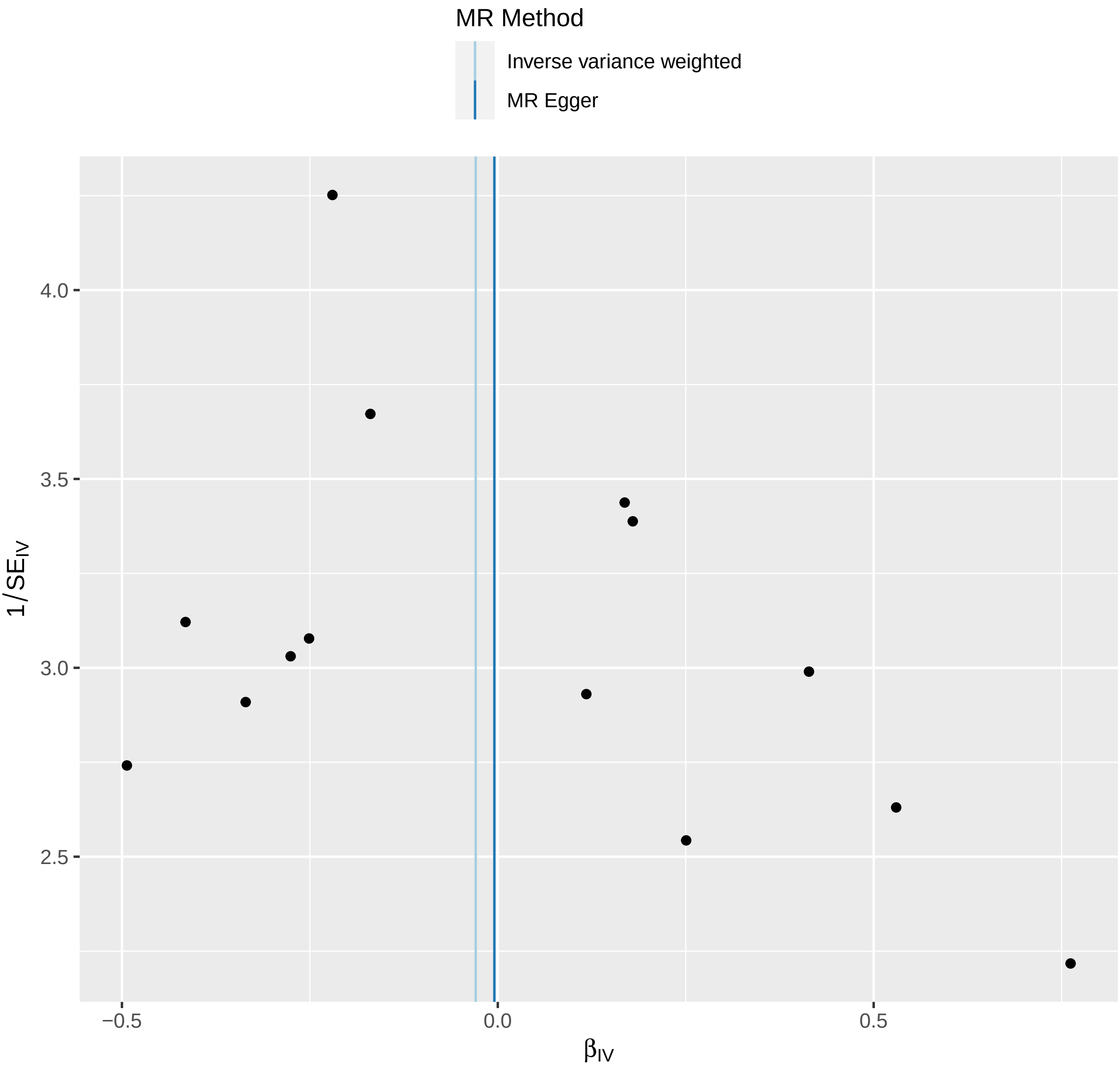

Batch 1−90 : Gut microbiota abundance (genus Howardella id.2000) on Diabetic nephropathy (more control exclusions) with a threshold of 1e−05

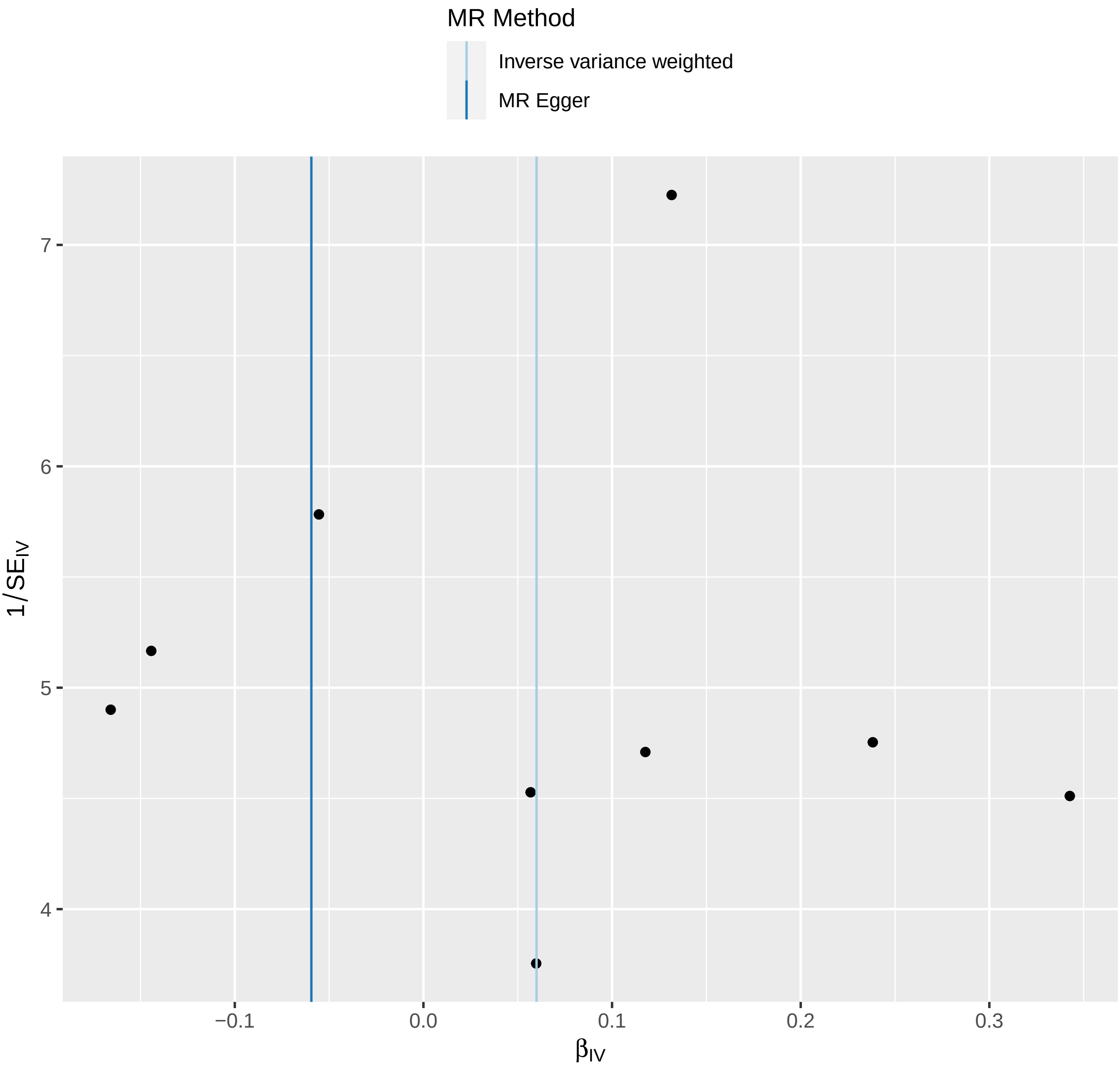

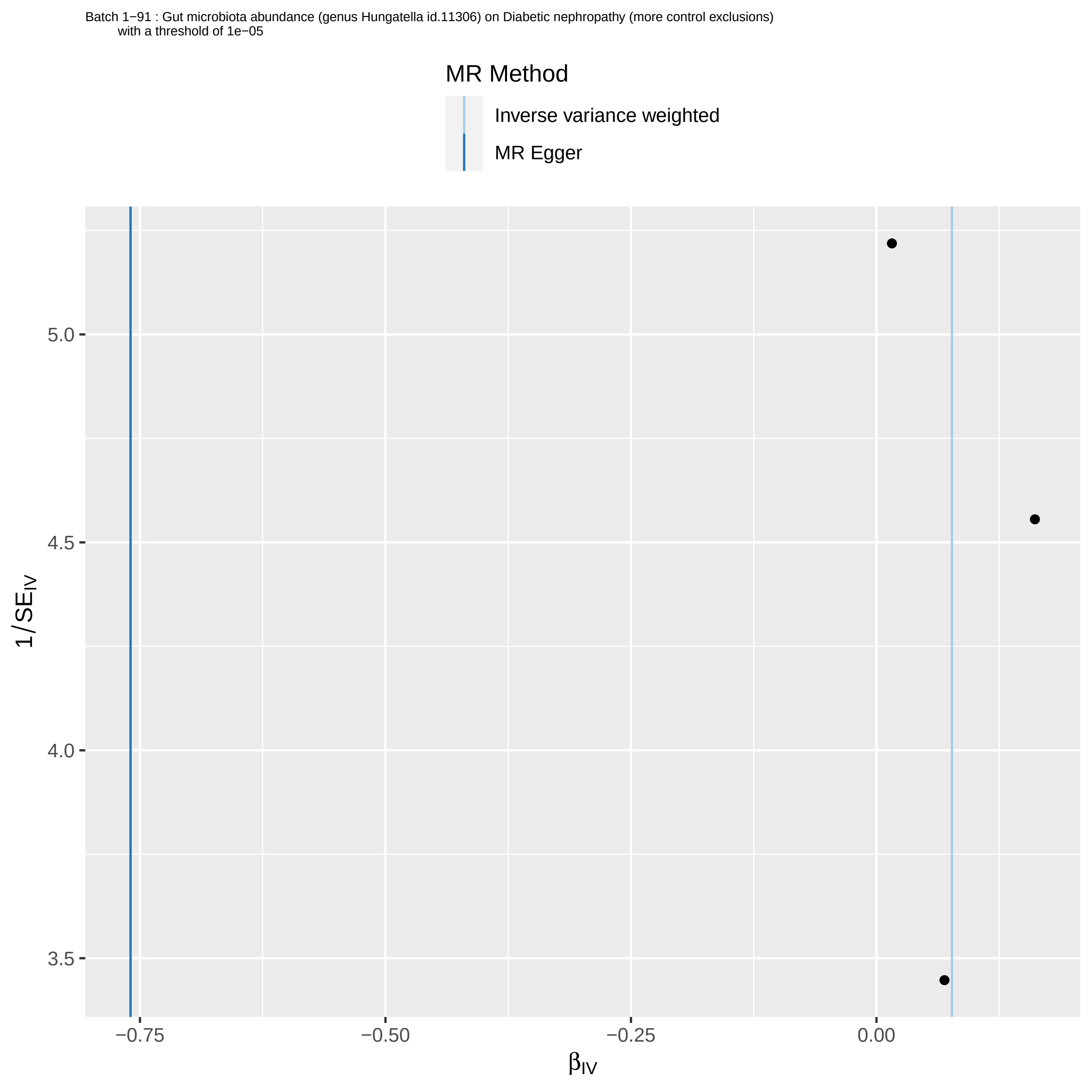
Batch 1−91 : Gut microbiota abundance (genus Hungatella id.11306) on Diabetic nephropathy (more control exclusions)
with a threshold of 1e−05
MR Method
Inverse variance weighted
MR Egger
5.0
4.5
4.0
3.5
1/SE_IV
−0.75
−0.50
−0.25
0.00
β_IV

Batch 1−92 : Gut microbiota abundance (genus Intestinibacter id.11345) on Diabetic nephropathy (more control exclusions) with a threshold of 1e−05

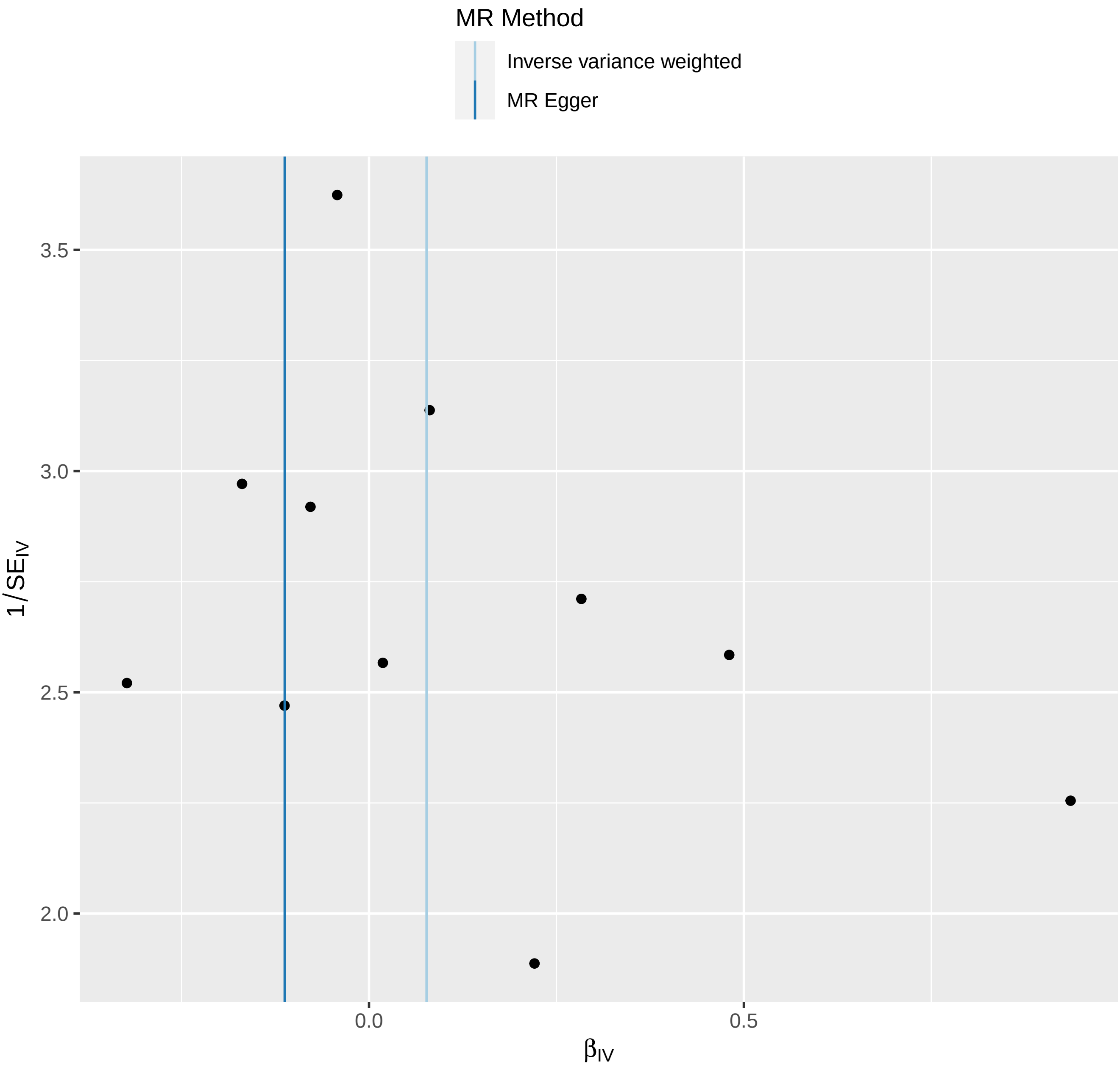

Batch 1−93 : Gut microbiota abundance (genus Intestinimonas id.2062) on Diabetic nephropathy (more control exclusions) with a threshold of 1e−05

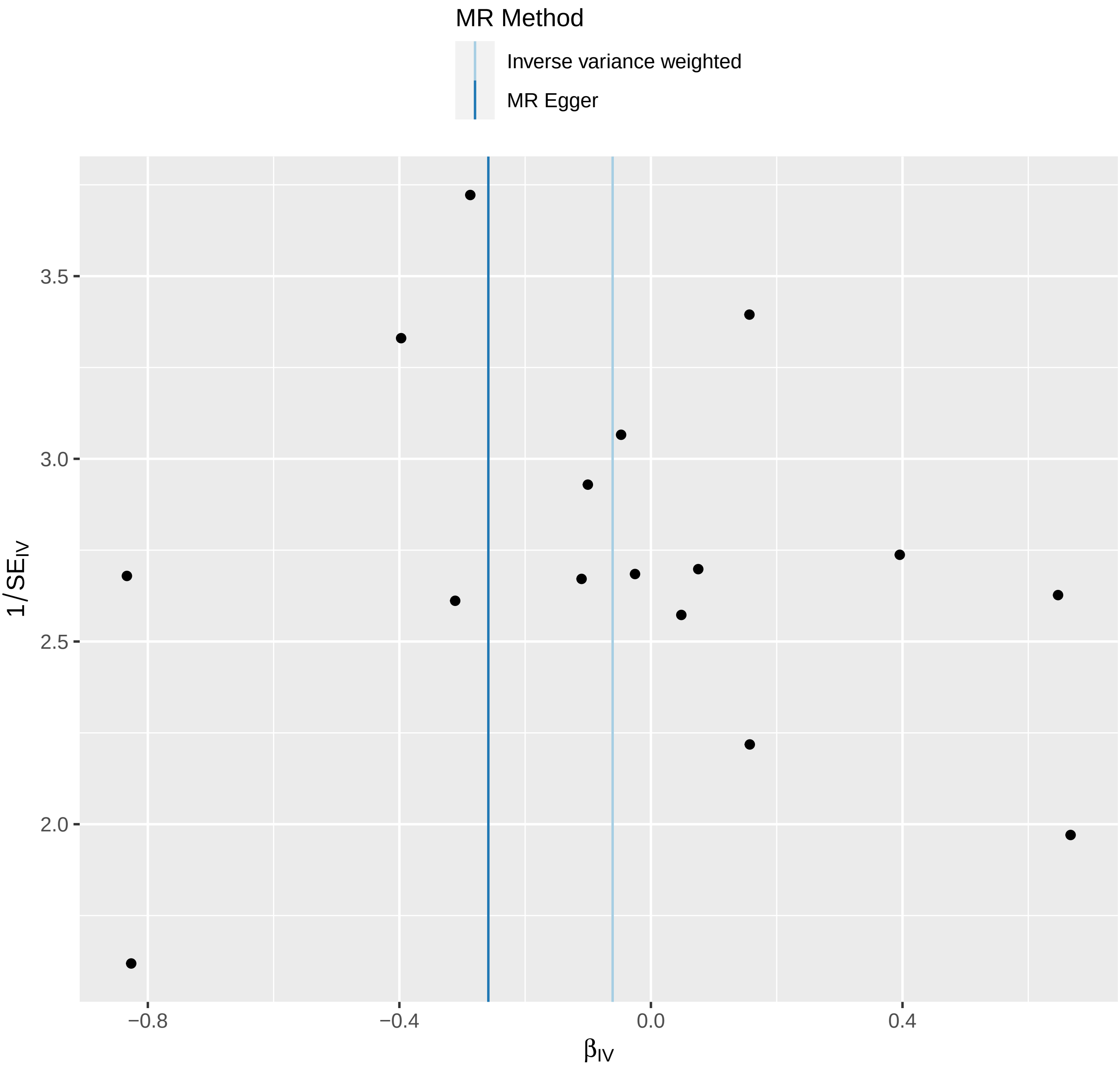

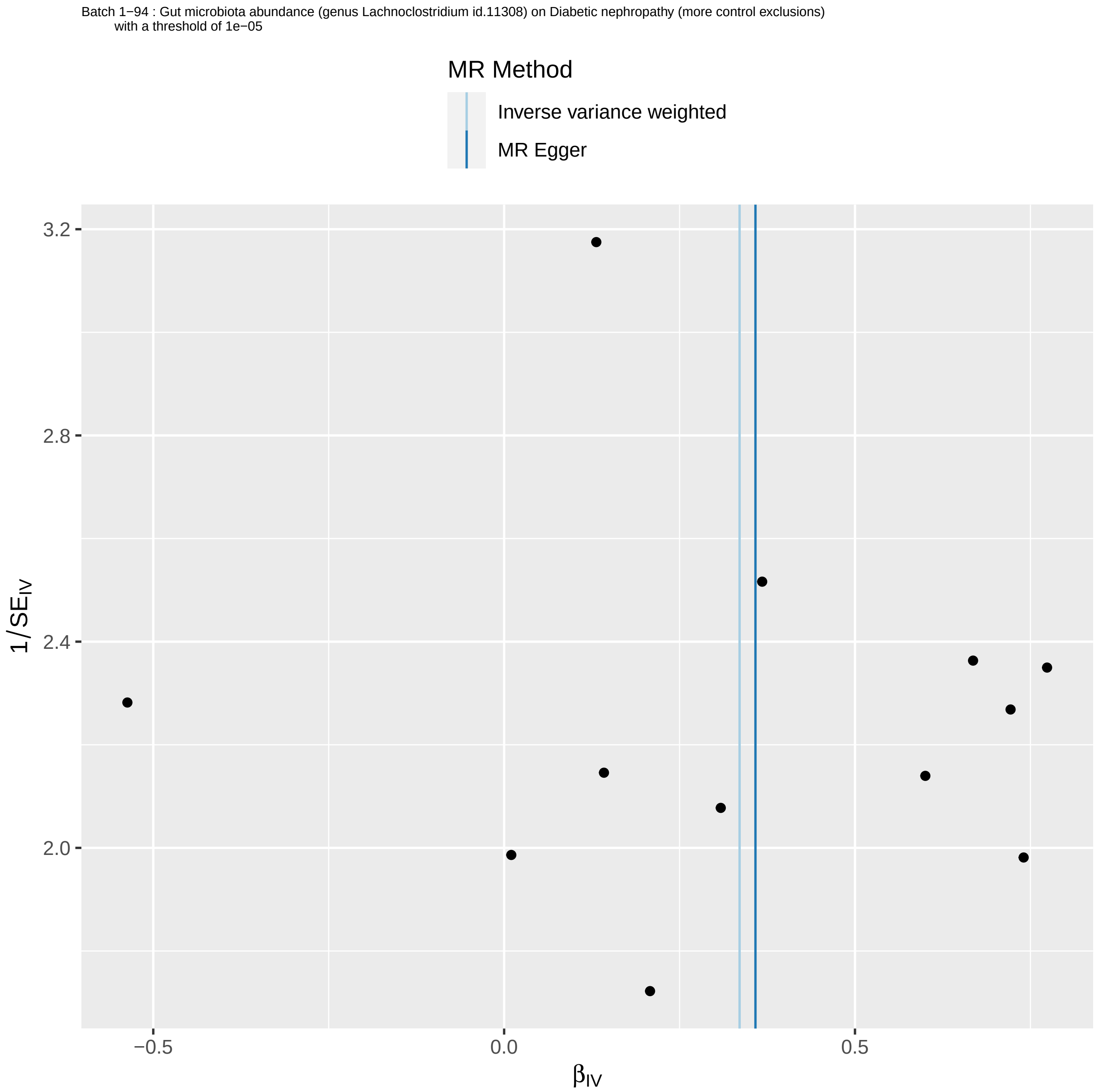
Batch 1−94 : Gut microbiota abundance (genus Lachnoclostridium id.11308) on Diabetic nephropathy (more control exclusions)
with a threshold of 1e−05
MR Method
Inverse variance weighted
MR Egger
3.2
2.8
2.4
2.0
1/SE_IV
−0.5
0.0
0.5
β_IV

Batch 1−96 : Gut microbiota abundance (genus Lachnospiraceae FCS020 group id.11314) on Diabetic nephropathy (more control exclusions) with a threshold of 1e−05

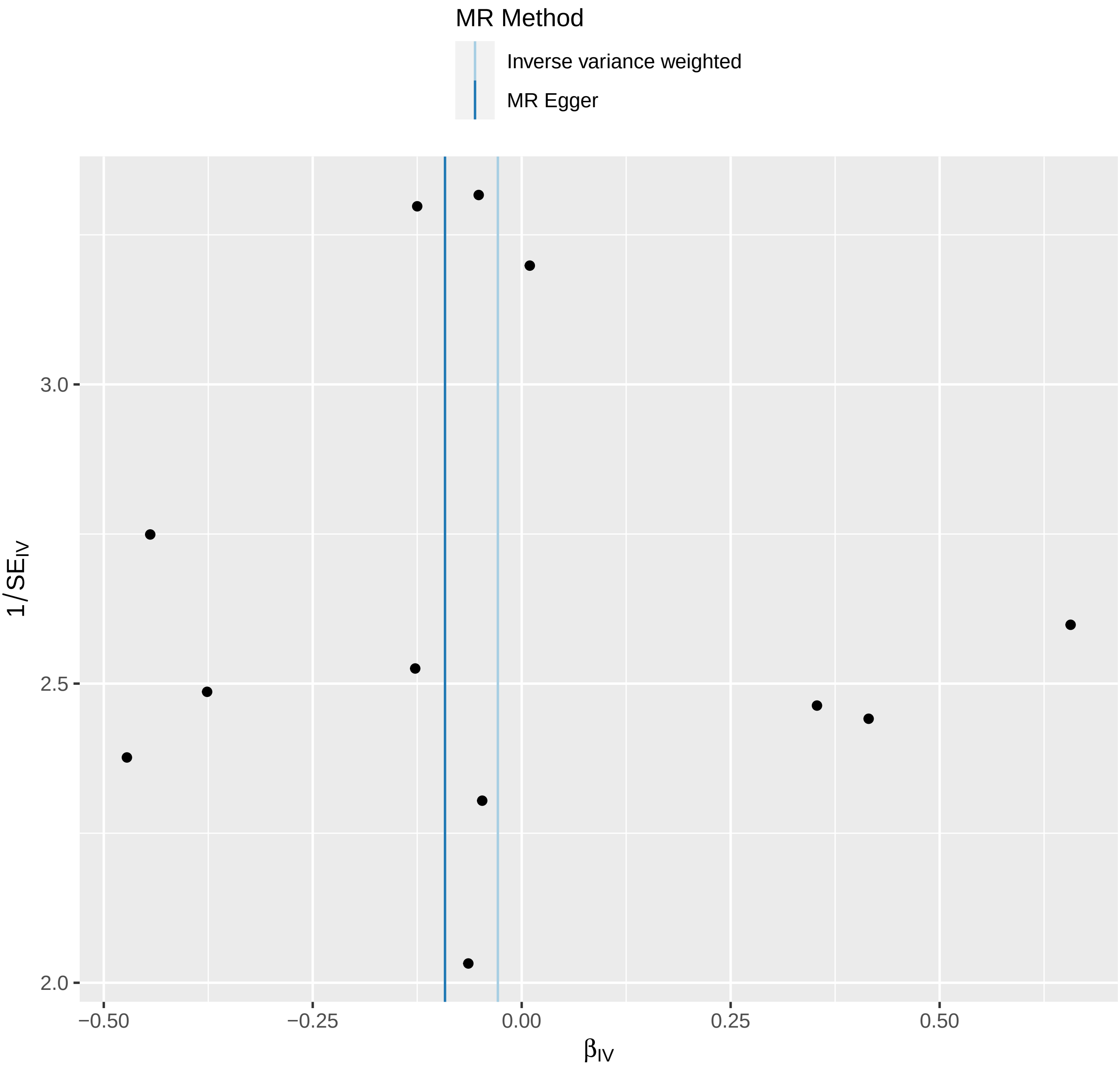

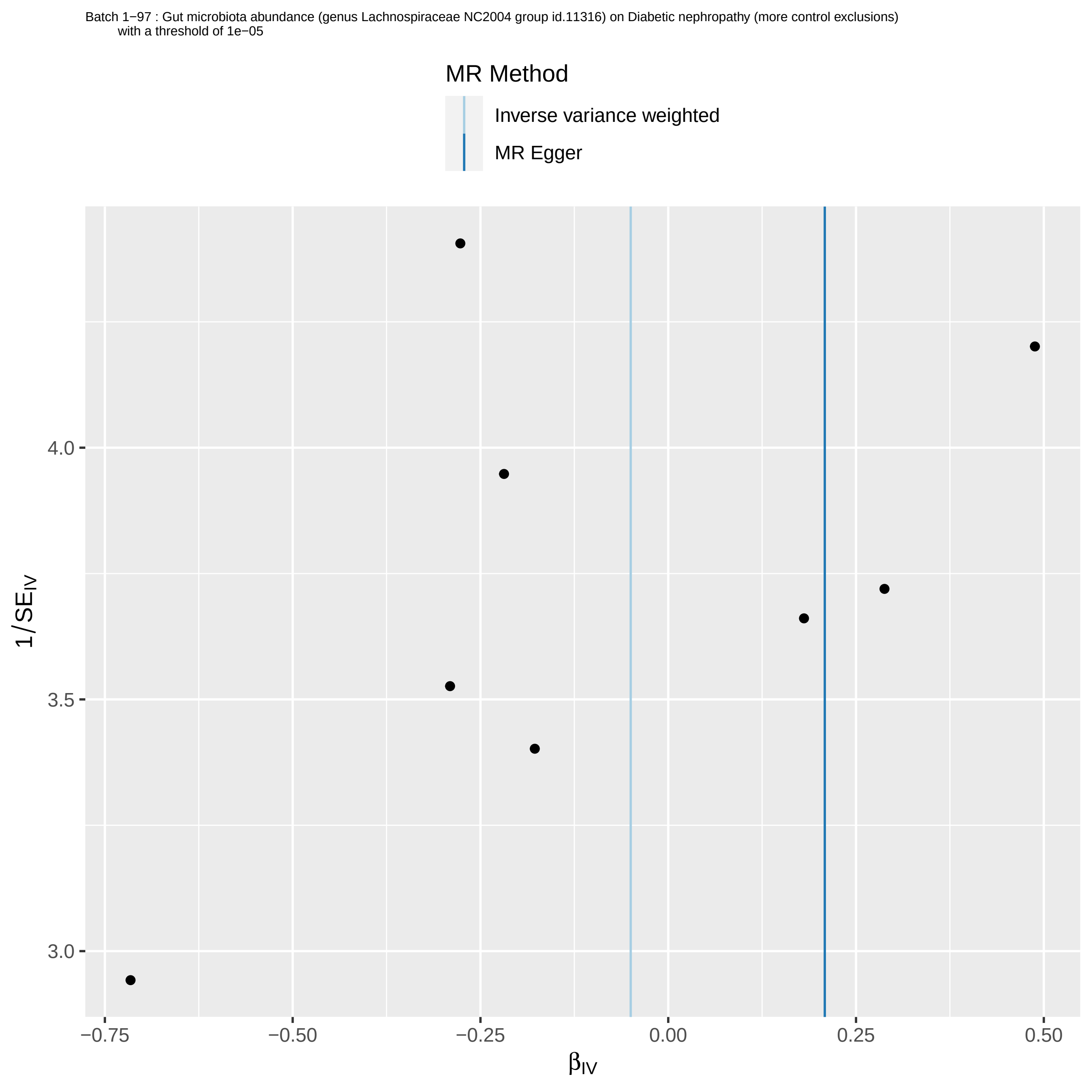

Batch 1−97 : Gut microbiota abundance (genus Lachnospiraceae NC2004 group id.11316) on Diabetic nephropathy (more control exclusions)
with a threshold of 1e−05
MR Method
Inverse variance weighted
MR Egger
4.0
3.5
3.0
1/SE_IV
−0.75
−0.50
−0.25
0.00
0.25
0.50
β_IV

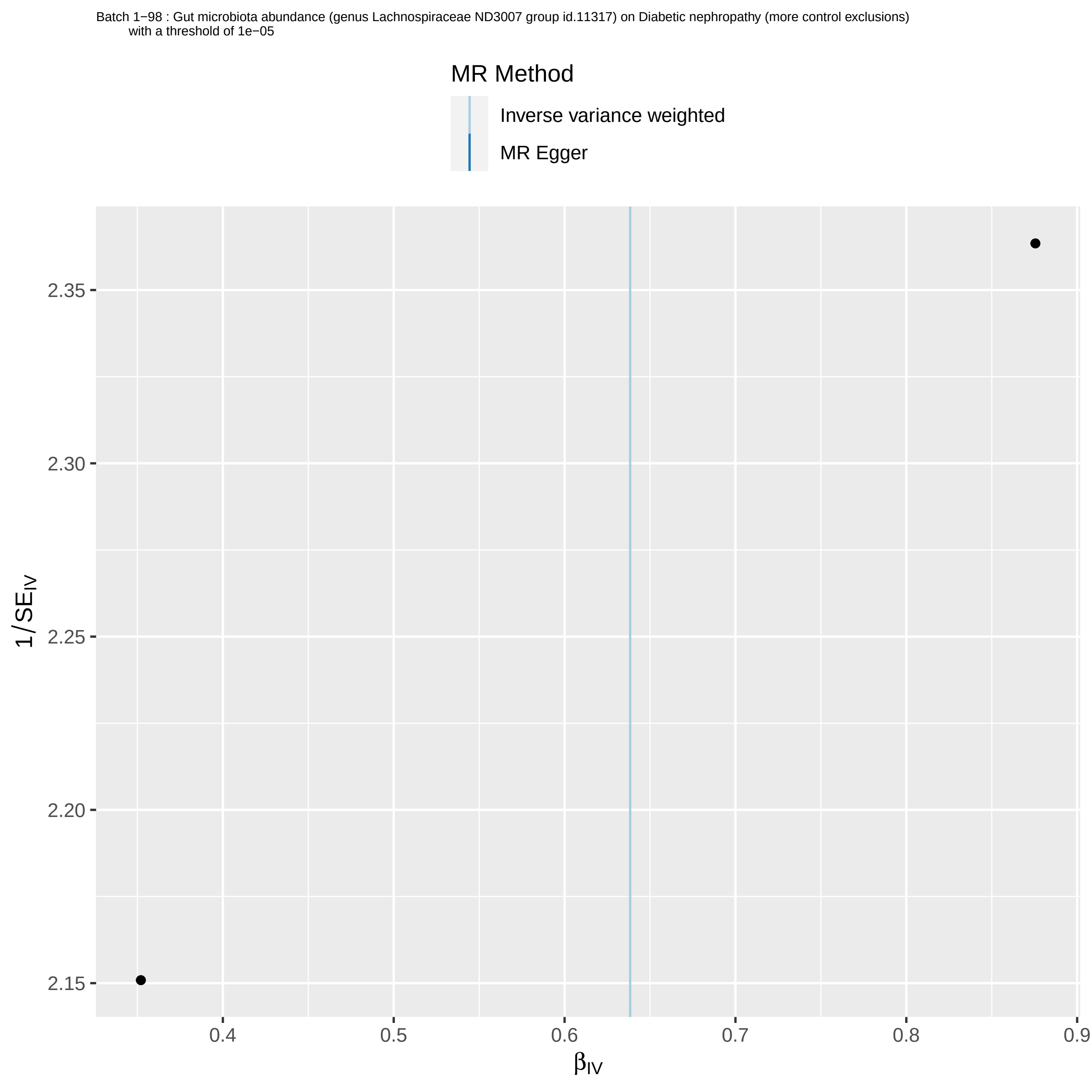

Batch 1−98 : Gut microbiota abundance (genus Lachnospiraceae ND3007 group id.11317) on Diabetic nephropathy (more control exclusions)
with a threshold of 1e−05
MR Method
Inverse variance weighted
MR Egger
2.35
2.30
2.25
2.20
2.15
0.4
0.5
0.6
0.7
0.8
0.9
$1/SE_{IV}$
β_{IV}

Batch 1−99 : Gut microbiota abundance (genus Lachnospiraceae NK4A136 group id.11319) on Diabetic nephropathy (more control exclusions) with a threshold of 1e−05

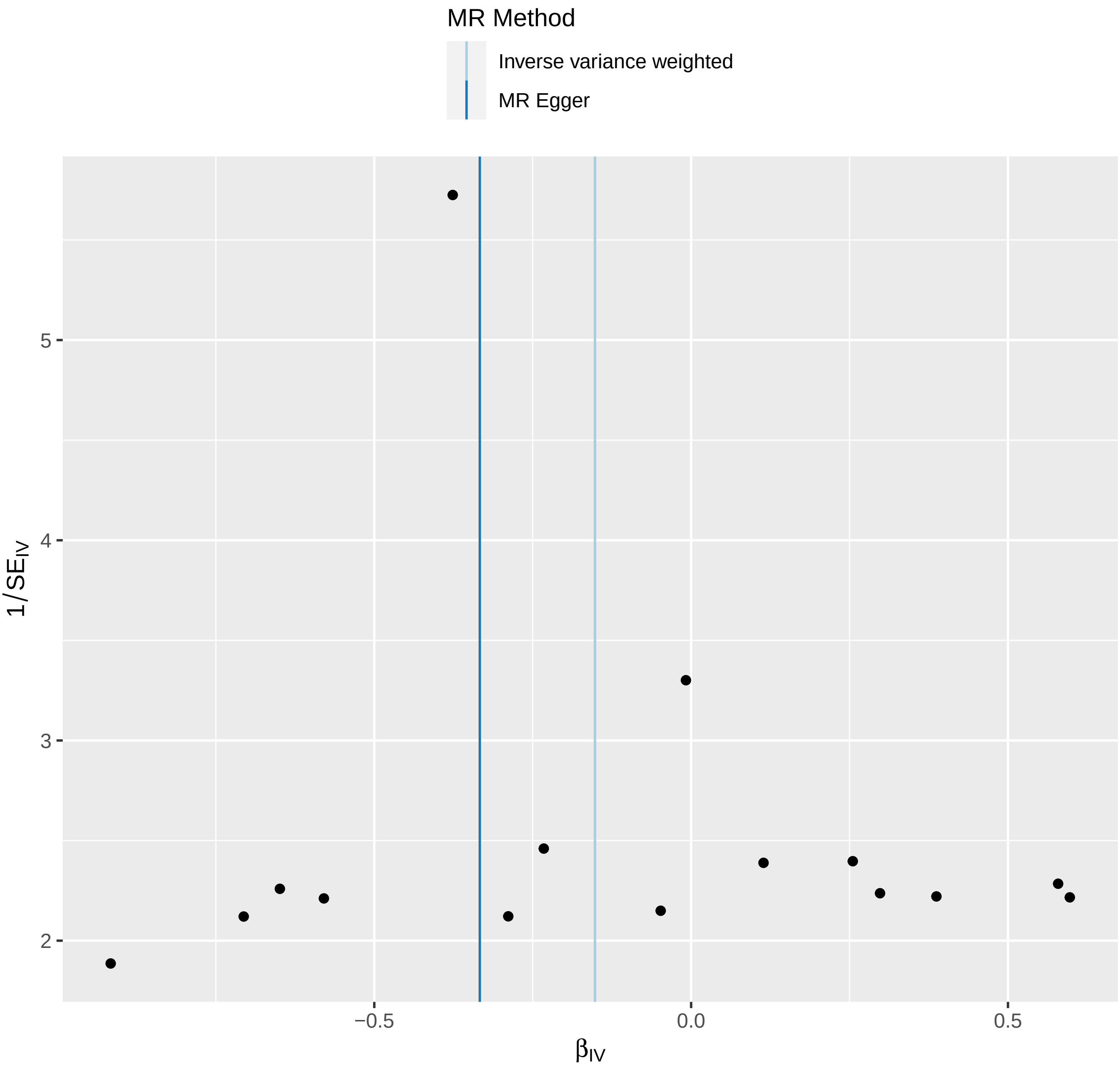

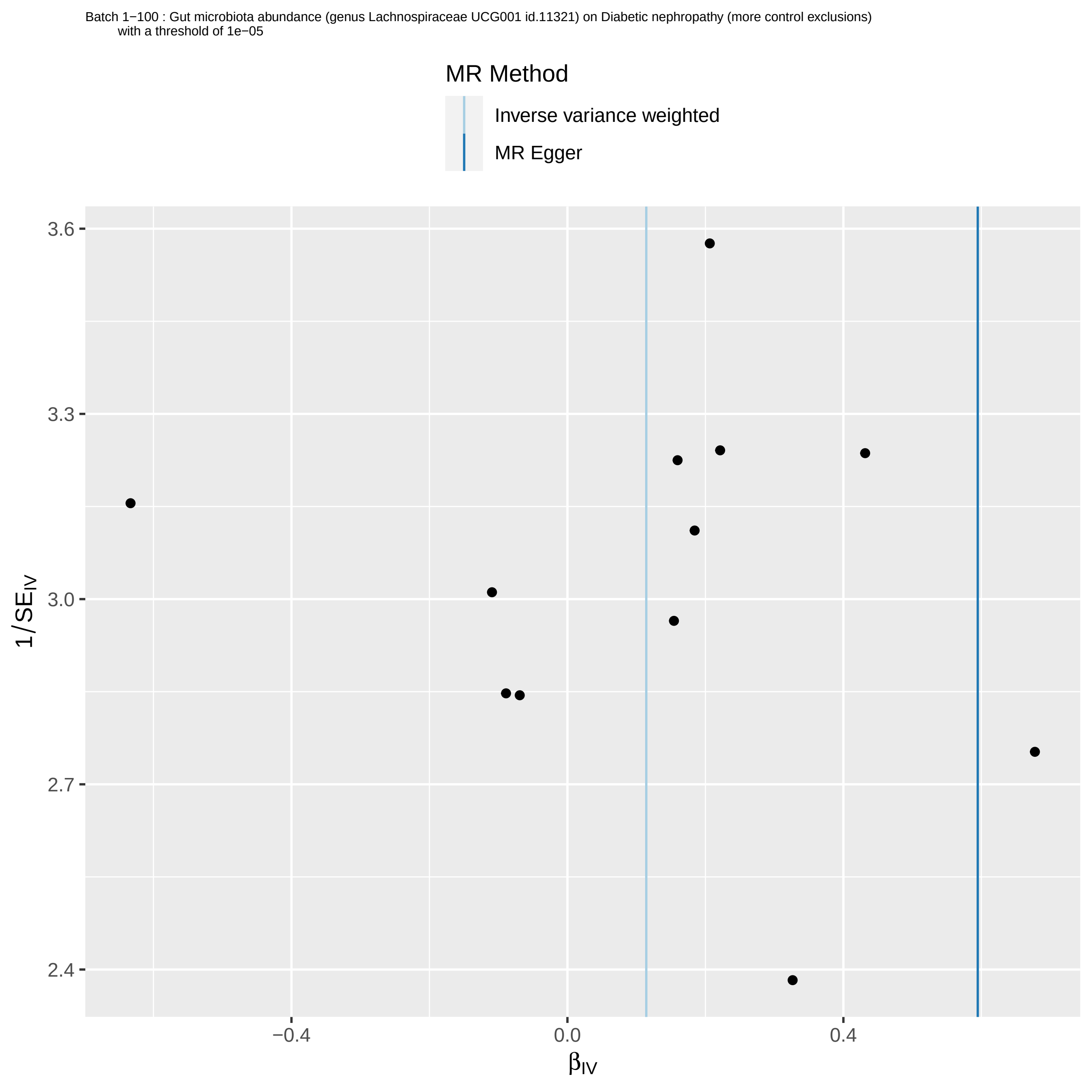

Batch 1−100 : Gut microbiota abundance (genus Lachnospiraceae UCG001 id.11321) on Diabetic nephropathy (more control exclusions)
with a threshold of 1e−05
MR Method
Inverse variance weighted
MR Egger
3.6
3.3
3.0
2.7
2.4
1/SE_IV
−0.4
0.0
0.4
β_IV

Batch 1−101 : Gut microbiota abundance (genus Lachnospiraceae UCG004 id.11324) on Diabetic nephropathy (more control exclusions) with a threshold of 1e−05

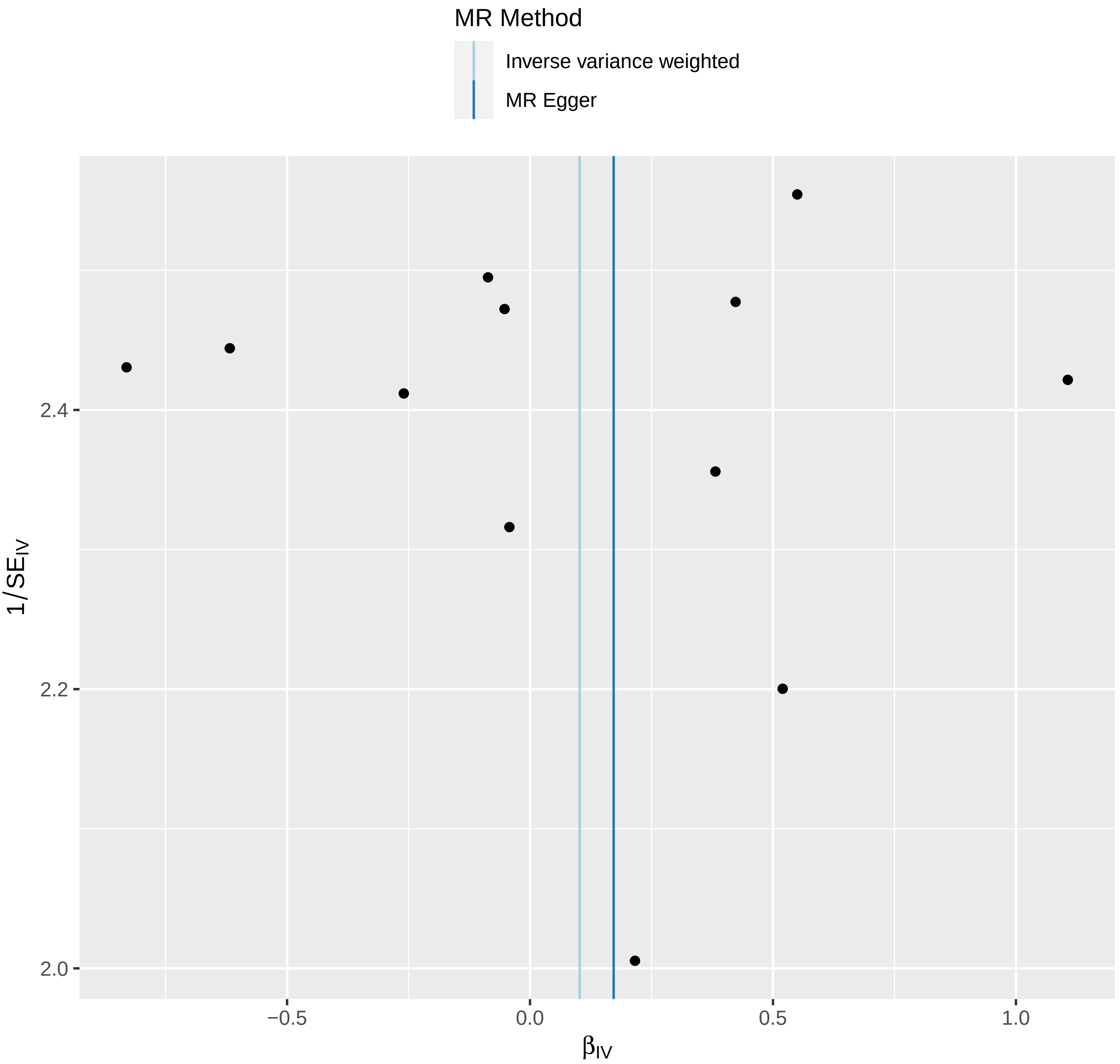

Batch 1−102 : Gut microbiota abundance (genus Lachnospiraceae UCG008 id.11328) on Diabetic nephropathy (more control exclusions) with a threshold of 1e−05

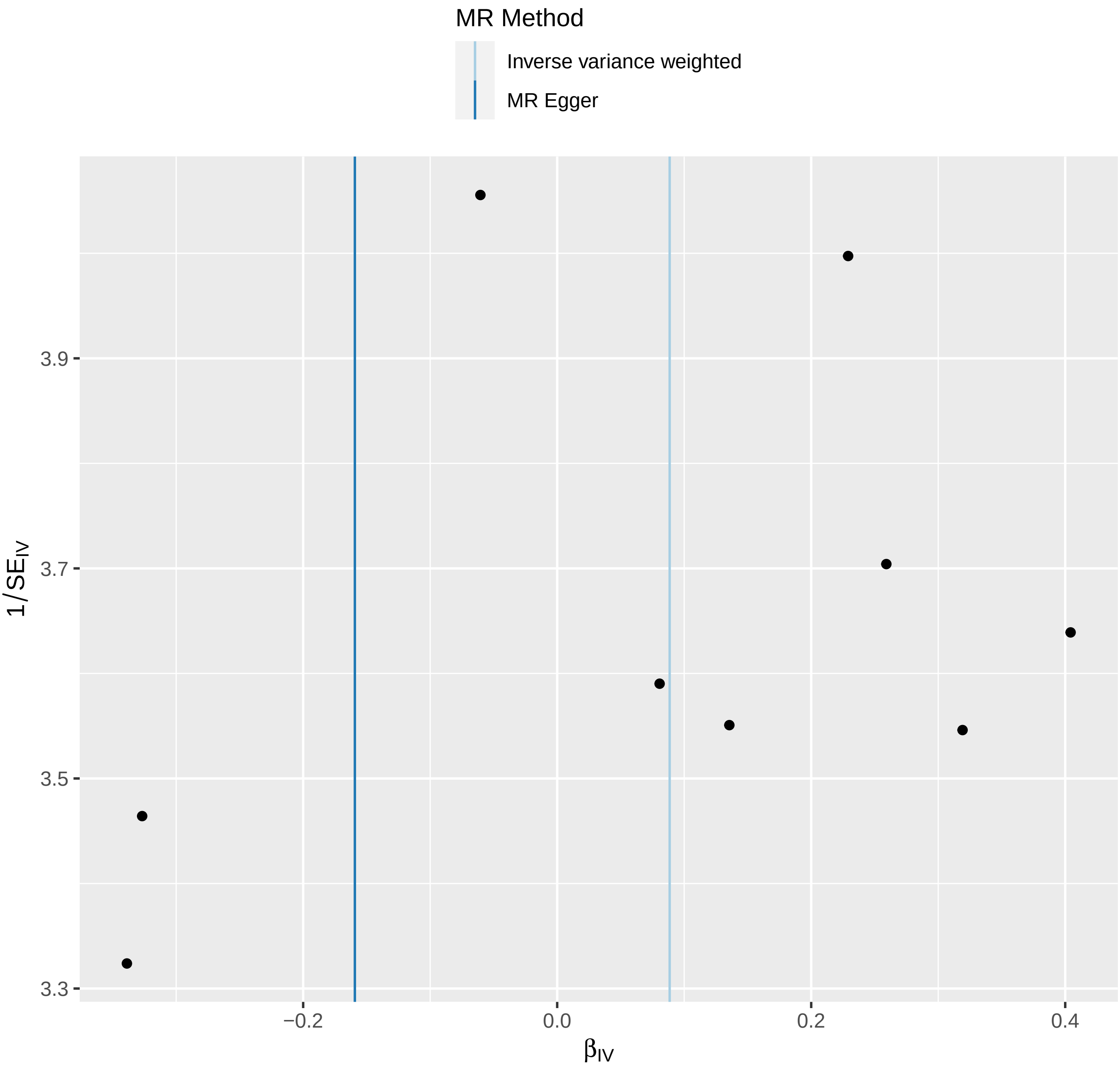

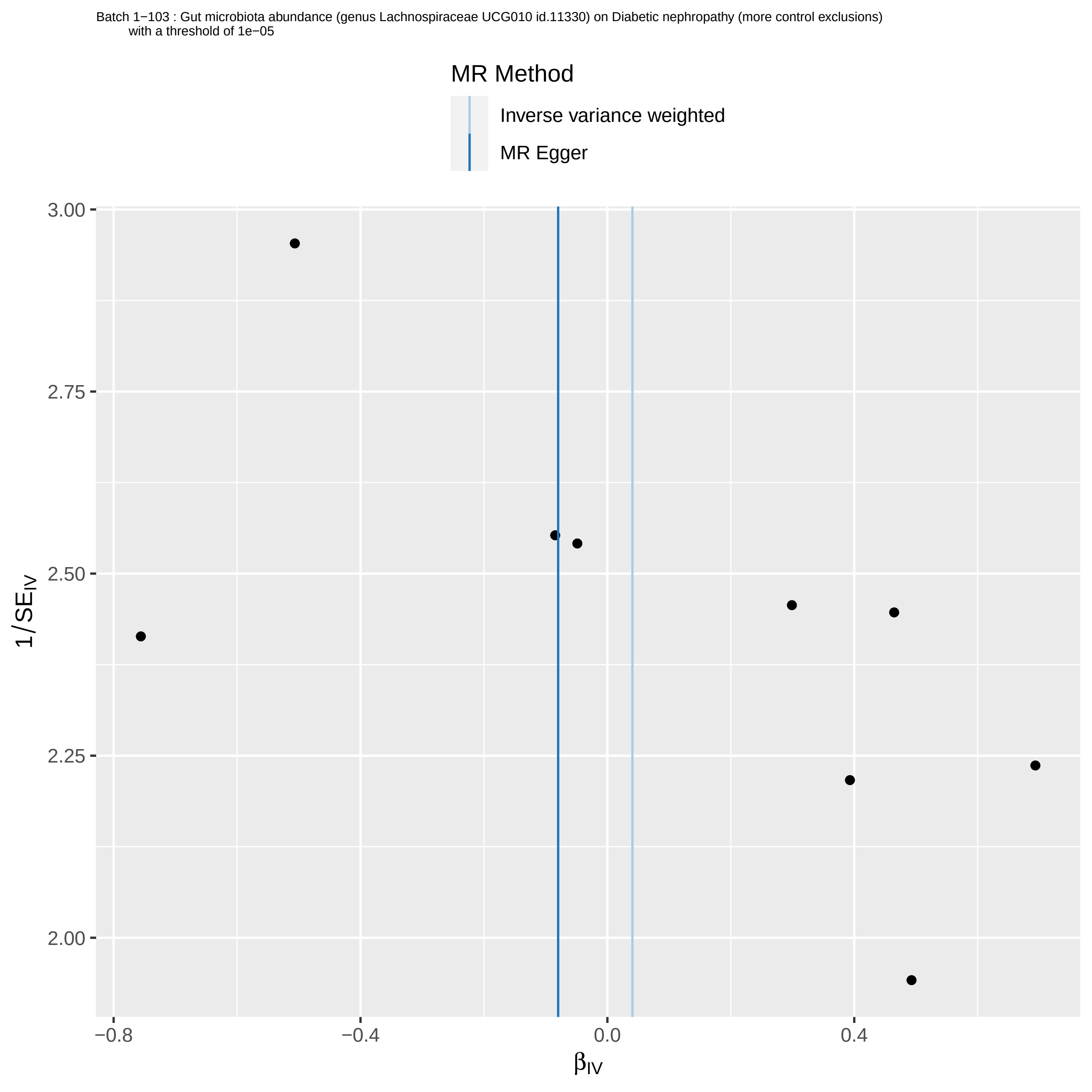

Batch 1−103 : Gut microbiota abundance (genus Lachnospiraceae UCG010 id.11330) on Diabetic nephropathy (more control exclusions)
with a threshold of 1e−05
MR Method
Inverse variance weighted
MR Egger
3.00
2.75
2.50
2.25
2.00
1/SE_IV
−0.8
−0.4
0.0
0.4
β_IV

Batch 1−104 : Gut microbiota abundance (genus Lactobacillus id.1837) on Diabetic nephropathy (more control exclusions)
with a threshold of 1e−05

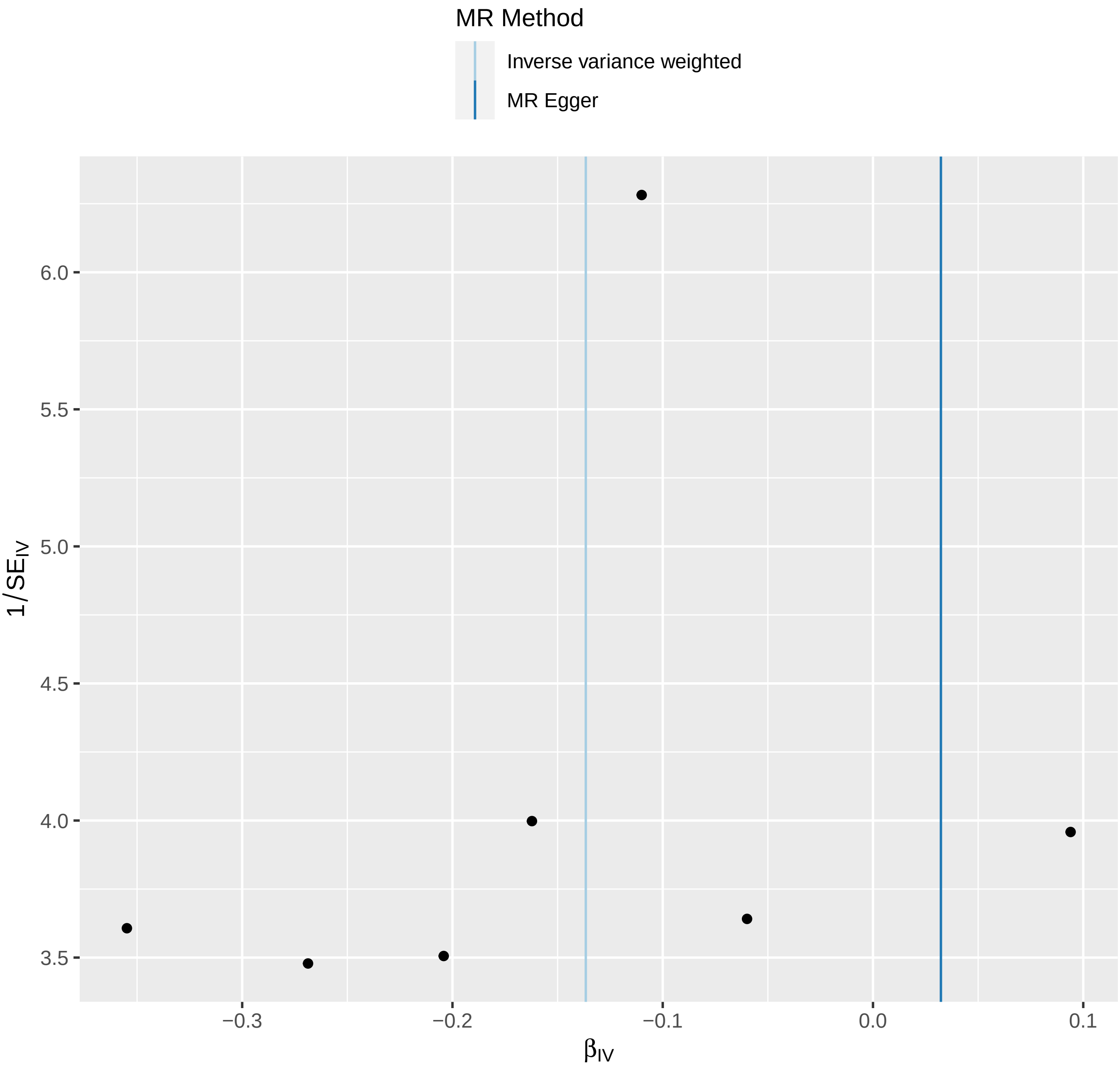

Batch 1−105 : Gut microbiota abundance (genus Lactococcus id.1851) on Diabetic nephropathy (more control exclusions) with a threshold of 1e−05

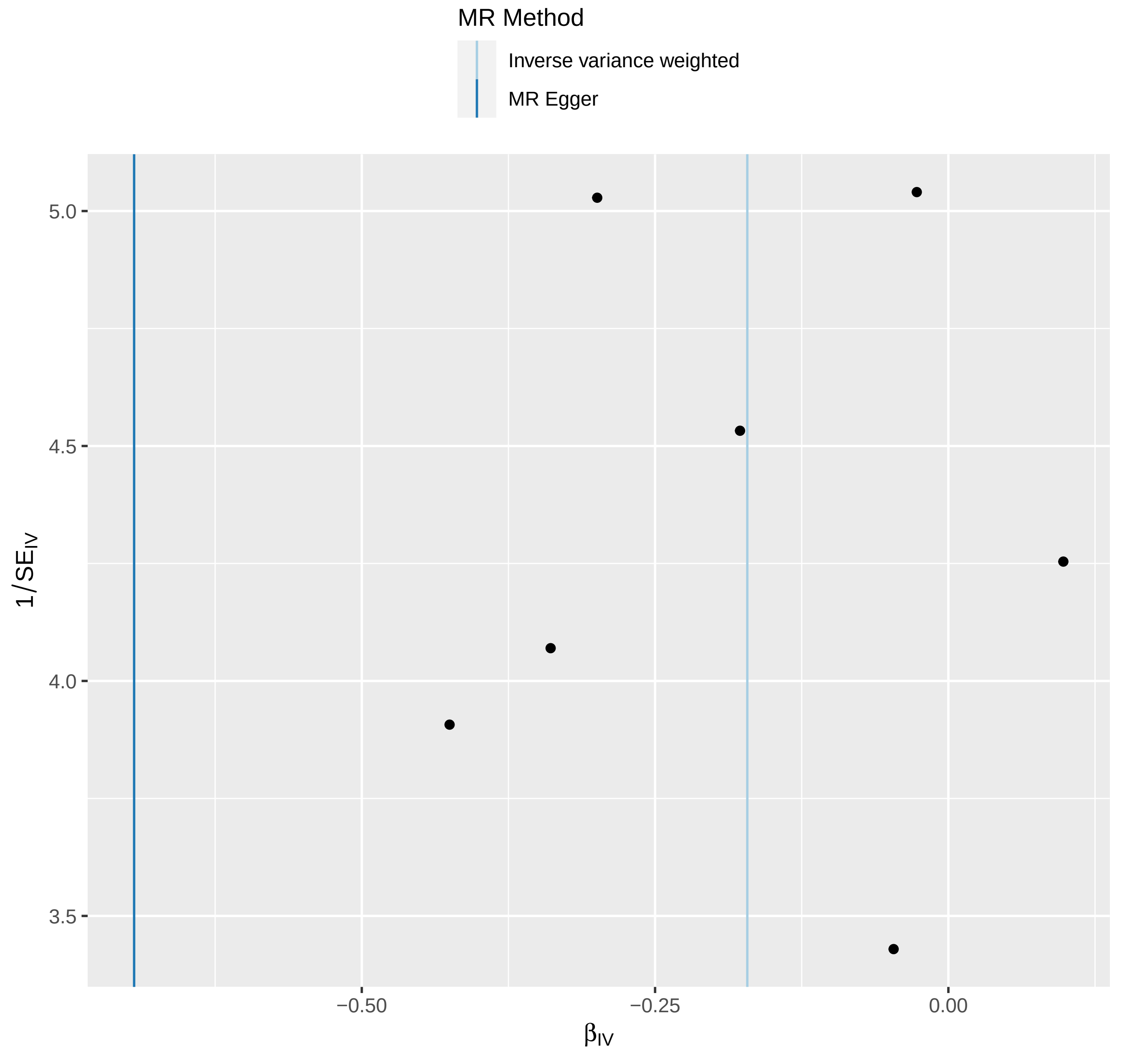

Batch 1−106 : Gut microbiota abundance (genus Marvinbryantia id.2005) on Diabetic nephropathy (more control exclusions) with a threshold of 1e−05

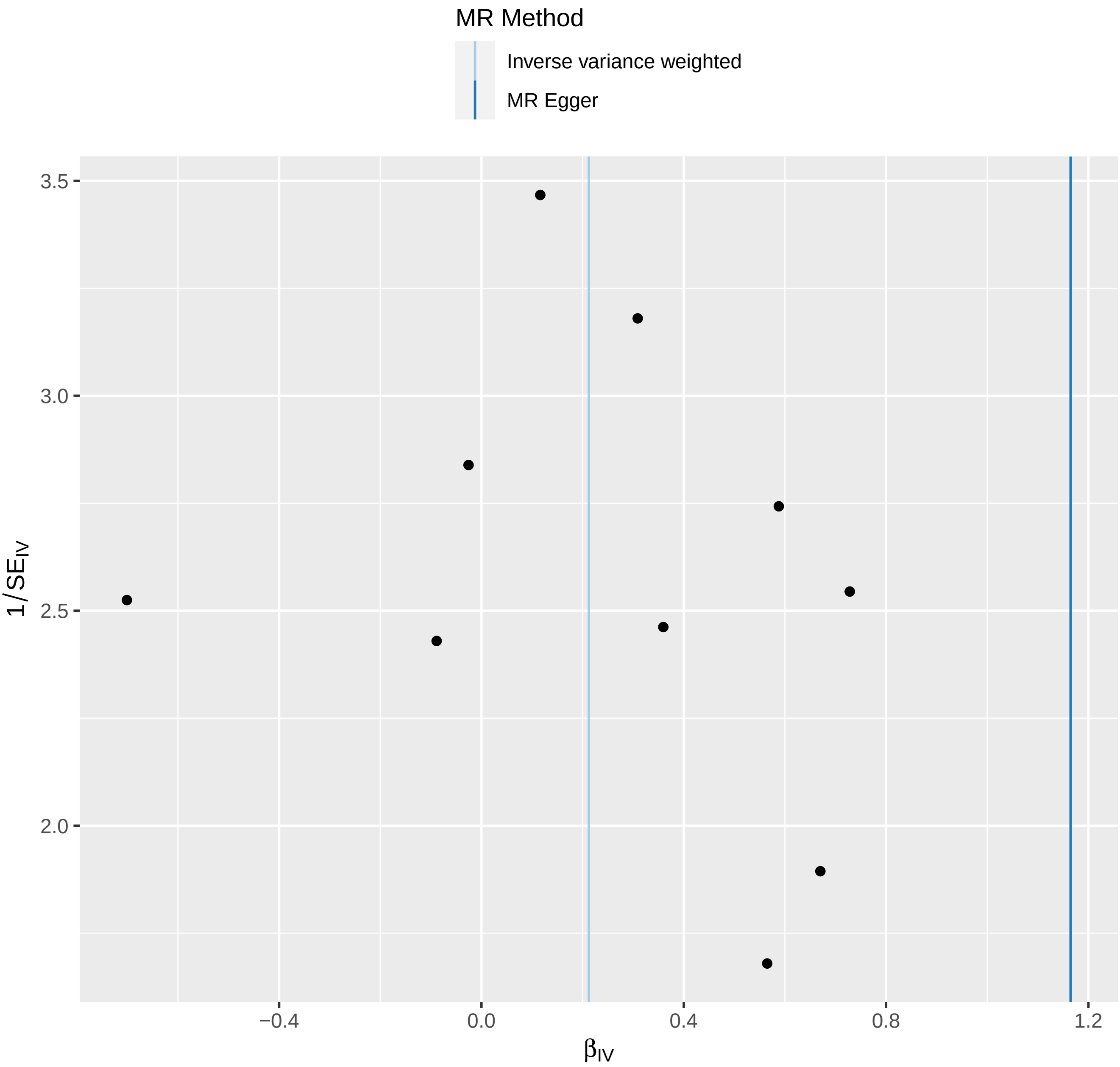

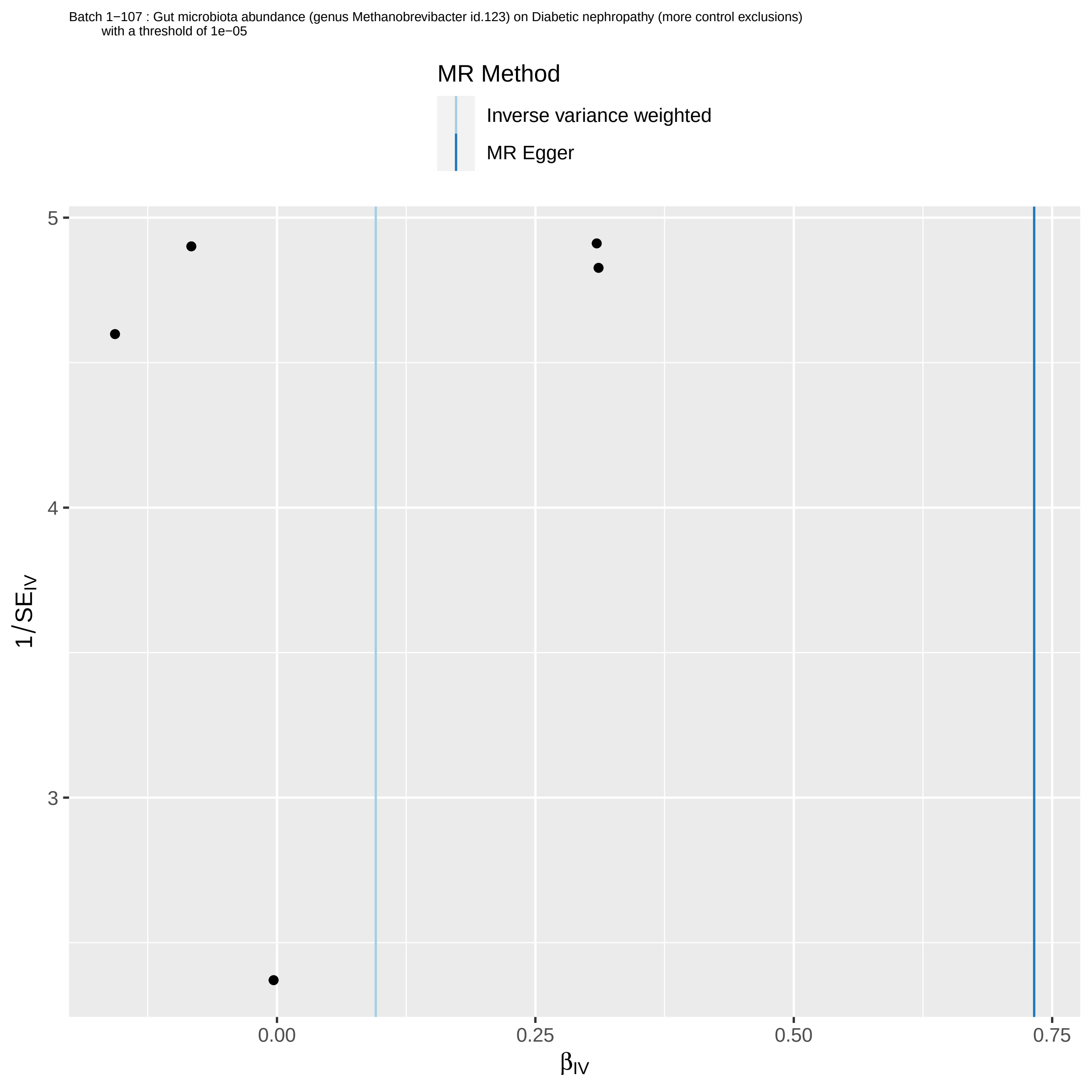

Batch 1−107 : Gut microbiota abundance (genus Methanobrevibacter id.123) on Diabetic nephropathy (more control exclusions)
with a threshold of 1e−05
MR Method
Inverse variance weighted
MR Egger
5
4
3
1/SE_IV
0.00
0.25
0.50
0.75
β_IV

Batch 1−108 : Gut microbiota abundance (genus Odoribacter id.952) on Diabetic nephropathy (more control exclusions)
with a threshold of 1e−05

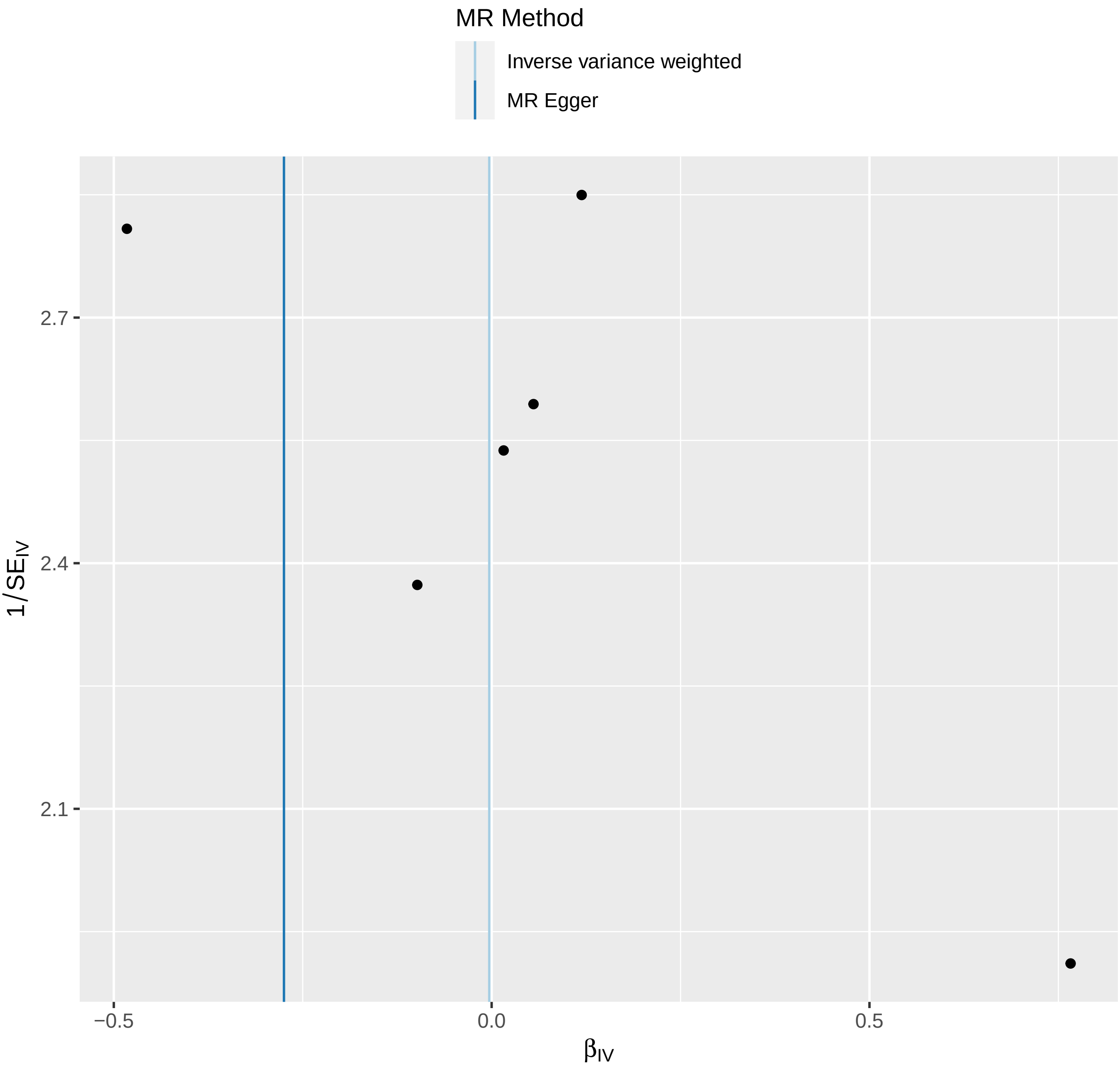

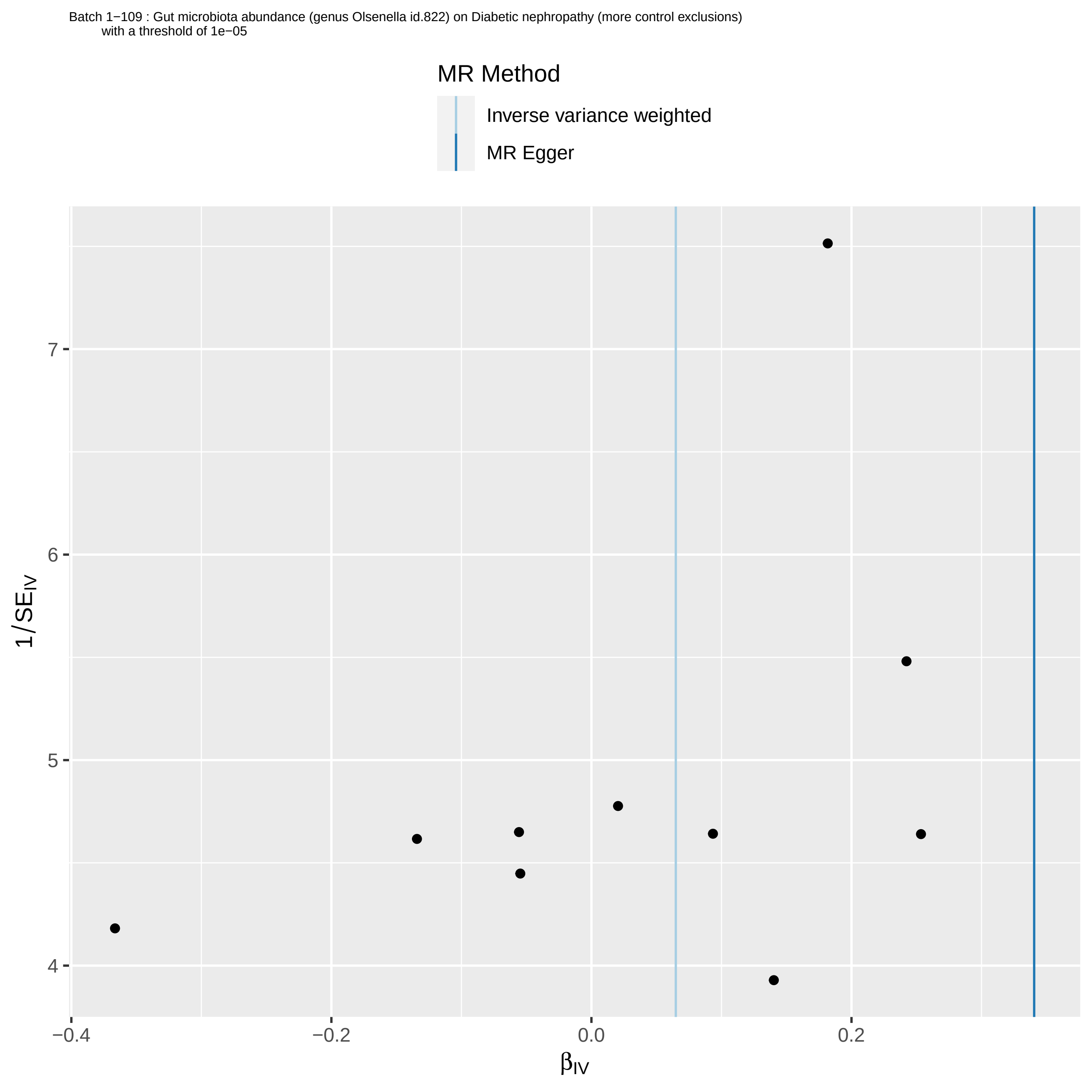

Batch 1−109 : Gut microbiota abundance (genus Olsenella id.822) on Diabetic nephropathy (more control exclusions)
with a threshold of 1e−05
MR Method
Inverse variance weighted
MR Egger
7
6
5
4
1/SE_IV
−0.4
−0.2
0.0
0.2
β_IV

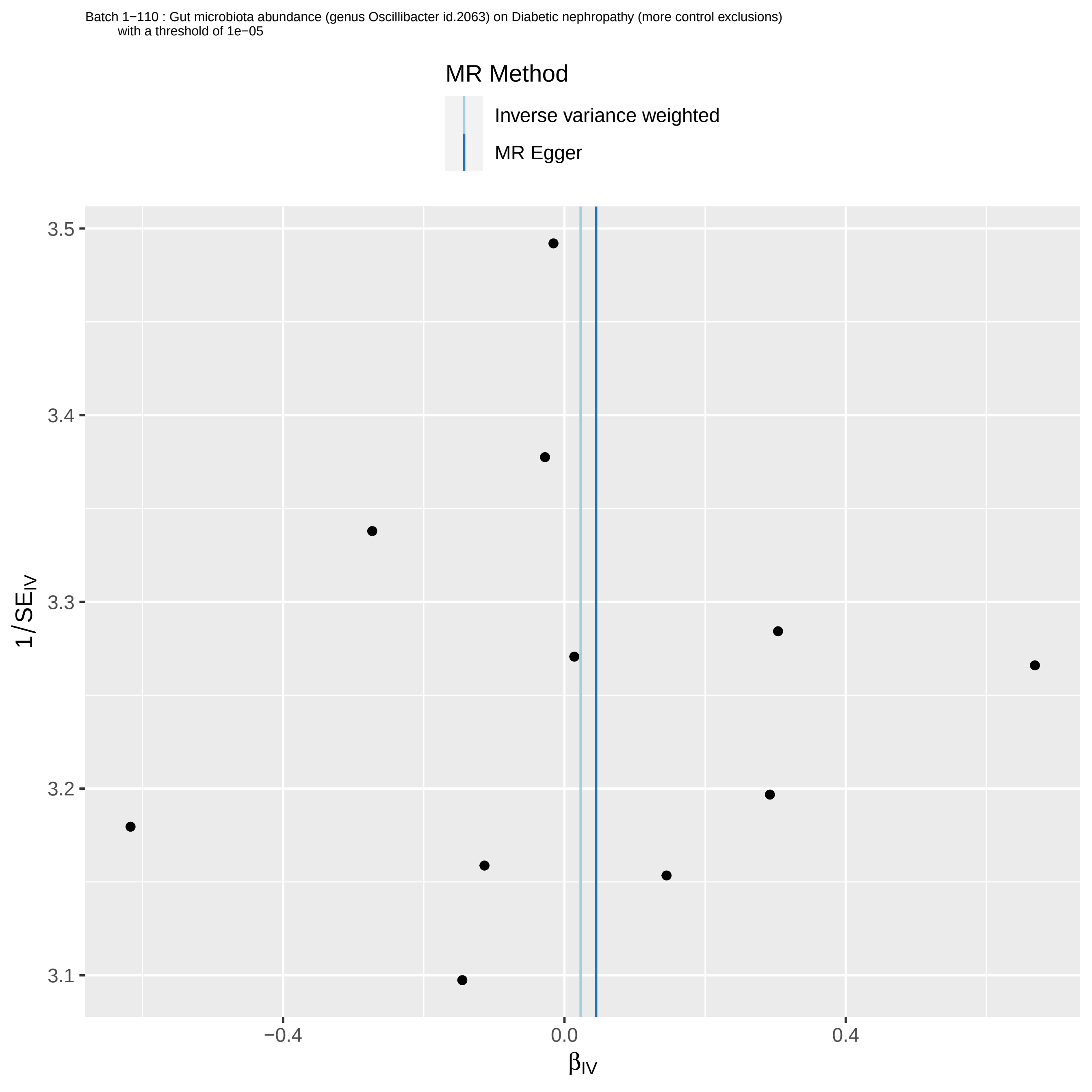
Batch 1−110 : Gut microbiota abundance (genus Oscillibacter id.2063) on Diabetic nephropathy (more control exclusions)
with a threshold of 1e−05
MR Method
Inverse variance weighted
MR Egger
3.5
3.4
3.3
3.2
3.1
1/SE$_{IV}$
−0.4
0.0
0.4
β$_{IV}$

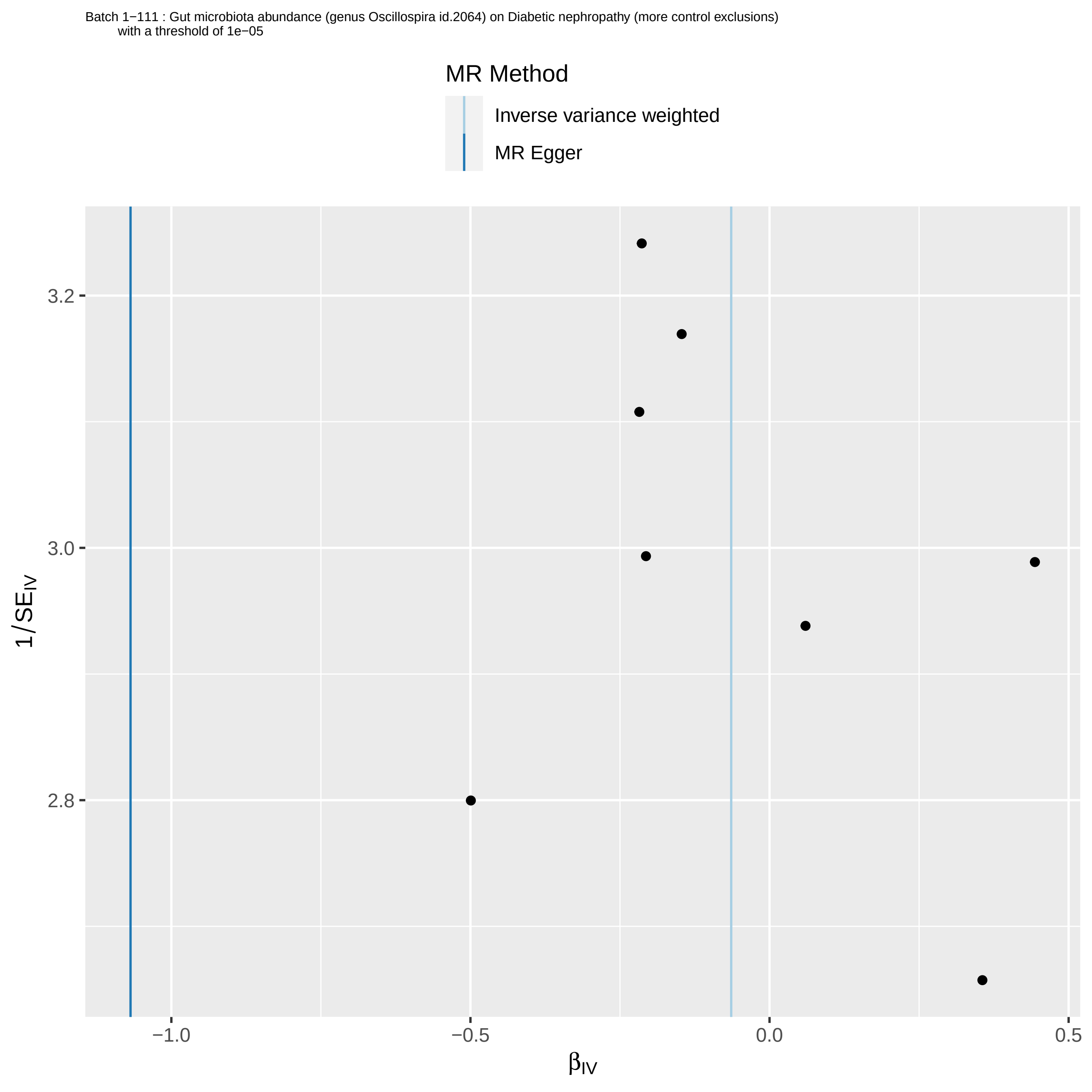

Batch 1−111 : Gut microbiota abundance (genus Oscillospira id.2064) on Diabetic nephropathy (more control exclusions)
with a threshold of 1e−05
MR Method
Inverse variance weighted
MR Egger
3.2
3.0
2.8
1/SE_IV
−1.0
−0.5
0.0
0.5
β_IV

Batch 1−112 : Gut microbiota abundance (genus Oxalobacter id.2978) on Diabetic nephropathy (more control exclusions) with a threshold of 1e−05

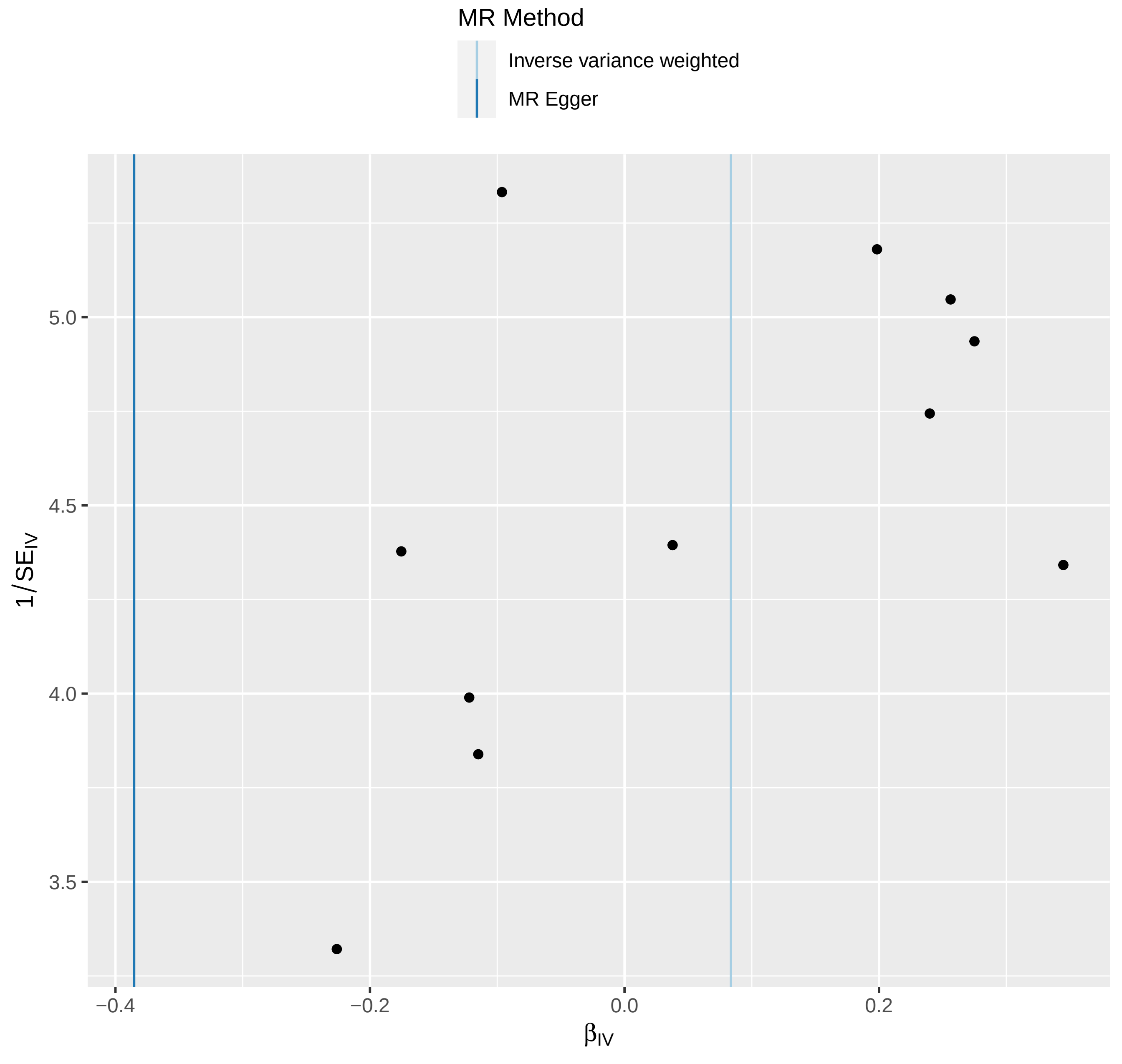

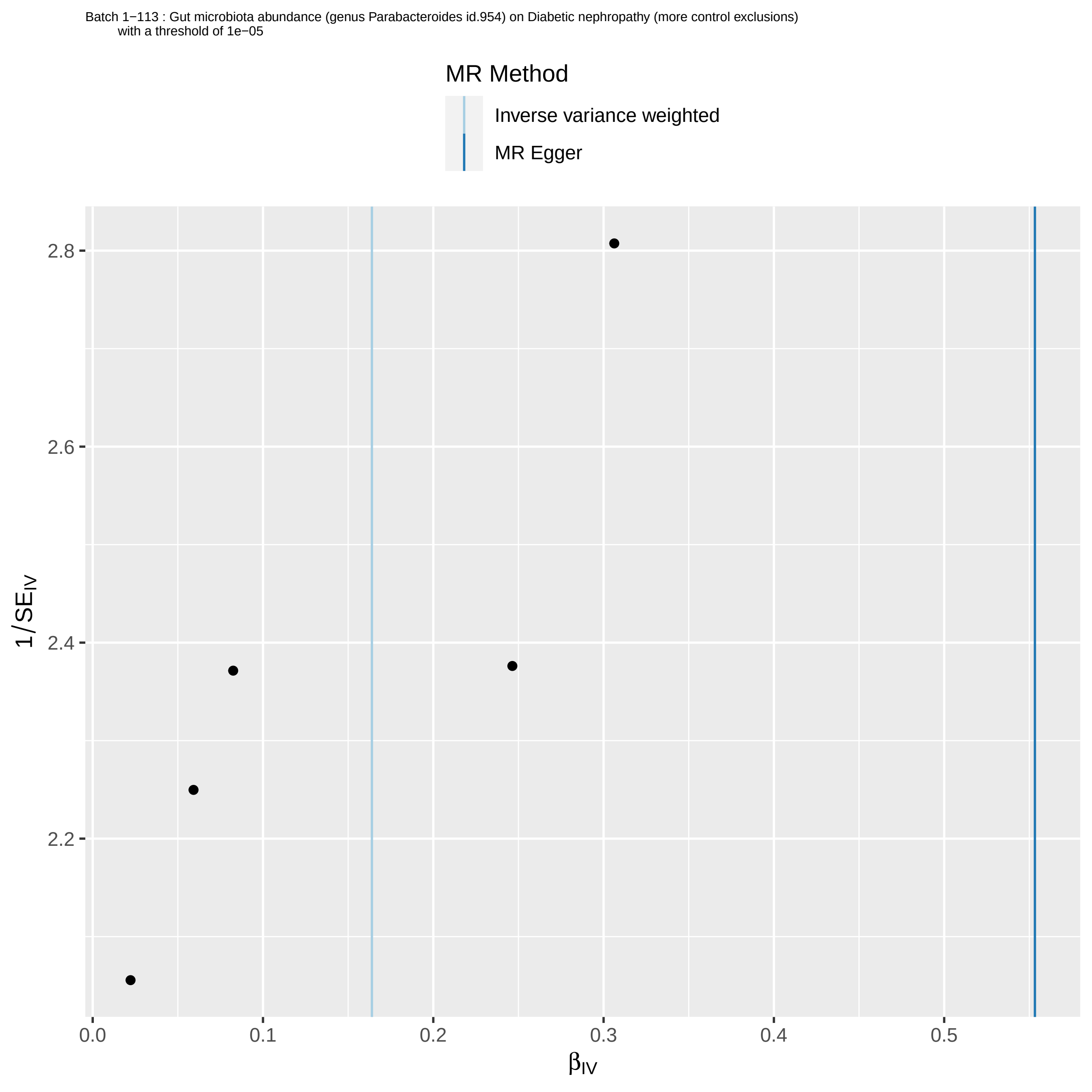

Batch 1−113 : Gut microbiota abundance (genus Parabacteroides id.954) on Diabetic nephropathy (more control exclusions)
with a threshold of 1e−05
MR Method
Inverse variance weighted
MR Egger
2.8
2.6
2.4
2.2
1/SE_IV
0.0
0.1
0.2
0.3
0.4
0.5
β_IV

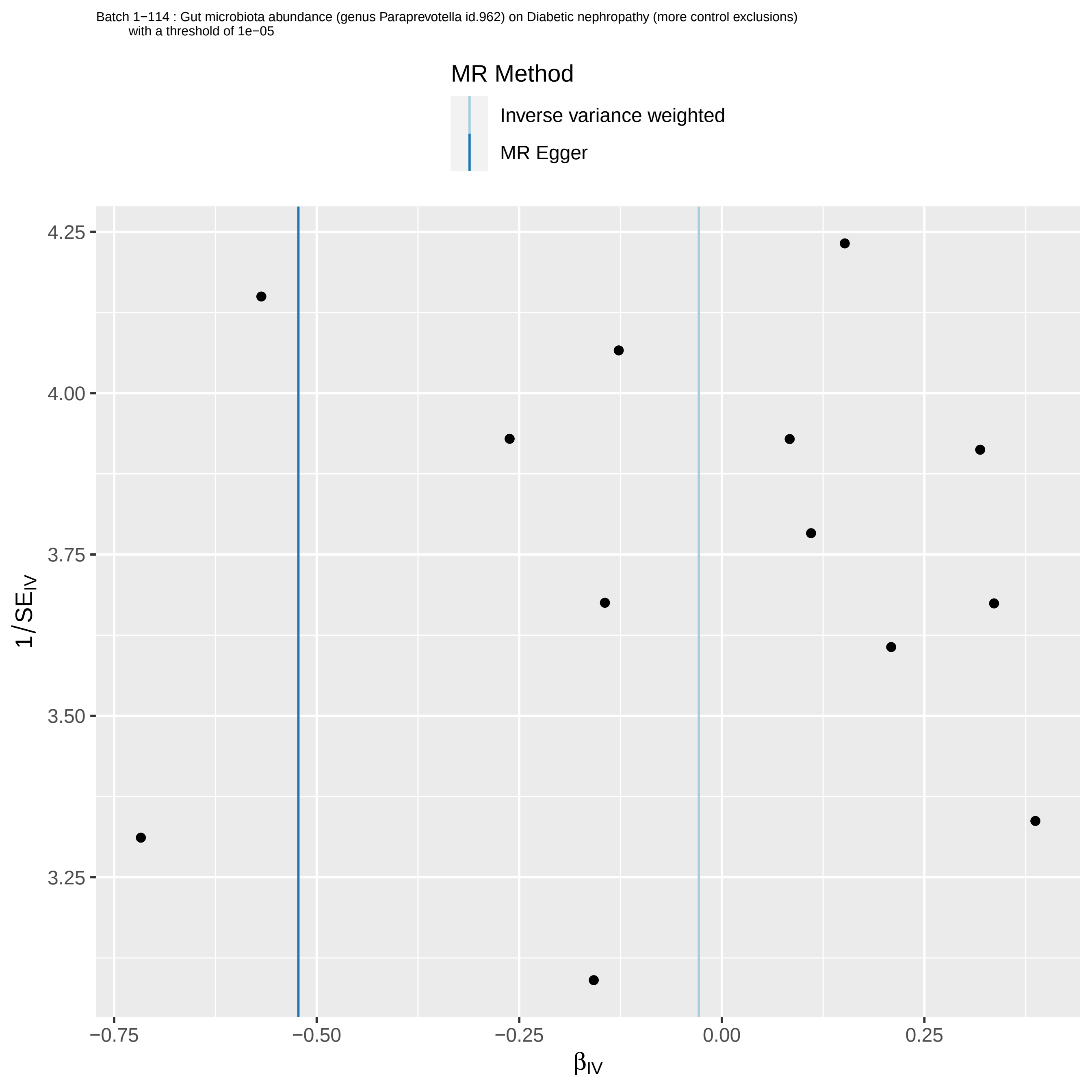

Batch 1−114 : Gut microbiota abundance (genus Paraprevotella id.962) on Diabetic nephropathy (more control exclusions)
with a threshold of 1e−05
MR Method
Inverse variance weighted
MR Egger
4.25
4.00
3.75
3.50
3.25
1/SE_IV
−0.75
−0.50
−0.25
0.00
0.25
β_IV

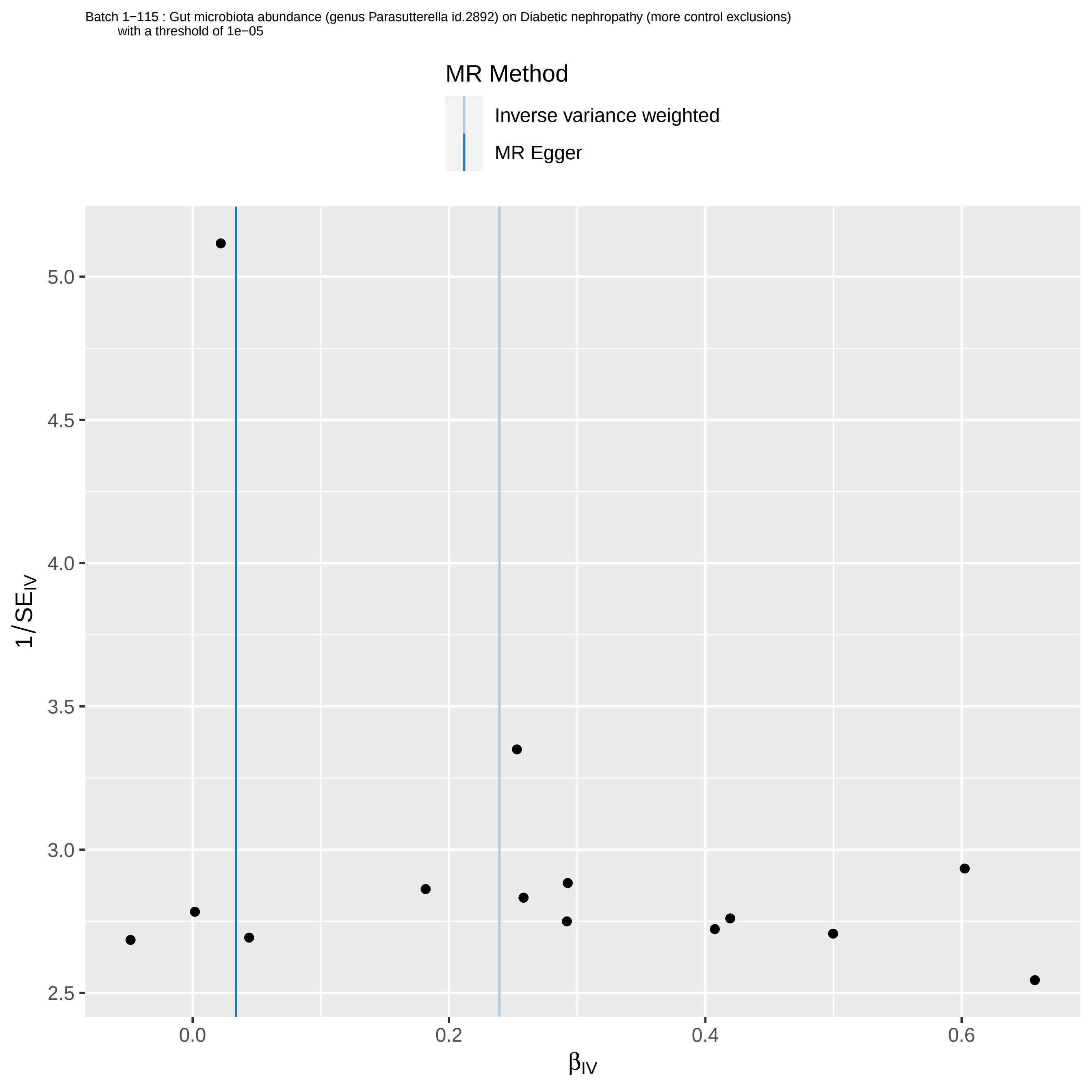

Batch 1−115 : Gut microbiota abundance (genus Parasutterella id.2892) on Diabetic nephropathy (more control exclusions) with a threshold of 1e−05
MR Method
Inverse variance weighted
MR Egger
5.0
4.5
4.0
3.5
3.0
2.5
1/SE_IV
0.0
0.2
0.4
0.6
β_IV

Batch 1−116 : Gut microbiota abundance (genus Peptococcus id.2037) on Diabetic nephropathy (more control exclusions)
with a threshold of 1e−05

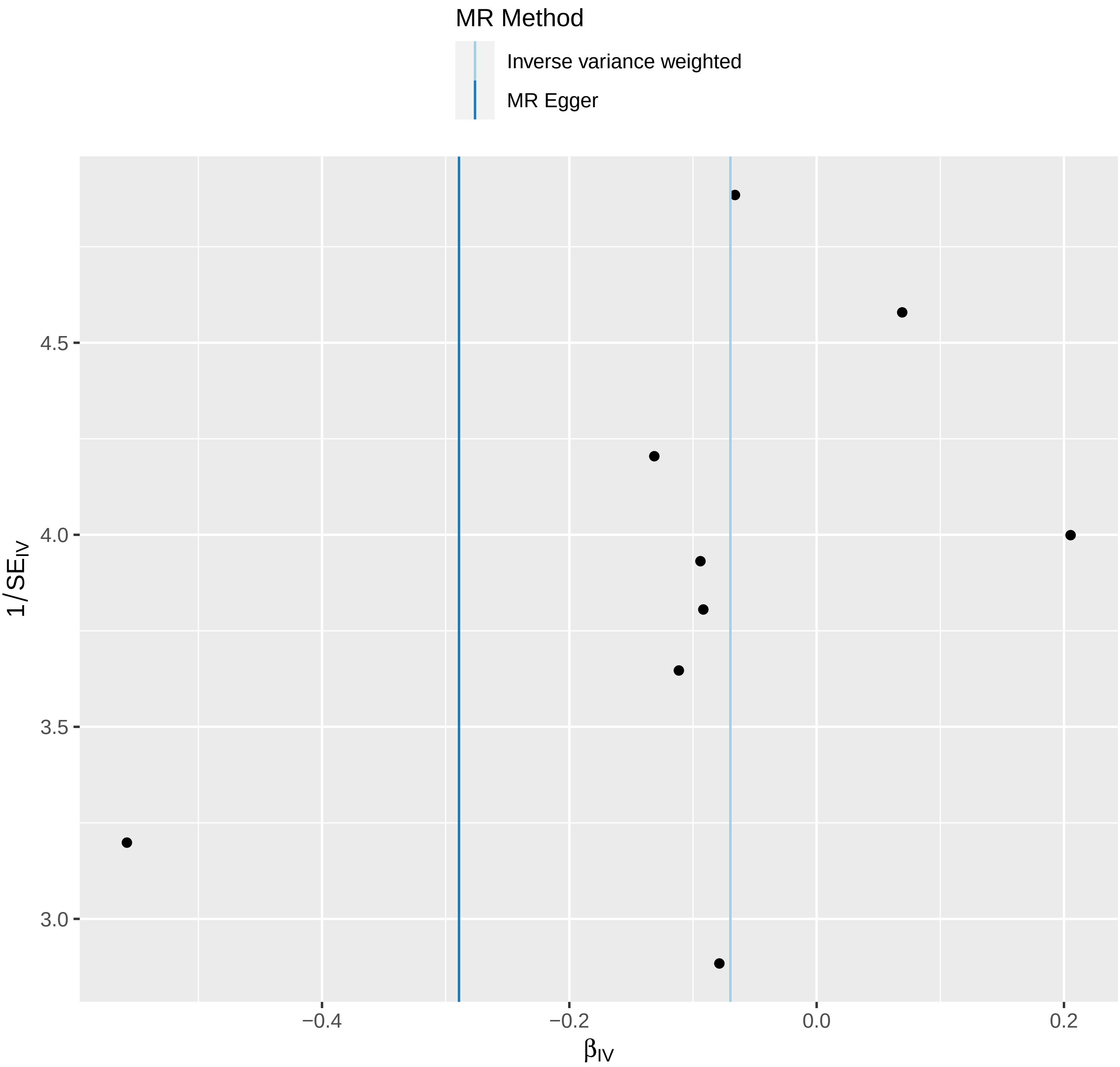

Batch 1−117 : Gut microbiota abundance (genus Phascolarctobacterium id.2168) on Diabetic nephropathy (more control exclusions) with a threshold of 1e−05

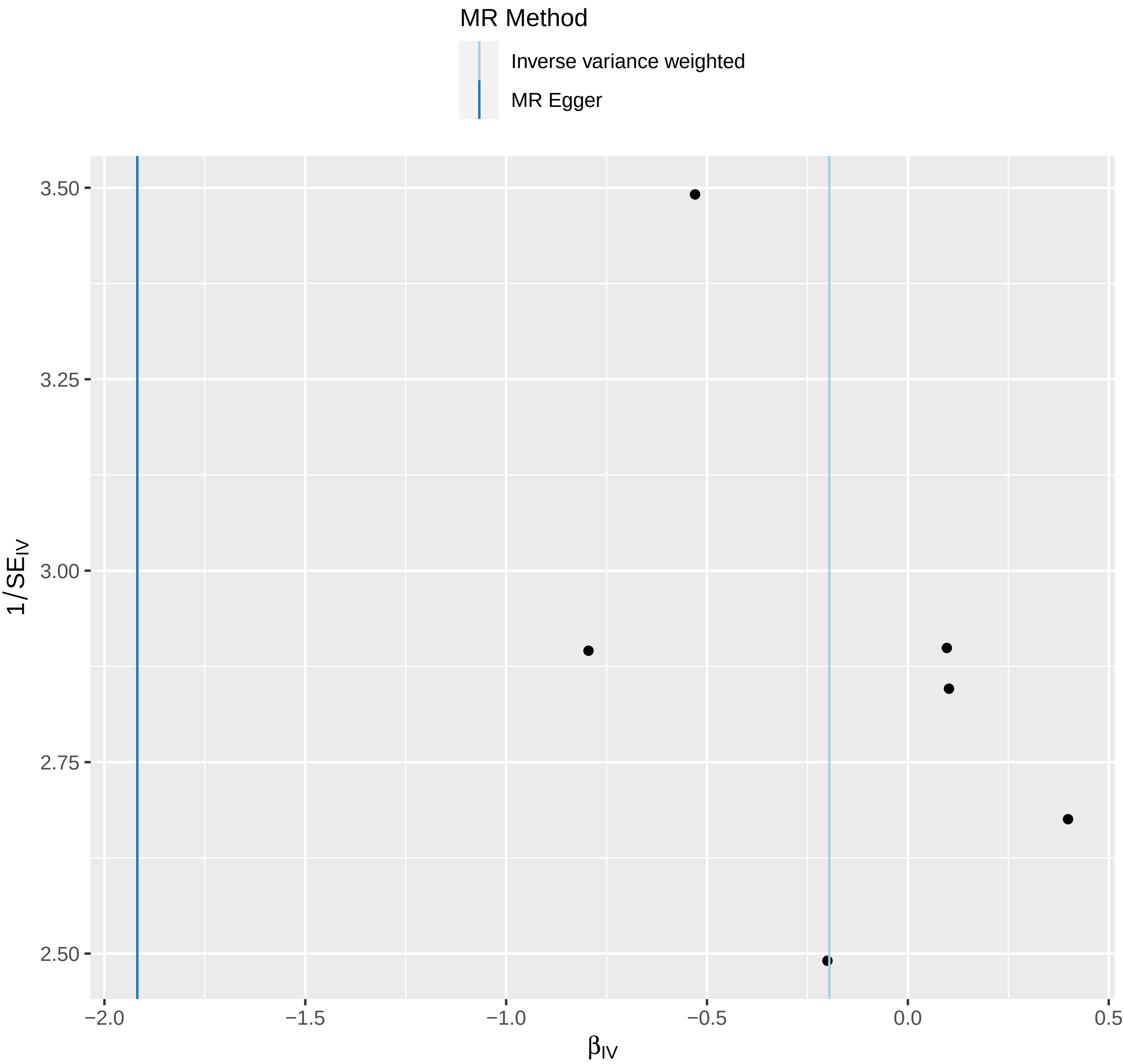

Batch 1−118 : Gut microbiota abundance (genus Prevotella7 id.11182) on Diabetic nephropathy (more control exclusions)
with a threshold of 1e−05

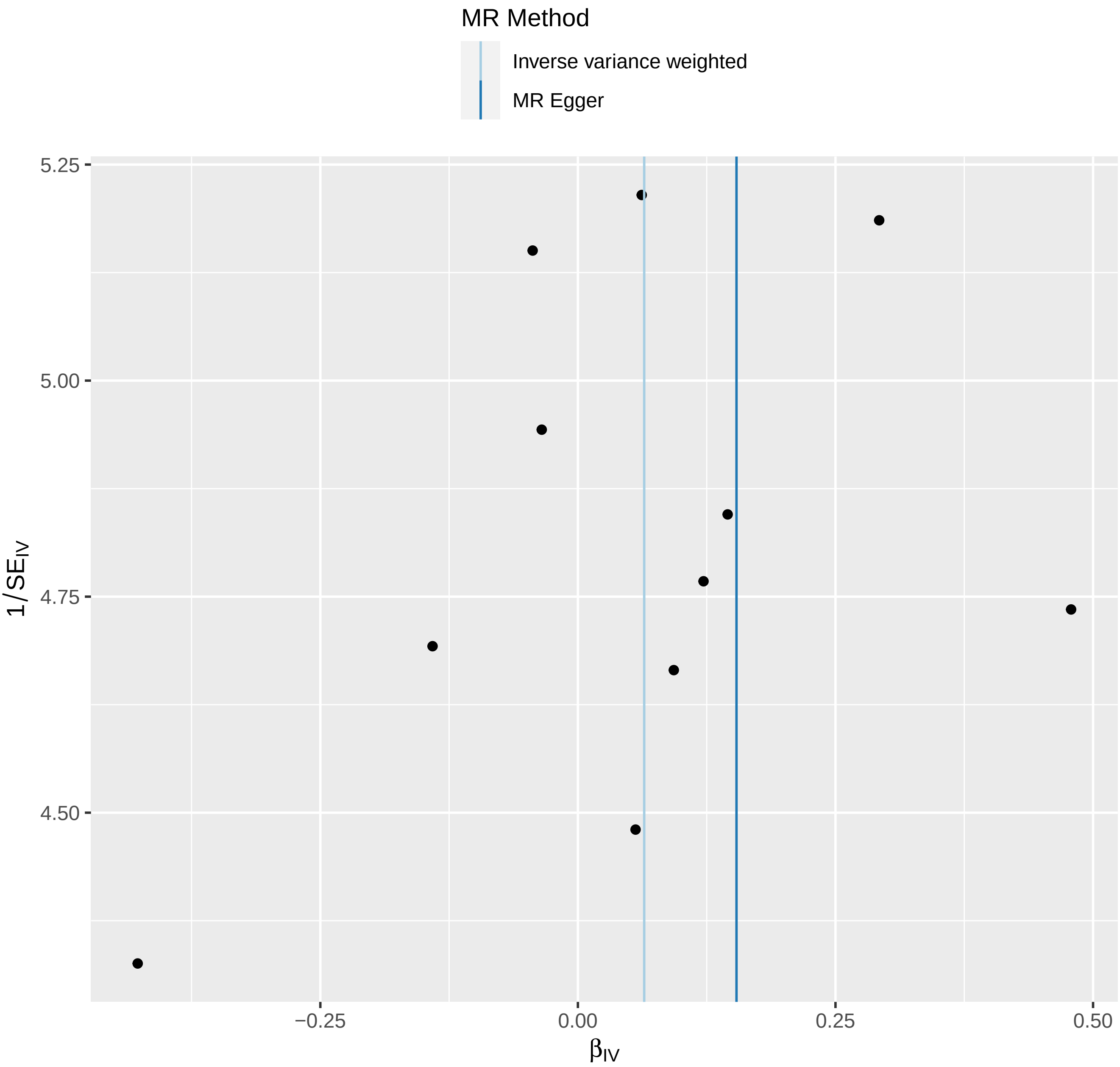

Batch 1−119 : Gut microbiota abundance (genus Prevotella9 id.11183) on Diabetic nephropathy (more control exclusions) with a threshold of 1e−05

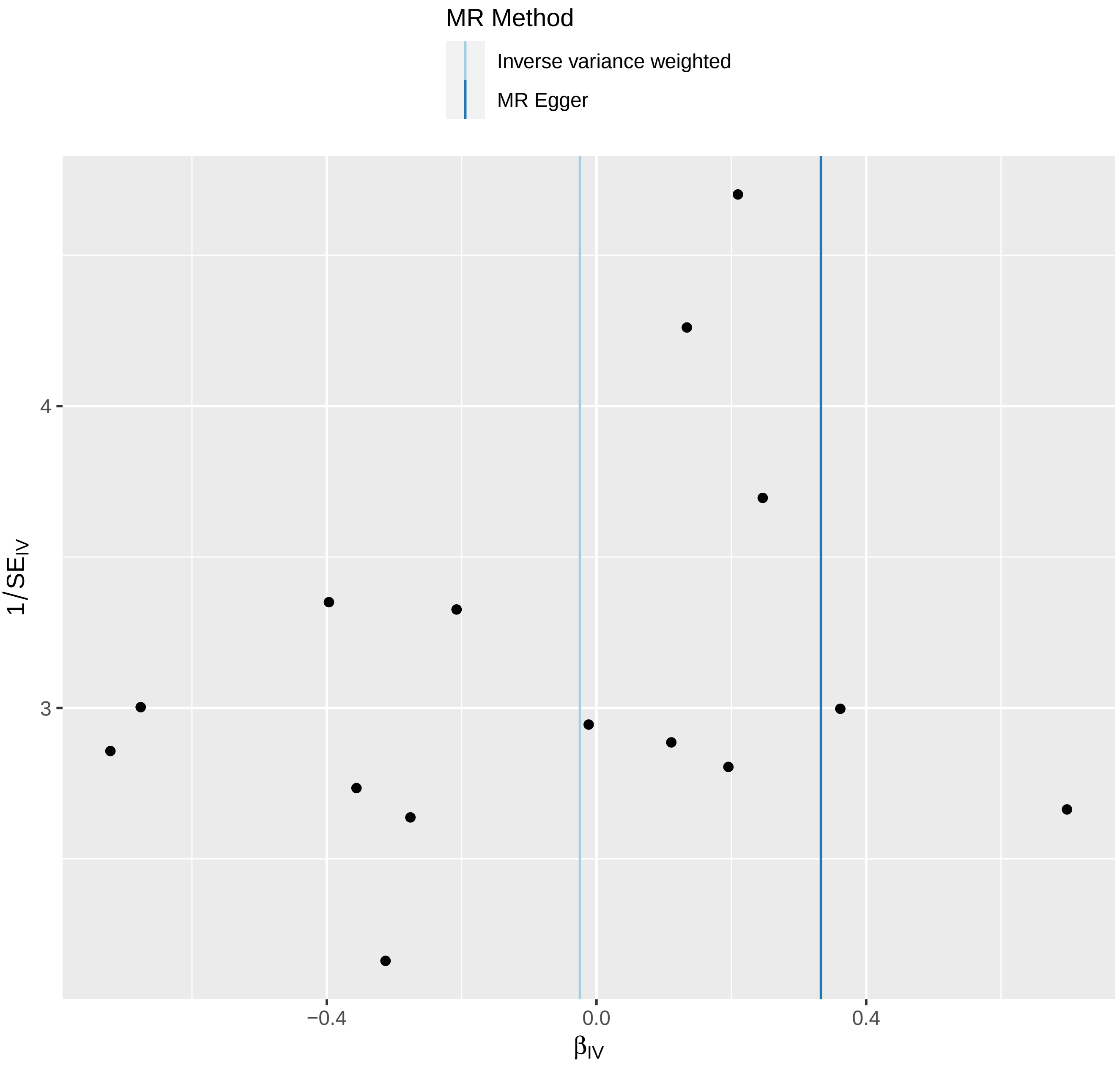

Batch 1−120 : Gut microbiota abundance (genus Rikenellaceae RC9 gut group id.11191) on Diabetic nephropathy (more control exclusions) with a threshold of 1e−05

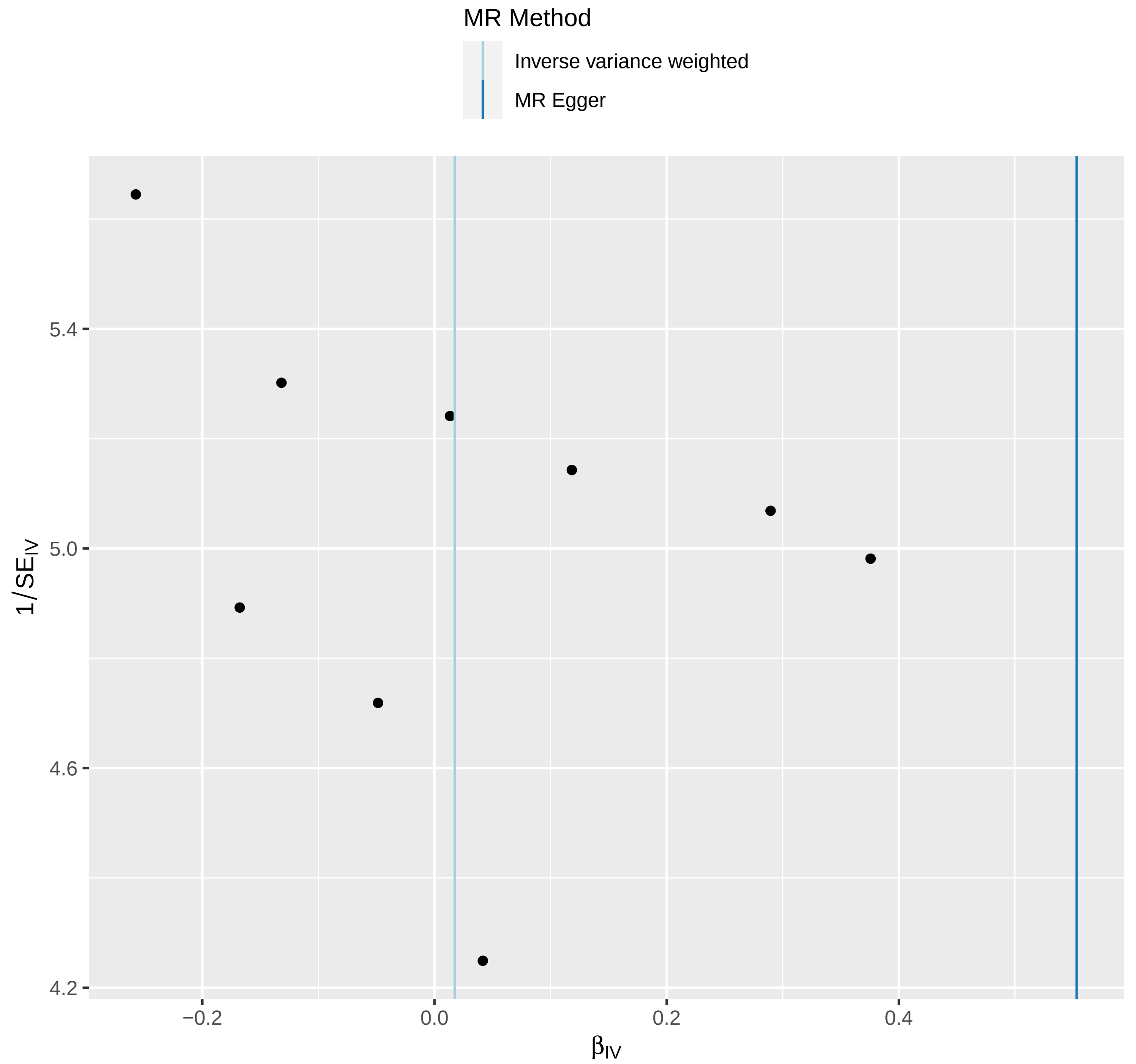

Batch 1−121 : Gut microbiota abundance (genus Romboutsia id.11347) on Diabetic nephropathy (more control exclusions) with a threshold of 1e−05

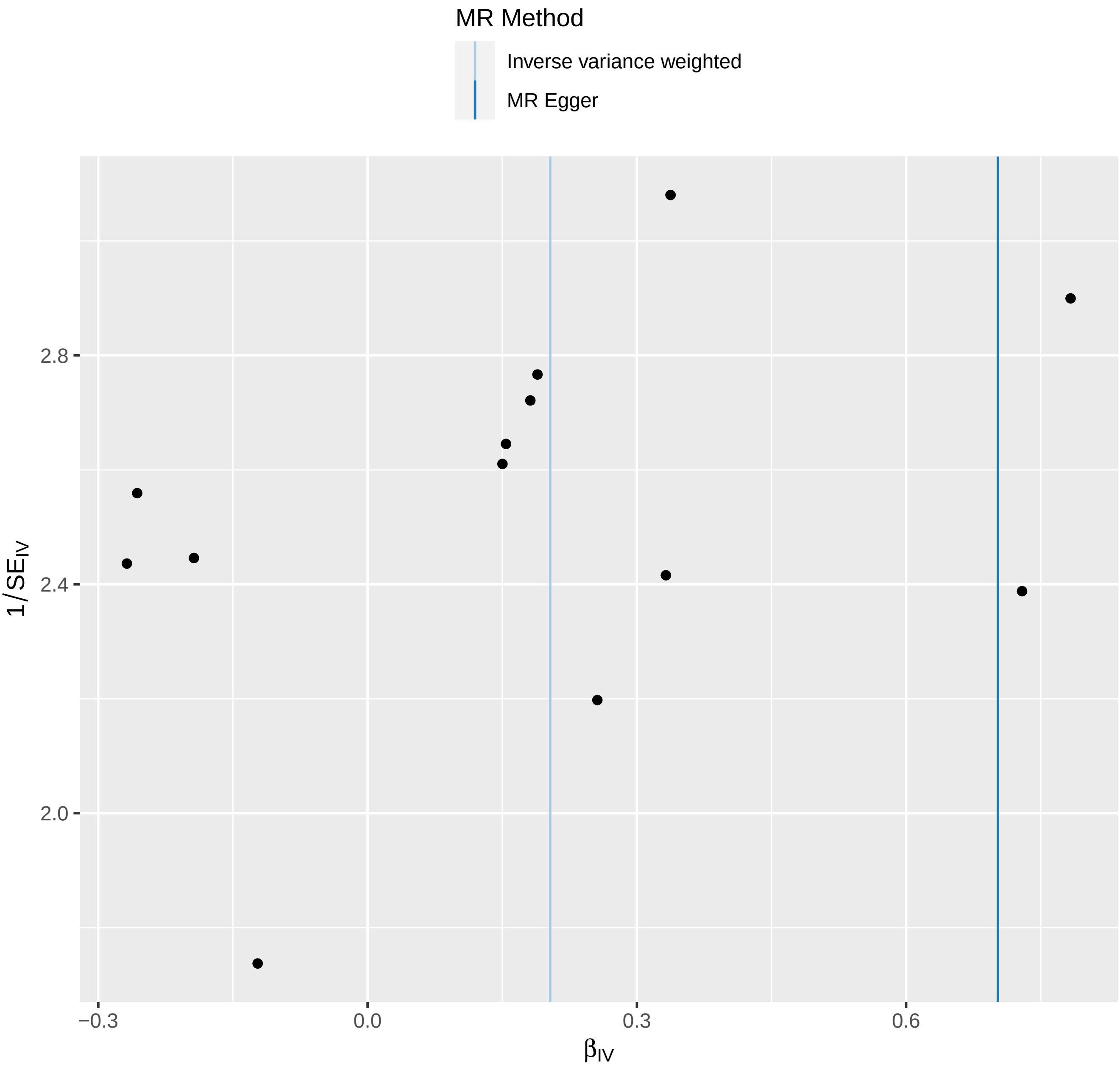

Batch 1−122 : Gut microbiota abundance (genus Roseburia id.2012) on Diabetic nephropathy (more control exclusions)
with a threshold of 1e−05

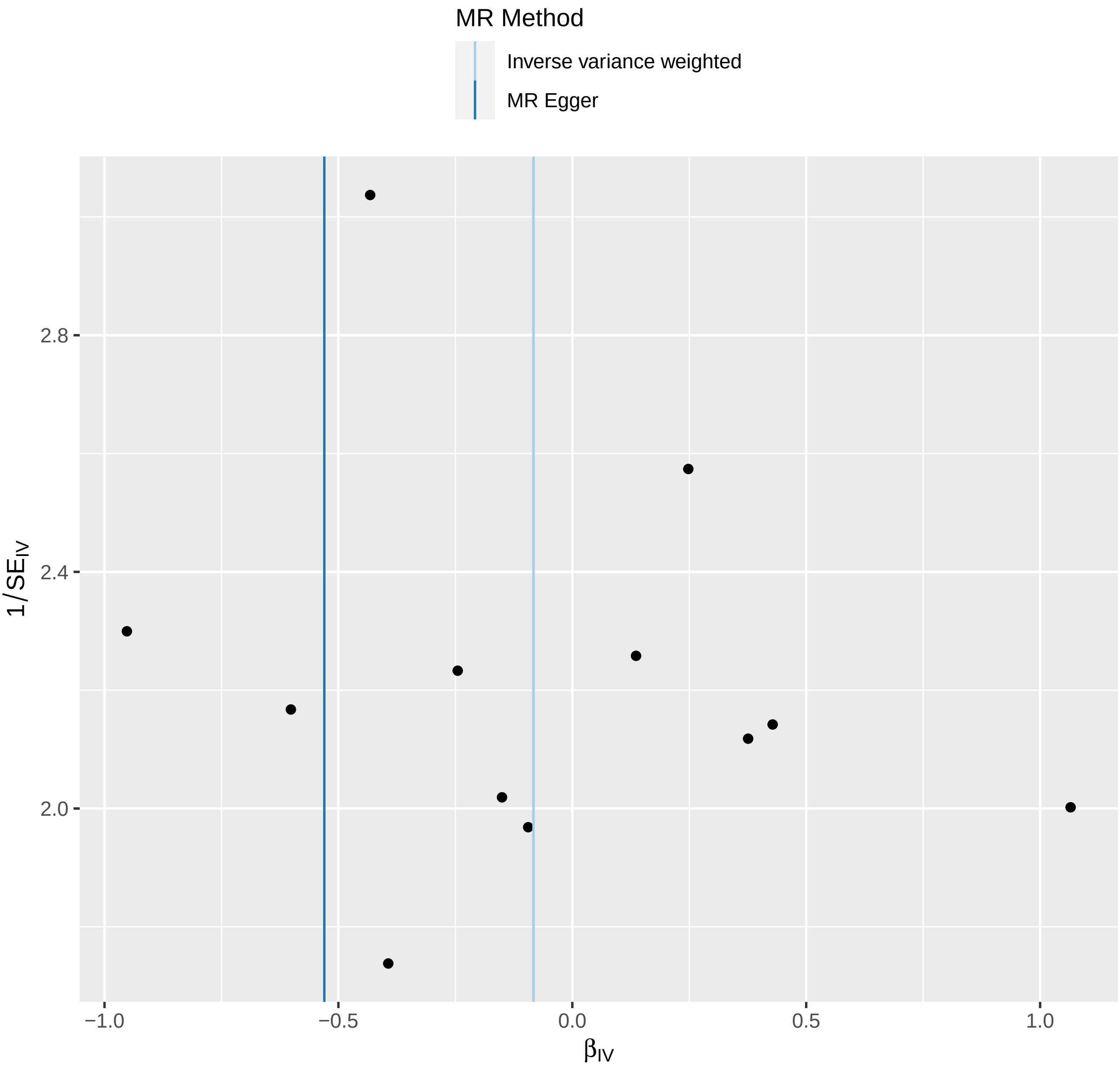

Batch 1−123 : Gut microbiota abundance (genus Ruminiclostridium5 id.11355) on Diabetic nephropathy (more control exclusions)
with a threshold of 1e−05

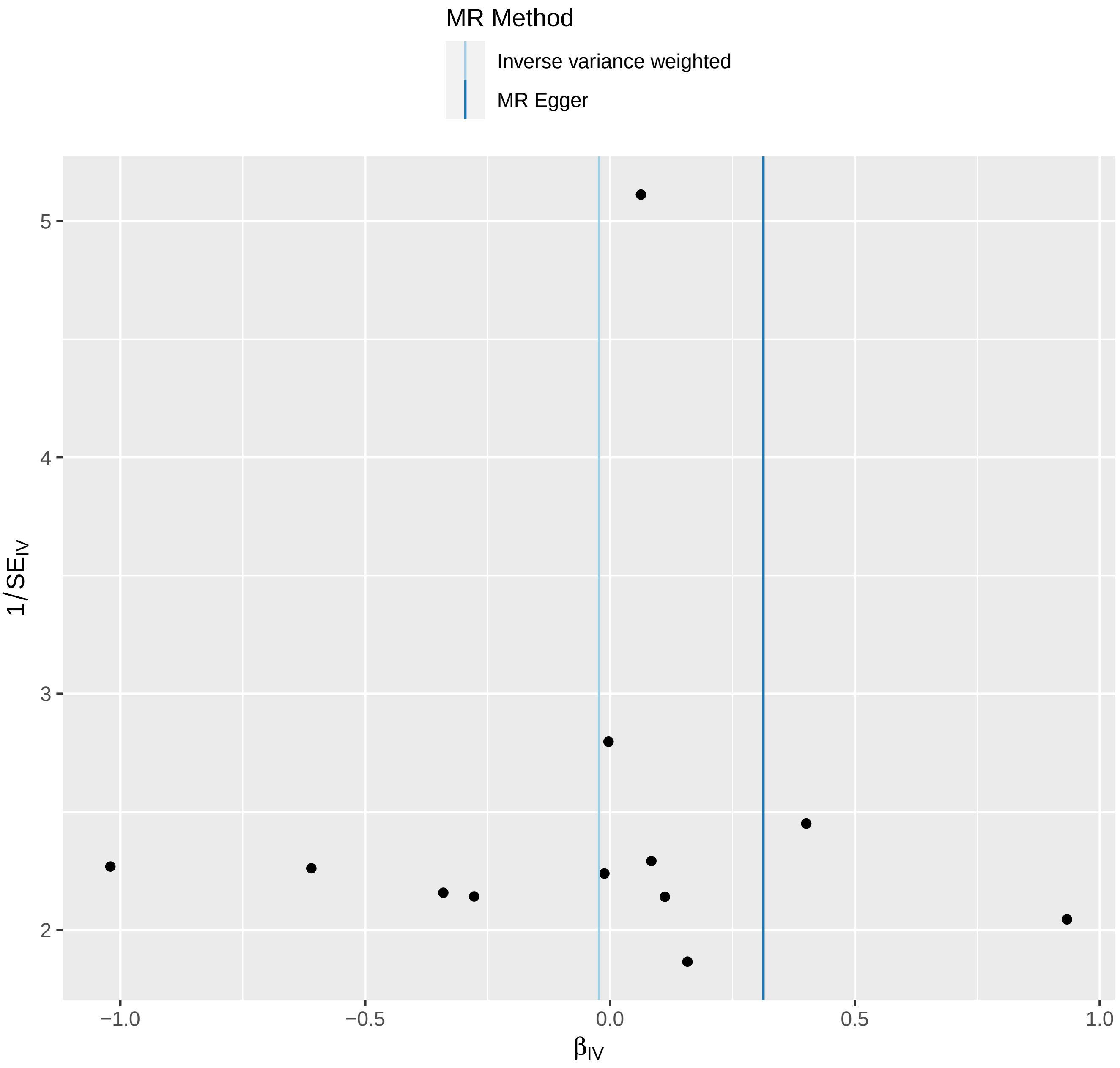

Batch 1−124 : Gut microbiota abundance (genus Ruminiclostridium6 id.11356) on Diabetic nephropathy (more control exclusions) with a threshold of 1e−05

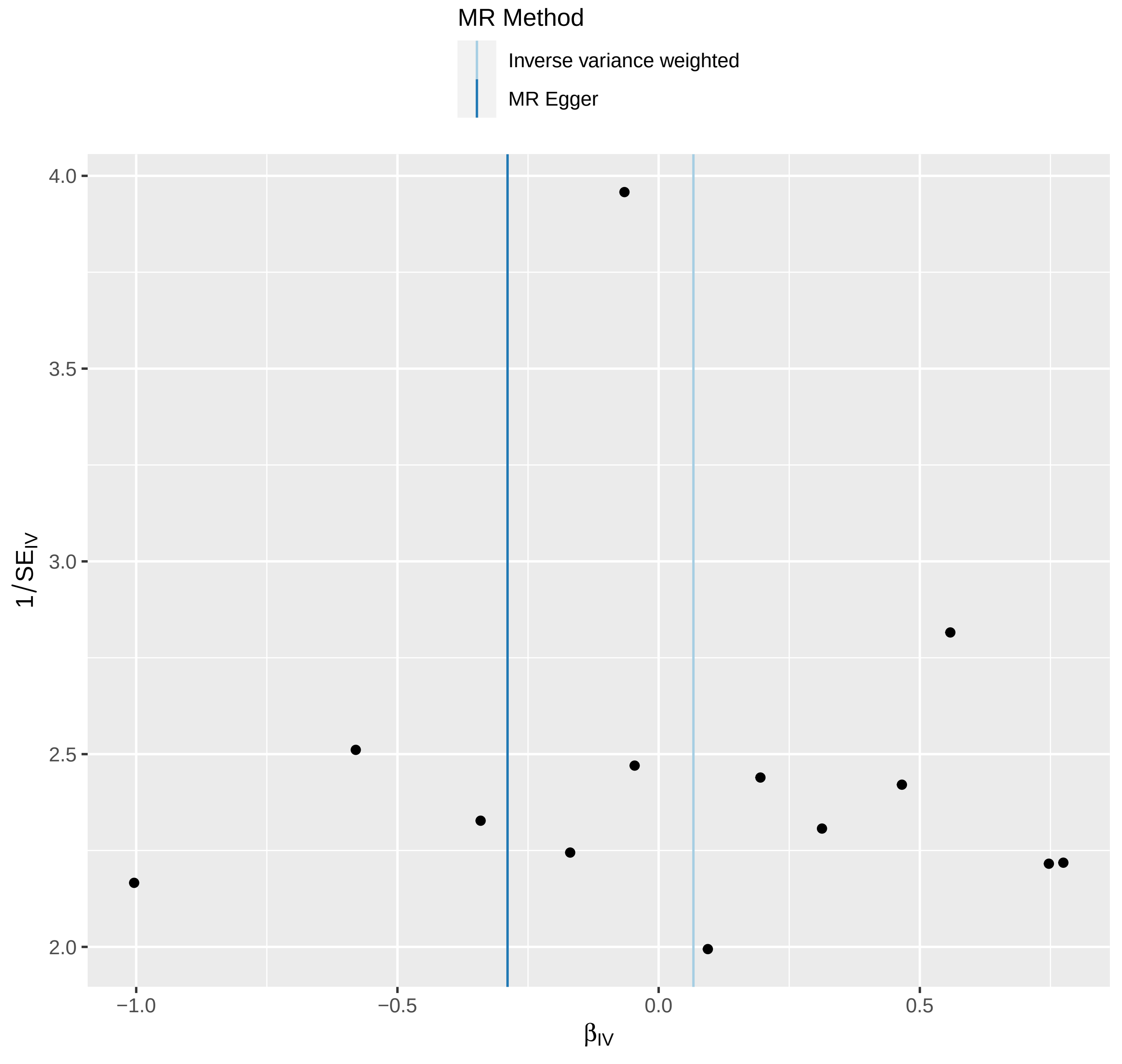

Batch 1−125 : Gut microbiota abundance (genus Ruminiclostridium9 id.11357) on Diabetic nephropathy (more control exclusions) with a threshold of 1e−05

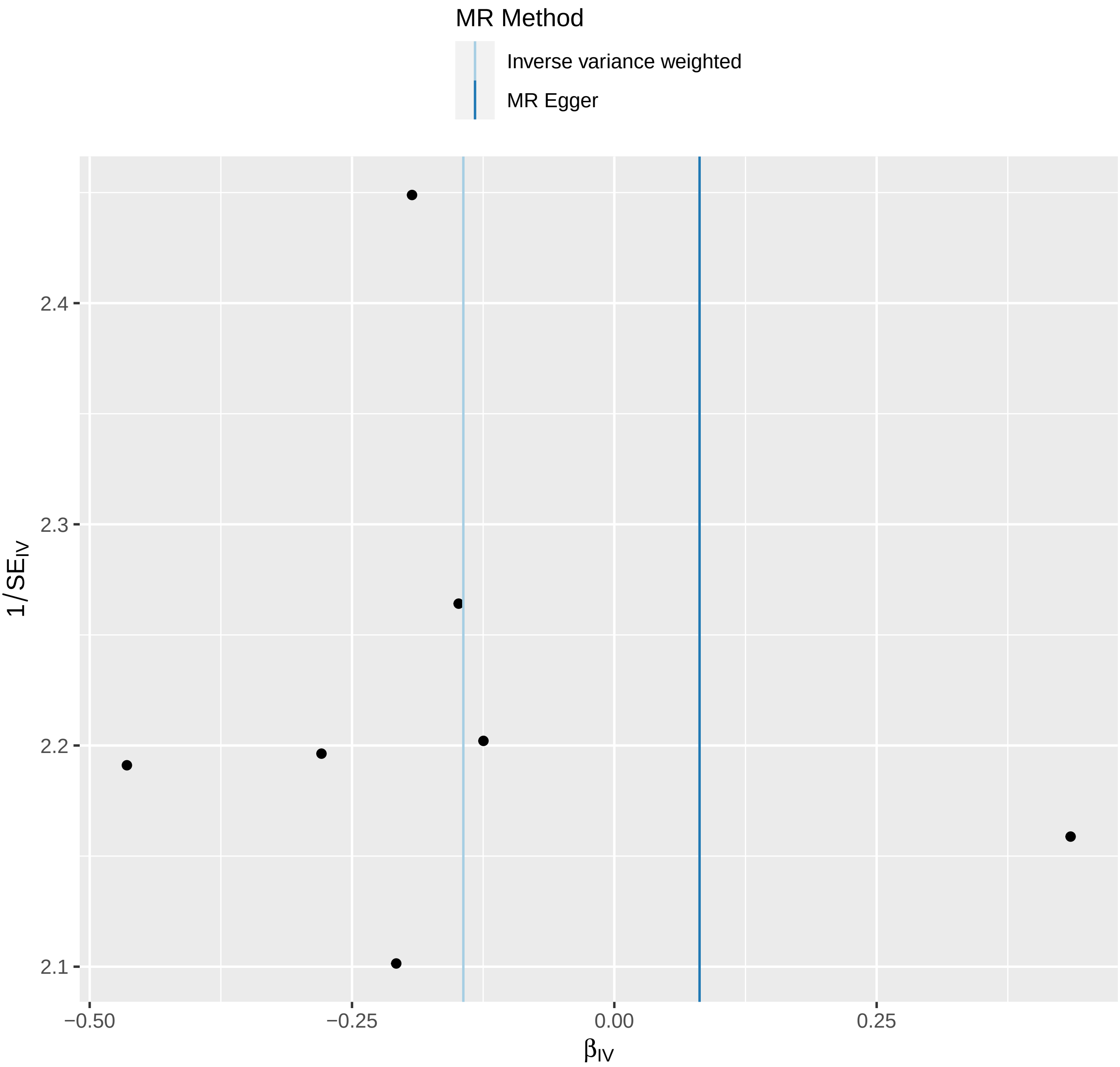

Batch 1−126 : Gut microbiota abundance (genus Ruminococcaceae NK4A214 group id.11358) on Diabetic nephropathy (more control exclusions) with a threshold of 1e−05

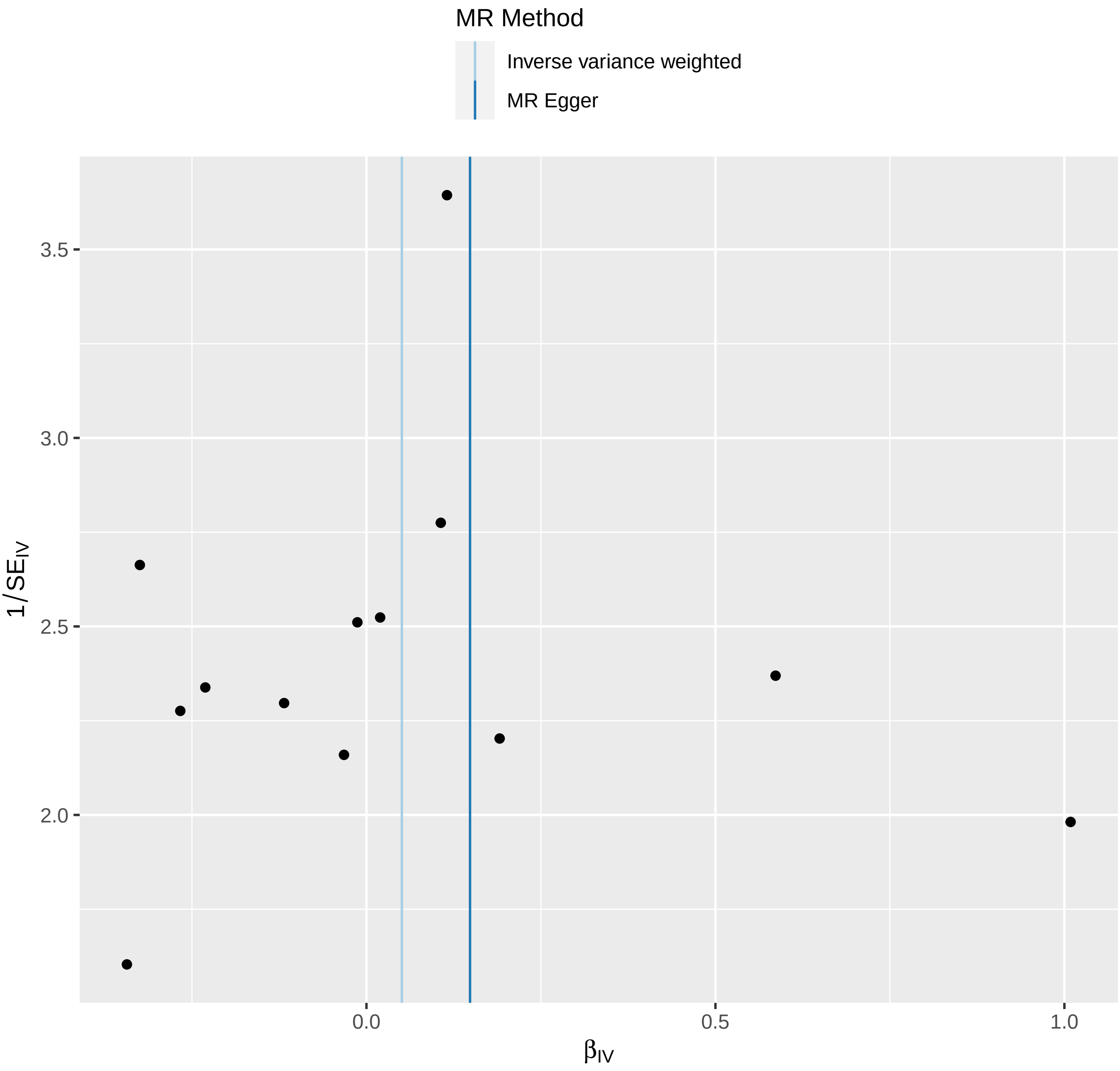

Batch 1−127 : Gut microbiota abundance (genus Ruminococcaceae UCG002 id.11360) on Diabetic nephropathy (more control exclusions) with a threshold of 1e−05

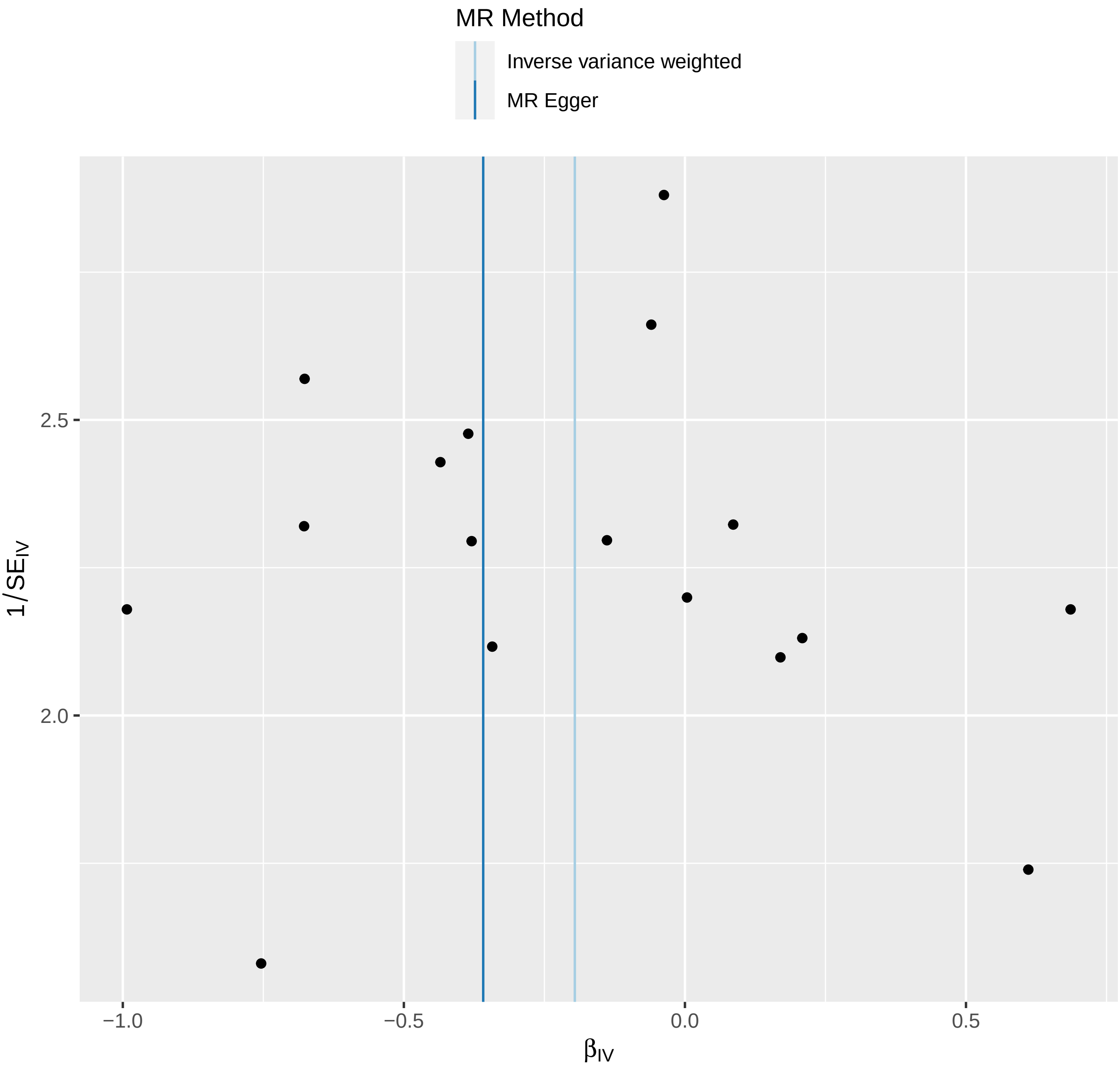

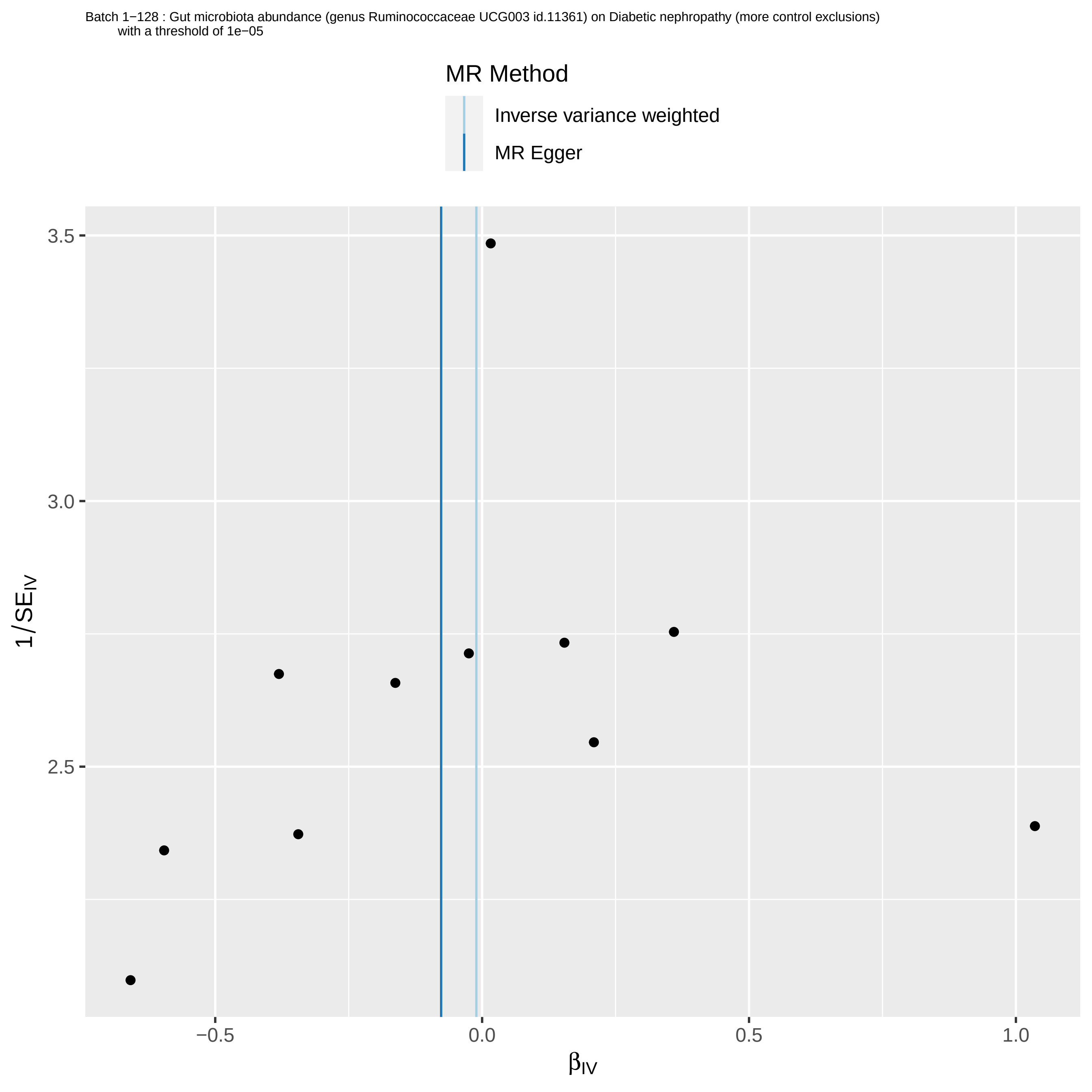
Batch 1−128 : Gut microbiota abundance (genus Ruminococcaceae UCG003 id.11361) on Diabetic nephropathy (more control exclusions) with a threshold of 1e−05
MR Method
Inverse variance weighted
MR Egger
3.5
3.0
2.5
1/SE_IV
−0.5
0.0
0.5
1.0
β_IV

Batch 1−129 : Gut microbiota abundance (genus Ruminococcaceae UCG004 id.11362) on Diabetic nephropathy (more control exclusions) with a threshold of 1e−05

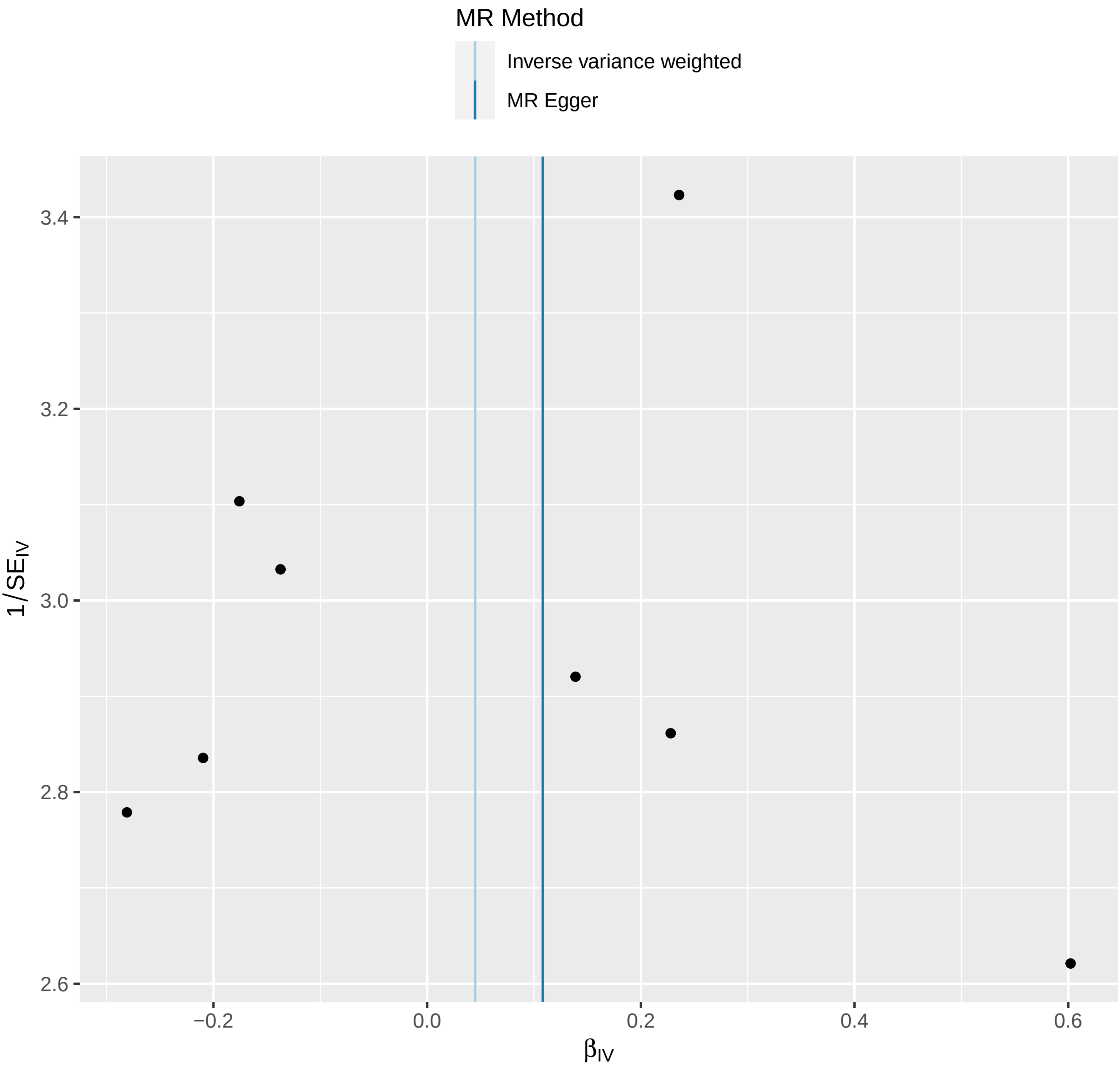

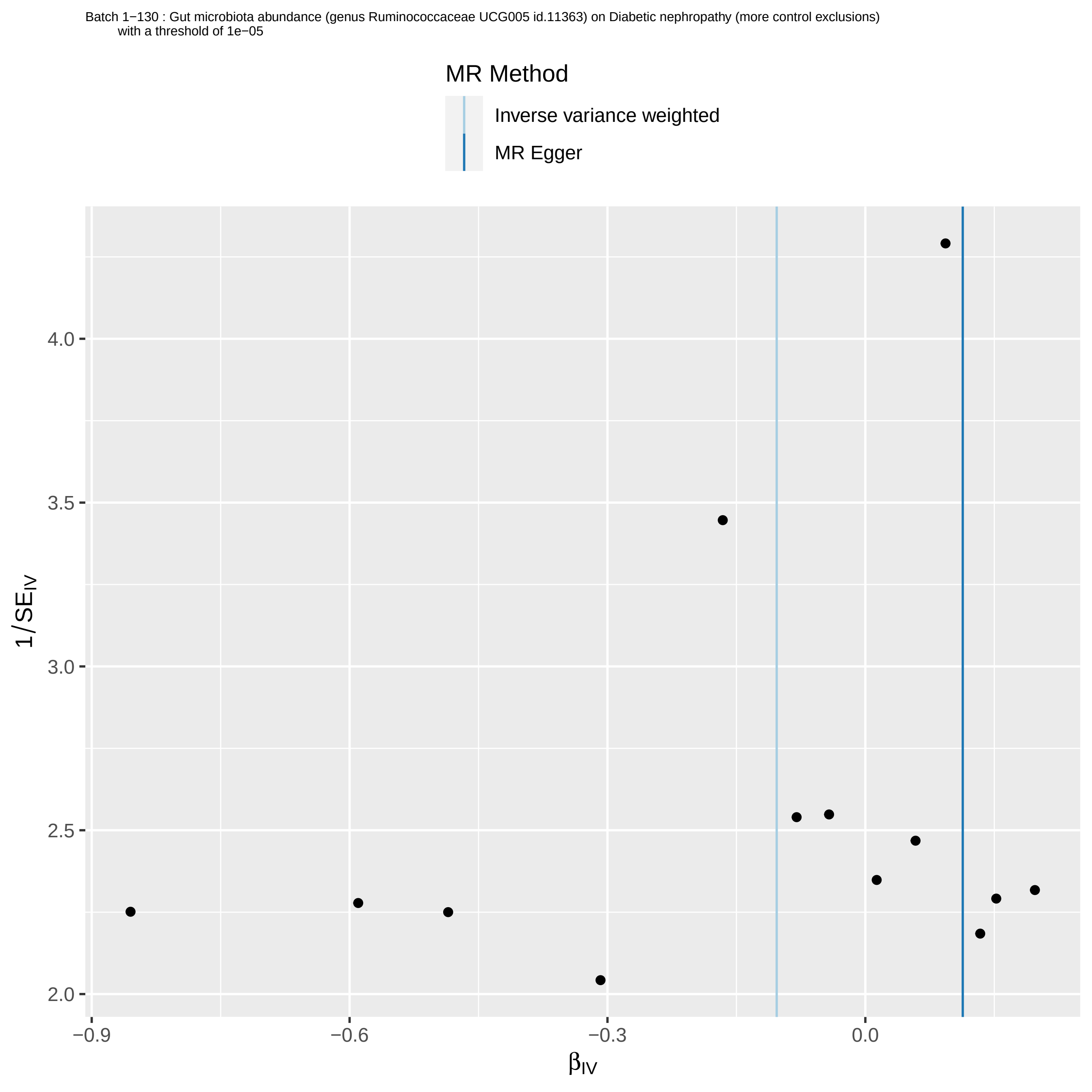

Batch 1−130 : Gut microbiota abundance (genus Ruminococcaceae UCG005 id.11363) on Diabetic nephropathy (more control exclusions)
with a threshold of 1e−05
MR Method
Inverse variance weighted
MR Egger
4.0
3.5
3.0
2.5
2.0
1/SE$_{IV}$
−0.9
−0.6
−0.3
0.0
β_{IV}

Batch 1−131 : Gut microbiota abundance (genus Ruminococcaceae UCG009 id.11366) on Diabetic nephropathy (more control exclusions) with a threshold of 1e−05

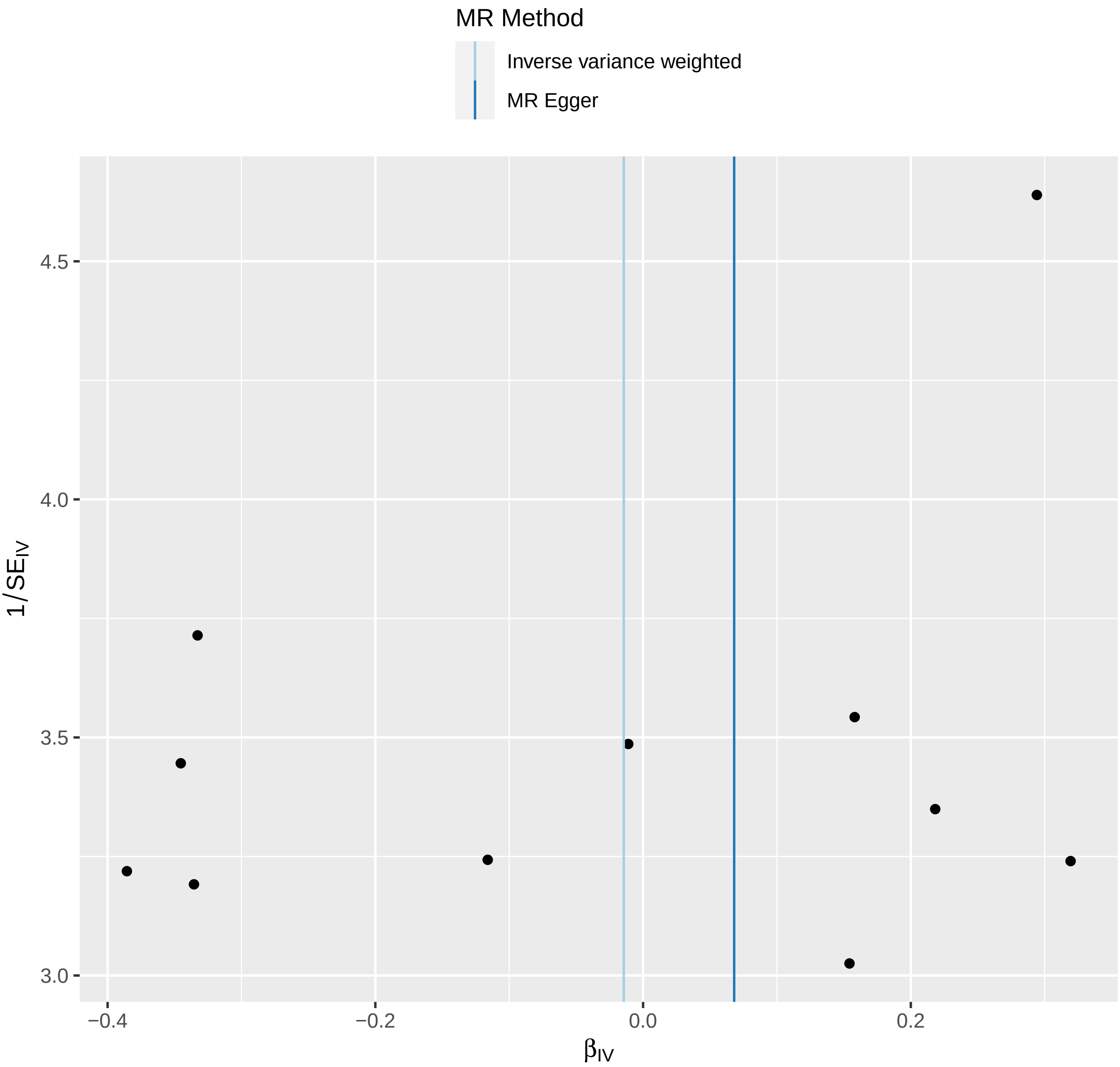

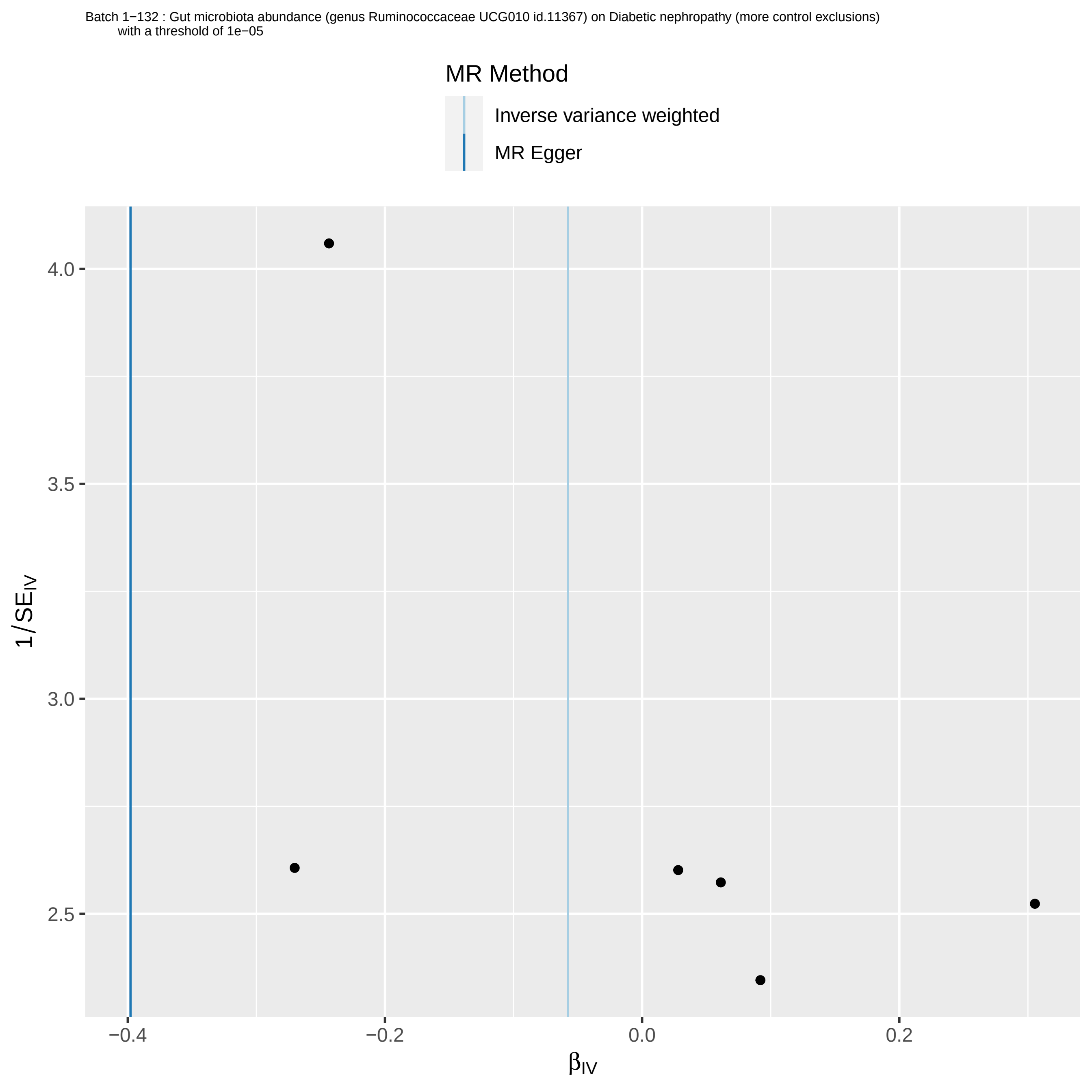

Batch 1−132 : Gut microbiota abundance (genus Ruminococcaceae UCG010 id.11367) on Diabetic nephropathy (more control exclusions) with a threshold of 1e−05
MR Method
Inverse variance weighted
MR Egger
4.0
3.5
3.0
2.5
−0.4
−0.2
0.0
0.2
$1/SE_{IV}$
β_{IV}

Batch 1−133 : Gut microbiota abundance (genus Ruminococcaceae UCG011 id.11368) on Diabetic nephropathy (more control exclusions) with a threshold of 1e−05

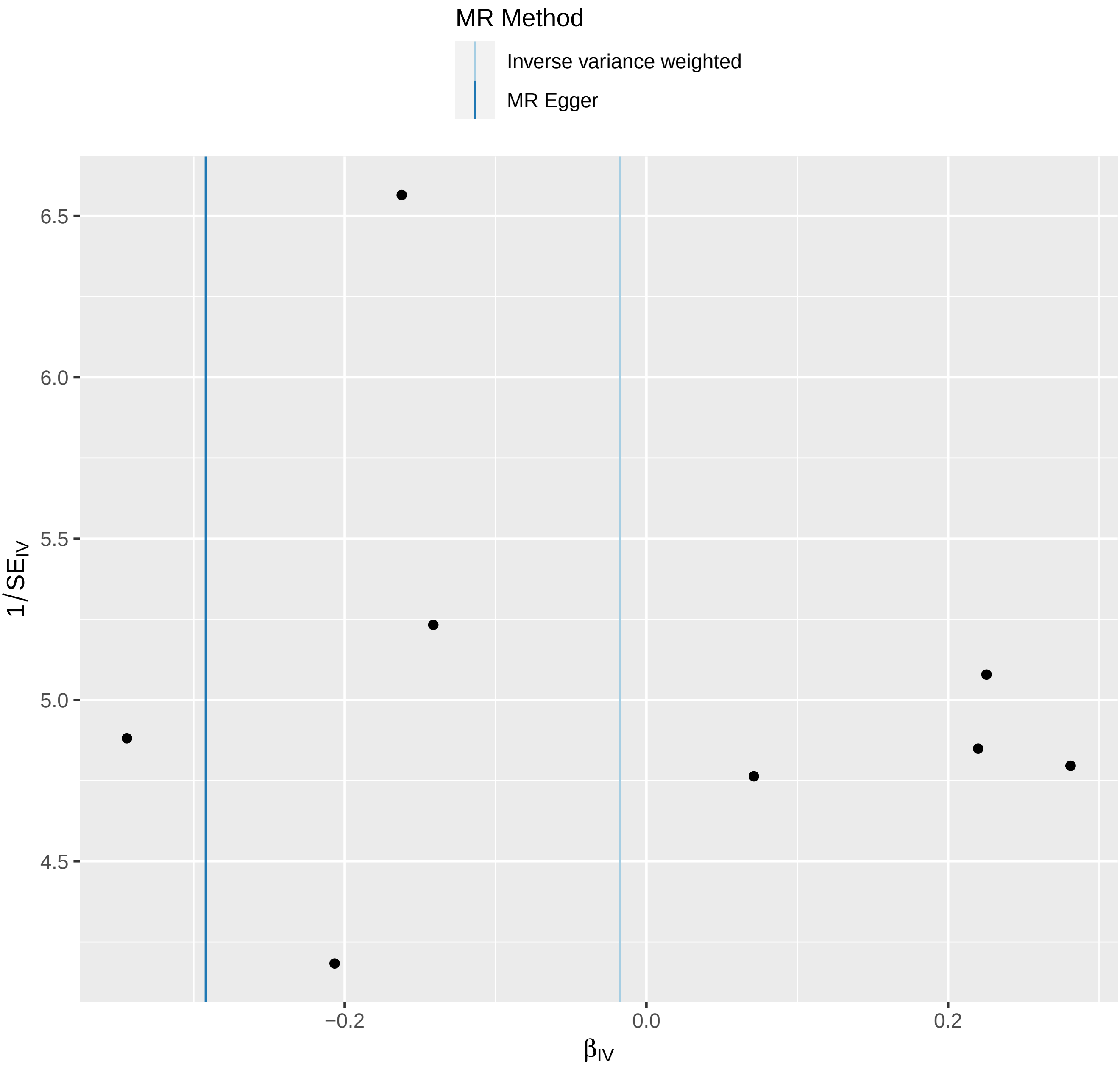

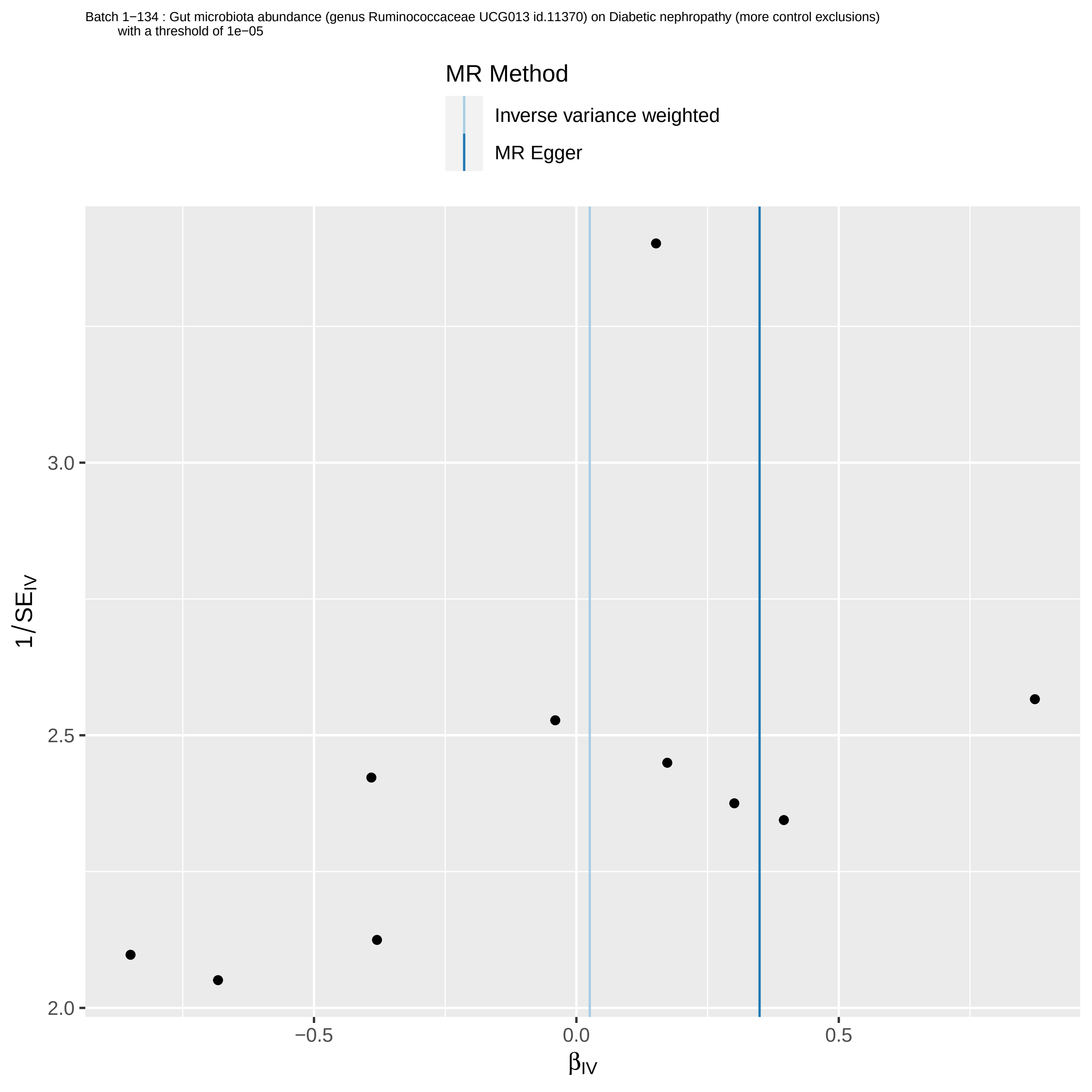

Batch 1−134 : Gut microbiota abundance (genus Ruminococcaceae UCG013 id.11370) on Diabetic nephropathy (more control exclusions)
with a threshold of 1e−05
MR Method
Inverse variance weighted
MR Egger
3.0
2.5
2.0
1/SE$_{IV}$
−0.5
0.0
0.5
β$_{IV}$

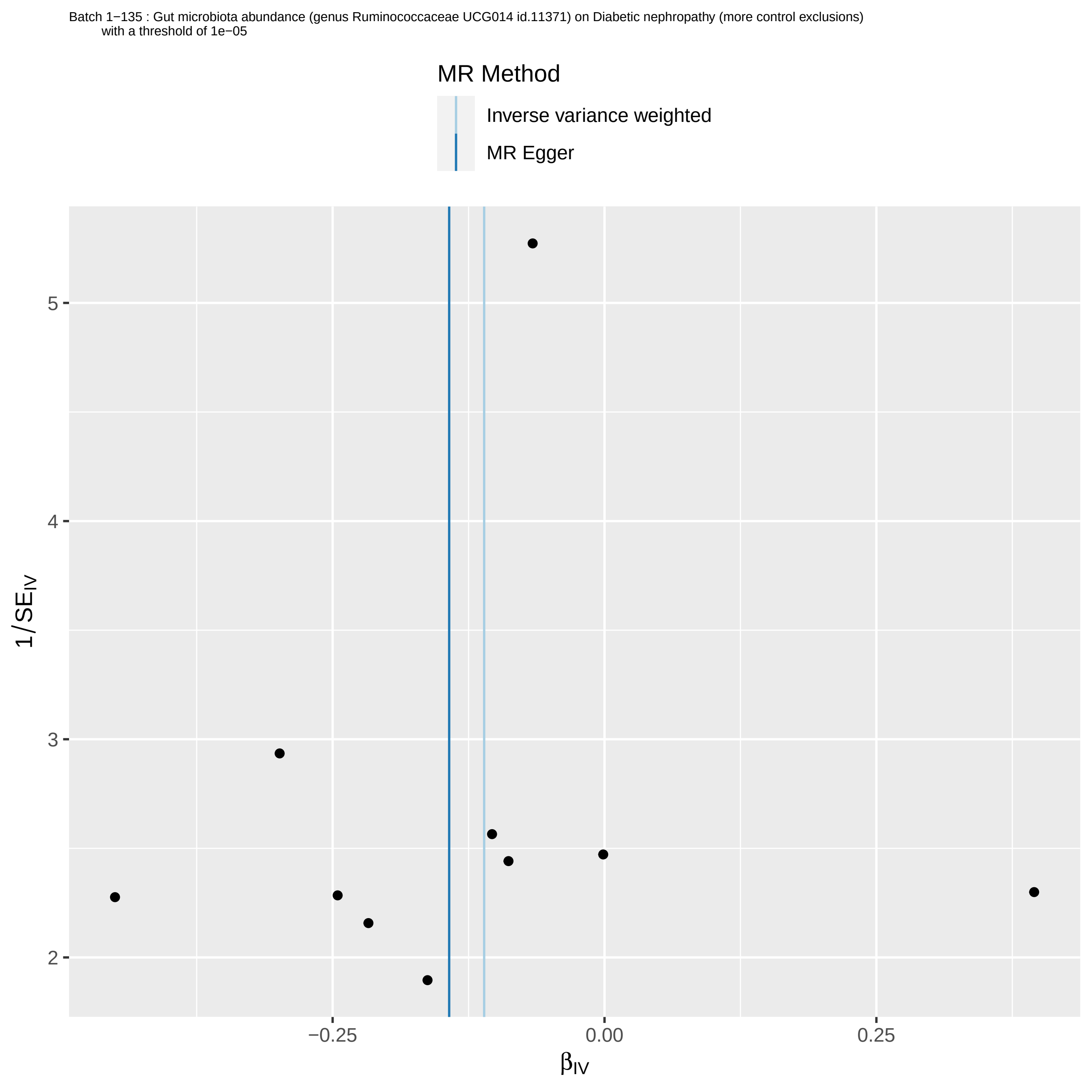

Batch 1−135 : Gut microbiota abundance (genus Ruminococcaceae UCG014 id.11371) on Diabetic nephropathy (more control exclusions)
with a threshold of 1e−05
MR Method
Inverse variance weighted
MR Egger
5
4
3
2
1/SE_IV
−0.25
0.00
0.25
β_IV

Batch 1−136 : Gut microbiota abundance (genus Ruminococcus gauvreauii group id.11342) on Diabetic nephropathy (more control exclusions) with a threshold of 1e−05

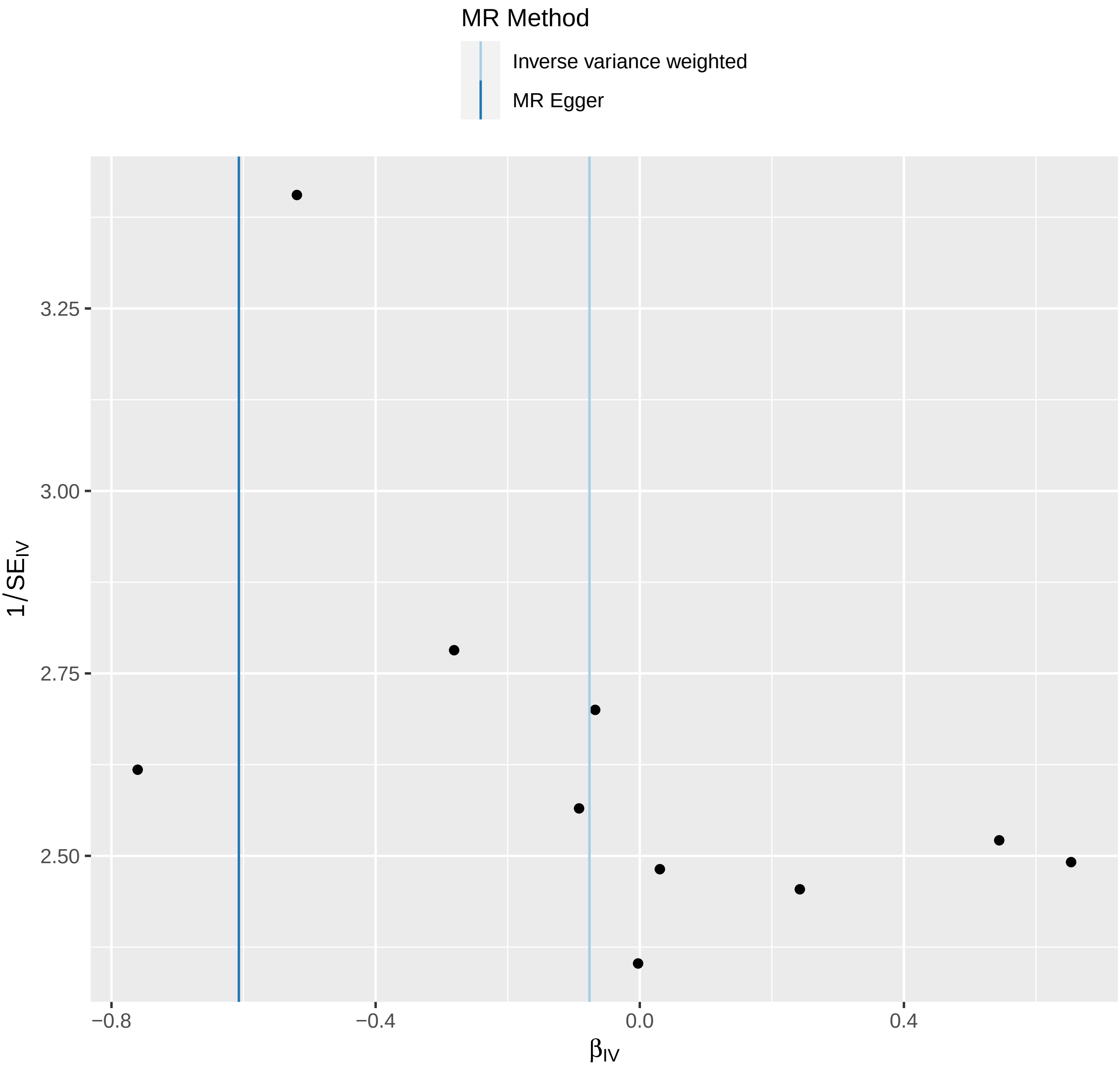

Batch 1−137 : Gut microbiota abundance (genus Ruminococcus gnavus group id.14376) on Diabetic nephropathy (more control exclusions) with a threshold of 1e−05

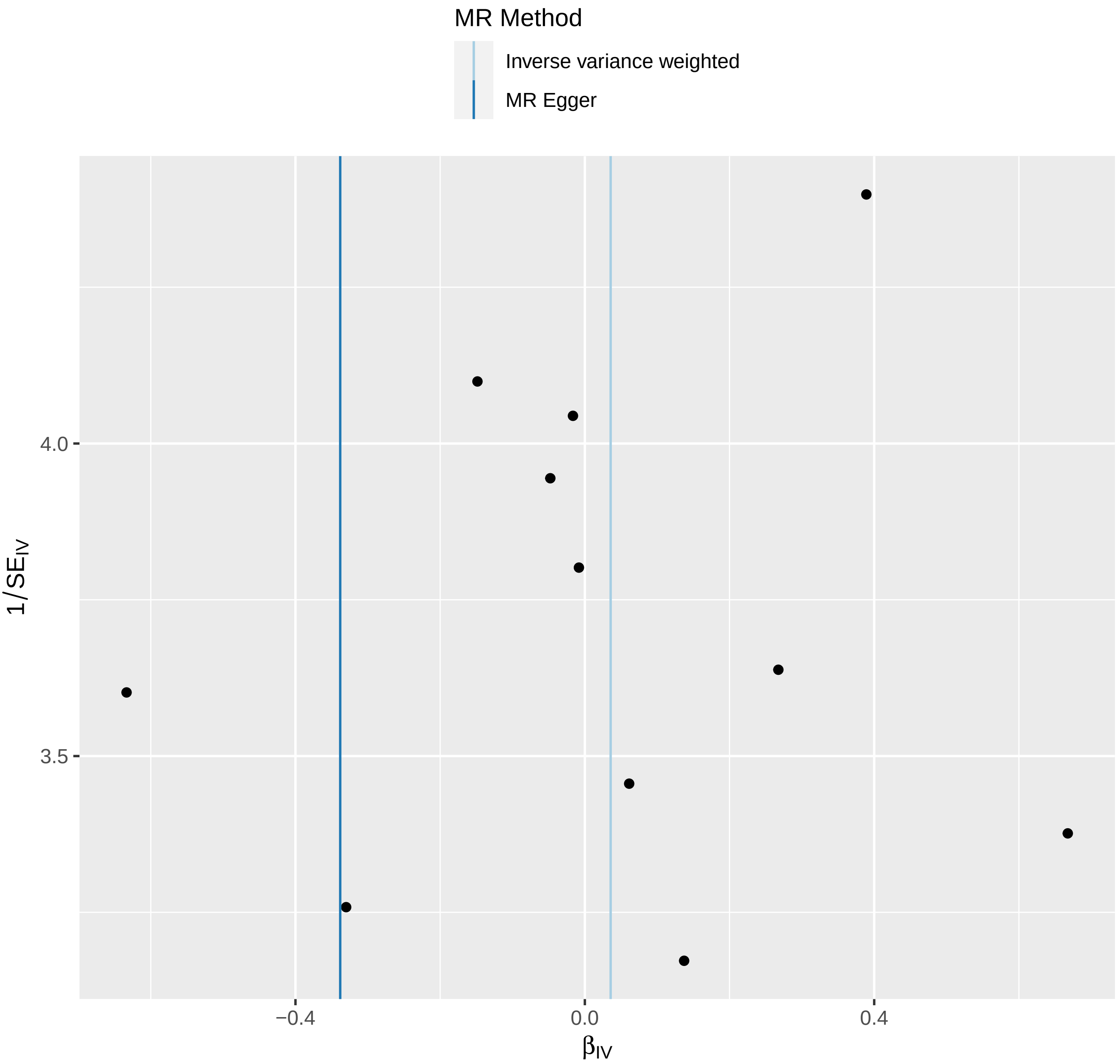

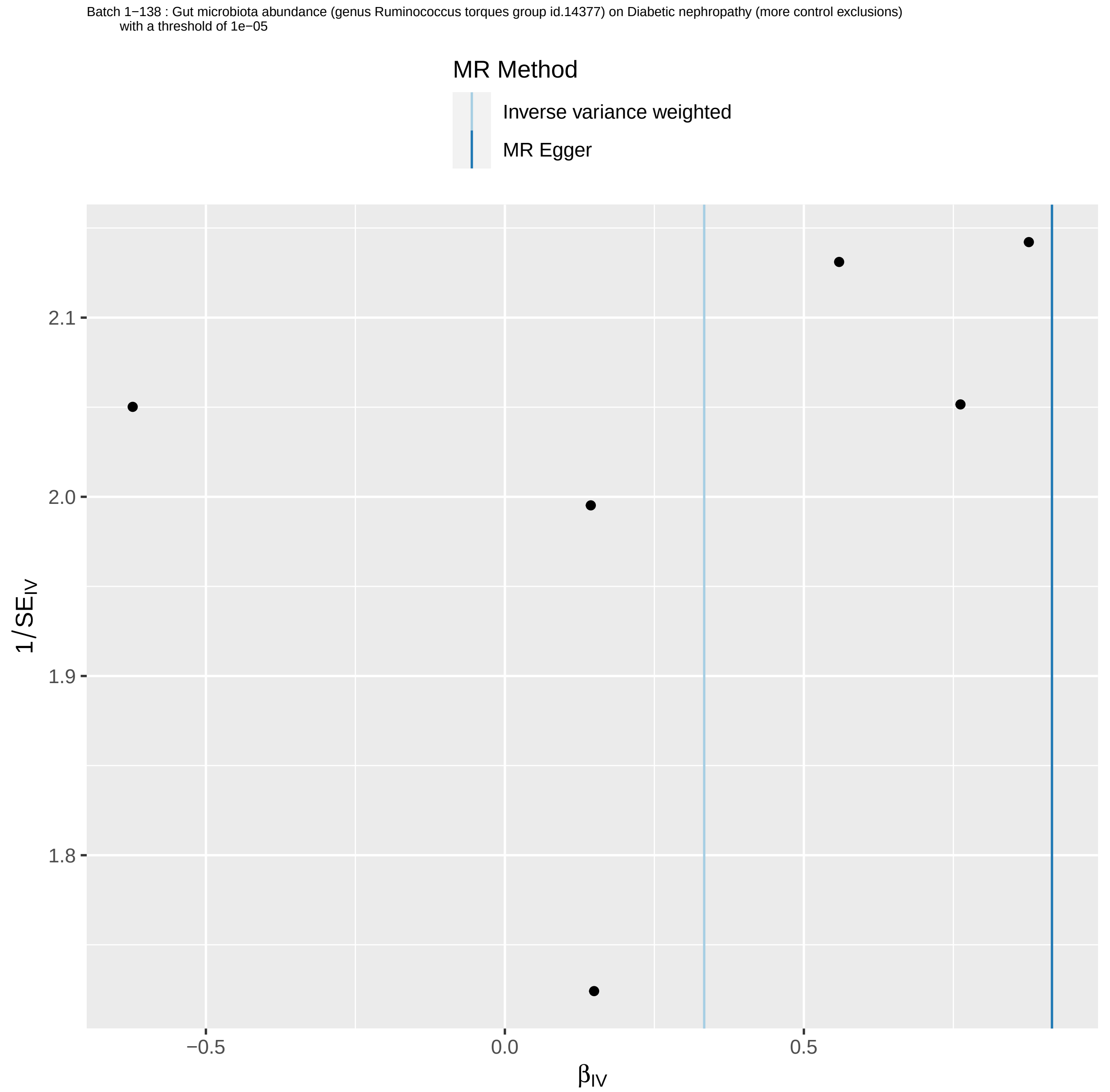
Batch 1−138 : Gut microbiota abundance (genus Ruminococcus torques group id.14377) on Diabetic nephropathy (more control exclusions) with a threshold of 1e−05
MR Method
Inverse variance weighted
MR Egger
2.1
2.0
1.9
1.8
1/SE_IV
−0.5
0.0
0.5
β_IV

Batch 1−139 : Gut microbiota abundance (genus Ruminococcus1 id.11373) on Diabetic nephropathy (more control exclusions) with a threshold of 1e−05

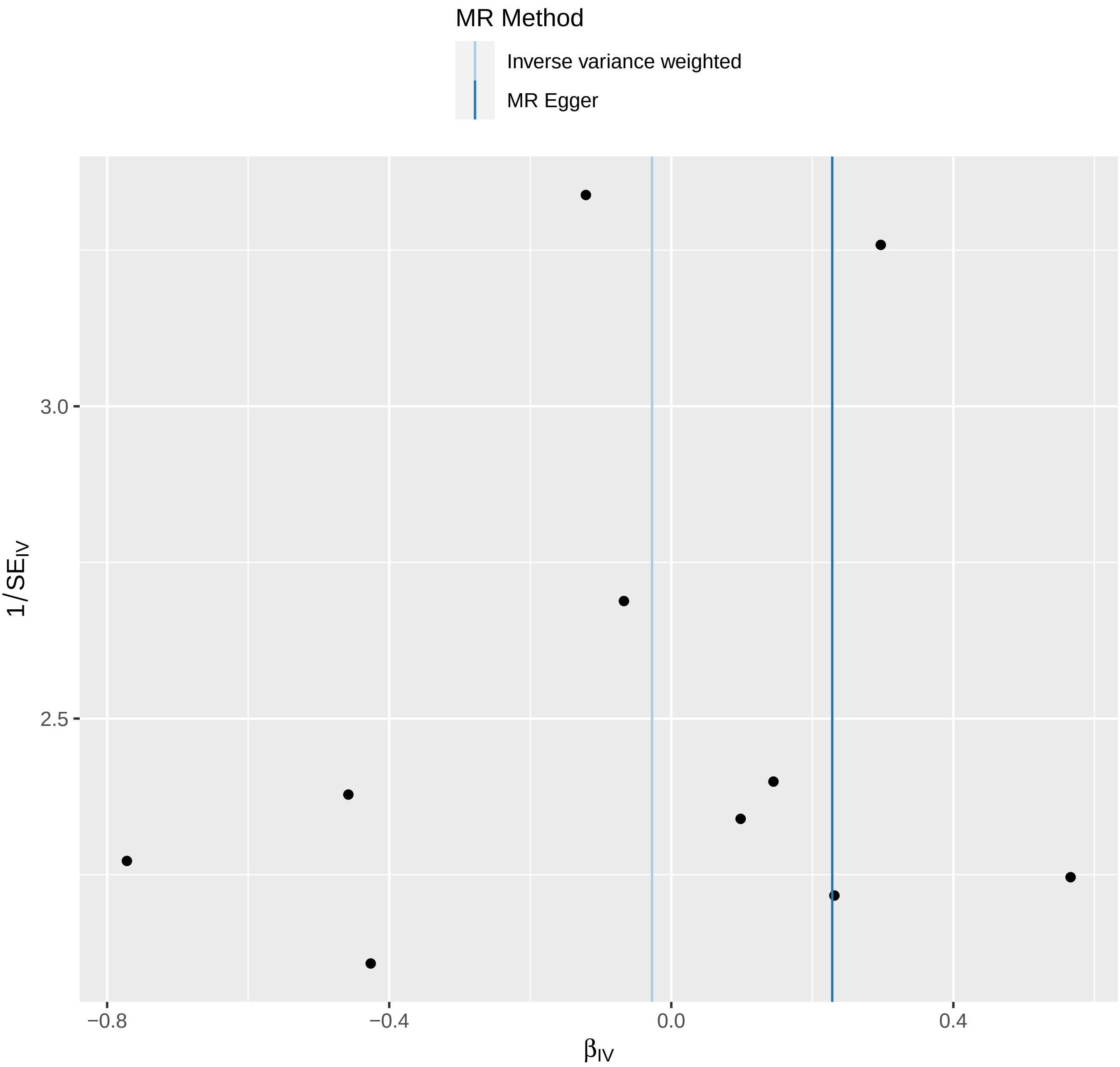

Batch 1−140 : Gut microbiota abundance (genus Ruminococcus2 id.11374) on Diabetic nephropathy (more control exclusions) with a threshold of 1e−05

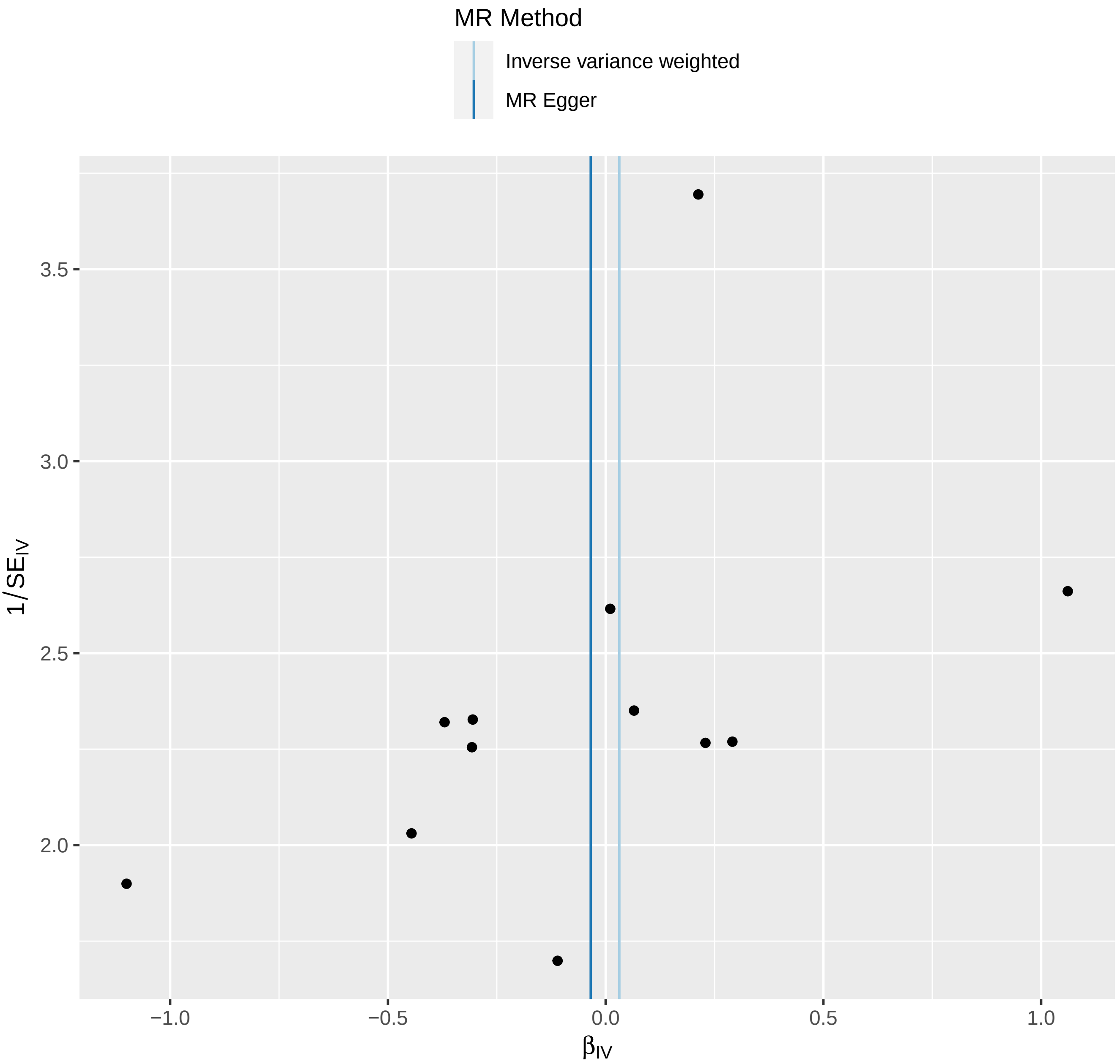

Batch 1−141 : Gut microbiota abundance (genus Sellimonas id.14369) on Diabetic nephropathy (more control exclusions) with a threshold of 1e−05

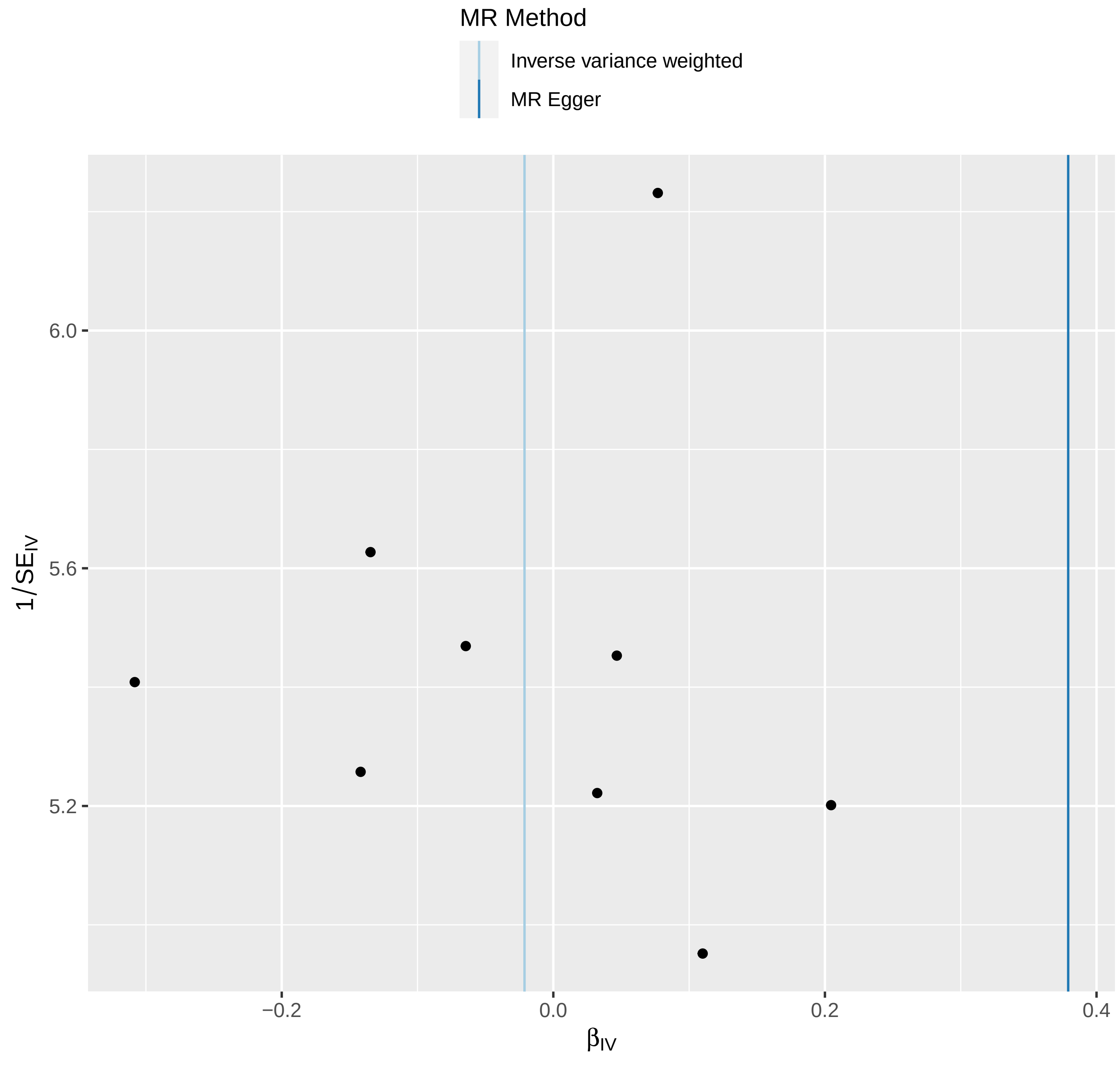

Batch 1−142 : Gut microbiota abundance (genus Senegalimassilia id.11160) on Diabetic nephropathy (more control exclusions) with a threshold of 1e−05

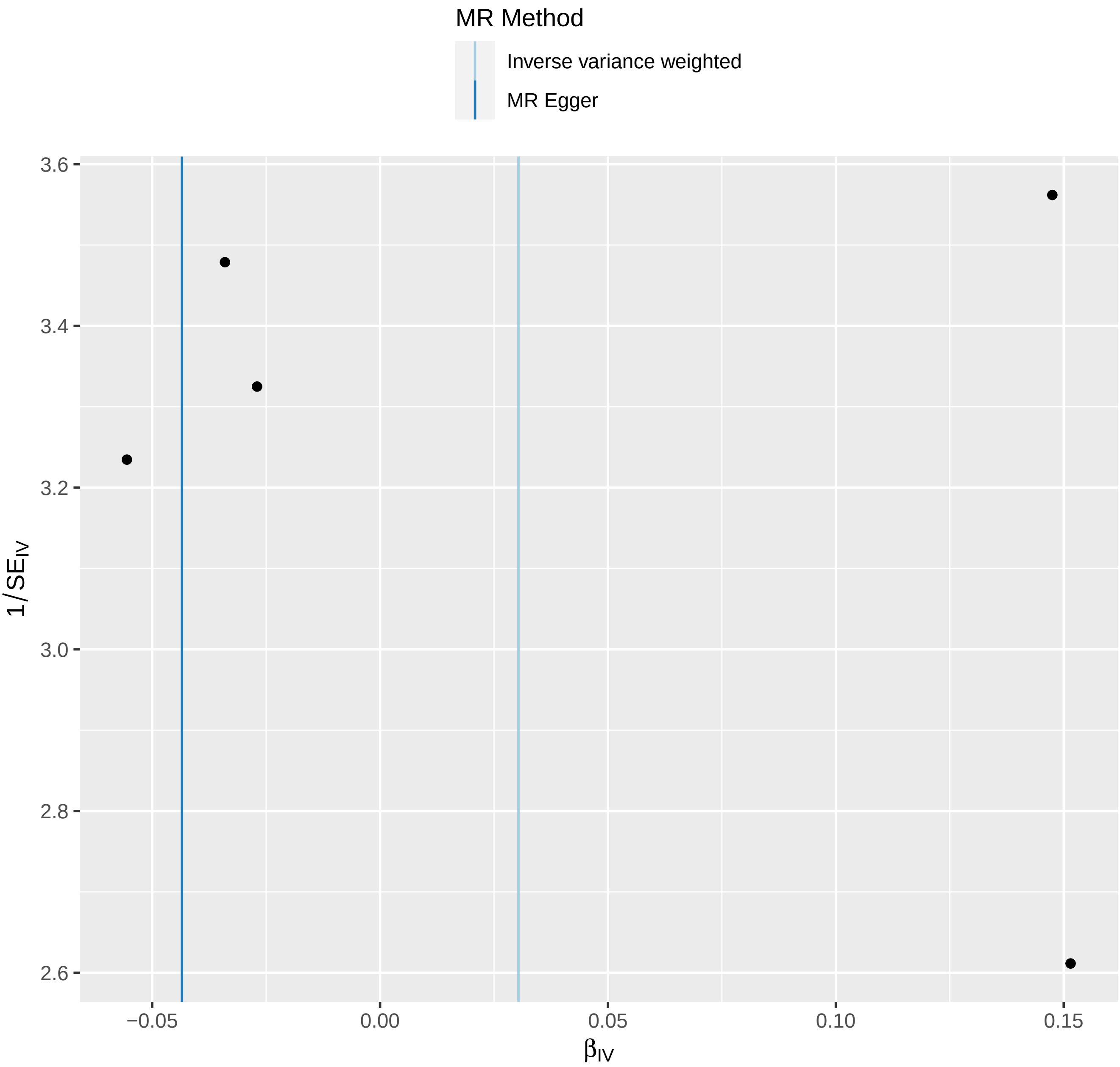

Batch 1−143 : Gut microbiota abundance (genus Slackia id.825) on Diabetic nephropathy (more control exclusions) with a threshold of 1e−05

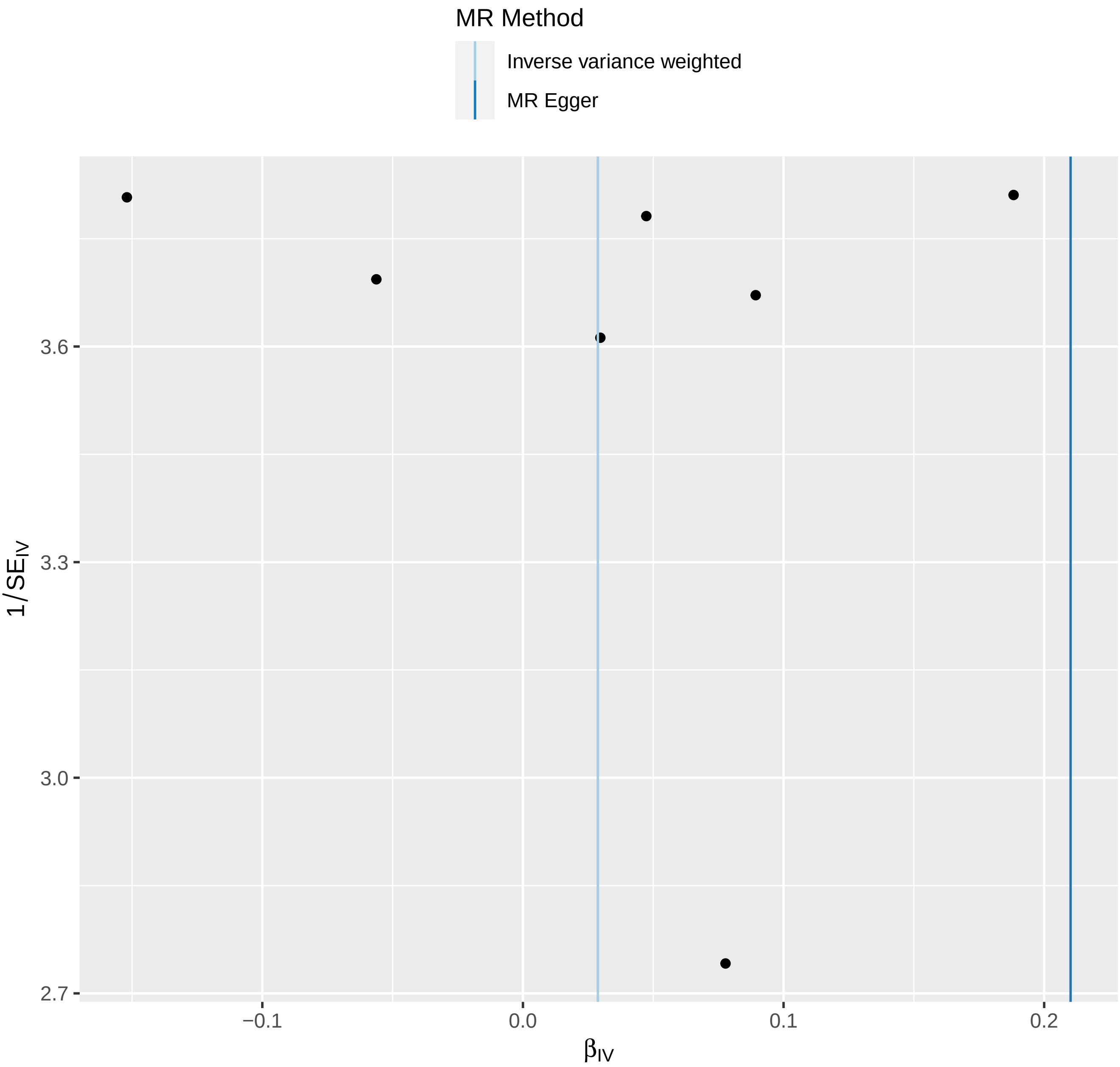

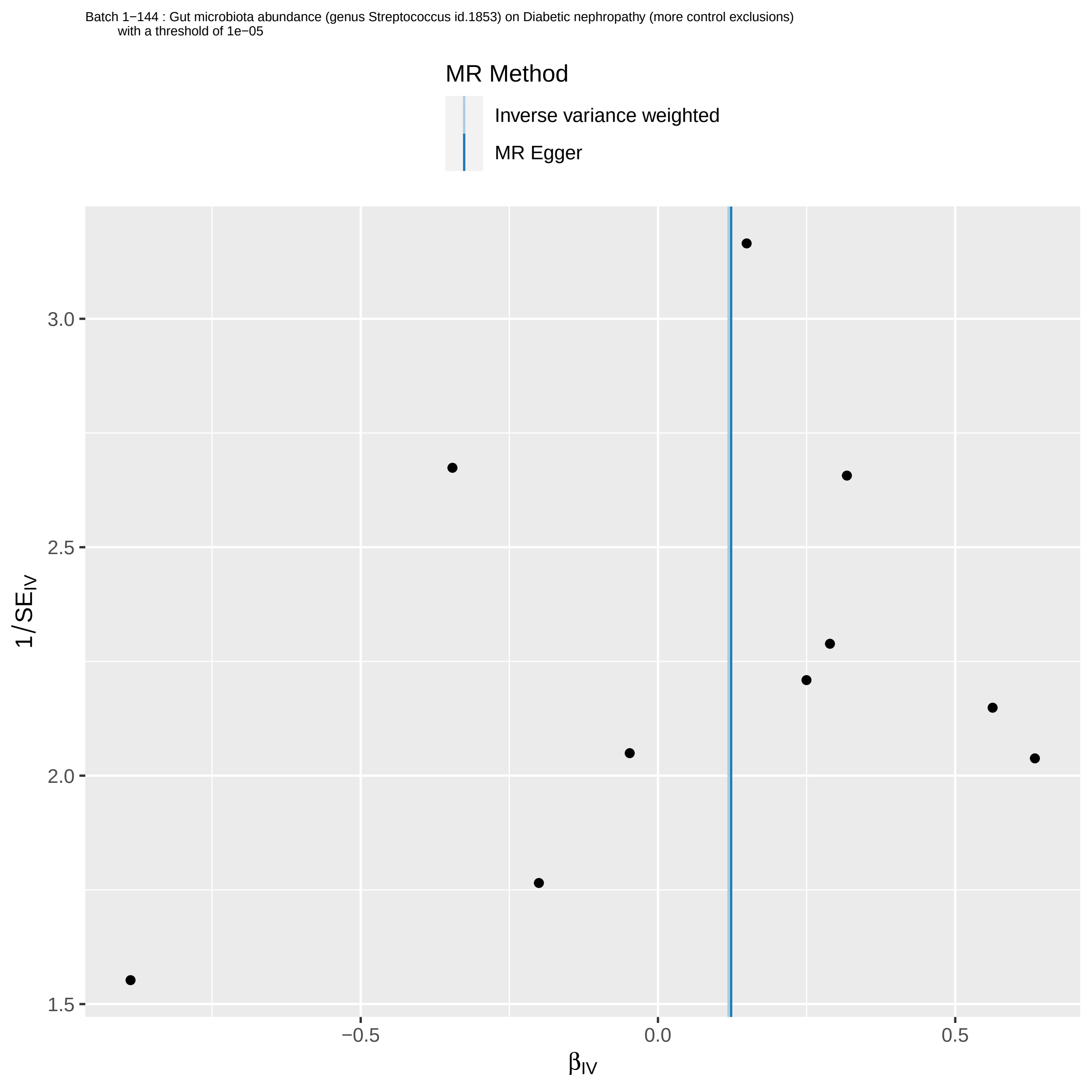

Batch 1−144 : Gut microbiota abundance (genus Streptococcus id.1853) on Diabetic nephropathy (more control exclusions) with a threshold of 1e−05
MR Method
Inverse variance weighted
MR Egger
3.0
2.5
2.0
1.5
1/SE_IV
−0.5
0.0
0.5
β_IV

Batch 1−145 : Gut microbiota abundance (genus Subdoligranulum id.2070) on Diabetic nephropathy (more control exclusions) with a threshold of 1e−05

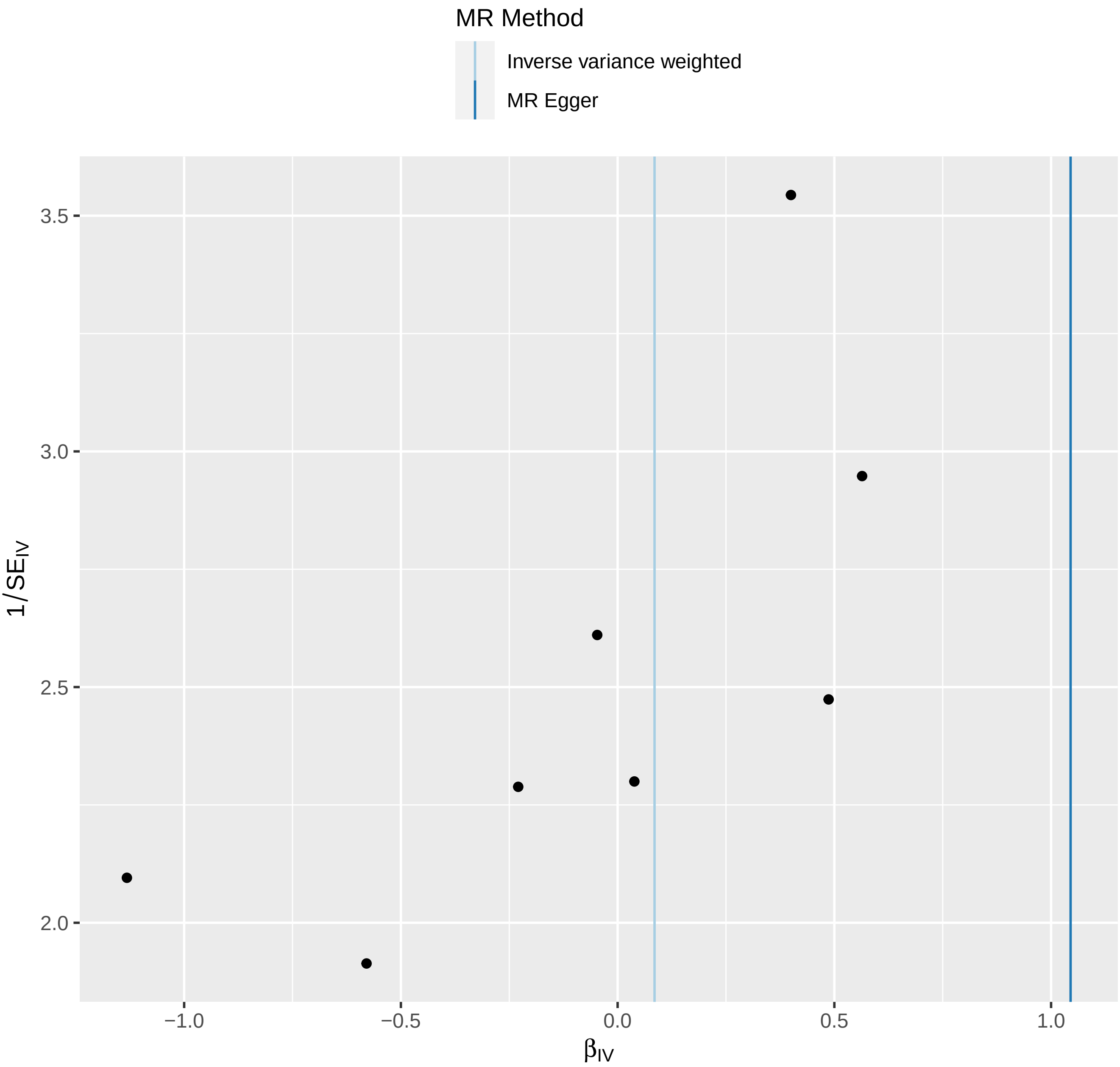

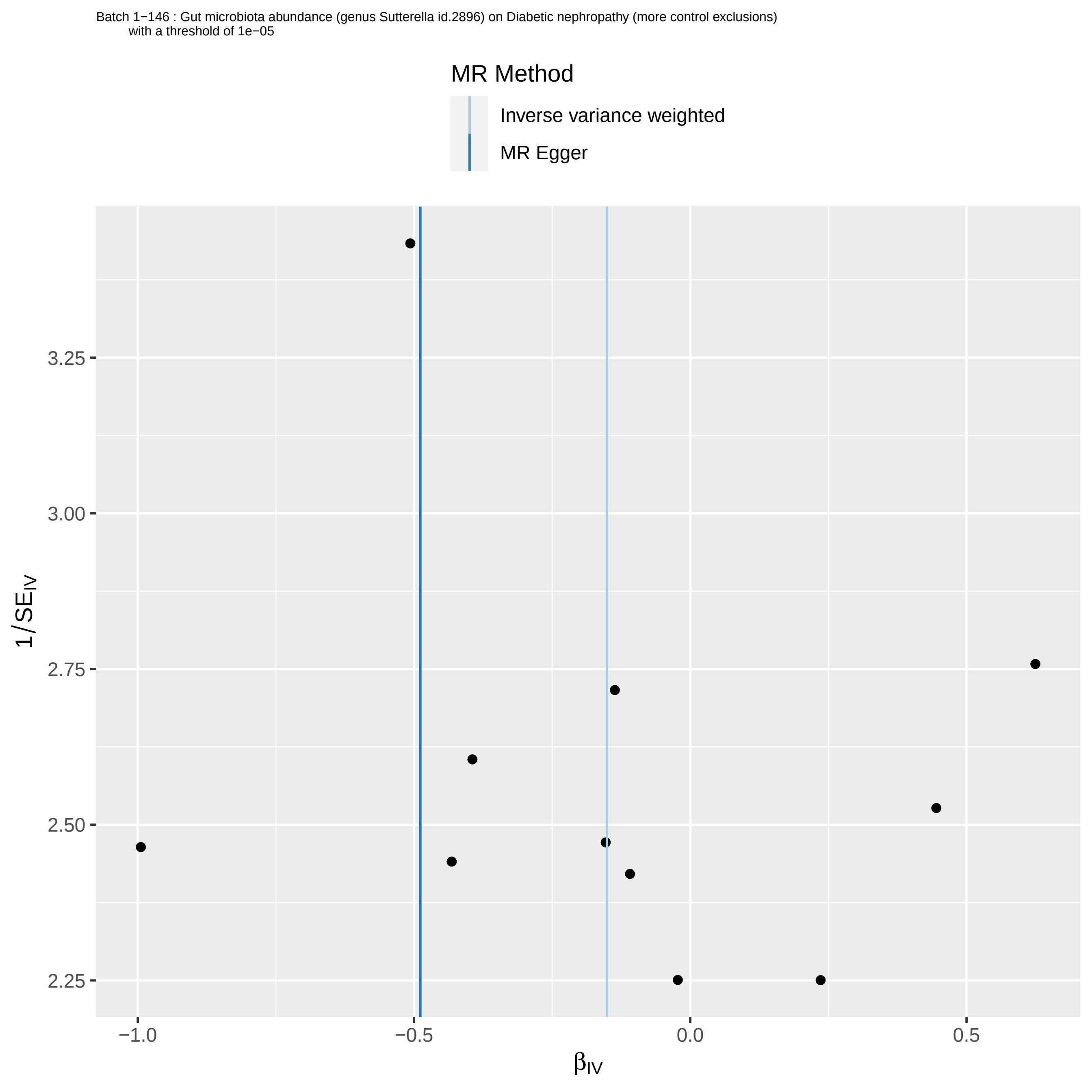

Batch 1−146 : Gut microbiota abundance (genus Sutterella id.2896) on Diabetic nephropathy (more control exclusions)
with a threshold of 1e−05
MR Method
Inverse variance weighted
MR Egger
1/SE_IV
3.25
3.00
2.75
2.50
2.25
−1.0
−0.5
0.0
0.5
β_IV

Batch 1−147 : Gut microbiota abundance (genus Terrisporobacter id.11348) on Diabetic nephropathy (more control exclusions) with a threshold of 1e−05

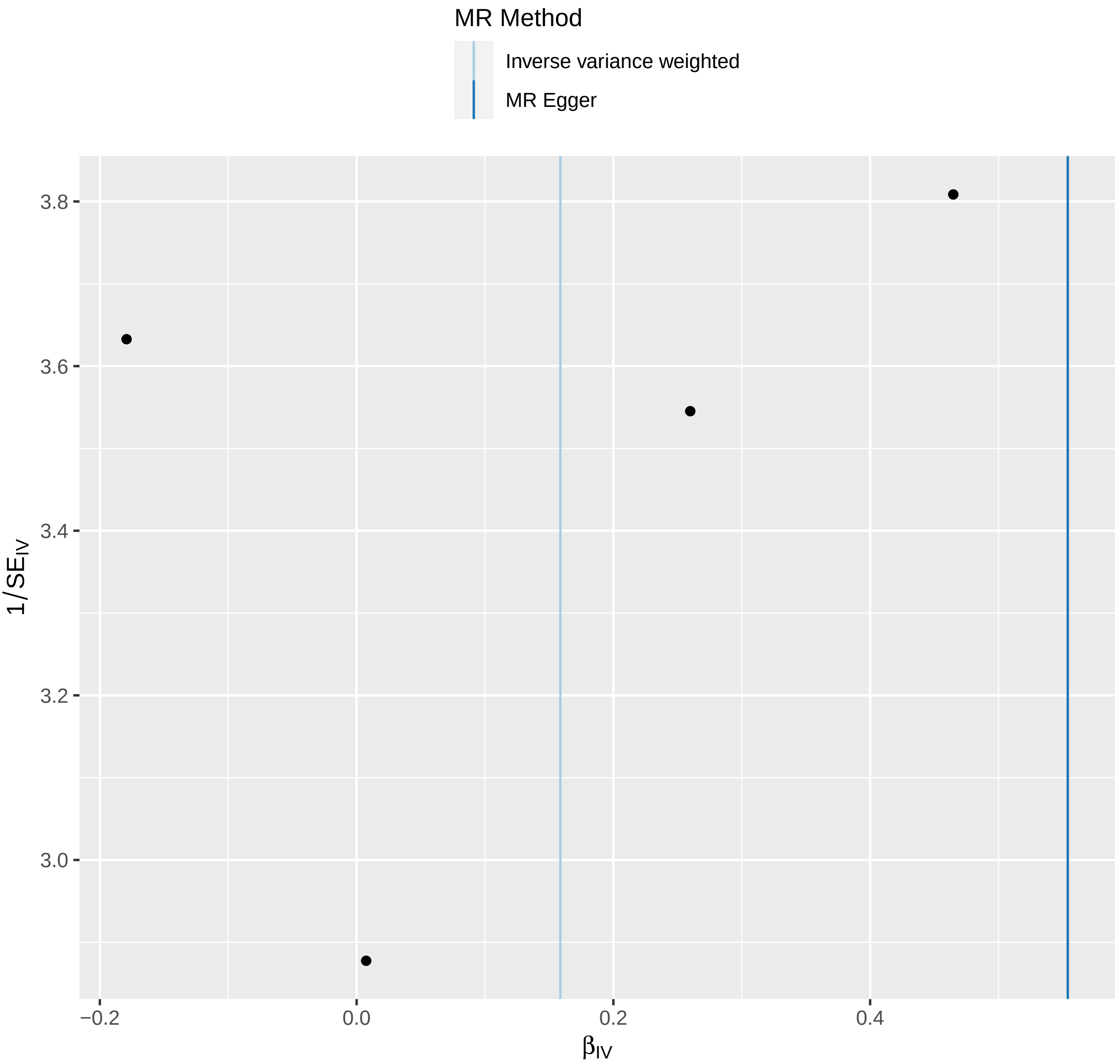

Batch 1−148 : Gut microbiota abundance (genus Turicibacter id.2162) on Diabetic nephropathy (more control exclusions) with a threshold of 1e−05

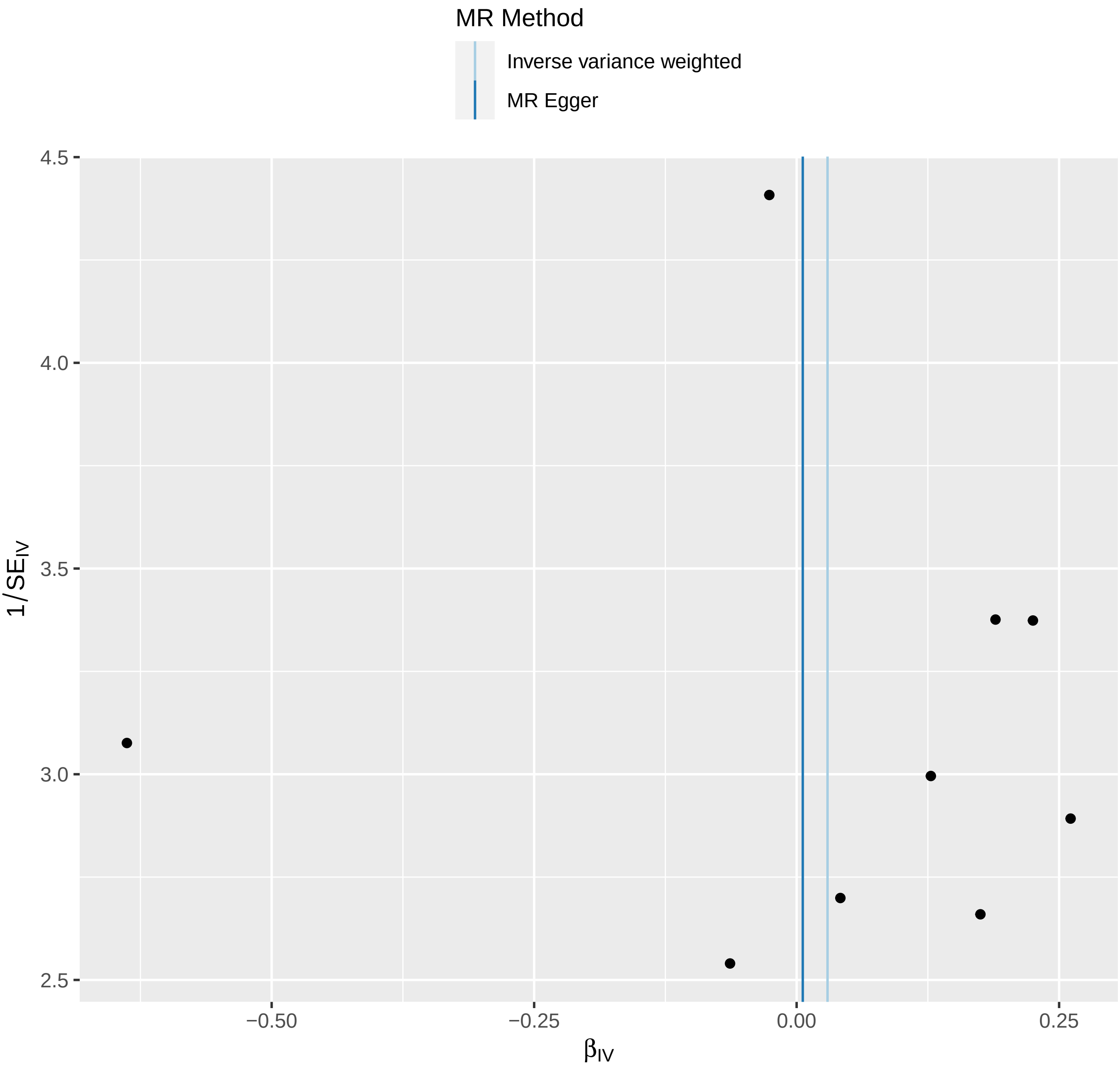

Batch 1−149 : Gut microbiota abundance (genus Tyzzerella3 id.11335) on Diabetic nephropathy (more control exclusions) with a threshold of 1e−05

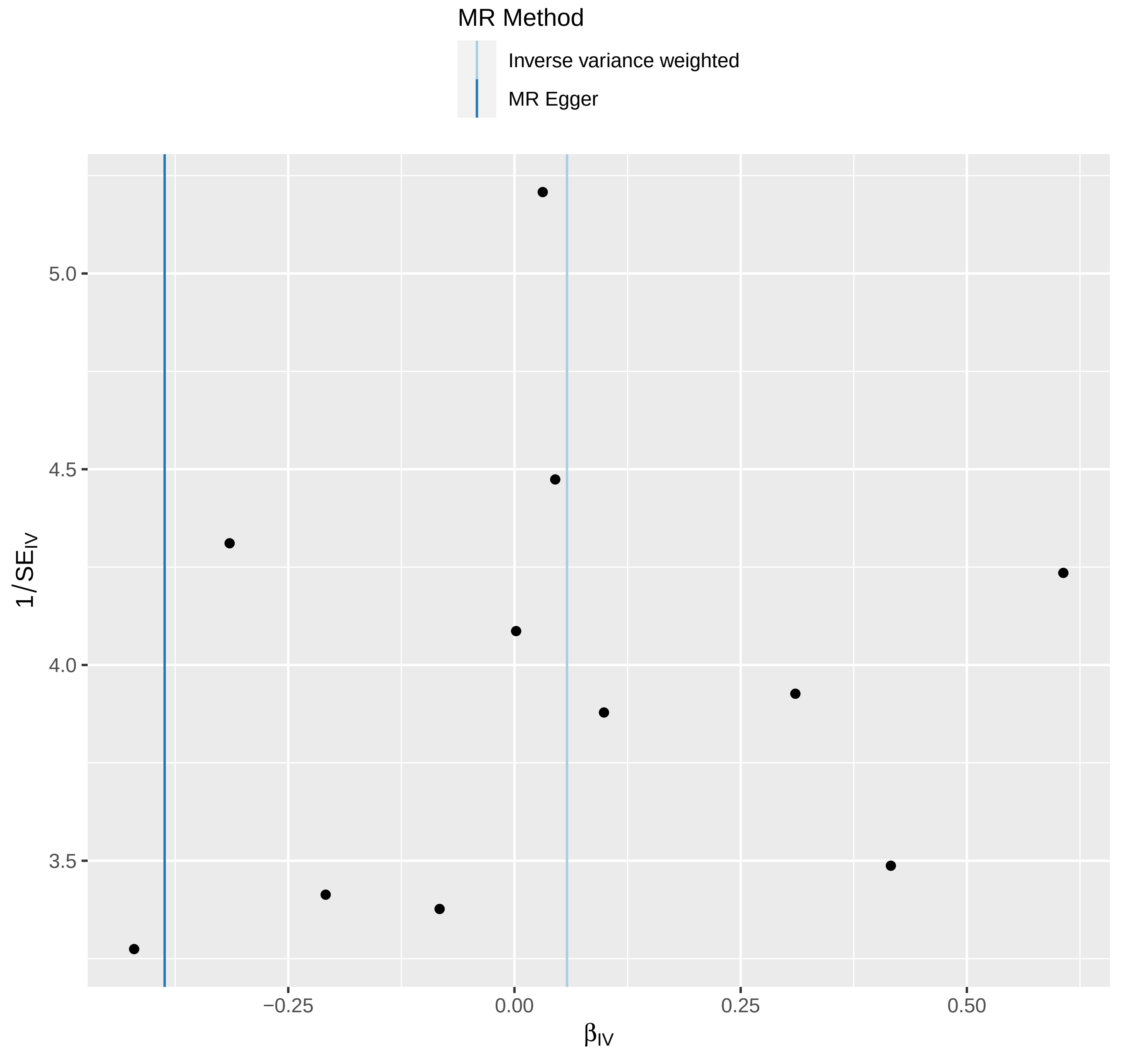

Batch 1−150 : Gut microbiota abundance (genus Veillonella id.2198) on Diabetic nephropathy (more control exclusions) with a threshold of 1e−05

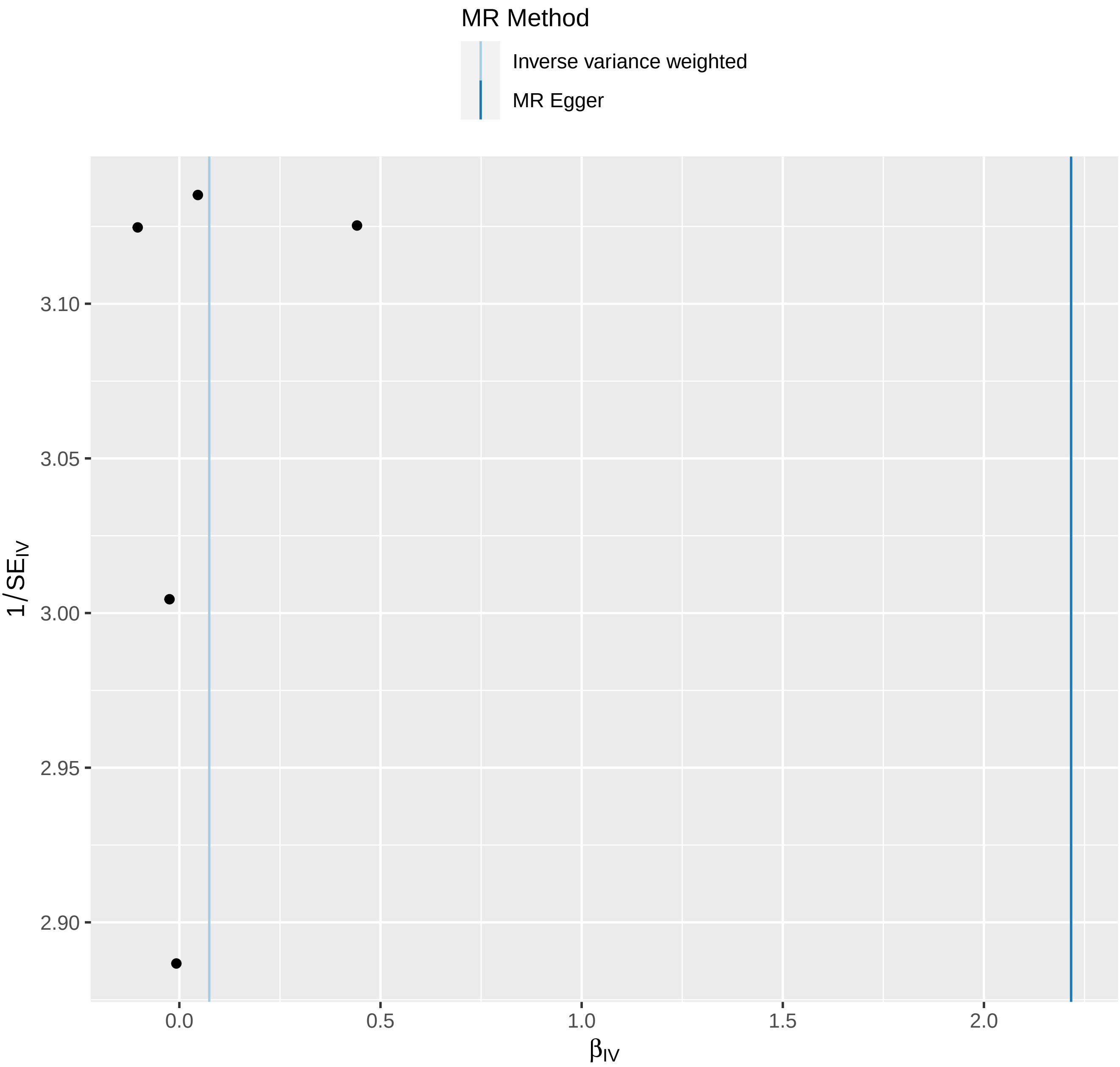

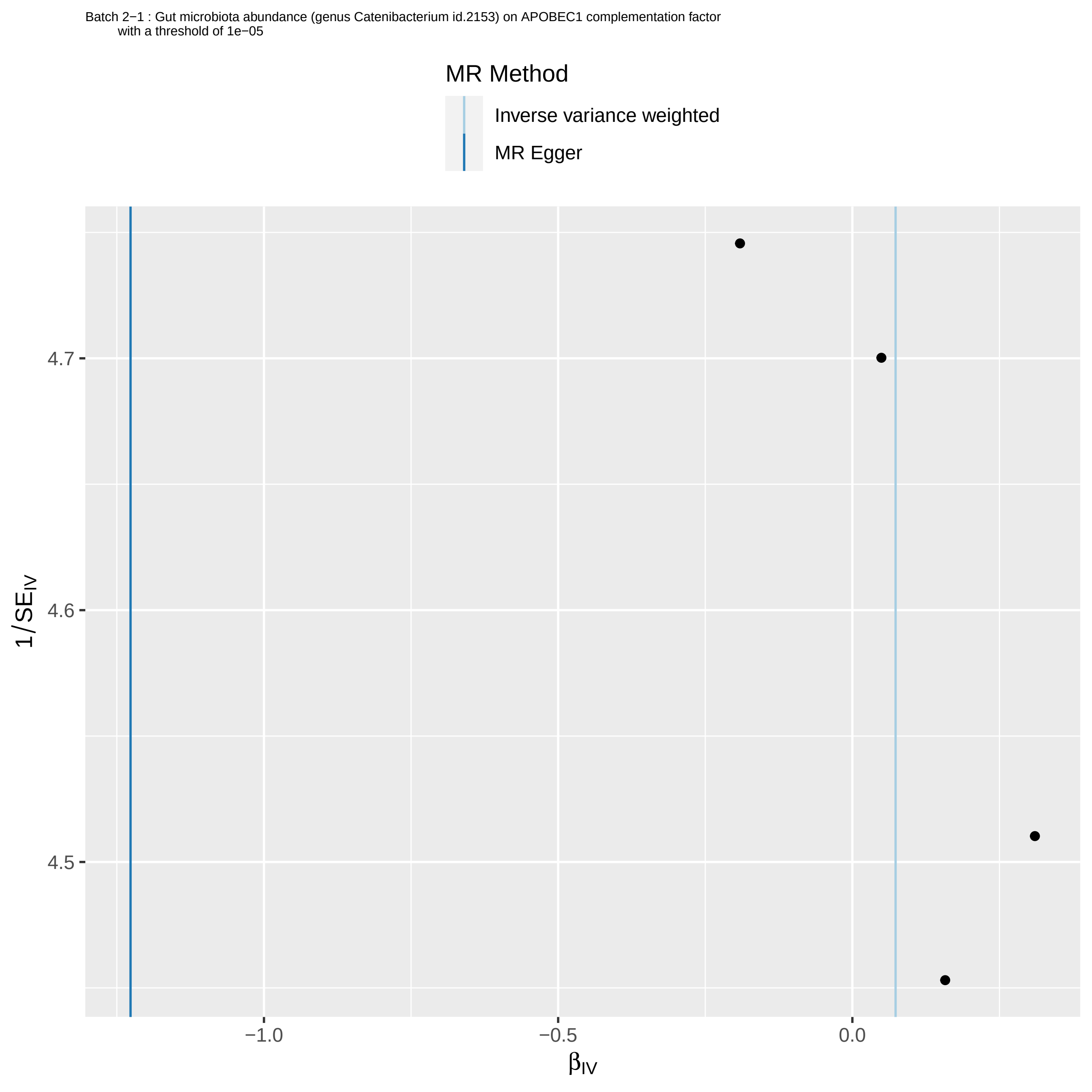

Batch 2−1 : Gut microbiota abundance (genus Catenibacterium id.2153) on APOBEC1 complementation factor
with a threshold of 1e−05
MR Method
Inverse variance weighted
MR Egger
4.7
4.6
4.5
1/SE_IV
−1.0
−0.5
0.0
β_IV

Batch 2−2 : Gut microbiota abundance (genus Parasutterella id.2892) on APOBEC1 complementation factor
with a threshold of 1e−05

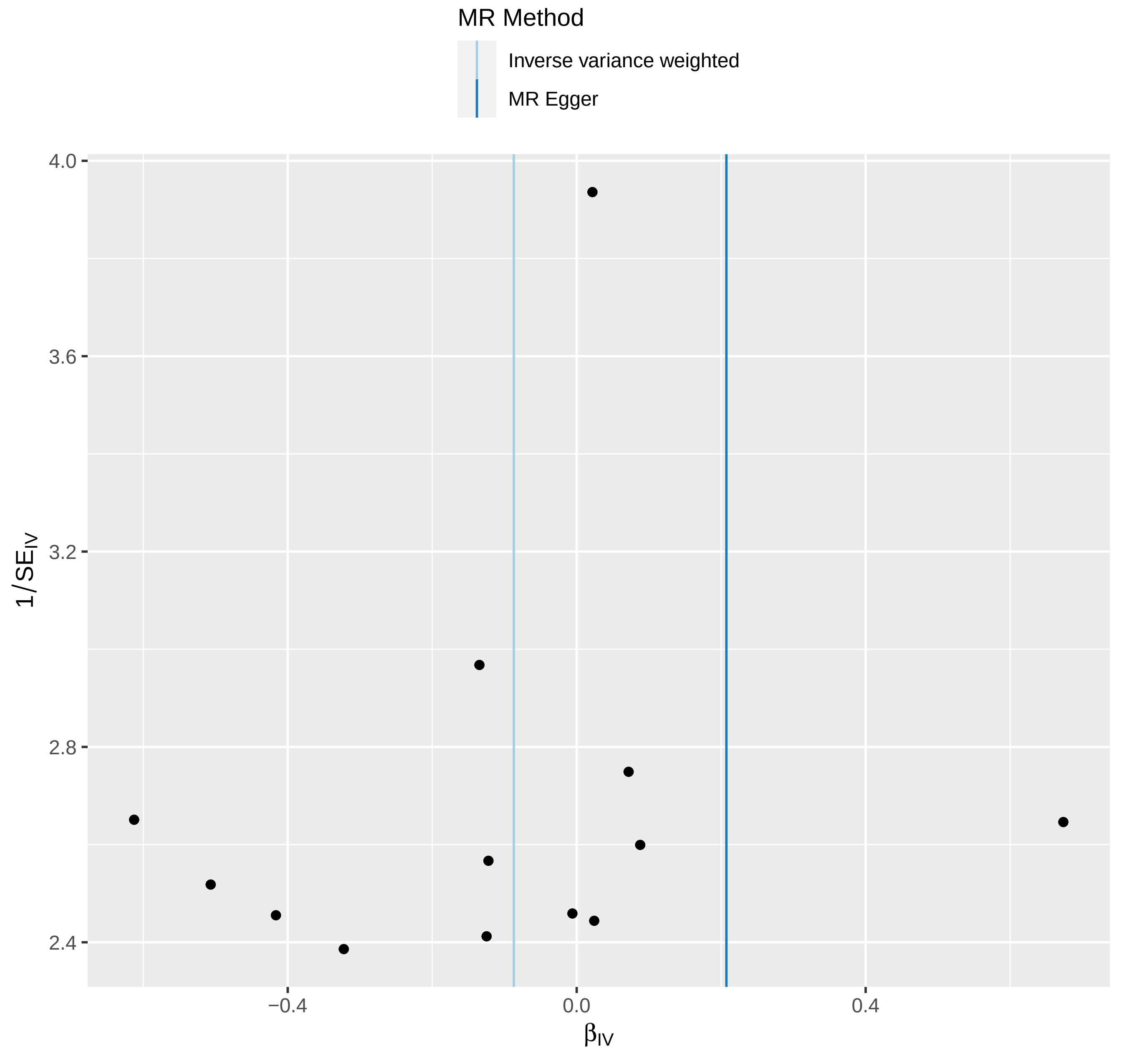

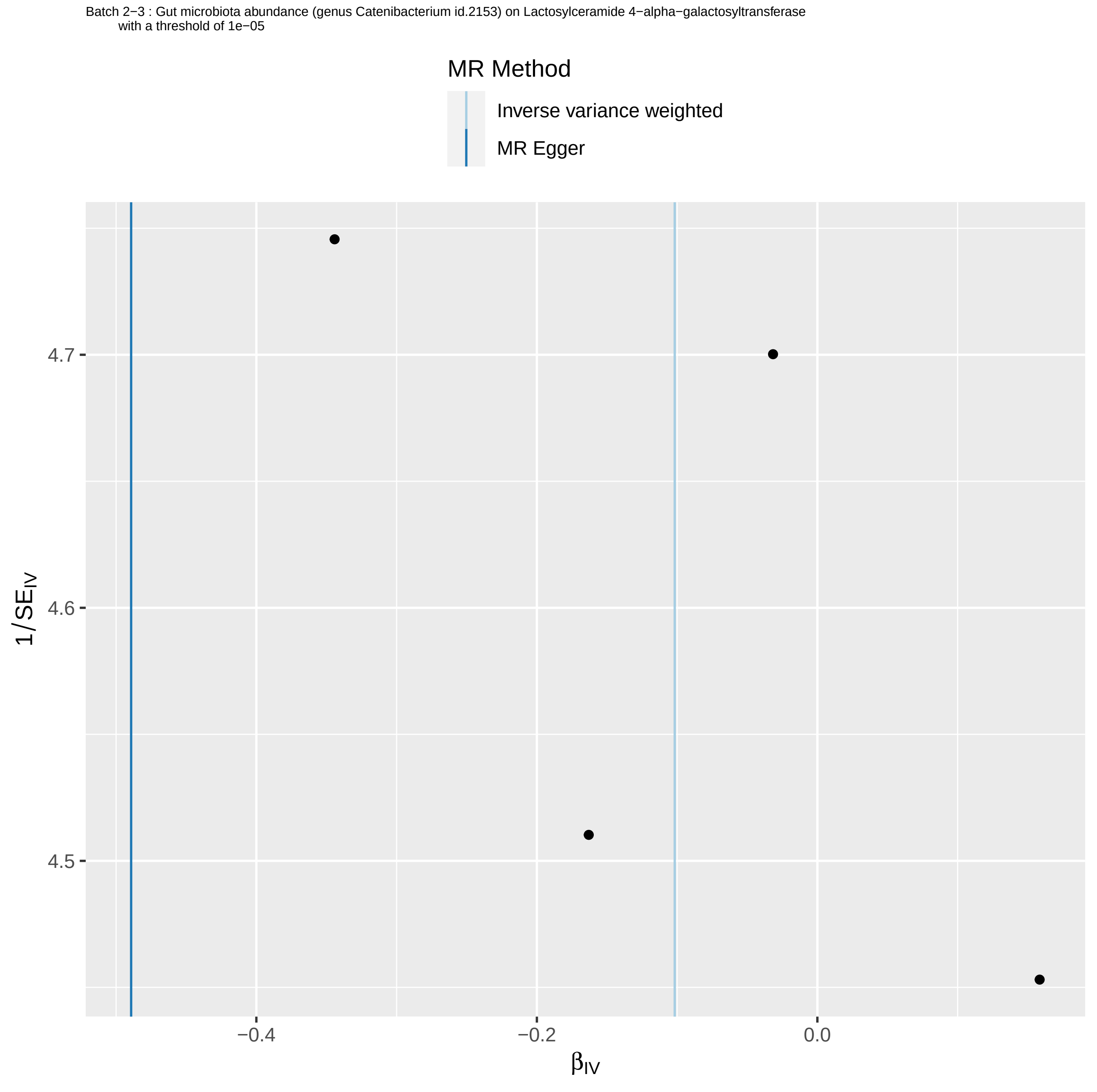
Batch 2−3 : Gut microbiota abundance (genus Catenibacterium id.2153) on Lactosylceramide 4−alpha−galactosyltransferase
with a threshold of 1e−05
MR Method
Inverse variance weighted
MR Egger
4.7
4.6
4.5
1/SEIV
−0.4
−0.2
0.0
βIV

Batch 2−4 : Gut microbiota abundance (genus Parasutterella id.2892) on Lactosylceramide 4−alpha−galactosyltransferase
with a threshold of 1e−05

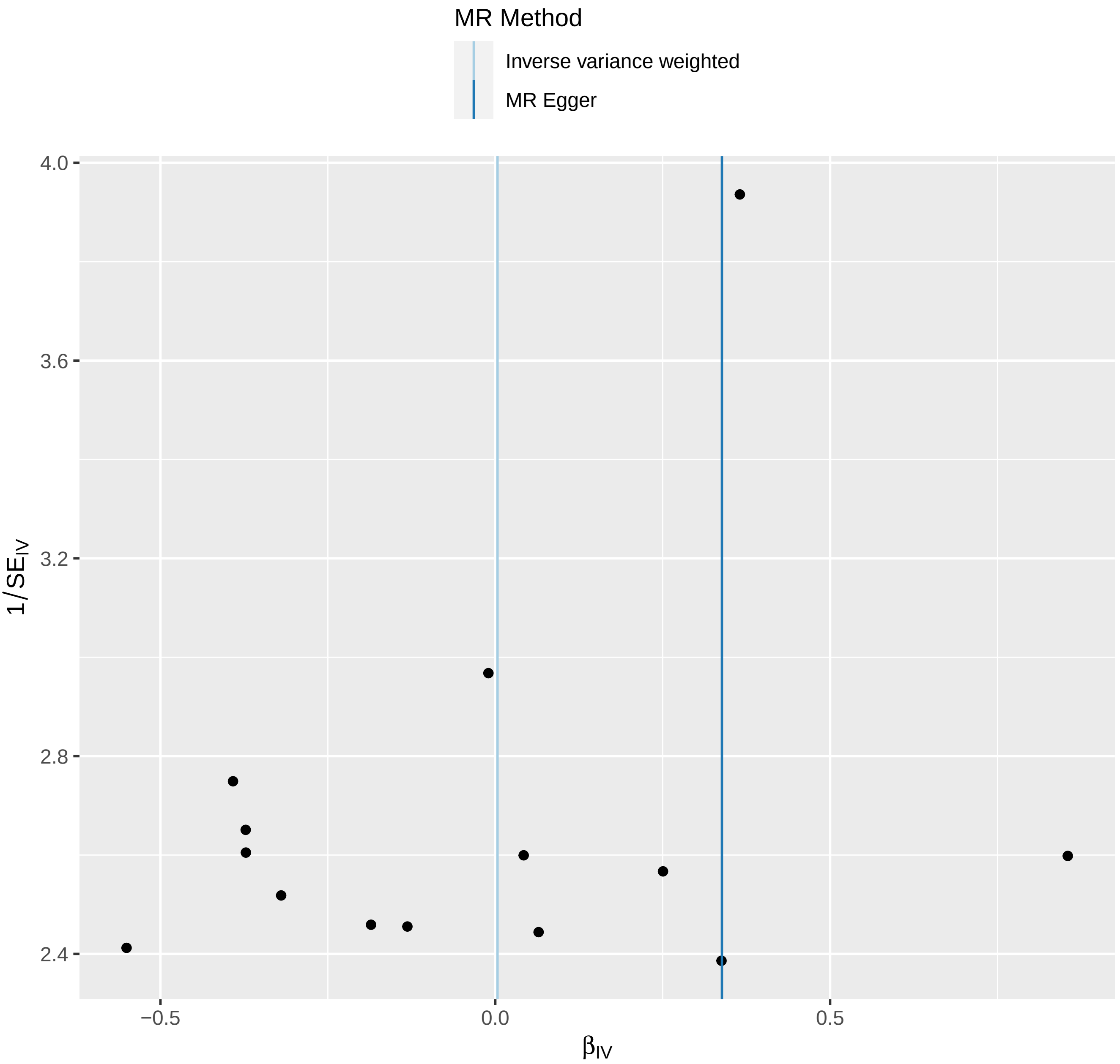

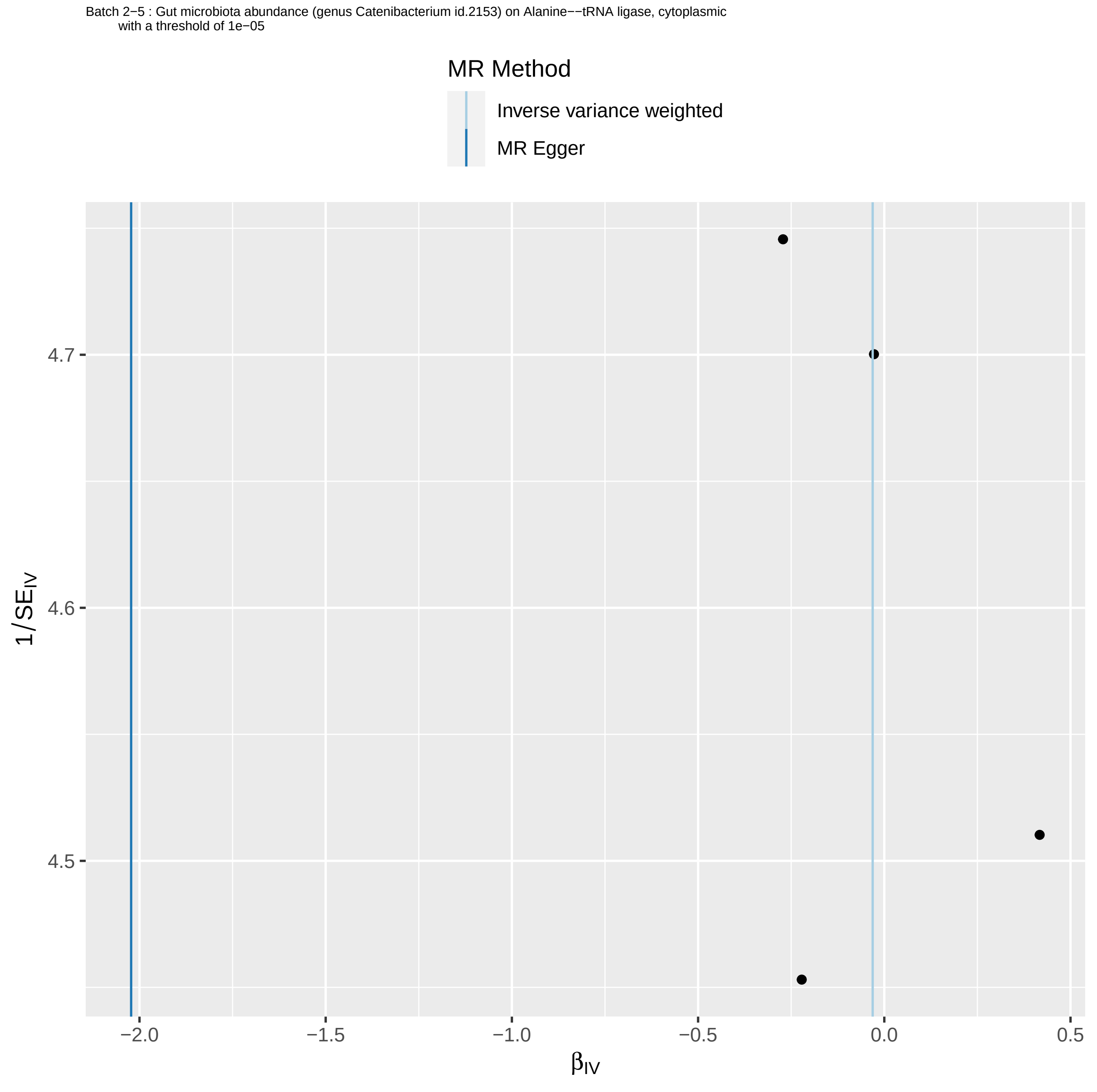
Batch 2−5 : Gut microbiota abundance (genus Catenibacterium id.2153) on Alanine−−tRNA ligase, cytoplasmic
with a threshold of 1e−05
MR Method
Inverse variance weighted
MR Egger
4.7
4.6
4.5
1/SE_IV
−2.0
−1.5
−1.0
−0.5
0.0
0.5
β_IV

Batch 2−6 : Gut microbiota abundance (genus Parasutterella id.2892) on Alanine−−tRNA ligase, cytoplasmic
with a threshold of 1e−05

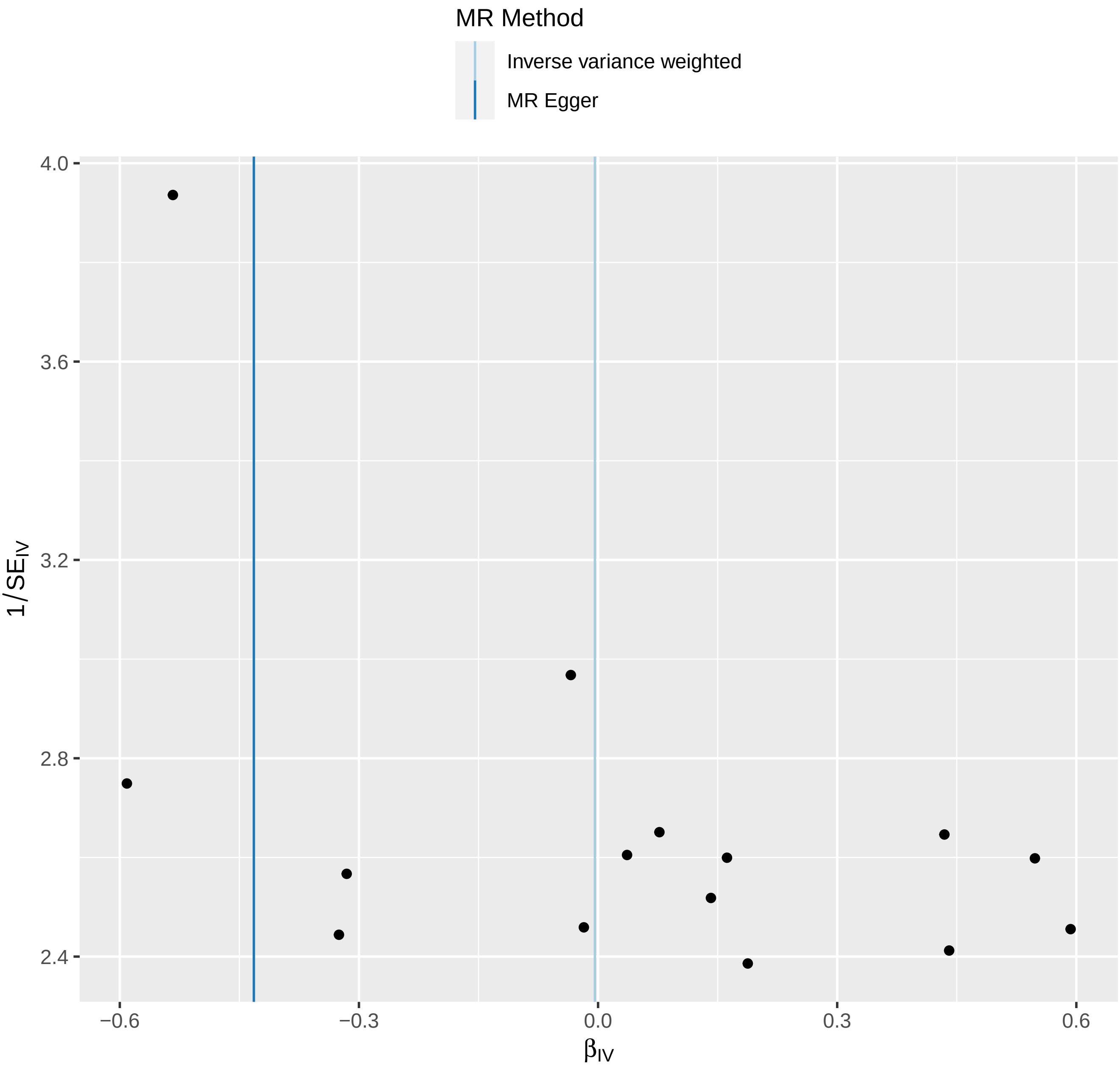

Batch 2−7 : Gut microbiota abundance (genus Catenibacterium id.2153) on Multidrug resistance−associated protein 6
with a threshold of 1e−05

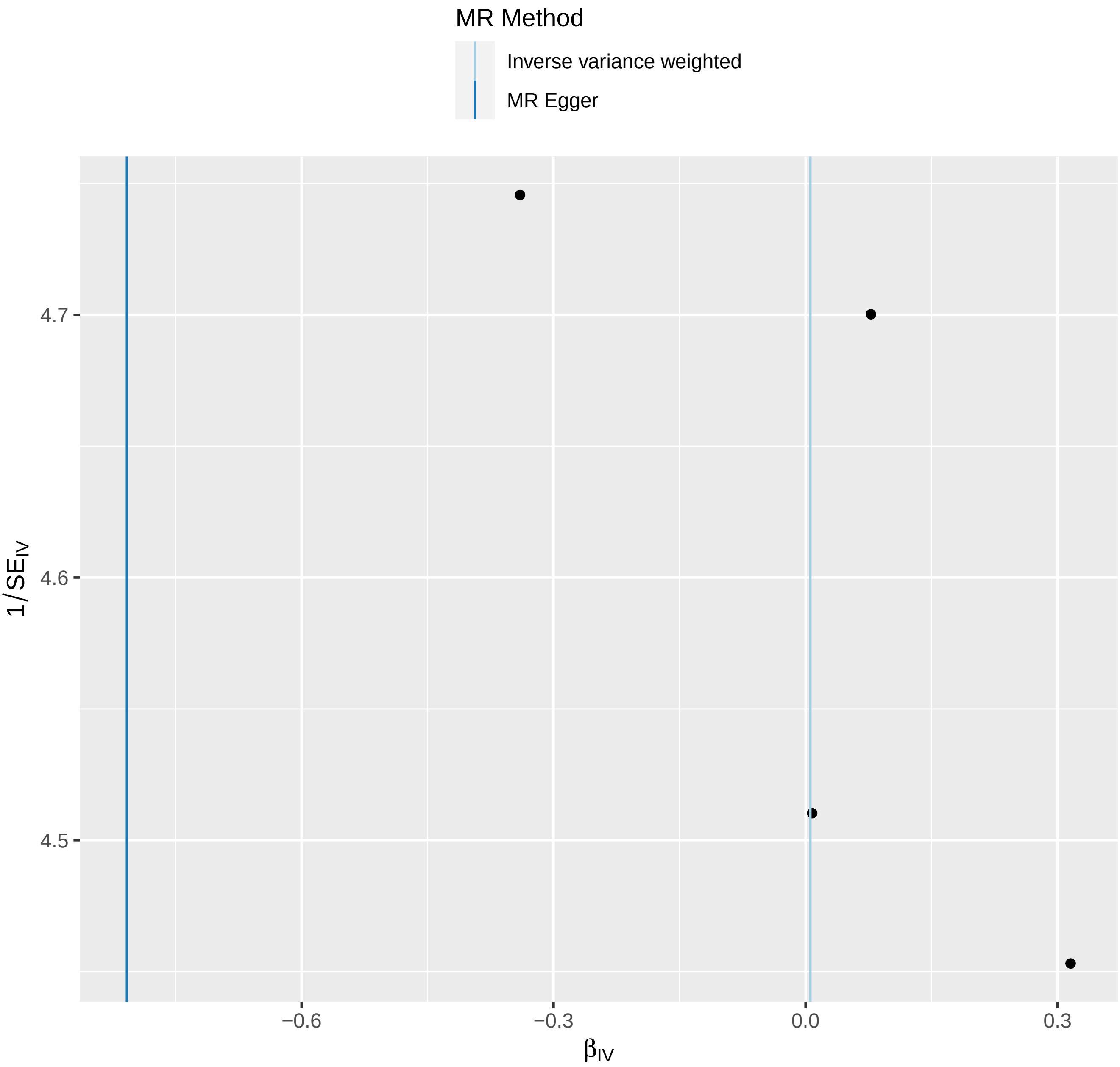

Batch 2−8 : Gut microbiota abundance (genus Parasutterella id.2892) on Multidrug resistance−associated protein 6
with a threshold of 1e−05

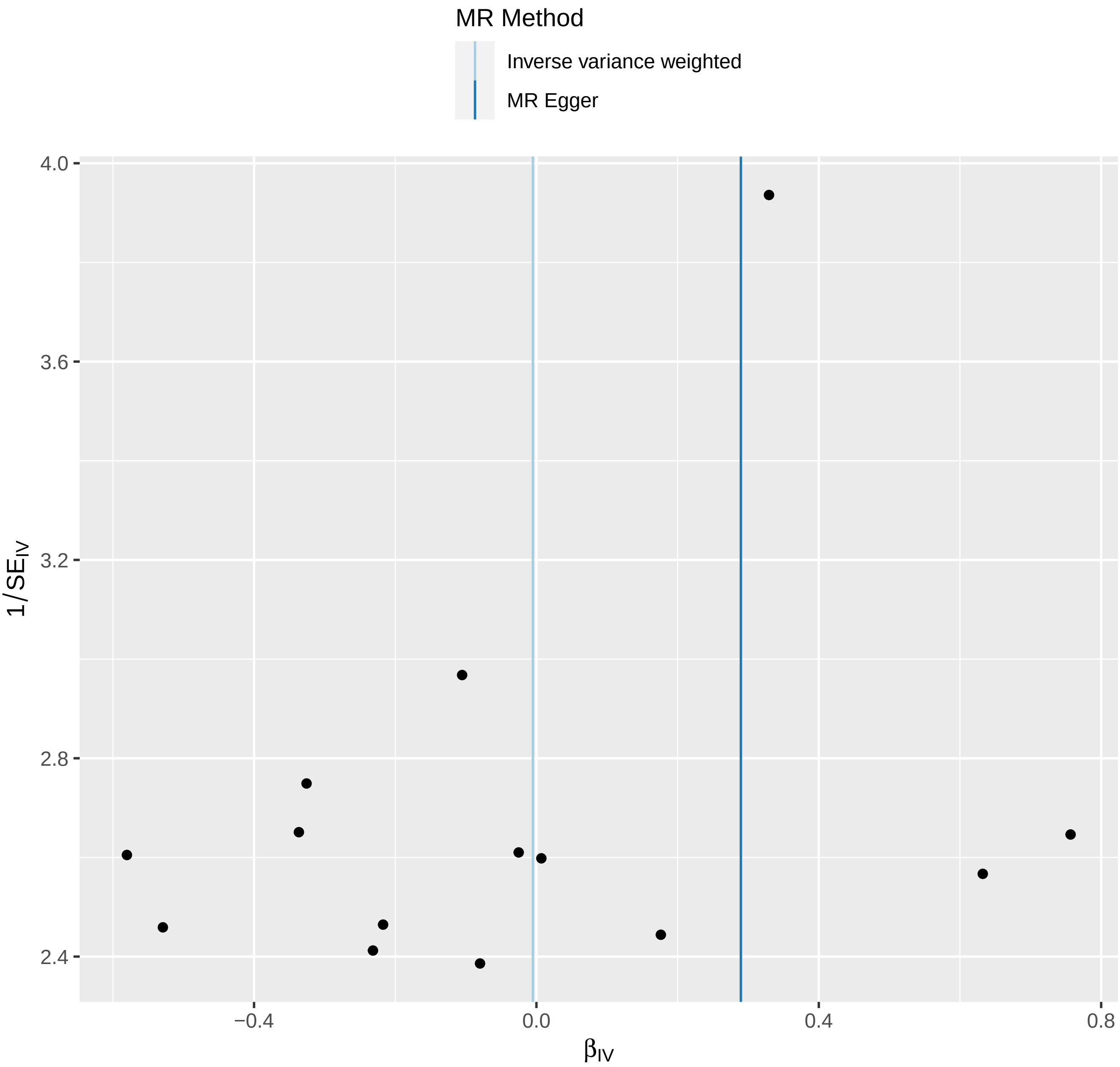

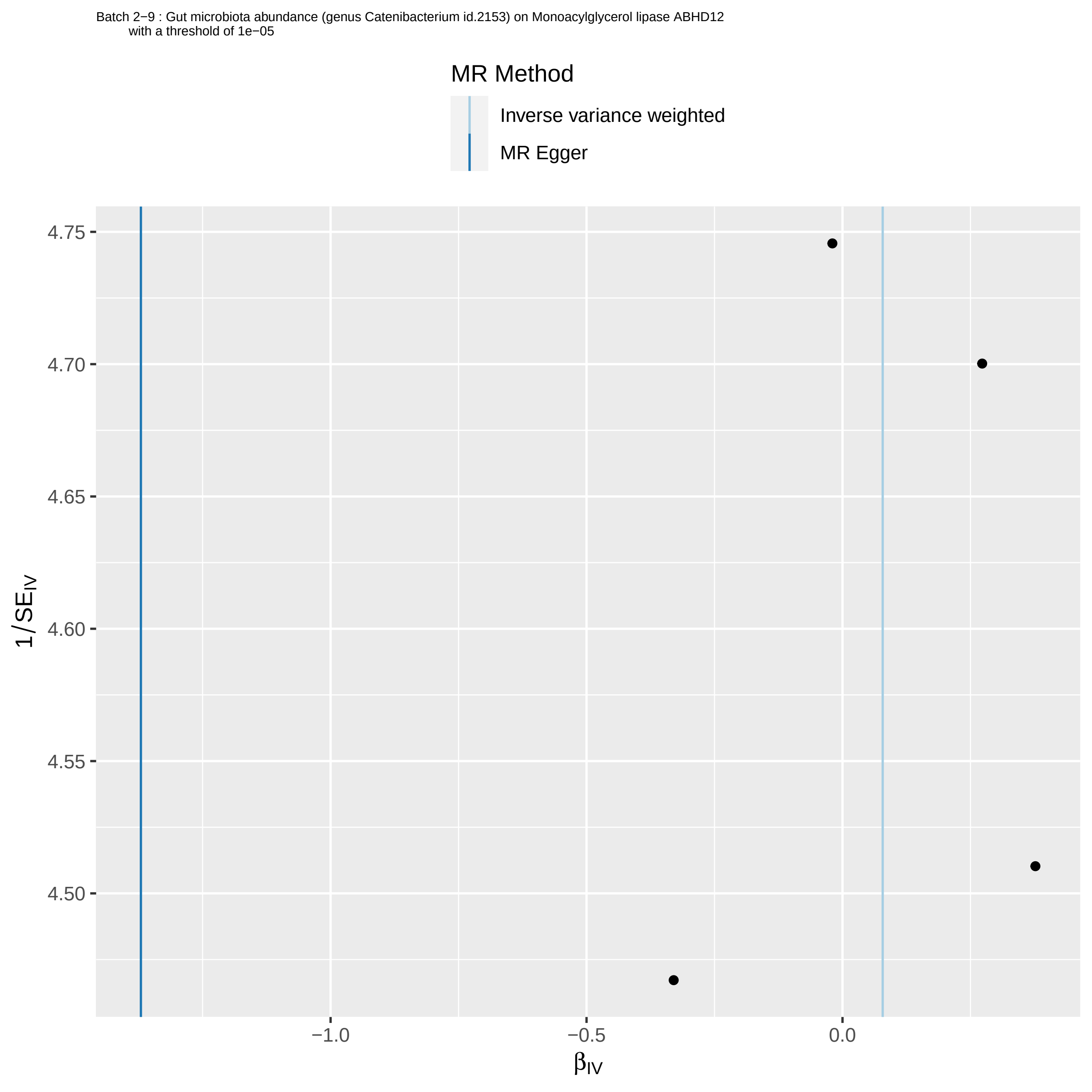

Batch 2−9 : Gut microbiota abundance (genus Catenibacterium id.2153) on Monoacylglycerol lipase ABHD12
with a threshold of 1e−05
MR Method
Inverse variance weighted
MR Egger
4.75
4.70
4.65
4.60
4.55
4.50
1/SE_IV
−1.0
−0.5
0.0
β_IV

Batch 2−10 : Gut microbiota abundance (genus Parasutterella id.2892) on Monoacylglycerol lipase ABHD12
with a threshold of 1e−05

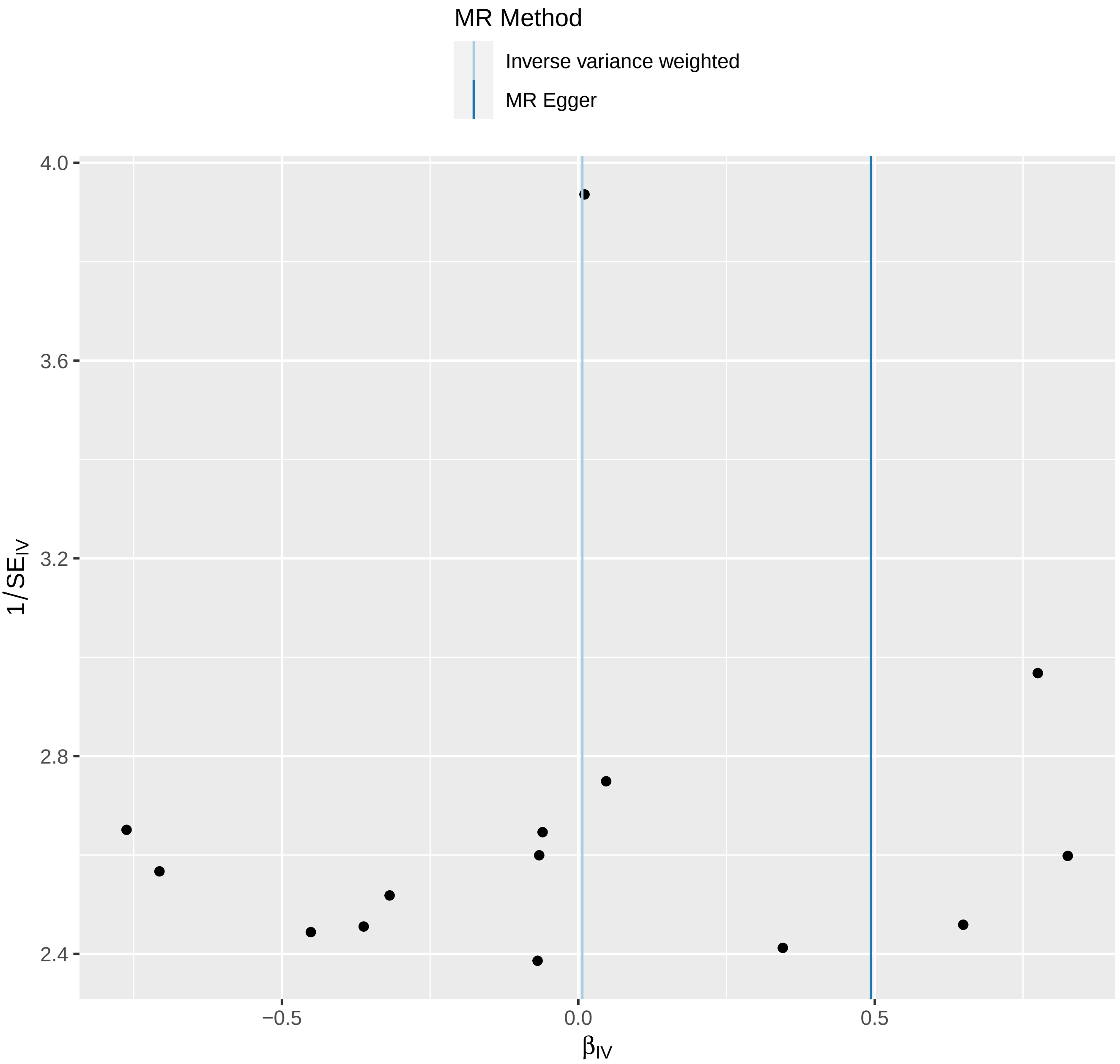

Batch 2−11 : Gut microbiota abundance (genus Catenibacterium id.2153) on Alpha/beta hydrolase domain−containing protein 14A
with a threshold of 1e−05

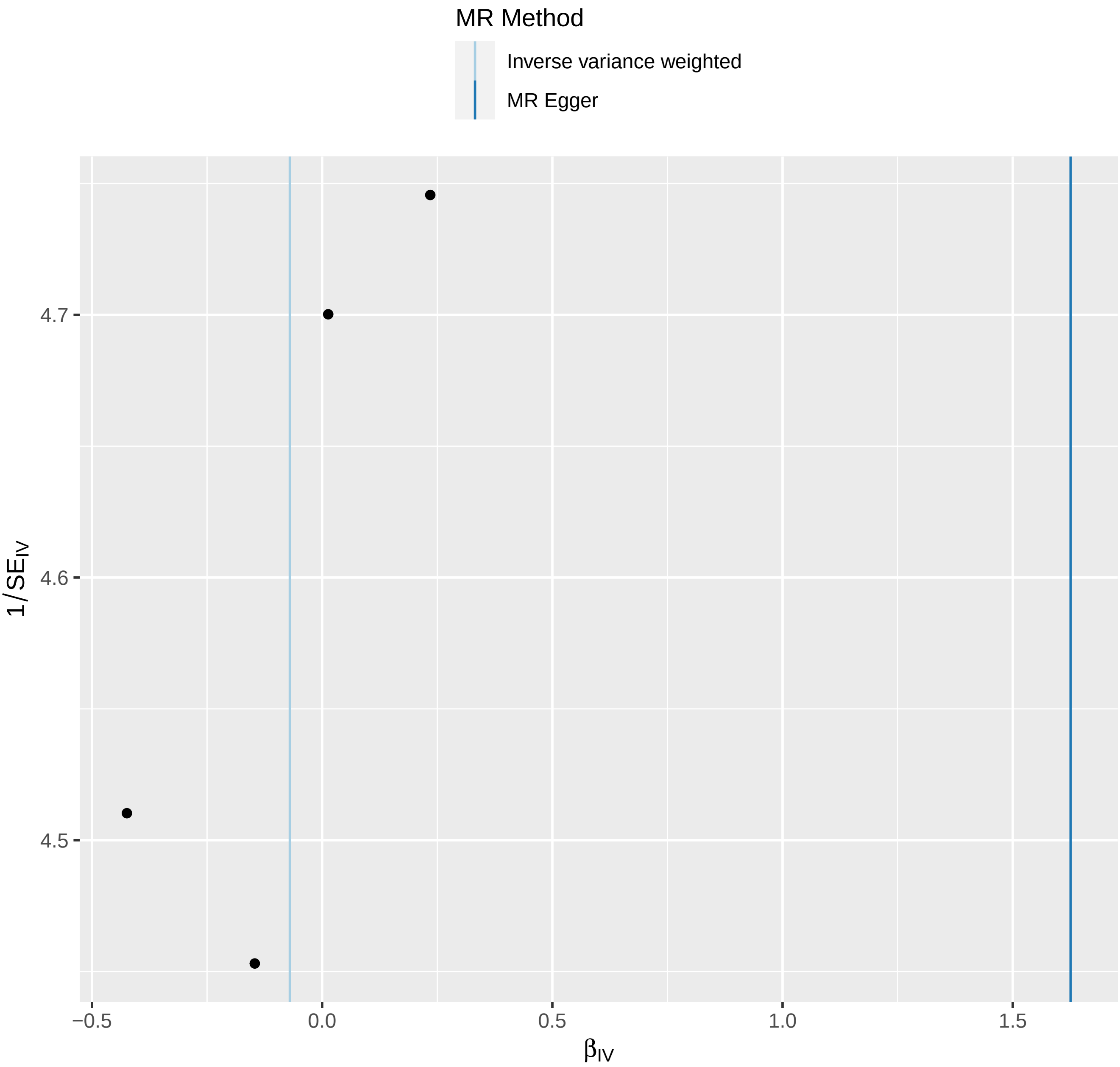

Batch 2−12 : Gut microbiota abundance (genus Parasutterella id.2892) on Alpha/beta hydrolase domain−containing protein 14A
with a threshold of 1e−05

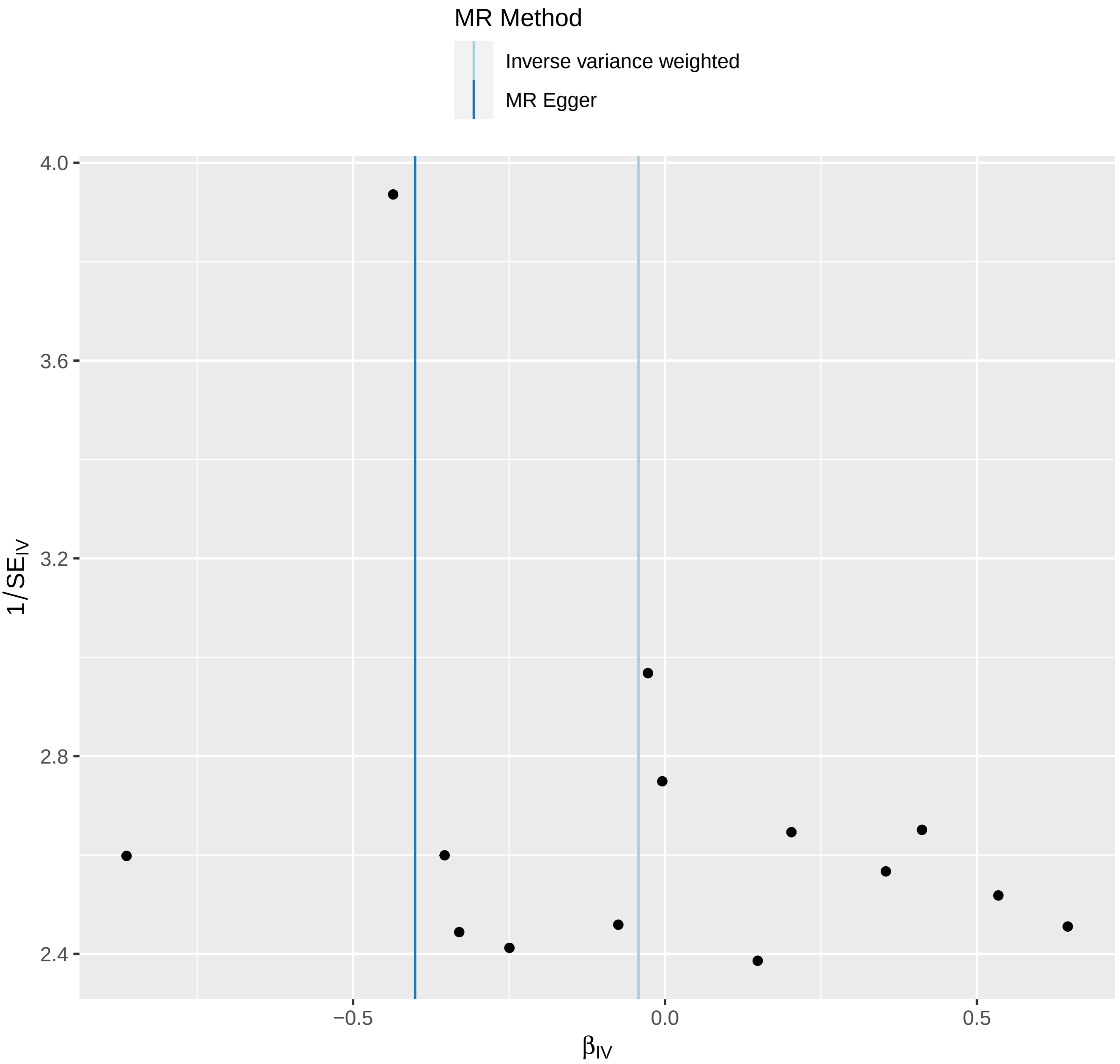

Batch 2−13 : Gut microbiota abundance (genus Catenibacterium id.2153) on Tyrosine−protein kinase ABL1
with a threshold of 1e−05

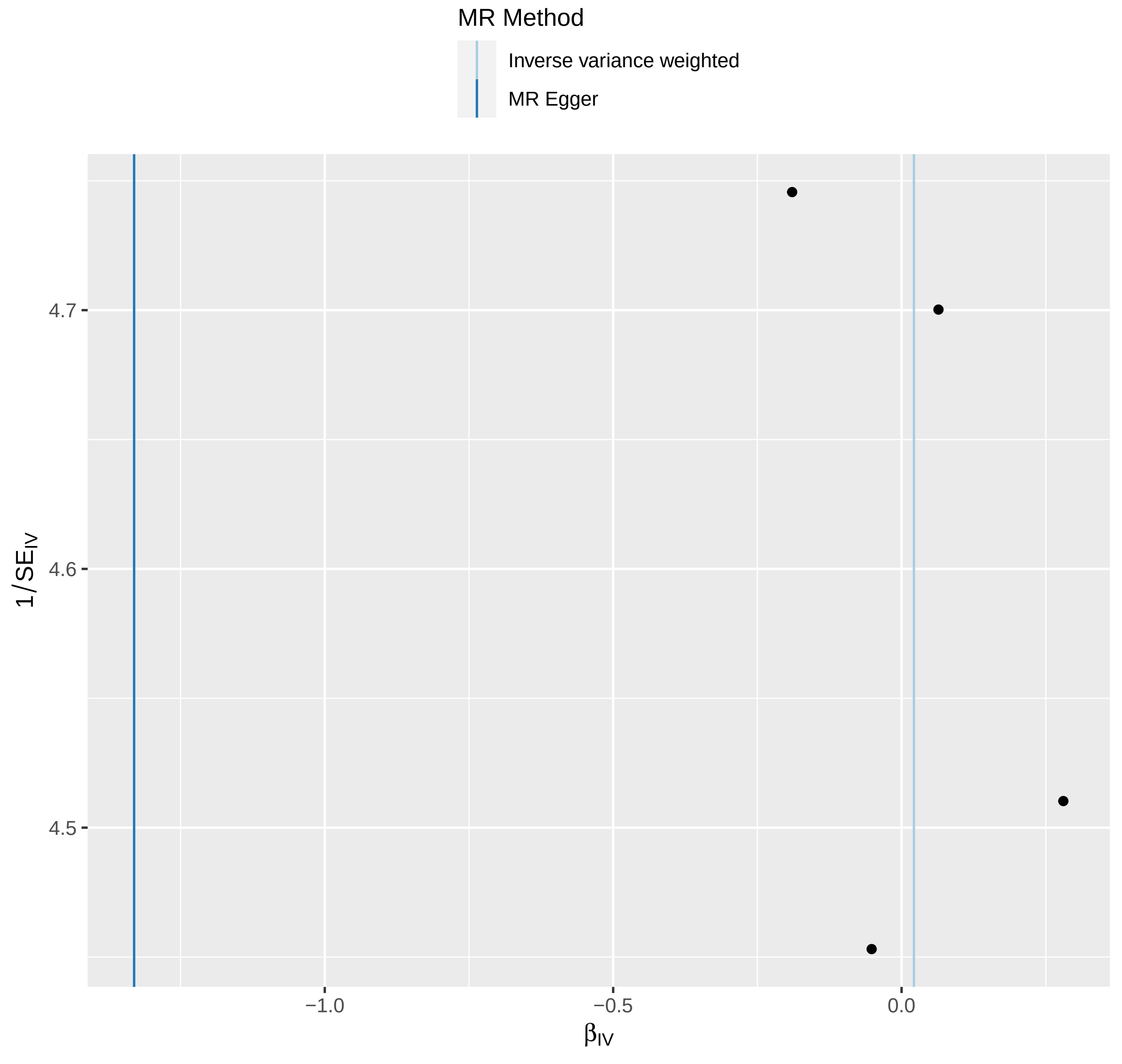

Batch 2−14 : Gut microbiota abundance (genus Parasutterella id.2892) on Tyrosine−protein kinase ABL1
with a threshold of 1e−05

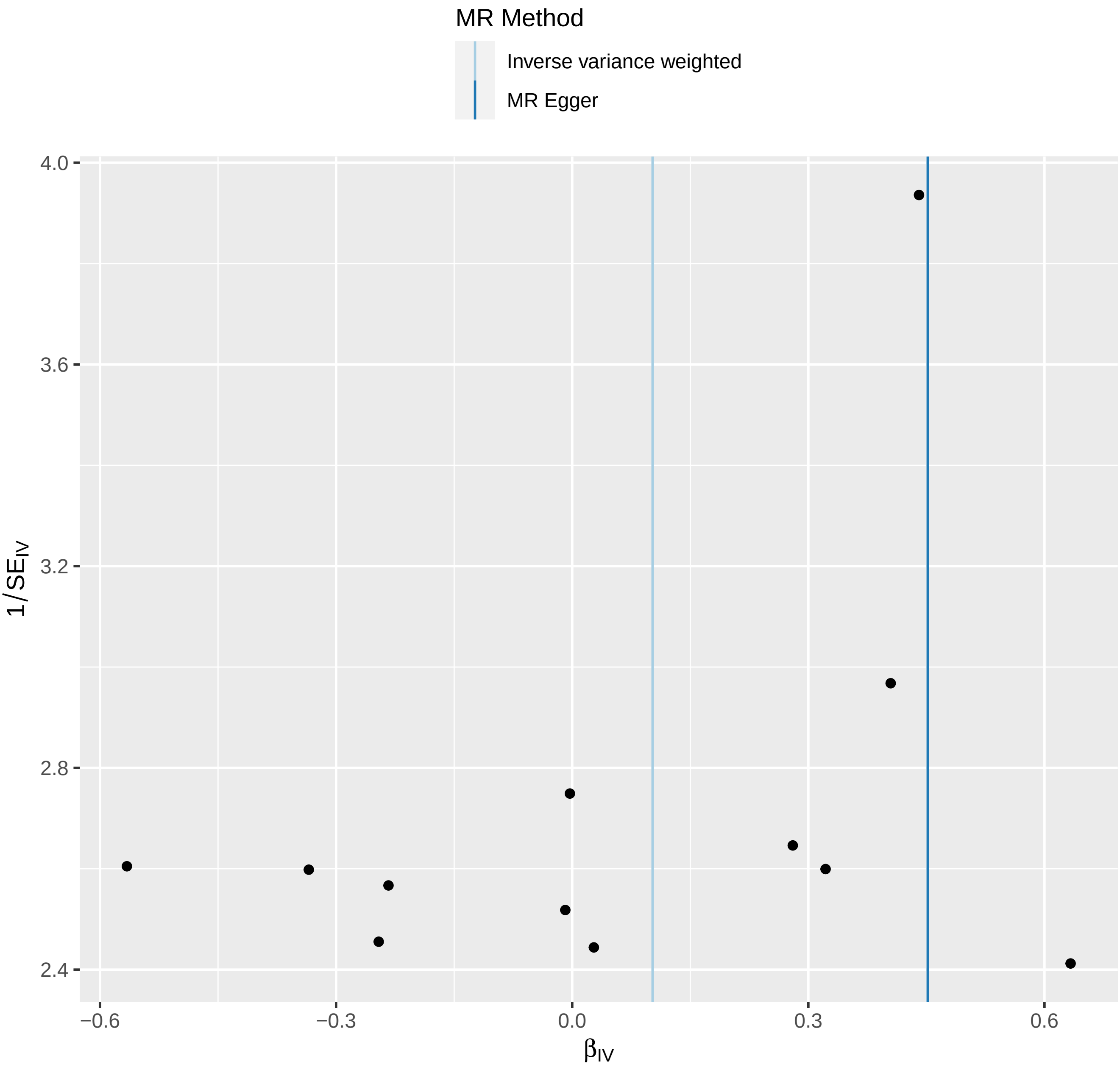

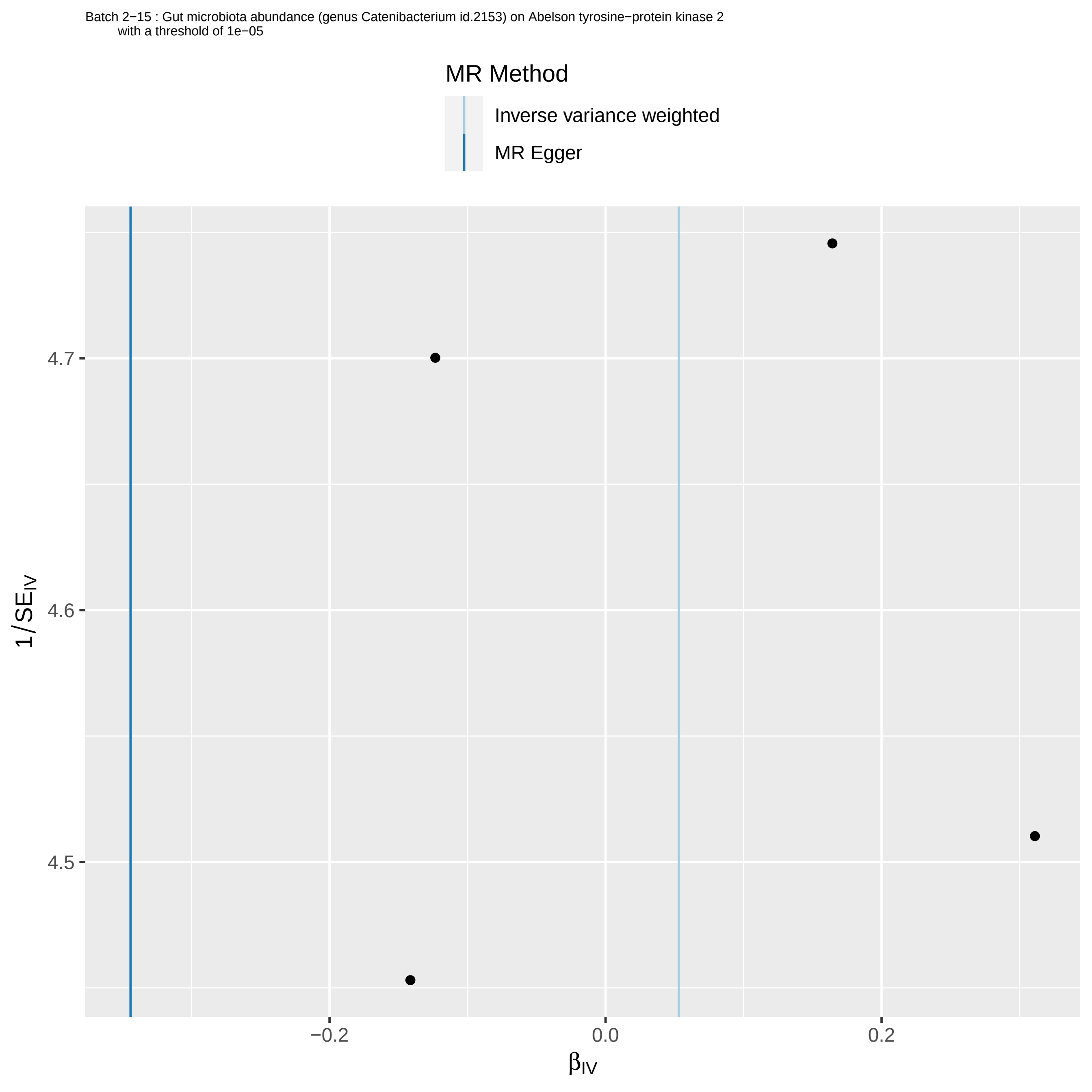

Batch 2−15 : Gut microbiota abundance (genus Catenibacterium id.2153) on Abelson tyrosine−protein kinase 2
with a threshold of 1e−05
MR Method
Inverse variance weighted
MR Egger
4.7
4.6
4.5
1/SE_IV
−0.2
0.0
0.2
β_IV

Batch 2−16 : Gut microbiota abundance (genus Parasutterella id.2892) on Abelson tyrosine−protein kinase 2
with a threshold of 1e−05

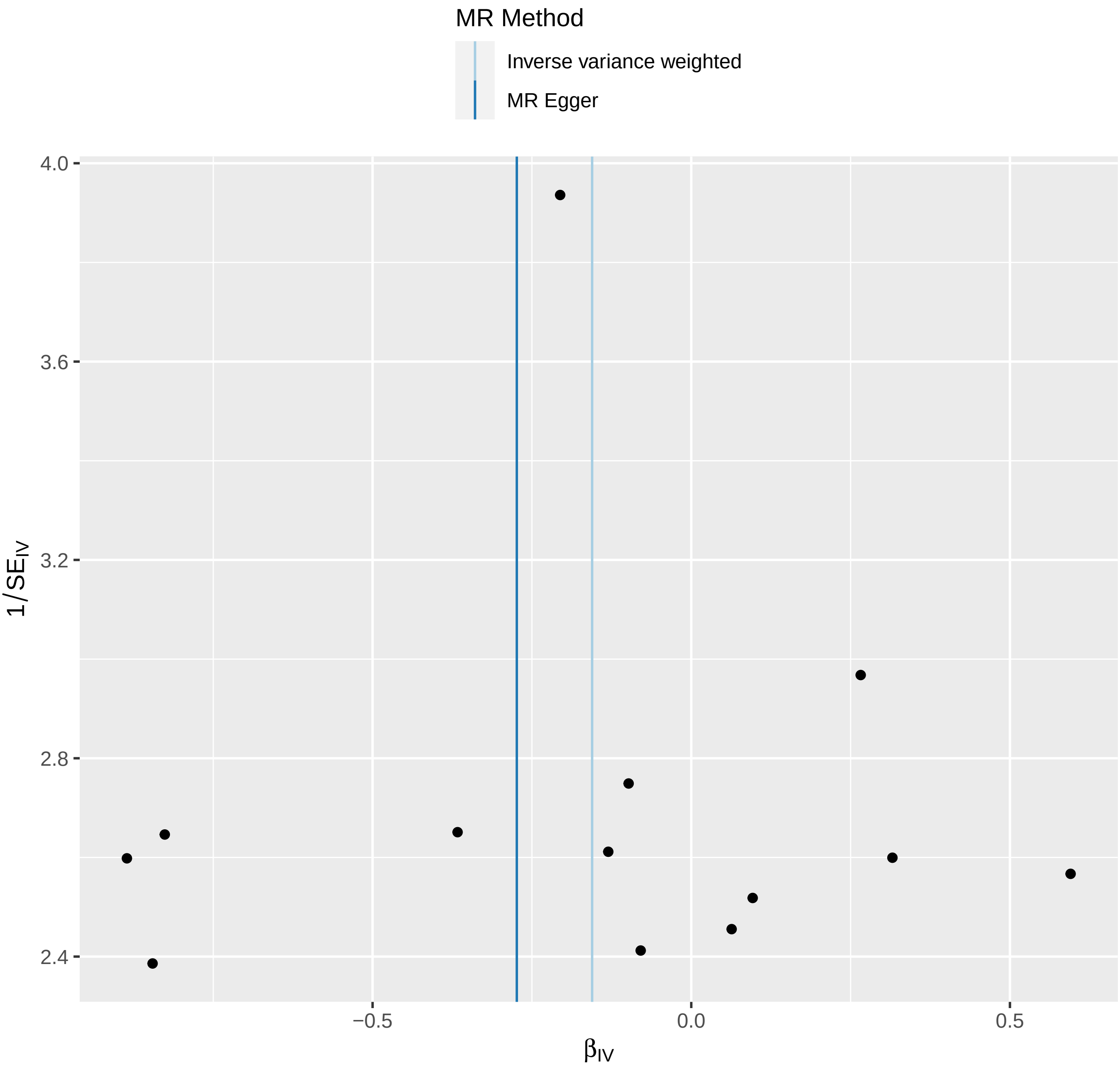

Batch 2−17 : Gut microbiota abundance (genus Catenibacterium id.2153) on Actin−binding LIM protein 3
with a threshold of 1e−05

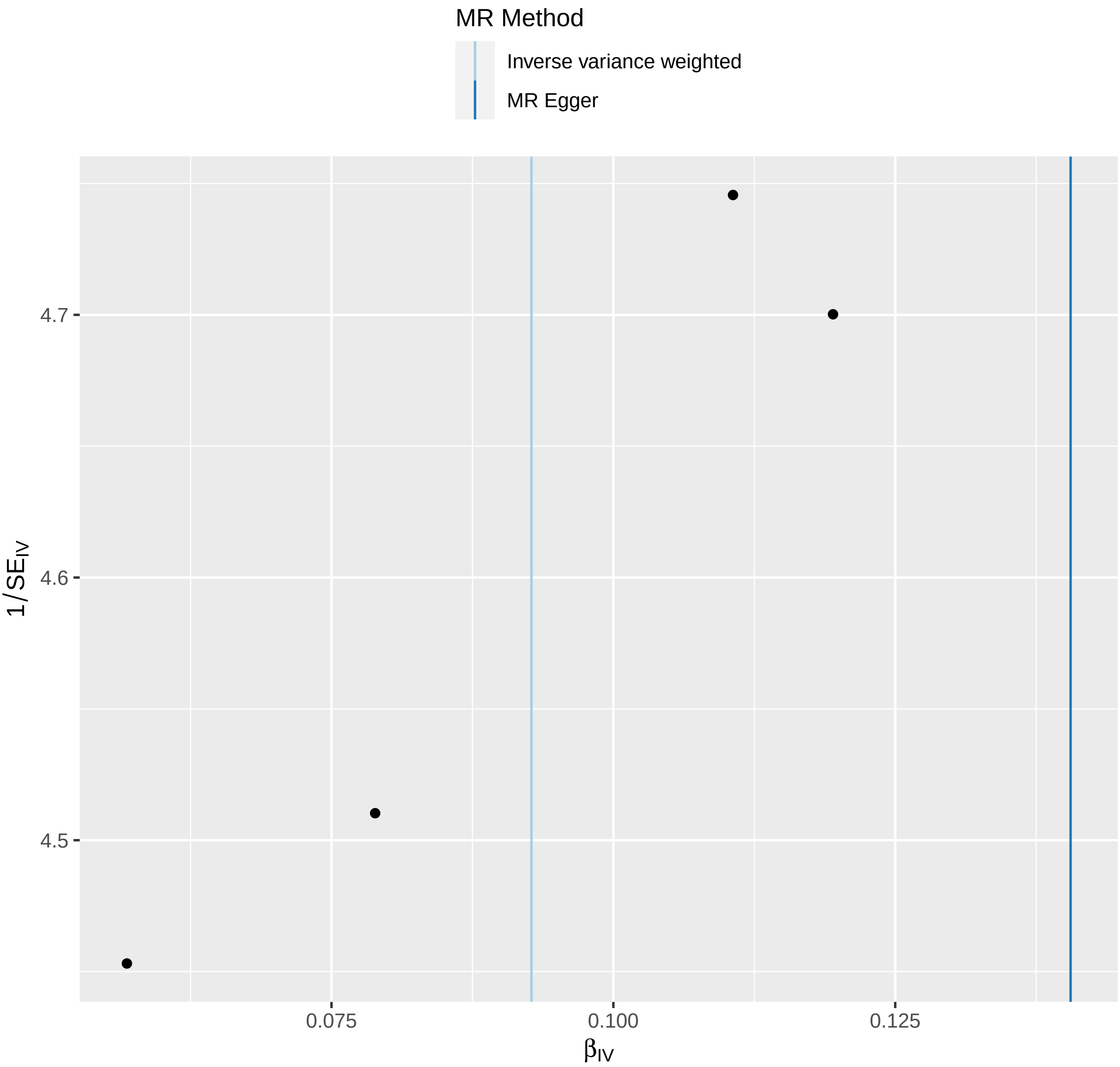

Batch 2−18 : Gut microbiota abundance (genus Parasutterella id.2892) on Actin−binding LIM protein 3
with a threshold of 1e−05

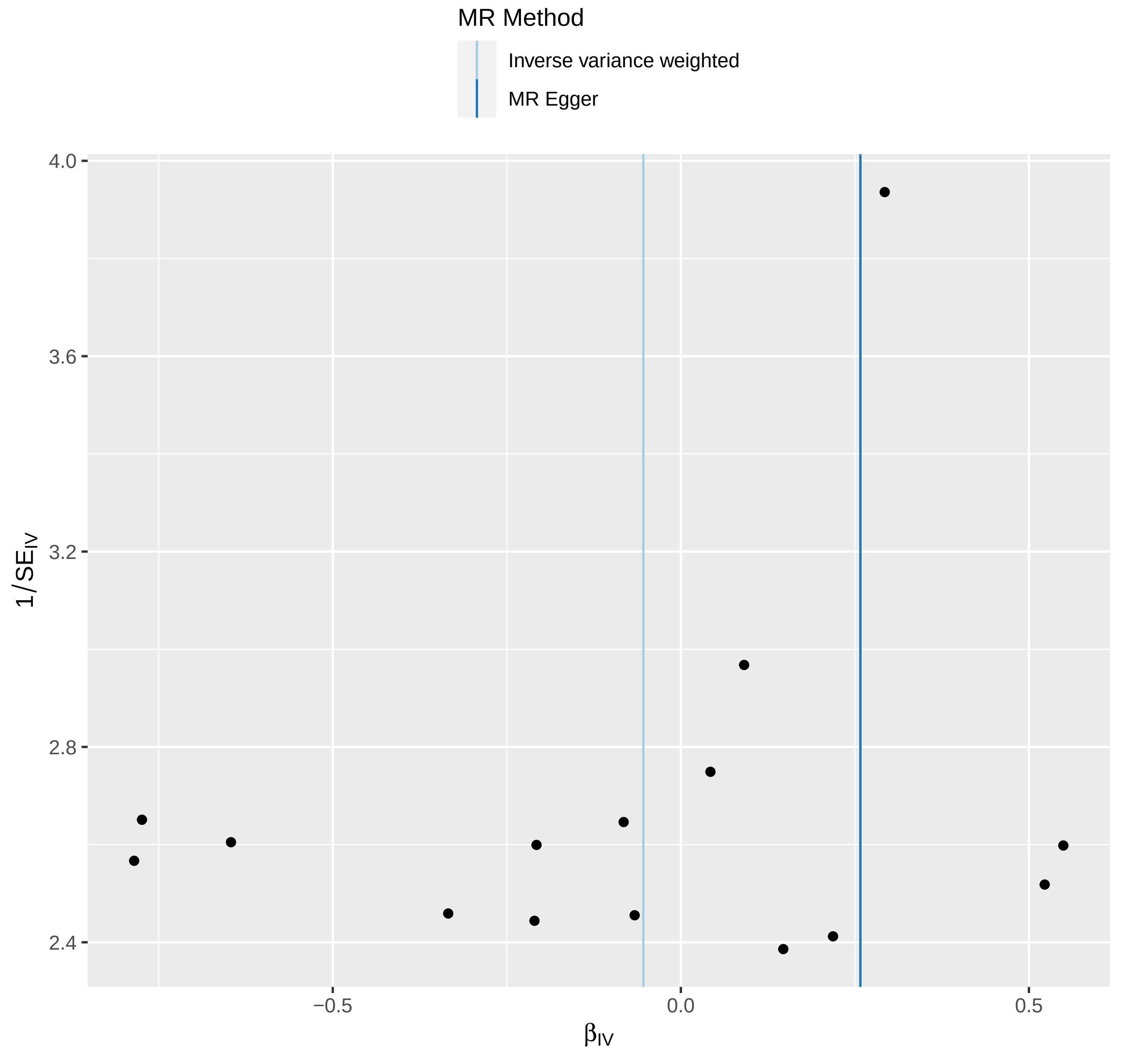

Batch 2−19 : Gut microbiota abundance (genus Catenibacterium id.2153) on Histo−blood group ABO system transferase with a threshold of 1e−05

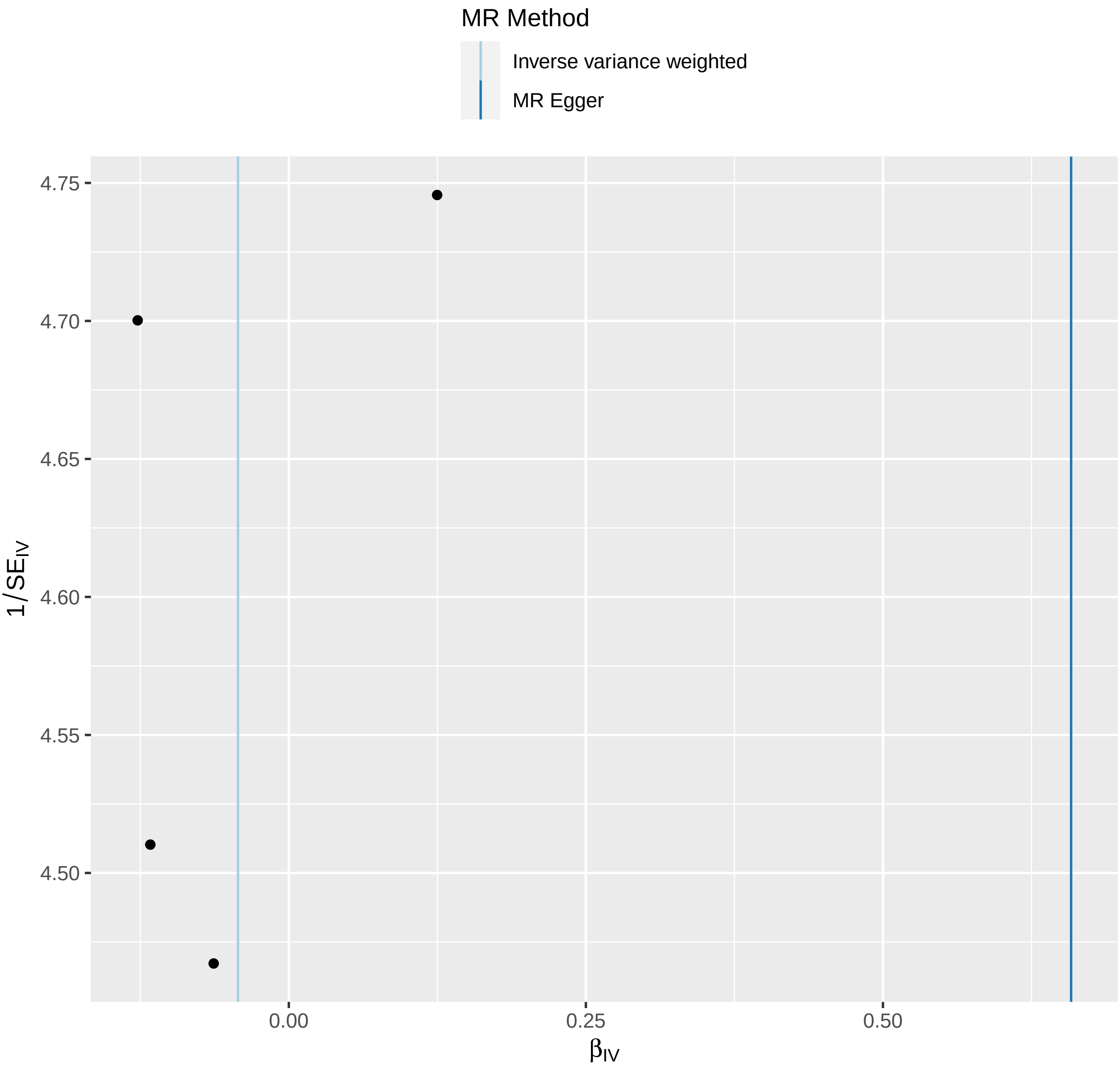

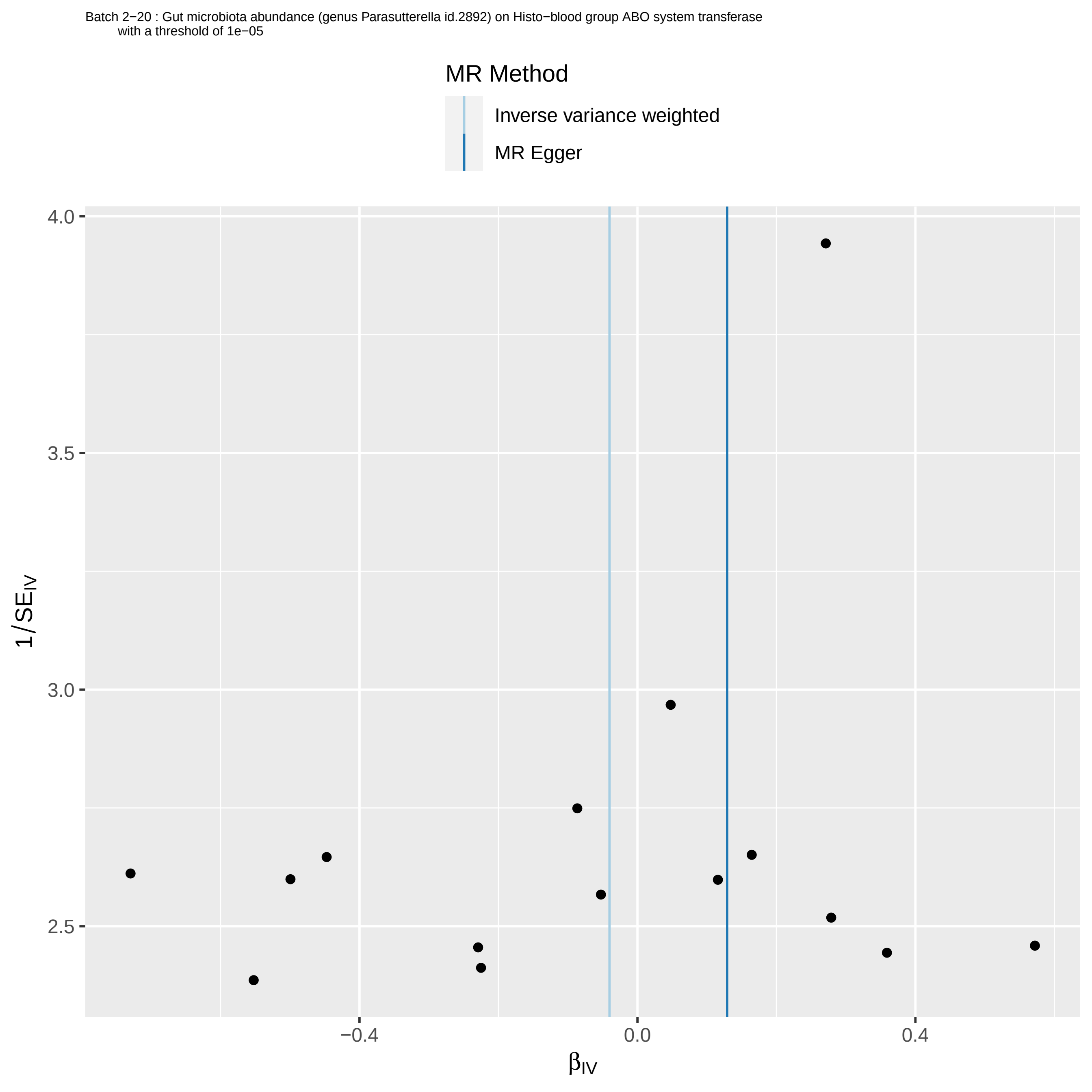

Batch 2−20 : Gut microbiota abundance (genus Parasutterella id.2892) on Histo−blood group ABO system transferase
with a threshold of 1e−05
MR Method
Inverse variance weighted
MR Egger
4.0
3.5
3.0
2.5
1/SE_IV
−0.4
0.0
0.4
β_IV

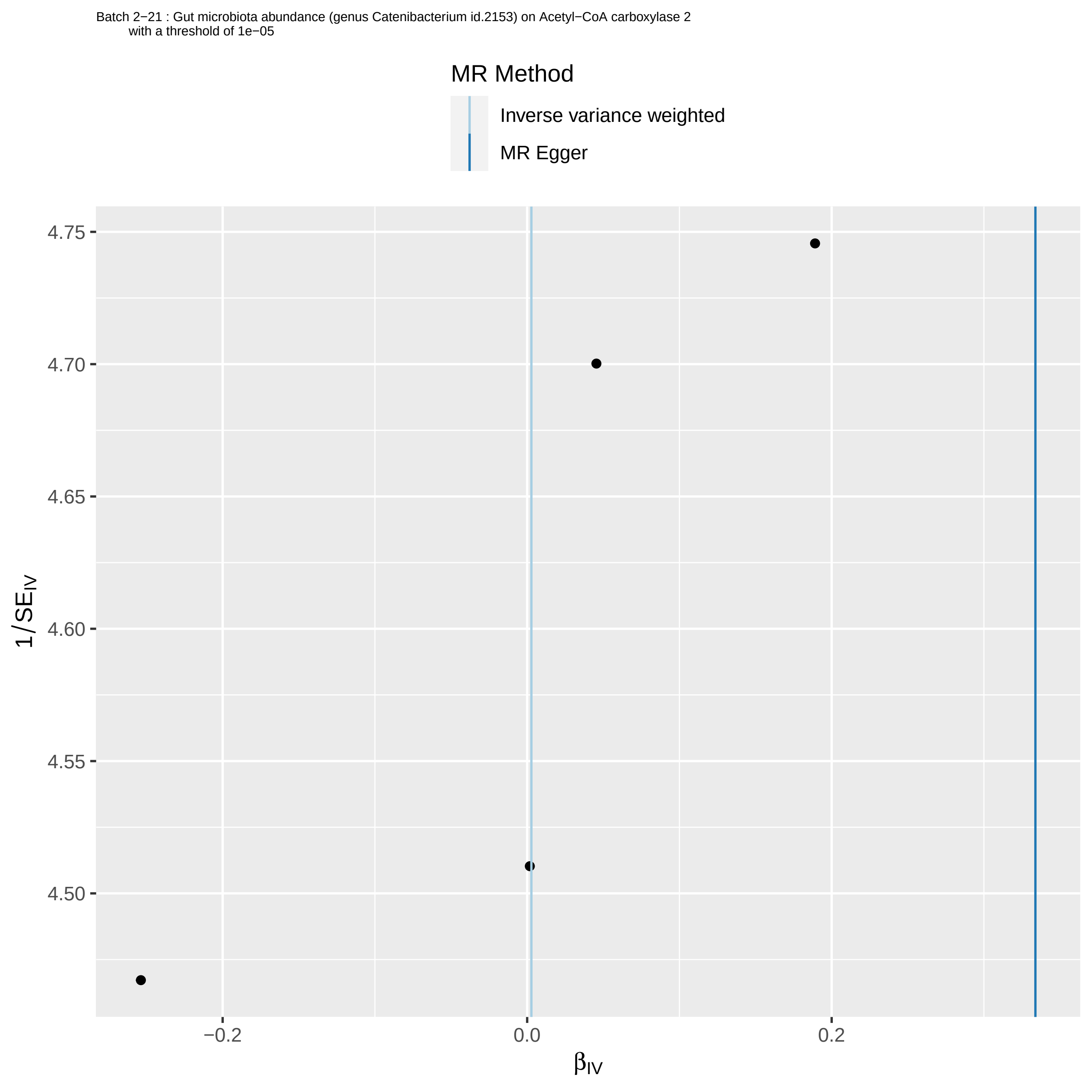
Batch 2−21 : Gut microbiota abundance (genus Catenibacterium id.2153) on Acetyl−CoA carboxylase 2
with a threshold of 1e−05
MR Method
Inverse variance weighted
MR Egger
4.75
4.70
4.65
4.60
4.55
4.50
1/SE_IV
−0.2
0.0
0.2
β_IV

Batch 2−22 : Gut microbiota abundance (genus Parasutterella id.2892) on Acetyl−CoA carboxylase 2
with a threshold of 1e−05

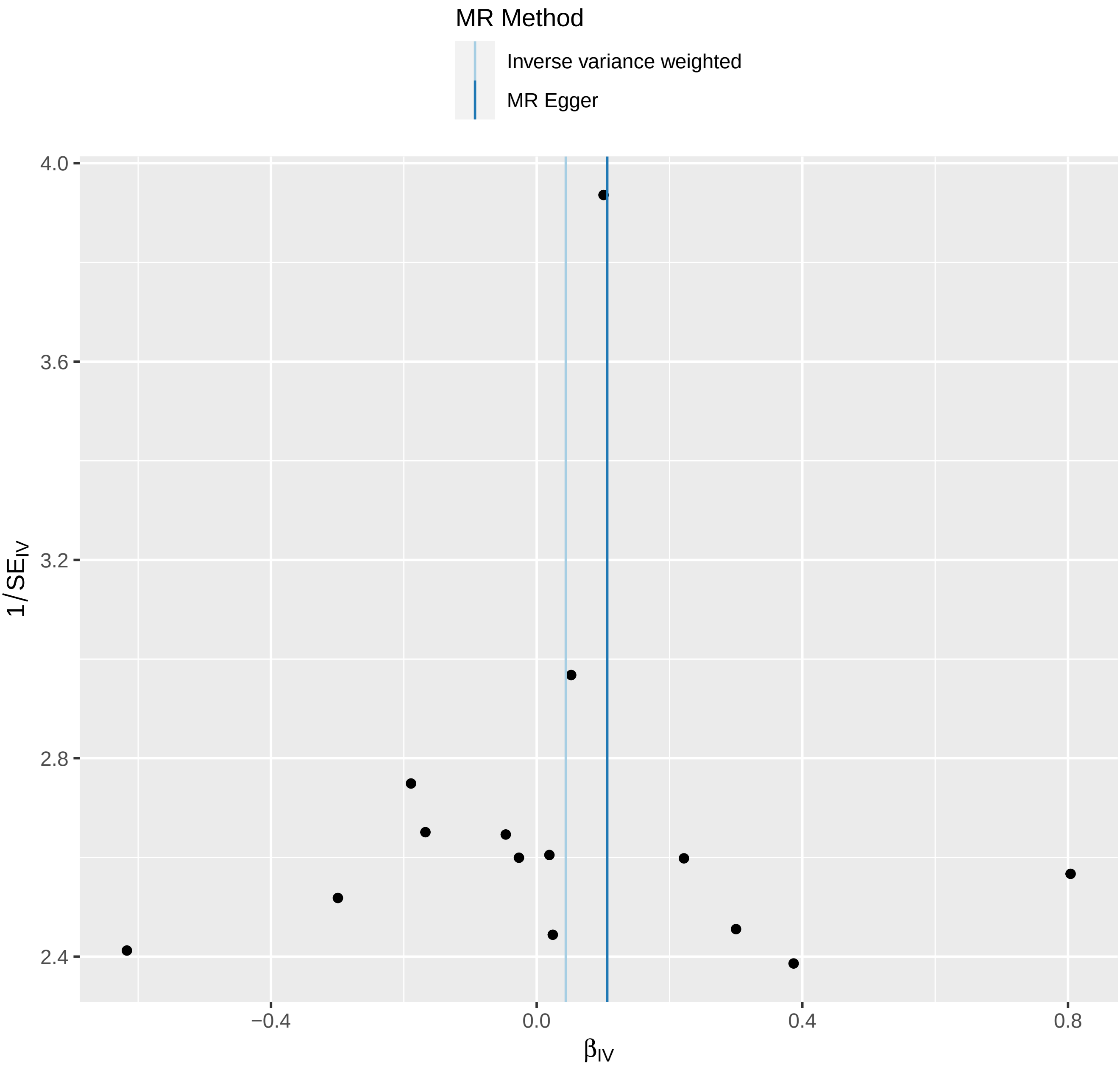

Batch 2−23 : Gut microbiota abundance (genus Catenibacterium id.2153) on Aggrecan core protein
with a threshold of 1e−05

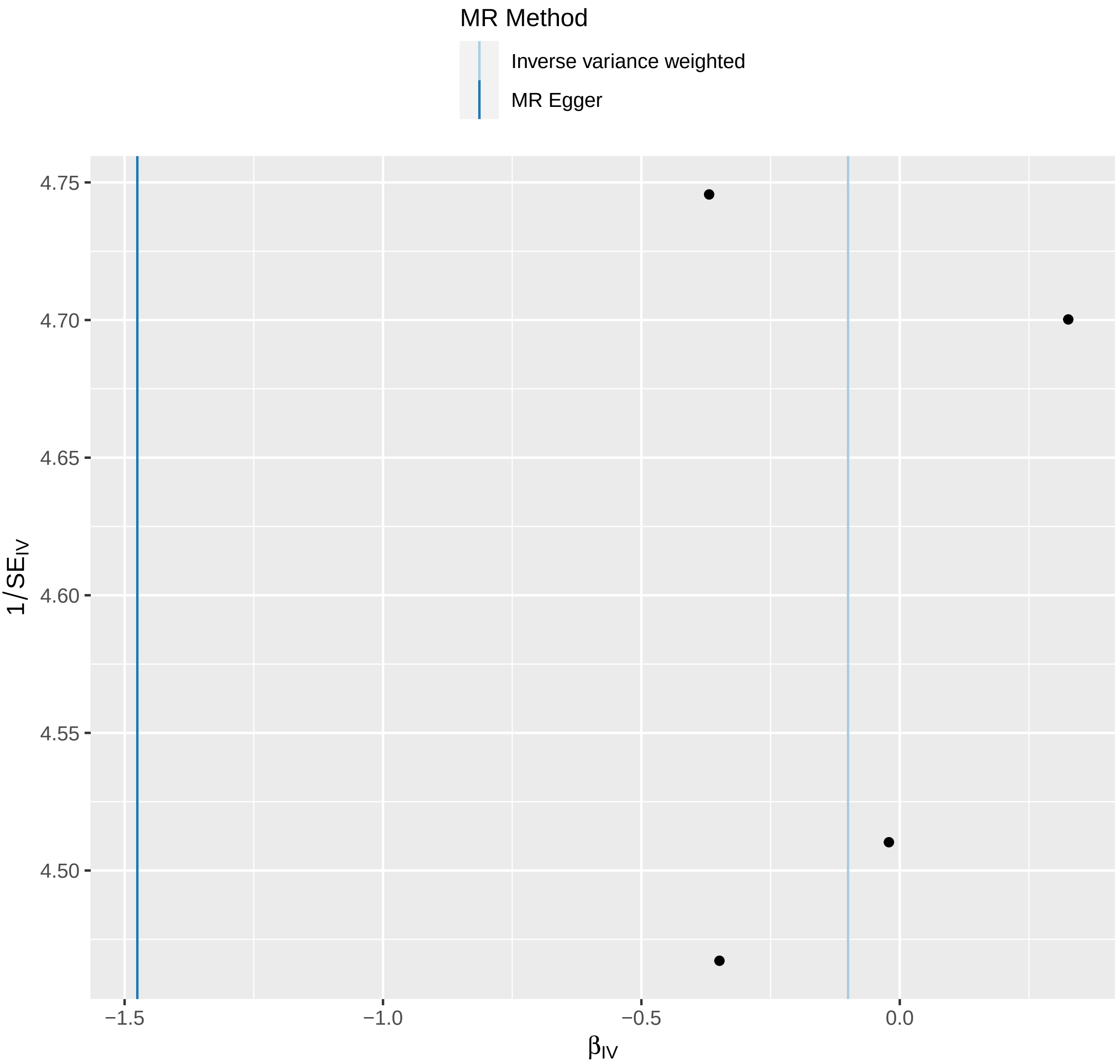

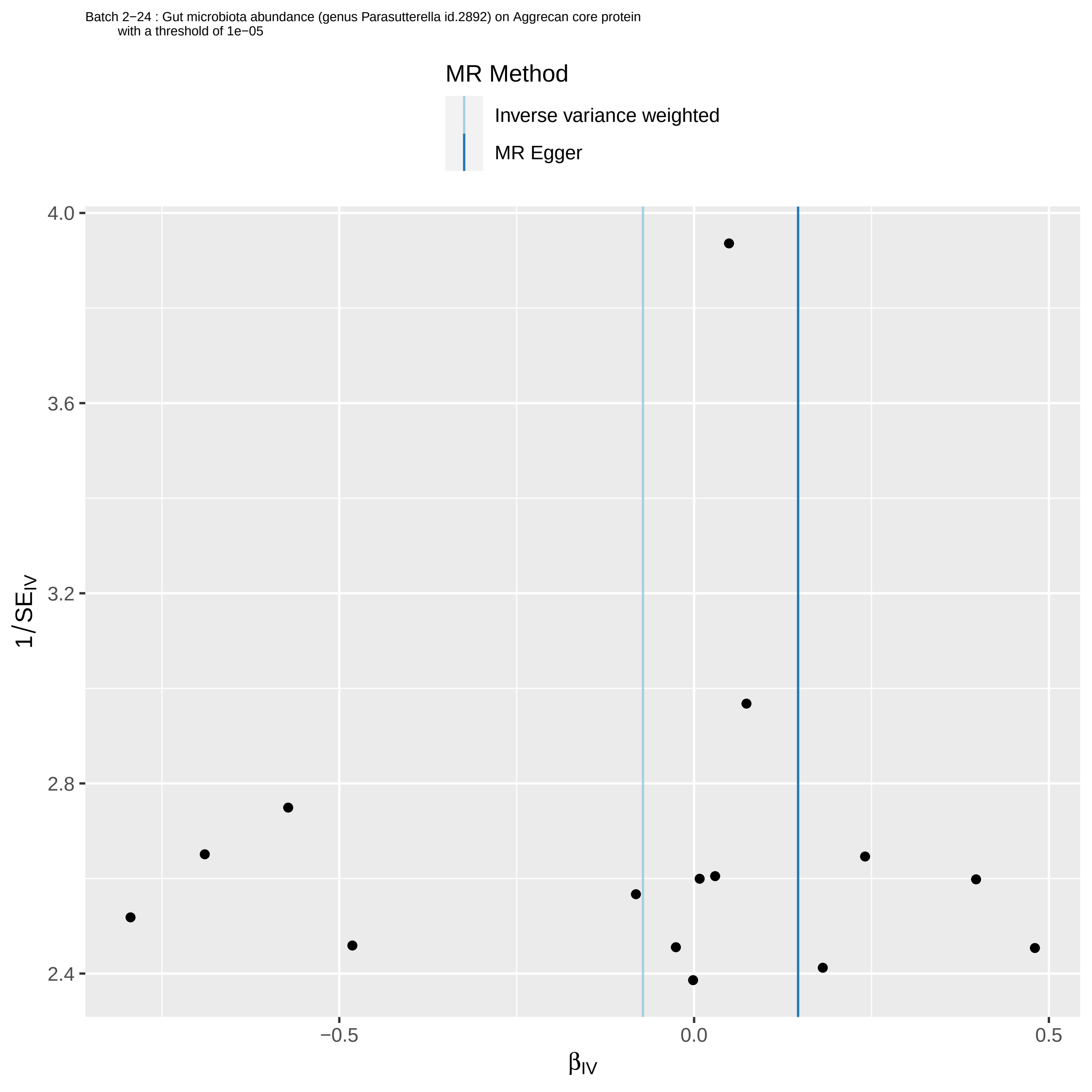

Batch 2−24 : Gut microbiota abundance (genus Parasutterella id.2892) on Aggrecan core protein
with a threshold of 1e−05
MR Method
Inverse variance weighted
MR Egger
4.0
3.6
3.2
2.8
2.4
1/SE$_{IV}$
−0.5
0.0
0.5
β$_{IV}$

Batch 2−25 : Gut microbiota abundance (genus Catenibacterium id.2153) on Arf−GAP with coiled−coil, ANK repeat and PH domain−containing protein 2 with a threshold of 1e−05

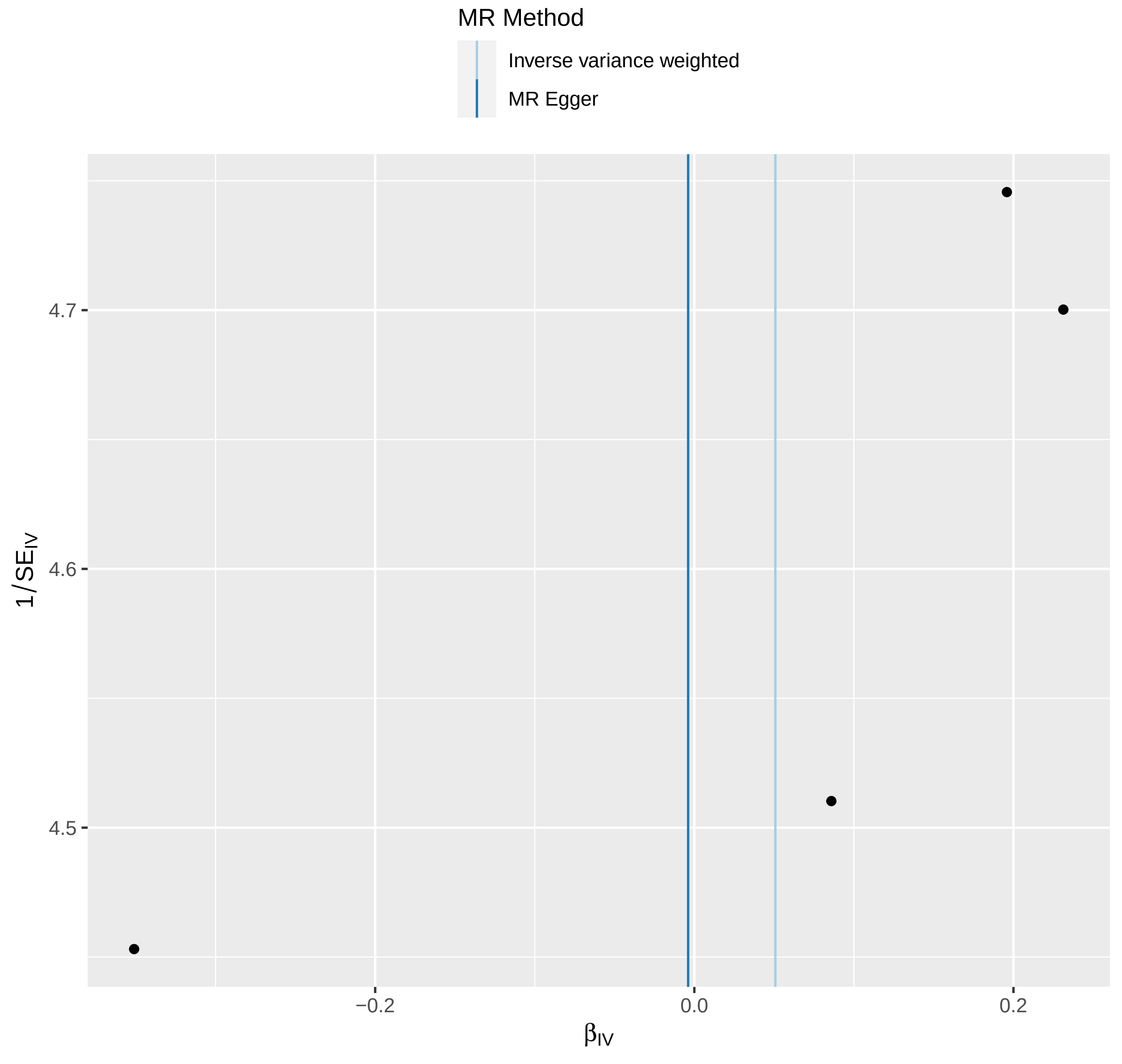

Batch 2−26 : Gut microbiota abundance (genus Parasutterella id.2892) on Arf−GAP with coiled−coil, ANK repeat and PH domain−containing protein 2 with a threshold of 1e−05

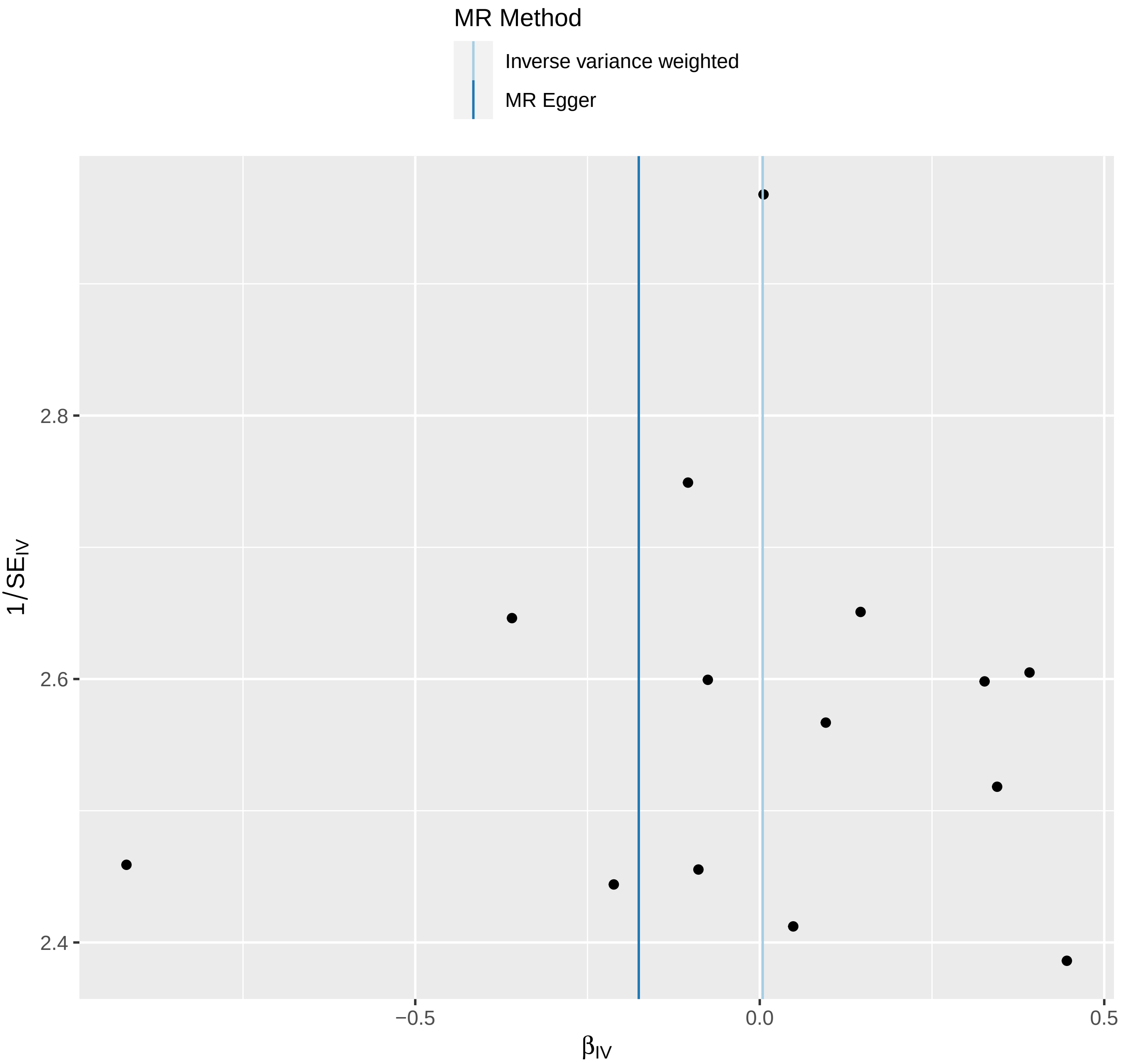

Batch 2−27 : Gut microbiota abundance (genus Catenibacterium id.2153) on Acyl−CoA−binding domain−containing protein 6
with a threshold of 1e−05

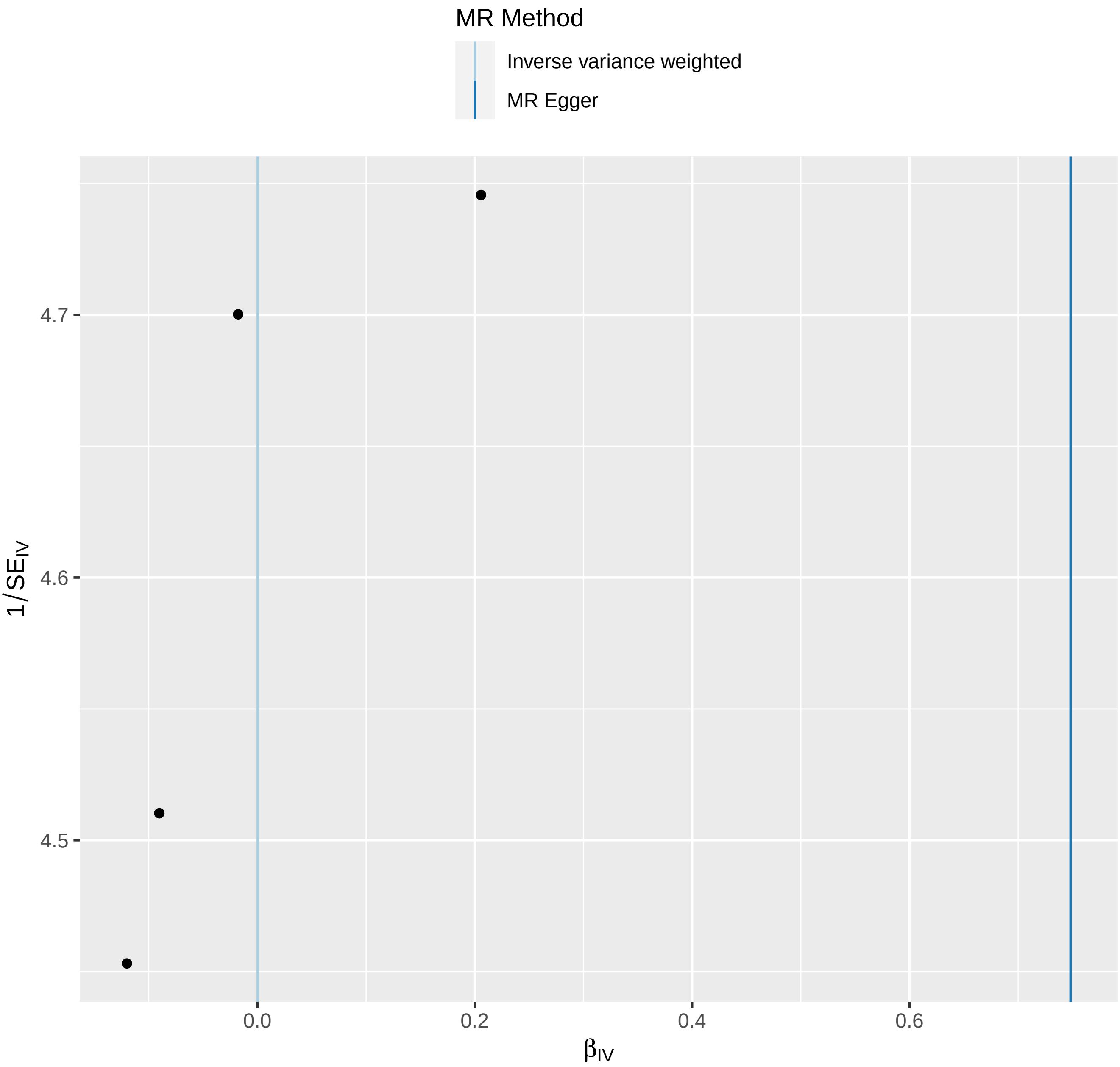

Batch 2−28 : Gut microbiota abundance (genus Parasutterella id.2892) on Acyl−CoA−binding domain−containing protein 6 with a threshold of 1e−05

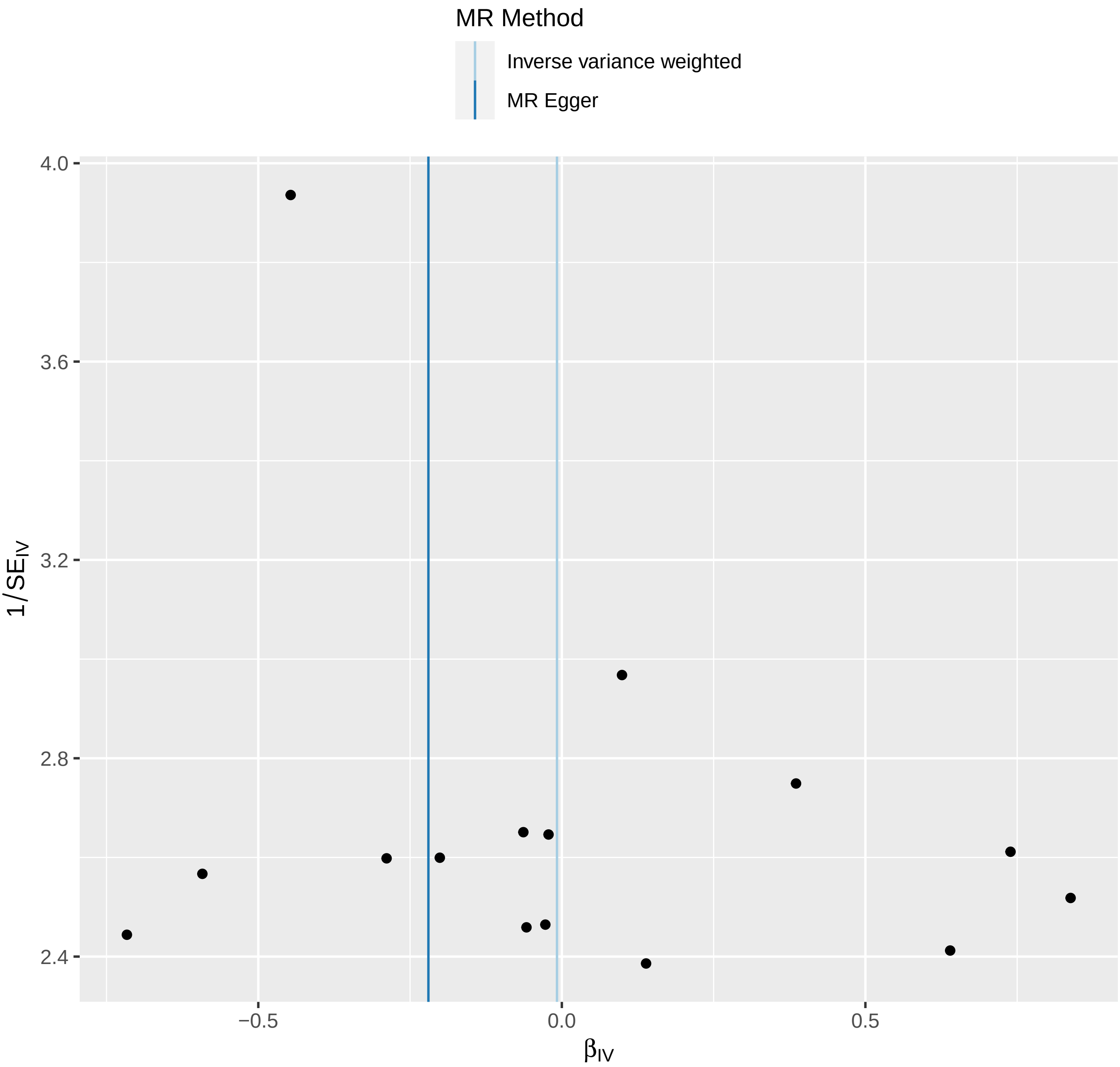

Batch 2−29 : Gut microbiota abundance (genus Catenibacterium id.2153) on Acyl−CoA−binding domain−containing protein 7
with a threshold of 1e−05

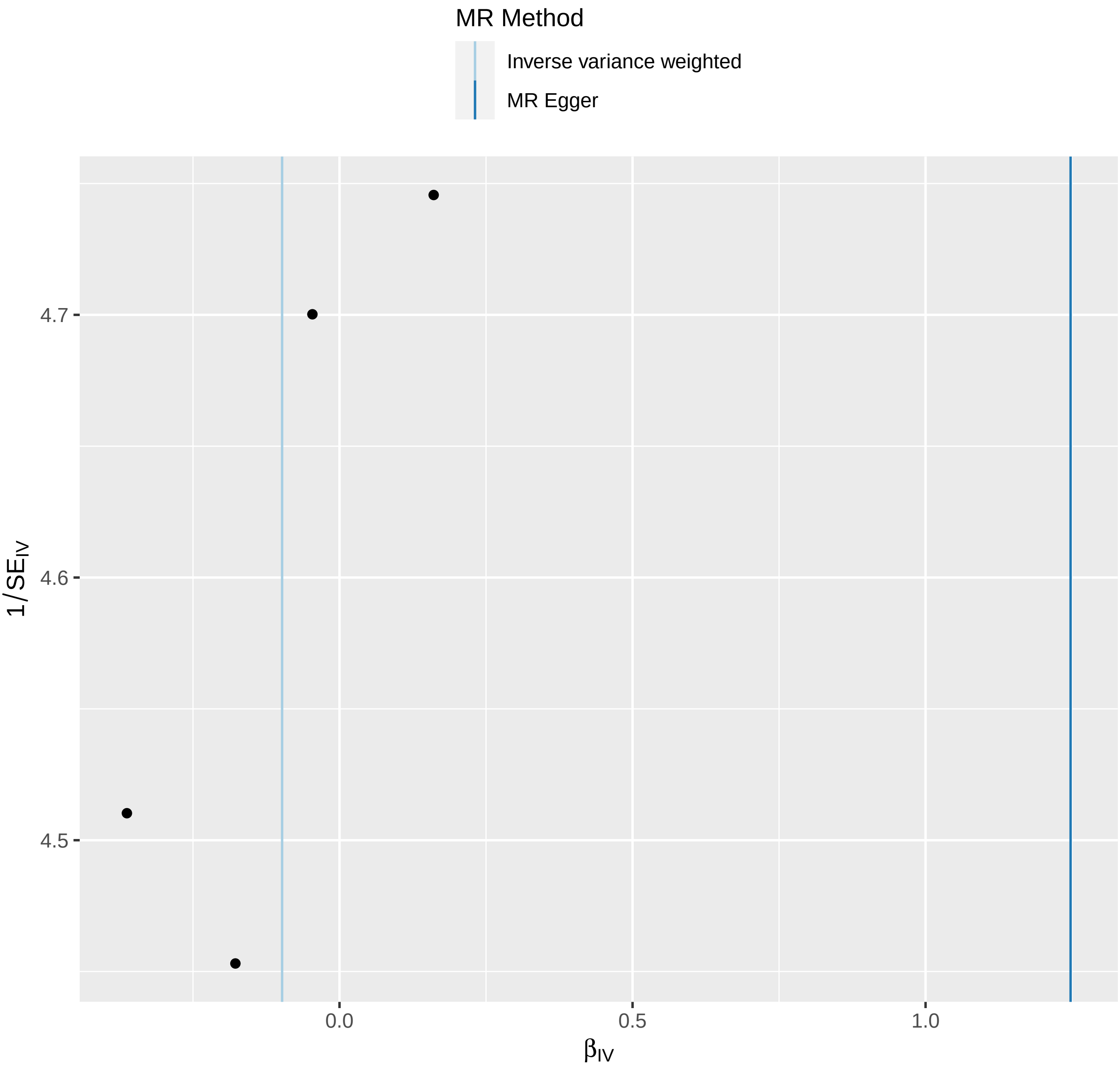

Batch 2−30 : Gut microbiota abundance (genus Parasutterella id.2892) on Acyl−CoA−binding domain−containing protein 7
with a threshold of 1e−05

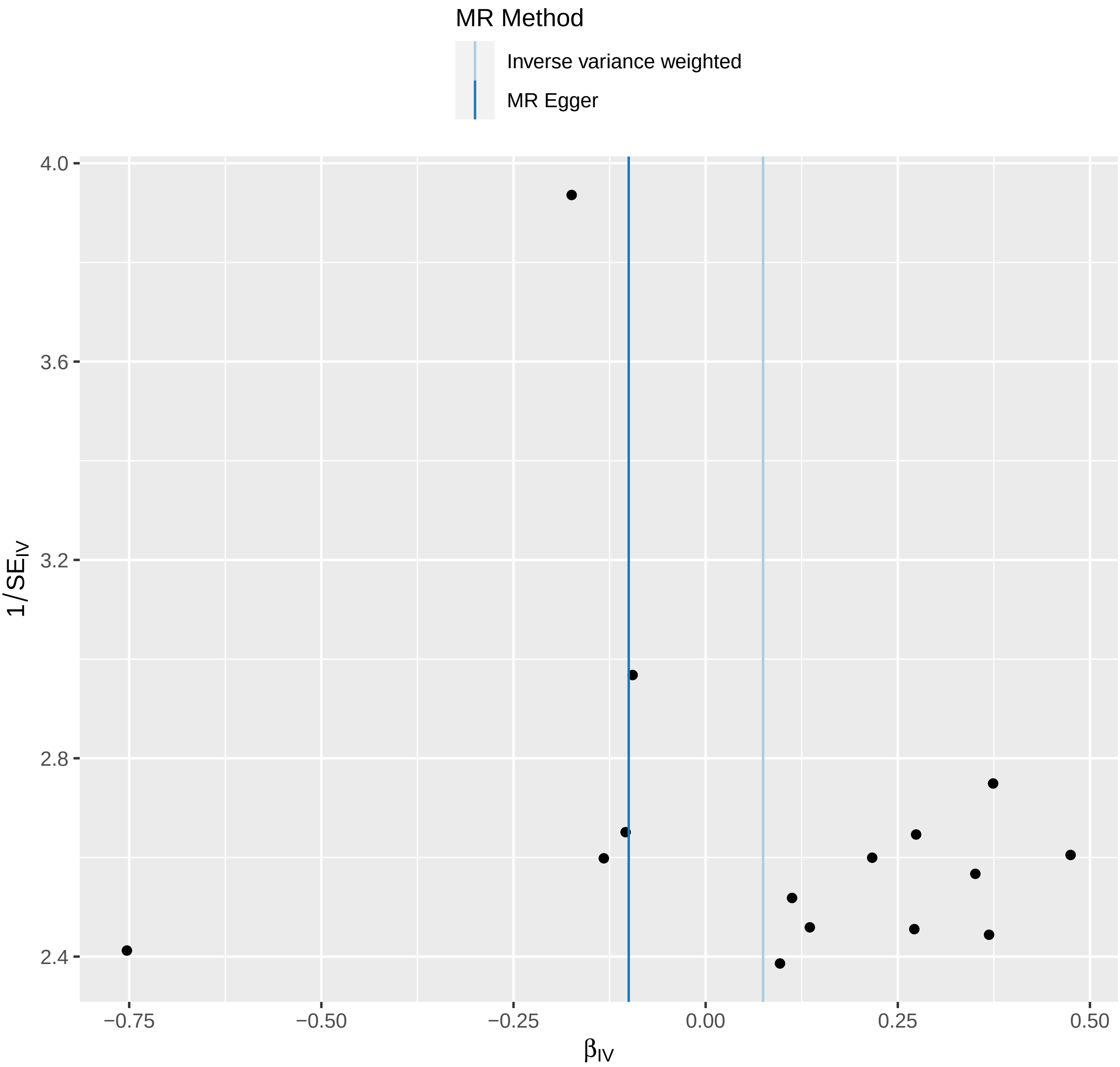

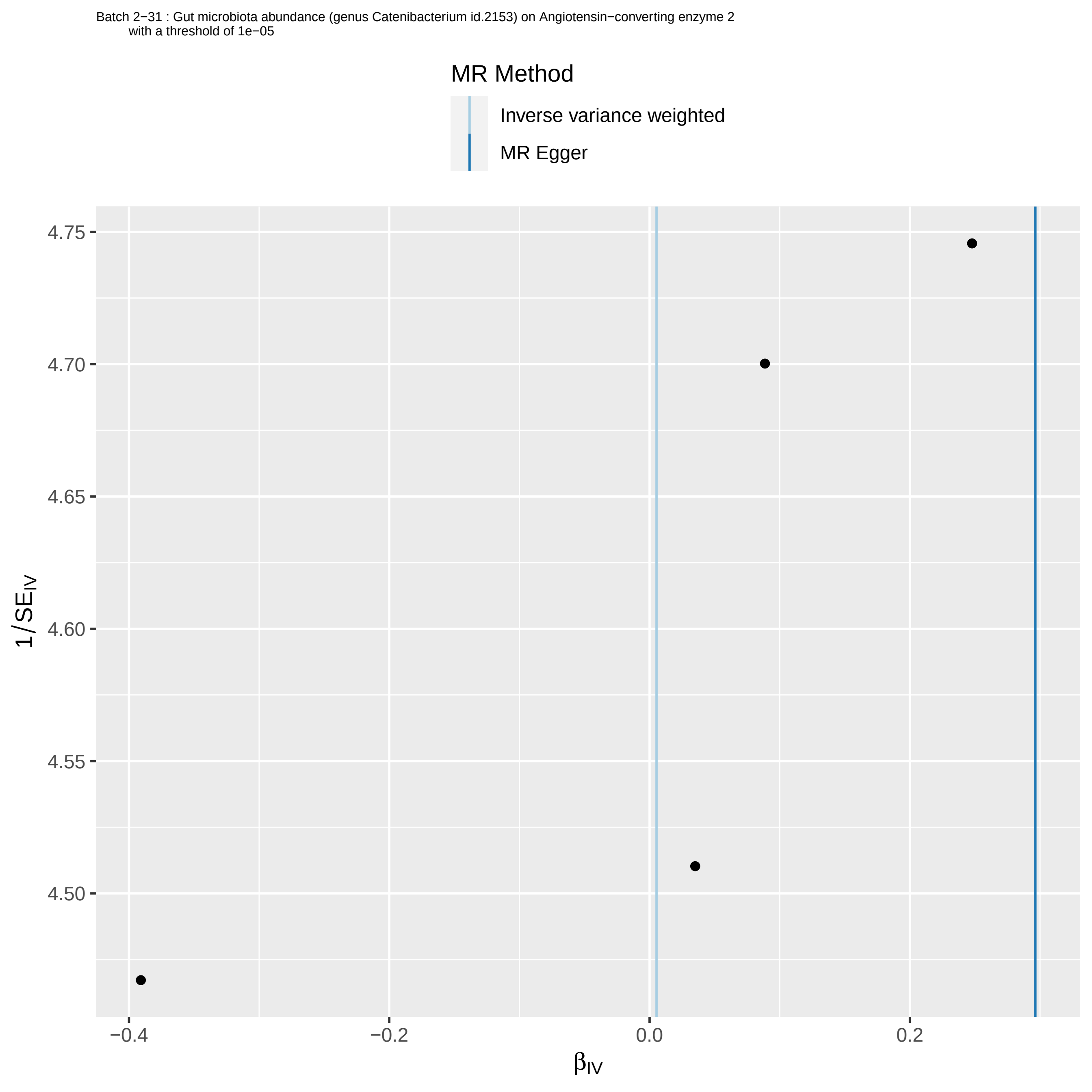
Batch 2−31 : Gut microbiota abundance (genus Catenibacterium id.2153) on Angiotensin−converting enzyme 2
with a threshold of 1e−05
MR Method
Inverse variance weighted
MR Egger
4.75
4.70
4.65
4.60
4.55
4.50
1/SE_IV
−0.4
−0.2
0.0
0.2
β_IV

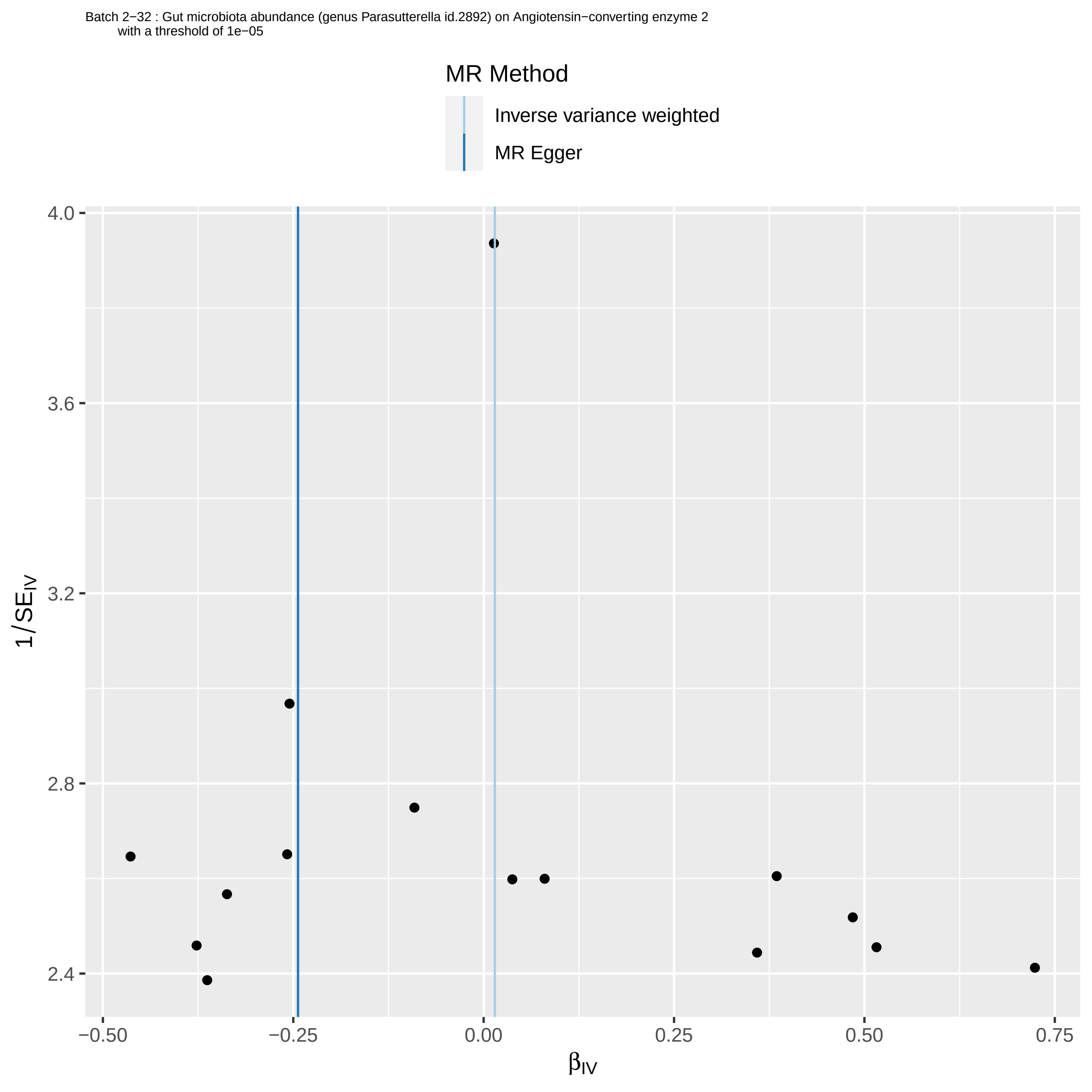

Batch 2−32 : Gut microbiota abundance (genus Parasutterella id.2892) on Angiotensin−converting enzyme 2
with a threshold of 1e−05
MR Method
Inverse variance weighted
MR Egger
4.0
3.6
3.2
2.8
2.4
1/SE$_{IV}$
−0.50
−0.25
0.00
0.25
0.50
0.75
β$_{IV}$

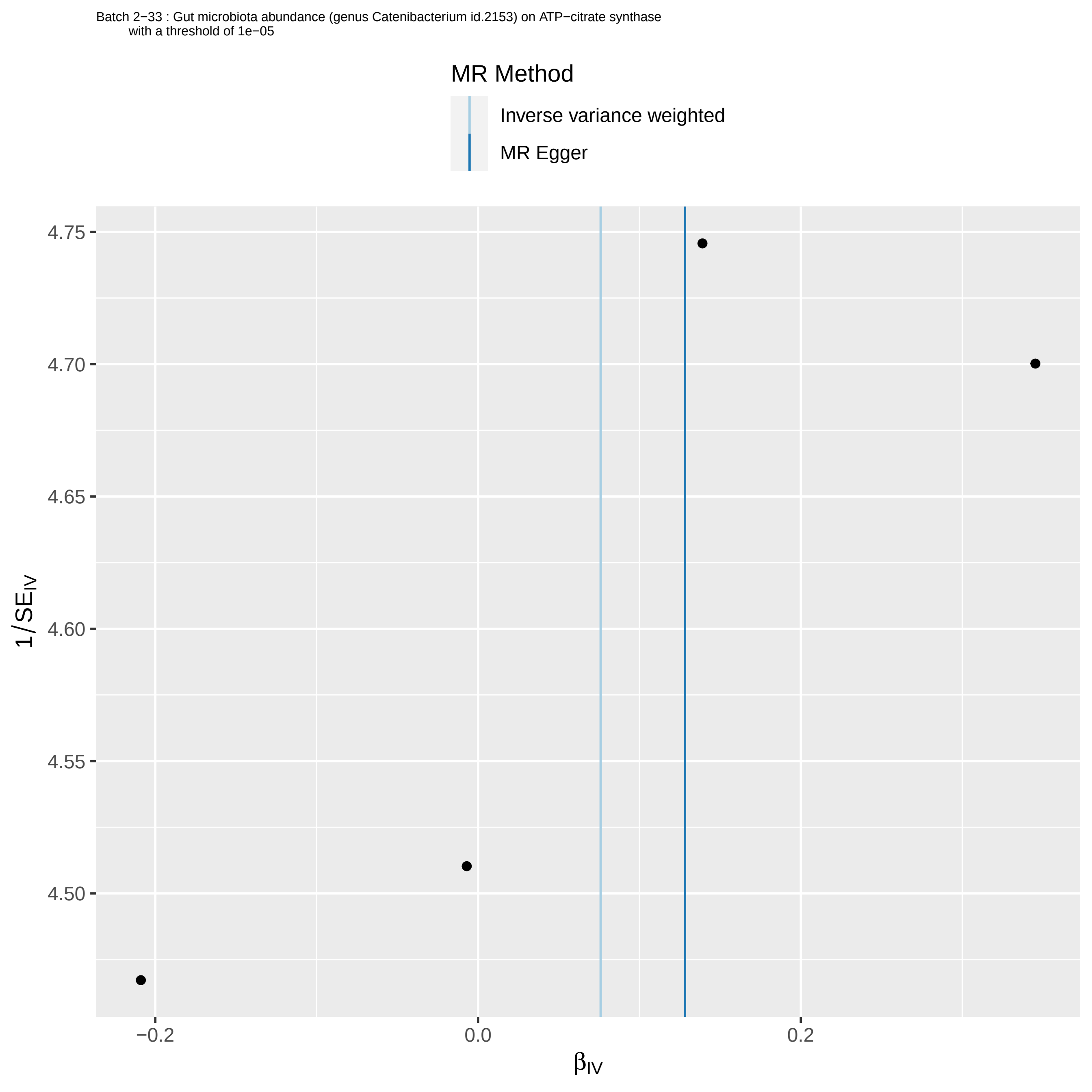

Batch 2−33 : Gut microbiota abundance (genus Catenibacterium id.2153) on ATP−citrate synthase
with a threshold of 1e−05
MR Method
Inverse variance weighted
MR Egger
4.75
4.70
4.65
4.60
4.55
4.50
1/SE_IV
−0.2
0.0
0.2
β_IV

Batch 2−34 : Gut microbiota abundance (genus Parasutterella id.2892) on ATP−citrate synthase
with a threshold of 1e−05

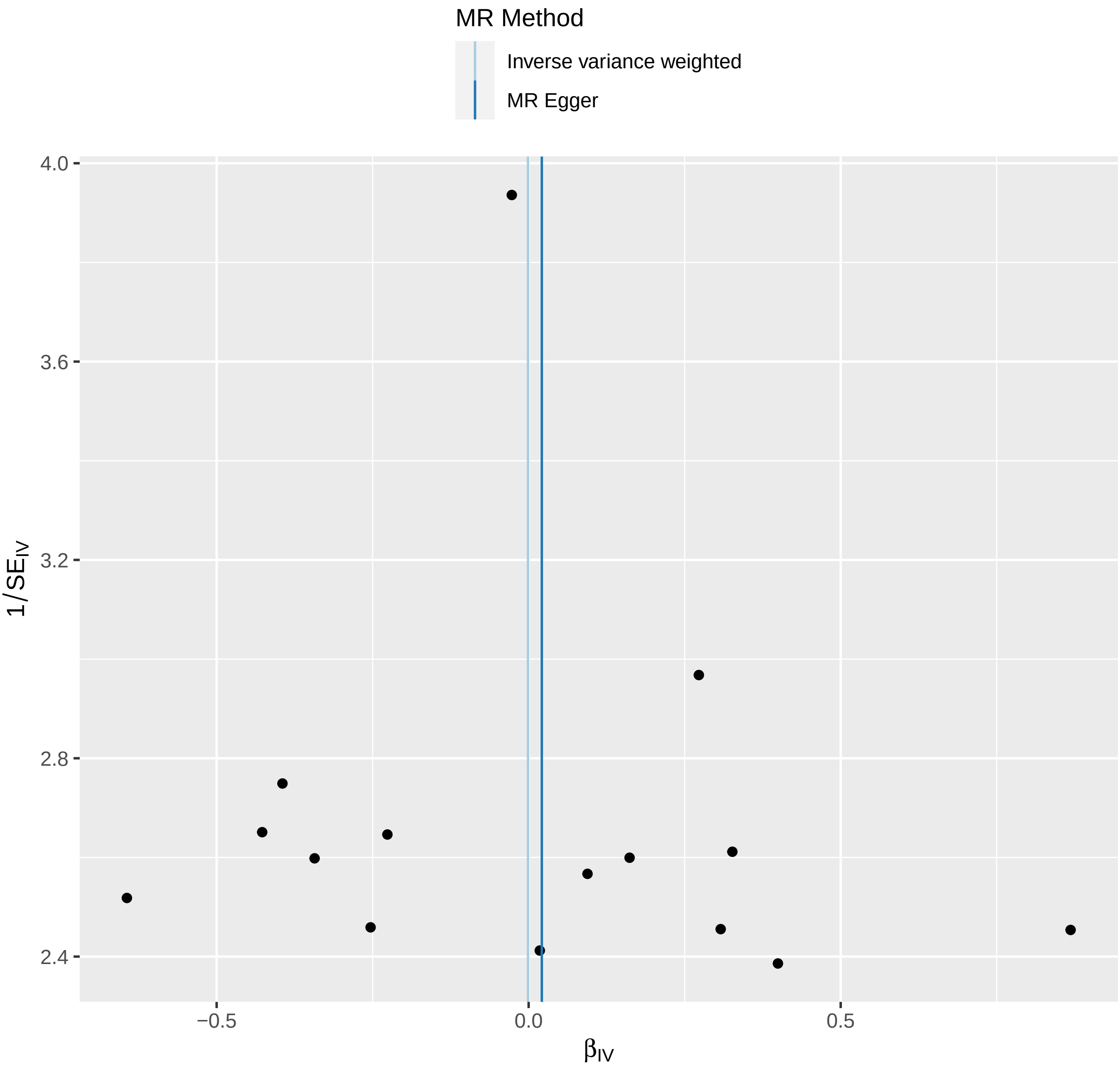

Batch 2−35 : Gut microbiota abundance (genus Catenibacterium id.2153) on Low molecular weight phosphotyrosine protein phosphatase with a threshold of 1e−05

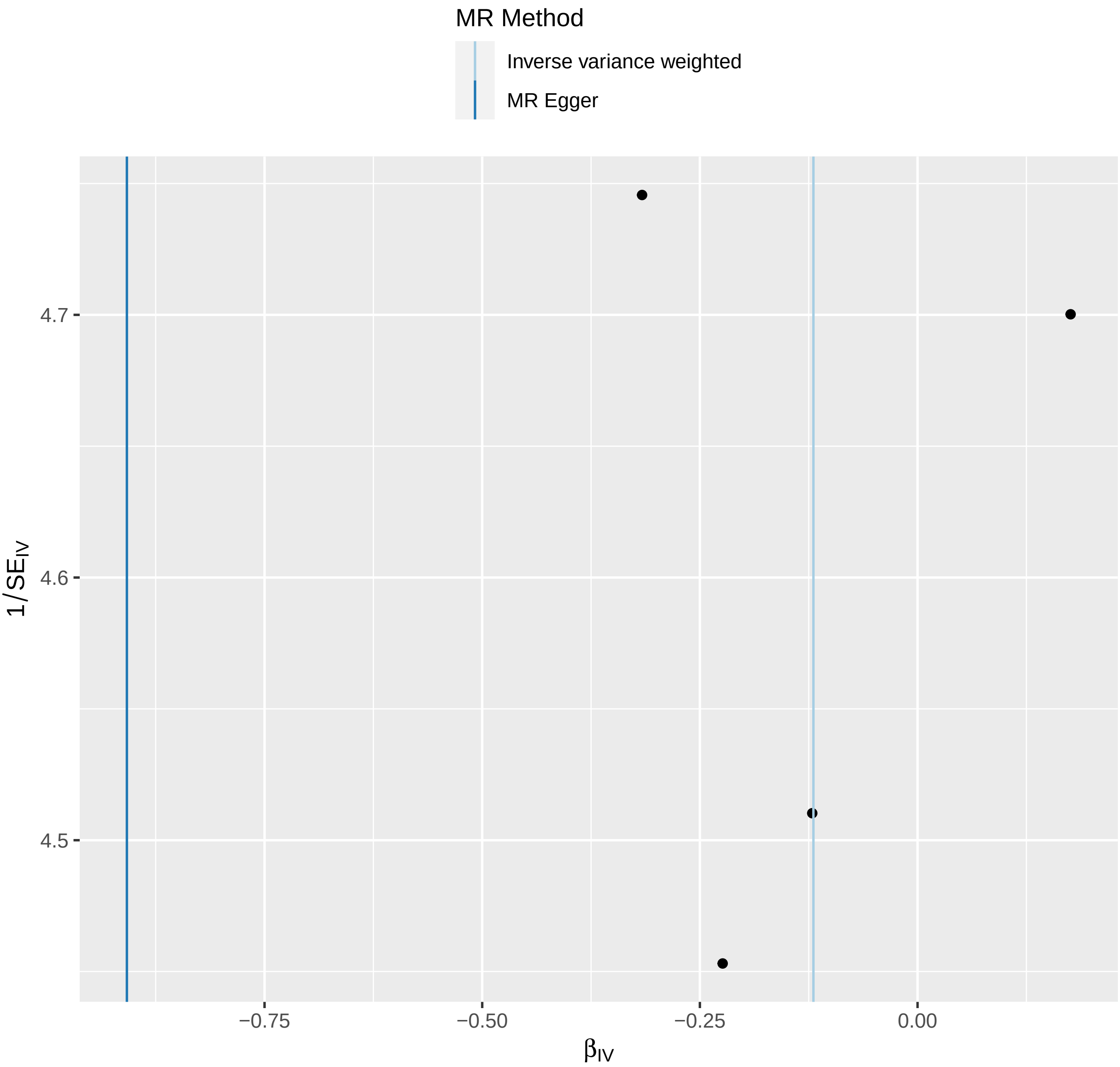

Batch 2−36 : Gut microbiota abundance (genus Parasutterella id.2892) on Low molecular weight phosphotyrosine protein phosphatase
with a threshold of 1e−05

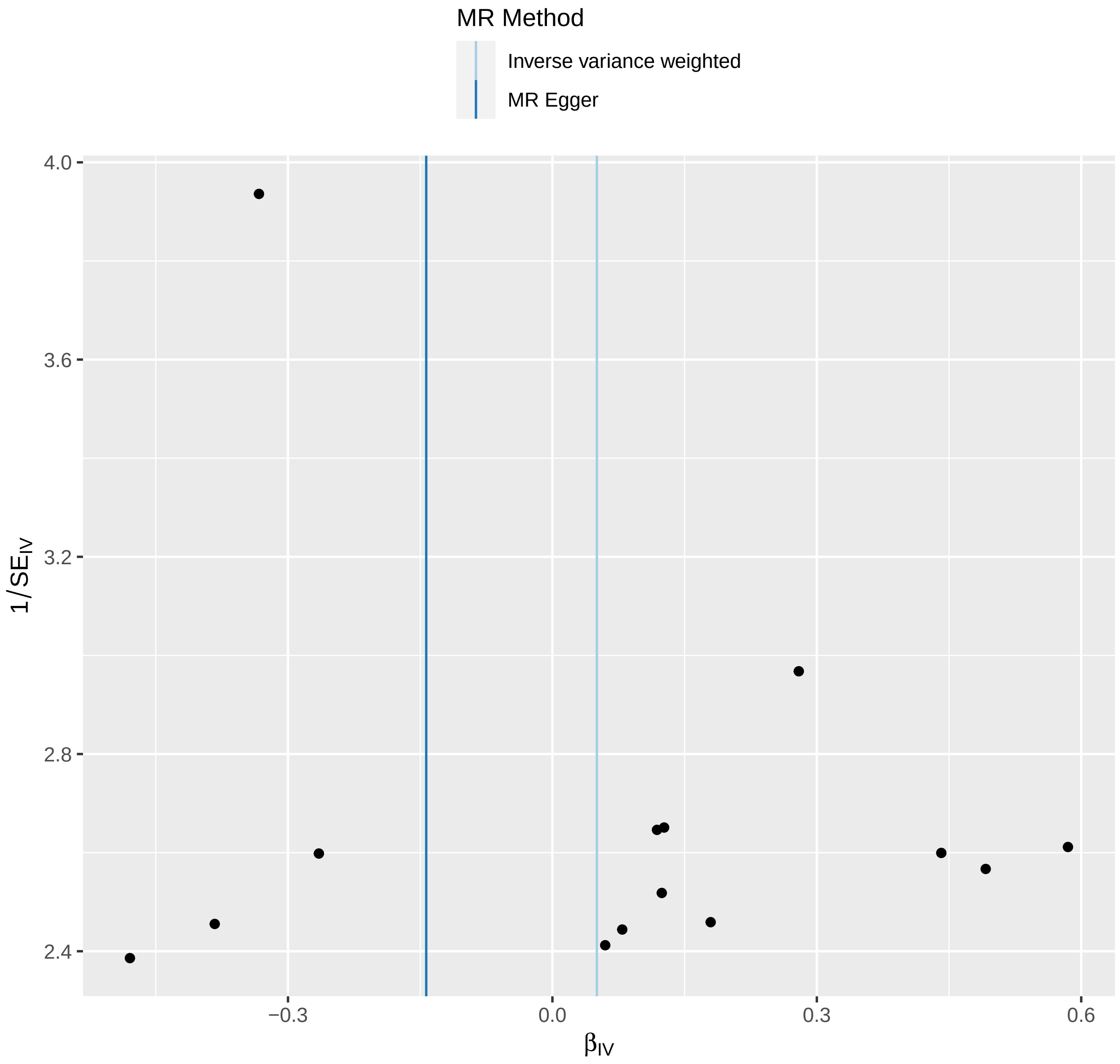

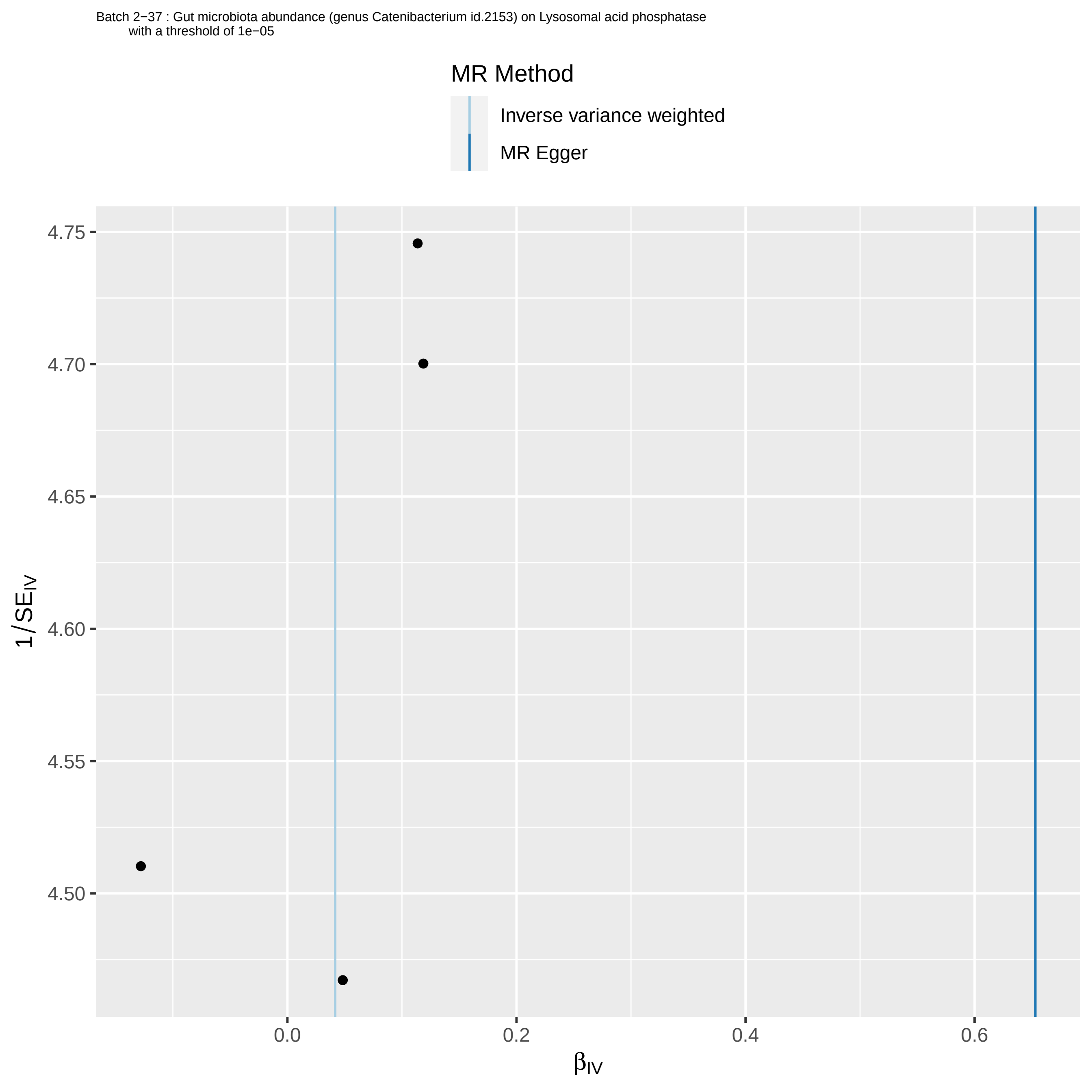
Batch 2−37 : Gut microbiota abundance (genus Catenibacterium id.2153) on Lysosomal acid phosphatase with a threshold of 1e−05
MR Method
Inverse variance weighted
MR Egger
4.75
4.70
4.65
4.60
4.55
4.50
1/SE_IV
0.0
0.2
0.4
0.6
β_IV

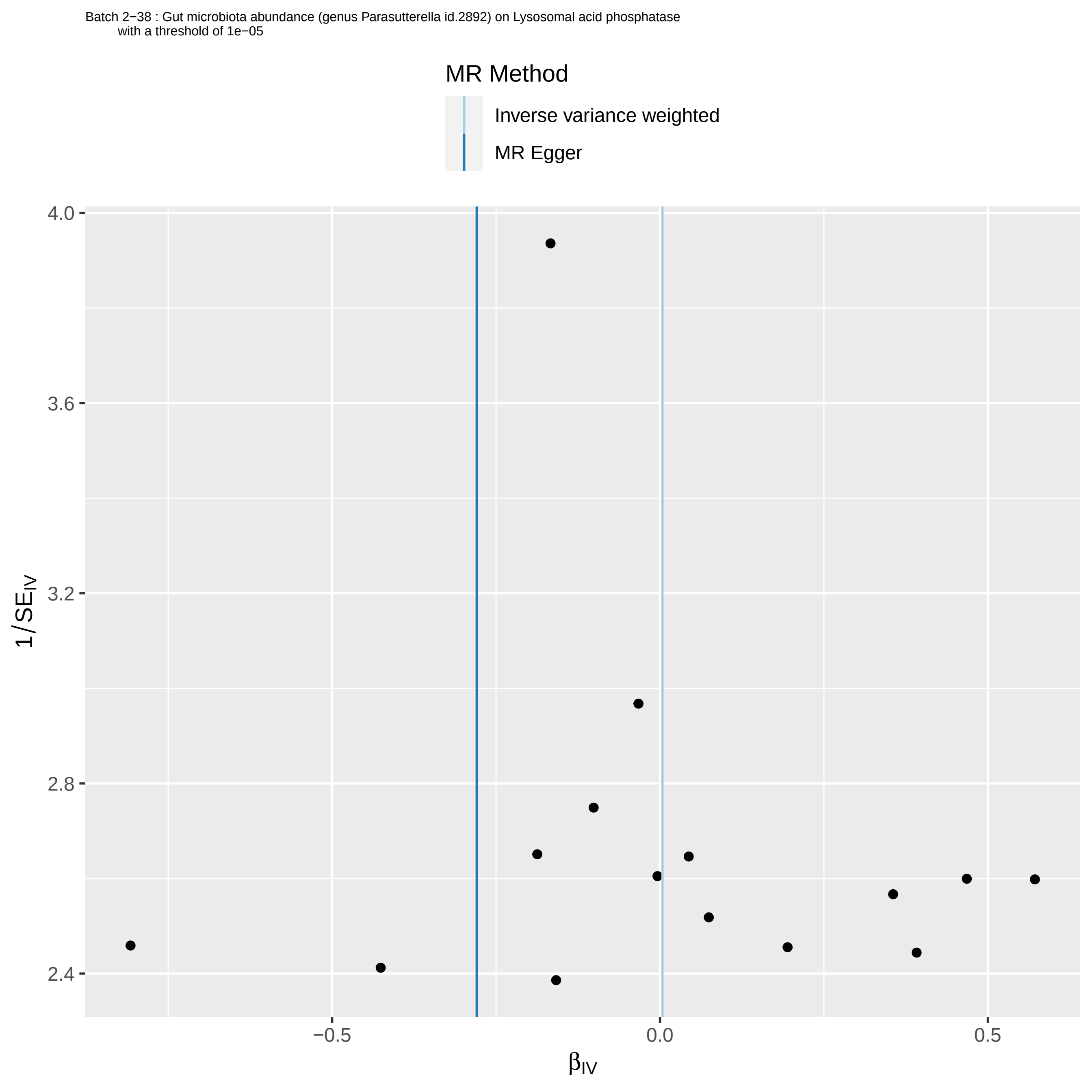
Batch 2−38 : Gut microbiota abundance (genus Parasutterella id.2892) on Lysosomal acid phosphatase
with a threshold of 1e−05
MR Method
Inverse variance weighted
MR Egger
4.0
3.6
3.2
2.8
2.4
1/SE_IV
−0.5
0.0
0.5
β_IV

Batch 2−39 : Gut microbiota abundance (genus Catenibacterium id.2153) on Tartrate−resistant acid phosphatase type 5
with a threshold of 1e−05

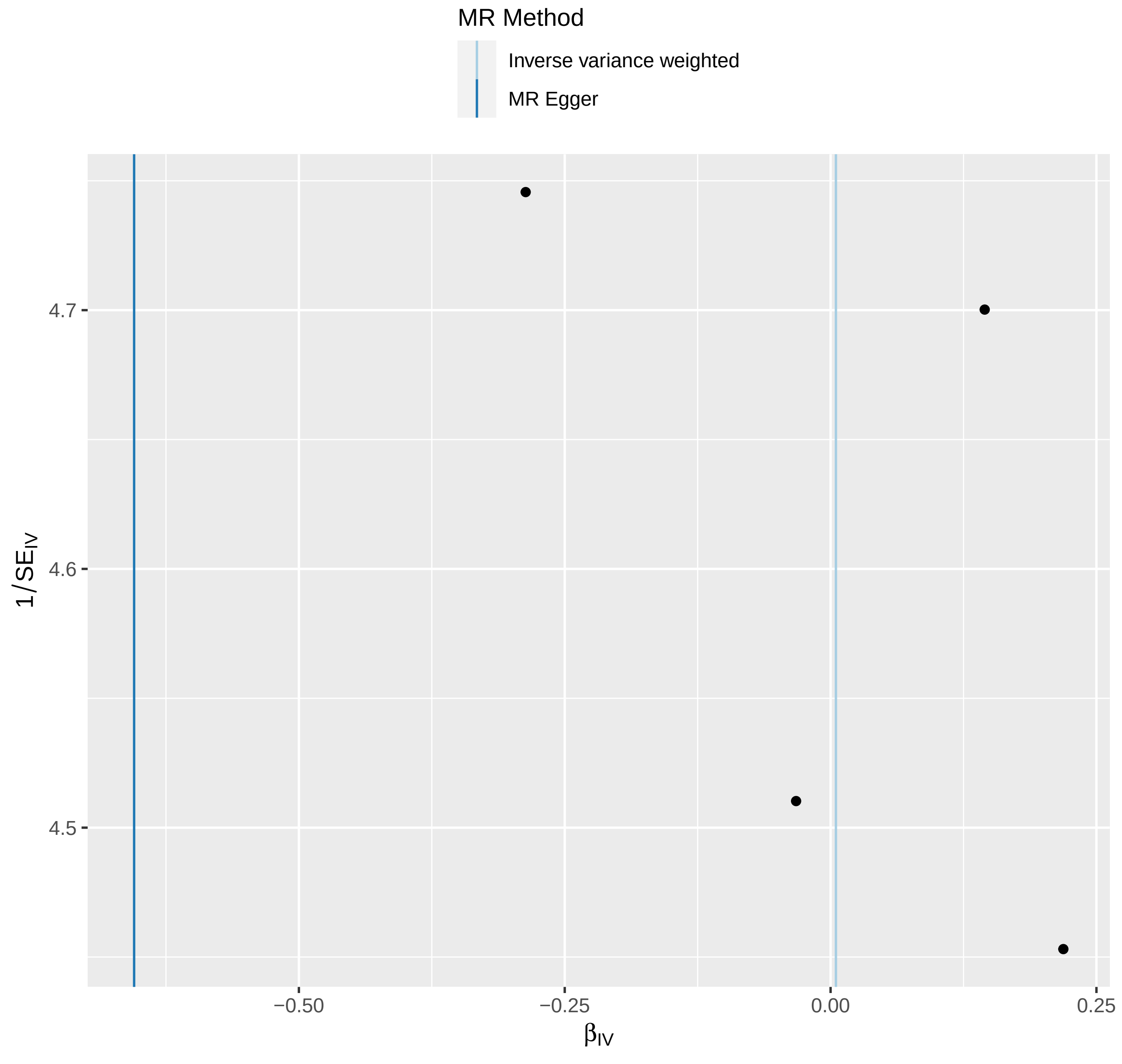

Batch 2−40 : Gut microbiota abundance (genus Parasutterella id.2892) on Tartrate−resistant acid phosphatase type 5
with a threshold of 1e−05

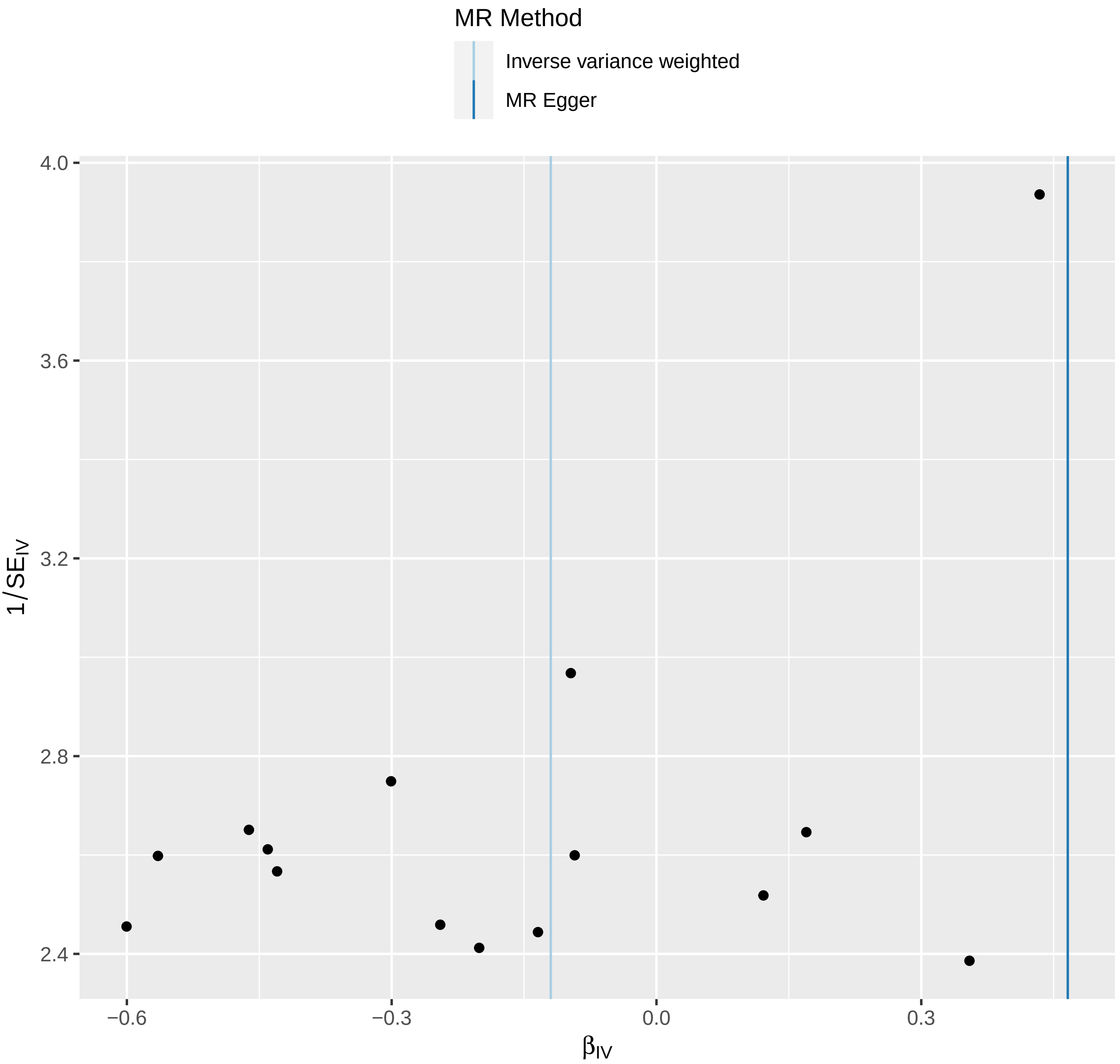

Batch 2−41 : Gut microbiota abundance (genus Catenibacterium id.2153) on 2−phosphoxylose phosphatase 1
with a threshold of 1e−05

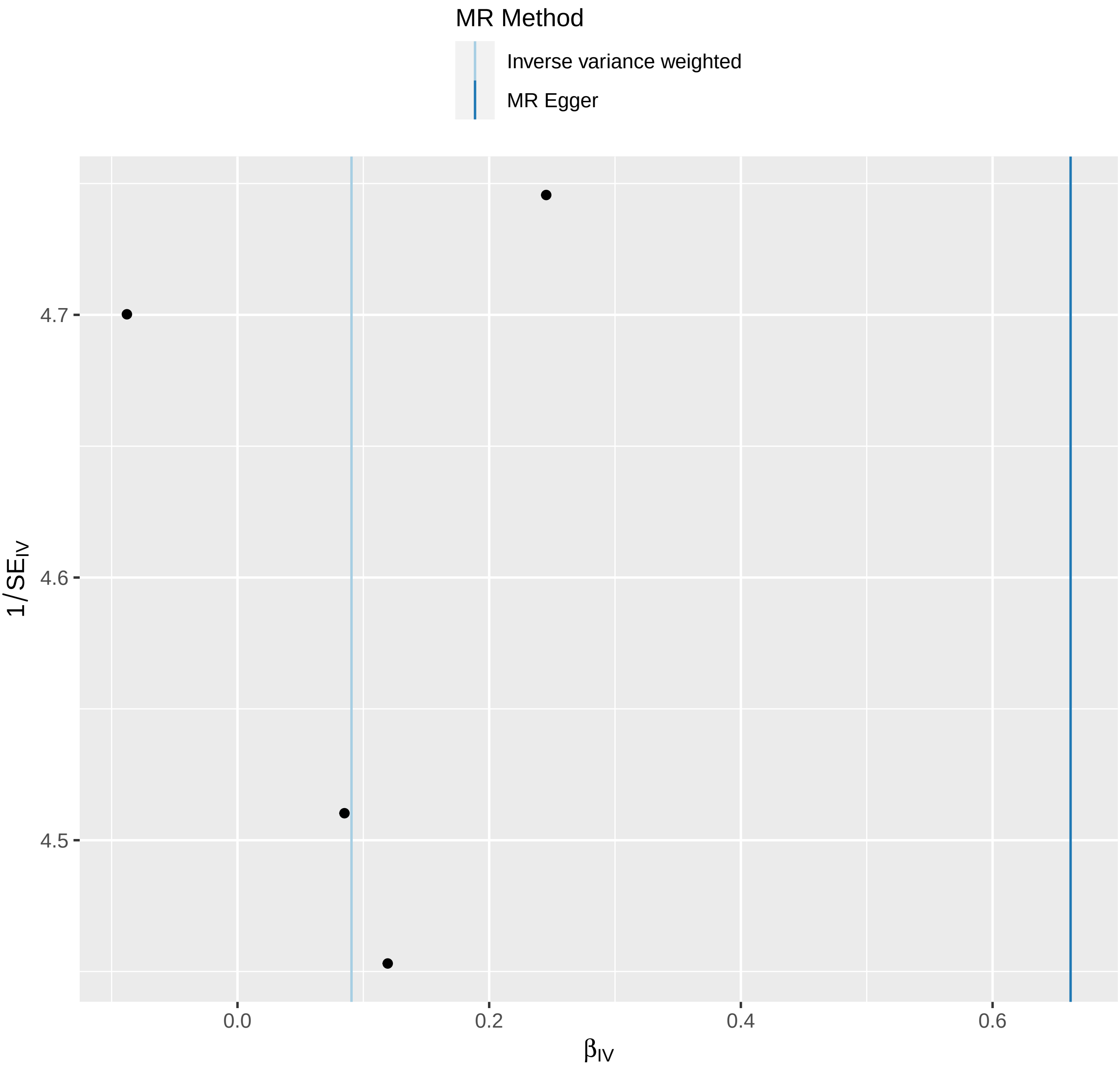

Batch 2−42 : Gut microbiota abundance (genus Parasutterella id.2892) on 2−phosphoxylose phosphatase 1
with a threshold of 1e−05

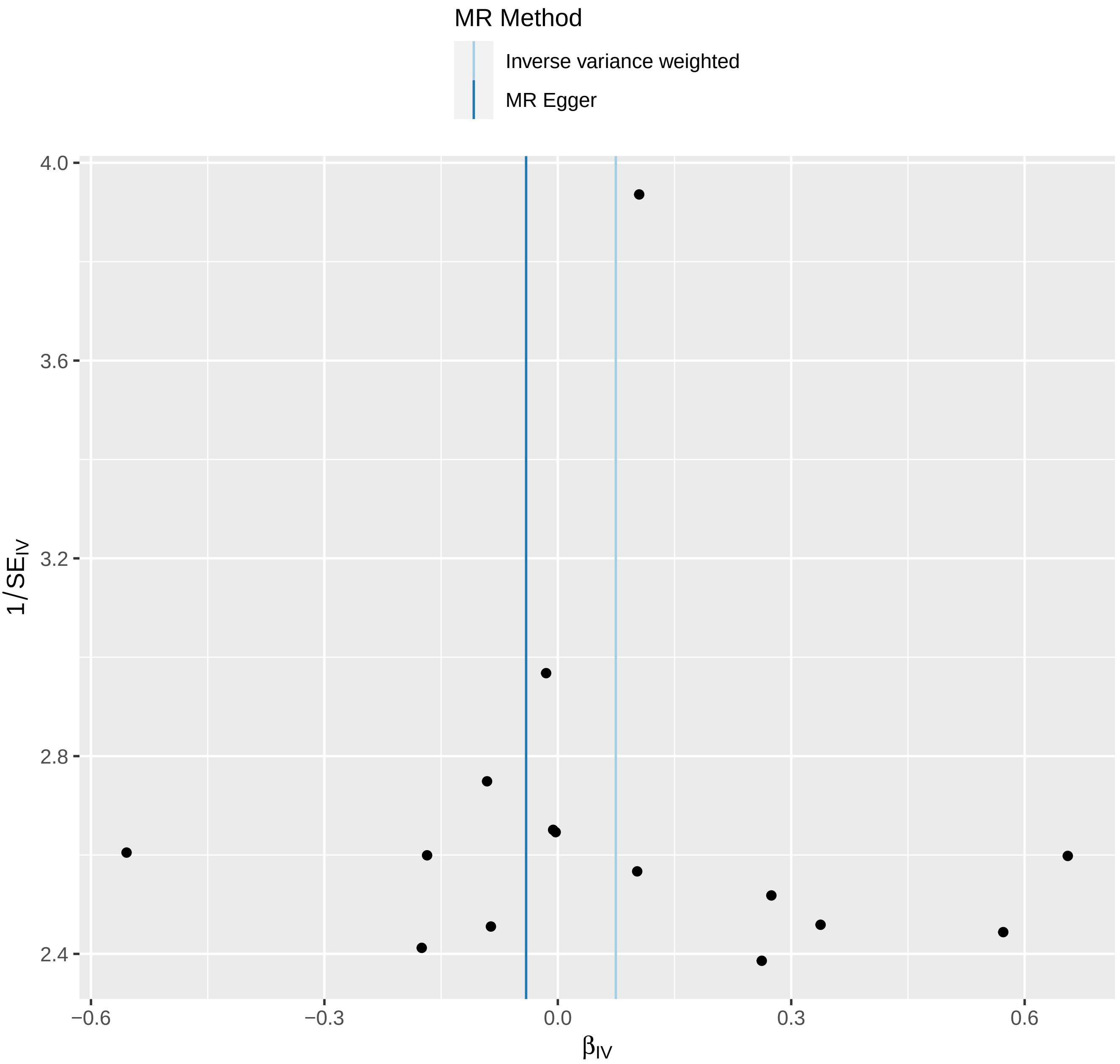

Batch 2−43 : Gut microbiota abundance (genus Catenibacterium id.2153) on Activin receptor type−1B
with a threshold of 1e−05

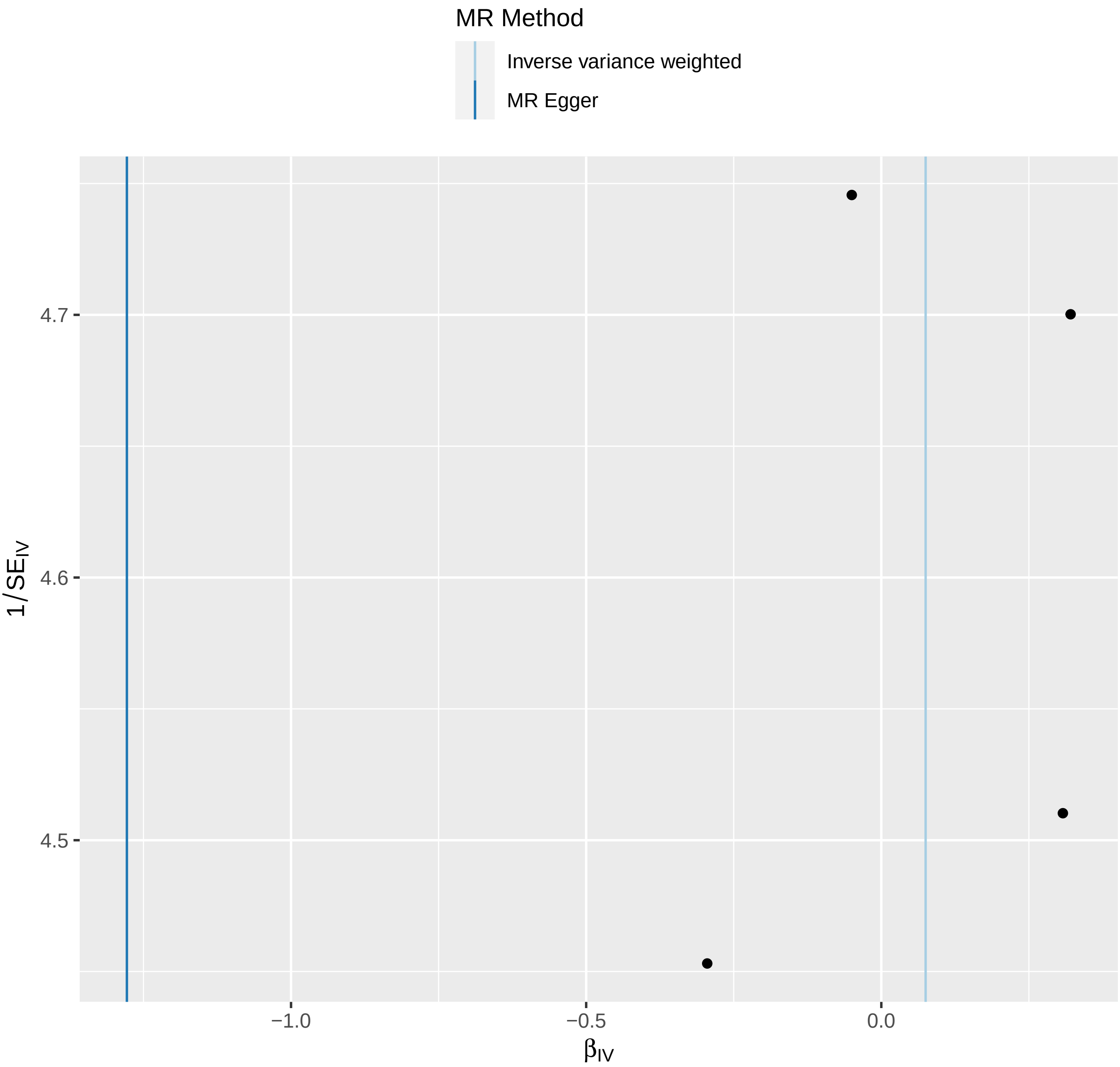

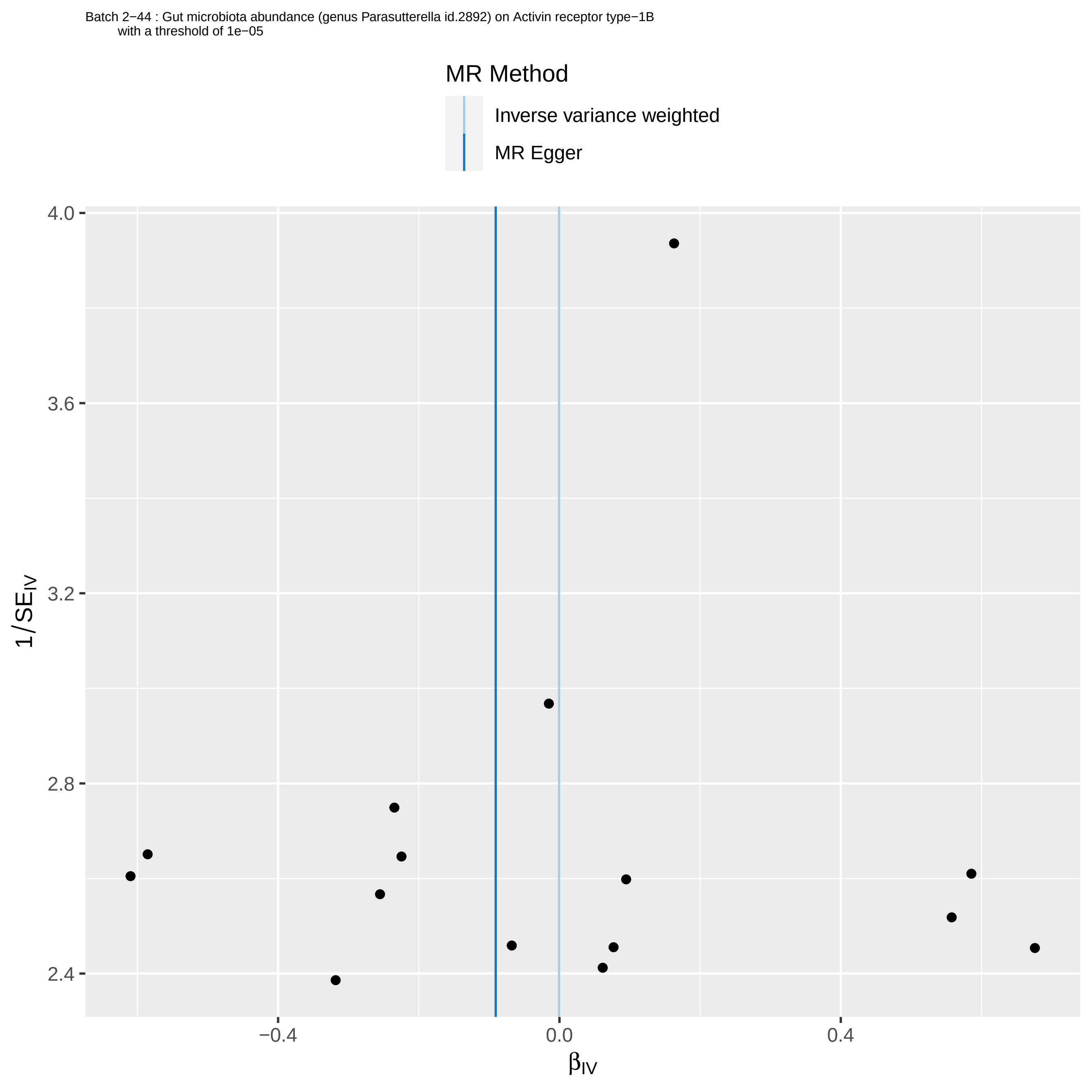

Batch 2−44 : Gut microbiota abundance (genus Parasutterella id.2892) on Activin receptor type−1B with a threshold of 1e−05
MR Method
Inverse variance weighted
MR Egger
4.0
3.6
3.2
2.8
2.4
1/SE_IV
−0.4
0.0
0.4
β_IV

Batch 2−45 : Gut microbiota abundance (genus Catenibacterium id.2153) on Serine/threonine−protein kinase receptor R3
with a threshold of 1e−05

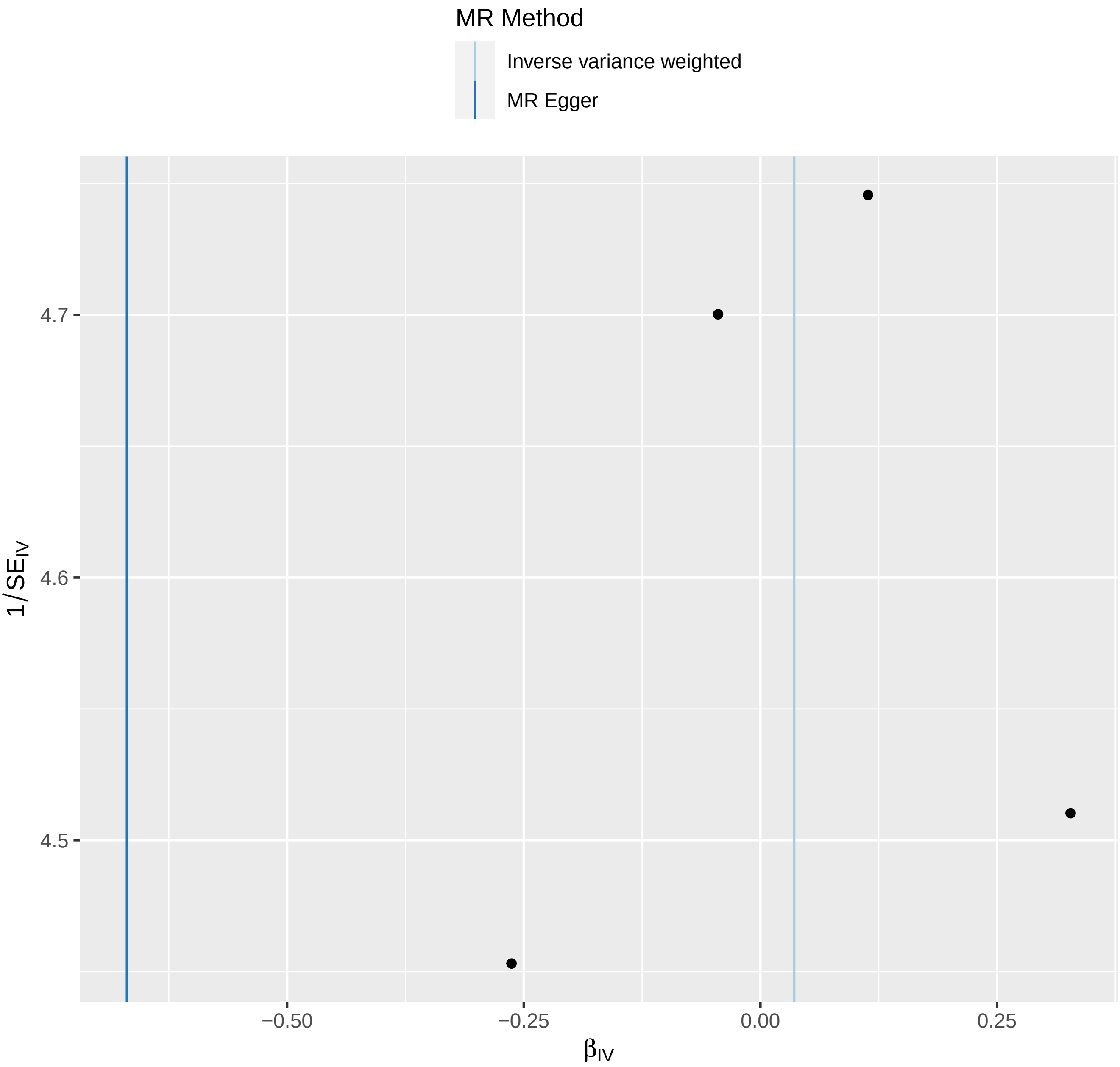

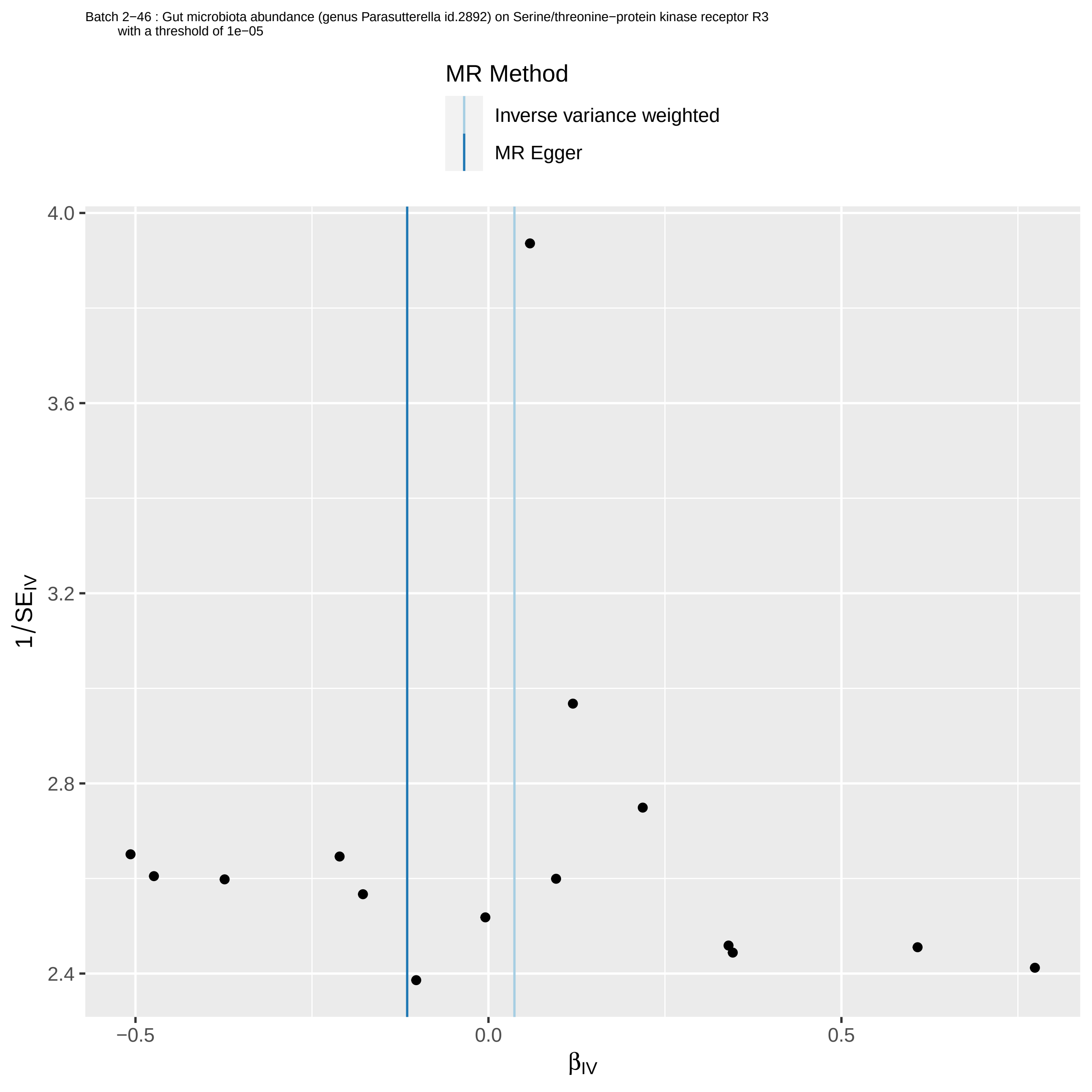
Batch 2−46 : Gut microbiota abundance (genus Parasutterella id.2892) on Serine/threonine−protein kinase receptor R3
with a threshold of 1e−05
MR Method
Inverse variance weighted
MR Egger
4.0
3.6
3.2
2.8
2.4
1/SE_IV
−0.5
0.0
0.5
β_IV

Batch 2−47 : Gut microbiota abundance (genus Catenibacterium id.2153) on Acylphosphatase−2
with a threshold of 1e−05

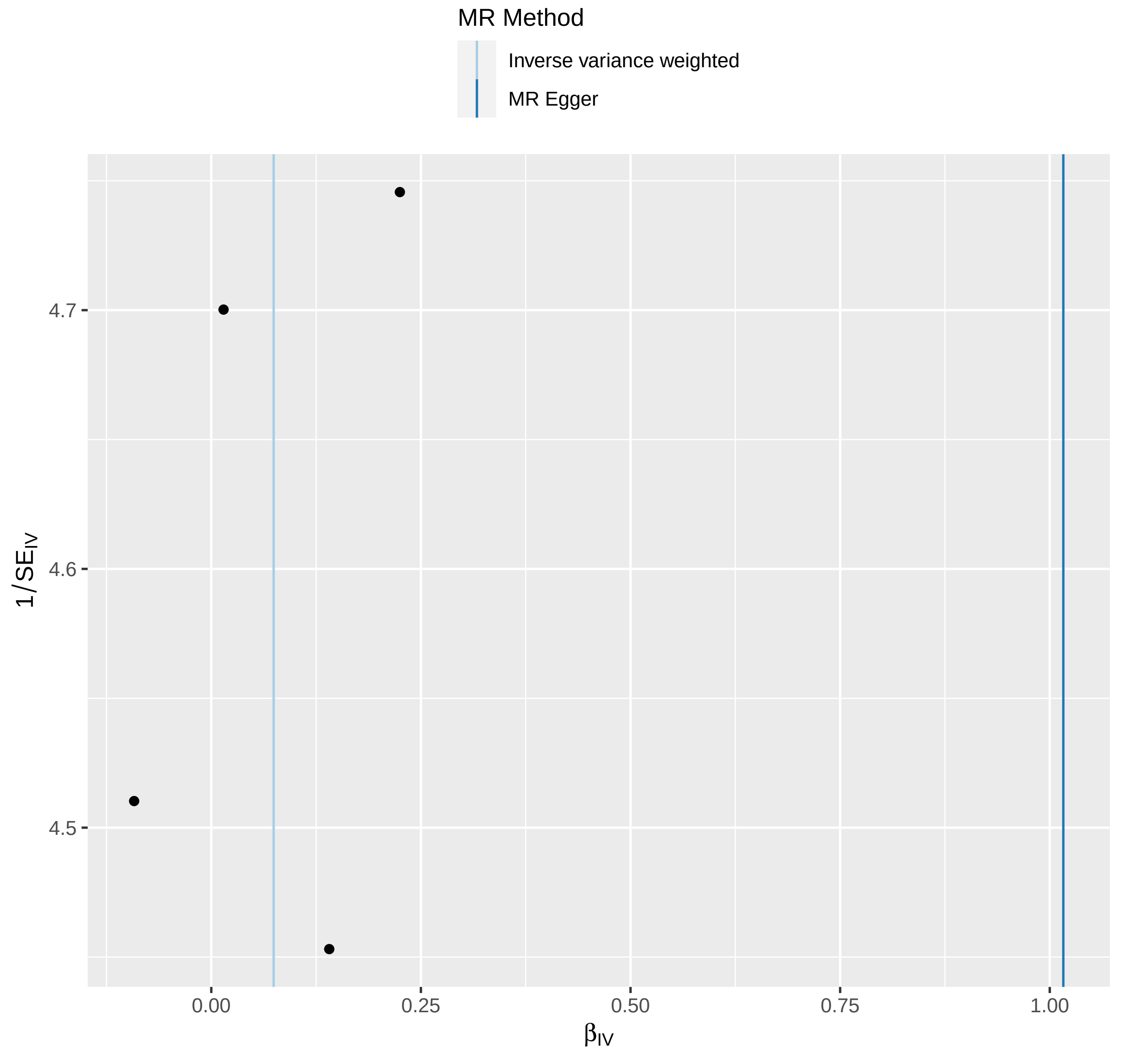

Batch 2−48 : Gut microbiota abundance (genus Parasutterella id.2892) on Acylphosphatase−2
with a threshold of 1e−05

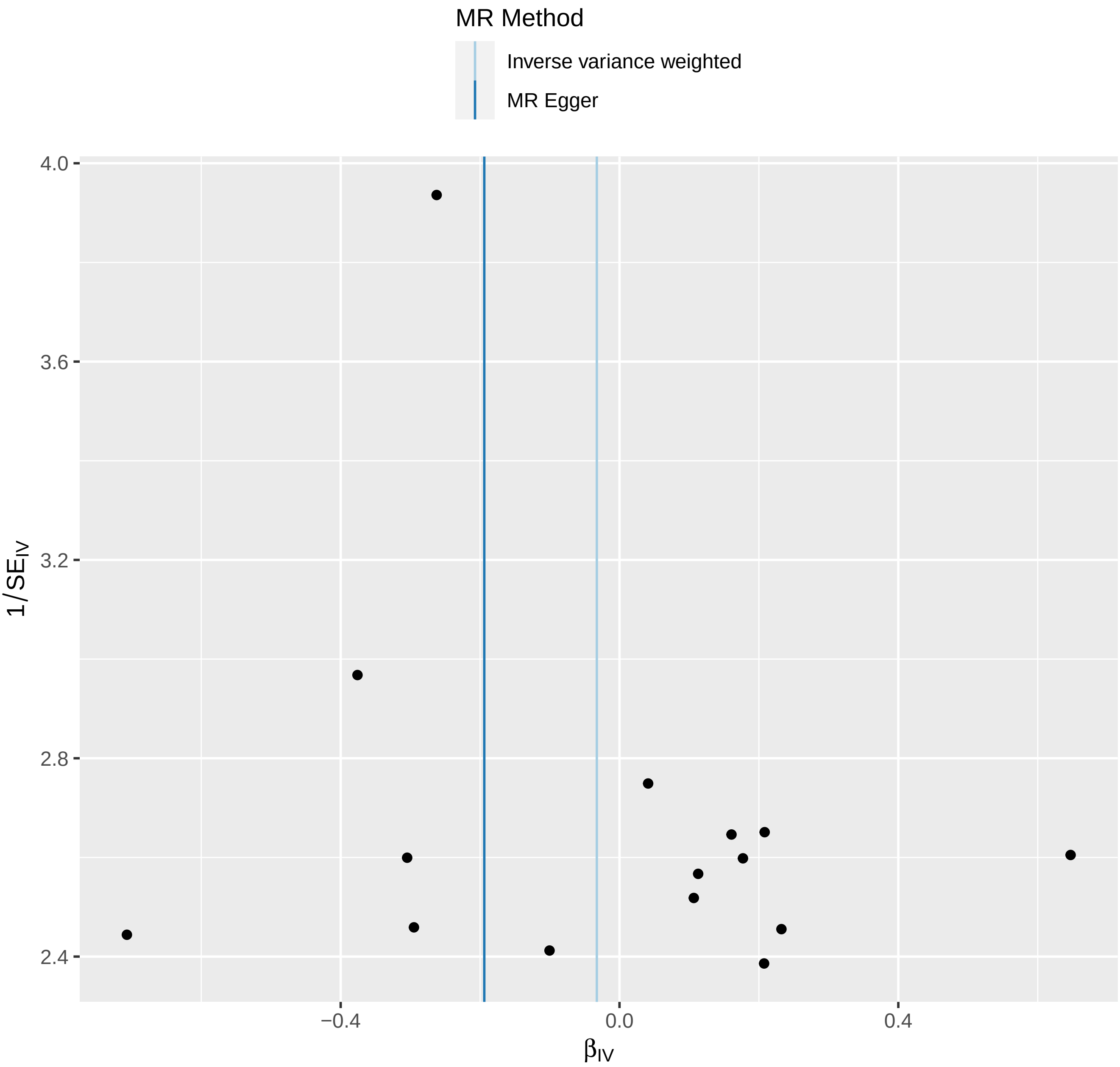

Batch 2−49 : Gut microbiota abundance (genus Catenibacterium id.2153) on Disintegrin and metalloproteinase domain−containing protein 11 with a threshold of 1e−05

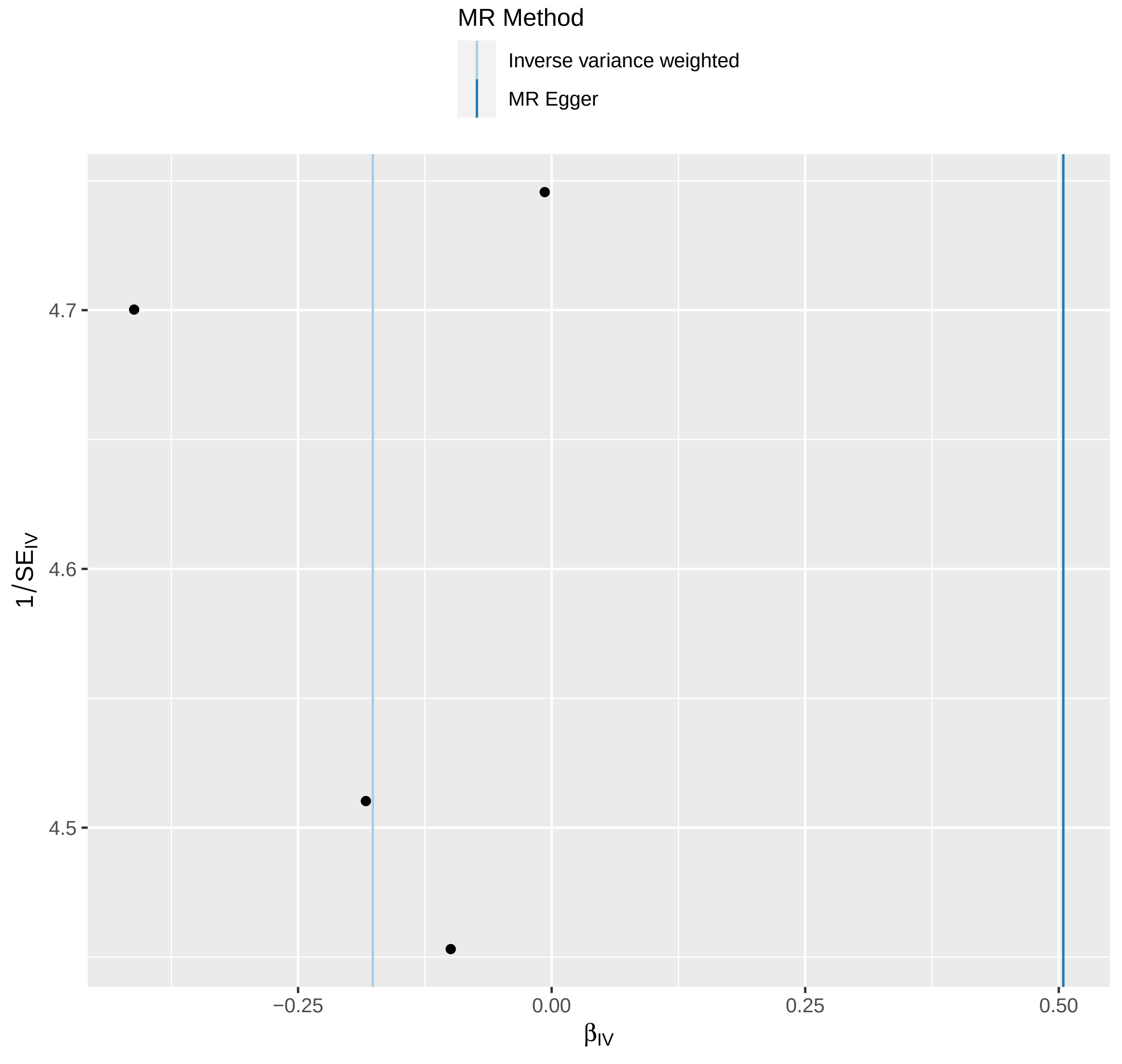

Batch 2−50 : Gut microbiota abundance (genus Parasutterella id.2892) on Disintegrin and metalloproteinase domain−containing protein 11 with a threshold of 1e−05

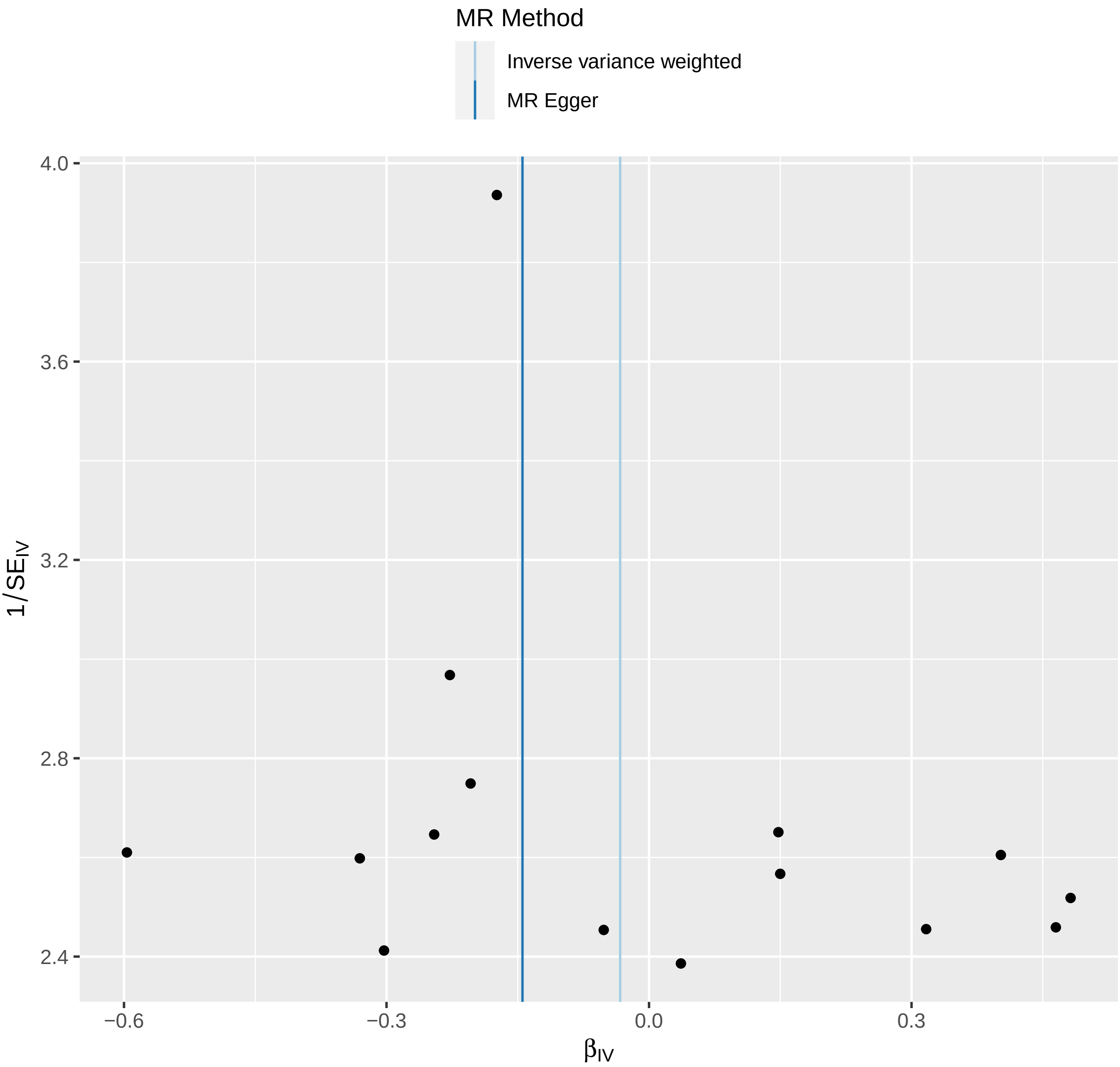

Batch 2−51 : Gut microbiota abundance (genus Catenibacterium id.2153) on Disintegrin and metalloproteinase domain−containing protein 12 with a threshold of 1e−05

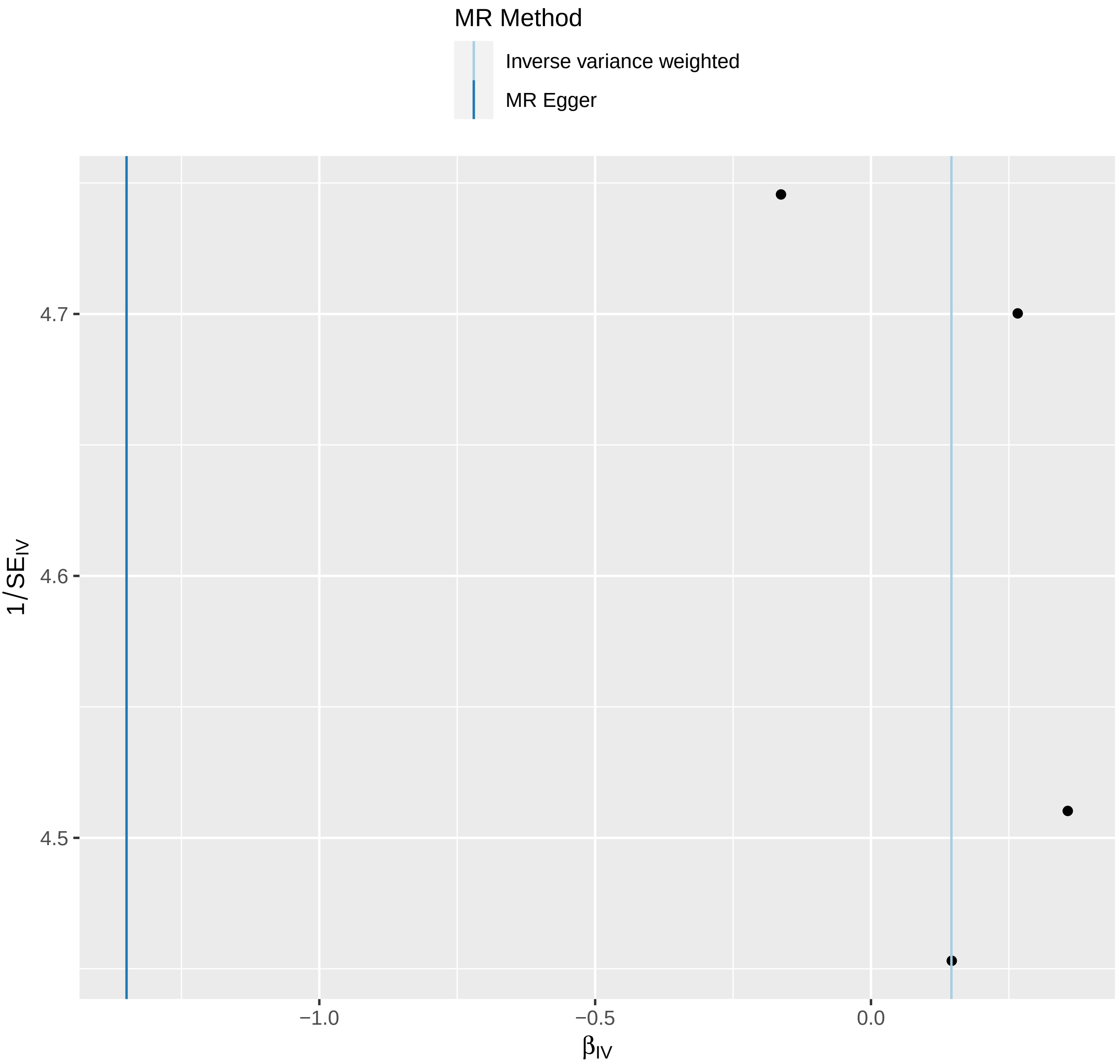

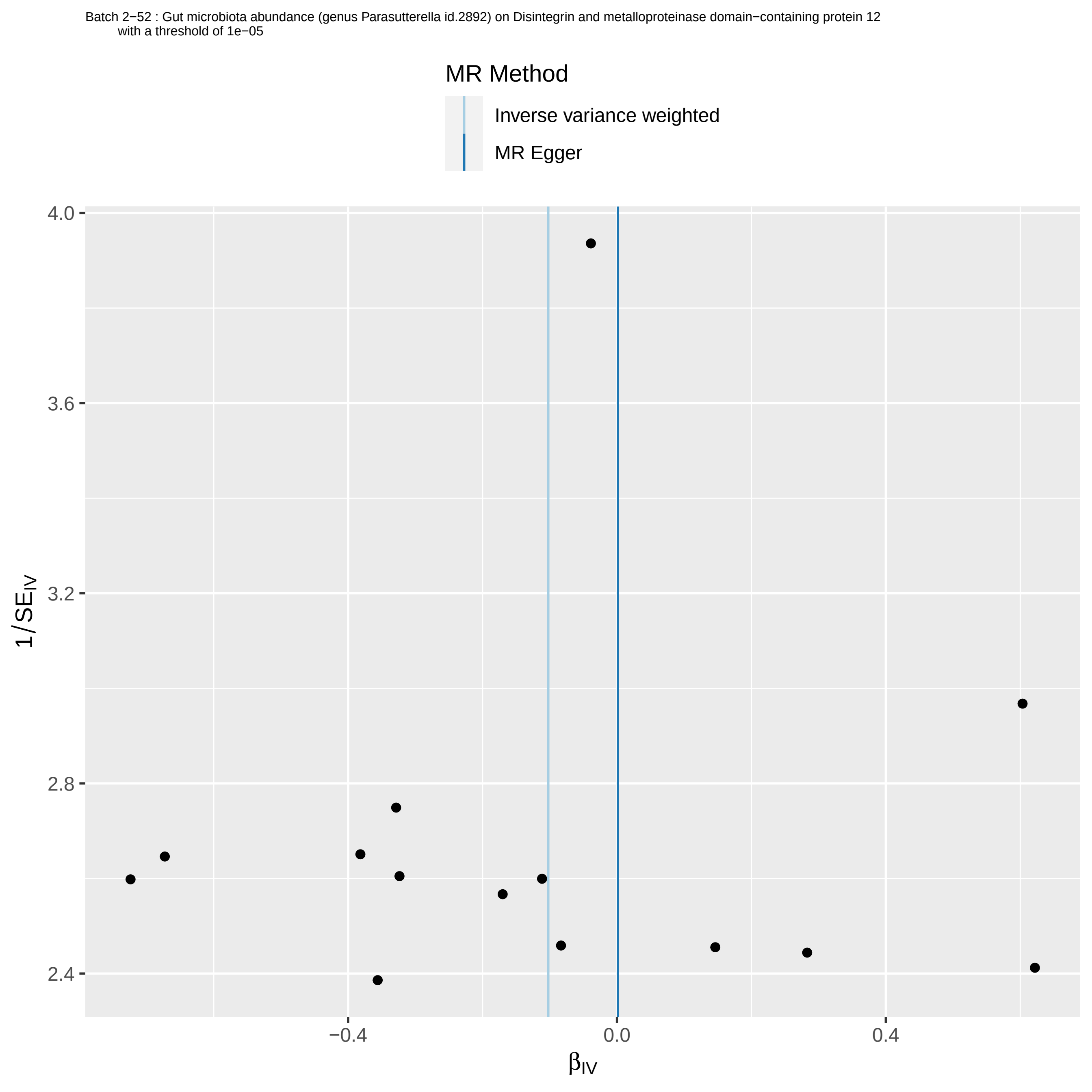

Batch 2−52 : Gut microbiota abundance (genus Parasutterella id.2892) on Disintegrin and metalloproteinase domain−containing protein 12
with a threshold of 1e−05
MR Method
Inverse variance weighted
MR Egger
4.0
3.6
3.2
2.8
2.4
1/SE$_{IV}$
−0.4
0.0
0.4
β$_{IV}$

Batch 2−53 : Gut microbiota abundance (genus Catenibacterium id.2153) on Disintegrin and metalloproteinase domain−containing protein 19 with a threshold of 1e−05

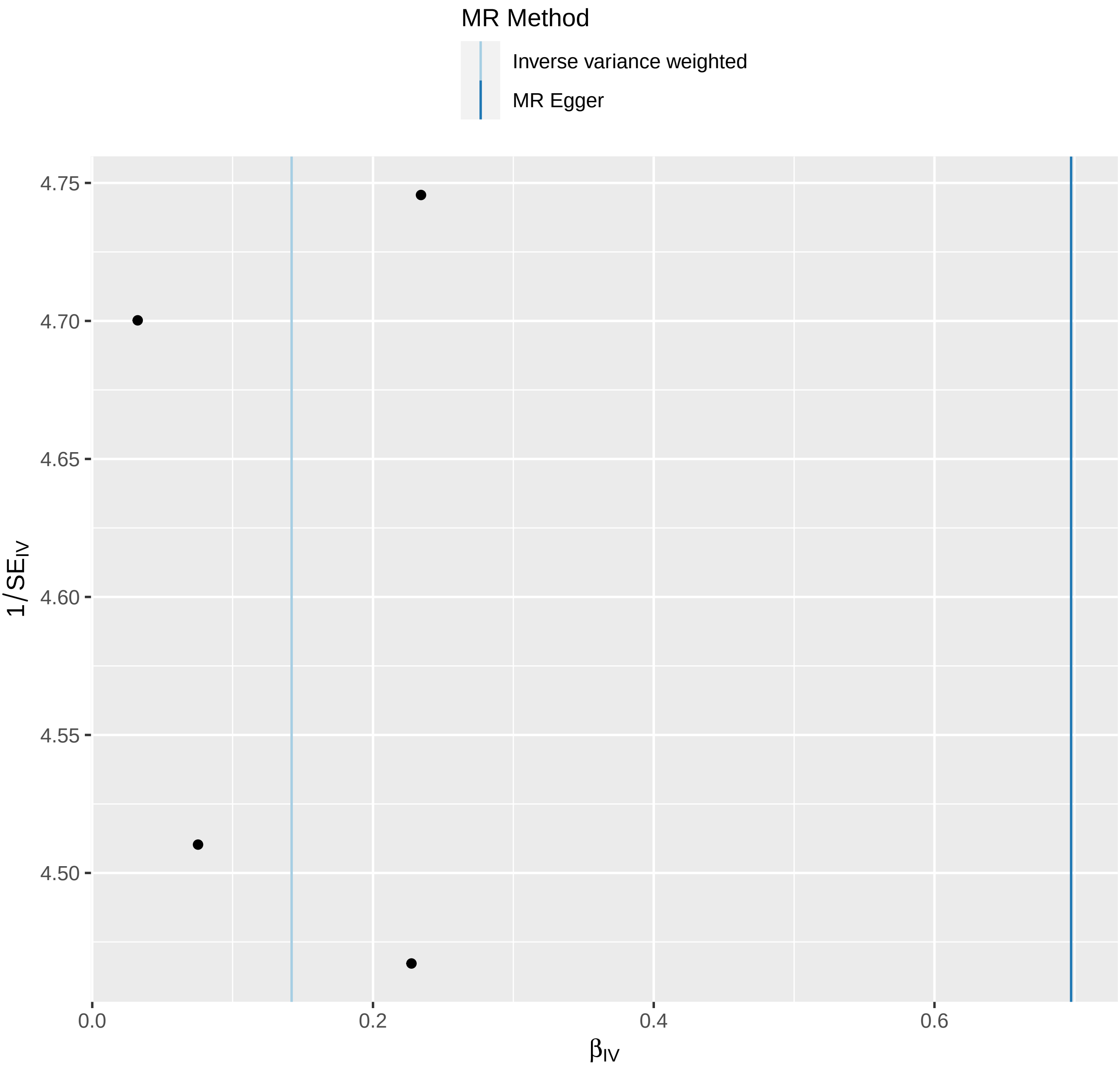

Batch 2−54 : Gut microbiota abundance (genus Parasutterella id.2892) on Disintegrin and metalloproteinase domain−containing protein 19
with a threshold of 1e−05

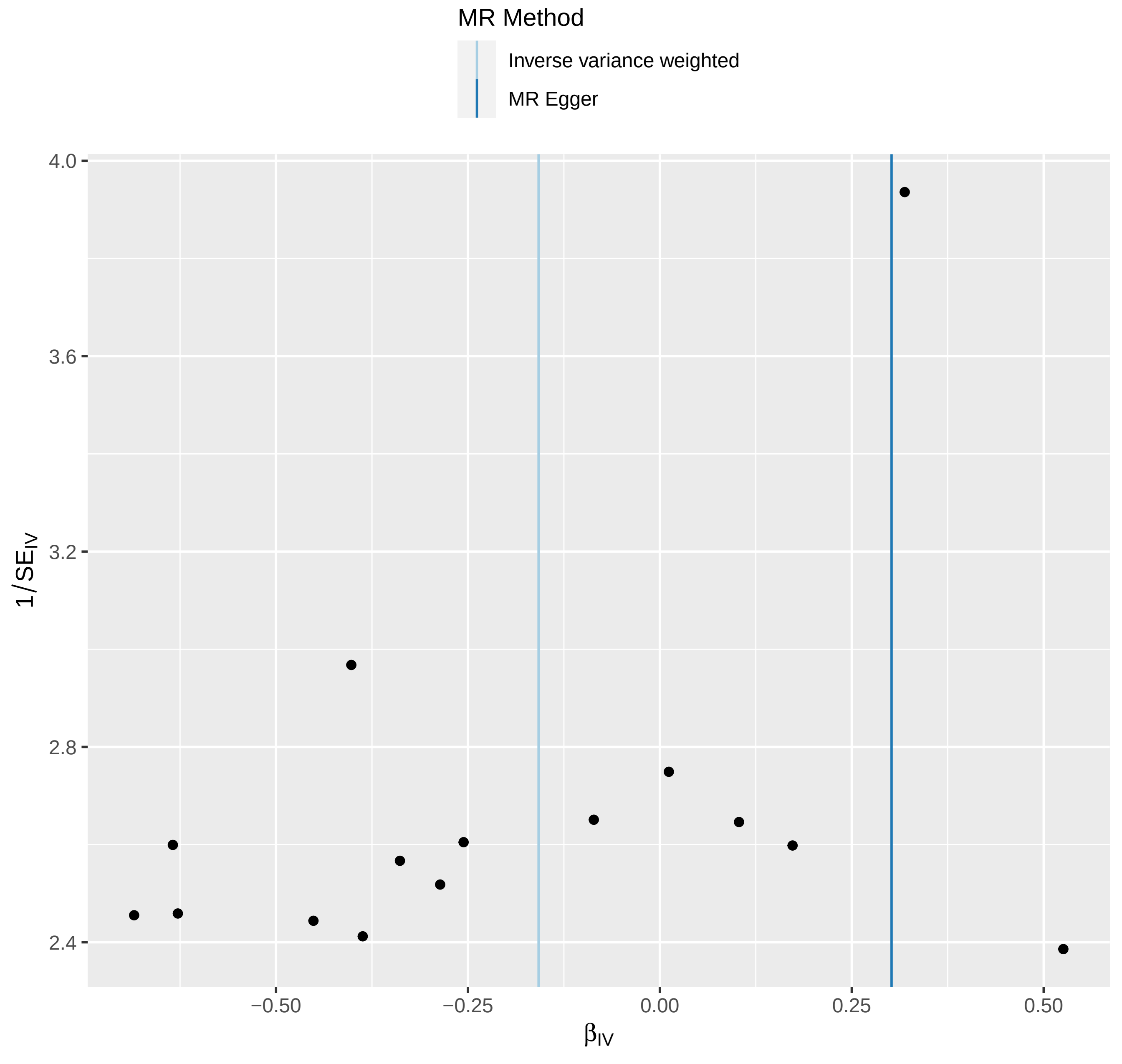

Batch 2−55 : Gut microbiota abundance (genus Catenibacterium id.2153) on Disintegrin and metalloproteinase domain−containing protein 23 with a threshold of 1e−05

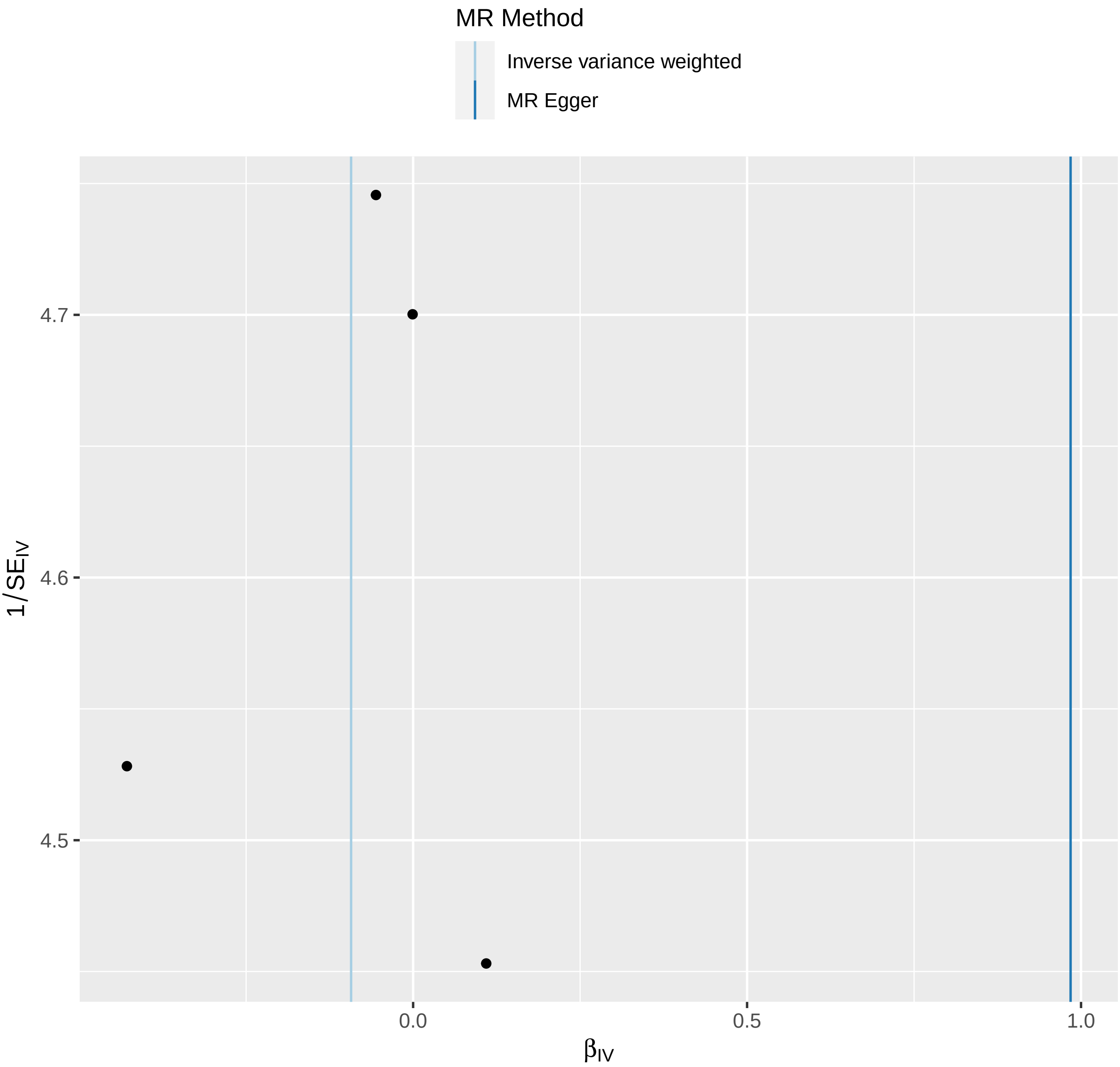

Batch 2−56 : Gut microbiota abundance (genus Parasutterella id.2892) on Disintegrin and metalloproteinase domain−containing protein 23 with a threshold of 1e−05

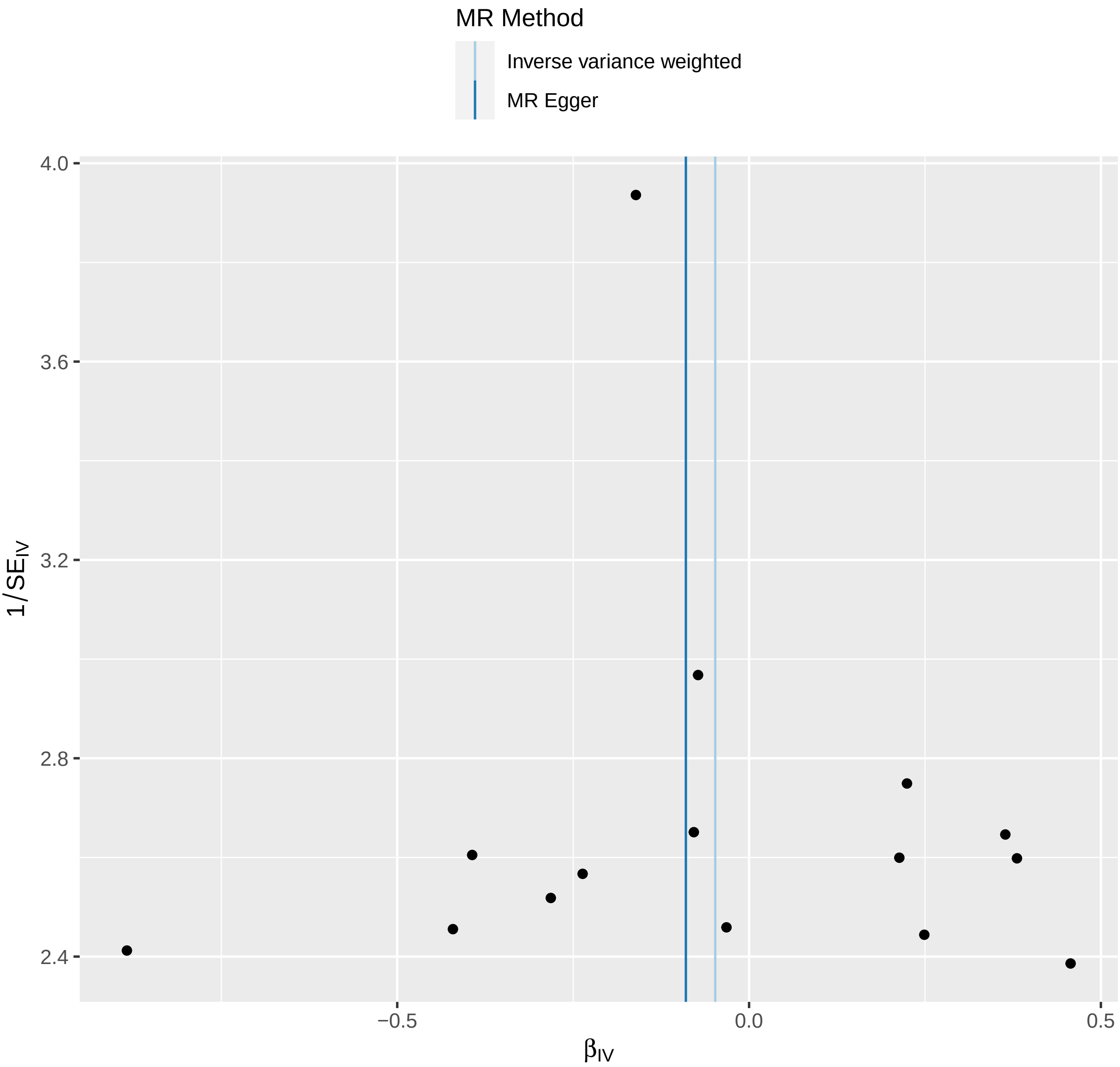

Batch 2−57 : Gut microbiota abundance (genus Catenibacterium id.2153) on Disintegrin and metalloproteinase domain−containing protein 29 with a threshold of 1e−05

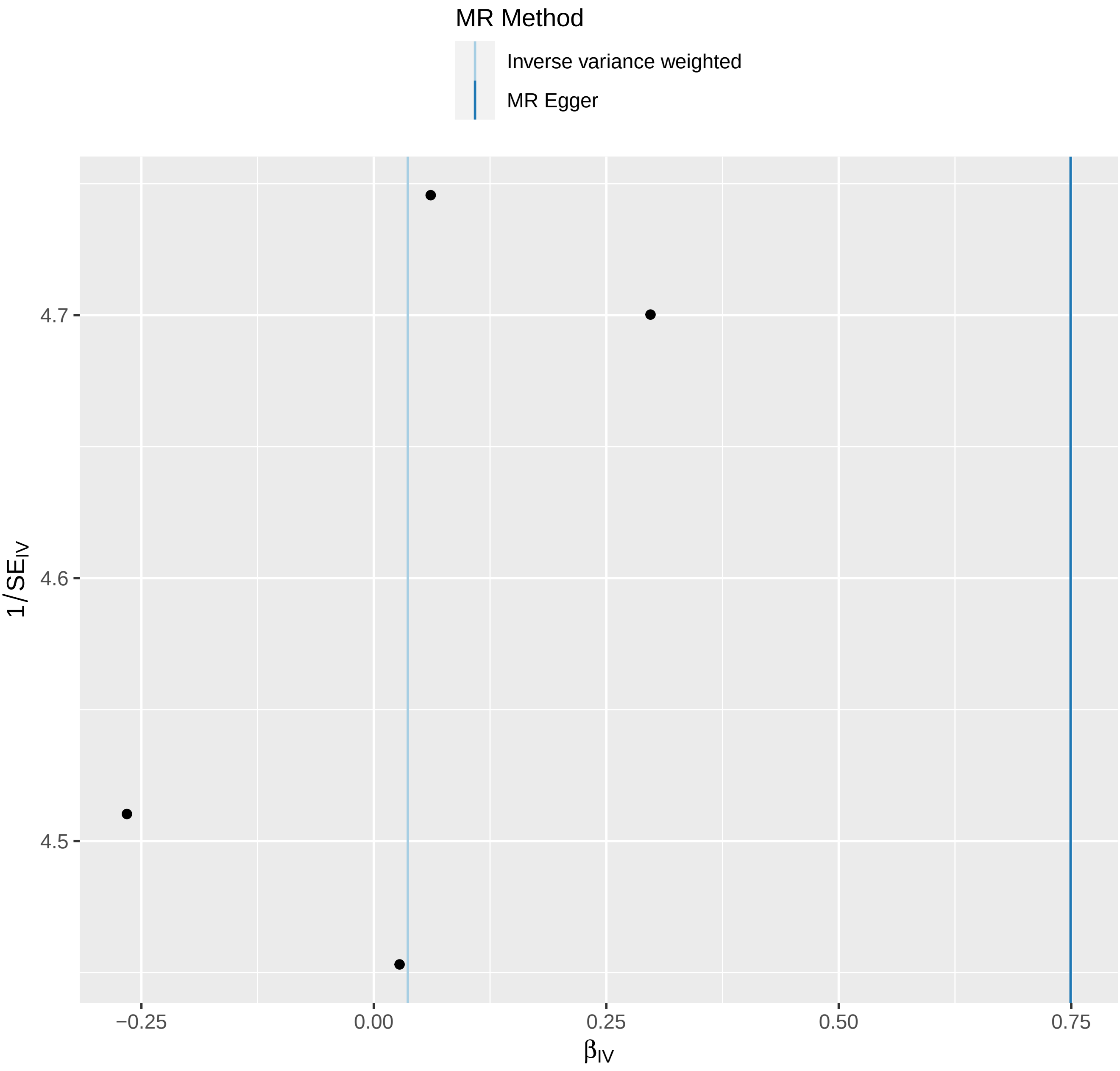

Batch 2−58 : Gut microbiota abundance (genus Parasutterella id.2892) on Disintegrin and metalloproteinase domain−containing protein 29 with a threshold of 1e−05

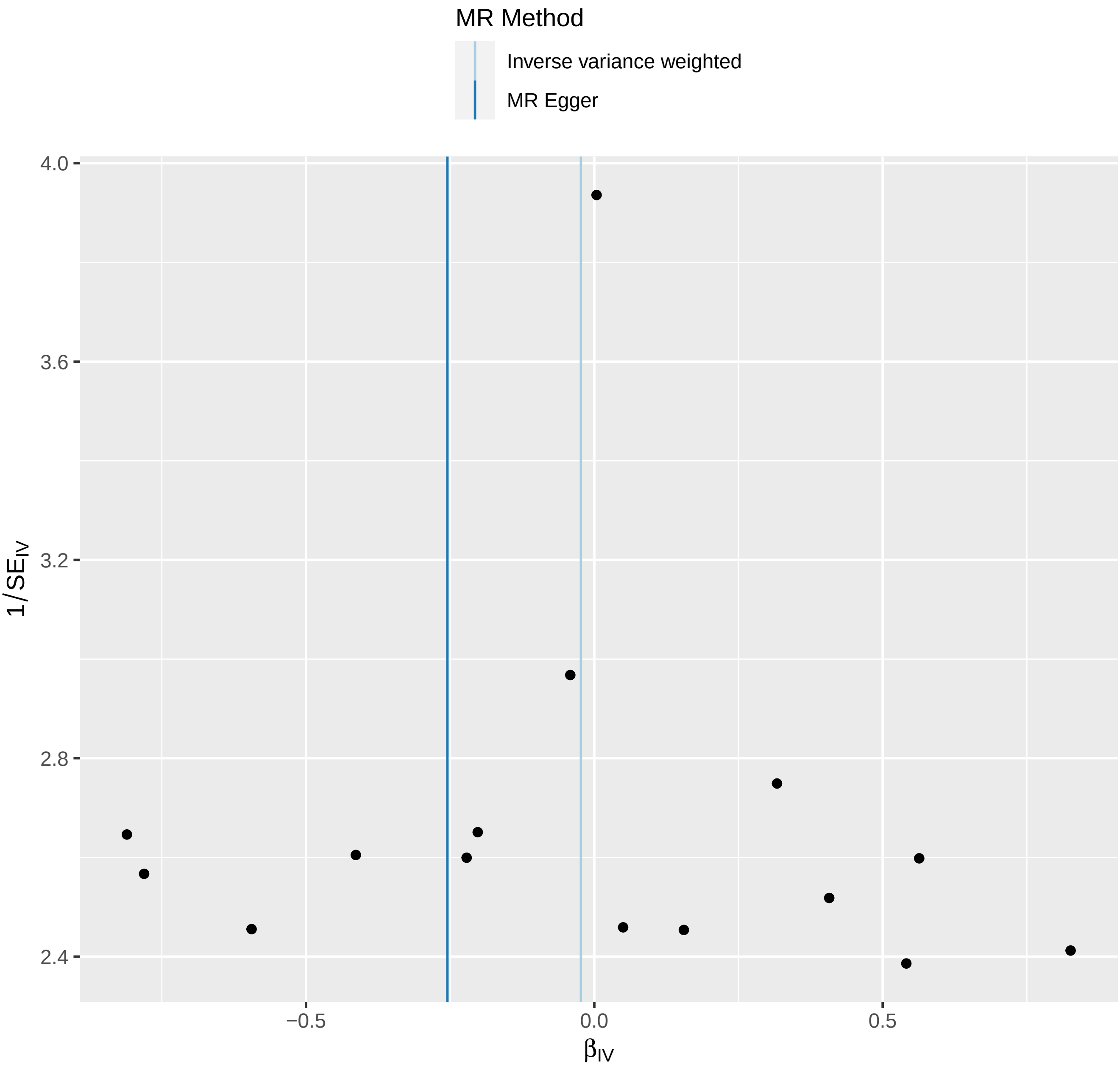

Batch 2−59 : Gut microbiota abundance (genus Catenibacterium id.2153) on Disintegrin and metalloproteinase domain−containing protein 9 with a threshold of 1e−05

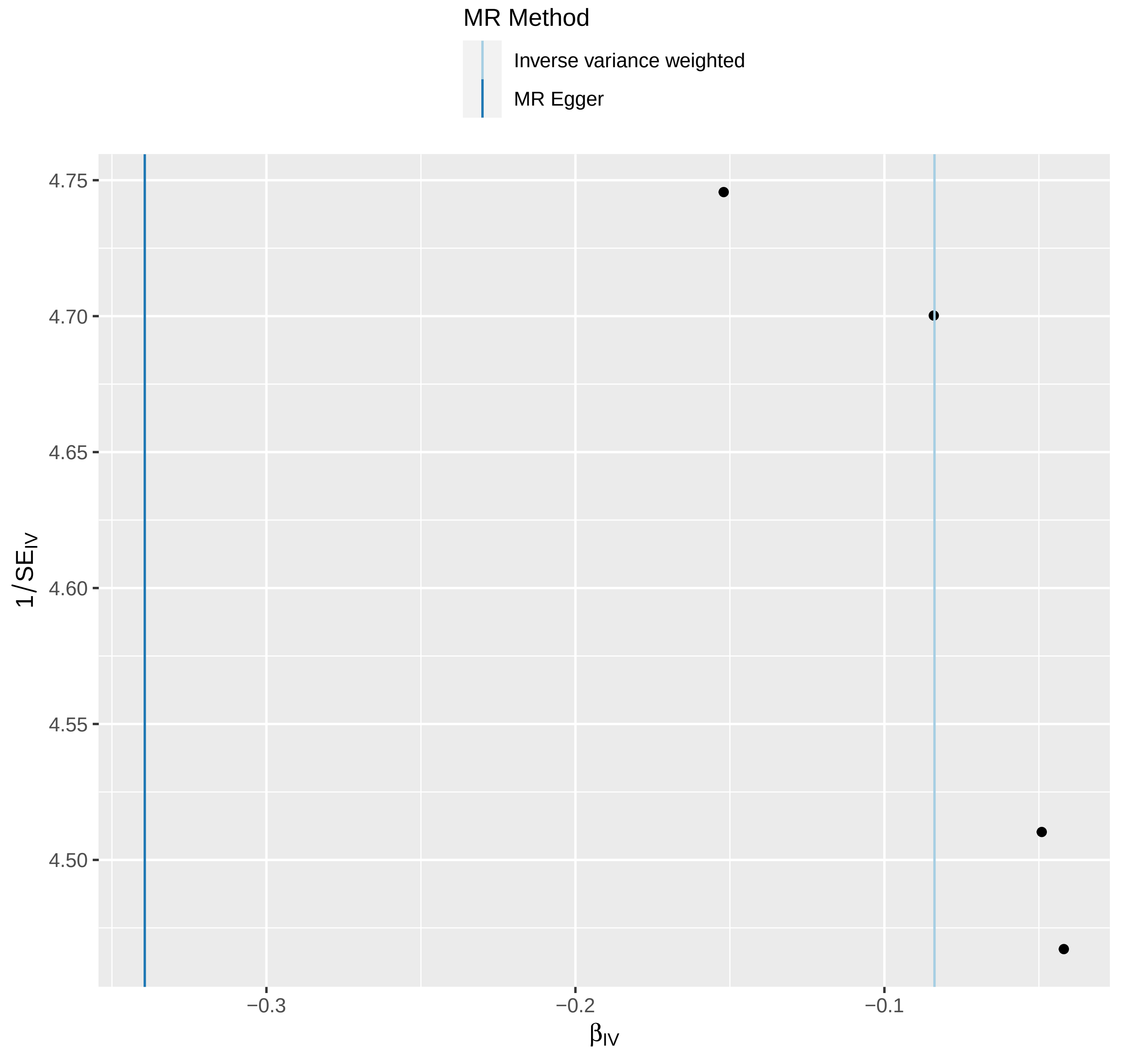

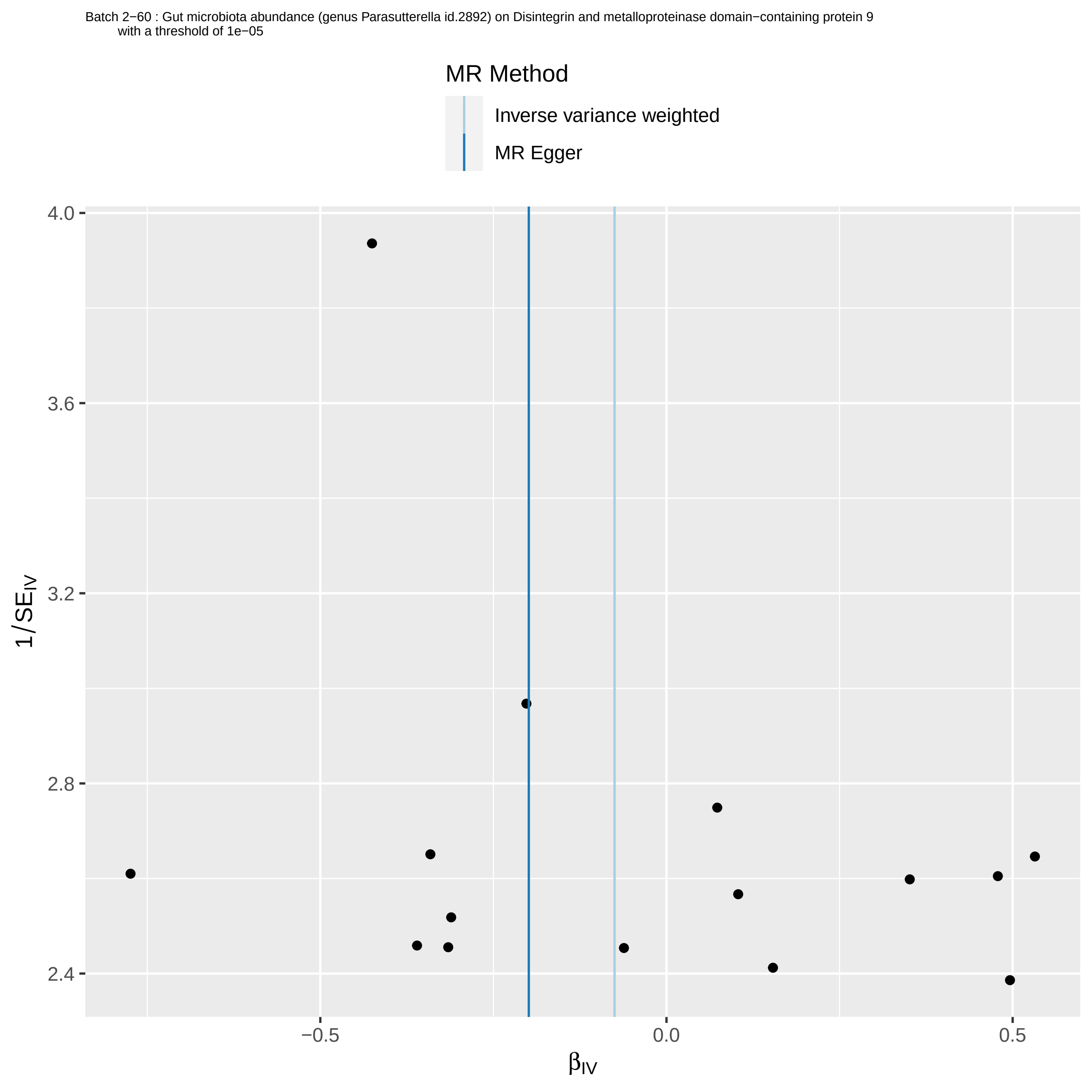

Batch 2−60 : Gut microbiota abundance (genus Parasutterella id.2892) on Disintegrin and metalloproteinase domain−containing protein 9
with a threshold of 1e−05
MR Method
Inverse variance weighted
MR Egger
4.0
3.6
3.2
2.8
2.4
1/SE_IV
−0.5
0.0
0.5
β_IV

Batch 2−61 : Gut microbiota abundance (genus Catenibacterium id.2153) on A disintegrin and metalloproteinase with thrombospondin motifs 1 with a threshold of 1e−05

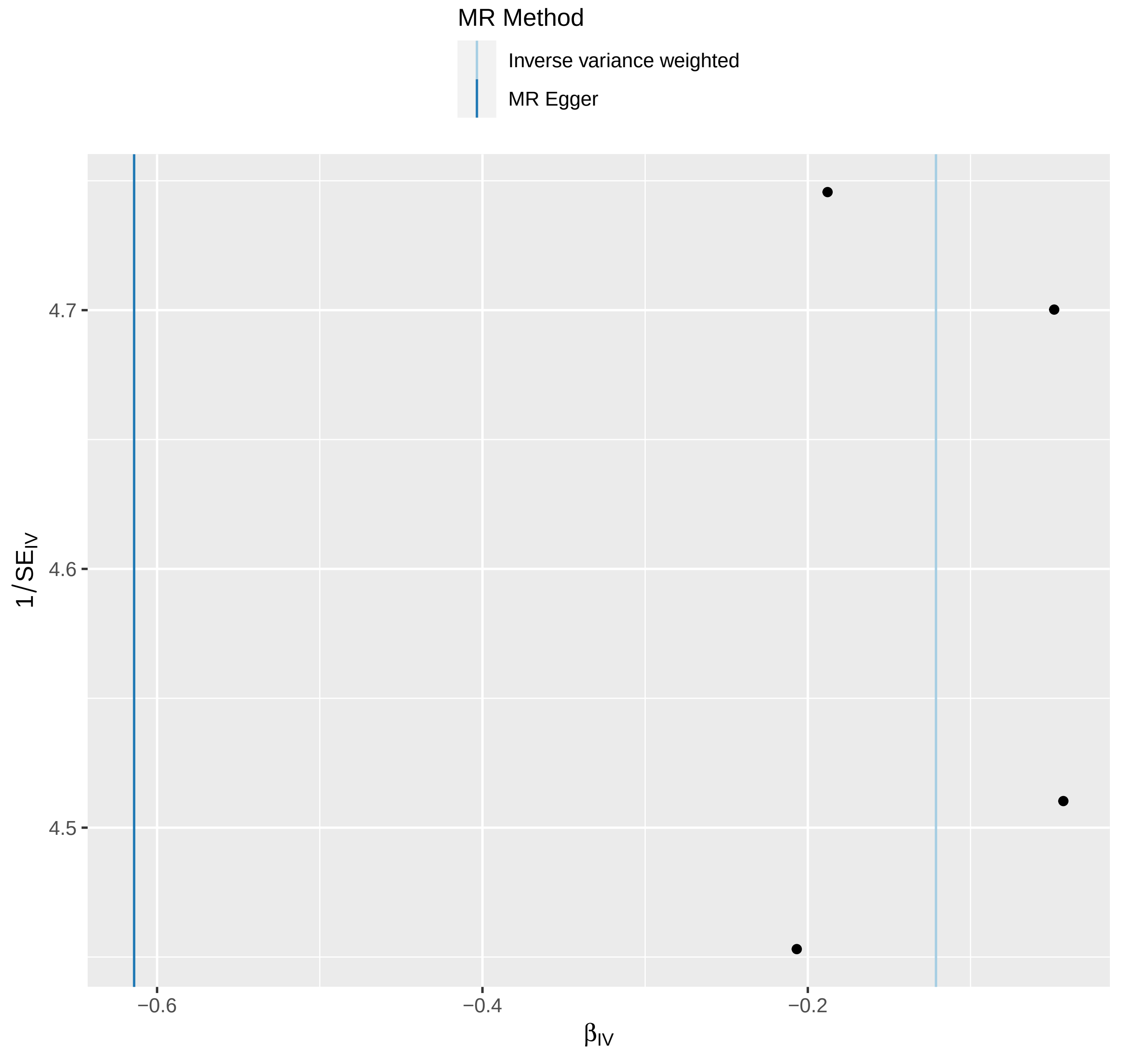

Batch 2−62 : Gut microbiota abundance (genus Parasutterella id.2892) on A disintegrin and metalloproteinase with thrombospondin motifs 1 with a threshold of 1e−05

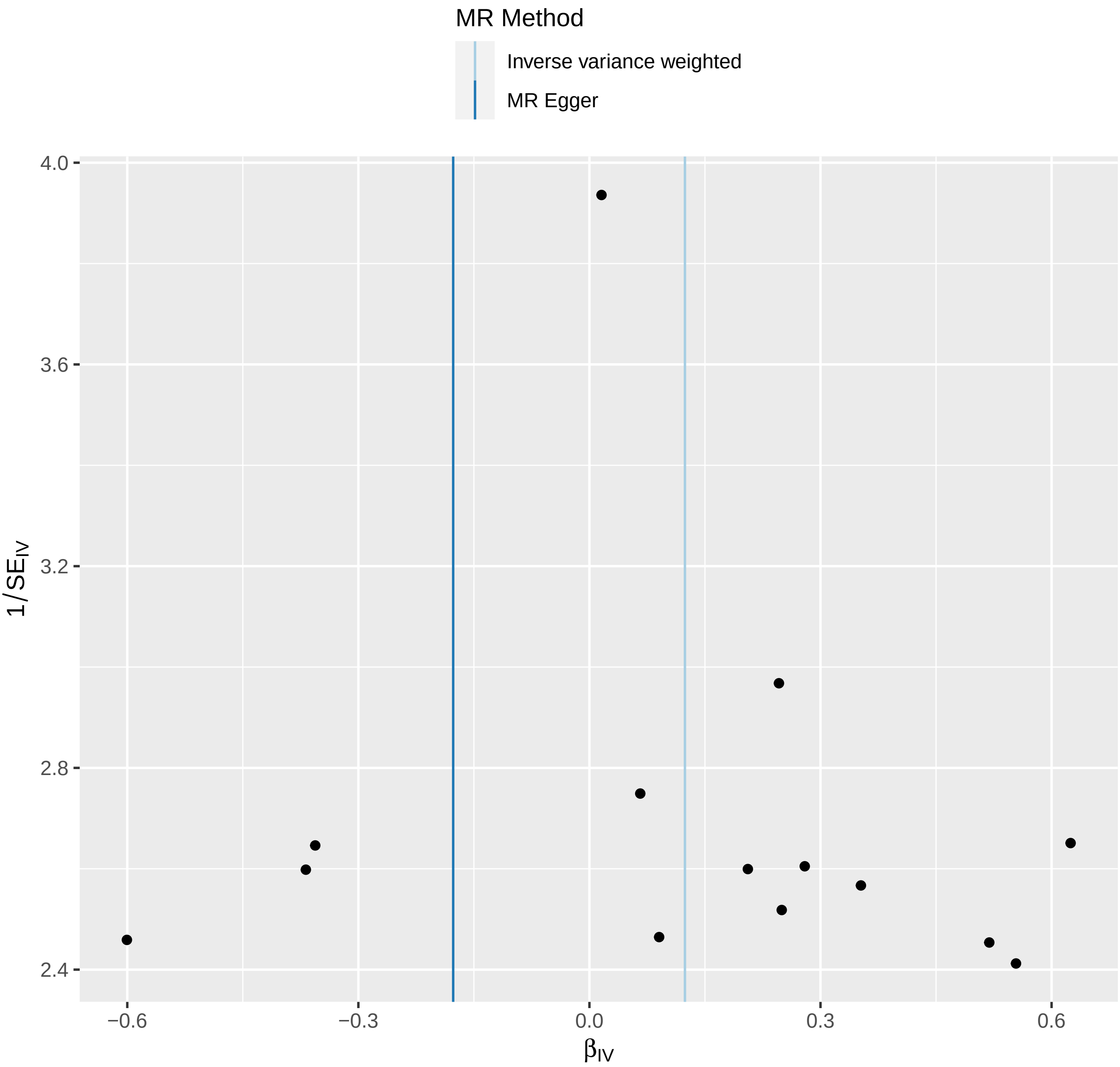

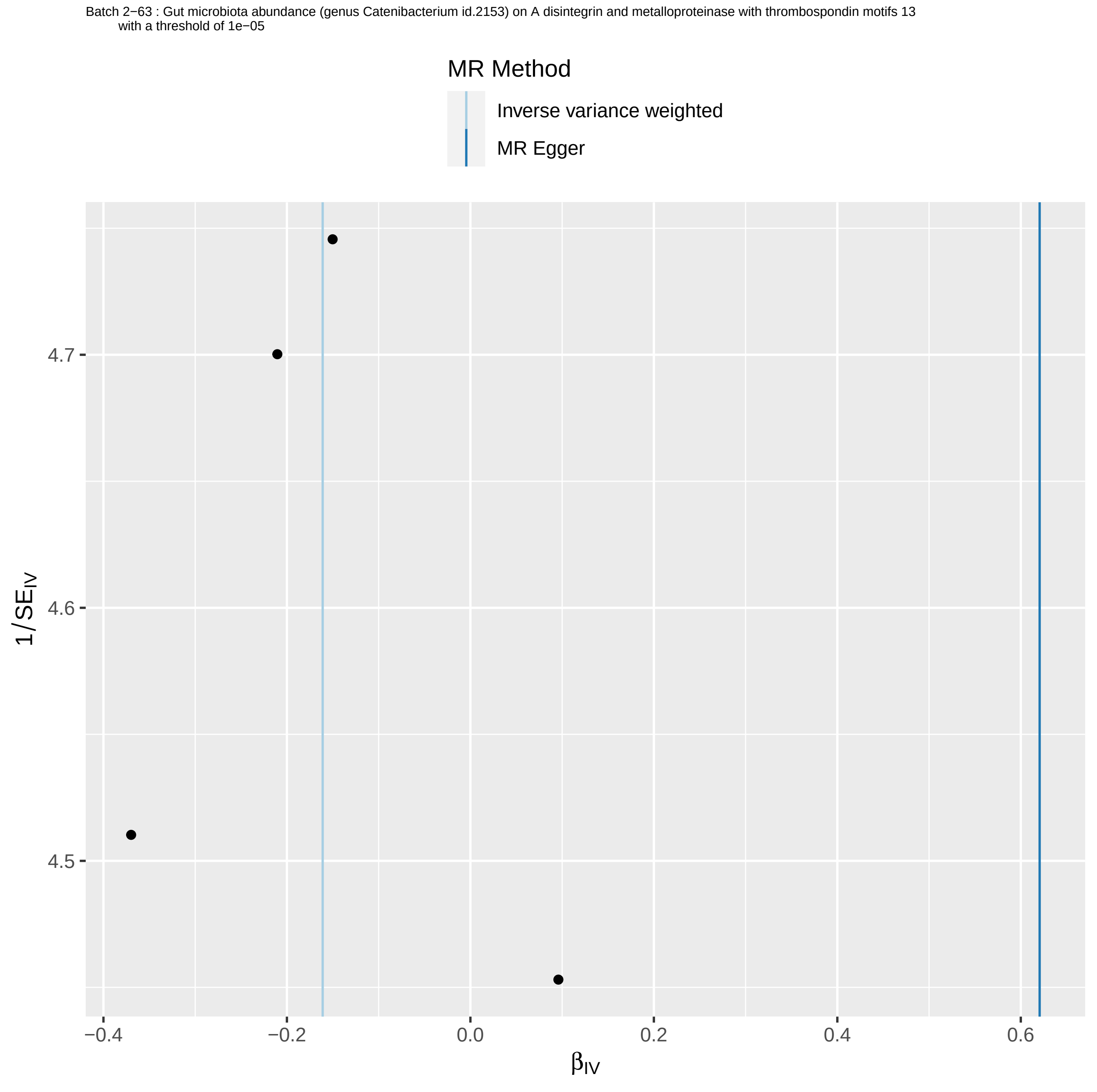
Batch 2−63 : Gut microbiota abundance (genus Catenibacterium id.2153) on A disintegrin and metalloproteinase with thrombospondin motifs 13 with a threshold of 1e−05
MR Method
Inverse variance weighted
MR Egger
4.7
4.6
4.5
1/SE_IV
−0.4
−0.2
0.0
0.2
0.4
0.6
β_IV

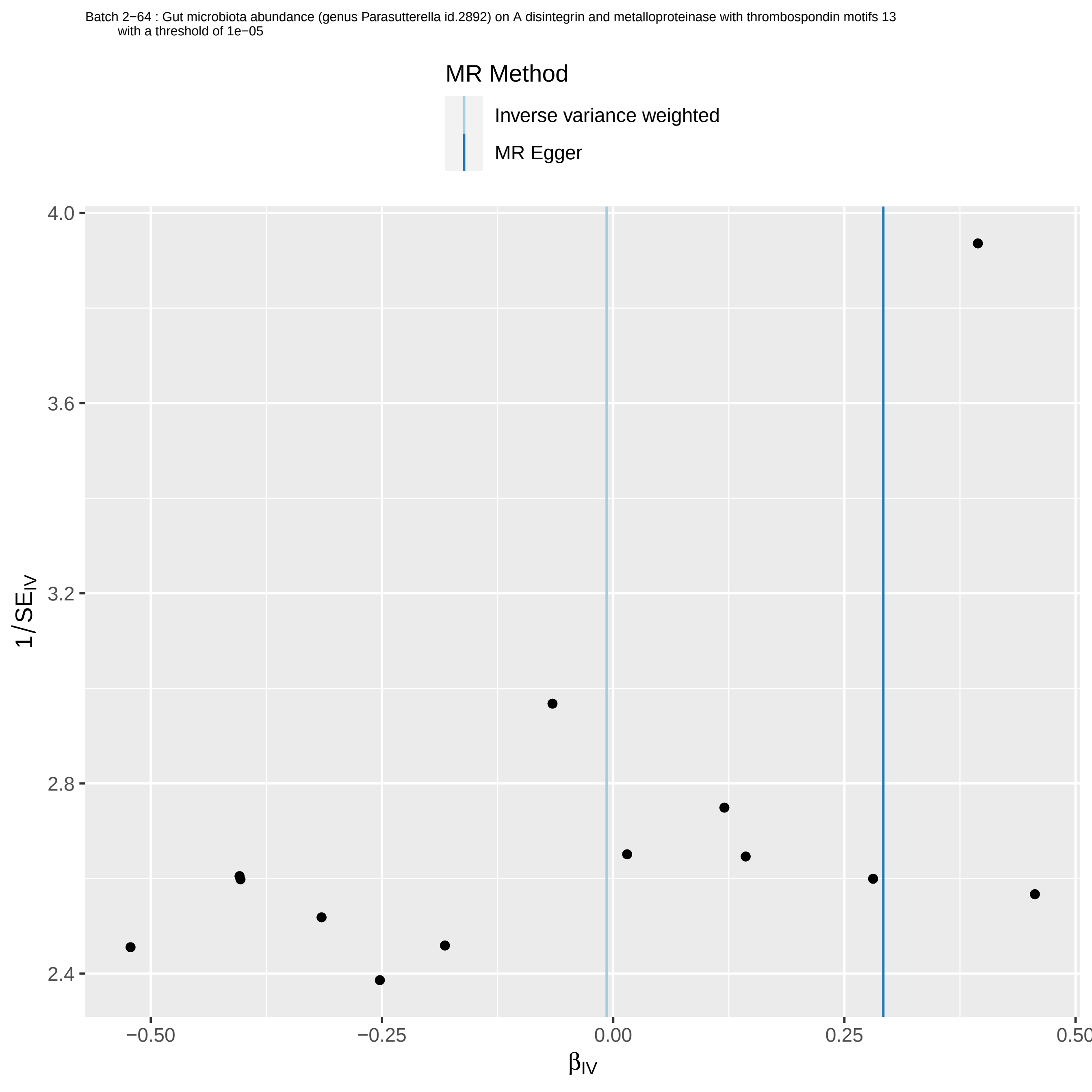

Batch 2−64 : Gut microbiota abundance (genus Parasutterella id.2892) on A disintegrin and metalloproteinase with thrombospondin motifs 13
with a threshold of 1e−05
MR Method
Inverse variance weighted
MR Egger
4.0
3.6
3.2
2.8
2.4
1/SE_IV
−0.50
−0.25
0.00
0.25
0.50
β_IV

Batch 2−65 : Gut microbiota abundance (genus Catenibacterium id.2153) on A disintegrin and metalloproteinase with thrombospondin motifs 15 with a threshold of 1e−05

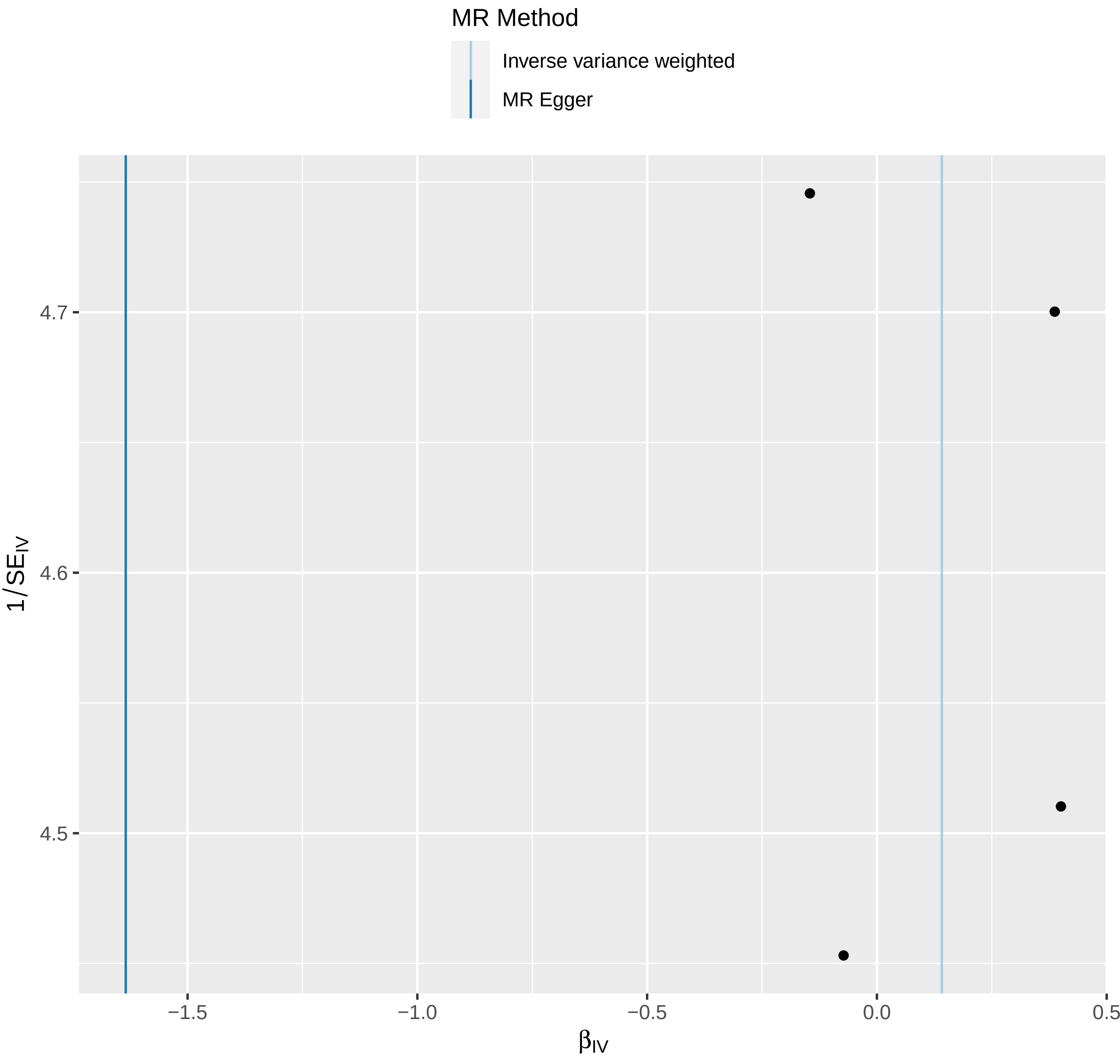

Batch 2−66 : Gut microbiota abundance (genus Parasutterella id.2892) on A disintegrin and metalloproteinase with thrombospondin motifs 15 with a threshold of 1e−05

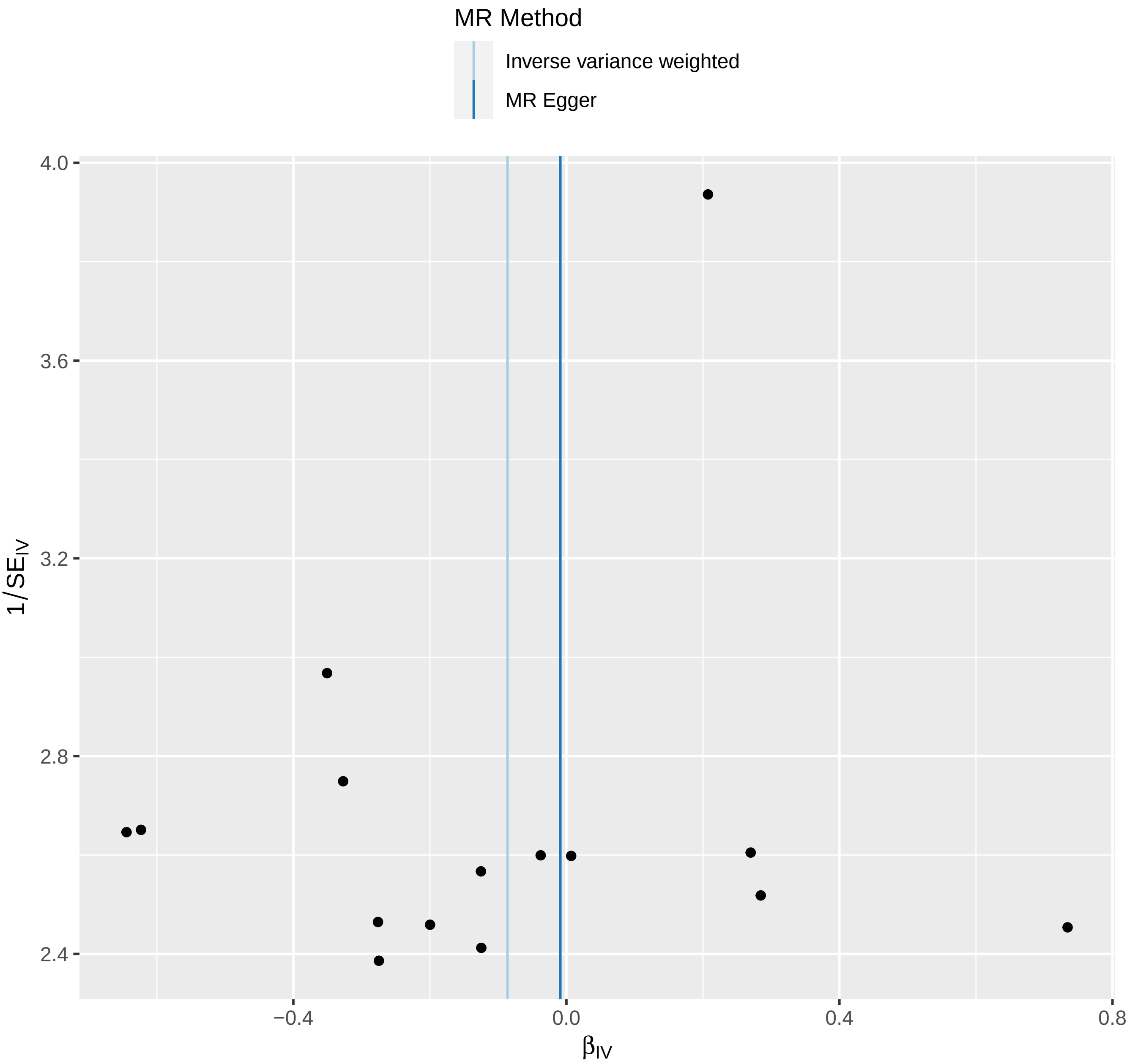

Batch 2−67 : Gut microbiota abundance (genus Catenibacterium id.2153) on A disintegrin and metalloproteinase with thrombospondin motifs 3 with a threshold of 1e−05

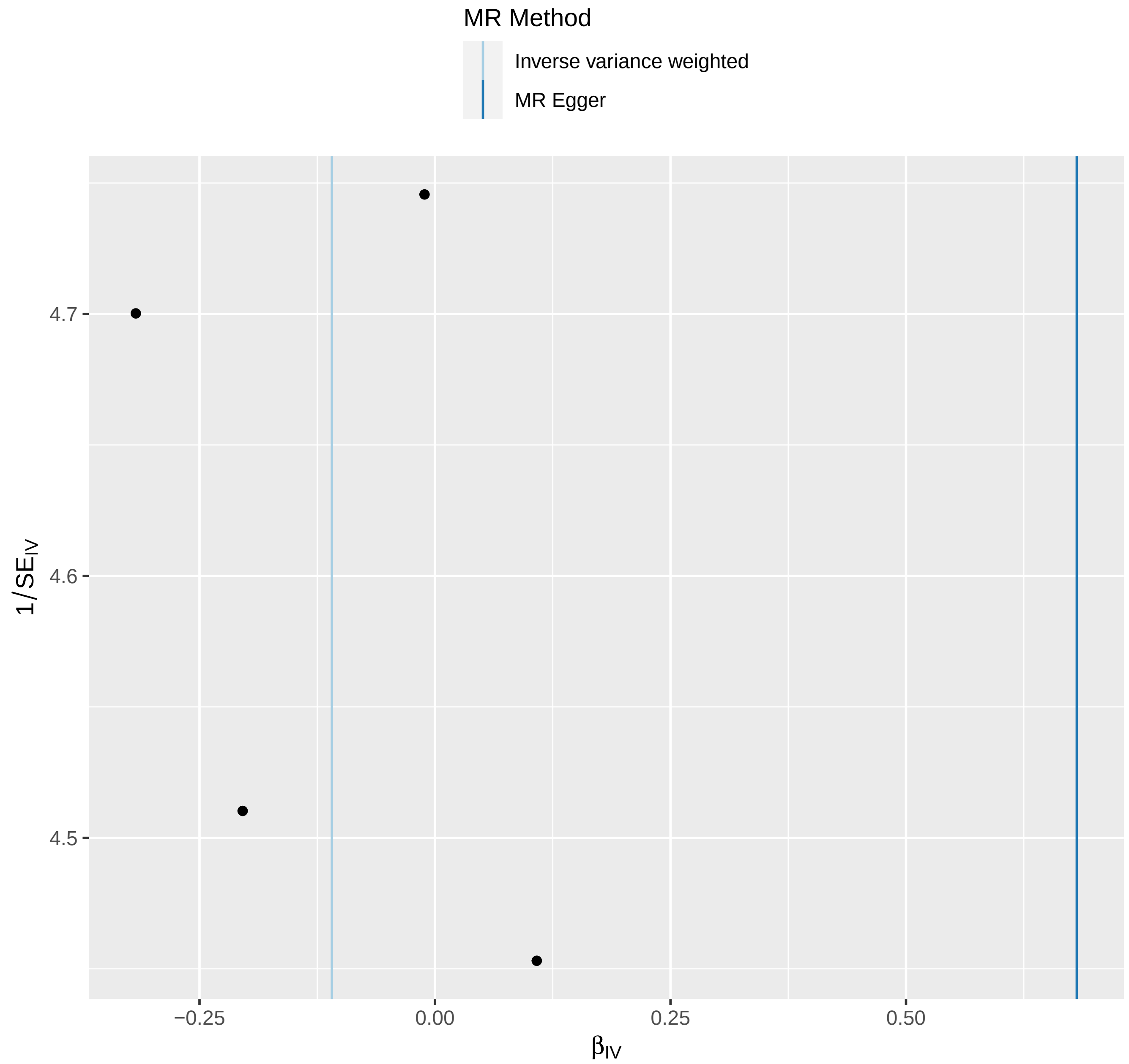

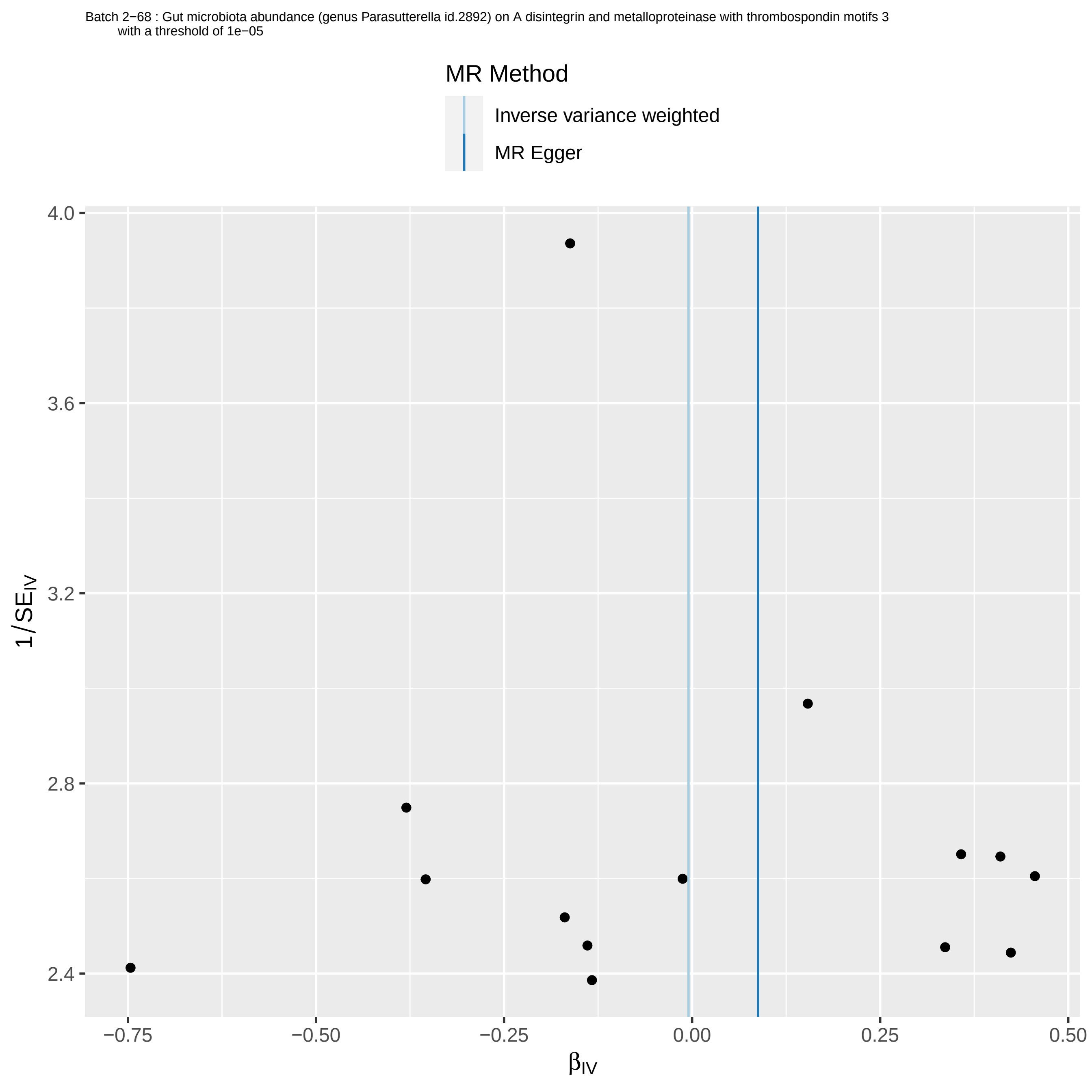

Batch 2−68 : Gut microbiota abundance (genus Parasutterella id.2892) on A disintegrin and metalloproteinase with thrombospondin motifs 3
with a threshold of 1e−05
MR Method
Inverse variance weighted
MR Egger
4.0
3.6
3.2
2.8
2.4
1/SE_IV
−0.75
−0.50
−0.25
0.00
0.25
0.50
β_IV

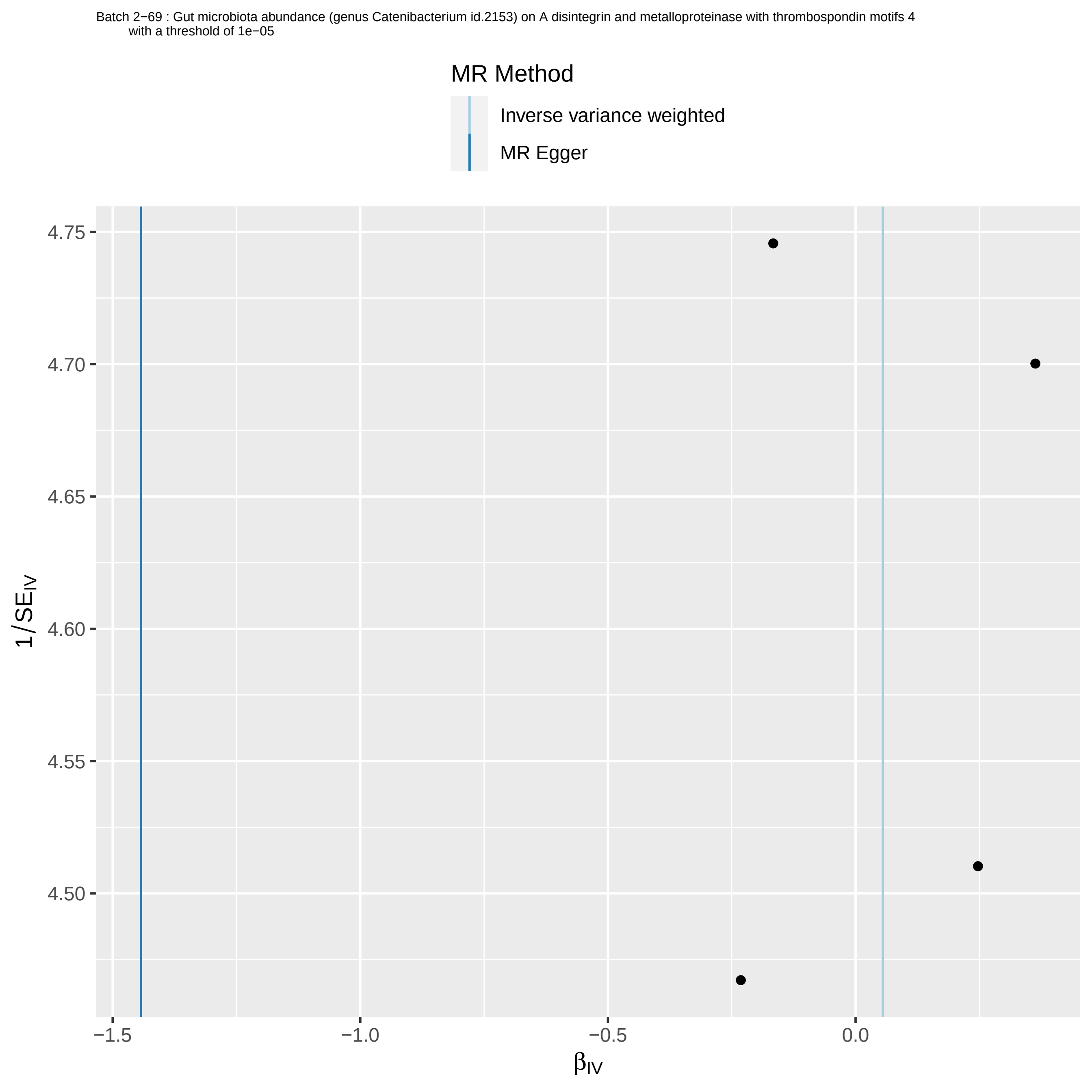
Batch 2−69 : Gut microbiota abundance (genus Catenibacterium id.2153) on A disintegrin and metalloproteinase with thrombospondin motifs 4 with a threshold of 1e−05
MR Method
Inverse variance weighted
MR Egger
4.75
4.70
4.65
4.60
4.55
4.50
1/SE_IV
−1.5
−1.0
−0.5
0.0
β_IV

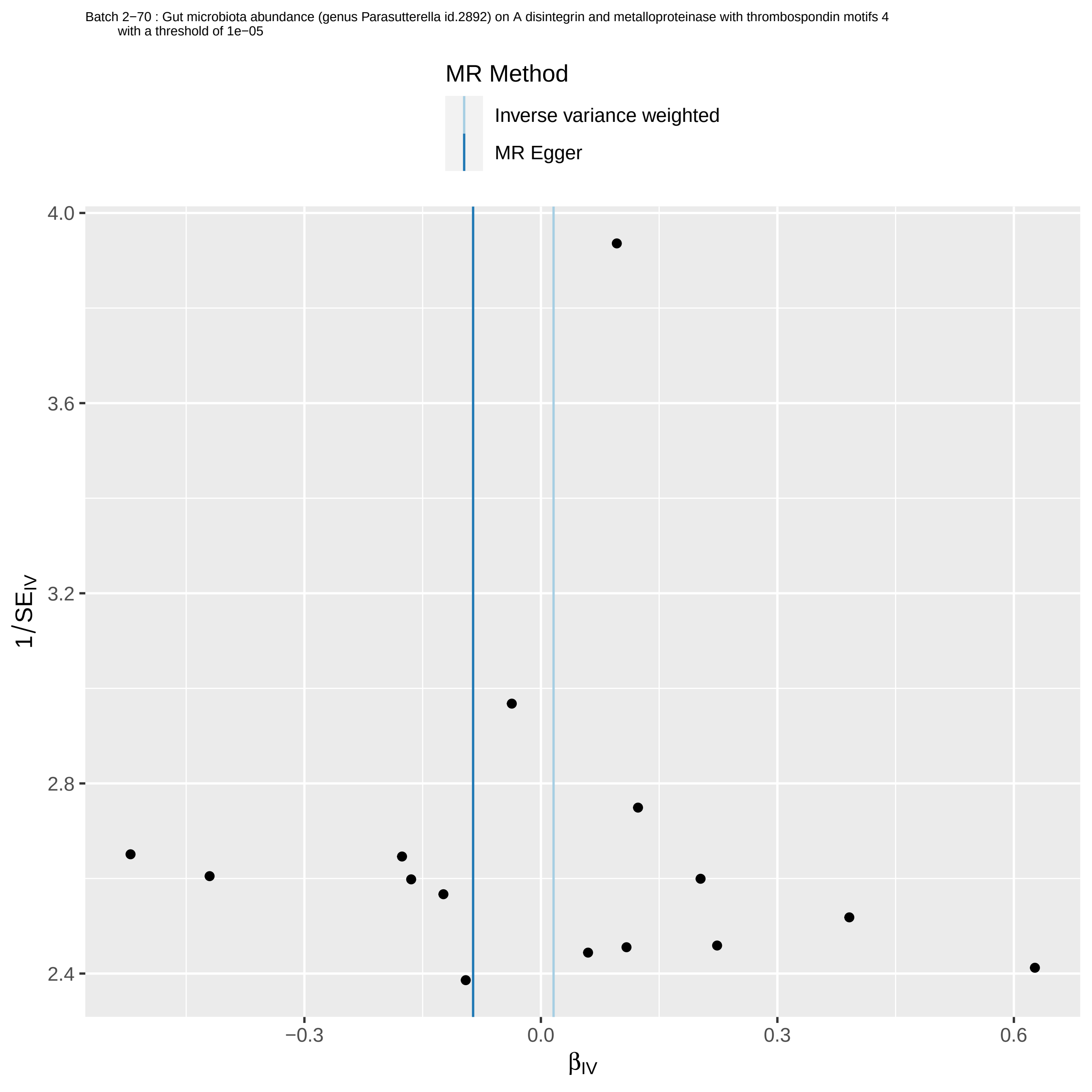
Batch 2−70 : Gut microbiota abundance (genus Parasutterella id.2892) on A disintegrin and metalloproteinase with thrombospondin motifs 4
with a threshold of 1e−05
MR Method
Inverse variance weighted
MR Egger
4.0
3.6
3.2
2.8
2.4
1/SE_IV
−0.3
0.0
0.3
0.6
β_IV

Batch 2−71 : Gut microbiota abundance (genus Catenibacterium id.2153) on A disintegrin and metalloproteinase with thrombospondin motifs 5 with a threshold of 1e−05

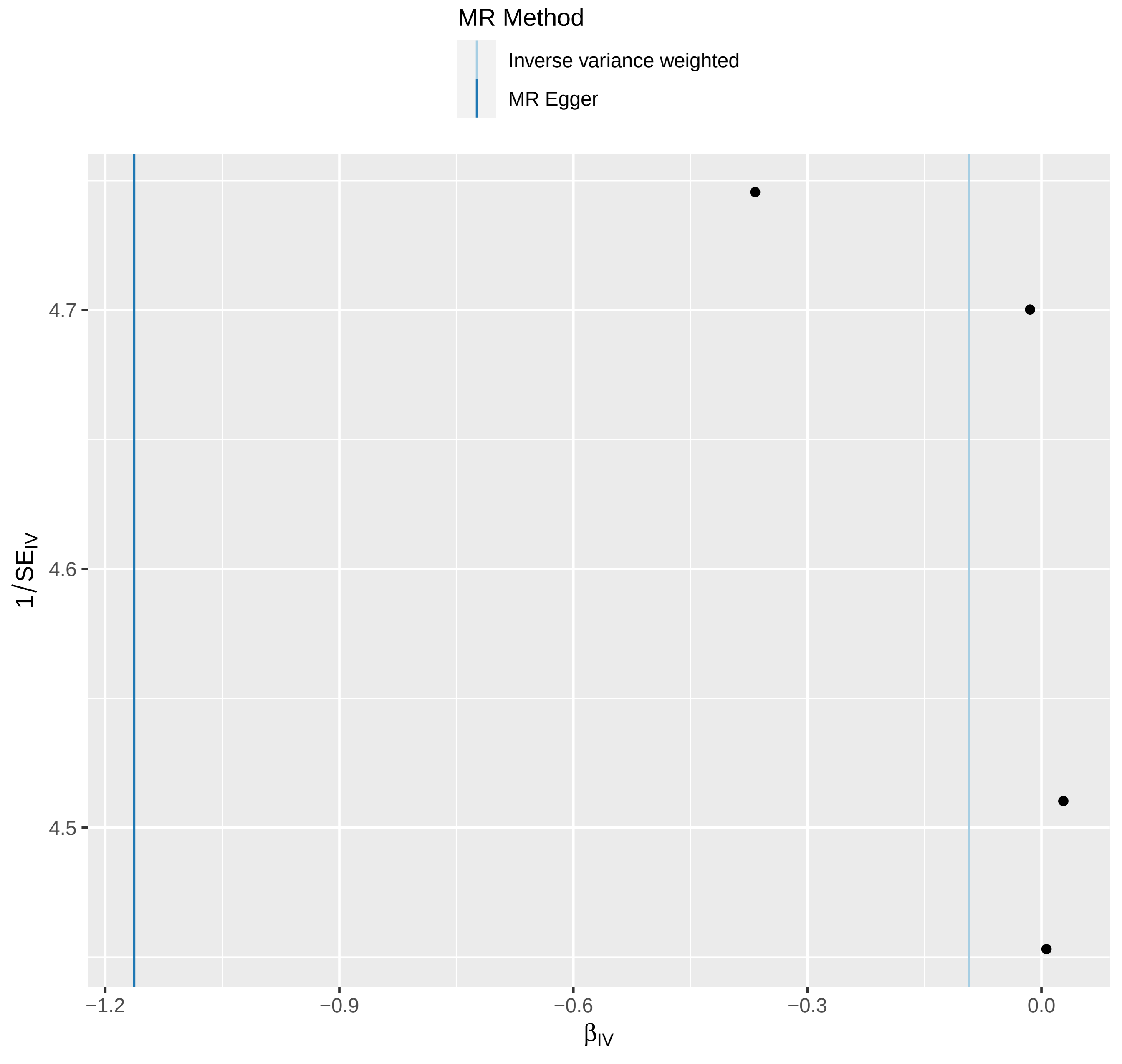

Batch 2−72 : Gut microbiota abundance (genus Parasutterella id.2892) on A disintegrin and metalloproteinase with thrombospondin motifs 5 with a threshold of 1e−05

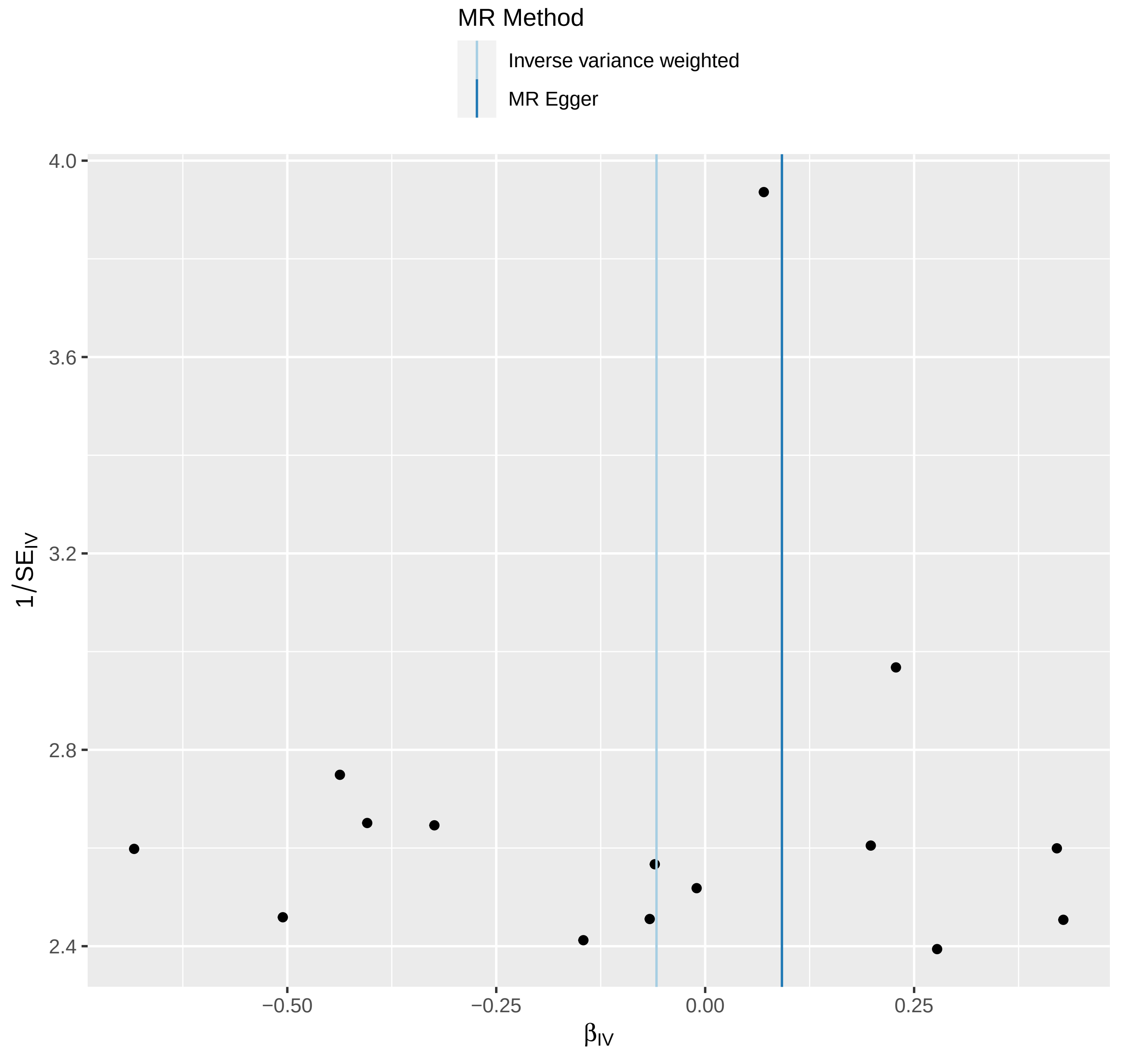

Batch 2−73 : Gut microbiota abundance (genus Catenibacterium id.2153) on A disintegrin and metalloproteinase with thrombospondin motifs 6 with a threshold of 1e−05

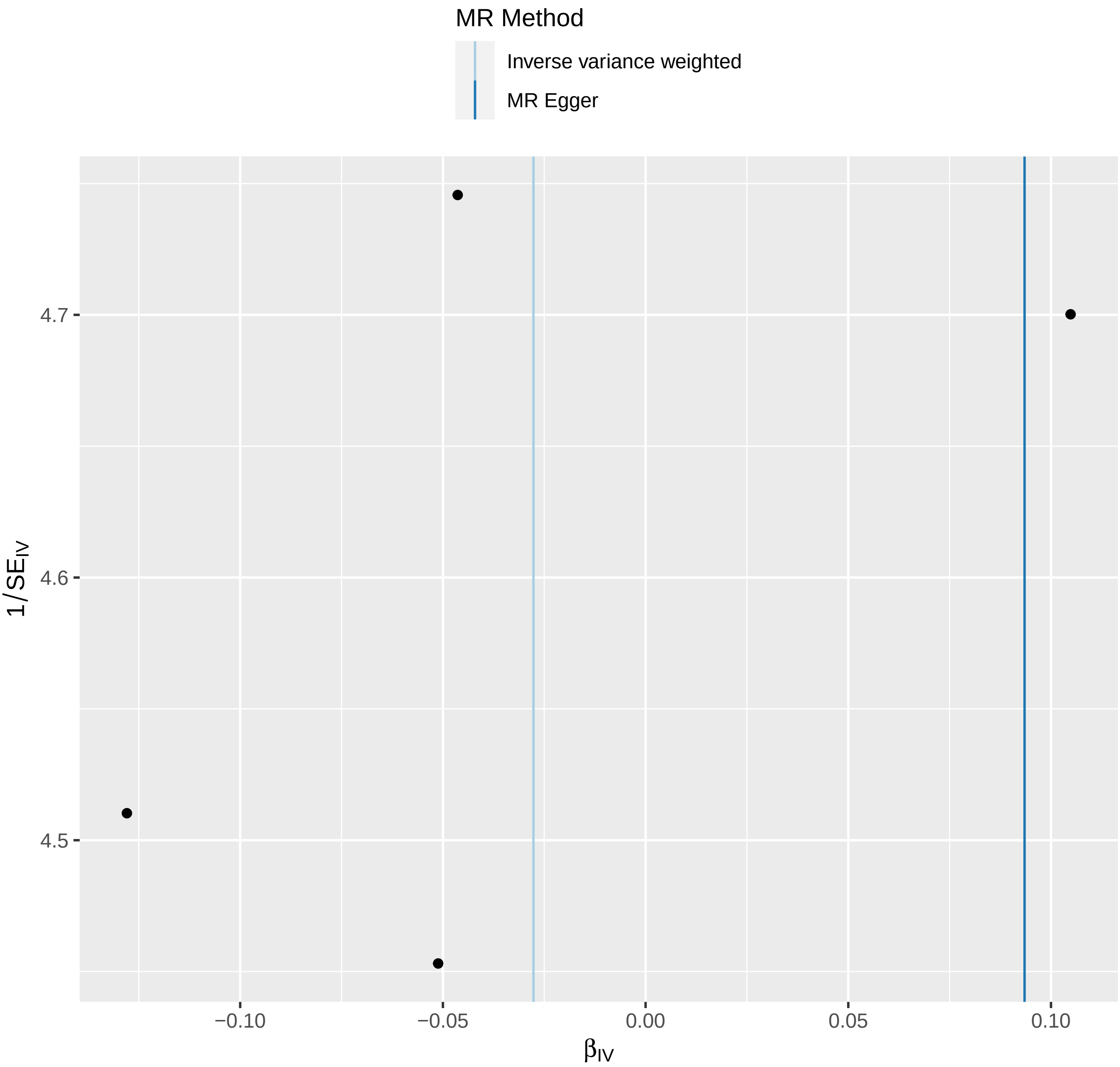

Batch 2−74 : Gut microbiota abundance (genus Parasutterella id.2892) on A disintegrin and metalloproteinase with thrombospondin motifs 6 with a threshold of 1e−05

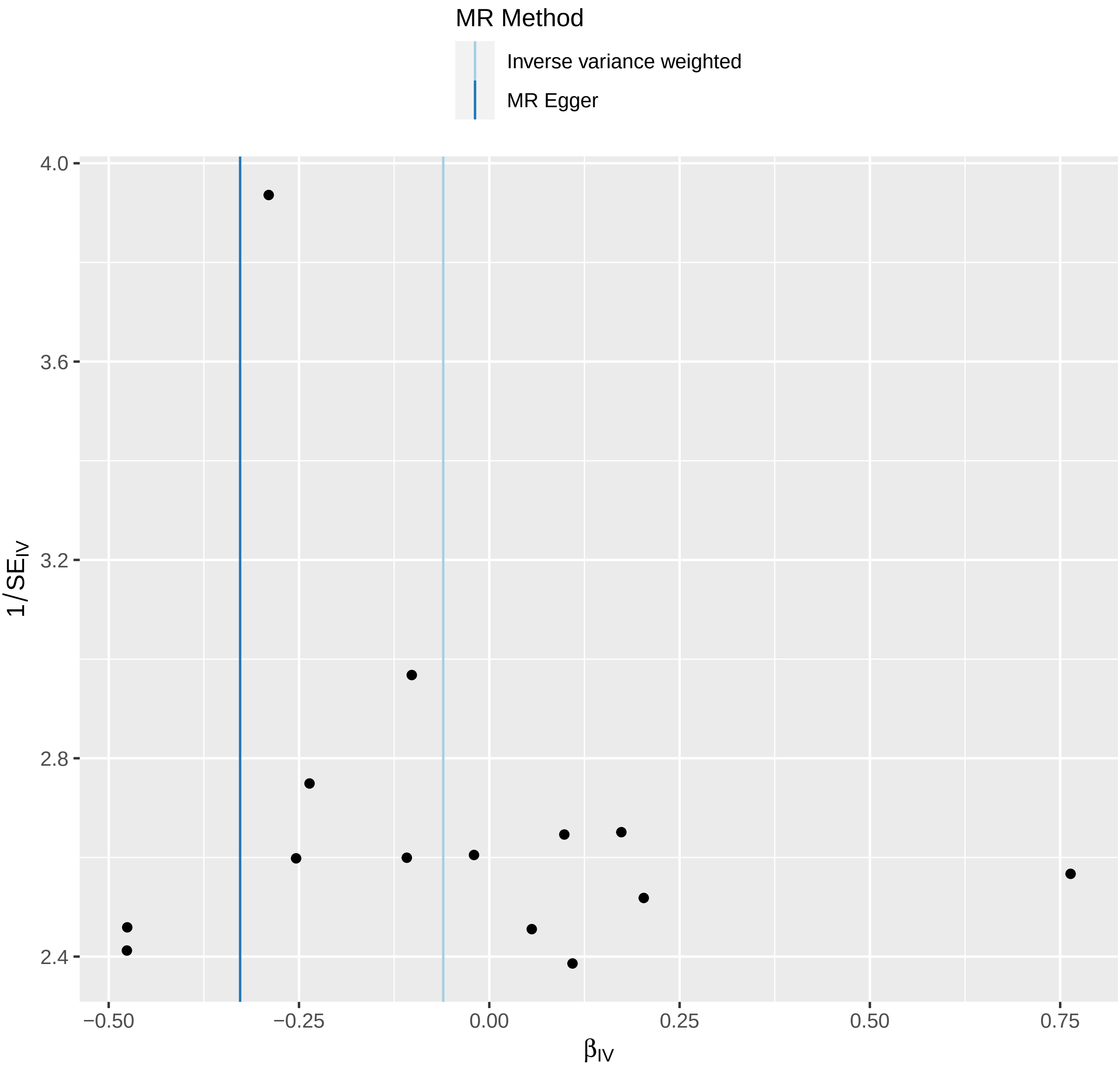

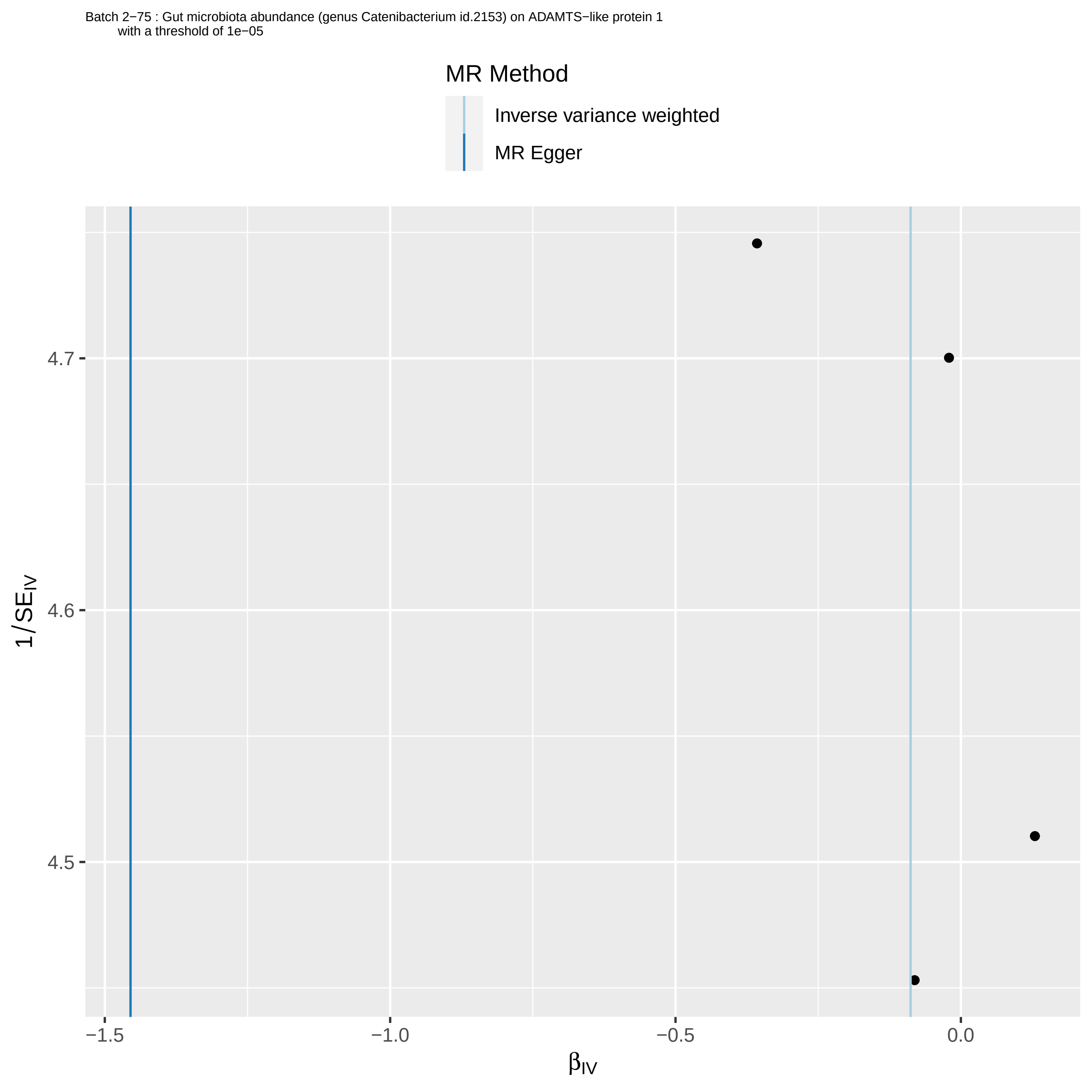

Batch 2−75 : Gut microbiota abundance (genus Catenibacterium id.2153) on ADAMTS−like protein 1
with a threshold of 1e−05
MR Method
Inverse variance weighted
MR Egger
4.7
4.6
4.5
1/SE$_{IV}$
−1.5
−1.0
−0.5
0.0
β_{IV}

Batch 2−76 : Gut microbiota abundance (genus Parasutterella id.2892) on ADAMTS−like protein 1
with a threshold of 1e−05

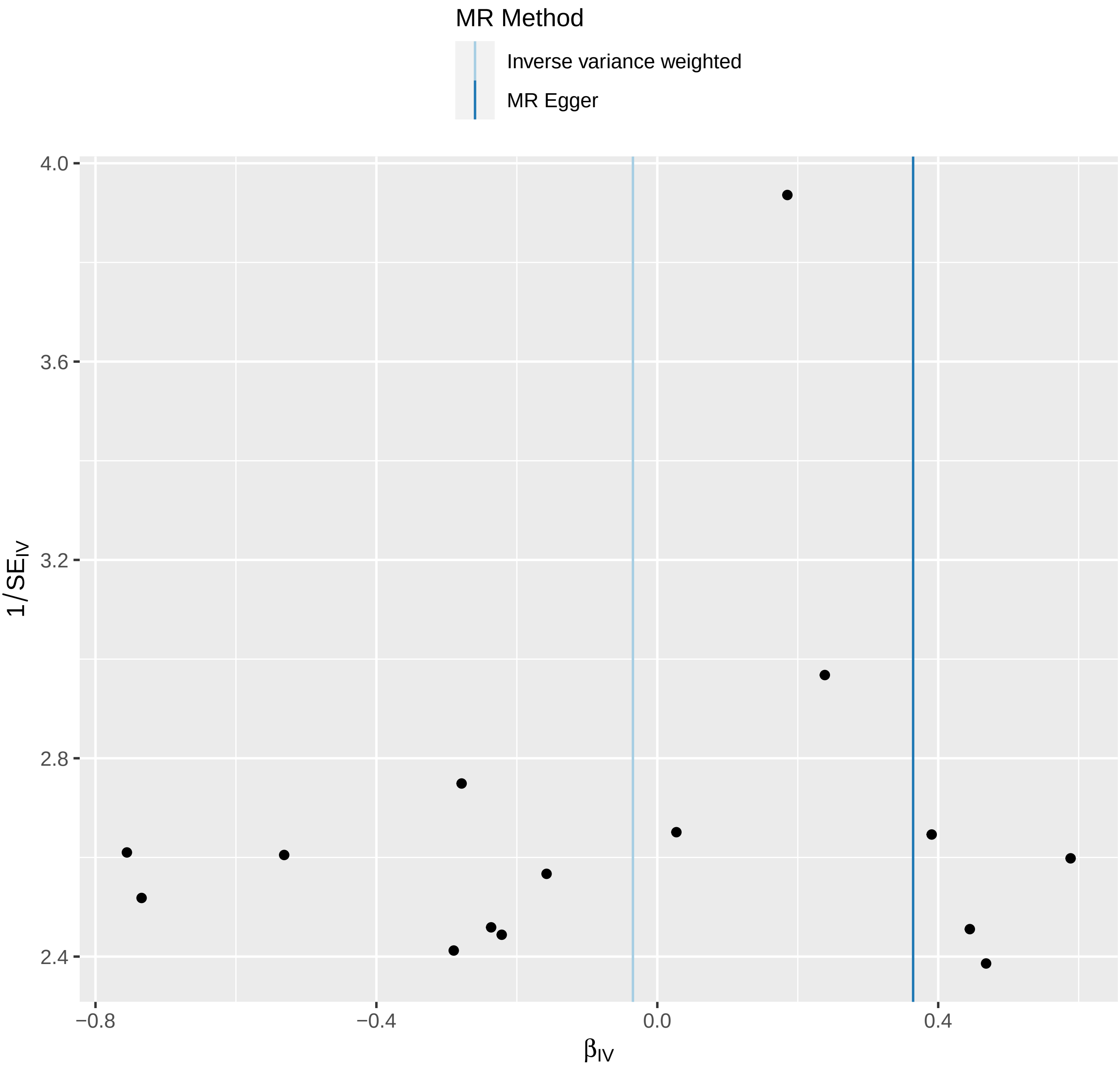

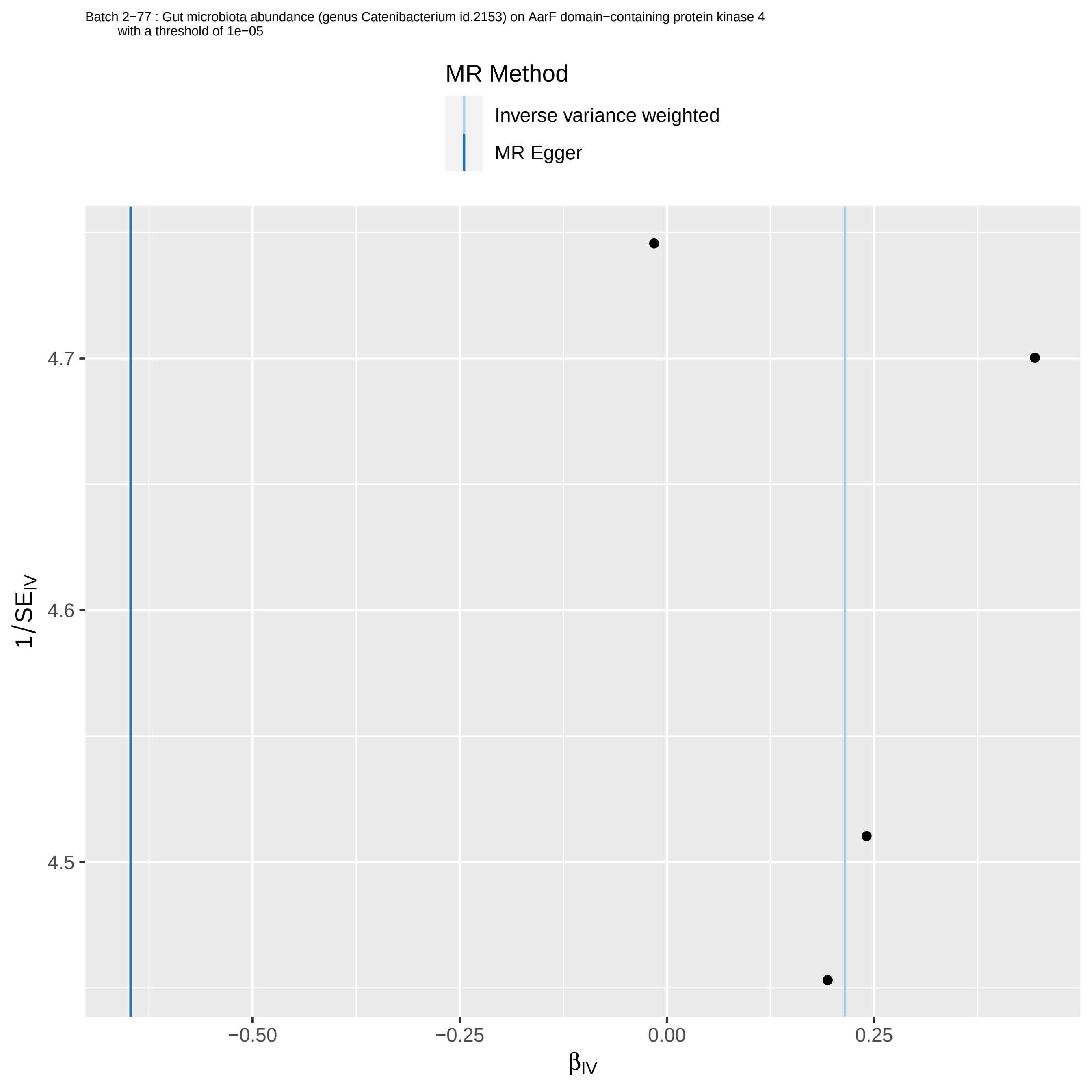

Batch 2−77 : Gut microbiota abundance (genus Catenibacterium id.2153) on AarF domain−containing protein kinase 4
with a threshold of 1e−05
MR Method
Inverse variance weighted
MR Egger
4.7
4.6
4.5
1/SE_IV
−0.50
−0.25
0.00
0.25
β_IV

Batch 2−78 : Gut microbiota abundance (genus Parasutterella id.2892) on AarF domain−containing protein kinase 4
with a threshold of 1e−05

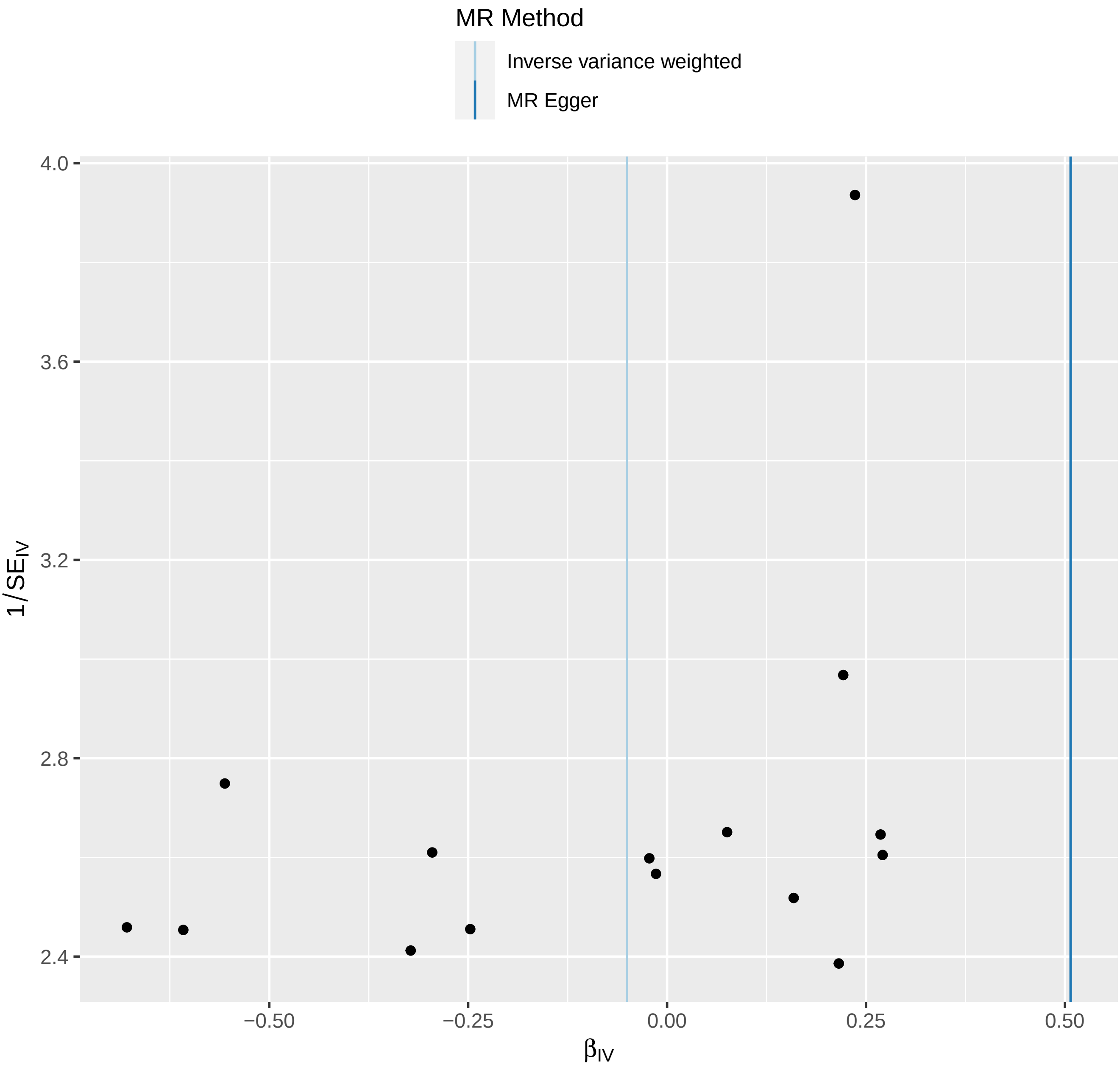

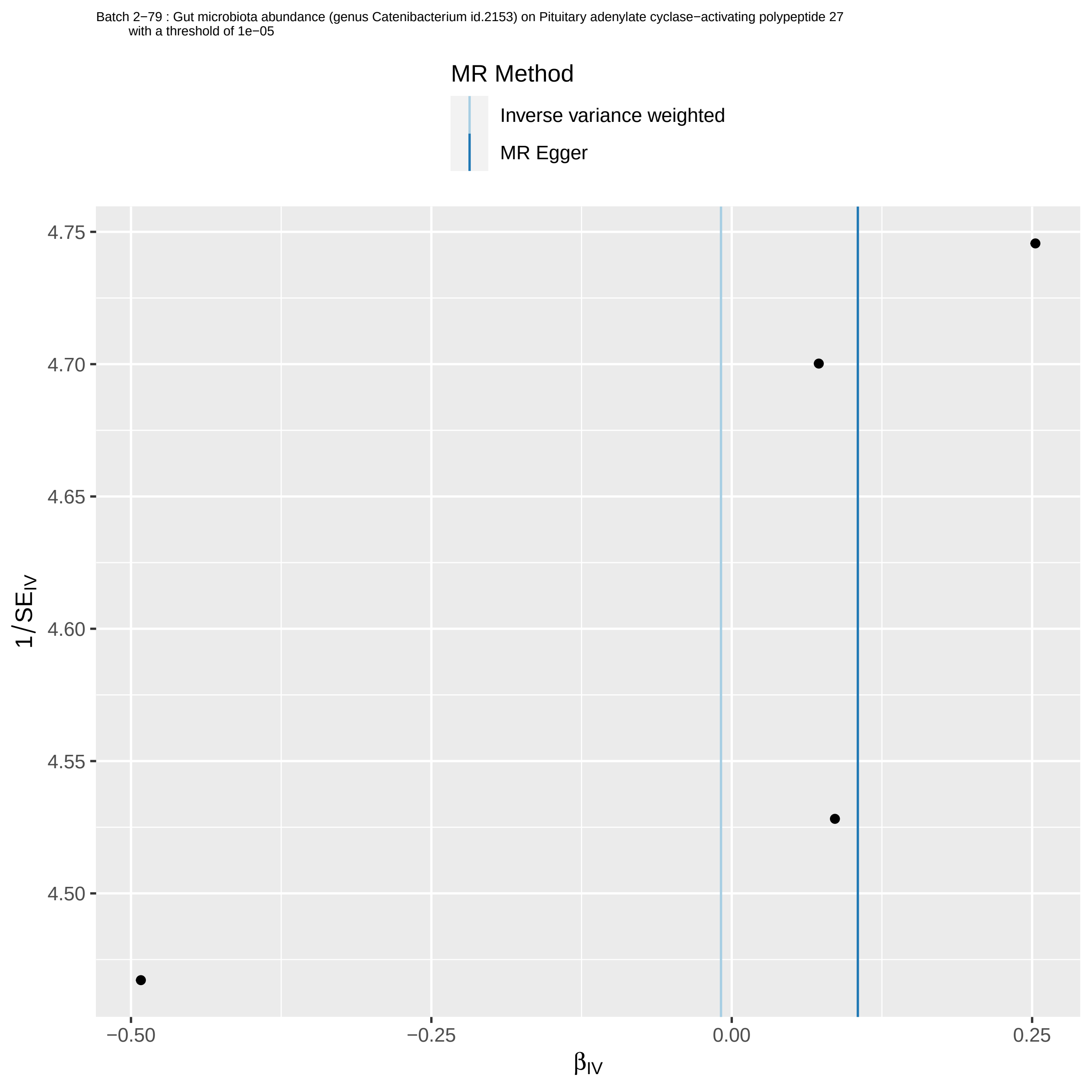
Batch 2−79 : Gut microbiota abundance (genus Catenibacterium id.2153) on Pituitary adenylate cyclase−activating polypeptide 27
with a threshold of 1e−05
MR Method
Inverse variance weighted
MR Egger
4.75
4.70
4.65
4.60
4.55
4.50
1/SE_IV
−0.50
−0.25
0.00
0.25
β_IV

Batch 2−80 : Gut microbiota abundance (genus Parasutterella id.2892) on Pituitary adenylate cyclase−activating polypeptide 27 with a threshold of 1e−05

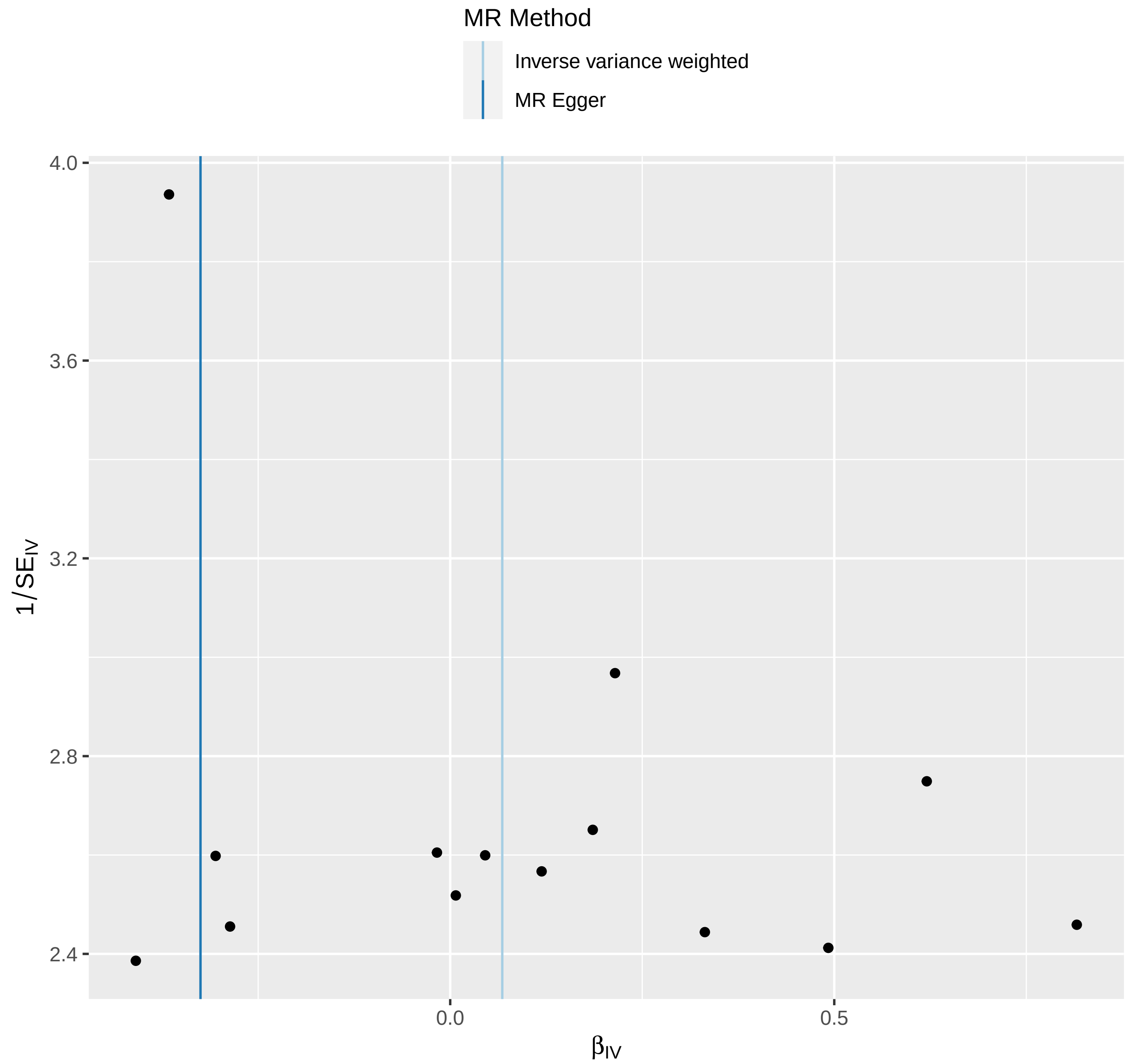

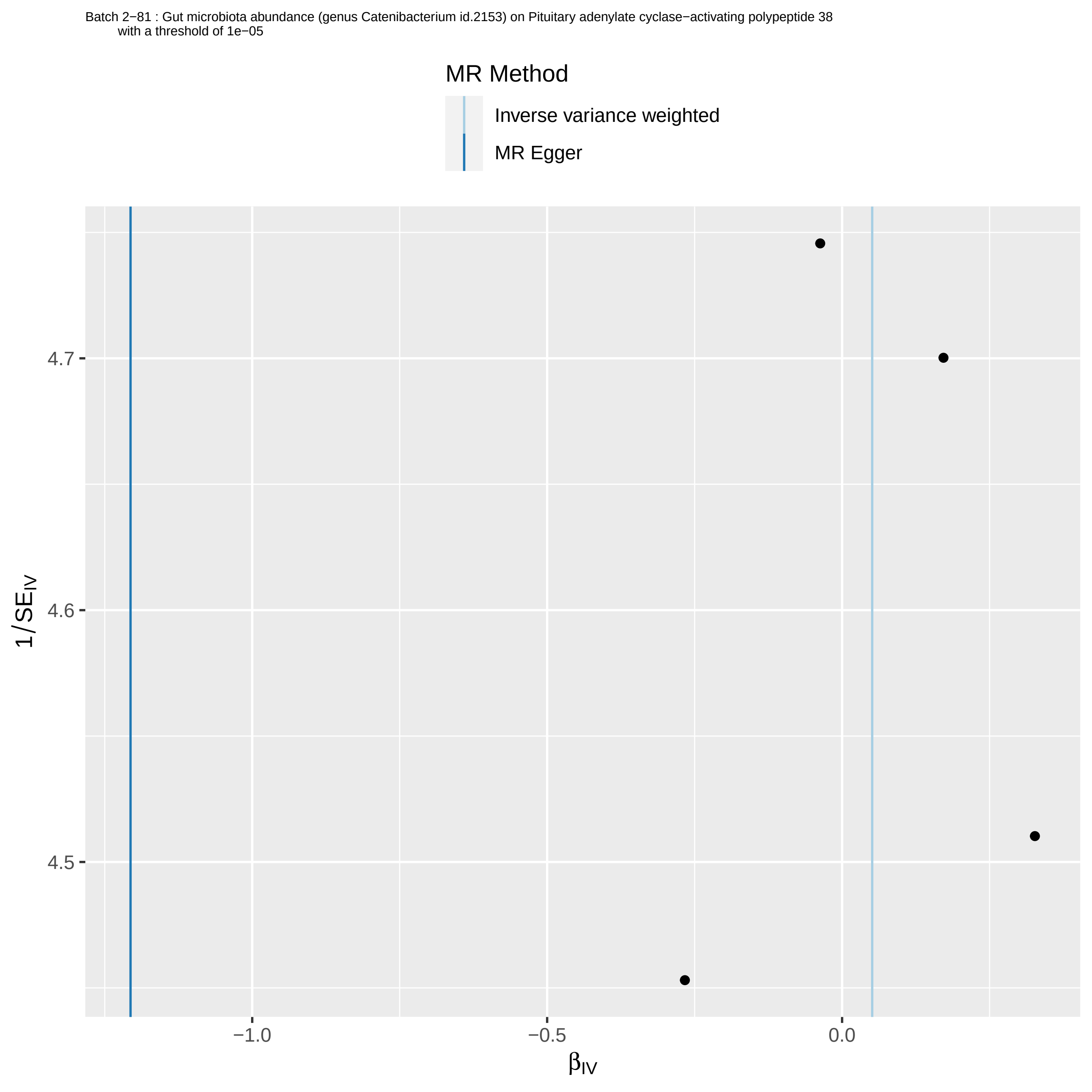
Batch 2−81 : Gut microbiota abundance (genus Catenibacterium id.2153) on Pituitary adenylate cyclase−activating polypeptide 38
with a threshold of 1e−05
MR Method
Inverse variance weighted
MR Egger
4.7
4.6
4.5
1/SE_IV
−1.0
−0.5
0.0
β_IV

Batch 2−82 : Gut microbiota abundance (genus Parasutterella id.2892) on Pituitary adenylate cyclase−activating polypeptide 38 with a threshold of 1e−05

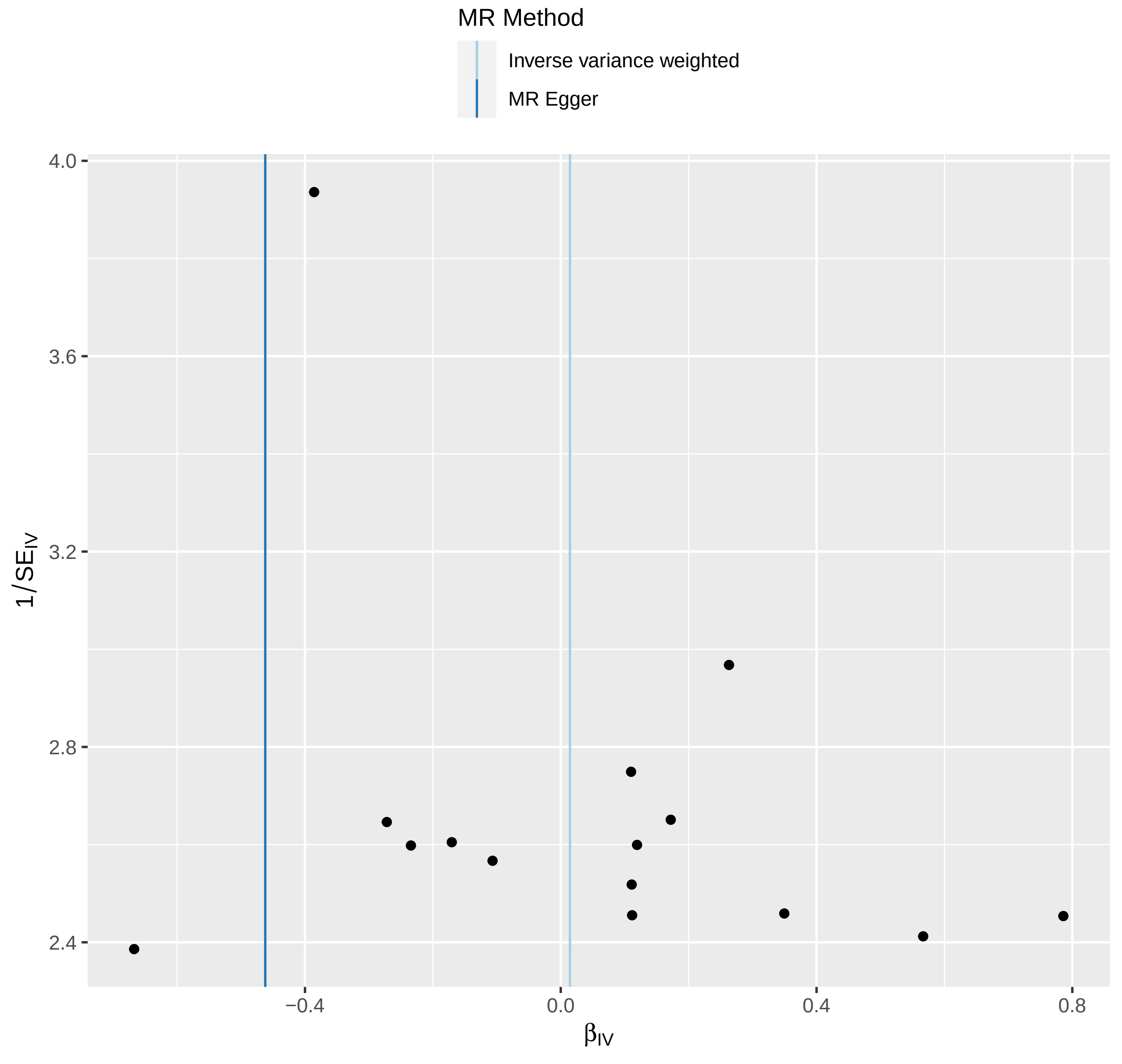

Batch 2−83 : Gut microbiota abundance (genus Catenibacterium id.2153) on Pituitary adenylate cyclase−activating polypeptide
with a threshold of 1e−05

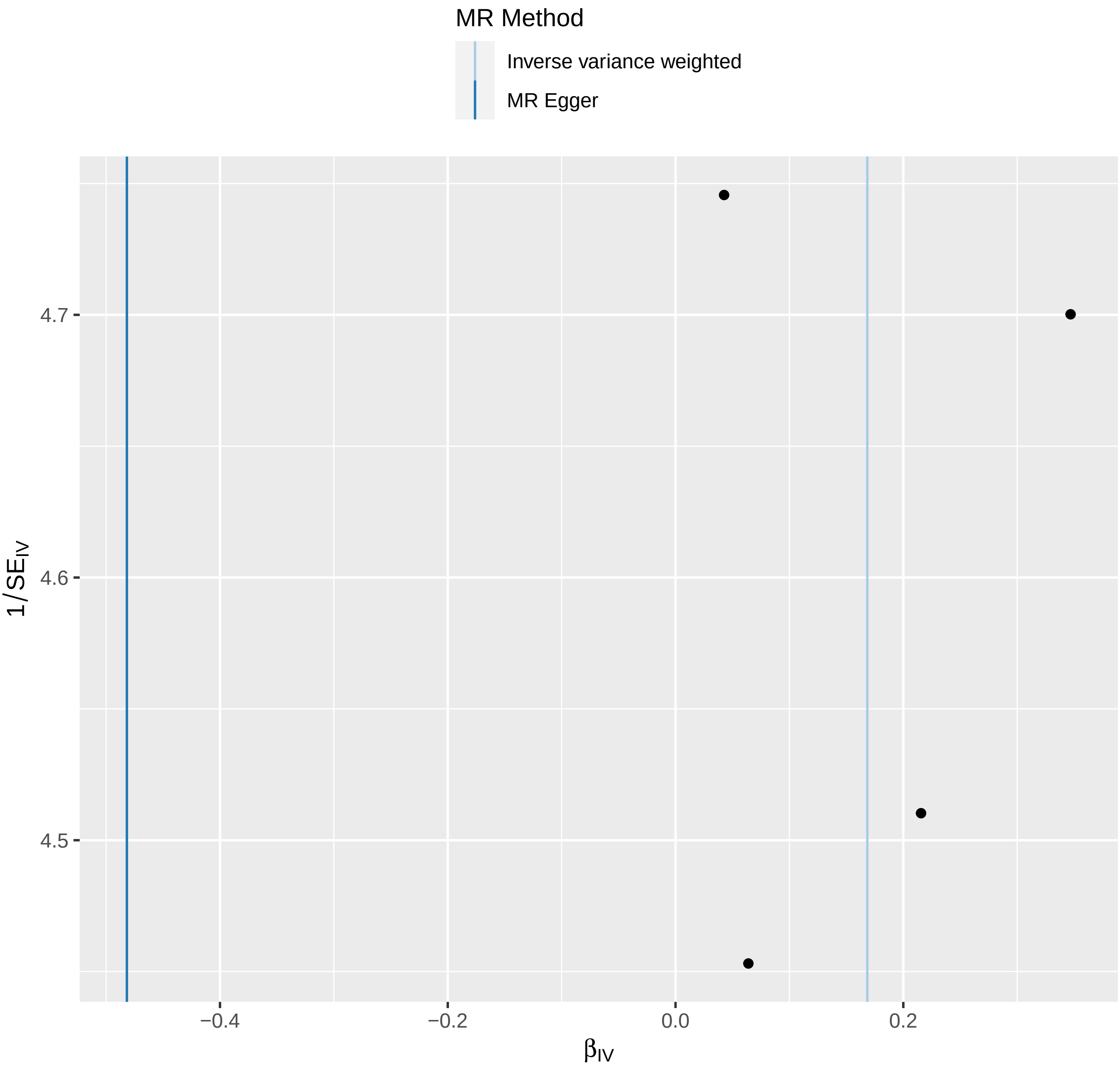

Batch 2−84 : Gut microbiota abundance (genus Parasutterella id.2892) on Pituitary adenylate cyclase−activating polypeptide with a threshold of 1e−05

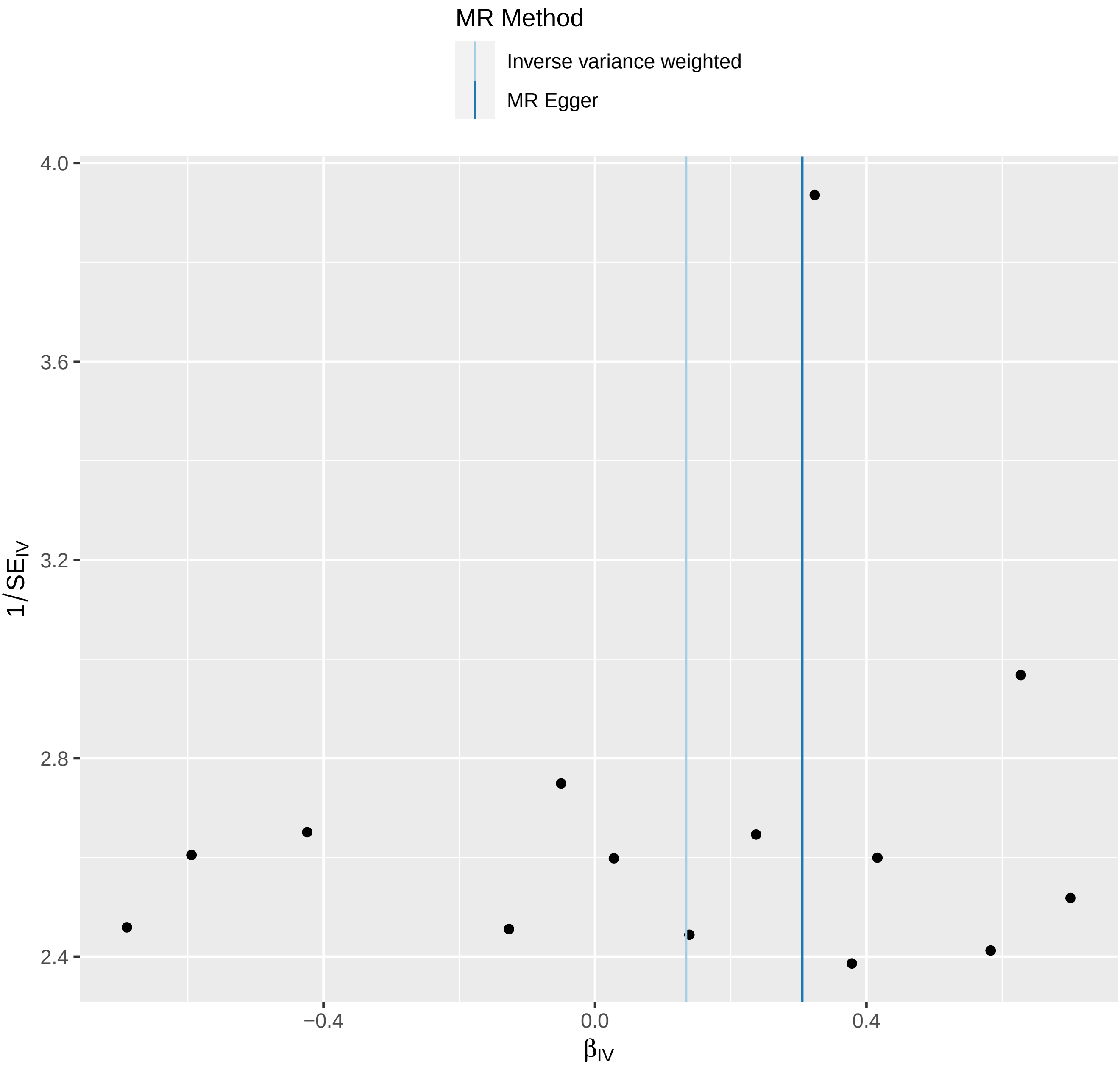

Batch 2−85 : Gut microbiota abundance (genus Catenibacterium id.2153) on Adhesion G−protein coupled receptor F1 with a threshold of 1e−05

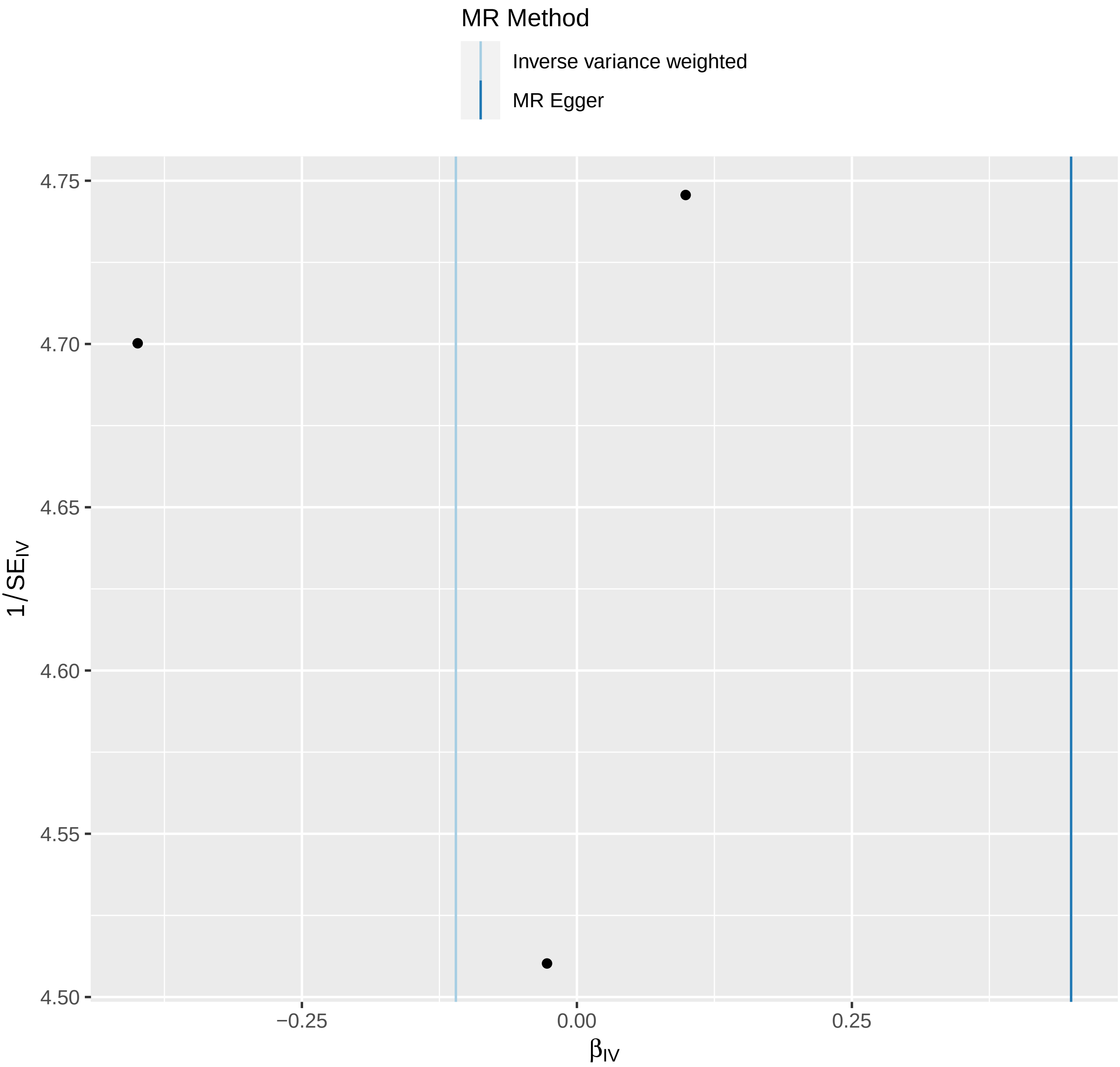

Batch 2−86 : Gut microbiota abundance (genus Parasutterella id.2892) on Adhesion G−protein coupled receptor F1
with a threshold of 1e−05

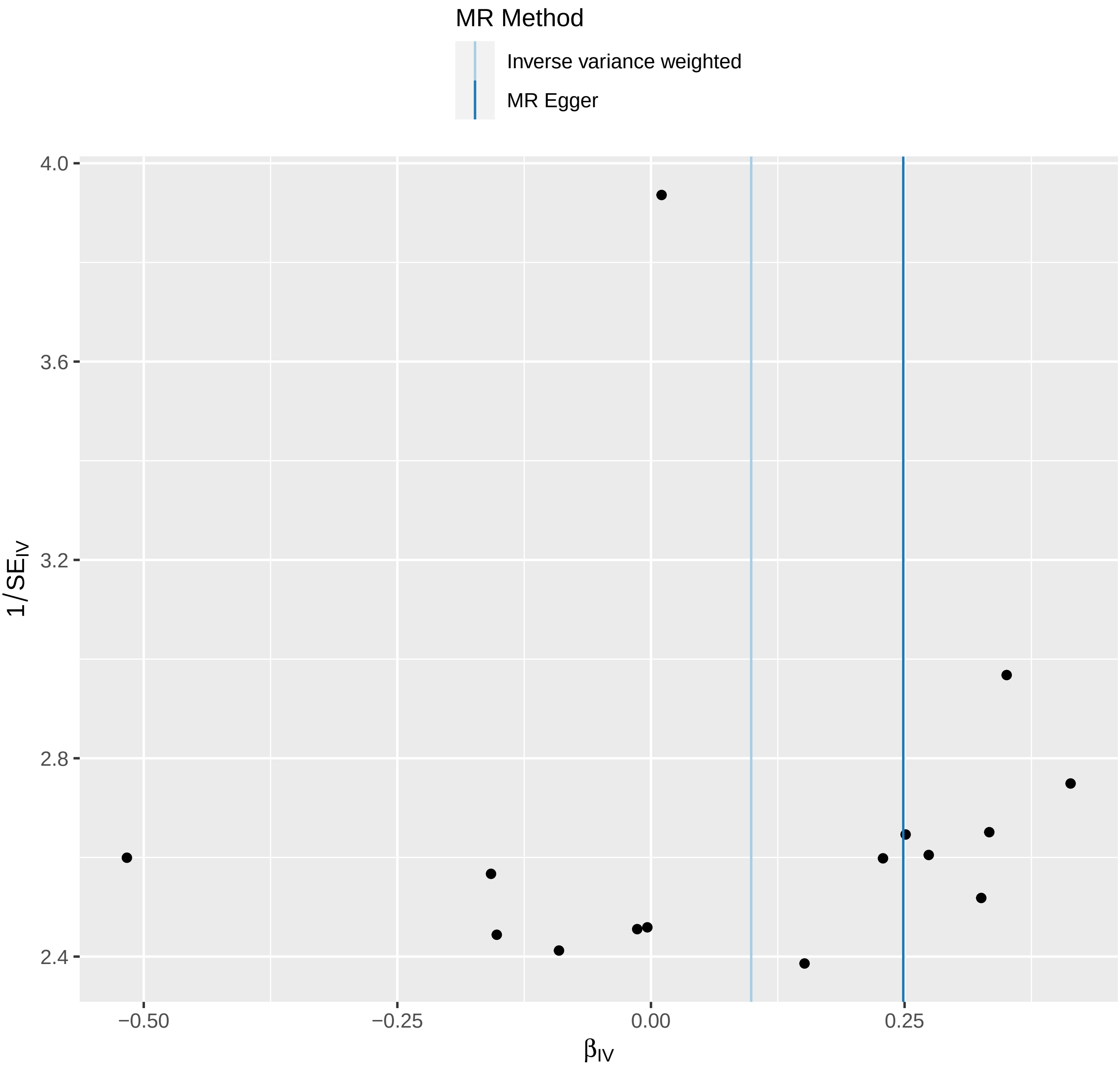

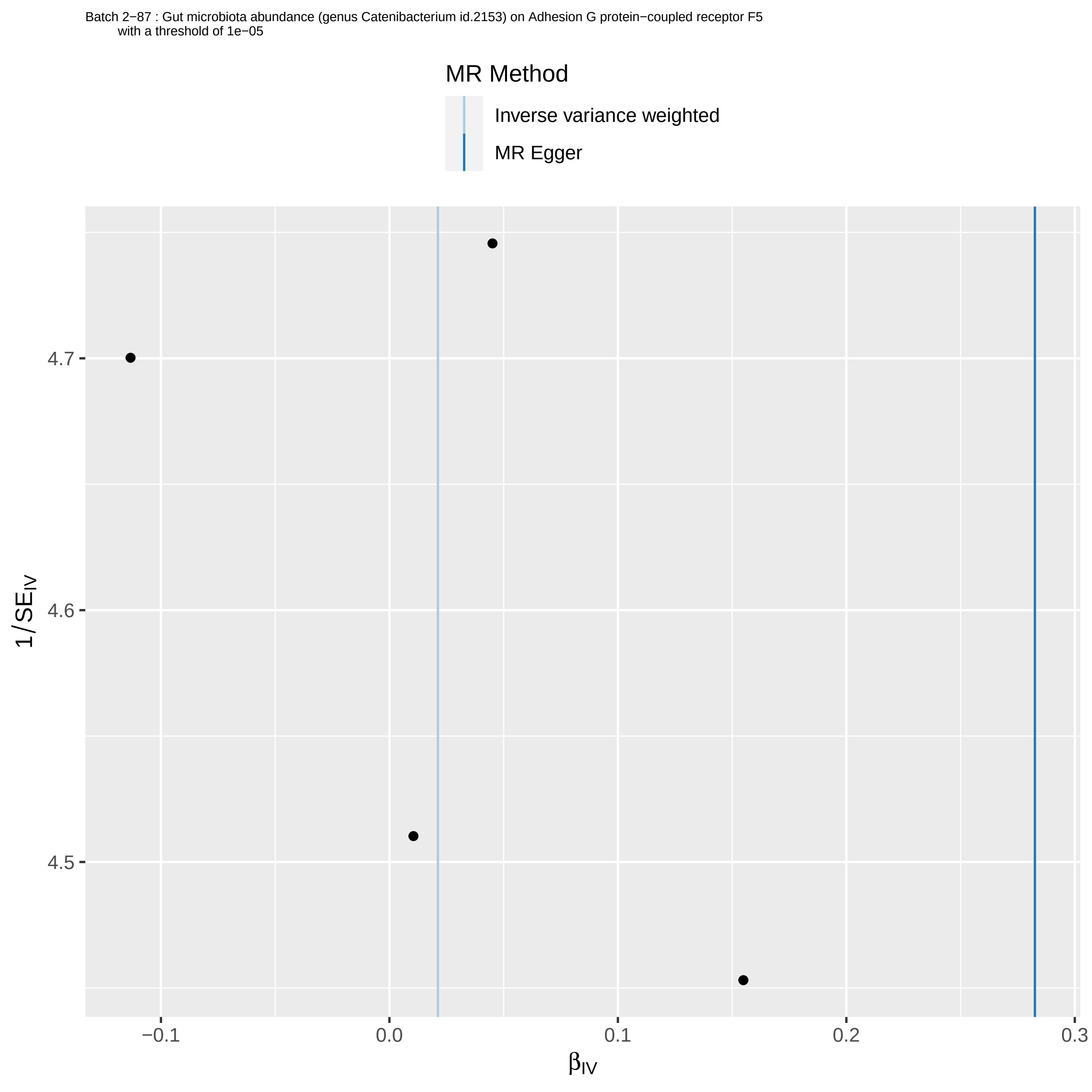
Batch 2−87 : Gut microbiota abundance (genus Catenibacterium id.2153) on Adhesion G protein−coupled receptor F5
with a threshold of 1e−05
MR Method
Inverse variance weighted
MR Egger
4.7
4.6
4.5
1/SE_IV
−0.1
0.0
0.1
0.2
0.3
β_IV

Batch 2−88 : Gut microbiota abundance (genus Parasutterella id.2892) on Adhesion G protein−coupled receptor F5
with a threshold of 1e−05

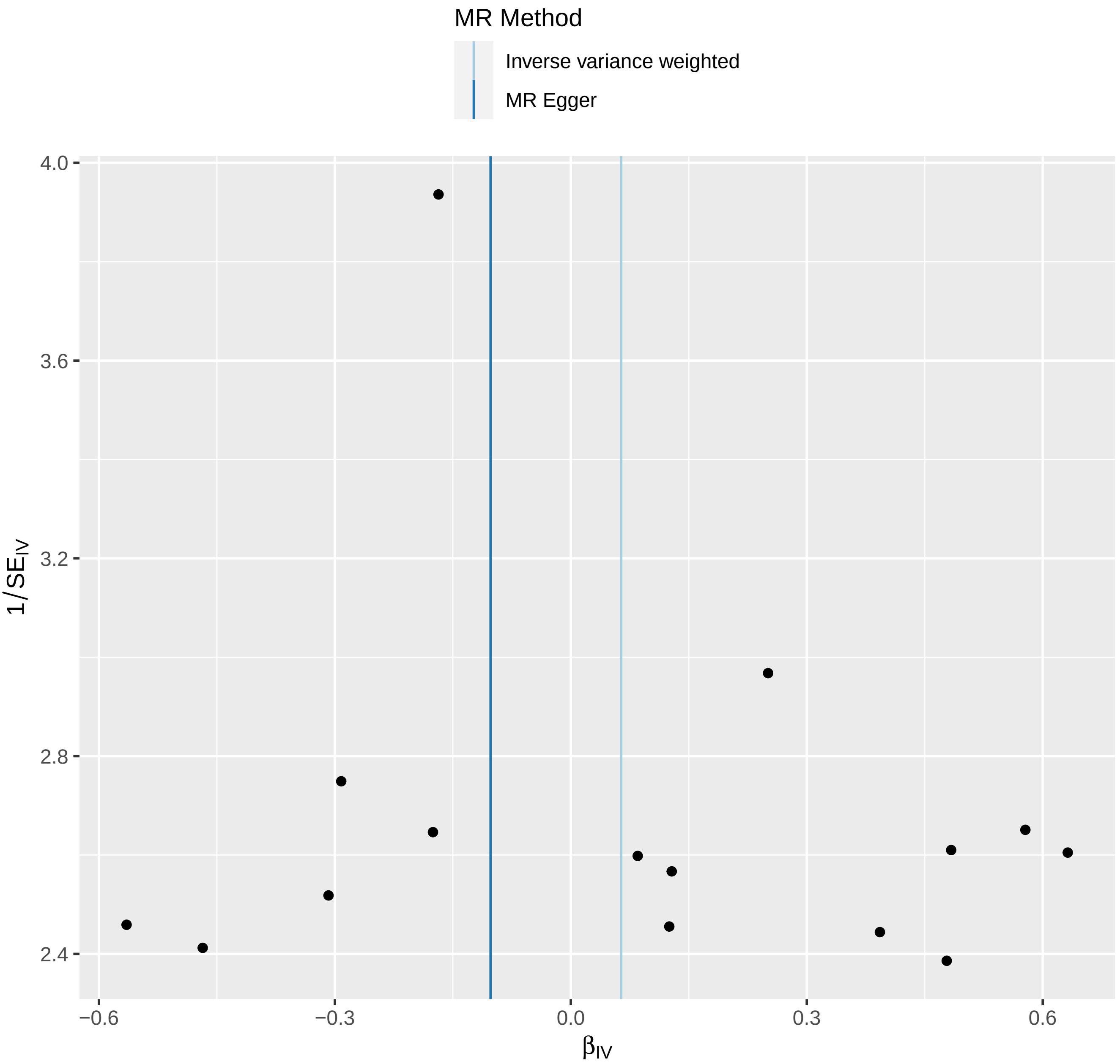

Batch 2−89 : Gut microbiota abundance (genus Catenibacterium id.2153) on Adhesion G−protein coupled receptor G5 with a threshold of 1e−05

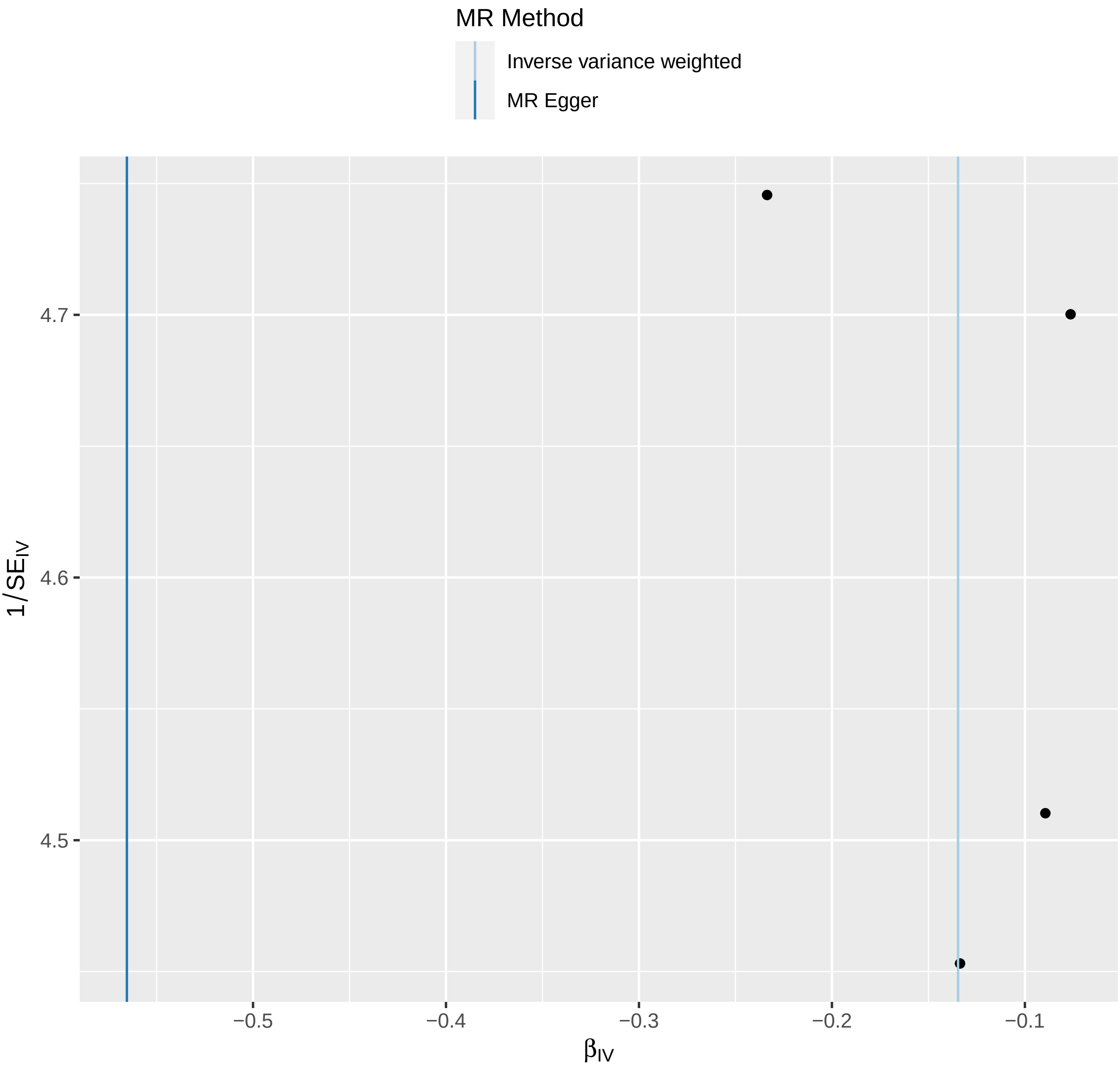

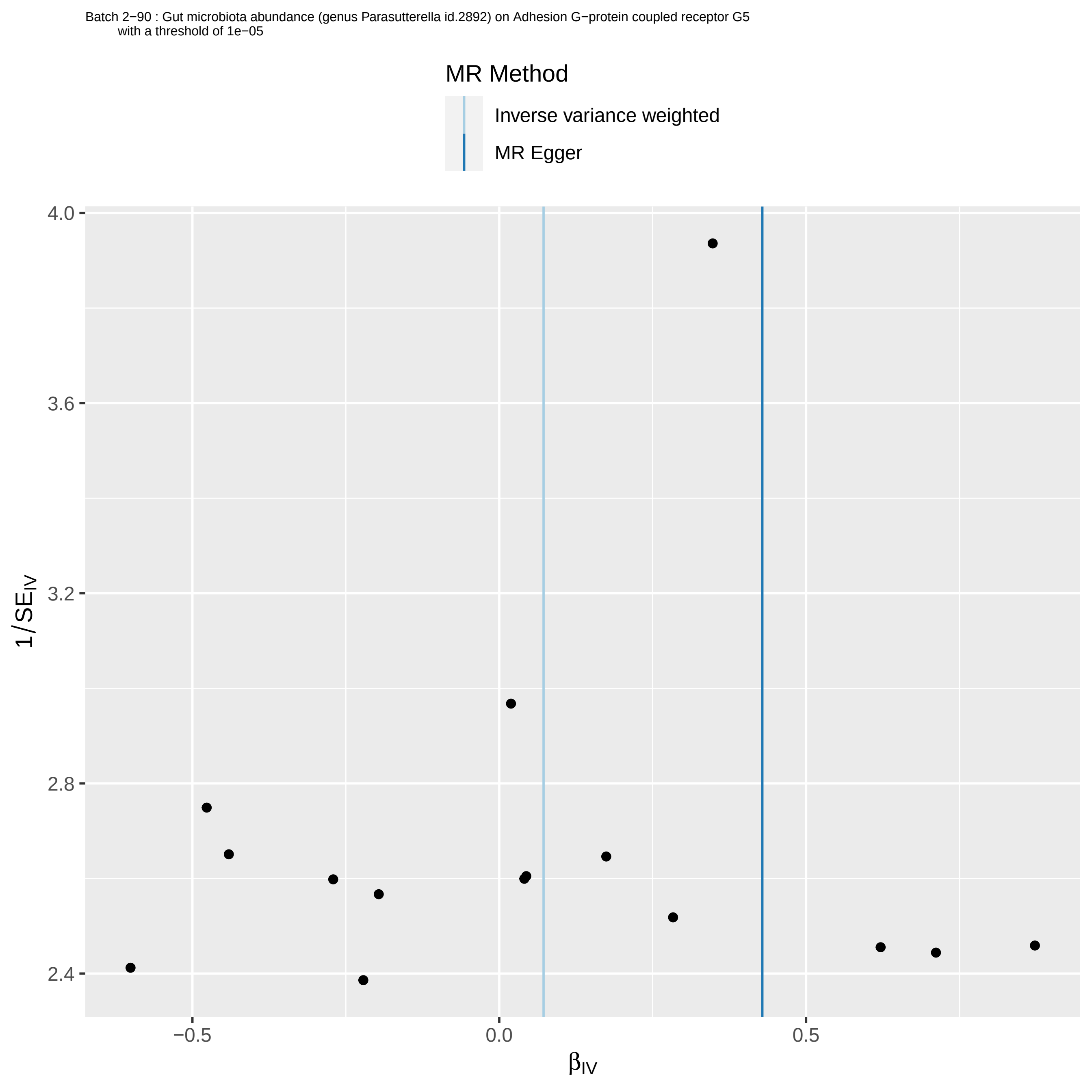

Batch 2−90 : Gut microbiota abundance (genus Parasutterella id.2892) on Adhesion G−protein coupled receptor G5
with a threshold of 1e−05
MR Method
Inverse variance weighted
MR Egger
4.0
3.6
3.2
2.8
2.4
1/SE_IV
−0.5
0.0
0.5
β_IV

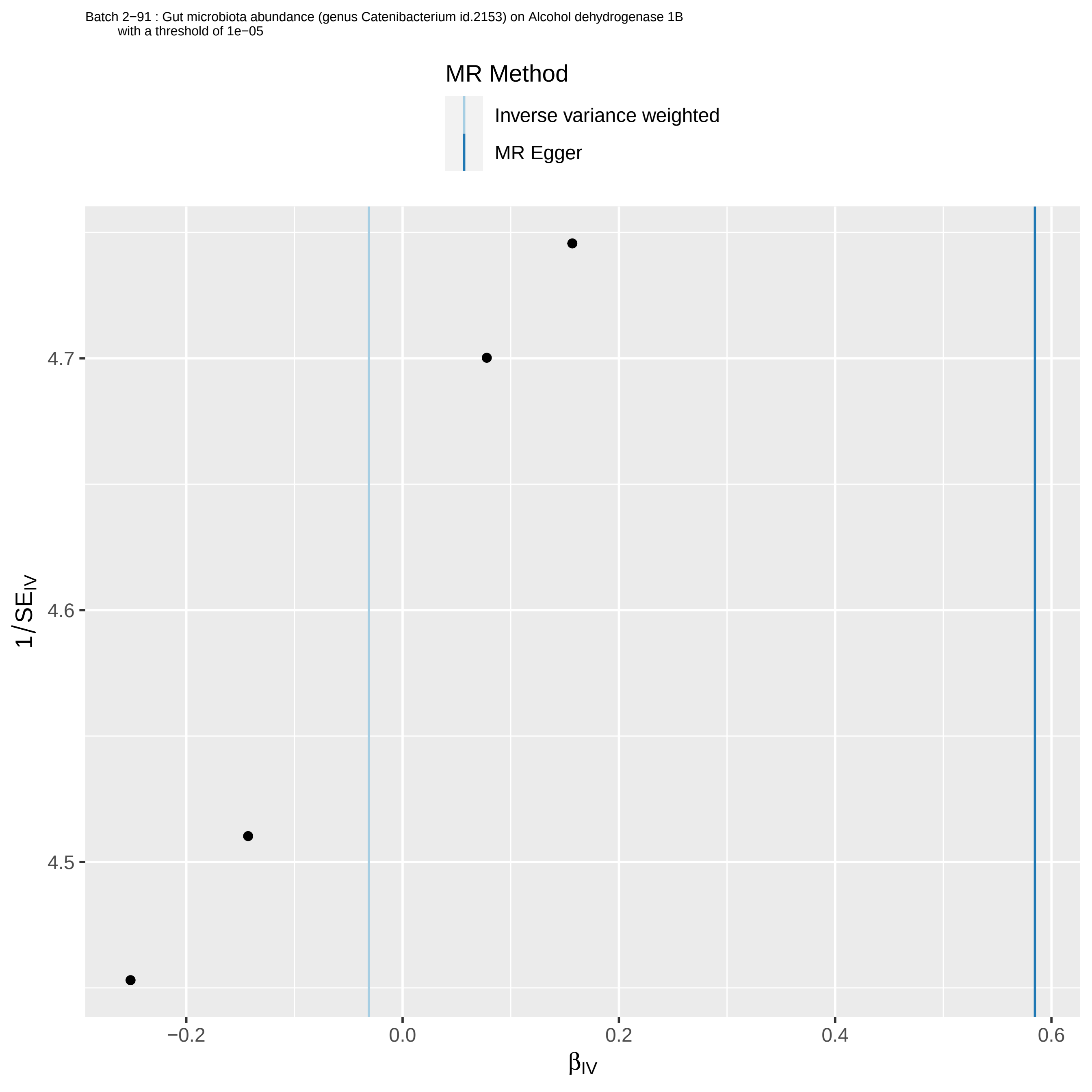
Batch 2−91 : Gut microbiota abundance (genus Catenibacterium id.2153) on Alcohol dehydrogenase 1B
with a threshold of 1e−05
MR Method
Inverse variance weighted
MR Egger
4.7
4.6
4.5
1/SE_IV
−0.2
0.0
0.2
0.4
0.6
β_IV

Batch 2−92 : Gut microbiota abundance (genus Parasutterella id.2892) on Alcohol dehydrogenase 1B
with a threshold of 1e−05

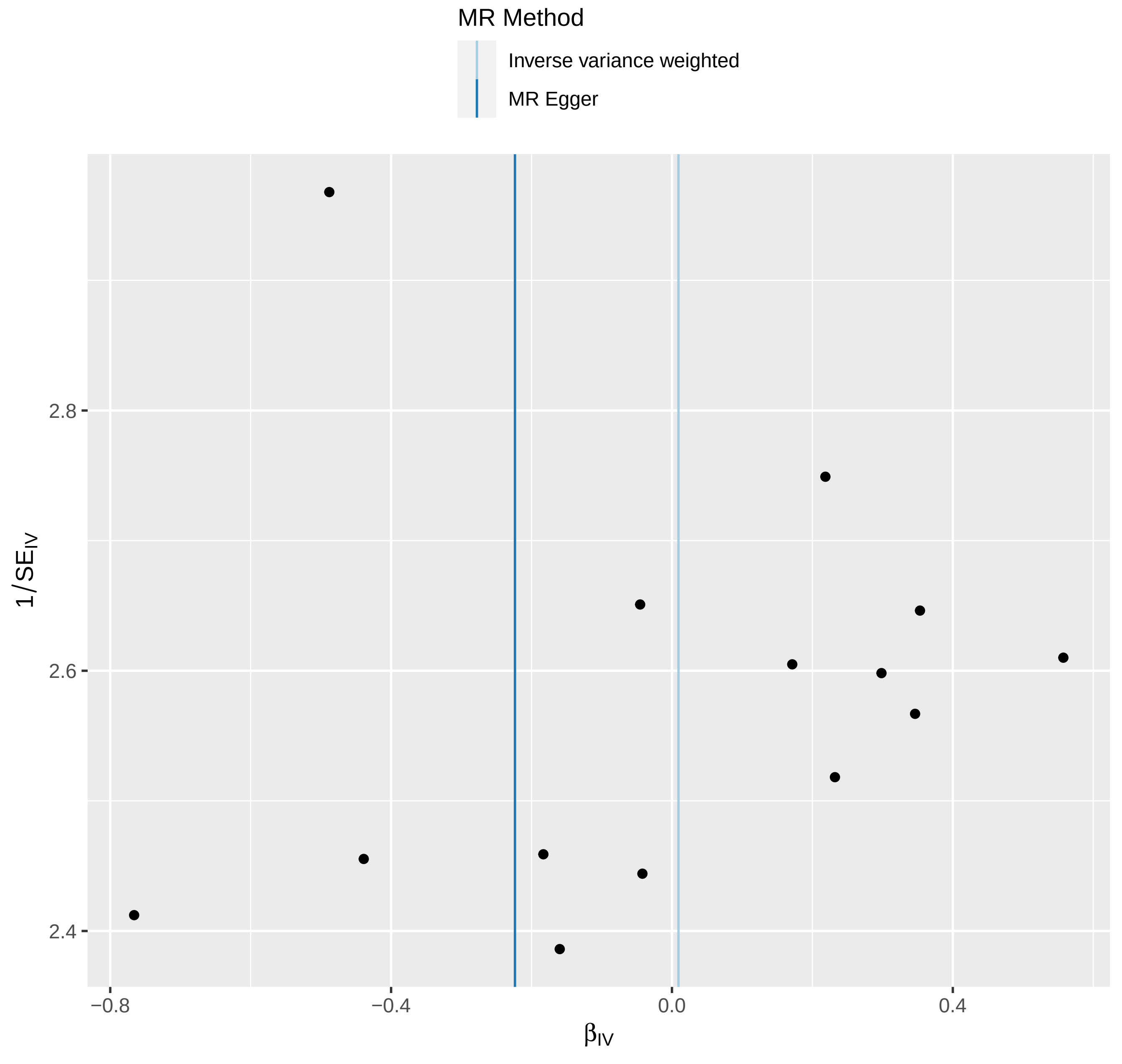

Batch 2−93 : Gut microbiota abundance (genus Catenibacterium id.2153) on Alcohol dehydrogenase 4 with a threshold of 1e−05

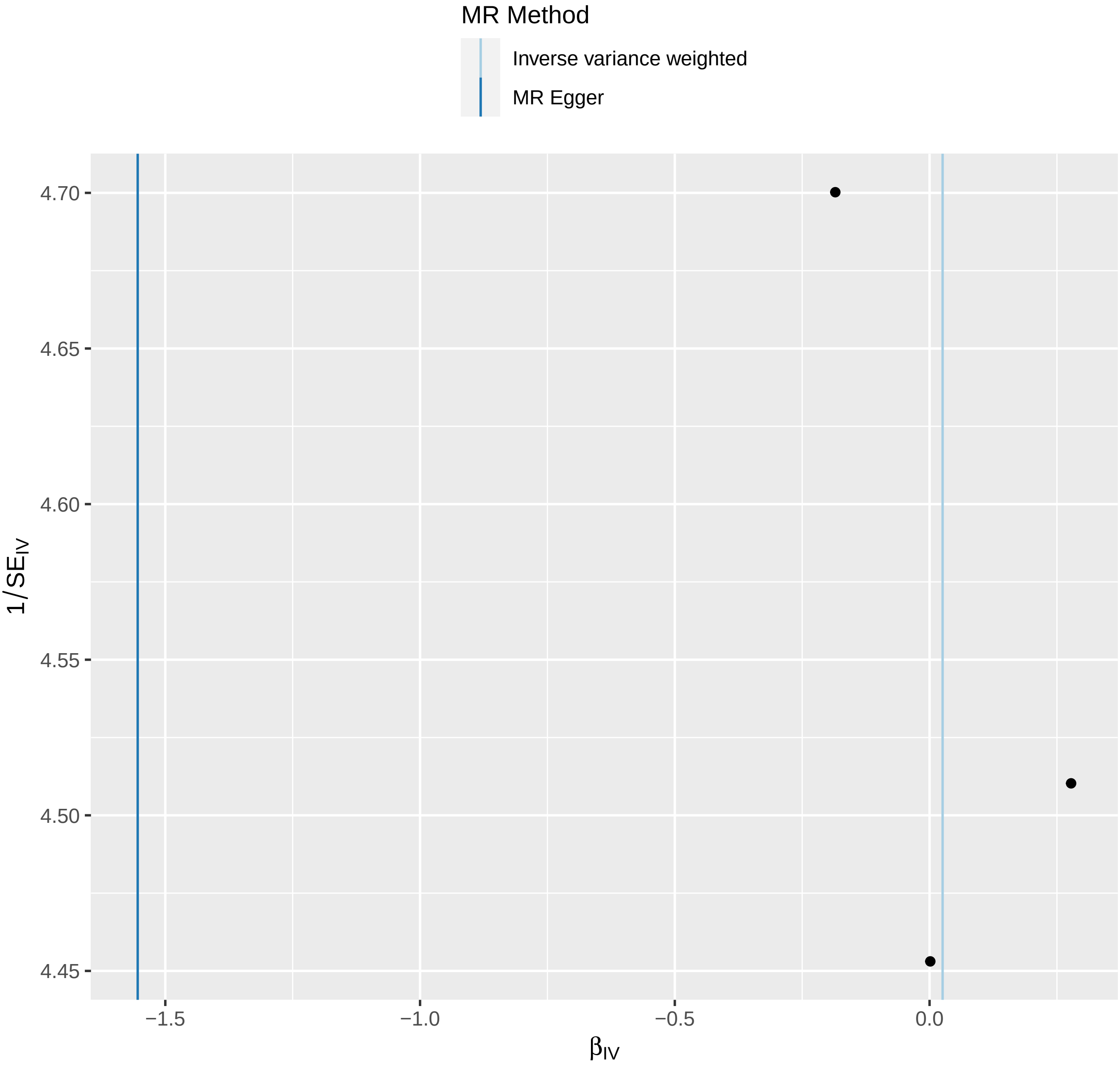

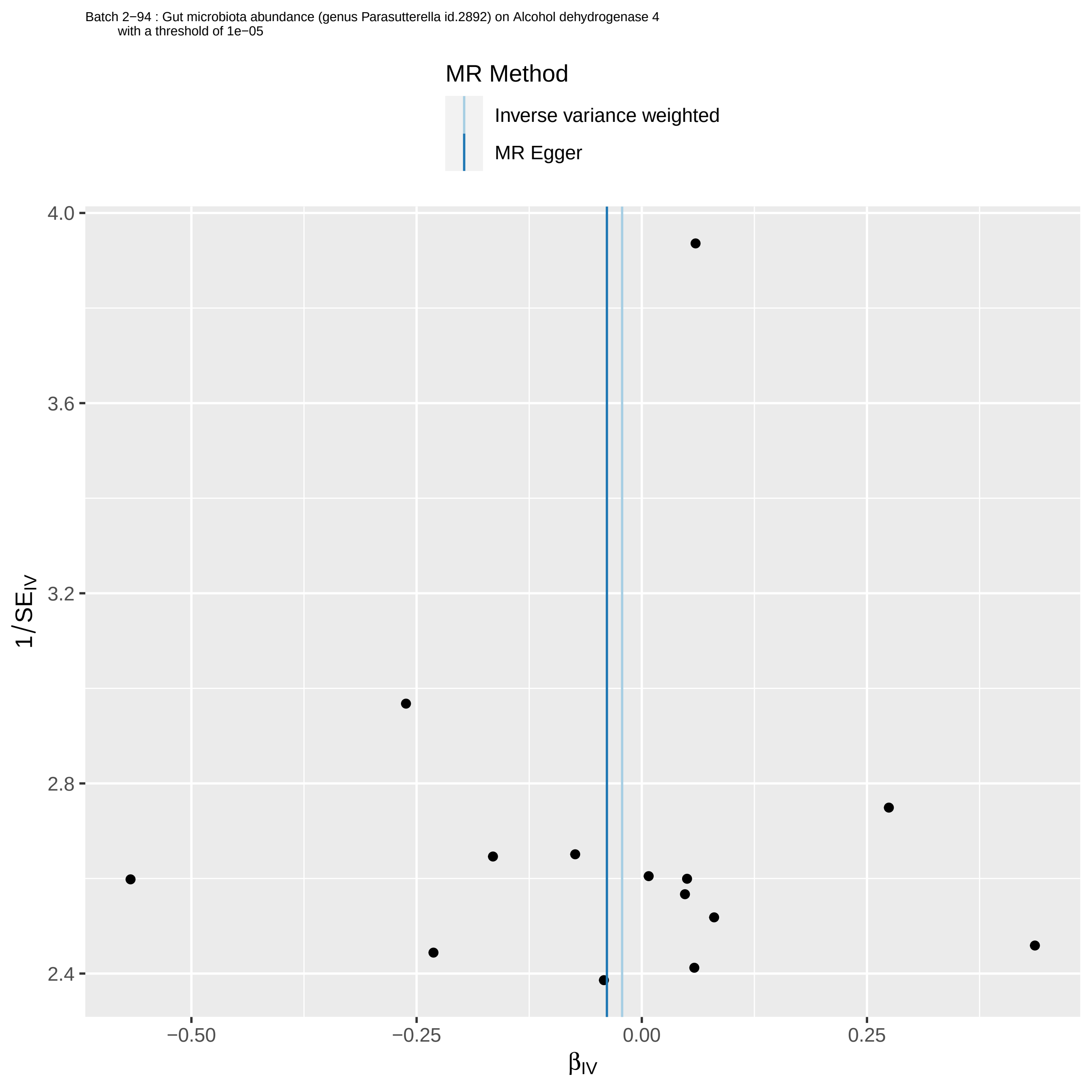

Batch 2−94 : Gut microbiota abundance (genus Parasutterella id.2892) on Alcohol dehydrogenase 4
with a threshold of 1e−05
MR Method
Inverse variance weighted
MR Egger
4.0
3.6
3.2
2.8
2.4
1/SE_IV
−0.50
−0.25
0.00
0.25
β_IV

Batch 2−95 : Gut microbiota abundance (genus Catenibacterium id.2153) on Adrenomedullin
with a threshold of 1e−05

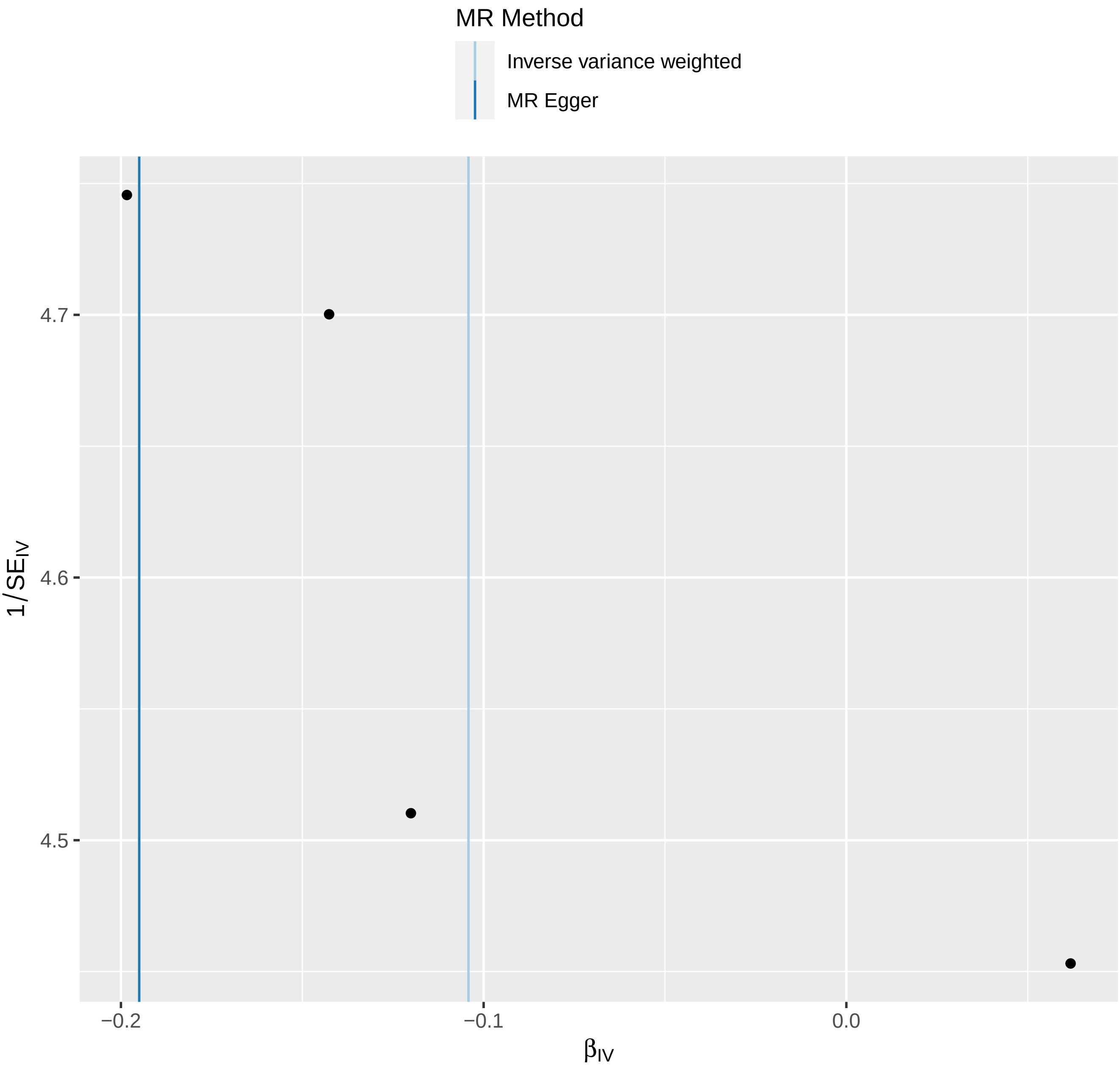

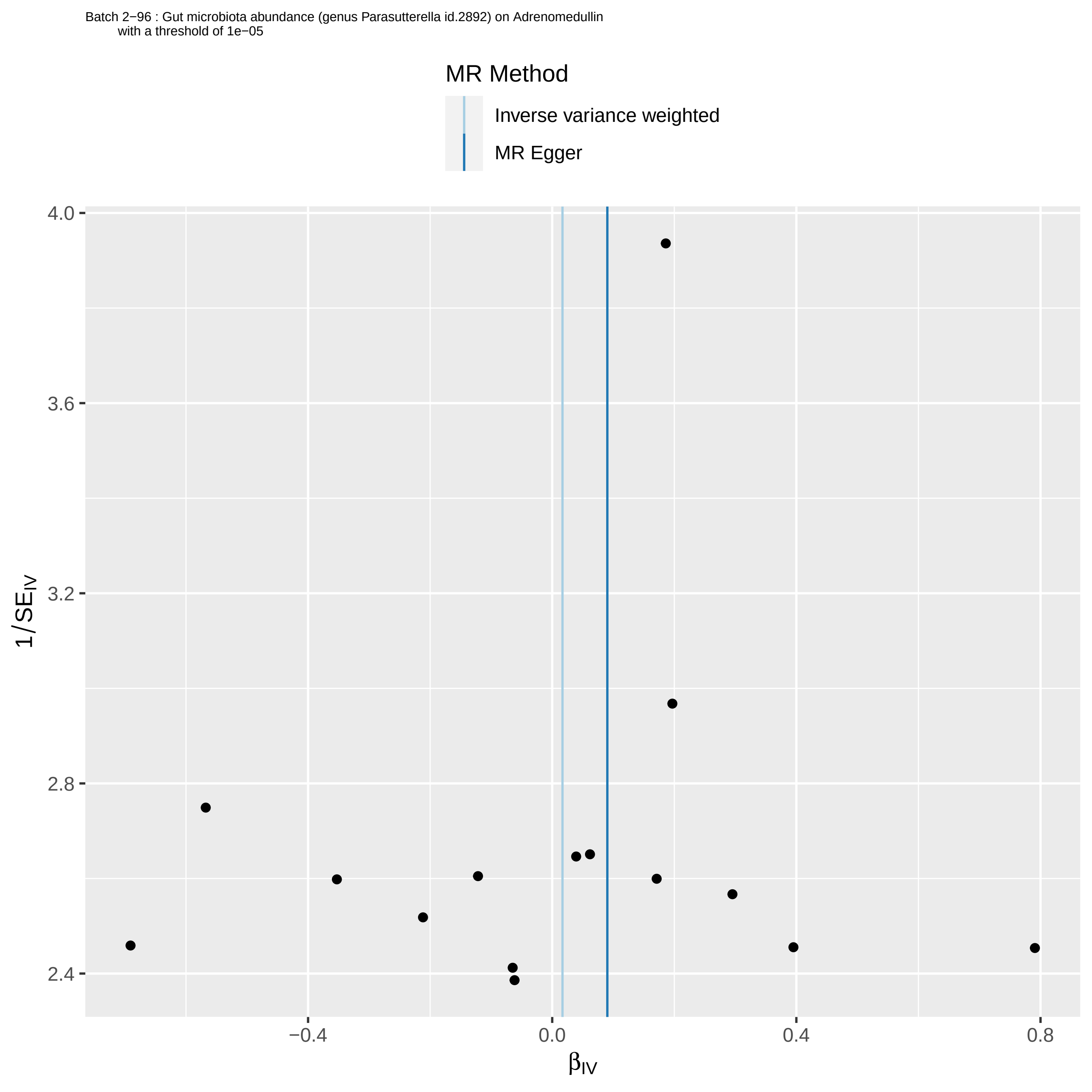
Batch 2−96 : Gut microbiota abundance (genus Parasutterella id.2892) on Adrenomedullin
with a threshold of 1e−05
MR Method
Inverse variance weighted
MR Egger
4.0
3.6
3.2
2.8
2.4
1/SE_IV
−0.4
0.0
0.4
0.8
β_IV

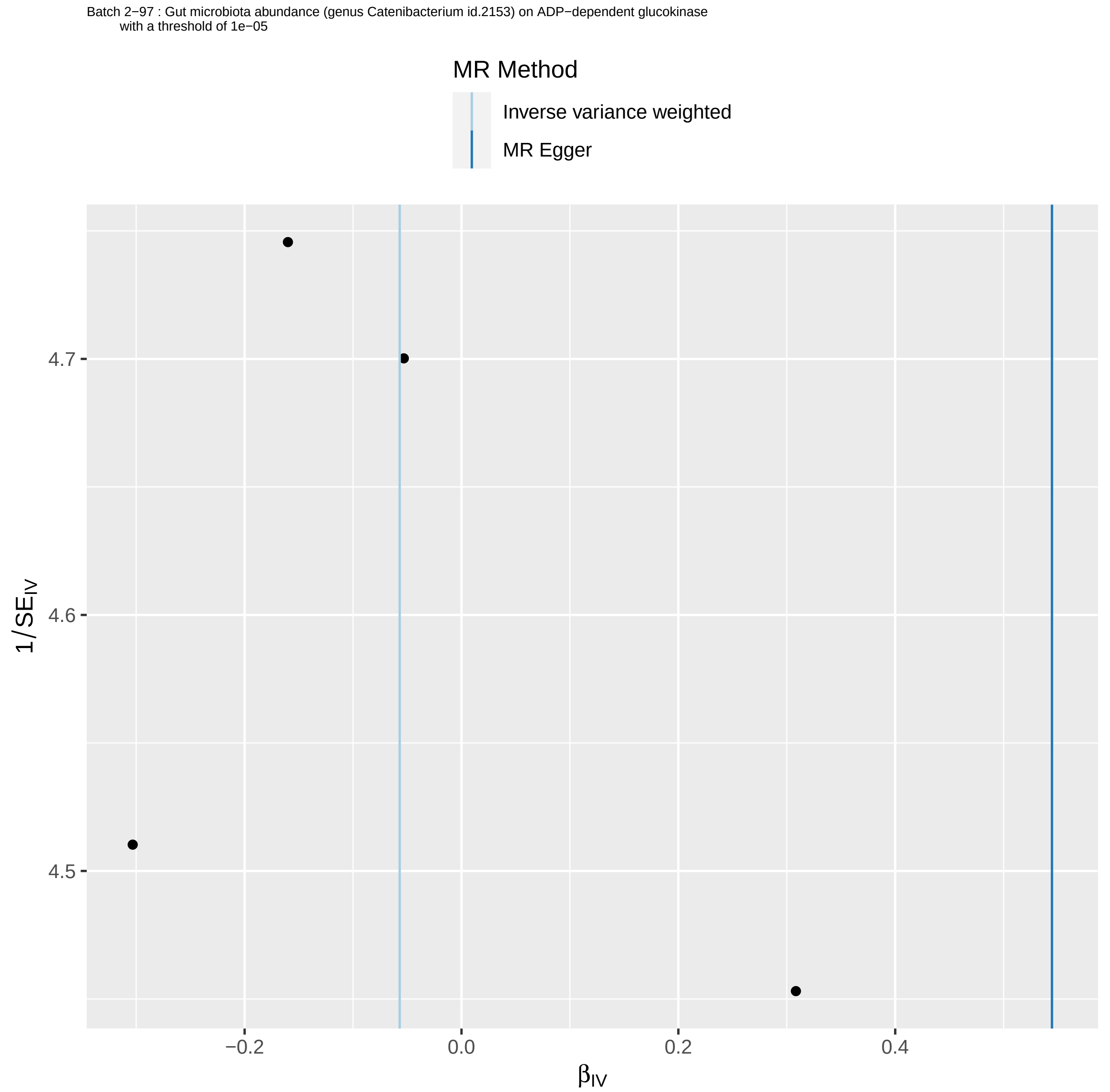

Batch 2−97 : Gut microbiota abundance (genus Catenibacterium id.2153) on ADP−dependent glucokinase
with a threshold of 1e−05
MR Method
Inverse variance weighted
MR Egger
4.7
4.6
4.5
1/SE$_{IV}$
−0.2
0.0
0.2
0.4
β$_{IV}$

Batch 2−98 : Gut microbiota abundance (genus Parasutterella id.2892) on ADP−dependent glucokinase
with a threshold of 1e−05

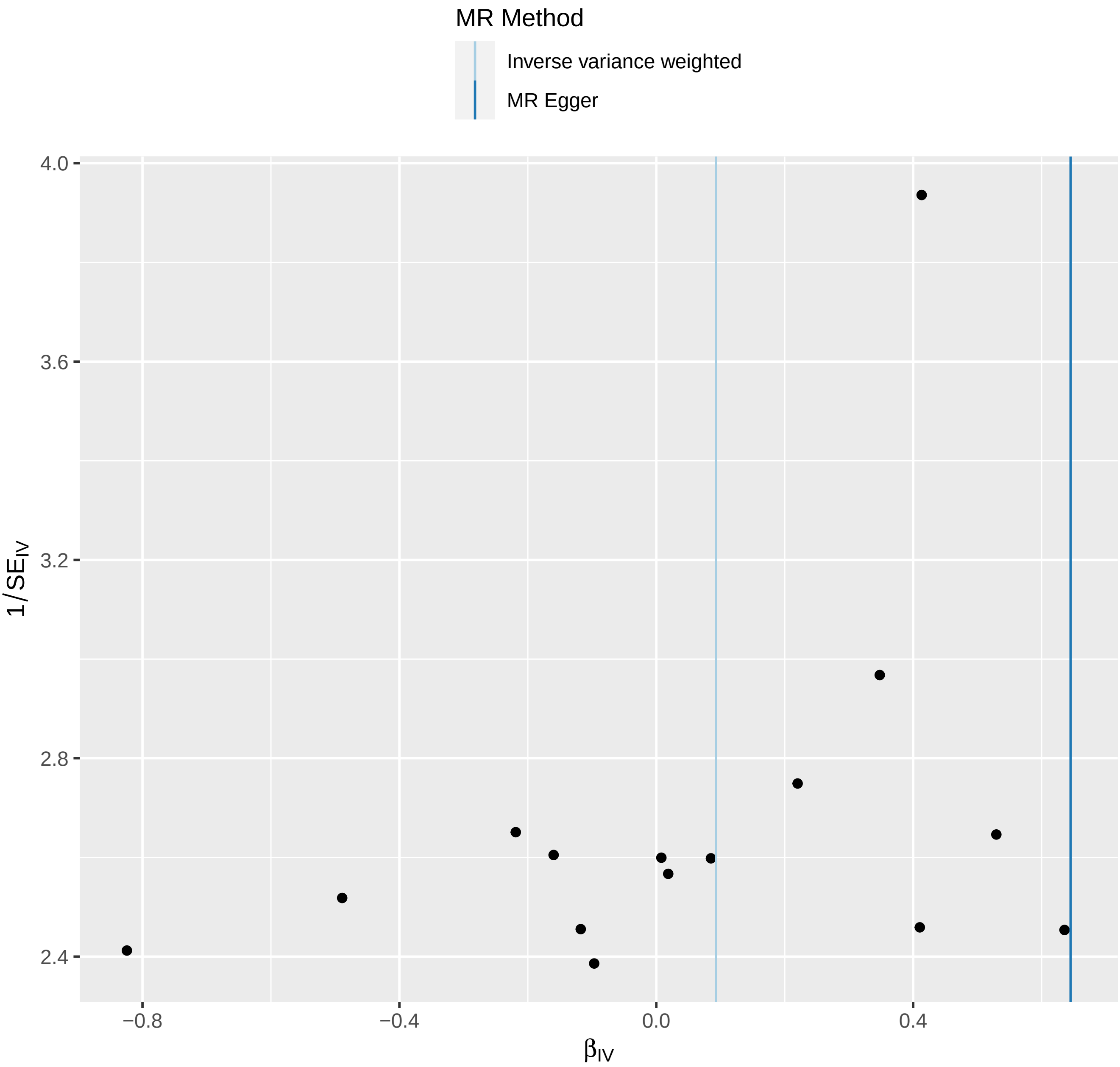

Batch 2−99 : Gut microbiota abundance (genus Catenibacterium id.2153) on beta−adrenergic receptor kinase 1
with a threshold of 1e−05

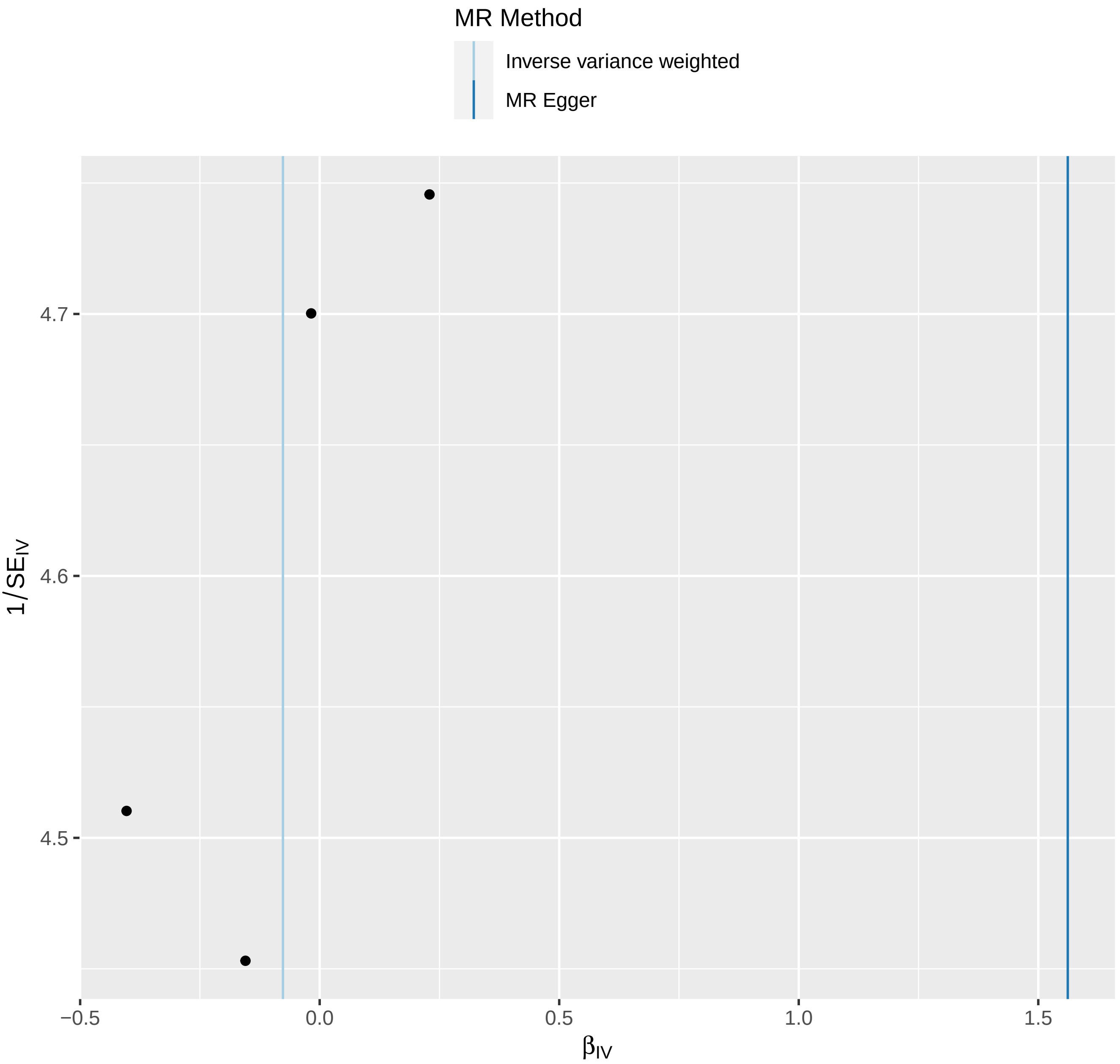

Batch 2−100 : Gut microbiota abundance (genus Parasutterella id.2892) on beta−adrenergic receptor kinase 1
with a threshold of 1e−05

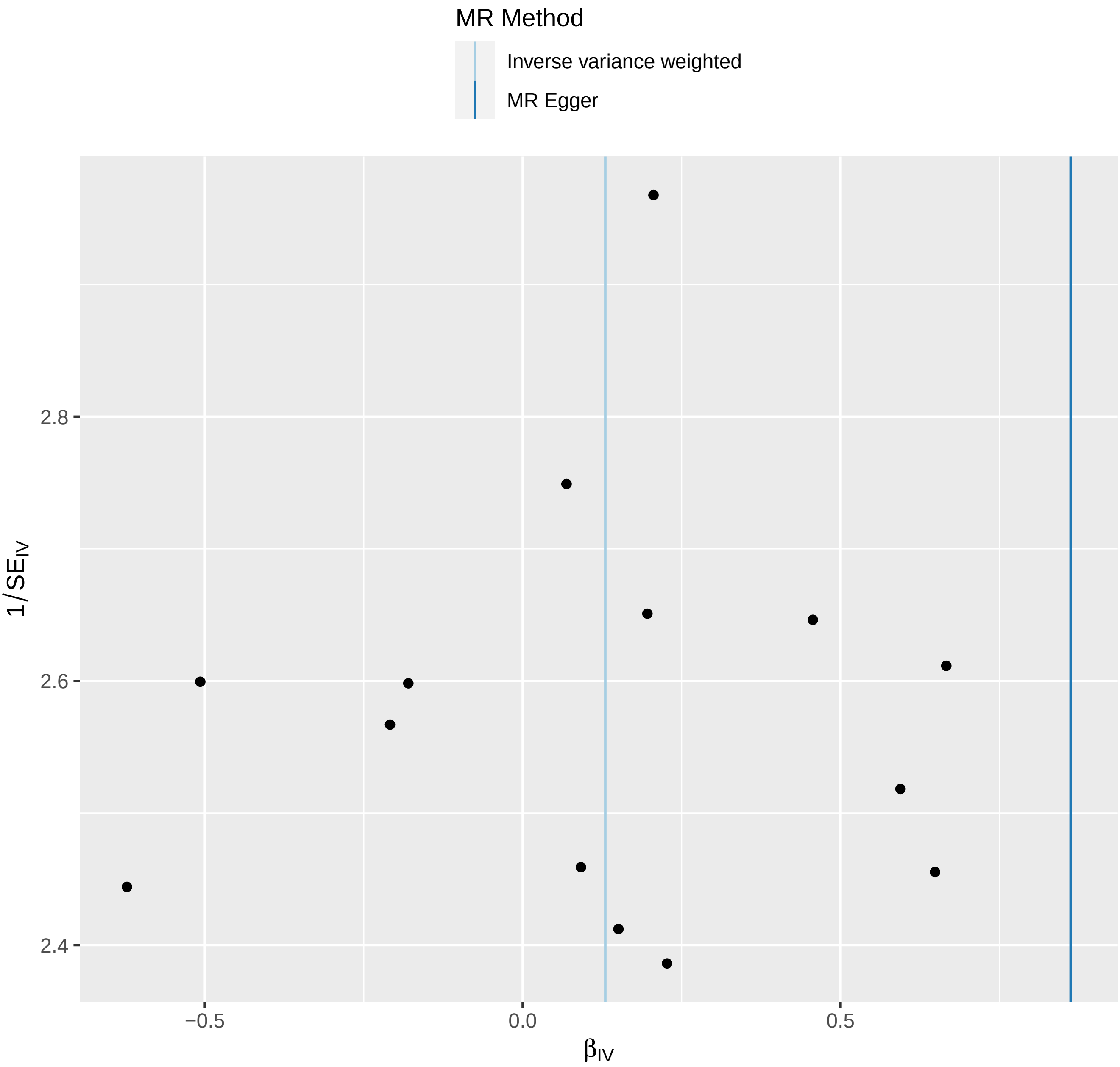

Batch 2−101 : Gut microbiota abundance (genus Catenibacterium id.2153) on Adenylosuccinate synthetase isozyme 2
with a threshold of 1e−05

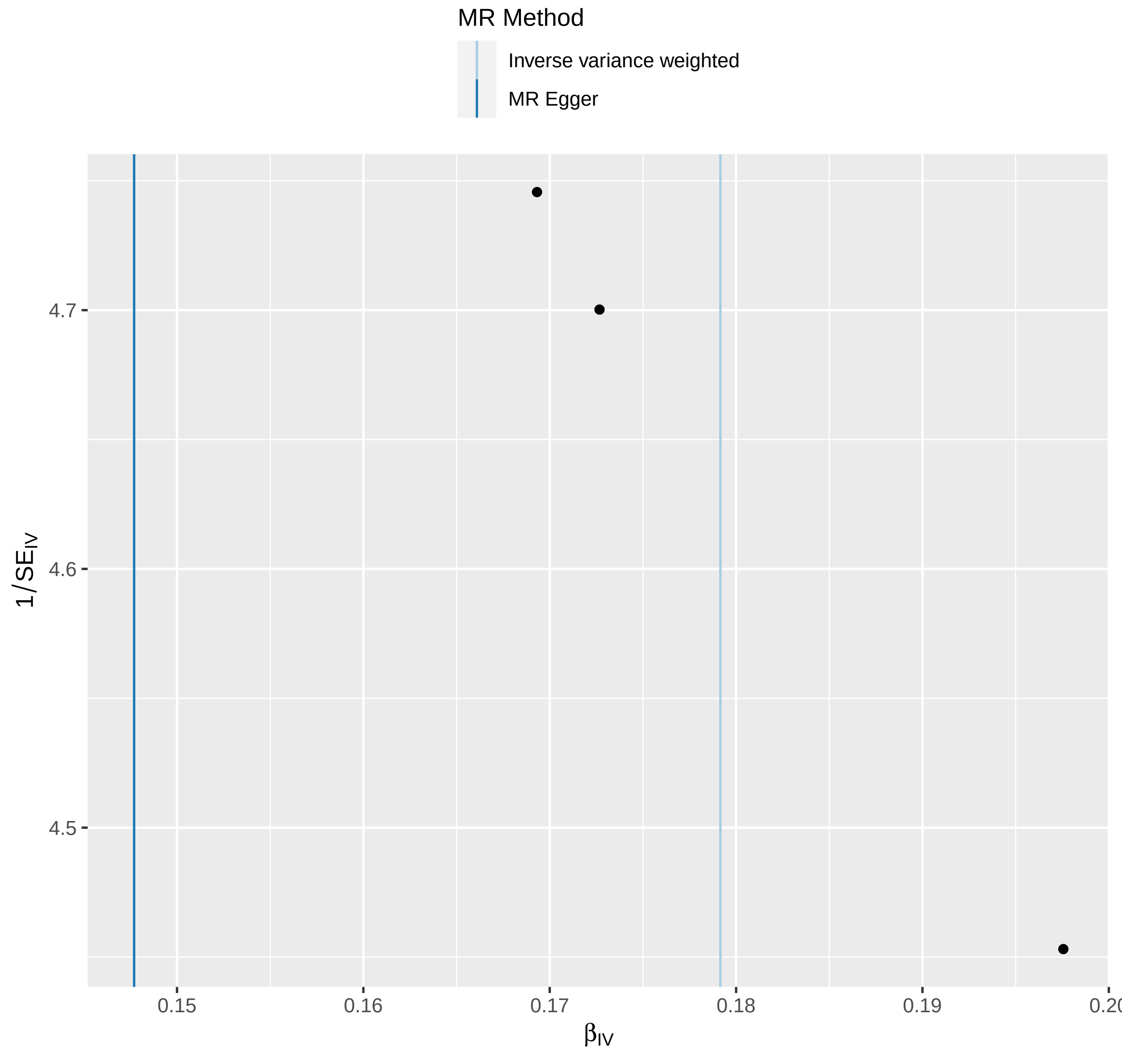

Batch 2−102 : Gut microbiota abundance (genus Parasutterella id.2892) on Adenylosuccinate synthetase isozyme 2
with a threshold of 1e−05

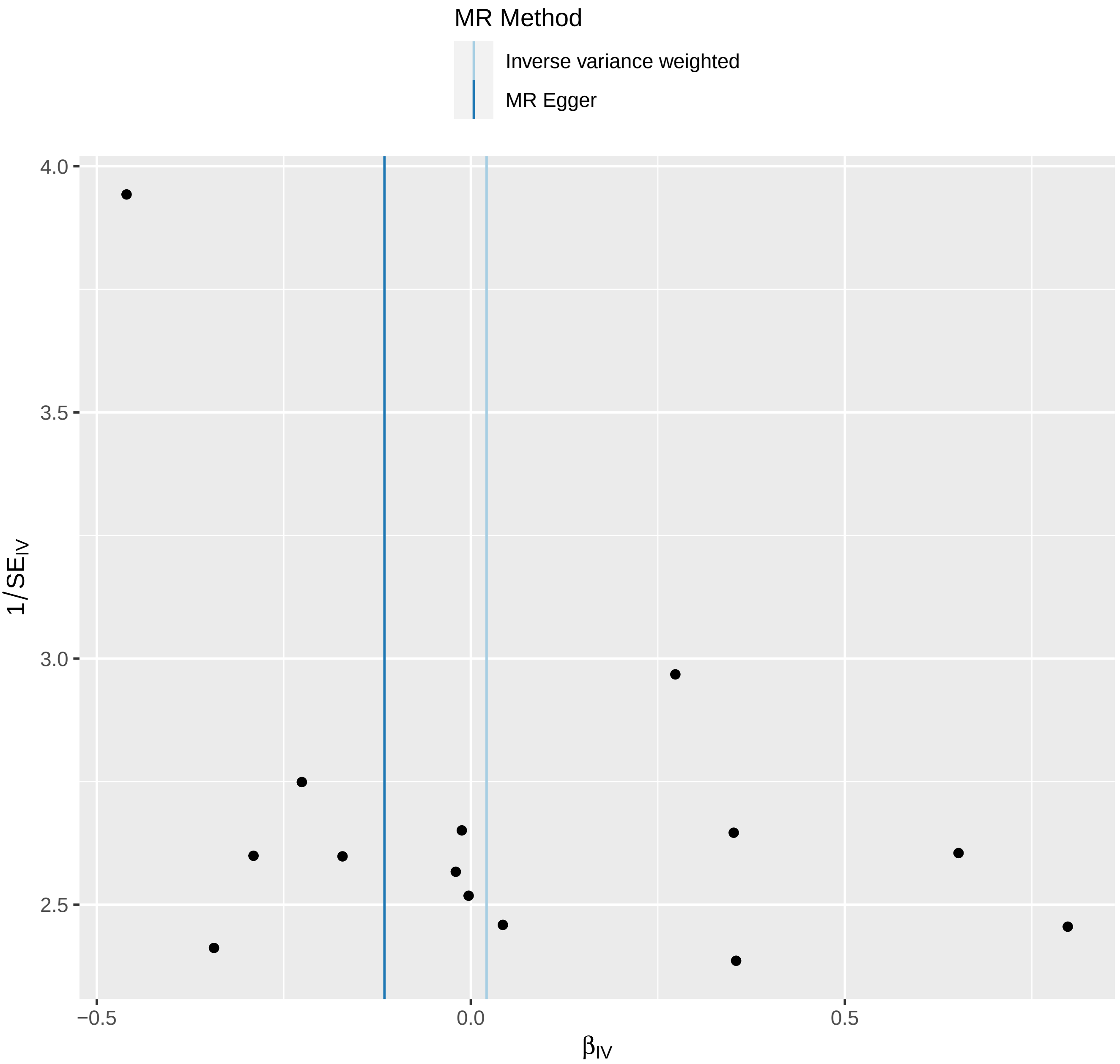

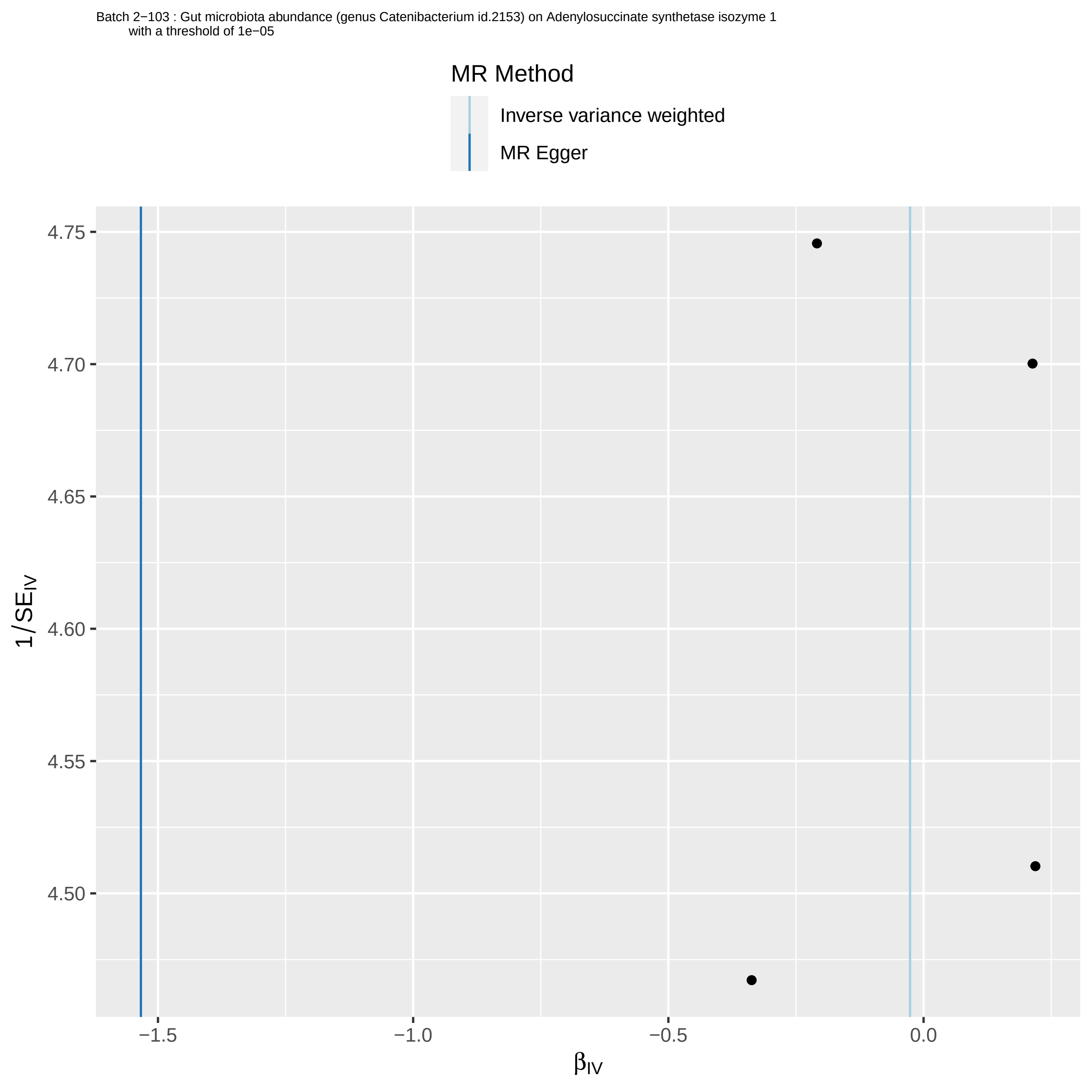

Batch 2−103 : Gut microbiota abundance (genus Catenibacterium id.2153) on Adenylosuccinate synthetase isozyme 1
with a threshold of 1e−05
MR Method
Inverse variance weighted
MR Egger
4.75
4.70
4.65
4.60
4.55
4.50
1/SE_IV
−1.5
−1.0
−0.5
0.0
β_IV

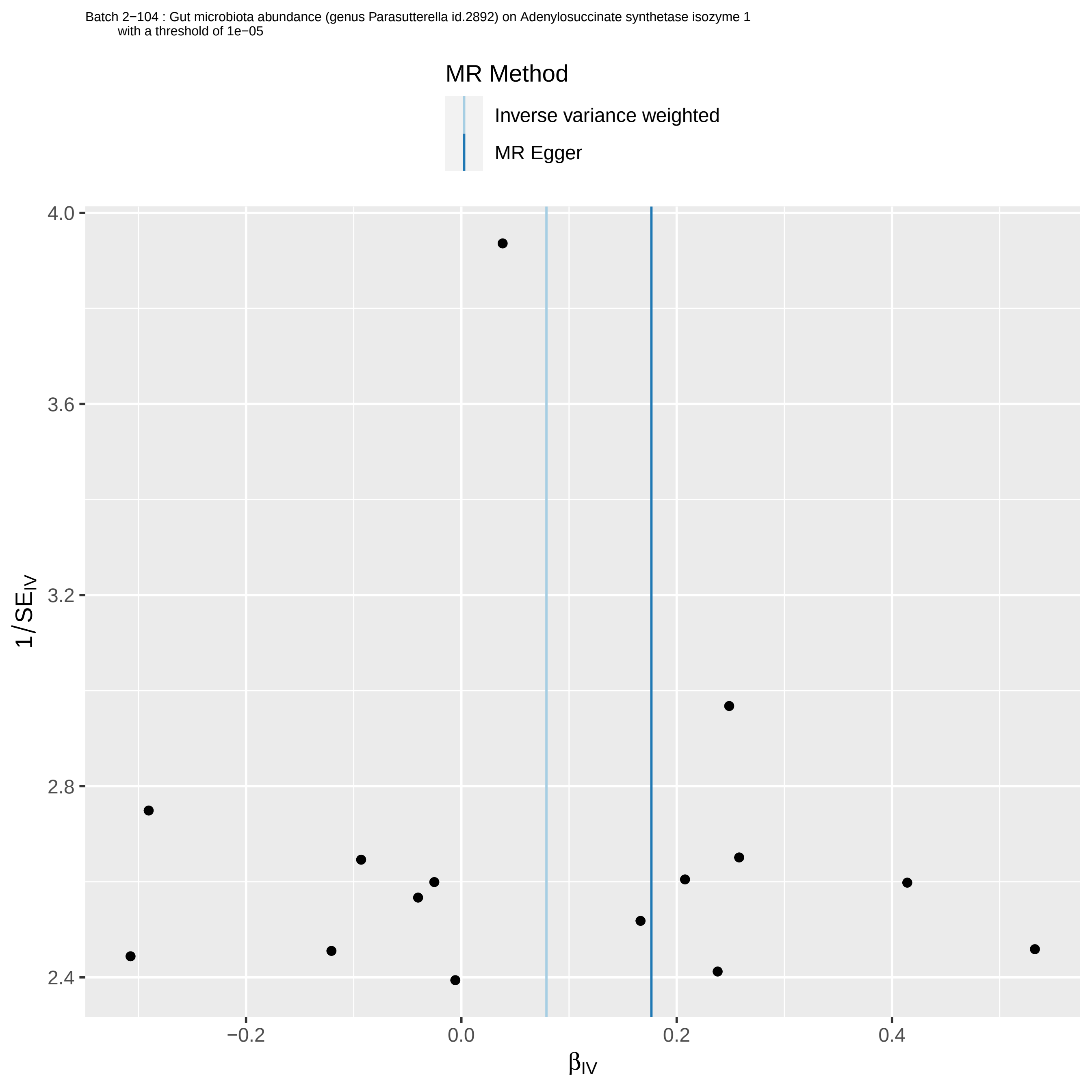
Batch 2−104 : Gut microbiota abundance (genus Parasutterella id.2892) on Adenylosuccinate synthetase isozyme 1
with a threshold of 1e−05
MR Method
Inverse variance weighted
MR Egger
4.0
3.6
3.2
2.8
2.4
1/SE_IV
−0.2
0.0
0.2
0.4
β_IV

Batch 2−105 : Gut microbiota abundance (genus Catenibacterium id.2153) on alpha−Fetoprotein
with a threshold of 1e−05

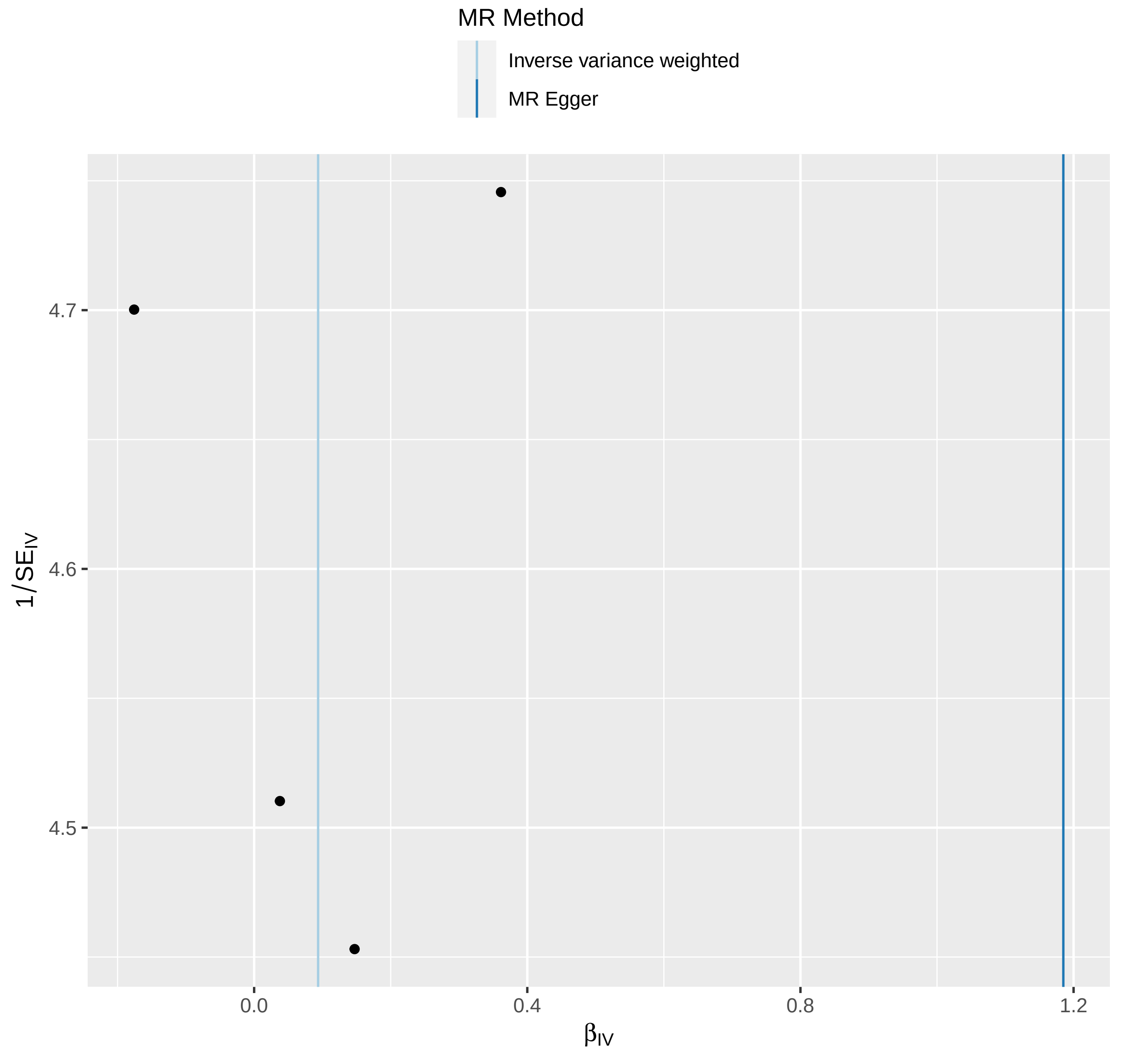

Batch 2−106 : Gut microbiota abundance (genus Parasutterella id.2892) on alpha−Fetoprotein
with a threshold of 1e−05

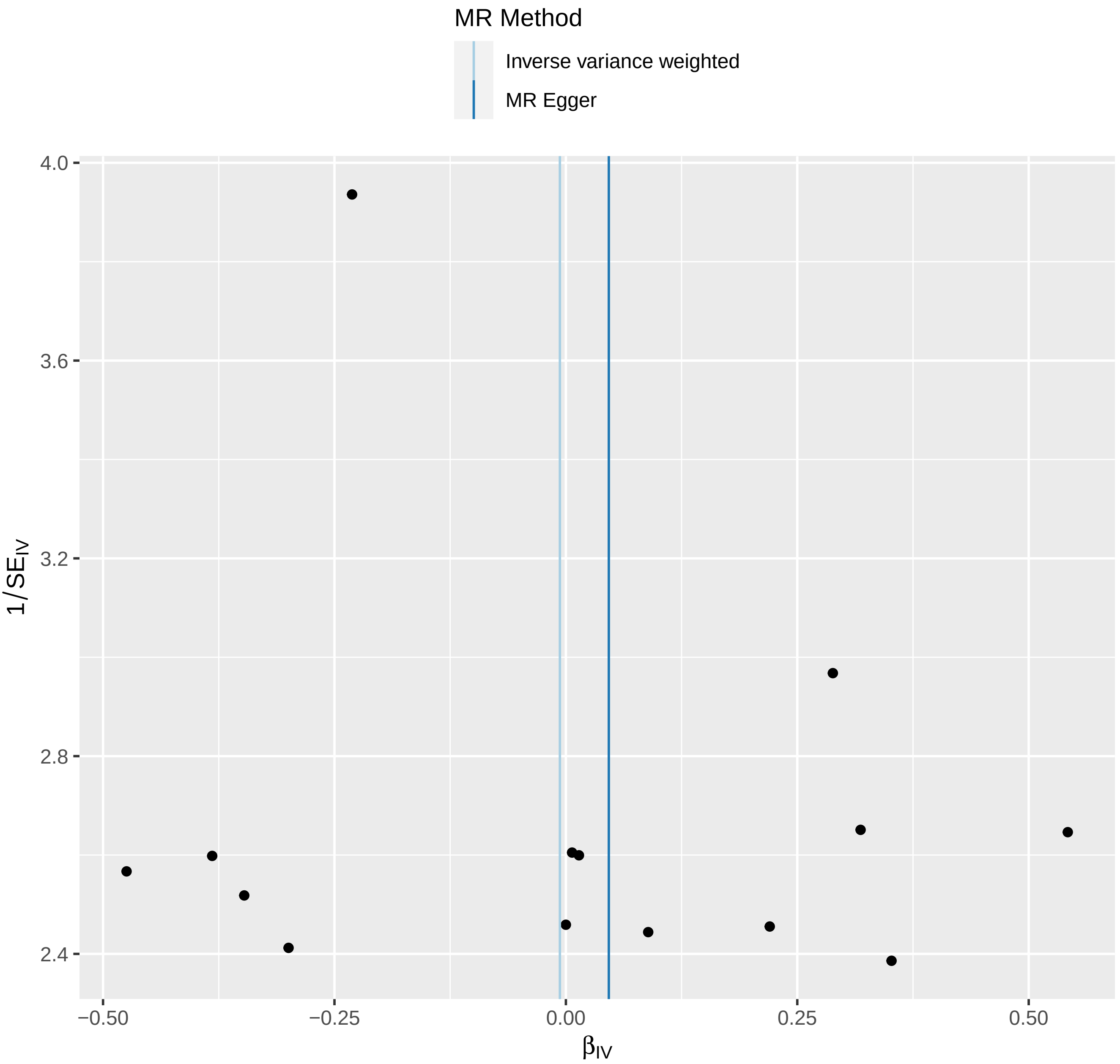

Batch 2−107 : Gut microbiota abundance (genus Catenibacterium id.2153) on Advanced glycosylation end product−specific receptor, soluble with a threshold of 1e−05

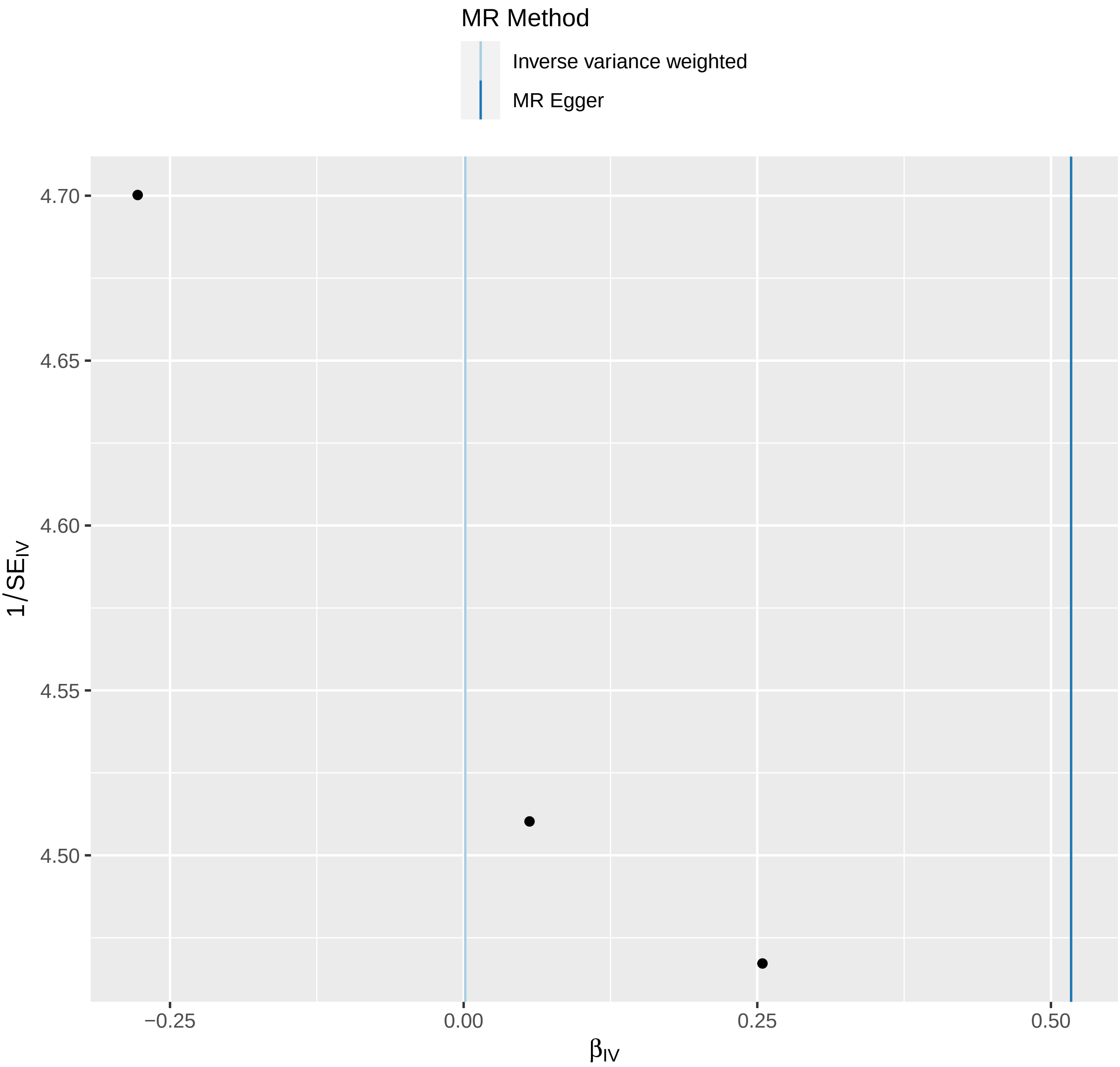

Batch 2−108 : Gut microbiota abundance (genus Parasutterella id.2892) on Advanced glycosylation end product−specific receptor, soluble with a threshold of 1e−05

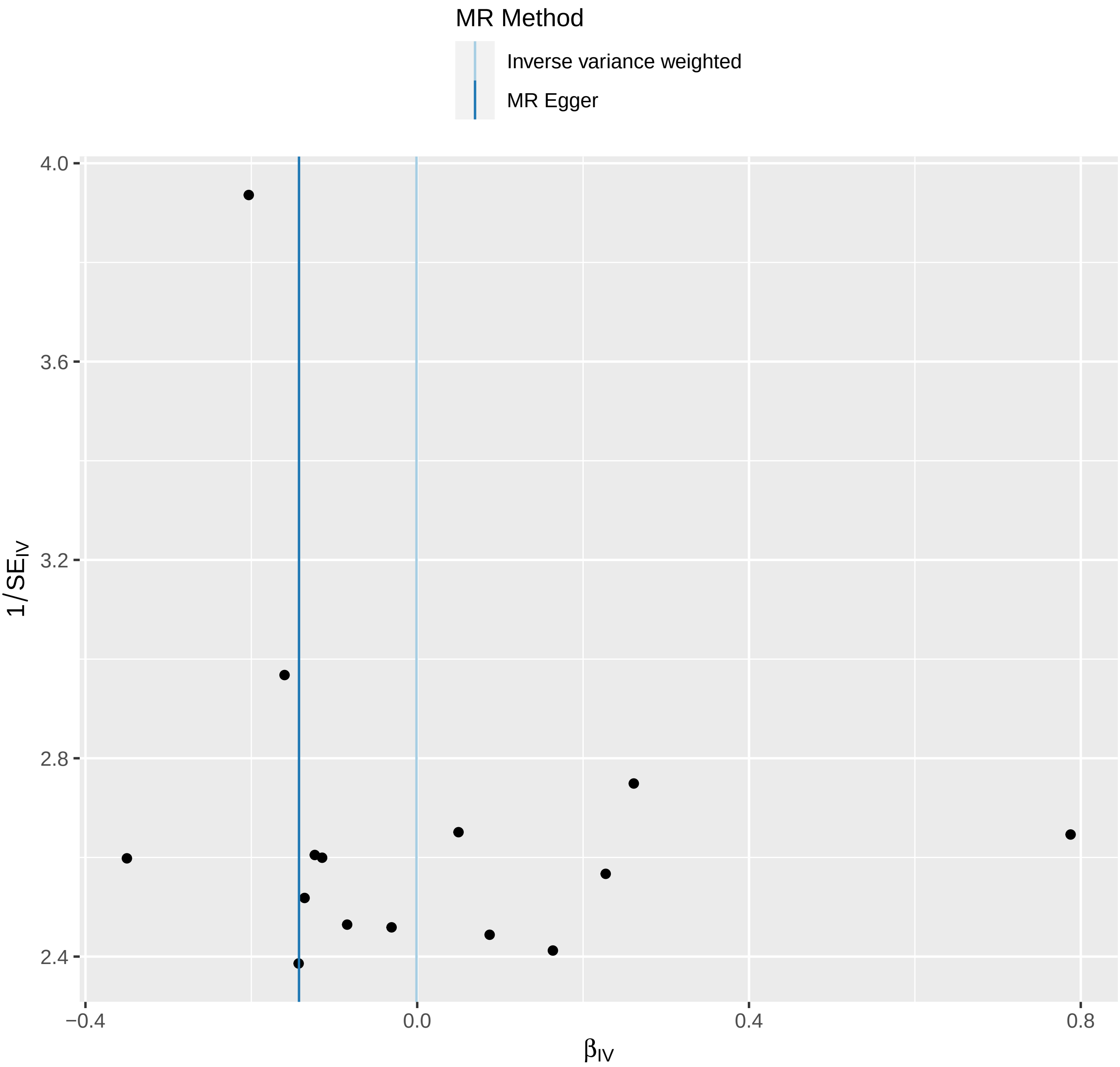

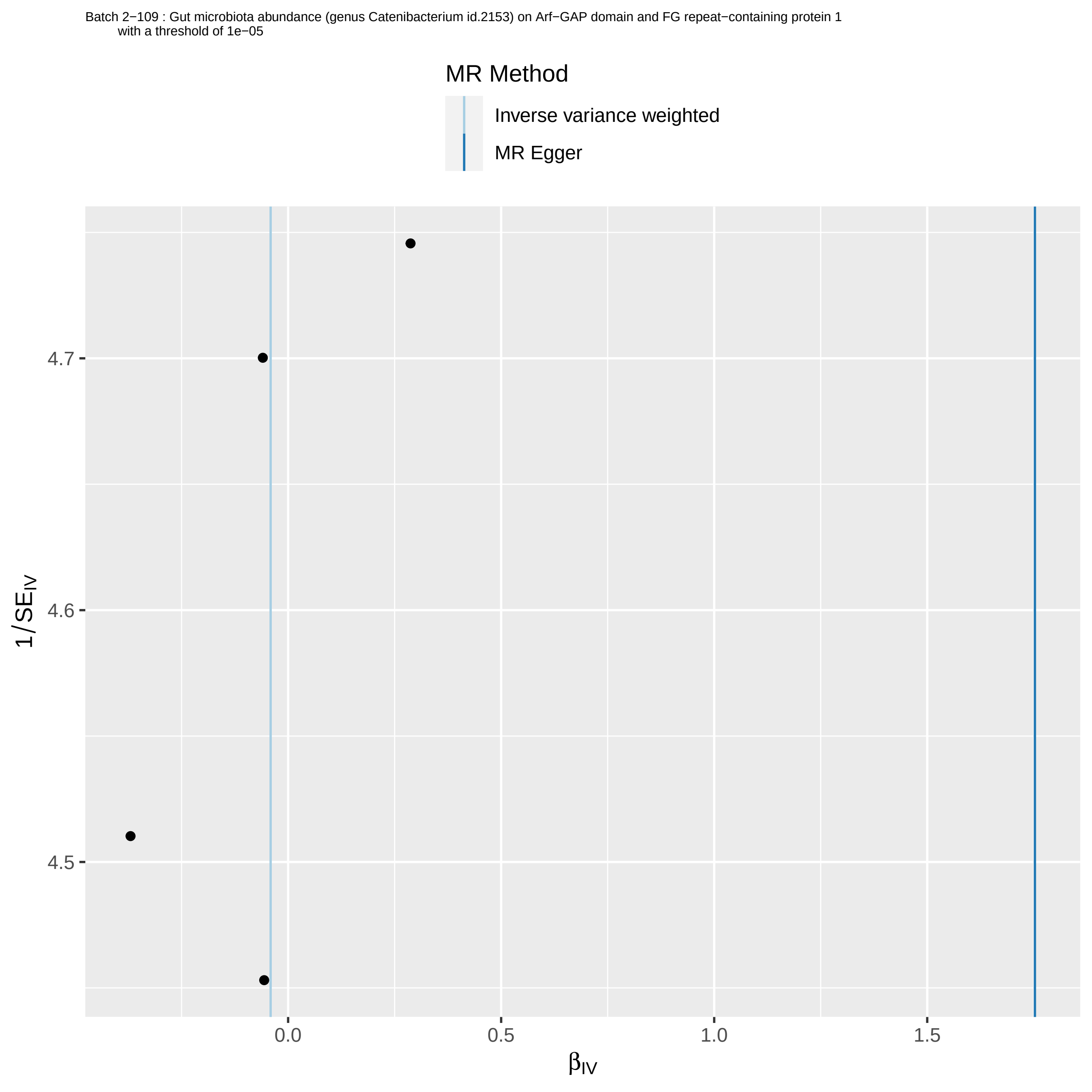
Batch 2−109 : Gut microbiota abundance (genus Catenibacterium id.2153) on Arf−GAP domain and FG repeat−containing protein 1
with a threshold of 1e−05
MR Method
Inverse variance weighted
MR Egger
4.7
4.6
4.5
1/SE_IV
0.0
0.5
1.0
1.5
β_IV

Batch 2−110 : Gut microbiota abundance (genus Parasutterella id.2892) on Arf−GAP domain and FG repeat−containing protein 1
with a threshold of 1e−05

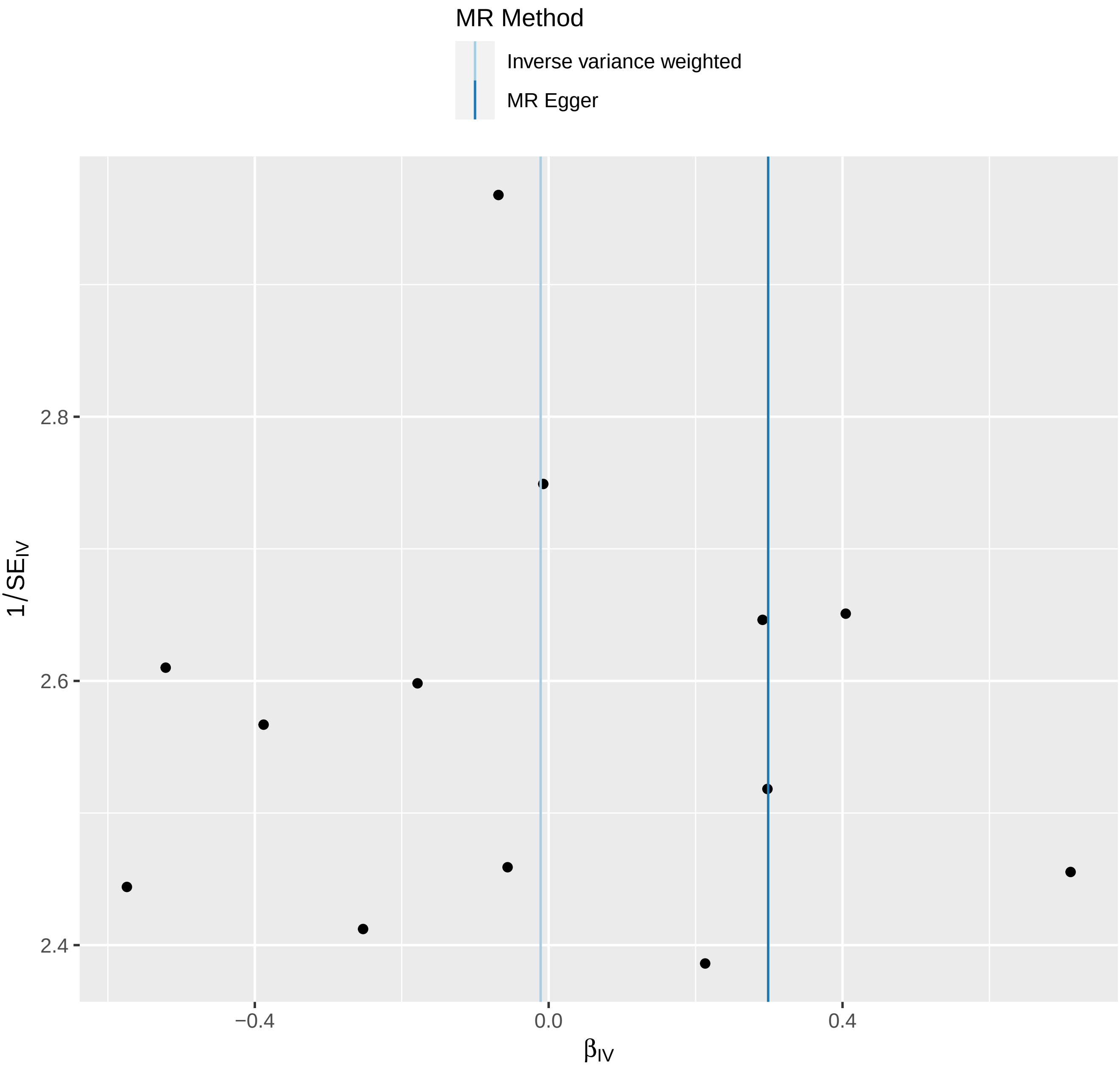

Batch 2−111 : Gut microbiota abundance (genus Catenibacterium id.2153) on Angiogenic factor with G patch and FHA domains 1 with a threshold of 1e−05

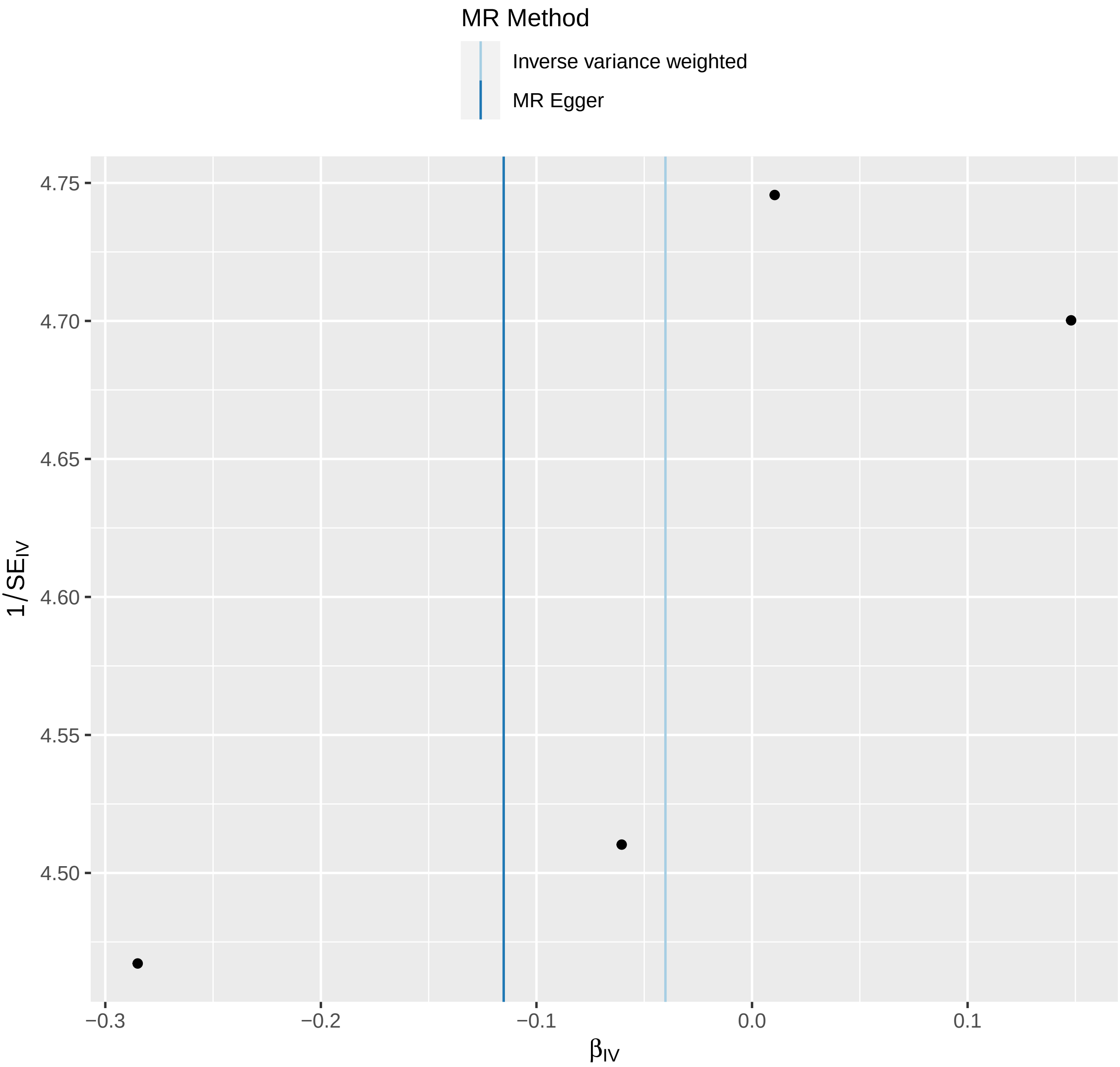

Batch 2−112 : Gut microbiota abundance (genus Parasutterella id.2892) on Angiogenic factor with G patch and FHA domains 1 with a threshold of 1e−05

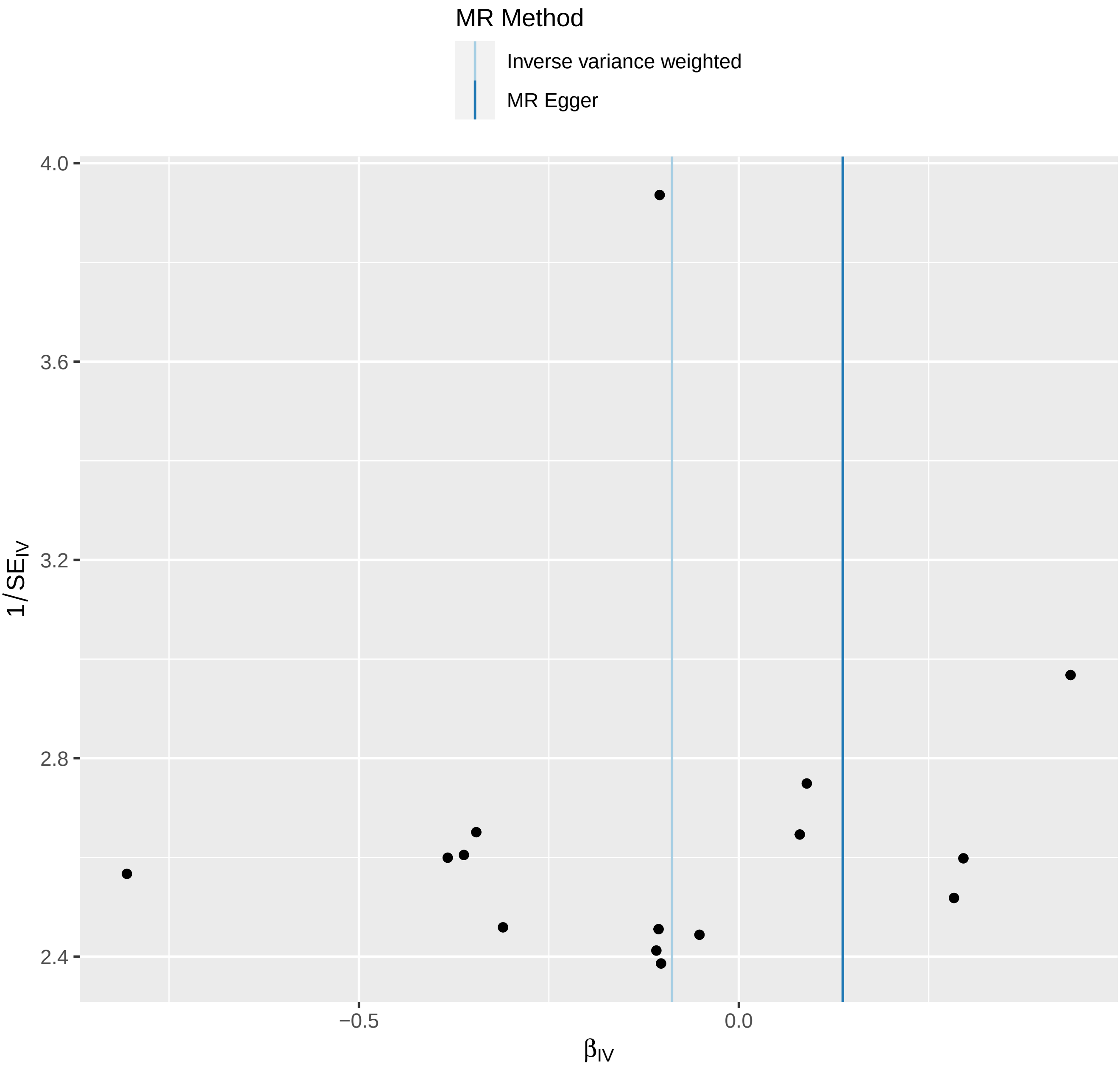

Batch 2−113 : Gut microbiota abundance (genus Catenibacterium id.2153) on Protein argonaute−1
with a threshold of 1e−05

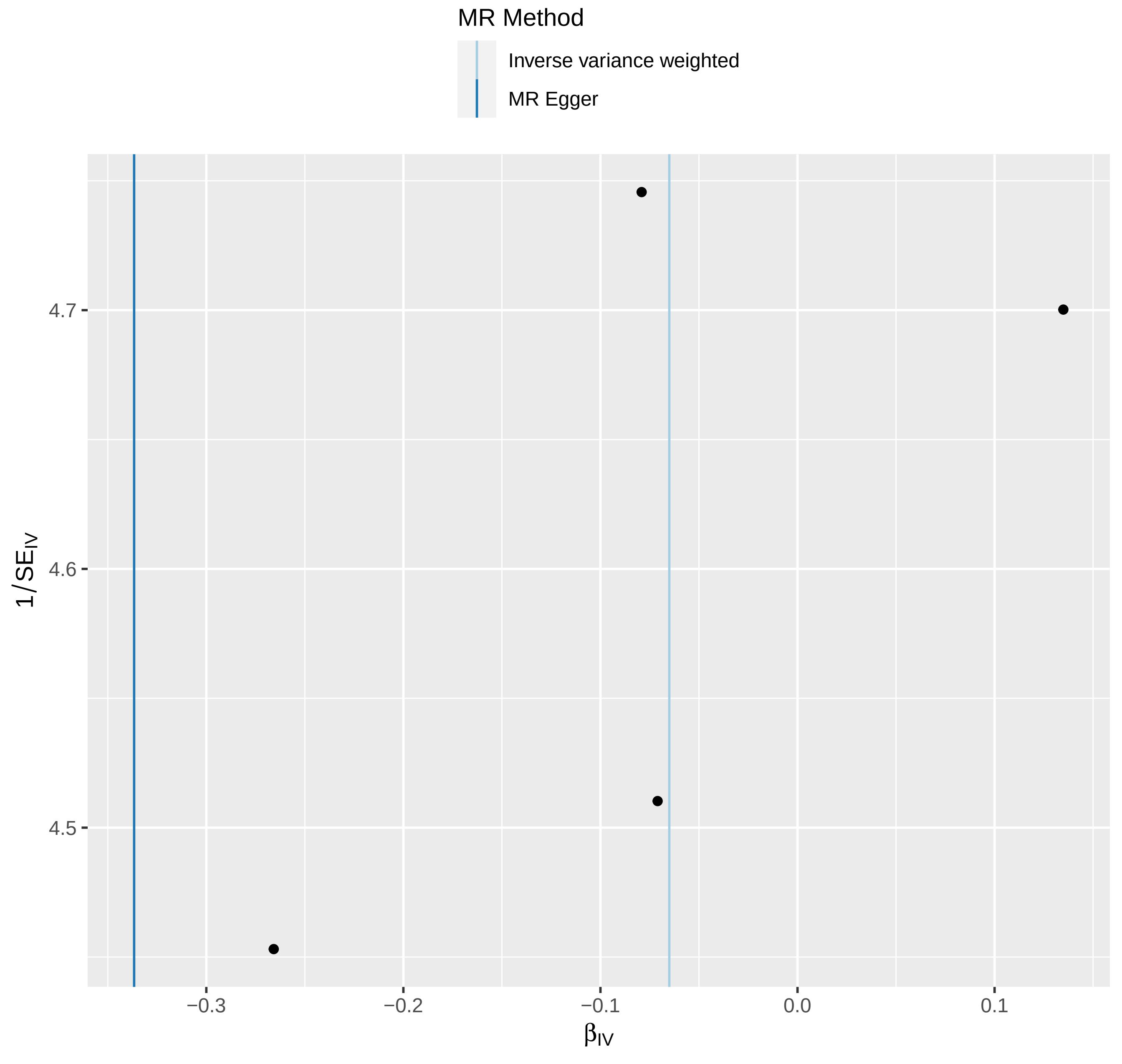

Batch 2−114 : Gut microbiota abundance (genus Parasutterella id.2892) on Protein argonaute−1 with a threshold of 1e−05

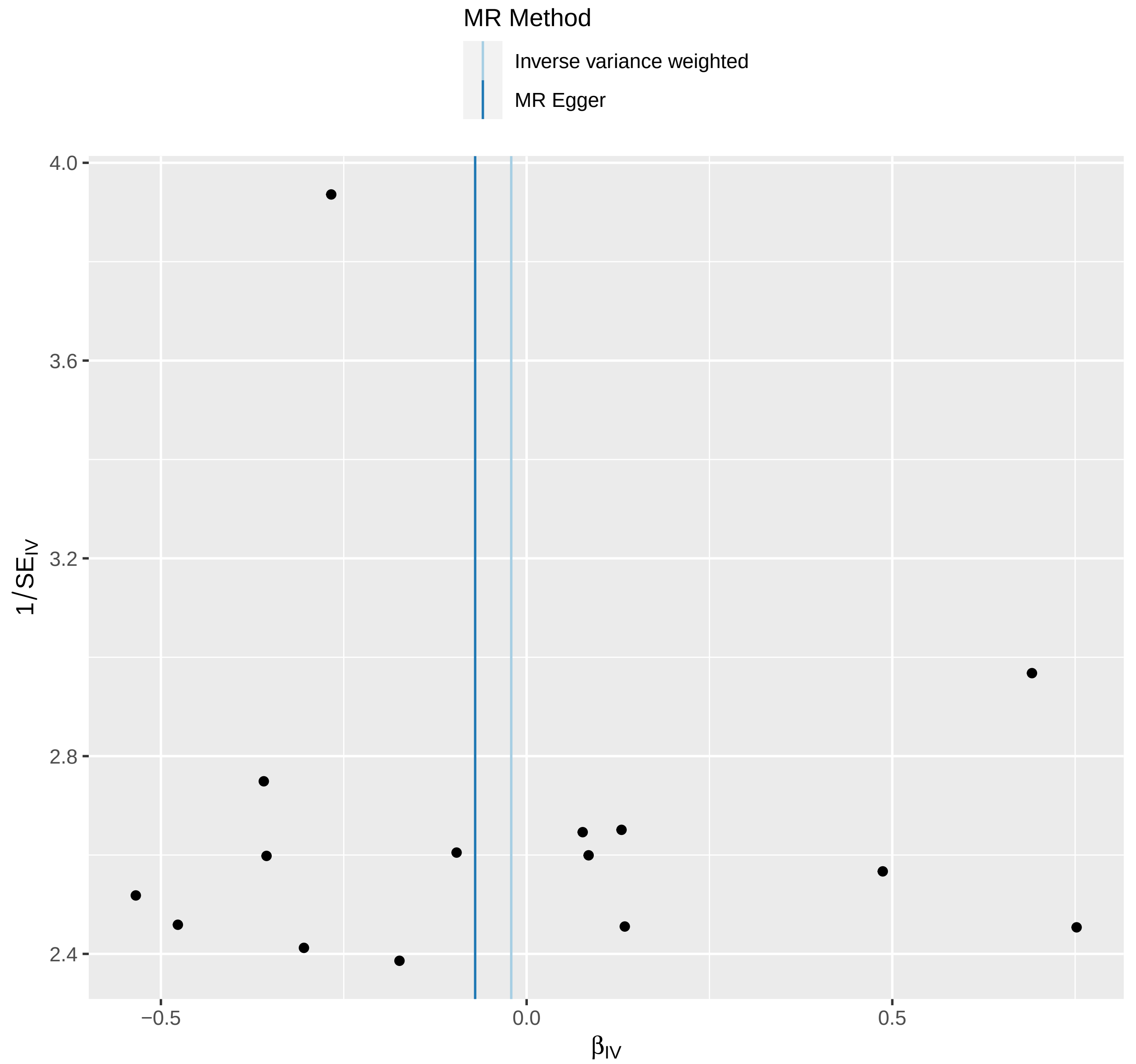

Batch 2−115 : Gut microbiota abundance (genus Catenibacterium id.2153) on Anterior gradient protein 2 homolog
with a threshold of 1e−05

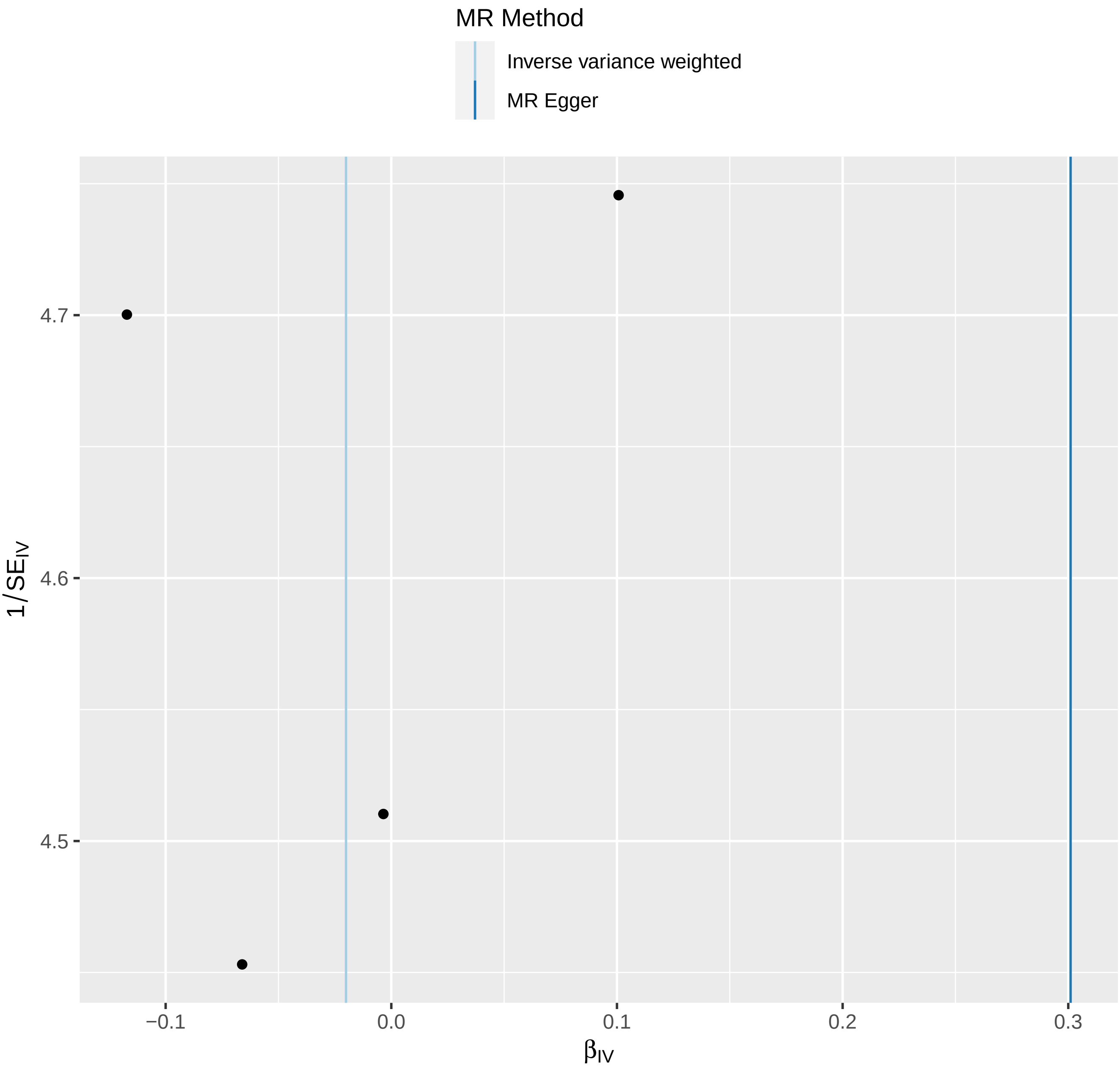

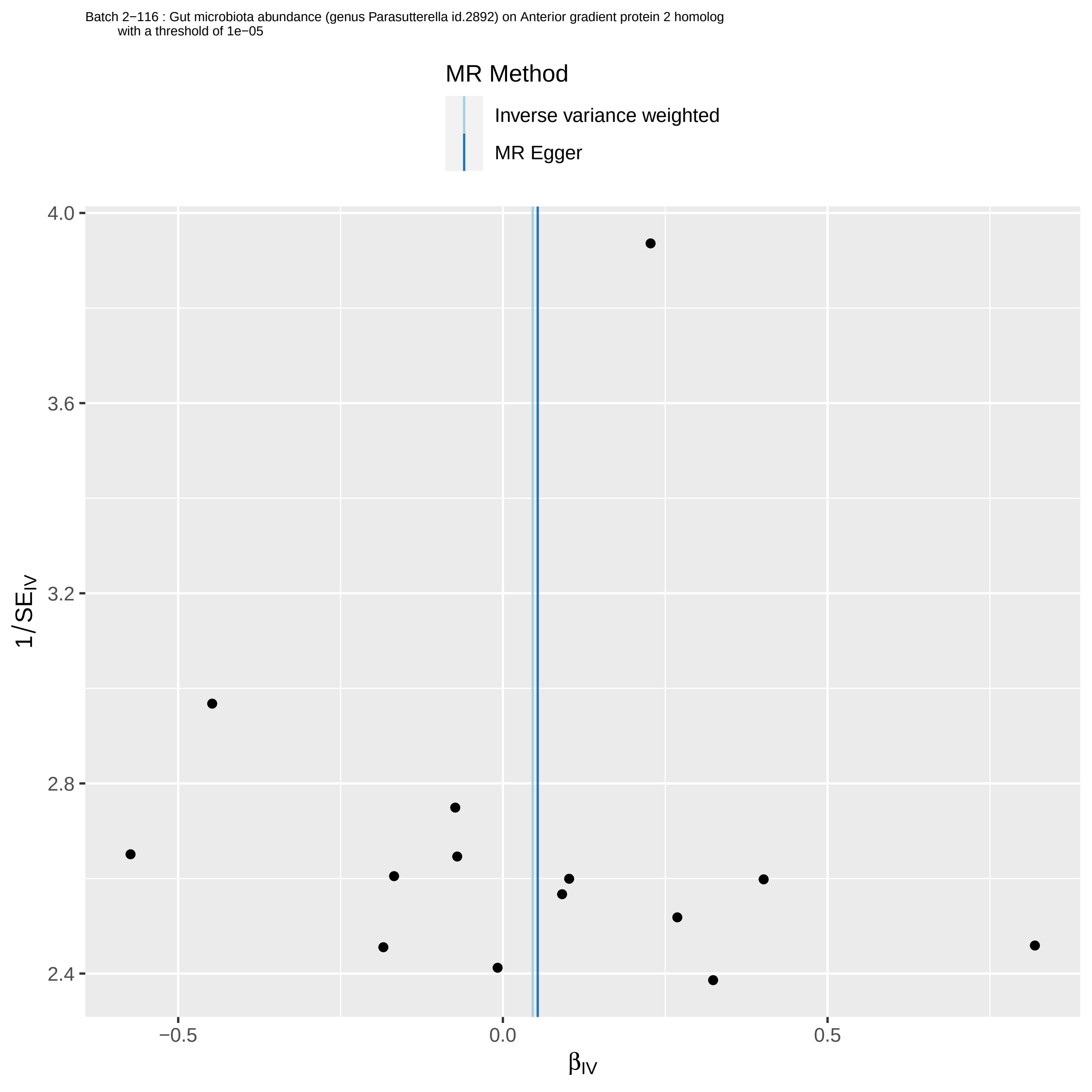

Batch 2−116 : Gut microbiota abundance (genus Parasutterella id.2892) on Anterior gradient protein 2 homolog
with a threshold of 1e−05
MR Method
Inverse variance weighted
MR Egger
4.0
3.6
3.2
2.8
2.4
1/SE_IV
−0.5
0.0
0.5
β_IV

Batch 2−117 : Gut microbiota abundance (genus Catenibacterium id.2153) on Agouti−related protein with a threshold of 1e−05

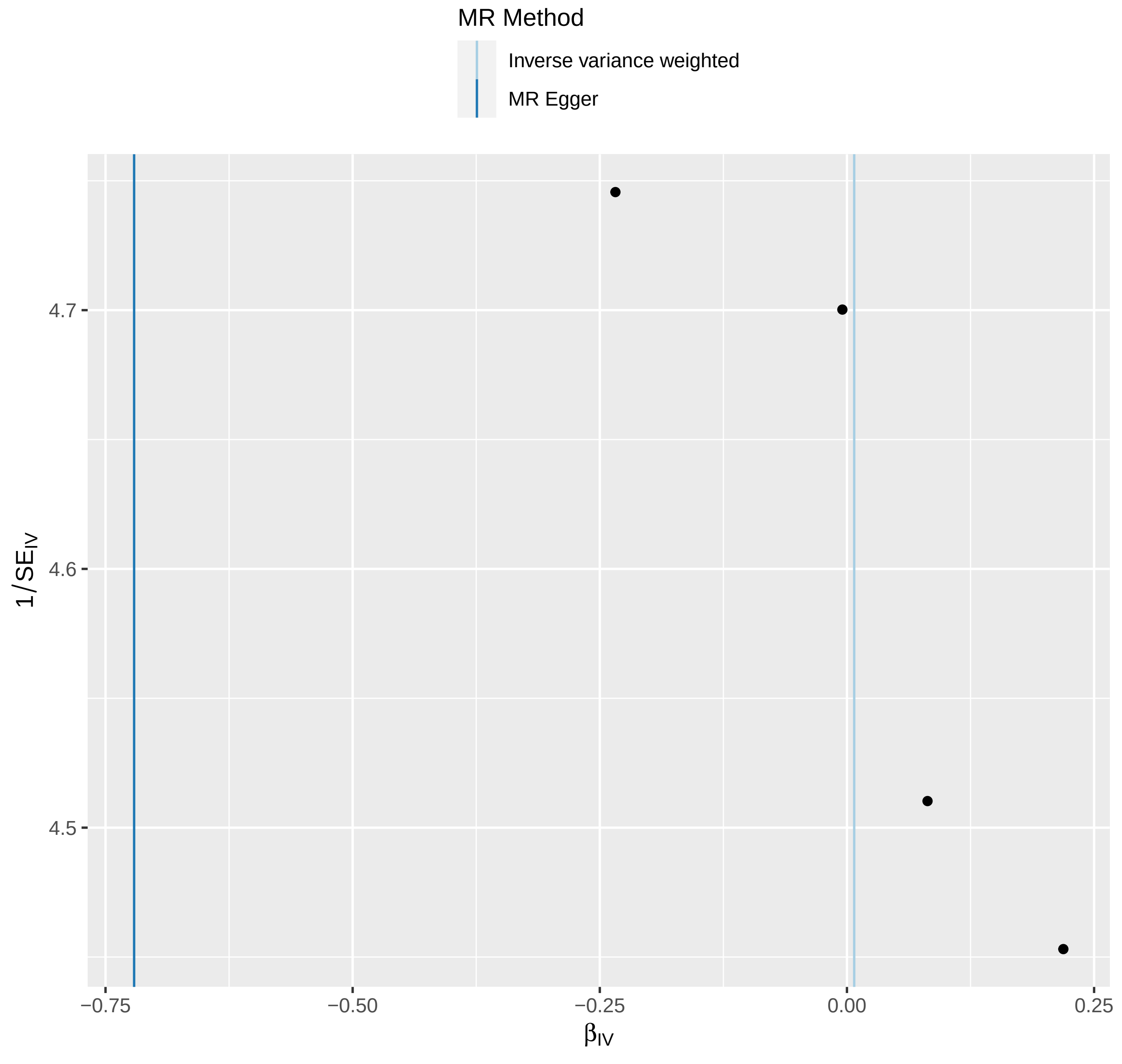

Batch 2−118 : Gut microbiota abundance (genus Parasutterella id.2892) on Agouti−related protein
with a threshold of 1e−05

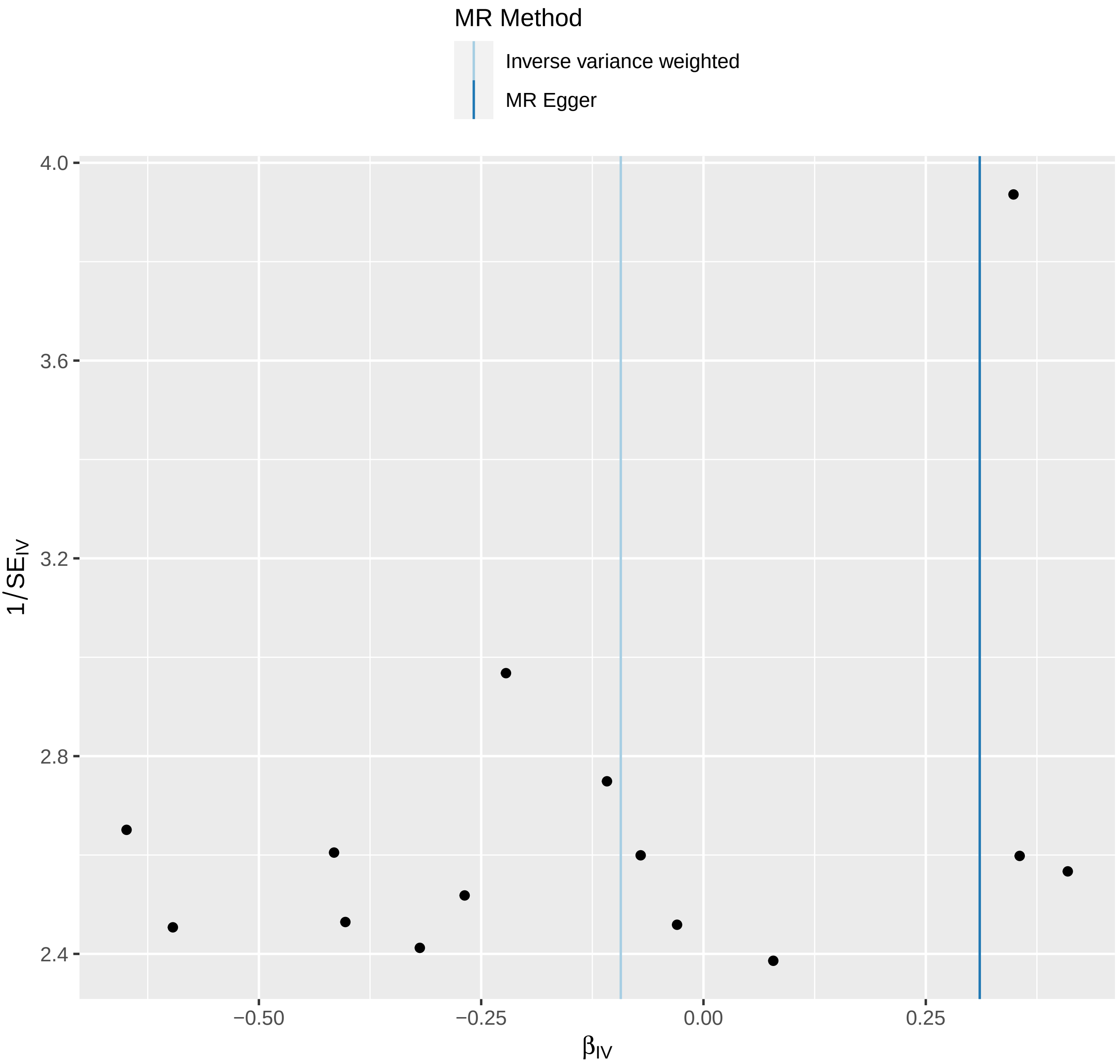

Batch 2−119 : Gut microbiota abundance (genus Catenibacterium id.2153) on Activator of 90 kDa heat shock protein ATPase homolog 1 with a threshold of 1e−05

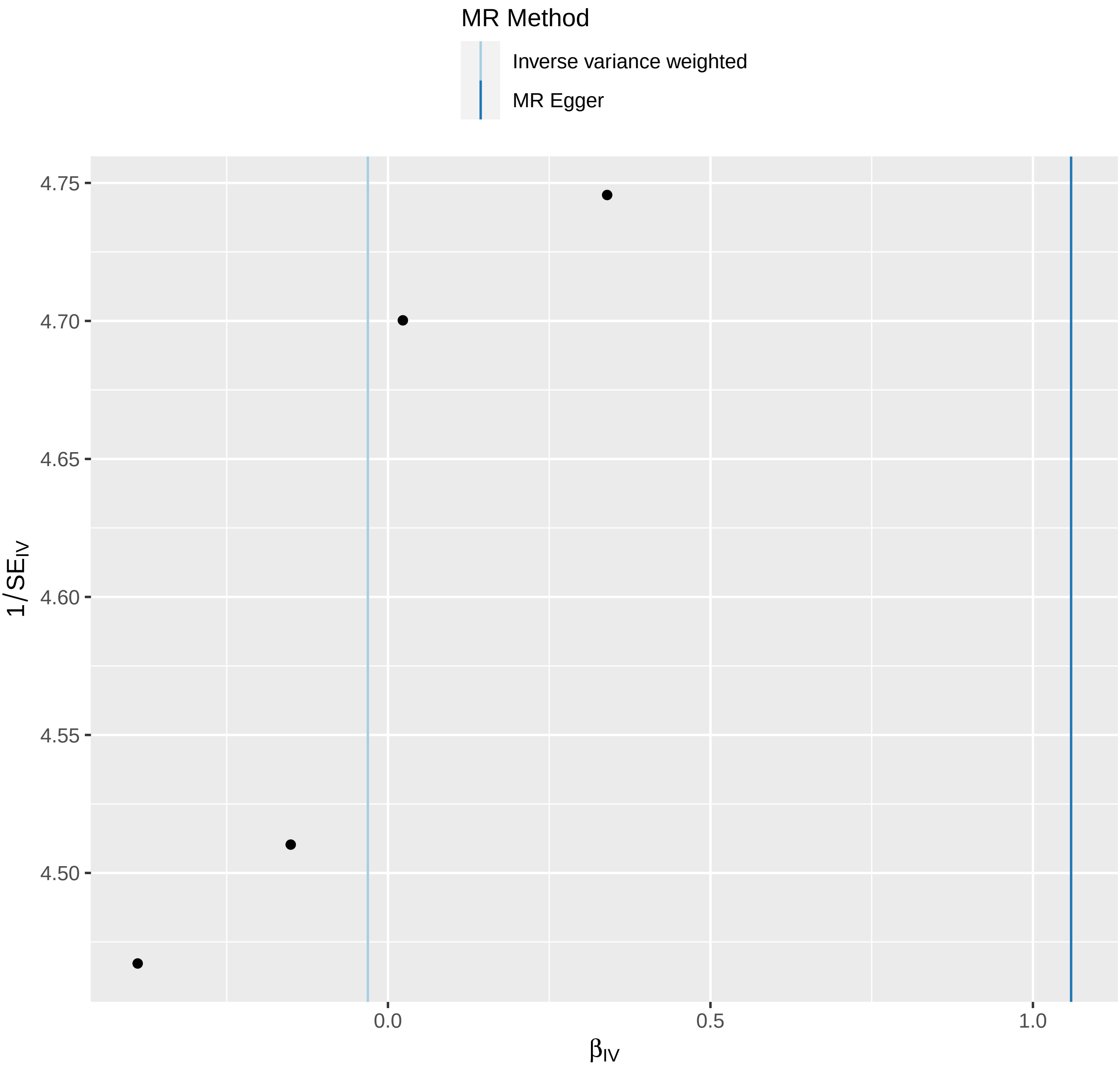

Batch 2−120 : Gut microbiota abundance (genus Parasutterella id.2892) on Activator of 90 kDa heat shock protein ATPase homolog 1 with a threshold of 1e−05

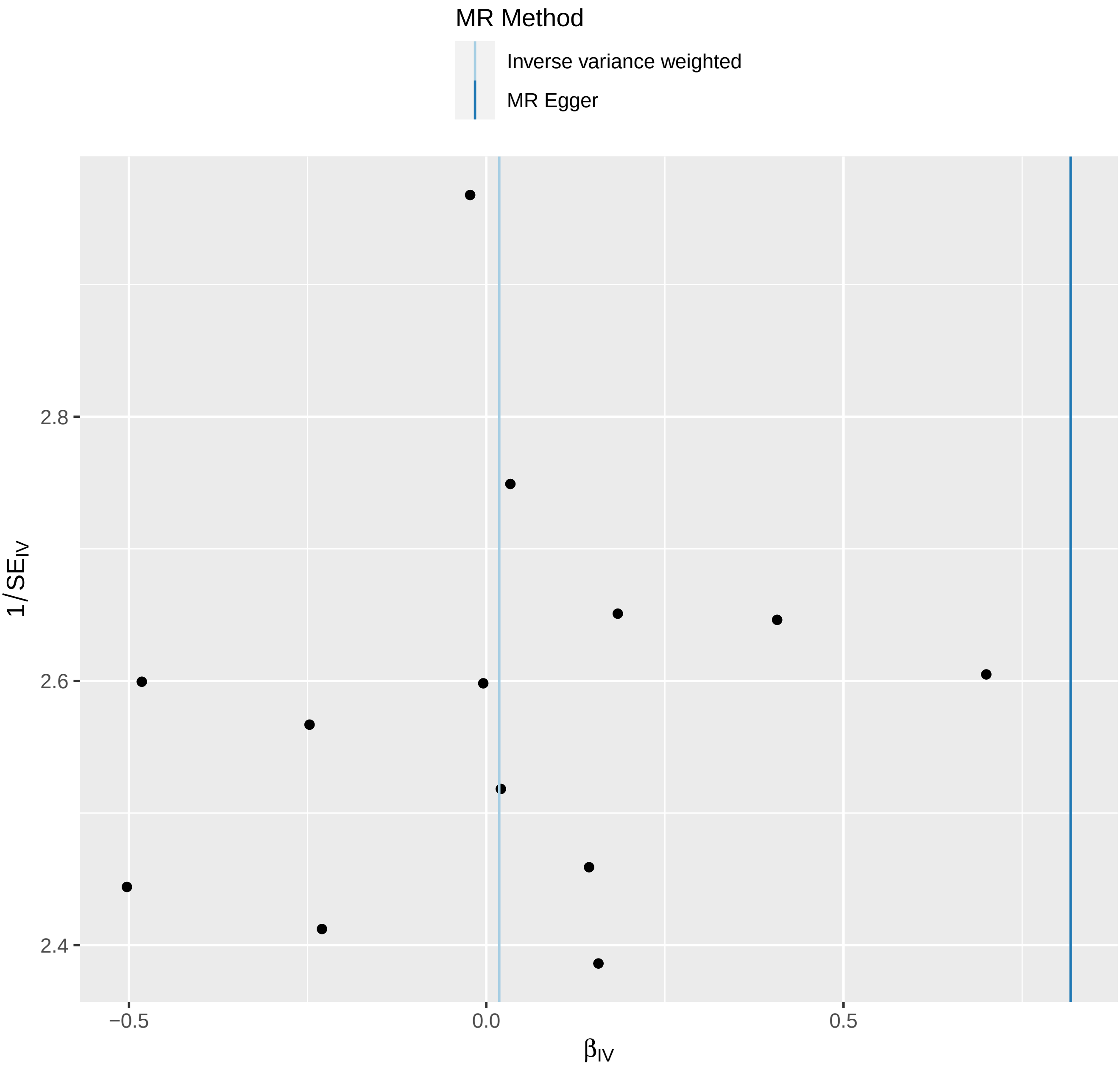

Batch 2−121 : Gut microbiota abundance (genus Catenibacterium id.2153) on Alpha−2−HS−glycoprotein
with a threshold of 1e−05

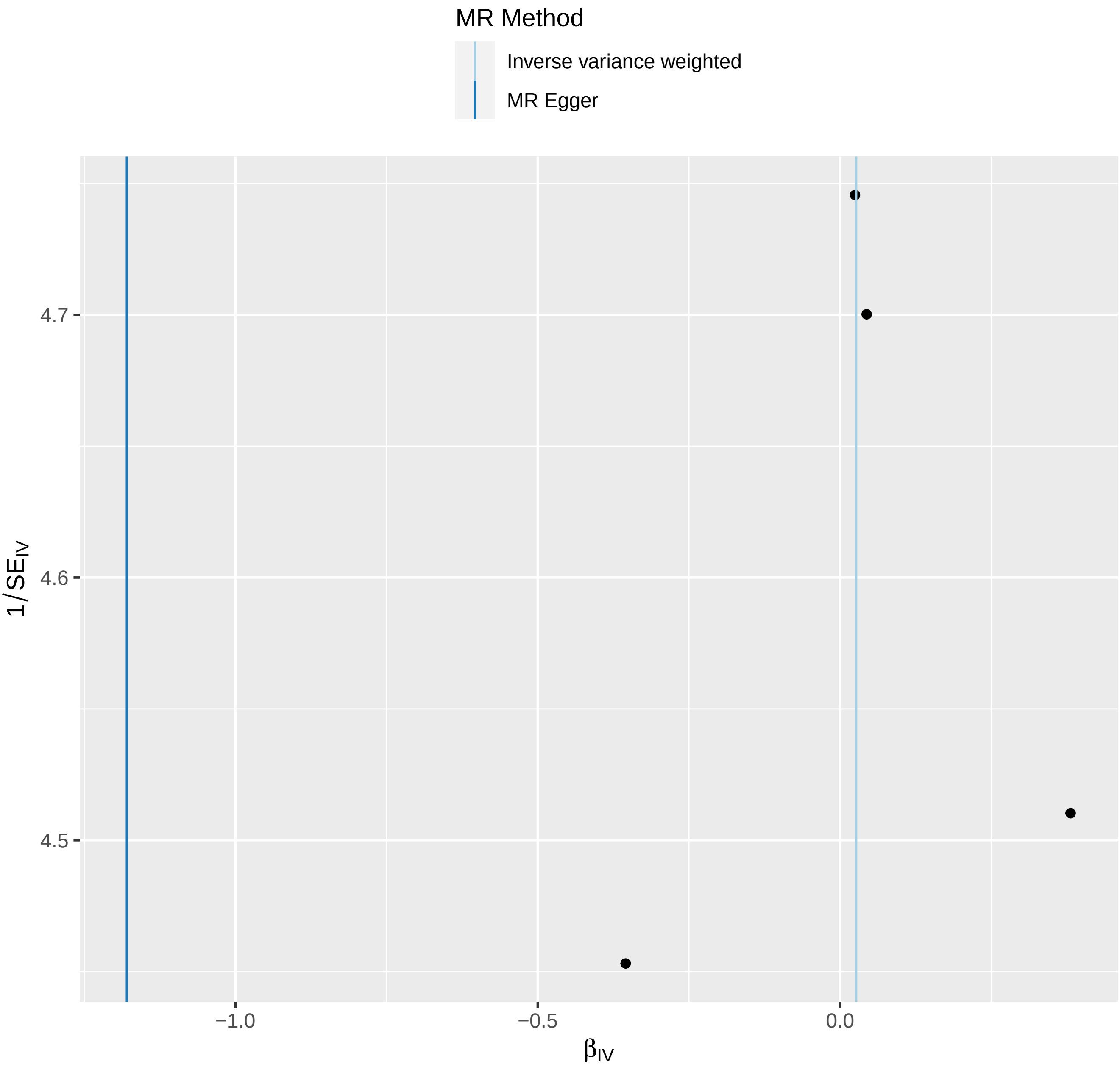

Batch 2−122 : Gut microbiota abundance (genus Parasutterella id.2892) on Alpha−2−HS−glycoprotein
with a threshold of 1e−05

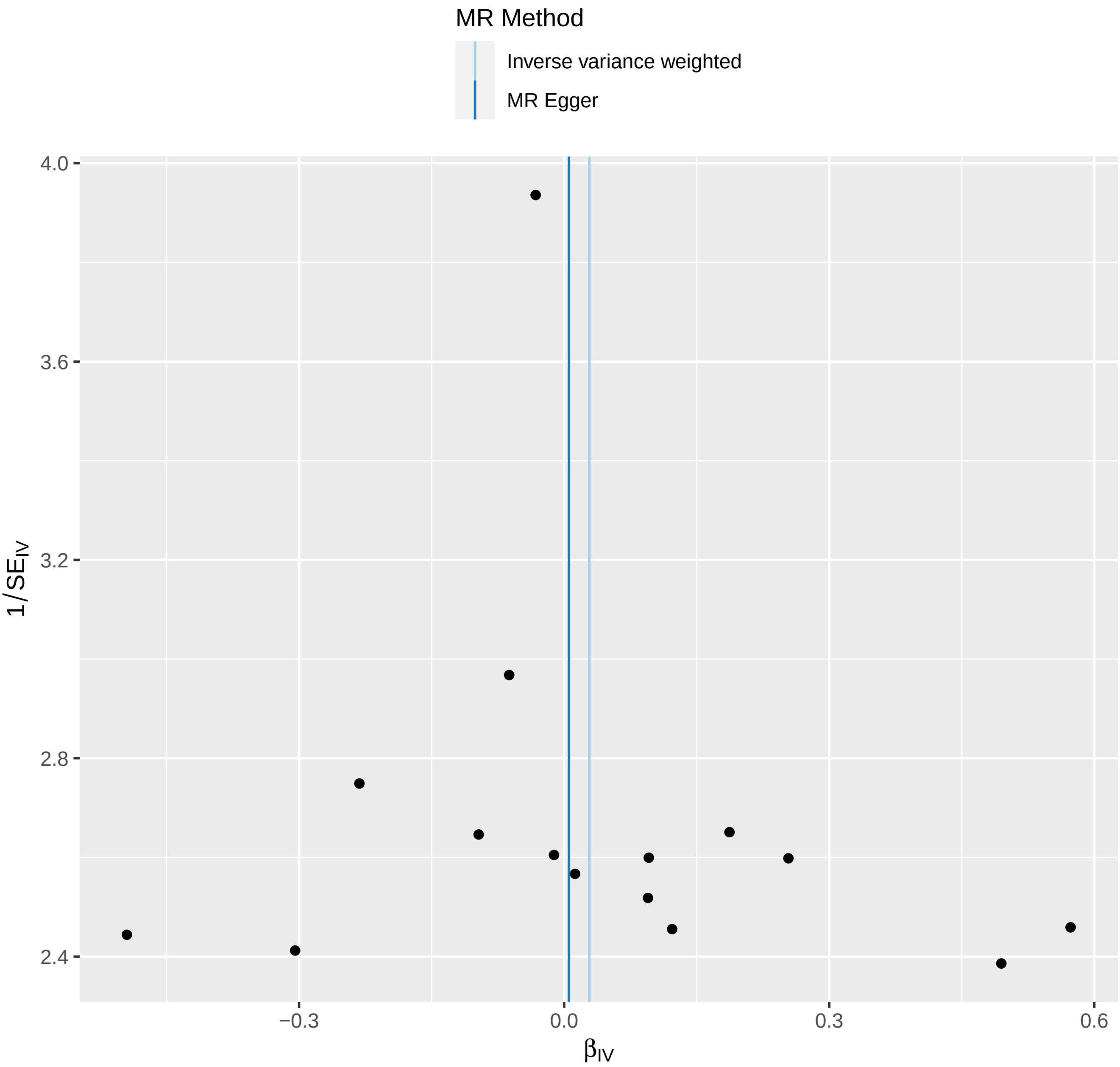

Batch 2−123 : Gut microbiota abundance (genus Catenibacterium id.2153) on Allograft inflammatory factor 1 with a threshold of 1e−05

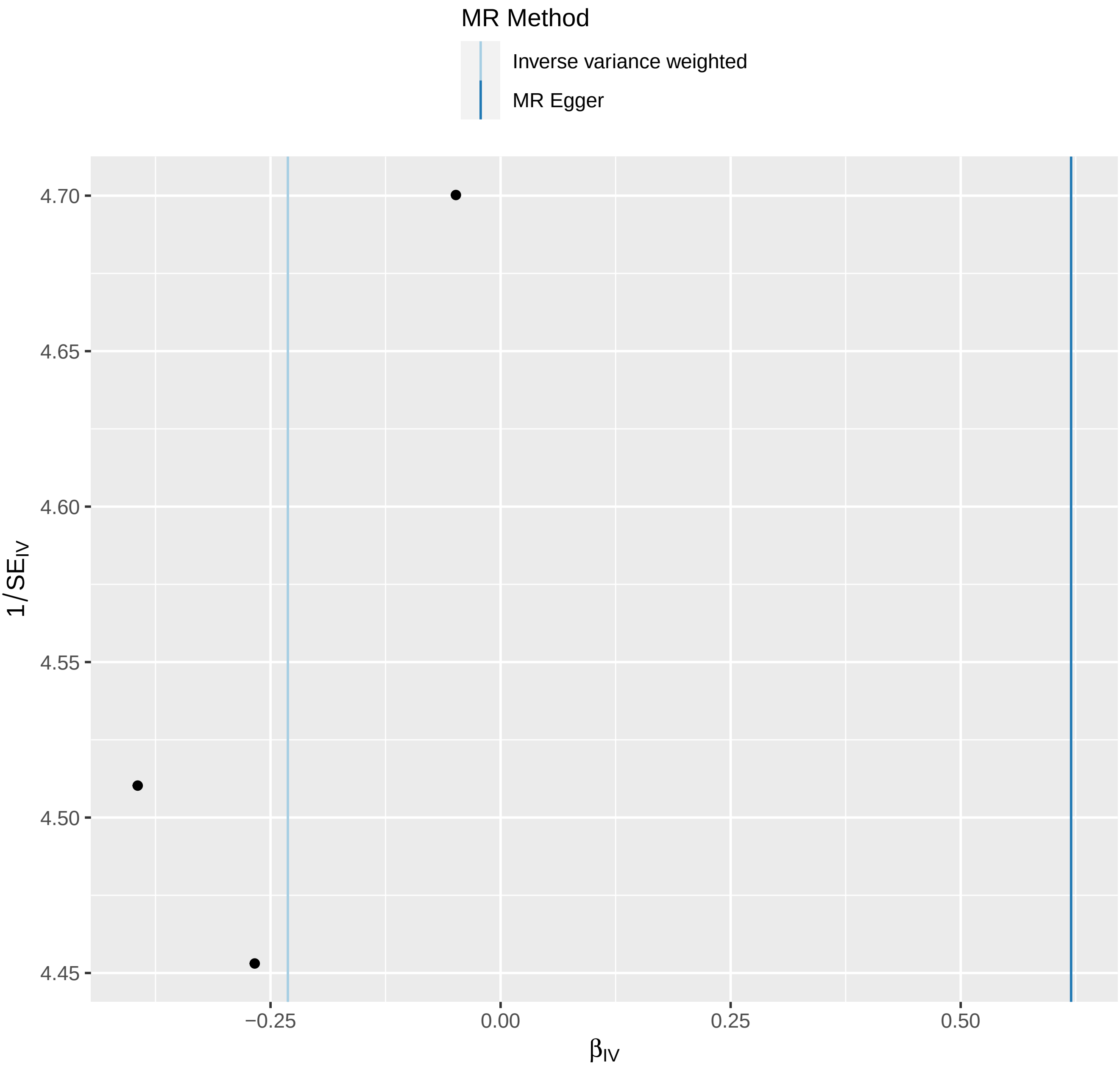

Batch 2−124 : Gut microbiota abundance (genus Parasutterella id.2892) on Allograft inflammatory factor 1
with a threshold of 1e−05

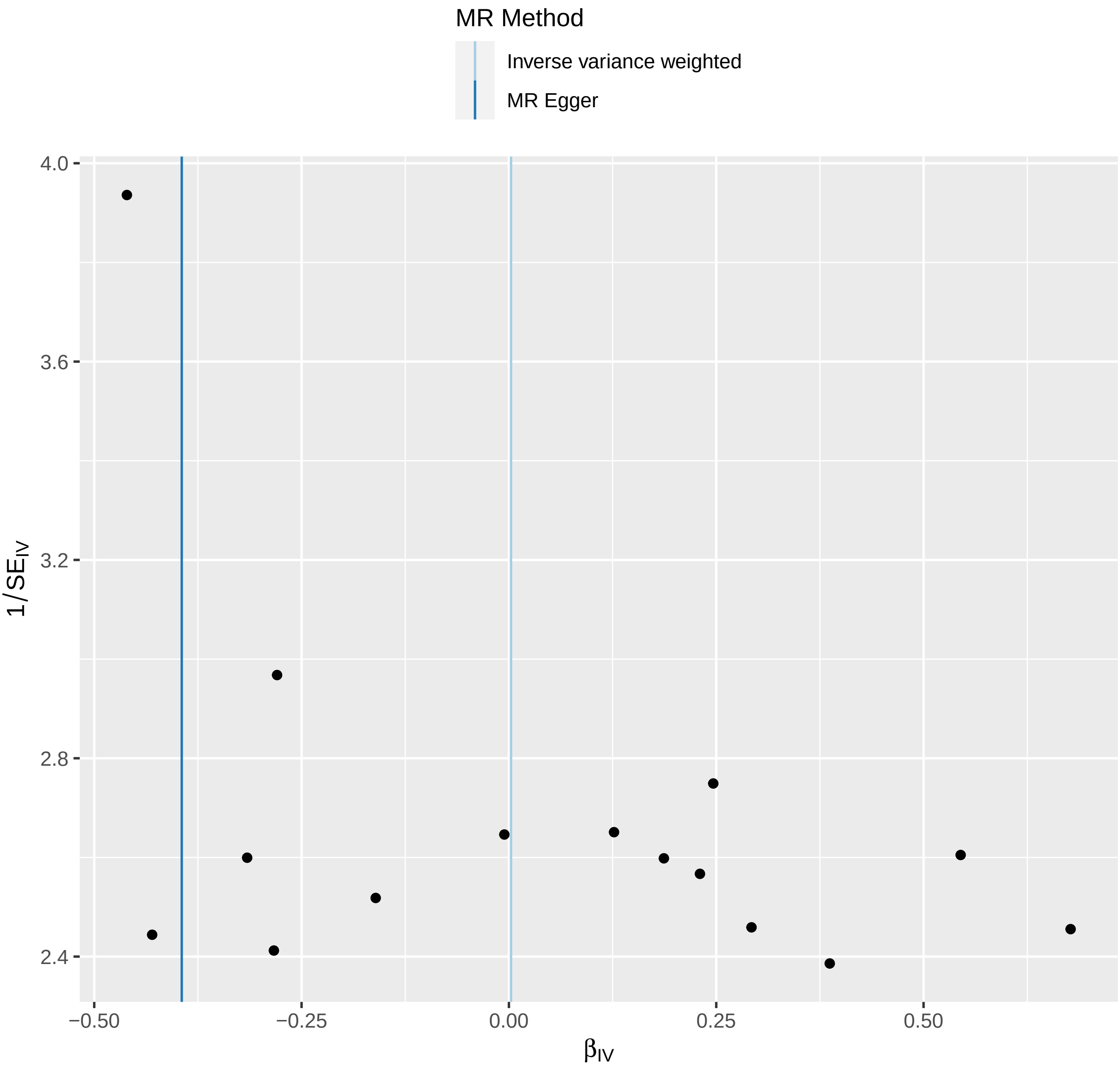

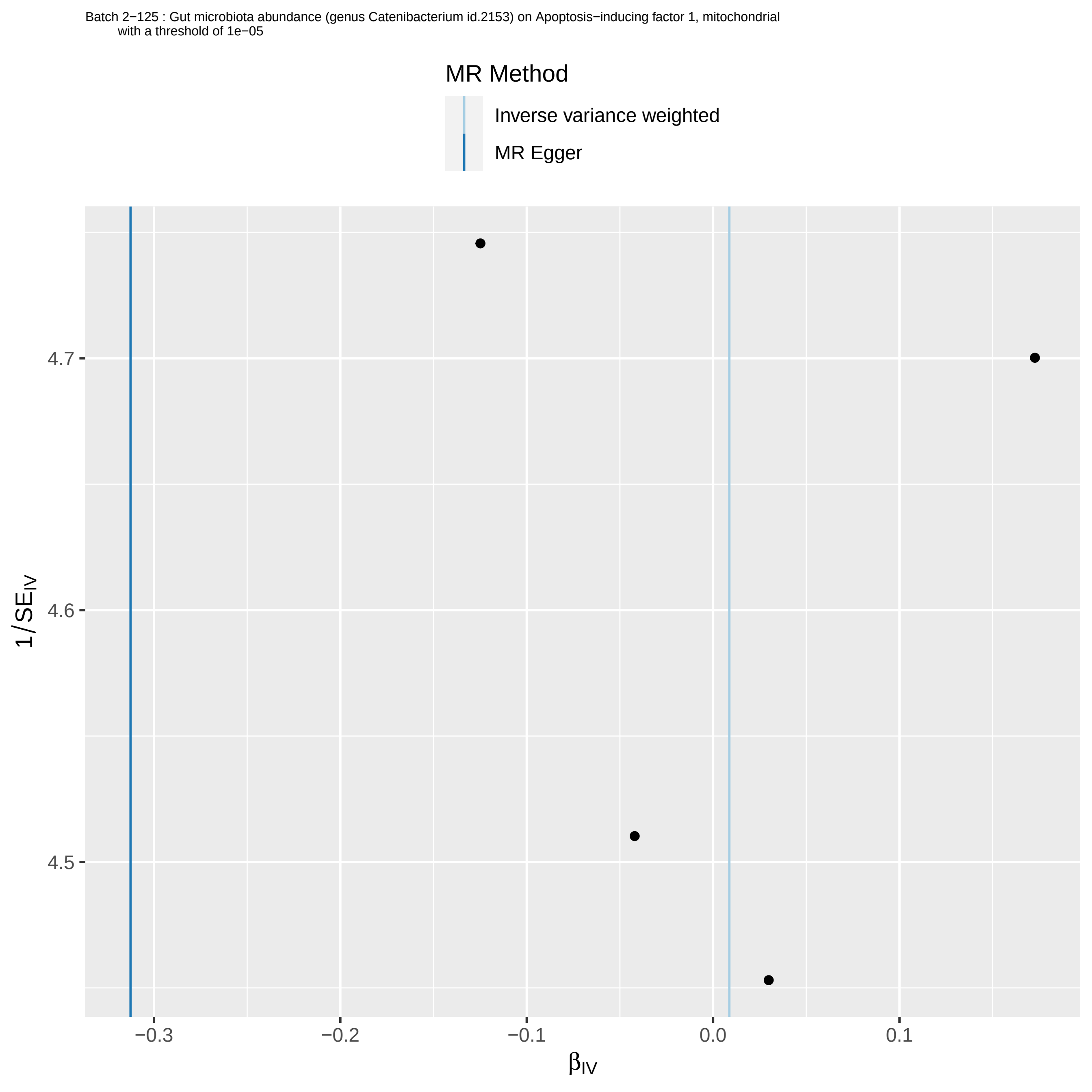
Batch 2−125 : Gut microbiota abundance (genus Catenibacterium id.2153) on Apoptosis−inducing factor 1, mitochondrial
with a threshold of 1e−05
MR Method
Inverse variance weighted
MR Egger
4.7
4.6
4.5
1/SE_IV
−0.3
−0.2
−0.1
0.0
0.1
β_IV

Batch 2−126 : Gut microbiota abundance (genus Parasutterella id.2892) on Apoptosis−inducing factor 1, mitochondrial
with a threshold of 1e−05

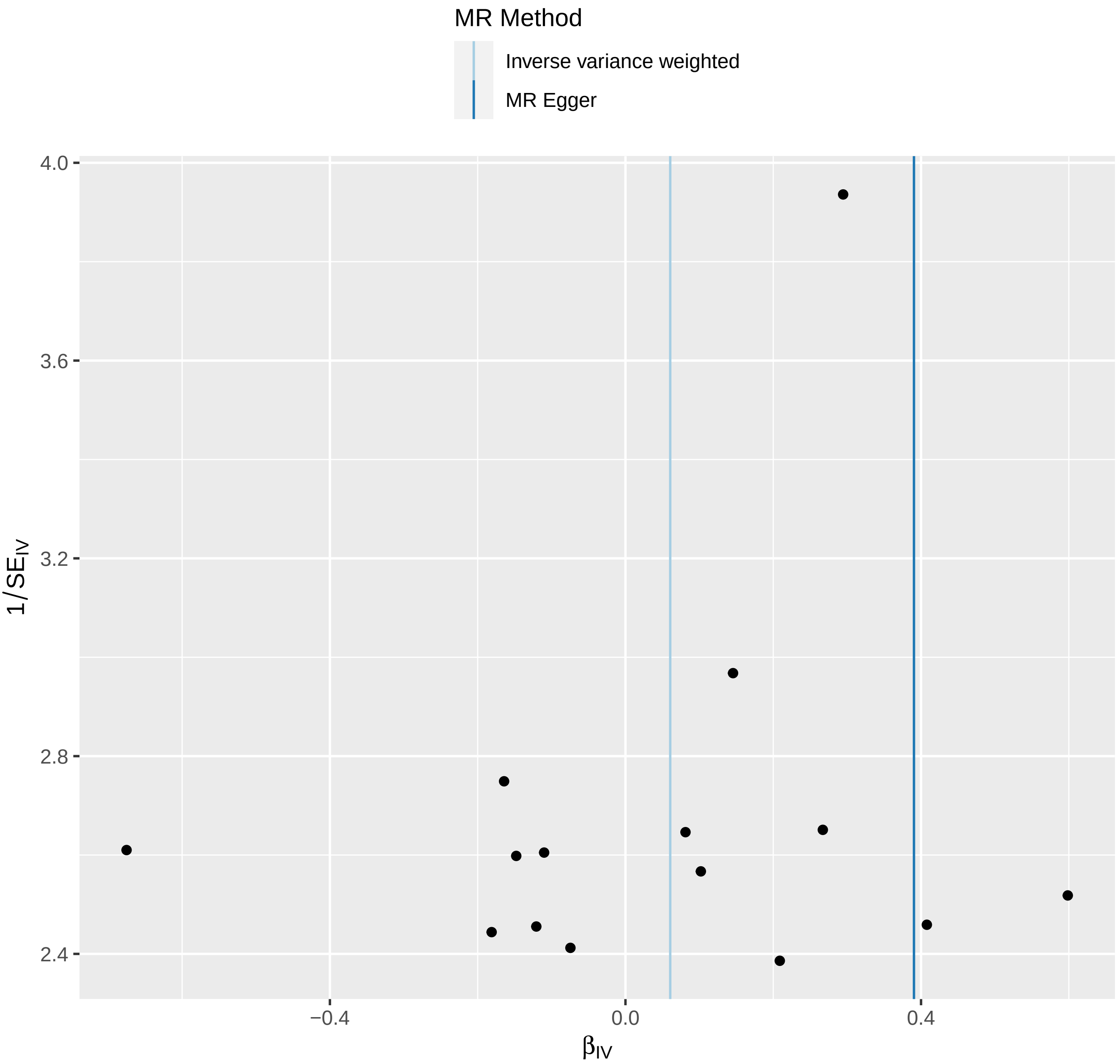

Batch 2−127 : Gut microbiota abundance (genus Catenibacterium id.2153) on Apoptosis−inducing factor 1, mitochondrial
with a threshold of 1e−05

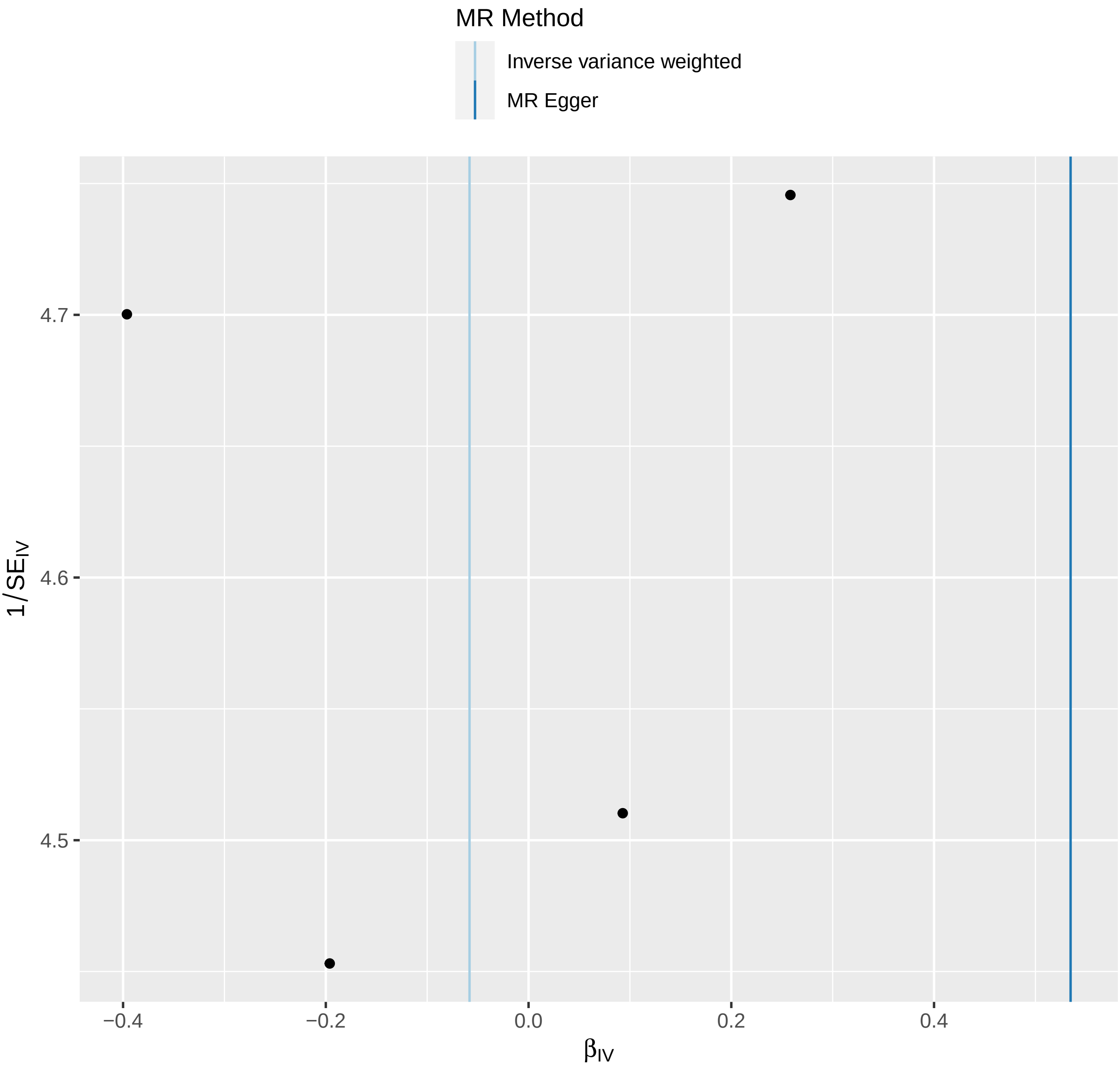

Batch 2−128 : Gut microbiota abundance (genus Parasutterella id.2892) on Apoptosis−inducing factor 1, mitochondrial
with a threshold of 1e−05

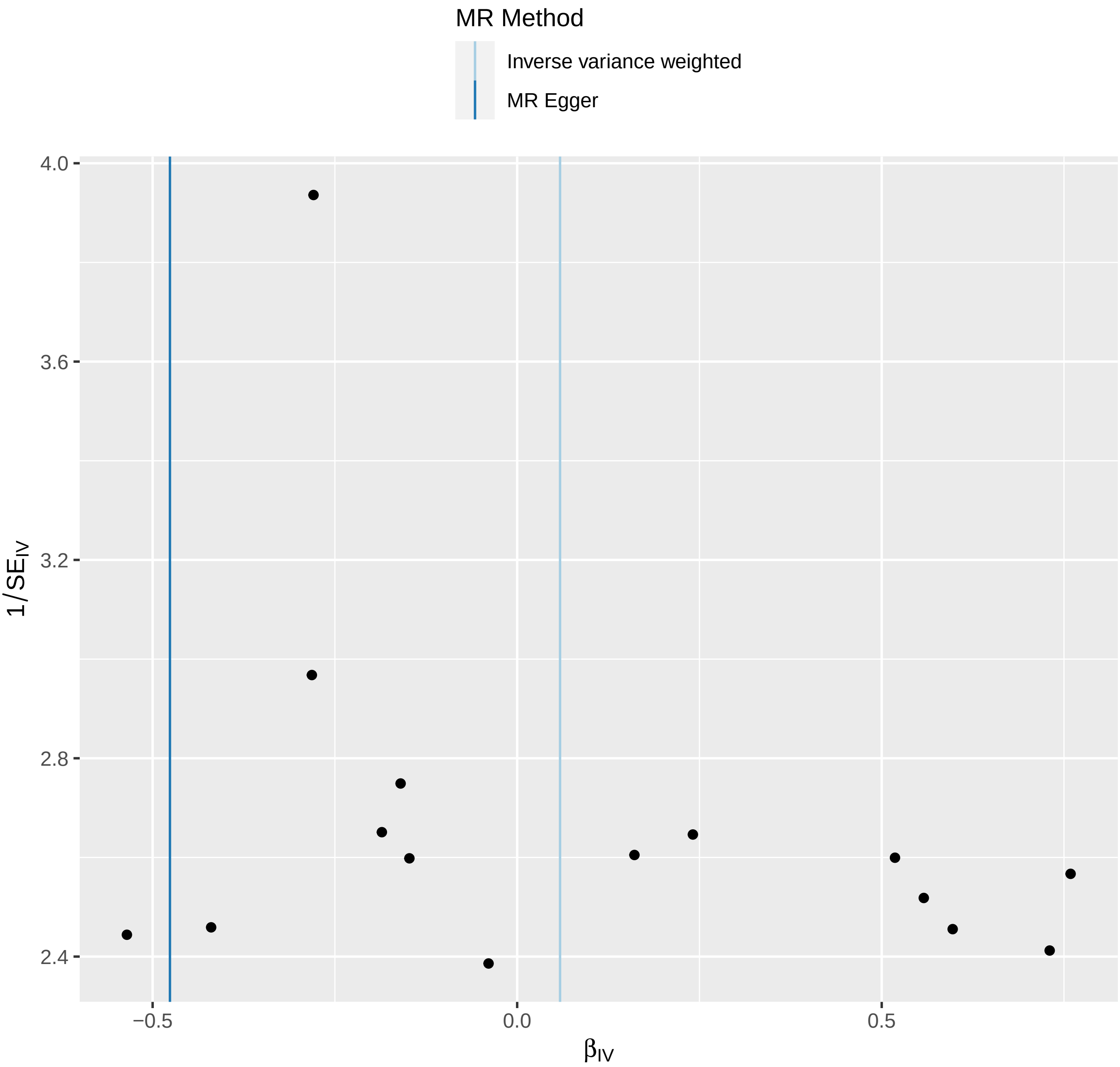

Batch 2−129 : Gut microbiota abundance (genus Catenibacterium id.2153) on Endothelial monocyte−activating polypeptide 2
with a threshold of 1e−05

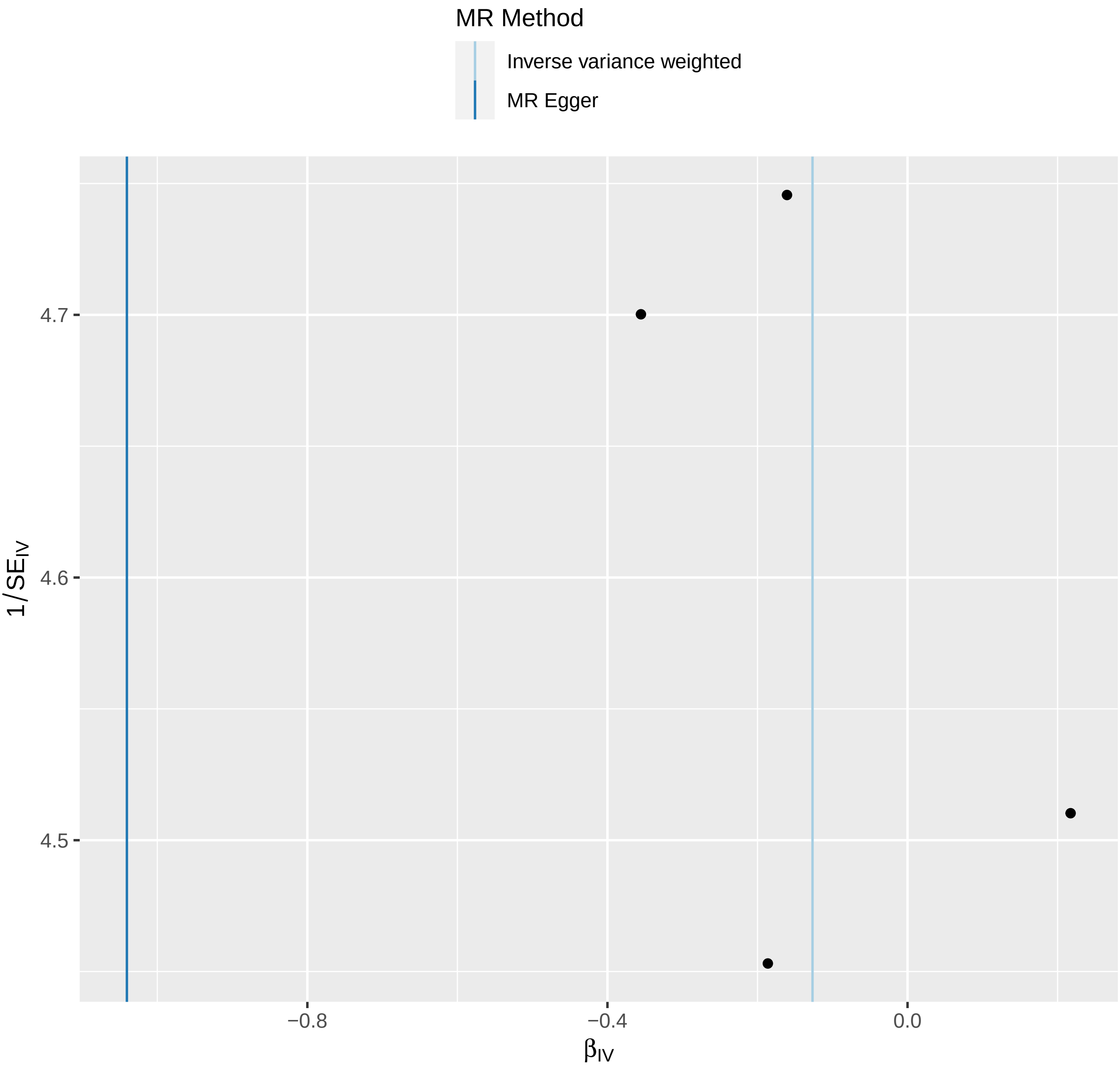

Batch 2−130 : Gut microbiota abundance (genus Parasutterella id.2892) on Endothelial monocyte−activating polypeptide 2 with a threshold of 1e−05

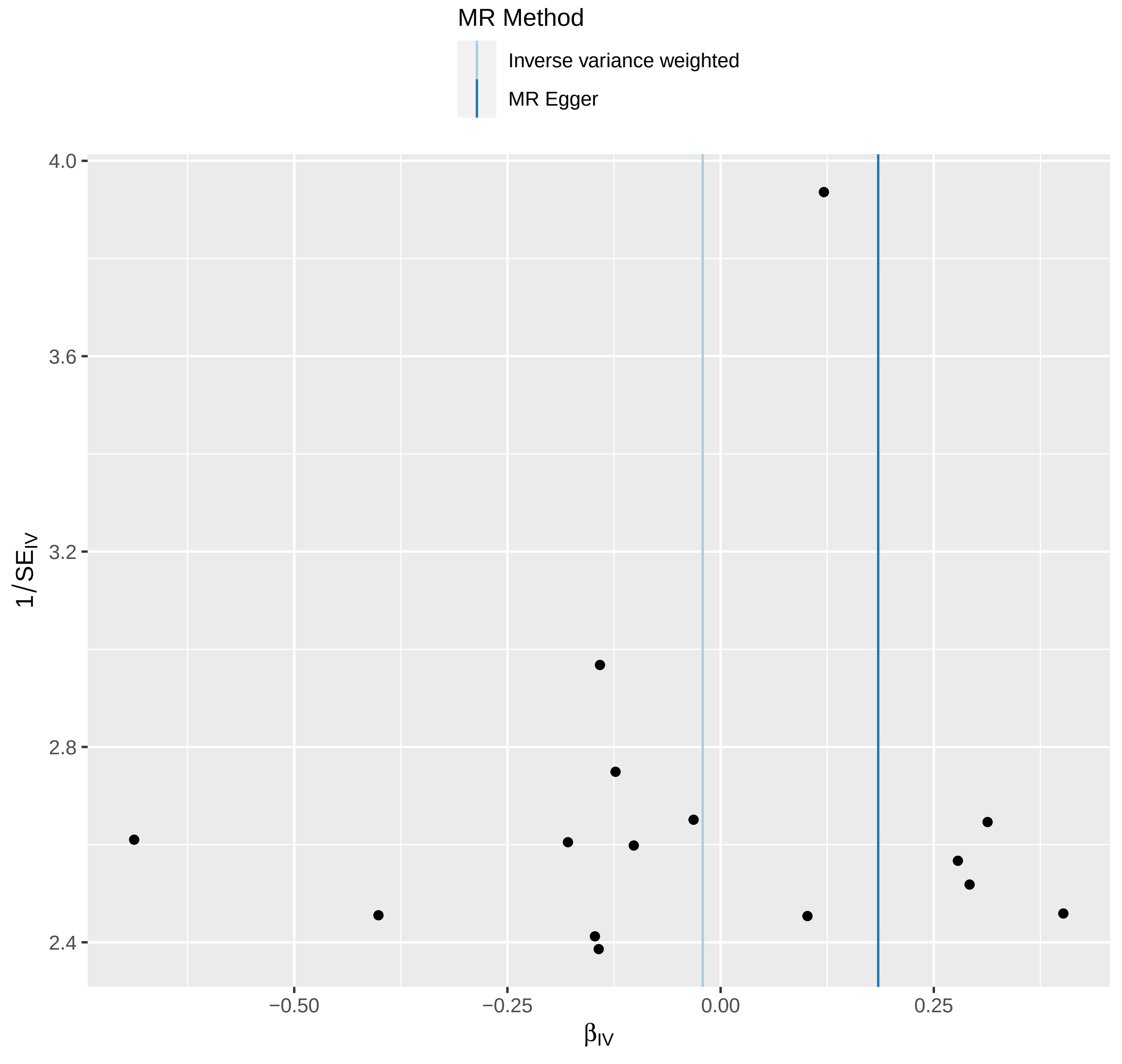

Batch 2−131 : Gut microbiota abundance (genus Catenibacterium id.2153) on Adenylate kinase isoenzyme 1
with a threshold of 1e−05

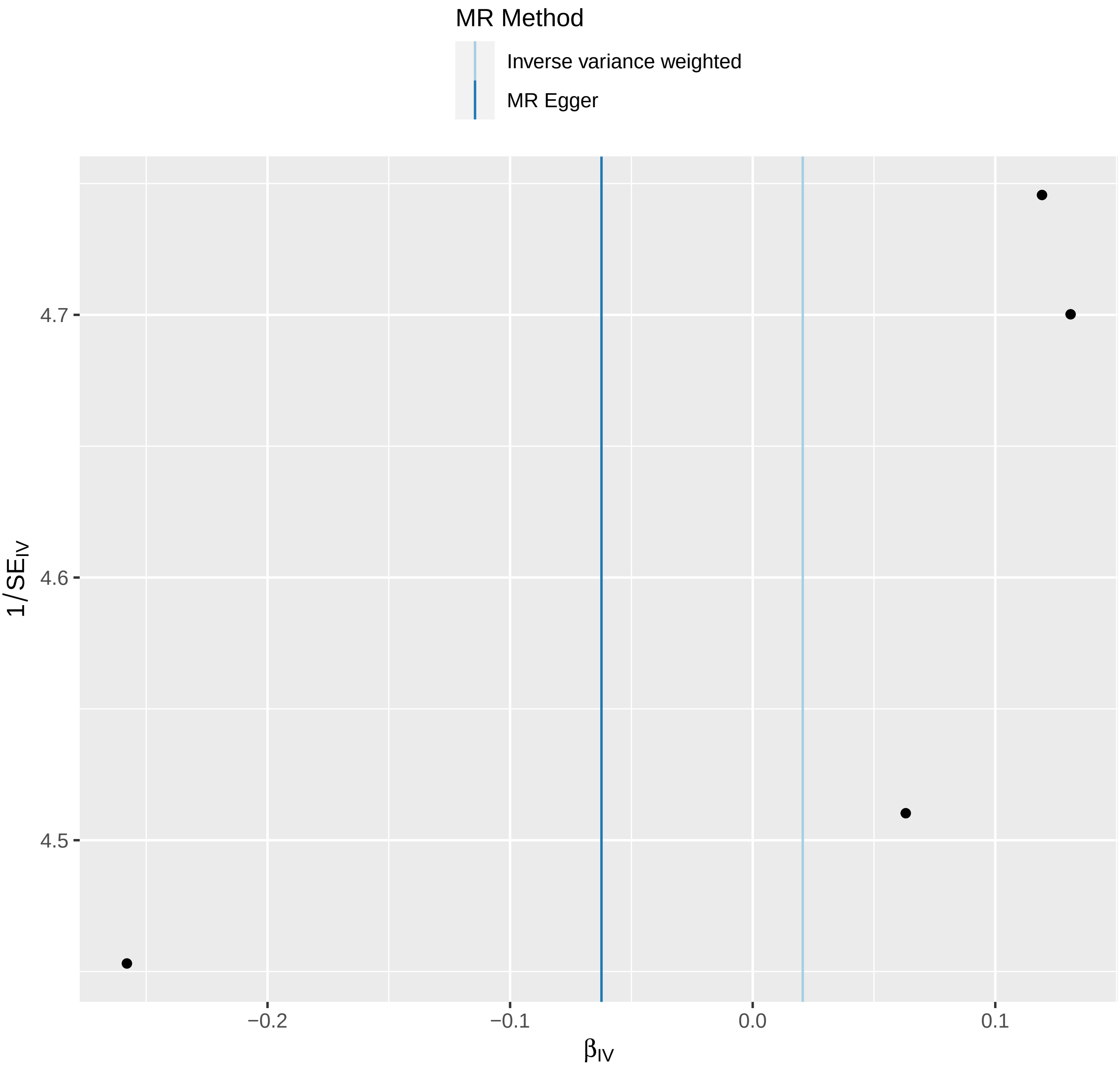

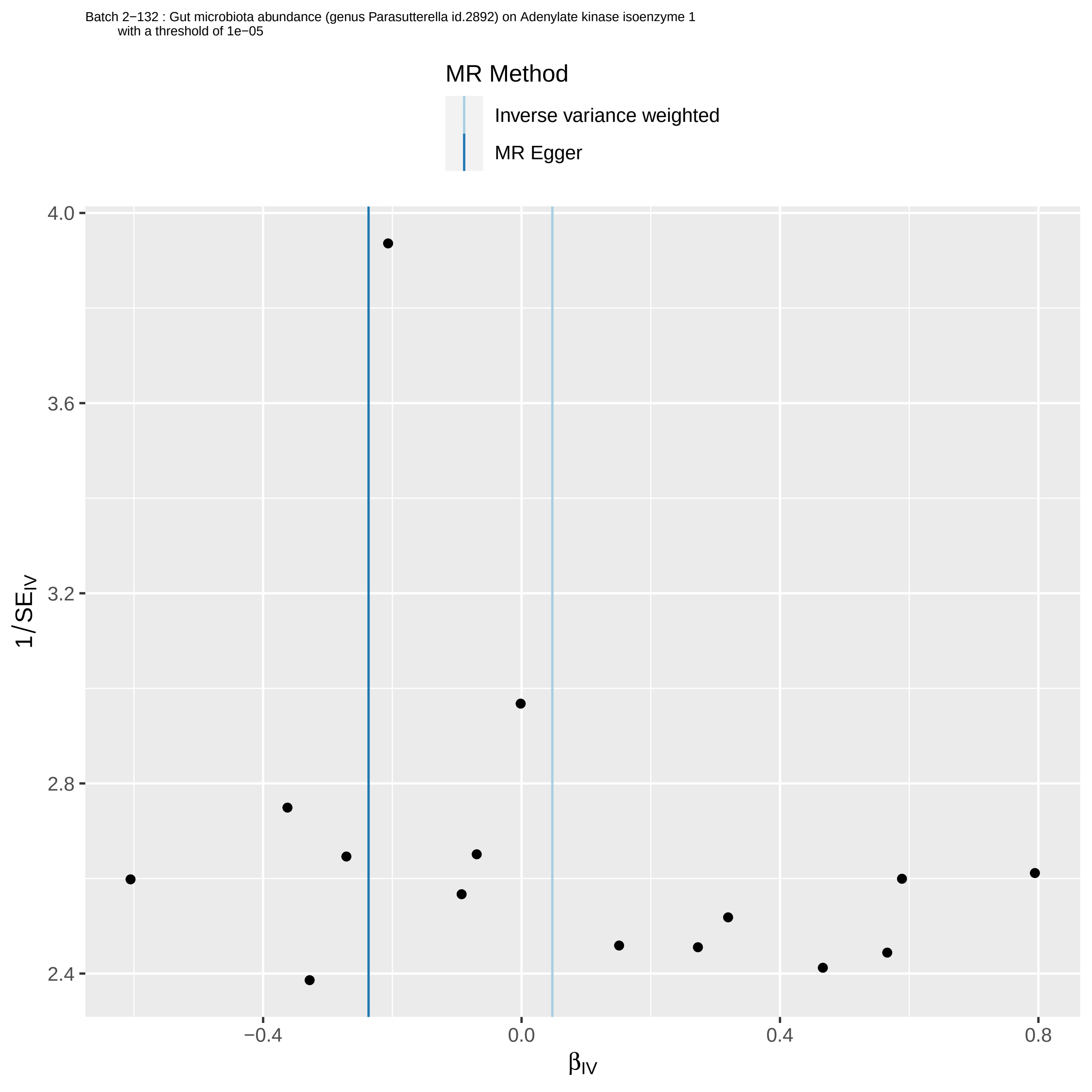

Batch 2−132 : Gut microbiota abundance (genus Parasutterella id.2892) on Adenylate kinase isoenzyme 1
with a threshold of 1e−05
MR Method
Inverse variance weighted
MR Egger
4.0
3.6
3.2
2.8
2.4
1/SE_IV
−0.4
0.0
0.4
0.8
β_IV

Batch 2−133 : Gut microbiota abundance (genus Catenibacterium id.2153) on Alcohol dehydrogenase [NADP(+)] with a threshold of 1e−05

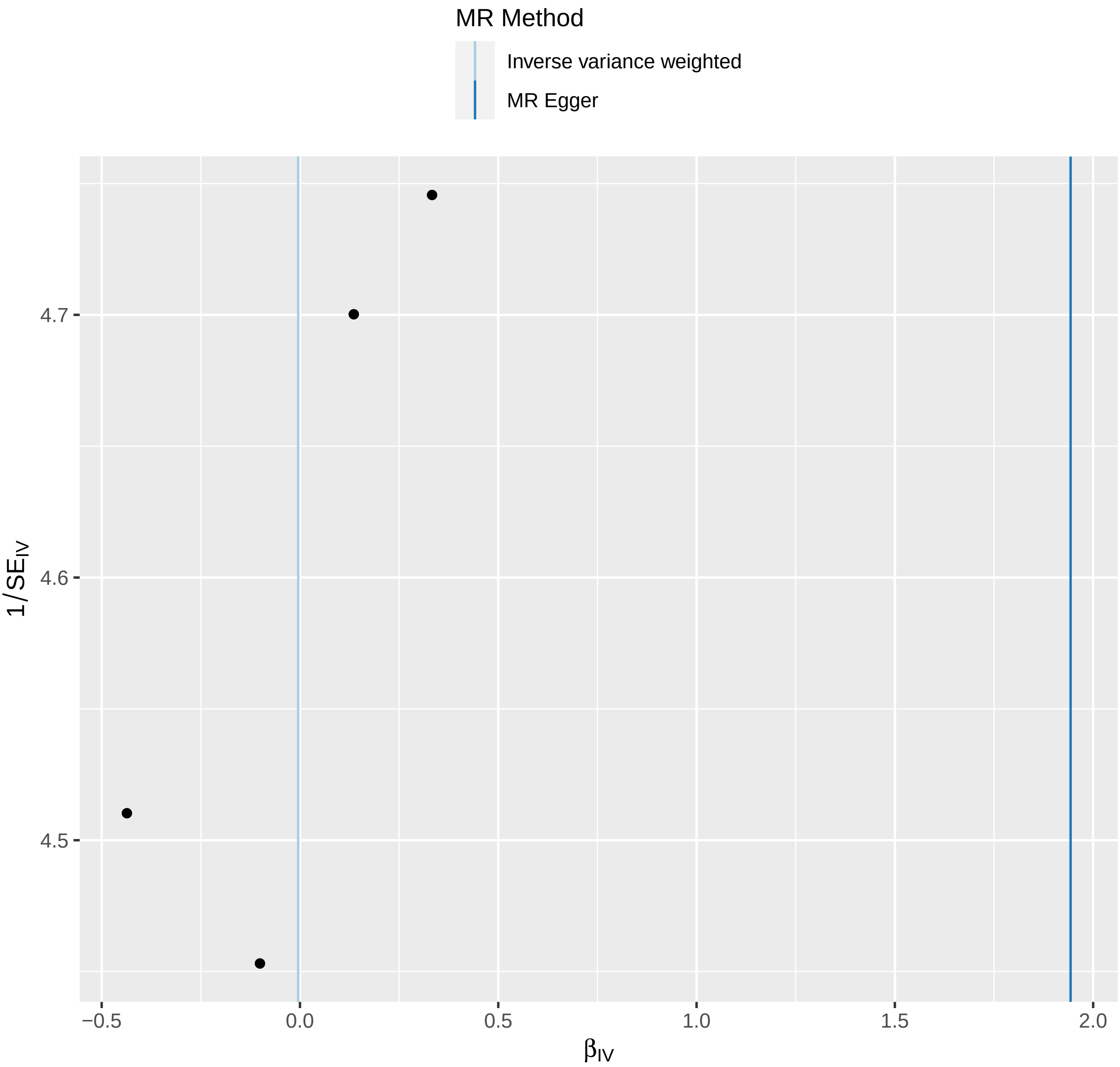

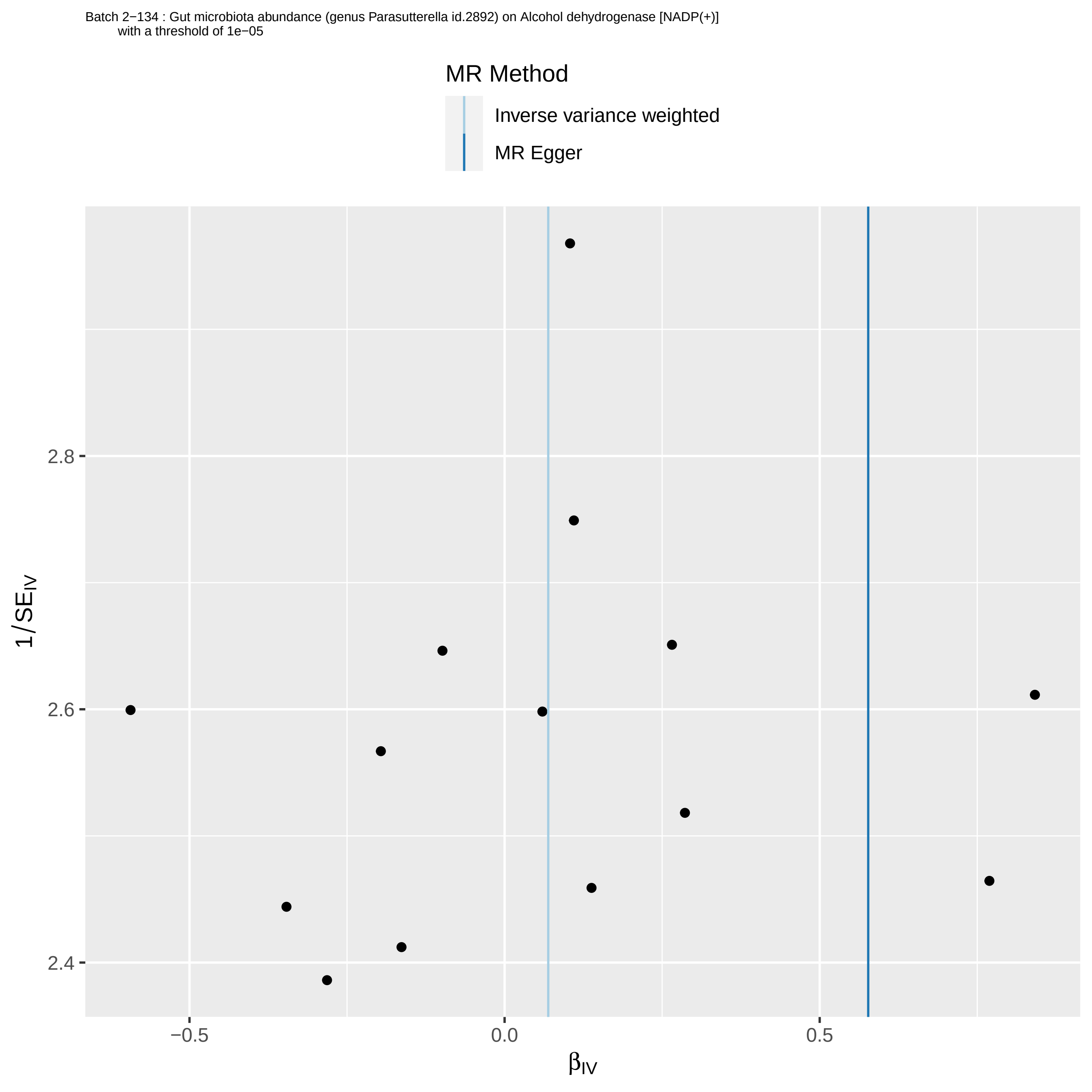

Batch 2−134 : Gut microbiota abundance (genus Parasutterella id.2892) on Alcohol dehydrogenase [NADP(+)]
with a threshold of 1e−05
MR Method
Inverse variance weighted
MR Egger
2.8
2.6
2.4
−0.5
0.0
0.5
1/SE
IV
β
IV

Batch 2−135 : Gut microbiota abundance (genus Catenibacterium id.2153) on Aldose reductase
with a threshold of 1e−05

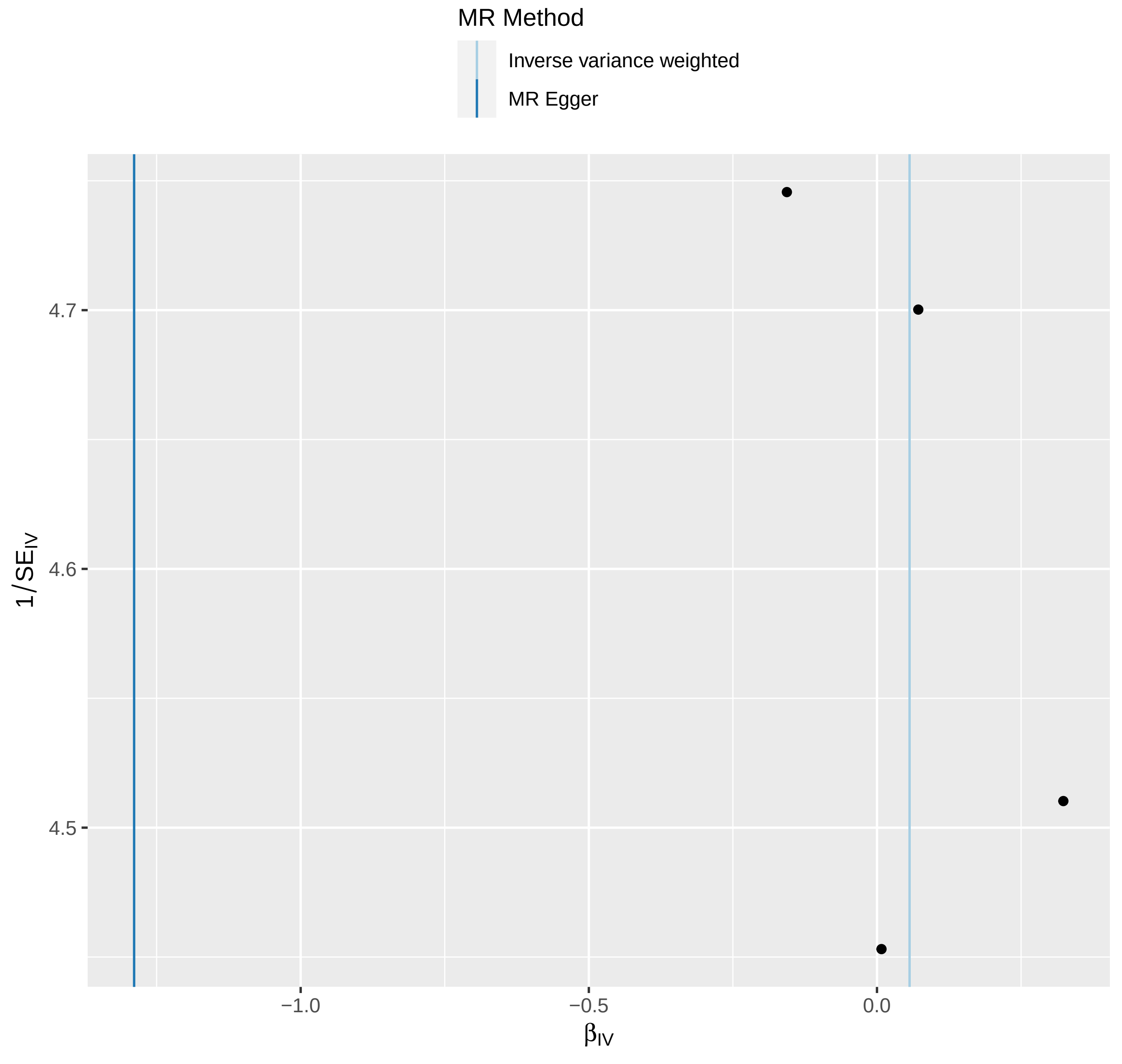

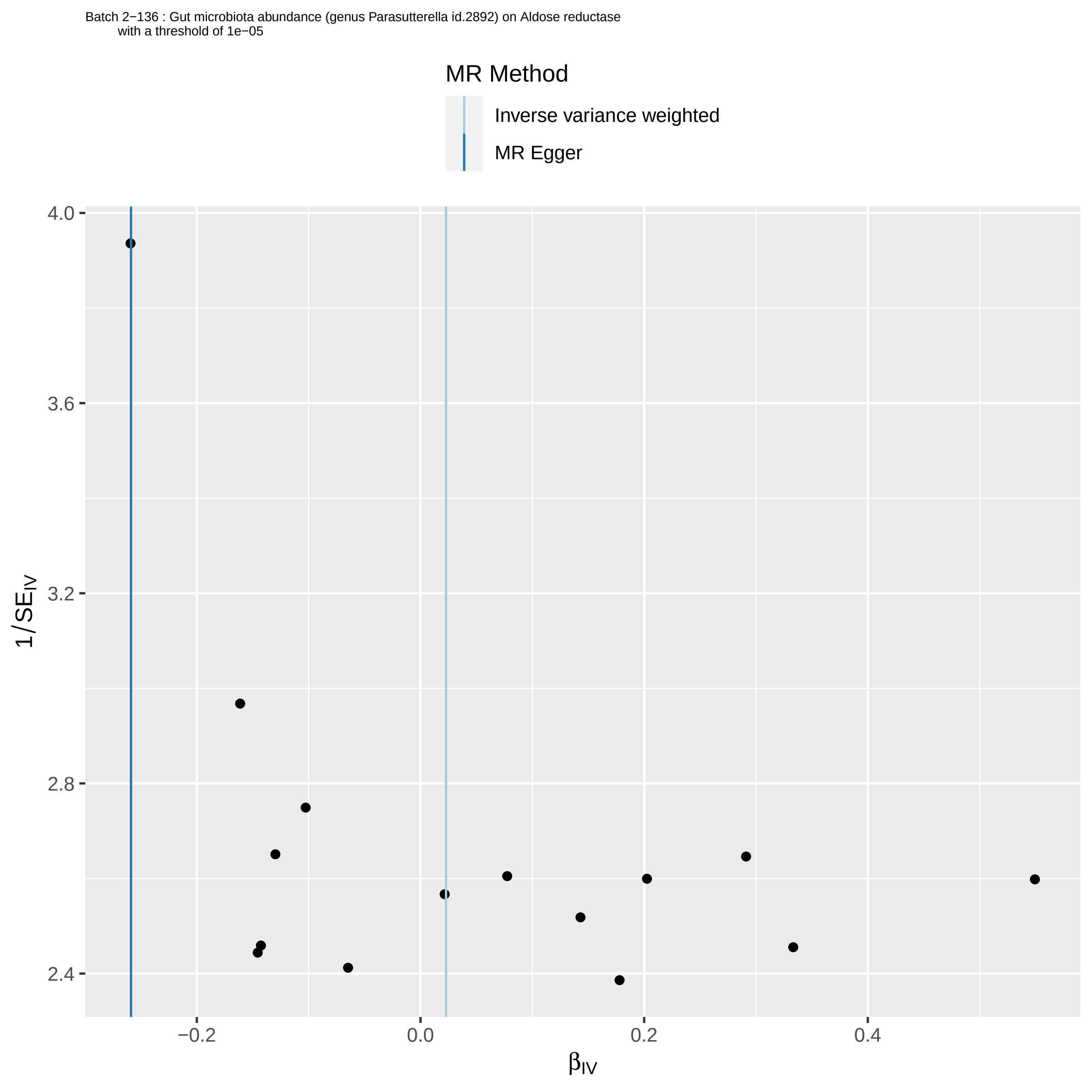

Batch 2−136 : Gut microbiota abundance (genus Parasutterella id.2892) on Aldose reductase
with a threshold of 1e−05
MR Method
Inverse variance weighted
MR Egger
4.0
3.6
3.2
2.8
2.4
1/SE_IV
−0.2
0.0
0.2
0.4
β_IV

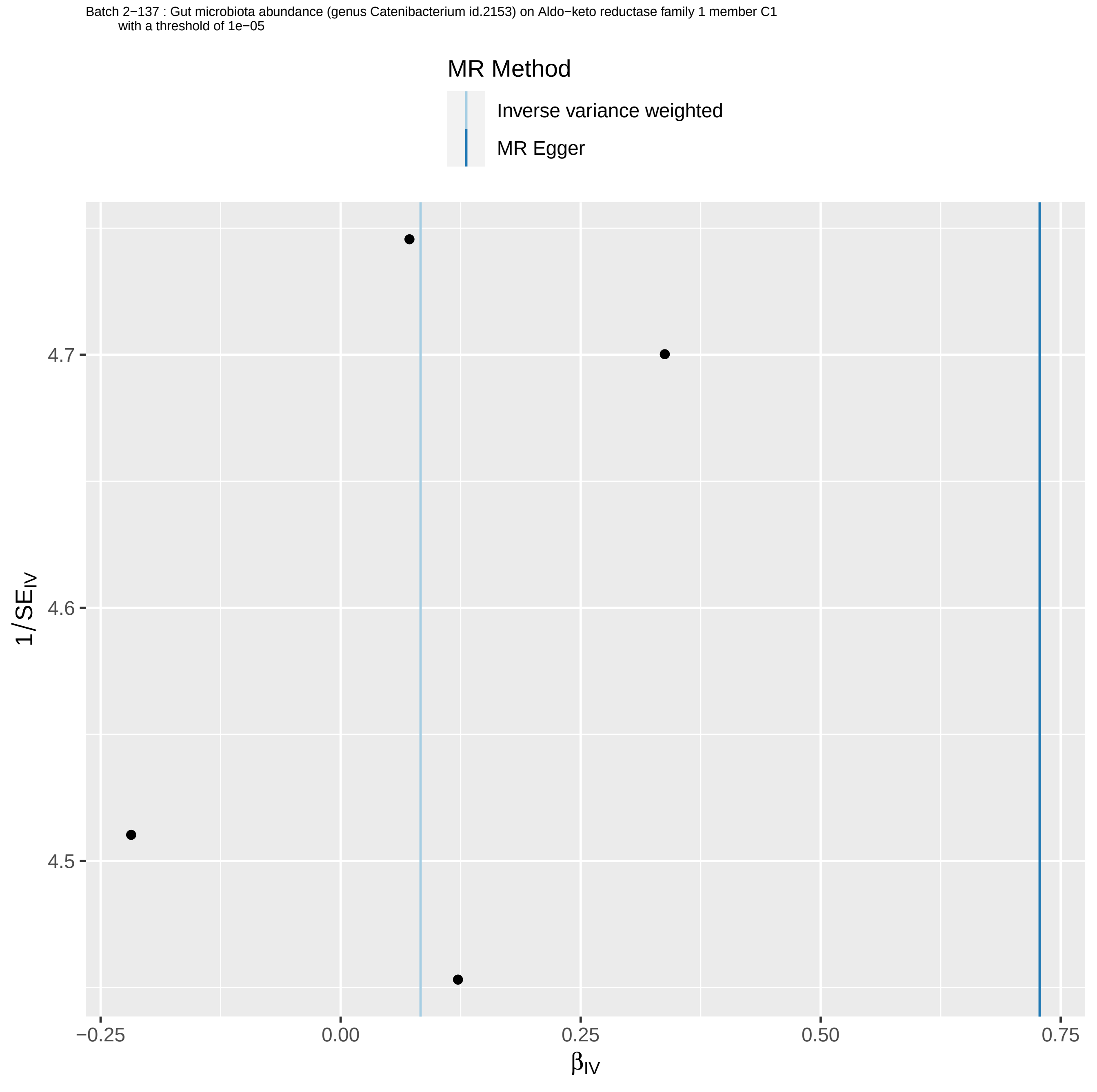

Batch 2−137 : Gut microbiota abundance (genus Catenibacterium id.2153) on Aldo−keto reductase family 1 member C1
with a threshold of 1e−05
MR Method
Inverse variance weighted
MR Egger
4.7
4.6
4.5
1/SE_IV
−0.25
0.00
0.25
0.50
0.75
β_IV

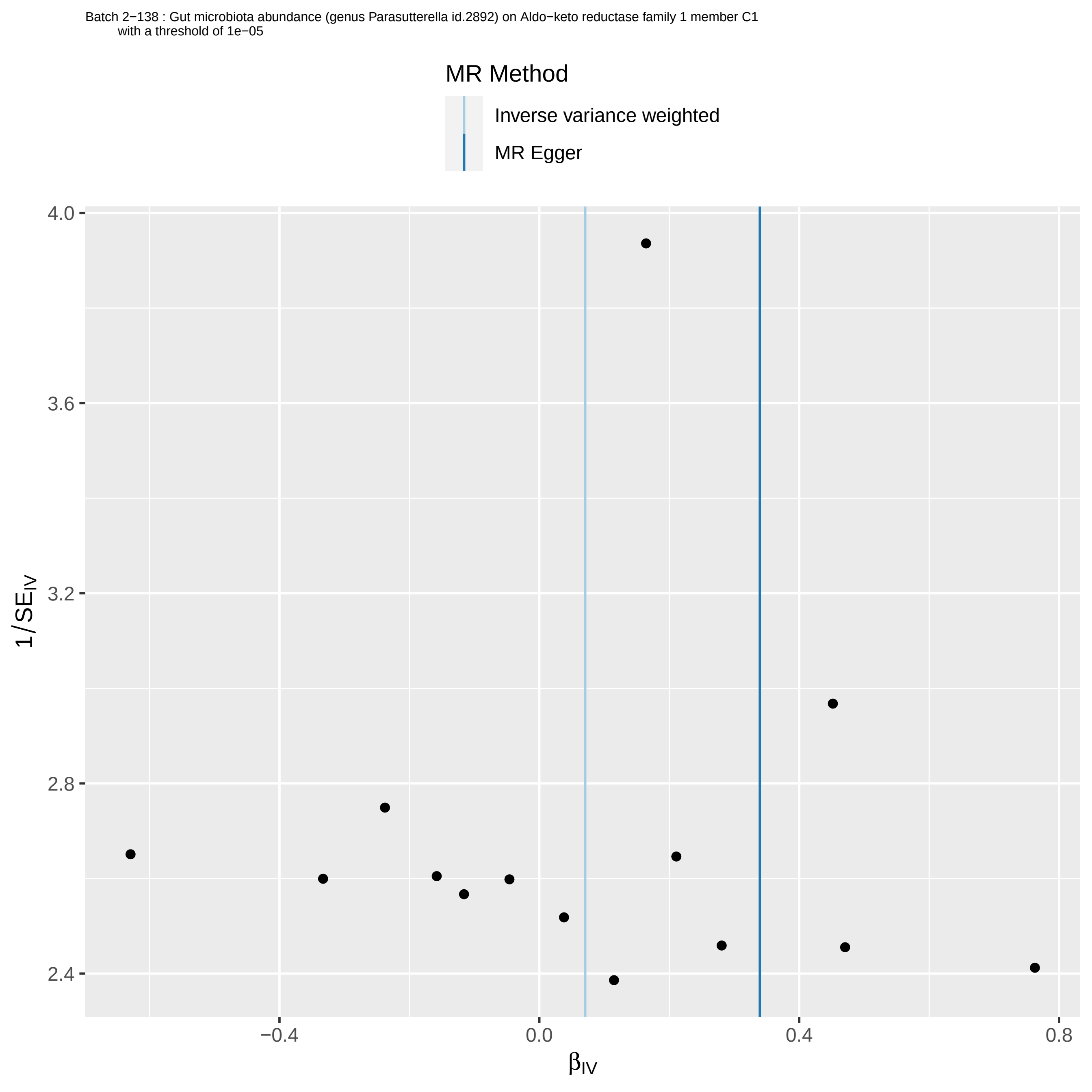

Batch 2−138 : Gut microbiota abundance (genus Parasutterella id.2892) on Aldo−keto reductase family 1 member C1
with a threshold of 1e−05
MR Method
Inverse variance weighted
MR Egger
4.0
3.6
3.2
2.8
2.4
1/SE_IV
−0.4
0.0
0.4
0.8
β_IV

Batch 2−139 : Gut microbiota abundance (genus Catenibacterium id.2153) on RAC−alpha/beta/gamma serine/threonine−protein kinase
with a threshold of 1e−05

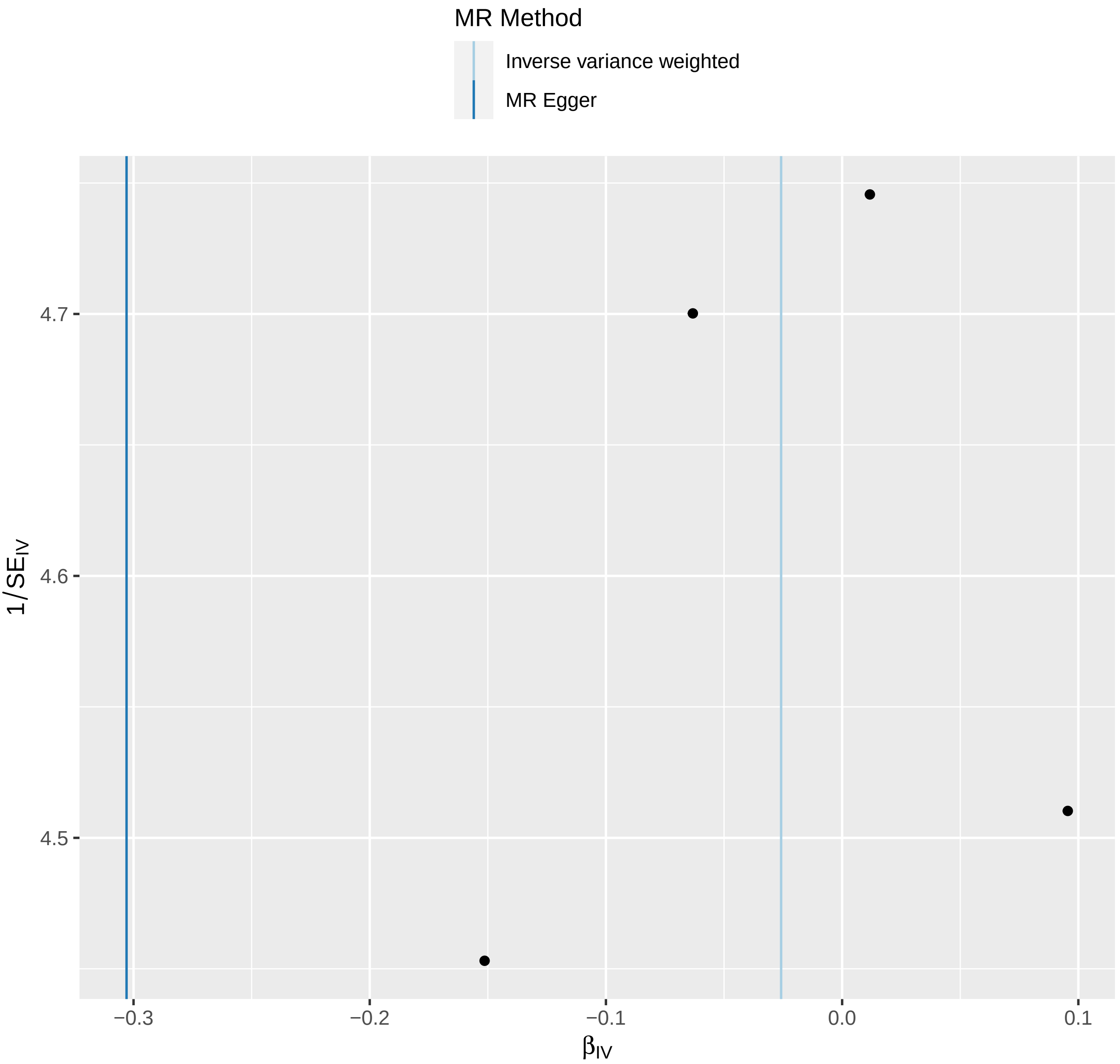

Batch 2−140 : Gut microbiota abundance (genus Parasutterella id.2892) on RAC−alpha/beta/gamma serine/threonine−protein kinase with a threshold of 1e−05

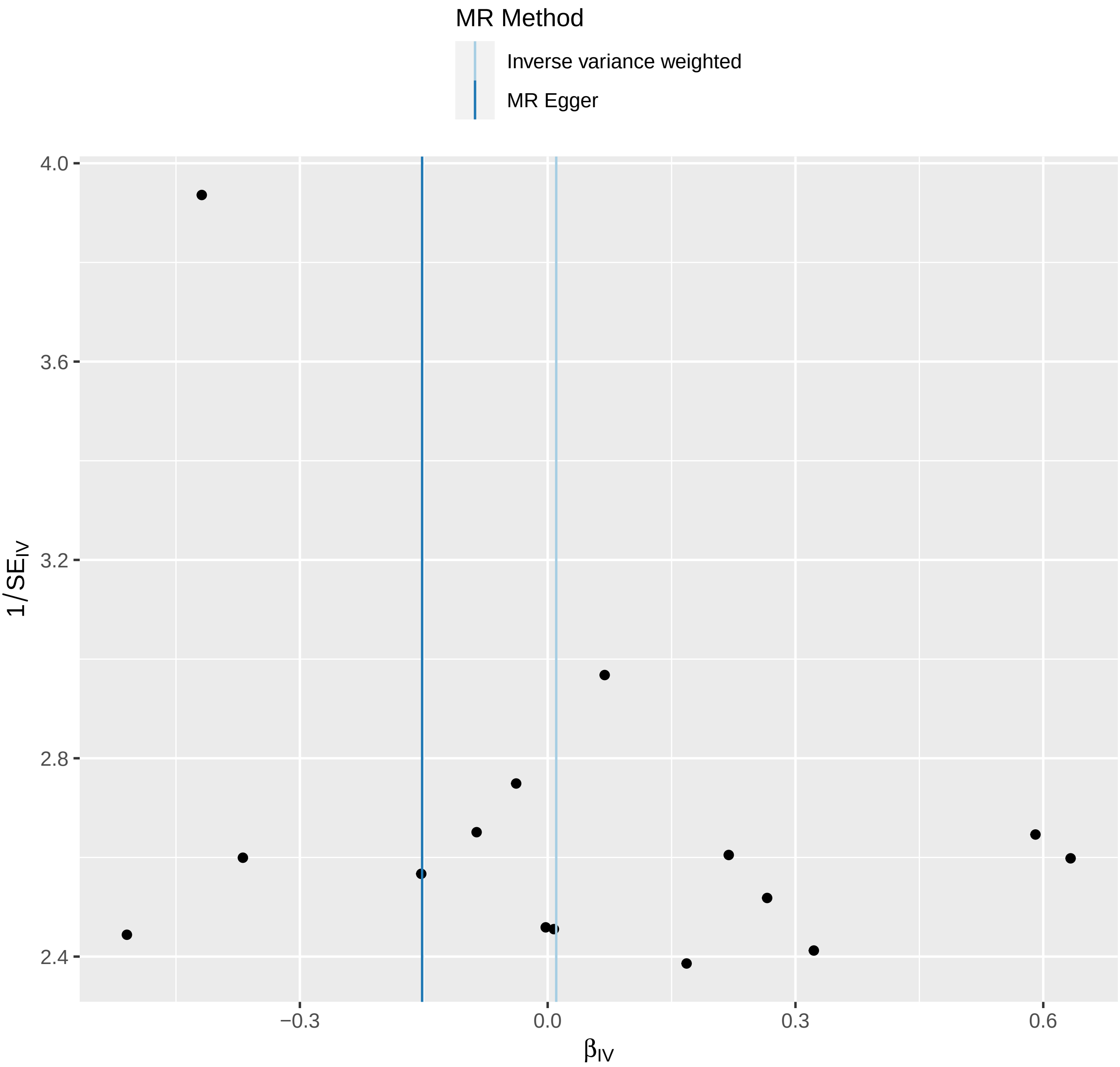

Batch 2−141 : Gut microbiota abundance (genus Catenibacterium id.2153) on RAC−alpha/beta/gamma serine/threonine−protein kinase with a threshold of 1e−05

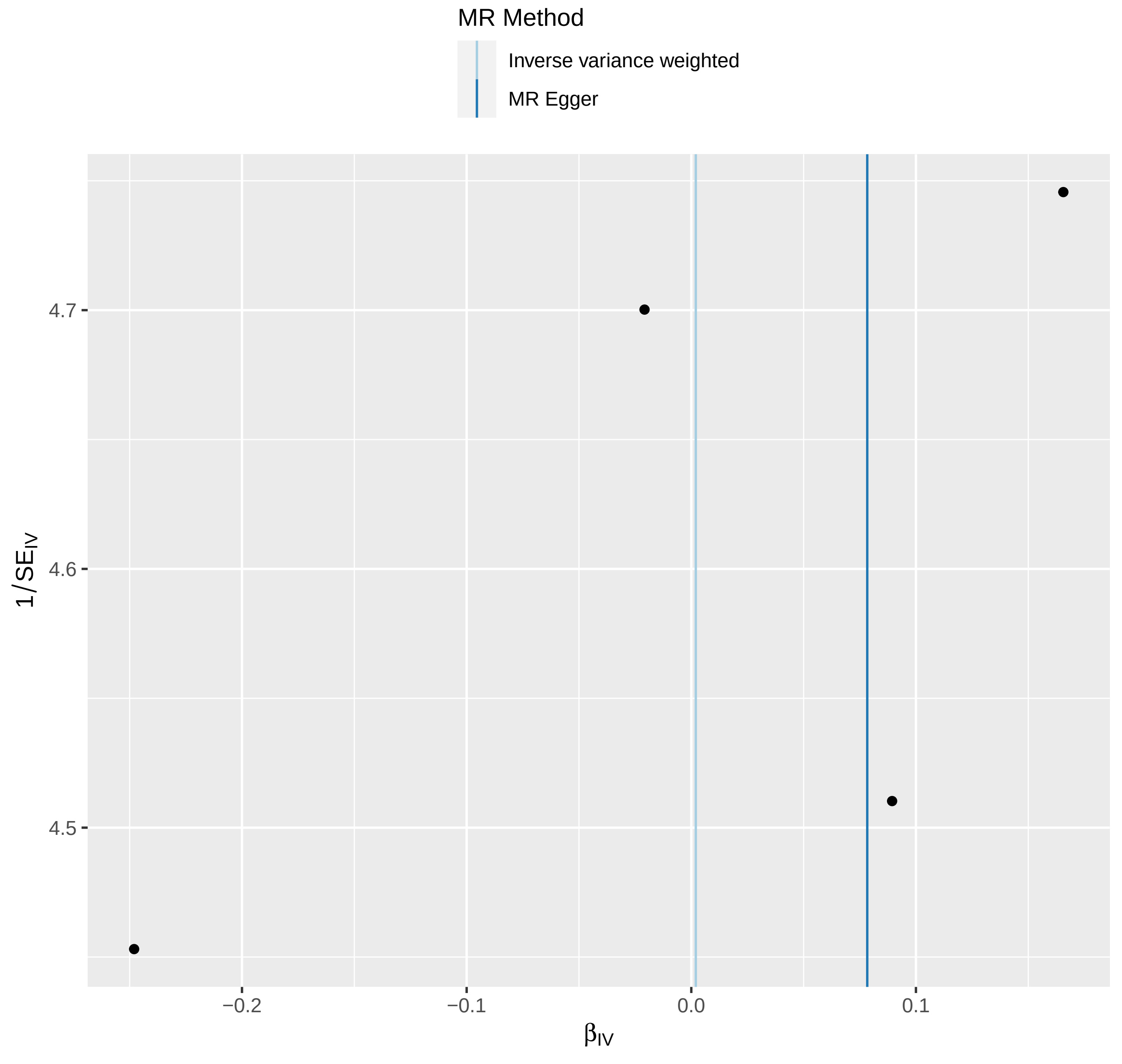

Batch 2−142 : Gut microbiota abundance (genus Parasutterella id.2892) on RAC−alpha/beta/gamma serine/threonine−protein kinase with a threshold of 1e−05

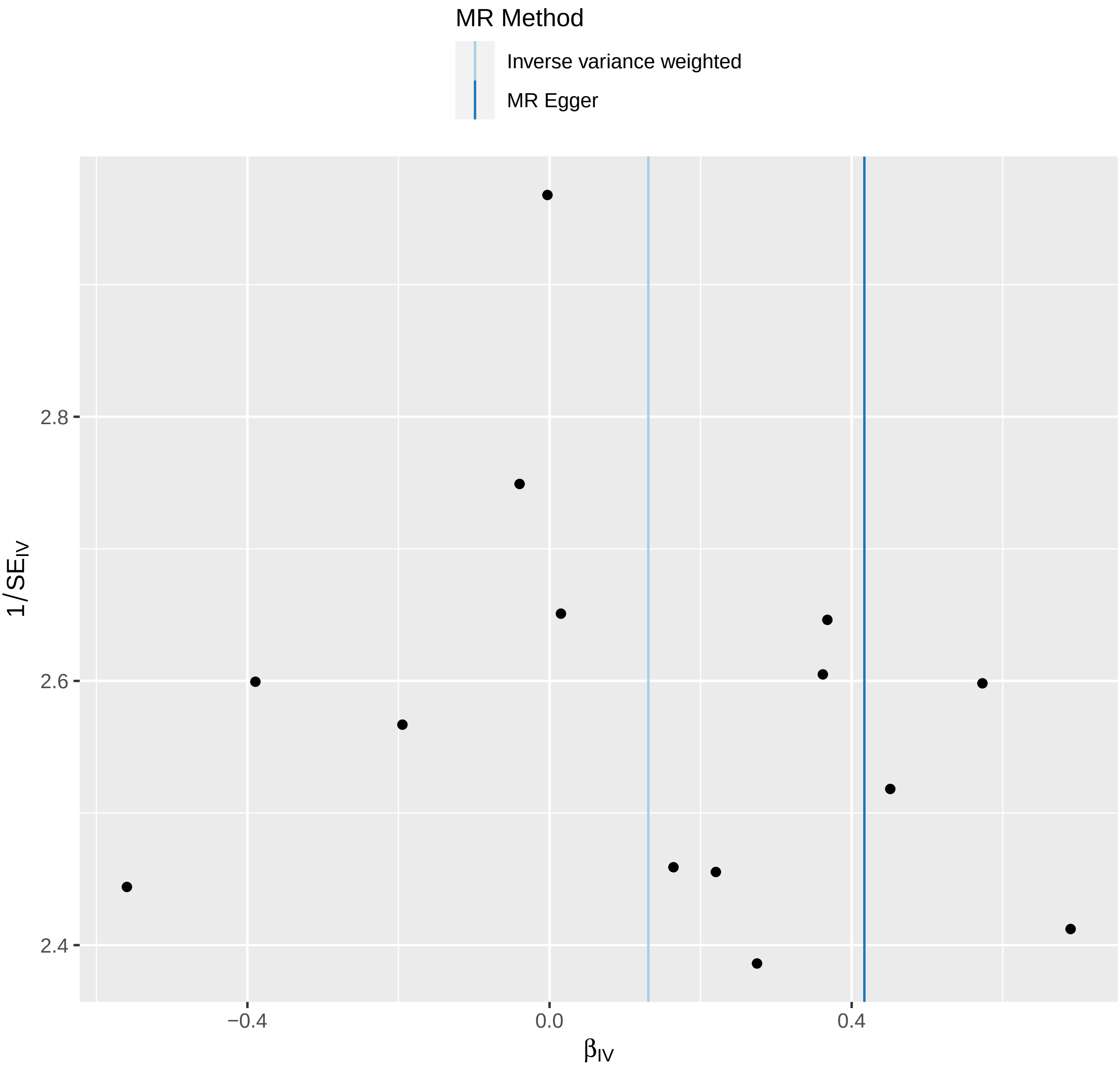

Batch 2−143 : Gut microbiota abundance (genus Catenibacterium id.2153) on RAC−beta serine/threonine−protein kinase
with a threshold of 1e−05

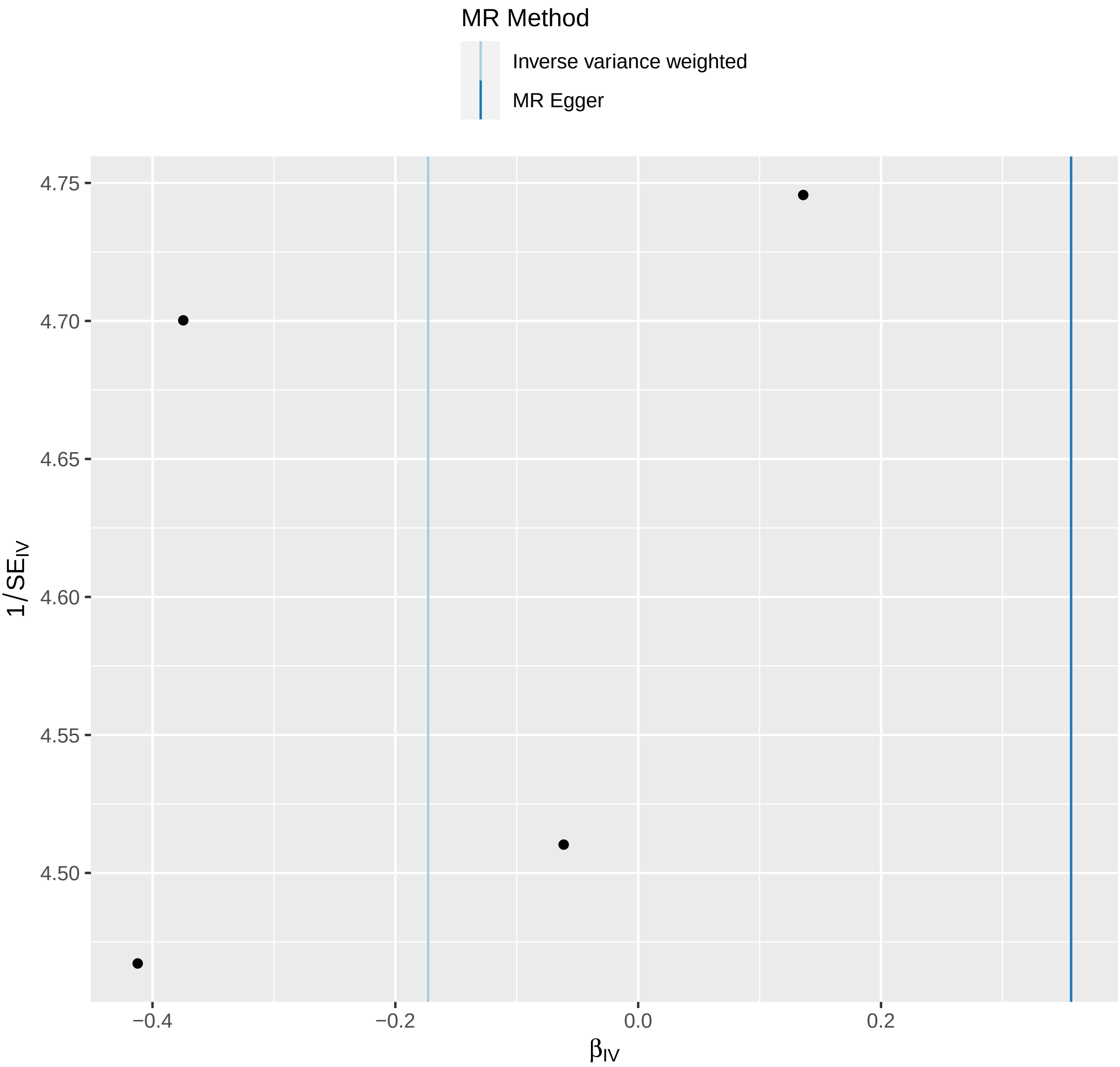

Batch 2−144 : Gut microbiota abundance (genus Parasutterella id.2892) on RAC−beta serine/threonine−protein kinase
with a threshold of 1e−05

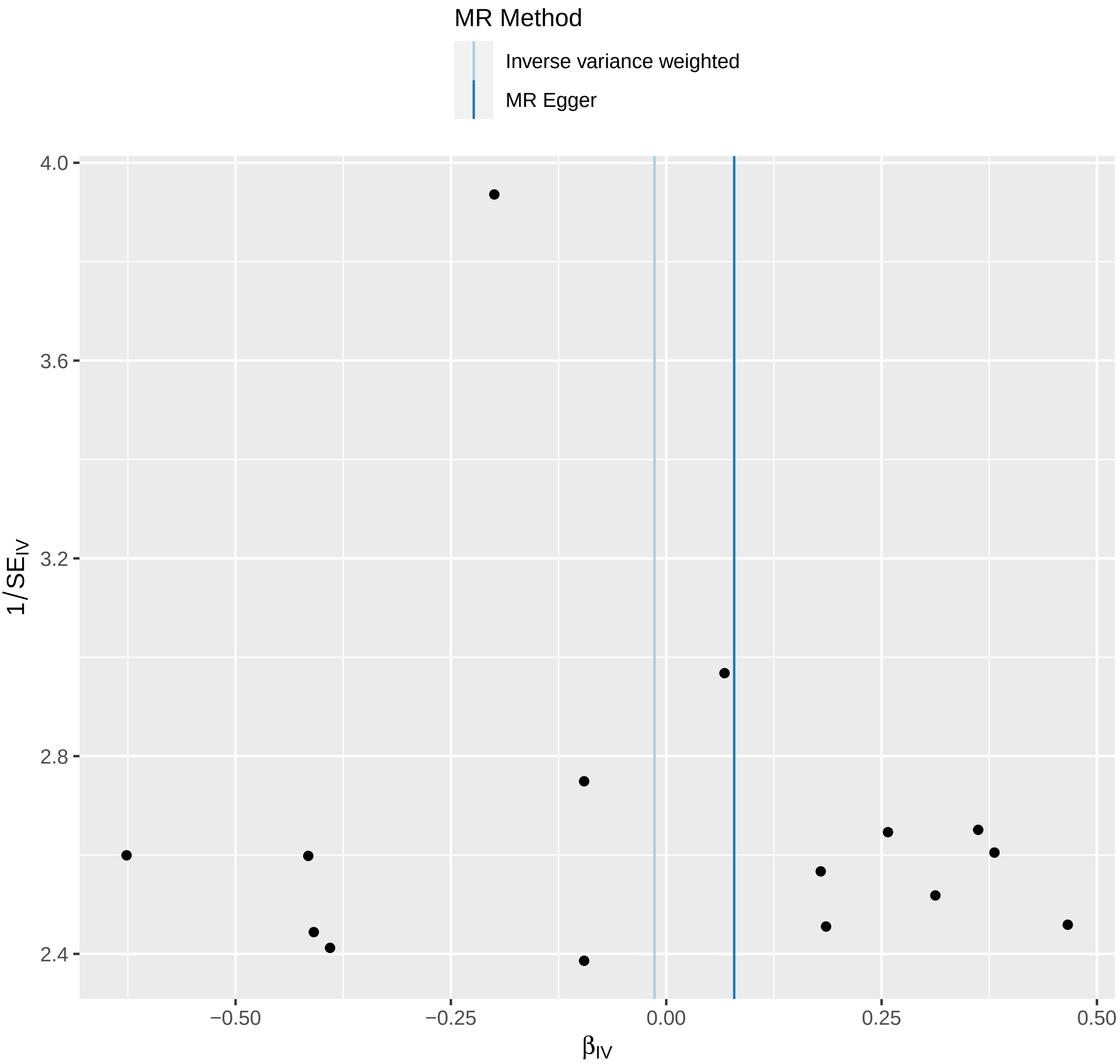

Batch 2−145 : Gut microbiota abundance (genus Catenibacterium id.2153) on RAC−beta serine/threonine−protein kinase
with a threshold of 1e−05

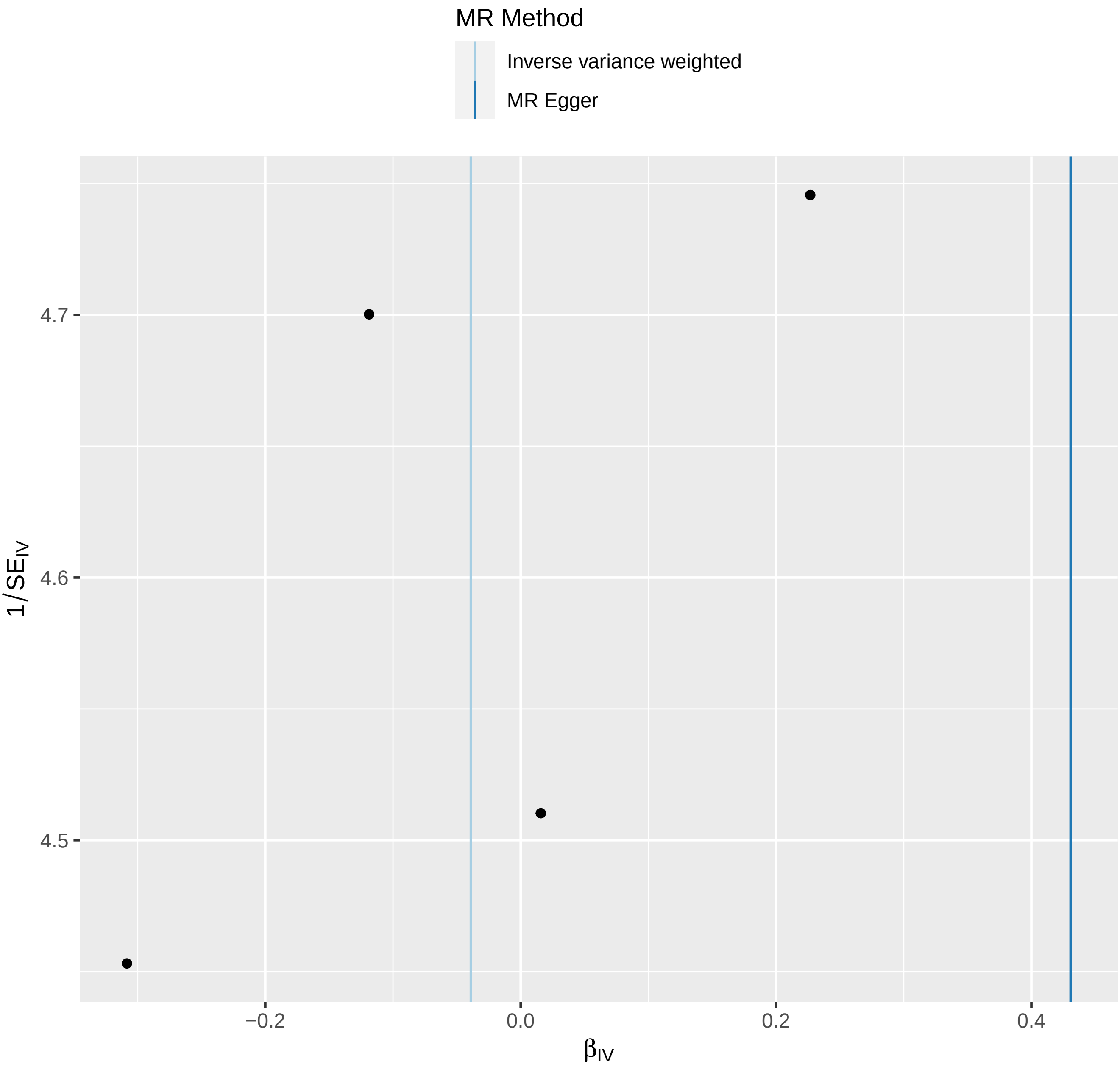

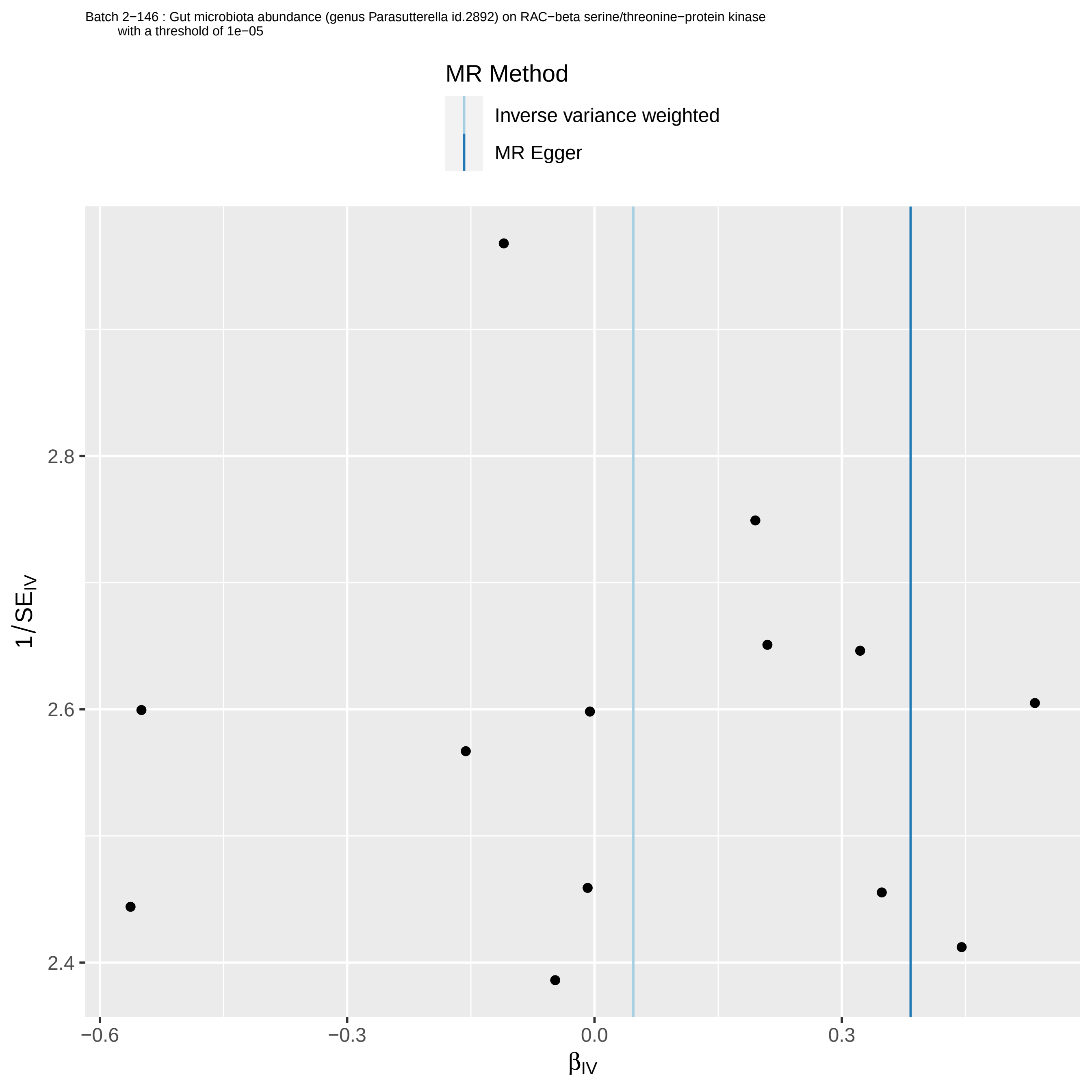
Batch 2−146 : Gut microbiota abundance (genus Parasutterella id.2892) on RAC−beta serine/threonine−protein kinase
with a threshold of 1e−05
MR Method
Inverse variance weighted
MR Egger
2.8
2.6
2.4
−0.6
−0.3
0.0
0.3
1/SE_IV
β_IV

Batch 2−147 : Gut microbiota abundance (genus Catenibacterium id.2153) on CD166 antigen
with a threshold of 1e−05

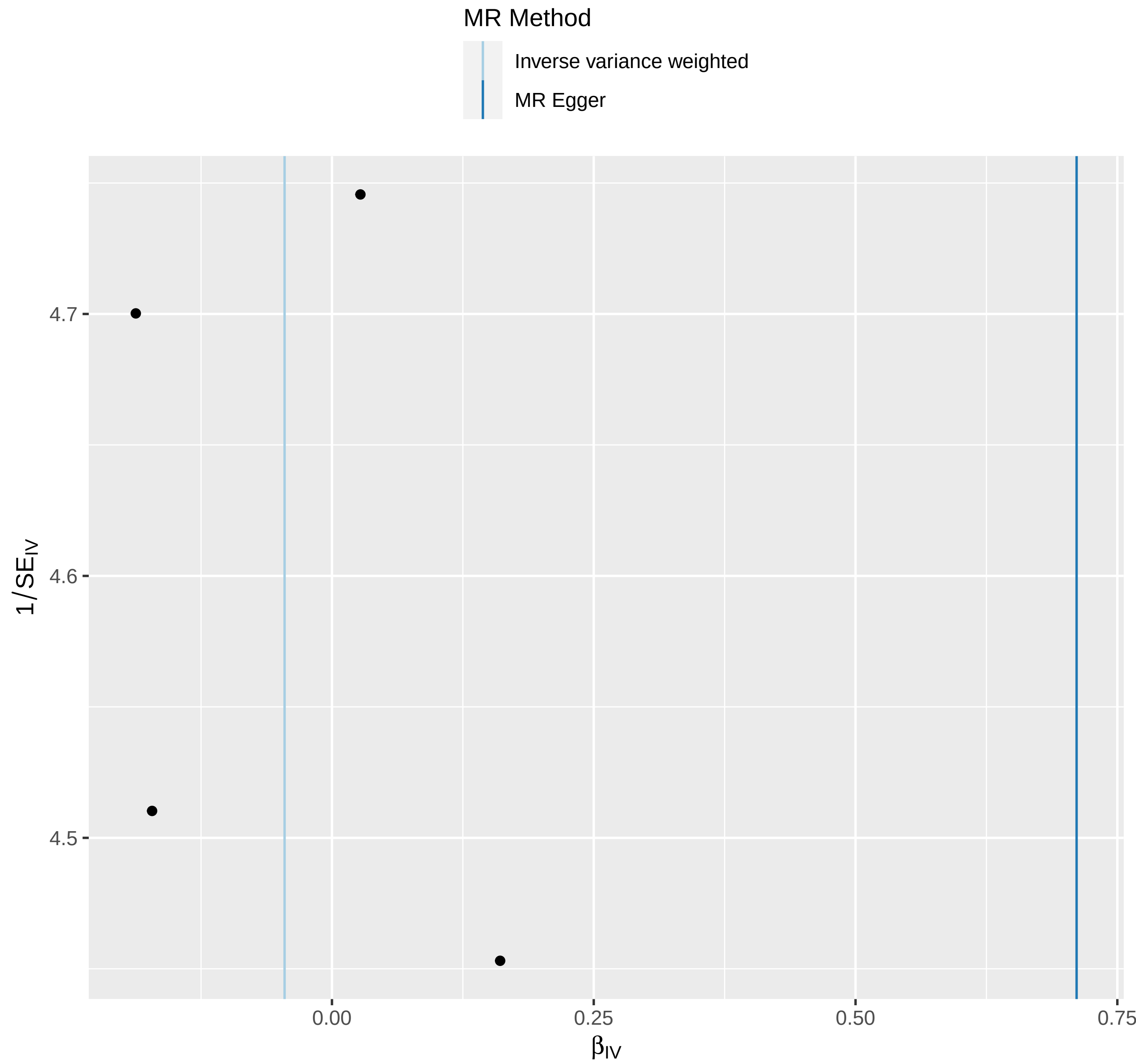

Batch 2−148 : Gut microbiota abundance (genus Parasutterella id.2892) on CD166 antigen
with a threshold of 1e−05

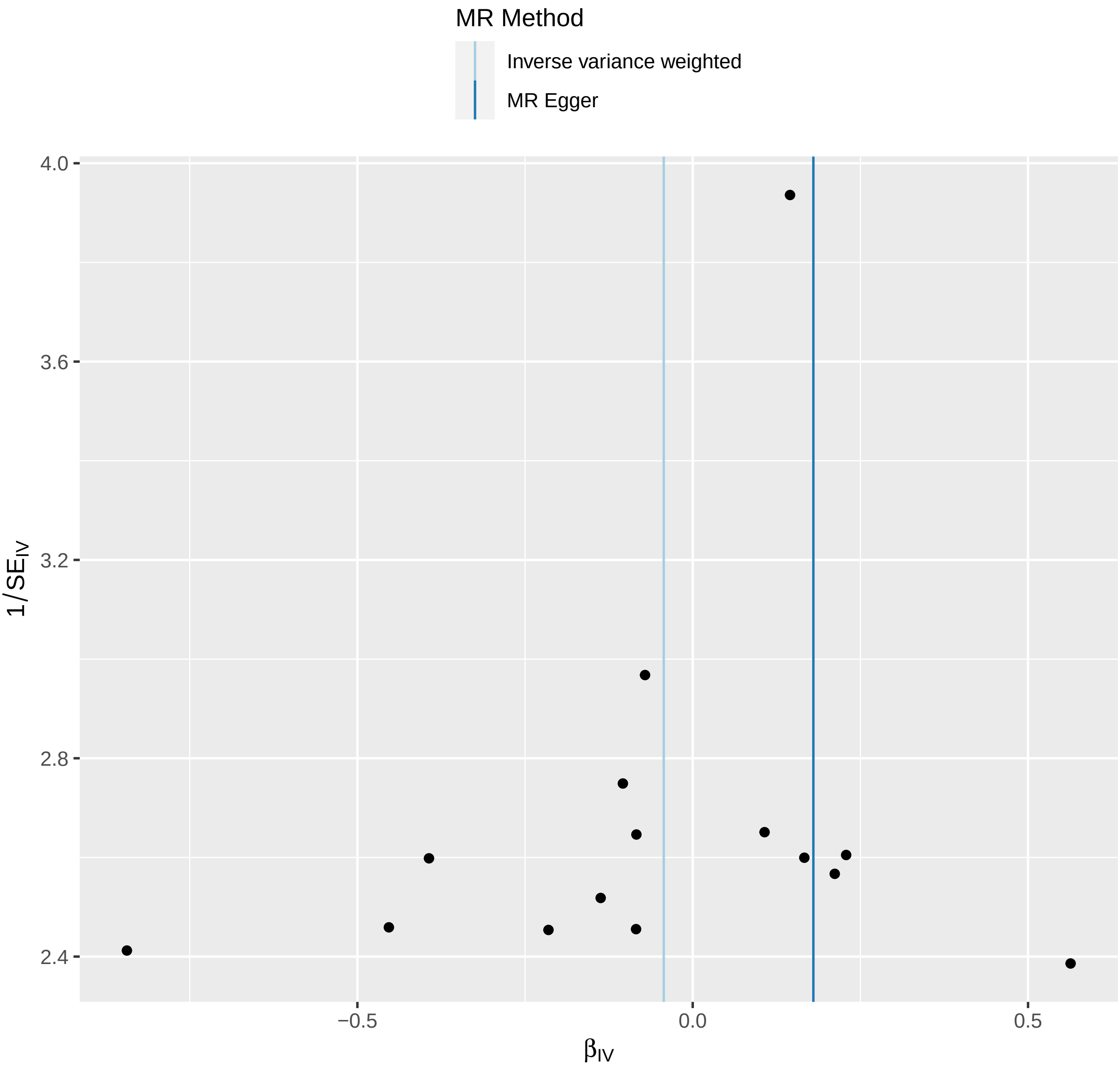

Batch 2−149 : Gut microbiota abundance (genus Catenibacterium id.2153) on Aldehyde dehydrogenase, dimeric NADP−preferring
with a threshold of 1e−05

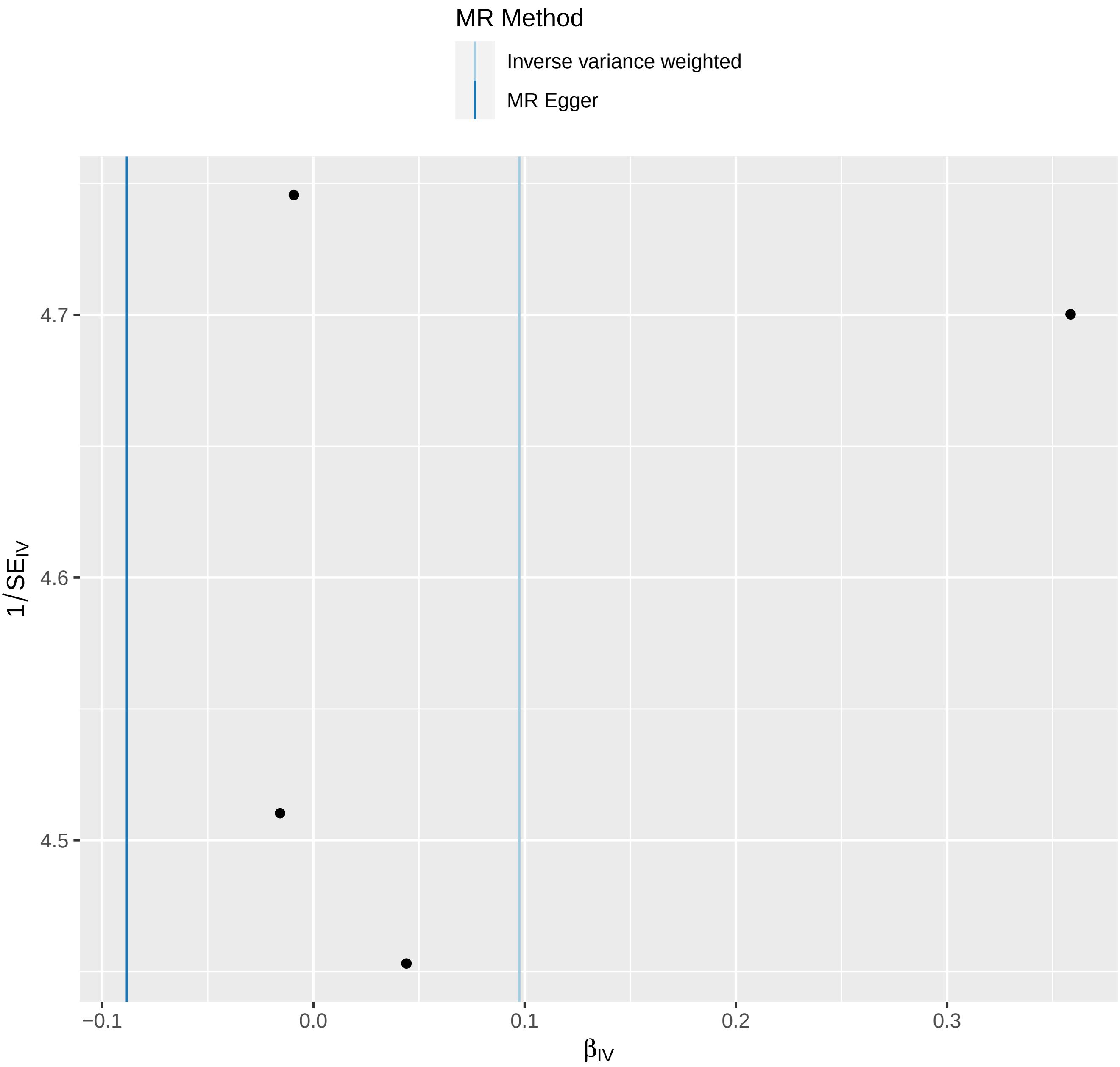

Batch 2−150 : Gut microbiota abundance (genus Parasutterella id.2892) on Aldehyde dehydrogenase, dimeric NADP−preferring with a threshold of 1e−05

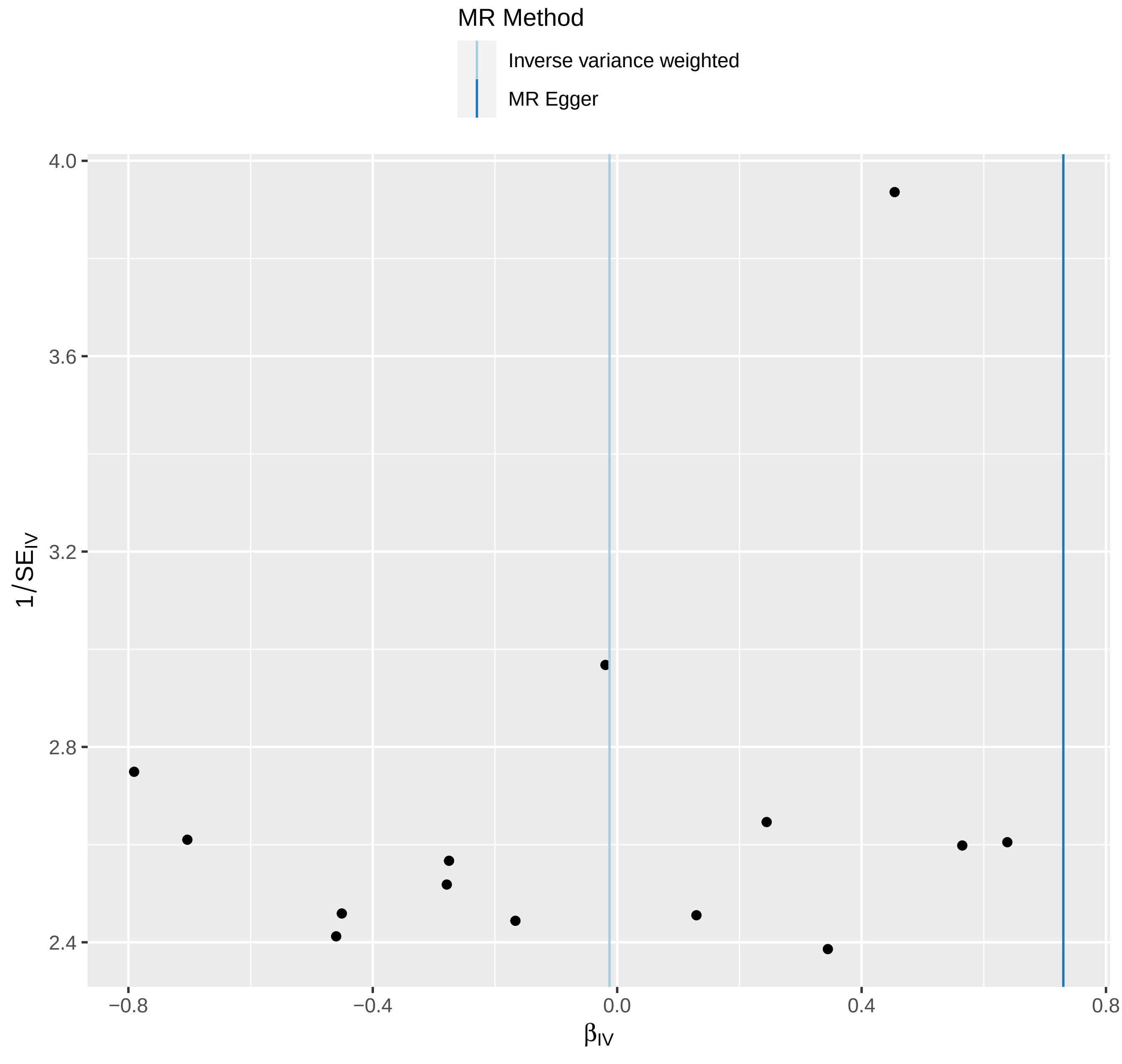

Batch 2−151 : Gut microbiota abundance (genus Catenibacterium id.2153) on Aldehyde dehydrogenase family 3 member B1
with a threshold of 1e−05

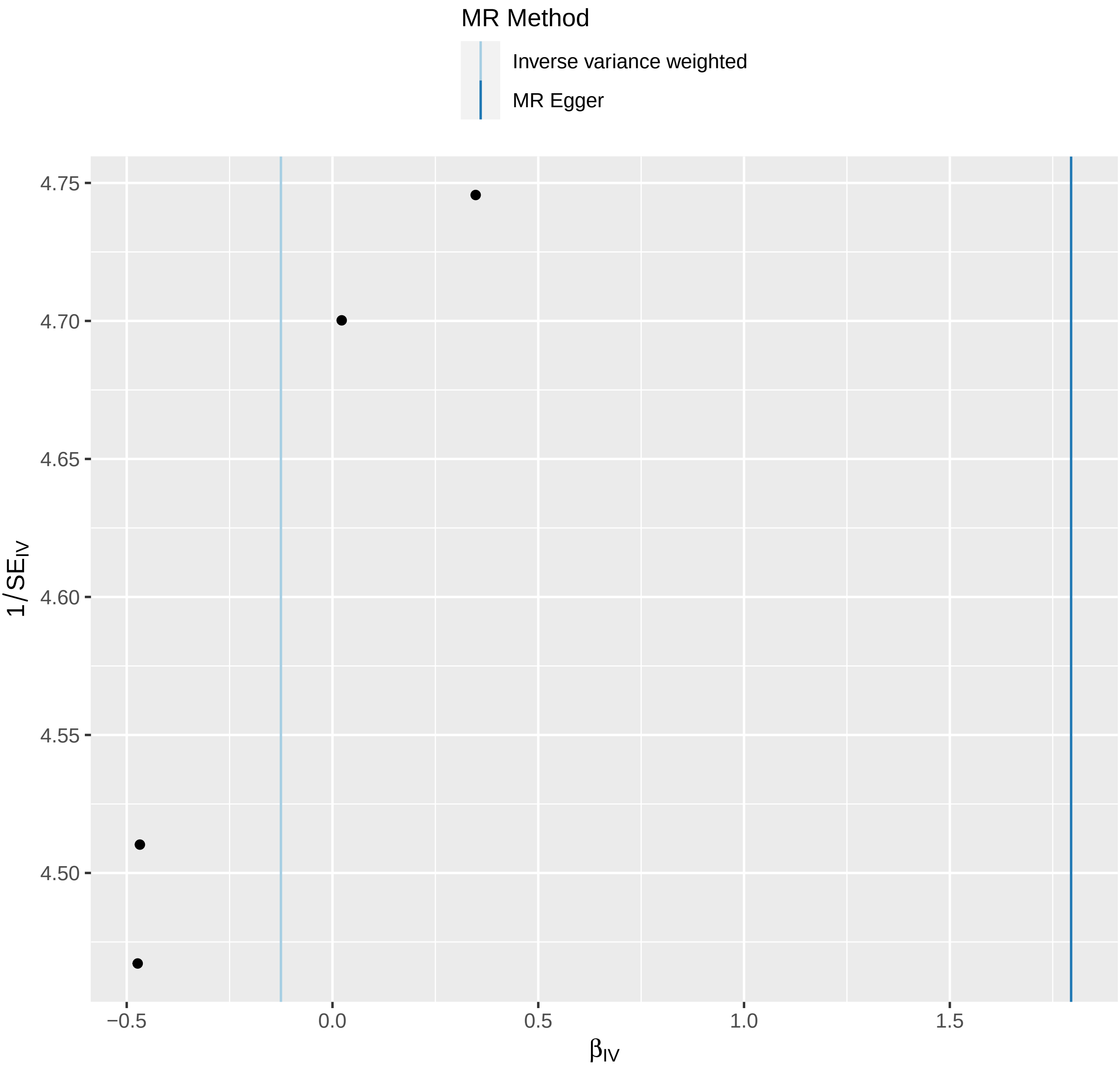

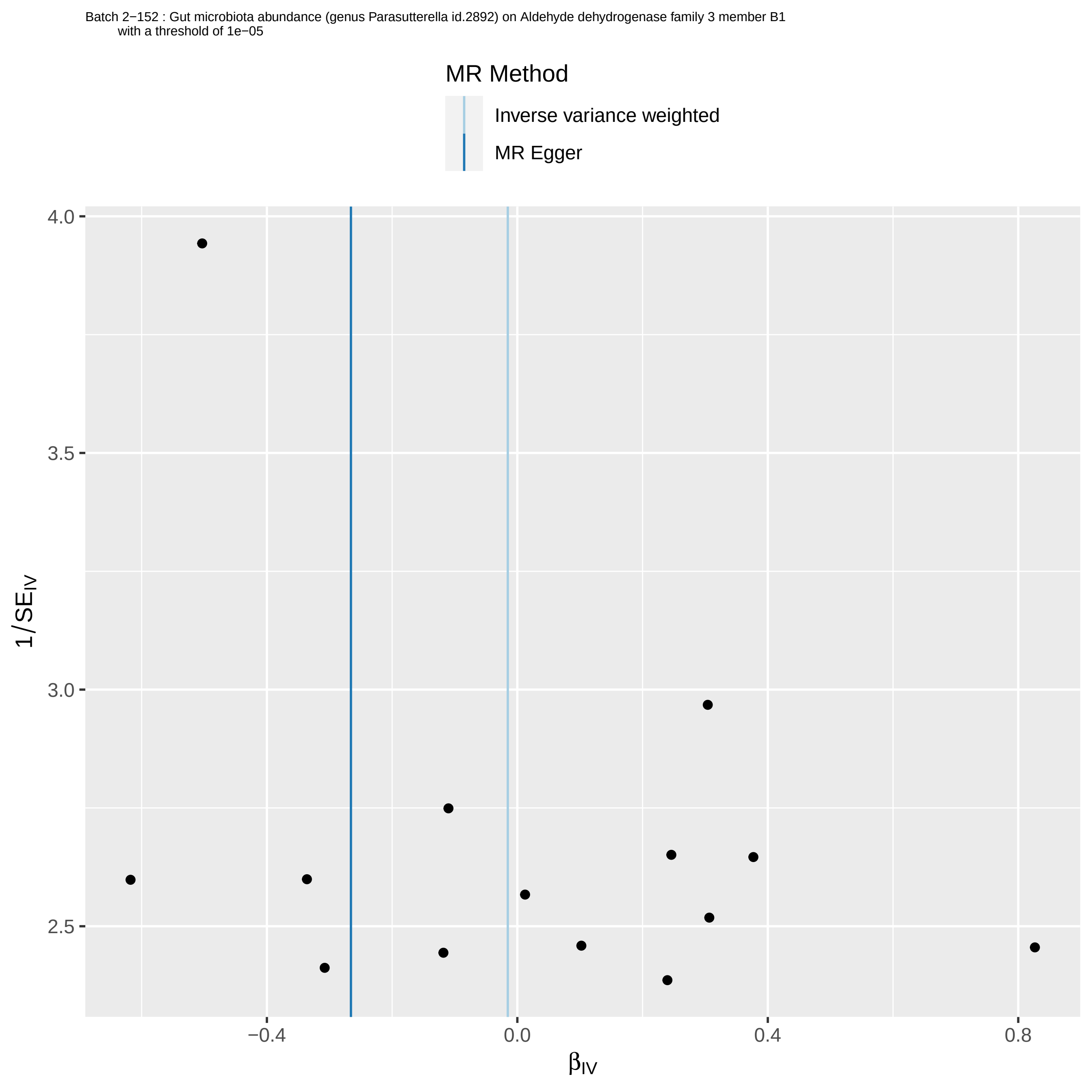
Batch 2−152 : Gut microbiota abundance (genus Parasutterella id.2892) on Aldehyde dehydrogenase family 3 member B1
with a threshold of 1e−05
MR Method
Inverse variance weighted
MR Egger
4.0
3.5
3.0
2.5
1/SE_IV
−0.4
0.0
0.4
0.8
β_IV

Batch 2−153 : Gut microbiota abundance (genus Catenibacterium id.2153) on Fructose−bisphosphate aldolase A with a threshold of 1e−05

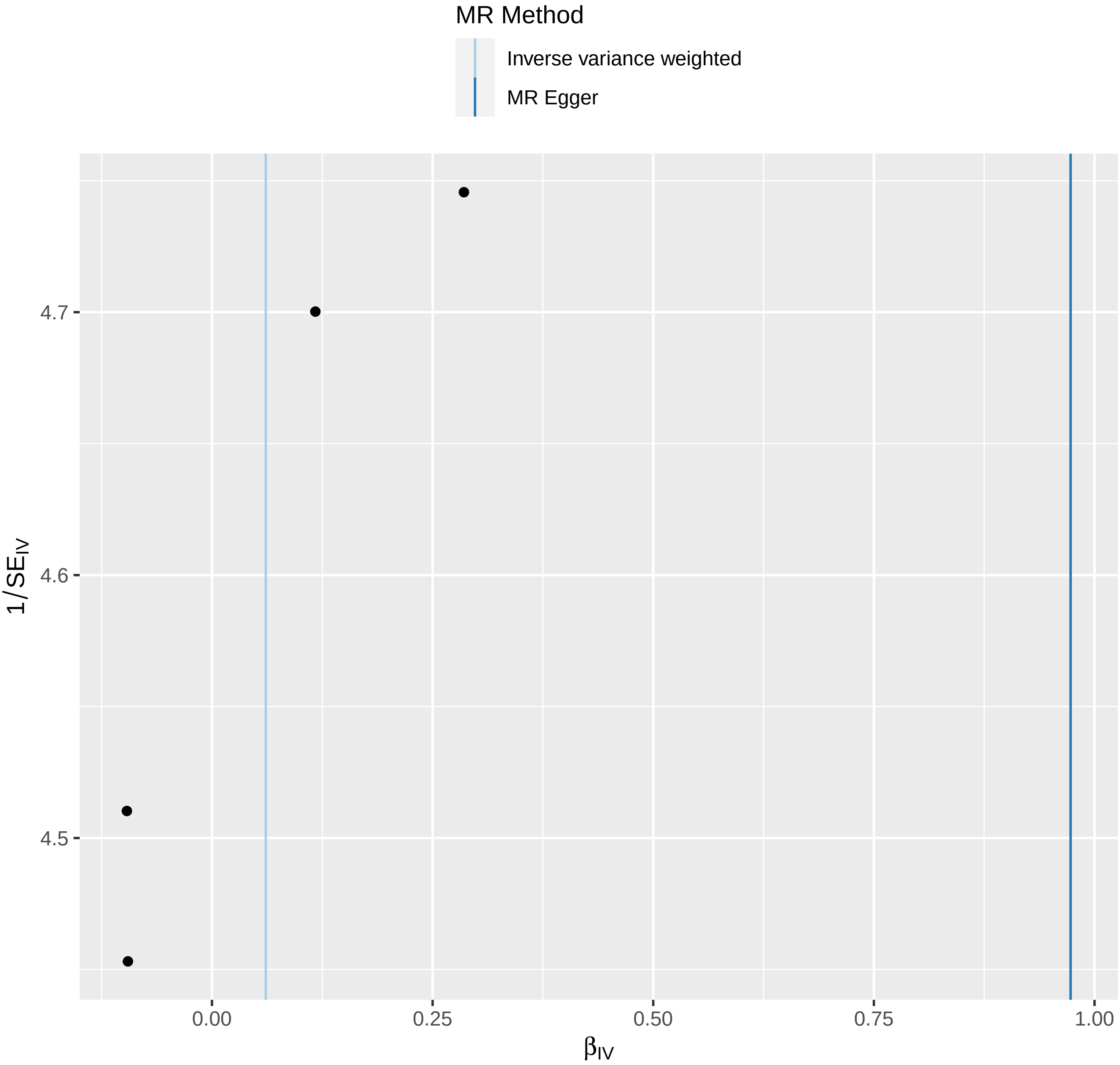

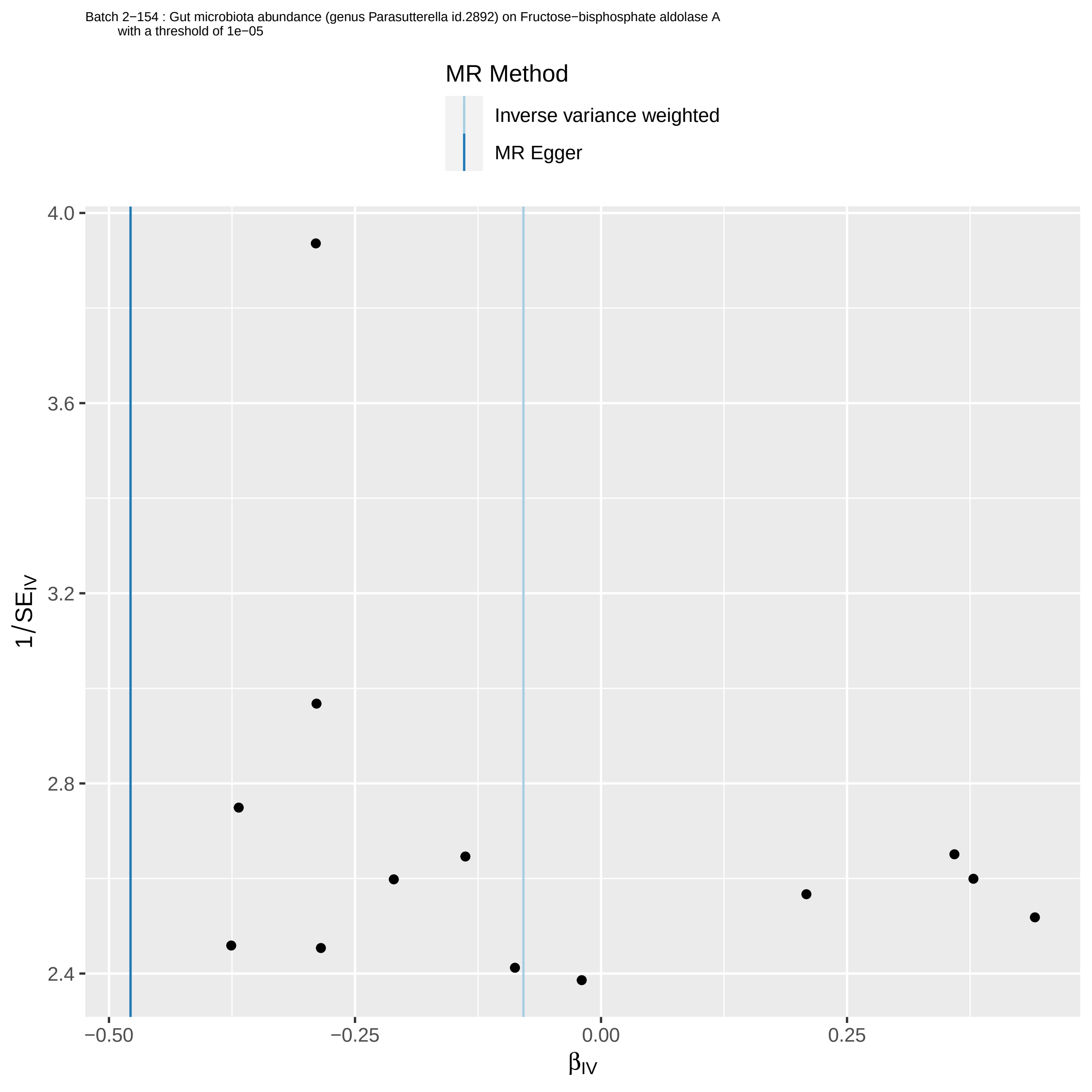

Batch 2−154 : Gut microbiota abundance (genus Parasutterella id.2892) on Fructose−bisphosphate aldolase A
with a threshold of 1e−05
MR Method
Inverse variance weighted
MR Egger
4.0
3.6
3.2
2.8
2.4
1/SE_IV
−0.50
−0.25
0.00
0.25
β_IV

Batch 2−155 : Gut microbiota abundance (genus Catenibacterium id.2153) on Fructose−bisphosphate aldolase C
with a threshold of 1e−05

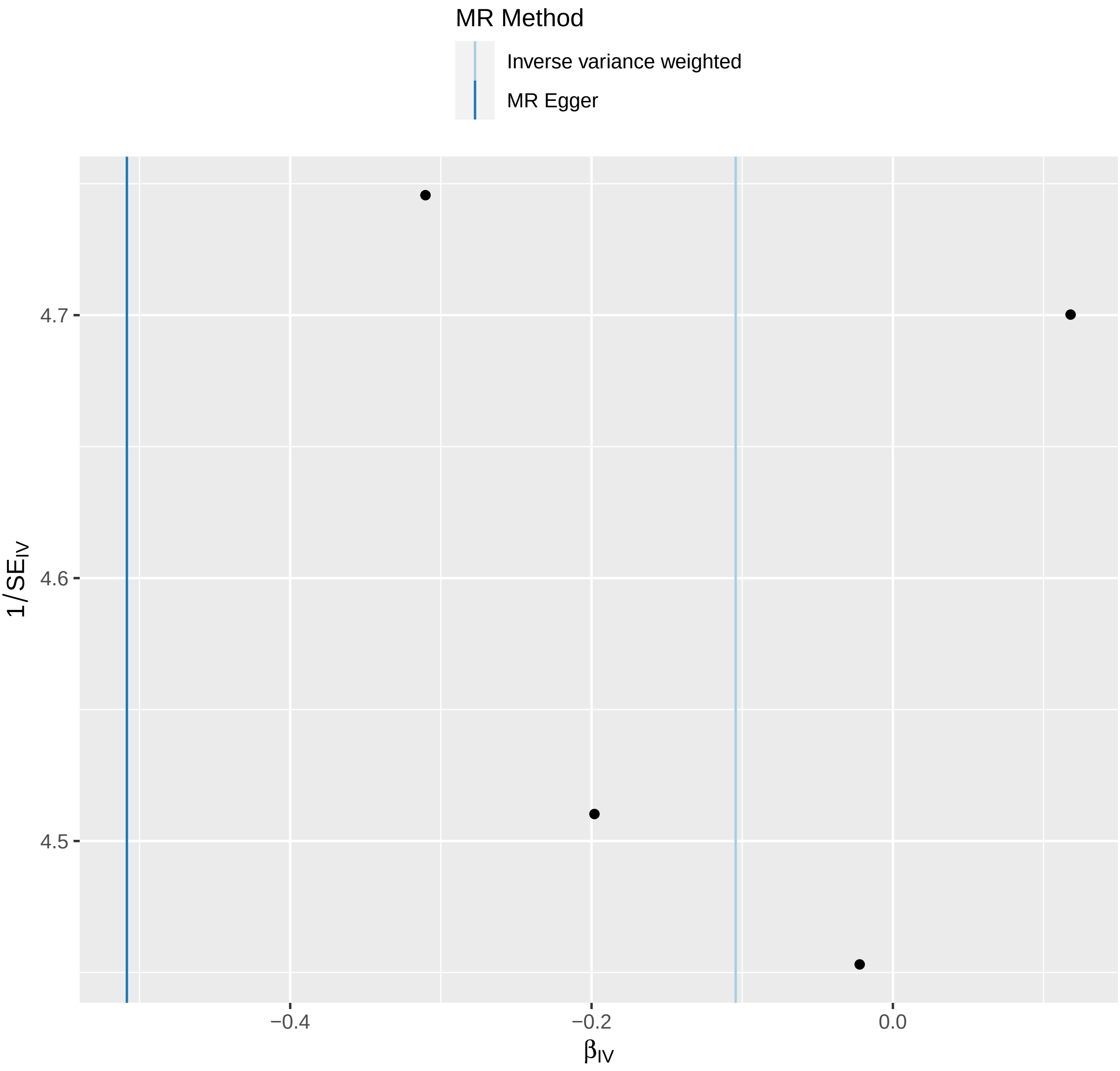

Batch 2−156 : Gut microbiota abundance (genus Parasutterella id.2892) on Fructose−bisphosphate aldolase C
with a threshold of 1e−05

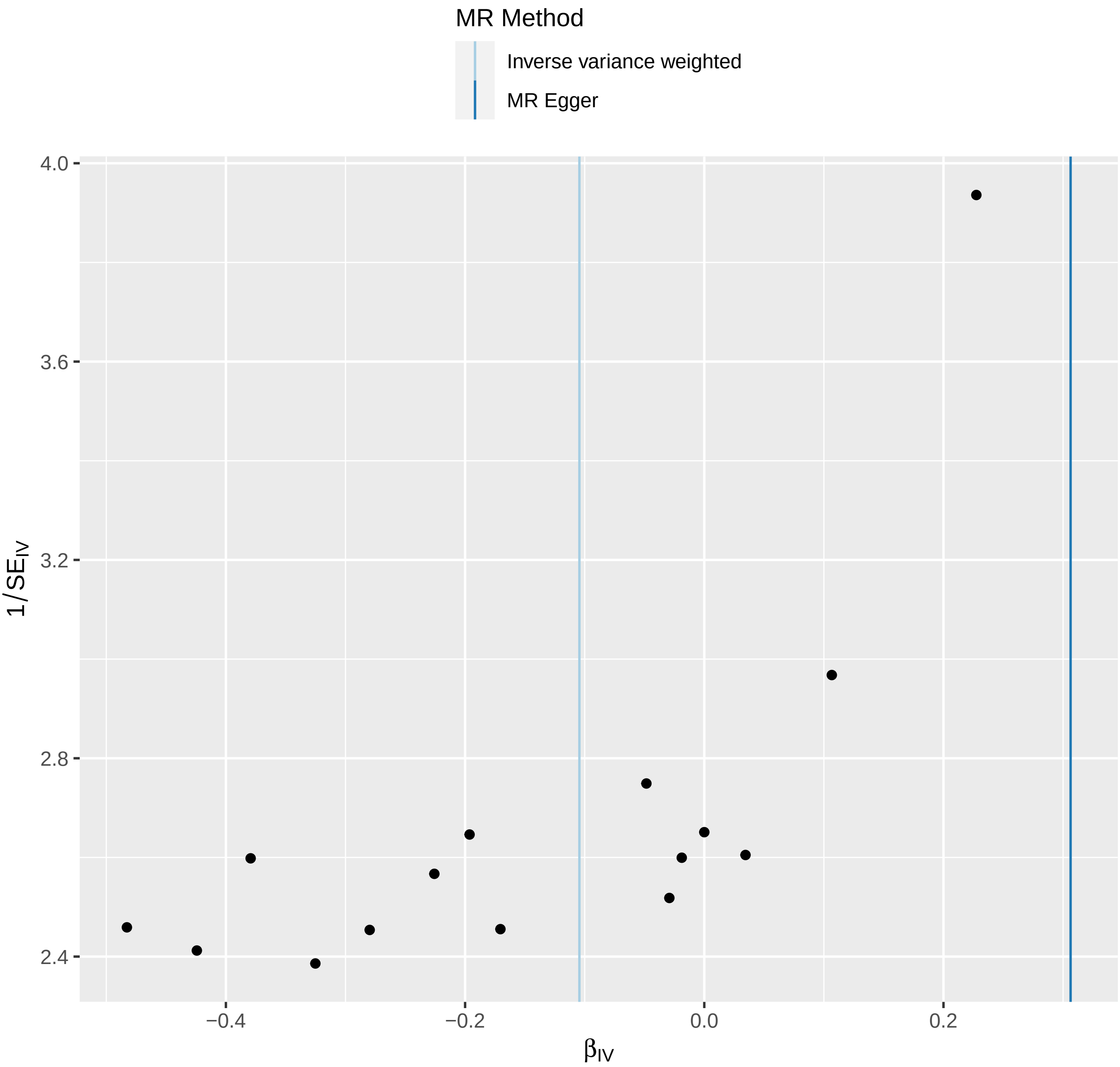

Batch 2−157 : Gut microbiota abundance (genus Catenibacterium id.2153) on RNA demethylase ALKBH5
with a threshold of 1e−05

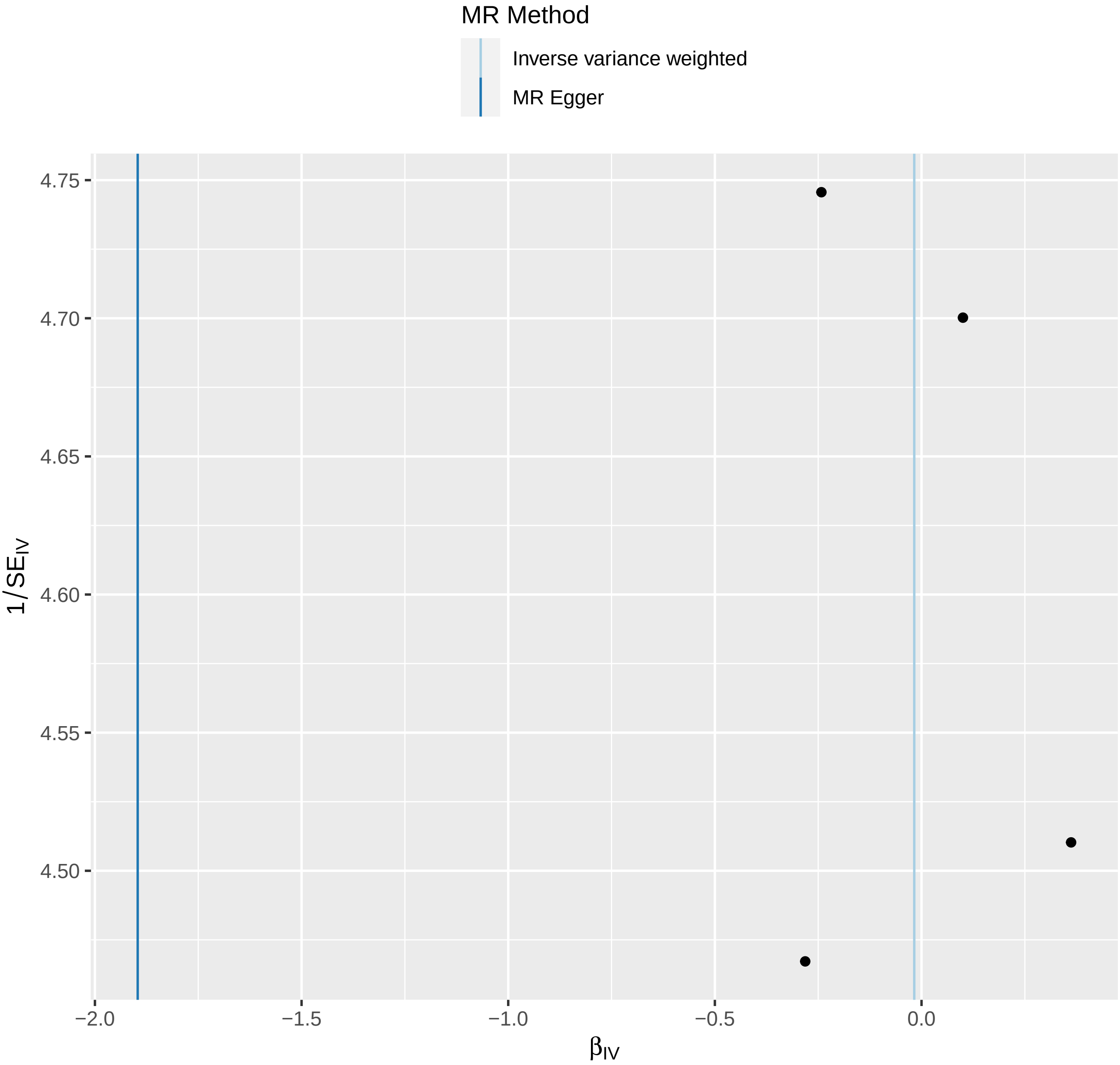

Batch 2−158 : Gut microbiota abundance (genus Parasutterella id.2892) on RNA demethylase ALKBH5
with a threshold of 1e−05

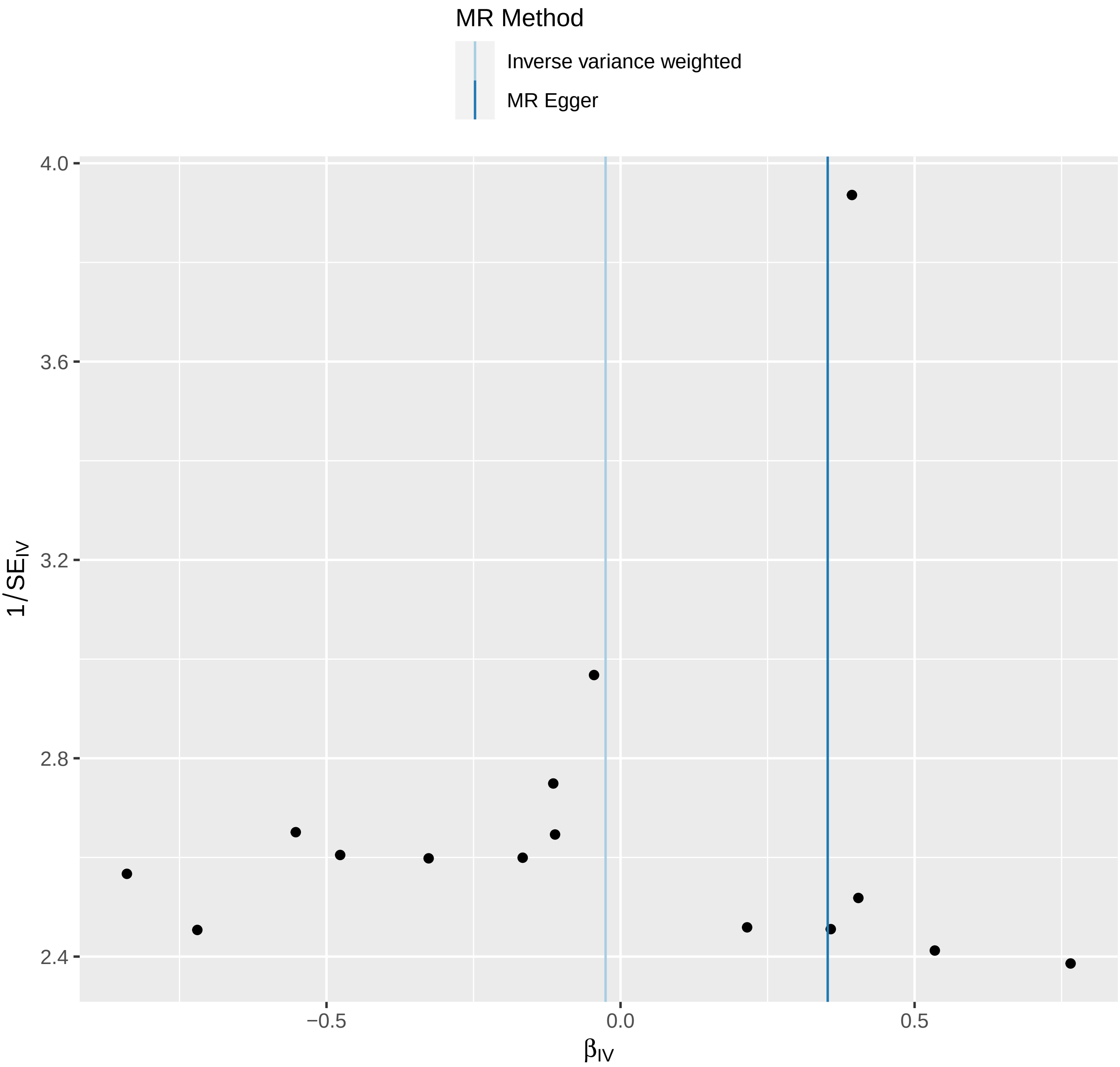

Batch 2−159 : Gut microbiota abundance (genus Catenibacterium id.2153) on Arachidonate 15−lipoxygenase B
with a threshold of 1e−05

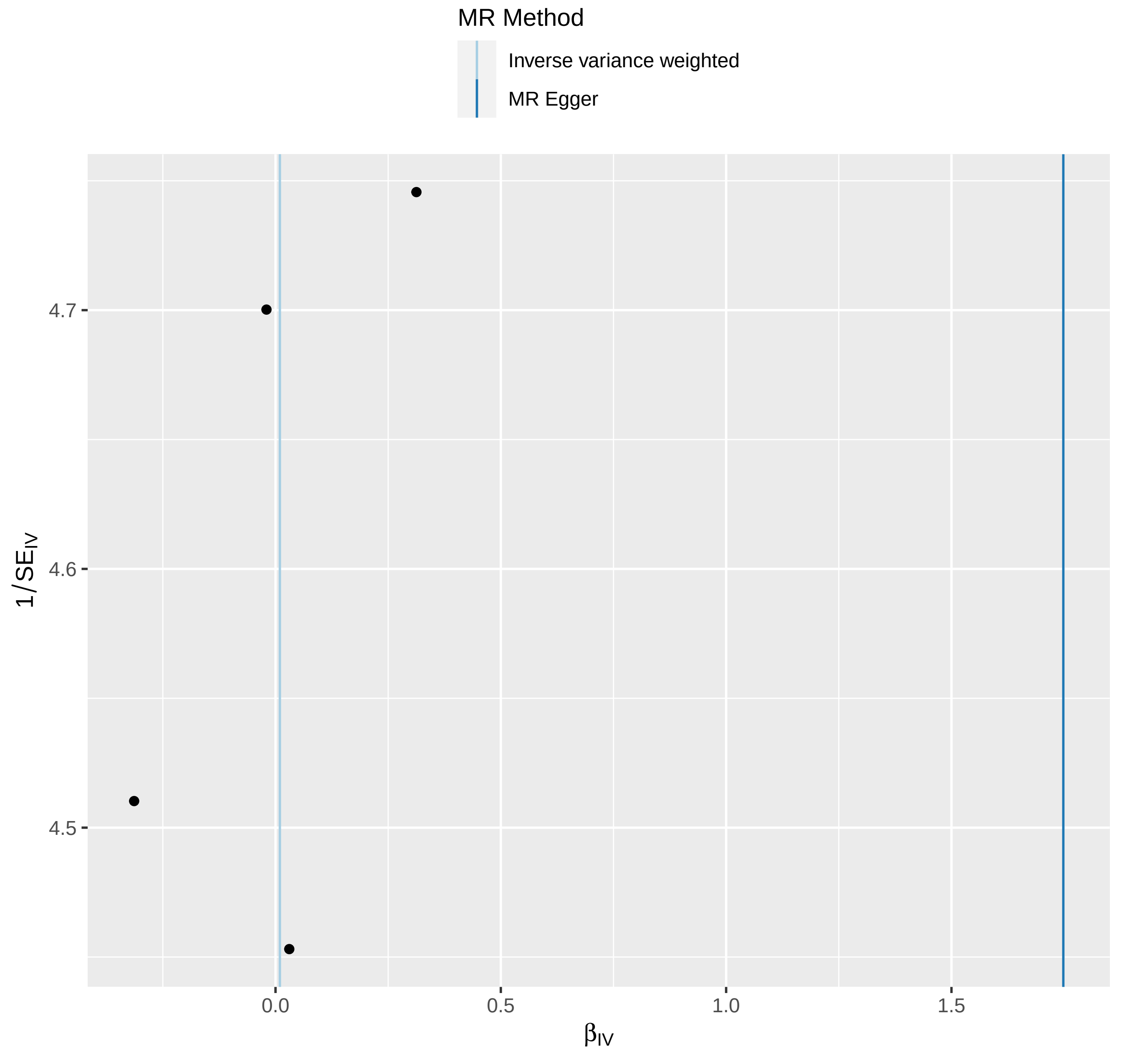

Batch 2−160 : Gut microbiota abundance (genus Parasutterella id.2892) on Arachidonate 15−lipoxygenase B
with a threshold of 1e−05

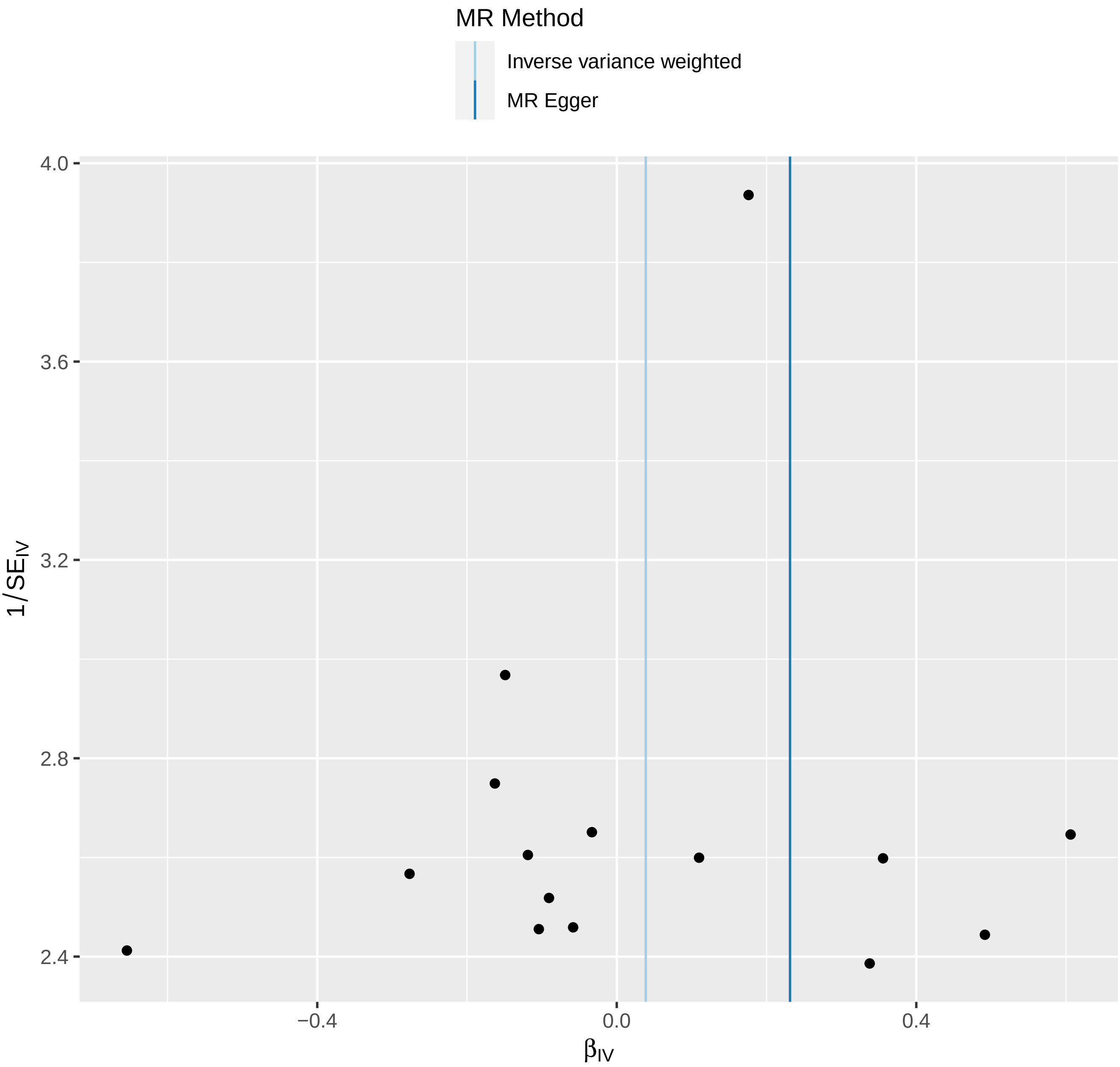

Batch 2−161 : Gut microbiota abundance (genus Catenibacterium id.2153) on Intestinal−type alkaline phosphatase
with a threshold of 1e−05

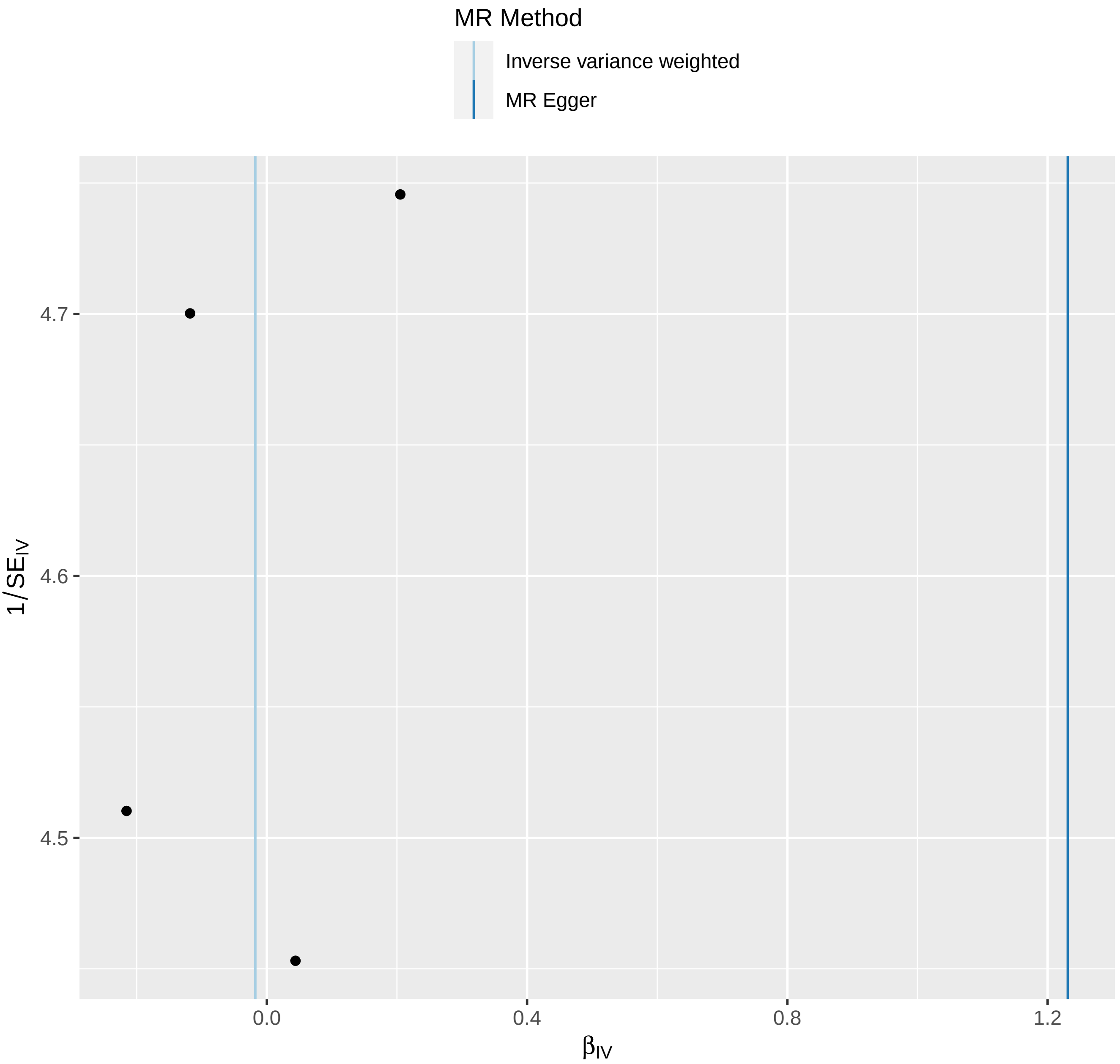

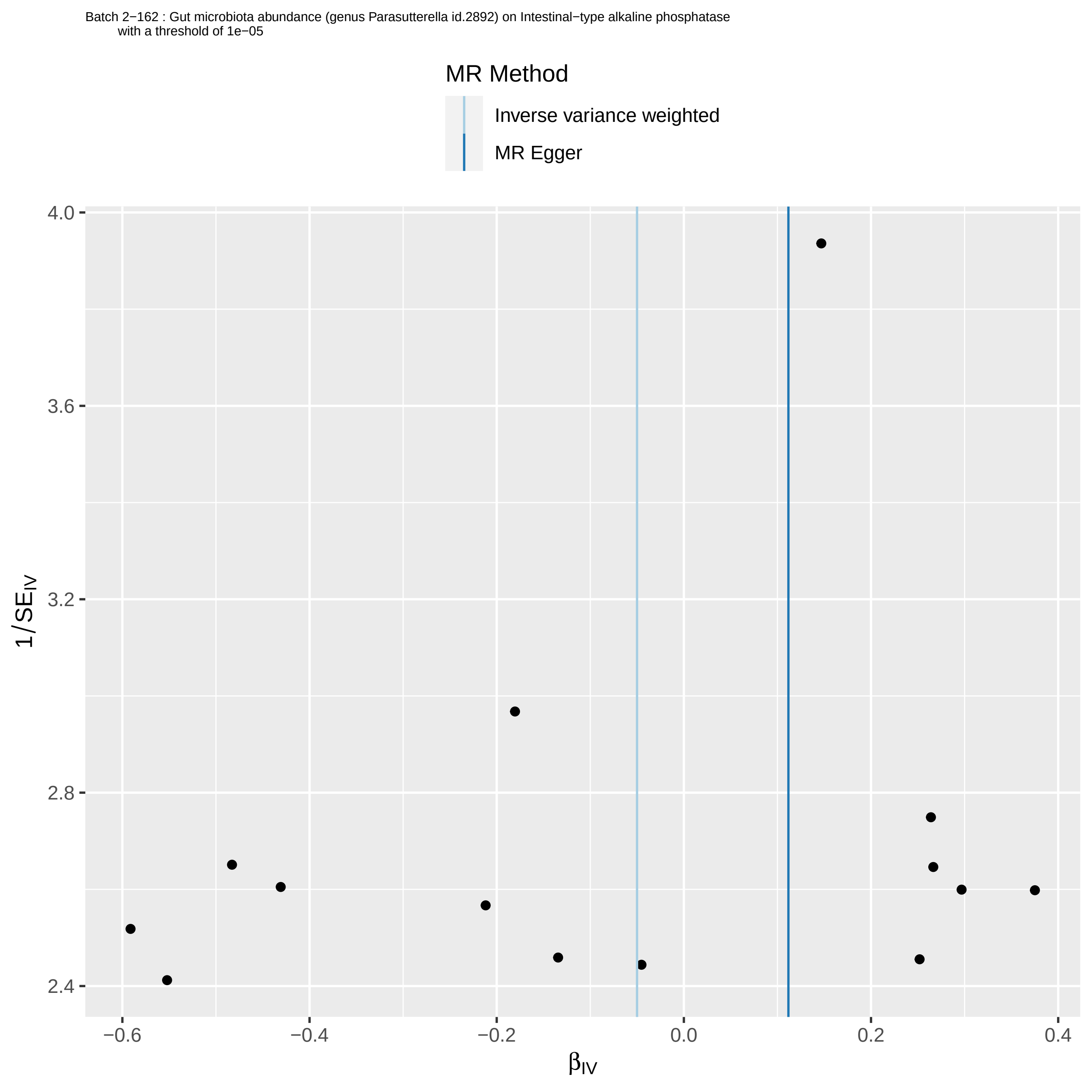

Batch 2−162 : Gut microbiota abundance (genus Parasutterella id.2892) on Intestinal−type alkaline phosphatase
with a threshold of 1e−05
MR Method
Inverse variance weighted
MR Egger
4.0
3.6
3.2
2.8
2.4
1/SE_IV
−0.6
−0.4
−0.2
0.0
0.2
0.4
β_IV

Batch 2−163 : Gut microbiota abundance (genus Catenibacterium id.2153) on Ameloblastin
with a threshold of 1e−05

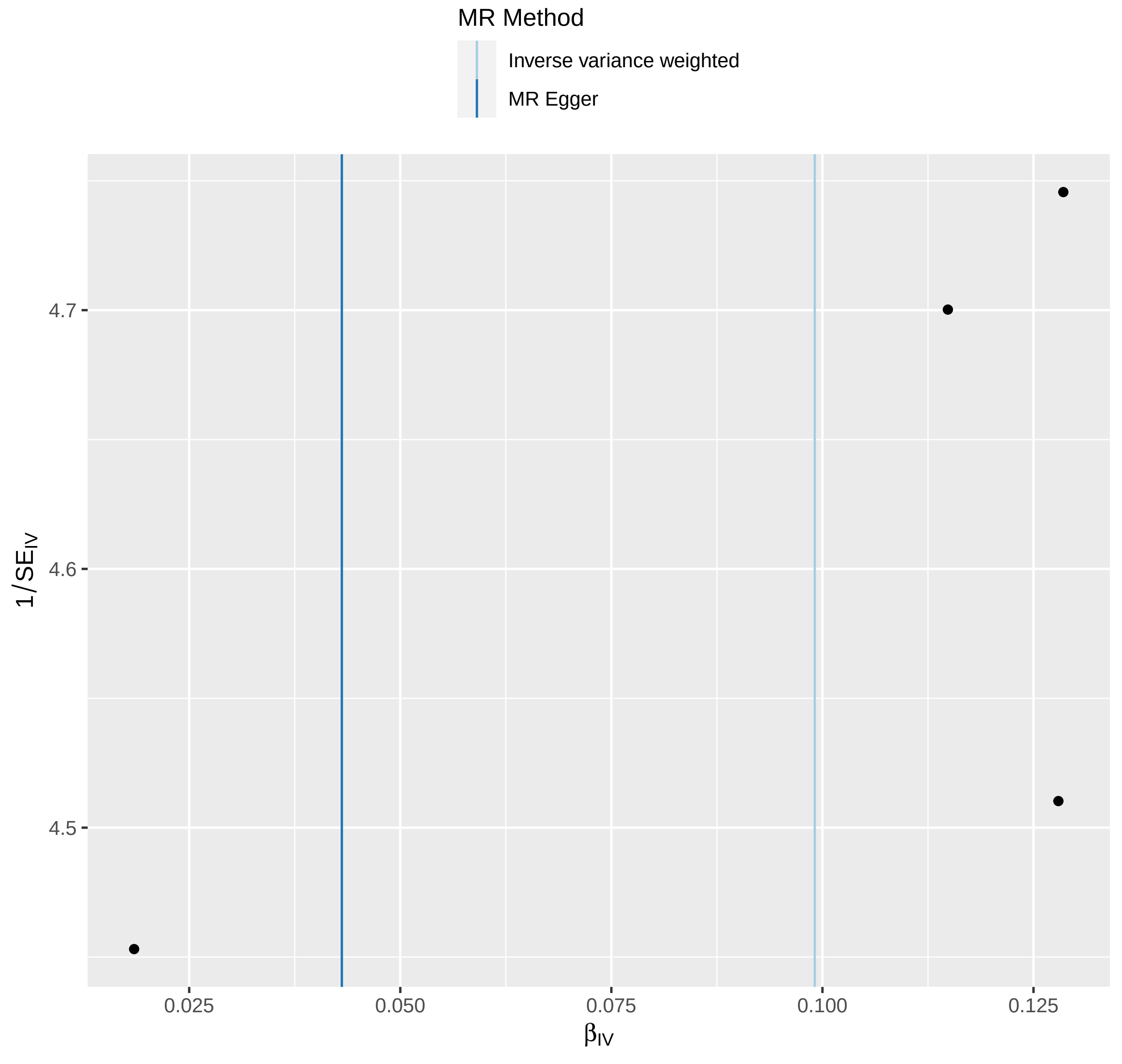

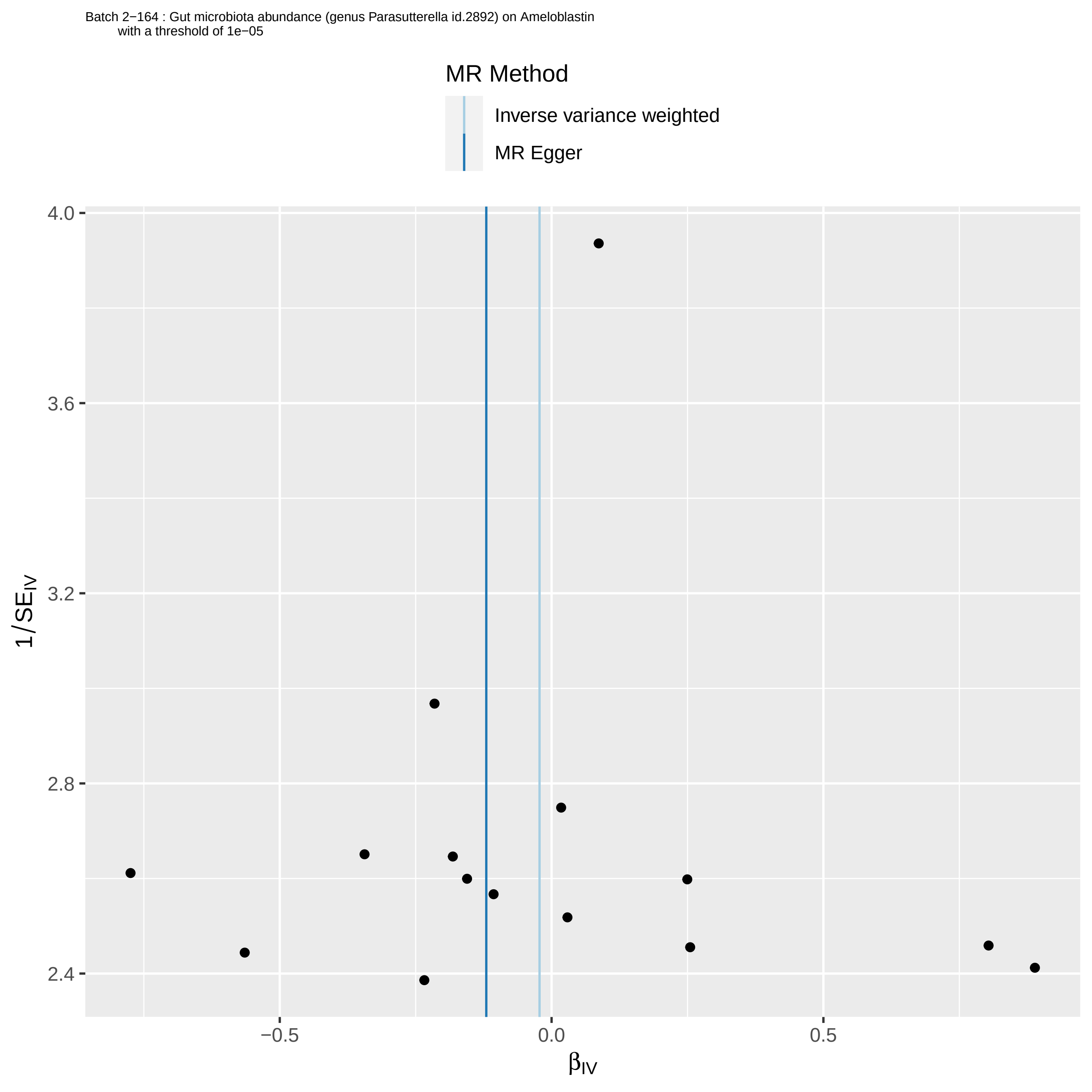

Batch 2−164 : Gut microbiota abundance (genus Parasutterella id.2892) on Ameloblastin
with a threshold of 1e−05
MR Method
Inverse variance weighted
MR Egger
4.0
3.6
3.2
2.8
2.4
1/SE_IV
−0.5
0.0
0.5
β_IV

Batch 2−165 : Gut microbiota abundance (genus Catenibacterium id.2153) on Amelogenin, X isoform
with a threshold of 1e−05

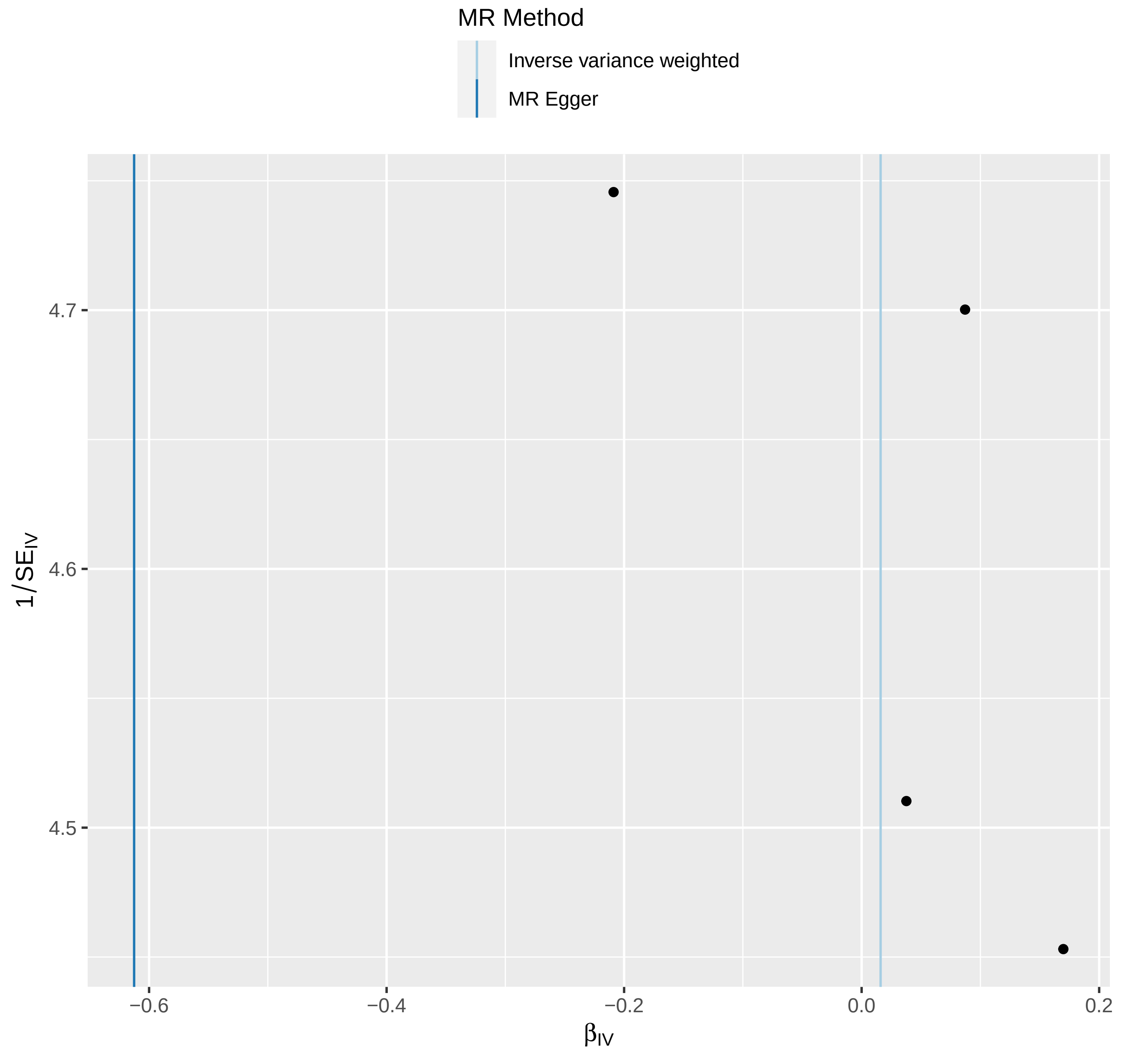

Batch 2−166 : Gut microbiota abundance (genus Parasutterella id.2892) on Amelogenin, X isoform
with a threshold of 1e−05

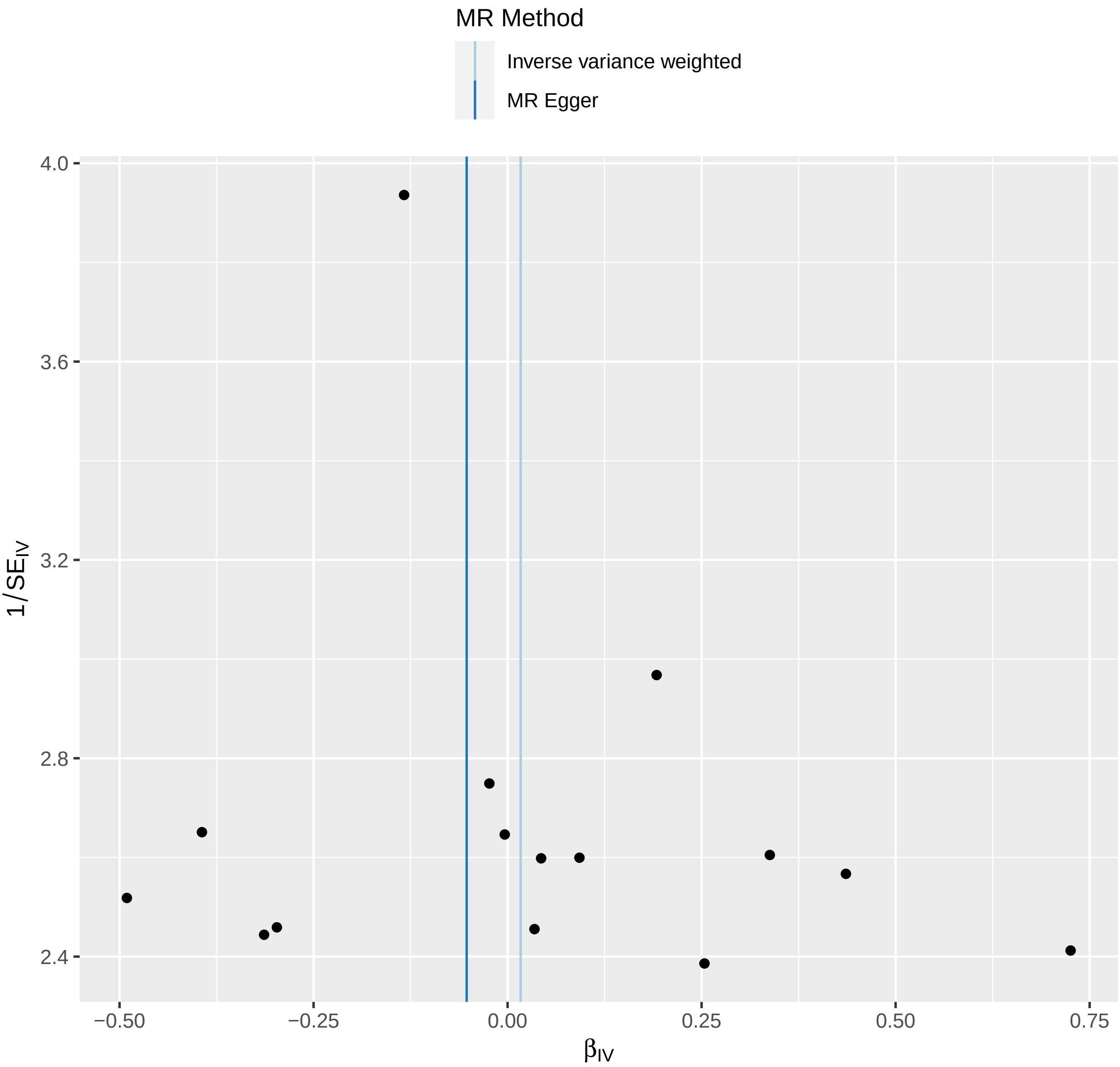

Batch 2−167 : Gut microbiota abundance (genus Catenibacterium id.2153) on Muellerian−inhibiting factor
with a threshold of 1e−05

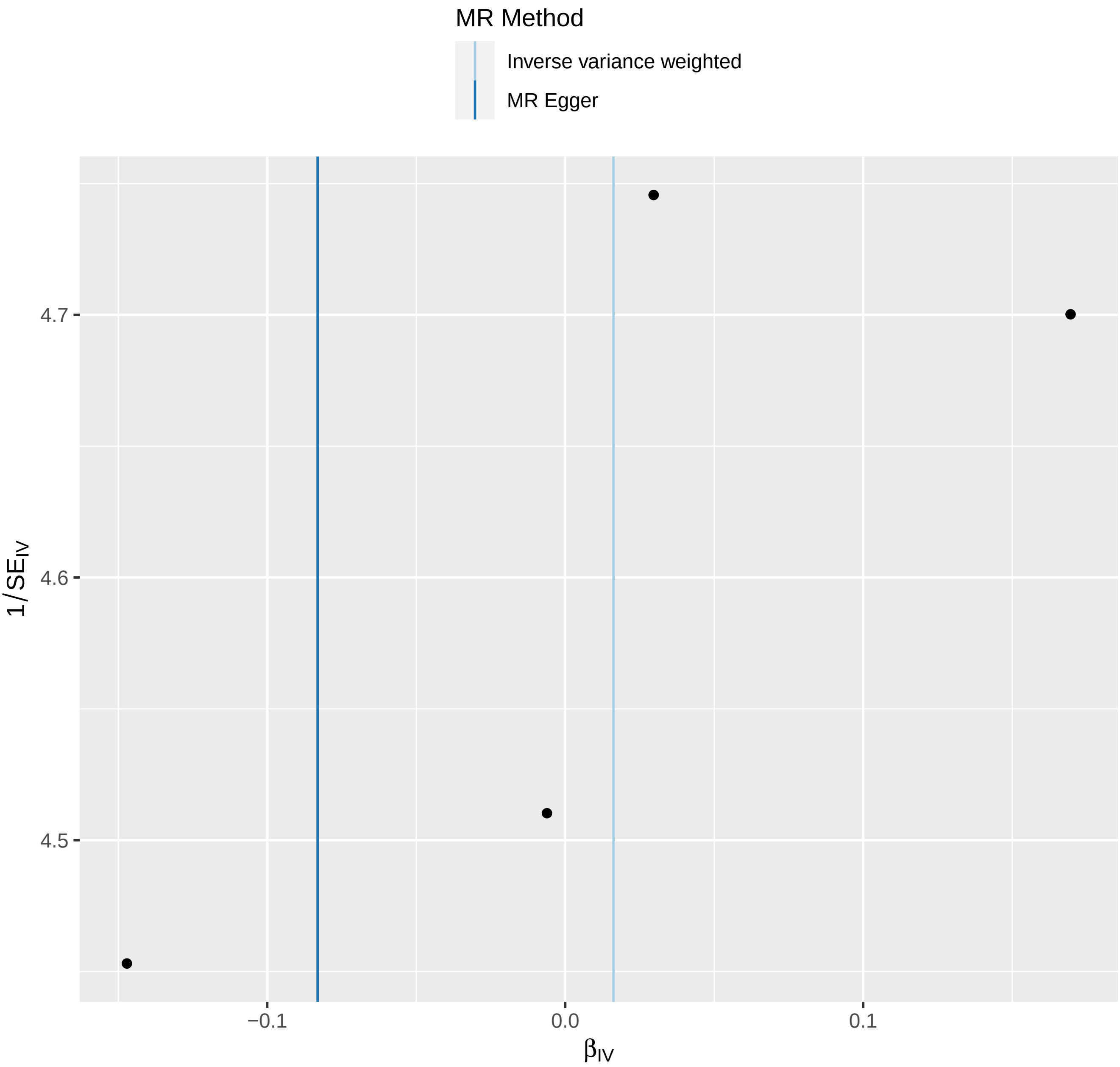

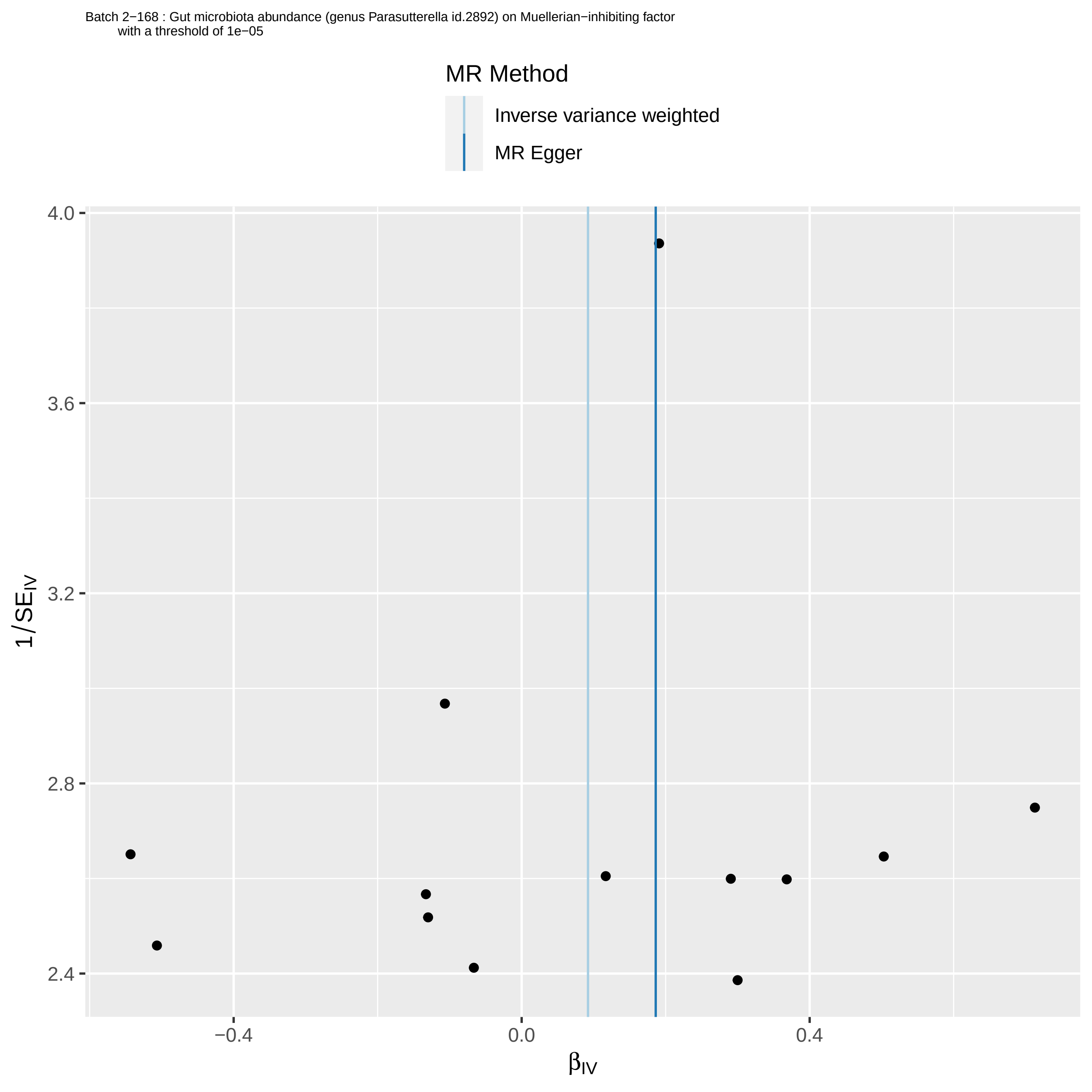

Batch 2−168 : Gut microbiota abundance (genus Parasutterella id.2892) on Muellerian−inhibiting factor
with a threshold of 1e−05
MR Method
Inverse variance weighted
MR Egger
4.0
3.6
3.2
2.8
2.4
1/SE_IV
−0.4
0.0
0.4
β_IV

Batch 2−169 : Gut microbiota abundance (genus Catenibacterium id.2153) on Junctional adhesion molecule−like
with a threshold of 1e−05

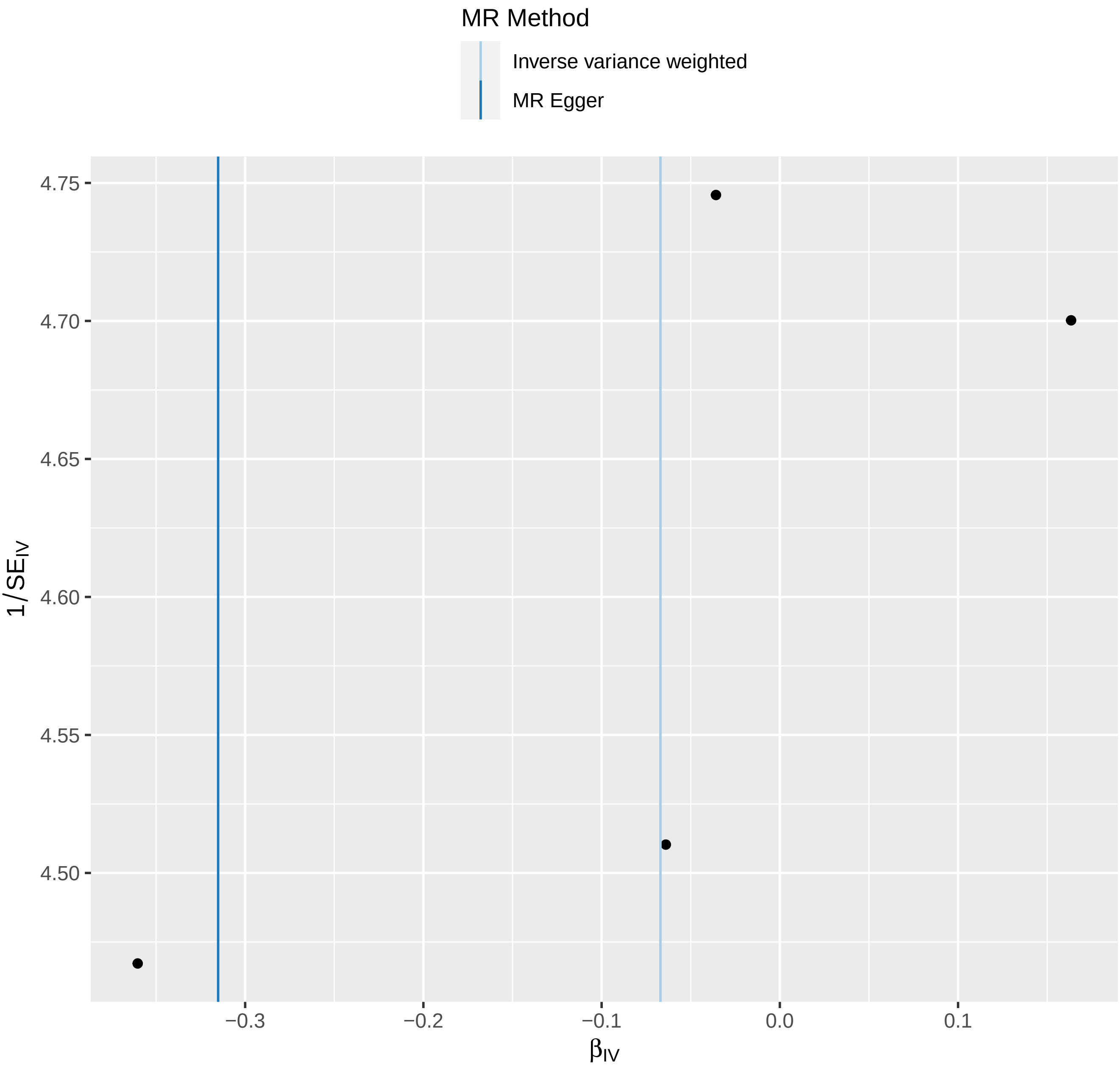

Batch 2−170 : Gut microbiota abundance (genus Parasutterella id.2892) on Junctional adhesion molecule−like with a threshold of 1e−05

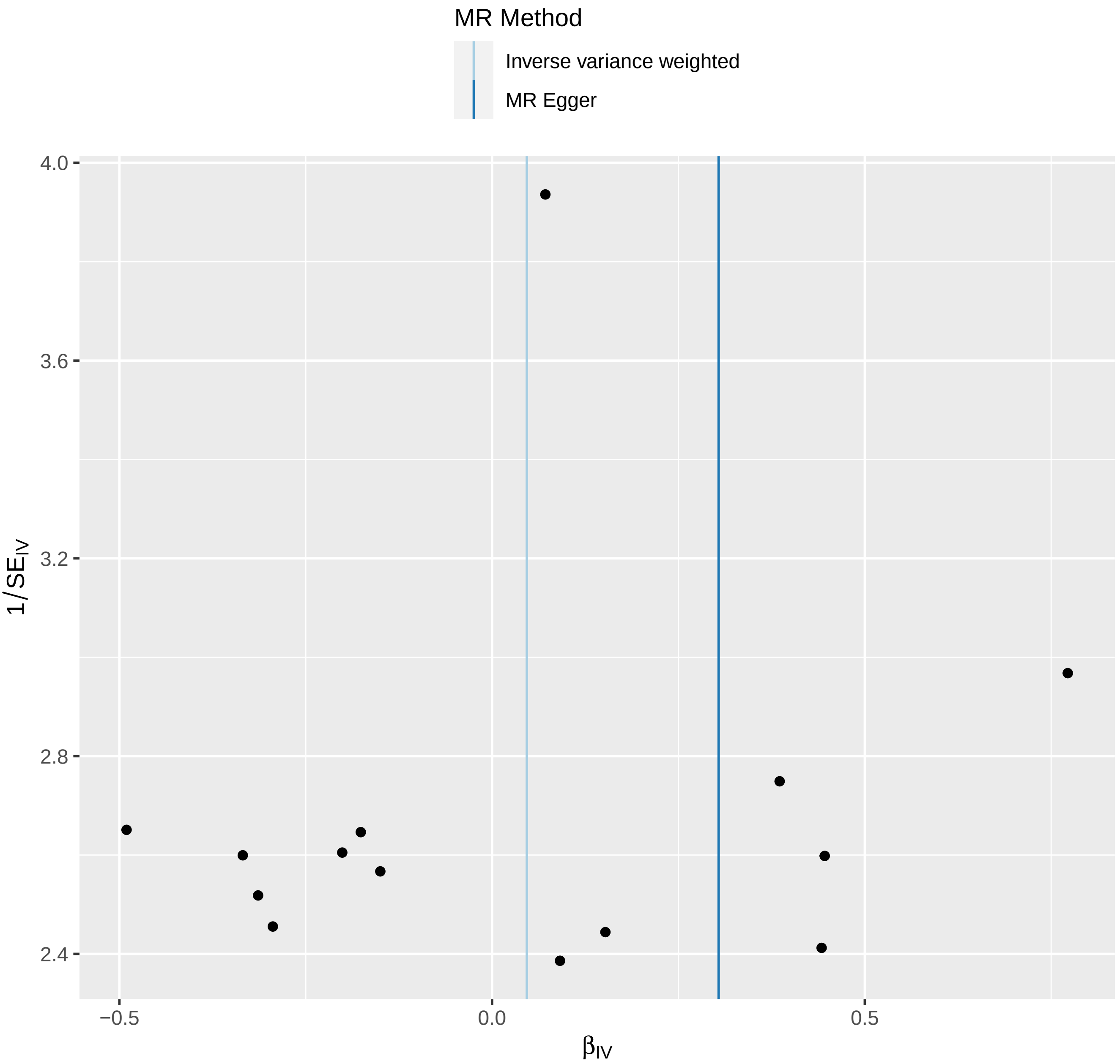

Batch 2−171 : Gut microbiota abundance (genus Catenibacterium id.2153) on Junctional adhesion molecule−like with a threshold of 1e−05

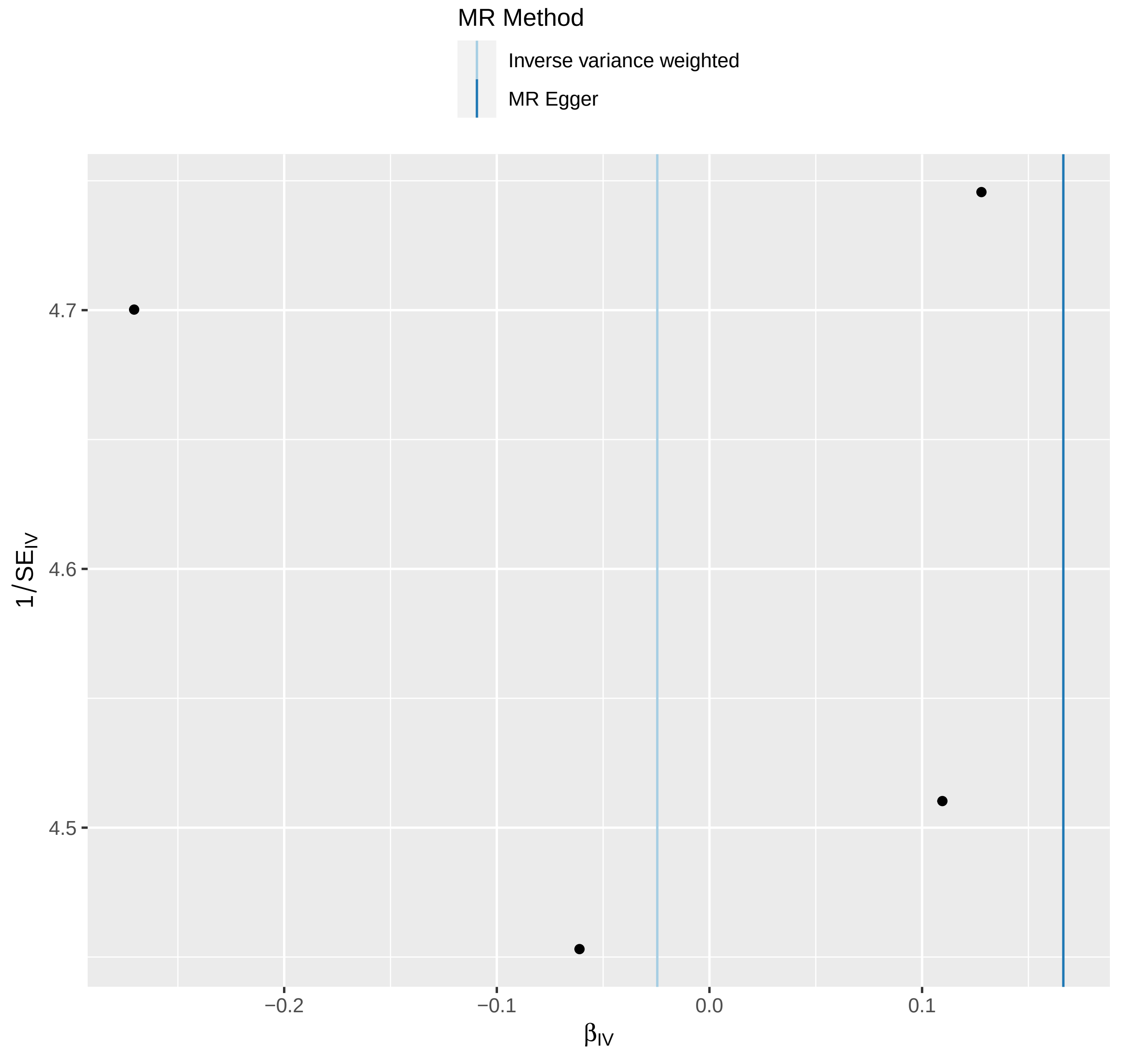

Batch 2−172 : Gut microbiota abundance (genus Parasutterella id.2892) on Junctional adhesion molecule−like
with a threshold of 1e−05

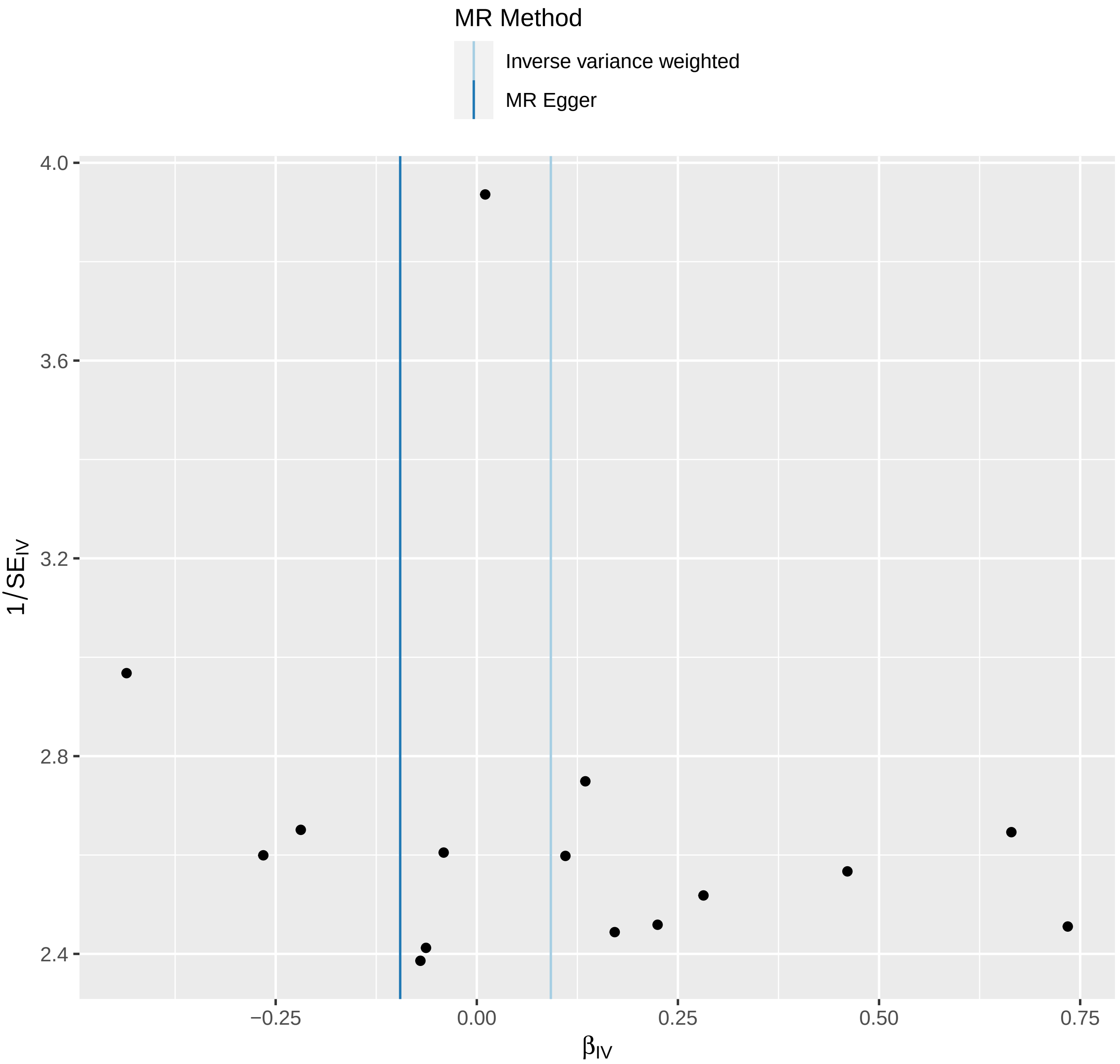

Batch 2−173 : Gut microbiota abundance (genus Catenibacterium id.2153) on Amphoterin−induced protein 1 with a threshold of 1e−05

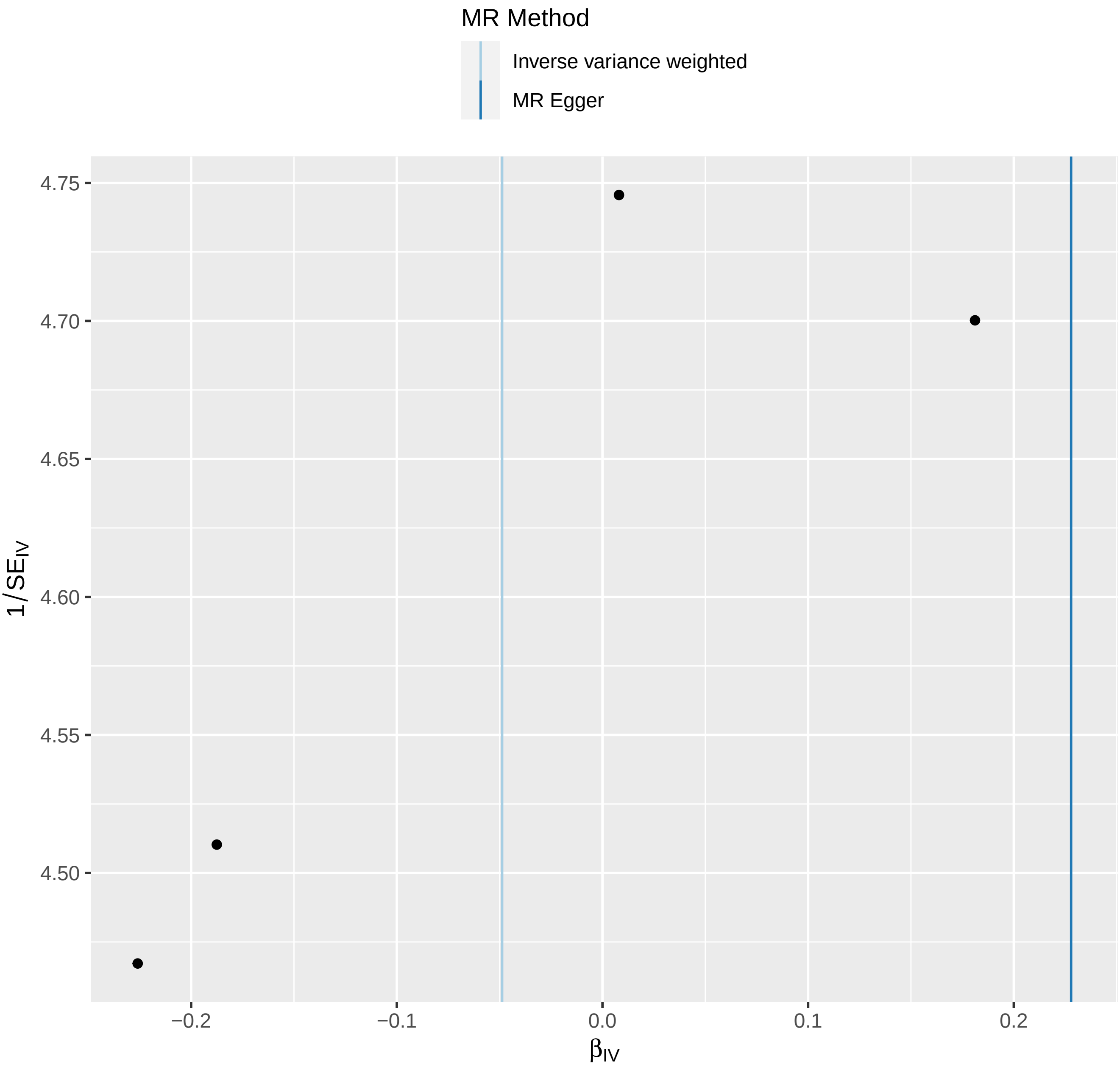

Batch 2−174 : Gut microbiota abundance (genus Parasutterella id.2892) on Amphoterin−induced protein 1
with a threshold of 1e−05

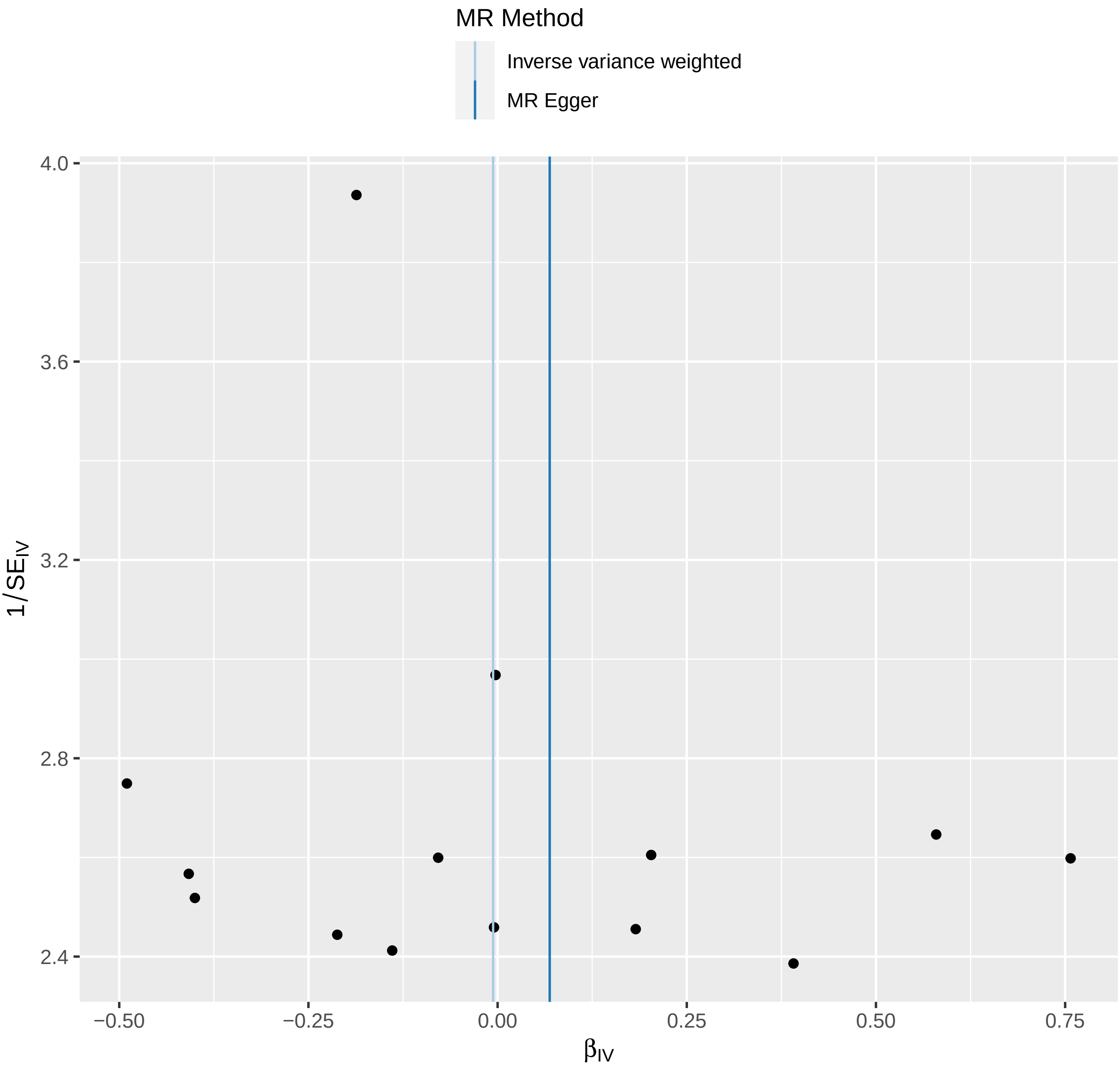

Batch 2−175 : Gut microbiota abundance (genus Catenibacterium id.2153) on Protein amnionless
with a threshold of 1e−05

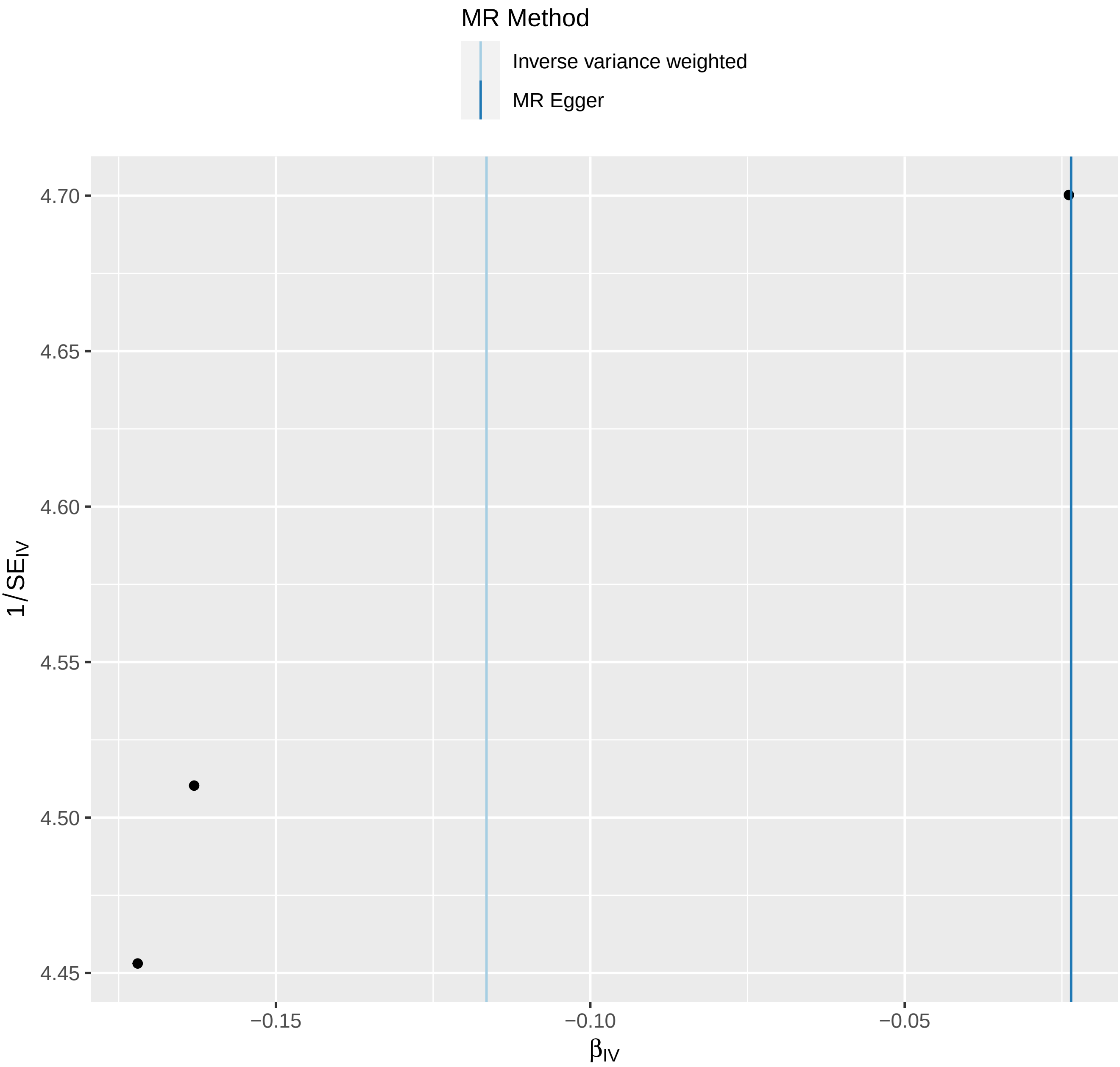

Batch 2−176 : Gut microbiota abundance (genus Parasutterella id.2892) on Protein amnionless
with a threshold of 1e−05

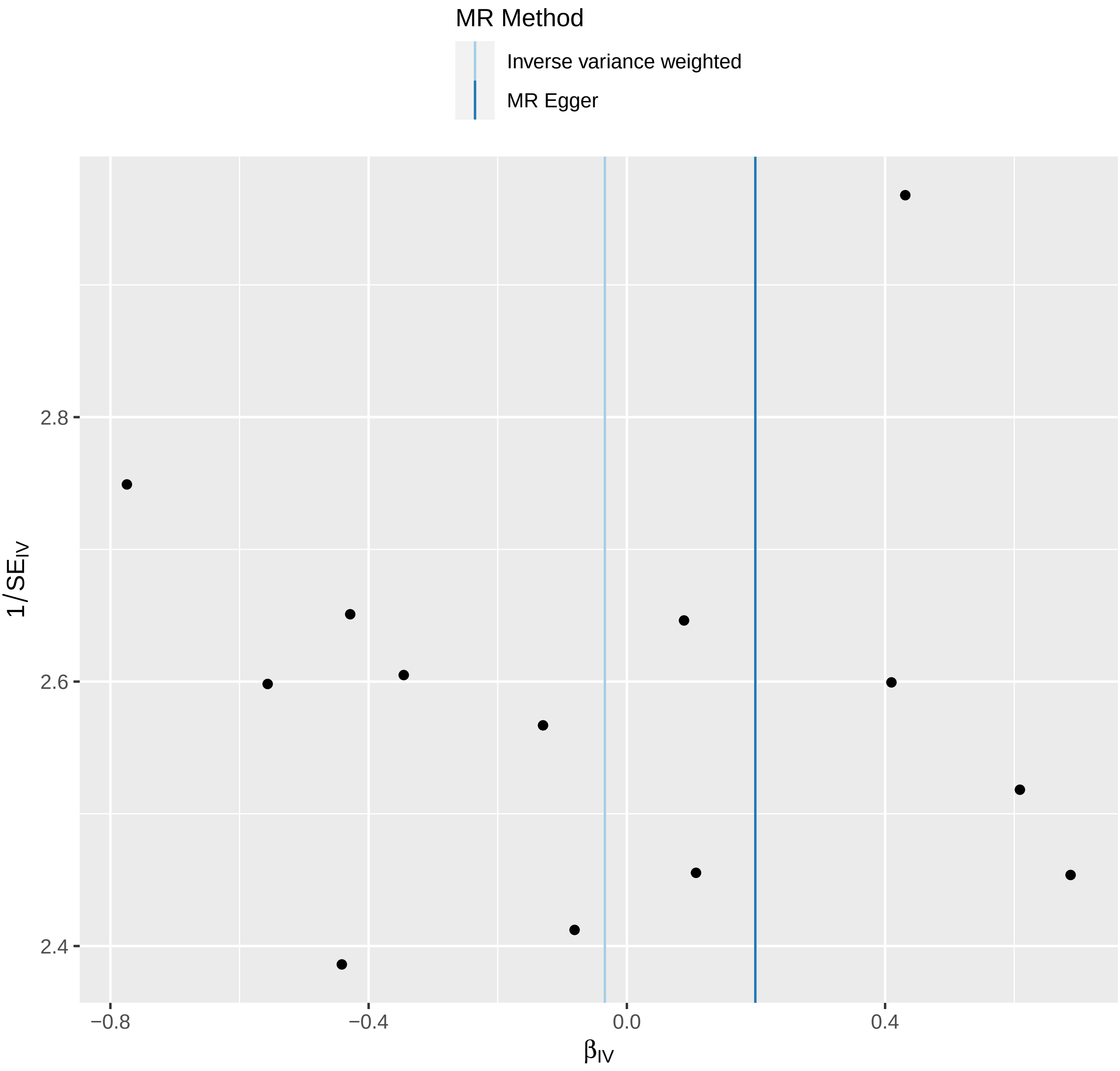

Batch 2−177 : Gut microbiota abundance (genus Catenibacterium id.2153) on Alpha−amylase 1
with a threshold of 1e−05

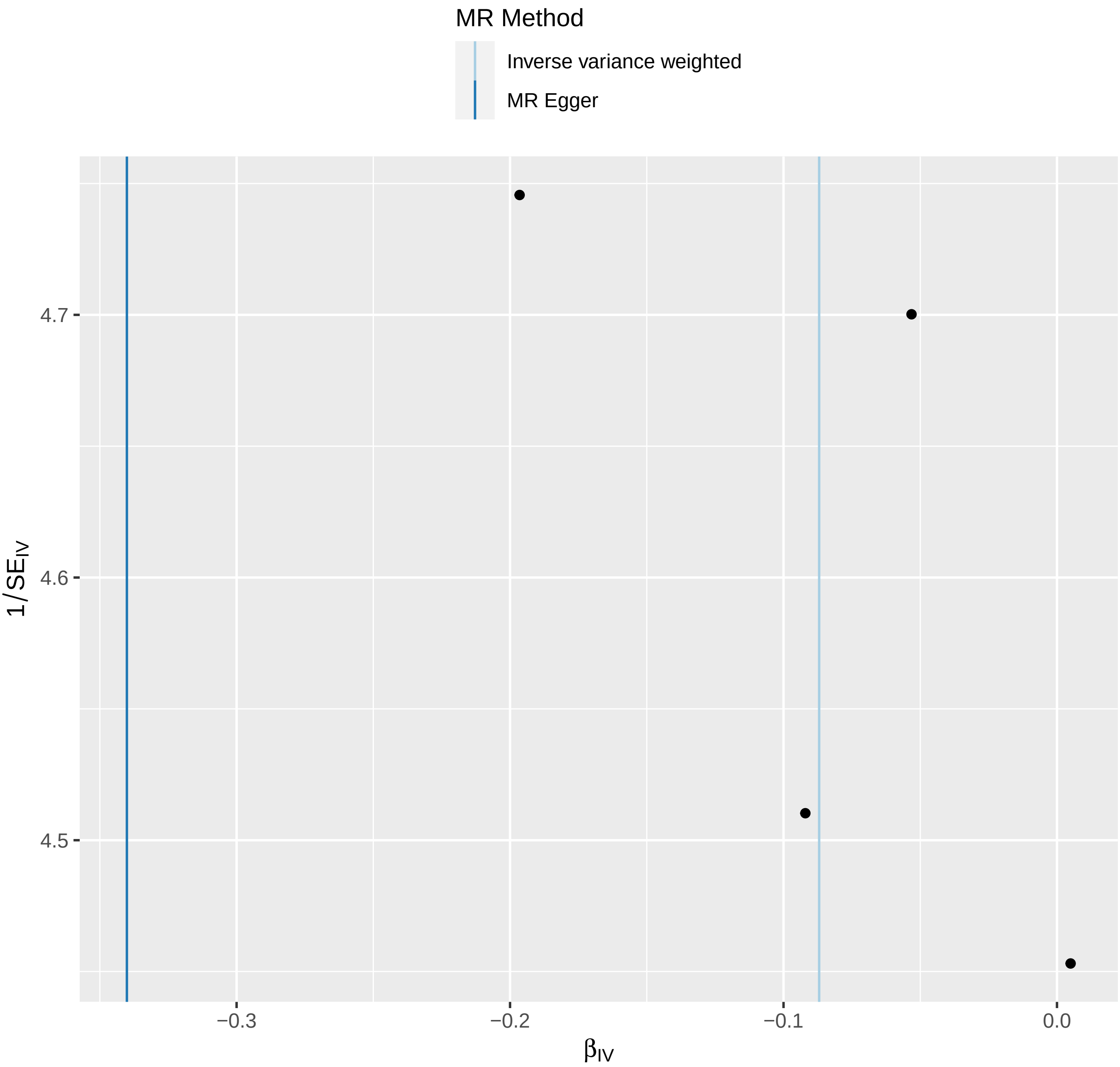

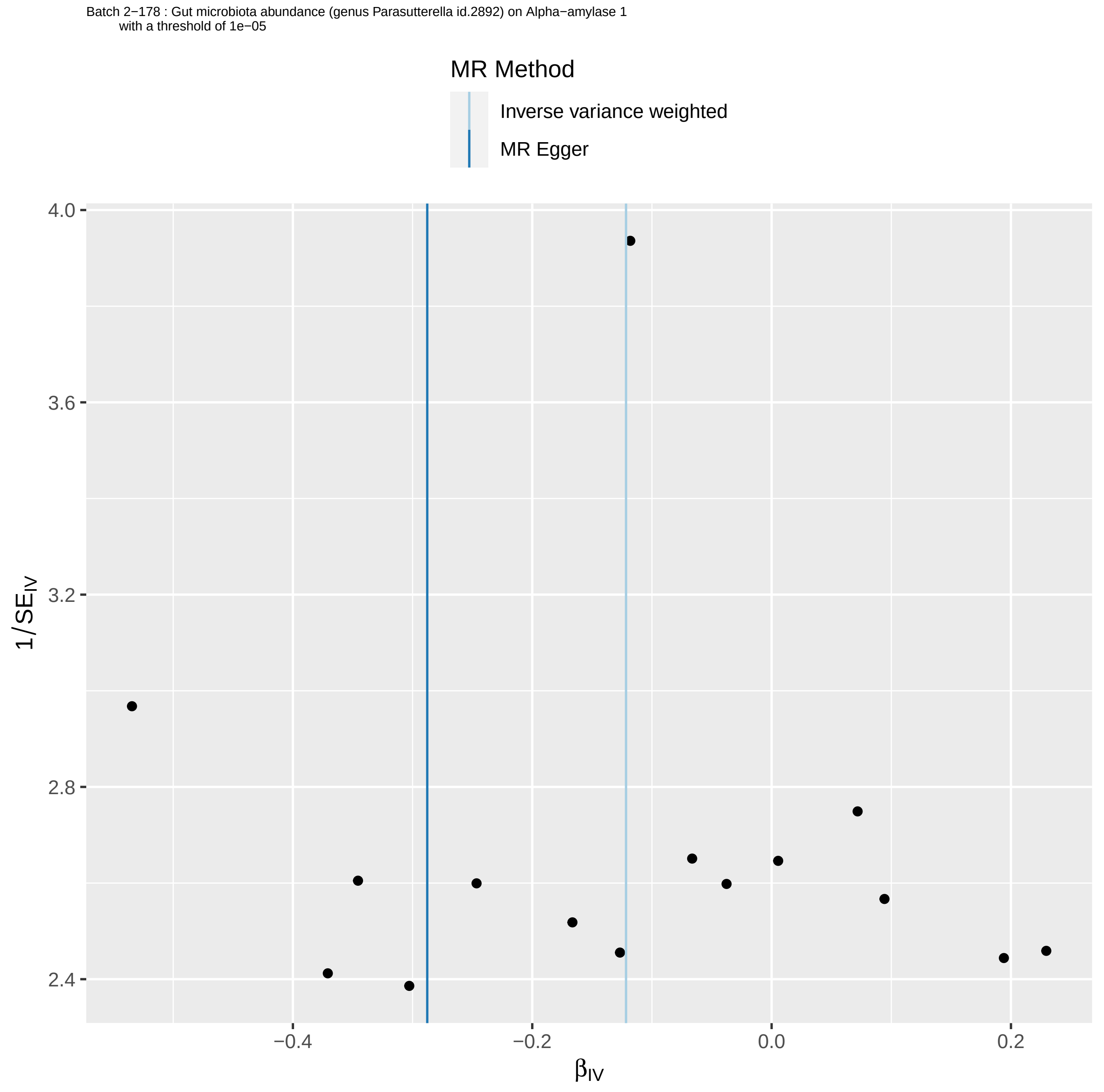
Batch 2−178 : Gut microbiota abundance (genus Parasutterella id.2892) on Alpha−amylase 1
with a threshold of 1e−05
MR Method
Inverse variance weighted
MR Egger
4.0
3.6
3.2
2.8
2.4
1/SE_IV
−0.4
−0.2
0.0
0.2
β_IV

Batch 2−179 : Gut microbiota abundance (genus Catenibacterium id.2153) on Anaphase−promoting complex subunit 10 with a threshold of 1e−05

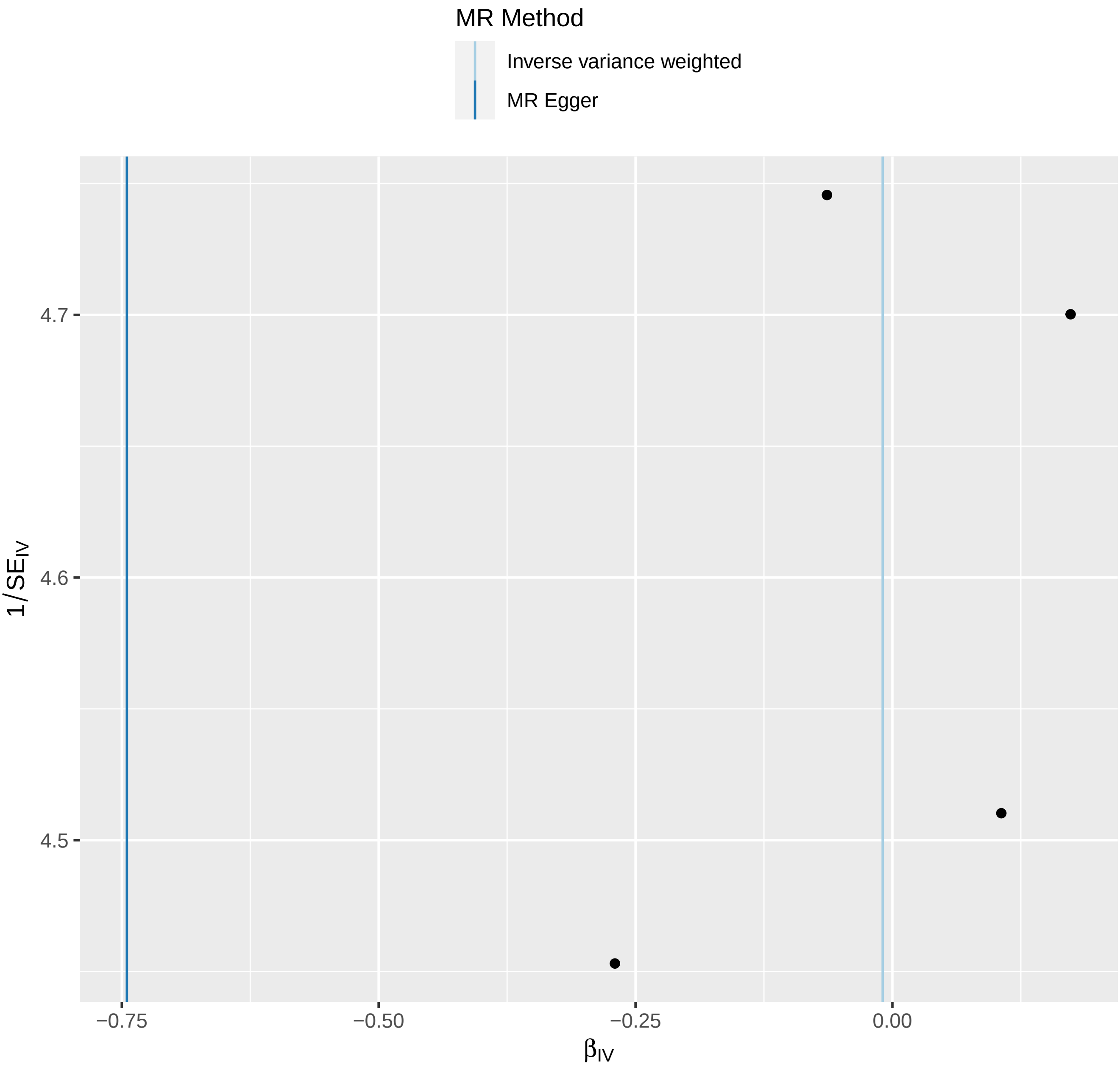

Batch 2−180 : Gut microbiota abundance (genus Parasutterella id.2892) on Anaphase−promoting complex subunit 10
with a threshold of 1e−05

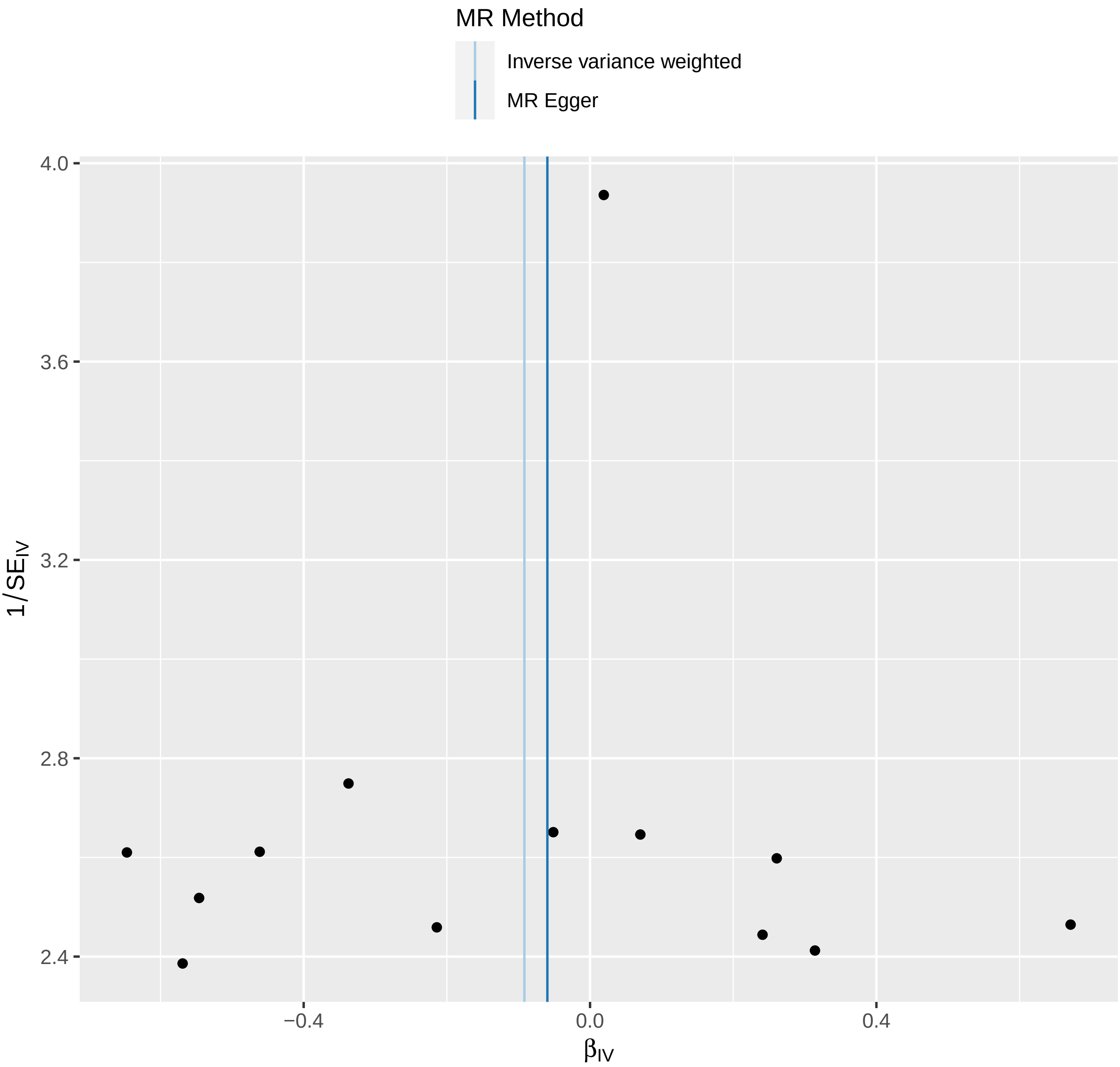

Batch 2−181 : Gut microbiota abundance (genus Catenibacterium id.2153) on Anaphase−promoting complex subunit 7
with a threshold of 1e−05

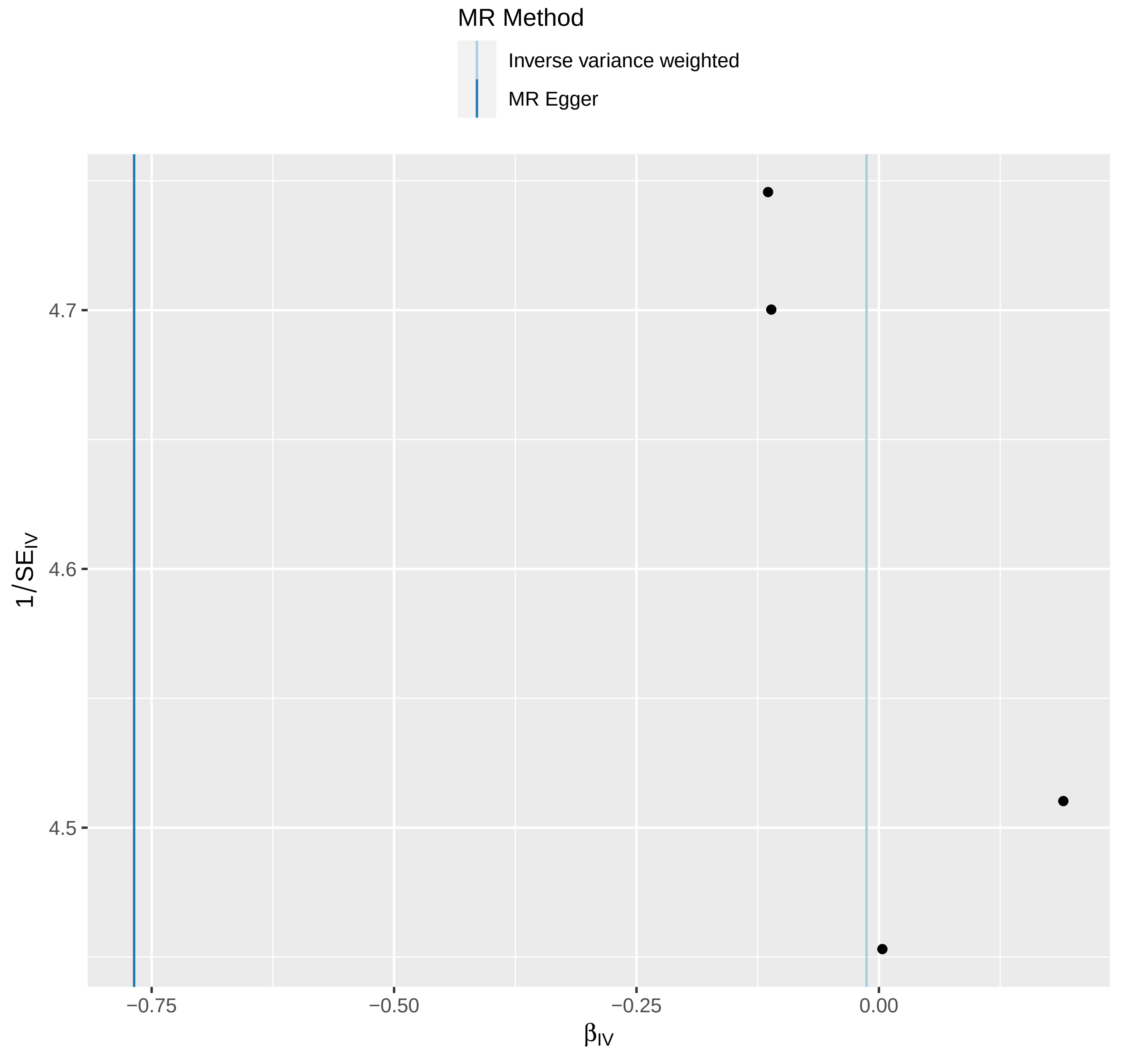

Batch 2−182 : Gut microbiota abundance (genus Parasutterella id.2892) on Anaphase−promoting complex subunit 7
with a threshold of 1e−05

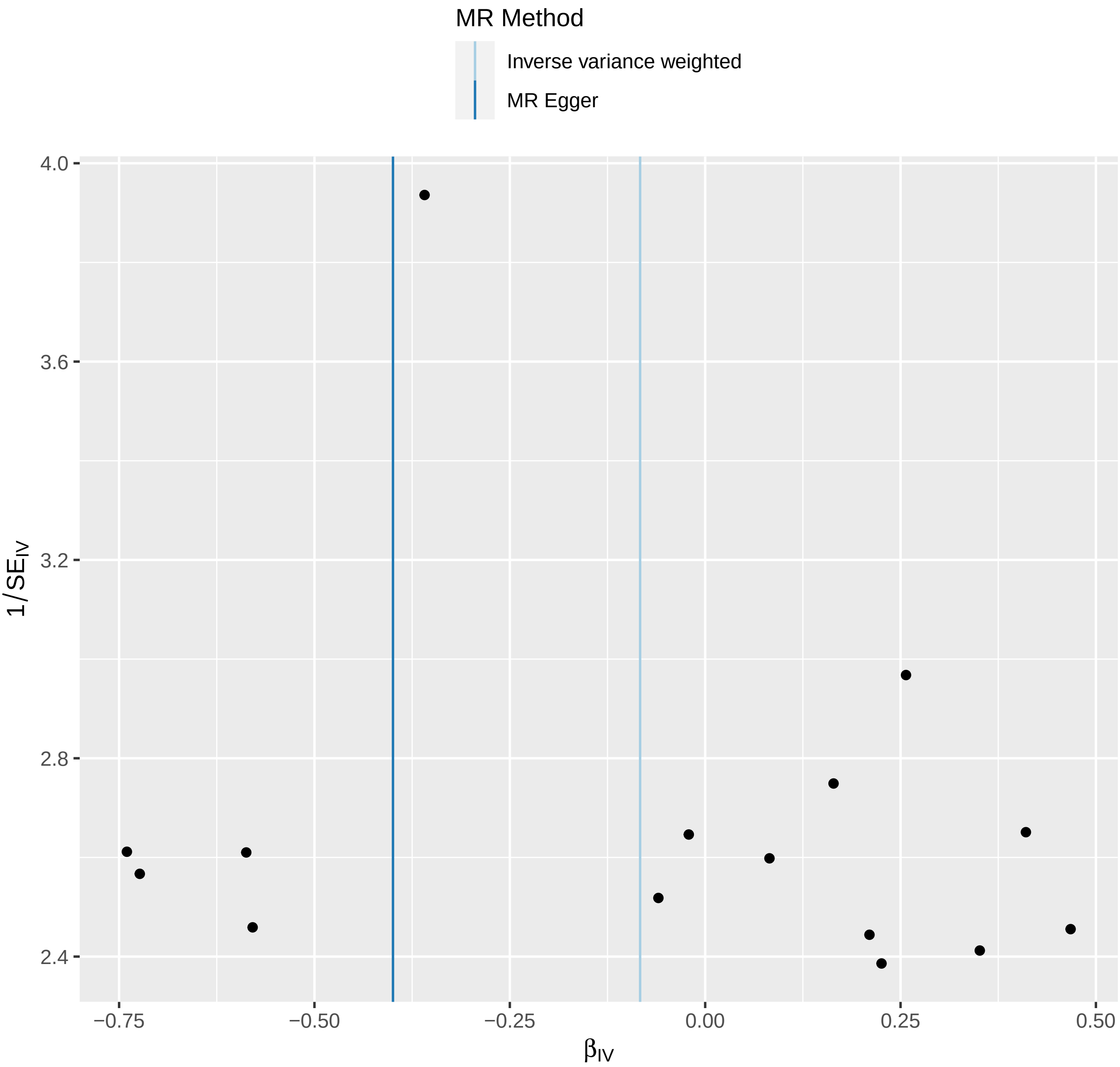

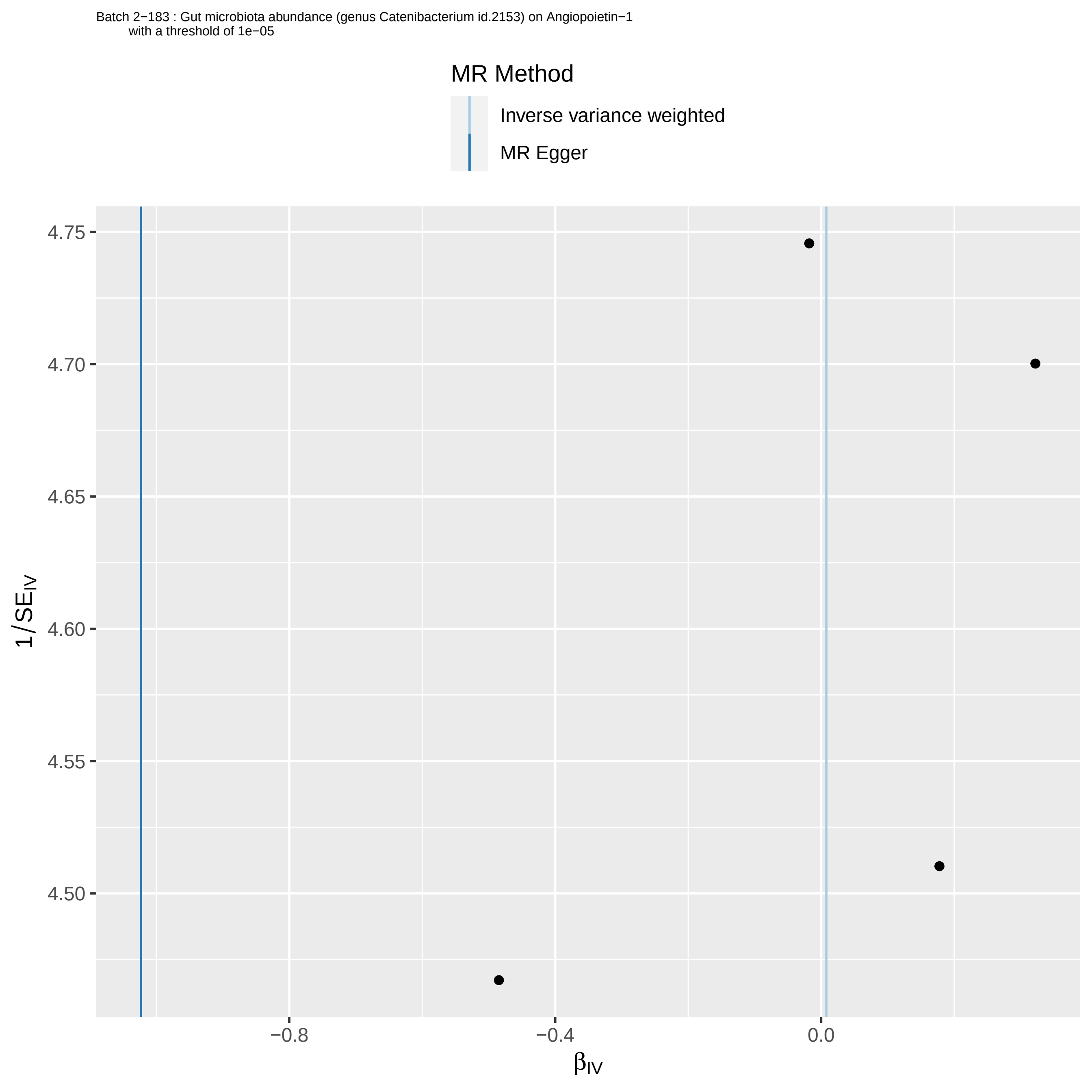

Batch 2−183 : Gut microbiota abundance (genus Catenibacterium id.2153) on Angiopoietin−1
with a threshold of 1e−05
MR Method
Inverse variance weighted
MR Egger
4.75
4.70
4.65
4.60
4.55
4.50
1/SE_IV
−0.8
−0.4
0.0
β_IV

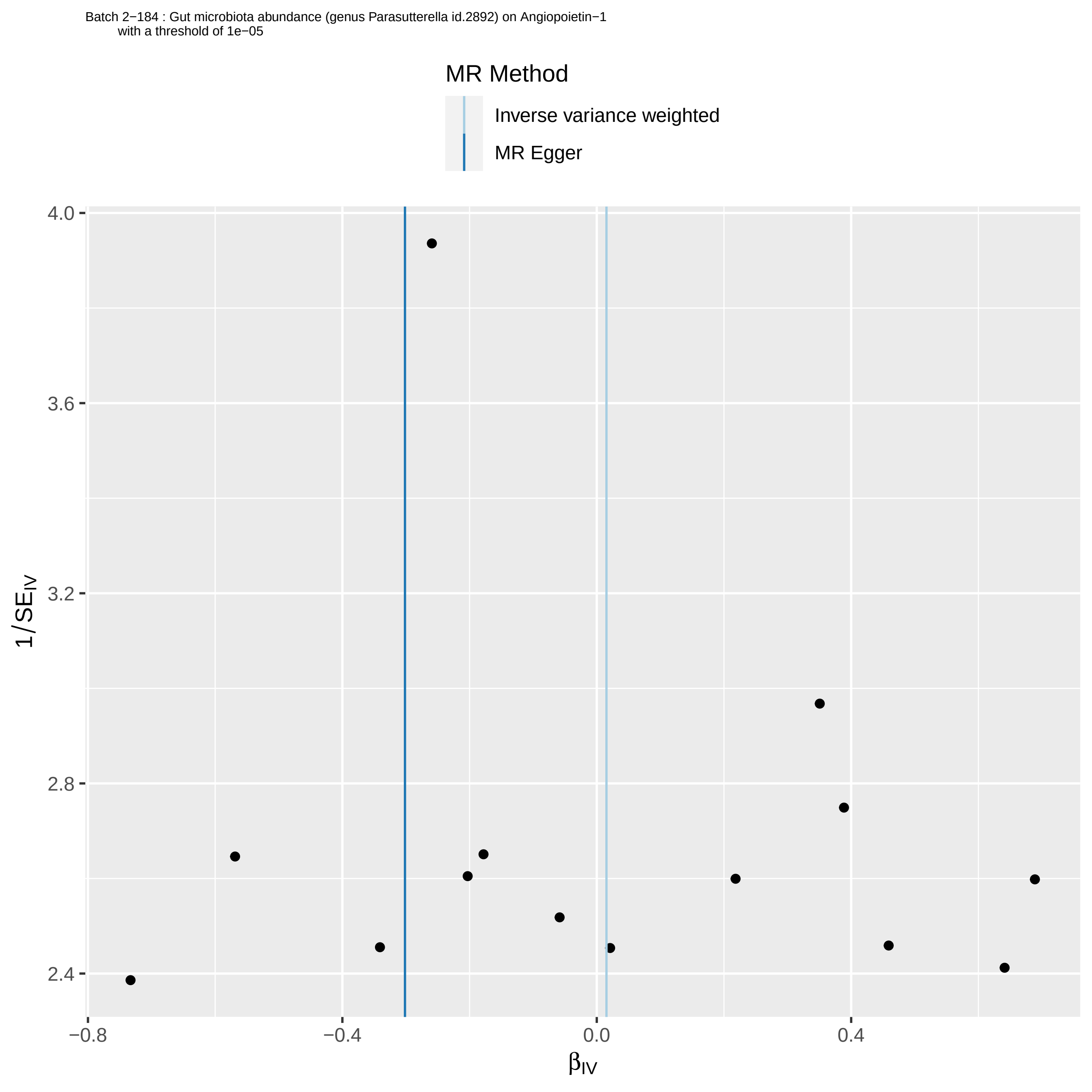

Batch 2−184 : Gut microbiota abundance (genus Parasutterella id.2892) on Angiopoietin−1
with a threshold of 1e−05
MR Method
Inverse variance weighted
MR Egger
4.0
3.6
3.2
2.8
2.4
1/SE_IV
−0.8
−0.4
0.0
0.4
β_IV

Batch 2−185 : Gut microbiota abundance (genus Catenibacterium id.2153) on Angiopoietin−2
with a threshold of 1e−05

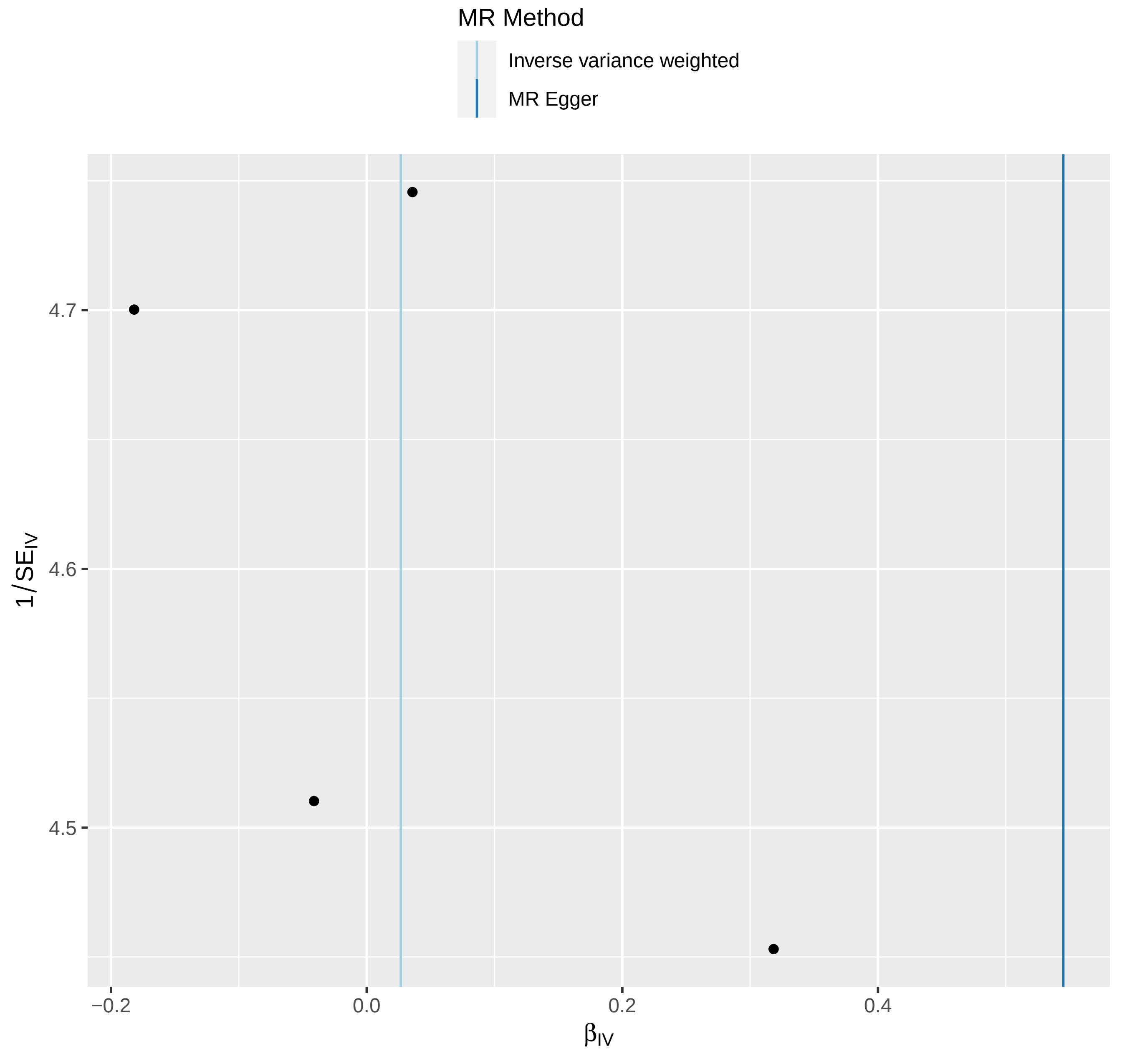

Batch 2−186 : Gut microbiota abundance (genus Parasutterella id.2892) on Angiopoietin−2
with a threshold of 1e−05

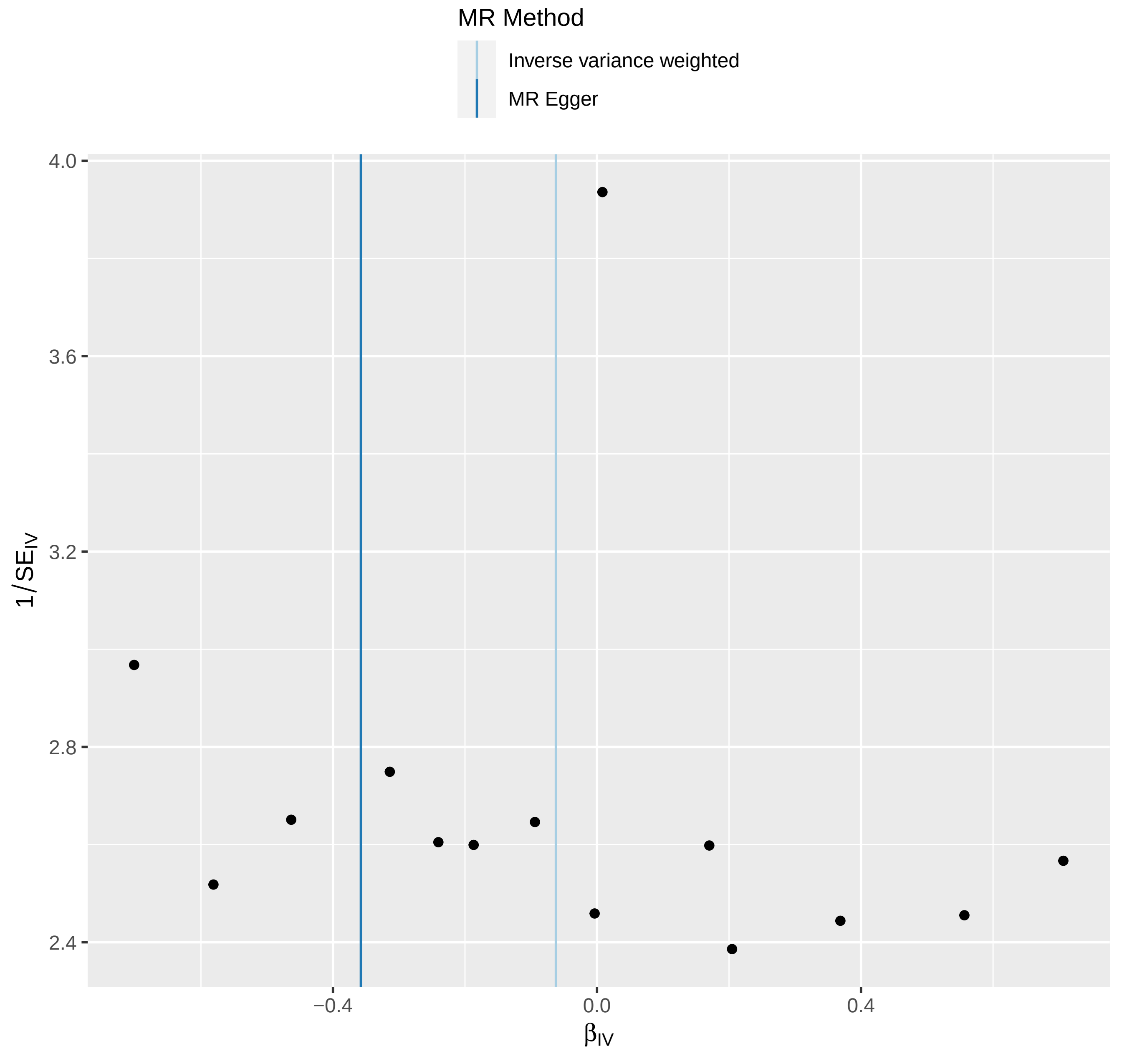

Batch 2−187 : Gut microbiota abundance (genus Catenibacterium id.2153) on Angiopoietin−2
with a threshold of 1e−05

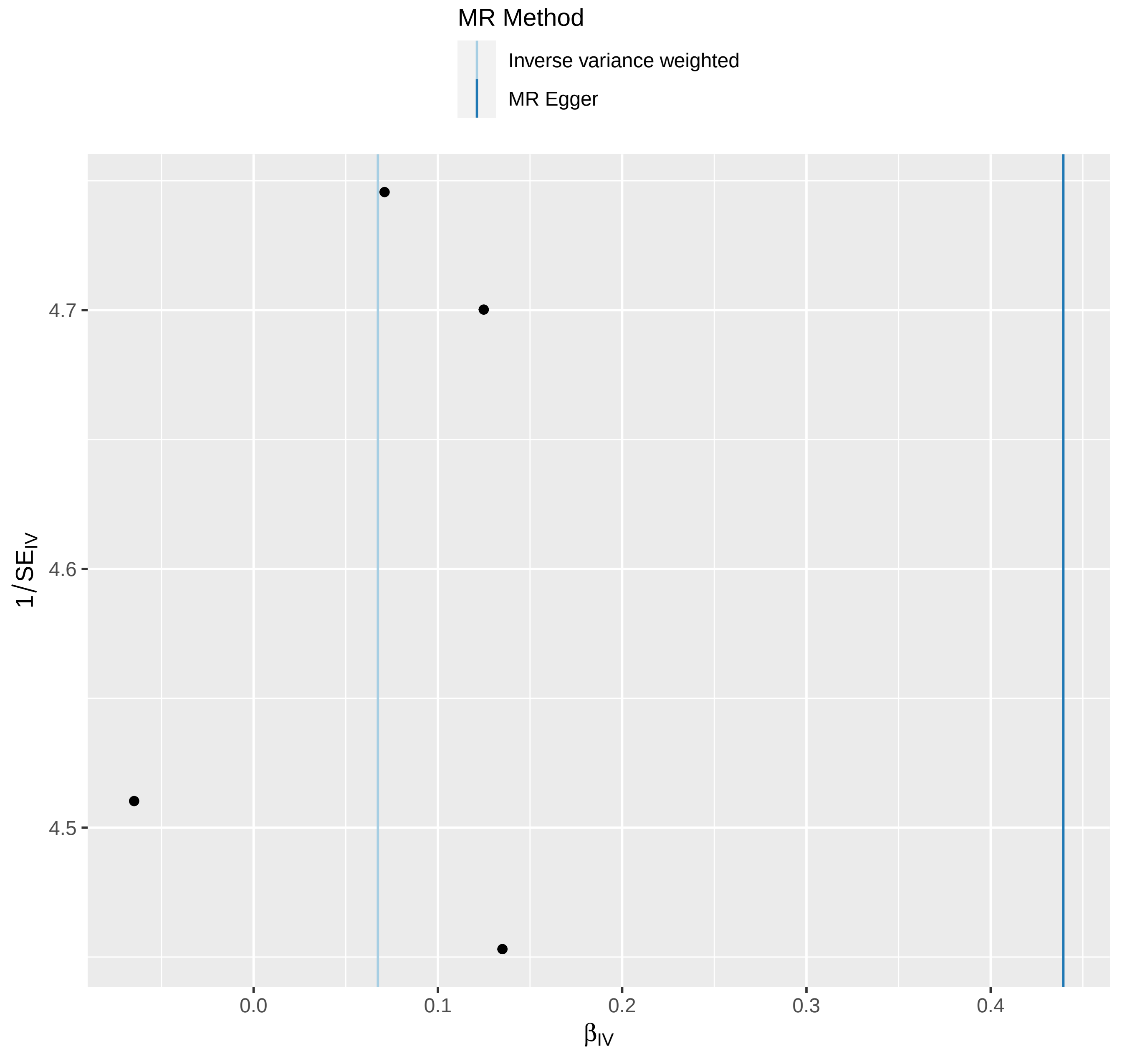

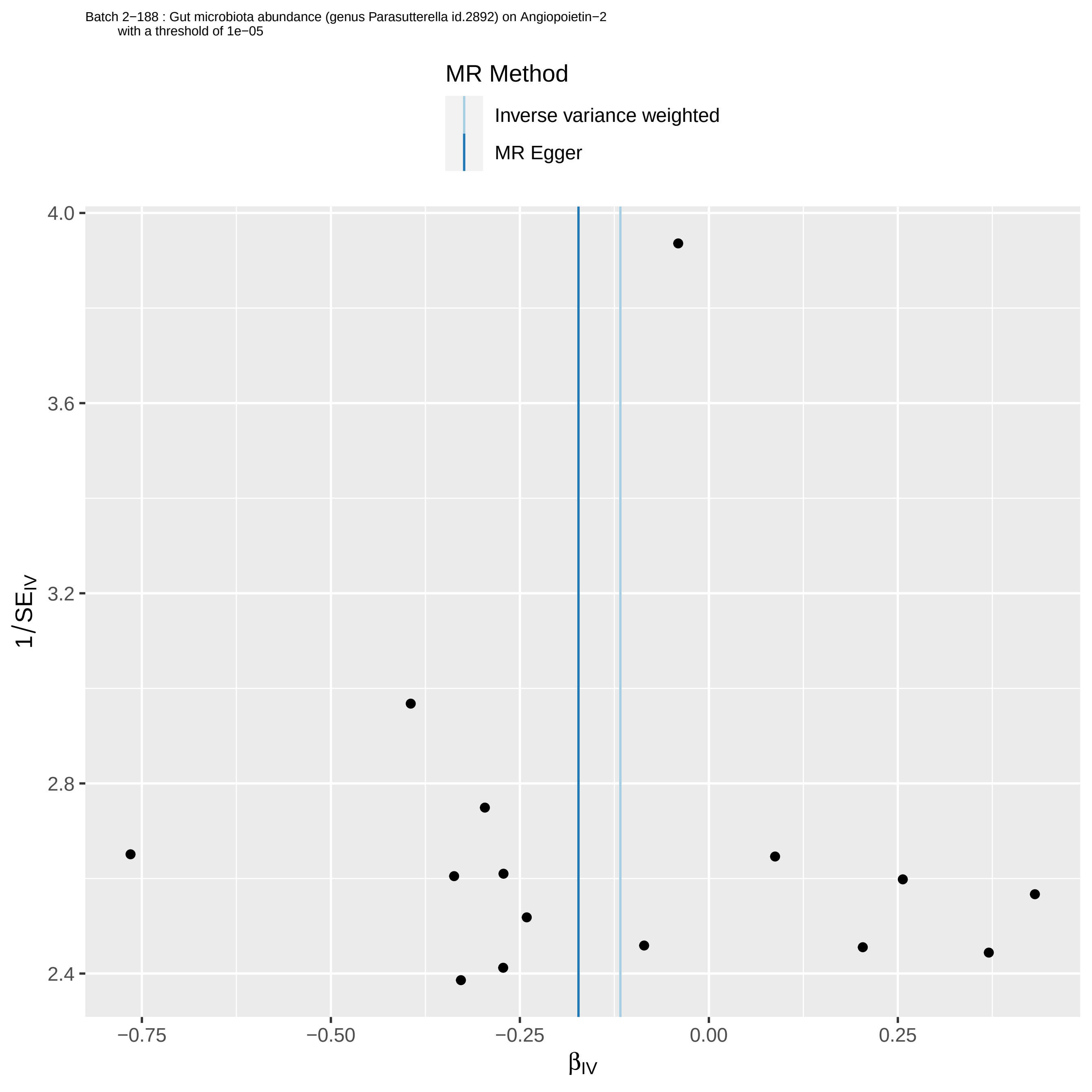
Batch 2−188 : Gut microbiota abundance (genus Parasutterella id.2892) on Angiopoietin−2
with a threshold of 1e−05
MR Method
Inverse variance weighted
MR Egger
4.0
3.6
3.2
2.8
2.4
1/SE_IV
−0.75
−0.50
−0.25
0.00
0.25
β_IV

Batch 2−189 : Gut microbiota abundance (genus Catenibacterium id.2153) on Angiopoietin−4
with a threshold of 1e−05

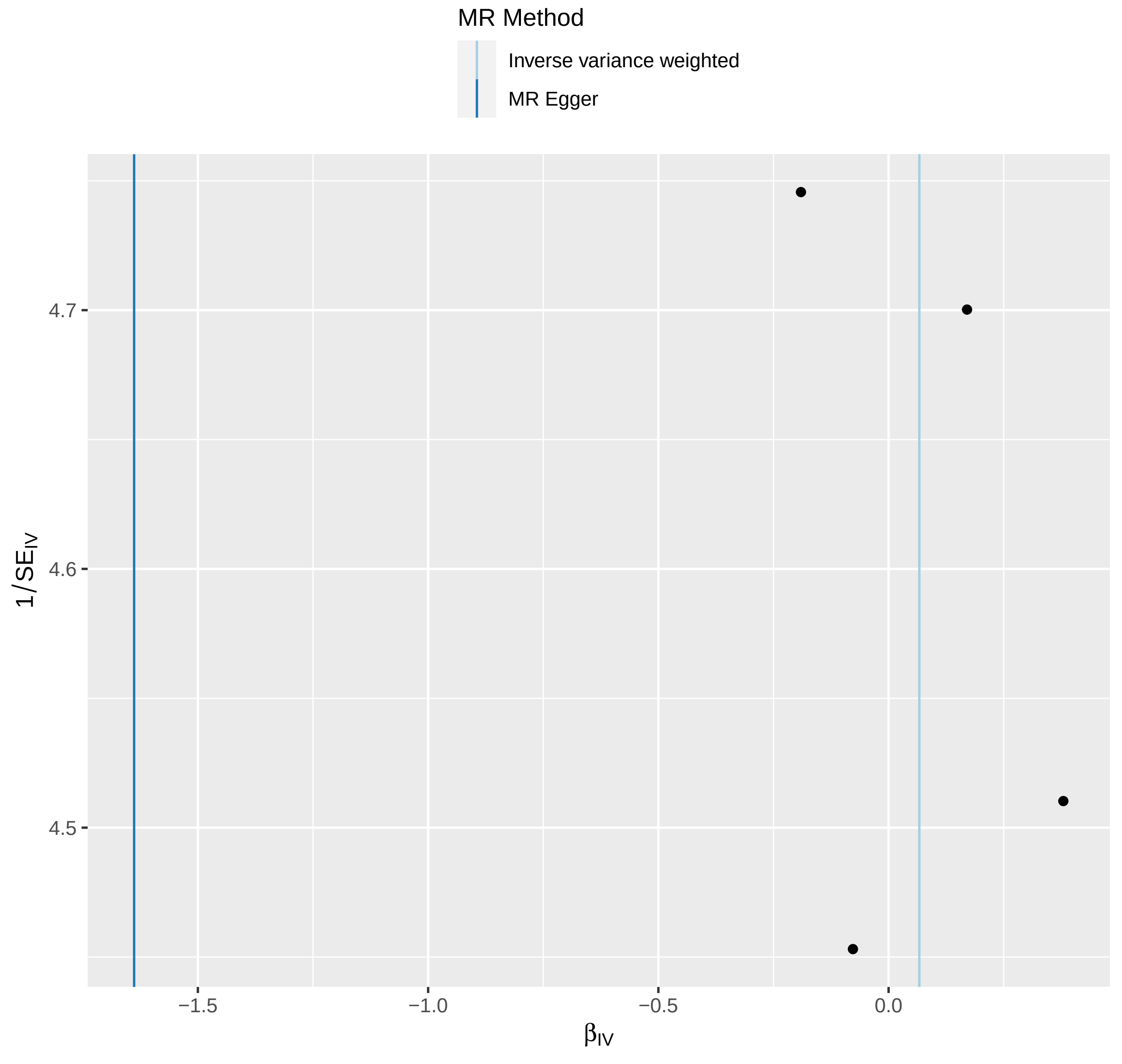

Batch 2−190 : Gut microbiota abundance (genus Parasutterella id.2892) on Angiopoietin−4
with a threshold of 1e−05

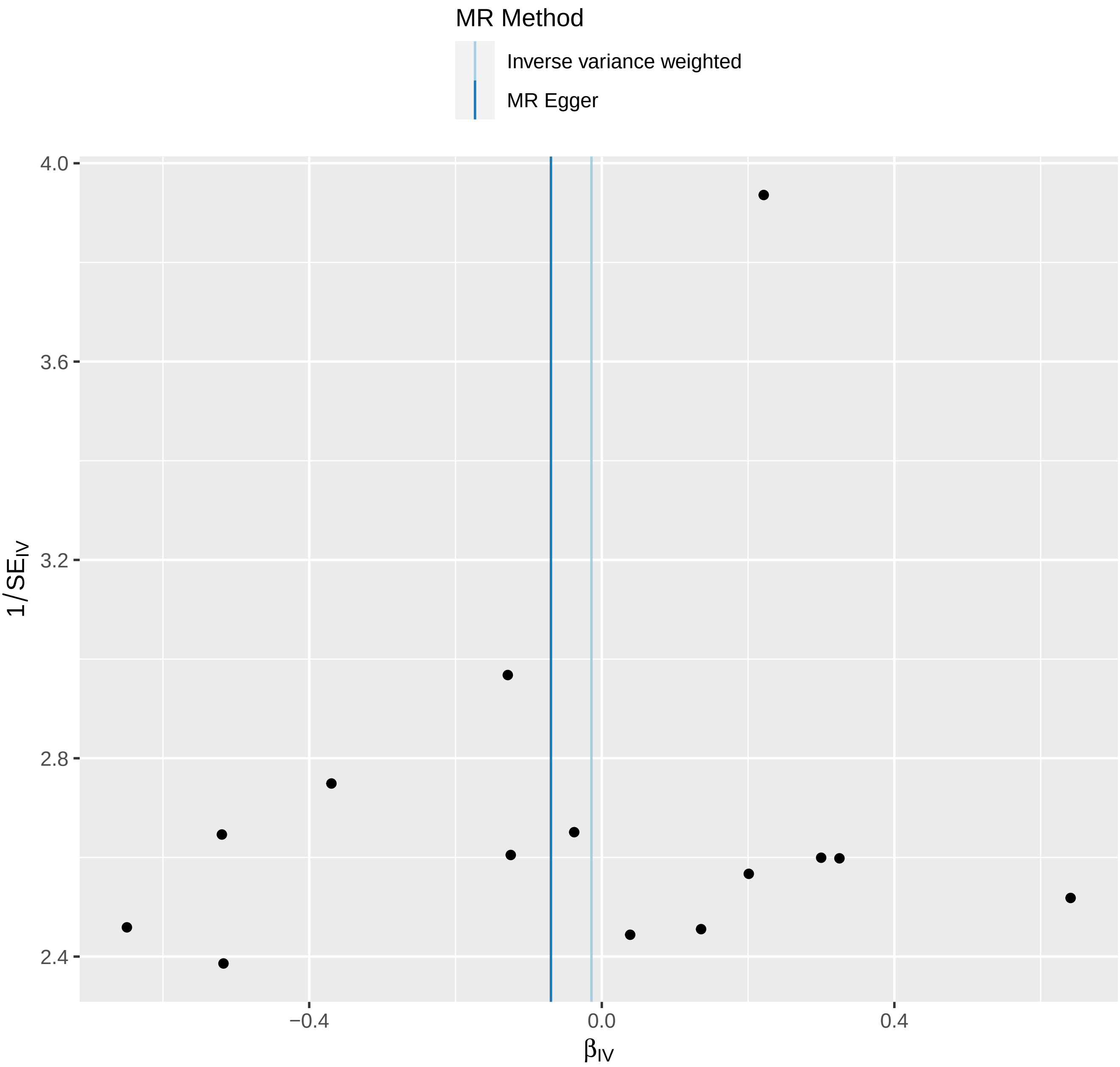

Batch 2−191 : Gut microbiota abundance (genus Catenibacterium id.2153) on Angiopoietin−related protein 1
with a threshold of 1e−05

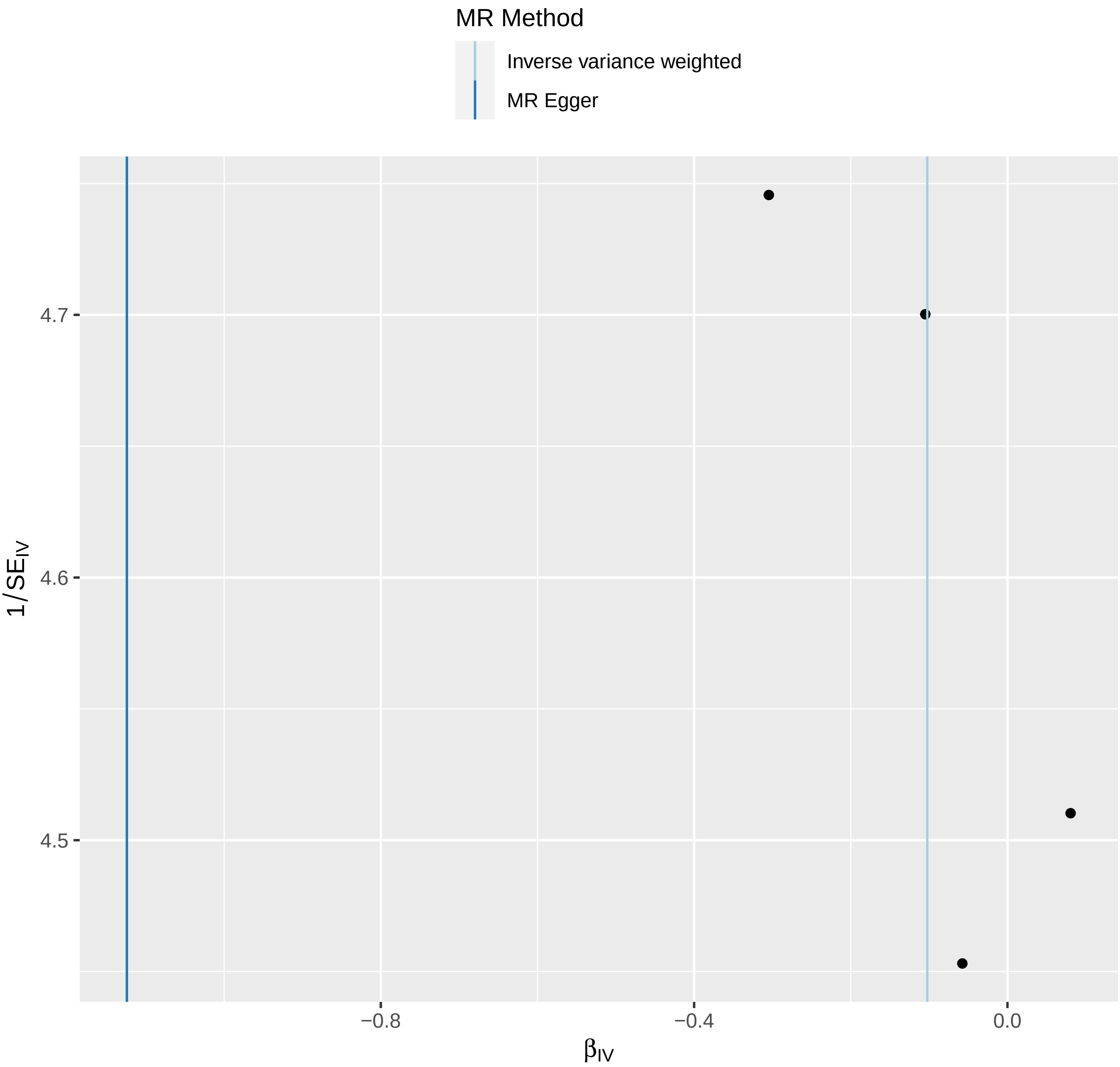

Batch 2−192 : Gut microbiota abundance (genus Parasutterella id.2892) on Angiopoietin−related protein 1
with a threshold of 1e−05

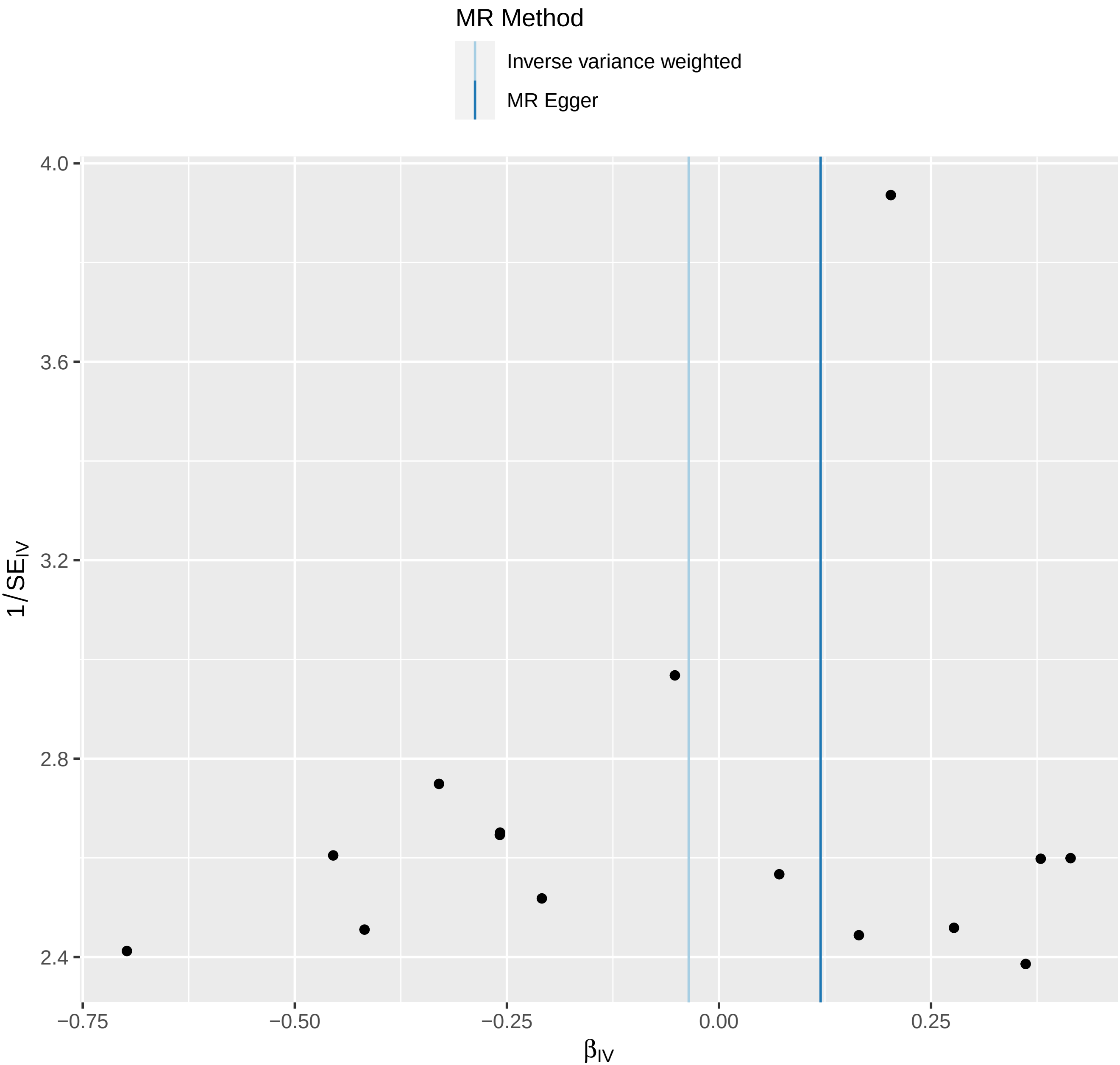

Batch 2−193 : Gut microbiota abundance (genus Catenibacterium id.2153) on Angiopoietin−related protein 1
with a threshold of 1e−05

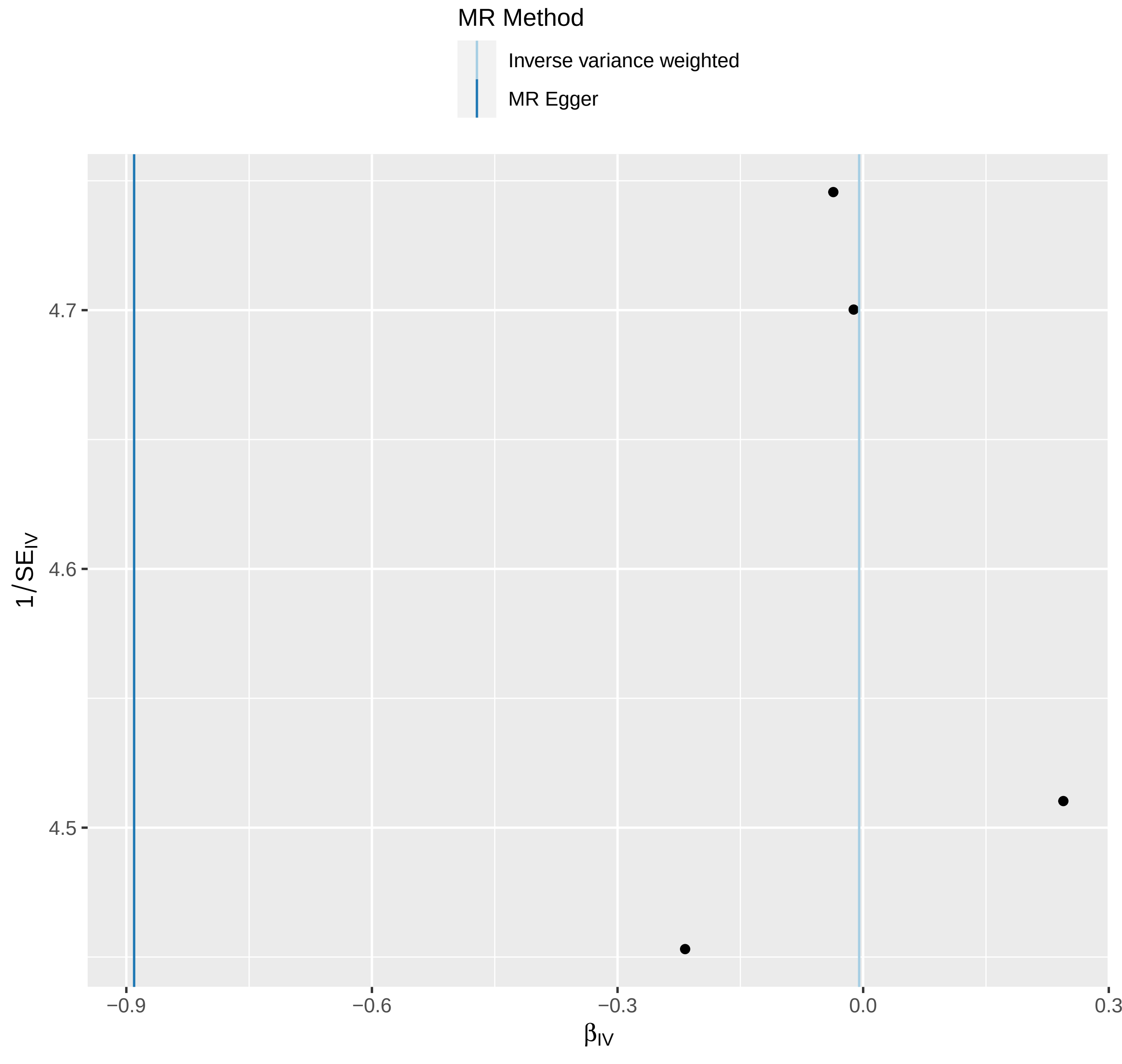

Batch 2−194 : Gut microbiota abundance (genus Parasutterella id.2892) on Angiopoietin−related protein 1
with a threshold of 1e−05

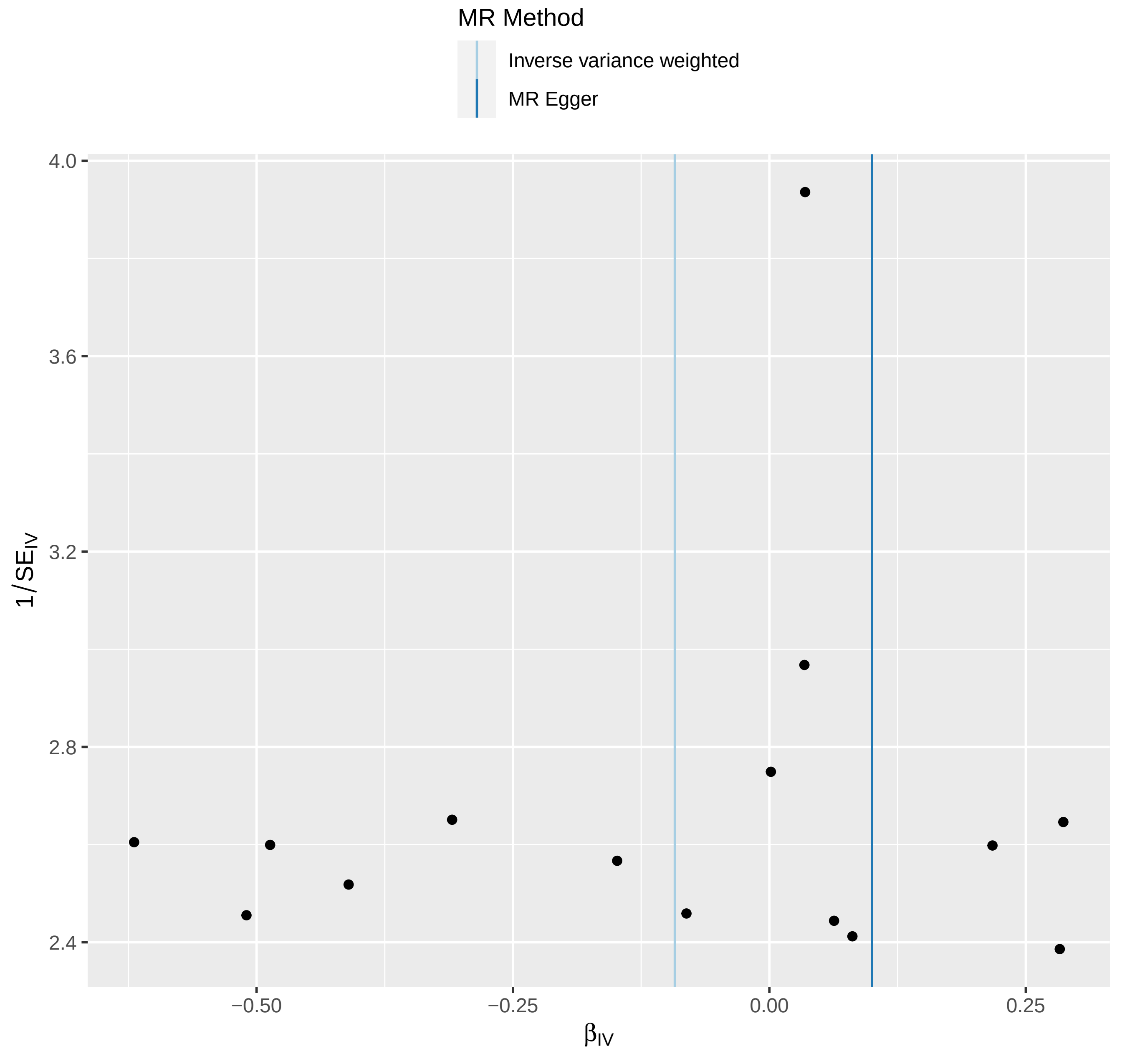

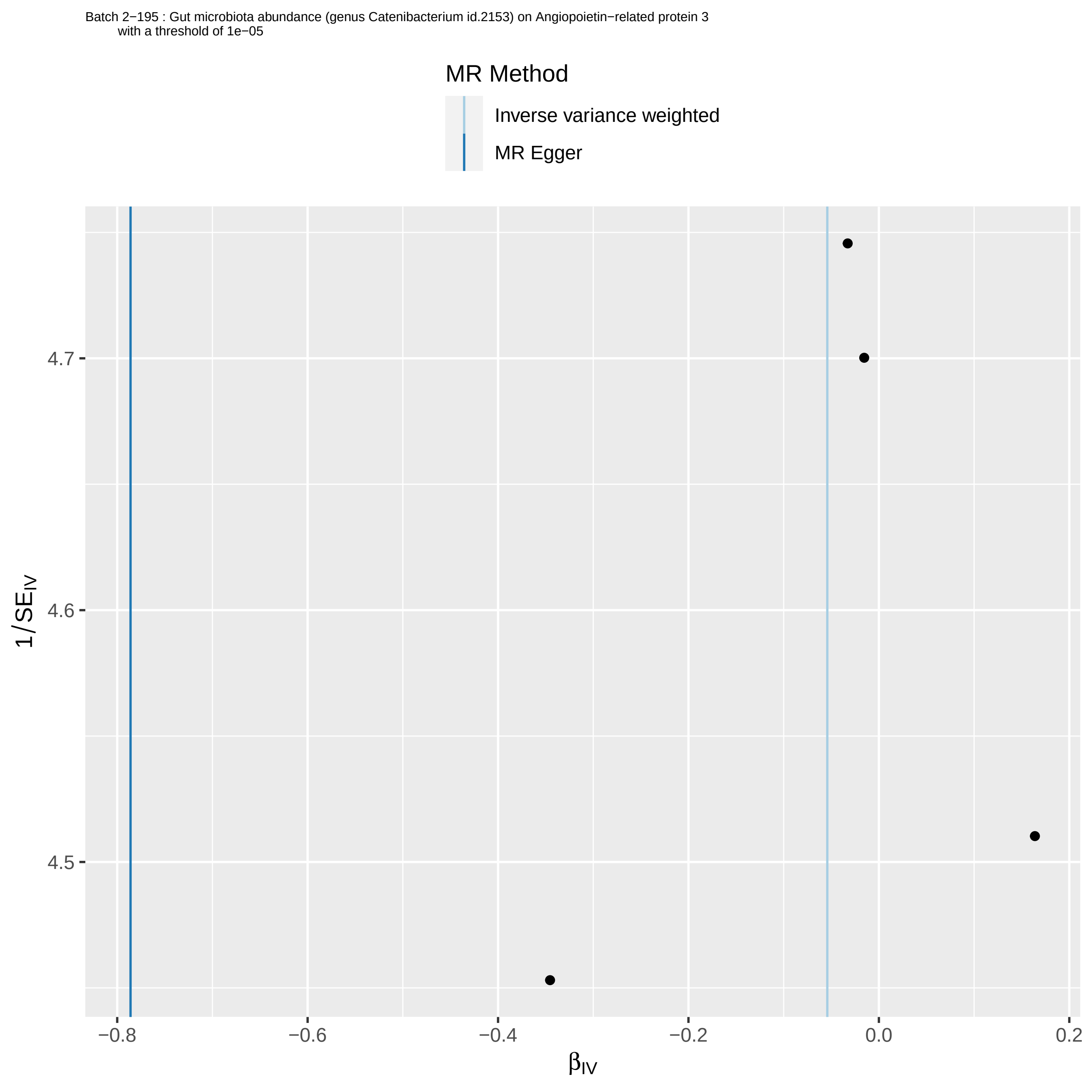

Batch 2−195 : Gut microbiota abundance (genus Catenibacterium id.2153) on Angiopoietin−related protein 3
with a threshold of 1e−05
MR Method
Inverse variance weighted
MR Egger
1/SE$_{IV}$
4.7
4.6
4.5
−0.8
−0.6
−0.4
−0.2
0.0
0.2
β$_{IV}$

Batch 2−196 : Gut microbiota abundance (genus Parasutterella id.2892) on Angiopoietin−related protein 3
with a threshold of 1e−05

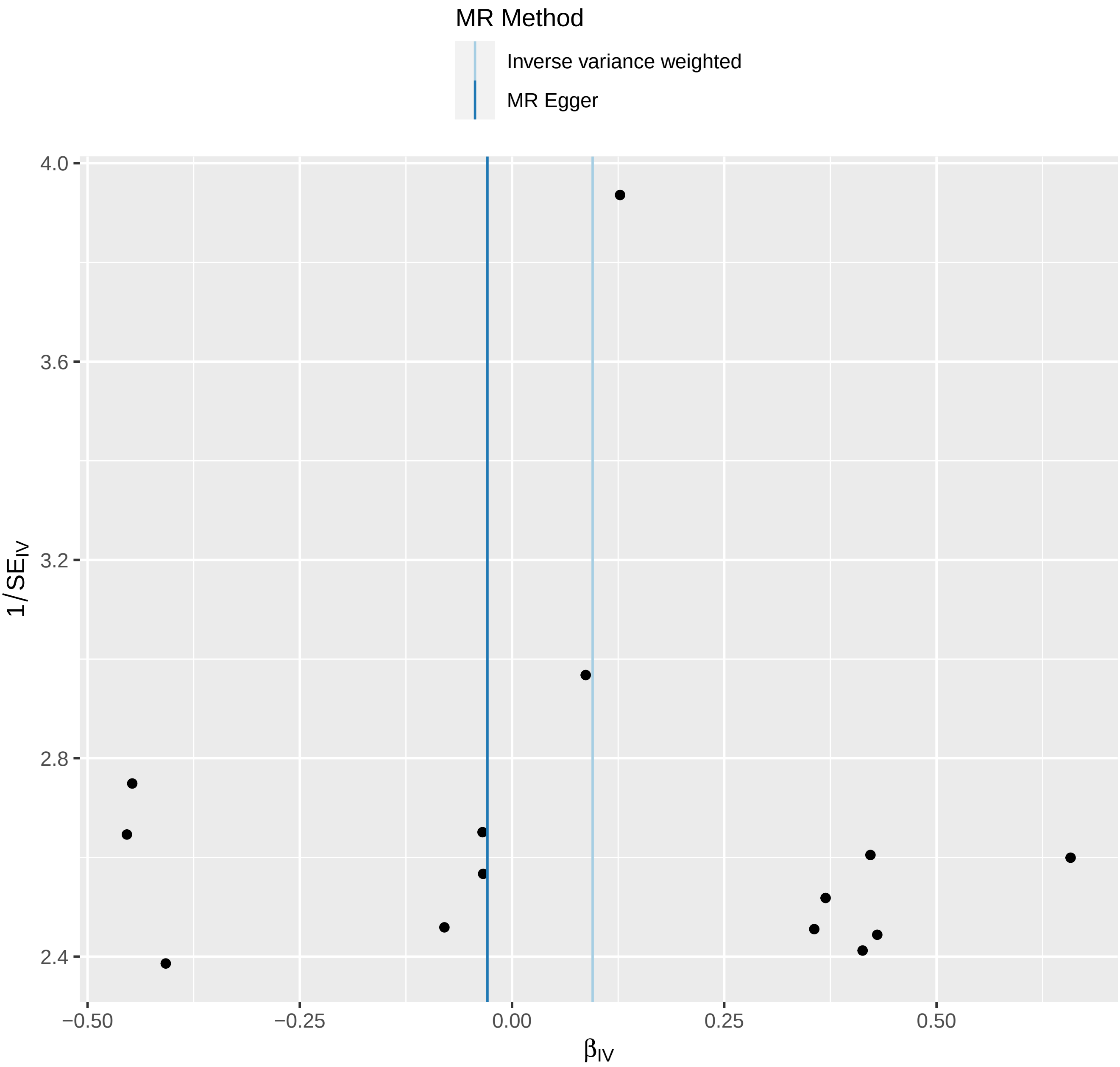

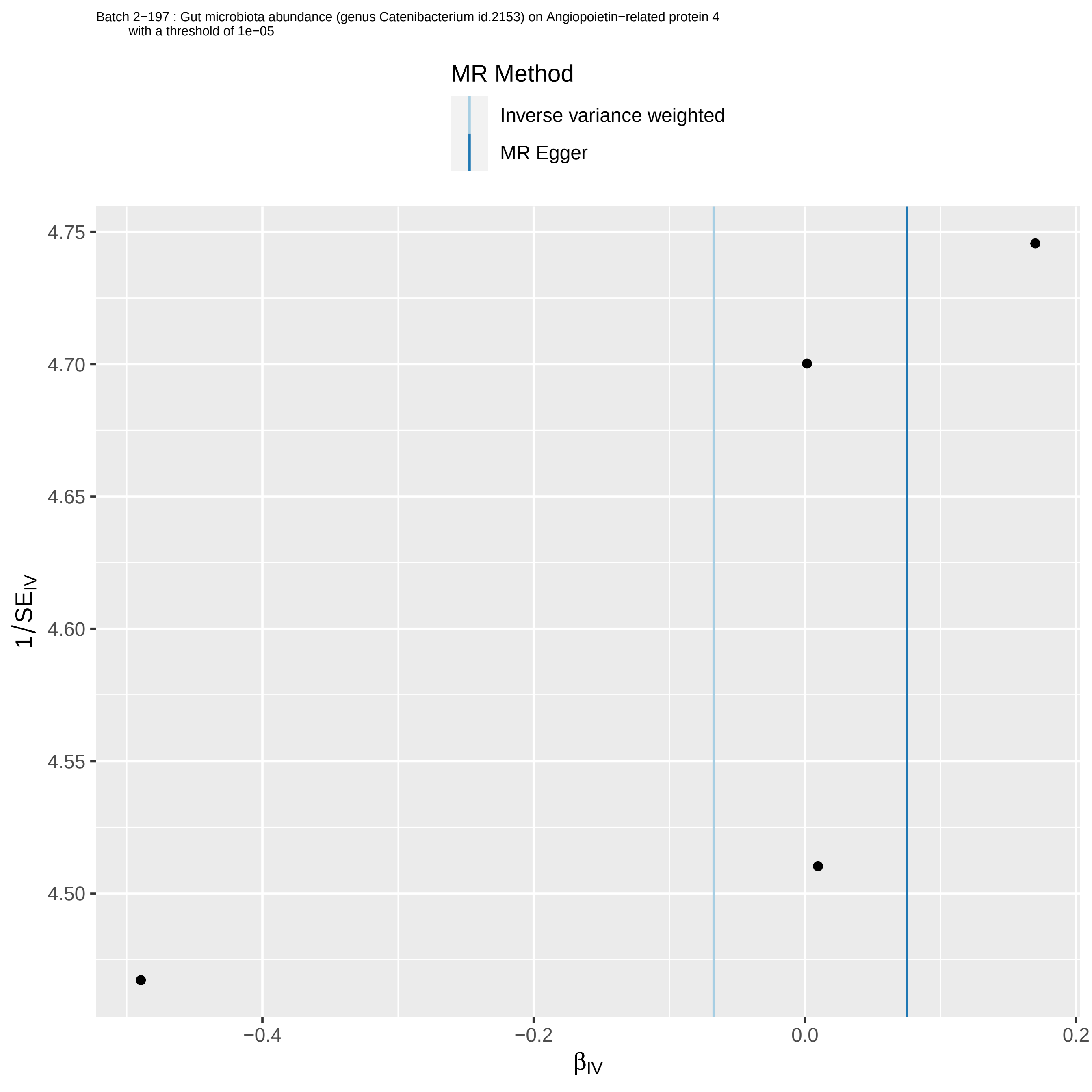

Batch 2−197 : Gut microbiota abundance (genus Catenibacterium id.2153) on Angiopoietin−related protein 4
with a threshold of 1e−05
MR Method
Inverse variance weighted
MR Egger
4.75
4.70
4.65
4.60
4.55
4.50
1/SE_IV
−0.4
−0.2
0.0
0.2
β_IV

Batch 2−198 : Gut microbiota abundance (genus Parasutterella id.2892) on Angiopoietin−related protein 4
with a threshold of 1e−05

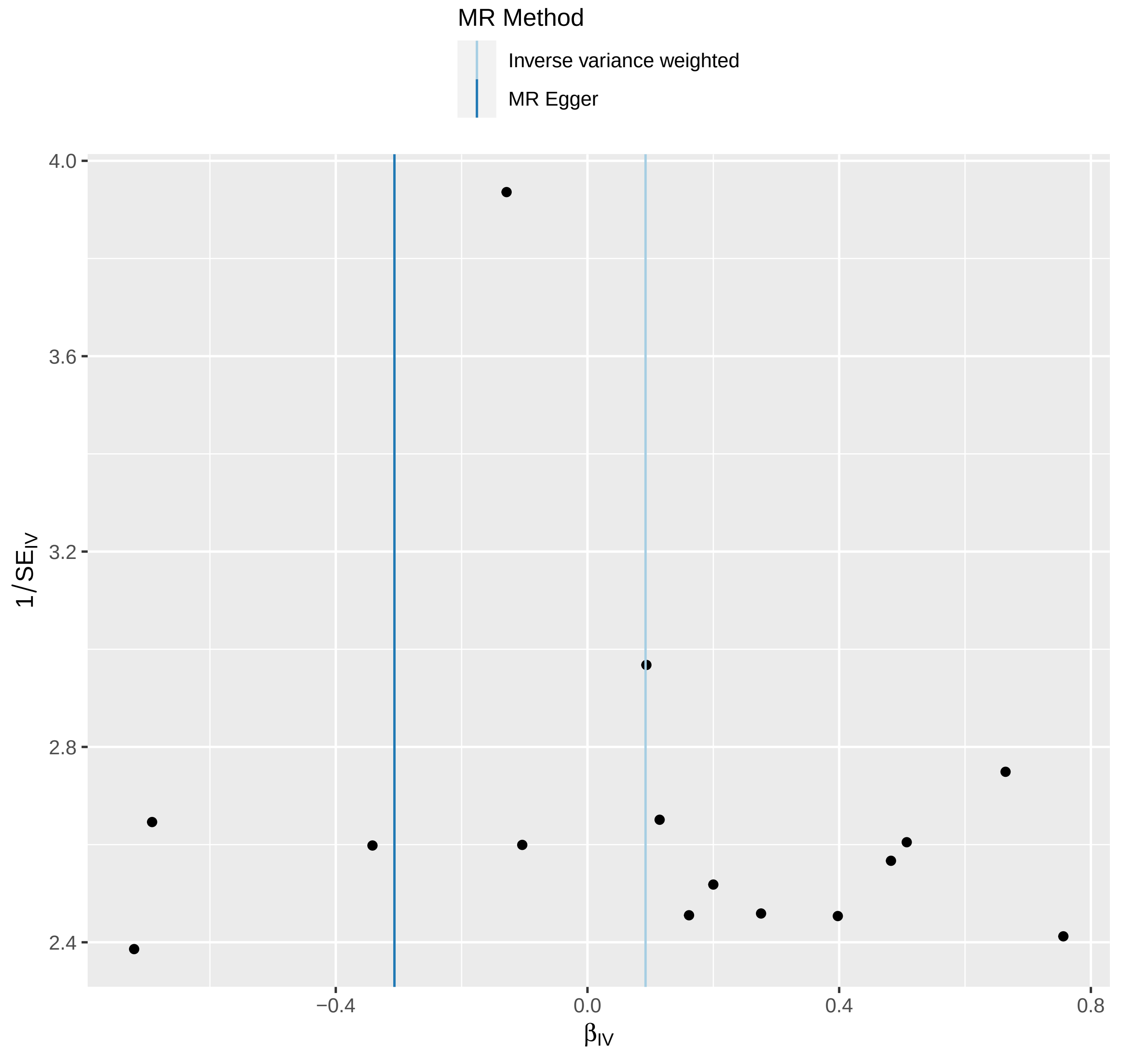

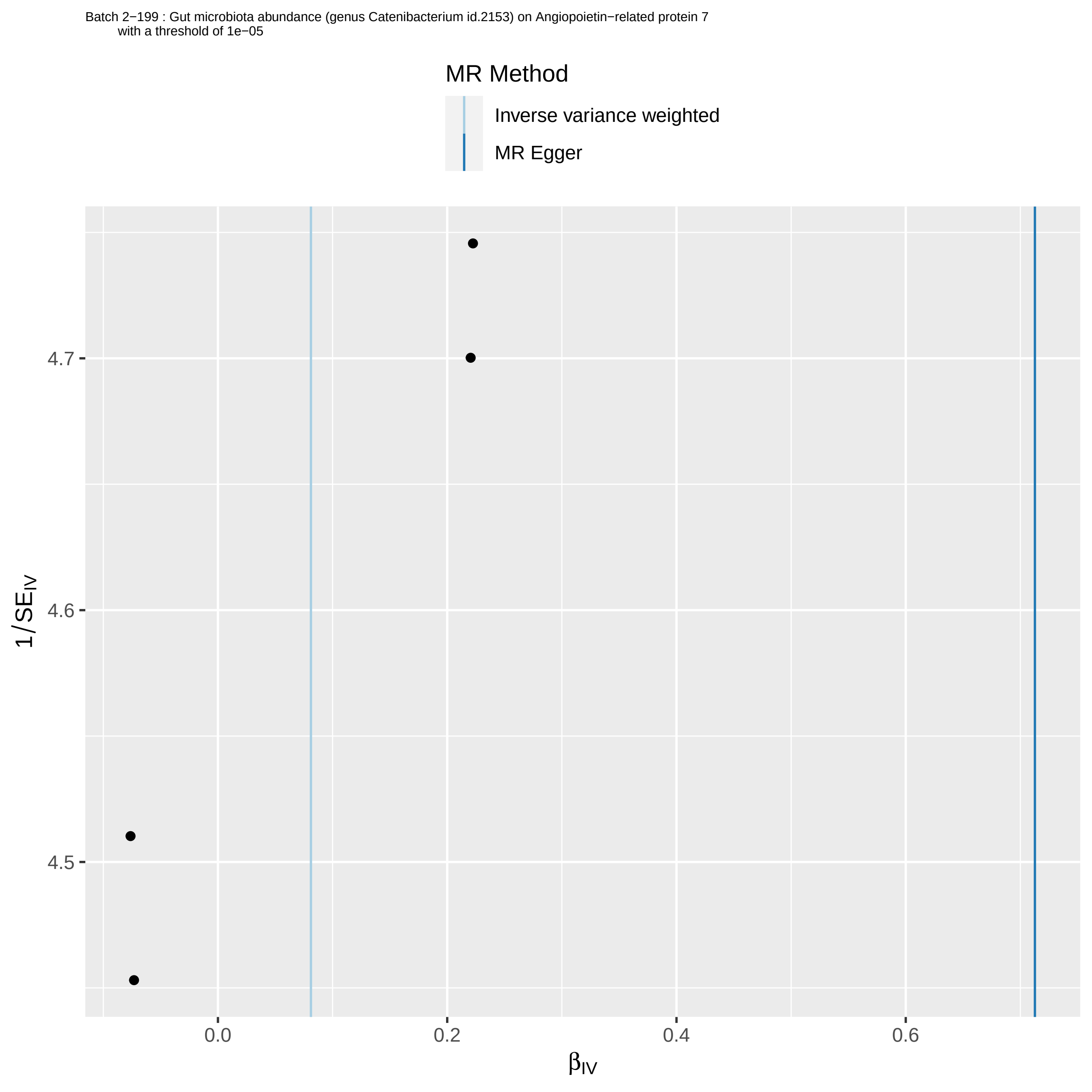
Batch 2−199 : Gut microbiota abundance (genus Catenibacterium id.2153) on Angiopoietin−related protein 7
with a threshold of 1e−05
MR Method
Inverse variance weighted
MR Egger
1/SE_IV
4.7
4.6
4.5
0.0
0.2
0.4
0.6
β_IV

Batch 2−200 : Gut microbiota abundance (genus Parasutterella id.2892) on Angiopoietin−related protein 7
with a threshold of 1e−05

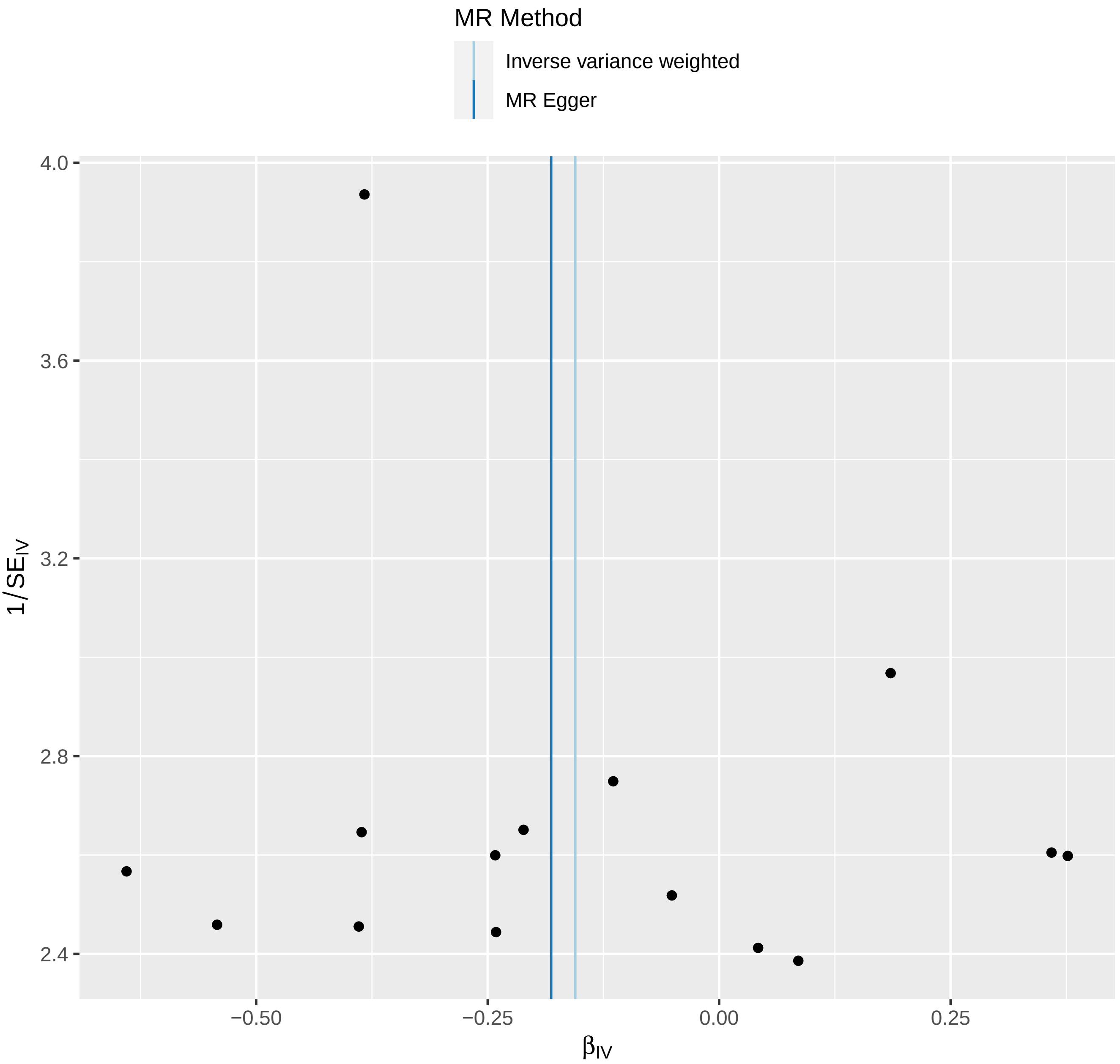

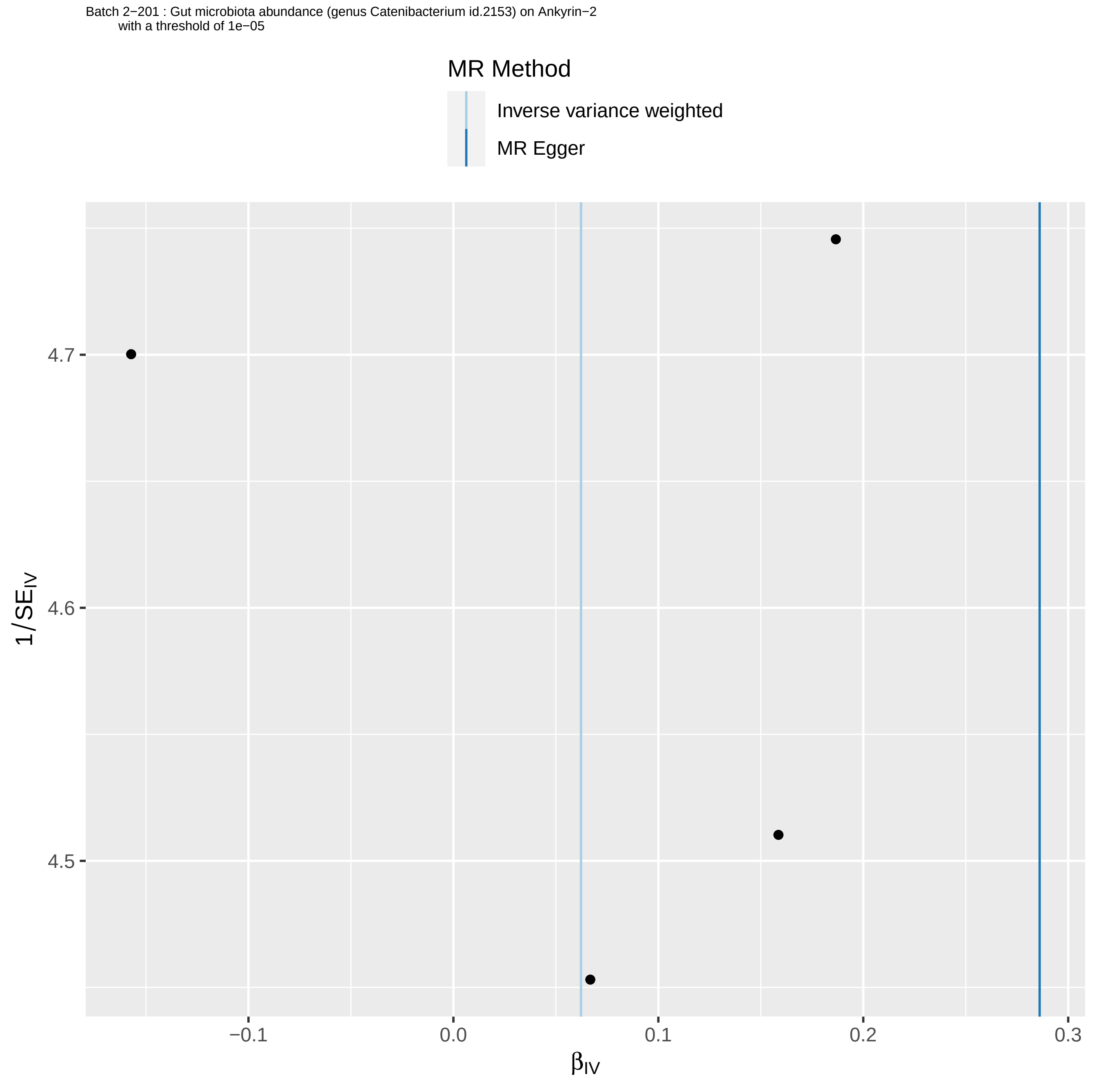
Batch 2−201 : Gut microbiota abundance (genus Catenibacterium id.2153) on Ankyrin−2
with a threshold of 1e−05
MR Method
Inverse variance weighted
MR Egger
4.7
4.6
4.5
1/SE_IV
−0.1
0.0
0.1
0.2
0.3
β_IV

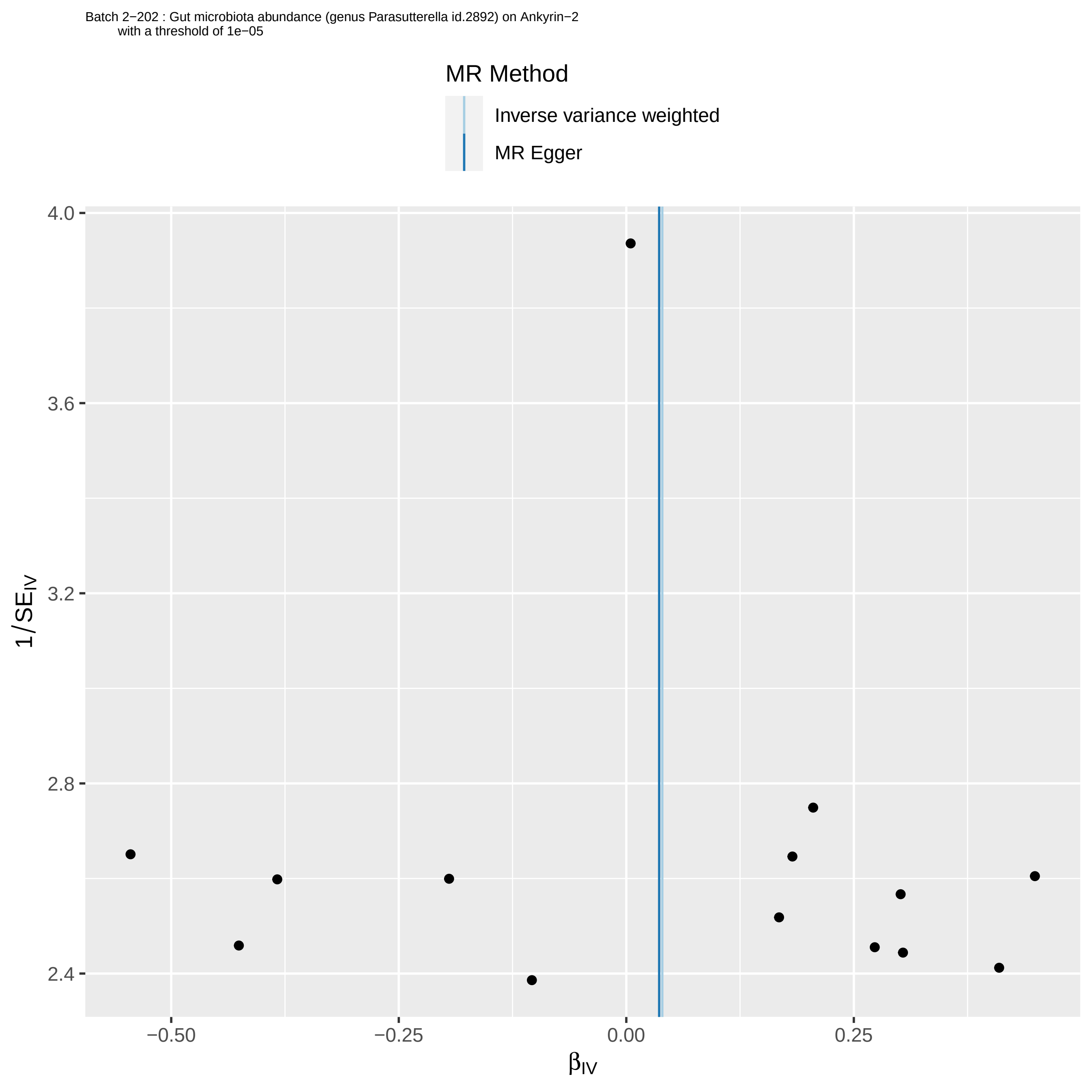

Batch 2−202 : Gut microbiota abundance (genus Parasutterella id.2892) on Ankyrin−2
with a threshold of 1e−05
MR Method
Inverse variance weighted
MR Egger
4.0
3.6
3.2
2.8
2.4
1/SE_IV
−0.50
−0.25
0.00
0.25
β_IV

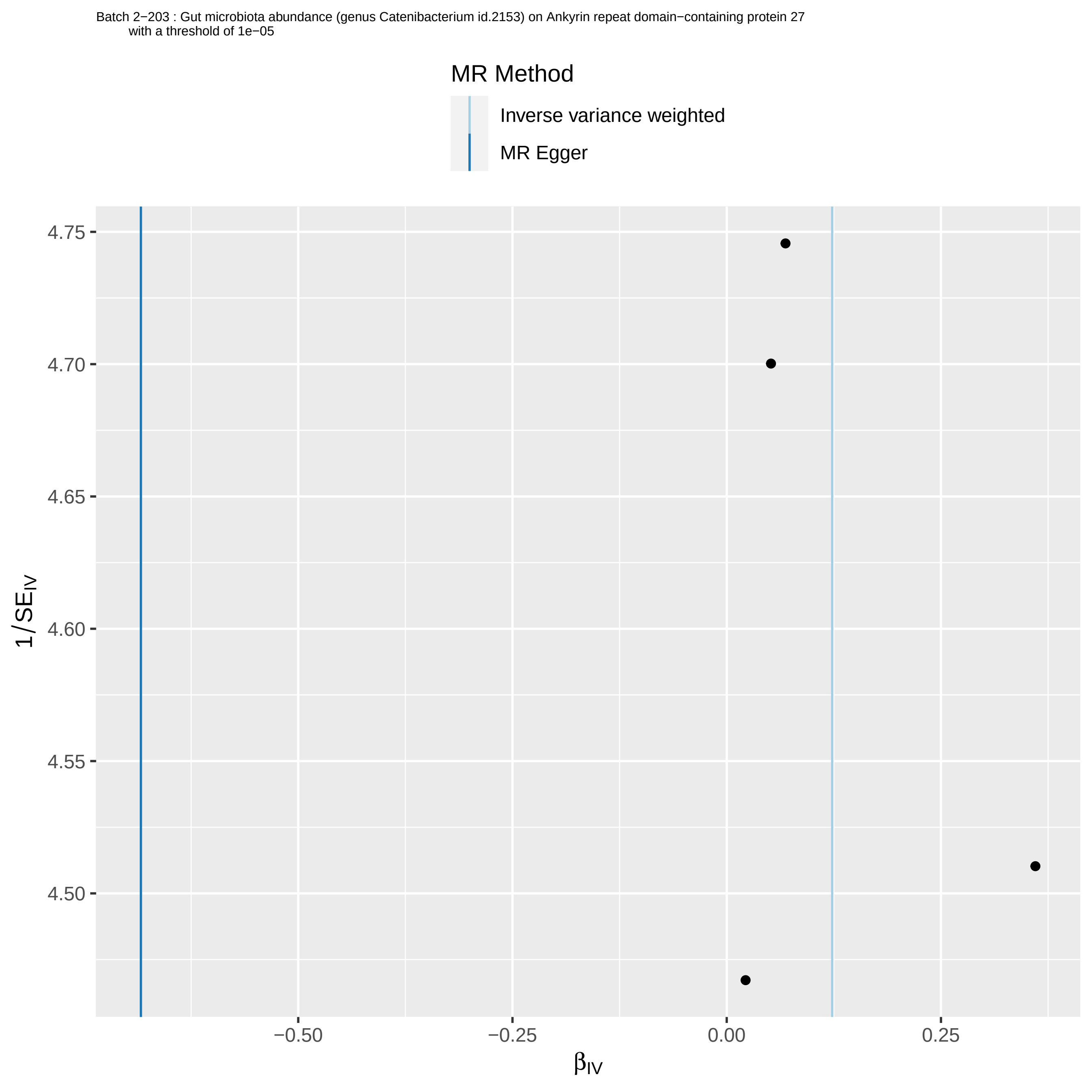
Batch 2−203 : Gut microbiota abundance (genus Catenibacterium id.2153) on Ankyrin repeat domain−containing protein 27
with a threshold of 1e−05
MR Method
Inverse variance weighted
MR Egger
4.75
4.70
4.65
4.60
4.55
4.50
1/SE_IV
−0.50
−0.25
0.00
0.25
β_IV

Batch 2−204 : Gut microbiota abundance (genus Parasutterella id.2892) on Ankyrin repeat domain−containing protein 27
with a threshold of 1e−05

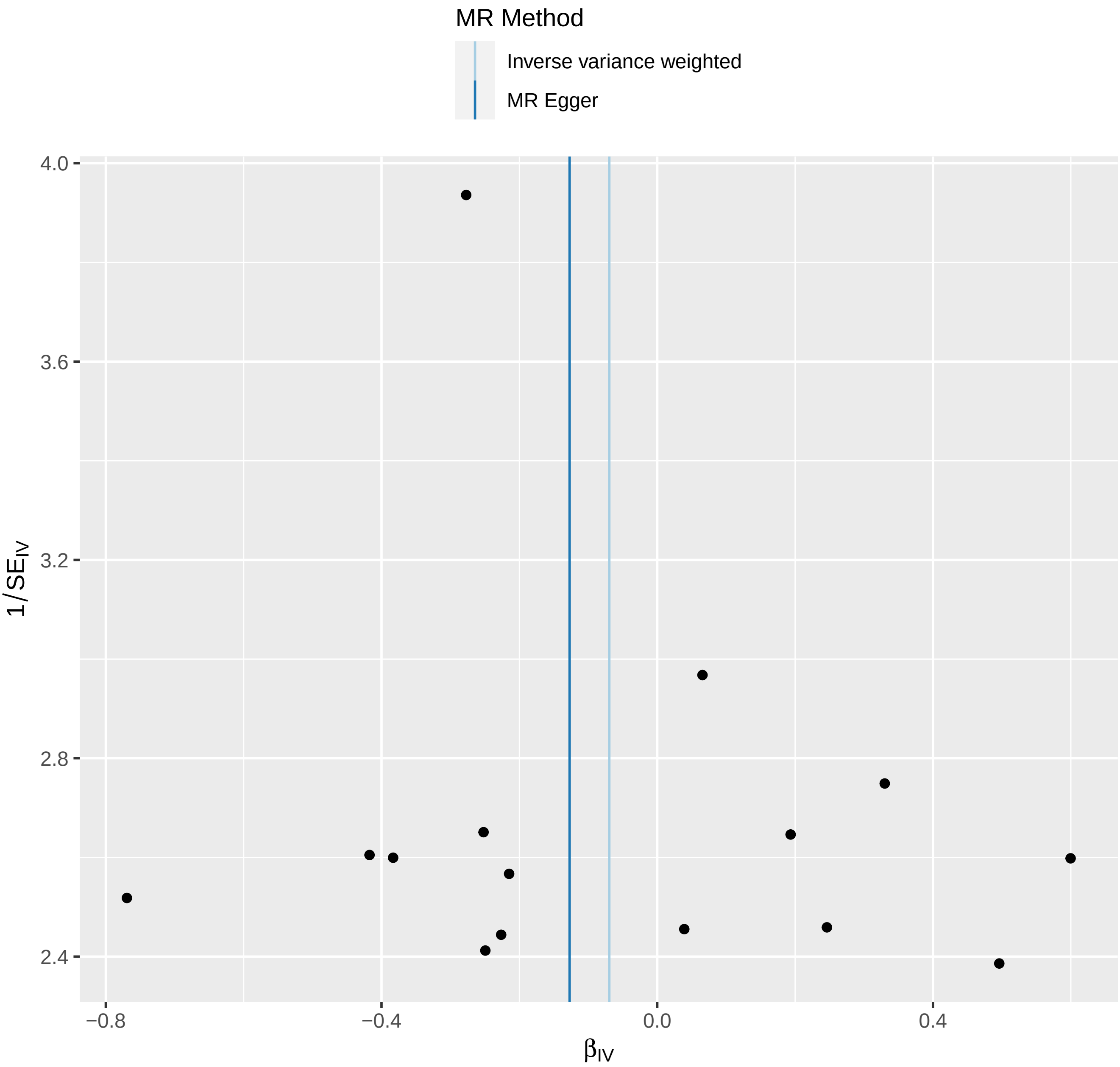

Batch 2−205 : Gut microbiota abundance (genus Catenibacterium id.2153) on Ankyrin repeat domain−containing protein 46
with a threshold of 1e−05

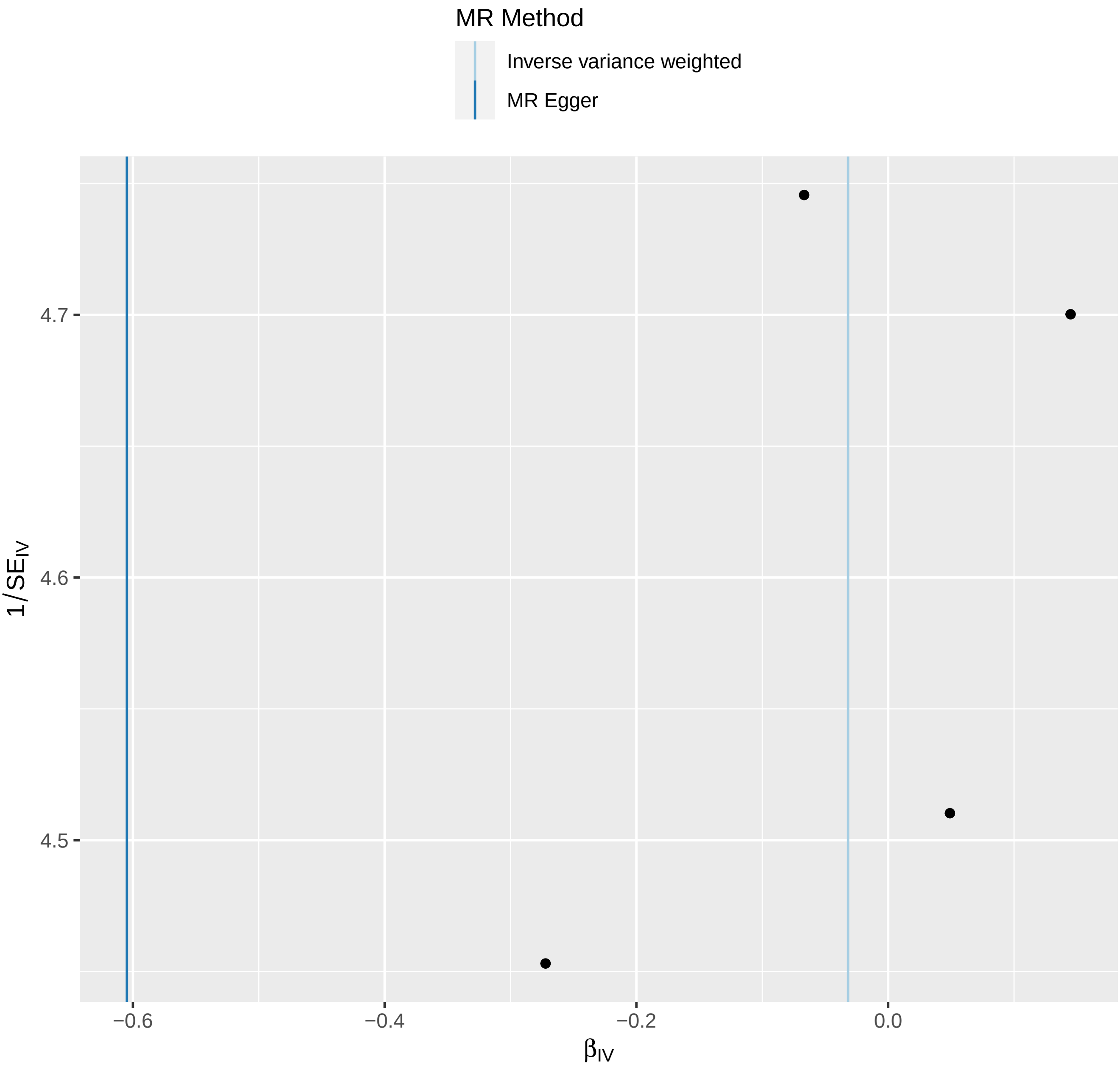

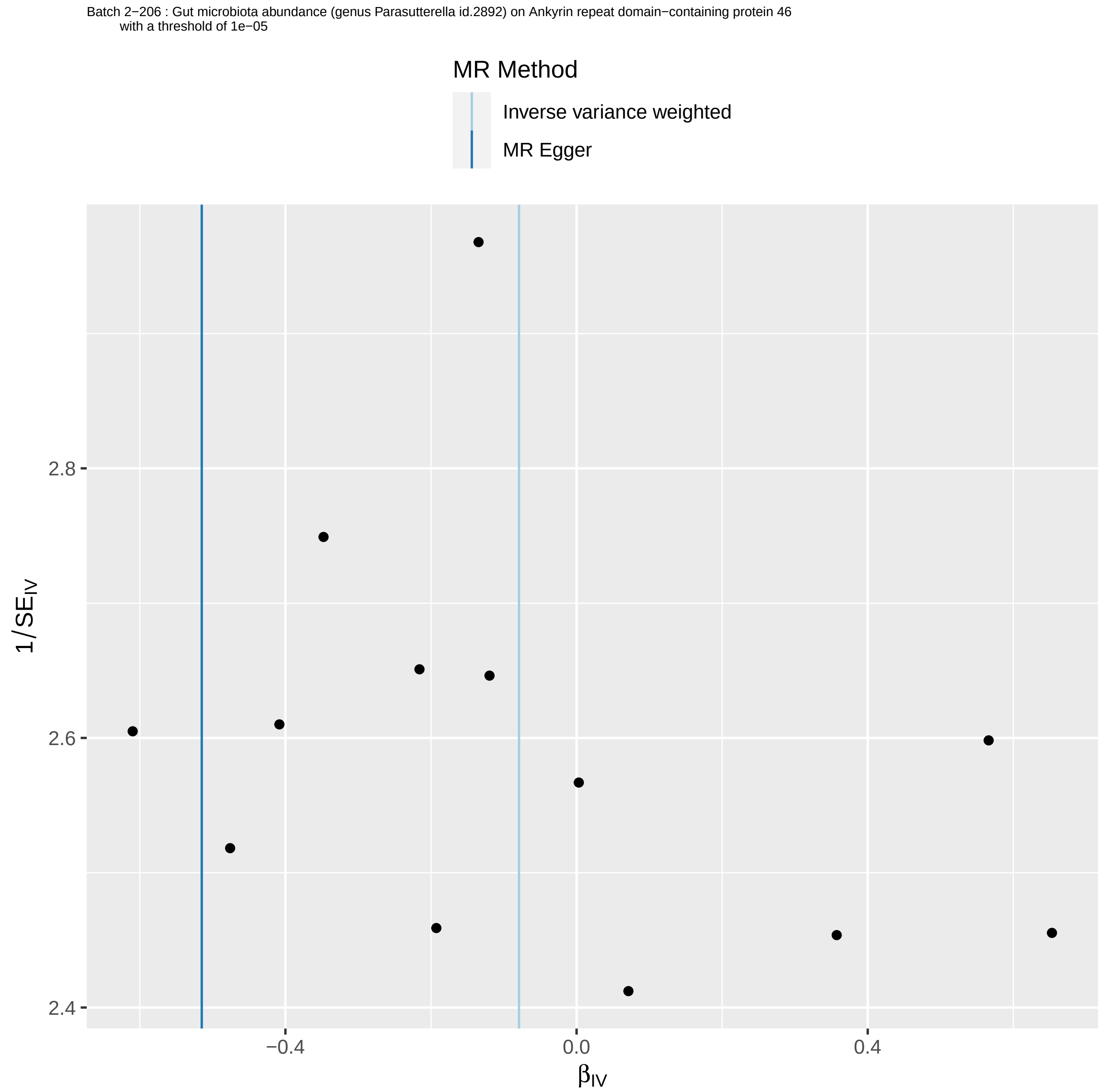
Batch 2−206 : Gut microbiota abundance (genus Parasutterella id.2892) on Ankyrin repeat domain−containing protein 46
with a threshold of 1e−05
MR Method
Inverse variance weighted
MR Egger
2.8
2.6
2.4
1/SE_IV
−0.4
0.0
0.4
β_IV

Batch 2−207 : Gut microbiota abundance (genus Catenibacterium id.2153) on Acidic leucine−rich nuclear phosphoprotein 32 family member A with a threshold of 1e−05

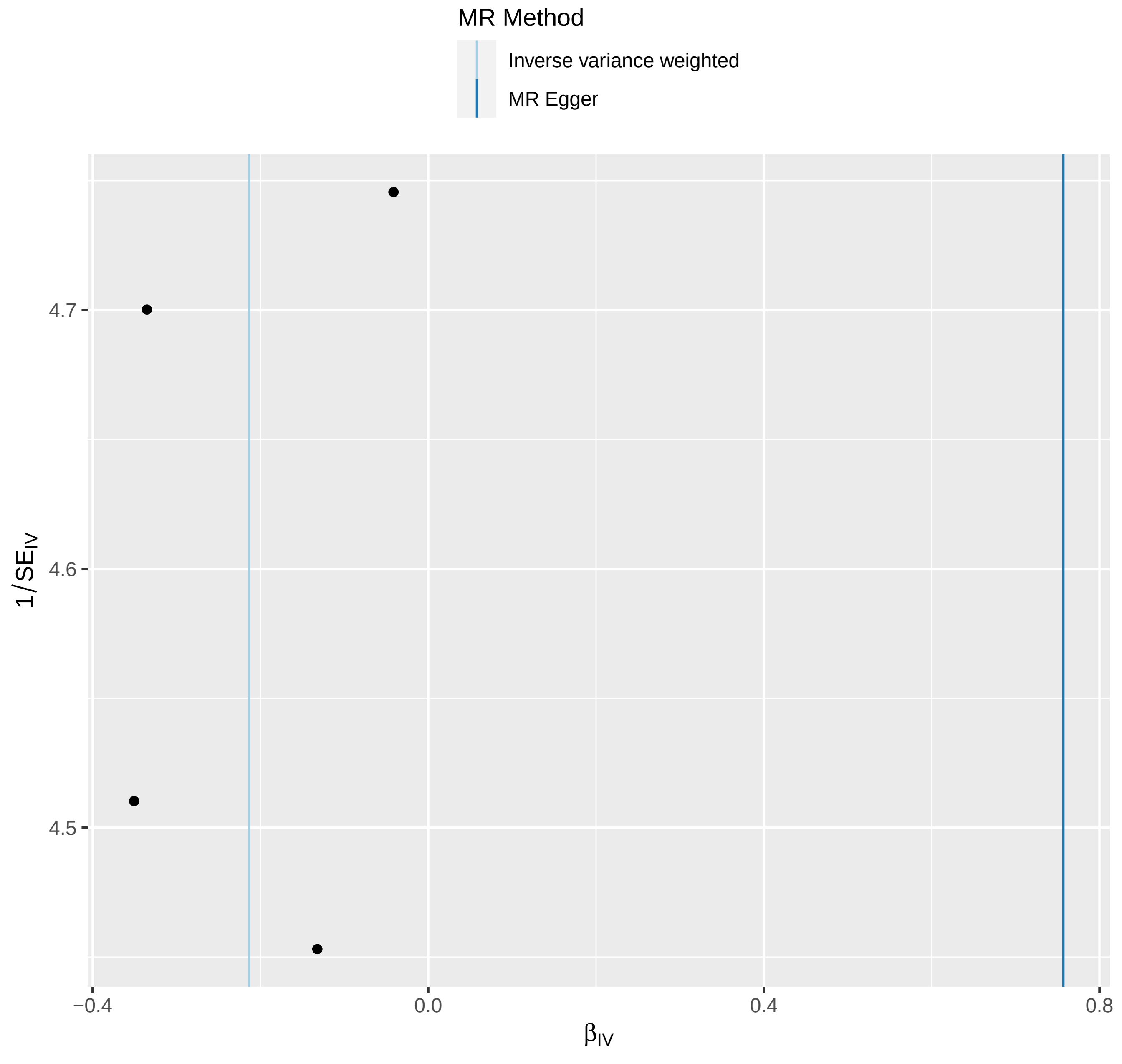

Batch 2−208 : Gut microbiota abundance (genus Parasutterella id.2892) on Acidic leucine−rich nuclear phosphoprotein 32 family member A
with a threshold of 1e−05

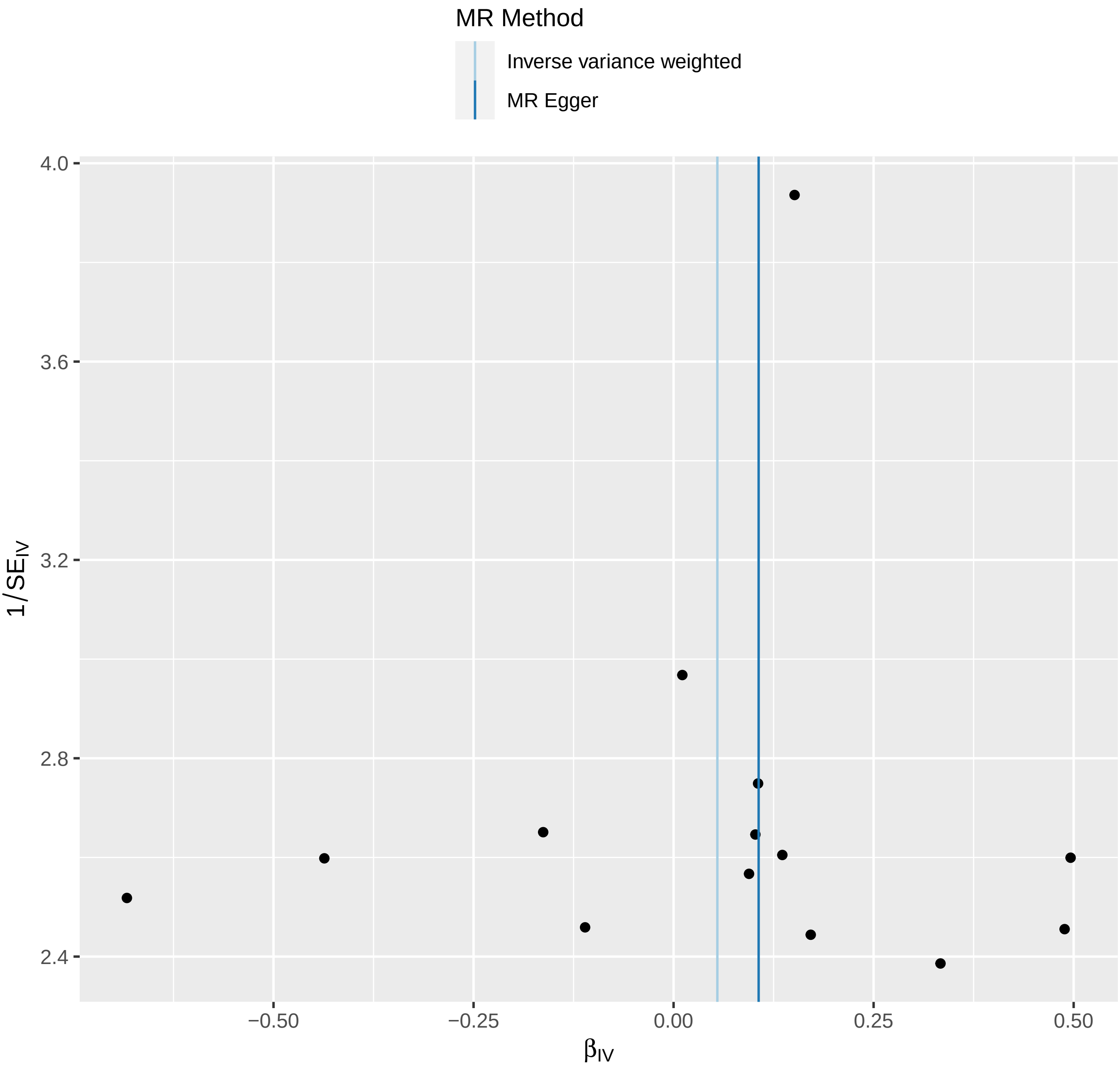

Batch 2−209 : Gut microbiota abundance (genus Catenibacterium id.2153) on Acidic leucine−rich nuclear phosphoprotein 32 family member B with a threshold of 1e−05

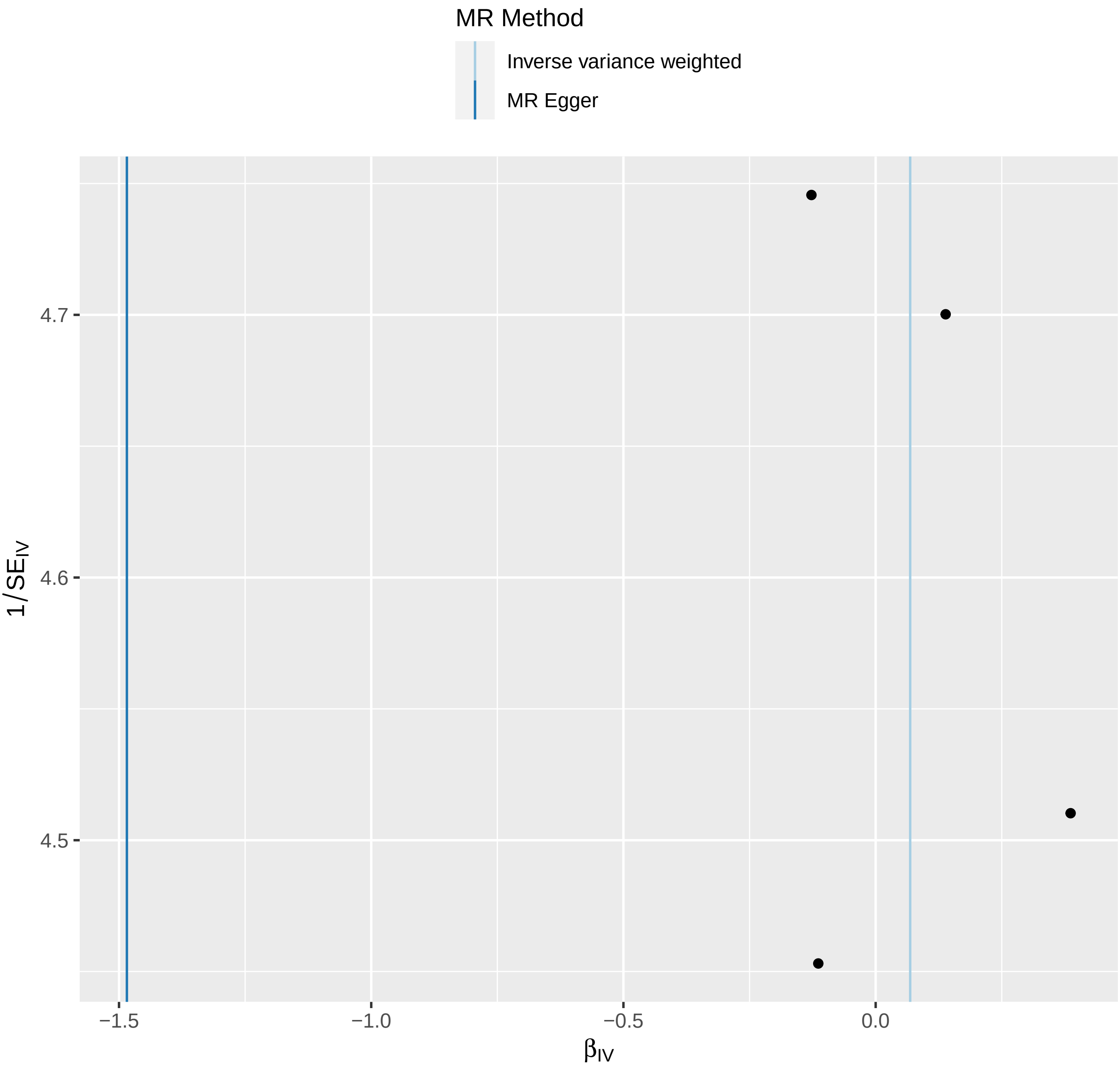

Batch 2−210 : Gut microbiota abundance (genus Parasutterella id.2892) on Acidic leucine−rich nuclear phosphoprotein 32 family member B with a threshold of 1e−05

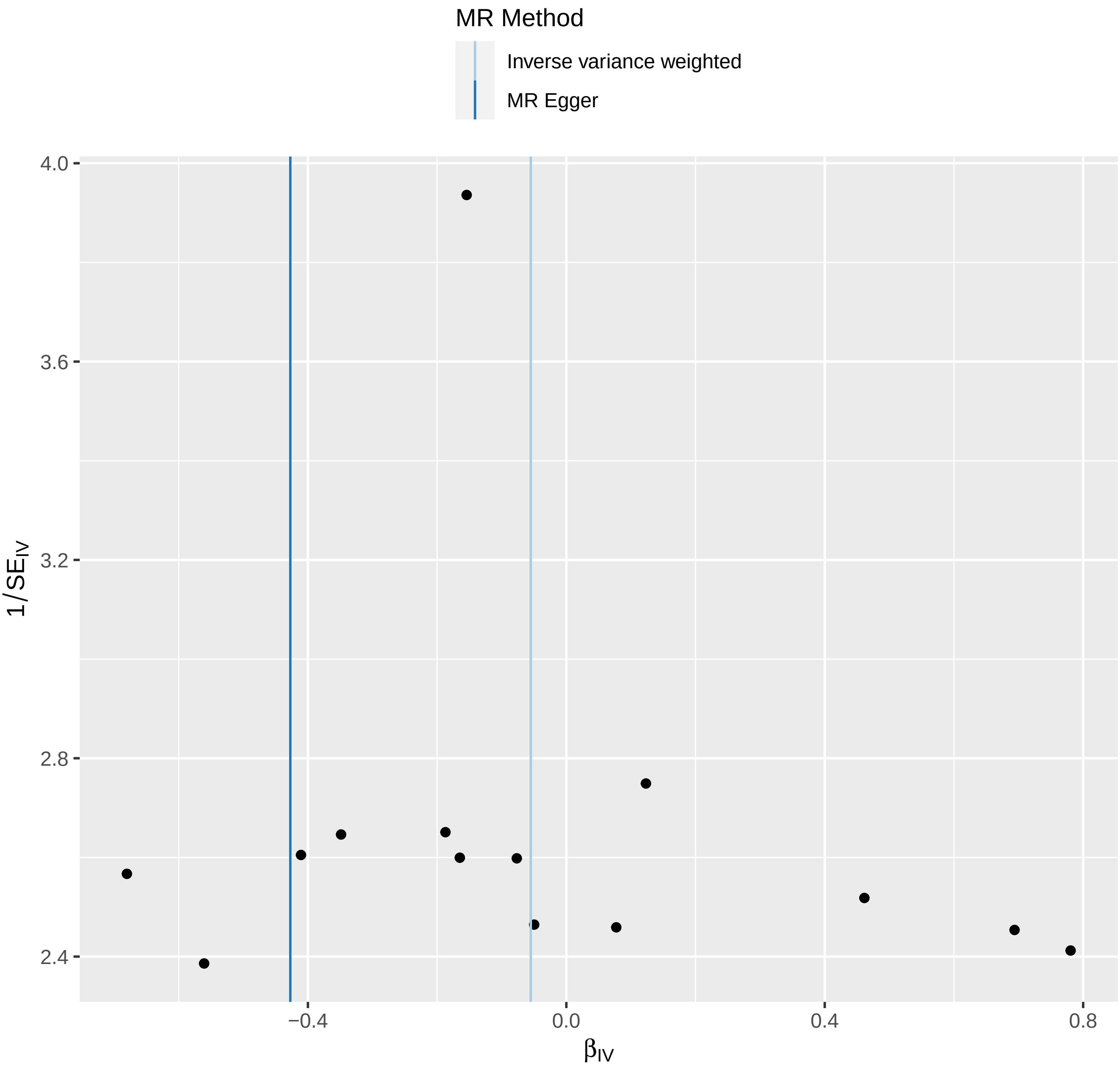

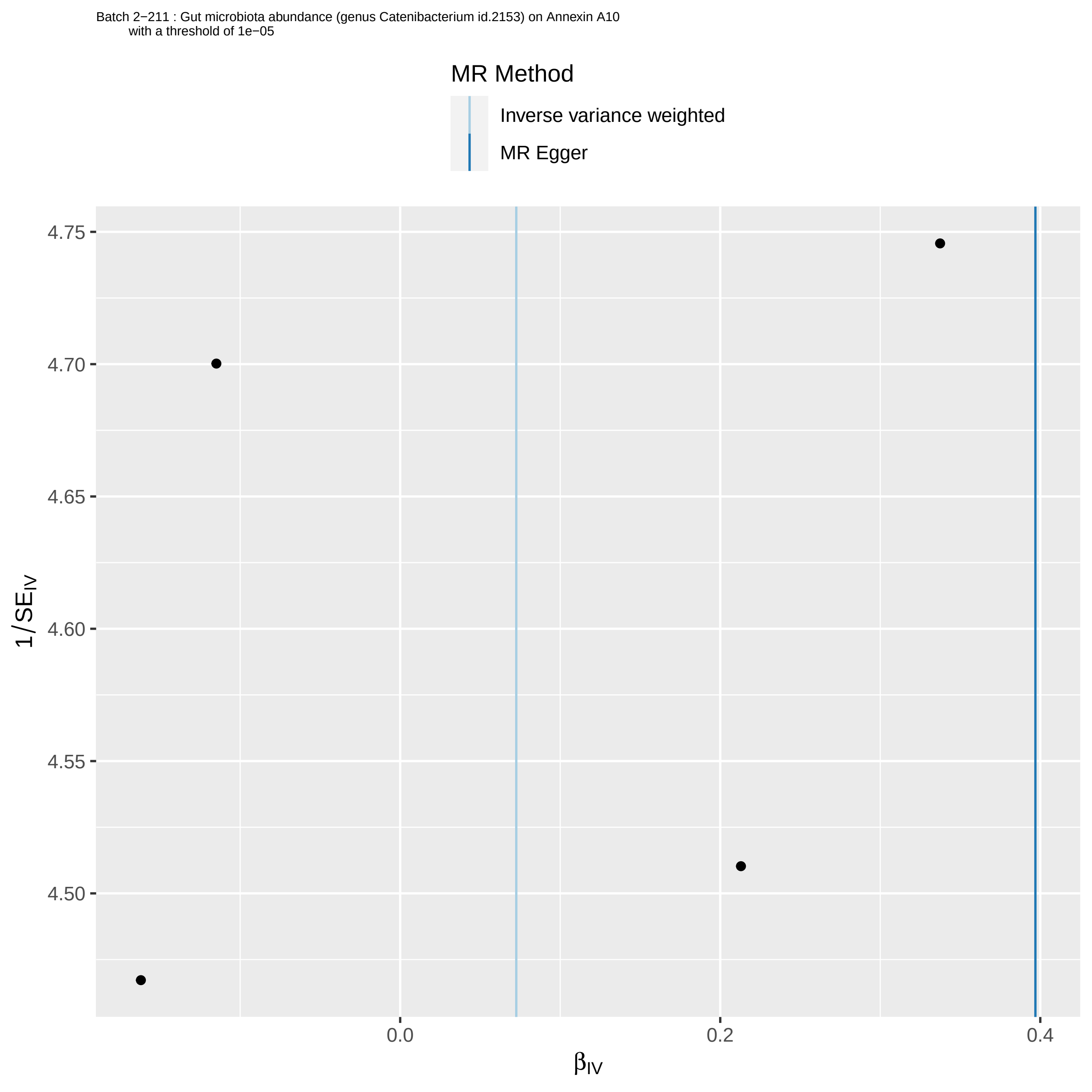

Batch 2−211 : Gut microbiota abundance (genus Catenibacterium id.2153) on Annexin A10
with a threshold of 1e−05
MR Method
Inverse variance weighted
MR Egger
4.75
4.70
4.65
4.60
4.55
4.50
1/SE_IV
0.0
0.2
0.4
β_IV

Batch 2−212 : Gut microbiota abundance (genus Parasutterella id.2892) on Annexin A10
with a threshold of 1e−05

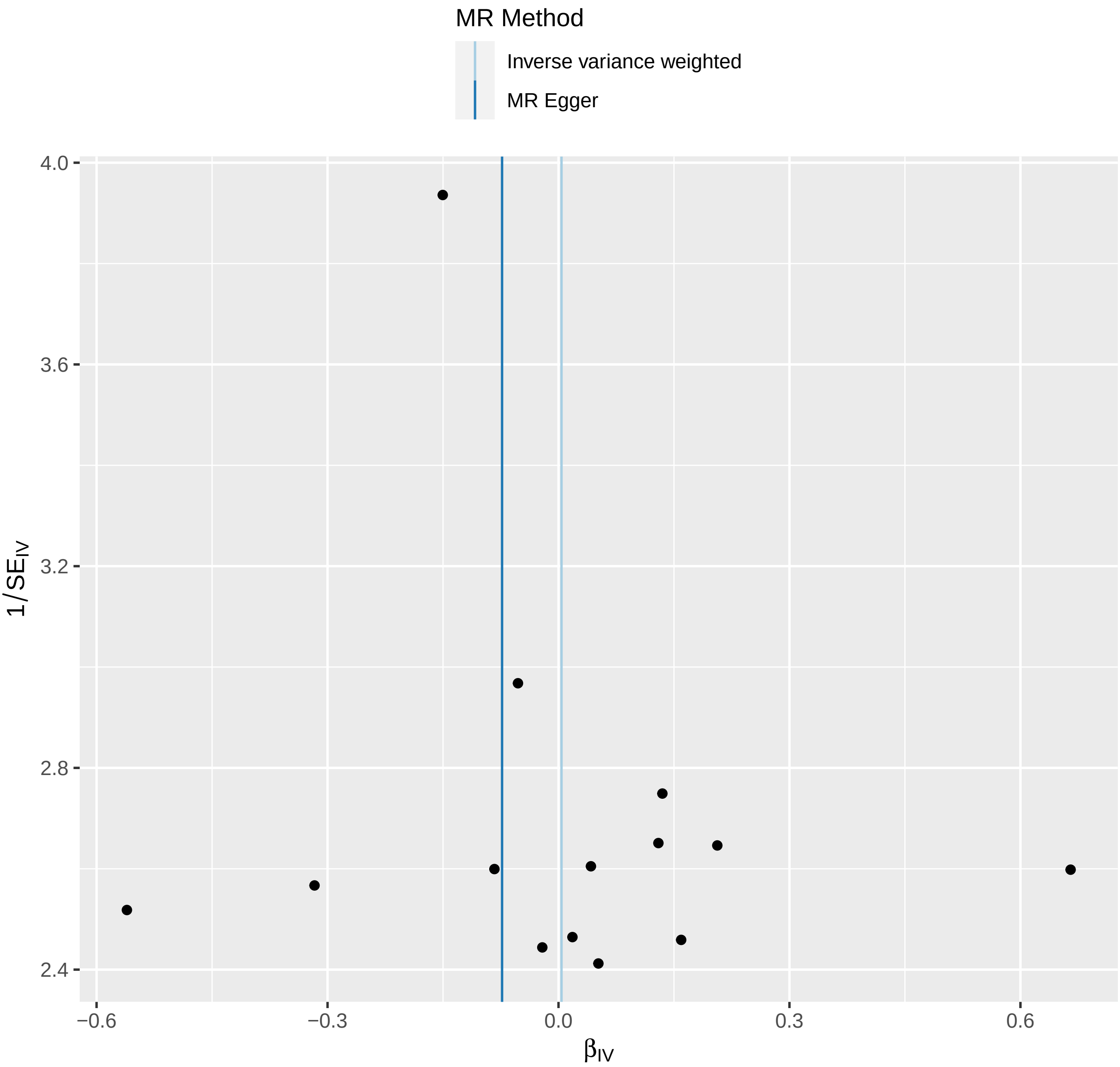

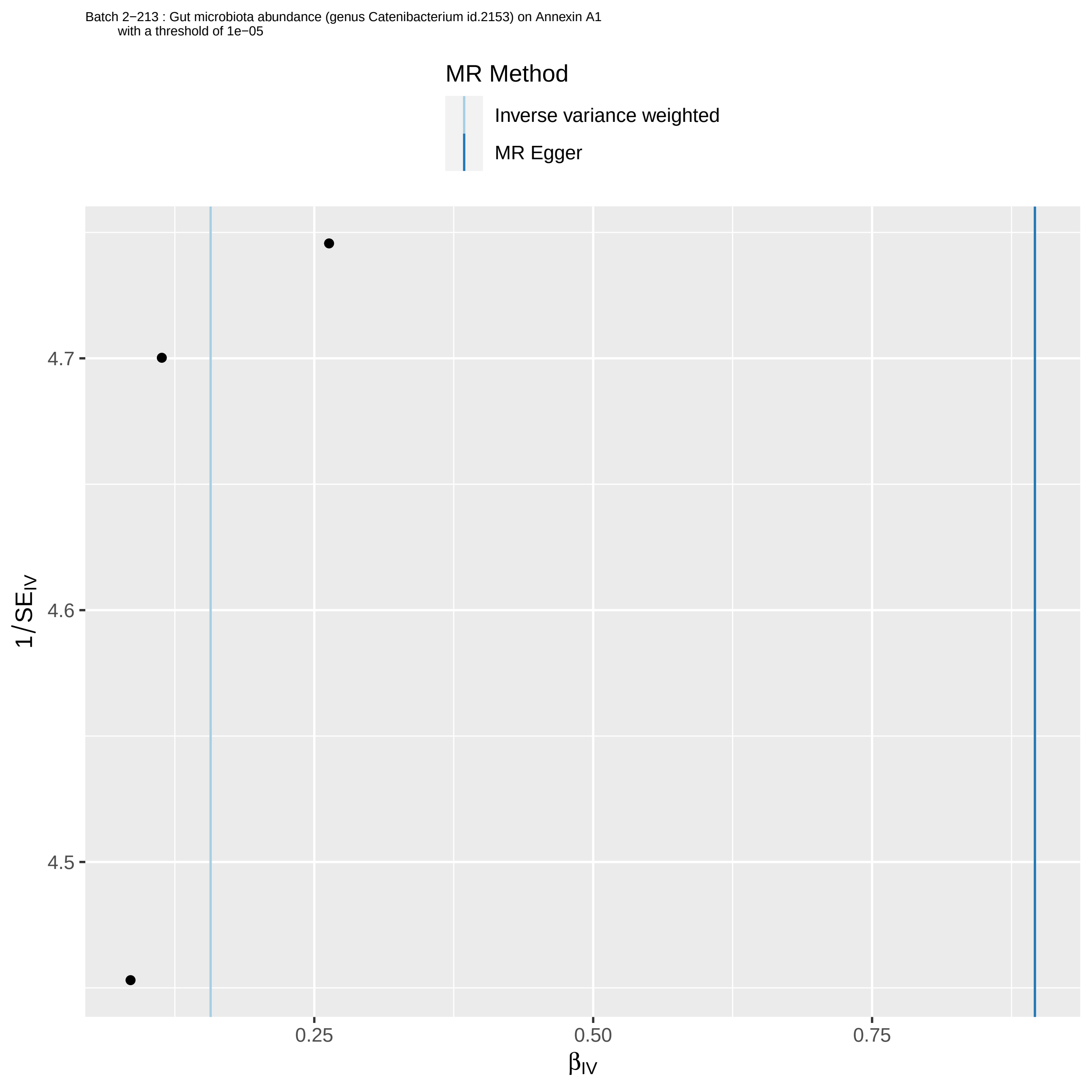

Batch 2−213 : Gut microbiota abundance (genus Catenibacterium id.2153) on Annexin A1
with a threshold of 1e−05
MR Method
Inverse variance weighted
MR Egger
4.7
4.6
4.5
$1/SE_{IV}$
0.25
0.50
0.75
β_{IV}

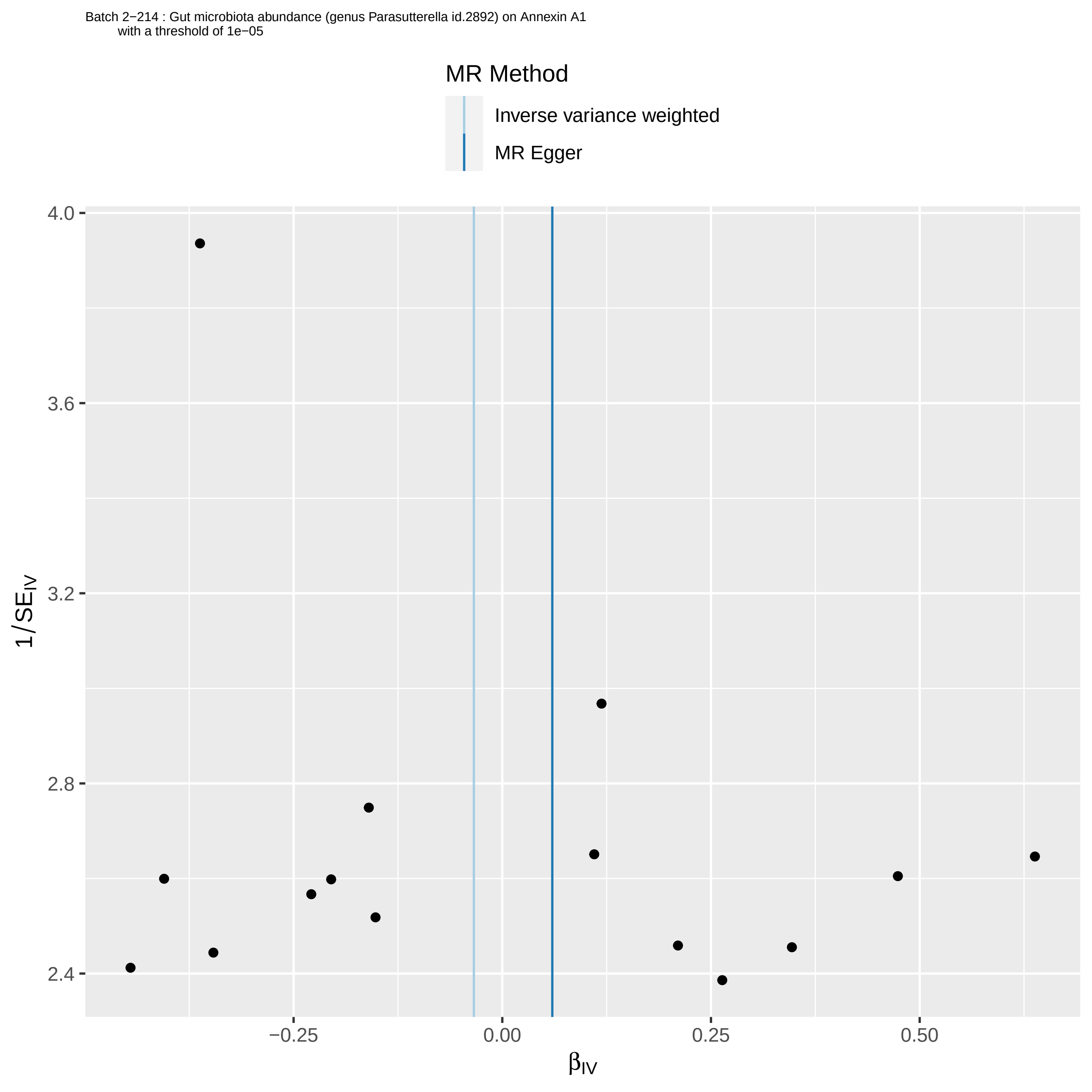

Batch 2−214 : Gut microbiota abundance (genus Parasutterella id.2892) on Annexin A1
with a threshold of 1e−05
MR Method
Inverse variance weighted
MR Egger
4.0
3.6
3.2
2.8
2.4
1/SE$_{IV}$
−0.25
0.00
0.25
0.50
β$_{IV}$

Batch 2−215 : Gut microbiota abundance (genus Catenibacterium id.2153) on Annexin A2
with a threshold of 1e−05

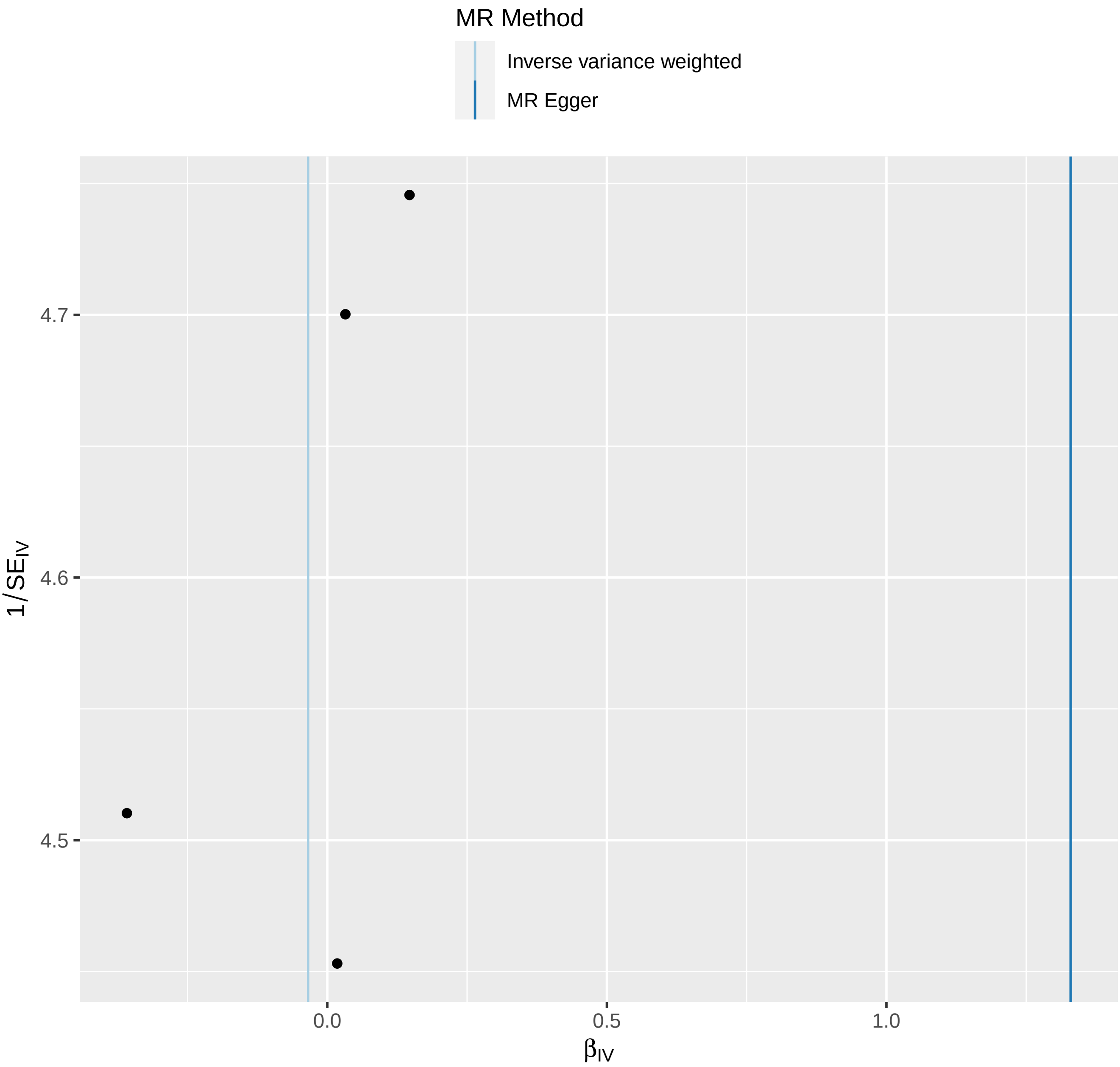

Batch 2−216 : Gut microbiota abundance (genus Parasutterella id.2892) on Annexin A2
with a threshold of 1e−05

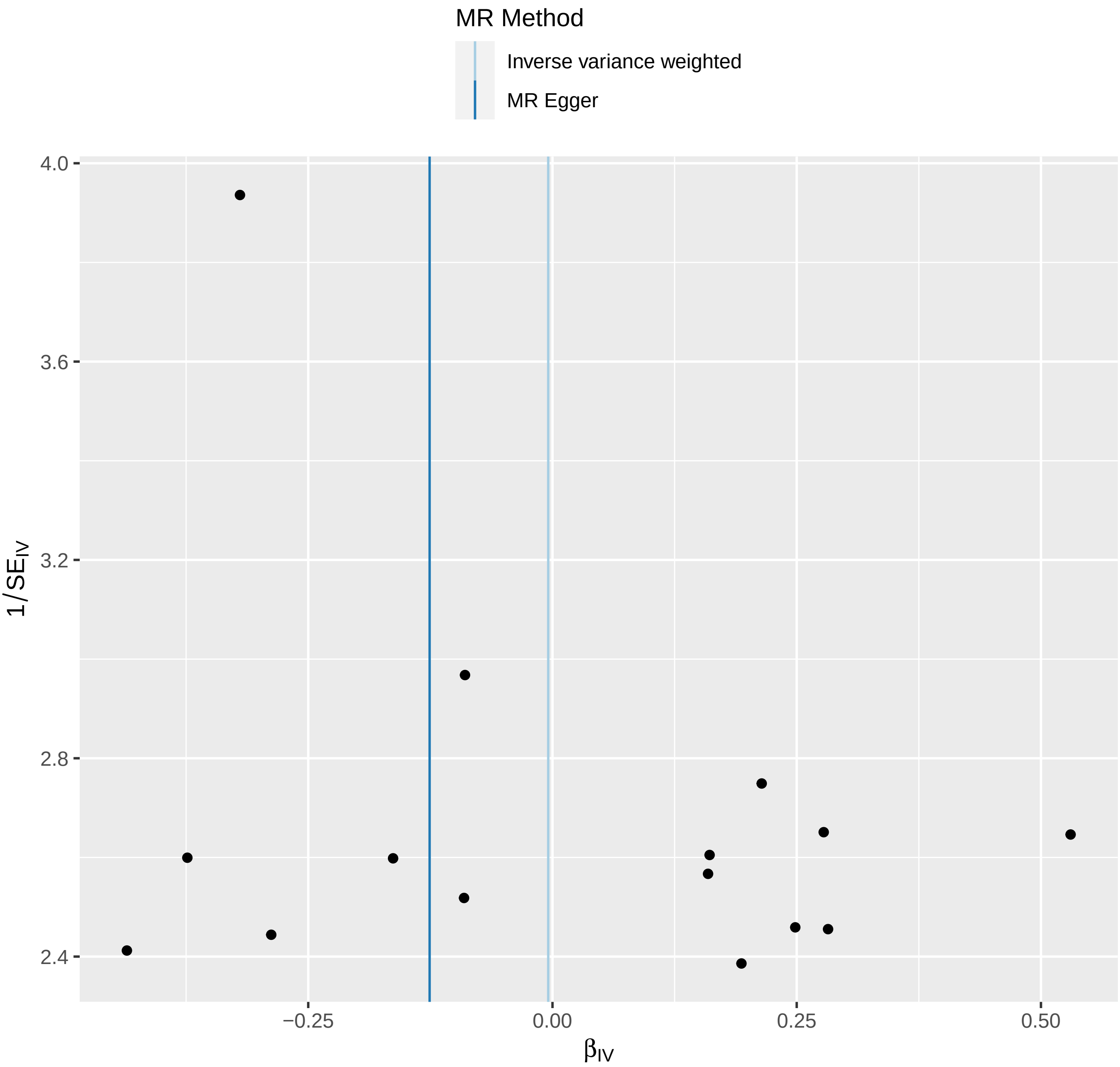

Batch 2−217 : Gut microbiota abundance (genus Catenibacterium id.2153) on Annexin A2
with a threshold of 1e−05

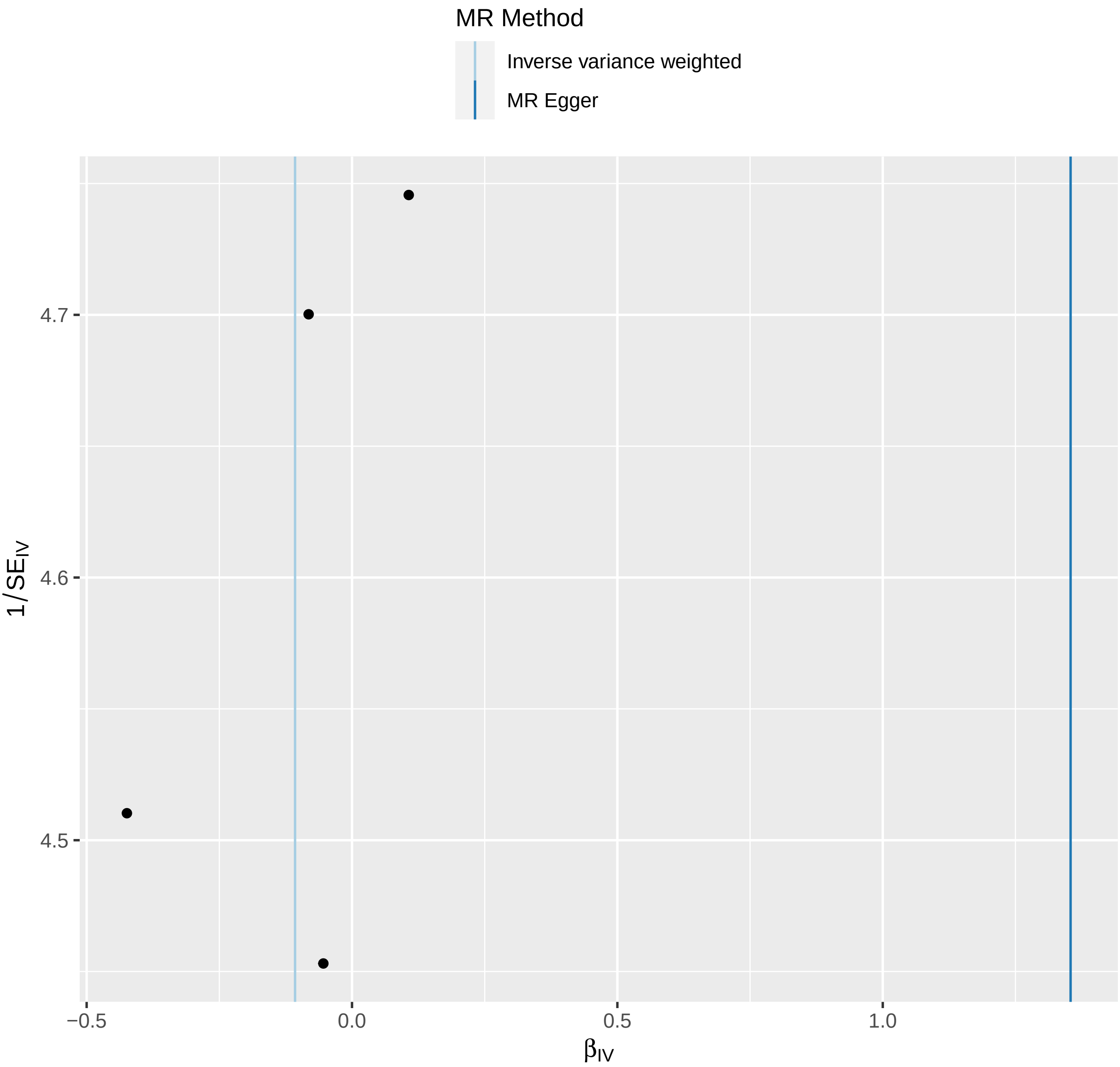

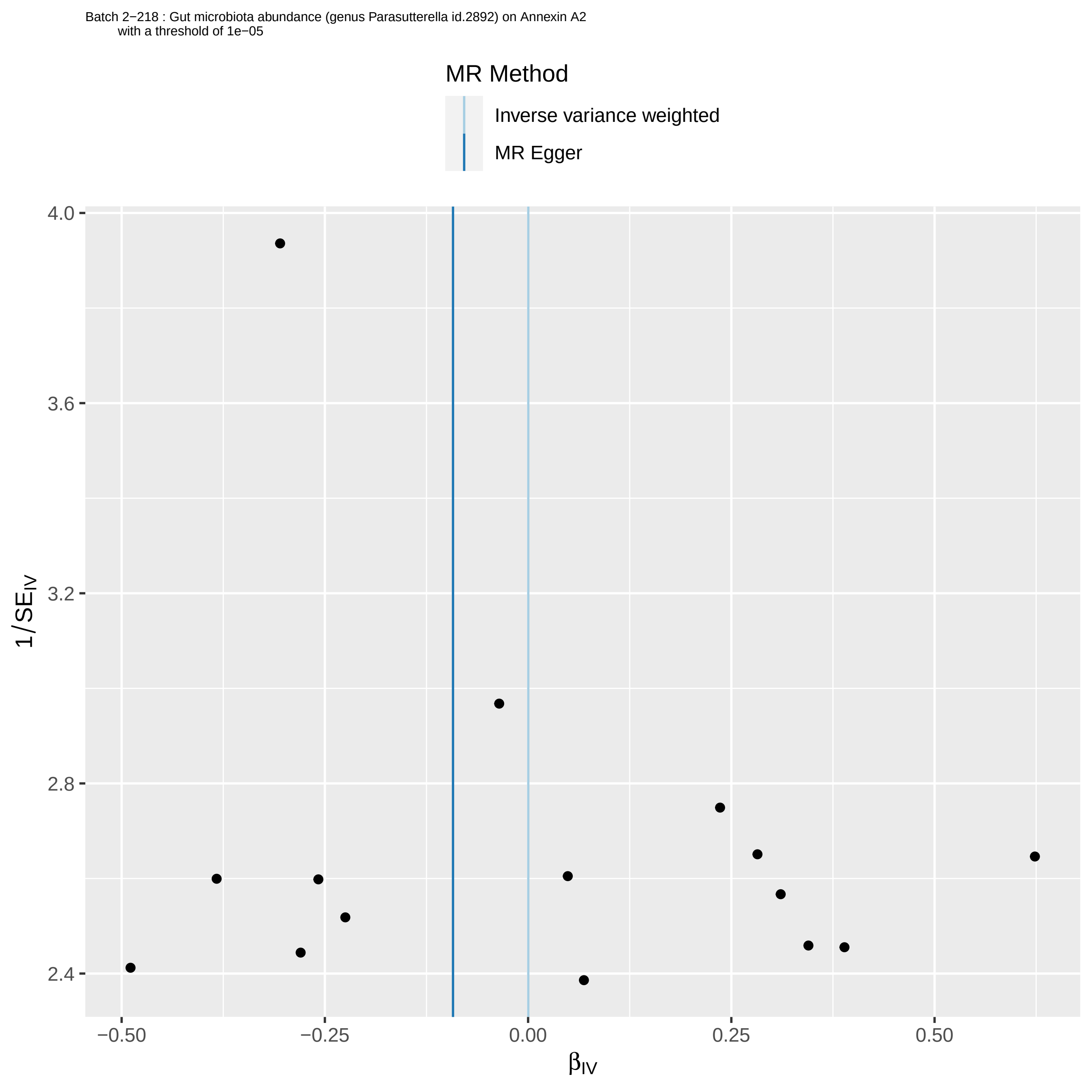

Batch 2−218 : Gut microbiota abundance (genus Parasutterella id.2892) on Annexin A2
with a threshold of 1e−05
MR Method
Inverse variance weighted
MR Egger
4.0
3.6
3.2
2.8
2.4
1/SE_IV
−0.50
−0.25
0.00
0.25
0.50
β_IV

Batch 2−219 : Gut microbiota abundance (genus Catenibacterium id.2153) on Annexin A9
with a threshold of 1e−05

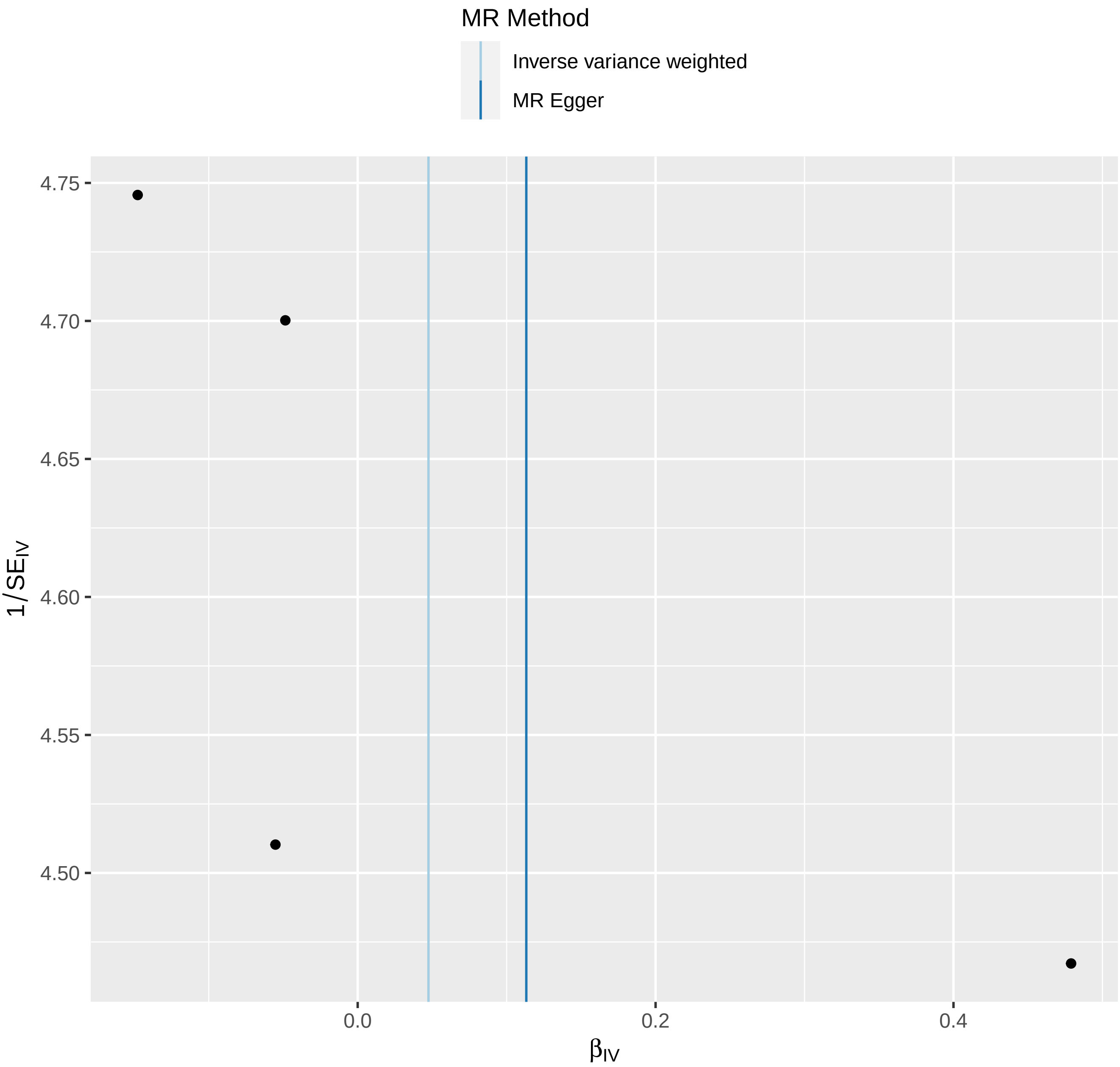

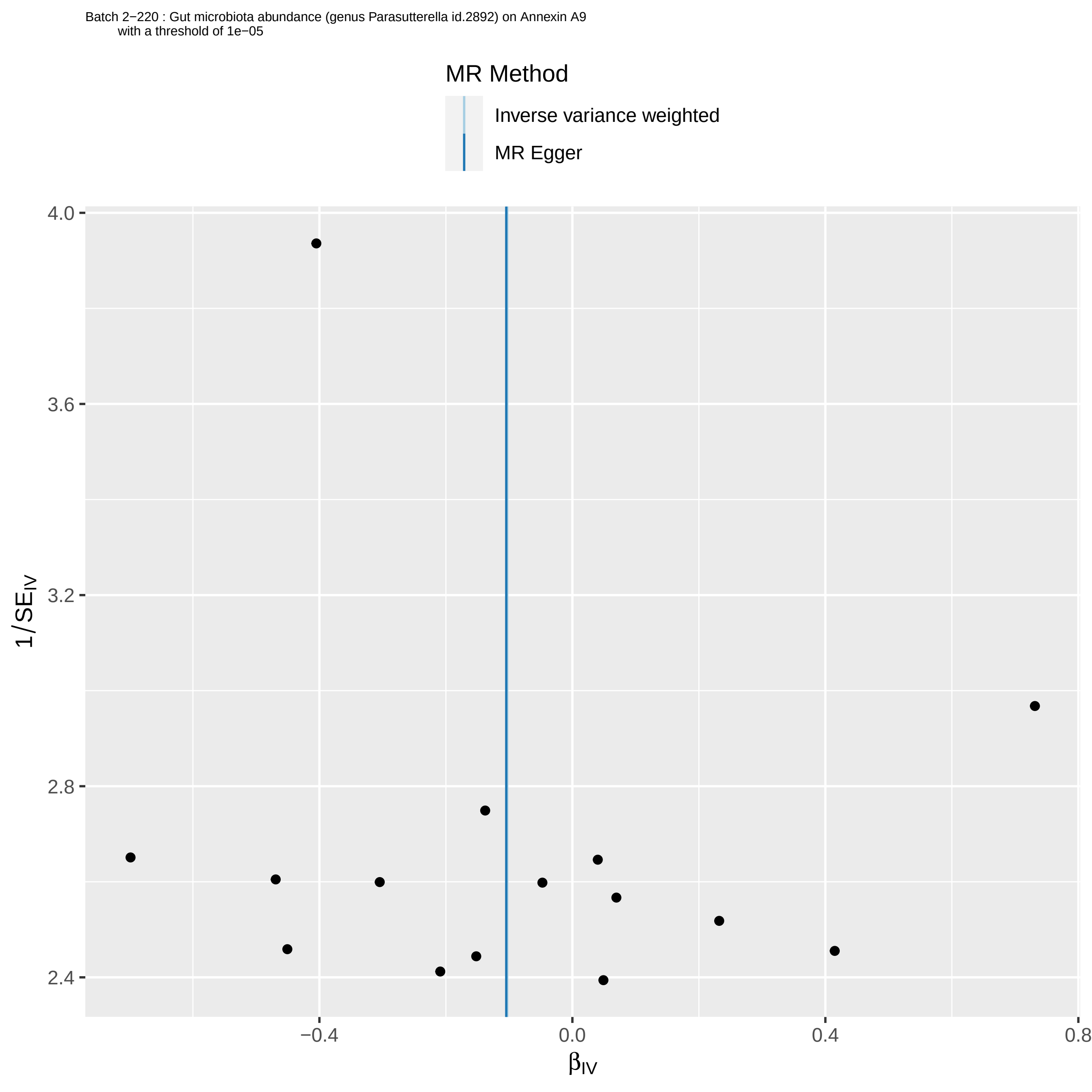
Batch 2−220 : Gut microbiota abundance (genus Parasutterella id.2892) on Annexin A9
with a threshold of 1e−05
MR Method
Inverse variance weighted
MR Egger
4.0
3.6
3.2
2.8
2.4
1/SE$_{IV}$
−0.4
0.0
0.4
0.8
β_{IV}

Batch 2−221 : Gut microbiota abundance (genus Catenibacterium id.2153) on Retina−specific copper amine oxidase
with a threshold of 1e−05

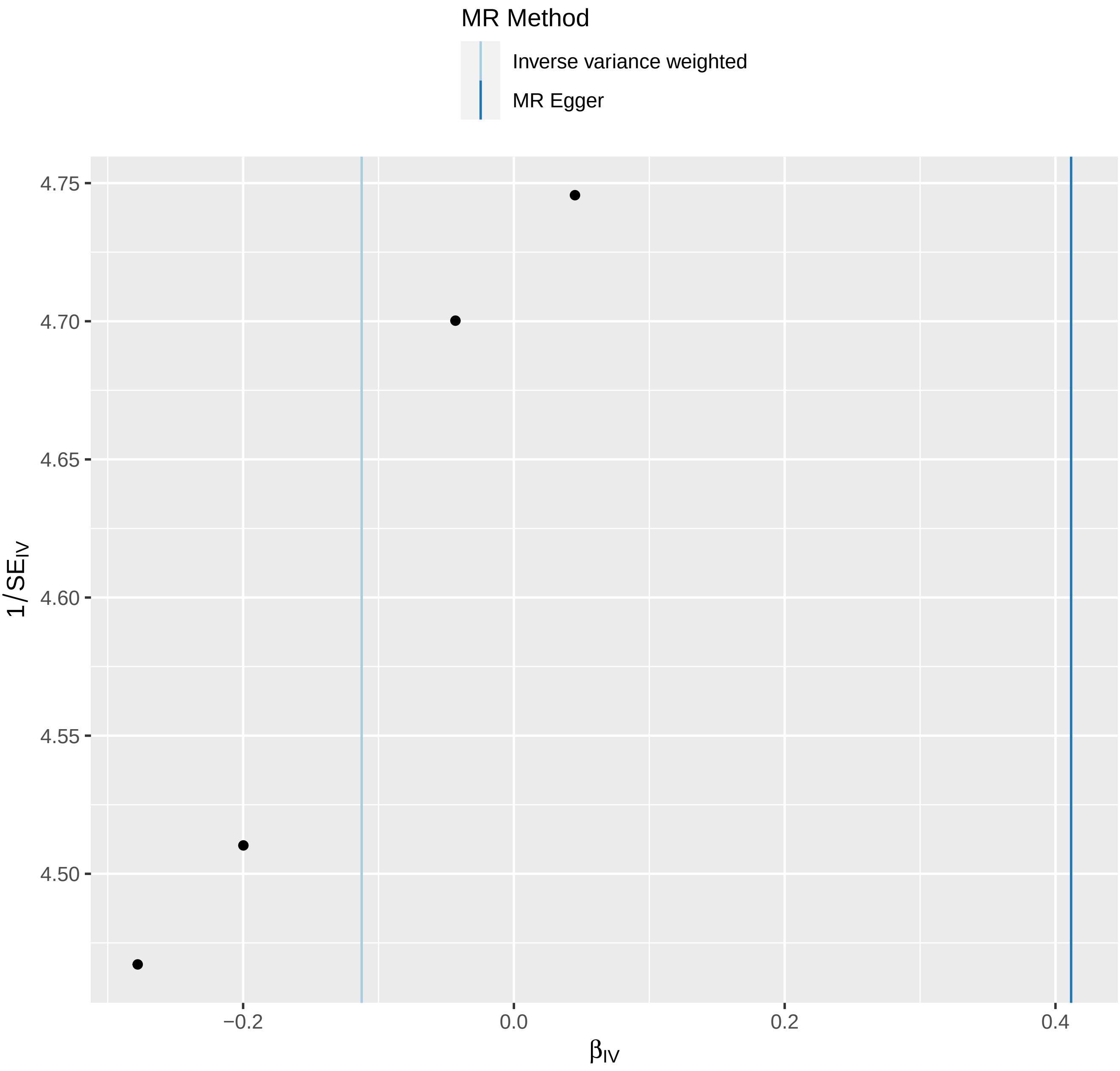

Batch 2−222 : Gut microbiota abundance (genus Parasutterella id.2892) on Retina−specific copper amine oxidase with a threshold of 1e−05

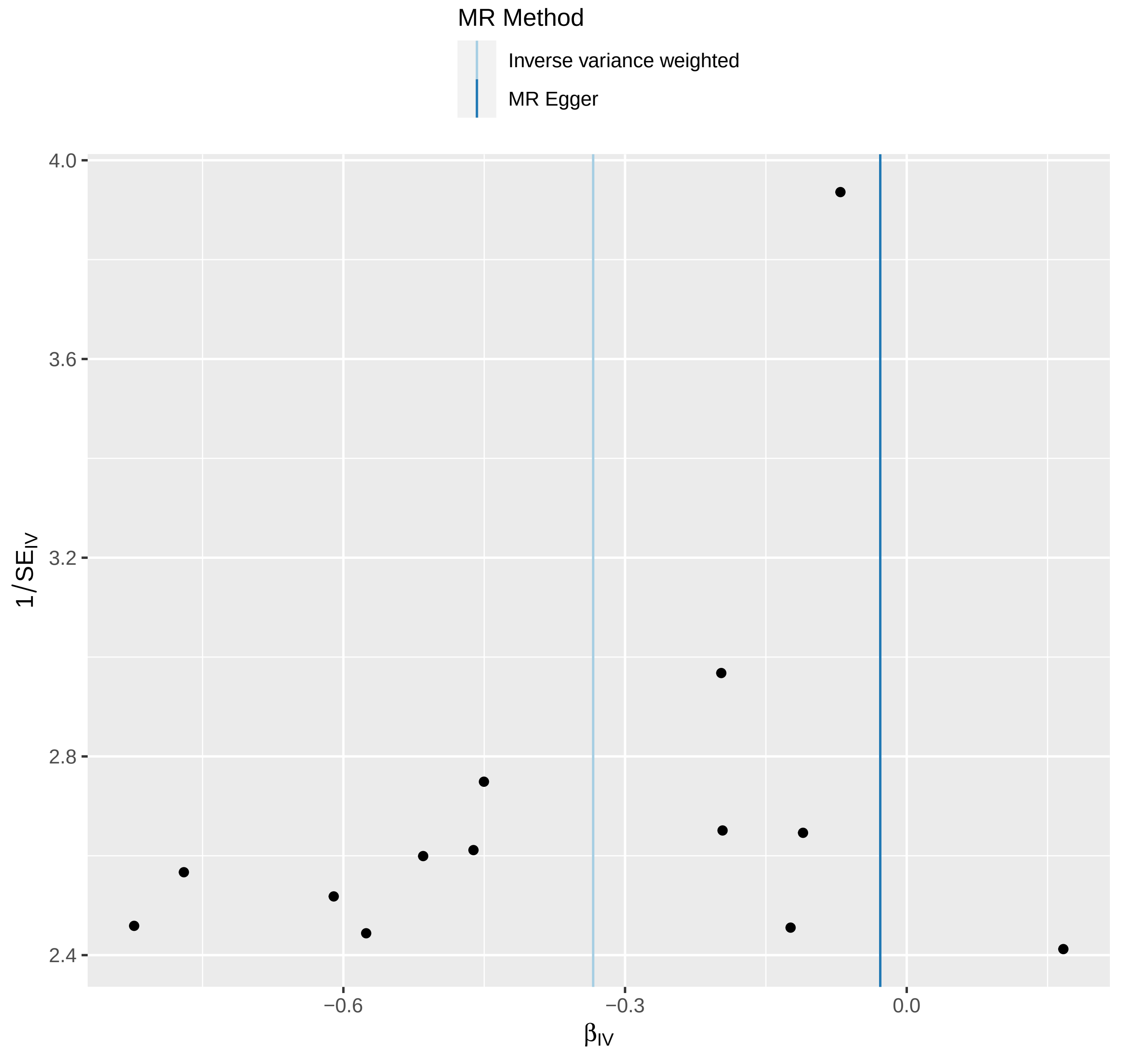

Batch 2−223 : Gut microbiota abundance (genus Catenibacterium id.2153) on AP−4 complex subunit mu−1
with a threshold of 1e−05

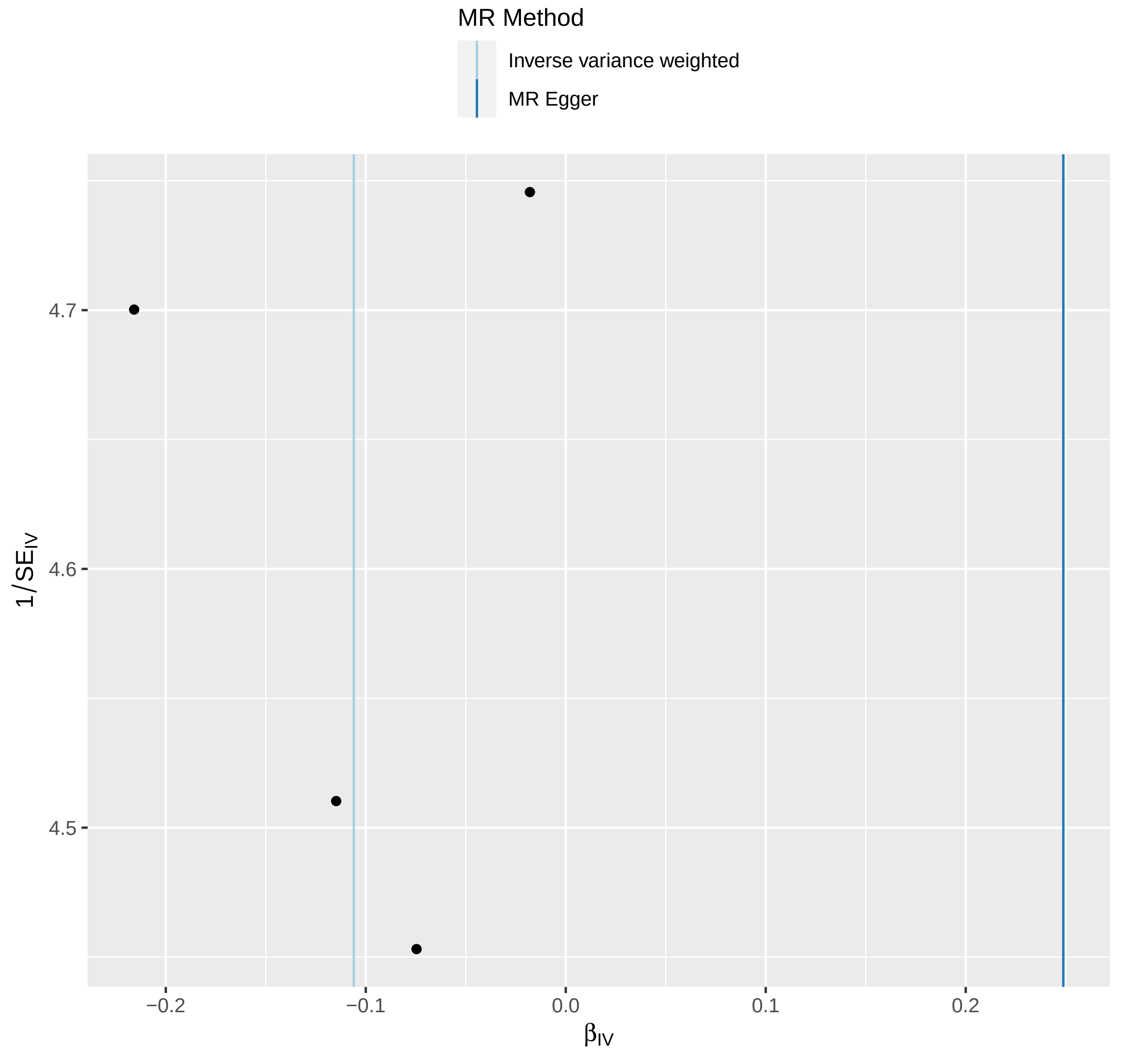

Batch 2−224 : Gut microbiota abundance (genus Parasutterella id.2892) on AP−4 complex subunit mu−1
with a threshold of 1e−05

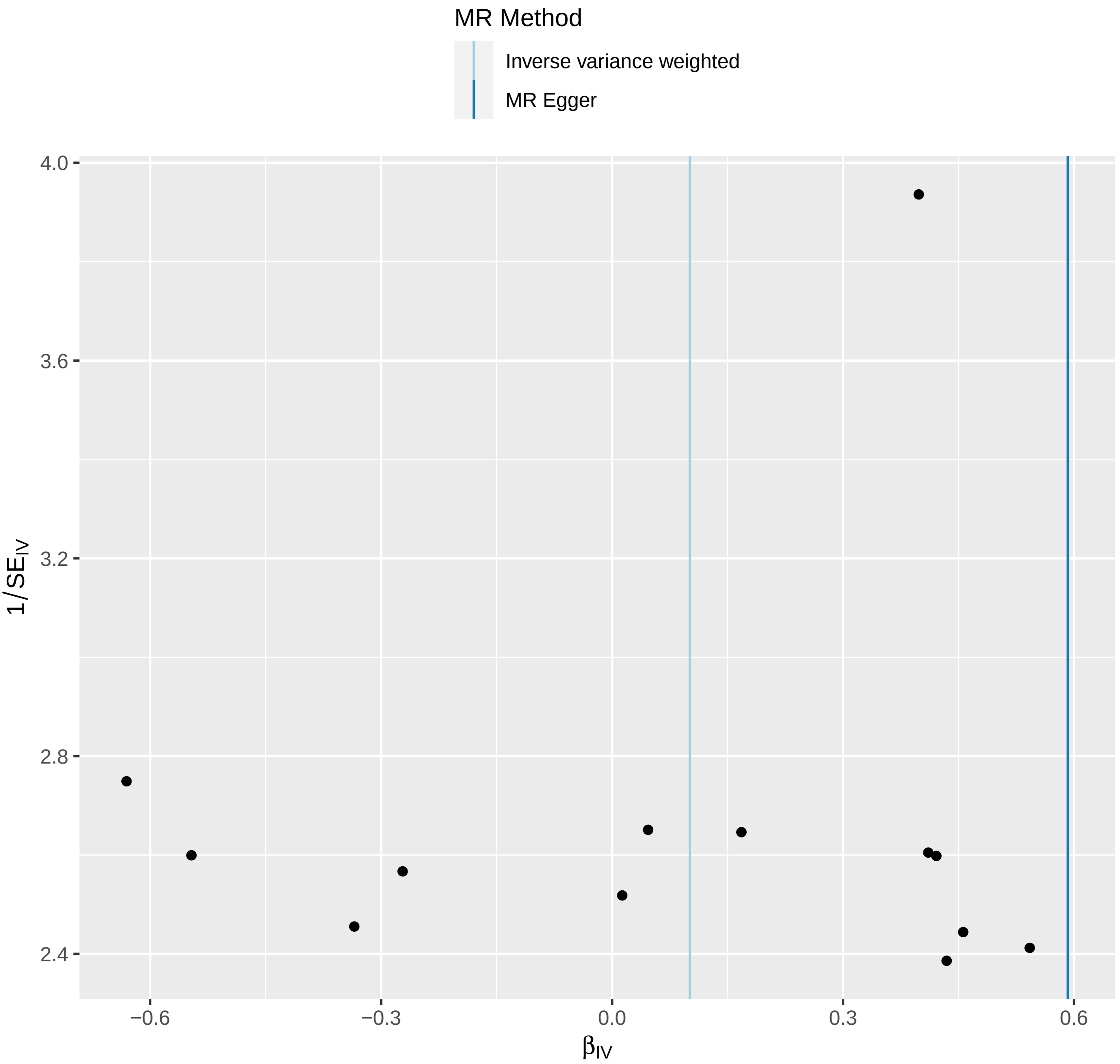

Batch 2−225 : Gut microbiota abundance (genus Catenibacterium id.2153) on Apoptotic protease−activating factor 1 with a threshold of 1e−05

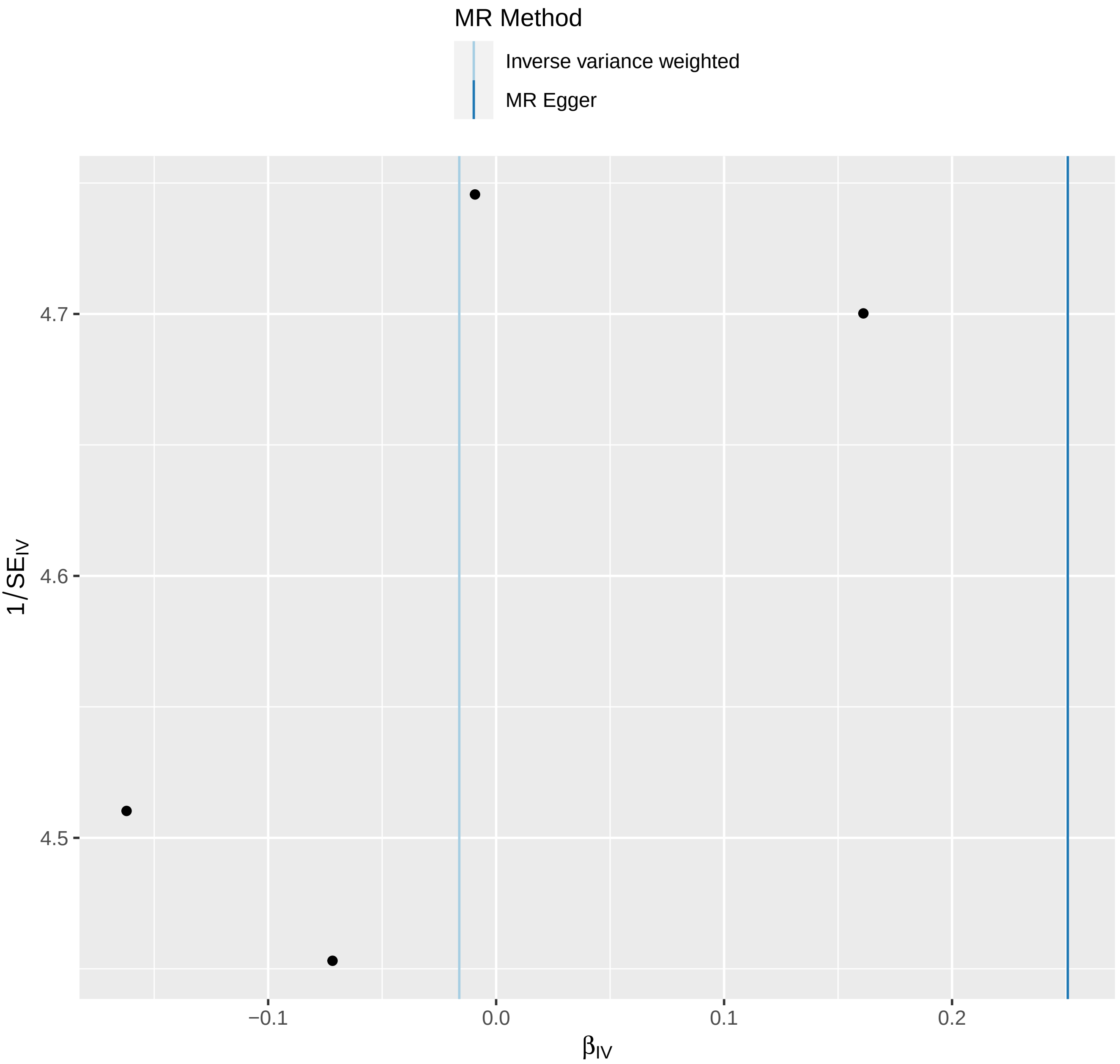

Batch 2−226 : Gut microbiota abundance (genus Parasutterella id.2892) on Apoptotic protease−activating factor 1
with a threshold of 1e−05

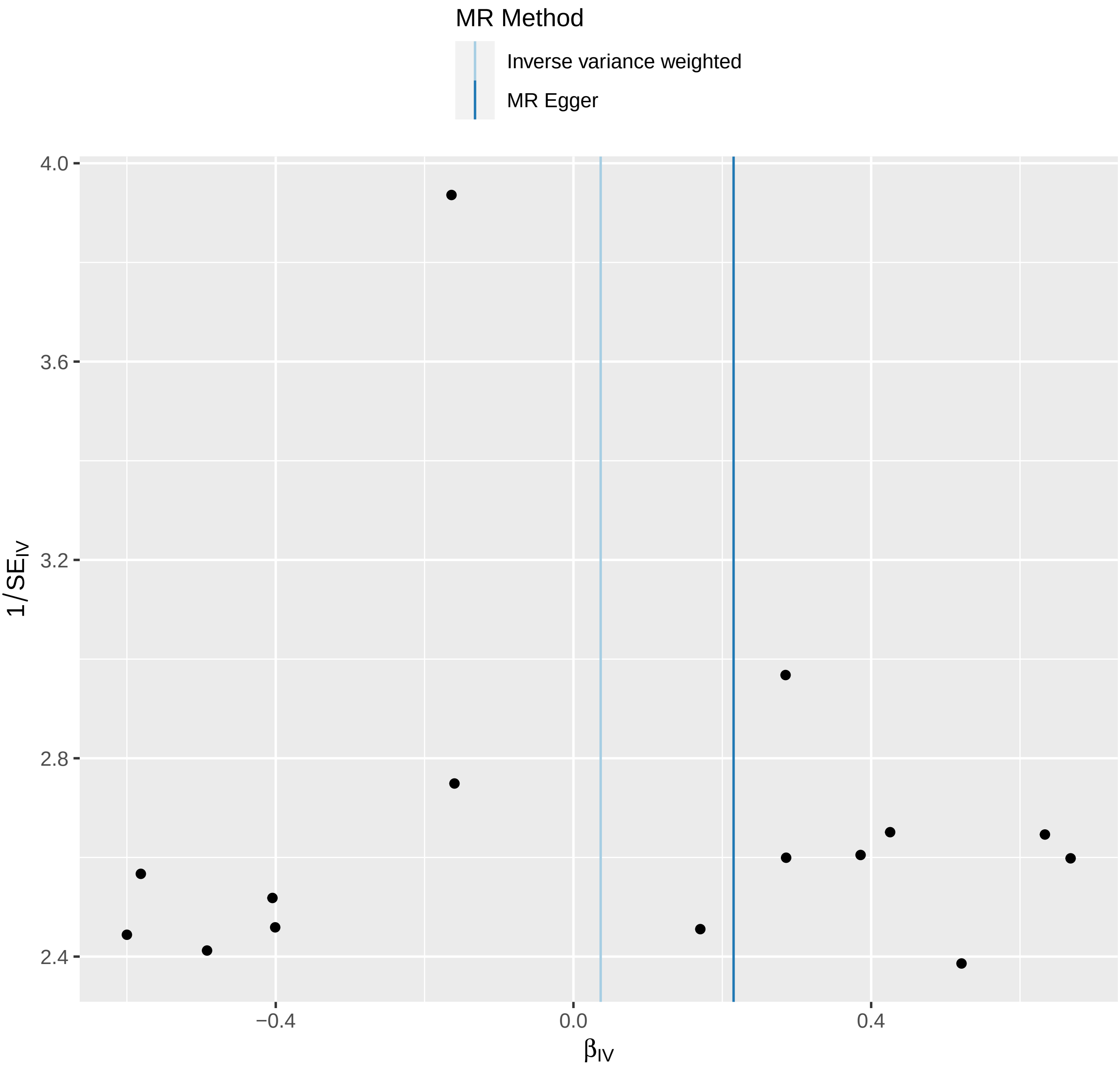

Batch 2−227 : Gut microbiota abundance (genus Catenibacterium id.2153) on Amyloid beta A4 precursor protein−binding family B member 1 with a threshold of 1e−05

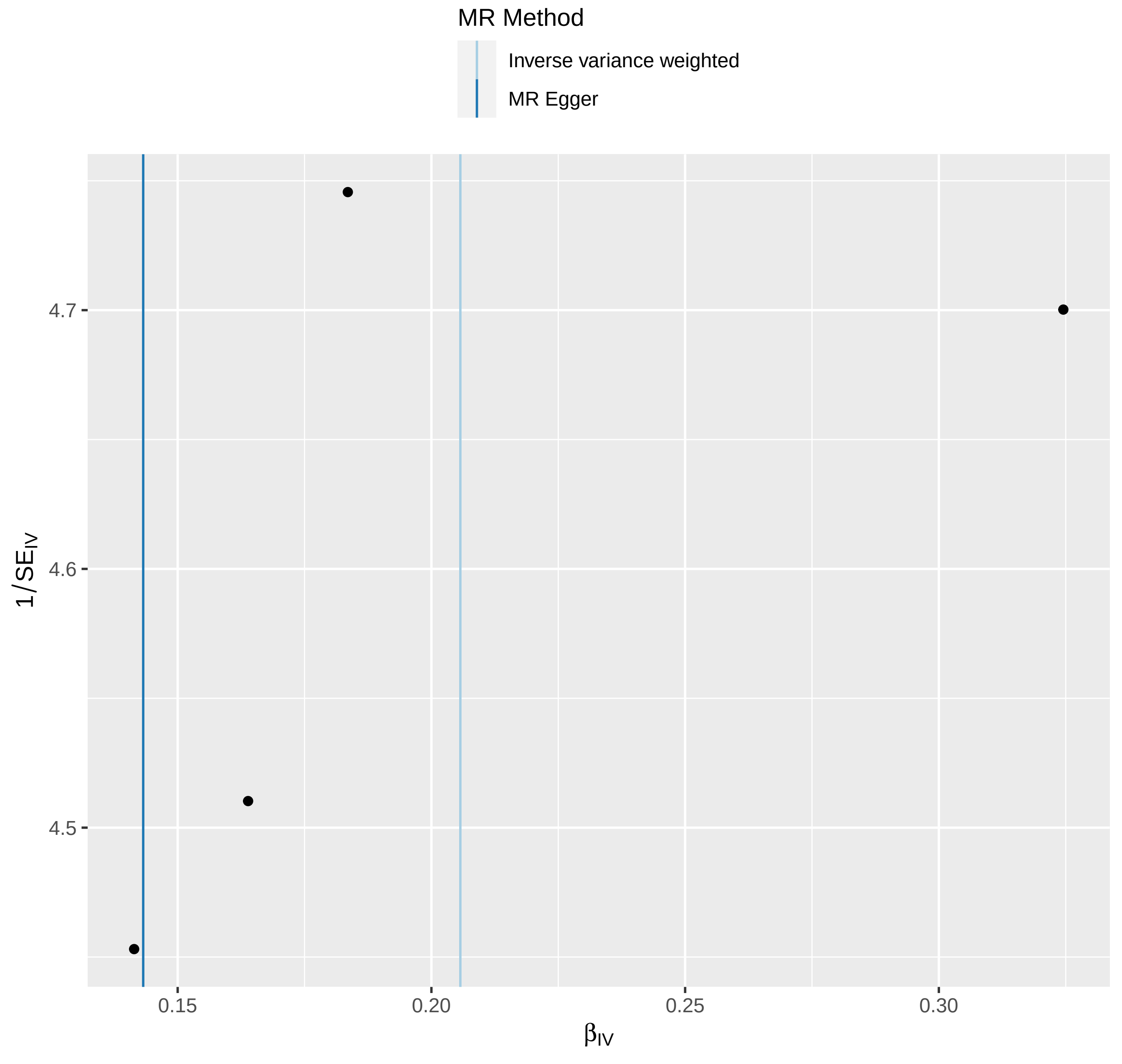

Batch 2−228 : Gut microbiota abundance (genus Parasutterella id.2892) on Amyloid beta A4 precursor protein−binding family B member 1 with a threshold of 1e−05

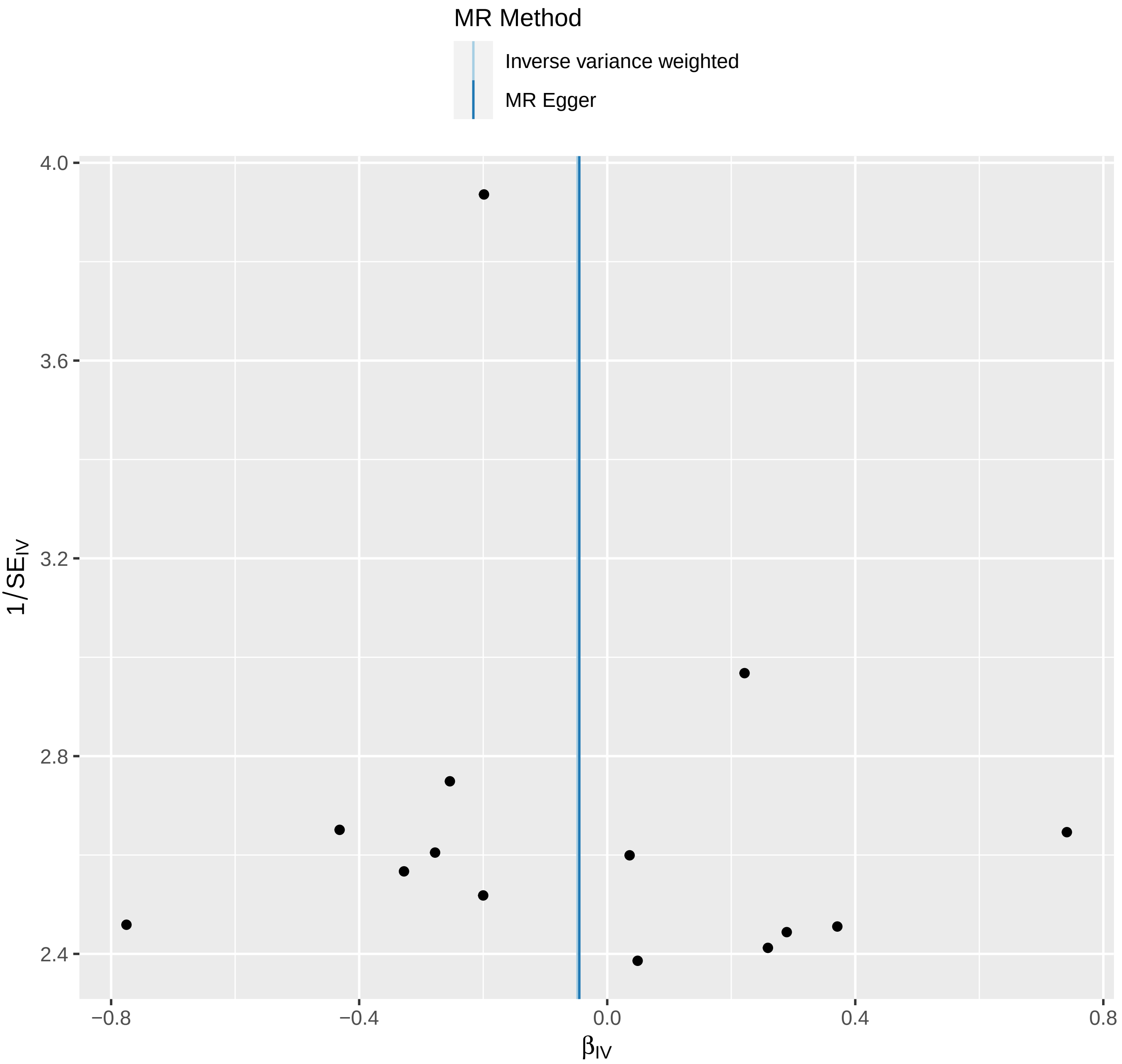

Batch 2−229 : Gut microbiota abundance (genus Catenibacterium id.2153) on Amyloid beta A4 precursor protein−binding family B member 1 with a threshold of 1e−05

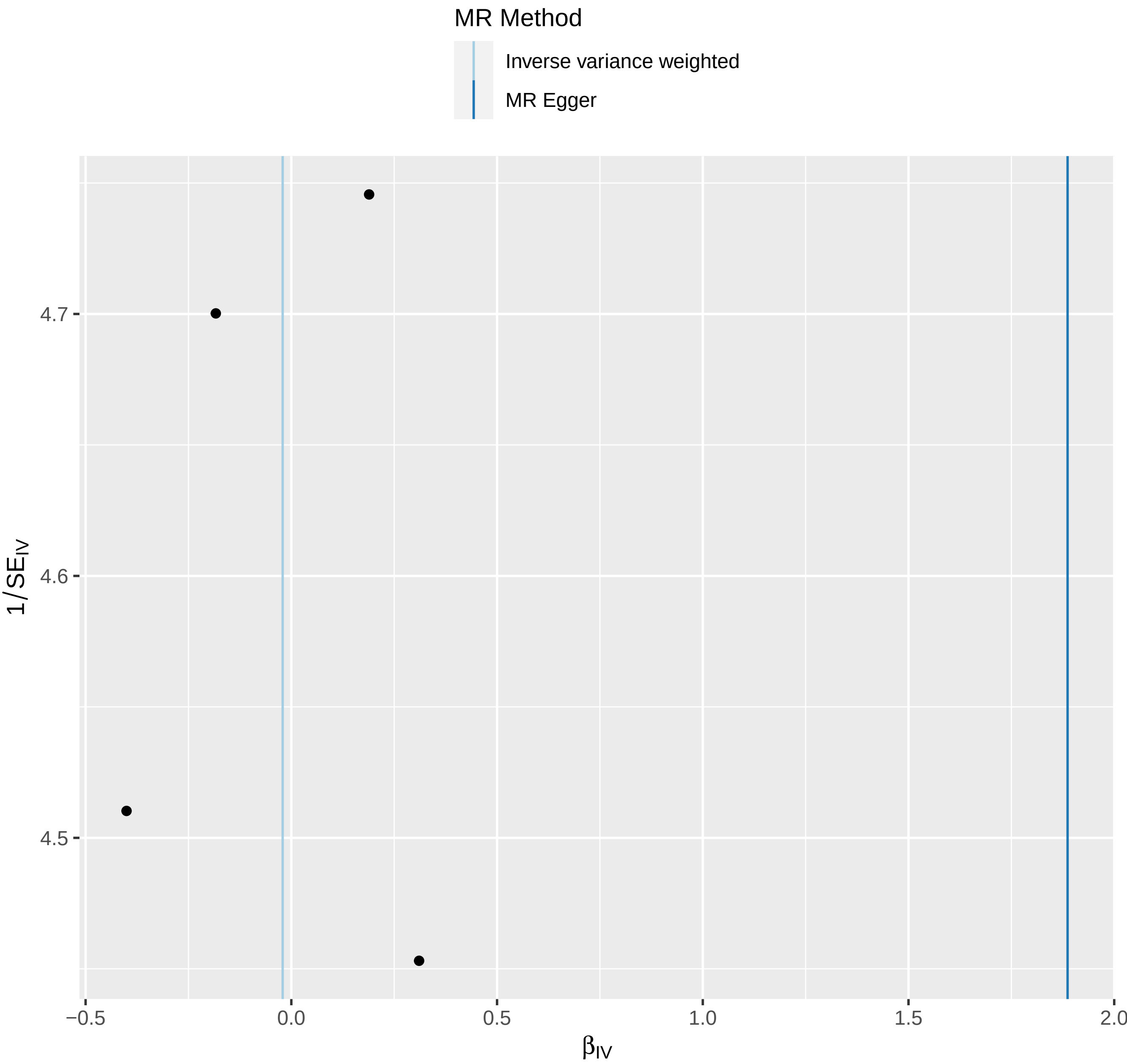

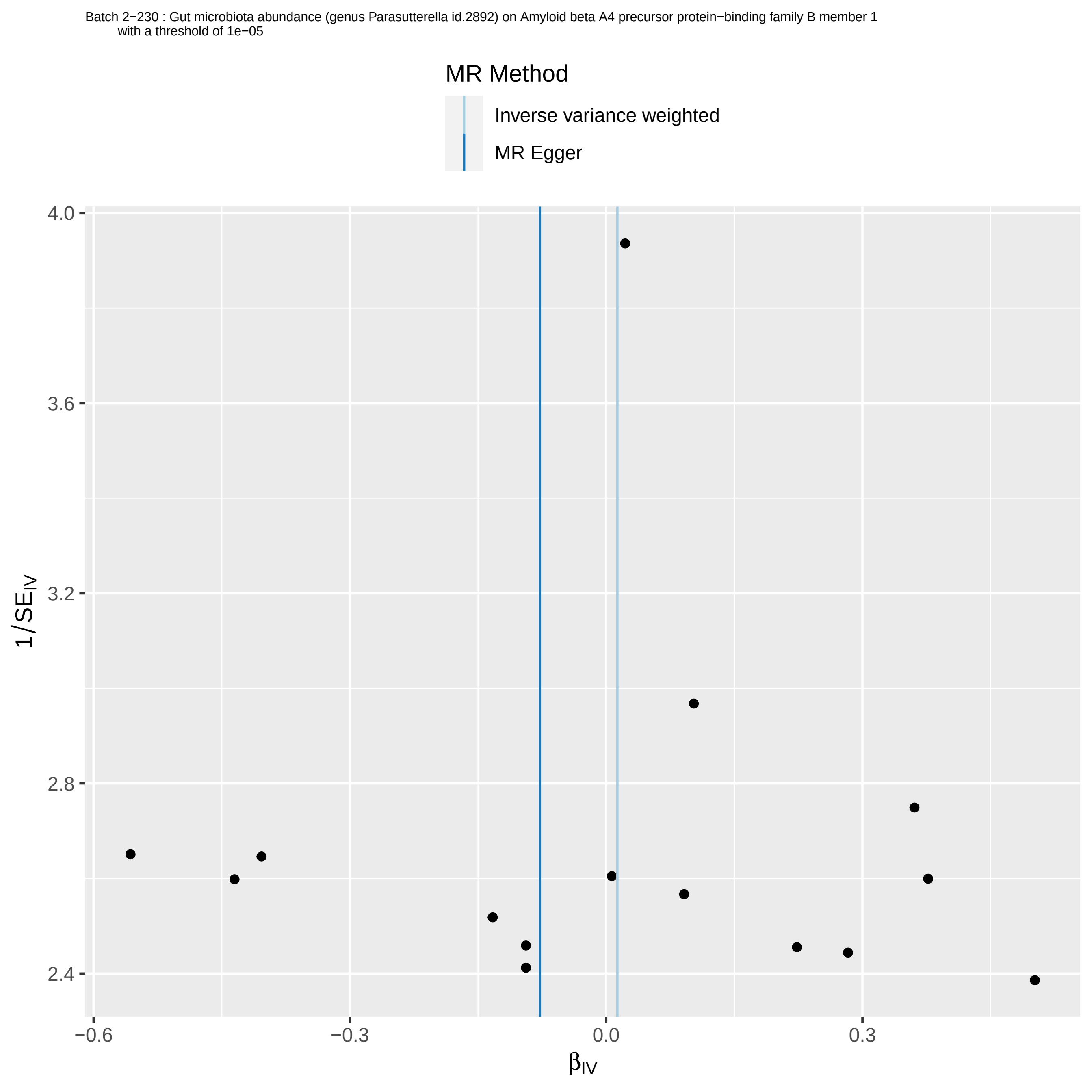

Batch 2−230 : Gut microbiota abundance (genus Parasutterella id.2892) on Amyloid beta A4 precursor protein−binding family B member 1
with a threshold of 1e−05
MR Method
Inverse variance weighted
MR Egger
4.0
3.6
3.2
2.8
2.4
1/SE_IV
−0.6
−0.3
0.0
0.3
β_IV

Batch 2−231 : Gut microbiota abundance (genus Catenibacterium id.2153) on Amyloid beta A4 precursor protein−binding family B member 2 with a threshold of 1e−05

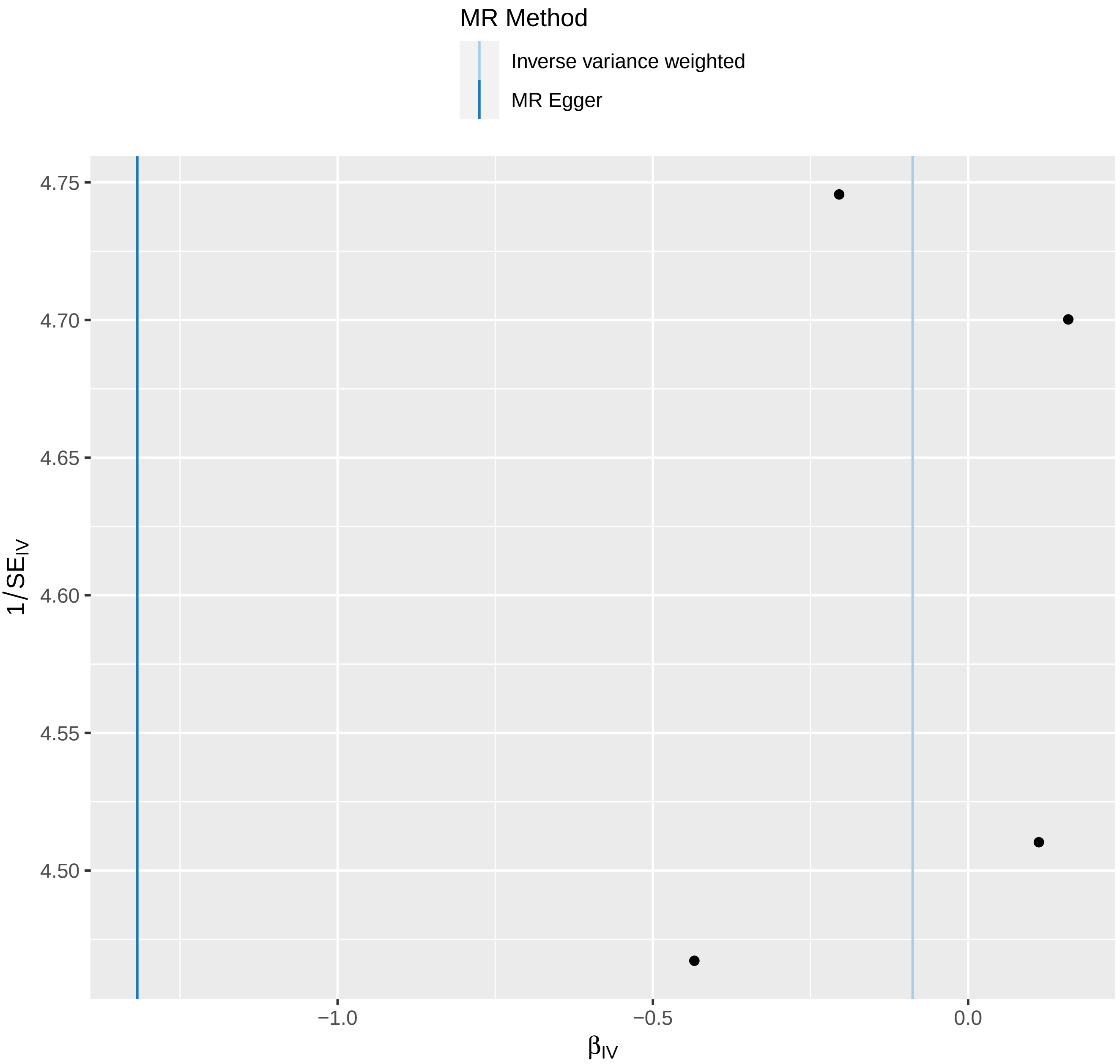

Batch 2−232 : Gut microbiota abundance (genus Parasutterella id.2892) on Amyloid beta A4 precursor protein−binding family B member 2 with a threshold of 1e−05

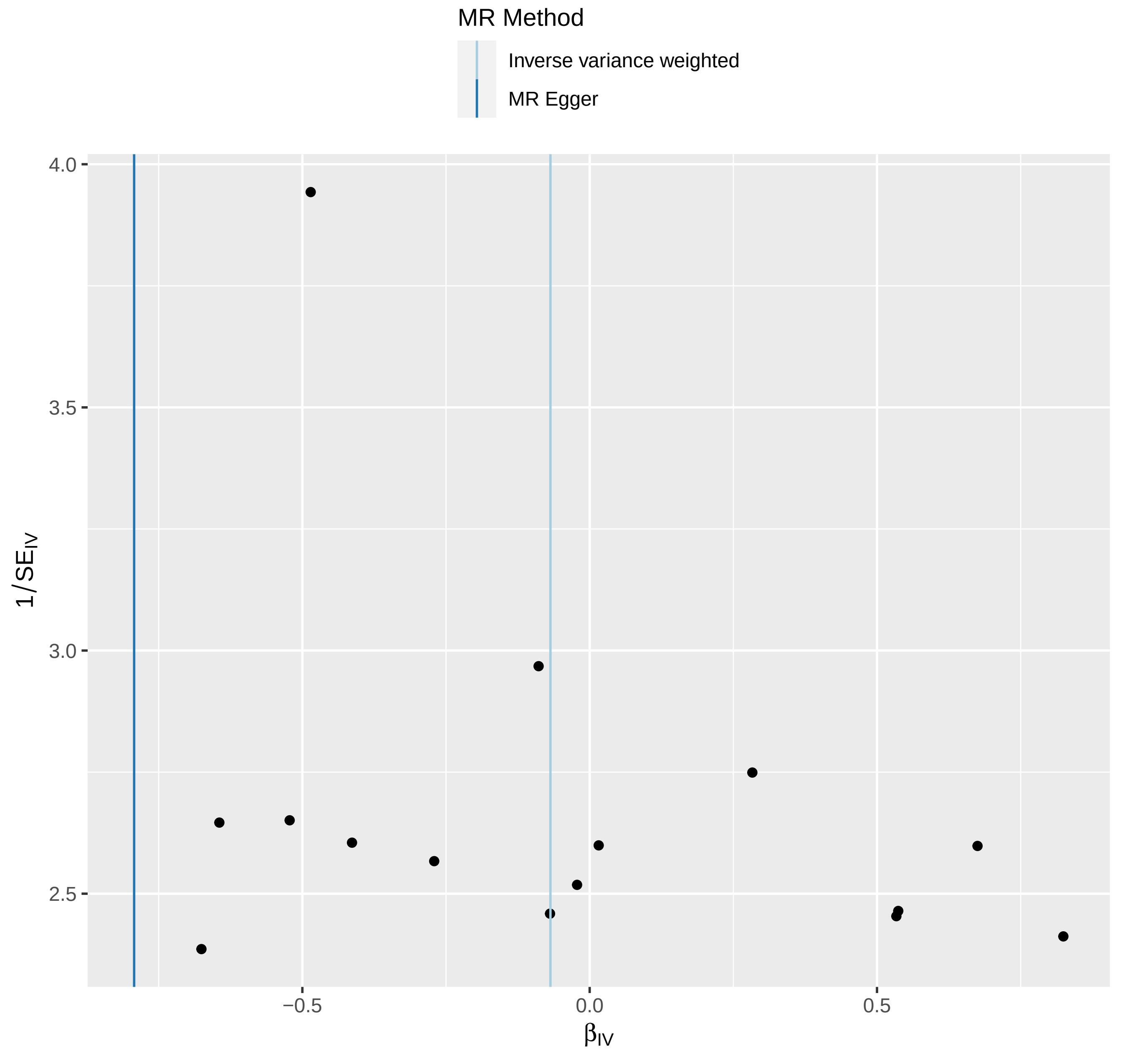

Batch 2−233 : Gut microbiota abundance (genus Catenibacterium id.2153) on Amyloid beta A4 precursor protein−binding family B member 2 with a threshold of 1e−05

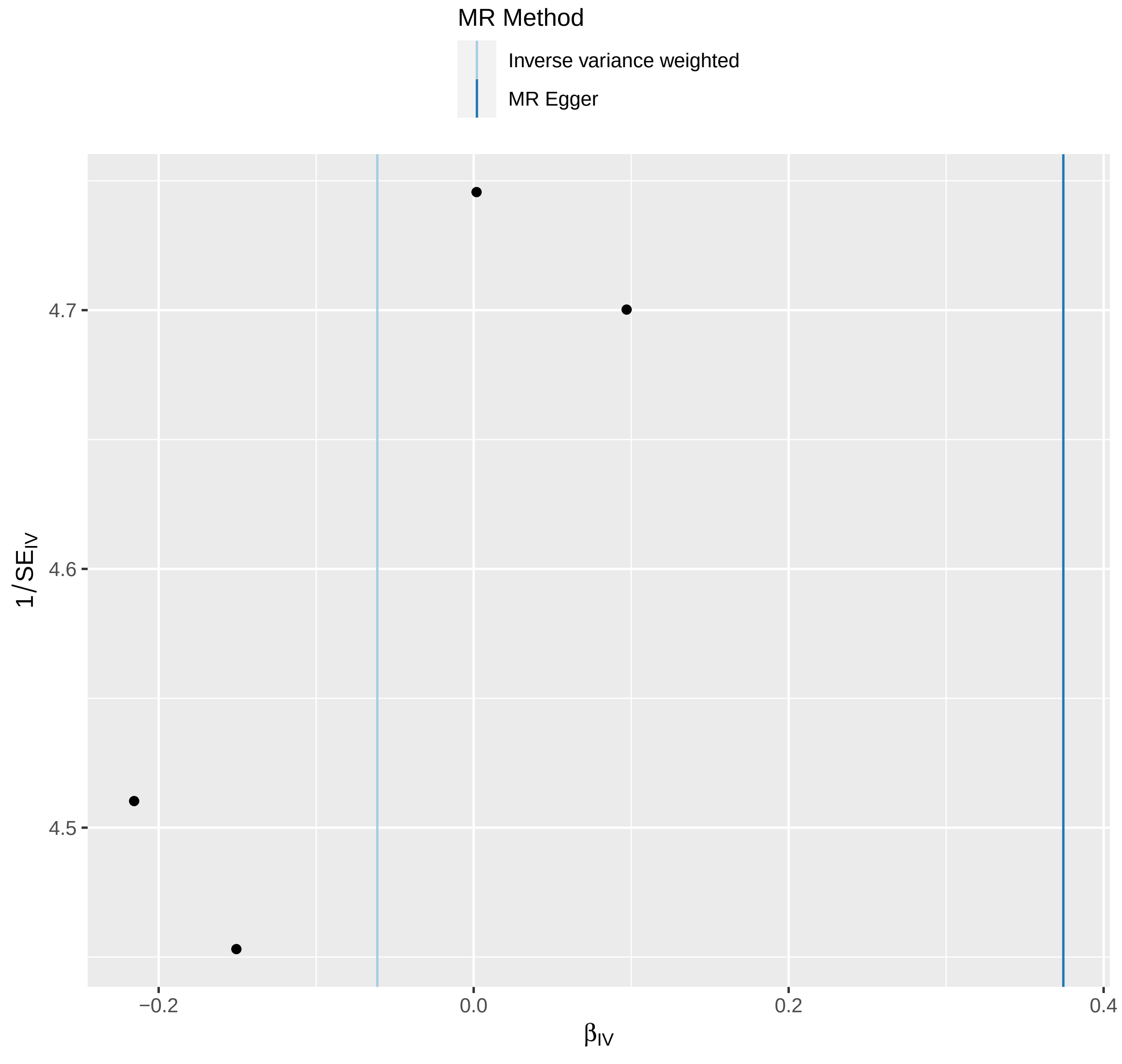

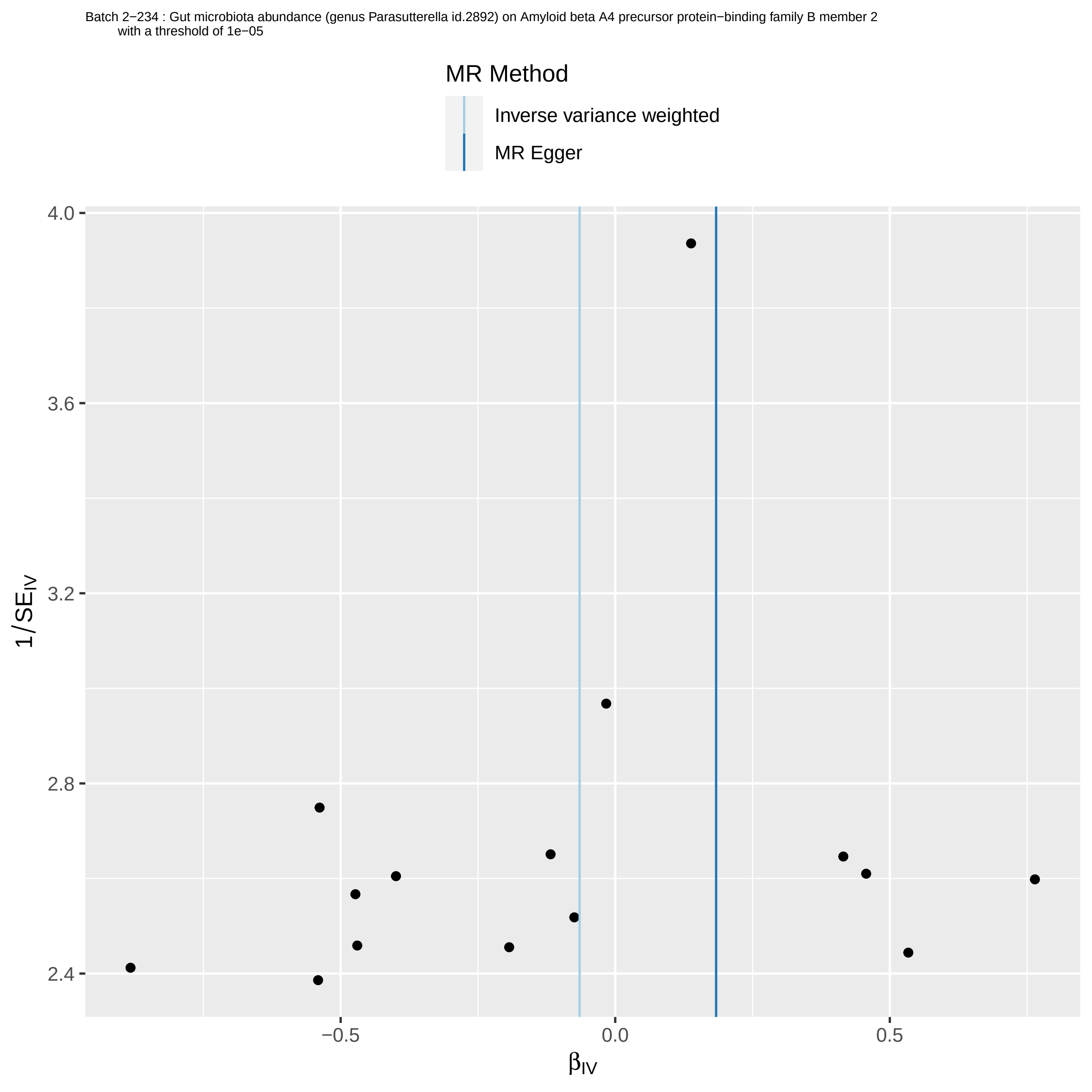
Batch 2−234 : Gut microbiota abundance (genus Parasutterella id.2892) on Amyloid beta A4 precursor protein−binding family B member 2
with a threshold of 1e−05
MR Method
Inverse variance weighted
MR Egger
4.0
3.6
3.2
2.8
2.4
1/SE_IV
−0.5
0.0
0.5
β_IV

Batch 2−235 : Gut microbiota abundance (genus Catenibacterium id.2153) on Amyloid beta A4 precursor protein−binding family B member 3 with a threshold of 1e−05

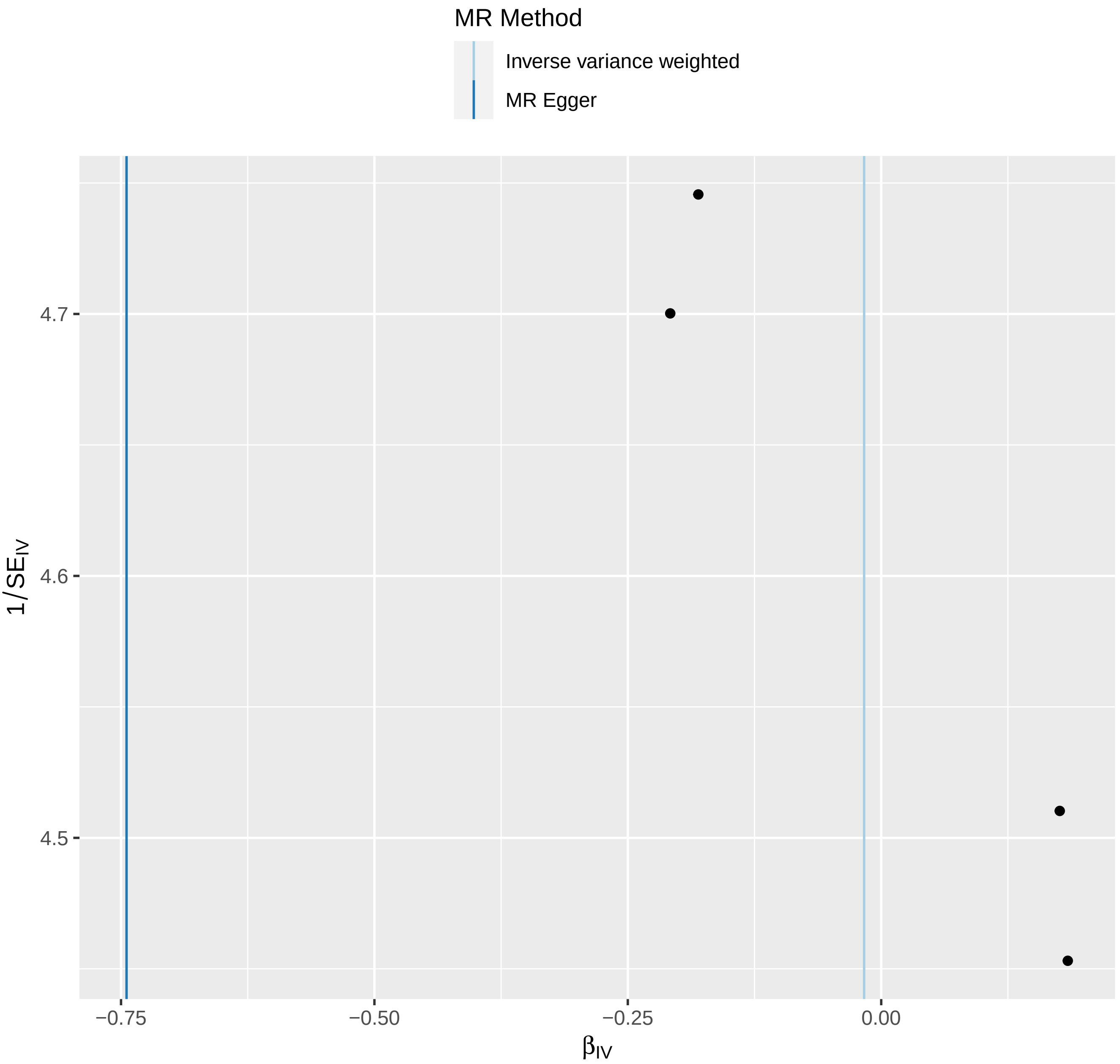

Batch 2−236 : Gut microbiota abundance (genus Parasutterella id.2892) on Amyloid beta A4 precursor protein−binding family B member 3
with a threshold of 1e−05

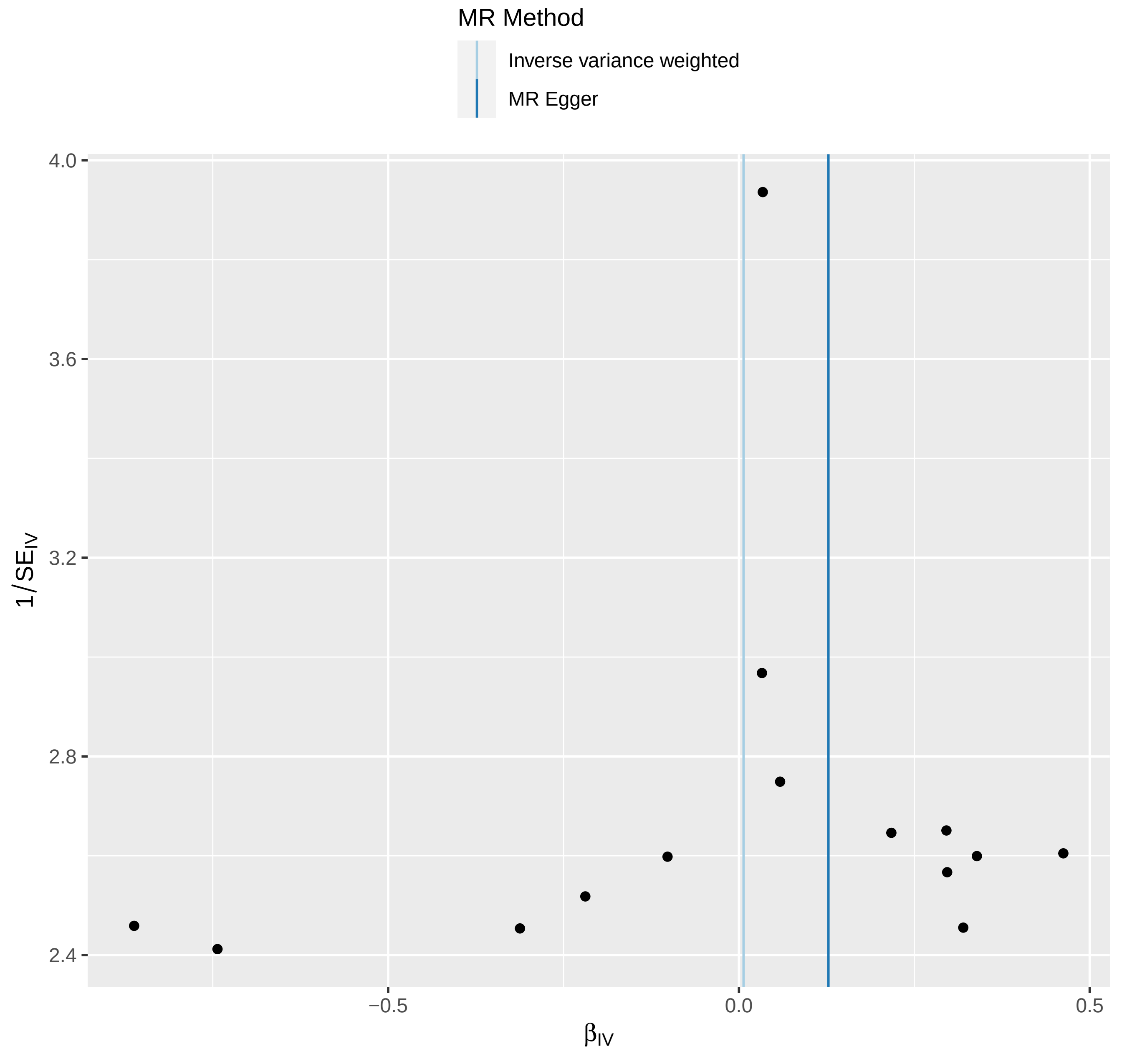

Batch 2−237 : Gut microbiota abundance (genus Catenibacterium id.2153) on Protein APCDD1
with a threshold of 1e−05

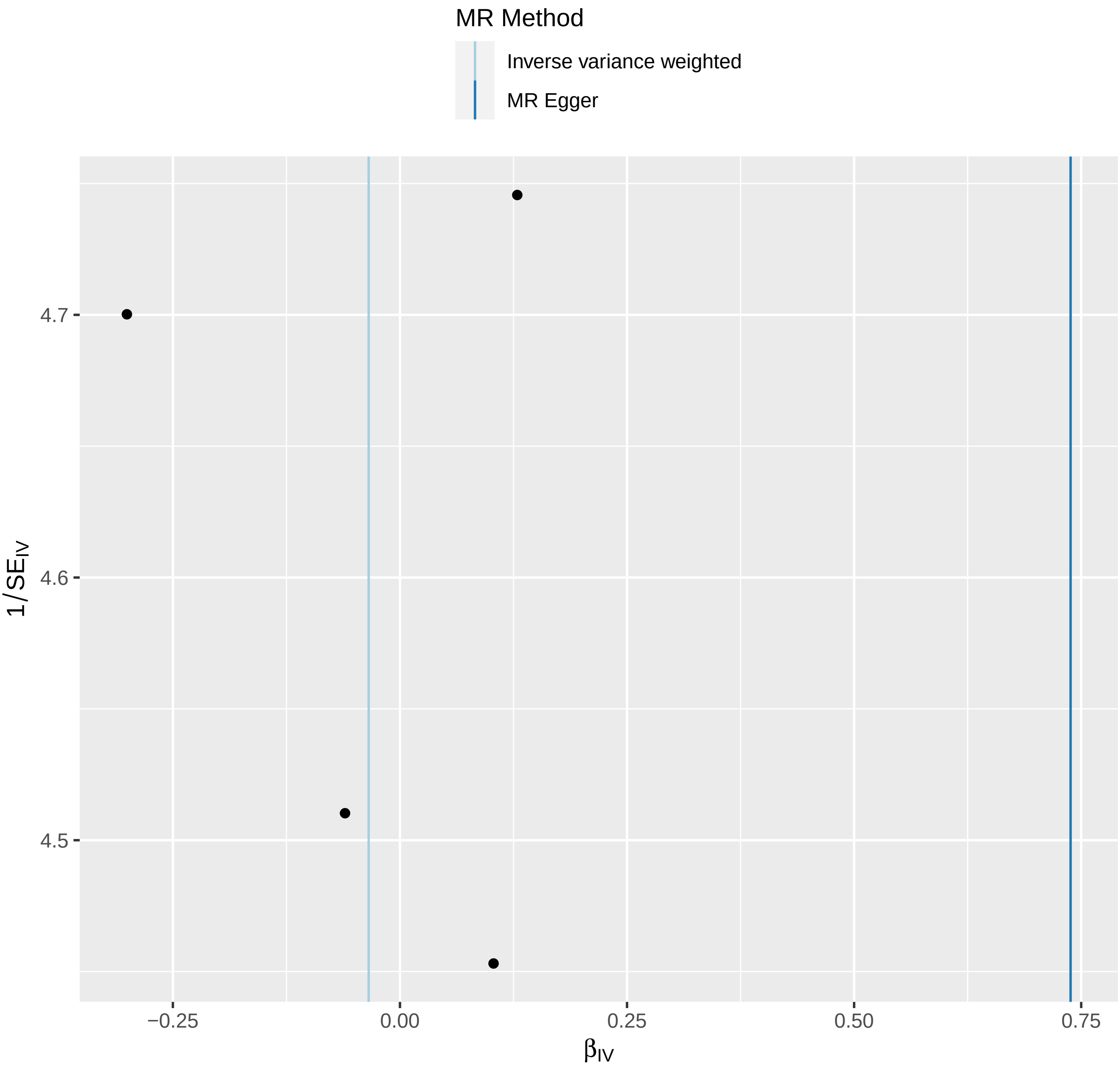

Batch 2−238 : Gut microbiota abundance (genus Parasutterella id.2892) on Protein APCDD1
with a threshold of 1e−05

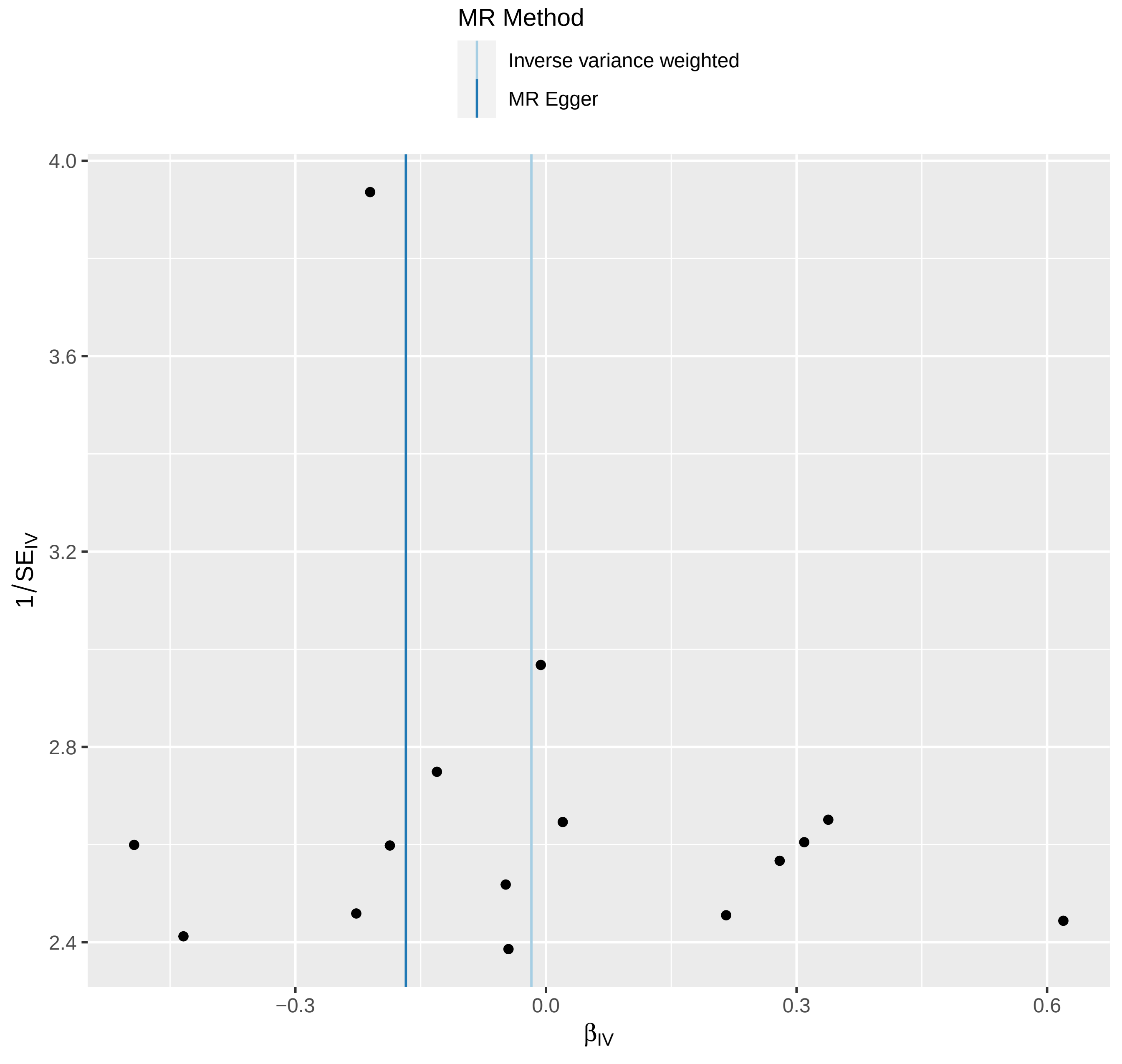

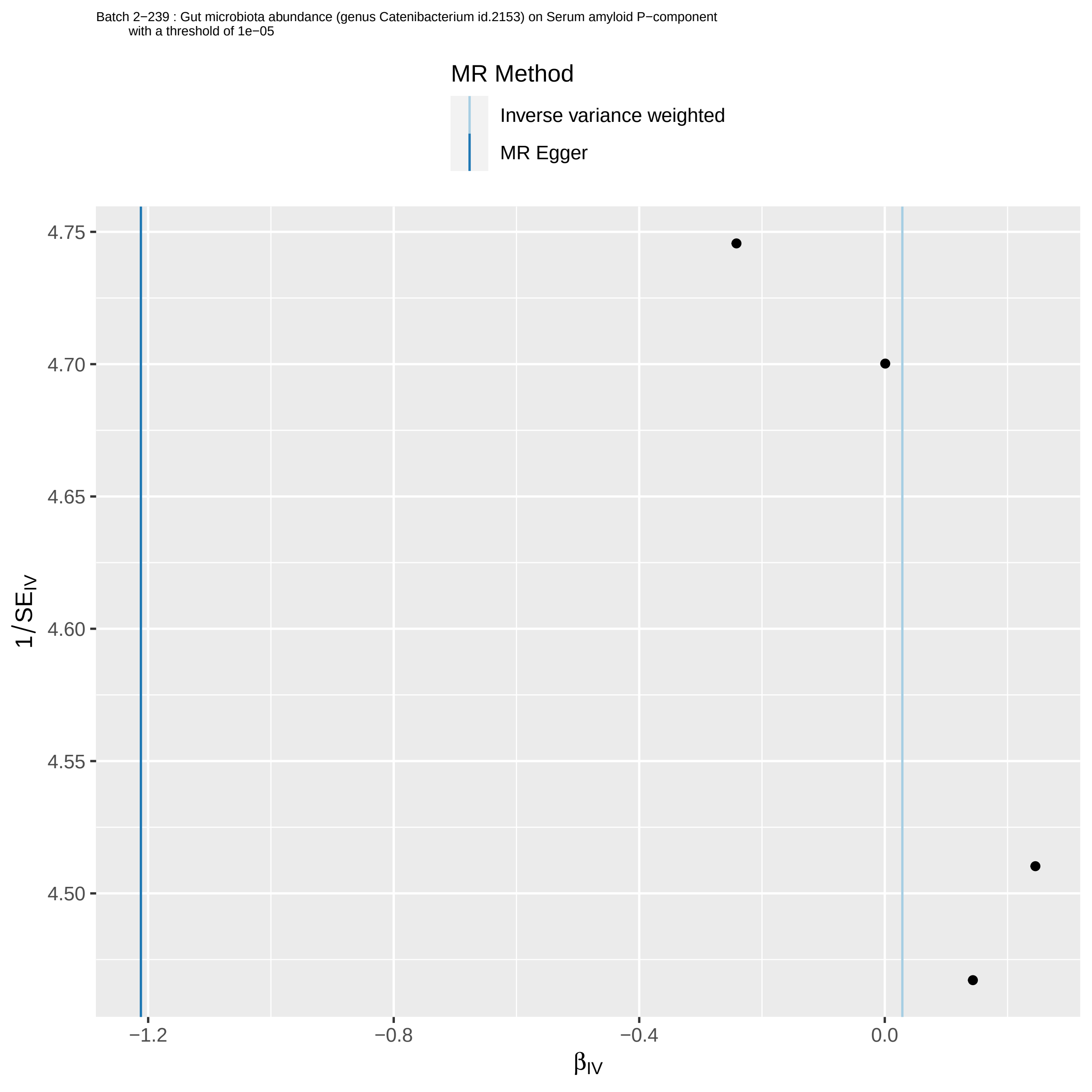

Batch 2−239 : Gut microbiota abundance (genus Catenibacterium id.2153) on Serum amyloid P−component with a threshold of 1e−05
MR Method
Inverse variance weighted
MR Egger
4.75
4.70
4.65
4.60
4.55
4.50
1/SE_IV
−1.2
−0.8
−0.4
0.0
β_IV

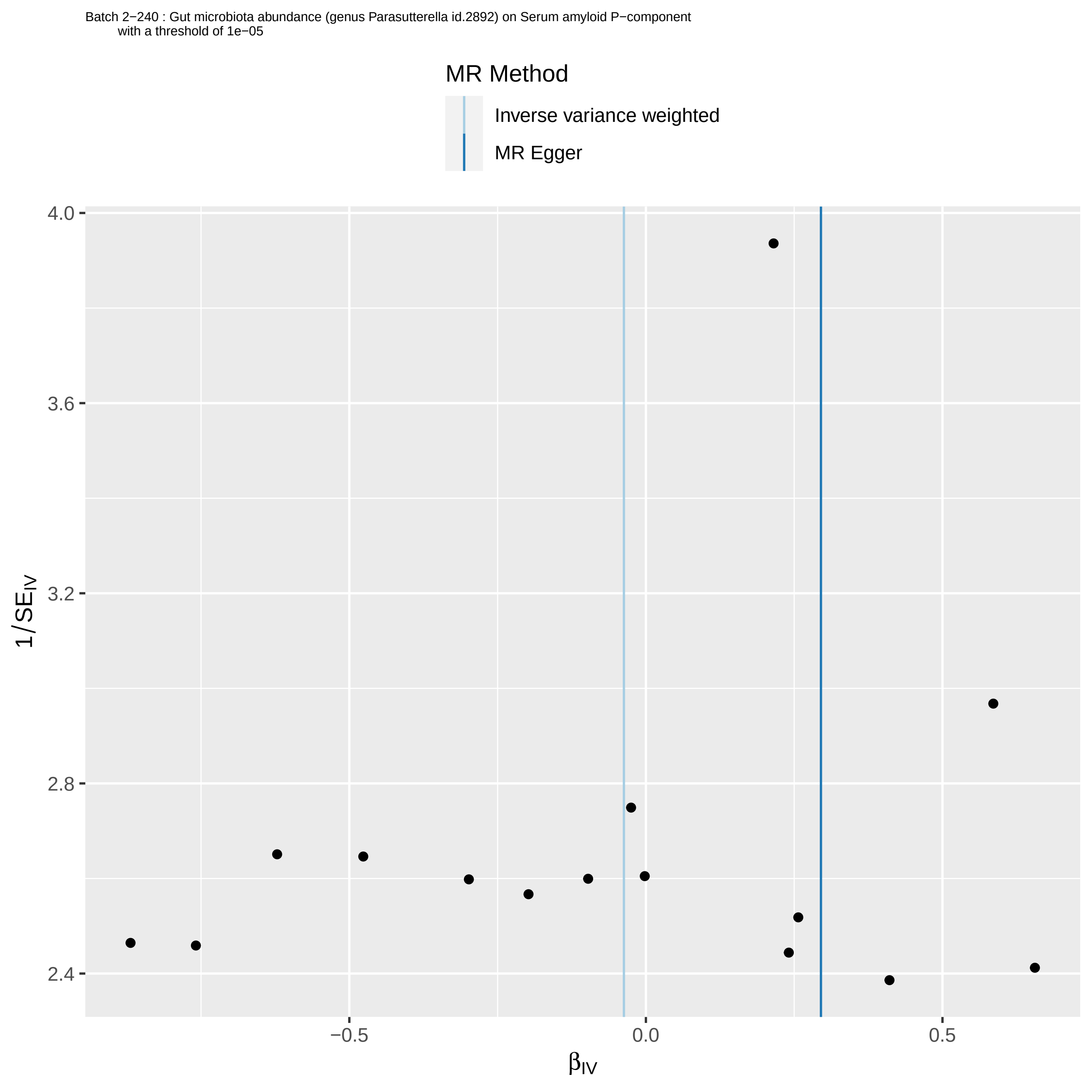

Batch 2−240 : Gut microbiota abundance (genus Parasutterella id.2892) on Serum amyloid P−component
with a threshold of 1e−05
MR Method
Inverse variance weighted
MR Egger
4.0
3.6
3.2
2.8
2.4
1/SE_IV
−0.5
0.0
0.5
β_IV

Batch 2−241 : Gut microbiota abundance (genus Catenibacterium id.2153) on DNA−(apurinic or apyrimidinic site) lyase
with a threshold of 1e−05

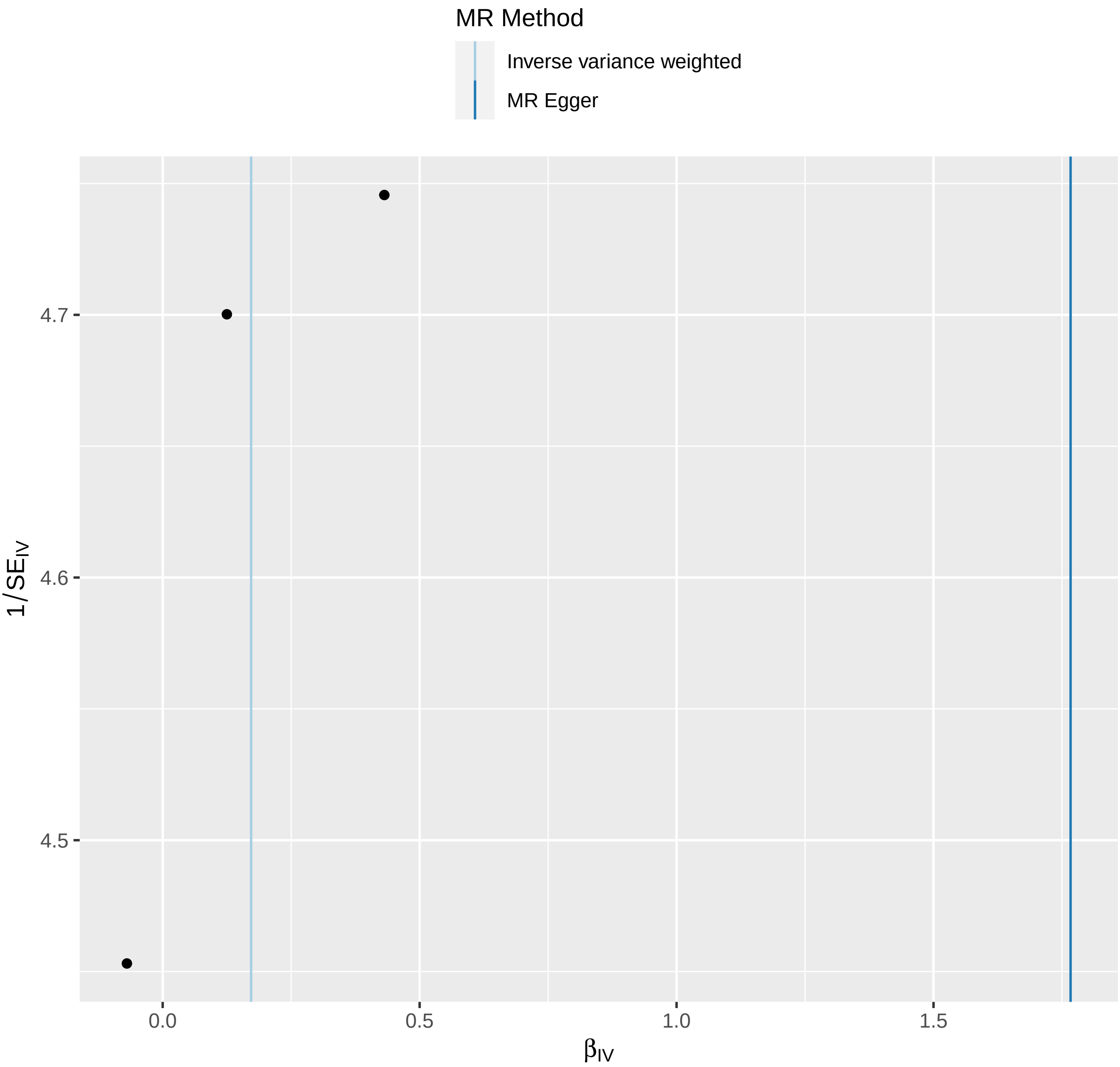

Batch 2−242 : Gut microbiota abundance (genus Parasutterella id.2892) on DNA−(apurinic or apyrimidinic site) lyase
with a threshold of 1e−05

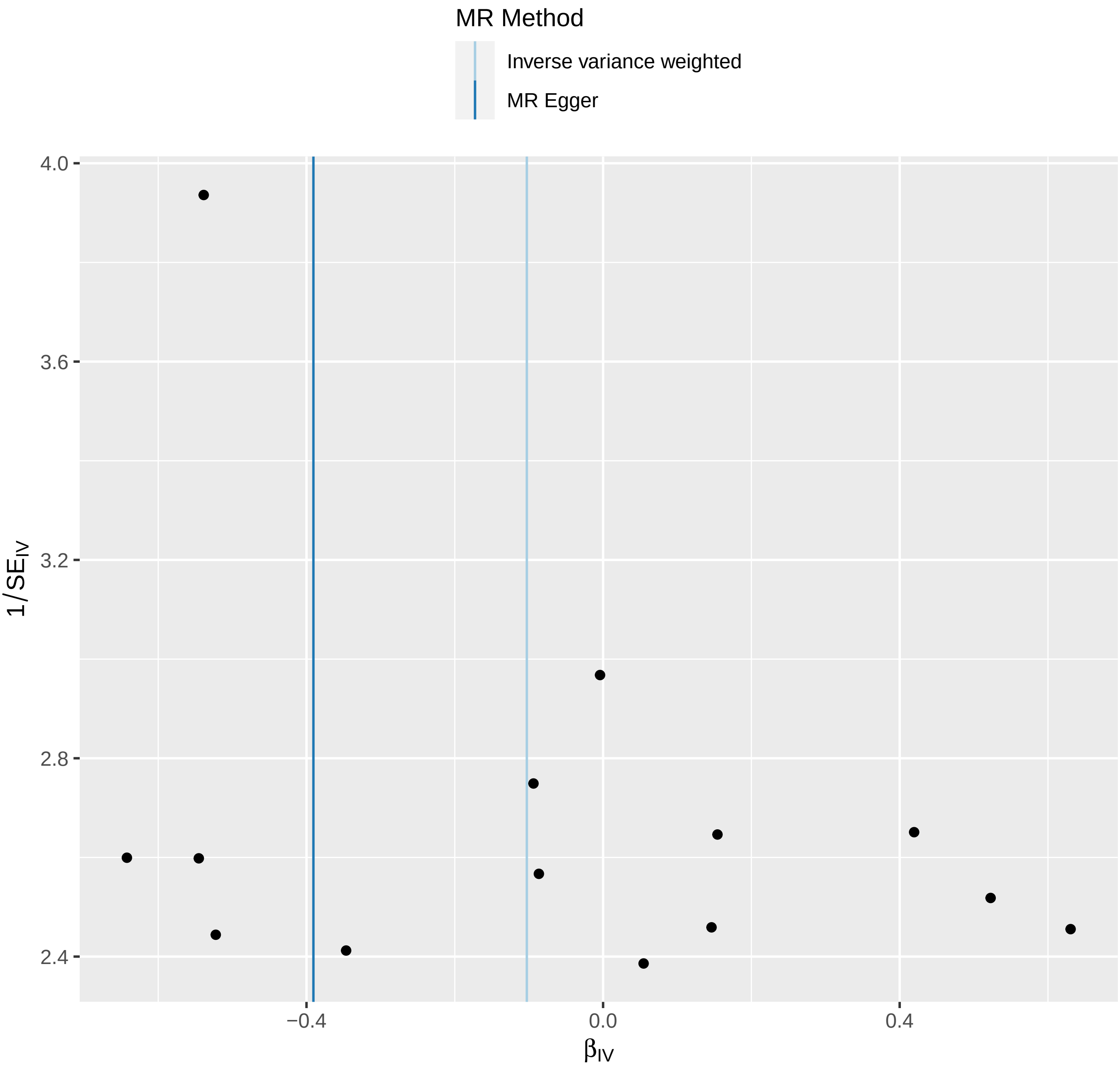

Batch 2−243 : Gut microbiota abundance (genus Catenibacterium id.2153) on Amyloid−like protein 2
with a threshold of 1e−05

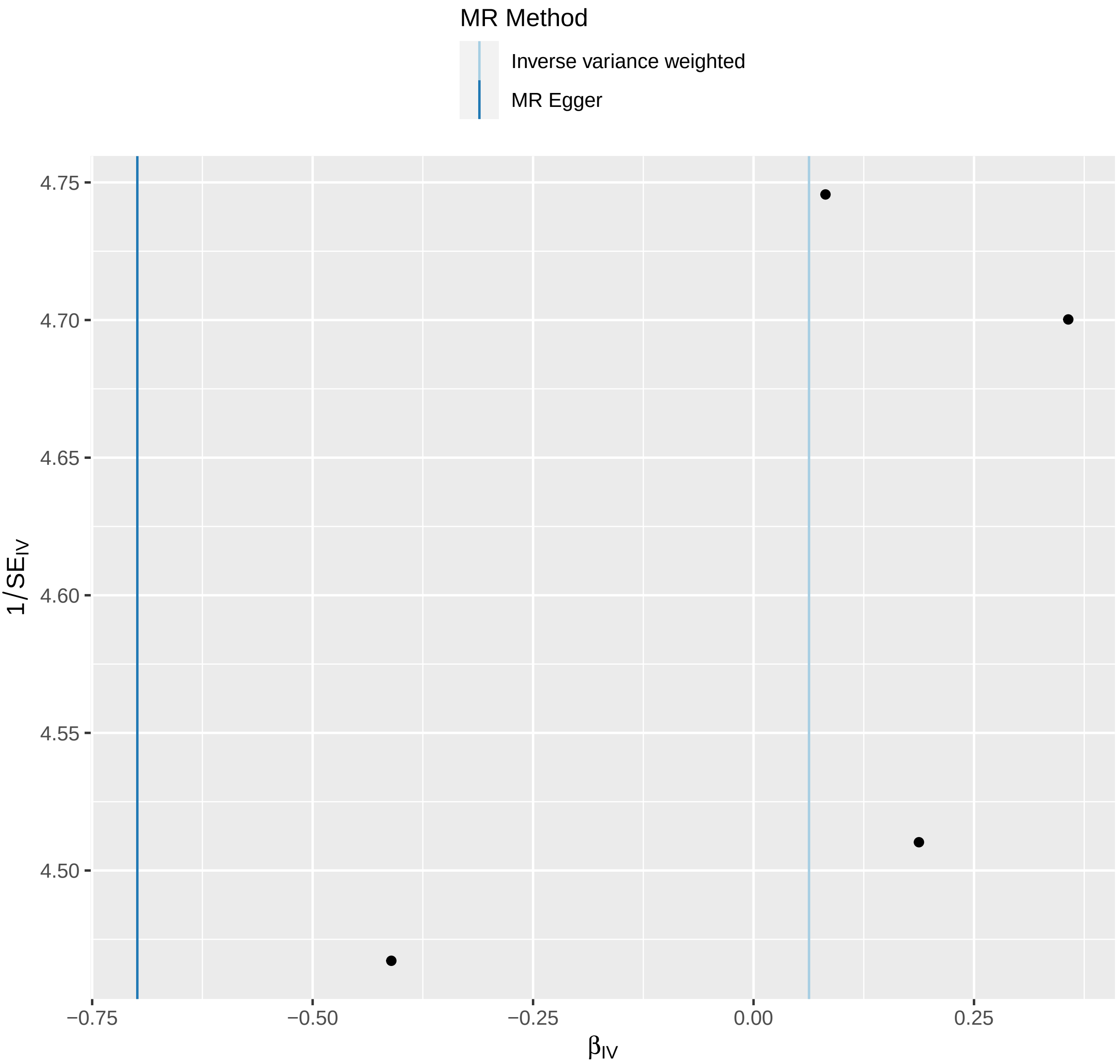

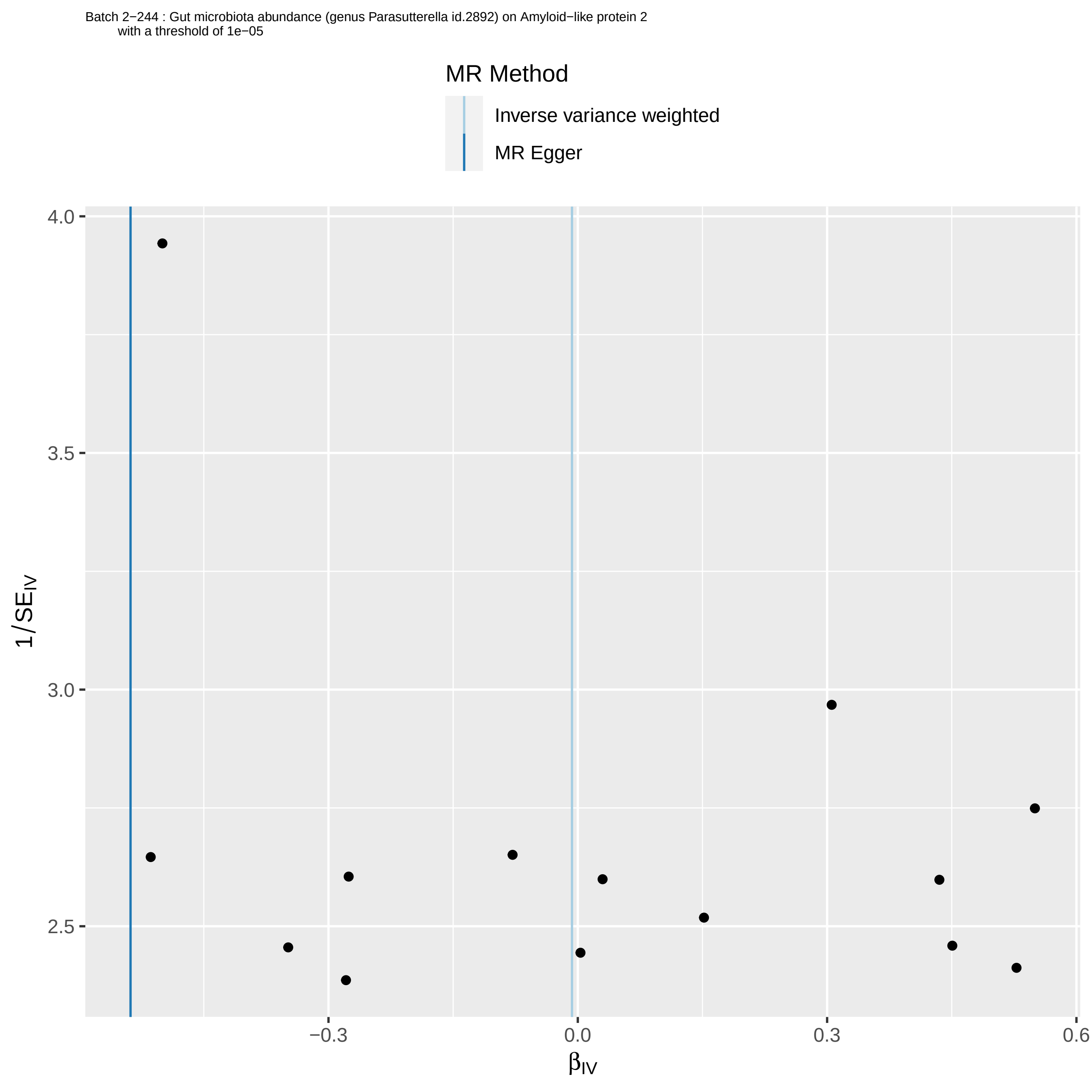

Batch 2−244 : Gut microbiota abundance (genus Parasutterella id.2892) on Amyloid−like protein 2
with a threshold of 1e−05
MR Method
Inverse variance weighted
MR Egger
4.0
3.5
3.0
2.5
1/SE_IV
−0.3
0.0
0.3
0.6
β_IV

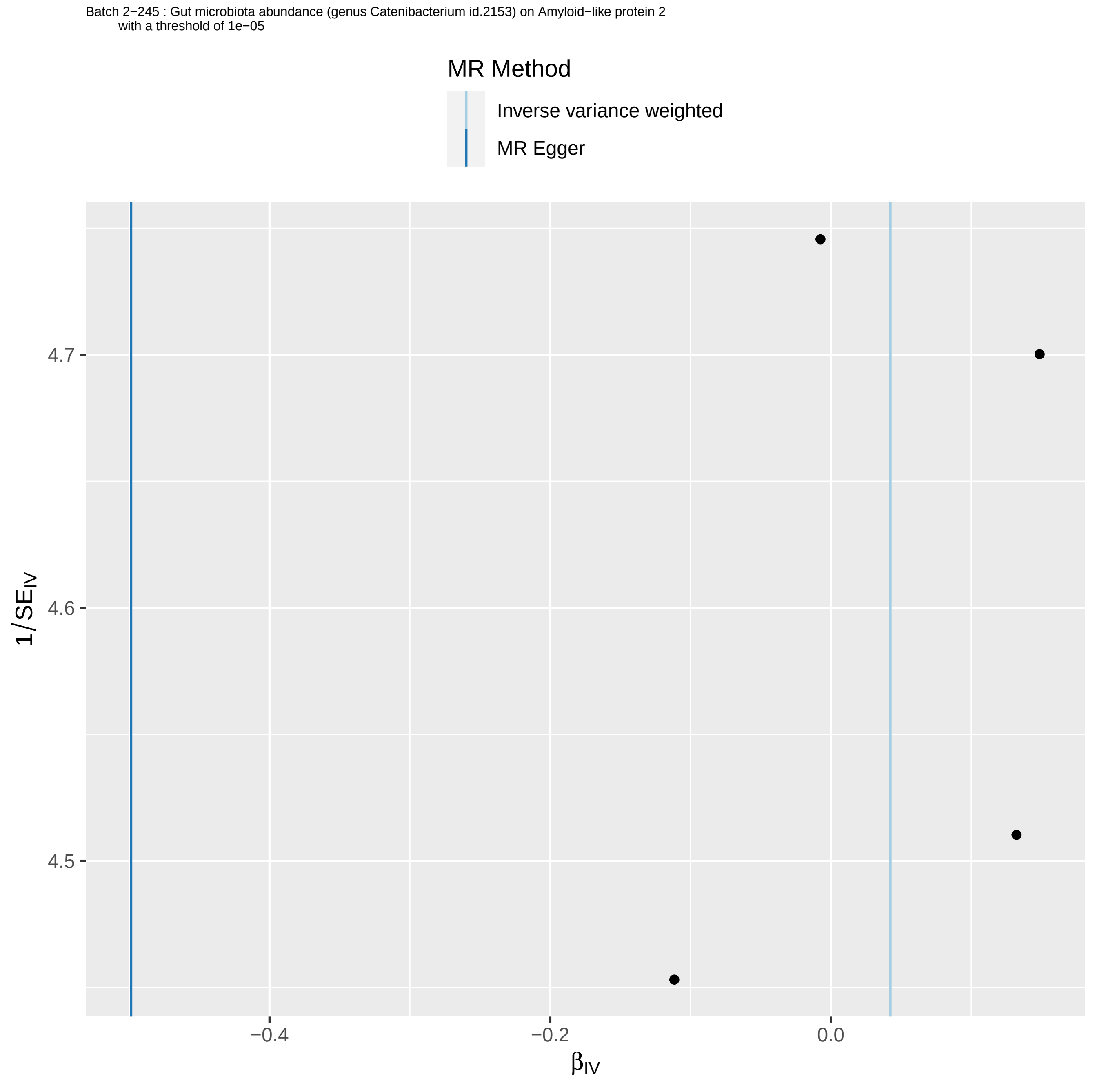

Batch 2−245 : Gut microbiota abundance (genus Catenibacterium id.2153) on Amyloid−like protein 2
with a threshold of 1e−05
MR Method
Inverse variance weighted
MR Egger
4.7
4.6
4.5
1/SE$_{IV}$
−0.4
−0.2
0.0
β$_{IV}$

Batch 2−246 : Gut microbiota abundance (genus Parasutterella id.2892) on Amyloid−like protein 2
with a threshold of 1e−05

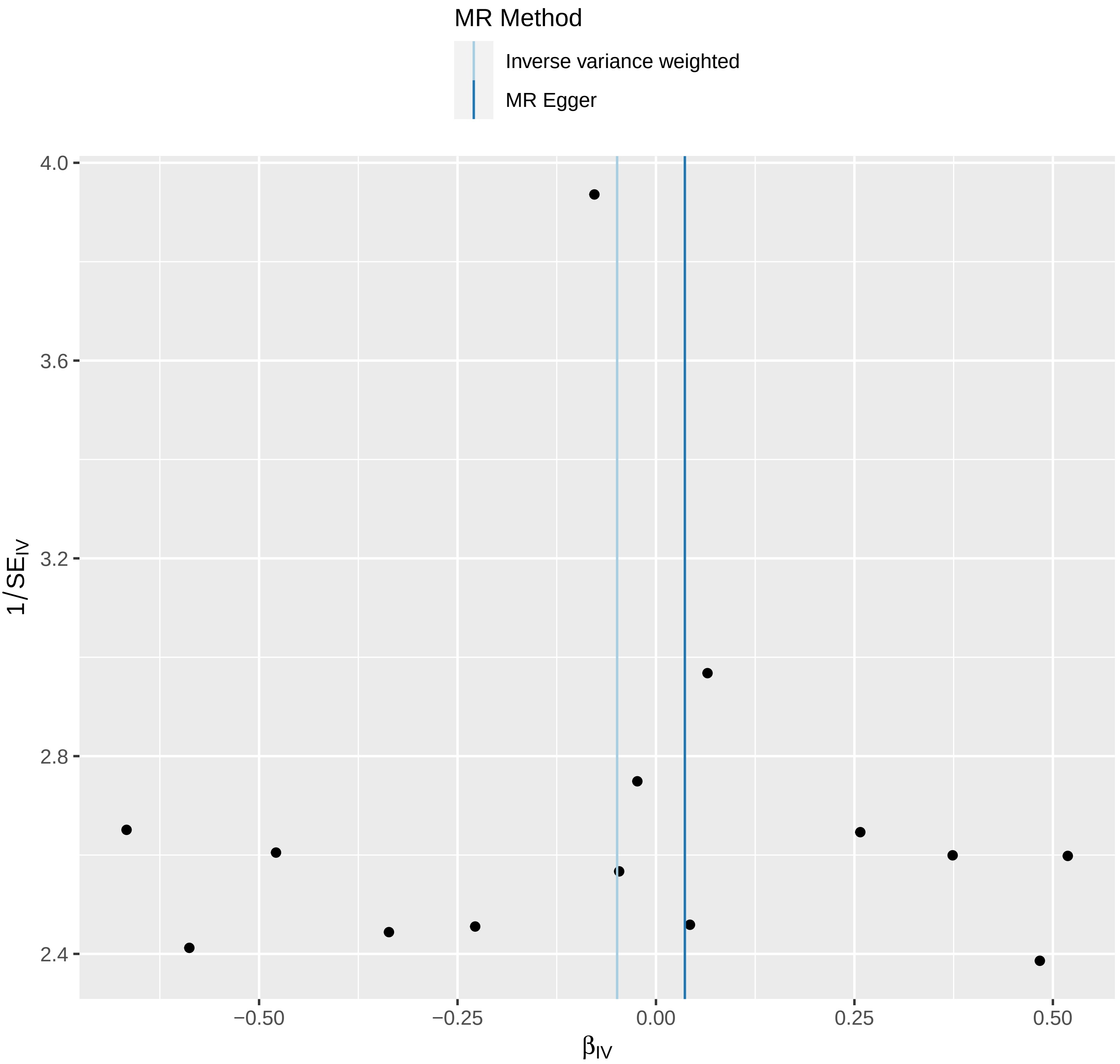

Batch 2−247 : Gut microbiota abundance (genus Catenibacterium id.2153) on Adipocyte plasma membrane−associated protein
with a threshold of 1e−05

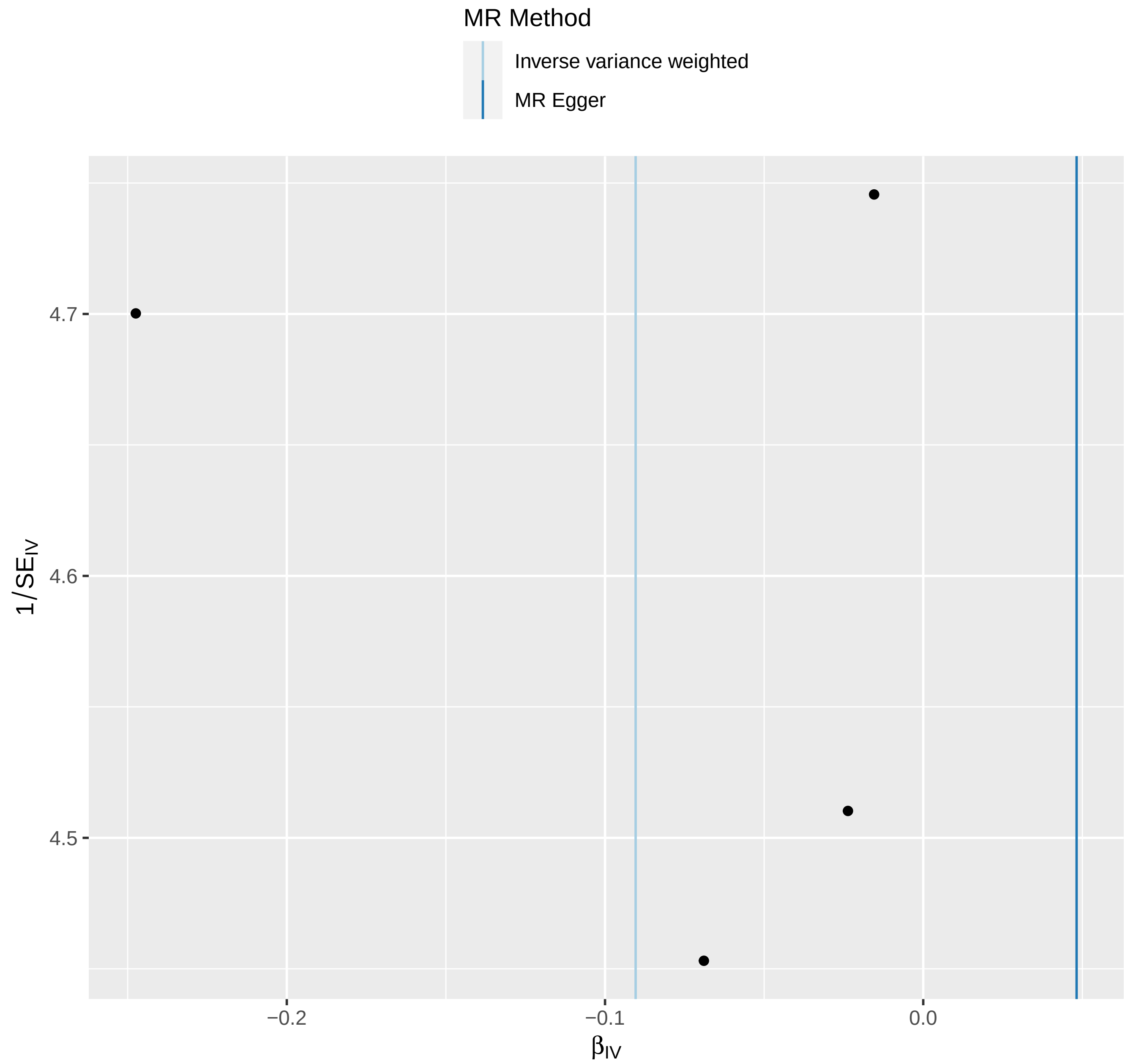

Batch 2−248 : Gut microbiota abundance (genus Parasutterella id.2892) on Adipocyte plasma membrane−associated protein with a threshold of 1e−05

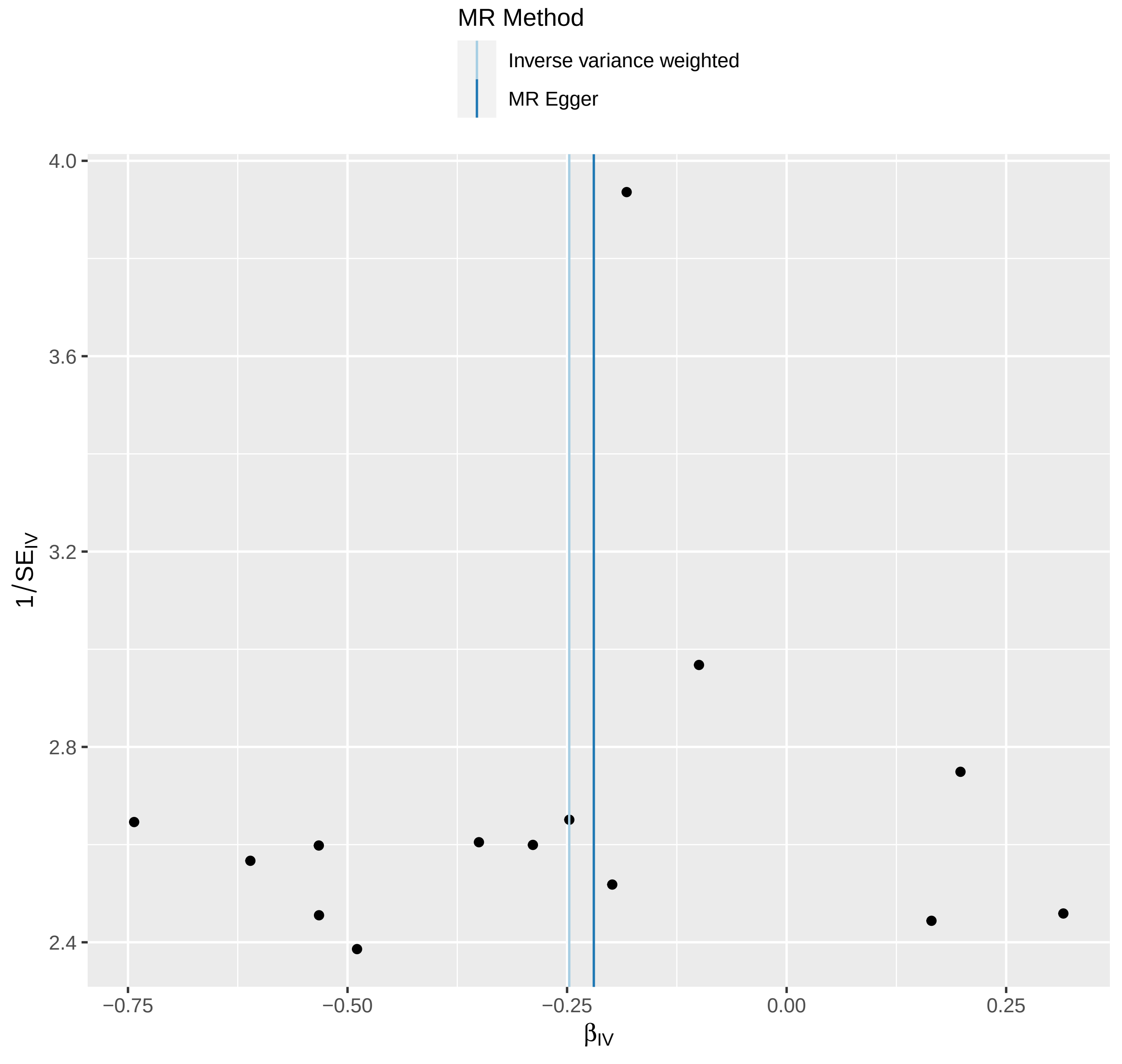

Batch 2−249 : Gut microbiota abundance (genus Catenibacterium id.2153) on Apolipoprotein A−V
with a threshold of 1e−05

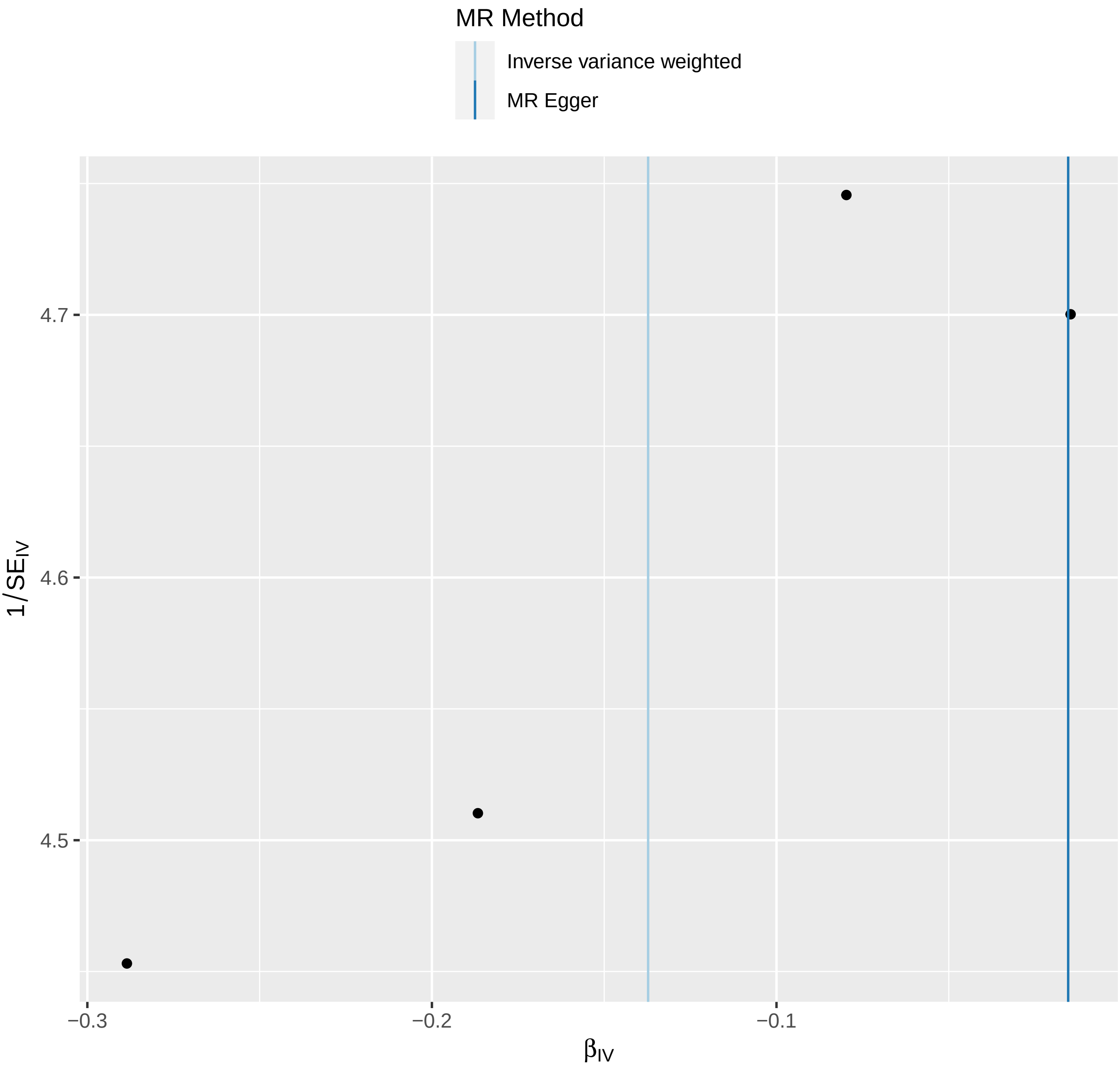

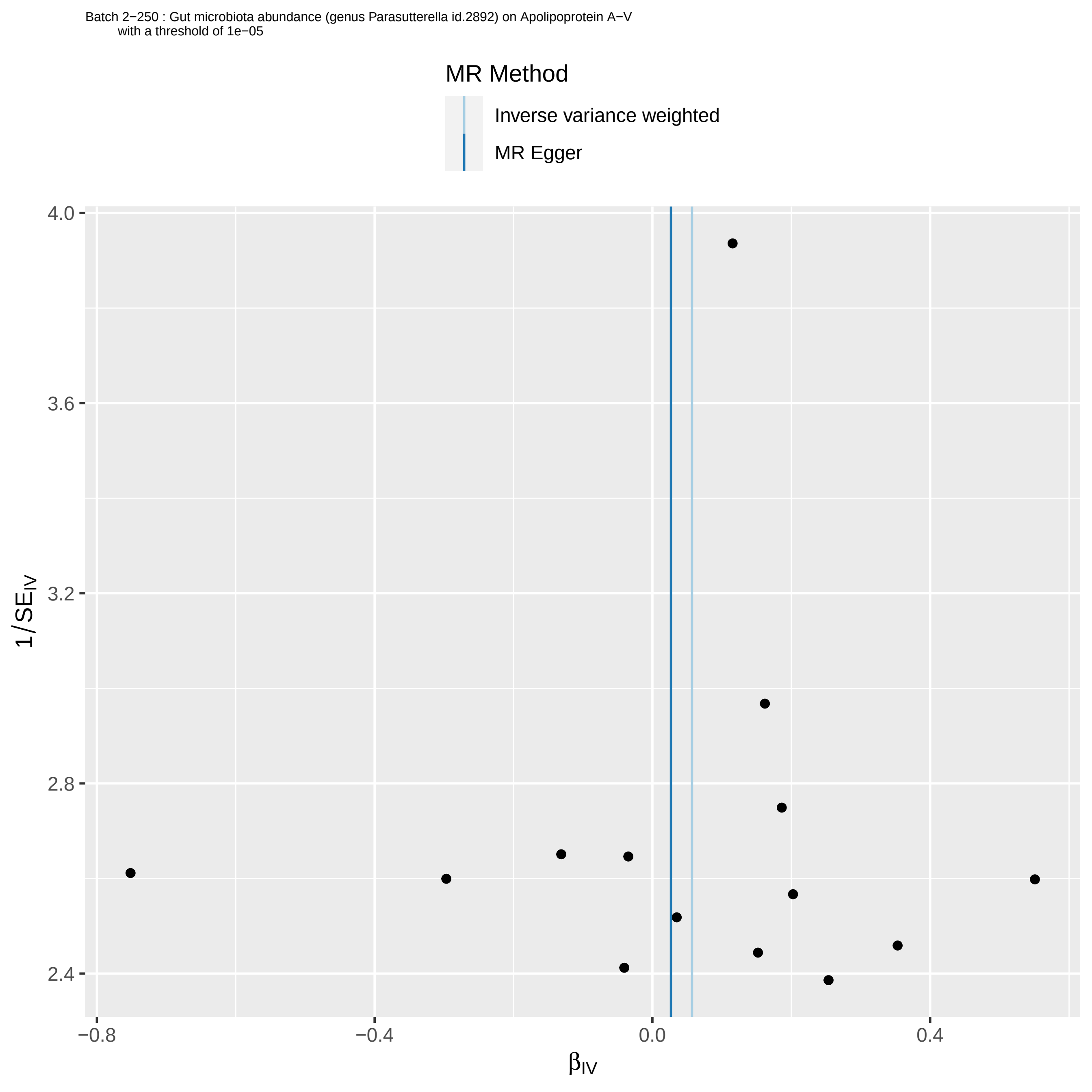

Batch 2−250 : Gut microbiota abundance (genus Parasutterella id.2892) on Apolipoprotein A−V
with a threshold of 1e−05
MR Method
Inverse variance weighted
MR Egger
4.0
3.6
3.2
2.8
2.4
1/SE_IV
−0.8
−0.4
0.0
0.4
β_IV

Batch 2−251 : Gut microbiota abundance (genus Catenibacterium id.2153) on Apolipoprotein A−V
with a threshold of 1e−05

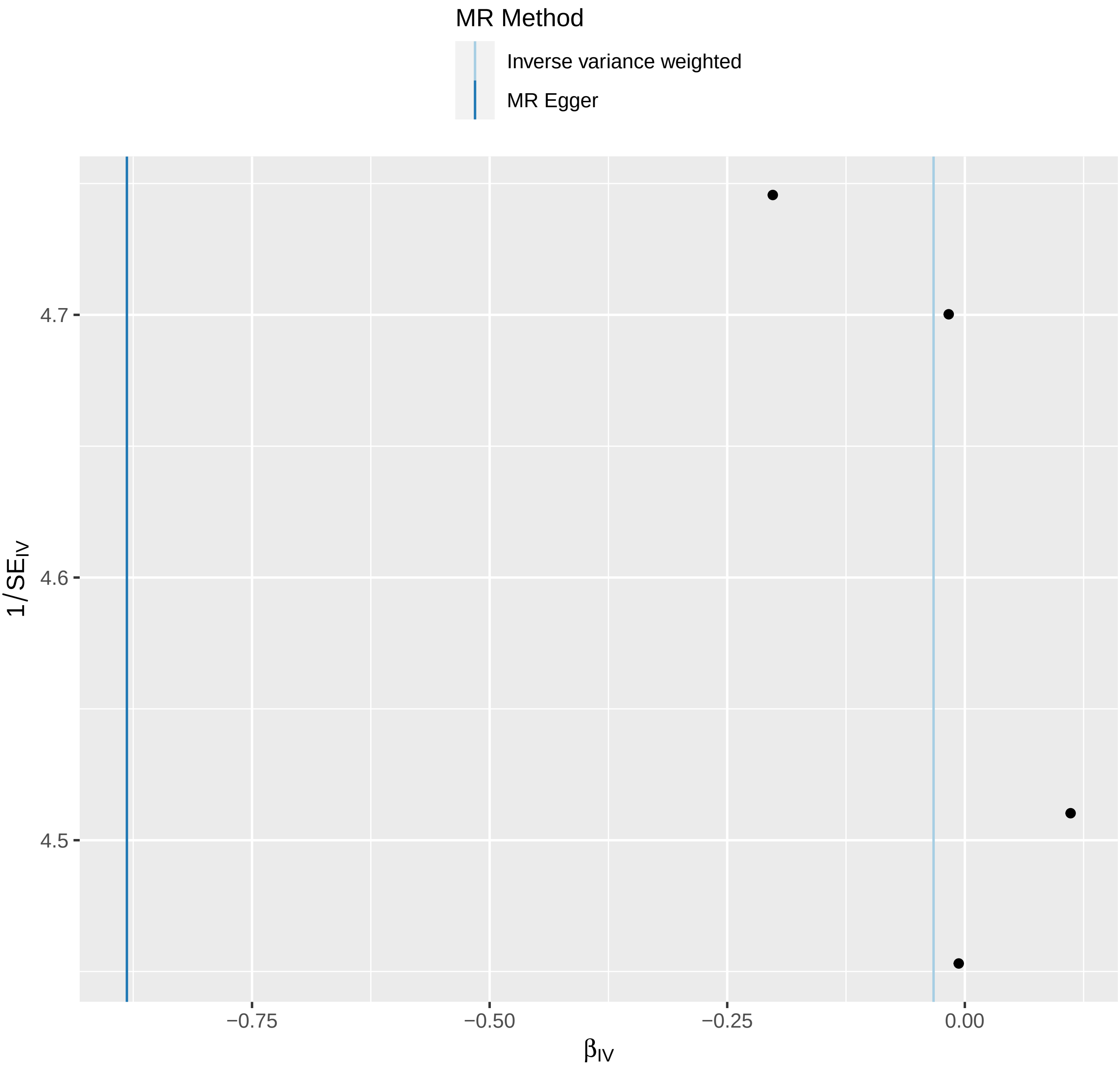

Batch 2−252 : Gut microbiota abundance (genus Parasutterella id.2892) on Apolipoprotein A−V
with a threshold of 1e−05

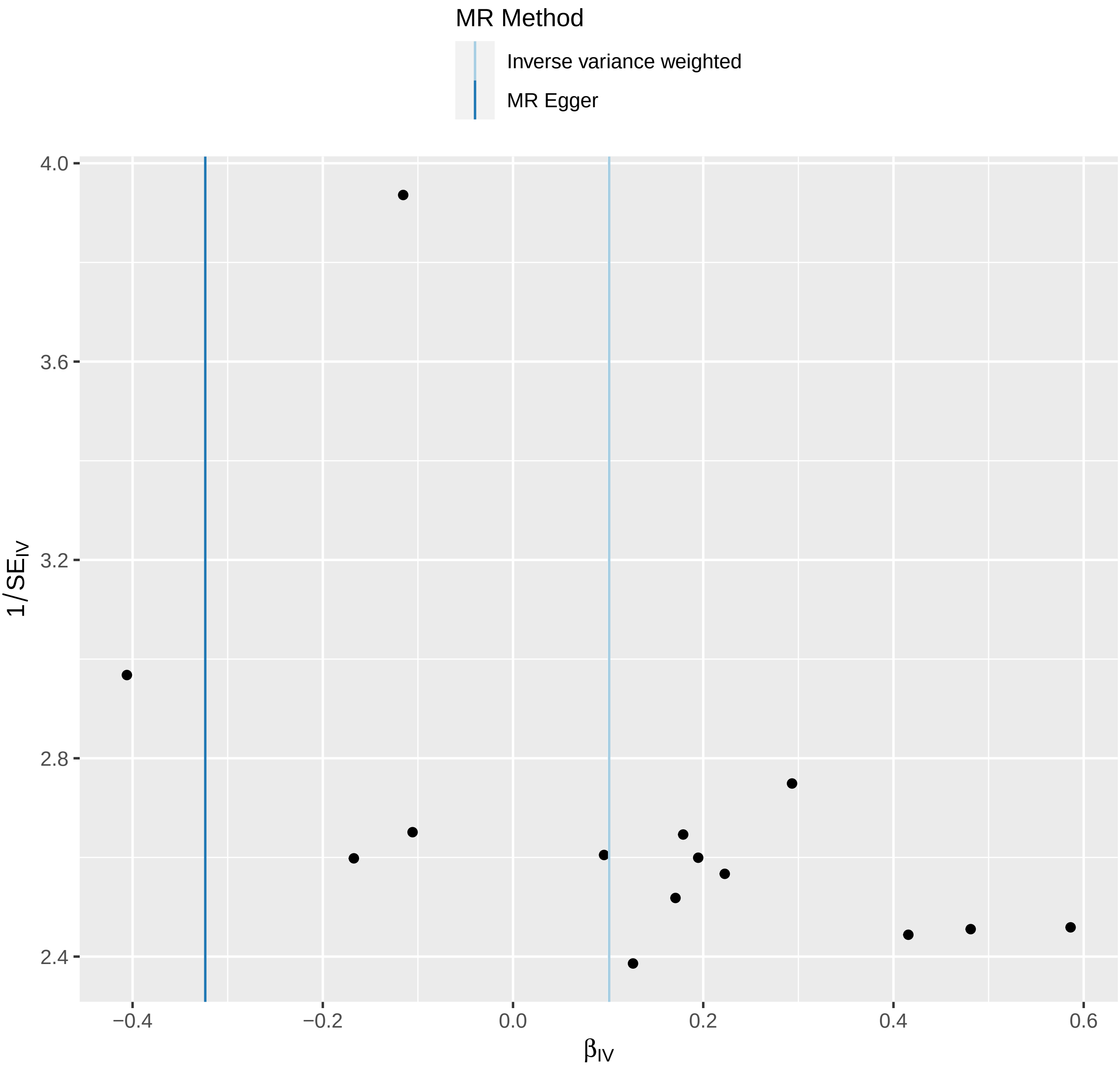

Batch 2−253 : Gut microbiota abundance (genus Catenibacterium id.2153) on Apolipoprotein B
with a threshold of 1e−05

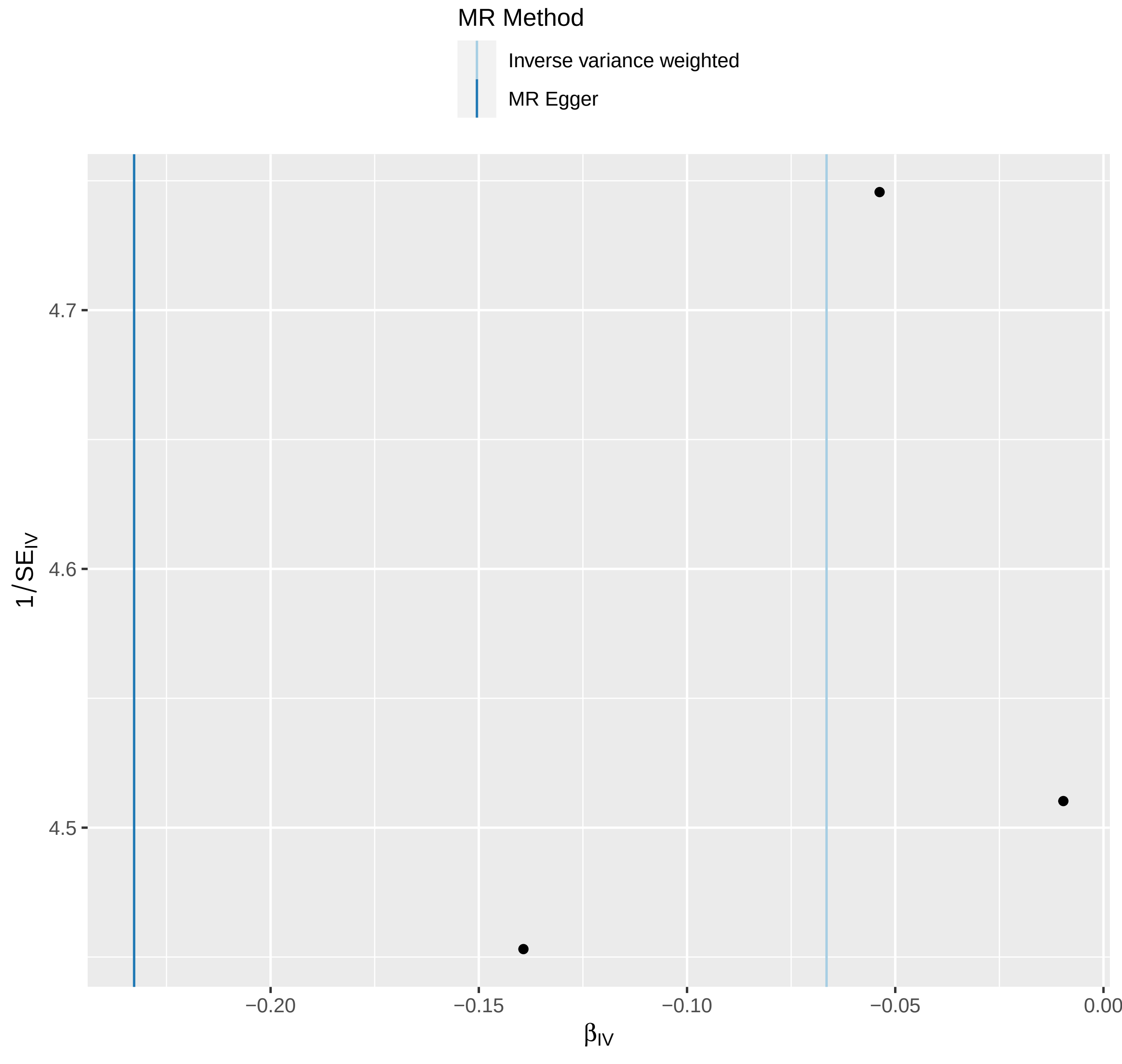

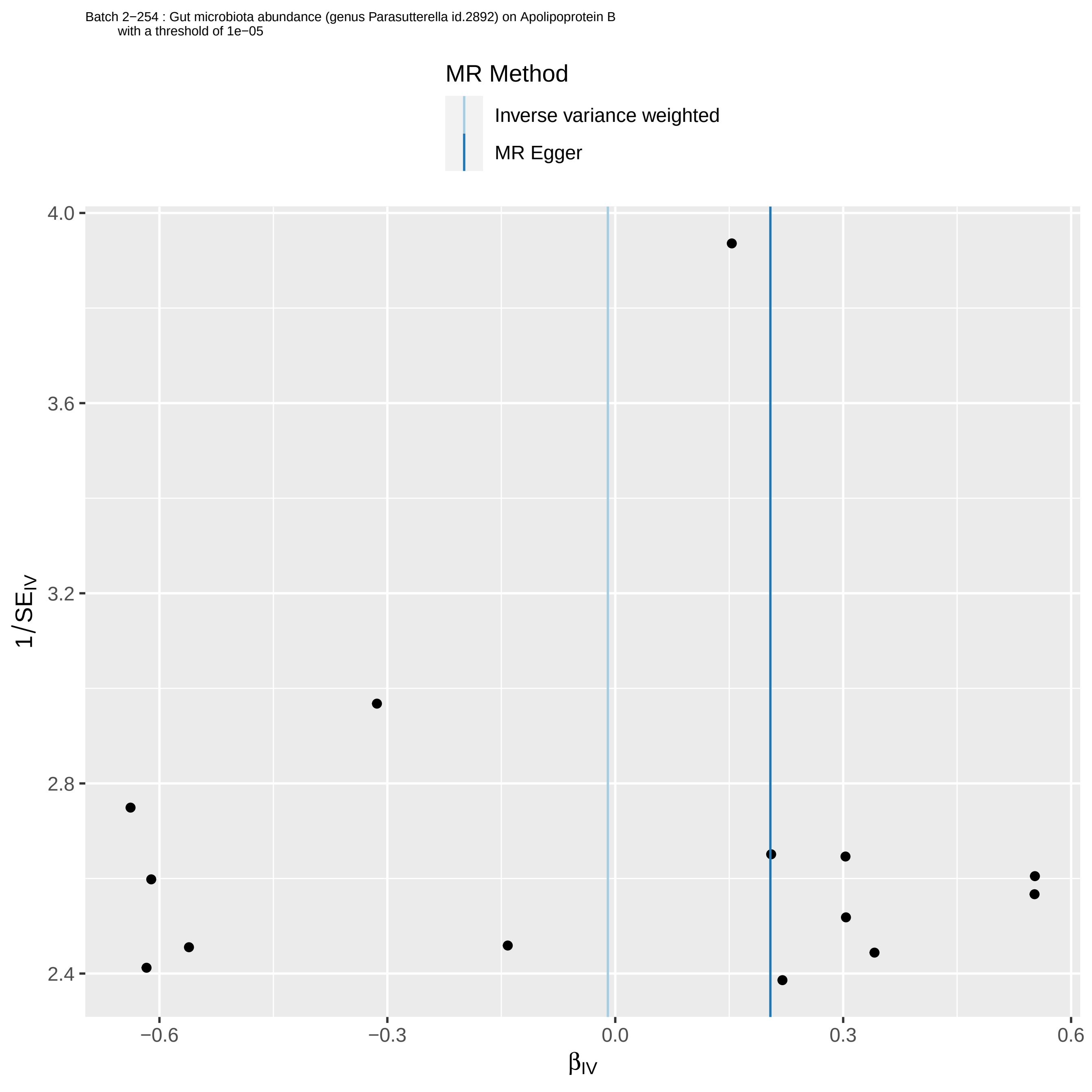

Batch 2−254 : Gut microbiota abundance (genus Parasutterella id.2892) on Apolipoprotein B
with a threshold of 1e−05
MR Method
Inverse variance weighted
MR Egger
4.0
3.6
3.2
2.8
2.4
1/SE_IV
−0.6
−0.3
0.0
0.3
0.6
β_IV

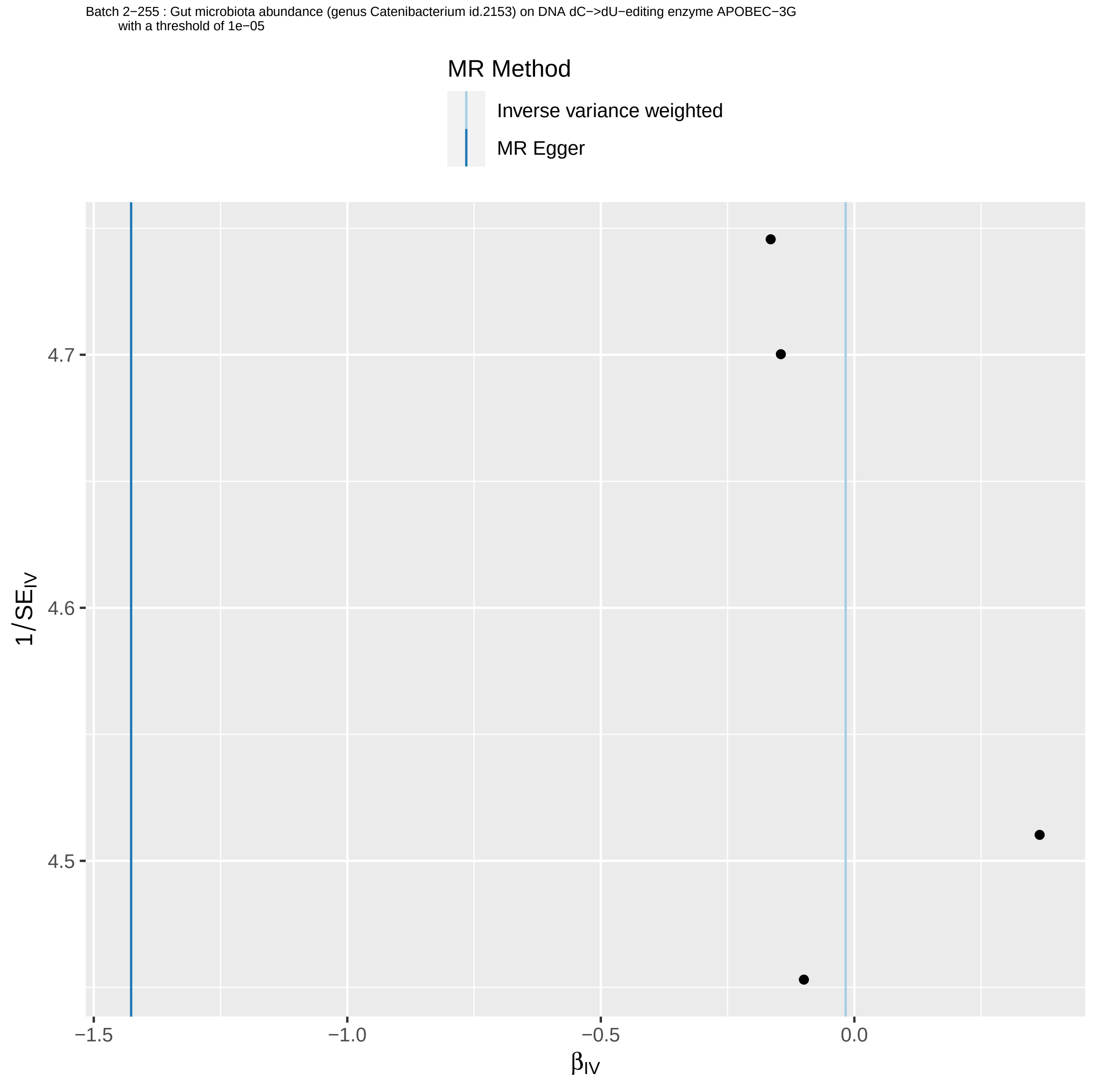

Batch 2−255 : Gut microbiota abundance (genus Catenibacterium id.2153) on DNA dC−>dU−editing enzyme APOBEC−3G
with a threshold of 1e−05
MR Method
Inverse variance weighted
MR Egger
4.7
4.6
4.5
1/SE_IV
−1.5
−1.0
−0.5
0.0
β_IV

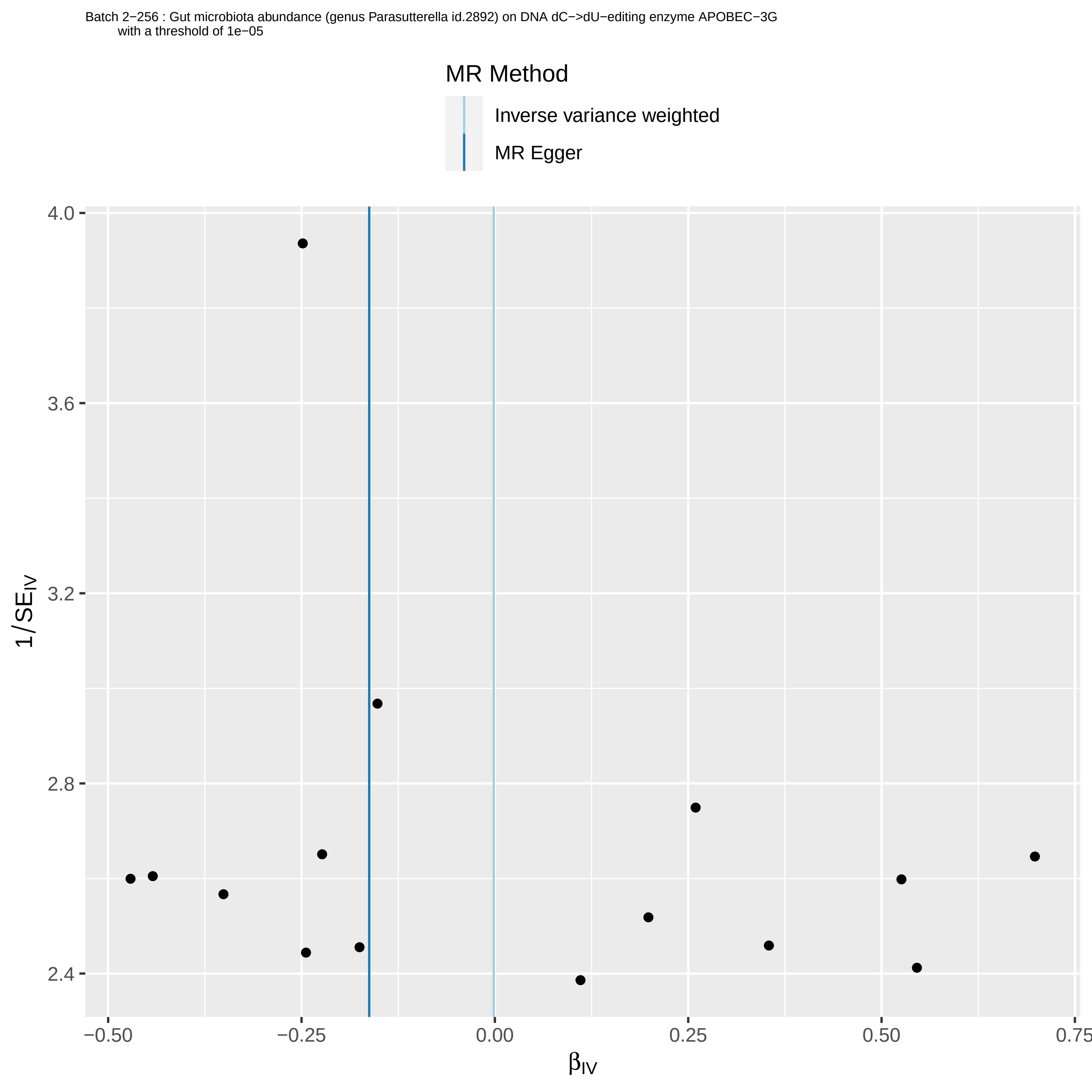
Batch 2−256 : Gut microbiota abundance (genus Parasutterella id.2892) on DNA dC−>dU−editing enzyme APOBEC−3G
with a threshold of 1e−05
MR Method
Inverse variance weighted
MR Egger
4.0
3.6
3.2
2.8
2.4
1/SE_IV
−0.50
−0.25
0.00
0.25
0.50
0.75
β_IV

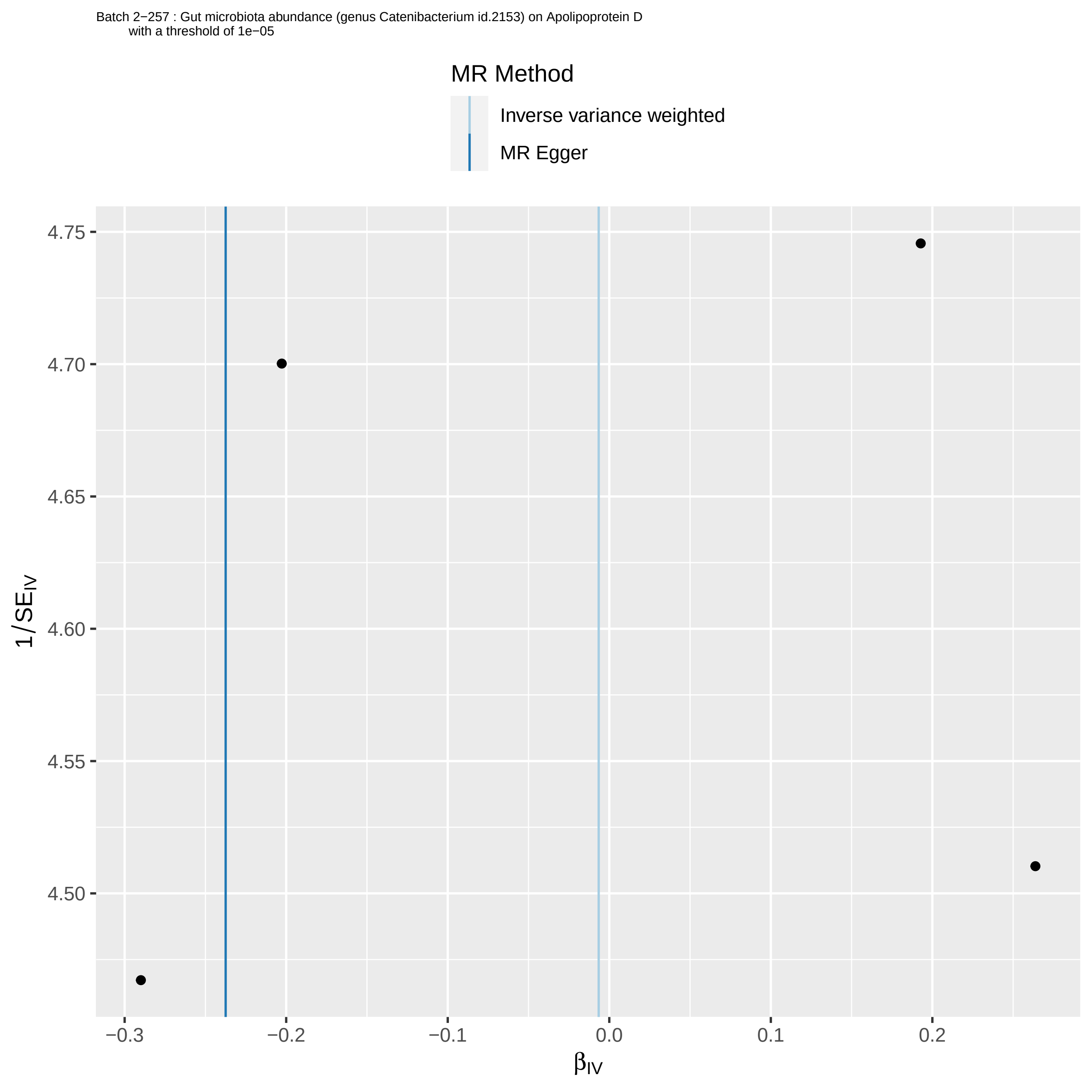
Batch 2−257 : Gut microbiota abundance (genus Catenibacterium id.2153) on Apolipoprotein D
with a threshold of 1e−05
MR Method
Inverse variance weighted
MR Egger
4.75
4.70
4.65
4.60
4.55
4.50
1/SE$_{IV}$
−0.3
−0.2
−0.1
0.0
0.1
0.2
β$_{IV}$

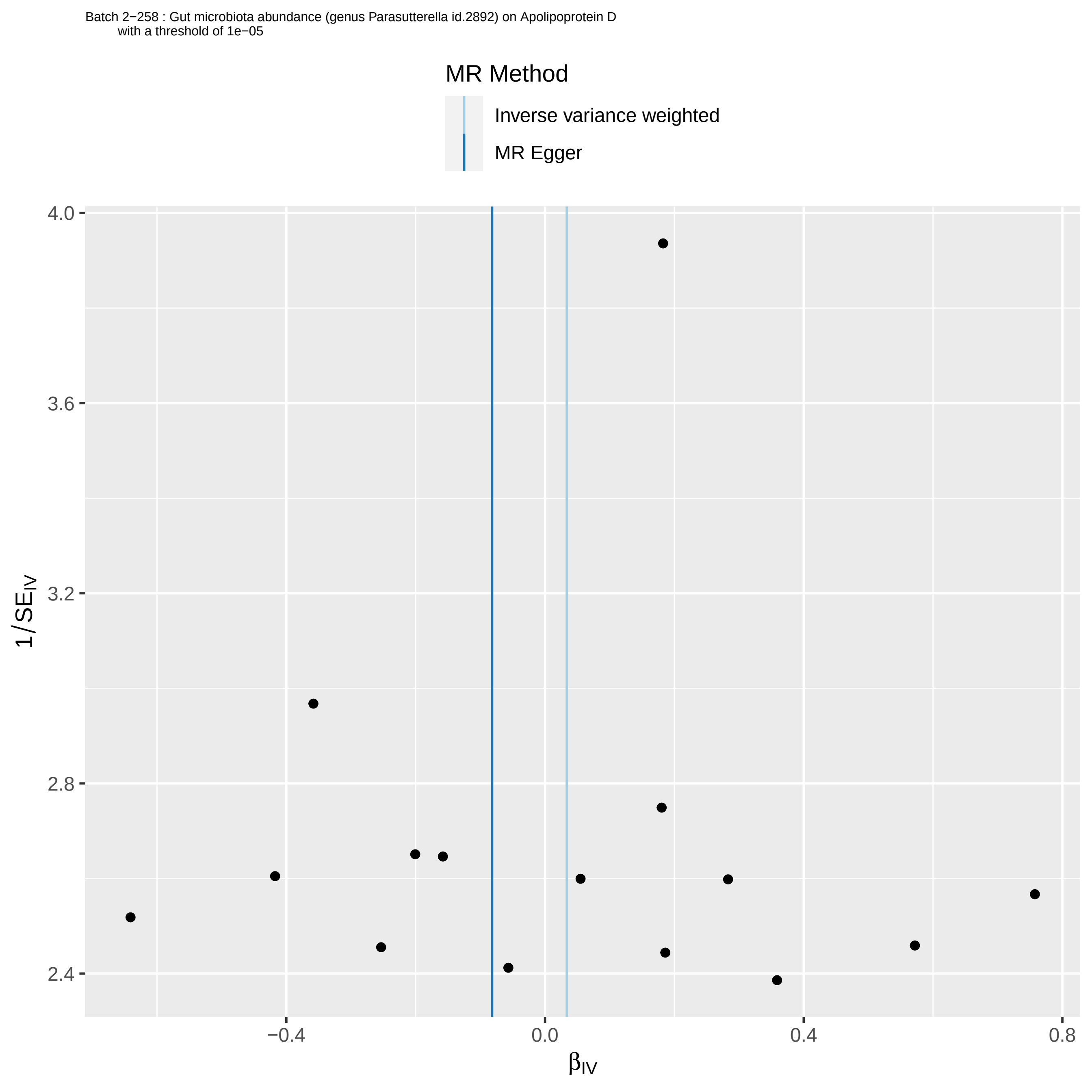
Batch 2−258 : Gut microbiota abundance (genus Parasutterella id.2892) on Apolipoprotein D
with a threshold of 1e−05
MR Method
Inverse variance weighted
MR Egger
1/SE_IV
β_IV
4.0
3.6
3.2
2.8
2.4
−0.4
0.0
0.4
0.8

Batch 2−259 : Gut microbiota abundance (genus Catenibacterium id.2153) on Apolipoprotein D
with a threshold of 1e−05

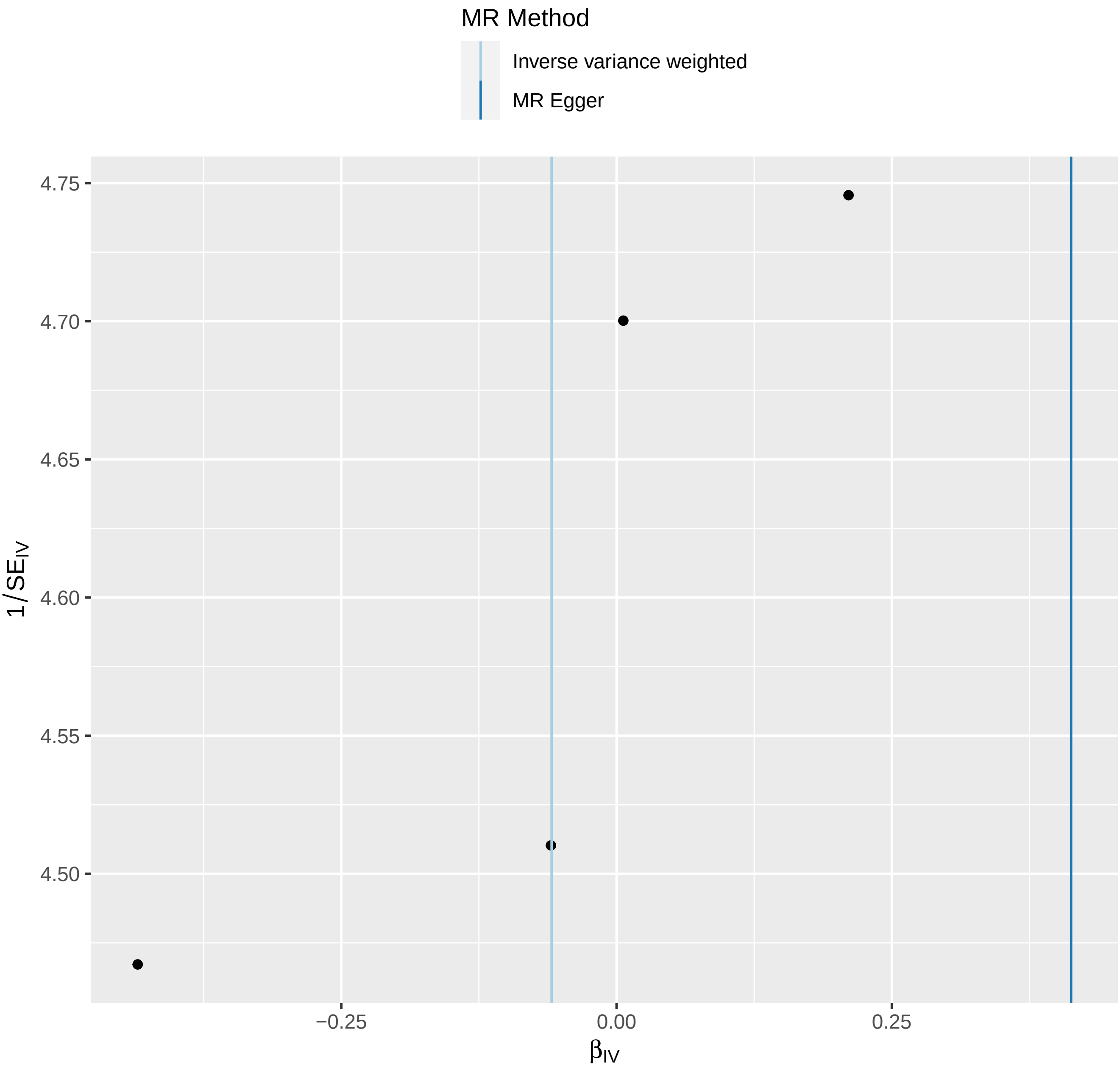

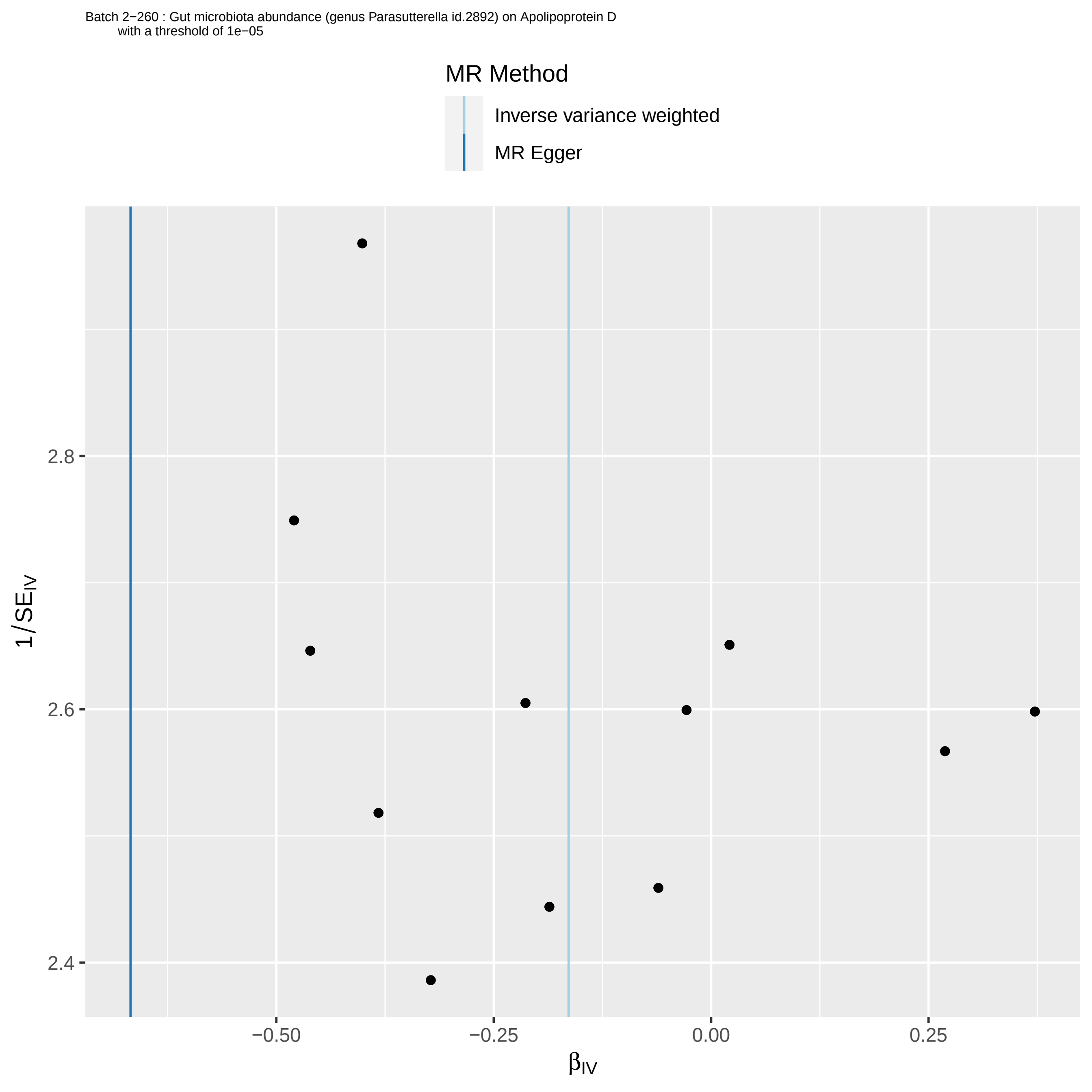
Batch 2−260 : Gut microbiota abundance (genus Parasutterella id.2892) on Apolipoprotein D
with a threshold of 1e−05
MR Method
Inverse variance weighted
MR Egger
2.8
2.6
2.4
1/SE$_{IV}$
−0.50
−0.25
0.00
0.25
β_{IV}

Batch 2−261 : Gut microbiota abundance (genus Catenibacterium id.2153) on Apolipoprotein E (isoform E3) with a threshold of 1e−05

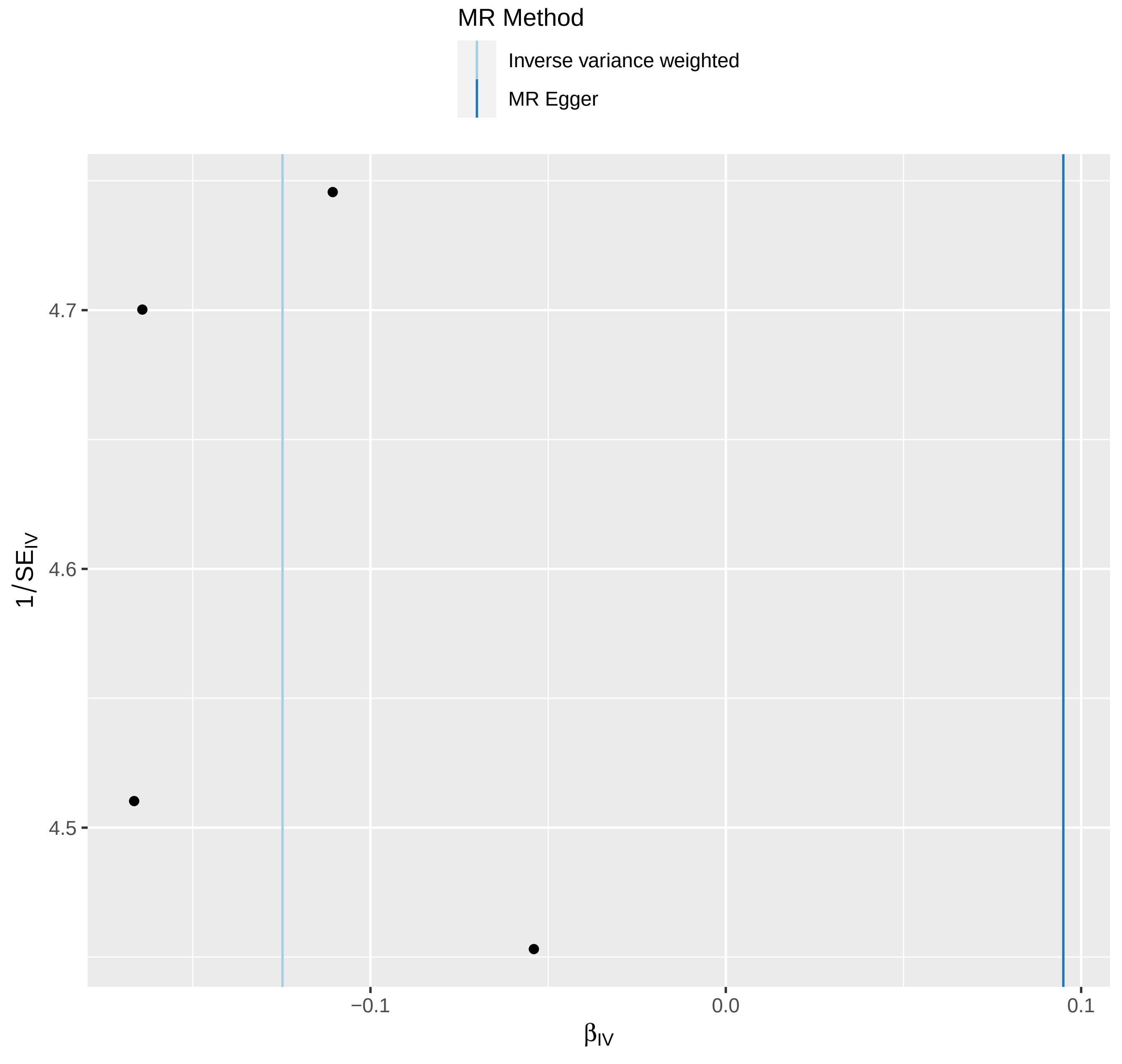

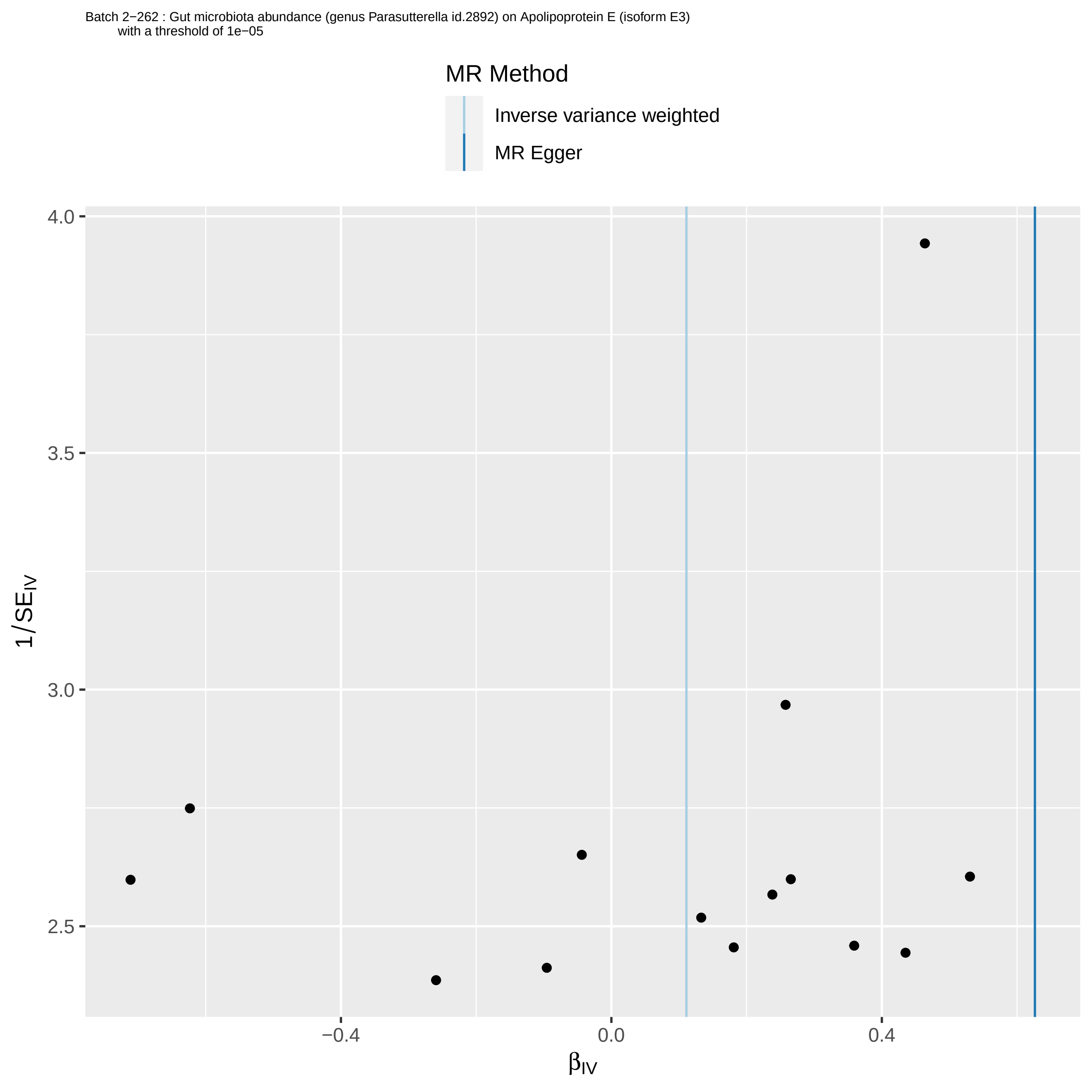
Batch 2−262 : Gut microbiota abundance (genus Parasutterella id.2892) on Apolipoprotein E (isoform E3)
with a threshold of 1e−05
MR Method
Inverse variance weighted
MR Egger
4.0
3.5
3.0
2.5
1/SE_IV
−0.4
0.0
0.4
β_IV

Batch 2−263 : Gut microbiota abundance (genus Catenibacterium id.2153) on Apolipoprotein E (isoform E2)
with a threshold of 1e−05

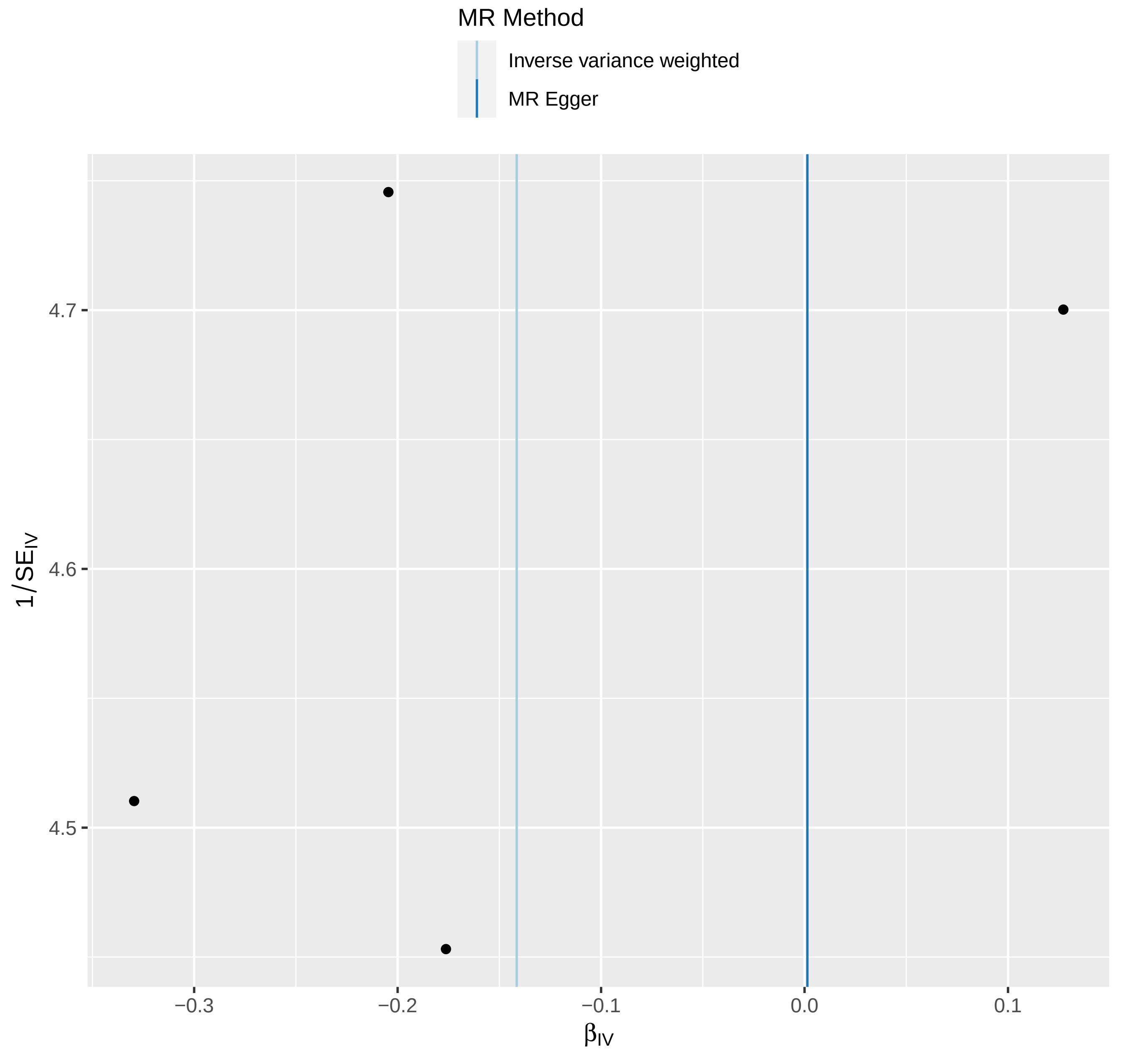

Batch 2−264 : Gut microbiota abundance (genus Parasutterella id.2892) on Apolipoprotein E (isoform E2) with a threshold of 1e−05

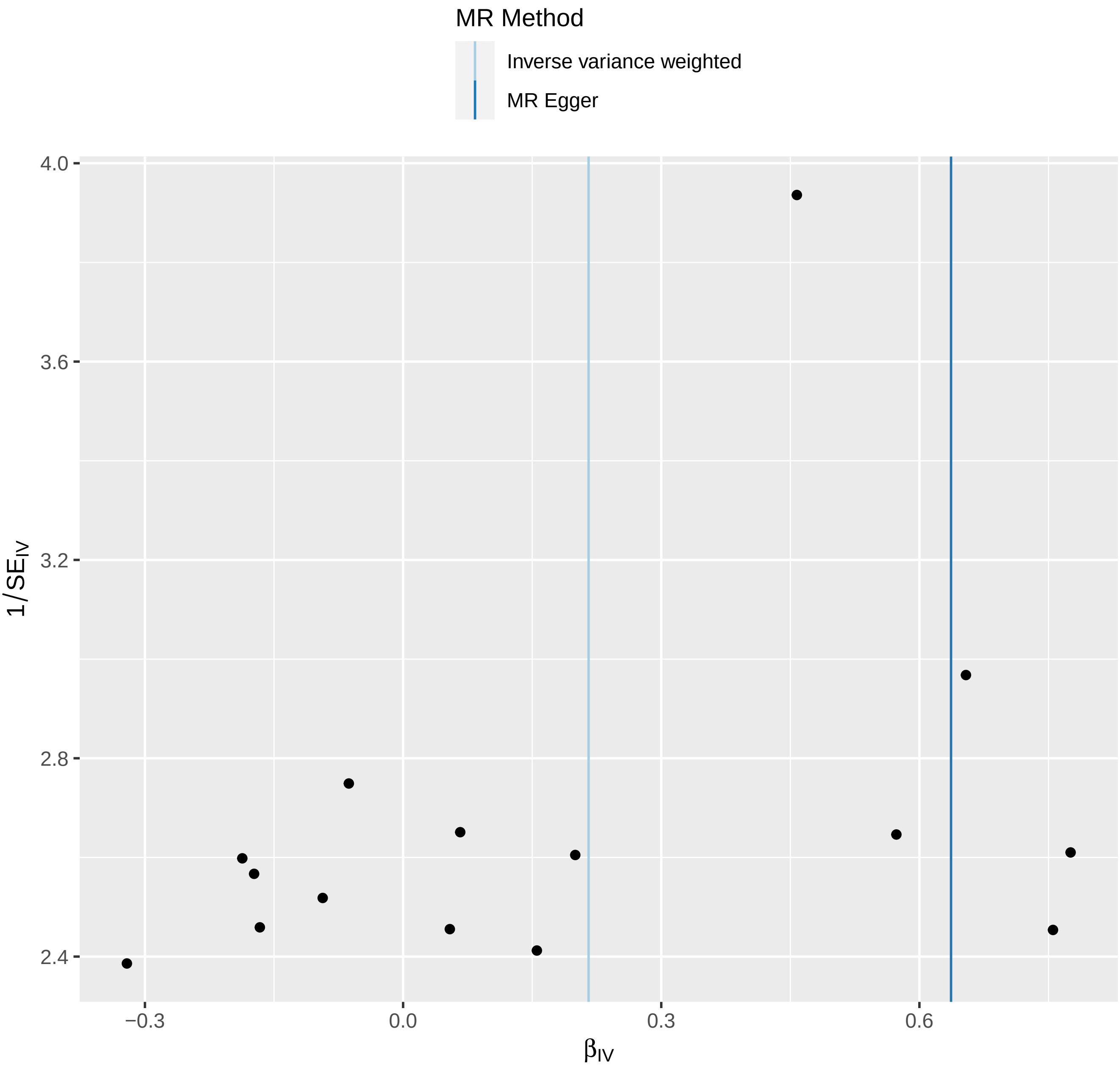

Batch 2−265 : Gut microbiota abundance (genus Catenibacterium id.2153) on Apolipoprotein F
with a threshold of 1e−05

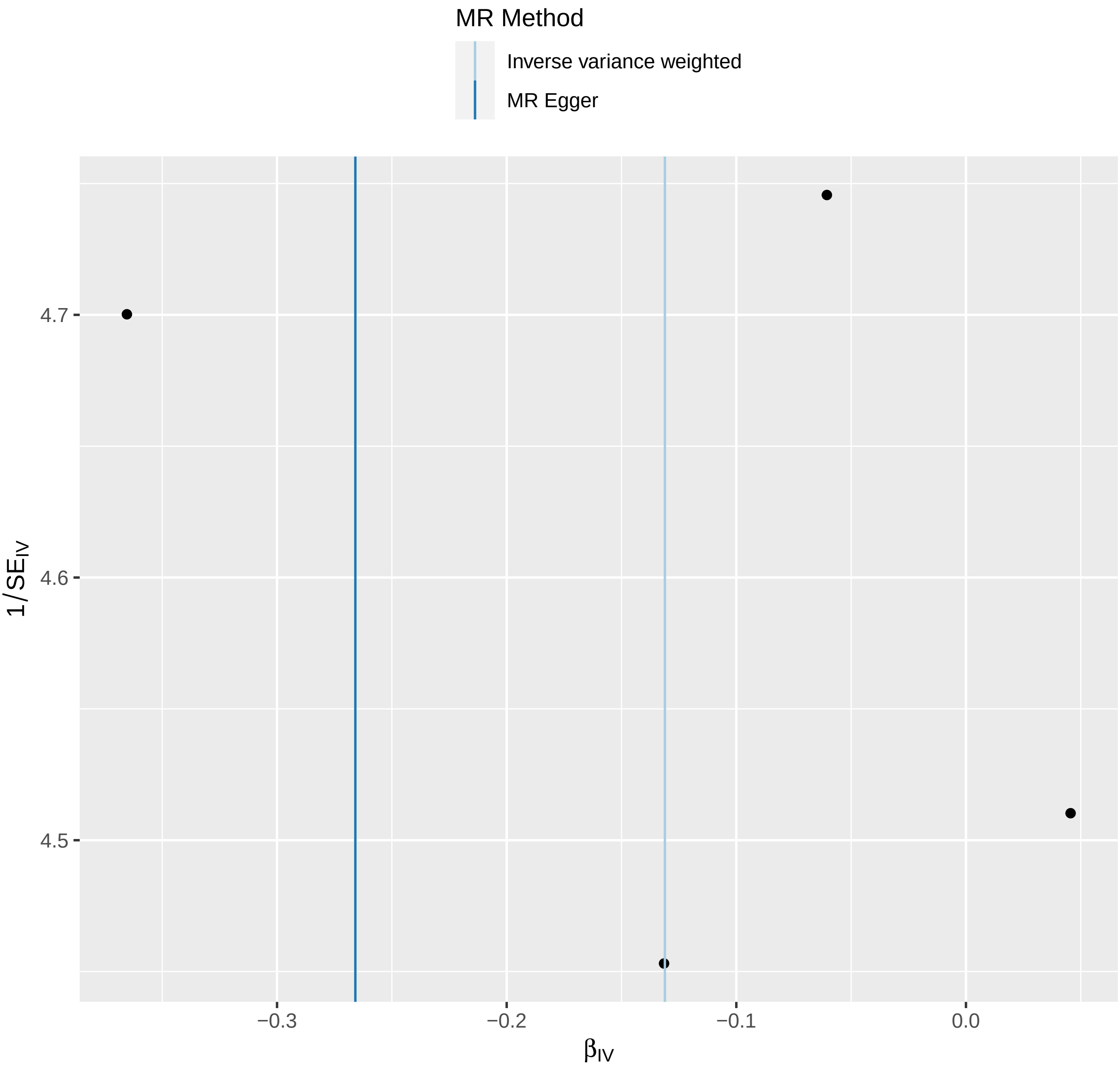

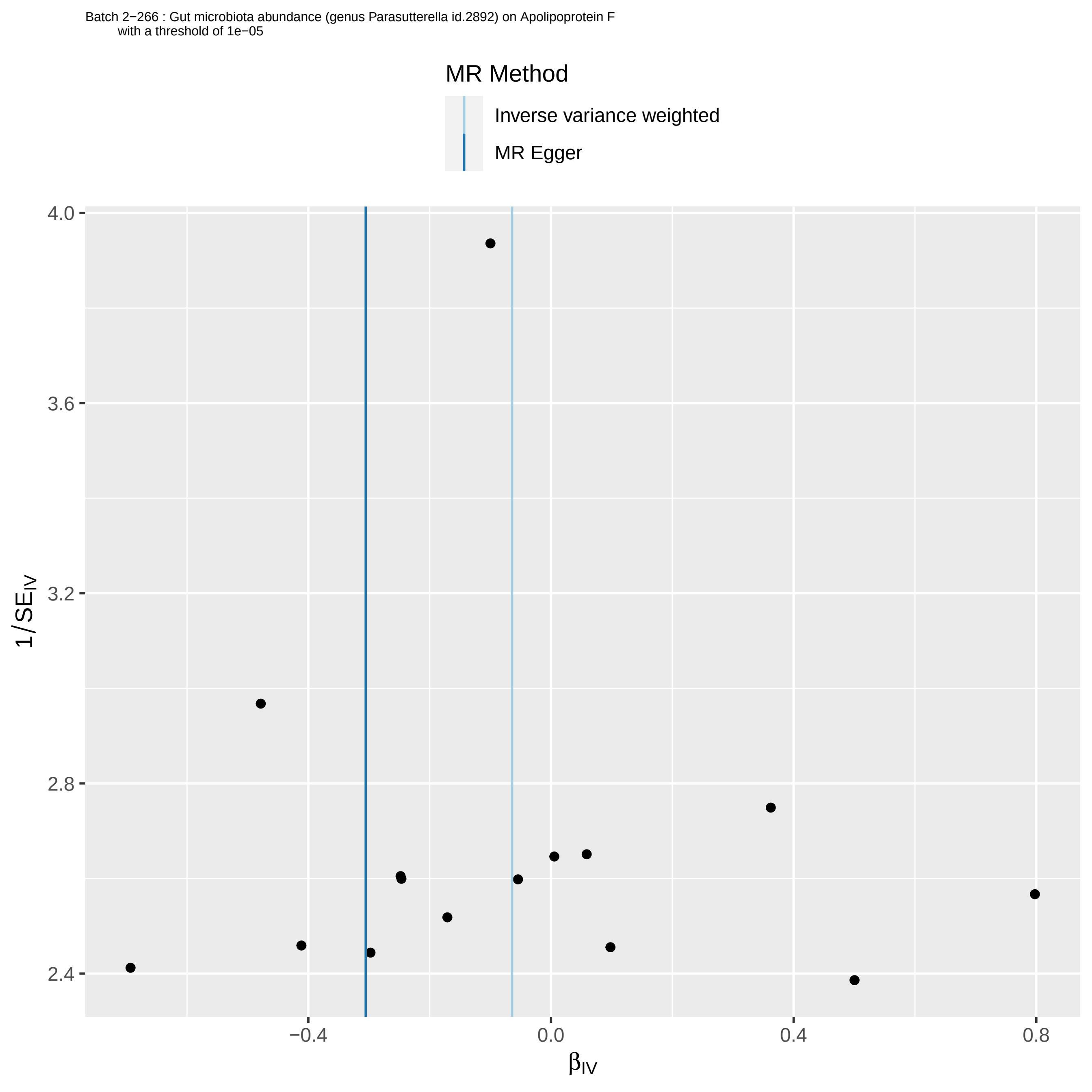
Batch 2−266 : Gut microbiota abundance (genus Parasutterella id.2892) on Apolipoprotein F
with a threshold of 1e−05
MR Method
Inverse variance weighted
MR Egger
4.0
3.6
3.2
2.8
2.4
1/SE_IV
−0.4
0.0
0.4
0.8
β_IV

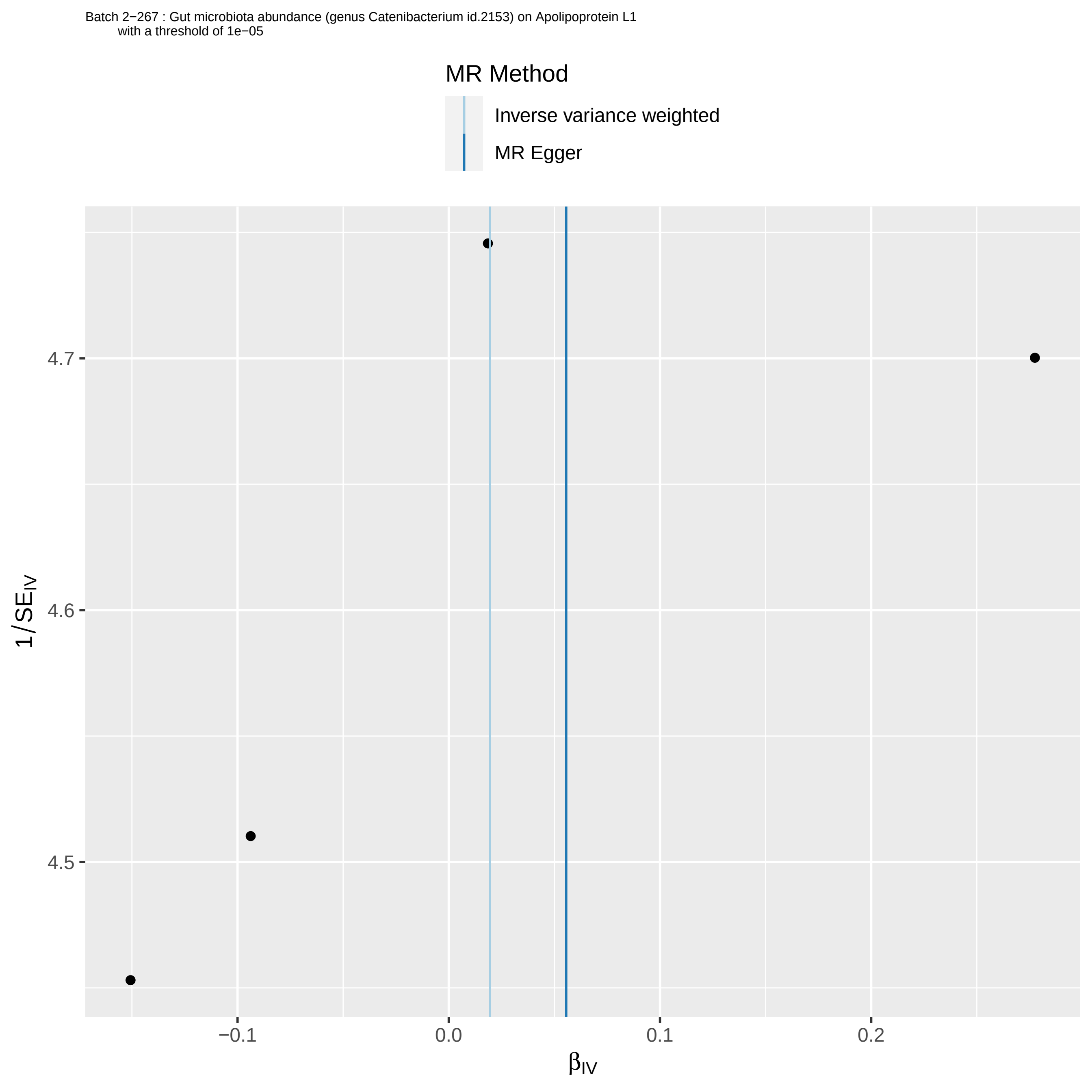
Batch 2−267 : Gut microbiota abundance (genus Catenibacterium id.2153) on Apolipoprotein L1
with a threshold of 1e−05
MR Method
Inverse variance weighted
MR Egger
4.7
4.6
4.5
$1/SE_{IV}$
−0.1
0.0
0.1
0.2
β_{IV}

Batch 2−268 : Gut microbiota abundance (genus Parasutterella id.2892) on Apolipoprotein L1
with a threshold of 1e−05

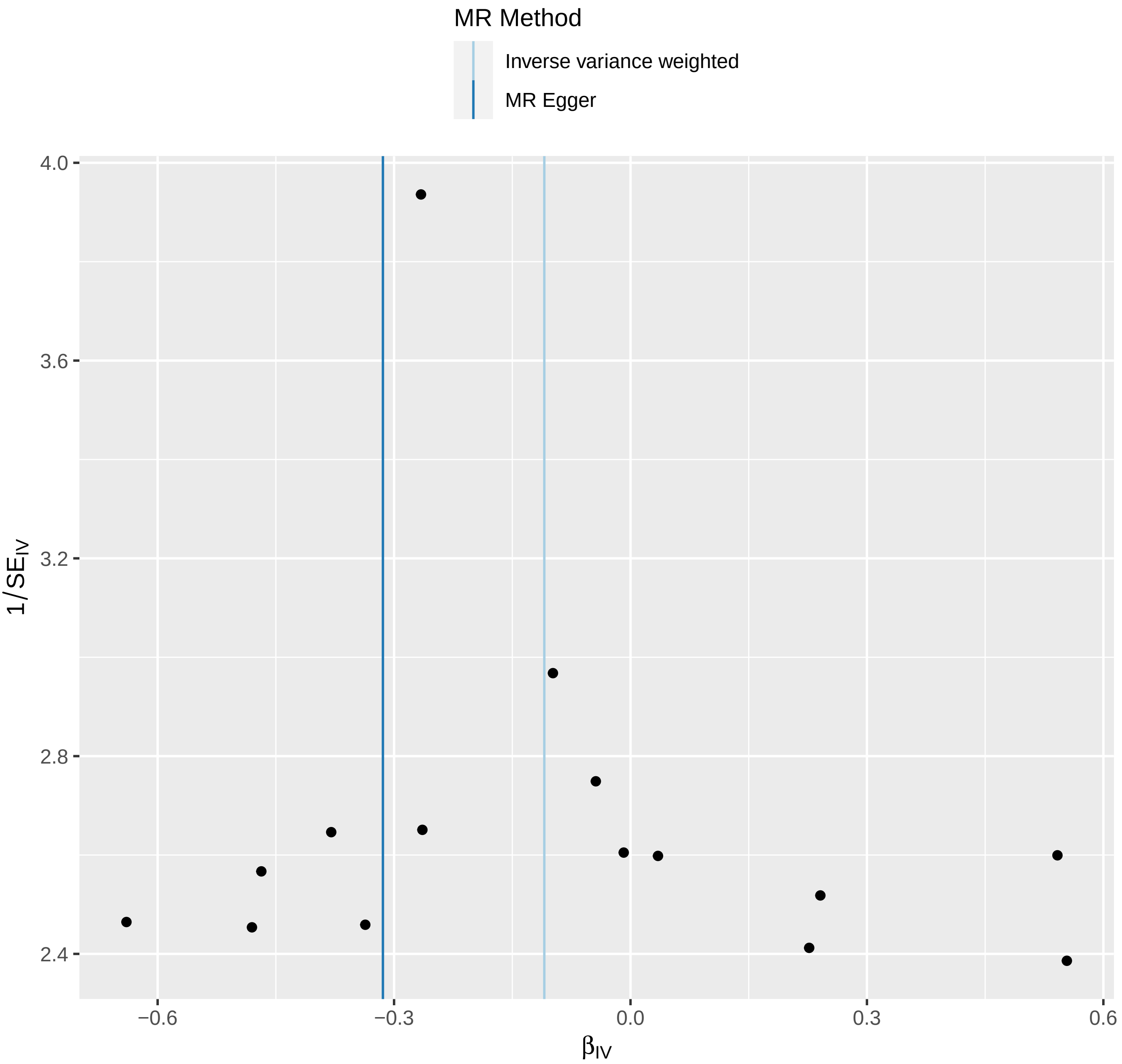

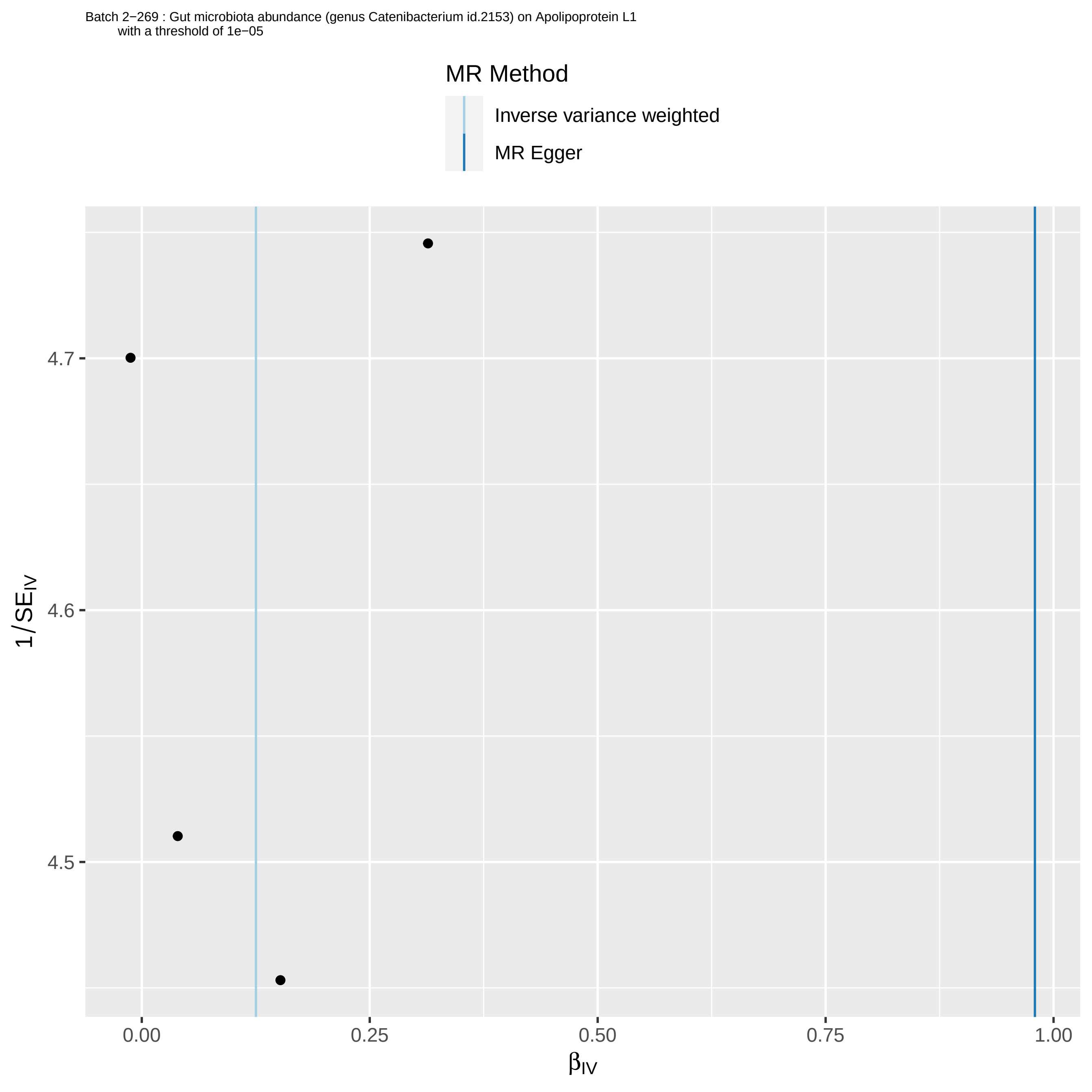
Batch 2−269 : Gut microbiota abundance (genus Catenibacterium id.2153) on Apolipoprotein L1
with a threshold of 1e−05
MR Method
Inverse variance weighted
MR Egger
4.7
4.6
4.5
$1/SE_{IV}$
0.00
0.25
0.50
0.75
1.00
β_{IV}

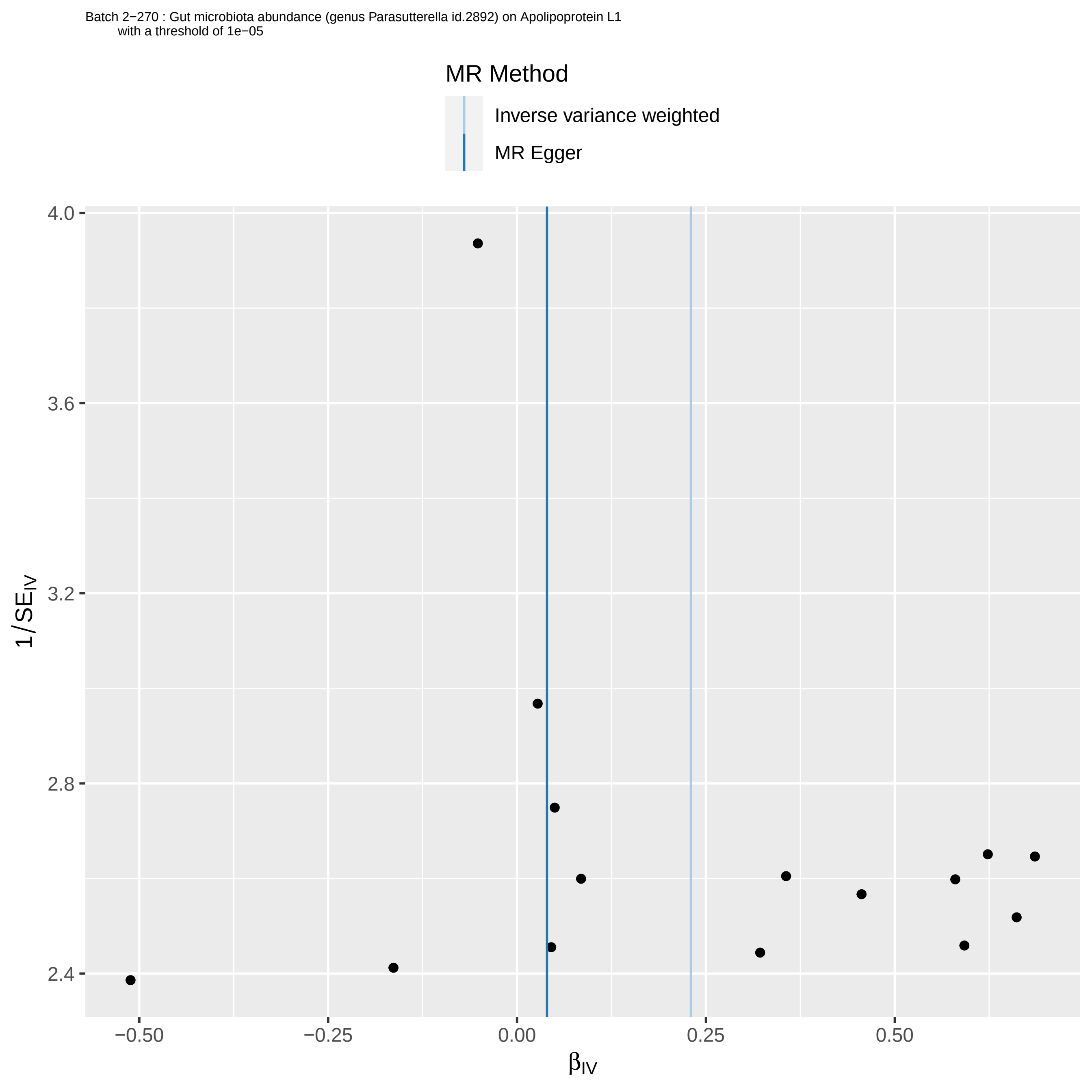
Batch 2−270 : Gut microbiota abundance (genus Parasutterella id.2892) on Apolipoprotein L1
with a threshold of 1e−05
MR Method
Inverse variance weighted
MR Egger
4.0
3.6
3.2
2.8
2.4
1/SE_IV
−0.50
−0.25
0.00
0.25
0.50
β_IV

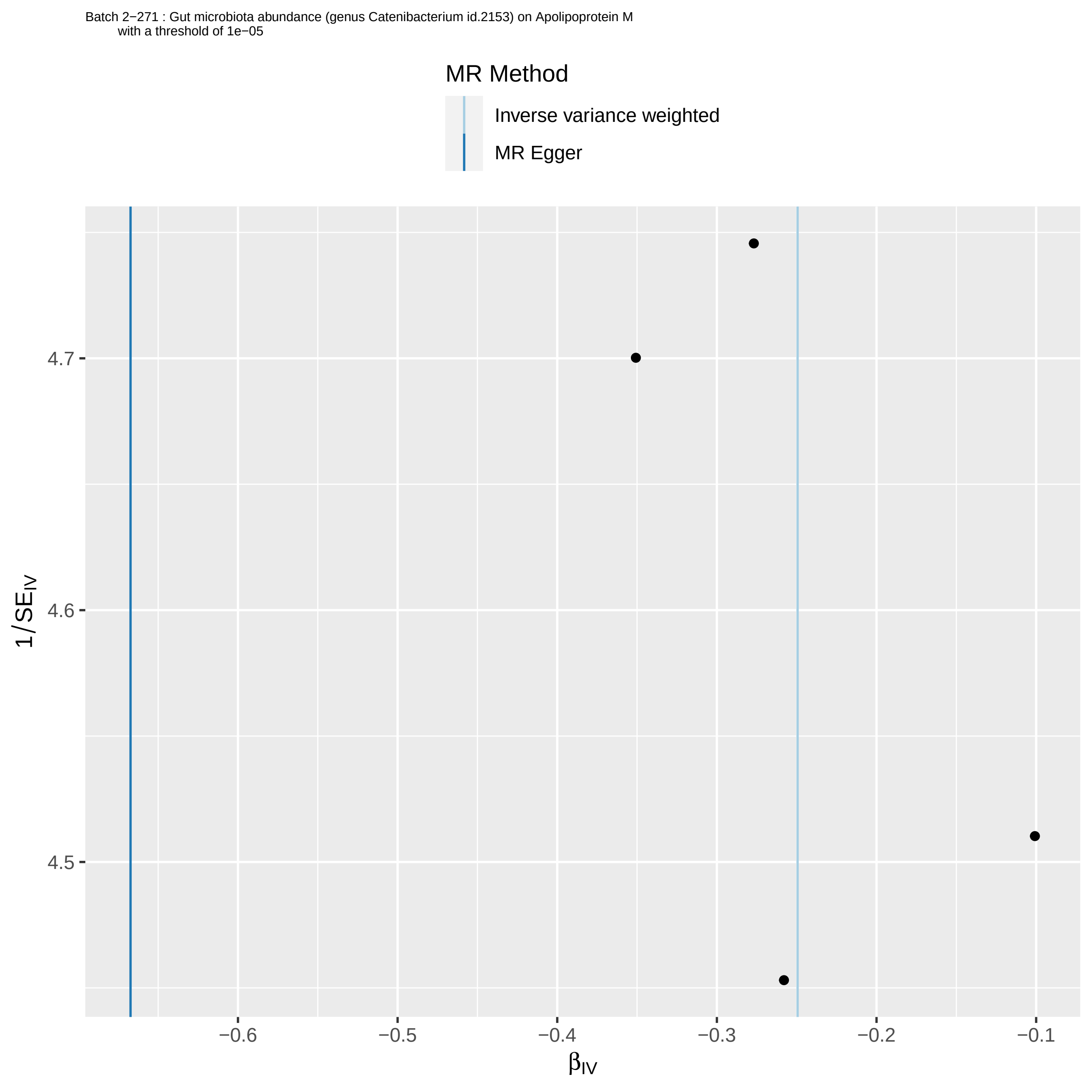
Batch 2−271 : Gut microbiota abundance (genus Catenibacterium id.2153) on Apolipoprotein M
with a threshold of 1e−05
MR Method
Inverse variance weighted
MR Egger
4.7
4.6
4.5
1/SE$_{IV}$
−0.6
−0.5
−0.4
−0.3
−0.2
−0.1
β$_{IV}$

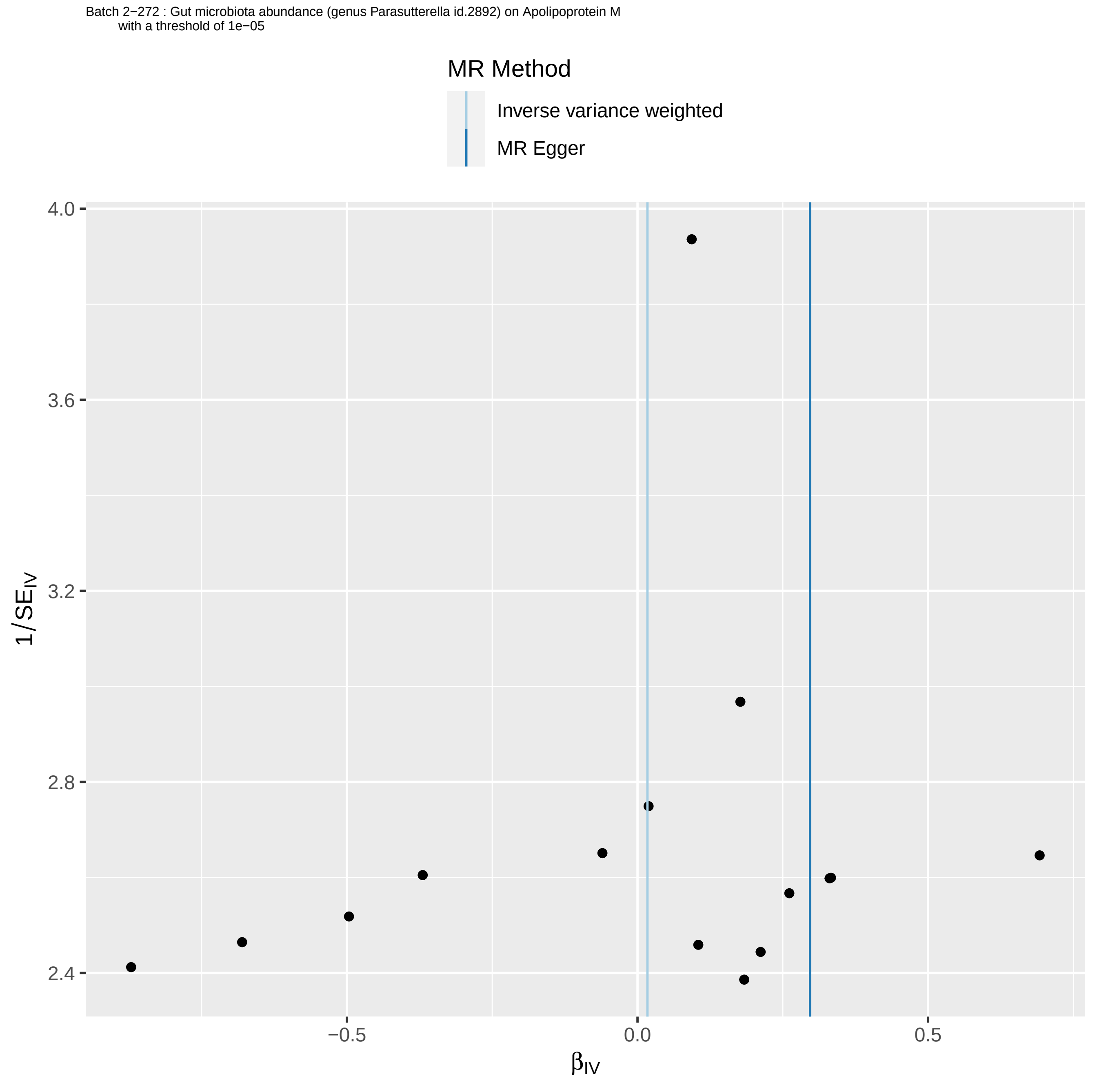

Batch 2−272 : Gut microbiota abundance (genus Parasutterella id.2892) on Apolipoprotein M
with a threshold of 1e−05
MR Method
Inverse variance weighted
MR Egger
4.0
3.6
3.2
2.8
2.4
1/SE_IV
−0.5
0.0
0.5
β_IV

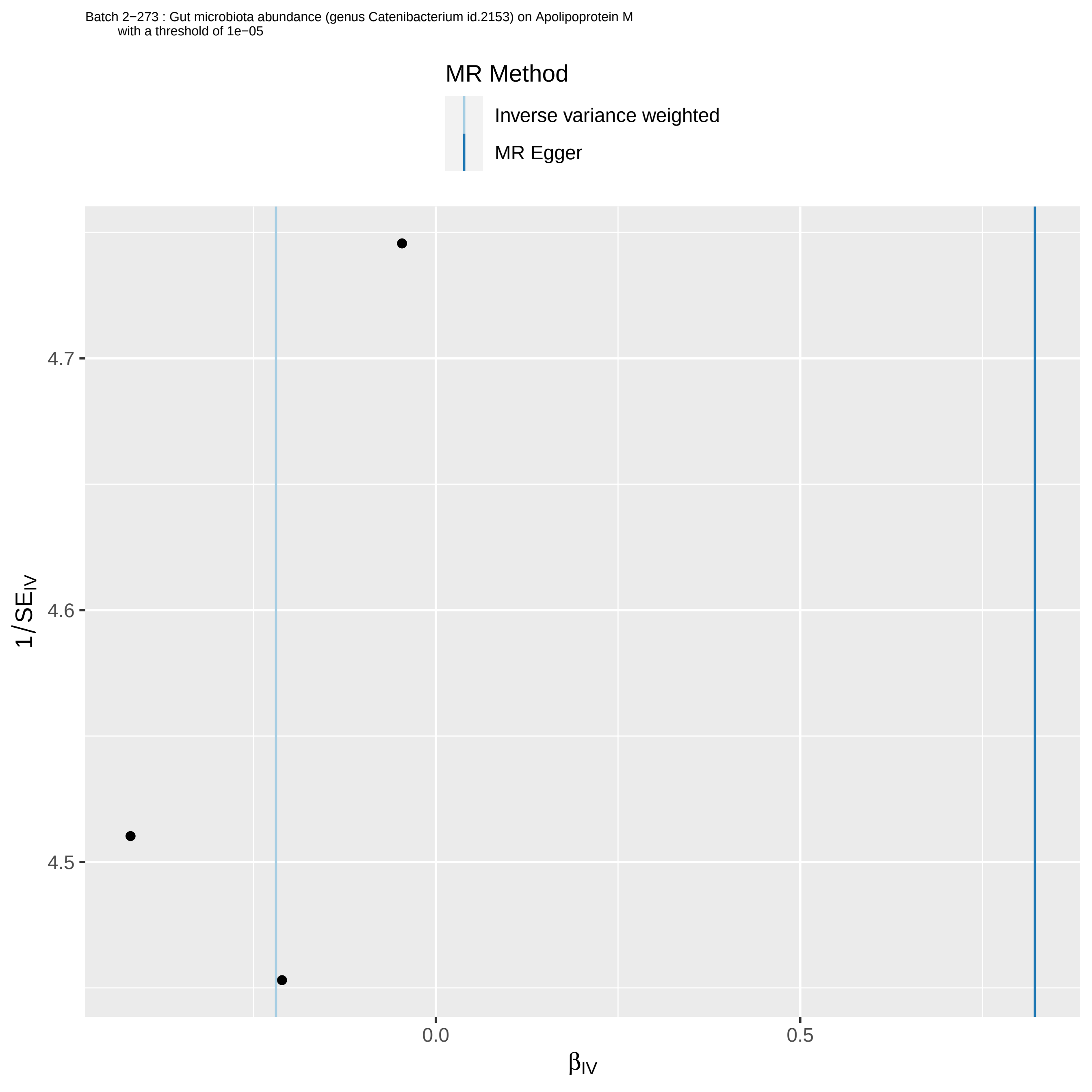

Batch 2−273 : Gut microbiota abundance (genus Catenibacterium id.2153) on Apolipoprotein M
with a threshold of 1e−05
MR Method
Inverse variance weighted
MR Egger
4.7
4.6
4.5
1/SE_IV
0.0
0.5
β_IV

Batch 2−274 : Gut microbiota abundance (genus Parasutterella id.2892) on Apolipoprotein M
with a threshold of 1e−05

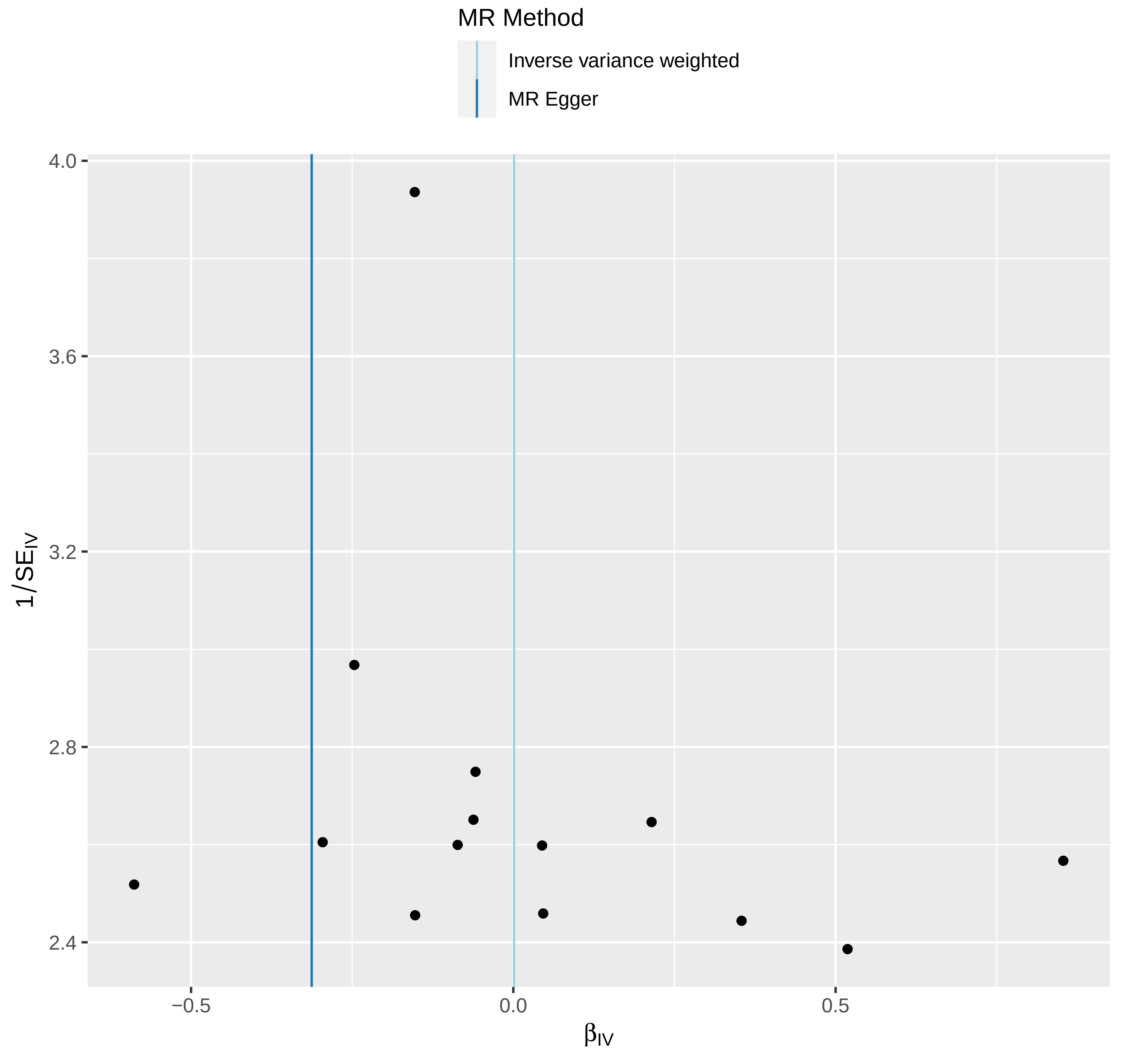

Batch 2−275 : Gut microbiota abundance (genus Catenibacterium id.2153) on MICOS complex subunit MIC26
with a threshold of 1e−05

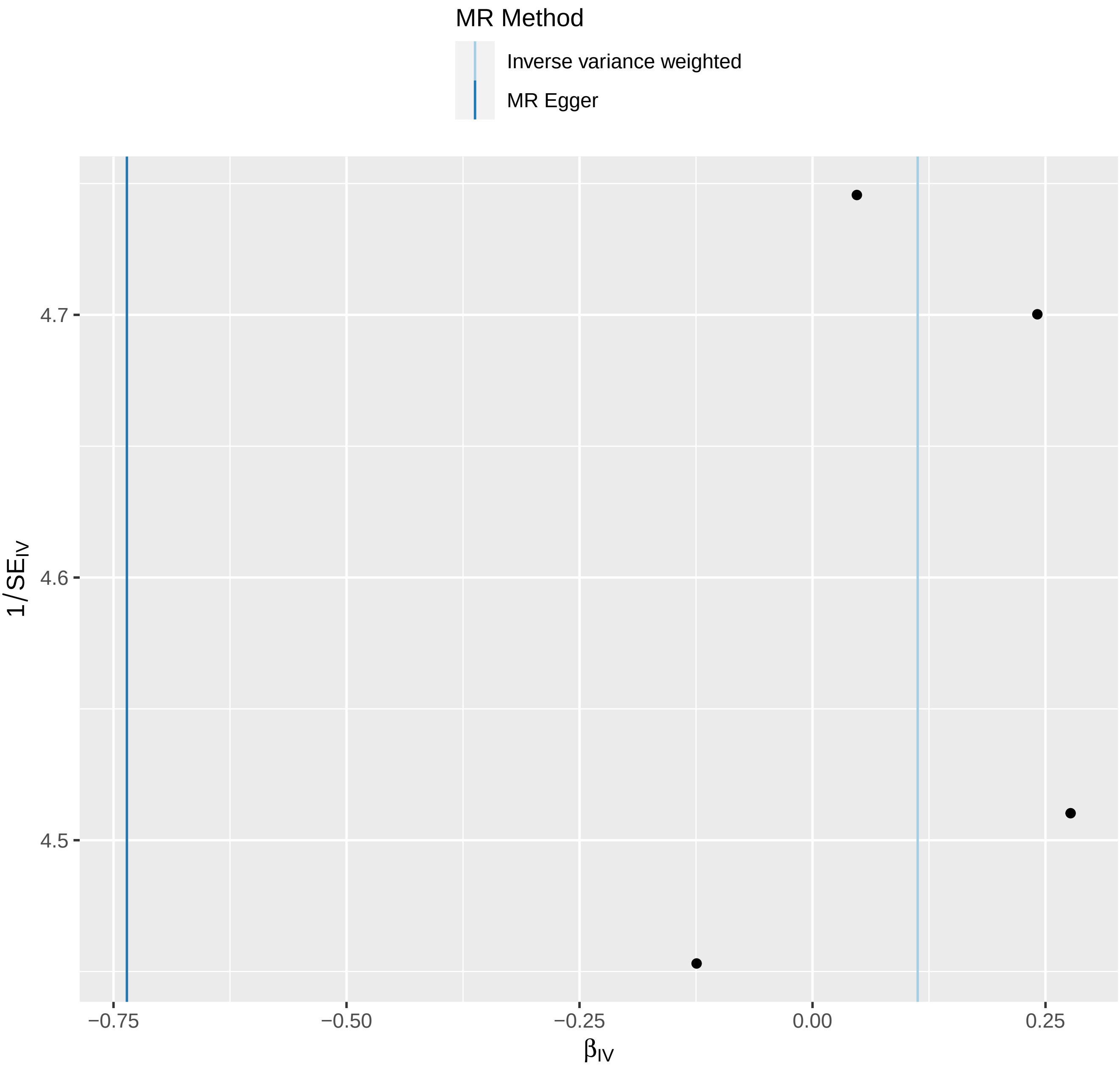

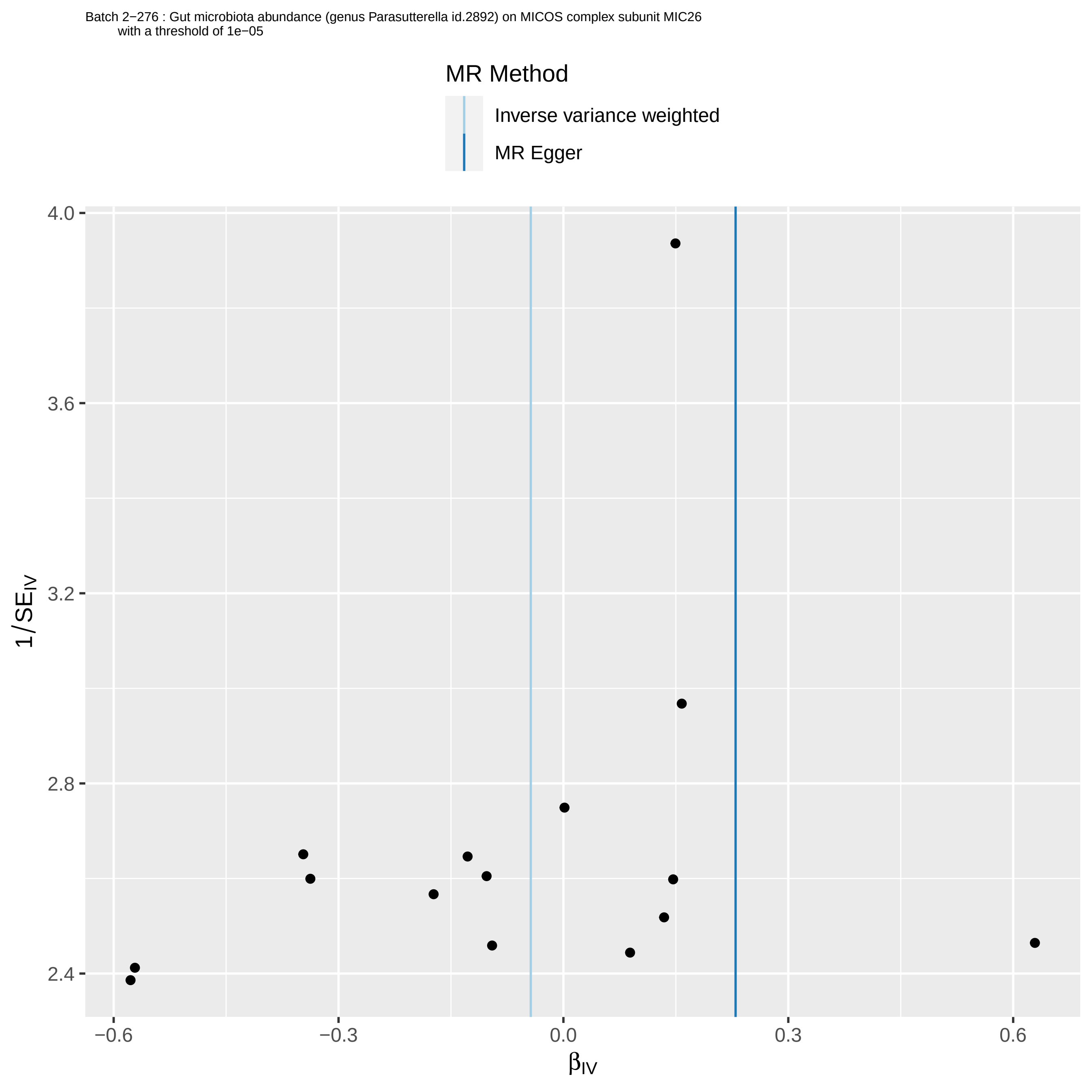

Batch 2−276 : Gut microbiota abundance (genus Parasutterella id.2892) on MICOS complex subunit MIC26
with a threshold of 1e−05
MR Method
Inverse variance weighted
MR Egger
4.0
3.6
3.2
2.8
2.4
1/SE$_{IV}$
−0.6
−0.3
0.0
0.3
0.6
β_{IV}

Batch 2−277 : Gut microbiota abundance (genus Catenibacterium id.2153) on Amyloid beta A4 protein
with a threshold of 1e−05

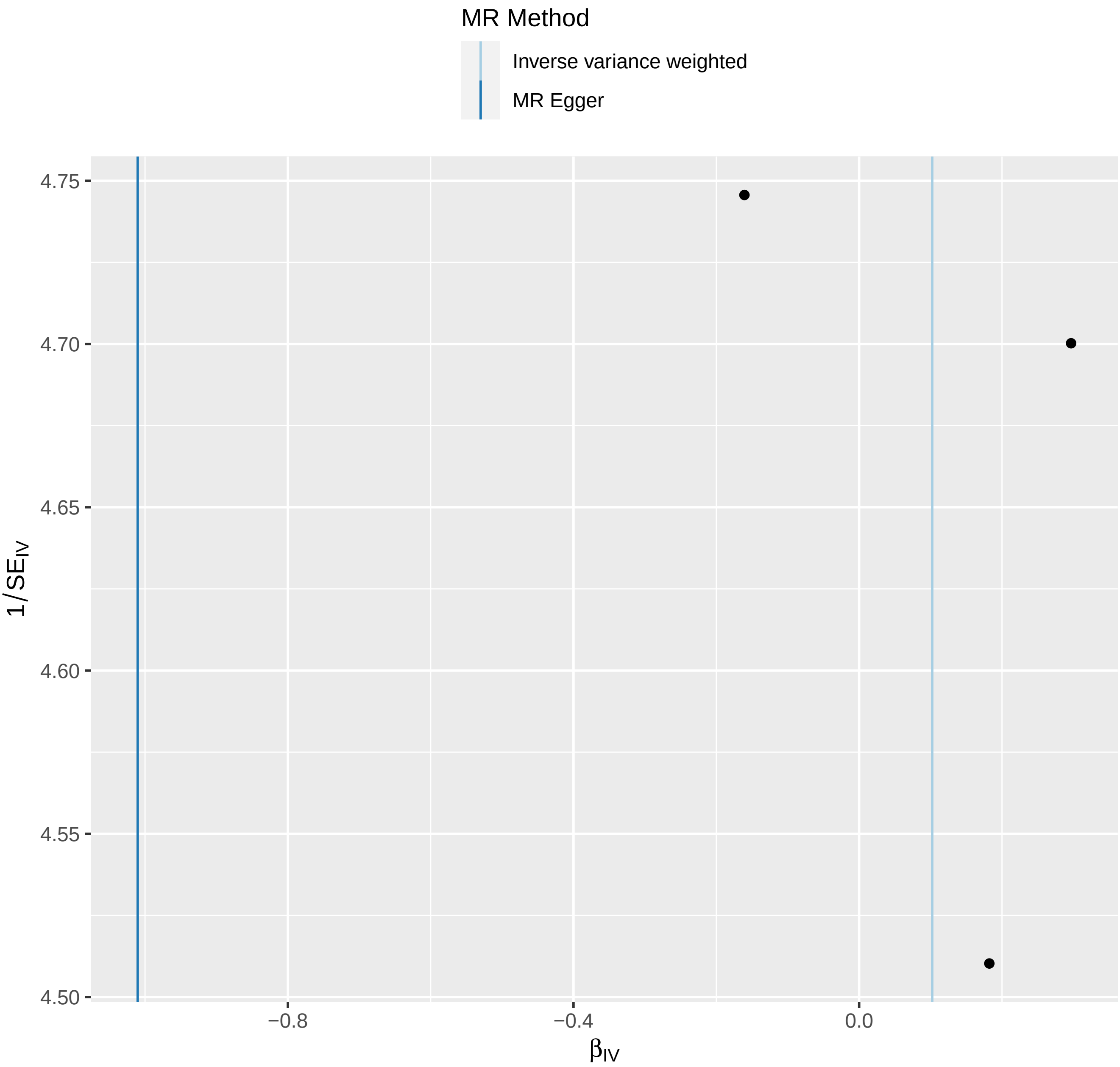

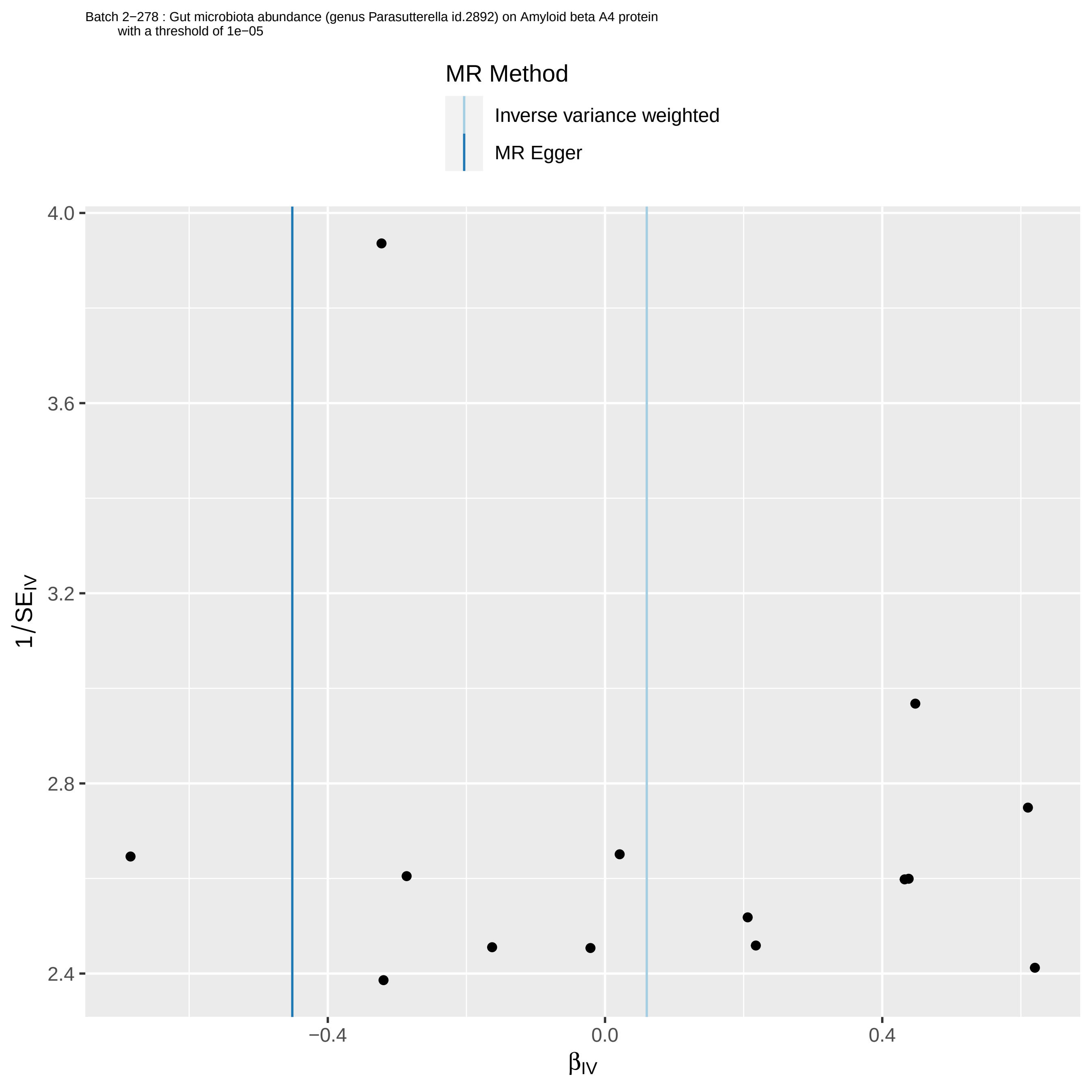

Batch 2−278 : Gut microbiota abundance (genus Parasutterella id.2892) on Amyloid beta A4 protein with a threshold of 1e−05
MR Method
Inverse variance weighted
MR Egger
4.0
3.6
3.2
2.8
2.4
1/SE$_{IV}$
−0.4
0.0
0.4
β$_{IV}$

Batch 2−279 : Gut microbiota abundance (genus Catenibacterium id.2153) on DCC−interacting protein 13−alpha
with a threshold of 1e−05

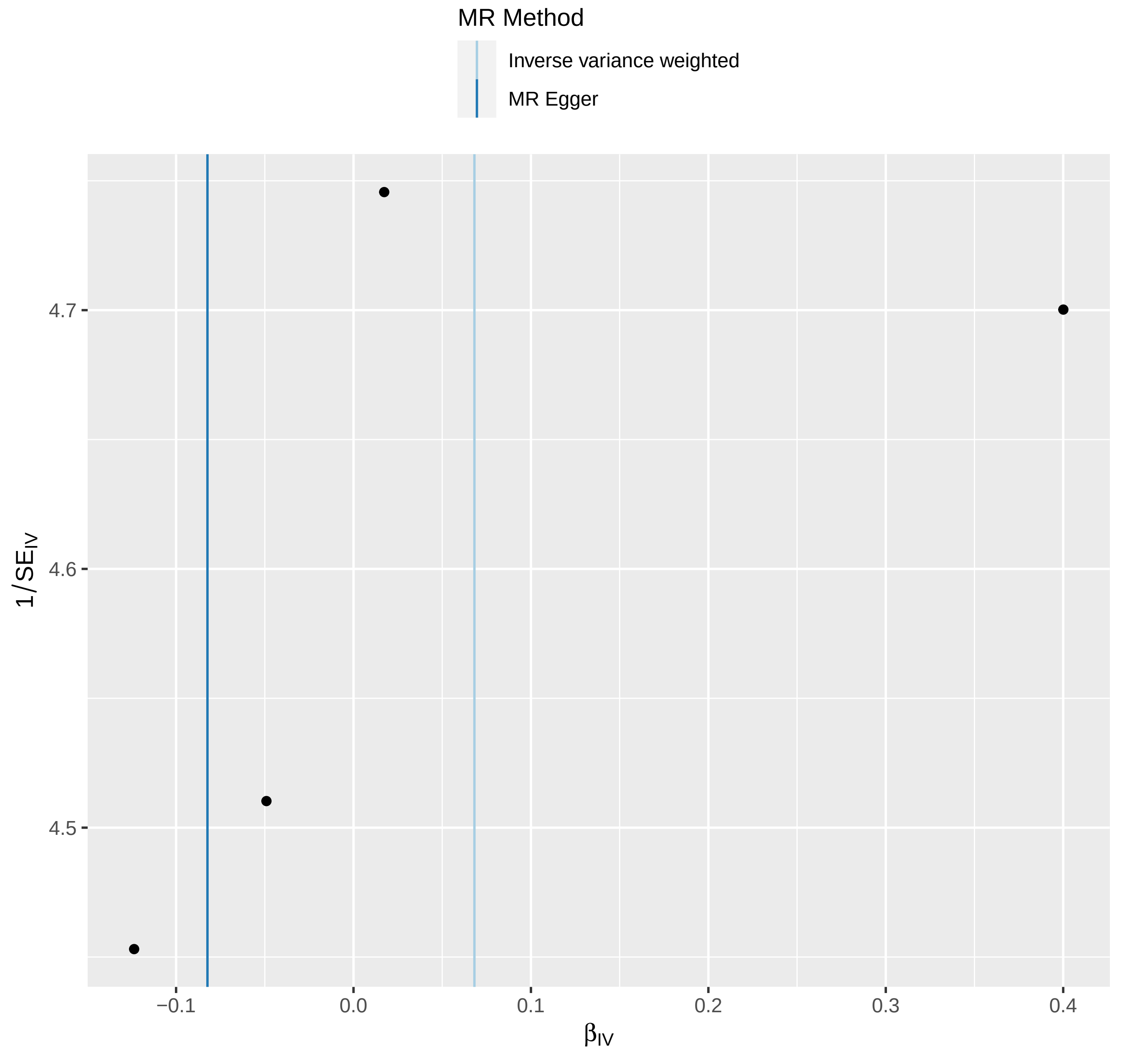

Batch 2−280 : Gut microbiota abundance (genus Parasutterella id.2892) on DCC−interacting protein 13−alpha
with a threshold of 1e−05

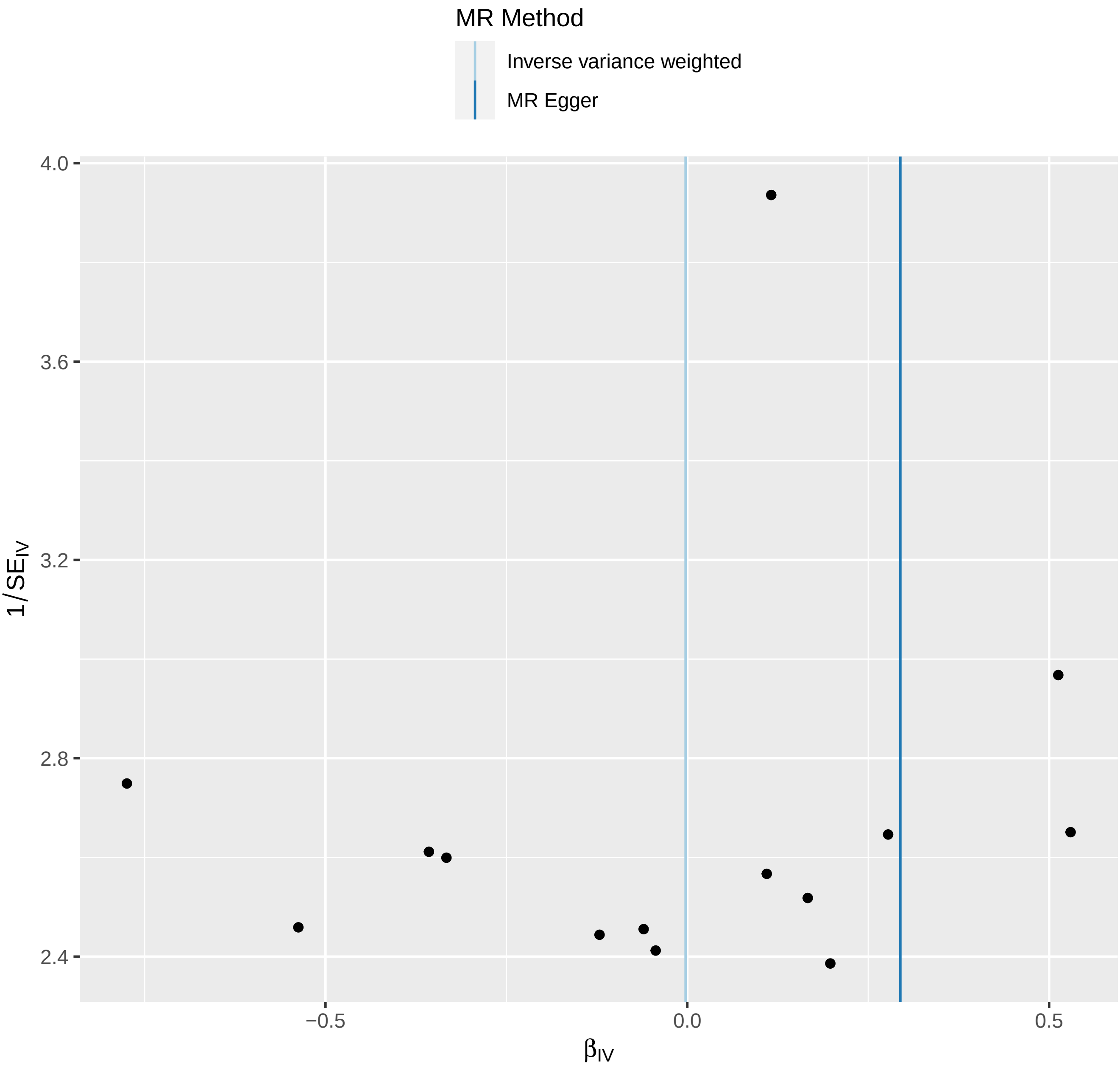

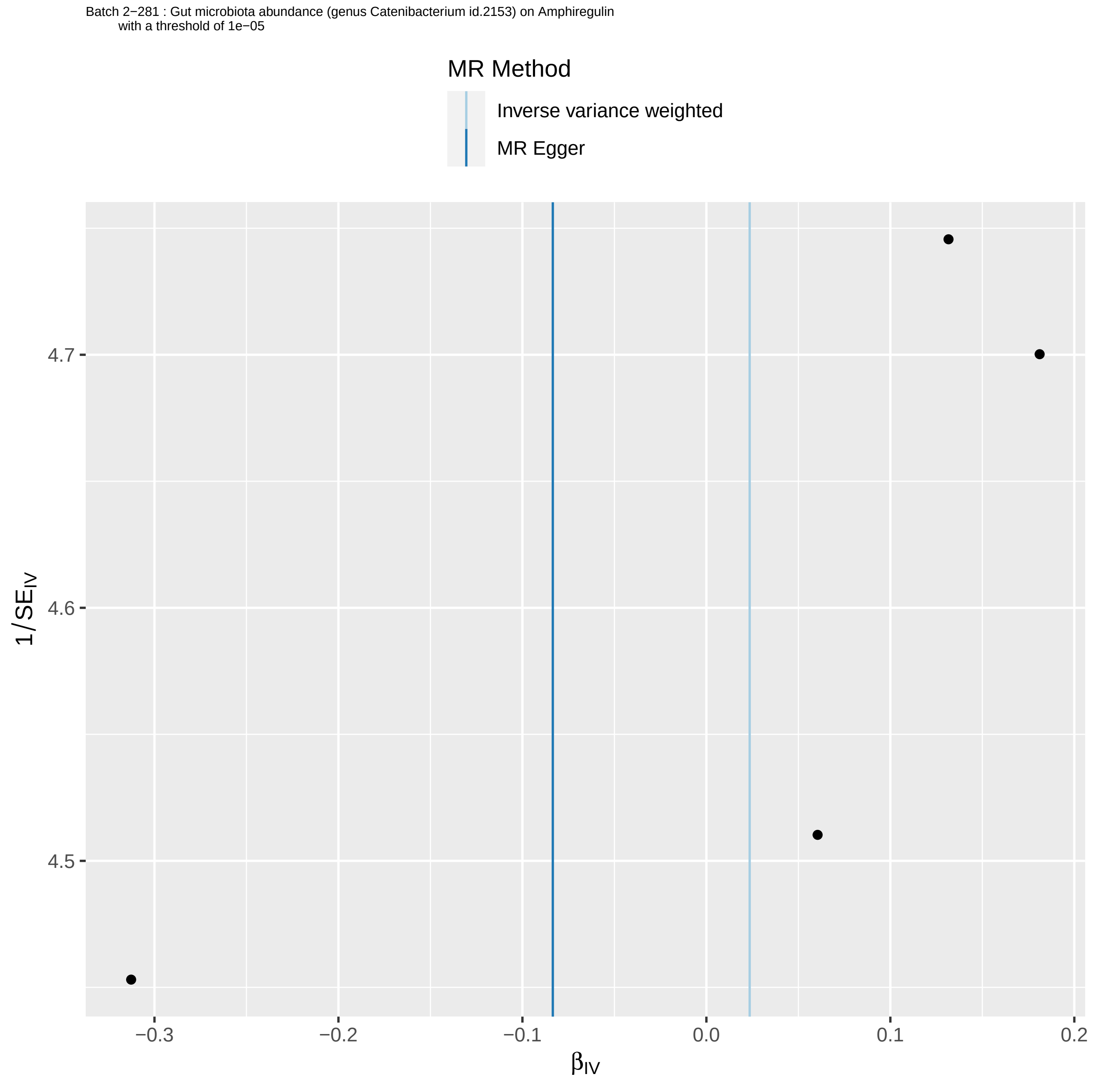

Batch 2−281 : Gut microbiota abundance (genus Catenibacterium id.2153) on Amphiregulin
with a threshold of 1e−05
MR Method
Inverse variance weighted
MR Egger
4.7
4.6
4.5
1/SE$_{IV}$
−0.3
−0.2
−0.1
0.0
0.1
0.2
β_{IV}

Batch 2−282 : Gut microbiota abundance (genus Parasutterella id.2892) on Amphiregulin
with a threshold of 1e−05

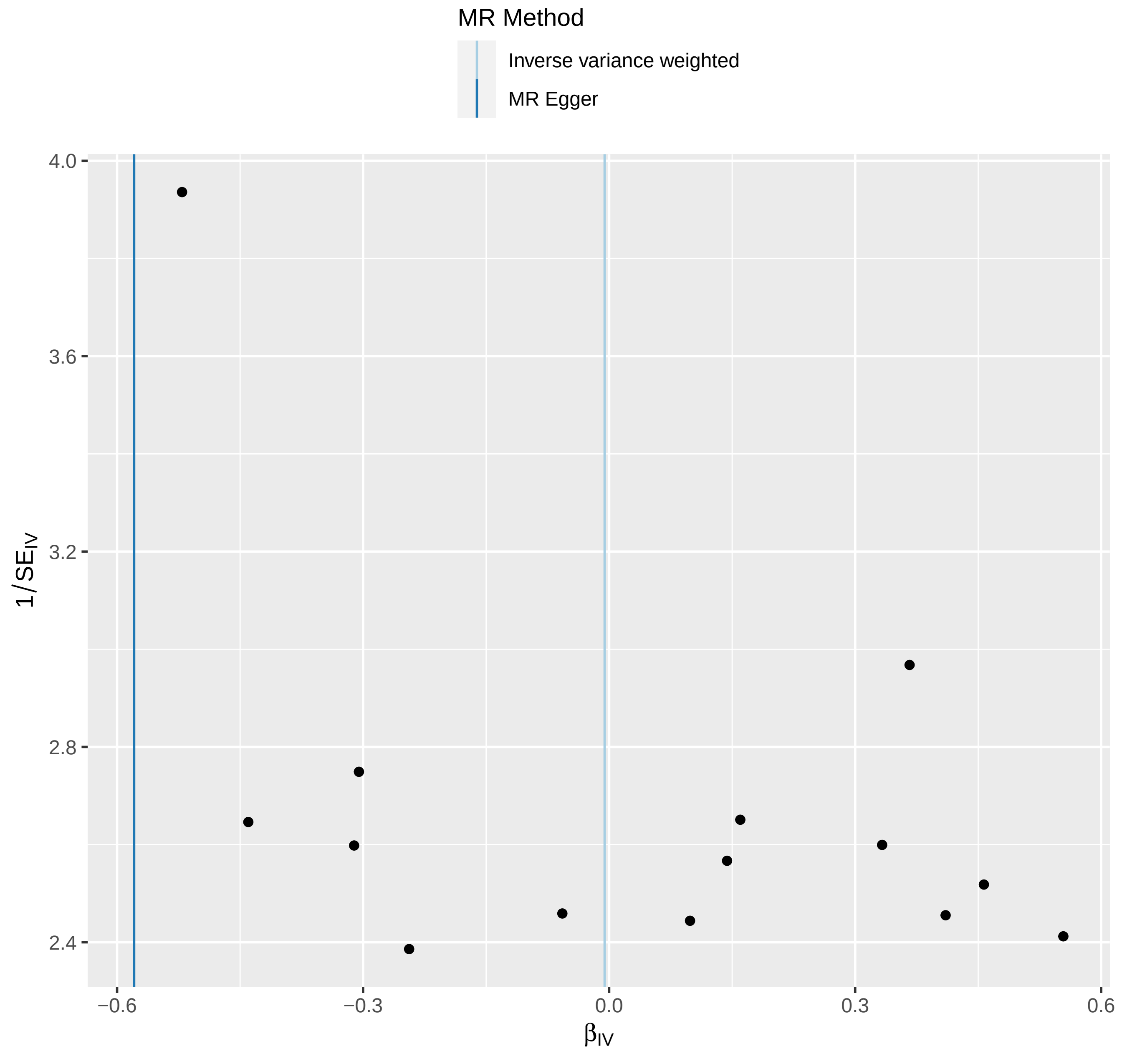

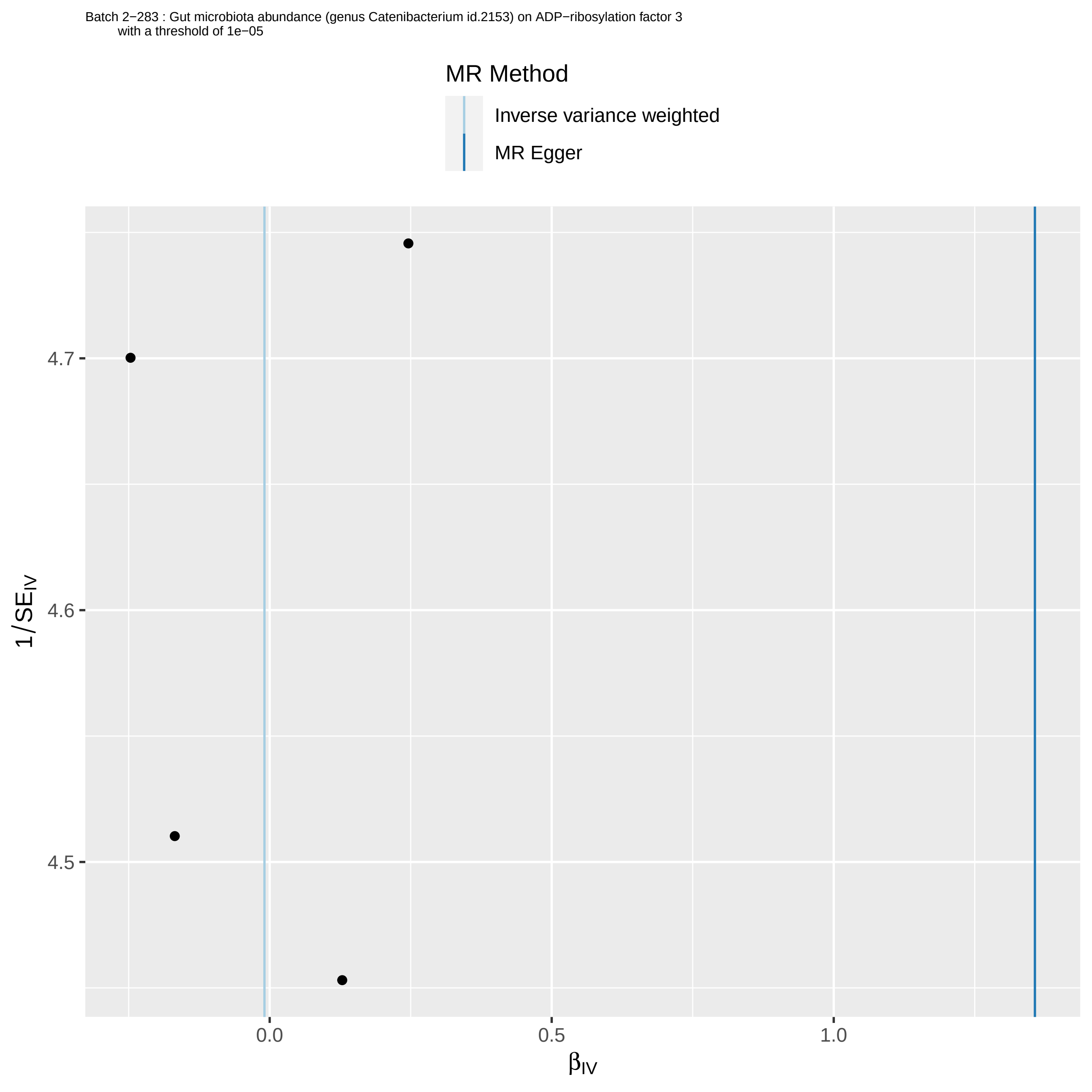

Batch 2−283 : Gut microbiota abundance (genus Catenibacterium id.2153) on ADP−ribosylation factor 3
with a threshold of 1e−05
MR Method
Inverse variance weighted
MR Egger
4.7
4.6
4.5
$1/SE_{IV}$
0.0
0.5
1.0
β_{IV}

Batch 2−284 : Gut microbiota abundance (genus Parasutterella id.2892) on ADP−ribosylation factor 3
with a threshold of 1e−05

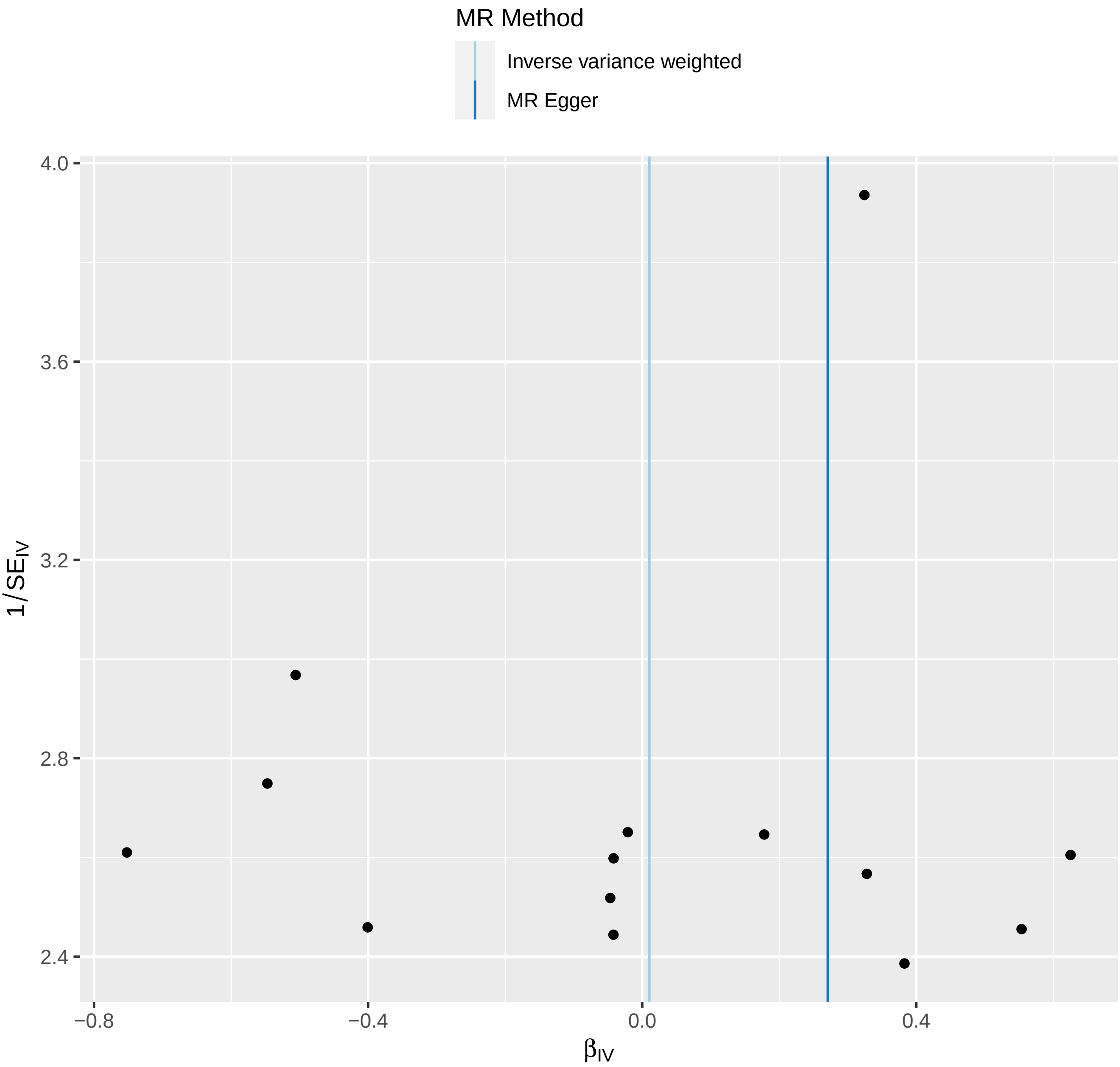

Batch 2−285 : Gut microbiota abundance (genus Catenibacterium id.2153) on ADP−ribosylation factor 6
with a threshold of 1e−05

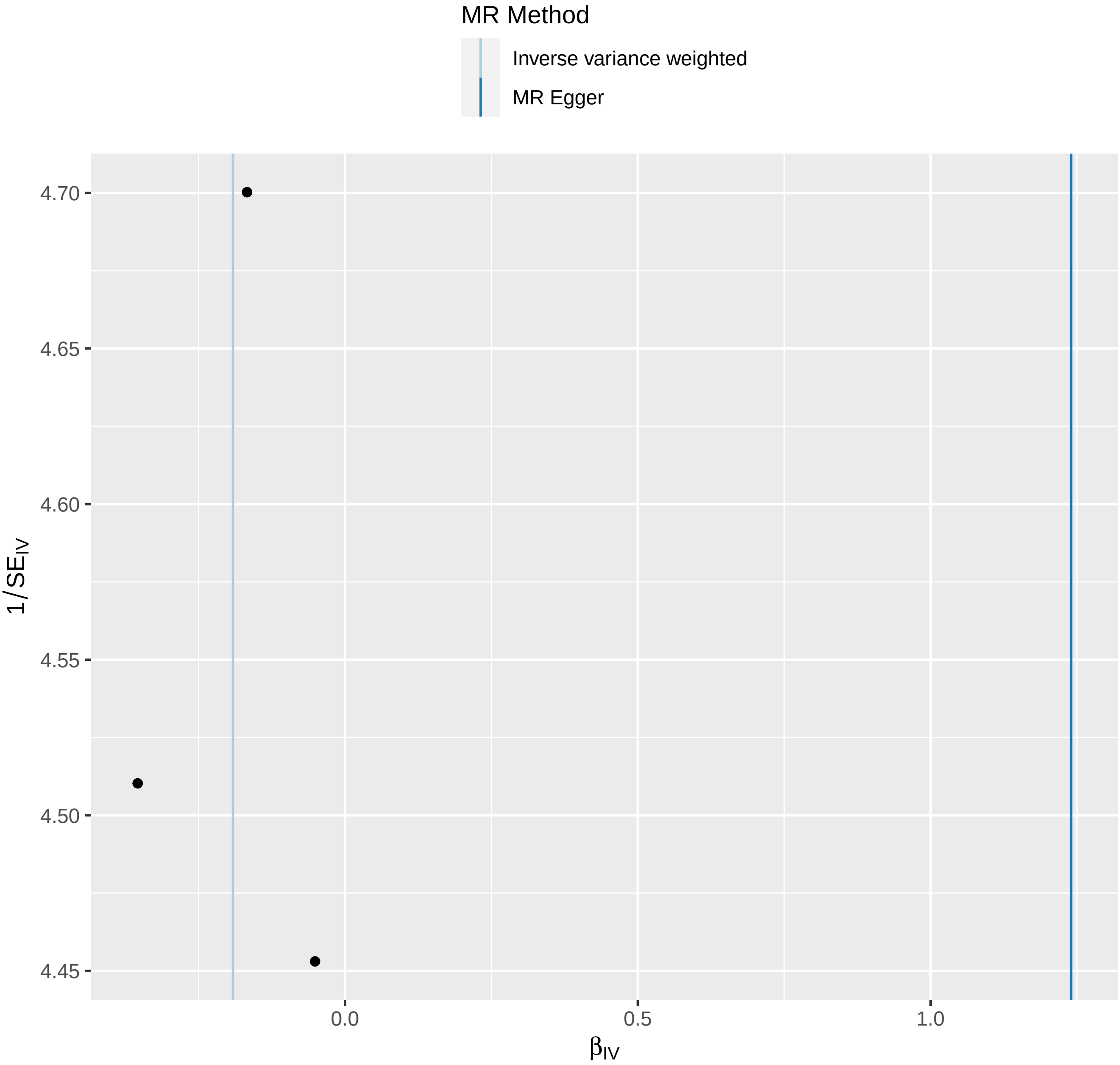

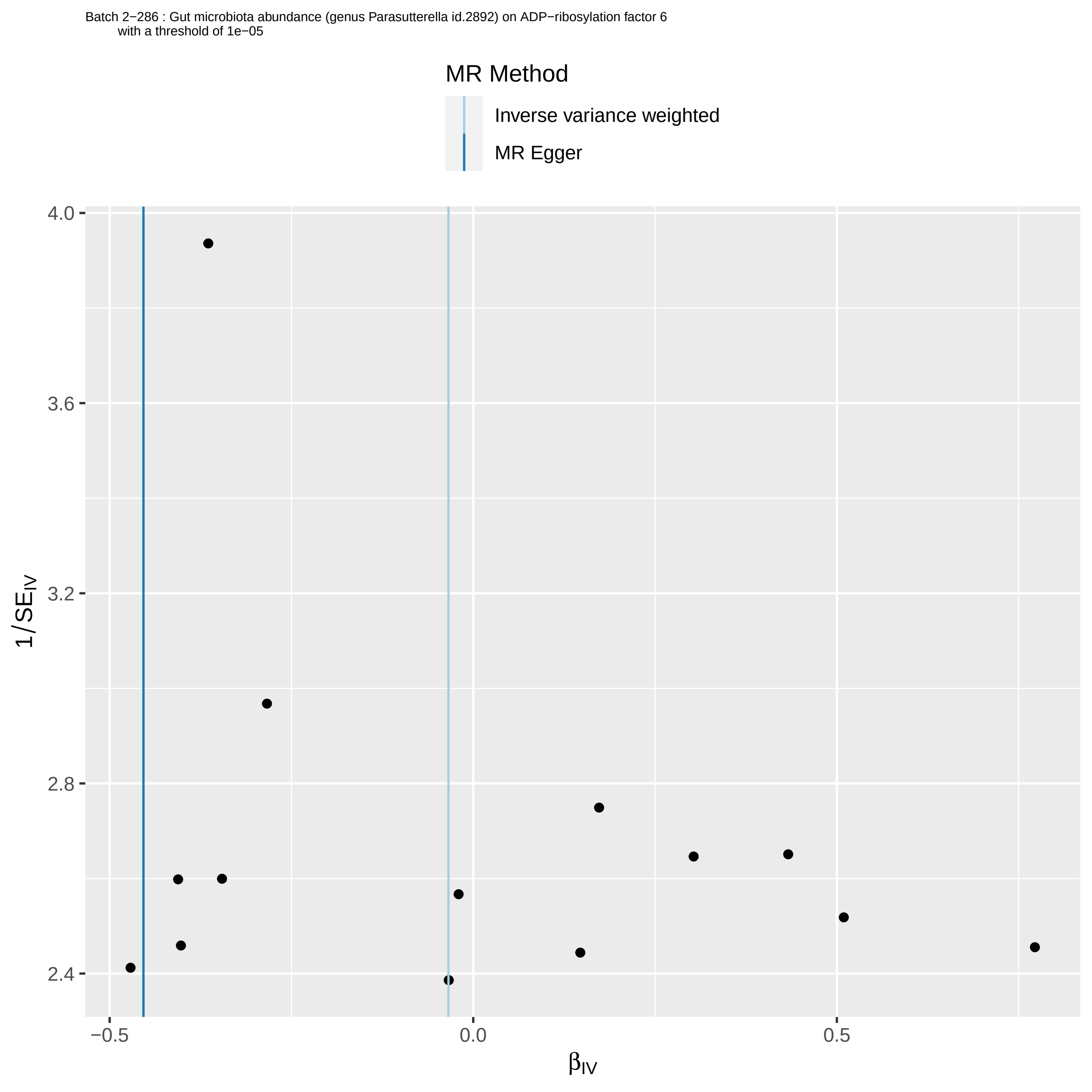
Batch 2−286 : Gut microbiota abundance (genus Parasutterella id.2892) on ADP−ribosylation factor 6
with a threshold of 1e−05
MR Method
Inverse variance weighted
MR Egger
4.0
3.6
3.2
2.8
2.4
1/SE_IV
−0.5
0.0
0.5
β_IV

Batch 2−287 : Gut microbiota abundance (genus Catenibacterium id.2153) on ADP−ribosylation factor GTPase−activating protein 2 with a threshold of 1e−05

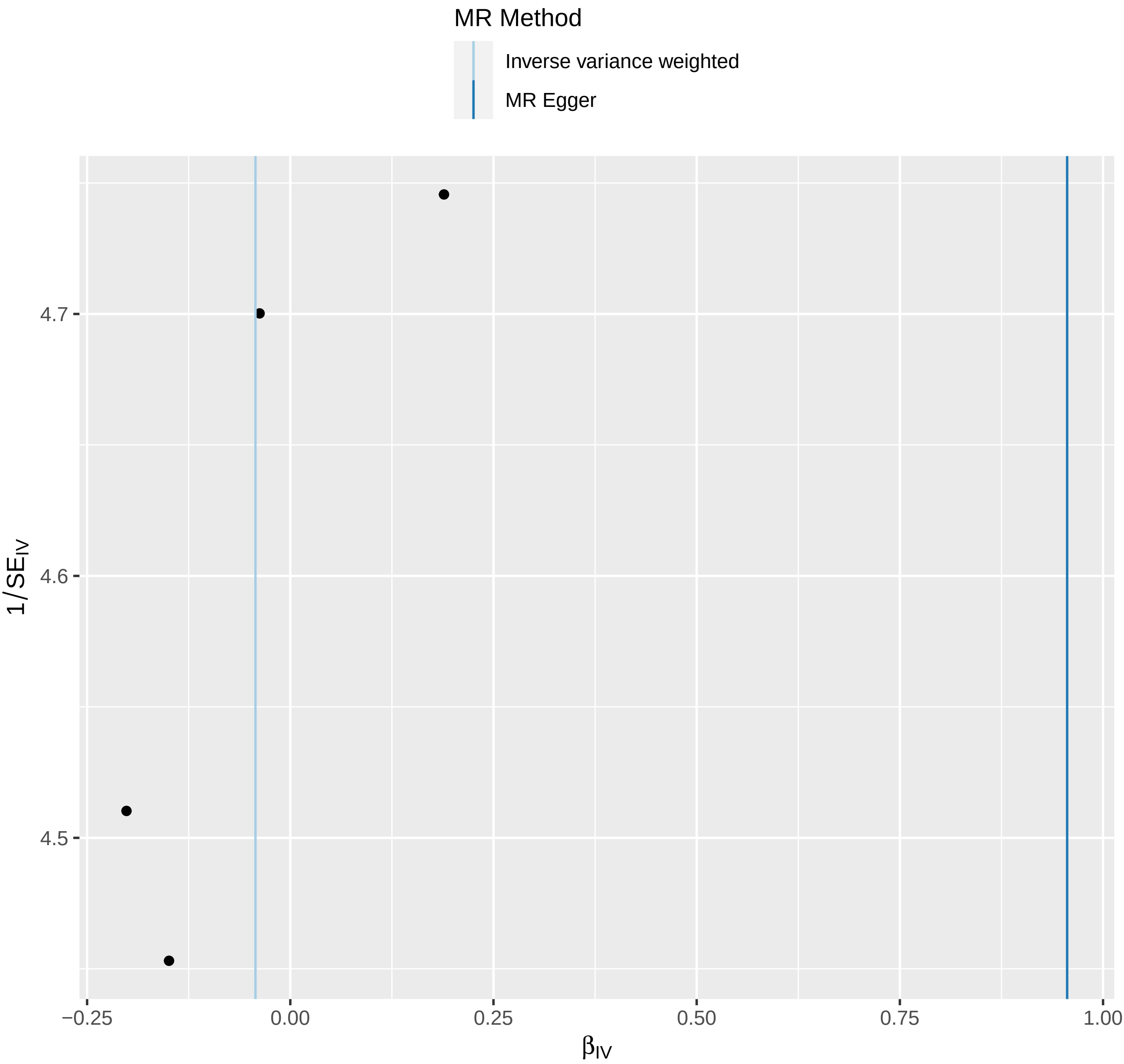

Batch 2−288 : Gut microbiota abundance (genus Parasutterella id.2892) on ADP−ribosylation factor GTPase−activating protein 2
with a threshold of 1e−05

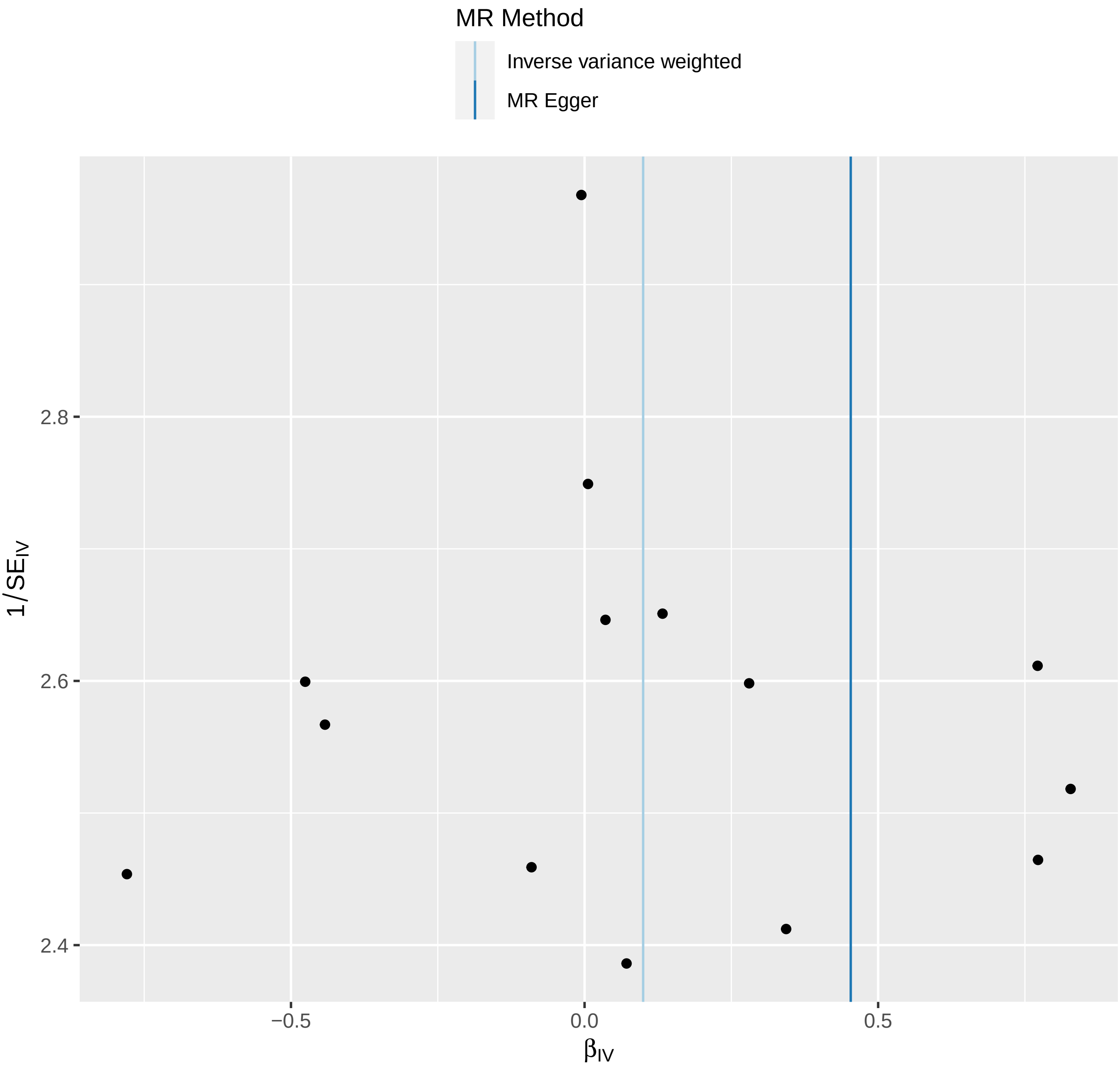

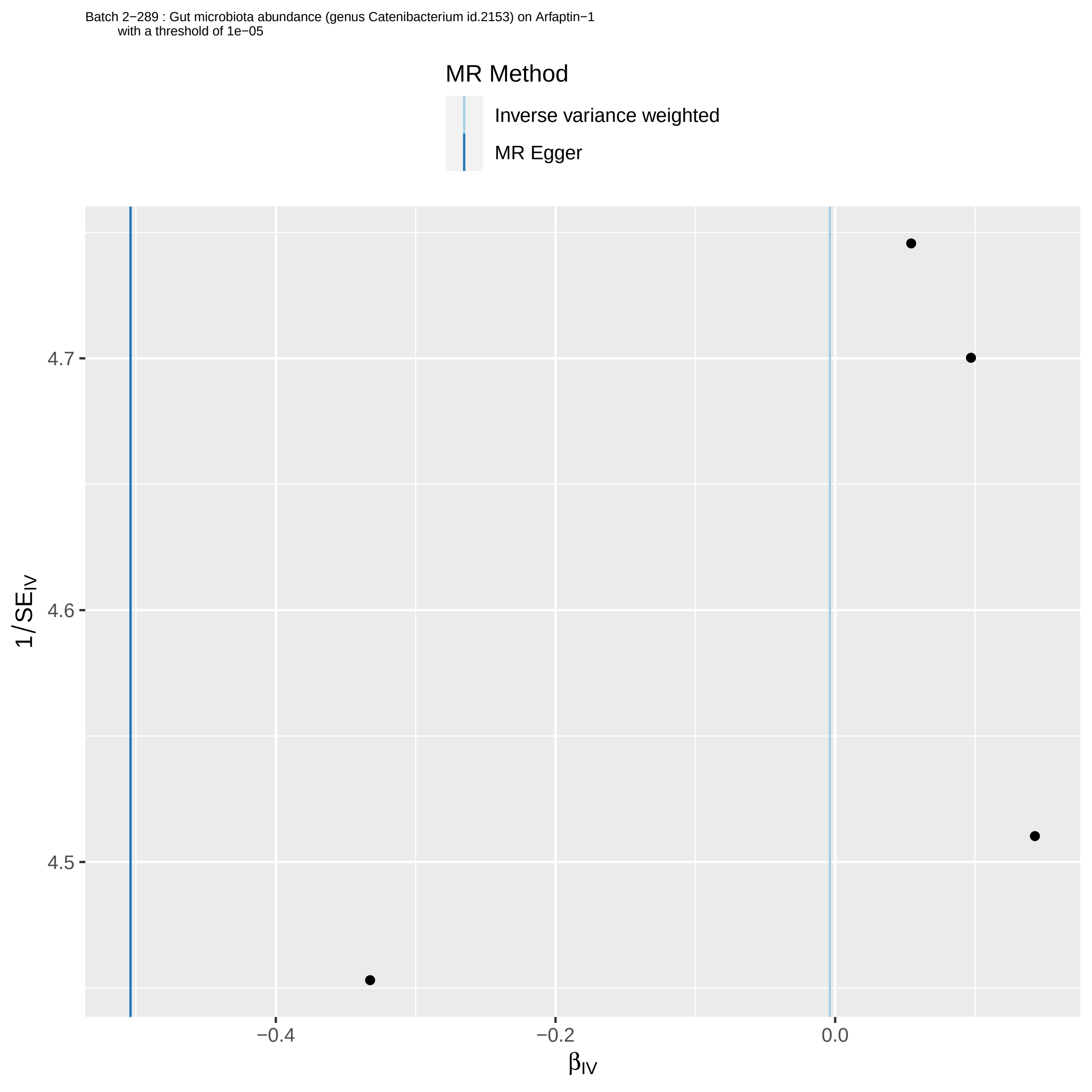

Batch 2−289 : Gut microbiota abundance (genus Catenibacterium id.2153) on Arfaptin−1
with a threshold of 1e−05
MR Method
Inverse variance weighted
MR Egger
4.7
4.6
4.5
1/SE$_{IV}$
−0.4
−0.2
0.0
β$_{IV}$

Batch 2−290 : Gut microbiota abundance (genus Parasutterella id.2892) on Arfaptin−1
with a threshold of 1e−05

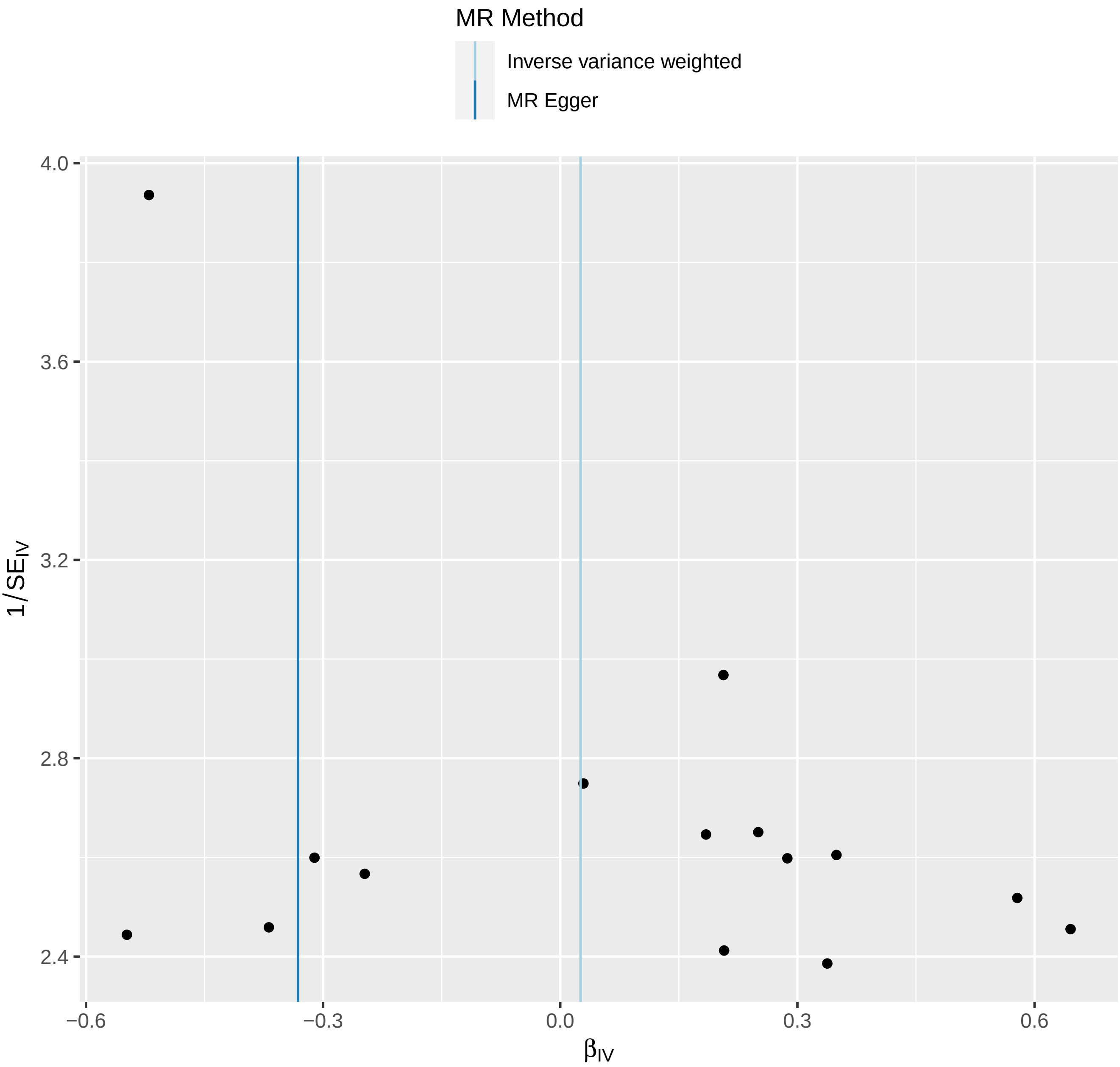

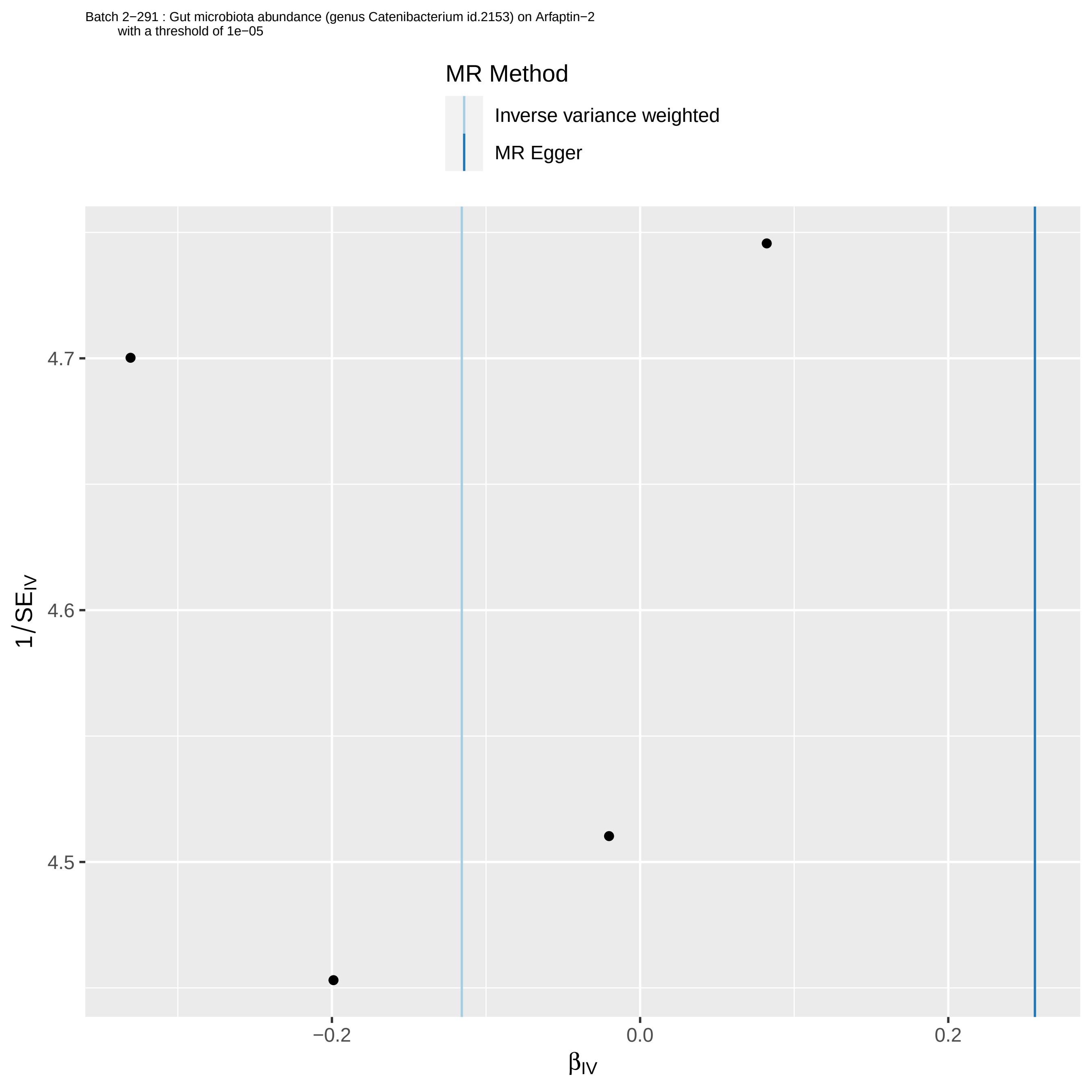

Batch 2−291 : Gut microbiota abundance (genus Catenibacterium id.2153) on Arfaptin−2
with a threshold of 1e−05
MR Method
Inverse variance weighted
MR Egger
4.7
4.6
4.5
1/SE_IV
−0.2
0.0
0.2
β_IV

Batch 2−292 : Gut microbiota abundance (genus Parasutterella id.2892) on Arfaptin−2
with a threshold of 1e−05

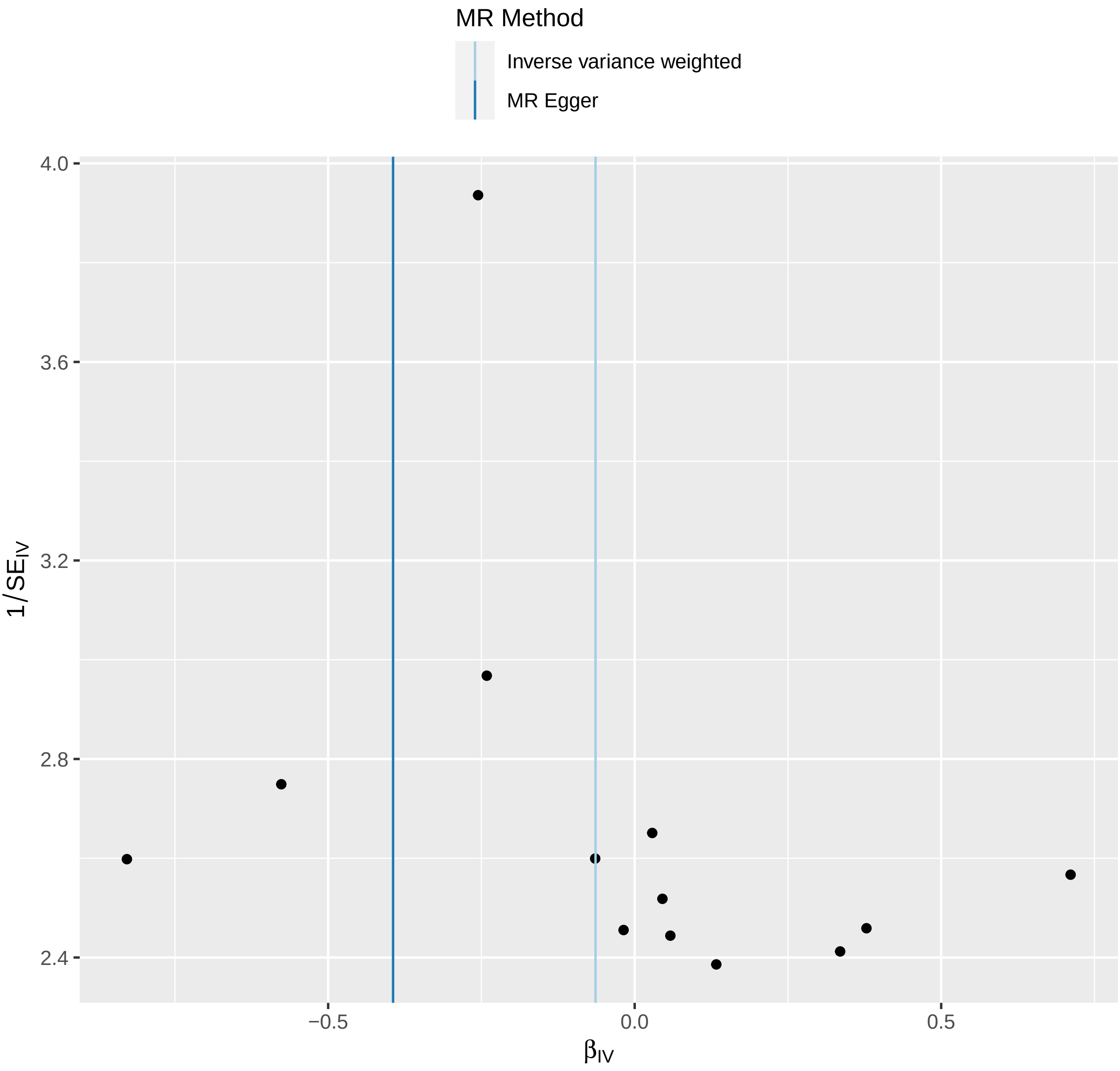

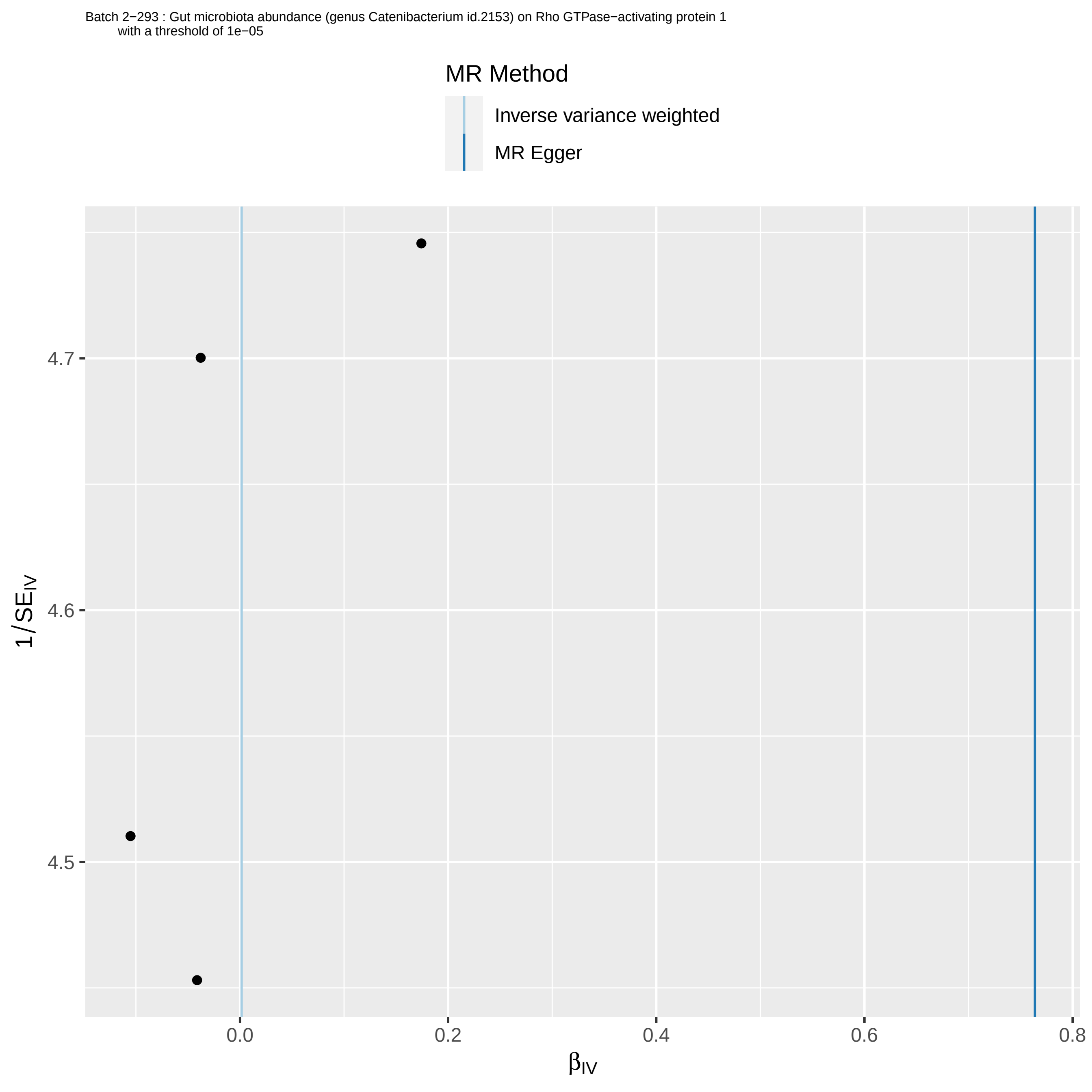
Batch 2−293 : Gut microbiota abundance (genus Catenibacterium id.2153) on Rho GTPase−activating protein 1
with a threshold of 1e−05
MR Method
Inverse variance weighted
MR Egger
4.7
4.6
4.5
$1/SE_{IV}$
0.0
0.2
0.4
0.6
0.8
β_{IV}

Batch 2−294 : Gut microbiota abundance (genus Parasutterella id.2892) on Rho GTPase−activating protein 1
with a threshold of 1e−05

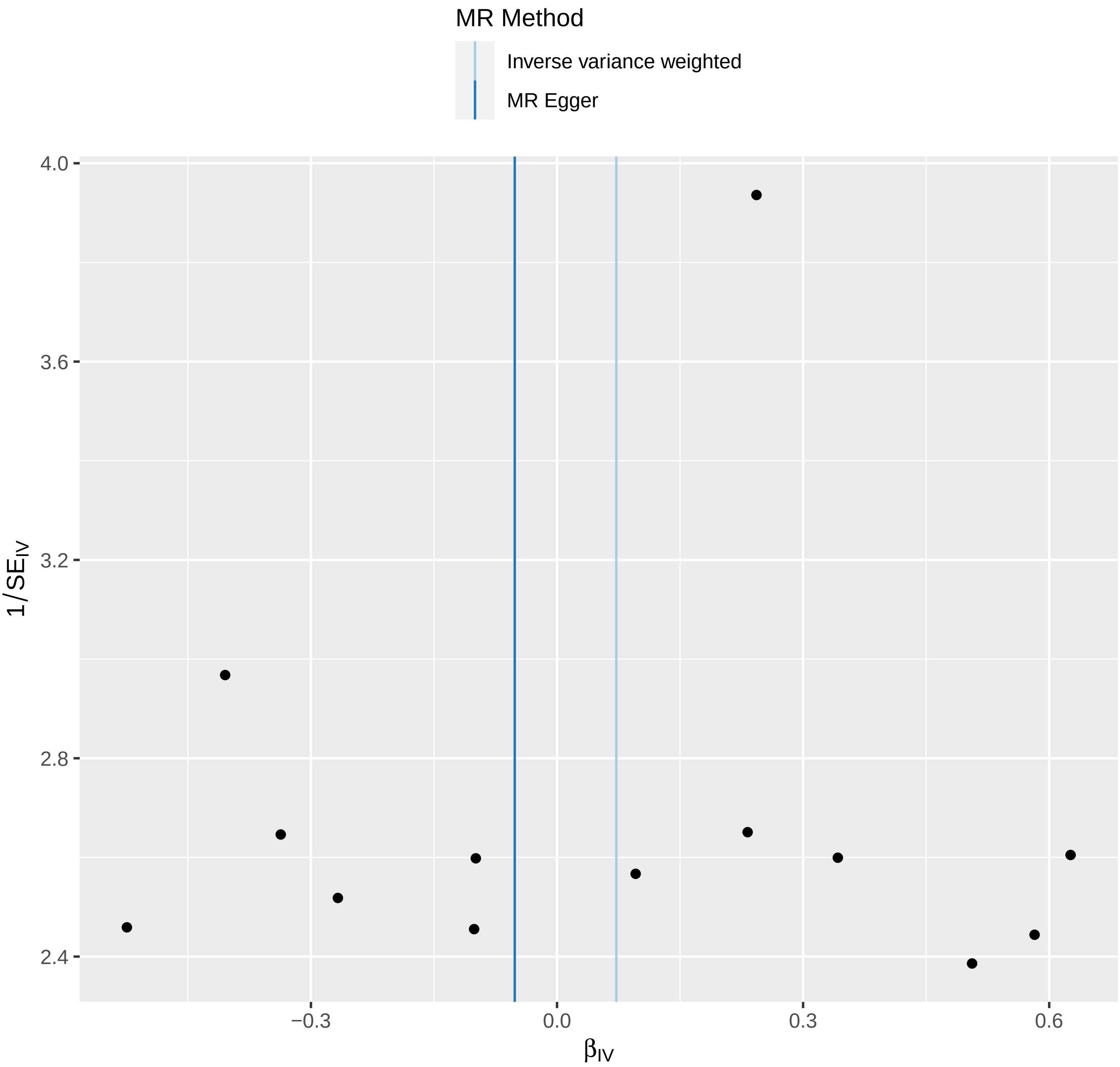

Batch 2−295 : Gut microbiota abundance (genus Catenibacterium id.2153) on Rho GTPase−activating protein 25
with a threshold of 1e−05

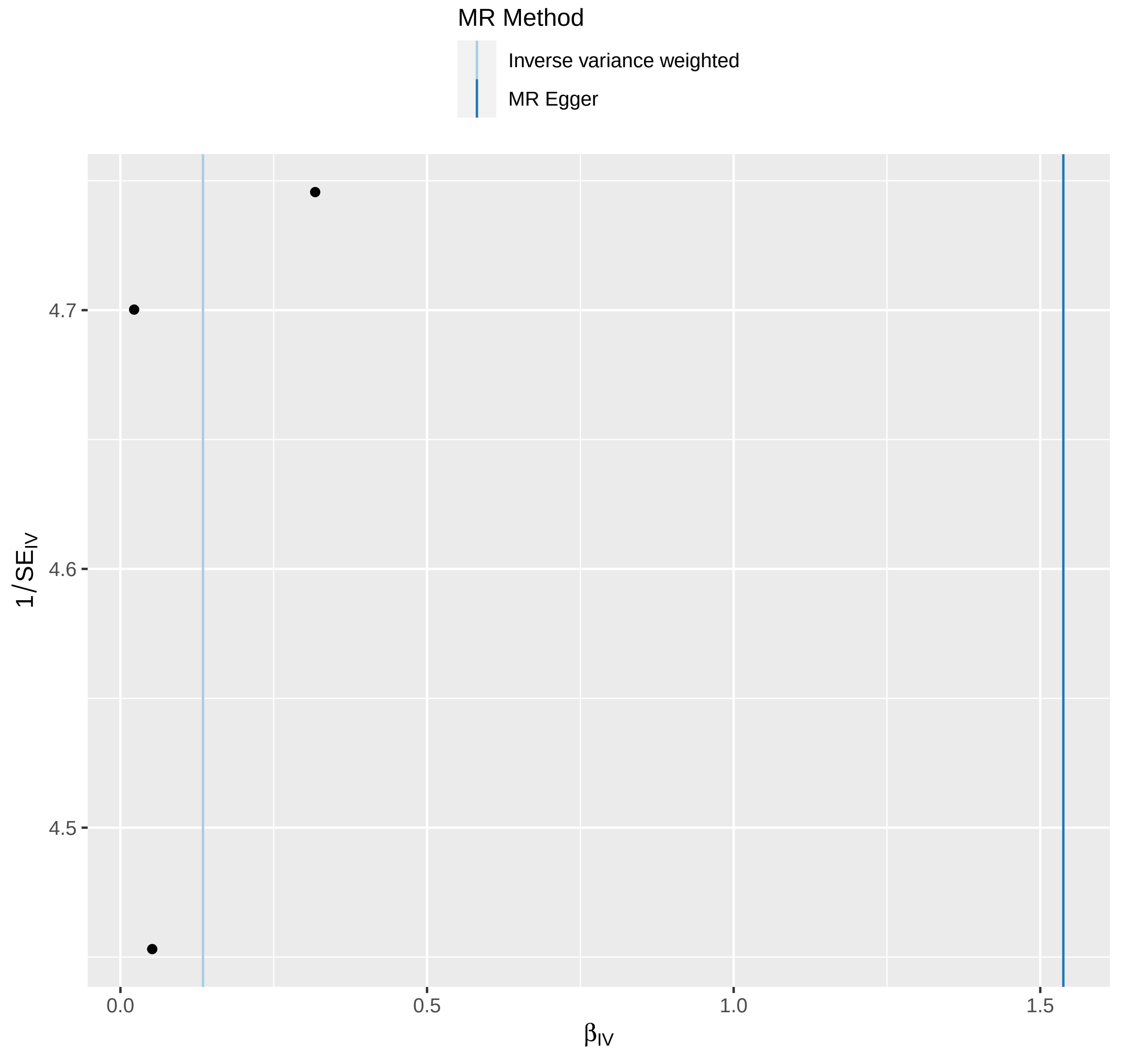

Batch 2−296 : Gut microbiota abundance (genus Parasutterella id.2892) on Rho GTPase−activating protein 25
with a threshold of 1e−05

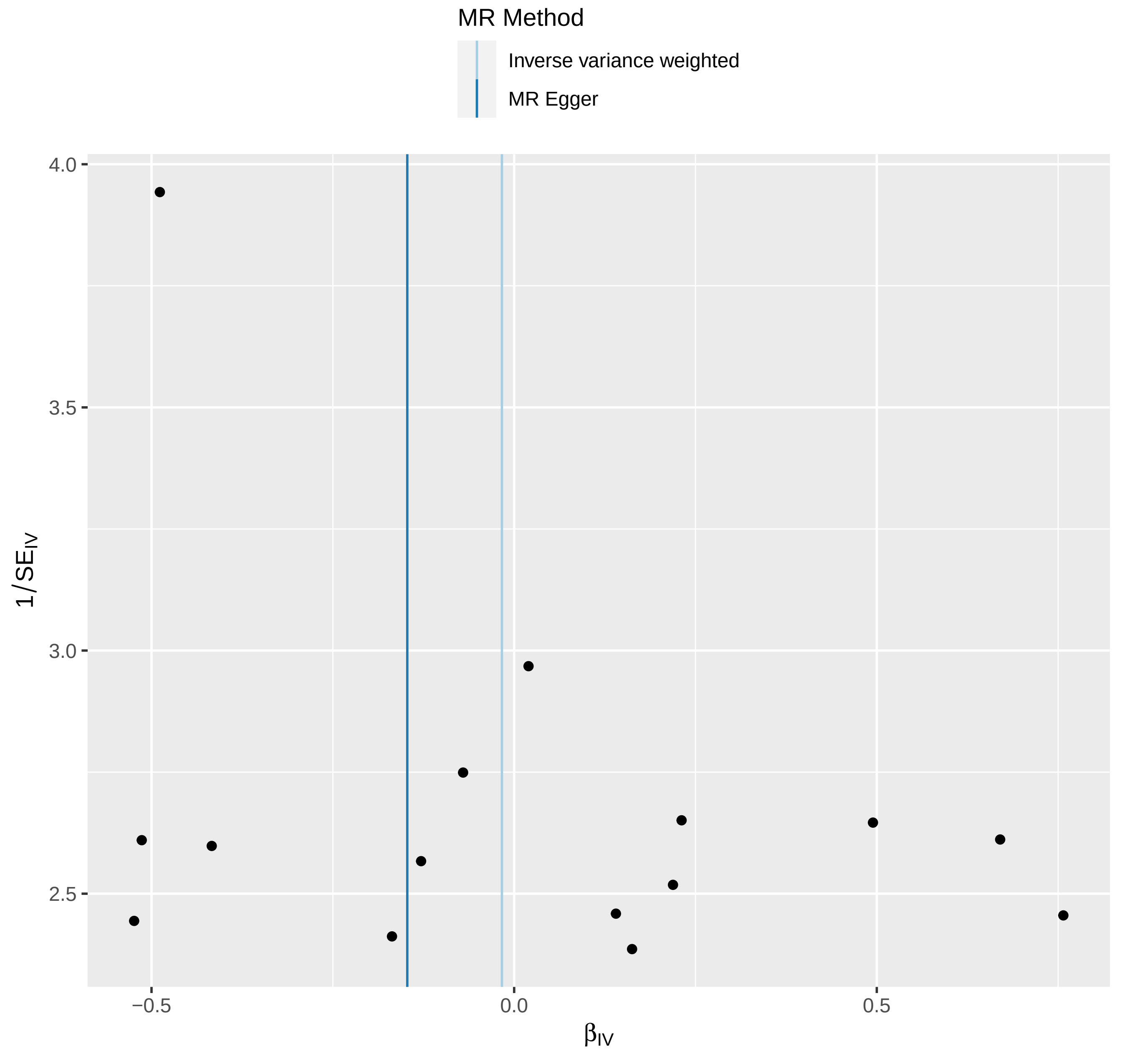

Batch 2−297 : Gut microbiota abundance (genus Catenibacterium id.2153) on Rho GTPase−activating protein 30
with a threshold of 1e−05

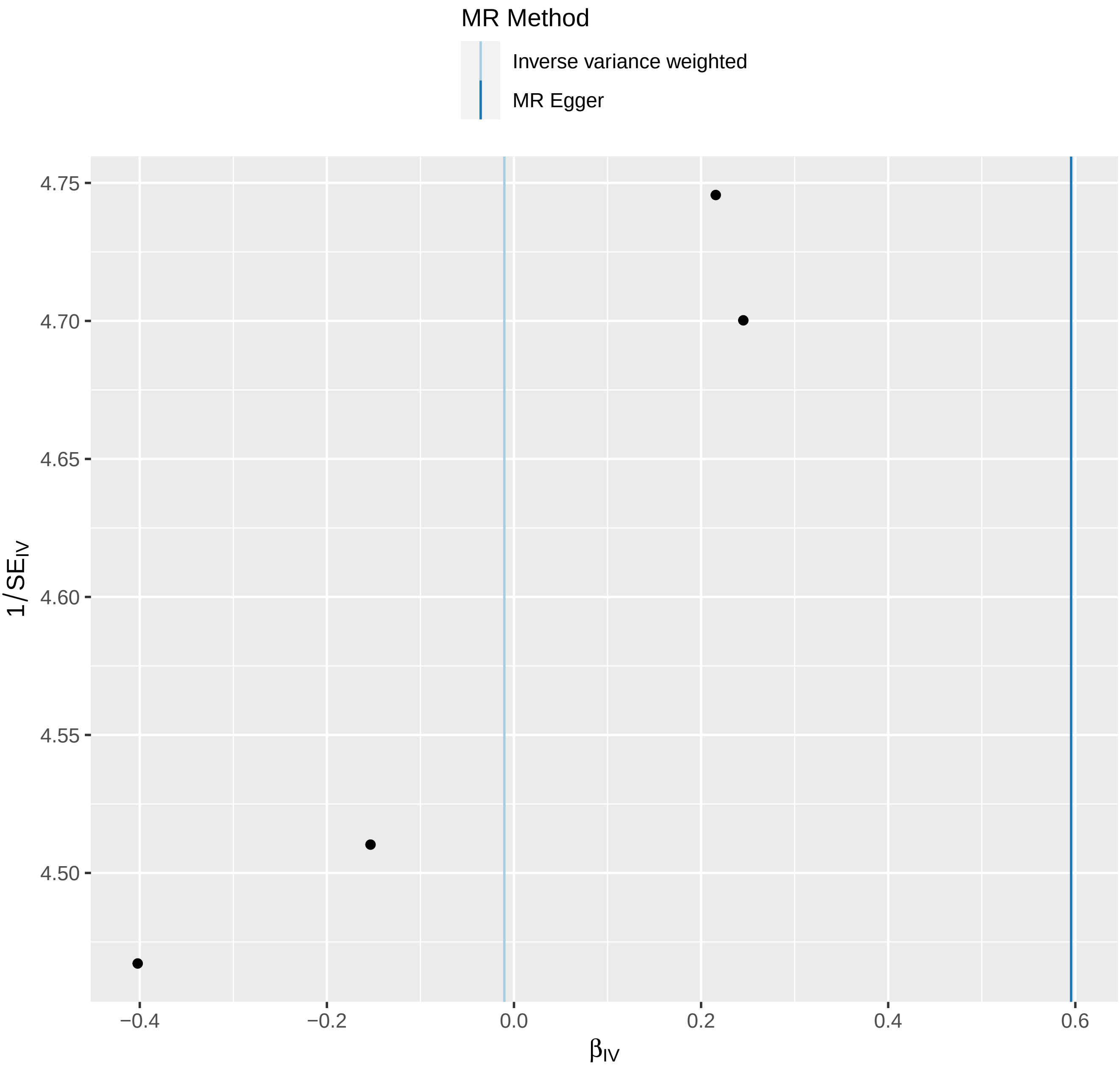

Batch 2−298 : Gut microbiota abundance (genus Parasutterella id.2892) on Rho GTPase−activating protein 30
with a threshold of 1e−05

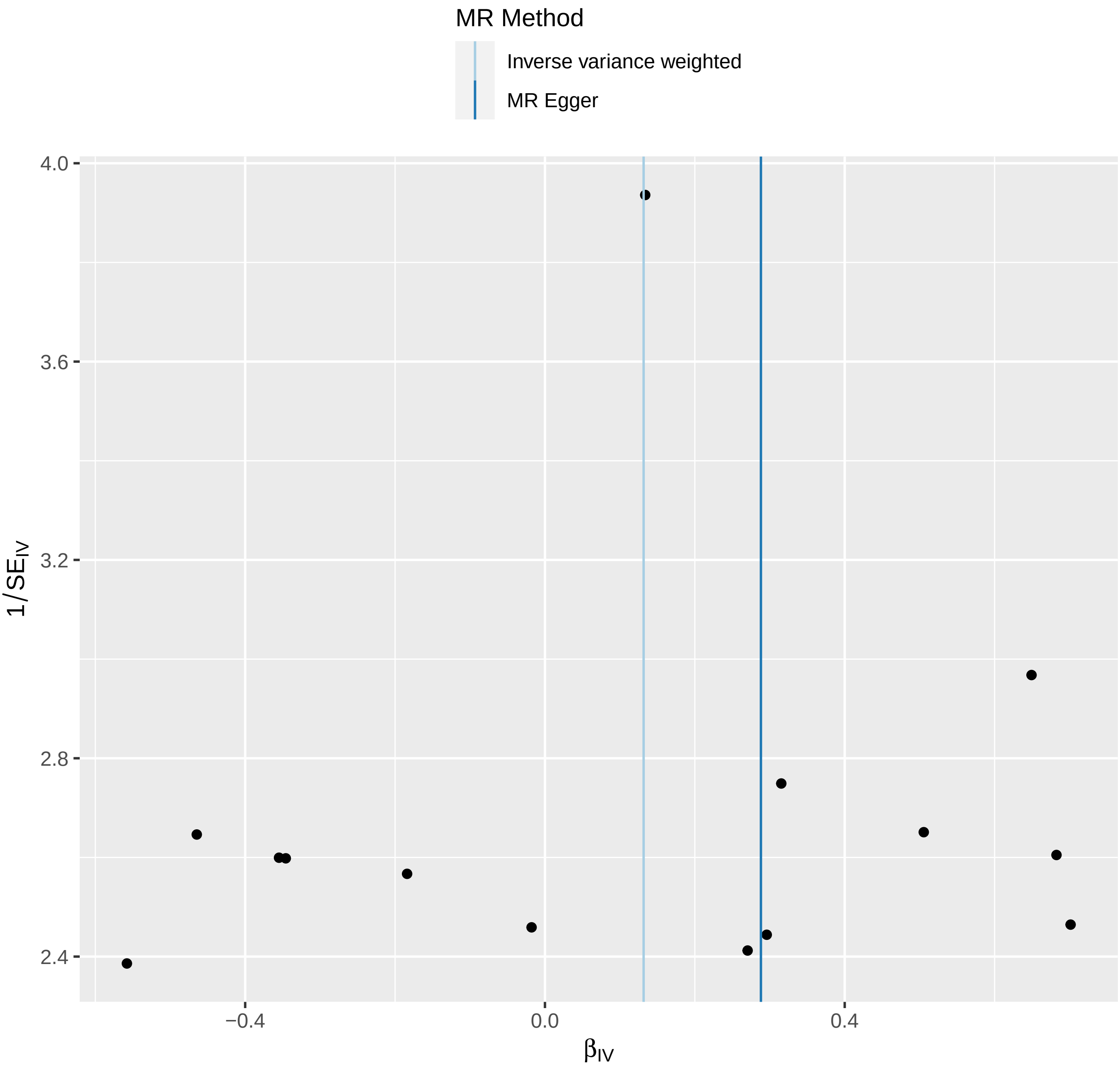

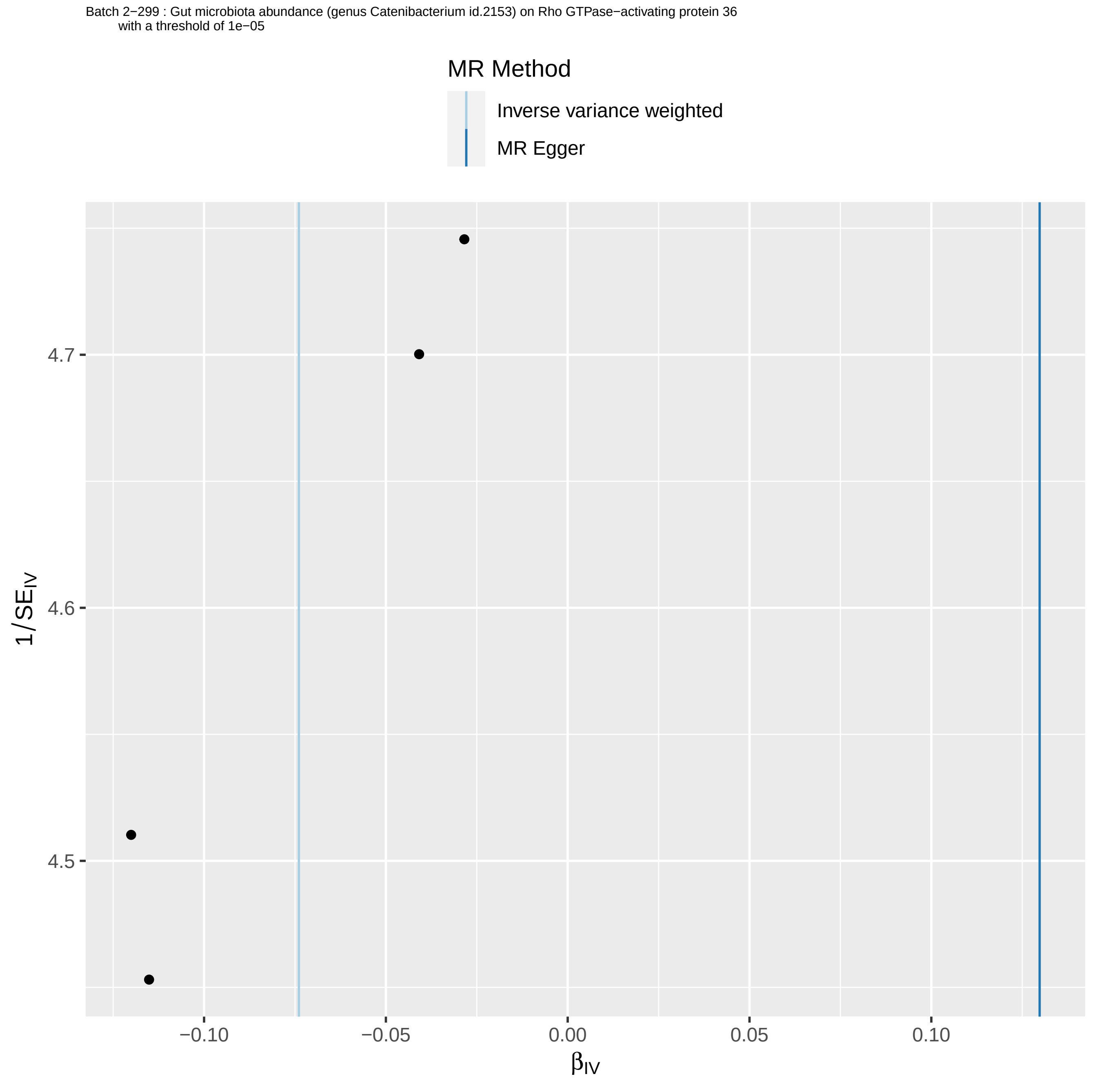
Batch 2−299 : Gut microbiota abundance (genus Catenibacterium id.2153) on Rho GTPase−activating protein 36
with a threshold of 1e−05
MR Method
Inverse variance weighted
MR Egger
4.7
4.6
4.5
1/SE_IV
−0.10
−0.05
0.00
0.05
0.10
β_IV

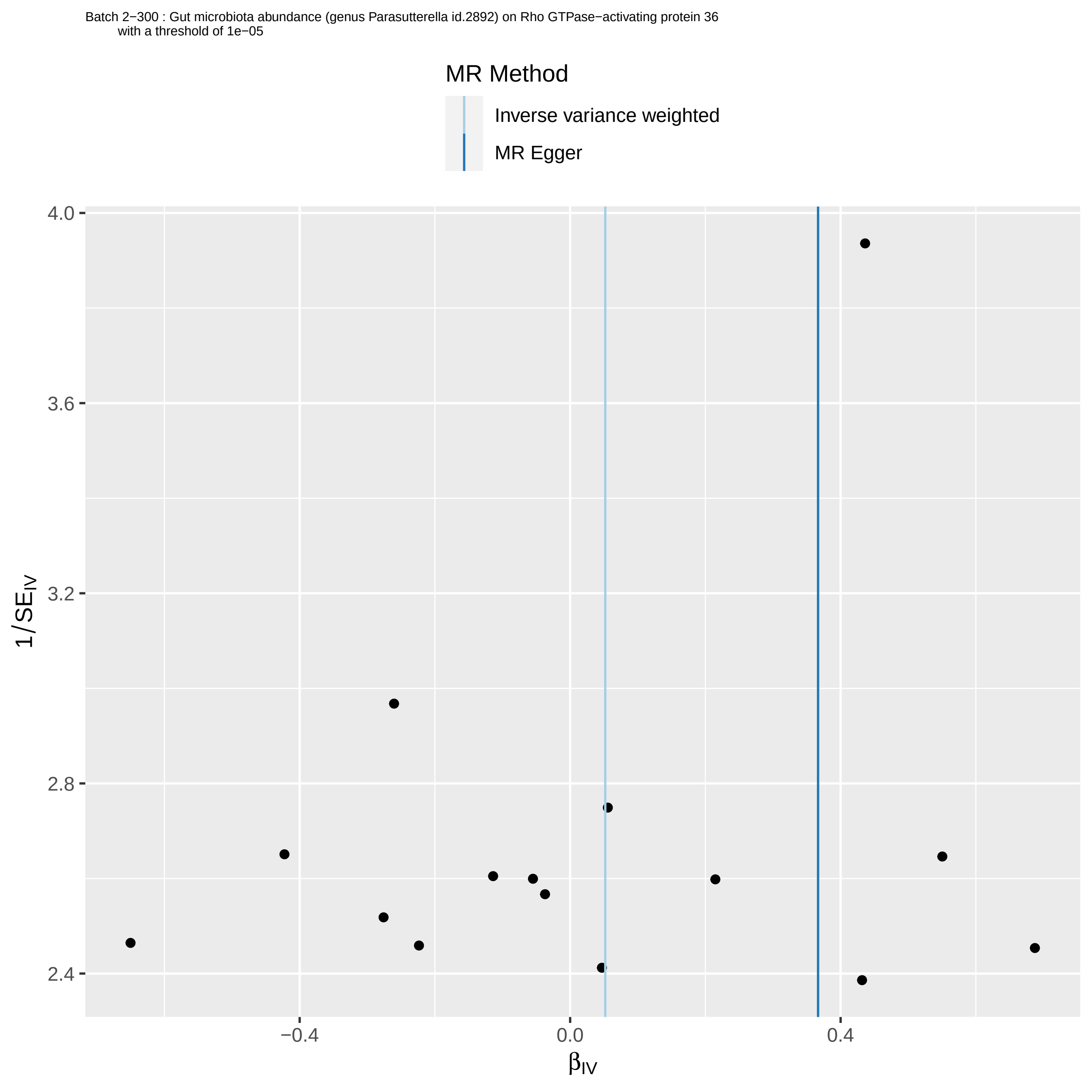
Batch 2−300 : Gut microbiota abundance (genus Parasutterella id.2892) on Rho GTPase−activating protein 36
with a threshold of 1e−05
MR Method
Inverse variance weighted
MR Egger
4.0
3.6
3.2
2.8
2.4
−0.4
0.0
0.4
1/SE_IV
β_IV

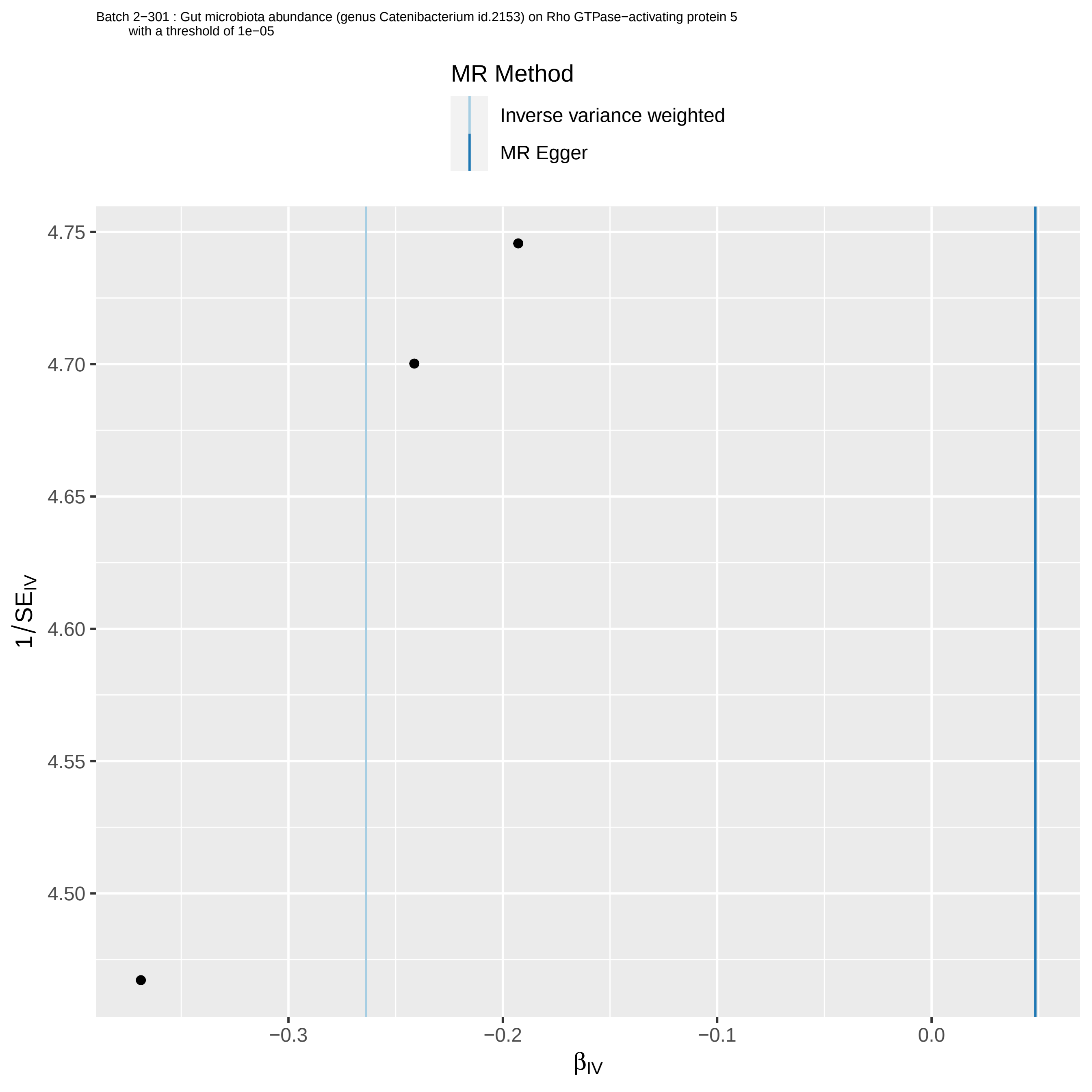
Batch 2−301 : Gut microbiota abundance (genus Catenibacterium id.2153) on Rho GTPase−activating protein 5
with a threshold of 1e−05
MR Method
Inverse variance weighted
MR Egger
4.75
4.70
4.65
4.60
4.55
4.50
1/SE_IV
−0.3
−0.2
−0.1
0.0
β_IV

Batch 2−302 : Gut microbiota abundance (genus Parasutterella id.2892) on Rho GTPase−activating protein 5
with a threshold of 1e−05

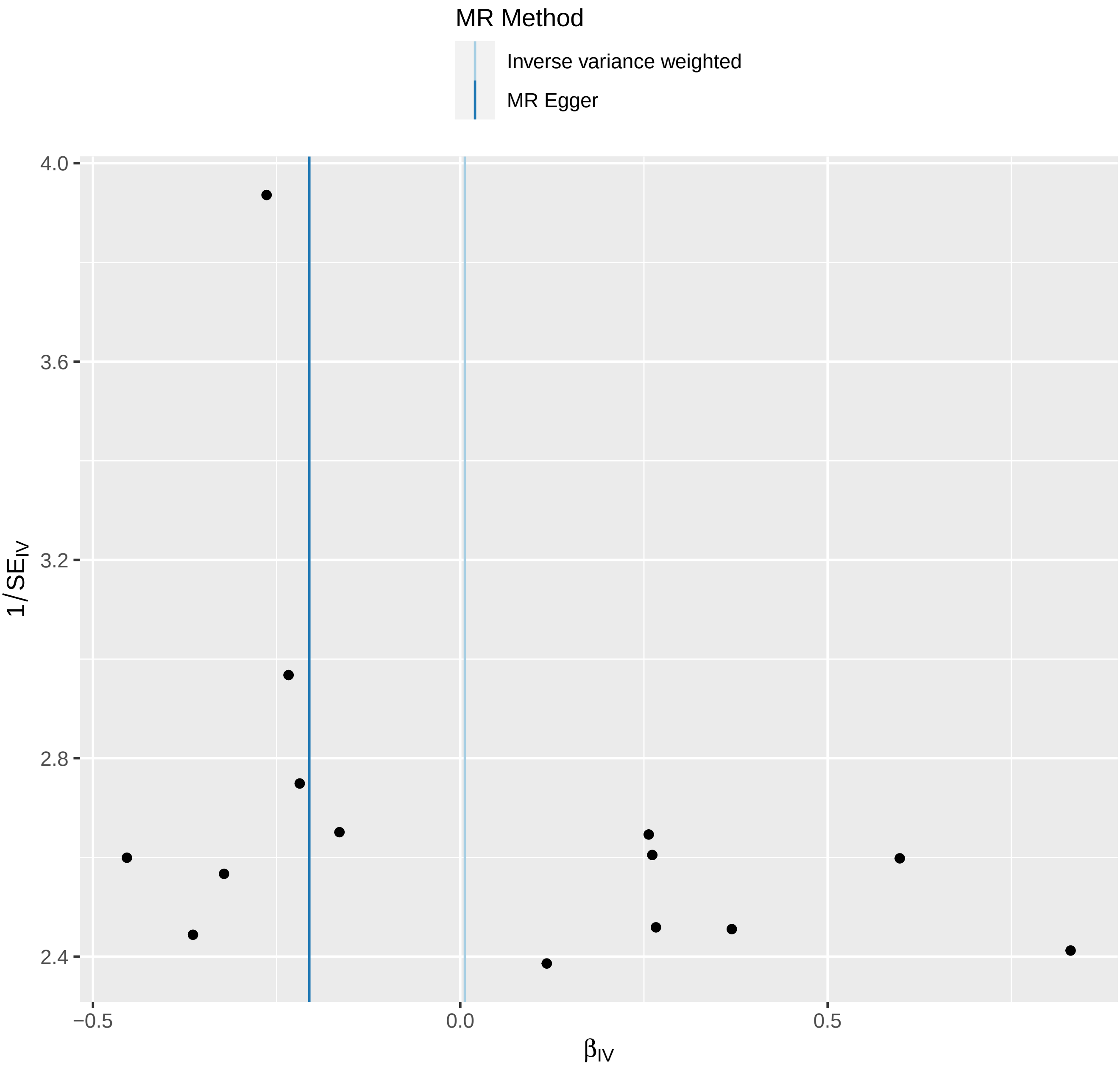

Batch 2−303 : Gut microbiota abundance (genus Catenibacterium id.2153) on Rho GDP−dissociation inhibitor 1
with a threshold of 1e−05

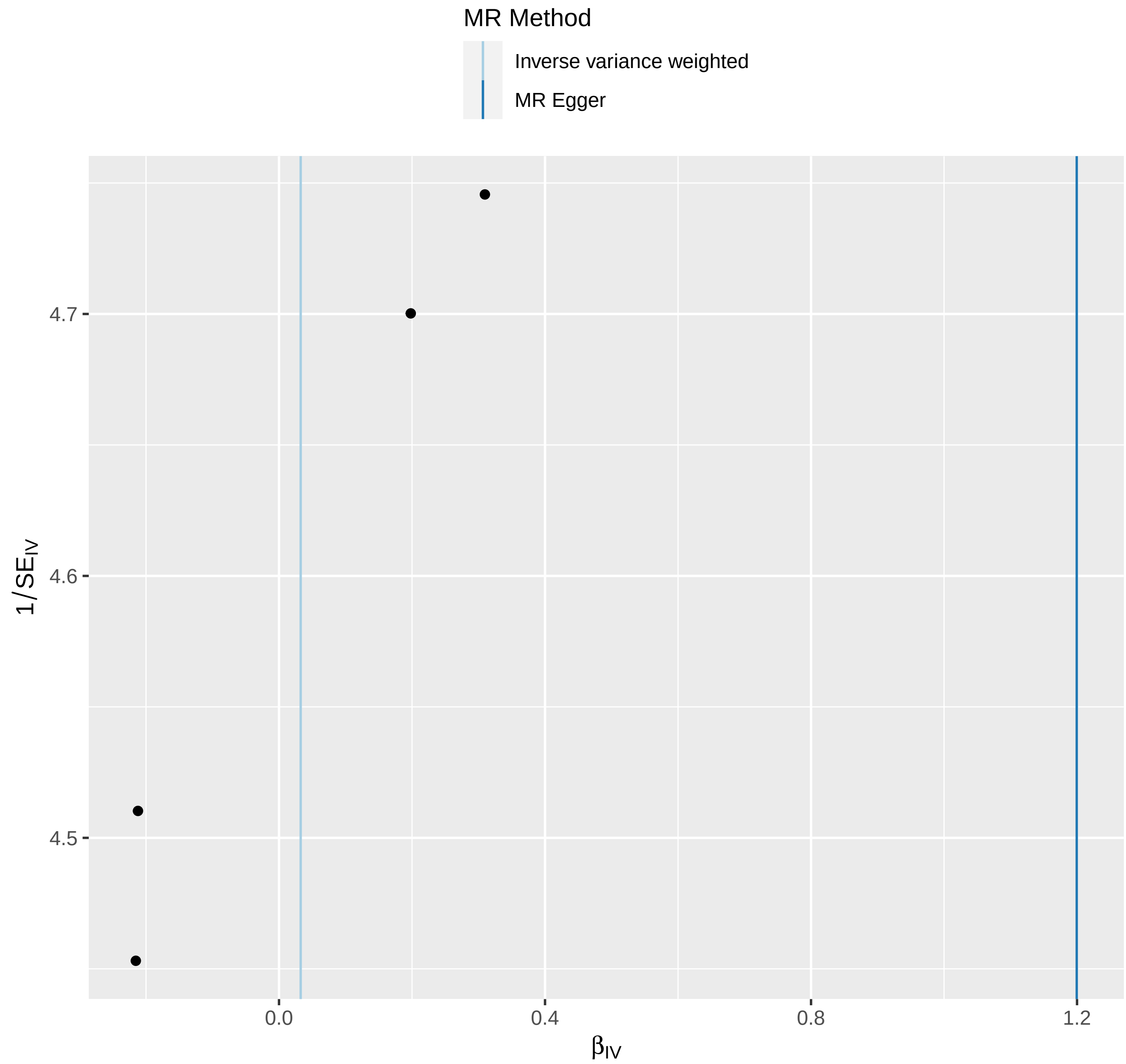

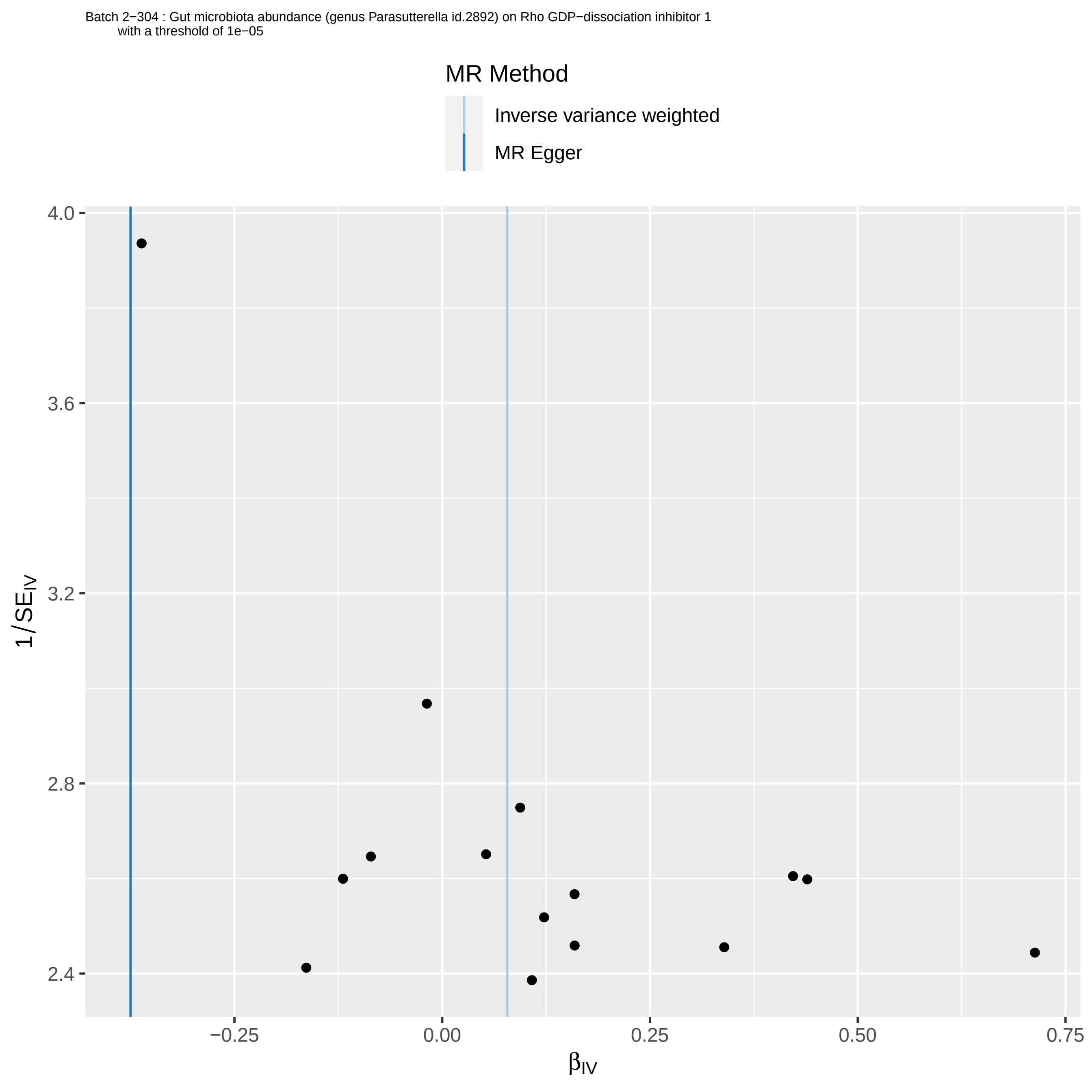

Batch 2−304 : Gut microbiota abundance (genus Parasutterella id.2892) on Rho GDP−dissociation inhibitor 1
with a threshold of 1e−05
MR Method
Inverse variance weighted
MR Egger
4.0
3.6
3.2
2.8
2.4
1/SE_IV
−0.25
0.00
0.25
0.50
0.75
β_IV

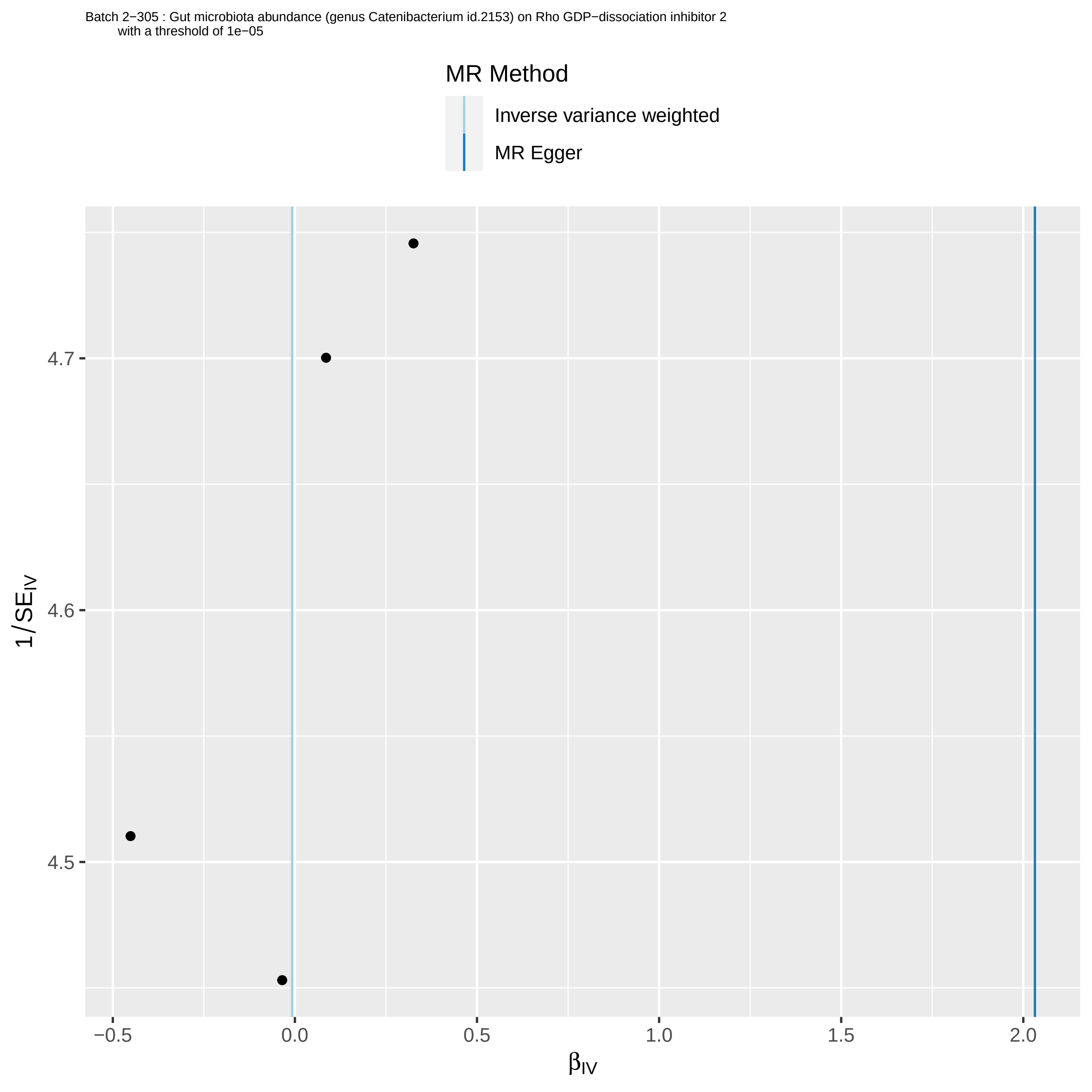

Batch 2−305 : Gut microbiota abundance (genus Catenibacterium id.2153) on Rho GDP−dissociation inhibitor 2
with a threshold of 1e−05
MR Method
Inverse variance weighted
MR Egger
4.7
4.6
4.5
−0.5
0.0
0.5
1.0
1.5
2.0
1/SE$_{IV}$
β$_{IV}$

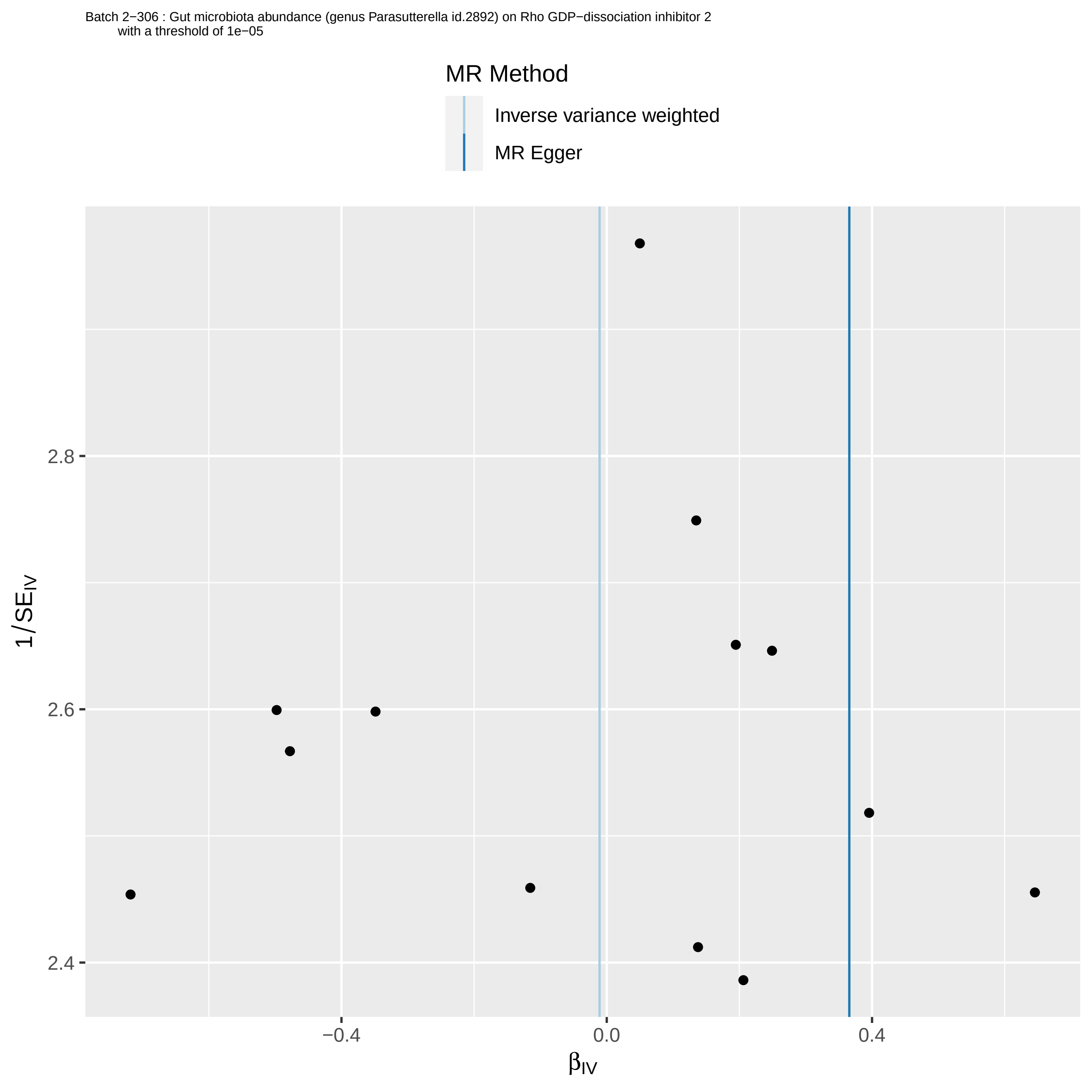
Batch 2−306 : Gut microbiota abundance (genus Parasutterella id.2892) on Rho GDP−dissociation inhibitor 2
with a threshold of 1e−05
MR Method
Inverse variance weighted
MR Egger
2.8
2.6
2.4
1/SE_IV
−0.4
0.0
0.4
β_IV

Batch 2−307 : Gut microbiota abundance (genus Catenibacterium id.2153) on Rho guanine nucleotide exchange factor 10 with a threshold of 1e−05

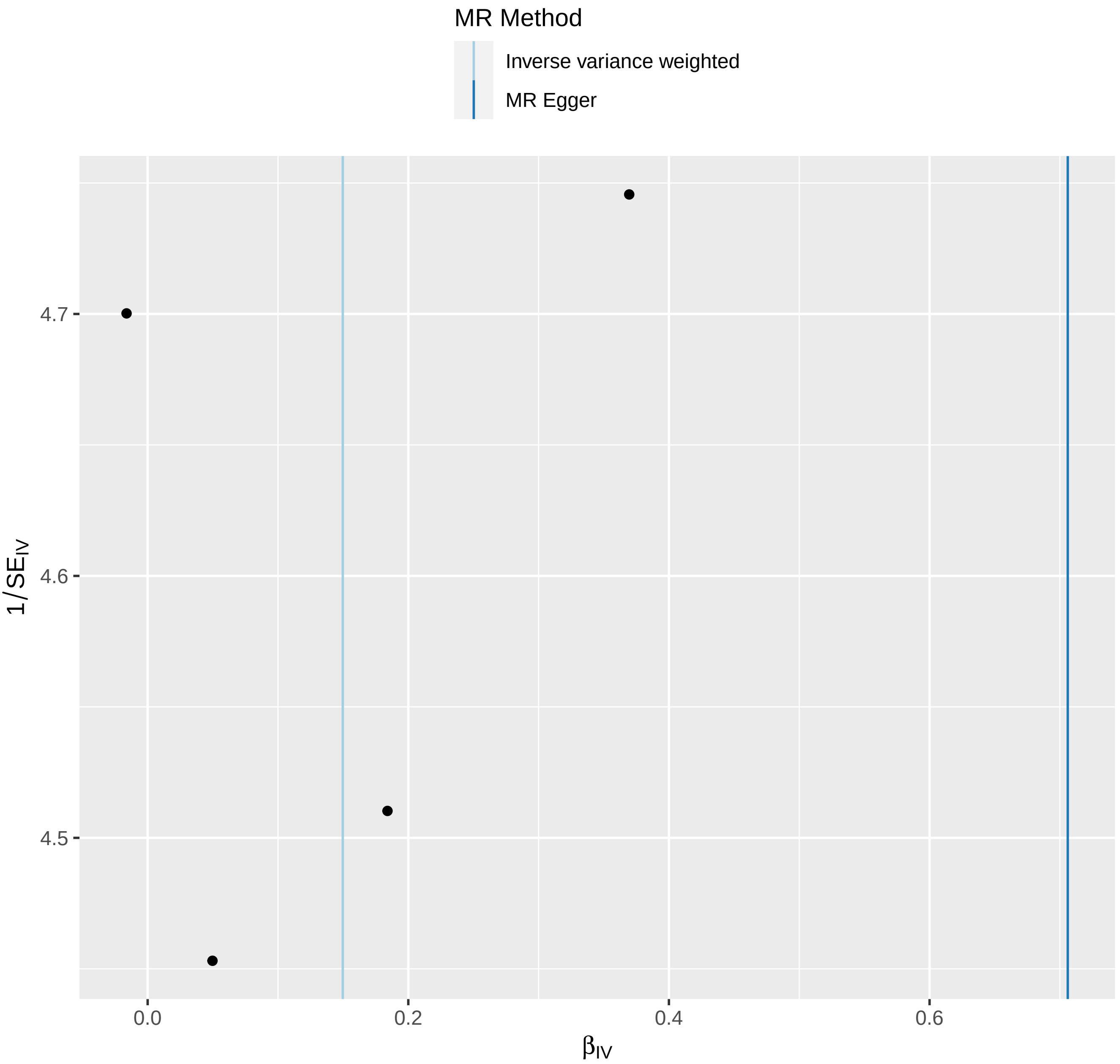

Batch 2−308 : Gut microbiota abundance (genus Parasutterella id.2892) on Rho guanine nucleotide exchange factor 10
with a threshold of 1e−05

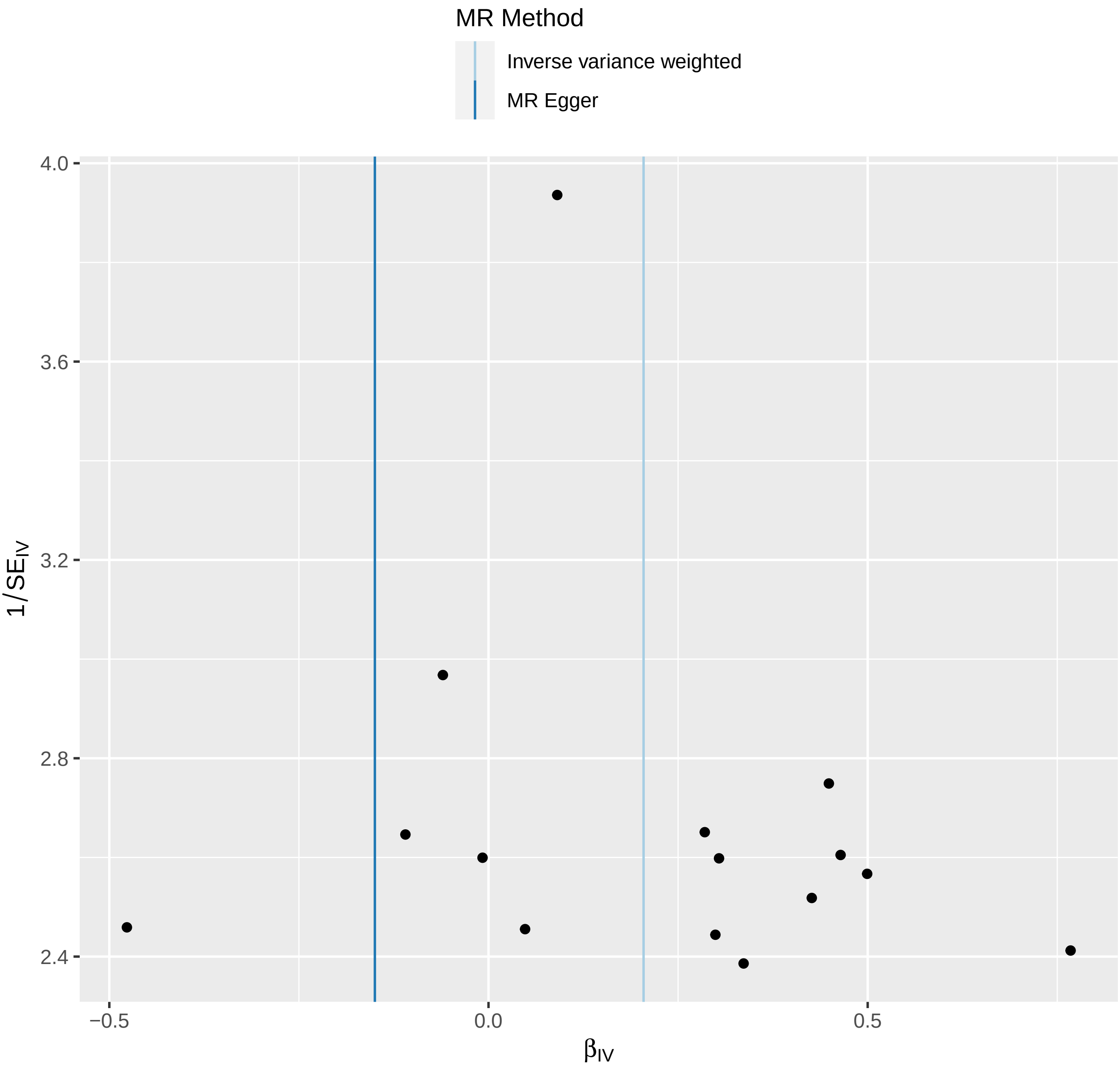

Batch 2−309 : Gut microbiota abundance (genus Catenibacterium id.2153) on Rho guanine nucleotide exchange factor 1 with a threshold of 1e−05

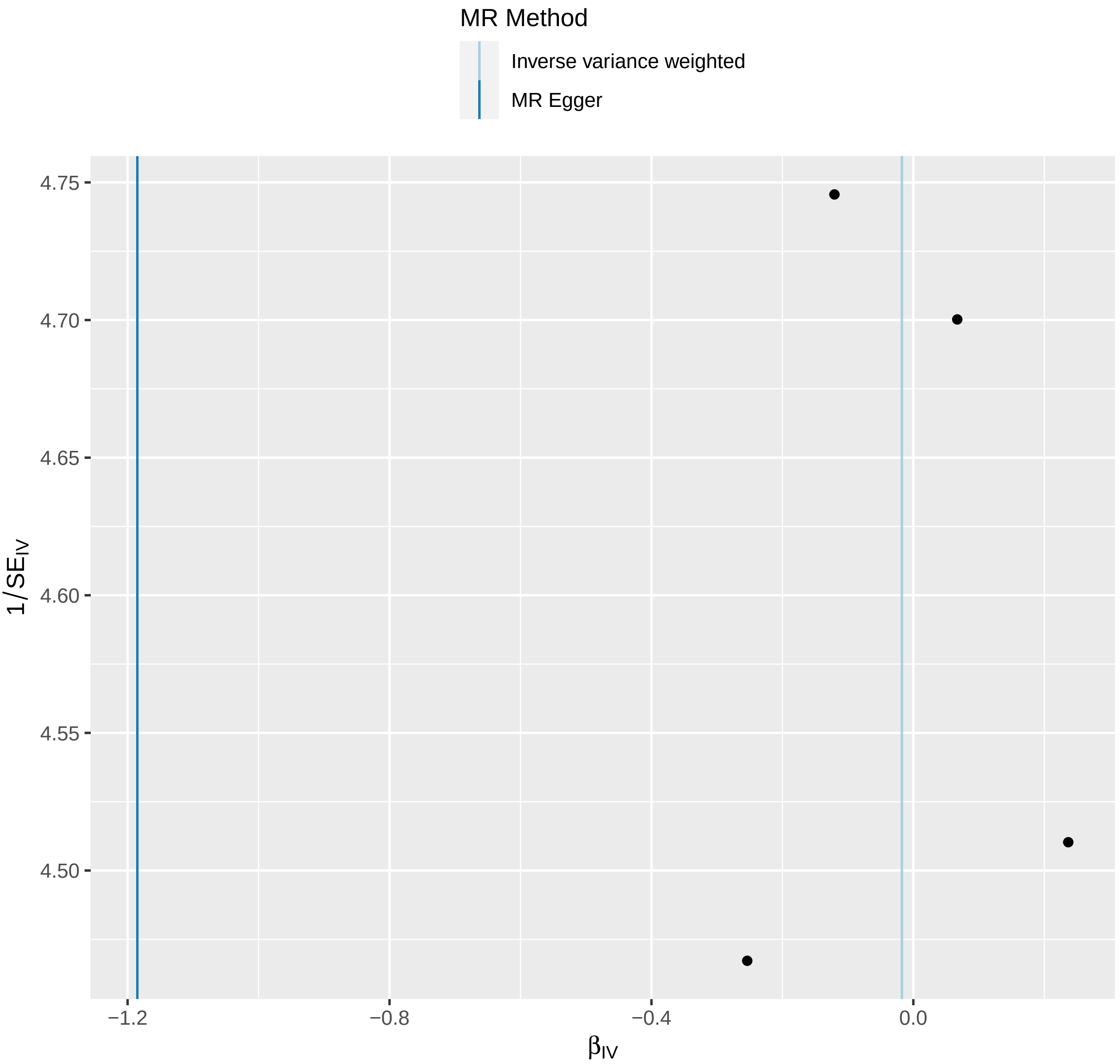

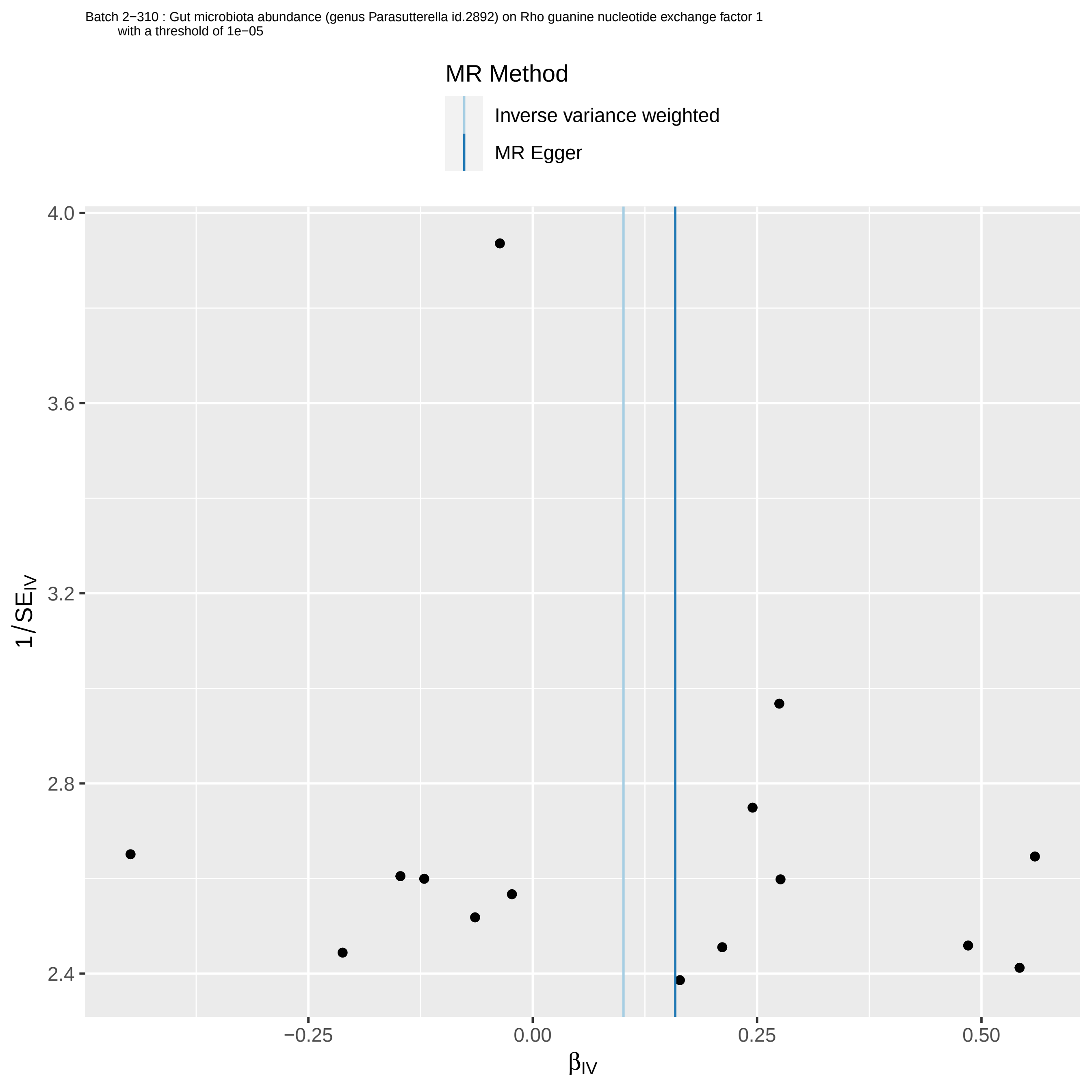
Batch 2−310 : Gut microbiota abundance (genus Parasutterella id.2892) on Rho guanine nucleotide exchange factor 1
with a threshold of 1e−05
MR Method
Inverse variance weighted
MR Egger
4.0
3.6
3.2
2.8
2.4
1/SE_IV
−0.25
0.00
0.25
0.50
β_IV

Batch 2−311 : Gut microbiota abundance (genus Catenibacterium id.2153) on Rho guanine nucleotide exchange factor 2 with a threshold of 1e−05

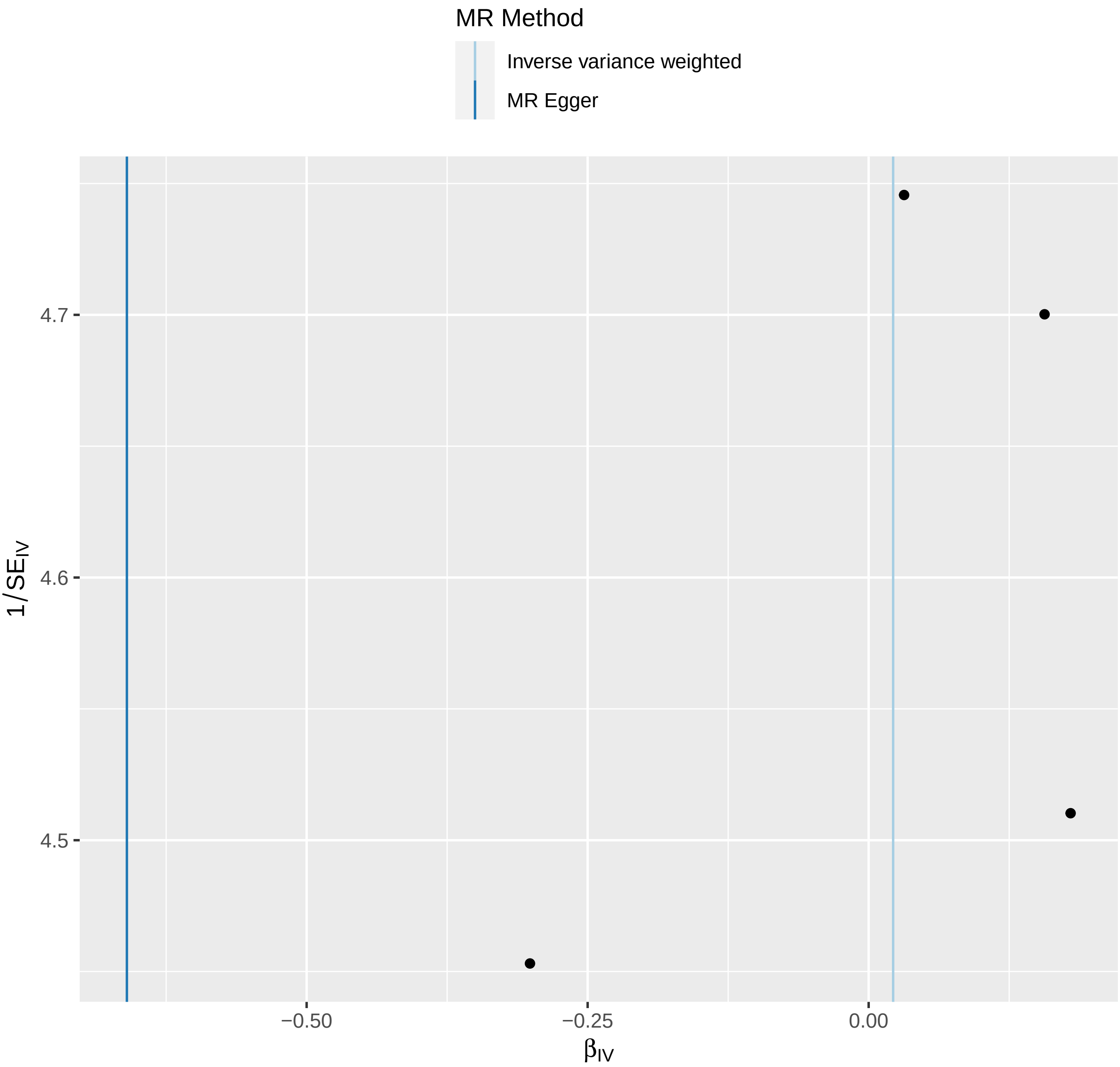

Batch 2−312 : Gut microbiota abundance (genus Parasutterella id.2892) on Rho guanine nucleotide exchange factor 2
with a threshold of 1e−05

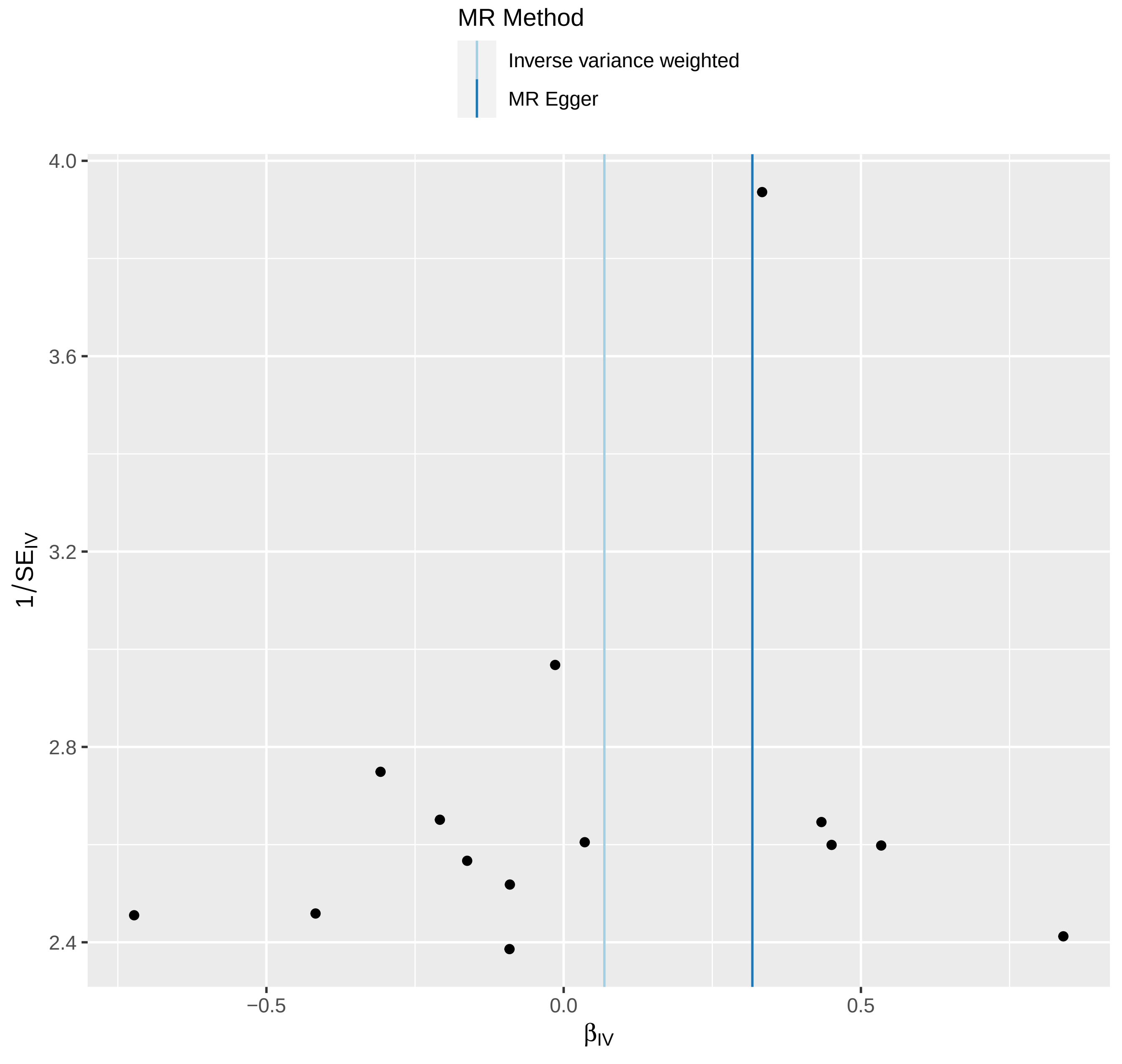

Batch 2−313 : Gut microbiota abundance (genus Catenibacterium id.2153) on Rho guanine nucleotide exchange factor 25 with a threshold of 1e−05

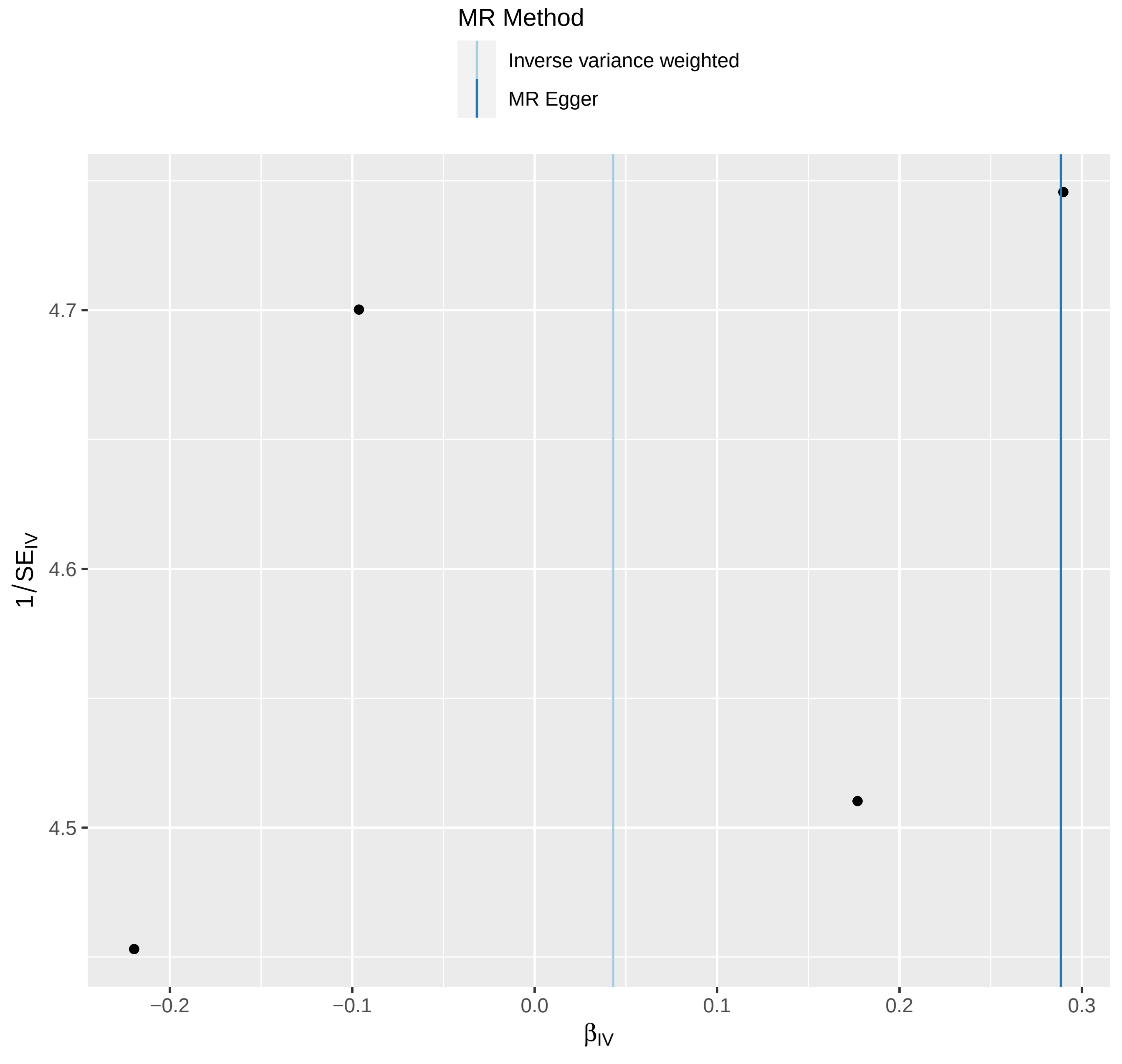

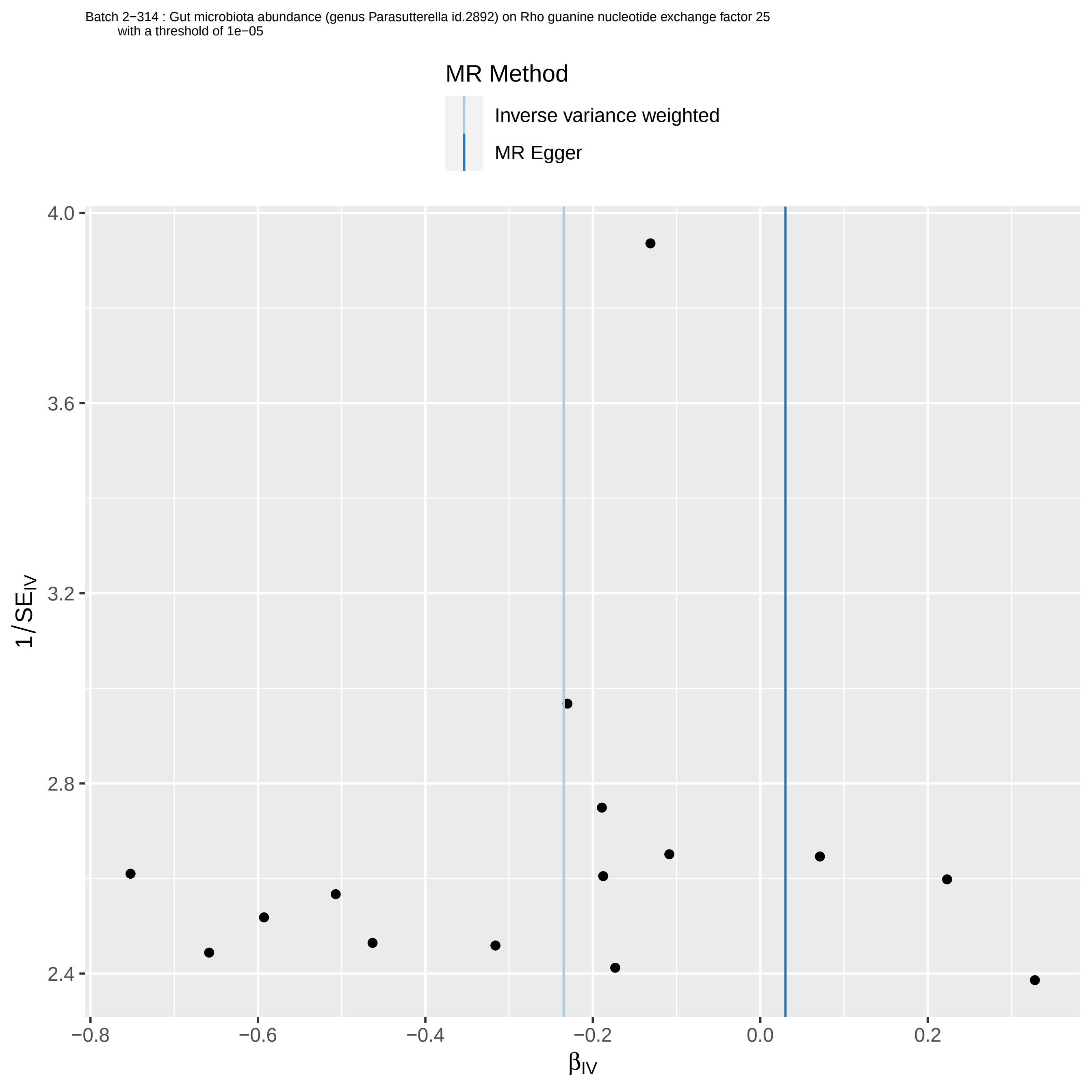
Batch 2−314 : Gut microbiota abundance (genus Parasutterella id.2892) on Rho guanine nucleotide exchange factor 25
with a threshold of 1e−05
MR Method
Inverse variance weighted
MR Egger
4.0
3.6
3.2
2.8
2.4
1/SE_IV
−0.8
−0.6
−0.4
−0.2
0.0
0.2
β_IV

Batch 2−315 : Gut microbiota abundance (genus Catenibacterium id.2153) on Rho guanine nucleotide exchange factor 7 with a threshold of 1e−05

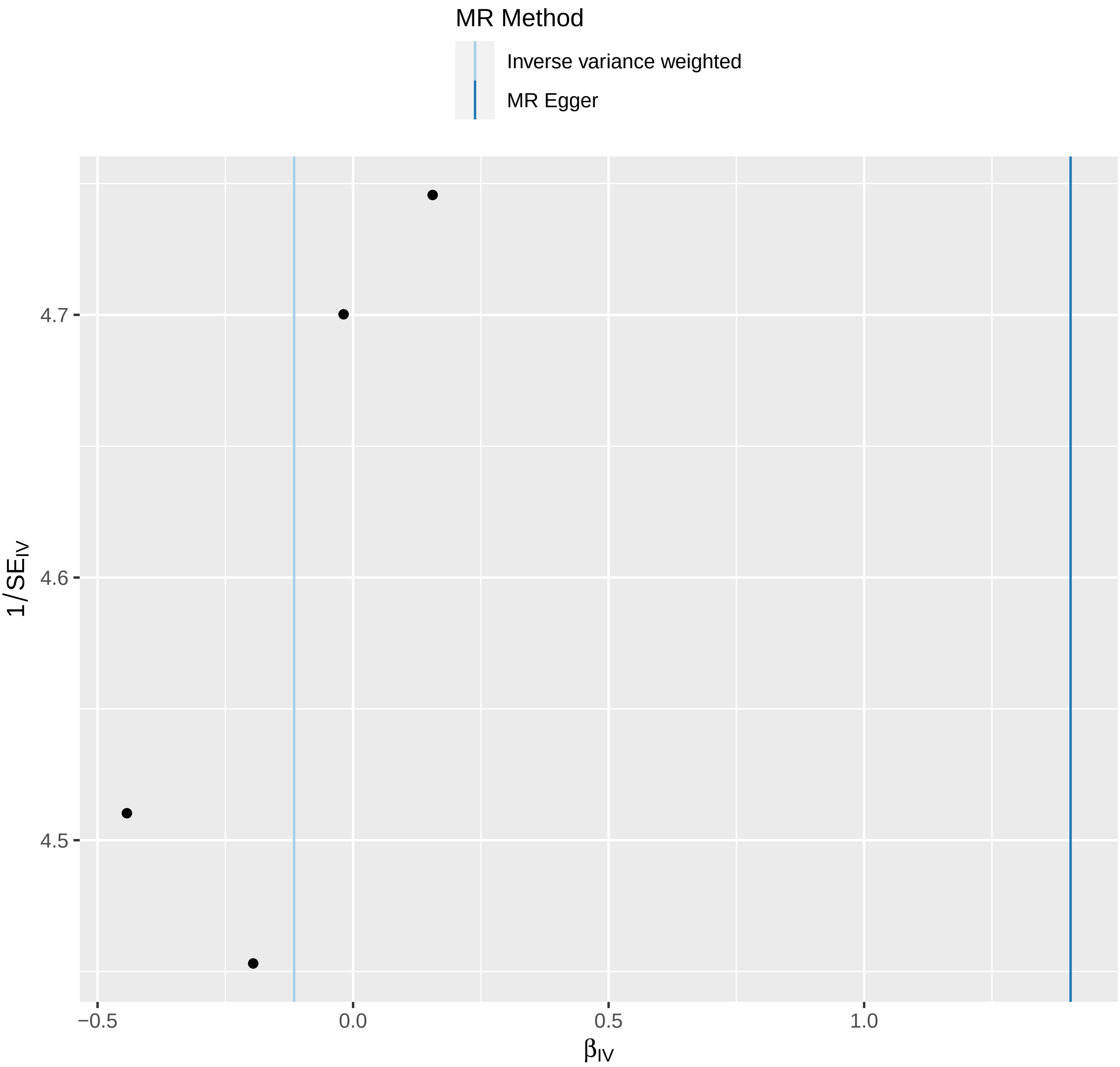

Batch 2−316 : Gut microbiota abundance (genus Parasutterella id.2892) on Rho guanine nucleotide exchange factor 7
with a threshold of 1e−05

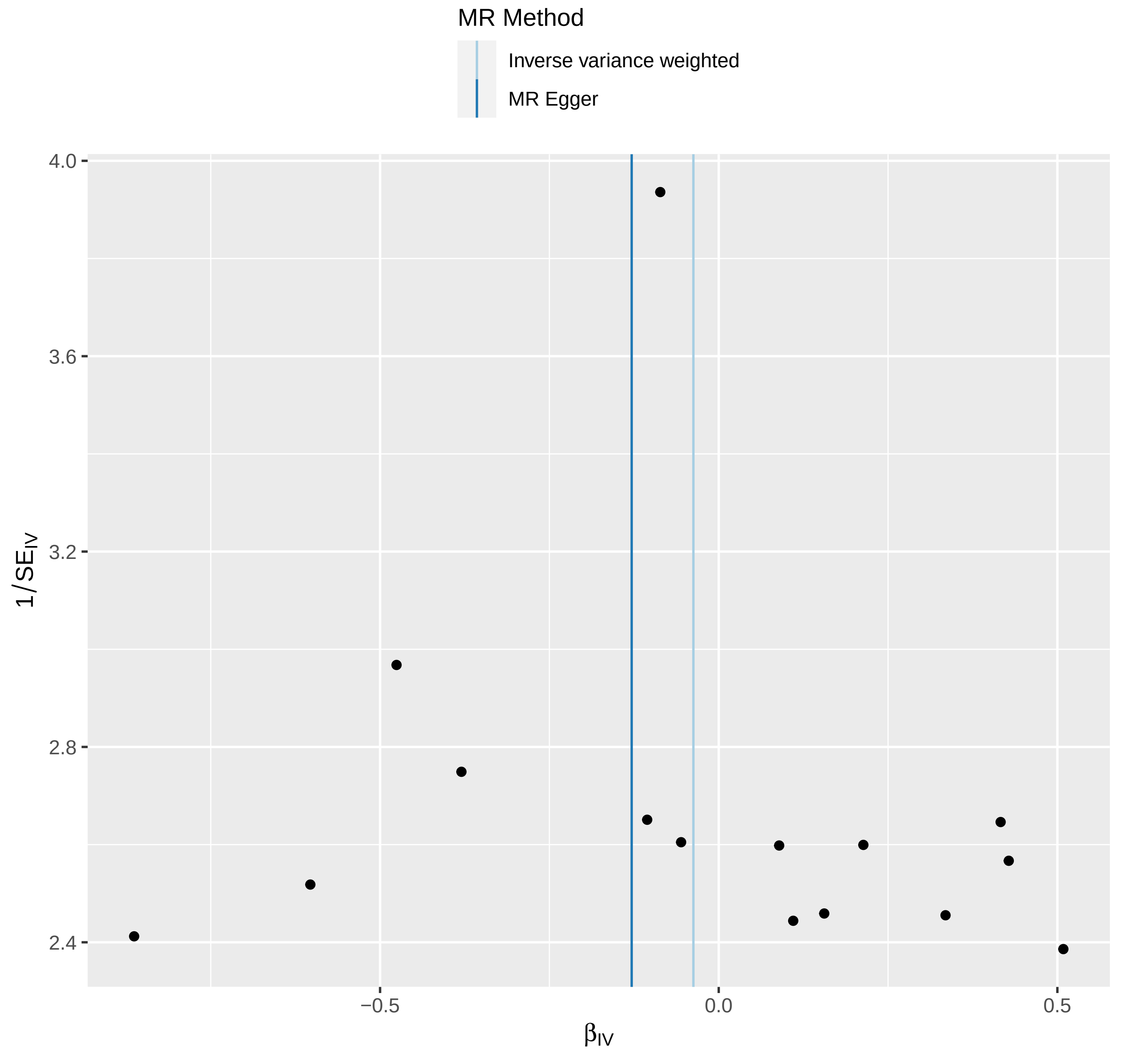

Batch 2−317 : Gut microbiota abundance (genus Catenibacterium id.2153) on AT−rich interactive domain−containing protein 3A with a threshold of 1e−05

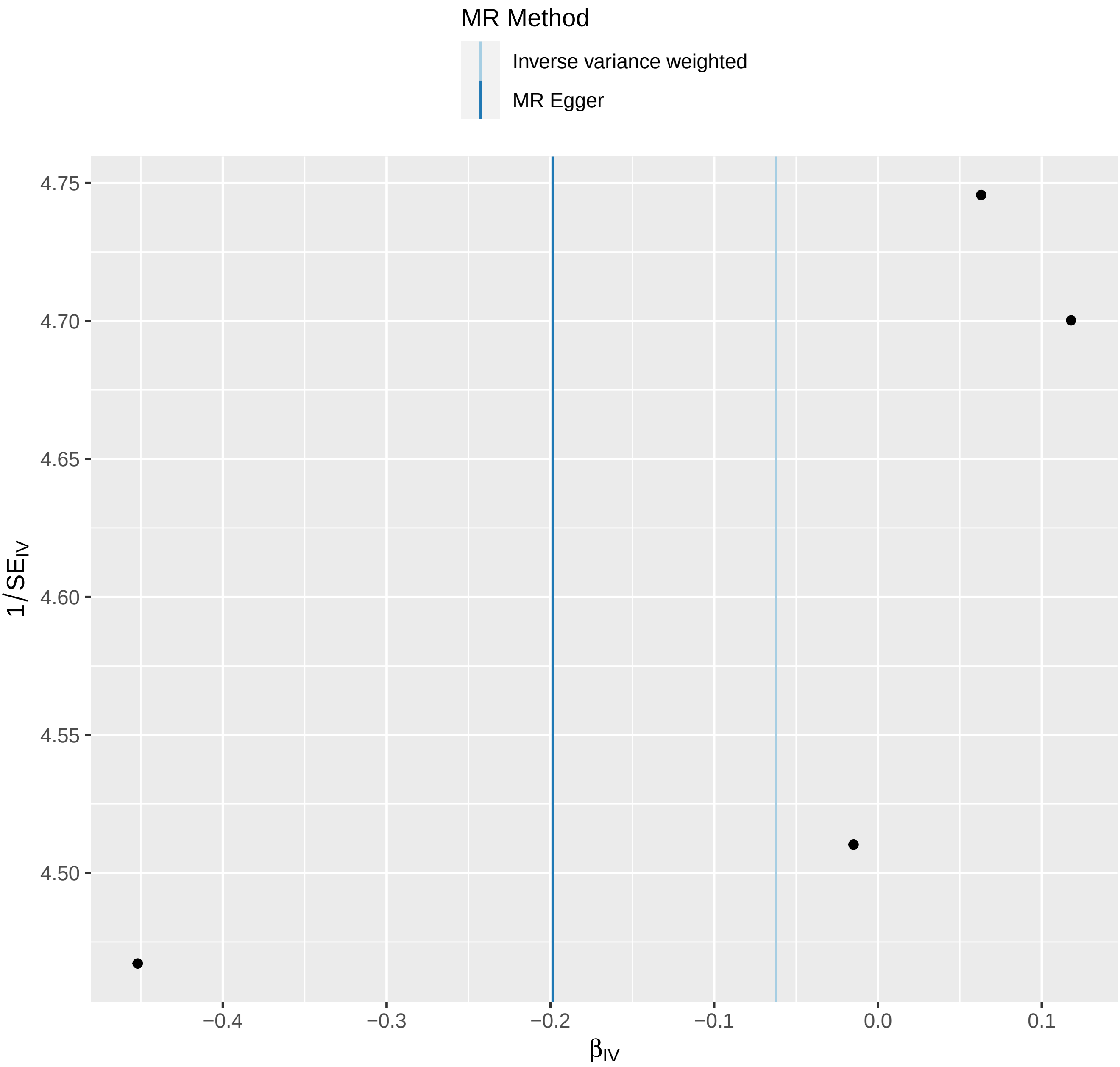

Batch 2−318 : Gut microbiota abundance (genus Parasutterella id.2892) on AT−rich interactive domain−containing protein 3A
with a threshold of 1e−05

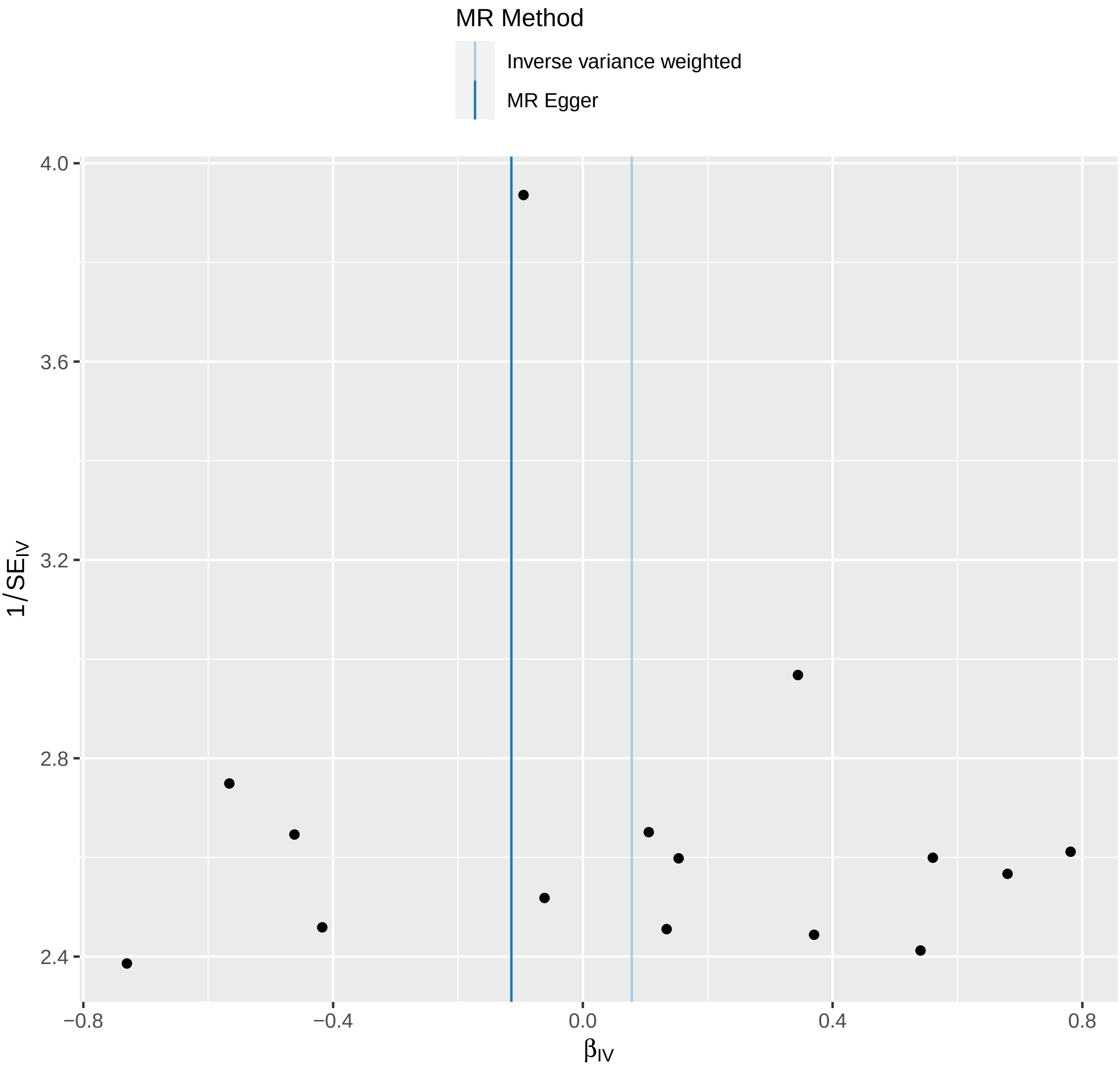

Batch 2−319 : Gut microbiota abundance (genus Catenibacterium id.2153) on ADP−ribosylation factor−like protein 11 with a threshold of 1e−05

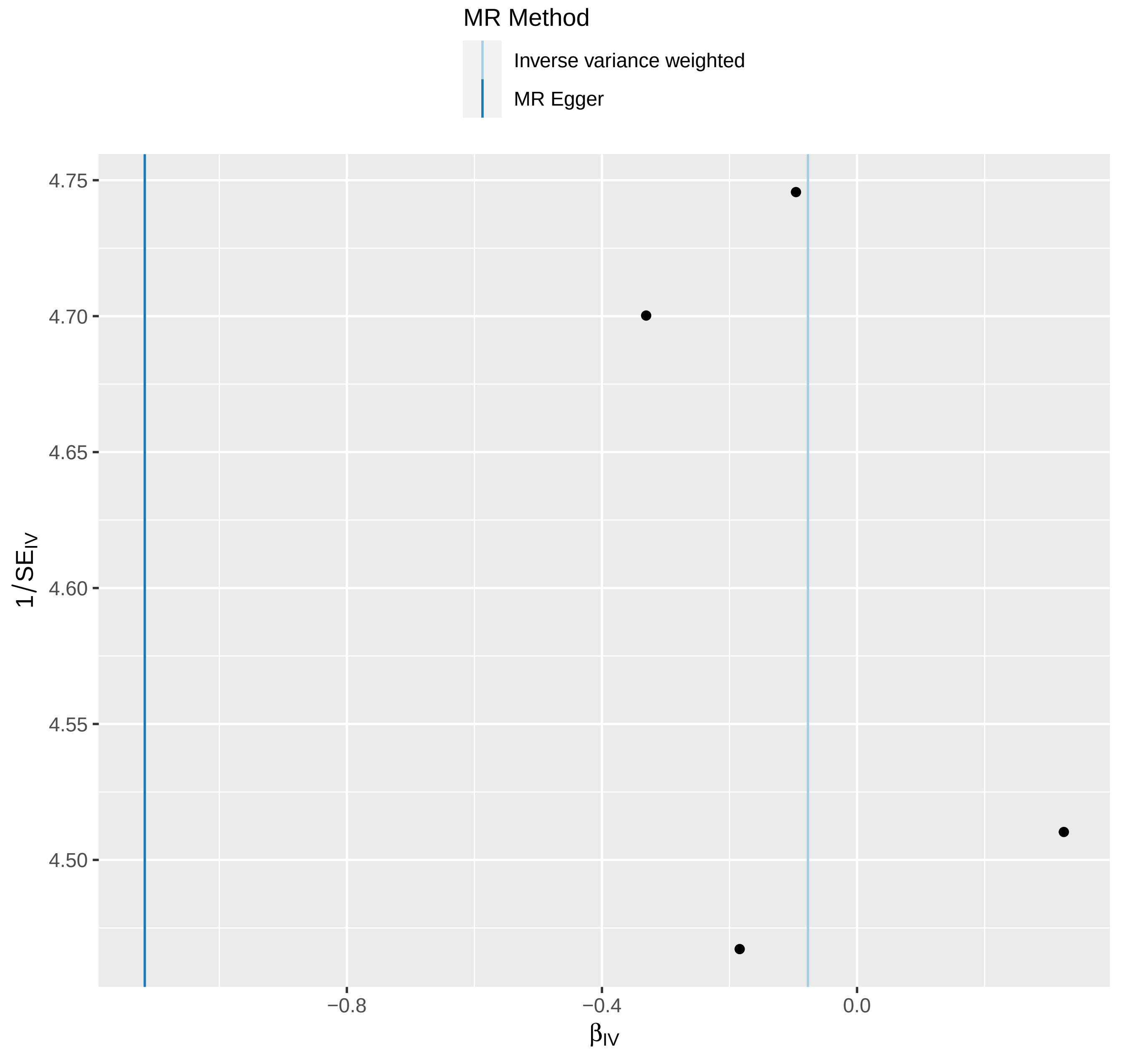

Batch 2−320 : Gut microbiota abundance (genus Parasutterella id.2892) on ADP−ribosylation factor−like protein 11
with a threshold of 1e−05

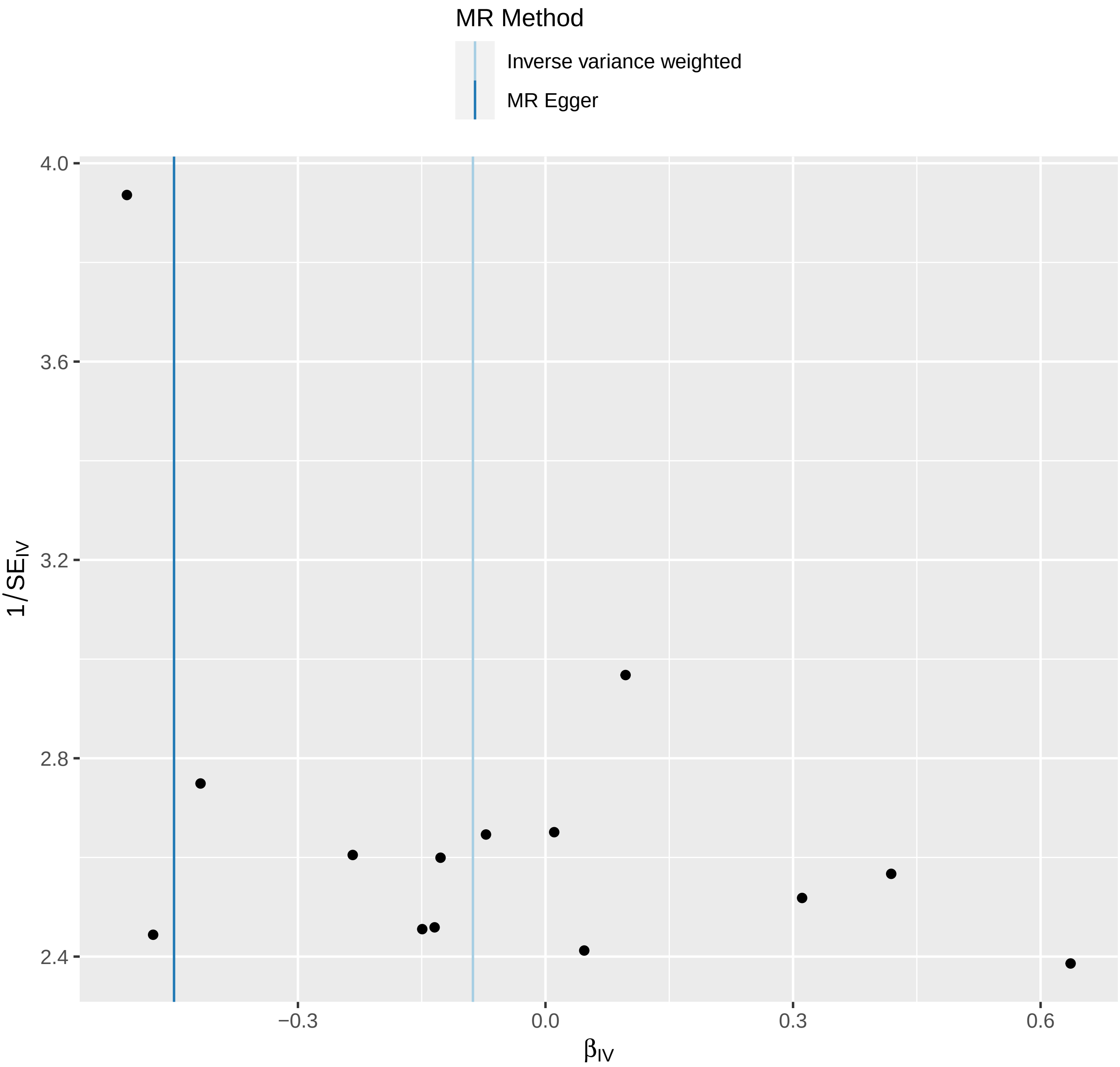

Batch 2−321 : Gut microbiota abundance (genus Catenibacterium id.2153) on ADP−ribosylation factor−like protein 1
with a threshold of 1e−05

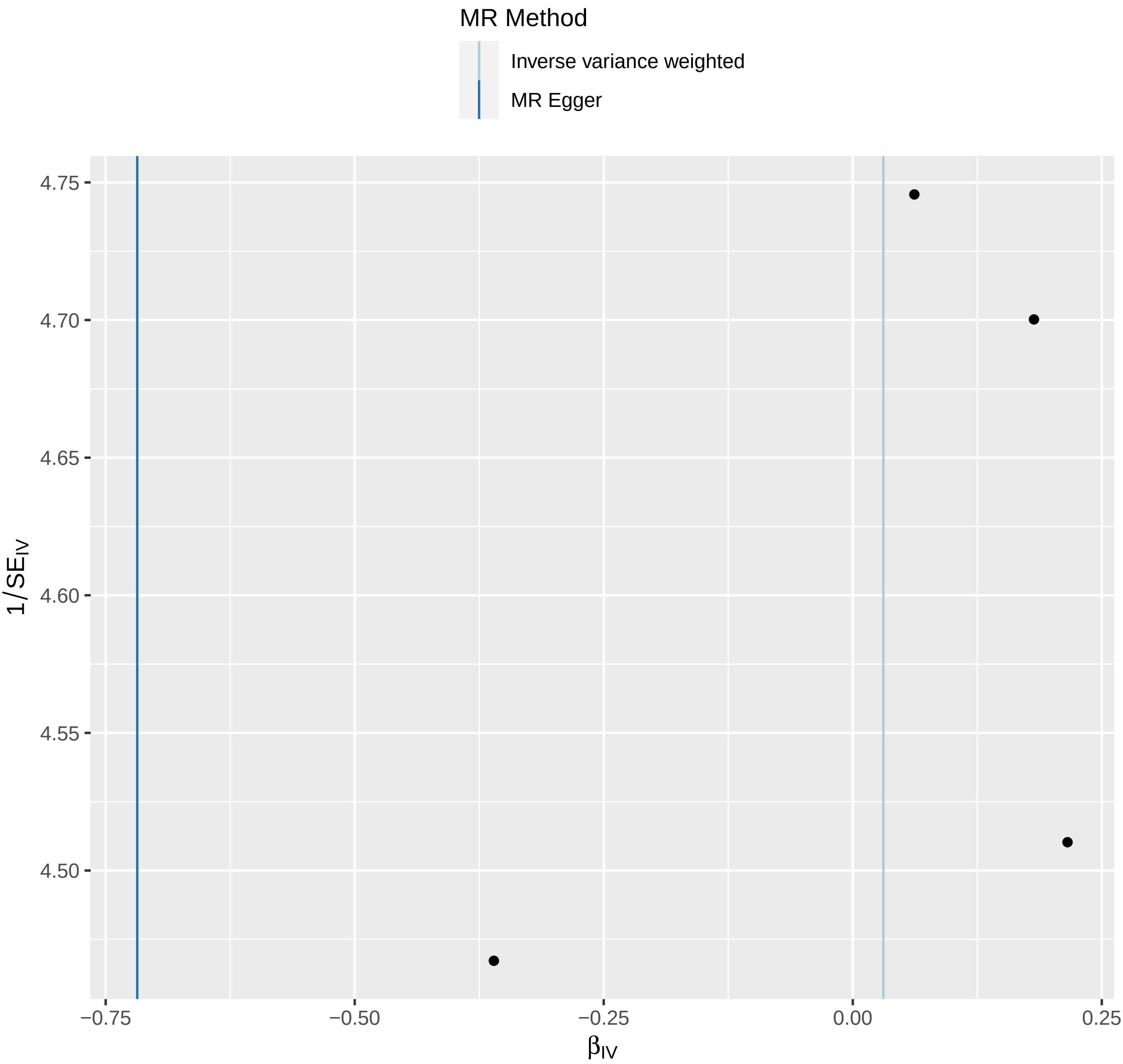

Batch 2−322 : Gut microbiota abundance (genus Parasutterella id.2892) on ADP−ribosylation factor−like protein 1 with a threshold of 1e−05

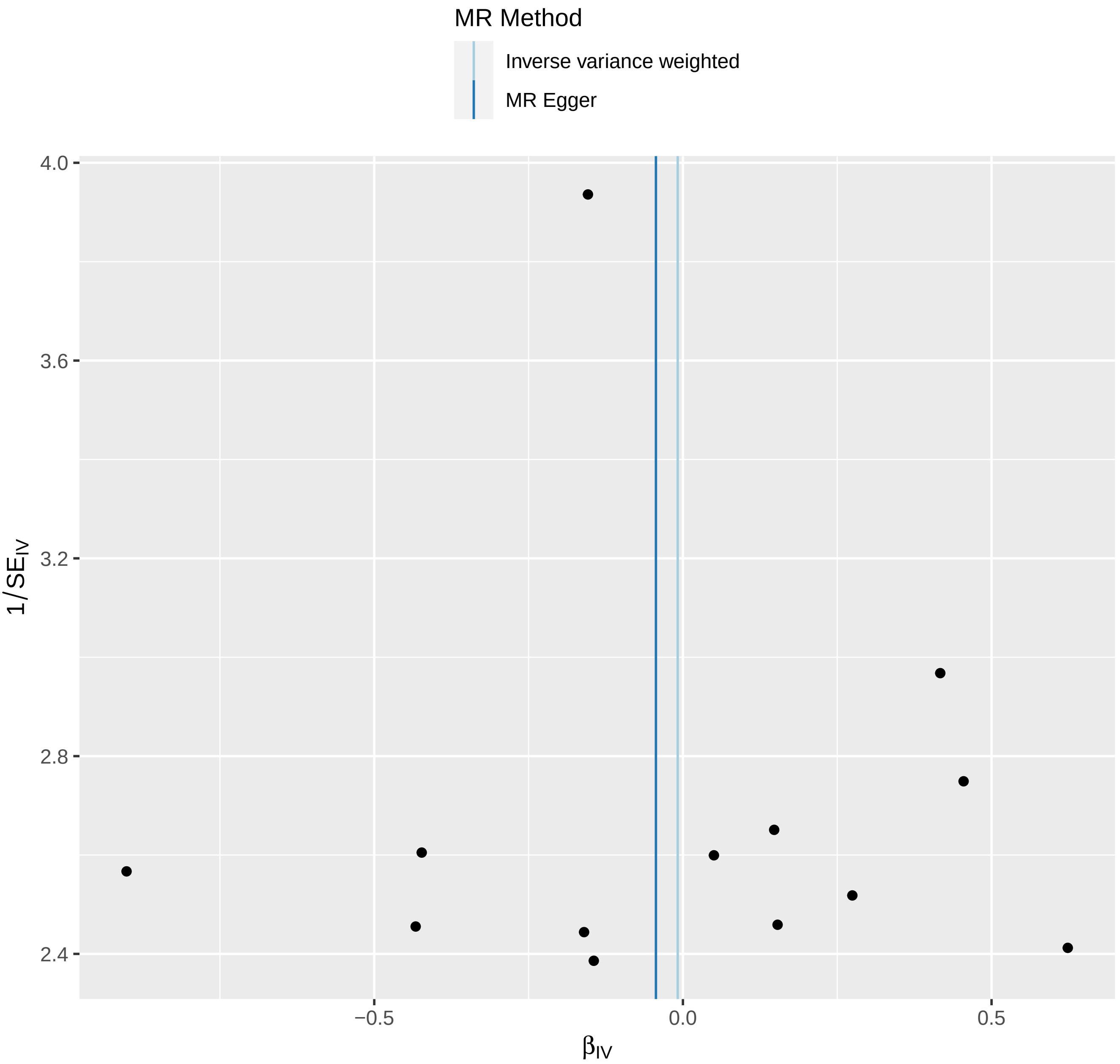

Batch 2−323 : Gut microbiota abundance (genus Catenibacterium id.2153) on ADP−ribosylation factor−like protein 3
with a threshold of 1e−05

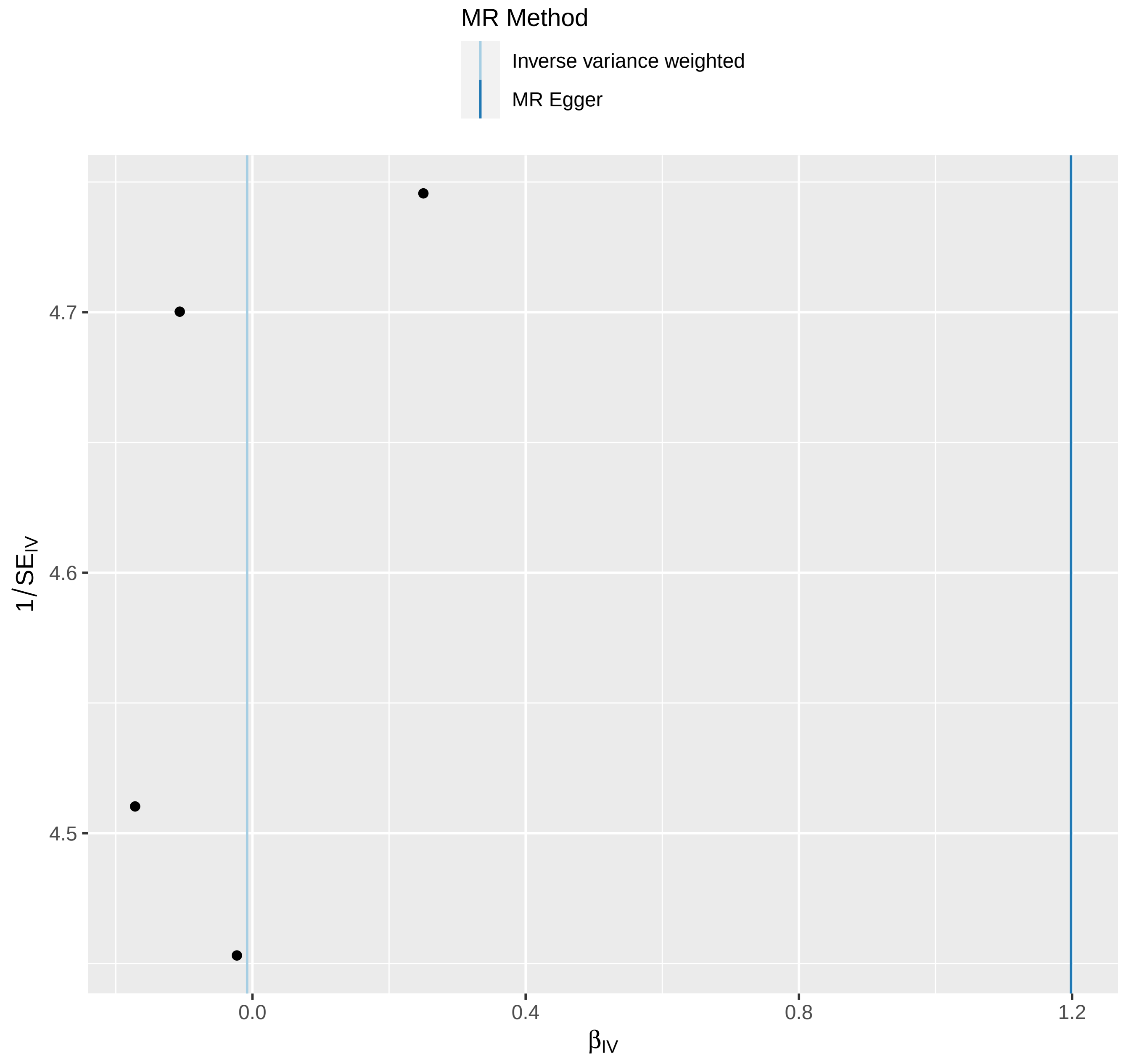

Batch 2−324 : Gut microbiota abundance (genus Parasutterella id.2892) on ADP−ribosylation factor−like protein 3
with a threshold of 1e−05

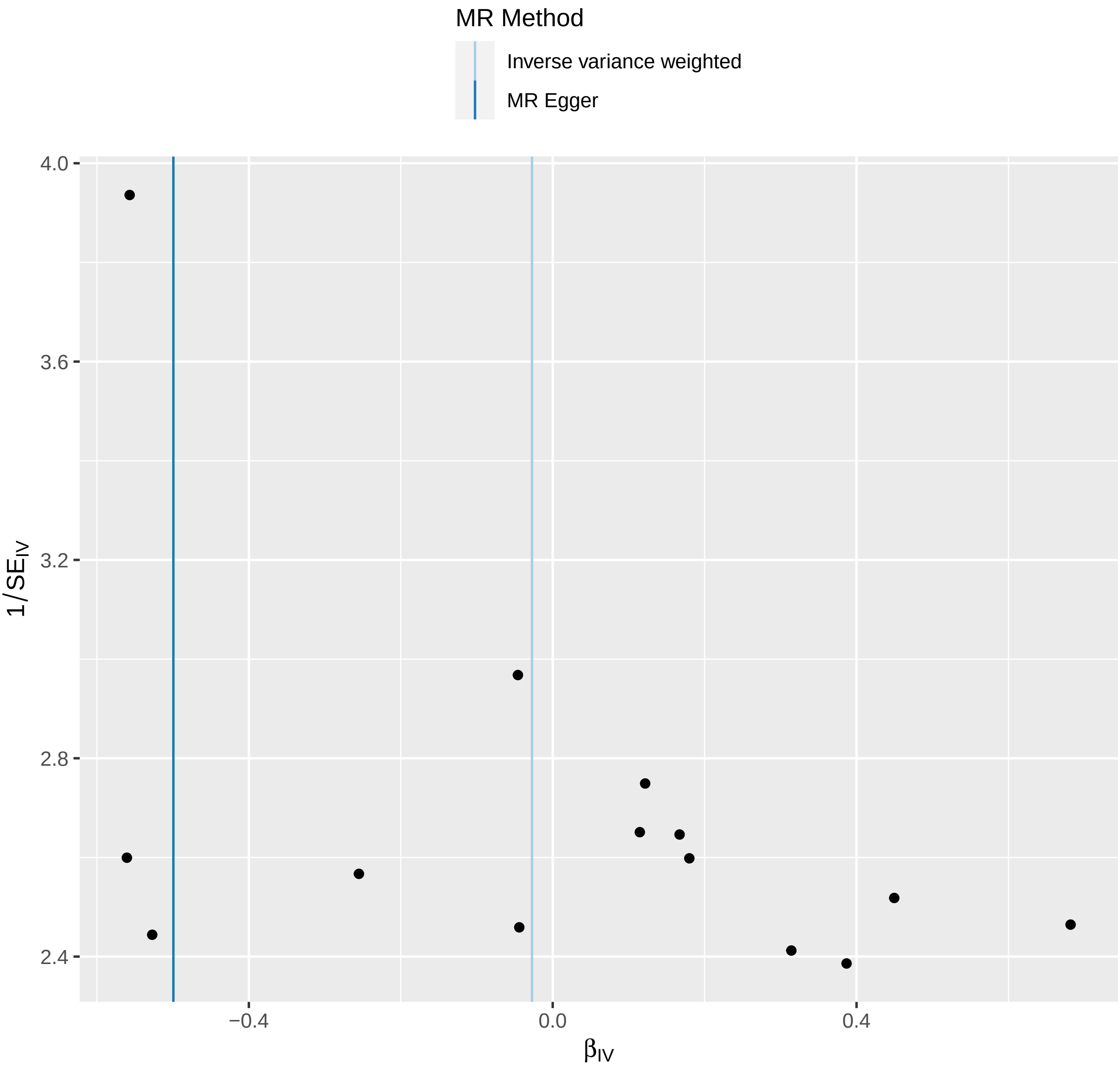

Batch 2−325 : Gut microbiota abundance (genus Catenibacterium id.2153) on Armadillo repeat−containing protein 5 with a threshold of 1e−05

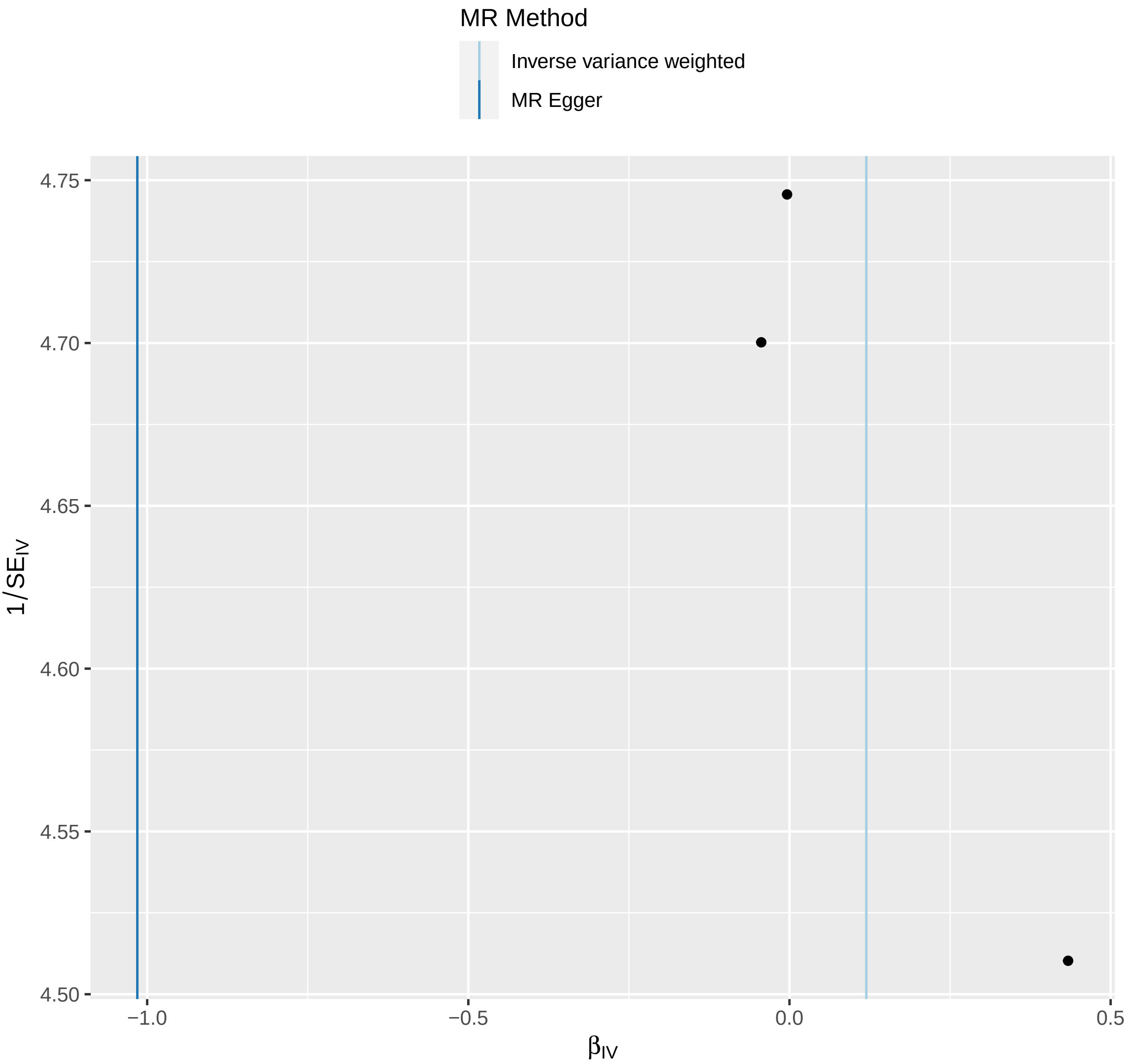

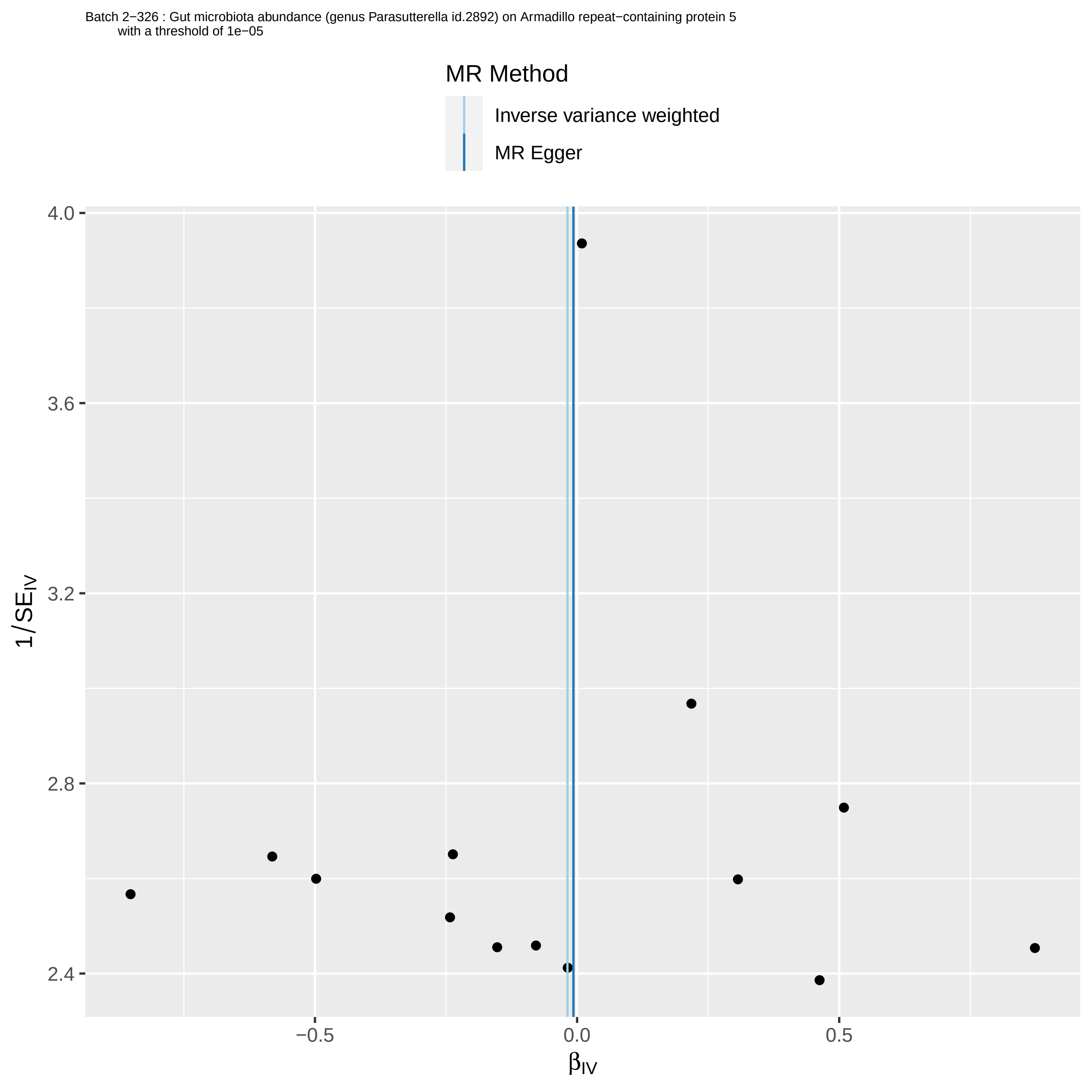

Batch 2−326 : Gut microbiota abundance (genus Parasutterella id.2892) on Armadillo repeat−containing protein 5
with a threshold of 1e−05
MR Method
Inverse variance weighted
MR Egger
4.0
3.6
3.2
2.8
2.4
1/SE_IV
−0.5
0.0
0.5
β_IV

Batch 2−327 : Gut microbiota abundance (genus Catenibacterium id.2153) on Armadillo repeat−containing protein 5
with a threshold of 1e−05

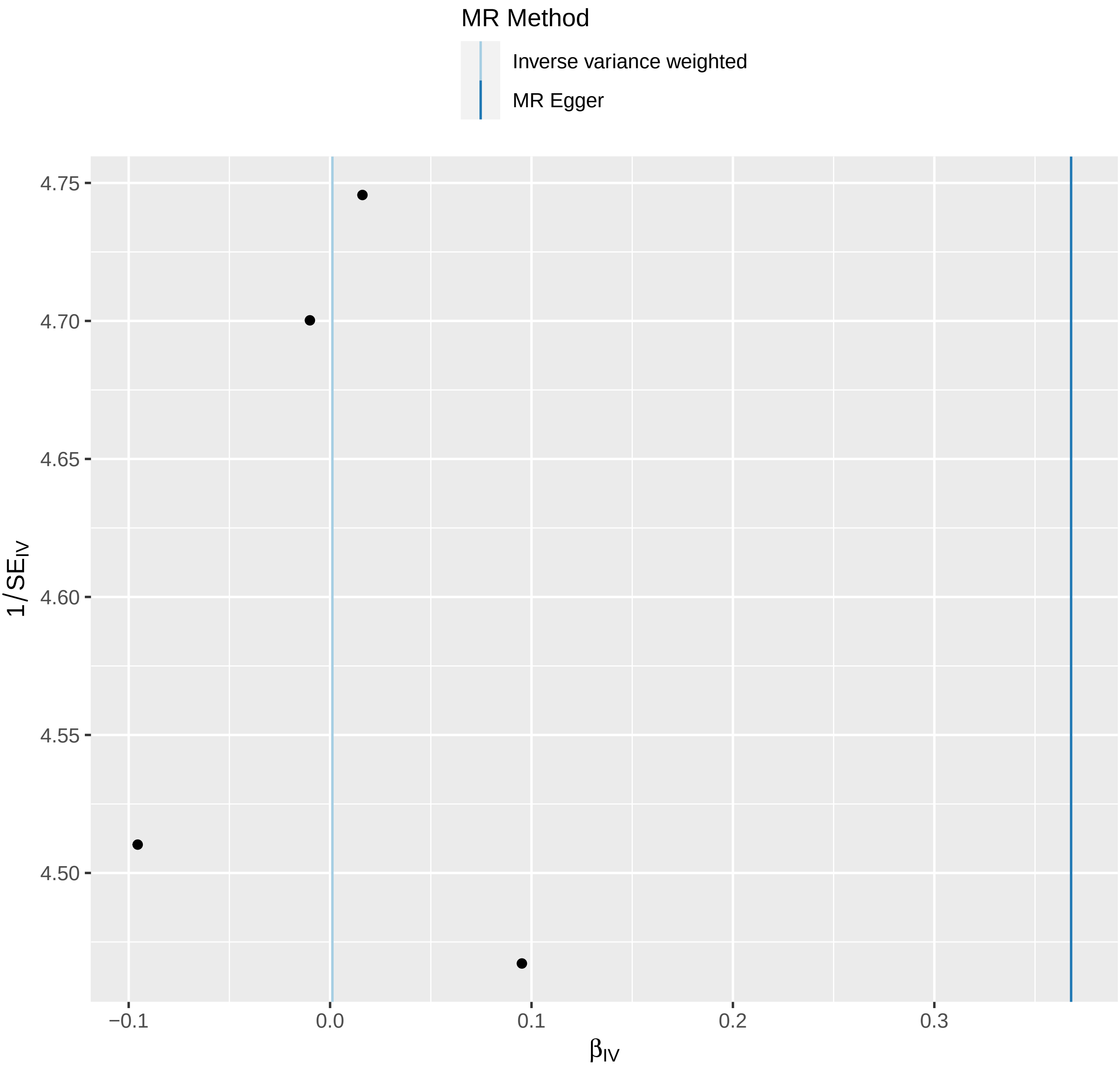

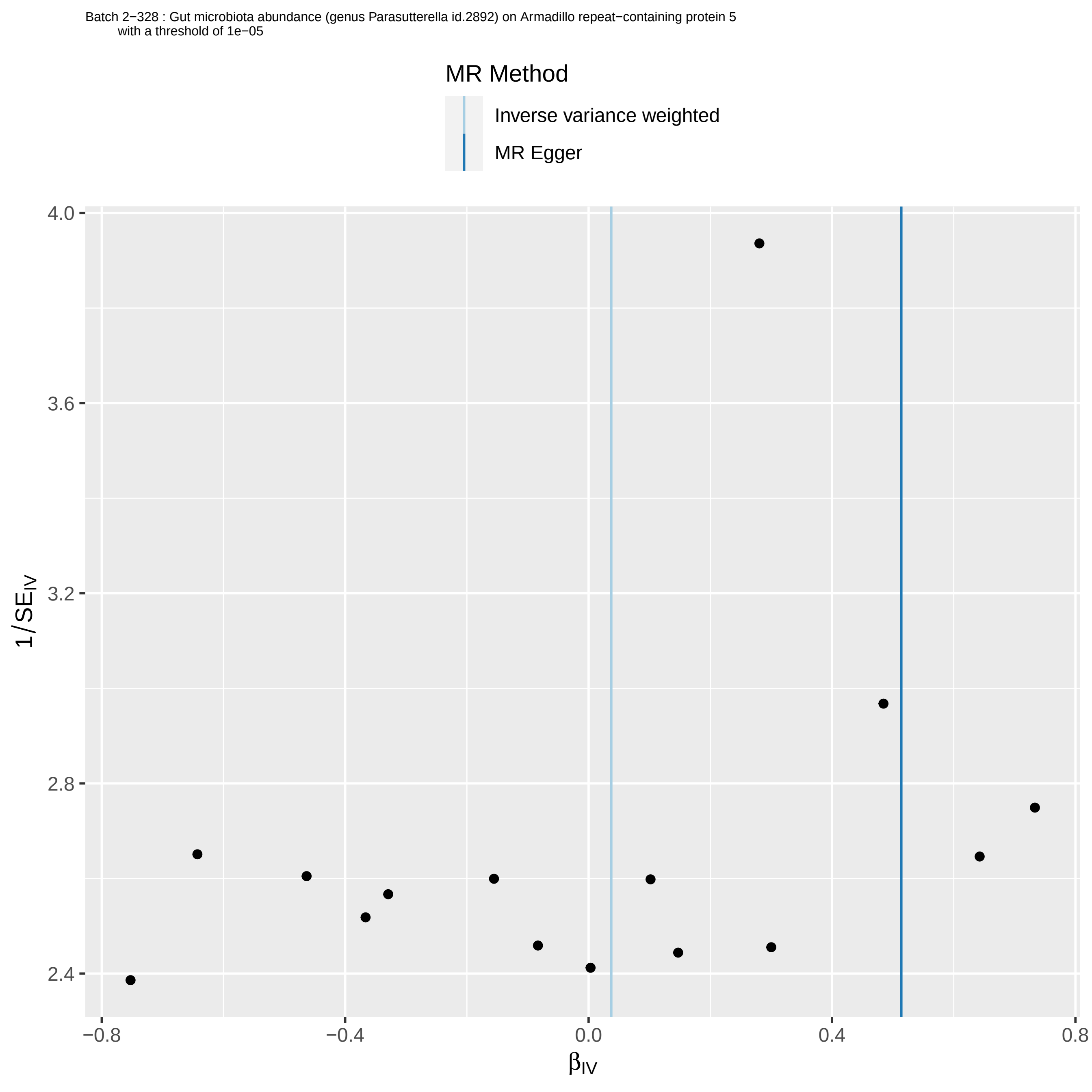
Batch 2−328 : Gut microbiota abundance (genus Parasutterella id.2892) on Armadillo repeat−containing protein 5
with a threshold of 1e−05
MR Method
Inverse variance weighted
MR Egger
1/SE_IV
β_IV
4.0
3.6
3.2
2.8
2.4
−0.8
−0.4
0.0
0.4
0.8

Batch 2−329 : Gut microbiota abundance (genus Catenibacterium id.2153) on Armadillo repeat−containing protein 5 with a threshold of 1e−05

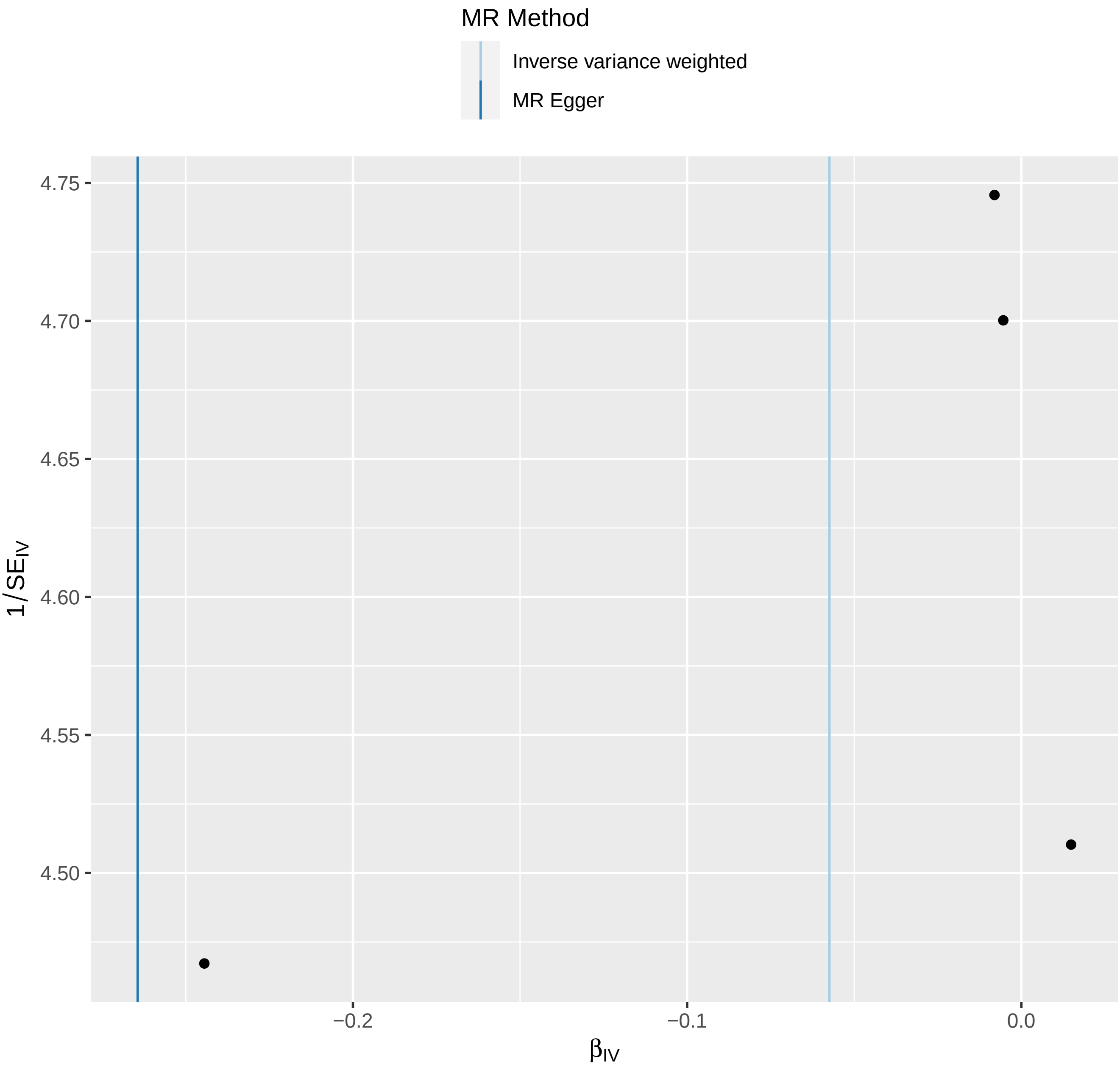

Batch 2−330 : Gut microbiota abundance (genus Parasutterella id.2892) on Armadillo repeat−containing protein 5
with a threshold of 1e−05

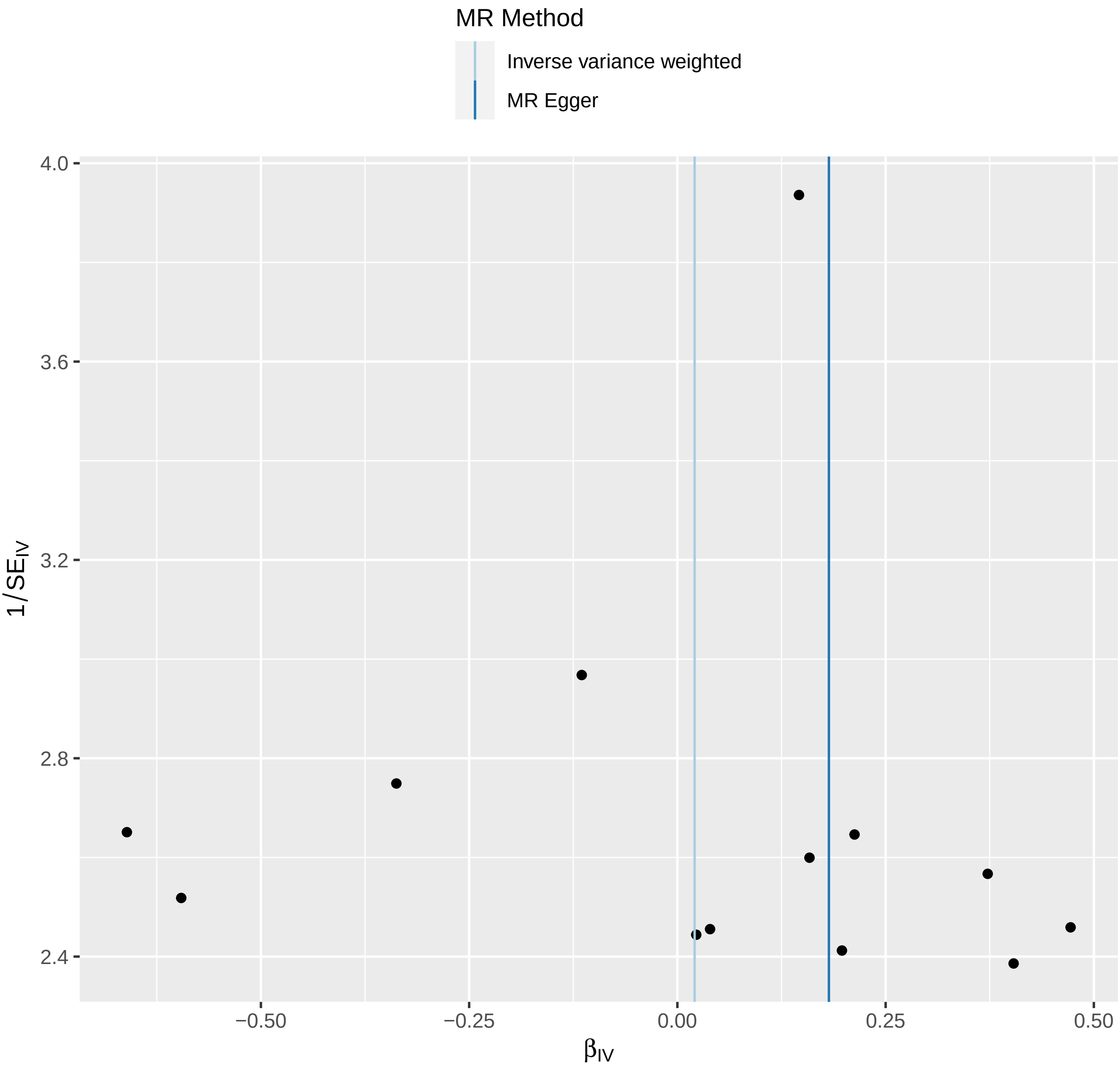

Batch 2−331 : Gut microbiota abundance (genus Catenibacterium id.2153) on Actin−related protein 2/3 complex subunit 3 with a threshold of 1e−05

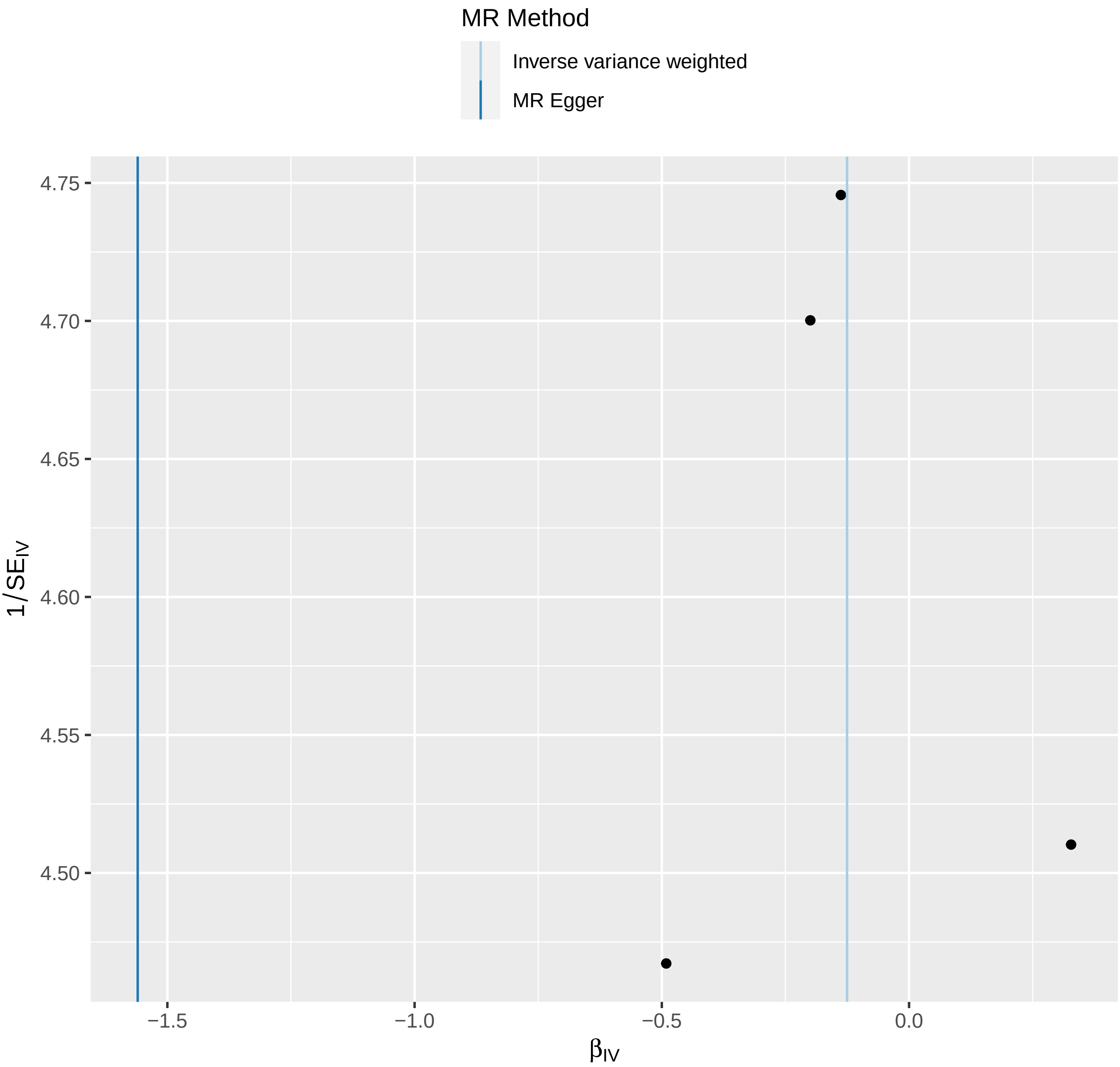

Batch 2−332 : Gut microbiota abundance (genus Parasutterella id.2892) on Actin−related protein 2/3 complex subunit 3 with a threshold of 1e−05

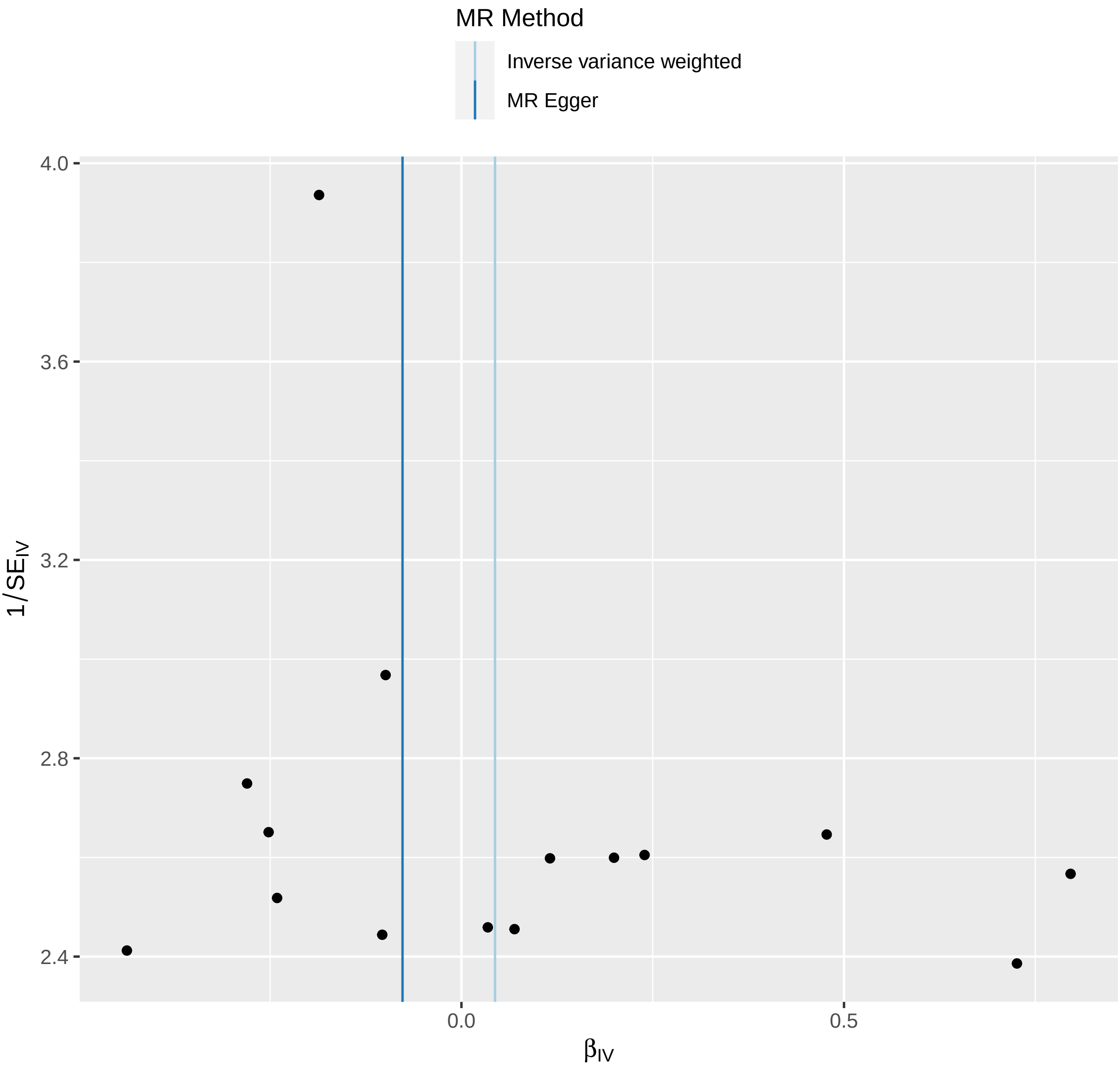

Batch 2−333 : Gut microbiota abundance (genus Catenibacterium id.2153) on cAMP−regulated phosphoprotein 19 with a threshold of 1e−05

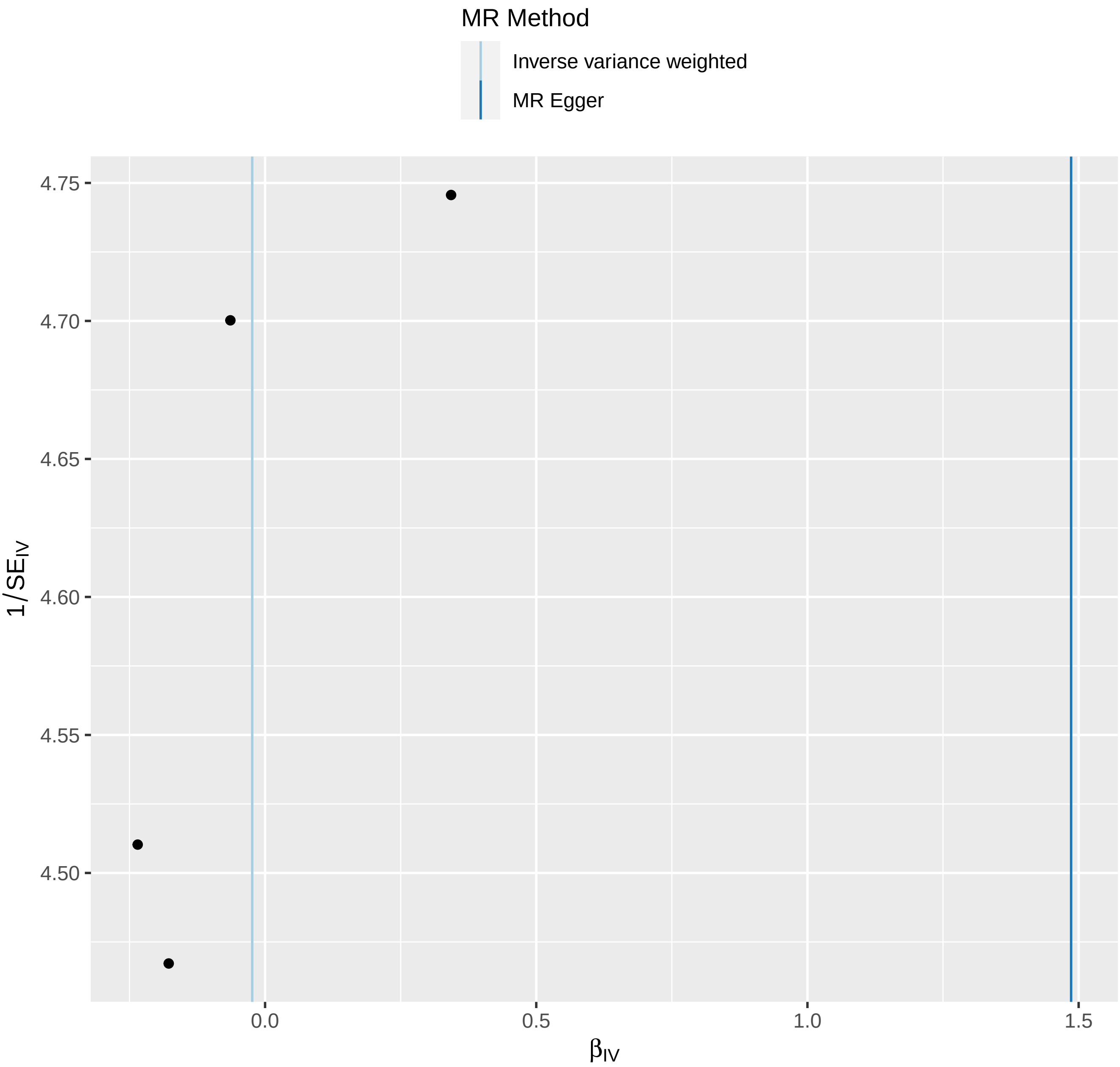

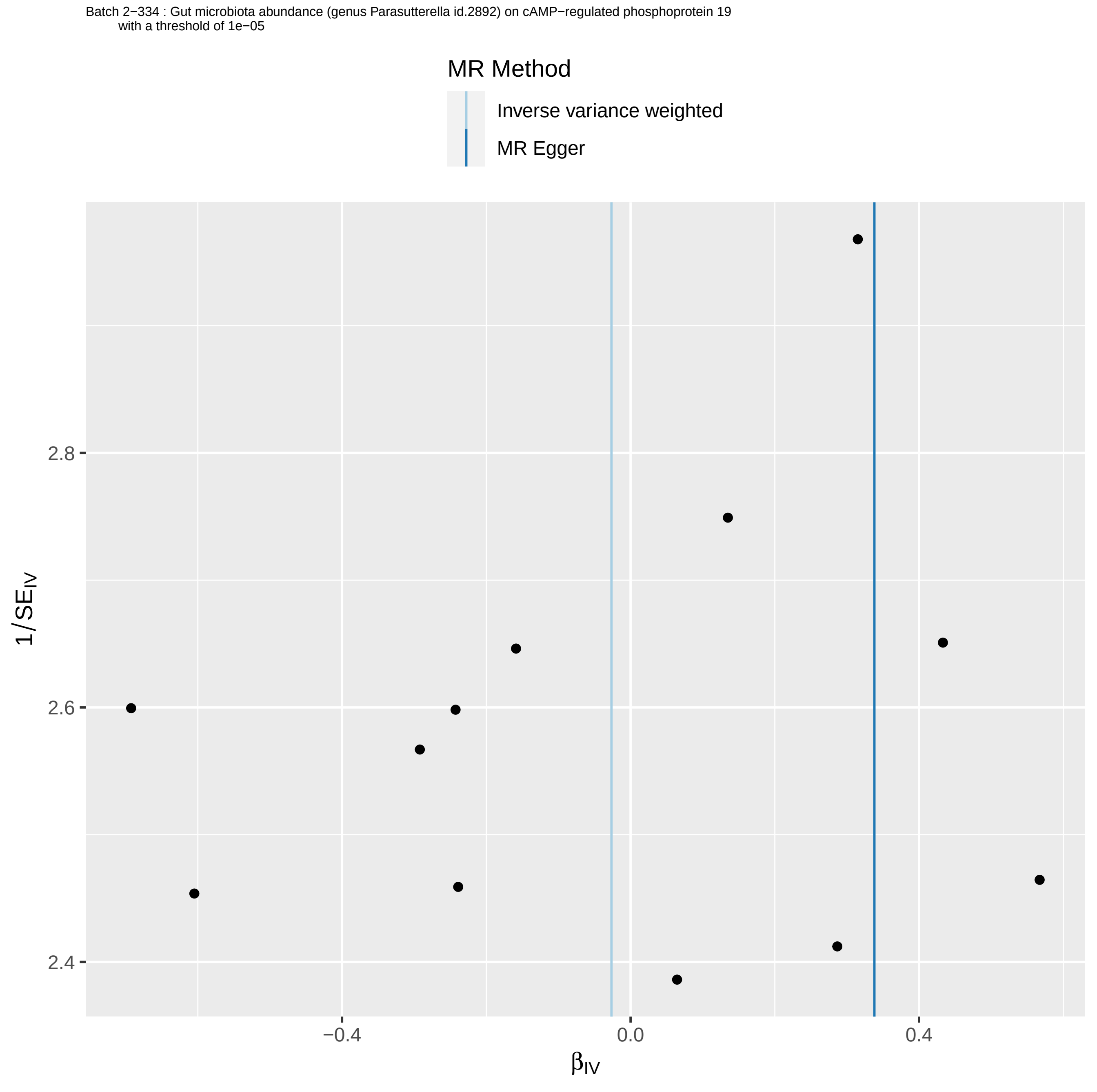
Batch 2−334 : Gut microbiota abundance (genus Parasutterella id.2892) on cAMP−regulated phosphoprotein 19
with a threshold of 1e−05
MR Method
Inverse variance weighted
MR Egger
2.8
2.6
2.4
1/SE_IV
−0.4
0.0
0.4
β_IV

Batch 2−335 : Gut microbiota abundance (genus Catenibacterium id.2153) on cAMP−regulated phosphoprotein 21
with a threshold of 1e−05

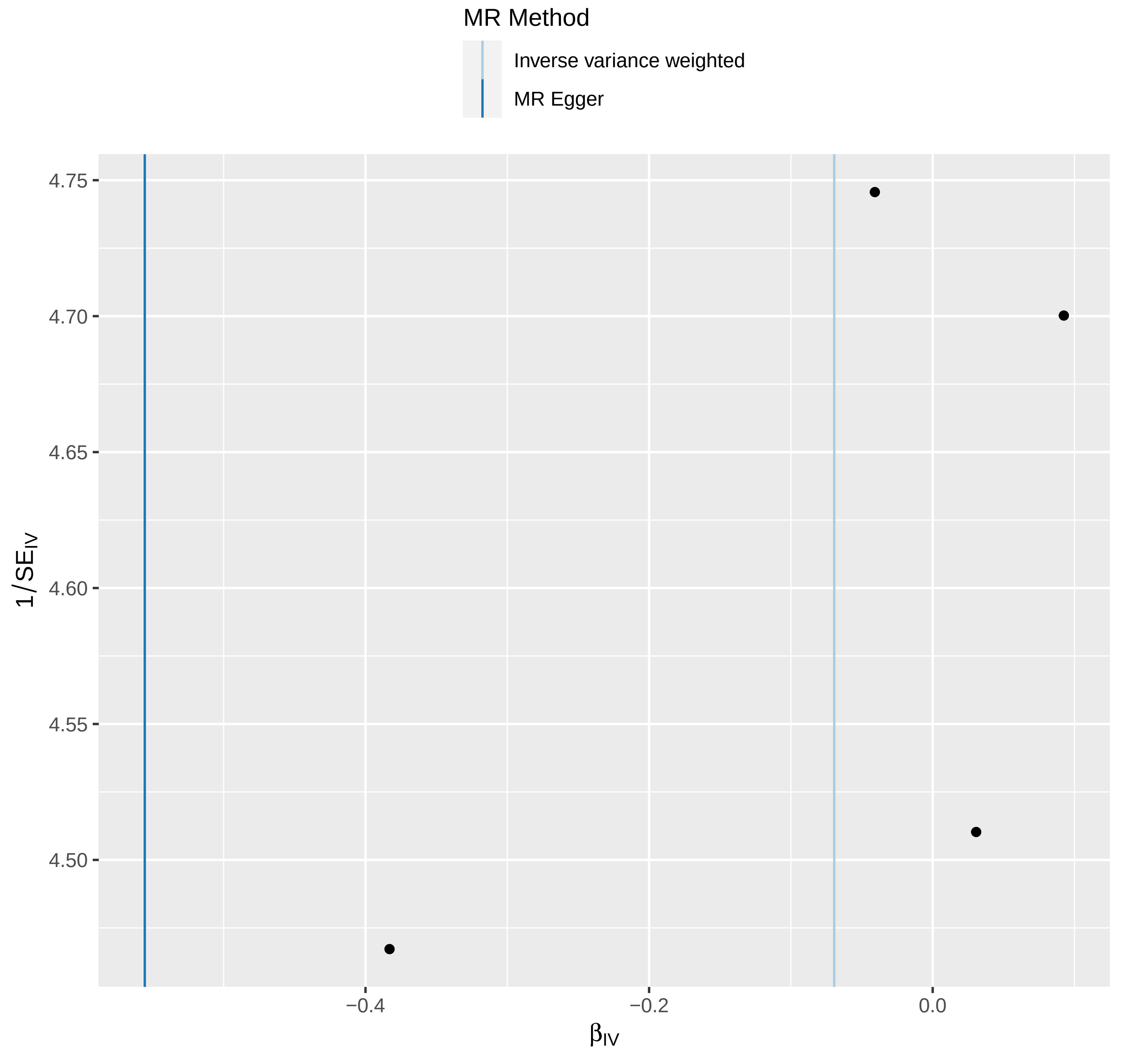

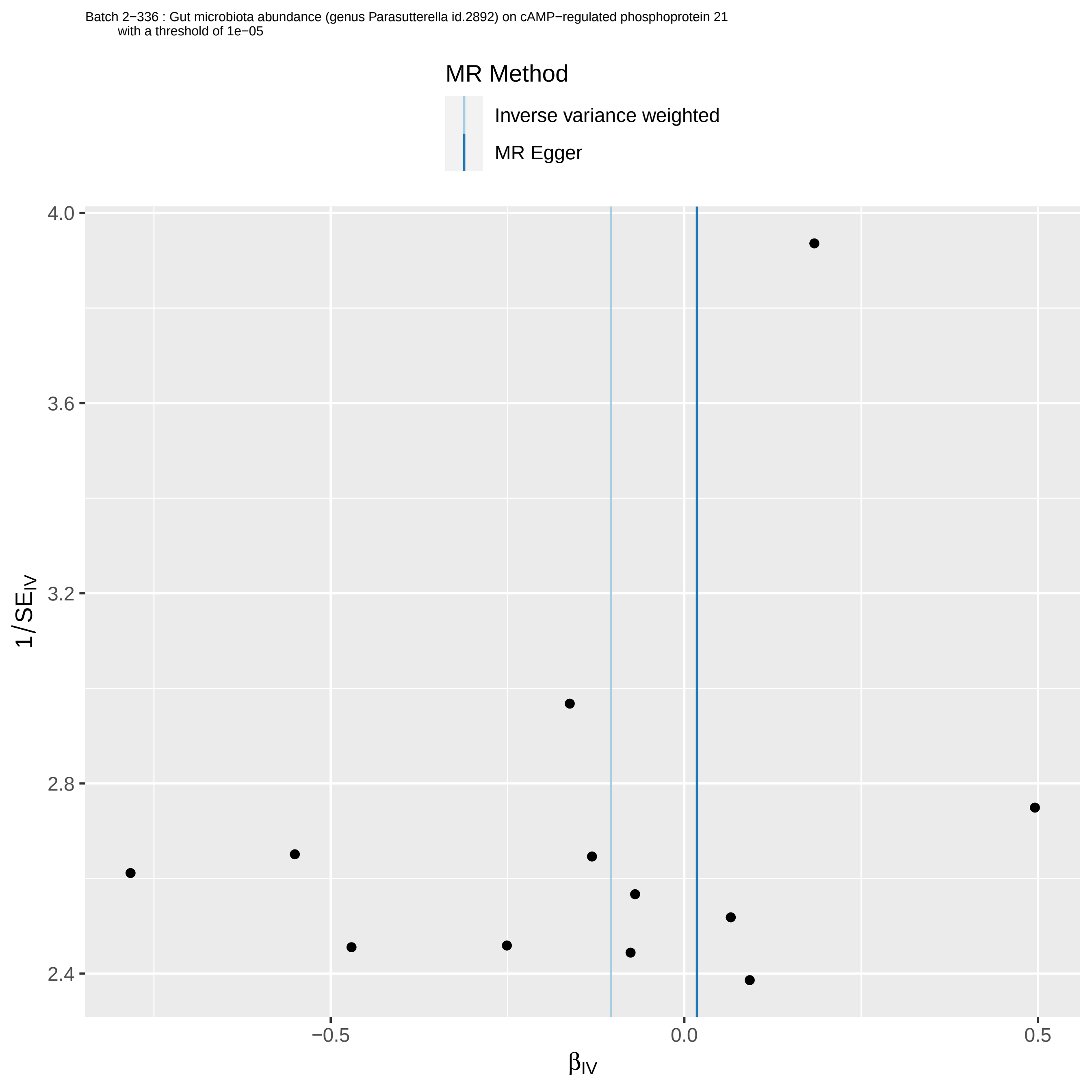
Batch 2−336 : Gut microbiota abundance (genus Parasutterella id.2892) on cAMP−regulated phosphoprotein 21
with a threshold of 1e−05
MR Method
Inverse variance weighted
MR Egger
4.0
3.6
3.2
2.8
2.4
1/SE_IV
−0.5
0.0
0.5
β_IV

Batch 2−337 : Gut microbiota abundance (genus Catenibacterium id.2153) on Beta−arrestin−1
with a threshold of 1e−05

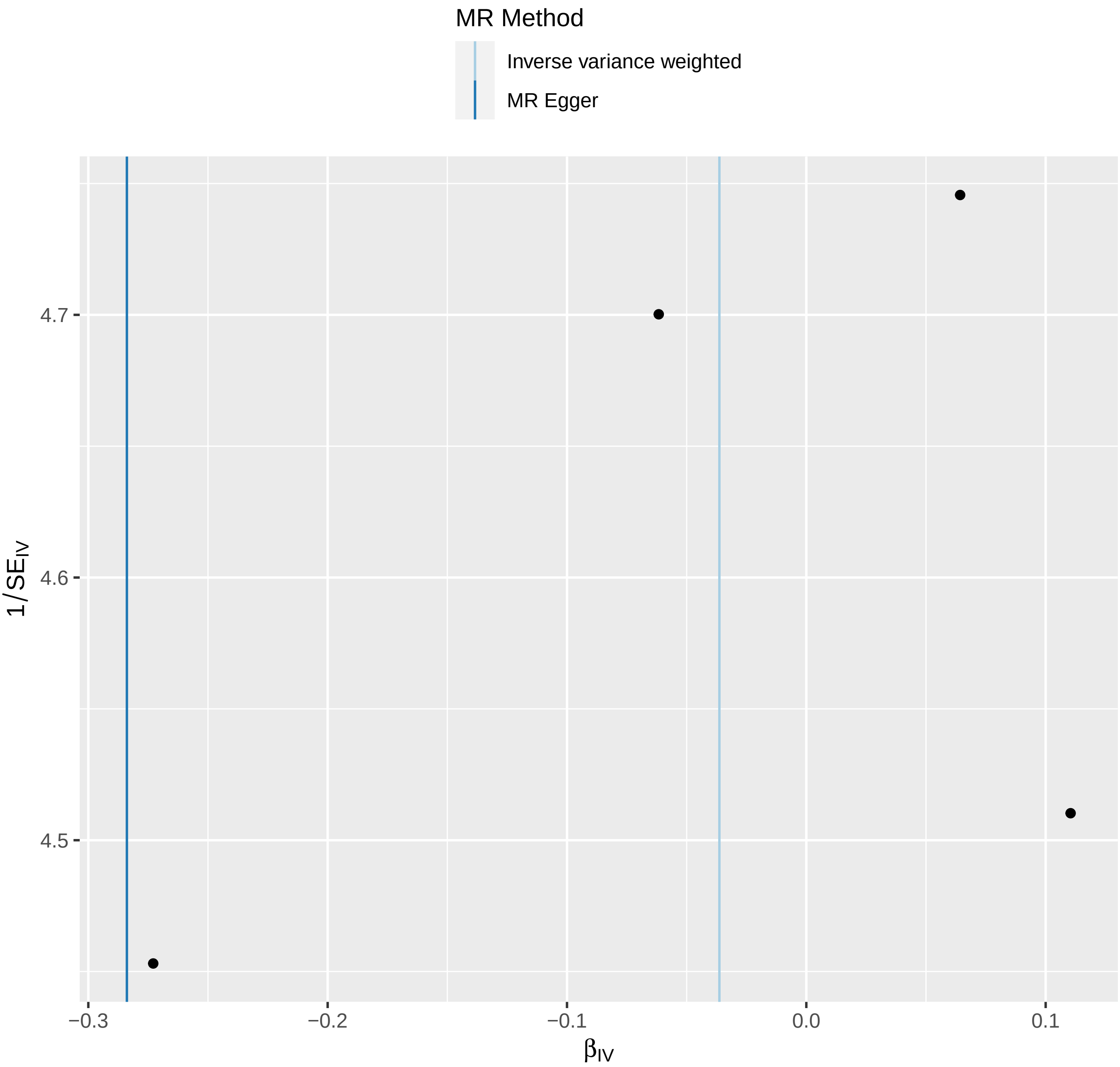

Batch 2−338 : Gut microbiota abundance (genus Parasutterella id.2892) on Beta−arrestin−1
with a threshold of 1e−05

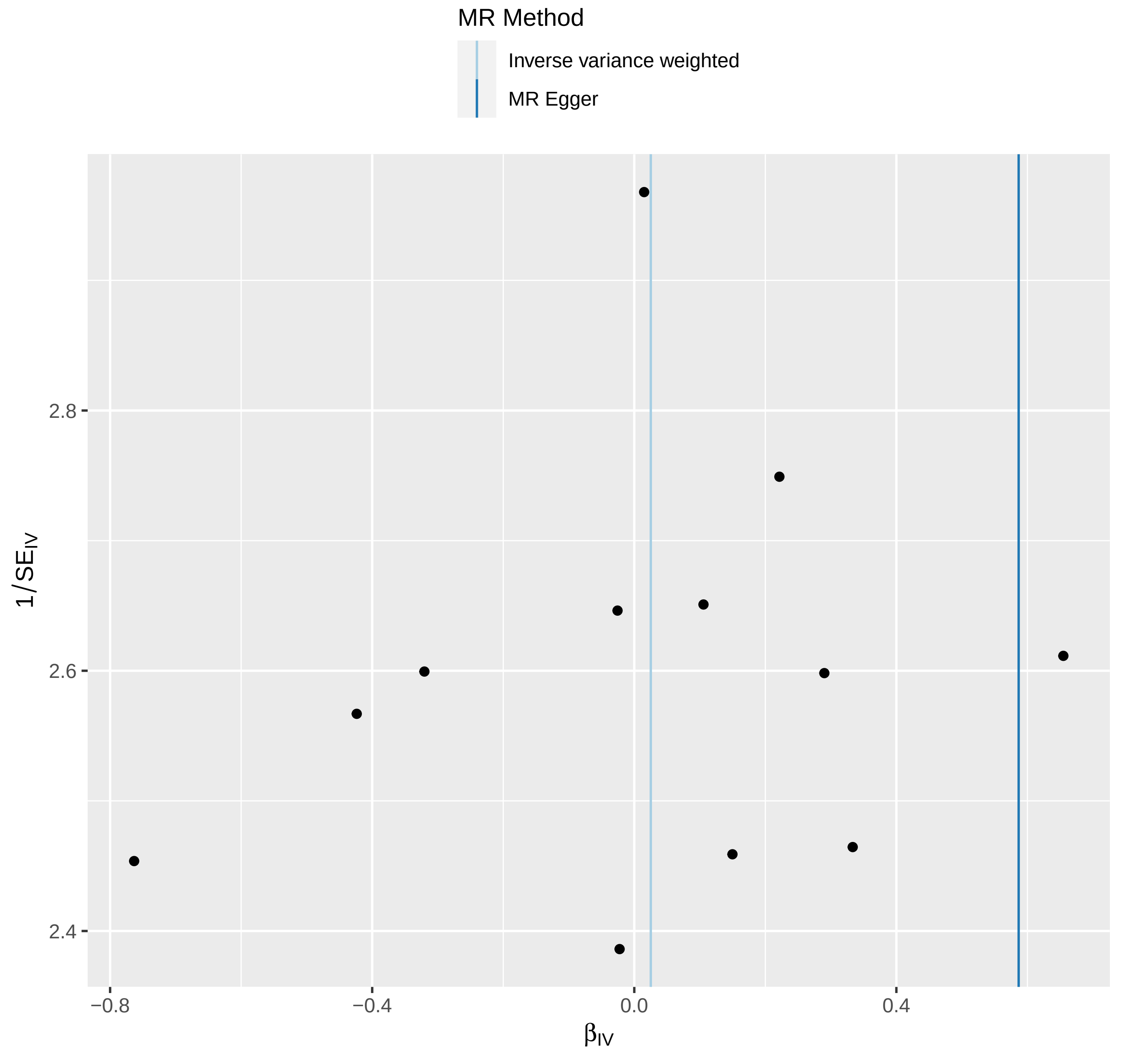

Batch 2−339 : Gut microbiota abundance (genus Catenibacterium id.2153) on Arrestin domain−containing protein 3 with a threshold of 1e−05

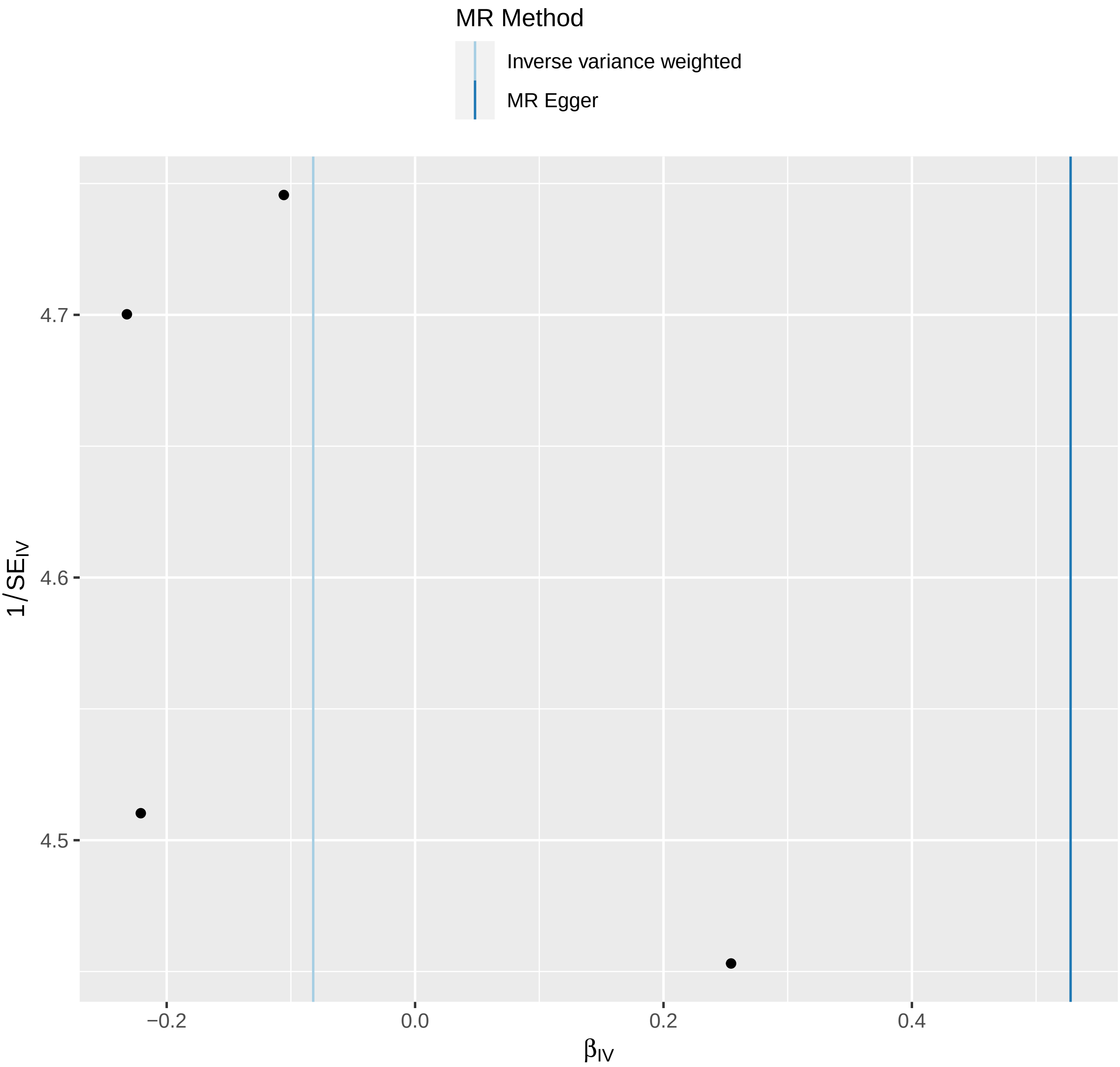

Batch 2−340 : Gut microbiota abundance (genus Parasutterella id.2892) on Arrestin domain−containing protein 3
with a threshold of 1e−05

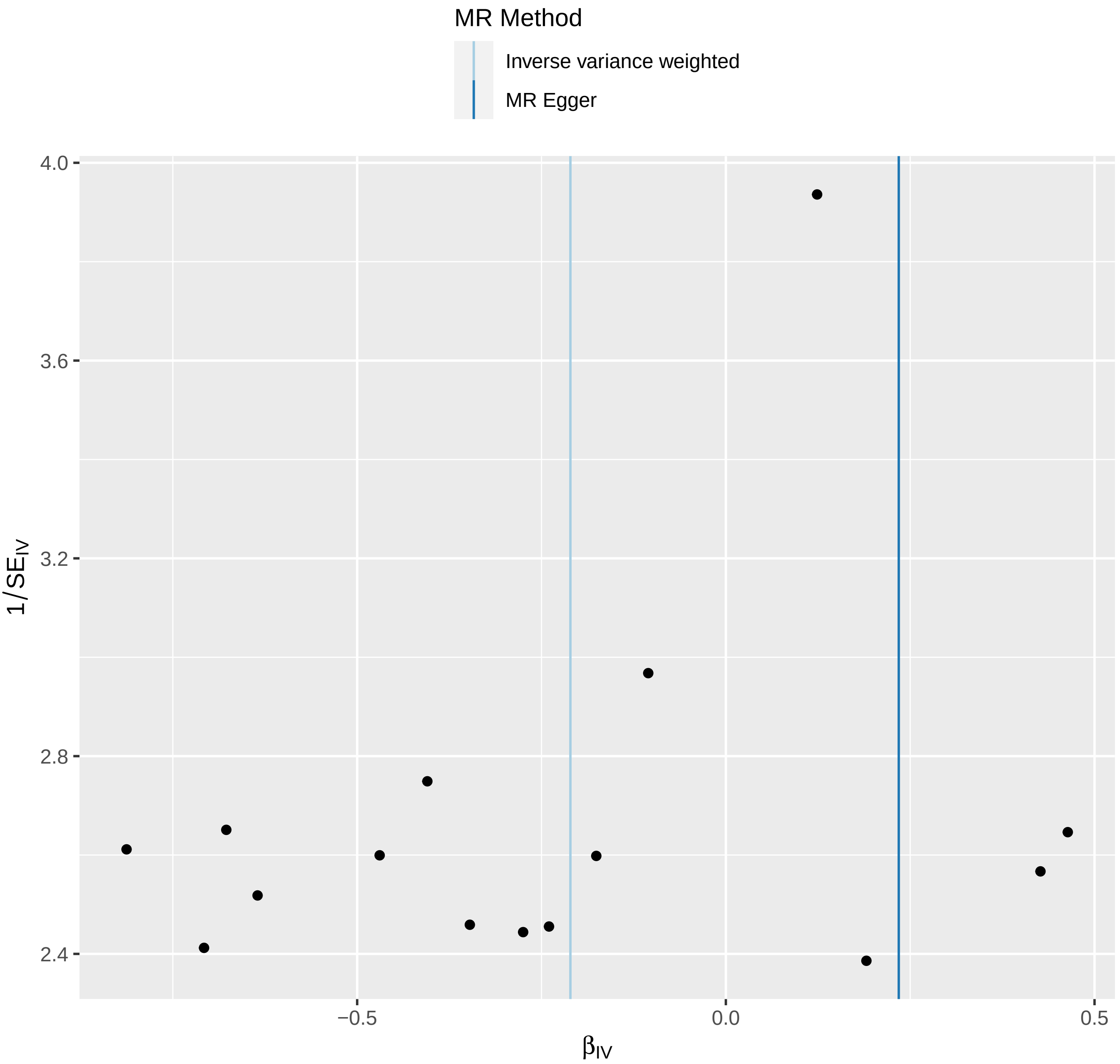

Batch 2−341 : Gut microbiota abundance (genus Catenibacterium id.2153) on Arylsulfatase A
with a threshold of 1e−05

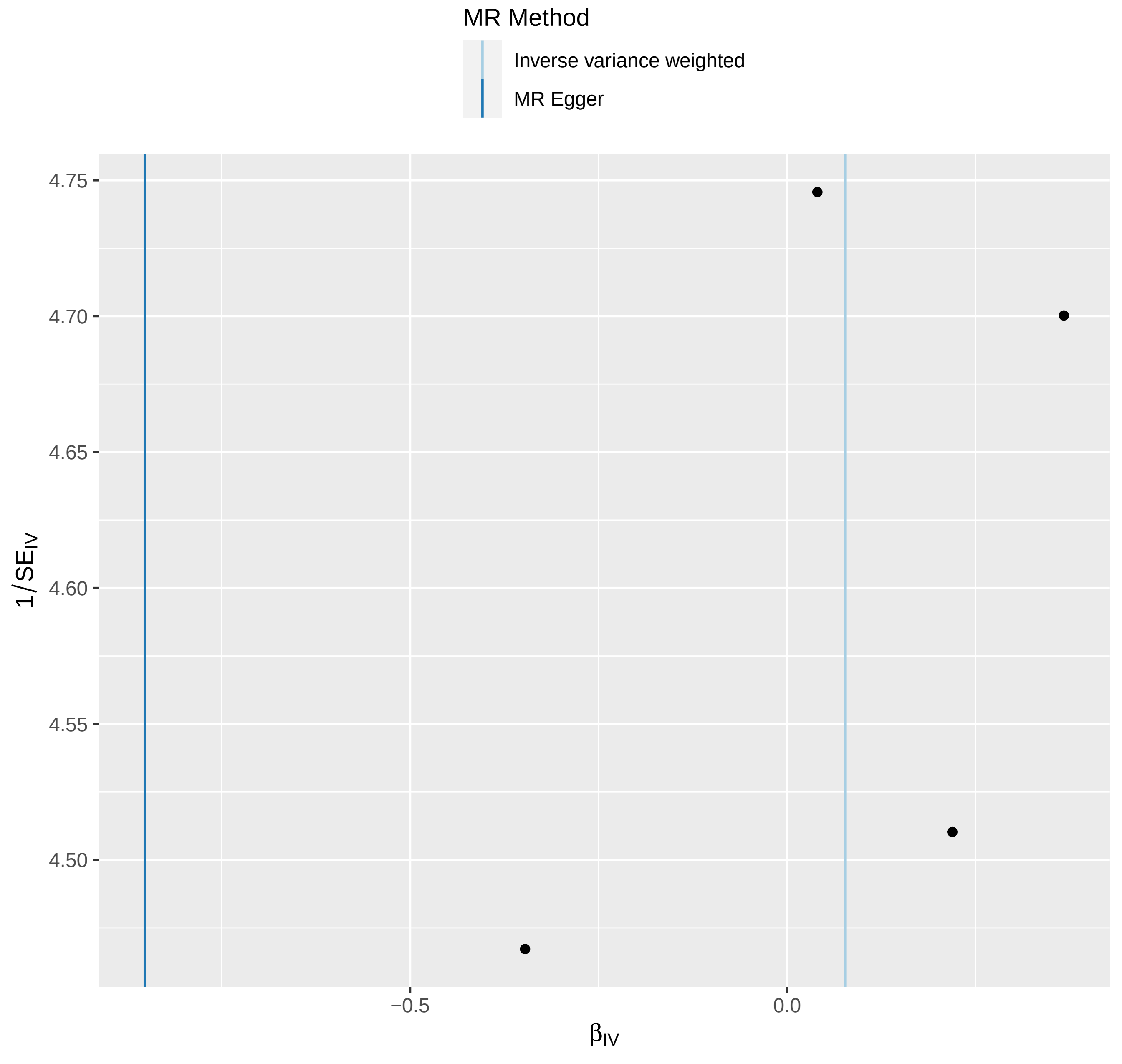

Batch 2−342 : Gut microbiota abundance (genus Parasutterella id.2892) on Arylsulfatase A
with a threshold of 1e−05

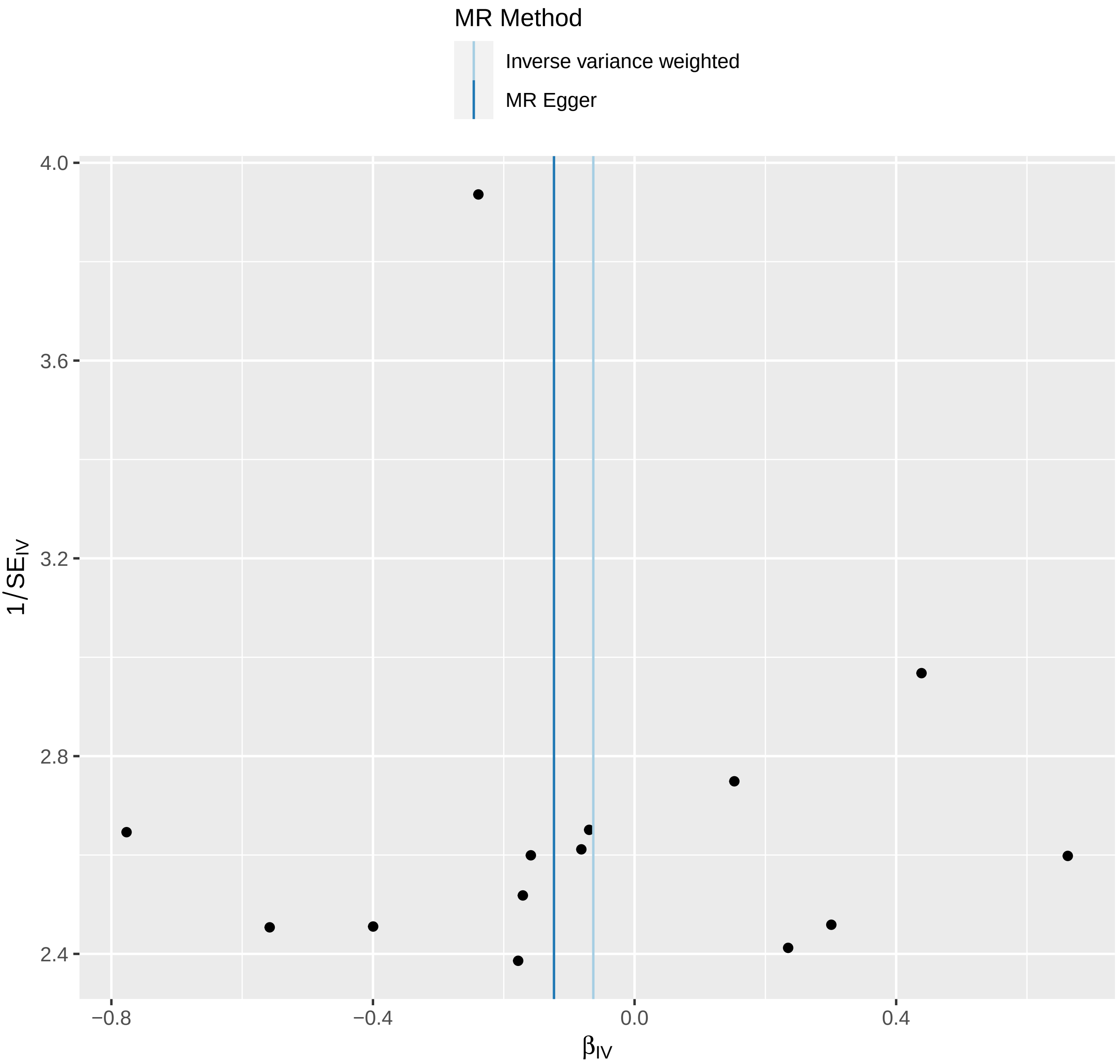

Batch 2−343 : Gut microbiota abundance (genus Catenibacterium id.2153) on Arylsulfatase B
with a threshold of 1e−05

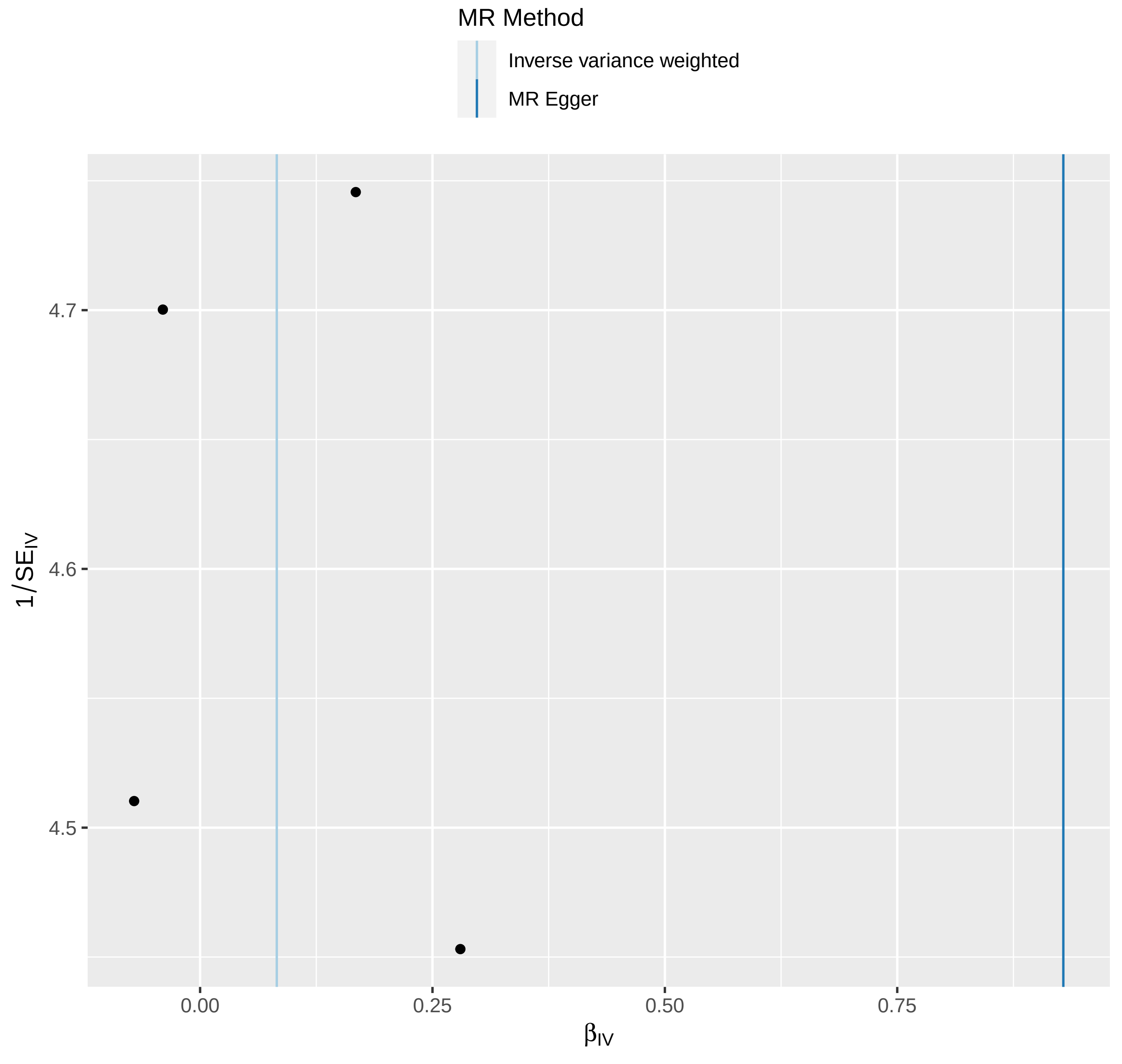

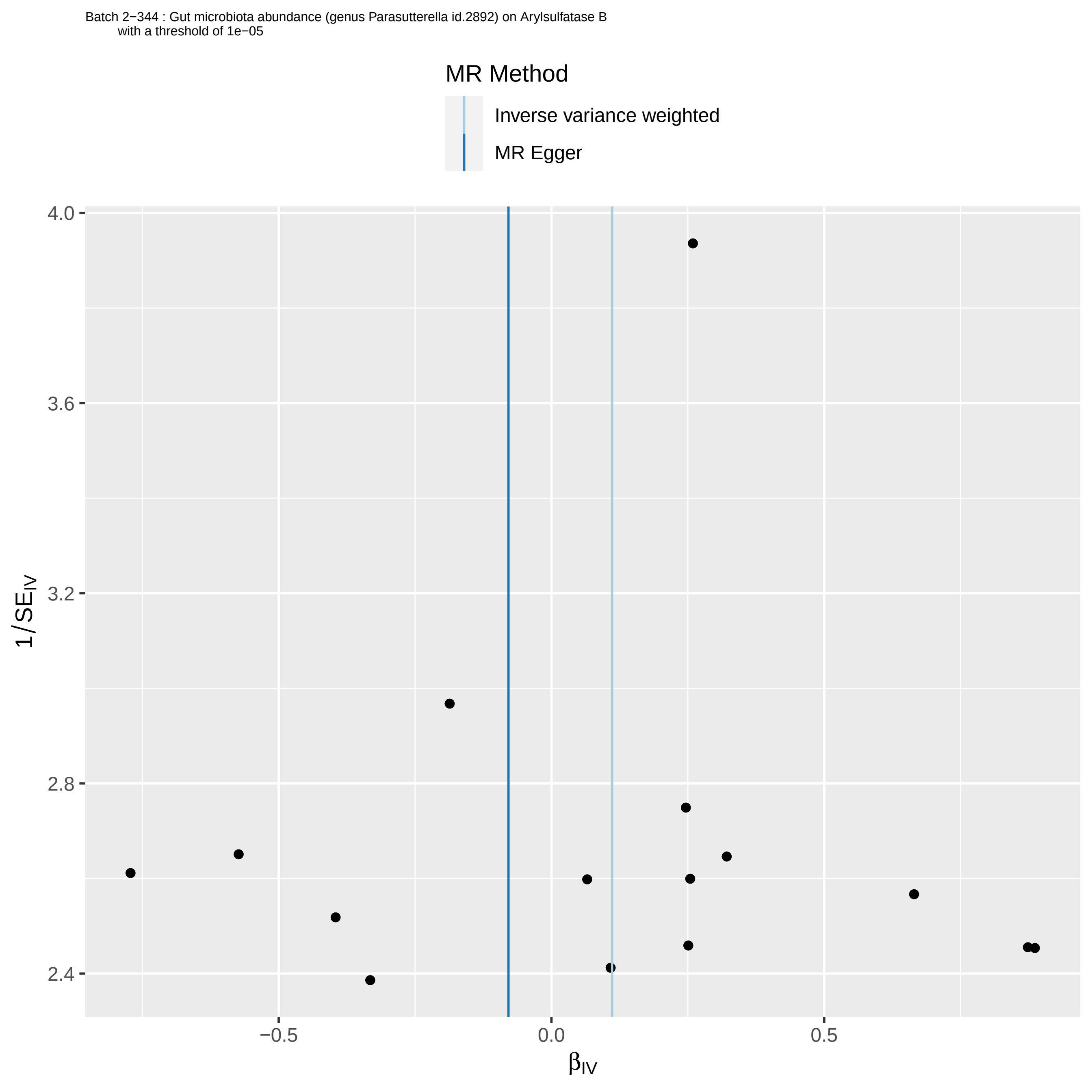

Batch 2−344 : Gut microbiota abundance (genus Parasutterella id.2892) on Arylsulfatase B
with a threshold of 1e−05
MR Method
Inverse variance weighted
MR Egger
4.0
3.6
3.2
2.8
2.4
1/SE_IV
−0.5
0.0
0.5
β_IV

Batch 2−345 : Gut microbiota abundance (genus Catenibacterium id.2153) on Arylsulfatase K
with a threshold of 1e−05

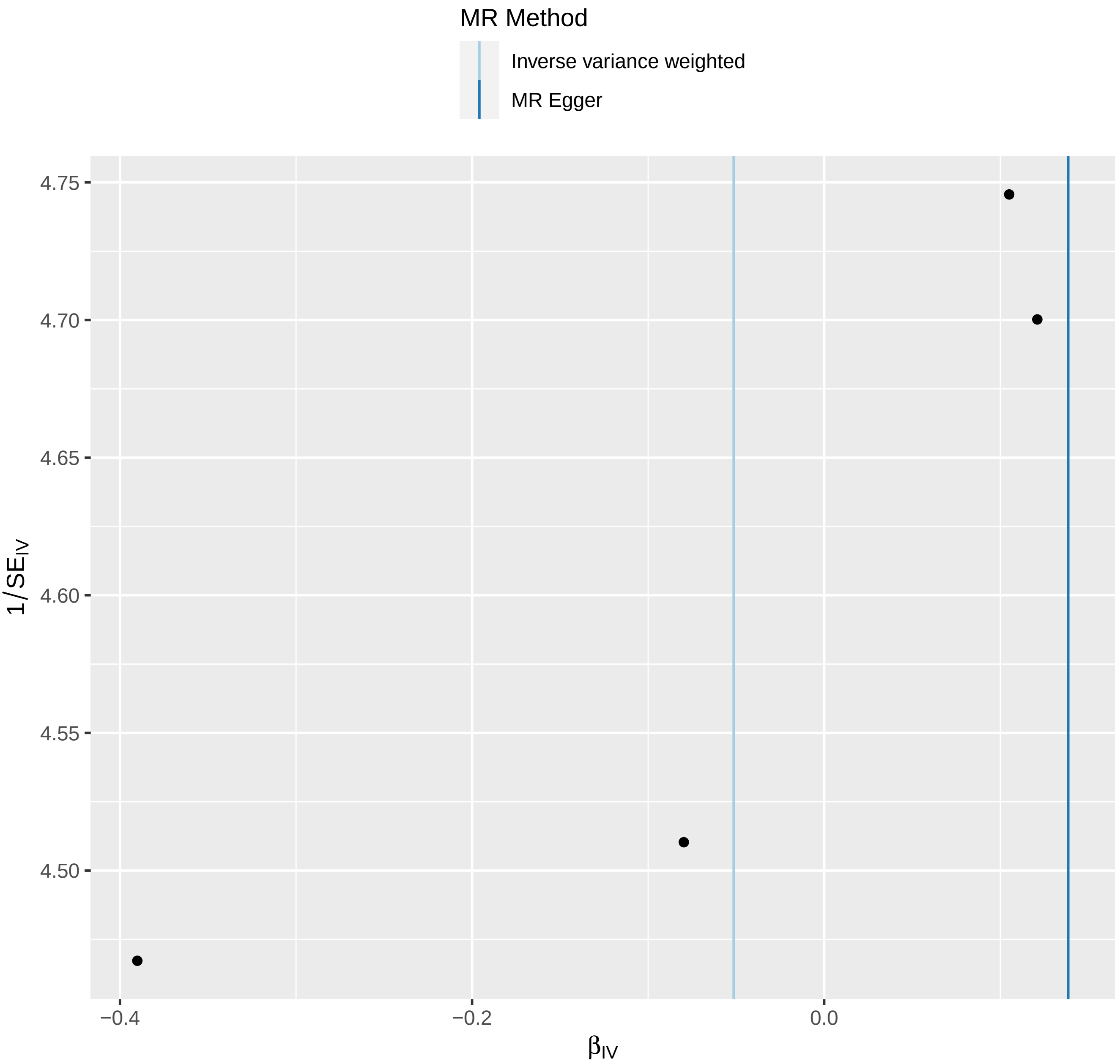

Batch 2−346 : Gut microbiota abundance (genus Parasutterella id.2892) on Arylsulfatase K
with a threshold of 1e−05

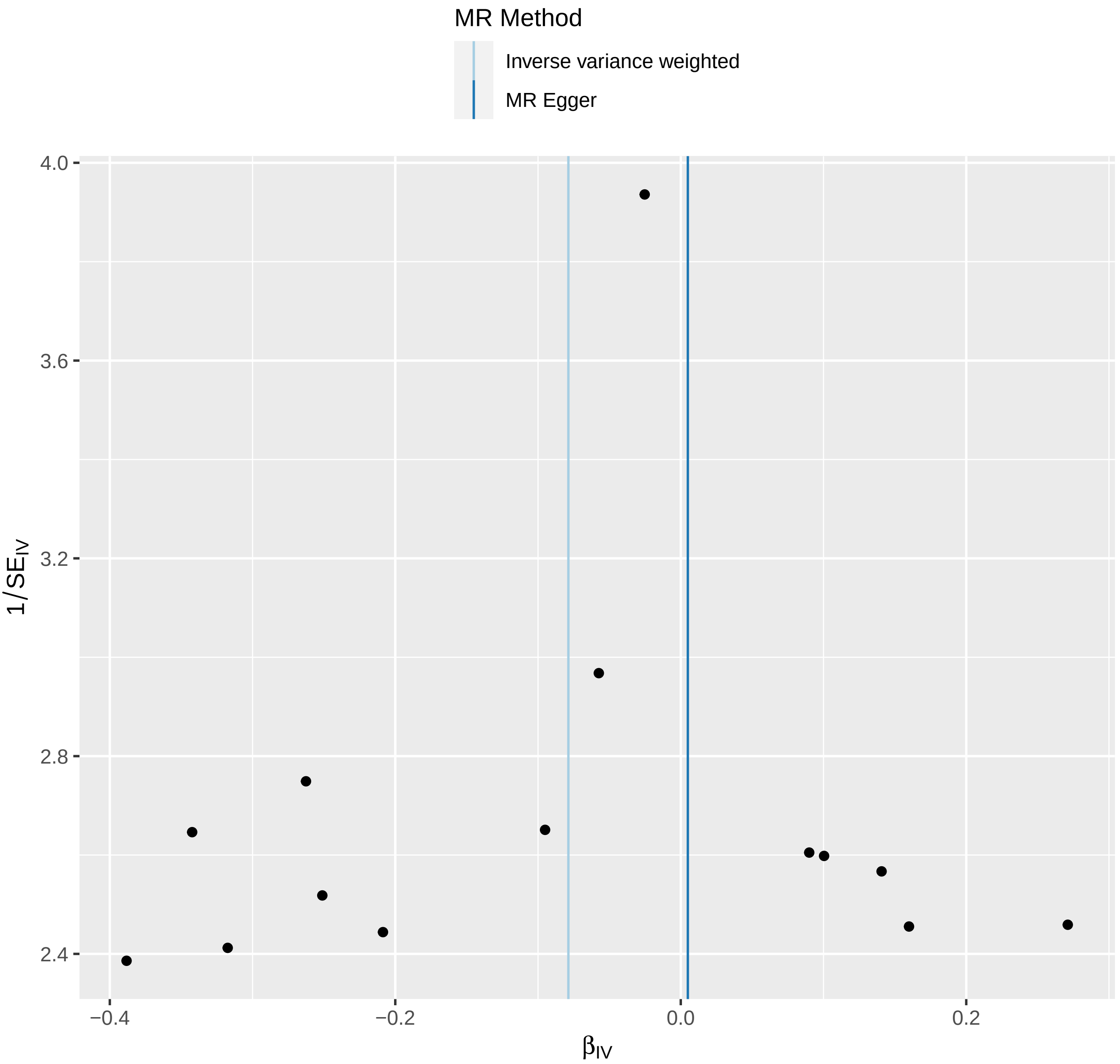

Batch 2−347 : Gut microbiota abundance (genus Catenibacterium id.2153) on Ecto−ADP−ribosyltransferase 3
with a threshold of 1e−05

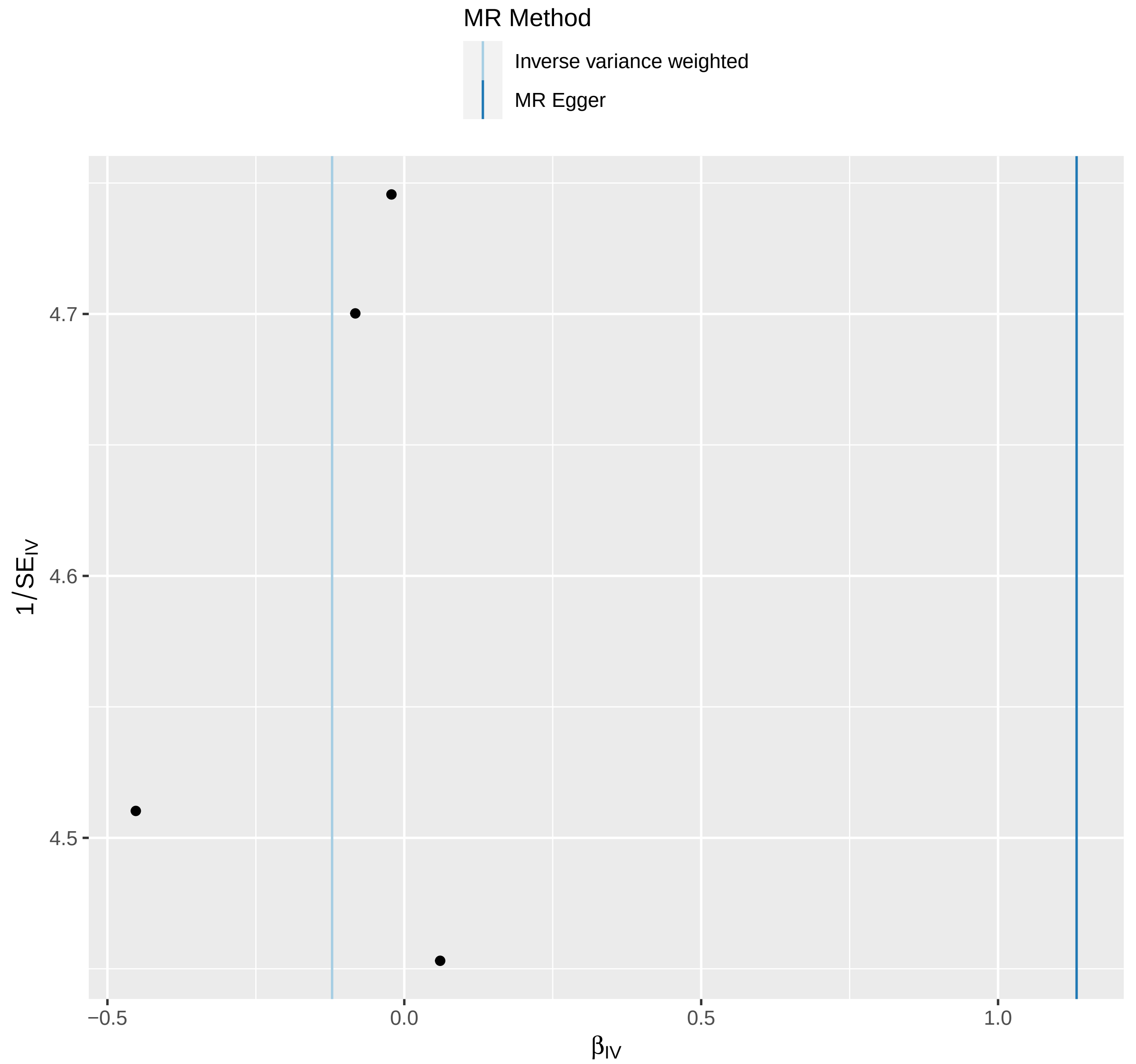

Batch 2−348 : Gut microbiota abundance (genus Parasutterella id.2892) on Ecto−ADP−ribosyltransferase 3
with a threshold of 1e−05

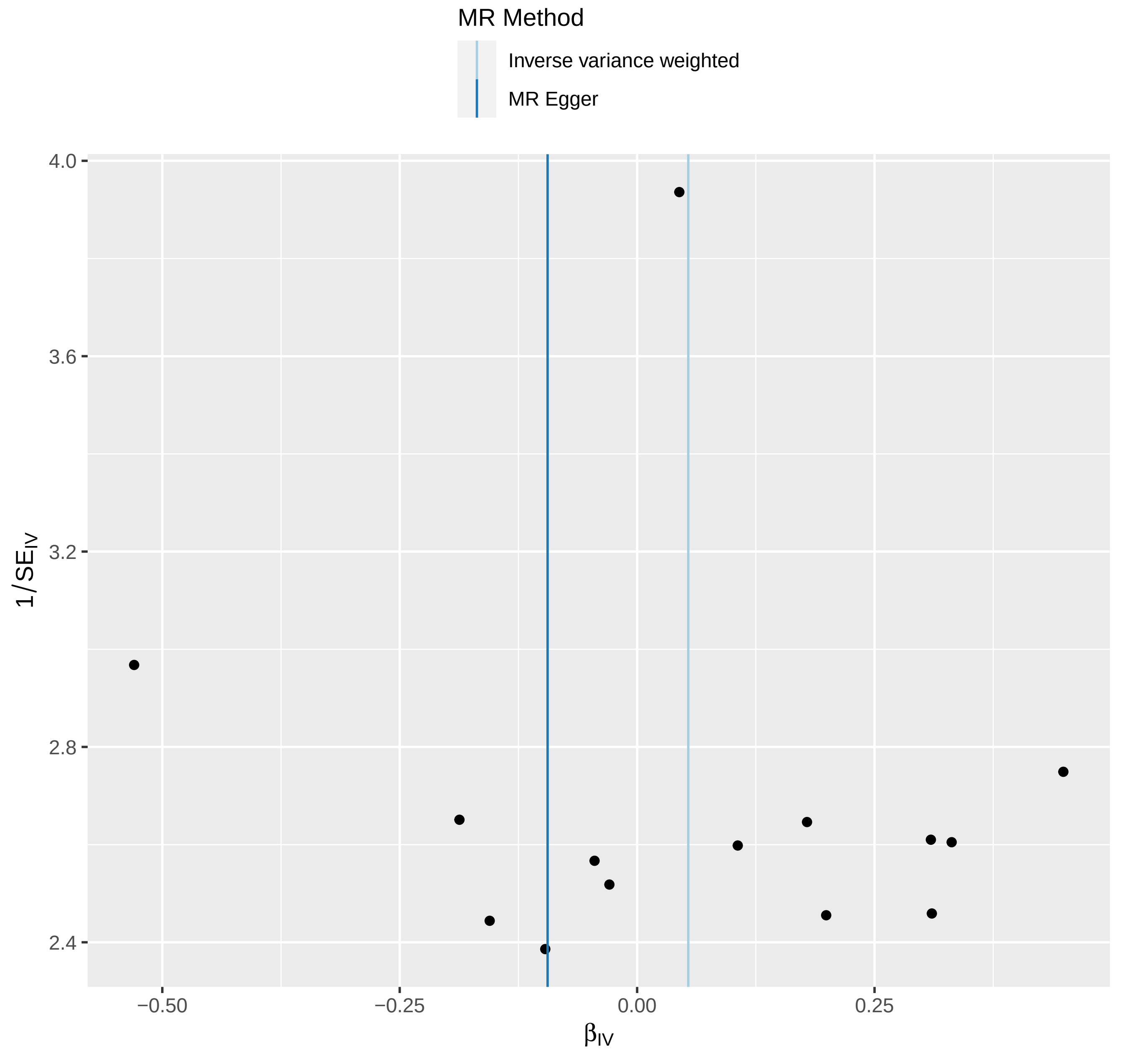

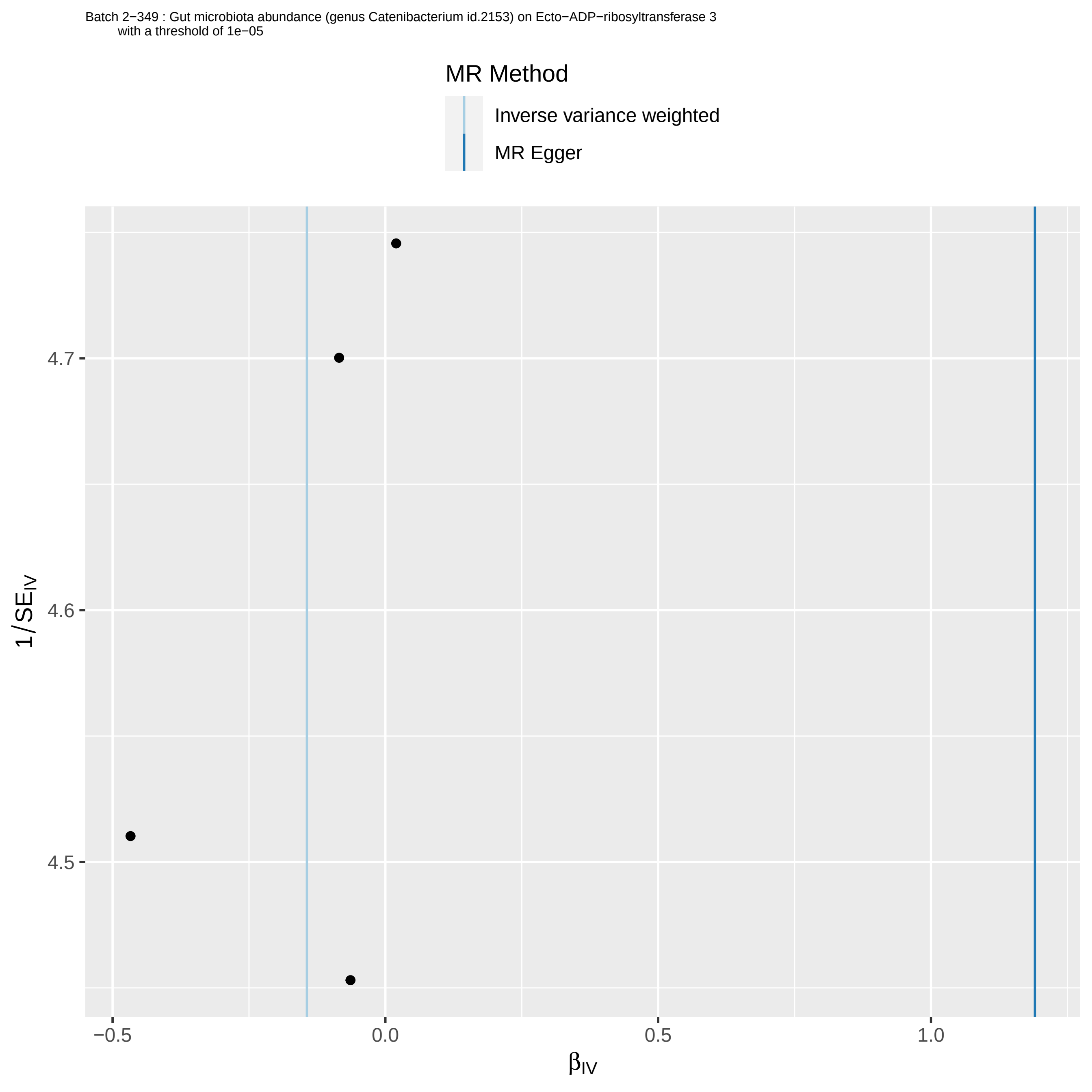

Batch 2−349 : Gut microbiota abundance (genus Catenibacterium id.2153) on Ecto−ADP−ribosyltransferase 3
with a threshold of 1e−05
MR Method
Inverse variance weighted
MR Egger
1/SE$_{IV}$
4.7
4.6
4.5
−0.5
0.0
0.5
1.0
β$_{IV}$

Batch 2−350 : Gut microbiota abundance (genus Parasutterella id.2892) on Ecto−ADP−ribosyltransferase 3
with a threshold of 1e−05

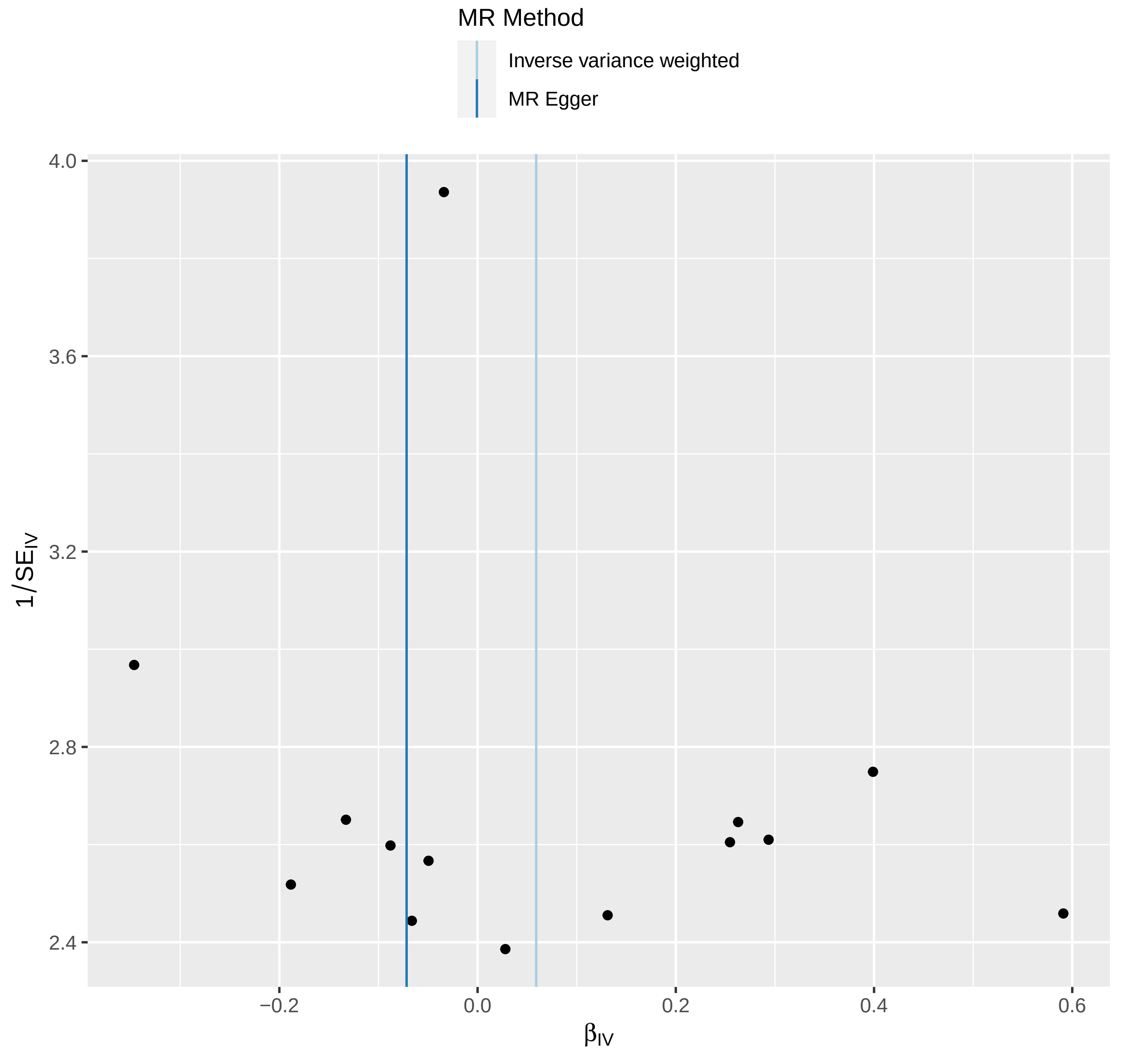

Batch 2−351 : Gut microbiota abundance (genus Catenibacterium id.2153) on Ecto−ADP−ribosyltransferase 4
with a threshold of 1e−05

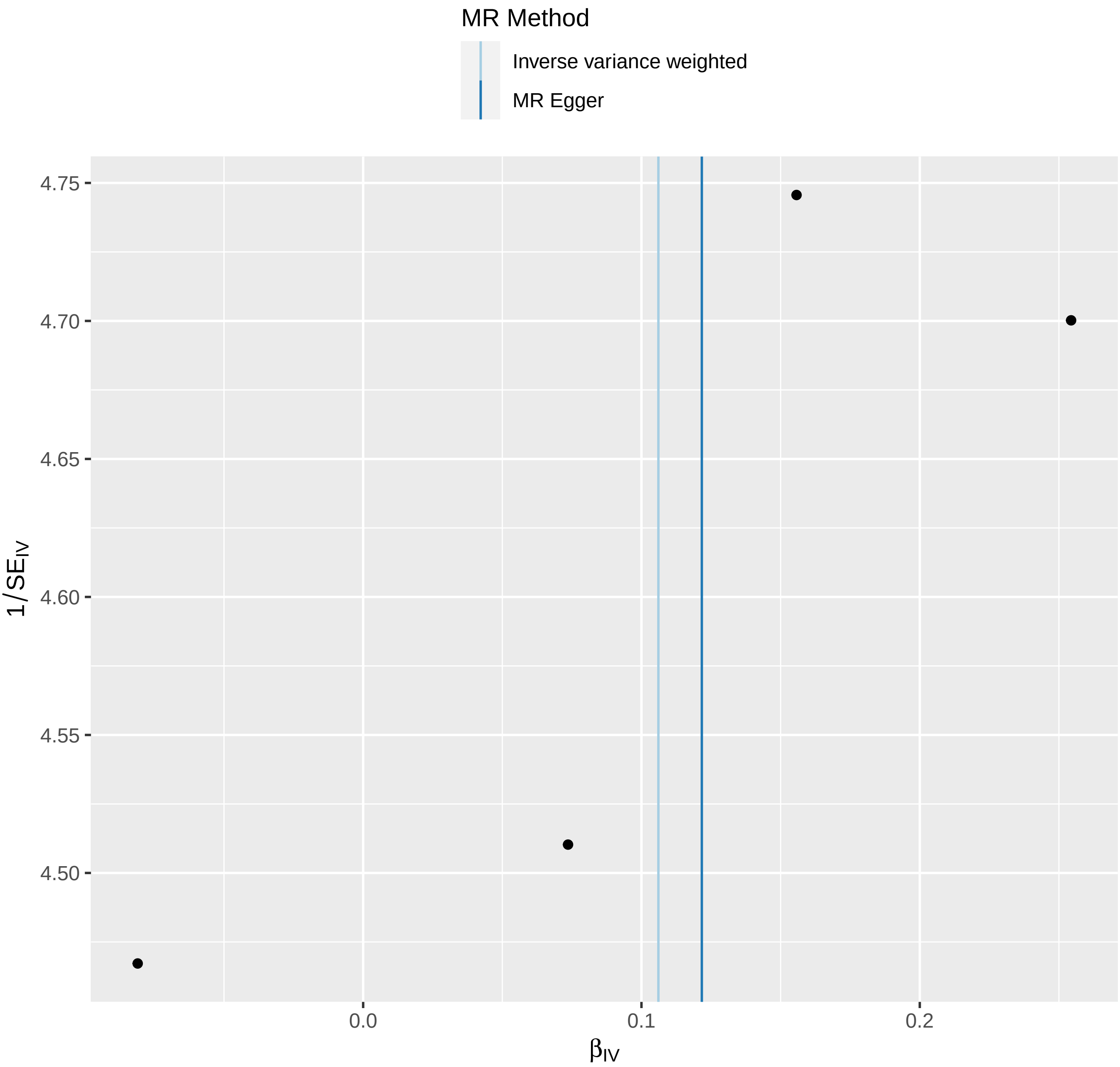

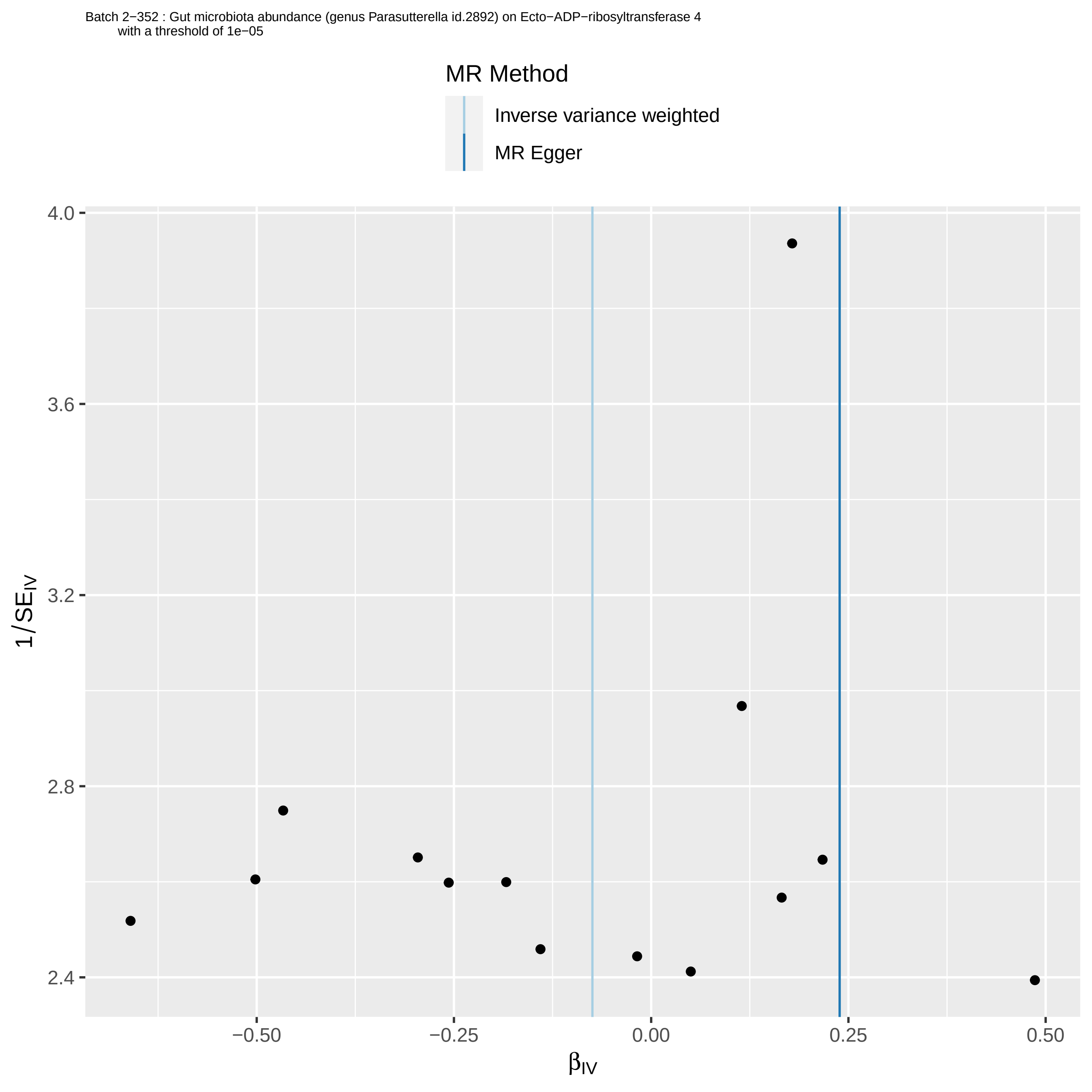

Batch 2−352 : Gut microbiota abundance (genus Parasutterella id.2892) on Ecto−ADP−ribosyltransferase 4
with a threshold of 1e−05
MR Method
Inverse variance weighted
MR Egger
4.0
3.6
3.2
2.8
2.4
1/SE_IV
−0.50
−0.25
0.00
0.25
0.50
β_IV

Batch 2−353 : Gut microbiota abundance (genus Catenibacterium id.2153) on Artemin
with a threshold of 1e−05

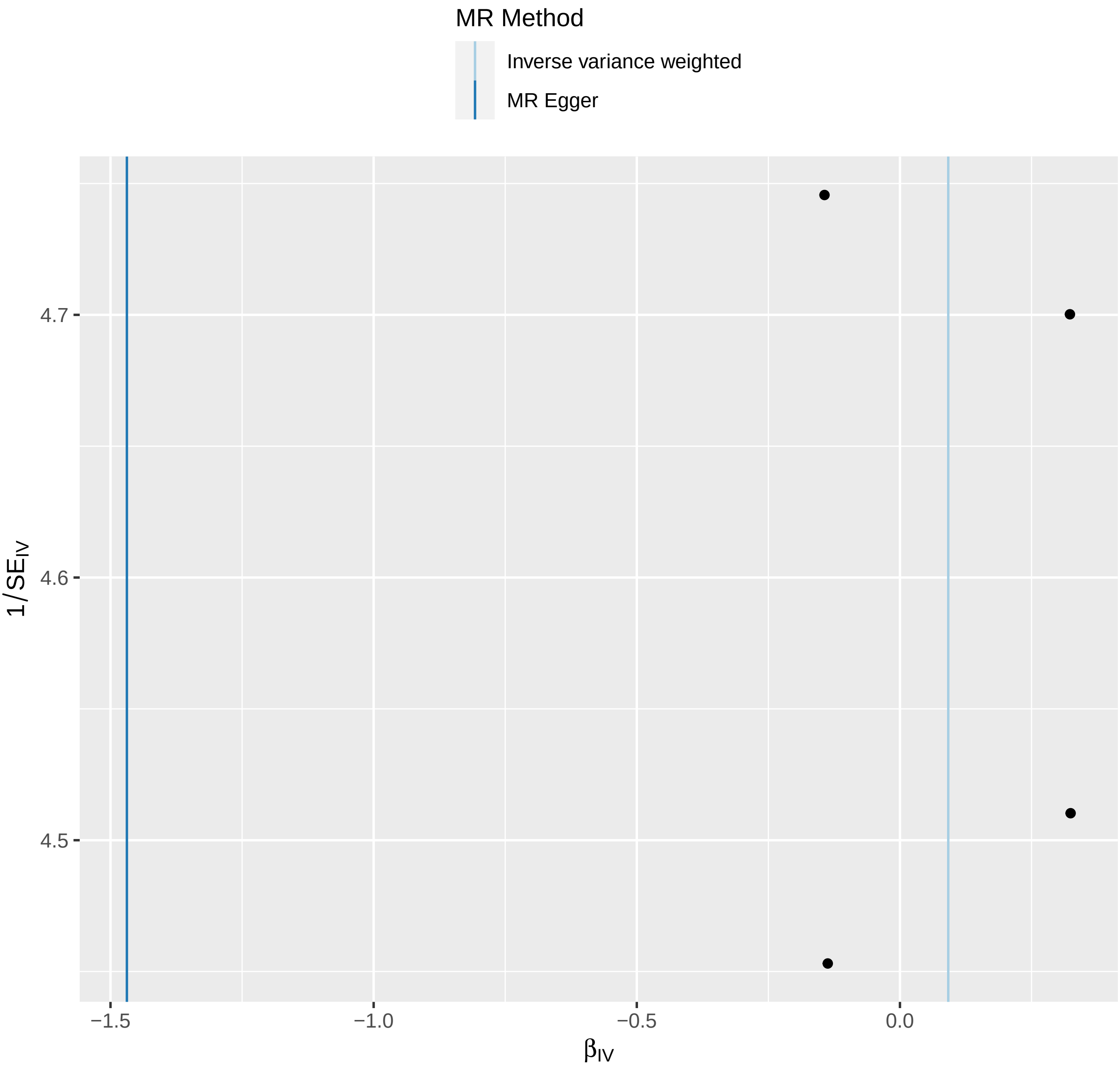

Batch 2−354 : Gut microbiota abundance (genus Parasutterella id.2892) on Artemin
with a threshold of 1e−05

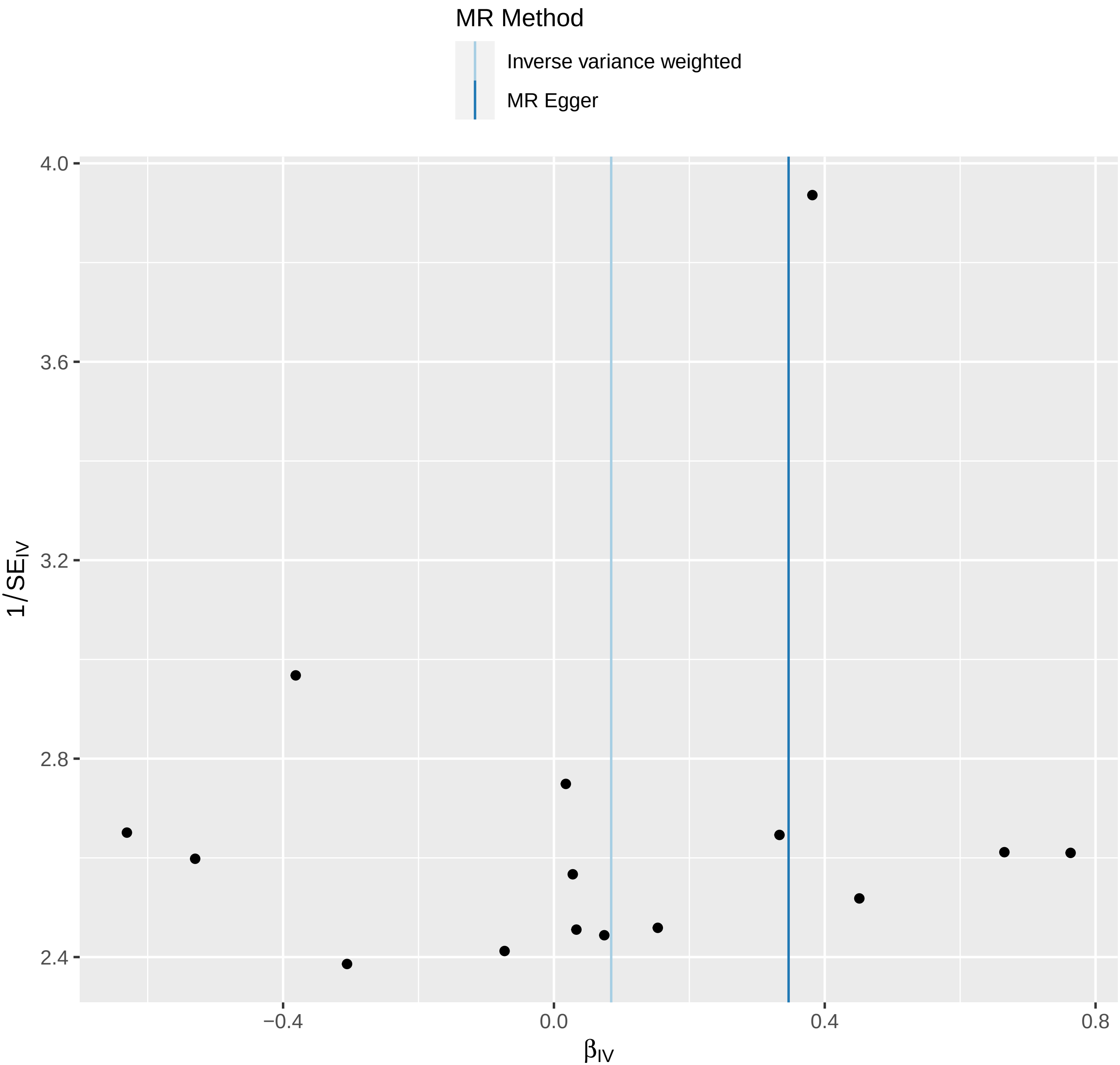

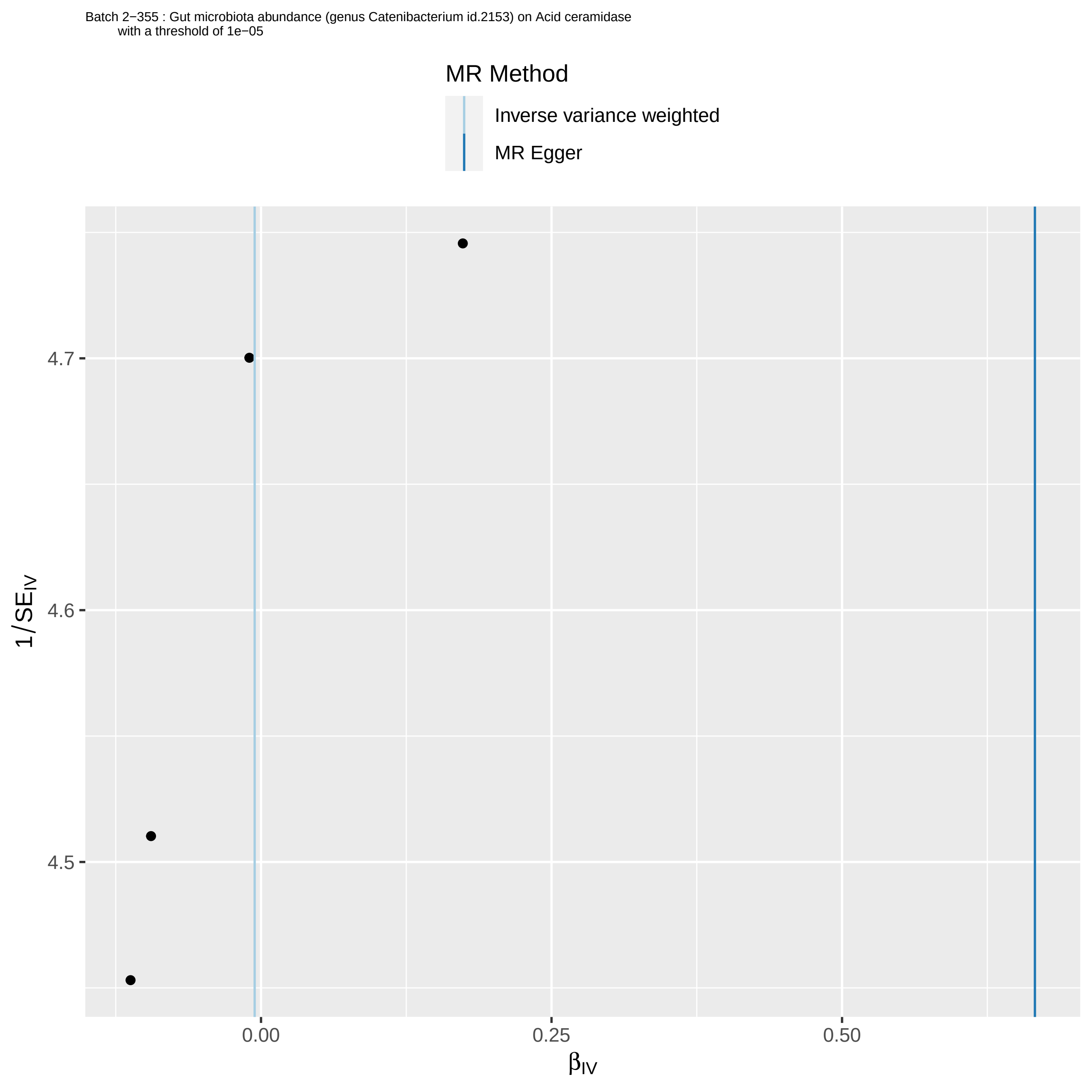
Batch 2−355 : Gut microbiota abundance (genus Catenibacterium id.2153) on Acid ceramidase
with a threshold of 1e−05
MR Method
Inverse variance weighted
MR Egger
4.7
4.6
4.5
$1/SE_{IV}$
0.00
0.25
0.50
β_{IV}

Batch 2−356 : Gut microbiota abundance (genus Parasutterella id.2892) on Acid ceramidase
with a threshold of 1e−05

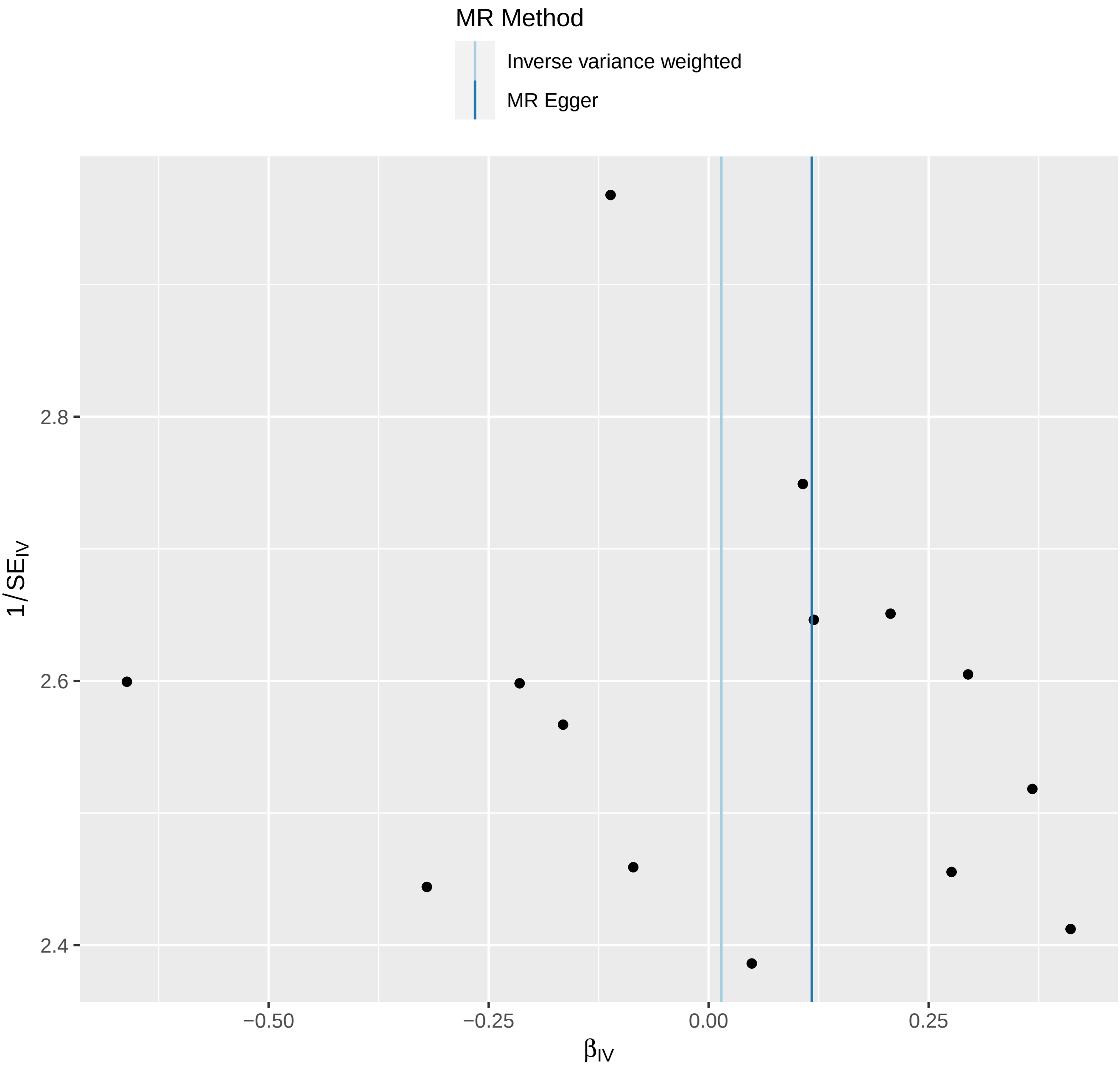

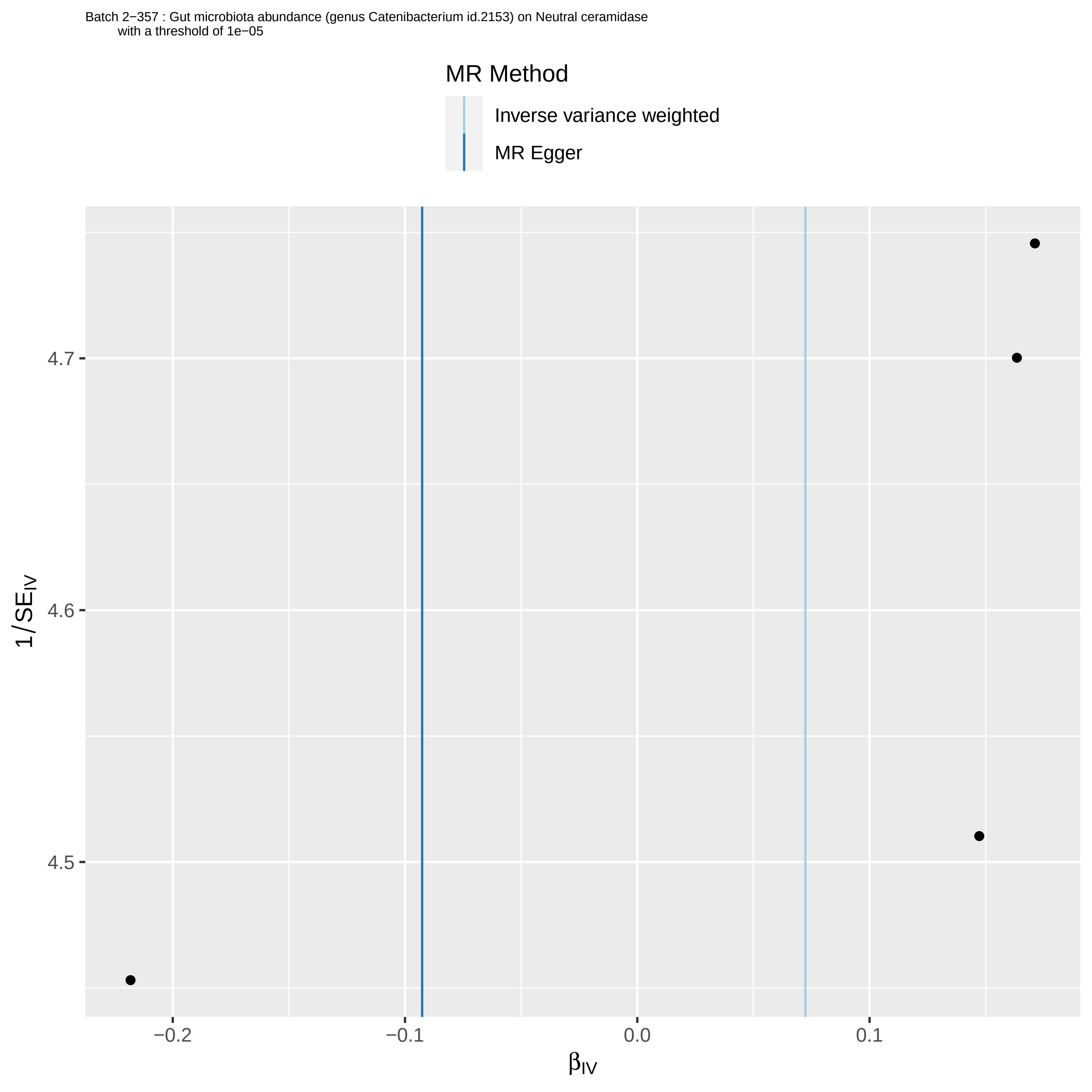
Batch 2−357 : Gut microbiota abundance (genus Catenibacterium id.2153) on Neutral ceramidase
with a threshold of 1e−05
MR Method
Inverse variance weighted
MR Egger
4.7
4.6
4.5
1/SE_IV
−0.2
−0.1
0.0
0.1
β_IV

Batch 2−358 : Gut microbiota abundance (genus Parasutterella id.2892) on Neutral ceramidase
with a threshold of 1e−05

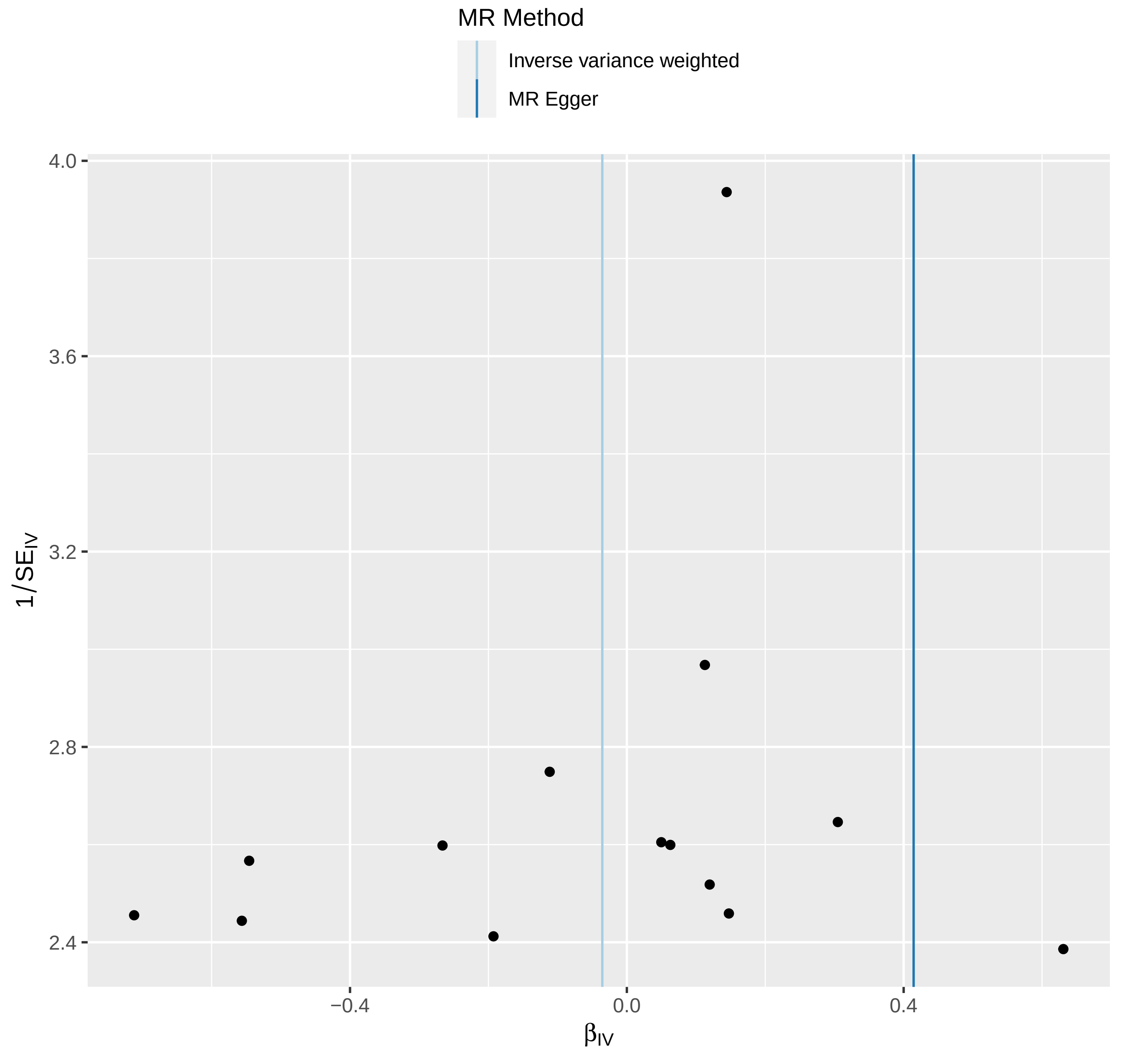

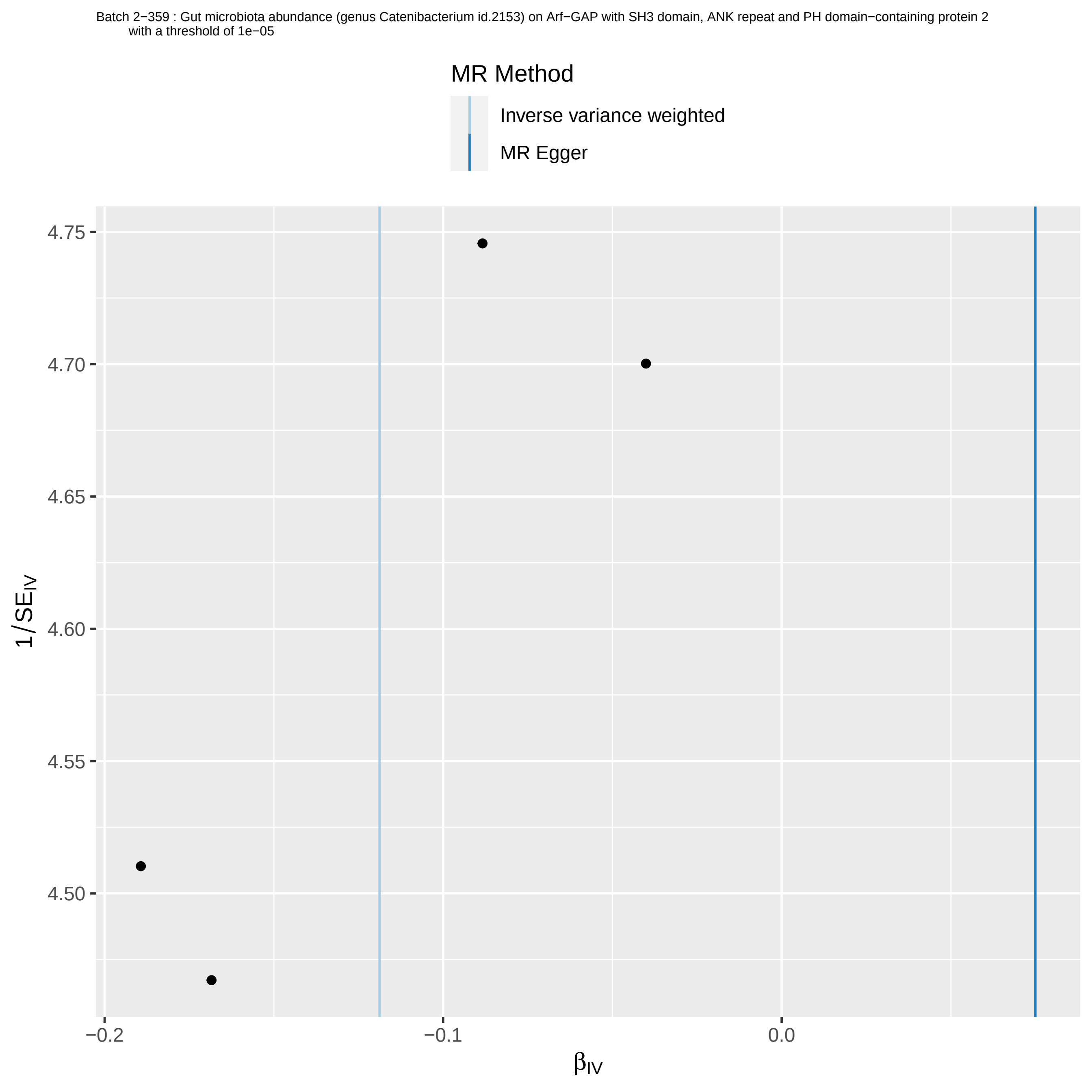
Batch 2−359 : Gut microbiota abundance (genus Catenibacterium id.2153) on Arf−GAP with SH3 domain, ANK repeat and PH domain−containing protein 2 with a threshold of 1e−05
MR Method
Inverse variance weighted
MR Egger
4.75
4.70
4.65
4.60
4.55
4.50
1/SE_IV
−0.2
−0.1
0.0
β_IV

Batch 2−360 : Gut microbiota abundance (genus Parasutterella id.2892) on Arf−GAP with SH3 domain, ANK repeat and PH domain−containing protein 2 with a threshold of 1e−05

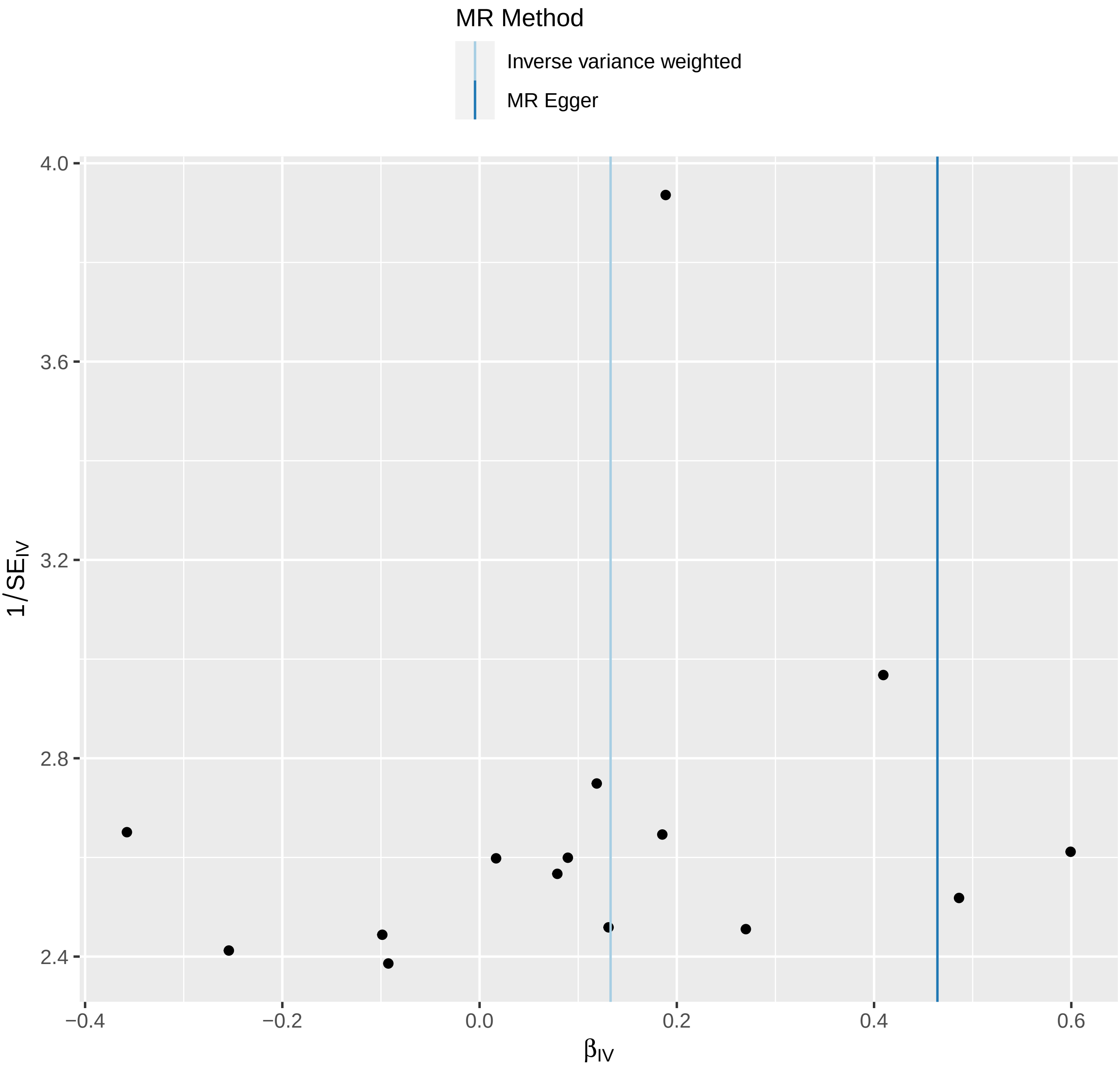

Batch 2−361 : Gut microbiota abundance (genus Catenibacterium id.2153) on Activating signal cointegrator 1 complex subunit 1 with a threshold of 1e−05

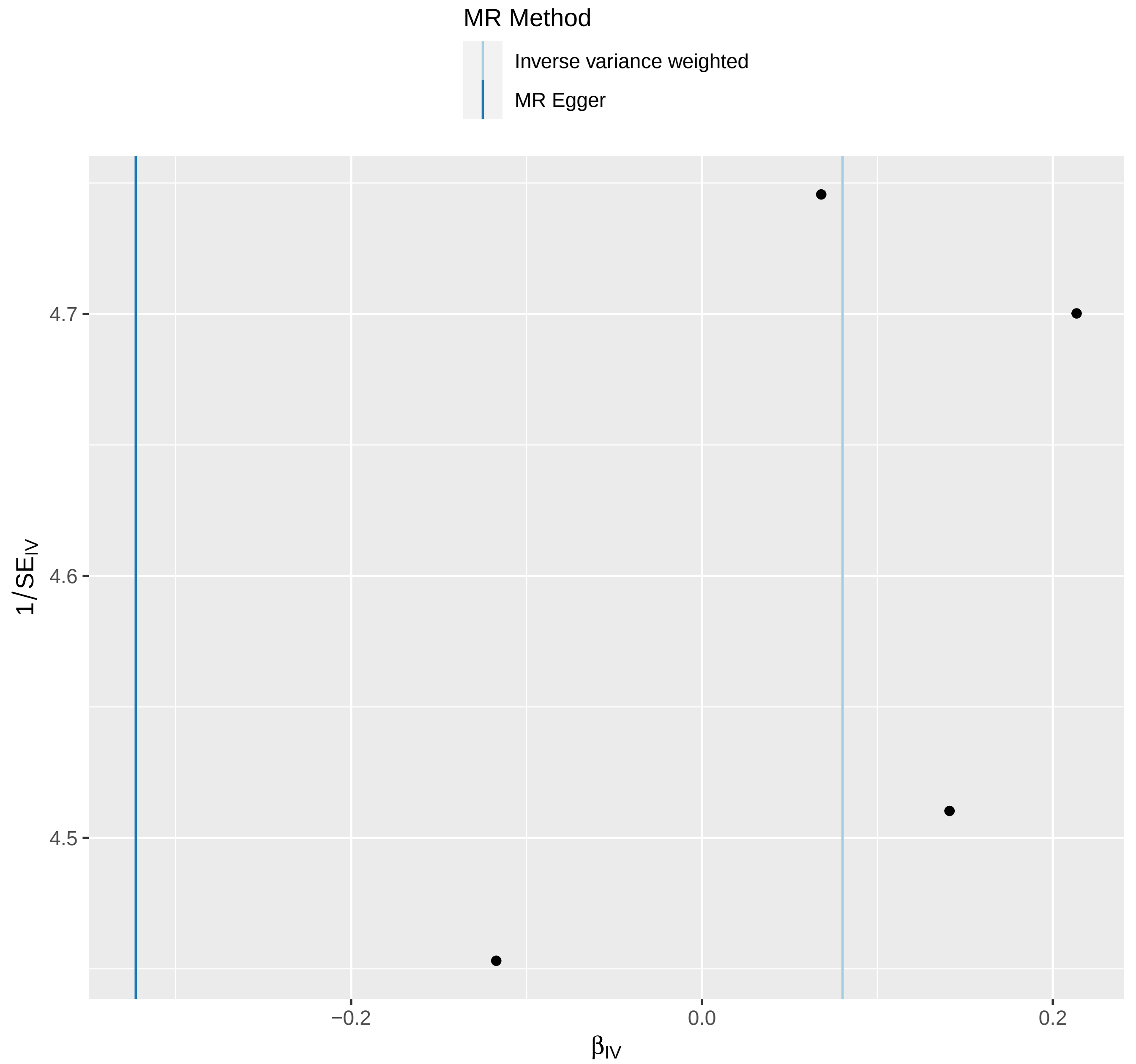

Batch 2−362 : Gut microbiota abundance (genus Parasutterella id.2892) on Activating signal cointegrator 1 complex subunit 1 with a threshold of 1e−05

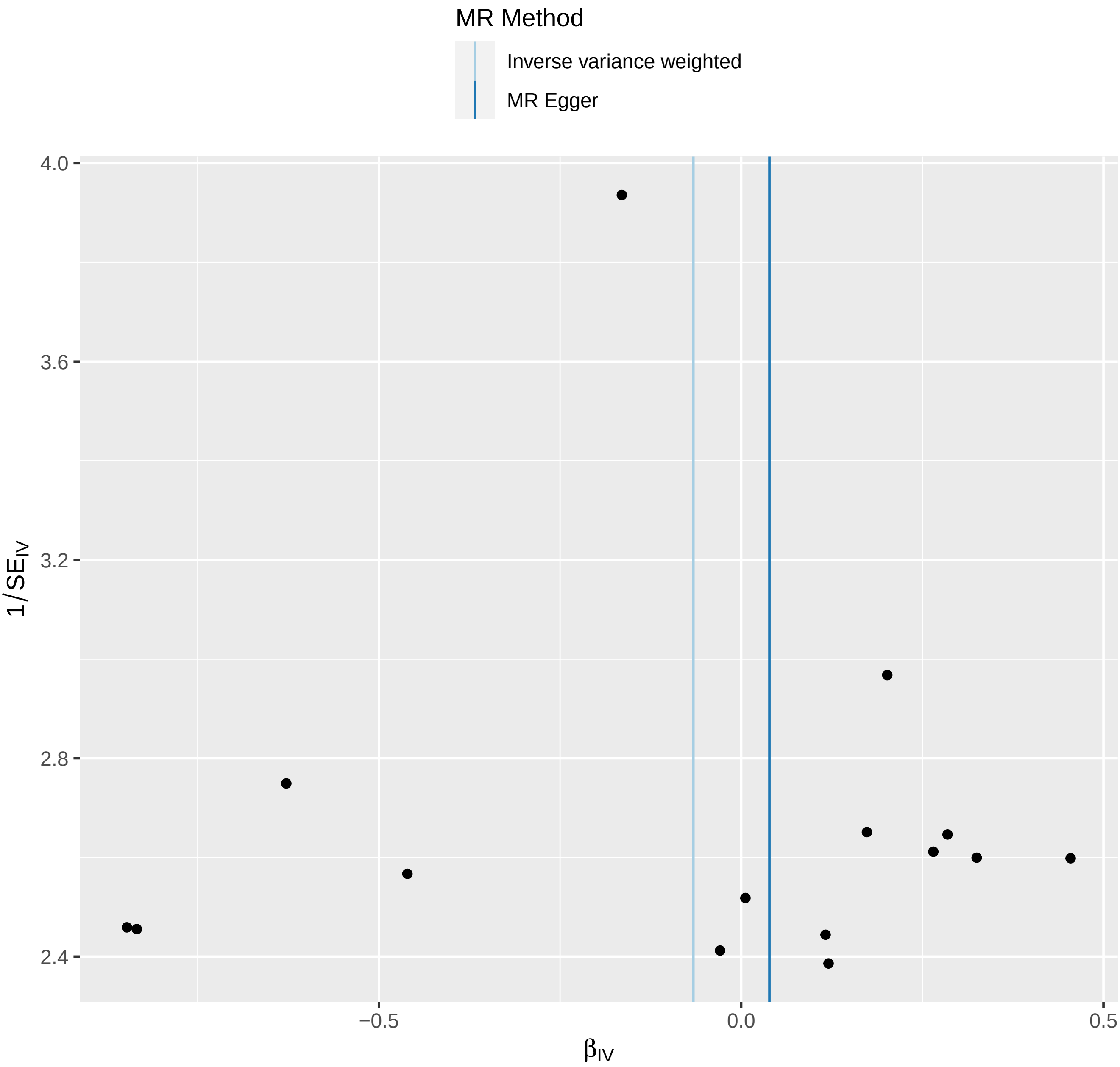

Batch 2−363 : Gut microbiota abundance (genus Catenibacterium id.2153) on Asialoglycoprotein receptor 1
with a threshold of 1e−05

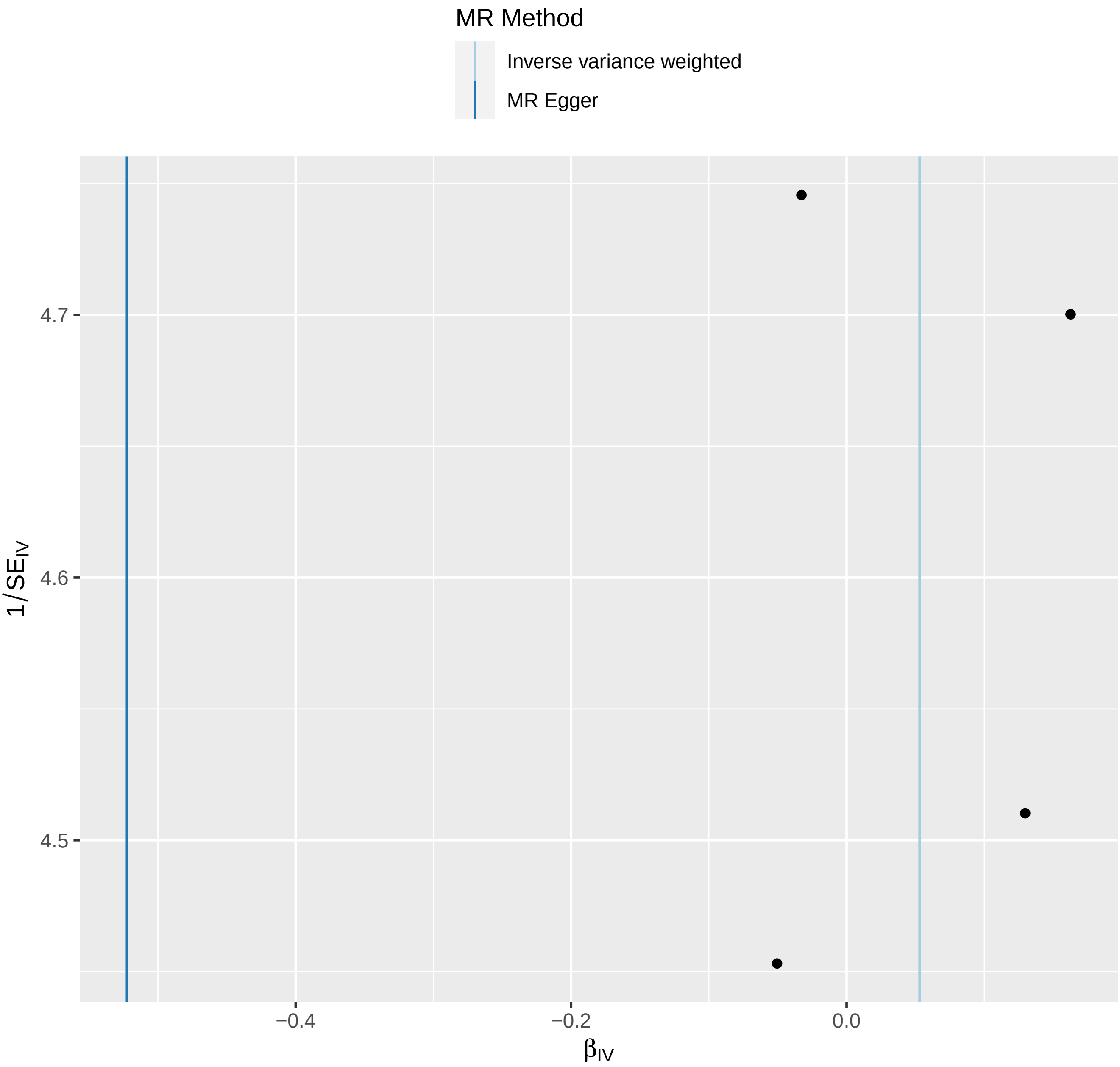

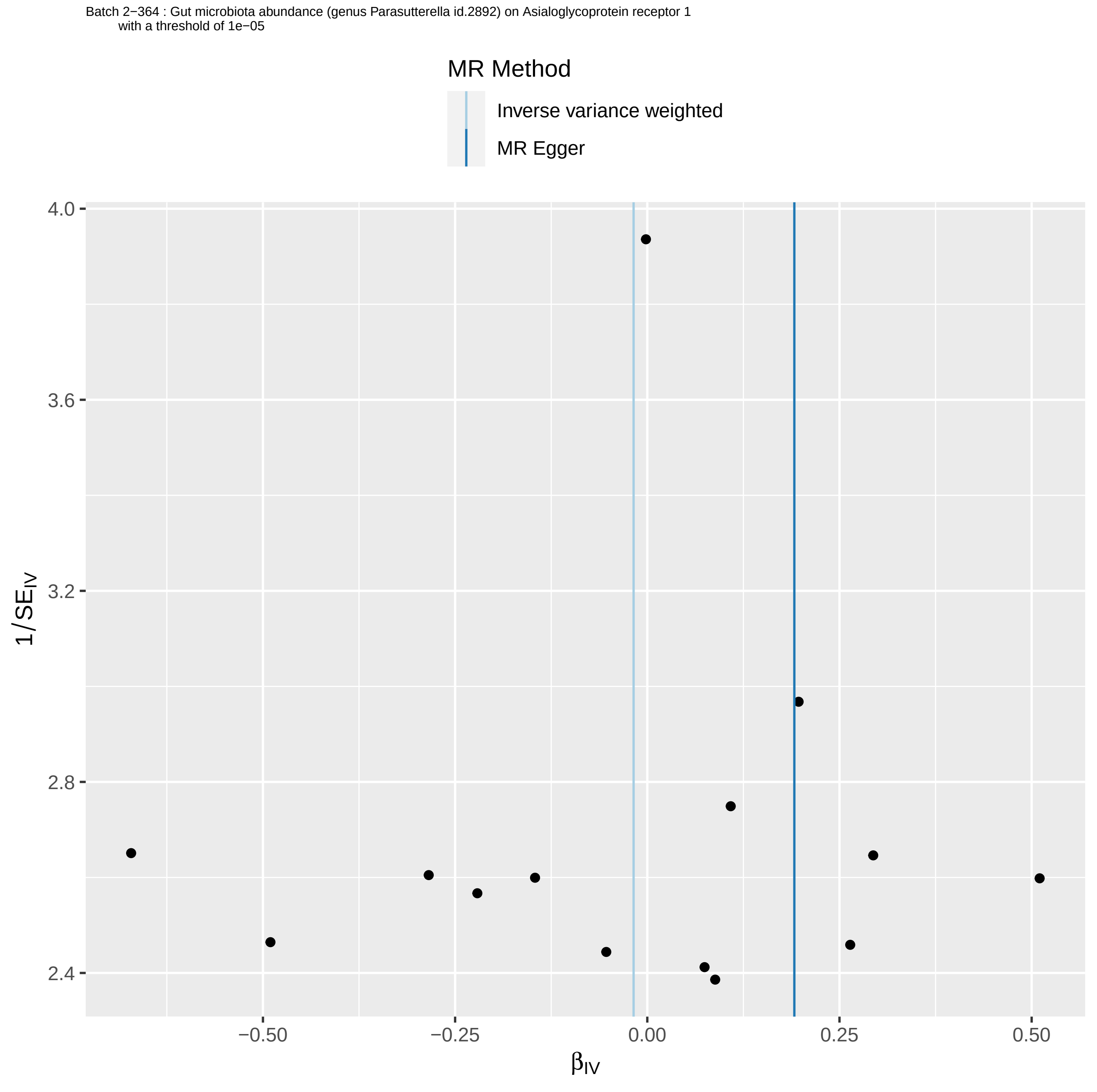

Batch 2−364 : Gut microbiota abundance (genus Parasutterella id.2892) on Asialoglycoprotein receptor 1
with a threshold of 1e−05
MR Method
Inverse variance weighted
MR Egger
4.0
3.6
3.2
2.8
2.4
1/SE_IV
−0.50
−0.25
0.00
0.25
0.50
β_IV

Batch 2−365 : Gut microbiota abundance (genus Catenibacterium id.2153) on Asialoglycoprotein receptor 2
with a threshold of 1e−05

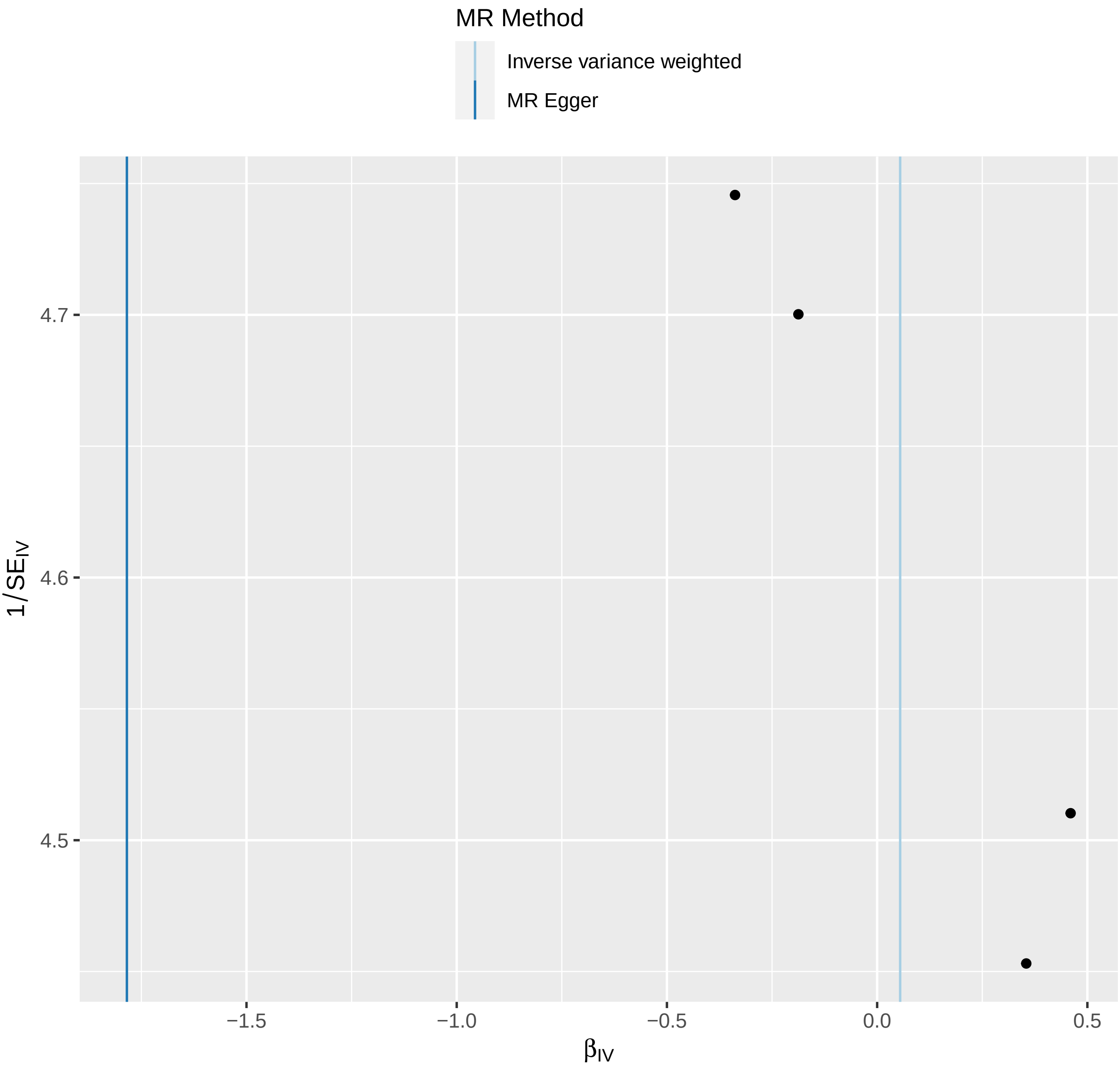

Batch 2−366 : Gut microbiota abundance (genus Parasutterella id.2892) on Asialoglycoprotein receptor 2 with a threshold of 1e−05

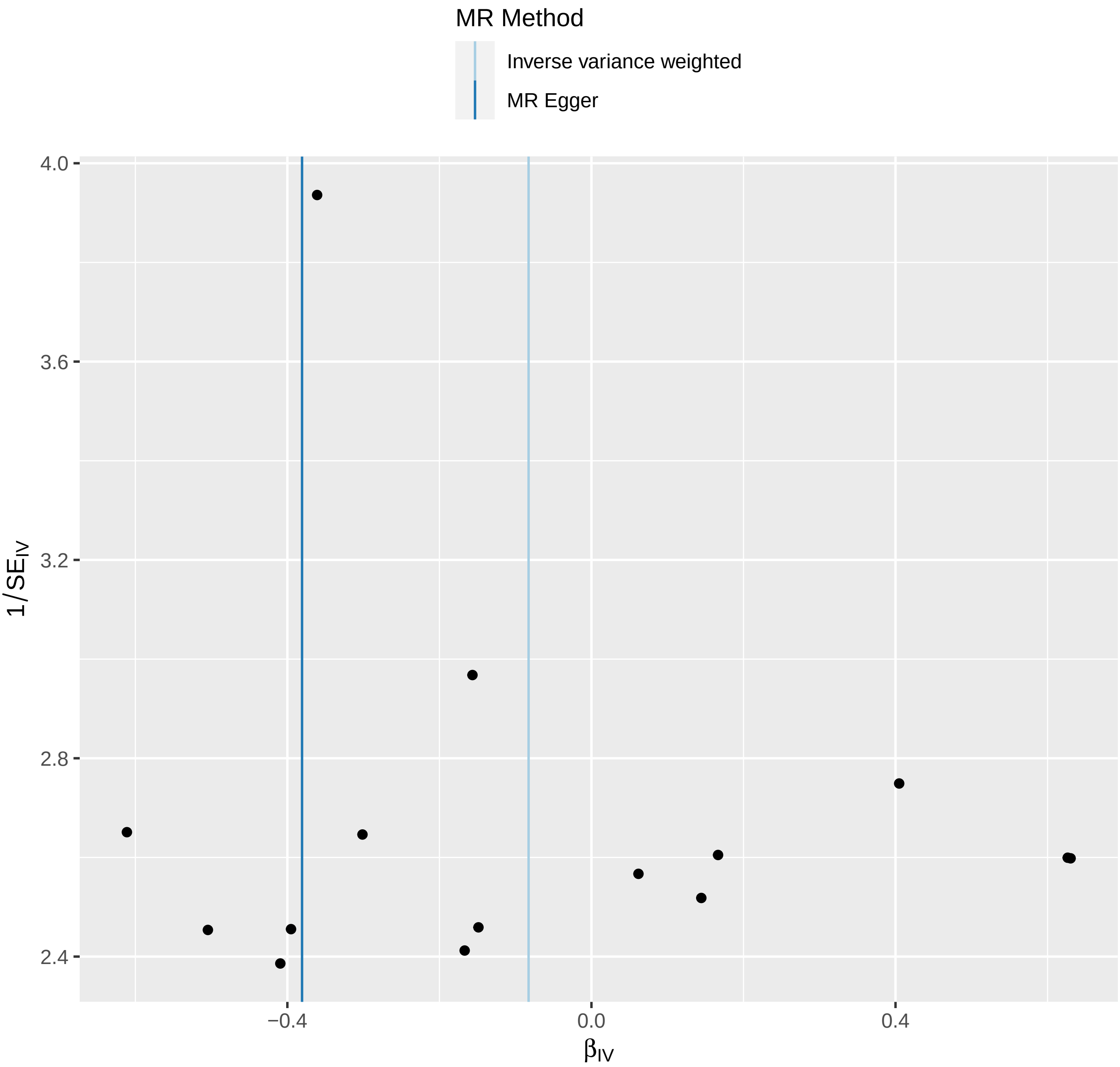

Batch 2−367 : Gut microbiota abundance (genus Catenibacterium id.2153) on Histone−lysine N−methyltransferase ASH1L
with a threshold of 1e−05

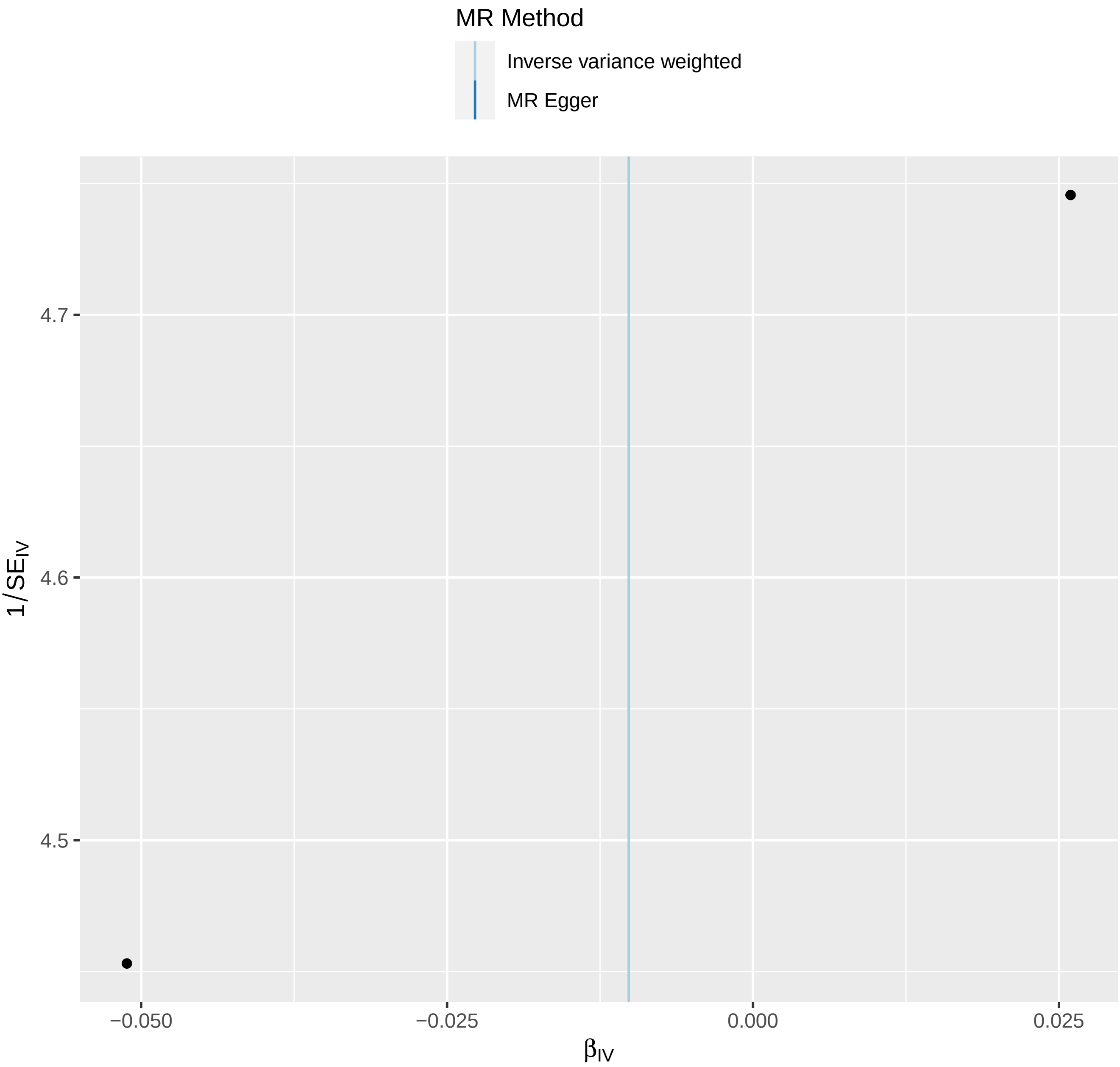

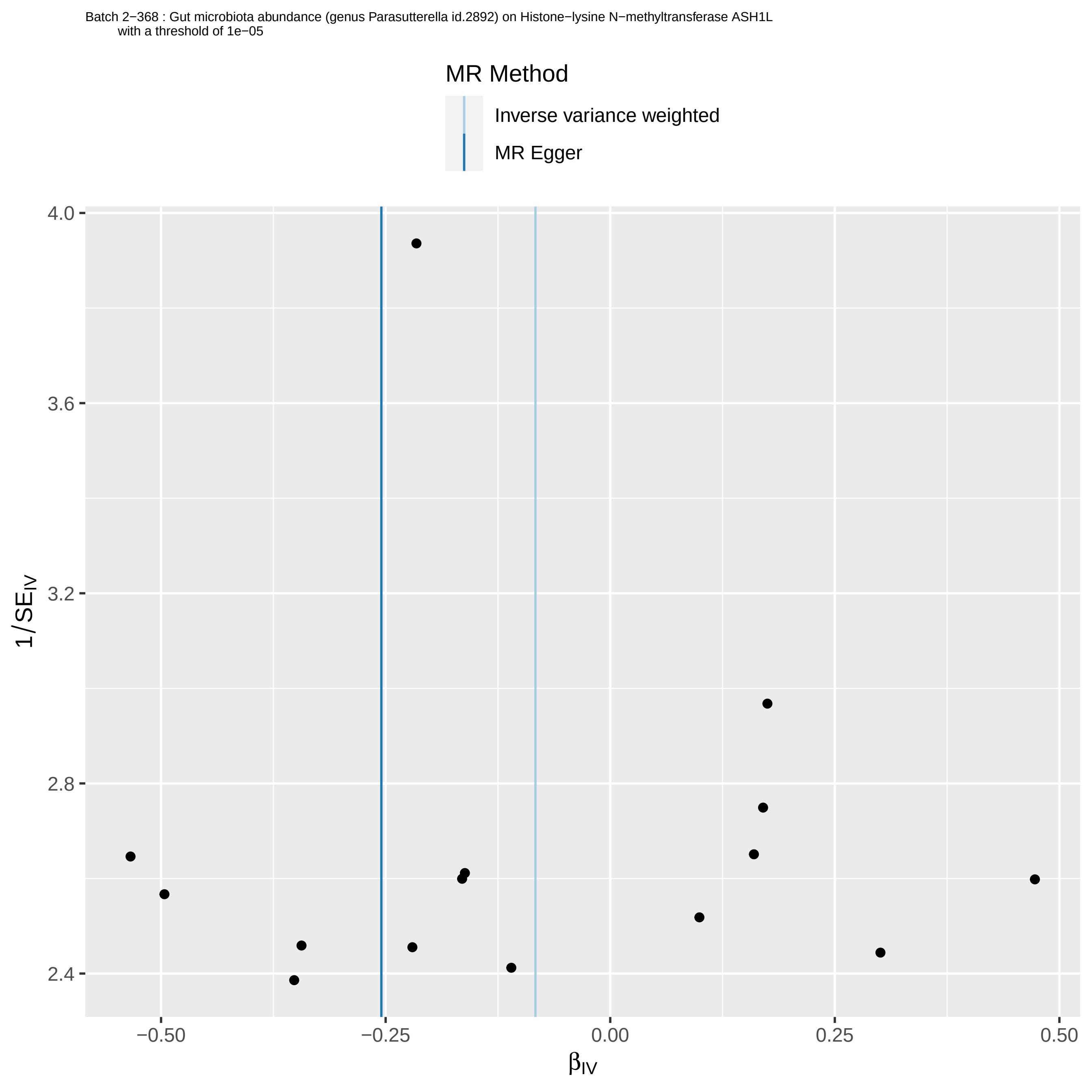
Batch 2−368 : Gut microbiota abundance (genus Parasutterella id.2892) on Histone−lysine N−methyltransferase ASH1L
with a threshold of 1e−05
MR Method
Inverse variance weighted
MR Egger
4.0
3.6
3.2
2.8
2.4
1/SE_IV
−0.50
−0.25
0.00
0.25
0.50
β_IV

Batch 2−369 : Gut microbiota abundance (genus Catenibacterium id.2153) on Set1/Ash2 histone methyltransferase complex subunit ASH2
with a threshold of 1e−05

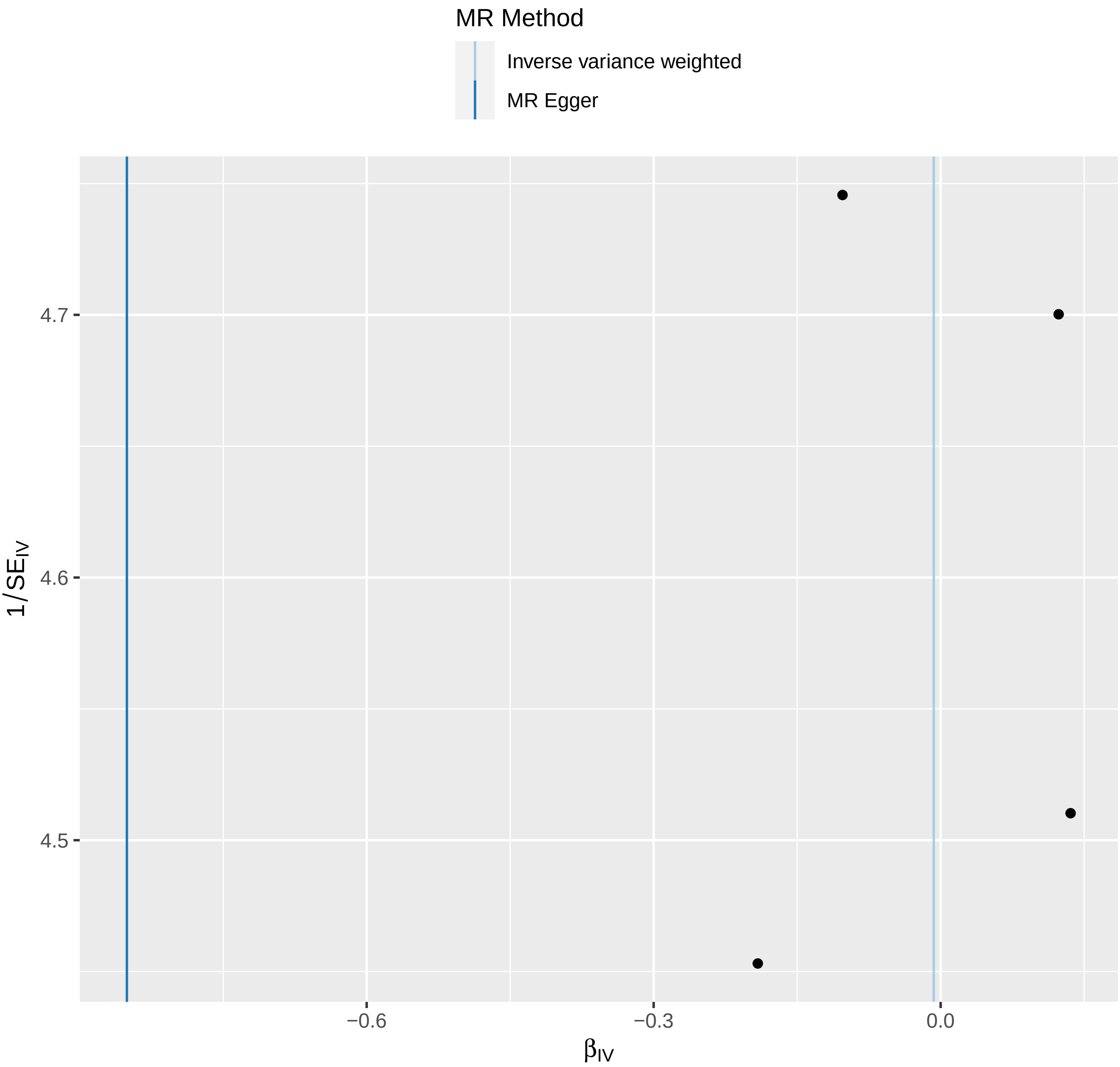

Batch 2−370 : Gut microbiota abundance (genus Parasutterella id.2892) on Set1/Ash2 histone methyltransferase complex subunit ASH2
with a threshold of 1e−05

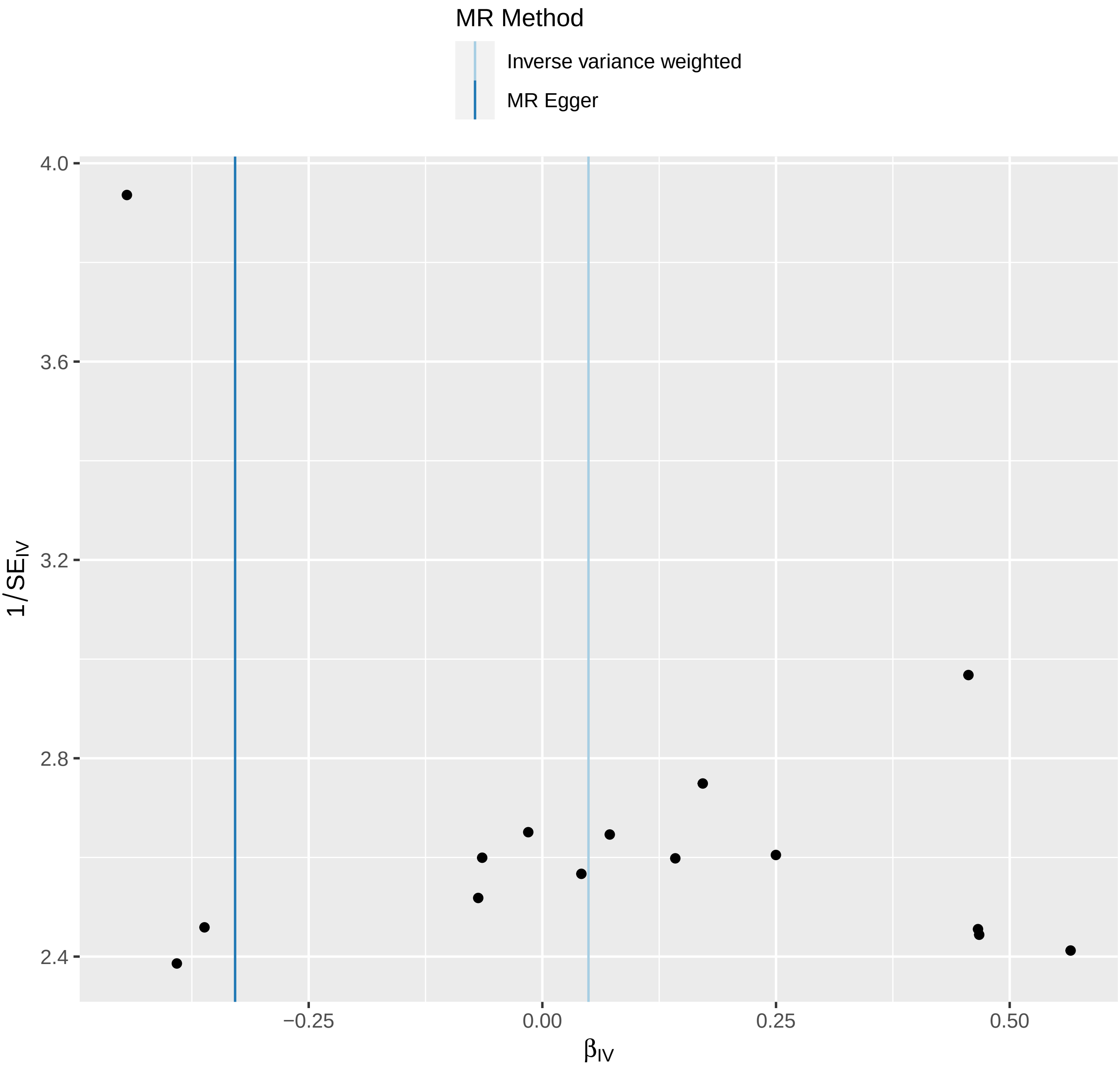

Batch 2−371 : Gut microbiota abundance (genus Catenibacterium id.2153) on Acid−sensing ion channel 4
with a threshold of 1e−05

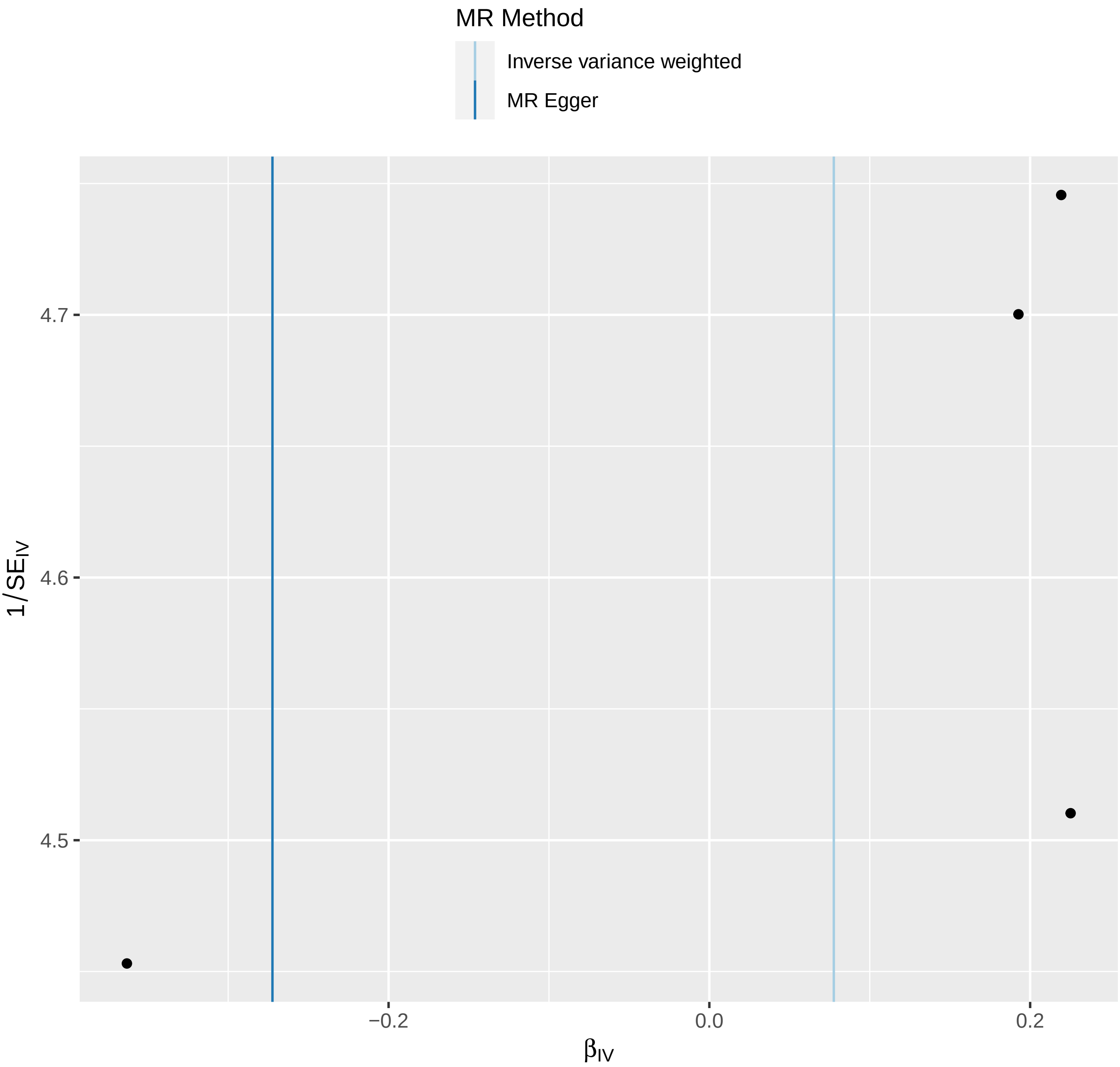

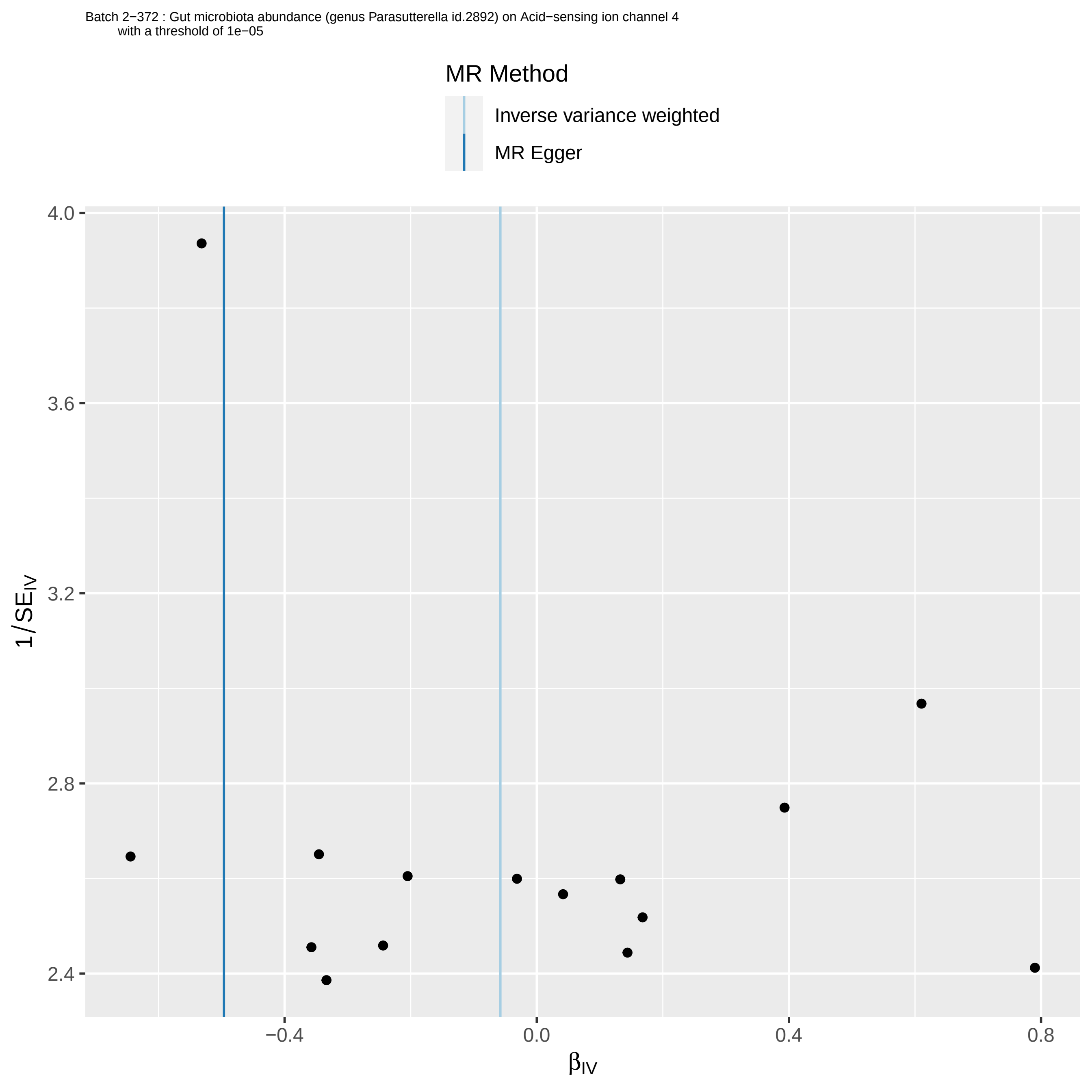
Batch 2−372 : Gut microbiota abundance (genus Parasutterella id.2892) on Acid−sensing ion channel 4
with a threshold of 1e−05
MR Method
Inverse variance weighted
MR Egger
4.0
3.6
3.2
2.8
2.4
1/SE_IV
−0.4
0.0
0.4
0.8
β_IV

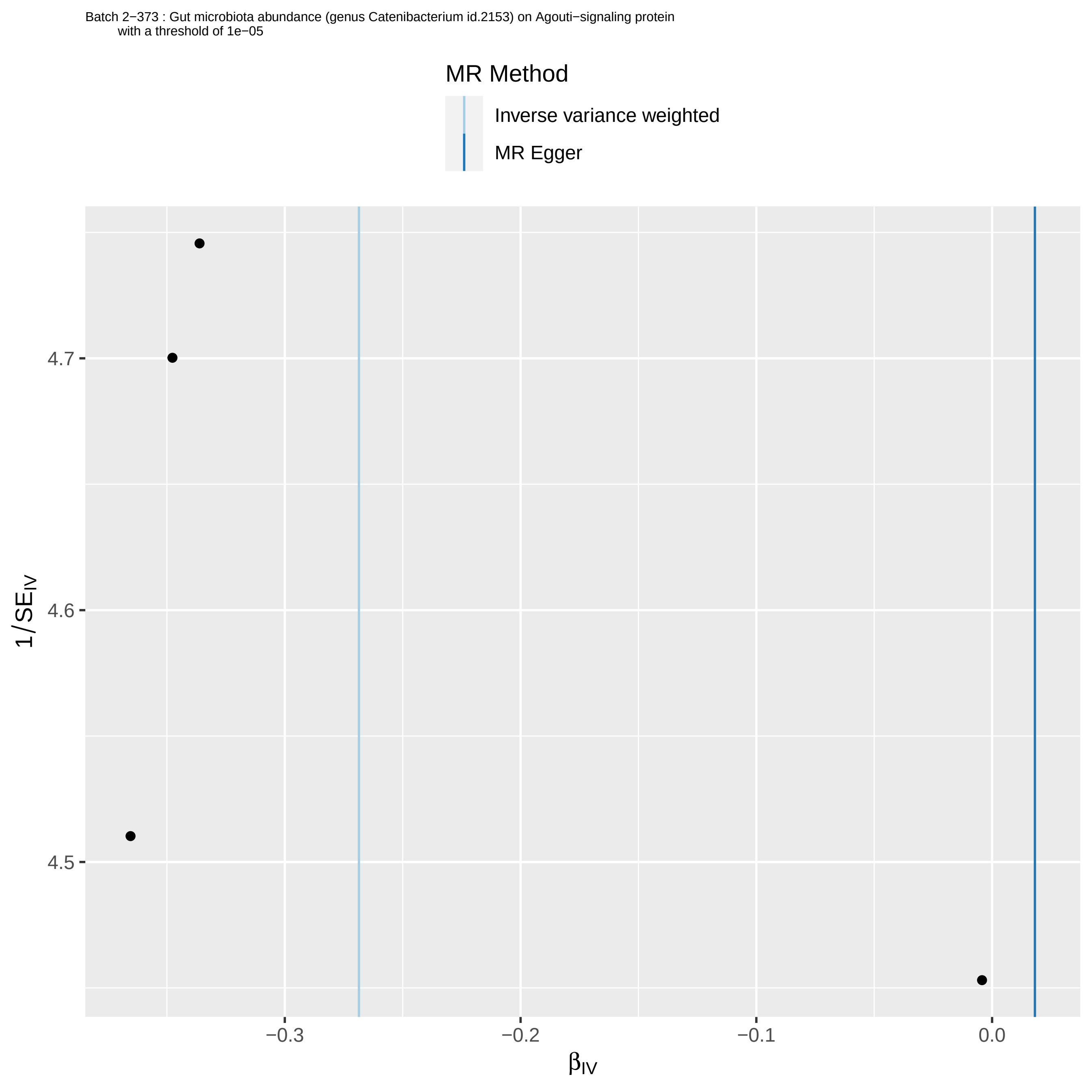

Batch 2−373 : Gut microbiota abundance (genus Catenibacterium id.2153) on Agouti−signaling protein
with a threshold of 1e−05
MR Method
Inverse variance weighted
MR Egger
4.7
4.6
4.5
1/SE_IV
−0.3
−0.2
−0.1
0.0
β_IV

Batch 2−374 : Gut microbiota abundance (genus Parasutterella id.2892) on Agouti−signaling protein
with a threshold of 1e−05

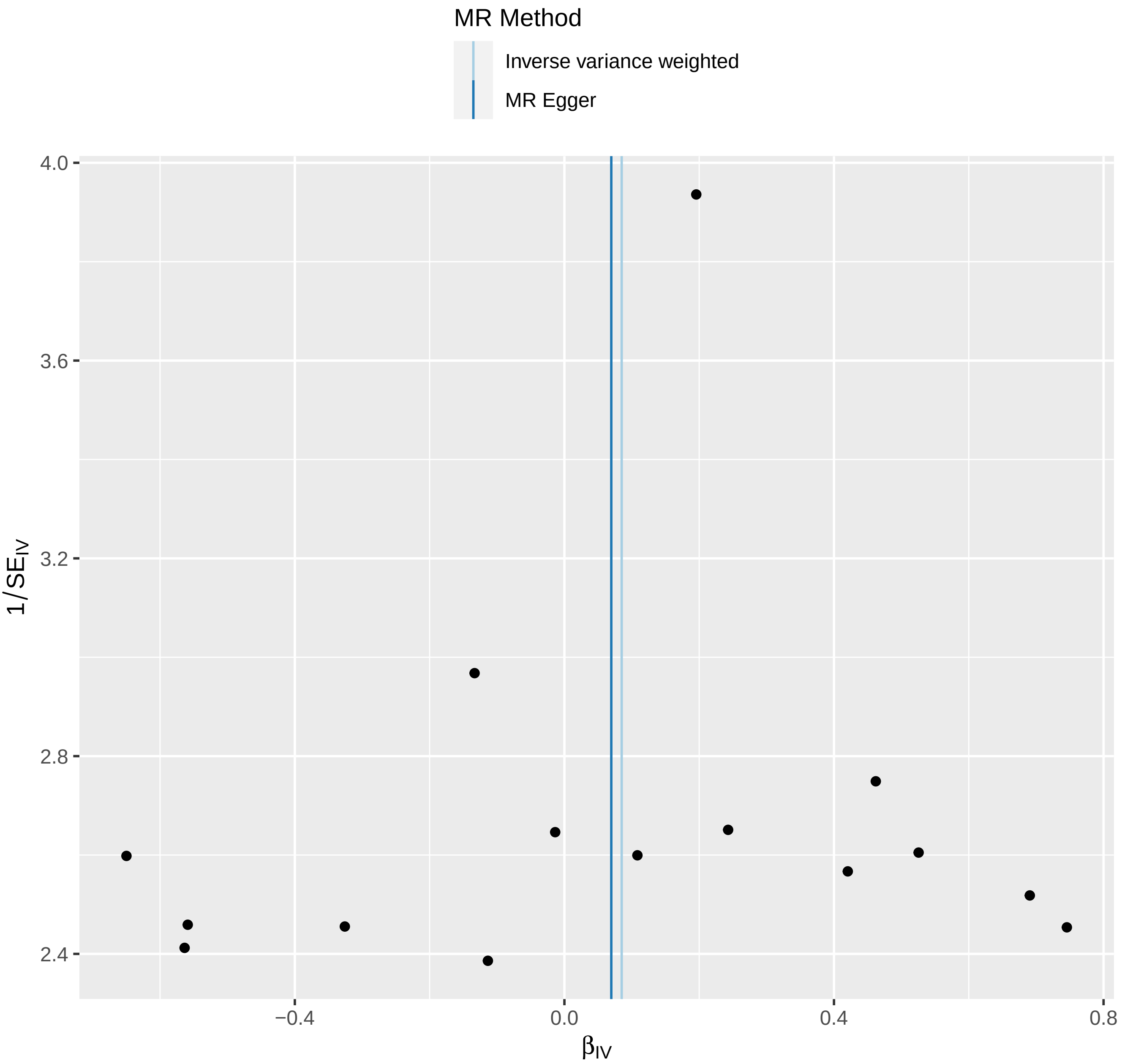

Batch 2−375 : Gut microbiota abundance (genus Catenibacterium id.2153) on Argininosuccinate lyase
with a threshold of 1e−05

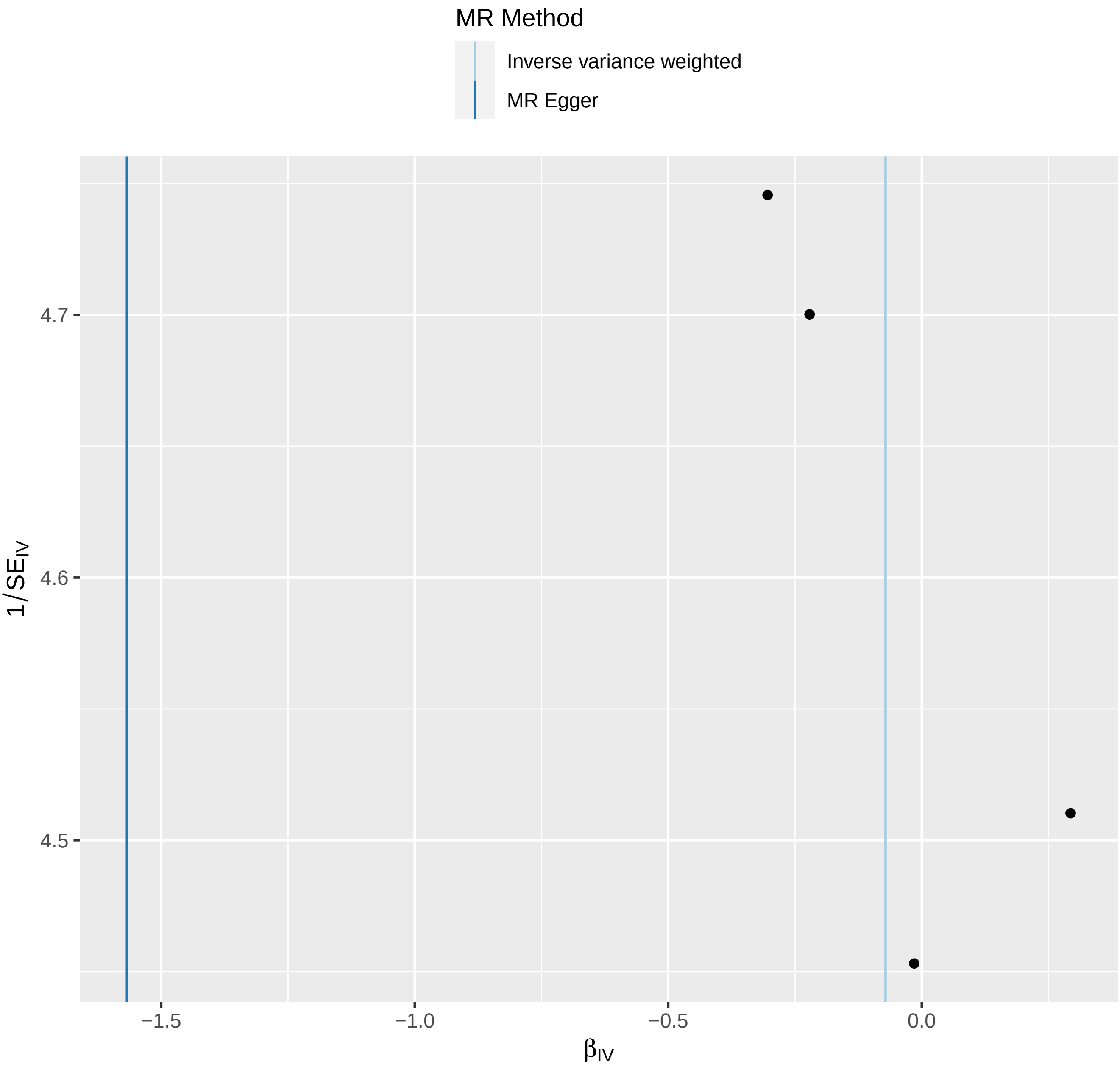

Batch 2−376 : Gut microbiota abundance (genus Parasutterella id.2892) on Argininosuccinate lyase
with a threshold of 1e−05

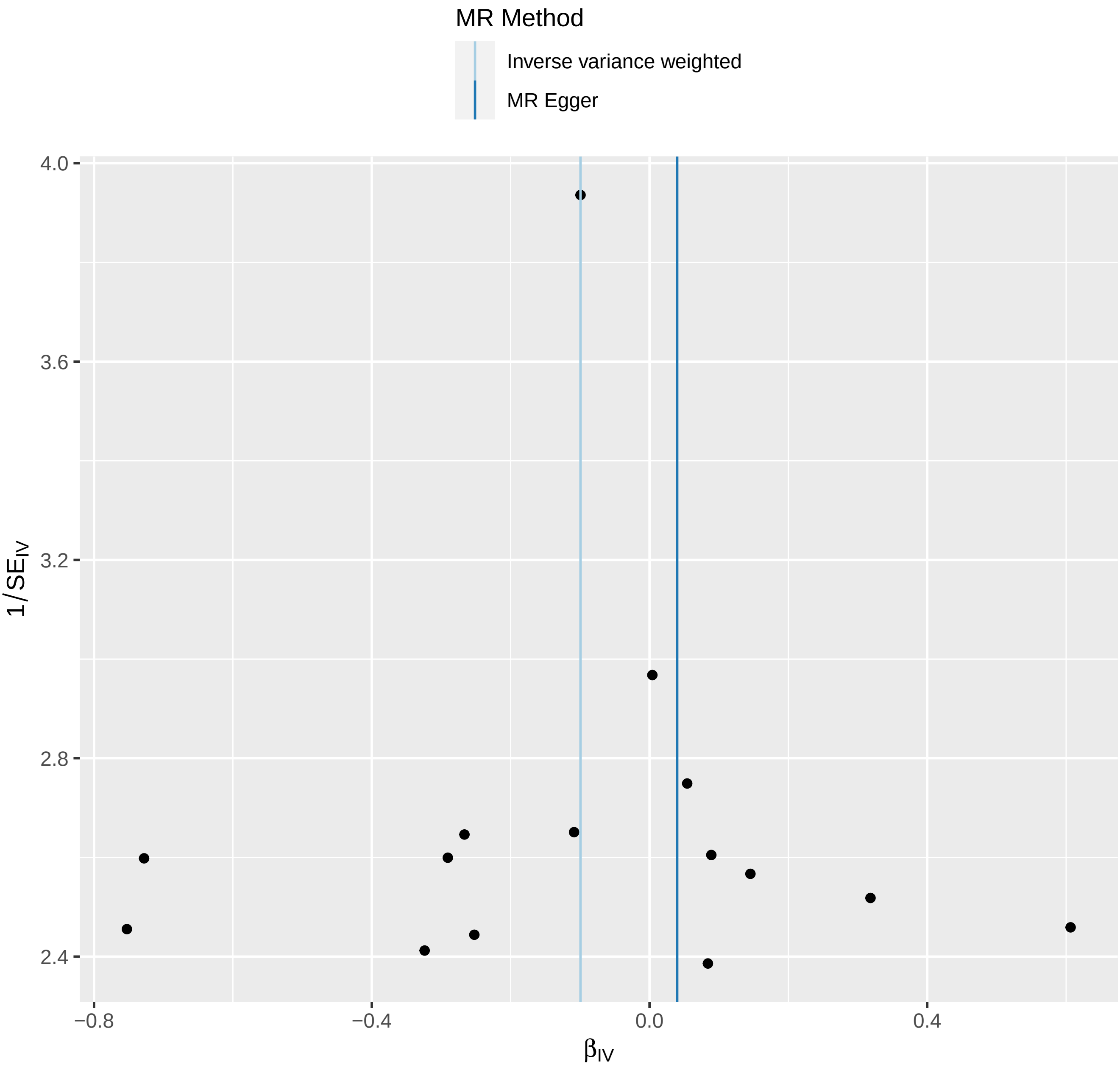

Batch 2−377 : Gut microbiota abundance (genus Catenibacterium id.2153) on N−acetylserotonin O−methyltransferase−like protein with a threshold of 1e−05

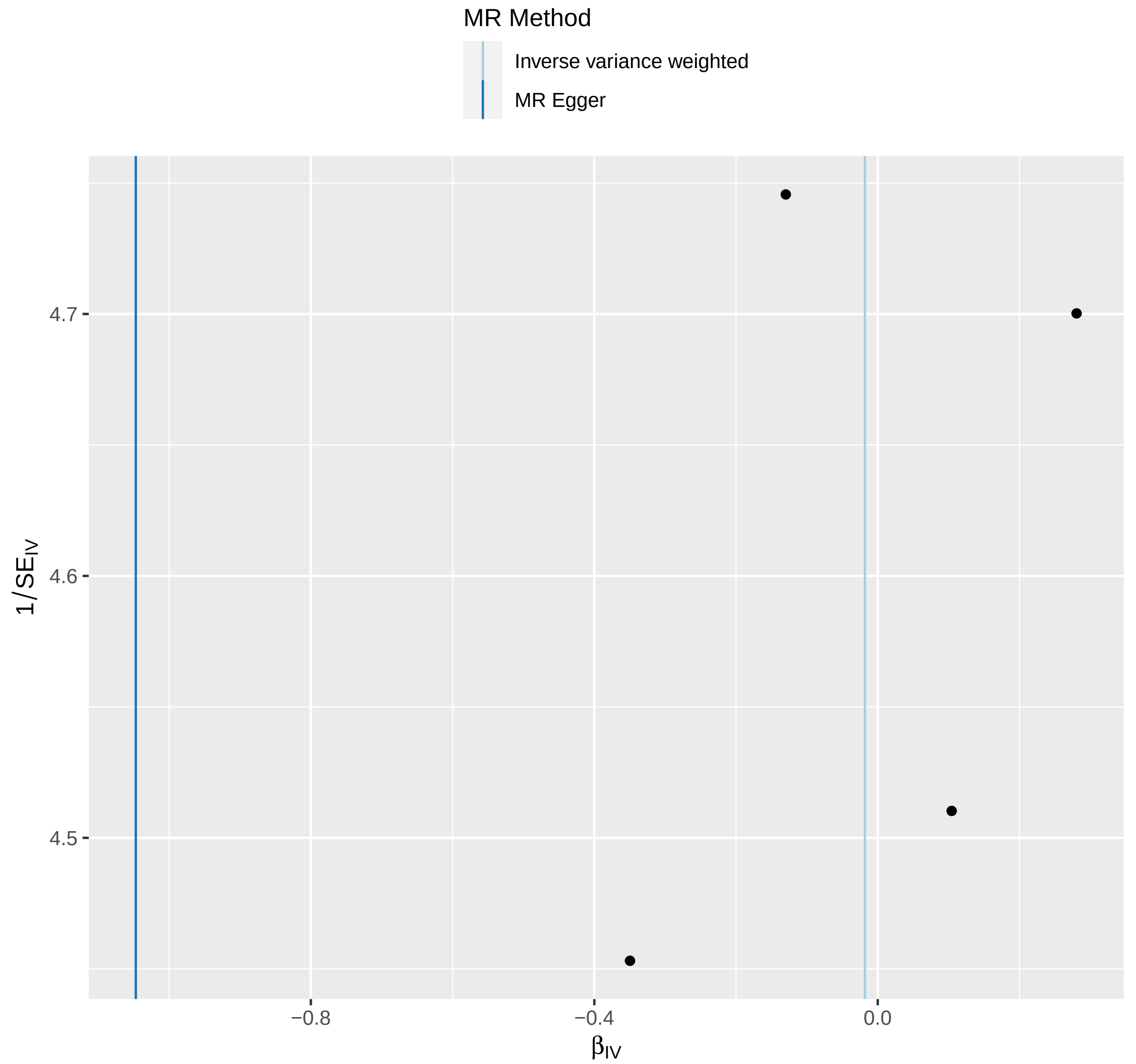

Batch 2−378 : Gut microbiota abundance (genus Parasutterella id.2892) on N−acetylserotonin O−methyltransferase−like protein with a threshold of 1e−05

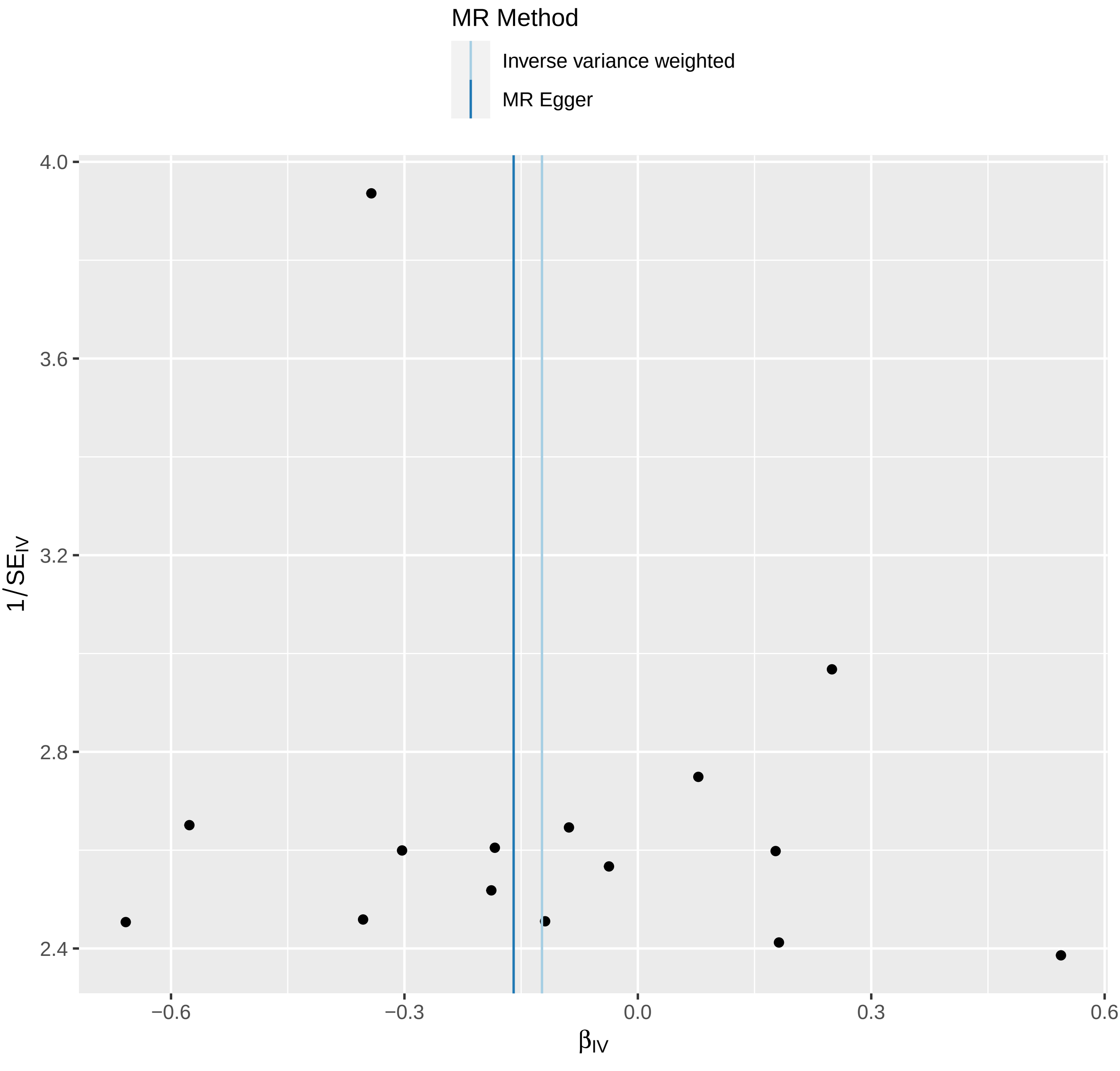

Batch 2−379 : Gut microbiota abundance (genus Catenibacterium id.2153) on ATPase ASNA1
with a threshold of 1e−05

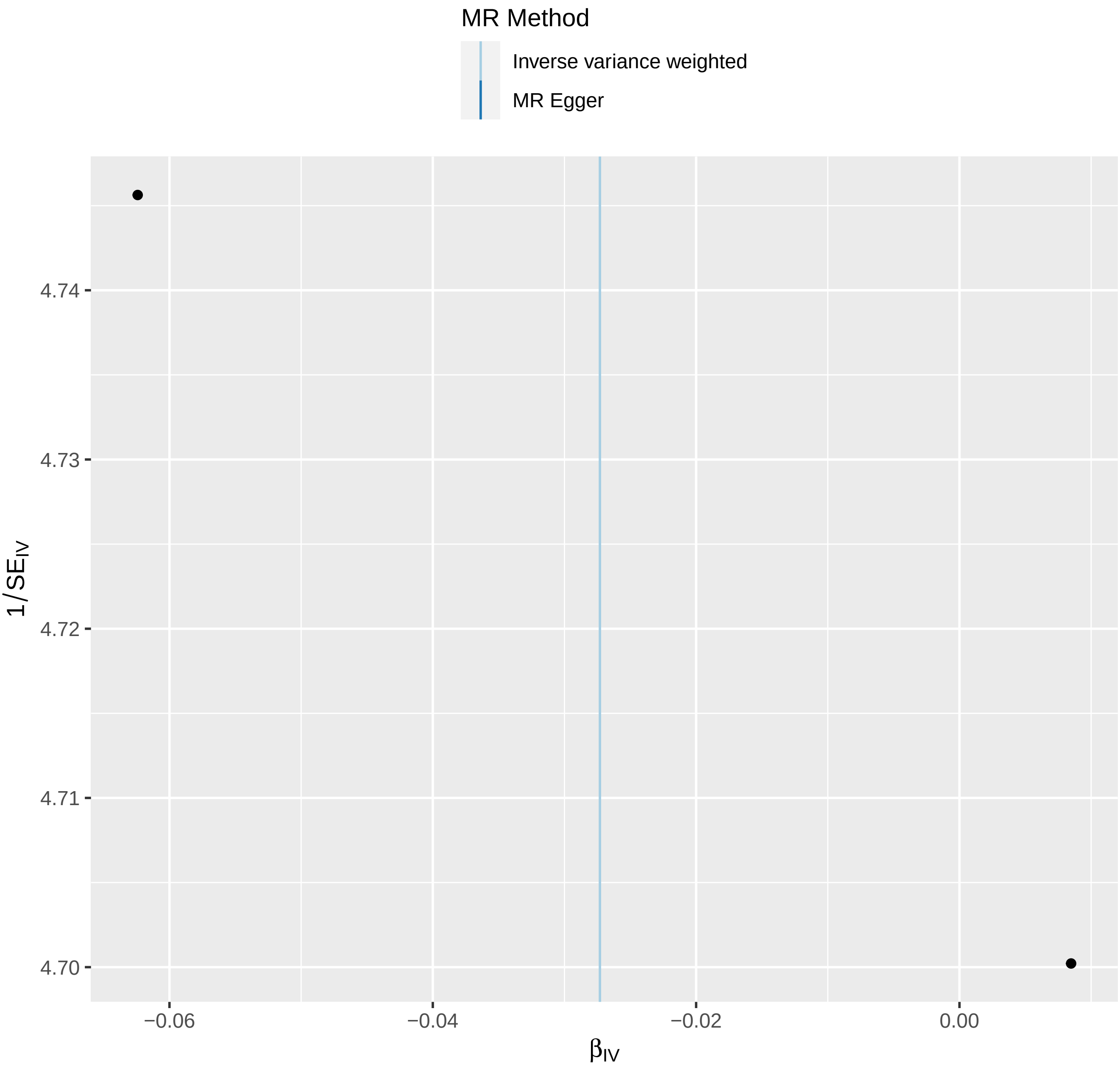

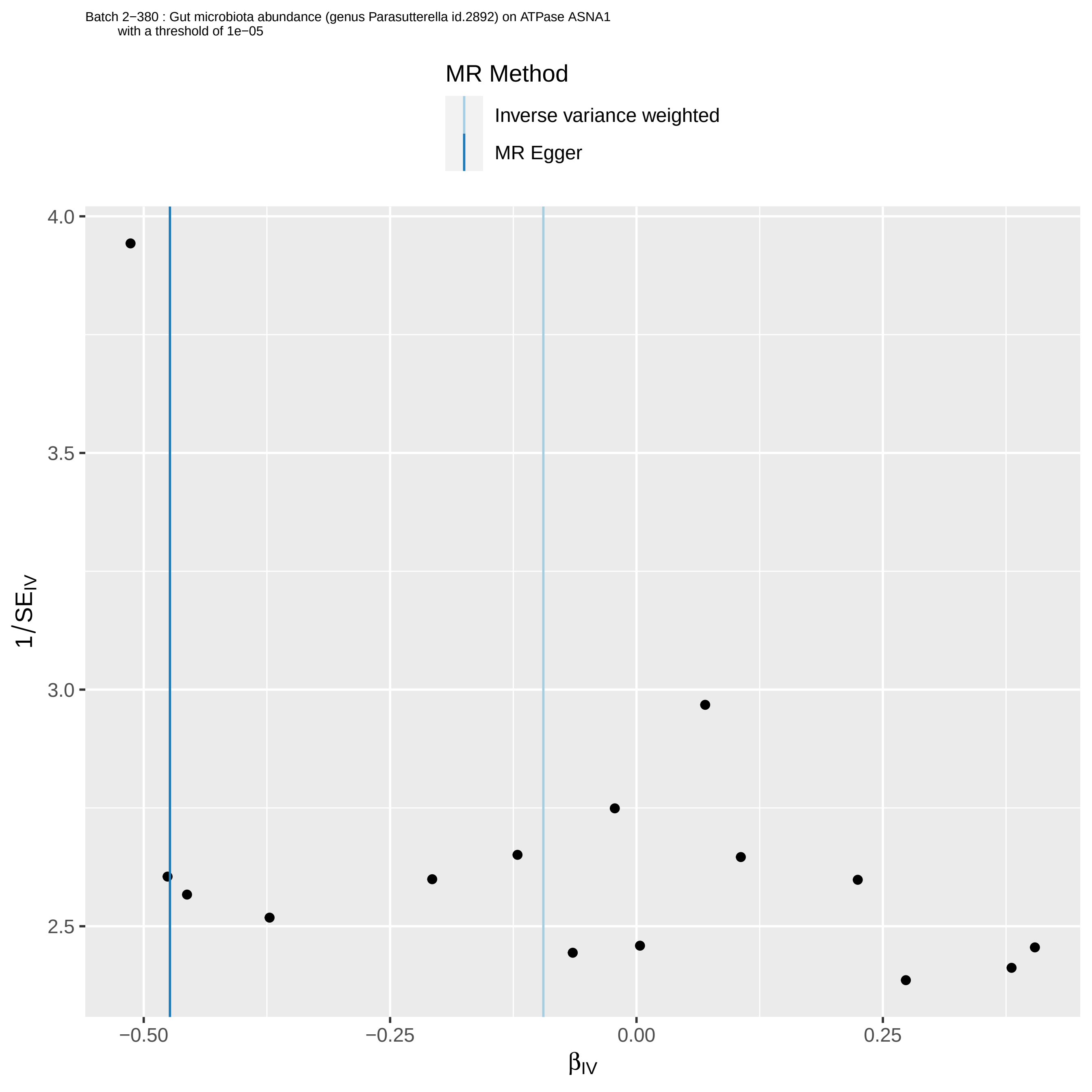

Batch 2−380 : Gut microbiota abundance (genus Parasutterella id.2892) on ATPase ASNA1
with a threshold of 1e−05
MR Method
Inverse variance weighted
MR Egger
4.0
3.5
3.0
2.5
1/SE_IV
−0.50
−0.25
0.00
0.25
β_IV

Batch 2−381 : Gut microbiota abundance (genus Catenibacterium id.2153) on Aspartyl/asparaginyl beta−hydroxylase with a threshold of 1e−05

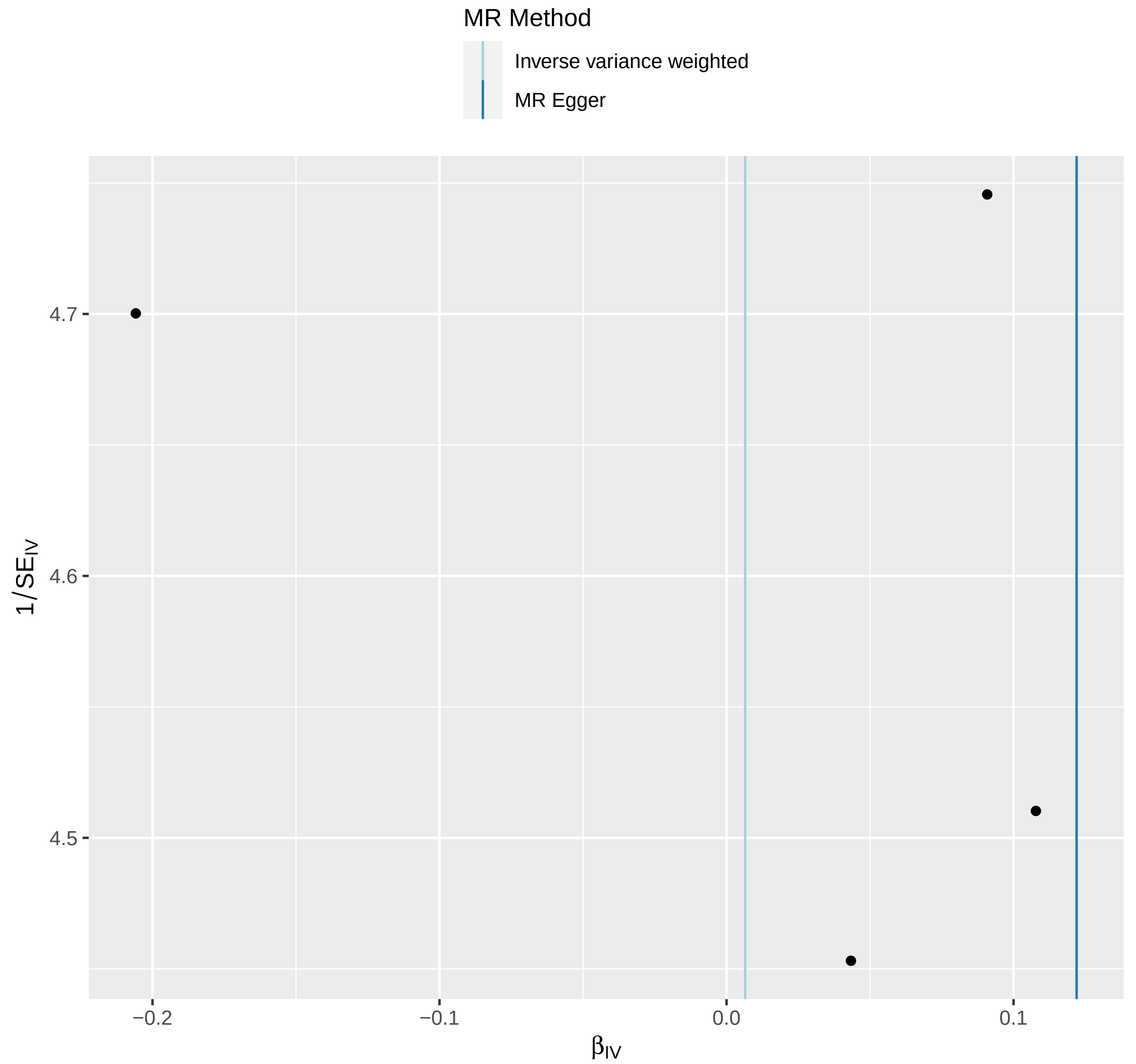

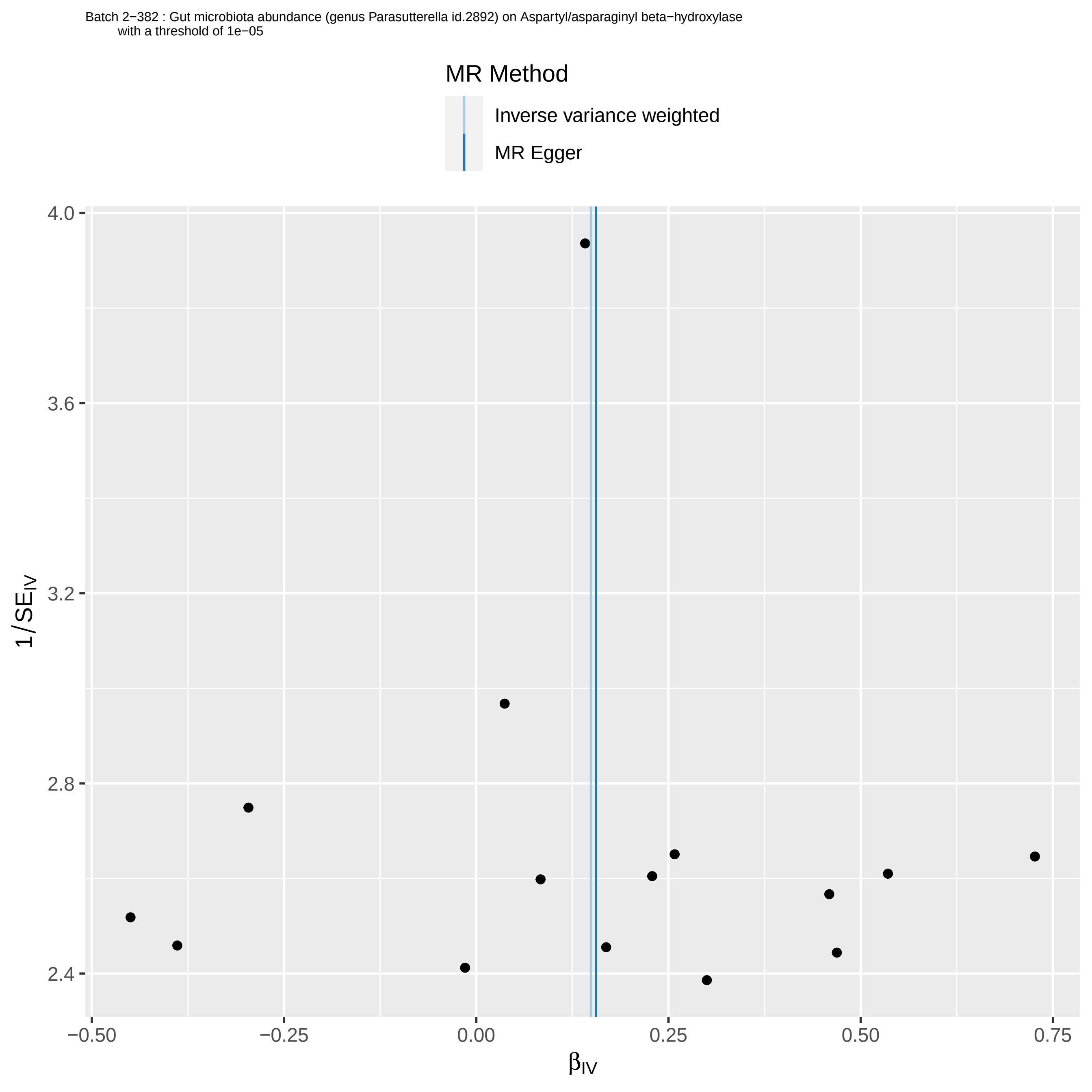
Batch 2−382 : Gut microbiota abundance (genus Parasutterella id.2892) on Aspartyl/asparaginyl beta−hydroxylase
with a threshold of 1e−05
MR Method
Inverse variance weighted
MR Egger
4.0
3.6
3.2
2.8
2.4
1/SE_IV
−0.50
−0.25
0.00
0.25
0.50
0.75
β_IV

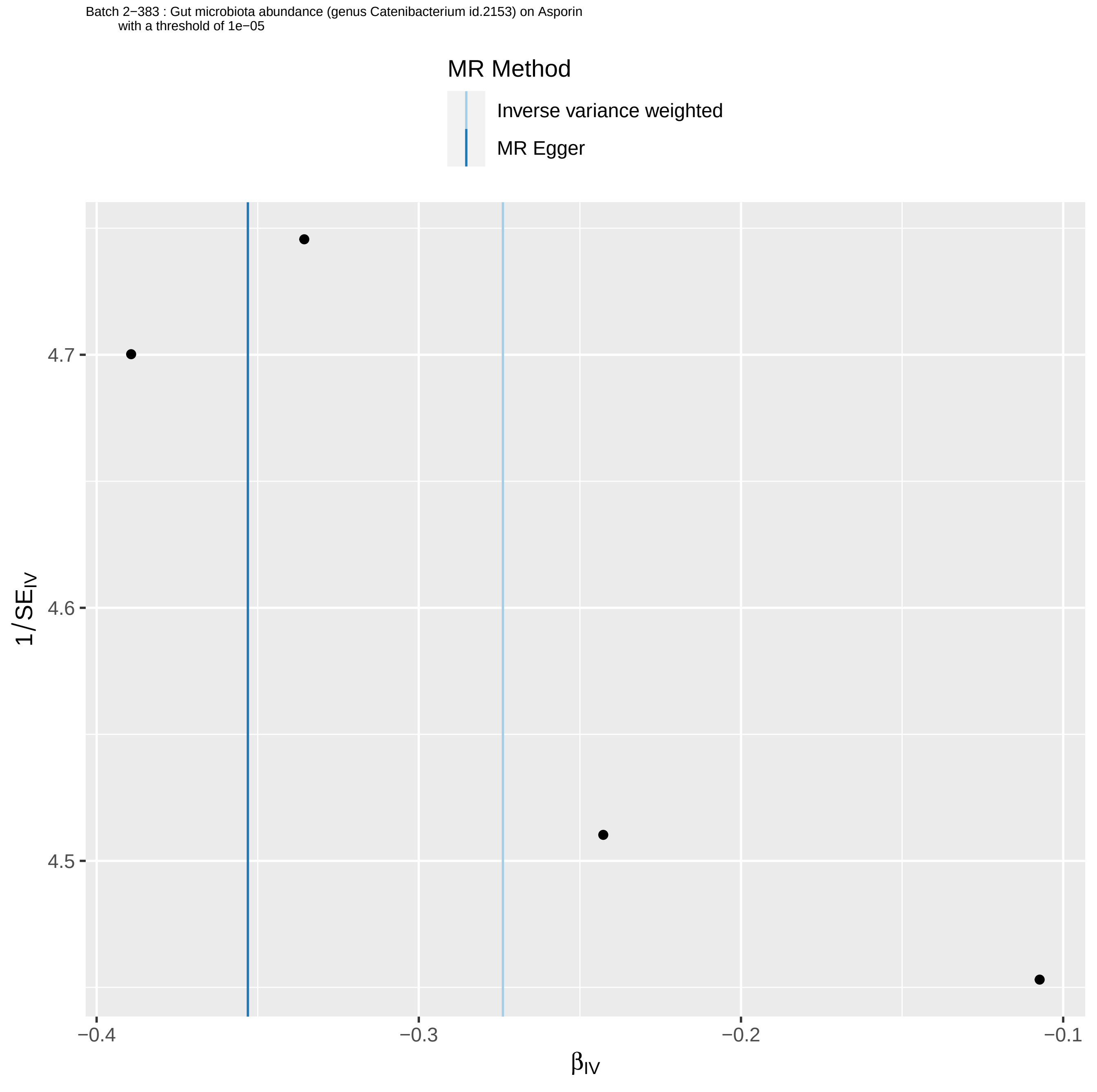

Batch 2−383 : Gut microbiota abundance (genus Catenibacterium id.2153) on Asporin
with a threshold of 1e−05
MR Method
Inverse variance weighted
MR Egger
4.7
4.6
4.5
1/SE_IV
−0.4
−0.3
−0.2
−0.1
β_IV

Batch 2−384 : Gut microbiota abundance (genus Parasutterella id.2892) on Asporin
with a threshold of 1e−05

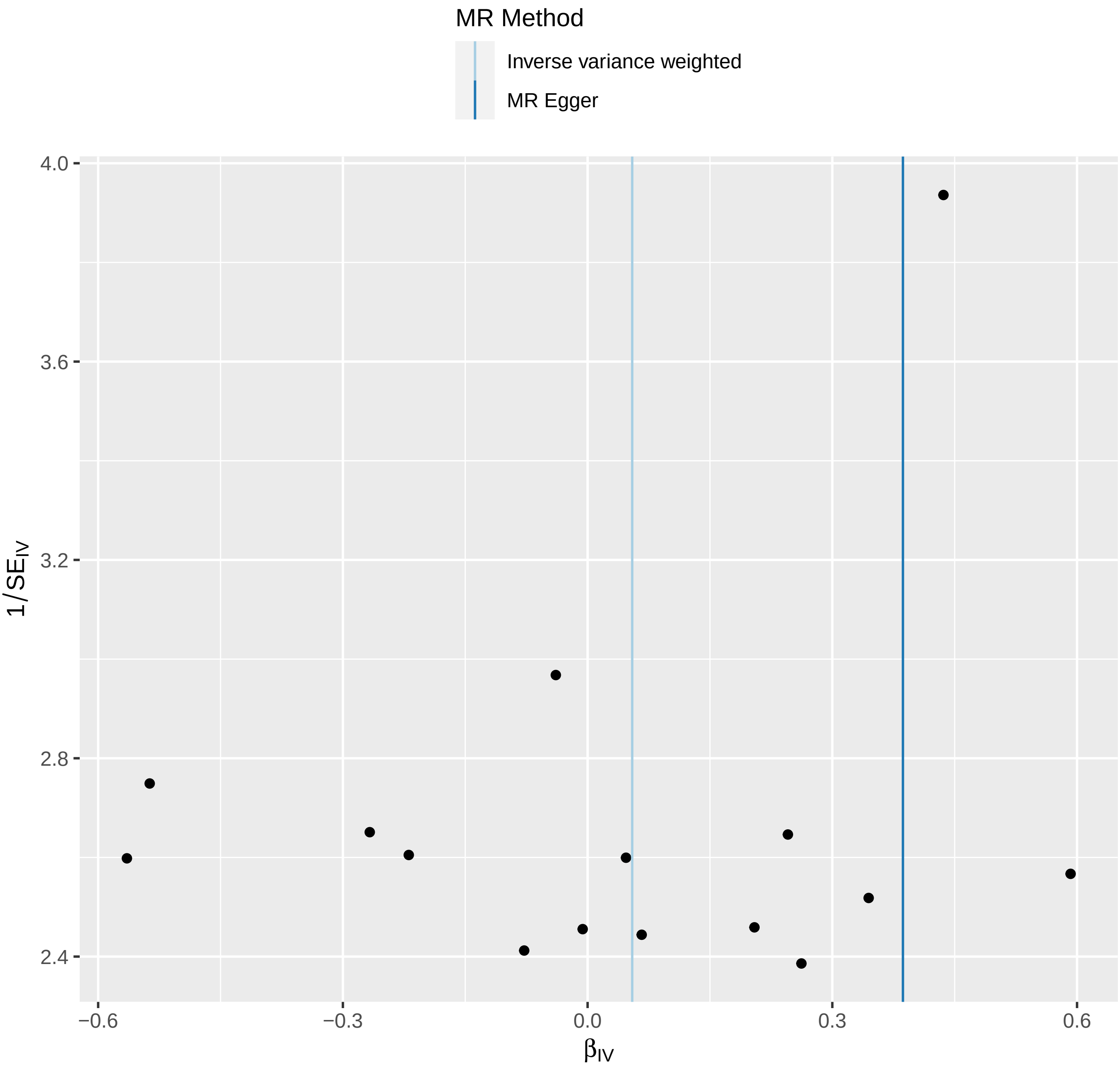

Batch 2−385 : Gut microbiota abundance (genus Catenibacterium id.2153) on Retroviral−like aspartic protease 1
with a threshold of 1e−05

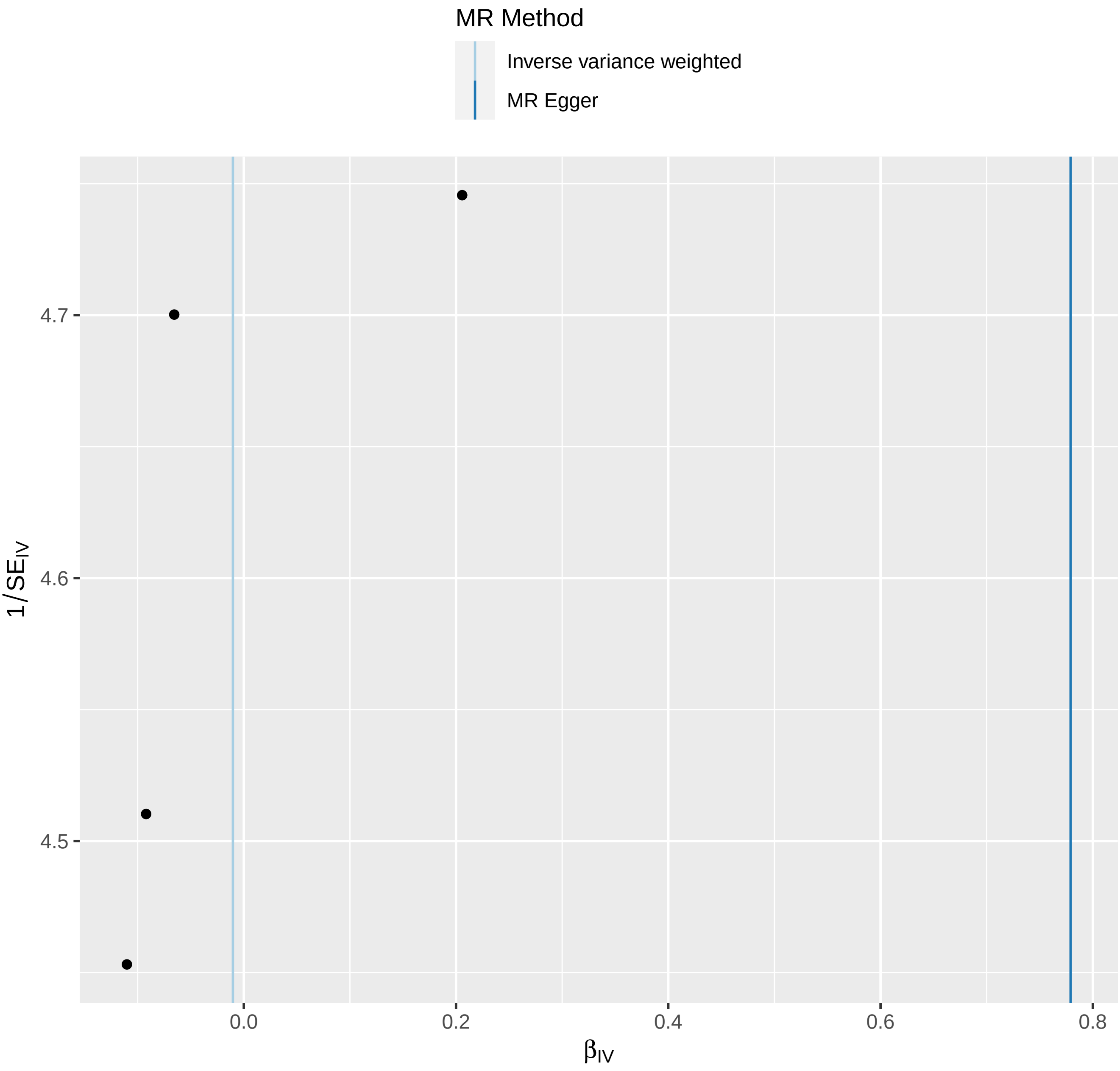

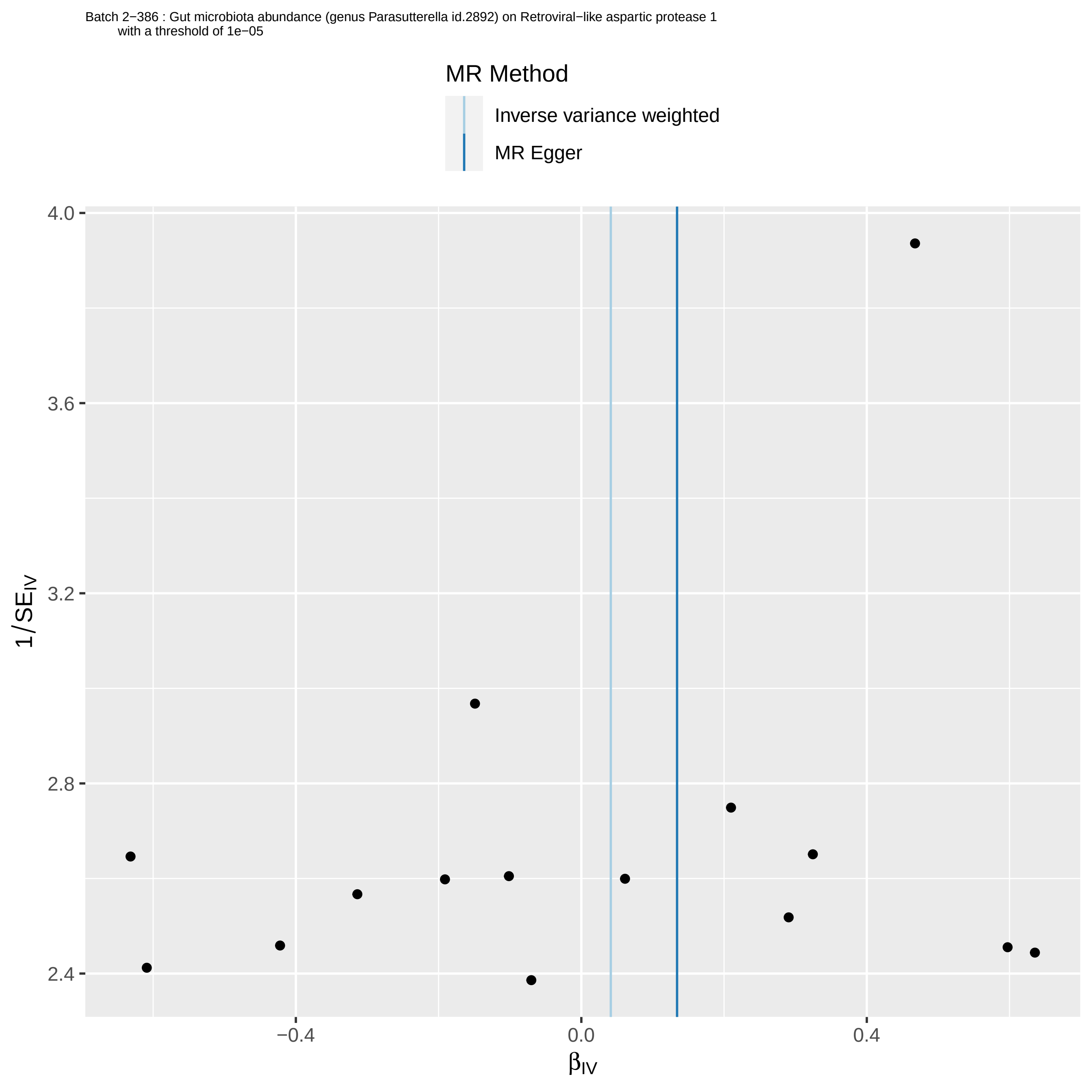

Batch 2−386 : Gut microbiota abundance (genus Parasutterella id.2892) on Retroviral−like aspartic protease 1
with a threshold of 1e−05
MR Method
Inverse variance weighted
MR Egger
4.0
3.6
3.2
2.8
2.4
1/SE_IV
−0.4
0.0
0.4
β_IV

Batch 2−387 : Gut microbiota abundance (genus Catenibacterium id.2153) on Astacin−like metalloendopeptidase with a threshold of 1e−05

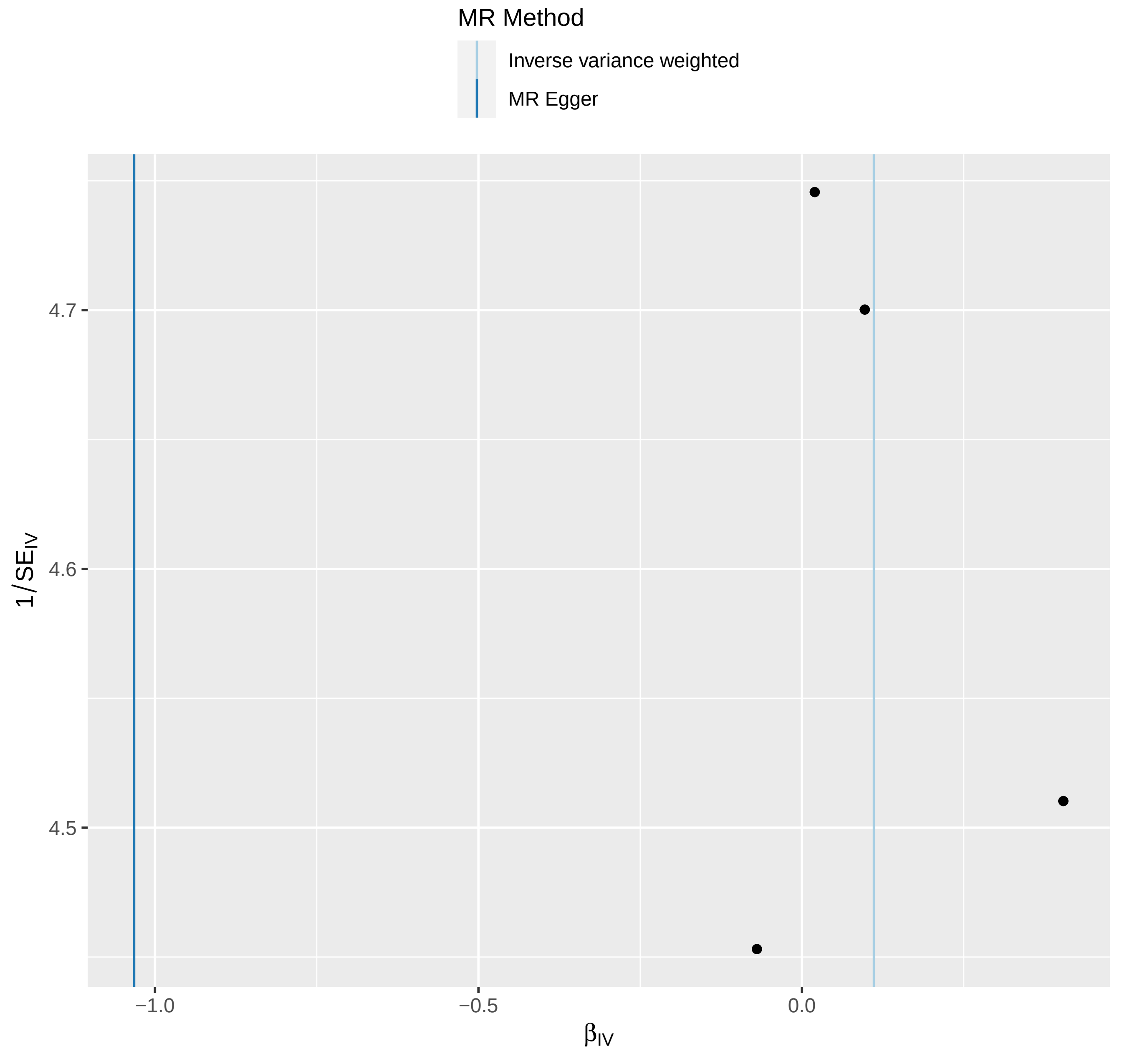

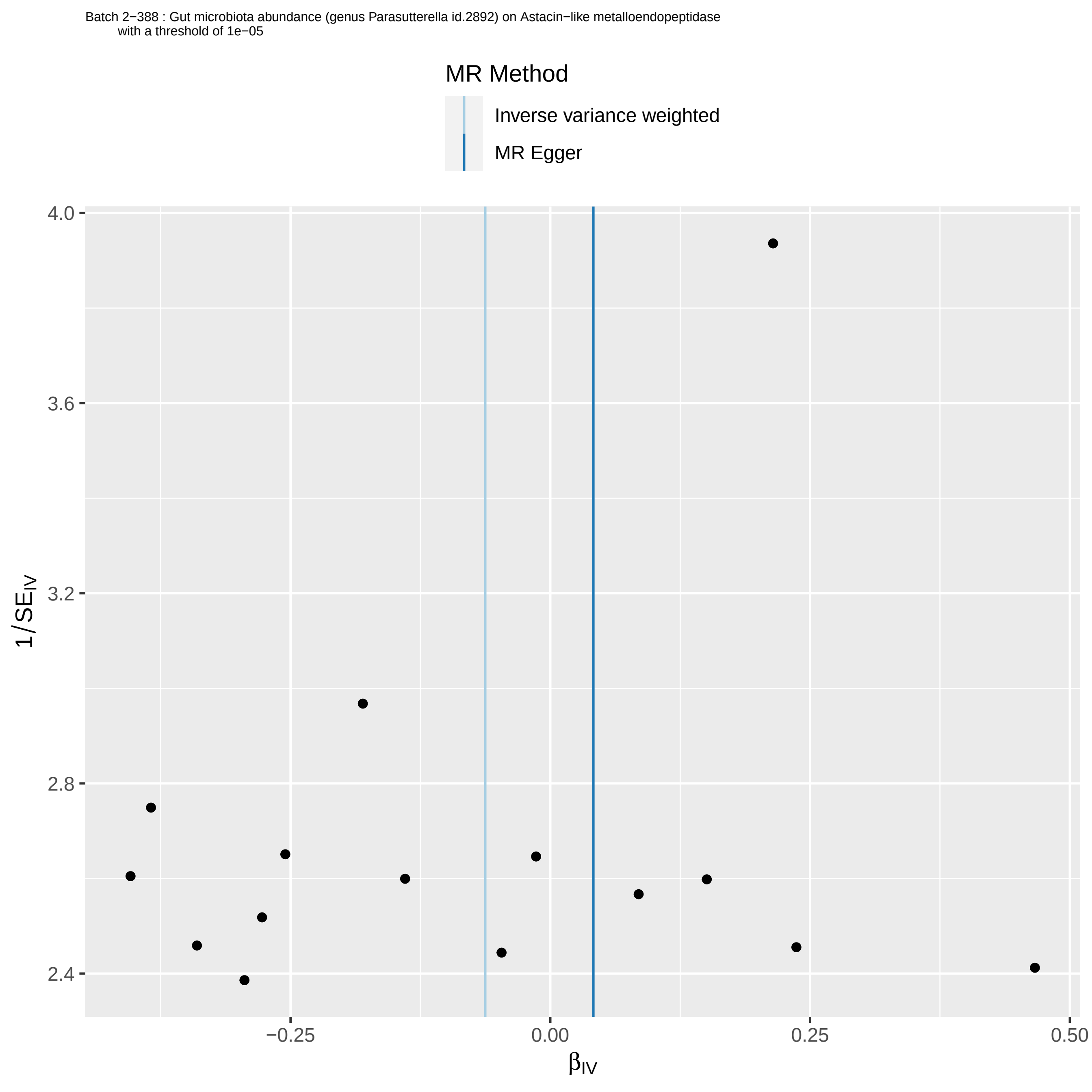

Batch 2−388 : Gut microbiota abundance (genus Parasutterella id.2892) on Astacin−like metalloendopeptidase
with a threshold of 1e−05
MR Method
Inverse variance weighted
MR Egger
4.0
3.6
3.2
2.8
2.4
1/SE$_{IV}$
−0.25
0.00
0.25
0.50
β$_{IV}$

Batch 2−389 : Gut microbiota abundance (genus Catenibacterium id.2153) on ATPase family AAA domain−containing protein 1
with a threshold of 1e−05

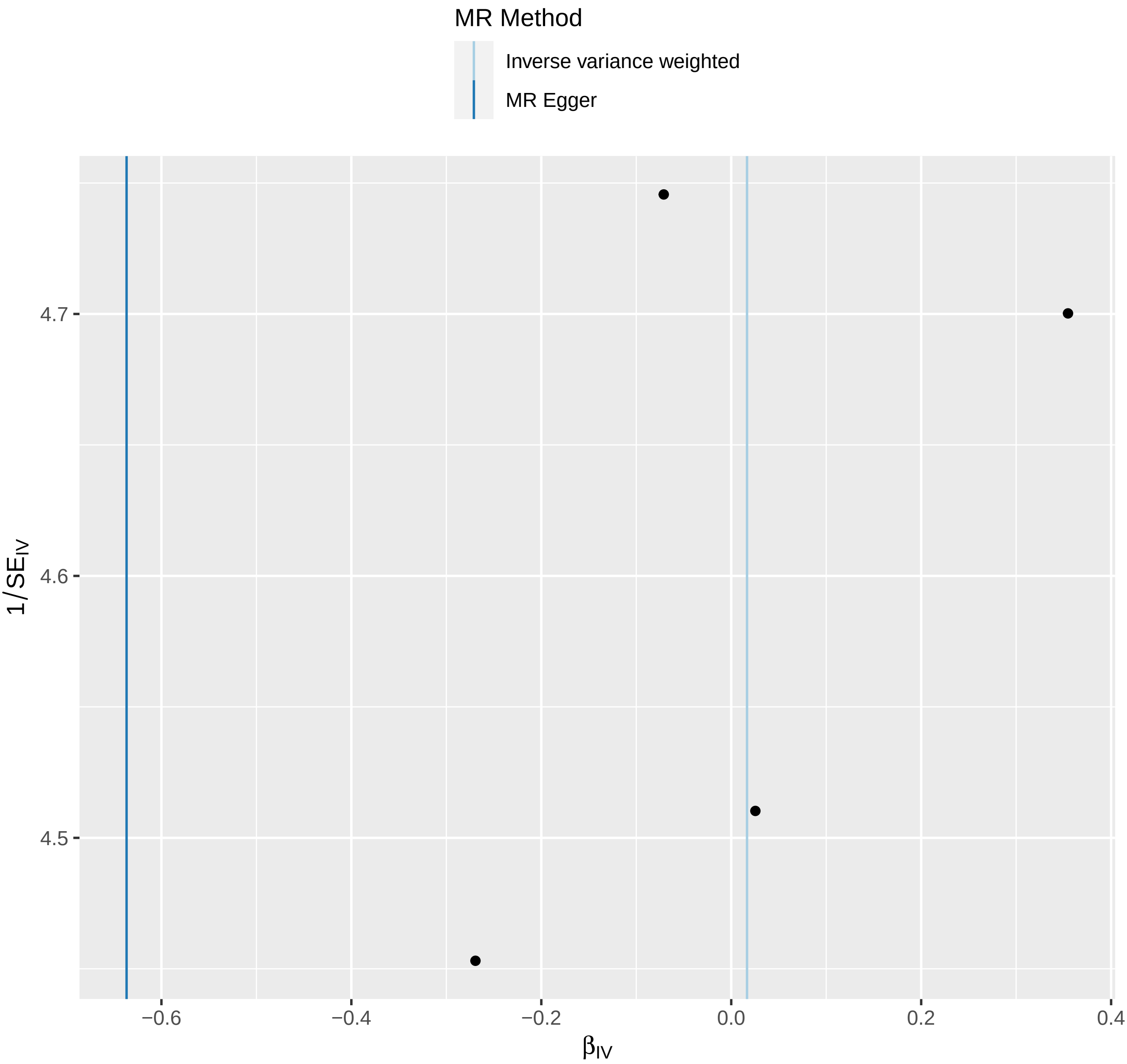

Batch 2−390 : Gut microbiota abundance (genus Parasutterella id.2892) on ATPase family AAA domain−containing protein 1
with a threshold of 1e−05

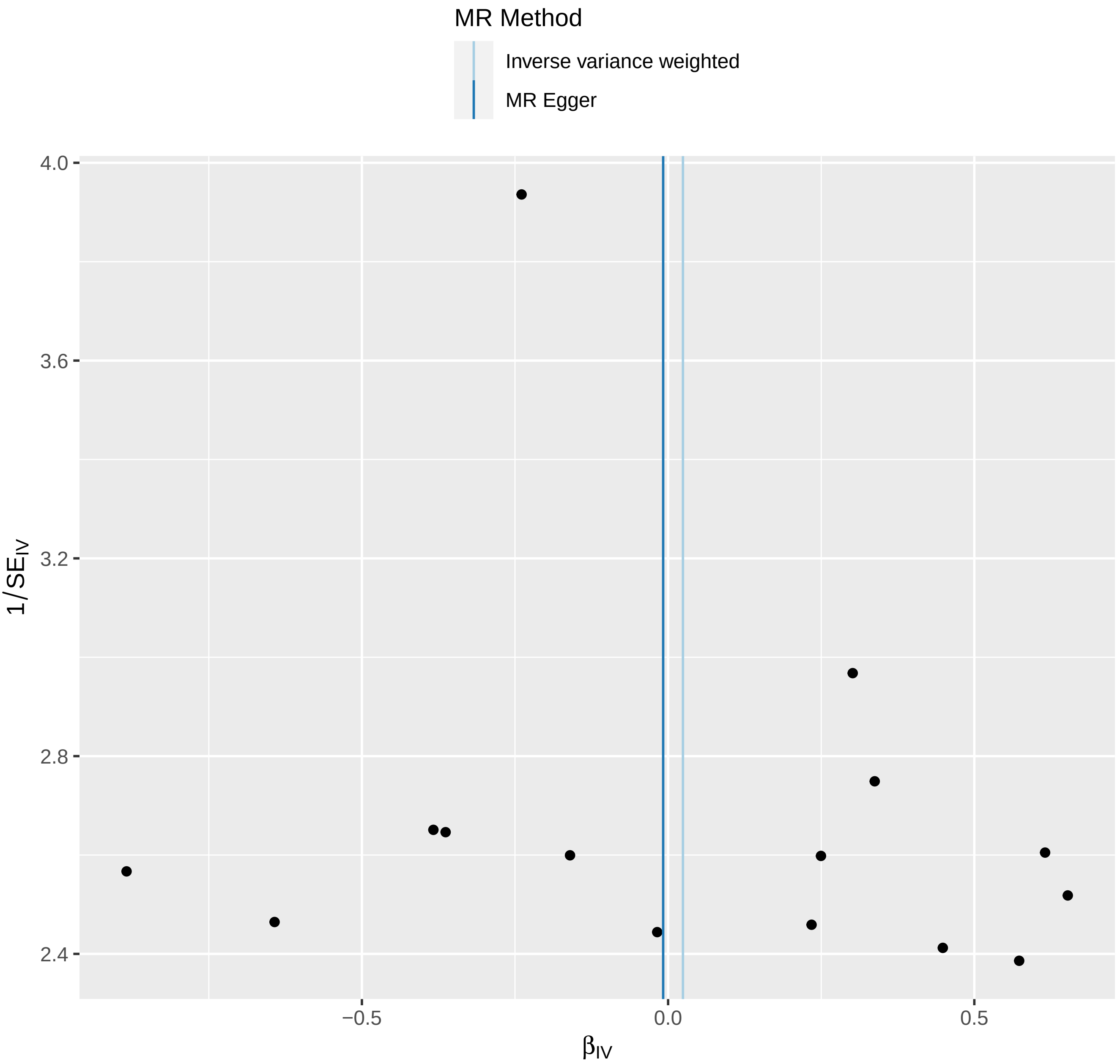

Batch 2−391 : Gut microbiota abundance (genus Catenibacterium id.2153) on ATPase family AAA domain−containing protein 2 with a threshold of 1e−05

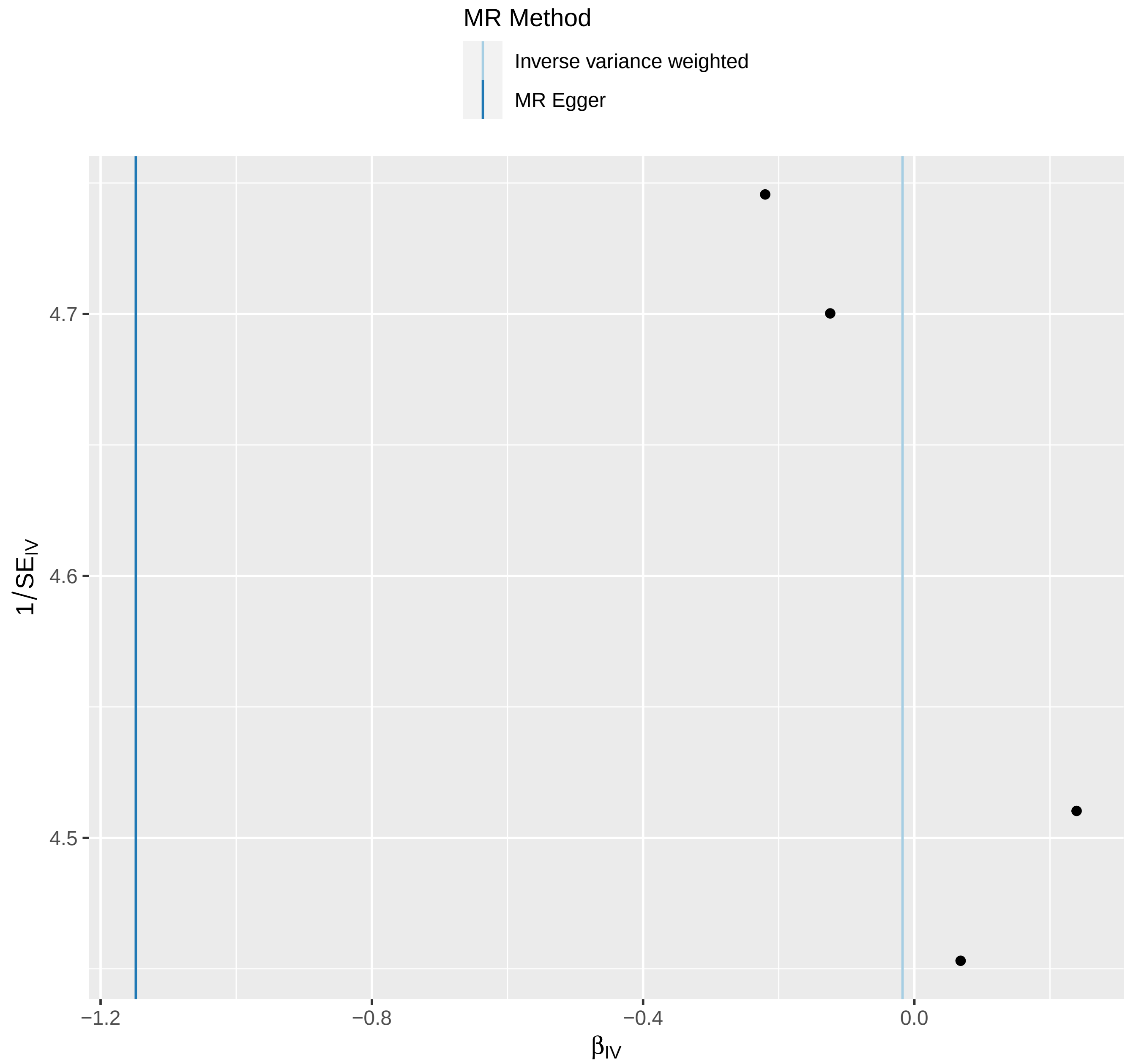

Batch 2−392 : Gut microbiota abundance (genus Parasutterella id.2892) on ATPase family AAA domain−containing protein 2 with a threshold of 1e−05

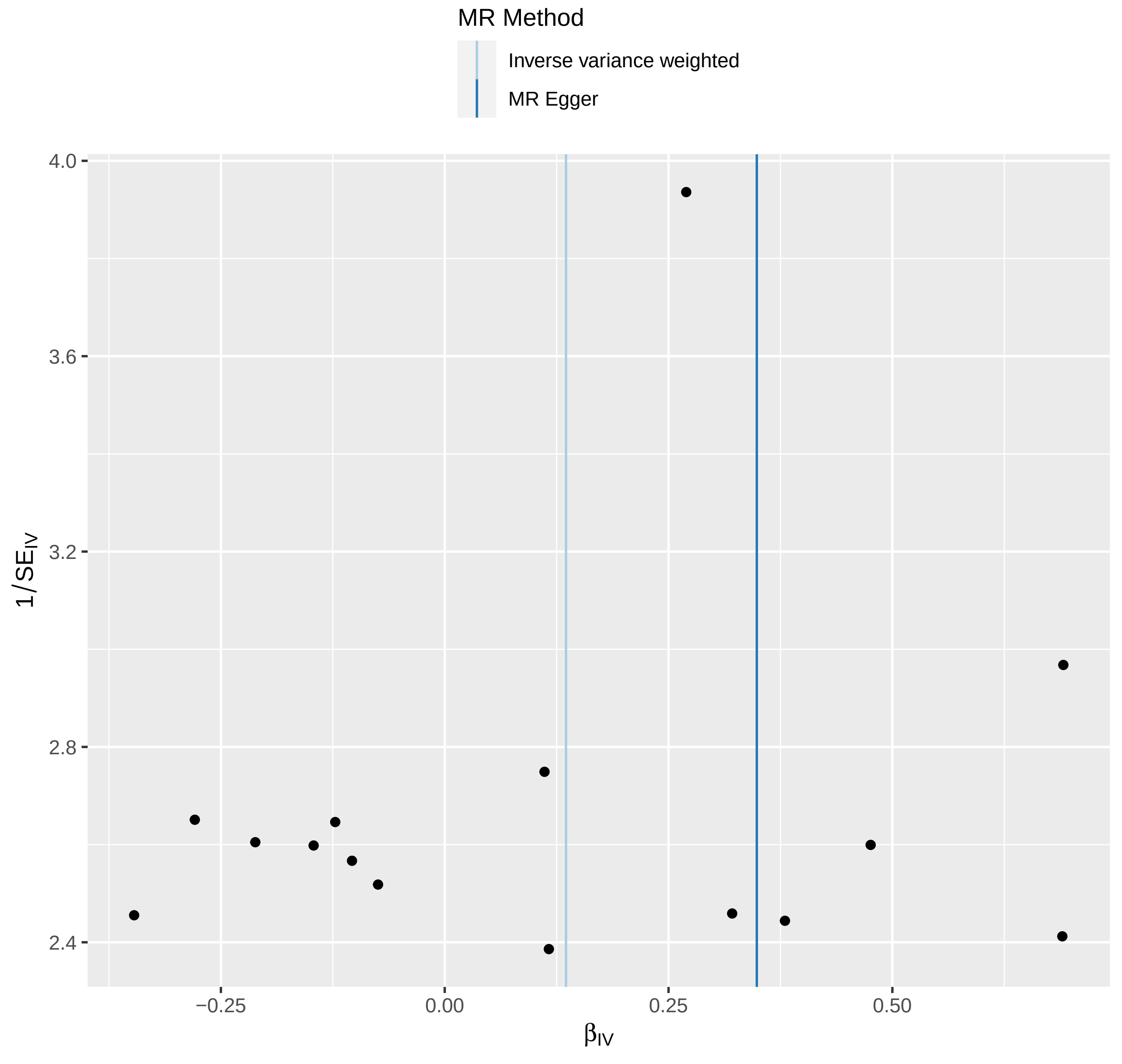

Batch 2−393 : Gut microbiota abundance (genus Catenibacterium id.2153) on Cyclic AMP−dependent transcription factor ATF−6 alpha with a threshold of 1e−05

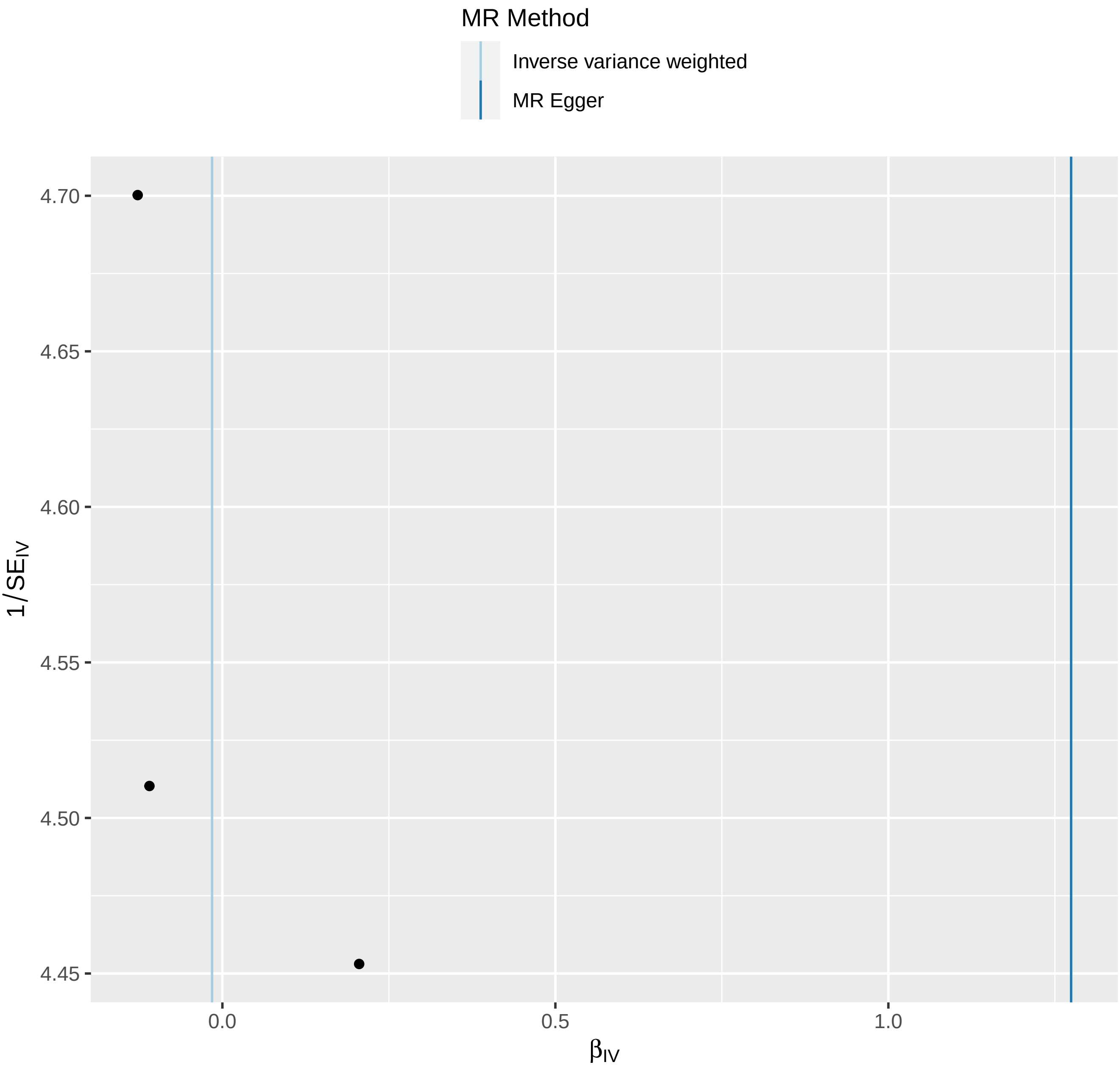

Batch 2−394 : Gut microbiota abundance (genus Parasutterella id.2892) on Cyclic AMP−dependent transcription factor ATF−6 alpha with a threshold of 1e−05

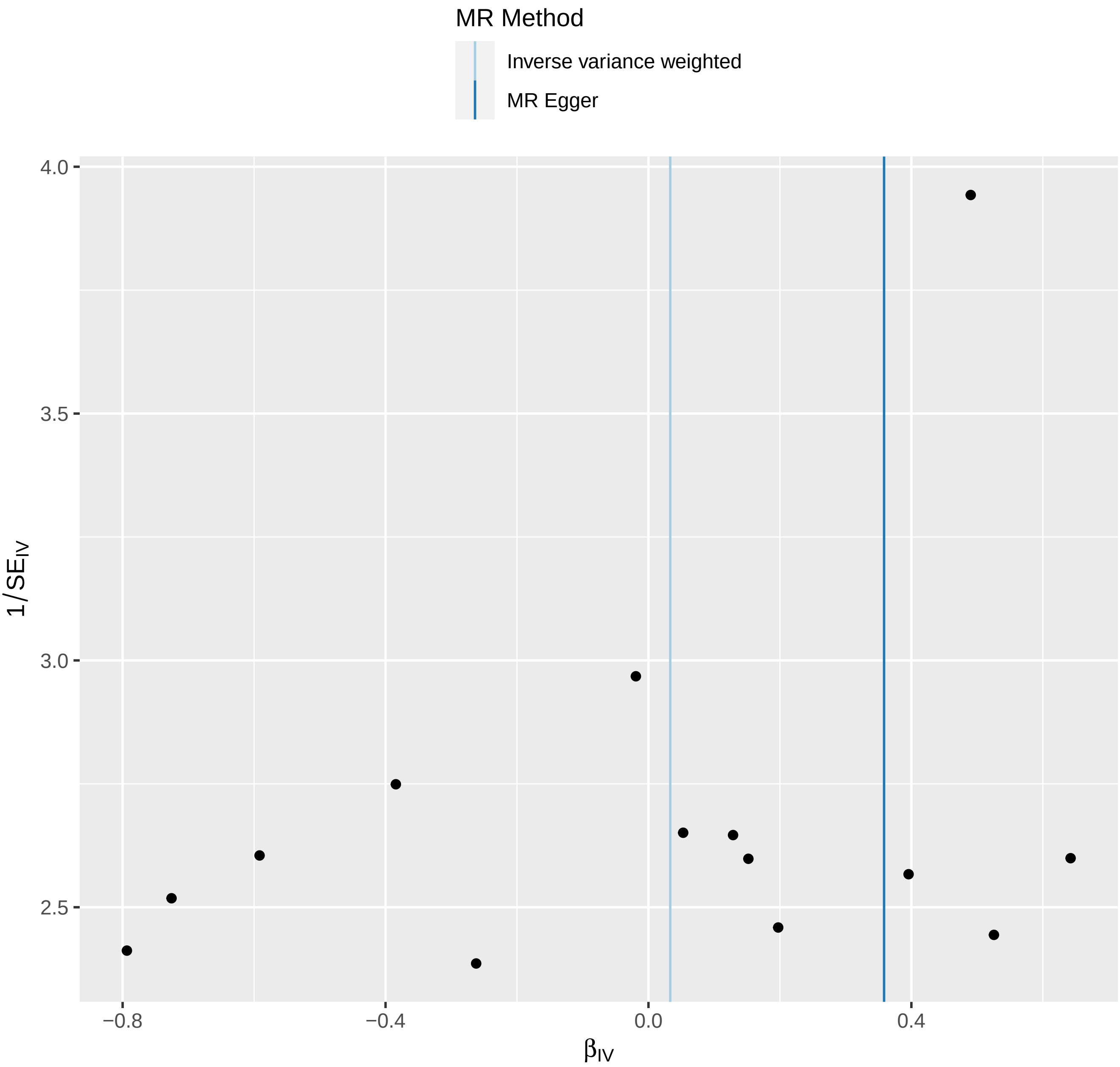

Batch 2−395 : Gut microbiota abundance (genus Catenibacterium id.2153) on Ubiquitin−like modifier−activating enzyme ATG7
with a threshold of 1e−05

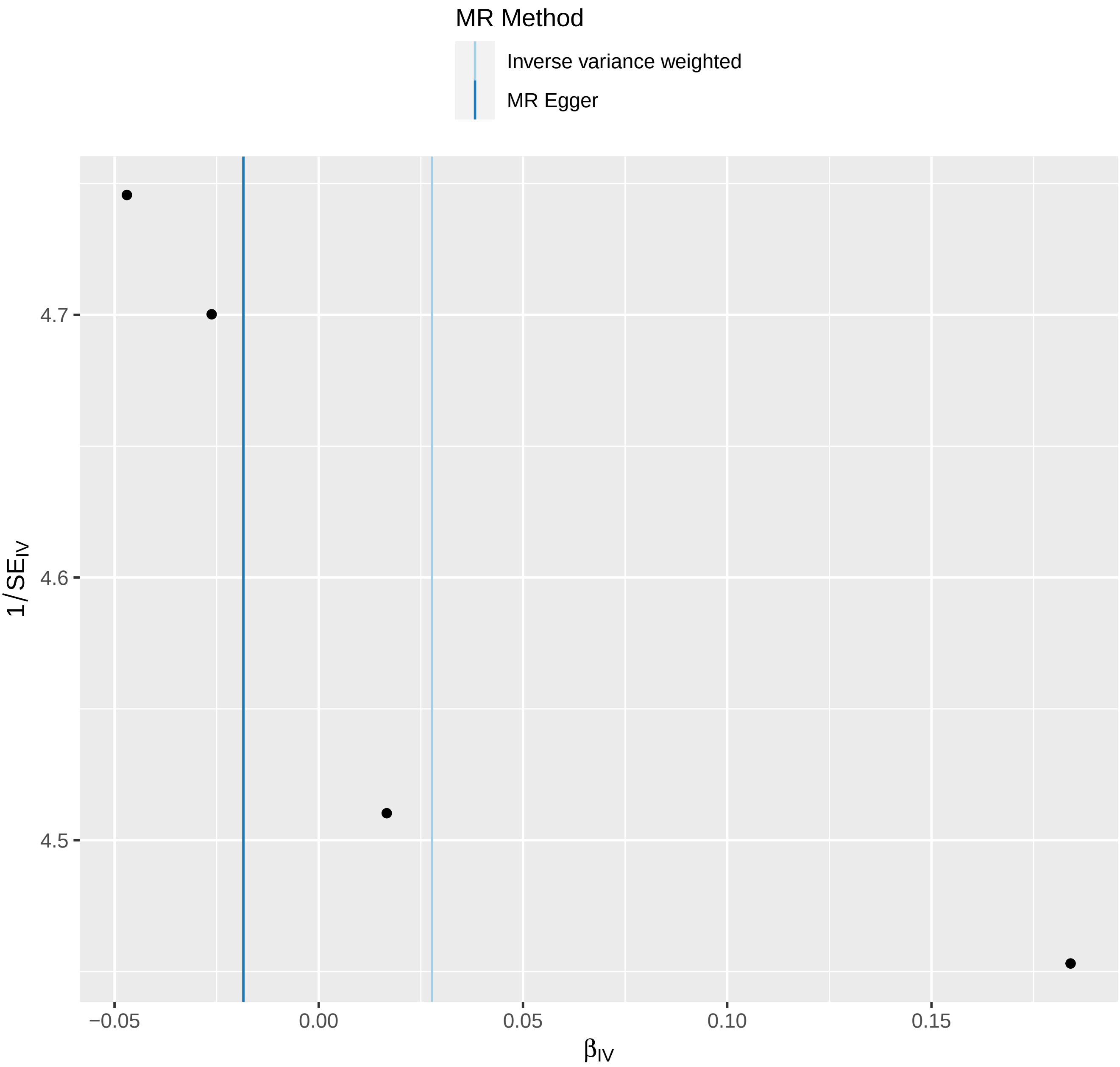

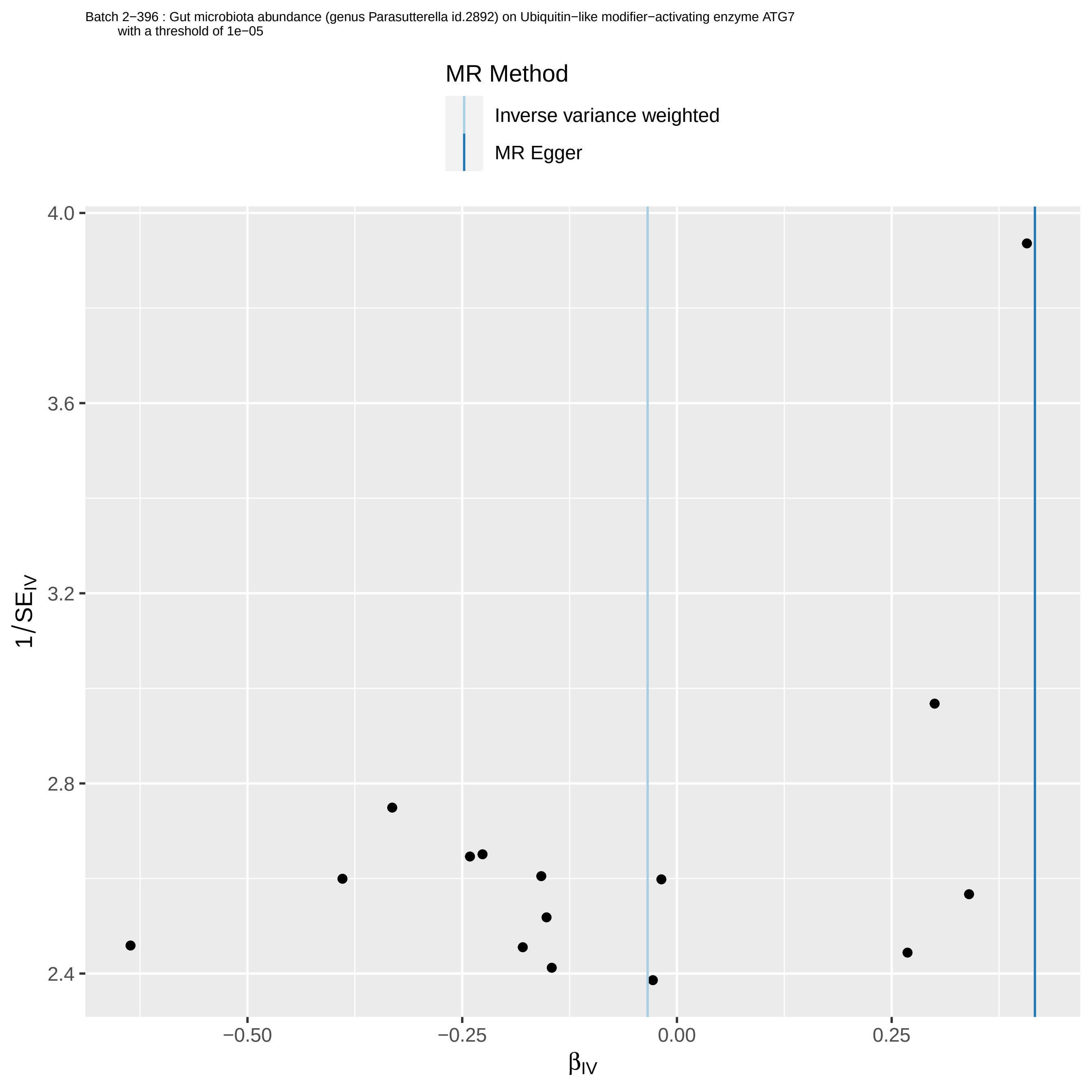

Batch 2−396 : Gut microbiota abundance (genus Parasutterella id.2892) on Ubiquitin−like modifier−activating enzyme ATG7
with a threshold of 1e−05
MR Method
Inverse variance weighted
MR Egger
4.0
3.6
3.2
2.8
2.4
1/SE_IV
−0.50
−0.25
0.00
0.25
β_IV

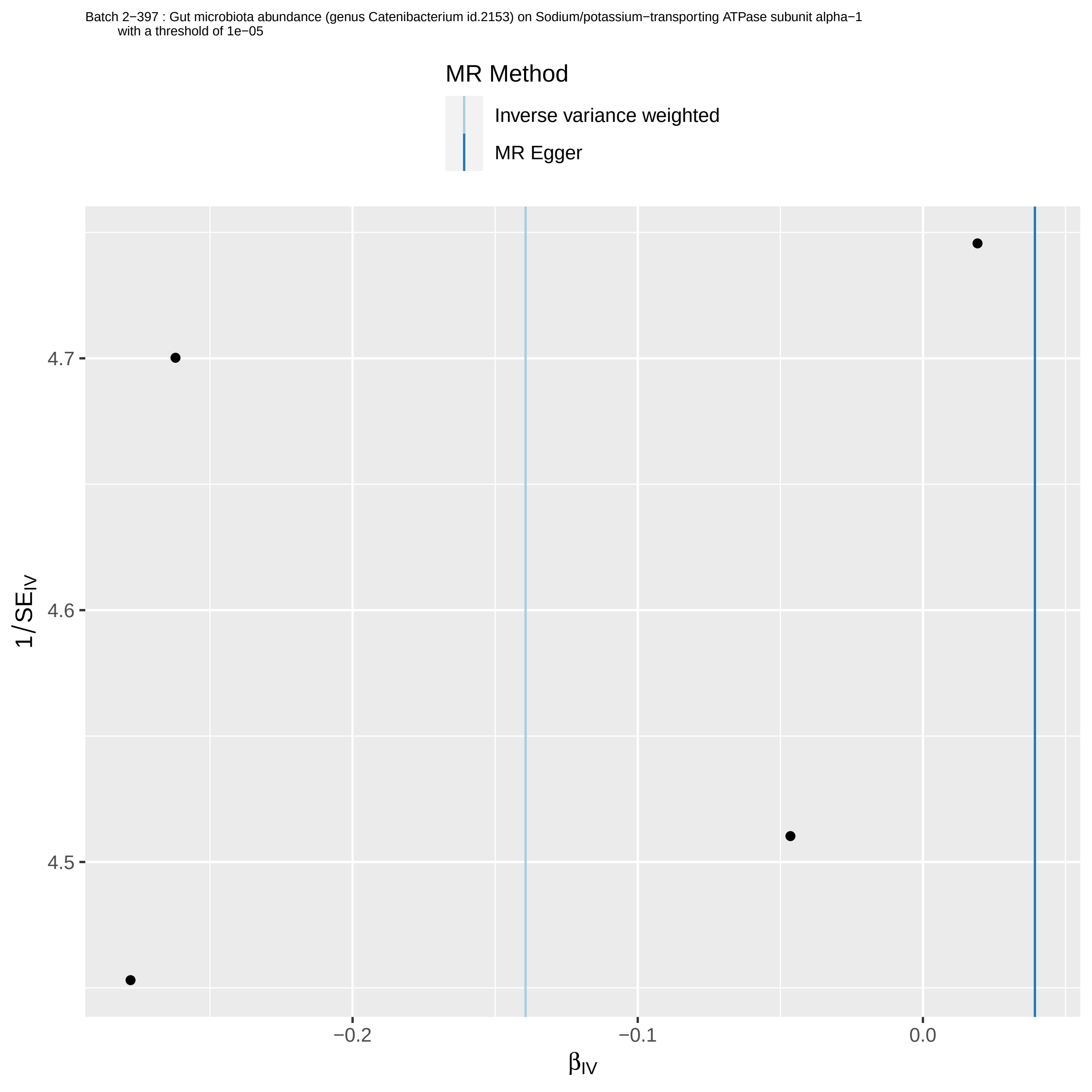

Batch 2−397 : Gut microbiota abundance (genus Catenibacterium id.2153) on Sodium/potassium−transporting ATPase subunit alpha−1
with a threshold of 1e−05
MR Method
Inverse variance weighted
MR Egger
4.7
4.6
4.5
1/SE$_{IV}$
−0.2
−0.1
0.0
β_{IV}

Batch 2−398 : Gut microbiota abundance (genus Parasutterella id.2892) on Sodium/potassium−transporting ATPase subunit alpha−1
with a threshold of 1e−05

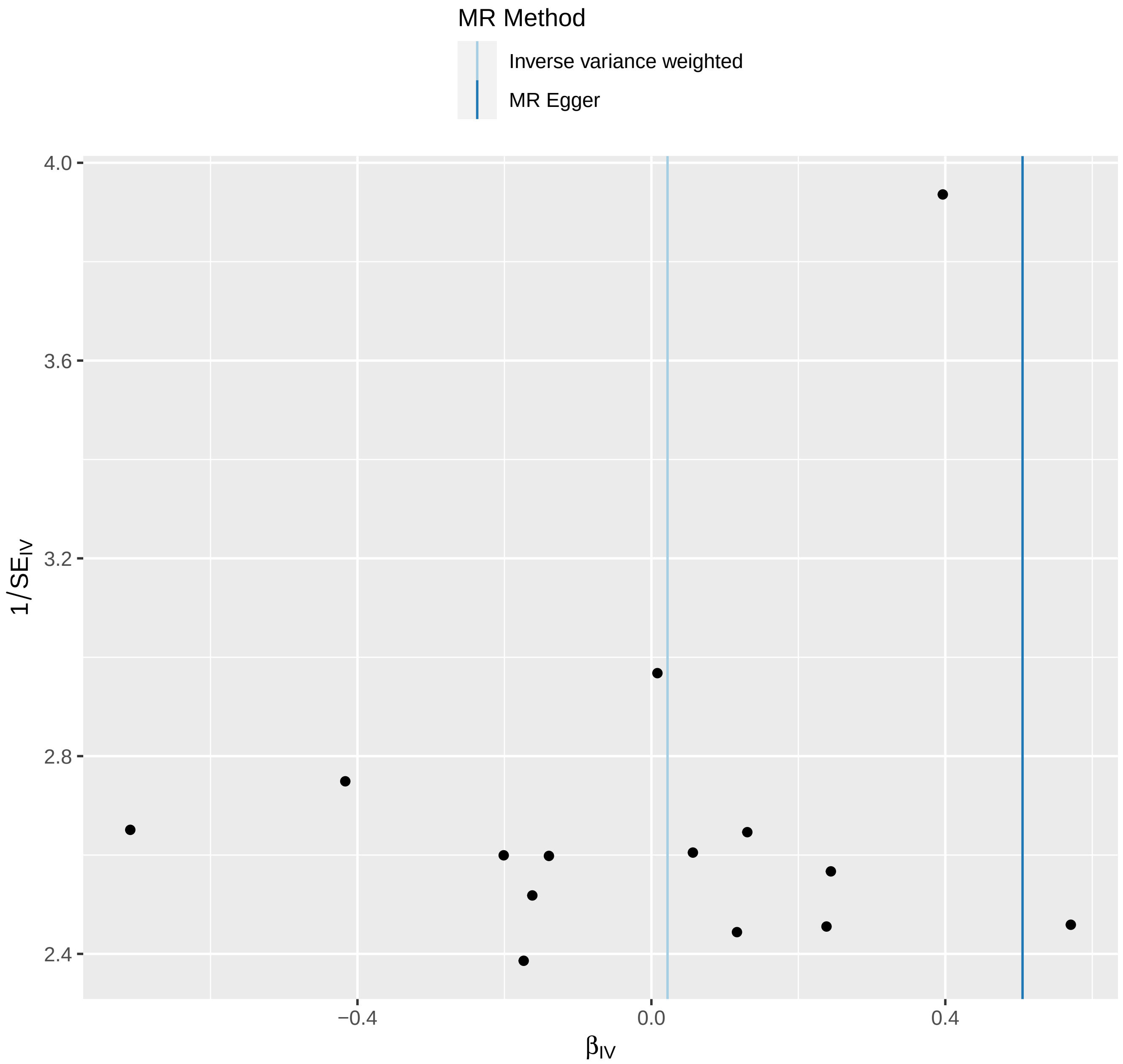

Batch 2−399 : Gut microbiota abundance (genus Catenibacterium id.2153) on Sodium/potassium−transporting ATPase subunit beta−2 with a threshold of 1e−05

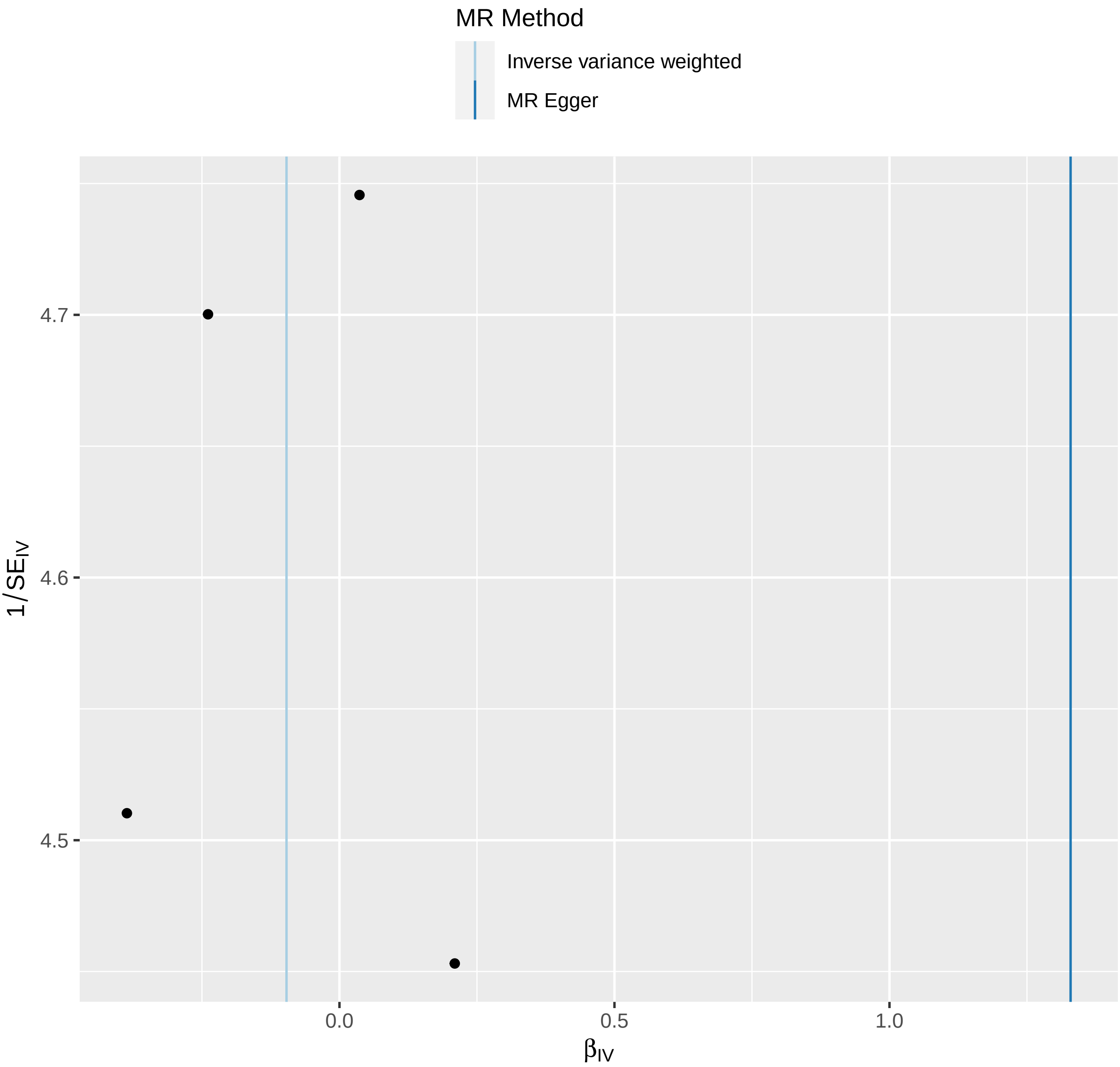

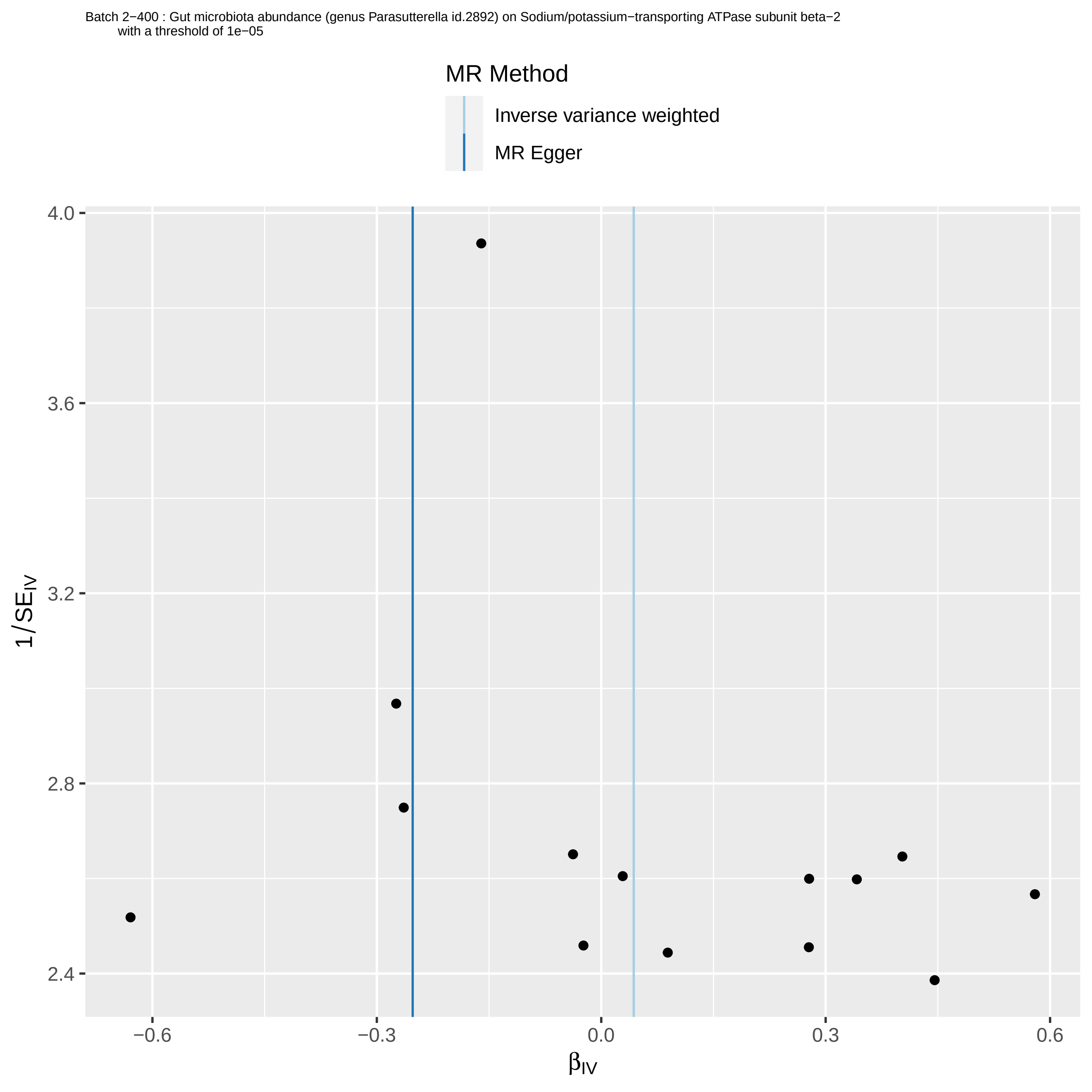

Batch 2−400 : Gut microbiota abundance (genus Parasutterella id.2892) on Sodium/potassium−transporting ATPase subunit beta−2
with a threshold of 1e−05
MR Method
Inverse variance weighted
MR Egger
4.0
3.6
3.2
2.8
2.4
1/SE_IV
−0.6
−0.3
0.0
0.3
0.6
β_IV

Batch 2−401 : Gut microbiota abundance (genus Catenibacterium id.2153) on Sarcoplasmic/endoplasmic reticulum calcium ATPase 3
with a threshold of 1e−05

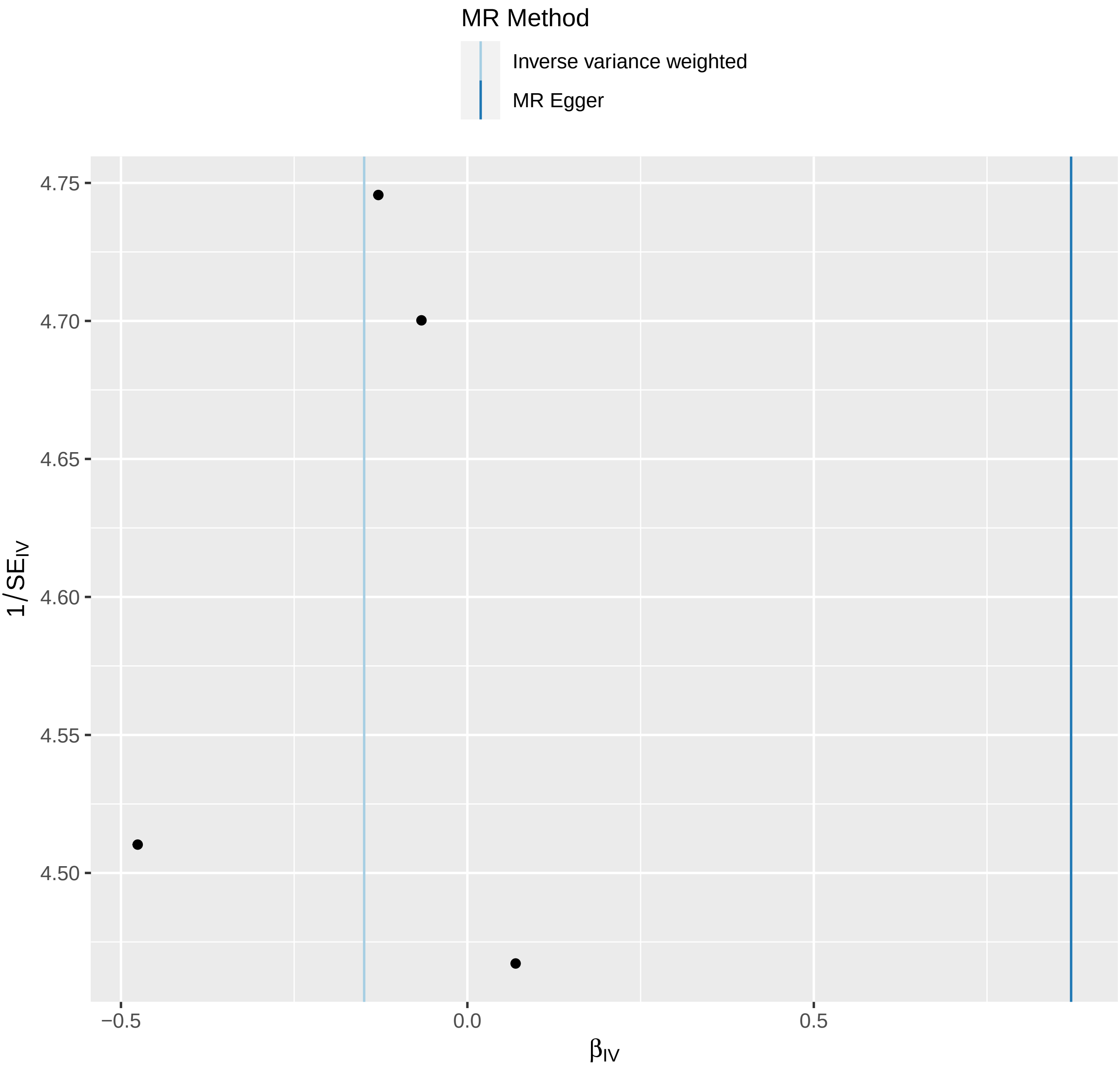

Batch 2−402 : Gut microbiota abundance (genus Parasutterella id.2892) on Sarcoplasmic/endoplasmic reticulum calcium ATPase 3
with a threshold of 1e−05

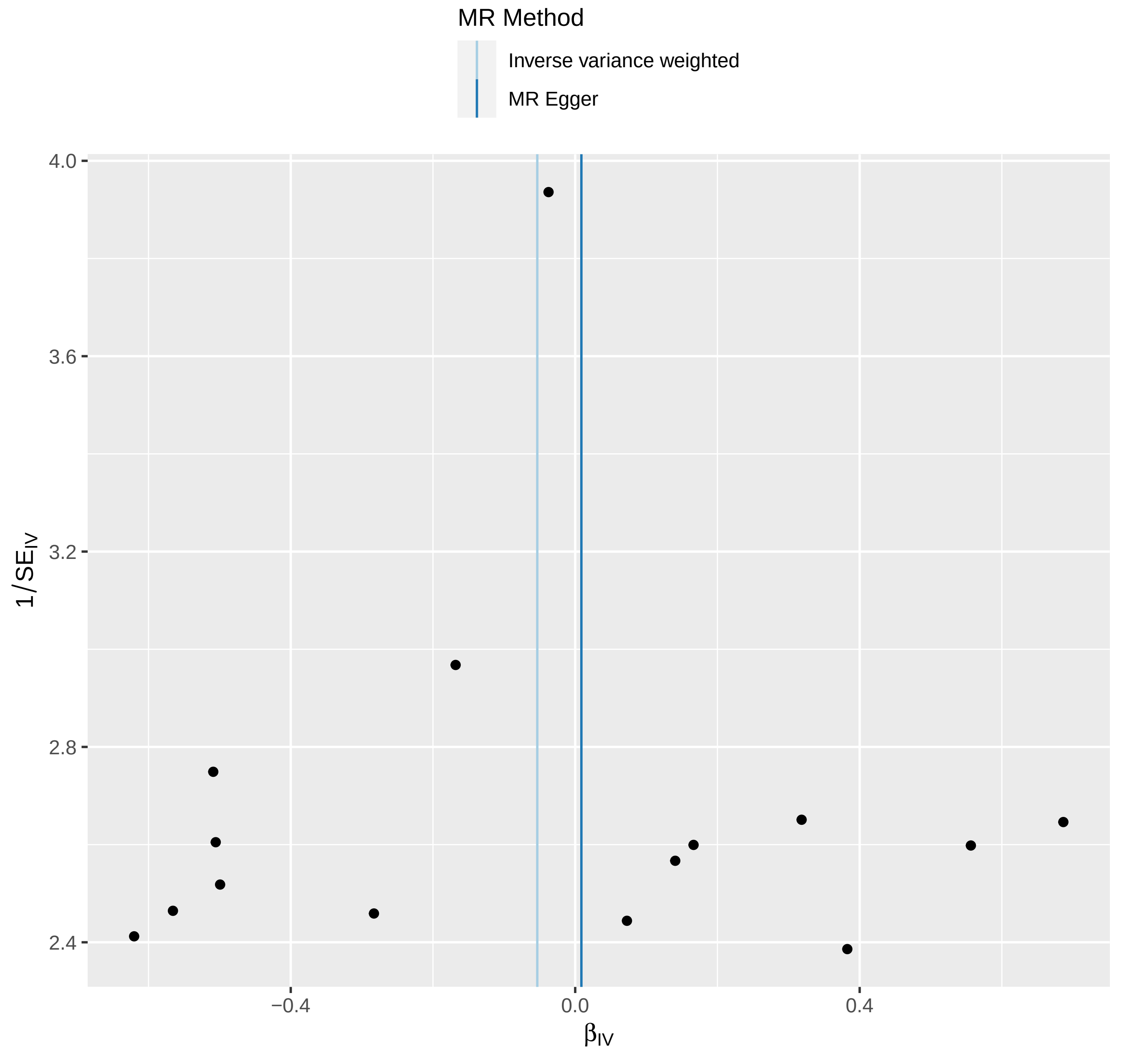

Batch 2−403 : Gut microbiota abundance (genus Catenibacterium id.2153) on Potassium−transporting ATPase subunit beta with a threshold of 1e−05

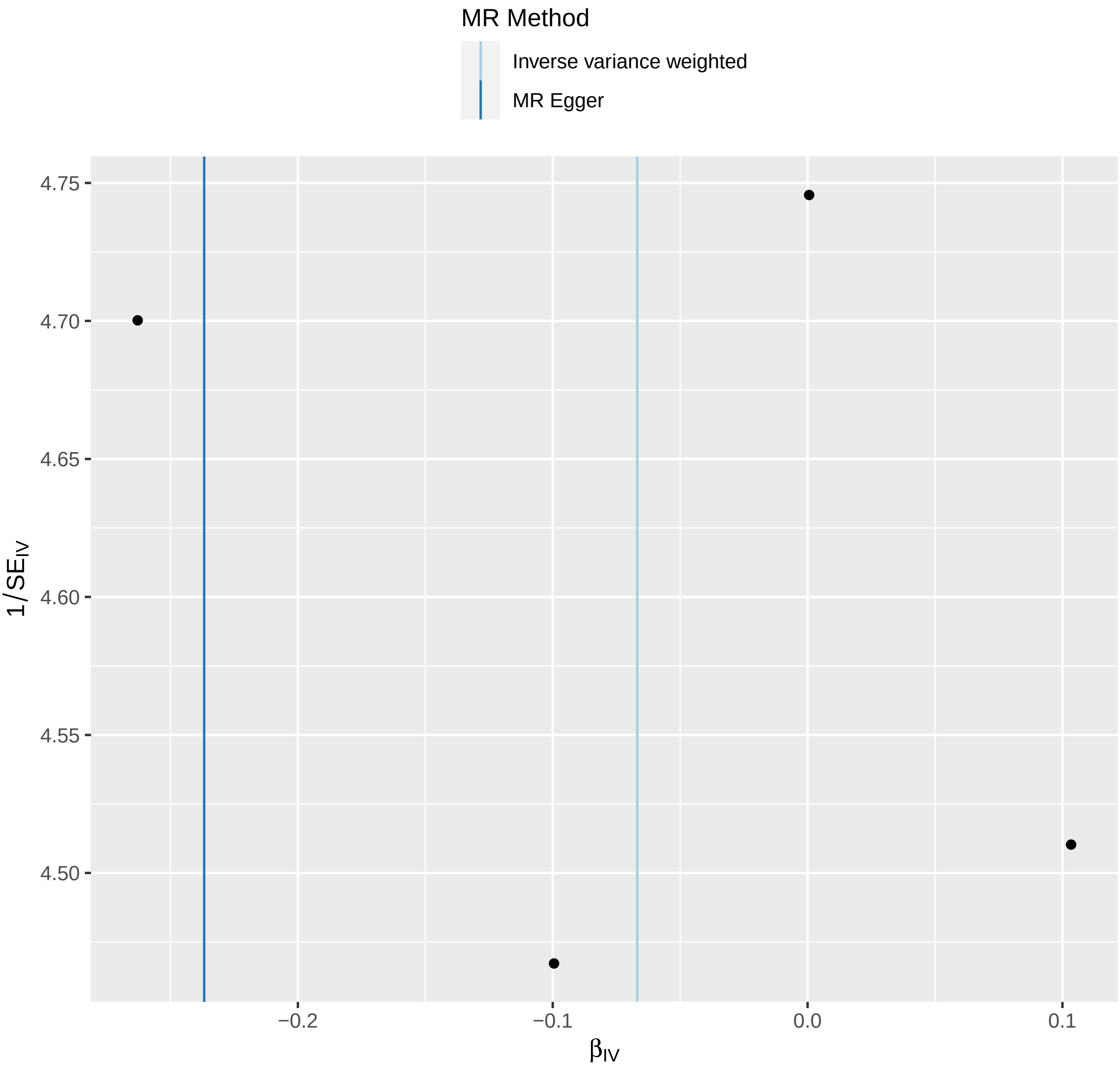

Batch 2−404 : Gut microbiota abundance (genus Parasutterella id.2892) on Potassium−transporting ATPase subunit beta
with a threshold of 1e−05

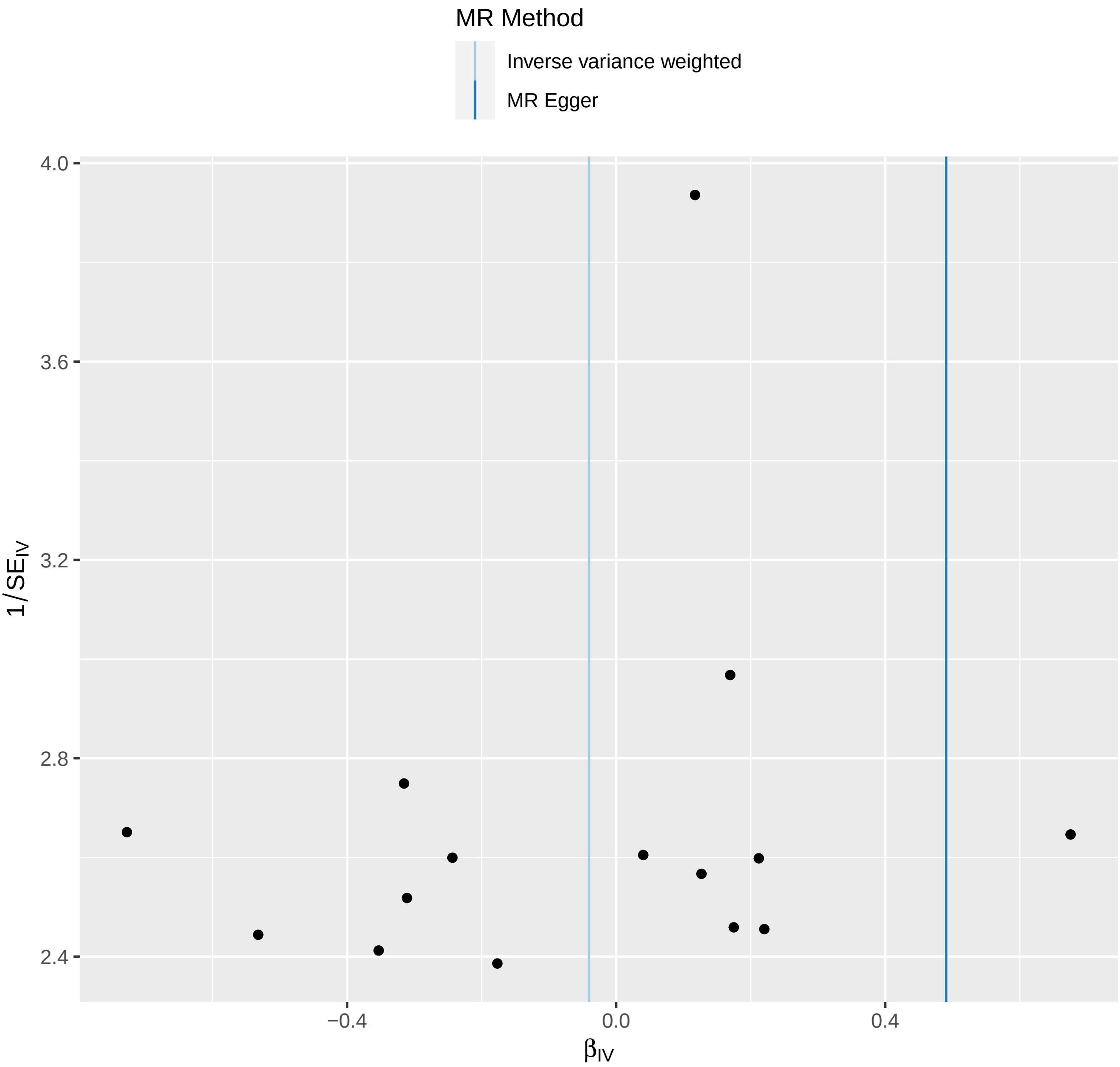

Batch 2−405 : Gut microbiota abundance (genus Catenibacterium id.2153) on ATP synthase subunit beta, mitochondrial
with a threshold of 1e−05

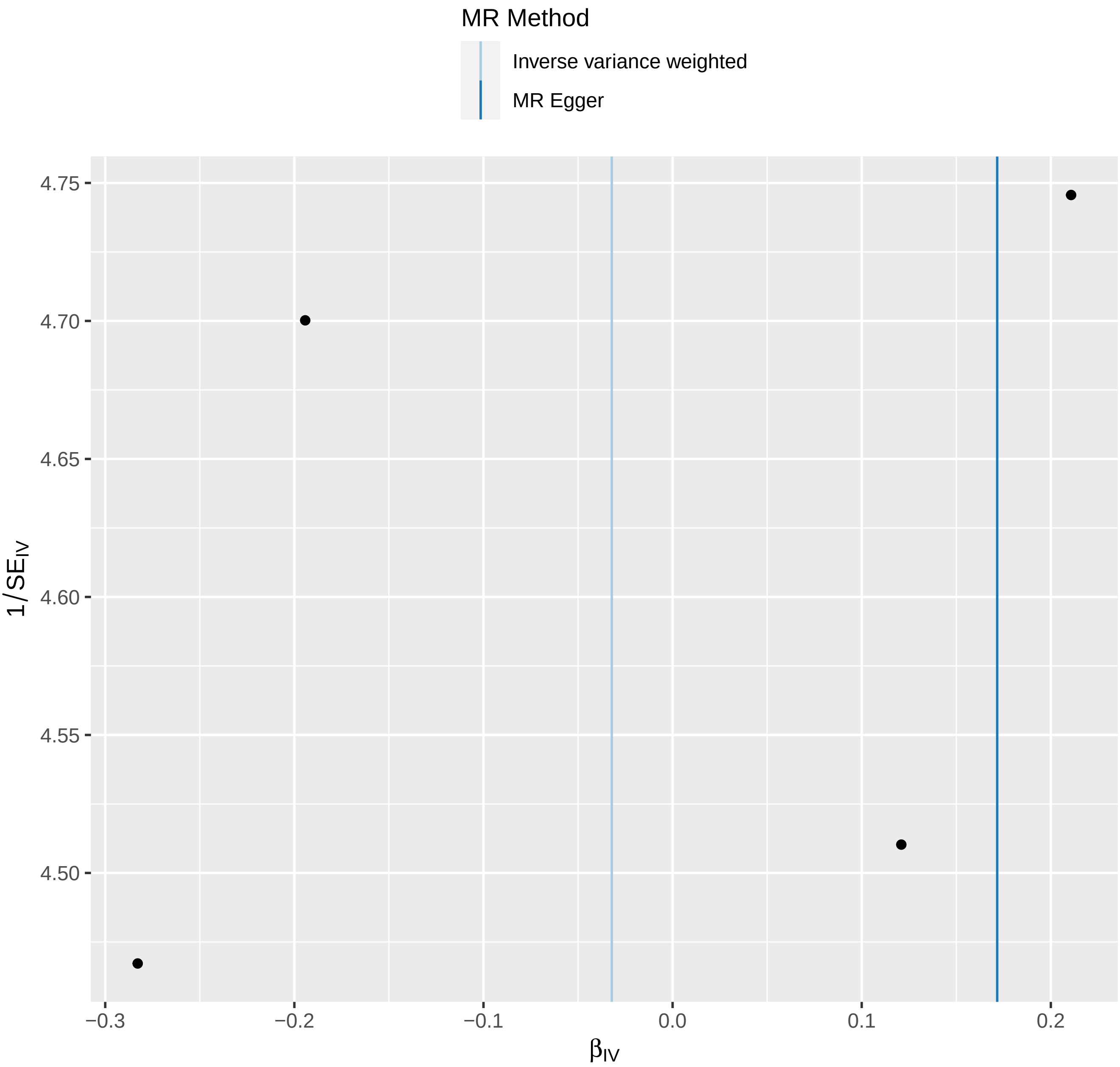

Batch 2−406 : Gut microbiota abundance (genus Parasutterella id.2892) on ATP synthase subunit beta, mitochondrial
with a threshold of 1e−05

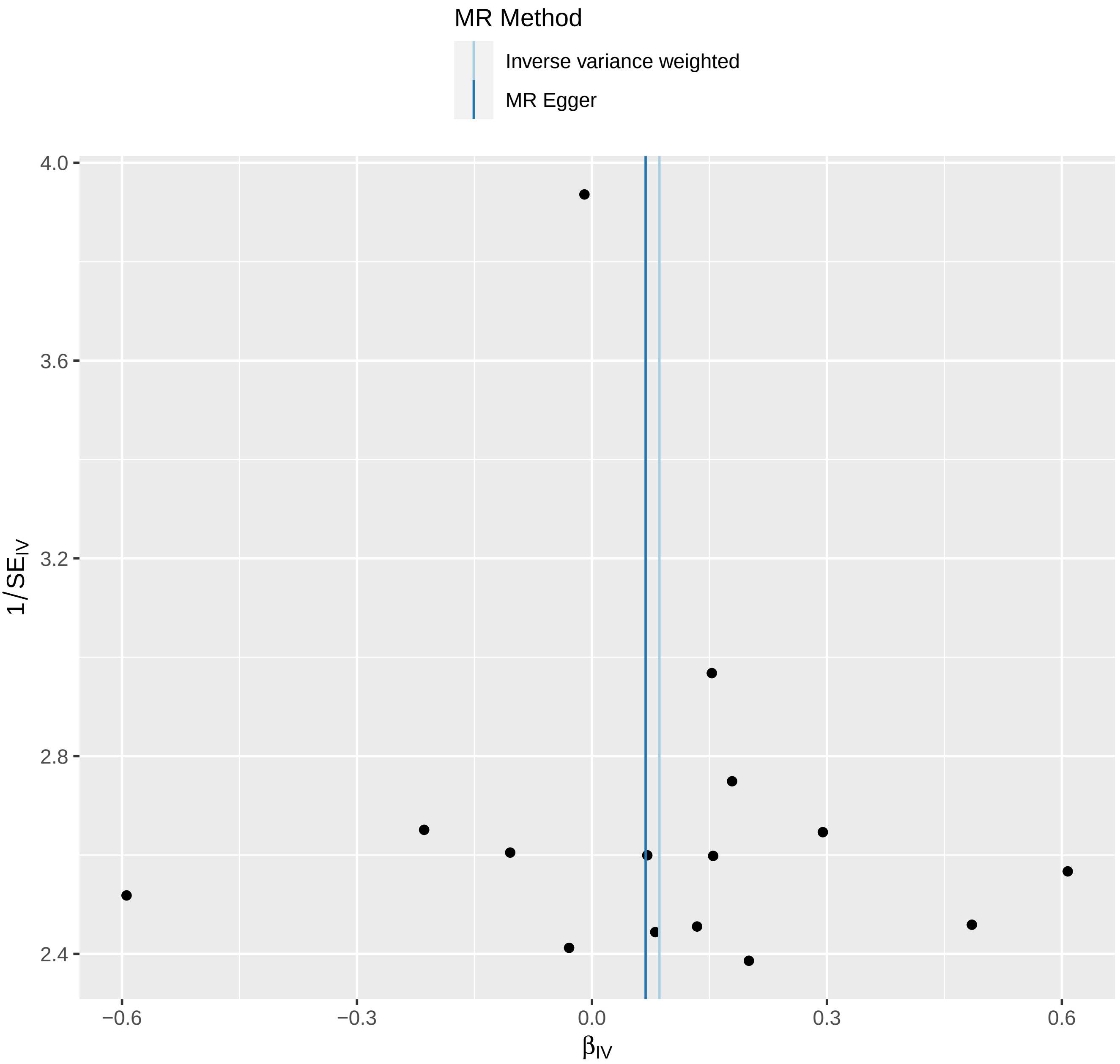

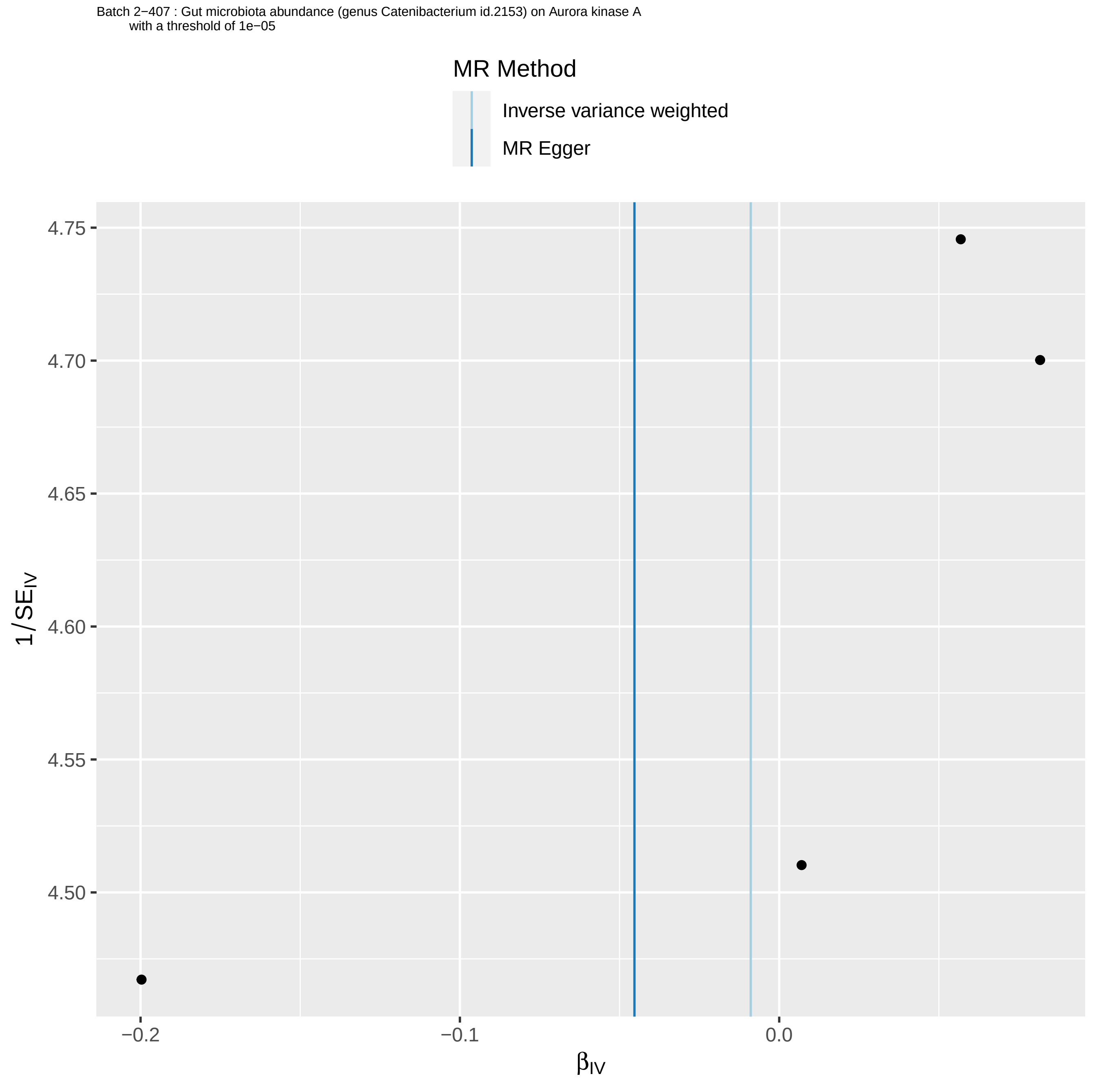

Batch 2−407 : Gut microbiota abundance (genus Catenibacterium id.2153) on Aurora kinase A
with a threshold of 1e−05
MR Method
Inverse variance weighted
MR Egger
4.75
4.70
4.65
4.60
4.55
4.50
1/SE_IV
−0.2
−0.1
0.0
β_IV

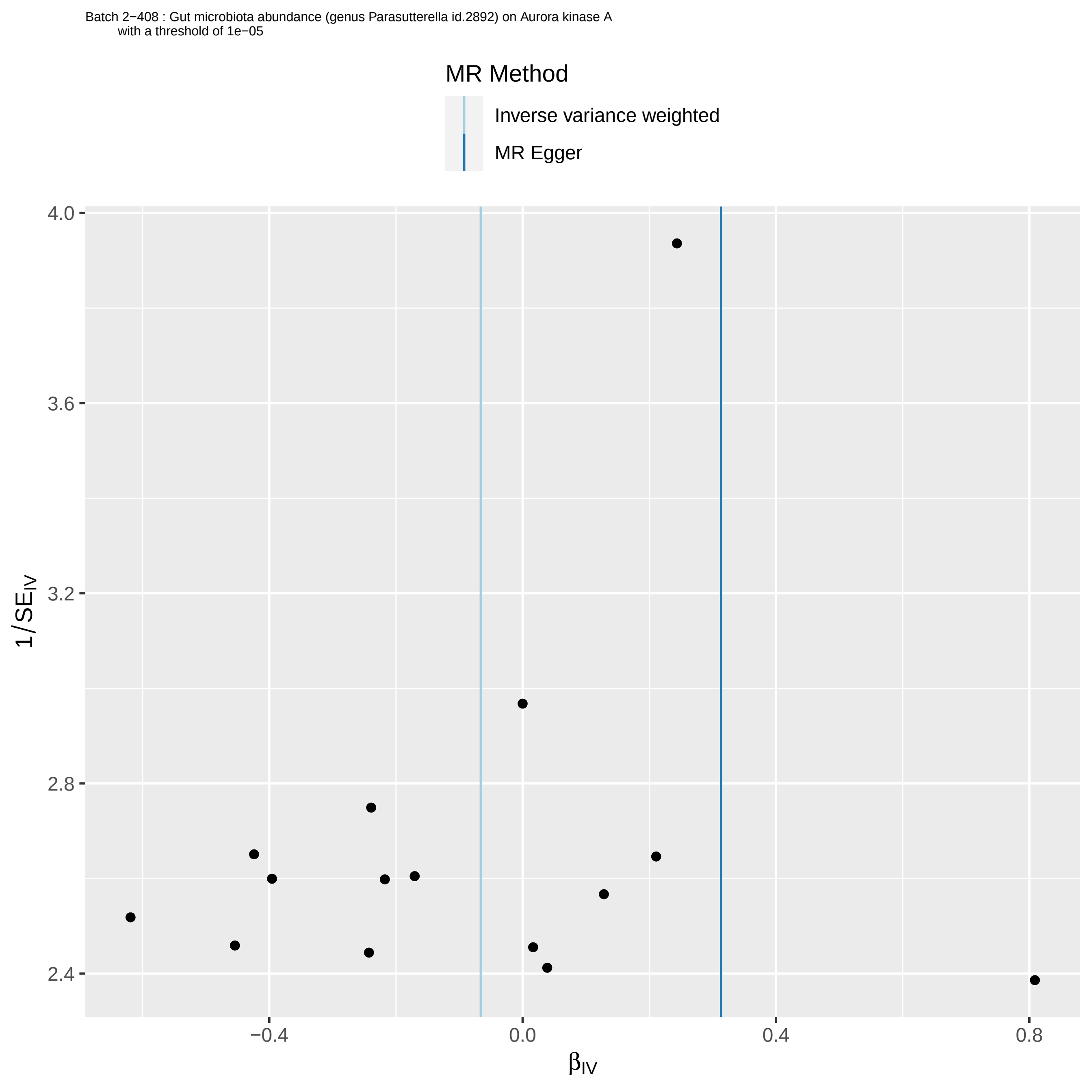
Batch 2−408 : Gut microbiota abundance (genus Parasutterella id.2892) on Aurora kinase A
with a threshold of 1e−05
MR Method
Inverse variance weighted
MR Egger
4.0
3.6
3.2
2.8
2.4
1/SE_IV
−0.4
0.0
0.4
0.8
β_IV

Batch 2−409 : Gut microbiota abundance (genus Catenibacterium id.2153) on Aurora kinase B
with a threshold of 1e−05

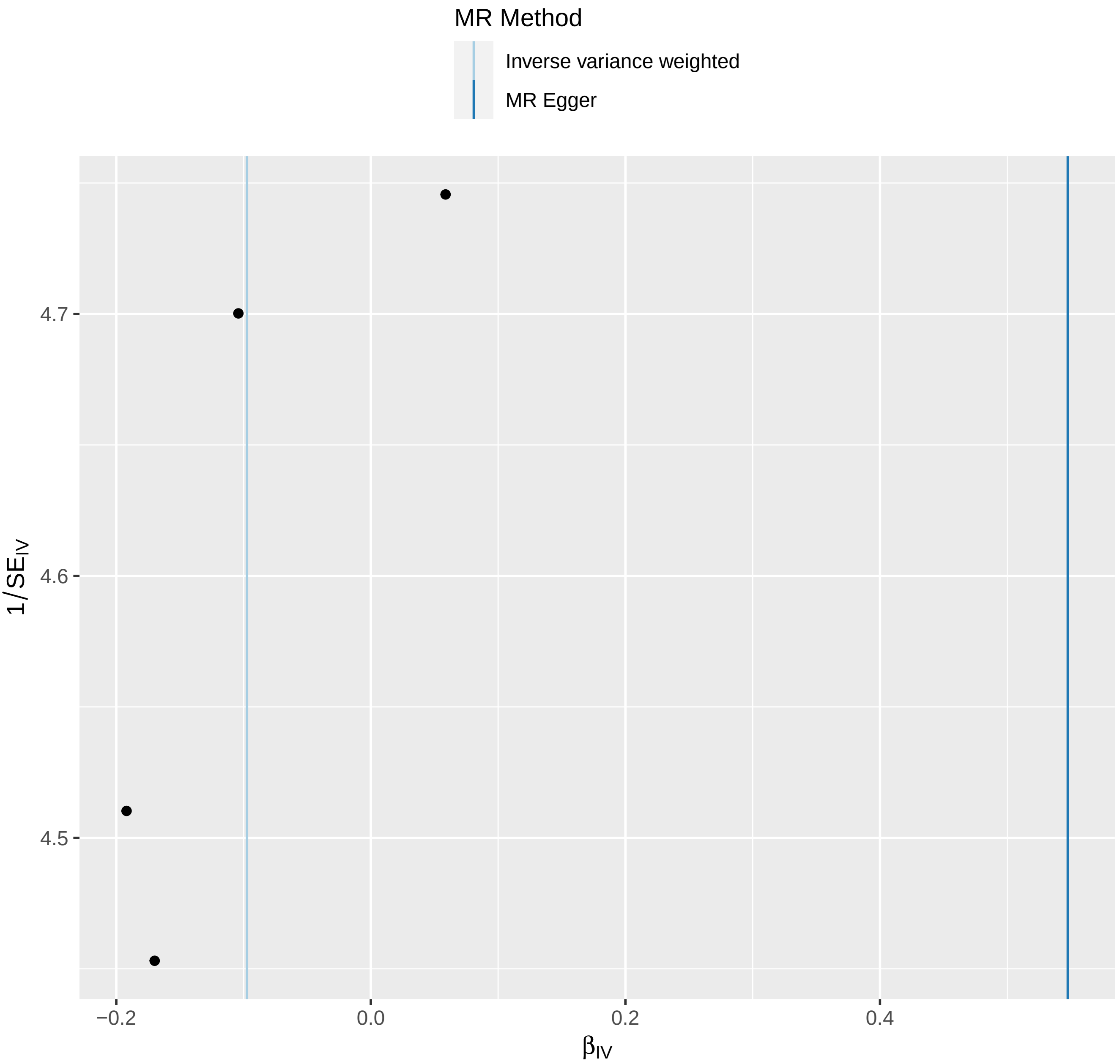

Batch 2−410 : Gut microbiota abundance (genus Parasutterella id.2892) on Aurora kinase B
with a threshold of 1e−05

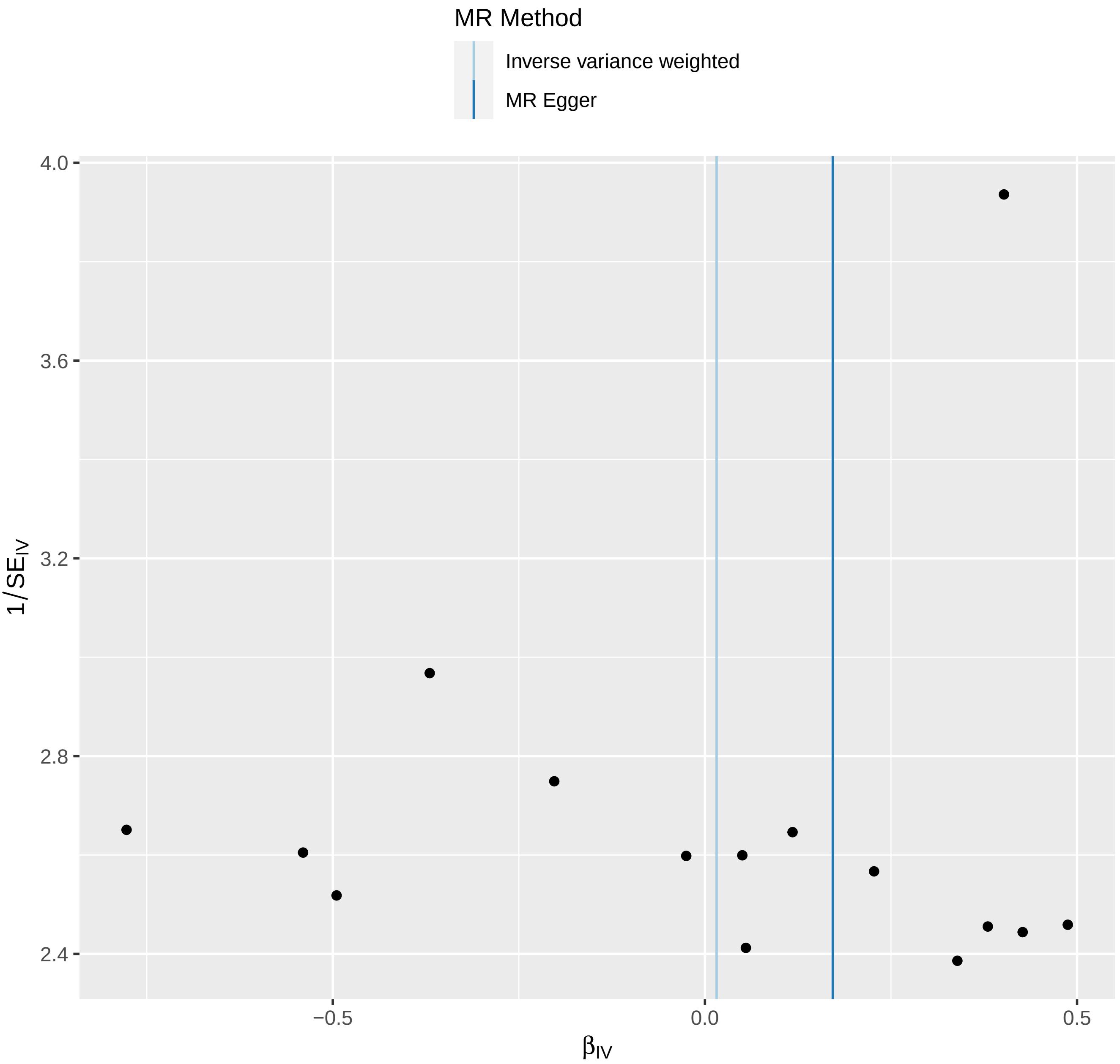

Batch 2−411 : Gut microbiota abundance (genus Catenibacterium id.2153) on Axin−2
with a threshold of 1e−05

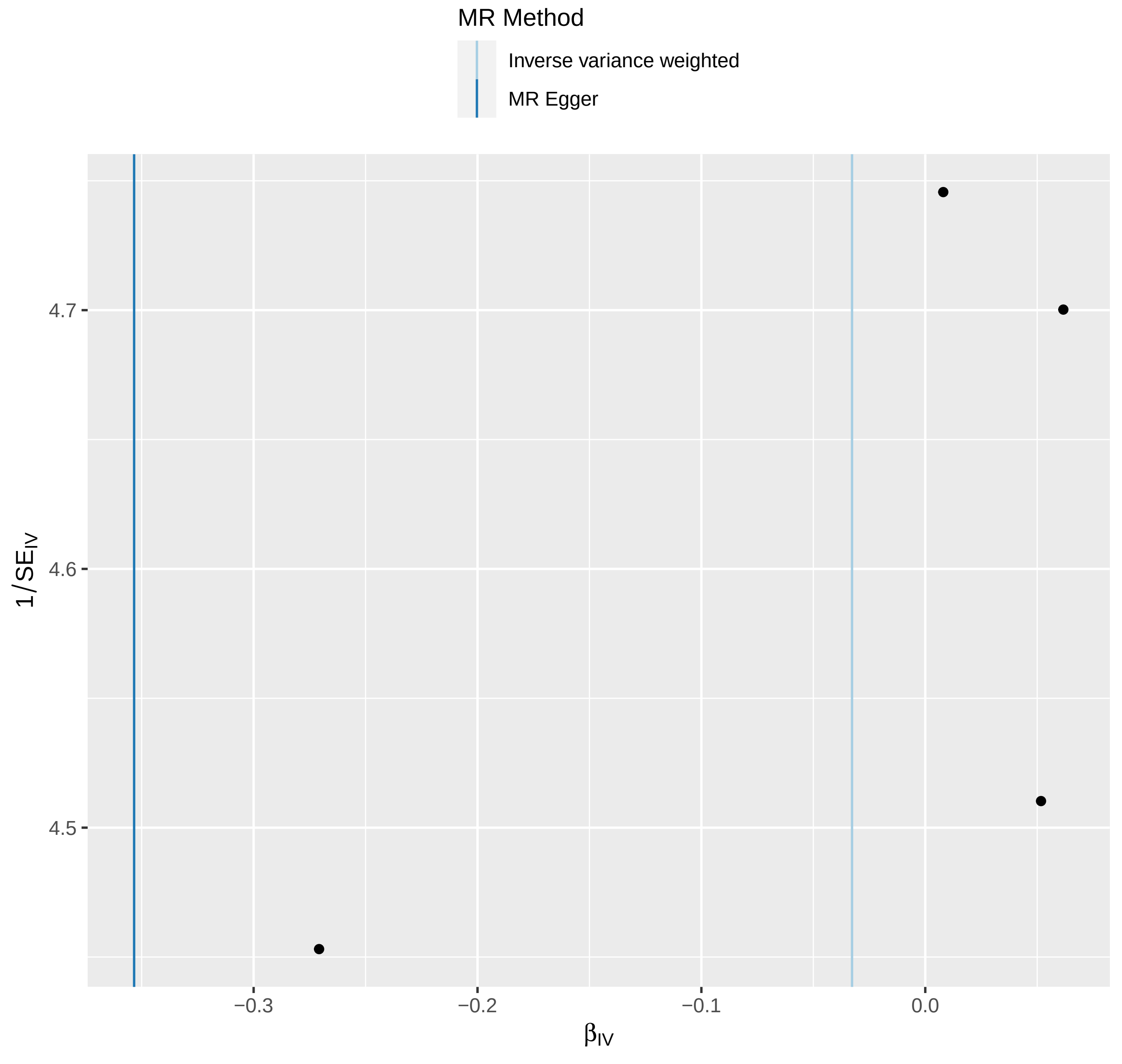

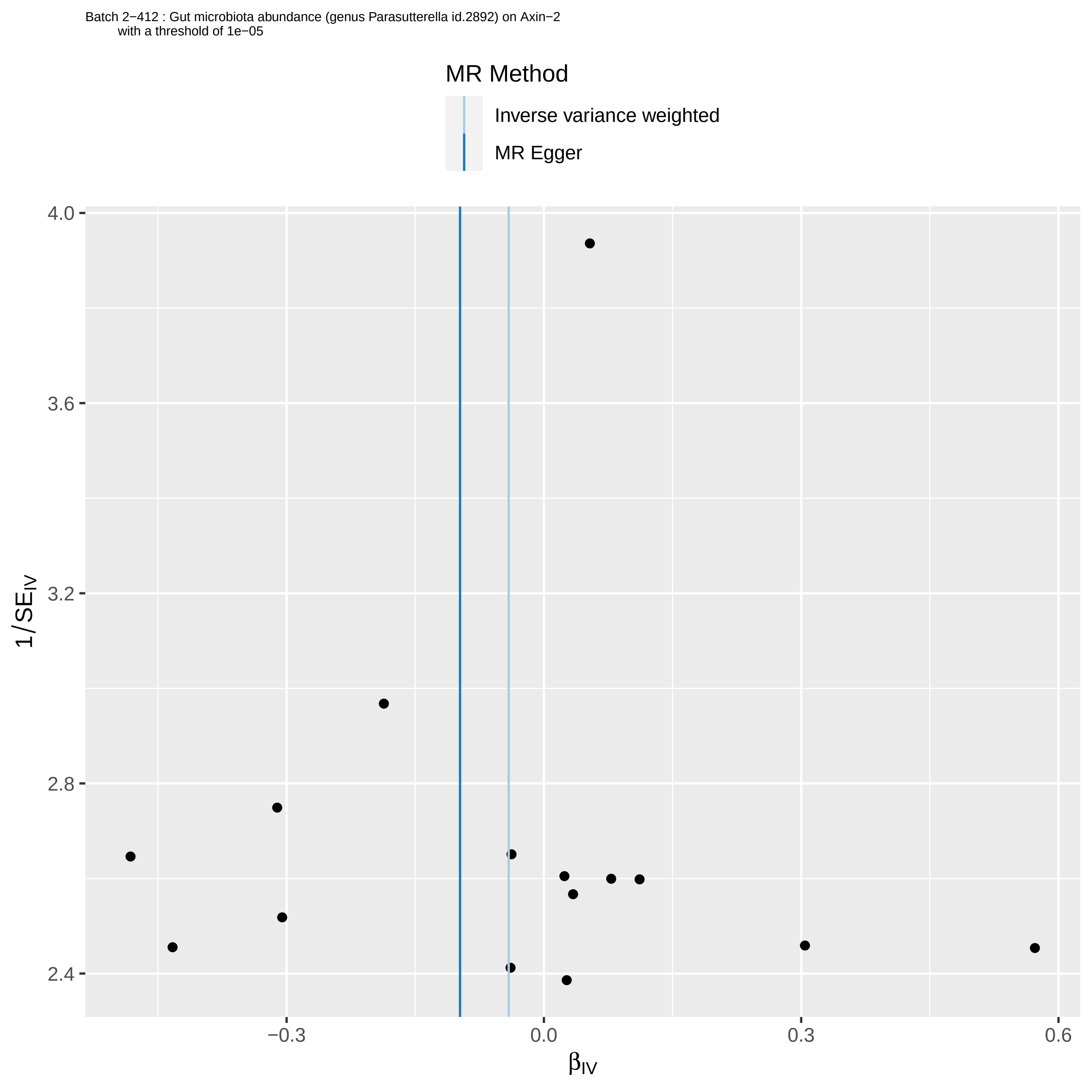
Batch 2−412 : Gut microbiota abundance (genus Parasutterella id.2892) on Axin−2
with a threshold of 1e−05
MR Method
Inverse variance weighted
MR Egger
4.0
3.6
3.2
2.8
2.4
1/SE_IV
−0.3
0.0
0.3
0.6
β_IV

Batch 2−413 : Gut microbiota abundance (genus Catenibacterium id.2153) on Axin−2
with a threshold of 1e−05

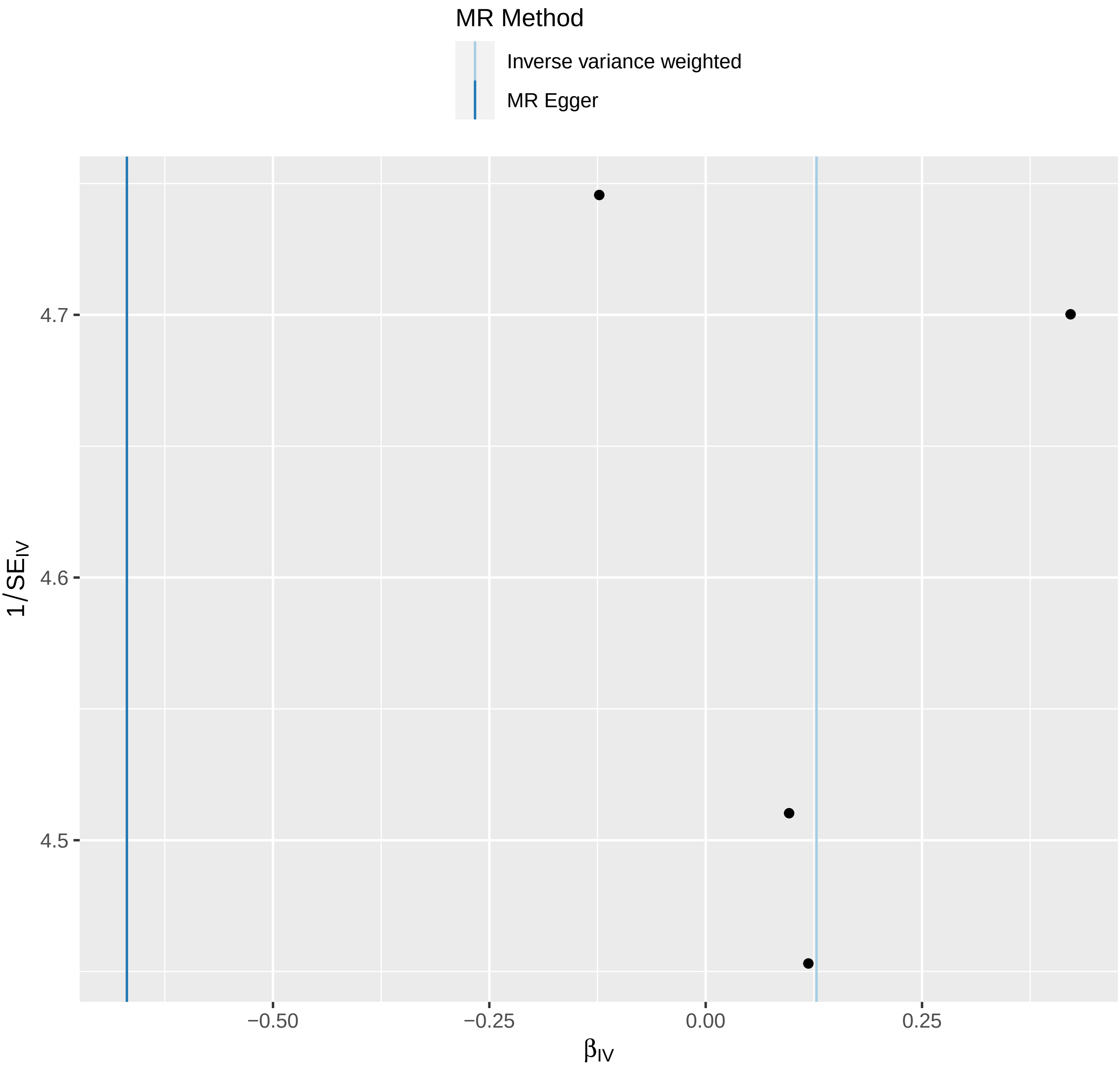

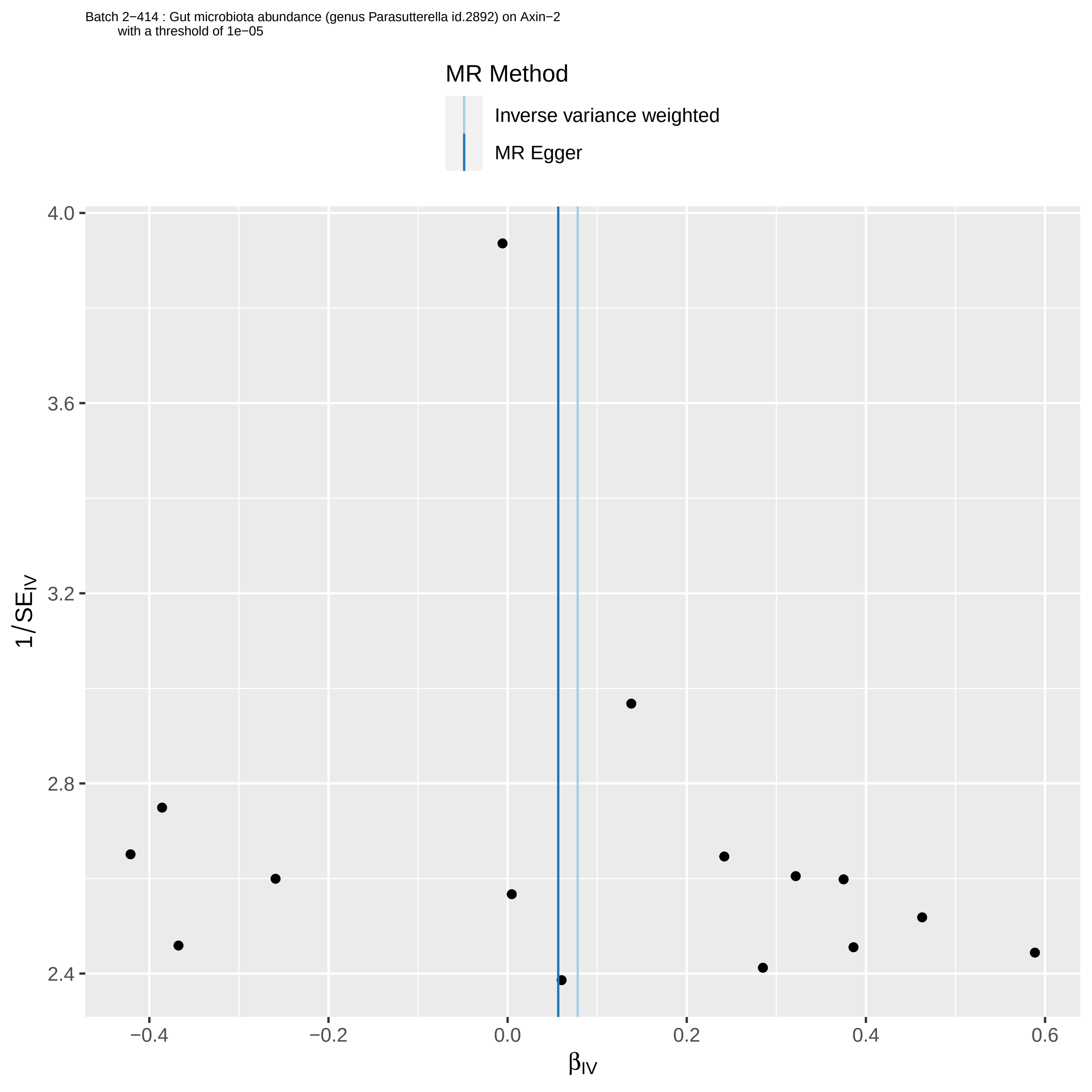

Batch 2−414 : Gut microbiota abundance (genus Parasutterella id.2892) on Axin−2
with a threshold of 1e−05
MR Method
Inverse variance weighted
MR Egger
4.0
3.6
3.2
2.8
2.4
1/SE$_{IV}$
−0.4
−0.2
0.0
0.2
0.4
0.6
β$_{IV}$

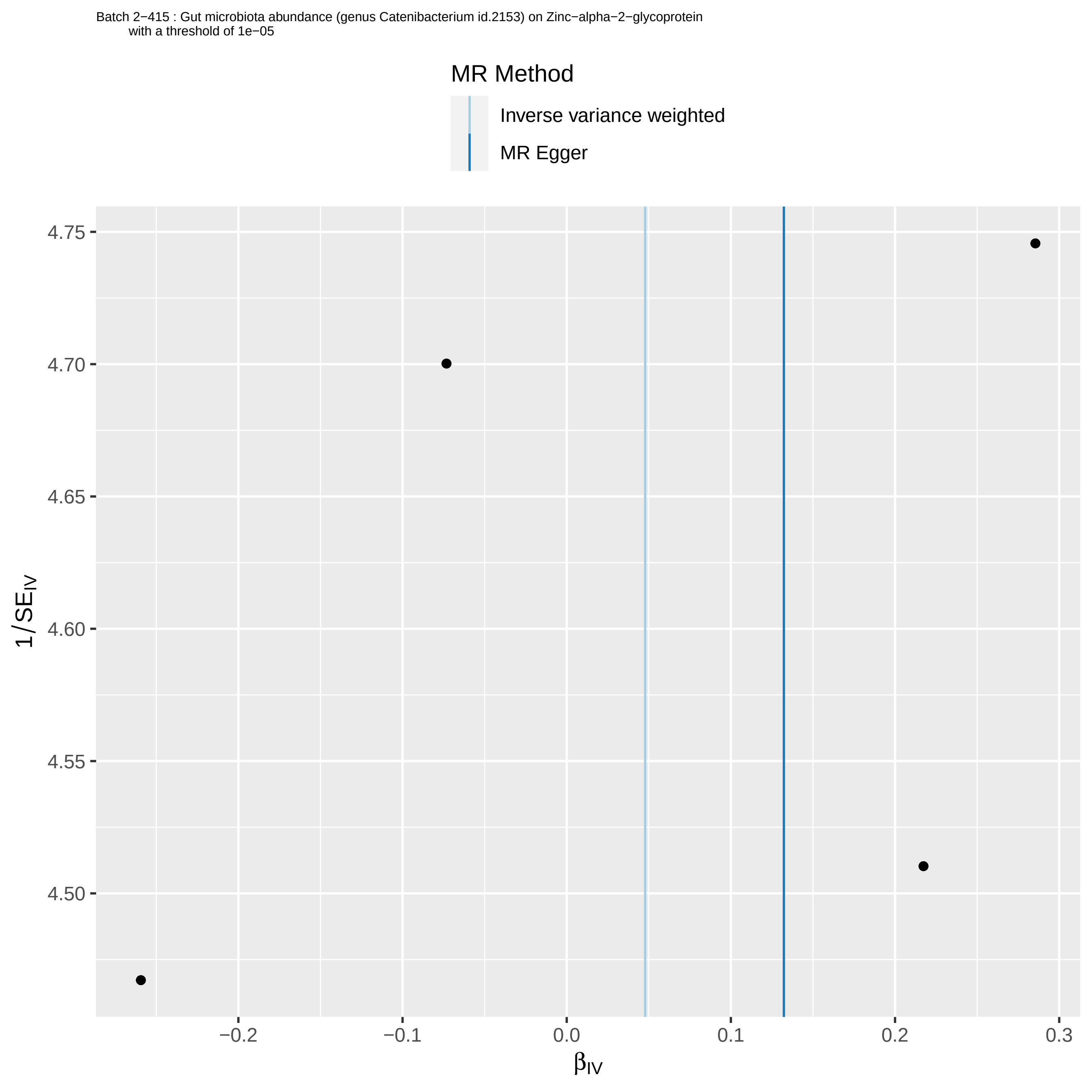

Batch 2−415 : Gut microbiota abundance (genus Catenibacterium id.2153) on Zinc−alpha−2−glycoprotein
with a threshold of 1e−05
MR Method
Inverse variance weighted
MR Egger
4.75
4.70
4.65
4.60
4.55
4.50
1/SE$_{IV}$
−0.2
−0.1
0.0
0.1
0.2
0.3
β$_{IV}$

Batch 2−416 : Gut microbiota abundance (genus Parasutterella id.2892) on Zinc−alpha−2−glycoprotein
with a threshold of 1e−05

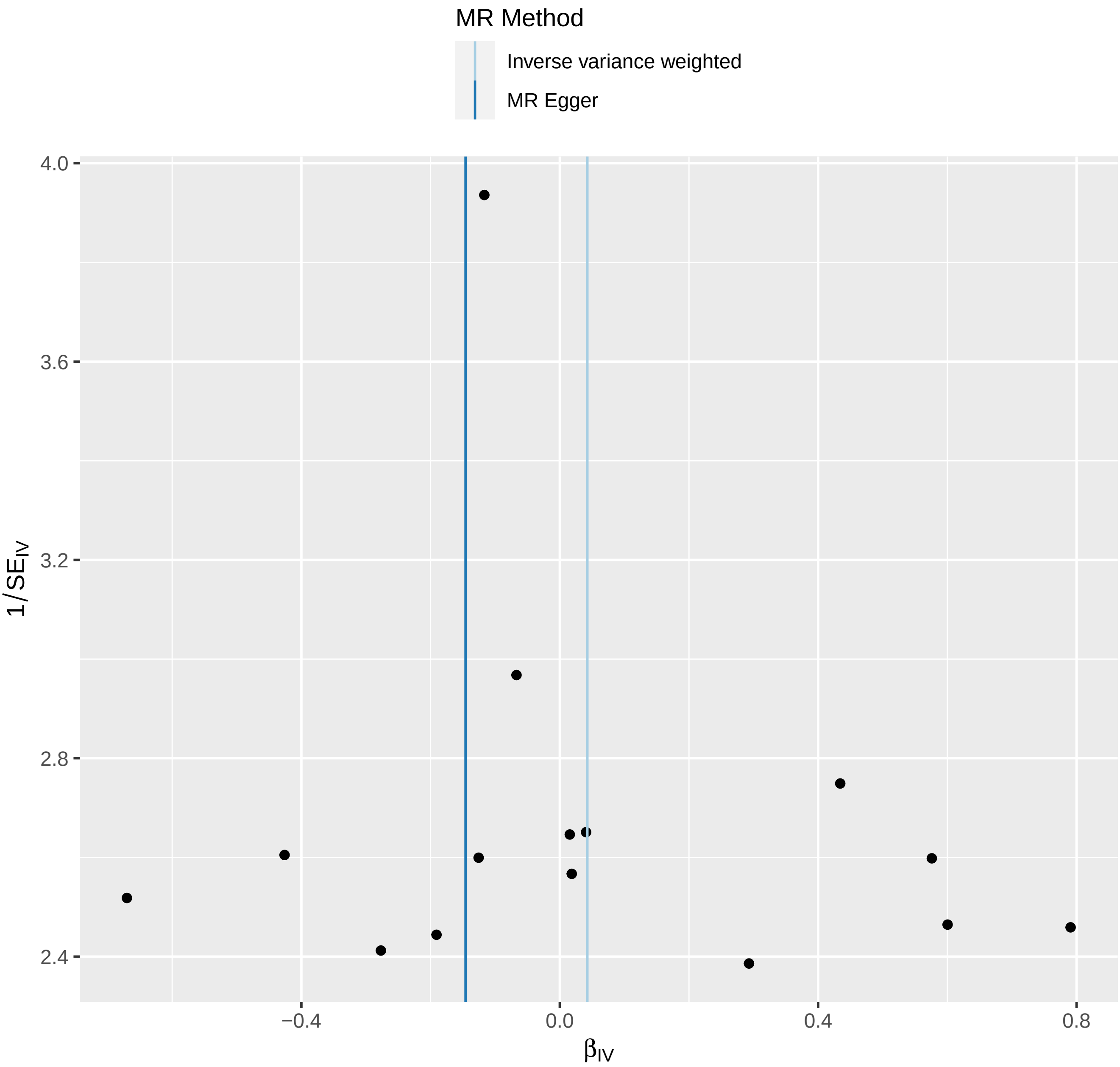

Batch 2−417 : Gut microbiota abundance (genus Catenibacterium id.2153) on Beta−1,3−galactosyltransferase 6
with a threshold of 1e−05

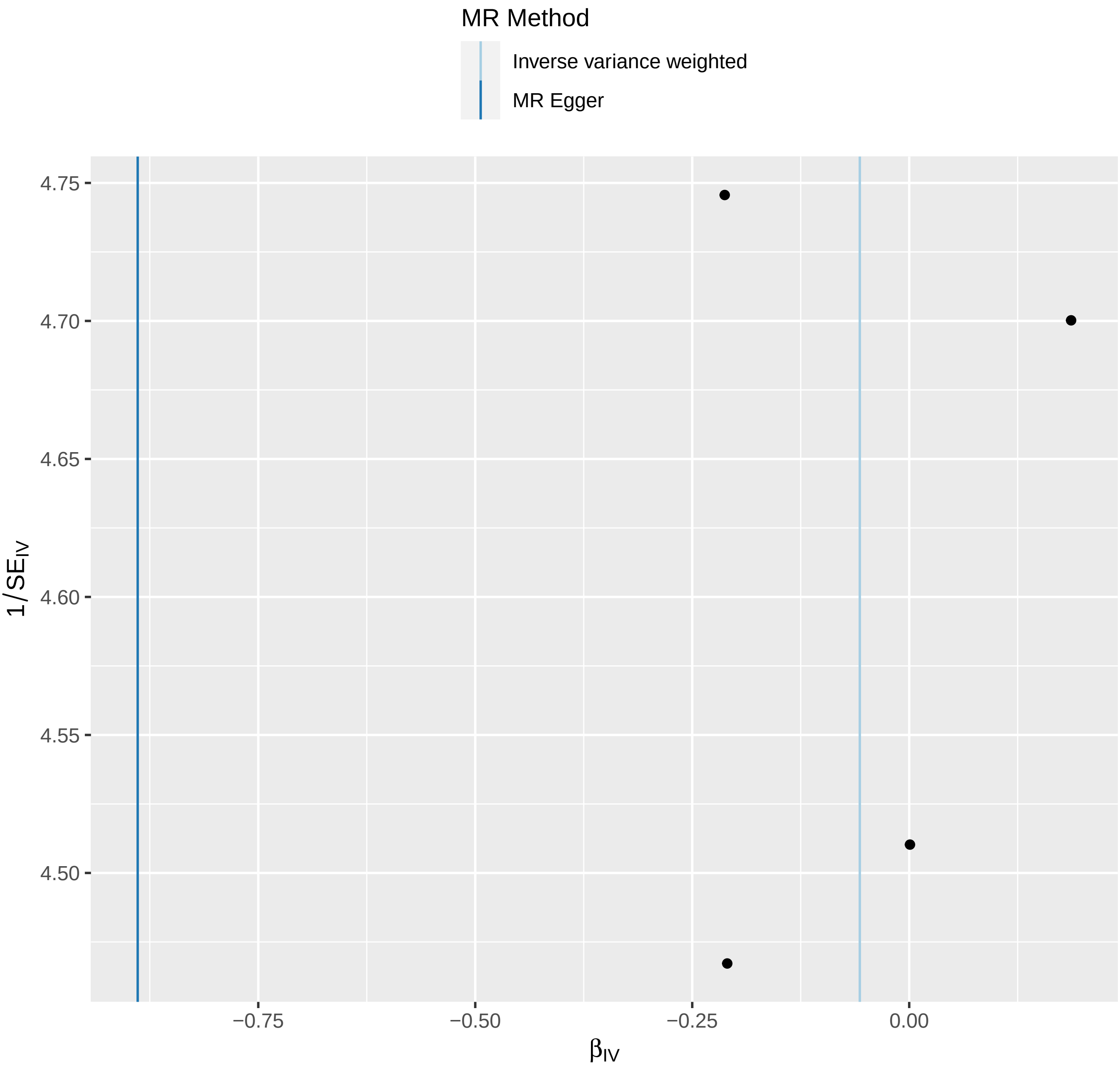

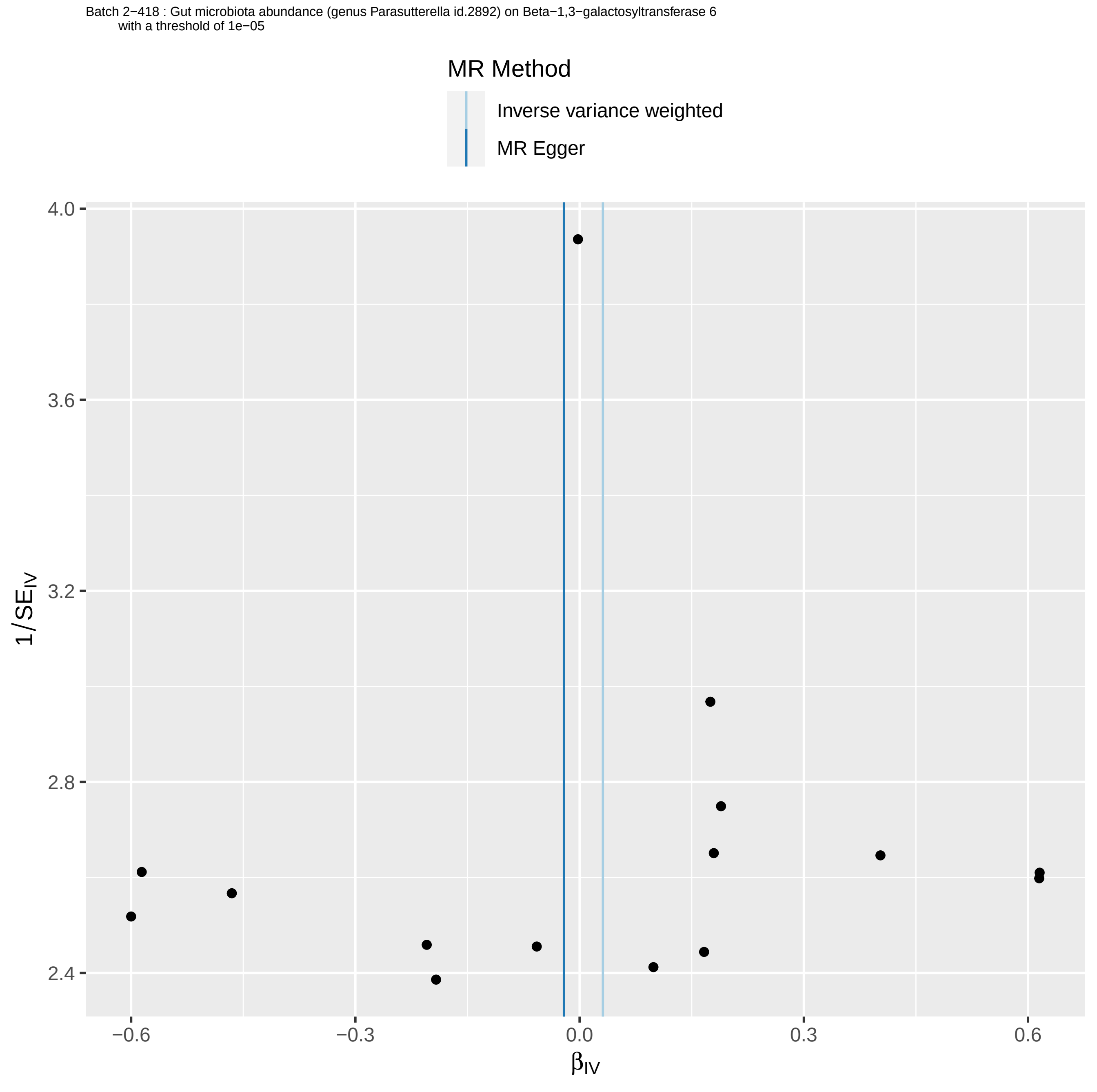

Batch 2−418 : Gut microbiota abundance (genus Parasutterella id.2892) on Beta−1,3−galactosyltransferase 6
with a threshold of 1e−05
MR Method
Inverse variance weighted
MR Egger
4.0
3.6
3.2
2.8
2.4
1/SE_IV
−0.6
−0.3
0.0
0.3
0.6
β_IV

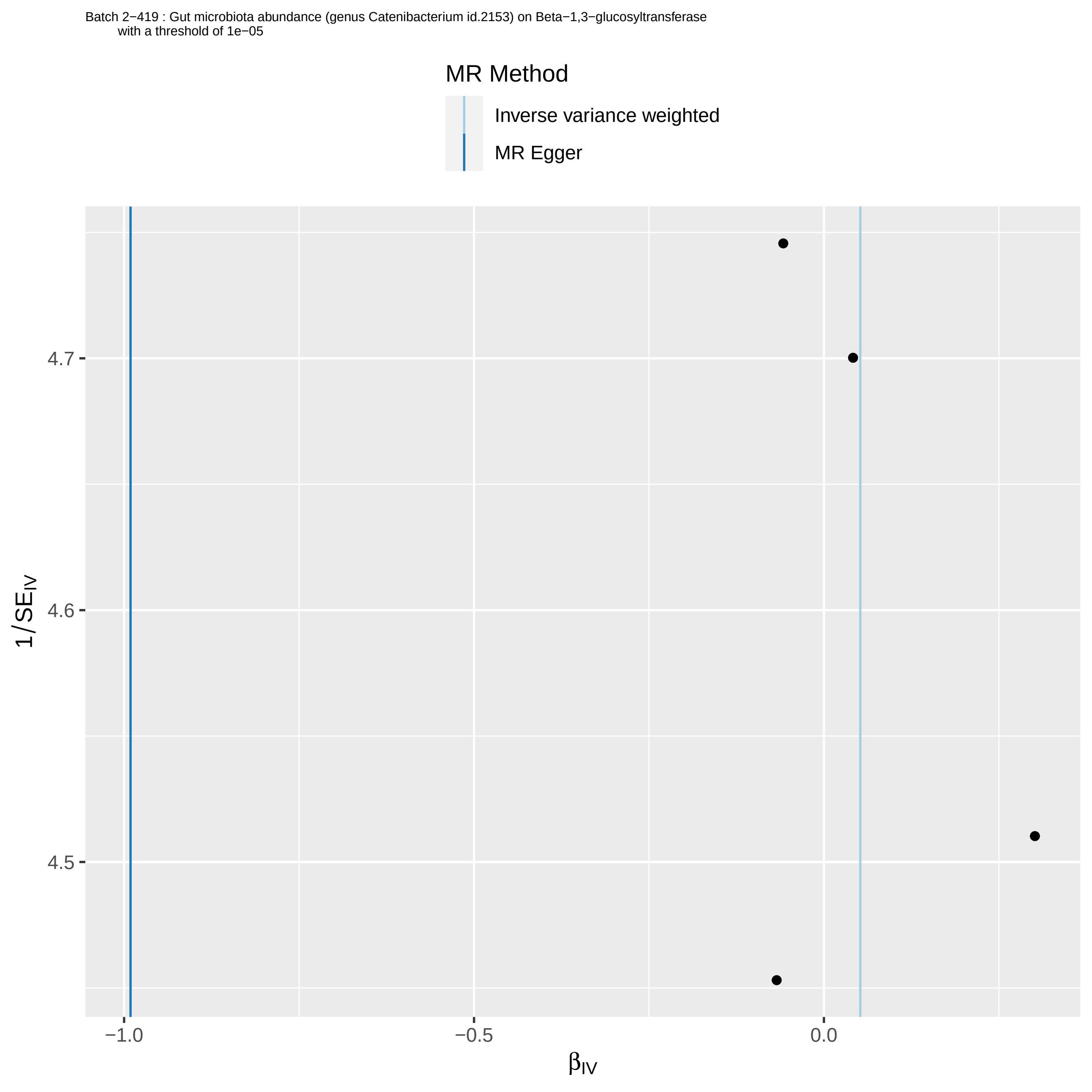

Batch 2−419 : Gut microbiota abundance (genus Catenibacterium id.2153) on Beta−1,3−glucosyltransferase
with a threshold of 1e−05
MR Method
Inverse variance weighted
MR Egger
4.7
4.6
4.5
1/SE_IV
−1.0
−0.5
0.0
β_IV

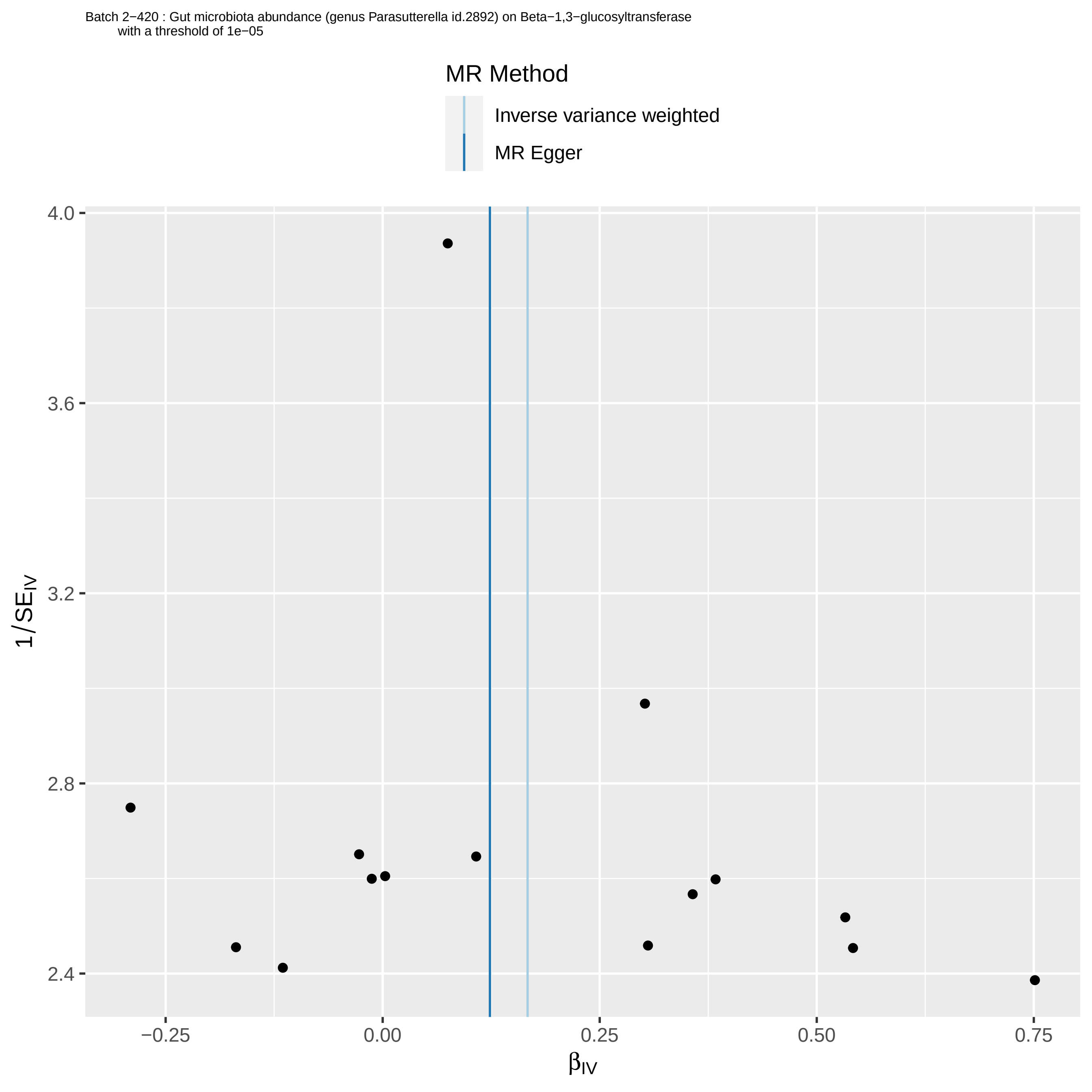
Batch 2−420 : Gut microbiota abundance (genus Parasutterella id.2892) on Beta−1,3−glucosyltransferase
with a threshold of 1e−05
MR Method
Inverse variance weighted
MR Egger
4.0
3.6
3.2
2.8
2.4
1/SE_IV
−0.25
0.00
0.25
0.50
0.75
β_IV

Batch 2−421 : Gut microbiota abundance (genus Catenibacterium id.2153) on Galactosylgalactosylxylosylprotein 3−beta−glucuronosyltransferase 3 with a threshold of 1e−05

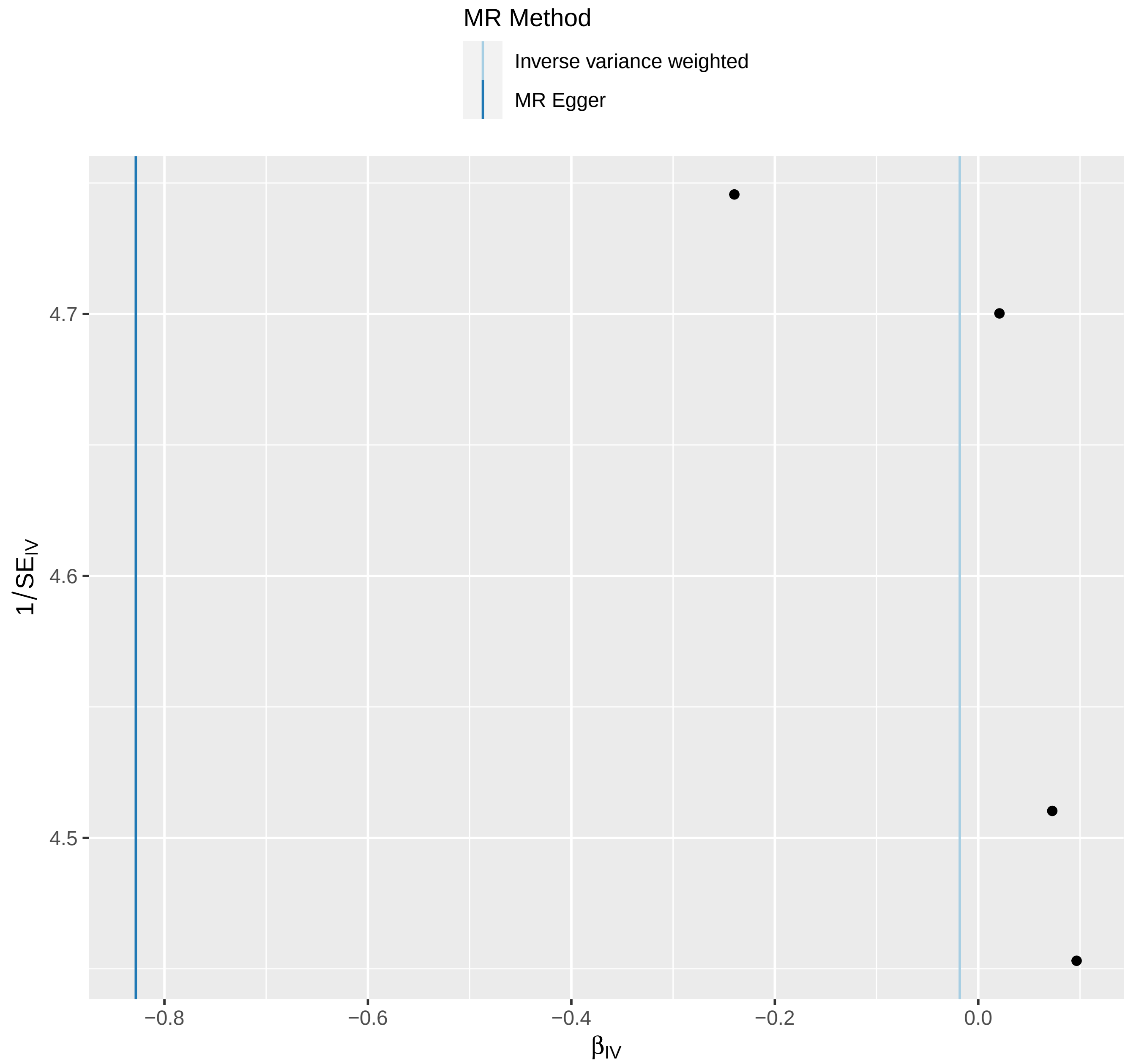

Batch 2−422 : Gut microbiota abundance (genus Parasutterella id.2892) on Galactosylgalactosylxylosylprotein 3−beta−glucuronosyltransferase 3
with a threshold of 1e−05

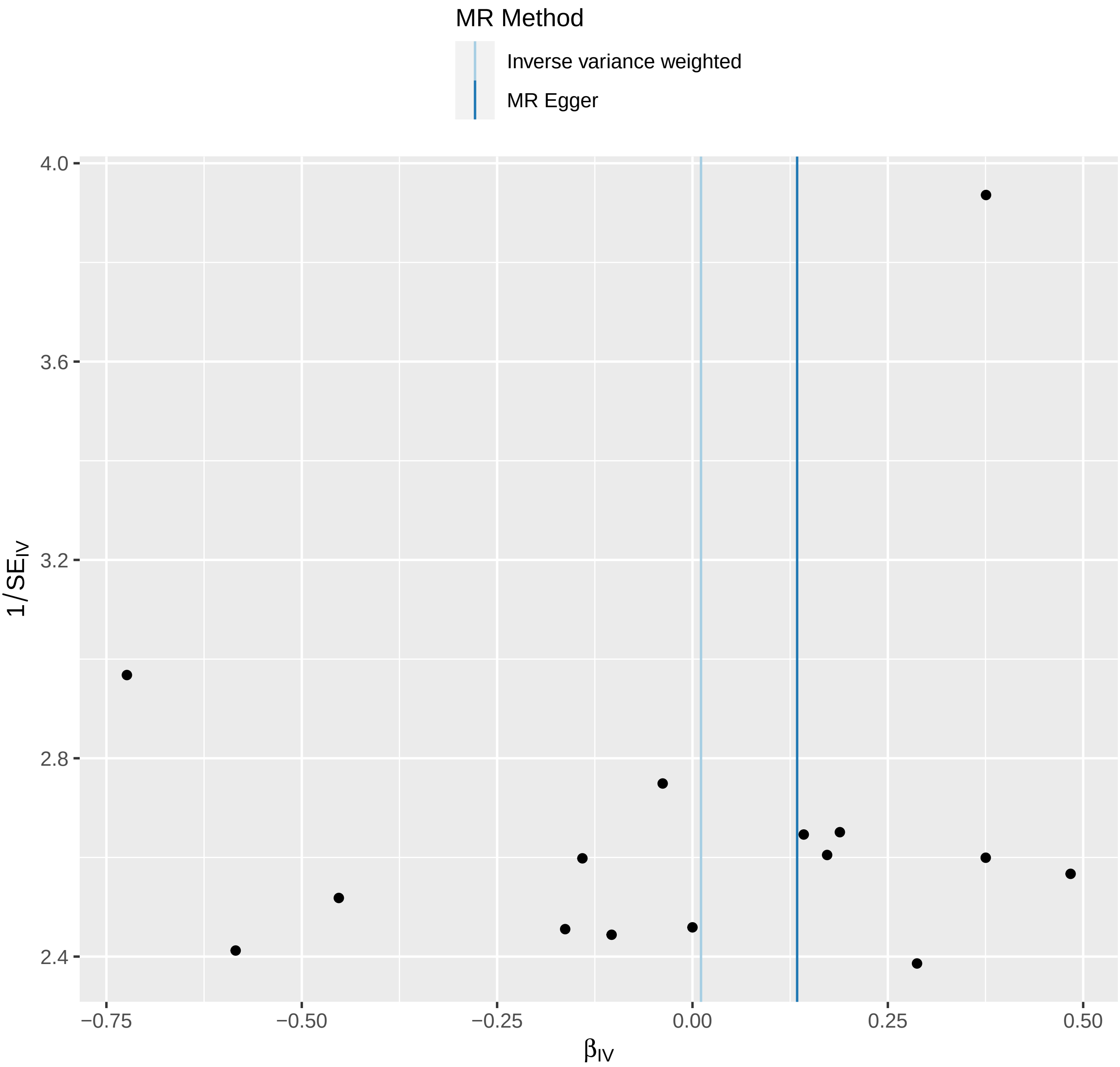

Batch 2−423 : Gut microbiota abundance (genus Catenibacterium id.2153) on Beta−1,4−glucuronyltransferase 1
with a threshold of 1e−05

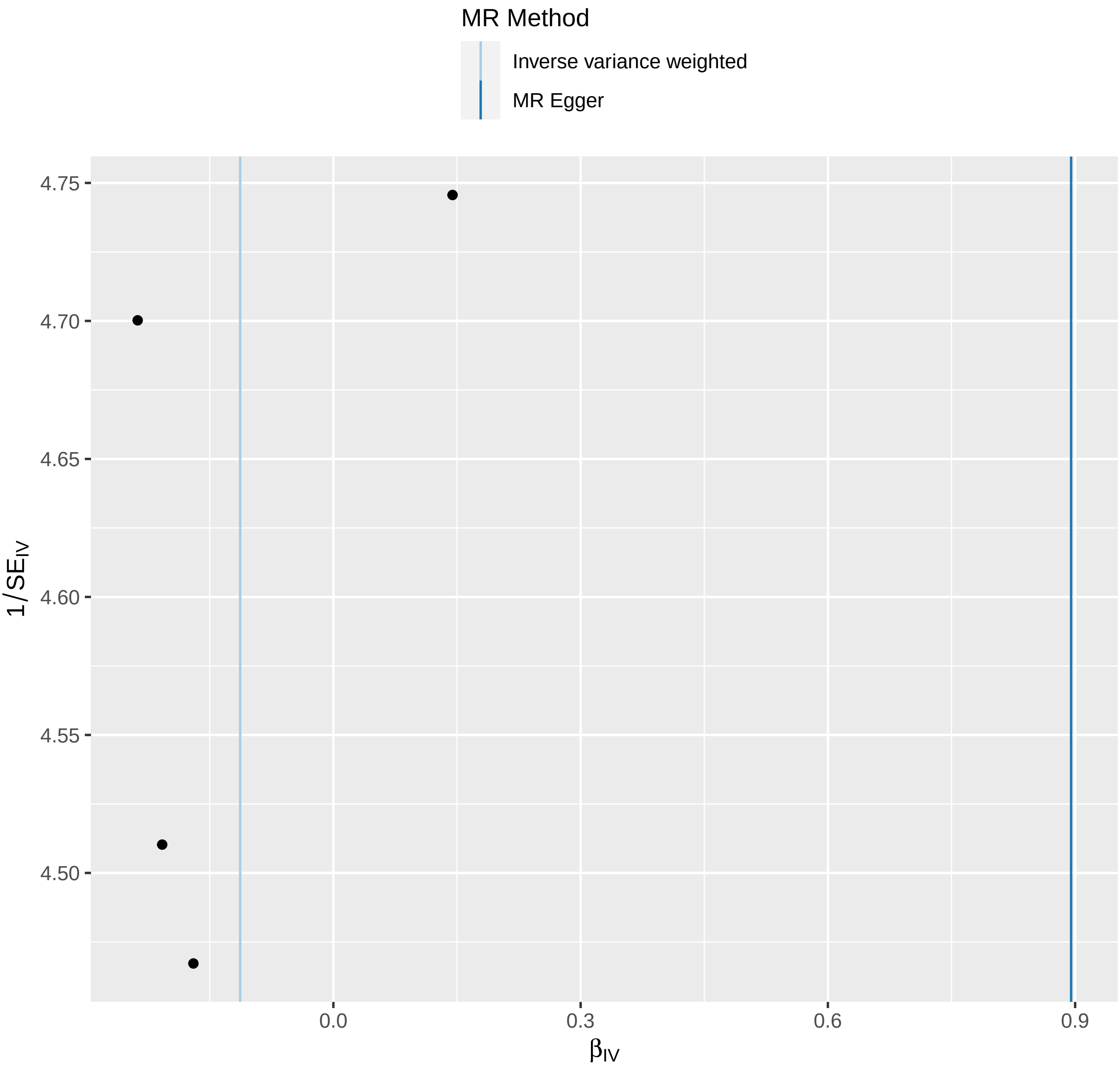

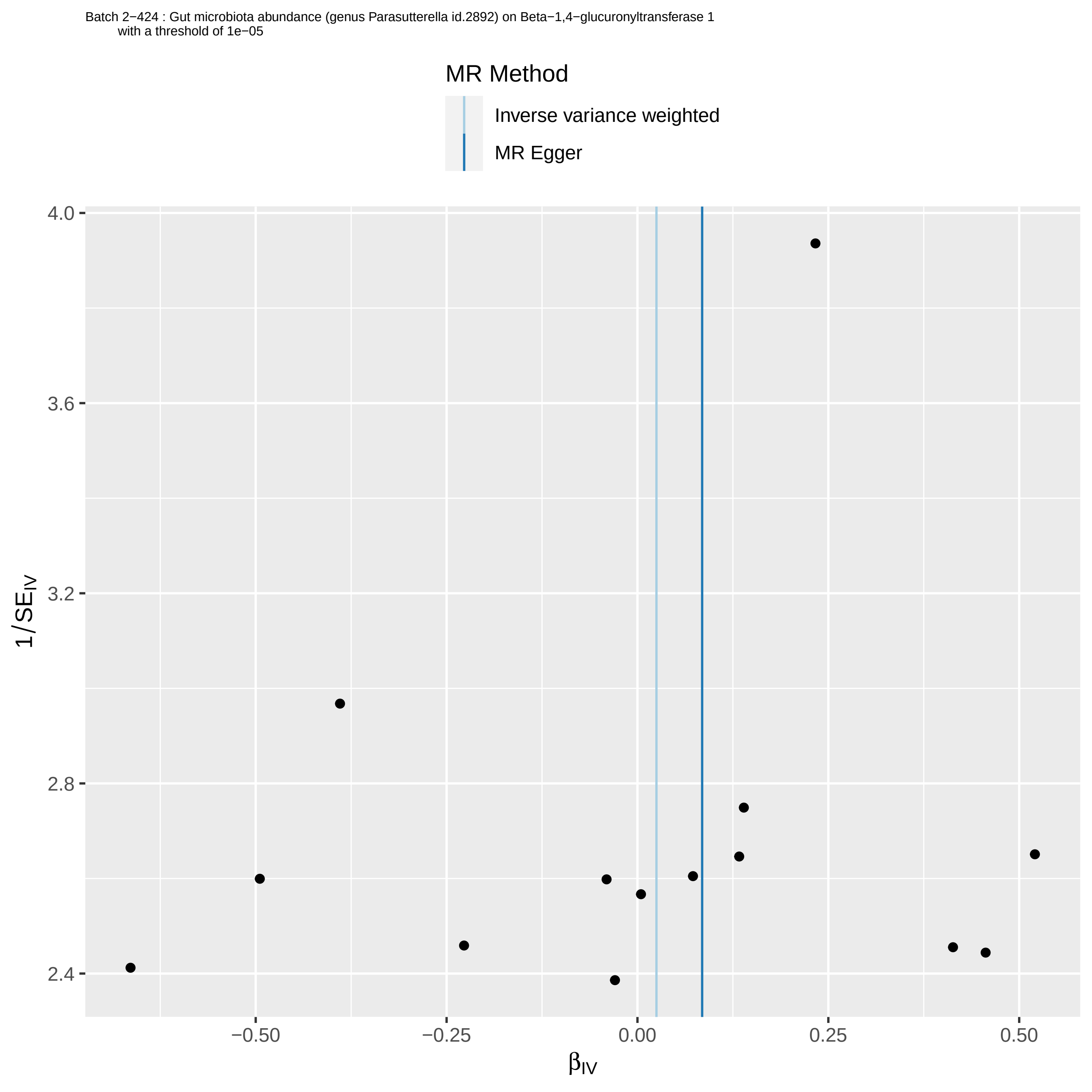

Batch 2−424 : Gut microbiota abundance (genus Parasutterella id.2892) on Beta−1,4−glucuronyltransferase 1
with a threshold of 1e−05
MR Method
Inverse variance weighted
MR Egger
4.0
3.6
3.2
2.8
2.4
1/SE$_{IV}$
−0.50
−0.25
0.00
0.25
0.50
β$_{IV}$

Batch 2−425 : Gut microbiota abundance (genus Catenibacterium id.2153) on N−acetyllactosaminide beta−1,3−N−acetylglucosaminyltransferase 2 with a threshold of 1e−05

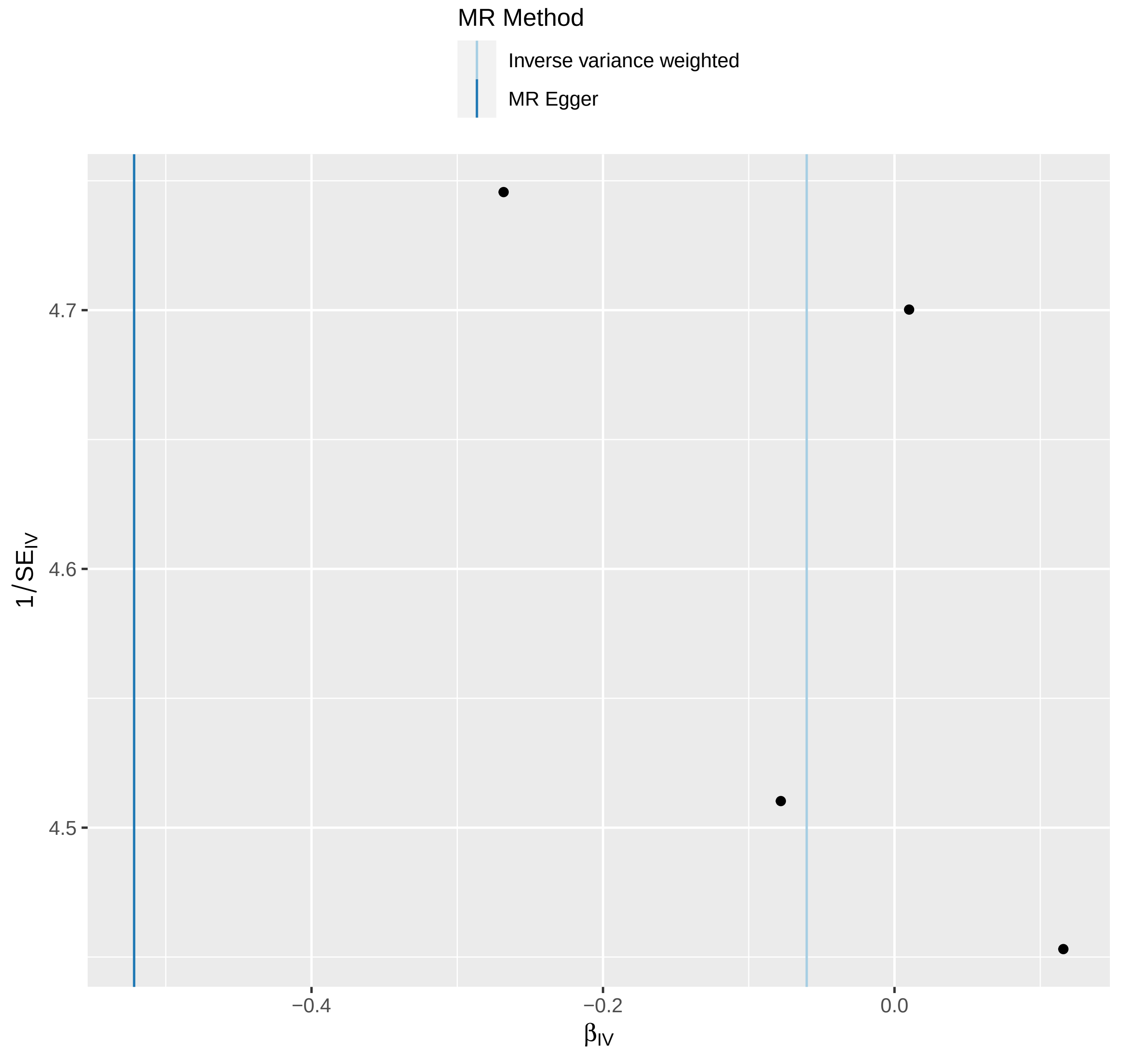

Batch 2−426 : Gut microbiota abundance (genus Parasutterella id.2892) on N−acetyllactosaminide beta−1,3−N−acetylglucosaminyltransferase 2
with a threshold of 1e−05

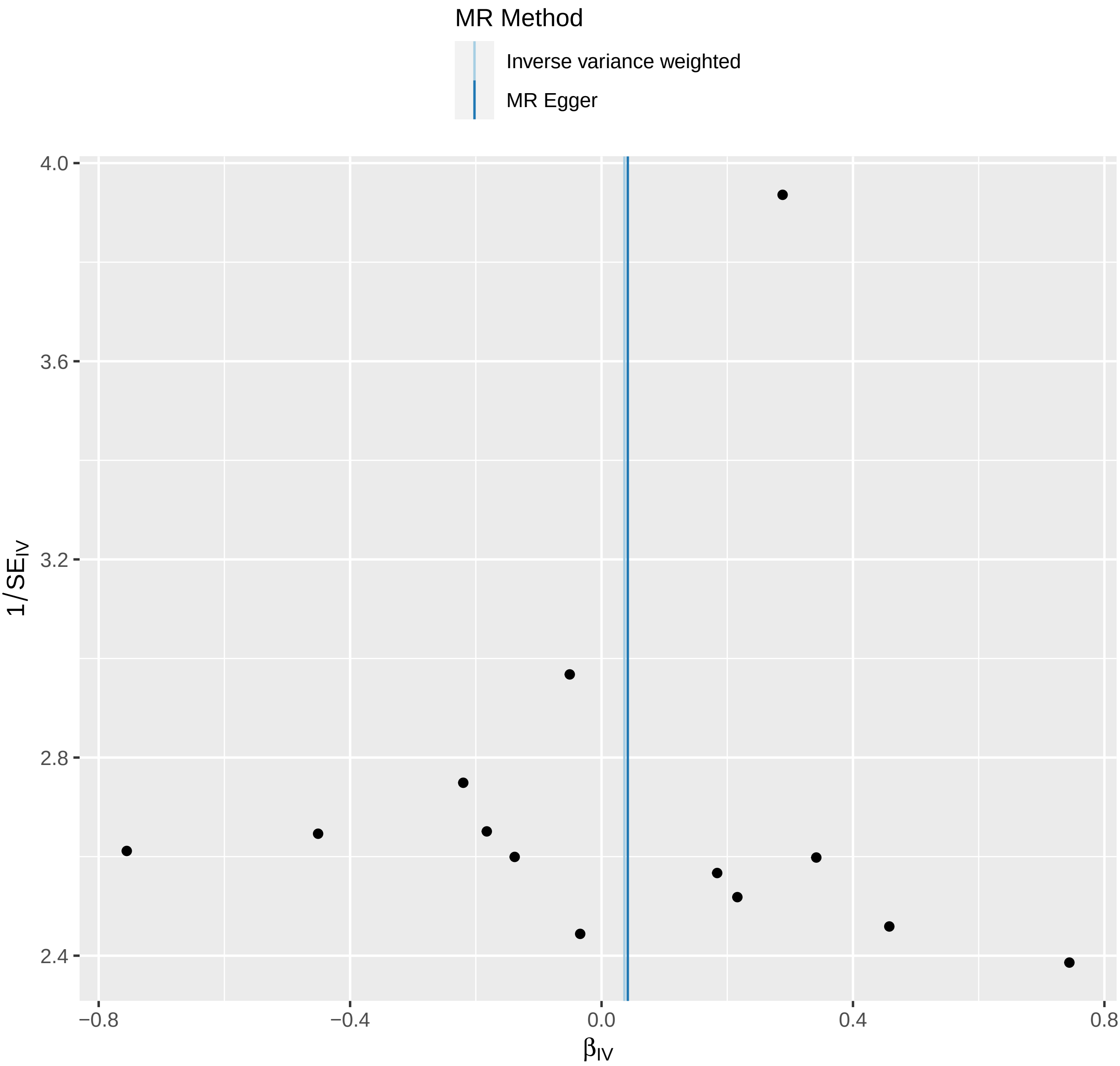

Batch 2−427 : Gut microbiota abundance (genus Catenibacterium id.2153) on UDP−GlcNAc:betaGal beta−1,3−N−acetylglucosaminyltransferase 6 with a threshold of 1e−05

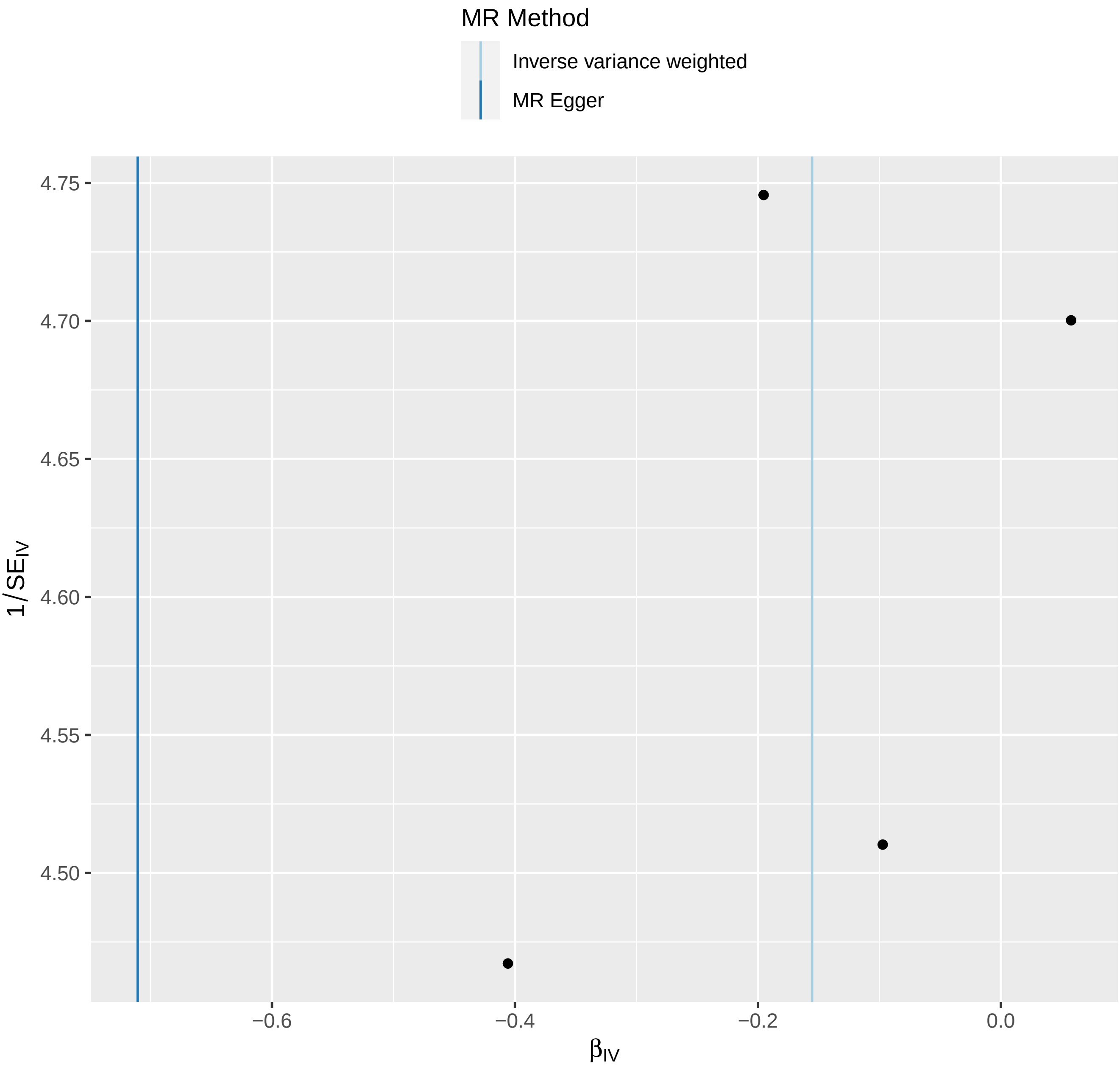

Batch 2−428 : Gut microbiota abundance (genus Parasutterella id.2892) on UDP−GlcNAc:betaGal beta−1,3−N−acetylglucosaminyltransferase 6 with a threshold of 1e−05

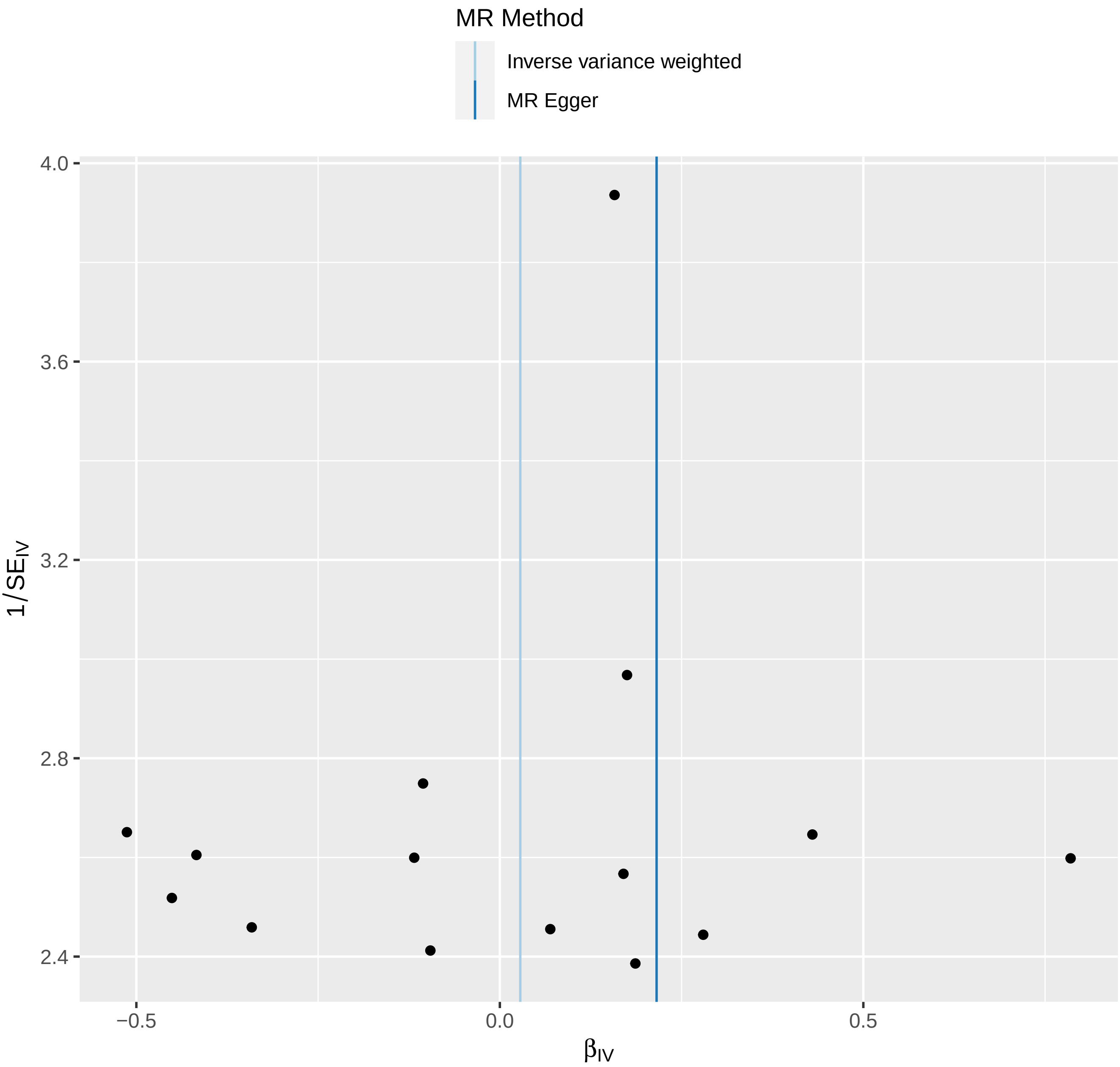

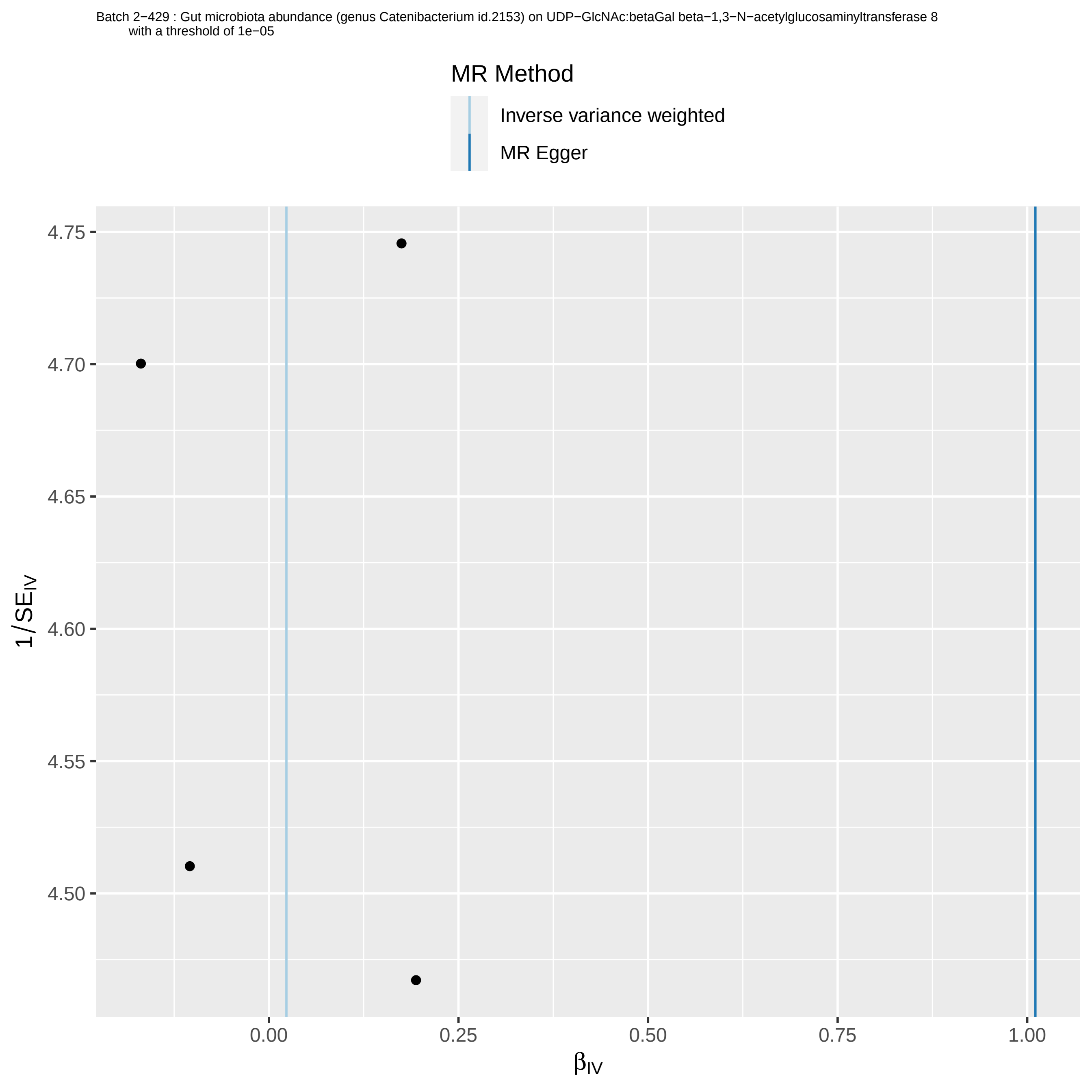

Batch 2−429 : Gut microbiota abundance (genus Catenibacterium id.2153) on UDP−GlcNAc:betaGal beta−1,3−N−acetylglucosaminyltransferase 8
with a threshold of 1e−05
MR Method
Inverse variance weighted
MR Egger
4.75
4.70
4.65
4.60
4.55
4.50
1/SE_IV
0.00
0.25
0.50
0.75
1.00
β_IV

Batch 2−430 : Gut microbiota abundance (genus Parasutterella id.2892) on UDP−GlcNAc:betaGal beta−1,3−N−acetylglucosaminyltransferase 8 with a threshold of 1e−05

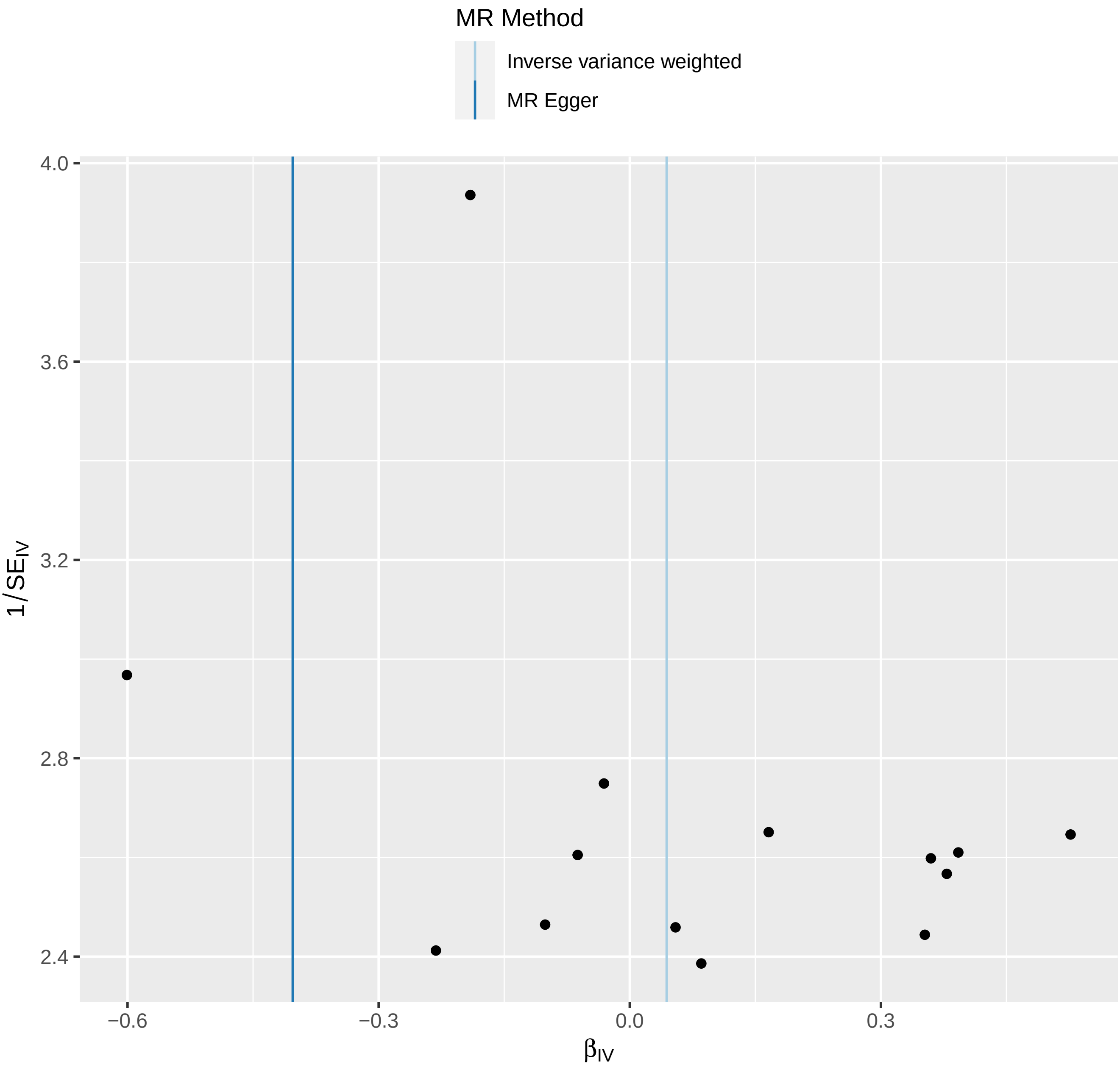

Batch 2−431 : Gut microbiota abundance (genus Catenibacterium id.2153) on Beta−1,4−galactosyltransferase 1
with a threshold of 1e−05

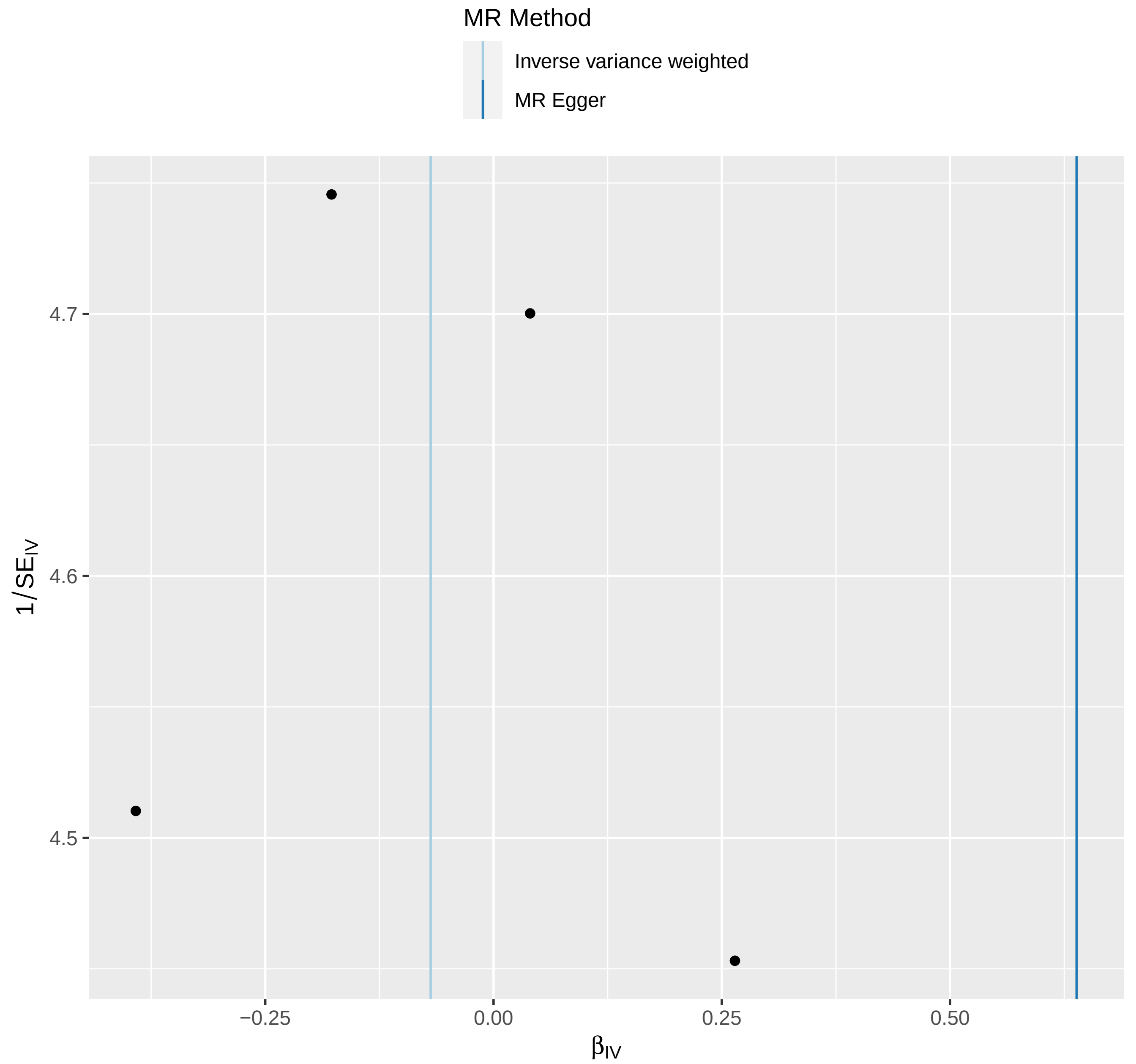

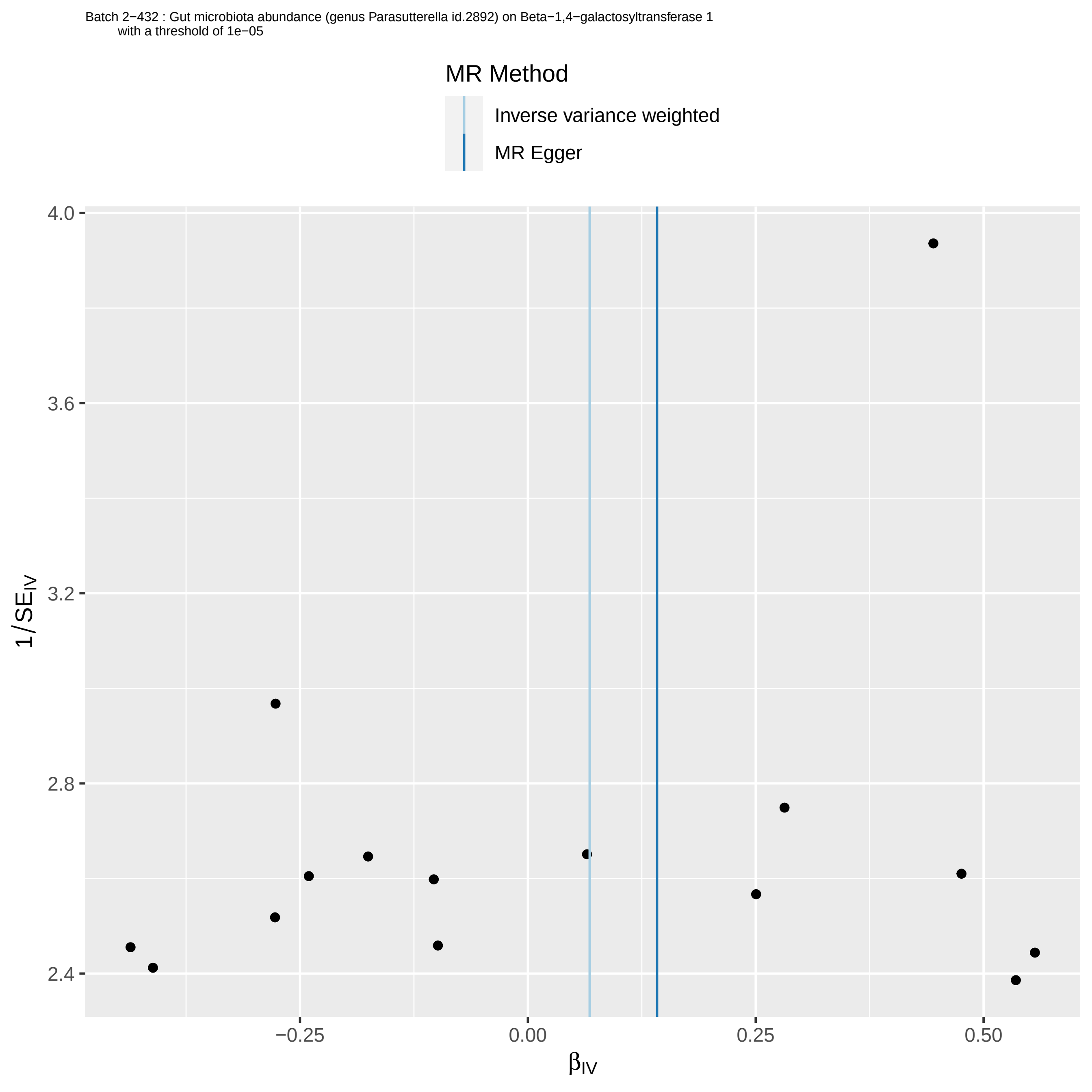

Batch 2−432 : Gut microbiota abundance (genus Parasutterella id.2892) on Beta−1,4−galactosyltransferase 1
with a threshold of 1e−05
MR Method
Inverse variance weighted
MR Egger
4.0
3.6
3.2
2.8
2.4
1/SE_IV
−0.25
0.00
0.25
0.50
β_IV

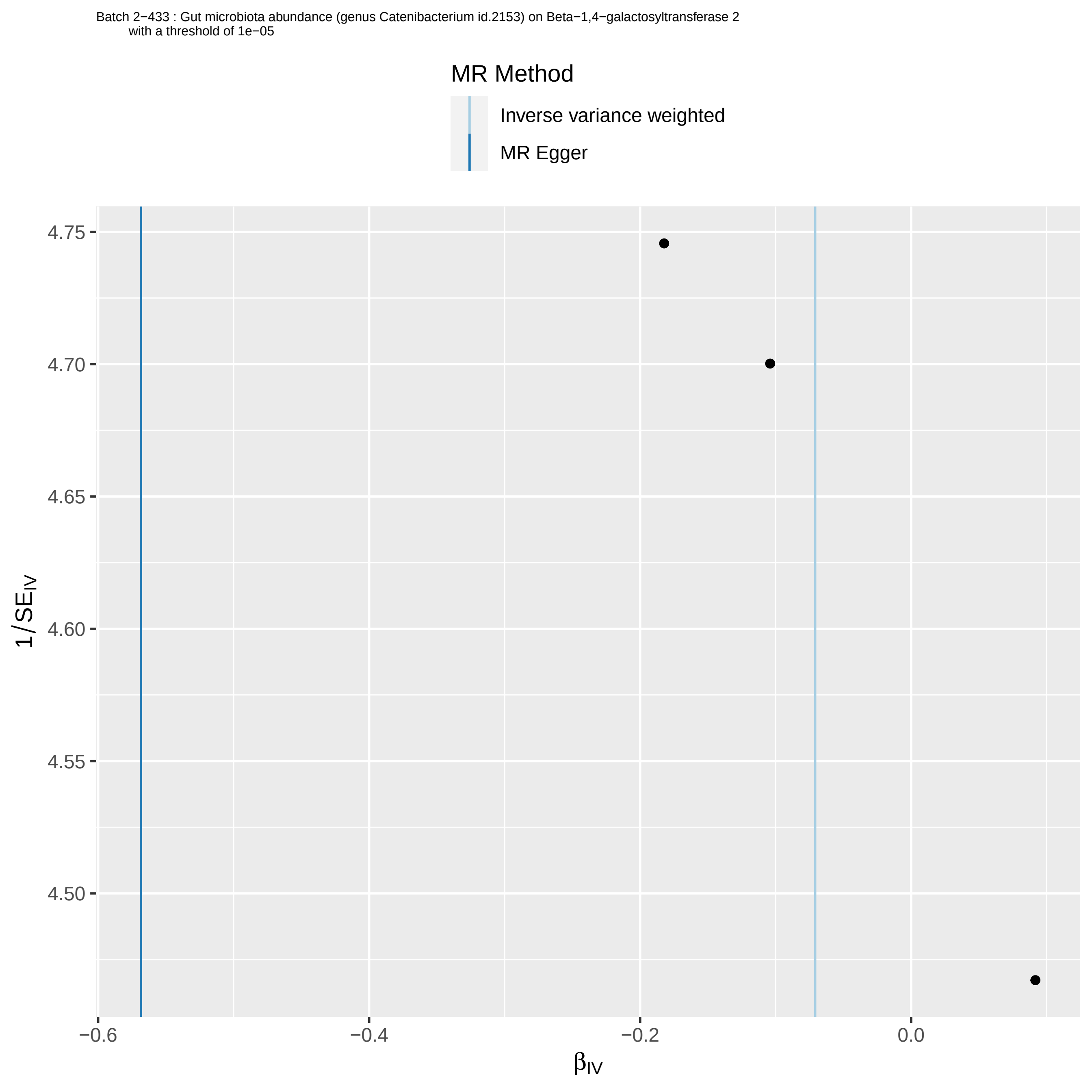

Batch 2−433 : Gut microbiota abundance (genus Catenibacterium id.2153) on Beta−1,4−galactosyltransferase 2
with a threshold of 1e−05
MR Method
Inverse variance weighted
MR Egger
4.75
4.70
4.65
4.60
4.55
4.50
1/SE$_{IV}$
−0.6
−0.4
−0.2
0.0
β$_{IV}$

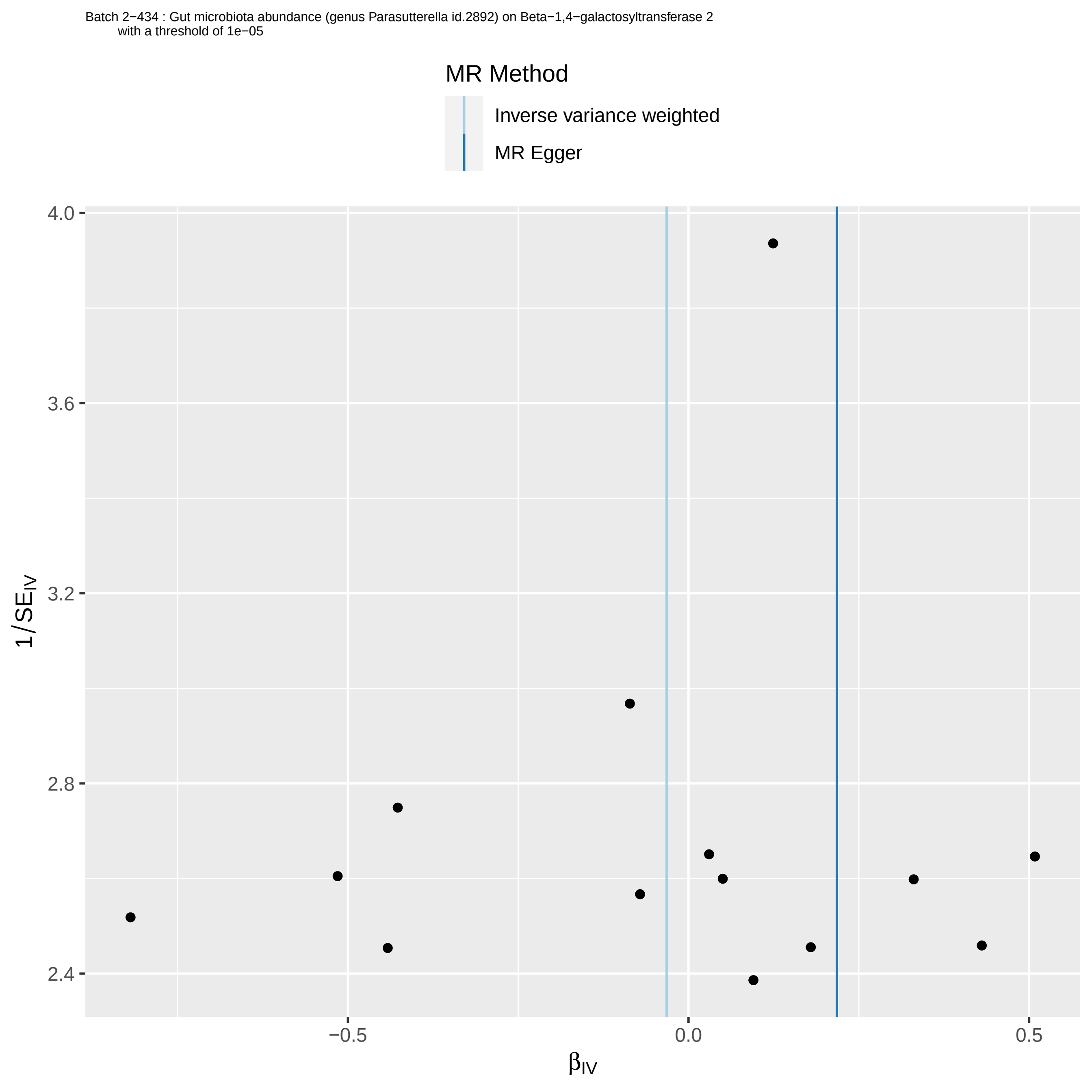

Batch 2−434 : Gut microbiota abundance (genus Parasutterella id.2892) on Beta−1,4−galactosyltransferase 2
with a threshold of 1e−05
MR Method
Inverse variance weighted
MR Egger
4.0
3.6
3.2
2.8
2.4
$1/SE_{IV}$
−0.5
0.0
0.5
β_{IV}

Batch 2−435 : Gut microbiota abundance (genus Catenibacterium id.2153) on Beta−1,4−galactosyltransferase 3 with a threshold of 1e−05

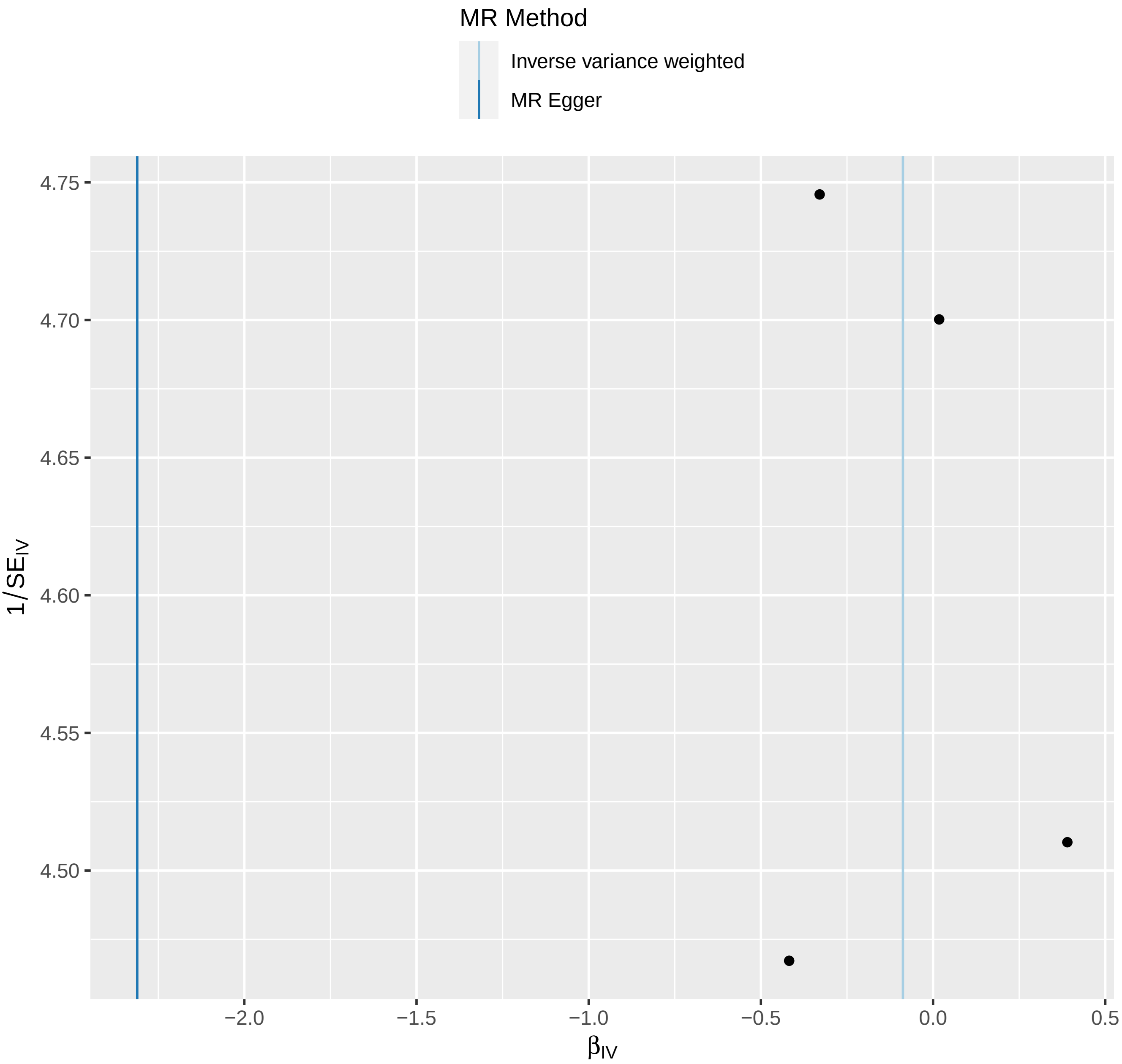

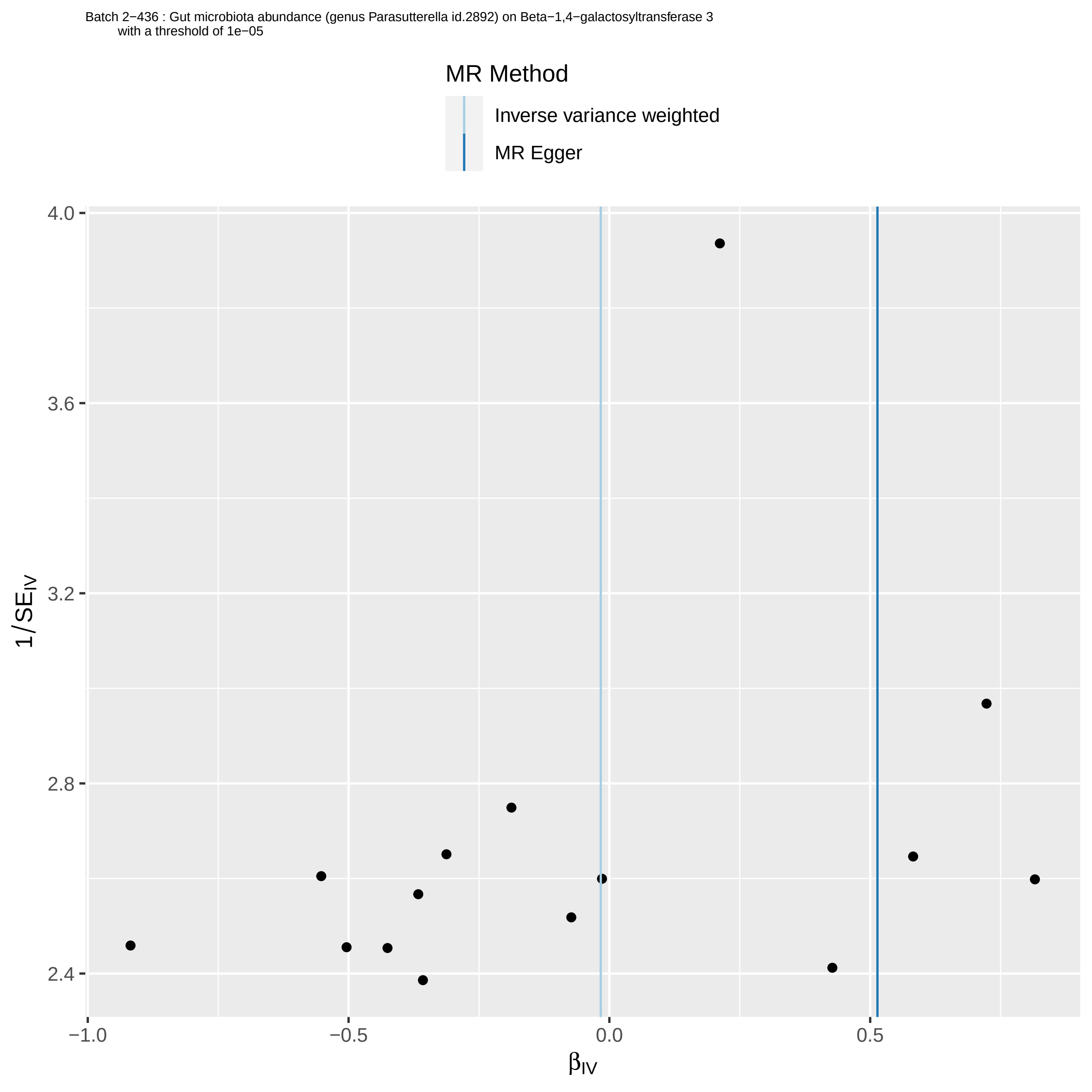
Batch 2−436 : Gut microbiota abundance (genus Parasutterella id.2892) on Beta−1,4−galactosyltransferase 3
with a threshold of 1e−05
MR Method
Inverse variance weighted
MR Egger
4.0
3.6
3.2
2.8
2.4
1/SE$_{IV}$
−1.0
−0.5
0.0
0.5
β$_{IV}$

Batch 2−437 : Gut microbiota abundance (genus Catenibacterium id.2153) on Beta−1,4−galactosyltransferase 5
with a threshold of 1e−05

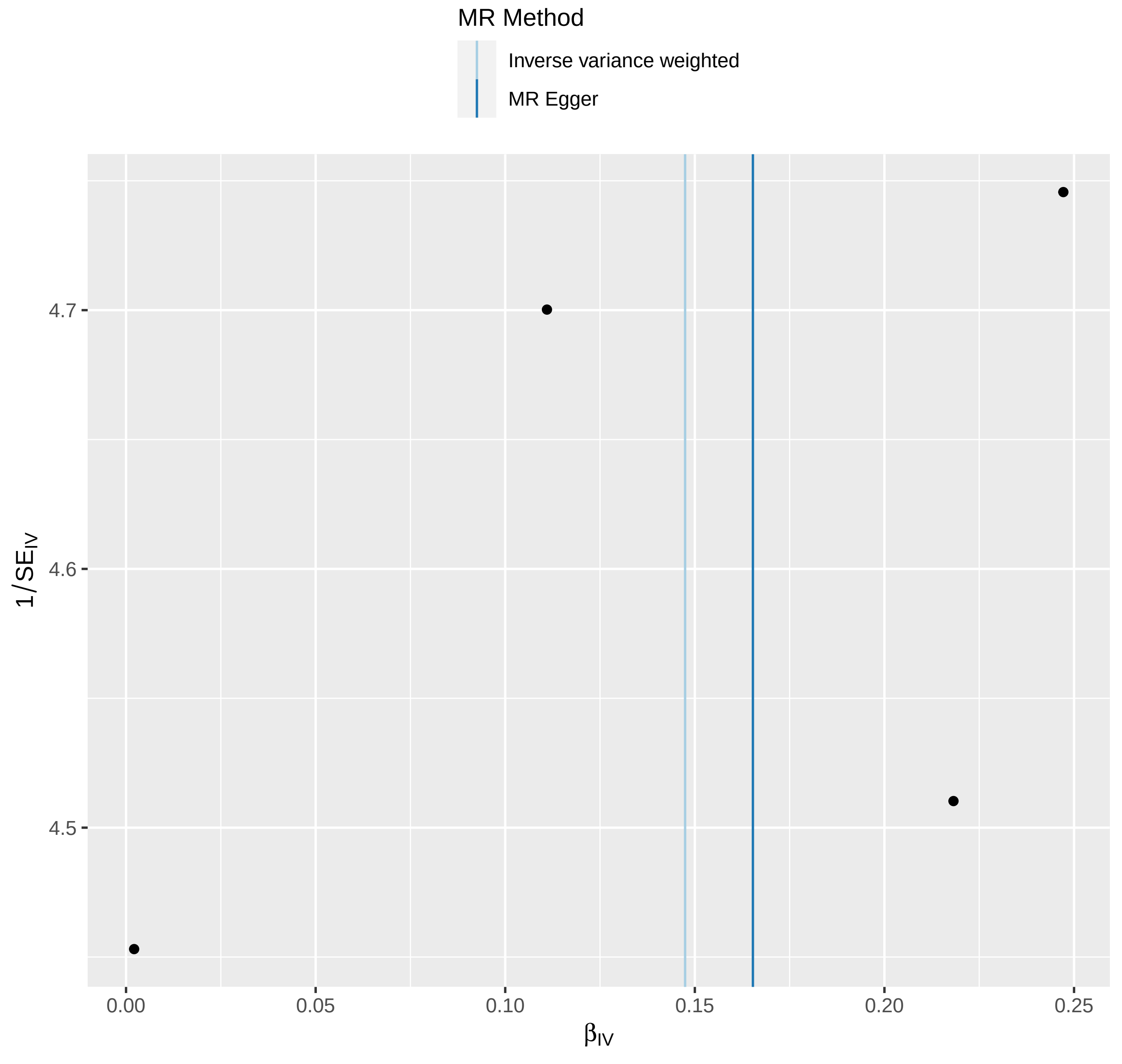

Batch 2−438 : Gut microbiota abundance (genus Parasutterella id.2892) on Beta−1,4−galactosyltransferase 5
with a threshold of 1e−05

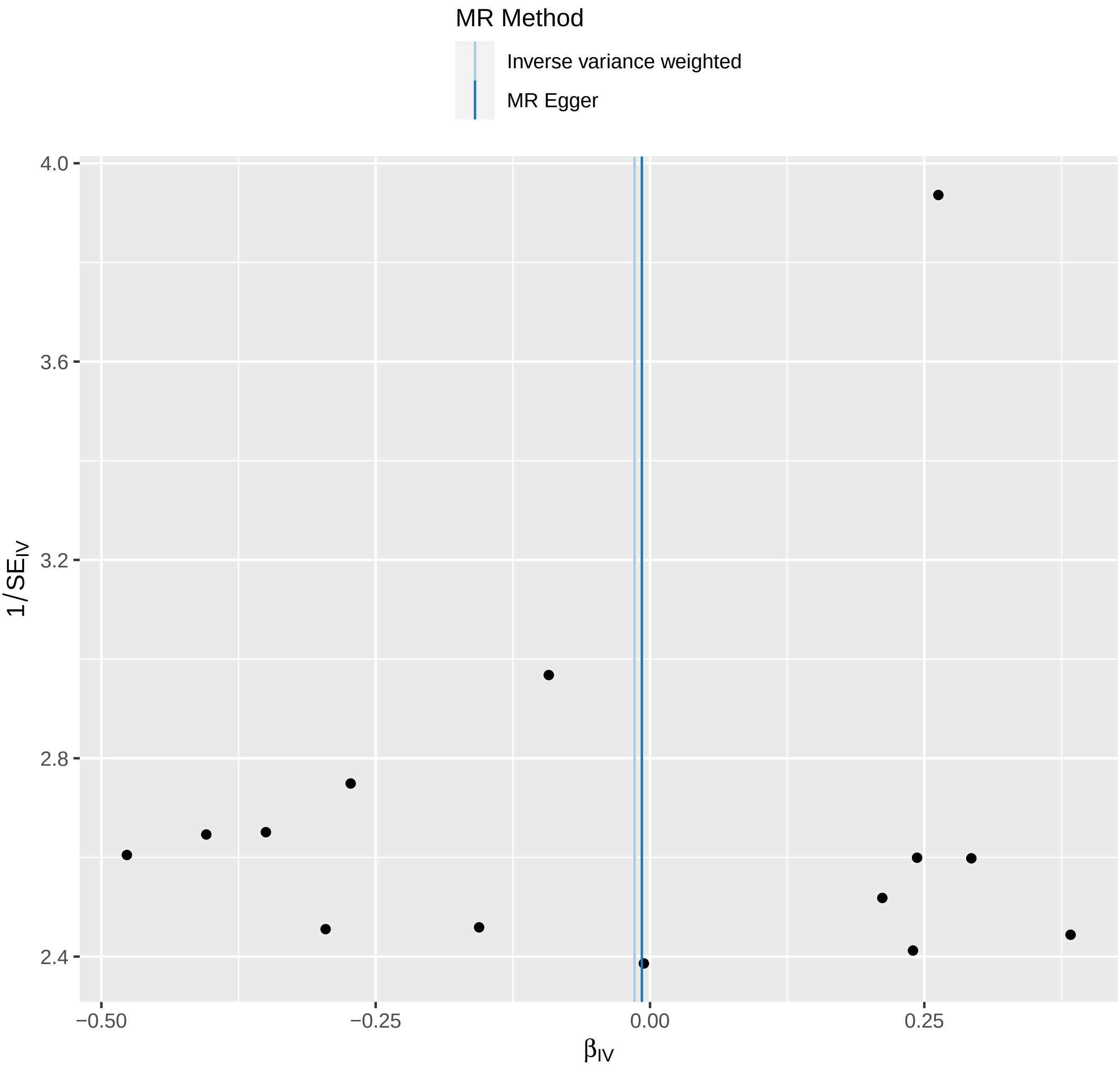

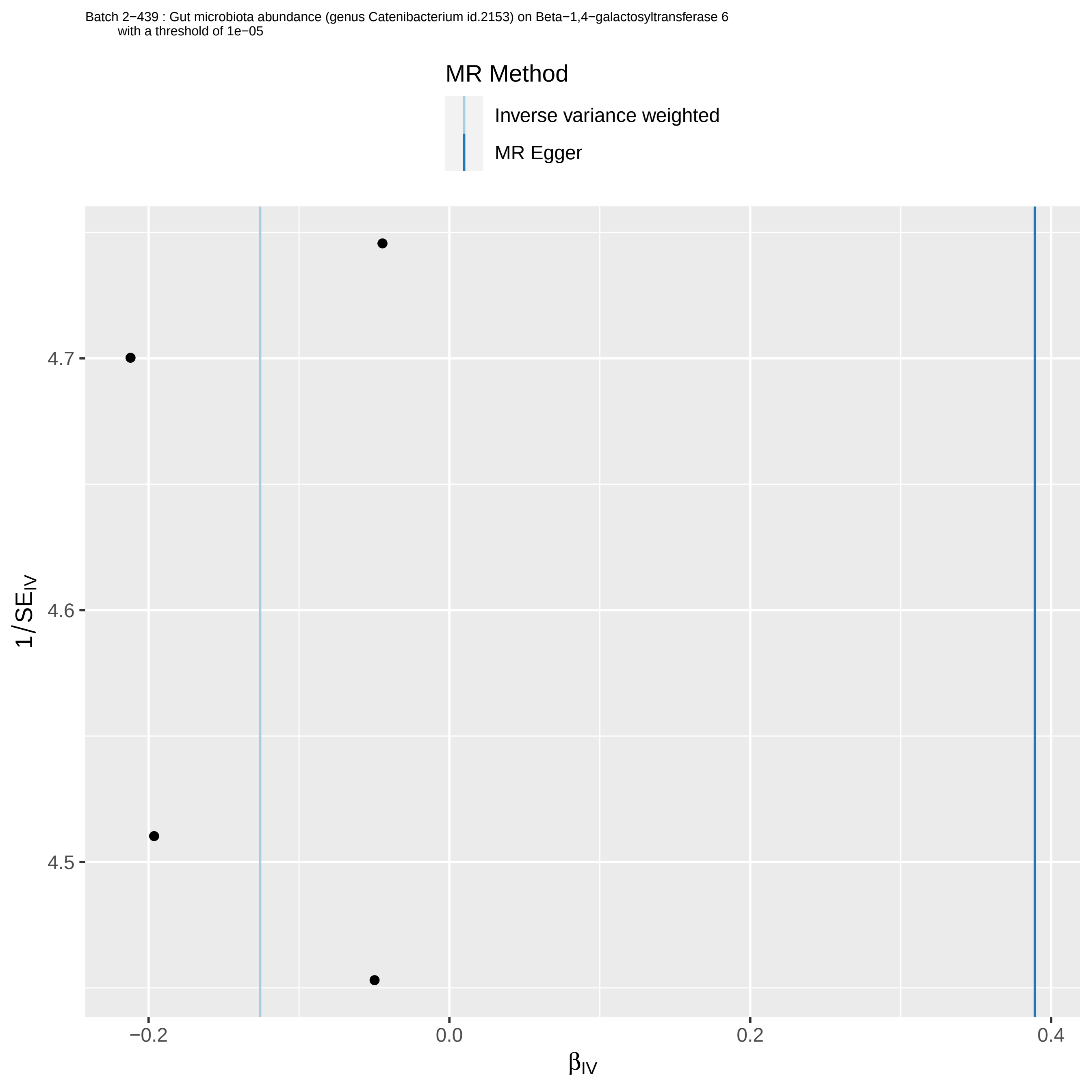
Batch 2−439 : Gut microbiota abundance (genus Catenibacterium id.2153) on Beta−1,4−galactosyltransferase 6
with a threshold of 1e−05
MR Method
Inverse variance weighted
MR Egger
4.7
4.6
4.5
1/SE_IV
−0.2
0.0
0.2
0.4
β_IV

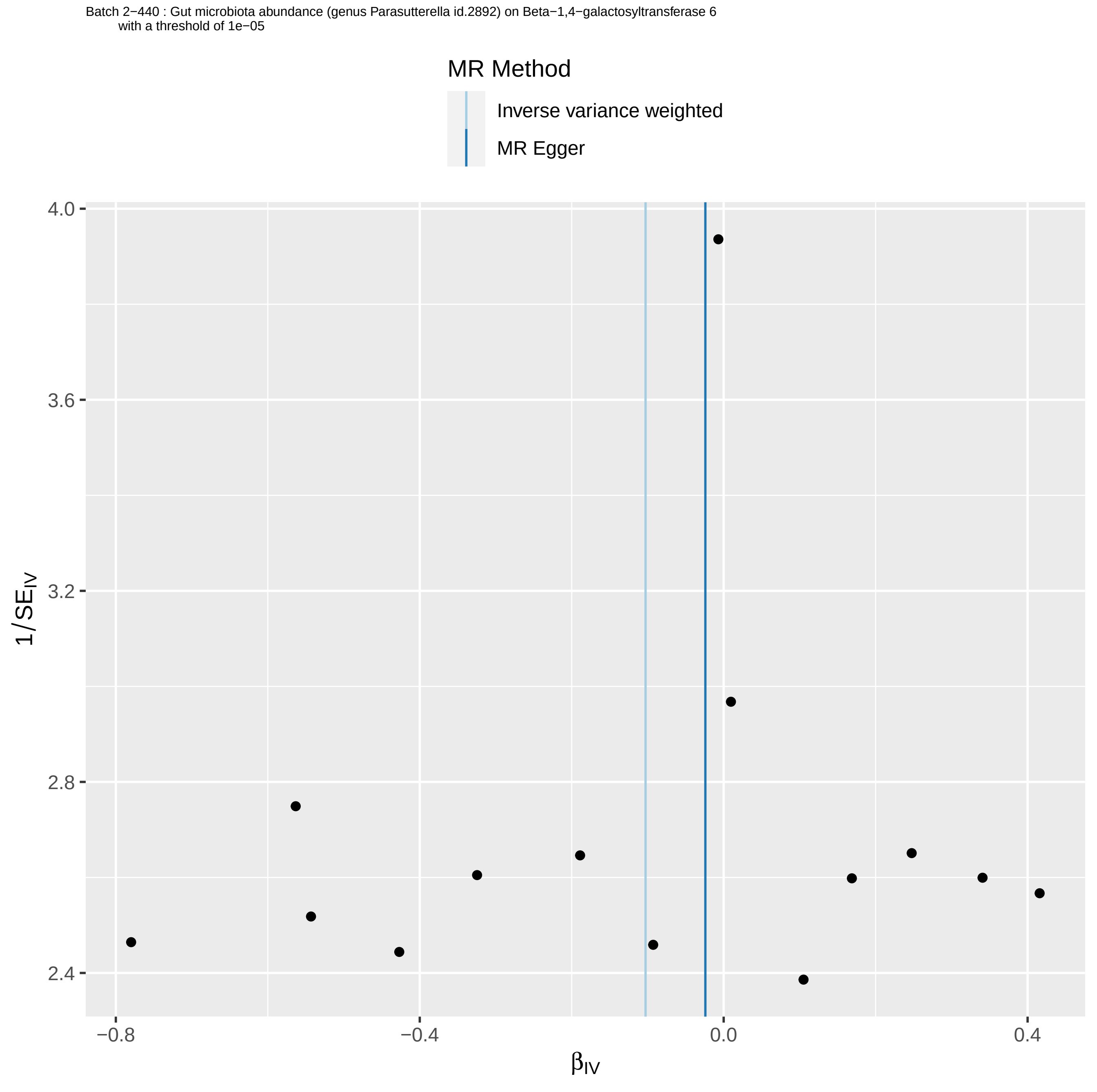

Batch 2−440 : Gut microbiota abundance (genus Parasutterella id.2892) on Beta−1,4−galactosyltransferase 6
with a threshold of 1e−05
MR Method
Inverse variance weighted
MR Egger
4.0
3.6
3.2
2.8
2.4
1/SE_IV
−0.8
−0.4
0.0
0.4
β_IV

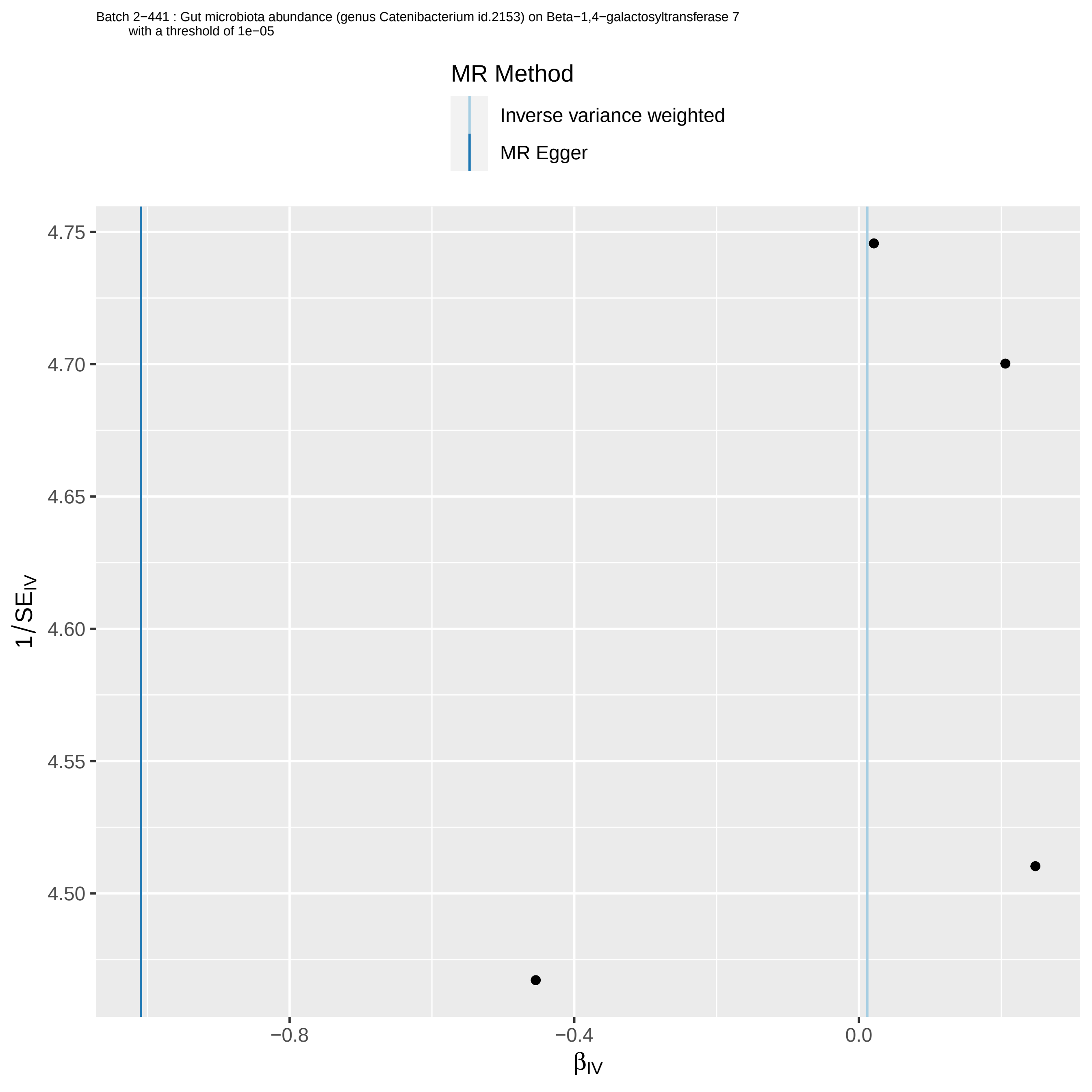
Batch 2−441 : Gut microbiota abundance (genus Catenibacterium id.2153) on Beta−1,4−galactosyltransferase 7
with a threshold of 1e−05
MR Method
Inverse variance weighted
MR Egger
4.75
4.70
4.65
4.60
4.55
4.50
1/SE_IV
−0.8
−0.4
0.0
β_IV

Batch 2−442 : Gut microbiota abundance (genus Parasutterella id.2892) on Beta−1,4−galactosyltransferase 7
with a threshold of 1e−05

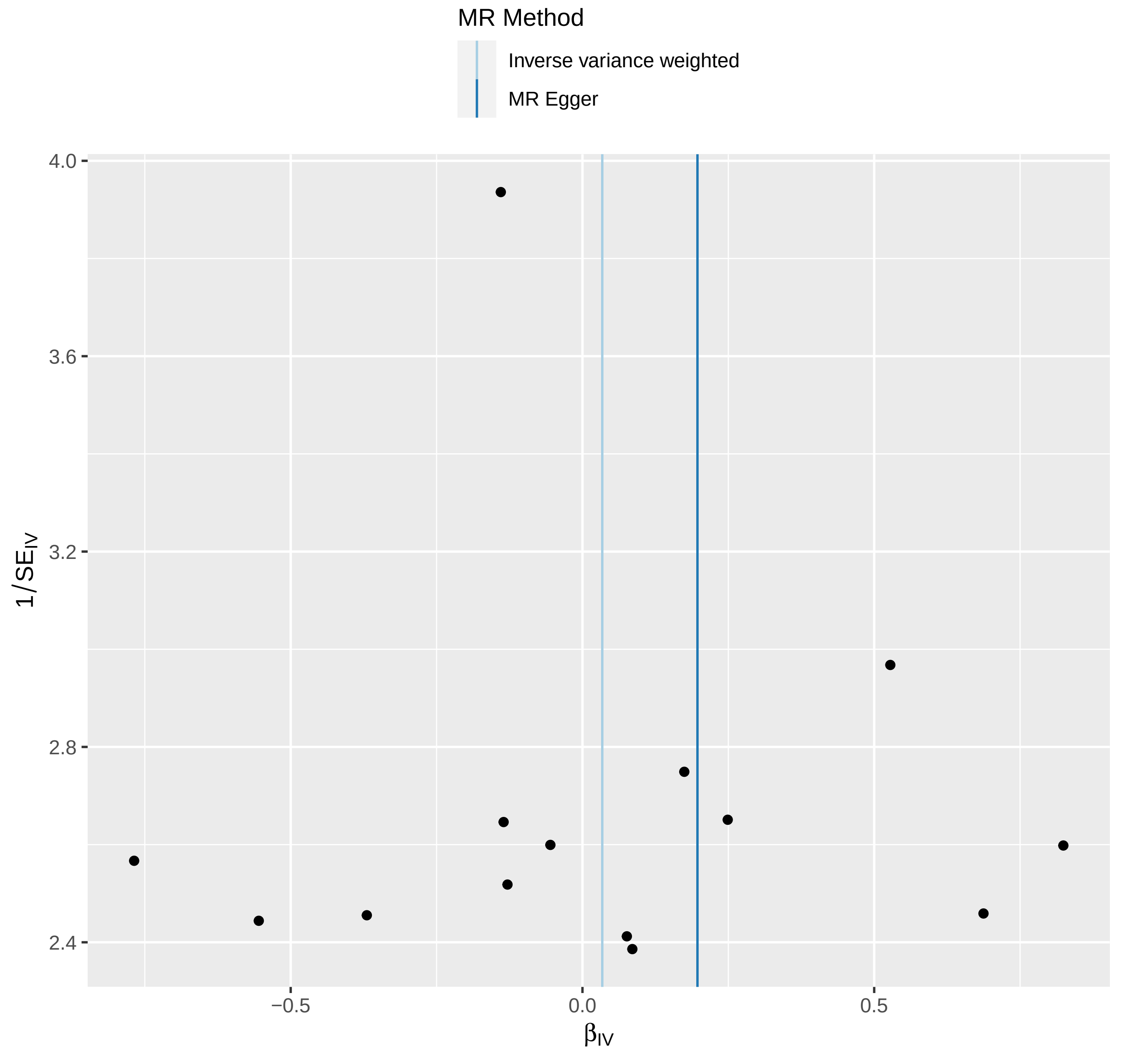

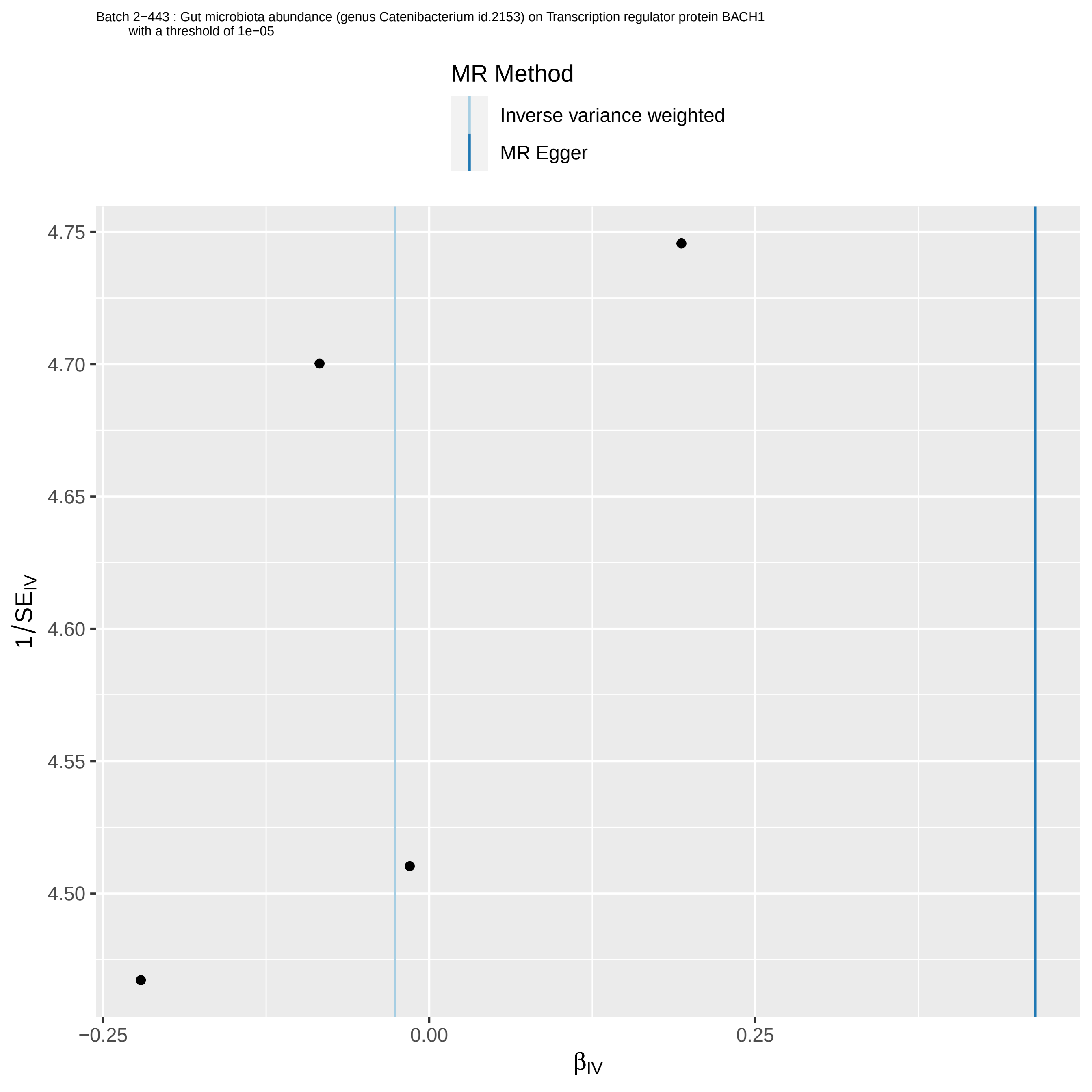

Batch 2−443 : Gut microbiota abundance (genus Catenibacterium id.2153) on Transcription regulator protein BACH1
with a threshold of 1e−05
MR Method
Inverse variance weighted
MR Egger
4.75
4.70
4.65
4.60
4.55
4.50
1/SE_IV
−0.25
0.00
0.25
β_IV

Batch 2−444 : Gut microbiota abundance (genus Parasutterella id.2892) on Transcription regulator protein BACH1
with a threshold of 1e−05

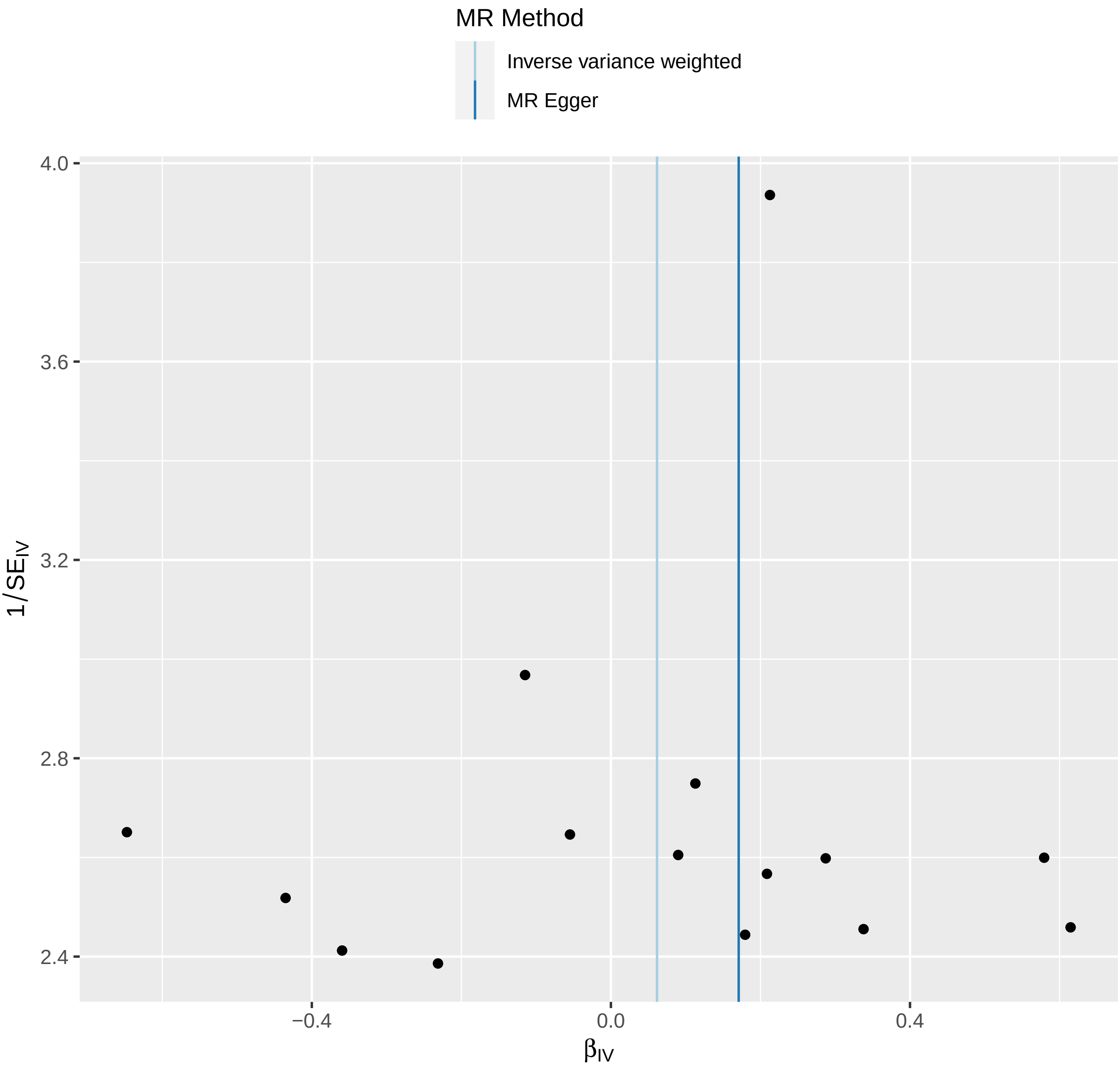

Batch 2−445 : Gut microbiota abundance (genus Catenibacterium id.2153) on Transcription regulator protein BACH2
with a threshold of 1e−05

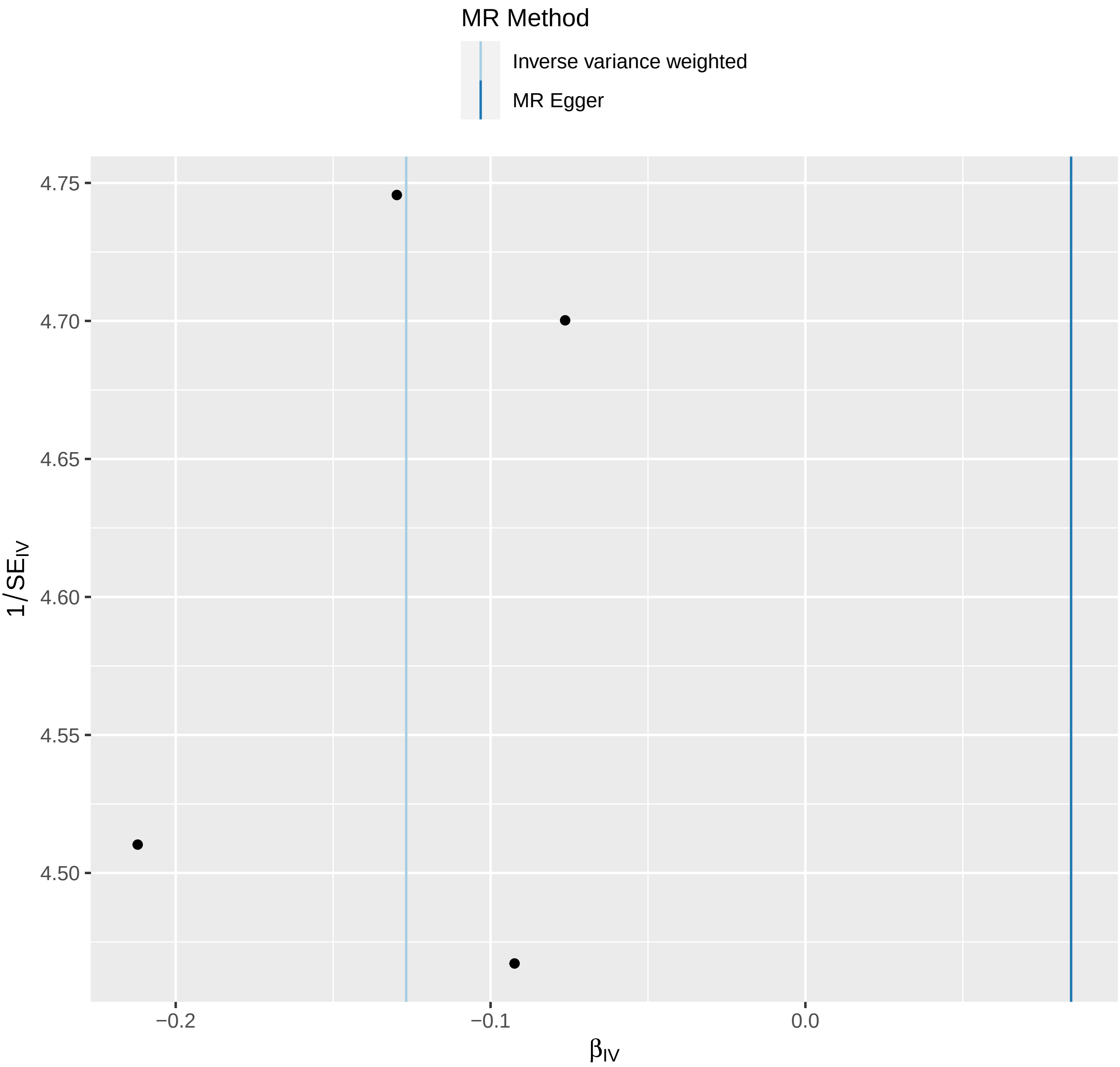

Batch 2−446 : Gut microbiota abundance (genus Parasutterella id.2892) on Transcription regulator protein BACH2
with a threshold of 1e−05

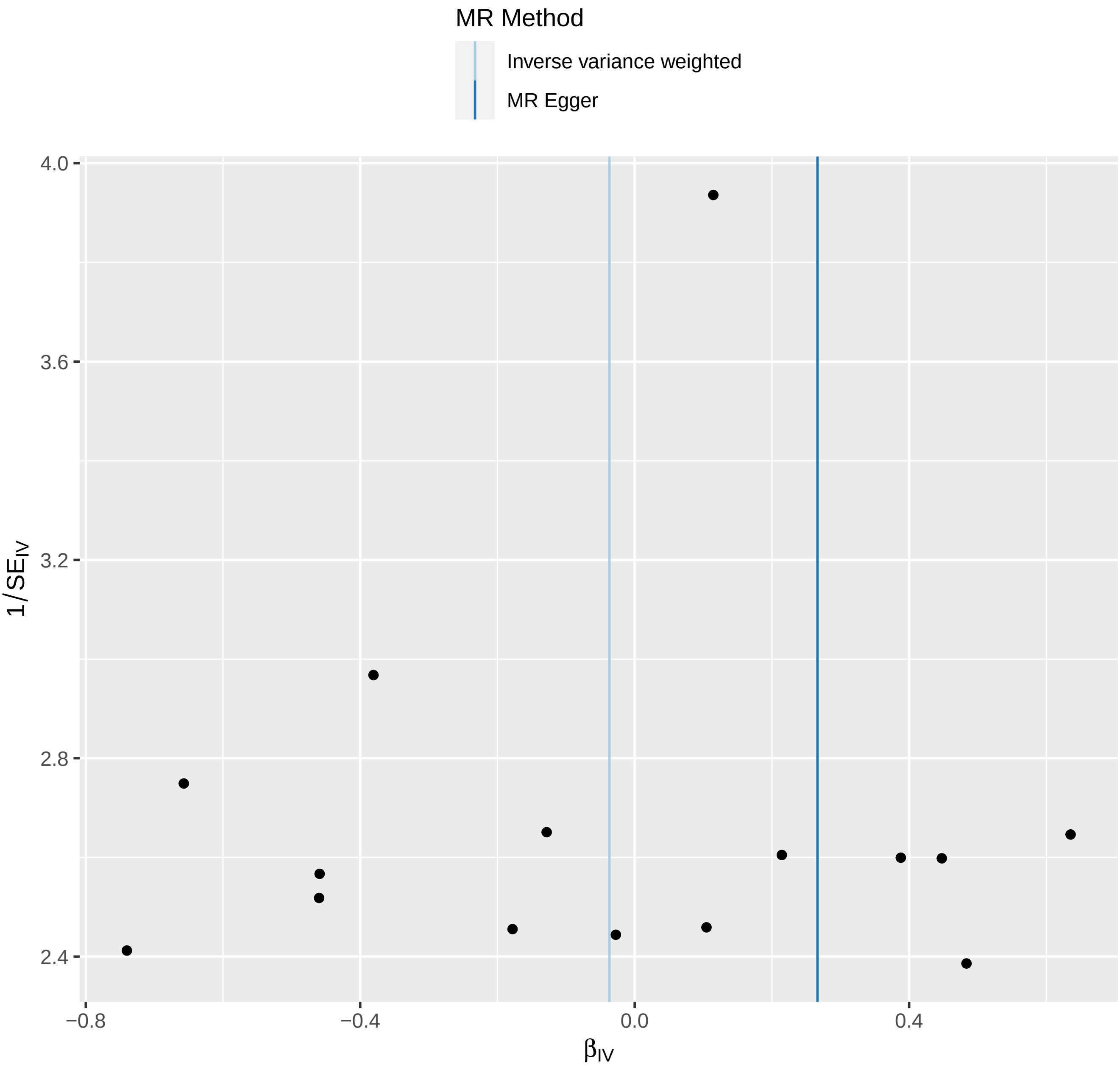

Batch 2−447 : Gut microbiota abundance (genus Catenibacterium id.2153) on Bcl2−associated agonist of cell death with a threshold of 1e−05

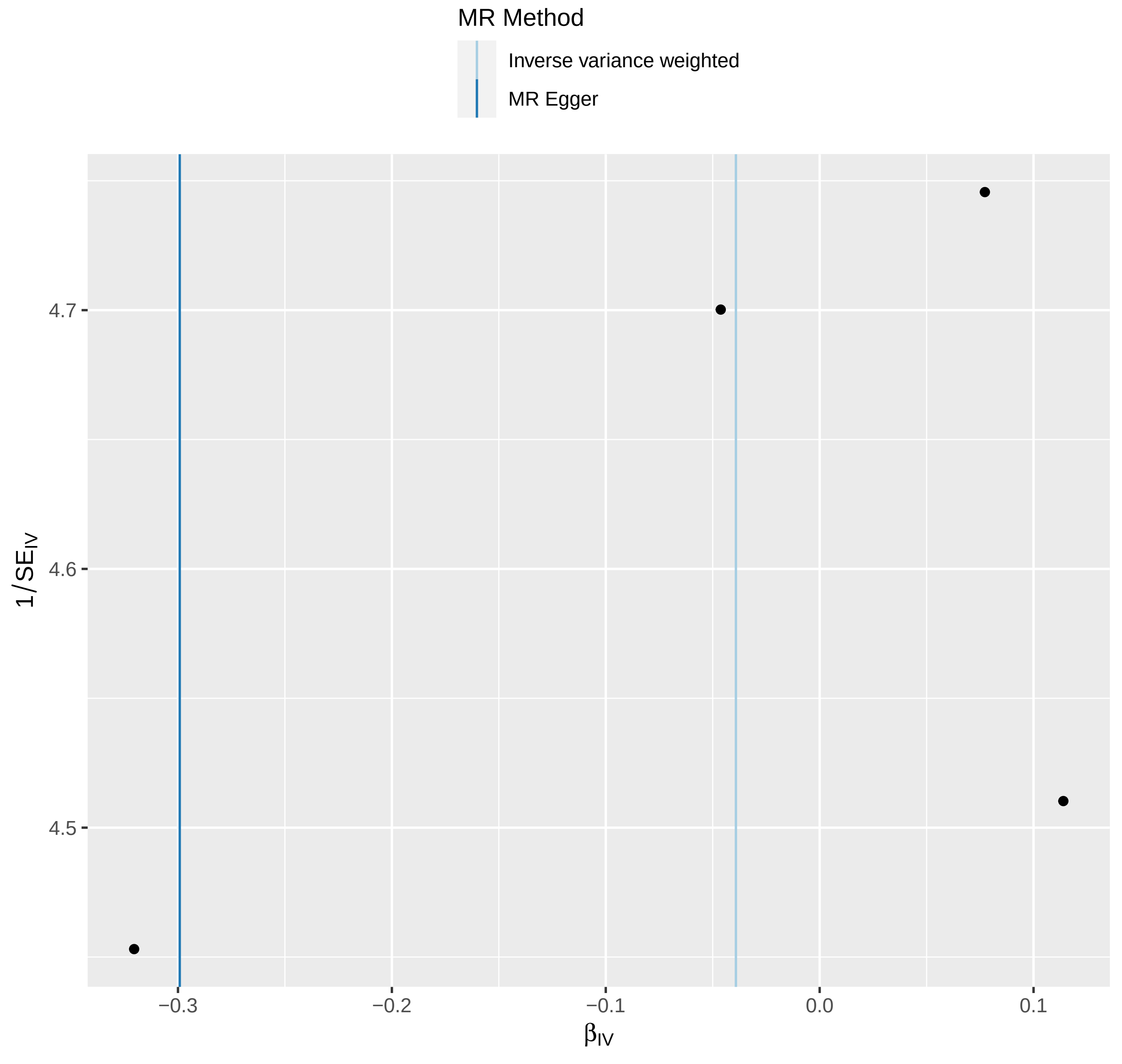

Batch 2−448 : Gut microbiota abundance (genus Parasutterella id.2892) on Bcl2−associated agonist of cell death
with a threshold of 1e−05

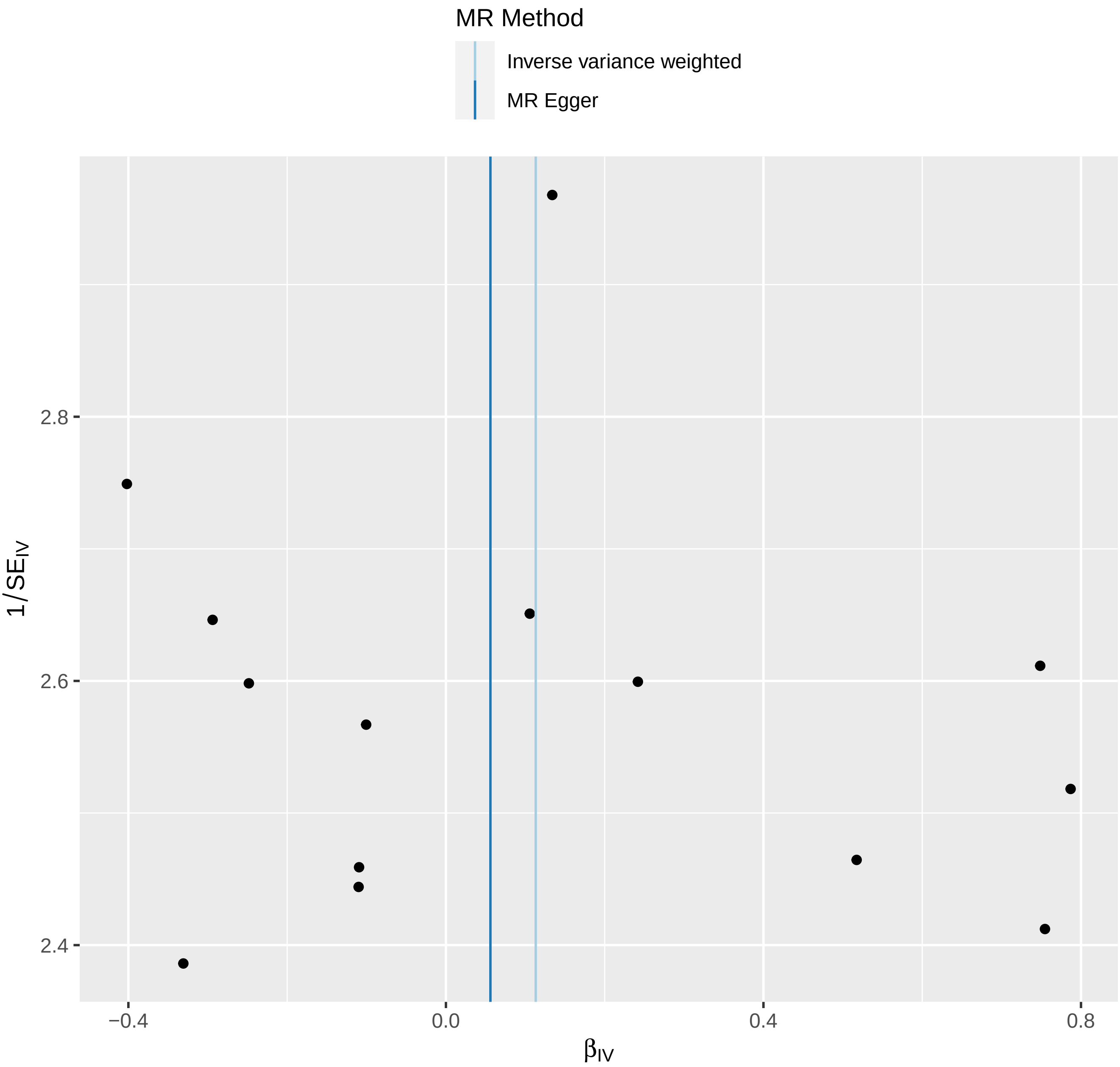

Batch 2−449 : Gut microbiota abundance (genus Catenibacterium id.2153) on BAG family molecular chaperone regulator 3 with a threshold of 1e−05

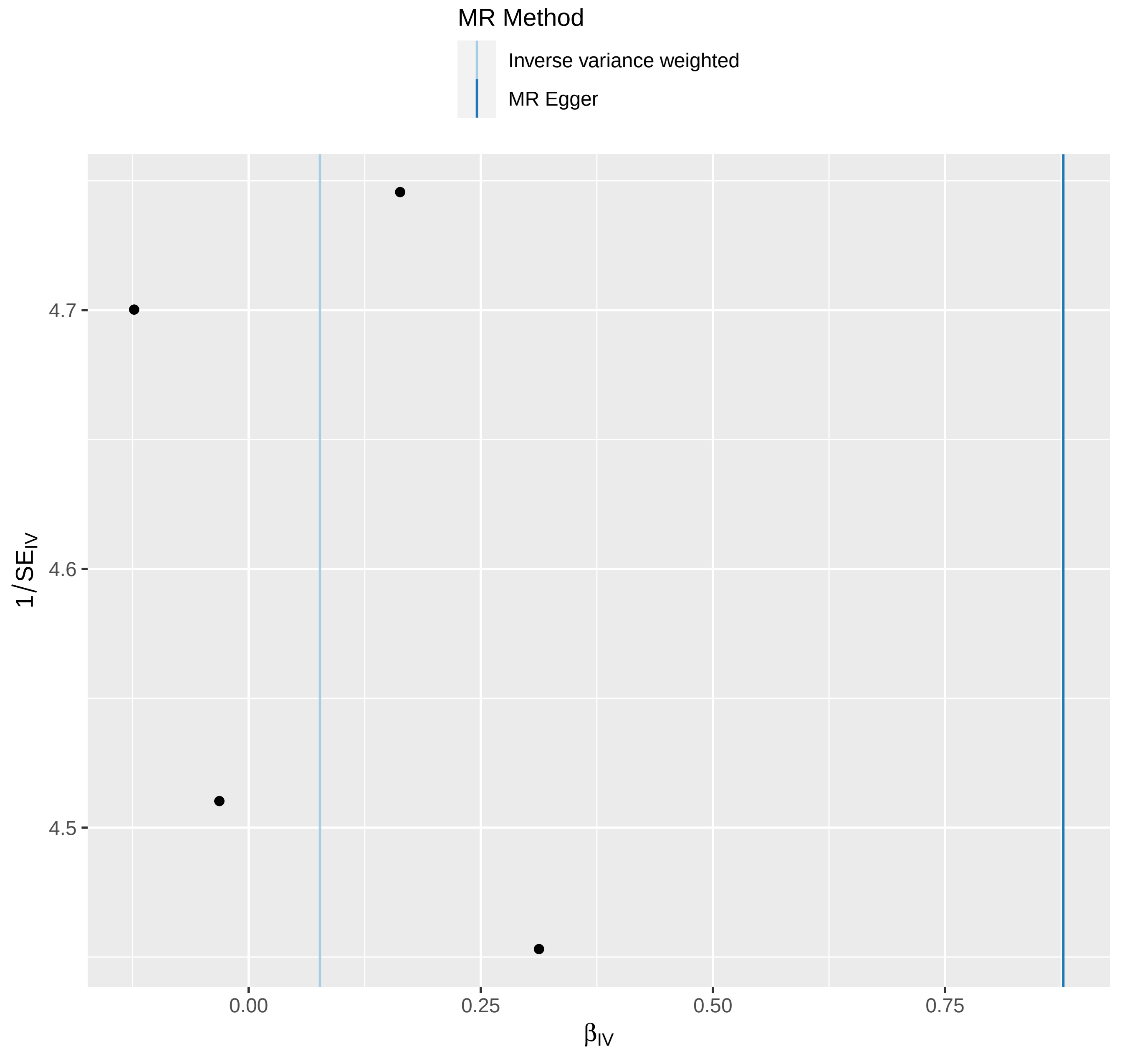

Batch 2−450 : Gut microbiota abundance (genus Parasutterella id.2892) on BAG family molecular chaperone regulator 3 with a threshold of 1e−05

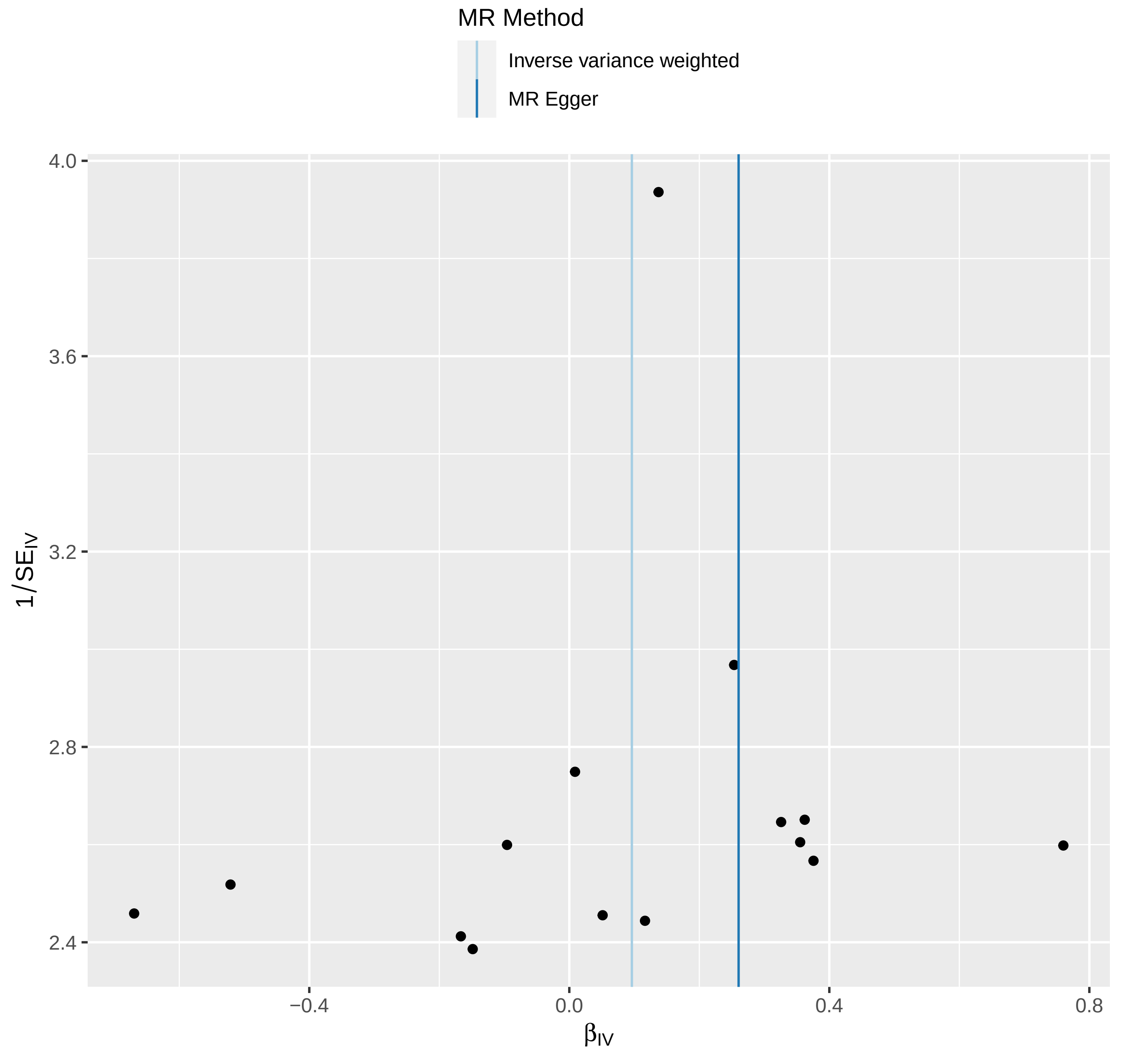

Batch 2−451 : Gut microbiota abundance (genus Catenibacterium id.2153) on BAG family molecular chaperone regulator 4 with a threshold of 1e−05

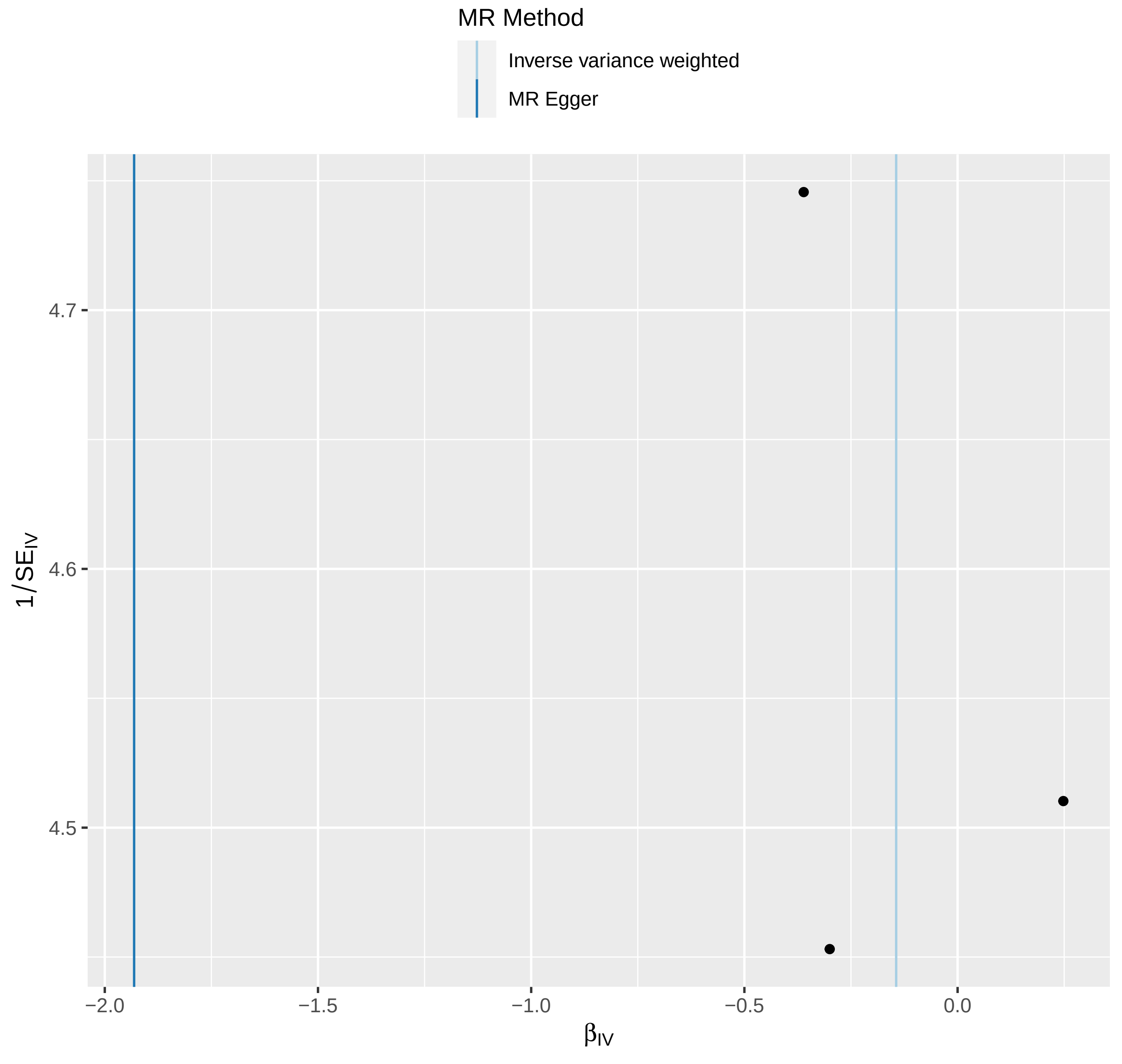

Batch 2−452 : Gut microbiota abundance (genus Parasutterella id.2892) on BAG family molecular chaperone regulator 4
with a threshold of 1e−05

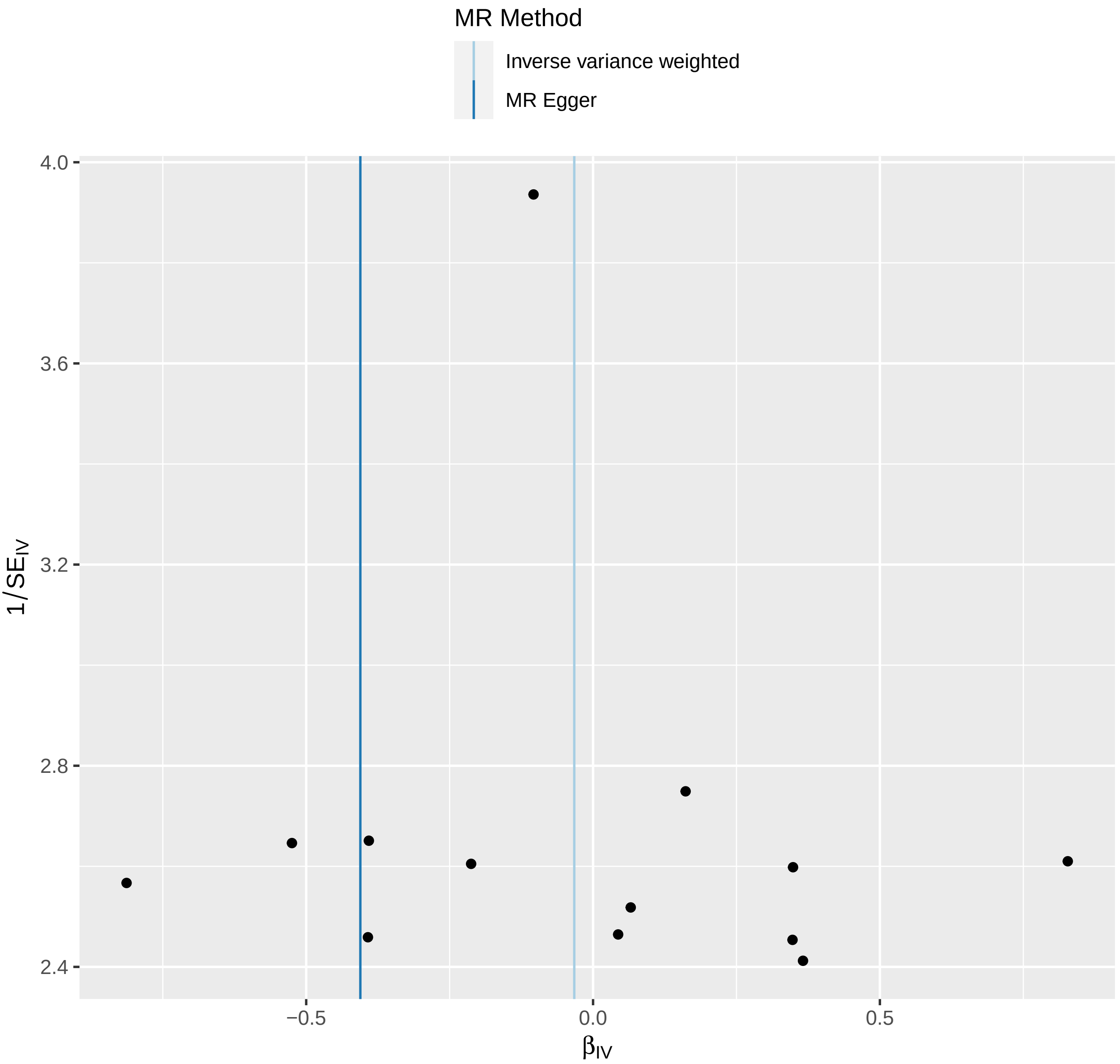

Batch 2−453 : Gut microbiota abundance (genus Catenibacterium id.2153) on B melanoma antigen 2
with a threshold of 1e−05

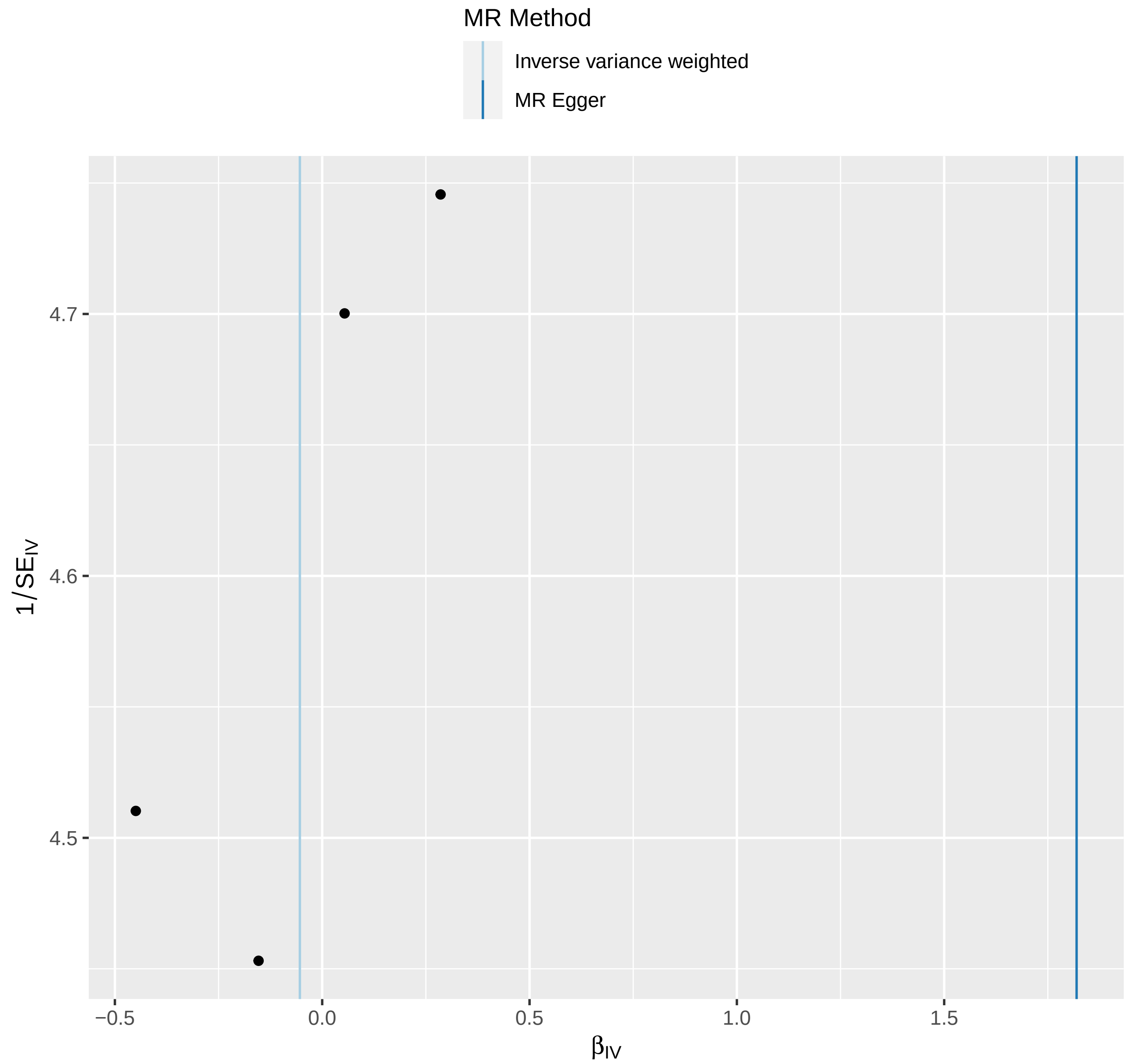

Batch 2−454 : Gut microbiota abundance (genus Parasutterella id.2892) on B melanoma antigen 2
with a threshold of 1e−05

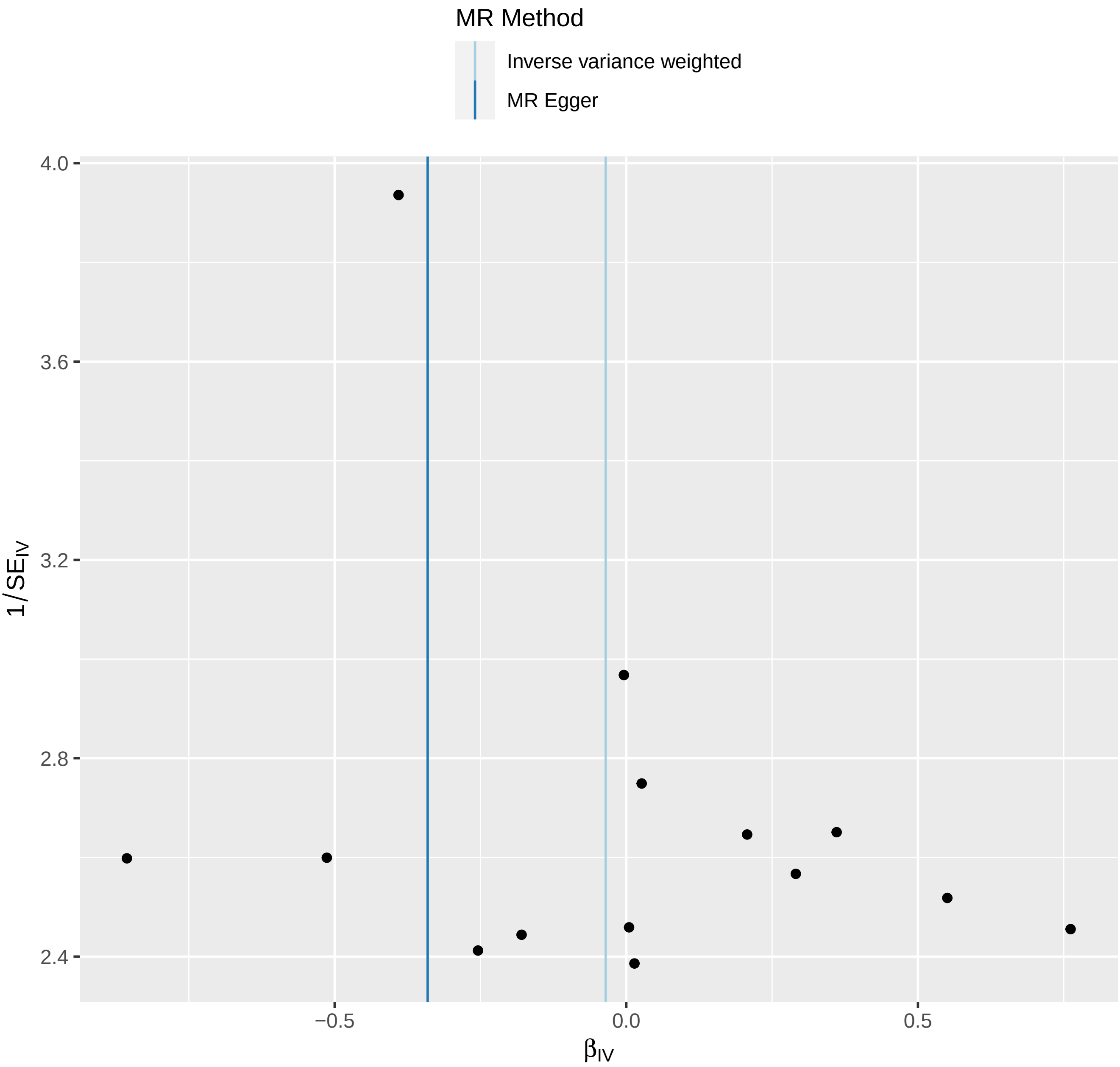

Batch 2−455 : Gut microbiota abundance (genus Catenibacterium id.2153) on B melanoma antigen 3
with a threshold of 1e−05

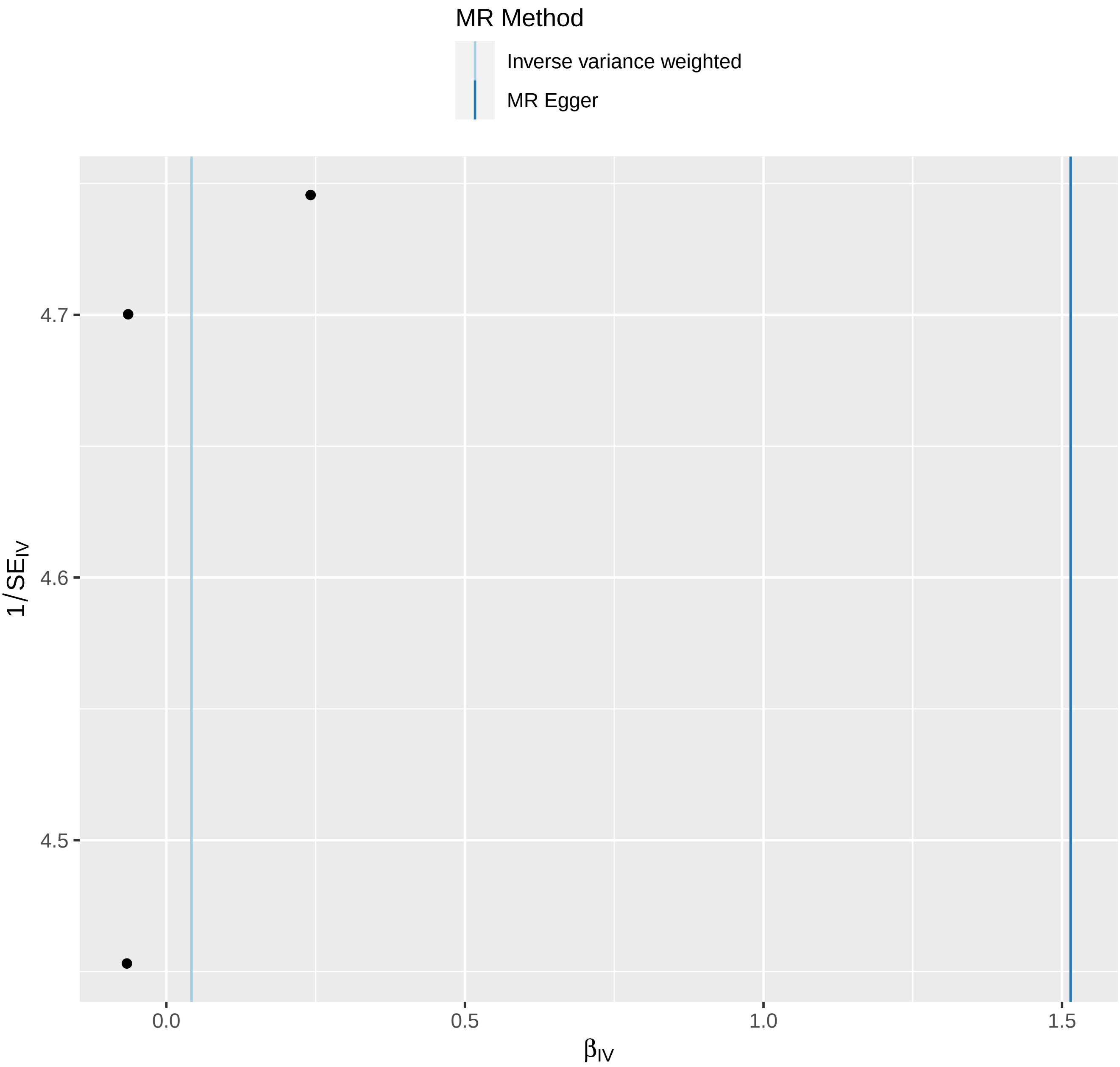

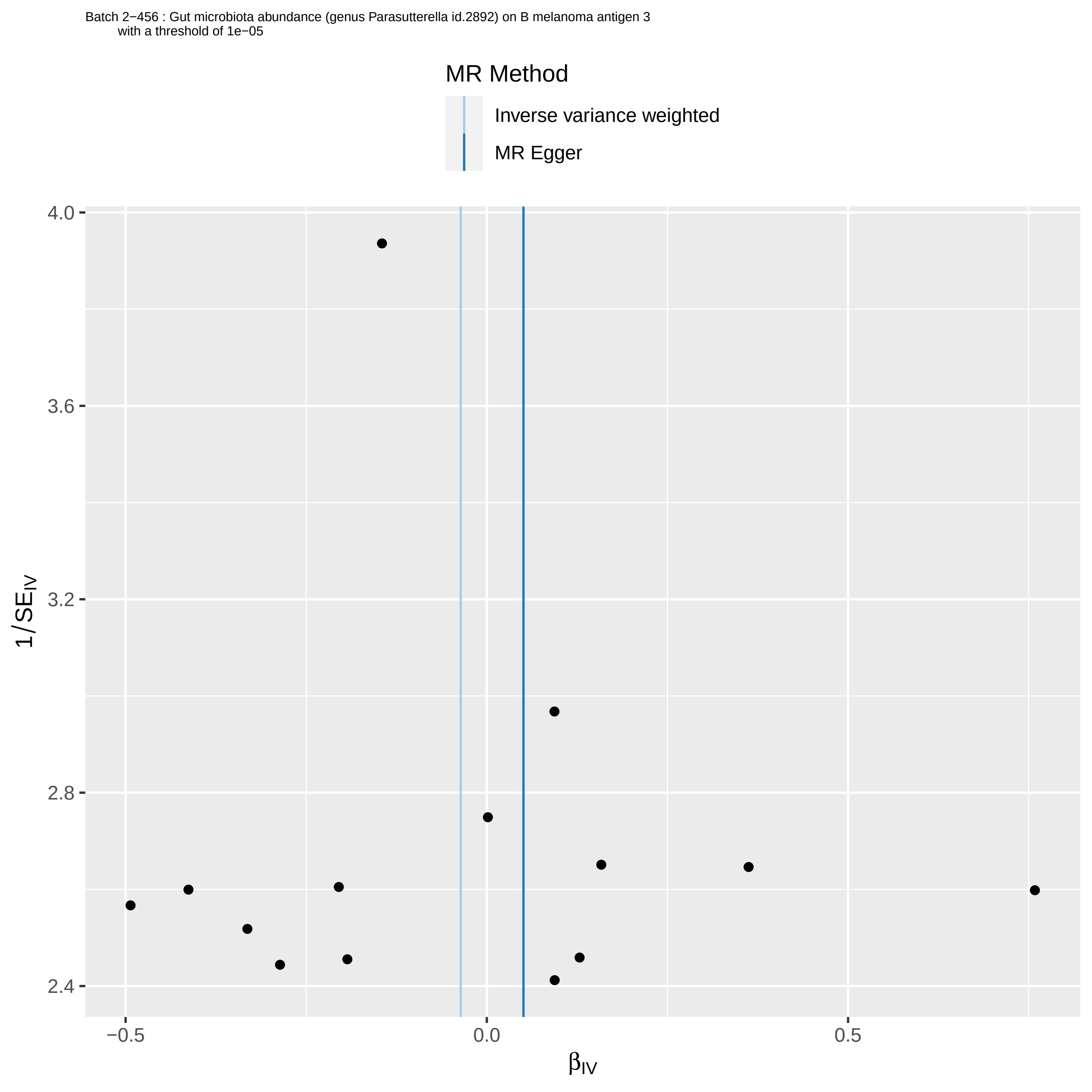

Batch 2−456 : Gut microbiota abundance (genus Parasutterella id.2892) on B melanoma antigen 3
with a threshold of 1e−05
MR Method
Inverse variance weighted
MR Egger
4.0
3.6
3.2
2.8
2.4
1/SE$_{IV}$
−0.5
0.0
0.5
β$_{IV}$

Batch 2−457 : Gut microbiota abundance (genus Catenibacterium id.2153) on BRCA1−associated RING domain protein 1
with a threshold of 1e−05

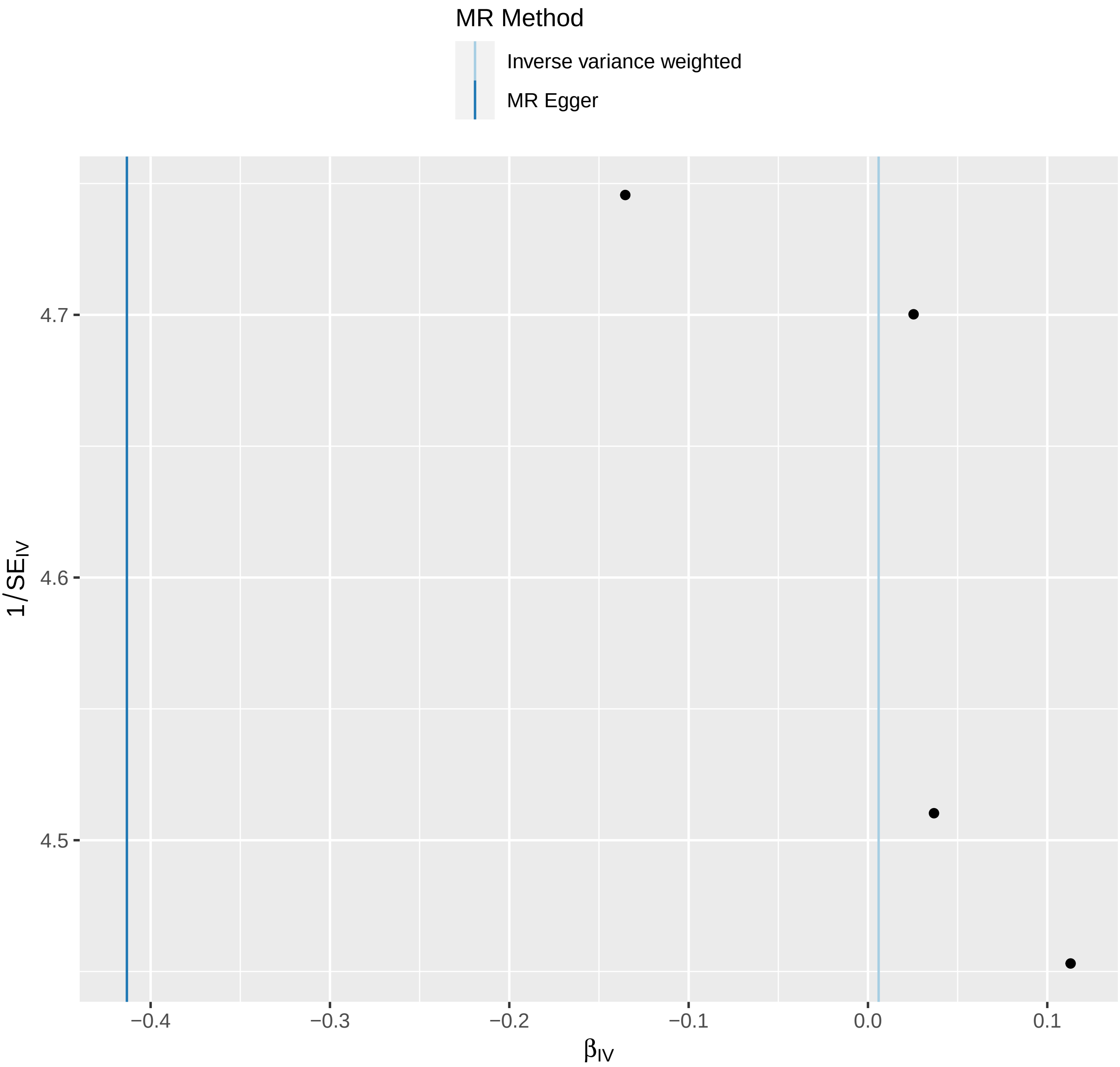

Batch 2−458 : Gut microbiota abundance (genus Parasutterella id.2892) on BRCA1−associated RING domain protein 1
with a threshold of 1e−05

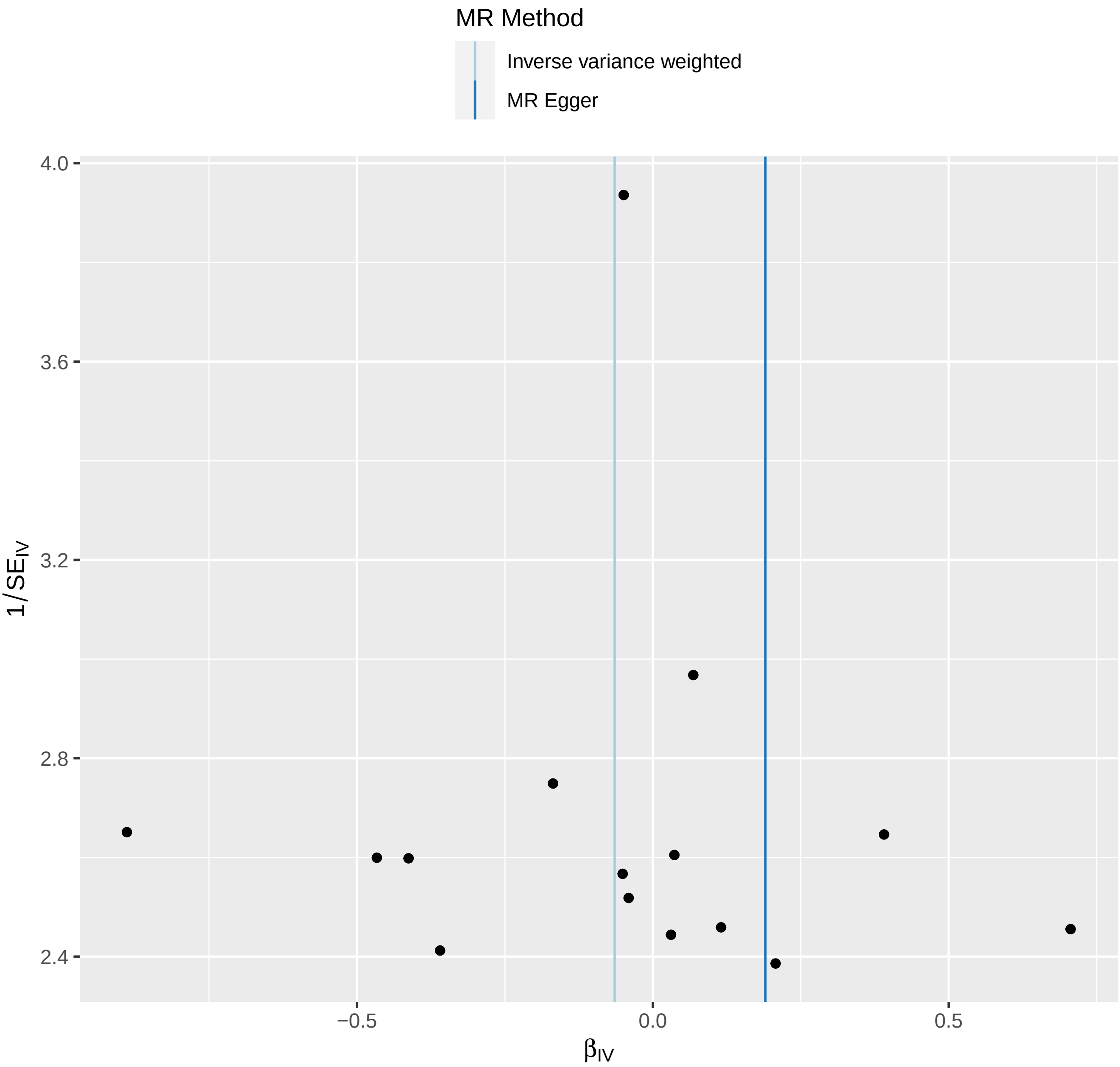

Batch 2−459 : Gut microbiota abundance (genus Catenibacterium id.2153) on Basic leucine zipper transcriptional factor ATF−like 3 with a threshold of 1e−05

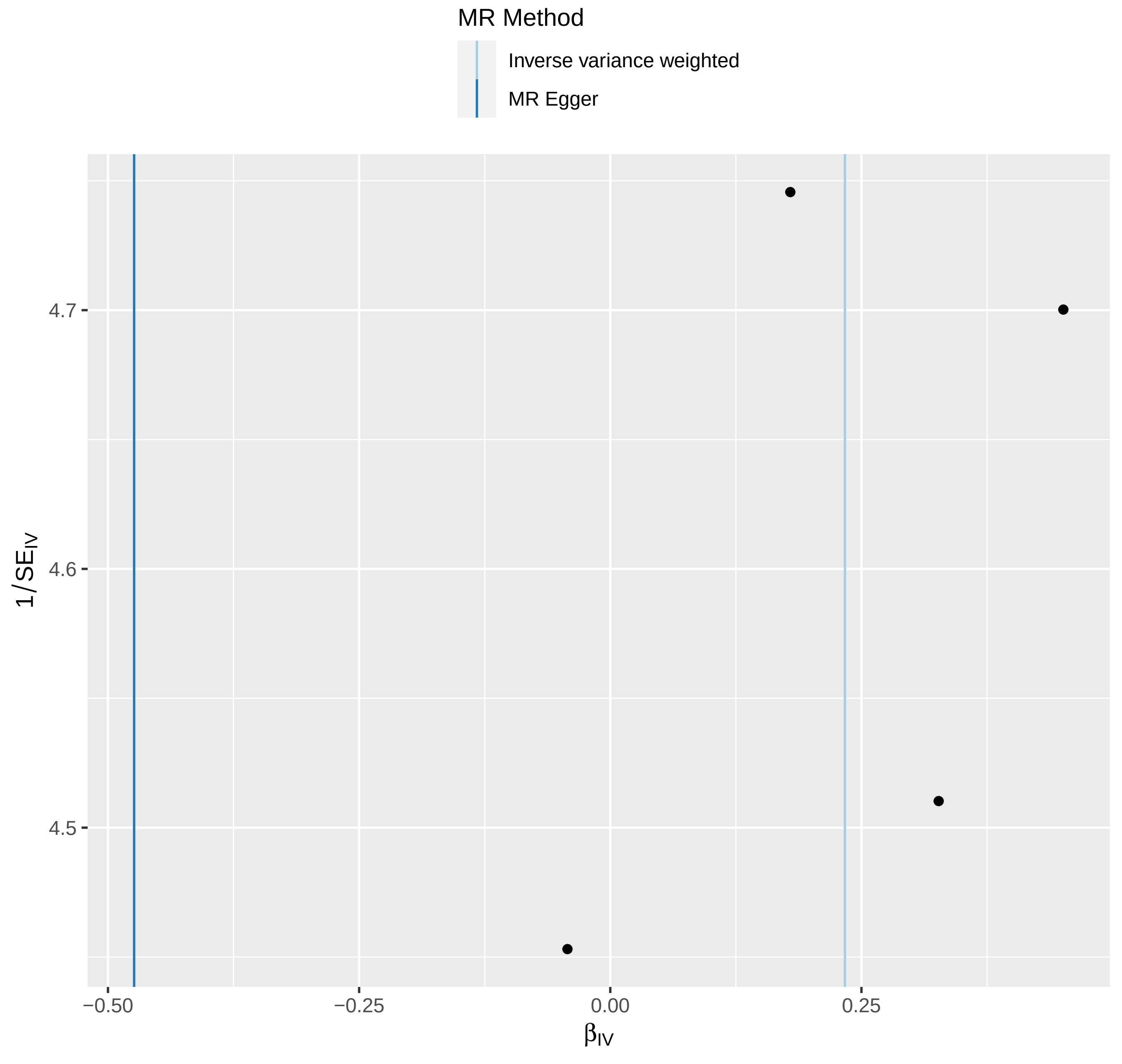

Batch 2−460 : Gut microbiota abundance (genus Parasutterella id.2892) on Basic leucine zipper transcriptional factor ATF−like 3 with a threshold of 1e−05

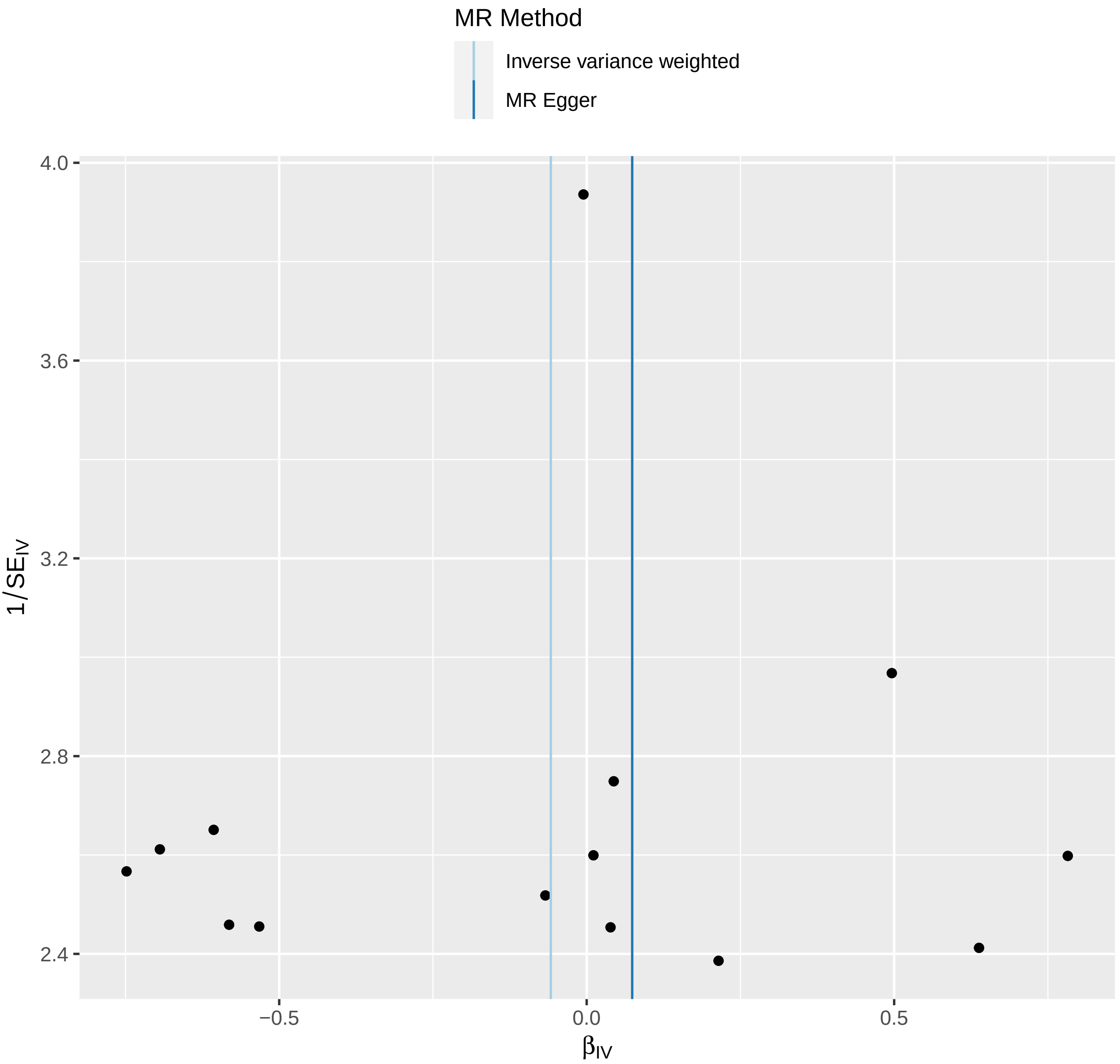

Batch 2−461 : Gut microbiota abundance (genus Catenibacterium id.2153) on Basal Cell Adhesion Molecule
with a threshold of 1e−05

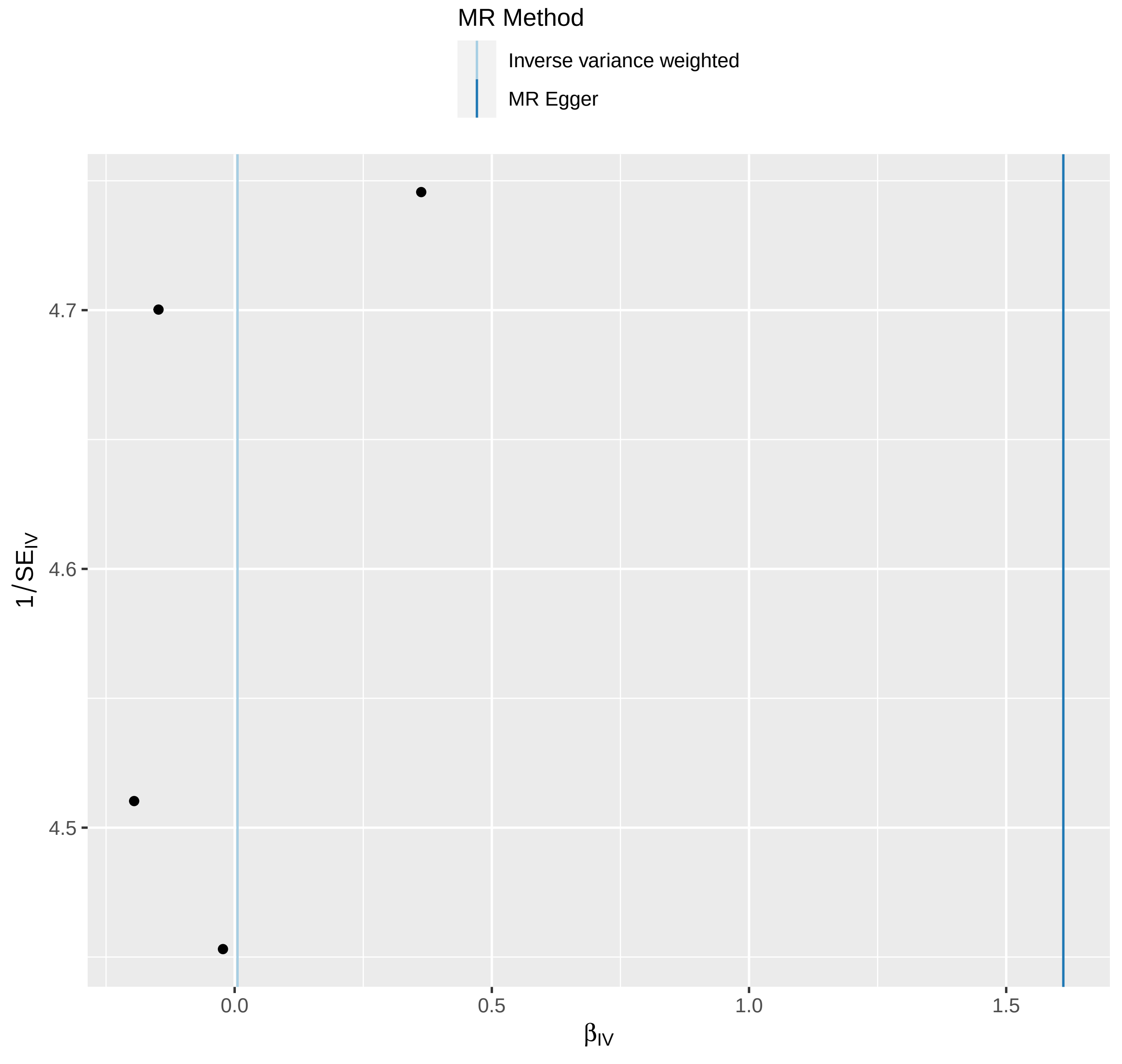

Batch 2−462 : Gut microbiota abundance (genus Parasutterella id.2892) on Basal Cell Adhesion Molecule
with a threshold of 1e−05

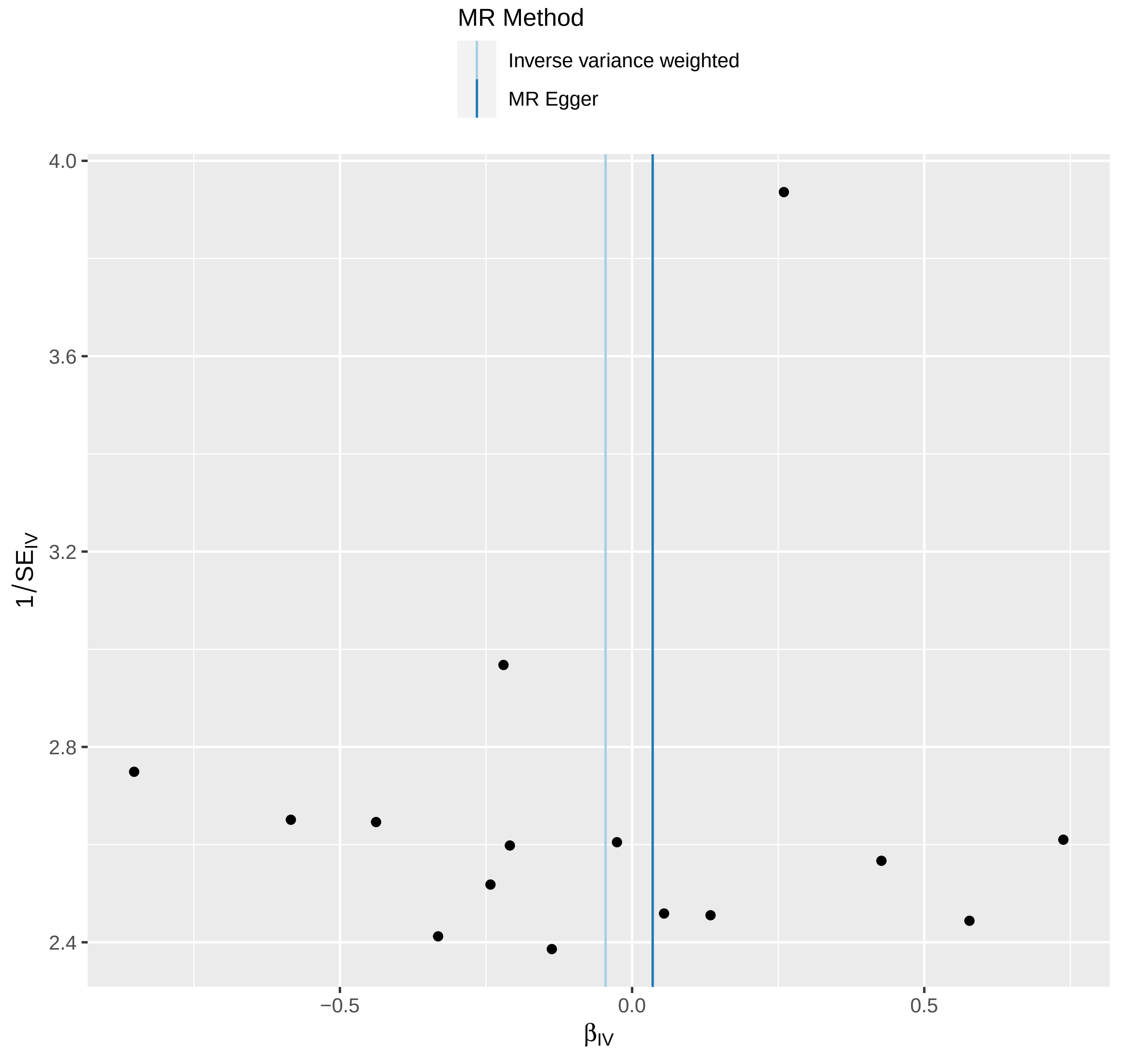

Batch 2−463 : Gut microbiota abundance (genus Catenibacterium id.2153) on Brevican core protein
with a threshold of 1e−05

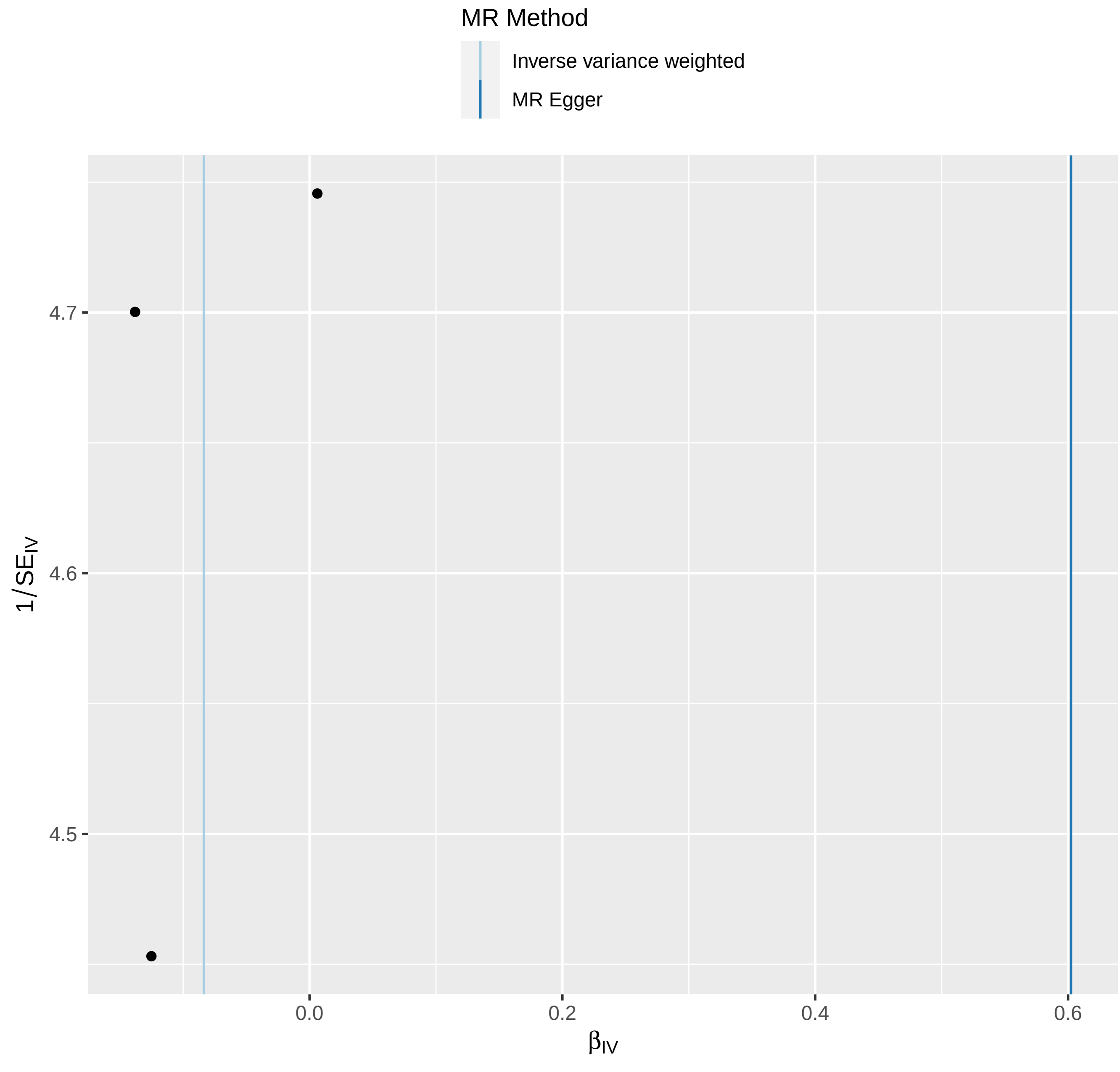

Batch 2−464 : Gut microbiota abundance (genus Parasutterella id.2892) on Brevican core protein
with a threshold of 1e−05

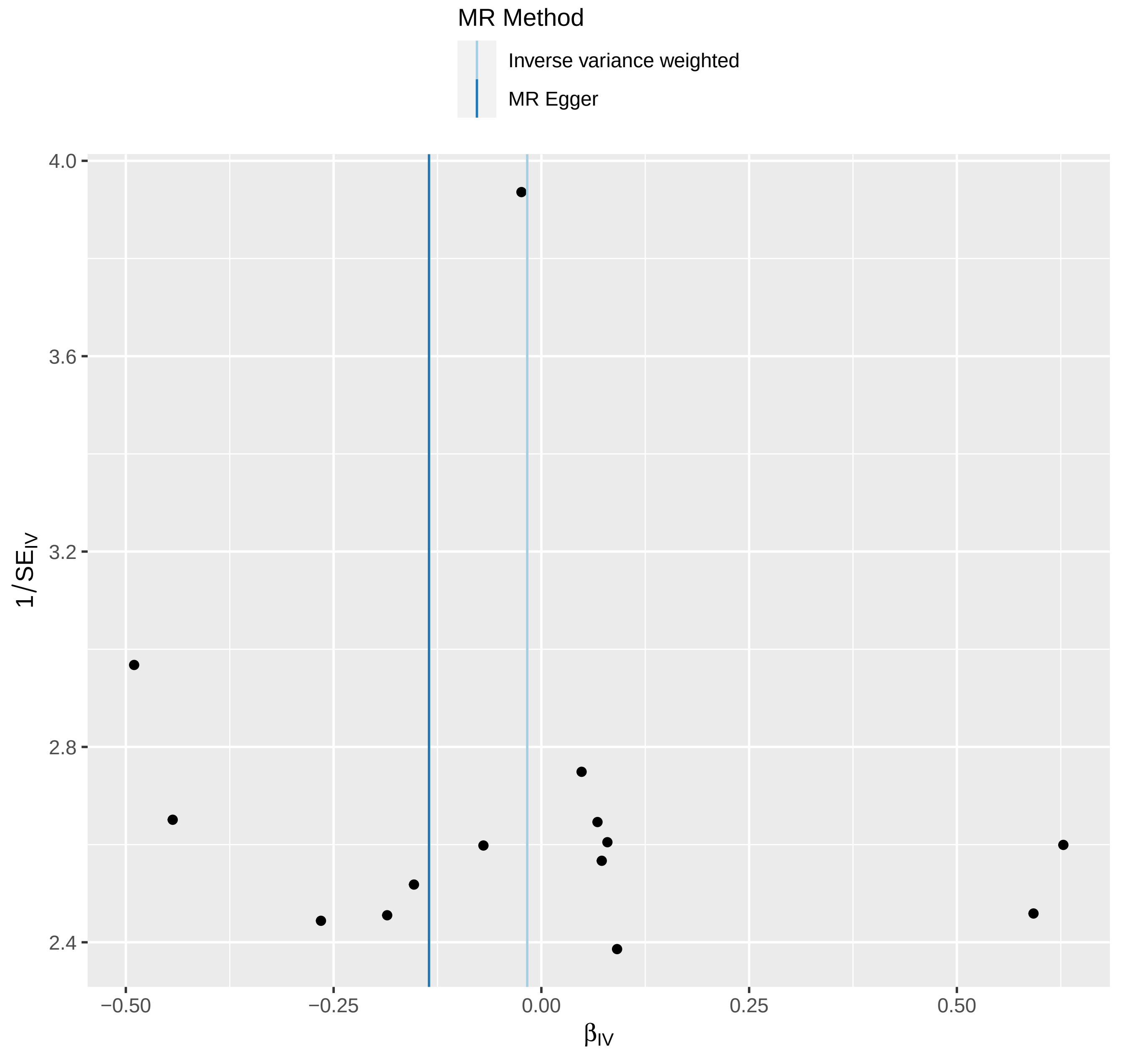

Batch 2−465 : Gut microbiota abundance (genus Catenibacterium id.2153) on B−cell receptor−associated protein 29
with a threshold of 1e−05

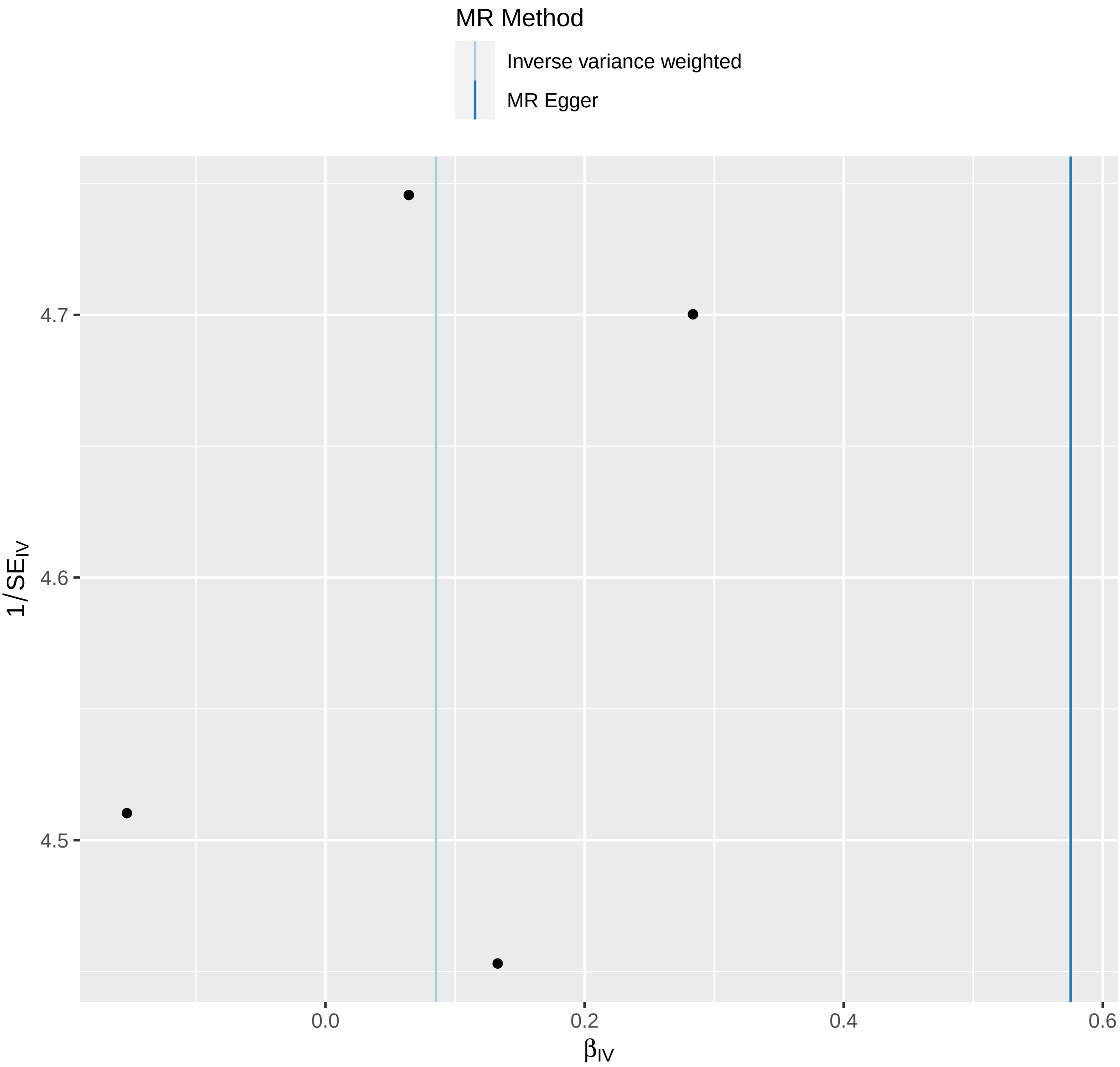

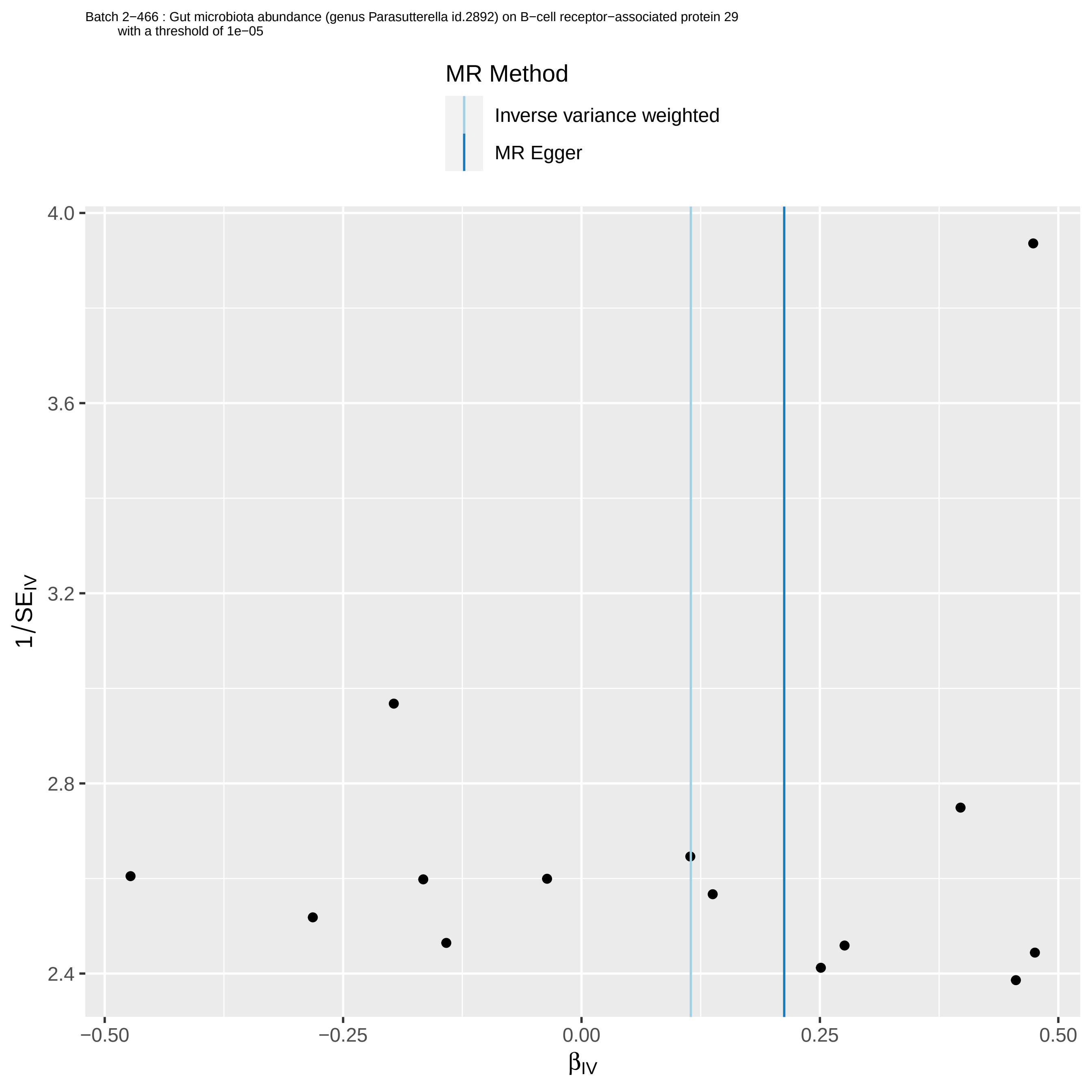
Batch 2−466 : Gut microbiota abundance (genus Parasutterella id.2892) on B−cell receptor−associated protein 29
with a threshold of 1e−05
MR Method
Inverse variance weighted
MR Egger
4.0
3.6
3.2
2.8
2.4
1/SE_IV
−0.50
−0.25
0.00
0.25
0.50
β_IV

Batch 2−467 : Gut microbiota abundance (genus Catenibacterium id.2153) on Breast cancer anti−estrogen resistance protein 3
with a threshold of 1e−05

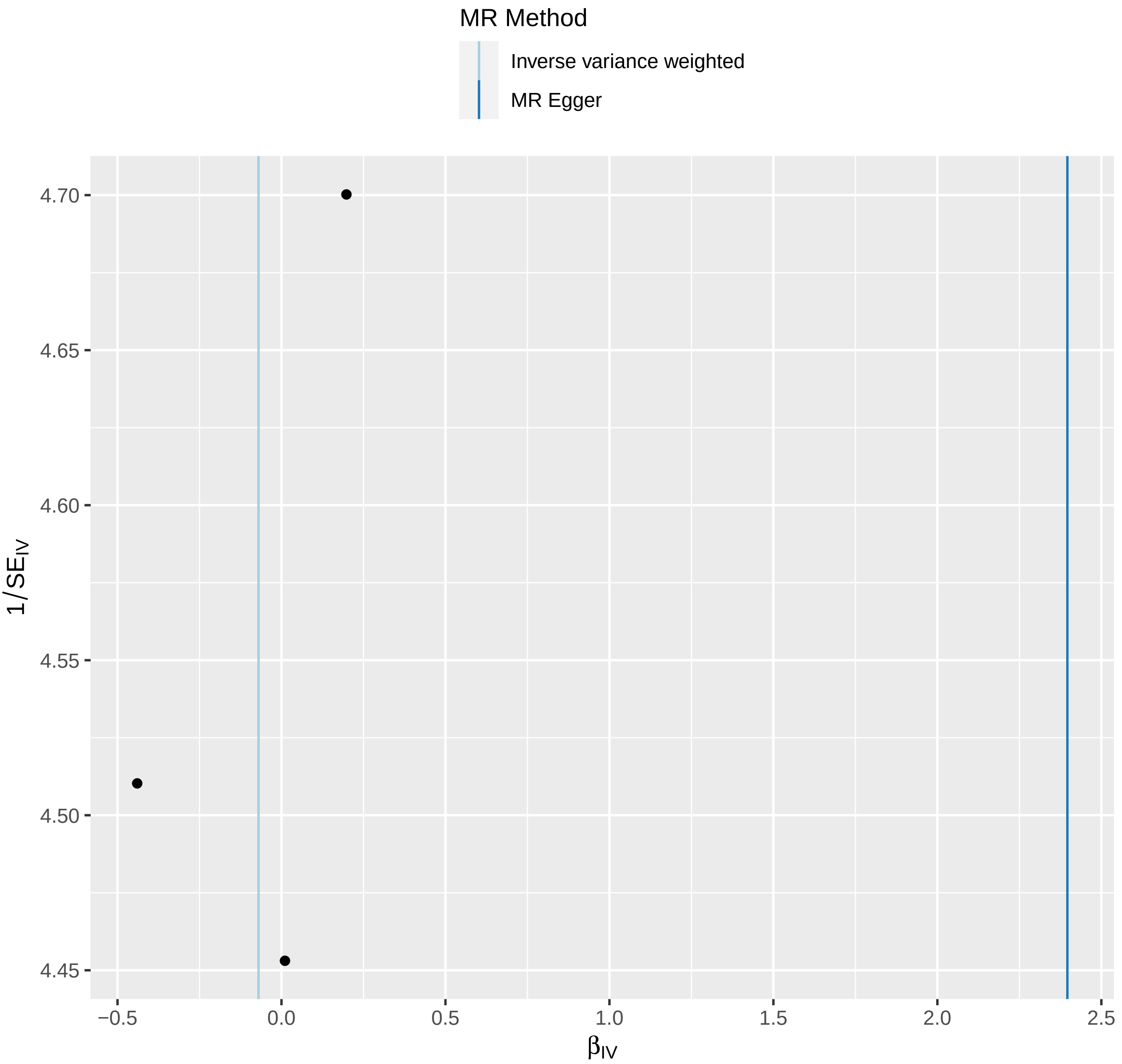

Batch 2−468 : Gut microbiota abundance (genus Parasutterella id.2892) on Breast cancer anti−estrogen resistance protein 3
with a threshold of 1e−05

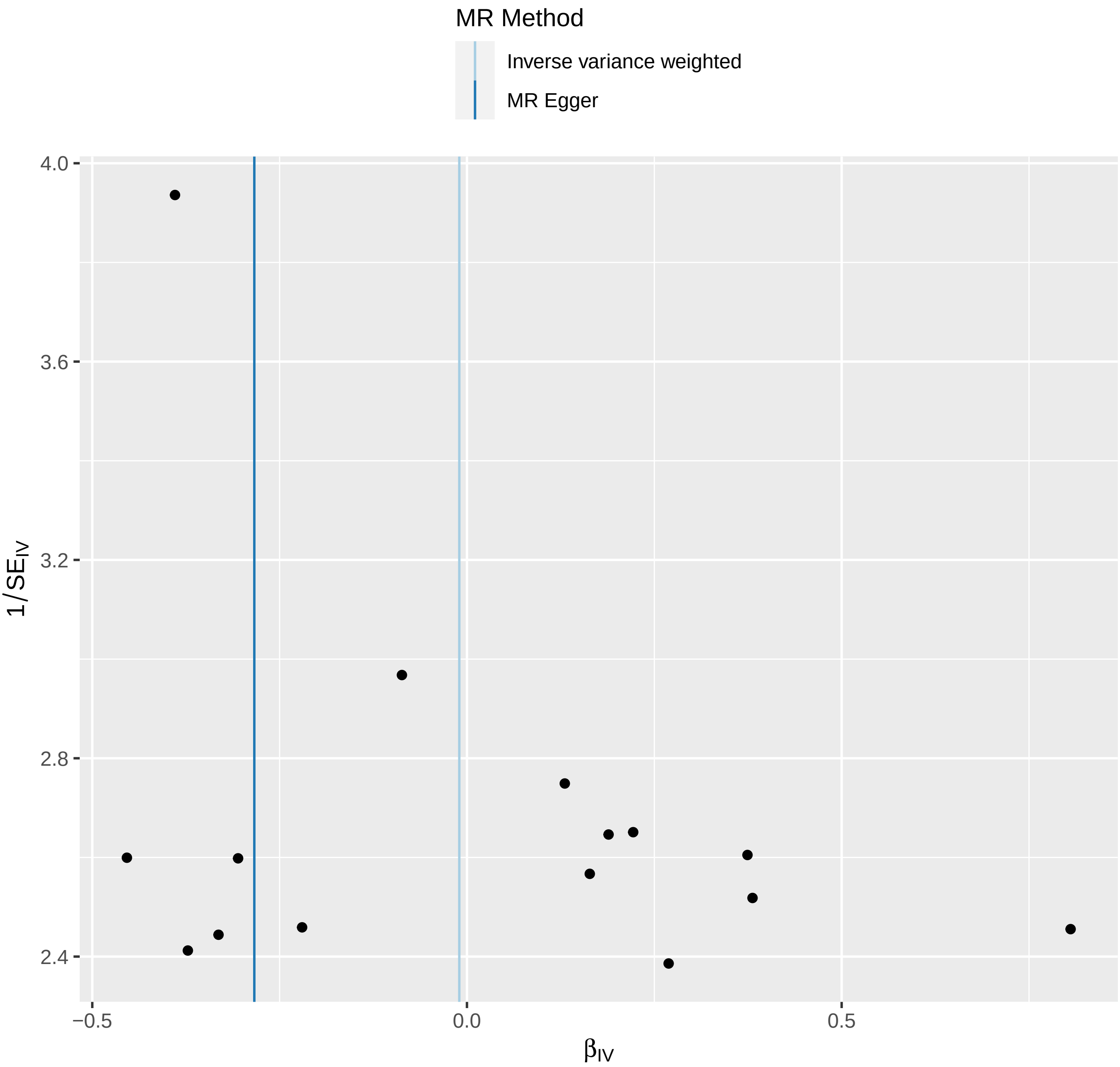

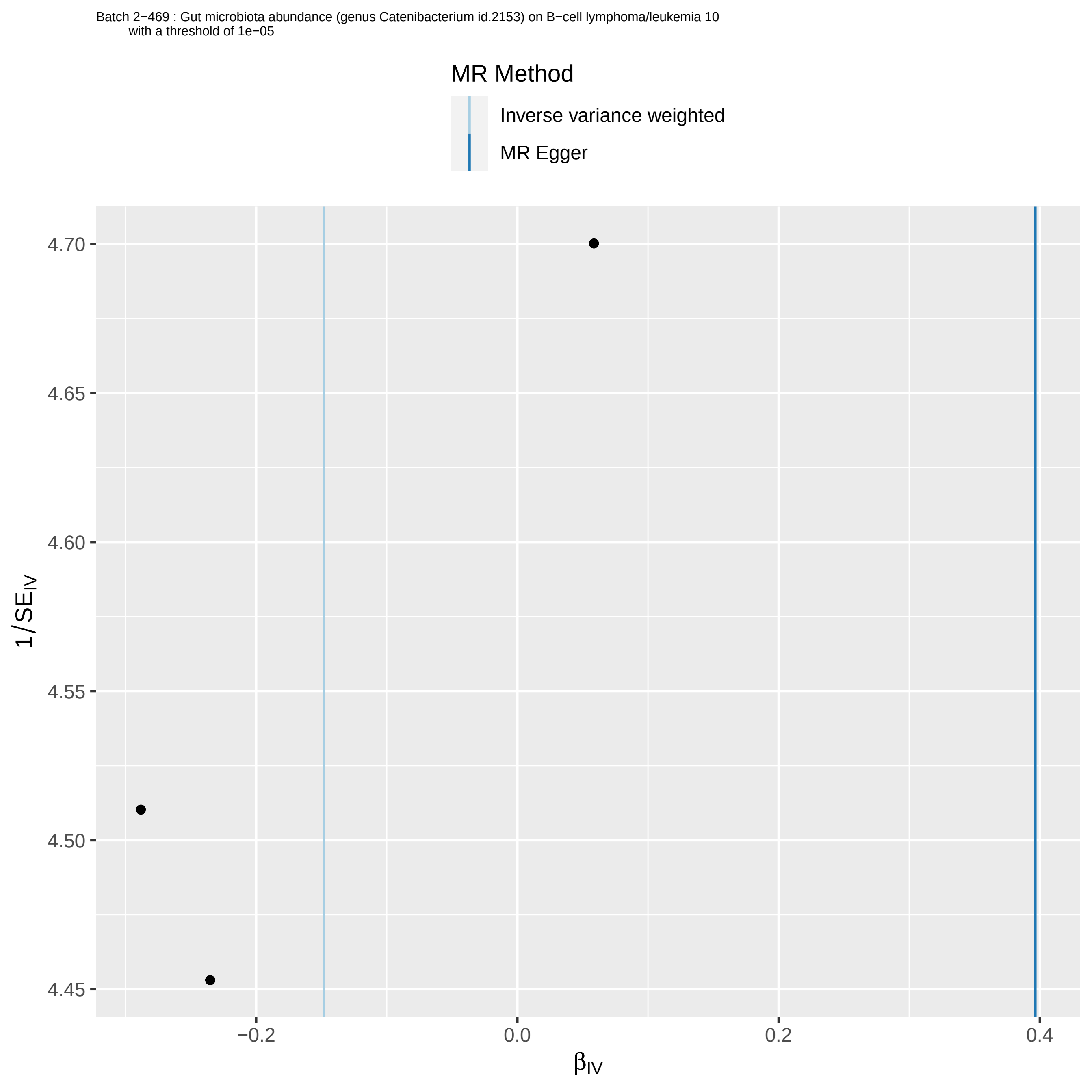

Batch 2−469 : Gut microbiota abundance (genus Catenibacterium id.2153) on B−cell lymphoma/leukemia 10
with a threshold of 1e−05
MR Method
Inverse variance weighted
MR Egger
4.70
4.65
4.60
4.55
4.50
4.45
1/SE_IV
−0.2
0.0
0.2
0.4
β_IV

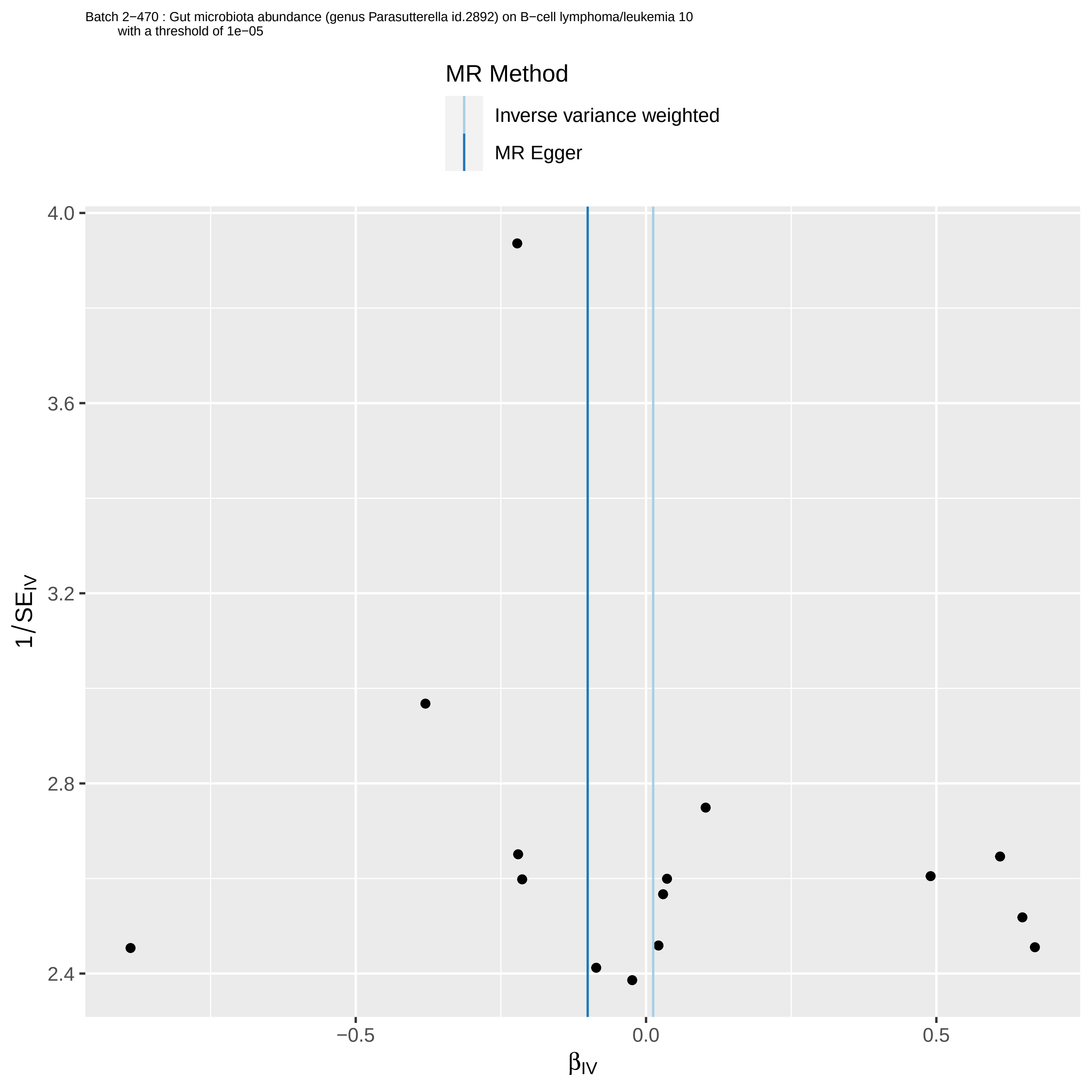
Batch 2−470 : Gut microbiota abundance (genus Parasutterella id.2892) on B−cell lymphoma/leukemia 10
with a threshold of 1e−05
MR Method
Inverse variance weighted
MR Egger
4.0
3.6
3.2
2.8
2.4
1/SE_IV
−0.5
0.0
0.5
β_IV

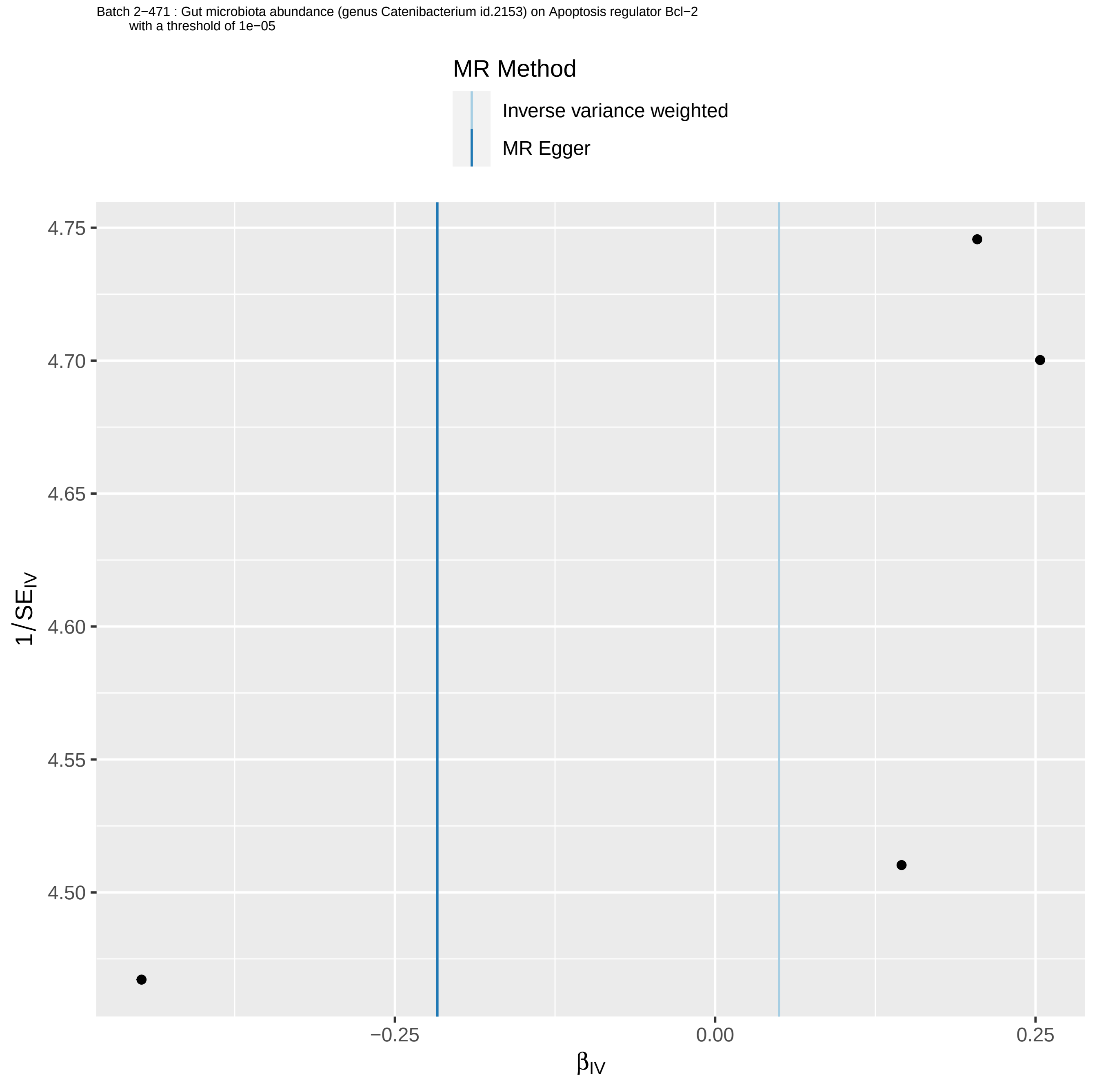

Batch 2−471 : Gut microbiota abundance (genus Catenibacterium id.2153) on Apoptosis regulator Bcl−2
with a threshold of 1e−05
MR Method
Inverse variance weighted
MR Egger
4.75
4.70
4.65
4.60
4.55
4.50
1/SEIV
−0.25
0.00
0.25
βIV

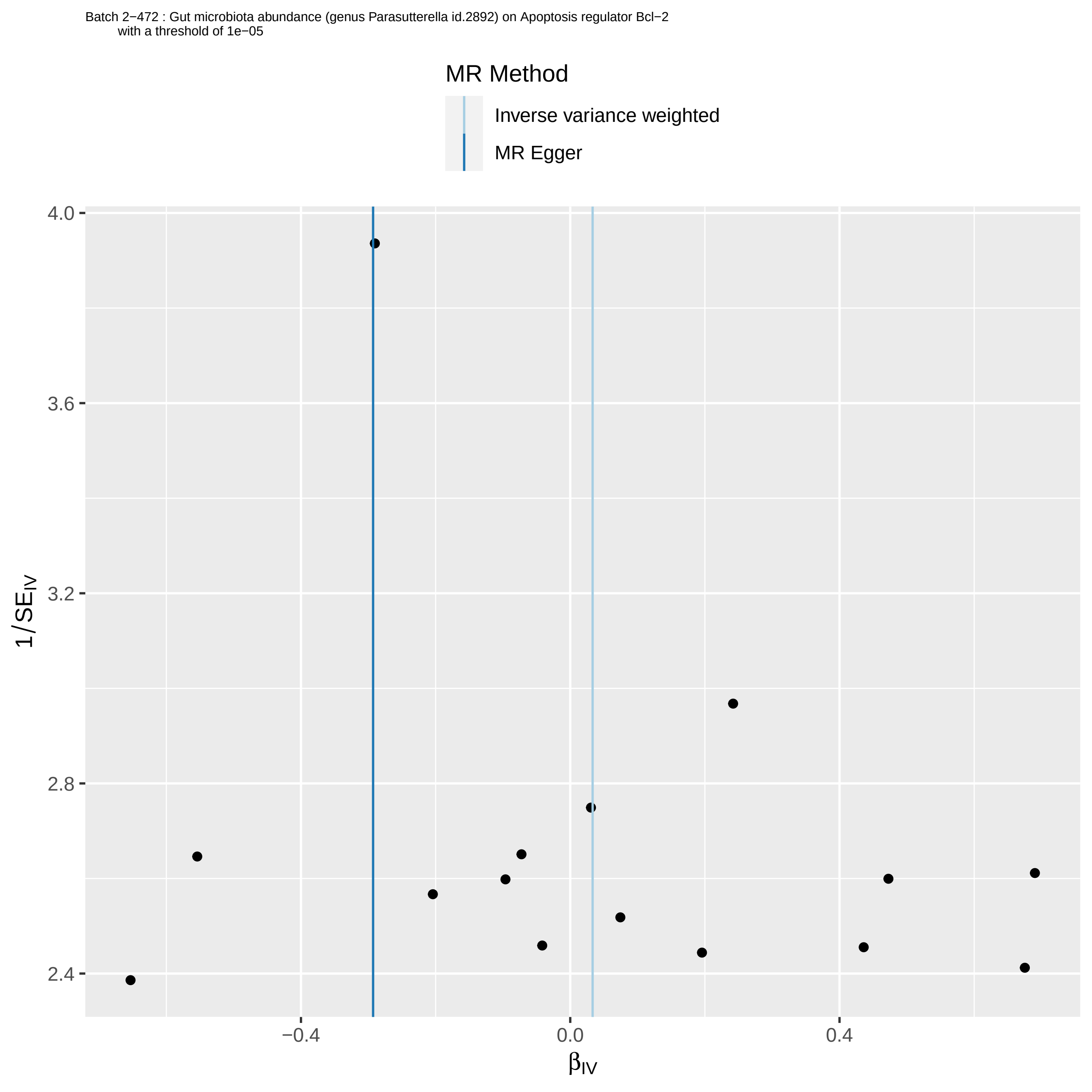

Batch 2−472 : Gut microbiota abundance (genus Parasutterella id.2892) on Apoptosis regulator Bcl−2
with a threshold of 1e−05
MR Method
Inverse variance weighted
MR Egger
4.0
3.6
3.2
2.8
2.4
$1/SE_{IV}$
−0.4
0.0
0.4
β_{IV}

Batch 2−473 : Gut microbiota abundance (genus Catenibacterium id.2153) on Bcl−2−related protein A1
with a threshold of 1e−05

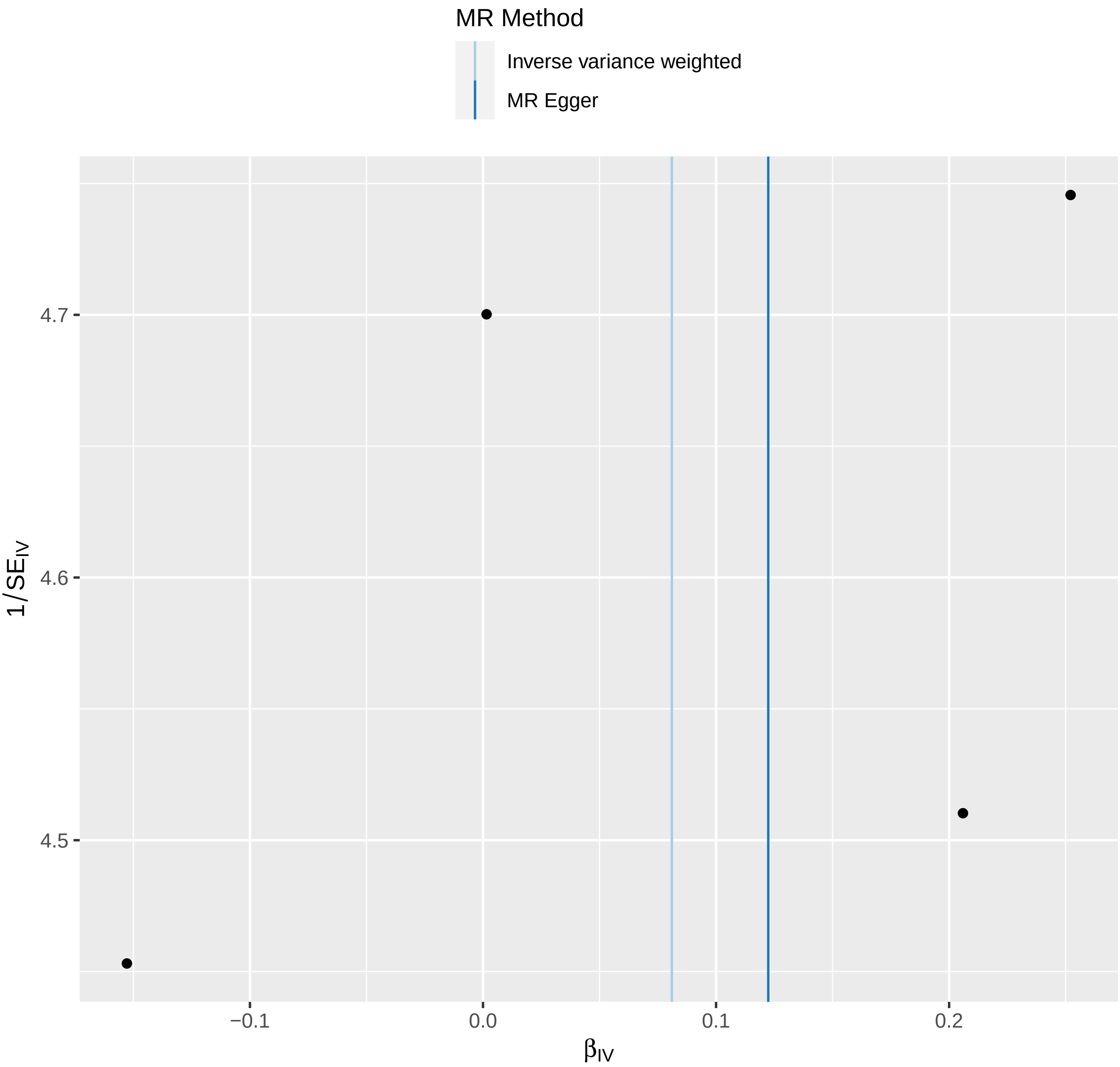

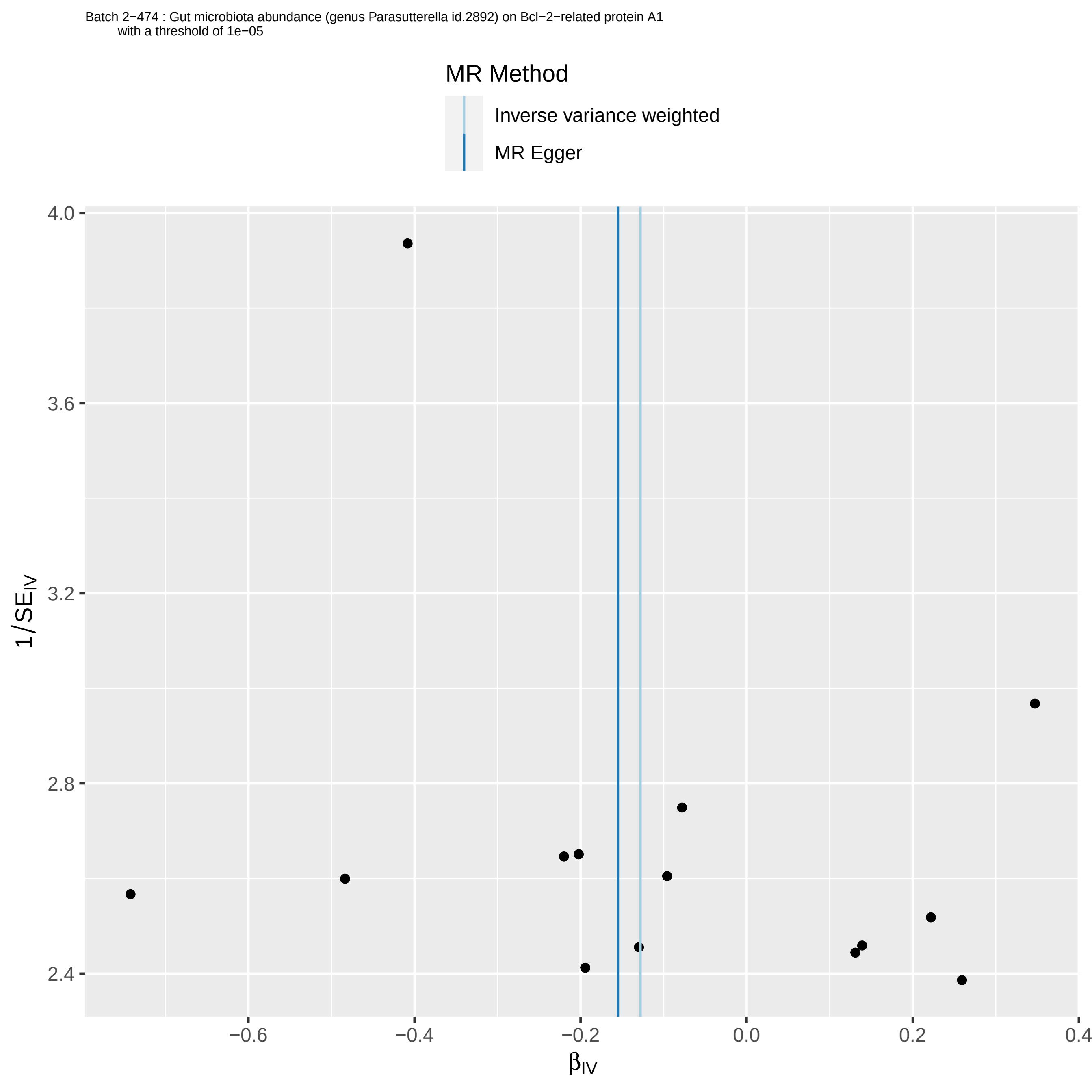

Batch 2−474 : Gut microbiota abundance (genus Parasutterella id.2892) on Bcl−2−related protein A1
with a threshold of 1e−05
MR Method
Inverse variance weighted
MR Egger
4.0
3.6
3.2
2.8
2.4
1/SE$_{IV}$
−0.6
−0.4
−0.2
0.0
0.2
0.4
β_{IV}

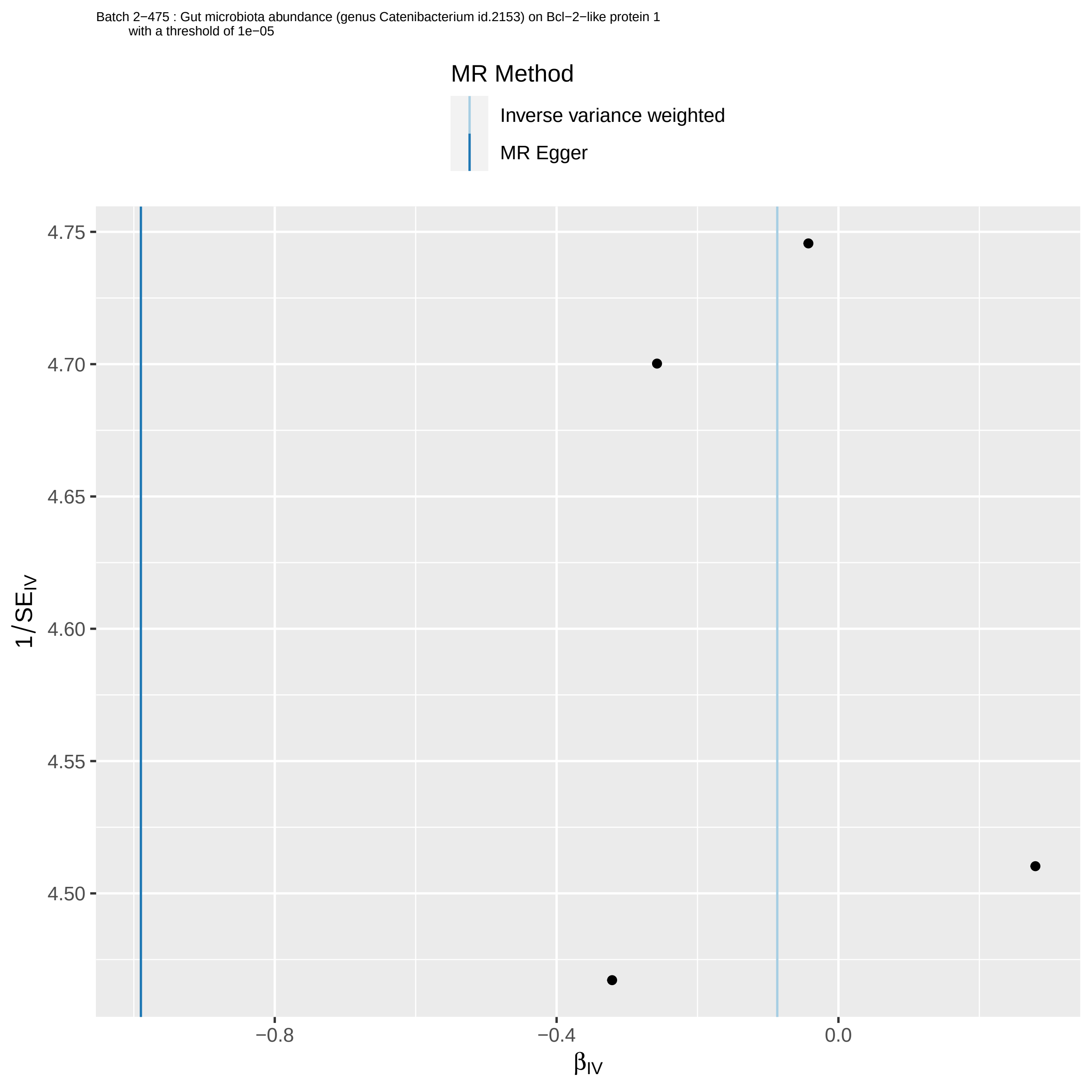

Batch 2−475 : Gut microbiota abundance (genus Catenibacterium id.2153) on Bcl−2−like protein 1
with a threshold of 1e−05
MR Method
Inverse variance weighted
MR Egger
4.75
4.70
4.65
4.60
4.55
4.50
1/SE_IV
−0.8
−0.4
0.0
β_IV

Batch 2−476 : Gut microbiota abundance (genus Parasutterella id.2892) on Bcl−2−like protein 1
with a threshold of 1e−05

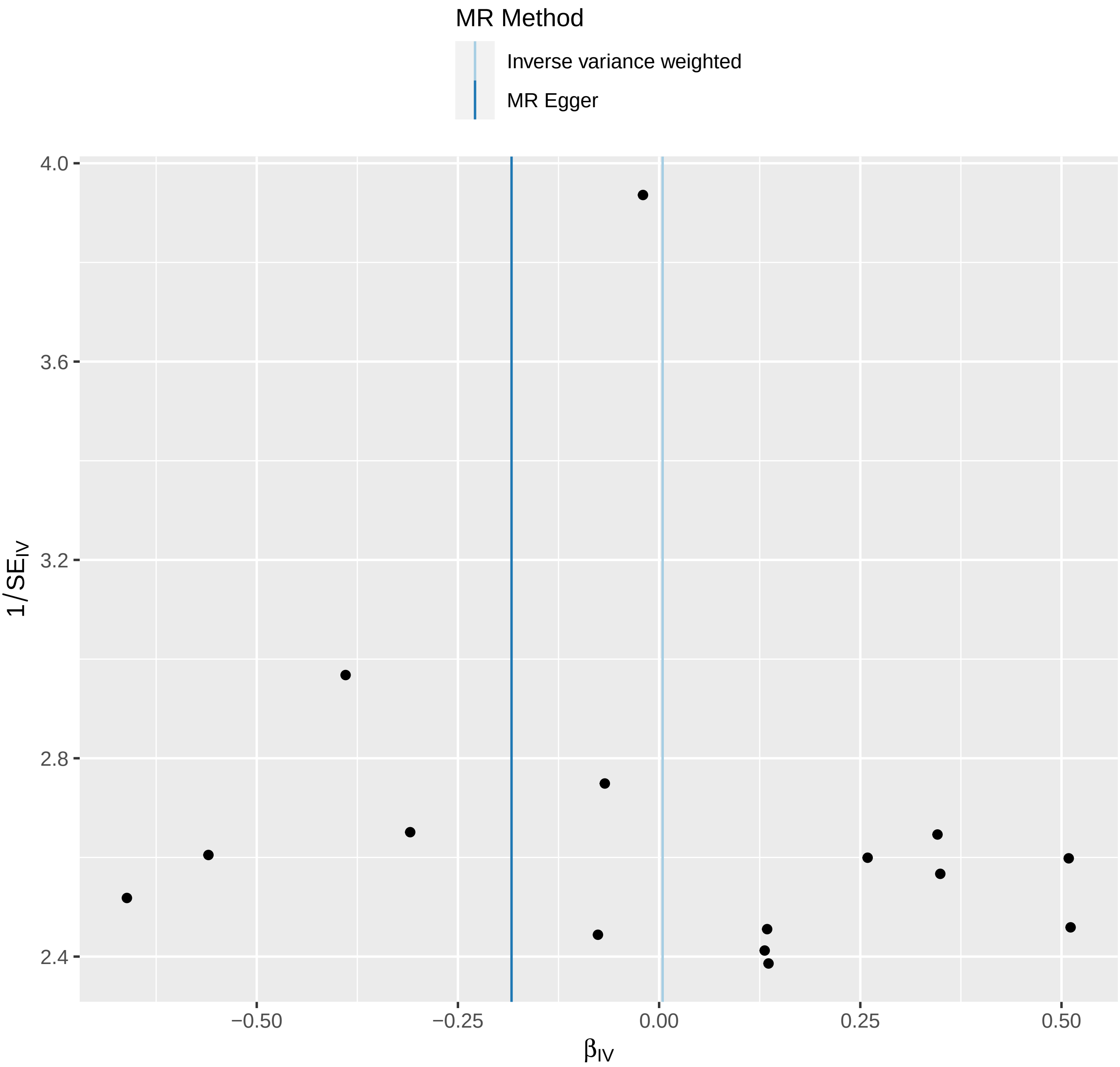

Batch 2−477 : Gut microbiota abundance (genus Catenibacterium id.2153) on Bcl−2−like protein 2
with a threshold of 1e−05

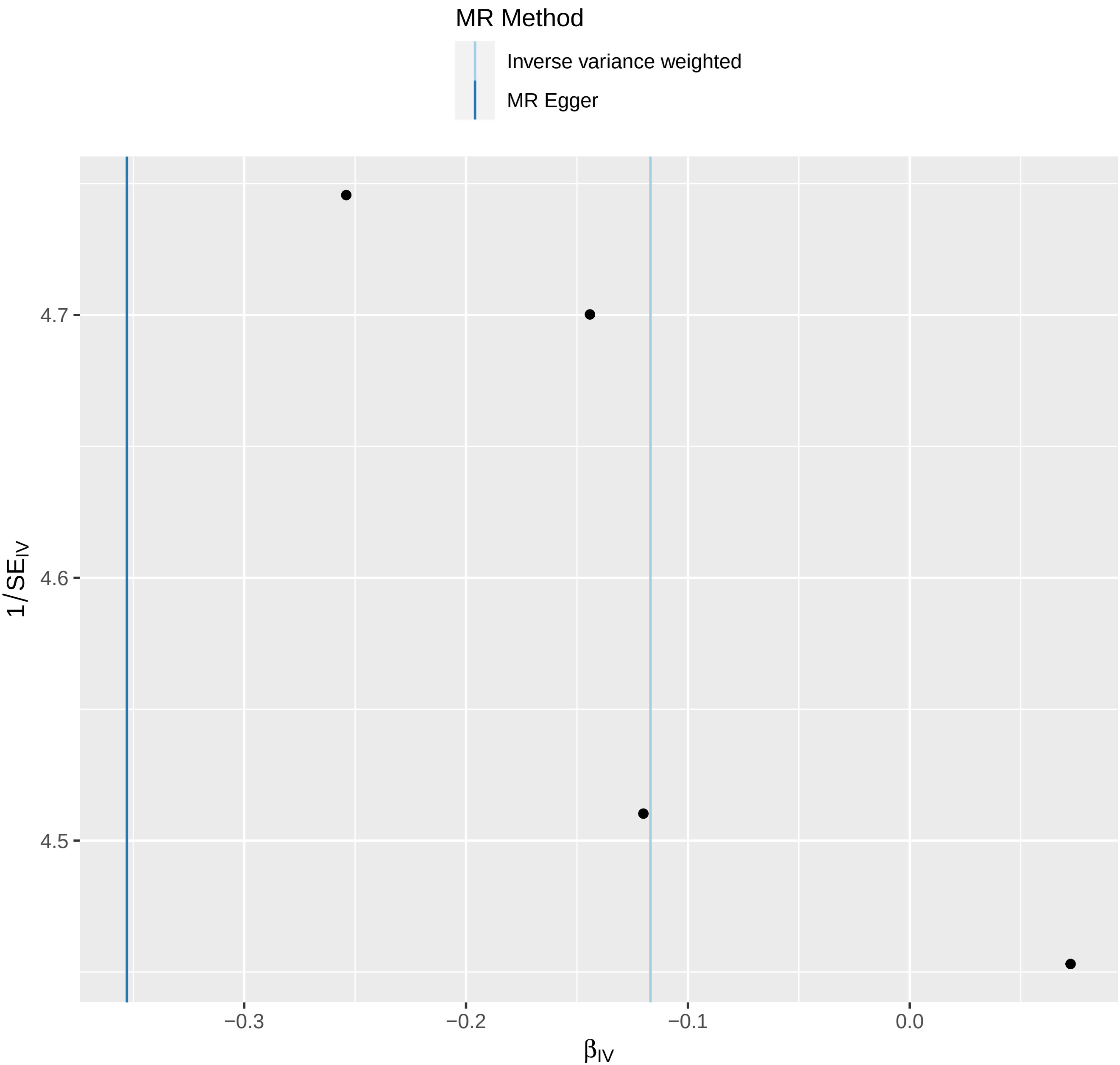

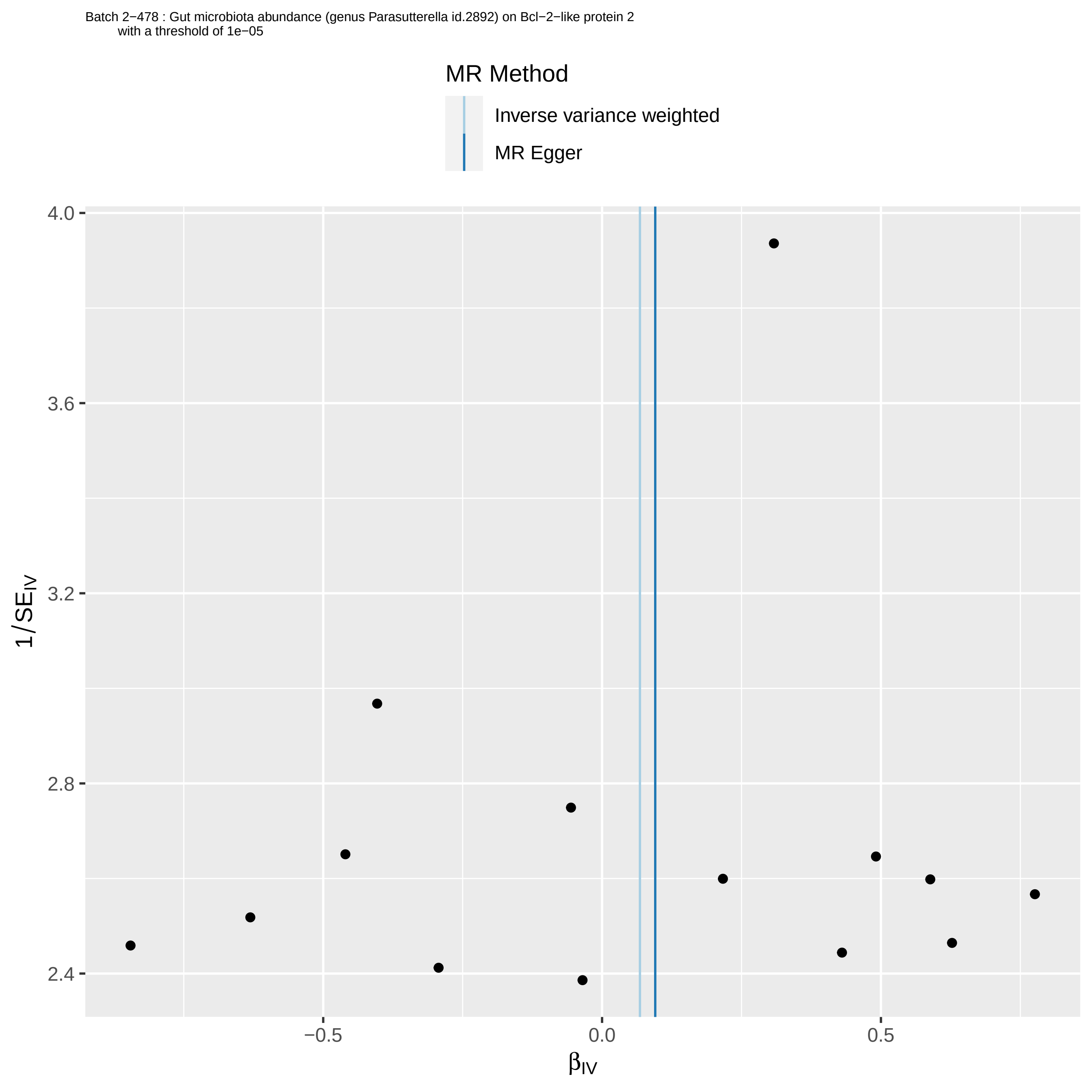

Batch 2−478 : Gut microbiota abundance (genus Parasutterella id.2892) on Bcl−2−like protein 2
with a threshold of 1e−05
MR Method
Inverse variance weighted
MR Egger
4.0
3.6
3.2
2.8
2.4
1/SE_IV
−0.5
0.0
0.5
β_IV

Batch 2−479 : Gut microbiota abundance (genus Catenibacterium id.2153) on Bcl−2−like protein 2
with a threshold of 1e−05

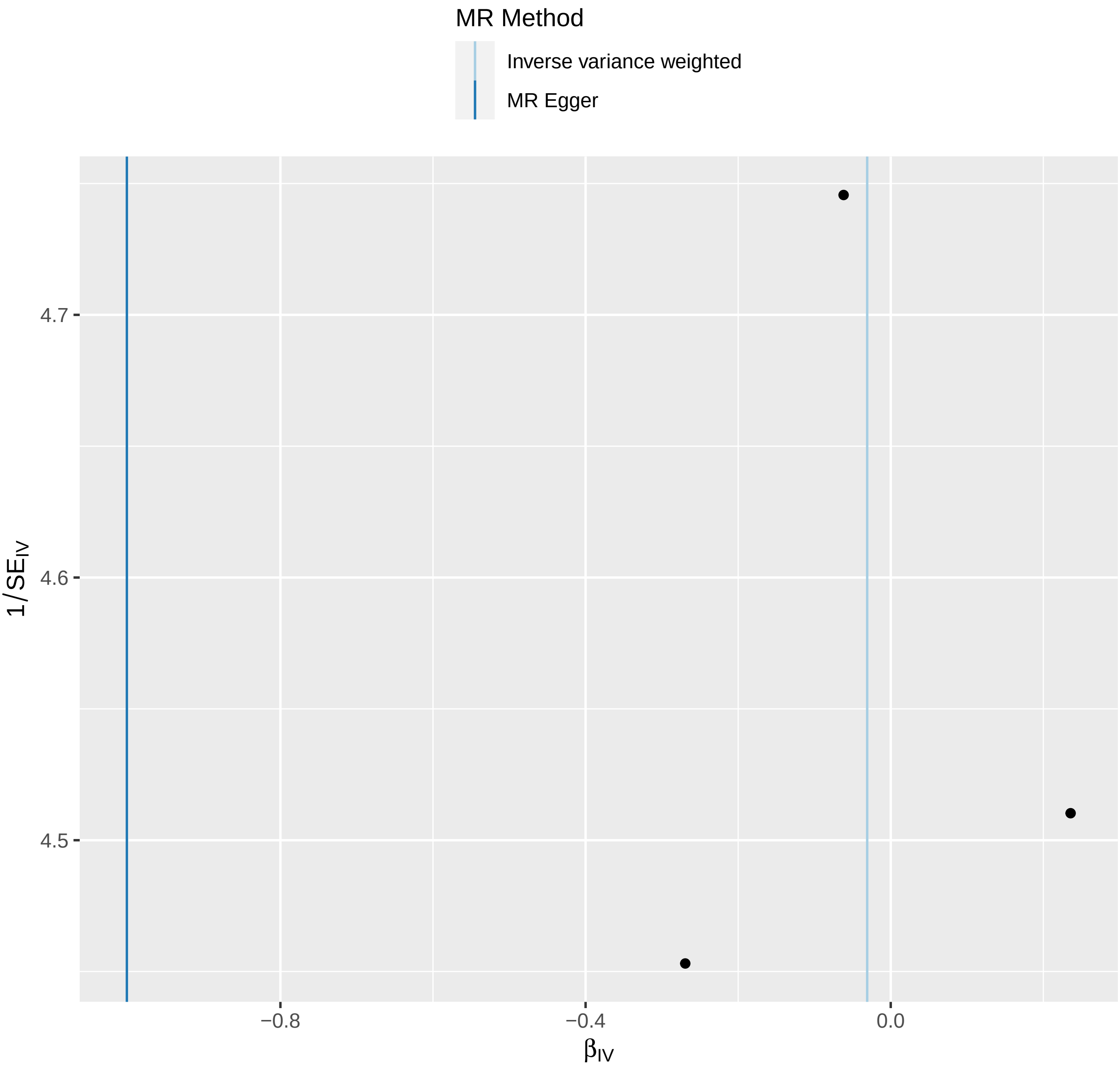

Batch 2−480 : Gut microbiota abundance (genus Parasutterella id.2892) on Bcl−2−like protein 2
with a threshold of 1e−05

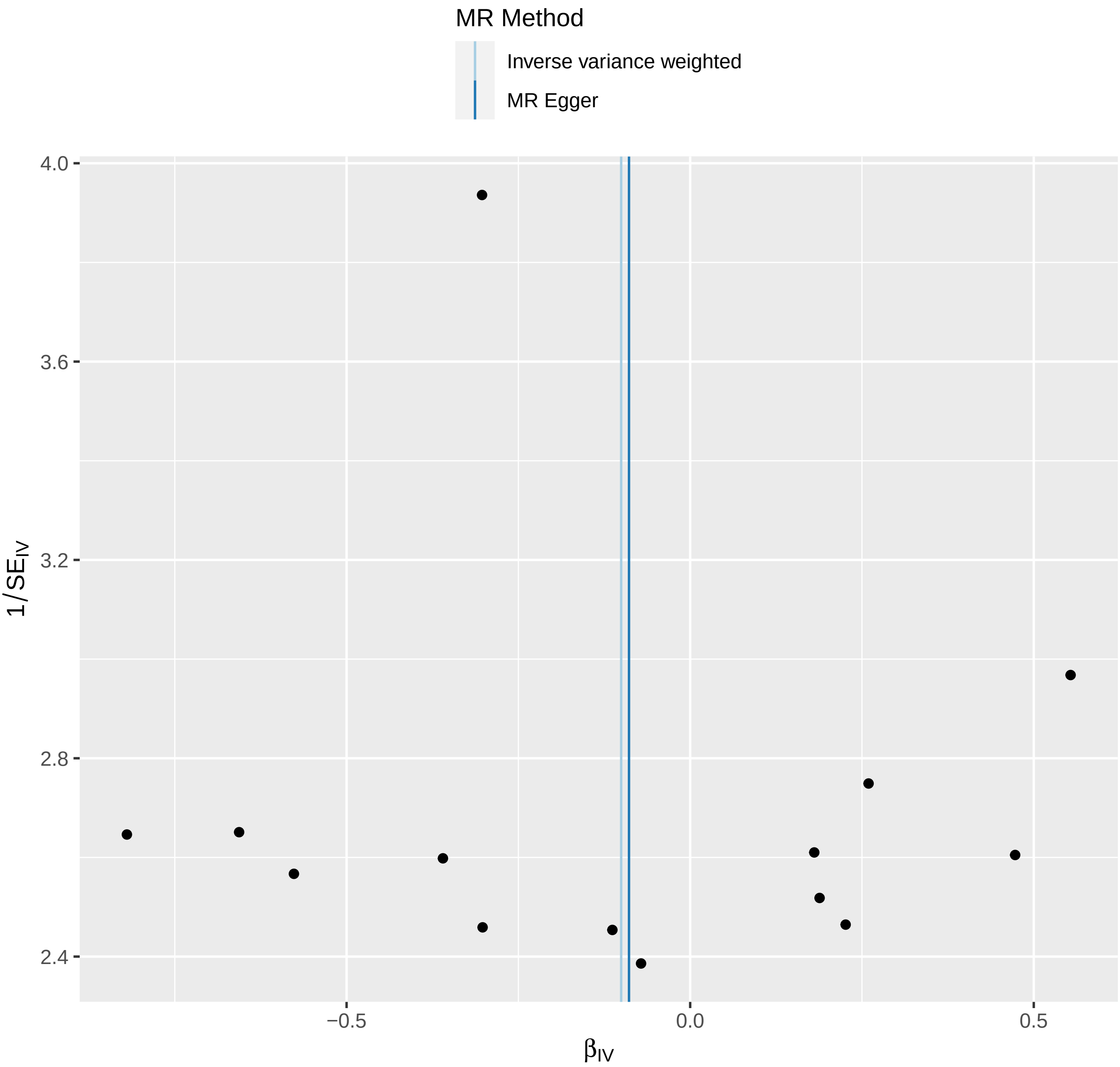

Batch 2−481 : Gut microbiota abundance (genus Catenibacterium id.2153) on B−cell lymphoma 6 protein
with a threshold of 1e−05

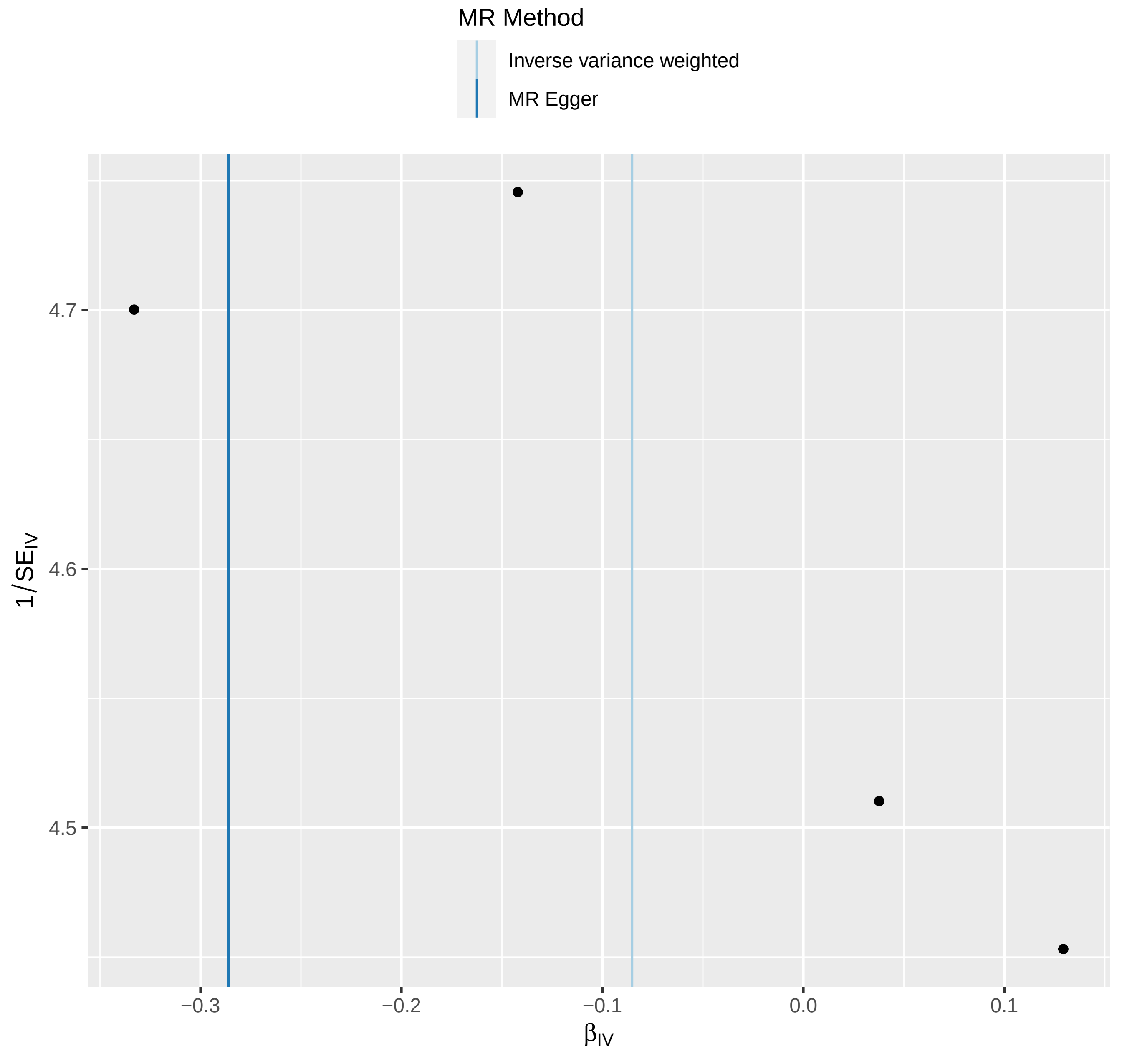

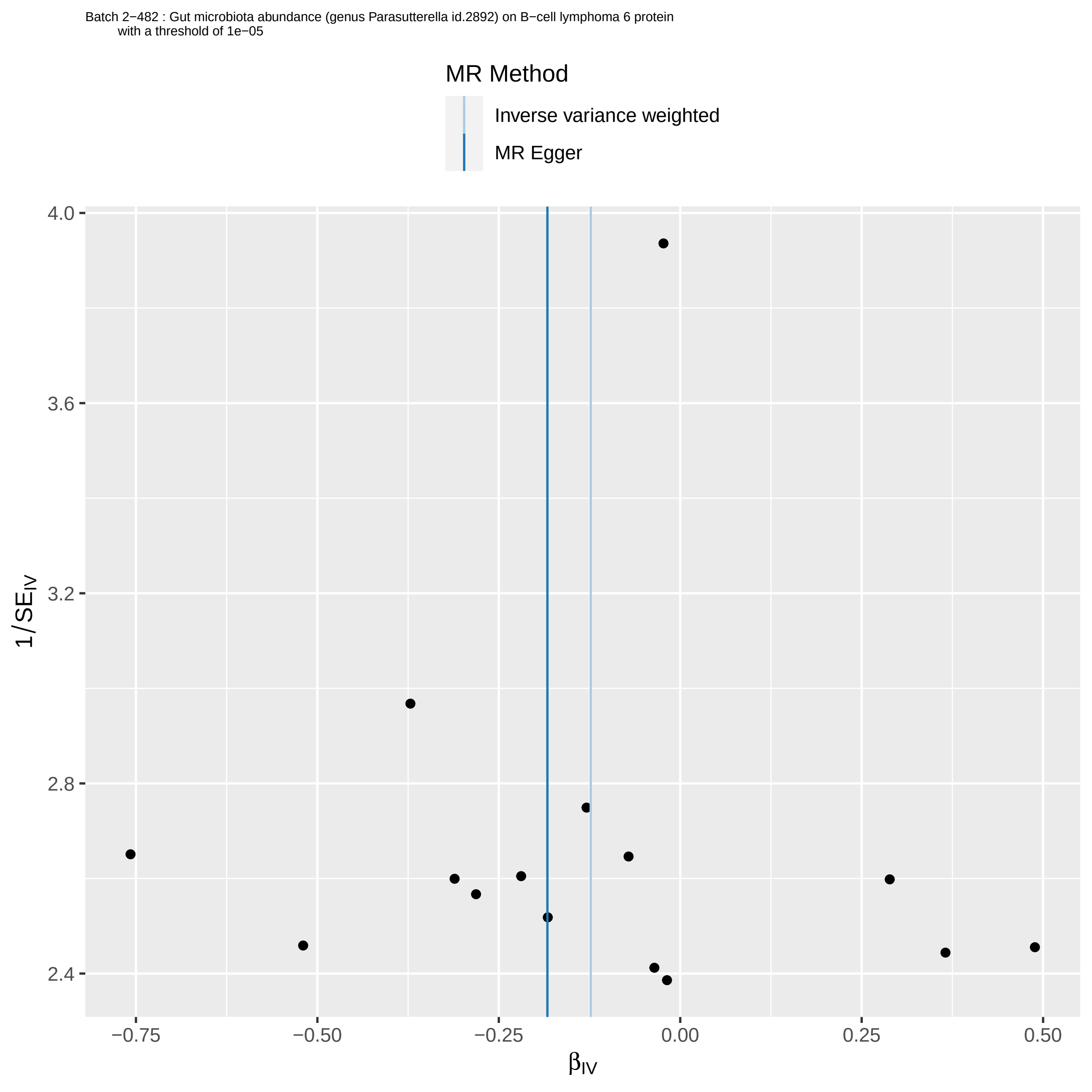
Batch 2−482 : Gut microbiota abundance (genus Parasutterella id.2892) on B−cell lymphoma 6 protein
with a threshold of 1e−05
MR Method
Inverse variance weighted
MR Egger
4.0
3.6
3.2
2.8
2.4
1/SE_IV
−0.75
−0.50
−0.25
0.00
0.25
0.50
β_IV

Batch 2−483 : Gut microbiota abundance (genus Catenibacterium id.2153) on Brain−derived neurotrophic factor
with a threshold of 1e−05

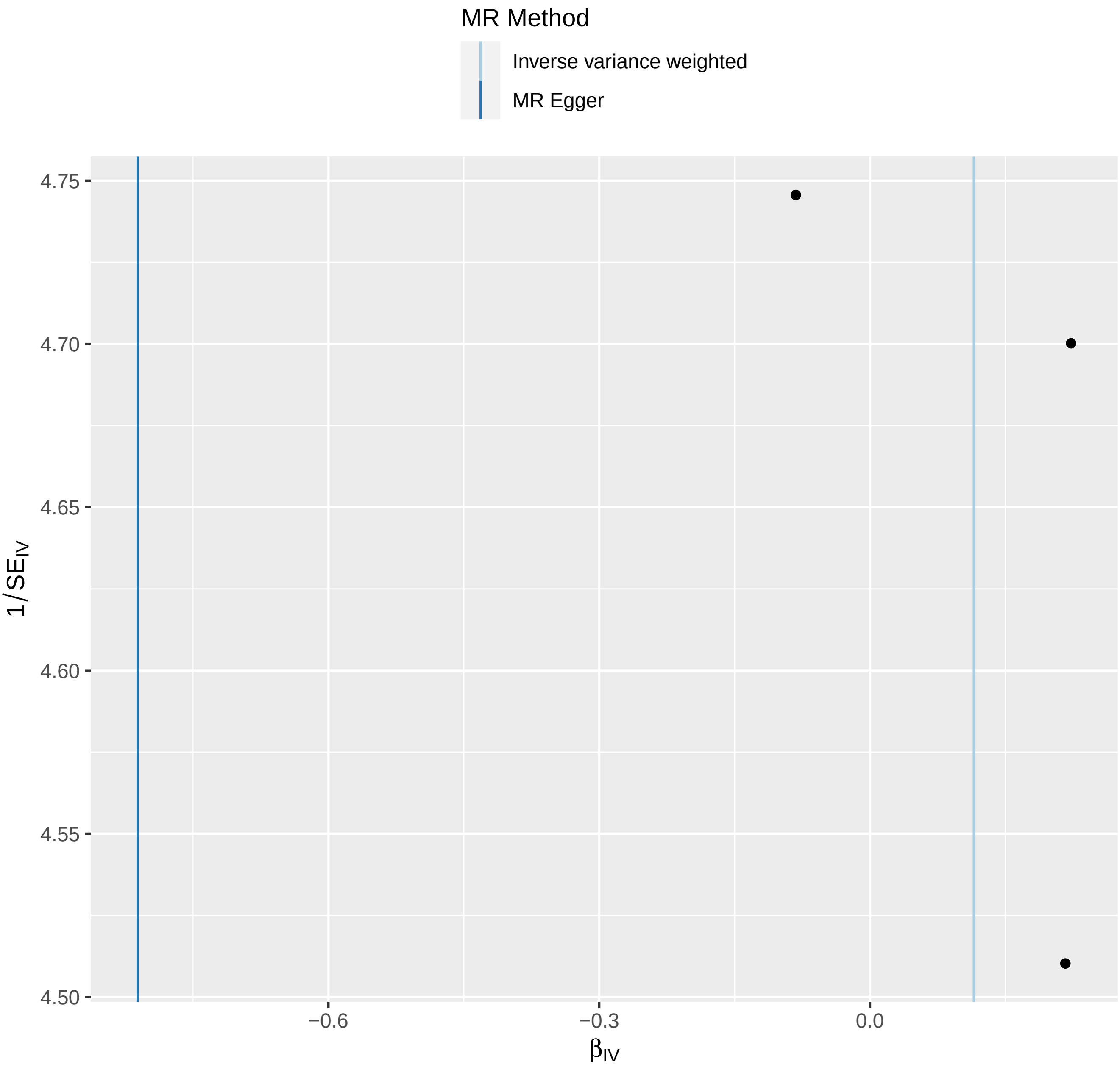

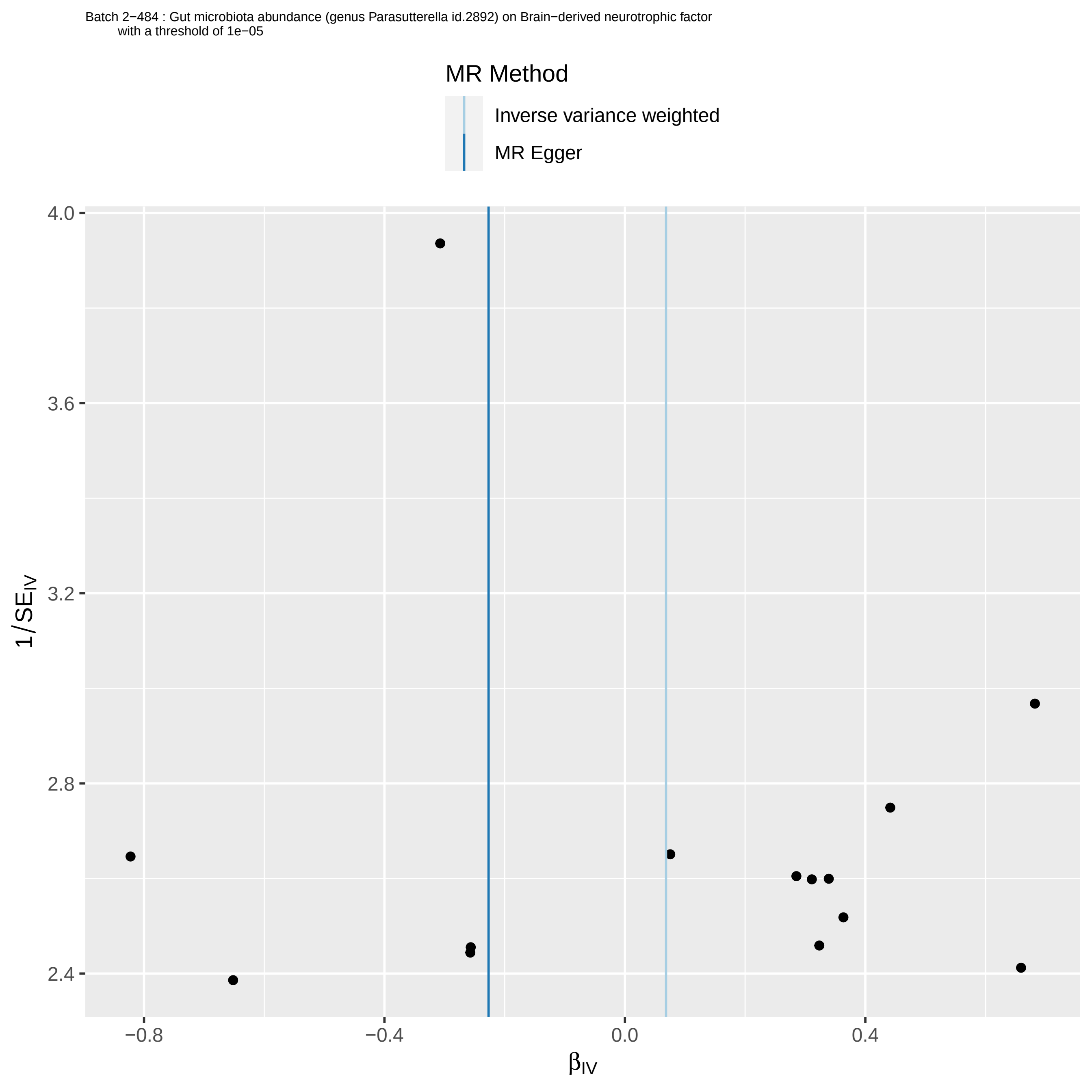

Batch 2−484 : Gut microbiota abundance (genus Parasutterella id.2892) on Brain−derived neurotrophic factor
with a threshold of 1e−05
MR Method
Inverse variance weighted
MR Egger
4.0
3.6
3.2
2.8
2.4
1/SE_IV
−0.8
−0.4
0.0
0.4
β_IV

Batch 2−485 : Gut microbiota abundance (genus Catenibacterium id.2153) on Transcription factor TFIIIB component B" homolog with a threshold of 1e−05

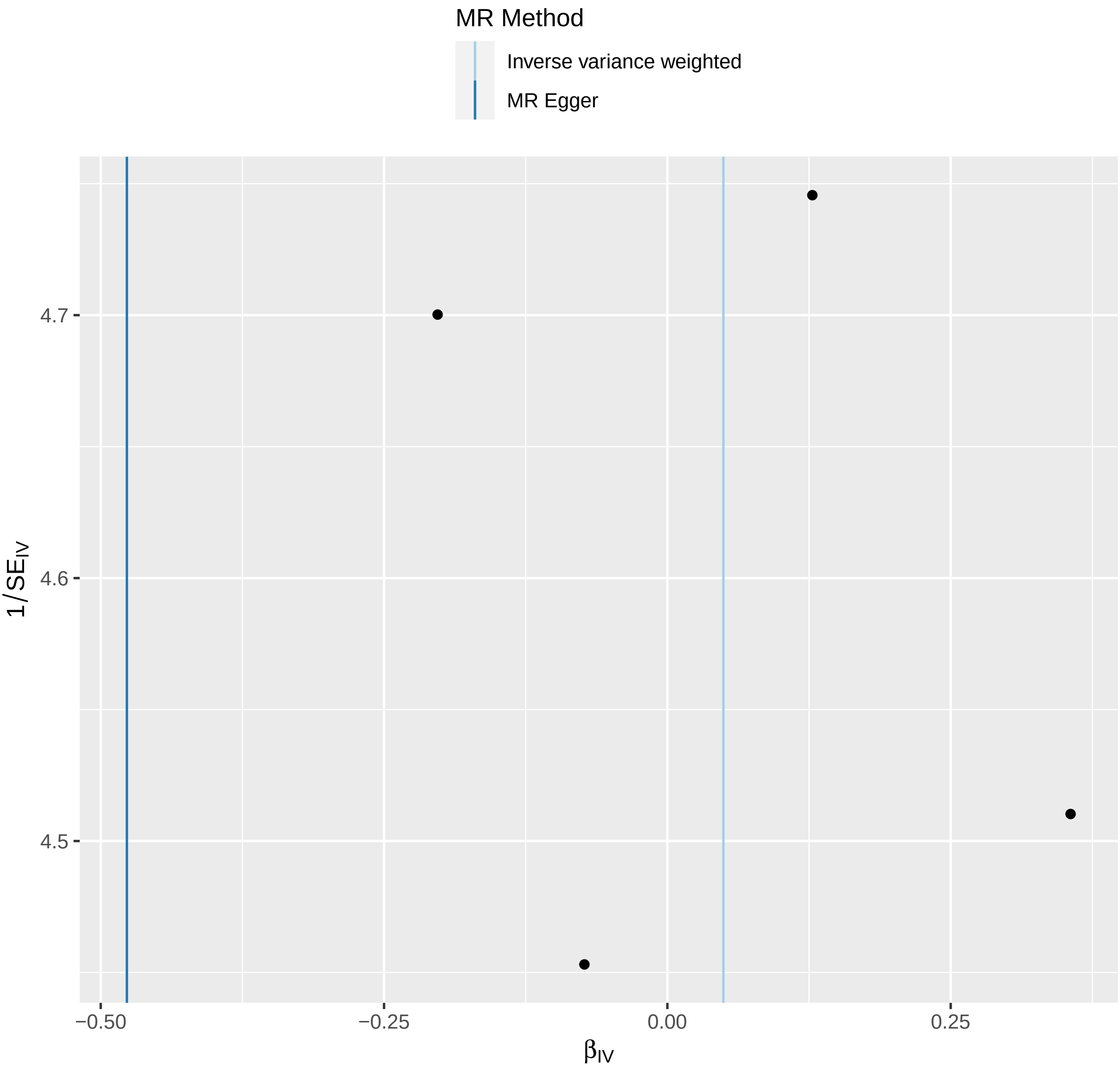

Batch 2−486 : Gut microbiota abundance (genus Parasutterella id.2892) on Transcription factor TFIIIB component B" homolog with a threshold of 1e−05

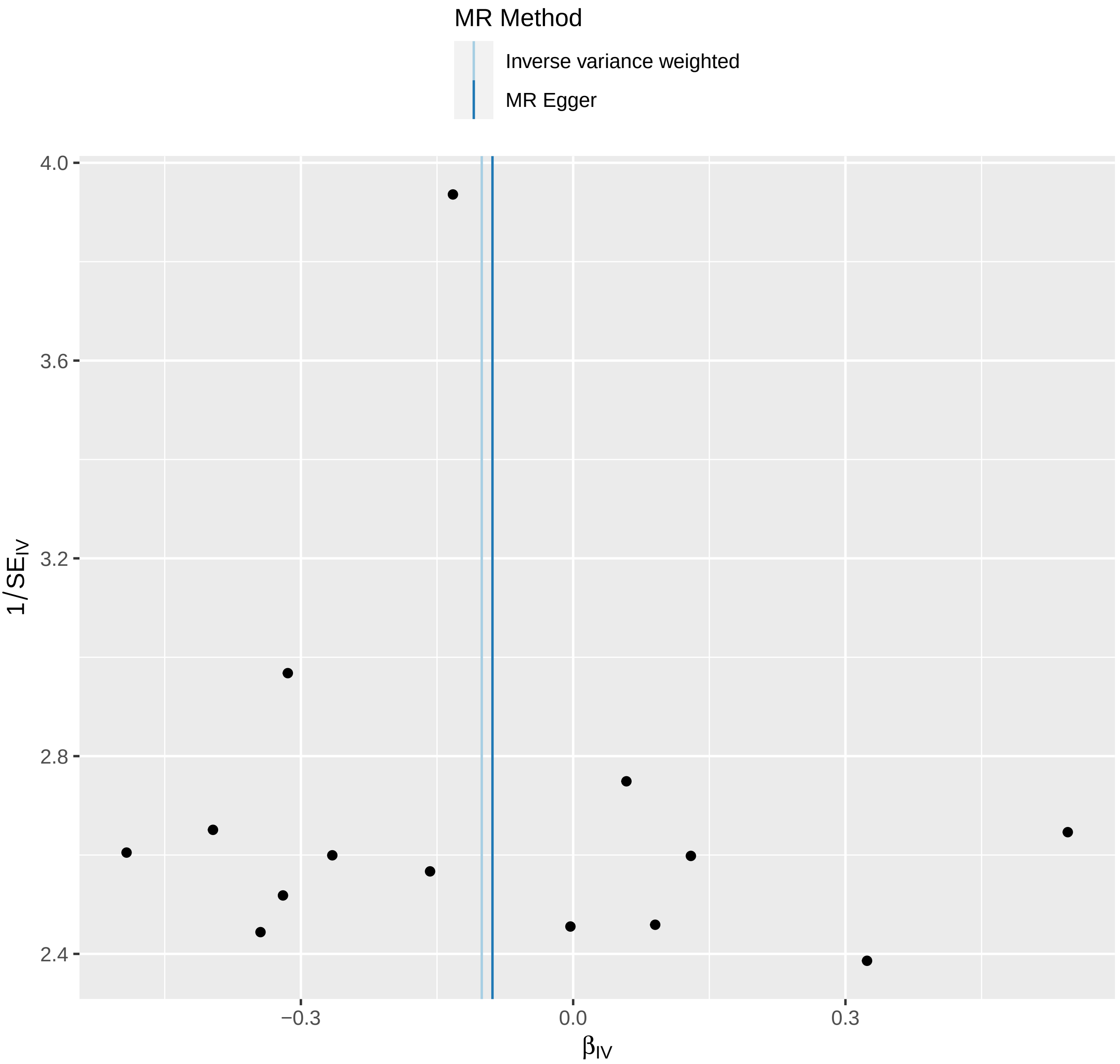

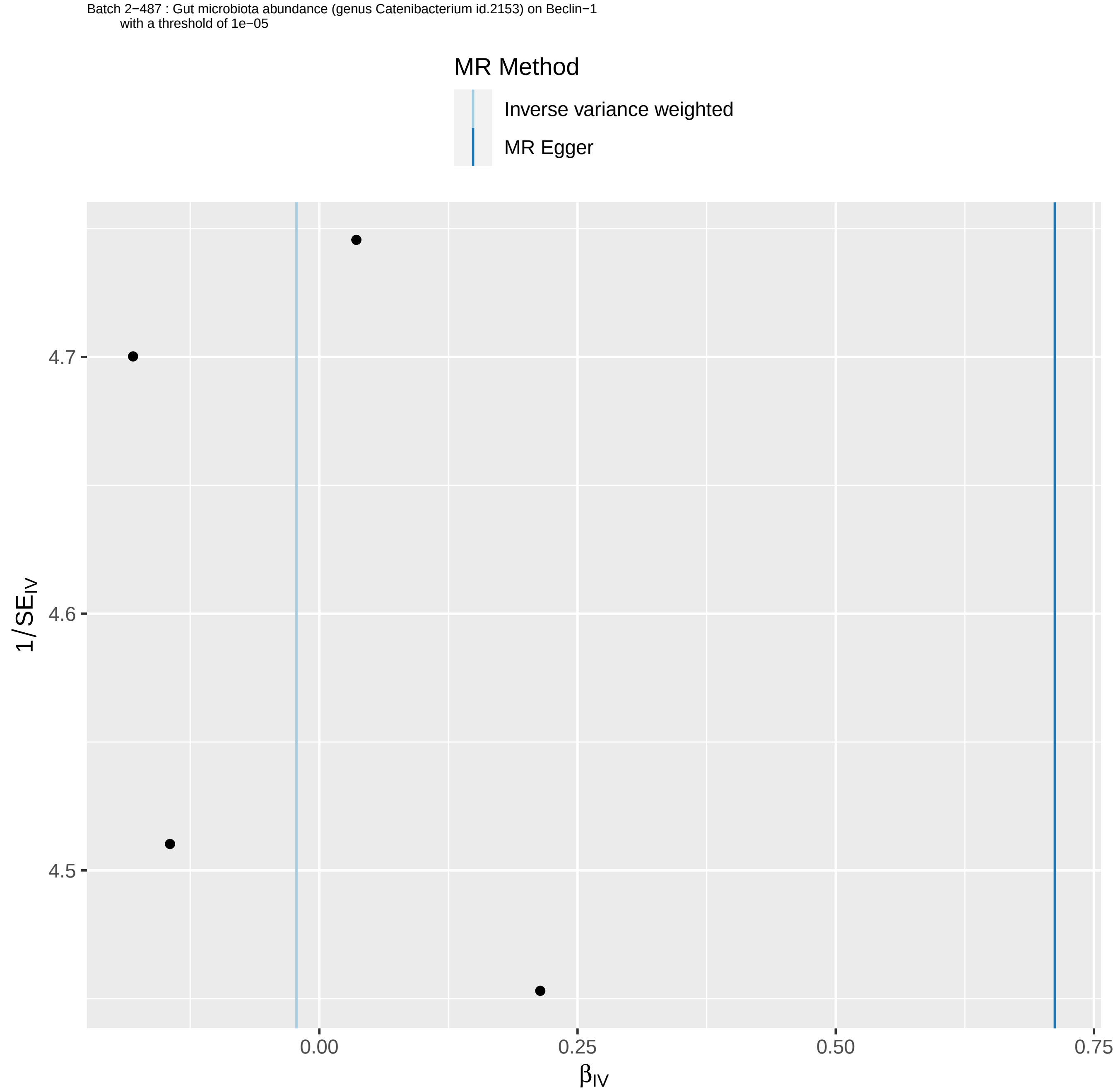
Batch 2−487 : Gut microbiota abundance (genus Catenibacterium id.2153) on Beclin−1
with a threshold of 1e−05
MR Method
Inverse variance weighted
MR Egger
4.7
4.6
4.5
1/SE$_{IV}$
0.00
0.25
0.50
0.75
β$_{IV}$

Batch 2−488 : Gut microbiota abundance (genus Parasutterella id.2892) on Beclin−1
with a threshold of 1e−05

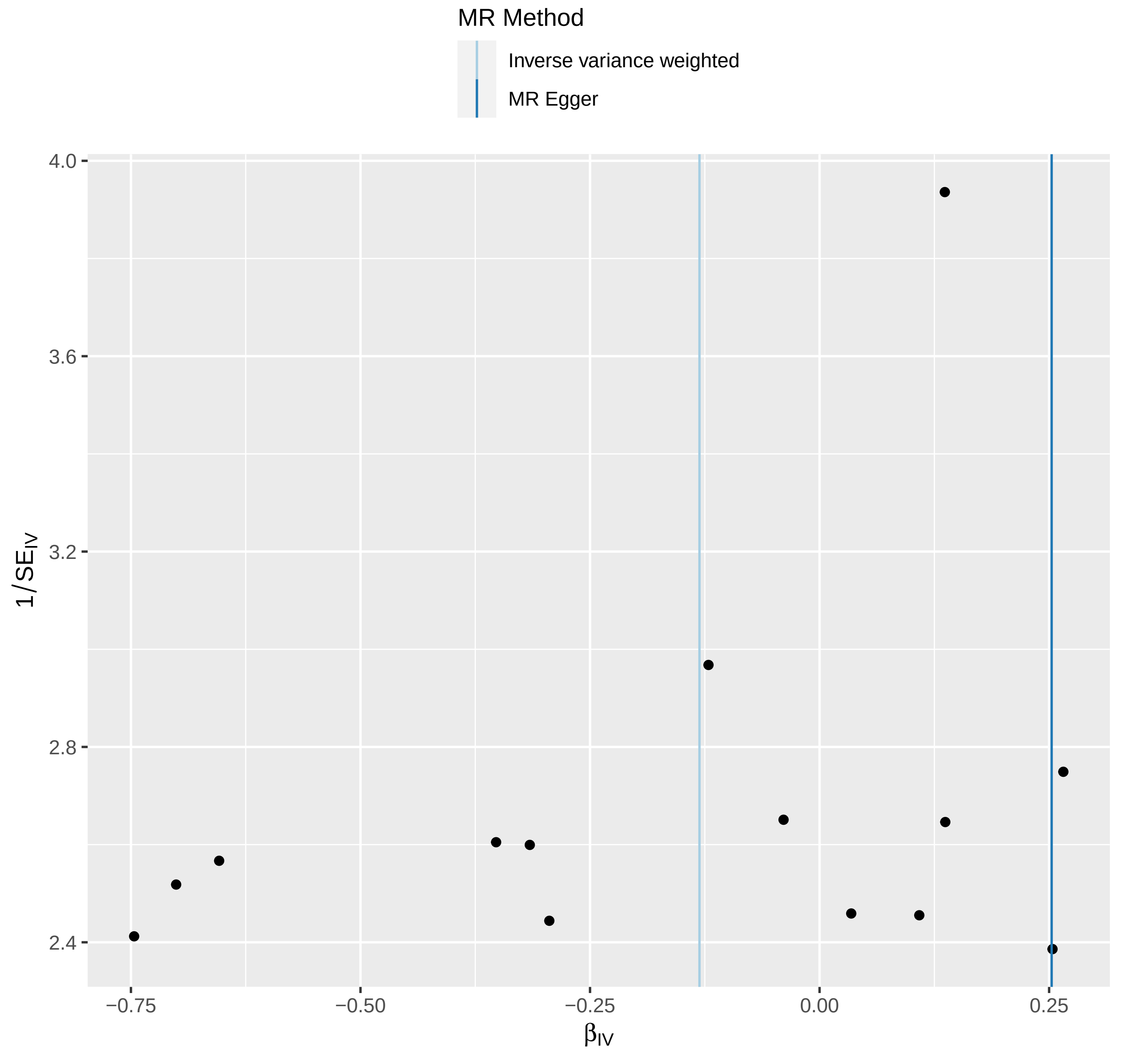

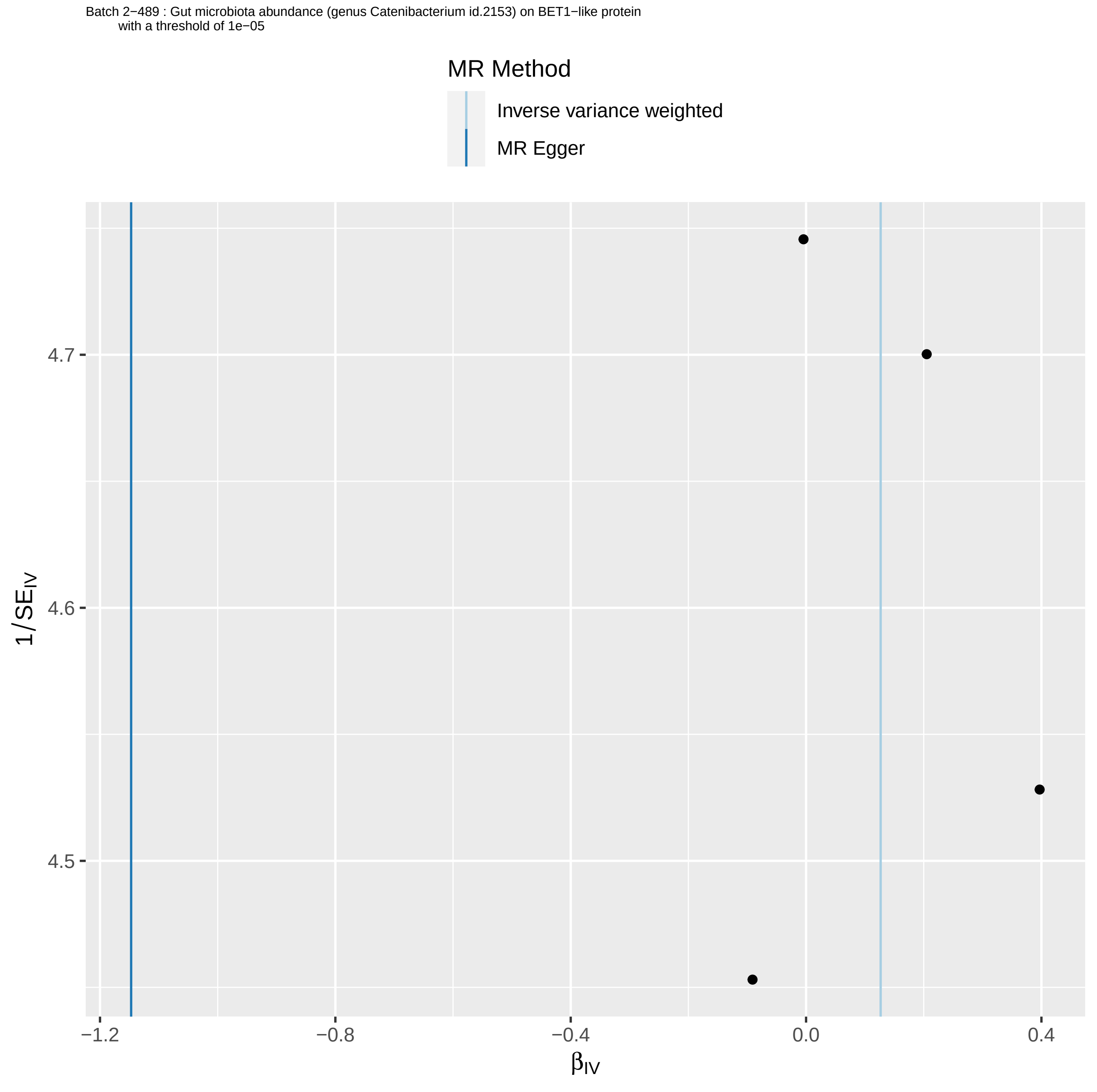
Batch 2−489 : Gut microbiota abundance (genus Catenibacterium id.2153) on BET1−like protein
with a threshold of 1e−05
MR Method
Inverse variance weighted
MR Egger
4.7
4.6
4.5
1/SE_IV
−1.2
−0.8
−0.4
0.0
0.4
β_IV

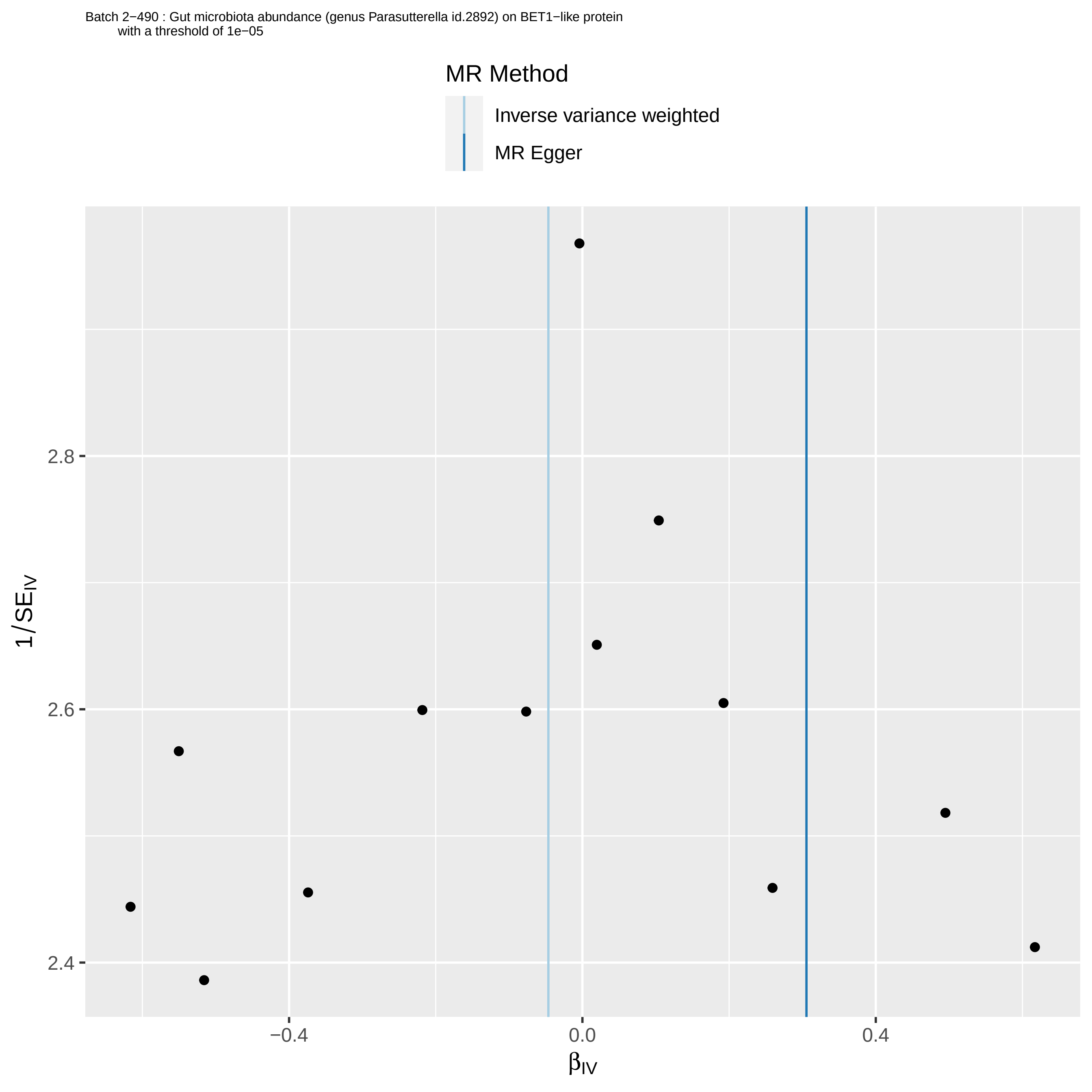
Batch 2−490 : Gut microbiota abundance (genus Parasutterella id.2892) on BET1−like protein
with a threshold of 1e−05
MR Method
Inverse variance weighted
MR Egger
2.8
2.6
2.4
−0.4
0.0
0.4
$1/SE_{IV}$
β_{IV}

Batch 2−491 : Gut microbiota abundance (genus Catenibacterium id.2153) on Osteocalcin
with a threshold of 1e−05

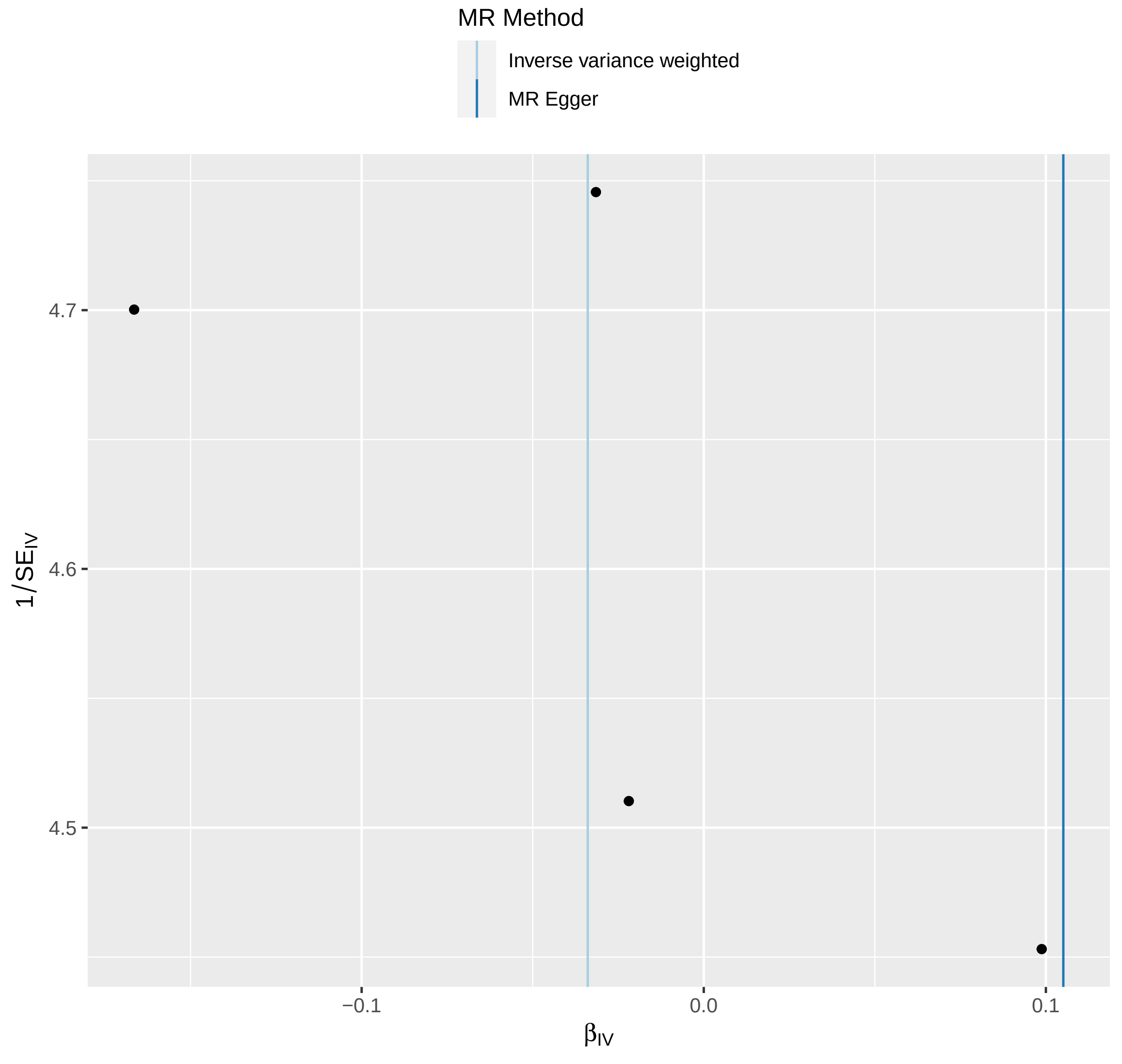

Batch 2−492 : Gut microbiota abundance (genus Parasutterella id.2892) on Osteocalcin
with a threshold of 1e−05

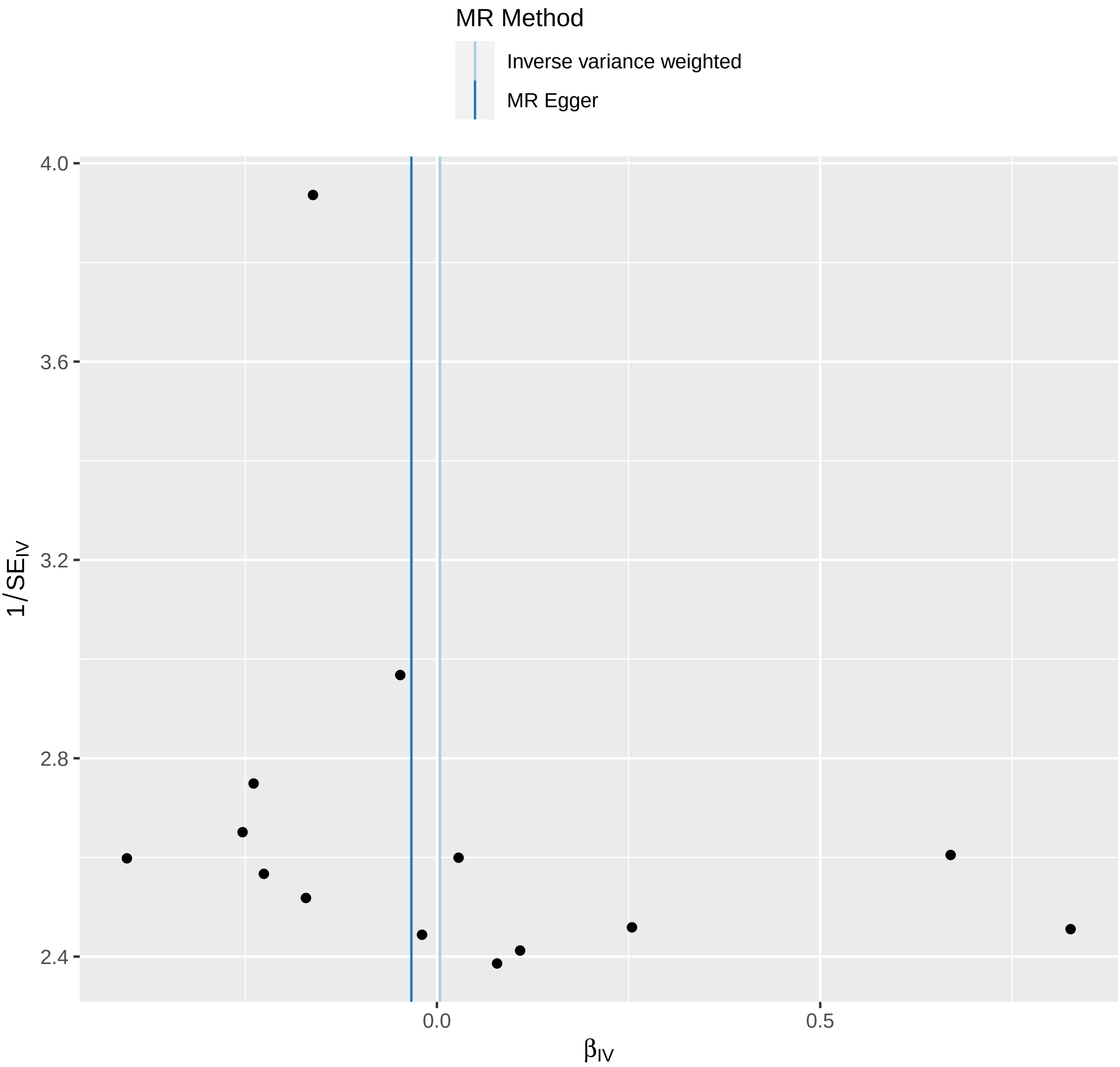

Batch 2−493 : Gut microbiota abundance (genus Catenibacterium id.2153) on Biglycan
with a threshold of 1e−05

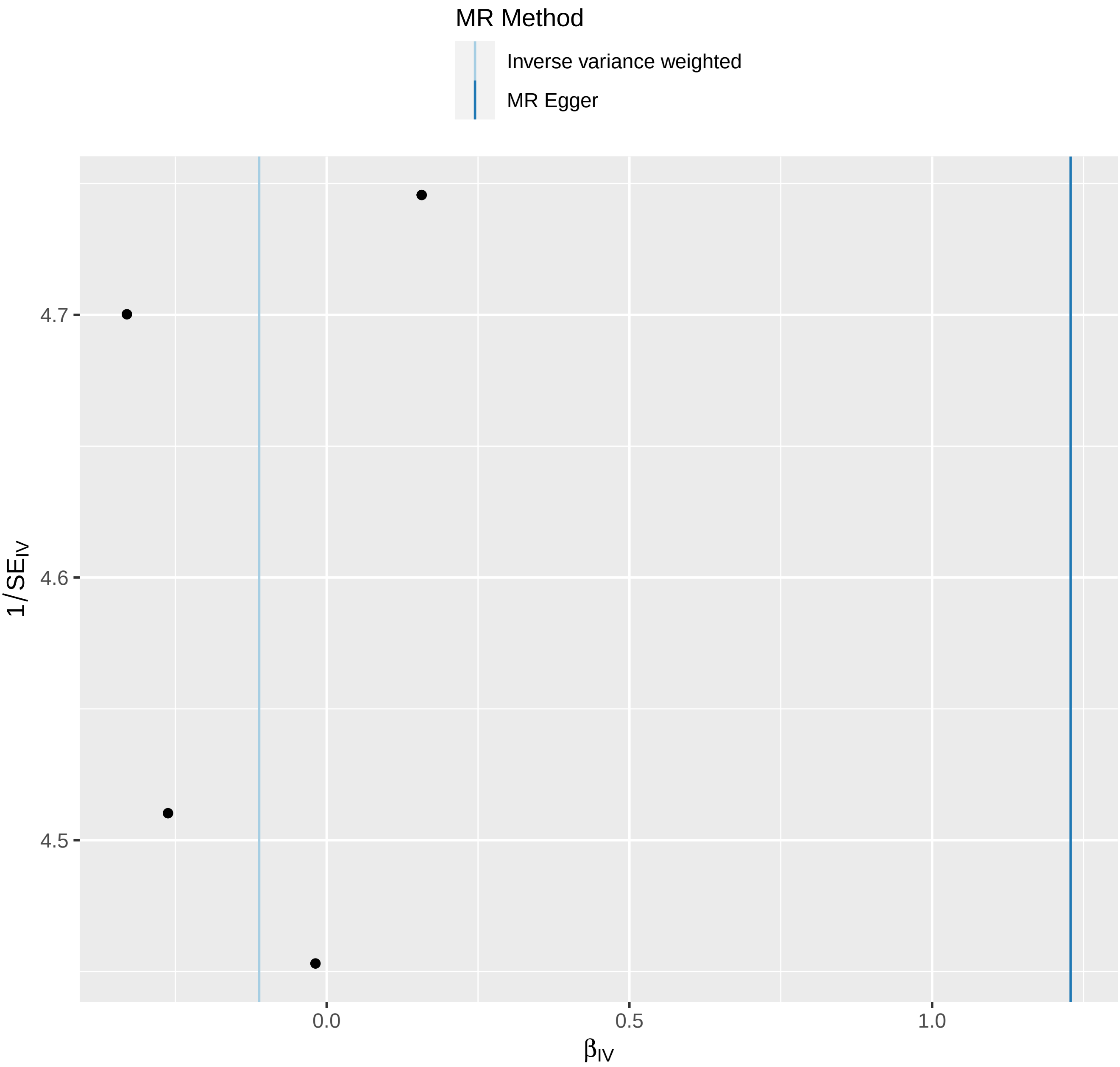

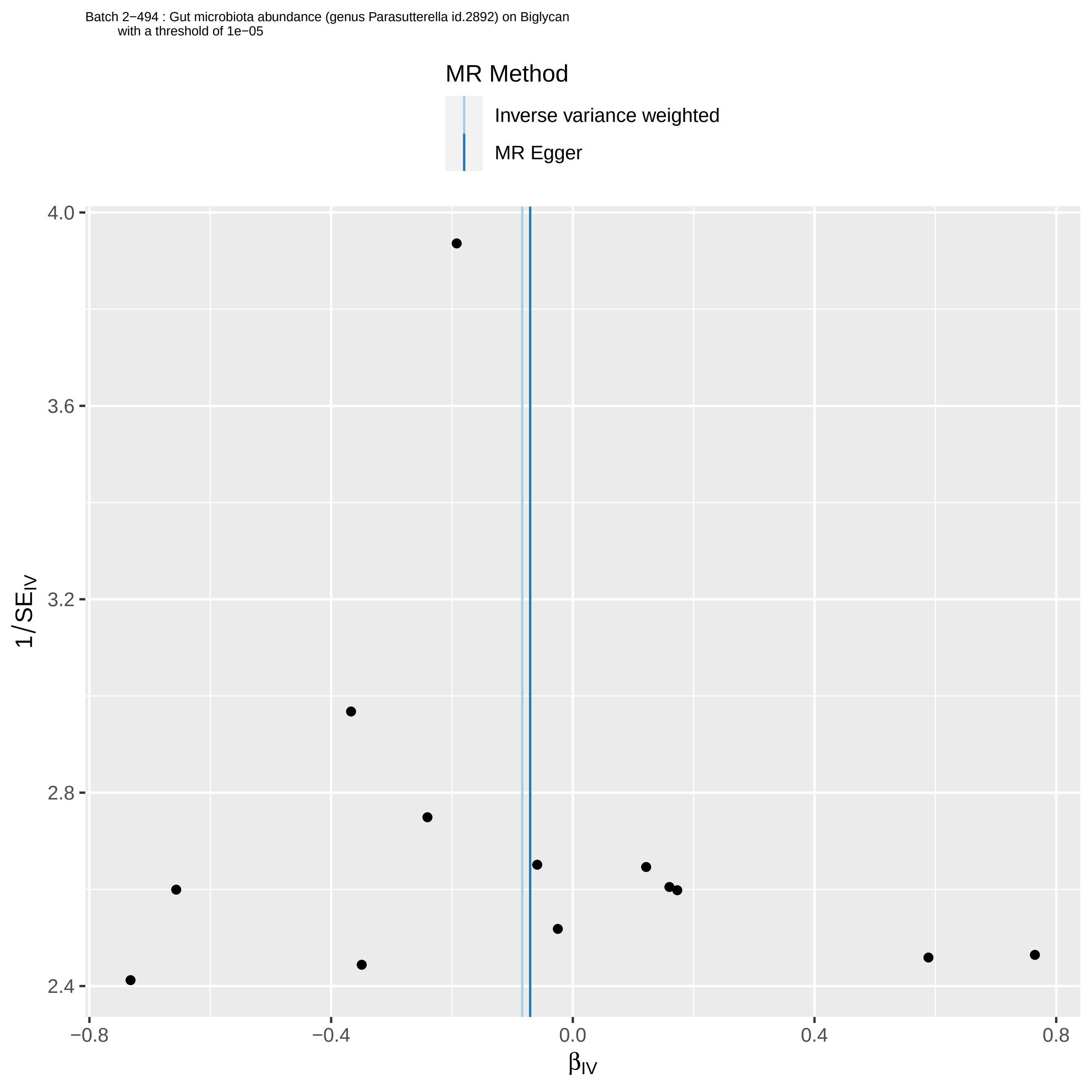
Batch 2−494 : Gut microbiota abundance (genus Parasutterella id.2892) on Biglycan
with a threshold of 1e−05
MR Method
Inverse variance weighted
MR Egger
4.0
3.6
3.2
2.8
2.4
1/SE_IV
−0.8
−0.4
0.0
0.4
0.8
β_IV

Batch 2−495 : Gut microbiota abundance (genus Catenibacterium id.2153) on Biglycan
with a threshold of 1e−05

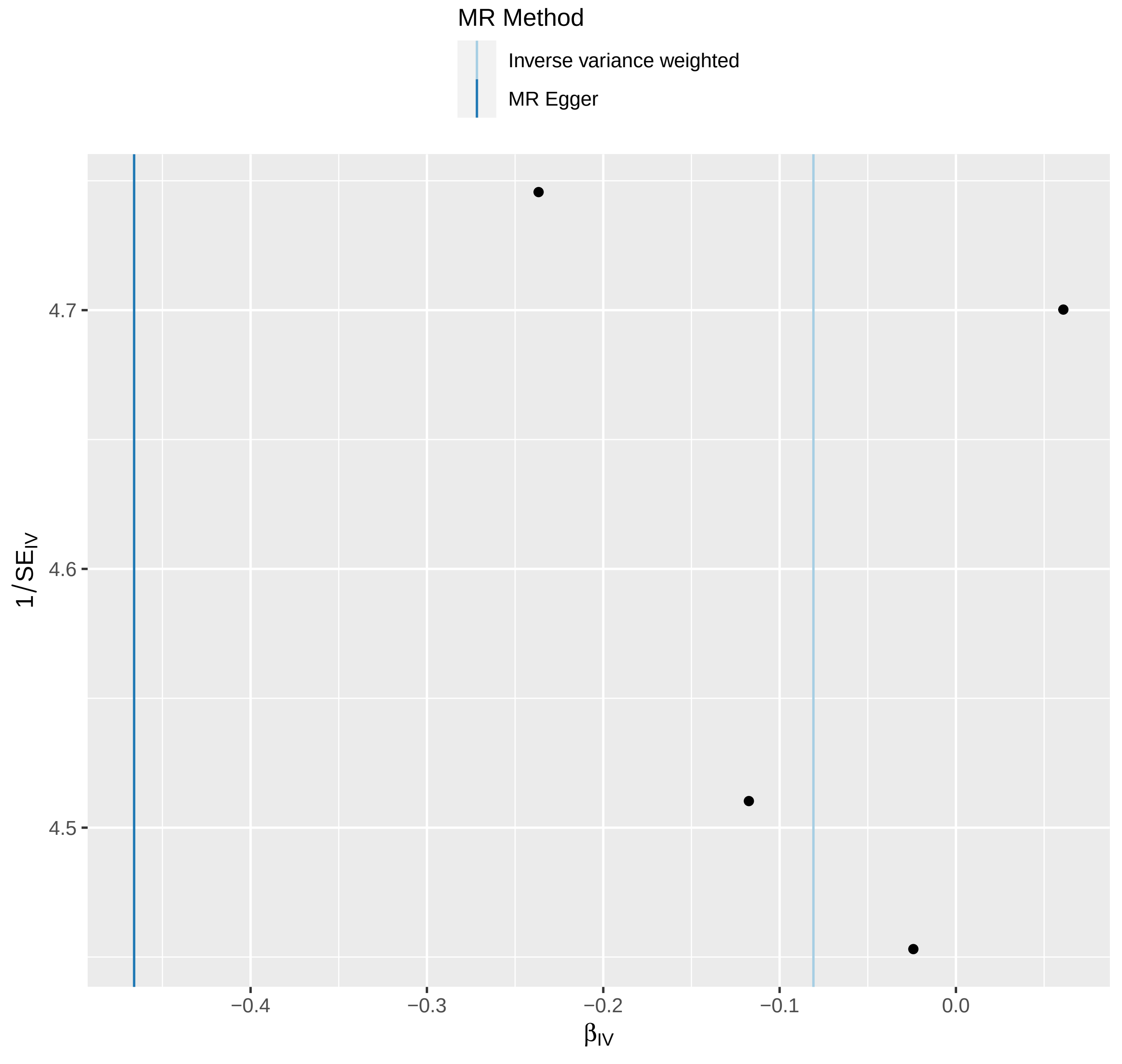

Batch 2−496 : Gut microbiota abundance (genus Parasutterella id.2892) on Biglycan
with a threshold of 1e−05

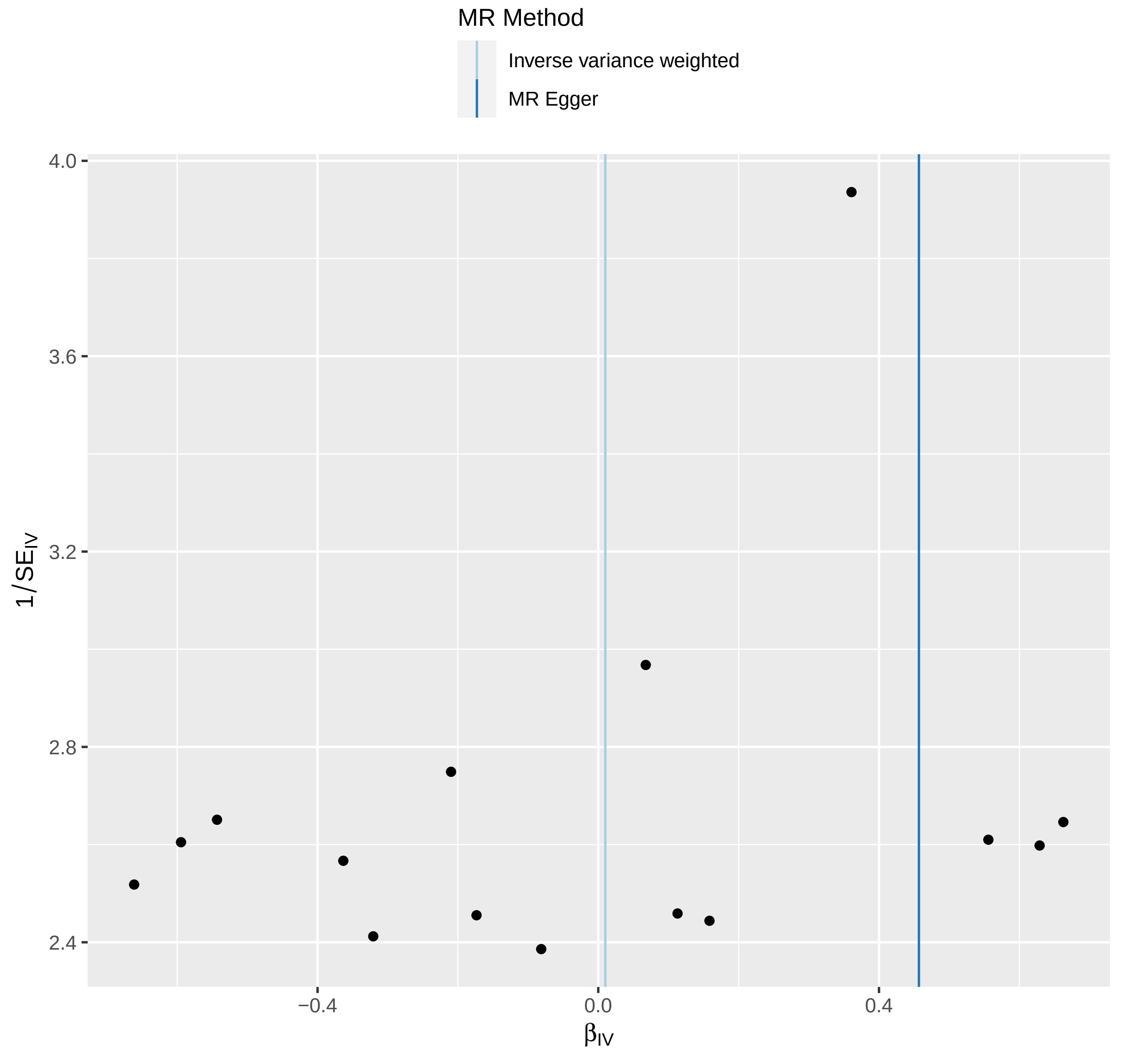

Batch 2−497 : Gut microbiota abundance (genus Catenibacterium id.2153) on BH3−interacting domain death agonist
with a threshold of 1e−05

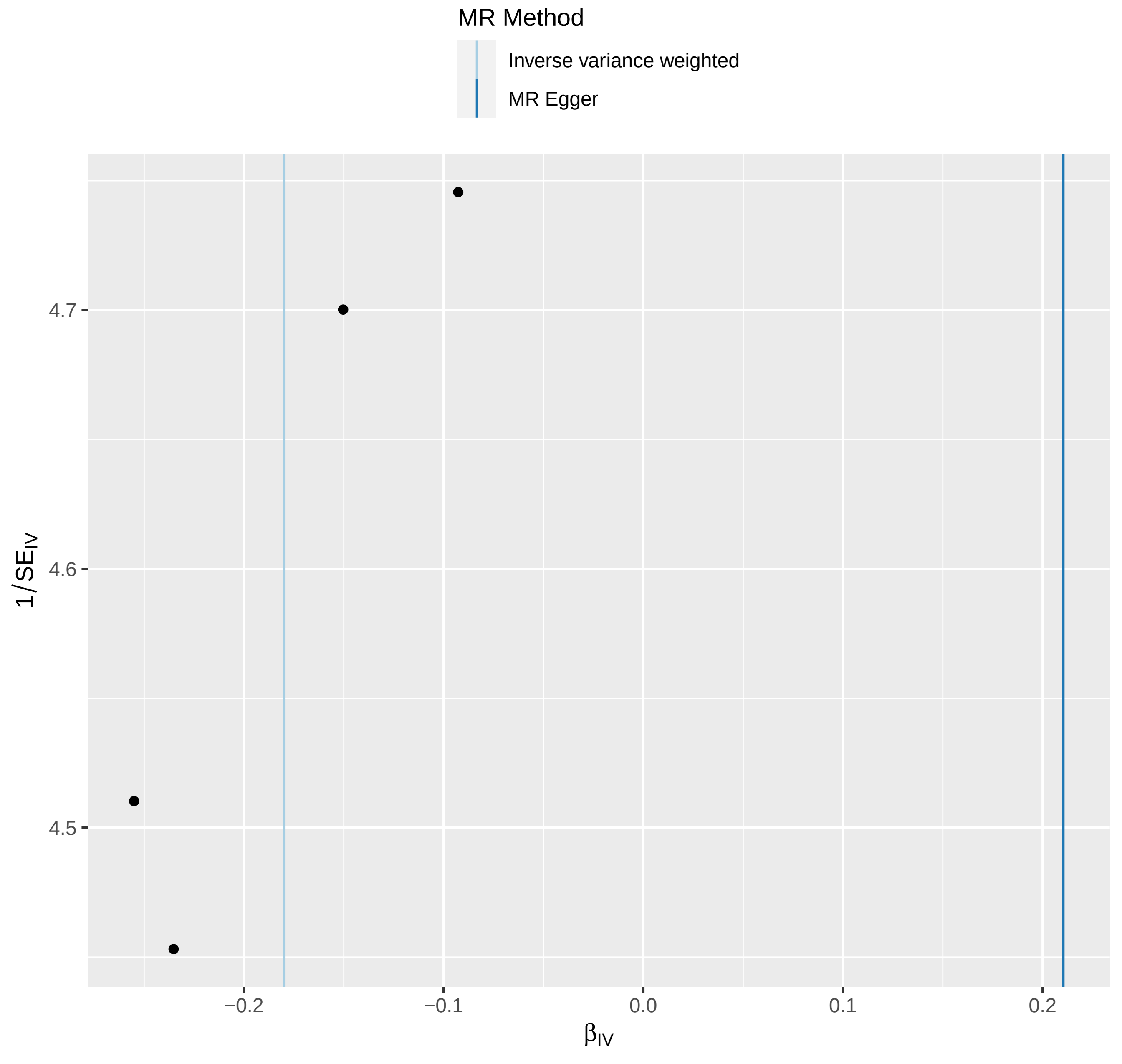

Batch 2−498 : Gut microbiota abundance (genus Parasutterella id.2892) on BH3−interacting domain death agonist with a threshold of 1e−05

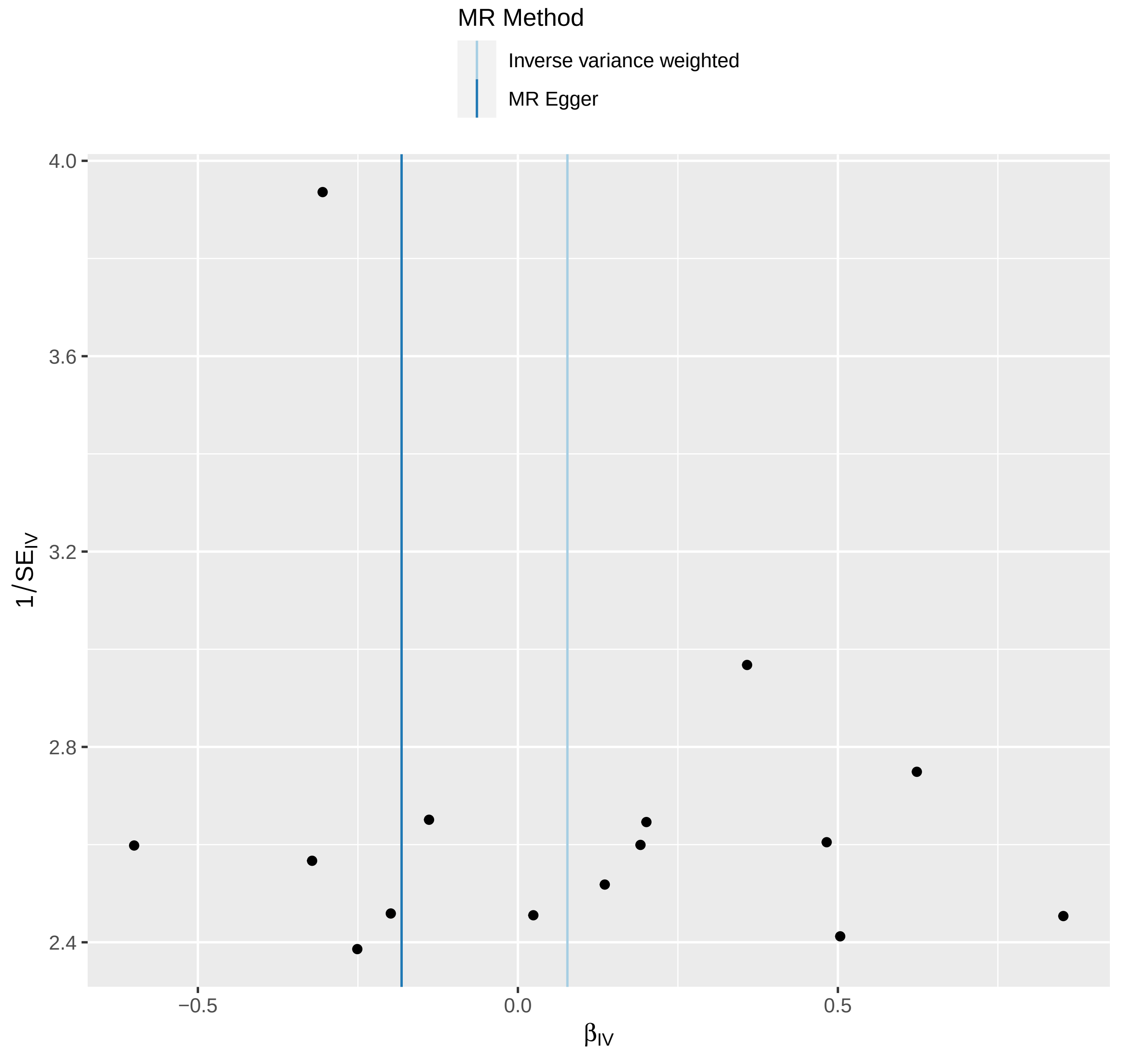

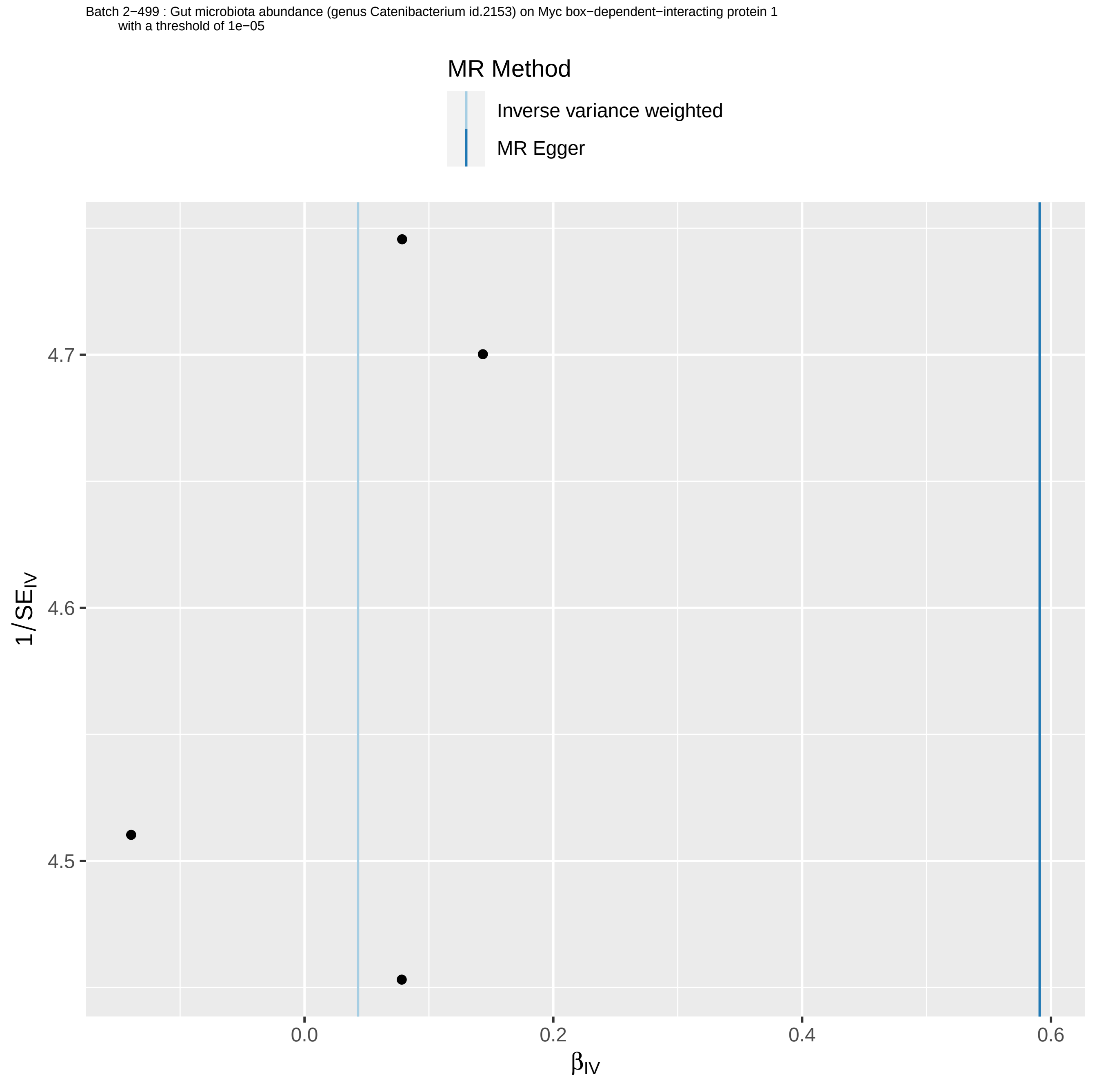
Batch 2−499 : Gut microbiota abundance (genus Catenibacterium id.2153) on Myc box−dependent−interacting protein 1
with a threshold of 1e−05
MR Method
Inverse variance weighted
MR Egger
4.7
4.6
4.5
1/SE_IV
0.0
0.2
0.4
0.6
β_IV

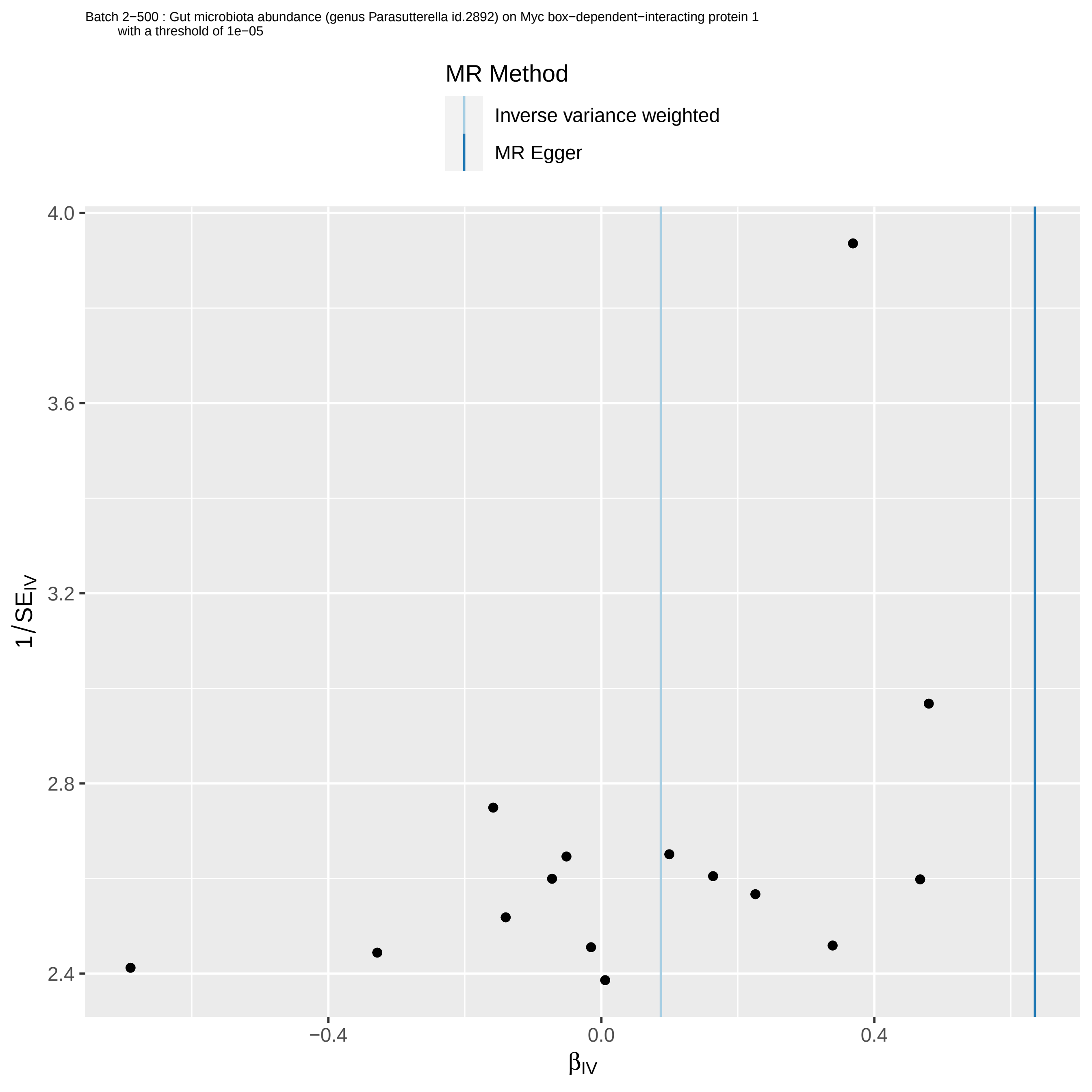
Batch 2−500 : Gut microbiota abundance (genus Parasutterella id.2892) on Myc box−dependent−interacting protein 1
with a threshold of 1e−05
MR Method
Inverse variance weighted
MR Egger
4.0
3.6
3.2
2.8
2.4
$1/SE_{IV}$
−0.4
0.0
0.4
β_{IV}

Batch 2−501 : Gut microbiota abundance (genus Catenibacterium id.2153) on Baculoviral IAP repeat−containing protein 3 with a threshold of 1e−05

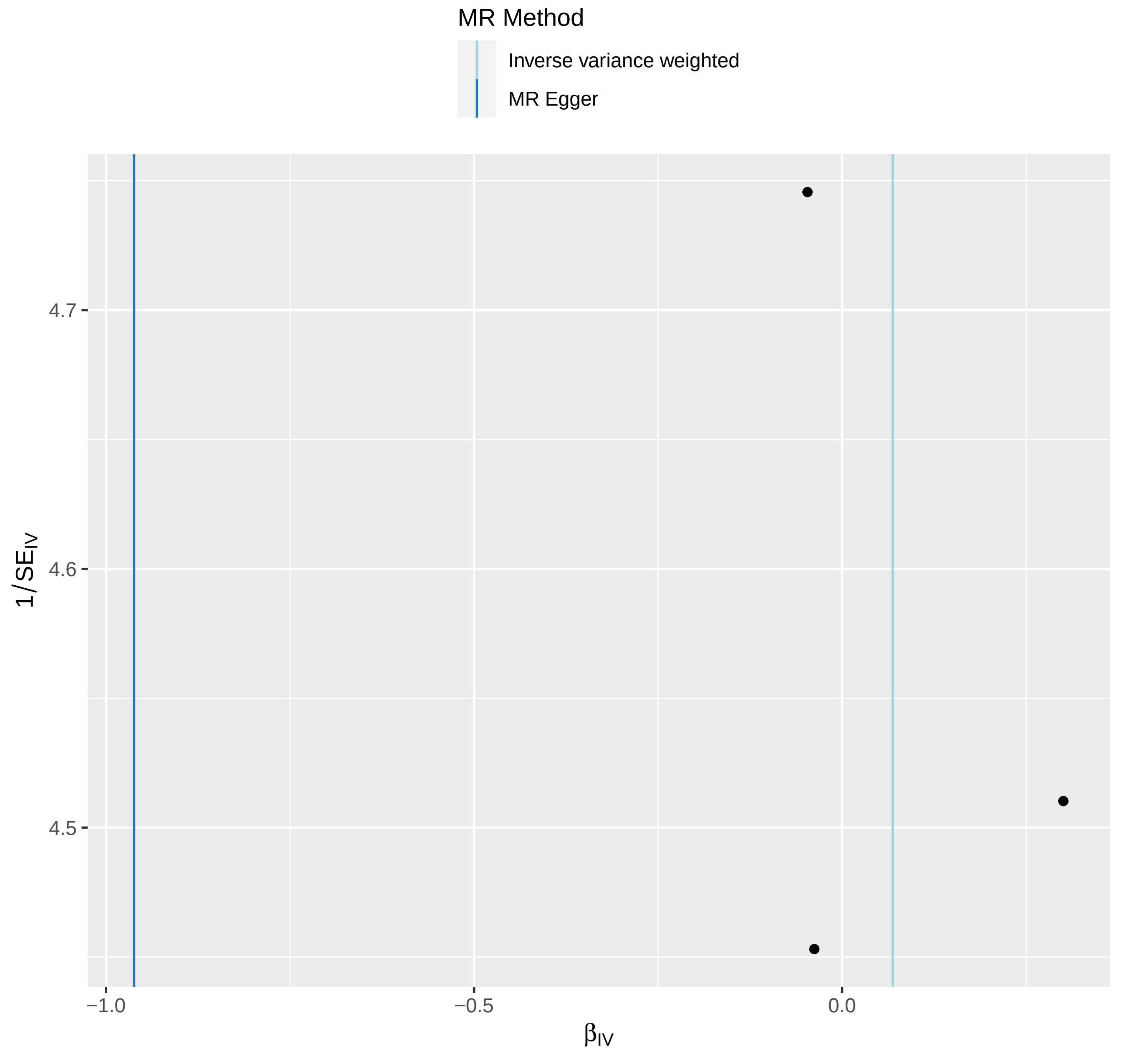

Batch 2−502 : Gut microbiota abundance (genus Parasutterella id.2892) on Baculoviral IAP repeat−containing protein 3
with a threshold of 1e−05

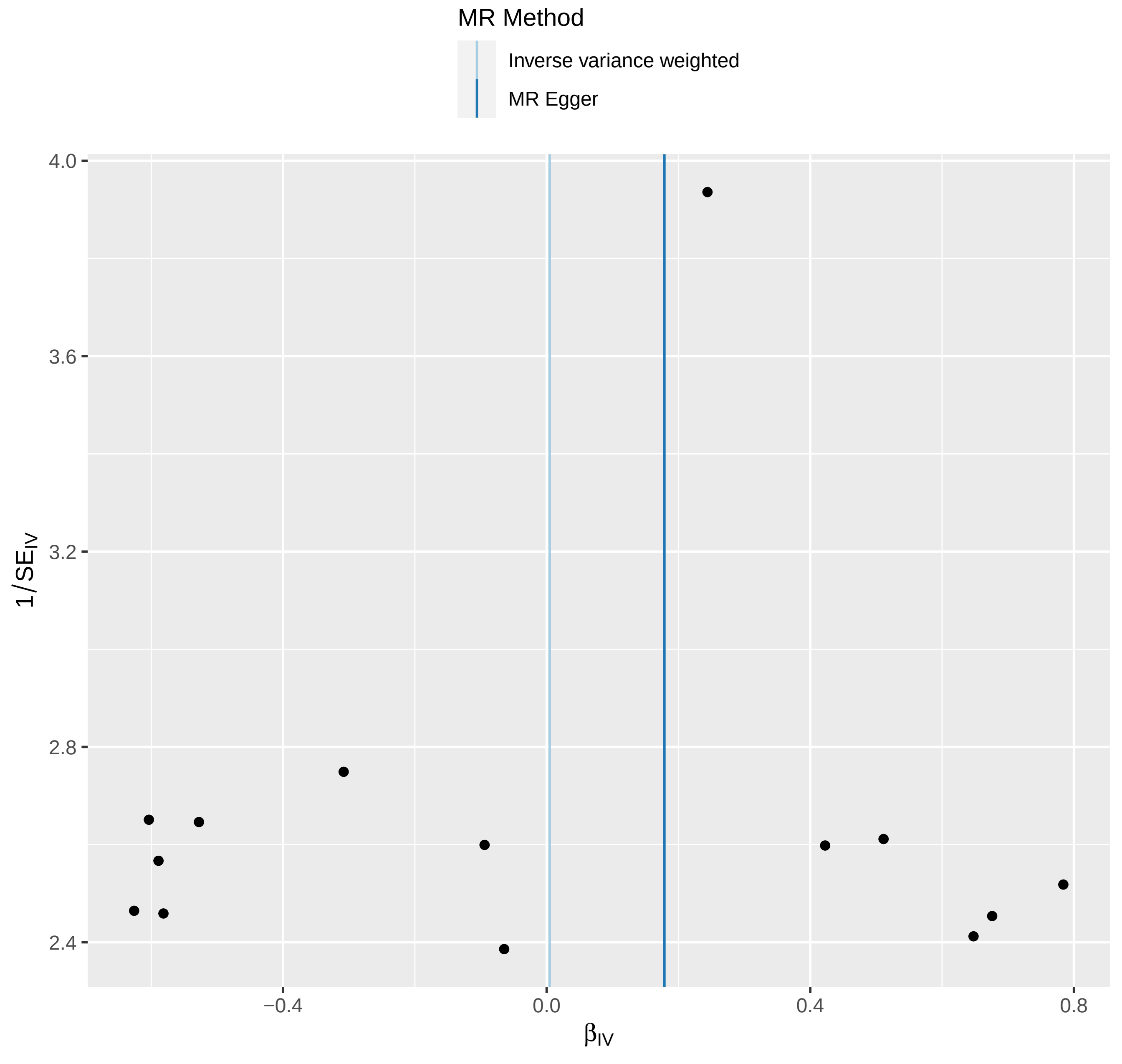

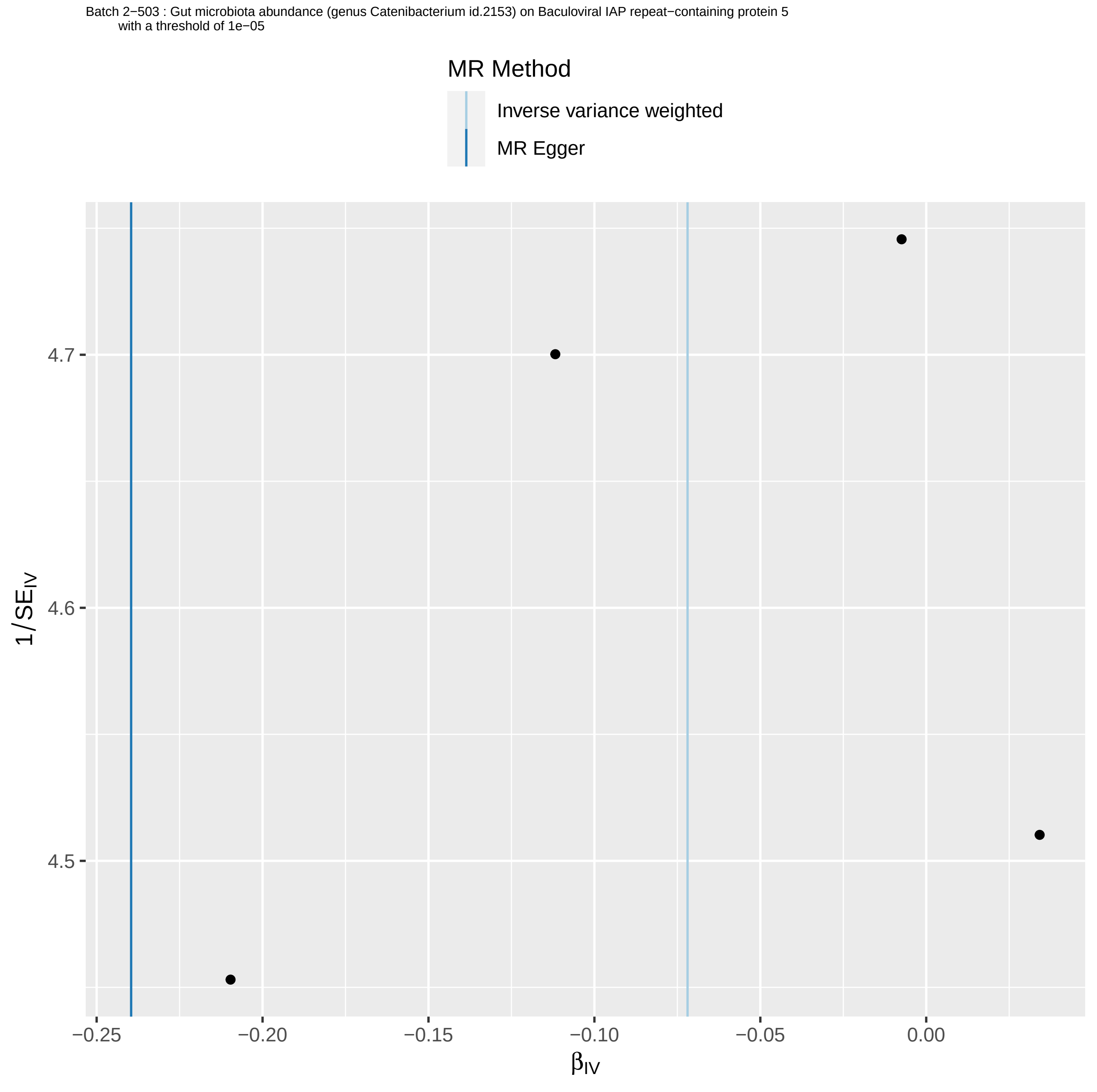
Batch 2−503 : Gut microbiota abundance (genus Catenibacterium id.2153) on Baculoviral IAP repeat−containing protein 5
with a threshold of 1e−05
MR Method
Inverse variance weighted
MR Egger
4.7
4.6
4.5
1/SE_IV
−0.25
−0.20
−0.15
−0.10
−0.05
0.00
β_IV

Batch 2−504 : Gut microbiota abundance (genus Parasutterella id.2892) on Baculoviral IAP repeat−containing protein 5 with a threshold of 1e−05

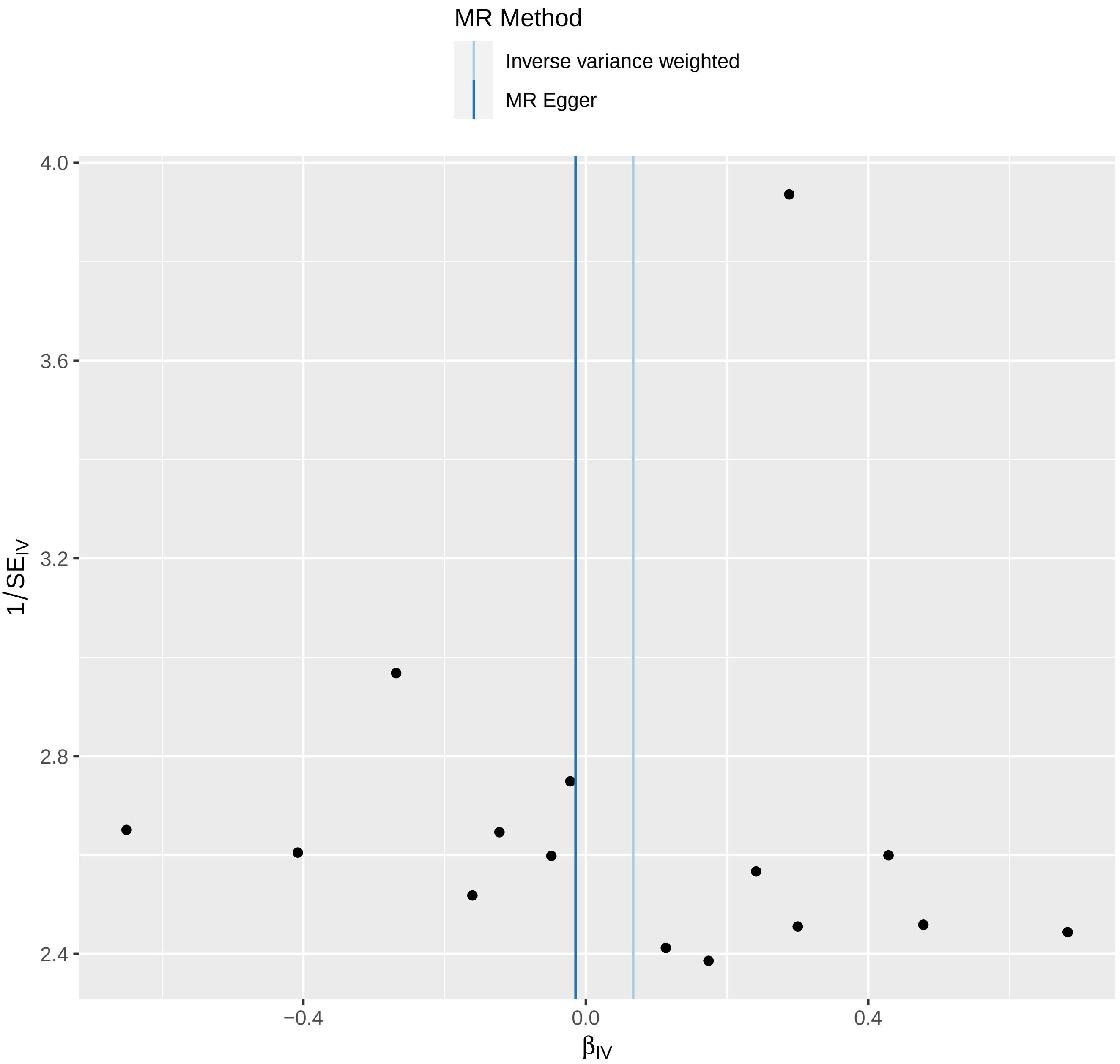

Batch 2−505 : Gut microbiota abundance (genus Catenibacterium id.2153) on Biliverdin reductase A
with a threshold of 1e−05

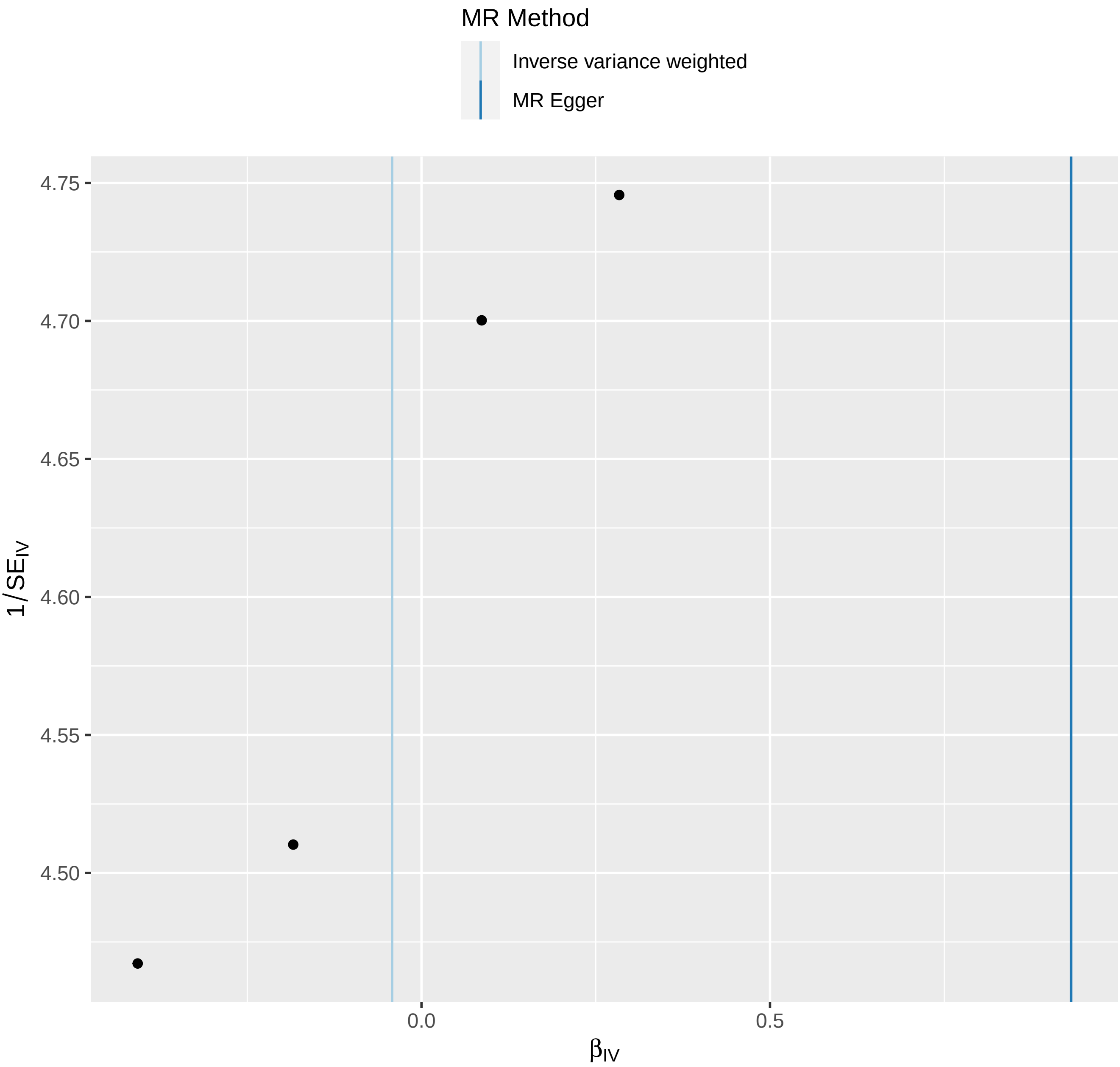

Batch 2−506 : Gut microbiota abundance (genus Parasutterella id.2892) on Biliverdin reductase A
with a threshold of 1e−05

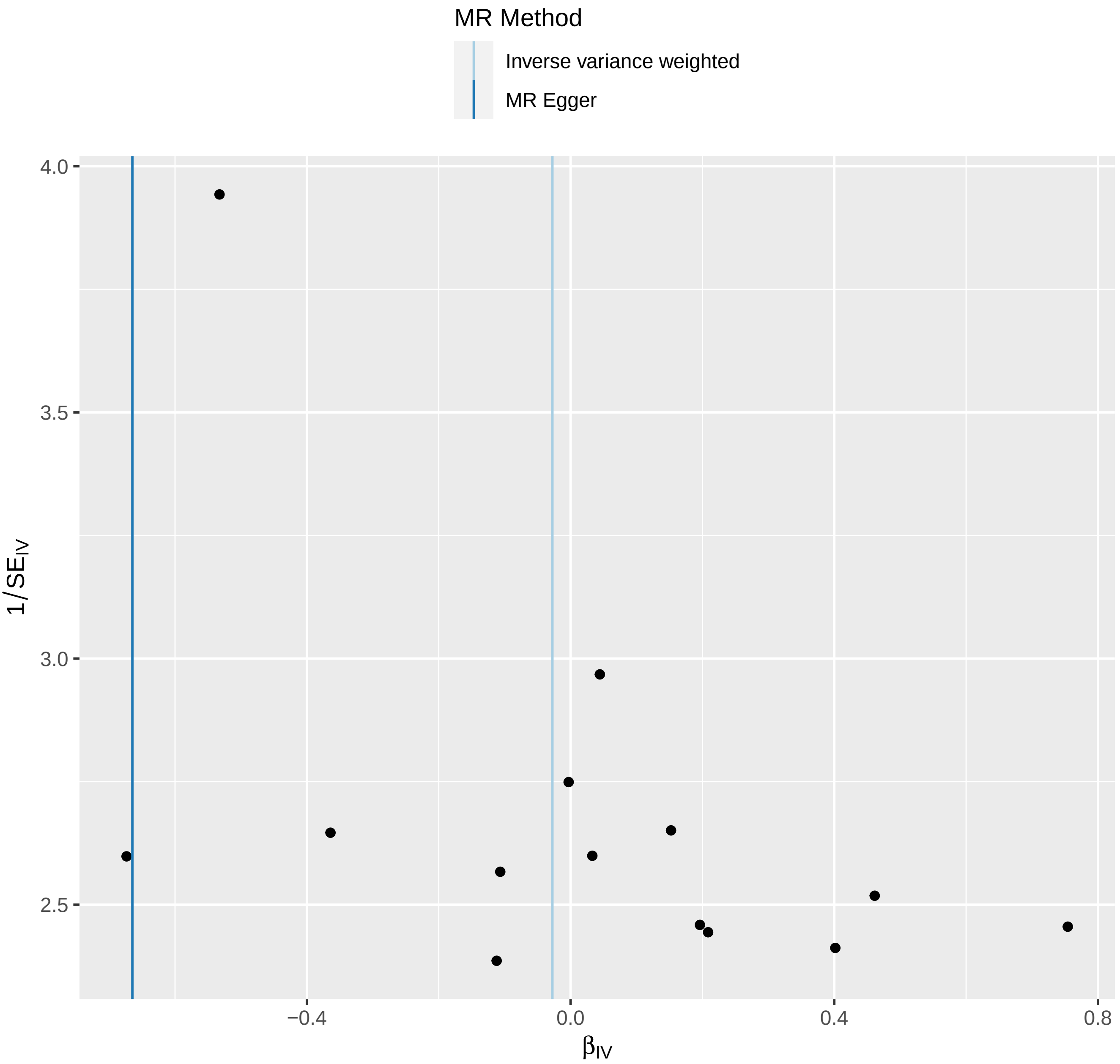

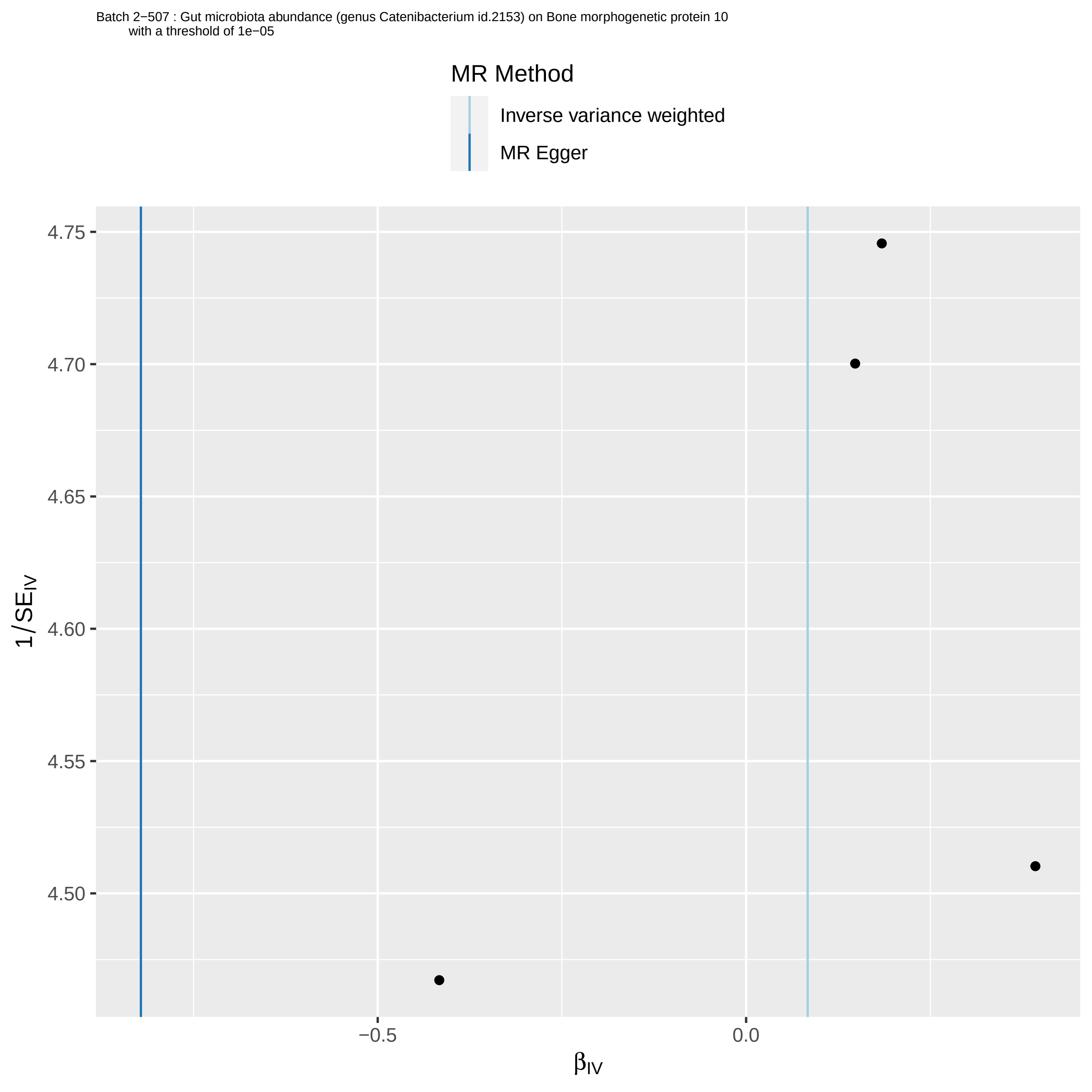

Batch 2−507 : Gut microbiota abundance (genus Catenibacterium id.2153) on Bone morphogenetic protein 10
with a threshold of 1e−05
MR Method
Inverse variance weighted
MR Egger
4.75
4.70
4.65
4.60
4.55
4.50
1/SE$_{IV}$
−0.5
0.0
β$_{IV}$

Batch 2−508 : Gut microbiota abundance (genus Parasutterella id.2892) on Bone morphogenetic protein 10
with a threshold of 1e−05

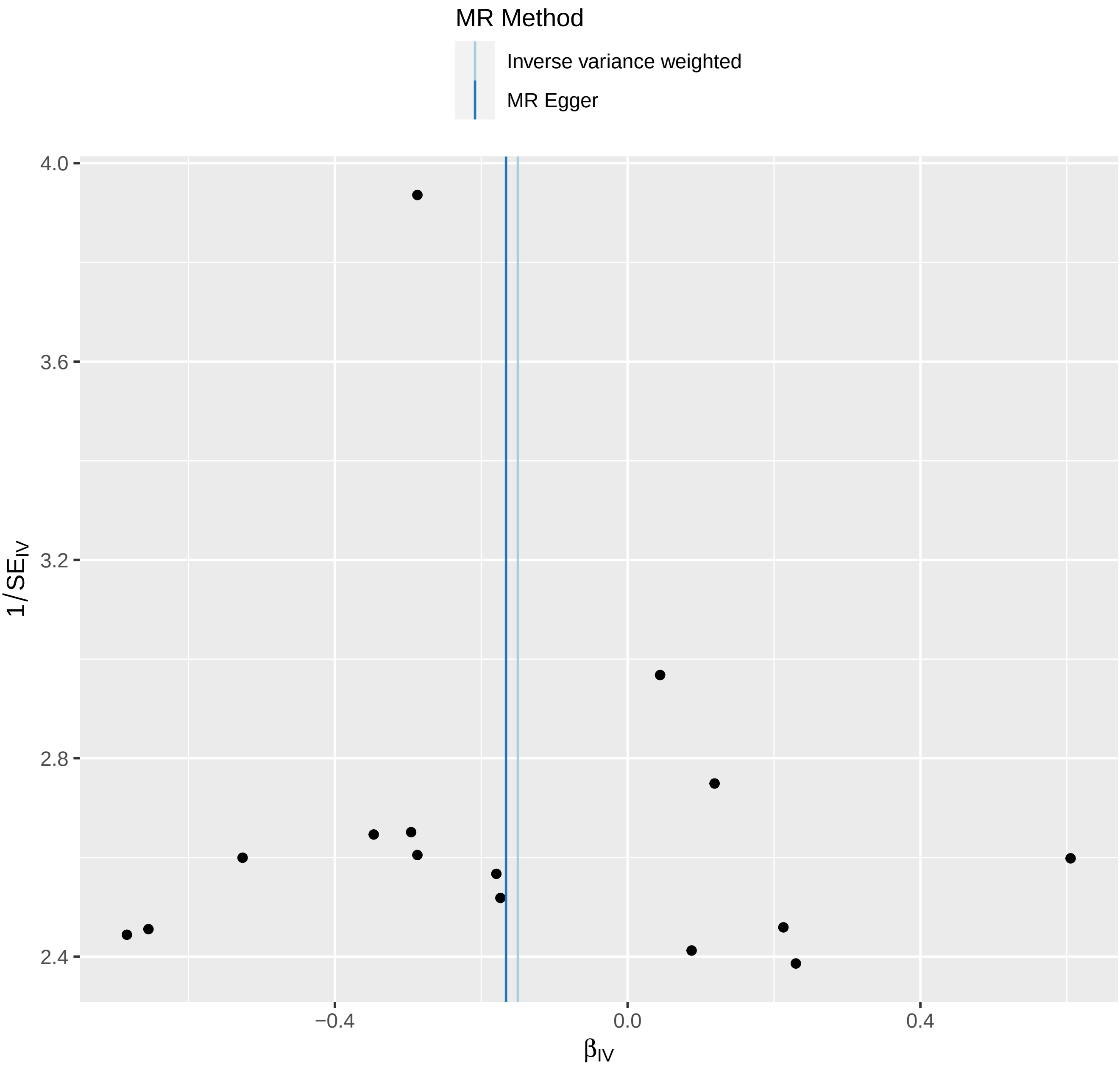

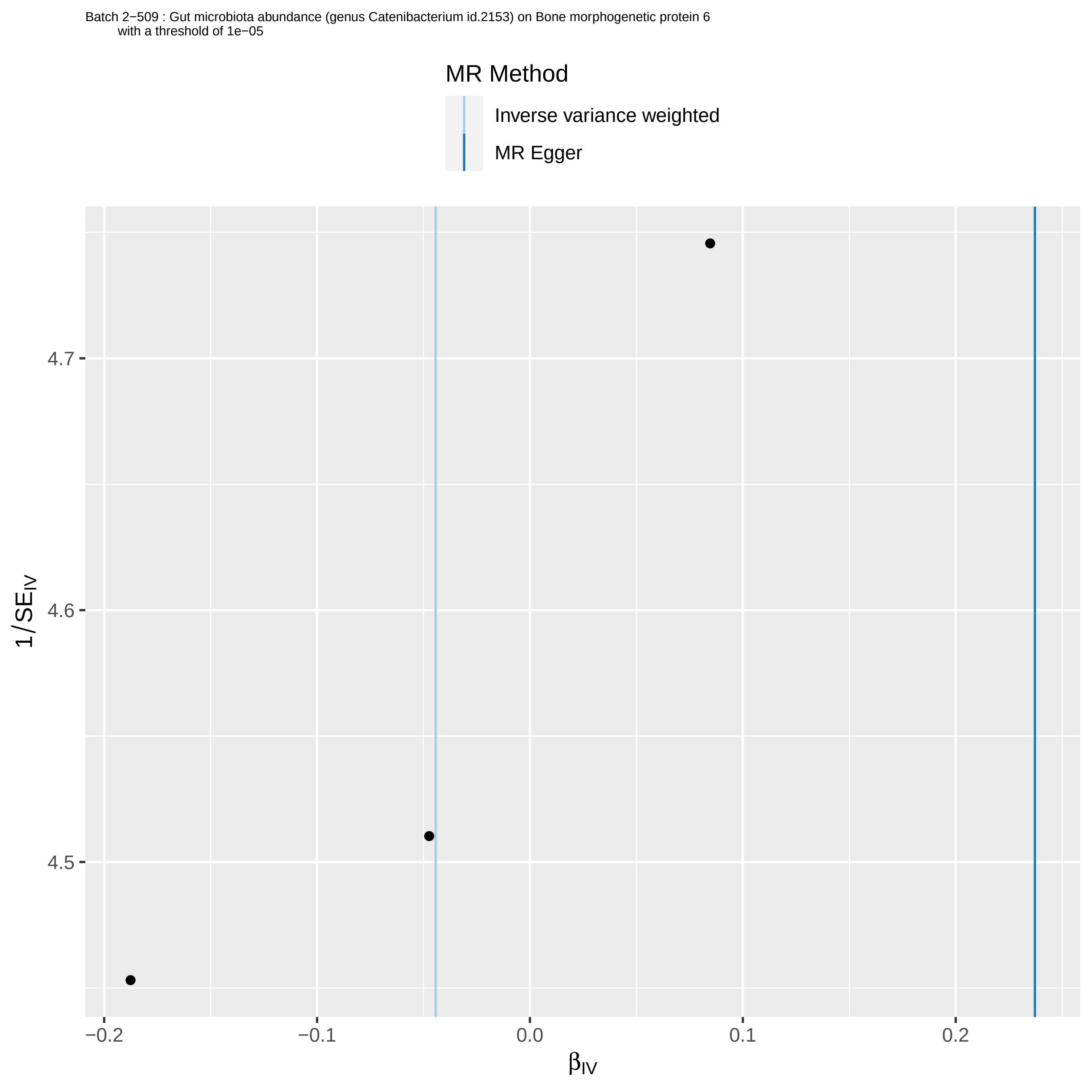

Batch 2−509 : Gut microbiota abundance (genus Catenibacterium id.2153) on Bone morphogenetic protein 6
with a threshold of 1e−05
MR Method
Inverse variance weighted
MR Egger
4.7
4.6
4.5
1/SE_IV
−0.2
−0.1
0.0
0.1
0.2
β_IV

Batch 2−510 : Gut microbiota abundance (genus Parasutterella id.2892) on Bone morphogenetic protein 6 with a threshold of 1e−05

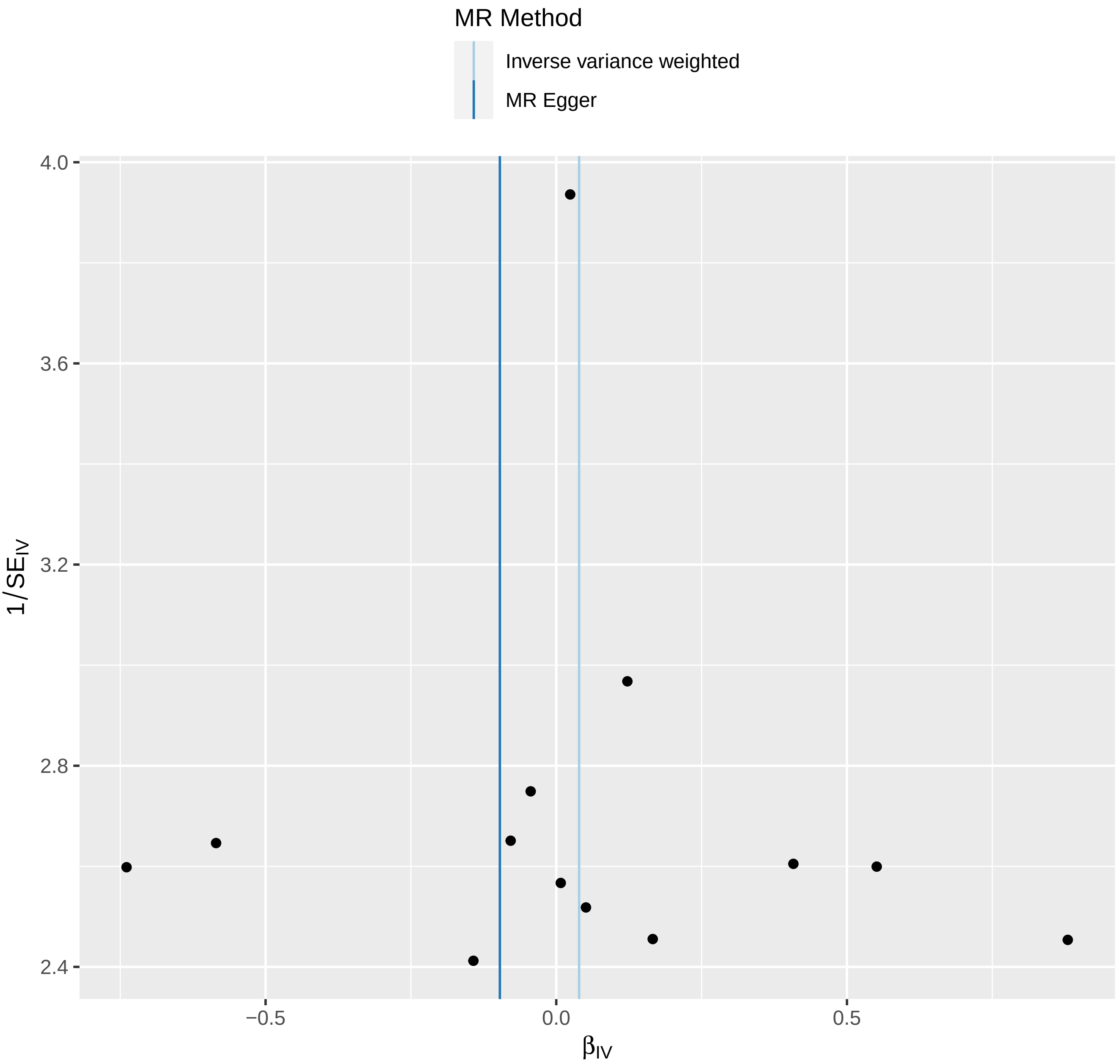

Batch 2−511 : Gut microbiota abundance (genus Catenibacterium id.2153) on Bone morphogenetic protein 6
with a threshold of 1e−05

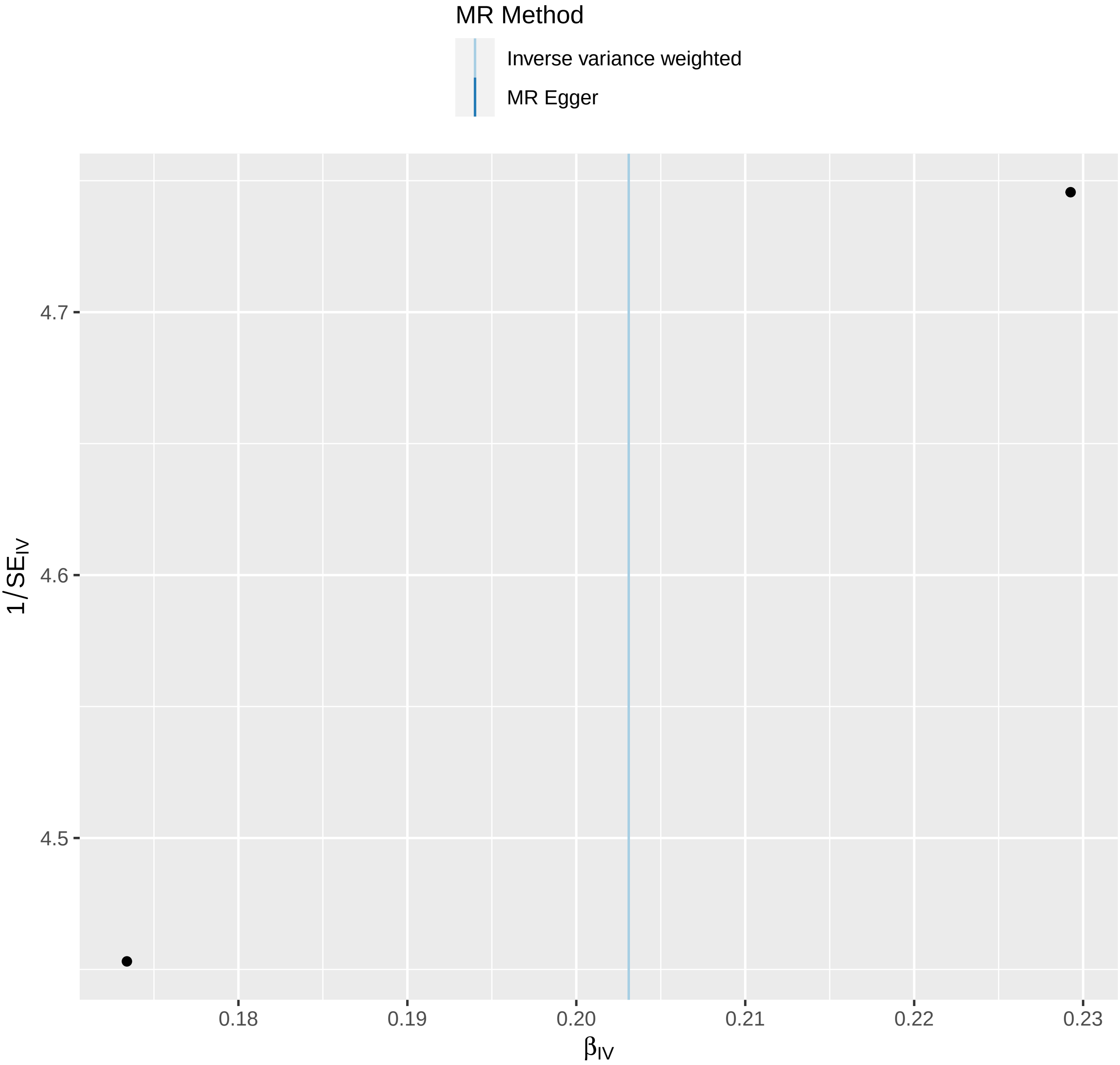

Batch 2−512 : Gut microbiota abundance (genus Parasutterella id.2892) on Bone morphogenetic protein 6
with a threshold of 1e−05

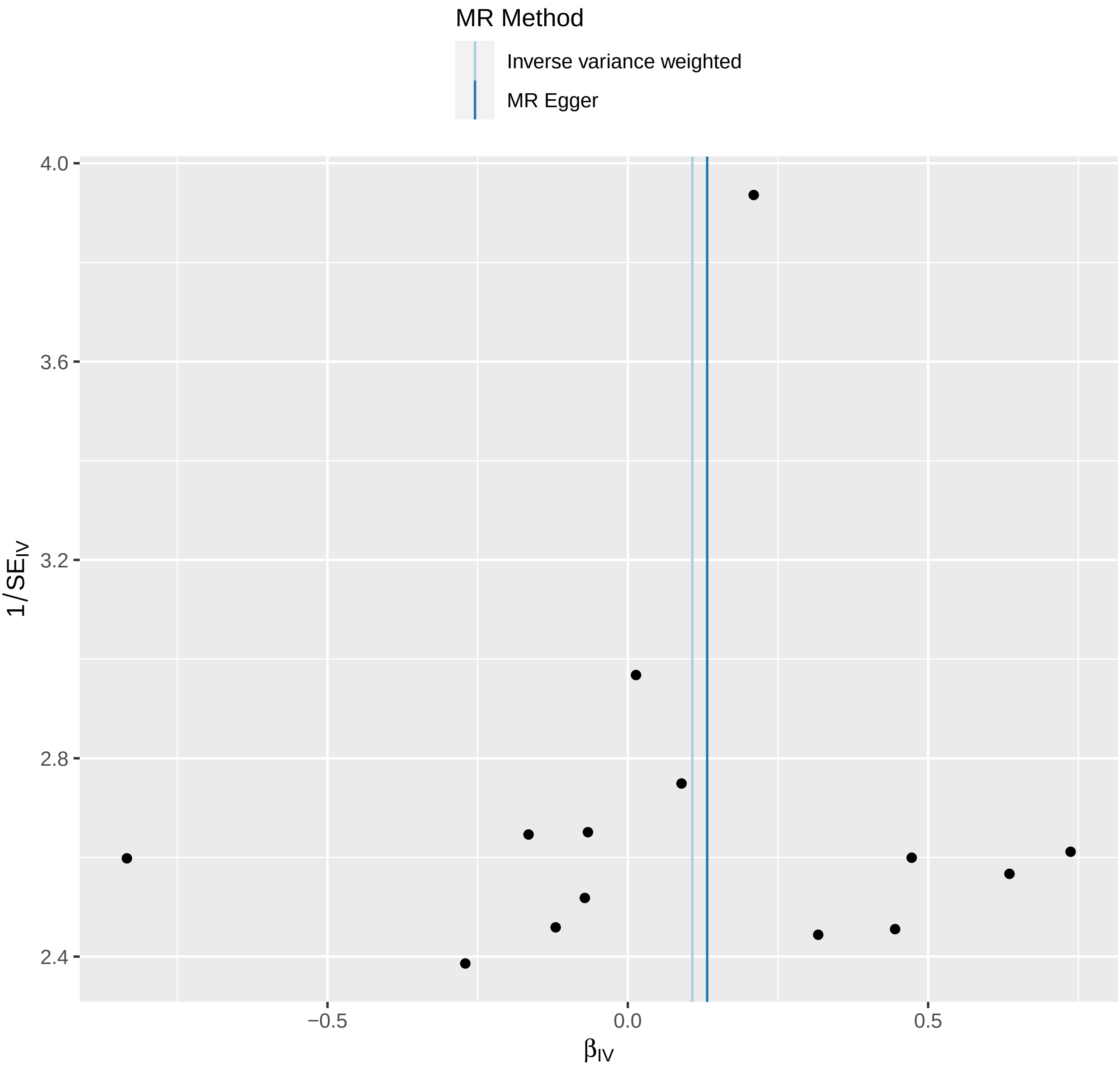

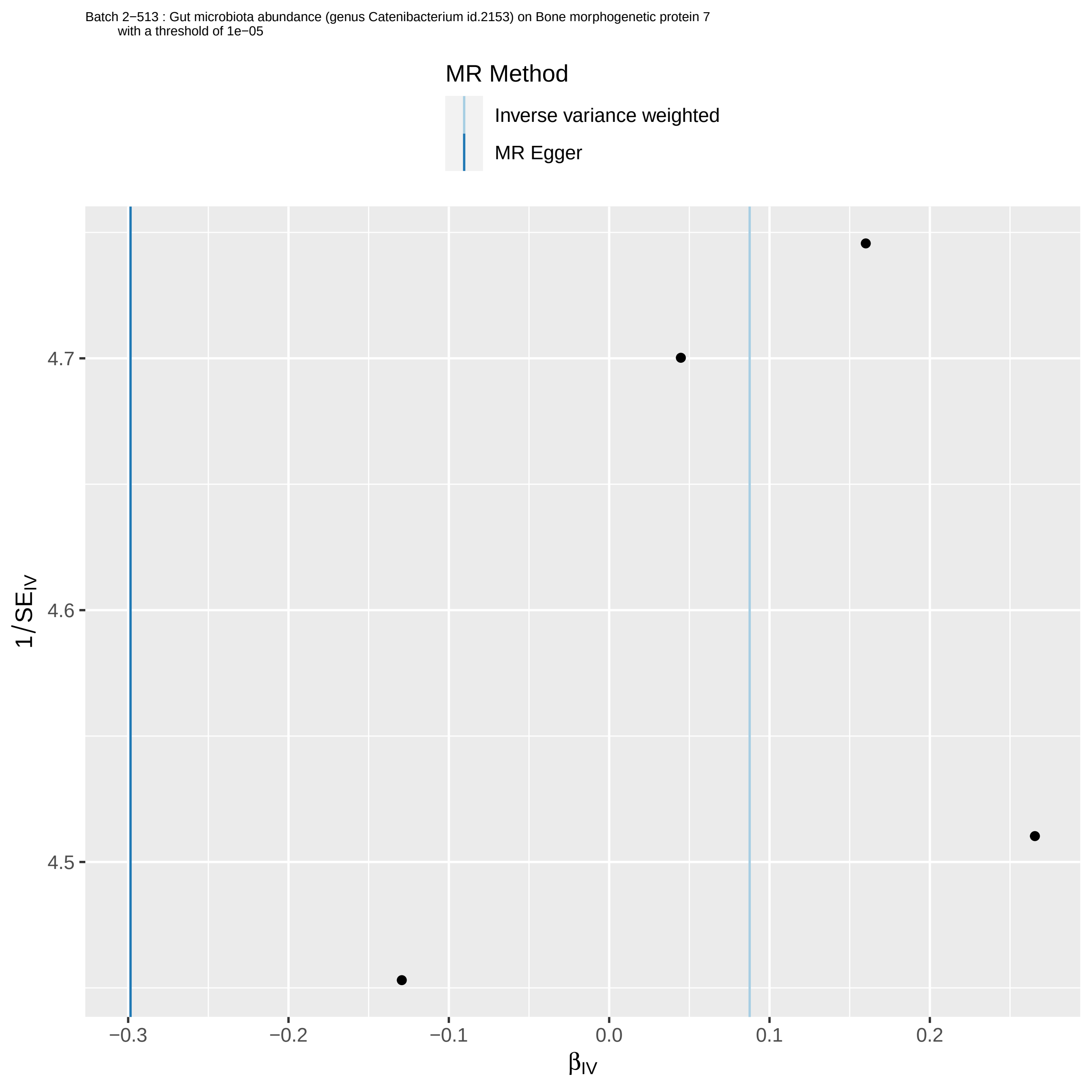

Batch 2−513 : Gut microbiota abundance (genus Catenibacterium id.2153) on Bone morphogenetic protein 7
with a threshold of 1e−05
MR Method
Inverse variance weighted
MR Egger
4.7
4.6
4.5
1/SE_IV
−0.3
−0.2
−0.1
0.0
0.1
0.2
β_IV

Batch 2−514 : Gut microbiota abundance (genus Parasutterella id.2892) on Bone morphogenetic protein 7
with a threshold of 1e−05

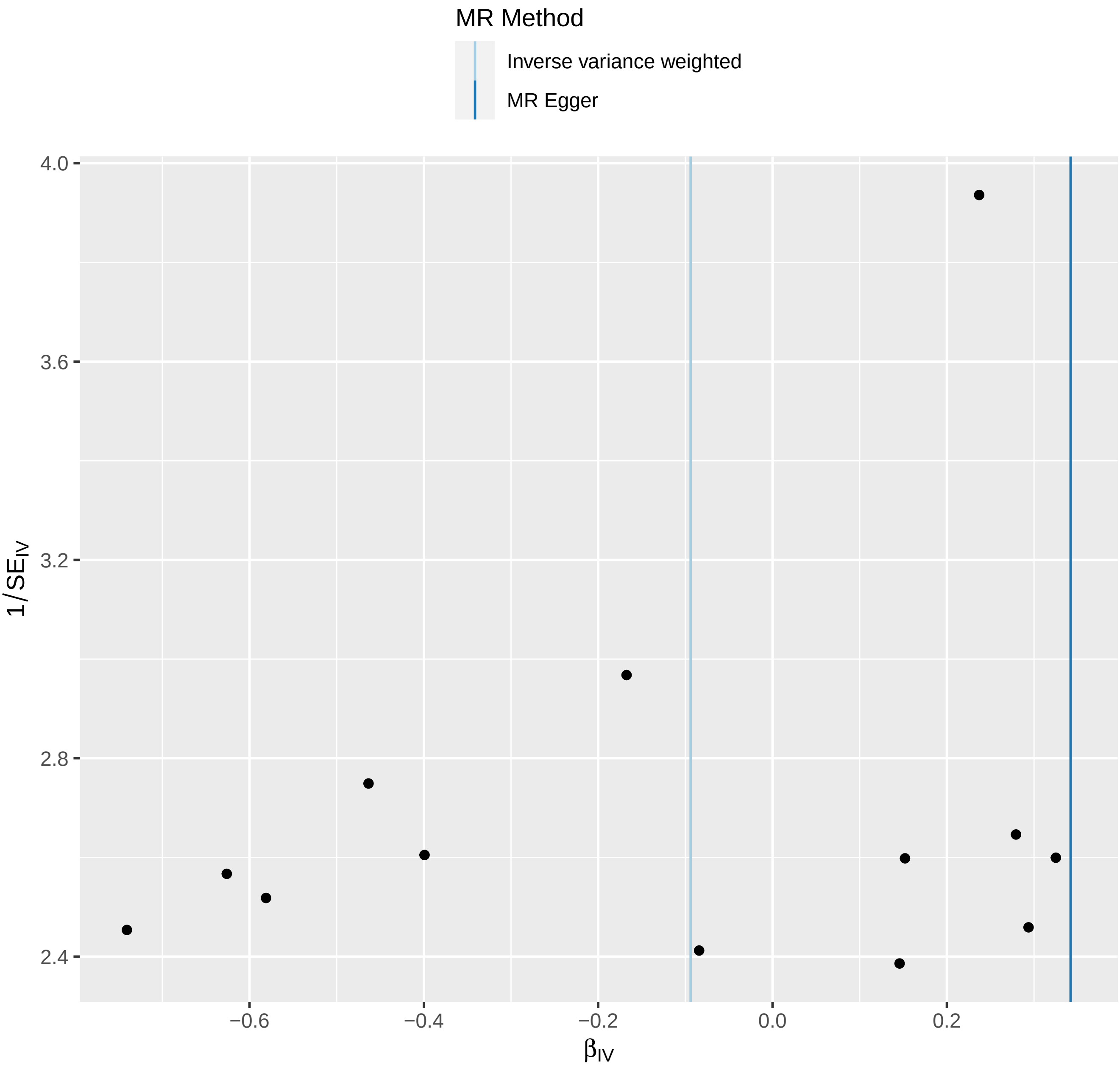

Batch 2−515 : Gut microbiota abundance (genus Catenibacterium id.2153) on BMP−binding endothelial regulator protein with a threshold of 1e−05

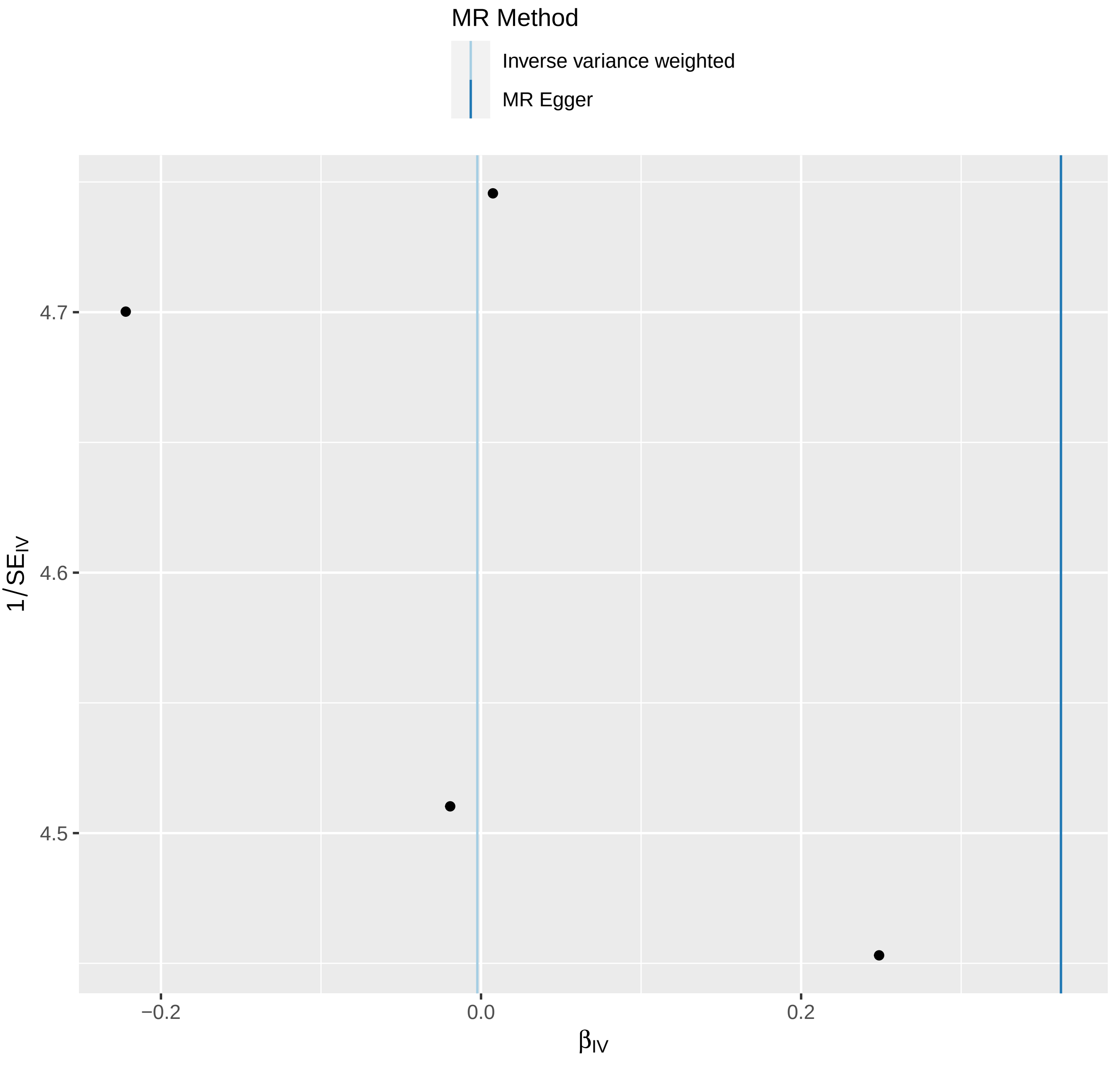

Batch 2−516 : Gut microbiota abundance (genus Parasutterella id.2892) on BMP−binding endothelial regulator protein
with a threshold of 1e−05

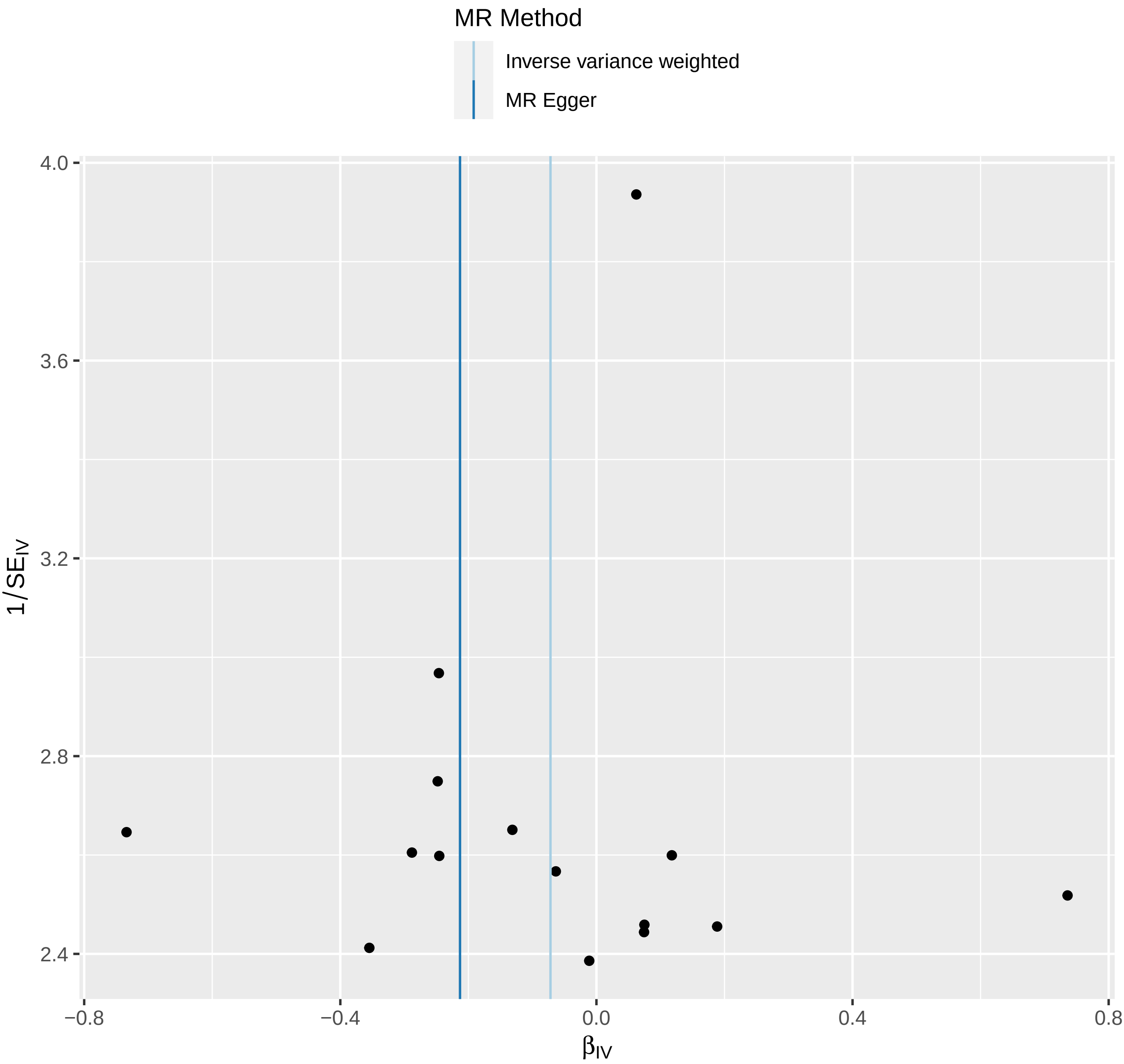

Batch 2−517 : Gut microbiota abundance (genus Catenibacterium id.2153) on Bone morphogenetic protein receptor type−1A with a threshold of 1e−05

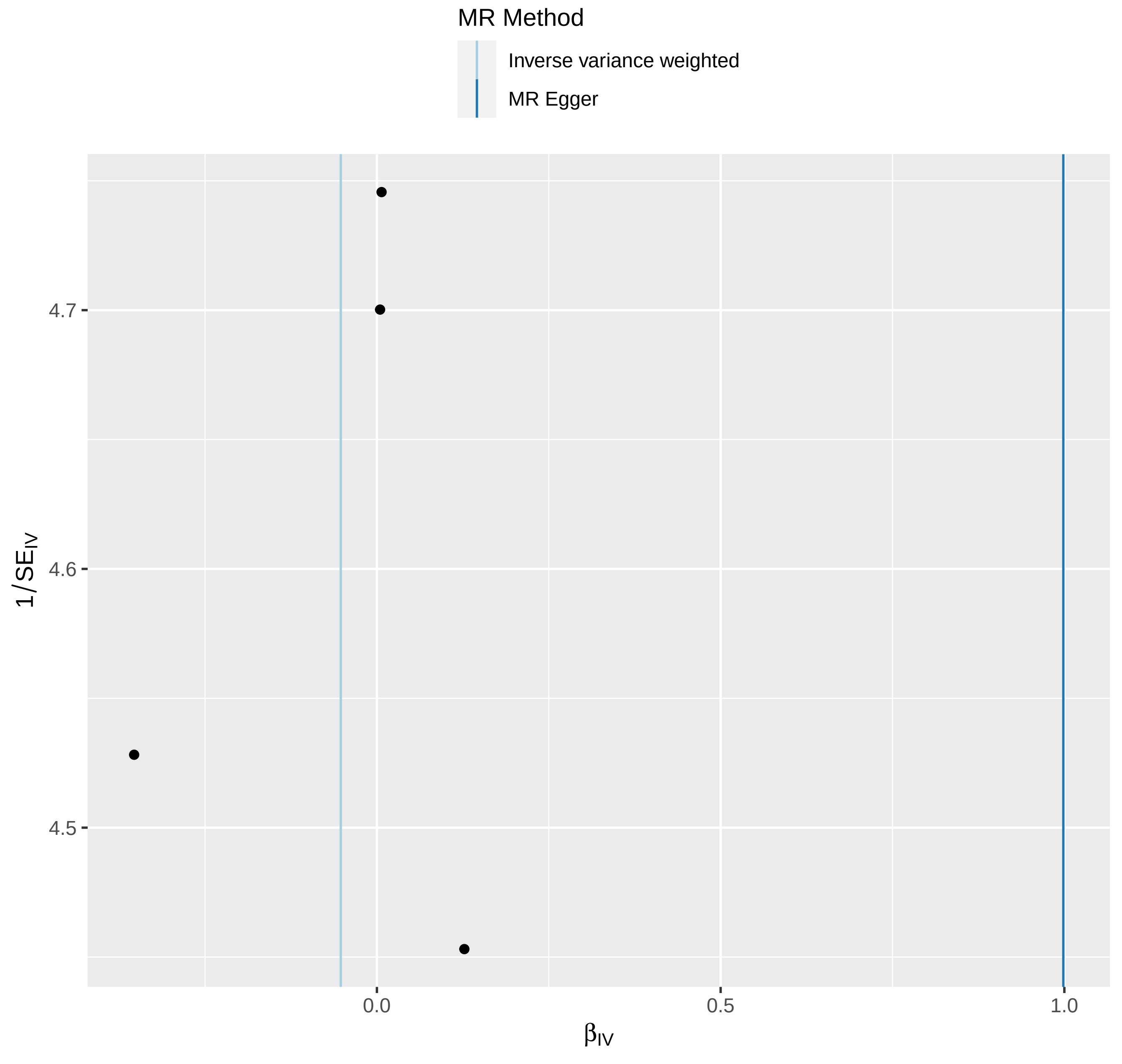

Batch 2−518 : Gut microbiota abundance (genus Parasutterella id.2892) on Bone morphogenetic protein receptor type−1A with a threshold of 1e−05

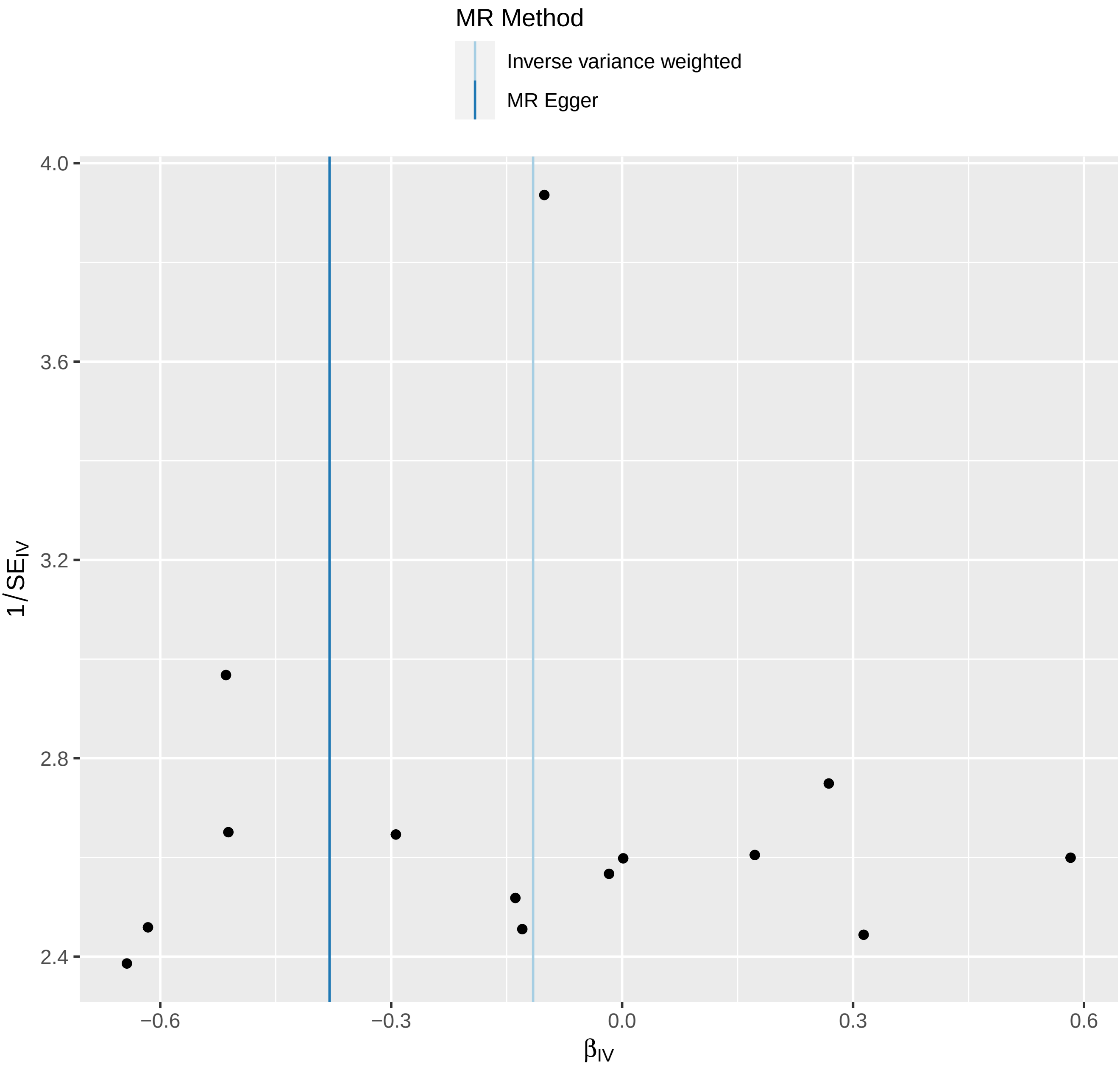

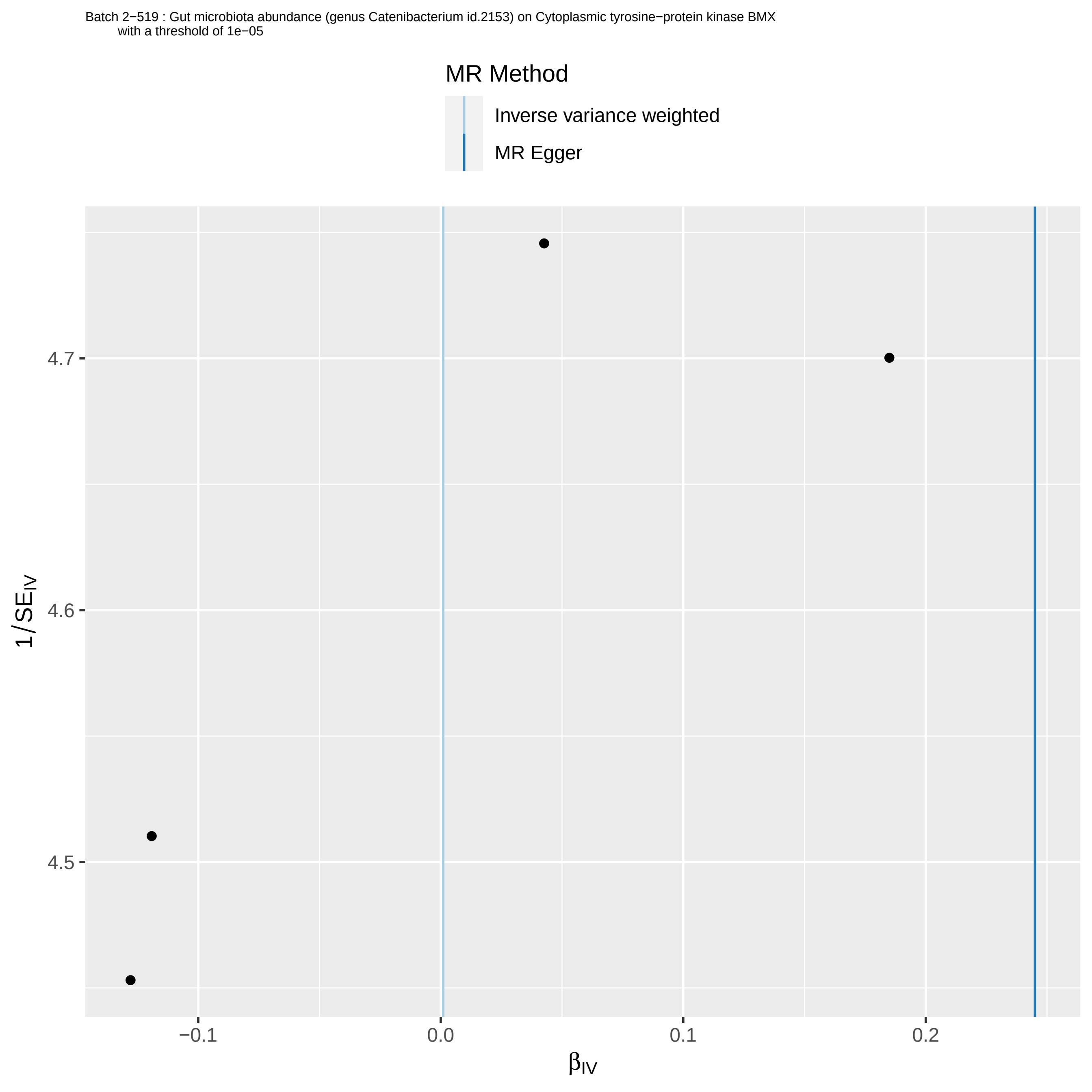
Batch 2−519 : Gut microbiota abundance (genus Catenibacterium id.2153) on Cytoplasmic tyrosine−protein kinase BMX
with a threshold of 1e−05
MR Method
Inverse variance weighted
MR Egger
4.7
4.6
4.5
1/SE_IV
−0.1
0.0
0.1
0.2
β_IV

Batch 2−520 : Gut microbiota abundance (genus Parasutterella id.2892) on Cytoplasmic tyrosine−protein kinase BMX
with a threshold of 1e−05

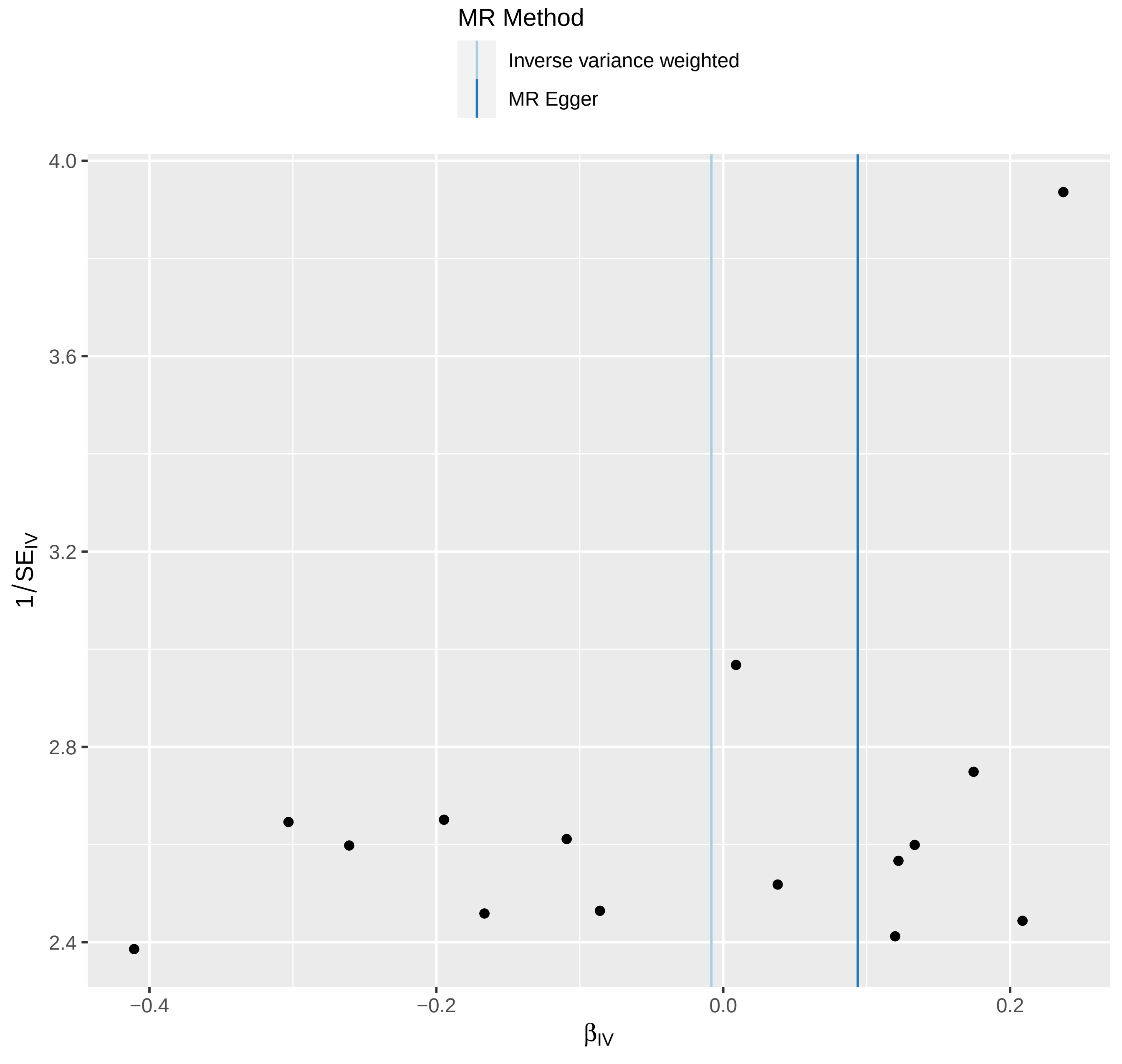

Batch 2−521 : Gut microbiota abundance (genus Catenibacterium id.2153) on BCL2/adenovirus E1B 19 kDa protein−interacting protein 3
with a threshold of 1e−05

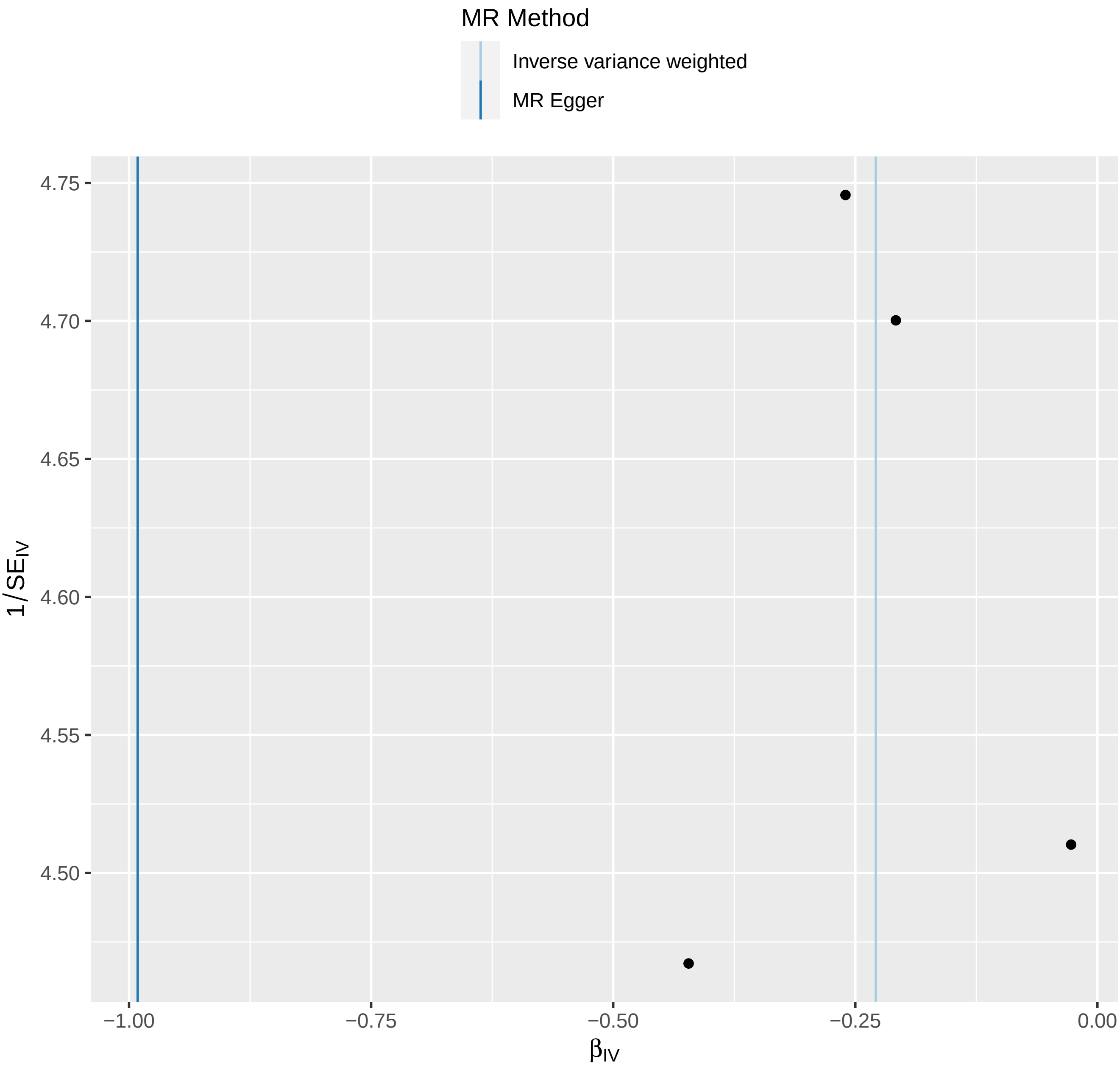

Batch 2−522 : Gut microbiota abundance (genus Parasutterella id.2892) on BCL2/adenovirus E1B 19 kDa protein−interacting protein 3 with a threshold of 1e−05

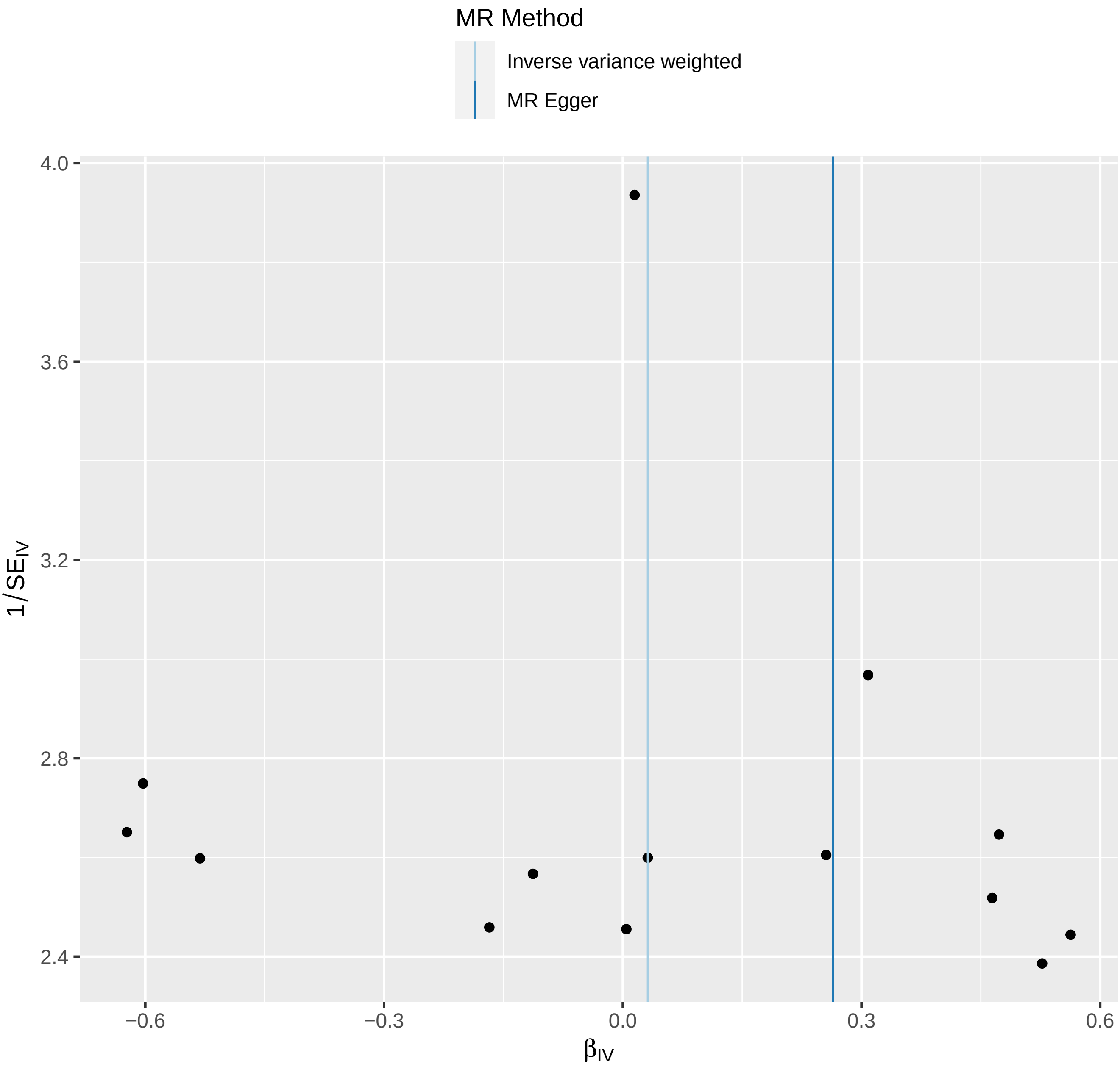

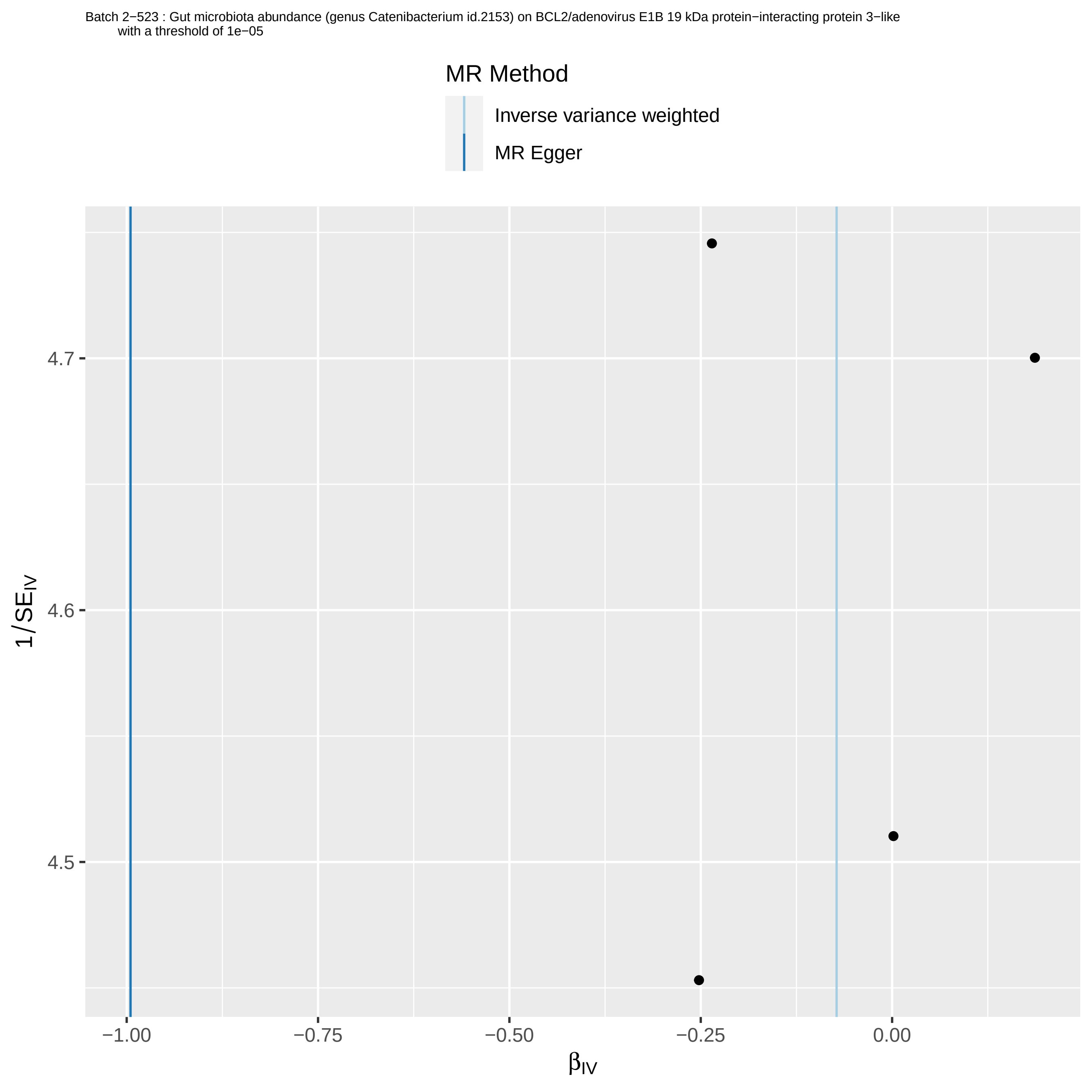
Batch 2−523 : Gut microbiota abundance (genus Catenibacterium id.2153) on BCL2/adenovirus E1B 19 kDa protein−interacting protein 3−like with a threshold of 1e−05
MR Method
Inverse variance weighted
MR Egger
4.7
4.6
4.5
1/SE_IV
−1.00
−0.75
−0.50
−0.25
0.00
β_IV

Batch 2−524 : Gut microbiota abundance (genus Parasutterella id.2892) on BCL2/adenovirus E1B 19 kDa protein−interacting protein 3−like
with a threshold of 1e−05

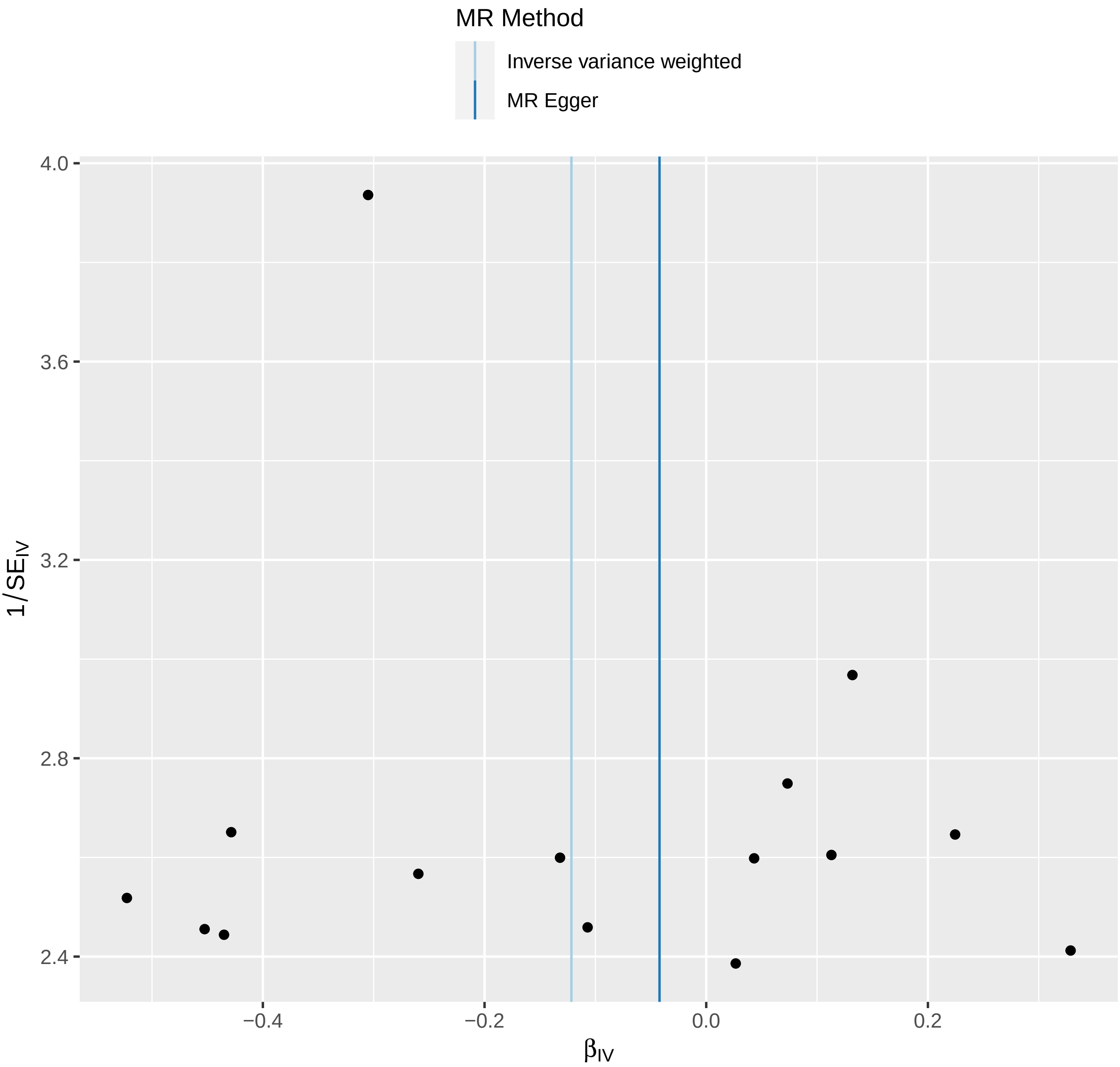

Batch 2−525 : Gut microbiota abundance (genus Catenibacterium id.2153) on Brother of CDO
with a threshold of 1e−05

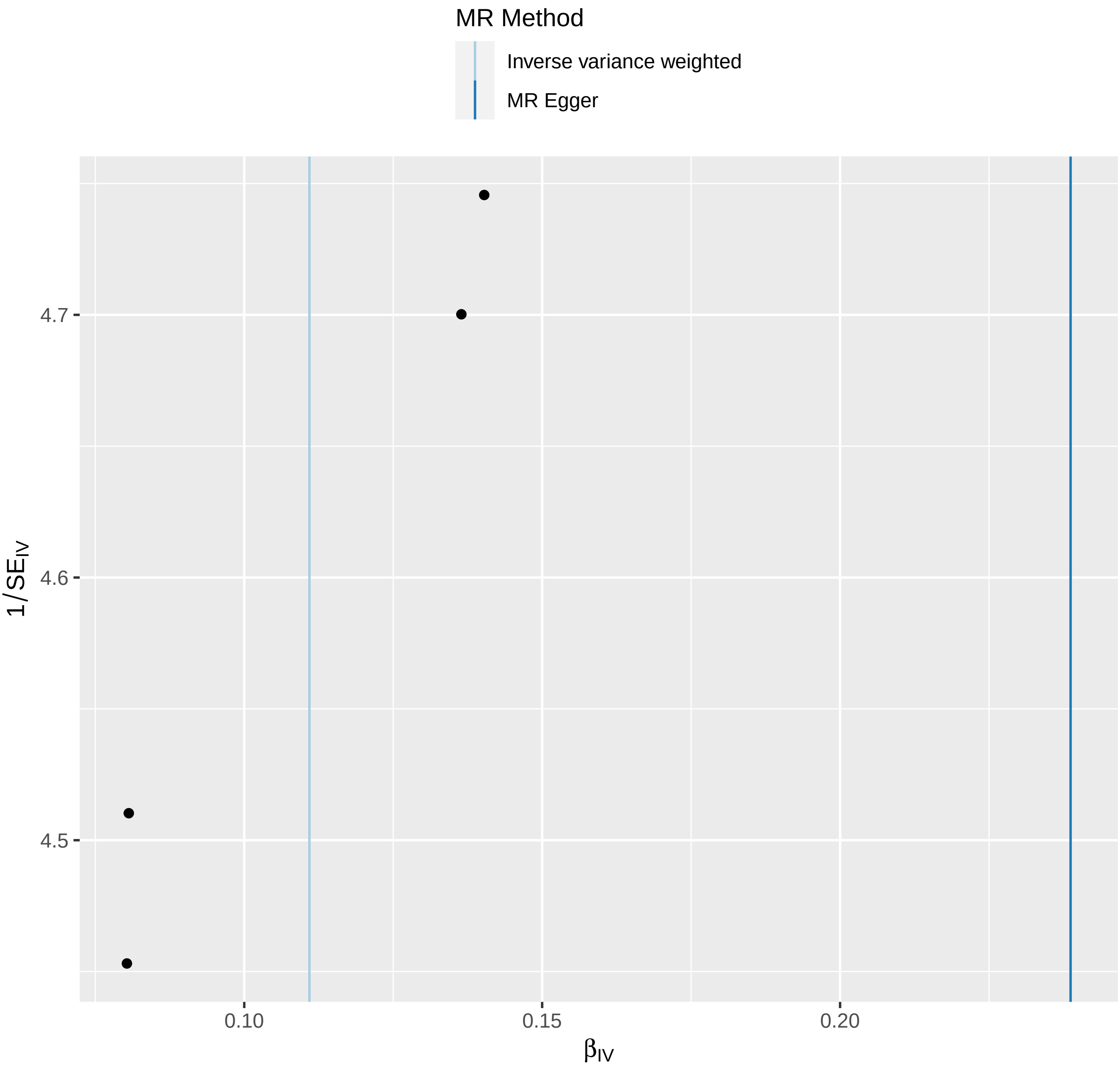

Batch 2−526 : Gut microbiota abundance (genus Parasutterella id.2892) on Brother of CDO
with a threshold of 1e−05

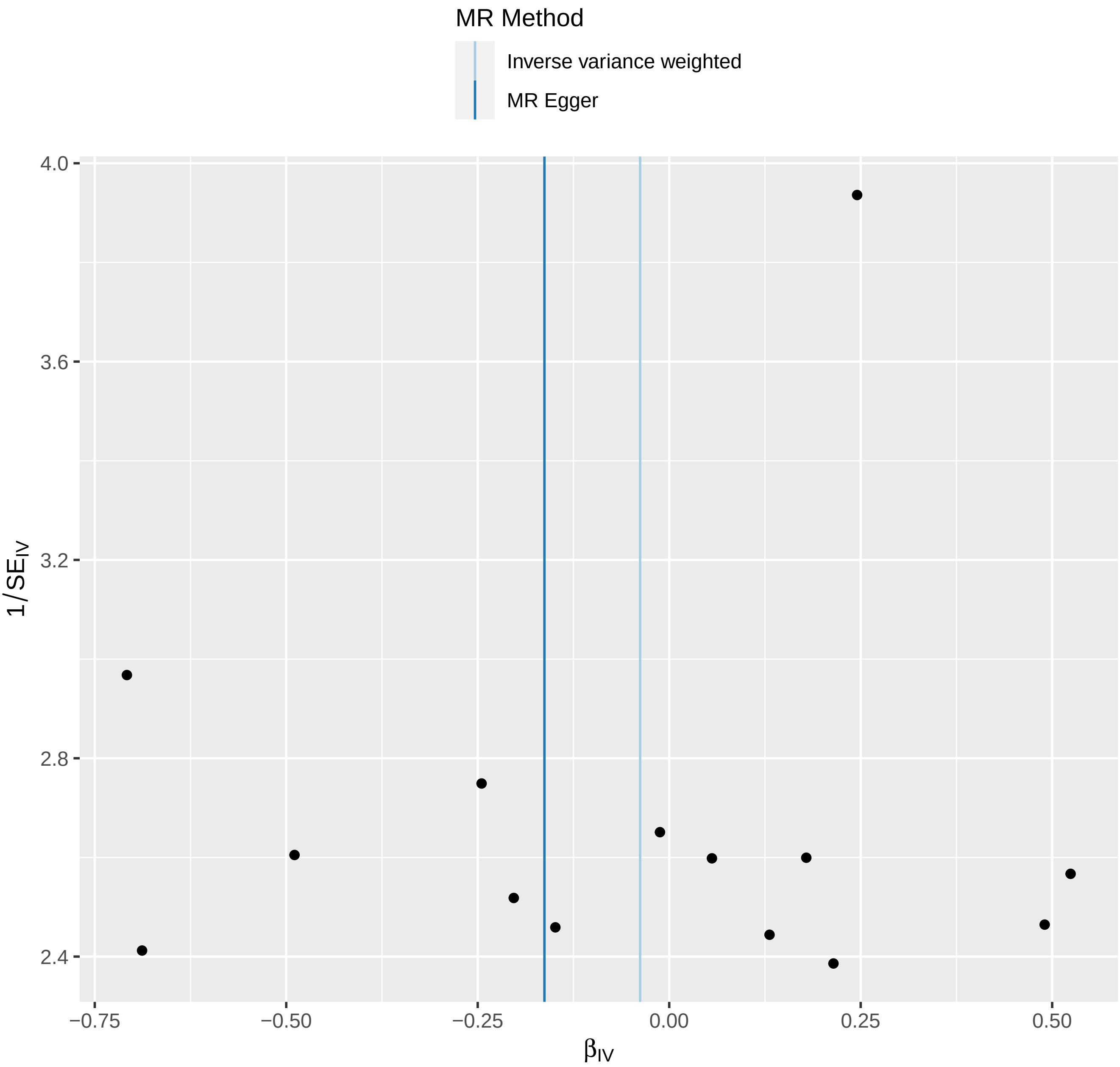

Batch 2−527 : Gut microbiota abundance (genus Catenibacterium id.2153) on BolA−like protein 2
with a threshold of 1e−05

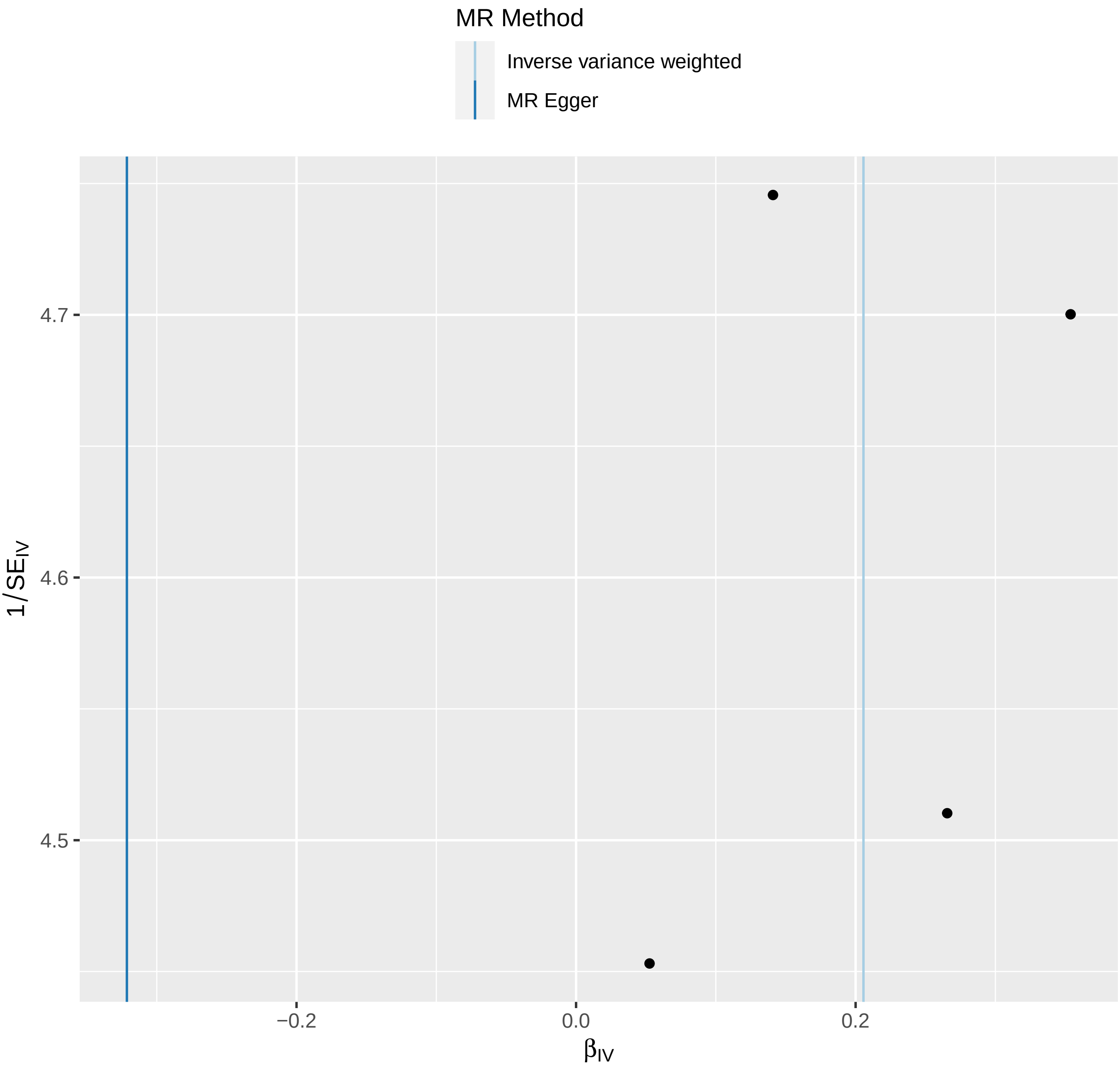

Batch 2−528 : Gut microbiota abundance (genus Parasutterella id.2892) on BolA−like protein 2
with a threshold of 1e−05

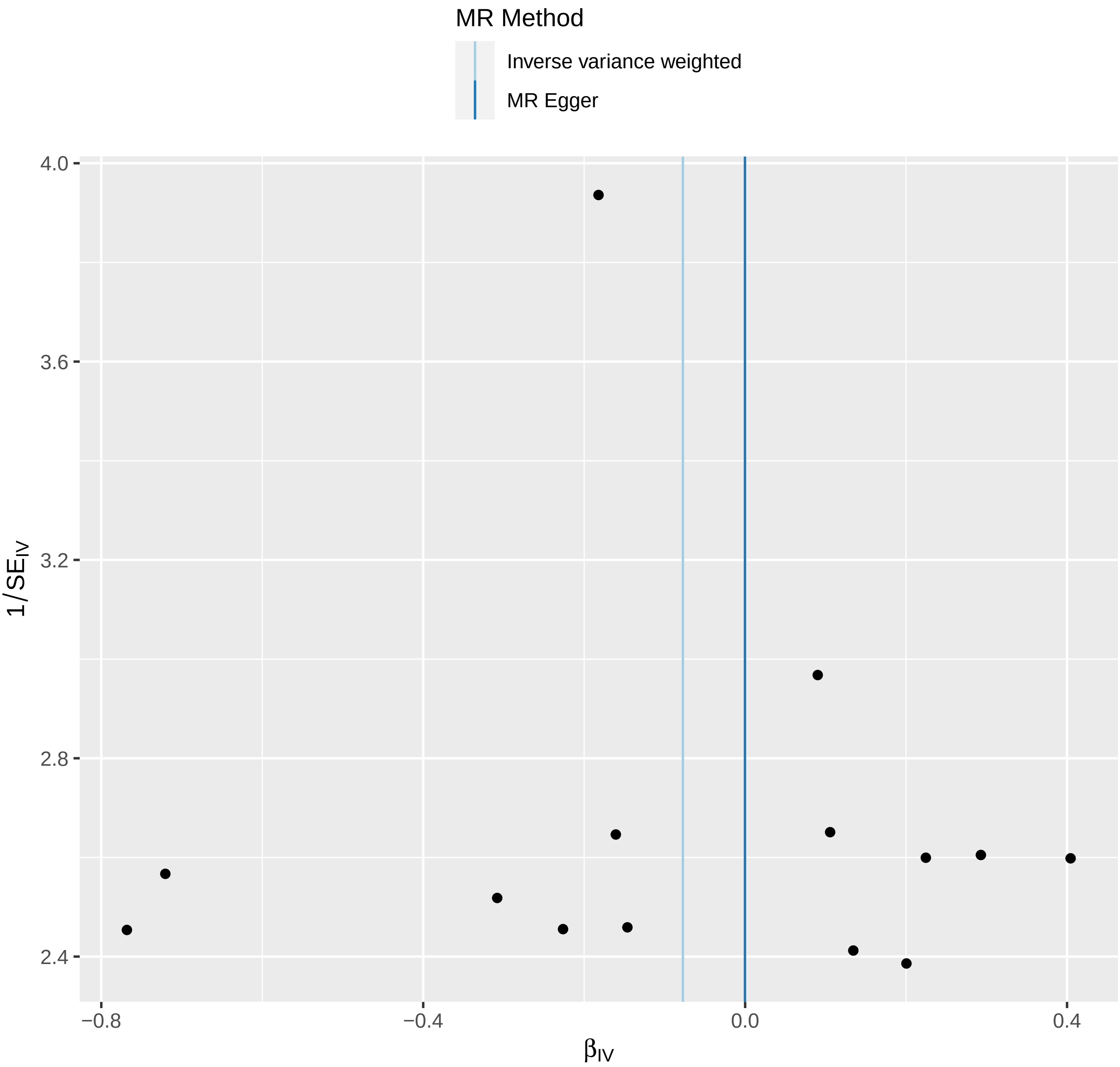

Batch 2−529 : Gut microbiota abundance (genus Catenibacterium id.2153) on Bisphosphoglycerate mutase with a threshold of 1e−05

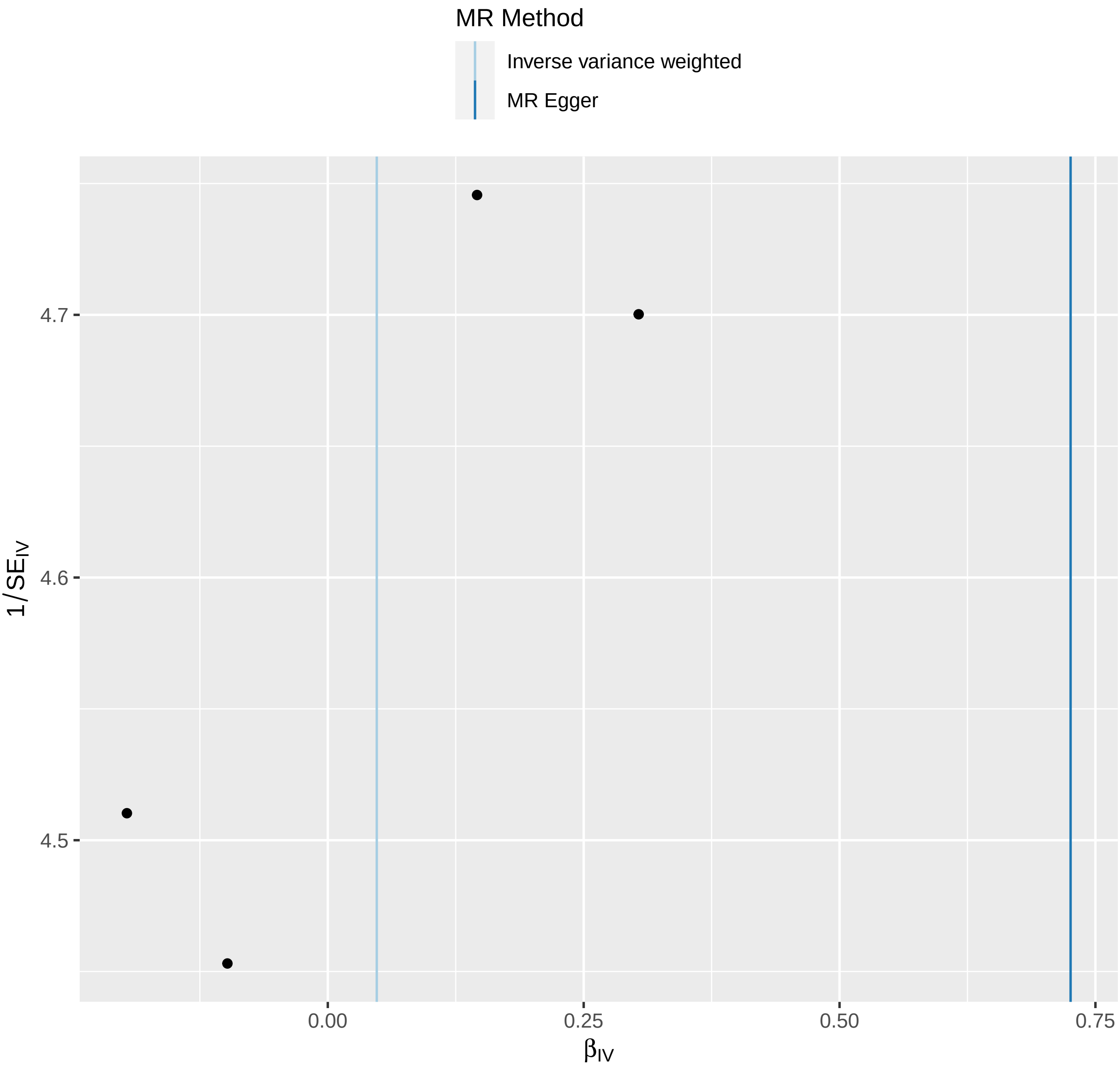

Batch 2−530 : Gut microbiota abundance (genus Parasutterella id.2892) on Bisphosphoglycerate mutase
with a threshold of 1e−05

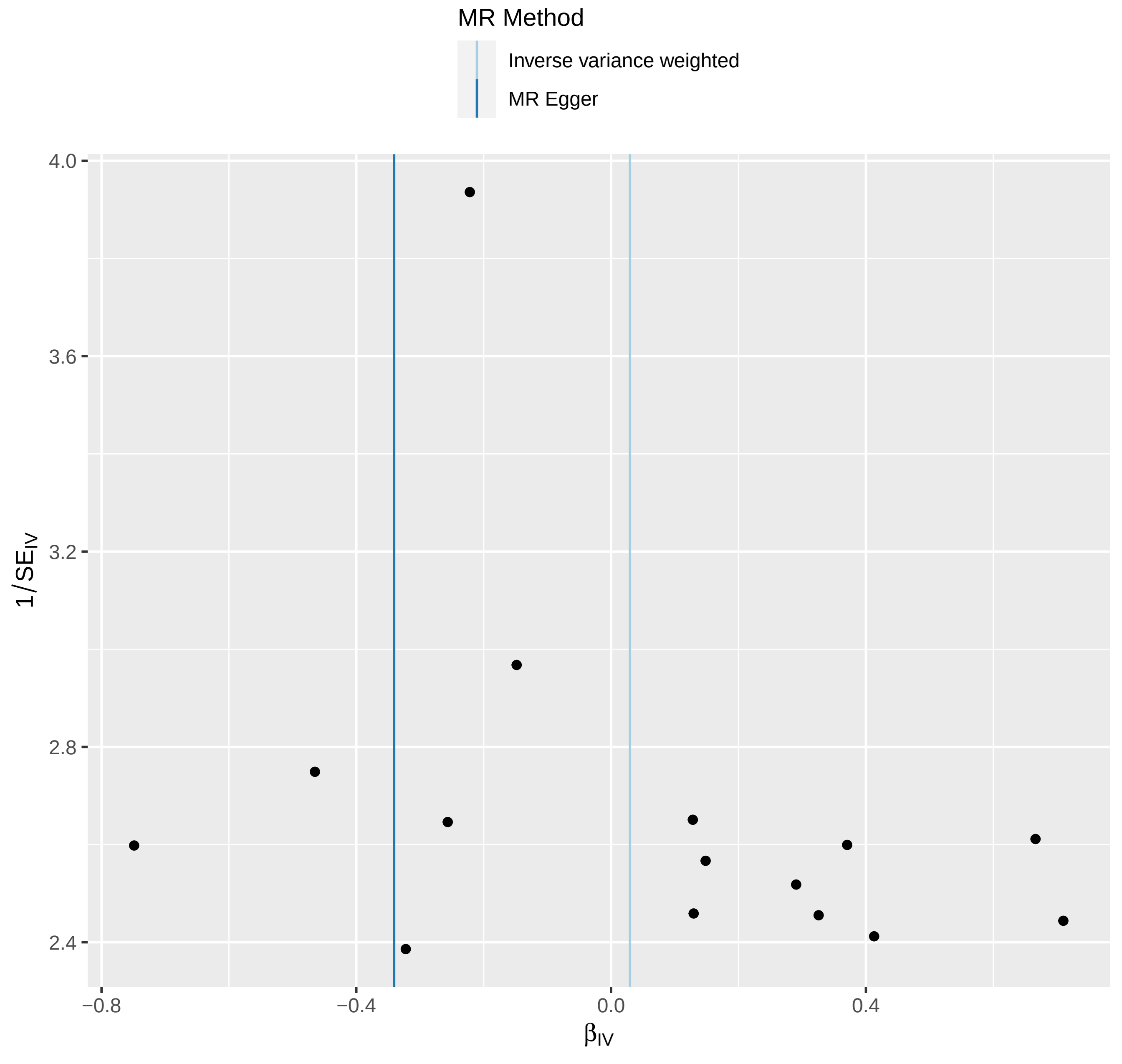

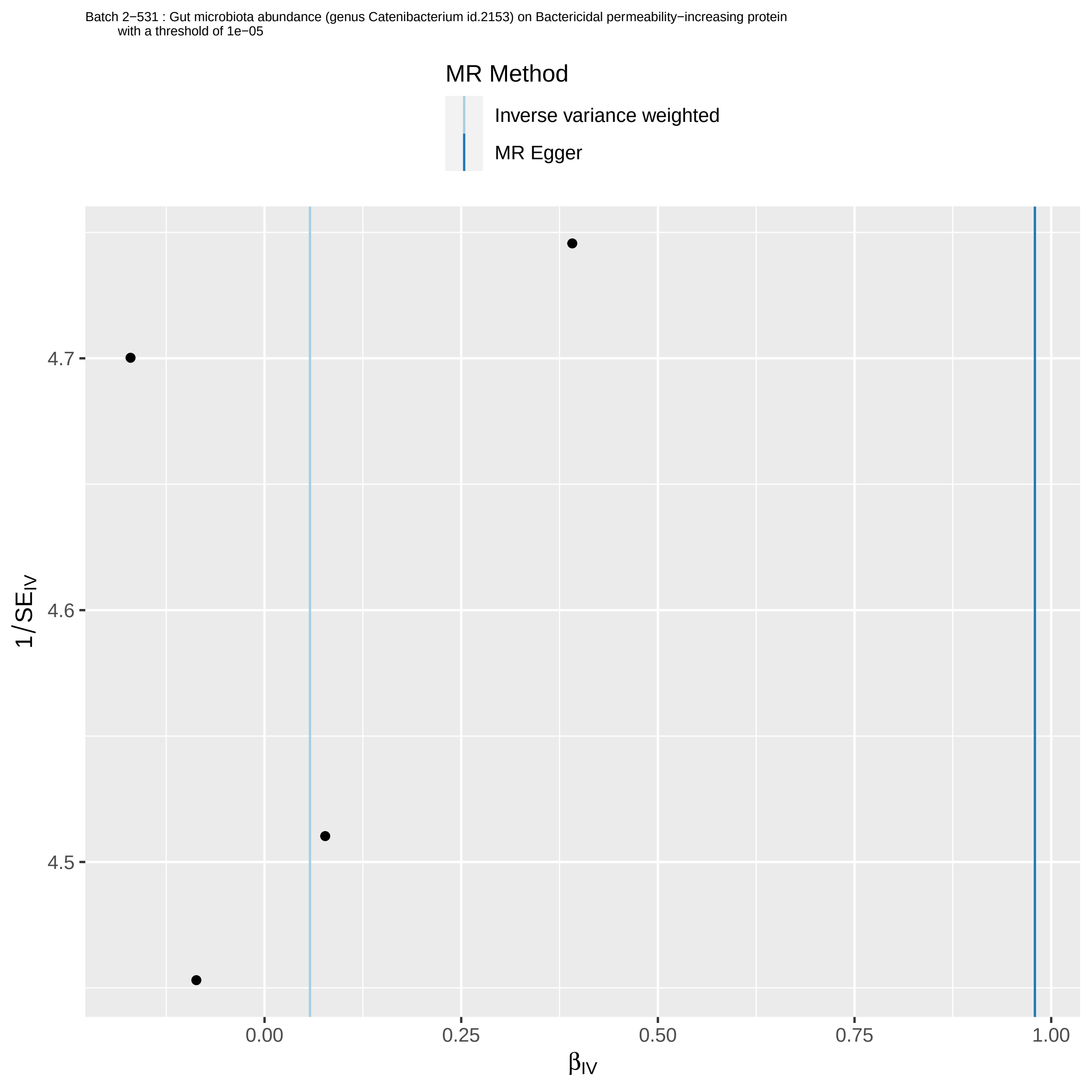

Batch 2−531 : Gut microbiota abundance (genus Catenibacterium id.2153) on Bactericidal permeability−increasing protein with a threshold of 1e−05
MR Method
Inverse variance weighted
MR Egger
4.7
4.6
4.5
1/SE_IV
0.00
0.25
0.50
0.75
1.00
β_IV

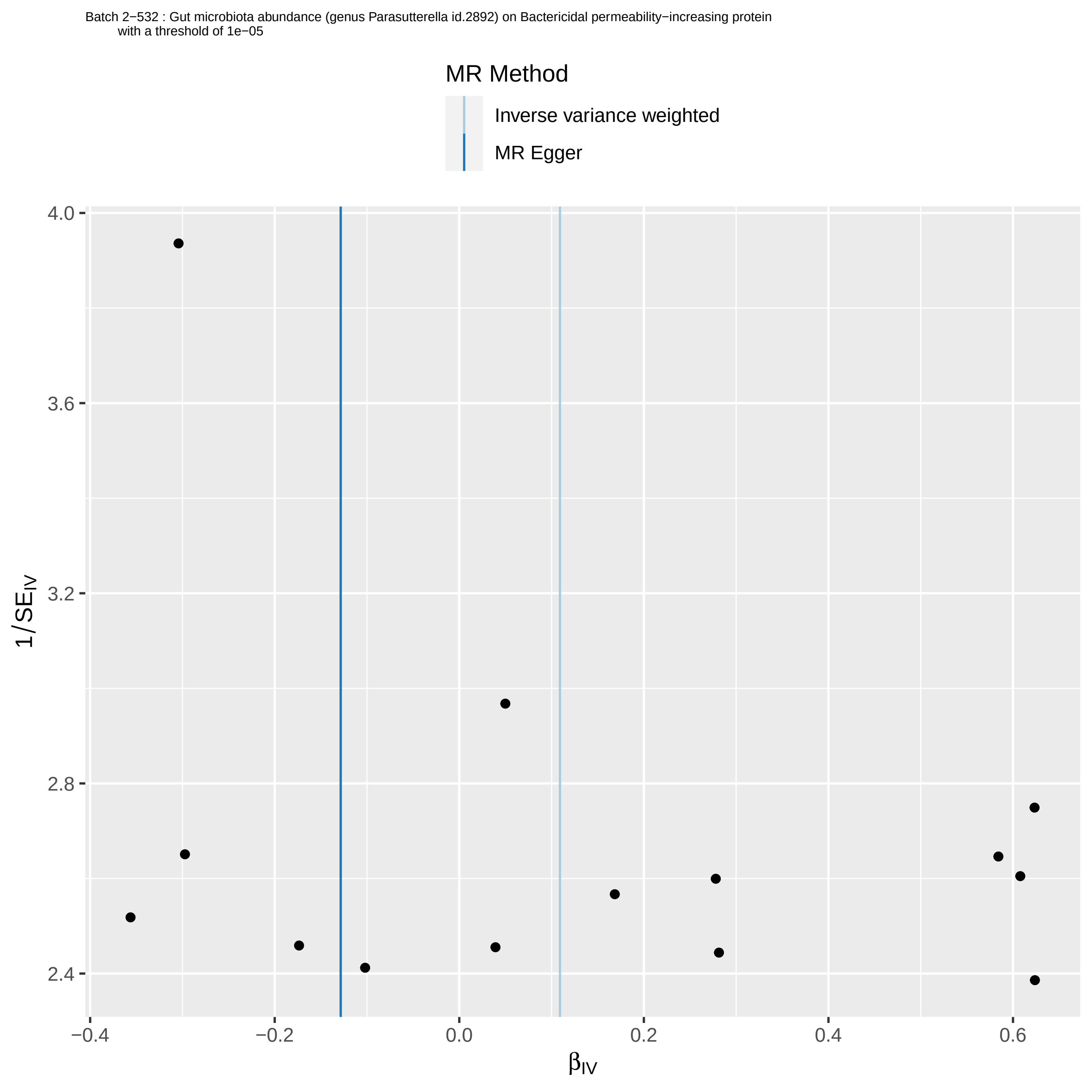

Batch 2−532 : Gut microbiota abundance (genus Parasutterella id.2892) on Bactericidal permeability−increasing protein
with a threshold of 1e−05
MR Method
Inverse variance weighted
MR Egger
4.0
3.6
3.2
2.8
2.4
1/SE_IV
−0.4
−0.2
0.0
0.2
0.4
0.6
β_IV

Batch 2−533 : Gut microbiota abundance (genus Catenibacterium id.2153) on BPI fold−containing family A member 1
with a threshold of 1e−05

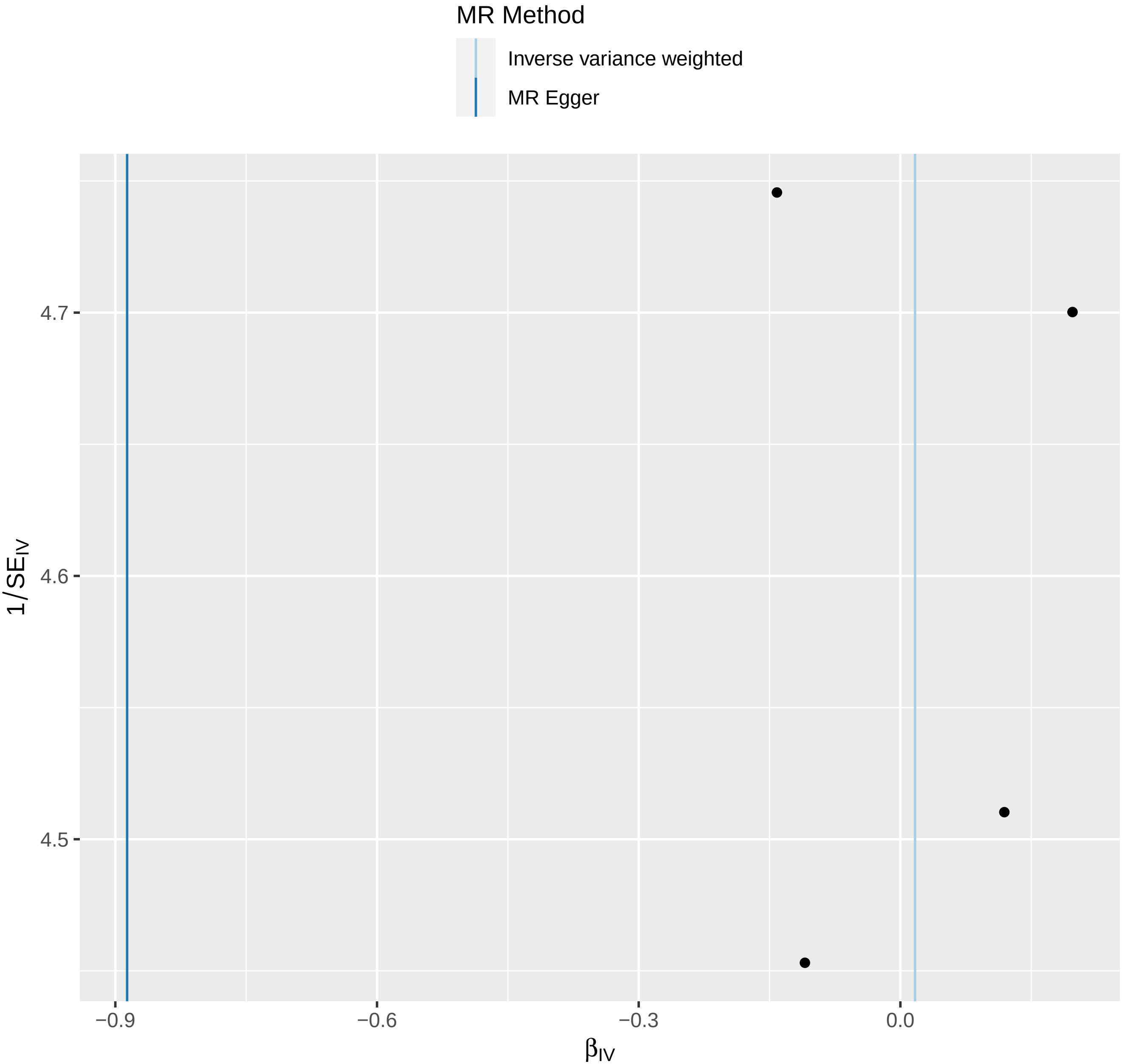

Batch 2−534 : Gut microbiota abundance (genus Parasutterella id.2892) on BPI fold−containing family A member 1
with a threshold of 1e−05

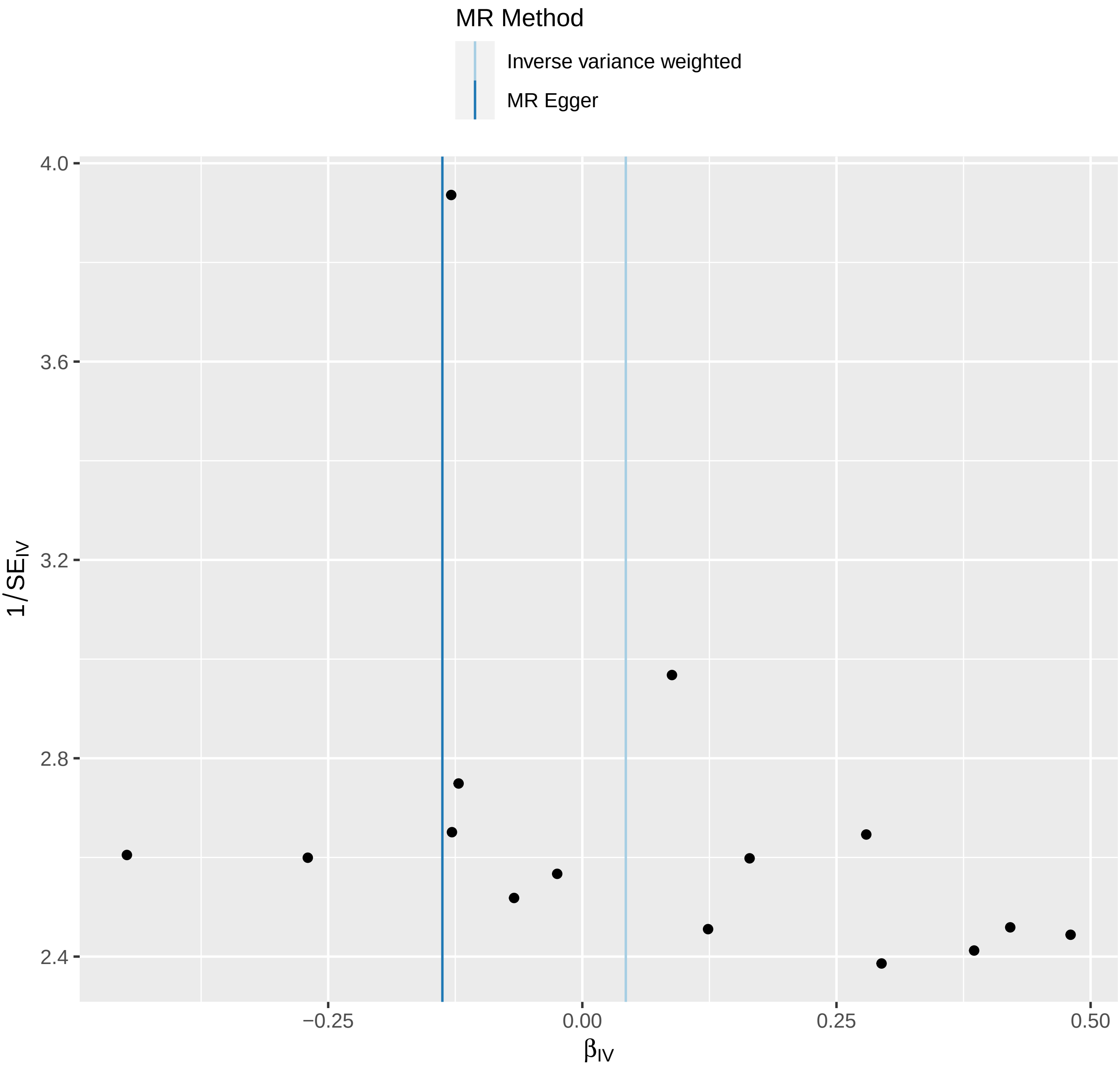

Batch 2−535 : Gut microbiota abundance (genus Catenibacterium id.2153) on BPI fold−containing family B member 1 with a threshold of 1e−05

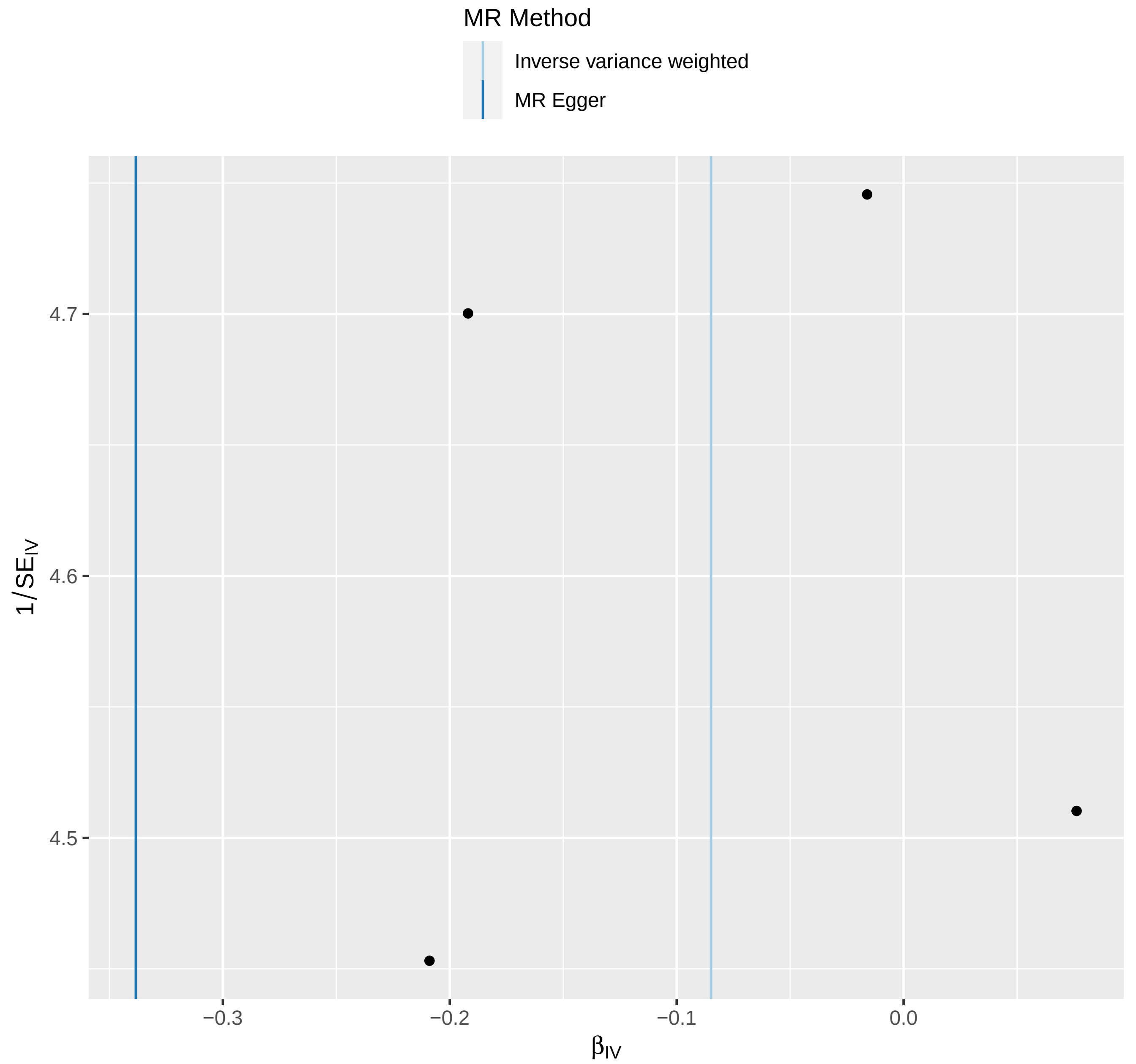

Batch 2−536 : Gut microbiota abundance (genus Parasutterella id.2892) on BPI fold−containing family B member 1
with a threshold of 1e−05

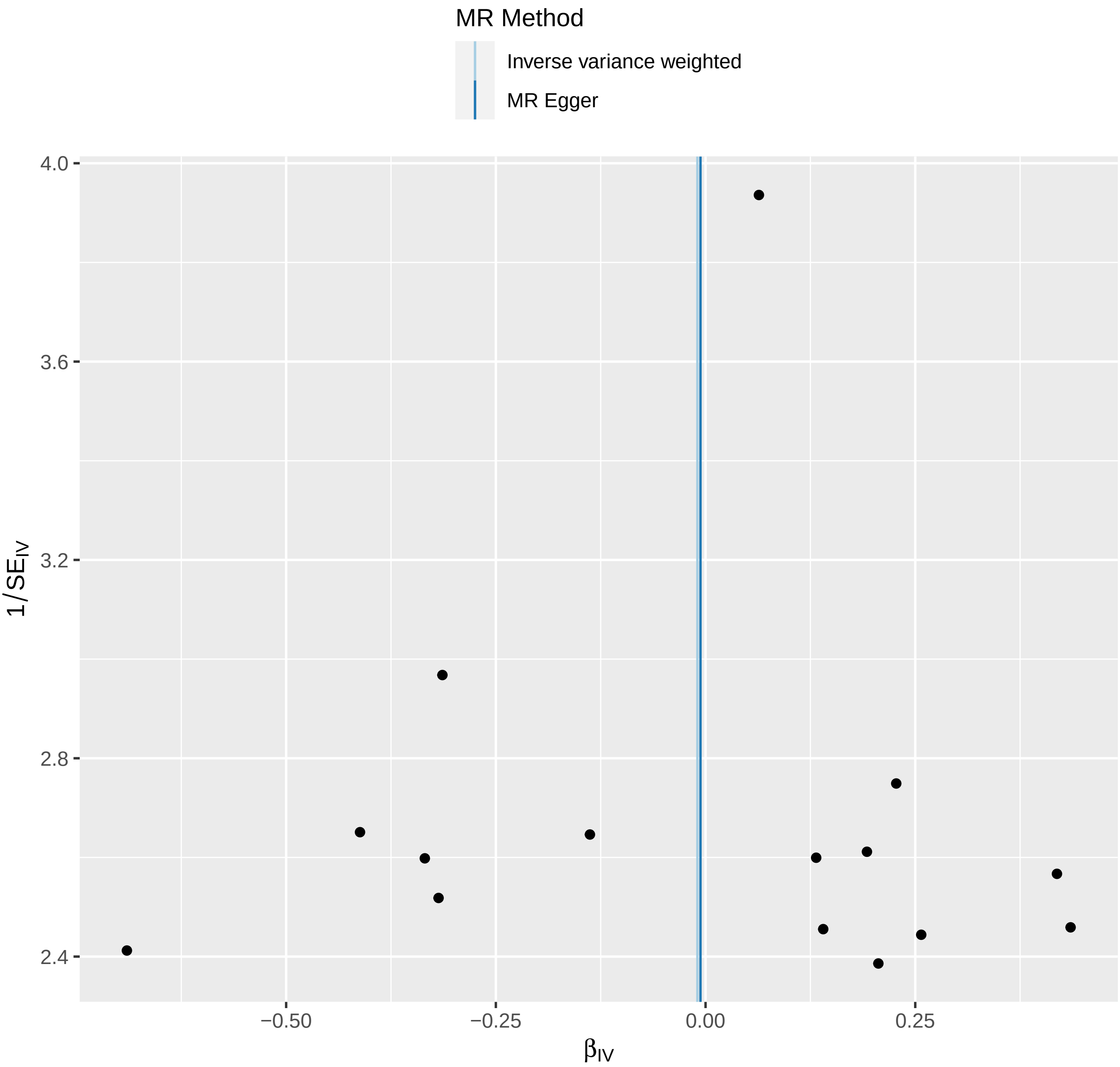

Batch 2−537 : Gut microbiota abundance (genus Catenibacterium id.2153) on Bromodomain−containing protein 1
with a threshold of 1e−05

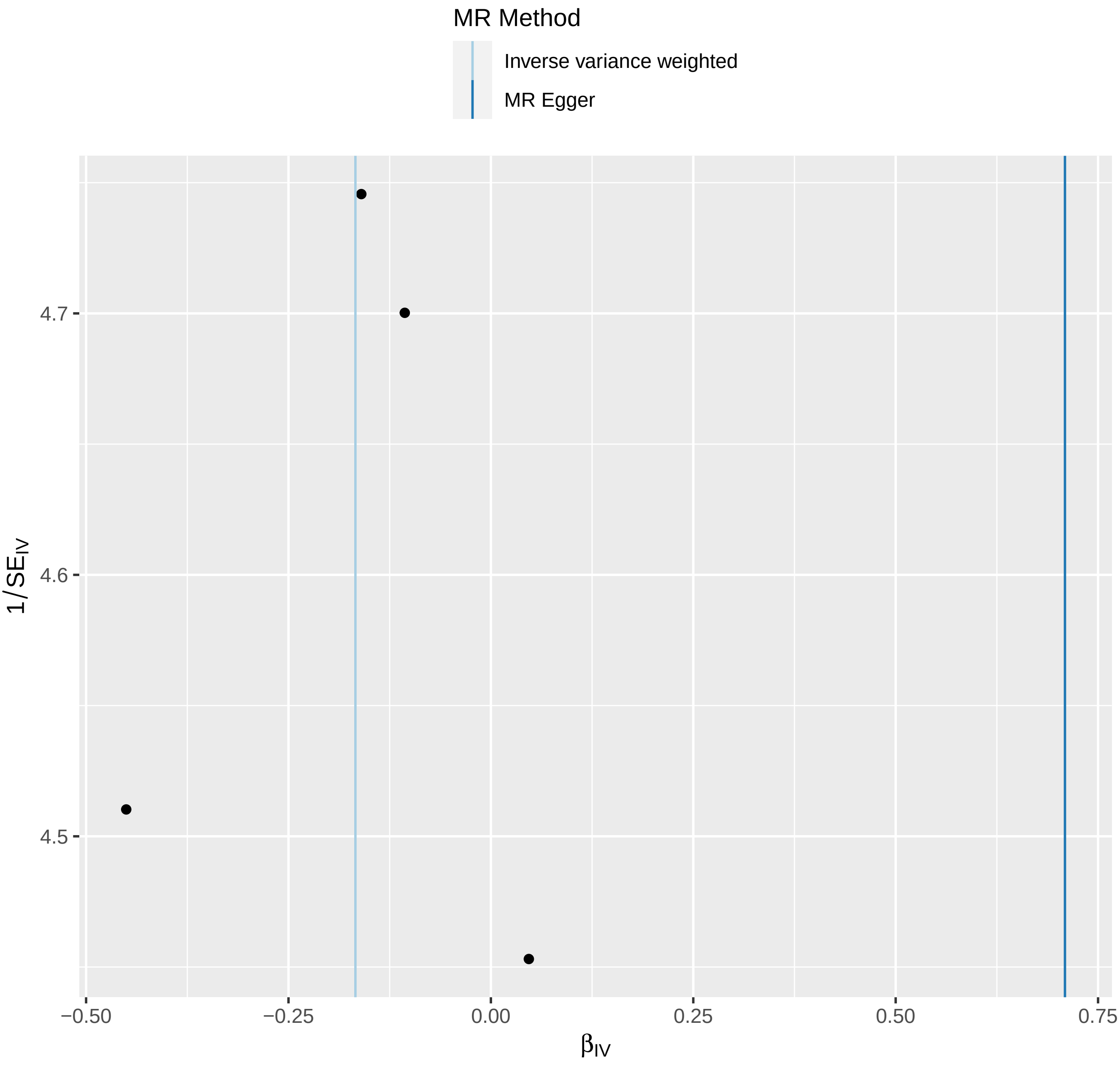

Batch 2−538 : Gut microbiota abundance (genus Parasutterella id.2892) on Bromodomain−containing protein 1
with a threshold of 1e−05

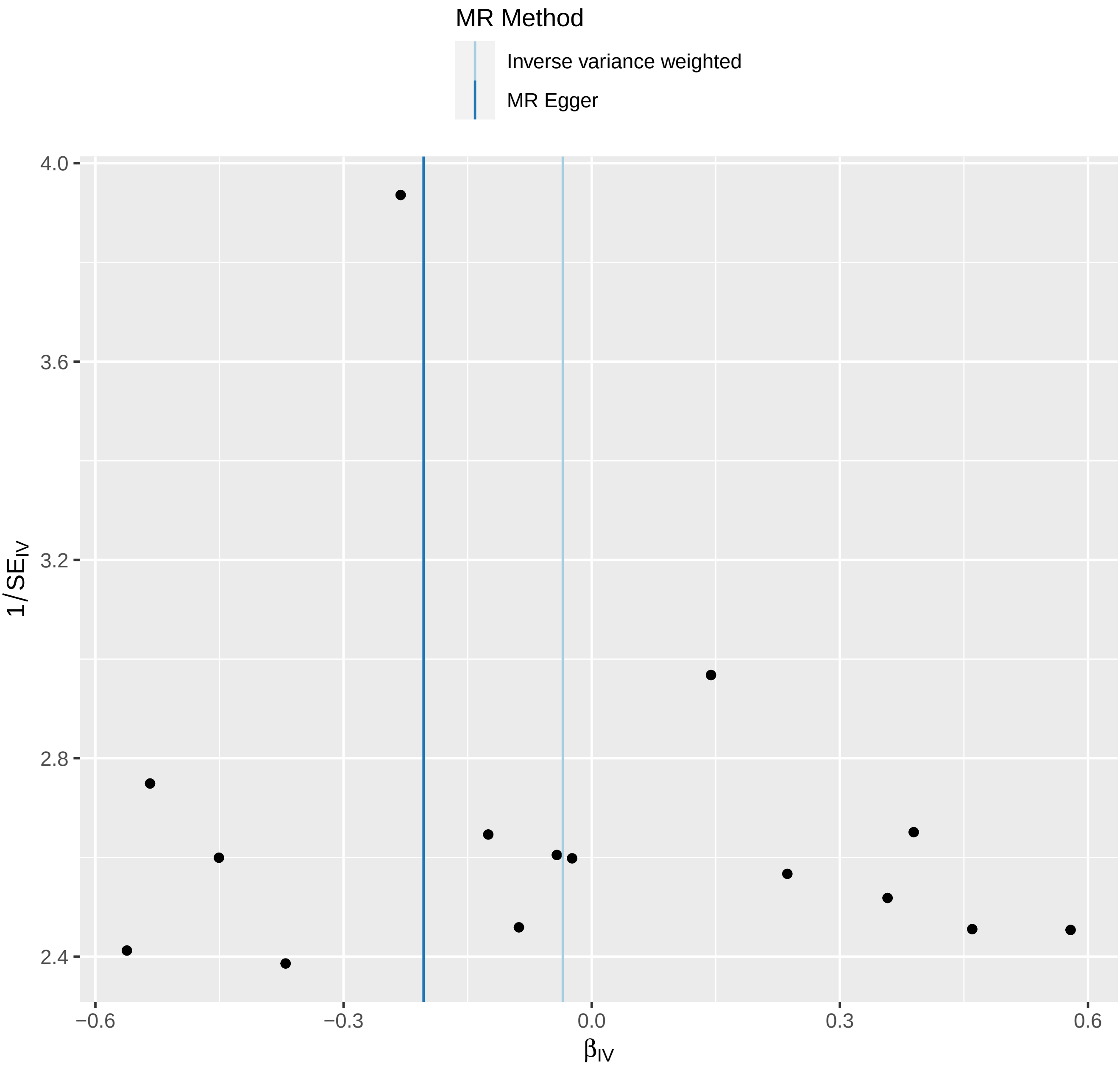

Batch 2−539 : Gut microbiota abundance (genus Catenibacterium id.2153) on Bromodomain testis−specific protein
with a threshold of 1e−05

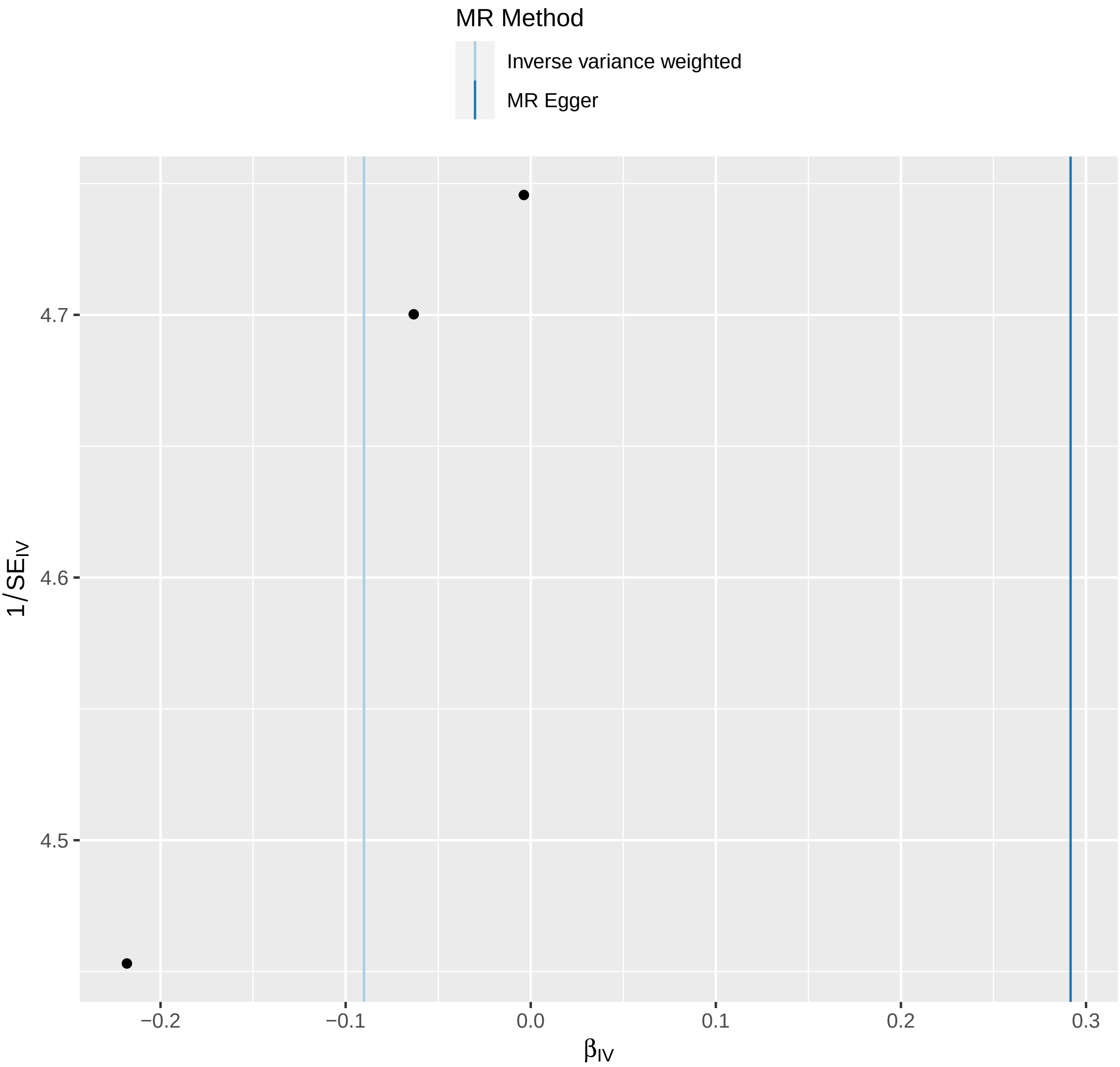

Batch 2−540 : Gut microbiota abundance (genus Parasutterella id.2892) on Bromodomain testis−specific protein
with a threshold of 1e−05

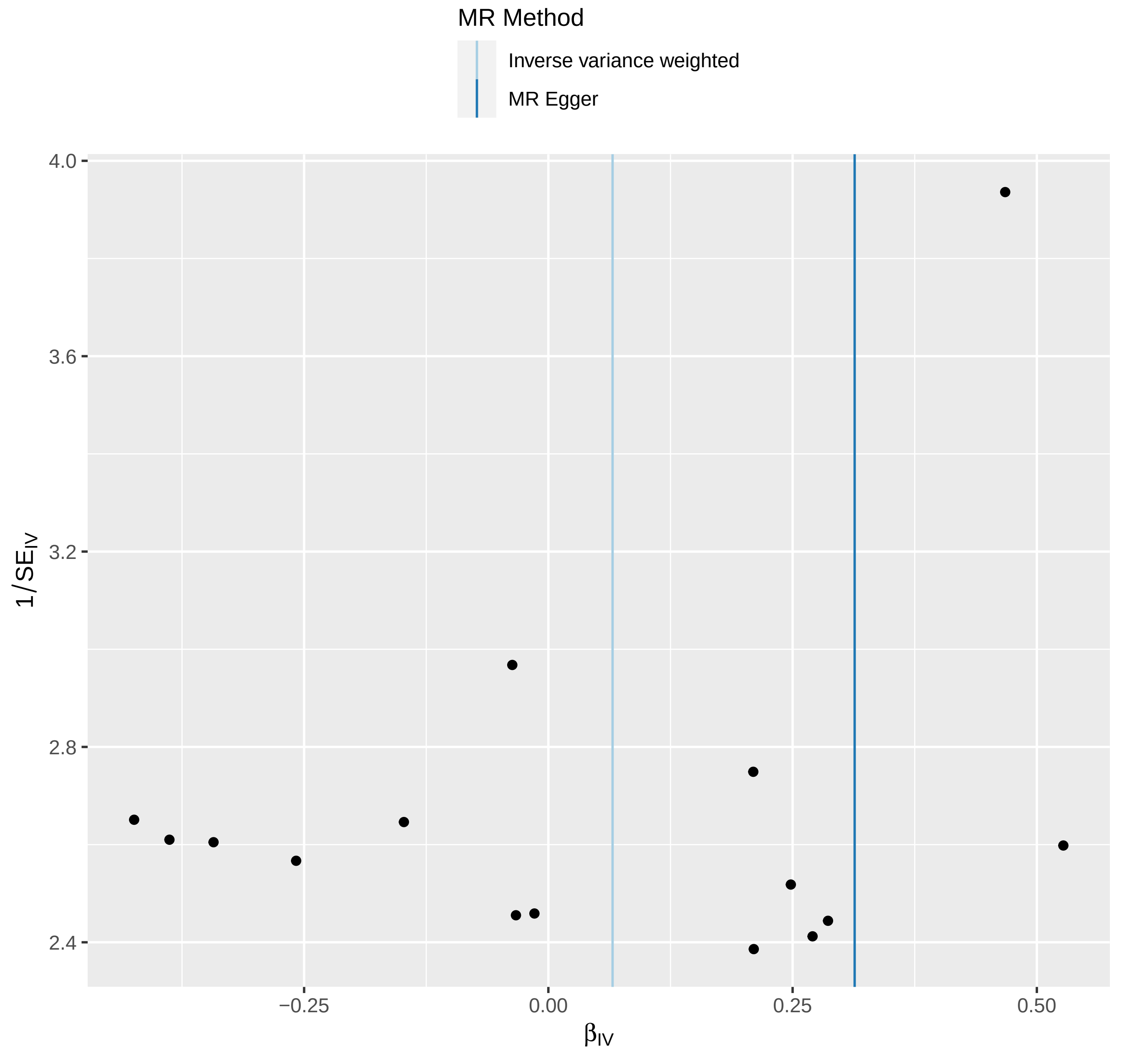

Batch 2−541 : Gut microbiota abundance (genus Catenibacterium id.2153) on Transcription factor IIIB 90 kDa subunit
with a threshold of 1e−05

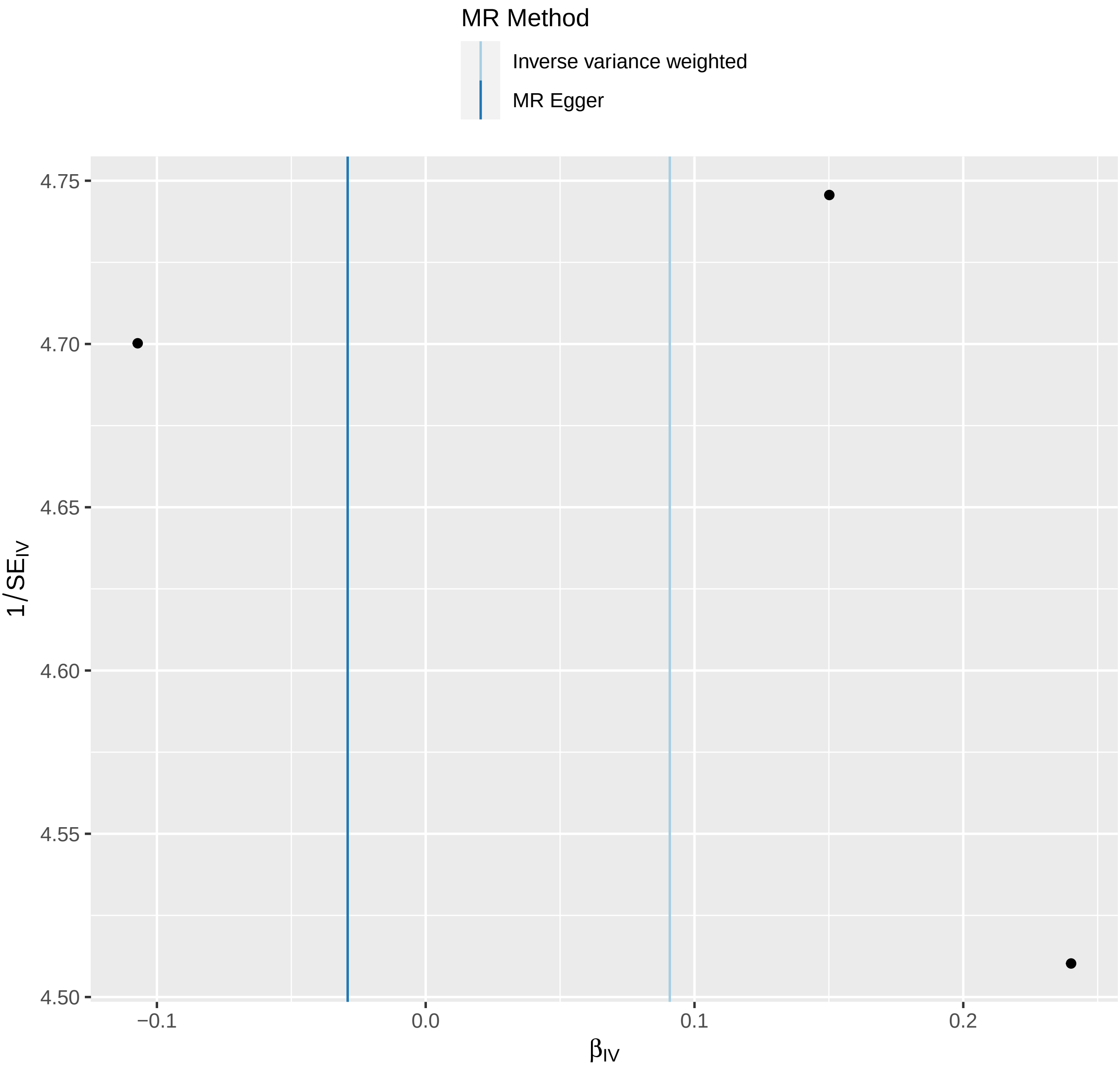

Batch 2−542 : Gut microbiota abundance (genus Parasutterella id.2892) on Transcription factor IIIB 90 kDa subunit
with a threshold of 1e−05

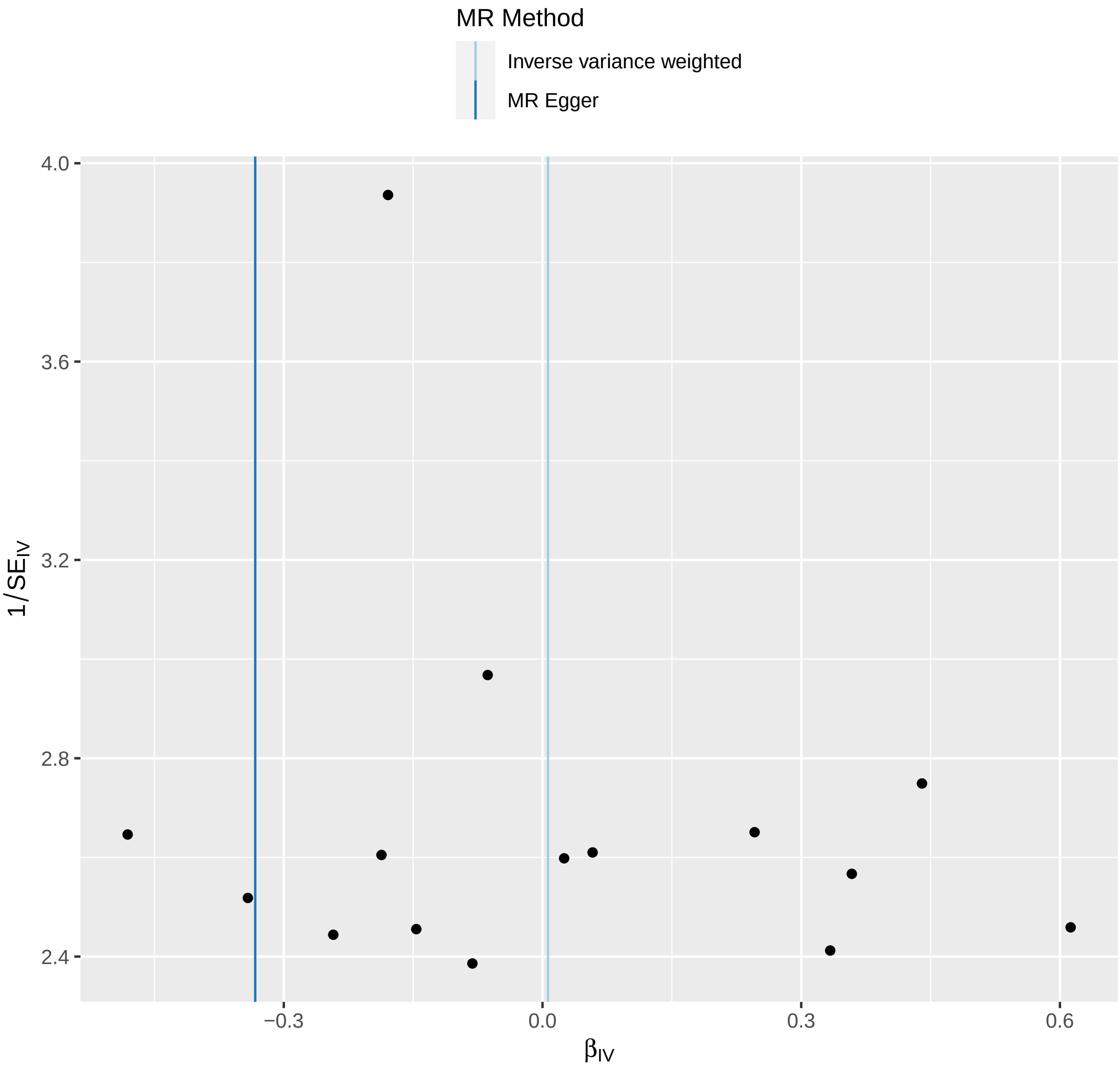

Batch 2−543 : Gut microbiota abundance (genus Catenibacterium id.2153) on BRICHOS domain−containing protein 5
with a threshold of 1e−05

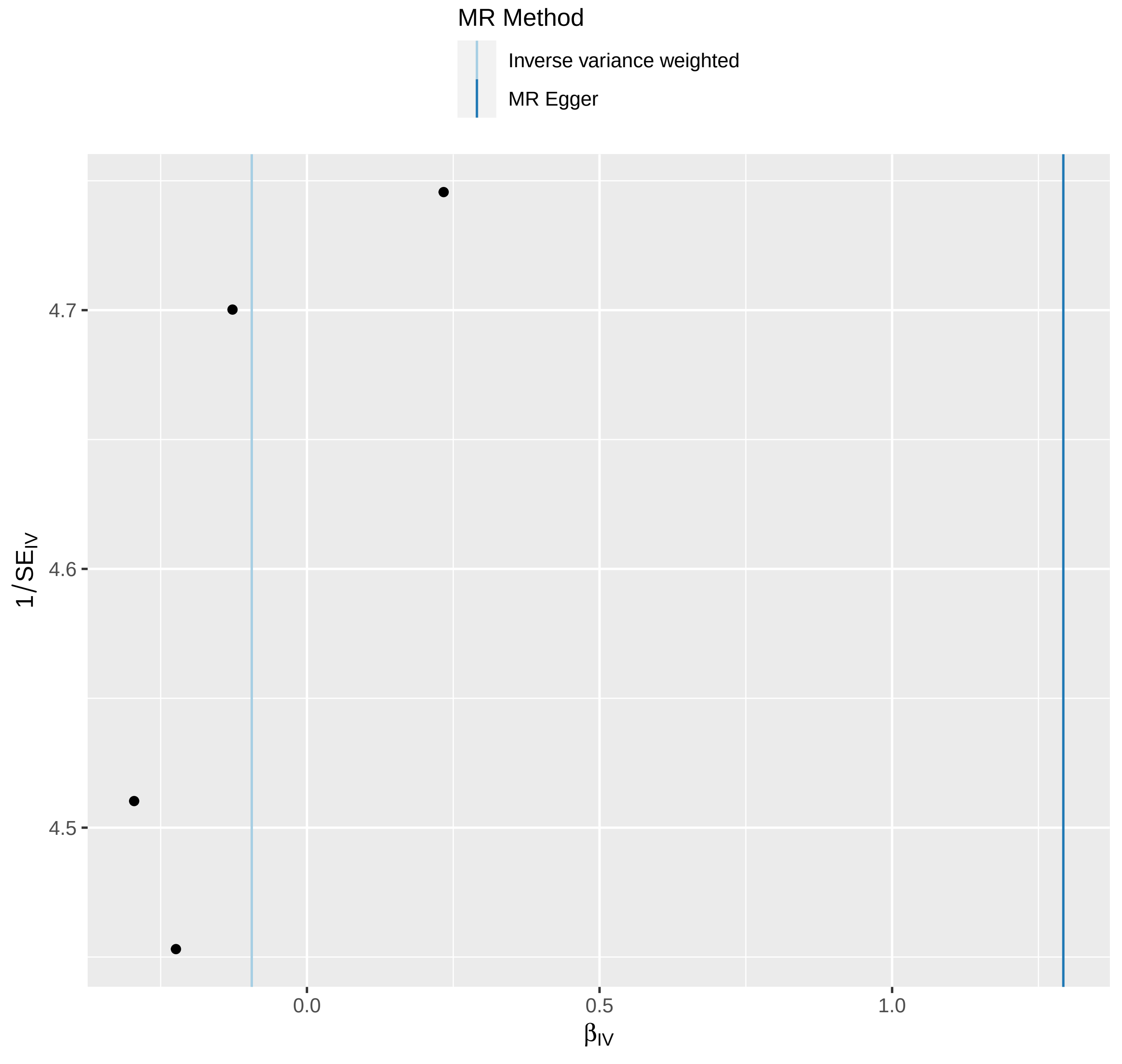

Batch 2−544 : Gut microbiota abundance (genus Parasutterella id.2892) on BRICHOS domain−containing protein 5
with a threshold of 1e−05

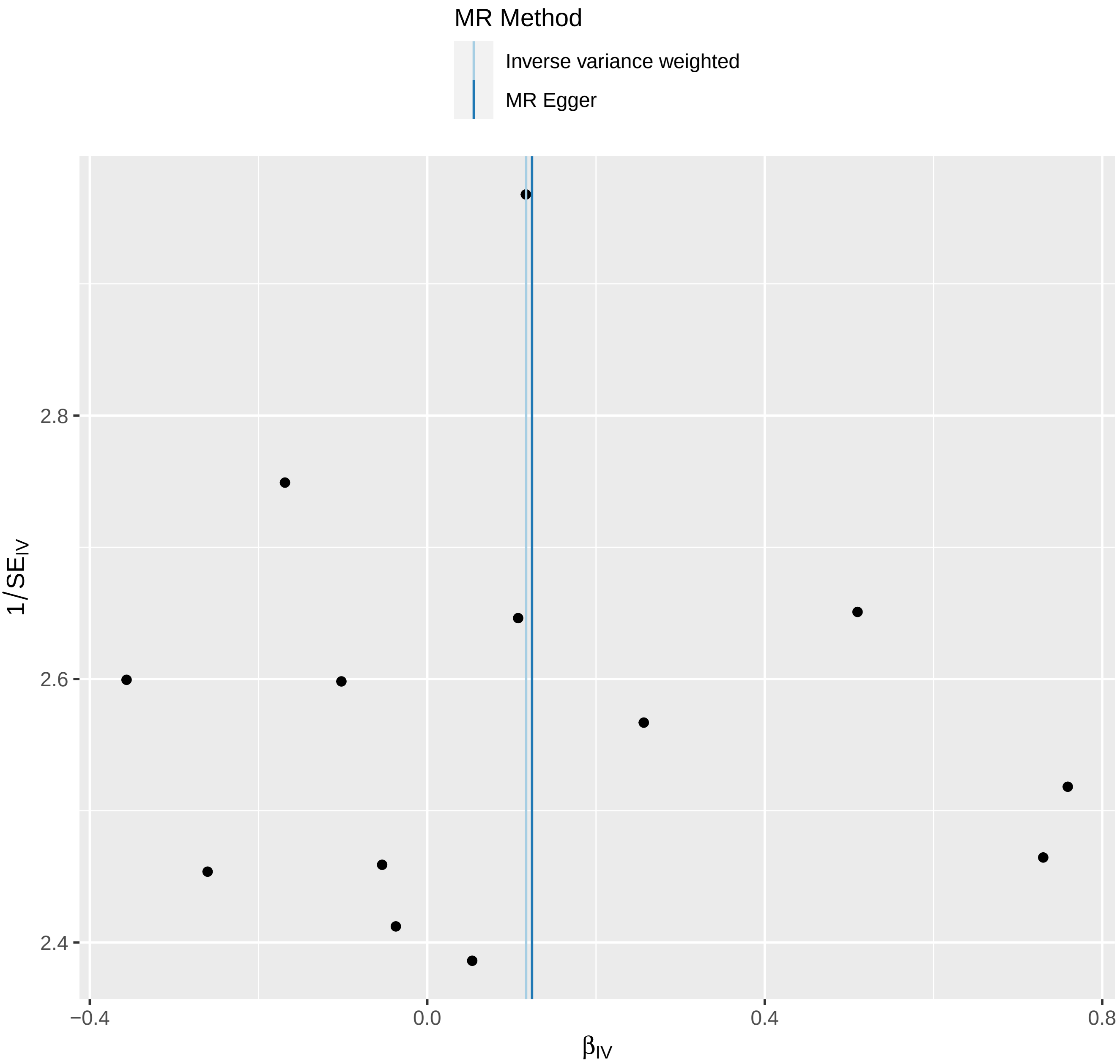

Batch 2−545 : Gut microbiota abundance (genus Catenibacterium id.2153) on Peregrin
with a threshold of 1e−05

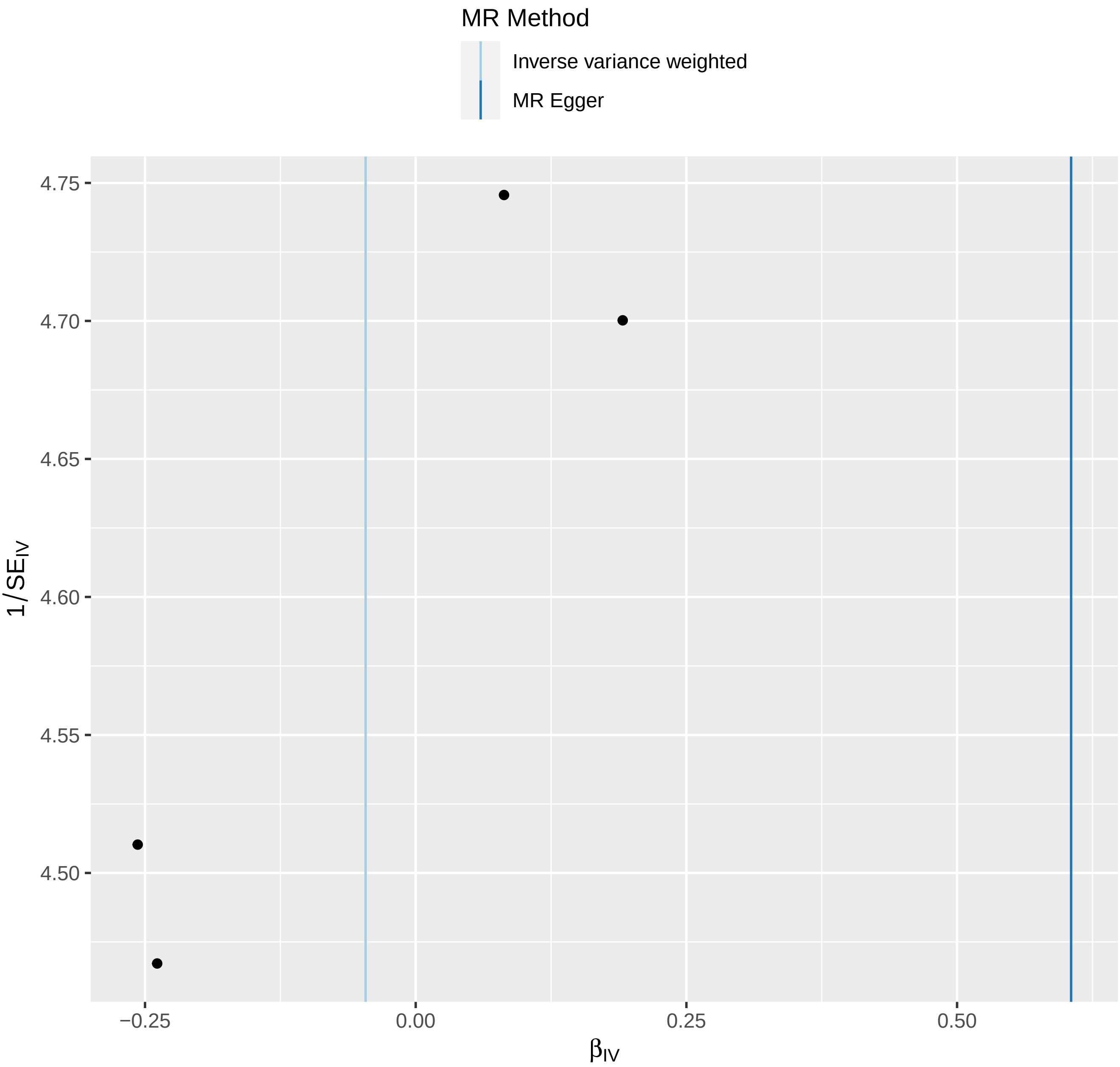

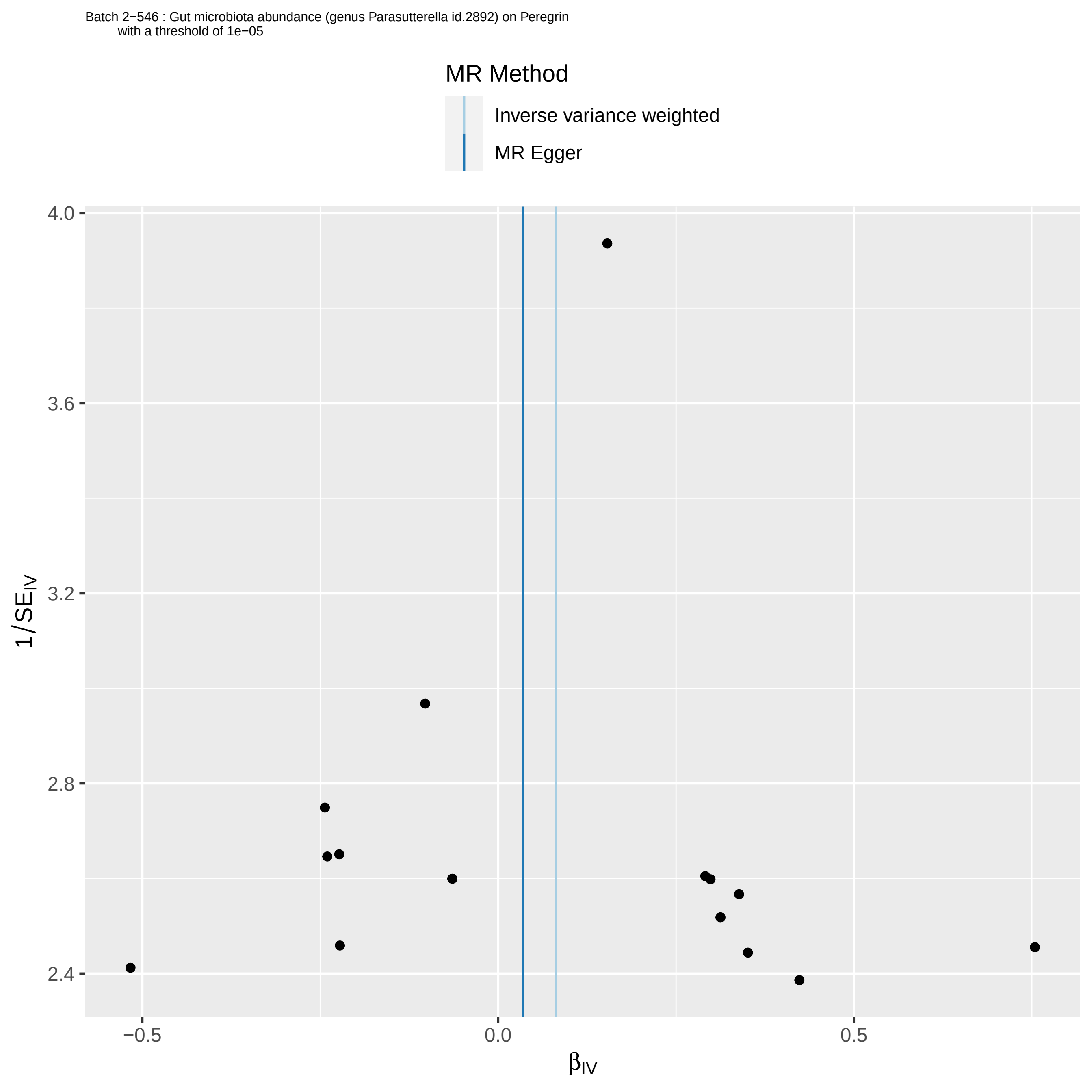

Batch 2−546 : Gut microbiota abundance (genus Parasutterella id.2892) on Peregrin
with a threshold of 1e−05
MR Method
Inverse variance weighted
MR Egger
4.0
3.6
3.2
2.8
2.4
1/SE_IV
−0.5
0.0
0.5
β_IV

Batch 2−547 : Gut microbiota abundance (genus Catenibacterium id.2153) on Serine/threonine−protein kinase BRSK2
with a threshold of 1e−05

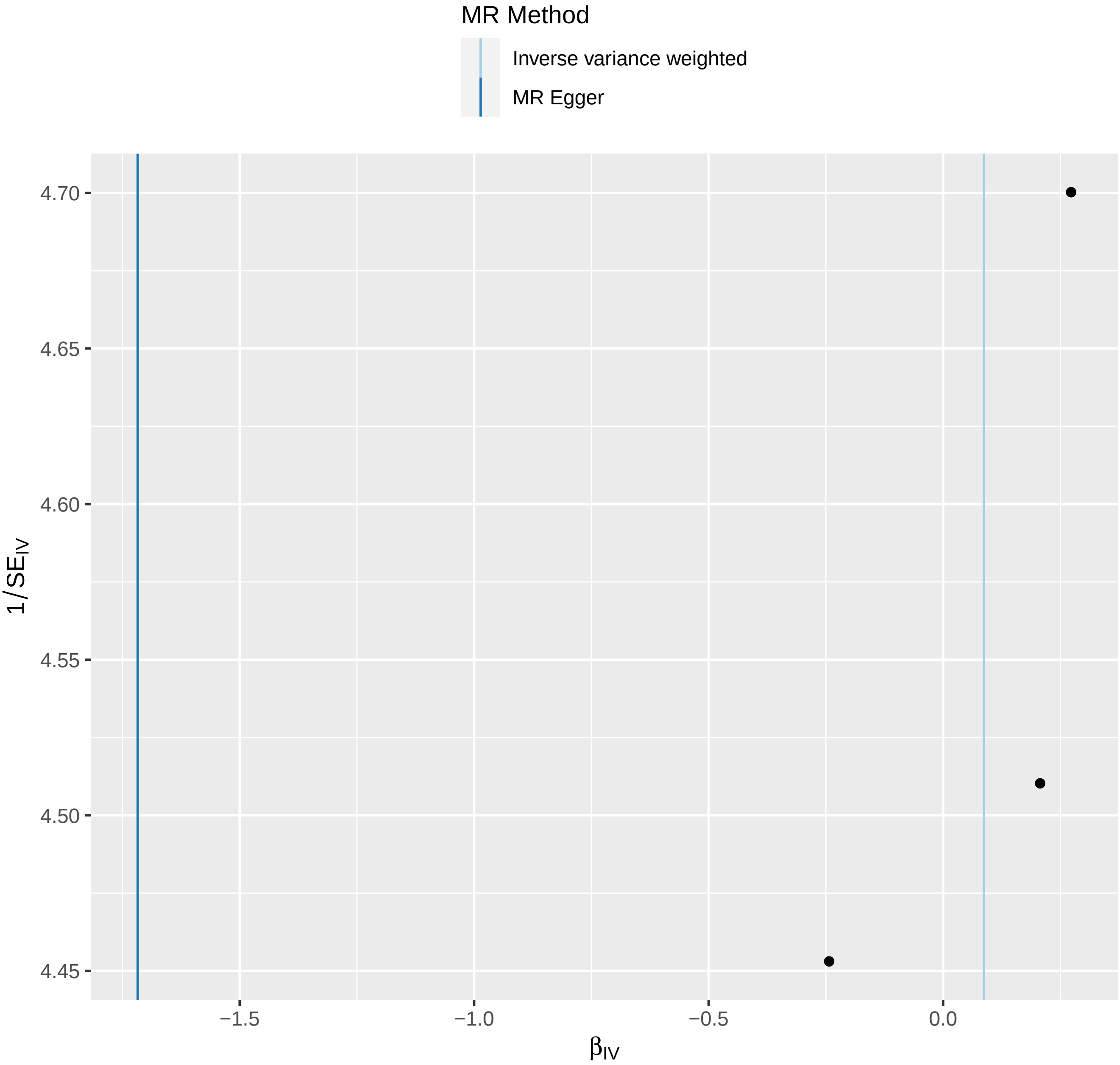

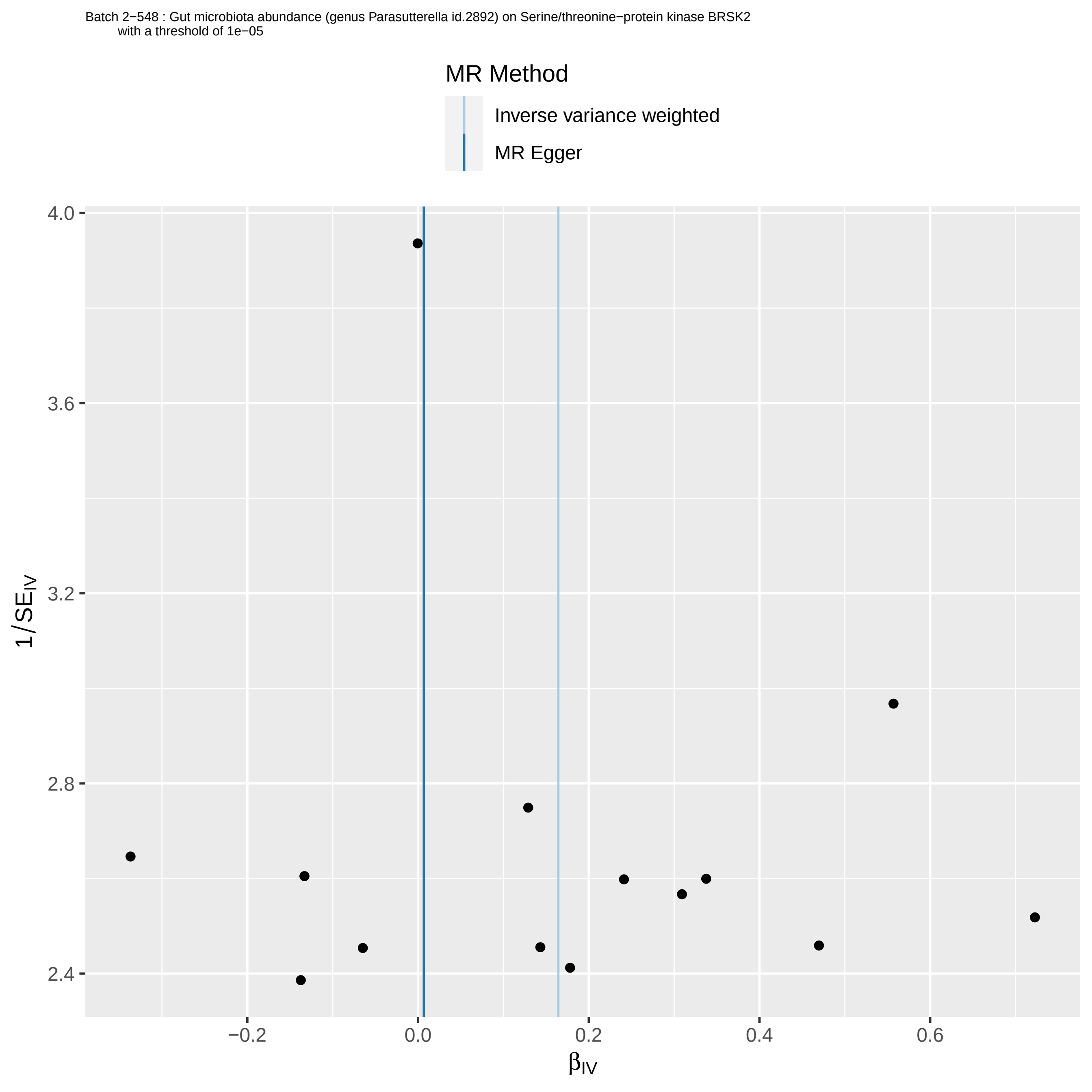

Batch 2−548 : Gut microbiota abundance (genus Parasutterella id.2892) on Serine/threonine−protein kinase BRSK2
with a threshold of 1e−05
MR Method
Inverse variance weighted
MR Egger
4.0
3.6
3.2
2.8
2.4
1/SE_IV
−0.2
0.0
0.2
0.4
0.6
β_IV

Batch 2−549 : Gut microbiota abundance (genus Catenibacterium id.2153) on Basigin
with a threshold of 1e−05

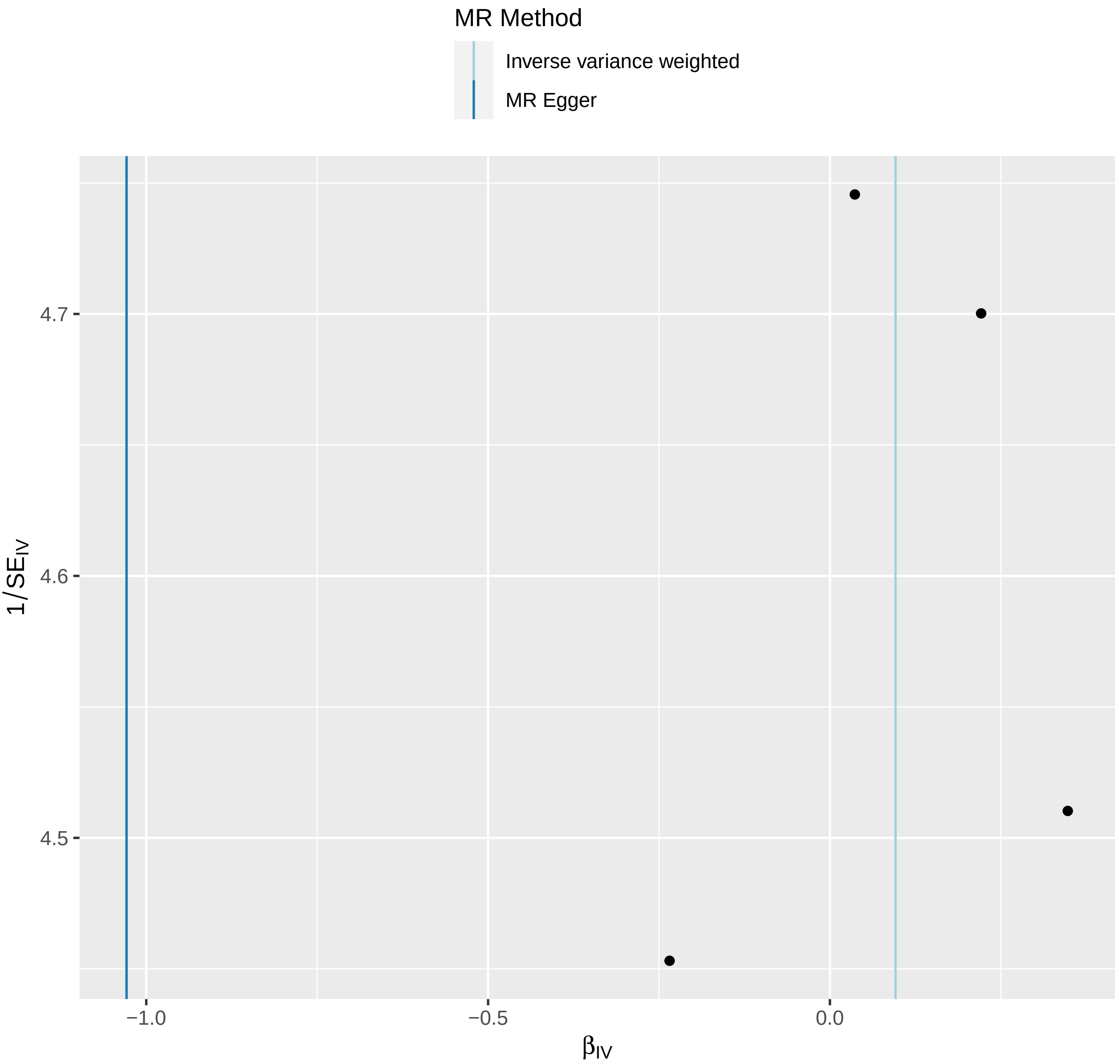

Batch 2−550 : Gut microbiota abundance (genus Parasutterella id.2892) on Basigin
with a threshold of 1e−05

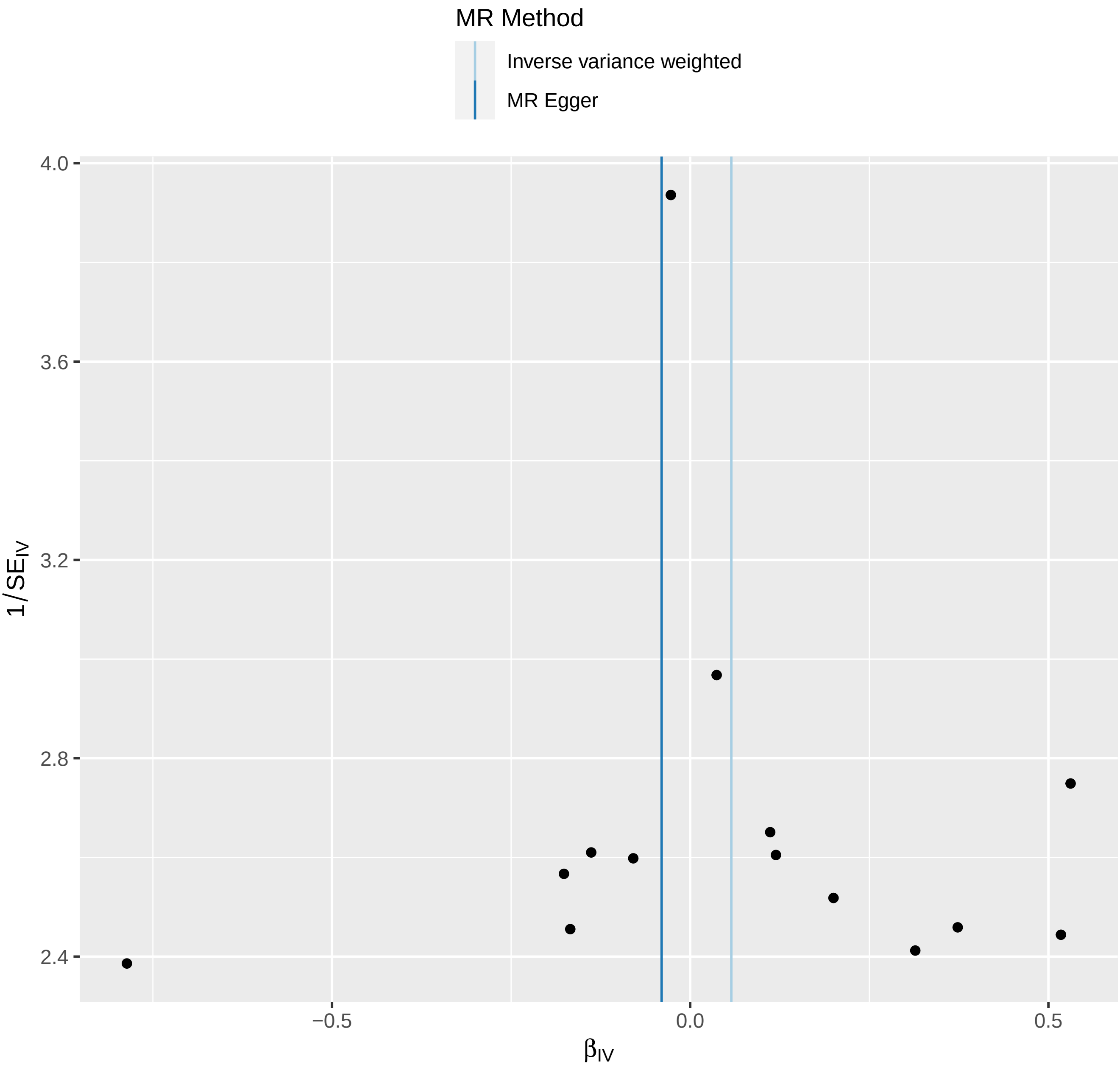

Batch 2−551 : Gut microbiota abundance (genus Catenibacterium id.2153) on ADP−ribosyl cyclase/cyclic ADP−ribose hydrolase 2
with a threshold of 1e−05

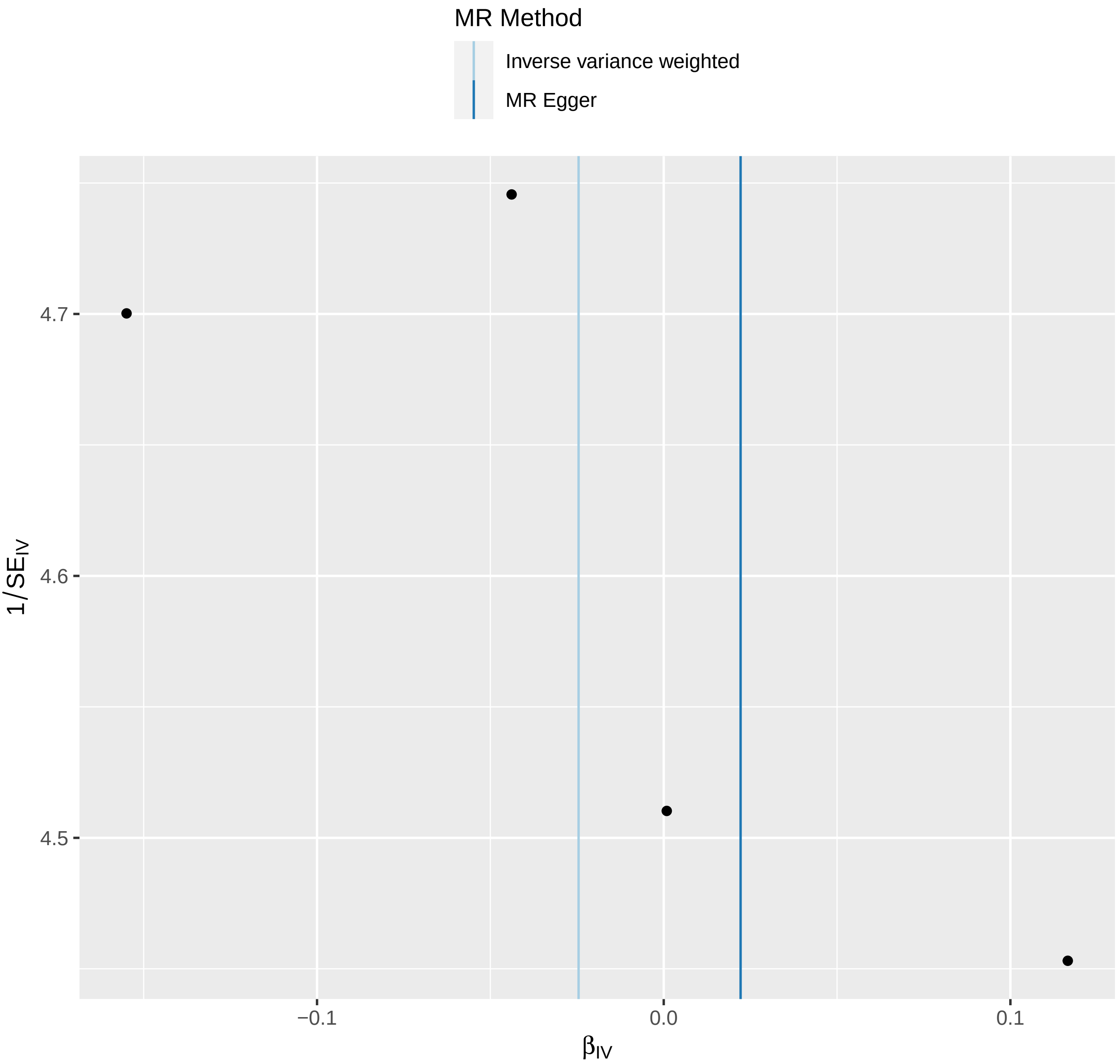

Batch 2−552 : Gut microbiota abundance (genus Parasutterella id.2892) on ADP−ribosyl cyclase/cyclic ADP−ribose hydrolase 2
with a threshold of 1e−05

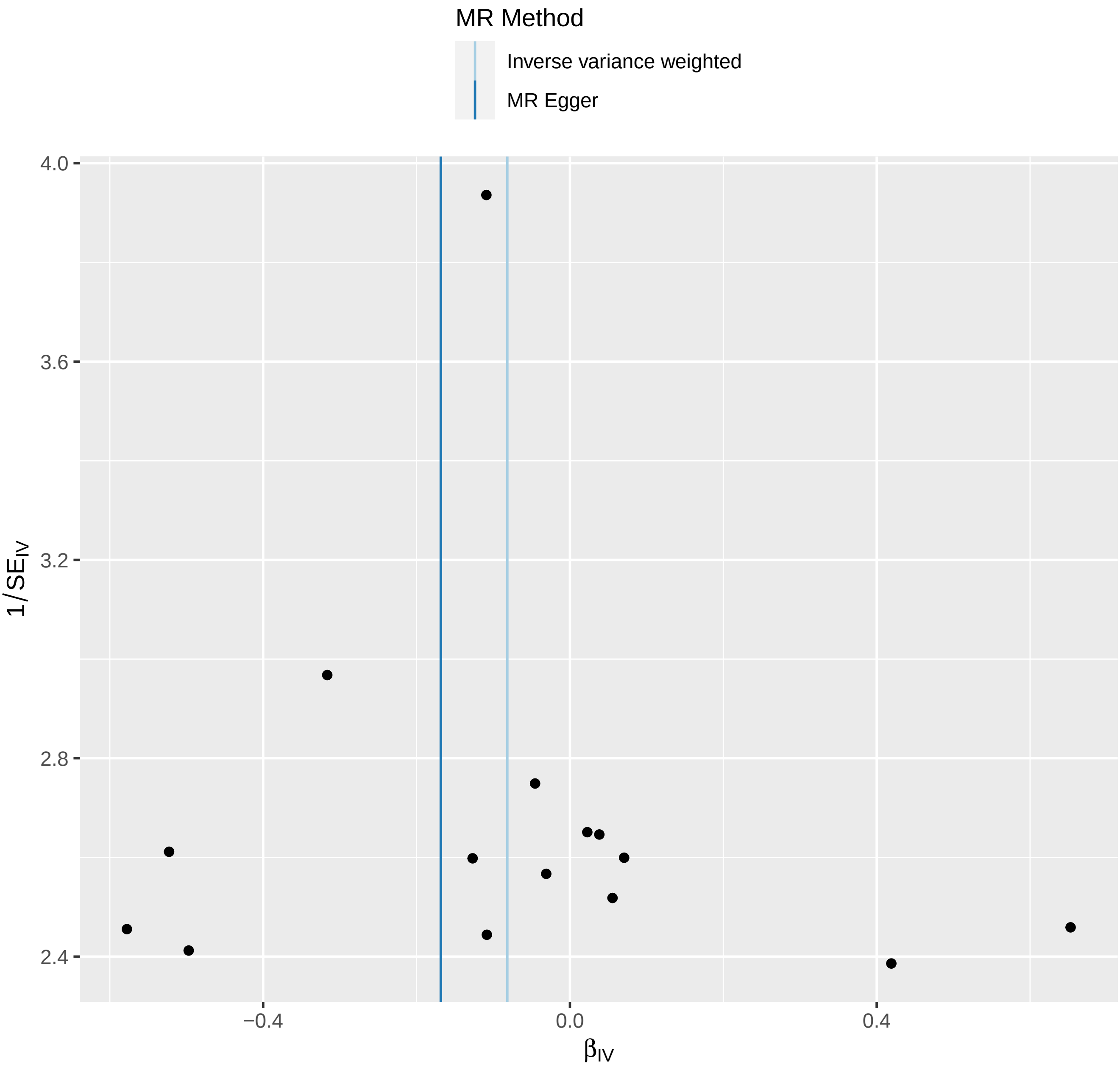

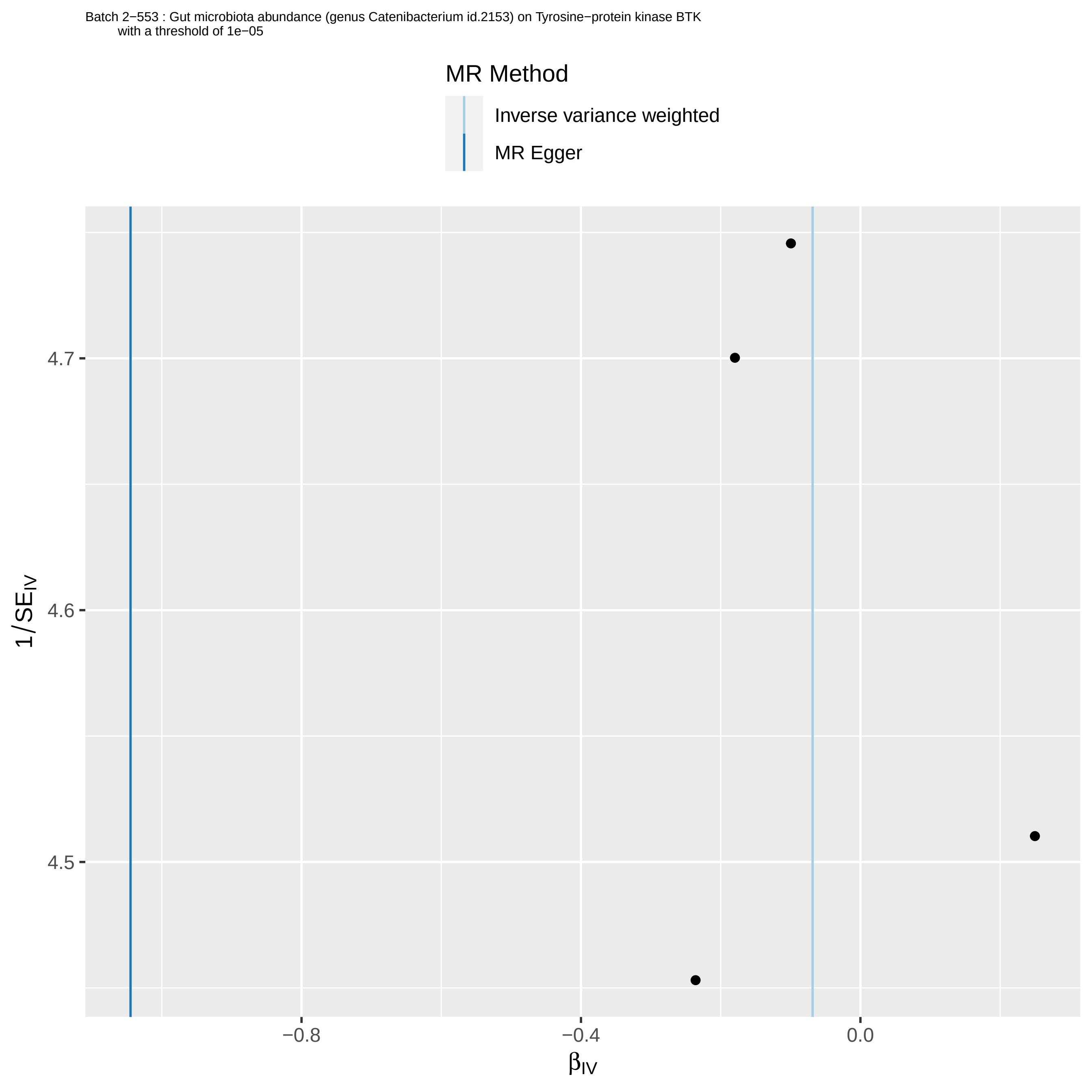
Batch 2−553 : Gut microbiota abundance (genus Catenibacterium id.2153) on Tyrosine−protein kinase BTK
with a threshold of 1e−05
MR Method
Inverse variance weighted
MR Egger
4.7
4.6
4.5
1/SE$_{IV}$
−0.8
−0.4
0.0
β_{IV}

Batch 2−554 : Gut microbiota abundance (genus Parasutterella id.2892) on Tyrosine−protein kinase BTK
with a threshold of 1e−05

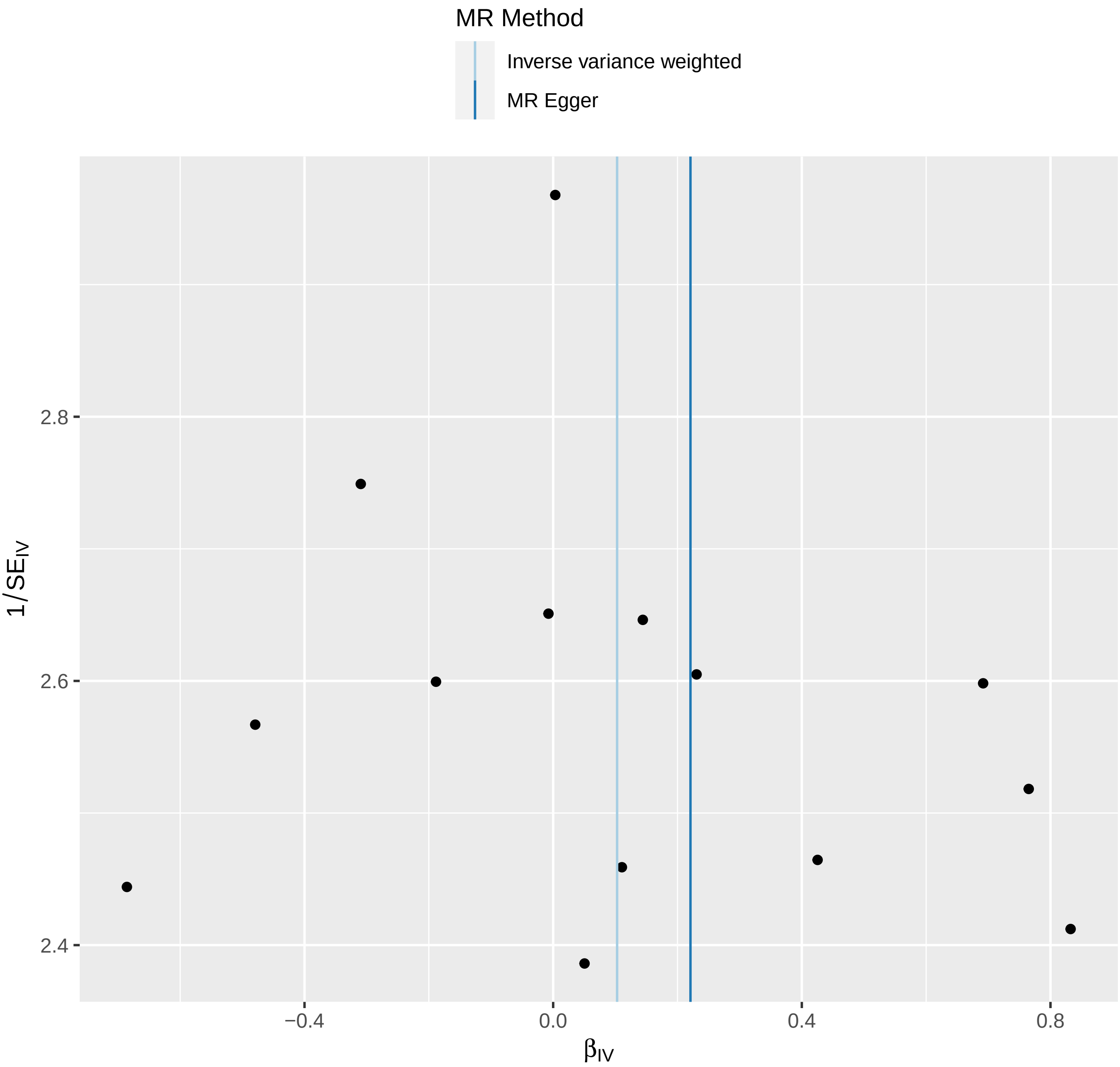

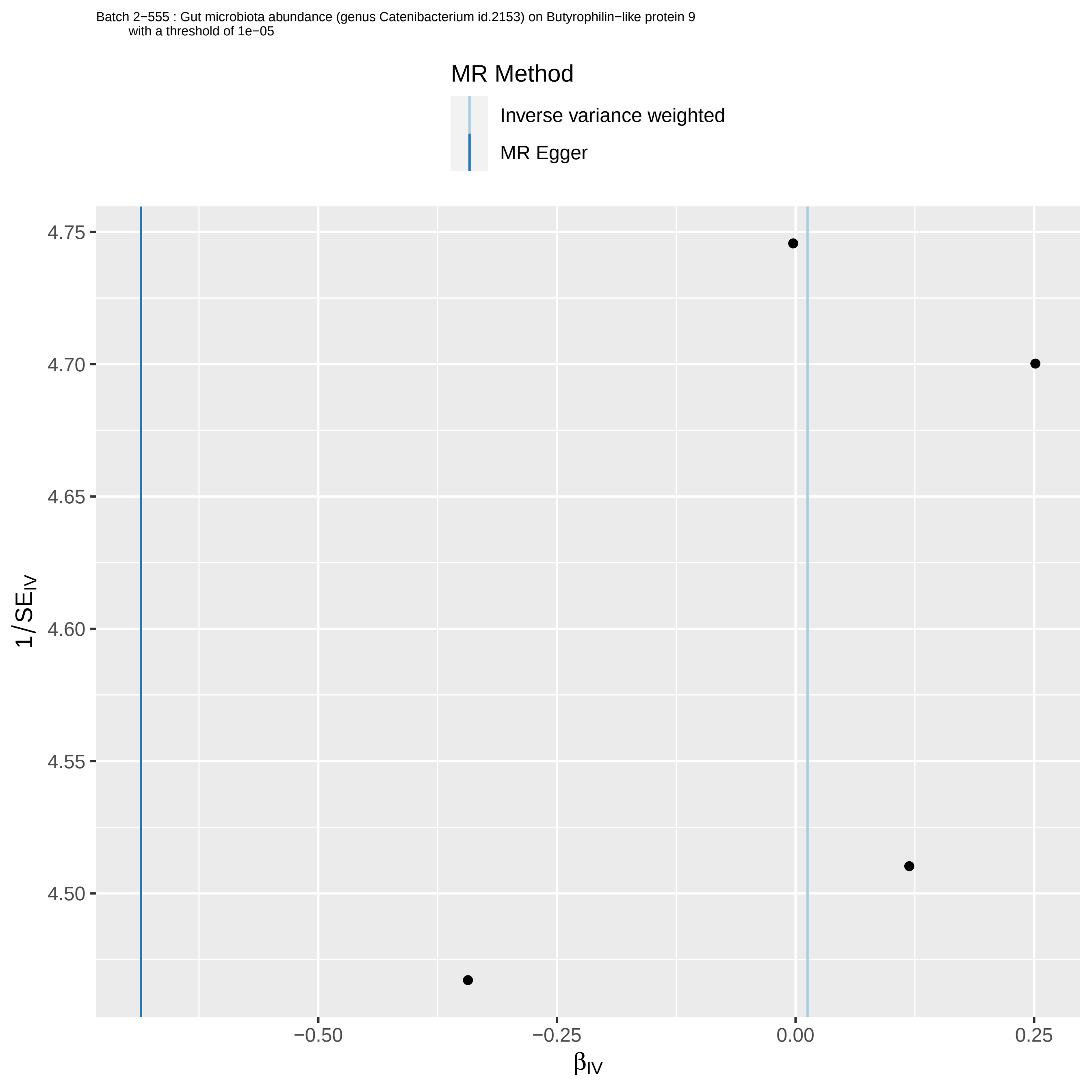
Batch 2−555 : Gut microbiota abundance (genus Catenibacterium id.2153) on Butyrophilin−like protein 9
with a threshold of 1e−05
MR Method
Inverse variance weighted
MR Egger
4.75
4.70
4.65
4.60
4.55
4.50
1/SE$_{IV}$
−0.50
−0.25
0.00
0.25
β$_{IV}$

Batch 2−556 : Gut microbiota abundance (genus Parasutterella id.2892) on Butyrophilin−like protein 9
with a threshold of 1e−05

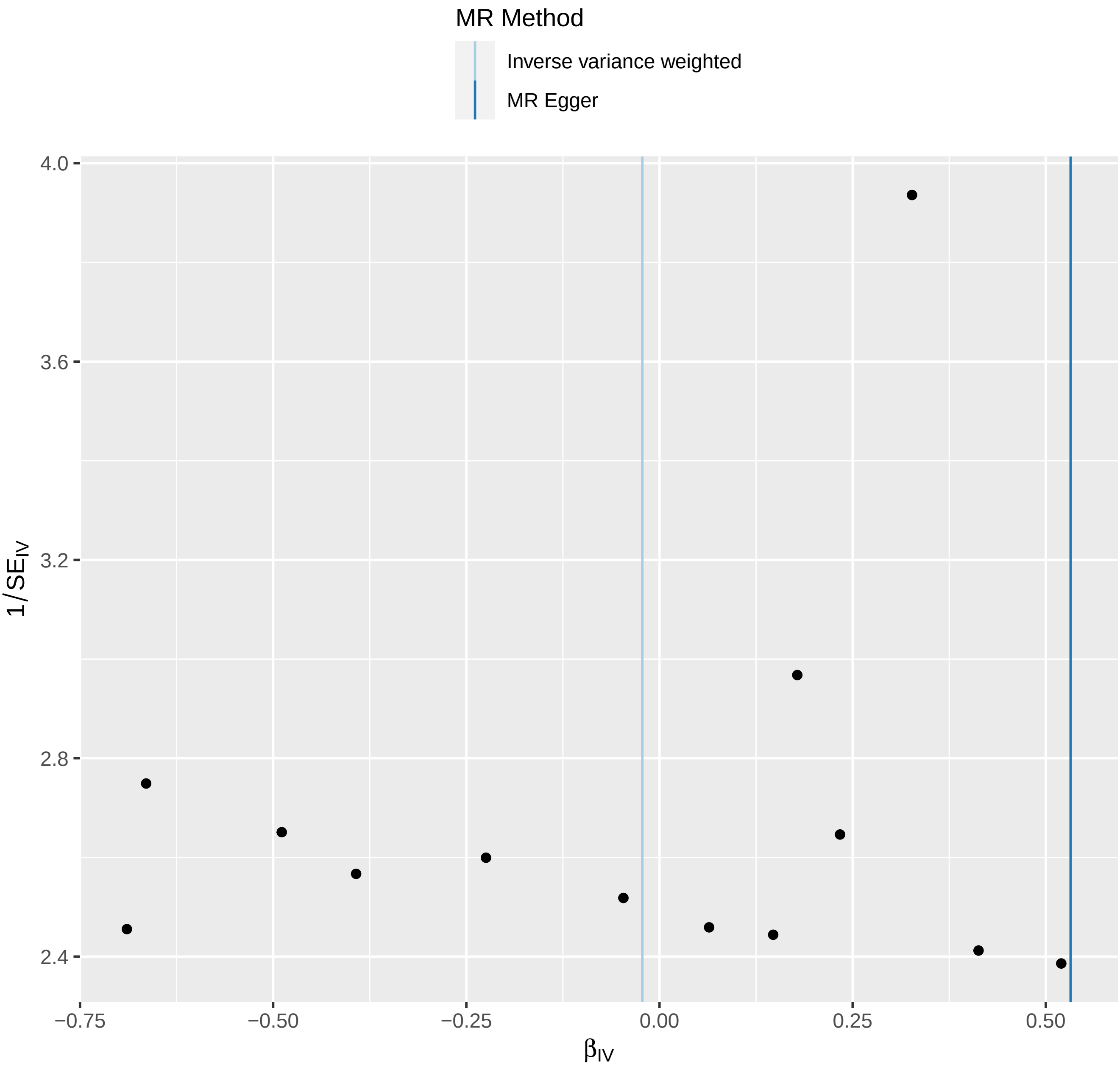

Batch 2−557 : Gut microbiota abundance (genus Catenibacterium id.2153) on Mitotic checkpoint serine/threonine−protein kinase BUB1 with a threshold of 1e−05

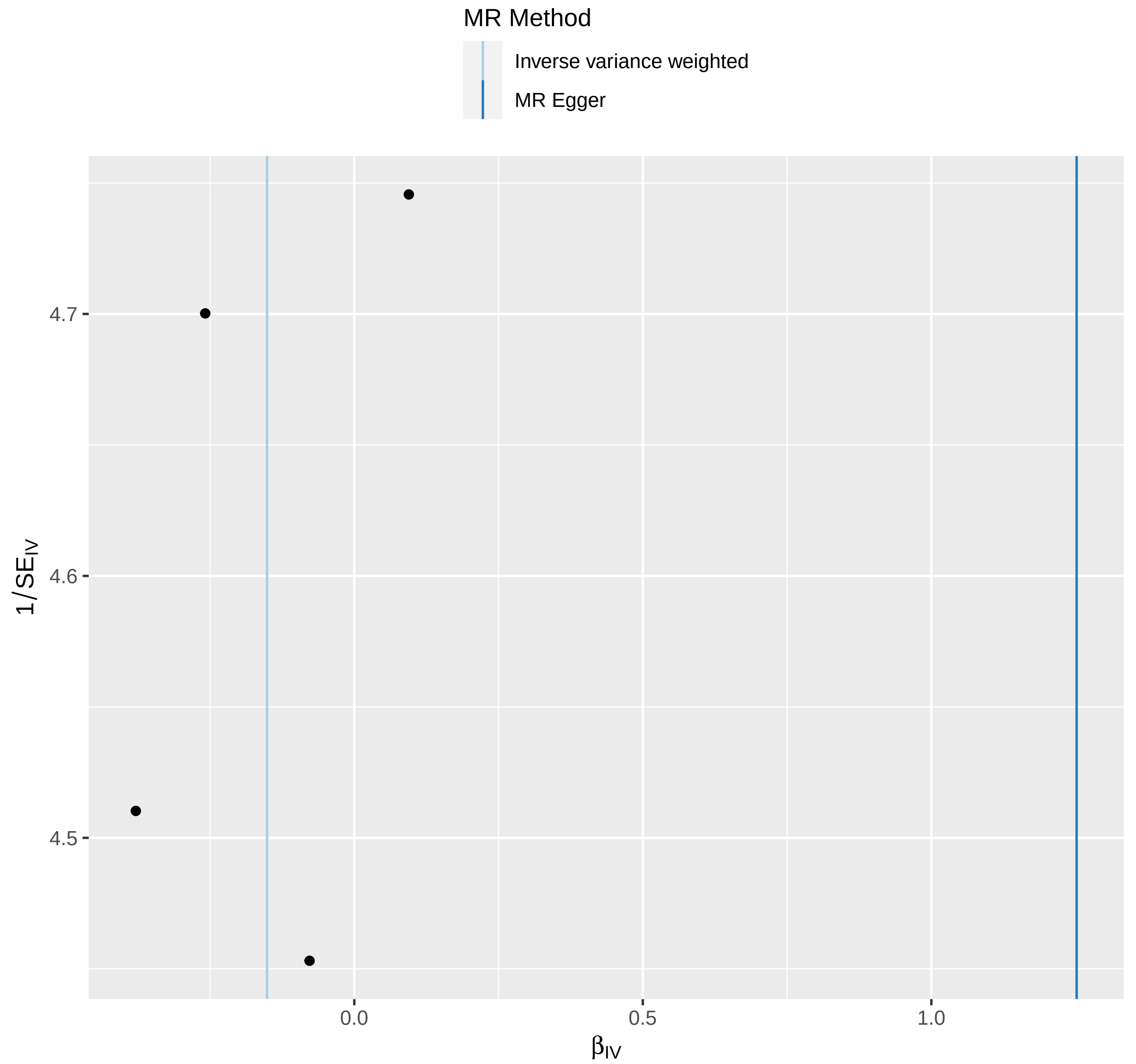

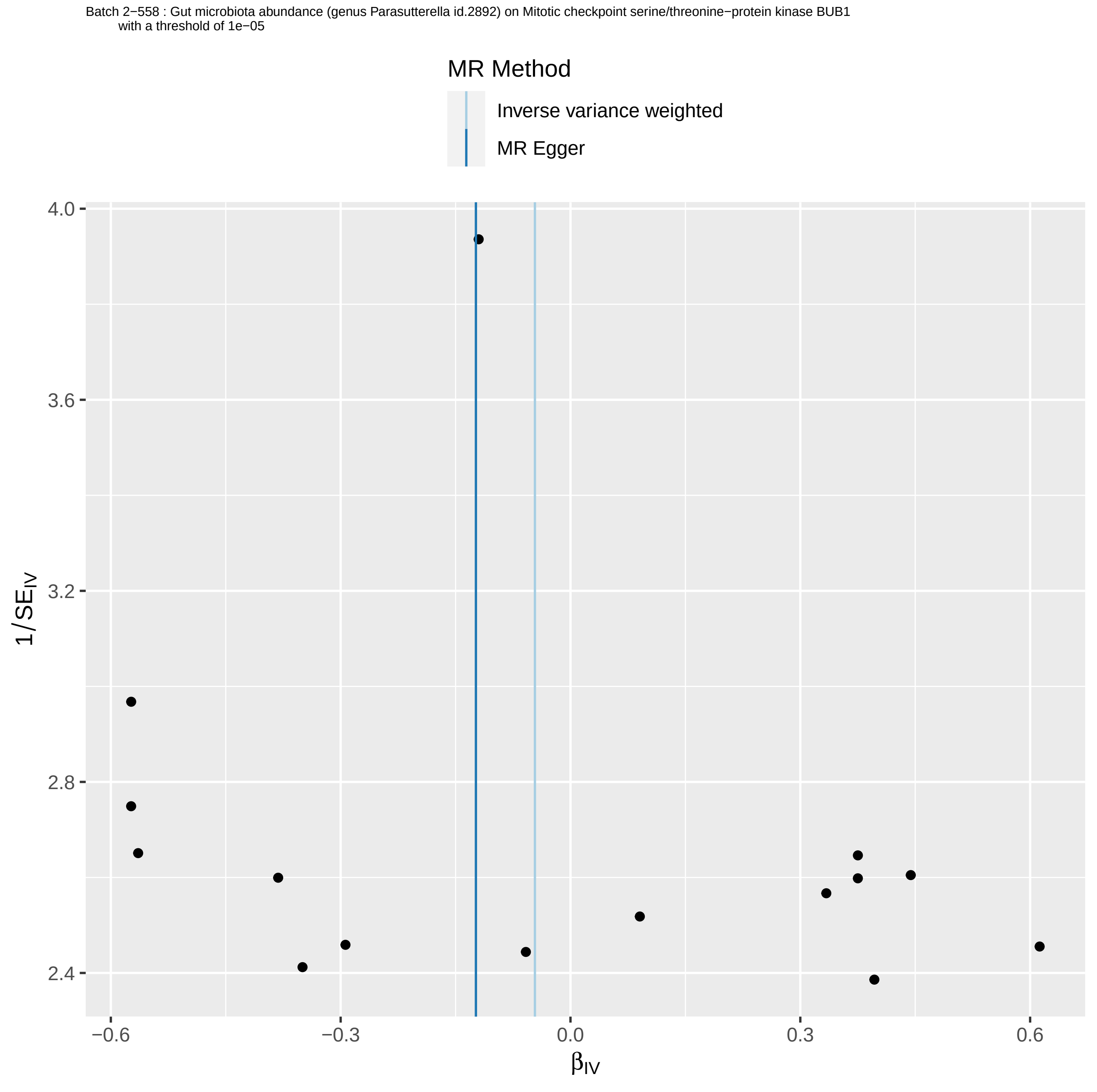
Batch 2−558 : Gut microbiota abundance (genus Parasutterella id.2892) on Mitotic checkpoint serine/threonine−protein kinase BUB1 with a threshold of 1e−05
MR Method
Inverse variance weighted
MR Egger
4.0
3.6
3.2
2.8
2.4
1/SE_IV
−0.6
−0.3
0.0
0.3
0.6
β_IV

Batch 2−559 : Gut microbiota abundance (genus Catenibacterium id.2153) on Uncharacterized protein C10orf35
with a threshold of 1e−05

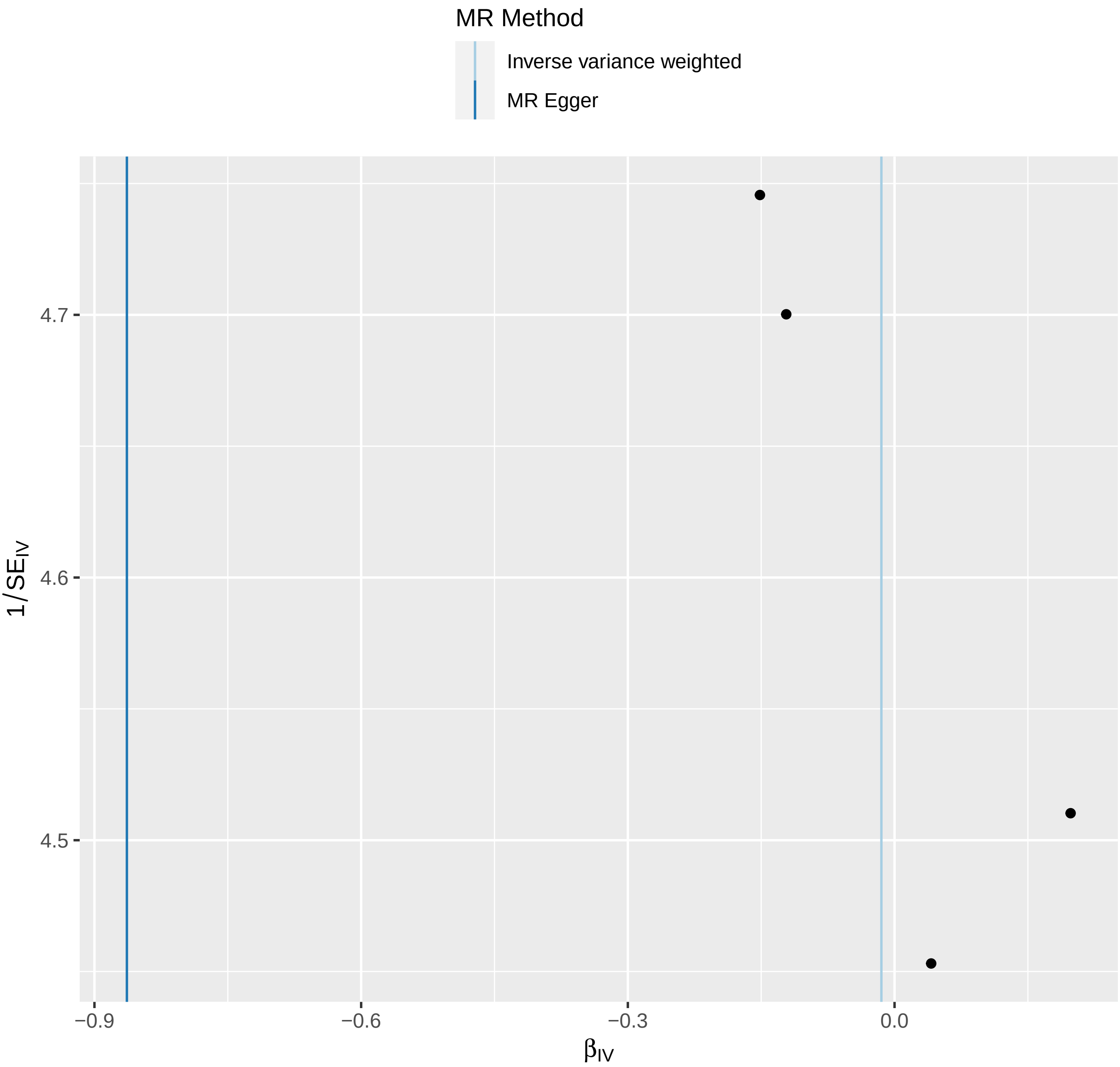

Batch 2−560 : Gut microbiota abundance (genus Parasutterella id.2892) on Uncharacterized protein C10orf35
with a threshold of 1e−05

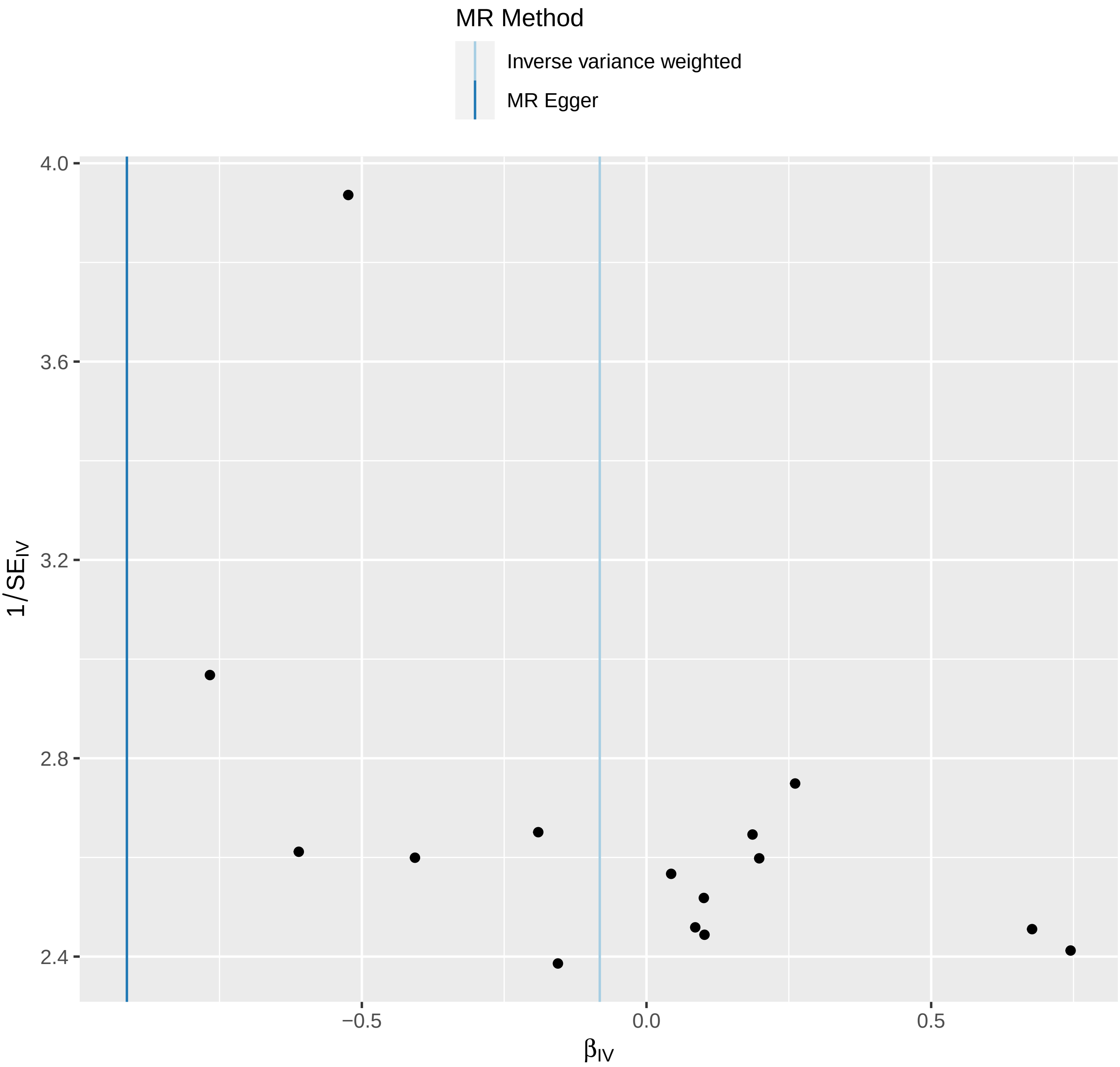

Batch 2−561 : Gut microbiota abundance (genus Catenibacterium id.2153) on Platelet receptor Gi24
with a threshold of 1e−05

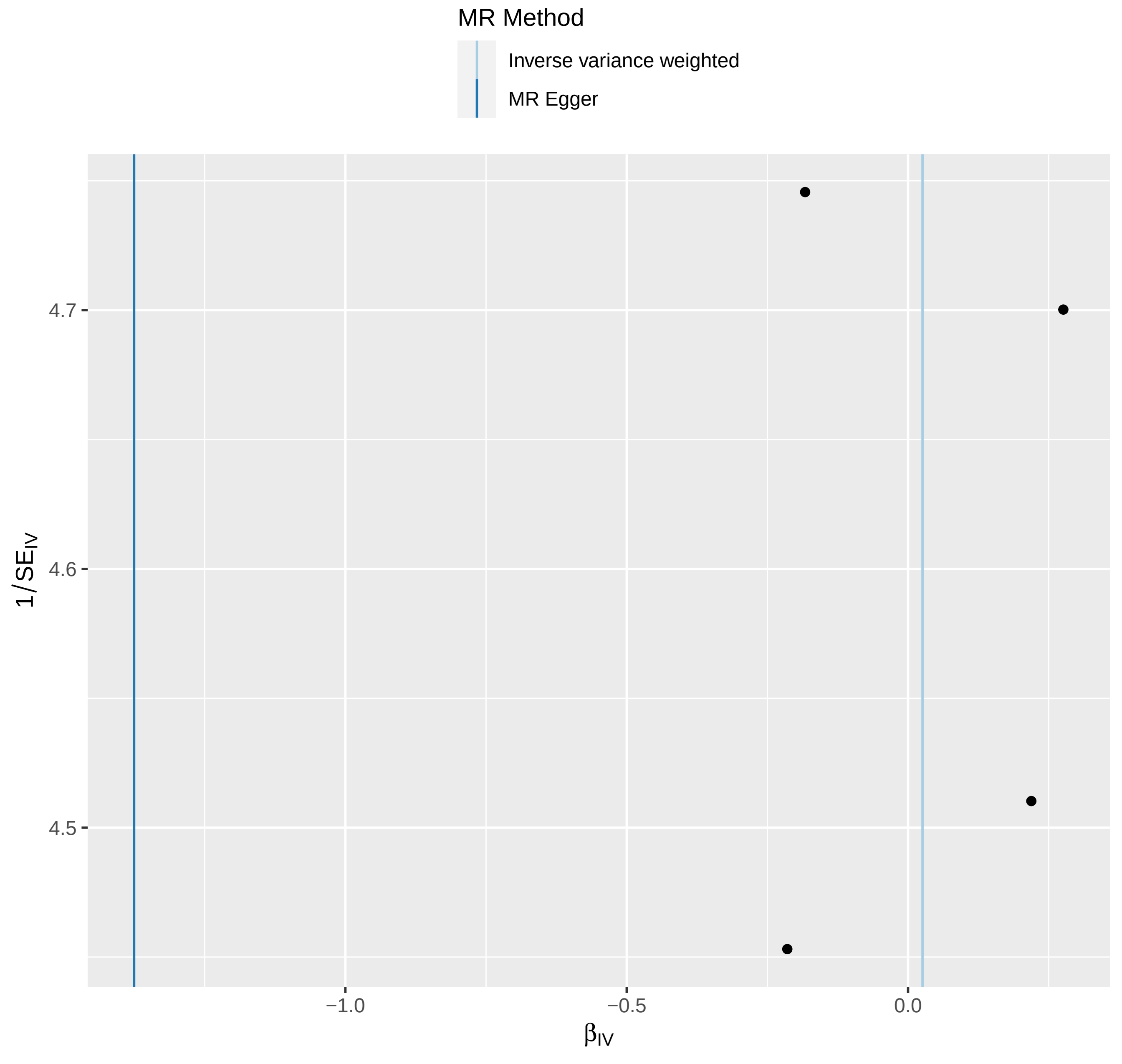

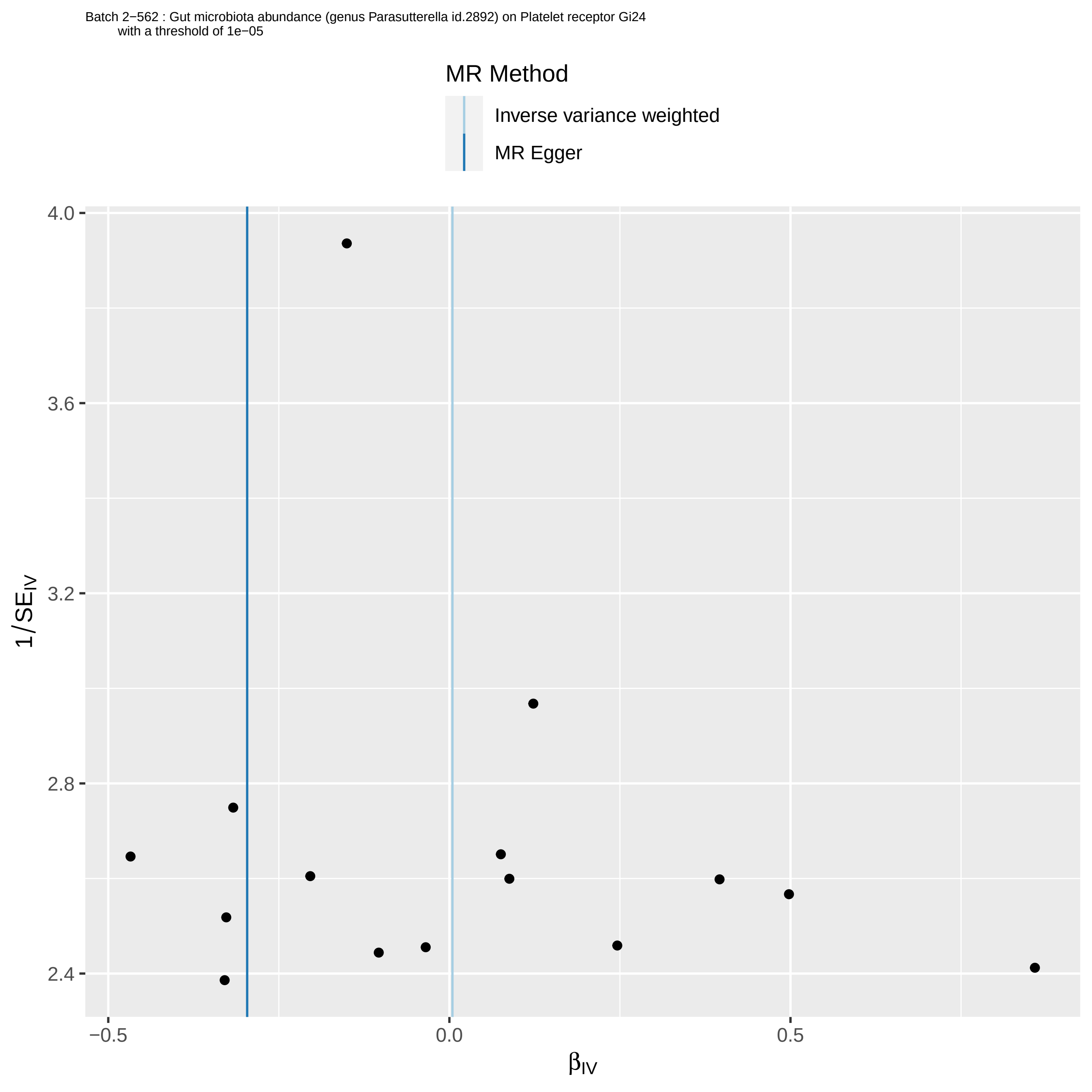

Batch 2−562 : Gut microbiota abundance (genus Parasutterella id.2892) on Platelet receptor Gi24
with a threshold of 1e−05
MR Method
Inverse variance weighted
MR Egger
4.0
3.6
3.2
2.8
2.4
1/SE_IV
−0.5
0.0
0.5
β_IV

Batch 2−563 : Gut microbiota abundance (genus Catenibacterium id.2153) on UPF0696 protein C11orf68
with a threshold of 1e−05

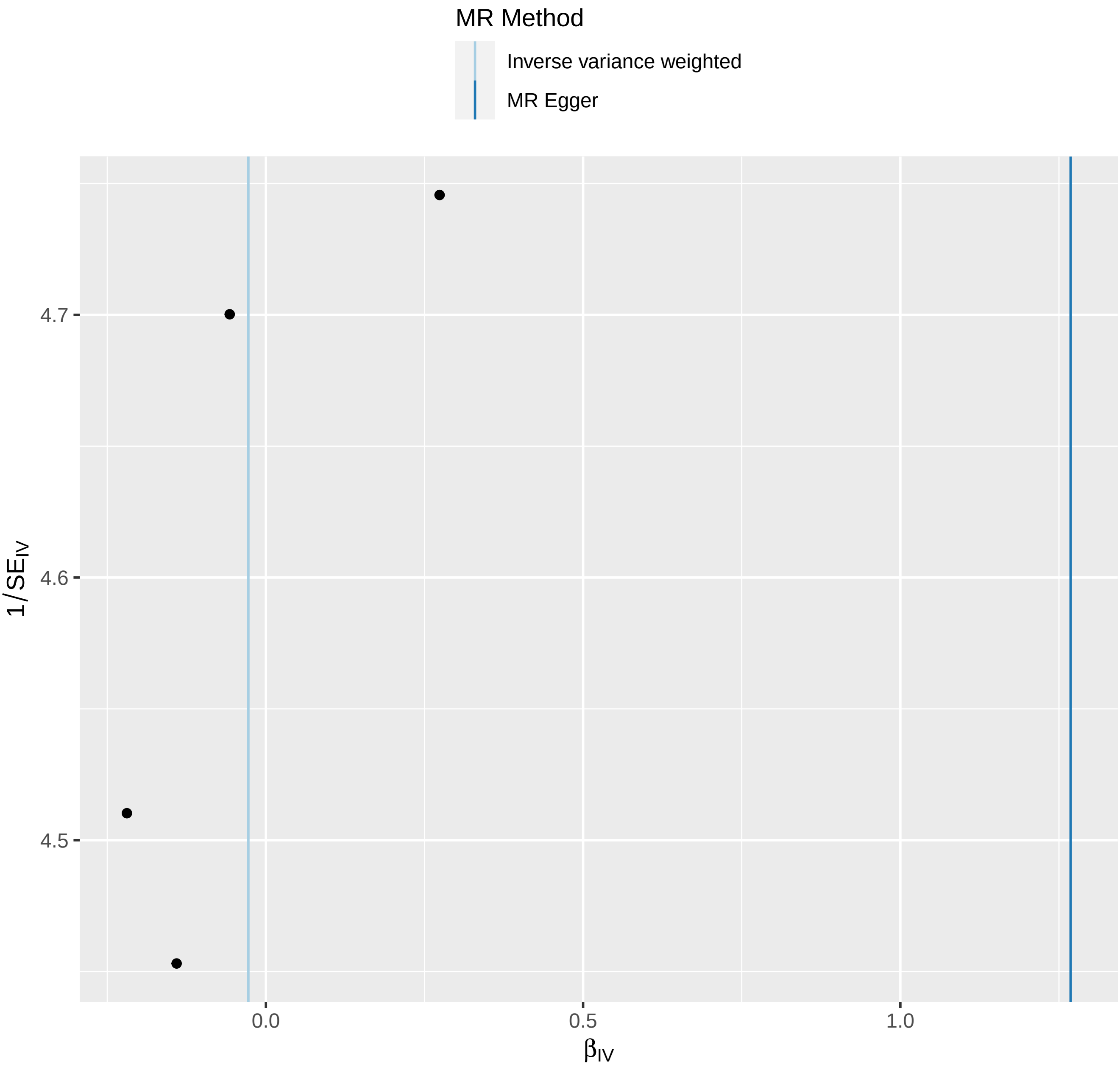

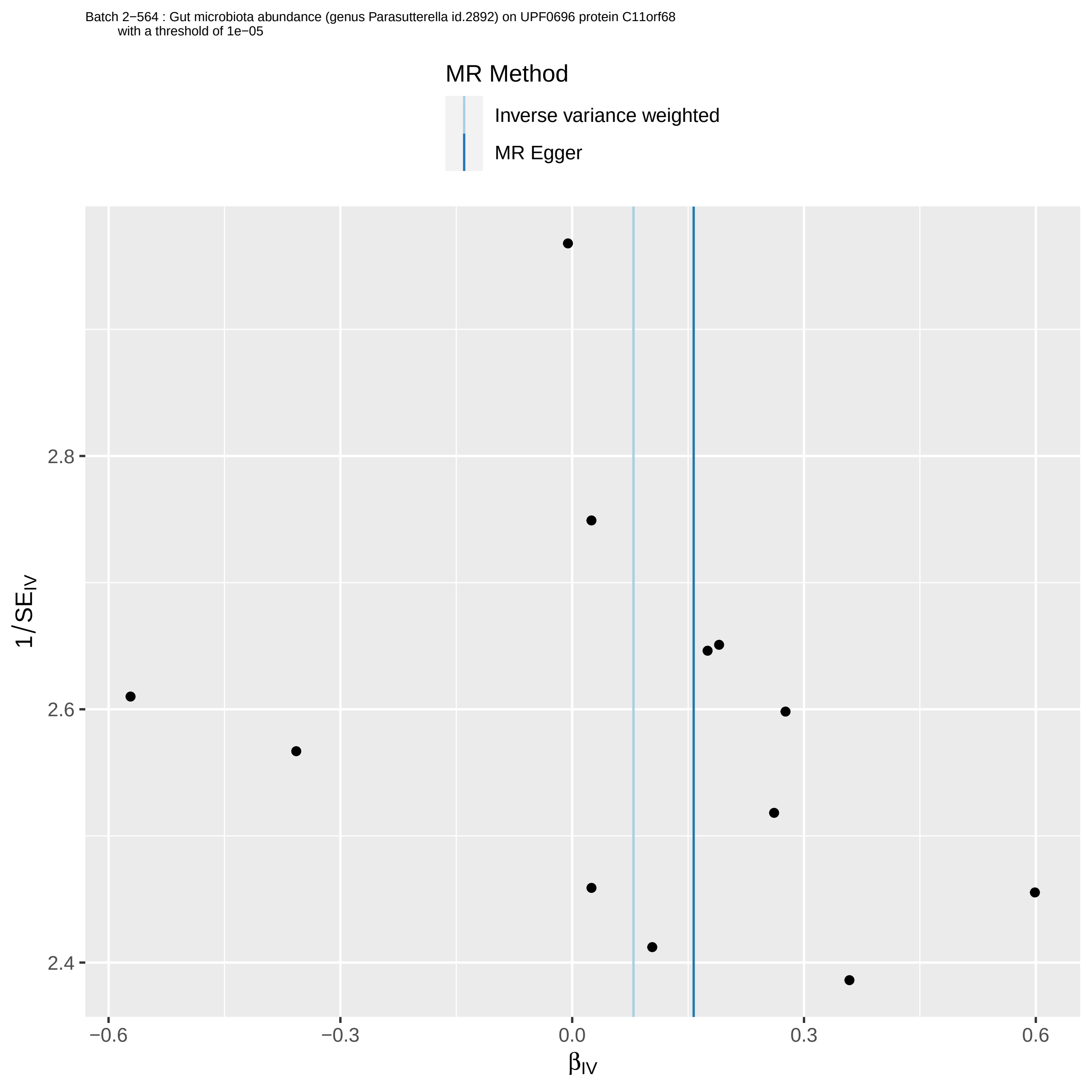

Batch 2−564 : Gut microbiota abundance (genus Parasutterella id.2892) on UPF0696 protein C11orf68
with a threshold of 1e−05
MR Method
Inverse variance weighted
MR Egger
2.8
2.6
2.4
1/SE_IV
−0.6
−0.3
0.0
0.3
0.6
β_IV

Batch 2−565 : Gut microbiota abundance (genus Catenibacterium id.2153) on UPF0454 protein C12orf49 with a threshold of 1e−05

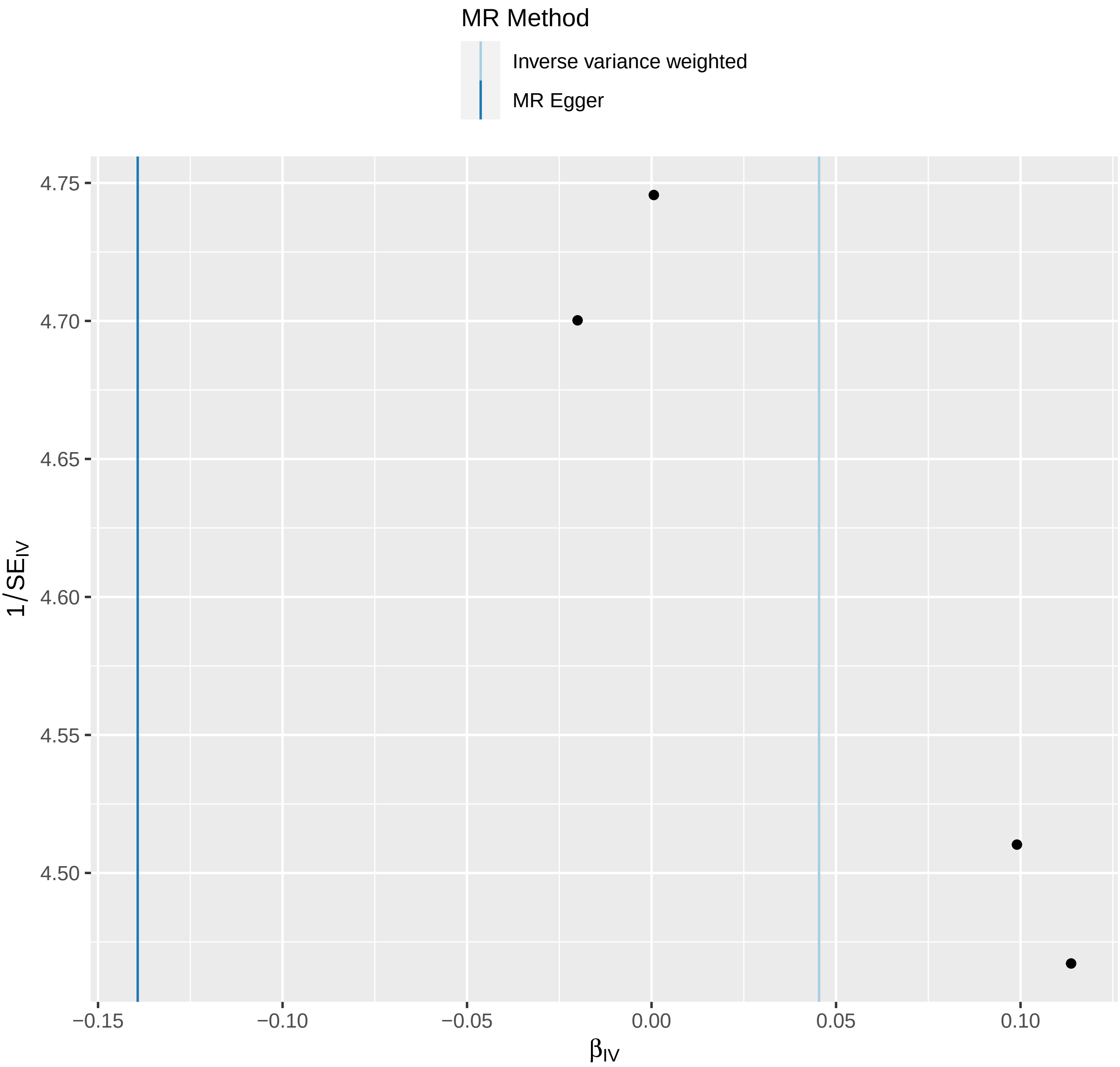

Batch 2−566 : Gut microbiota abundance (genus Parasutterella id.2892) on UPF0454 protein C12orf49
with a threshold of 1e−05

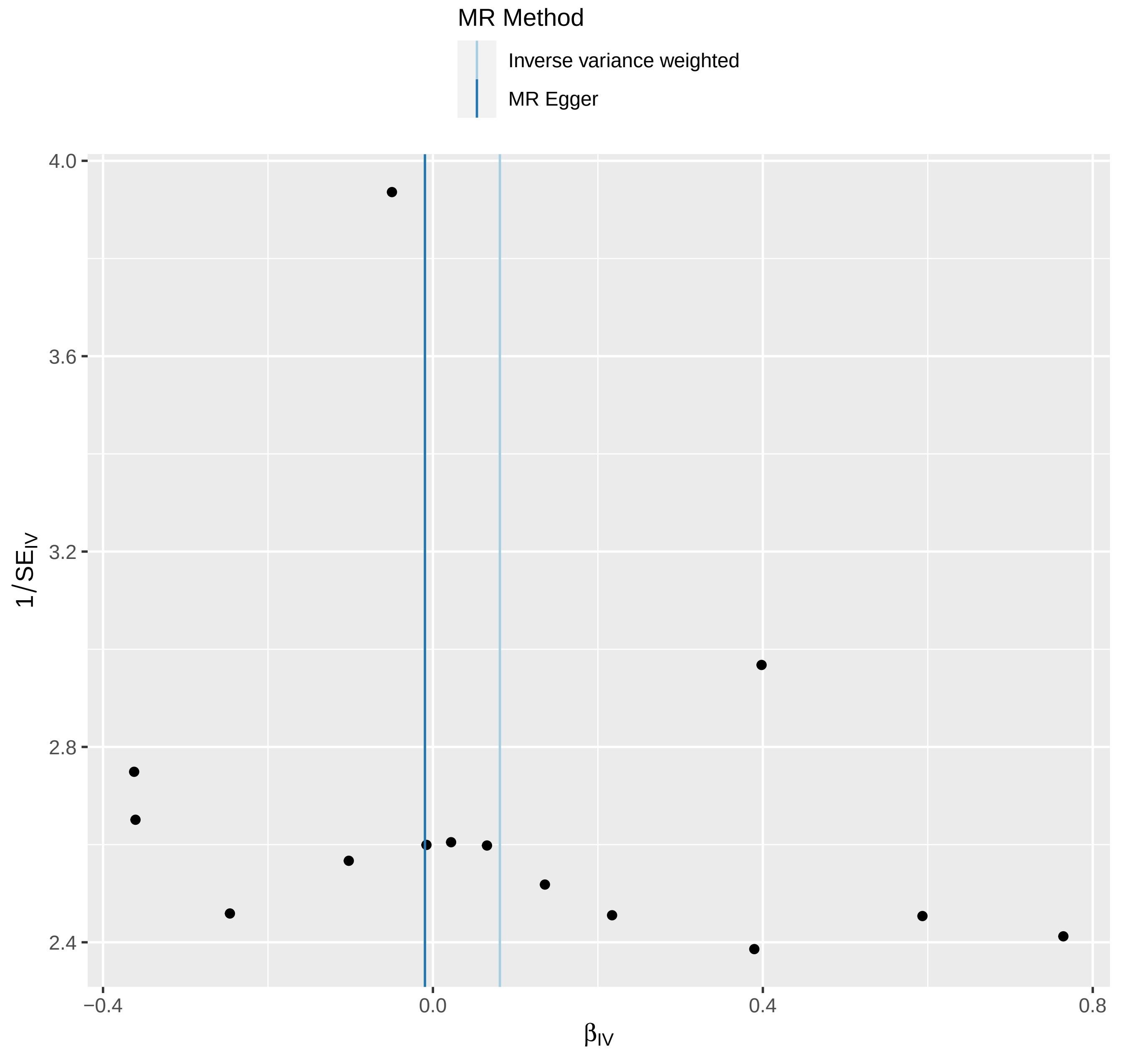

Batch 2−567 : Gut microbiota abundance (genus Catenibacterium id.2153) on Uncharacterized protein C14orf93
with a threshold of 1e−05

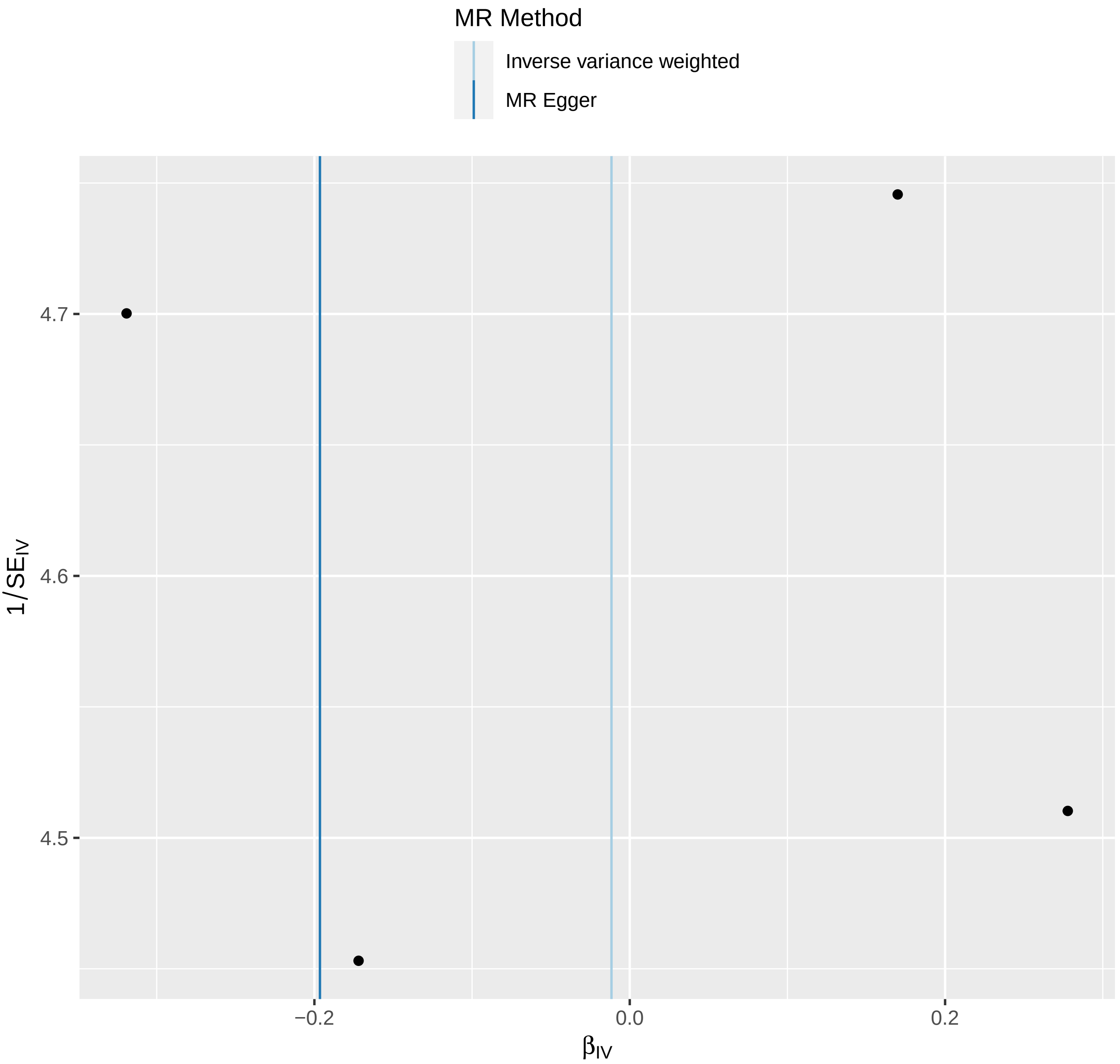

Batch 2−568 : Gut microbiota abundance (genus Parasutterella id.2892) on Uncharacterized protein C14orf93
with a threshold of 1e−05

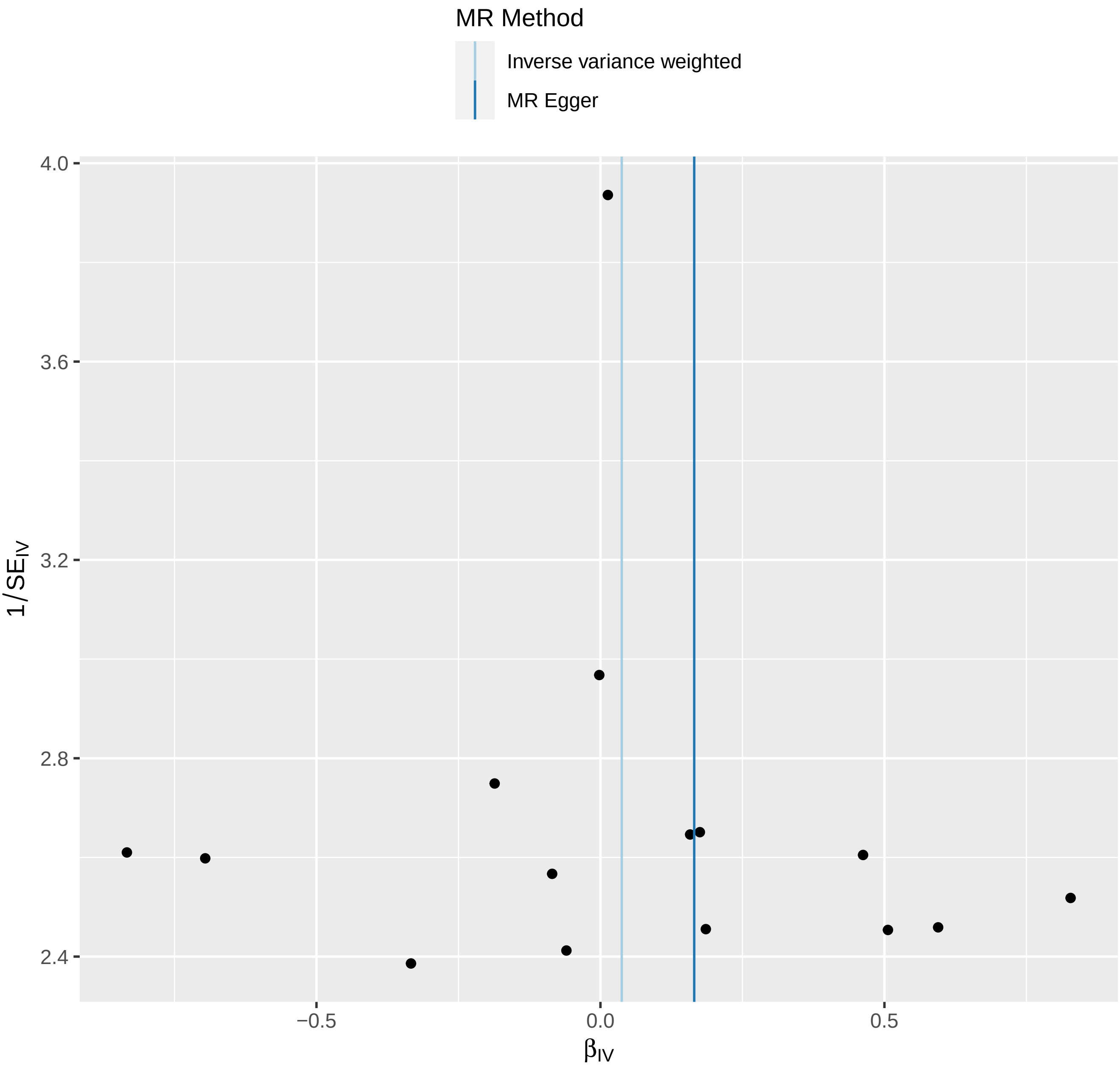

Batch 2−569 : Gut microbiota abundance (genus Catenibacterium id.2153) on Transmembrane protein C16orf54
with a threshold of 1e−05

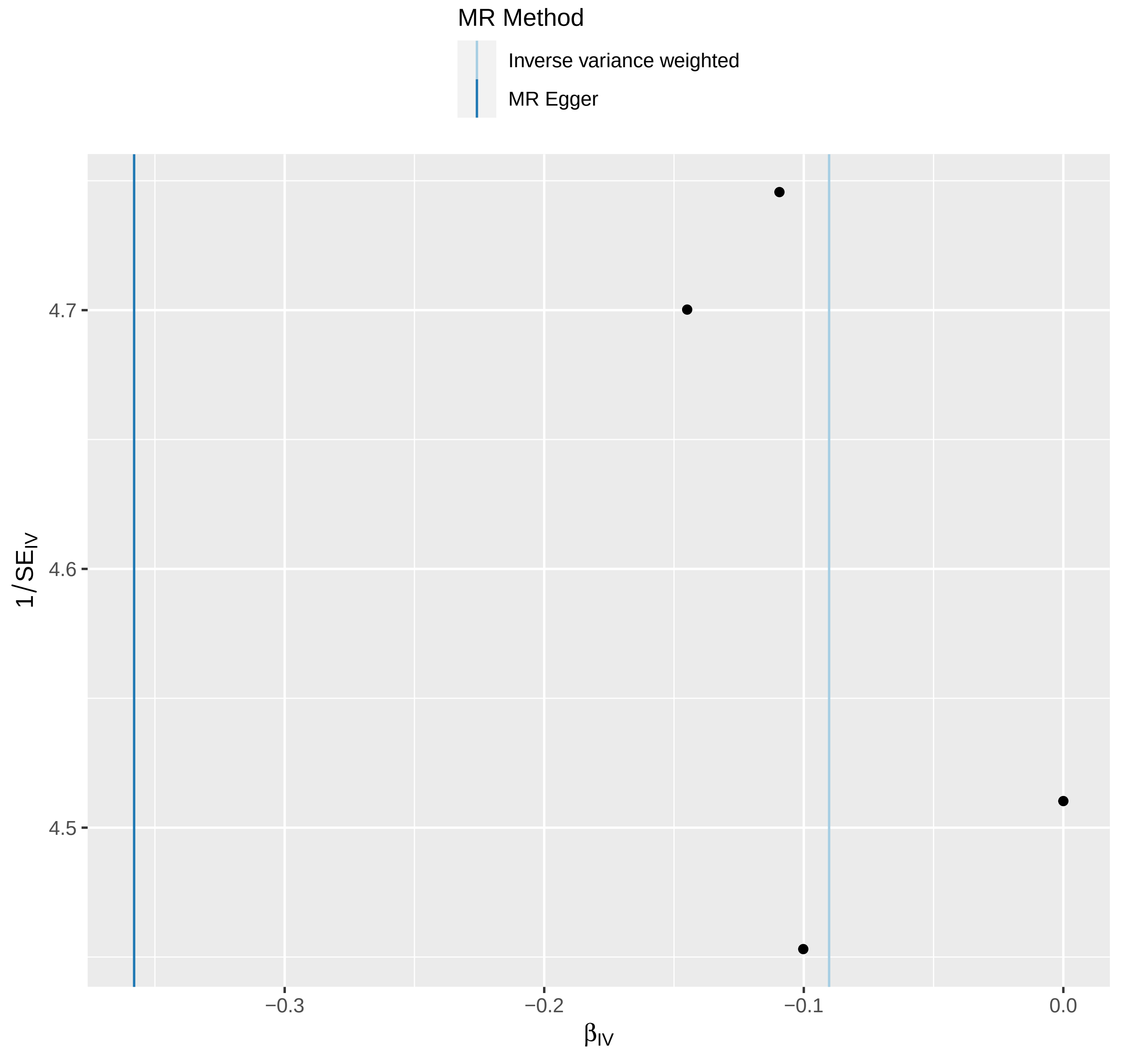

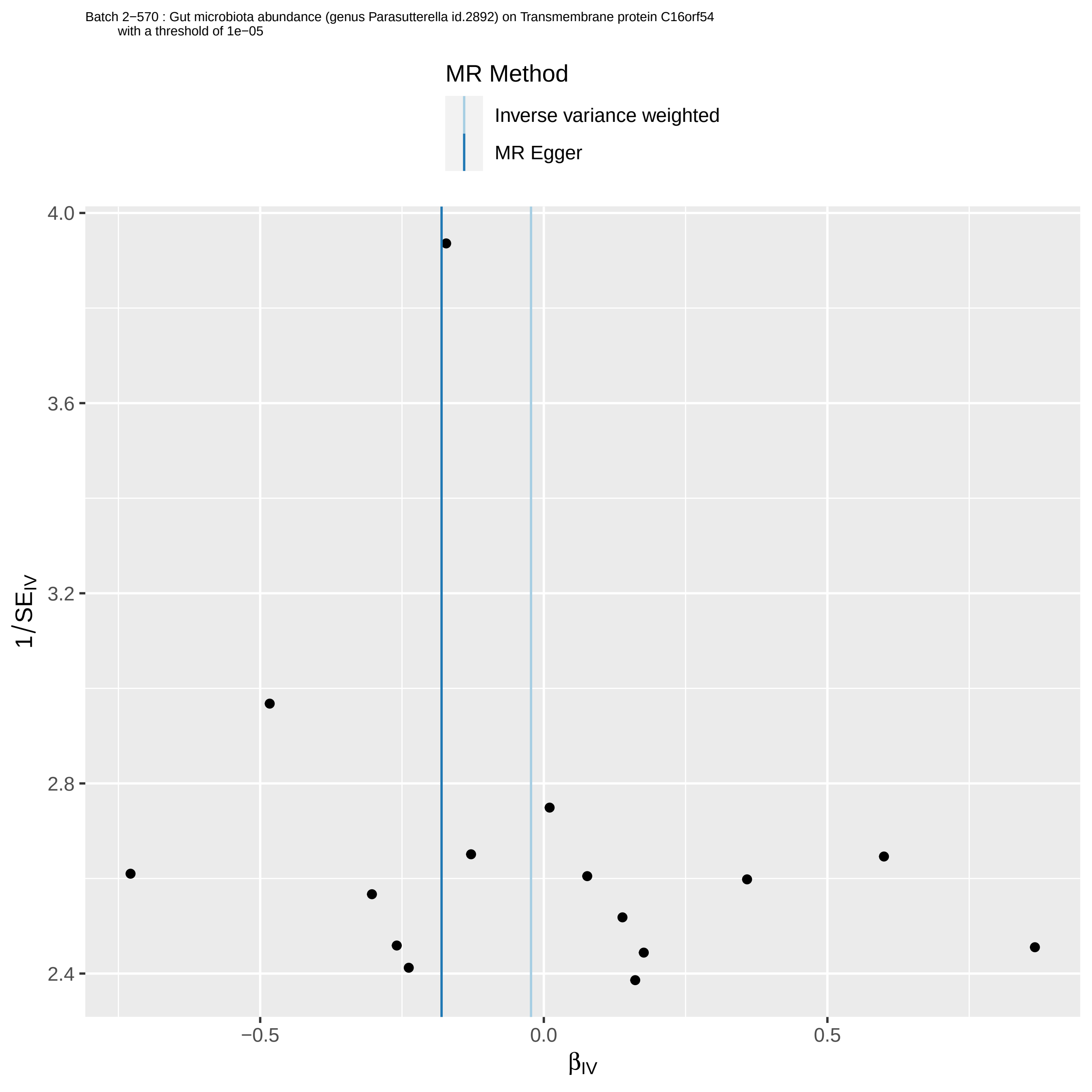
Batch 2−570 : Gut microbiota abundance (genus Parasutterella id.2892) on Transmembrane protein C16orf54
with a threshold of 1e−05
MR Method
Inverse variance weighted
MR Egger
4.0
3.6
3.2
2.8
2.4
1/SE_IV
−0.5
0.0
0.5
β_IV

Batch 2−571 : Gut microbiota abundance (genus Catenibacterium id.2153) on Uncharacterized protein C17orf67
with a threshold of 1e−05

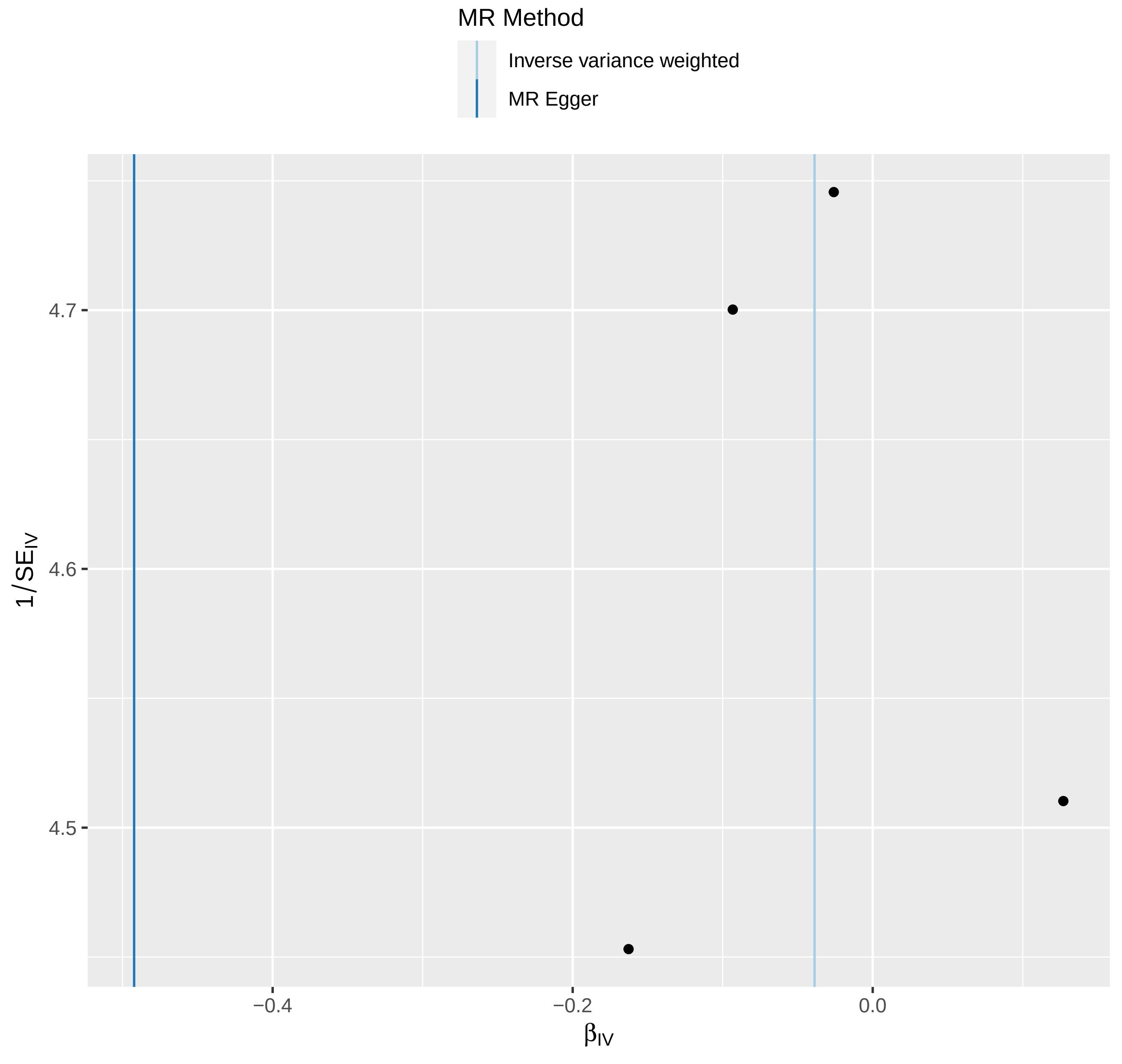

Batch 2−572 : Gut microbiota abundance (genus Parasutterella id.2892) on Uncharacterized protein C17orf67
with a threshold of 1e−05

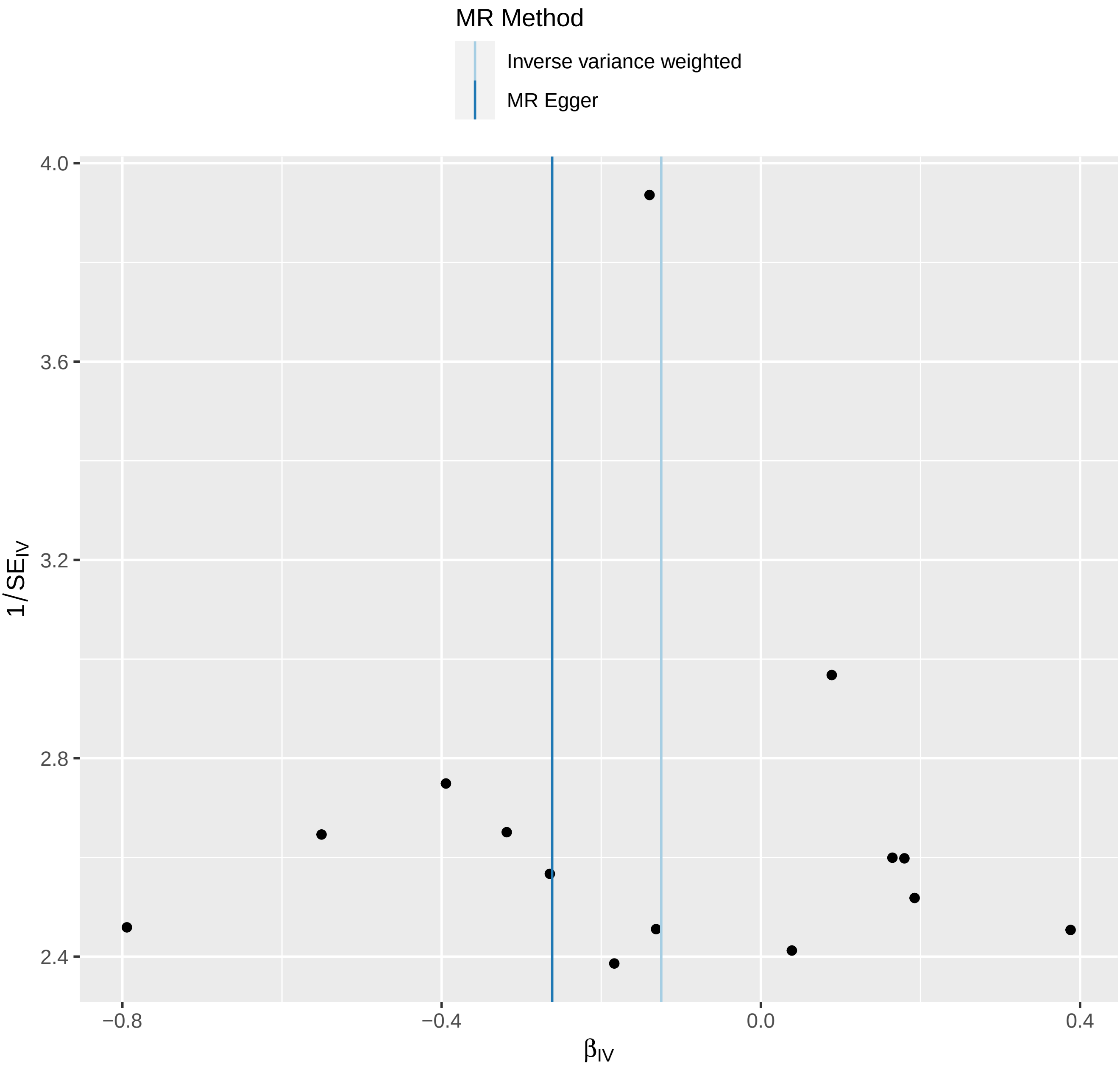

Batch 2−573 : Gut microbiota abundance (genus Catenibacterium id.2153) on Uncharacterized protein C17orf78
with a threshold of 1e−05

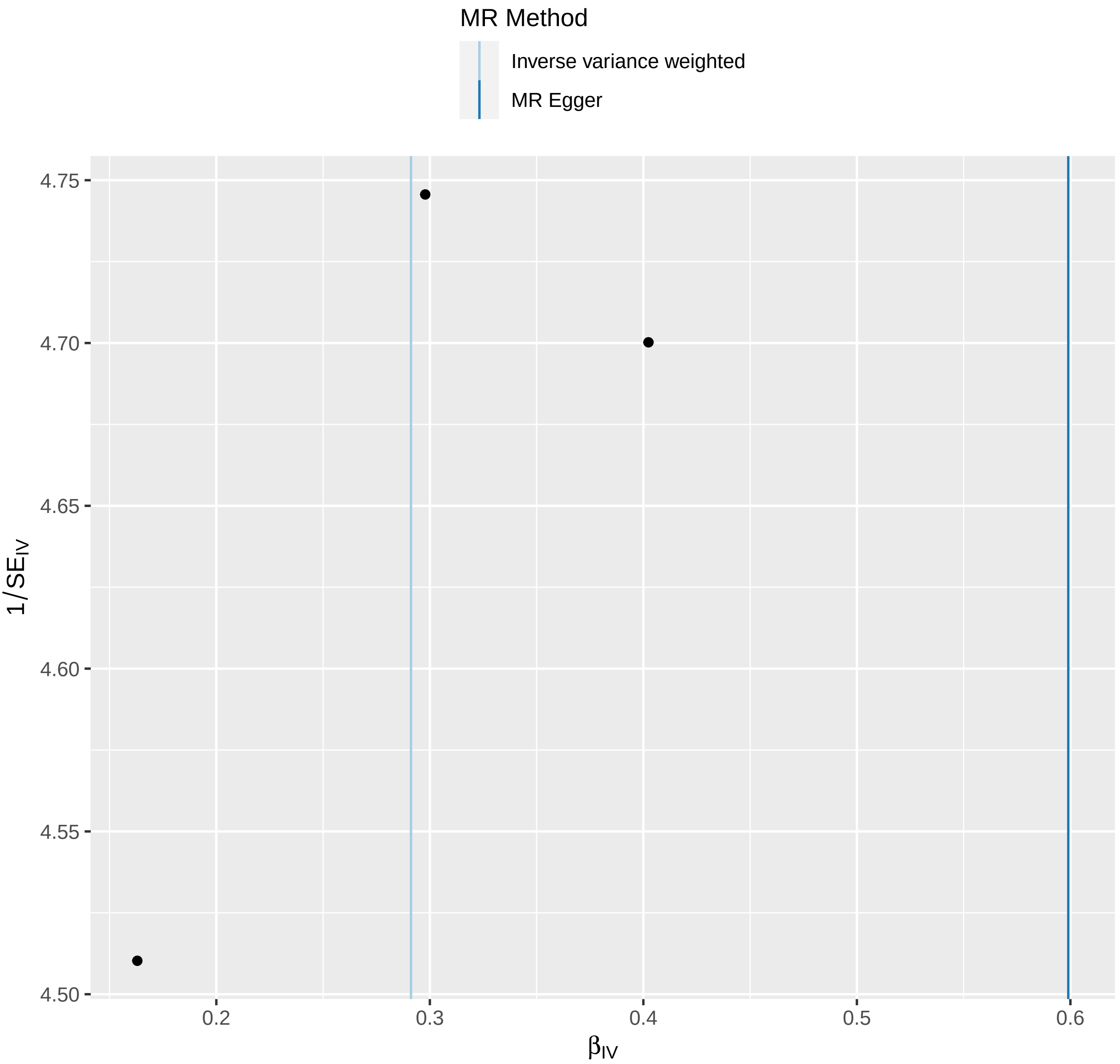

Batch 2−574 : Gut microbiota abundance (genus Parasutterella id.2892) on Uncharacterized protein C17orf78
with a threshold of 1e−05

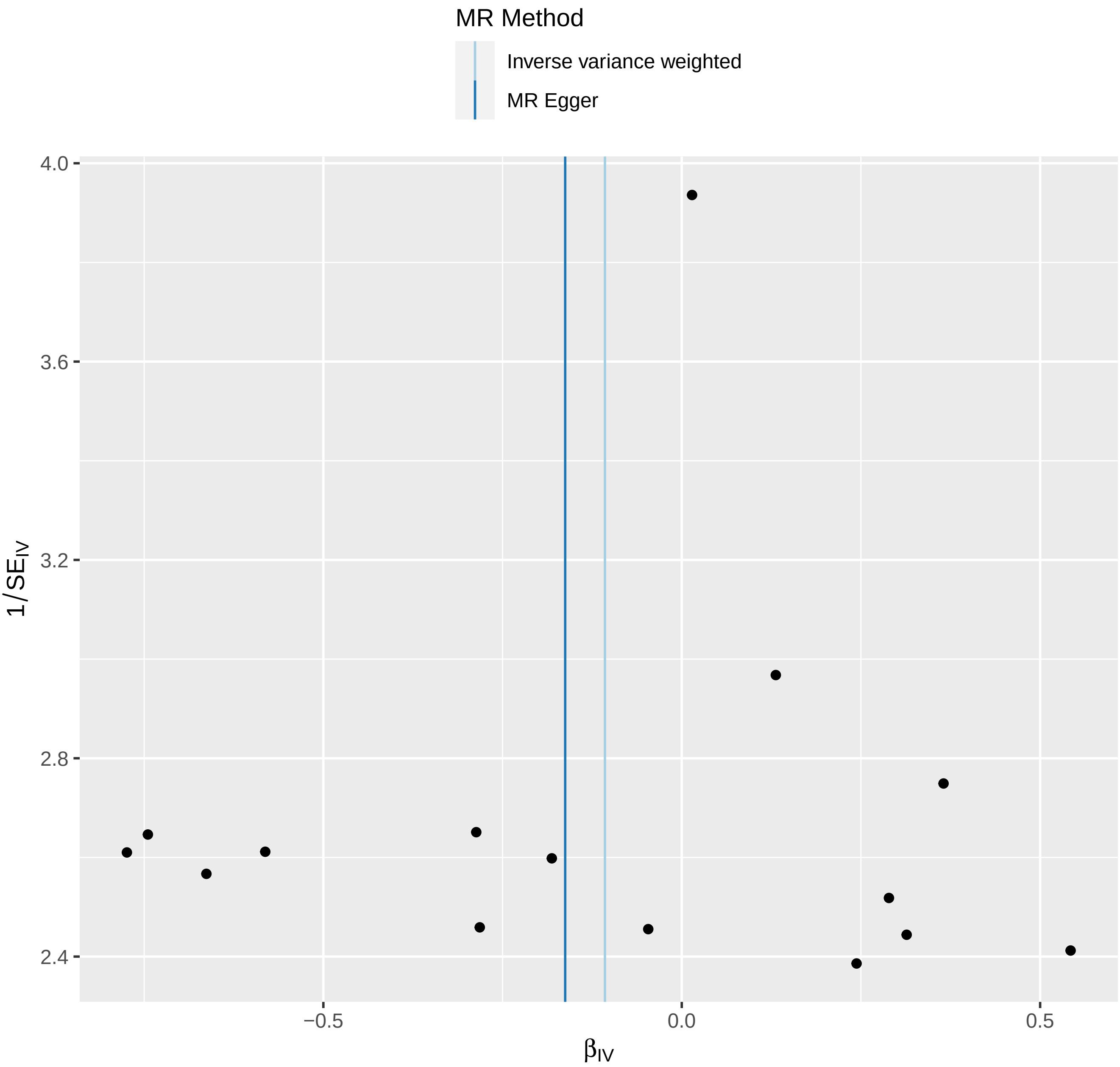

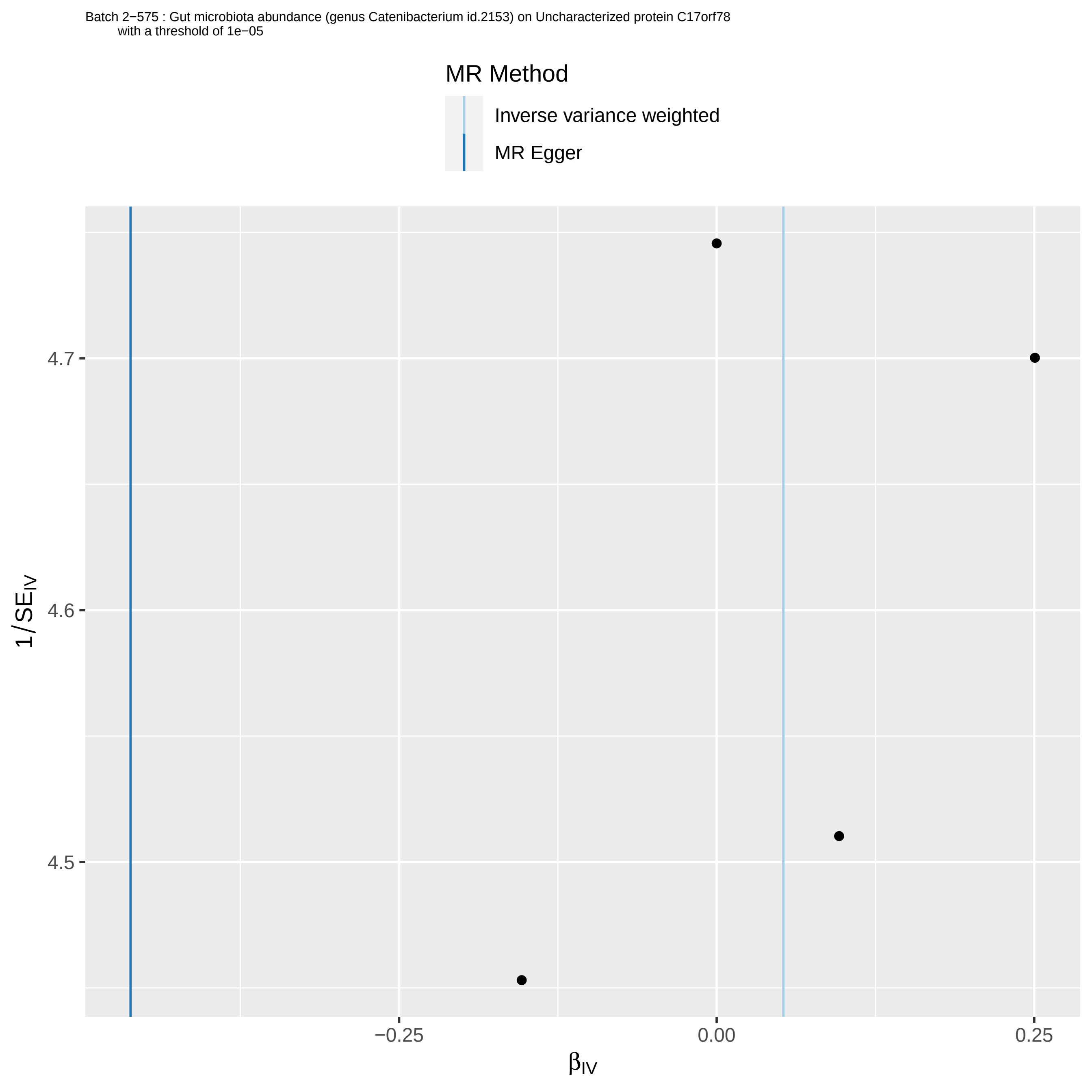
Batch 2−575 : Gut microbiota abundance (genus Catenibacterium id.2153) on Uncharacterized protein C17orf78
with a threshold of 1e−05
MR Method
Inverse variance weighted
MR Egger
4.7
4.6
4.5
1/SE_IV
−0.25
0.00
0.25
β_IV

Batch 2−576 : Gut microbiota abundance (genus Parasutterella id.2892) on Uncharacterized protein C17orf78
with a threshold of 1e−05

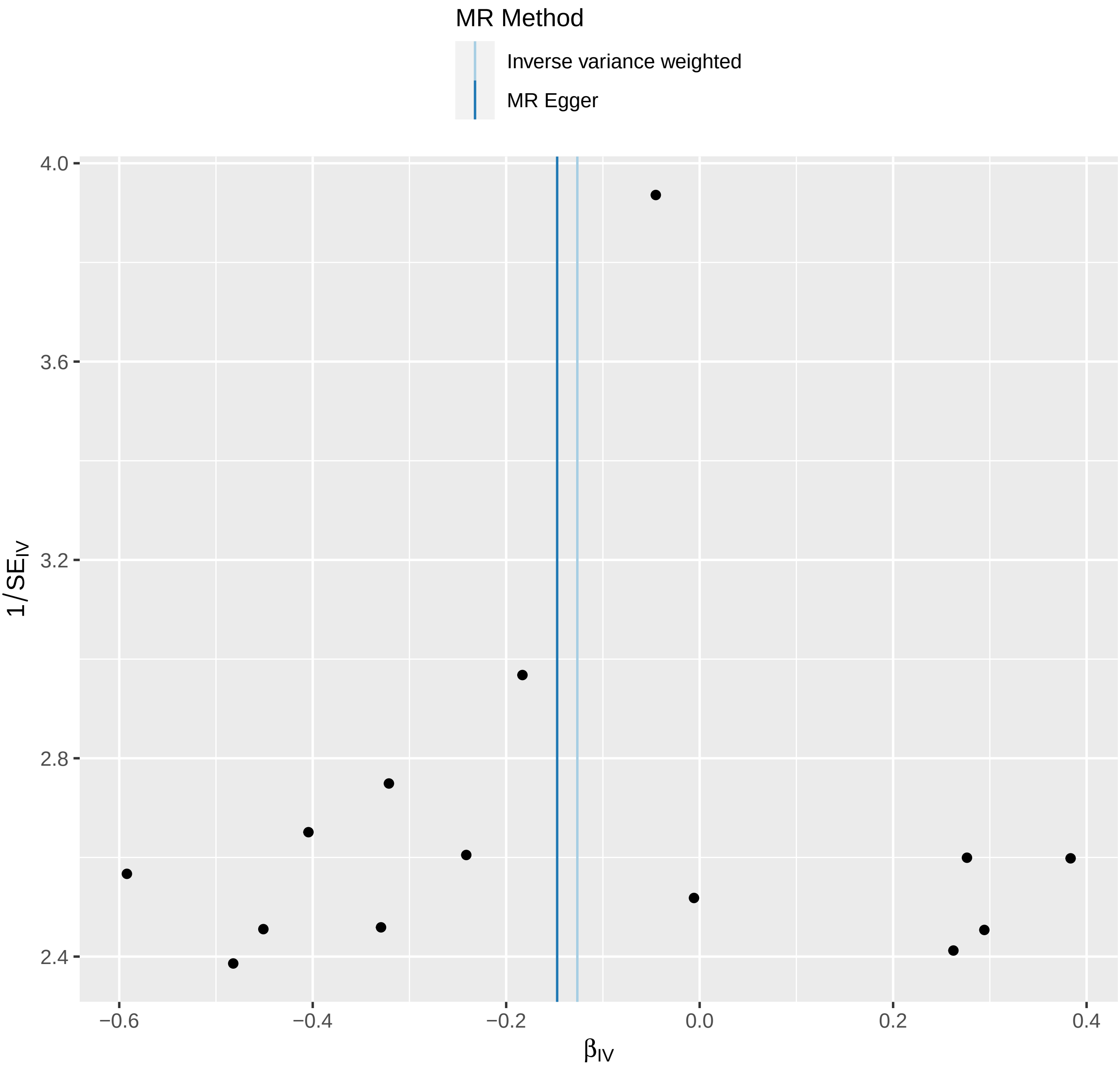

Batch 2−577 : Gut microbiota abundance (genus Catenibacterium id.2153) on Uncharacterized protein C17orf89
with a threshold of 1e−05

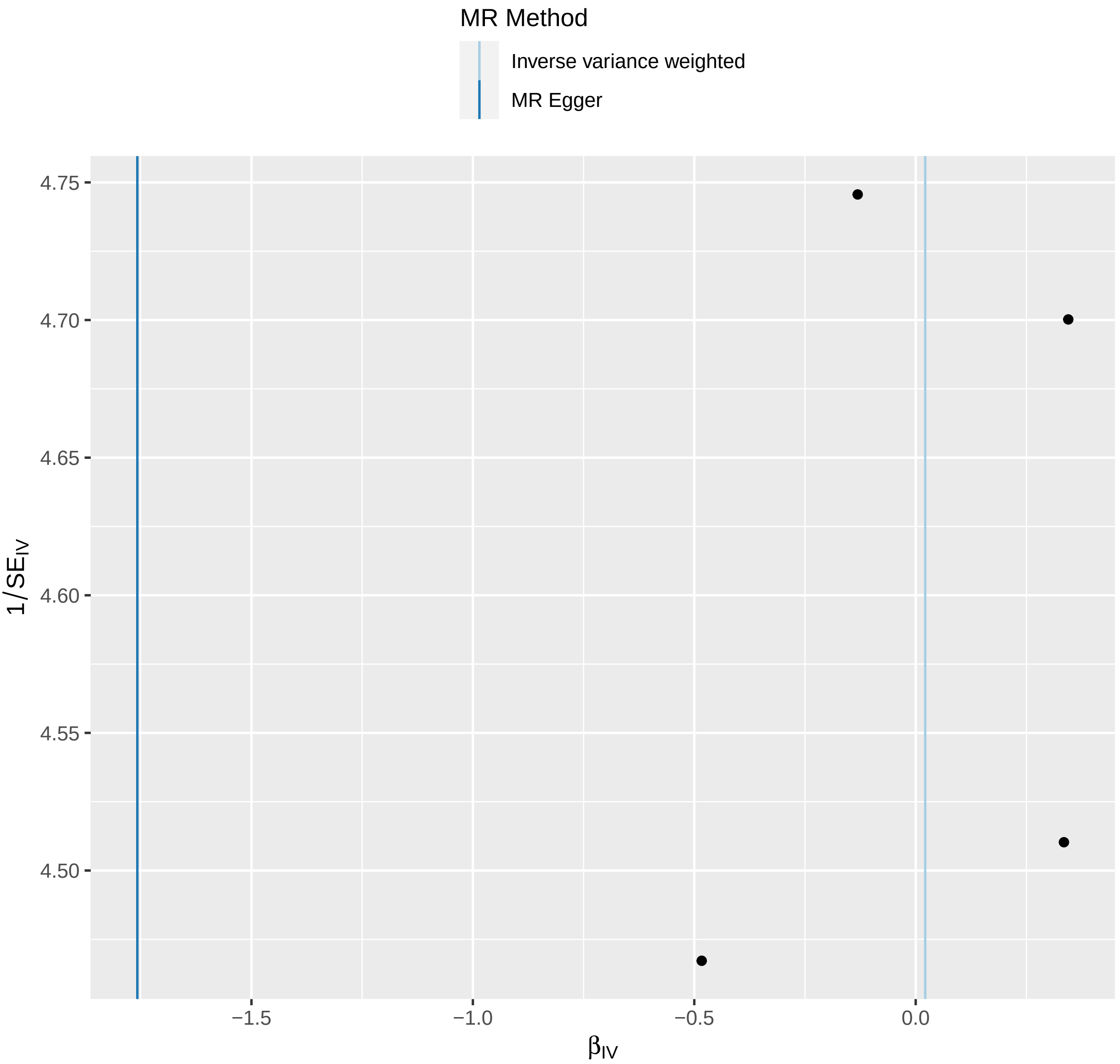

Batch 2−578 : Gut microbiota abundance (genus Parasutterella id.2892) on Uncharacterized protein C17orf89
with a threshold of 1e−05

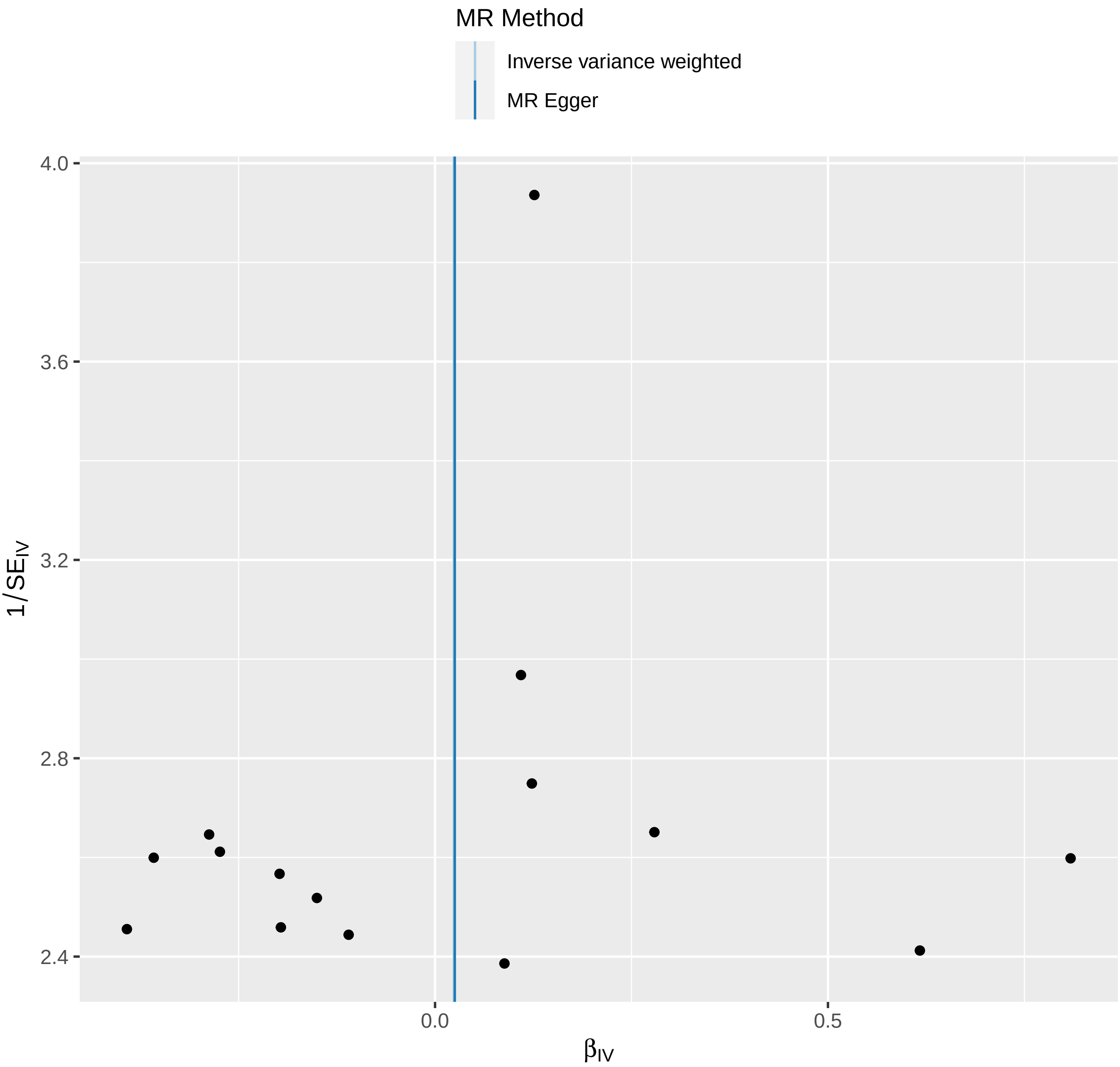

Batch 2−579 : Gut microbiota abundance (genus Catenibacterium id.2153) on UPF0729 protein C18orf32
with a threshold of 1e−05

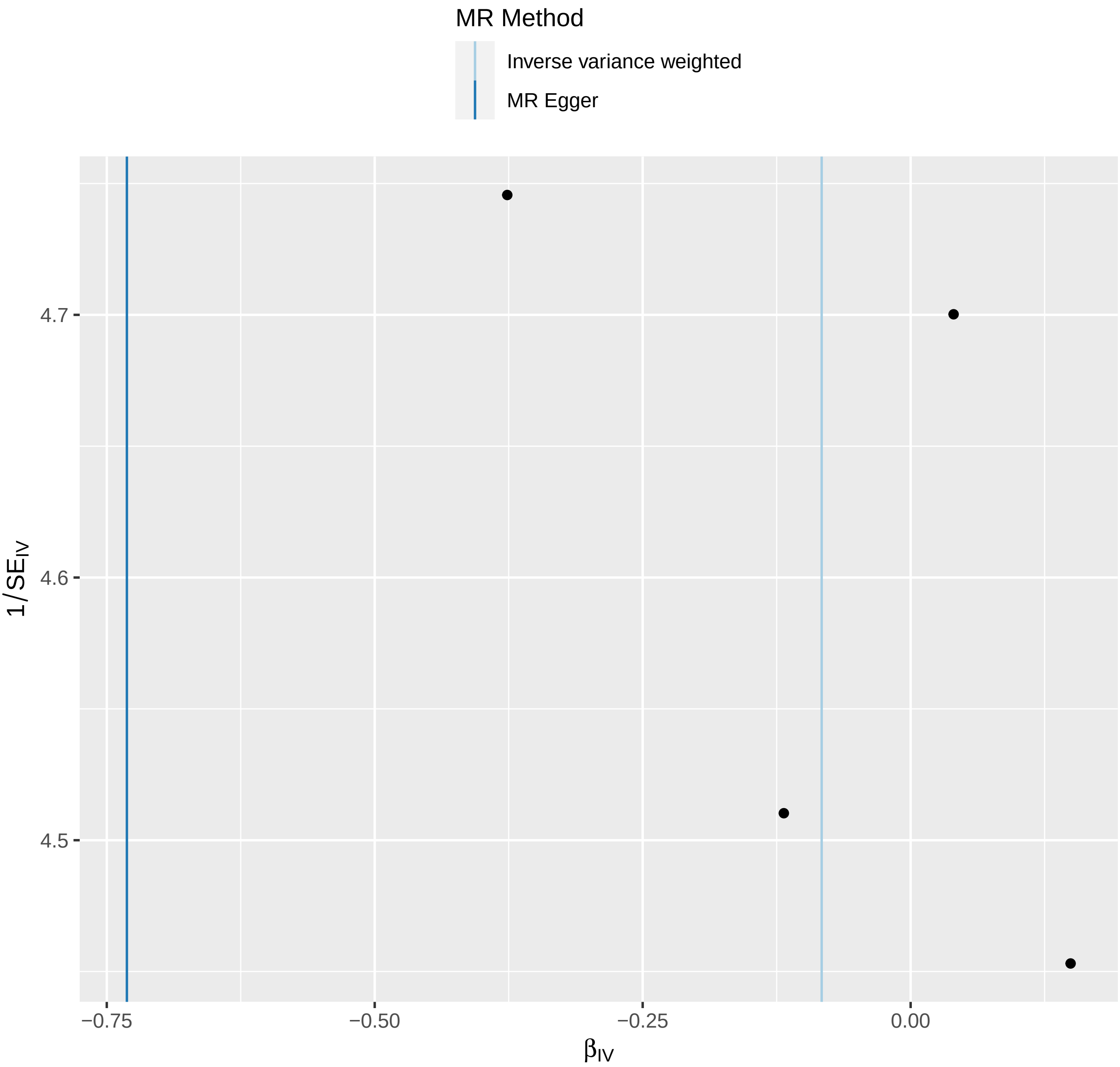

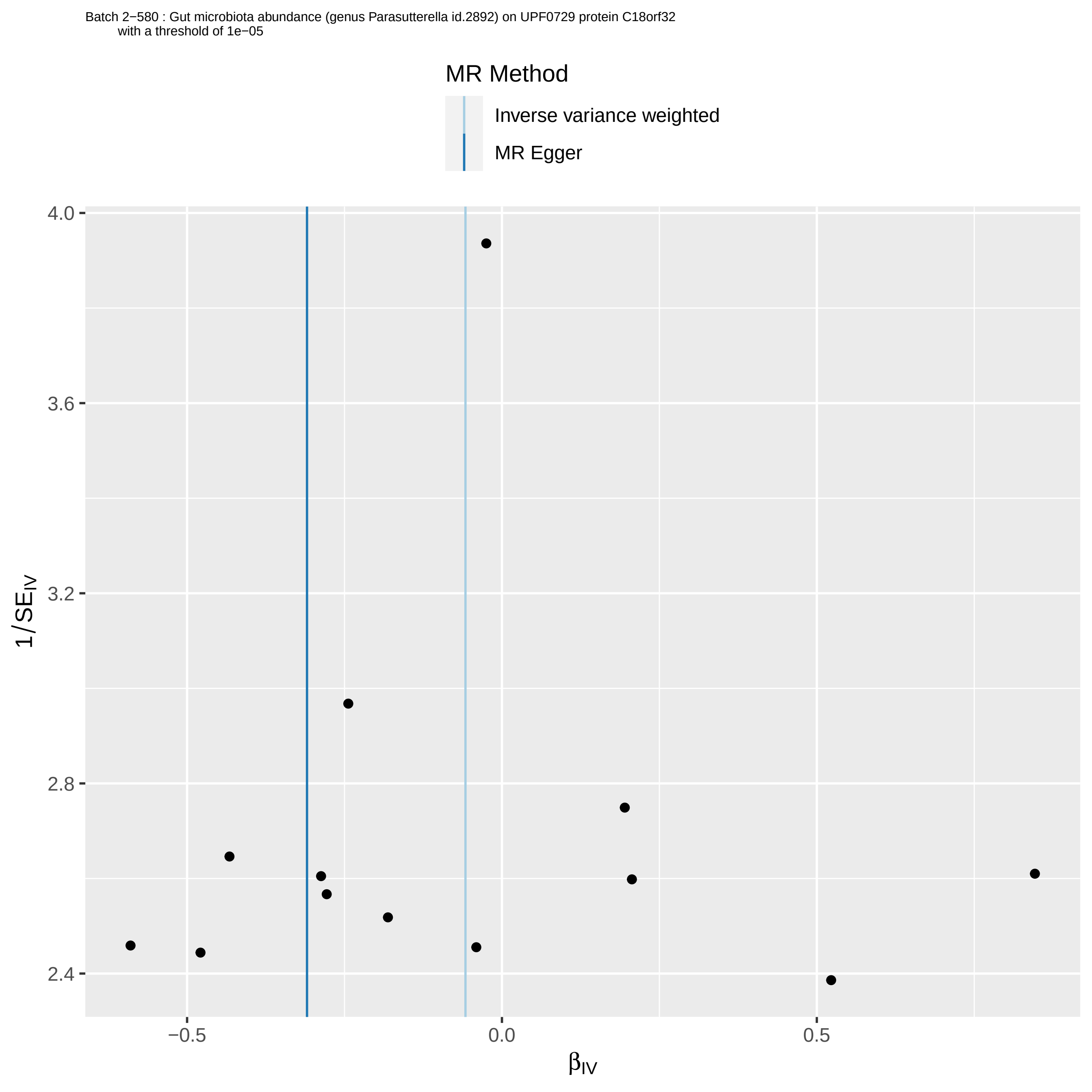

Batch 2−580 : Gut microbiota abundance (genus Parasutterella id.2892) on UPF0729 protein C18orf32
with a threshold of 1e−05
MR Method
Inverse variance weighted
MR Egger
4.0
3.6
3.2
2.8
2.4
1/SE_IV
−0.5
0.0
0.5
β_IV

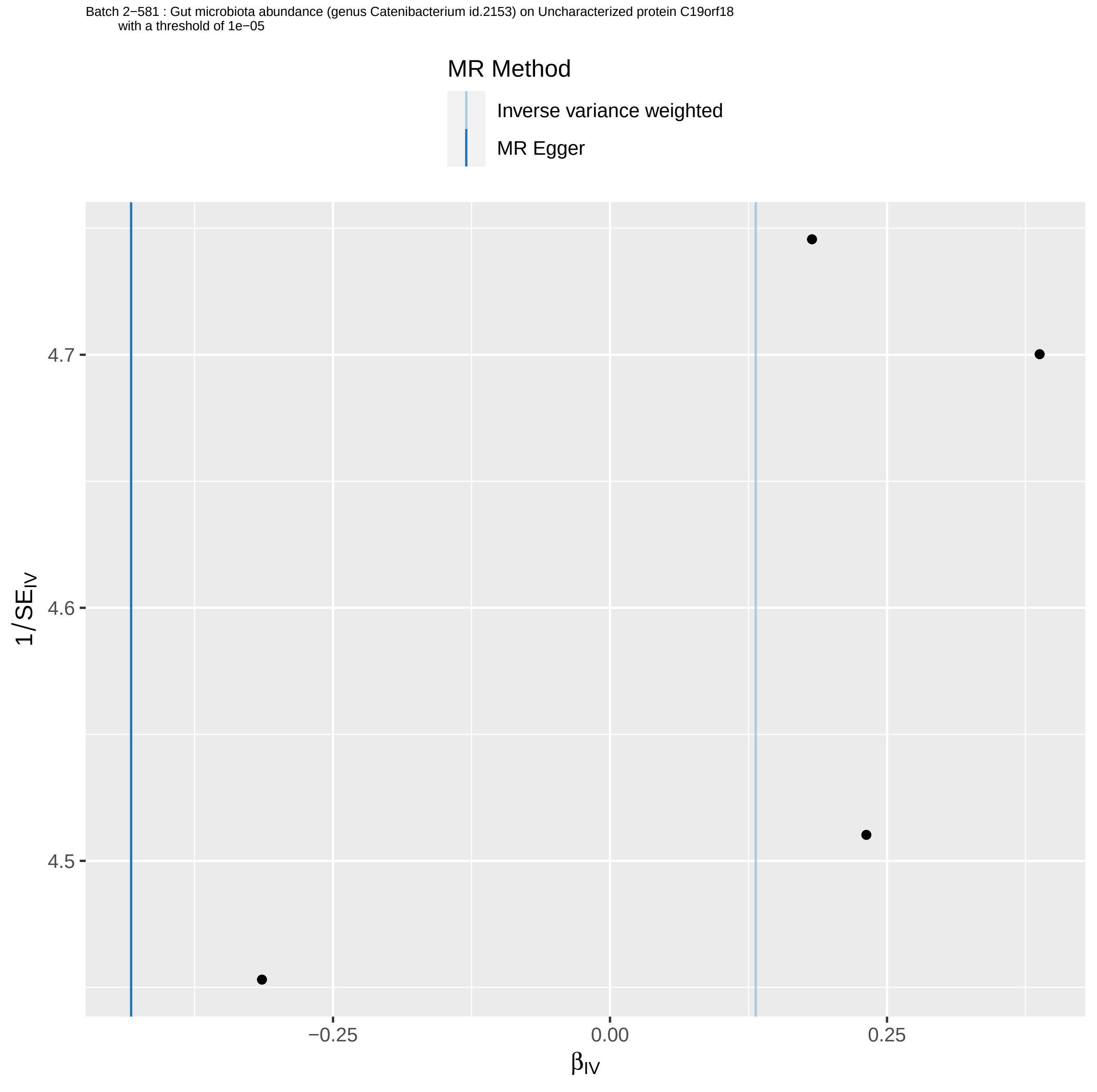

Batch 2−581 : Gut microbiota abundance (genus Catenibacterium id.2153) on Uncharacterized protein C19orf18
with a threshold of 1e−05
MR Method
Inverse variance weighted
MR Egger
4.7
4.6
4.5
1/SE$_{IV}$
−0.25
0.00
0.25
β$_{IV}$

Batch 2−582 : Gut microbiota abundance (genus Parasutterella id.2892) on Uncharacterized protein C19orf18
with a threshold of 1e−05

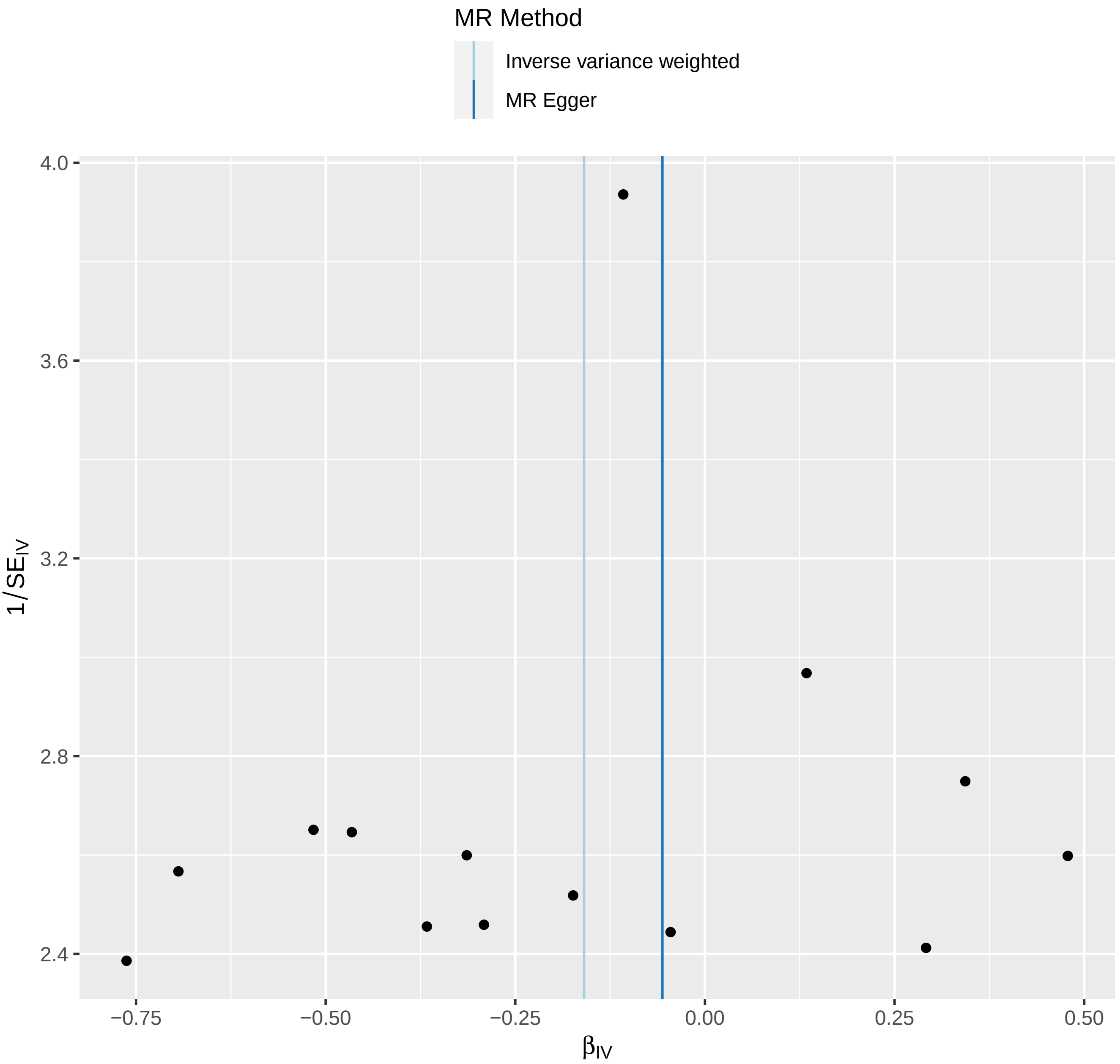

Batch 2−583 : Gut microbiota abundance (genus Catenibacterium id.2153) on Angiopoietin−like protein 8
with a threshold of 1e−05

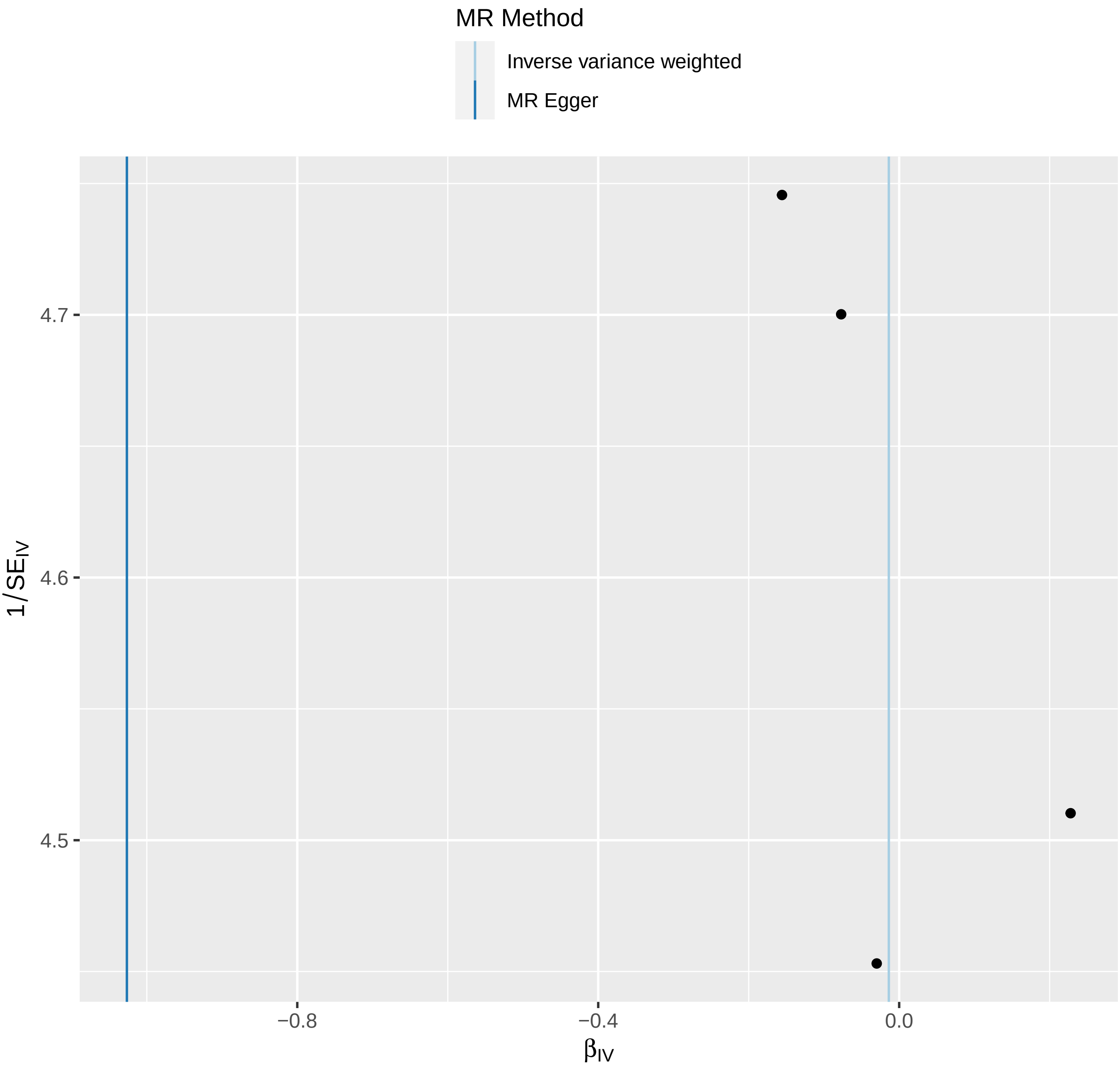

Batch 2−584 : Gut microbiota abundance (genus Parasutterella id.2892) on Angiopoietin−like protein 8
with a threshold of 1e−05

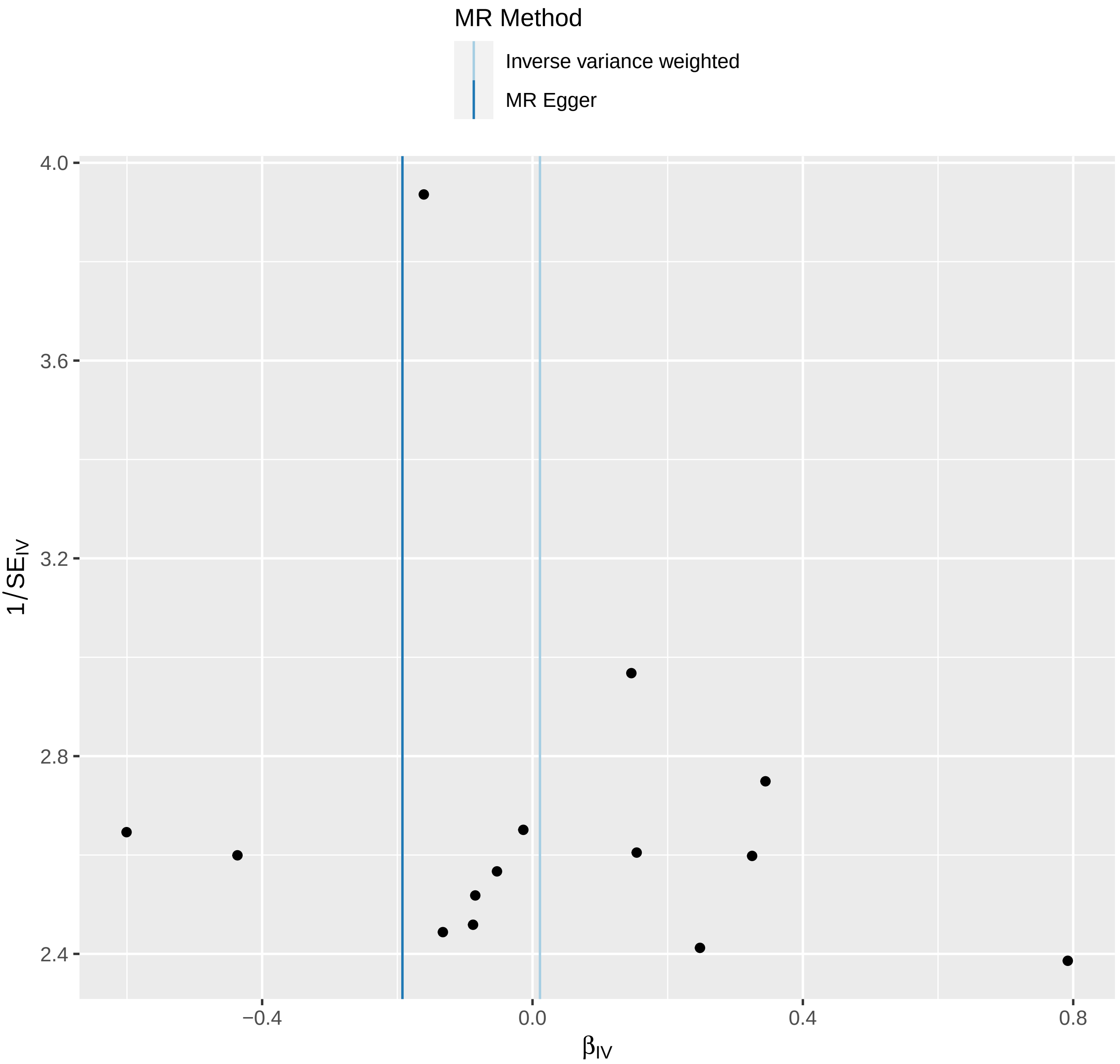

Batch 2−585 : Gut microbiota abundance (genus Catenibacterium id.2153) on Nuclear nucleic acid−binding protein C1D
with a threshold of 1e−05

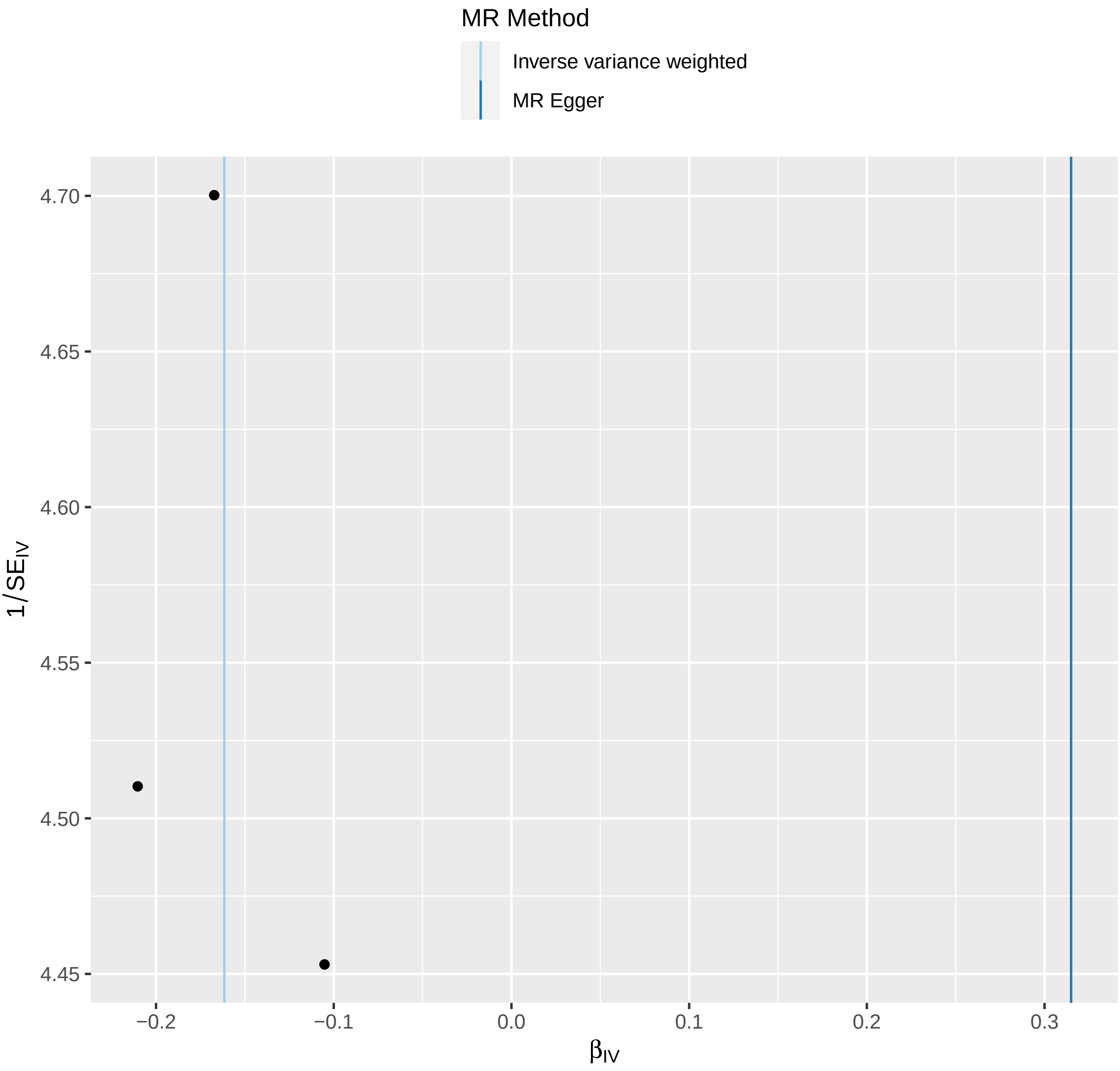

Batch 2−586 : Gut microbiota abundance (genus Parasutterella id.2892) on Nuclear nucleic acid−binding protein C1D
with a threshold of 1e−05

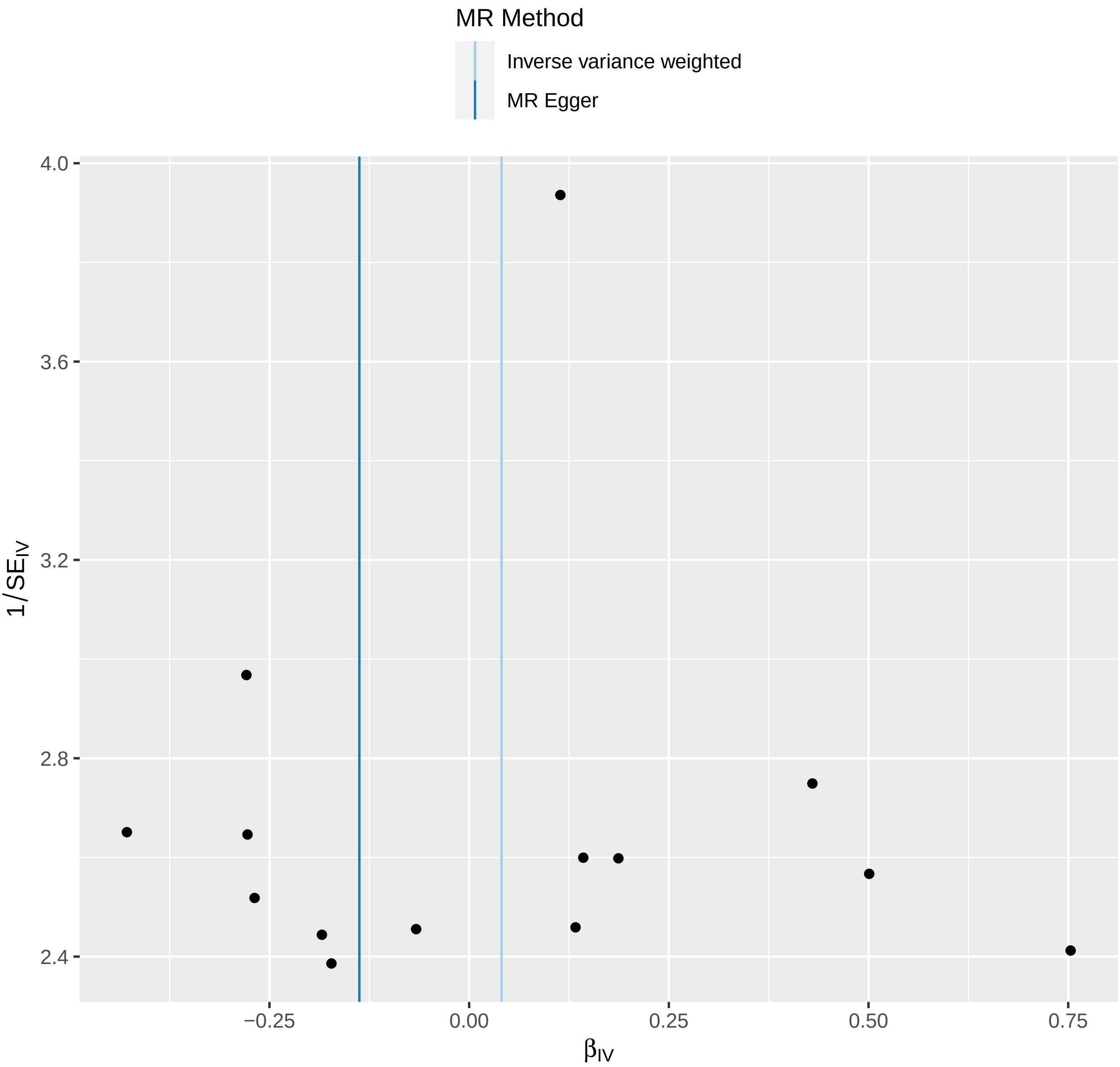

Batch 2−587 : Gut microbiota abundance (genus Catenibacterium id.2153) on C1GALT1−specific chaperone 1 with a threshold of 1e−05

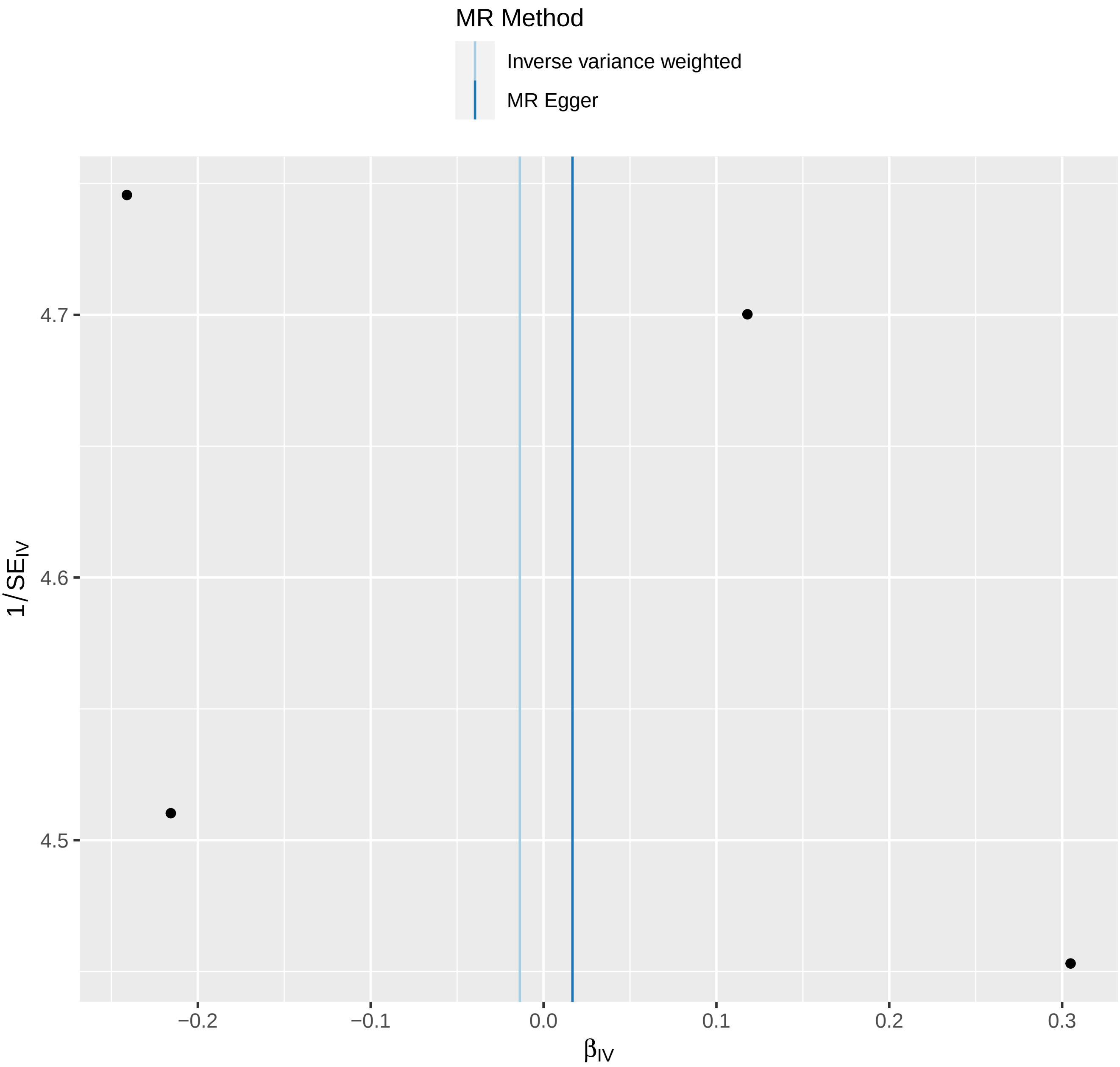

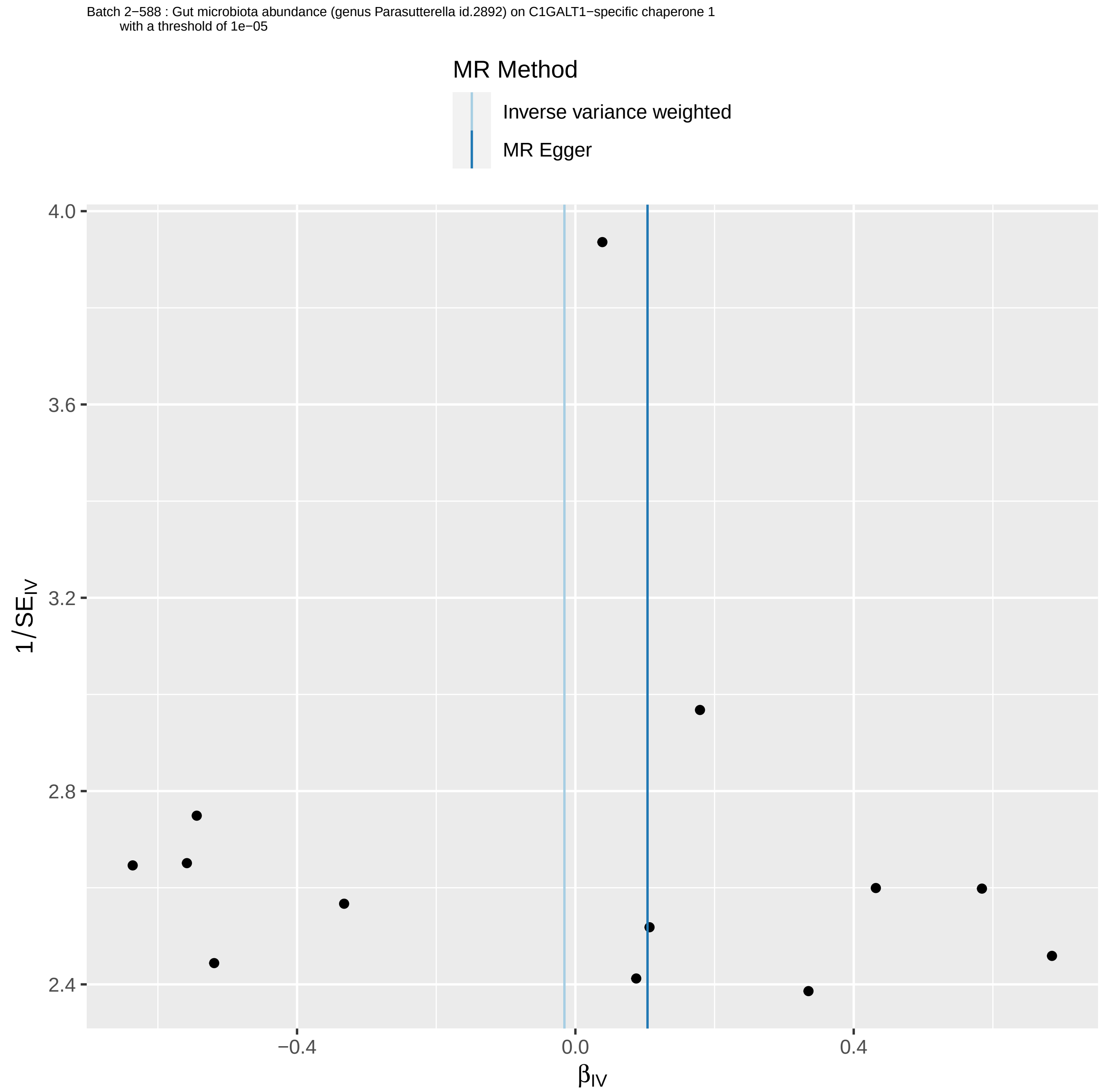
Batch 2−588 : Gut microbiota abundance (genus Parasutterella id.2892) on C1GALT1−specific chaperone 1
with a threshold of 1e−05
MR Method
Inverse variance weighted
MR Egger
4.0
3.6
3.2
2.8
2.4
1/SE_IV
−0.4
0.0
0.4
β_IV

Batch 2−589 : Gut microbiota abundance (genus Catenibacterium id.2153) on Uncharacterized protein C1orf115
with a threshold of 1e−05

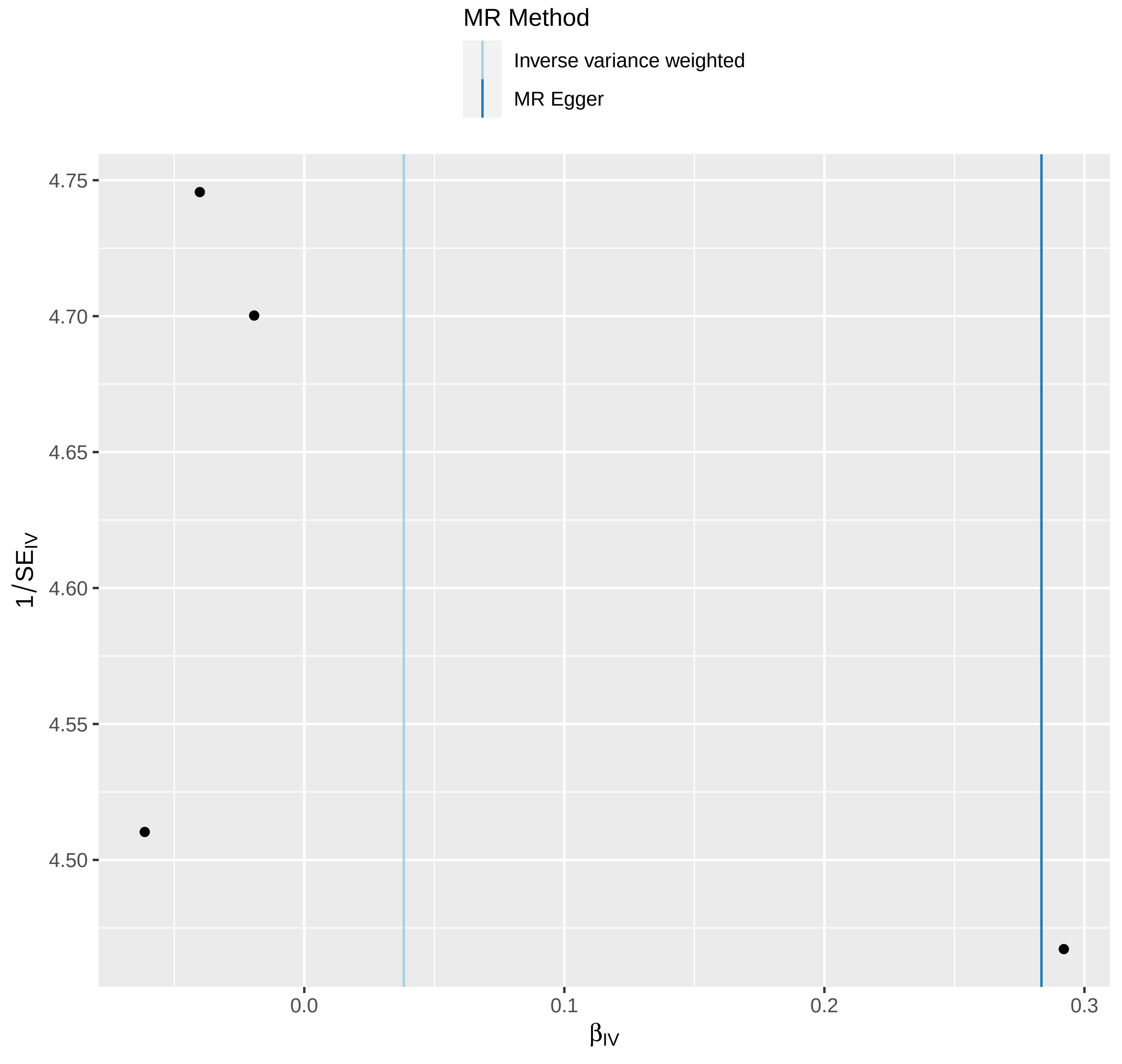

Batch 2−590 : Gut microbiota abundance (genus Parasutterella id.2892) on Uncharacterized protein C1orf115
with a threshold of 1e−05

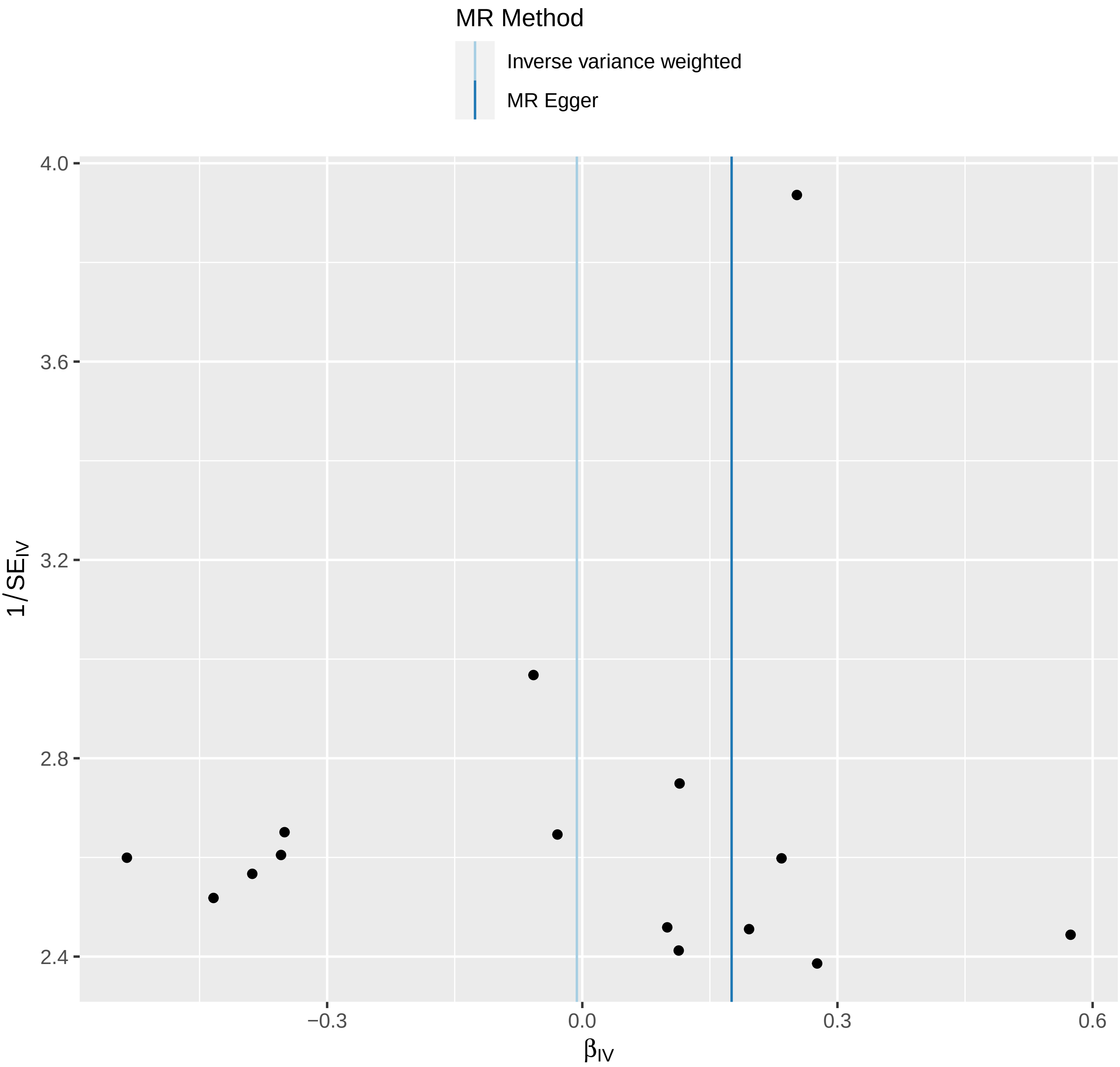

Batch 2−591 : Gut microbiota abundance (genus Catenibacterium id.2153) on Uncharacterized protein C1orf185
with a threshold of 1e−05

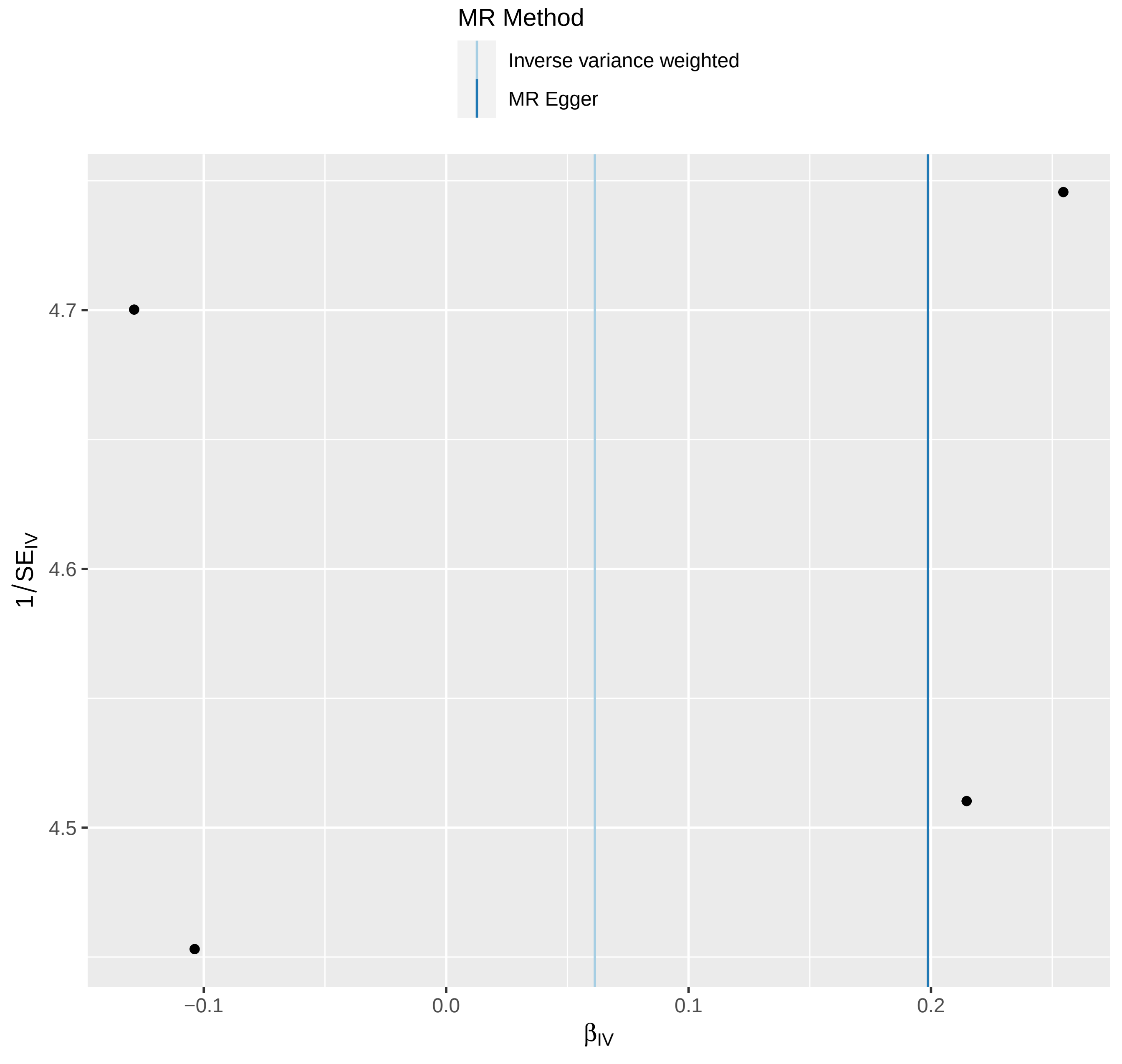

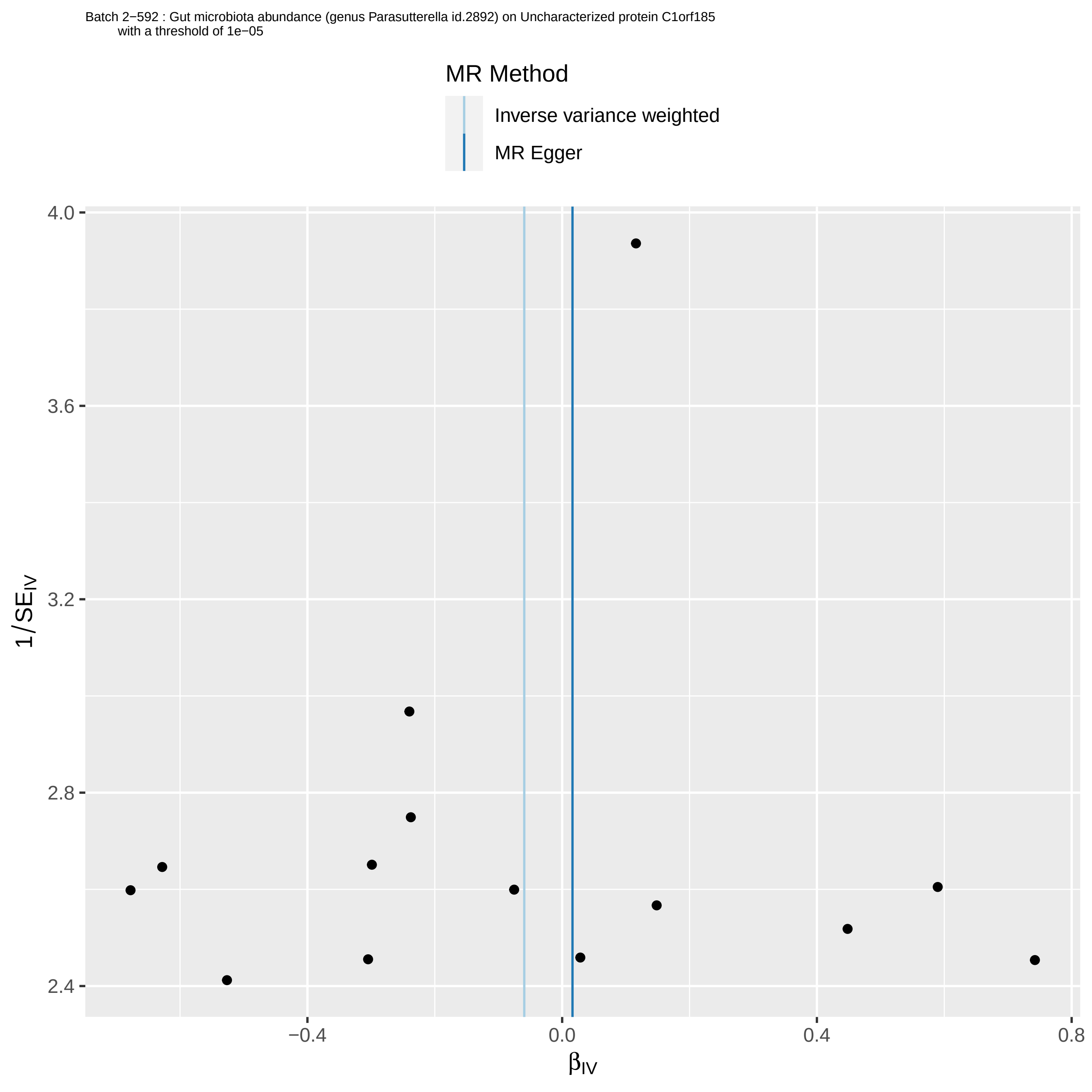
Batch 2−592 : Gut microbiota abundance (genus Parasutterella id.2892) on Uncharacterized protein C1orf185
with a threshold of 1e−05
MR Method
Inverse variance weighted
MR Egger
4.0
3.6
3.2
2.8
2.4
1/SE_IV
−0.4
0.0
0.4
0.8
β_IV

Batch 2−593 : Gut microbiota abundance (genus Catenibacterium id.2153) on Uncharacterized protein C1orf198
with a threshold of 1e−05

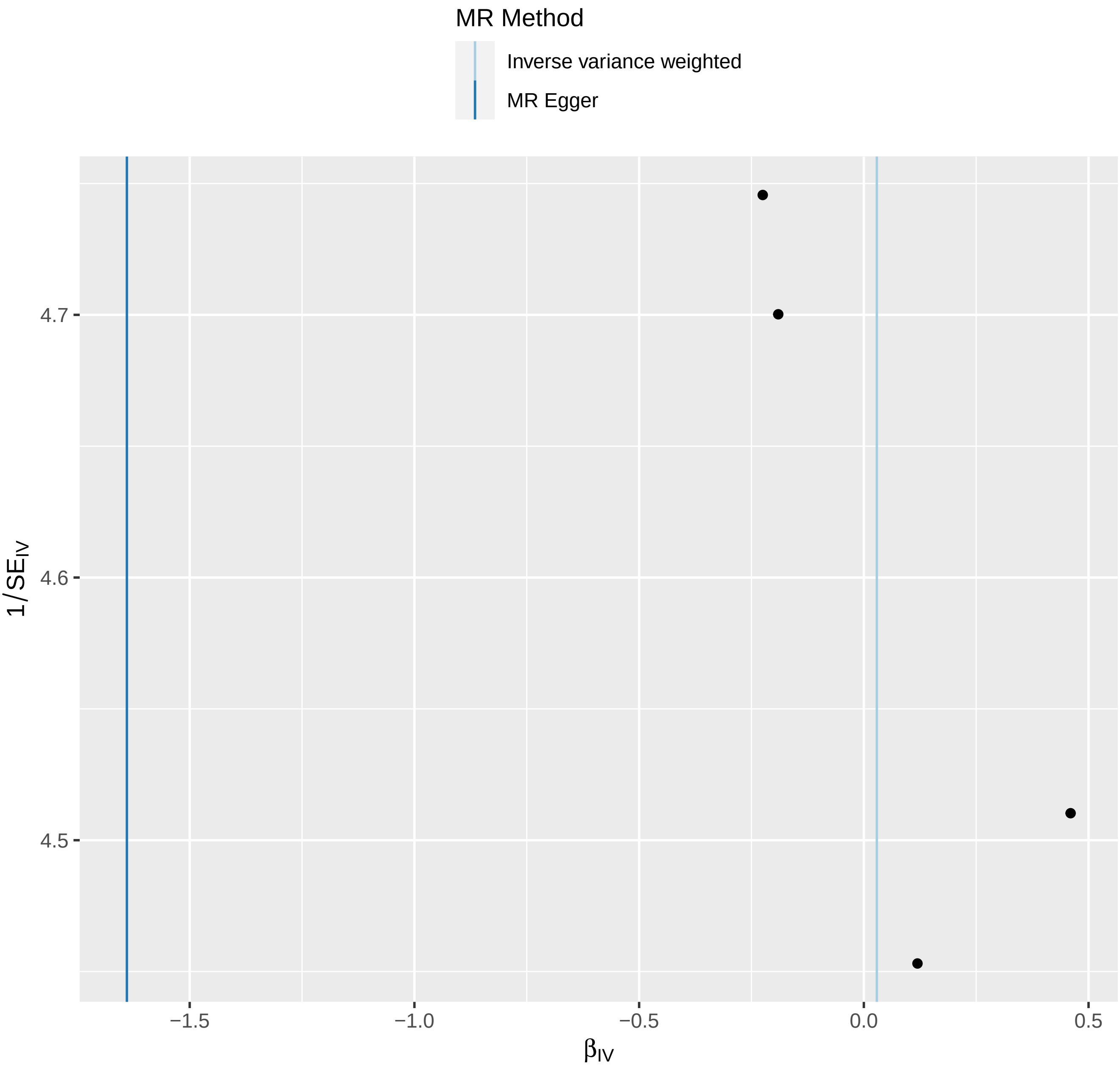

Batch 2−594 : Gut microbiota abundance (genus Parasutterella id.2892) on Uncharacterized protein C1orf198 with a threshold of 1e−05

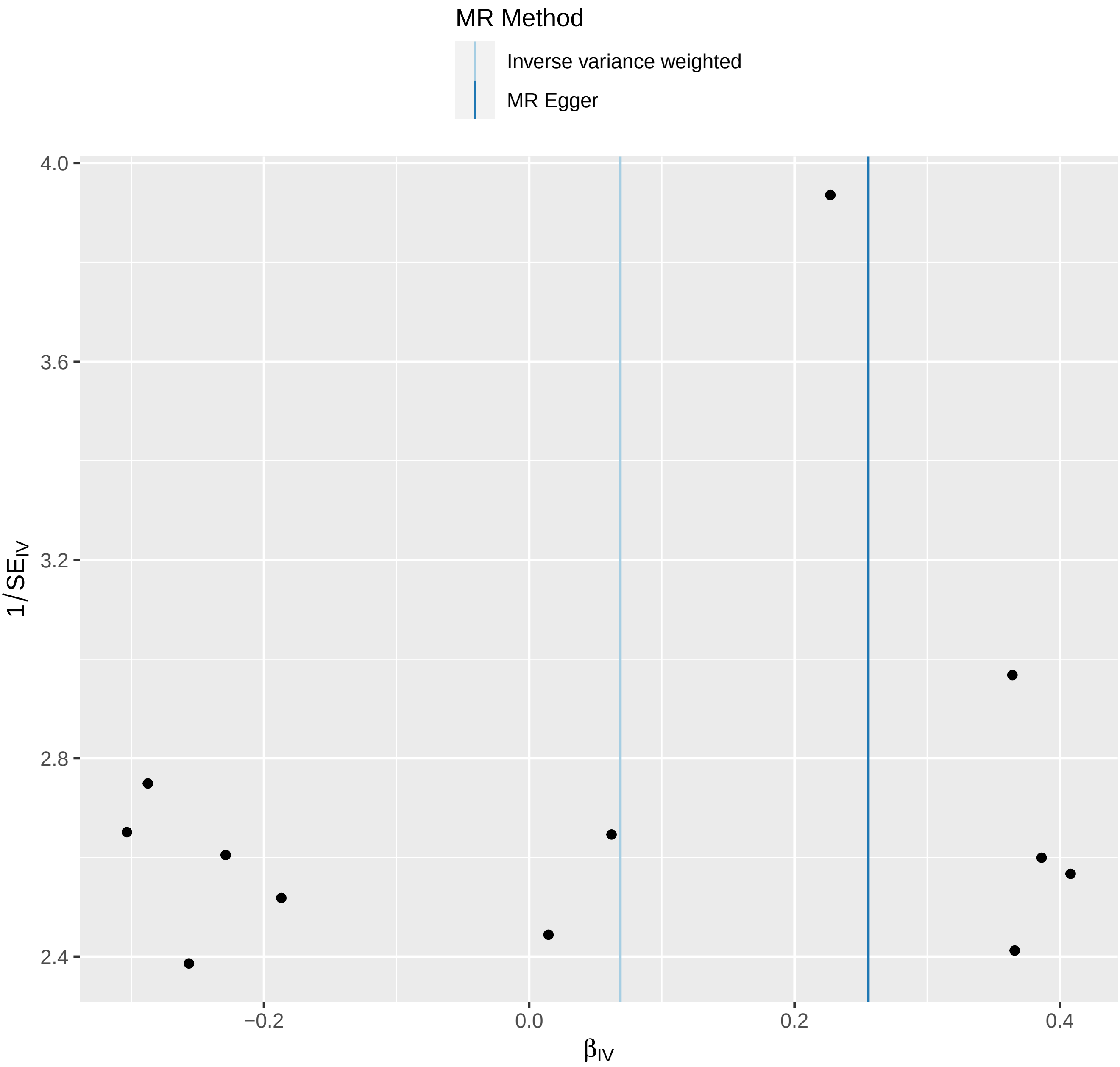

Batch 2−595 : Gut microbiota abundance (genus Catenibacterium id.2153) on Type III endosome membrane protein TEMP with a threshold of 1e−05

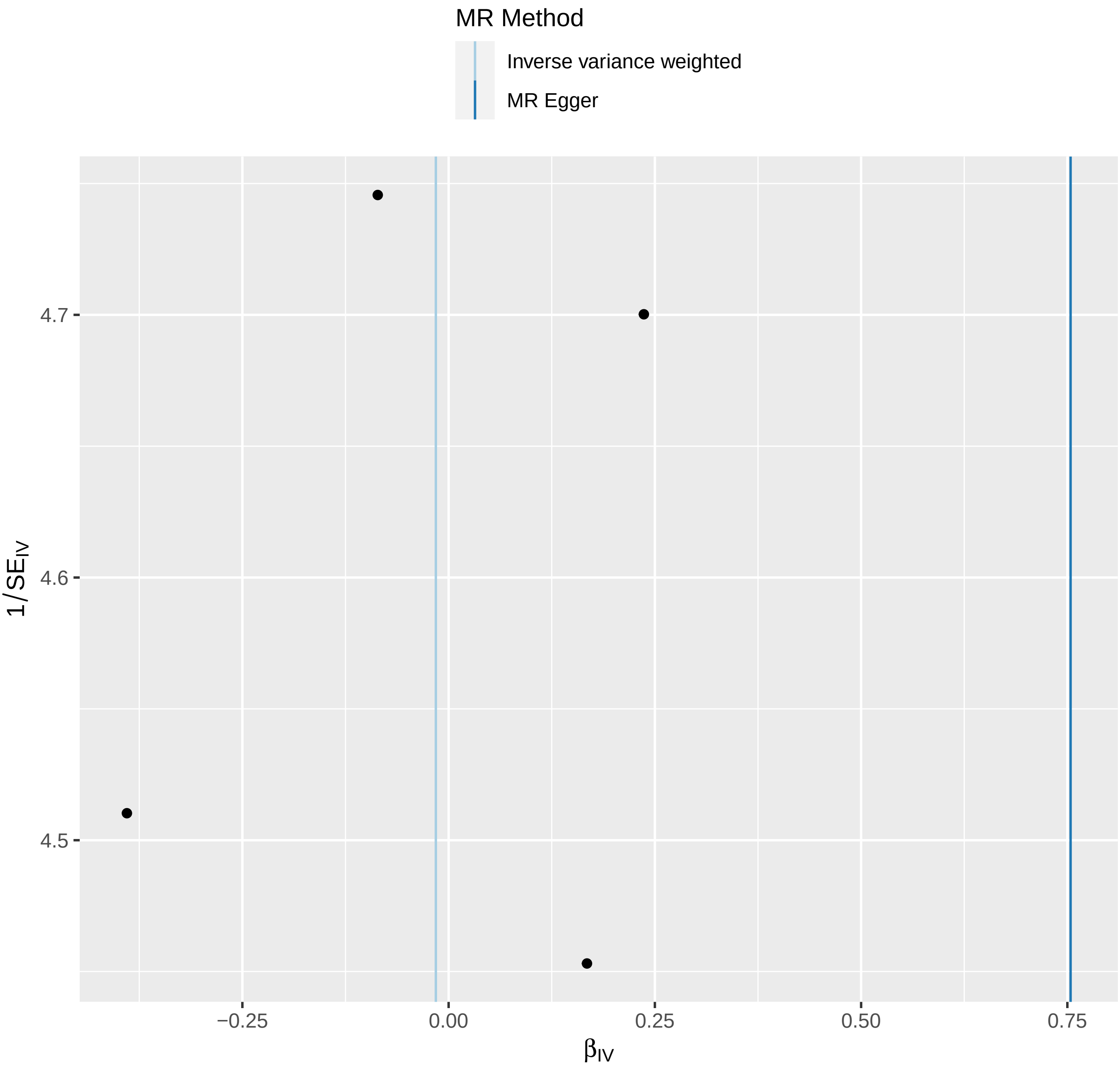

Batch 2−596 : Gut microbiota abundance (genus Parasutterella id.2892) on Type III endosome membrane protein TEMP
with a threshold of 1e−05

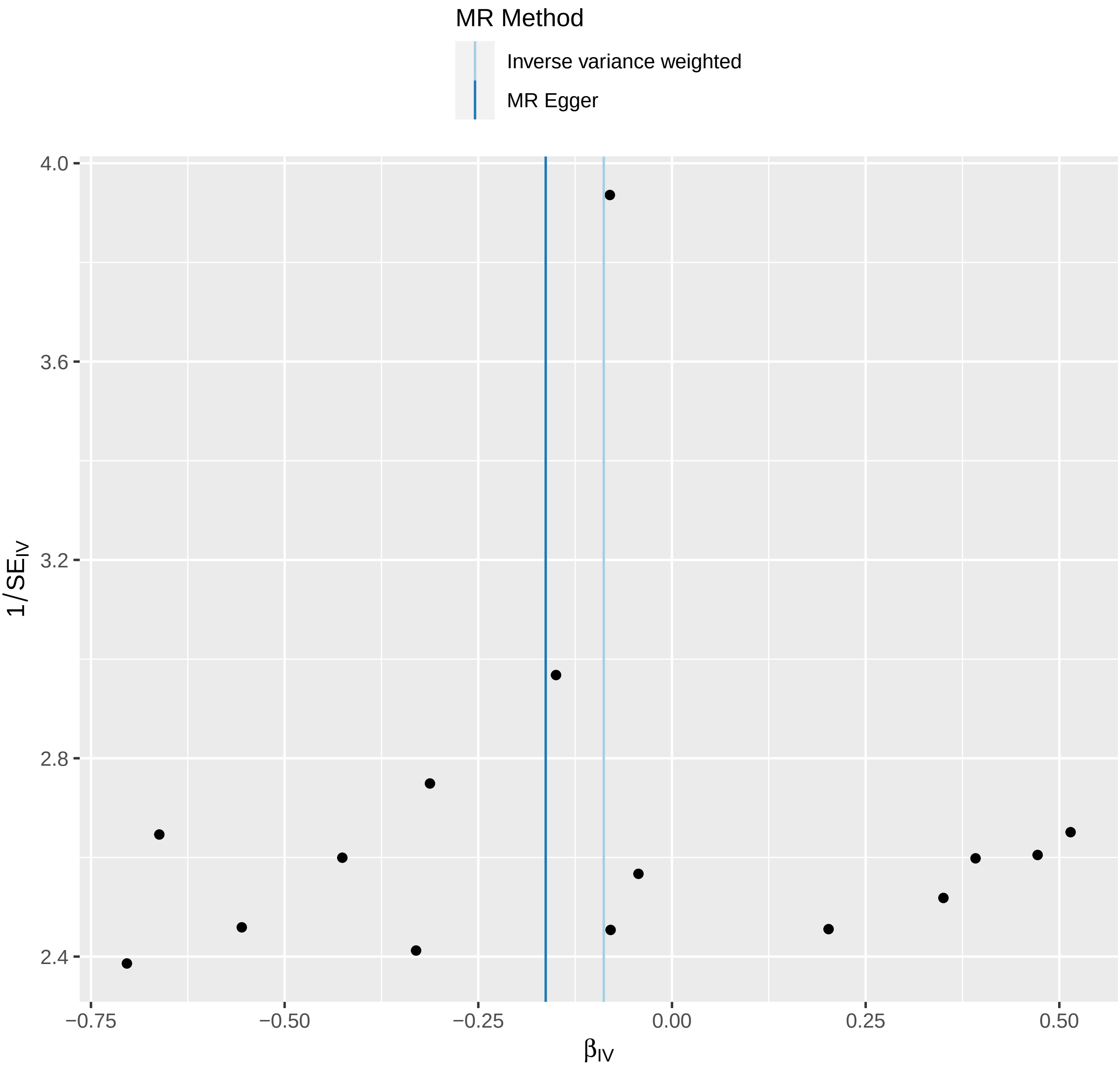

Batch 2−597 : Gut microbiota abundance (genus Catenibacterium id.2153) on Uncharacterized protein C1orf226
with a threshold of 1e−05

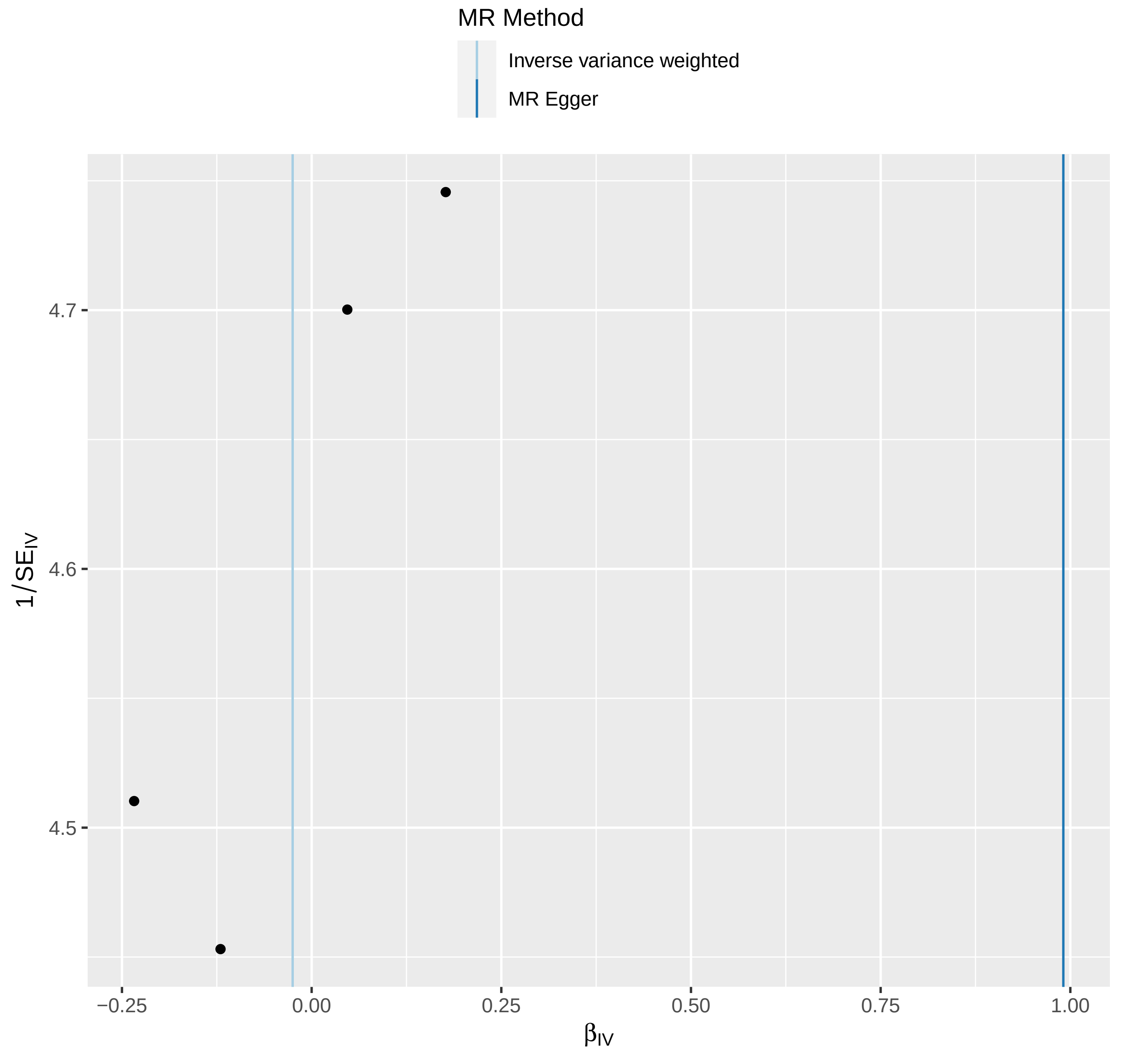

Batch 2−598 : Gut microbiota abundance (genus Parasutterella id.2892) on Uncharacterized protein C1orf226
with a threshold of 1e−05

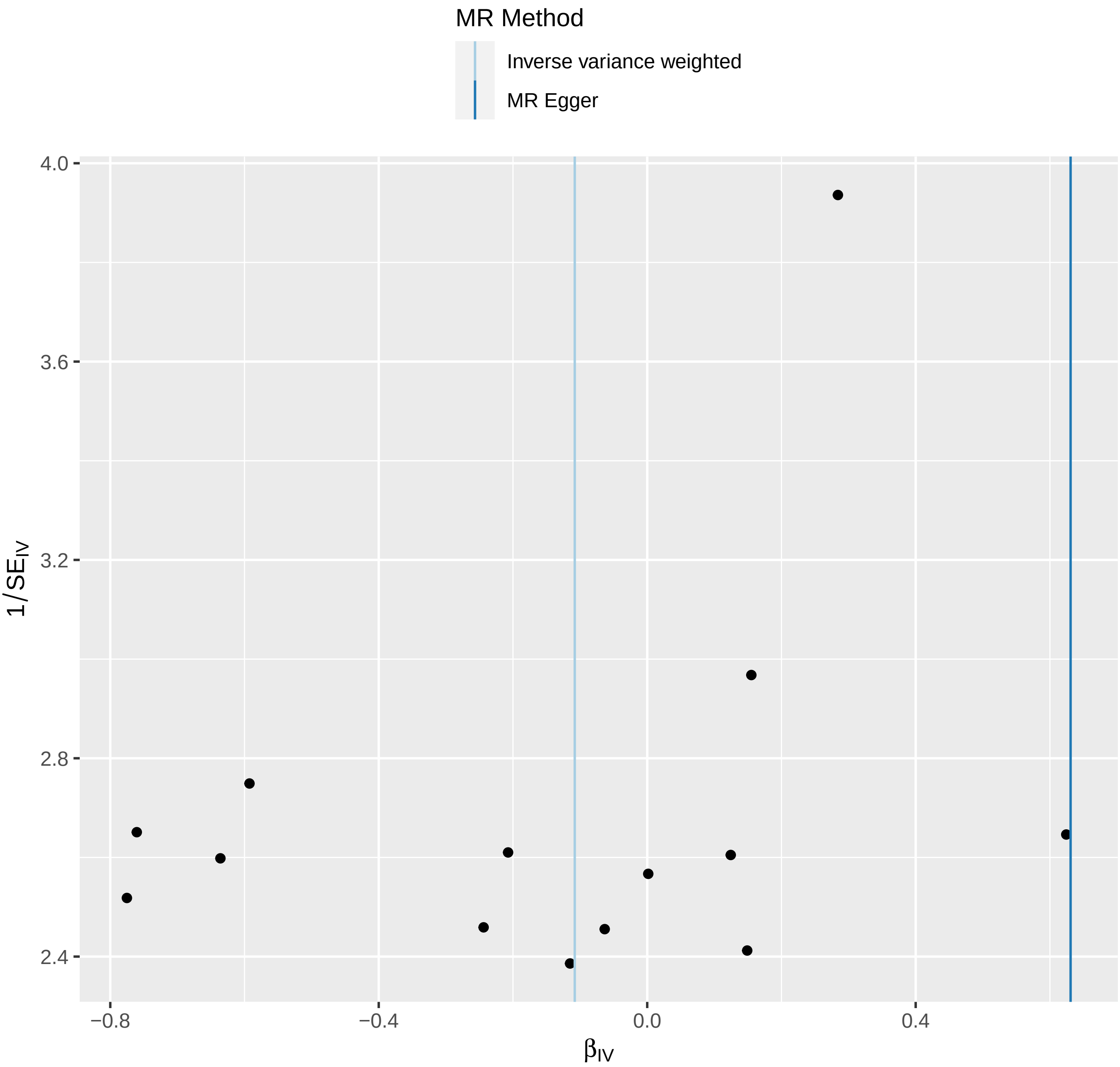

Batch 2−599 : Gut microbiota abundance (genus Catenibacterium id.2153) on Complement component 1 Q subcomponent−binding protein, mitochondrial with a threshold of 1e−05

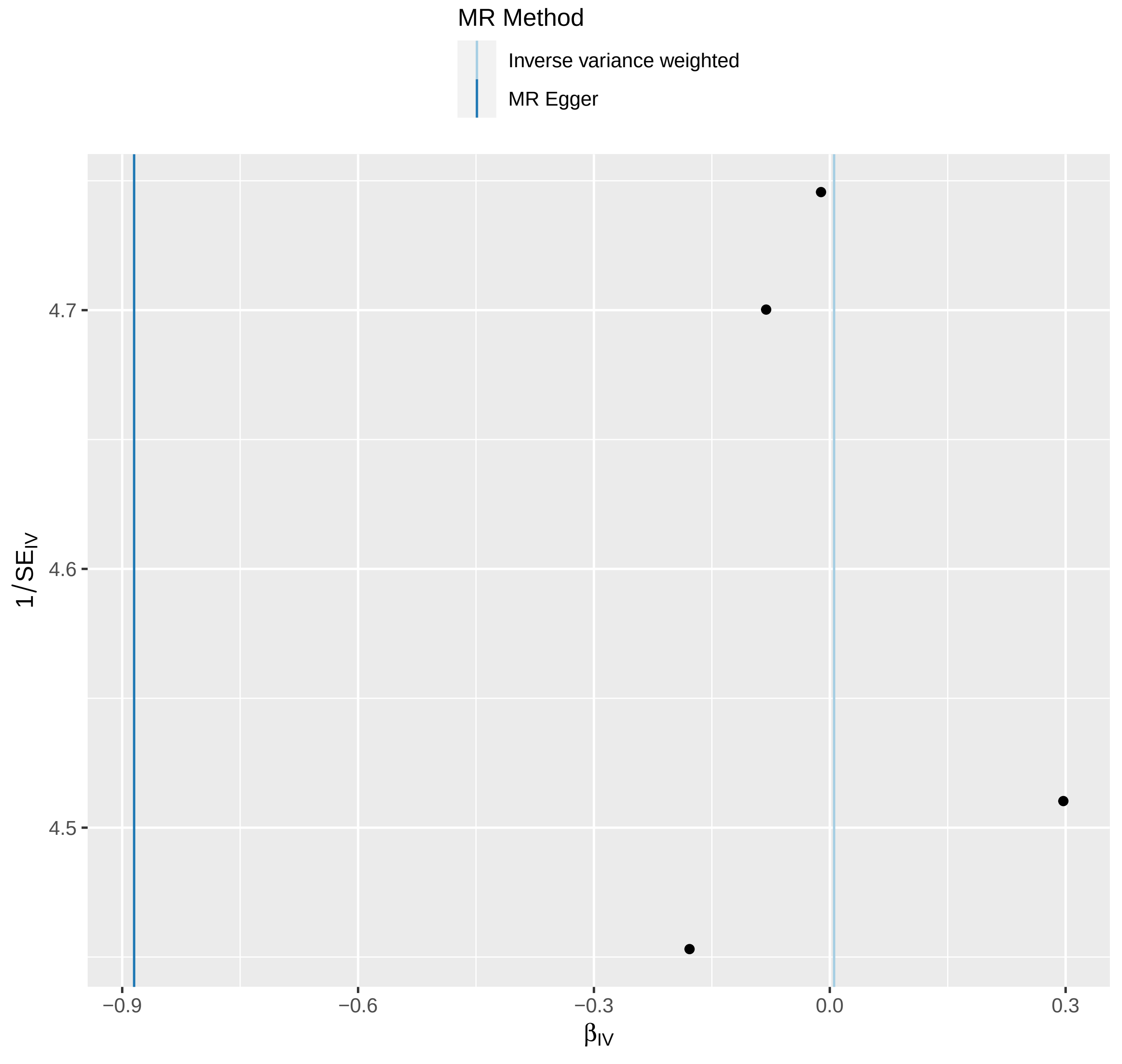

Batch 2−600 : Gut microbiota abundance (genus Parasutterella id.2892) on Complement component 1 Q subcomponent−binding protein, mitochondrial with a threshold of 1e−05

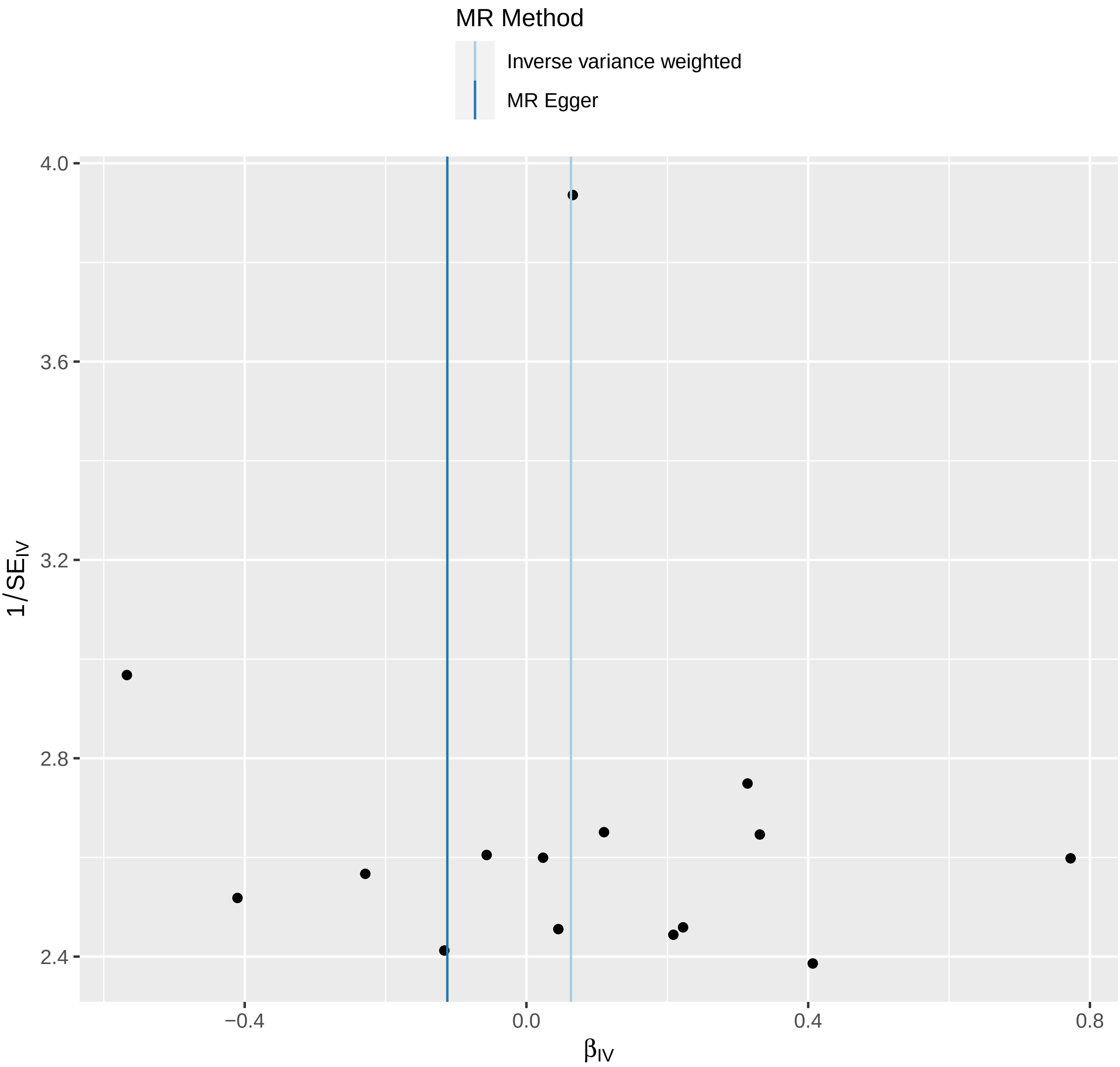

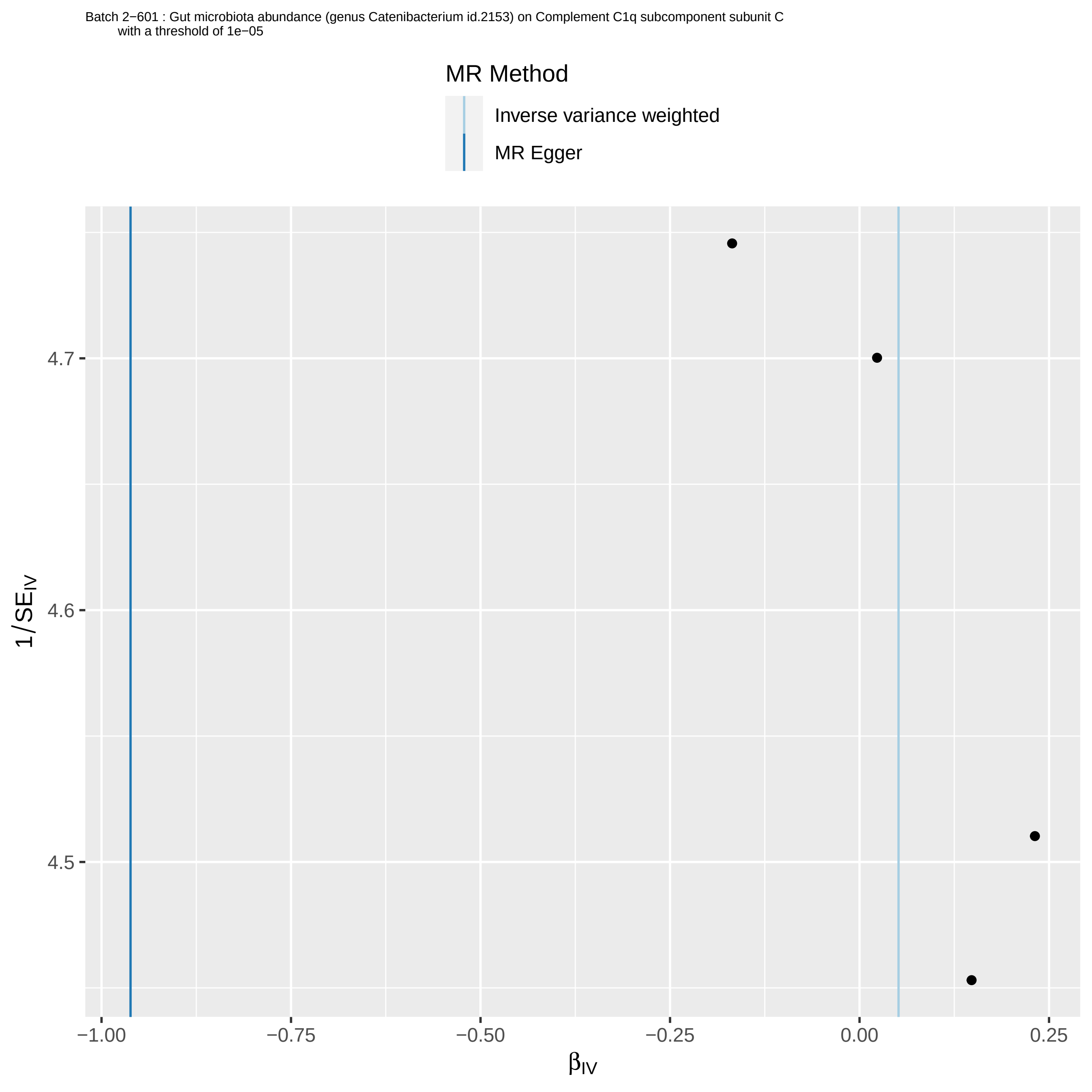

Batch 2−601 : Gut microbiota abundance (genus Catenibacterium id.2153) on Complement C1q subcomponent subunit C
with a threshold of 1e−05
MR Method
Inverse variance weighted
MR Egger
4.7
4.6
4.5
1/SE_IV
−1.00
−0.75
−0.50
−0.25
0.00
0.25
β_IV

Batch 2−602 : Gut microbiota abundance (genus Parasutterella id.2892) on Complement C1q subcomponent subunit C
with a threshold of 1e−05

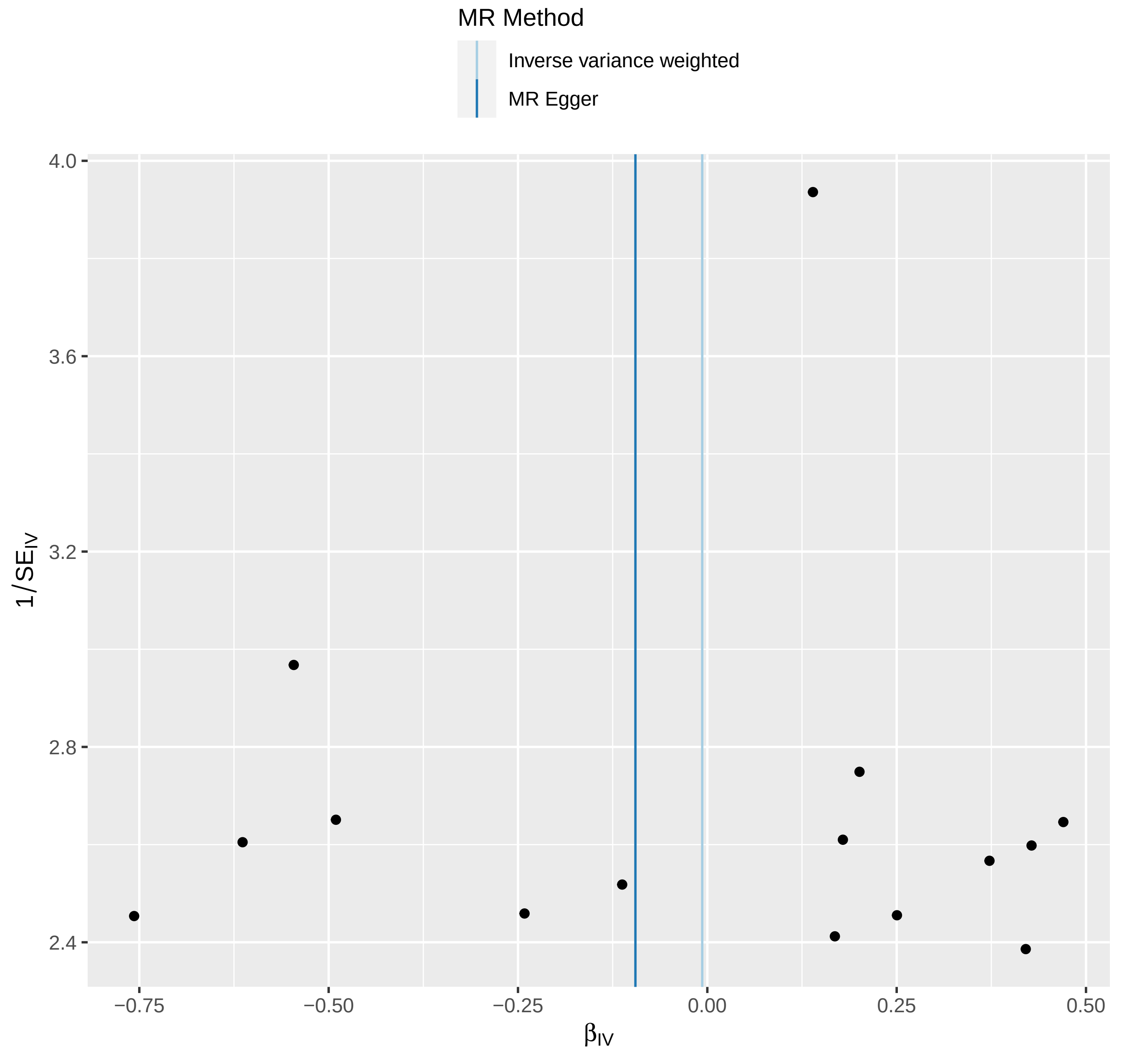

Batch 2−603 : Gut microbiota abundance (genus Catenibacterium id.2153) on Complement C1q−like protein 4 with a threshold of 1e−05

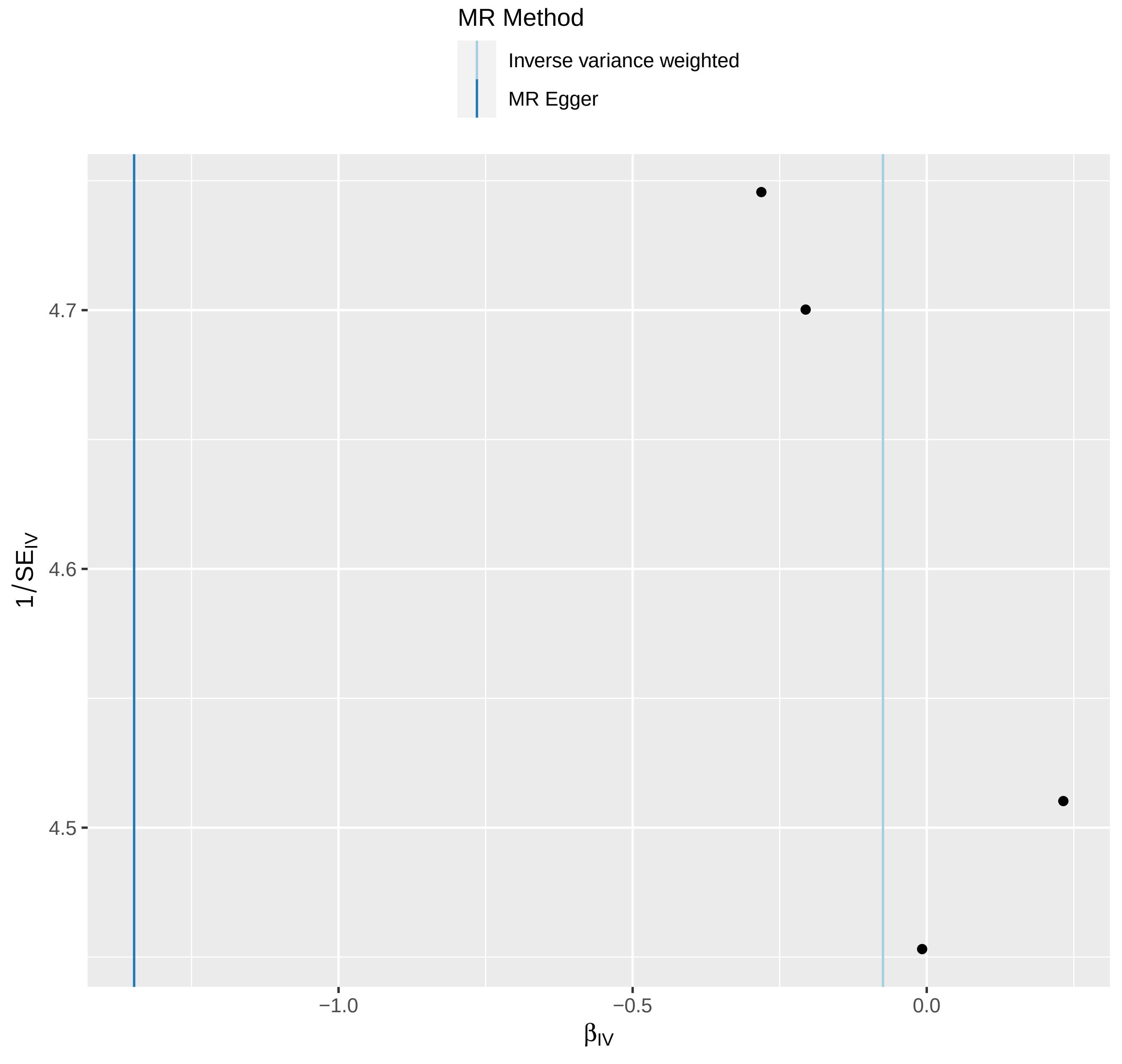

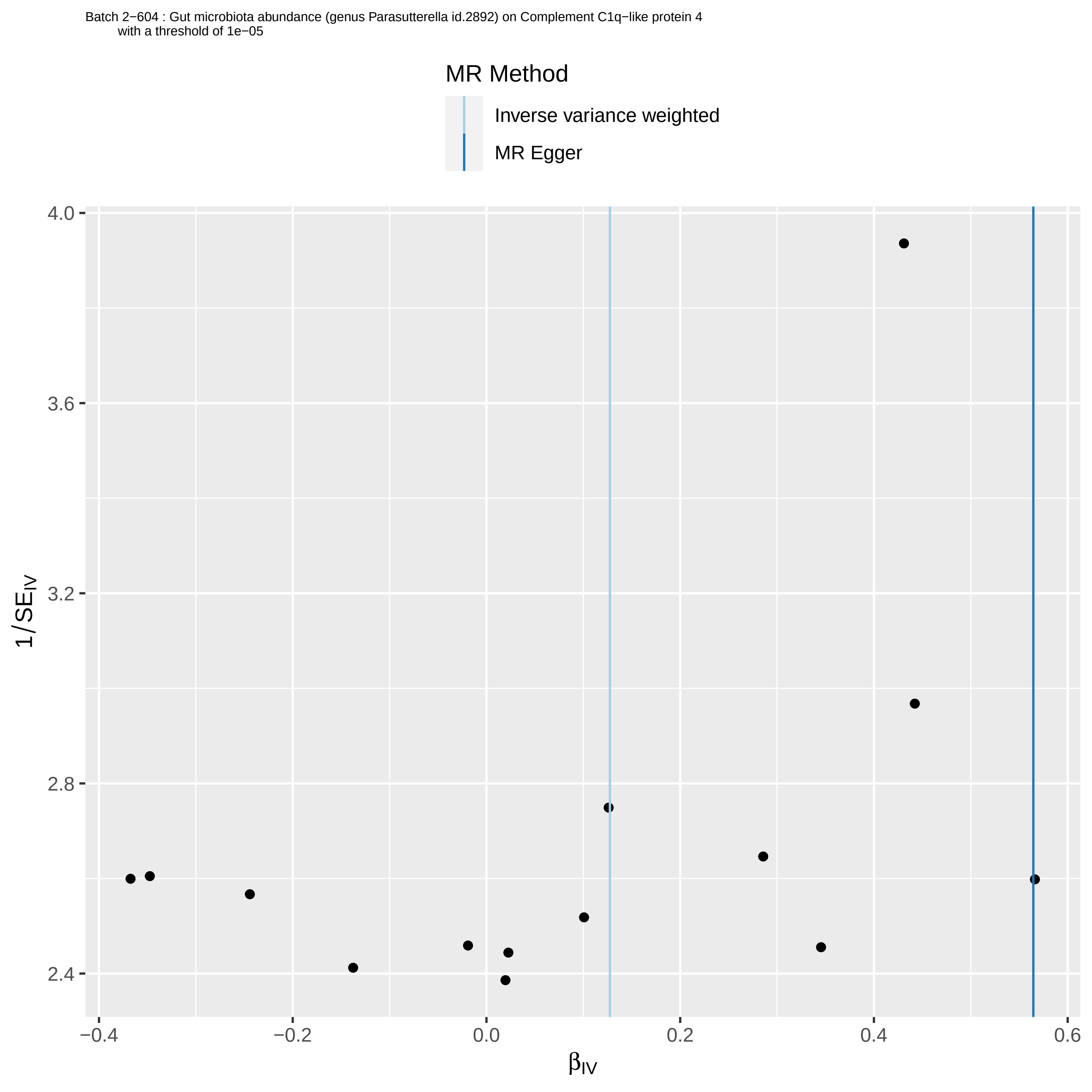

Batch 2−604 : Gut microbiota abundance (genus Parasutterella id.2892) on Complement C1q−like protein 4
with a threshold of 1e−05
MR Method
Inverse variance weighted
MR Egger
4.0
3.6
3.2
2.8
2.4
$1/SE_{IV}$
−0.4
−0.2
0.0
0.2
0.4
0.6
β_{IV}

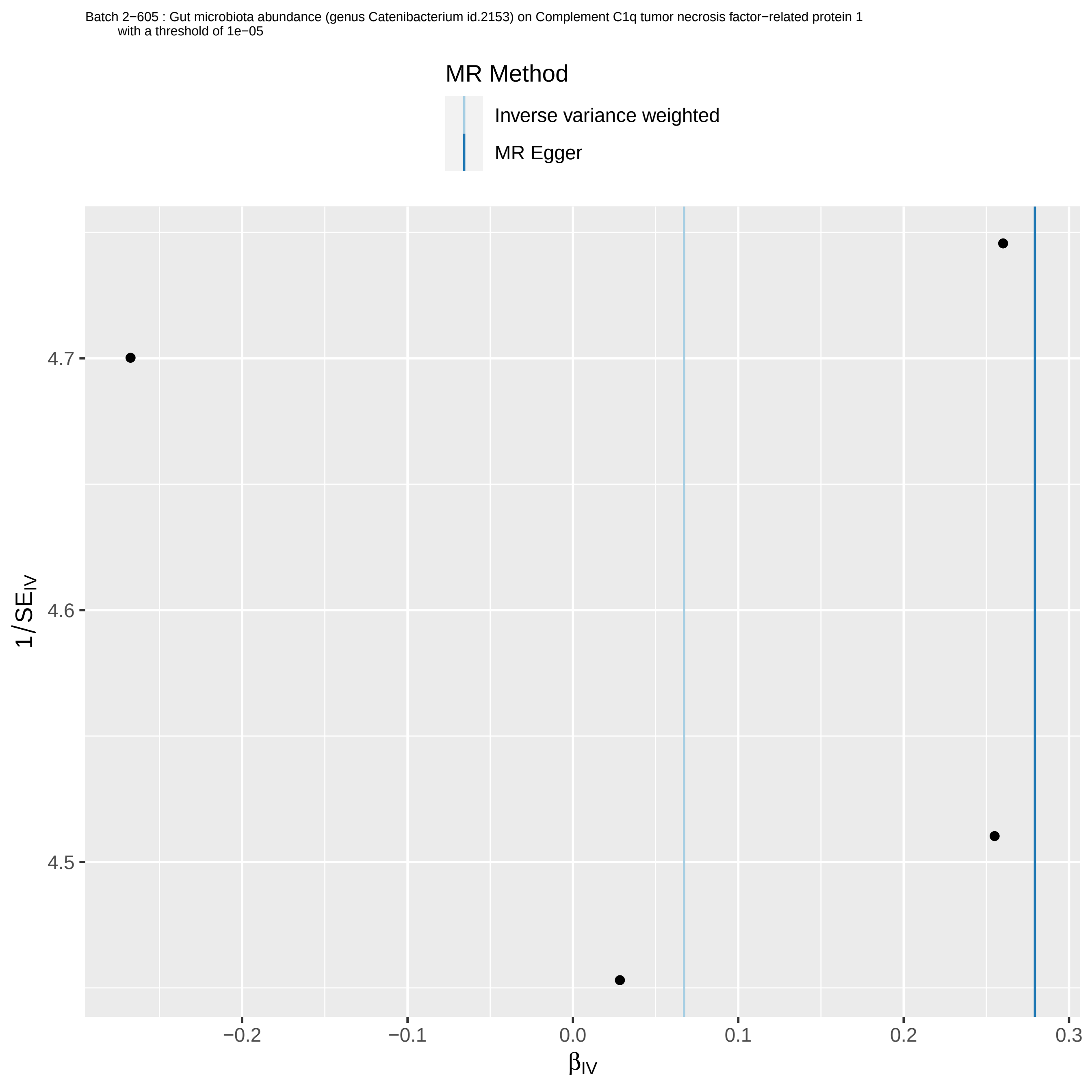

Batch 2−605 : Gut microbiota abundance (genus Catenibacterium id.2153) on Complement C1q tumor necrosis factor−related protein 1
with a threshold of 1e−05
MR Method
Inverse variance weighted
MR Egger
4.7
4.6
4.5
1/SE_IV
−0.2
−0.1
0.0
0.1
0.2
0.3
β_IV

Batch 2−606 : Gut microbiota abundance (genus Parasutterella id.2892) on Complement C1q tumor necrosis factor−related protein 1
with a threshold of 1e−05

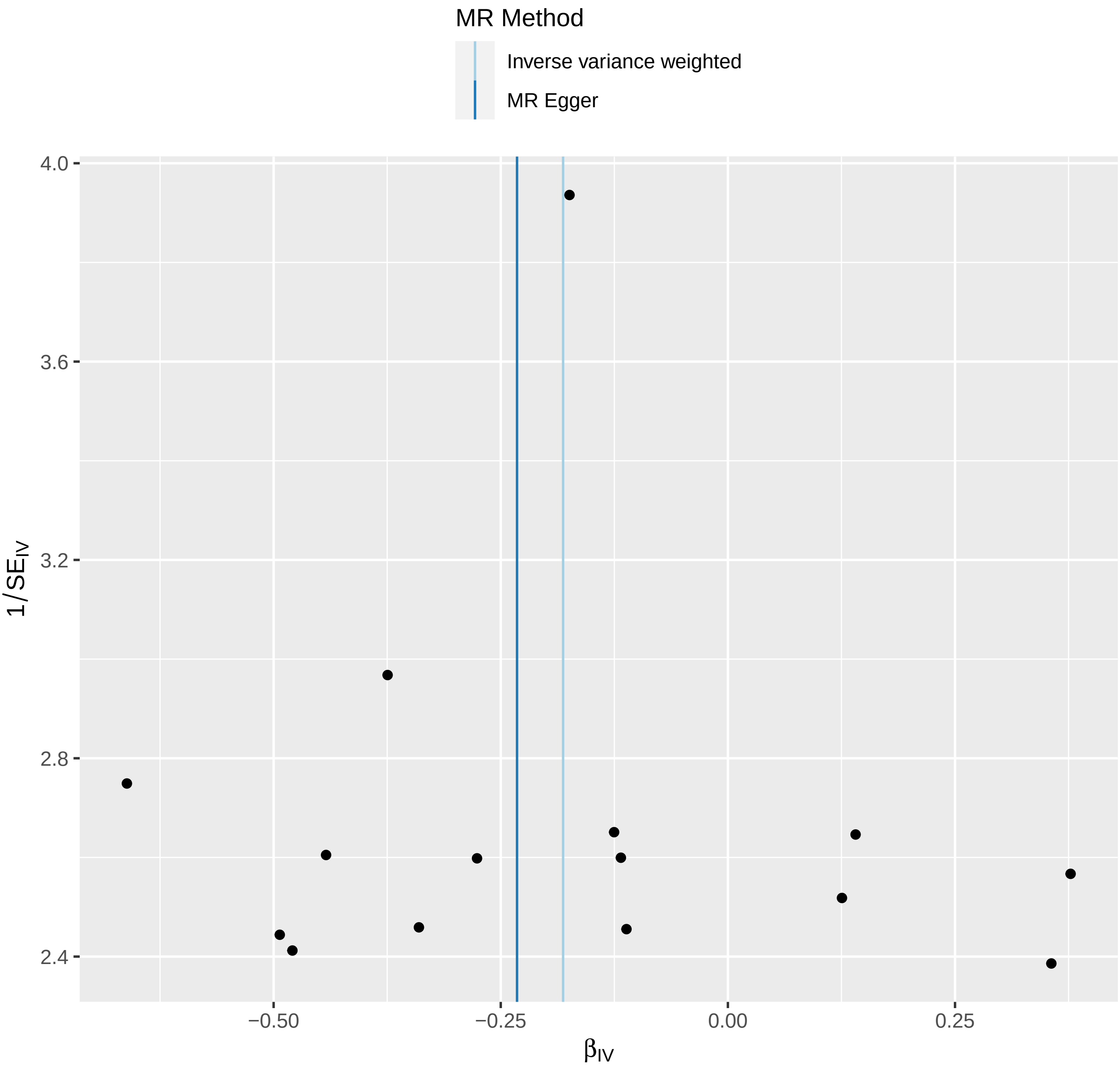

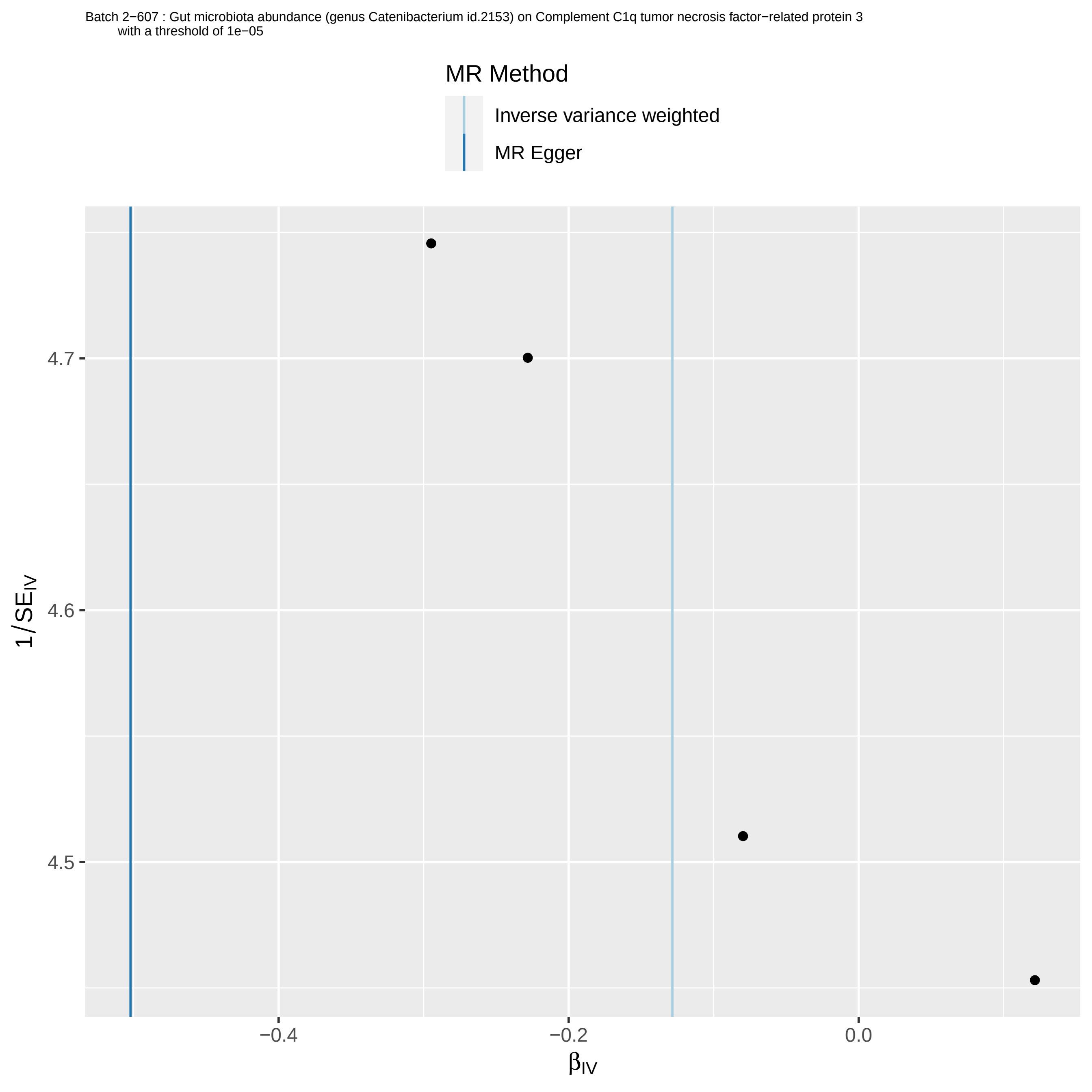

Batch 2−607 : Gut microbiota abundance (genus Catenibacterium id.2153) on Complement C1q tumor necrosis factor−related protein 3
with a threshold of 1e−05
MR Method
Inverse variance weighted
MR Egger
4.7
4.6
4.5
1/SE$_{IV}$
−0.4
−0.2
0.0
β_{IV}

Batch 2−608 : Gut microbiota abundance (genus Parasutterella id.2892) on Complement C1q tumor necrosis factor−related protein 3 with a threshold of 1e−05

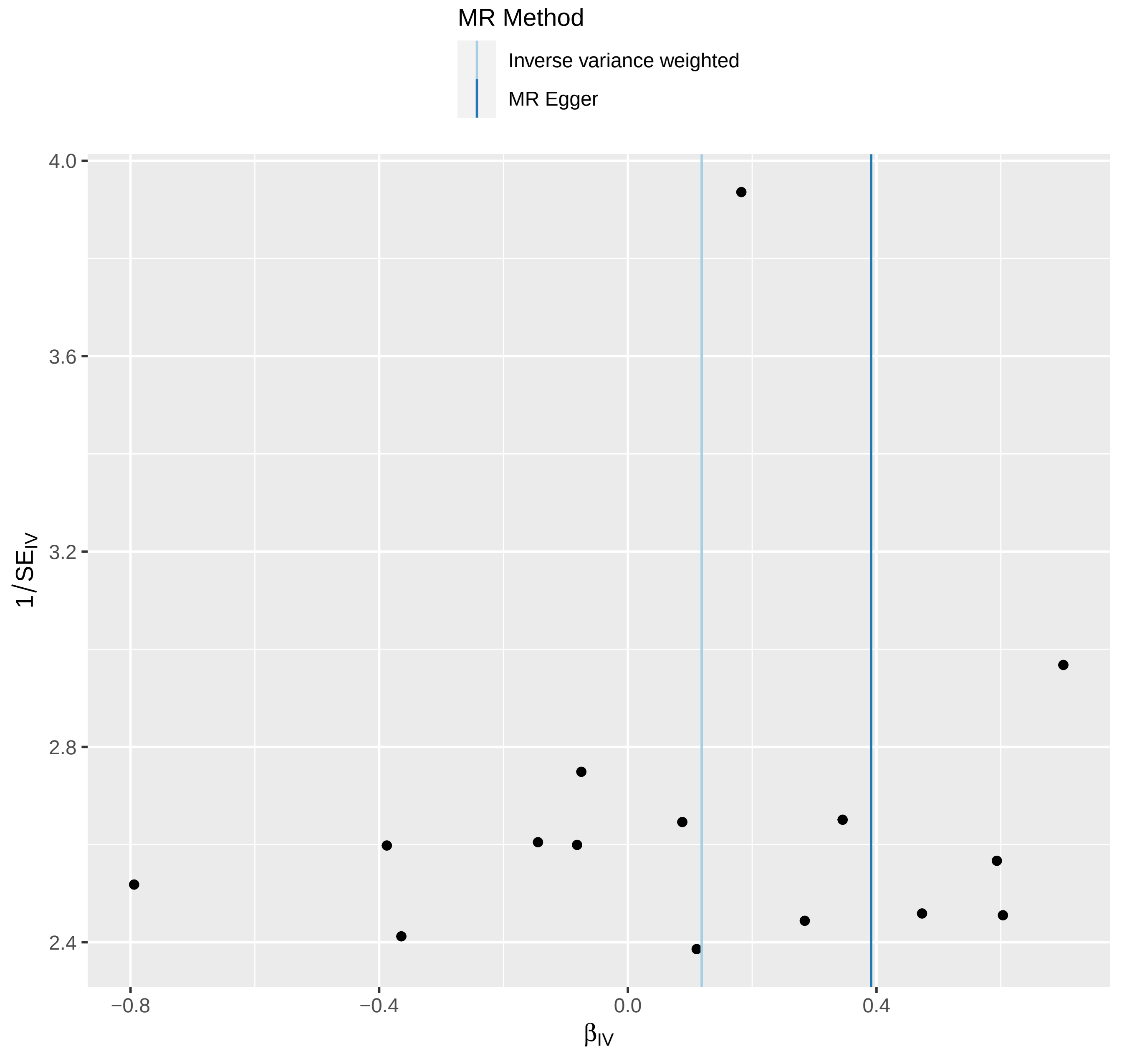

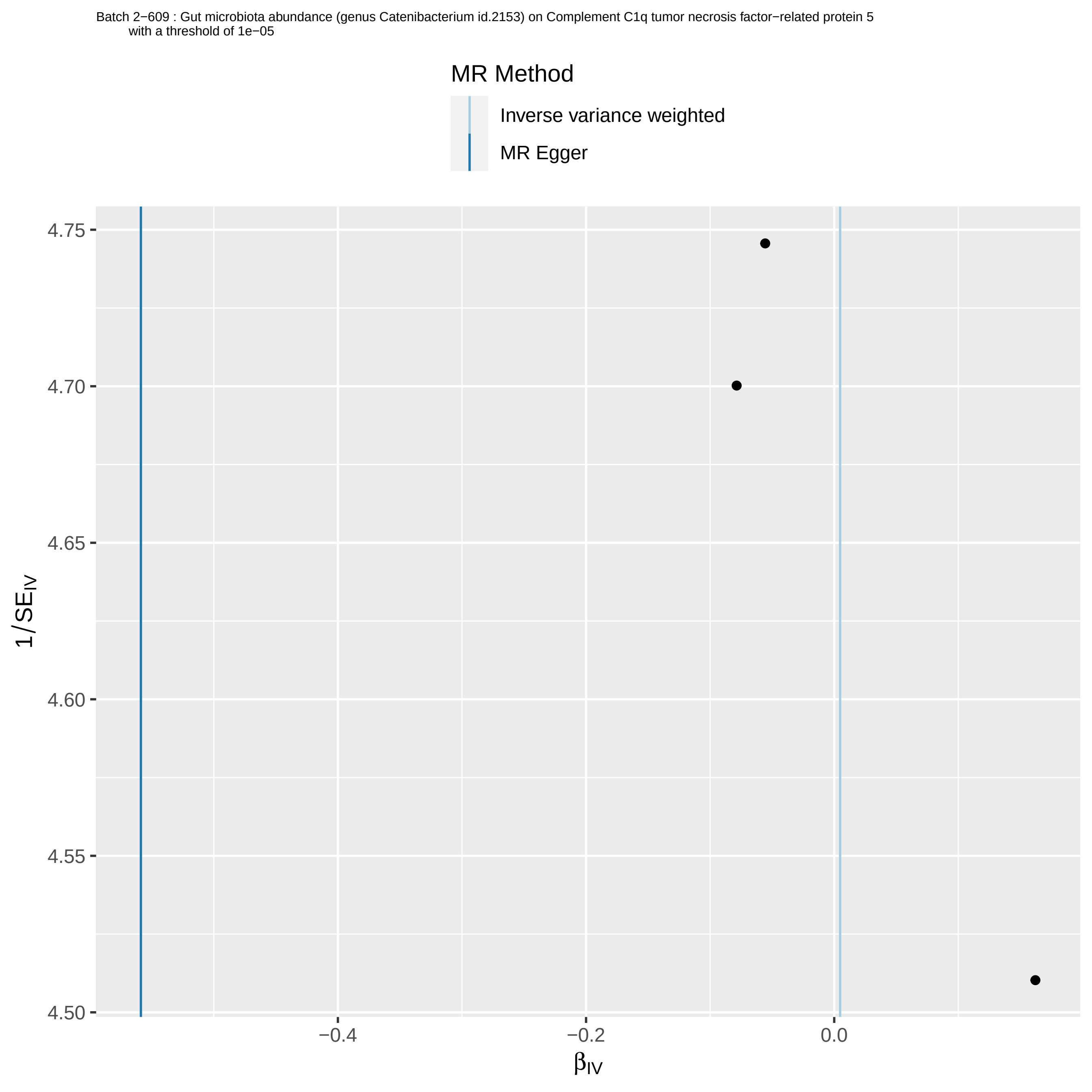
Batch 2−609 : Gut microbiota abundance (genus Catenibacterium id.2153) on Complement C1q tumor necrosis factor−related protein 5
with a threshold of 1e−05
MR Method
Inverse variance weighted
MR Egger
4.75
4.70
4.65
4.60
4.55
4.50
1/SE_IV
−0.4
−0.2
0.0
β_IV

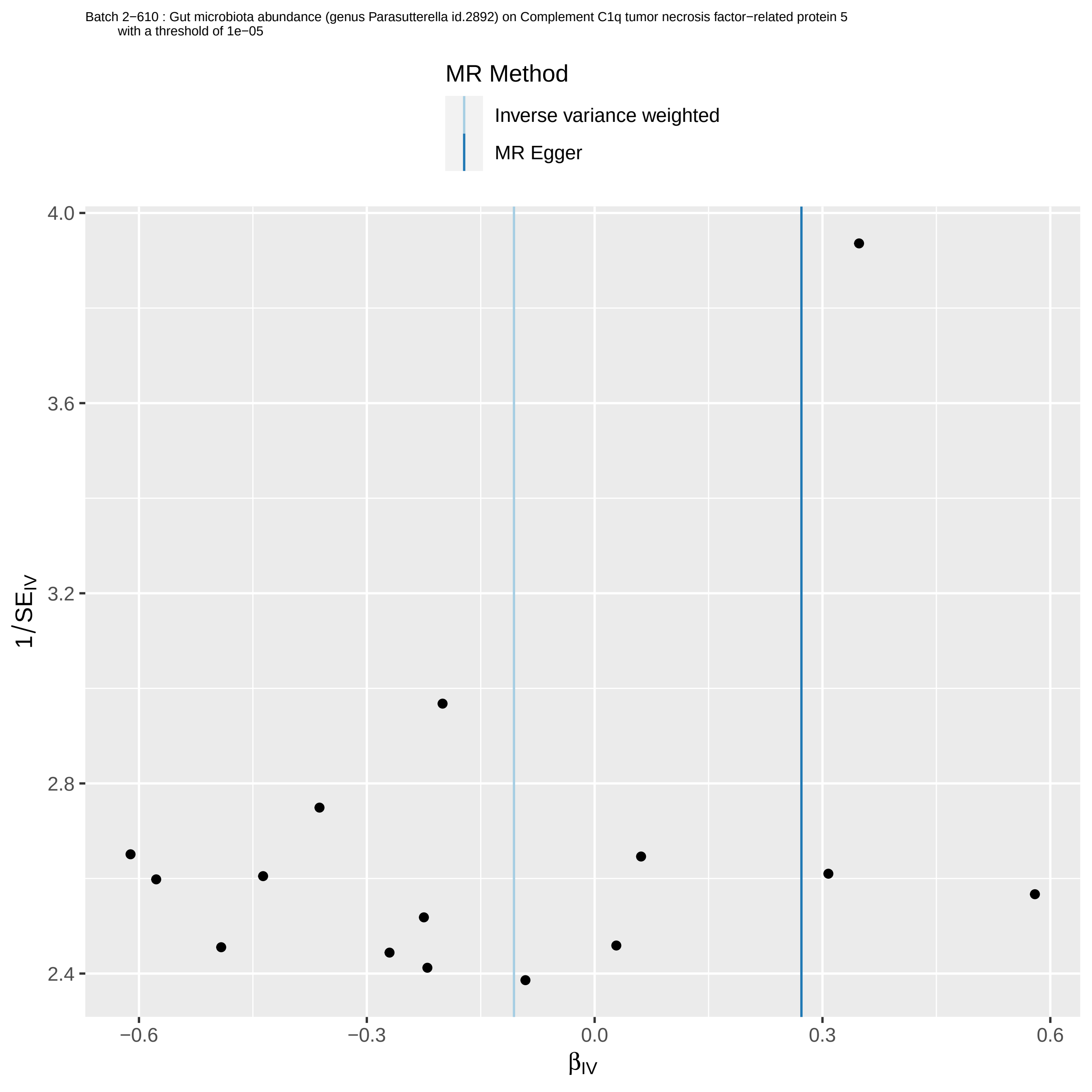
Batch 2−610 : Gut microbiota abundance (genus Parasutterella id.2892) on Complement C1q tumor necrosis factor−related protein 5
with a threshold of 1e−05
MR Method
Inverse variance weighted
MR Egger
4.0
3.6
3.2
2.8
2.4
1/SE_IV
−0.6
−0.3
0.0
0.3
0.6
β_IV

Batch 2−611 : Gut microbiota abundance (genus Catenibacterium id.2153) on Complement C1q and tumor necrosis factor−related protein 9A with a threshold of 1e−05

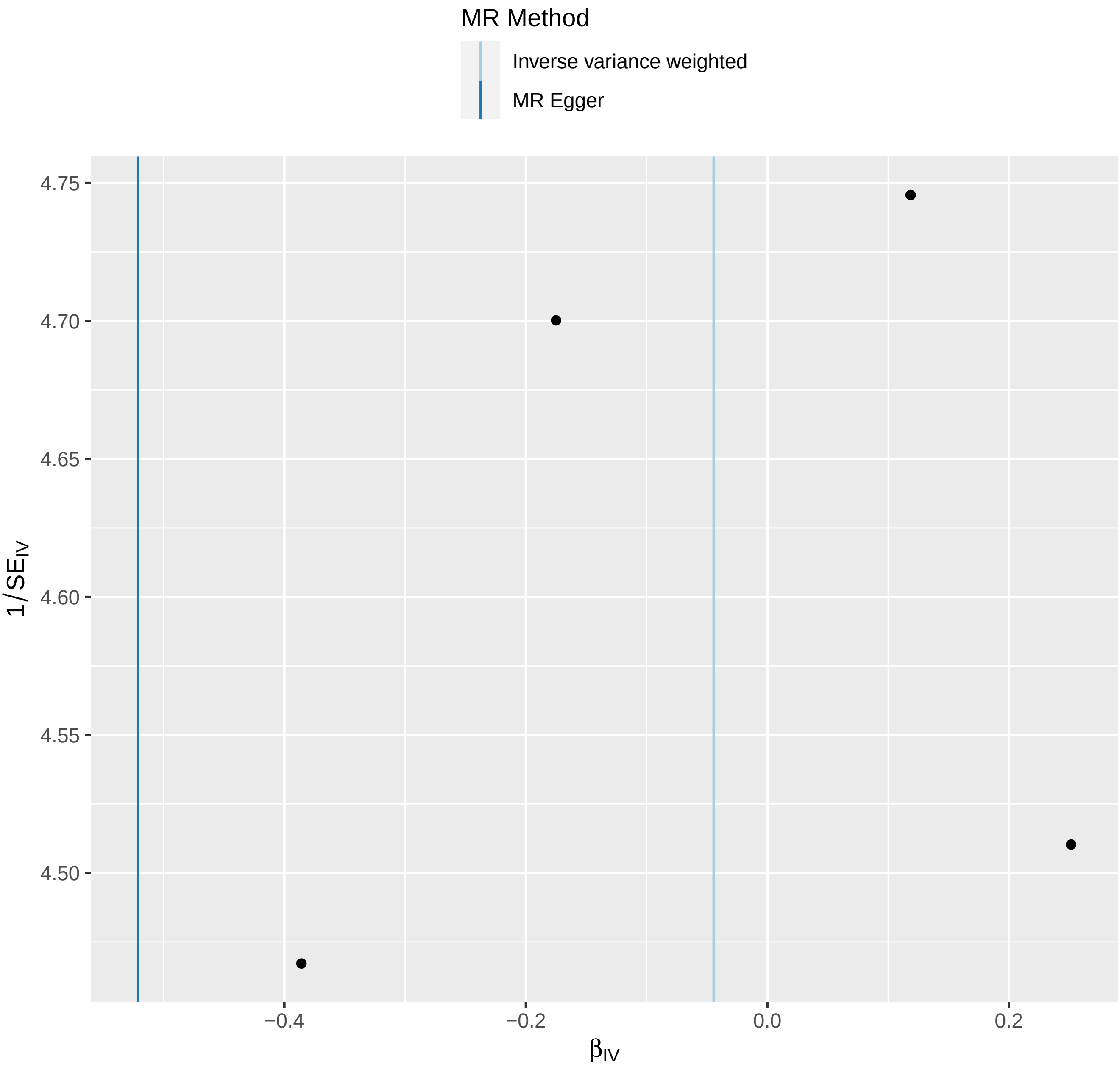

Batch 2−612 : Gut microbiota abundance (genus Parasutterella id.2892) on Complement C1q and tumor necrosis factor−related protein 9A
with a threshold of 1e−05

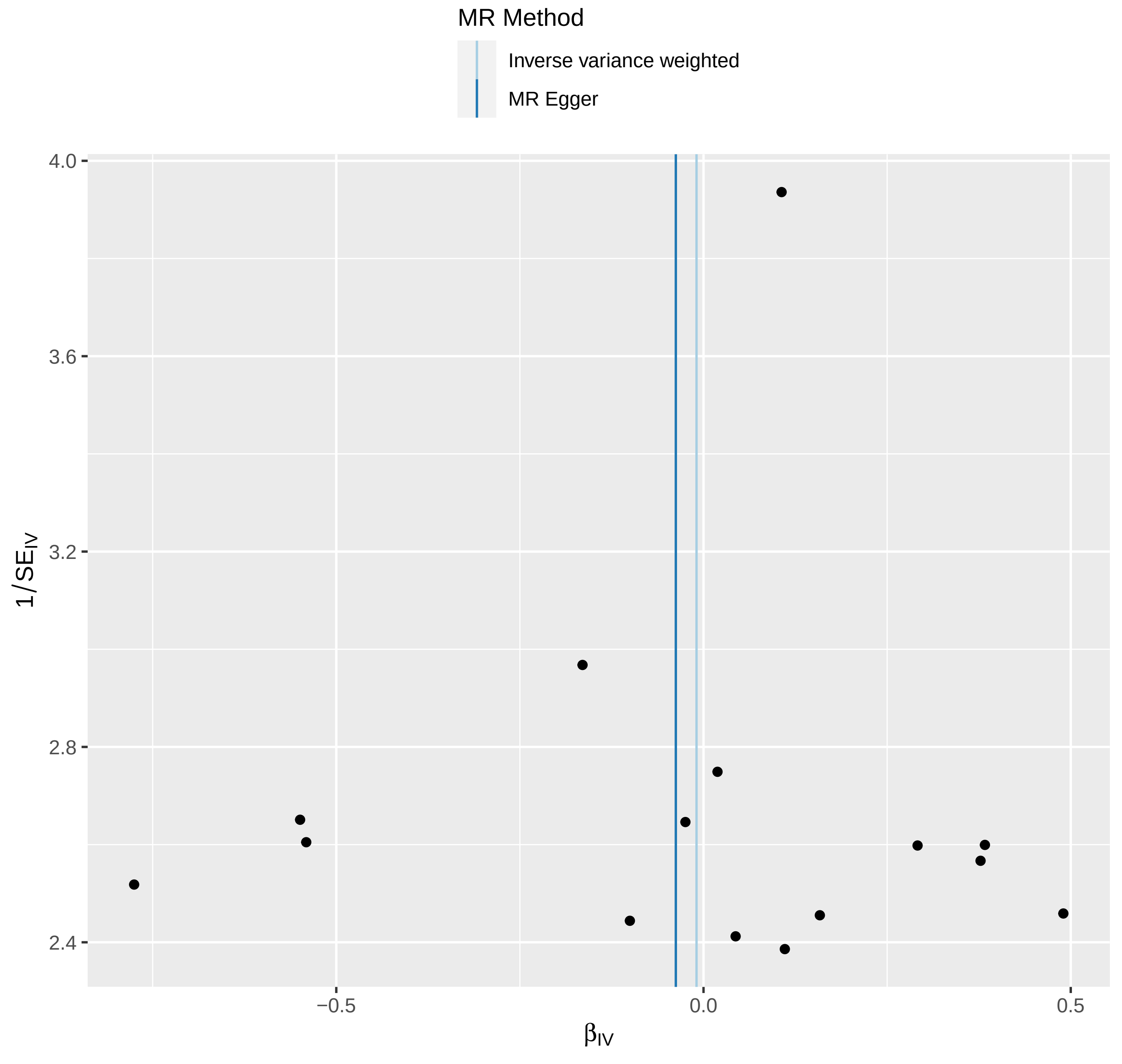

Batch 2−613 : Gut microbiota abundance (genus Catenibacterium id.2153) on Complement C1r subcomponent−like protein
with a threshold of 1e−05

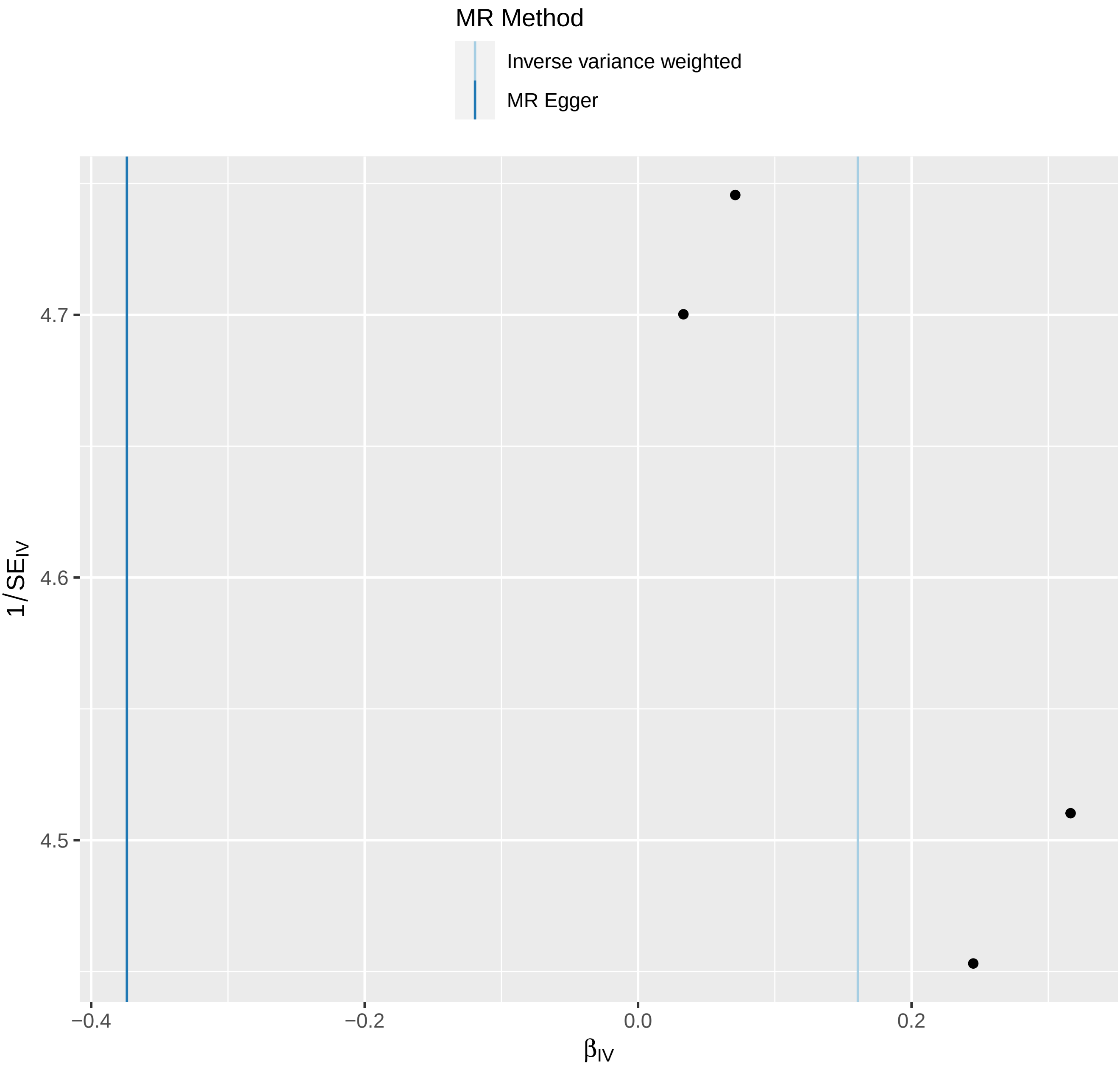

Batch 2−614 : Gut microbiota abundance (genus Parasutterella id.2892) on Complement C1r subcomponent−like protein
with a threshold of 1e−05

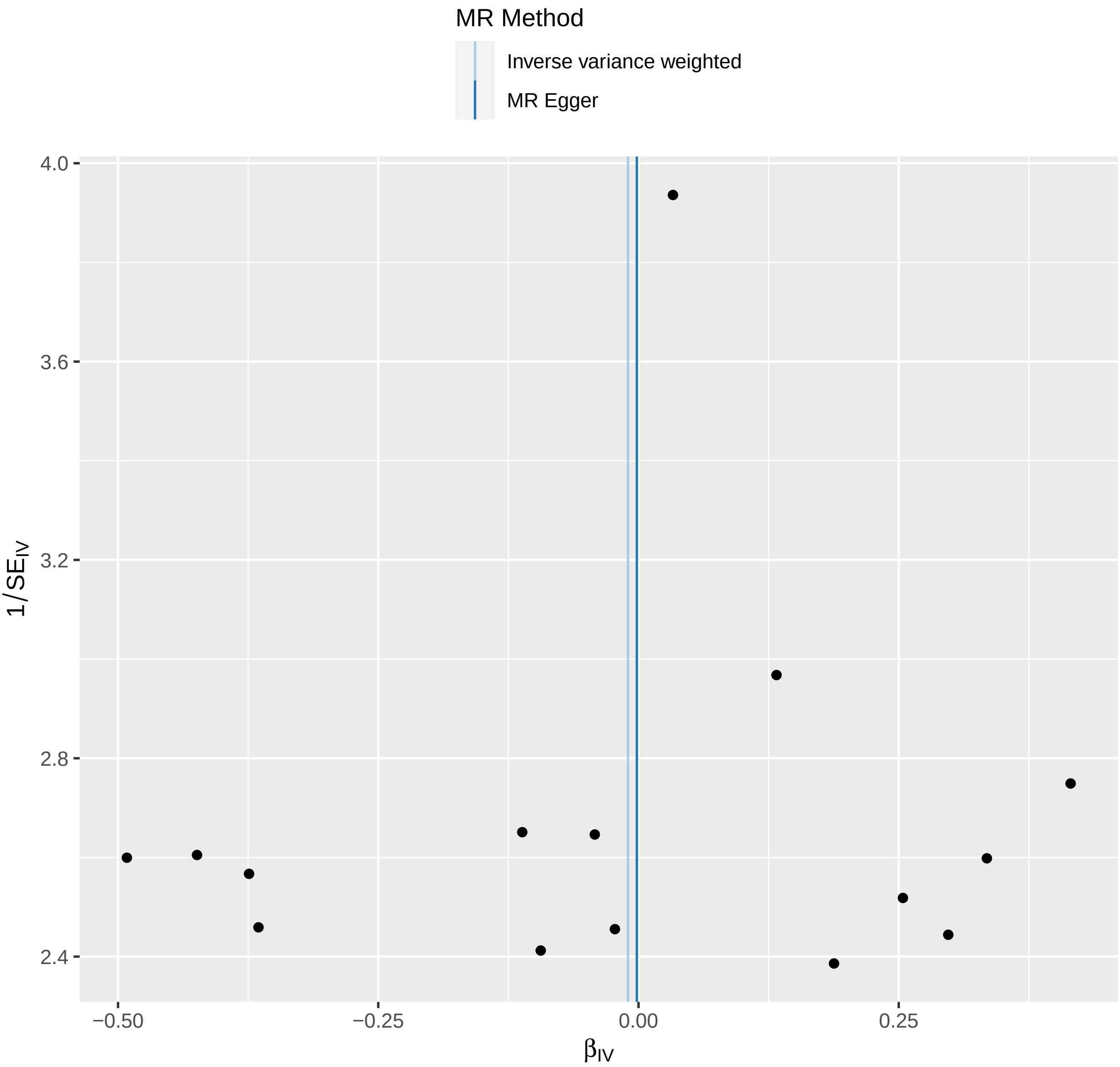

Batch 2−615 : Gut microbiota abundance (genus Catenibacterium id.2153) on ES1 protein homolog, mitochondrial
with a threshold of 1e−05

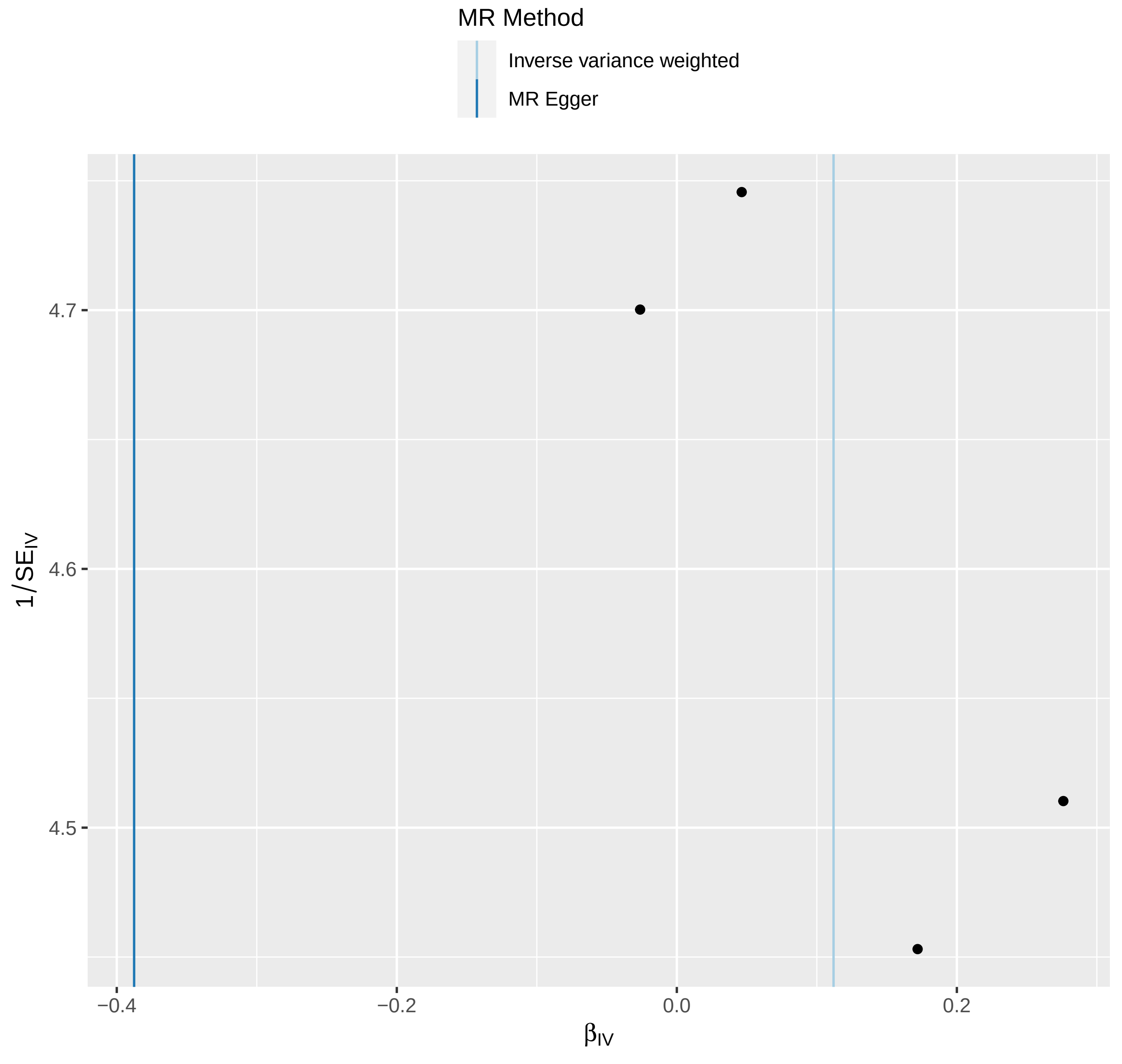

Batch 2−616 : Gut microbiota abundance (genus Parasutterella id.2892) on ES1 protein homolog, mitochondrial
with a threshold of 1e−05

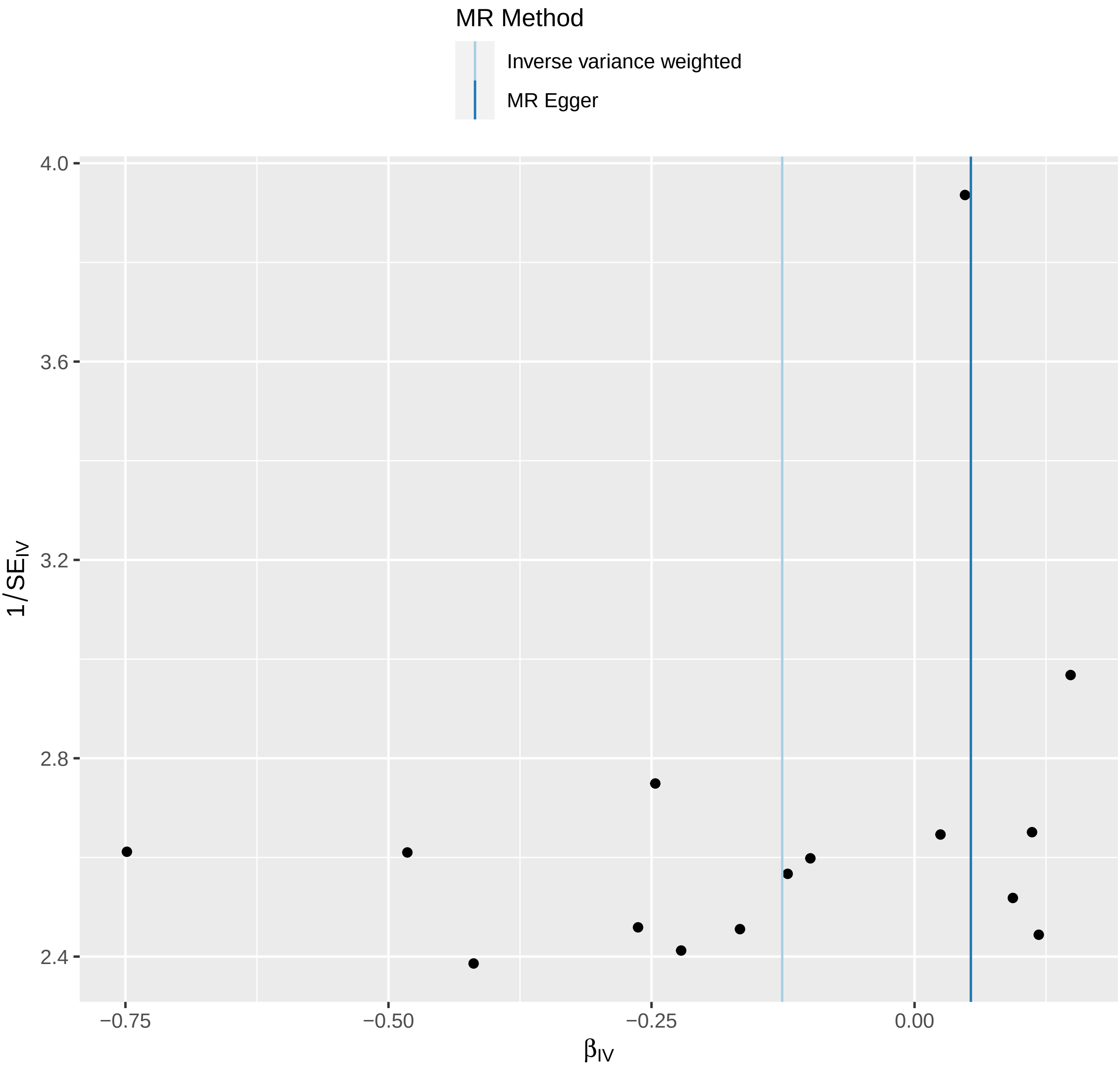

Batch 2−617 : Gut microbiota abundance (genus Catenibacterium id.2153) on Uncharacterized protein C22orf15
with a threshold of 1e−05

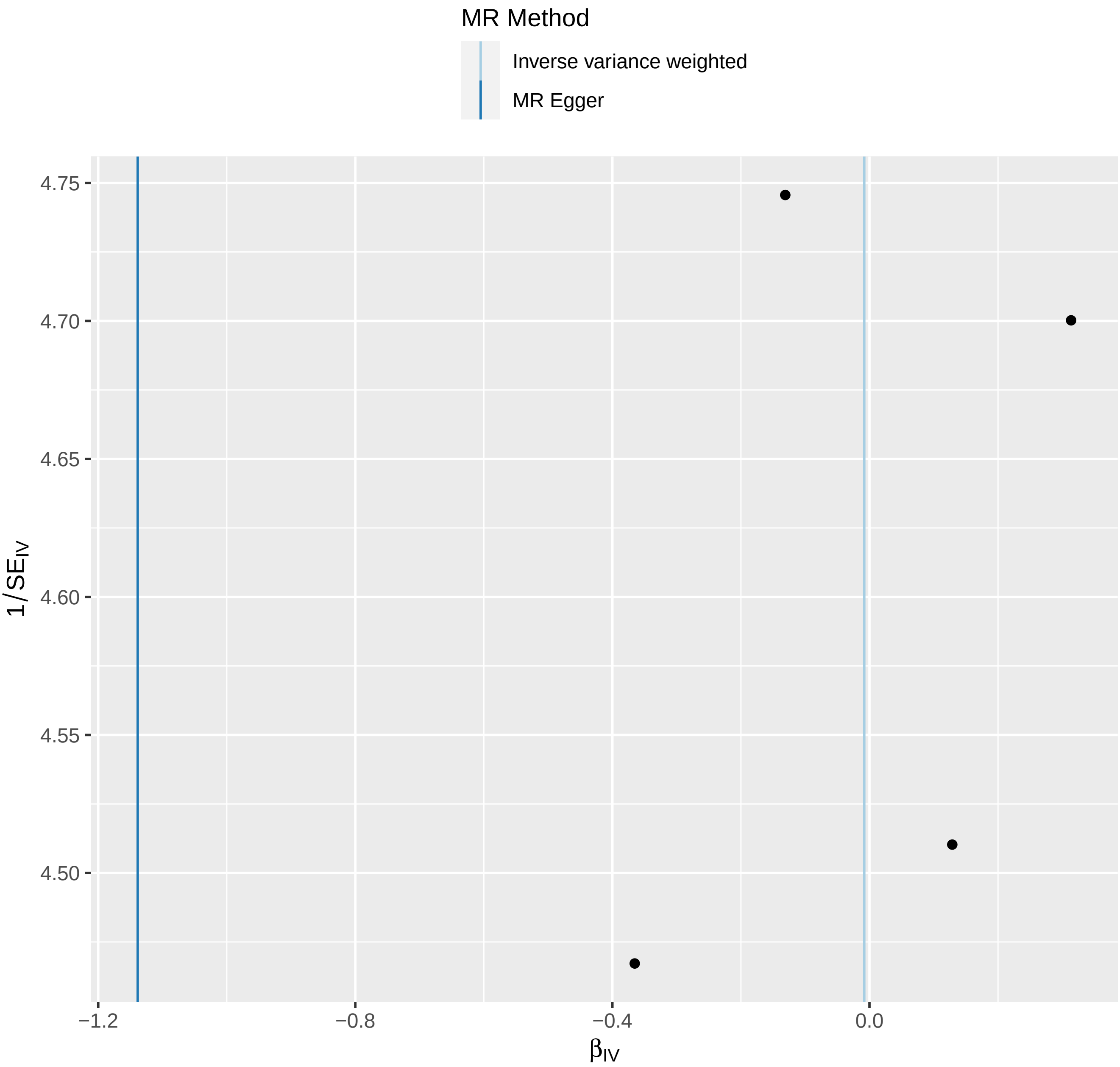

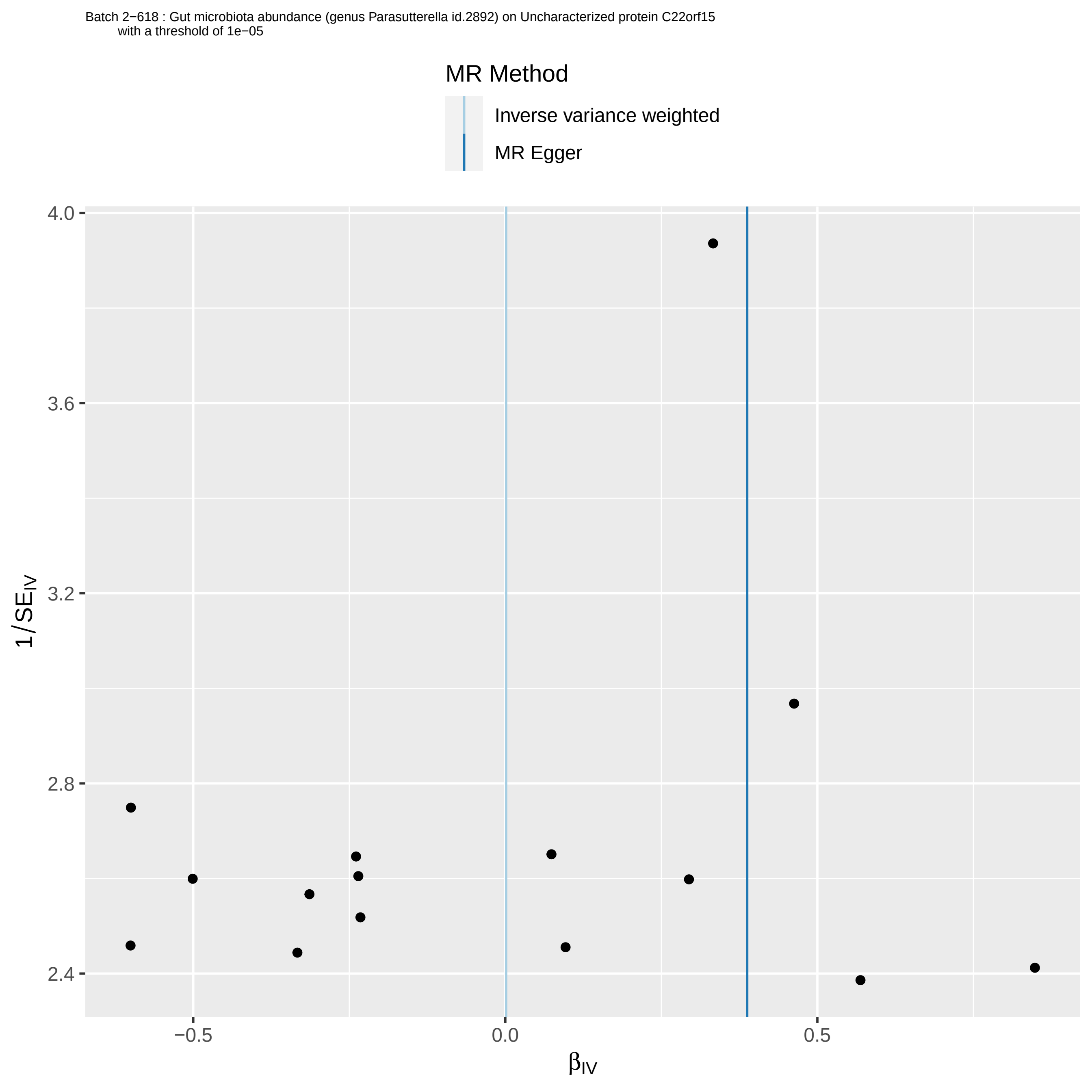

Batch 2−618 : Gut microbiota abundance (genus Parasutterella id.2892) on Uncharacterized protein C22orf15
with a threshold of 1e−05
MR Method
Inverse variance weighted
MR Egger
4.0
3.6
3.2
2.8
2.4
1/SE_IV
−0.5
0.0
0.5
β_IV

Batch 2−619 : Gut microbiota abundance (genus Catenibacterium id.2153) on Augurin
with a threshold of 1e−05

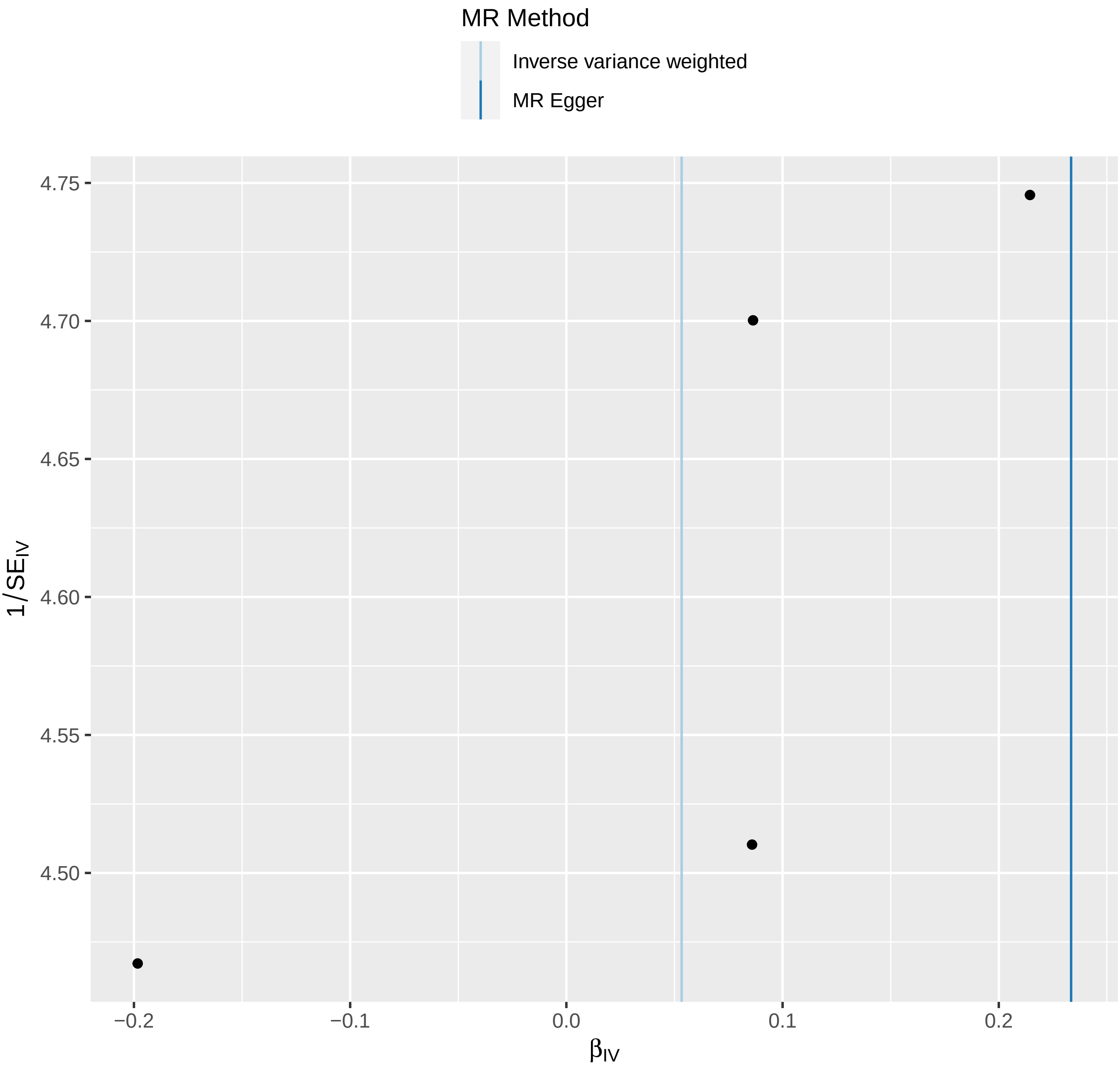

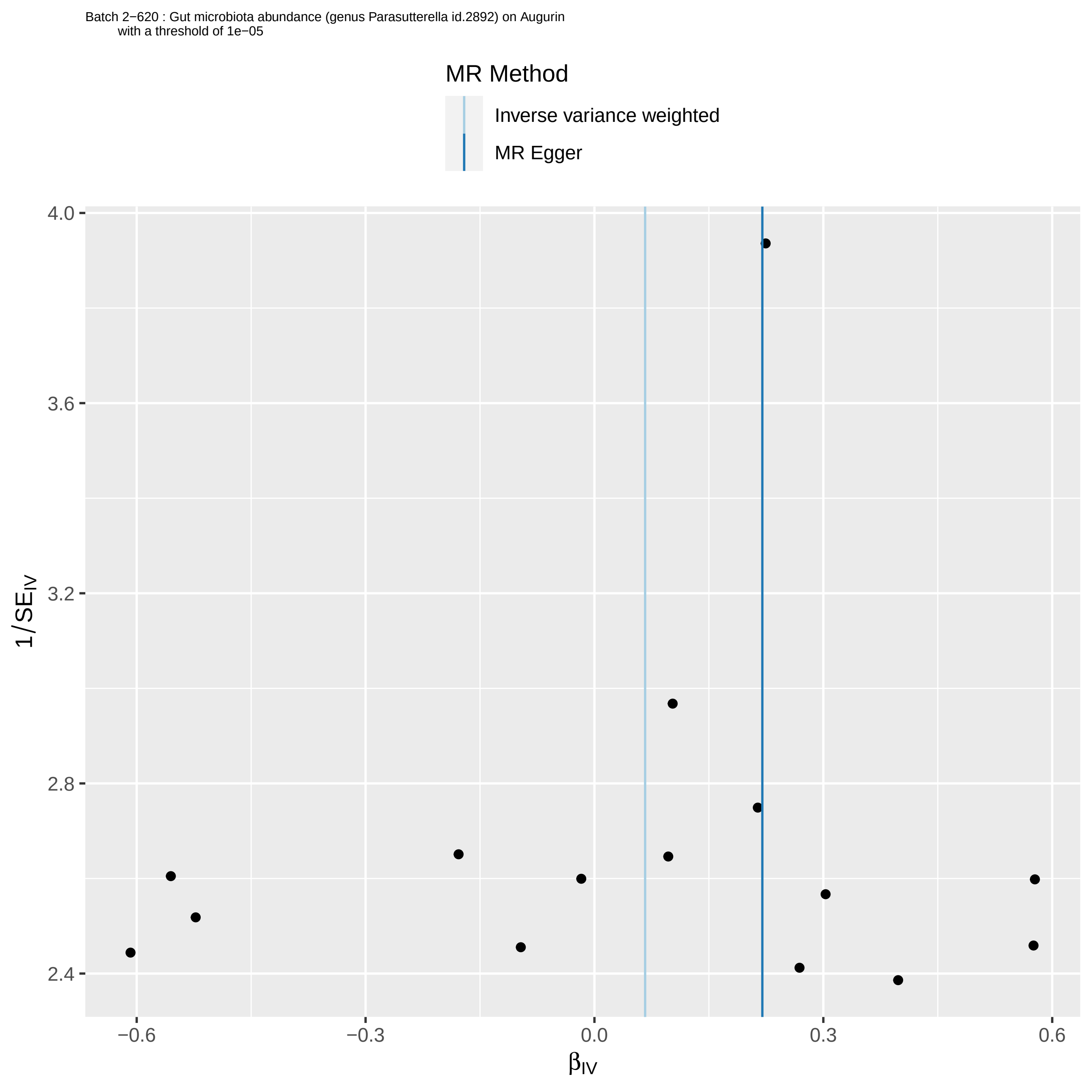

Batch 2−620 : Gut microbiota abundance (genus Parasutterella id.2892) on Augurin
with a threshold of 1e−05
MR Method
Inverse variance weighted
MR Egger
4.0
3.6
3.2
2.8
2.4
1/SE$_{IV}$
−0.6
−0.3
0.0
0.3
0.6
β$_{IV}$

Batch 2−621 : Gut microbiota abundance (genus Catenibacterium id.2153) on Uncharacterized protein C2orf66
with a threshold of 1e−05

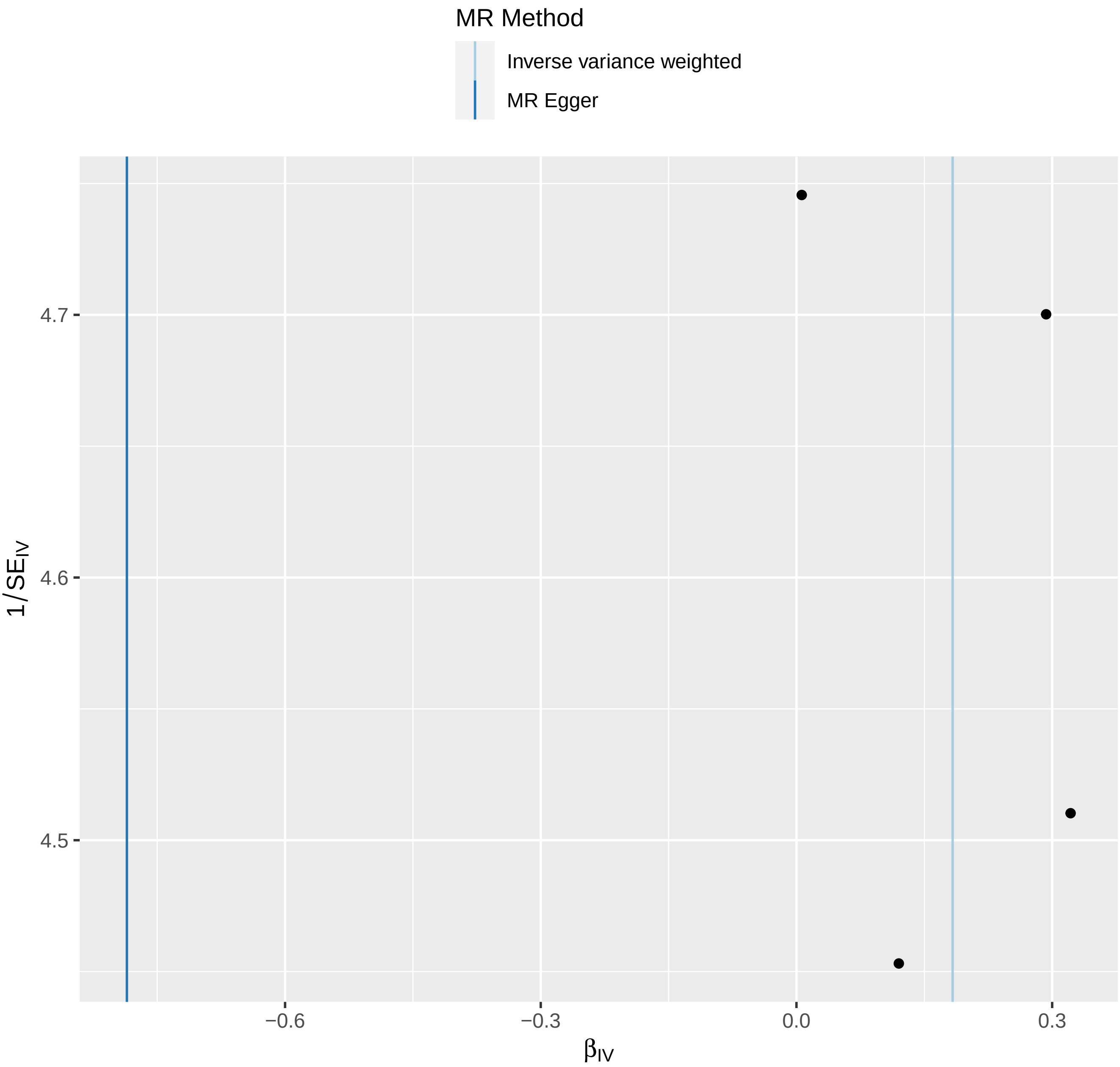

Batch 2−622 : Gut microbiota abundance (genus Parasutterella id.2892) on Uncharacterized protein C2orf66
with a threshold of 1e−05

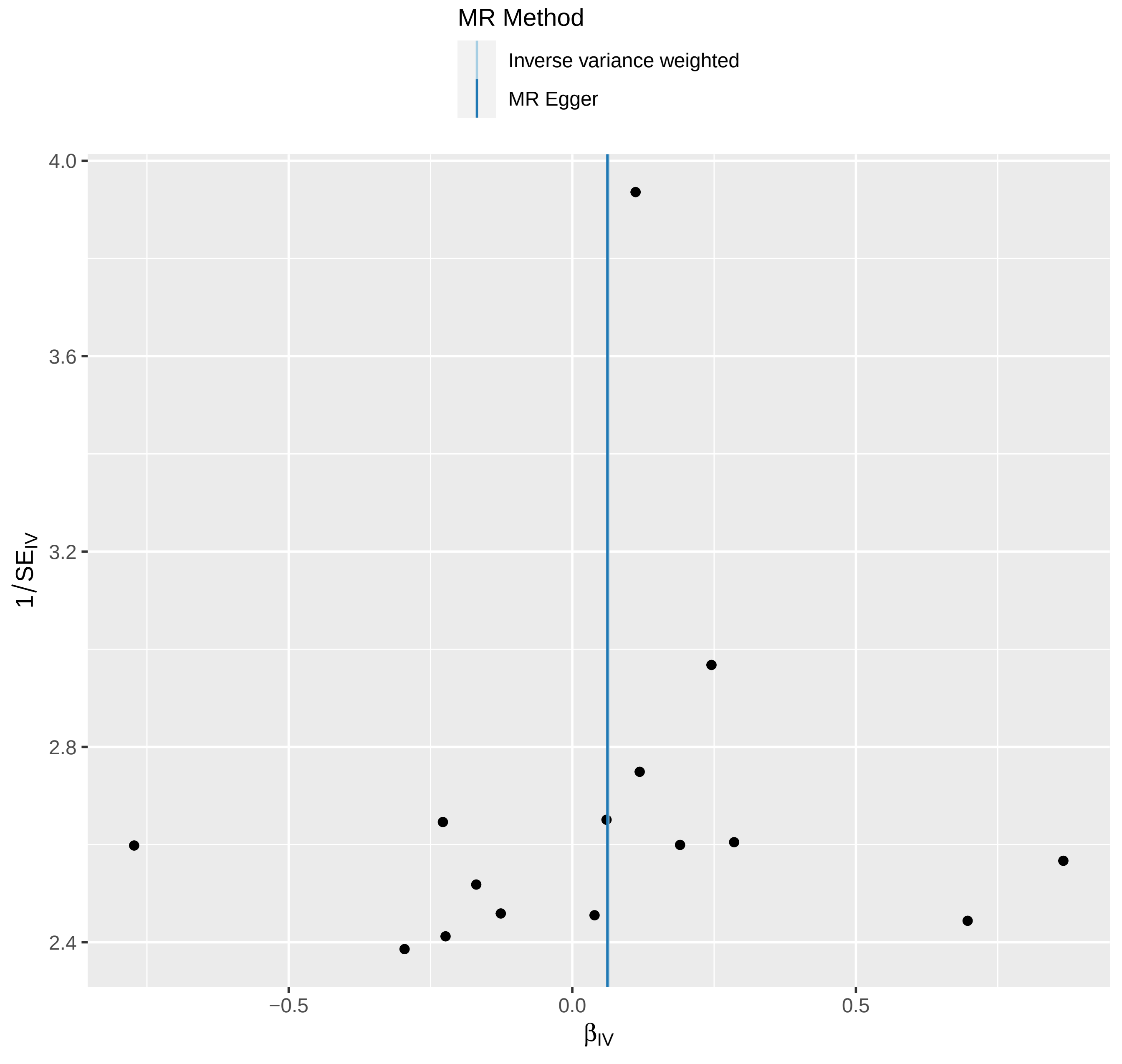

Batch 2−623 : Gut microbiota abundance (genus Catenibacterium id.2153) on Uncharacterized protein C2orf82
with a threshold of 1e−05

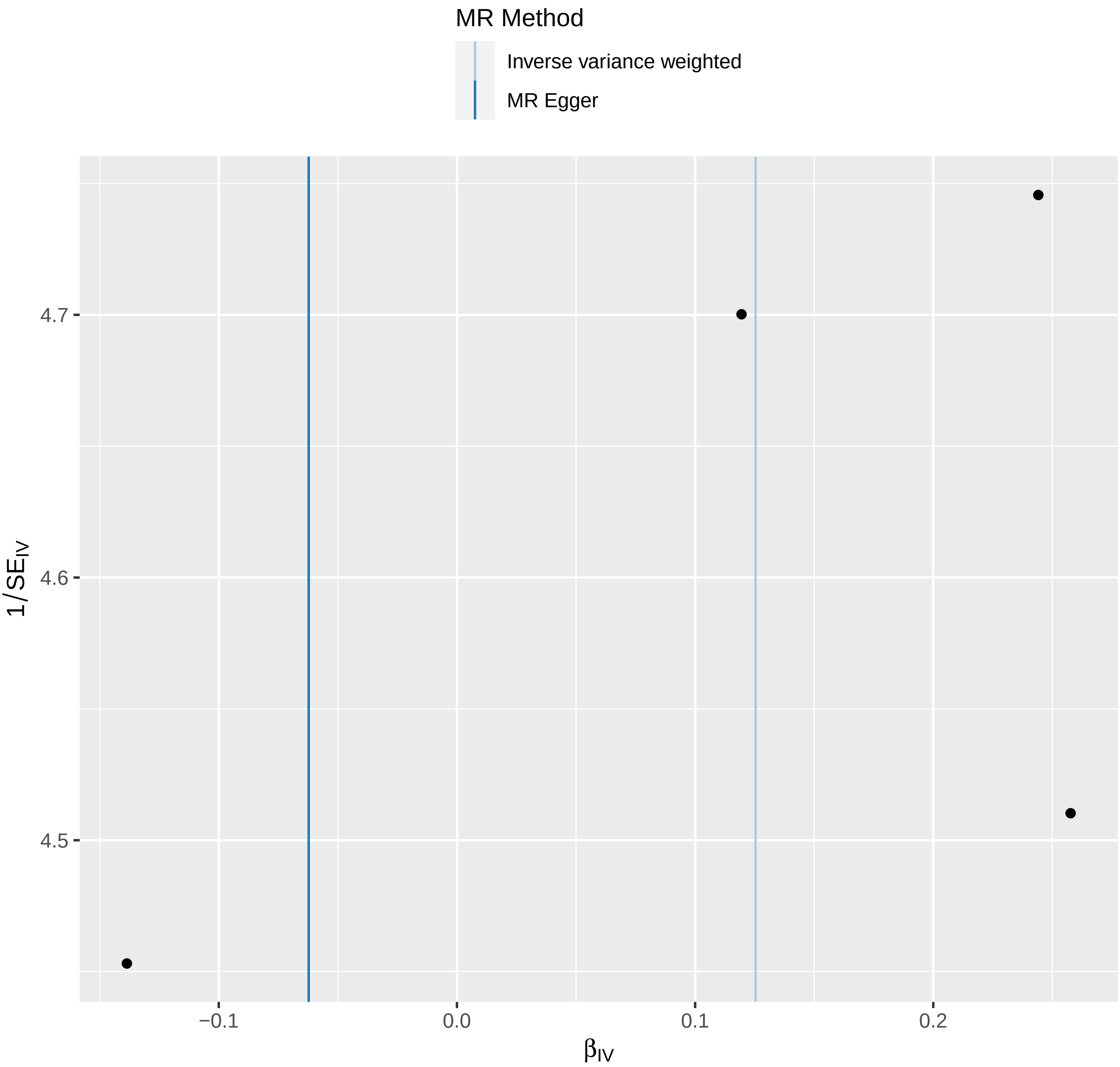

Batch 2−624 : Gut microbiota abundance (genus Parasutterella id.2892) on Uncharacterized protein C2orf82
with a threshold of 1e−05

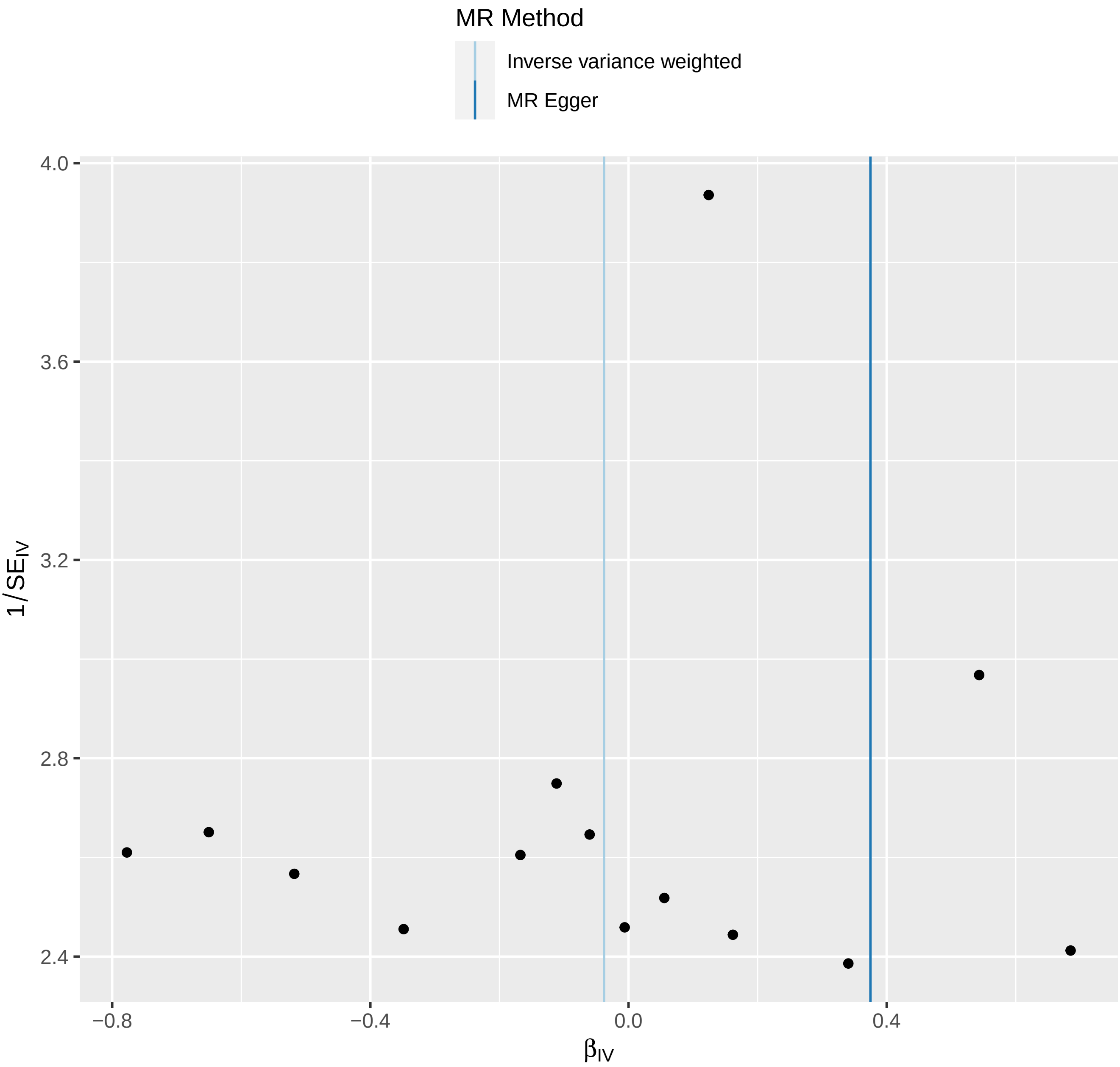

Batch 2−625 : Gut microbiota abundance (genus Catenibacterium id.2153) on C3a anaphylatoxin des Arginine
with a threshold of 1e−05

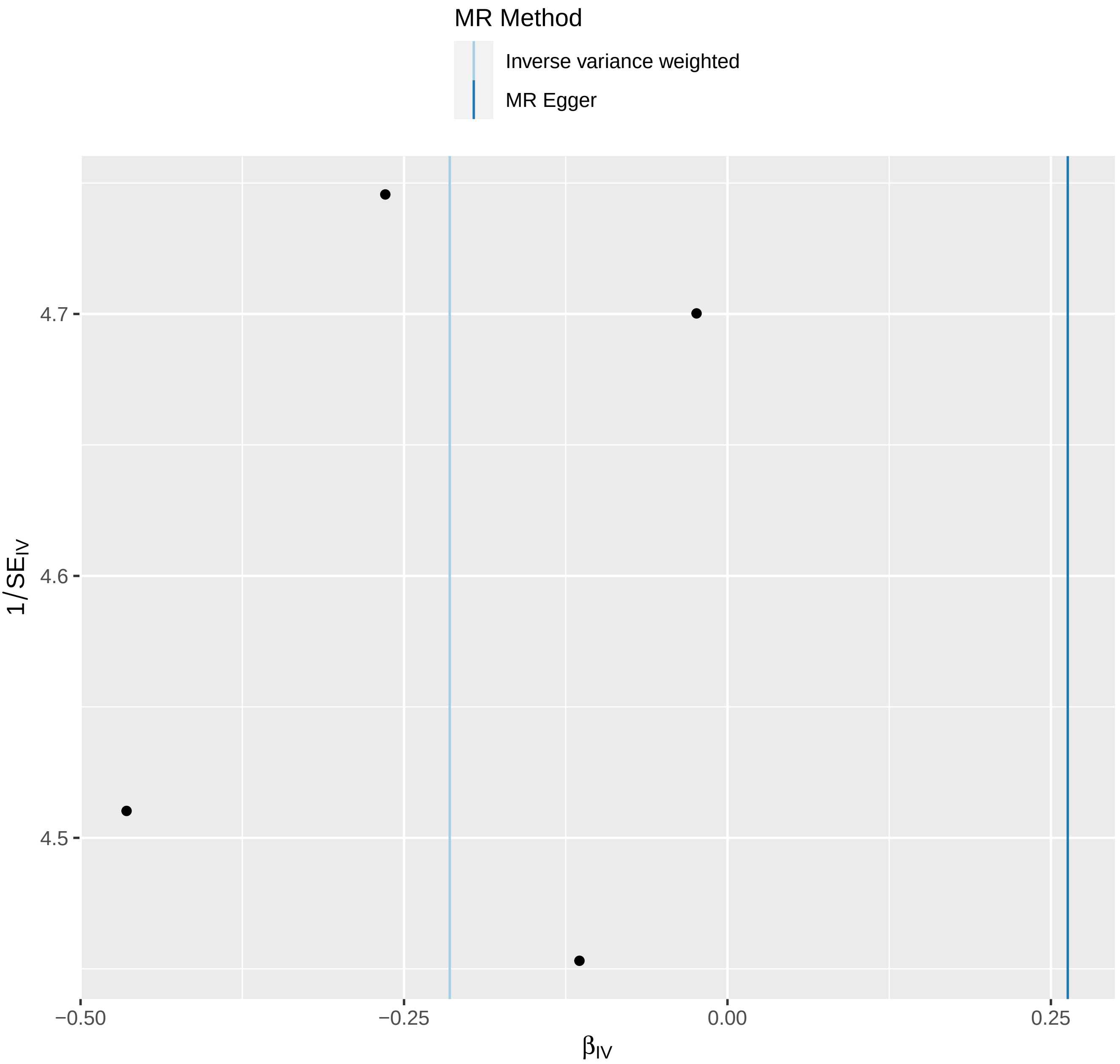

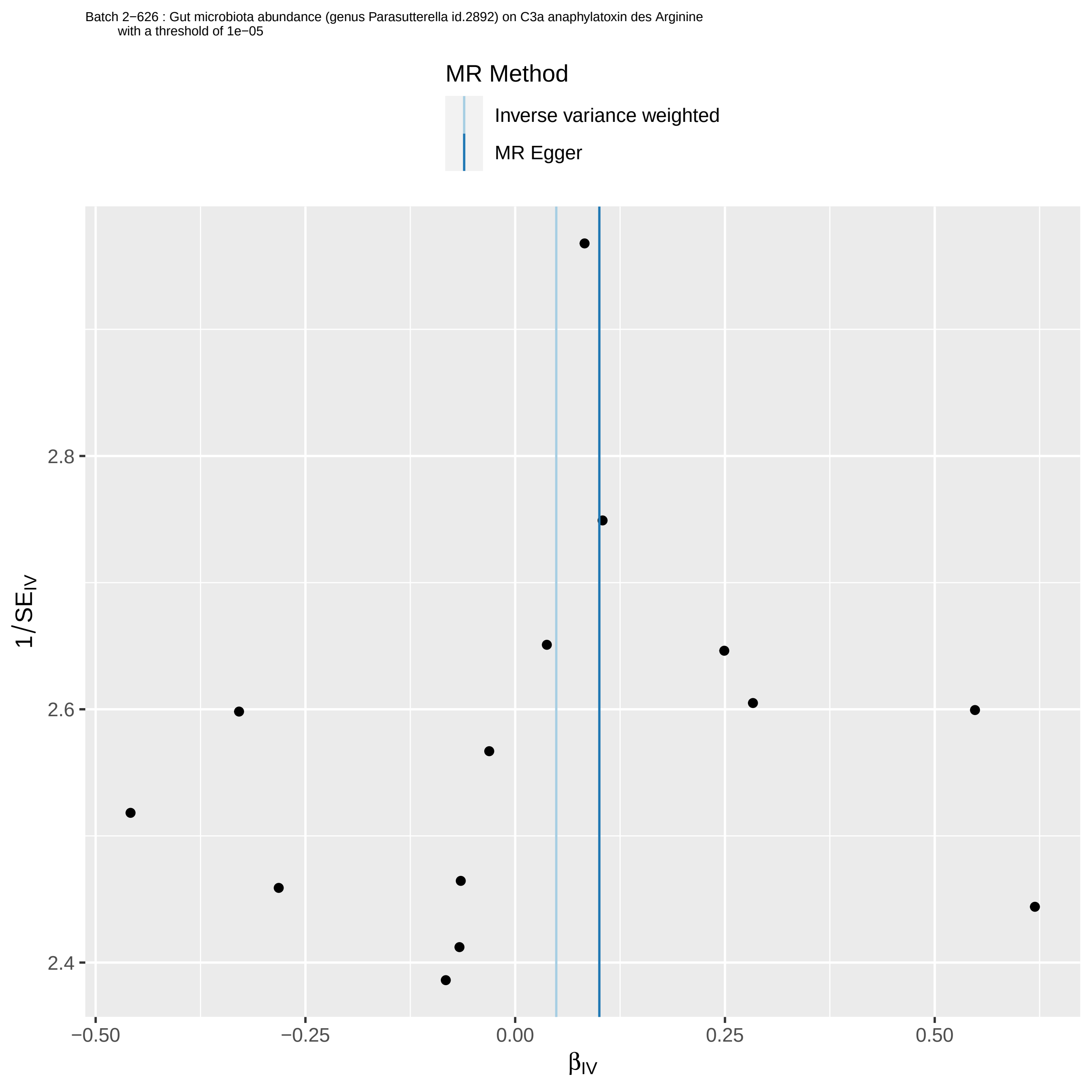

Batch 2−626 : Gut microbiota abundance (genus Parasutterella id.2892) on C3a anaphylatoxin des Arginine
with a threshold of 1e−05
MR Method
Inverse variance weighted
MR Egger
2.8
2.6
2.4
1/SE_IV
−0.50
−0.25
0.00
0.25
0.50
β_IV

Batch 2−627 : Gut microbiota abundance (genus Catenibacterium id.2153) on Uncharacterized protein C3orf18 with a threshold of 1e−05

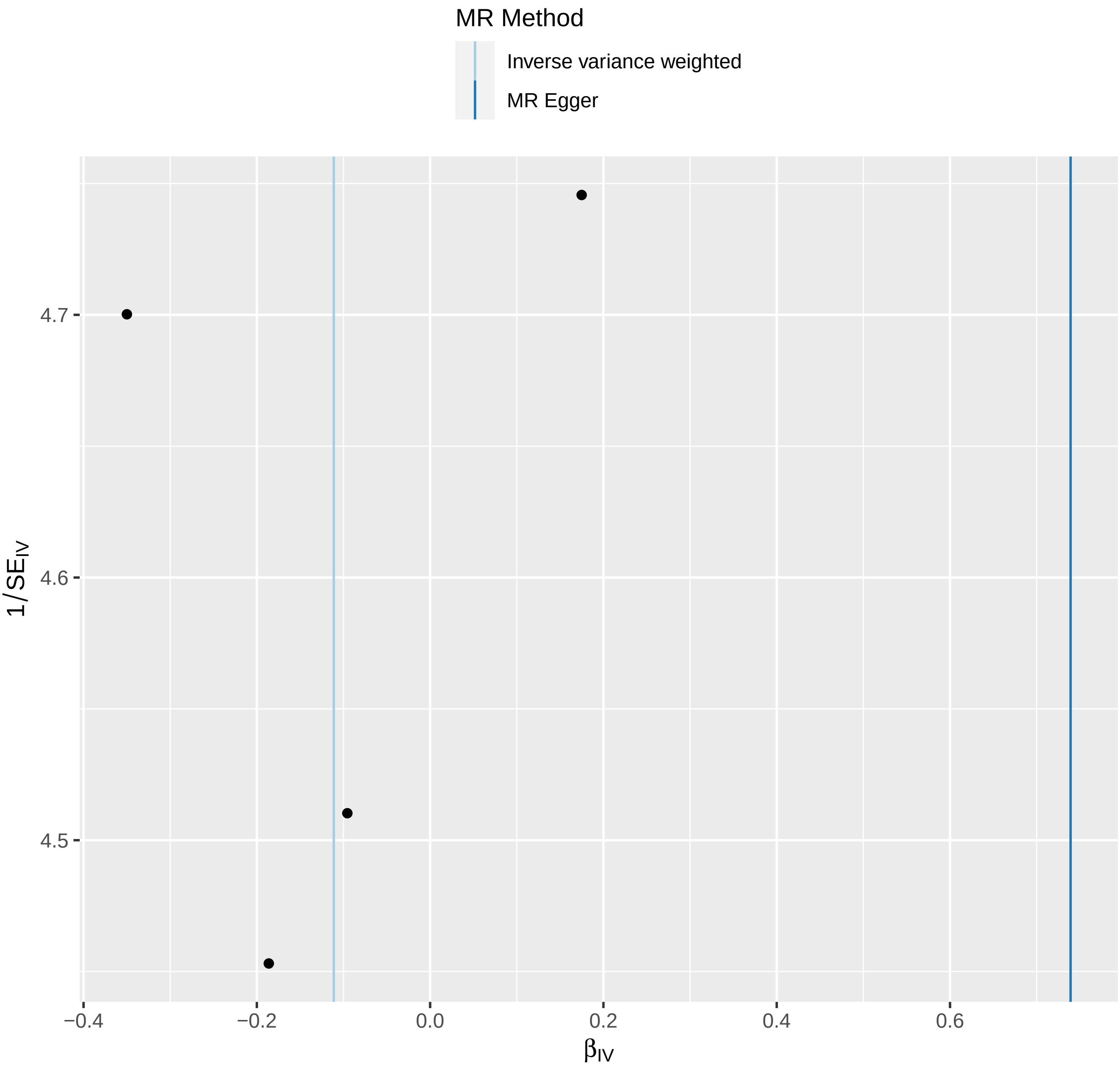

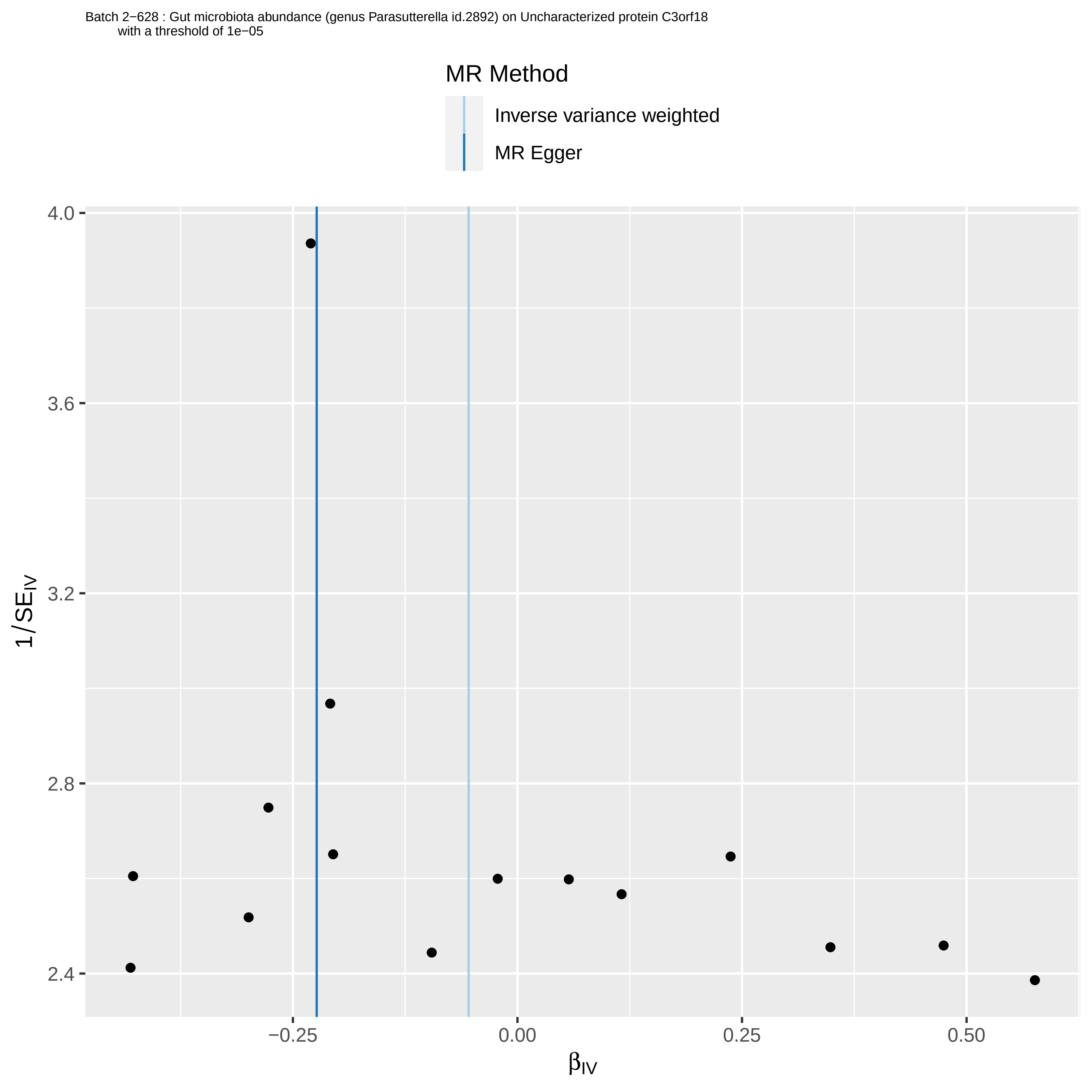
Batch 2−628 : Gut microbiota abundance (genus Parasutterella id.2892) on Uncharacterized protein C3orf18
with a threshold of 1e−05
MR Method
Inverse variance weighted
MR Egger
4.0
3.6
3.2
2.8
2.4
1/SE_IV
−0.25
0.00
0.25
0.50
β_IV

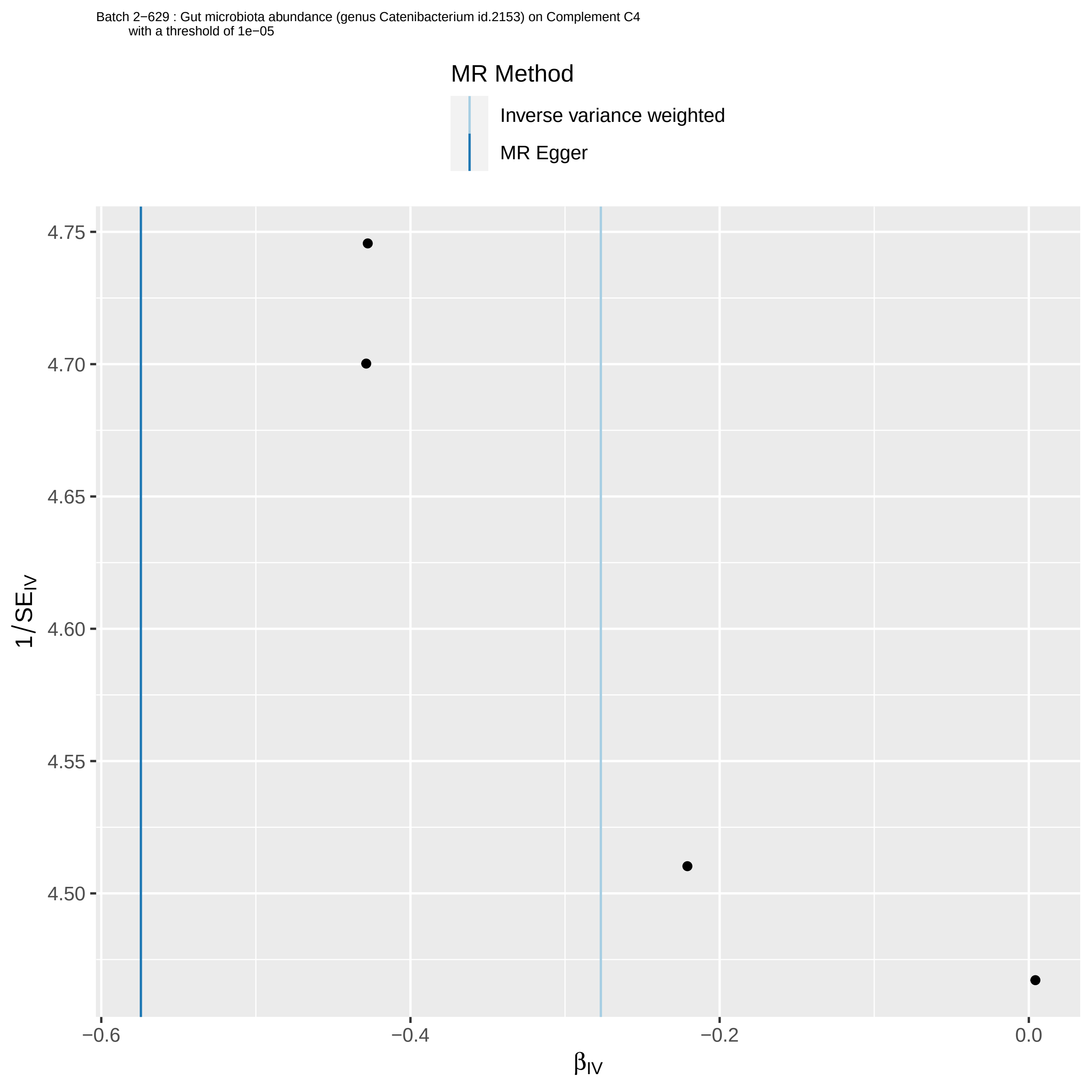
Batch 2−629 : Gut microbiota abundance (genus Catenibacterium id.2153) on Complement C4
with a threshold of 1e−05
MR Method
Inverse variance weighted
MR Egger
4.75
4.70
4.65
4.60
4.55
4.50
1/SE_IV
−0.6
−0.4
−0.2
0.0
β_IV

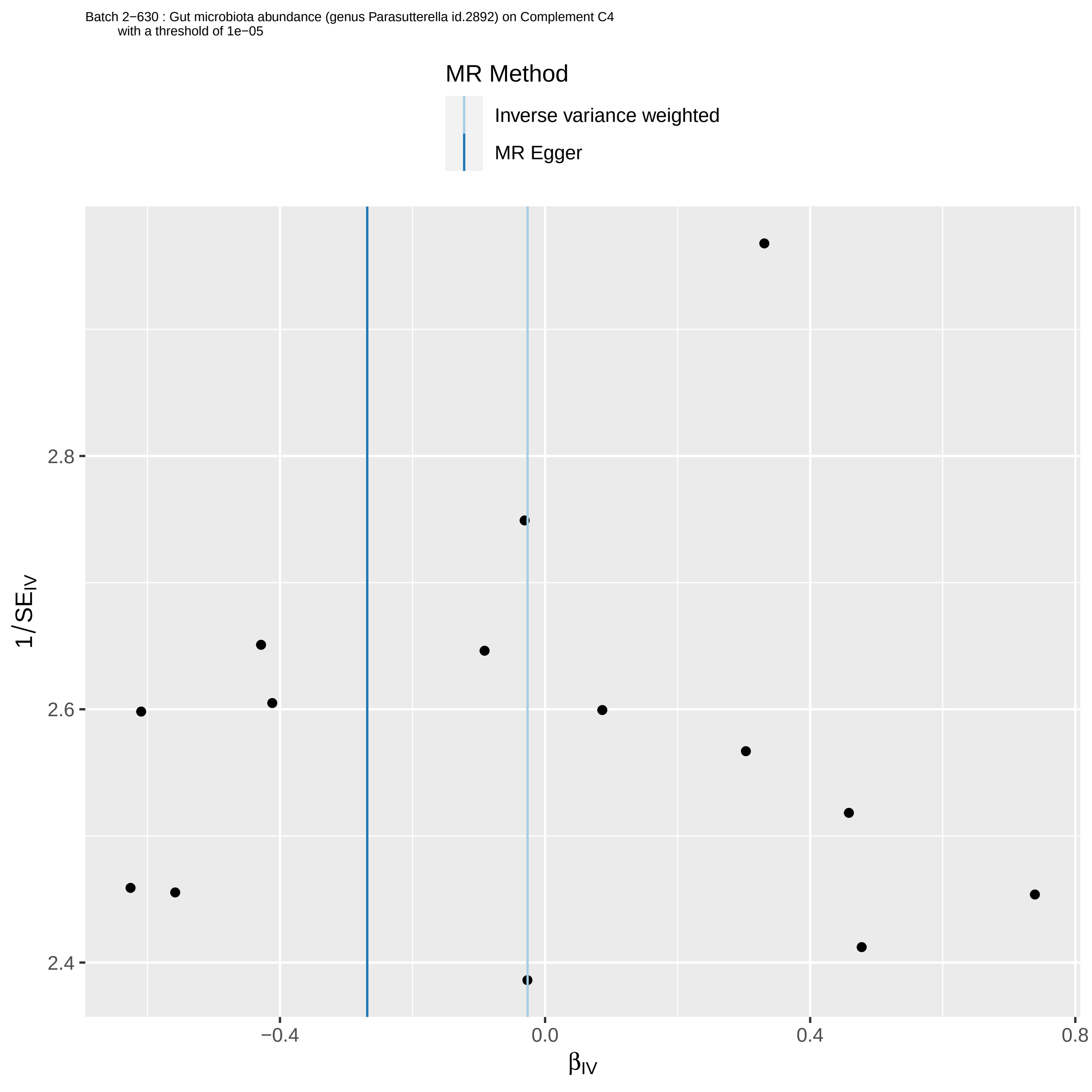

Batch 2−630 : Gut microbiota abundance (genus Parasutterella id.2892) on Complement C4
with a threshold of 1e−05
MR Method
Inverse variance weighted
MR Egger
2.8
2.6
2.4
1/SE_IV
−0.4
0.0
0.4
0.8
β_IV

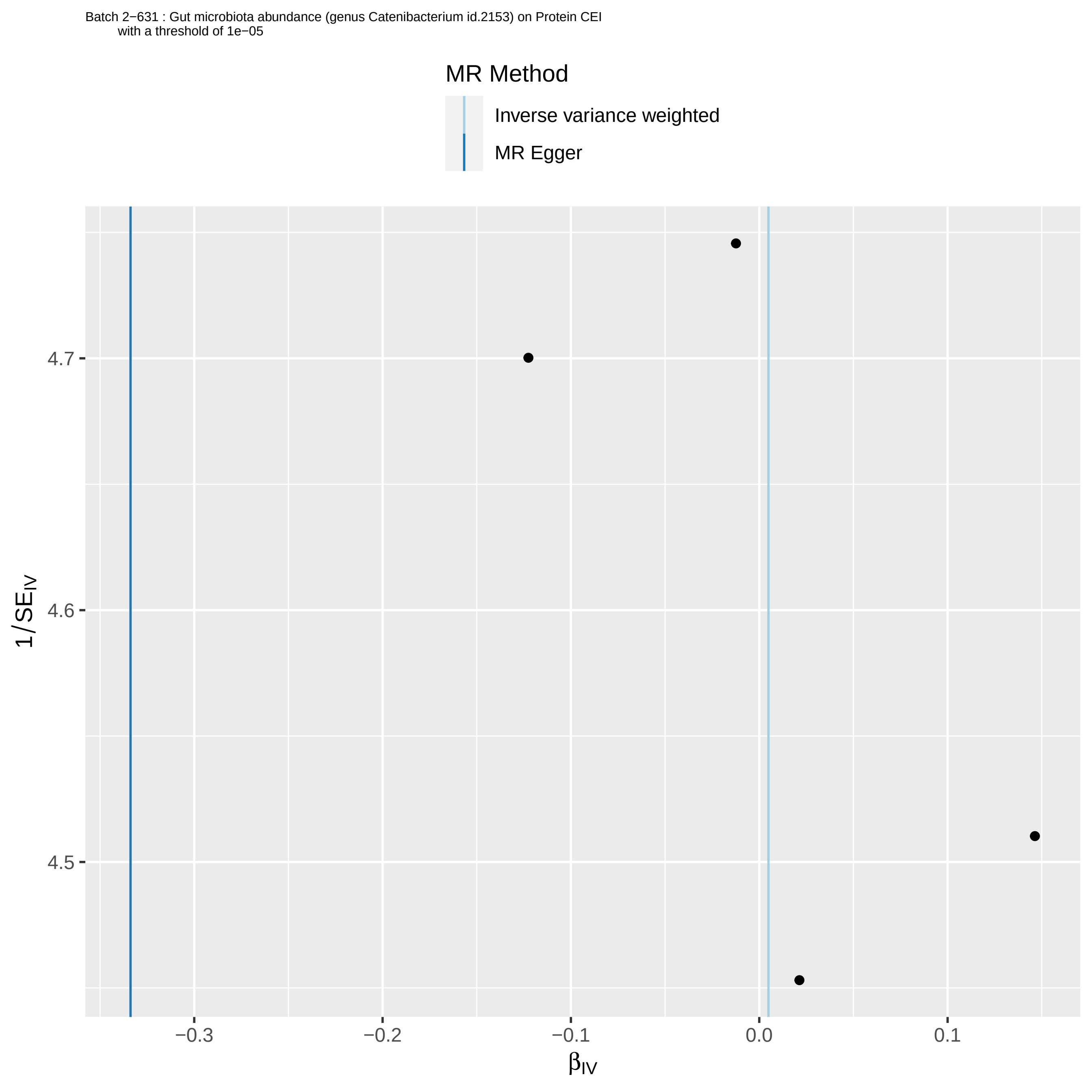
Batch 2−631 : Gut microbiota abundance (genus Catenibacterium id.2153) on Protein CEI
with a threshold of 1e−05
MR Method
Inverse variance weighted
MR Egger
4.7
4.6
4.5
1/SE_IV
−0.3
−0.2
−0.1
0.0
0.1
β_IV

Batch 2−632 : Gut microbiota abundance (genus Parasutterella id.2892) on Protein CEI
with a threshold of 1e−05

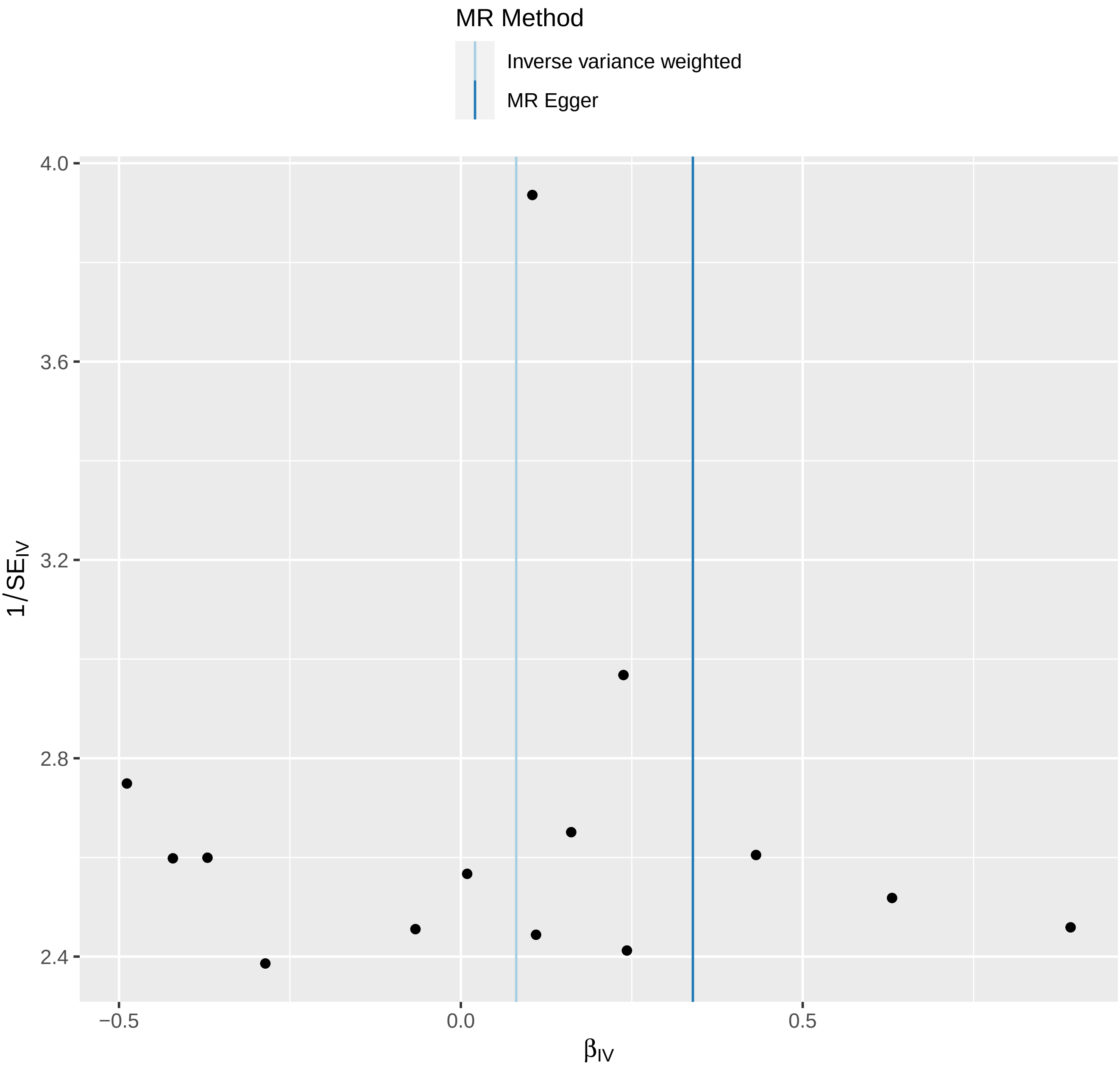

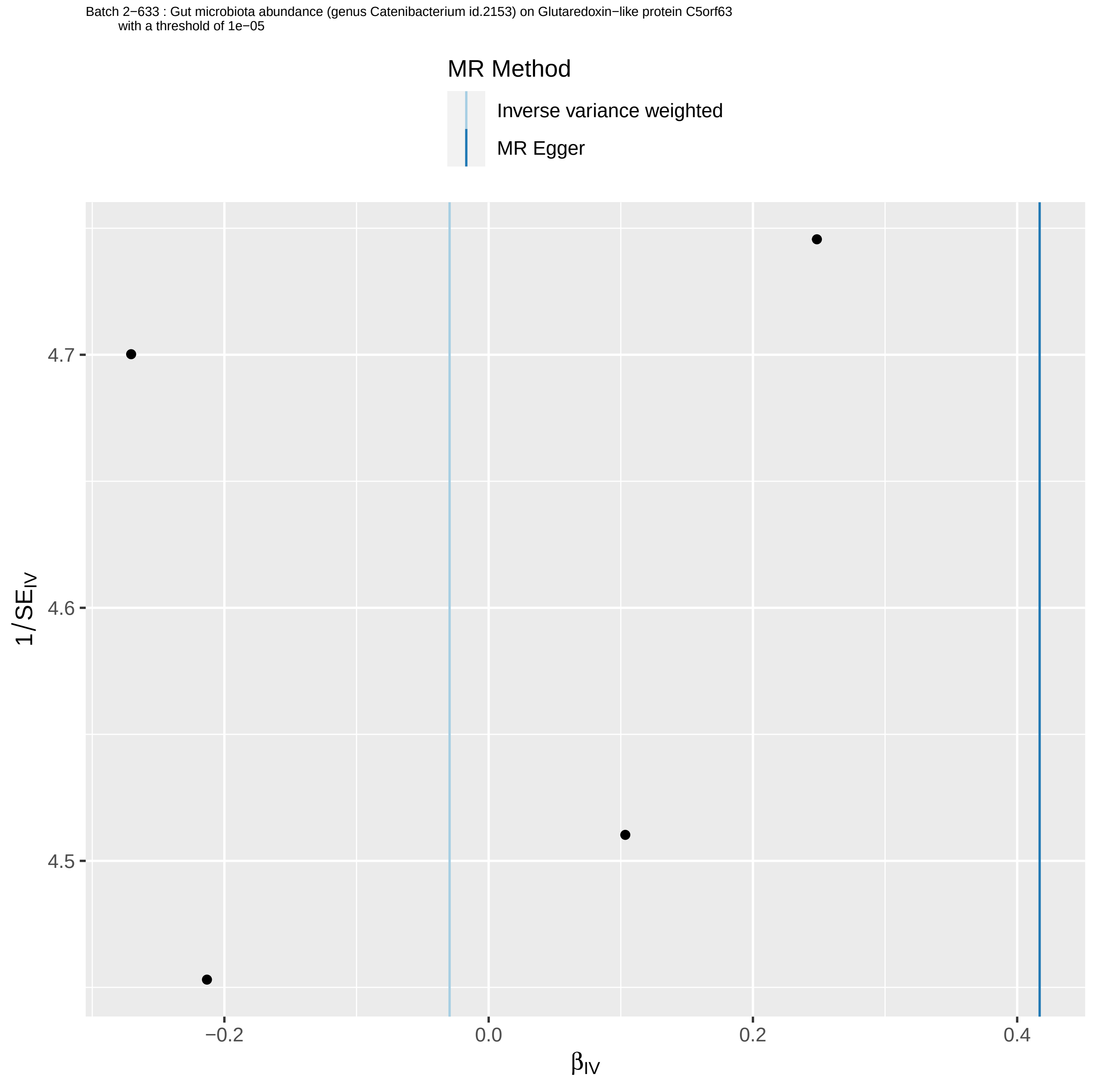

Batch 2−633 : Gut microbiota abundance (genus Catenibacterium id.2153) on Glutaredoxin−like protein C5orf63
with a threshold of 1e−05
MR Method
Inverse variance weighted
MR Egger
4.7
4.6
4.5
1/SE$_{IV}$
−0.2
0.0
0.2
0.4
β_{IV}

Batch 2−634 : Gut microbiota abundance (genus Parasutterella id.2892) on Glutaredoxin−like protein C5orf63
with a threshold of 1e−05

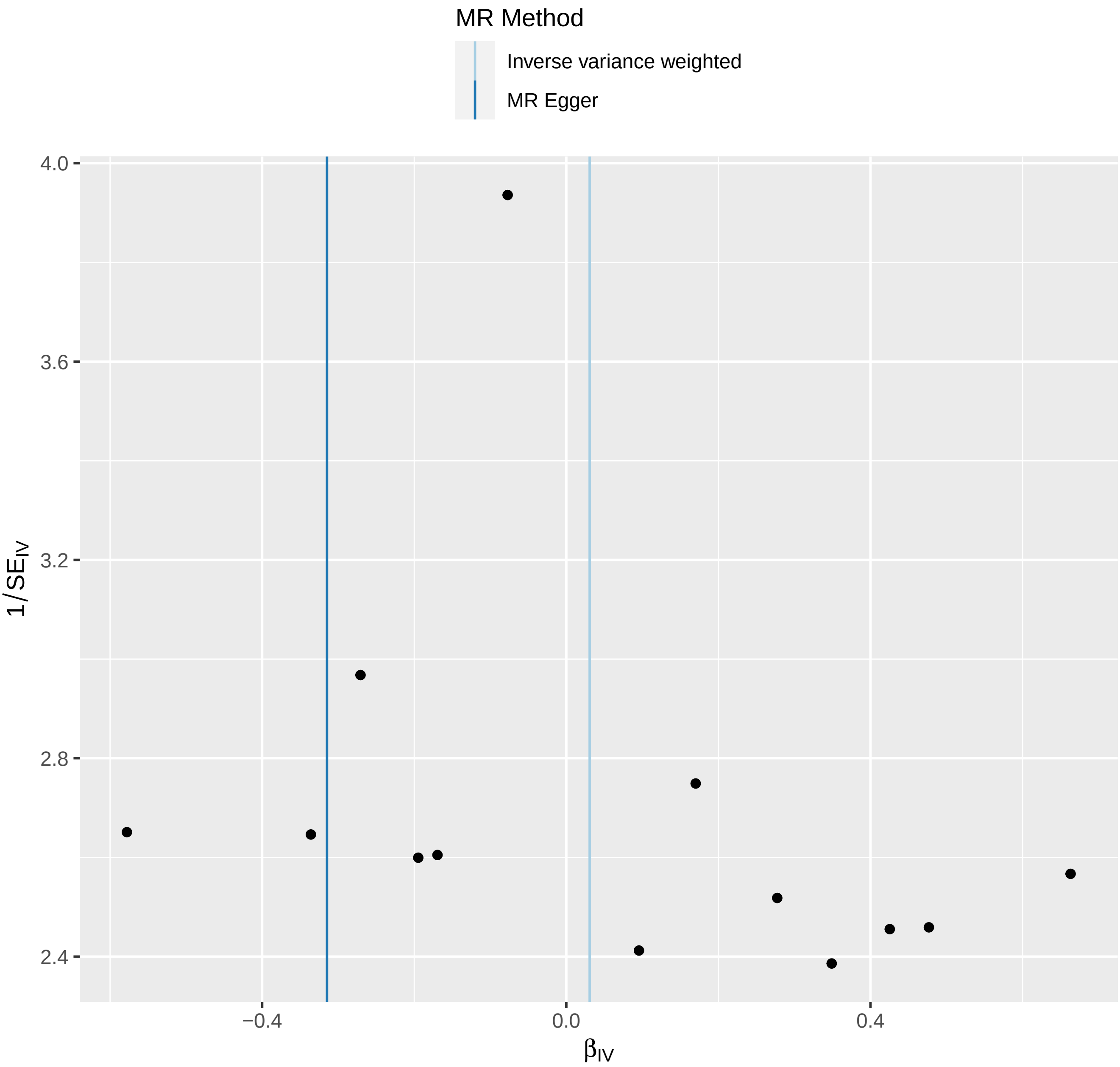

Batch 2−635 : Gut microbiota abundance (genus Catenibacterium id.2153) on Complement component C6
with a threshold of 1e−05

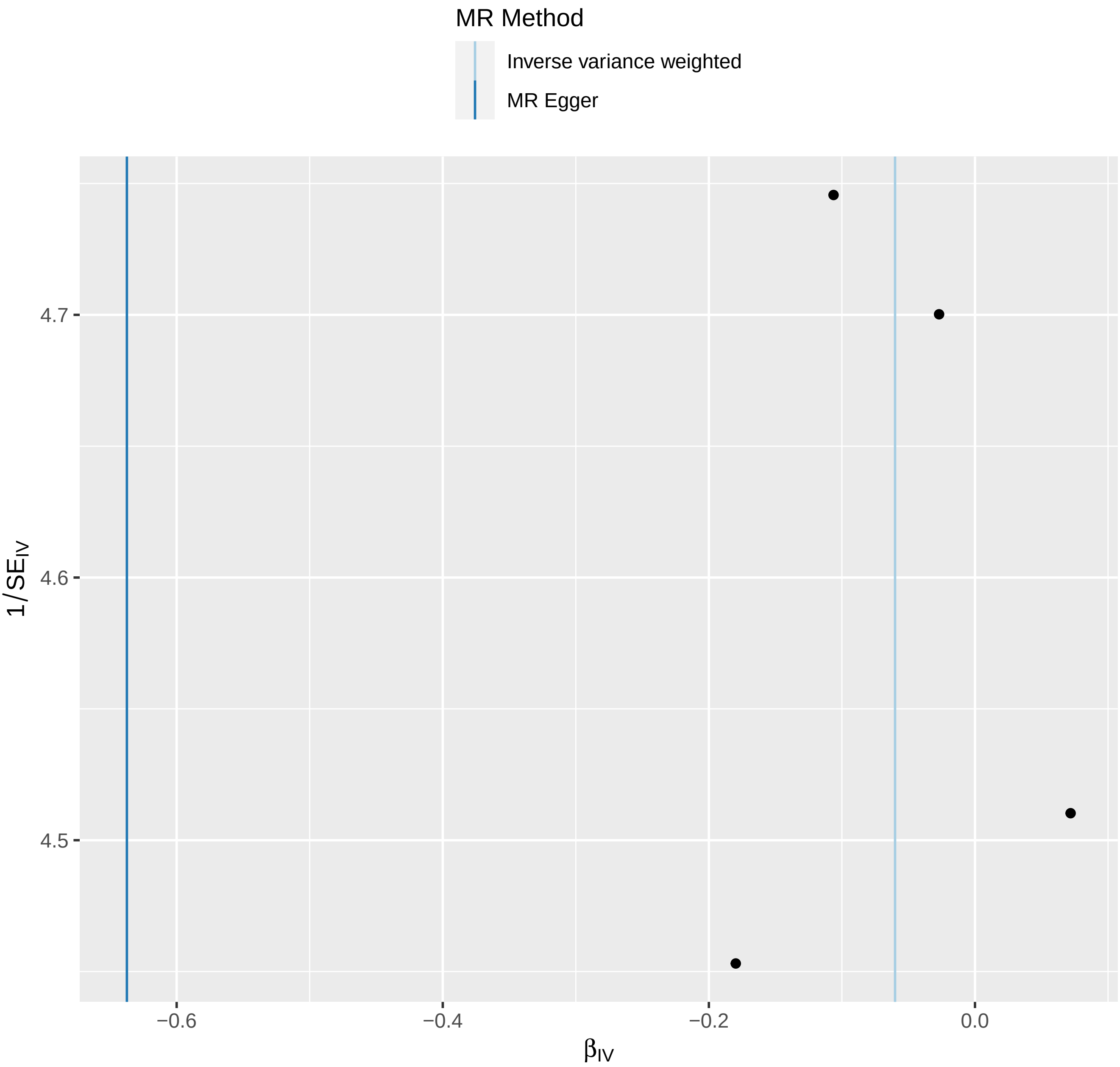

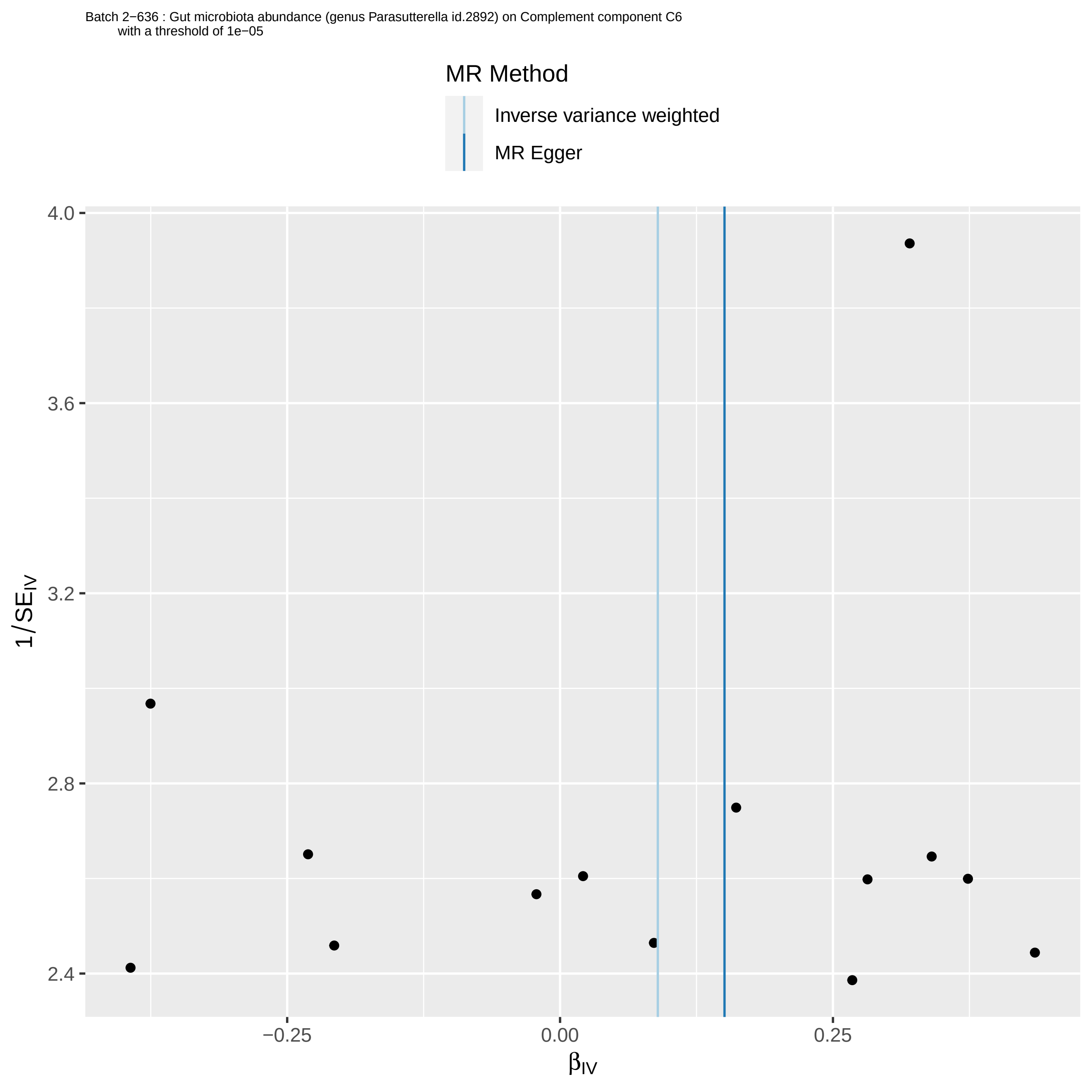

Batch 2−636 : Gut microbiota abundance (genus Parasutterella id.2892) on Complement component C6
with a threshold of 1e−05
MR Method
Inverse variance weighted
MR Egger
4.0
3.6
3.2
2.8
2.4
1/SE_IV
−0.25
0.00
0.25
β_IV

Batch 2−637 : Gut microbiota abundance (genus Catenibacterium id.2153) on Uncharacterized protein C6orf226
with a threshold of 1e−05

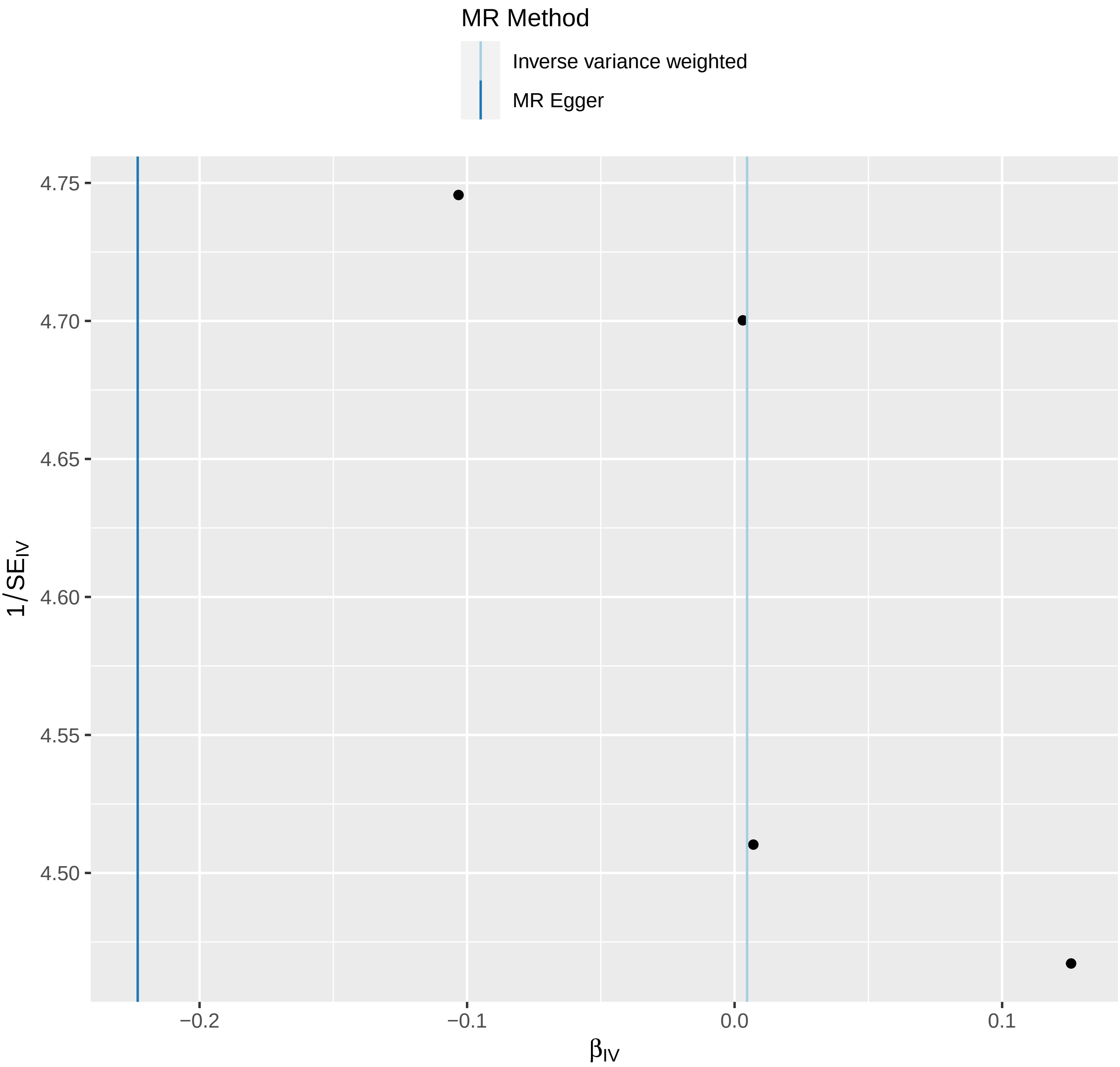

Batch 2−638 : Gut microbiota abundance (genus Parasutterella id.2892) on Uncharacterized protein C6orf226
with a threshold of 1e−05

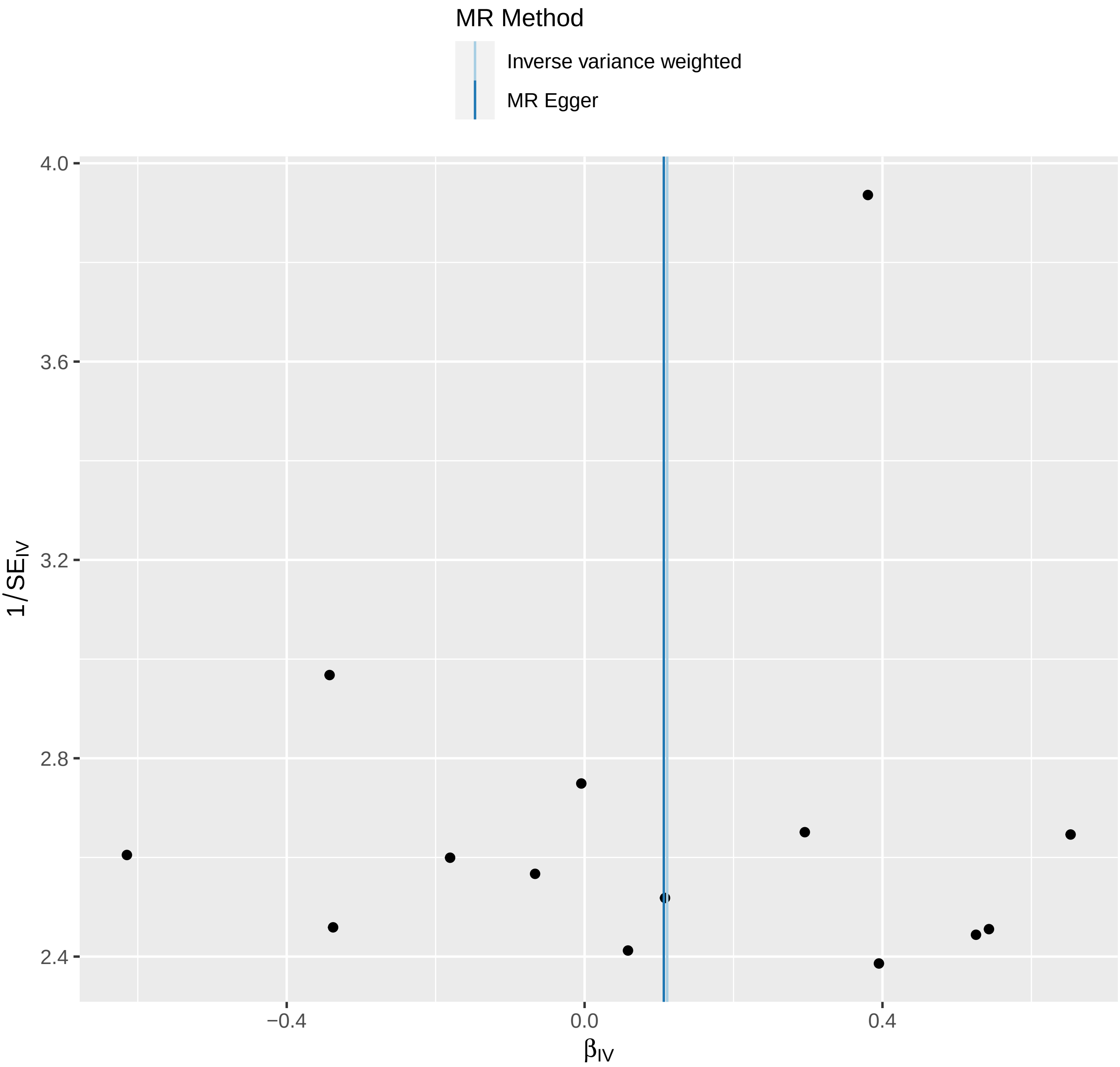

Batch 2−639 : Gut microbiota abundance (genus Catenibacterium id.2153) on Bombesin receptor−activated protein C6orf89 with a threshold of 1e−05

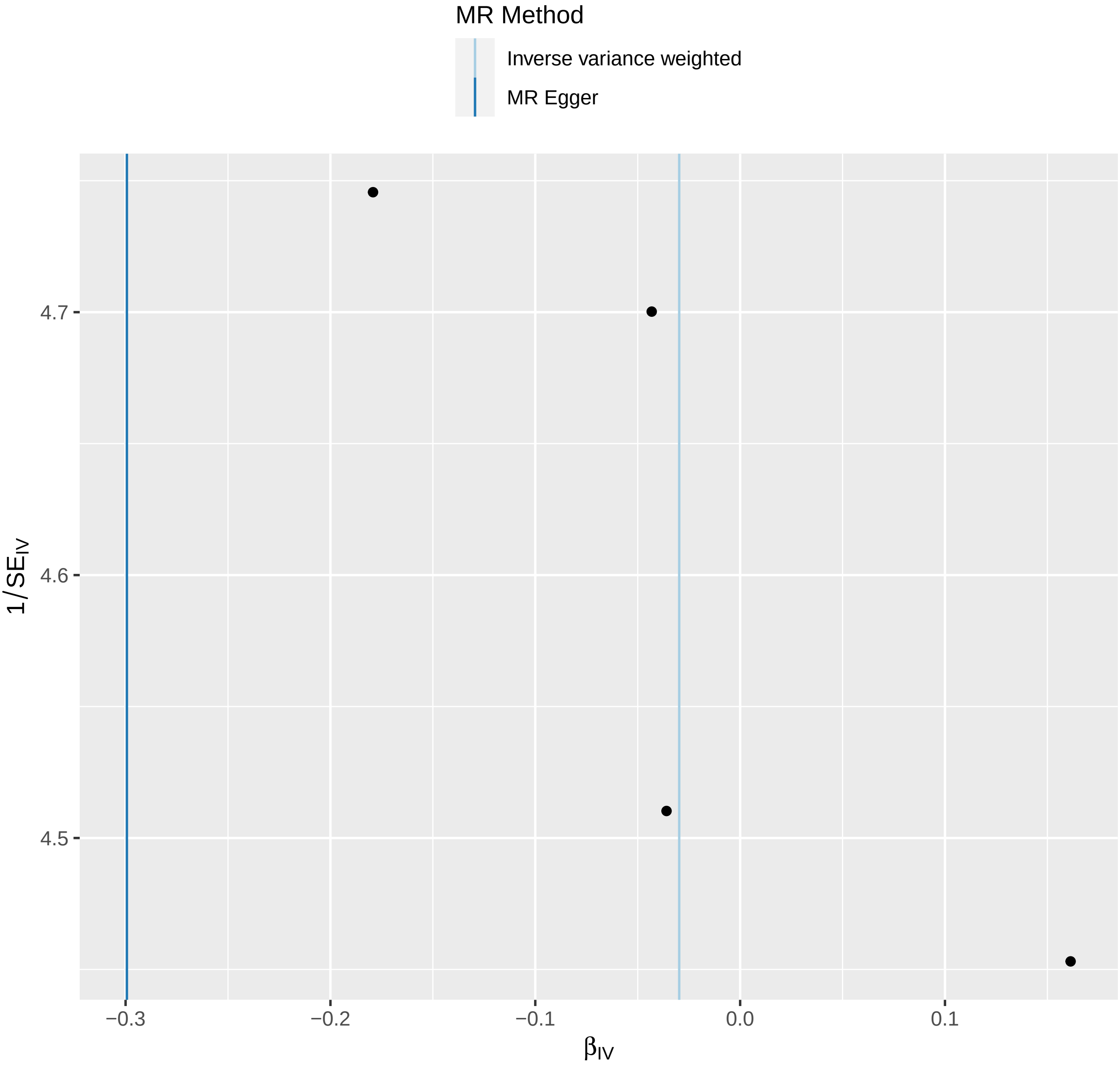

Batch 2−640 : Gut microbiota abundance (genus Parasutterella id.2892) on Bombesin receptor−activated protein C6orf89
with a threshold of 1e−05

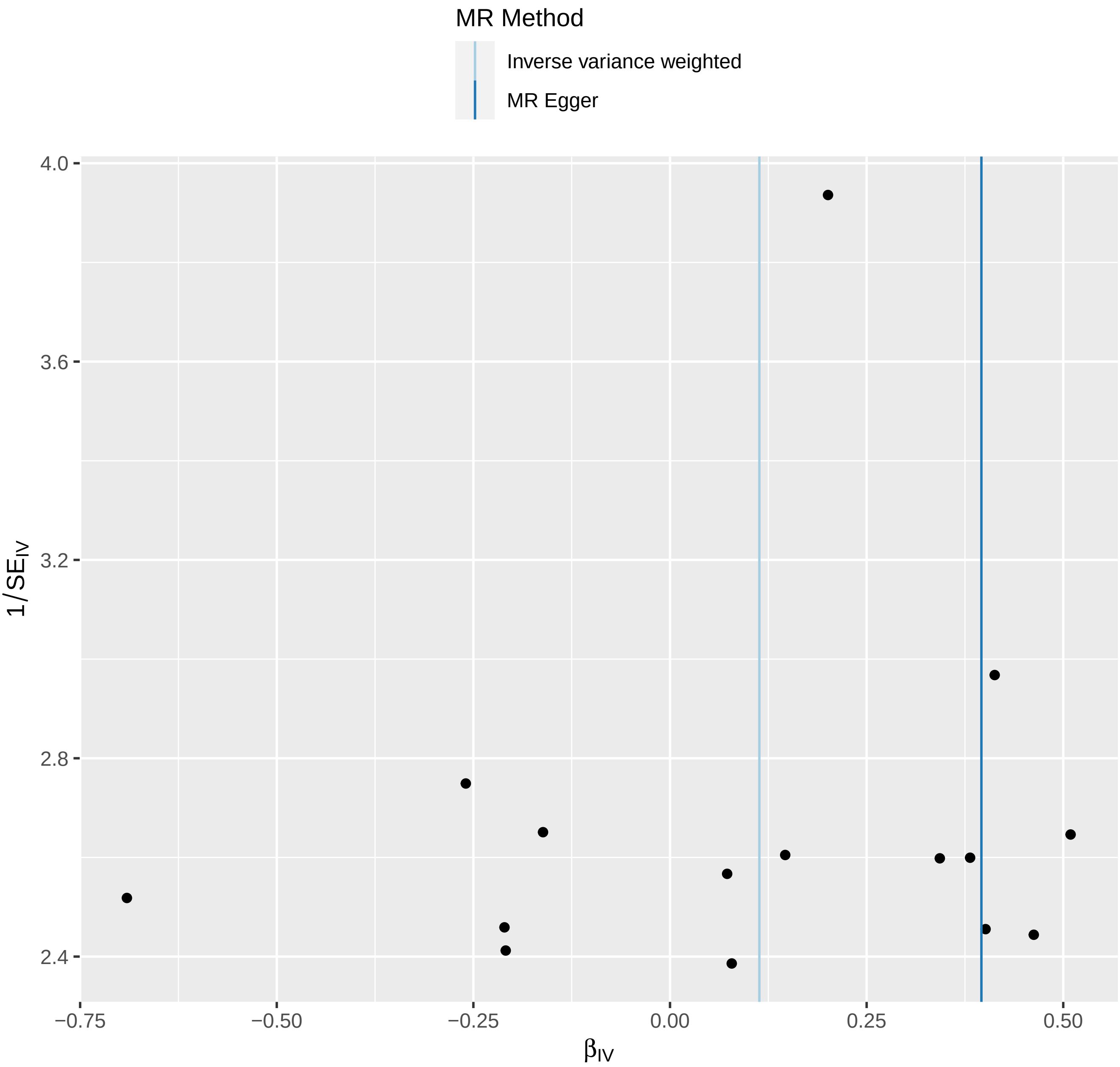

Batch 2−641 : Gut microbiota abundance (genus Catenibacterium id.2153) on Uncharacterized protein C7orf69
with a threshold of 1e−05

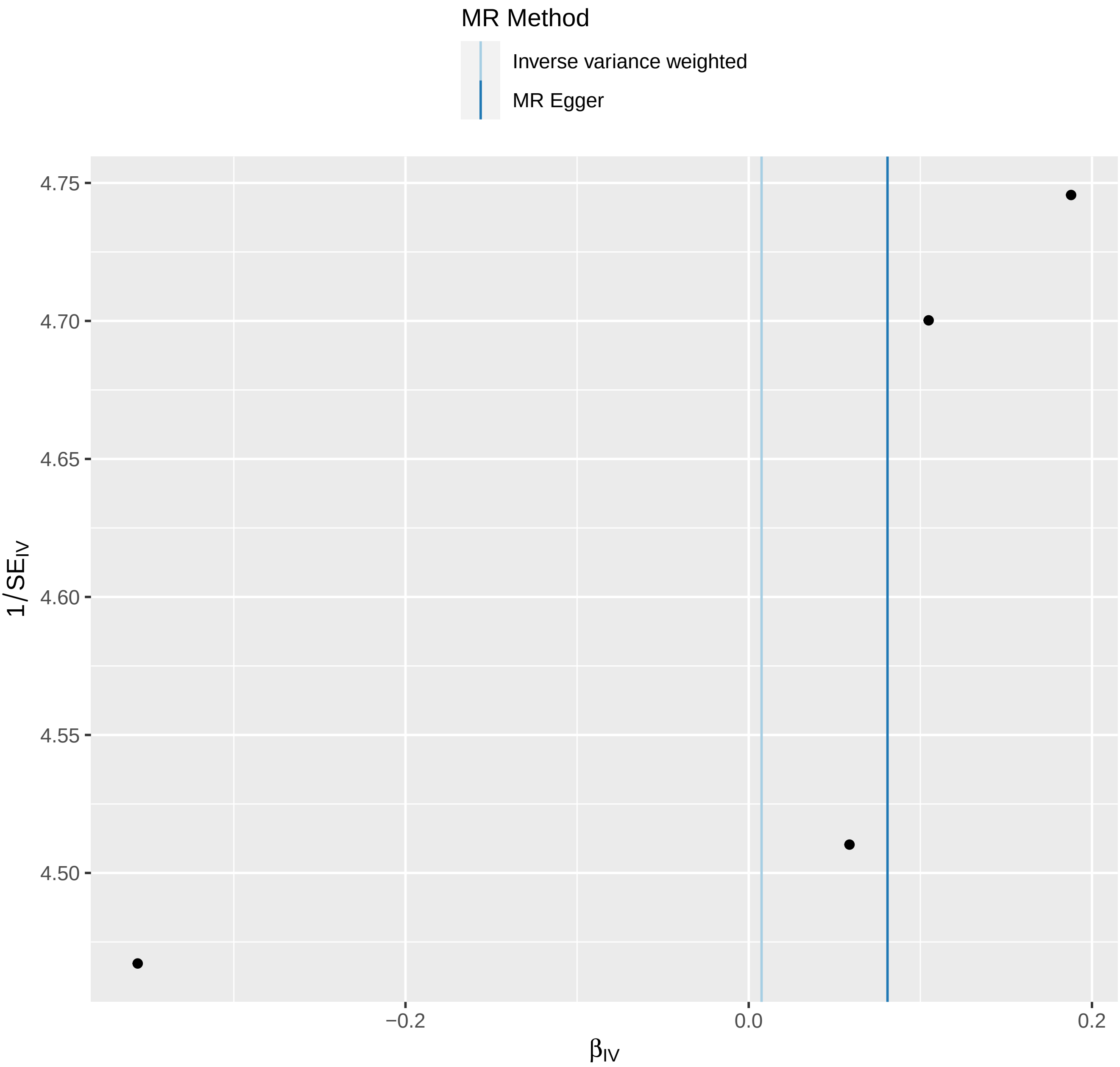

Batch 2−642 : Gut microbiota abundance (genus Parasutterella id.2892) on Uncharacterized protein C7orf69
with a threshold of 1e−05

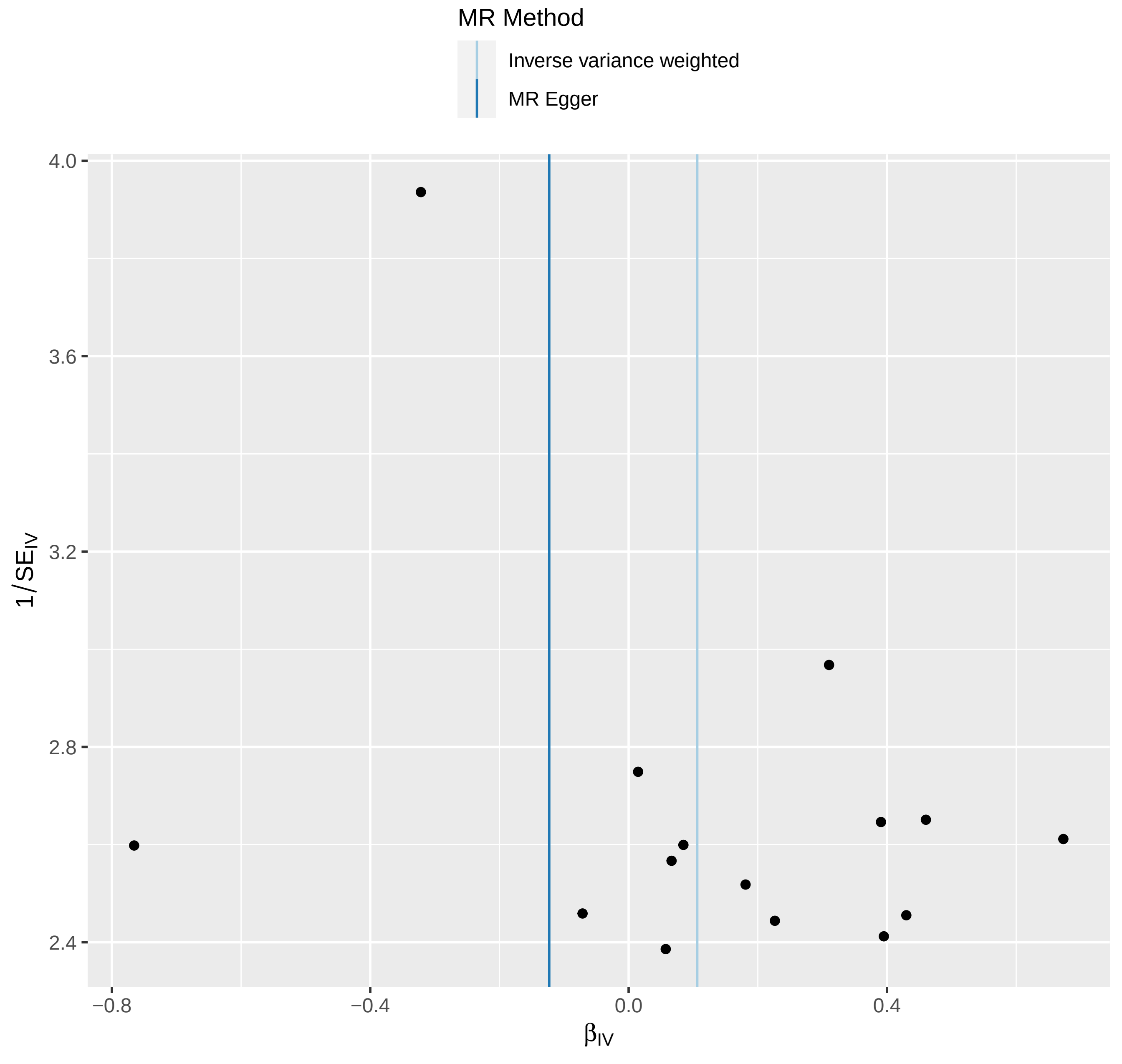

Batch 2−643 : Gut microbiota abundance (genus Catenibacterium id.2153) on Complement component C8
with a threshold of 1e−05

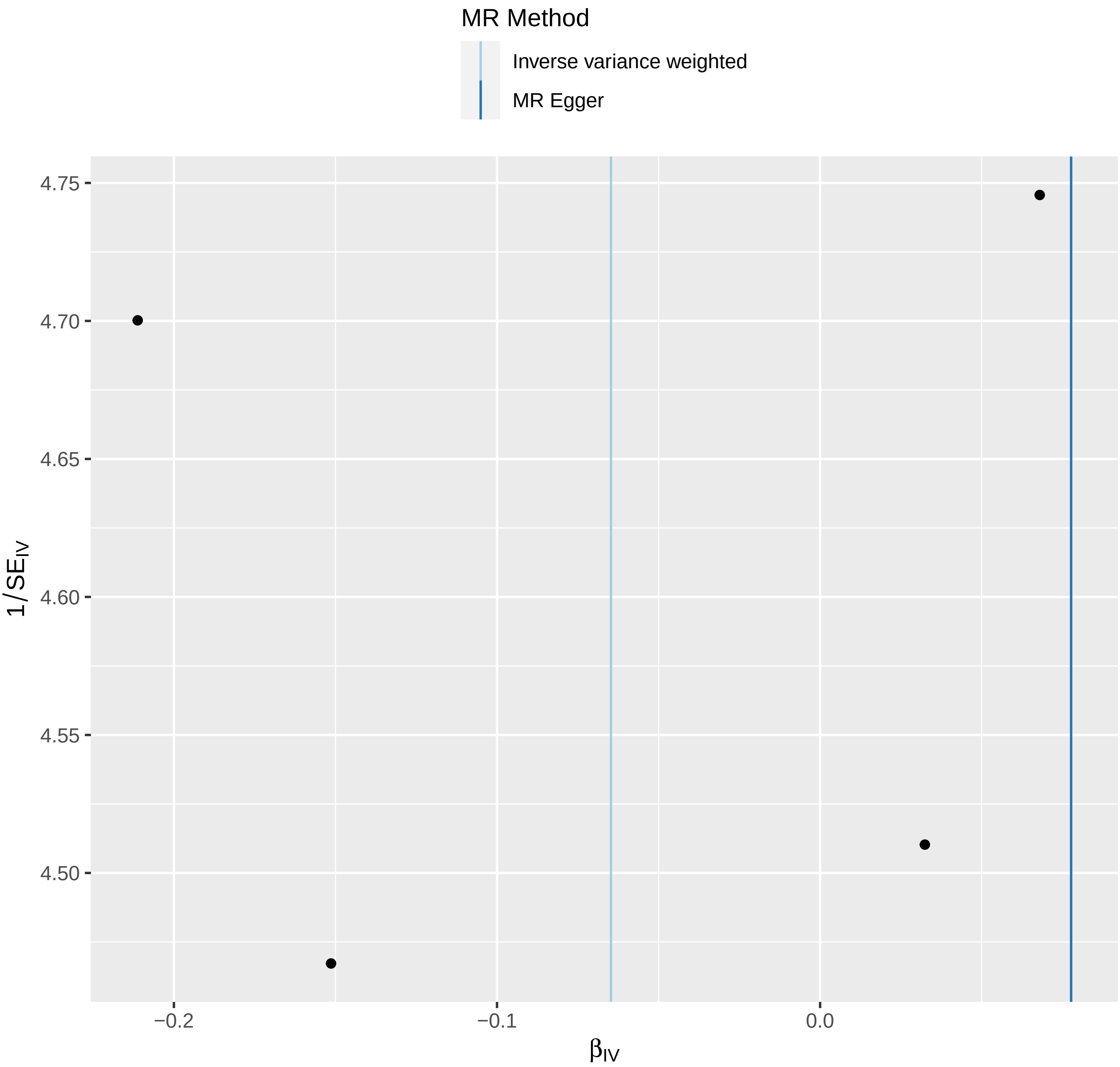

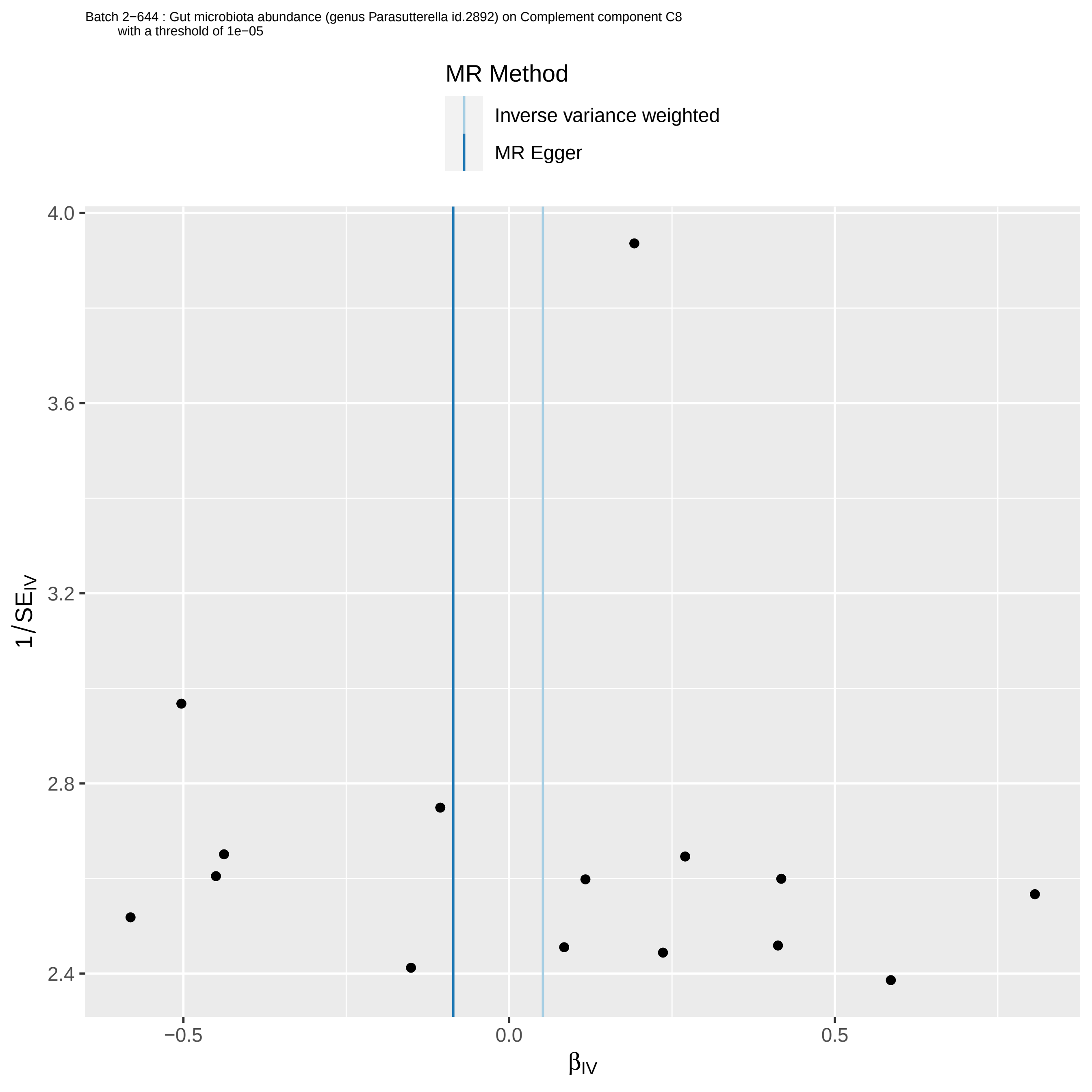

Batch 2−644 : Gut microbiota abundance (genus Parasutterella id.2892) on Complement component C8
with a threshold of 1e−05
MR Method
Inverse variance weighted
MR Egger
4.0
3.6
3.2
2.8
2.4
1/SE_IV
−0.5
0.0
0.5
β_IV

Batch 2−645 : Gut microbiota abundance (genus Catenibacterium id.2153) on UPF0488 protein C8orf33
with a threshold of 1e−05

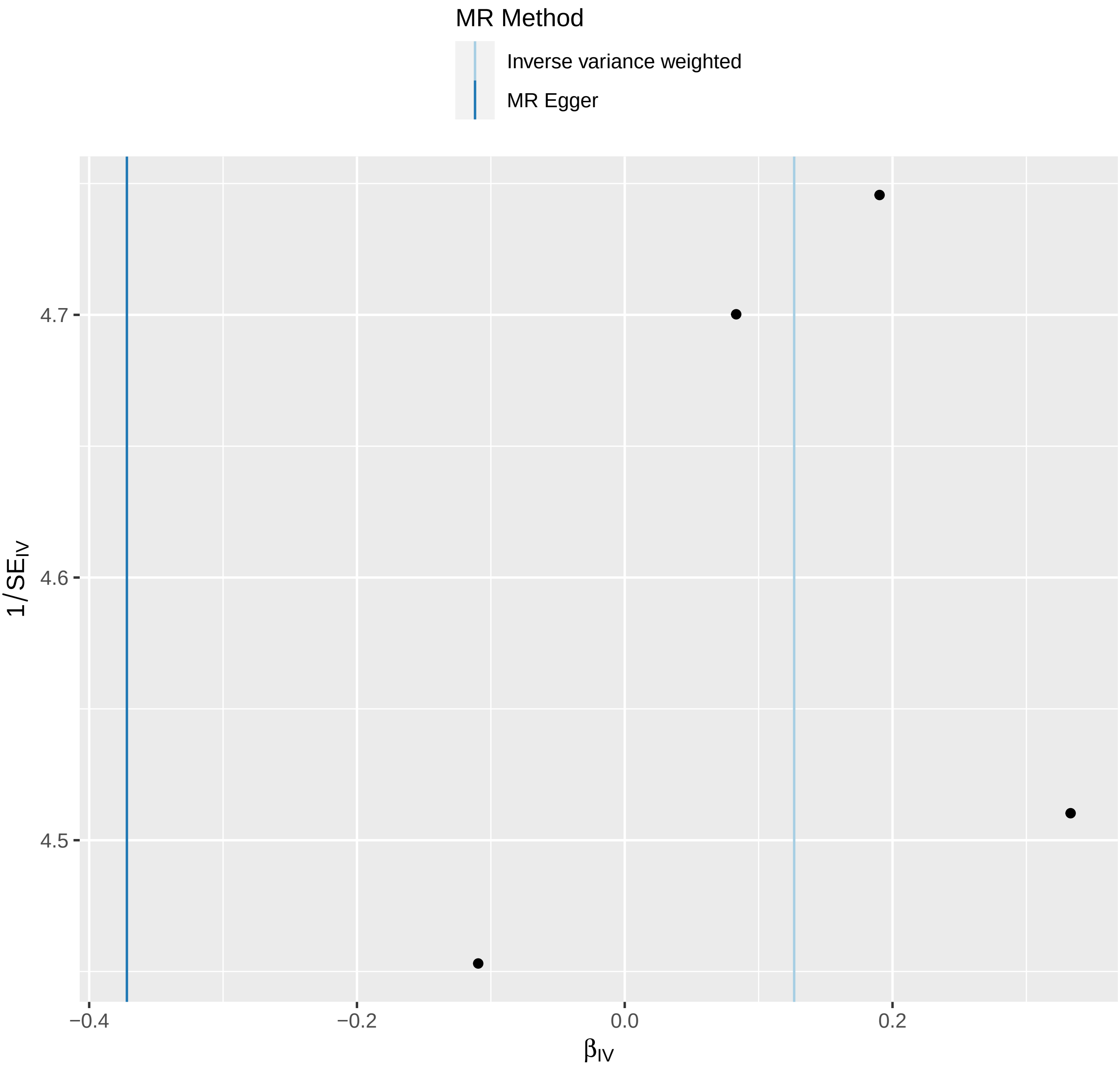

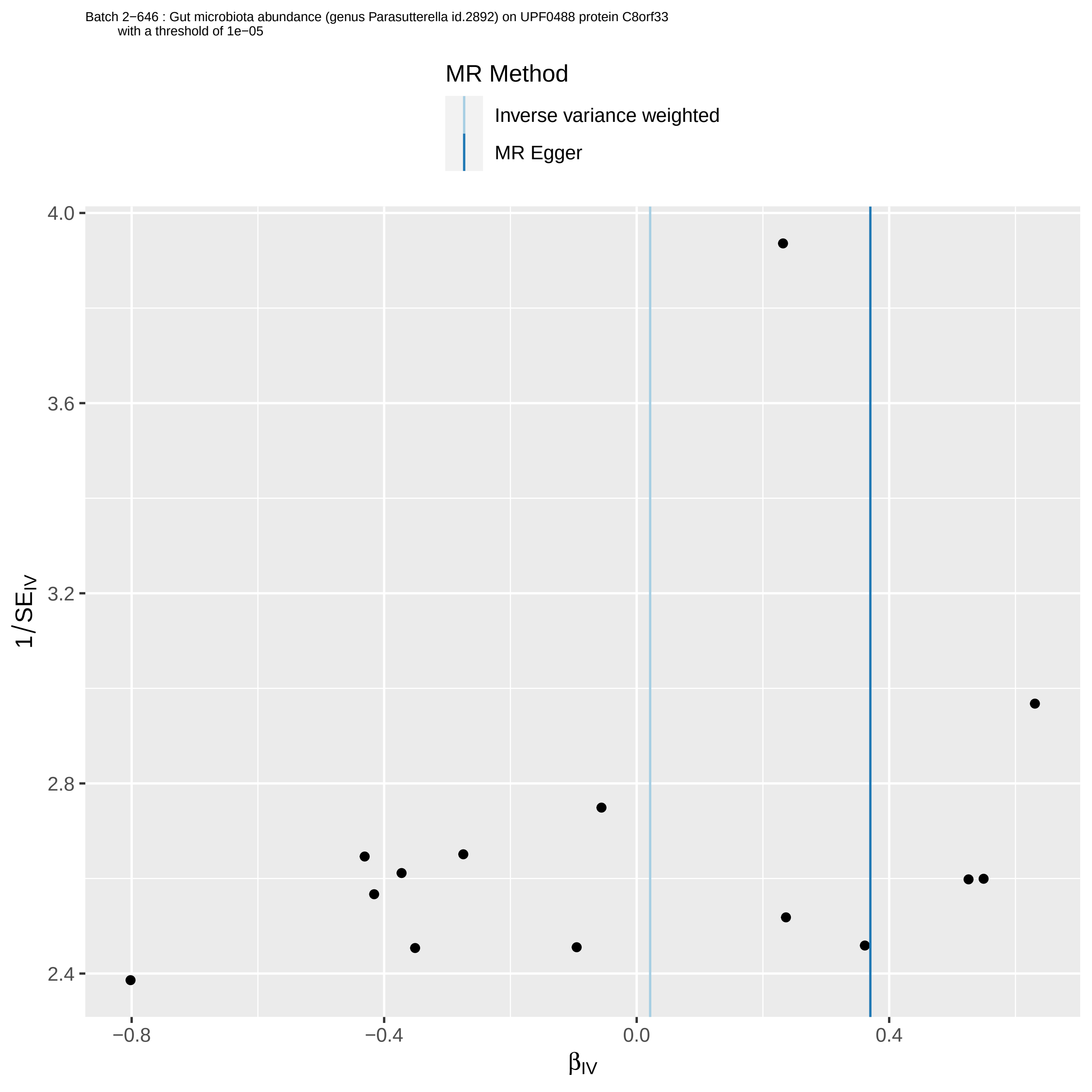

Batch 2−646 : Gut microbiota abundance (genus Parasutterella id.2892) on UPF0488 protein C8orf33
with a threshold of 1e−05
MR Method
Inverse variance weighted
MR Egger
4.0
3.6
3.2
2.8
2.4
1/SE$_{IV}$
−0.8
−0.4
0.0
0.4
β$_{IV}$

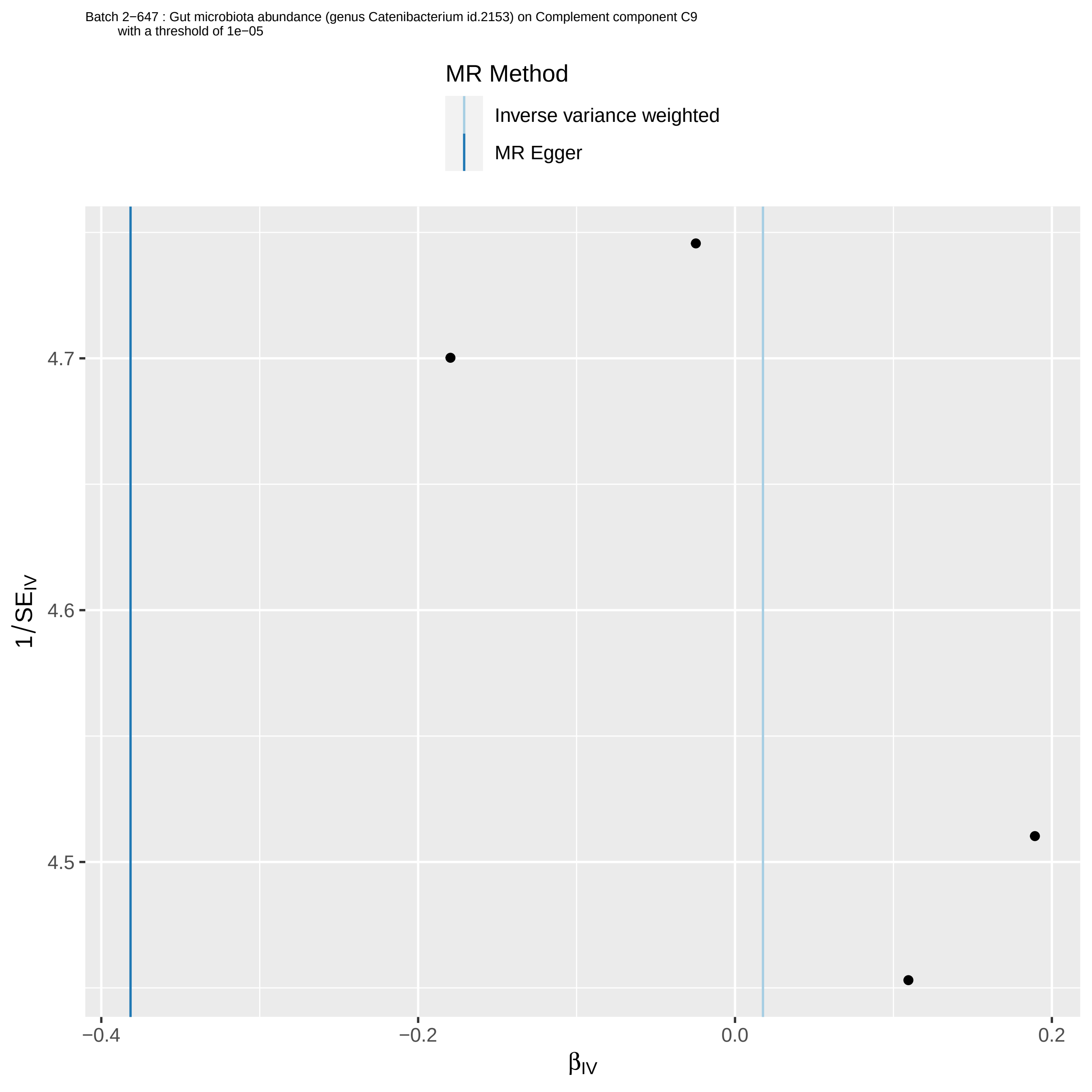

Batch 2−647 : Gut microbiota abundance (genus Catenibacterium id.2153) on Complement component C9
with a threshold of 1e−05
MR Method
Inverse variance weighted
MR Egger
4.7
4.6
4.5
1/SE_IV
−0.4
−0.2
0.0
0.2
β_IV

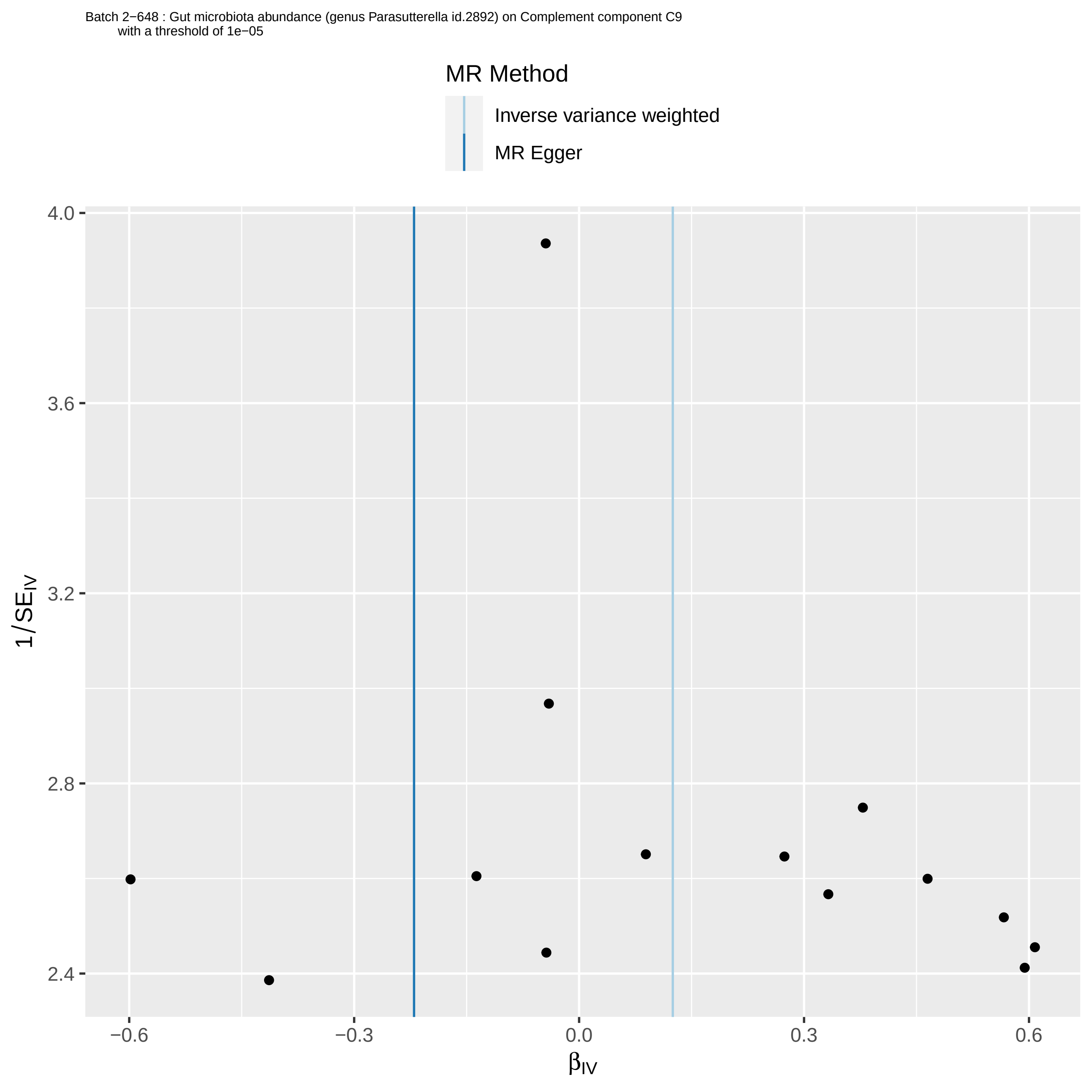

Batch 2−648 : Gut microbiota abundance (genus Parasutterella id.2892) on Complement component C9
with a threshold of 1e−05
MR Method
Inverse variance weighted
MR Egger
4.0
3.6
3.2
2.8
2.4
1/SE$_{IV}$
−0.6
−0.3
0.0
0.3
0.6
β$_{IV}$

Batch 2−649 : Gut microbiota abundance (genus Catenibacterium id.2153) on Bcl10−interacting CARD protein with a threshold of 1e−05

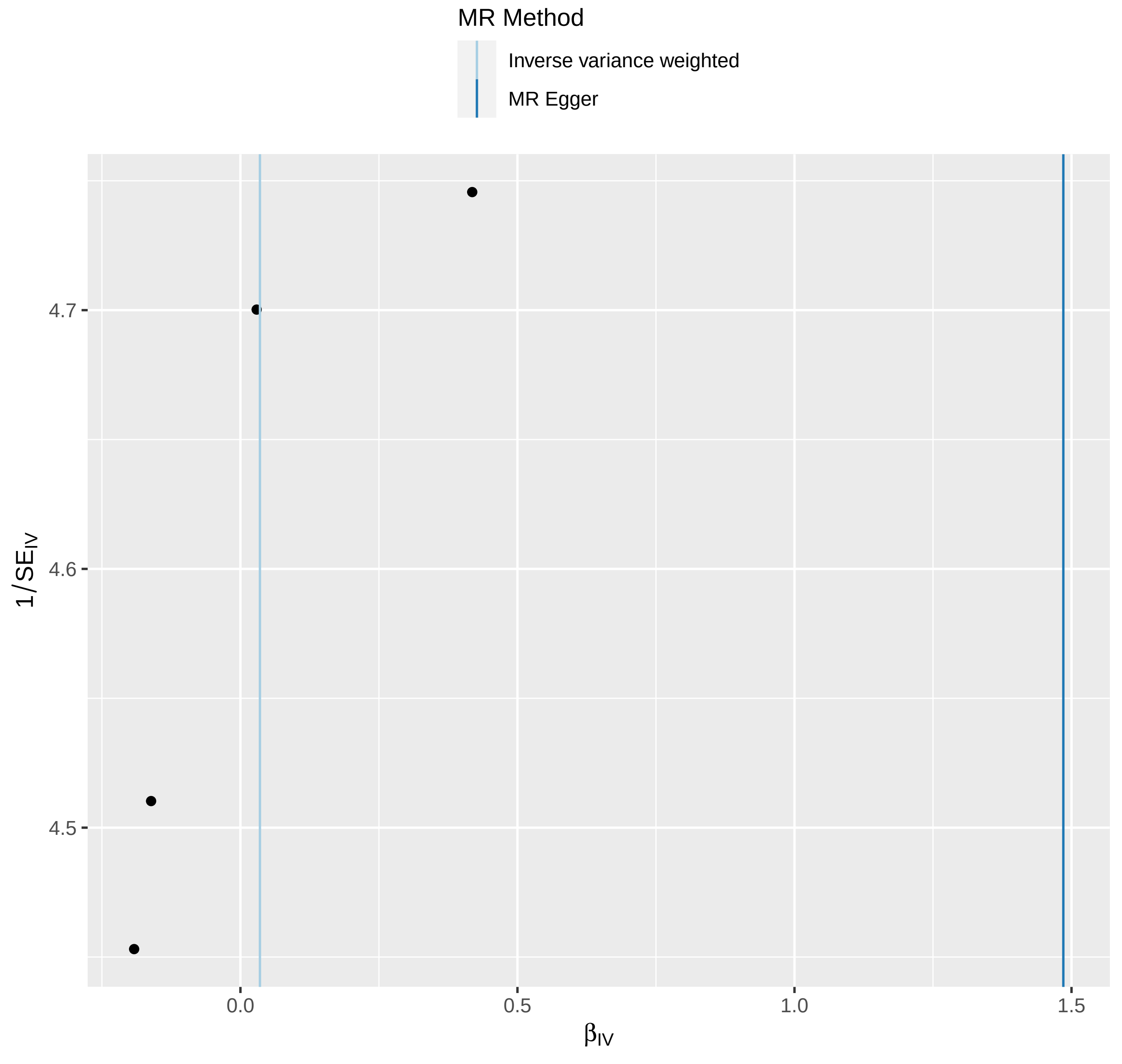

Batch 2−650 : Gut microbiota abundance (genus Parasutterella id.2892) on Bcl10−interacting CARD protein
with a threshold of 1e−05

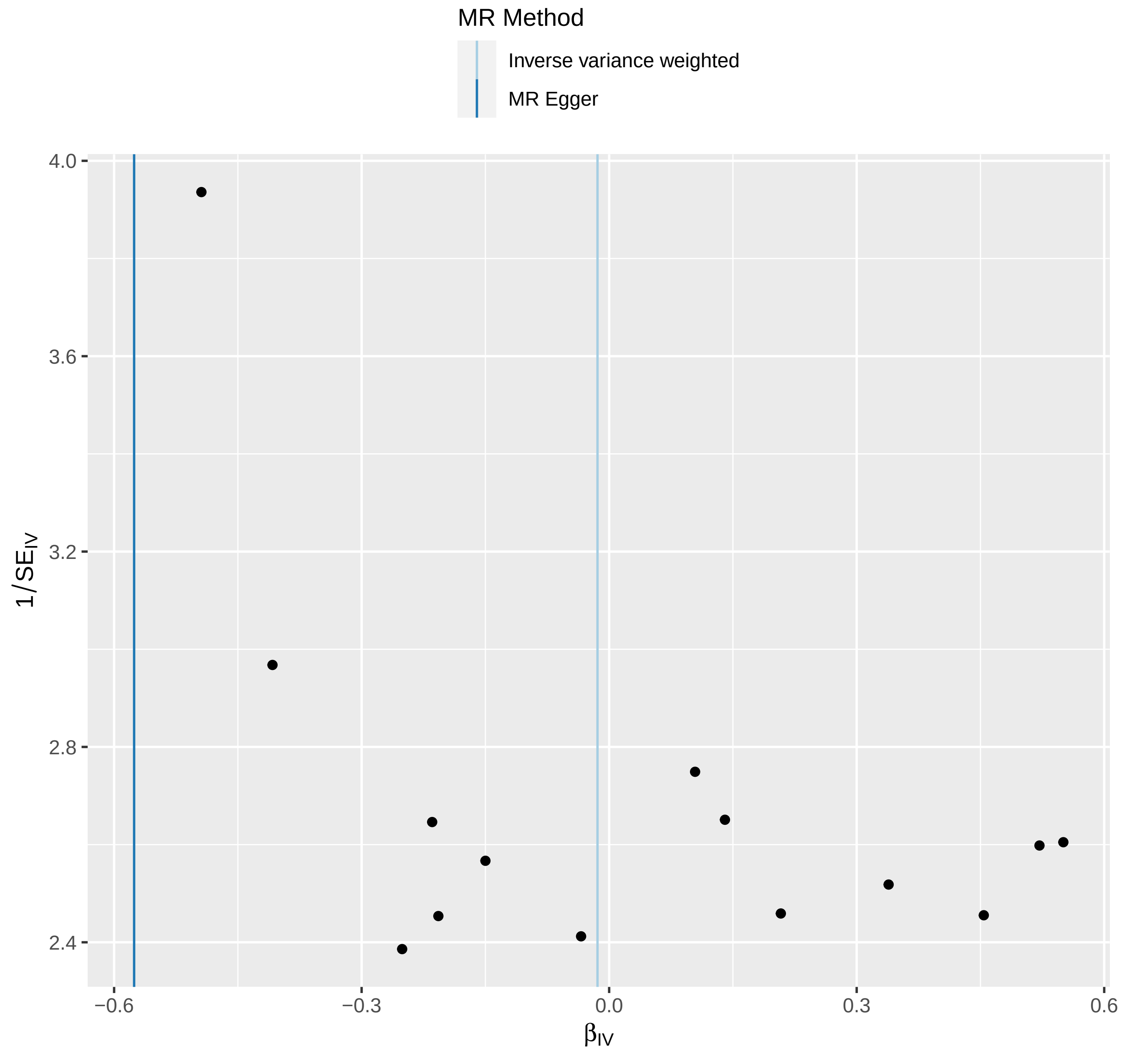

Batch 2−651 : Gut microbiota abundance (genus Catenibacterium id.2153) on Carbonic anhydrase−related protein 10
with a threshold of 1e−05

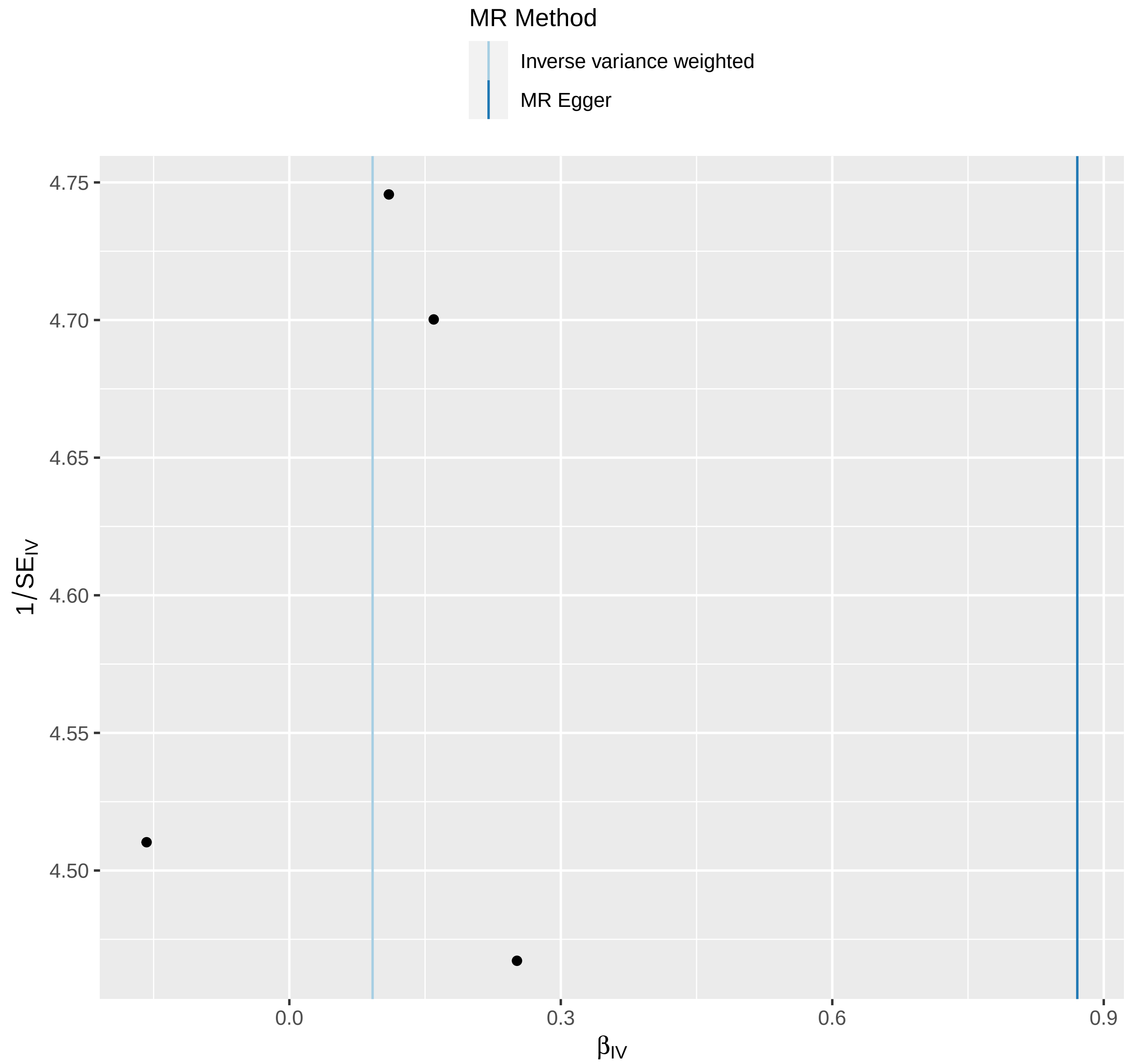

Batch 2−652 : Gut microbiota abundance (genus Parasutterella id.2892) on Carbonic anhydrase−related protein 10
with a threshold of 1e−05

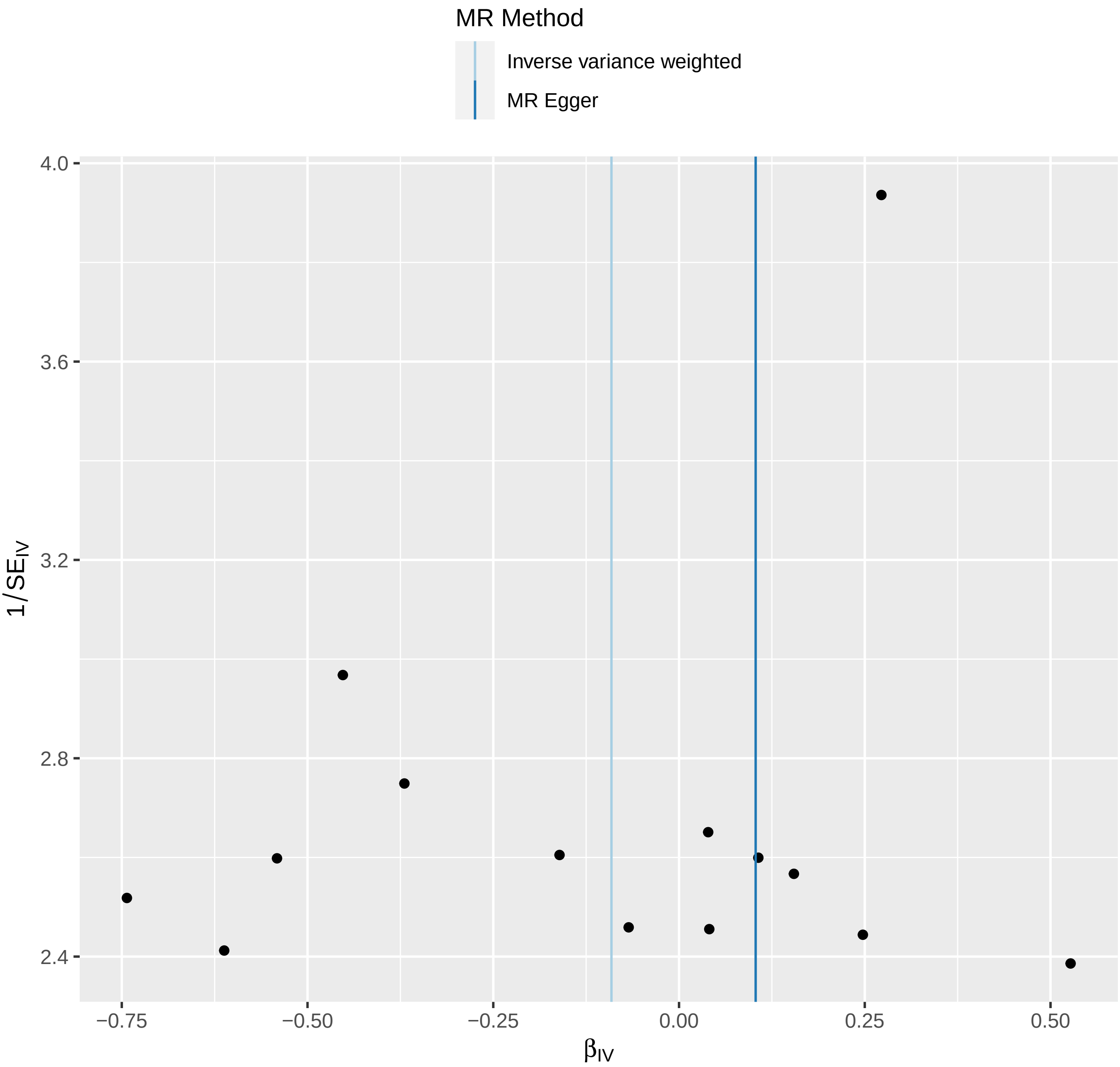

Batch 2−653 : Gut microbiota abundance (genus Catenibacterium id.2153) on Carbonic anhydrase−related protein 10 with a threshold of 1e−05

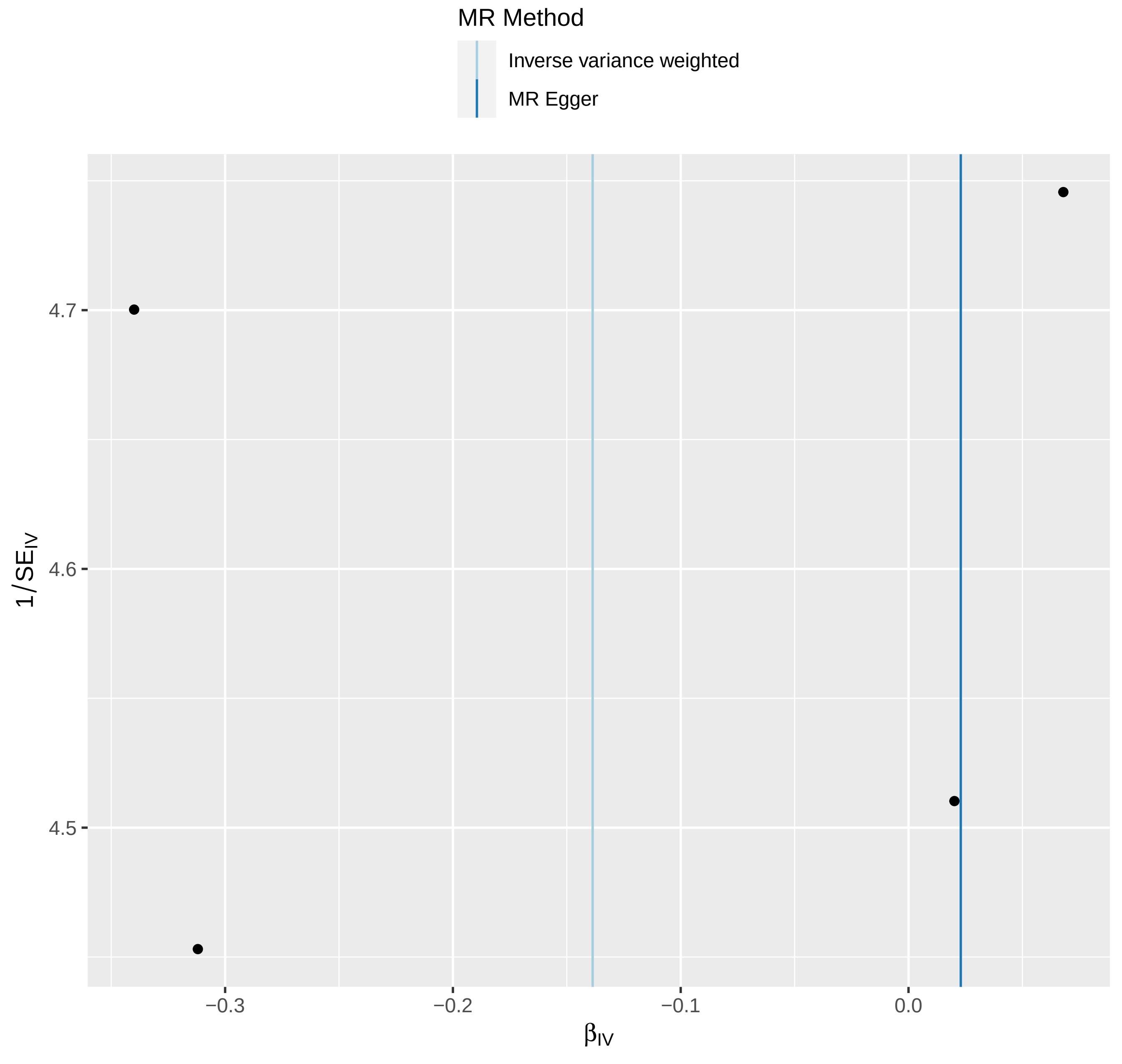

Batch 2−654 : Gut microbiota abundance (genus Parasutterella id.2892) on Carbonic anhydrase−related protein 10
with a threshold of 1e−05

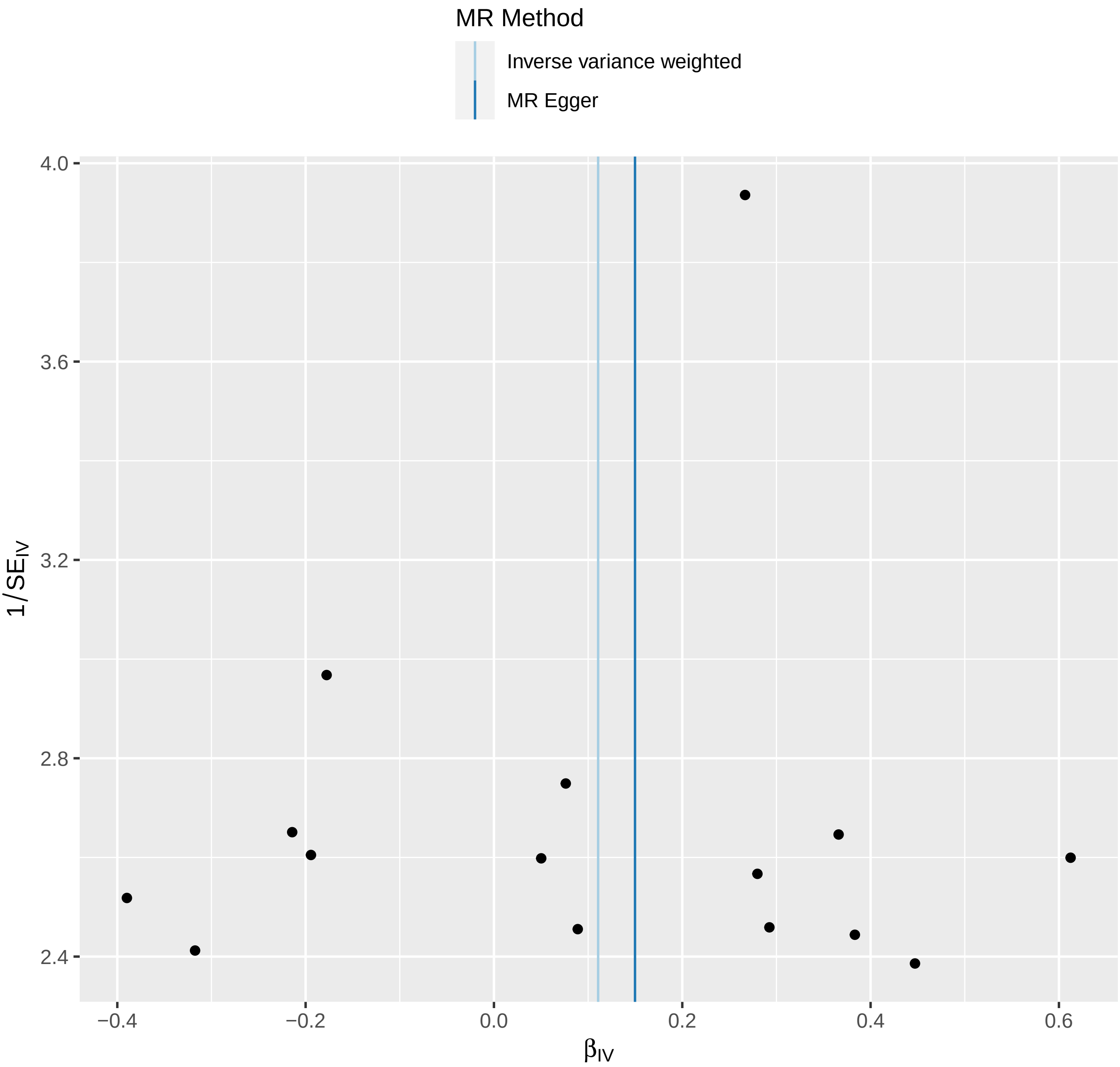

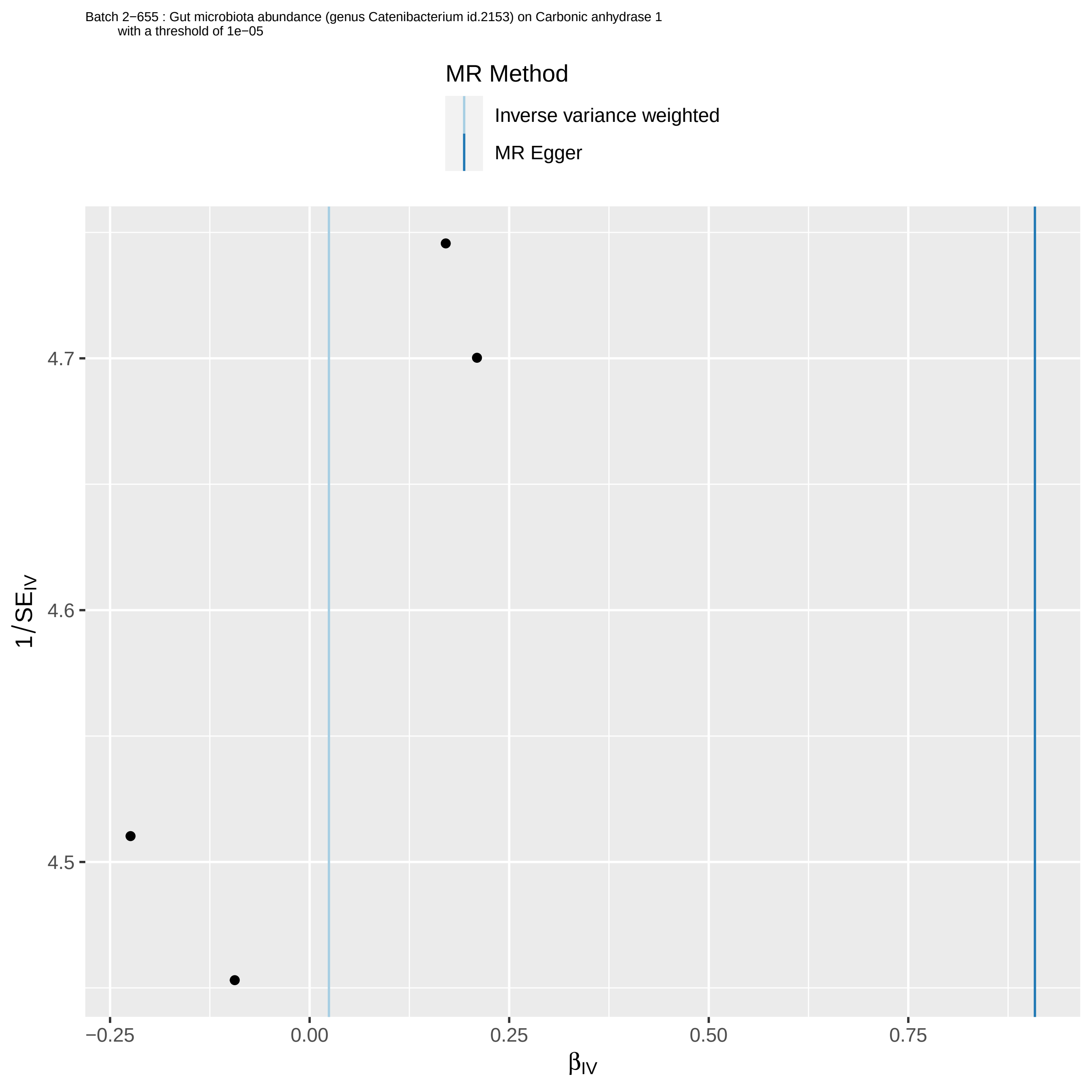
Batch 2−655 : Gut microbiota abundance (genus Catenibacterium id.2153) on Carbonic anhydrase 1
with a threshold of 1e−05
MR Method
Inverse variance weighted
MR Egger
4.7
4.6
4.5
1/SE$_{IV}$
−0.25
0.00
0.25
0.50
0.75
β_{IV}

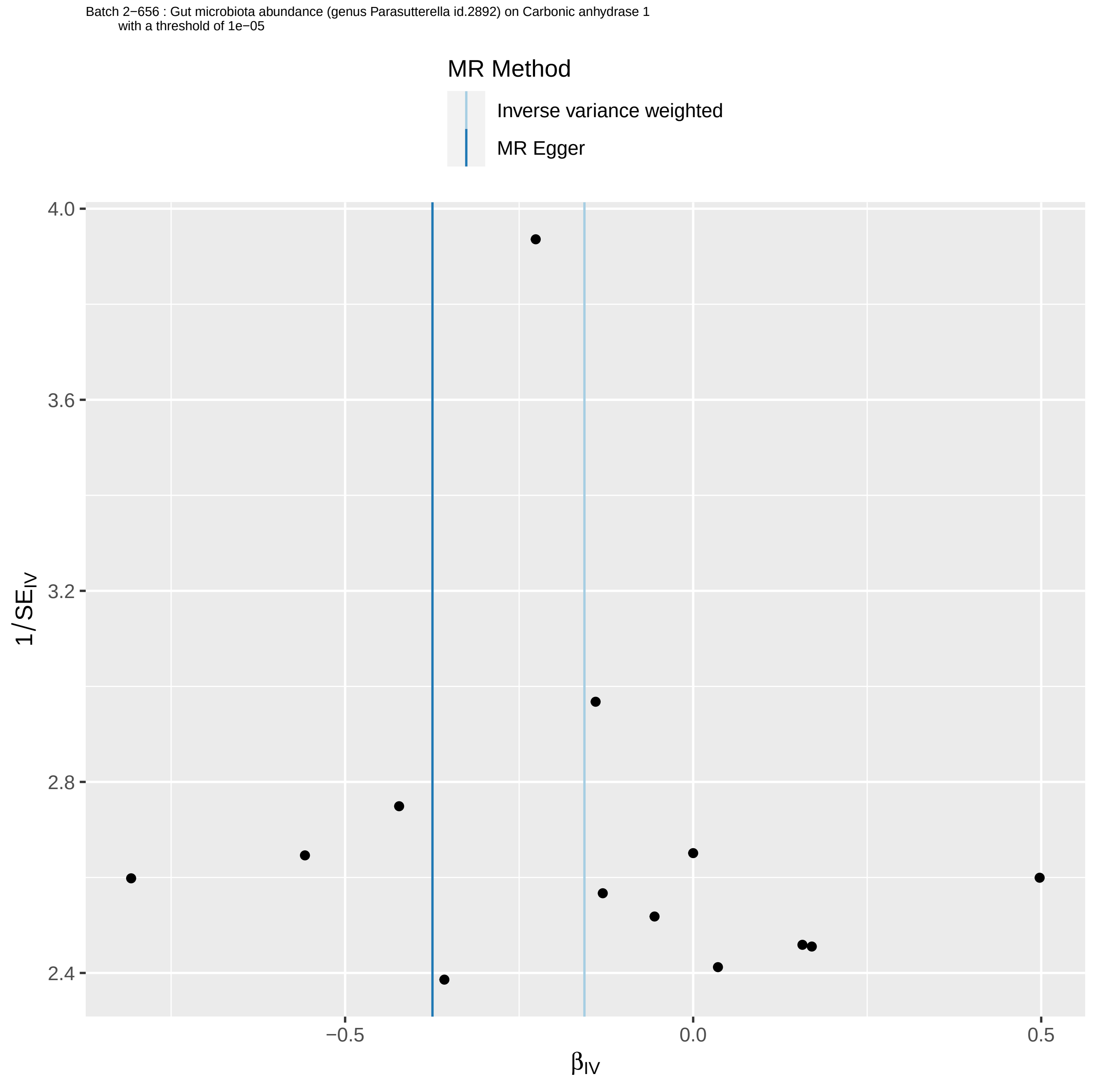
Batch 2−656 : Gut microbiota abundance (genus Parasutterella id.2892) on Carbonic anhydrase 1
with a threshold of 1e−05
MR Method
Inverse variance weighted
MR Egger
4.0
3.6
3.2
2.8
2.4
1/SE_IV
−0.5
0.0
0.5
β_IV

Batch 2−657 : Gut microbiota abundance (genus Catenibacterium id.2153) on Carbonic anhydrase 2
with a threshold of 1e−05

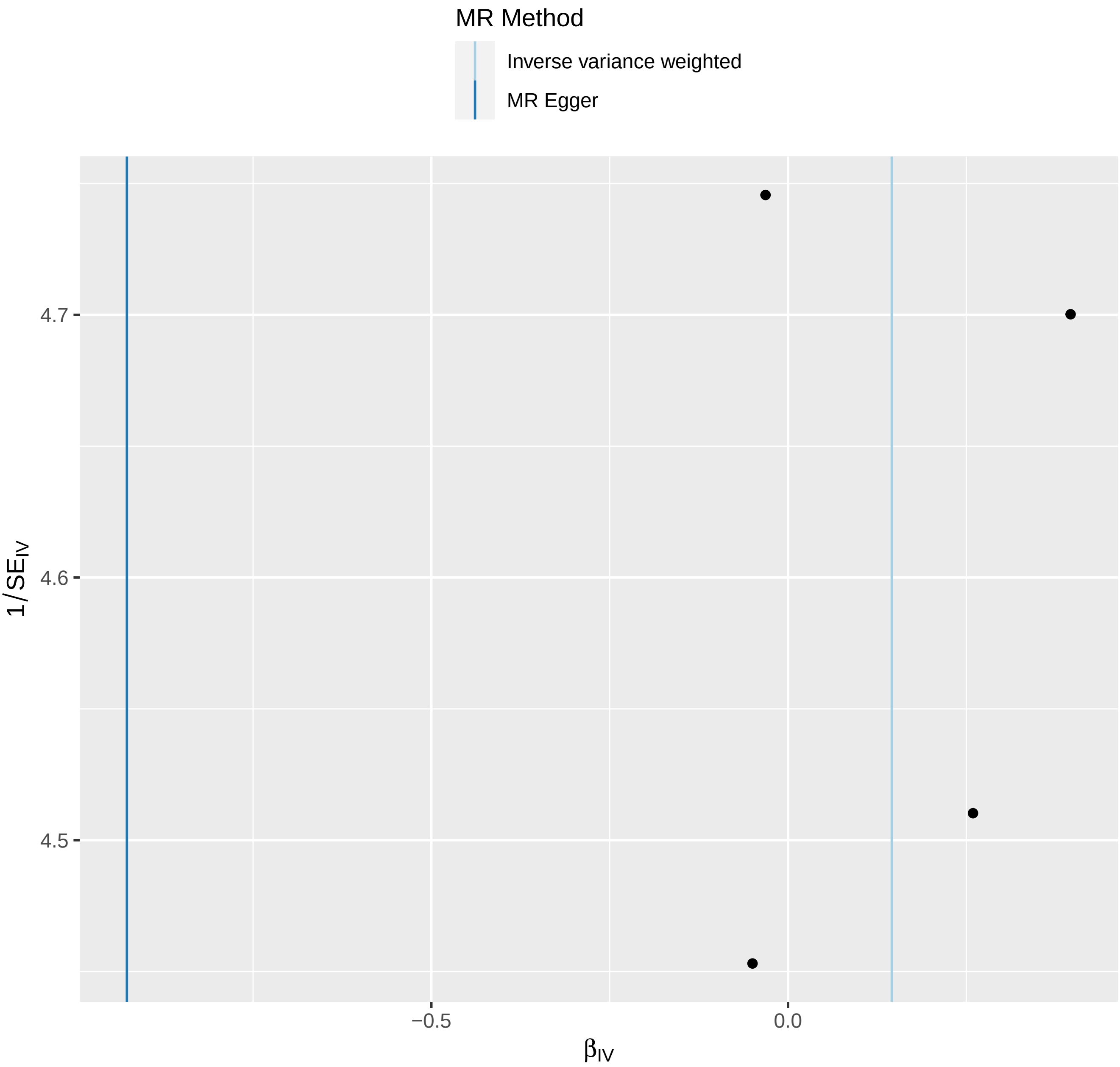

Batch 2−658 : Gut microbiota abundance (genus Parasutterella id.2892) on Carbonic anhydrase 2
with a threshold of 1e−05

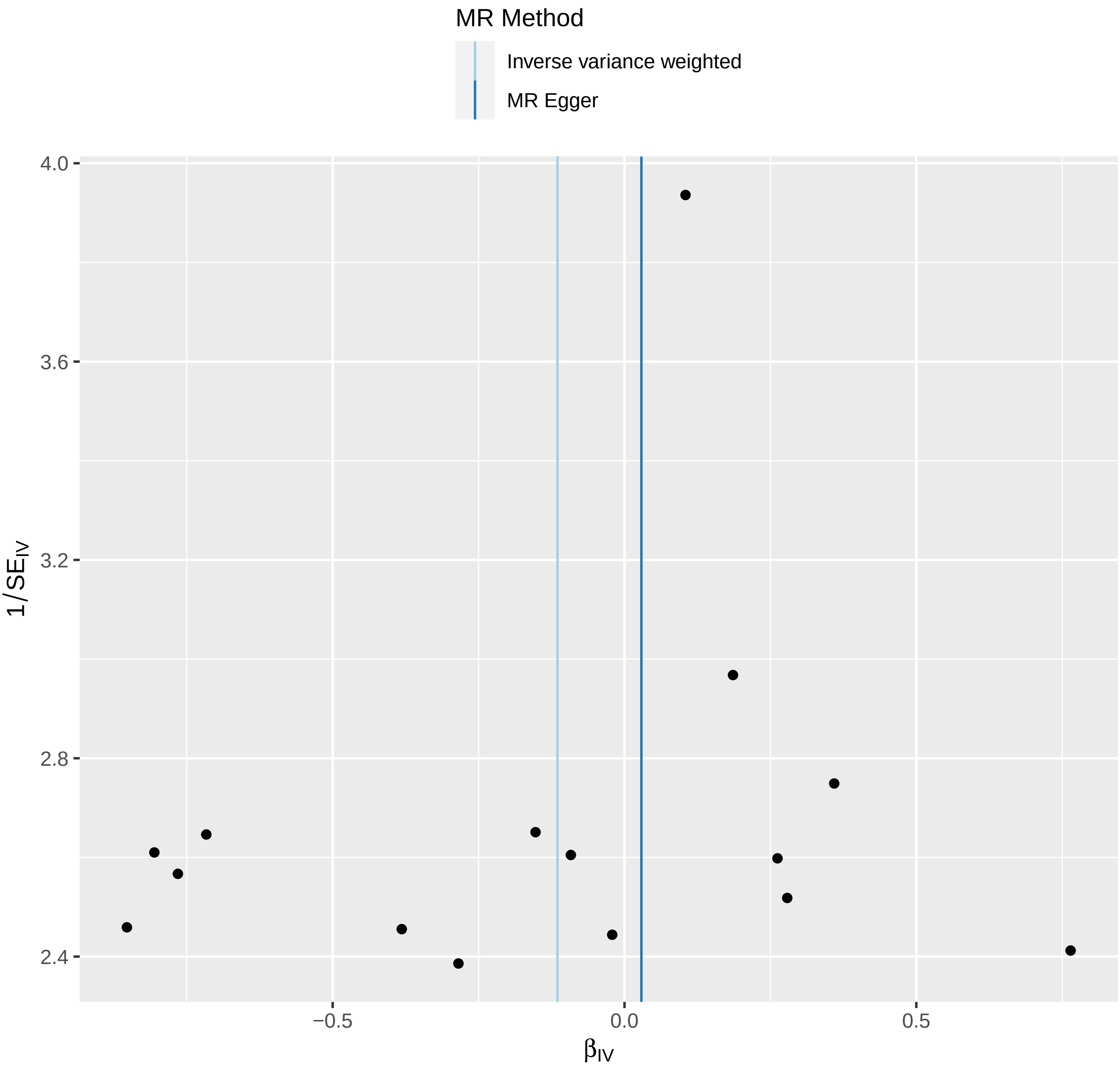

Batch 2−659 : Gut microbiota abundance (genus Catenibacterium id.2153) on Carbonic anhydrase 3
with a threshold of 1e−05

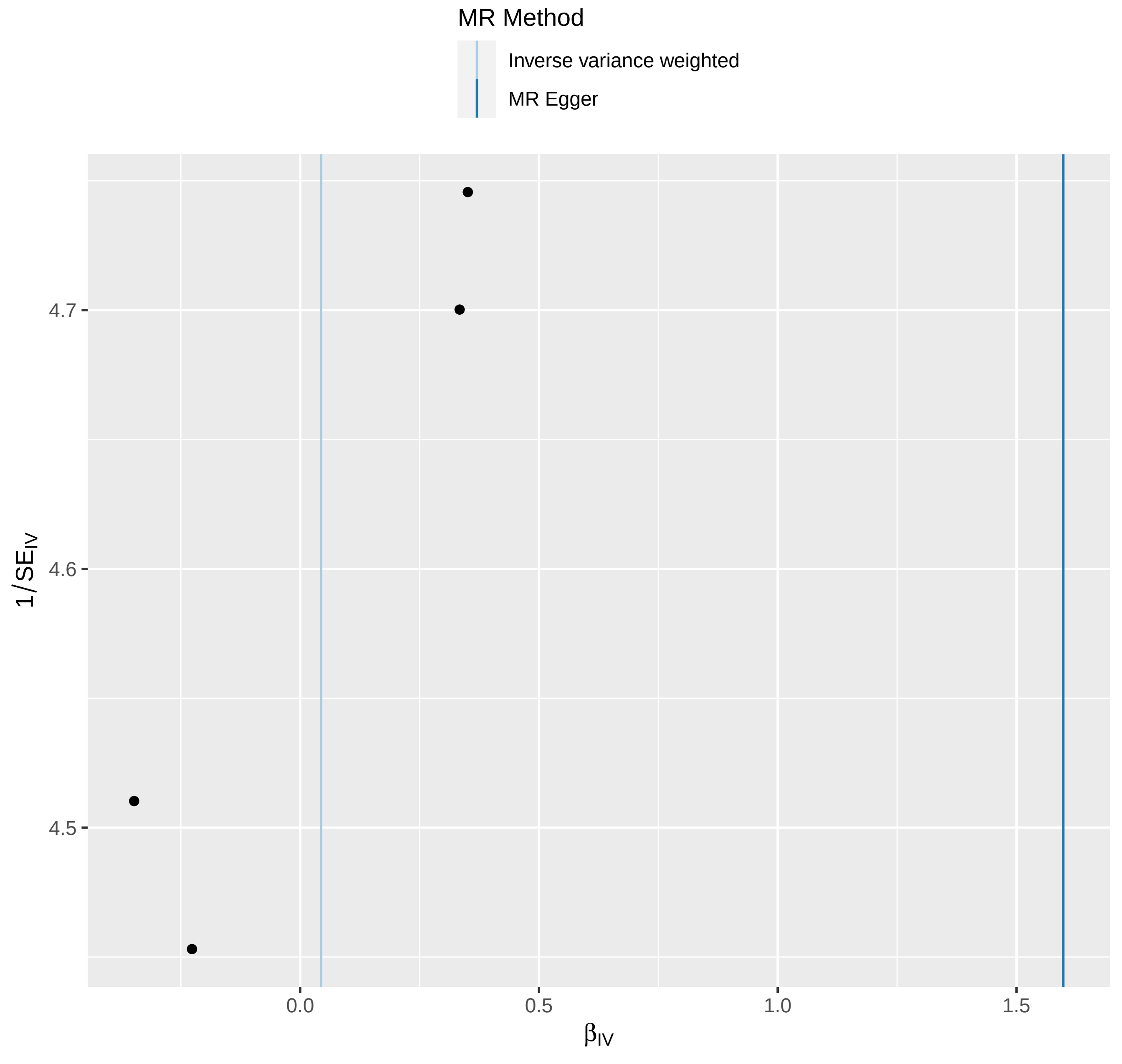

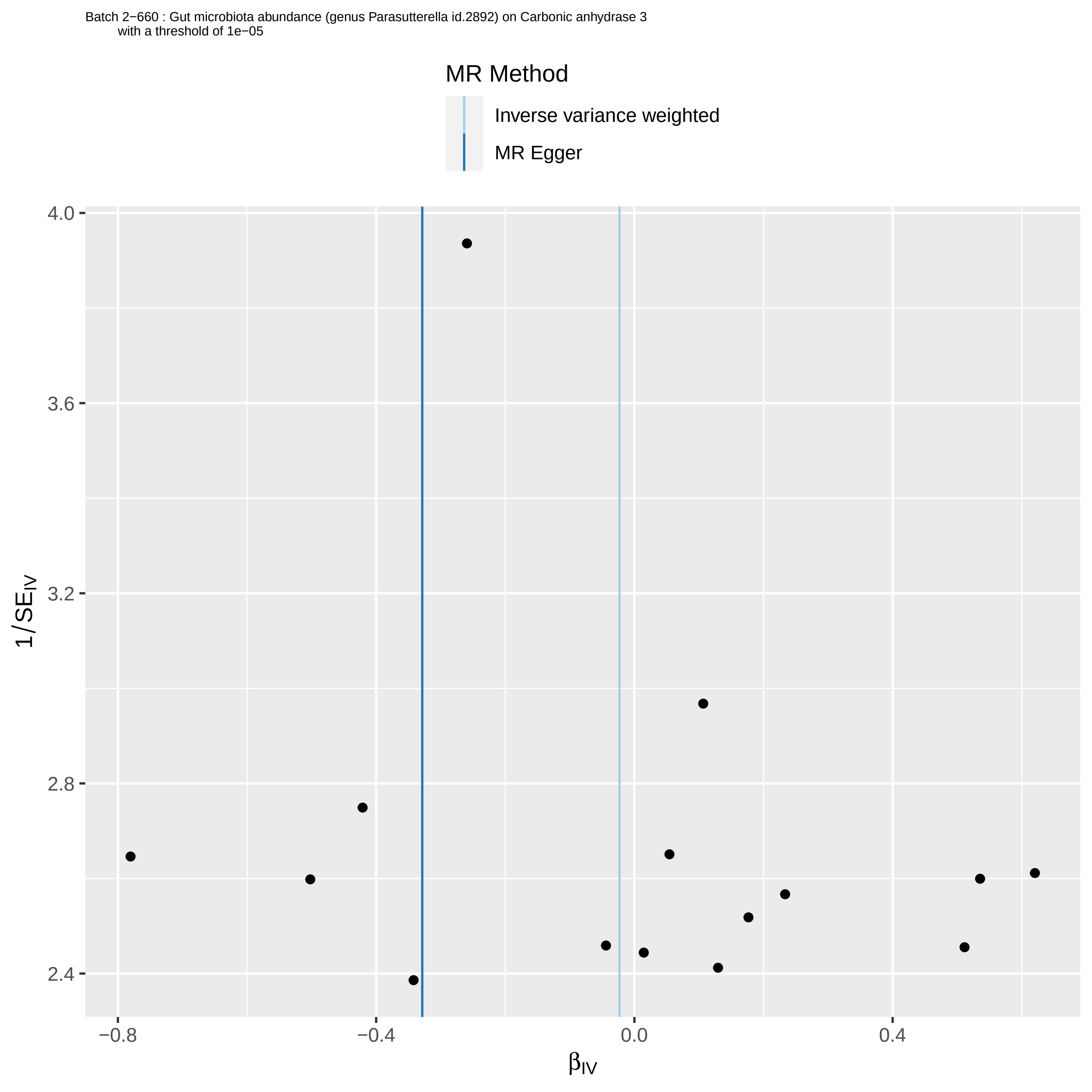
Batch 2−660 : Gut microbiota abundance (genus Parasutterella id.2892) on Carbonic anhydrase 3
with a threshold of 1e−05
MR Method
Inverse variance weighted
MR Egger
4.0
3.6
3.2
2.8
2.4
$1/SE_{IV}$
−0.8
−0.4
0.0
0.4
β_{IV}

Batch 2−661 : Gut microbiota abundance (genus Catenibacterium id.2153) on Carbonic anhydrase 4
with a threshold of 1e−05

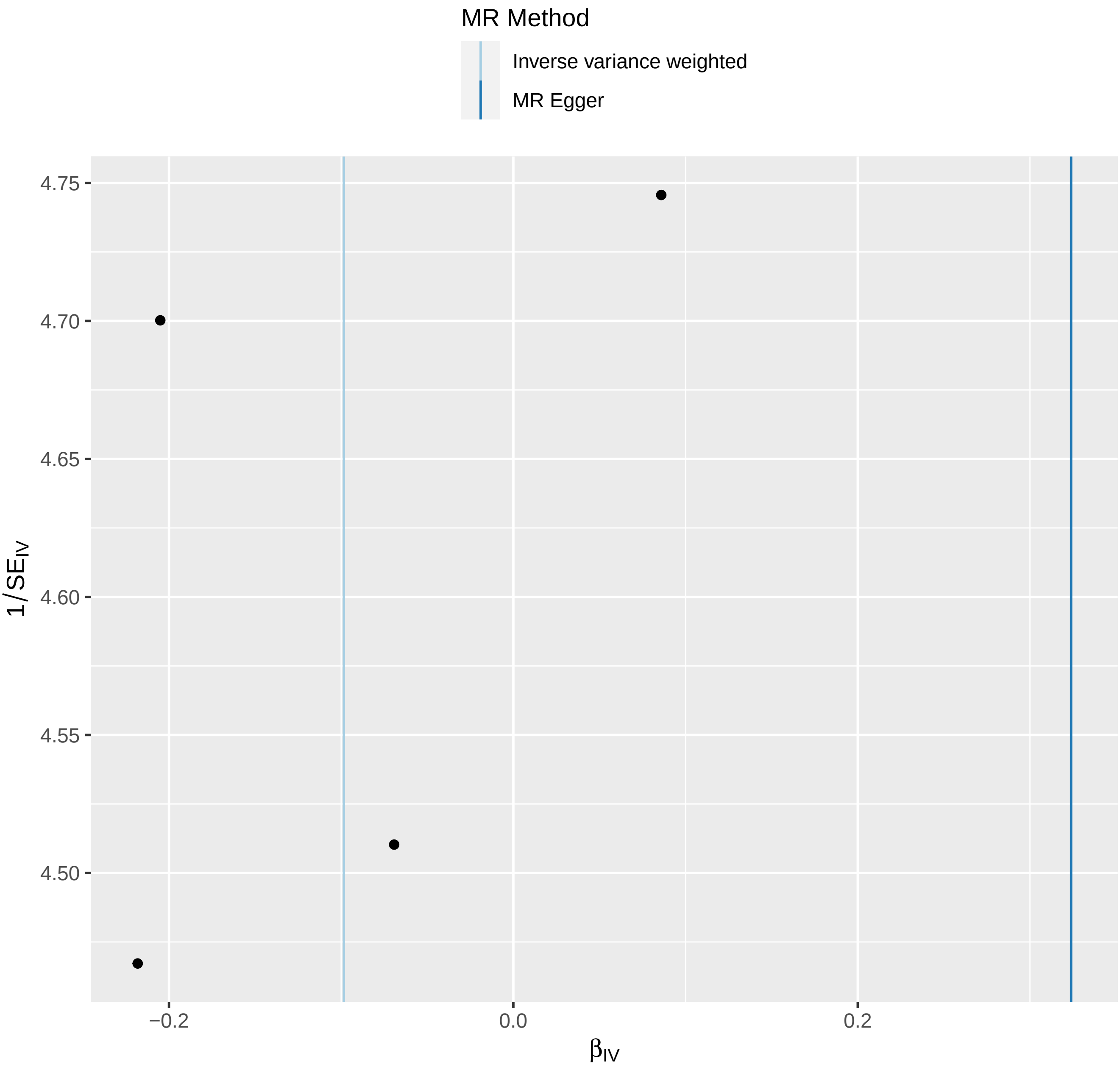

Batch 2−662 : Gut microbiota abundance (genus Parasutterella id.2892) on Carbonic anhydrase 4
with a threshold of 1e−05

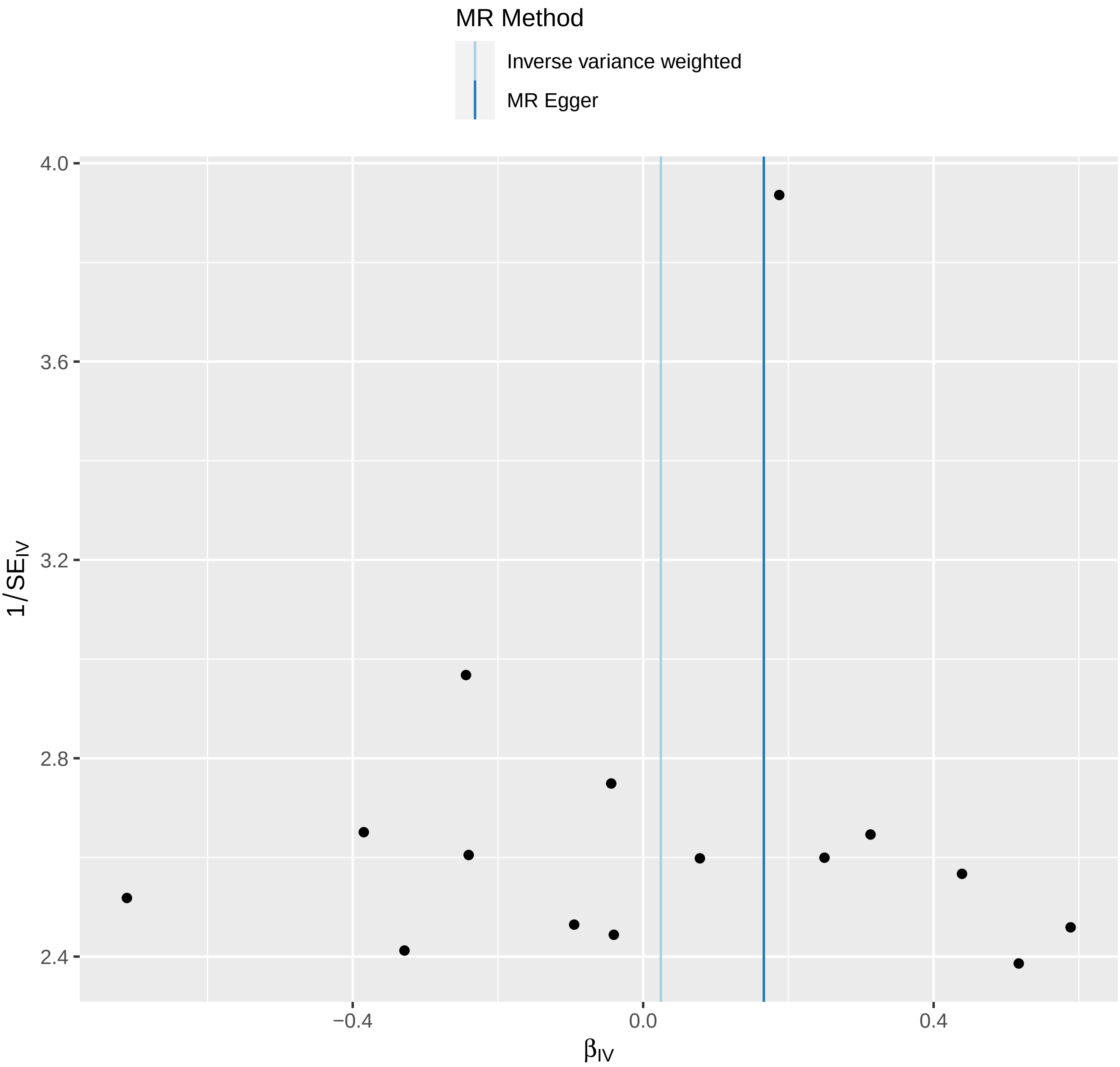

Batch 2−663 : Gut microbiota abundance (genus Catenibacterium id.2153) on Carbonic anhydrase 5A, mitochondrial
with a threshold of 1e−05

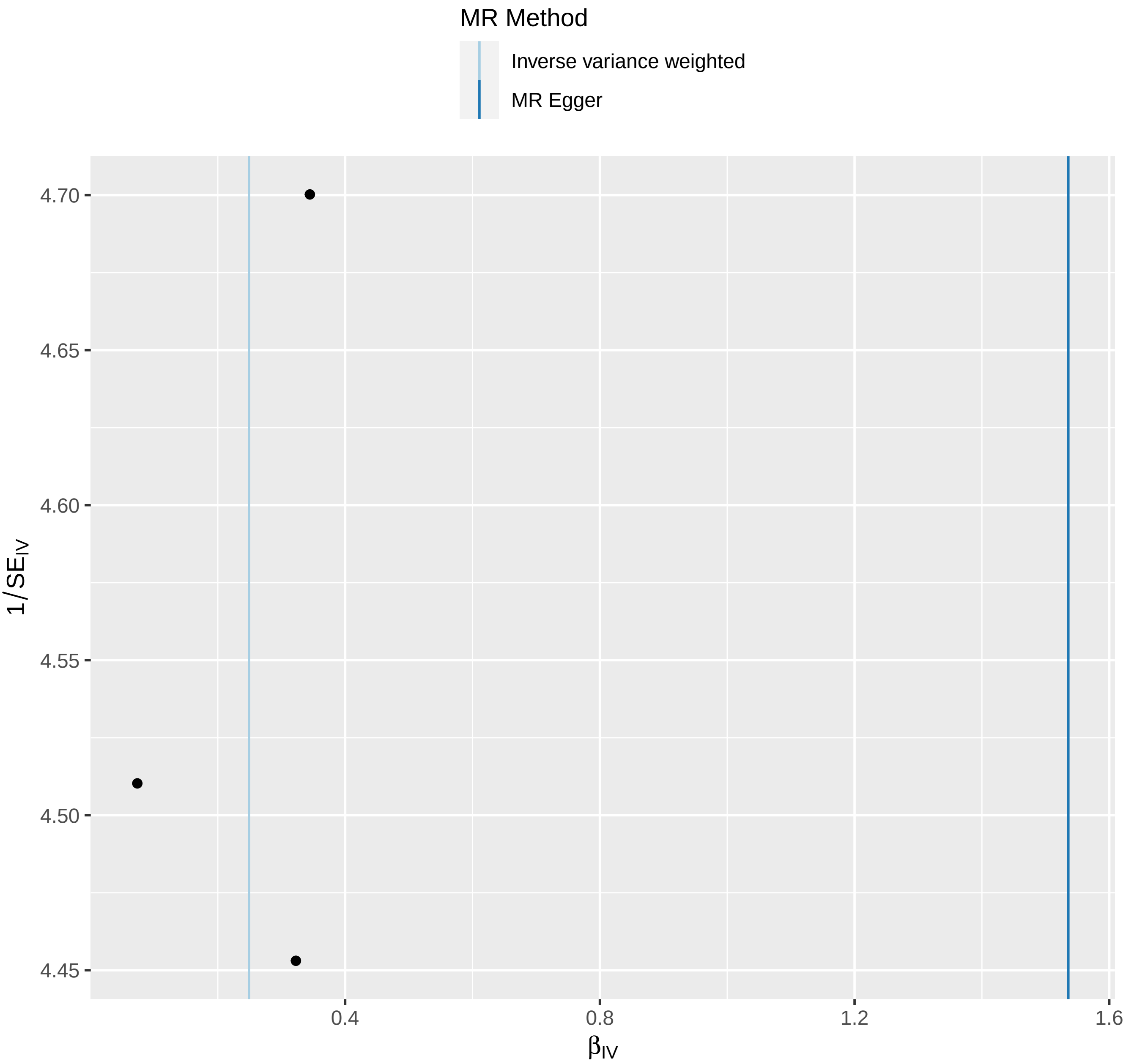

Batch 2−664 : Gut microbiota abundance (genus Parasutterella id.2892) on Carbonic anhydrase 5A, mitochondrial
with a threshold of 1e−05

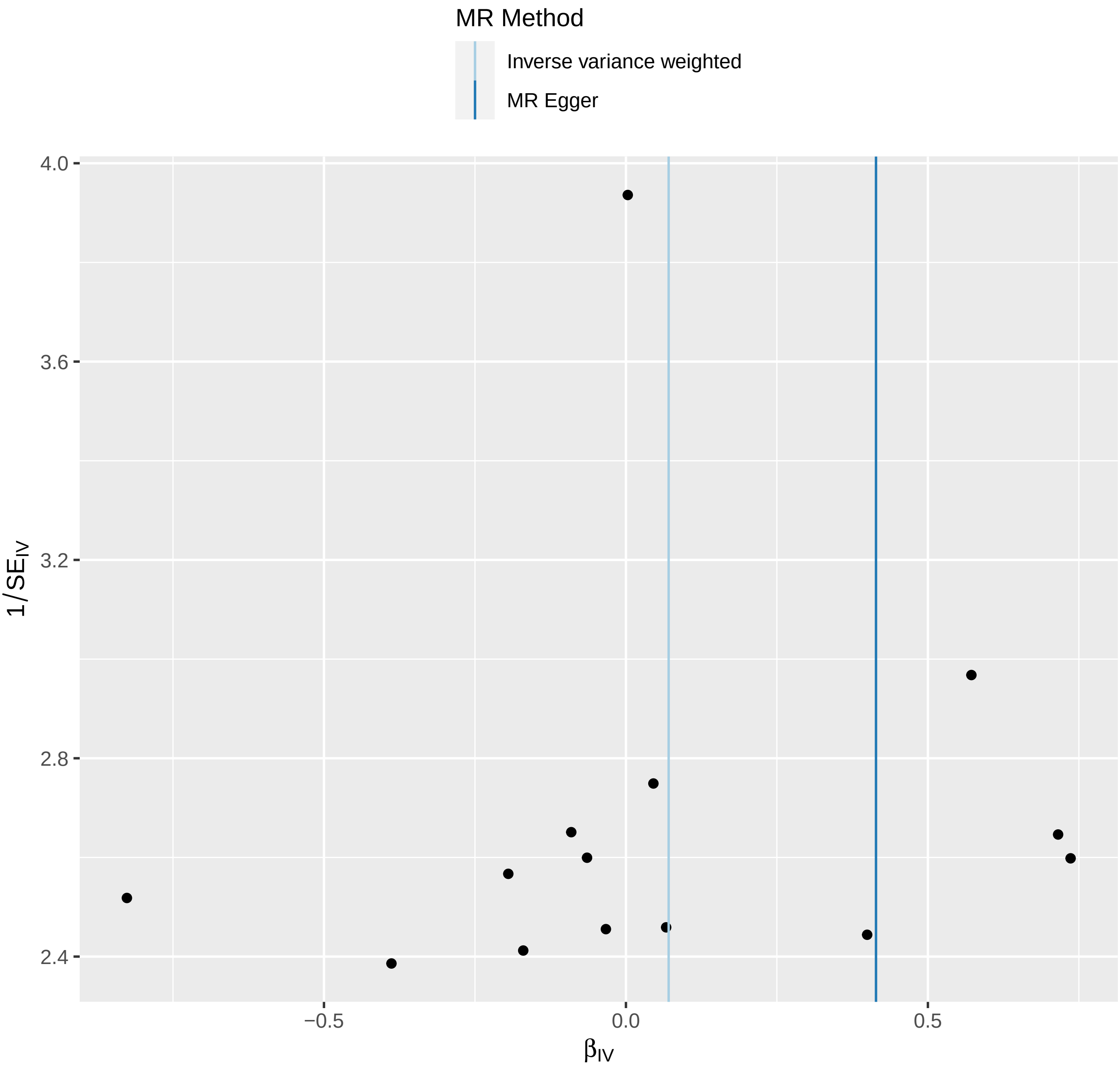

Batch 2−665 : Gut microbiota abundance (genus Catenibacterium id.2153) on Carbonic anhydrase 6
with a threshold of 1e−05

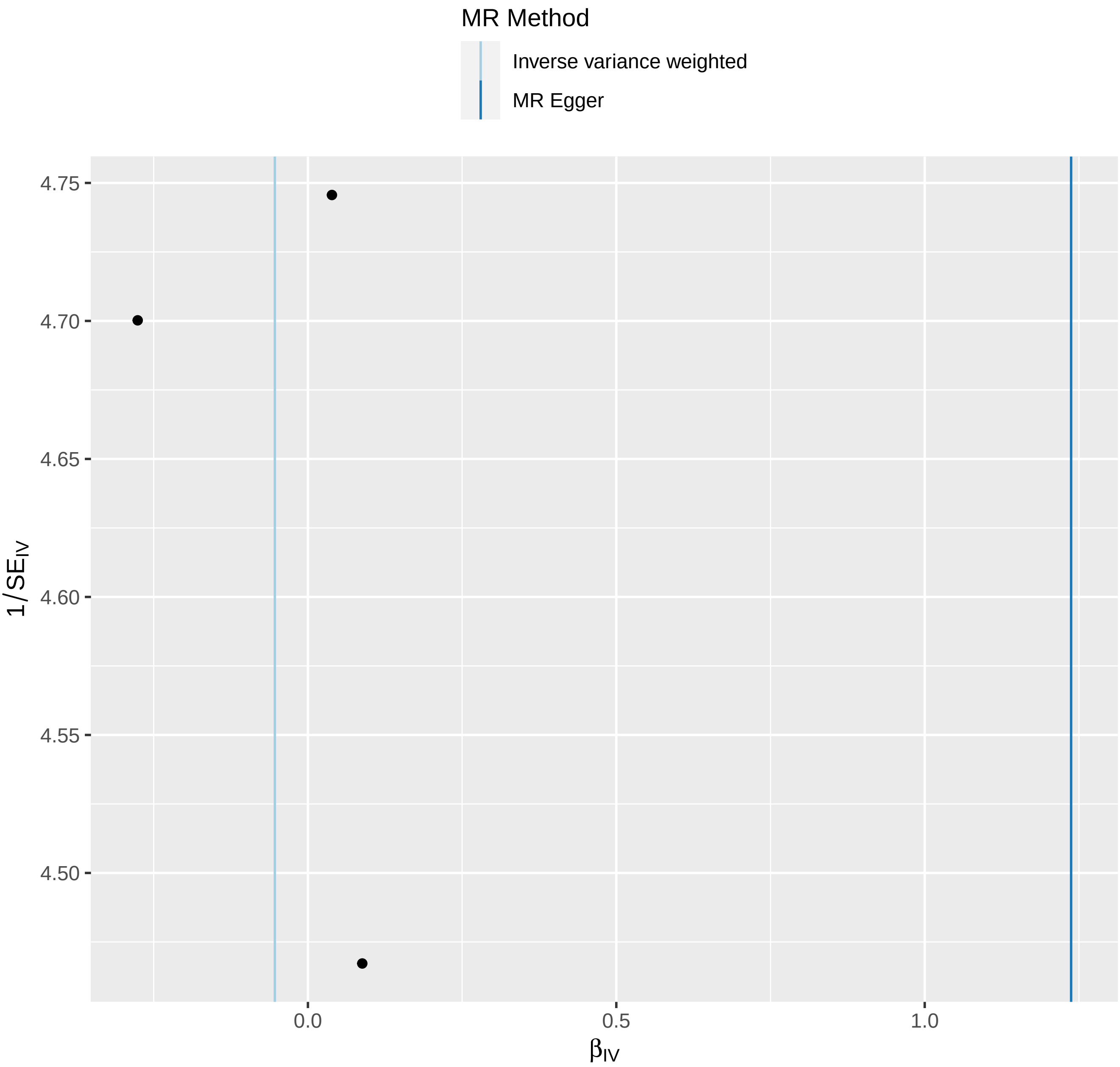

Batch 2−666 : Gut microbiota abundance (genus Parasutterella id.2892) on Carbonic anhydrase 6
with a threshold of 1e−05

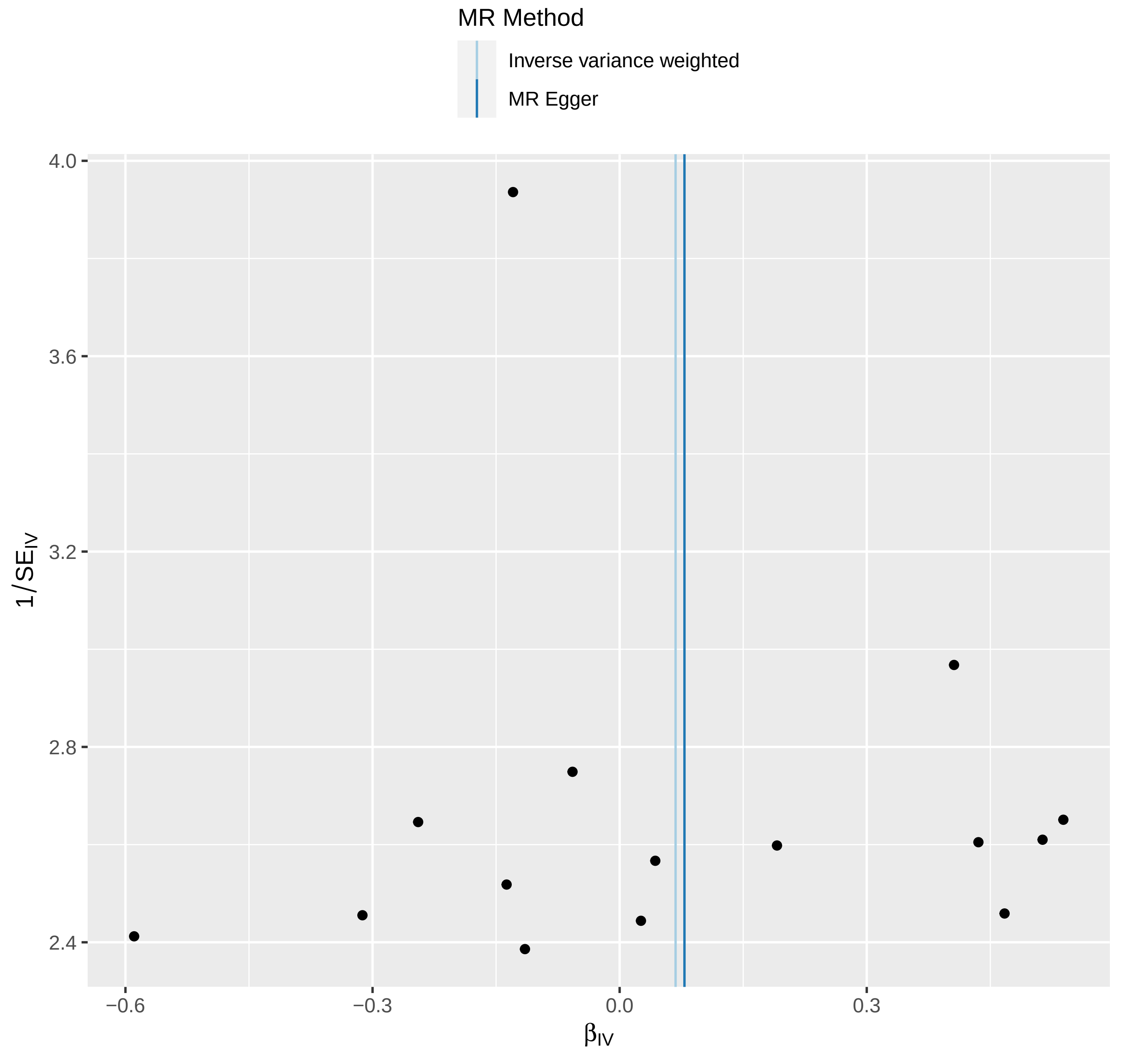

Batch 2−667 : Gut microbiota abundance (genus Catenibacterium id.2153) on Carbonic anhydrase 9 with a threshold of 1e−05

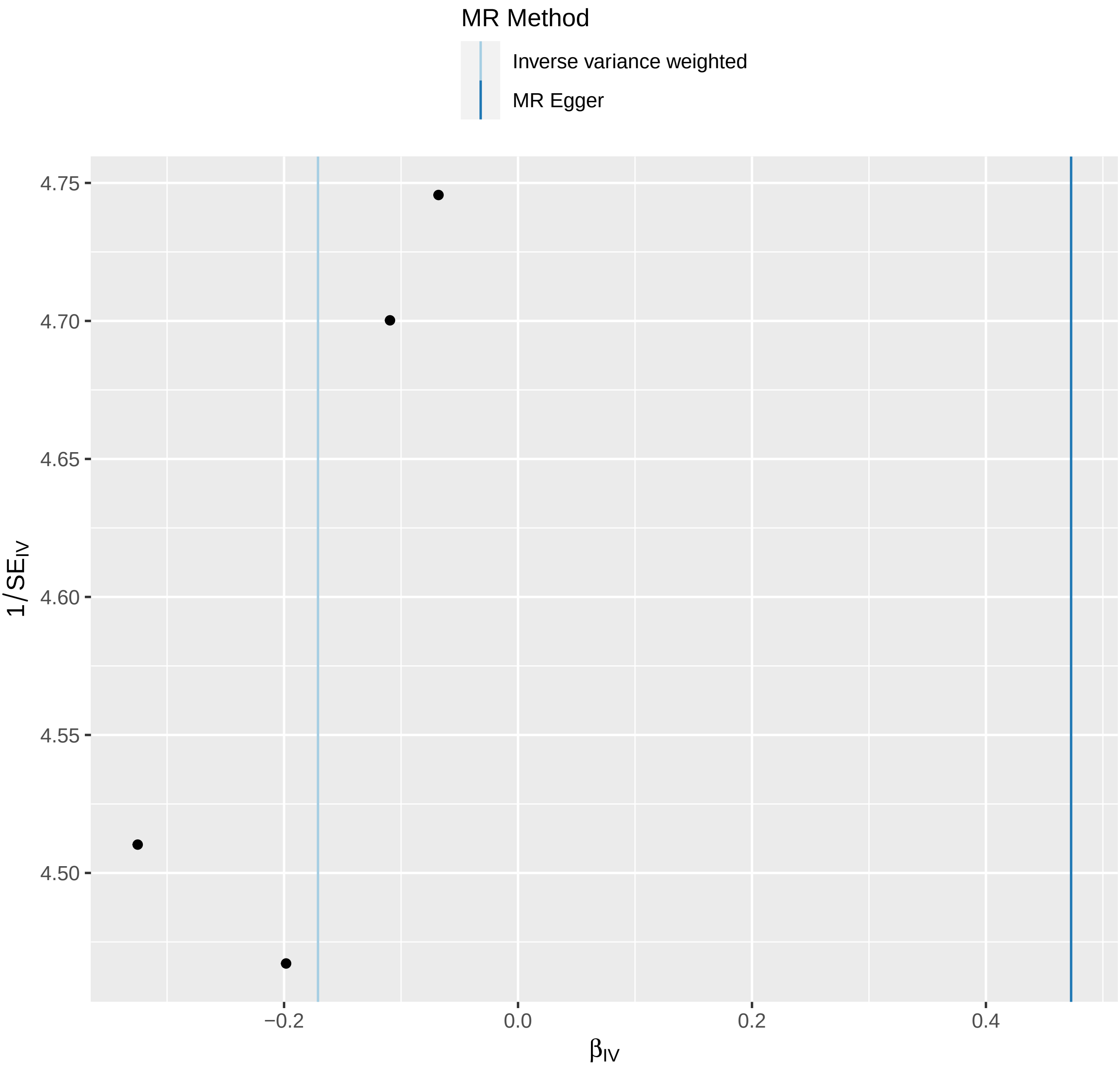

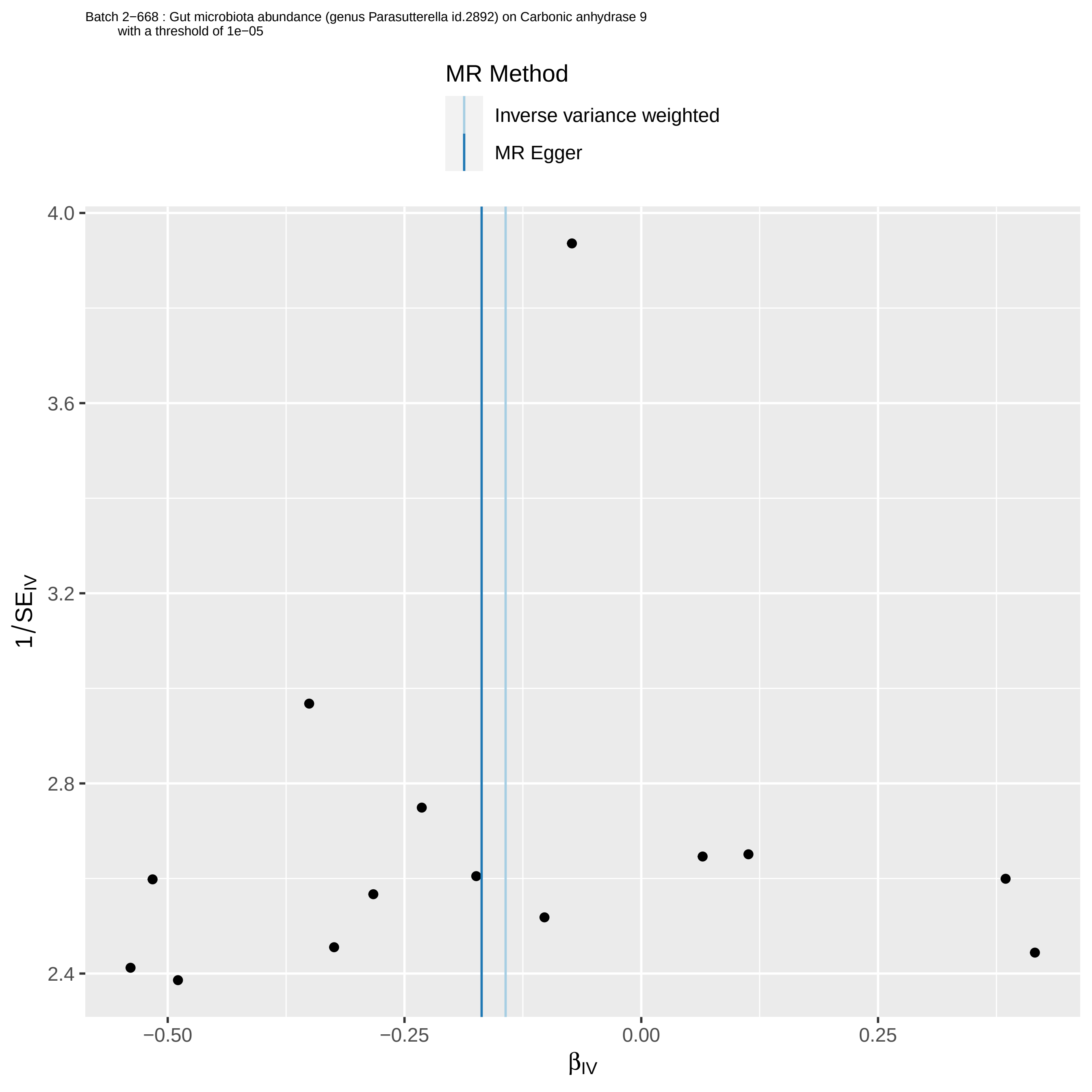
Batch 2−668 : Gut microbiota abundance (genus Parasutterella id.2892) on Carbonic anhydrase 9
with a threshold of 1e−05
MR Method
Inverse variance weighted
MR Egger
4.0
3.6
3.2
2.8
2.4
1/SE$_{IV}$
−0.50
−0.25
0.00
0.25
β$_{IV}$

Batch 2−669 : Gut microbiota abundance (genus Catenibacterium id.2153) on Voltage−dependent calcium channel subunit alpha−2/delta−3 with a threshold of 1e−05

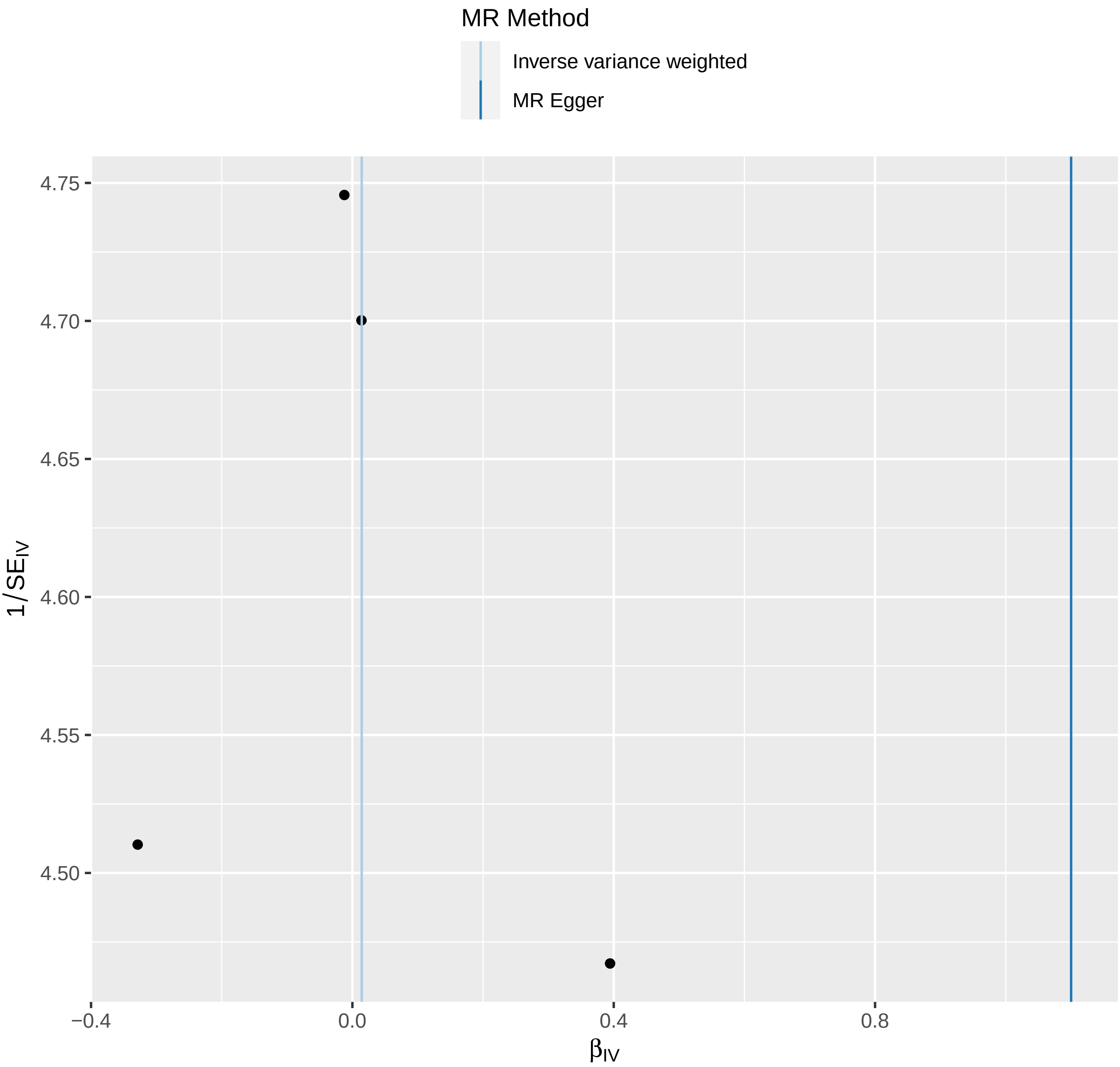

Batch 2−670 : Gut microbiota abundance (genus Parasutterella id.2892) on Voltage−dependent calcium channel subunit alpha−2/delta−3
with a threshold of 1e−05

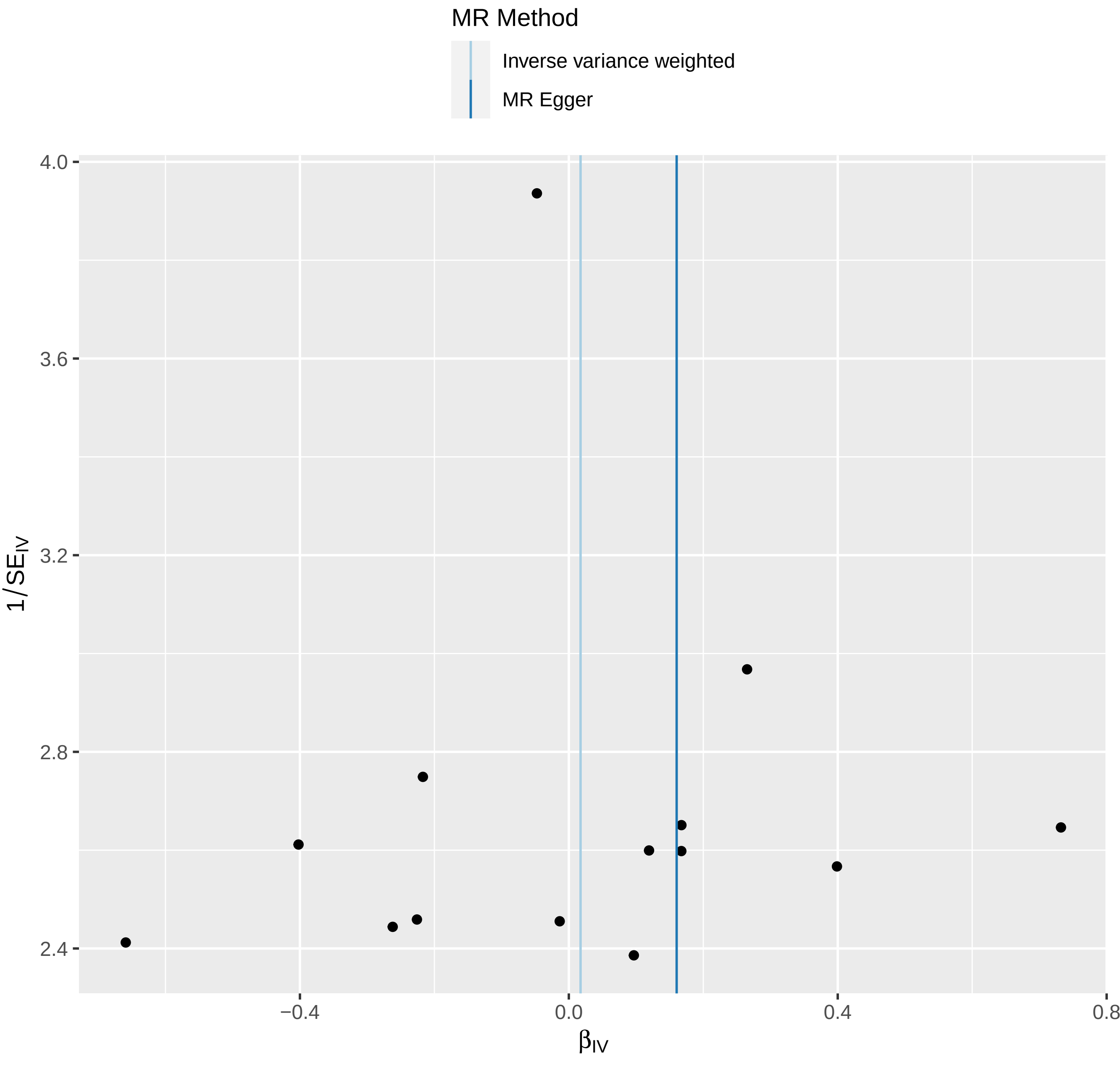

Batch 2−671 : Gut microbiota abundance (genus Catenibacterium id.2153) on Calcyclin−binding protein with a threshold of 1e−05

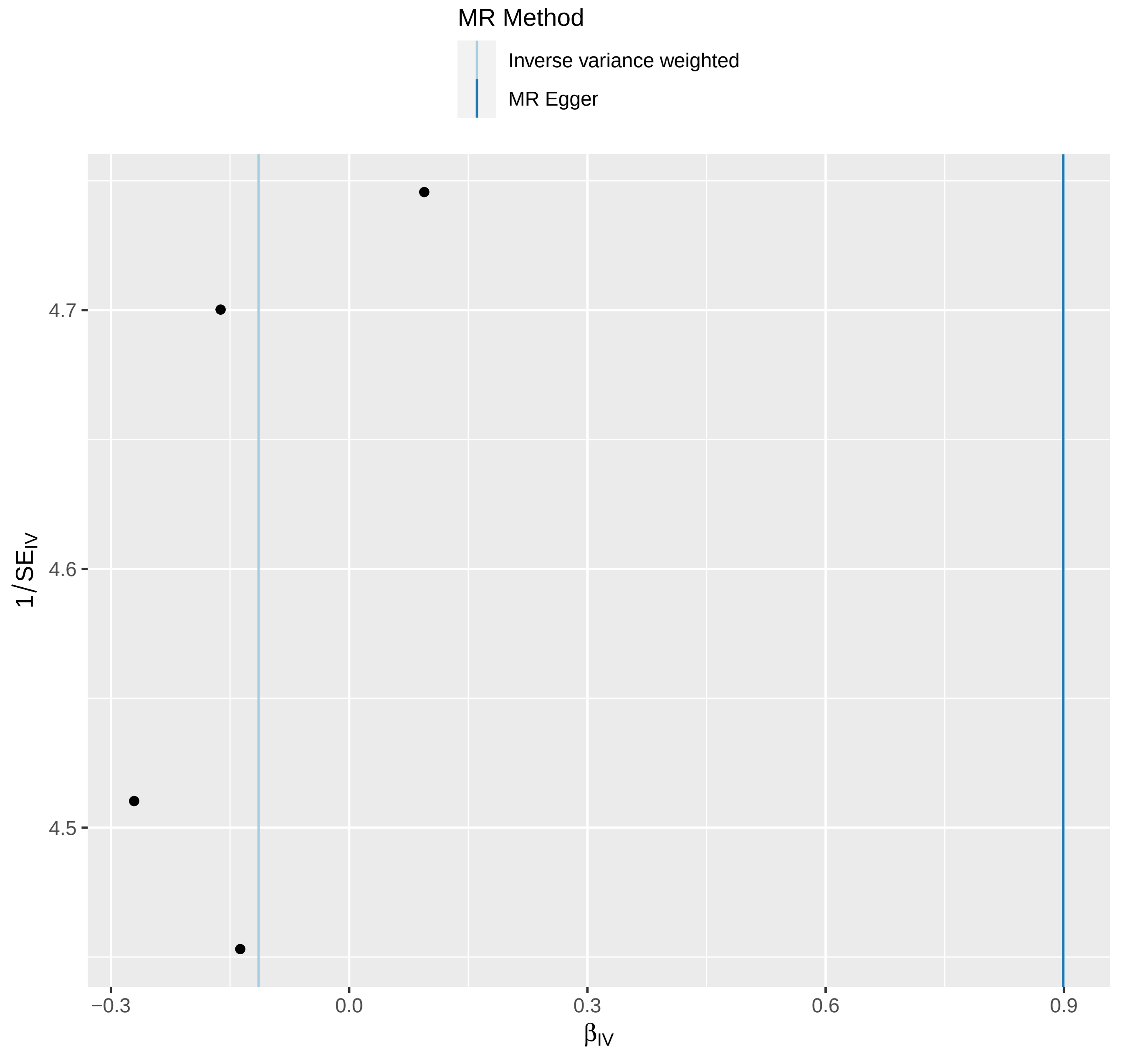

Batch 2−672 : Gut microbiota abundance (genus Parasutterella id.2892) on Calcyclin−binding protein
with a threshold of 1e−05

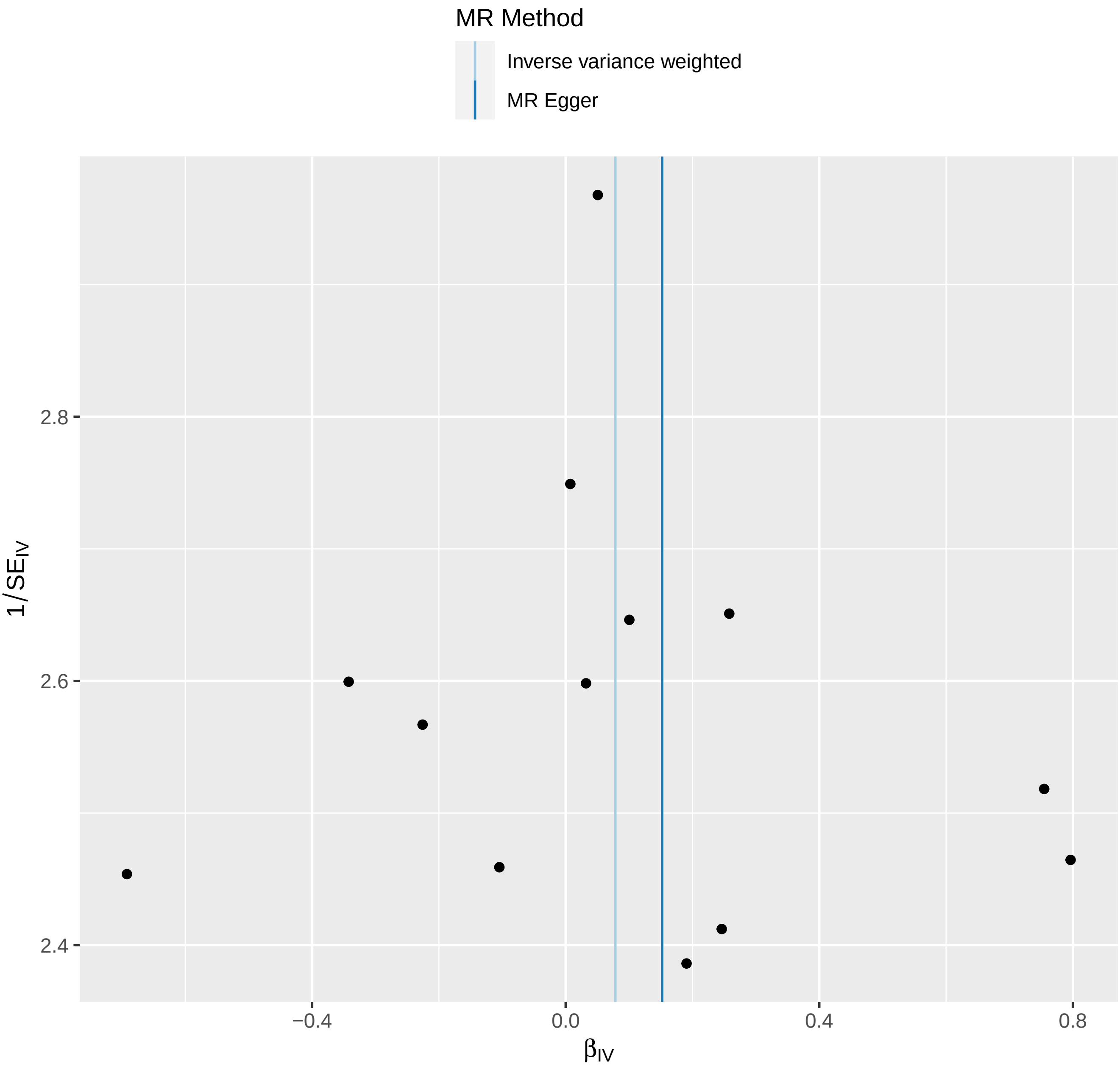

Batch 2−673 : Gut microbiota abundance (genus Catenibacterium id.2153) on Cell adhesion molecule 1
with a threshold of 1e−05

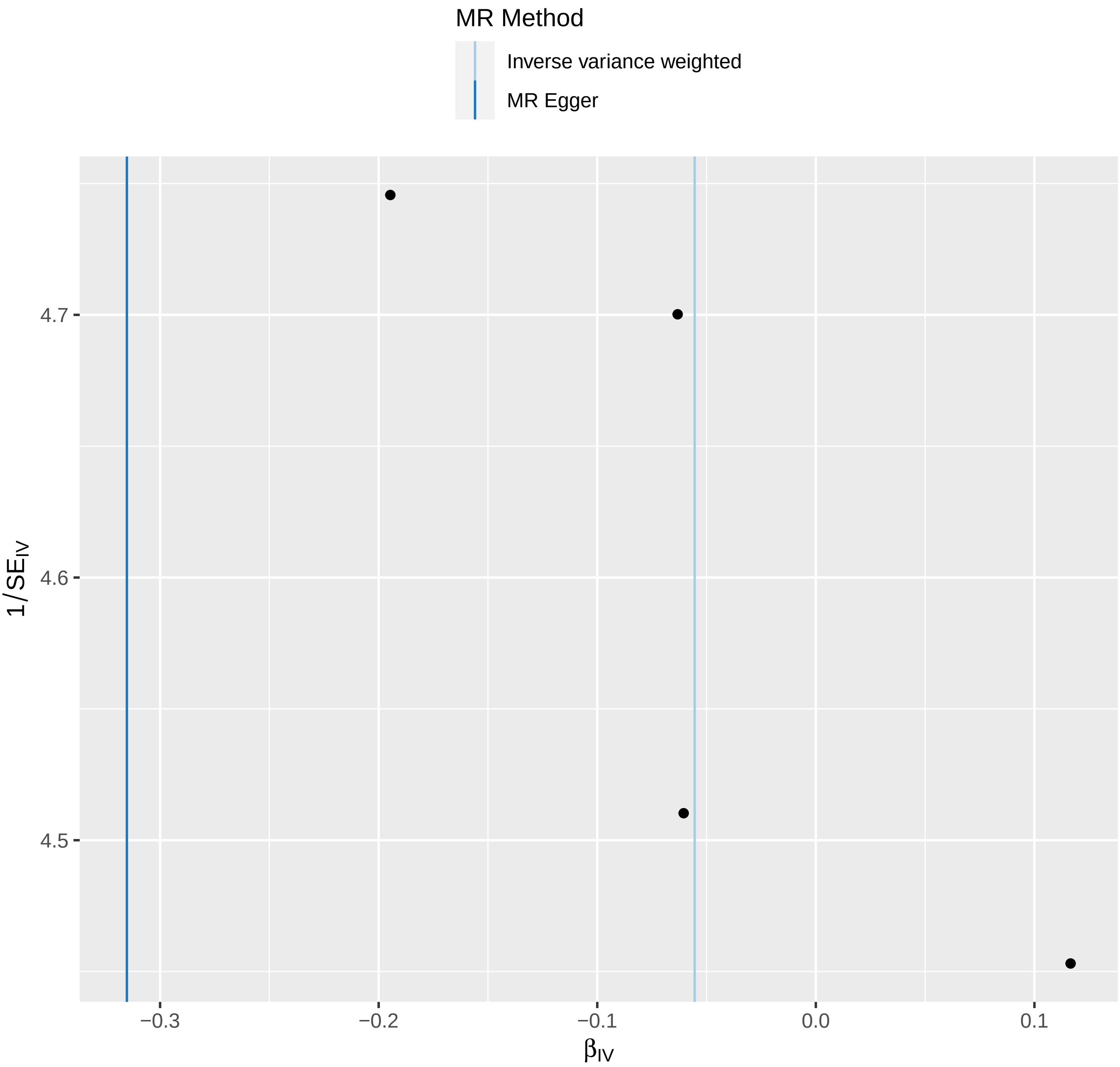

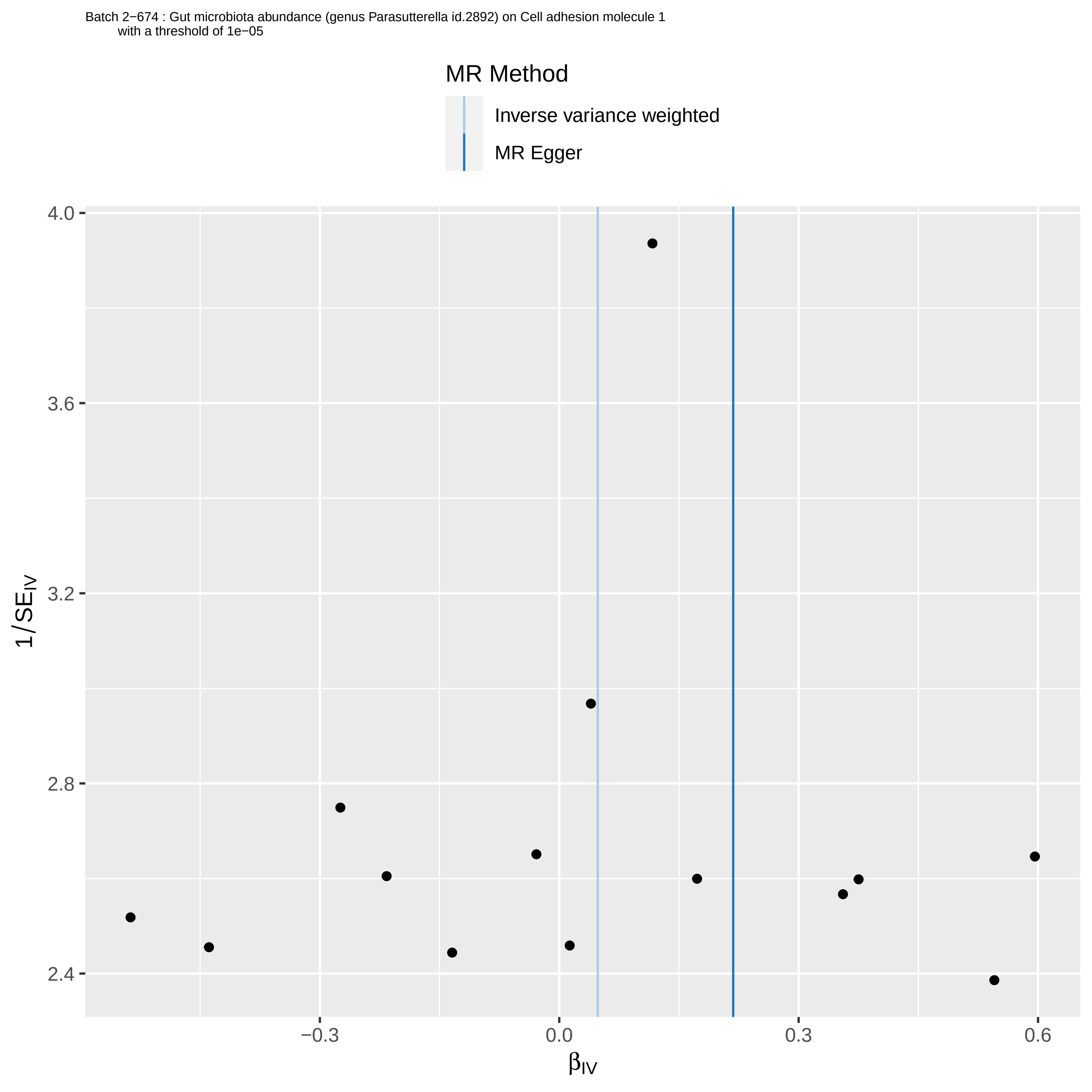
Batch 2−674 : Gut microbiota abundance (genus Parasutterella id.2892) on Cell adhesion molecule 1
with a threshold of 1e−05
MR Method
Inverse variance weighted
MR Egger
4.0
3.6
3.2
2.8
2.4
1/SE_IV
−0.3
0.0
0.3
0.6
β_IV

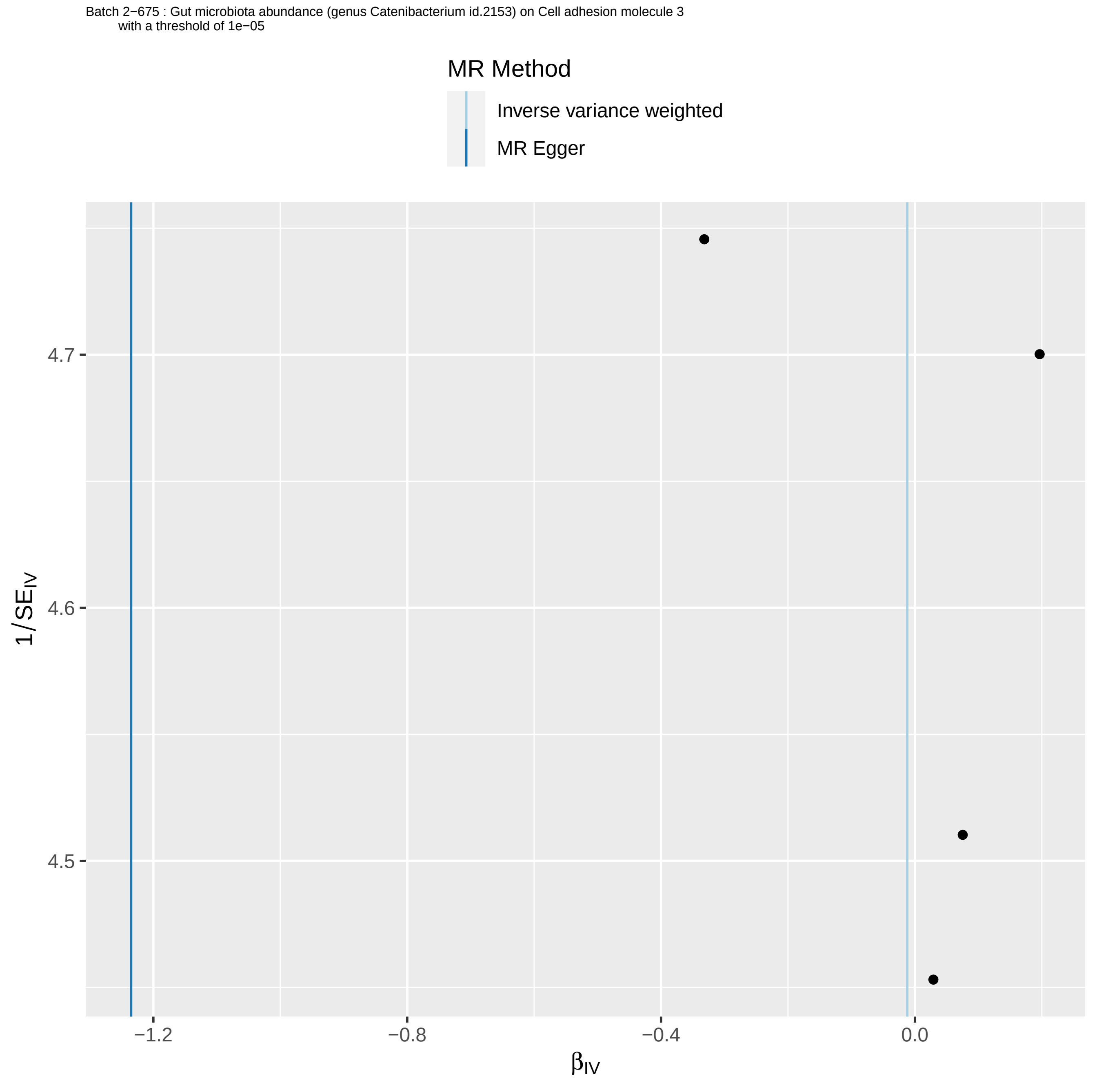
Batch 2−675 : Gut microbiota abundance (genus Catenibacterium id.2153) on Cell adhesion molecule 3
with a threshold of 1e−05
MR Method
Inverse variance weighted
MR Egger
4.7
4.6
4.5
1/SE_IV
−1.2
−0.8
−0.4
0.0
β_IV

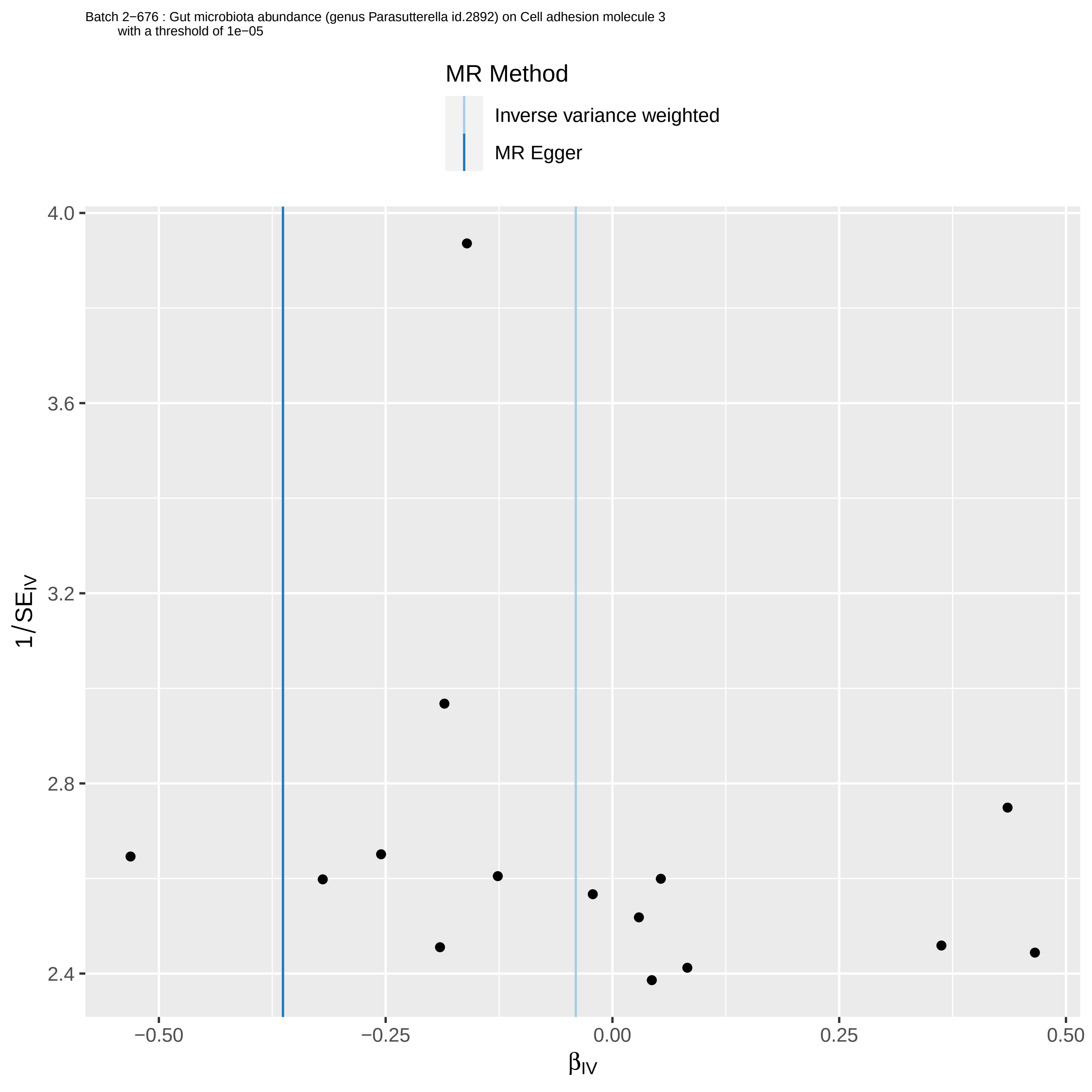
Batch 2−676 : Gut microbiota abundance (genus Parasutterella id.2892) on Cell adhesion molecule 3
with a threshold of 1e−05
MR Method
Inverse variance weighted
MR Egger
4.0
3.6
3.2
2.8
2.4
1/SE_IV
−0.50
−0.25
0.00
0.25
0.50
β_IV

Batch 2−677 : Gut microbiota abundance (genus Catenibacterium id.2153) on Cell adhesion molecule 4
with a threshold of 1e−05

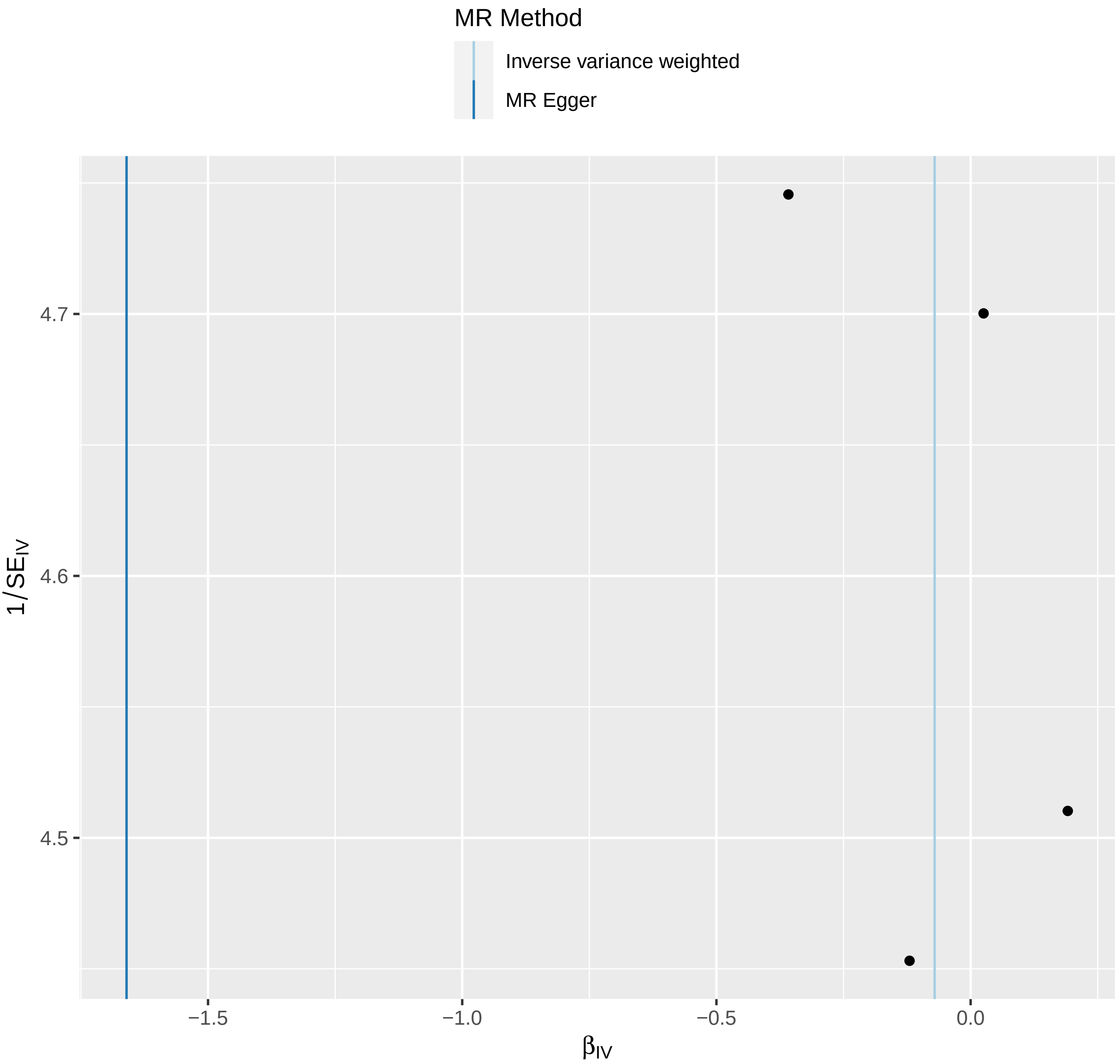

Batch 2−678 : Gut microbiota abundance (genus Parasutterella id.2892) on Cell adhesion molecule 4
with a threshold of 1e−05

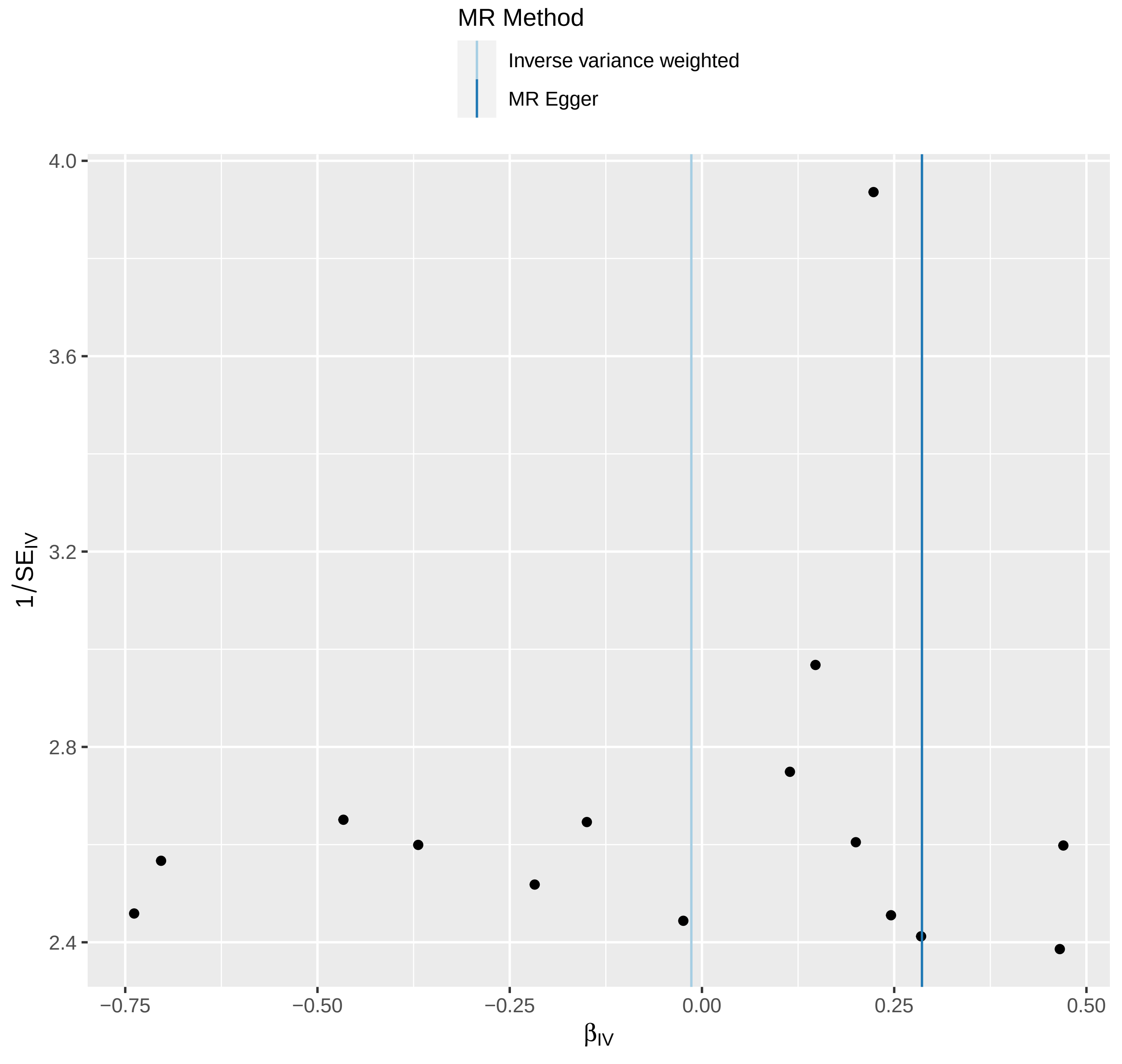

Batch 2−679 : Gut microbiota abundance (genus Catenibacterium id.2153) on Calbindin
with a threshold of 1e−05

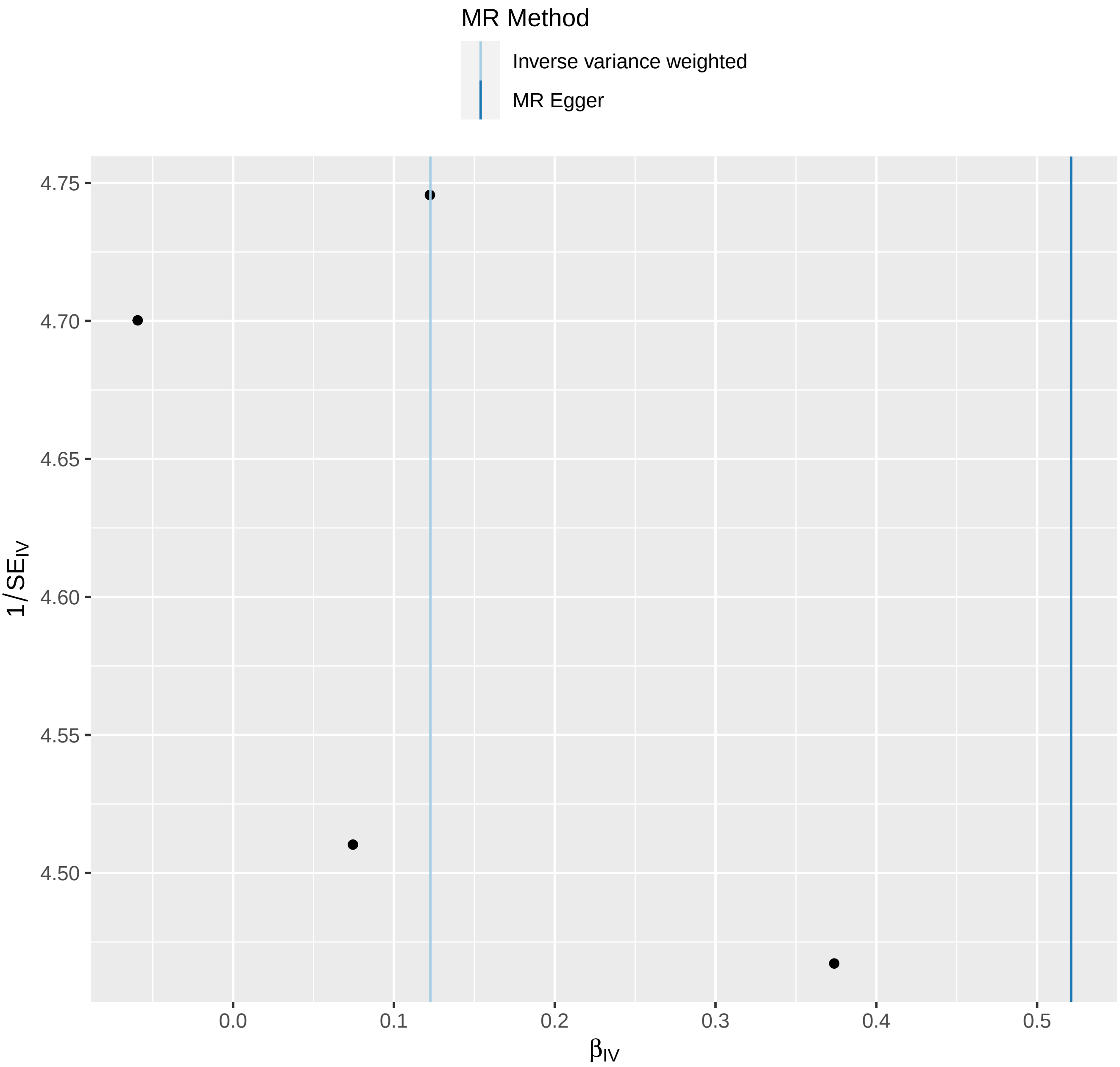

Batch 2−680 : Gut microbiota abundance (genus Parasutterella id.2892) on Calbindin
with a threshold of 1e−05

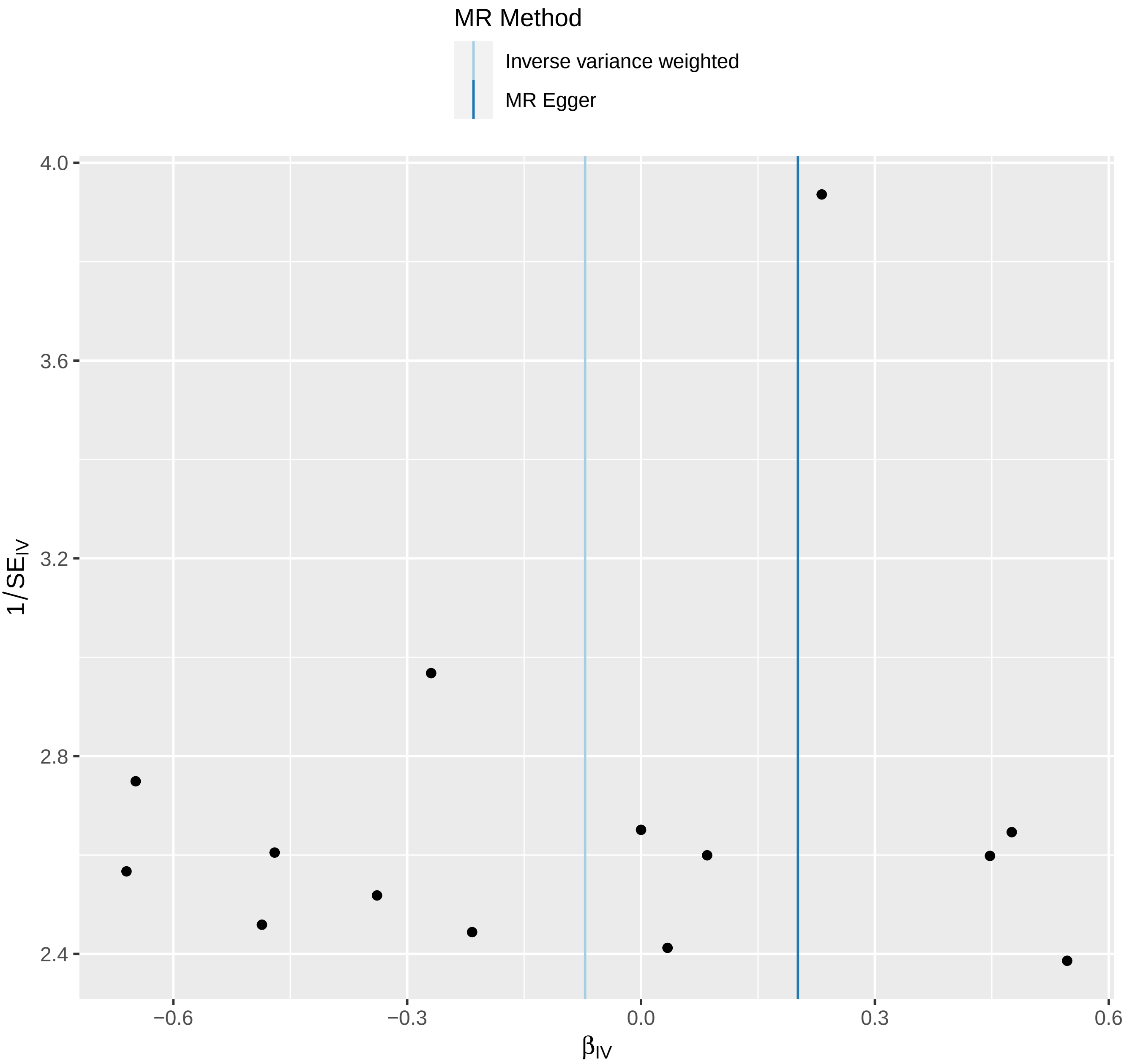

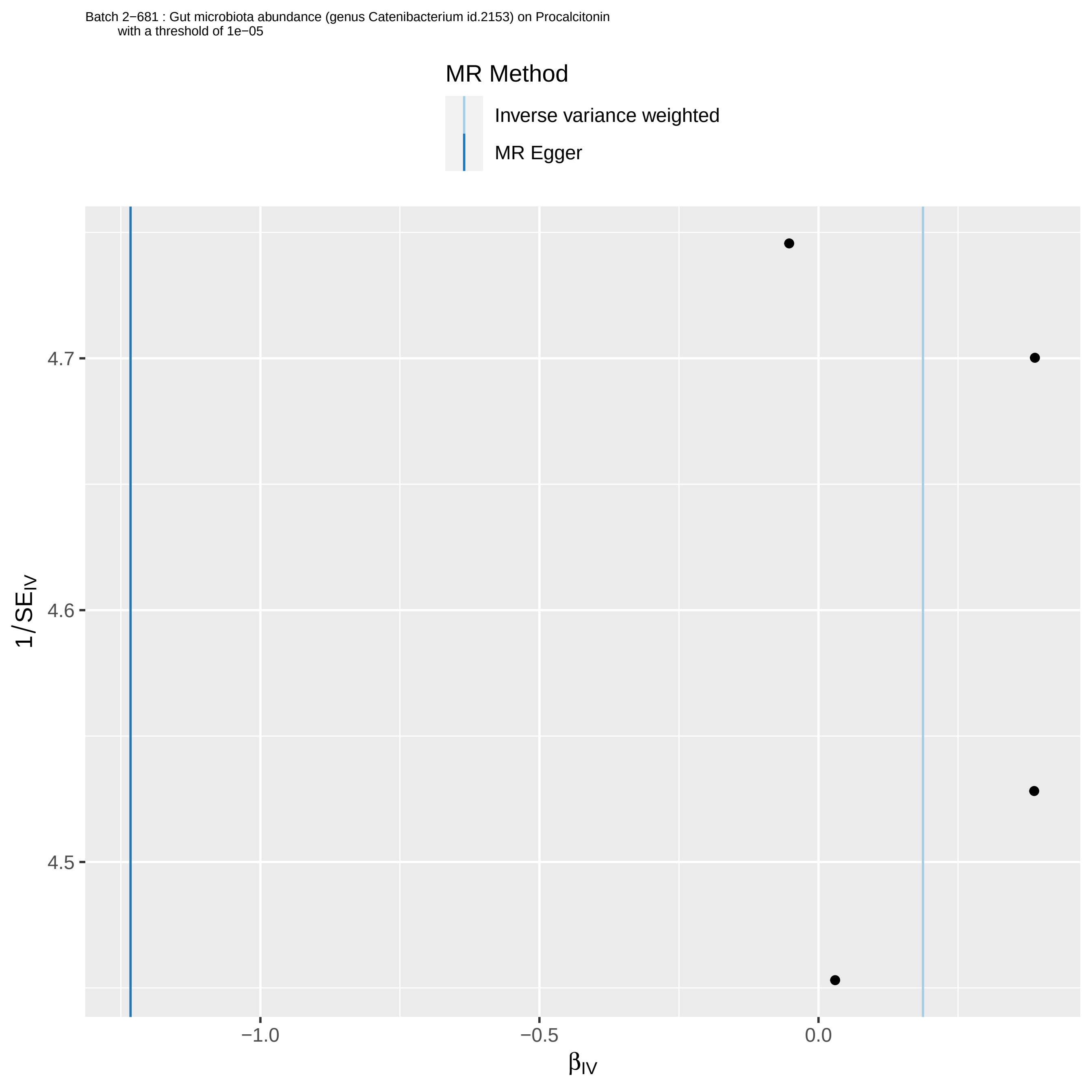

Batch 2−681 : Gut microbiota abundance (genus Catenibacterium id.2153) on Procalcitonin
with a threshold of 1e−05
MR Method
Inverse variance weighted
MR Egger
4.7
4.6
4.5
1/SE_IV
−1.0
−0.5
0.0
β_IV

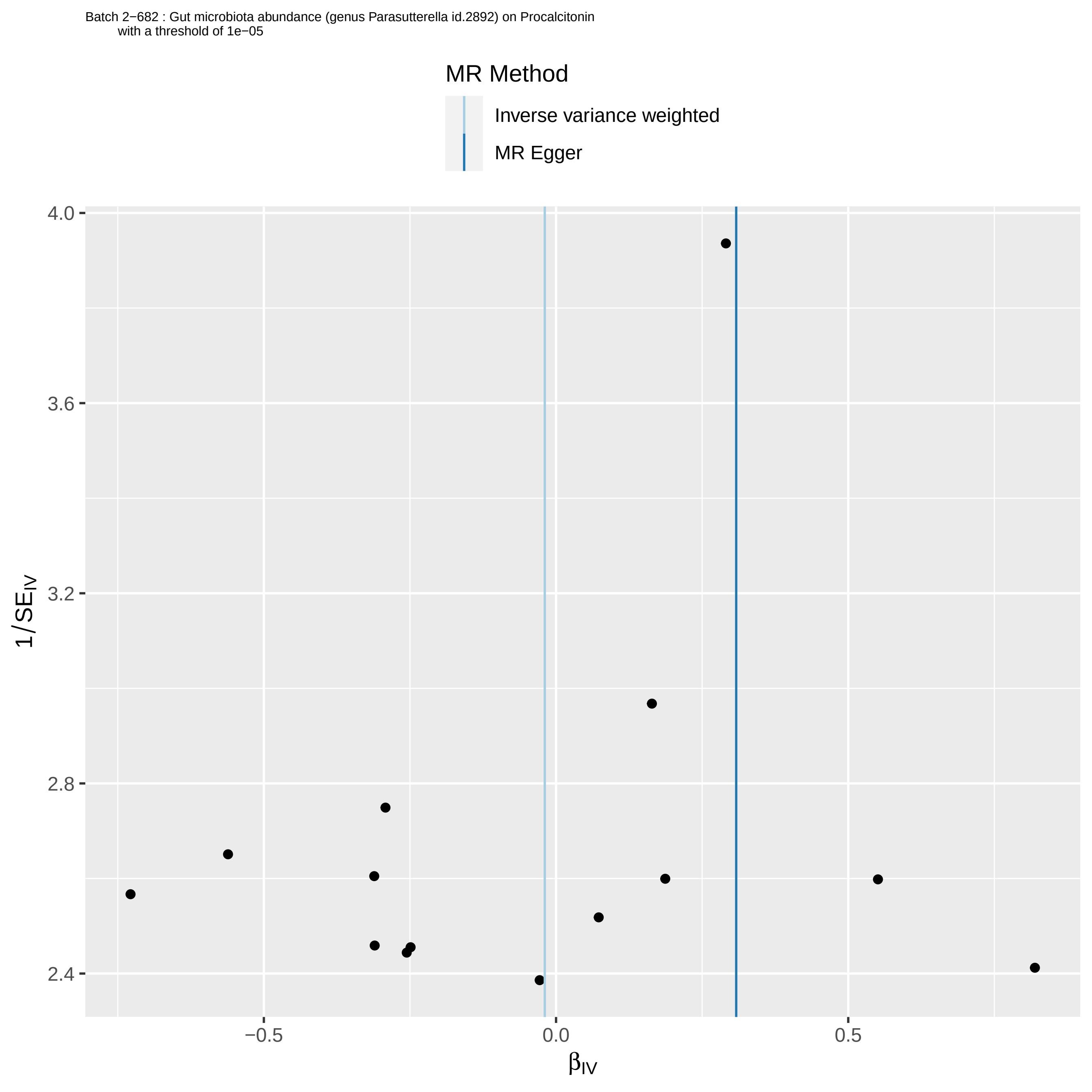

Batch 2−682 : Gut microbiota abundance (genus Parasutterella id.2892) on Procalcitonin
with a threshold of 1e−05
MR Method
Inverse variance weighted
MR Egger
4.0
3.6
3.2
2.8
2.4
1/SE_IV
−0.5
0.0
0.5
β_IV

Batch 2−683 : Gut microbiota abundance (genus Catenibacterium id.2153) on Calcium−binding and coiled−coil domain−containing protein 2 with a threshold of 1e−05

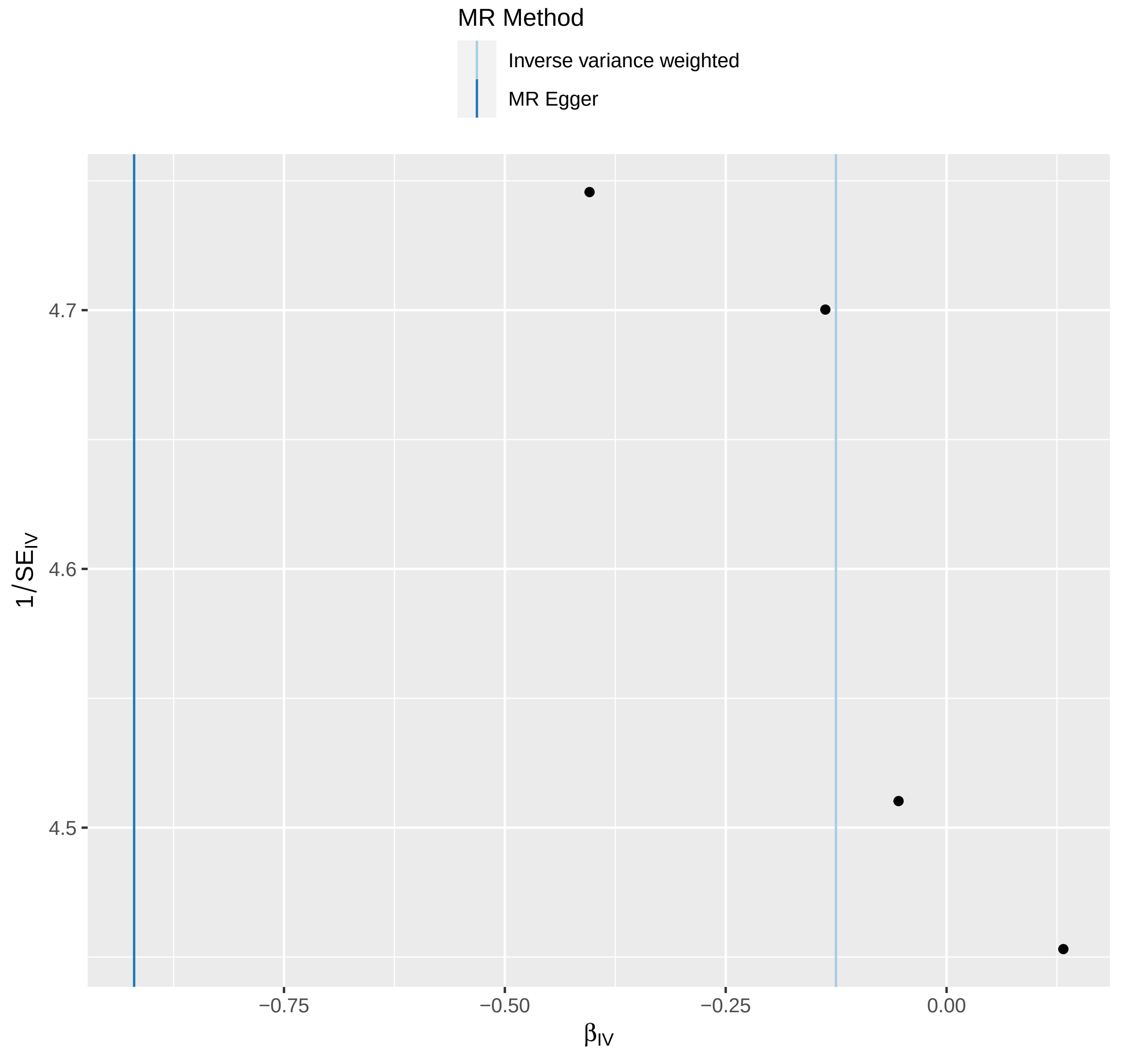

Batch 2−684 : Gut microbiota abundance (genus Parasutterella id.2892) on Calcium−binding and coiled−coil domain−containing protein 2
with a threshold of 1e−05

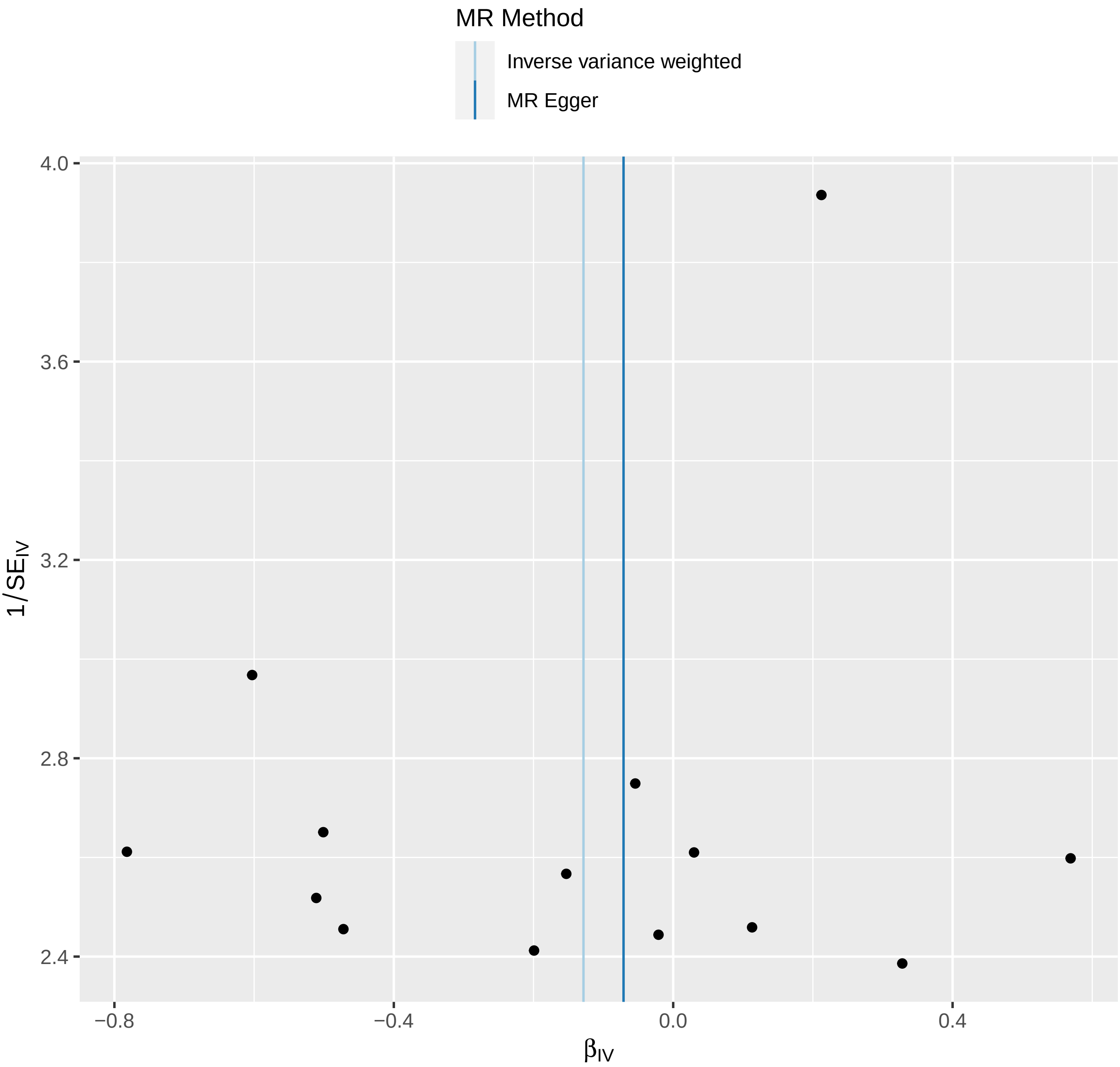

Batch 2−685 : Gut microbiota abundance (genus Catenibacterium id.2153) on Calcium−binding protein 8
with a threshold of 1e−05

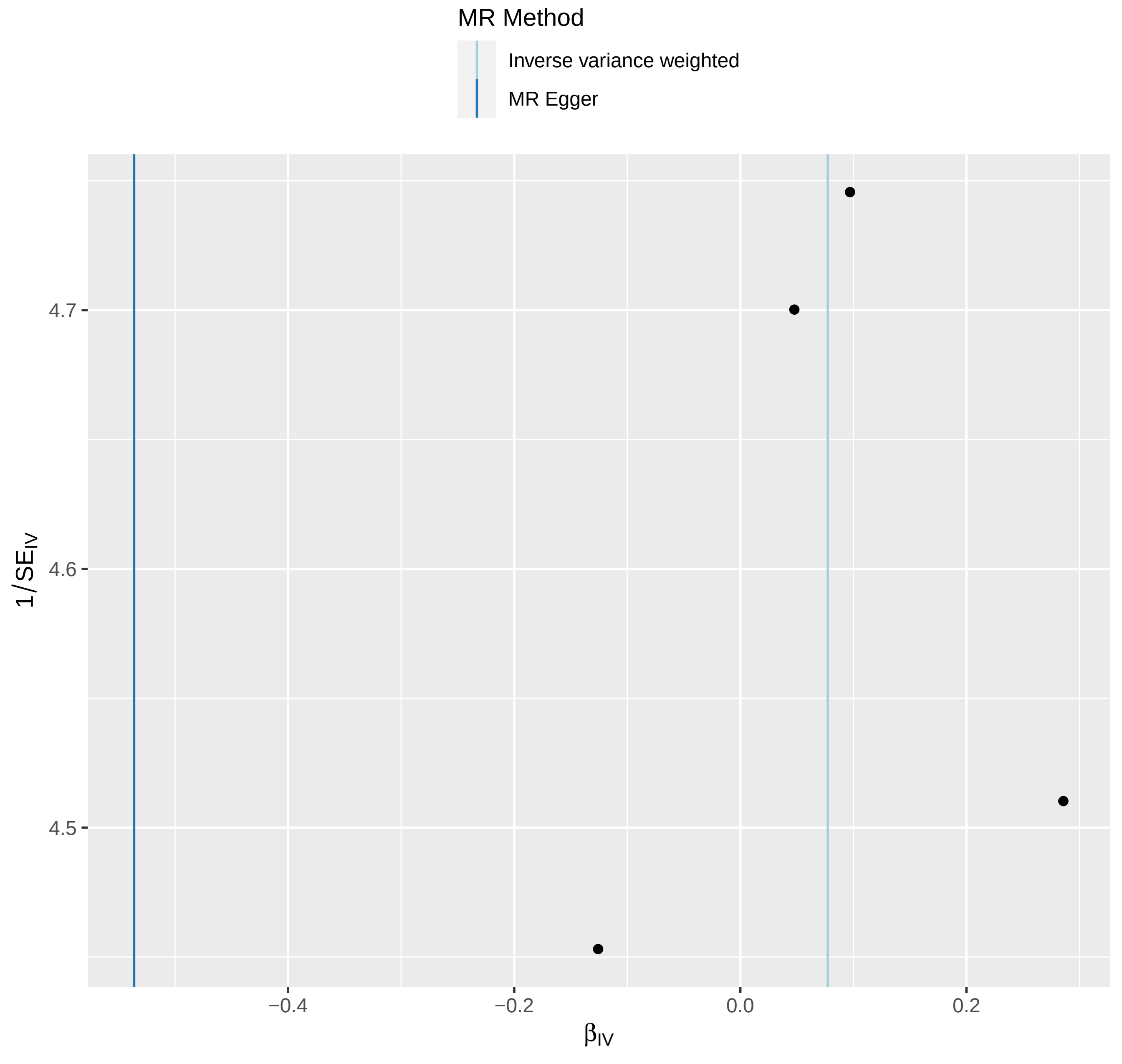

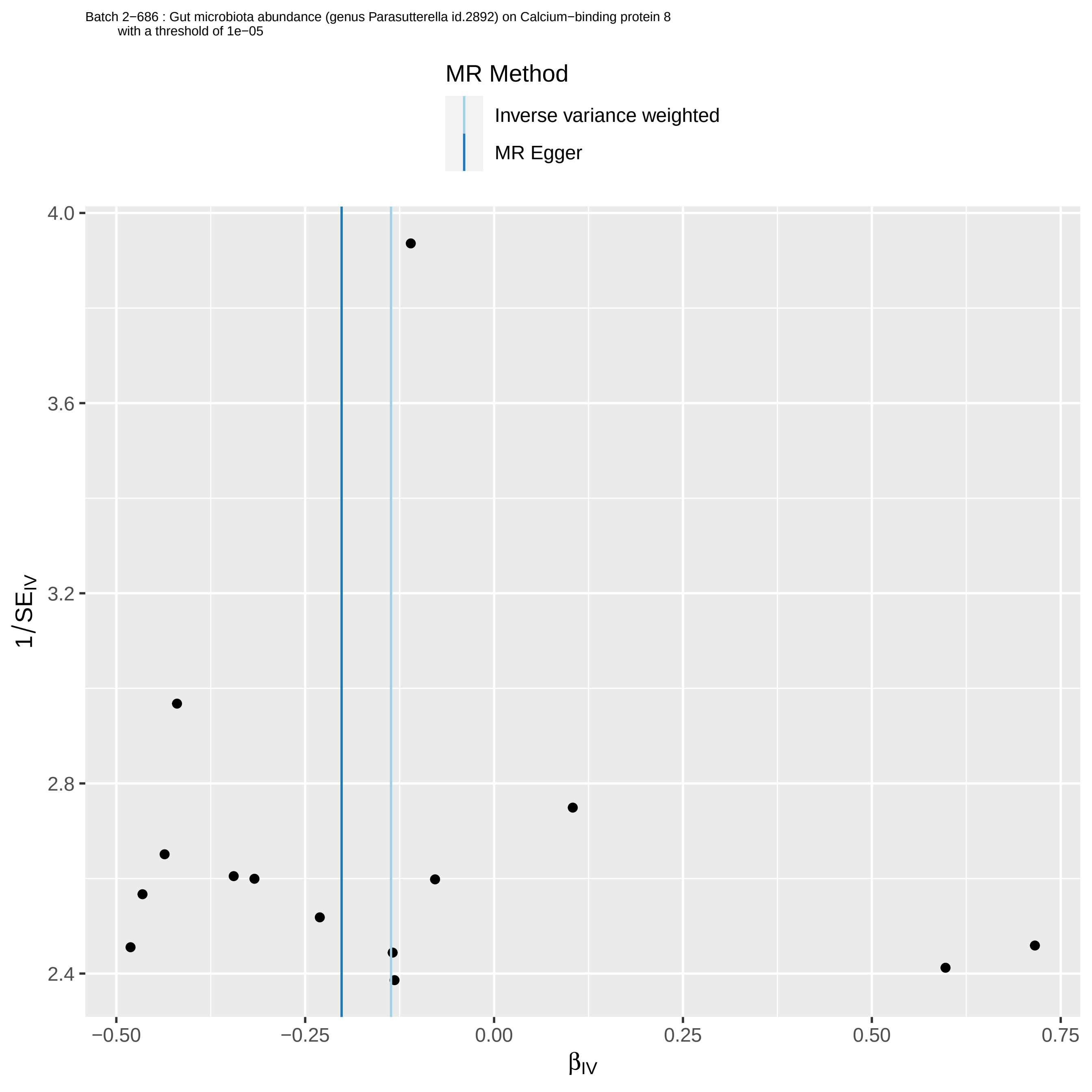

Batch 2−686 : Gut microbiota abundance (genus Parasutterella id.2892) on Calcium−binding protein 8
with a threshold of 1e−05
MR Method
Inverse variance weighted
MR Egger
4.0
3.6
3.2
2.8
2.4
1/SE_IV
−0.50
−0.25
0.00
0.25
0.50
0.75
β_IV

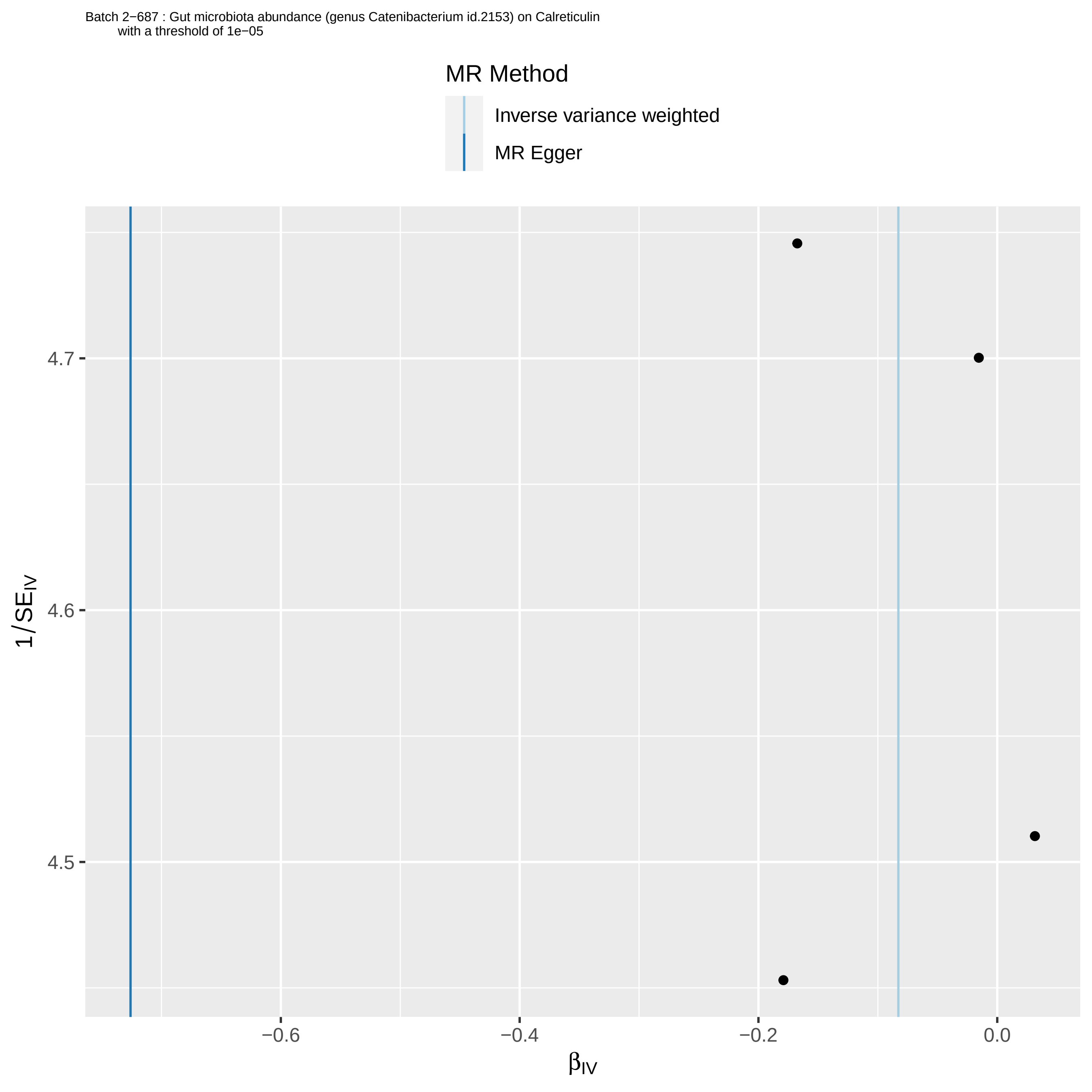

Batch 2−687 : Gut microbiota abundance (genus Catenibacterium id.2153) on Calreticulin
with a threshold of 1e−05
MR Method
Inverse variance weighted
MR Egger
4.7
4.6
4.5
1/SE$_{IV}$
−0.6
−0.4
−0.2
0.0
β$_{IV}$

Batch 2−688 : Gut microbiota abundance (genus Parasutterella id.2892) on Calreticulin
with a threshold of 1e−05

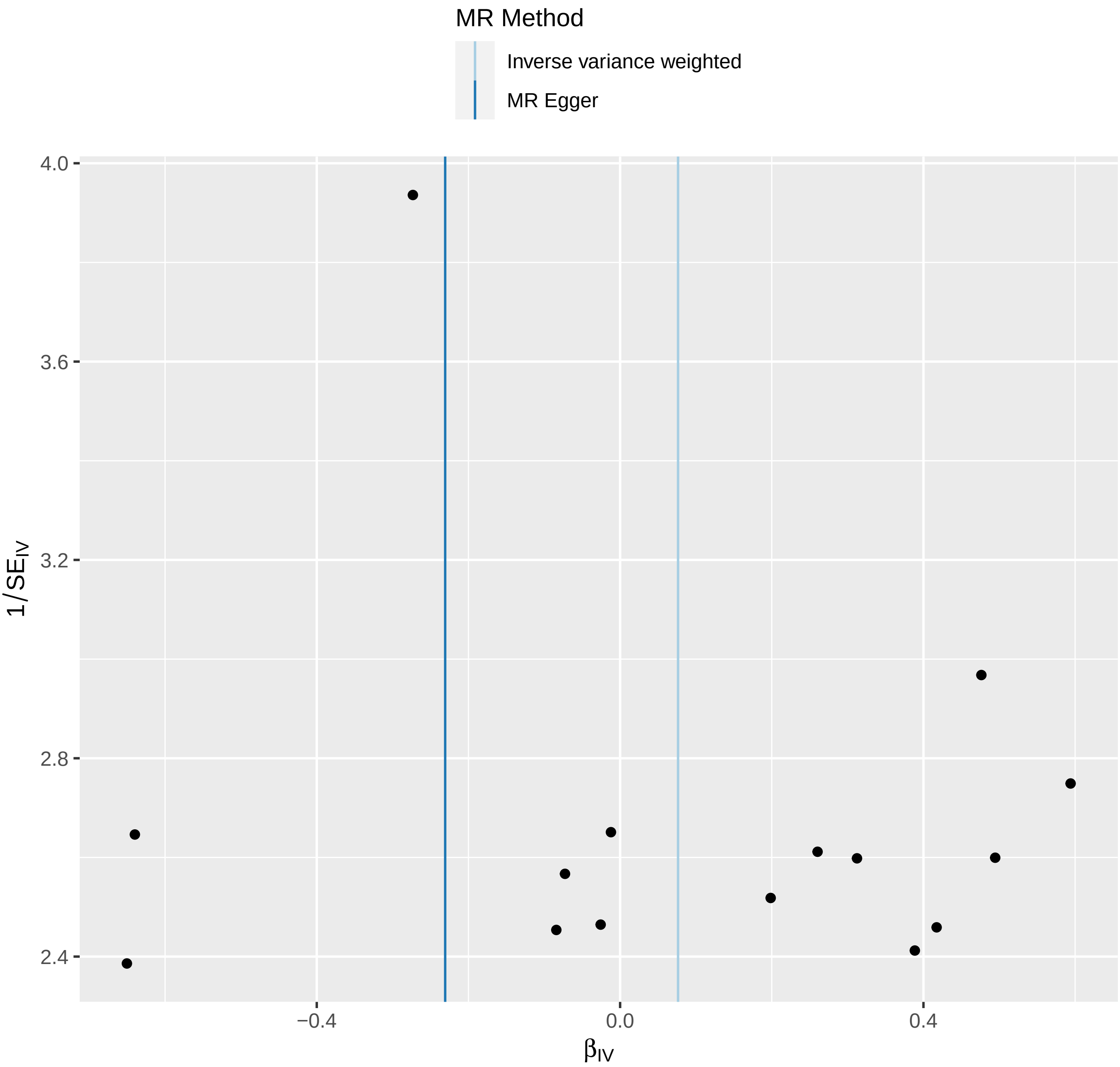

Batch 2−689 : Gut microbiota abundance (genus Catenibacterium id.2153) on Neuron−specific vesicular protein calcyon
with a threshold of 1e−05

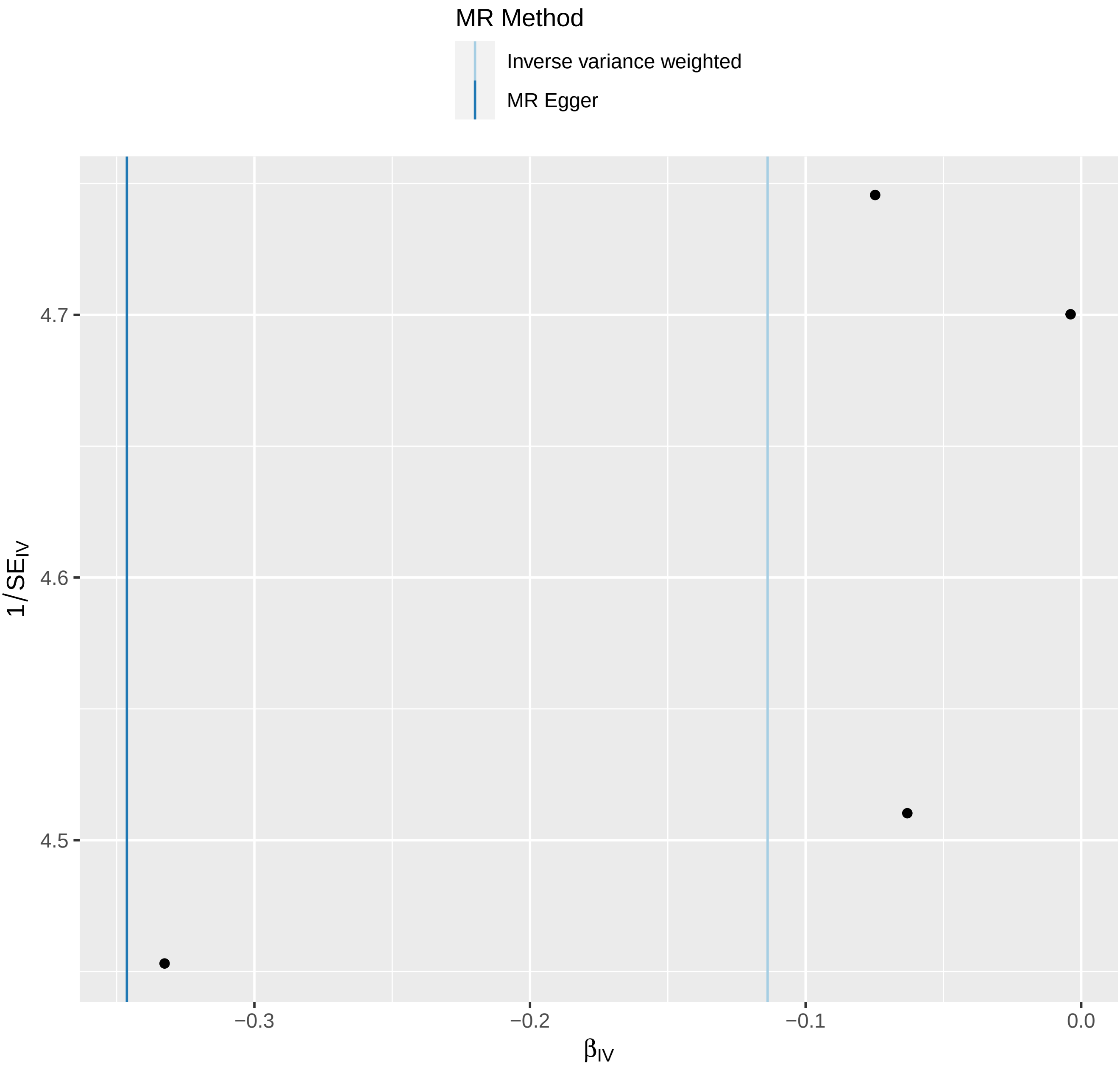

Batch 2−690 : Gut microbiota abundance (genus Parasutterella id.2892) on Neuron−specific vesicular protein calcyon
with a threshold of 1e−05

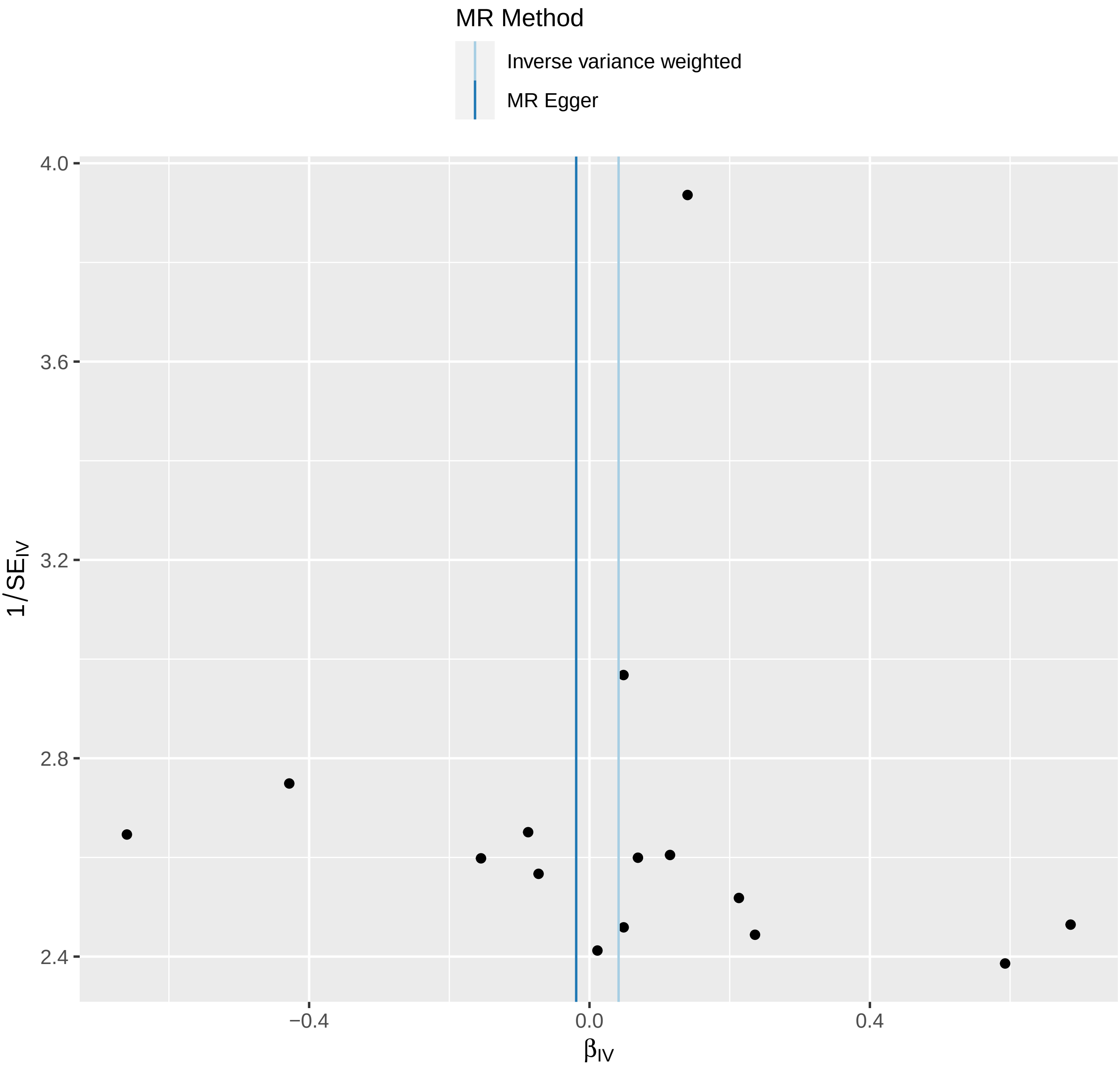

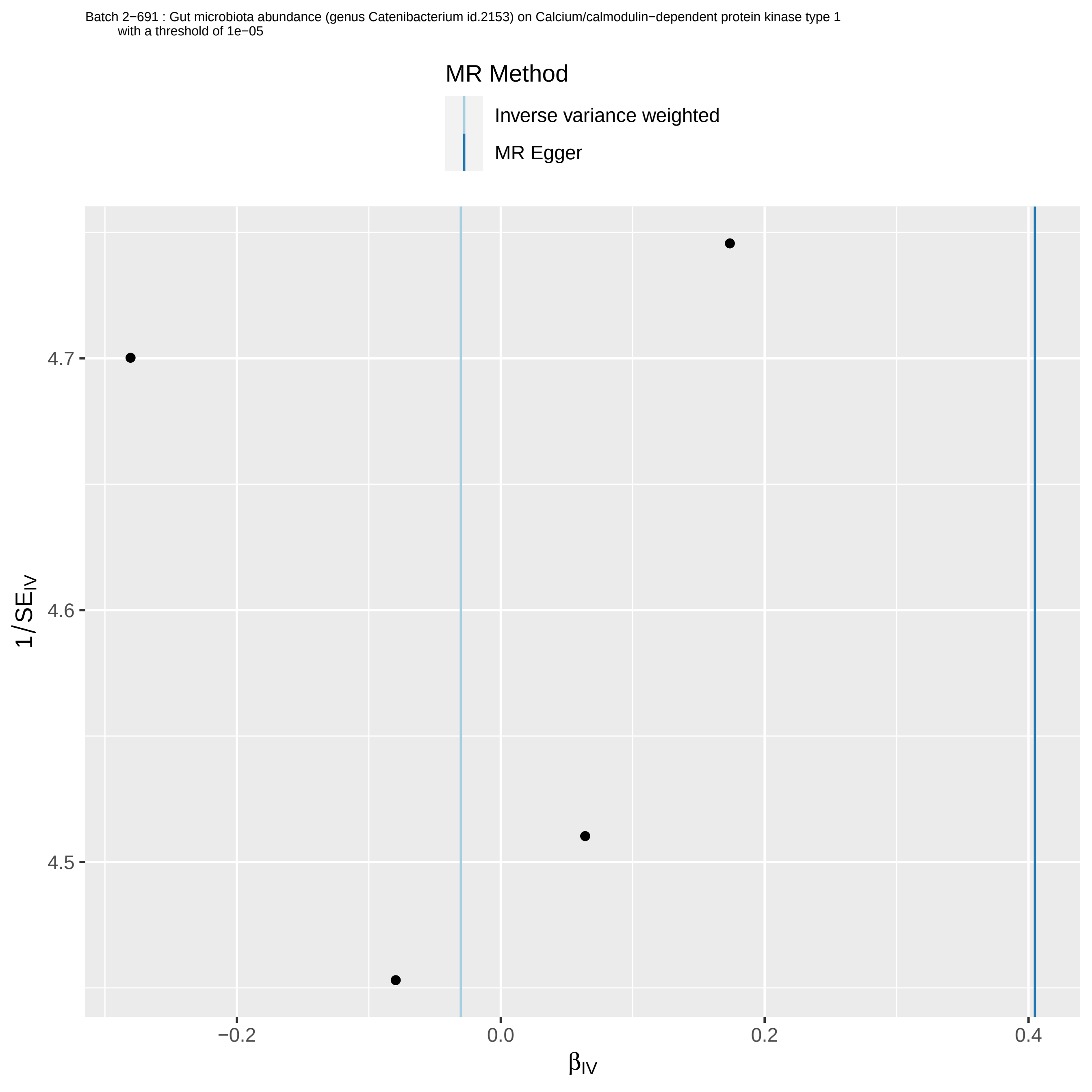

Batch 2−691 : Gut microbiota abundance (genus Catenibacterium id.2153) on Calcium/calmodulin−dependent protein kinase type 1
with a threshold of 1e−05
MR Method
Inverse variance weighted
MR Egger
4.7
4.6
4.5
1/SE_IV
−0.2
0.0
0.2
0.4
β_IV

Batch 2−692 : Gut microbiota abundance (genus Parasutterella id.2892) on Calcium/calmodulin−dependent protein kinase type 1 with a threshold of 1e−05

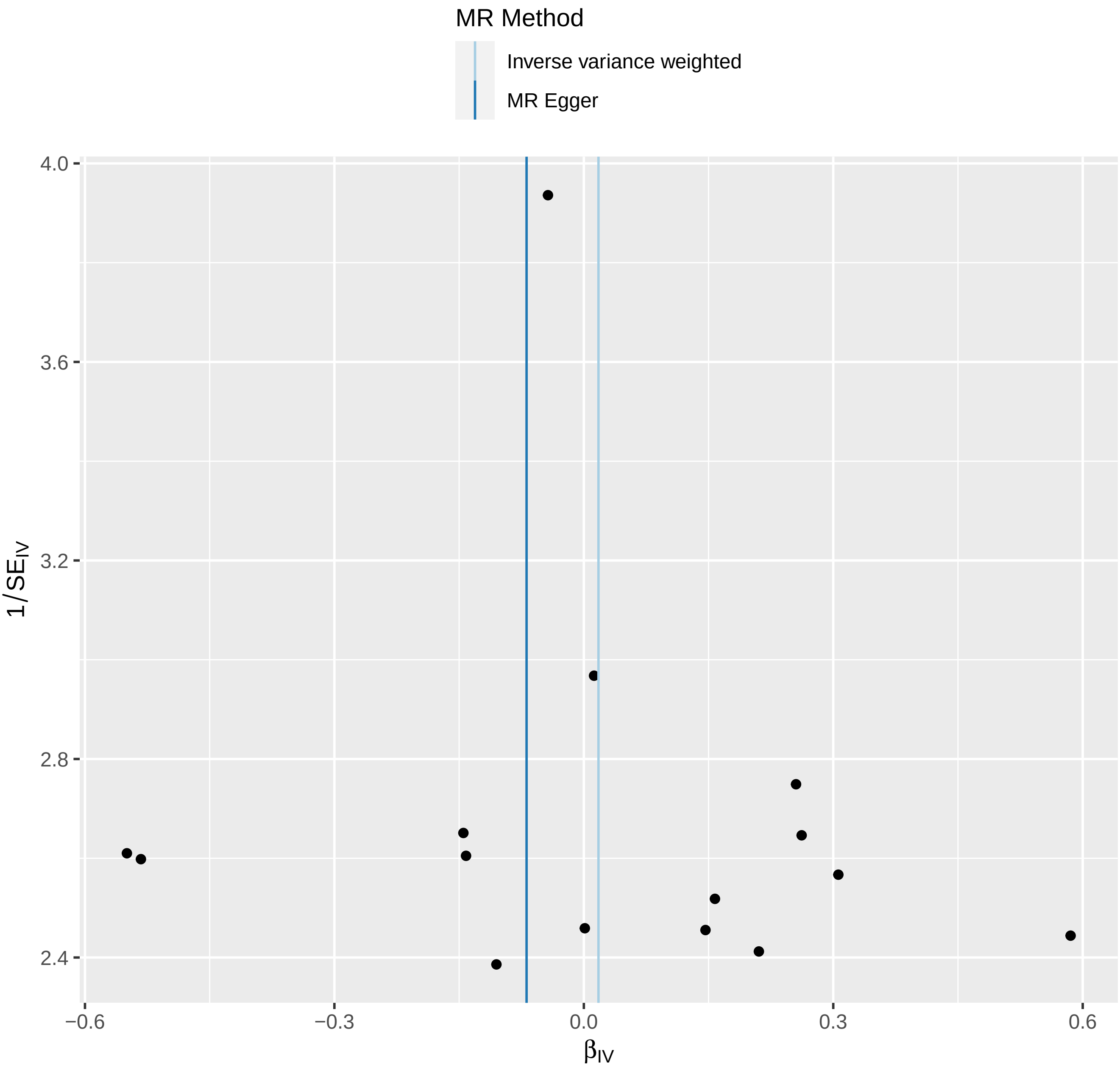

Batch 2−693 : Gut microbiota abundance (genus Catenibacterium id.2153) on Calcium/calmodulin−dependent protein kinase type 1D
with a threshold of 1e−05

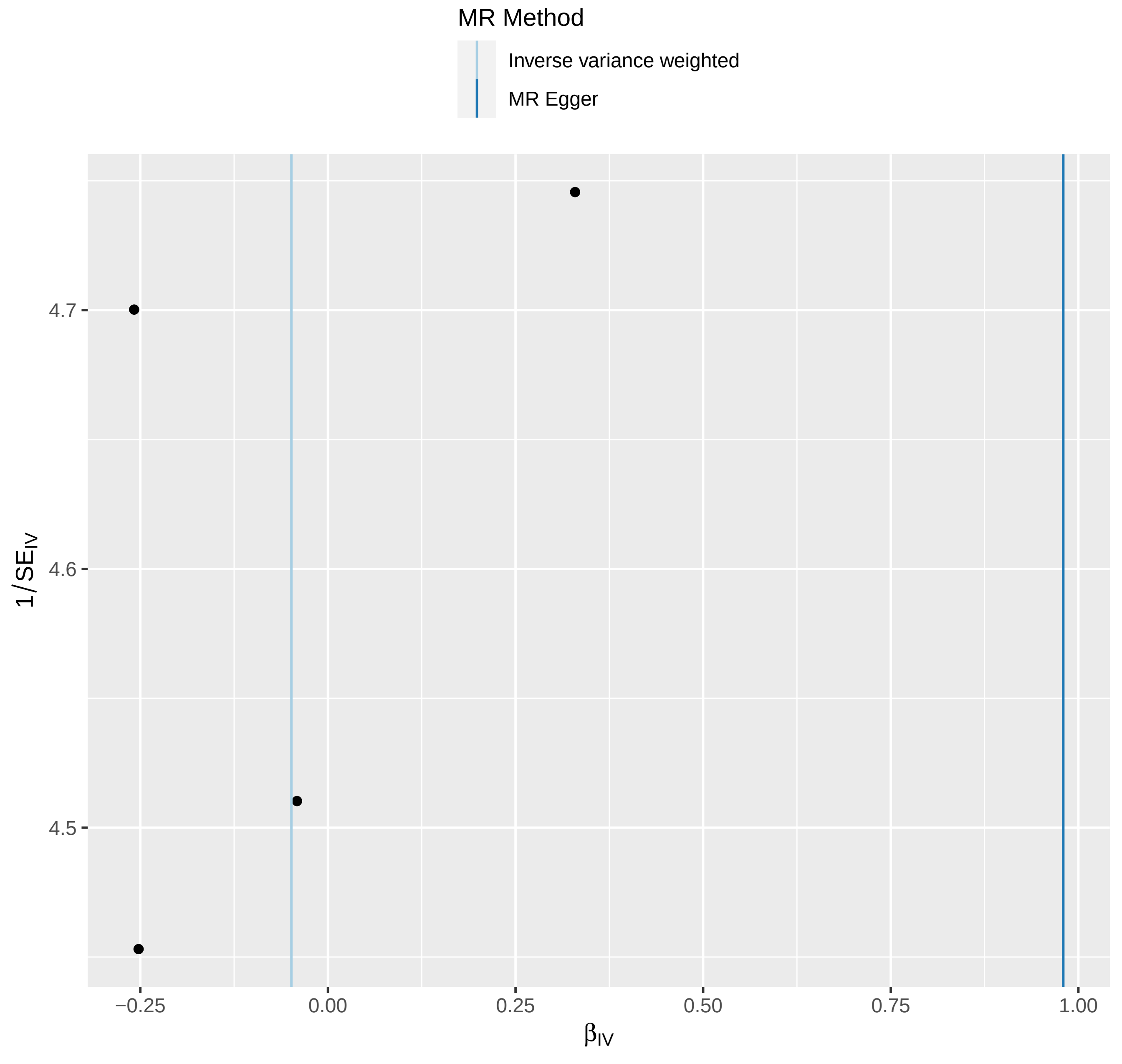

Batch 2−694 : Gut microbiota abundance (genus Parasutterella id.2892) on Calcium/calmodulin−dependent protein kinase type 1D
with a threshold of 1e−05

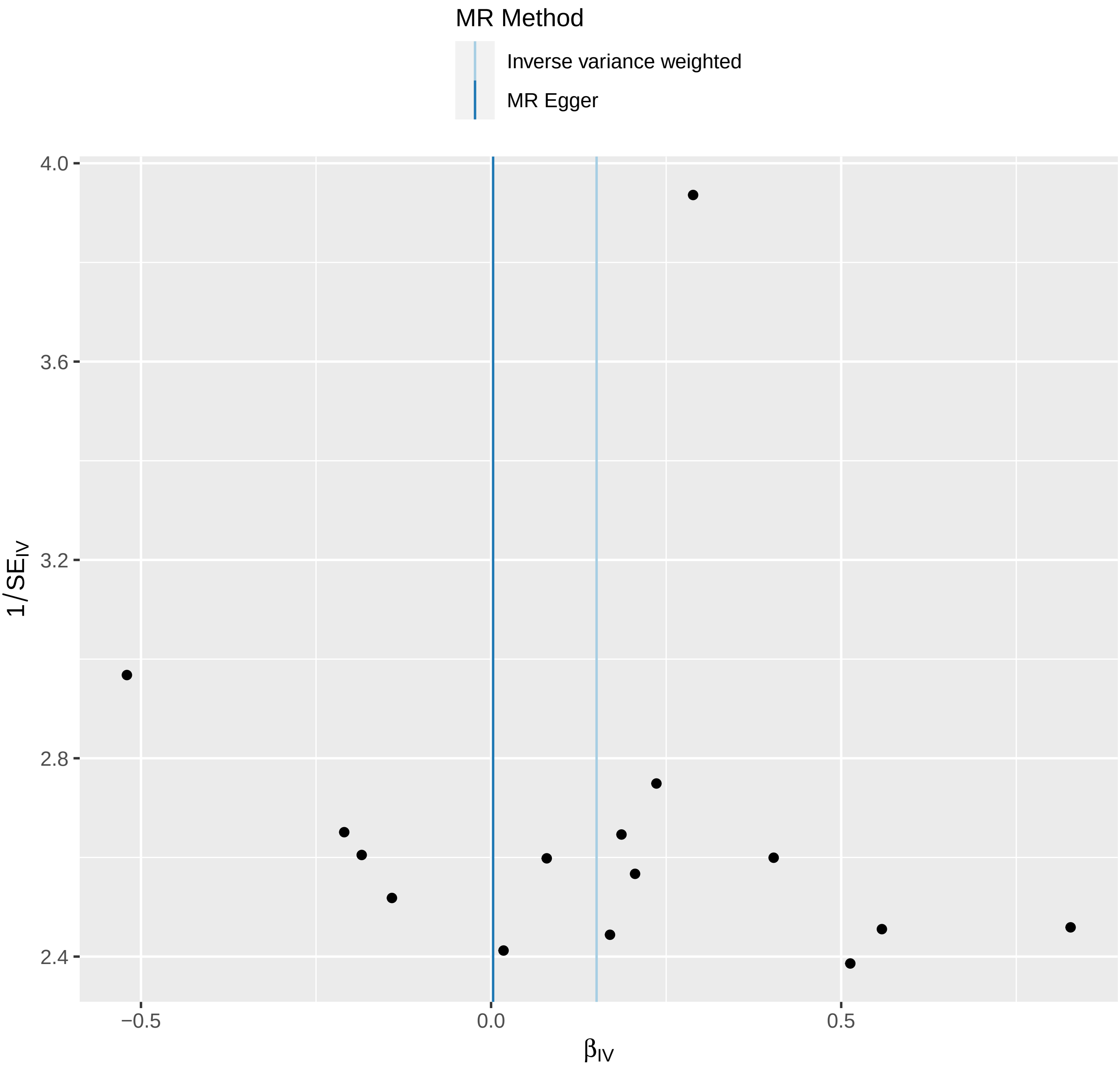

Batch 2−695 : Gut microbiota abundance (genus Catenibacterium id.2153) on Calcium/calmodulin−dependent protein kinase type II subunit alpha with a threshold of 1e−05

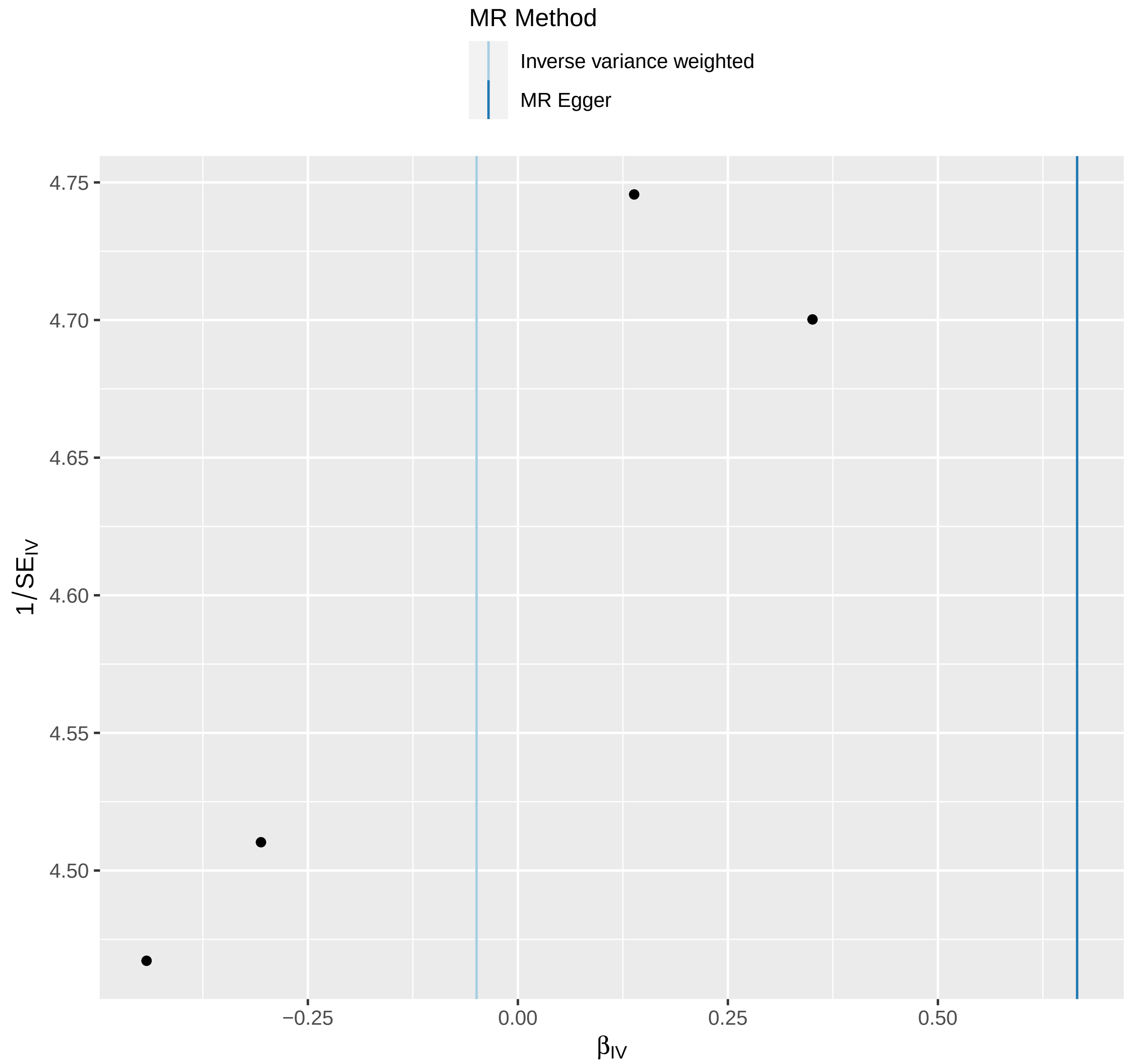

Batch 2−696 : Gut microbiota abundance (genus Parasutterella id.2892) on Calcium/calmodulin−dependent protein kinase type II subunit alpha
with a threshold of 1e−05

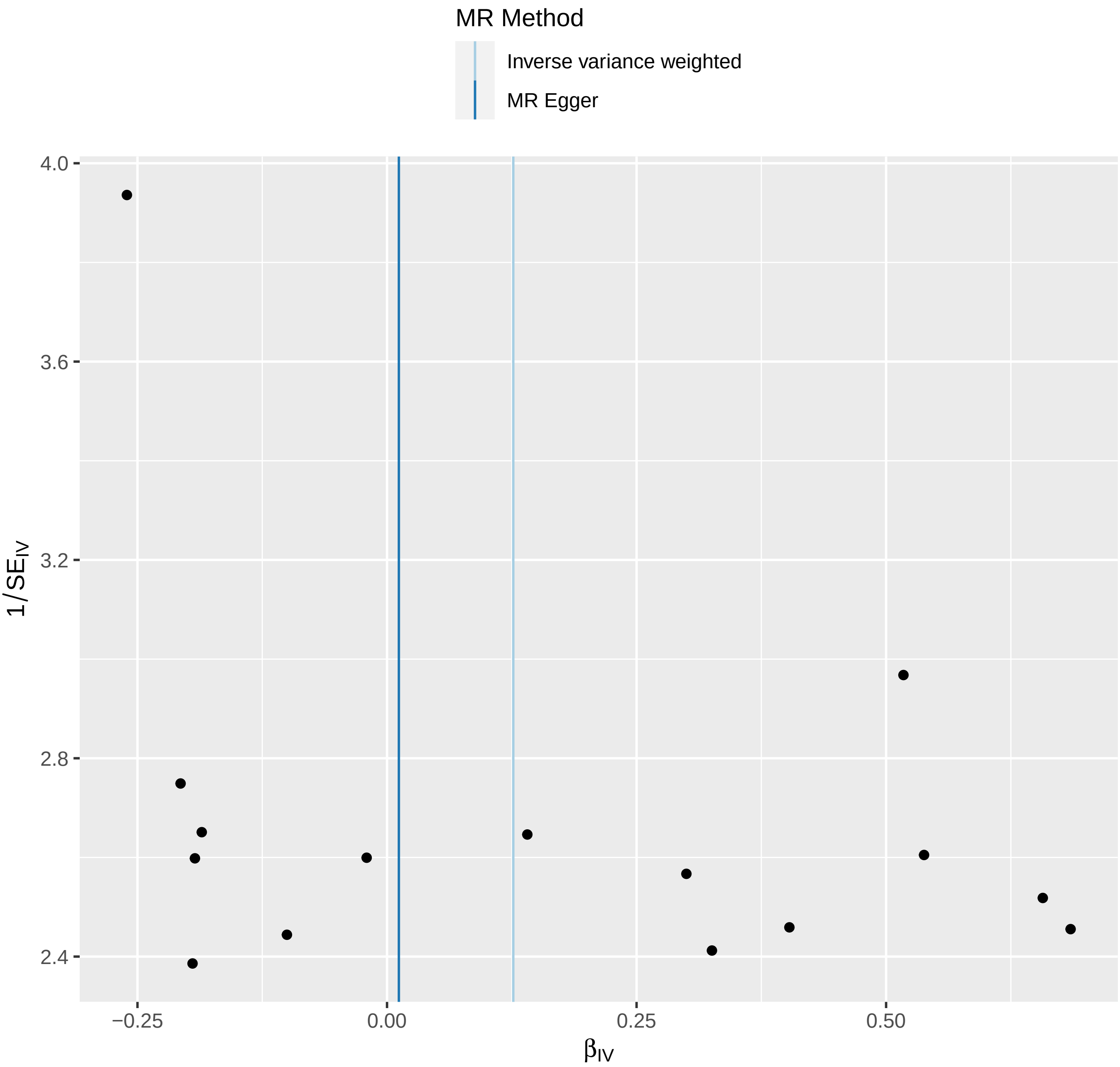

Batch 2−697 : Gut microbiota abundance (genus Catenibacterium id.2153) on Calcium/calmodulin−dependent protein kinase type II subunit beta with a threshold of 1e−05

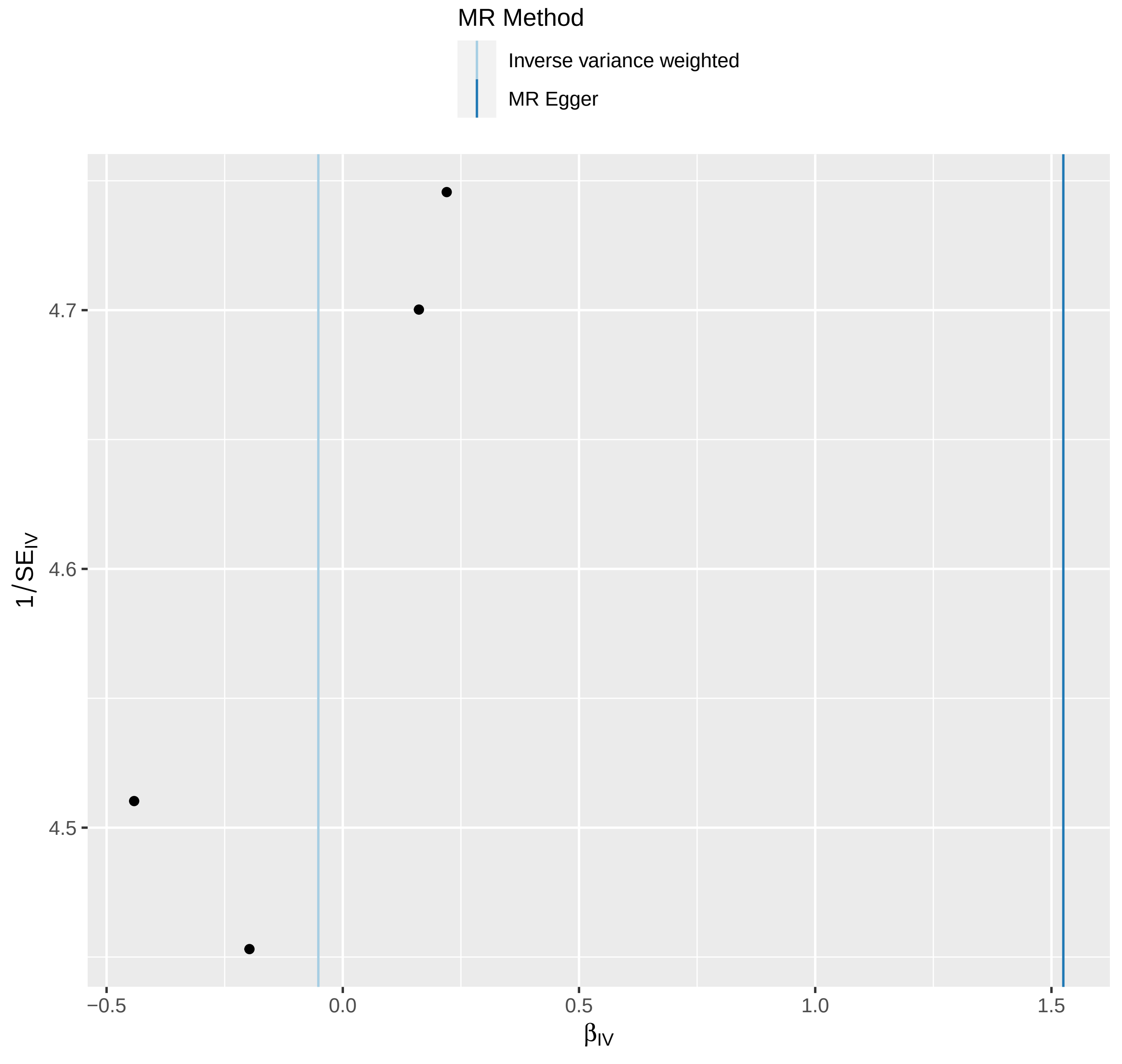

Batch 2−698 : Gut microbiota abundance (genus Parasutterella id.2892) on Calcium/calmodulin−dependent protein kinase type II subunit beta with a threshold of 1e−05

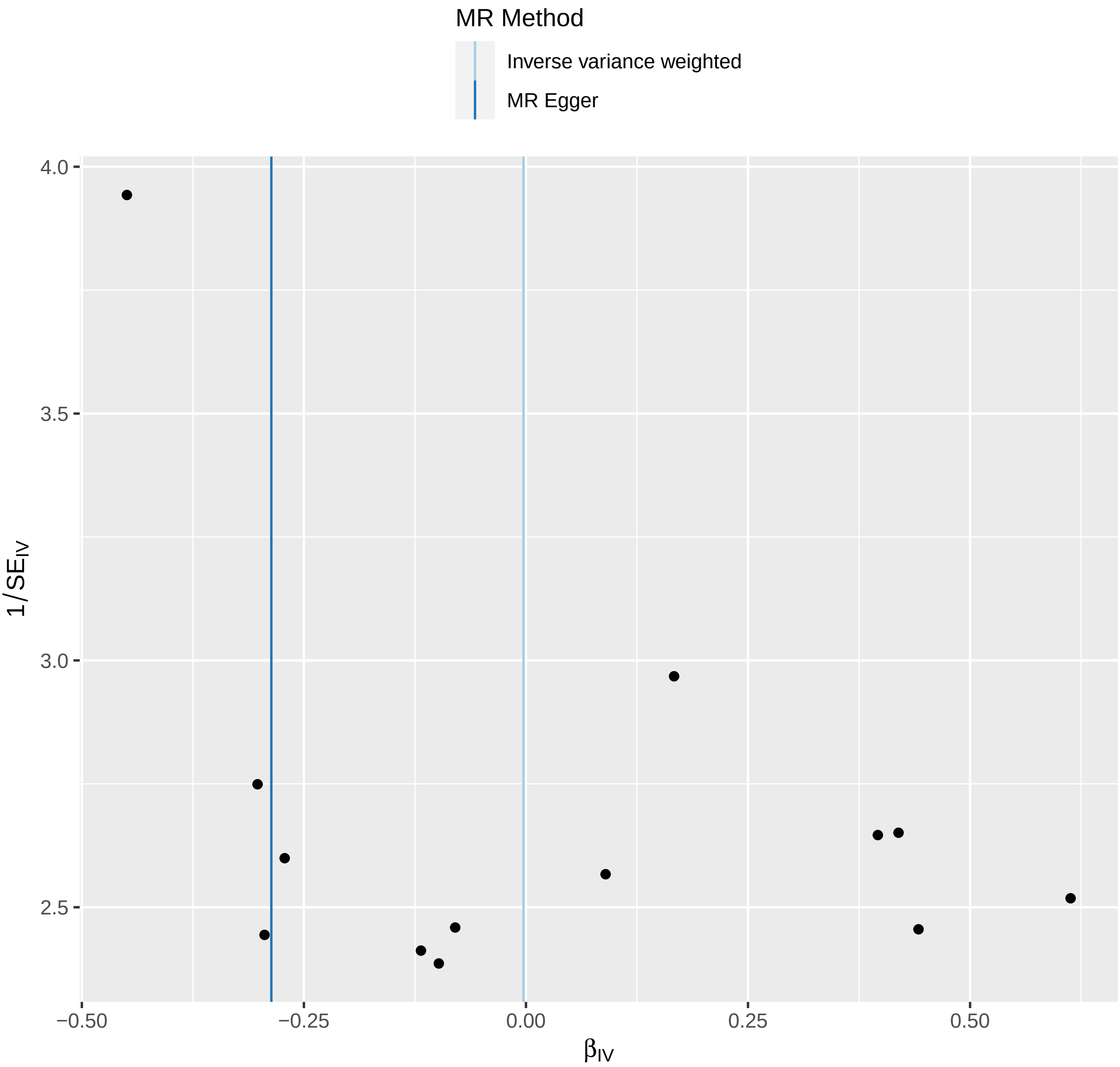

Batch 2−699 : Gut microbiota abundance (genus Catenibacterium id.2153) on Calcium/calmodulin−dependent protein kinase kinase 1 with a threshold of 1e−05

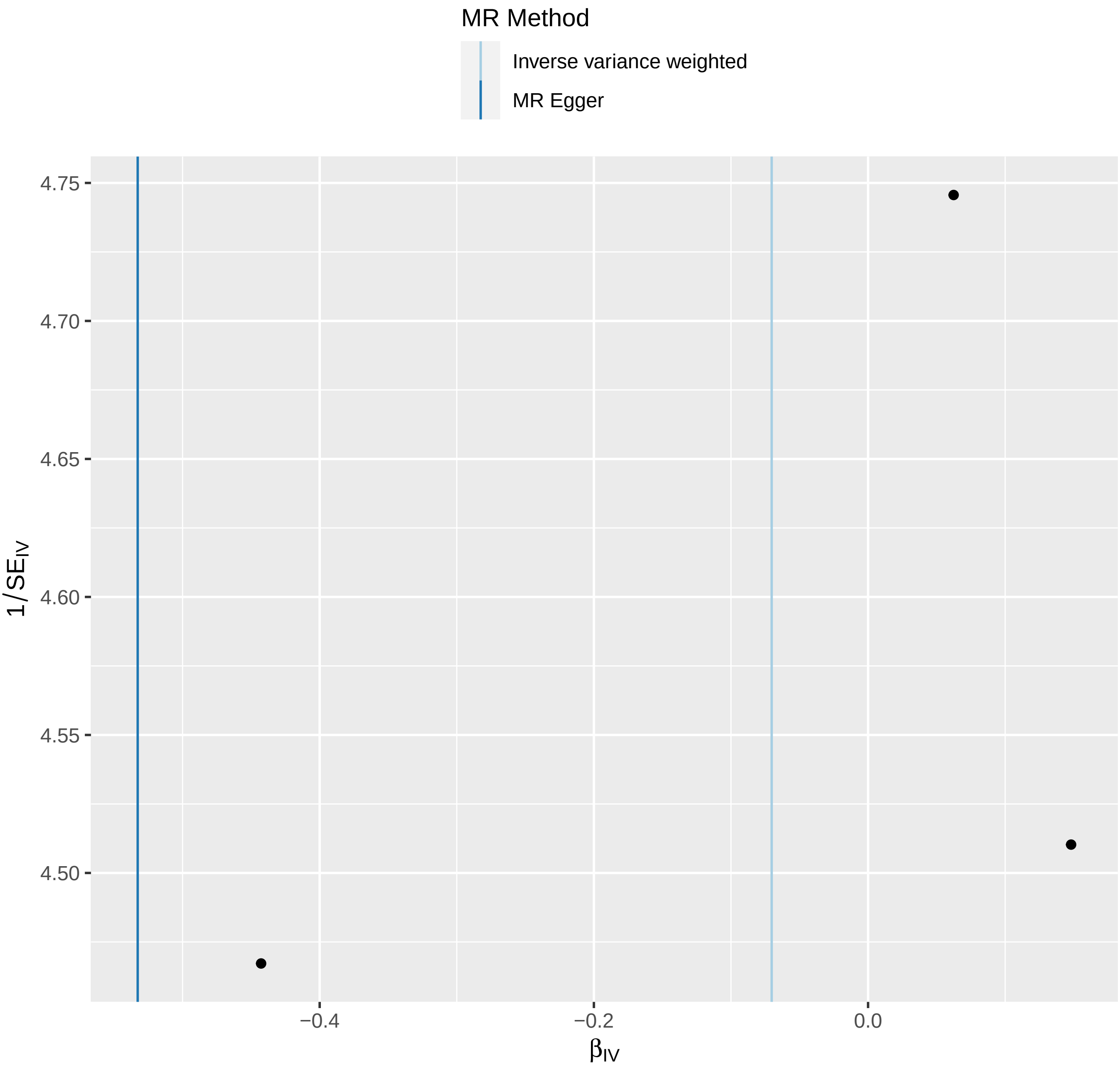

Batch 2−700 : Gut microbiota abundance (genus Parasutterella id.2892) on Calcium/calmodulin−dependent protein kinase kinase 1
with a threshold of 1e−05

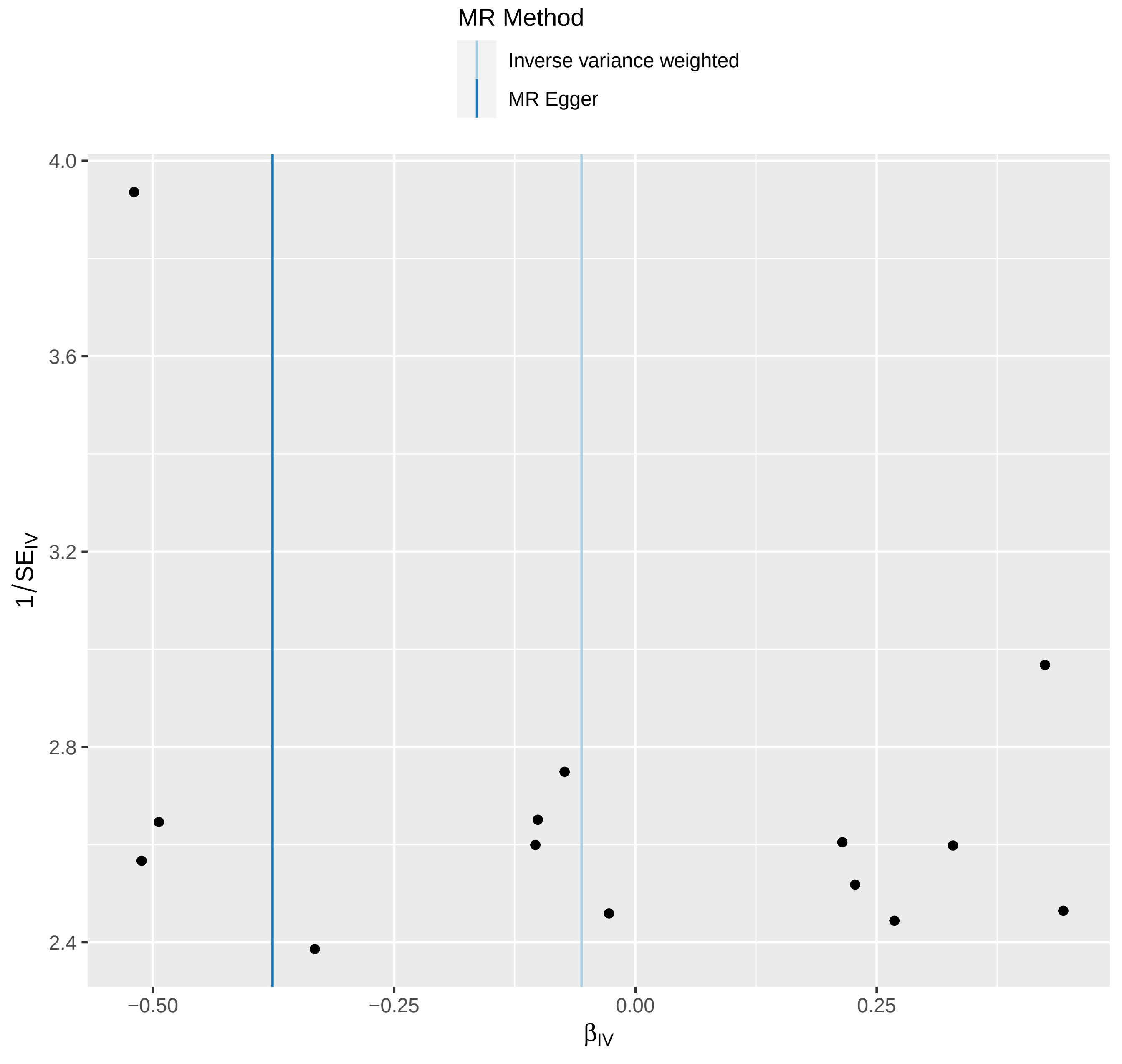

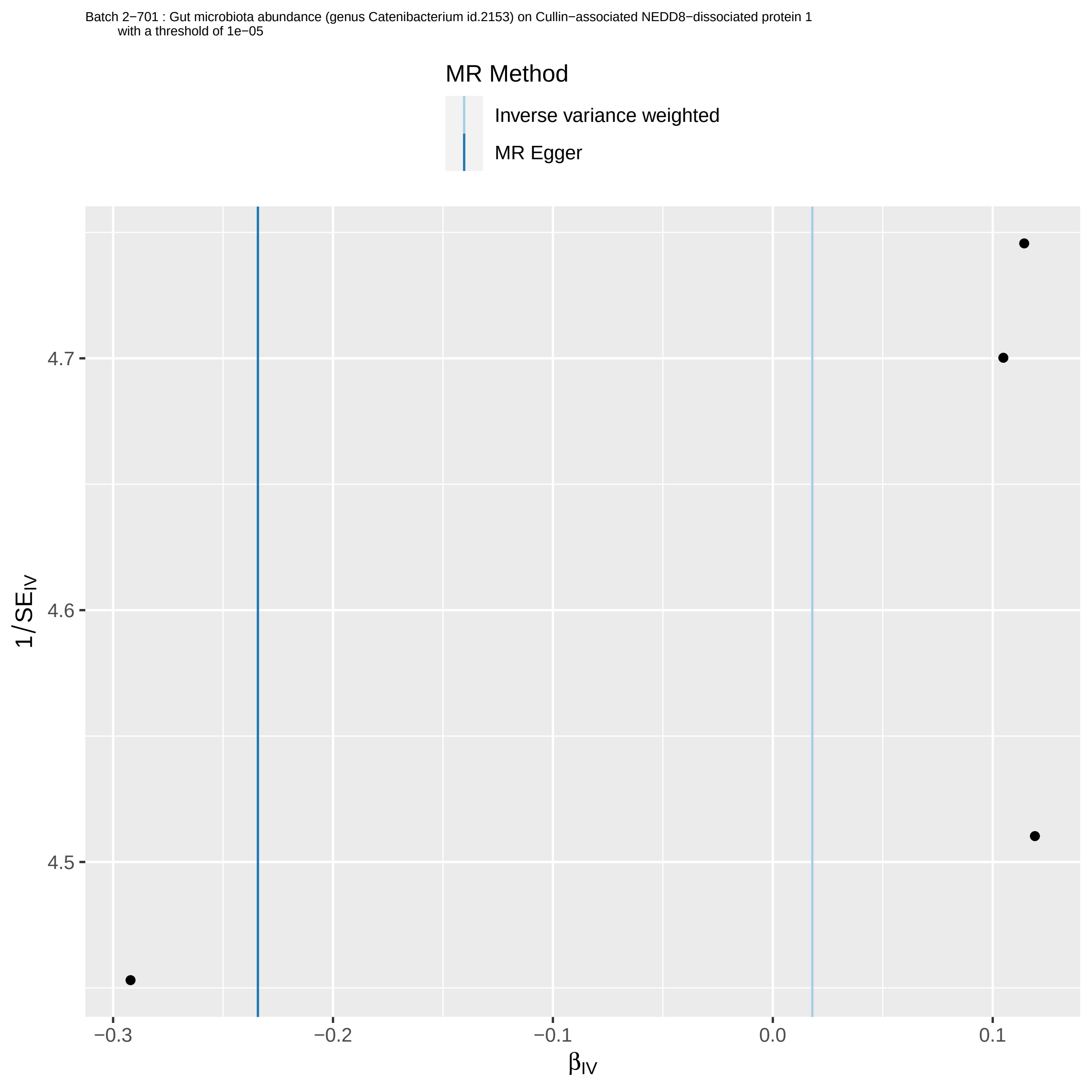
Batch 2−701 : Gut microbiota abundance (genus Catenibacterium id.2153) on Cullin−associated NEDD8−dissociated protein 1
with a threshold of 1e−05
MR Method
Inverse variance weighted
MR Egger
4.7
4.6
4.5
$1/SE_{IV}$
−0.3
−0.2
−0.1
0.0
0.1
β_{IV}

Batch 2−702 : Gut microbiota abundance (genus Parasutterella id.2892) on Cullin−associated NEDD8−dissociated protein 1 with a threshold of 1e−05

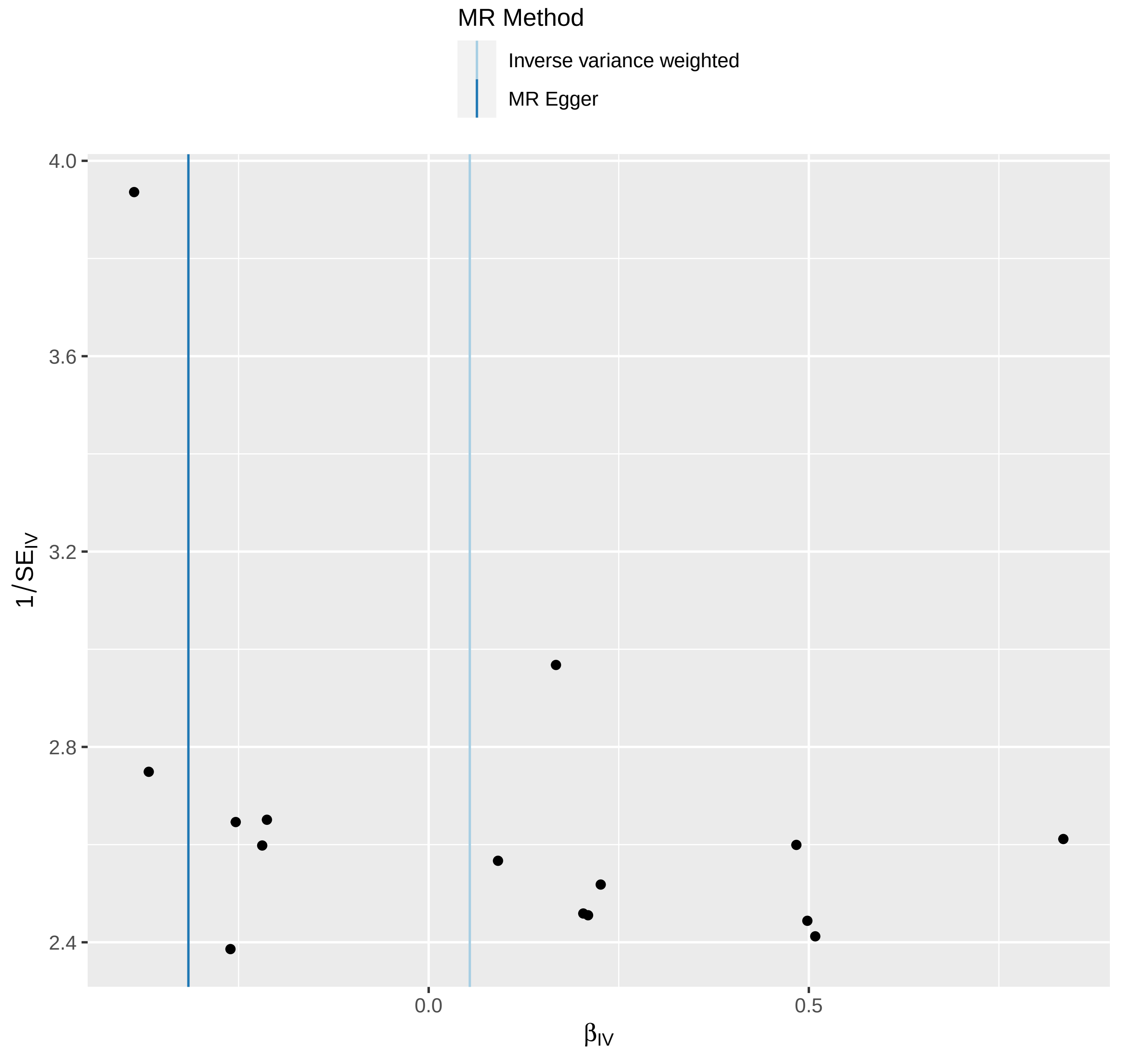

Batch 2−703 : Gut microbiota abundance (genus Catenibacterium id.2153) on Soluble calcium−activated nucleotidase 1
with a threshold of 1e−05

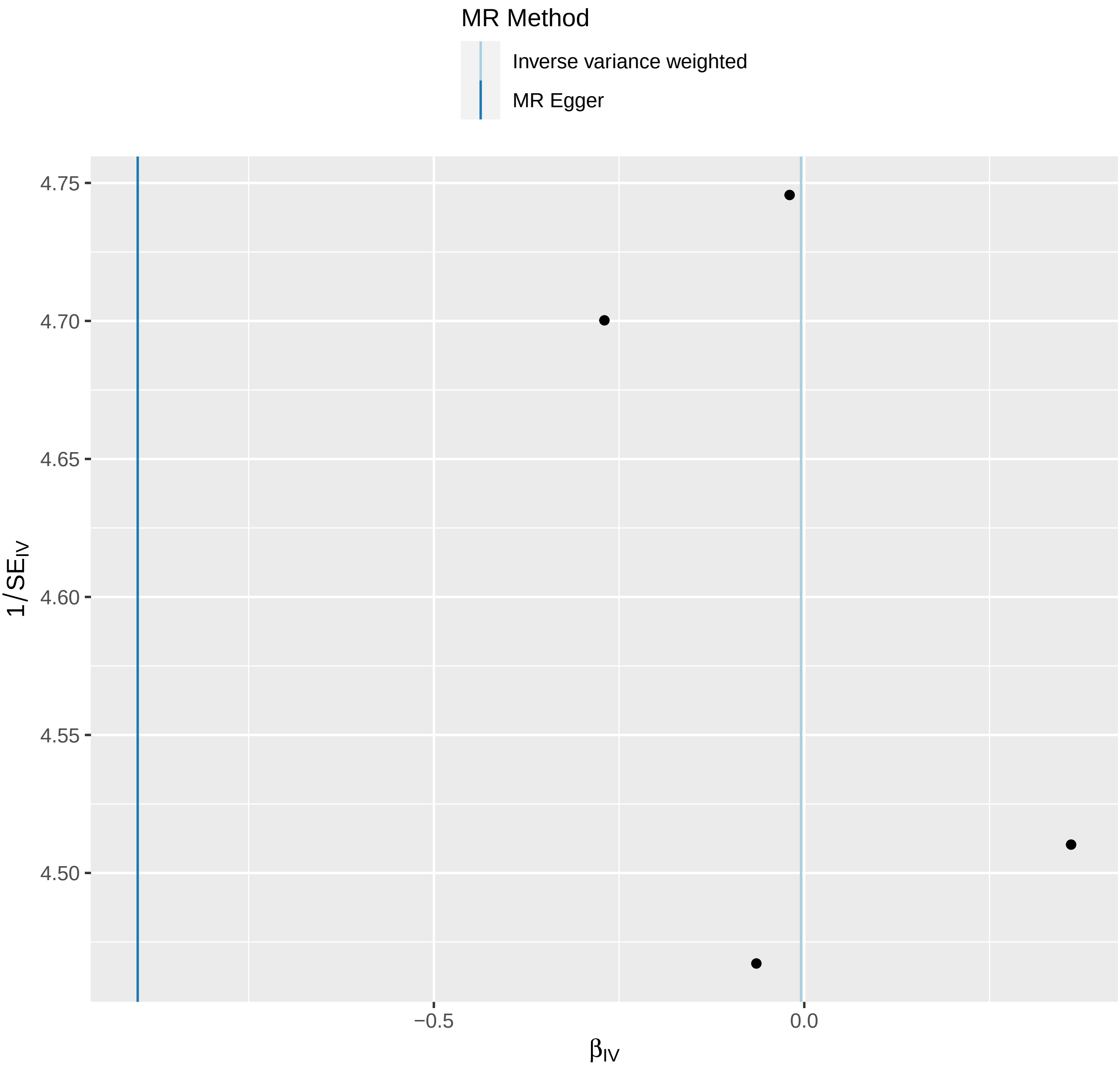

Batch 2−704 : Gut microbiota abundance (genus Parasutterella id.2892) on Soluble calcium−activated nucleotidase 1
with a threshold of 1e−05

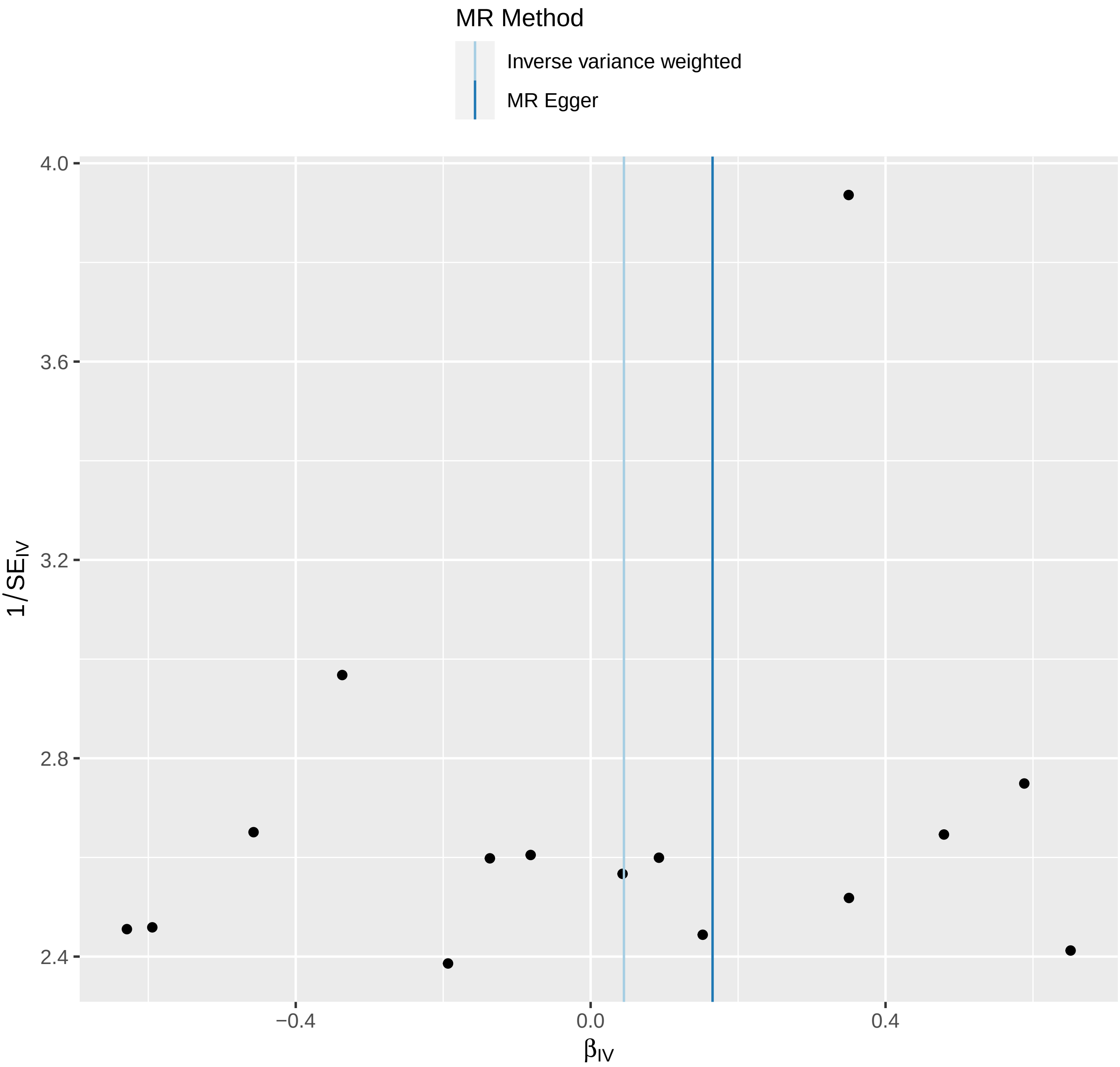

Batch 2−705 : Gut microbiota abundance (genus Catenibacterium id.2153) on Calnexin
with a threshold of 1e−05

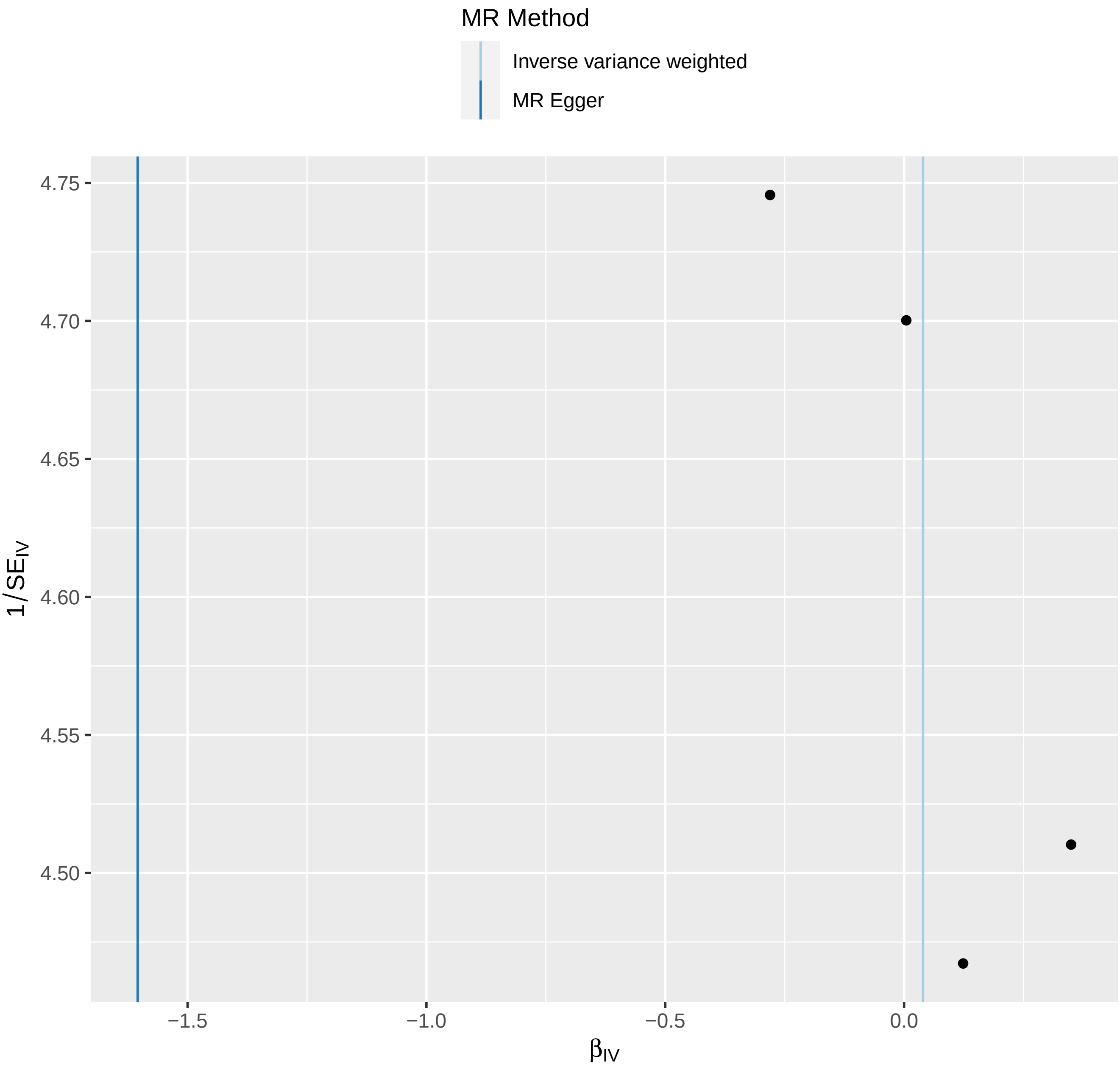

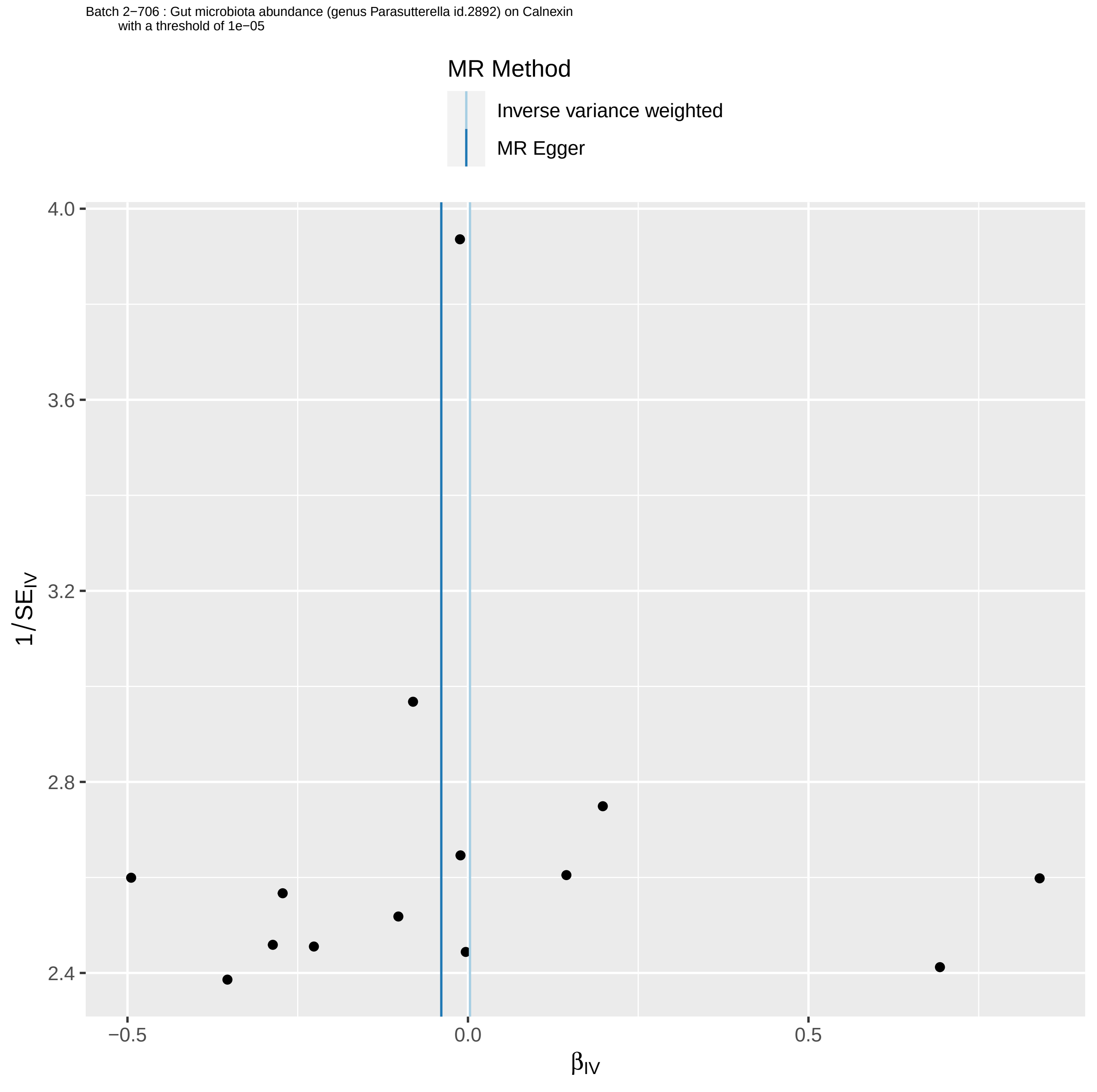
Batch 2−706 : Gut microbiota abundance (genus Parasutterella id.2892) on Calnexin
with a threshold of 1e−05
MR Method
Inverse variance weighted
MR Egger
4.0
3.6
3.2
2.8
2.4
1/SE_IV
−0.5
0.0
0.5
β_IV

Batch 2−707 : Gut microbiota abundance (genus Catenibacterium id.2153) on Calpain−2 catalytic subunit
with a threshold of 1e−05

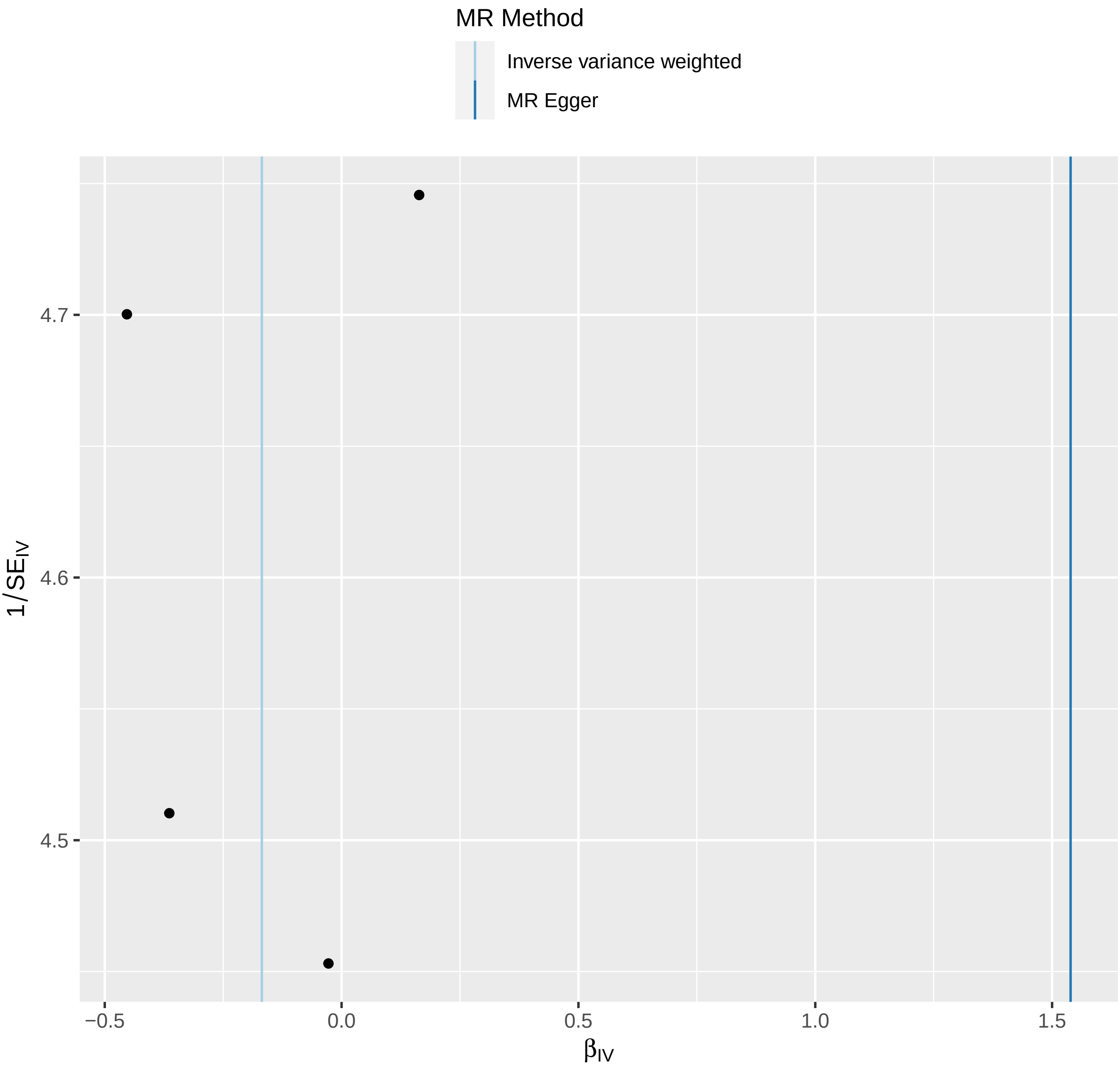

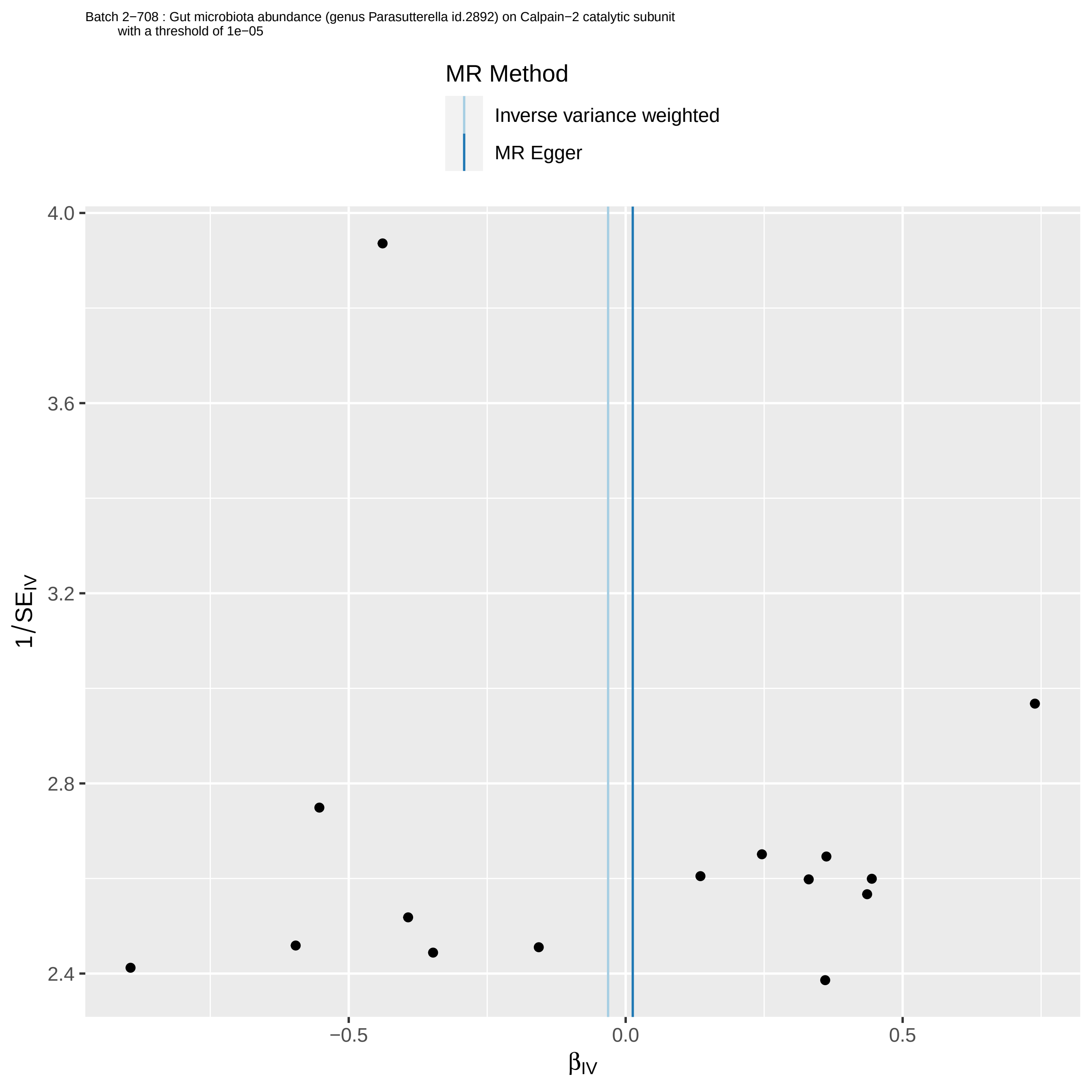

Batch 2−708 : Gut microbiota abundance (genus Parasutterella id.2892) on Calpain−2 catalytic subunit
with a threshold of 1e−05
MR Method
Inverse variance weighted
MR Egger
4.0
3.6
3.2
2.8
2.4
1/SE_IV
−0.5
0.0
0.5
β_IV

Batch 2−709 : Gut microbiota abundance (genus Catenibacterium id.2153) on Calpain−3
with a threshold of 1e−05

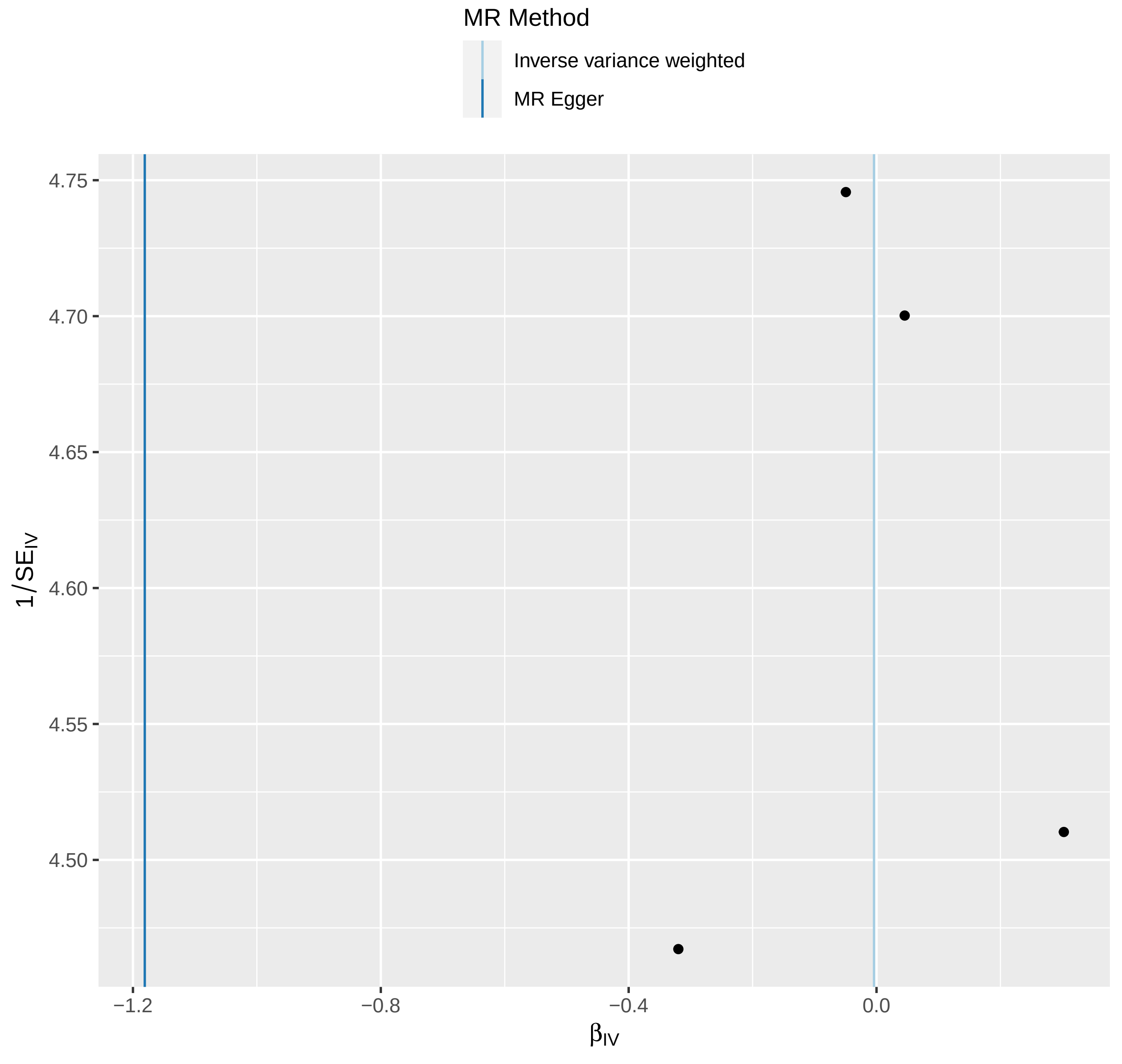

Batch 2−710 : Gut microbiota abundance (genus Parasutterella id.2892) on Calpain−3
with a threshold of 1e−05

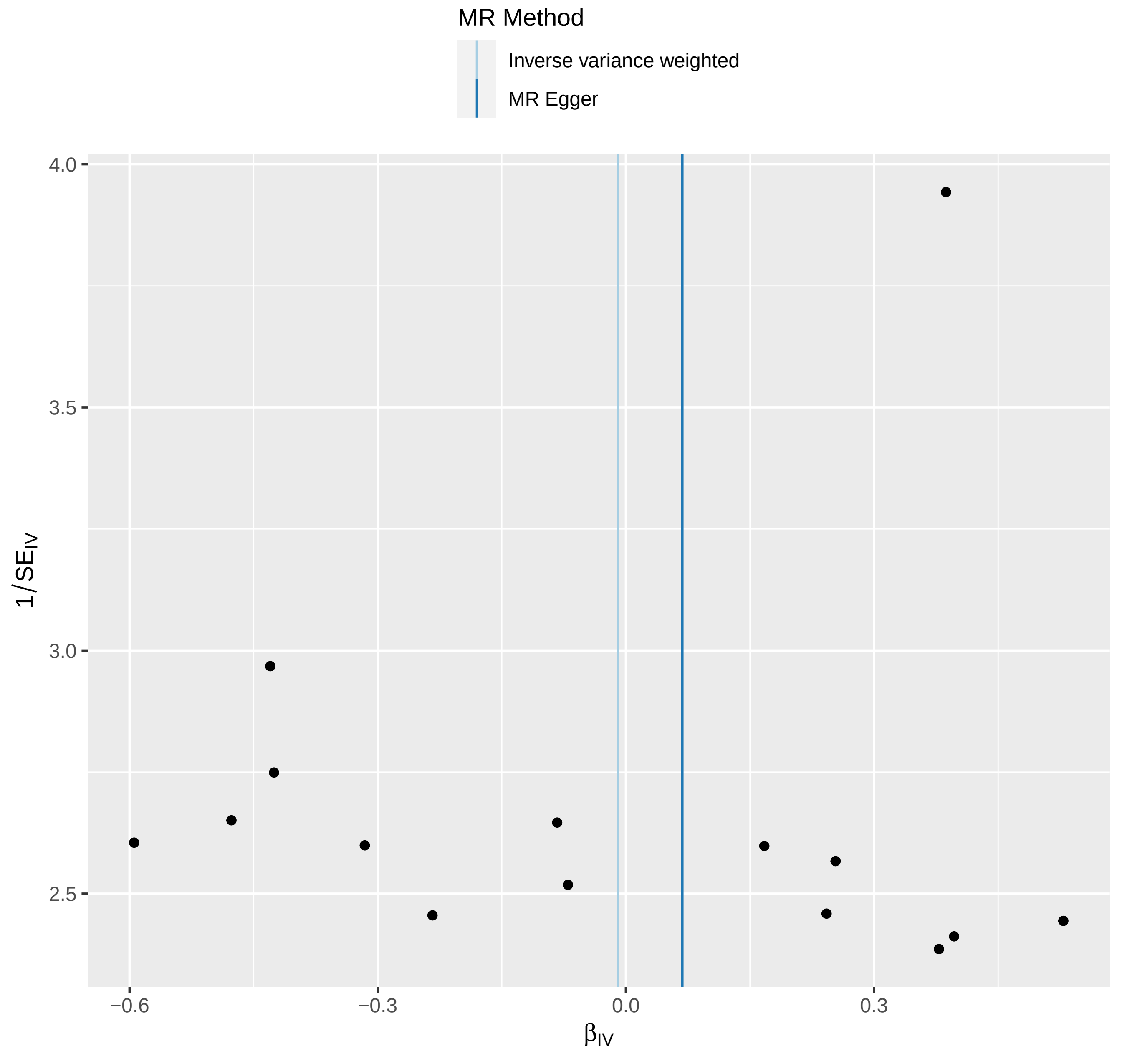

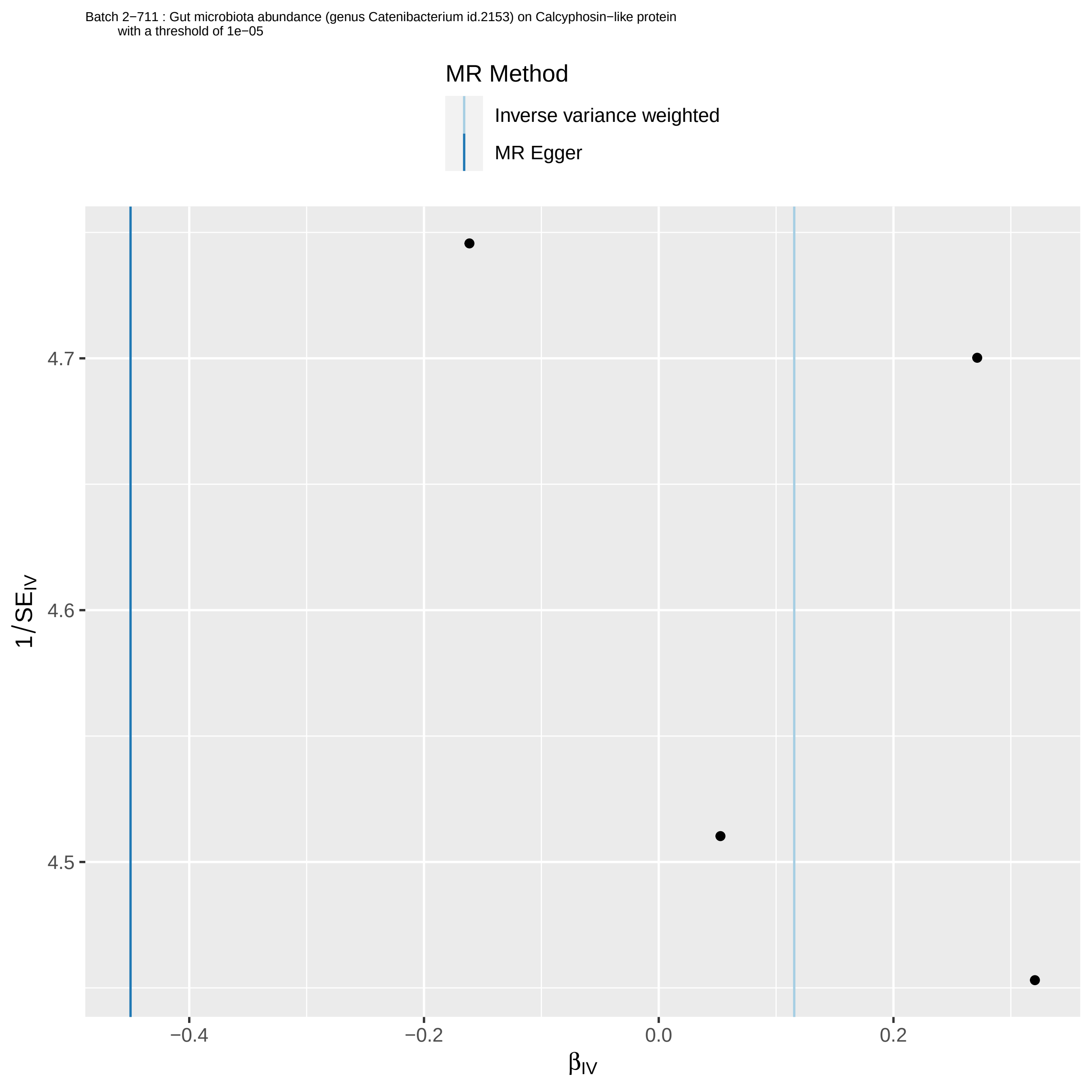
Batch 2−711 : Gut microbiota abundance (genus Catenibacterium id.2153) on Calcyphosin−like protein
with a threshold of 1e−05
MR Method
Inverse variance weighted
MR Egger
4.7
4.6
4.5
$1/SE_{IV}$
−0.4
−0.2
0.0
0.2
β_{IV}

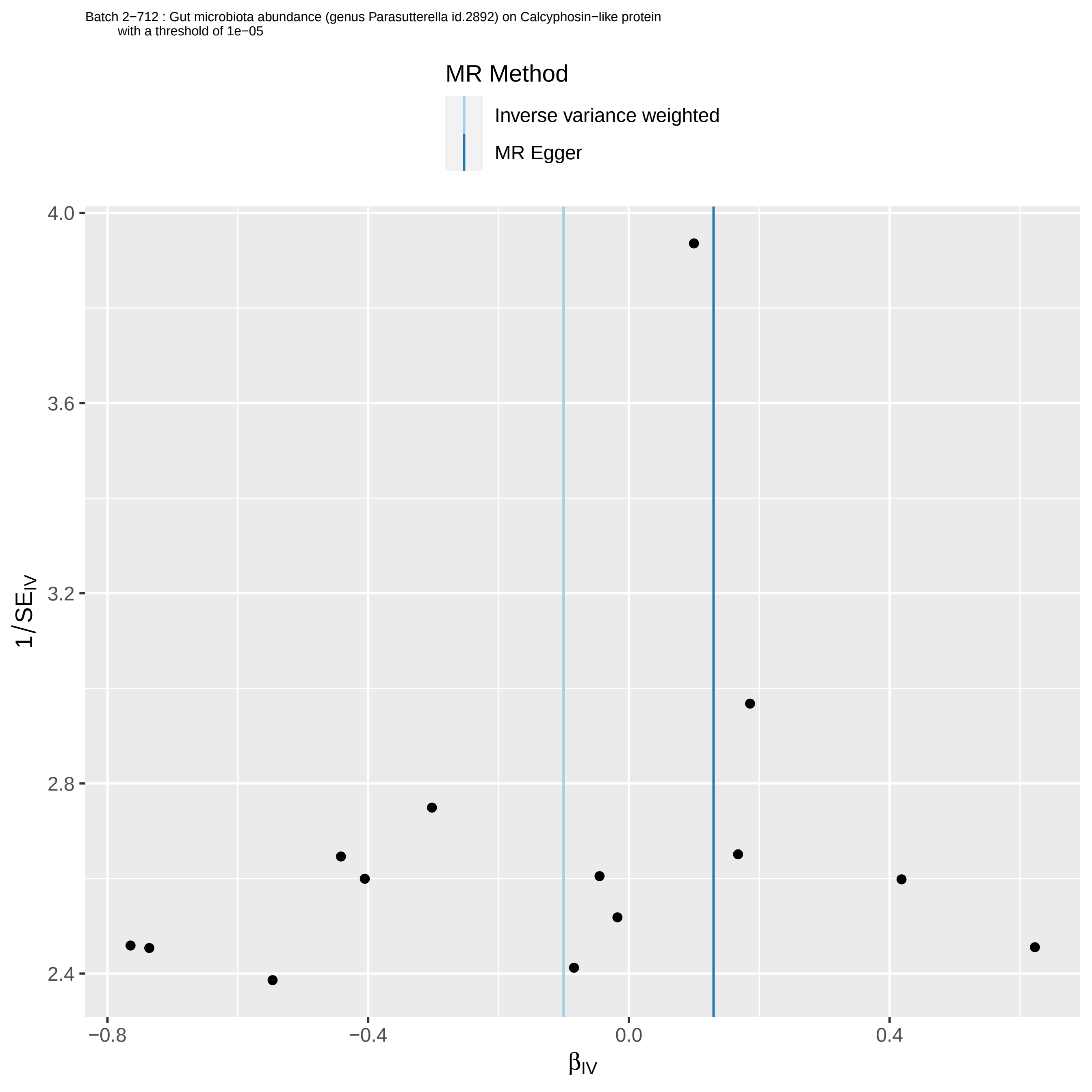

Batch 2−712 : Gut microbiota abundance (genus Parasutterella id.2892) on Calcyphosin−like protein
with a threshold of 1e−05
MR Method
Inverse variance weighted
MR Egger
4.0
3.6
3.2
2.8
2.4
1/SE_IV
−0.8
−0.4
0.0
0.4
β_IV

Batch 2−713 : Gut microbiota abundance (genus Catenibacterium id.2153) on Calcium−regulated heat stable protein 1
with a threshold of 1e−05

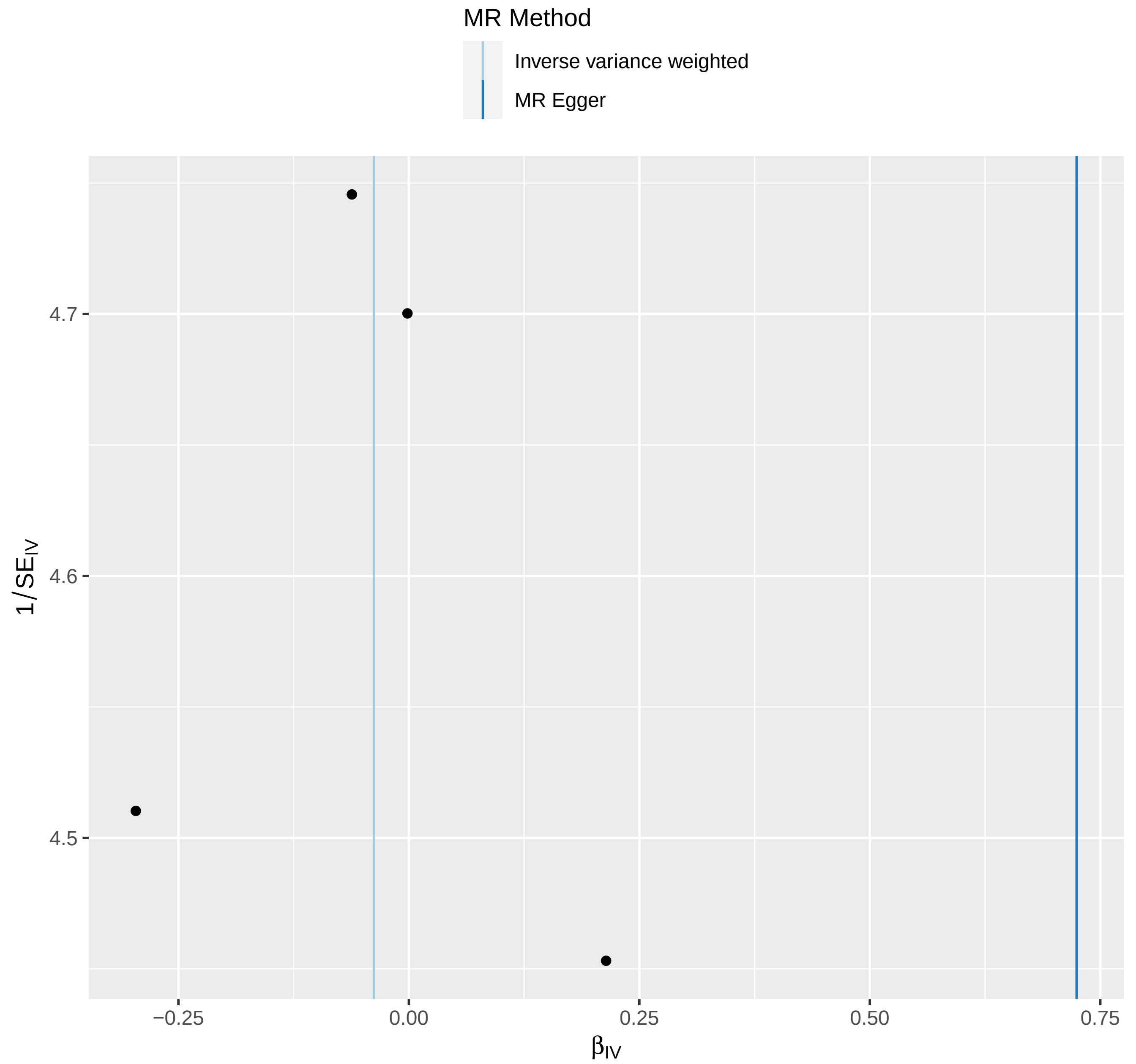

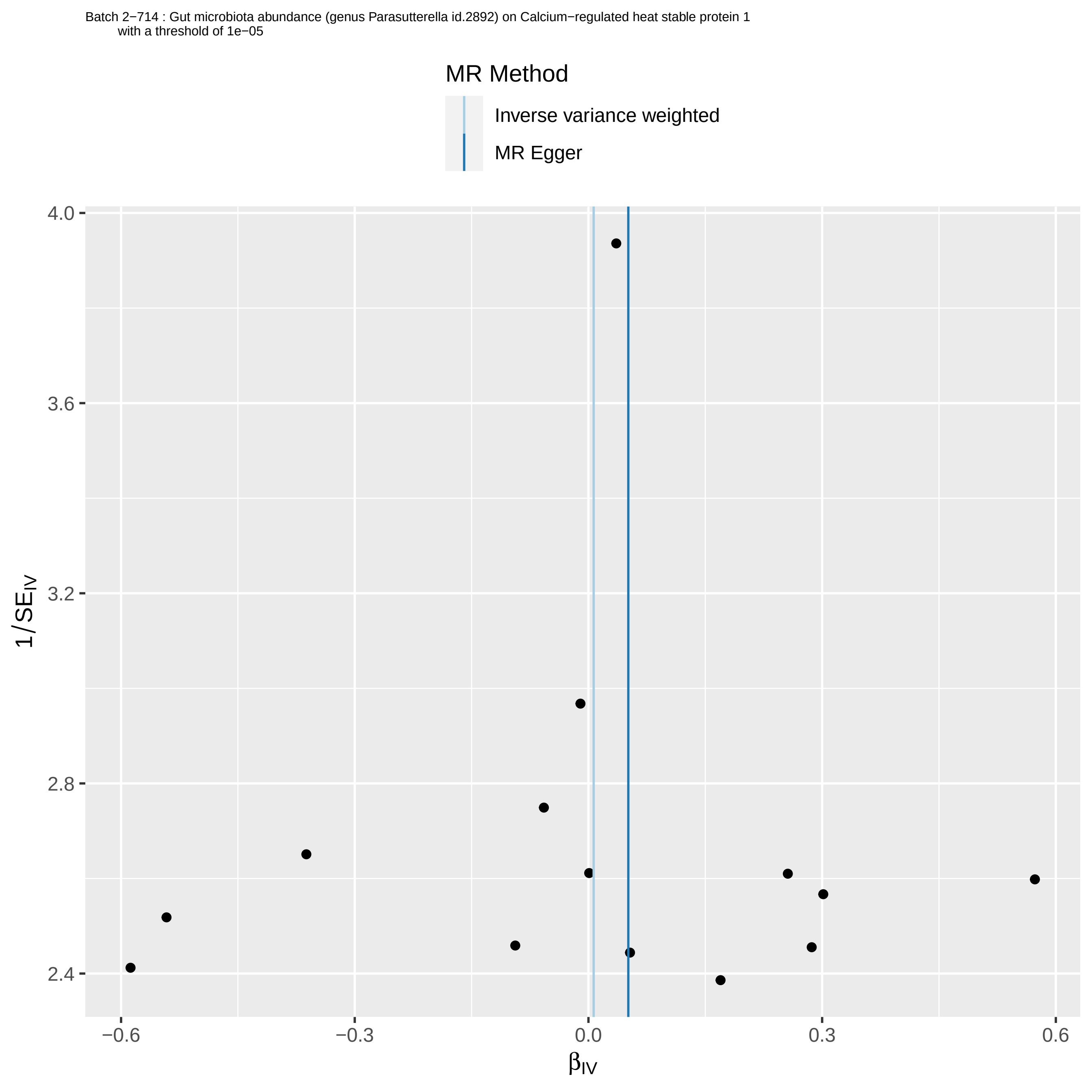
Batch 2−714 : Gut microbiota abundance (genus Parasutterella id.2892) on Calcium−regulated heat stable protein 1
with a threshold of 1e−05
MR Method
Inverse variance weighted
MR Egger
4.0
3.6
3.2
2.8
2.4
1/SE_IV
−0.6
−0.3
0.0
0.3
0.6
β_IV

Batch 2−715 : Gut microbiota abundance (genus Catenibacterium id.2153) on Cysteine−−tRNA ligase, cytoplasmic
with a threshold of 1e−05

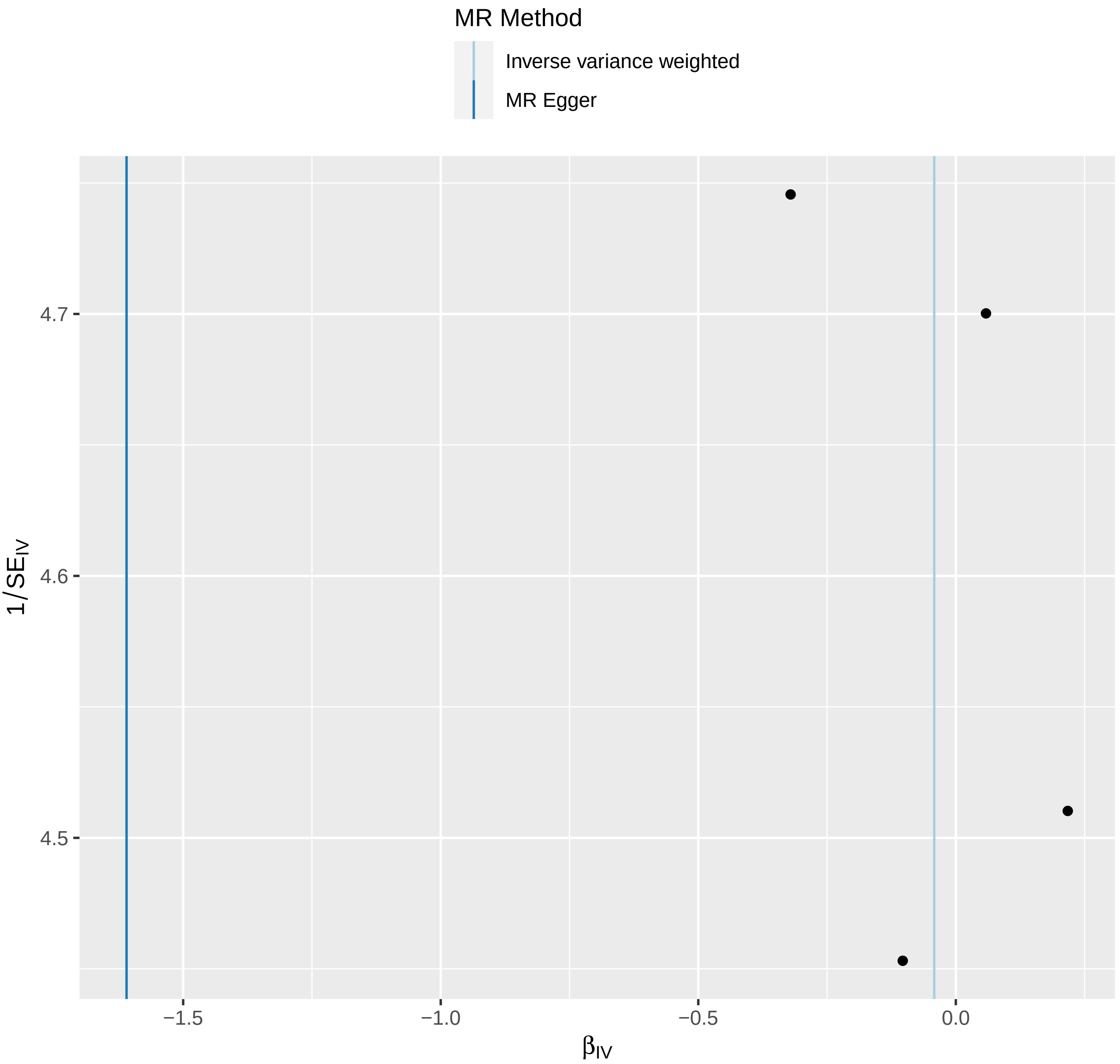

Batch 2−716 : Gut microbiota abundance (genus Parasutterella id.2892) on Cysteine−−tRNA ligase, cytoplasmic
with a threshold of 1e−05

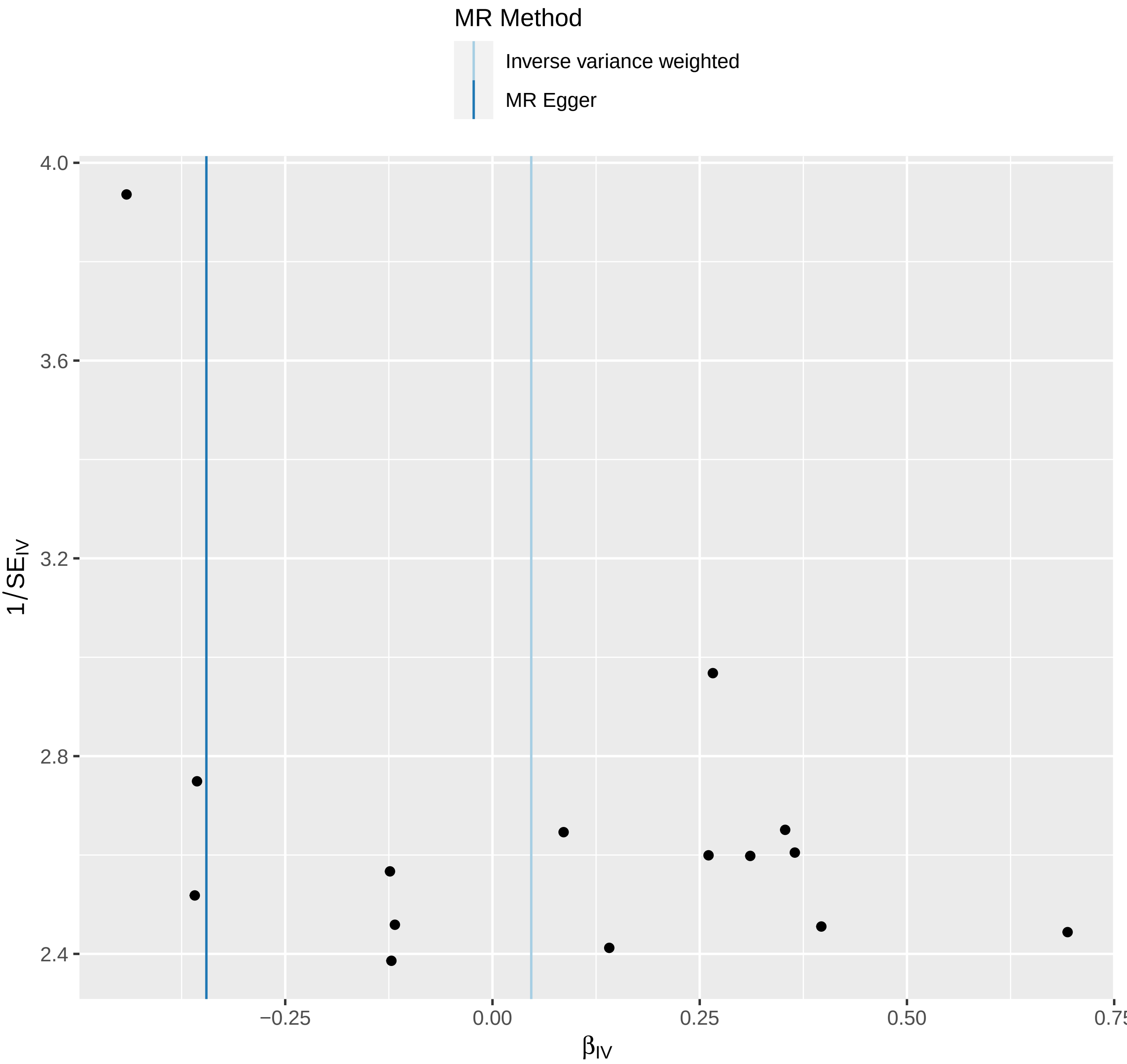

Batch 2−717 : Gut microbiota abundance (genus Catenibacterium id.2153) on Cocaine− and amphetamine−regulated transcript protein with a threshold of 1e−05

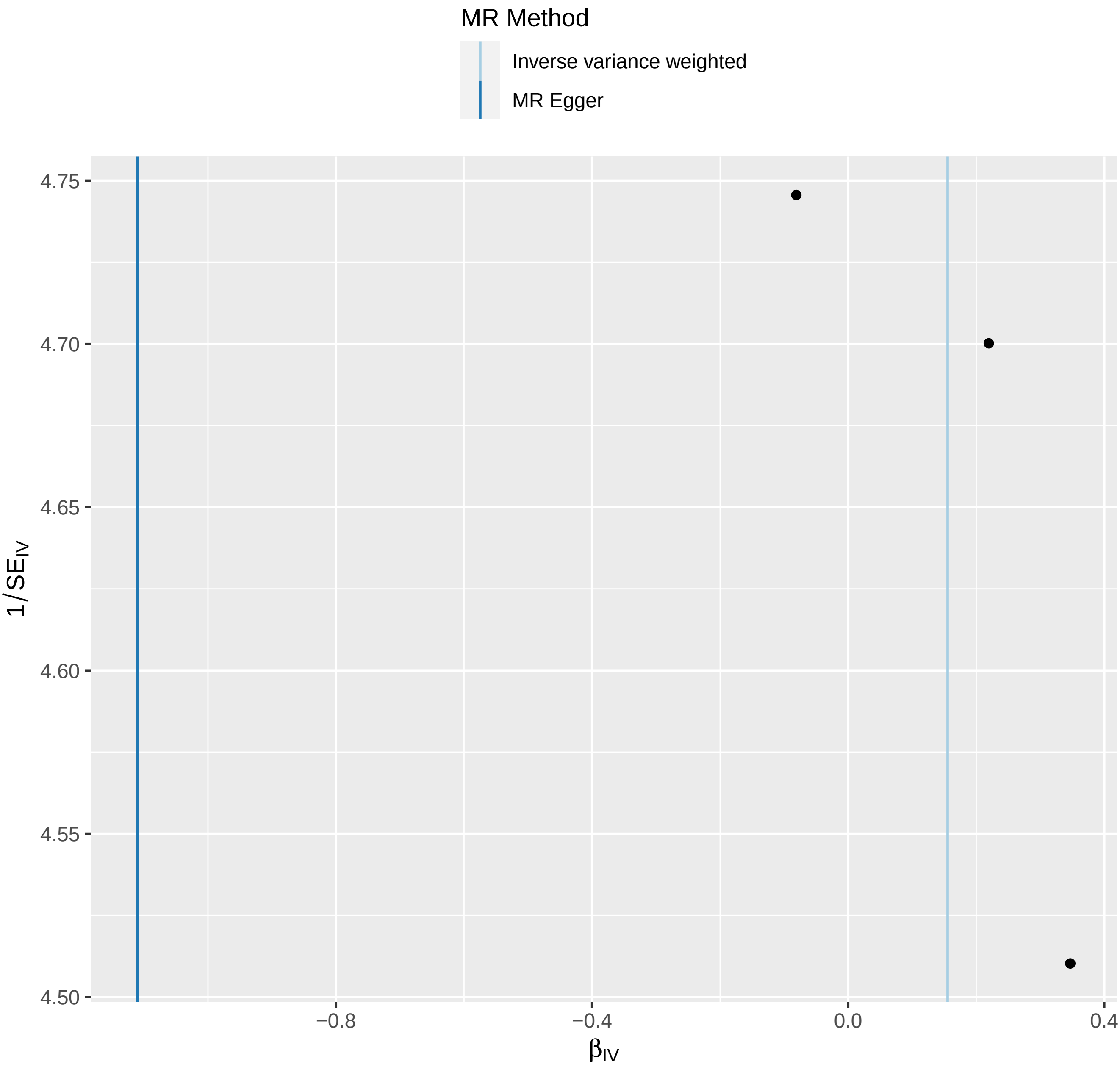

Batch 2−718 : Gut microbiota abundance (genus Parasutterella id.2892) on Cocaine− and amphetamine−regulated transcript protein with a threshold of 1e−05

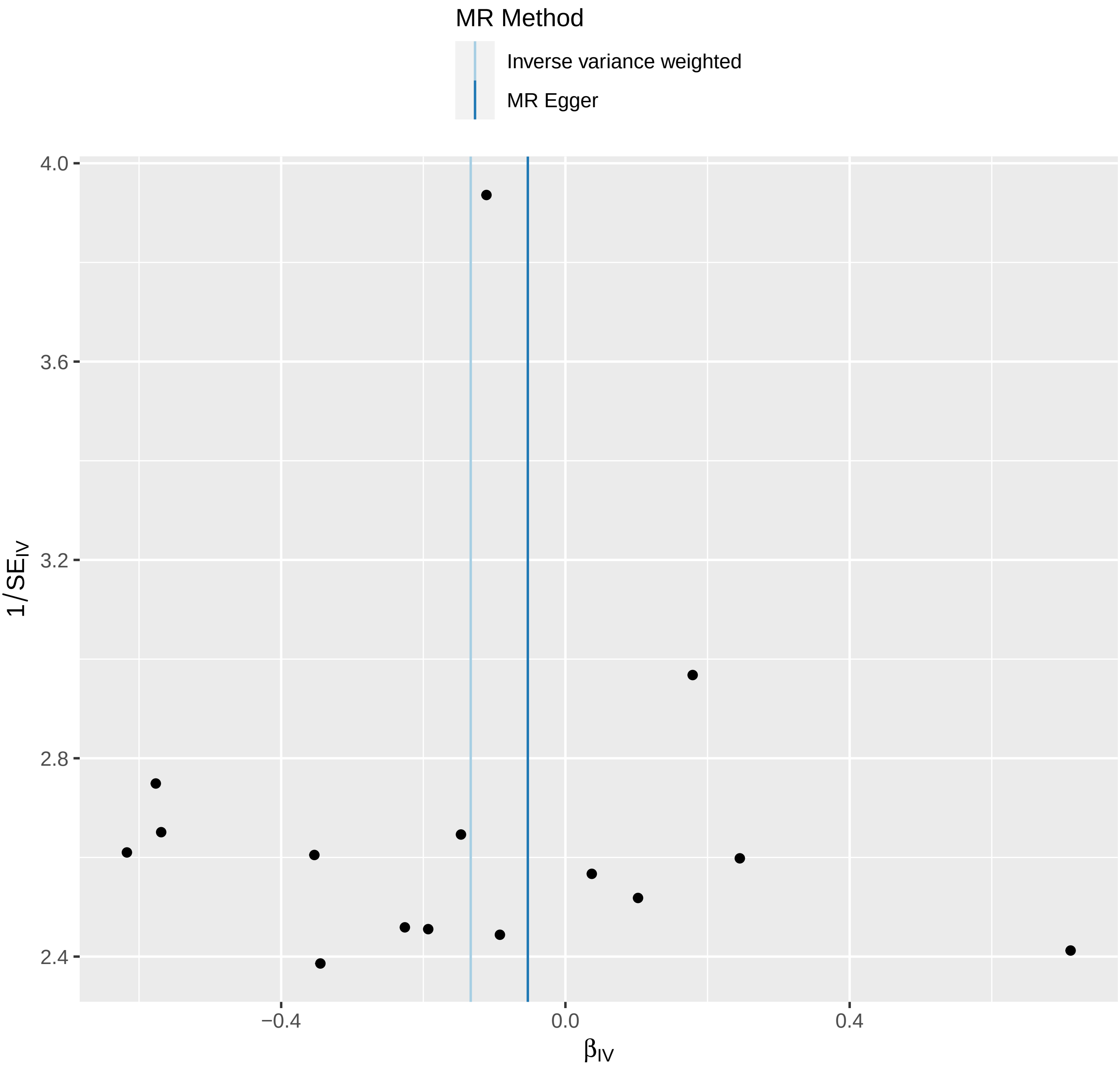

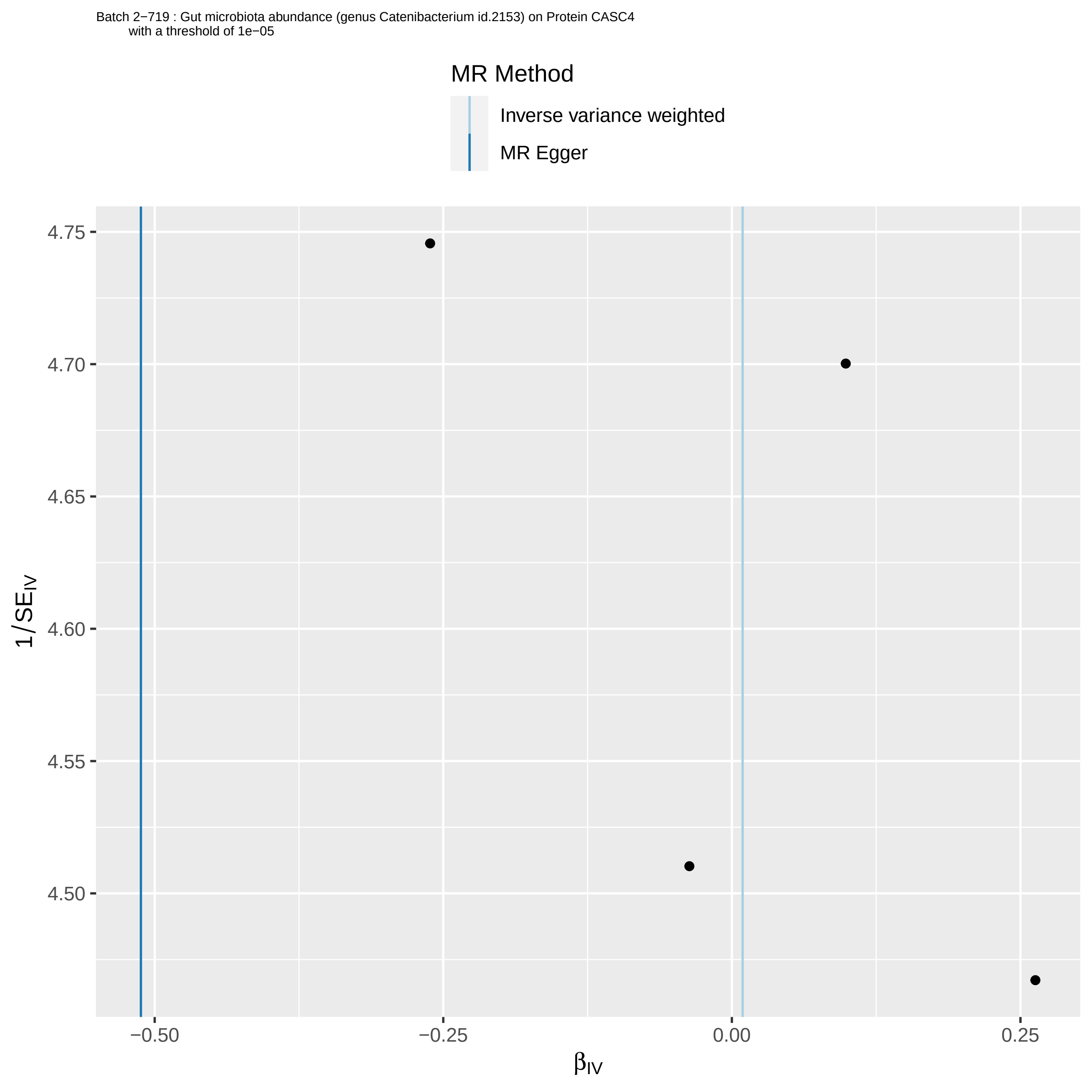

Batch 2−719 : Gut microbiota abundance (genus Catenibacterium id.2153) on Protein CASC4
with a threshold of 1e−05
MR Method
Inverse variance weighted
MR Egger
4.75
4.70
4.65
4.60
4.55
4.50
1/SE_IV
−0.50
−0.25
0.00
0.25
β_IV

Batch 2−720 : Gut microbiota abundance (genus Parasutterella id.2892) on Protein CASC4
with a threshold of 1e−05

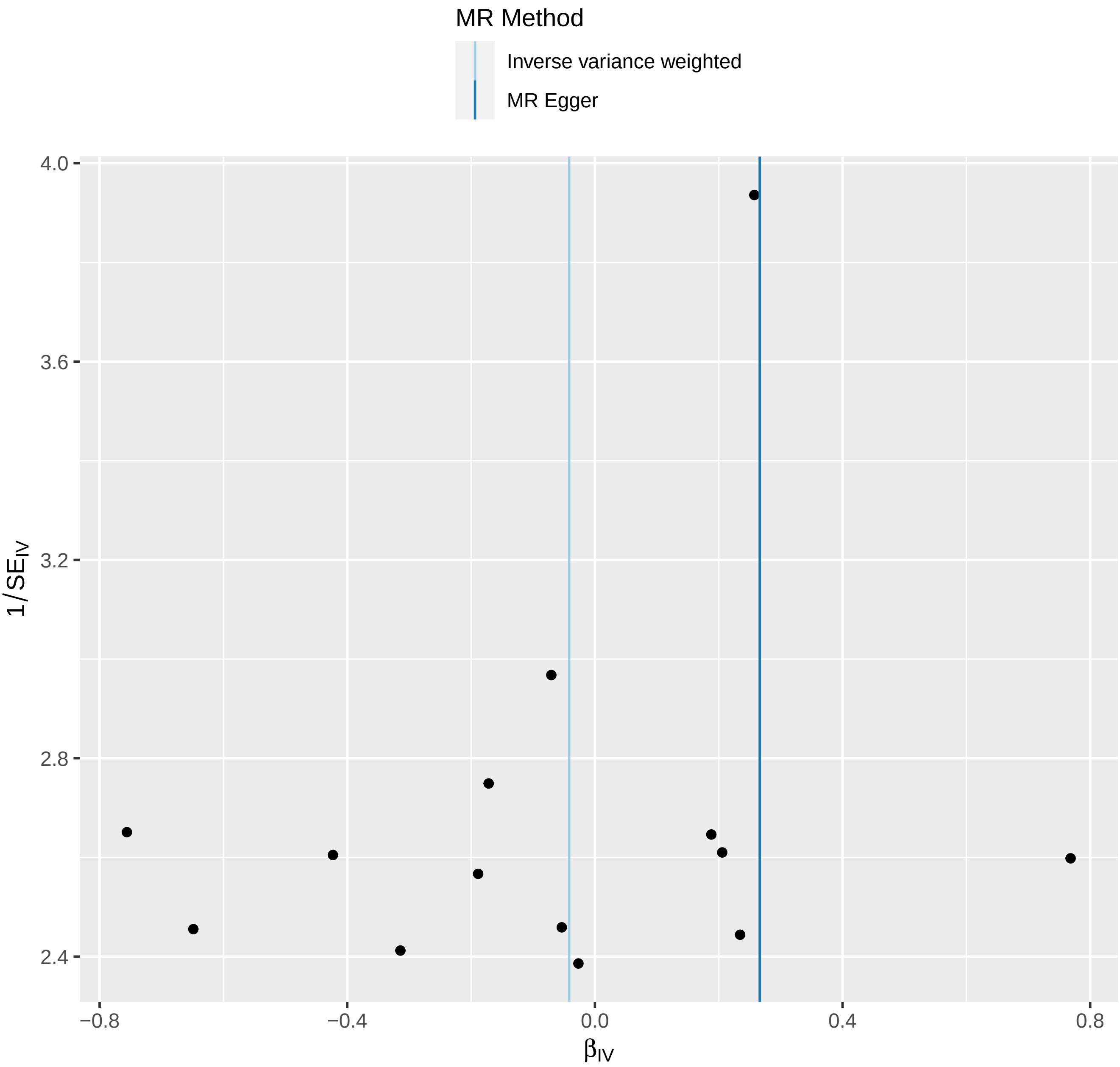

Batch 2−721 : Gut microbiota abundance (genus Catenibacterium id.2153) on Protein CASC4
with a threshold of 1e−05

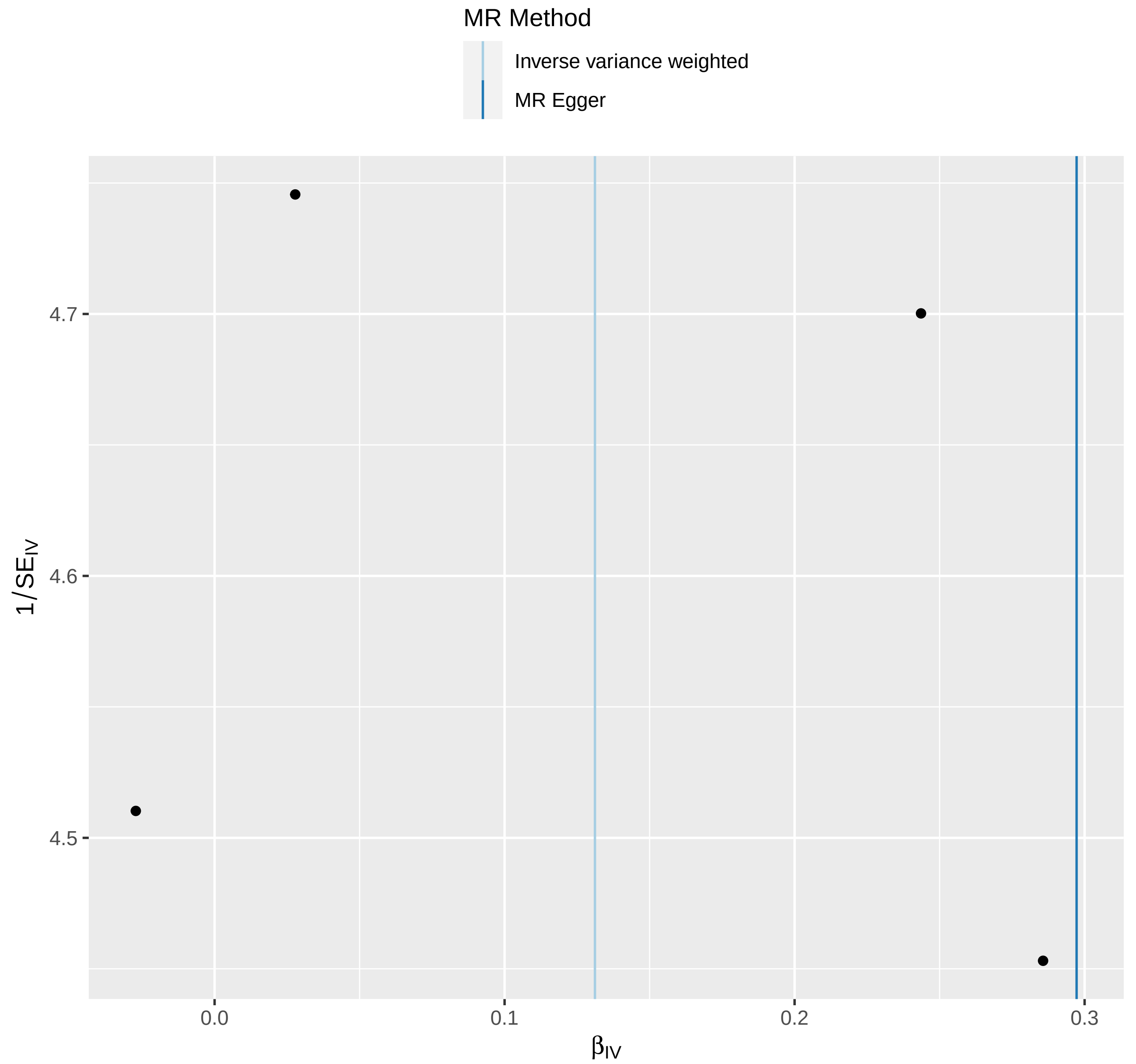

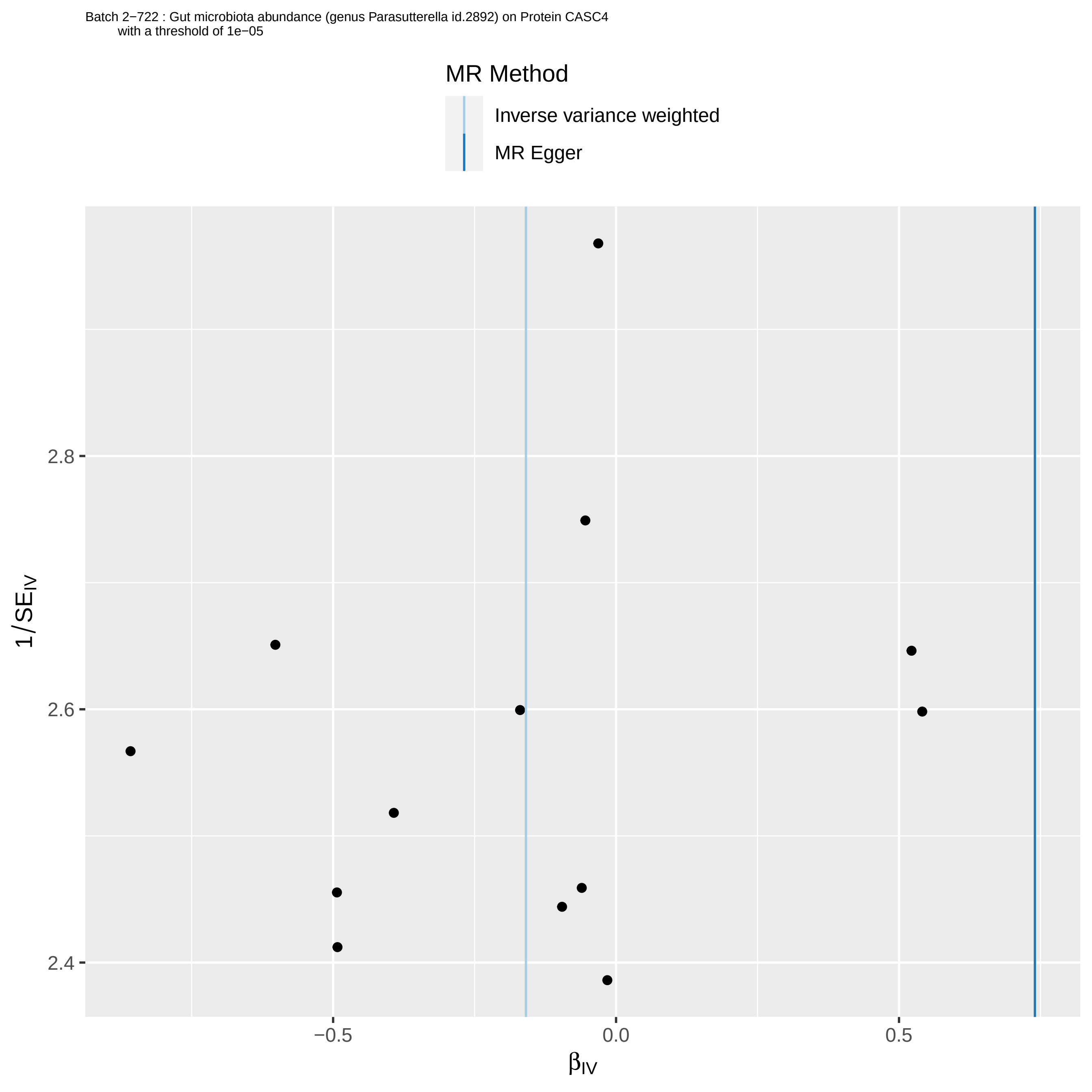

Batch 2−722 : Gut microbiota abundance (genus Parasutterella id.2892) on Protein CASC4
with a threshold of 1e−05
MR Method
Inverse variance weighted
MR Egger
2.8
2.6
2.4
1/SE_IV
−0.5
0.0
0.5
β_IV

Batch 2−723 : Gut microbiota abundance (genus Catenibacterium id.2153) on Caspase−10
with a threshold of 1e−05

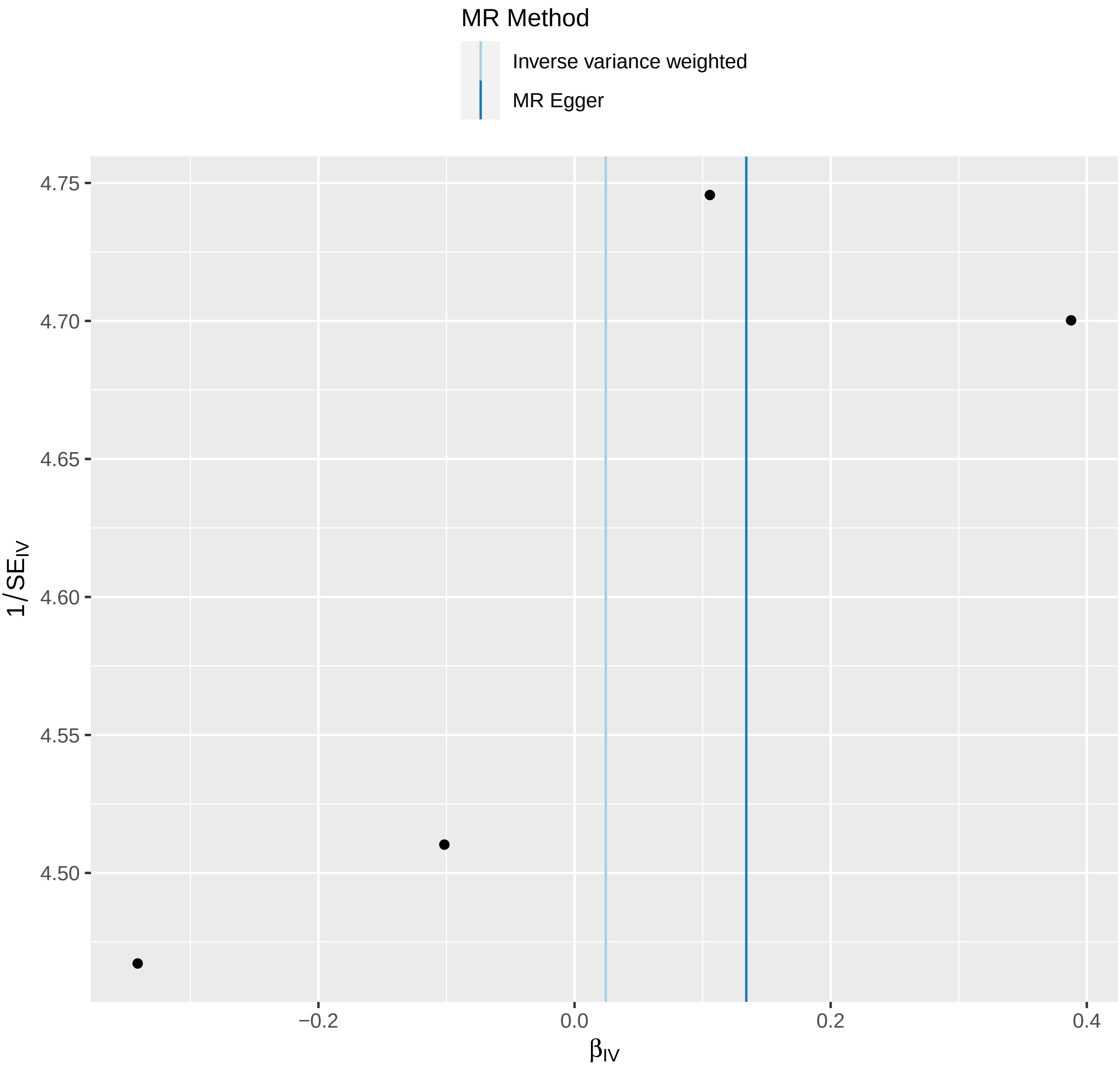

Batch 2−724 : Gut microbiota abundance (genus Parasutterella id.2892) on Caspase−10
with a threshold of 1e−05

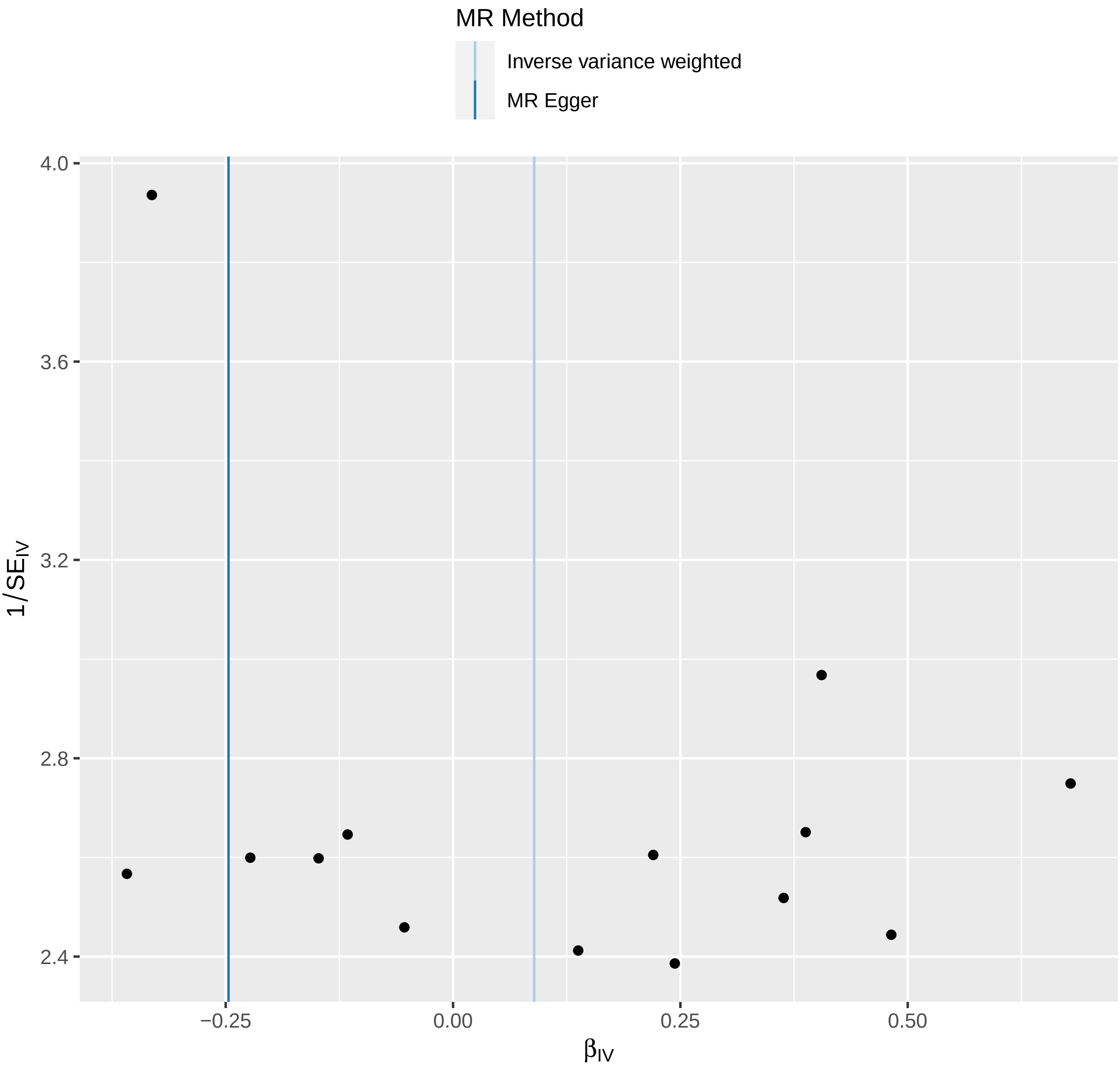

Batch 2−725 : Gut microbiota abundance (genus Catenibacterium id.2153) on Caspase−2
with a threshold of 1e−05

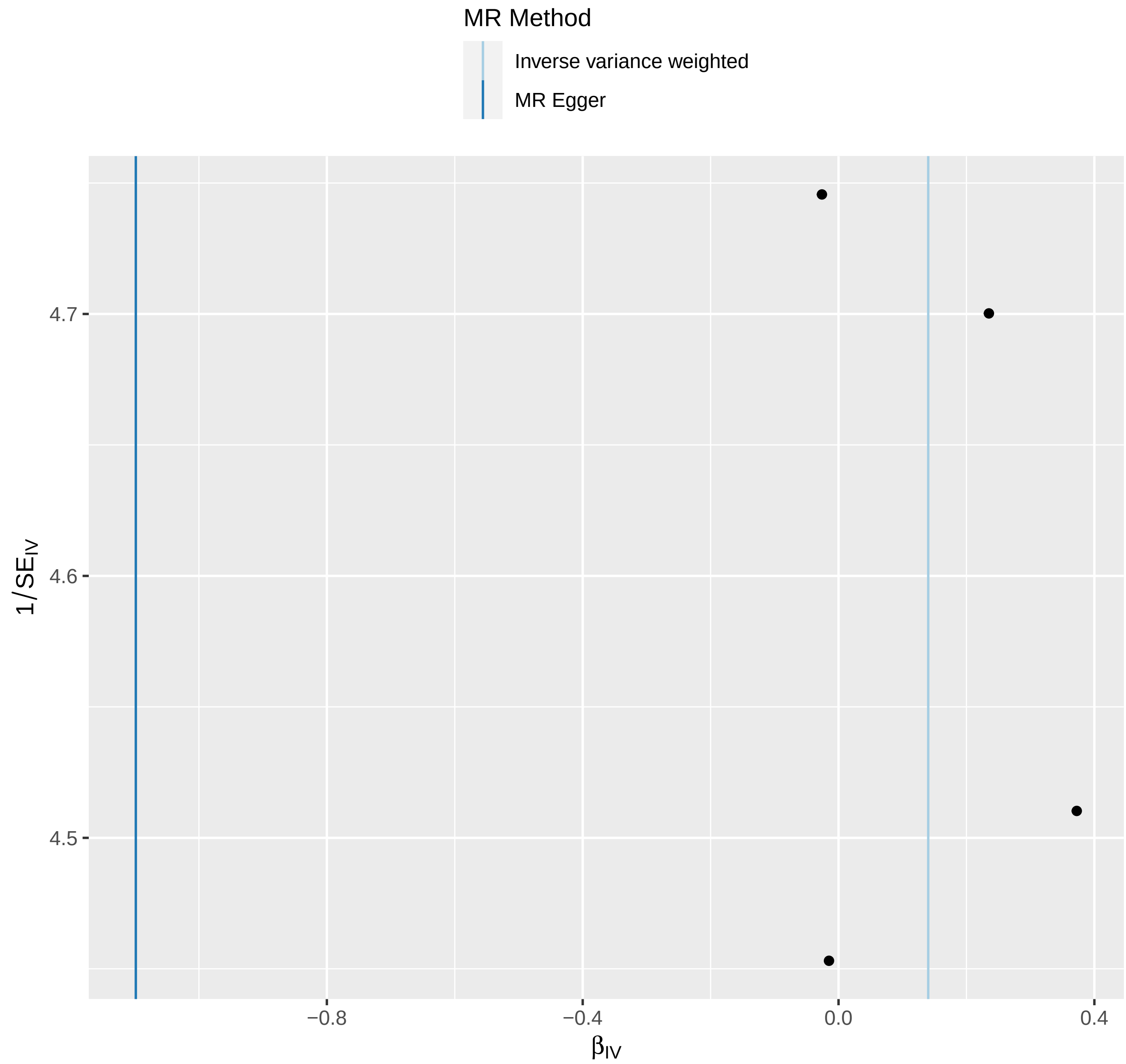

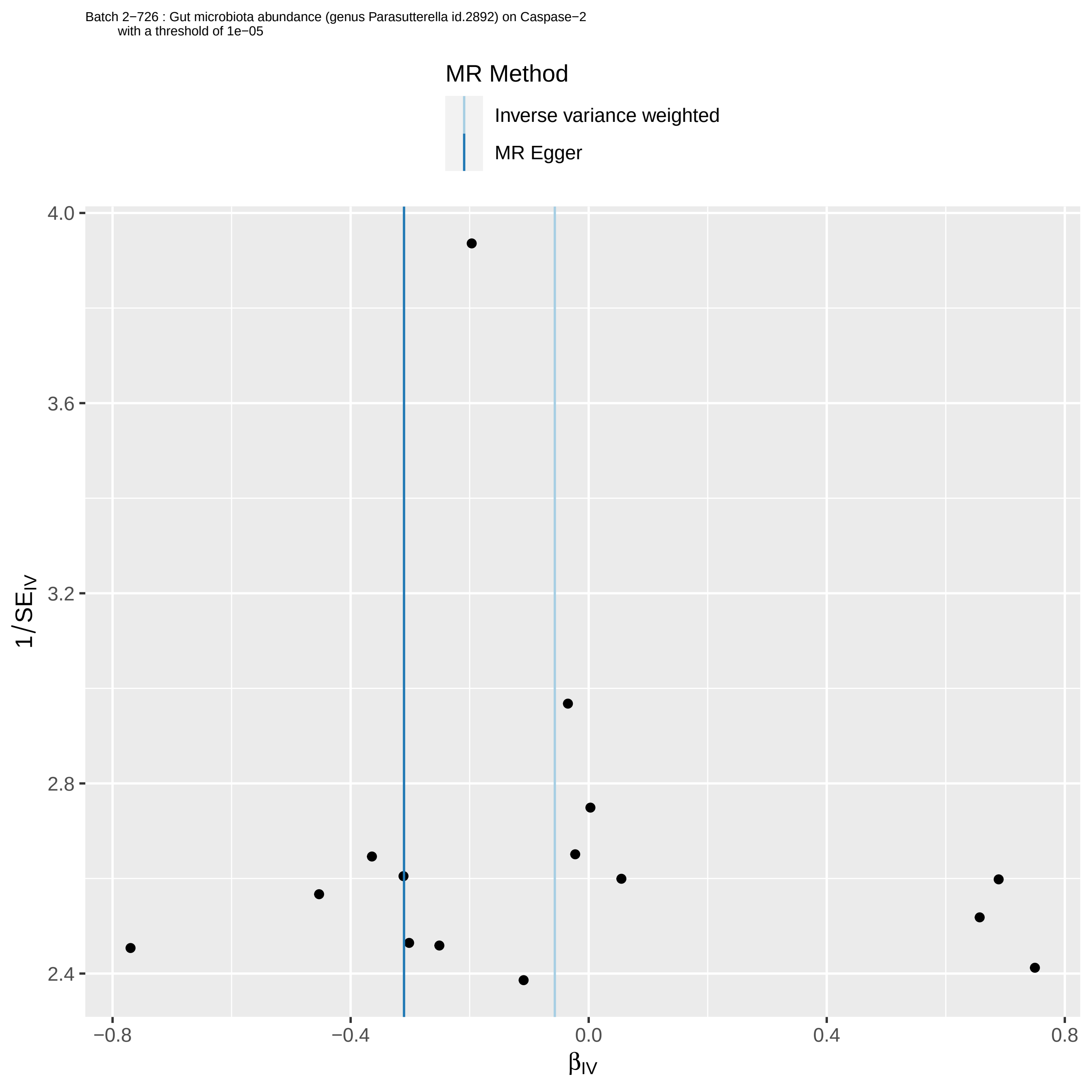

Batch 2−726 : Gut microbiota abundance (genus Parasutterella id.2892) on Caspase−2
with a threshold of 1e−05
MR Method
Inverse variance weighted
MR Egger
4.0
3.6
3.2
2.8
2.4
$1/SE_{IV}$
−0.8
−0.4
0.0
0.4
0.8
β_{IV}

Batch 2−727 : Gut microbiota abundance (genus Catenibacterium id.2153) on Caspase−3
with a threshold of 1e−05

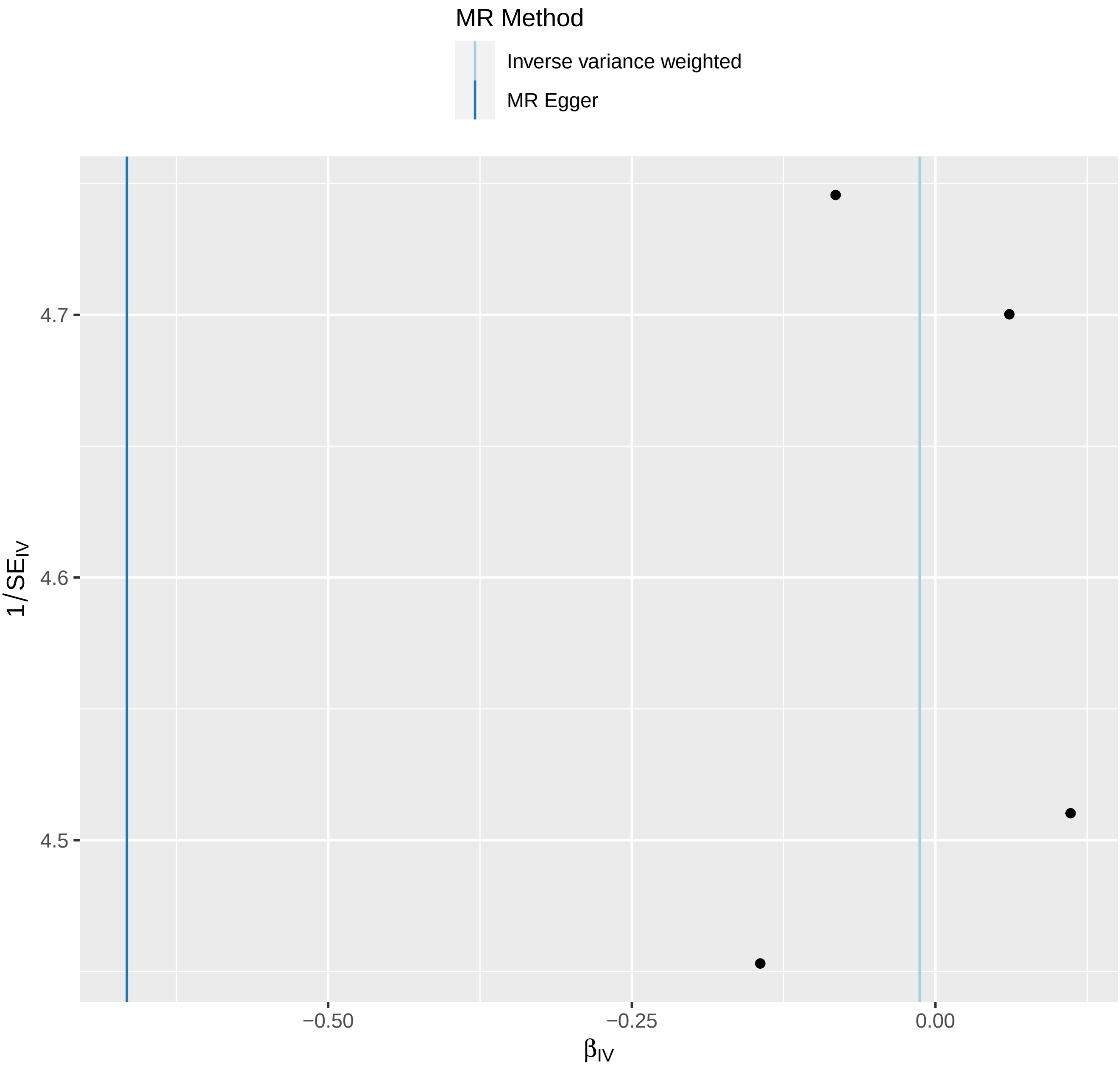

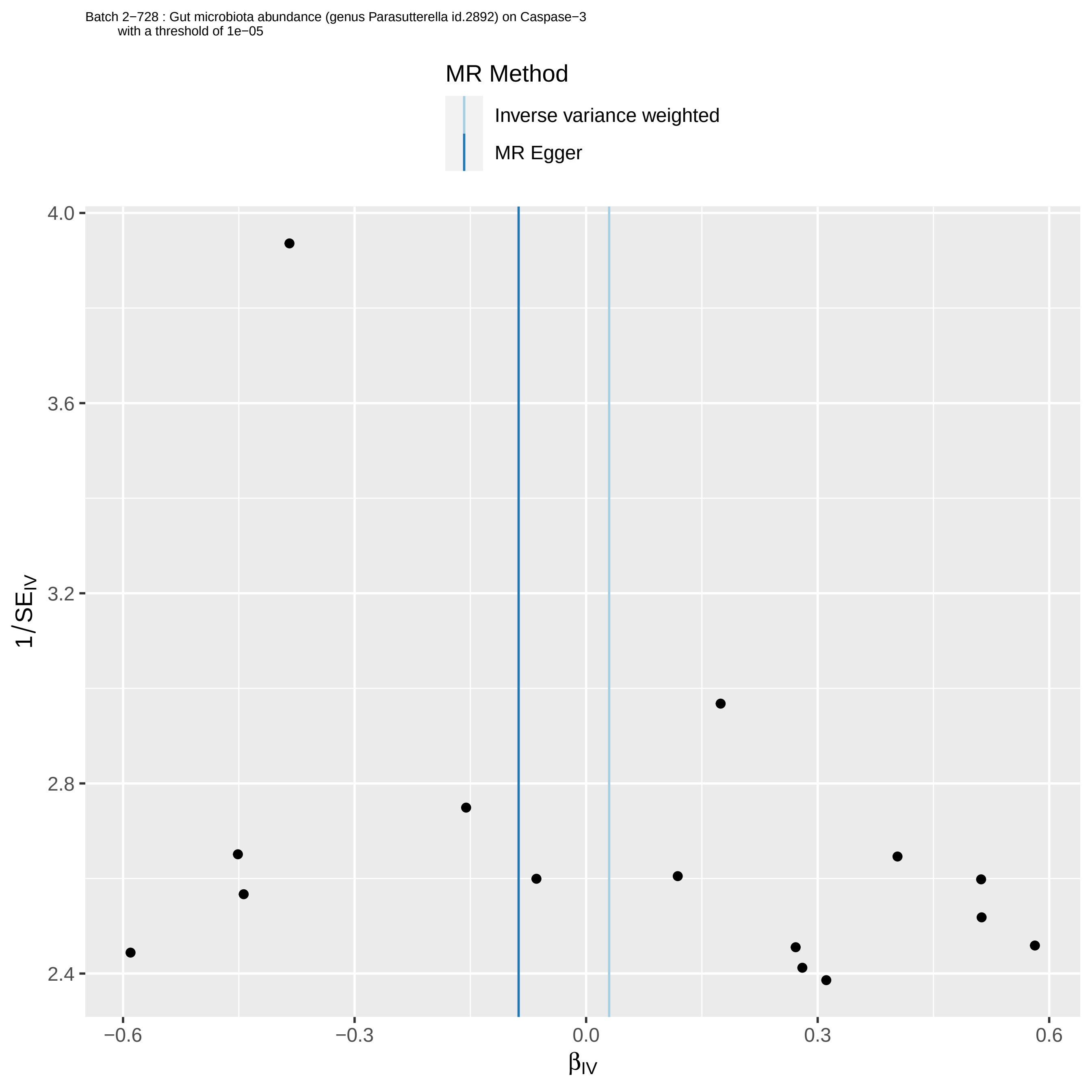
Batch 2−728 : Gut microbiota abundance (genus Parasutterella id.2892) on Caspase−3
with a threshold of 1e−05
MR Method
Inverse variance weighted
MR Egger
4.0
3.6
3.2
2.8
2.4
1/SE_IV
−0.6
−0.3
0.0
0.3
0.6
β_IV

Batch 2−729 : Gut microbiota abundance (genus Catenibacterium id.2153) on Cas scaffolding protein family member 4 with a threshold of 1e−05

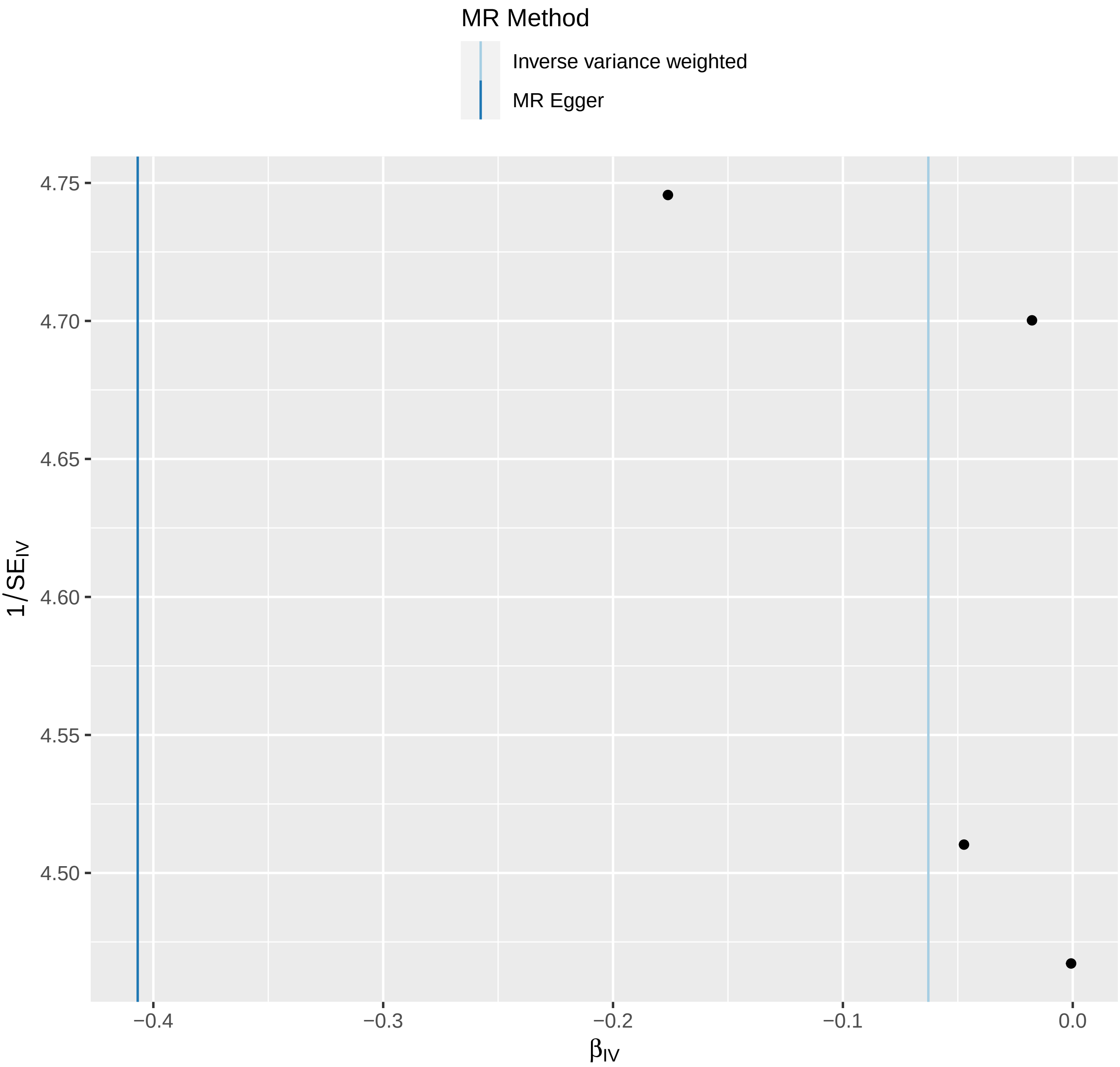

Batch 2−730 : Gut microbiota abundance (genus Parasutterella id.2892) on Cas scaffolding protein family member 4
with a threshold of 1e−05

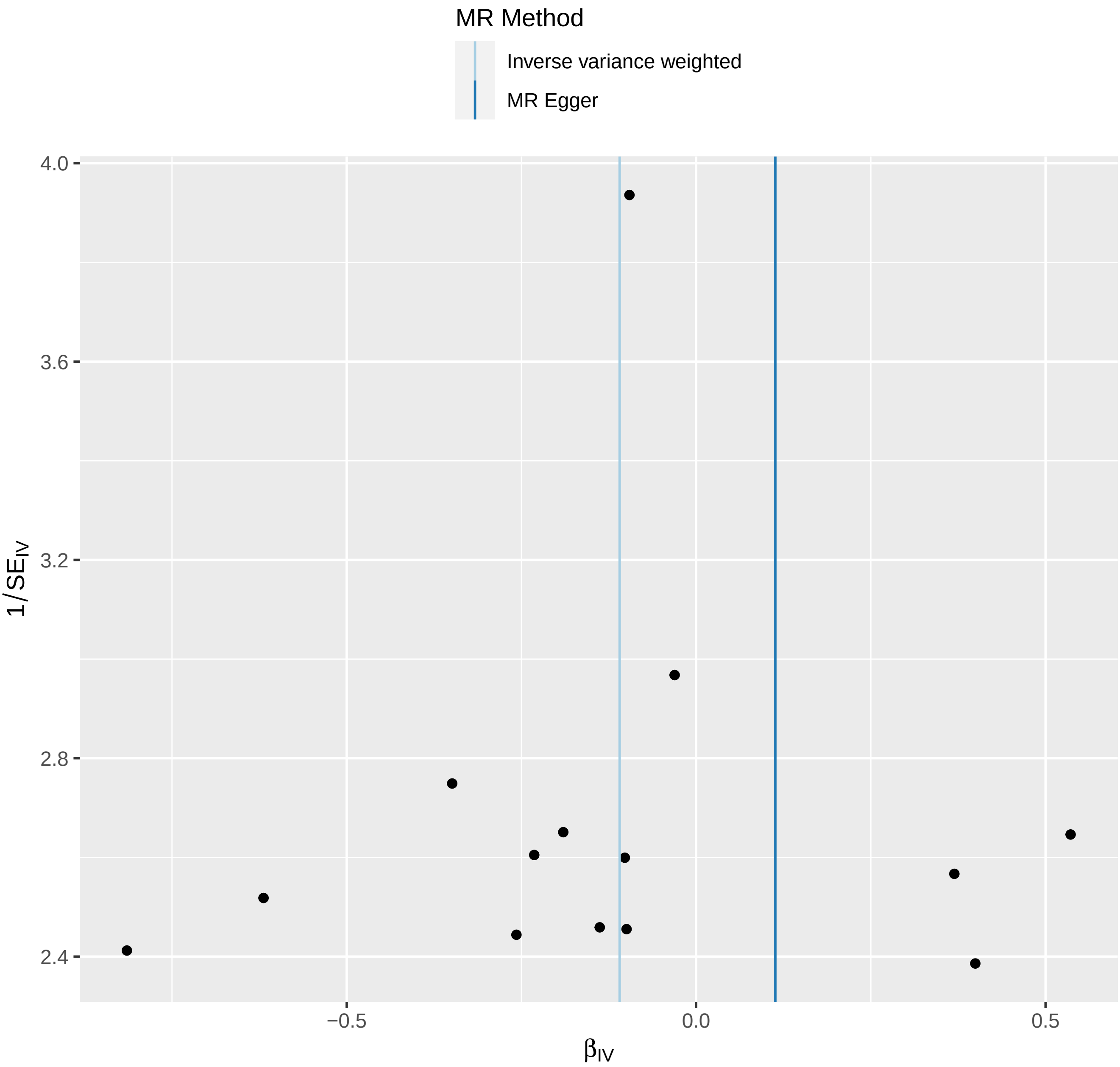

Batch 2−731 : Gut microbiota abundance (genus Catenibacterium id.2153) on Calpastatin
with a threshold of 1e−05

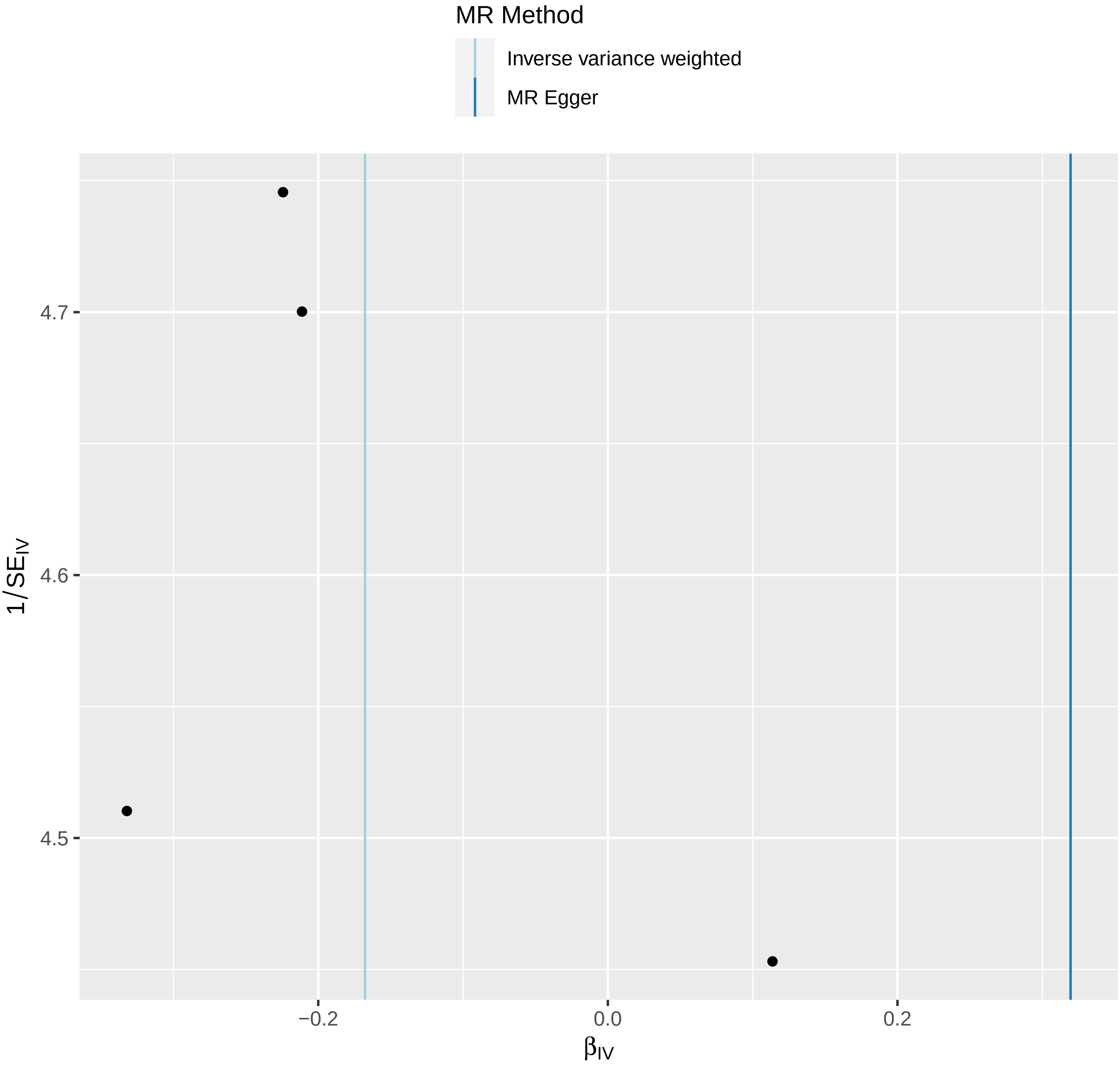

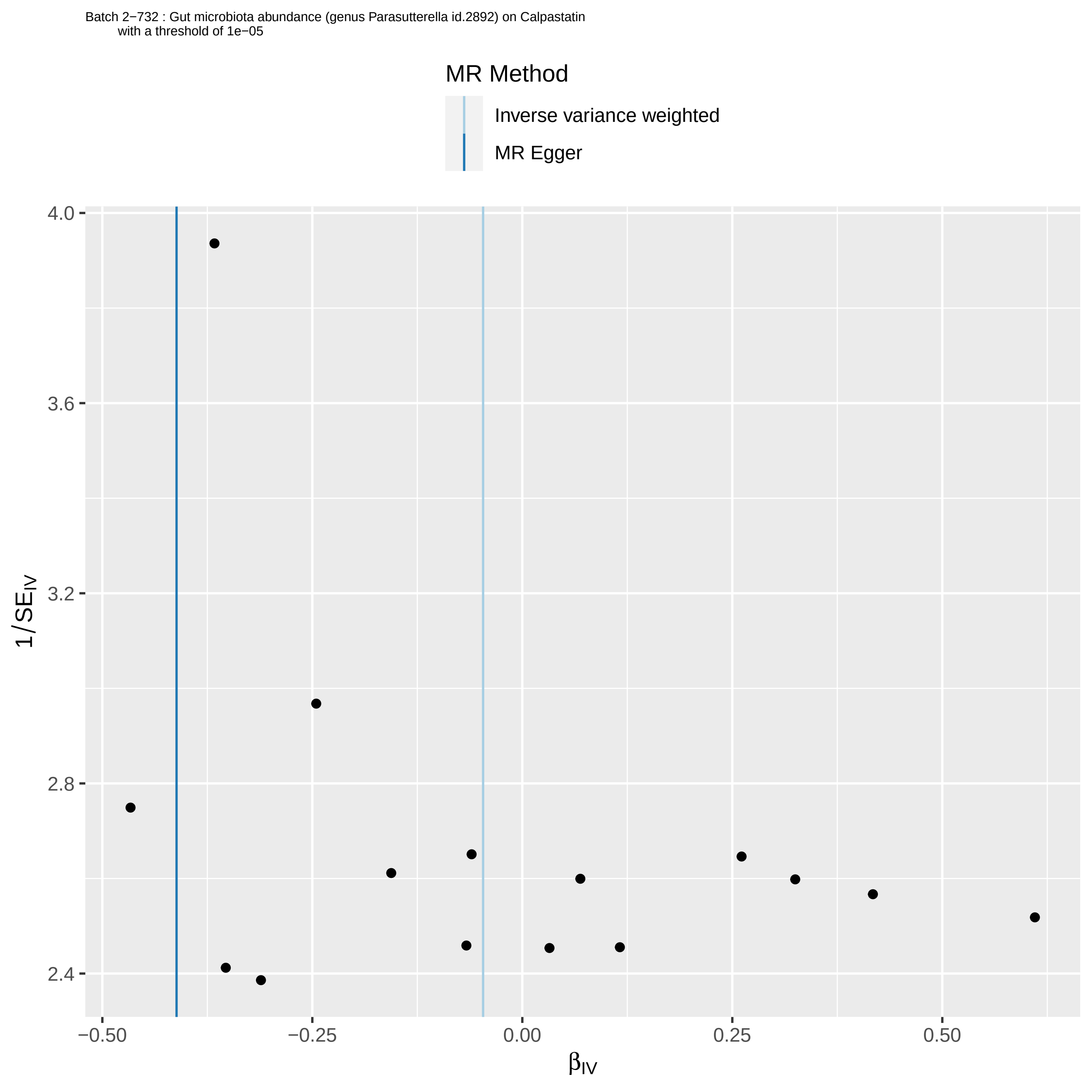

Batch 2−732 : Gut microbiota abundance (genus Parasutterella id.2892) on Calpastatin
with a threshold of 1e−05
MR Method
Inverse variance weighted
MR Egger
4.0
3.6
3.2
2.8
2.4
1/SE_IV
−0.50
−0.25
0.00
0.25
0.50
β_IV

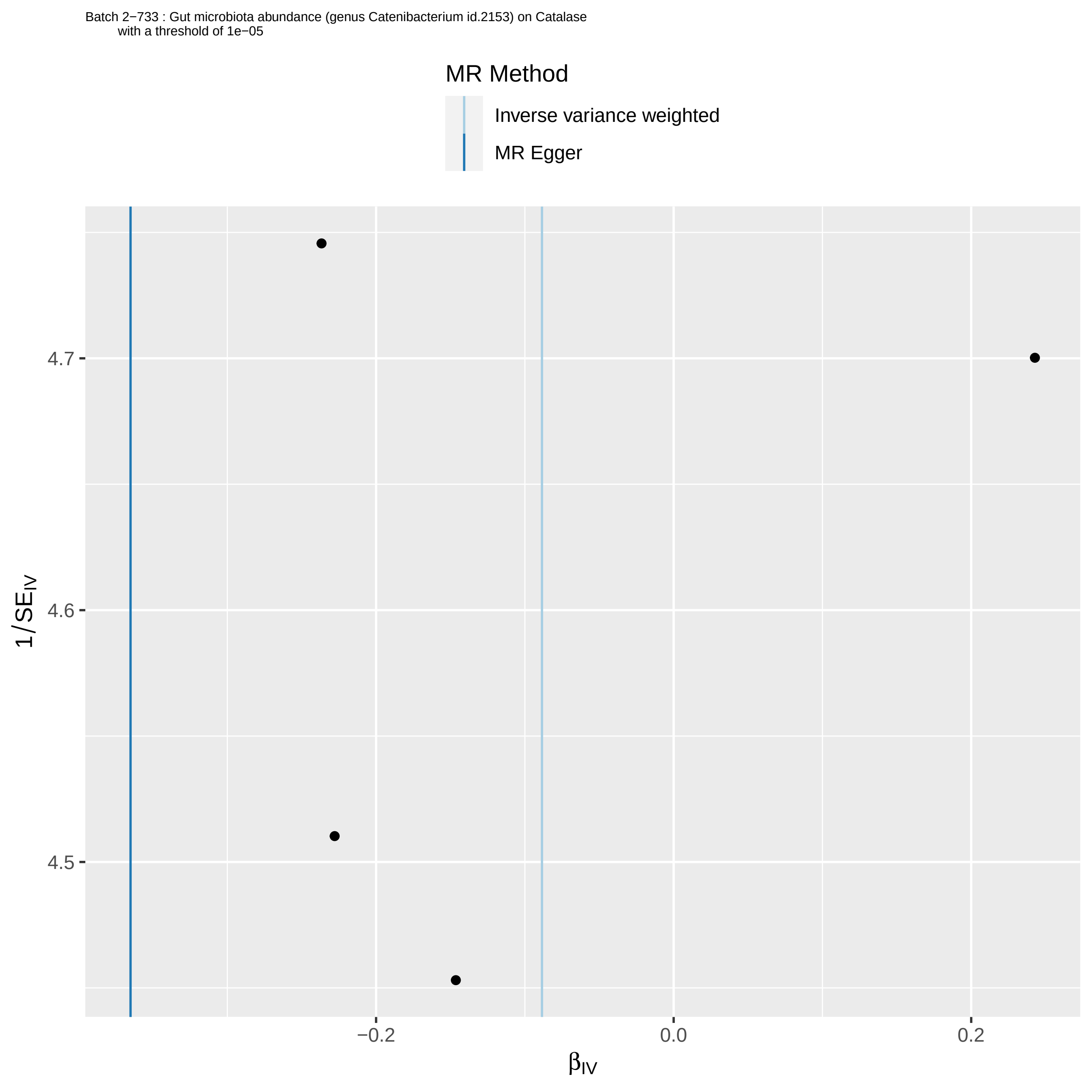
Batch 2−733 : Gut microbiota abundance (genus Catenibacterium id.2153) on Catalase
with a threshold of 1e−05
MR Method
Inverse variance weighted
MR Egger
4.7
4.6
4.5
1/SE$_{IV}$
−0.2
0.0
0.2
β_{IV}

Batch 2−734 : Gut microbiota abundance (genus Parasutterella id.2892) on Catalase
with a threshold of 1e−05

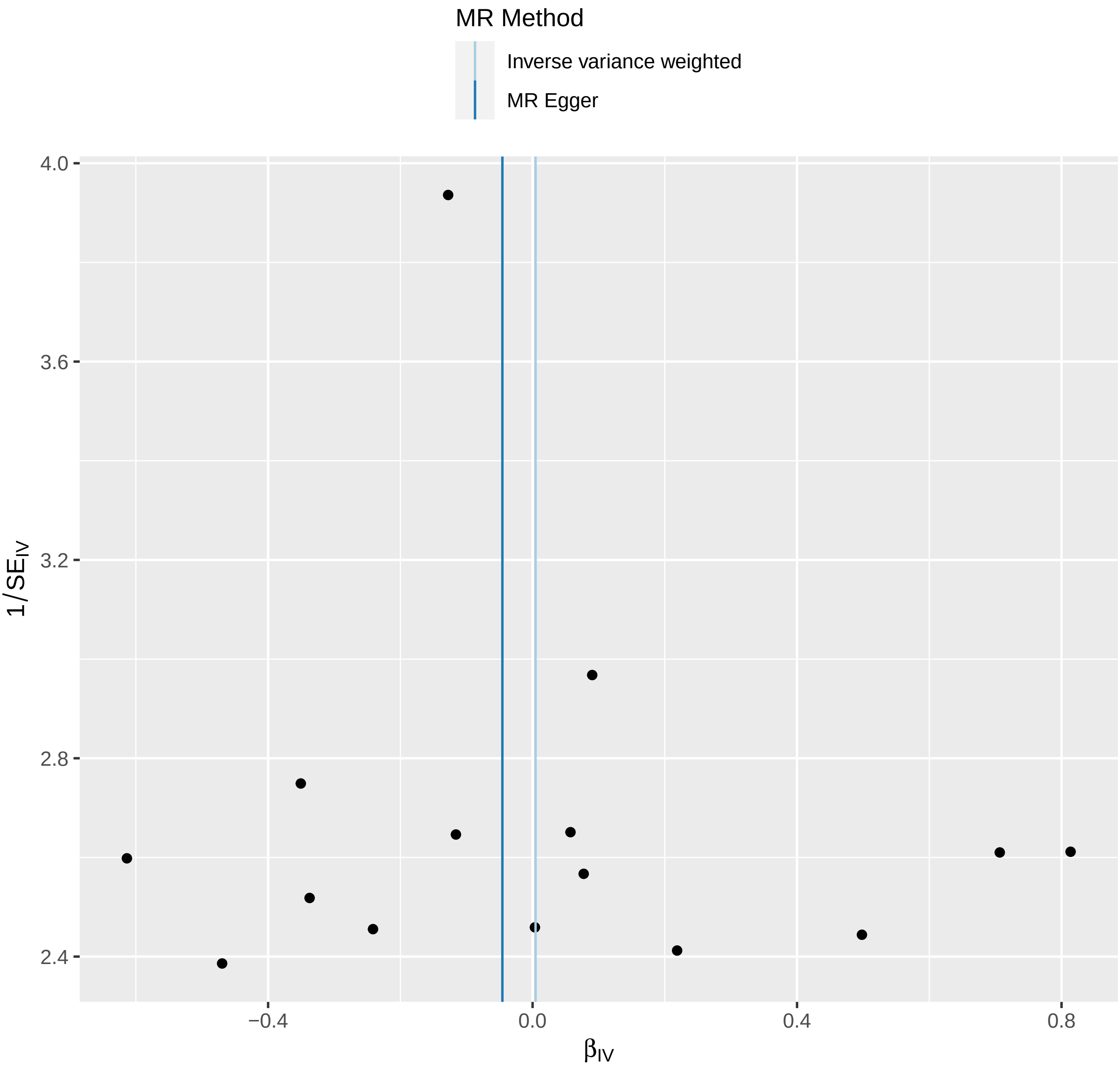

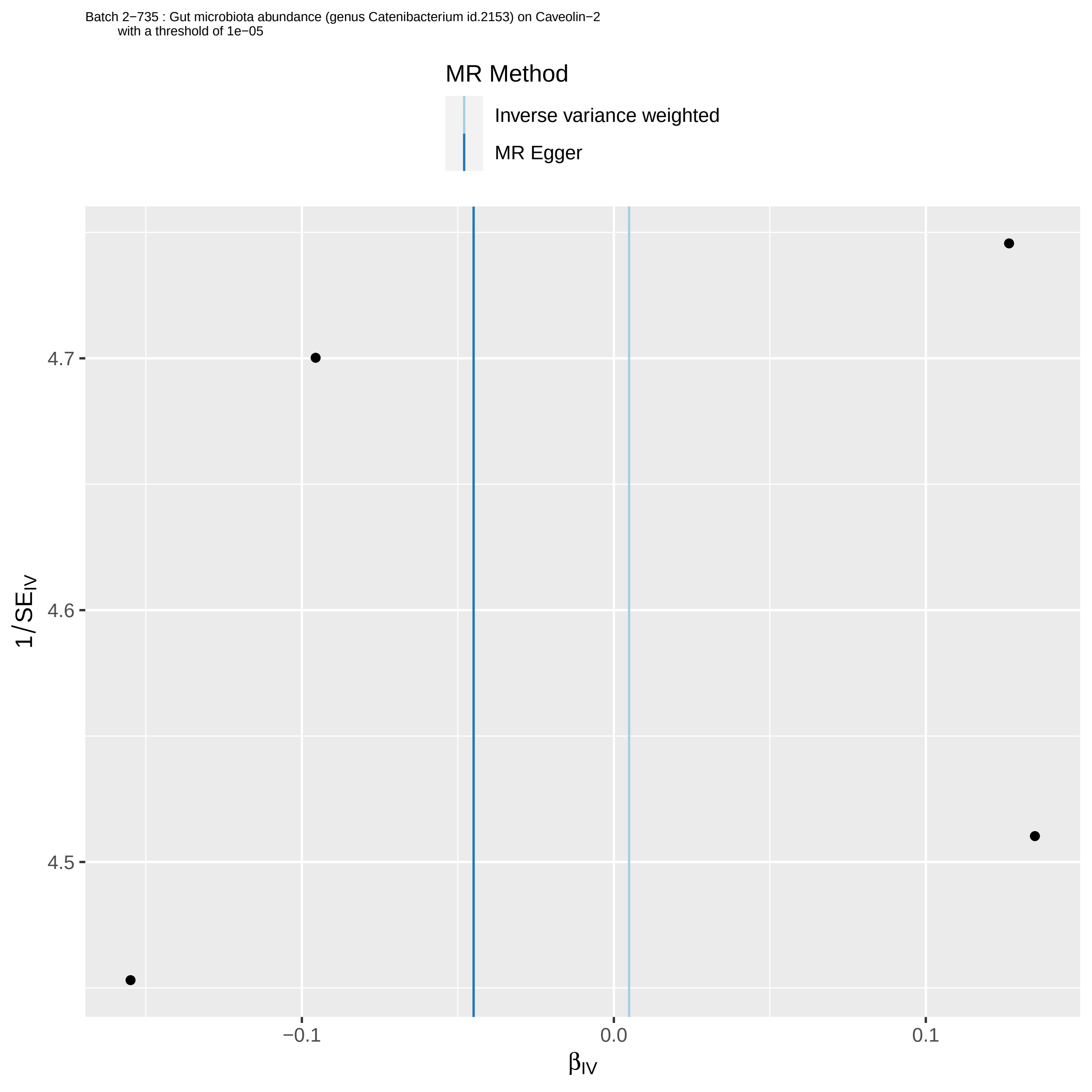
Batch 2−735 : Gut microbiota abundance (genus Catenibacterium id.2153) on Caveolin−2
with a threshold of 1e−05
MR Method
Inverse variance weighted
MR Egger
4.7
4.6
4.5
1/SEIV
−0.1
0.0
0.1
βIV

Batch 2−736 : Gut microbiota abundance (genus Parasutterella id.2892) on Caveolin−2
with a threshold of 1e−05

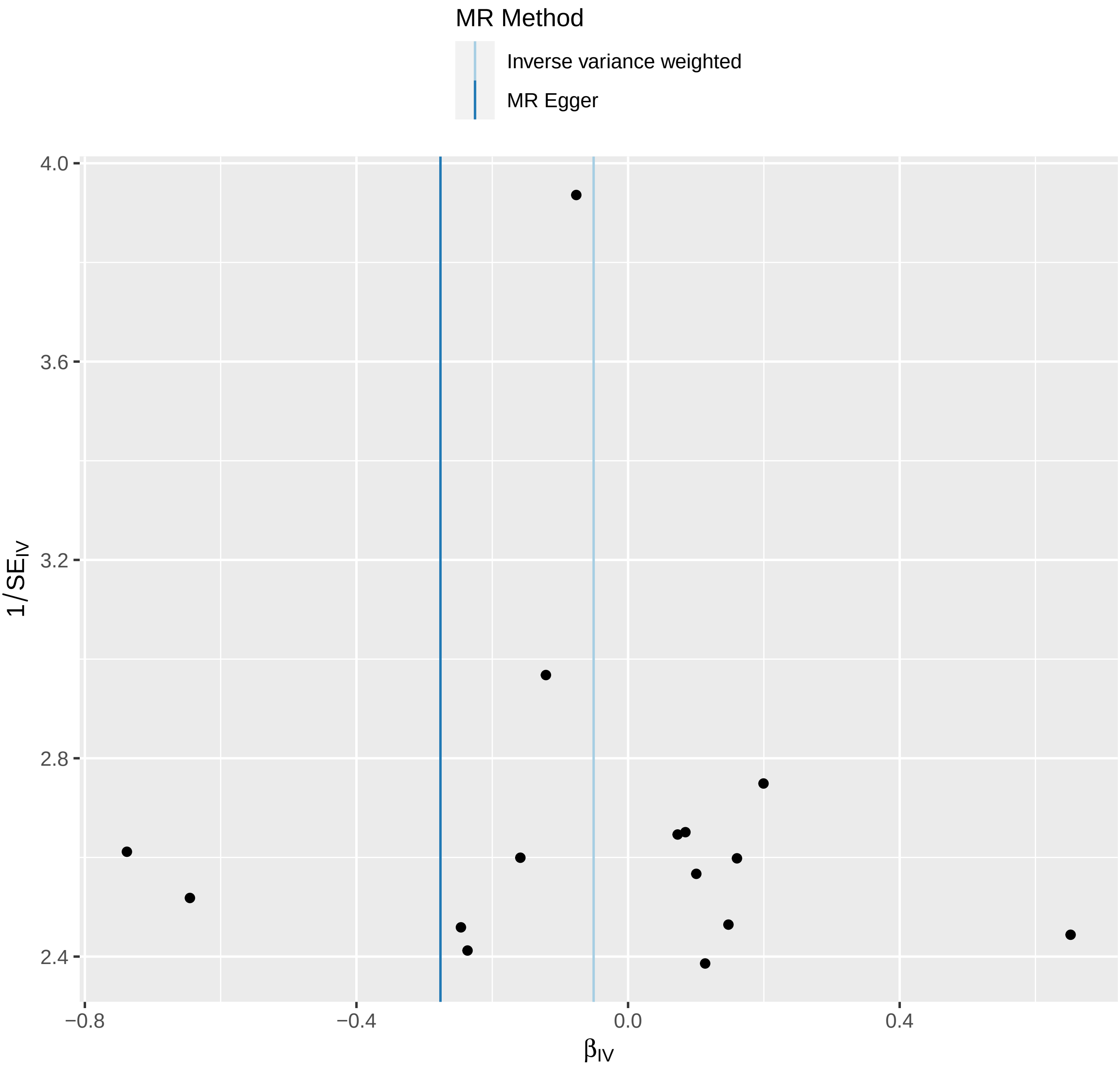

Batch 2−737 : Gut microbiota abundance (genus Catenibacterium id.2153) on Core−binding factor subunit beta
with a threshold of 1e−05

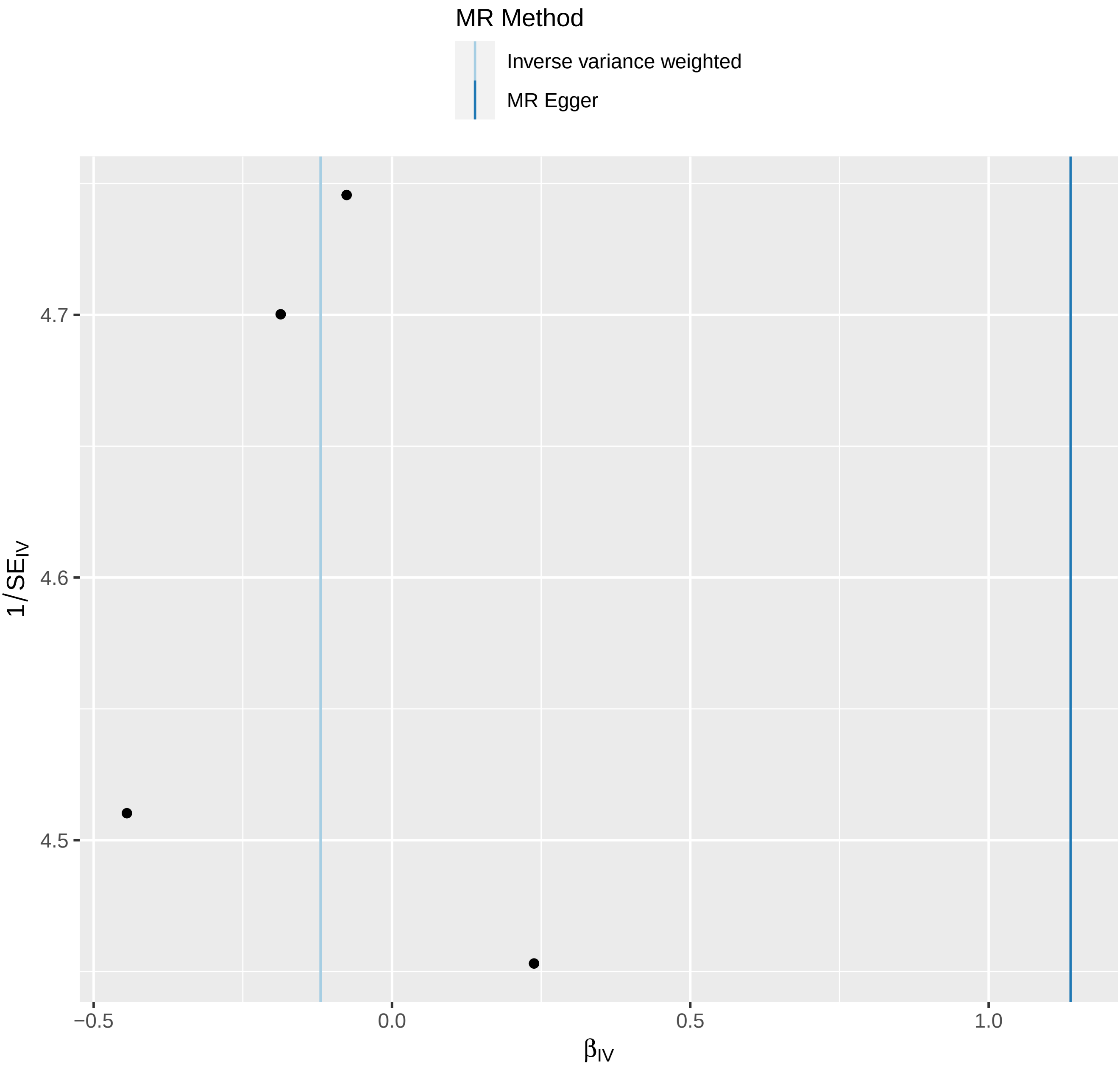

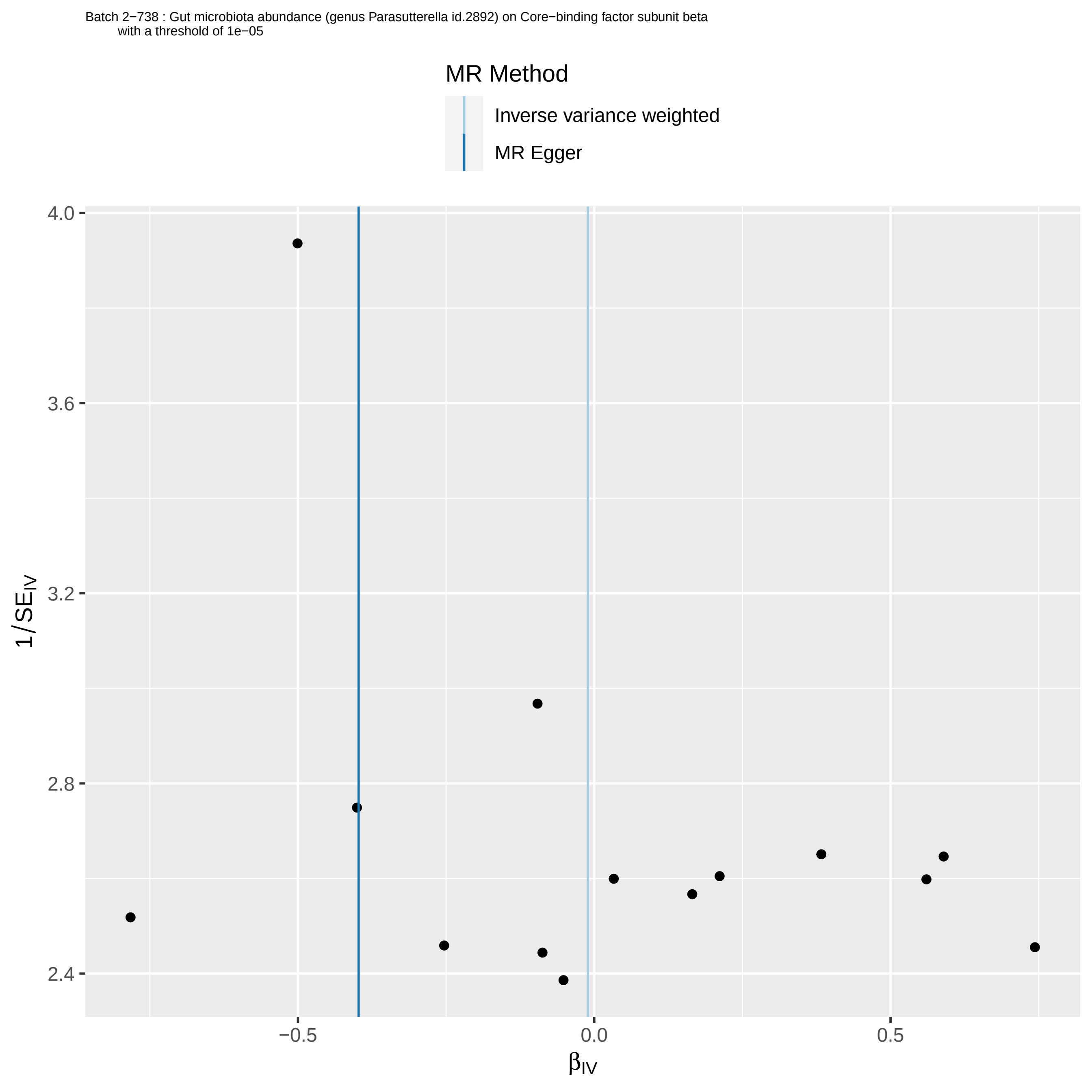
Batch 2−738 : Gut microbiota abundance (genus Parasutterella id.2892) on Core−binding factor subunit beta
with a threshold of 1e−05
MR Method
Inverse variance weighted
MR Egger
4.0
3.6
3.2
2.8
2.4
1/SE_IV
−0.5
0.0
0.5
β_IV

Batch 2−739 : Gut microbiota abundance (genus Catenibacterium id.2153) on E3 ubiquitin−protein ligase CBL
with a threshold of 1e−05

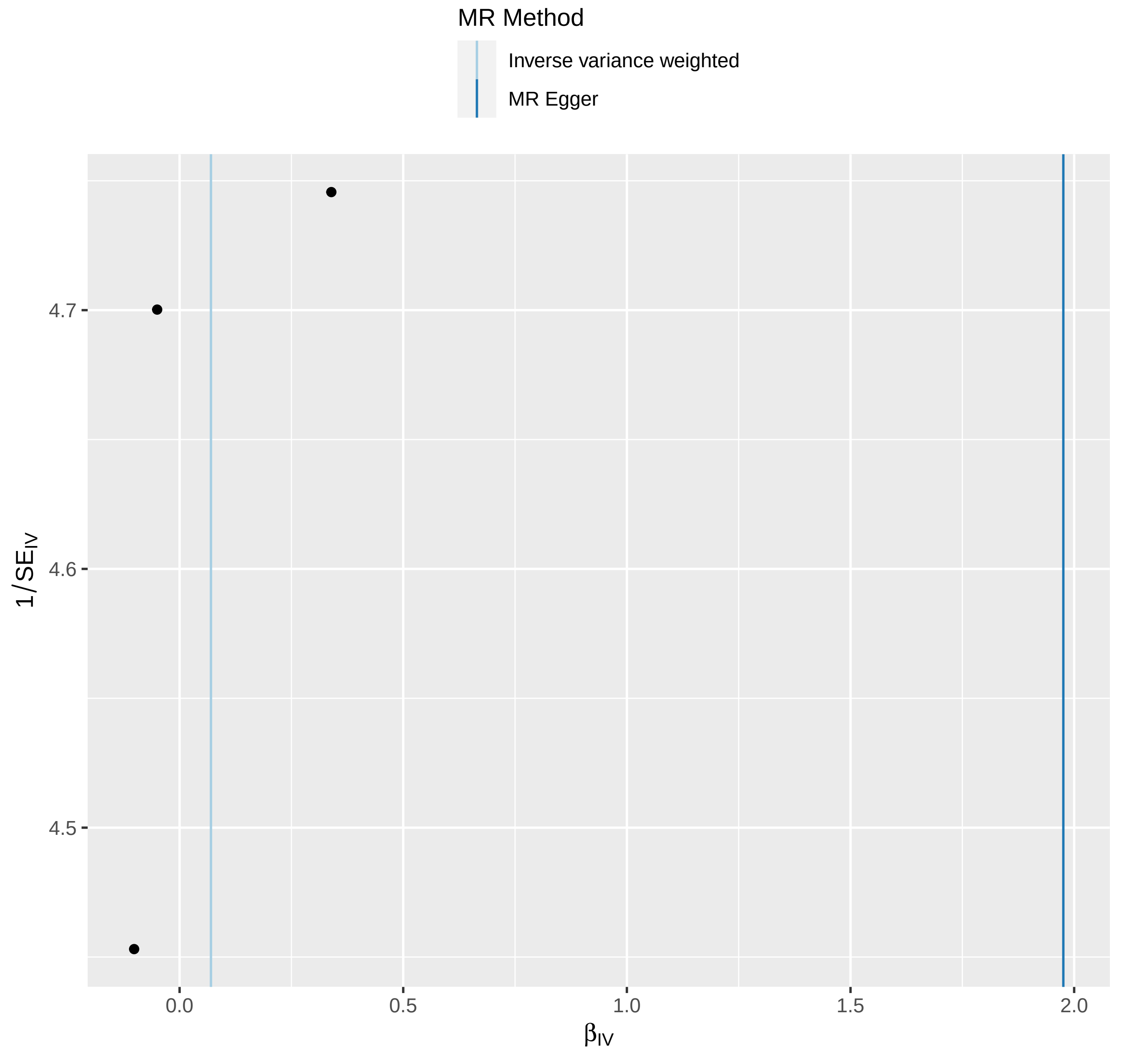

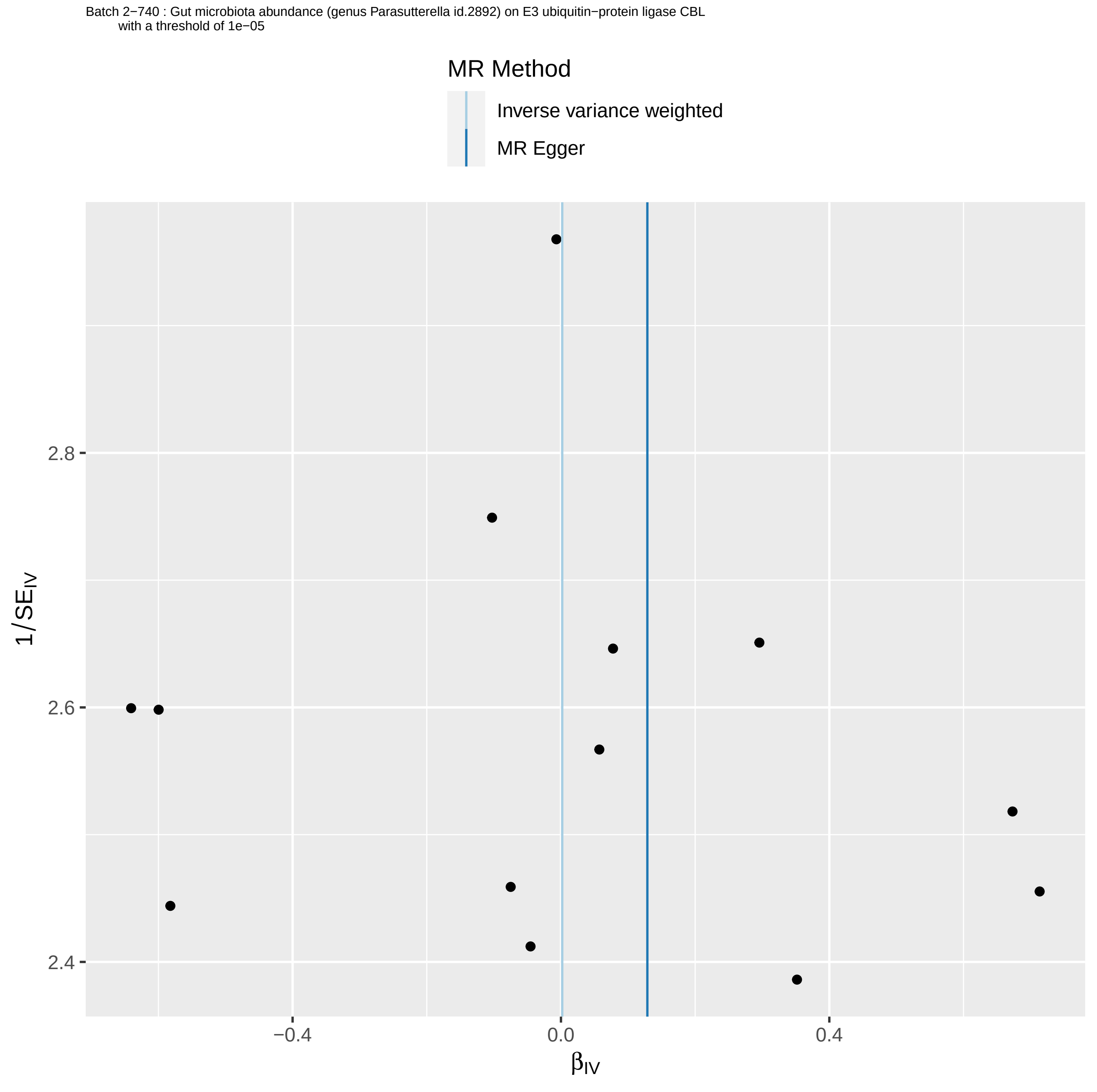

Batch 2−740 : Gut microbiota abundance (genus Parasutterella id.2892) on E3 ubiquitin−protein ligase CBL
with a threshold of 1e−05
MR Method
Inverse variance weighted
MR Egger
2.8
2.6
2.4
1/SE$_{IV}$
−0.4
0.0
0.4
β$_{IV}$

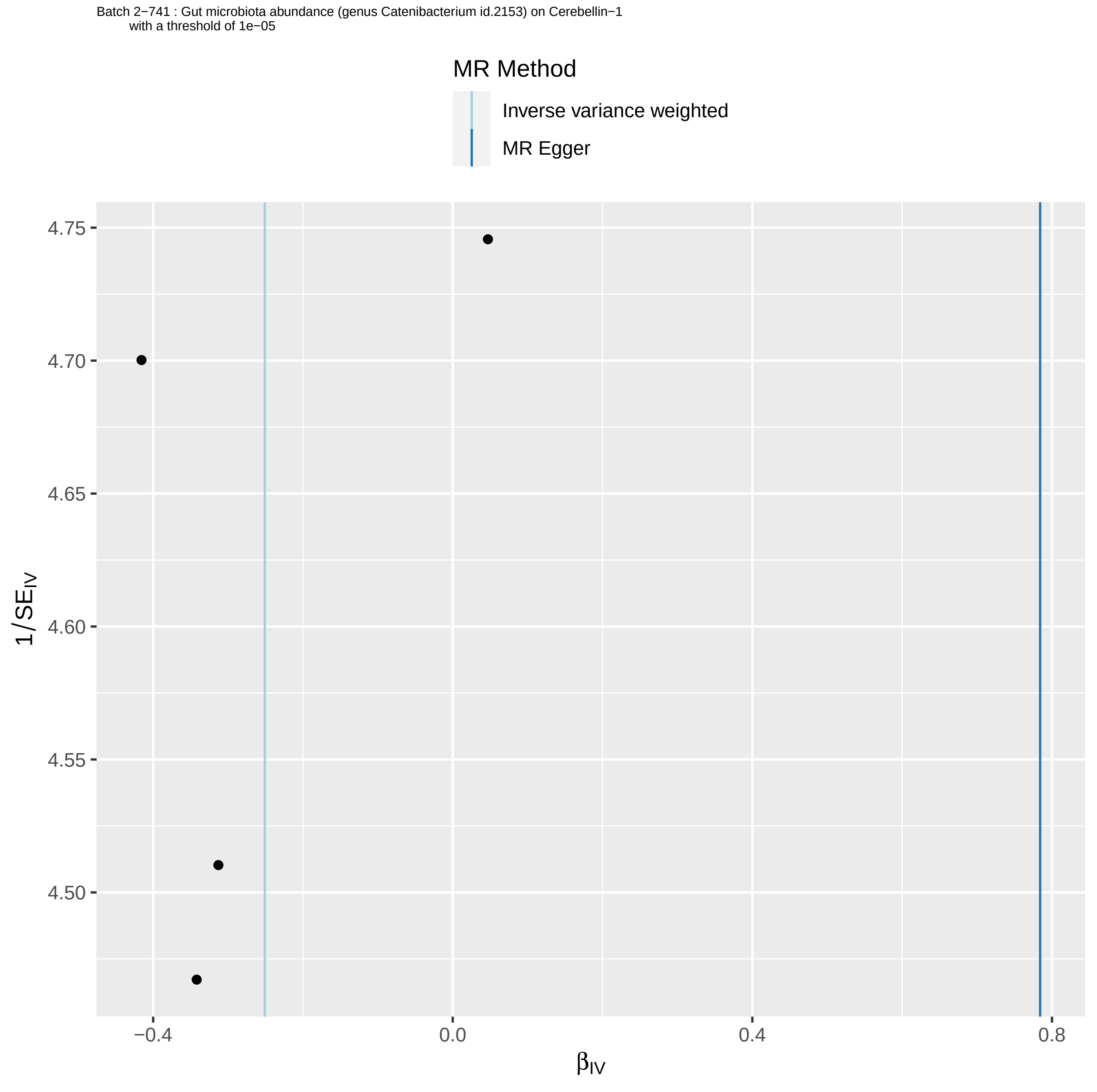

Batch 2−741 : Gut microbiota abundance (genus Catenibacterium id.2153) on Cerebellin−1
with a threshold of 1e−05
MR Method
Inverse variance weighted
MR Egger
4.75
4.70
4.65
4.60
4.55
4.50
1/SE_IV
−0.4
0.0
0.4
0.8
β_IV

Batch 2−742 : Gut microbiota abundance (genus Parasutterella id.2892) on Cerebellin−1
with a threshold of 1e−05

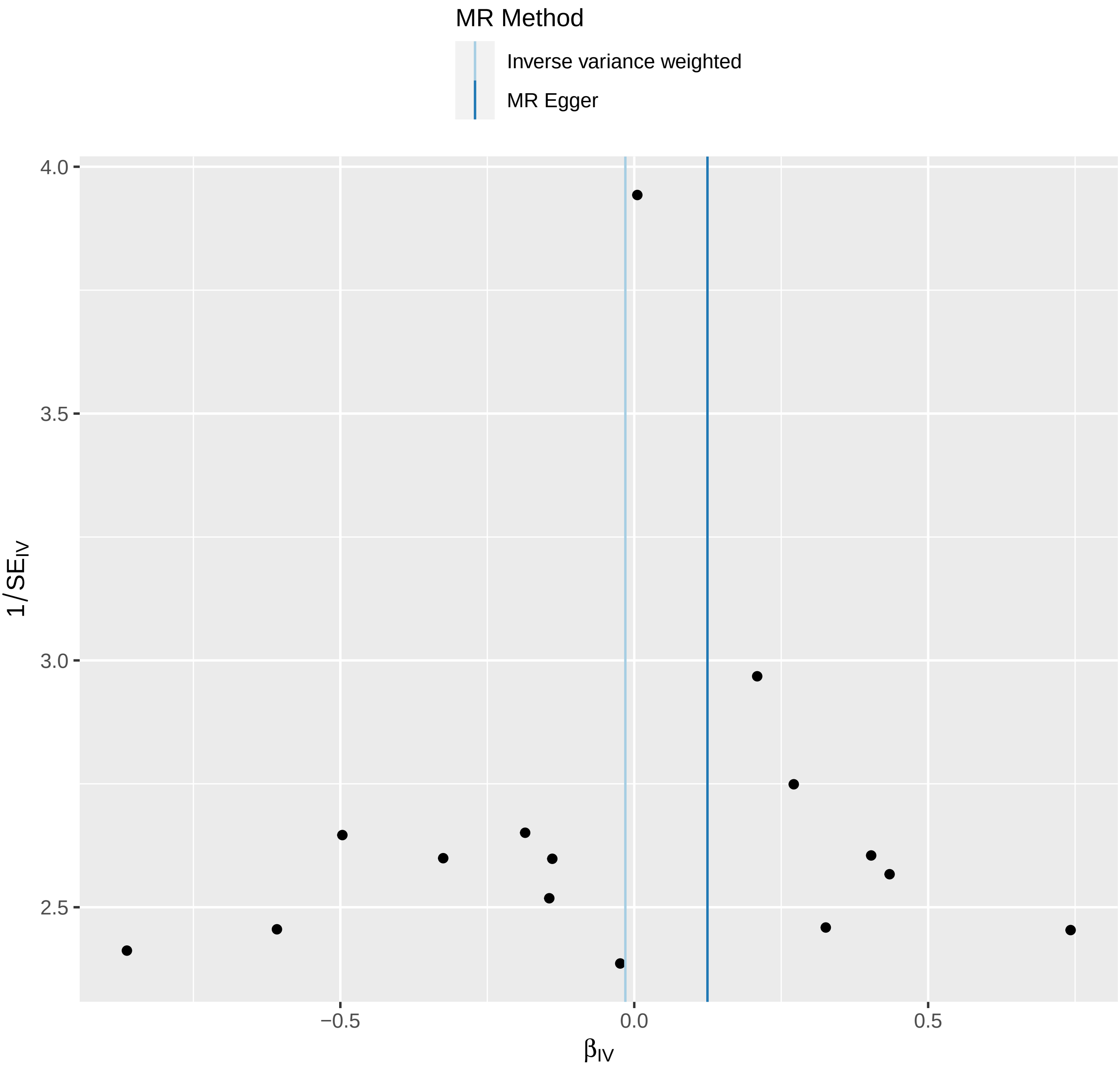

Batch 2−743 : Gut microbiota abundance (genus Catenibacterium id.2153) on Cerebellin−4
with a threshold of 1e−05

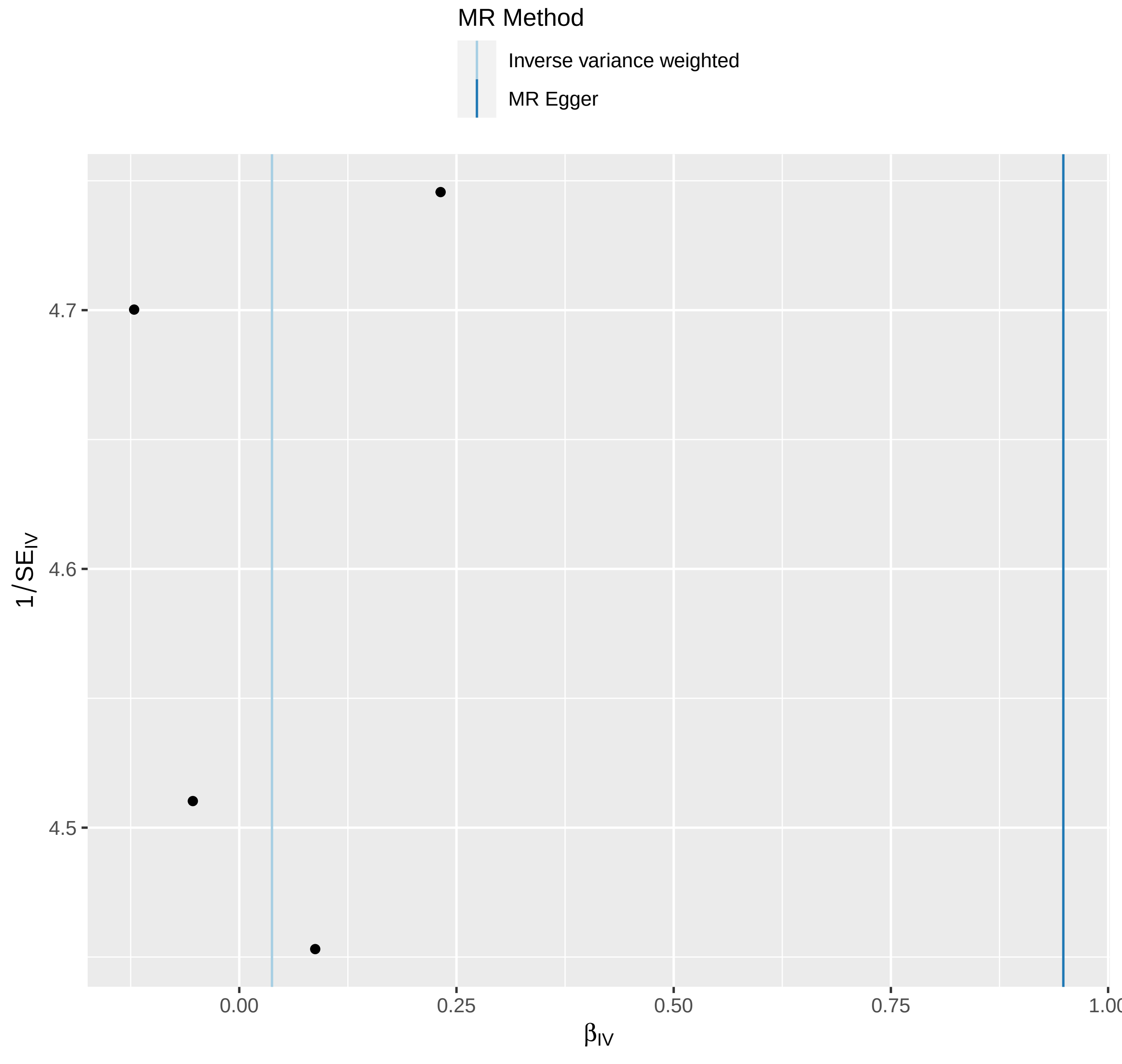

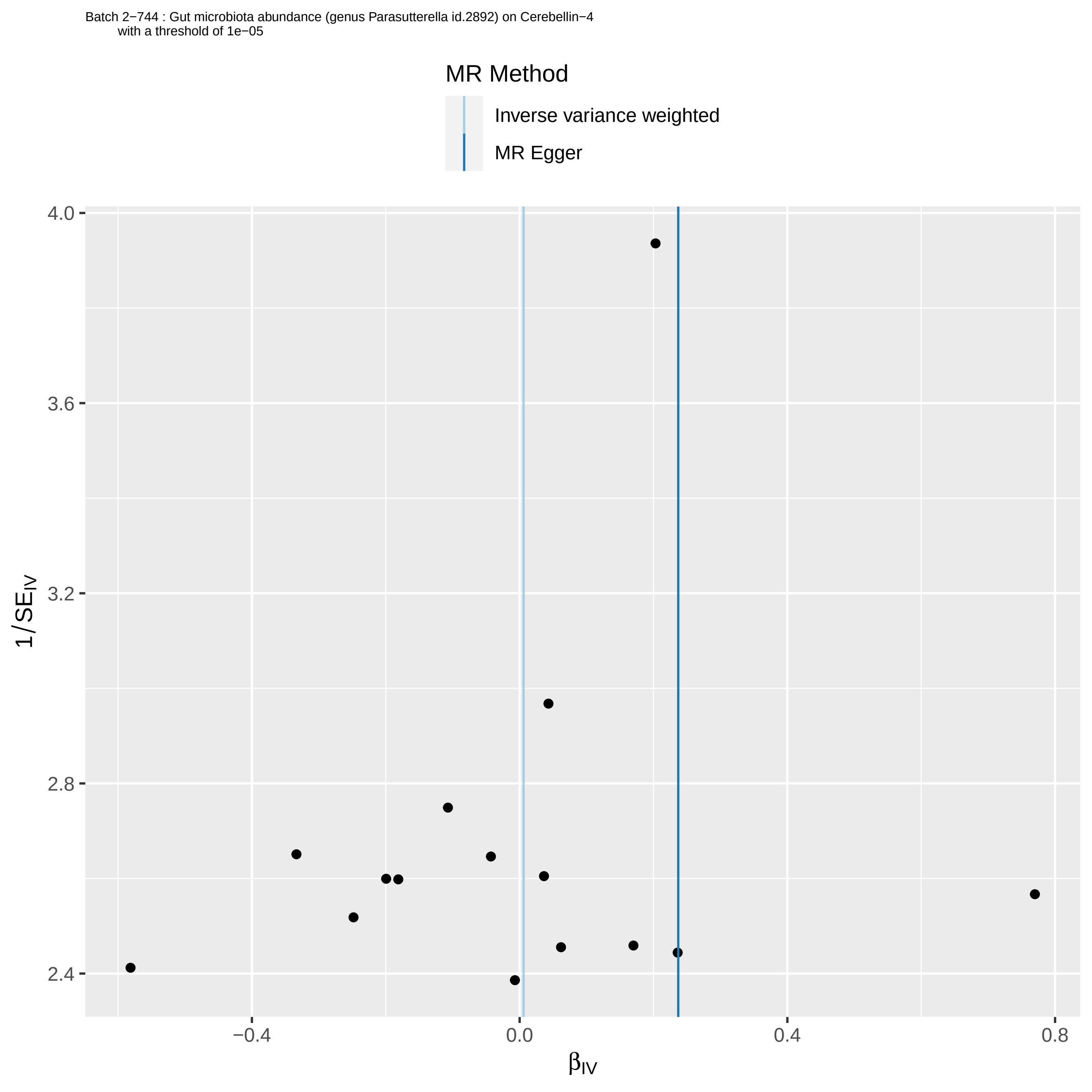

Batch 2−744 : Gut microbiota abundance (genus Parasutterella id.2892) on Cerebellin−4
with a threshold of 1e−05
MR Method
Inverse variance weighted
MR Egger
4.0
3.6
3.2
2.8
2.4
1/SE_IV
−0.4
0.0
0.4
0.8
β_IV

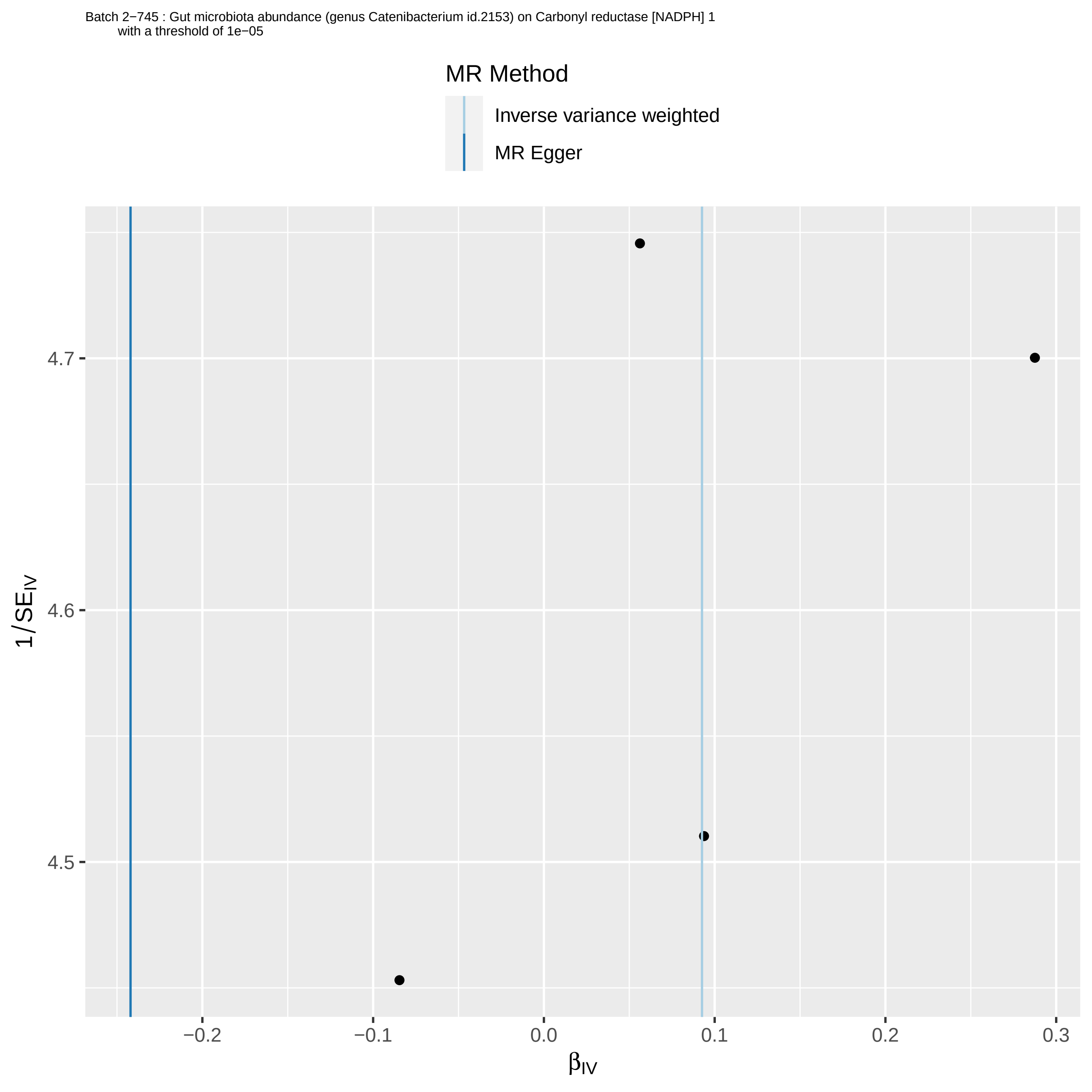
Batch 2−745 : Gut microbiota abundance (genus Catenibacterium id.2153) on Carbonyl reductase [NADPH] 1
with a threshold of 1e−05
MR Method
Inverse variance weighted
MR Egger
4.7
4.6
4.5
1/SE_IV
−0.2
−0.1
0.0
0.1
0.2
0.3
β_IV

Batch 2−746 : Gut microbiota abundance (genus Parasutterella id.2892) on Carbonyl reductase [NADPH] 1
with a threshold of 1e−05

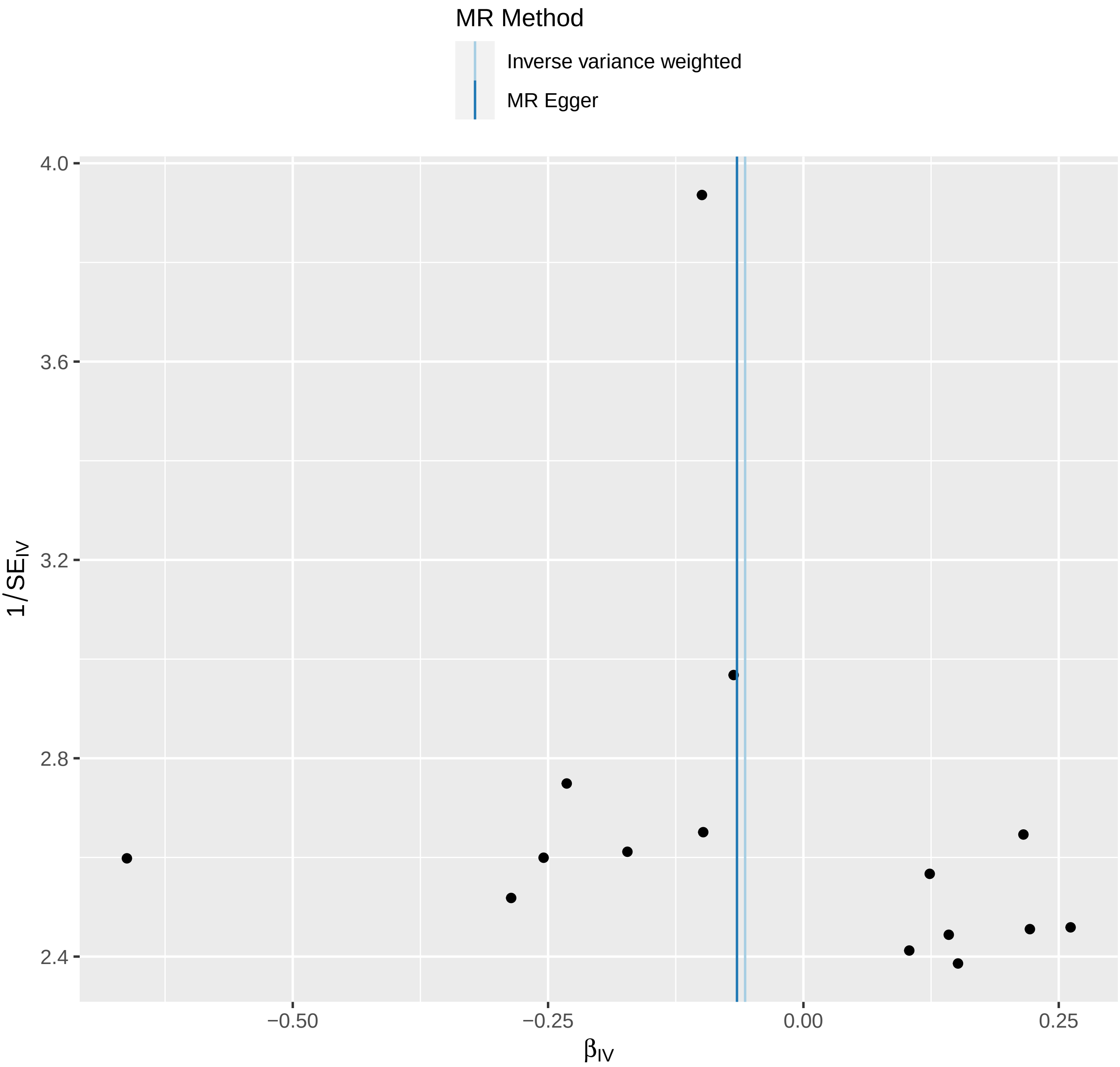

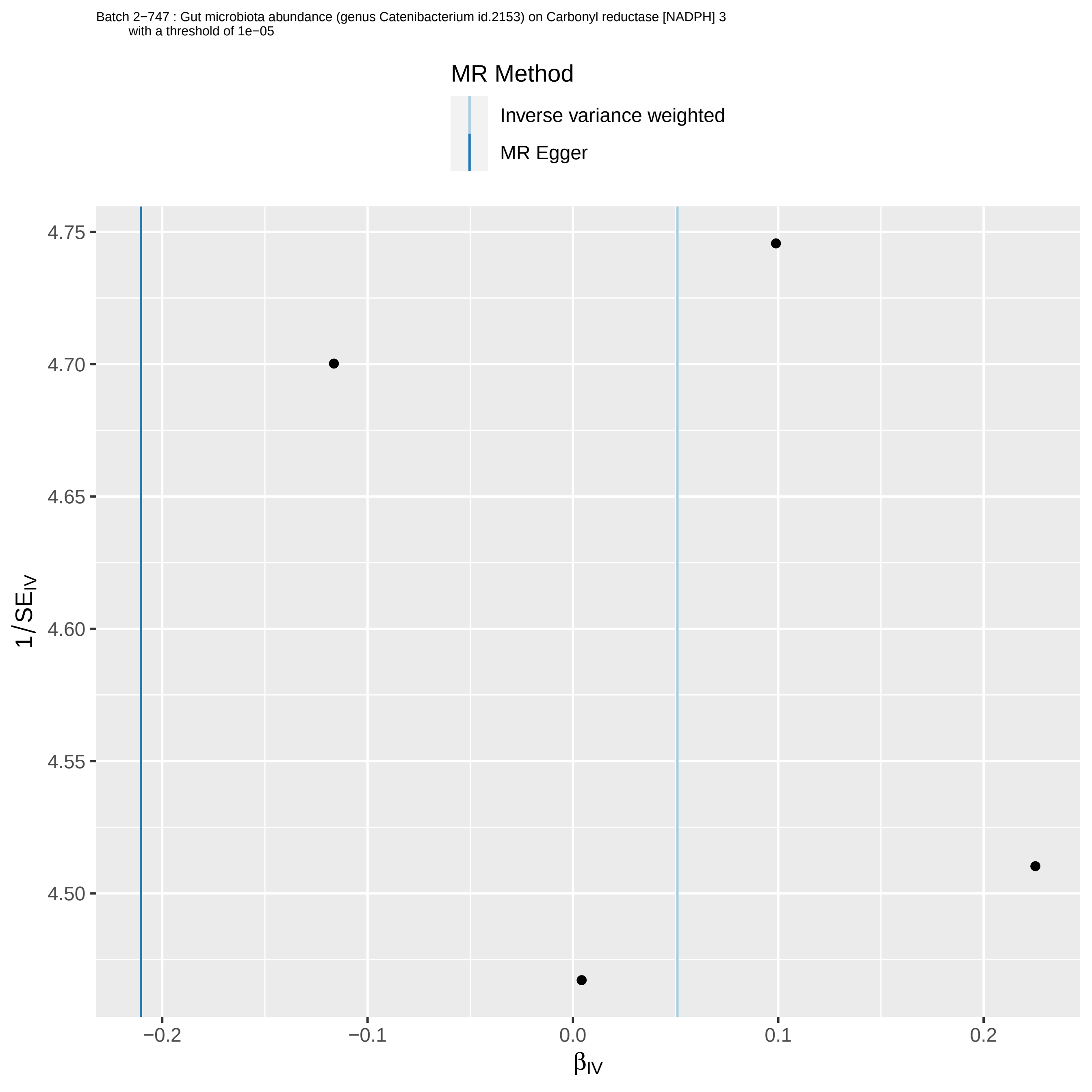
Batch 2−747 : Gut microbiota abundance (genus Catenibacterium id.2153) on Carbonyl reductase [NADPH] 3
with a threshold of 1e−05
MR Method
Inverse variance weighted
MR Egger
4.75
4.70
4.65
4.60
4.55
4.50
1/SE_IV
−0.2
−0.1
0.0
0.1
0.2
β_IV

Batch 2−748 : Gut microbiota abundance (genus Parasutterella id.2892) on Carbonyl reductase [NADPH] 3
with a threshold of 1e−05

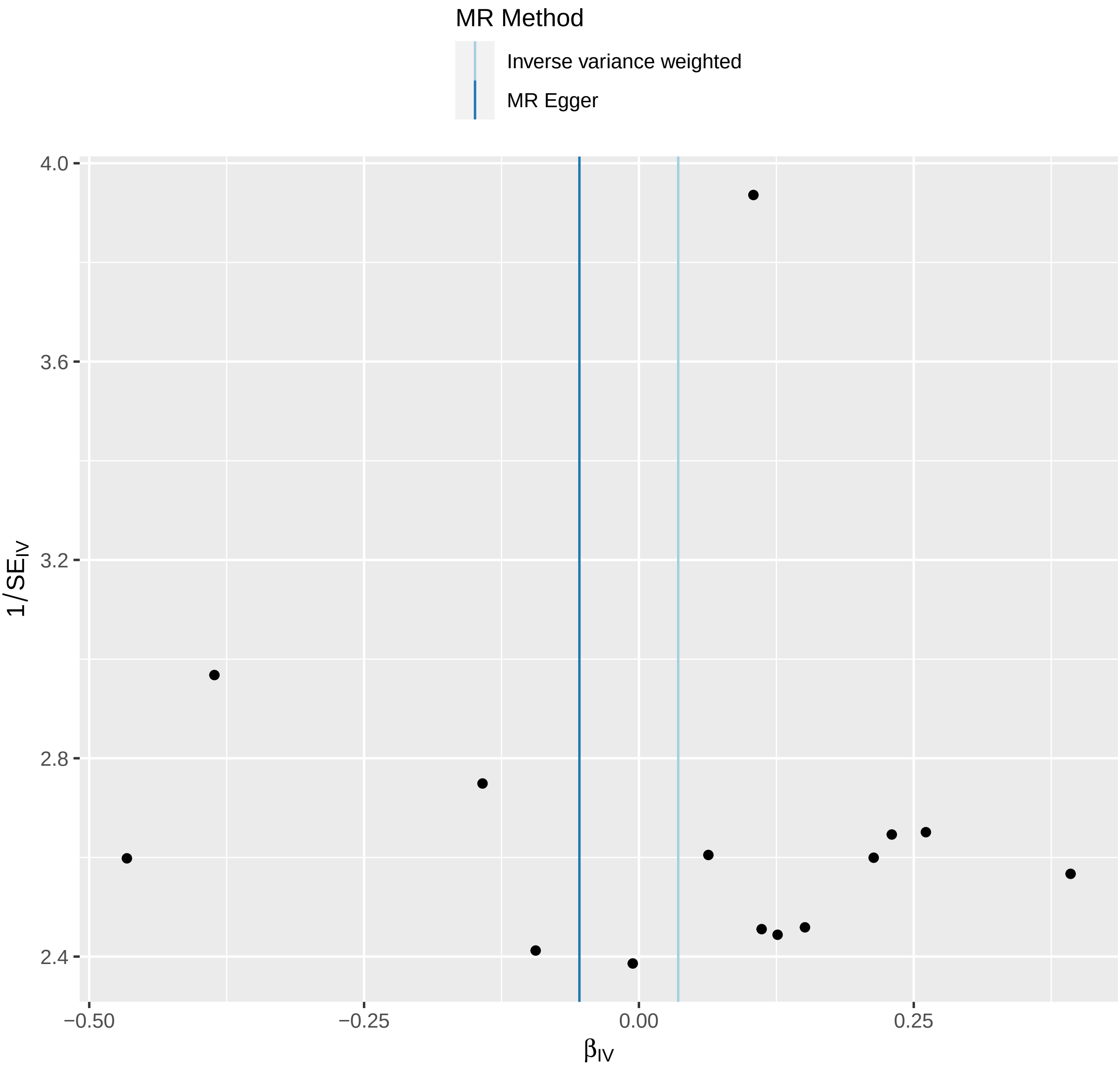

Batch 2−749 : Gut microbiota abundance (genus Catenibacterium id.2153) on Cystathionine beta−synthase
with a threshold of 1e−05

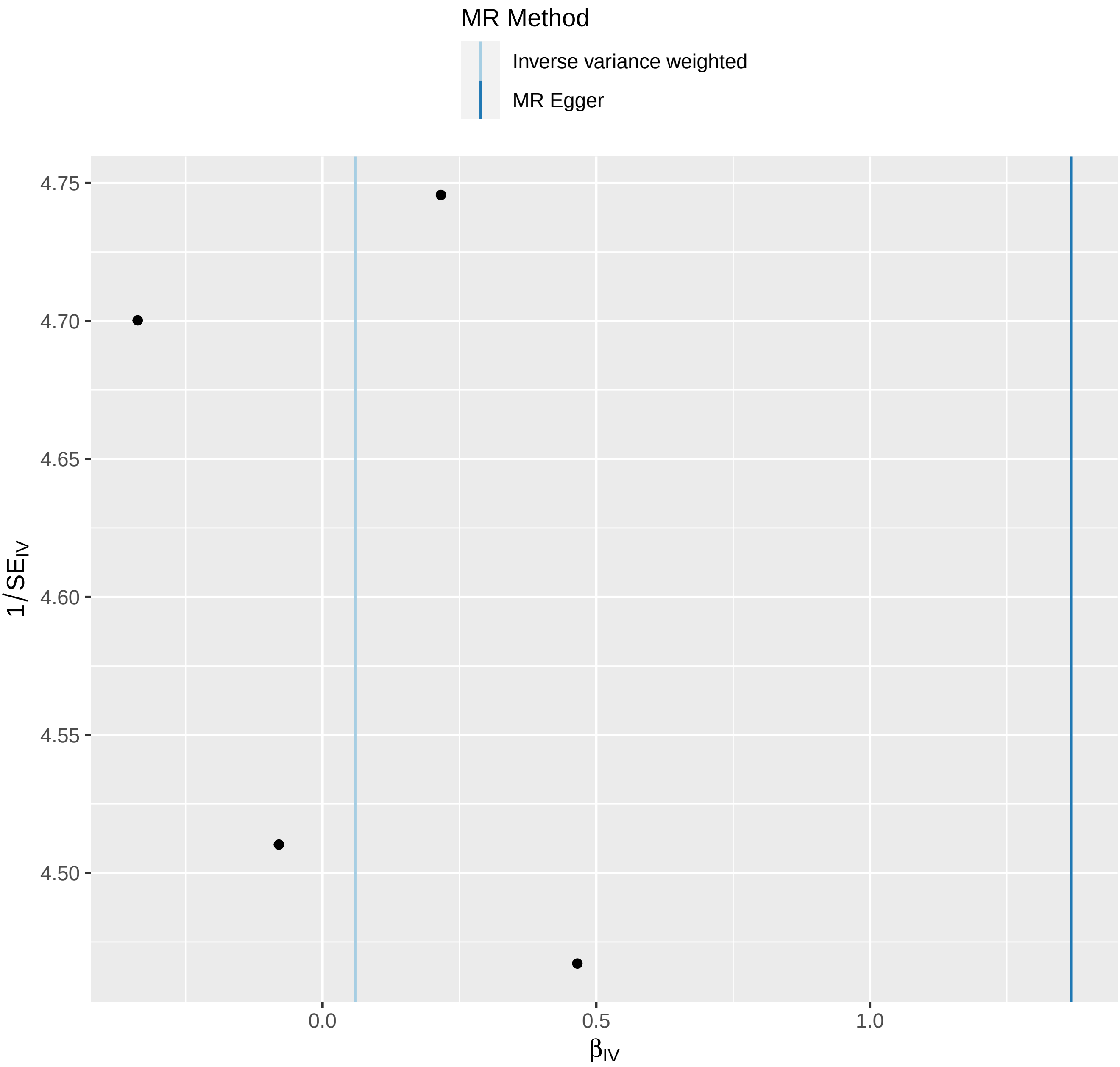

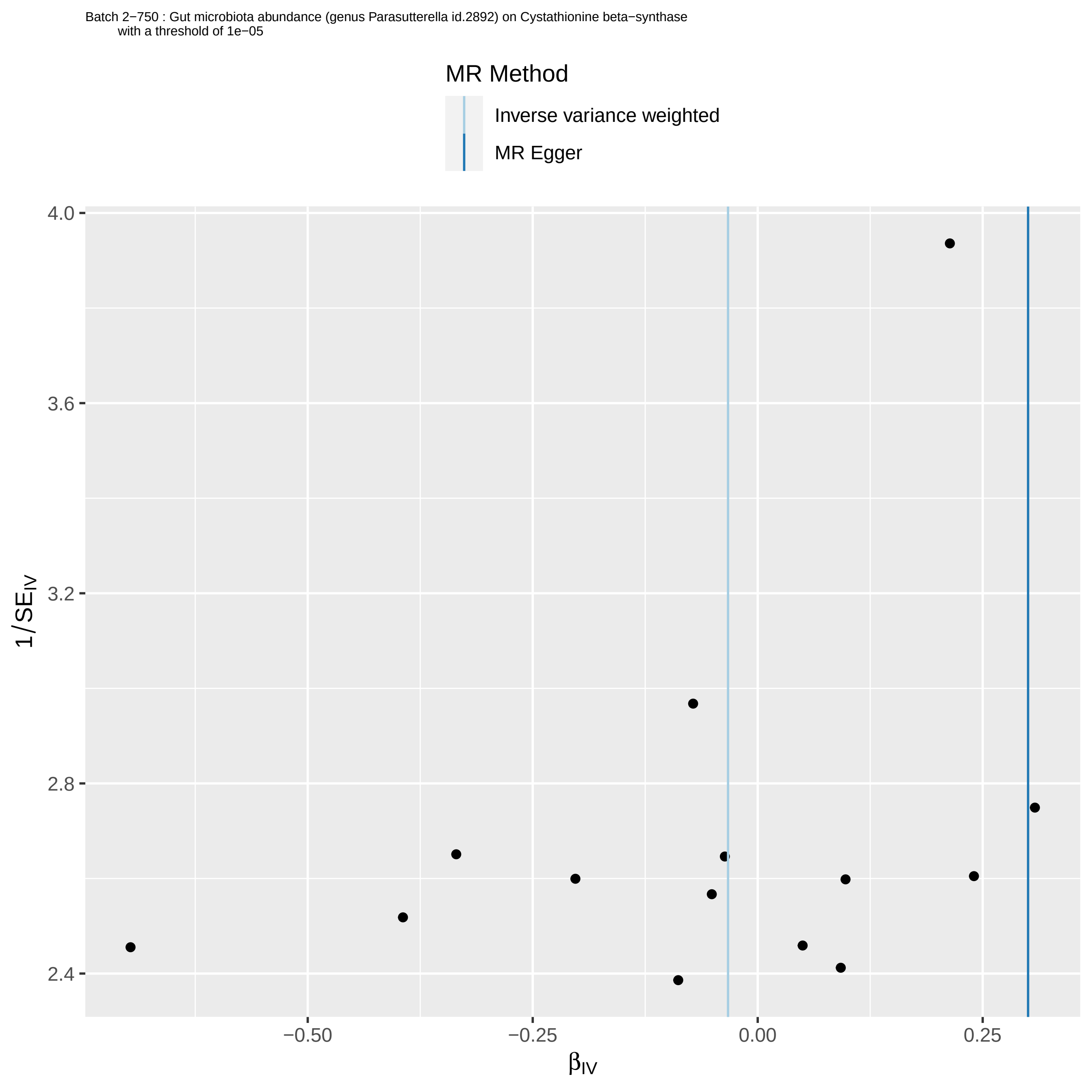

Batch 2−750 : Gut microbiota abundance (genus Parasutterella id.2892) on Cystathionine beta−synthase
with a threshold of 1e−05
MR Method
Inverse variance weighted
MR Egger
4.0
3.6
3.2
2.8
2.4
1/SE_IV
−0.50
−0.25
0.00
0.25
β_IV

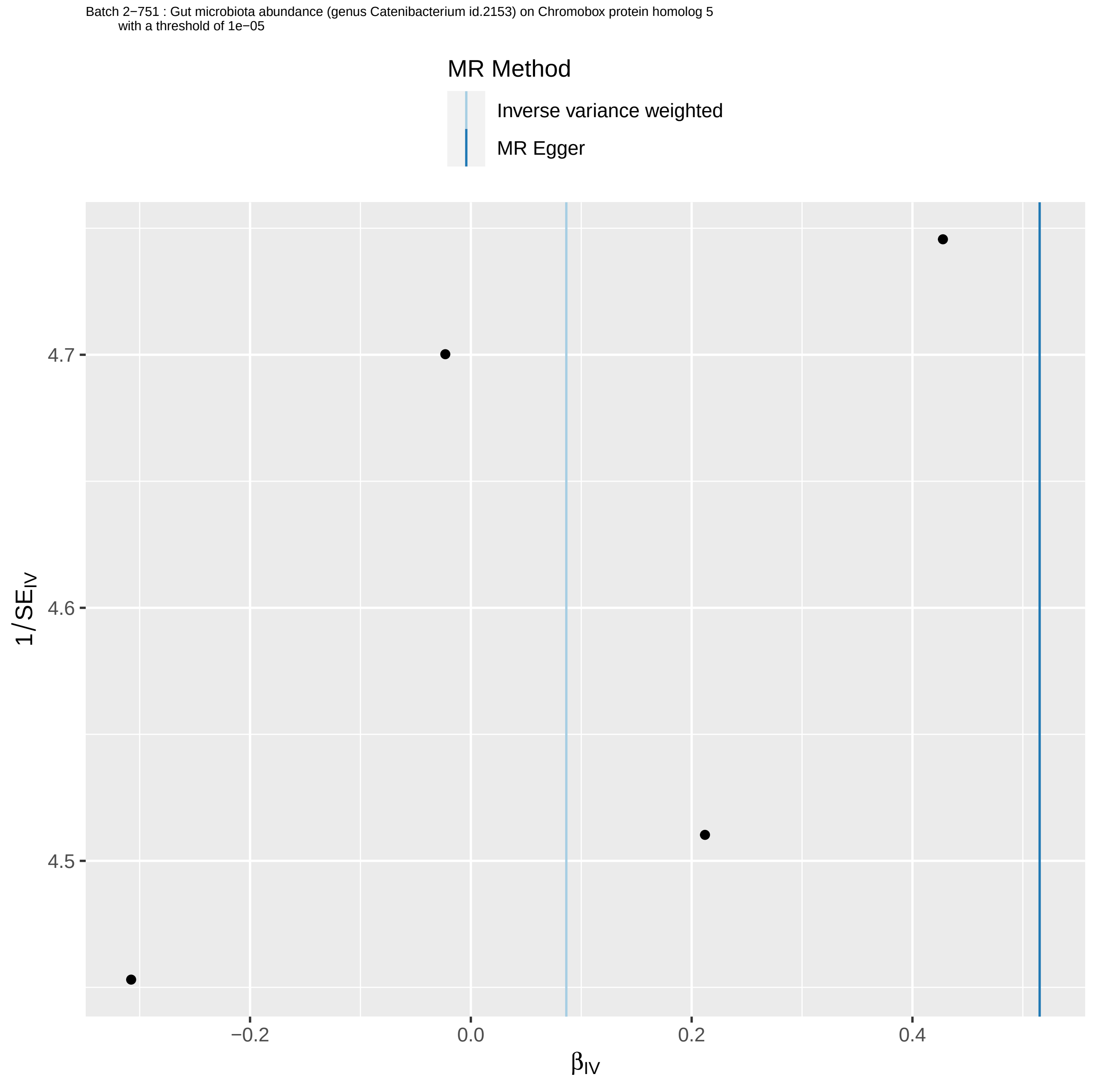
Batch 2−751 : Gut microbiota abundance (genus Catenibacterium id.2153) on Chromobox protein homolog 5
with a threshold of 1e−05
MR Method
Inverse variance weighted
MR Egger
4.7
4.6
4.5
1/SE_IV
−0.2
0.0
0.2
0.4
β_IV

Batch 2−752 : Gut microbiota abundance (genus Parasutterella id.2892) on Chromobox protein homolog 5
with a threshold of 1e−05

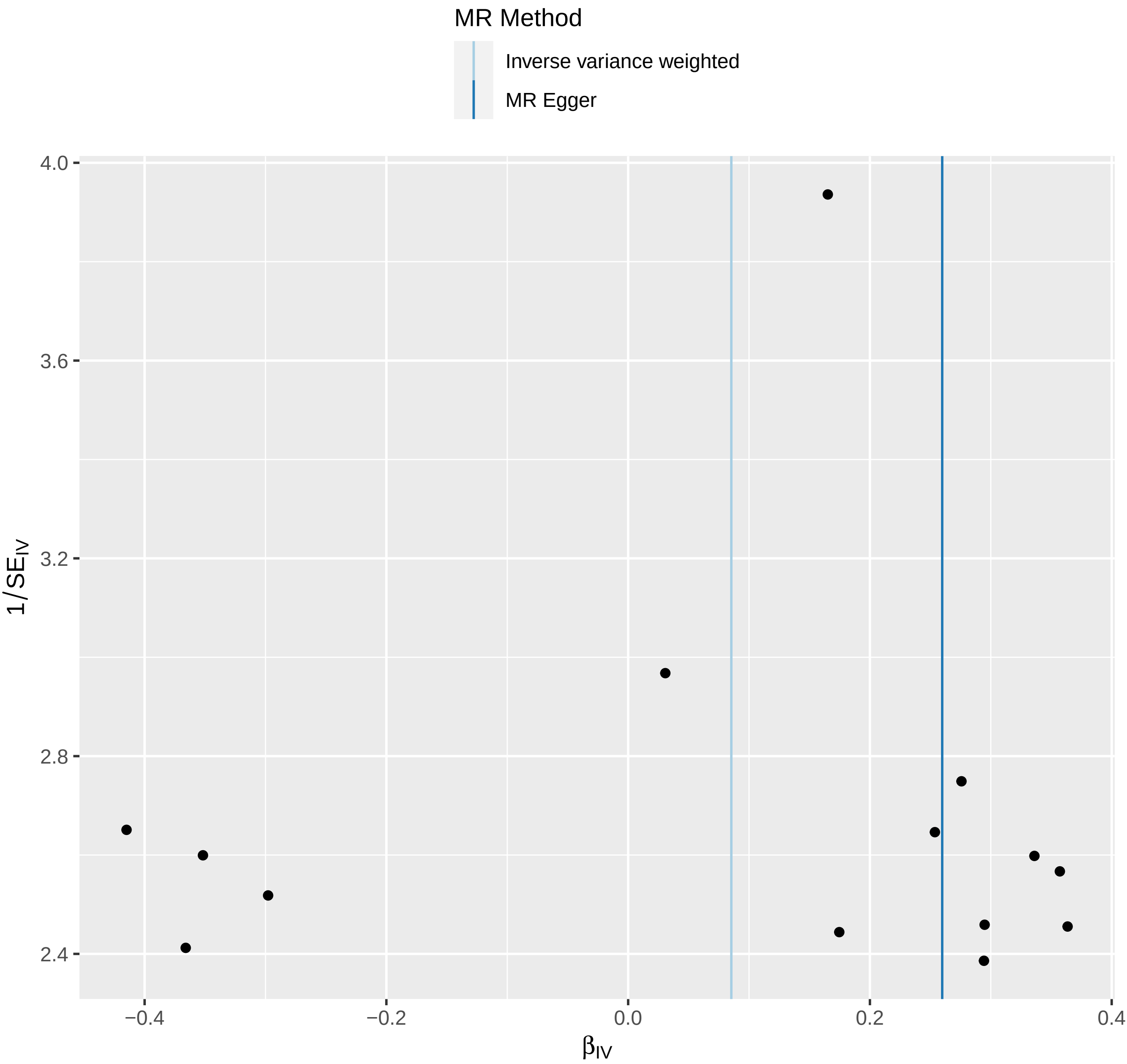

Batch 2−753 : Gut microbiota abundance (genus Catenibacterium id.2153) on Chromobox protein homolog 7
with a threshold of 1e−05

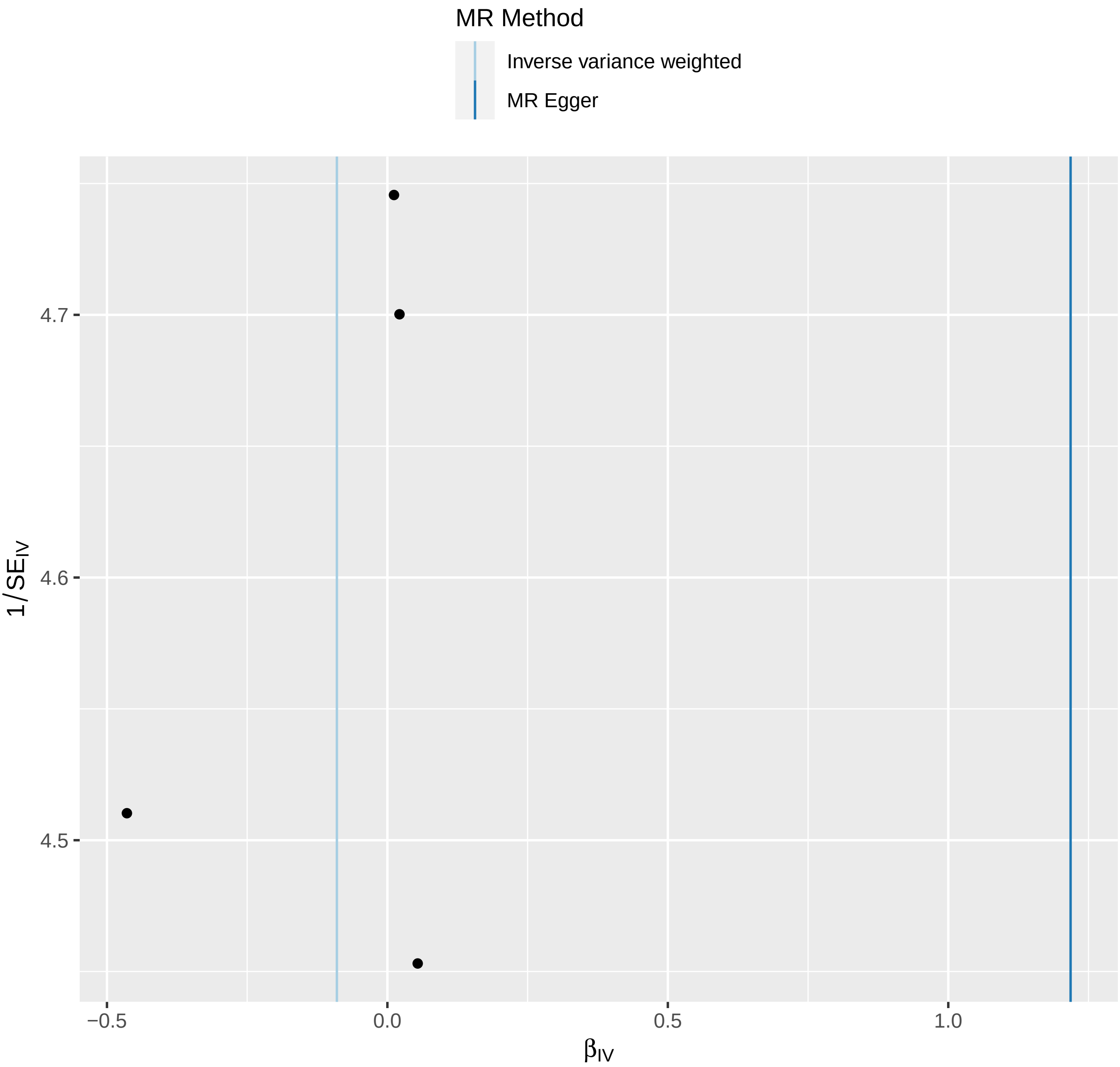

Batch 2−754 : Gut microbiota abundance (genus Parasutterella id.2892) on Chromobox protein homolog 7
with a threshold of 1e−05

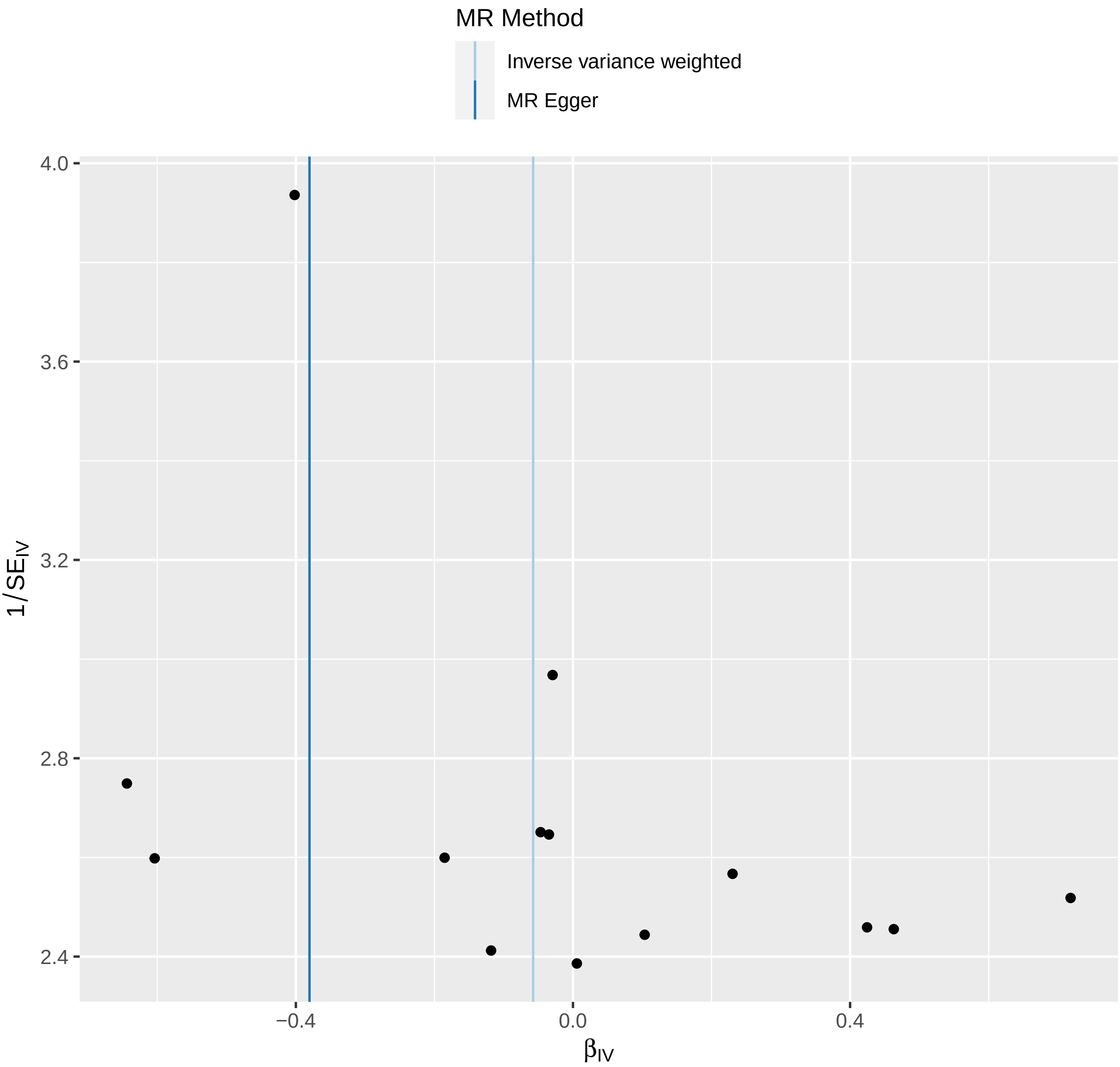

Batch 2−755 : Gut microbiota abundance (genus Catenibacterium id.2153) on Kynurenine−−oxoglutarate transaminase 3 with a threshold of 1e−05

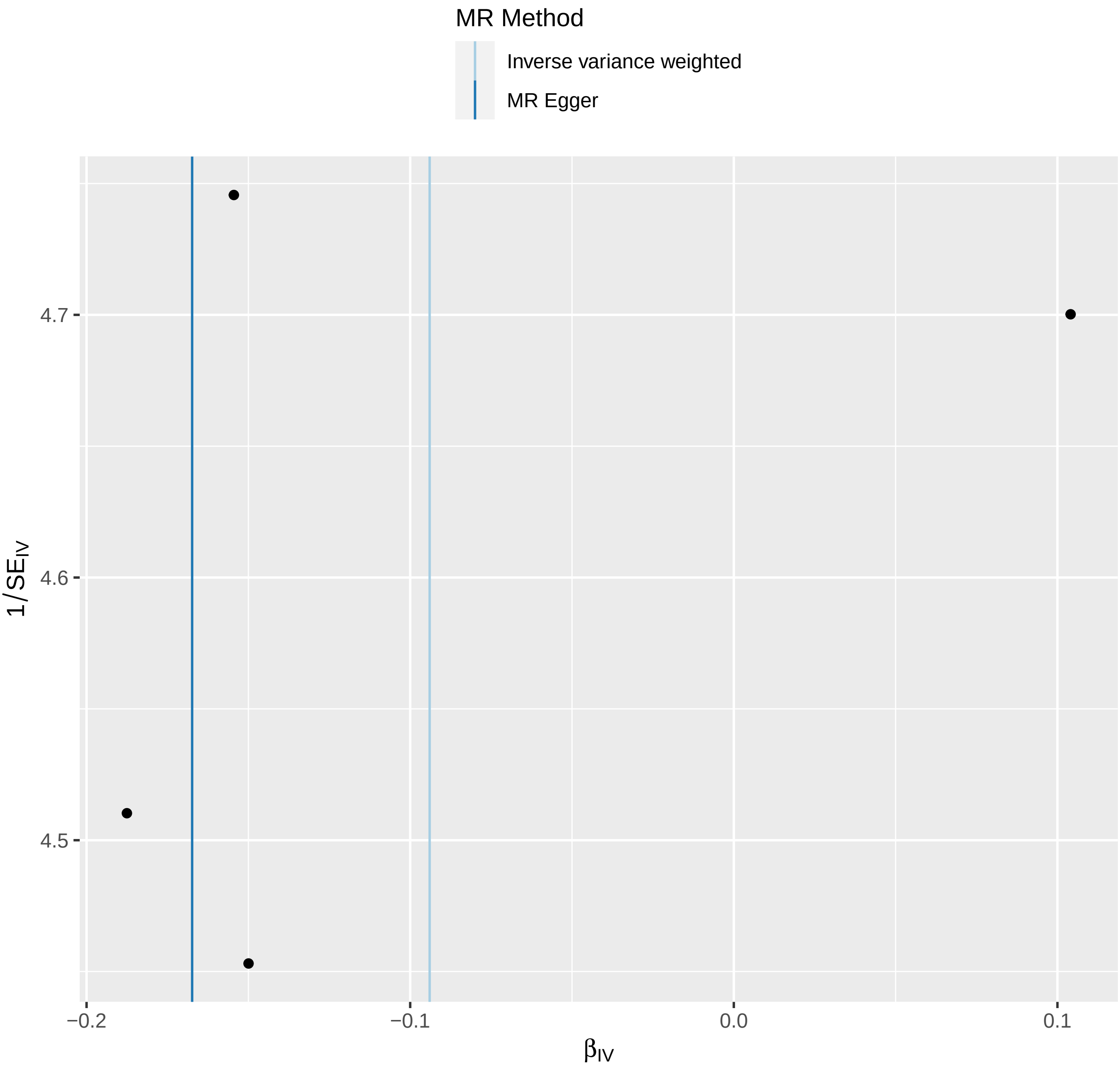

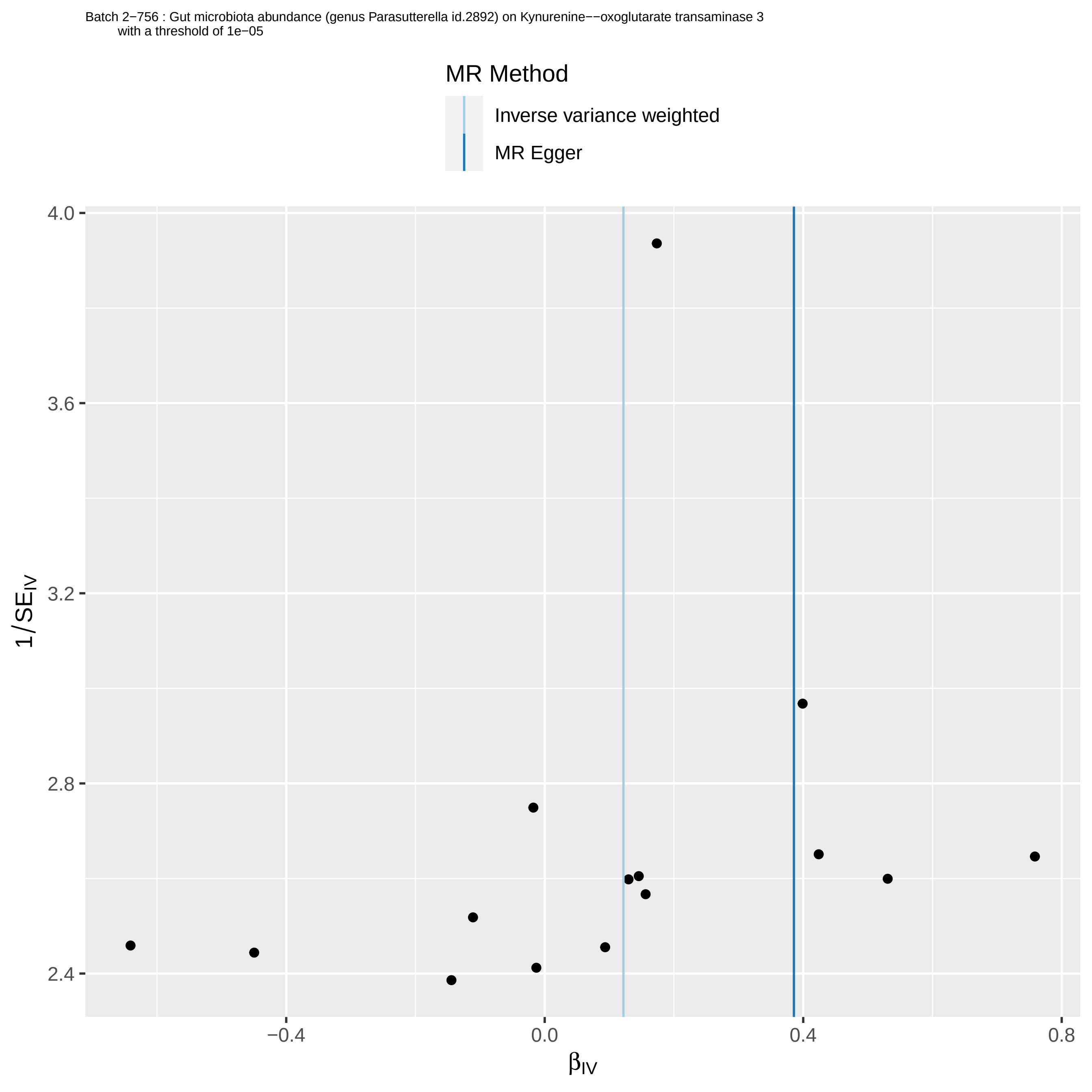

Batch 2−756 : Gut microbiota abundance (genus Parasutterella id.2892) on Kynurenine−−oxoglutarate transaminase 3
with a threshold of 1e−05
MR Method
Inverse variance weighted
MR Egger
4.0
3.6
3.2
2.8
2.4
1/SE_IV
−0.4
0.0
0.4
0.8
β_IV

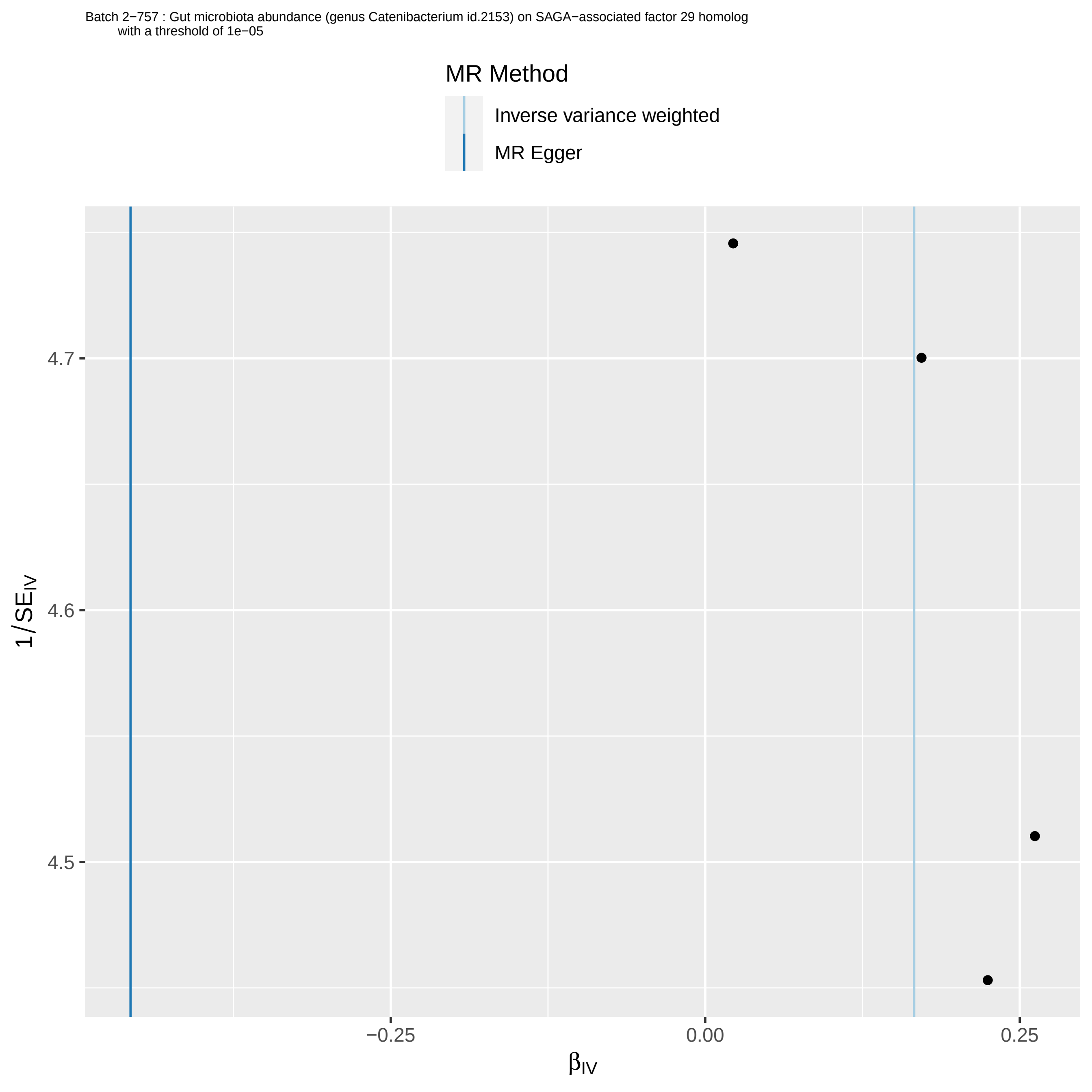
Batch 2−757 : Gut microbiota abundance (genus Catenibacterium id.2153) on SAGA−associated factor 29 homolog
with a threshold of 1e−05
MR Method
Inverse variance weighted
MR Egger
4.7
4.6
4.5
1/SE_IV
−0.25
0.00
0.25
β_IV

Batch 2−758 : Gut microbiota abundance (genus Parasutterella id.2892) on SAGA−associated factor 29 homolog with a threshold of 1e−05

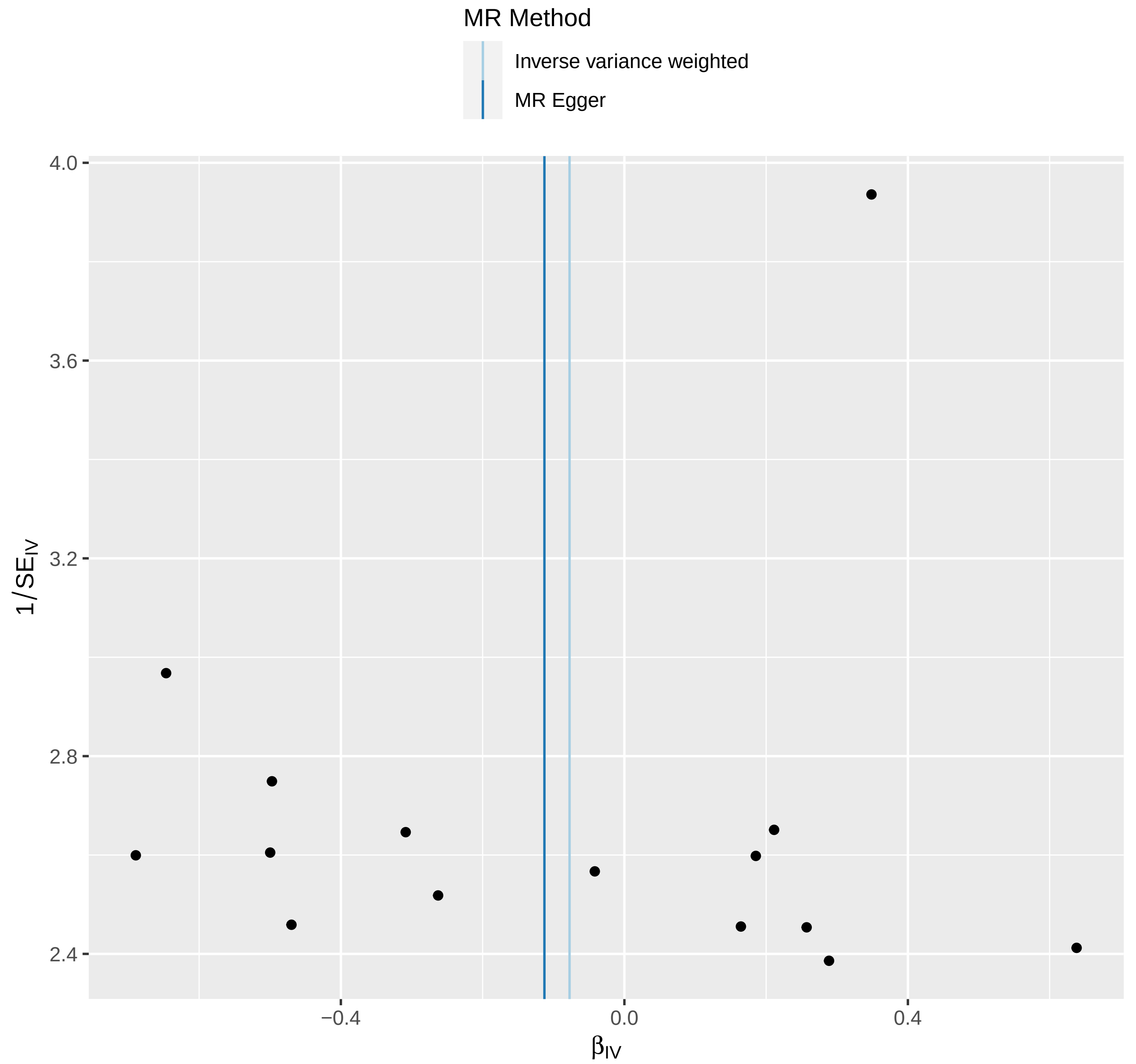

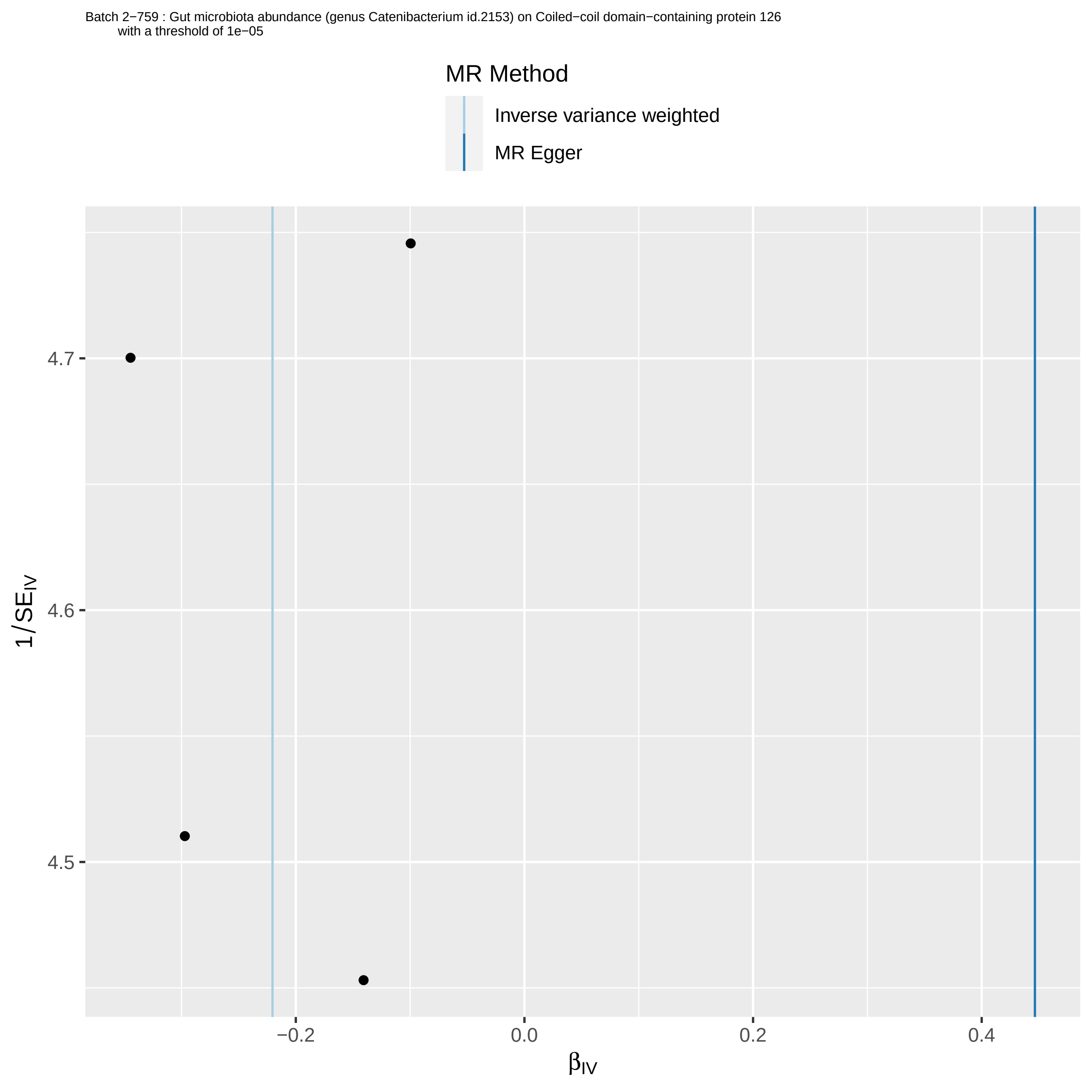

Batch 2−759 : Gut microbiota abundance (genus Catenibacterium id.2153) on Coiled−coil domain−containing protein 126
with a threshold of 1e−05
MR Method
Inverse variance weighted
MR Egger
4.7
4.6
4.5
1/SE_IV
−0.2
0.0
0.2
0.4
β_IV

Batch 2−760 : Gut microbiota abundance (genus Parasutterella id.2892) on Coiled−coil domain−containing protein 126
with a threshold of 1e−05

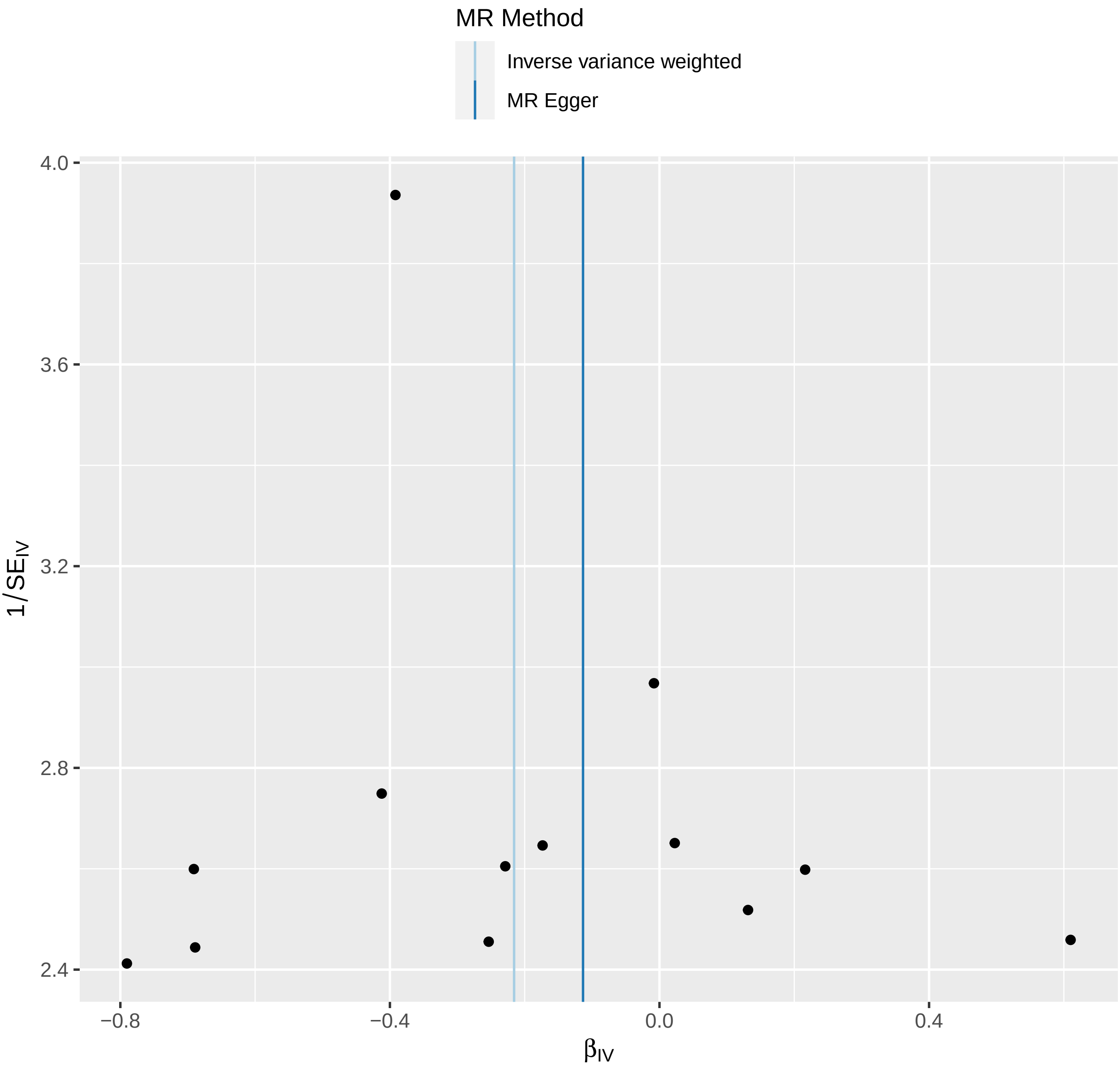

Batch 2−761 : Gut microbiota abundance (genus Catenibacterium id.2153) on Coiled−coil domain−containing protein 134
with a threshold of 1e−05

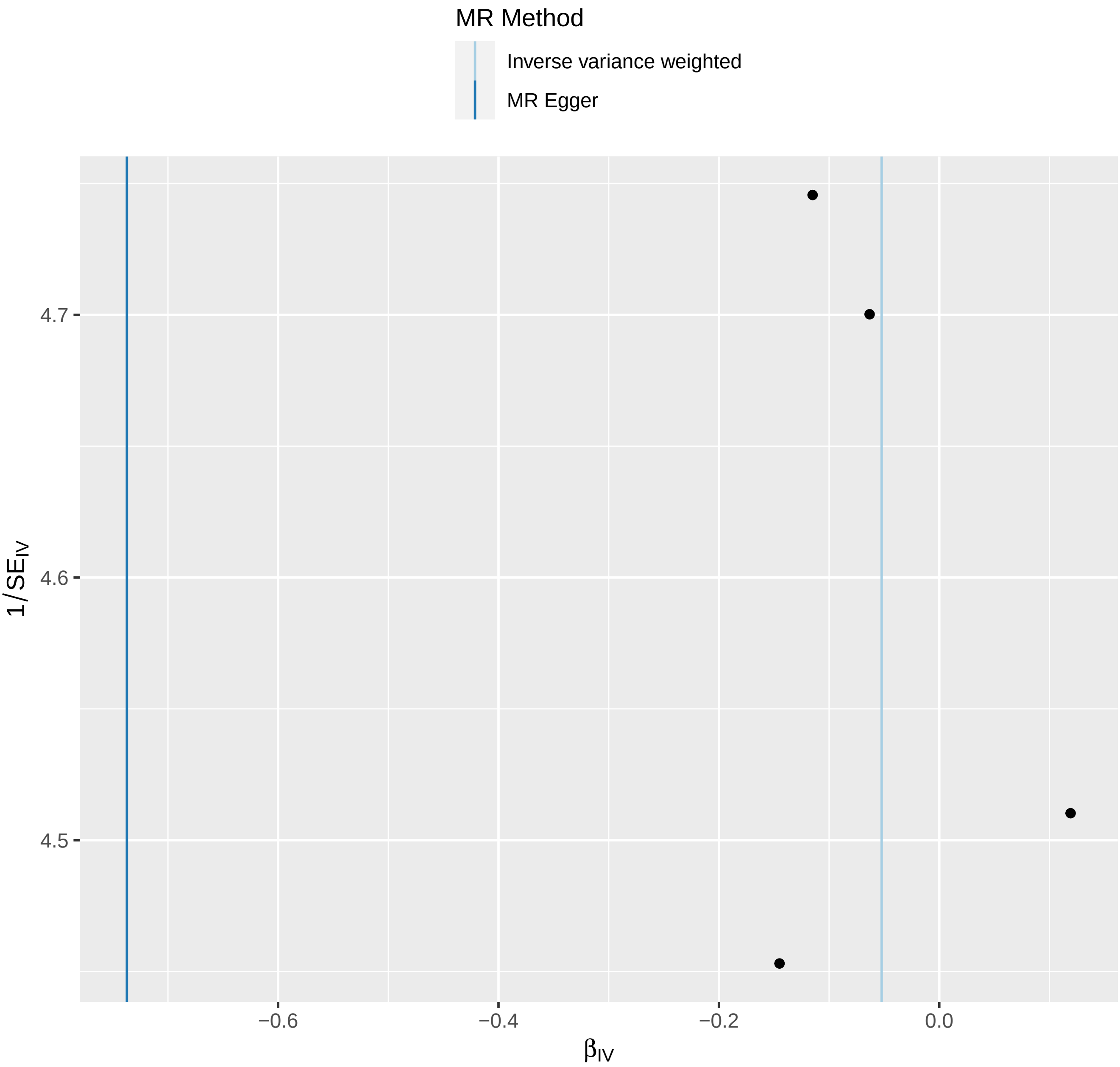

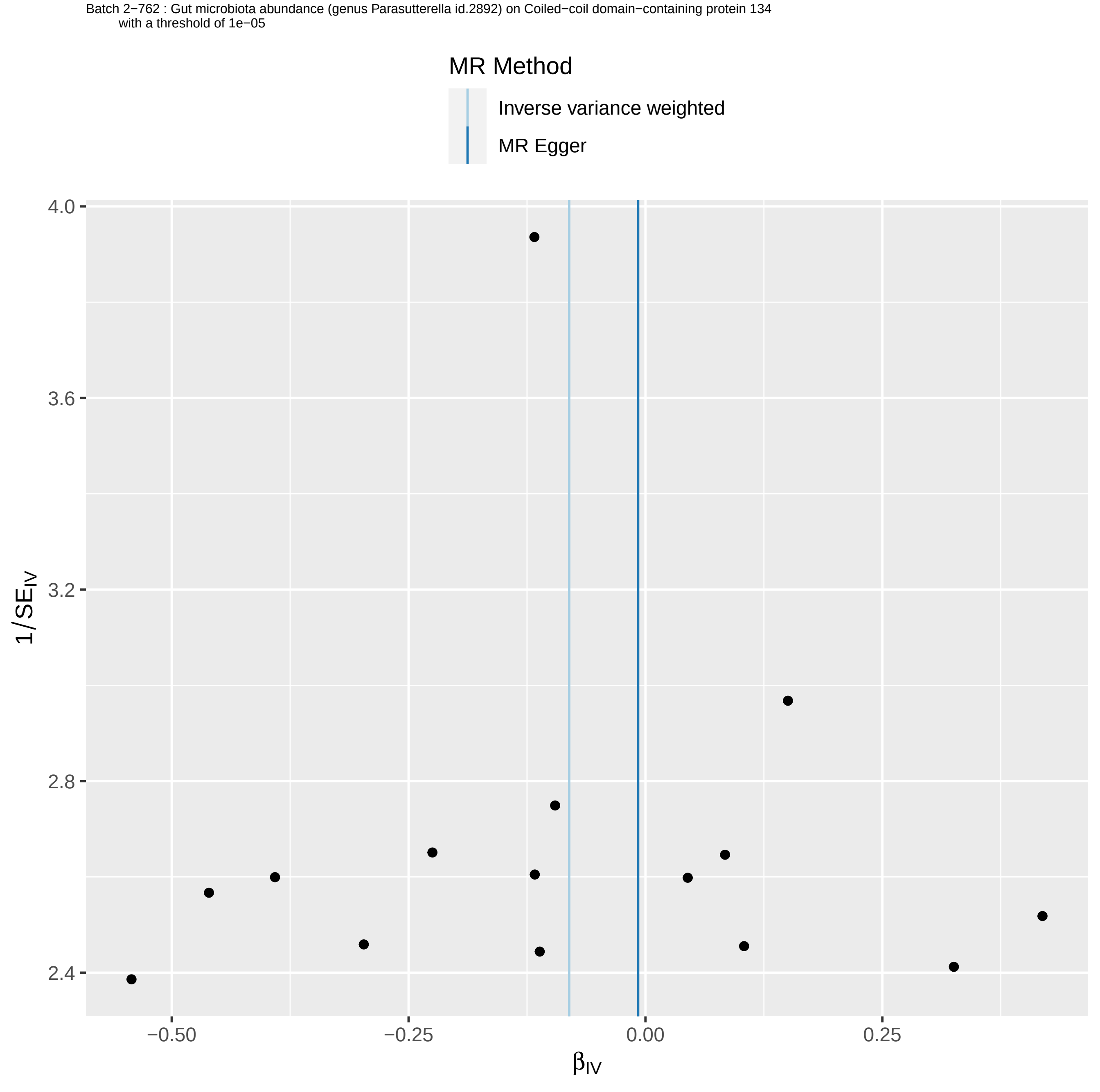
Batch 2−762 : Gut microbiota abundance (genus Parasutterella id.2892) on Coiled−coil domain−containing protein 134
with a threshold of 1e−05
MR Method
Inverse variance weighted
MR Egger
4.0
3.6
3.2
2.8
2.4
1/SE_IV
−0.50
−0.25
0.00
0.25
β_IV

Batch 2−763 : Gut microbiota abundance (genus Catenibacterium id.2153) on Coiled−coil domain−containing protein 167
with a threshold of 1e−05

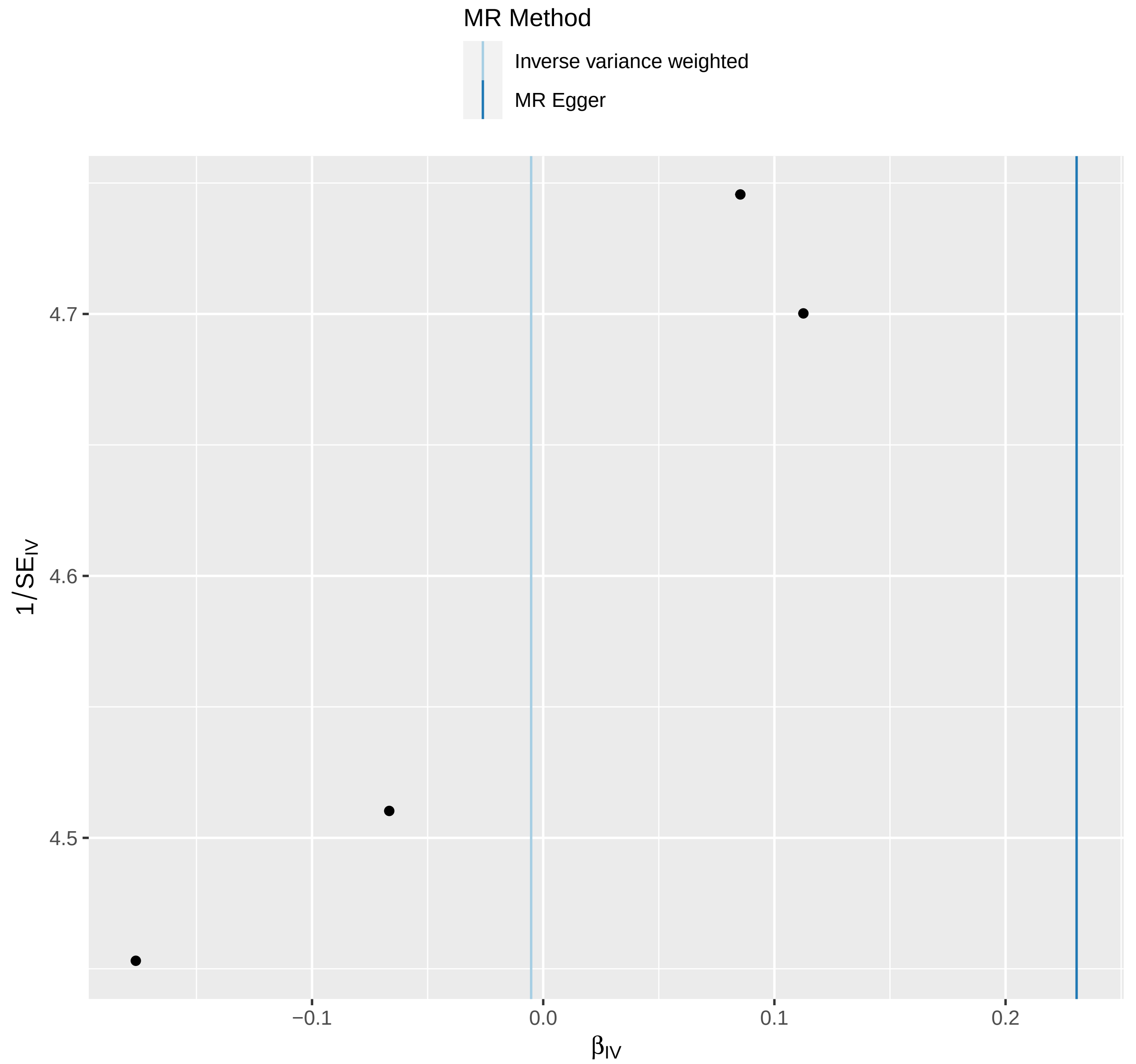

Batch 2−764 : Gut microbiota abundance (genus Parasutterella id.2892) on Coiled−coil domain−containing protein 167
with a threshold of 1e−05

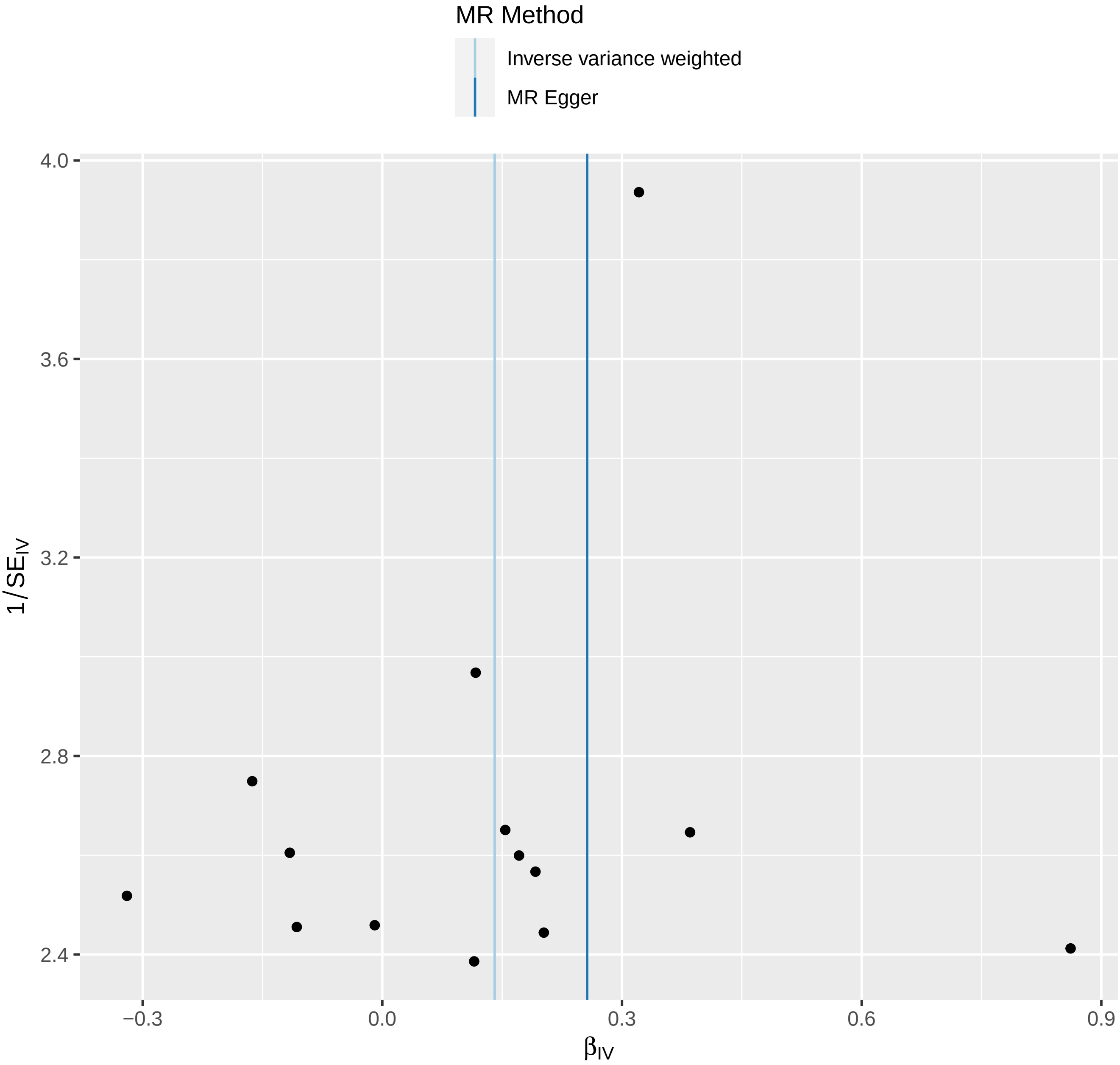

Batch 2−765 : Gut microbiota abundance (genus Catenibacterium id.2153) on Coiled−coil domain−containing protein 51
with a threshold of 1e−05

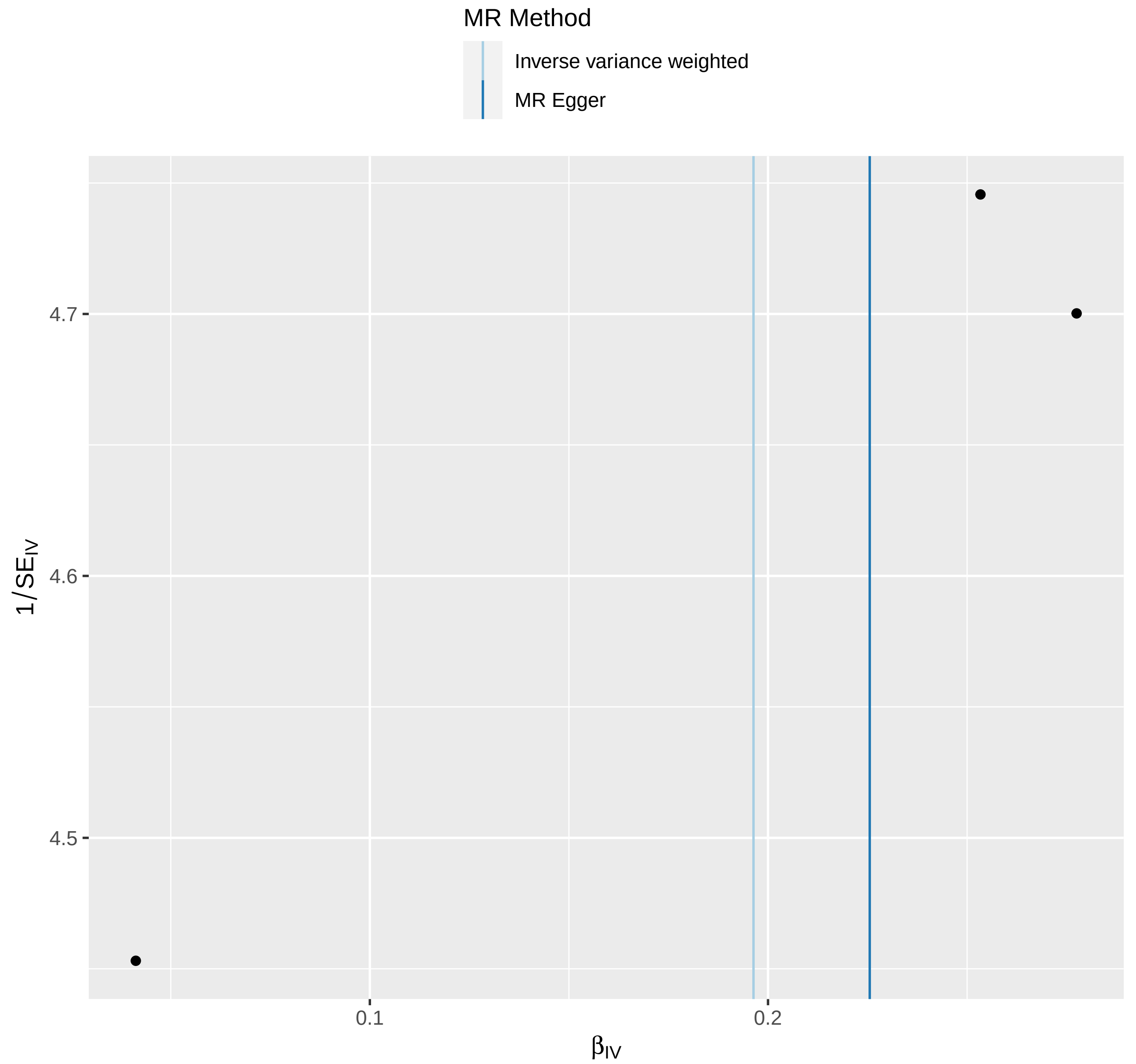

Batch 2−766 : Gut microbiota abundance (genus Parasutterella id.2892) on Coiled−coil domain−containing protein 51
with a threshold of 1e−05

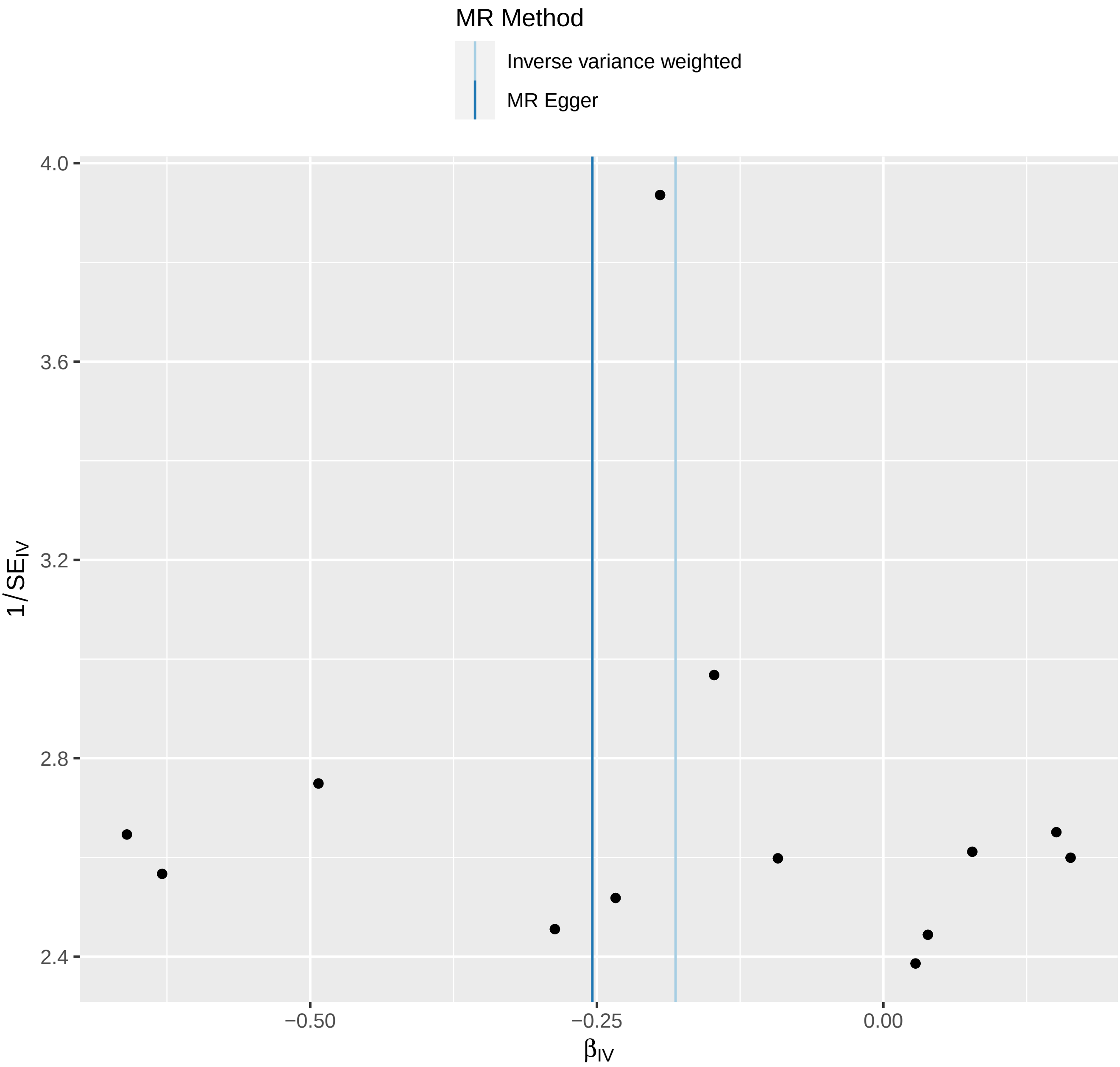

Batch 2−767 : Gut microbiota abundance (genus Catenibacterium id.2153) on Coiled−coil domain−containing protein 80 with a threshold of 1e−05

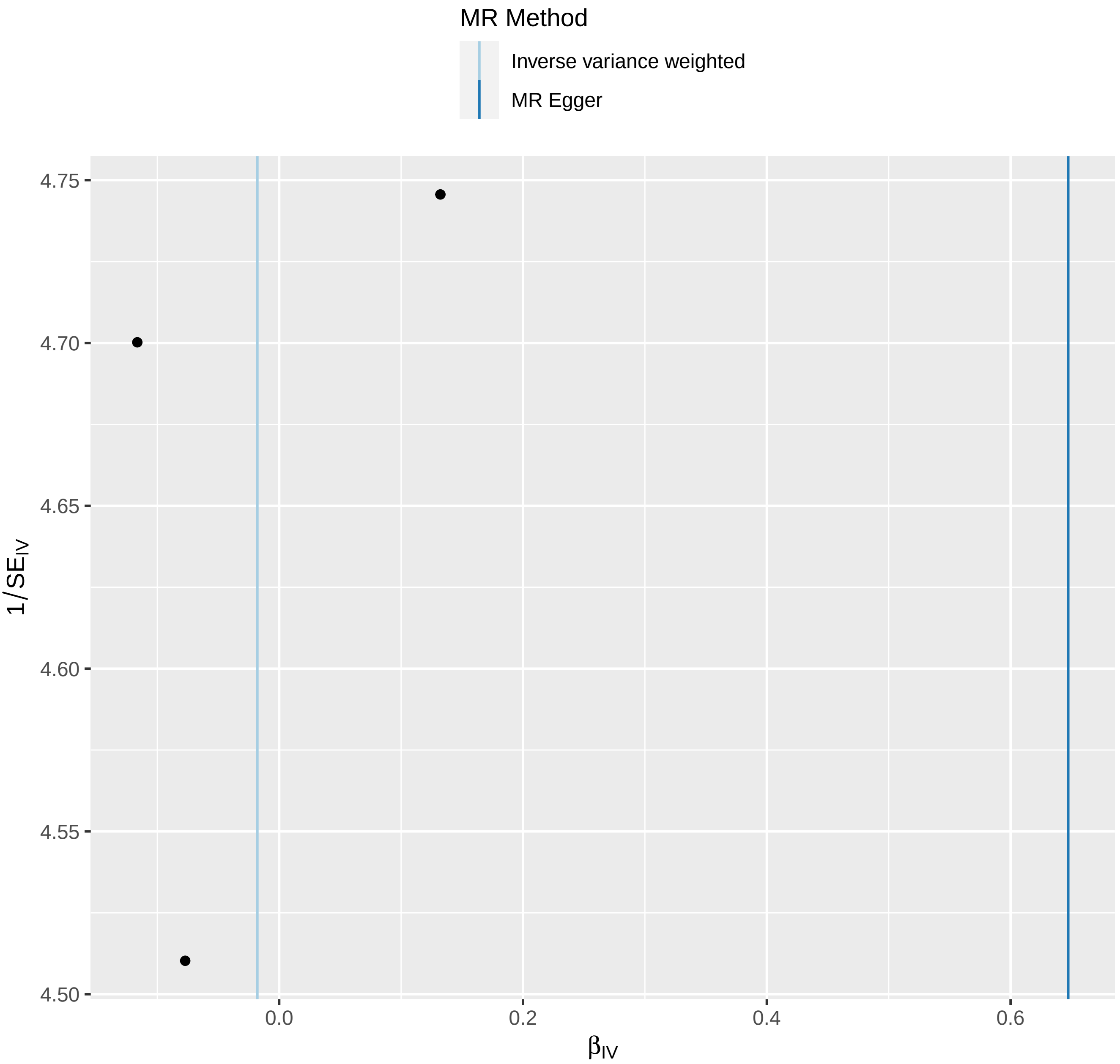

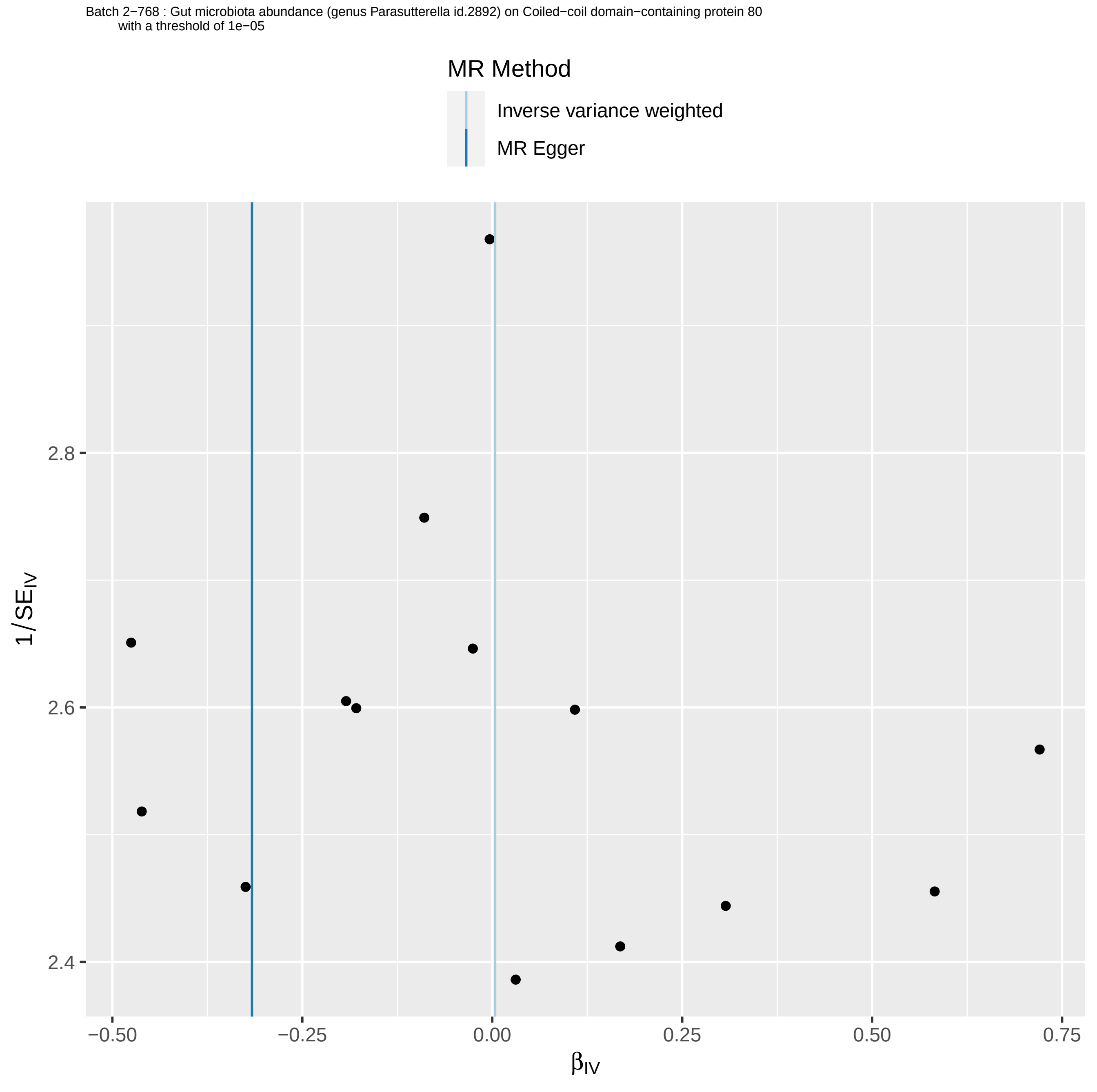

Batch 2−768 : Gut microbiota abundance (genus Parasutterella id.2892) on Coiled−coil domain−containing protein 80
with a threshold of 1e−05
MR Method
Inverse variance weighted
MR Egger
2.8
2.6
2.4
1/SE_IV
−0.50
−0.25
0.00
0.25
0.50
0.75
β_IV

Batch 2−769 : Gut microbiota abundance (genus Catenibacterium id.2153) on Coiled−coil domain−containing protein 90B, mitochondrial with a threshold of 1e−05

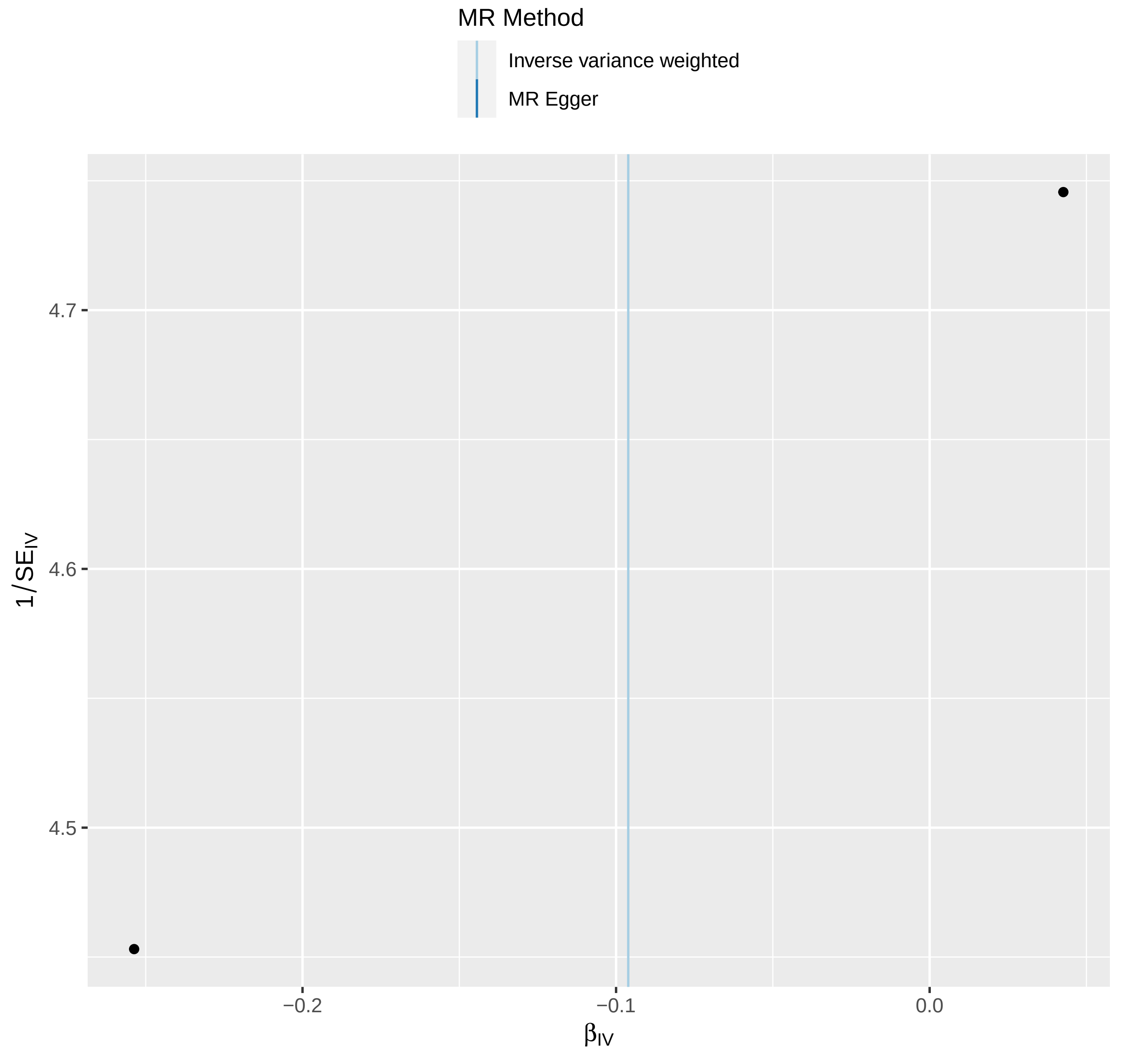

Batch 2−770 : Gut microbiota abundance (genus Parasutterella id.2892) on Coiled−coil domain−containing protein 90B, mitochondrial
with a threshold of 1e−05

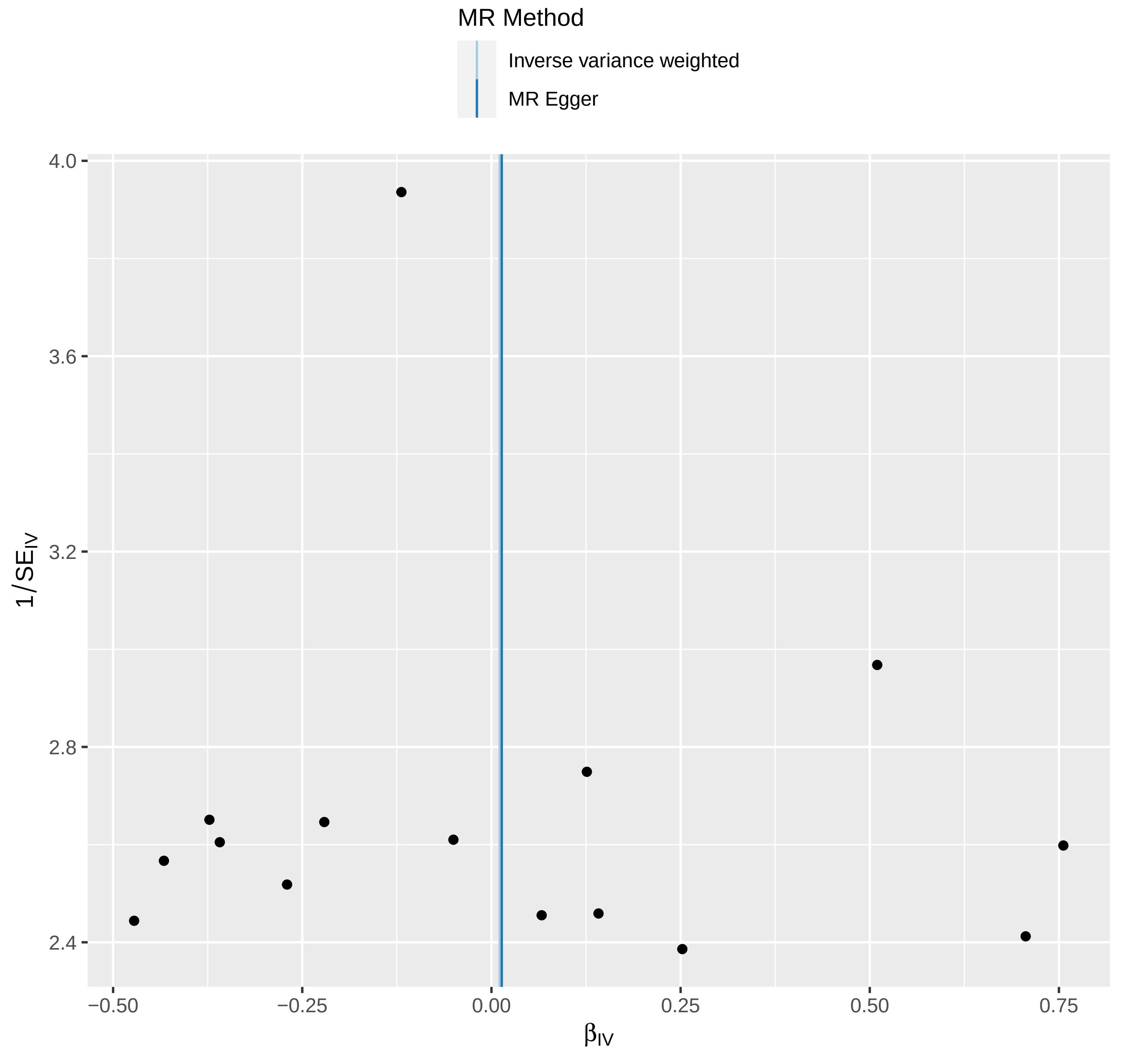

Batch 2−771 : Gut microbiota abundance (genus Catenibacterium id.2153) on C−C motif chemokine 1
with a threshold of 1e−05

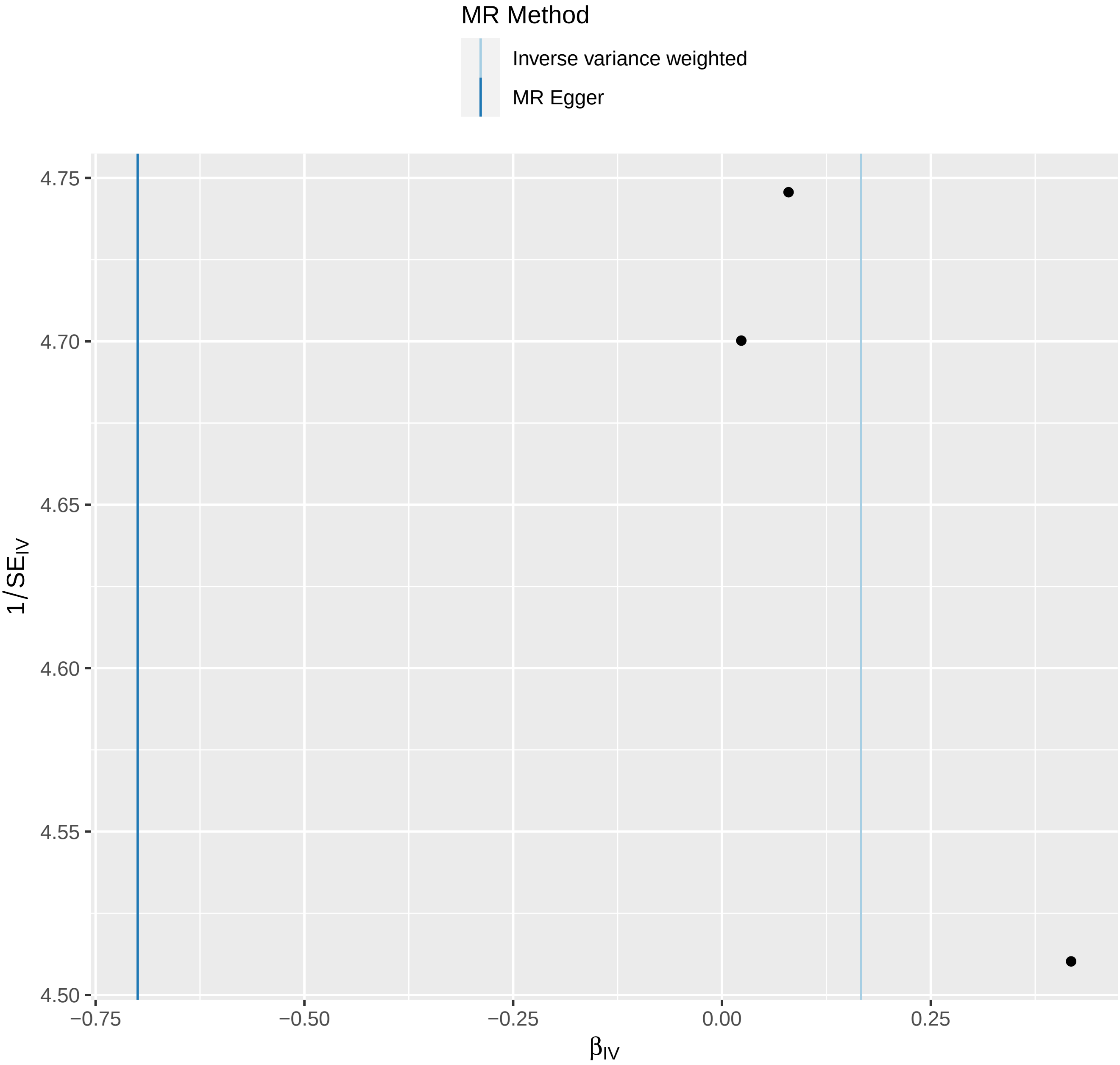

Batch 2−772 : Gut microbiota abundance (genus Parasutterella id.2892) on C−C motif chemokine 1
with a threshold of 1e−05

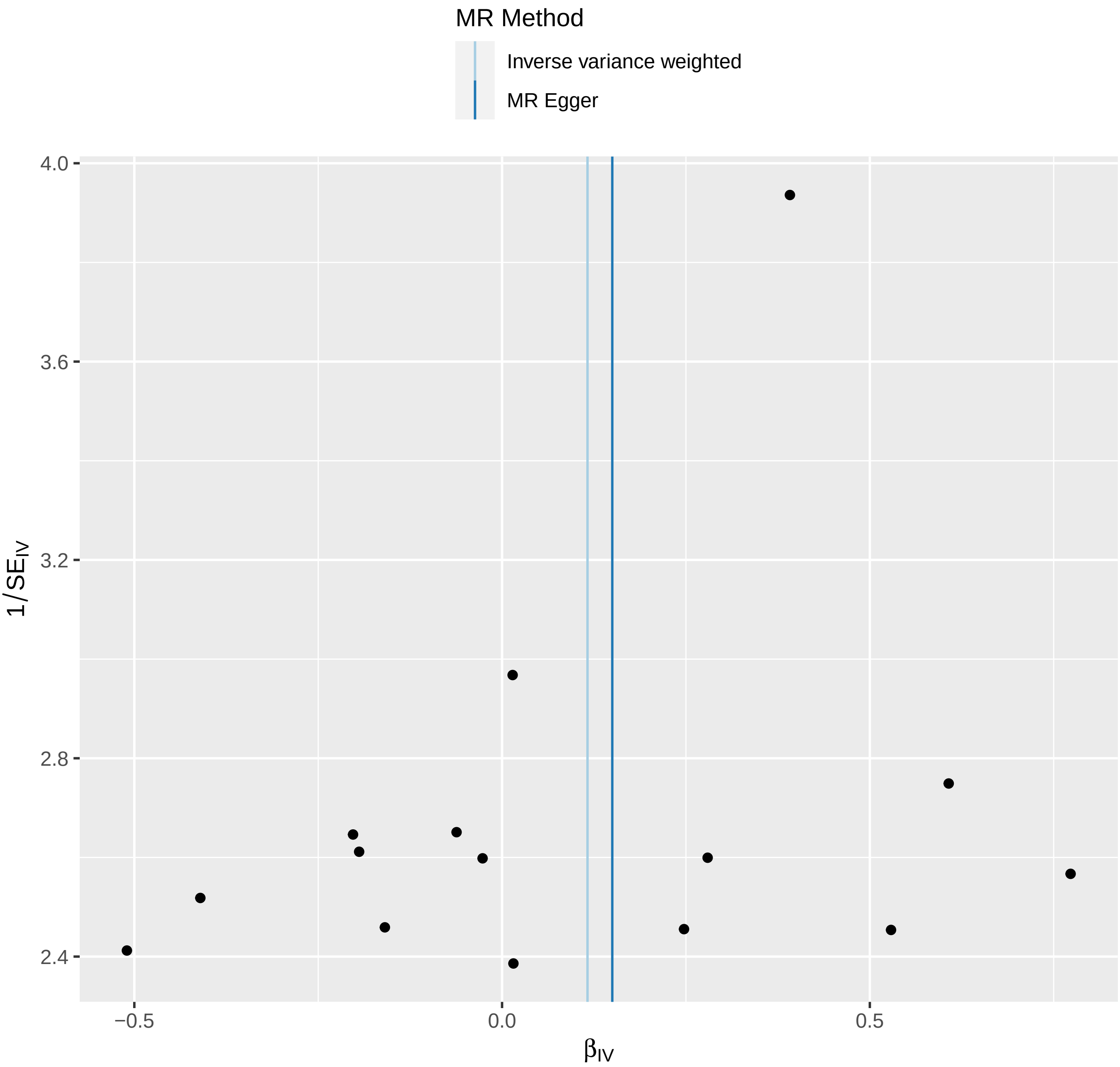

Batch 2−773 : Gut microbiota abundance (genus Catenibacterium id.2153) on Eotaxin
with a threshold of 1e−05

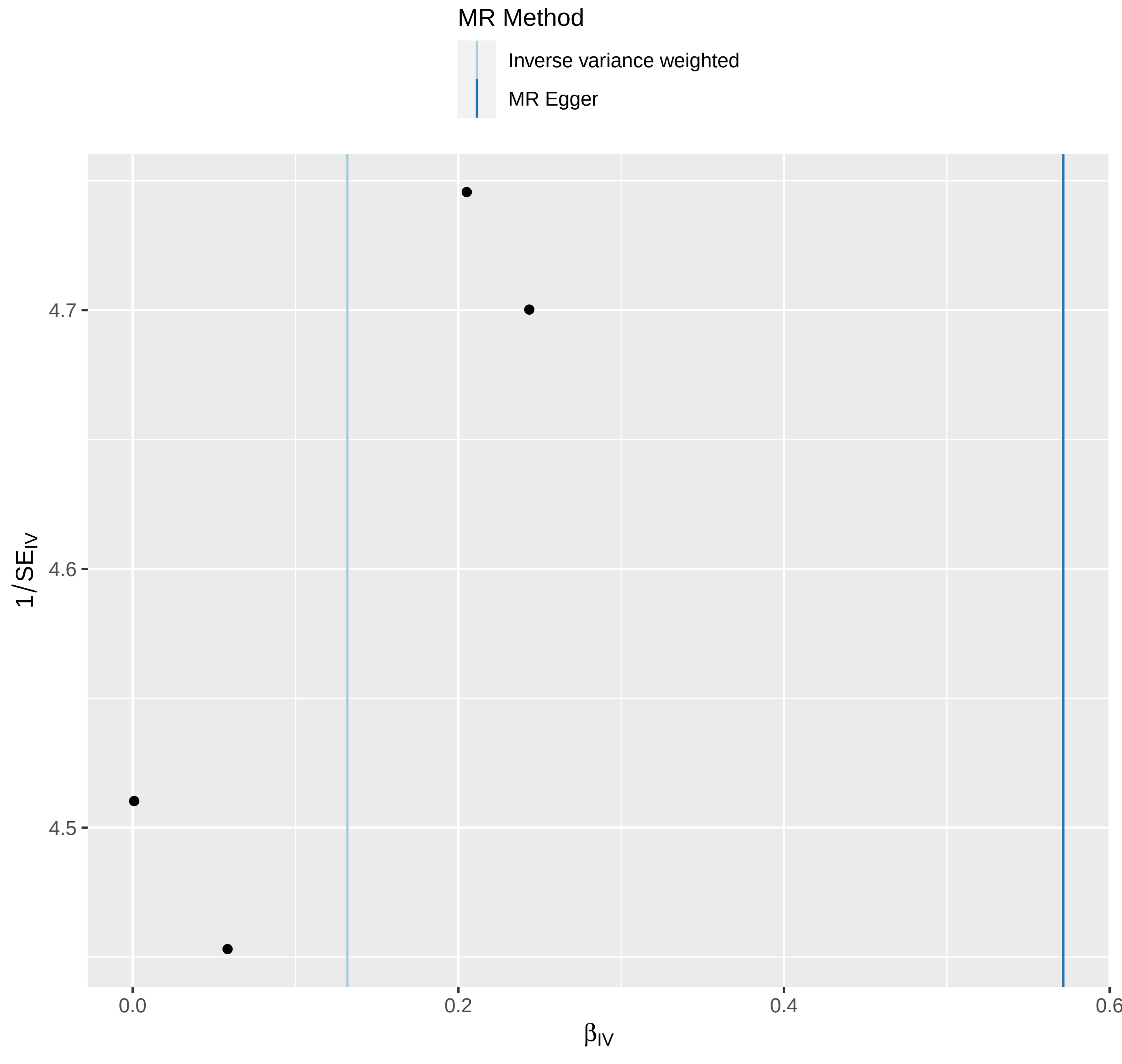

Batch 2−774 : Gut microbiota abundance (genus Parasutterella id.2892) on Eotaxin
with a threshold of 1e−05

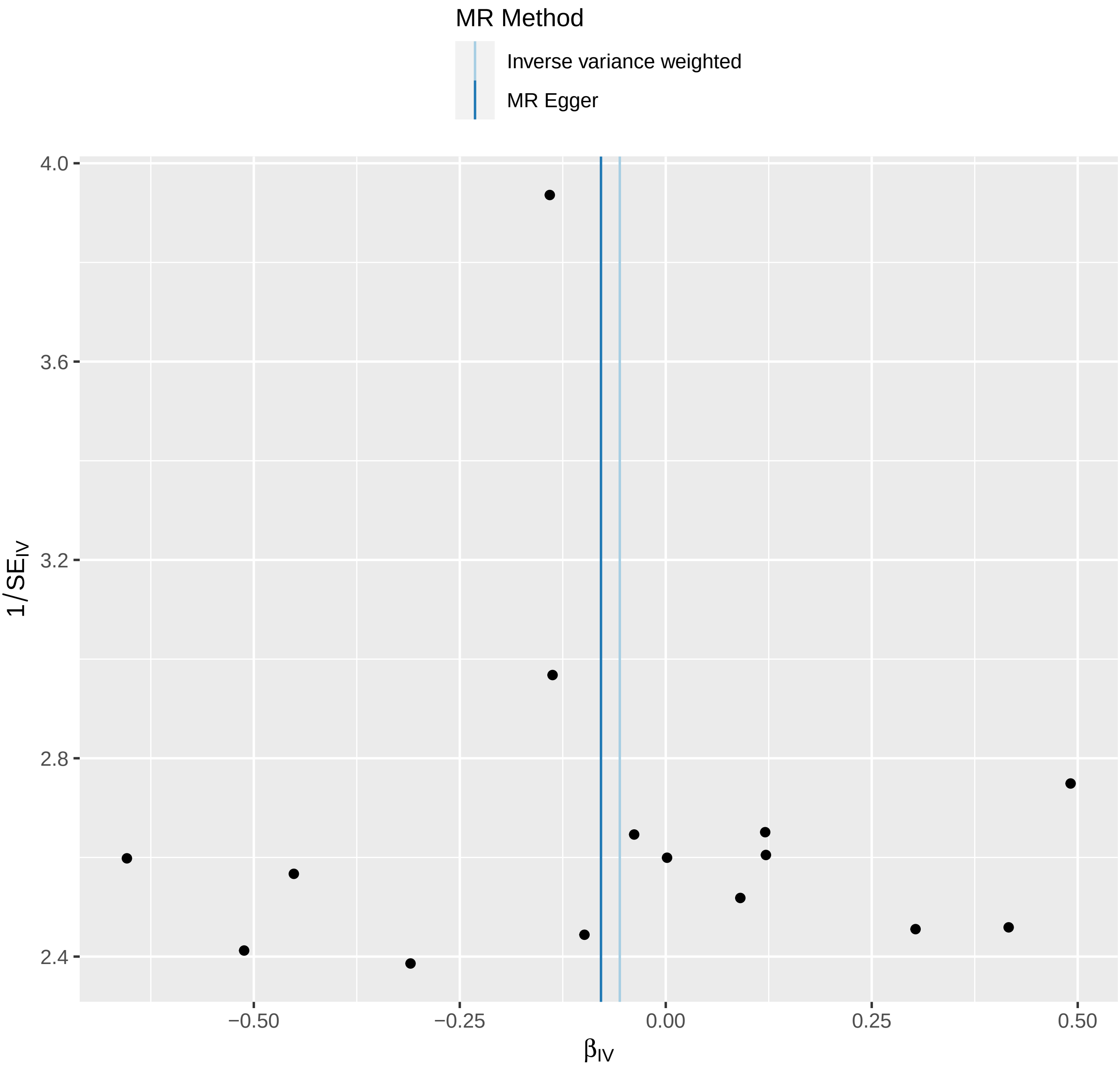

Batch 2−775 : Gut microbiota abundance (genus Catenibacterium id.2153) on C−C motif chemokine 1
with a threshold of 1e−05

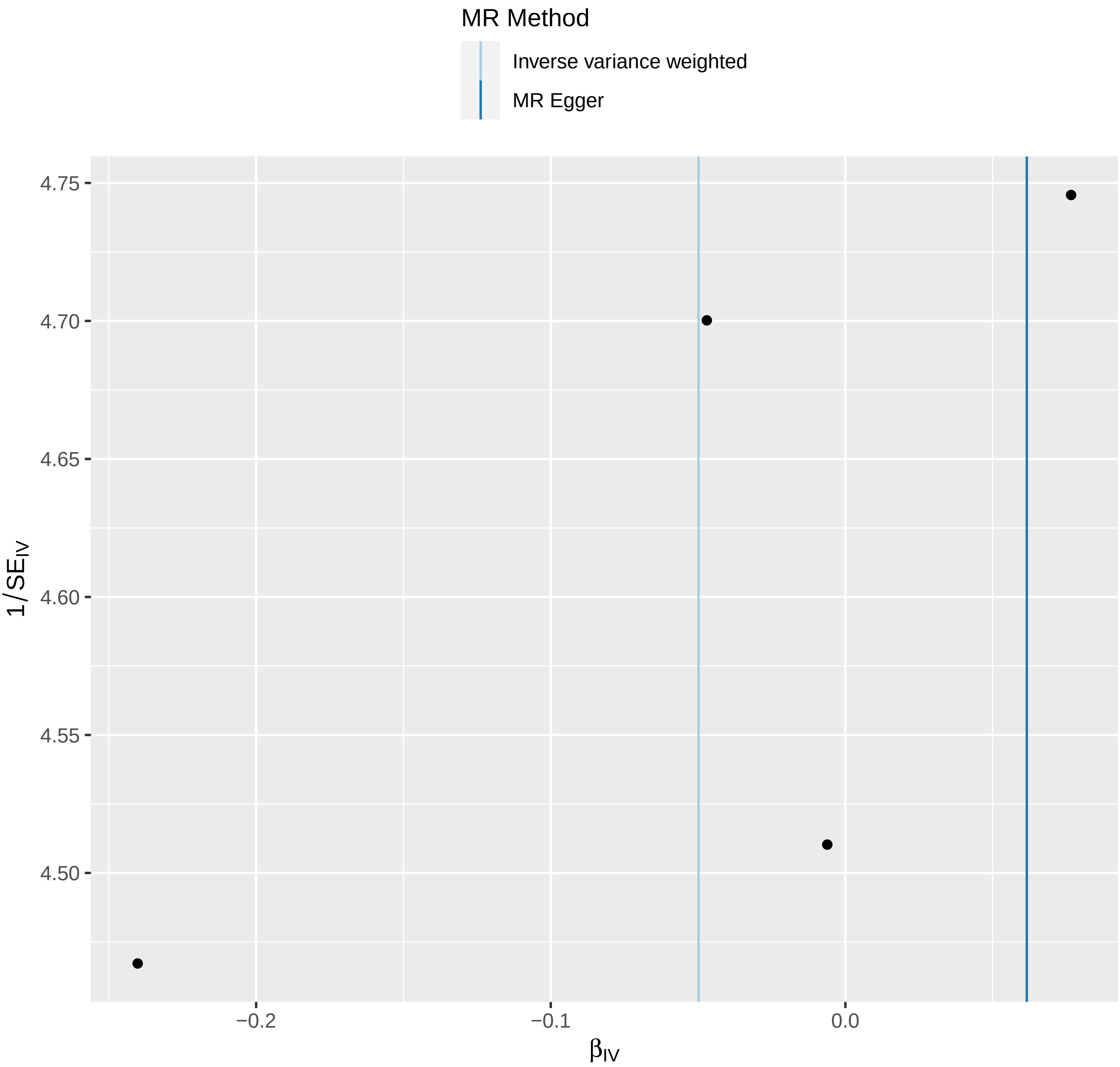

Batch 2−776 : Gut microbiota abundance (genus Parasutterella id.2892) on C−C motif chemokine 1
with a threshold of 1e−05

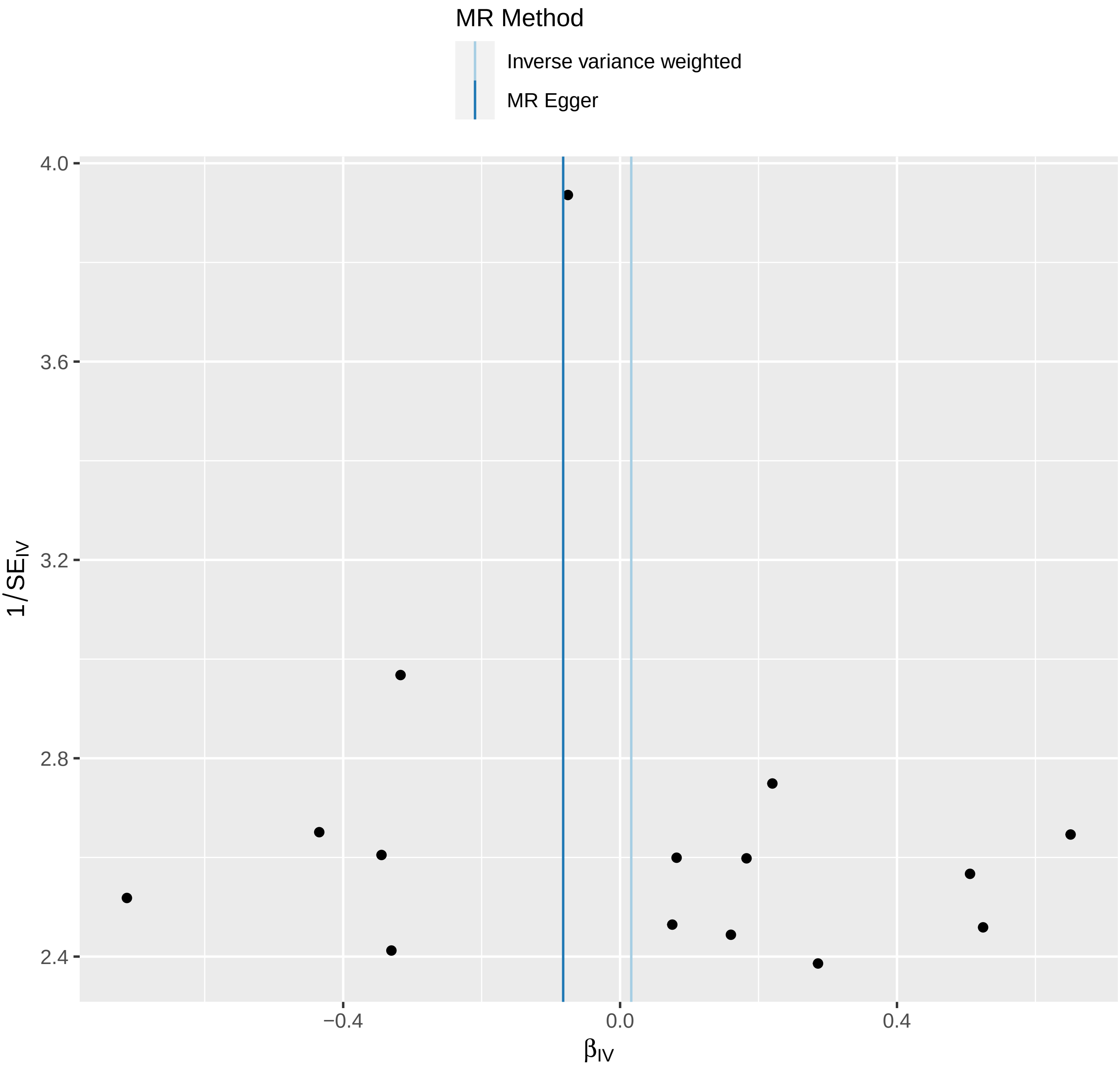

Batch 2−777 : Gut microbiota abundance (genus Catenibacterium id.2153) on C−C motif chemokine 13 with a threshold of 1e−05

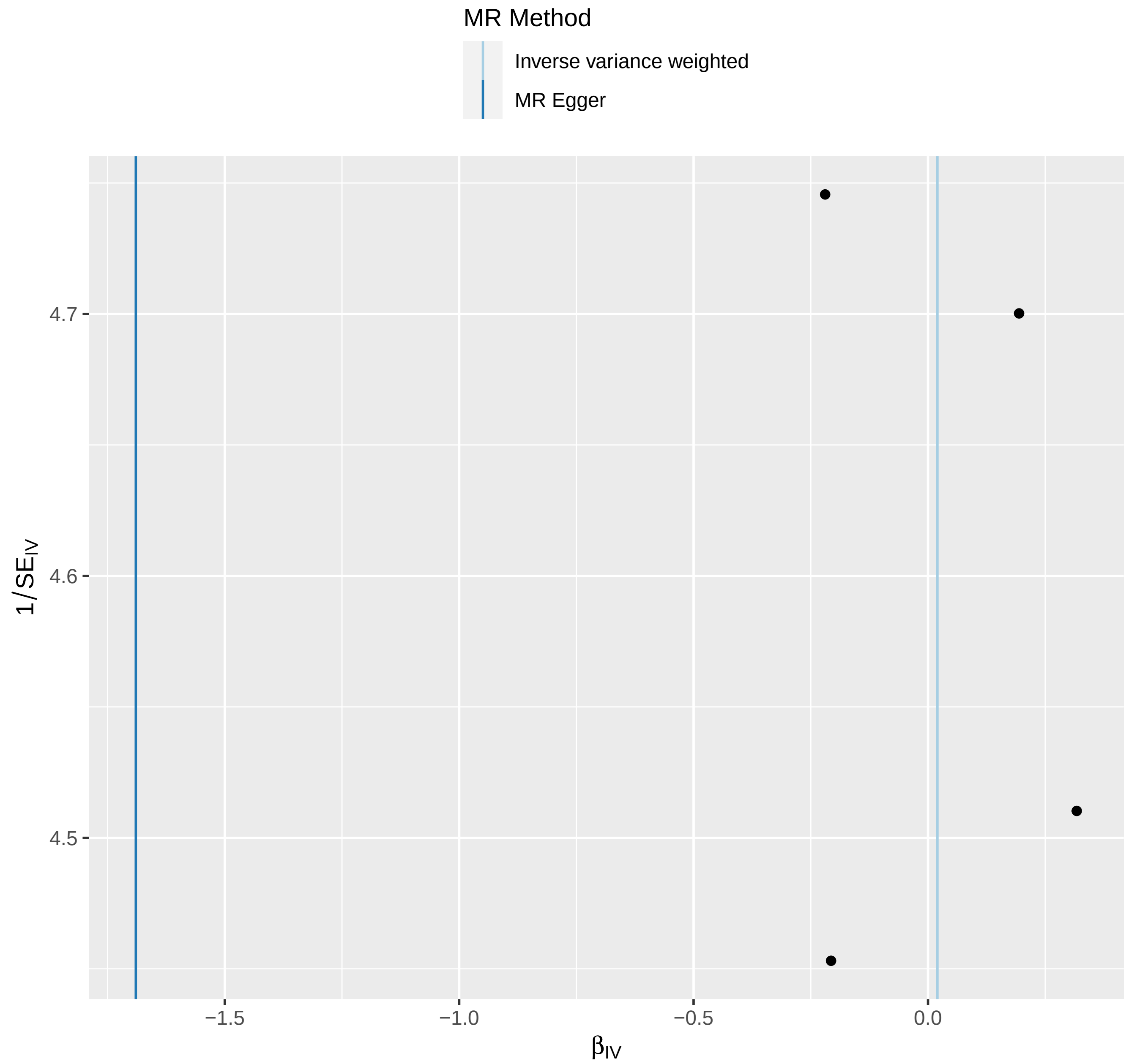

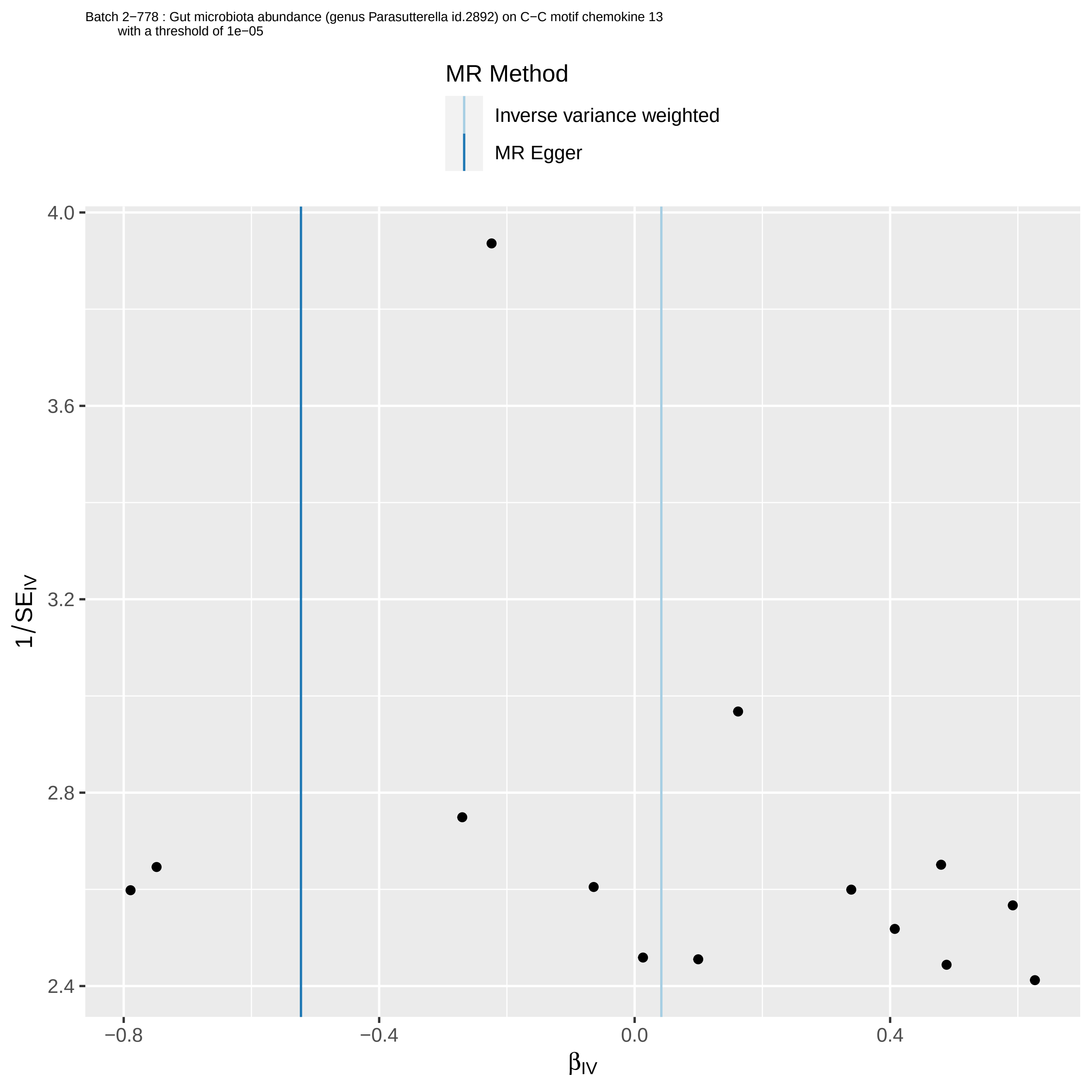

Batch 2−778 : Gut microbiota abundance (genus Parasutterella id.2892) on C−C motif chemokine 13
with a threshold of 1e−05
MR Method
Inverse variance weighted
MR Egger
4.0
3.6
3.2
2.8
2.4
1/SE$_{IV}$
−0.8
−0.4
0.0
0.4
β$_{IV}$

Batch 2−779 : Gut microbiota abundance (genus Catenibacterium id.2153) on C−C motif chemokine 14
with a threshold of 1e−05

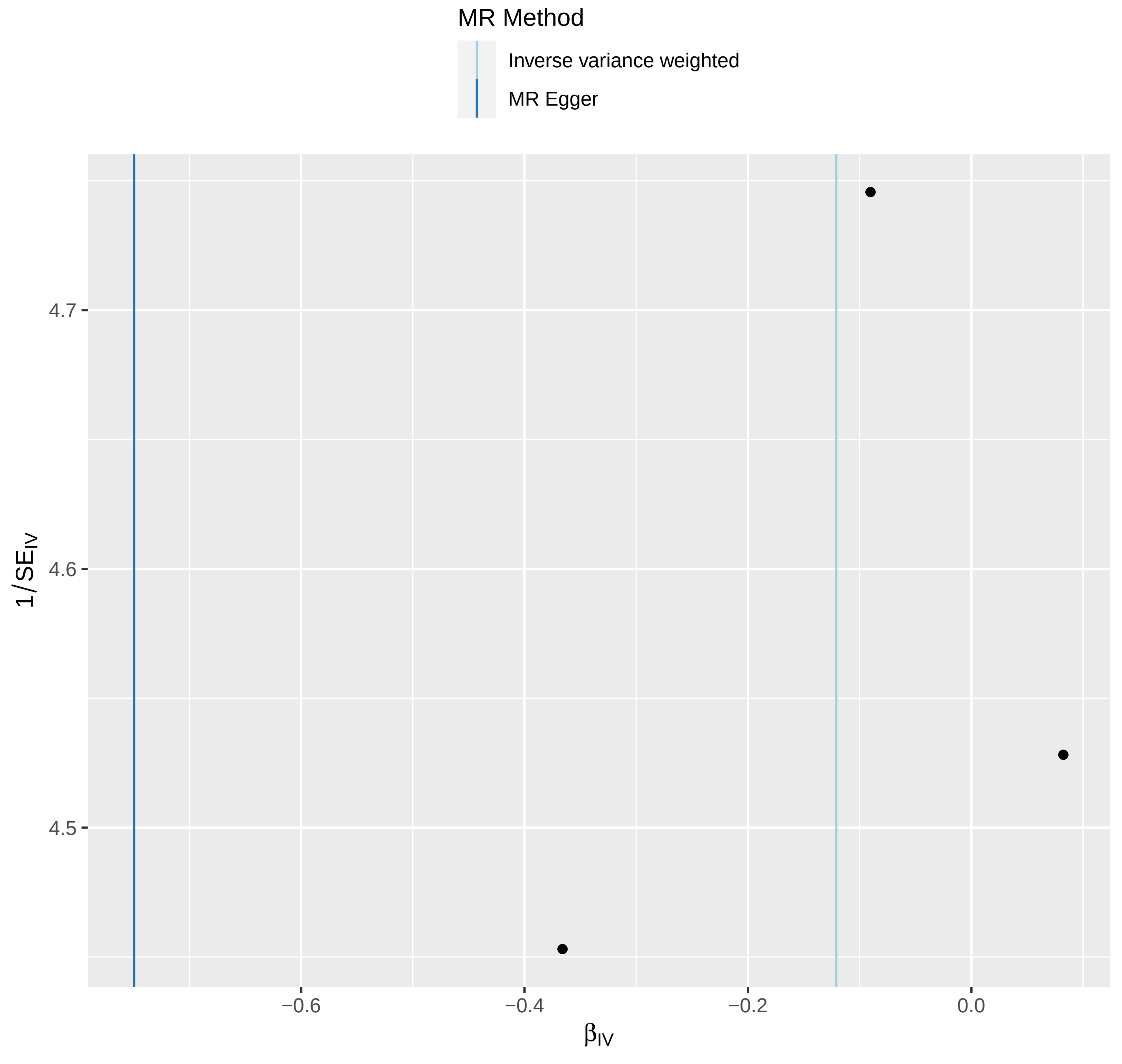

Batch 2−780 : Gut microbiota abundance (genus Parasutterella id.2892) on C−C motif chemokine 14
with a threshold of 1e−05

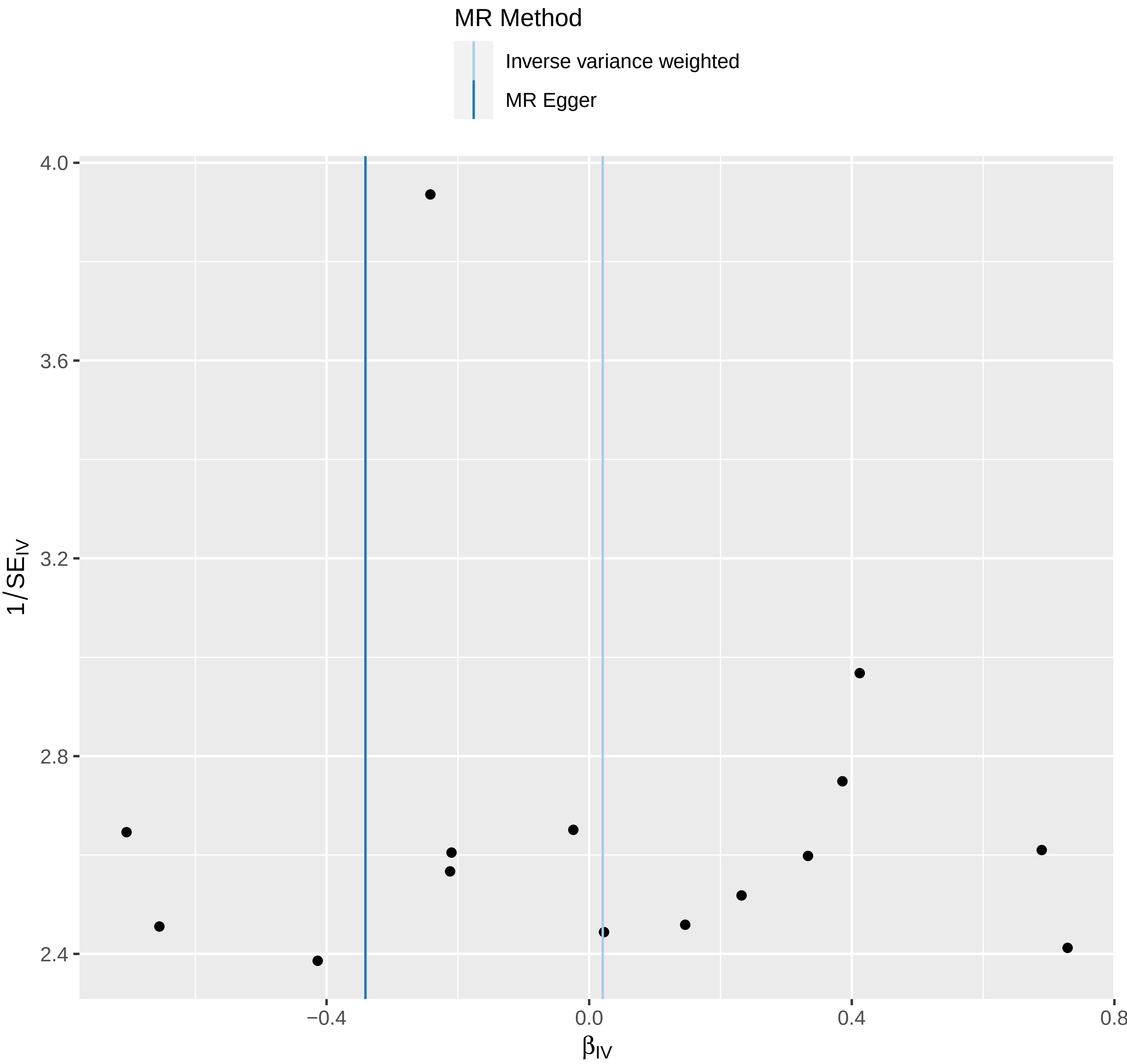

Batch 2−781 : Gut microbiota abundance (genus Catenibacterium id.2153) on C−C motif chemokine 15
with a threshold of 1e−05

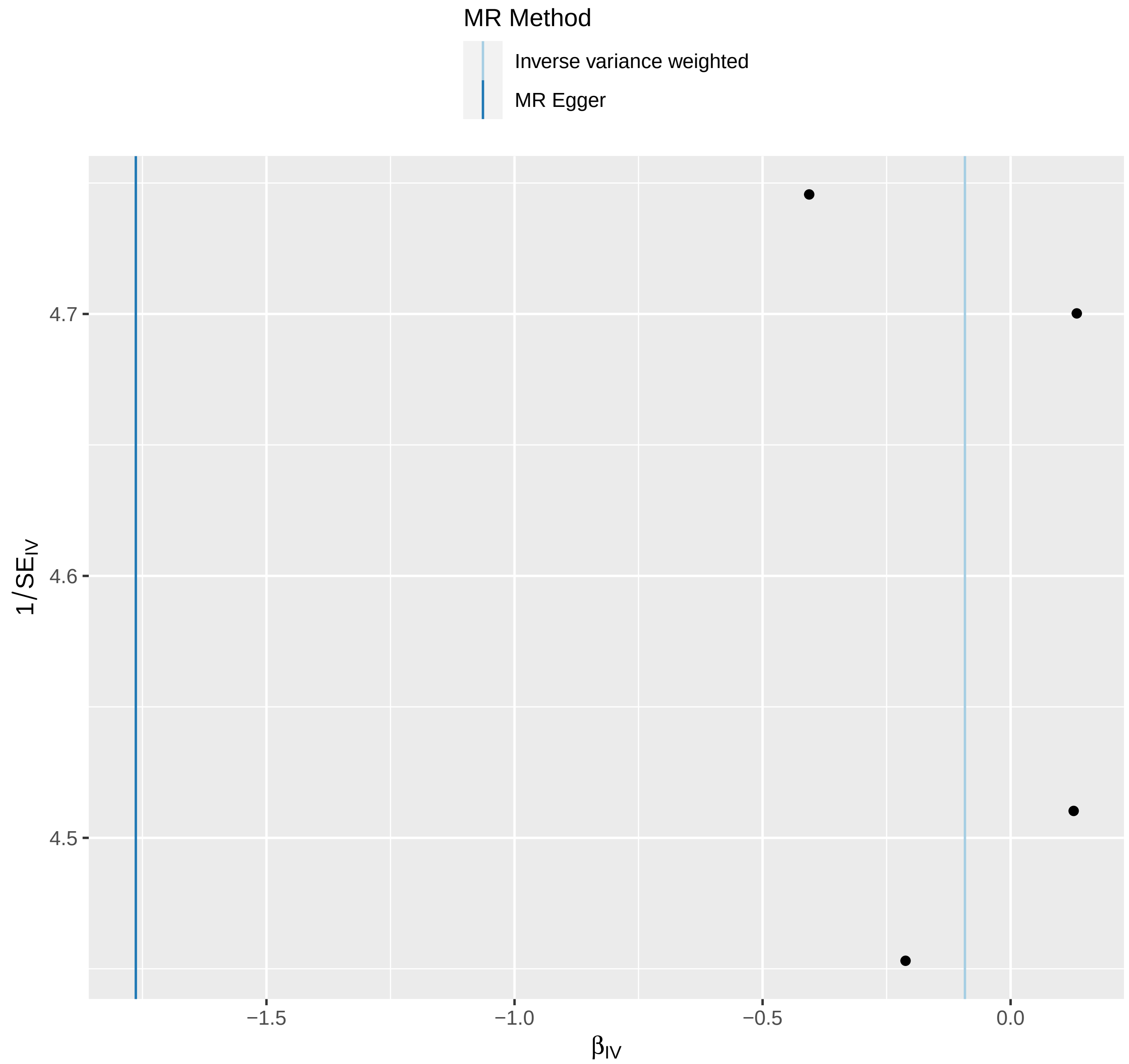

Batch 2−782 : Gut microbiota abundance (genus Parasutterella id.2892) on C−C motif chemokine 15
with a threshold of 1e−05

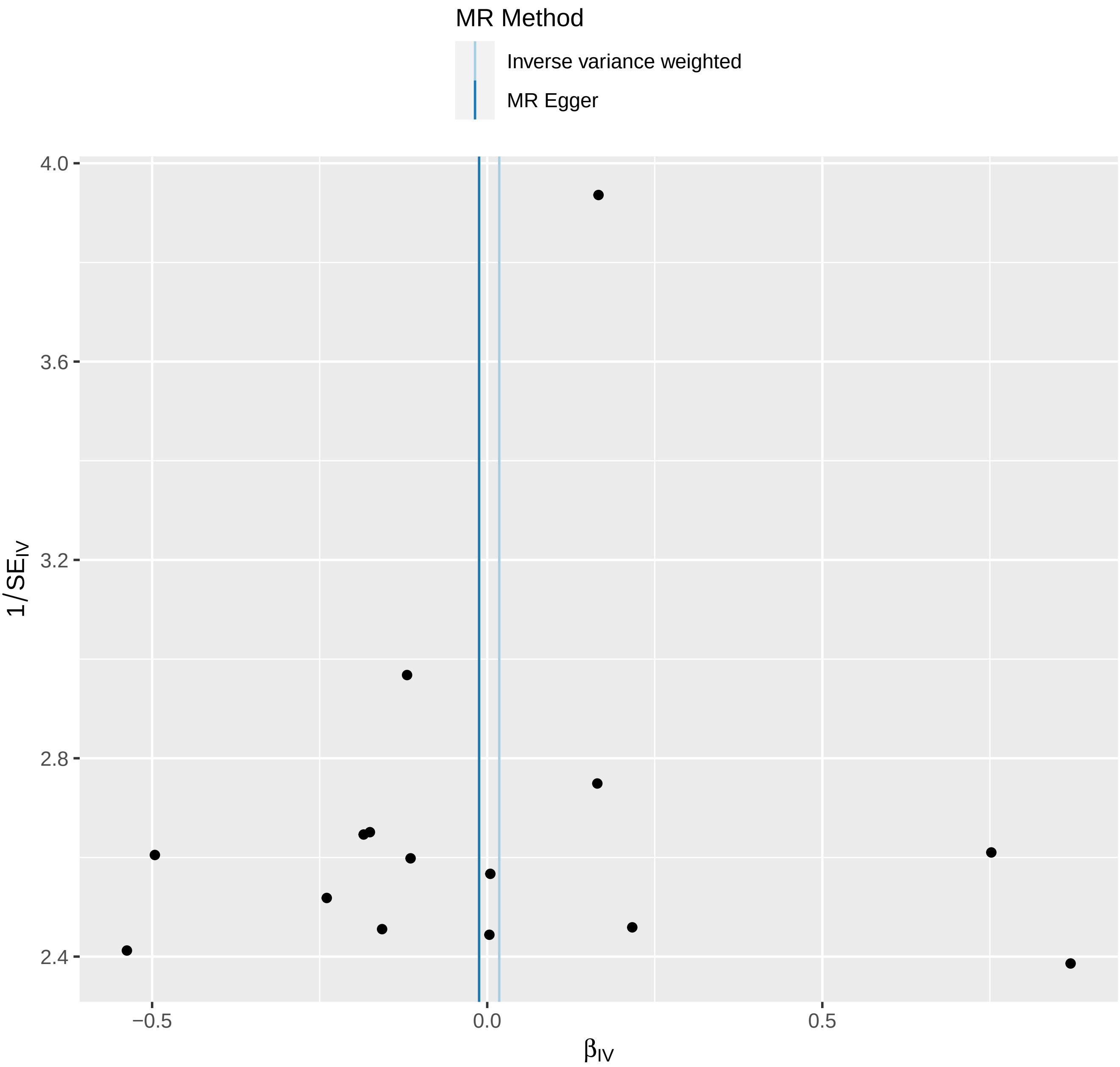

Batch 2−783 : Gut microbiota abundance (genus Catenibacterium id.2153) on C−C motif chemokine 15 with a threshold of 1e−05

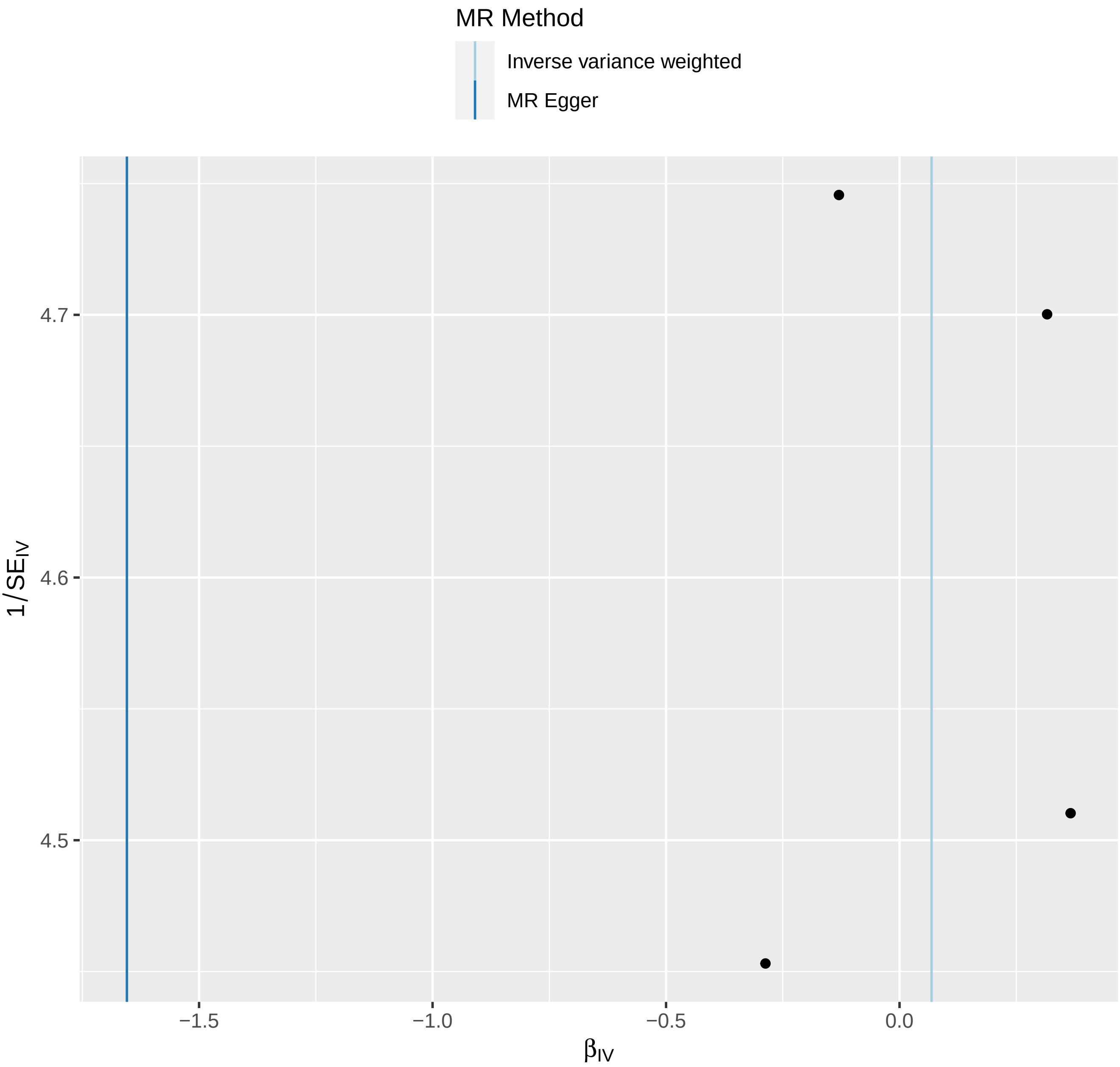

Batch 2−784 : Gut microbiota abundance (genus Parasutterella id.2892) on C−C motif chemokine 15
with a threshold of 1e−05

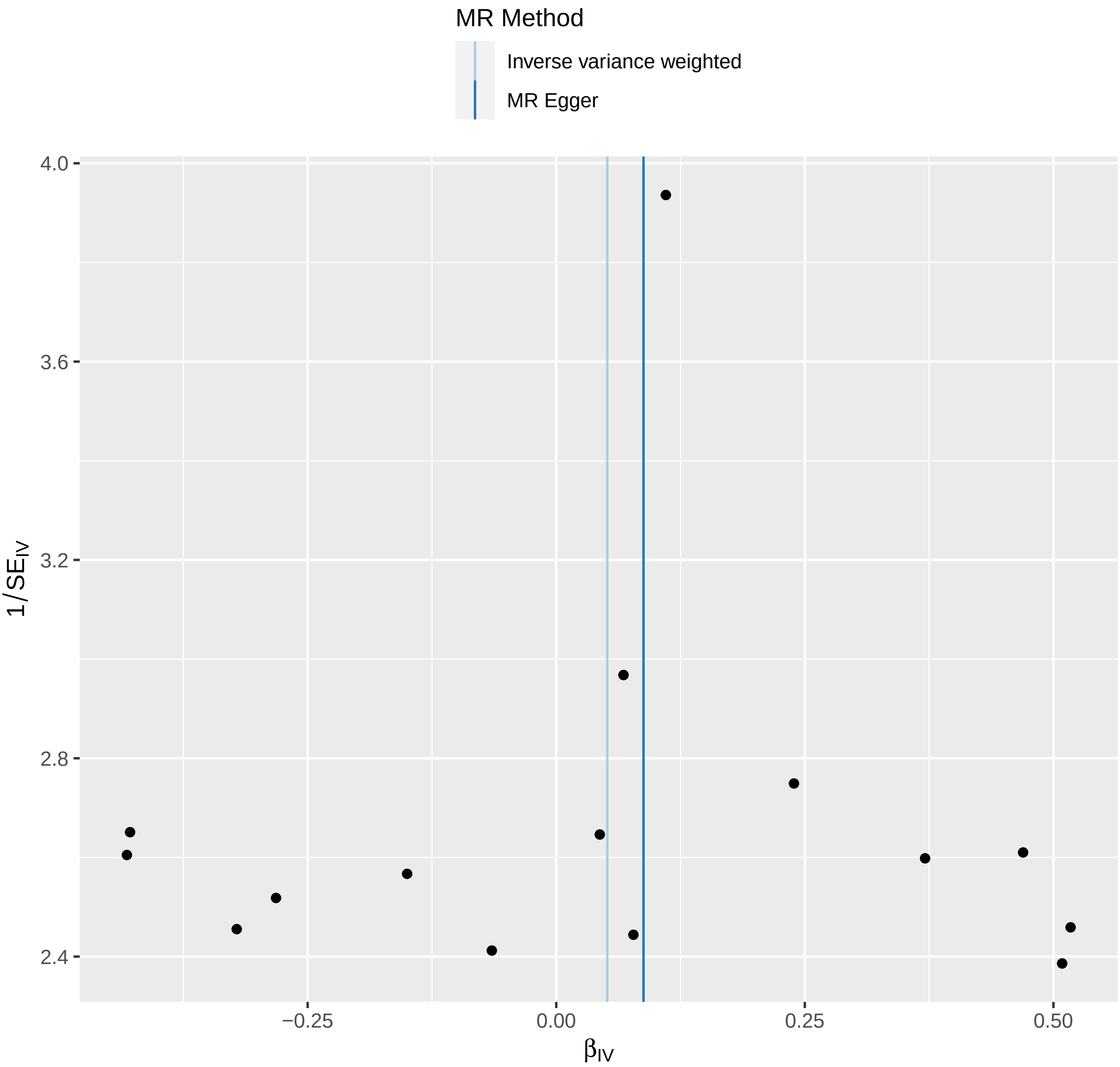

Batch 2−785 : Gut microbiota abundance (genus Catenibacterium id.2153) on C−C motif chemokine 16 with a threshold of 1e−05

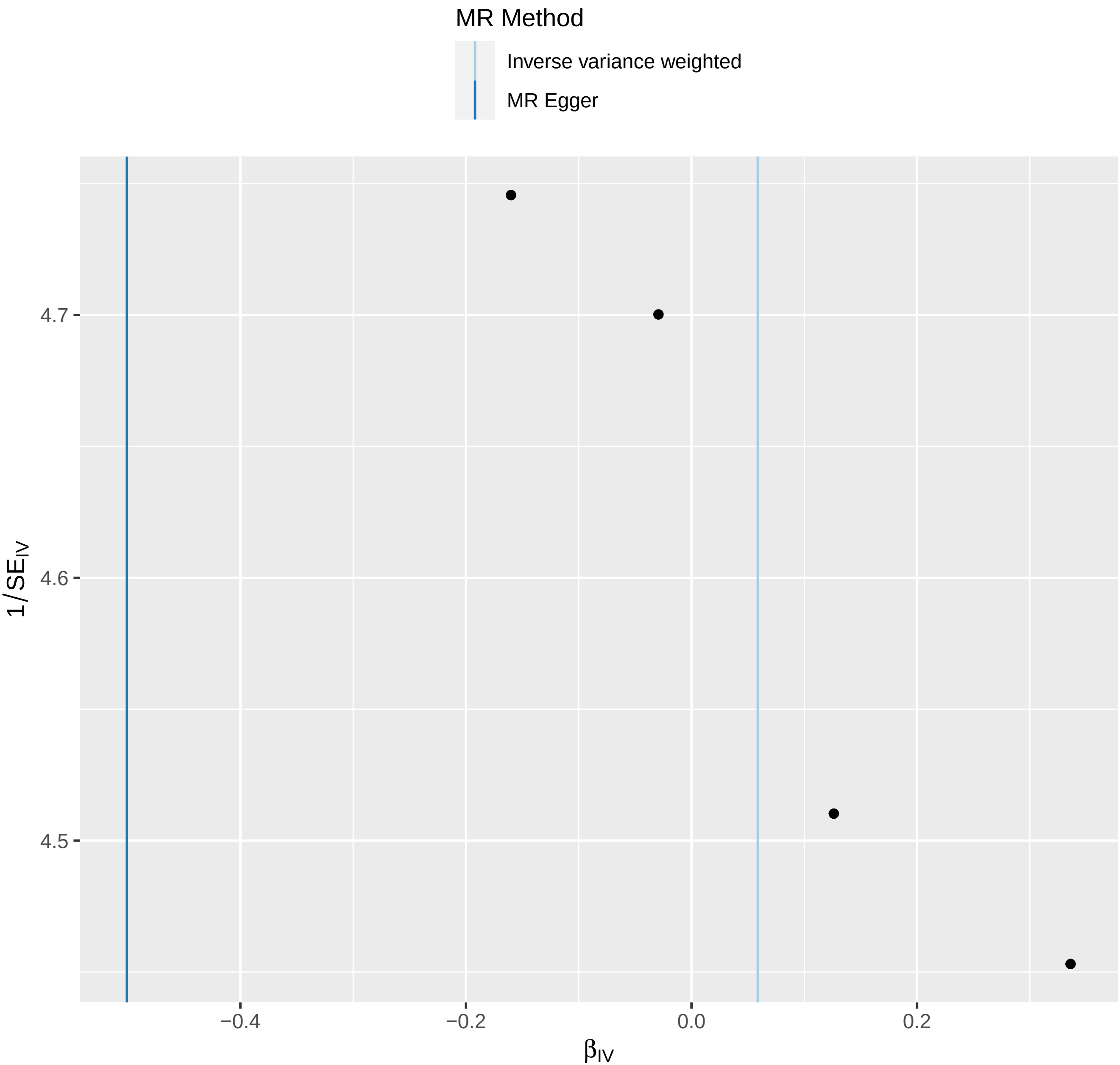

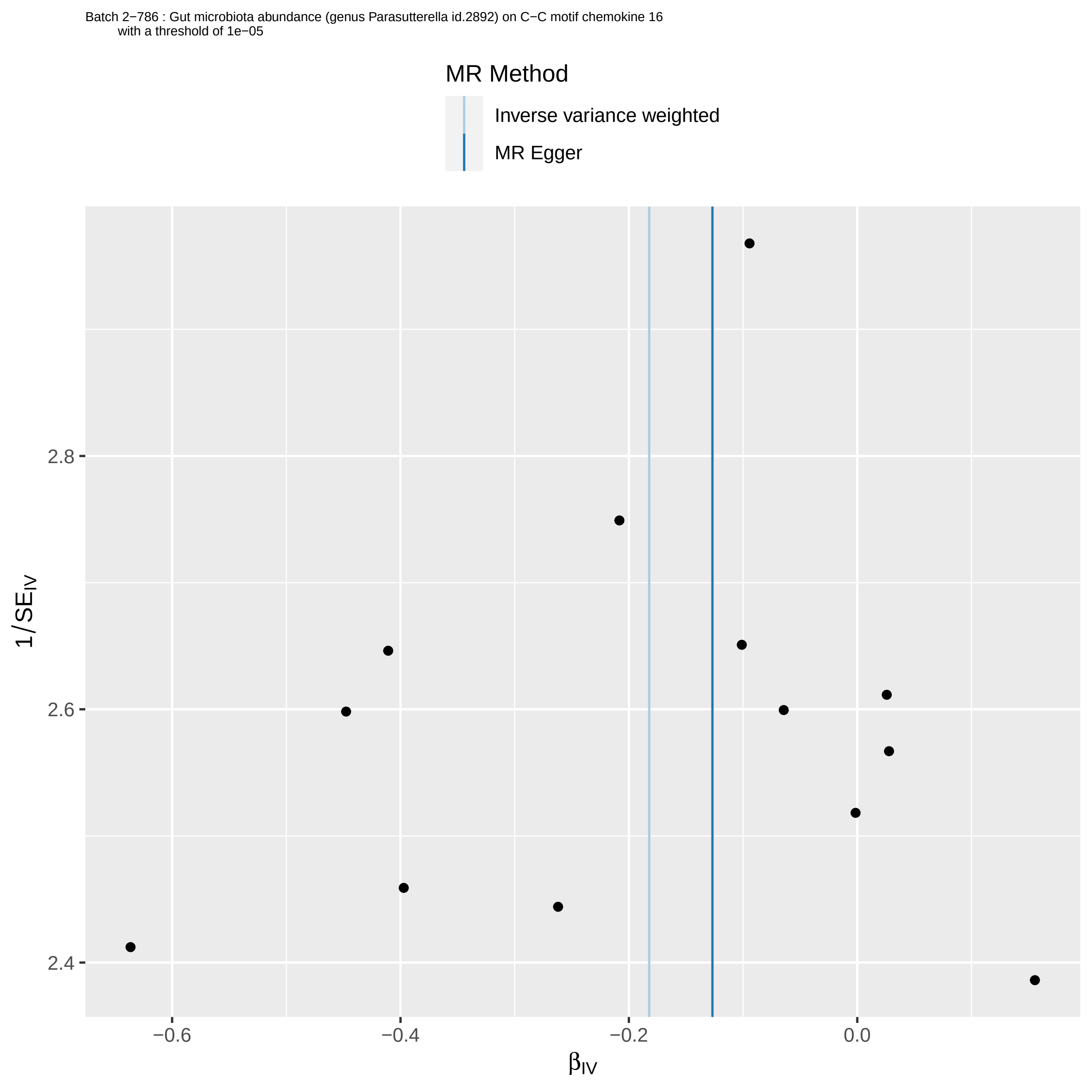
Batch 2−786 : Gut microbiota abundance (genus Parasutterella id.2892) on C−C motif chemokine 16
with a threshold of 1e−05
MR Method
Inverse variance weighted
MR Egger
2.8
2.6
2.4
1/SE_IV
−0.6
−0.4
−0.2
0.0
β_IV

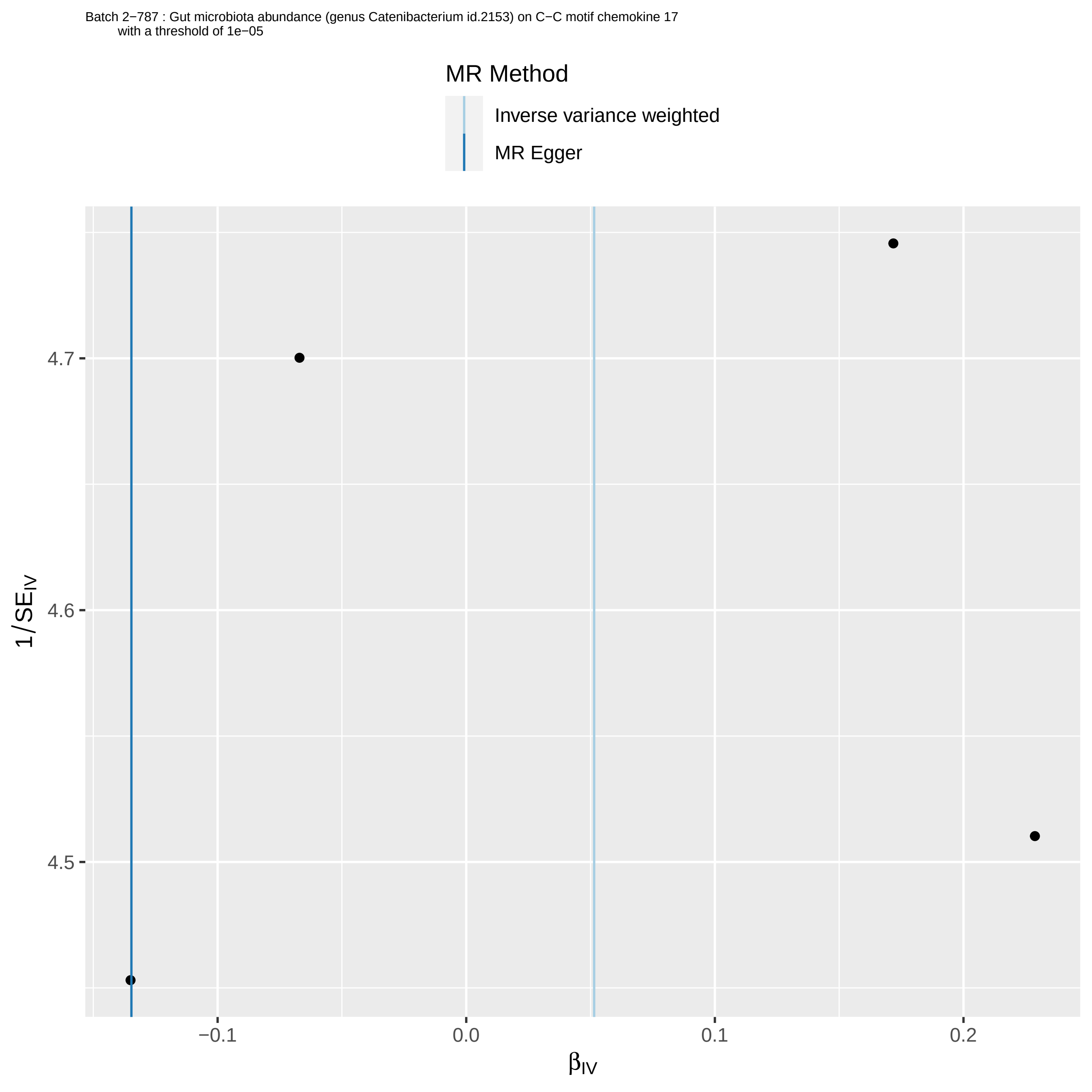
Batch 2−787 : Gut microbiota abundance (genus Catenibacterium id.2153) on C−C motif chemokine 17
with a threshold of 1e−05
MR Method
Inverse variance weighted
MR Egger
4.7
4.6
4.5
1/SE$_{IV}$
−0.1
0.0
0.1
0.2
β_{IV}

Batch 2−788 : Gut microbiota abundance (genus Parasutterella id.2892) on C−C motif chemokine 17
with a threshold of 1e−05

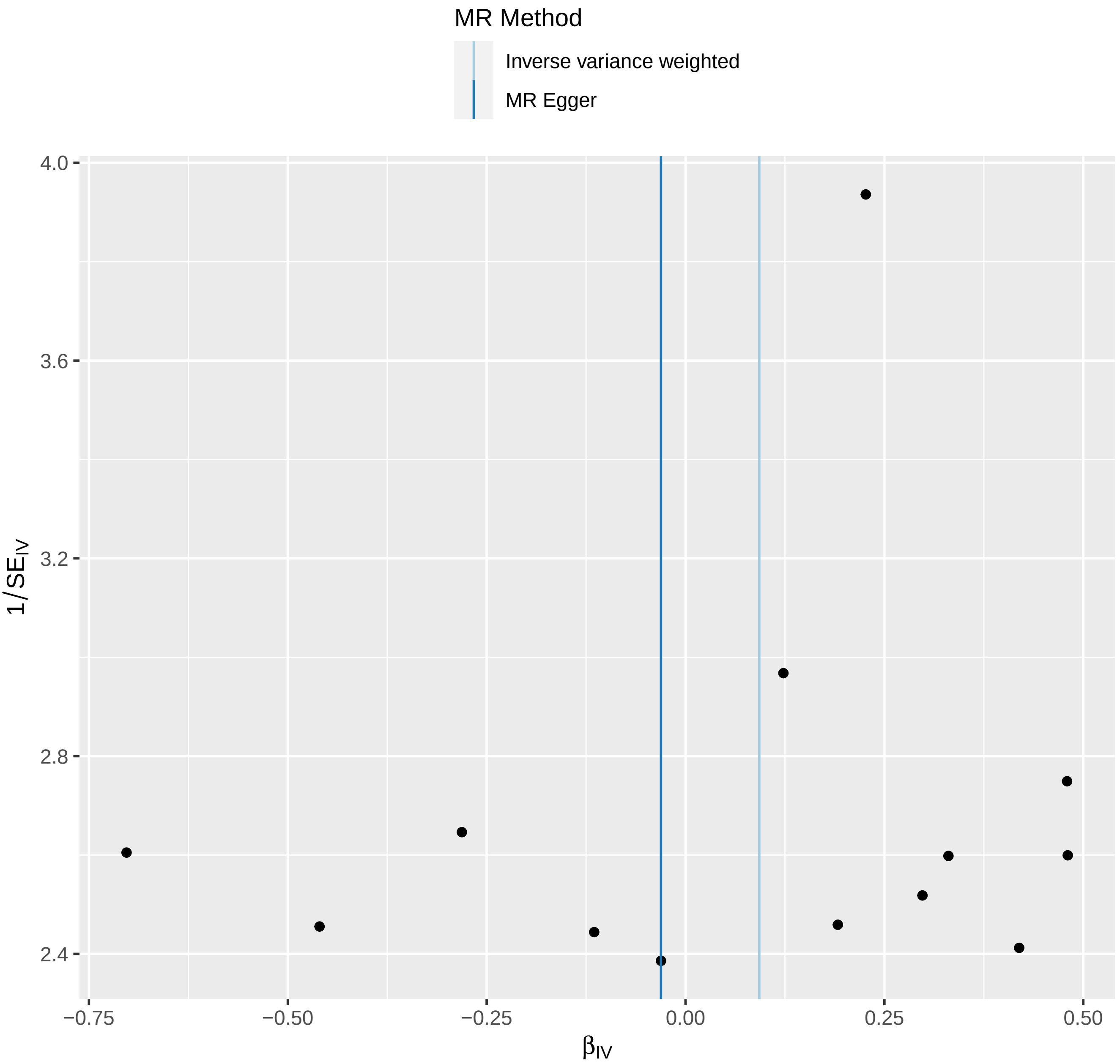

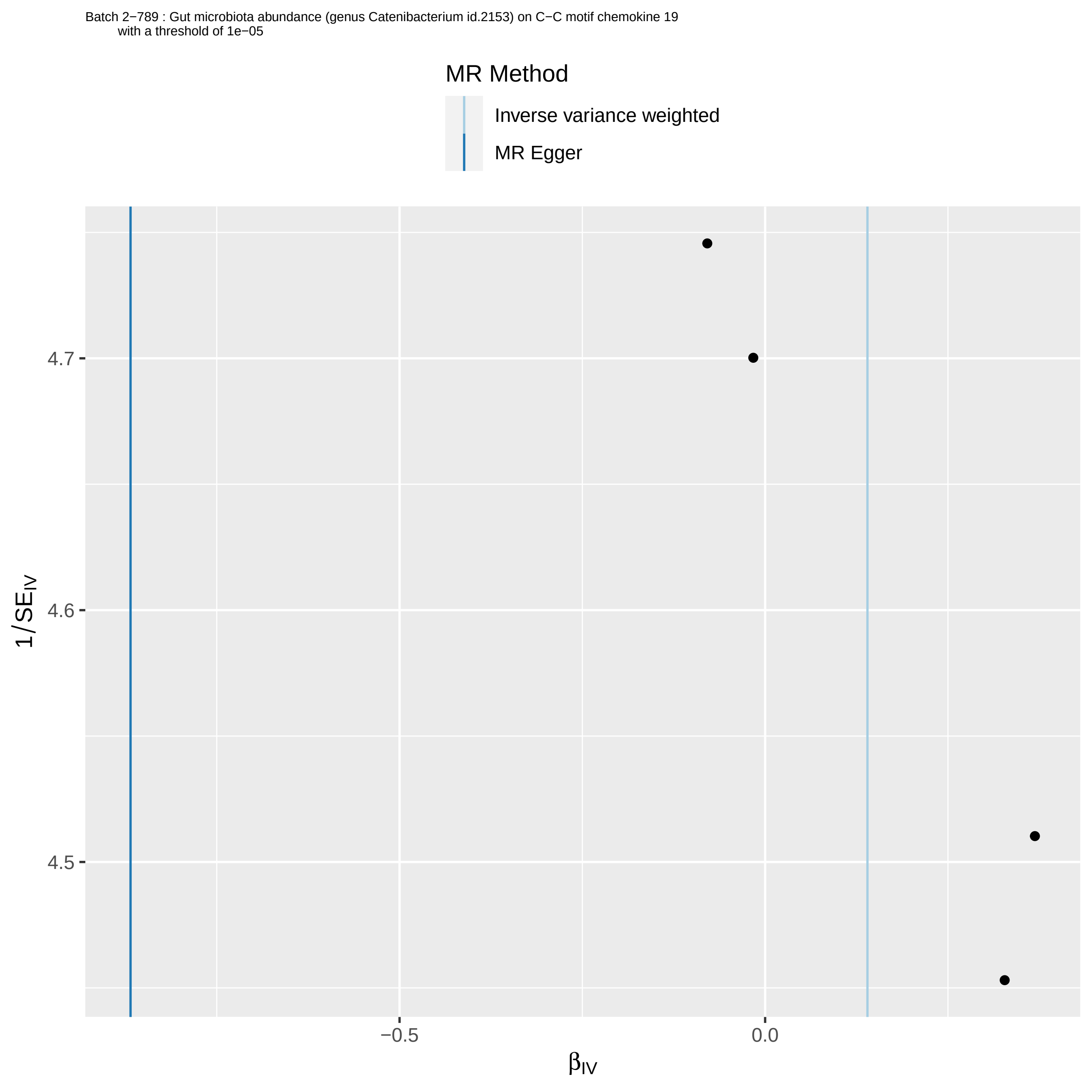
Batch 2−789 : Gut microbiota abundance (genus Catenibacterium id.2153) on C−C motif chemokine 19
with a threshold of 1e−05
MR Method
Inverse variance weighted
MR Egger
4.7
4.6
4.5
1/SE_IV
−0.5
0.0
β_IV

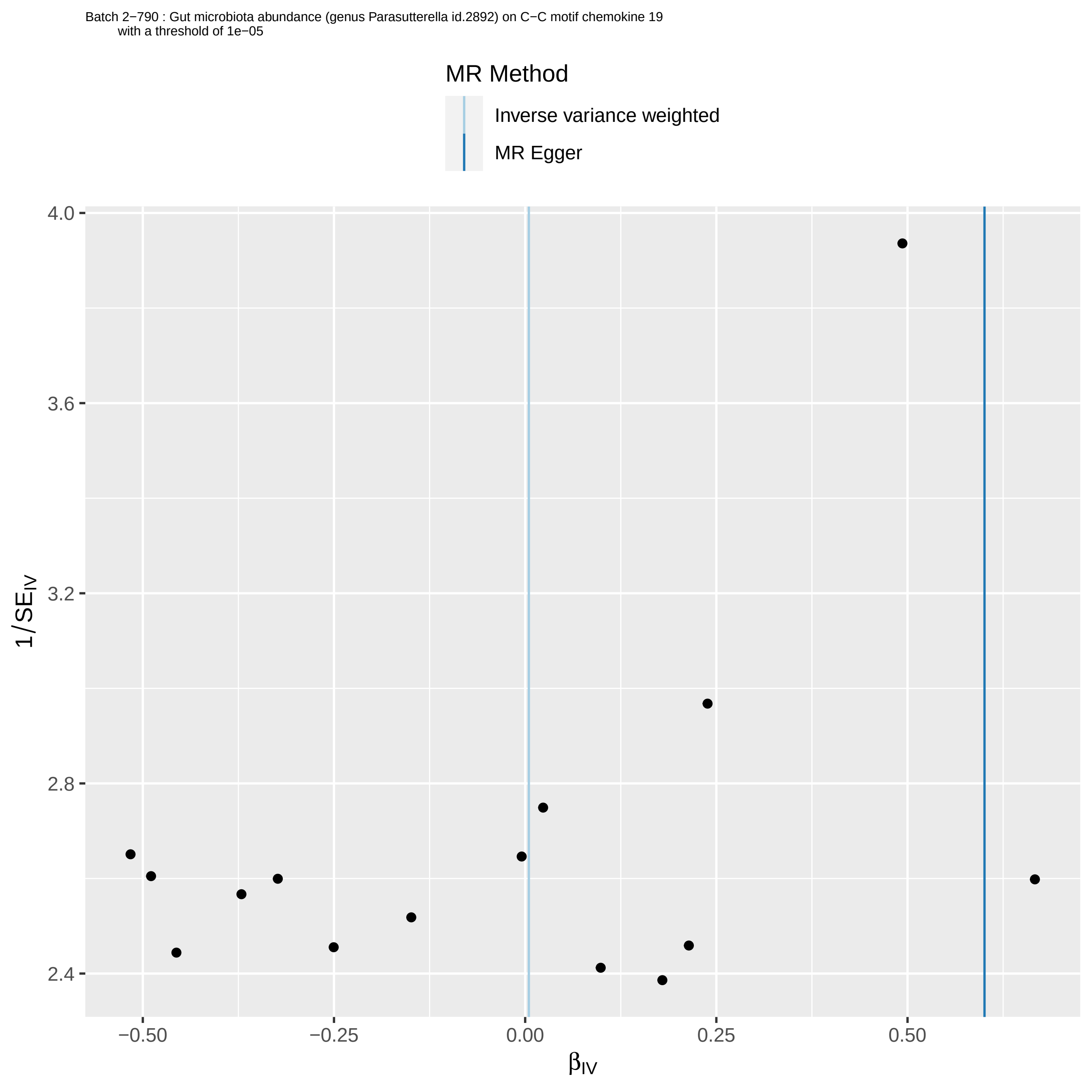
Batch 2−790 : Gut microbiota abundance (genus Parasutterella id.2892) on C−C motif chemokine 19
with a threshold of 1e−05
MR Method
Inverse variance weighted
MR Egger
4.0
3.6
3.2
2.8
2.4
1/SE_IV
−0.50
−0.25
0.00
0.25
0.50
β_IV

Batch 2−791 : Gut microbiota abundance (genus Catenibacterium id.2153) on C−C motif chemokine 20
with a threshold of 1e−05

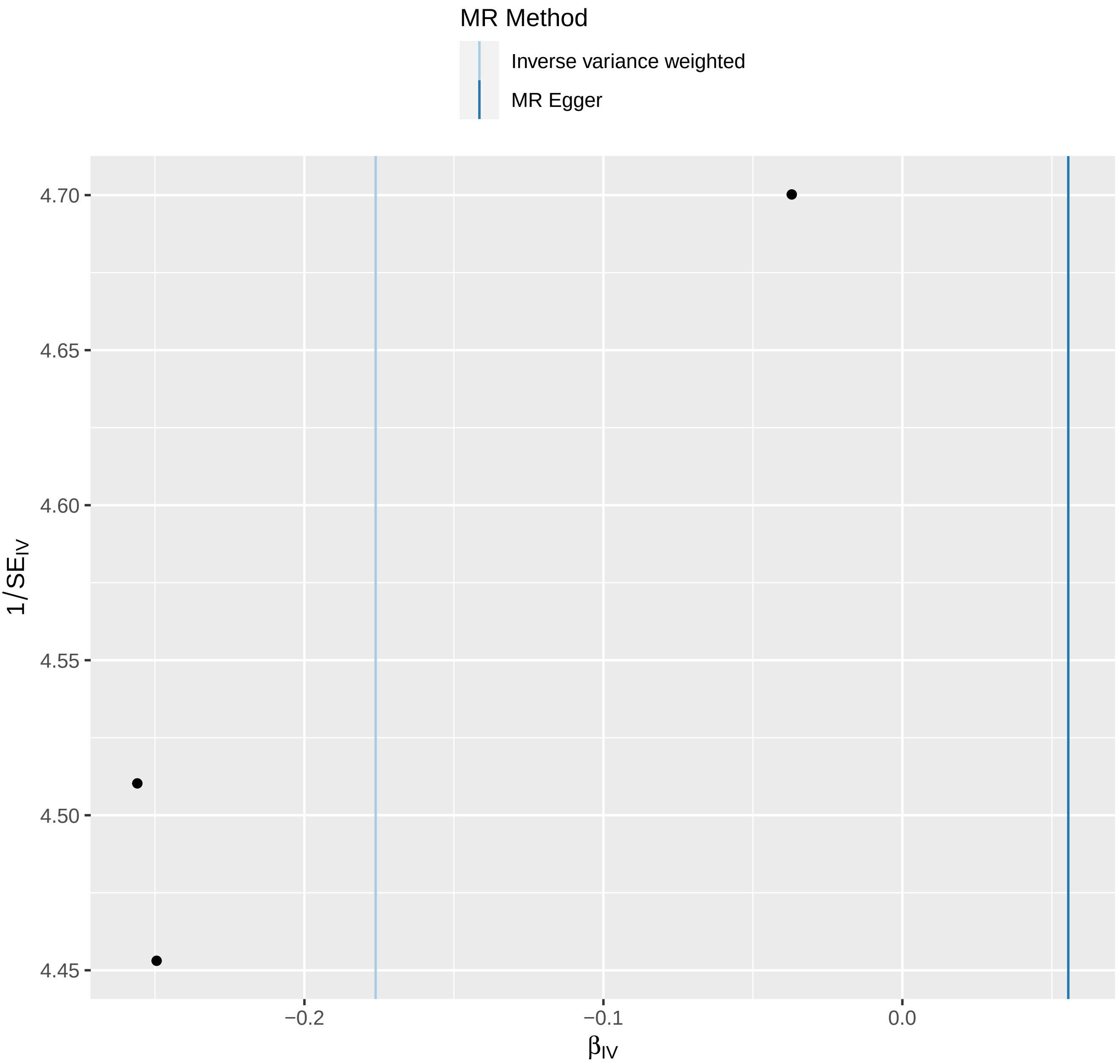

Batch 2−792 : Gut microbiota abundance (genus Parasutterella id.2892) on C−C motif chemokine 20 with a threshold of 1e−05

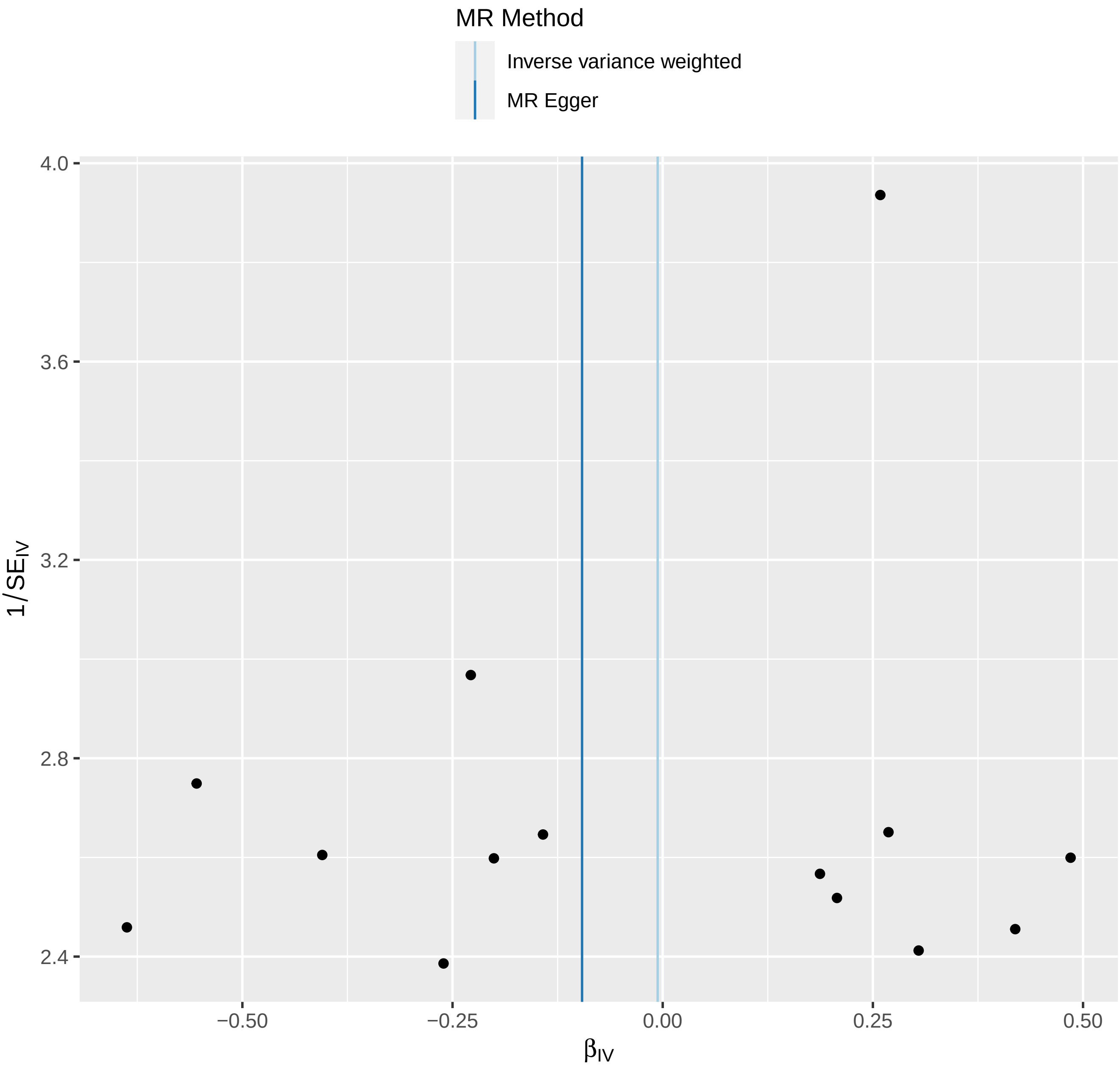

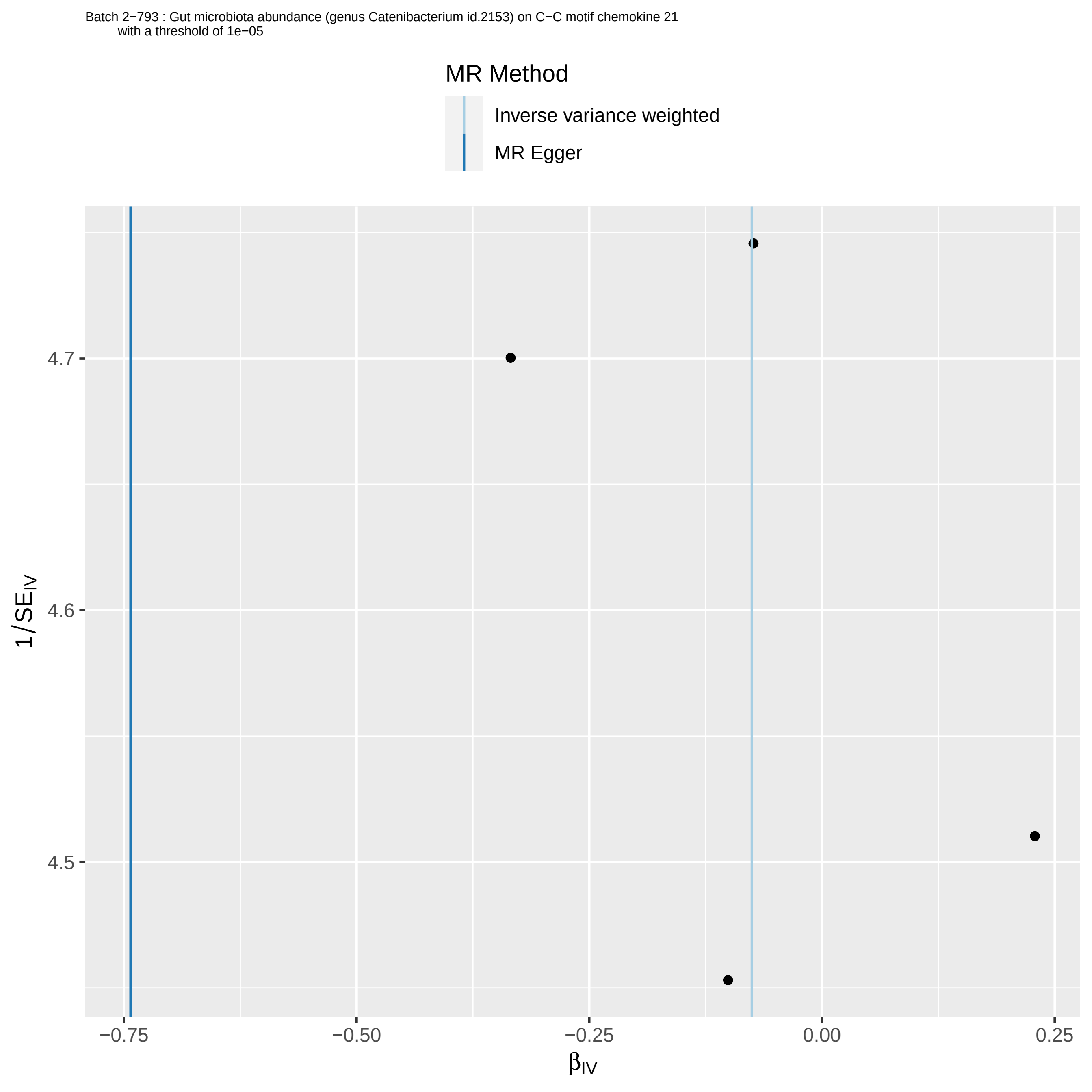
Batch 2−793 : Gut microbiota abundance (genus Catenibacterium id.2153) on C−C motif chemokine 21
with a threshold of 1e−05
MR Method
Inverse variance weighted
MR Egger
4.7
4.6
4.5
1/SE_IV
−0.75
−0.50
−0.25
0.00
0.25
β_IV

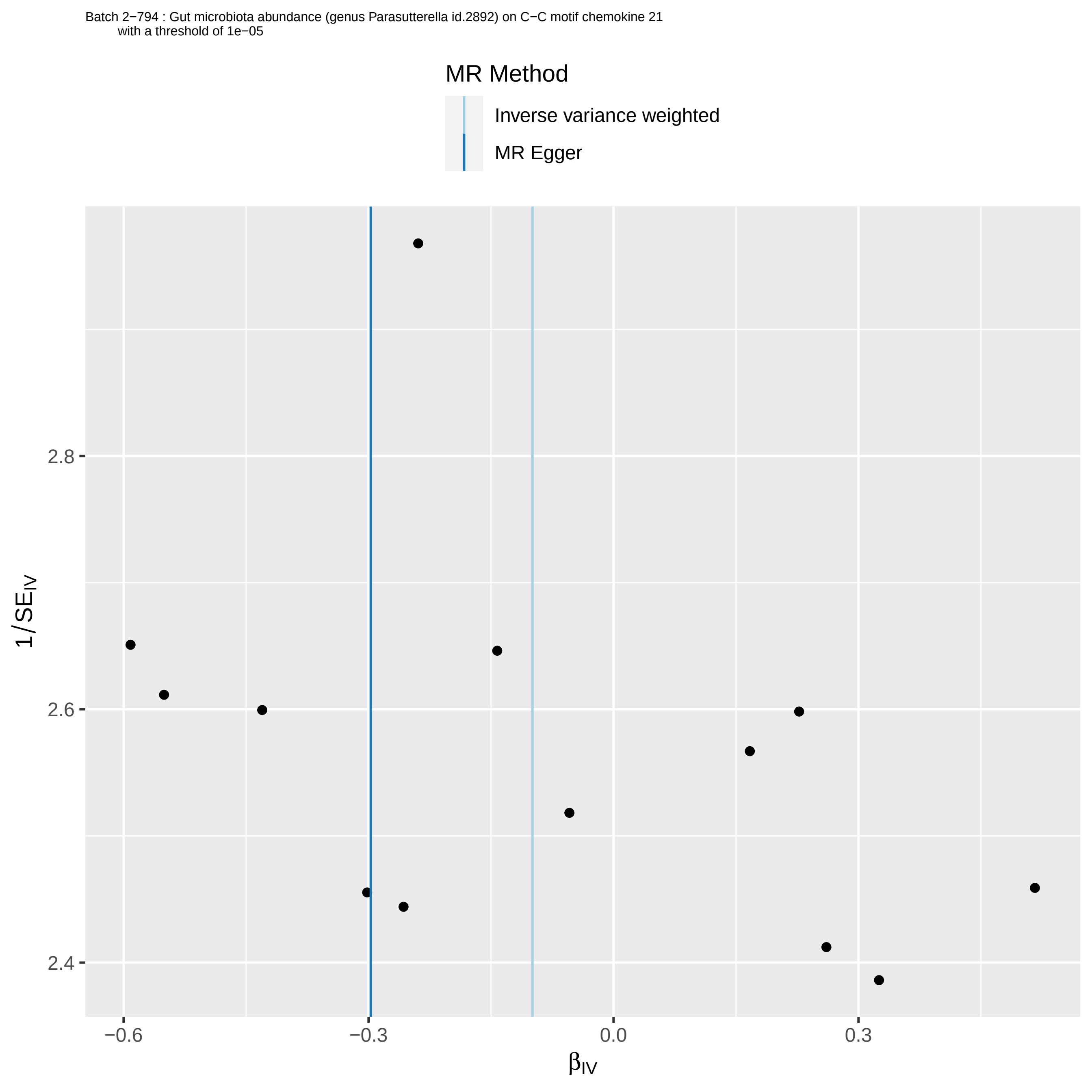

Batch 2−794 : Gut microbiota abundance (genus Parasutterella id.2892) on C−C motif chemokine 21
with a threshold of 1e−05
MR Method
Inverse variance weighted
MR Egger
2.8
2.6
2.4
1/SE_IV
−0.6
−0.3
0.0
0.3
β_IV

Batch 2−795 : Gut microbiota abundance (genus Catenibacterium id.2153) on C−C motif chemokine 22
with a threshold of 1e−05

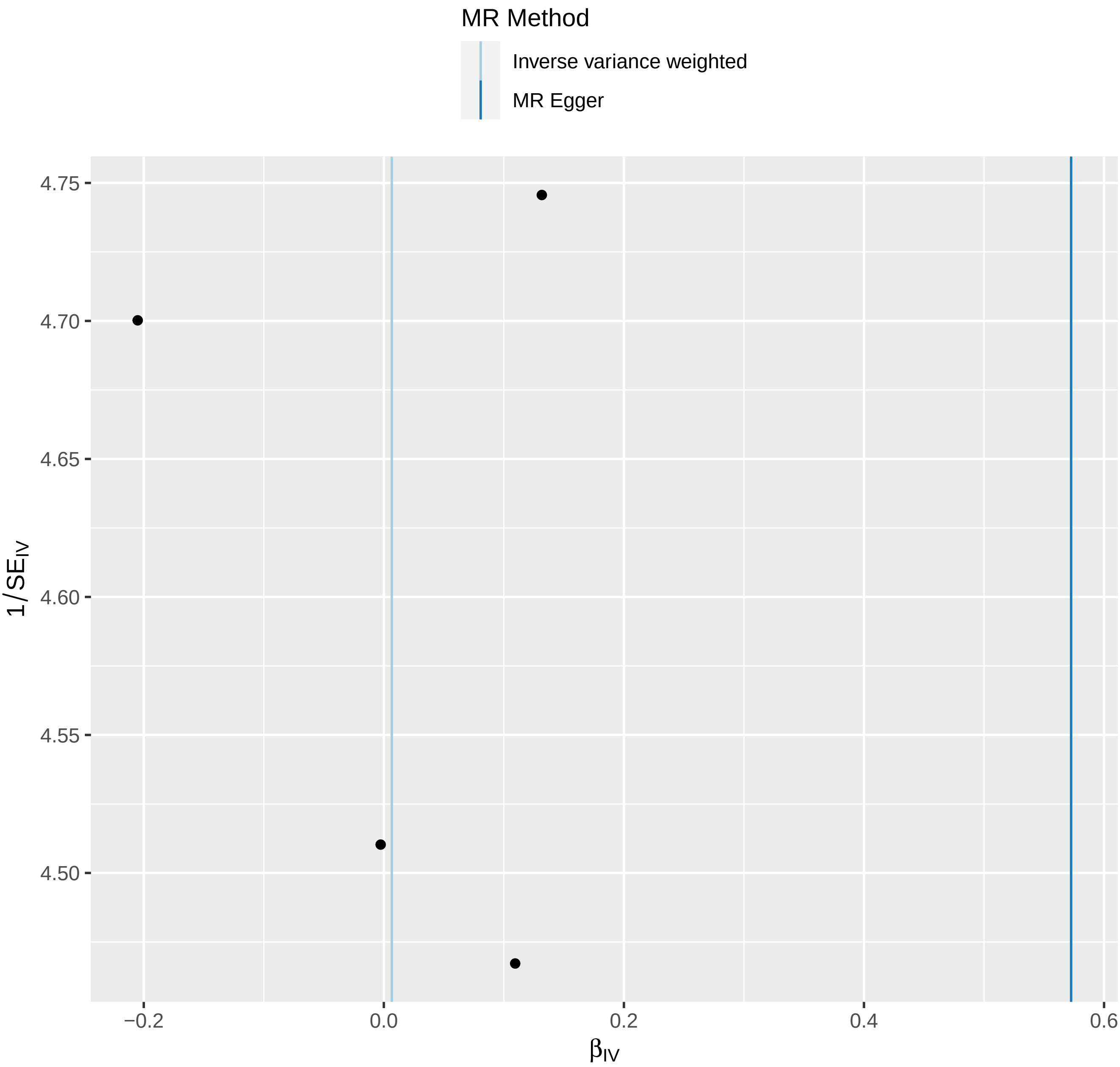

Batch 2−796 : Gut microbiota abundance (genus Parasutterella id.2892) on C−C motif chemokine 22
with a threshold of 1e−05

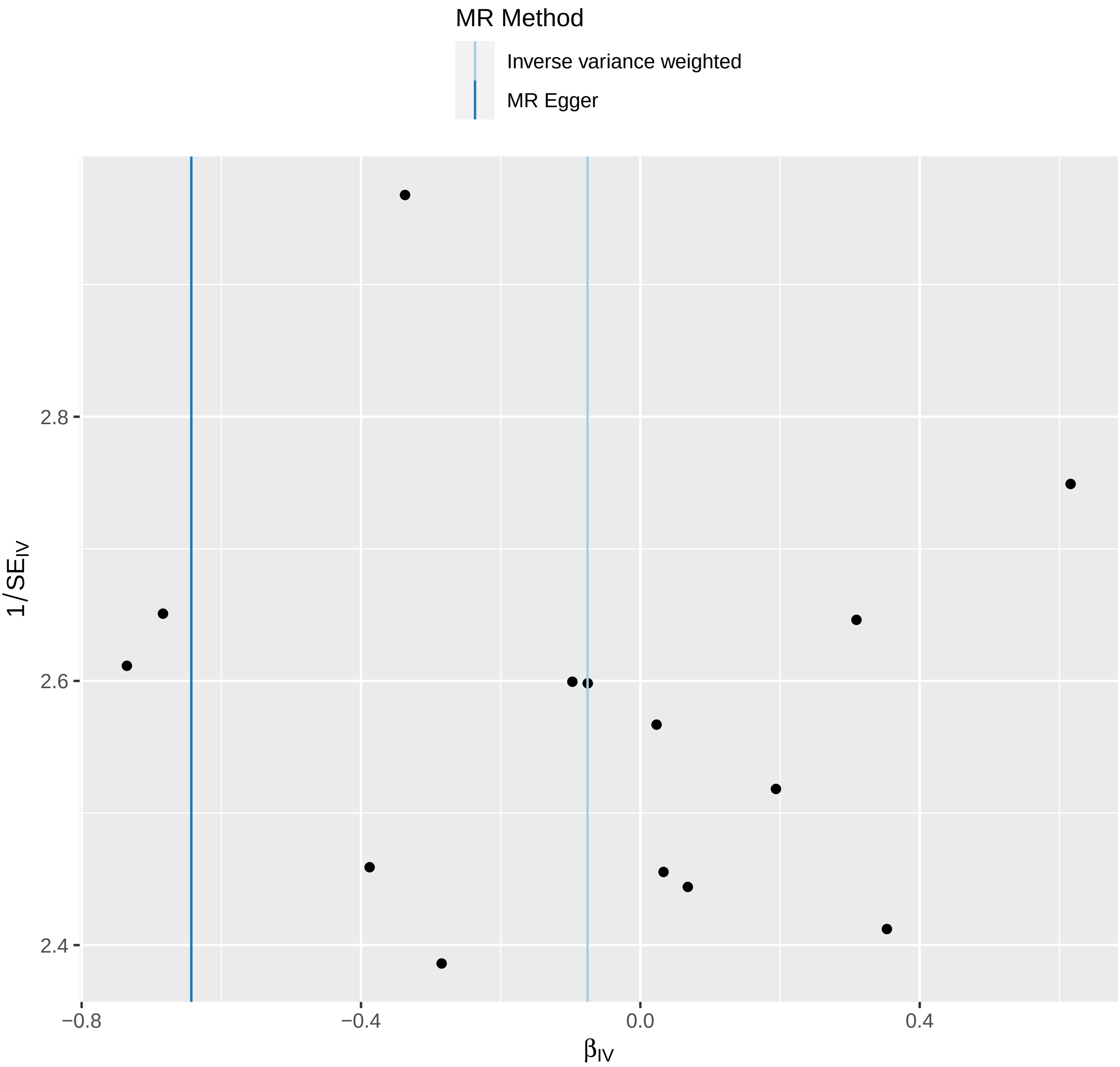

Batch 2−797 : Gut microbiota abundance (genus Catenibacterium id.2153) on C−C motif chemokine 23
with a threshold of 1e−05

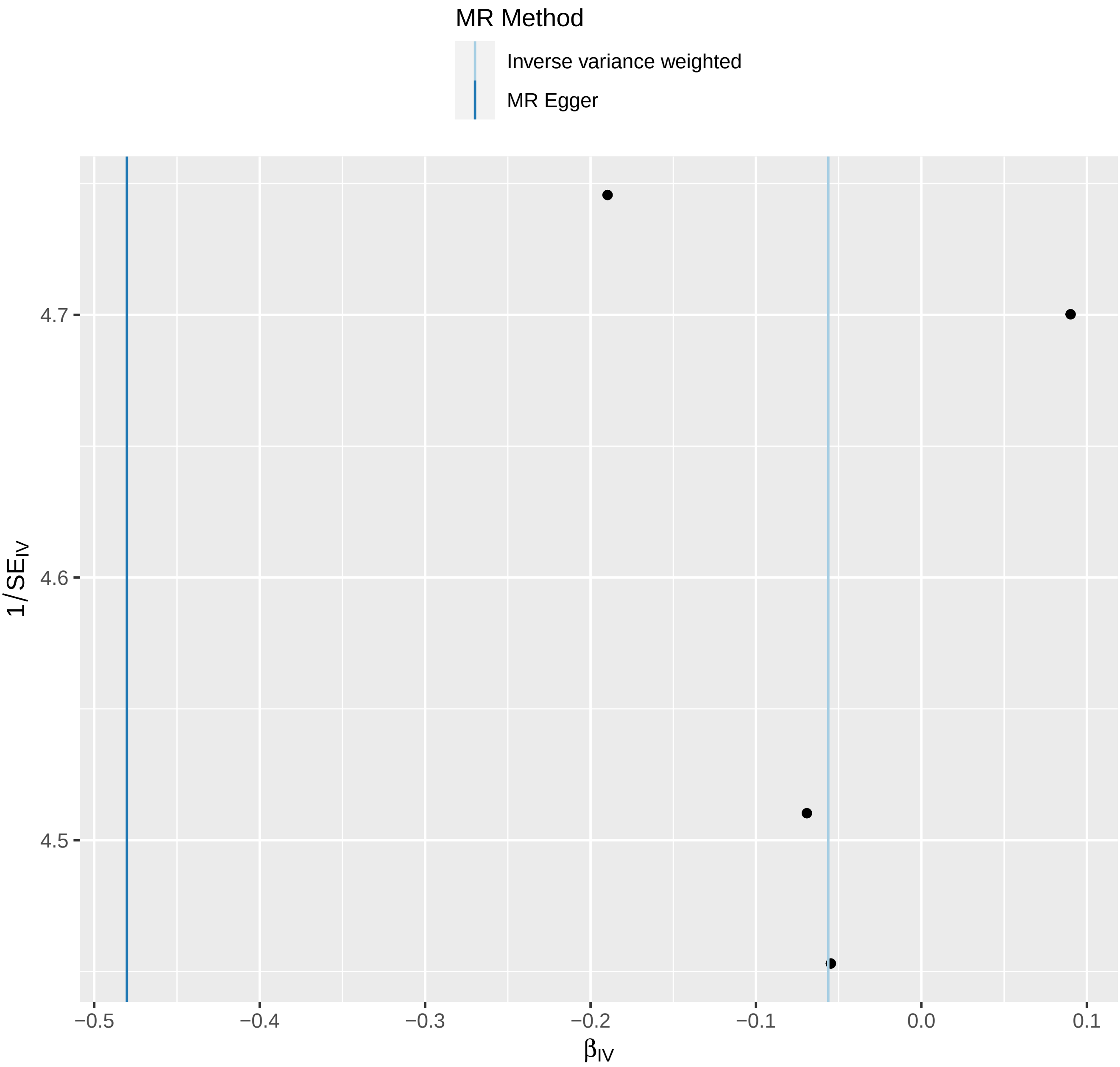

Batch 2−798 : Gut microbiota abundance (genus Parasutterella id.2892) on C−C motif chemokine 23
with a threshold of 1e−05

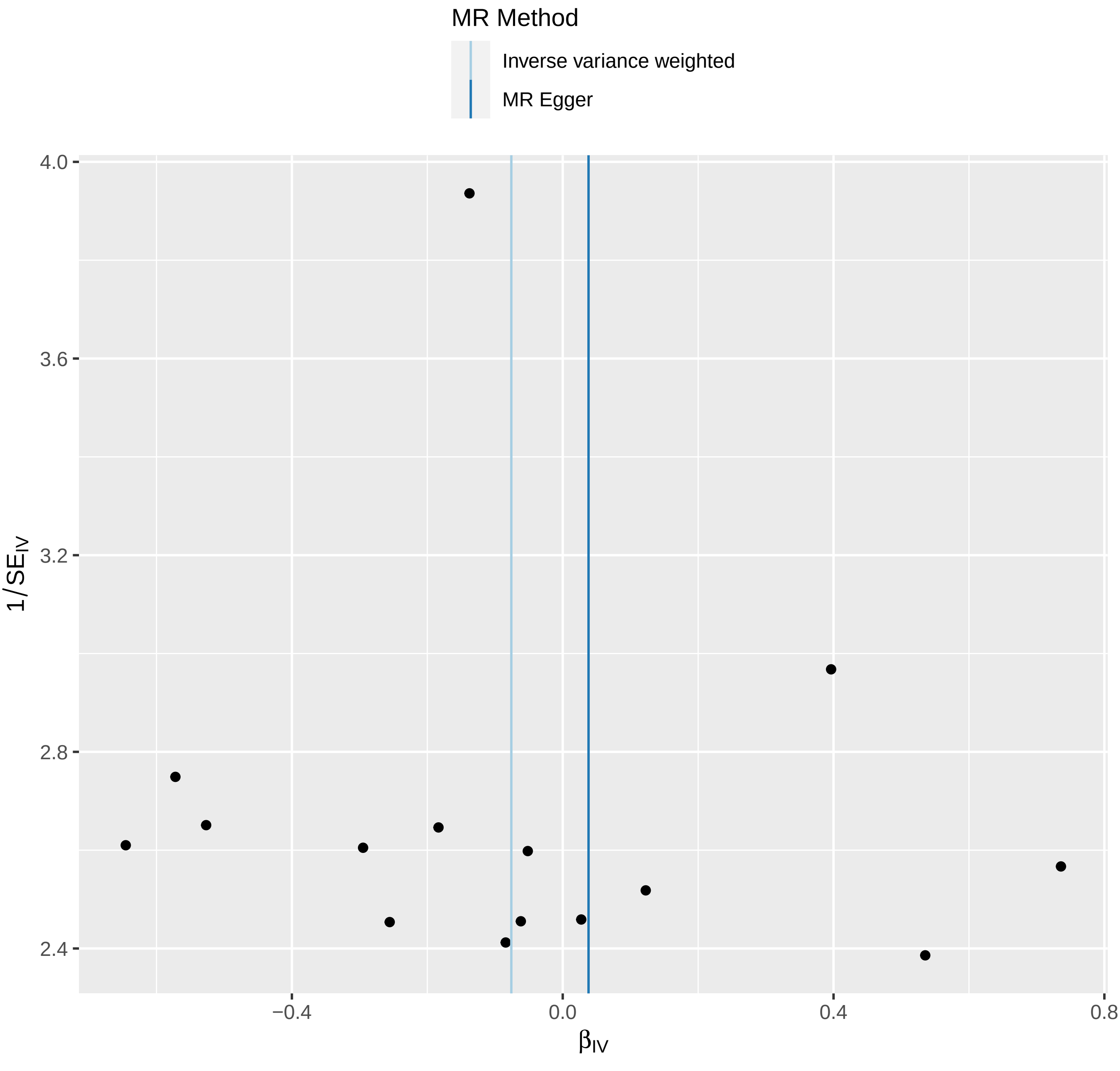

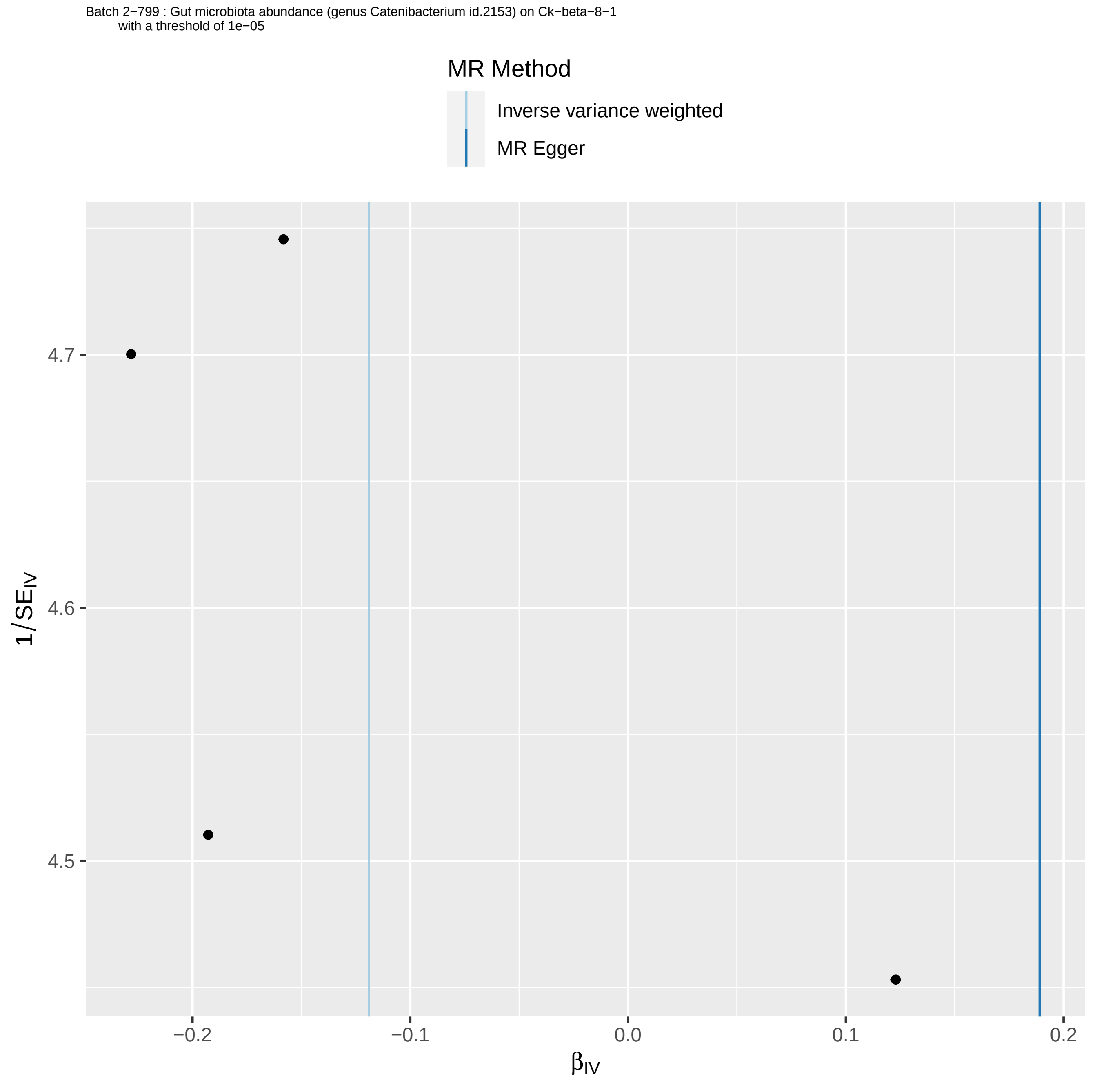

Batch 2−799 : Gut microbiota abundance (genus Catenibacterium id.2153) on Ck−beta−8−1
with a threshold of 1e−05
MR Method
Inverse variance weighted
MR Egger
4.7
4.6
4.5
1/SE_IV
−0.2
−0.1
0.0
0.1
0.2
β_IV

Batch 2−800 : Gut microbiota abundance (genus Parasutterella id.2892) on Ck−beta−8−1
with a threshold of 1e−05

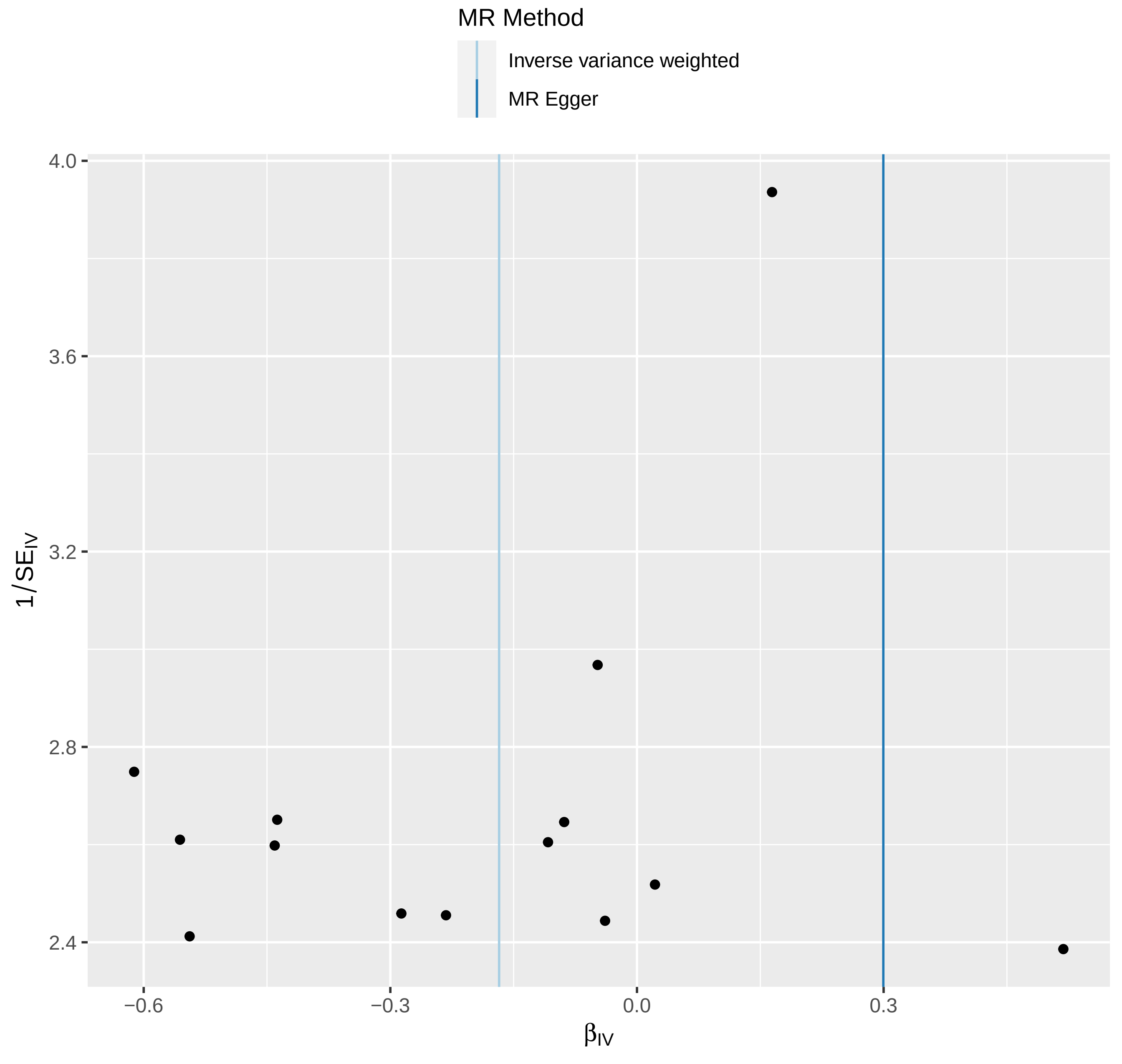

Batch 2−801 : Gut microbiota abundance (genus Catenibacterium id.2153) on C−C motif chemokine 24
with a threshold of 1e−05

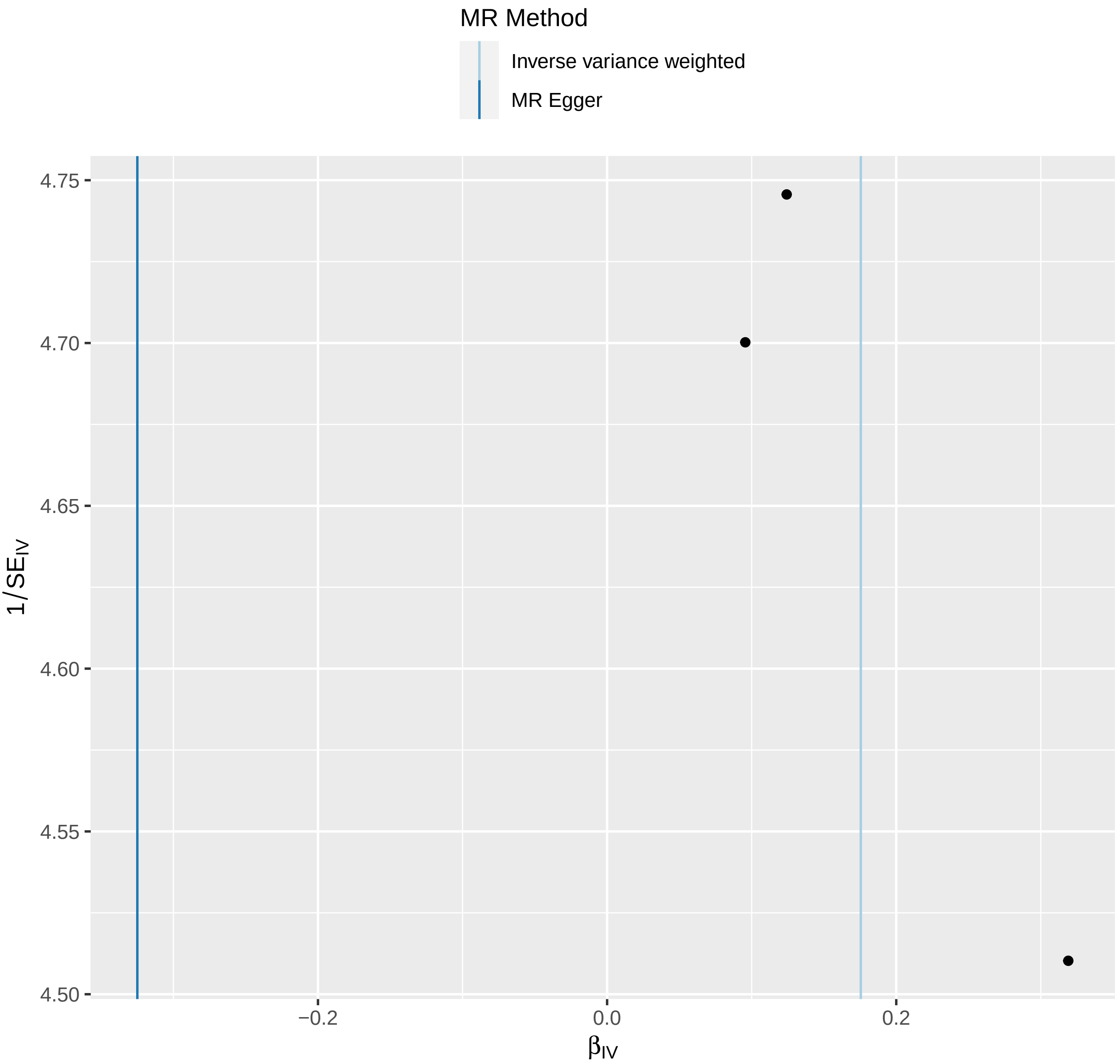

Batch 2−802 : Gut microbiota abundance (genus Parasutterella id.2892) on C−C motif chemokine 24
with a threshold of 1e−05

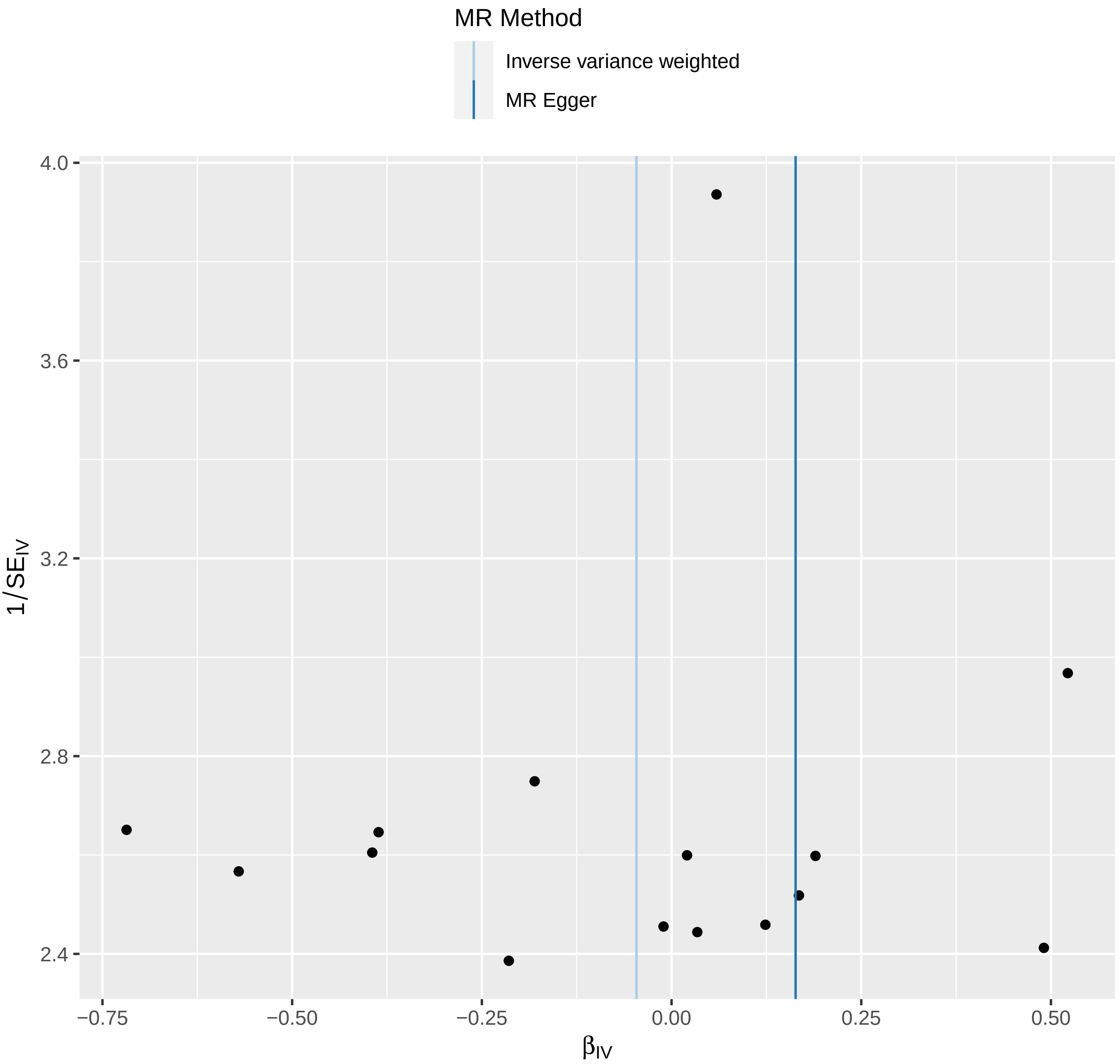

Batch 2−803 : Gut microbiota abundance (genus Catenibacterium id.2153) on C−C motif chemokine 25
with a threshold of 1e−05

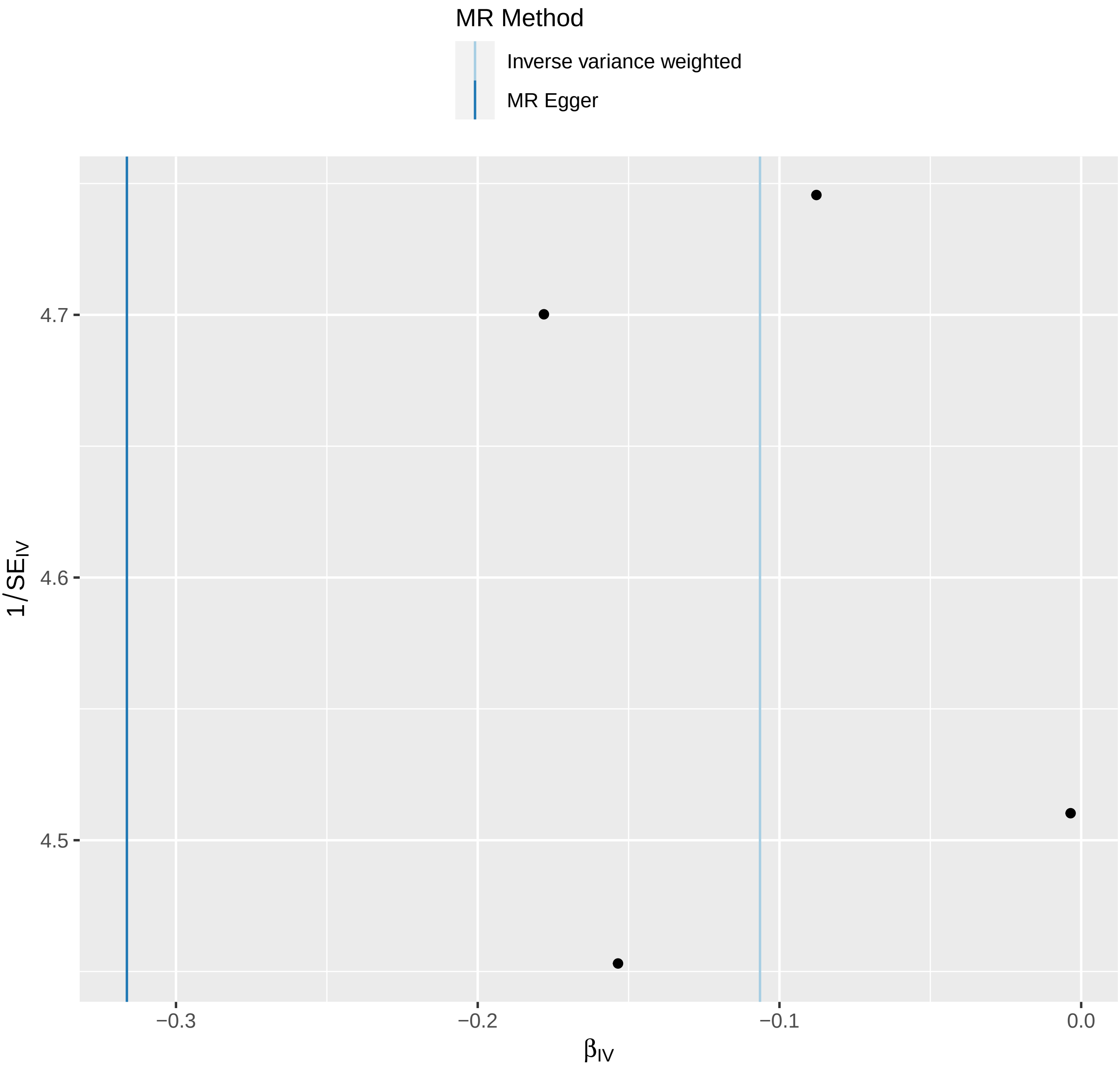

Batch 2−804 : Gut microbiota abundance (genus Parasutterella id.2892) on C−C motif chemokine 25
with a threshold of 1e−05

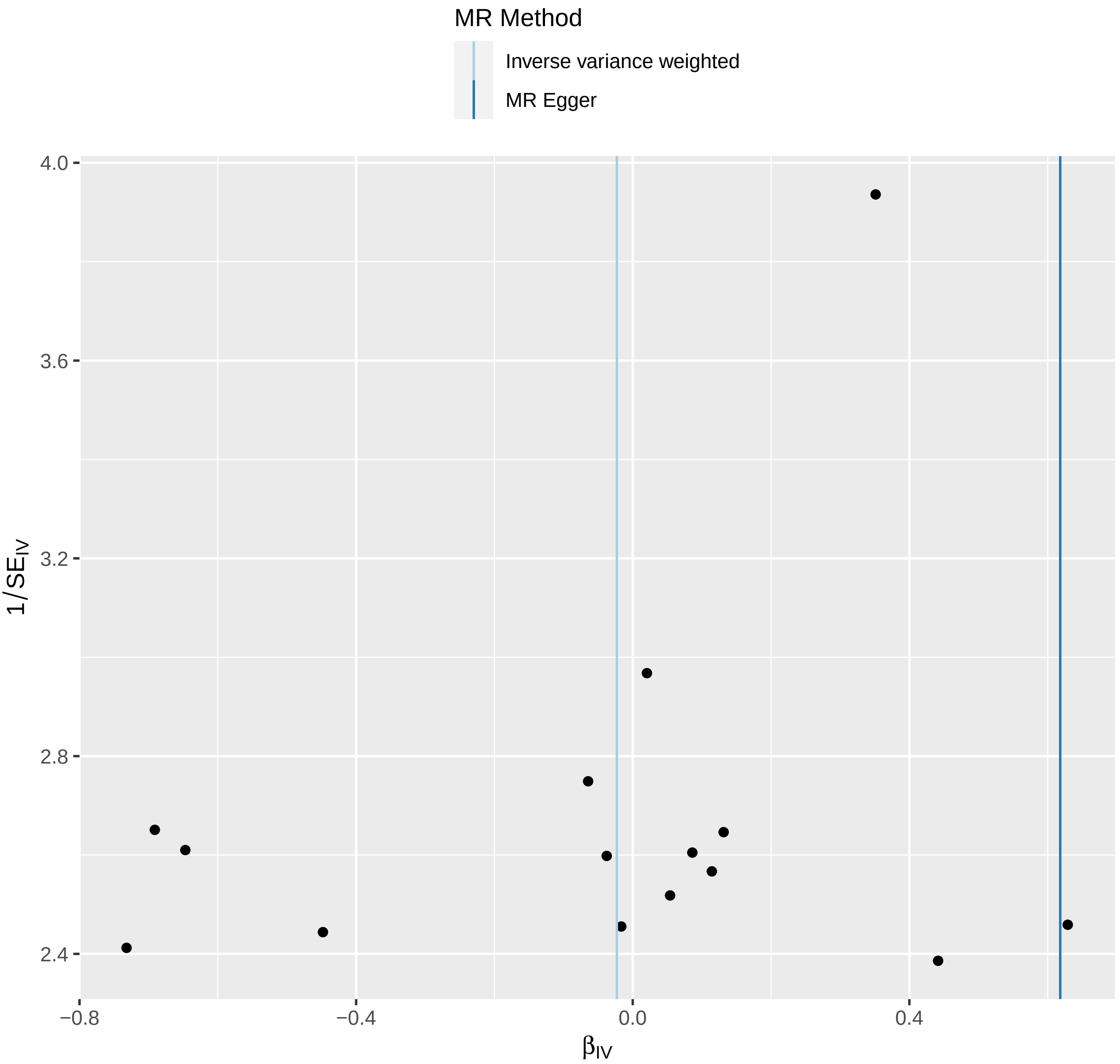

Batch 2−805 : Gut microbiota abundance (genus Catenibacterium id.2153) on C−C motif chemokine 25
with a threshold of 1e−05

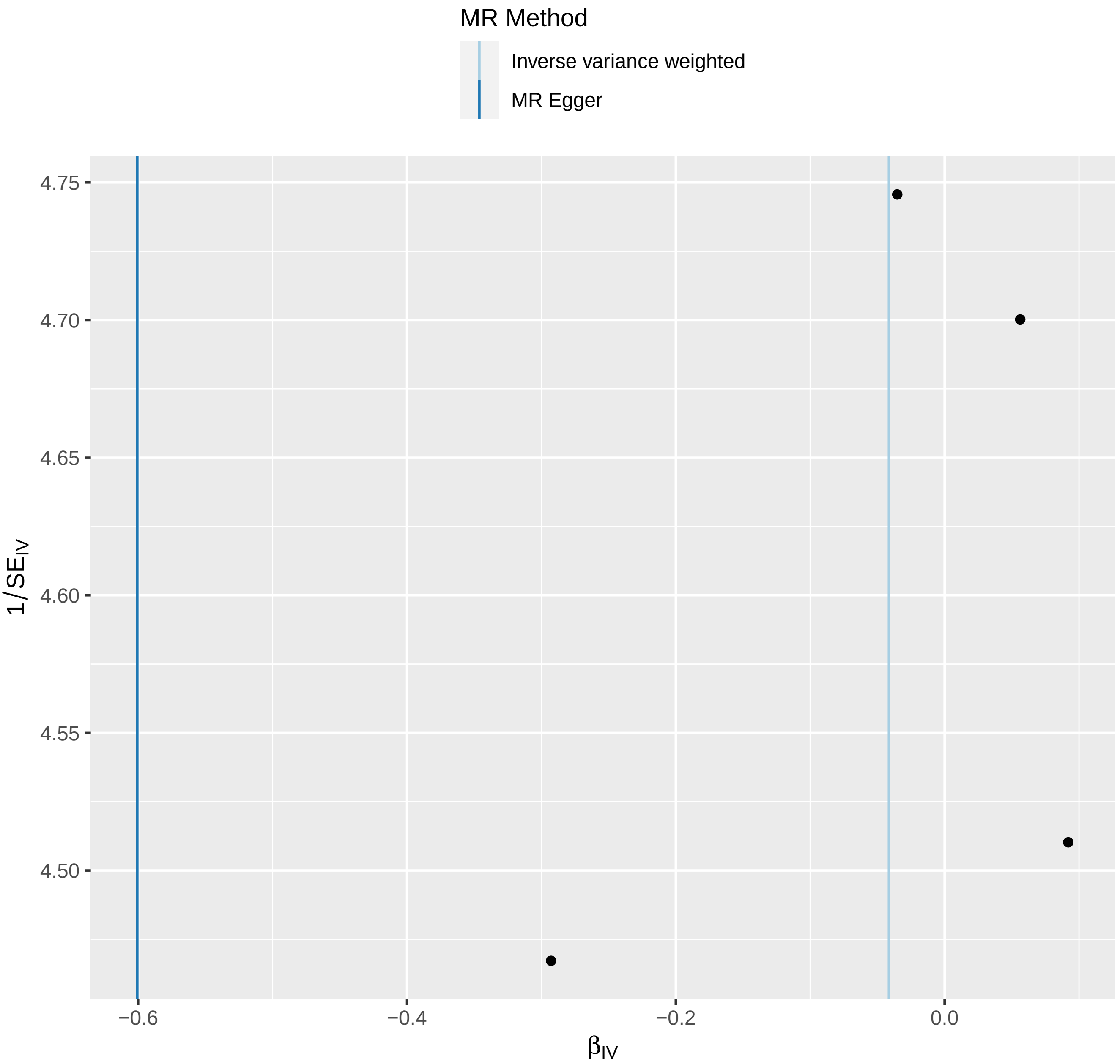

Batch 2−806 : Gut microbiota abundance (genus Parasutterella id.2892) on C−C motif chemokine 25 with a threshold of 1e−05

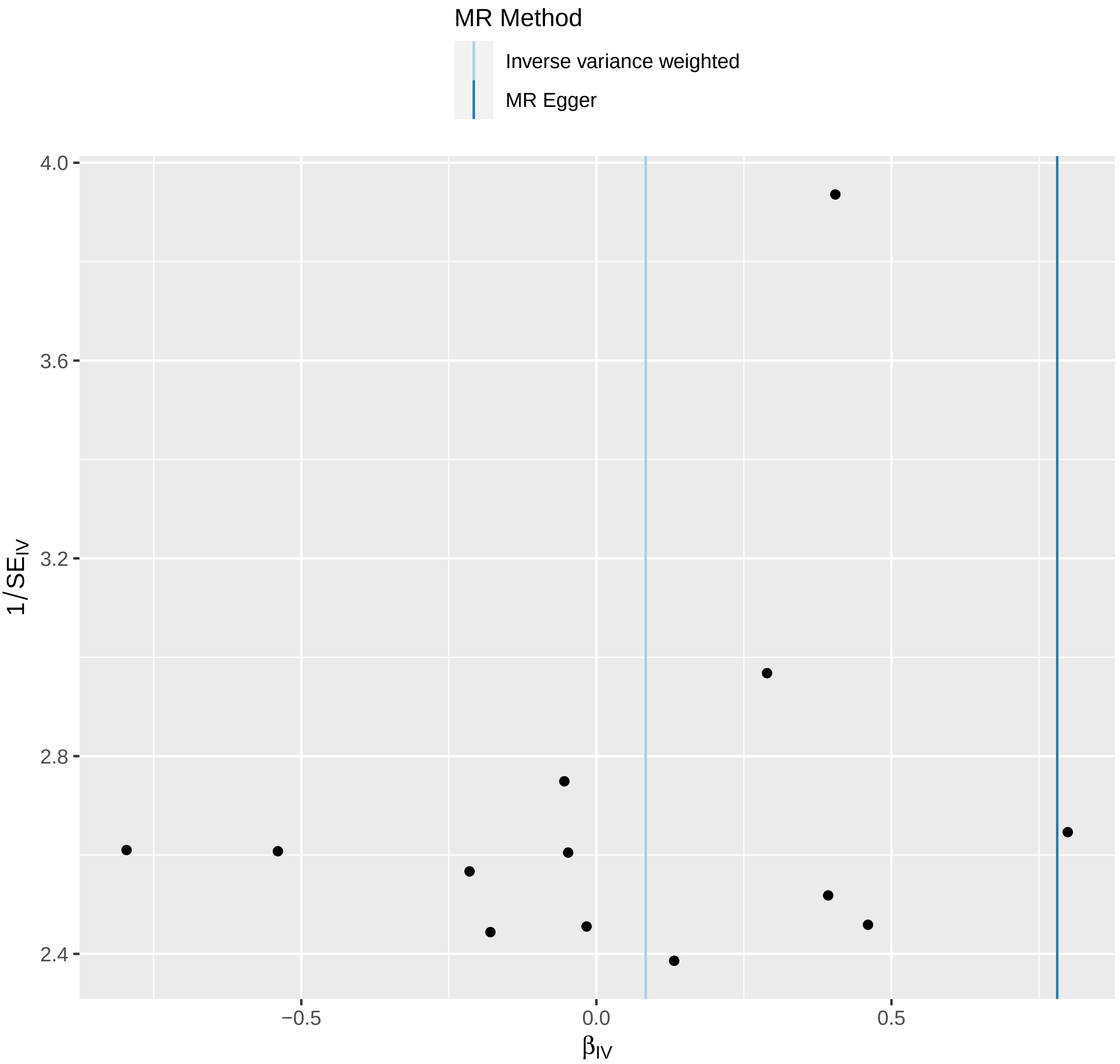

Batch 2−807 : Gut microbiota abundance (genus Catenibacterium id.2153) on C−C motif chemokine 26
with a threshold of 1e−05

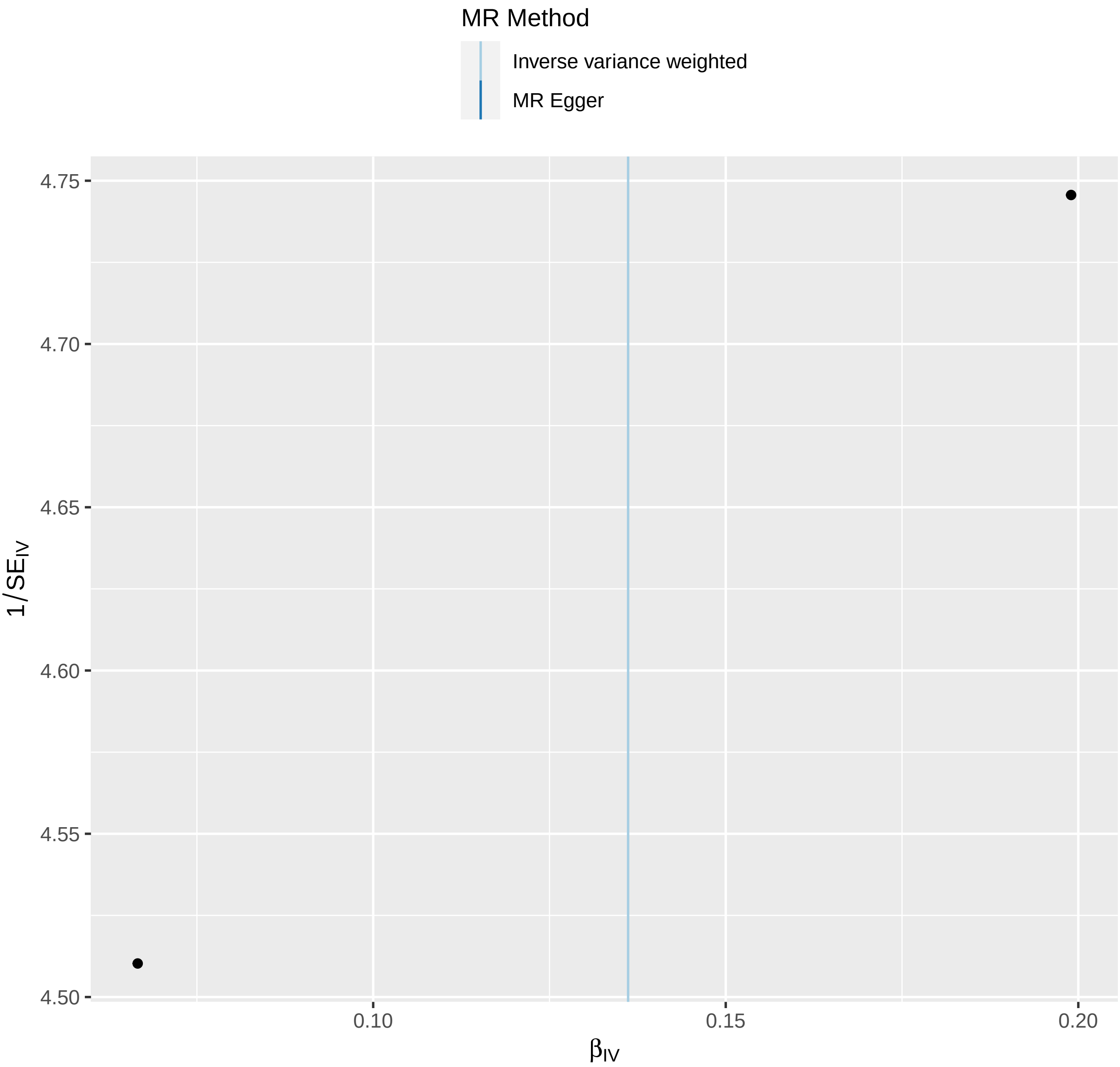

Batch 2−808 : Gut microbiota abundance (genus Parasutterella id.2892) on C−C motif chemokine 26
with a threshold of 1e−05

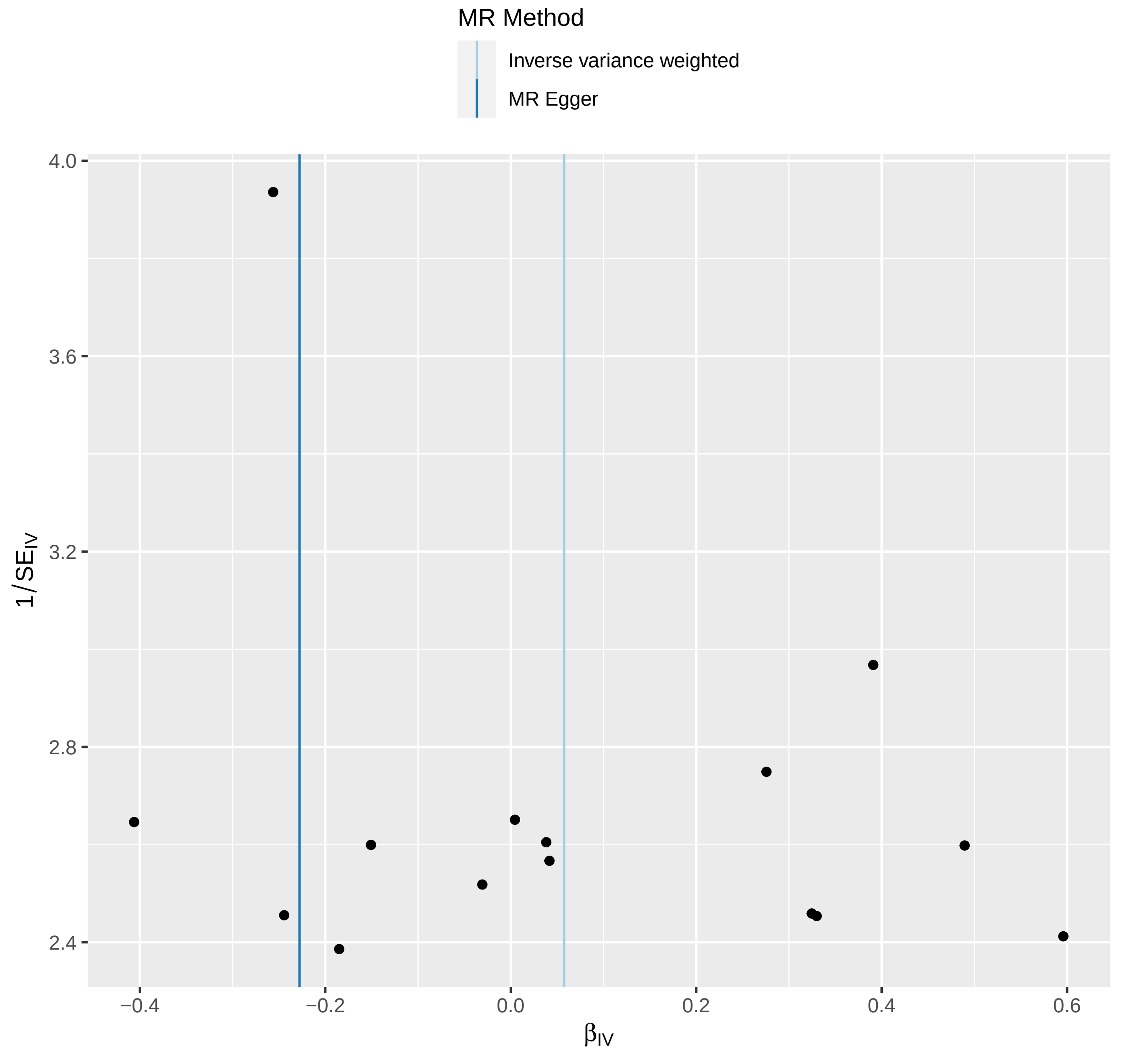

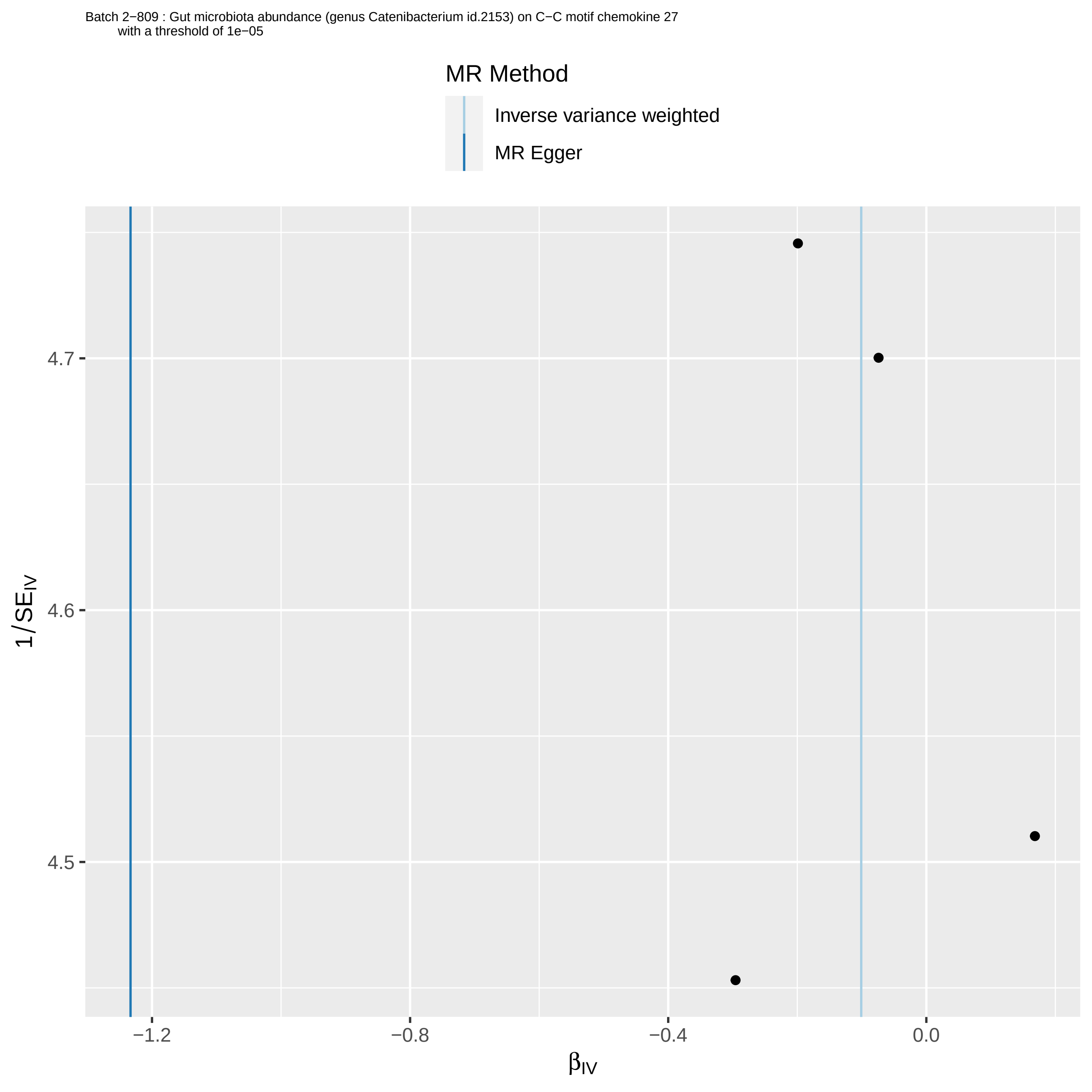

Batch 2−809 : Gut microbiota abundance (genus Catenibacterium id.2153) on C−C motif chemokine 27
with a threshold of 1e−05
MR Method
Inverse variance weighted
MR Egger
4.7
4.6
4.5
1/SE_IV
−1.2
−0.8
−0.4
0.0
β_IV

Batch 2−810 : Gut microbiota abundance (genus Parasutterella id.2892) on C−C motif chemokine 27
with a threshold of 1e−05

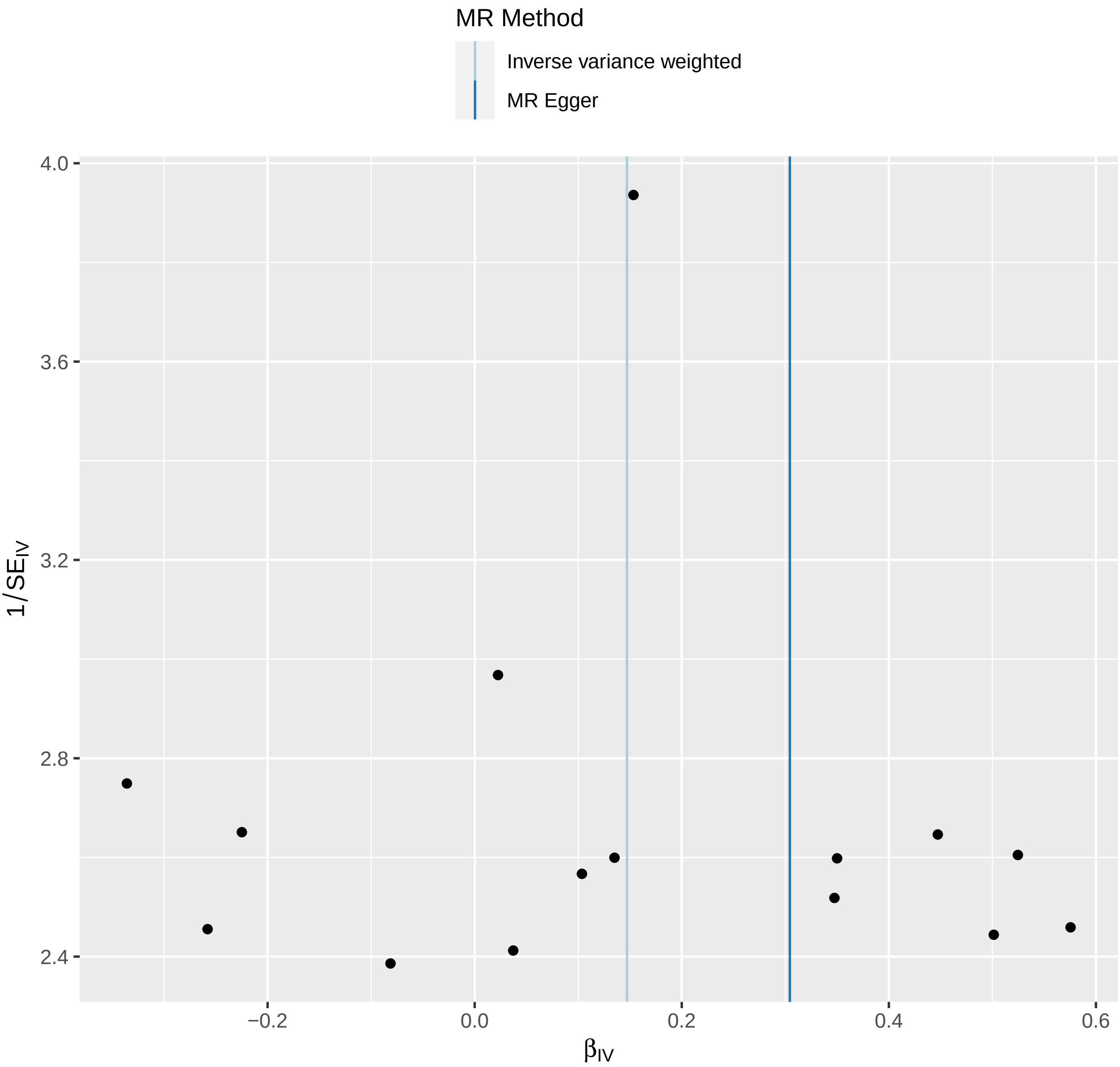

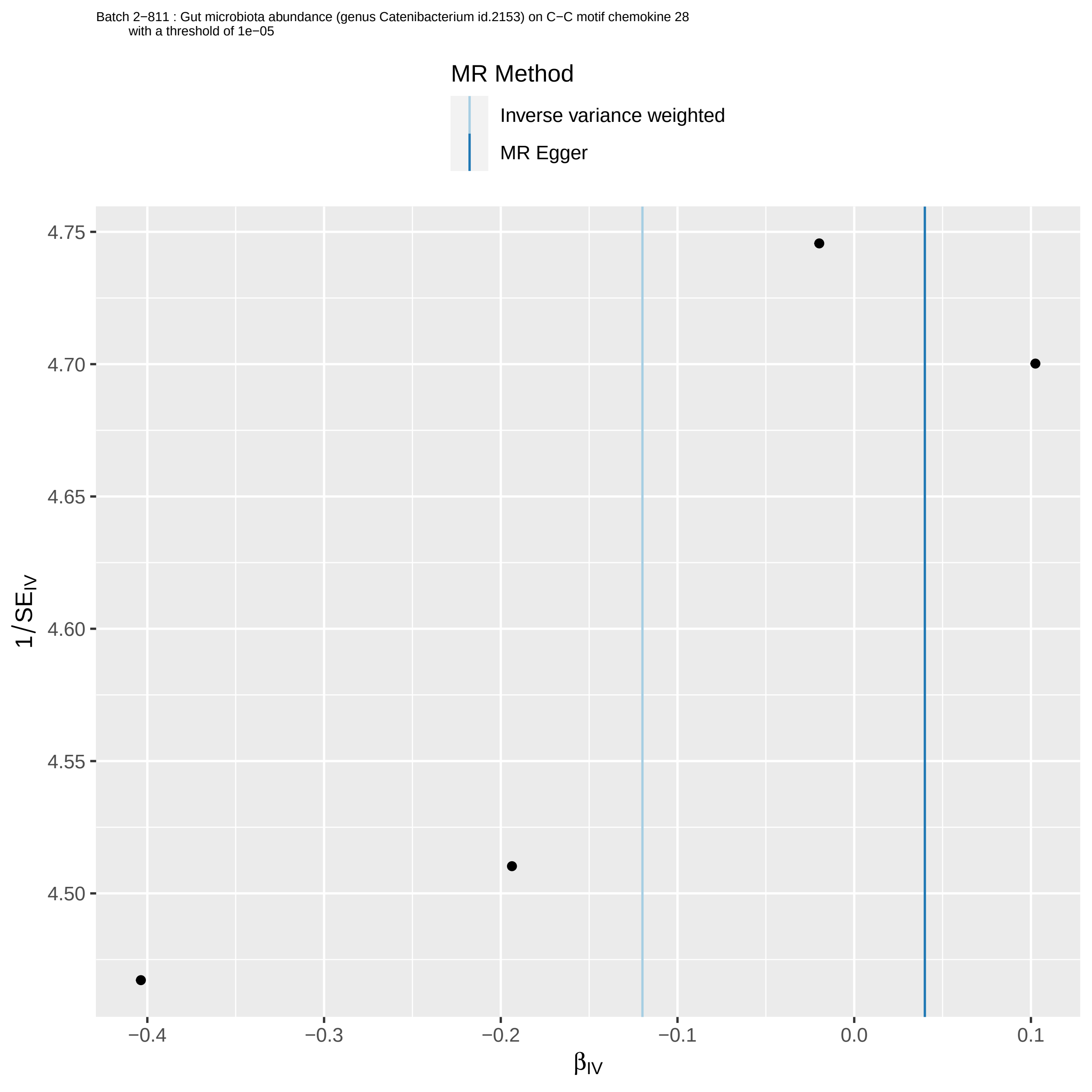

Batch 2−811 : Gut microbiota abundance (genus Catenibacterium id.2153) on C−C motif chemokine 28
with a threshold of 1e−05
MR Method
Inverse variance weighted
MR Egger
4.75
4.70
4.65
4.60
4.55
4.50
1/SE_IV
−0.4
−0.3
−0.2
−0.1
0.0
0.1
β_IV

Batch 2−812 : Gut microbiota abundance (genus Parasutterella id.2892) on C−C motif chemokine 28
with a threshold of 1e−05

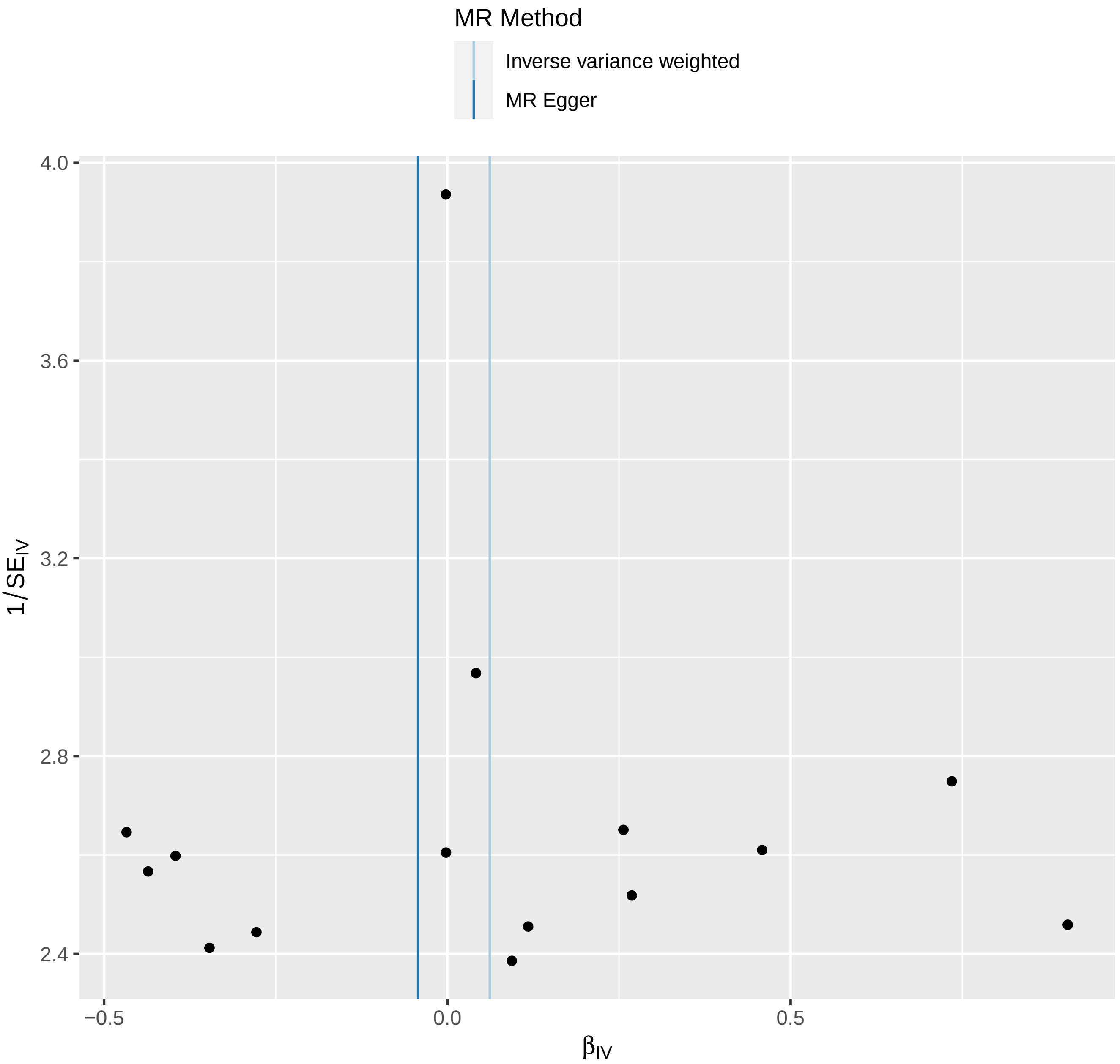

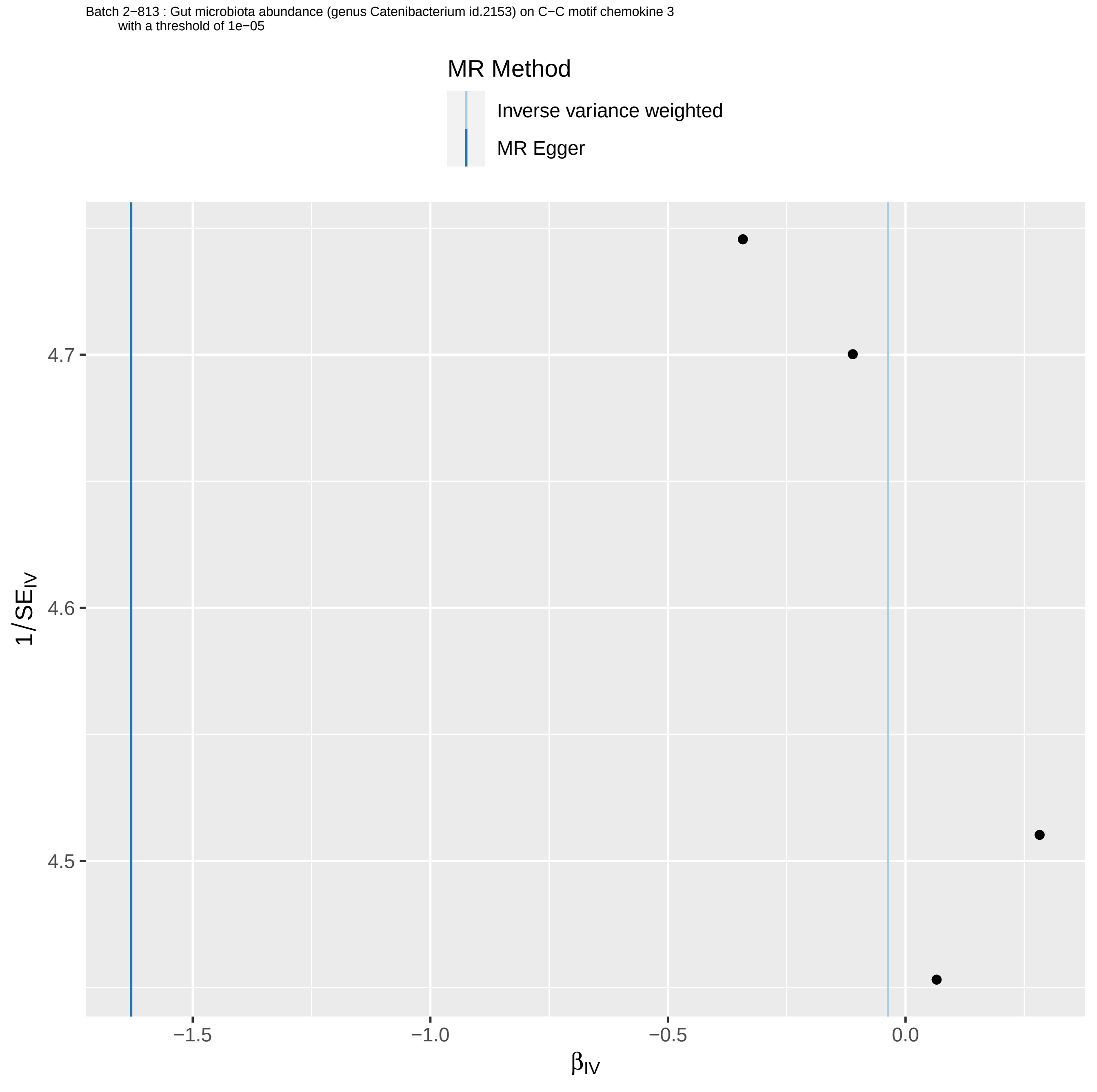

Batch 2−813 : Gut microbiota abundance (genus Catenibacterium id.2153) on C−C motif chemokine 3
with a threshold of 1e−05
MR Method
Inverse variance weighted
MR Egger
4.7
4.6
4.5
1/SE_IV
−1.5
−1.0
−0.5
0.0
β_IV

Batch 2−814 : Gut microbiota abundance (genus Parasutterella id.2892) on C−C motif chemokine 3
with a threshold of 1e−05

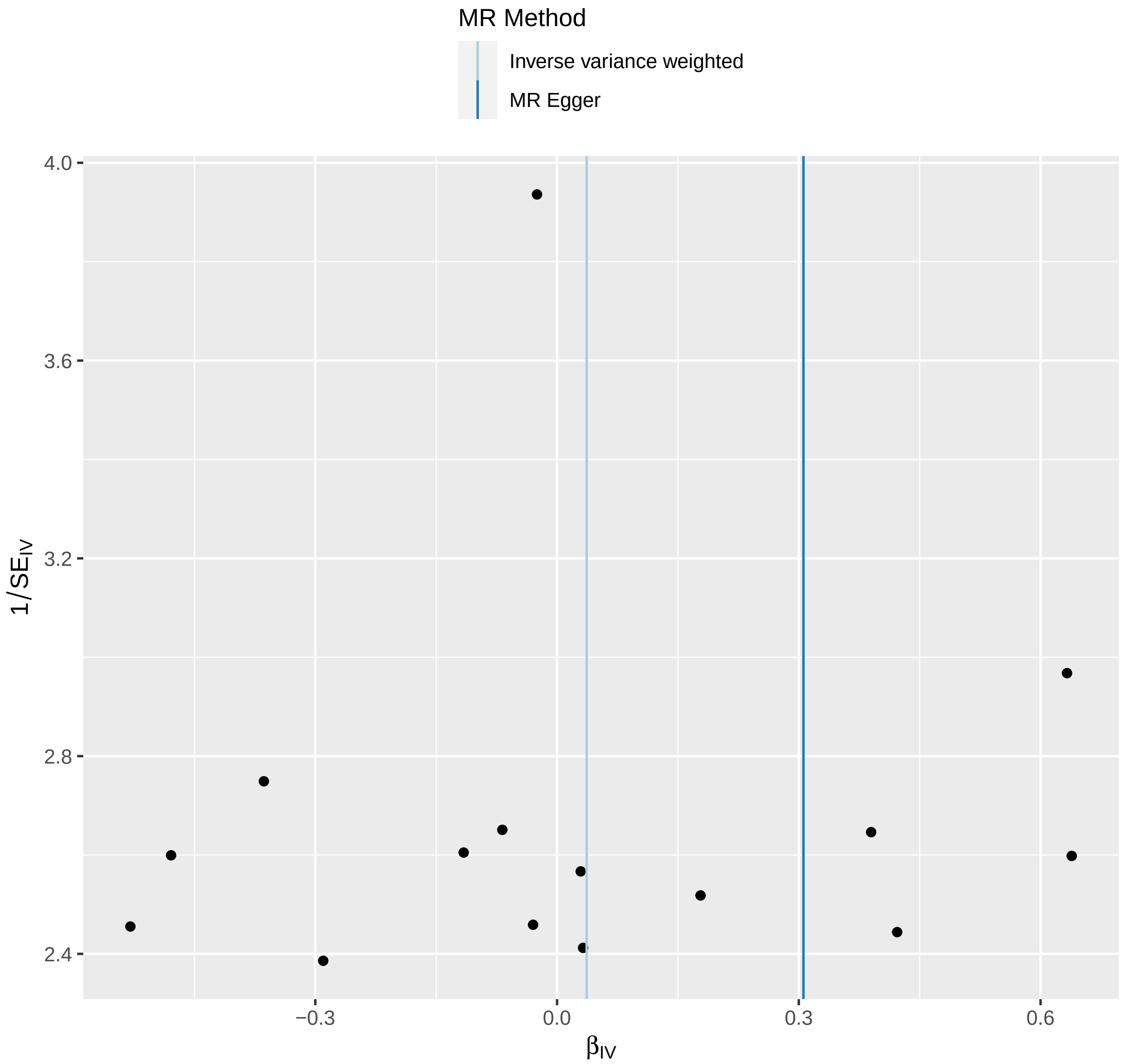

Batch 2−815 : Gut microbiota abundance (genus Catenibacterium id.2153) on C−C motif chemokine 3−like 1
with a threshold of 1e−05

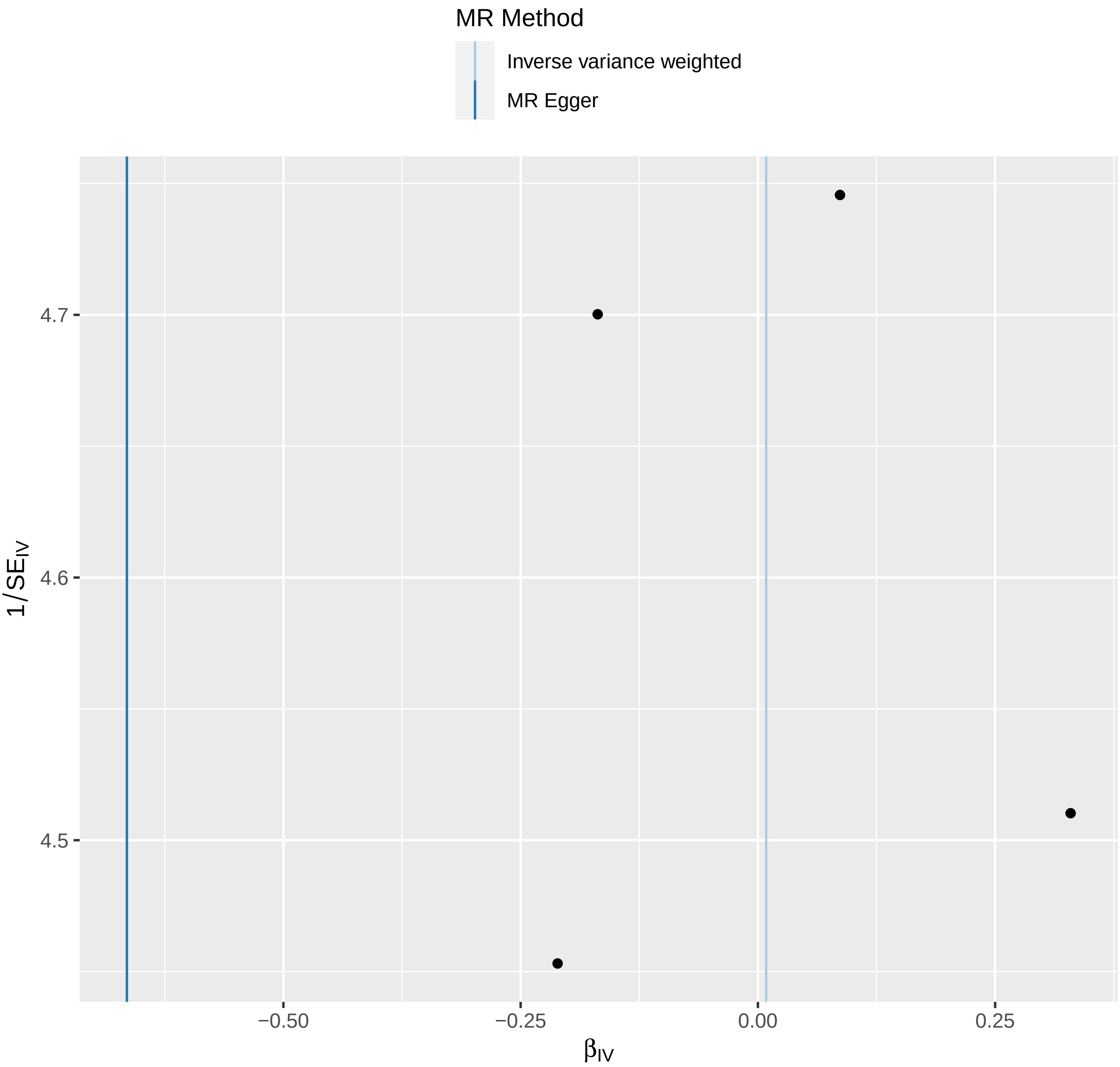

Batch 2−816 : Gut microbiota abundance (genus Parasutterella id.2892) on C−C motif chemokine 3−like 1
with a threshold of 1e−05

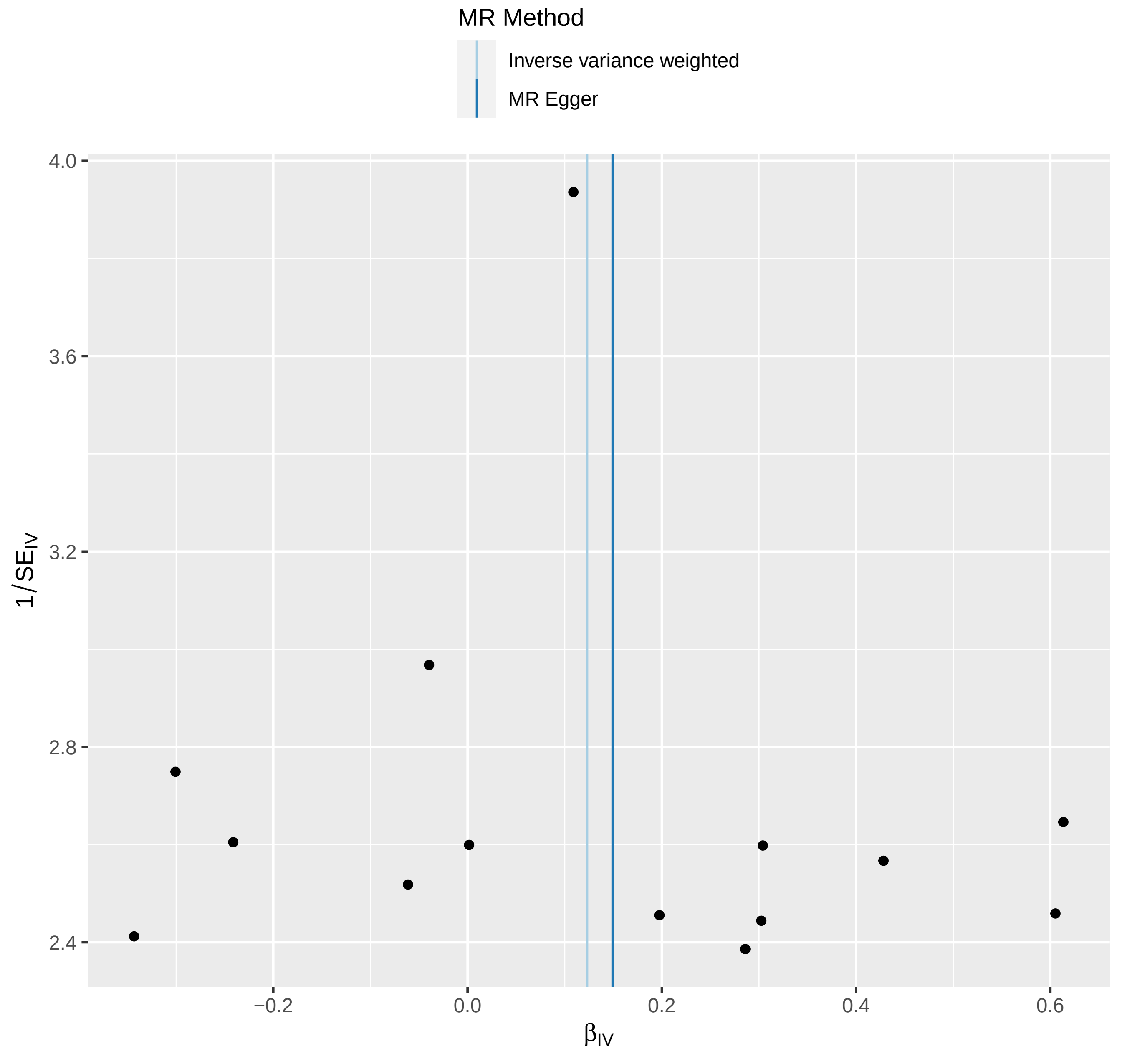

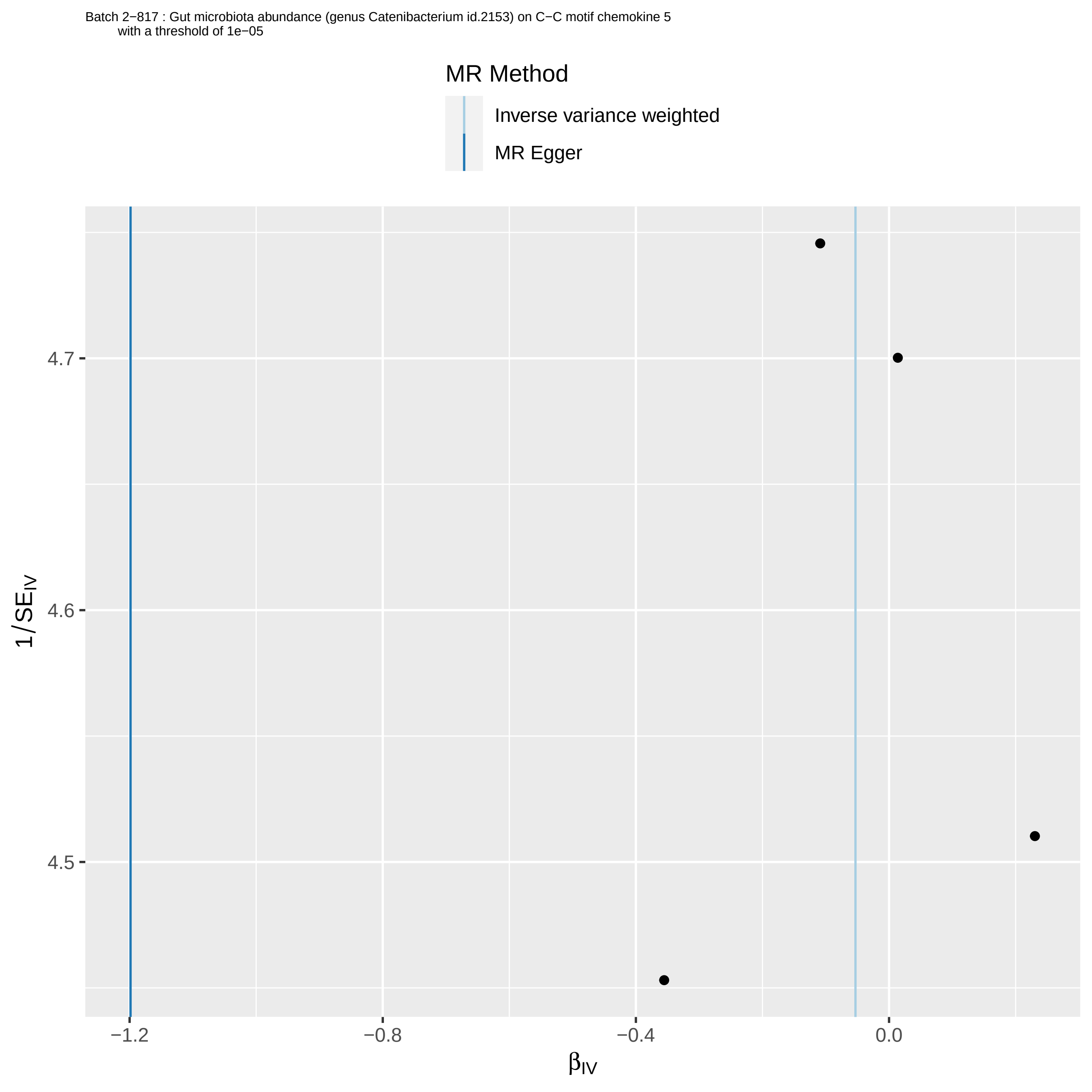

Batch 2−817 : Gut microbiota abundance (genus Catenibacterium id.2153) on C−C motif chemokine 5
with a threshold of 1e−05
MR Method
Inverse variance weighted
MR Egger
4.7
4.6
4.5
1/SE_IV
−1.2
−0.8
−0.4
0.0
β_IV

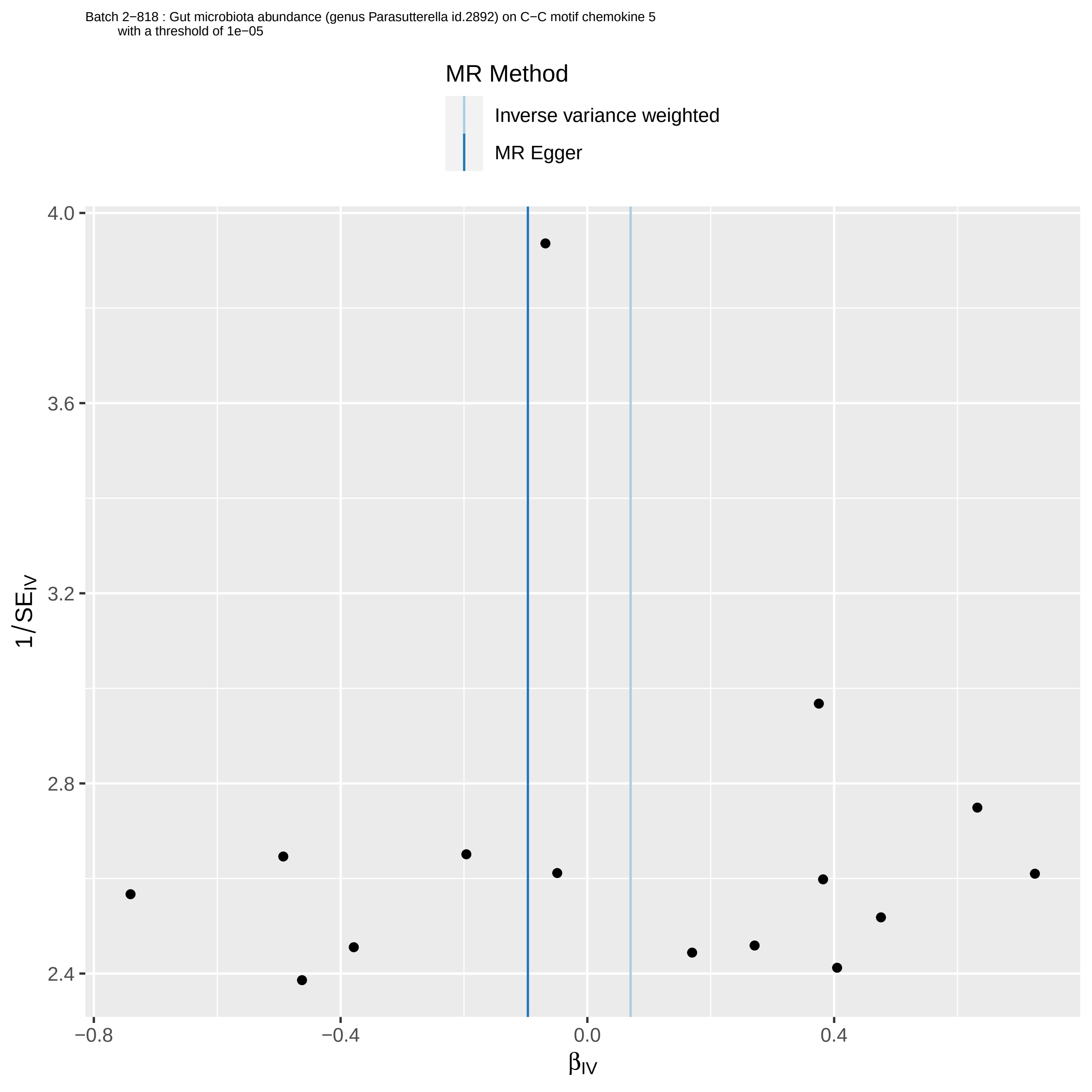
Batch 2−818 : Gut microbiota abundance (genus Parasutterella id.2892) on C−C motif chemokine 5
with a threshold of 1e−05
MR Method
Inverse variance weighted
MR Egger
4.0
3.6
3.2
2.8
2.4
$1/SE_{IV}$
−0.8
−0.4
0.0
0.4
β_{IV}

Batch 2−819 : Gut microbiota abundance (genus Catenibacterium id.2153) on C−C motif chemokine 7
with a threshold of 1e−05

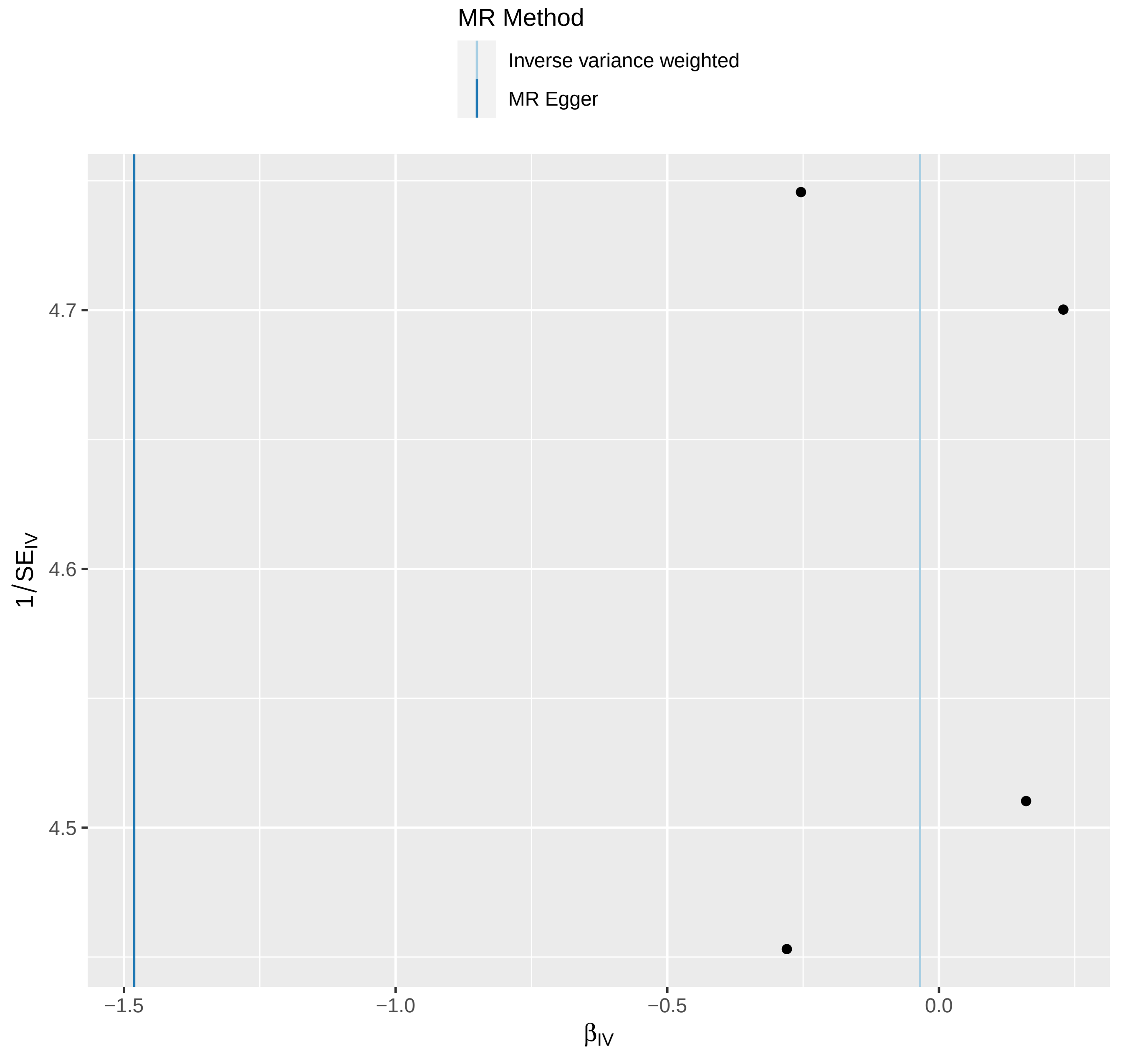

Batch 2−820 : Gut microbiota abundance (genus Parasutterella id.2892) on C−C motif chemokine 7
with a threshold of 1e−05

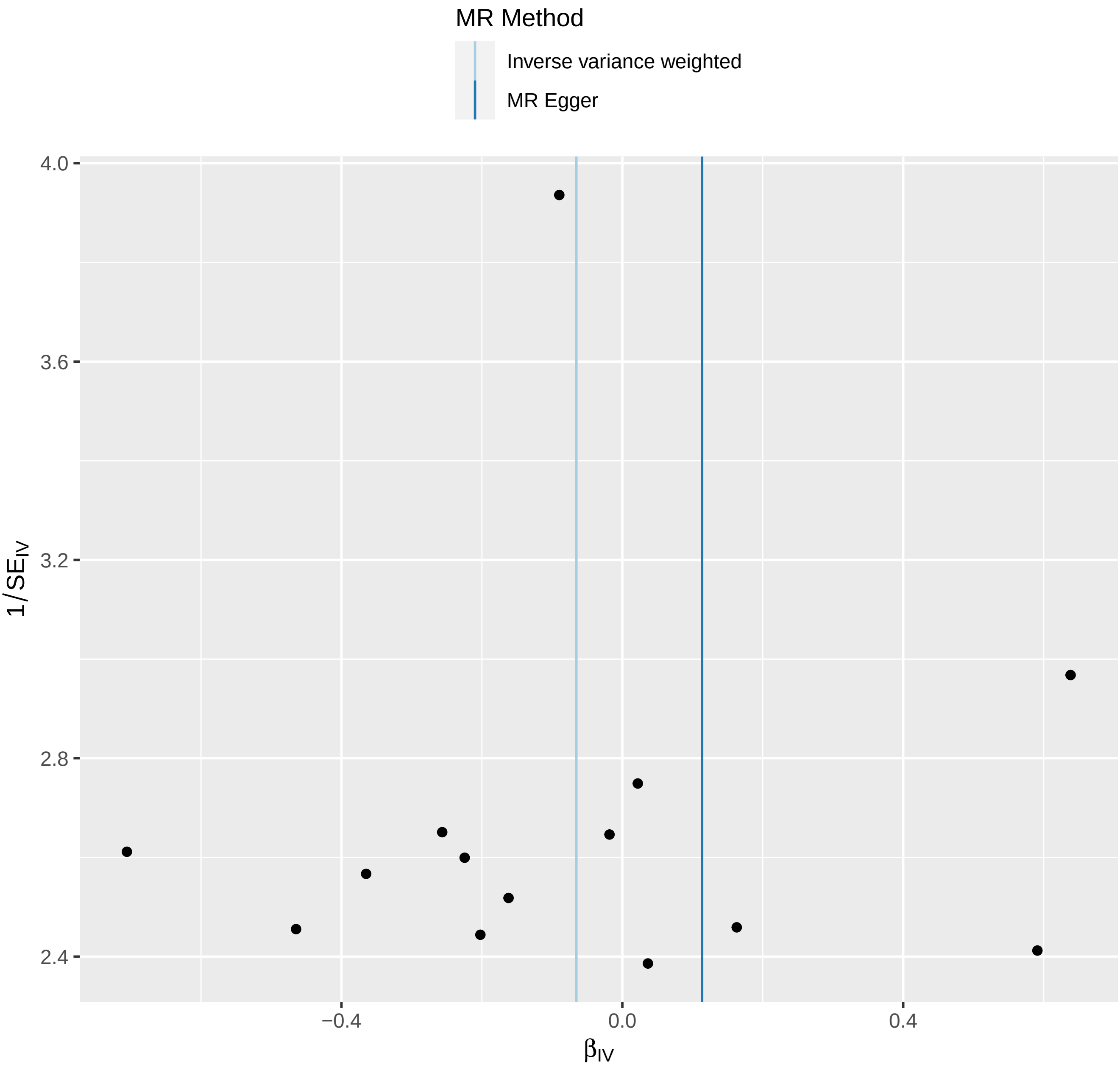

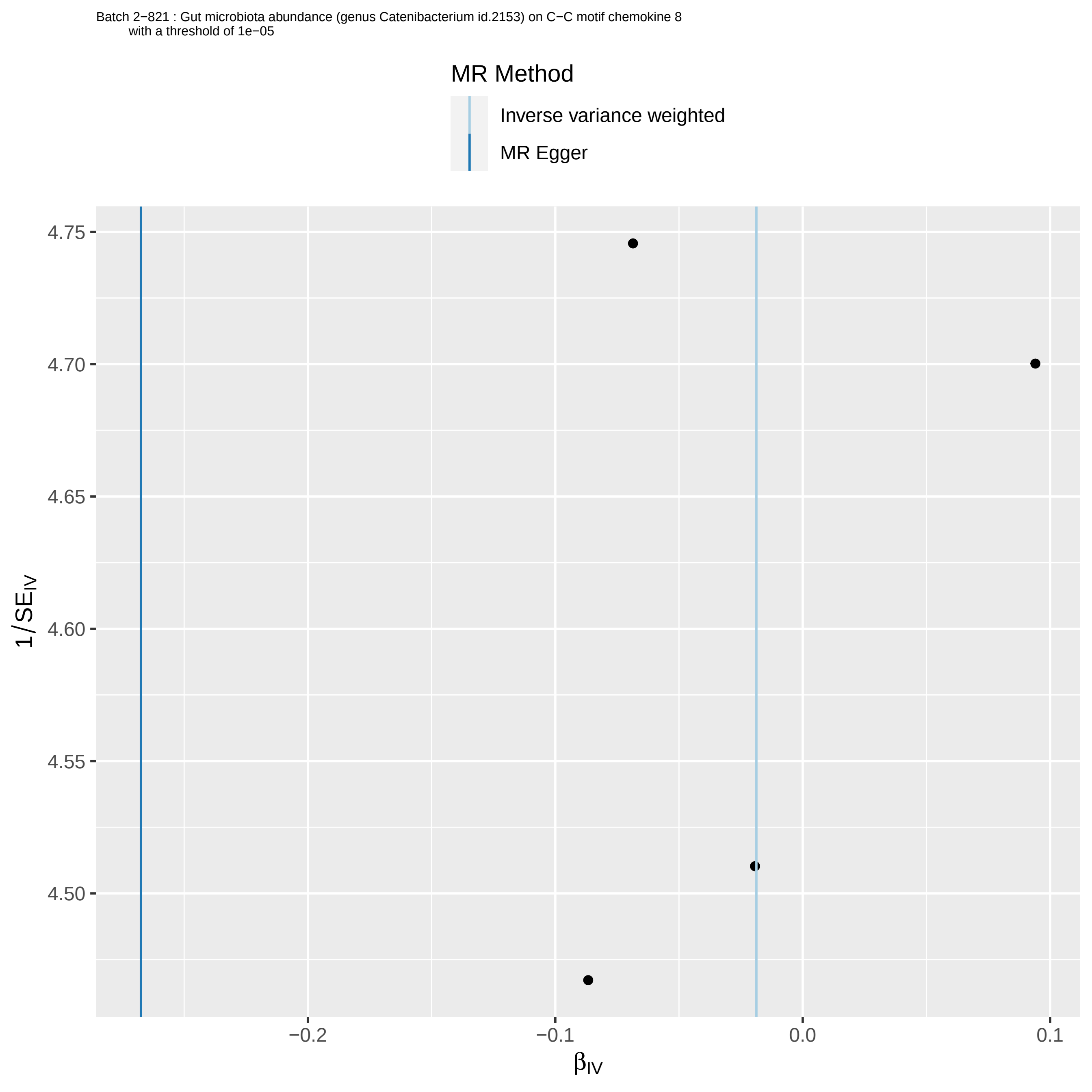
Batch 2−821 : Gut microbiota abundance (genus Catenibacterium id.2153) on C−C motif chemokine 8
with a threshold of 1e−05
MR Method
Inverse variance weighted
MR Egger
4.75
4.70
4.65
4.60
4.55
4.50
1/SE$_{IV}$
−0.2
−0.1
0.0
0.1
β$_{IV}$

Batch 2−822 : Gut microbiota abundance (genus Parasutterella id.2892) on C−C motif chemokine 8
with a threshold of 1e−05

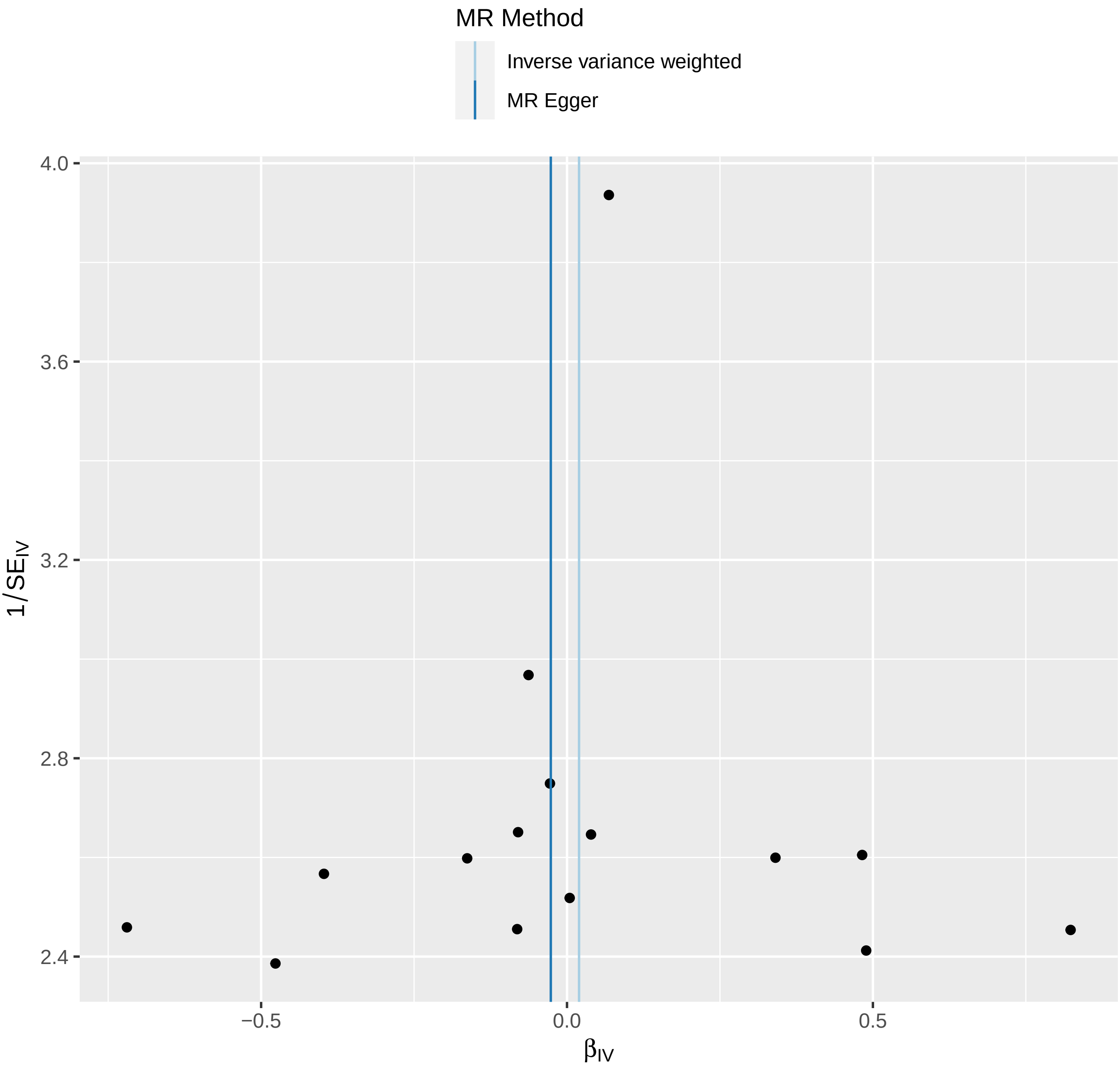

Batch 2−823 : Gut microbiota abundance (genus Catenibacterium id.2153) on Cerebral cavernous malformations 2 protein
with a threshold of 1e−05

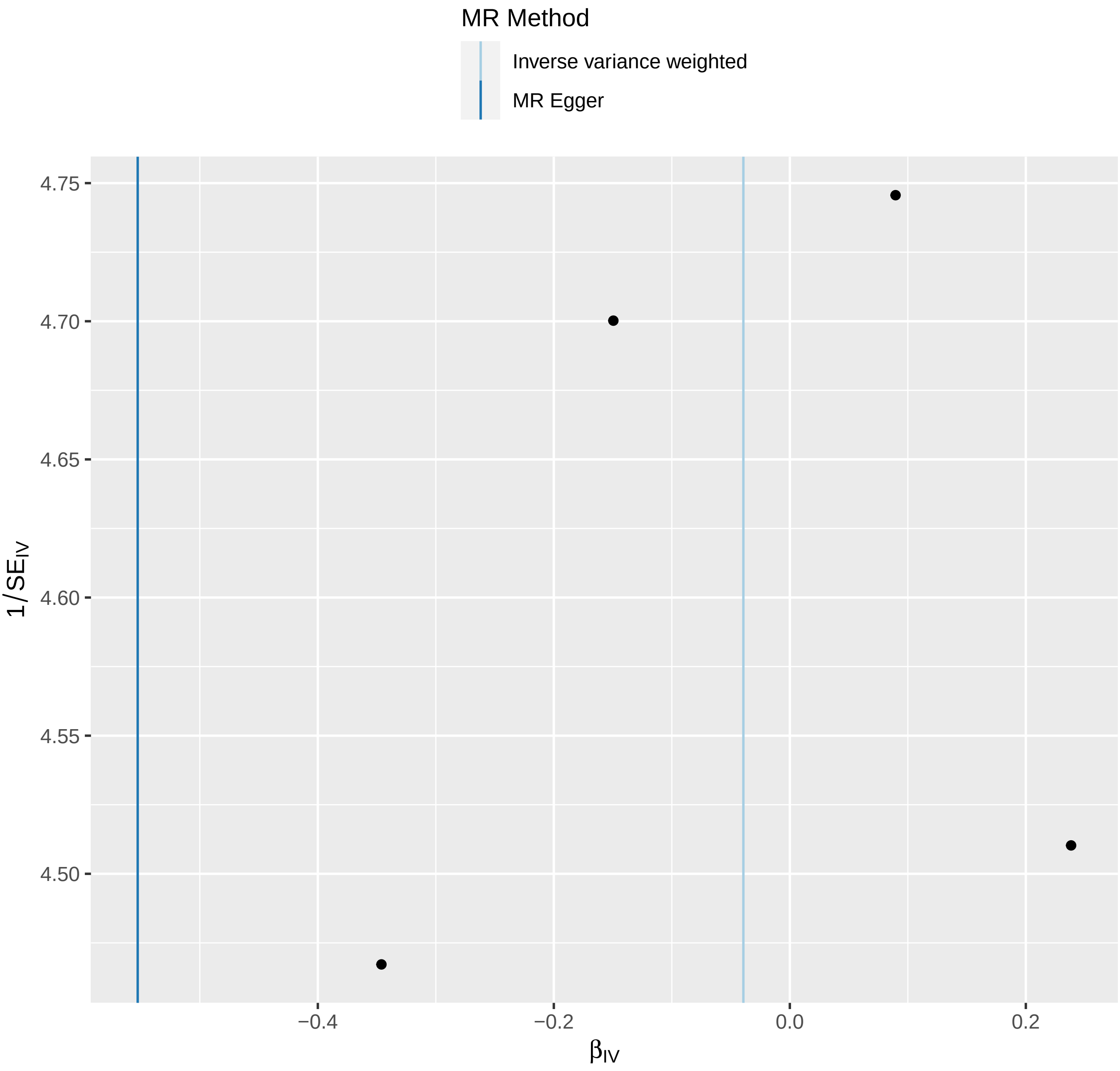

Batch 2−824 : Gut microbiota abundance (genus Parasutterella id.2892) on Cerebral cavernous malformations 2 protein
with a threshold of 1e−05

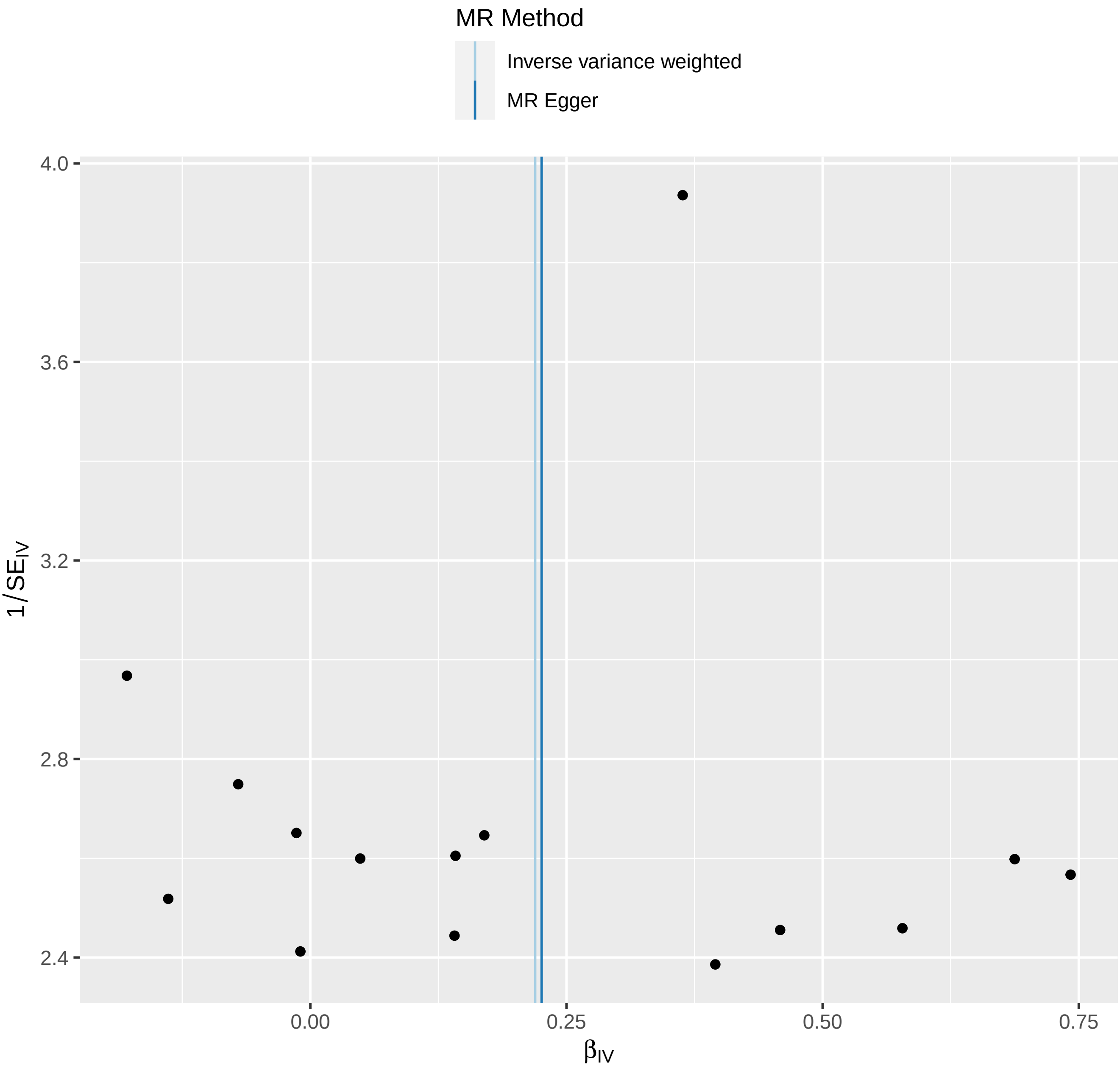

Batch 2−825 : Gut microbiota abundance (genus Catenibacterium id.2153) on G2/mitotic−specific cyclin−B1
with a threshold of 1e−05

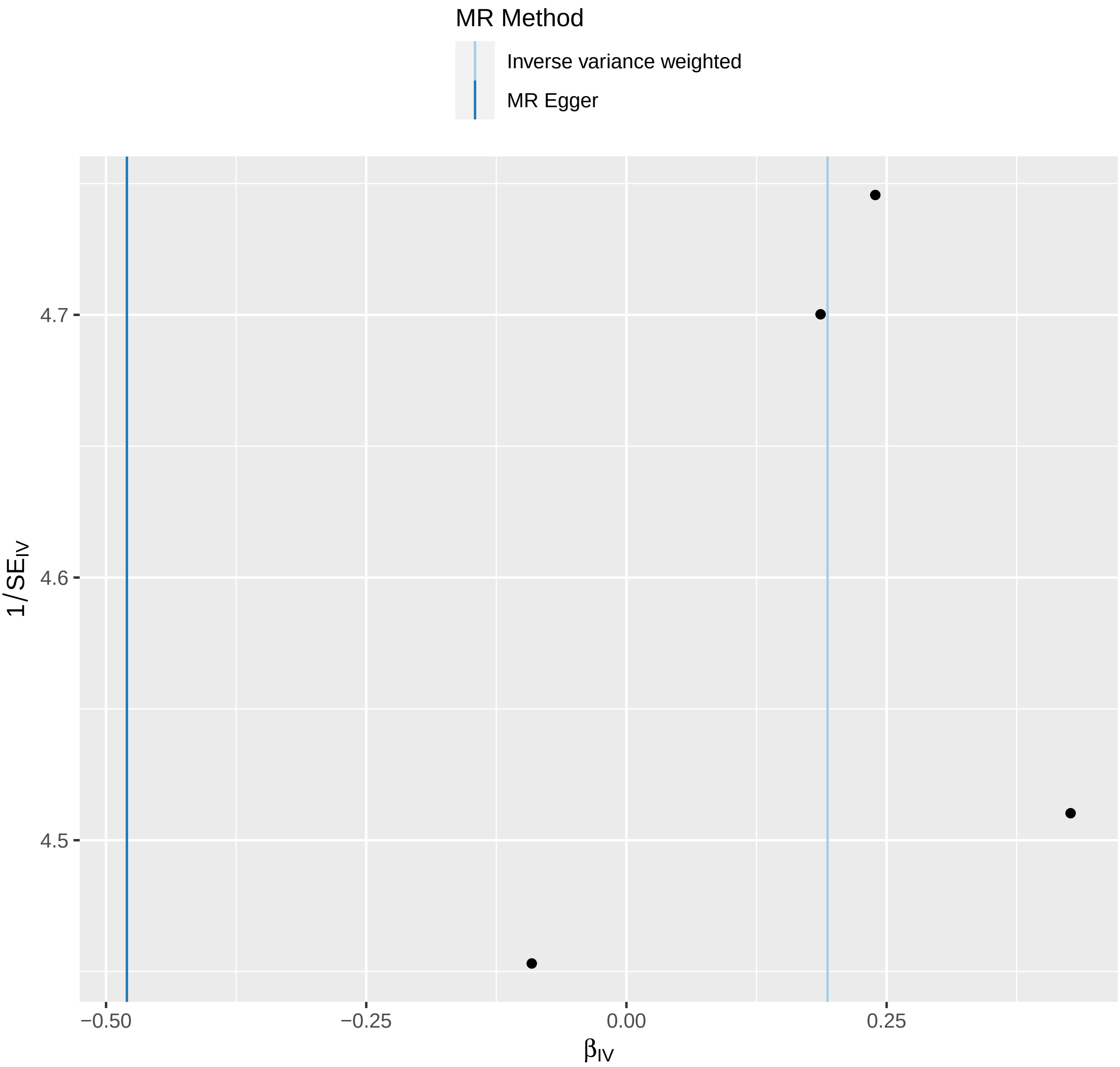

Batch 2−826 : Gut microbiota abundance (genus Parasutterella id.2892) on G2/mitotic−specific cyclin−B1
with a threshold of 1e−05

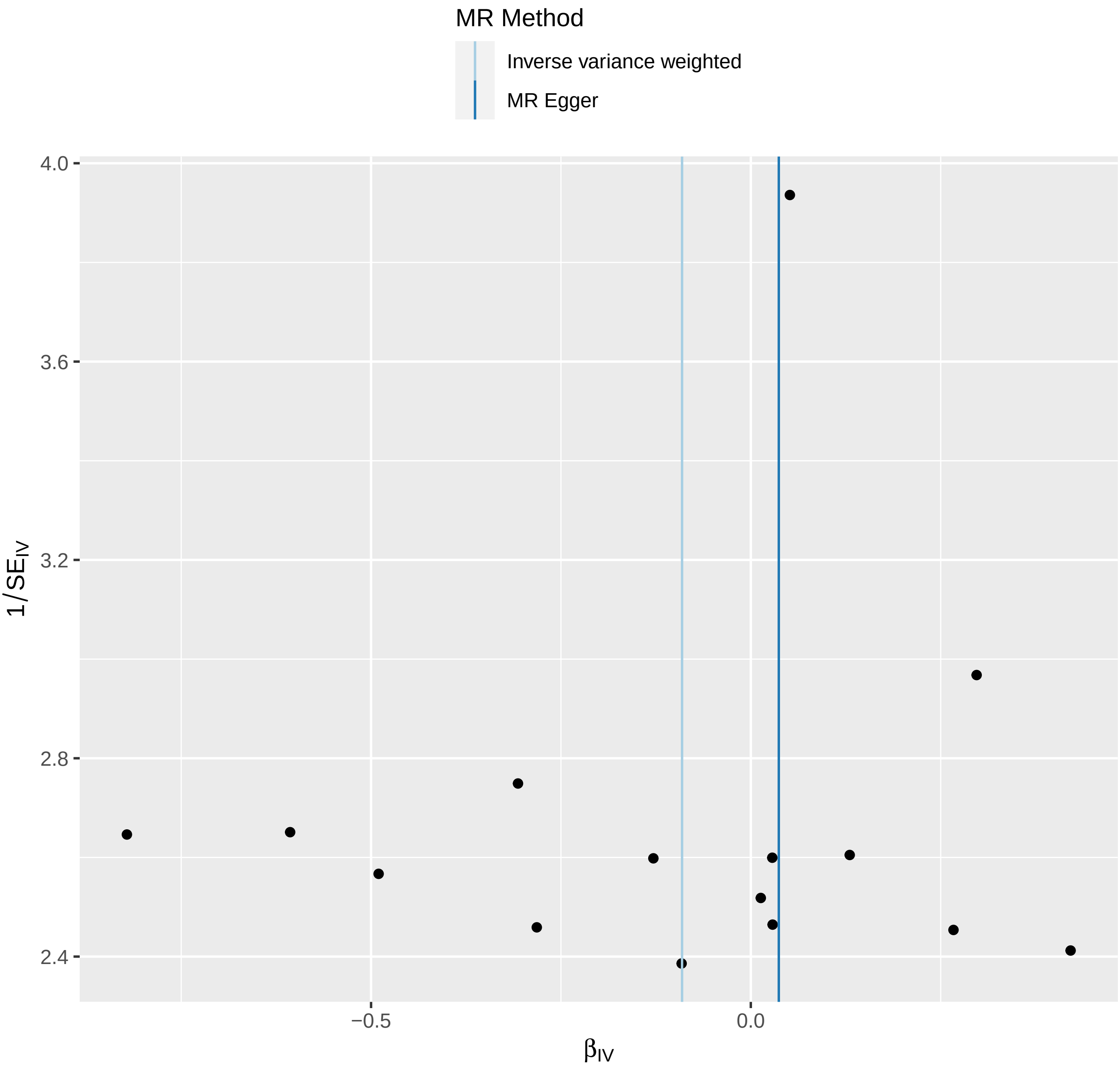

Batch 2−827 : Gut microbiota abundance (genus Catenibacterium id.2153) on E3 ubiquitin−protein ligase CCNB1IP1
with a threshold of 1e−05

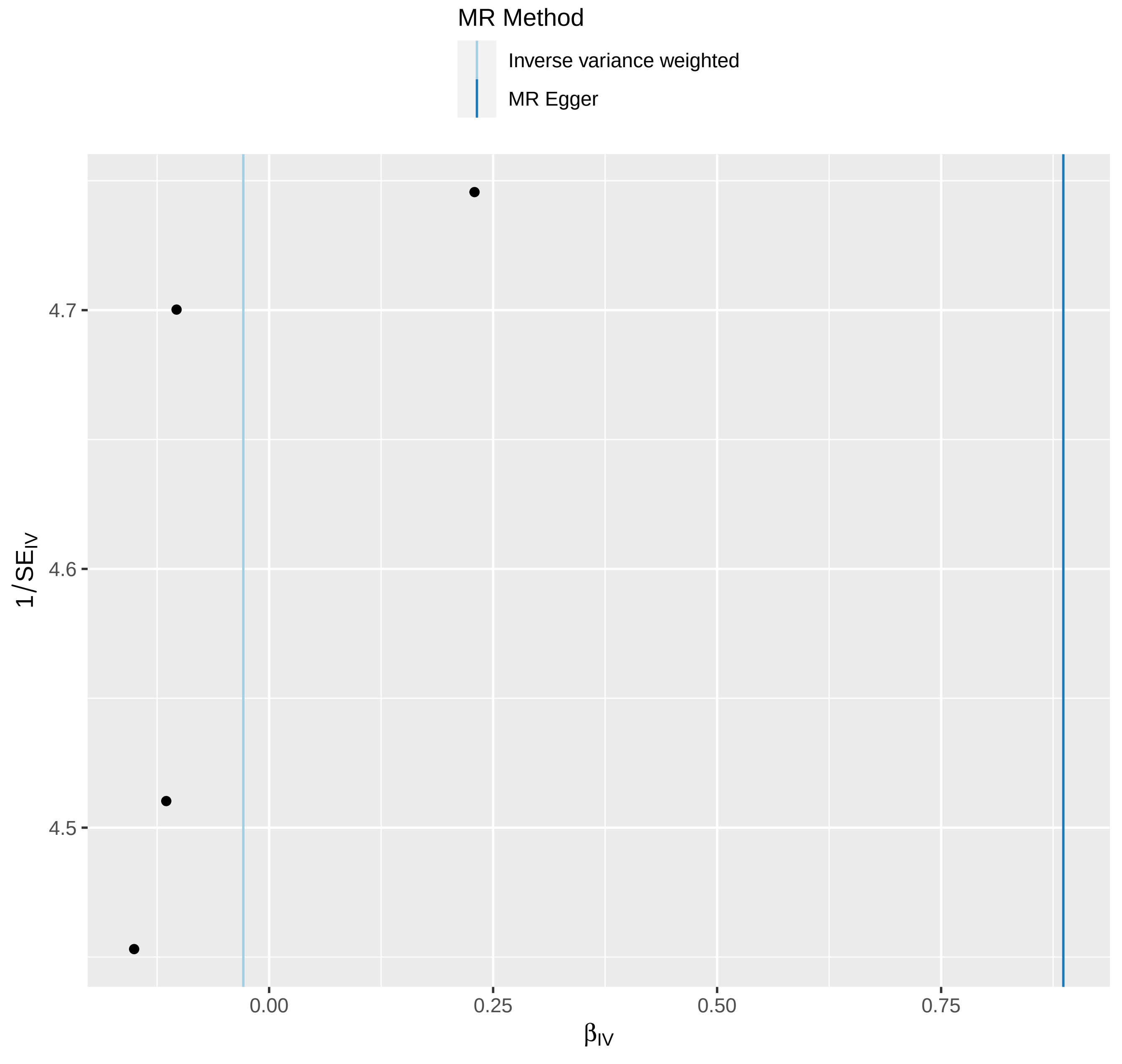

Batch 2−828 : Gut microbiota abundance (genus Parasutterella id.2892) on E3 ubiquitin−protein ligase CCNB1IP1
with a threshold of 1e−05

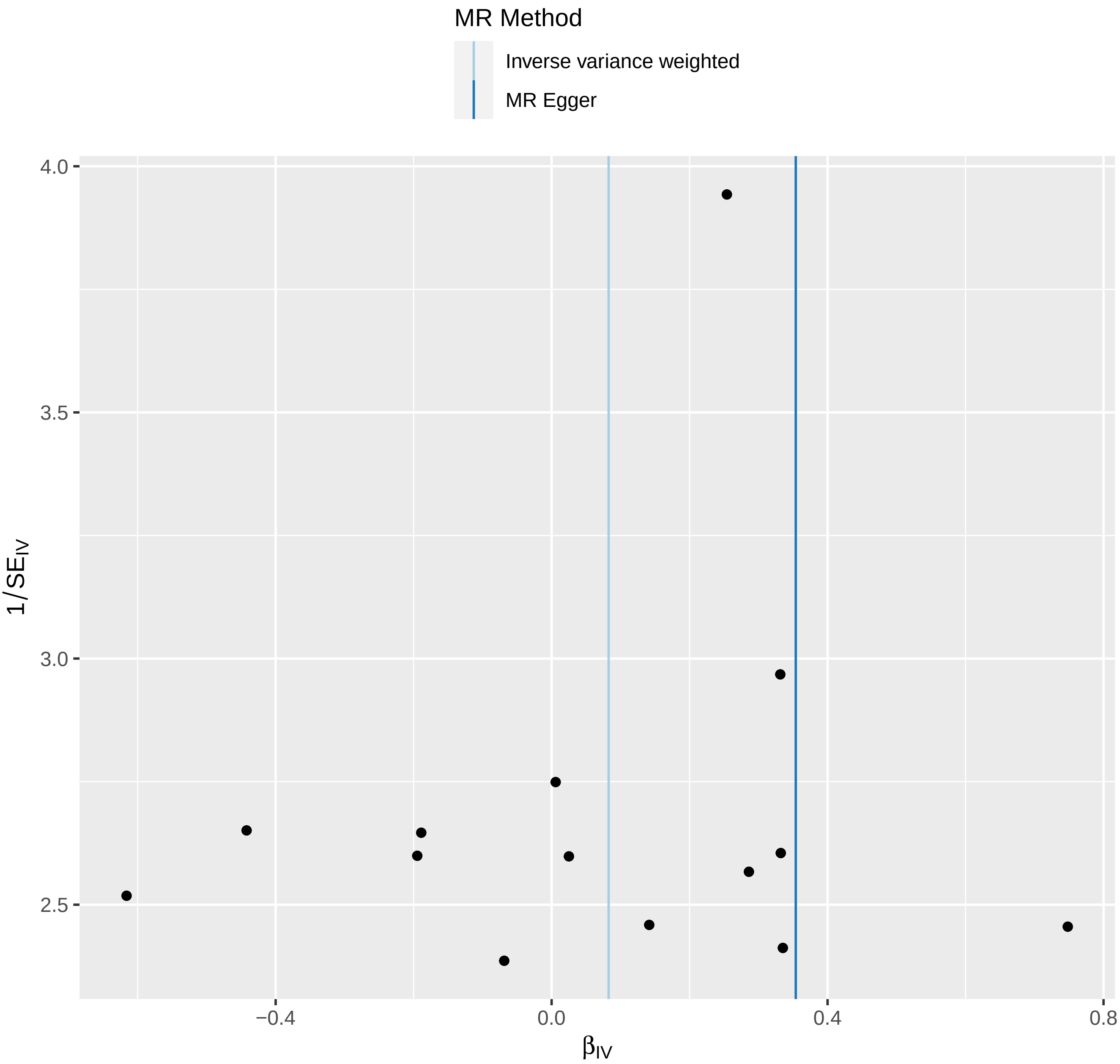

Batch 2−829 : Gut microbiota abundance (genus Catenibacterium id.2153) on Cyclin−C
with a threshold of 1e−05

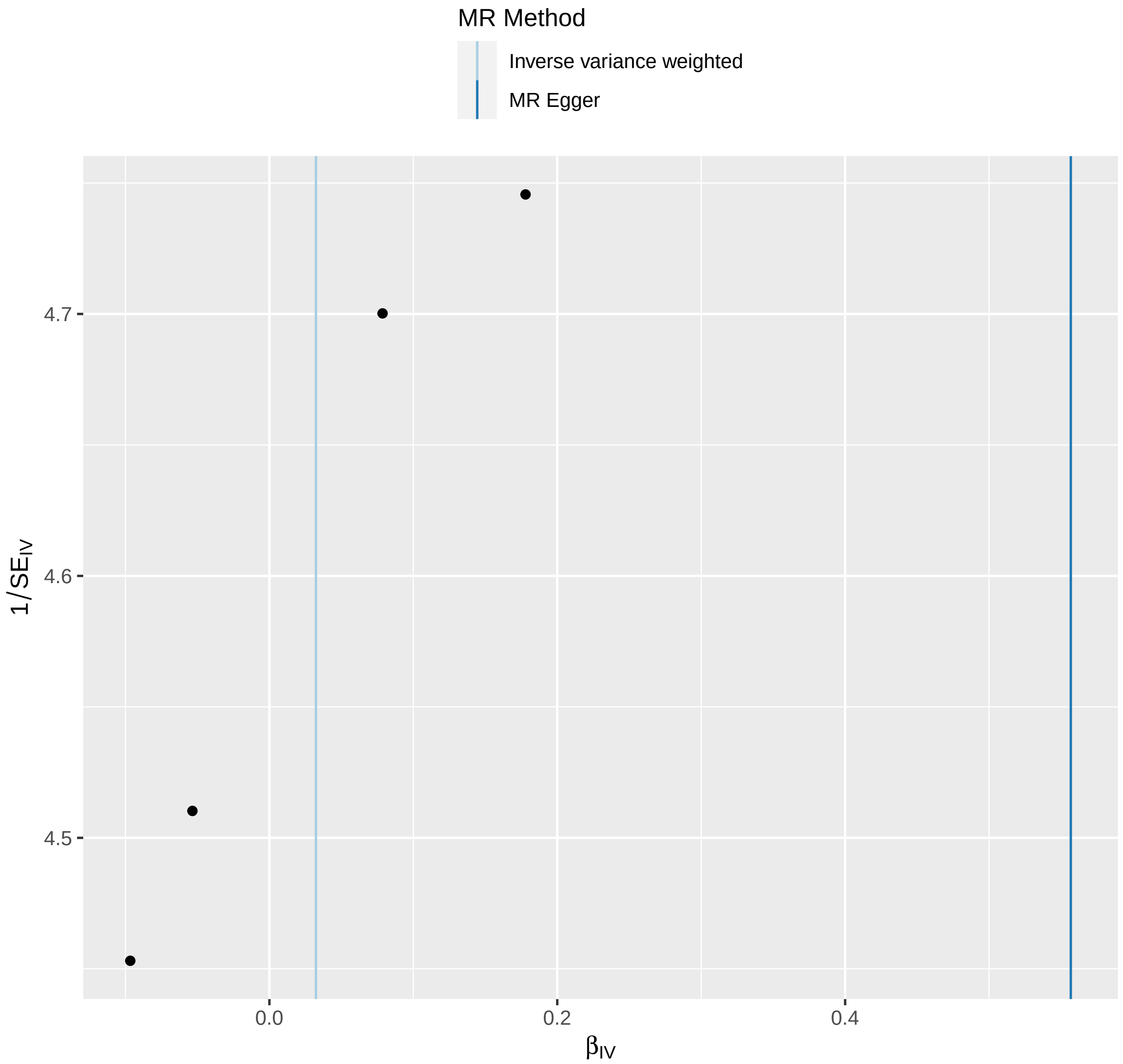

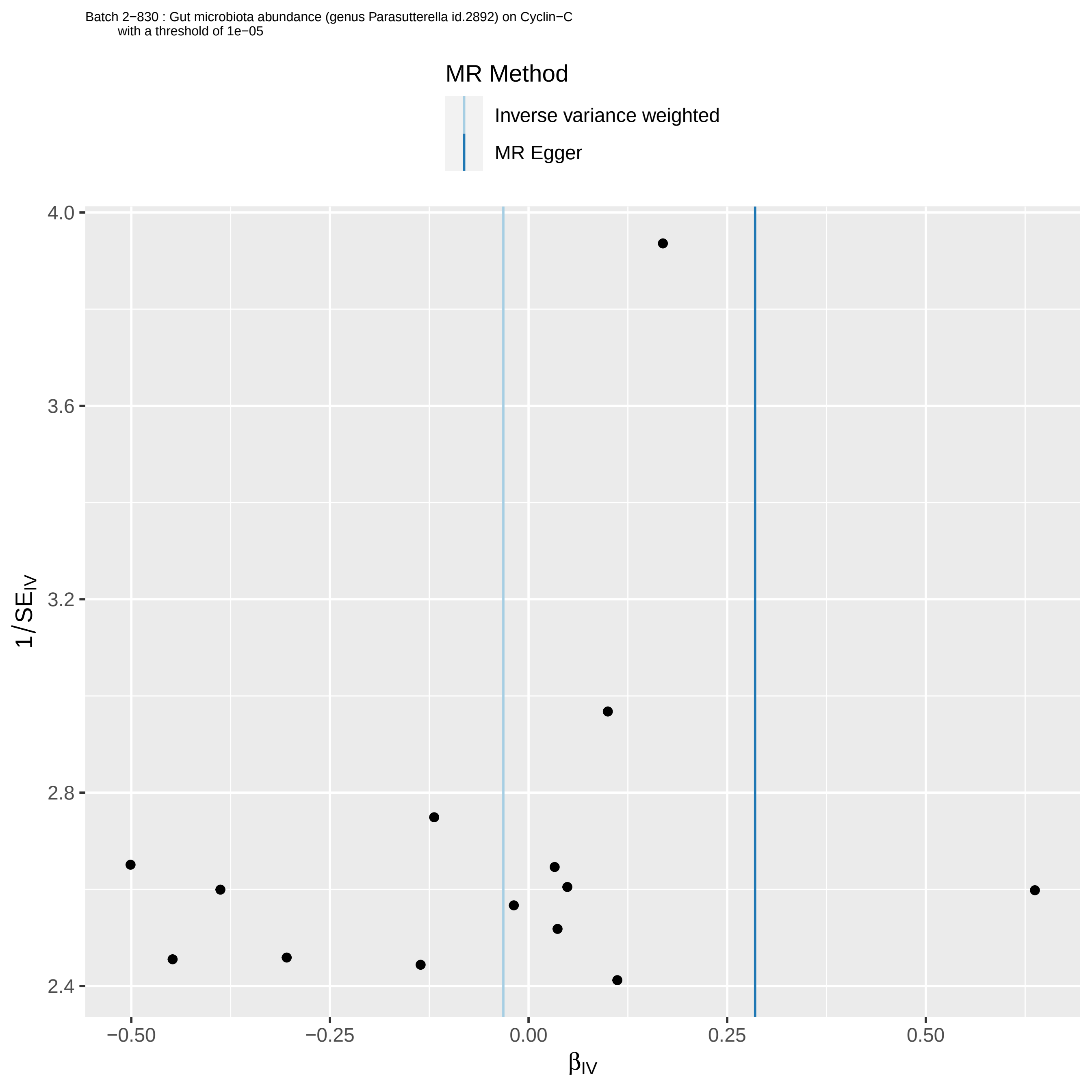

Batch 2−830 : Gut microbiota abundance (genus Parasutterella id.2892) on Cyclin−C
with a threshold of 1e−05
MR Method
Inverse variance weighted
MR Egger
4.0
3.6
3.2
2.8
2.4
1/SE_IV
−0.50
−0.25
0.00
0.25
0.50
β_IV

Batch 2−831 : Gut microbiota abundance (genus Catenibacterium id.2153) on Cyclin−H
with a threshold of 1e−05

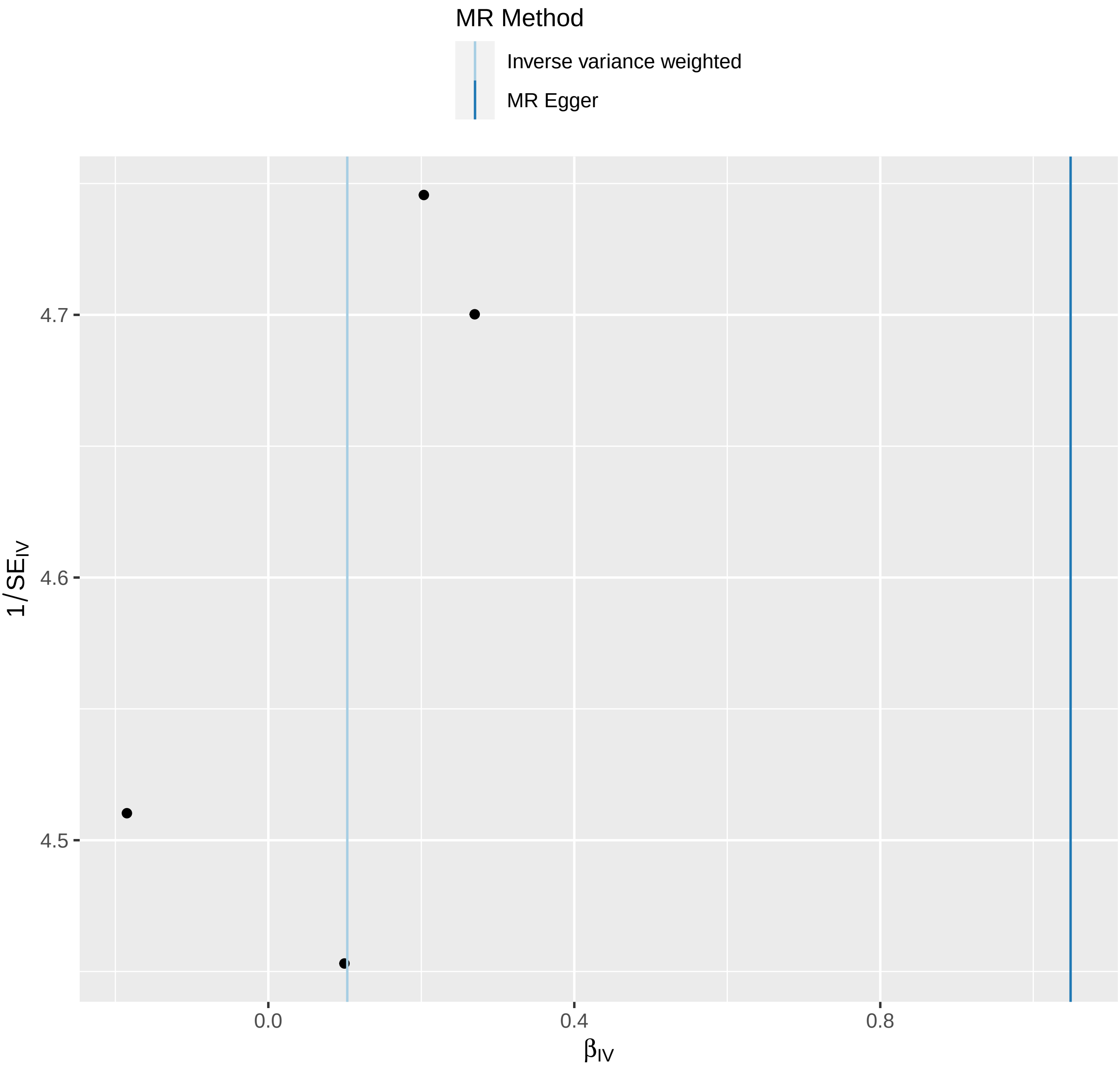

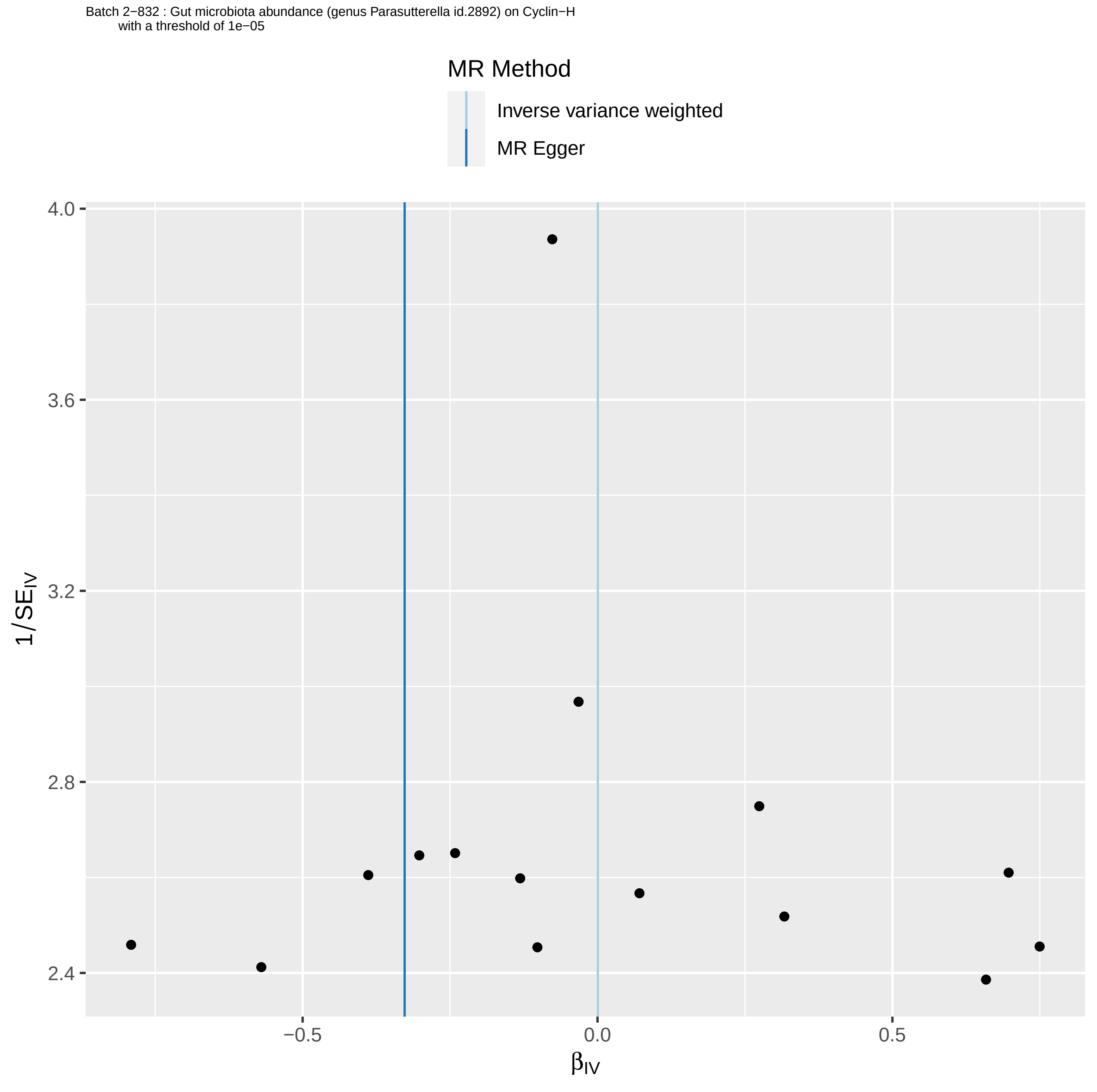

Batch 2−832 : Gut microbiota abundance (genus Parasutterella id.2892) on Cyclin−H
with a threshold of 1e−05
MR Method
Inverse variance weighted
MR Egger
4.0
3.6
3.2
2.8
2.4
1/SE_IV
−0.5
0.0
0.5
β_IV

Batch 2−833 : Gut microbiota abundance (genus Catenibacterium id.2153) on Cell cycle progression protein 1
with a threshold of 1e−05

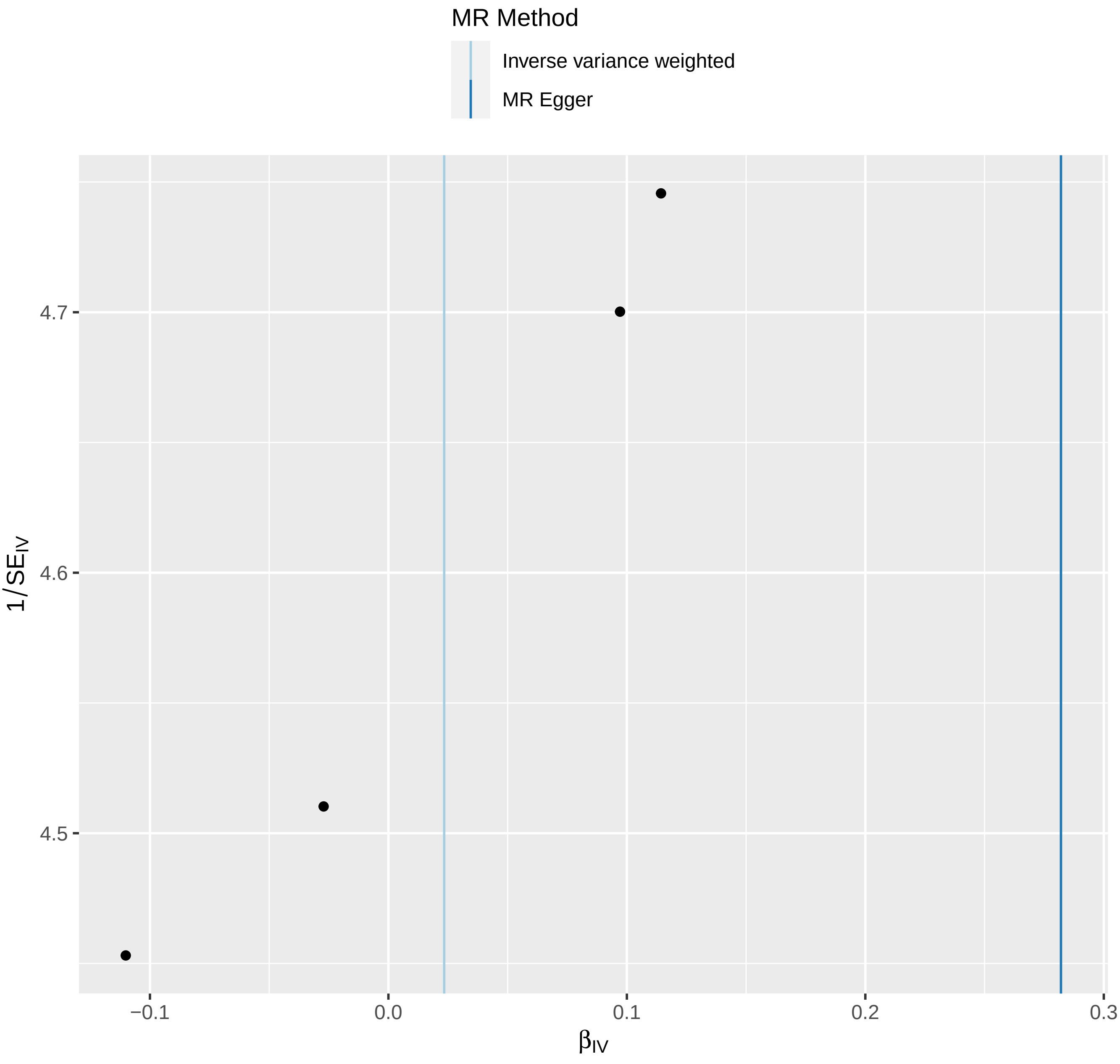

Batch 2−834 : Gut microbiota abundance (genus Parasutterella id.2892) on Cell cycle progression protein 1
with a threshold of 1e−05

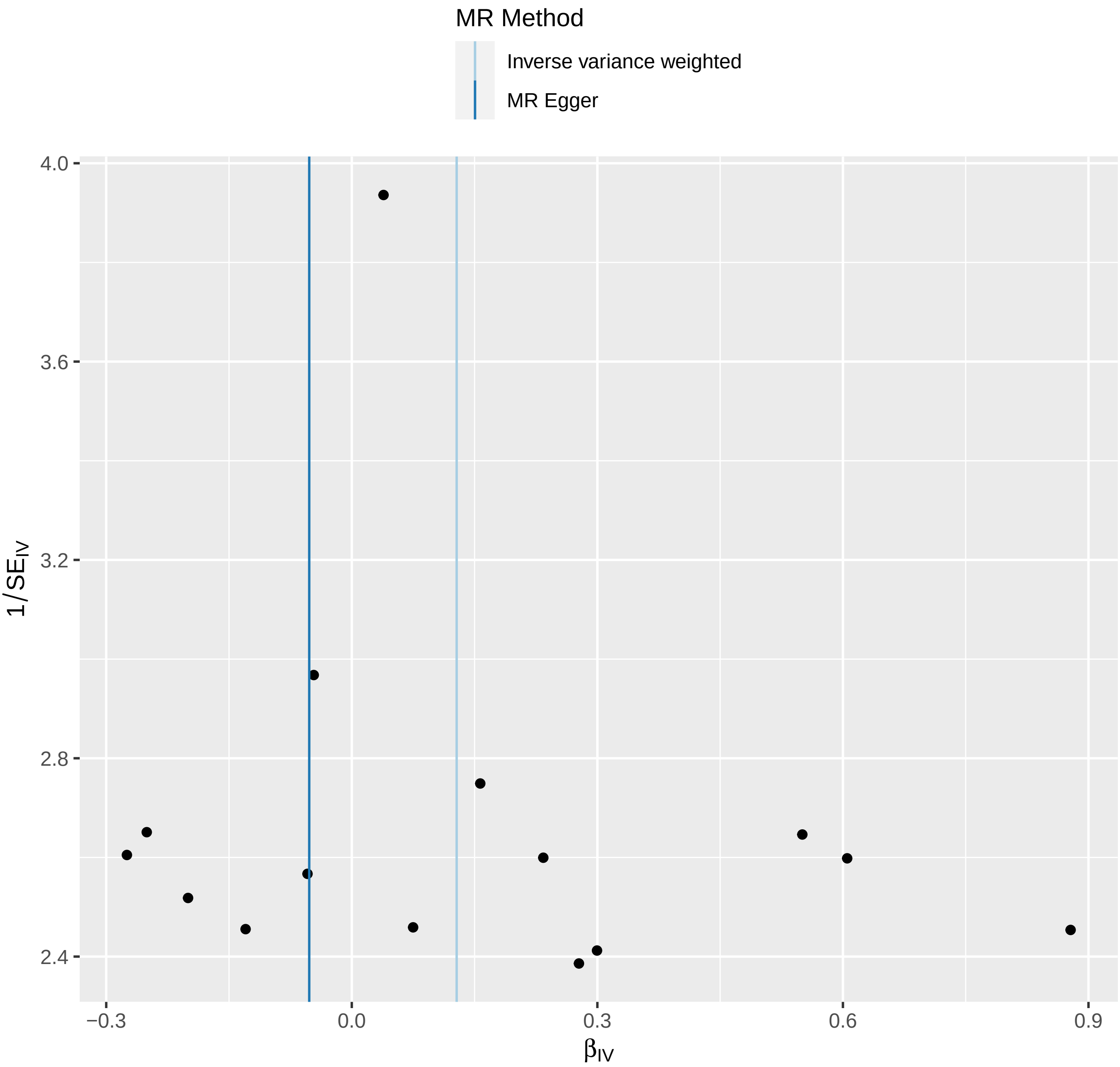

Batch 2−835 : Gut microbiota abundance (genus Catenibacterium id.2153) on CD109 antigen
with a threshold of 1e−05

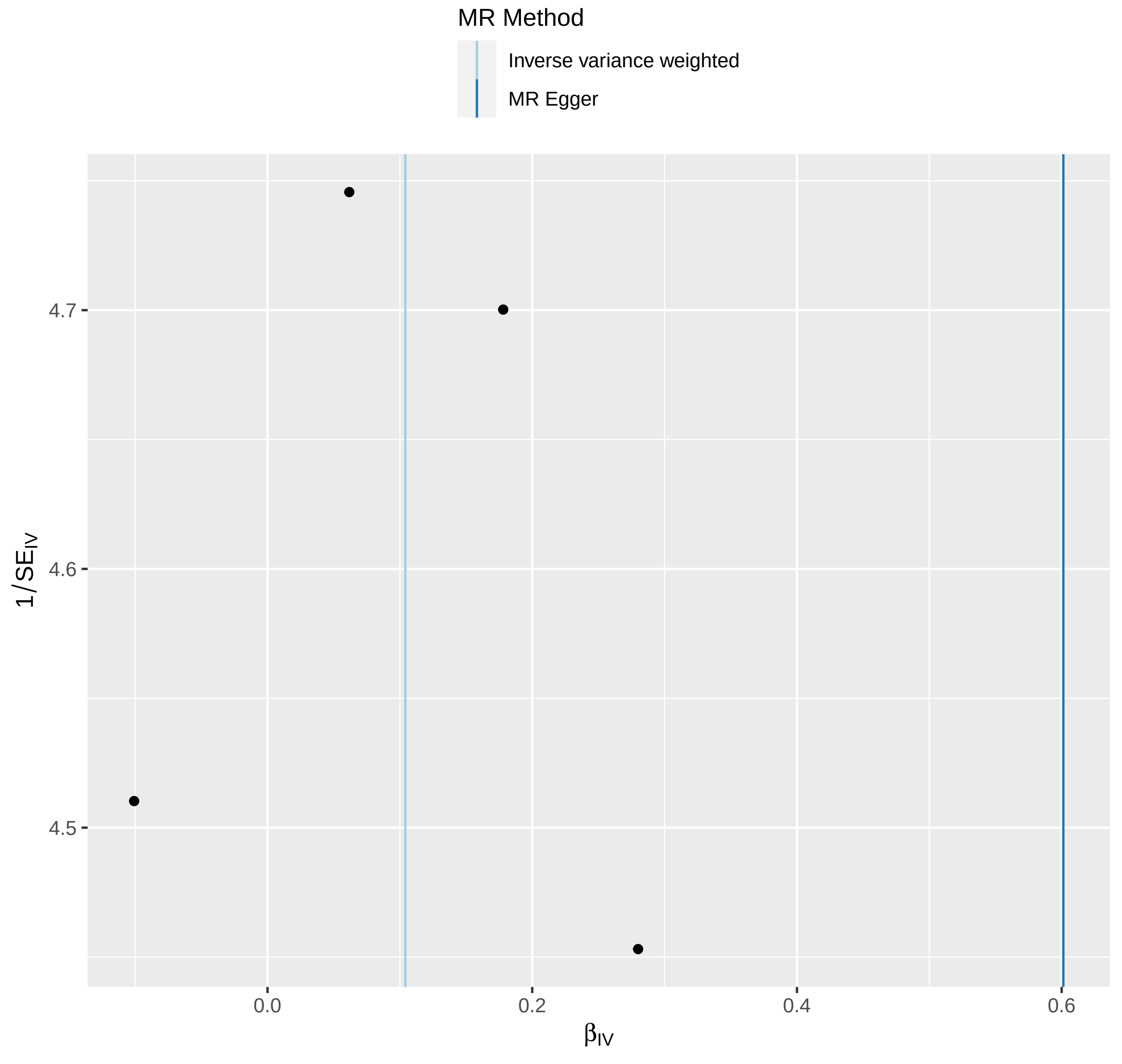

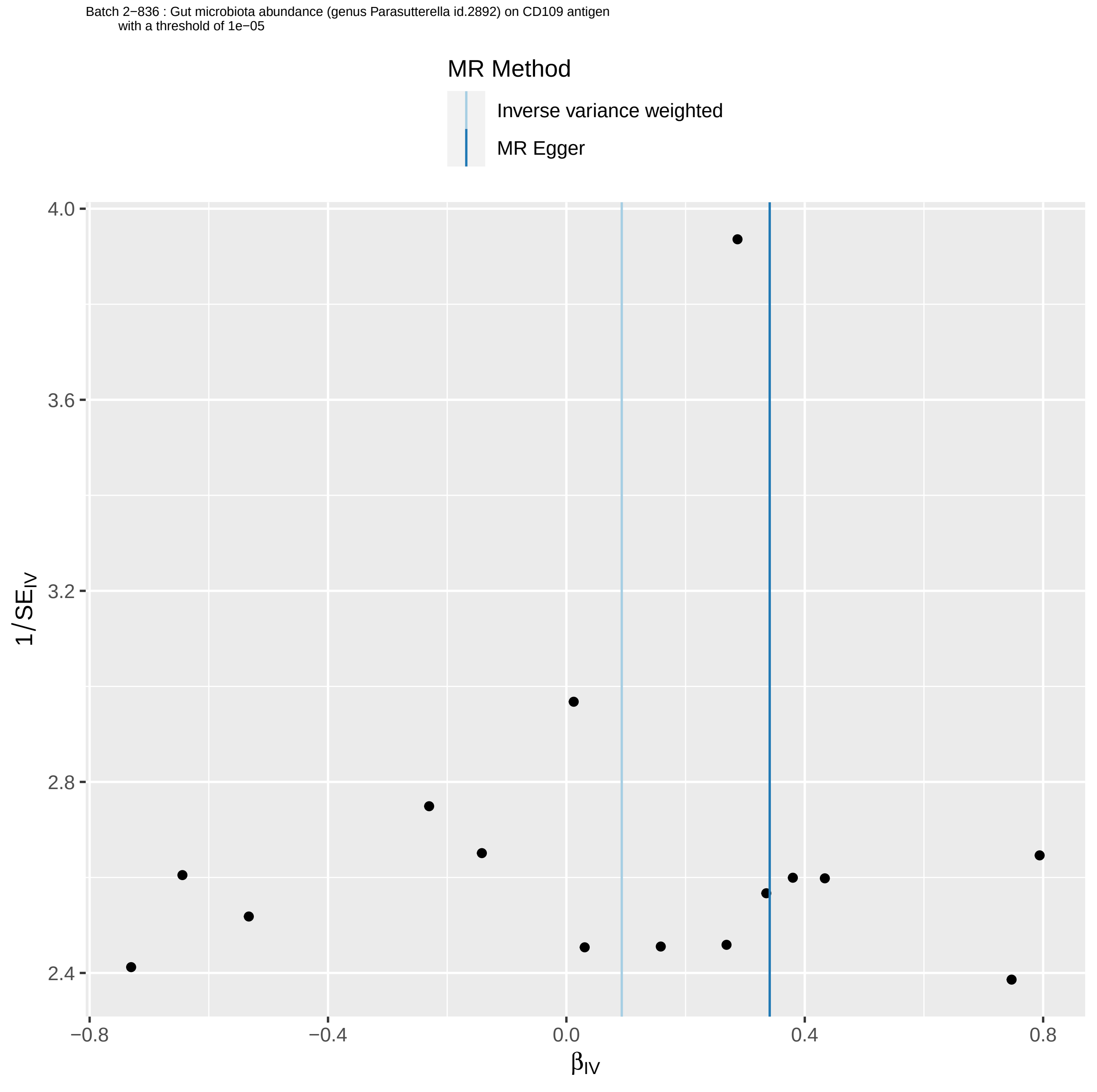
Batch 2−836 : Gut microbiota abundance (genus Parasutterella id.2892) on CD109 antigen
with a threshold of 1e−05
MR Method
Inverse variance weighted
MR Egger
4.0
3.6
3.2
2.8
2.4
1/SE_IV
−0.8
−0.4
0.0
0.4
0.8
β_IV

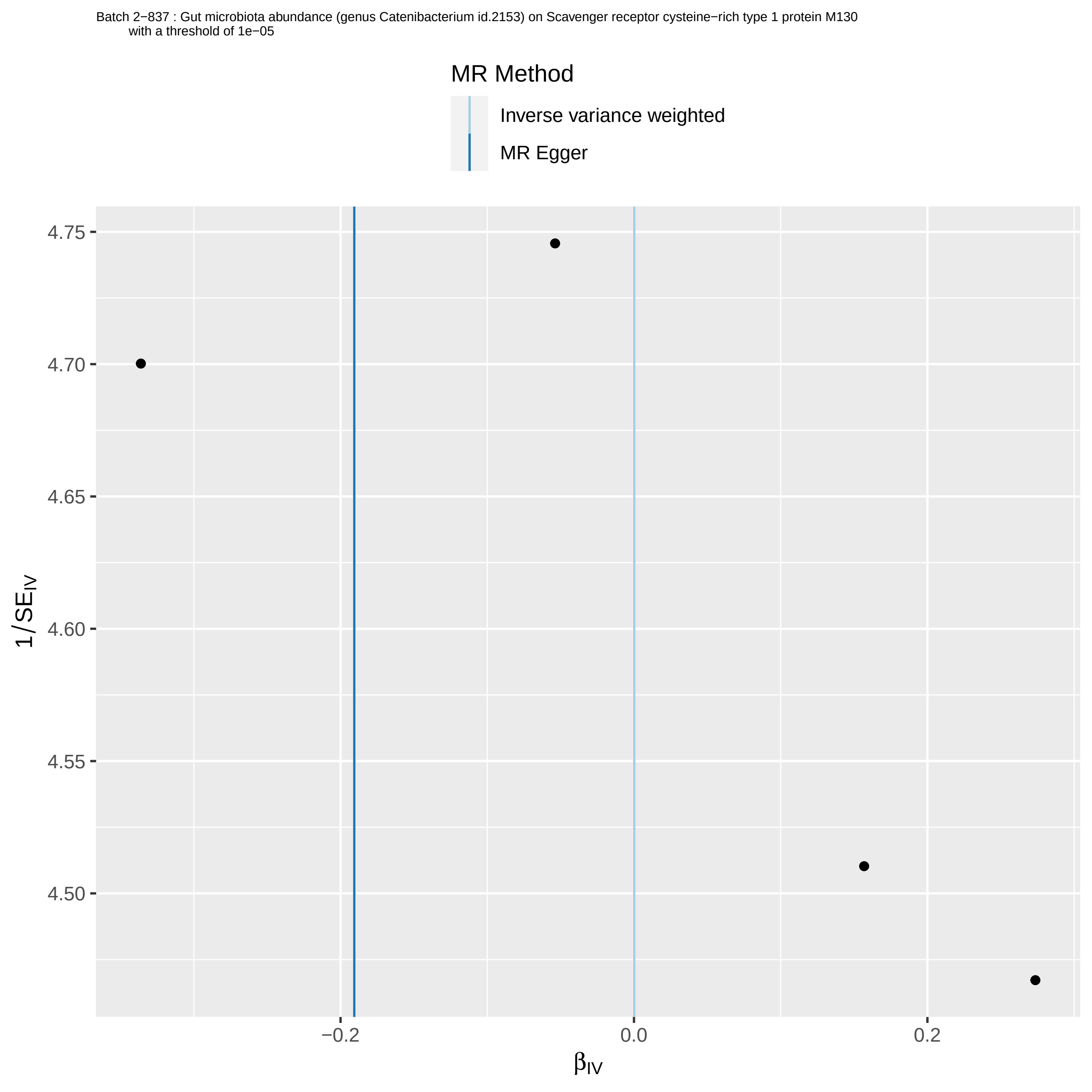

Batch 2−837 : Gut microbiota abundance (genus Catenibacterium id.2153) on Scavenger receptor cysteine−rich type 1 protein M130
with a threshold of 1e−05
MR Method
Inverse variance weighted
MR Egger
4.75
4.70
4.65
4.60
4.55
4.50
1/SE_IV
−0.2
0.0
0.2
β_IV

Batch 2−838 : Gut microbiota abundance (genus Parasutterella id.2892) on Scavenger receptor cysteine−rich type 1 protein M130
with a threshold of 1e−05

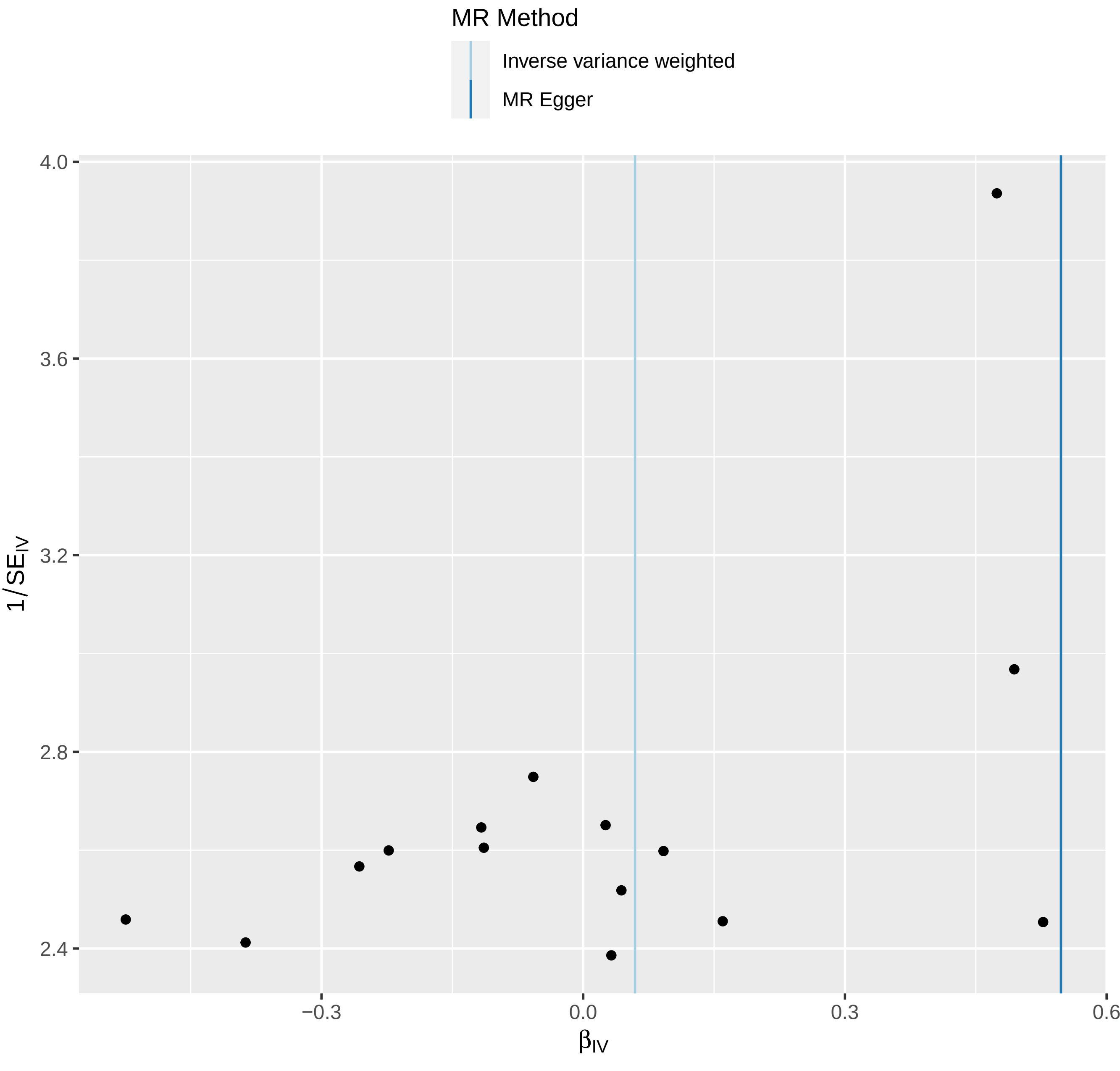

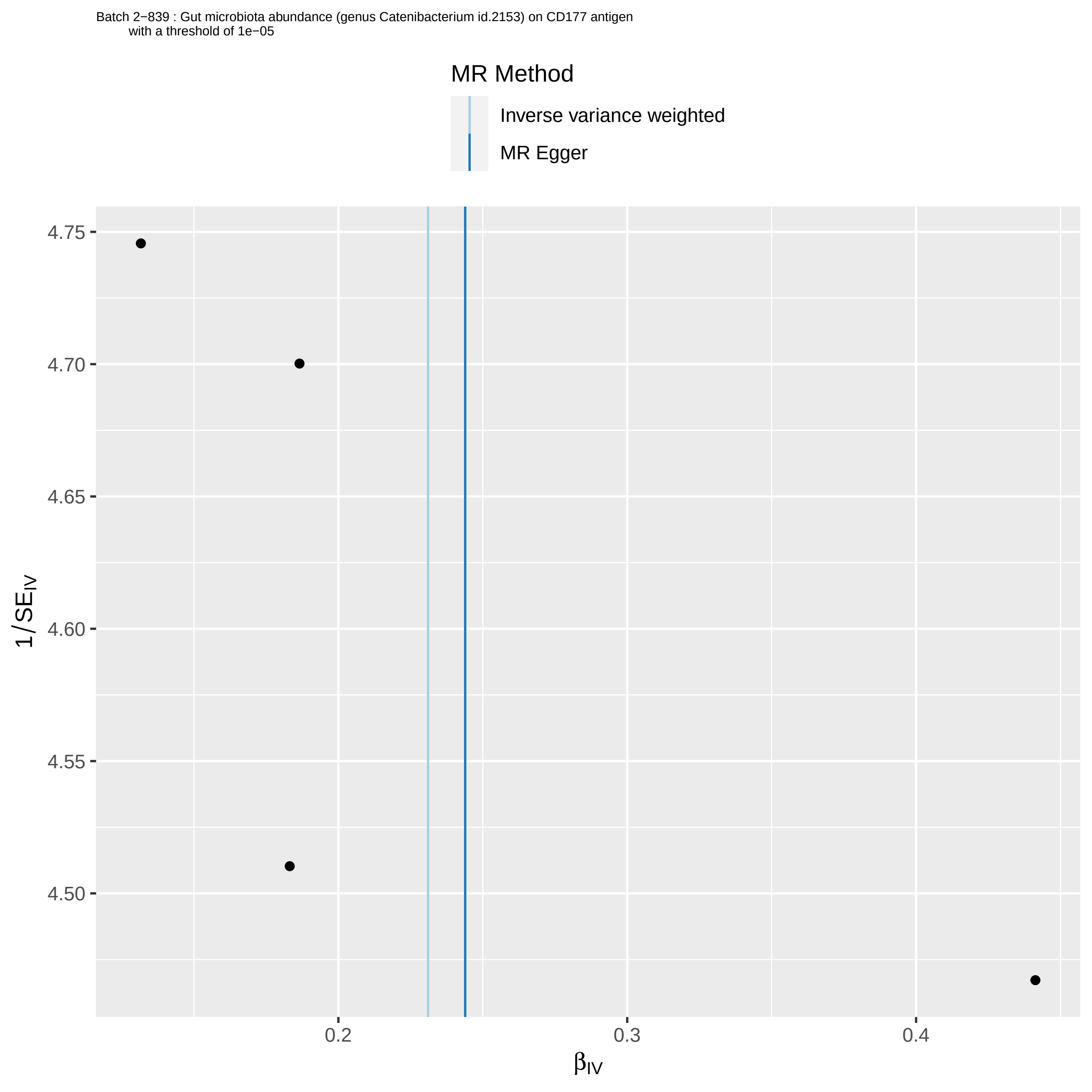
Batch 2−839 : Gut microbiota abundance (genus Catenibacterium id.2153) on CD177 antigen
with a threshold of 1e−05
MR Method
Inverse variance weighted
MR Egger
4.75
4.70
4.65
4.60
4.55
4.50
1/SE_IV
0.2
0.3
0.4
β_IV

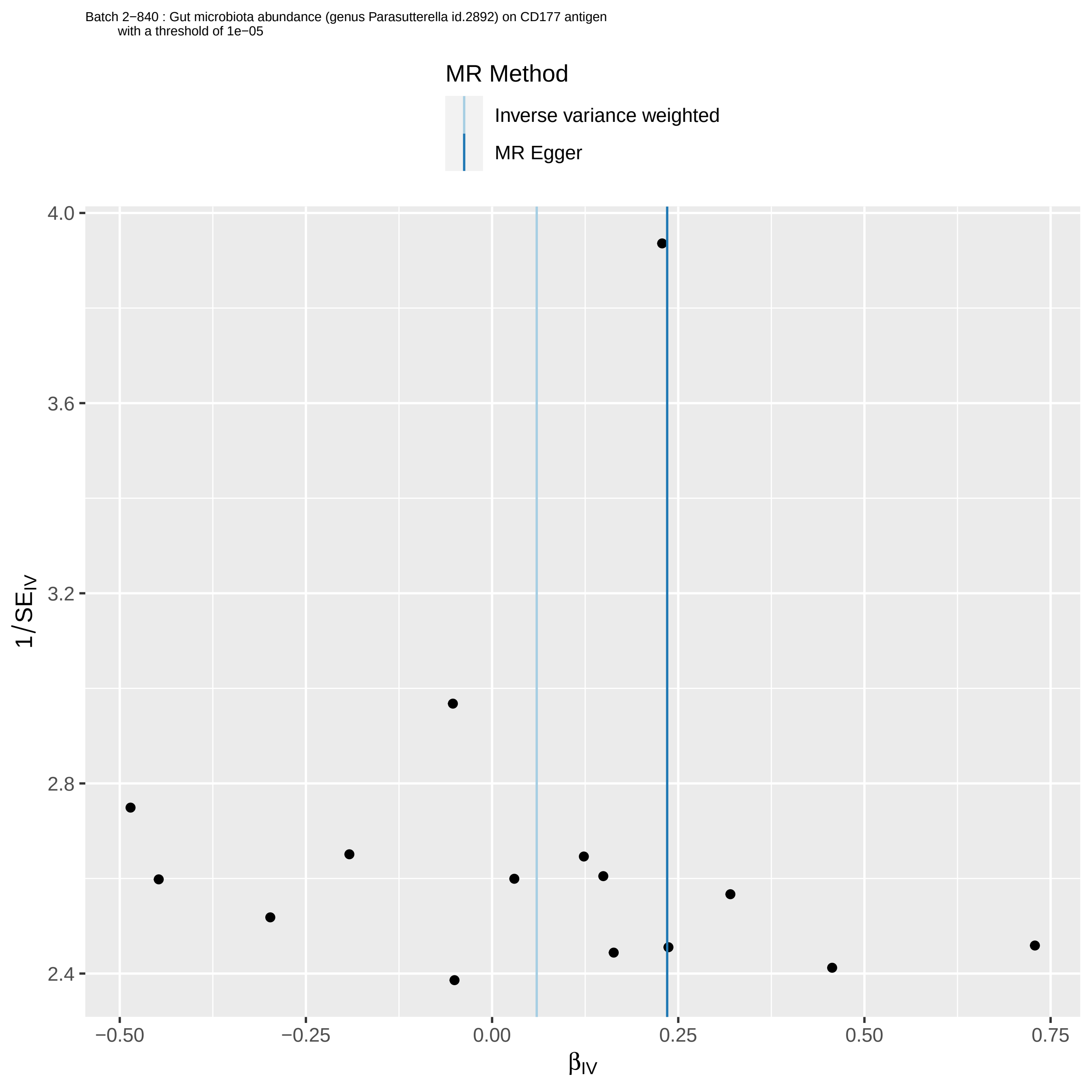

Batch 2−840 : Gut microbiota abundance (genus Parasutterella id.2892) on CD177 antigen
with a threshold of 1e−05
MR Method
Inverse variance weighted
MR Egger
4.0
3.6
3.2
2.8
2.4
1/SE_IV
−0.50
−0.25
0.00
0.25
0.50
0.75
β_IV

Batch 2−841 : Gut microbiota abundance (genus Catenibacterium id.2153) on Antigen−presenting glycoprotein CD1d
with a threshold of 1e−05

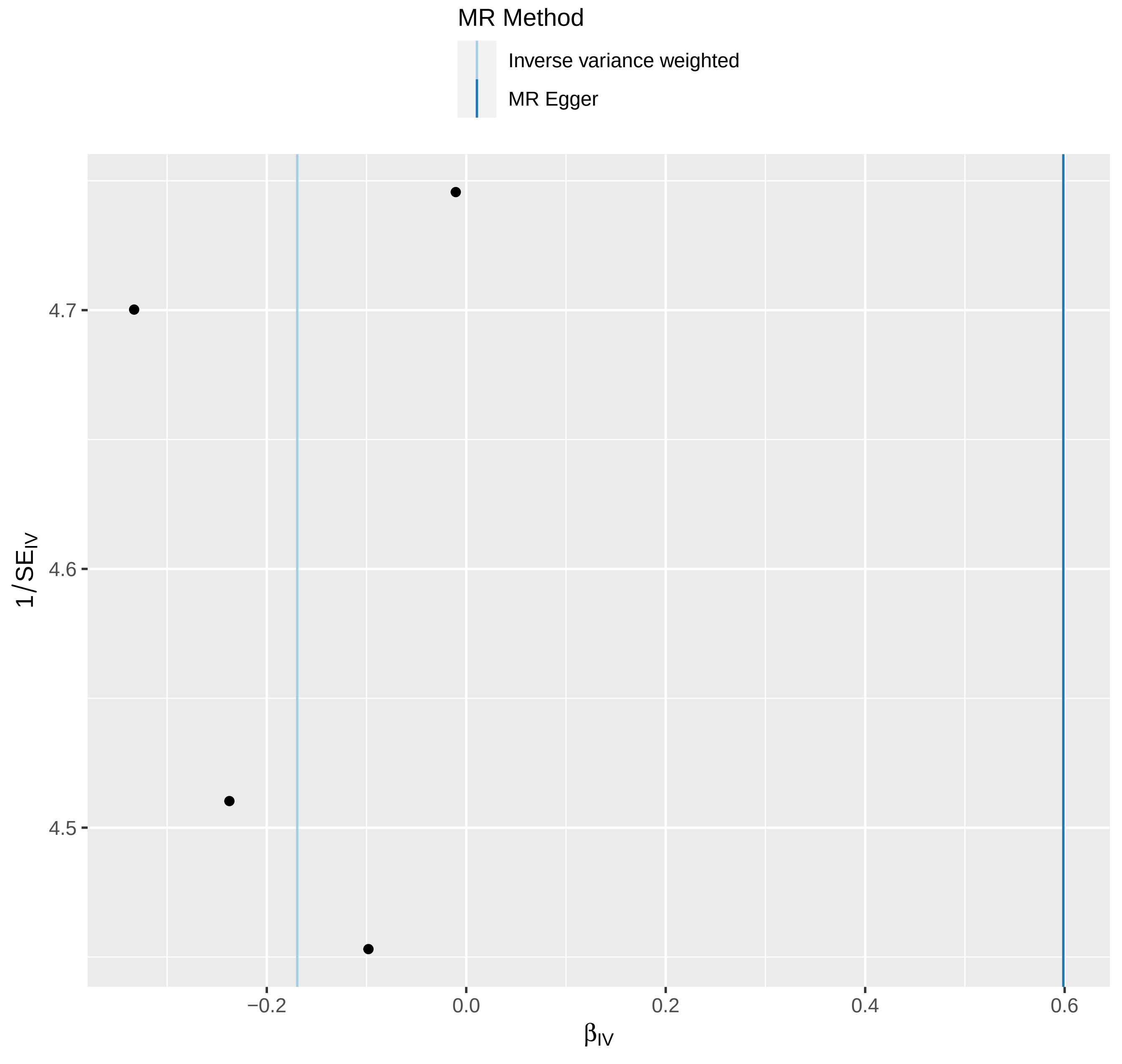

Batch 2−842 : Gut microbiota abundance (genus Parasutterella id.2892) on Antigen−presenting glycoprotein CD1d
with a threshold of 1e−05

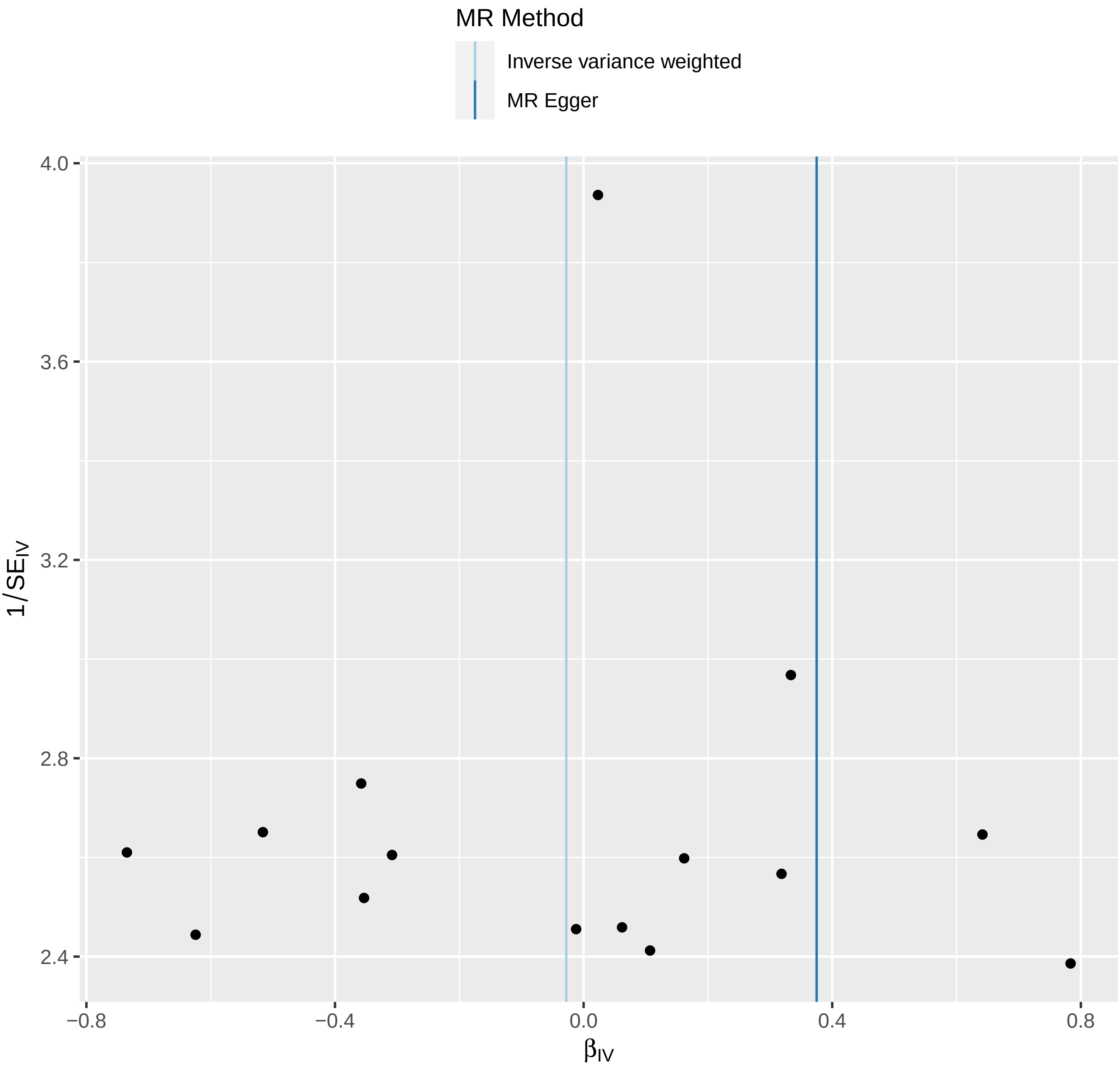

Batch 2−843 : Gut microbiota abundance (genus Catenibacterium id.2153) on OX−2 membrane glycoprotein
with a threshold of 1e−05

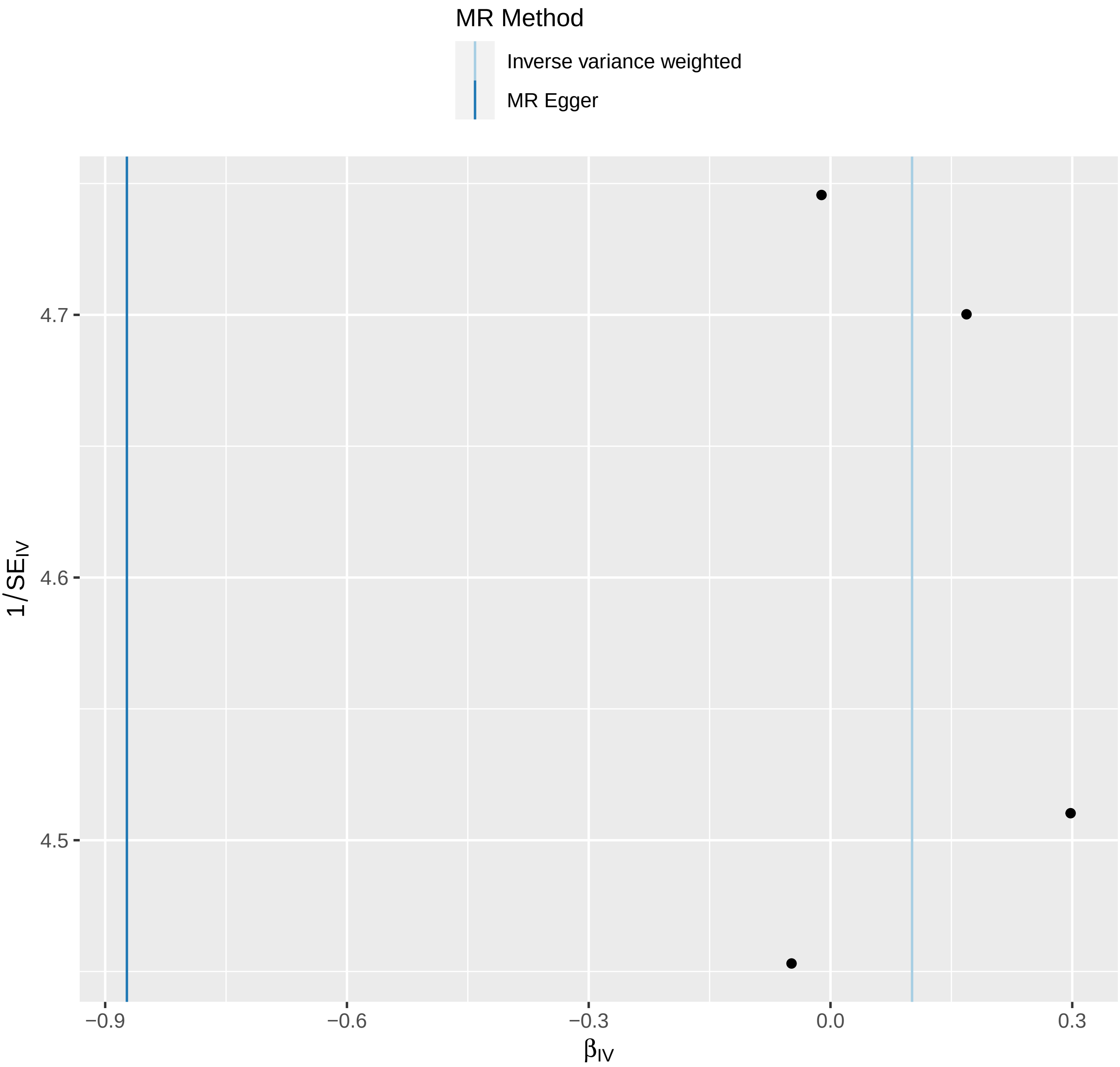

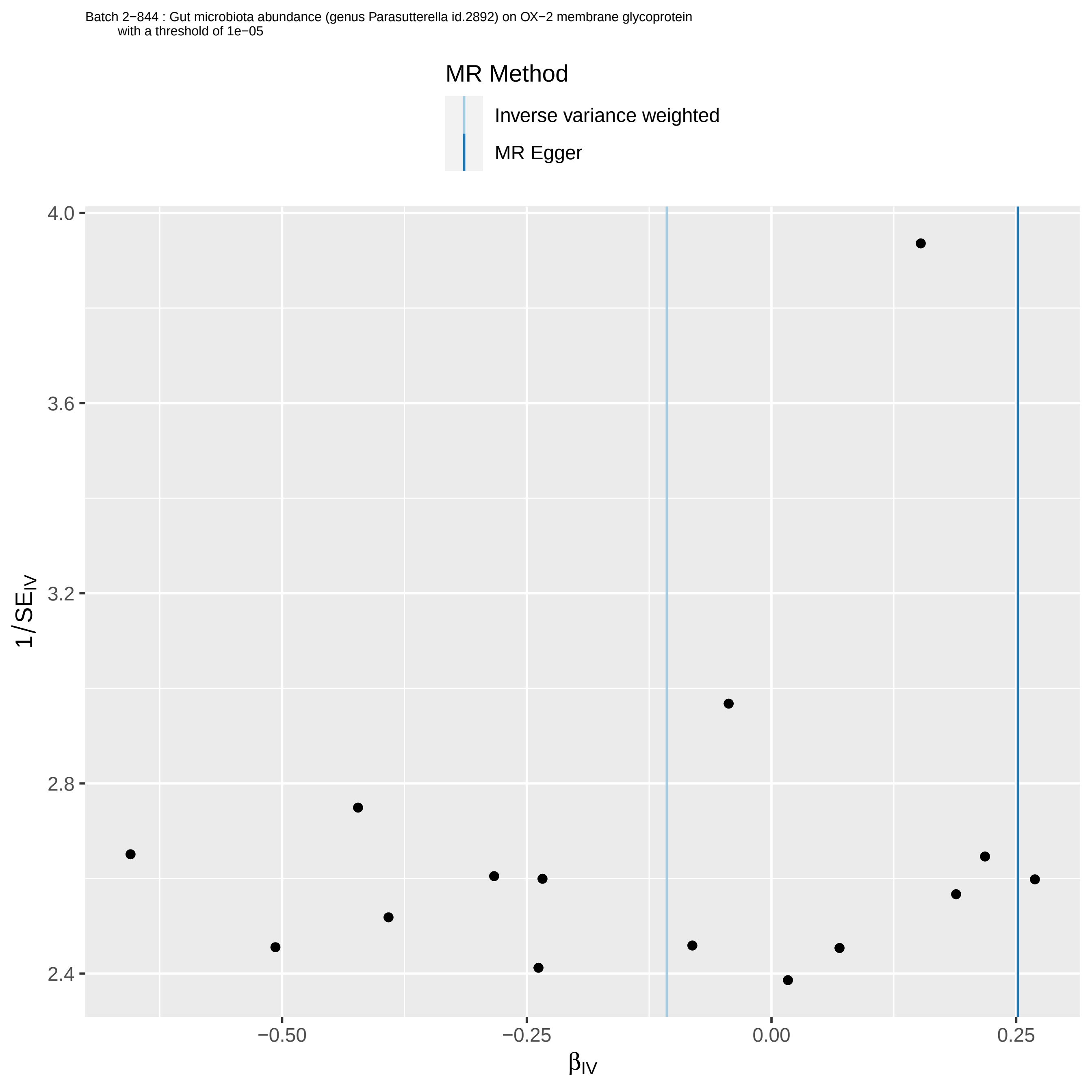
Batch 2−844 : Gut microbiota abundance (genus Parasutterella id.2892) on OX−2 membrane glycoprotein
with a threshold of 1e−05
MR Method
Inverse variance weighted
MR Egger
4.0
3.6
3.2
2.8
2.4
1/SE_IV
−0.50
−0.25
0.00
0.25
β_IV

Batch 2−845 : Gut microbiota abundance (genus Catenibacterium id.2153) on Cell surface glycoprotein CD200 receptor 1
with a threshold of 1e−05

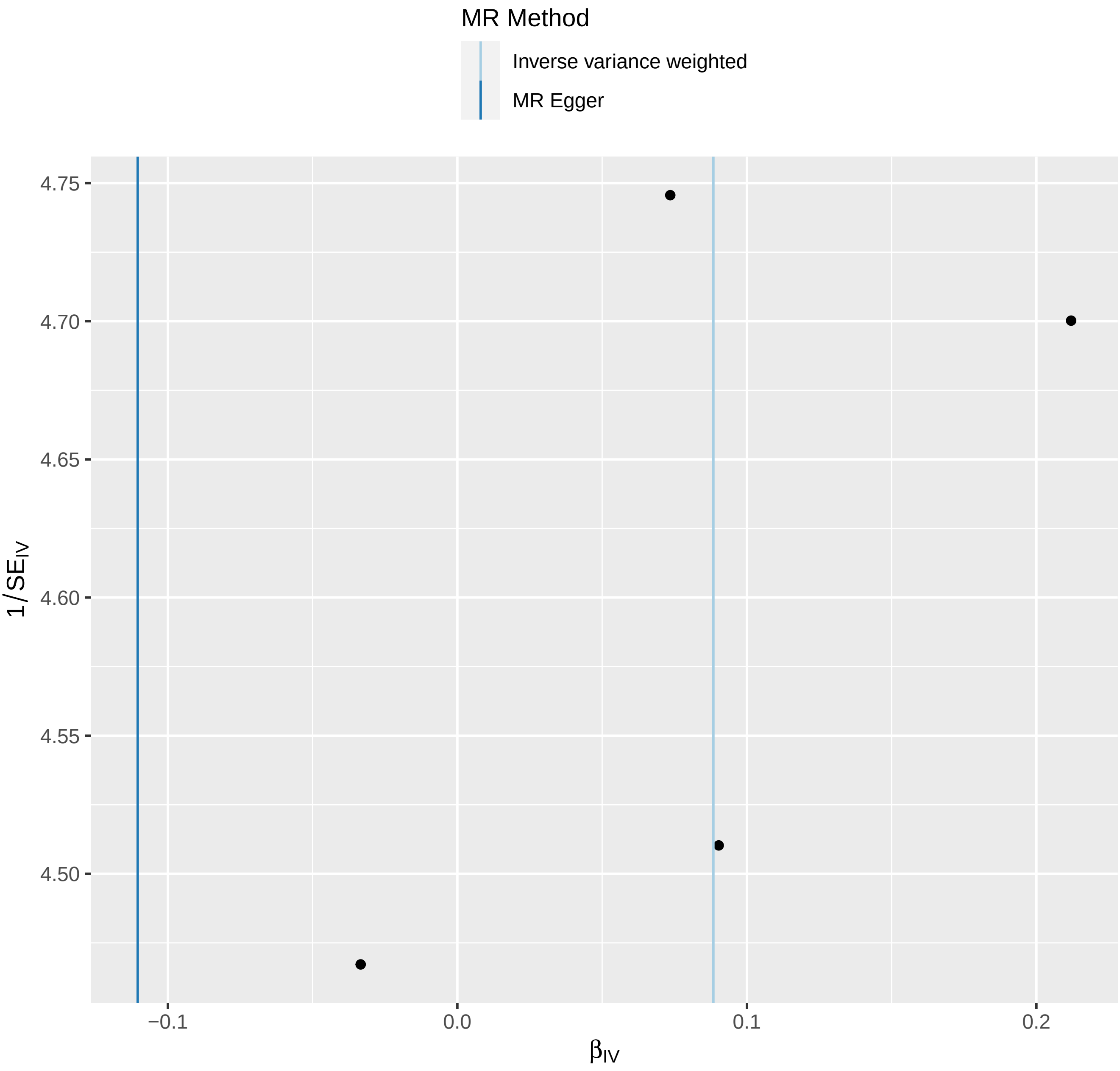

Batch 2−846 : Gut microbiota abundance (genus Parasutterella id.2892) on Cell surface glycoprotein CD200 receptor 1
with a threshold of 1e−05

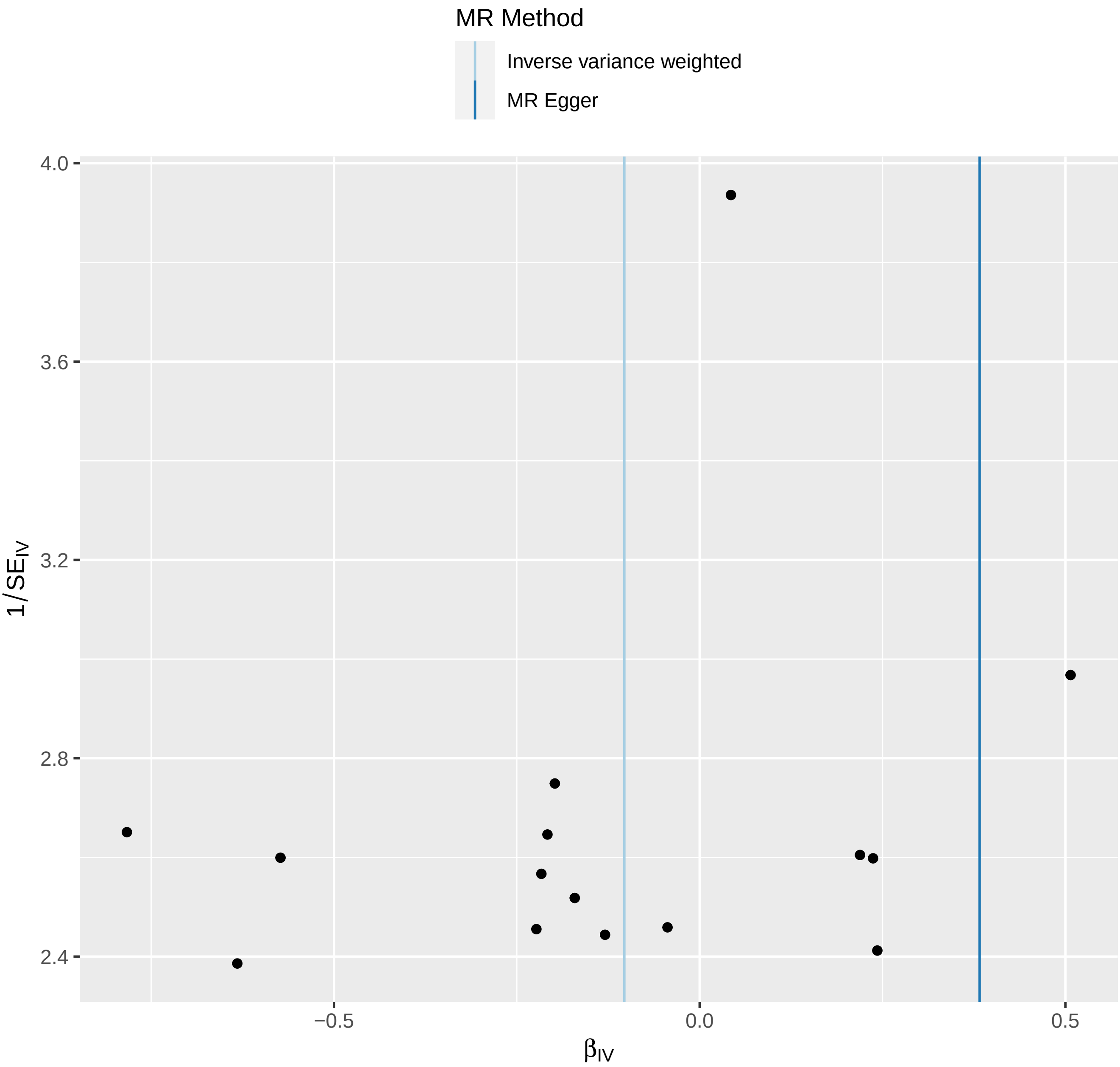

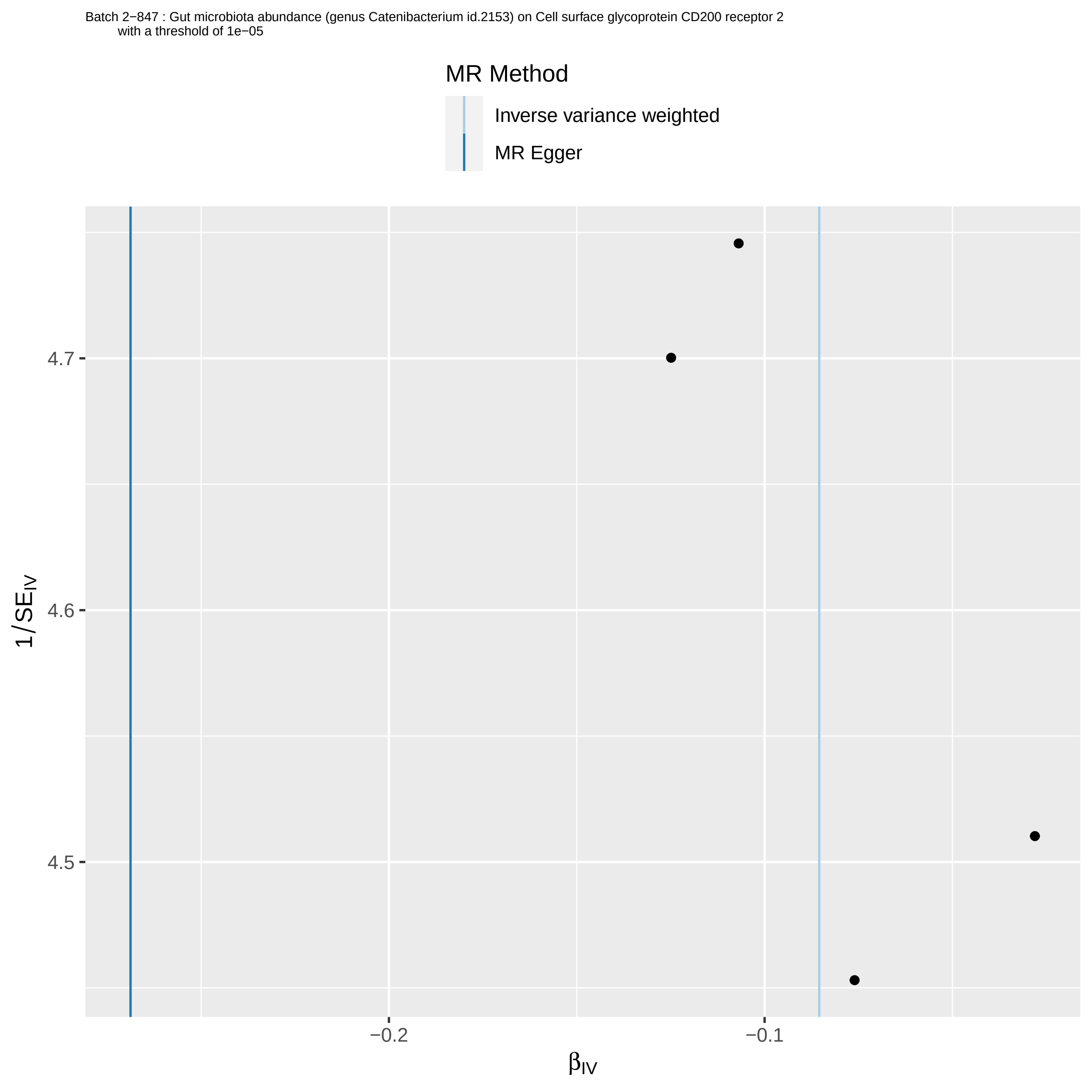
Batch 2−847 : Gut microbiota abundance (genus Catenibacterium id.2153) on Cell surface glycoprotein CD200 receptor 2
with a threshold of 1e−05
MR Method
Inverse variance weighted
MR Egger
4.7
4.6
4.5
1/SE_IV
−0.2
−0.1
β_IV

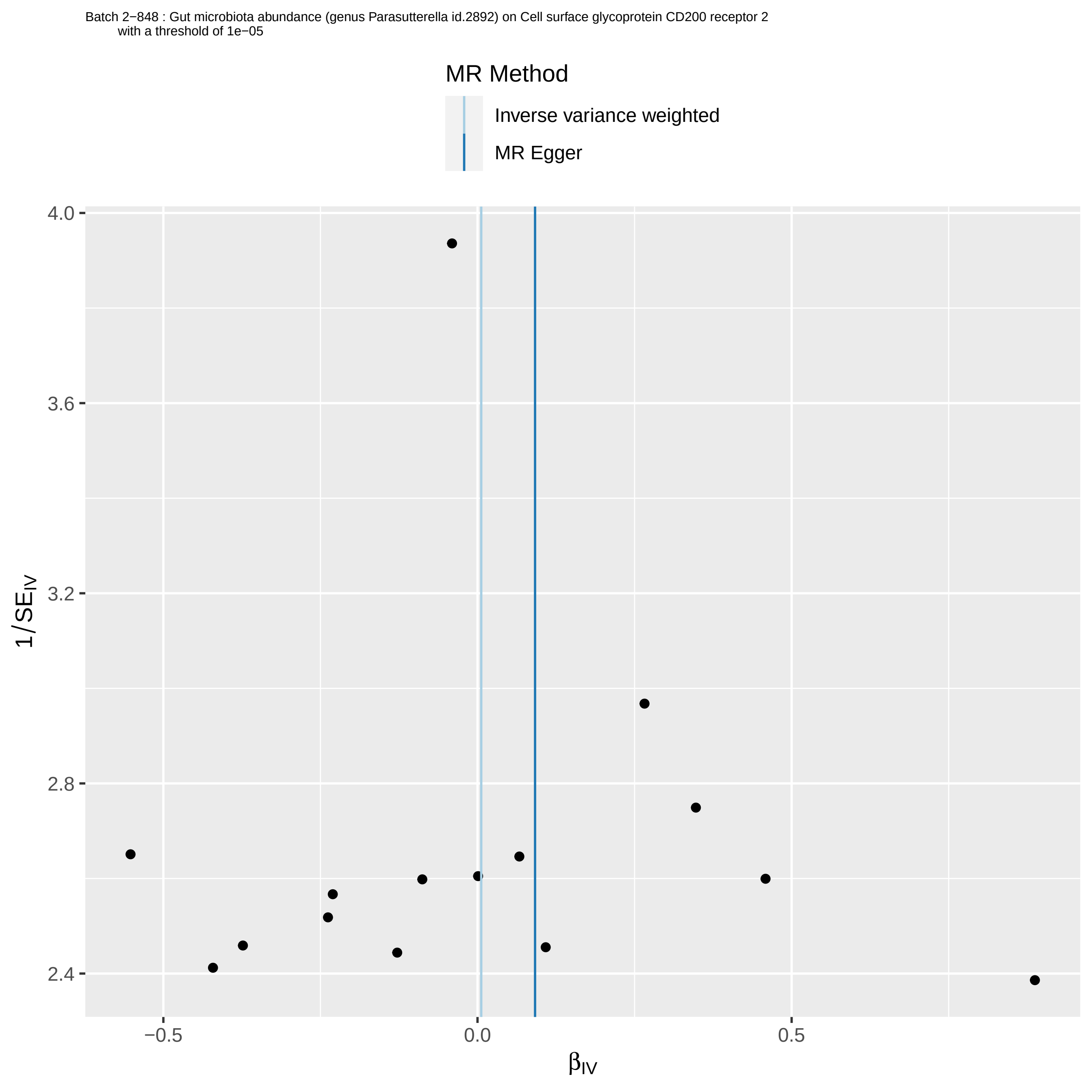

Batch 2−848 : Gut microbiota abundance (genus Parasutterella id.2892) on Cell surface glycoprotein CD200 receptor 2
with a threshold of 1e−05
MR Method
Inverse variance weighted
MR Egger
4.0
3.6
3.2
2.8
2.4
1/SE_IV
−0.5
0.0
0.5
β_IV

Batch 2−849 : Gut microbiota abundance (genus Catenibacterium id.2153) on C−type lectin domain family 4 member K with a threshold of 1e−05

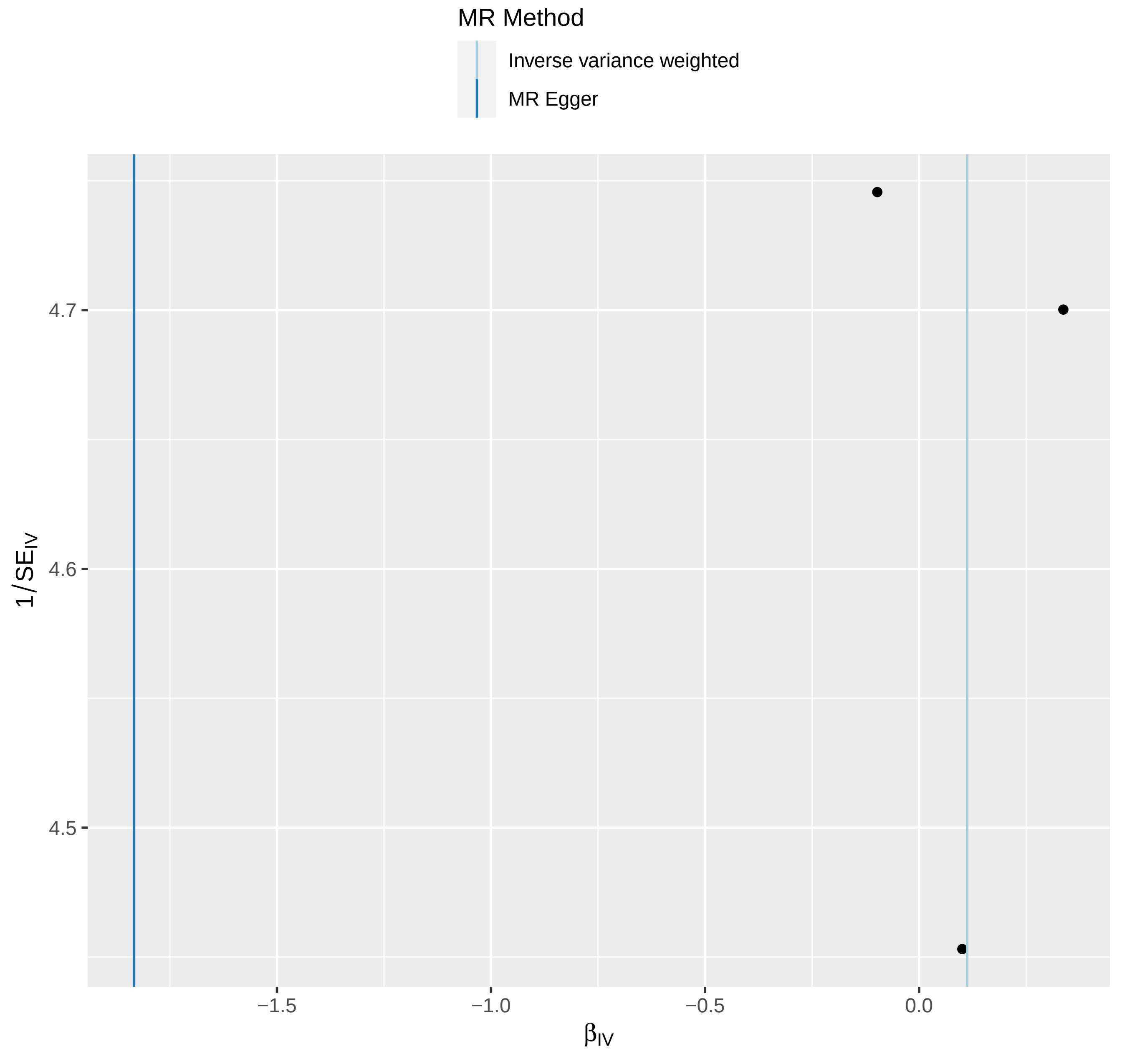

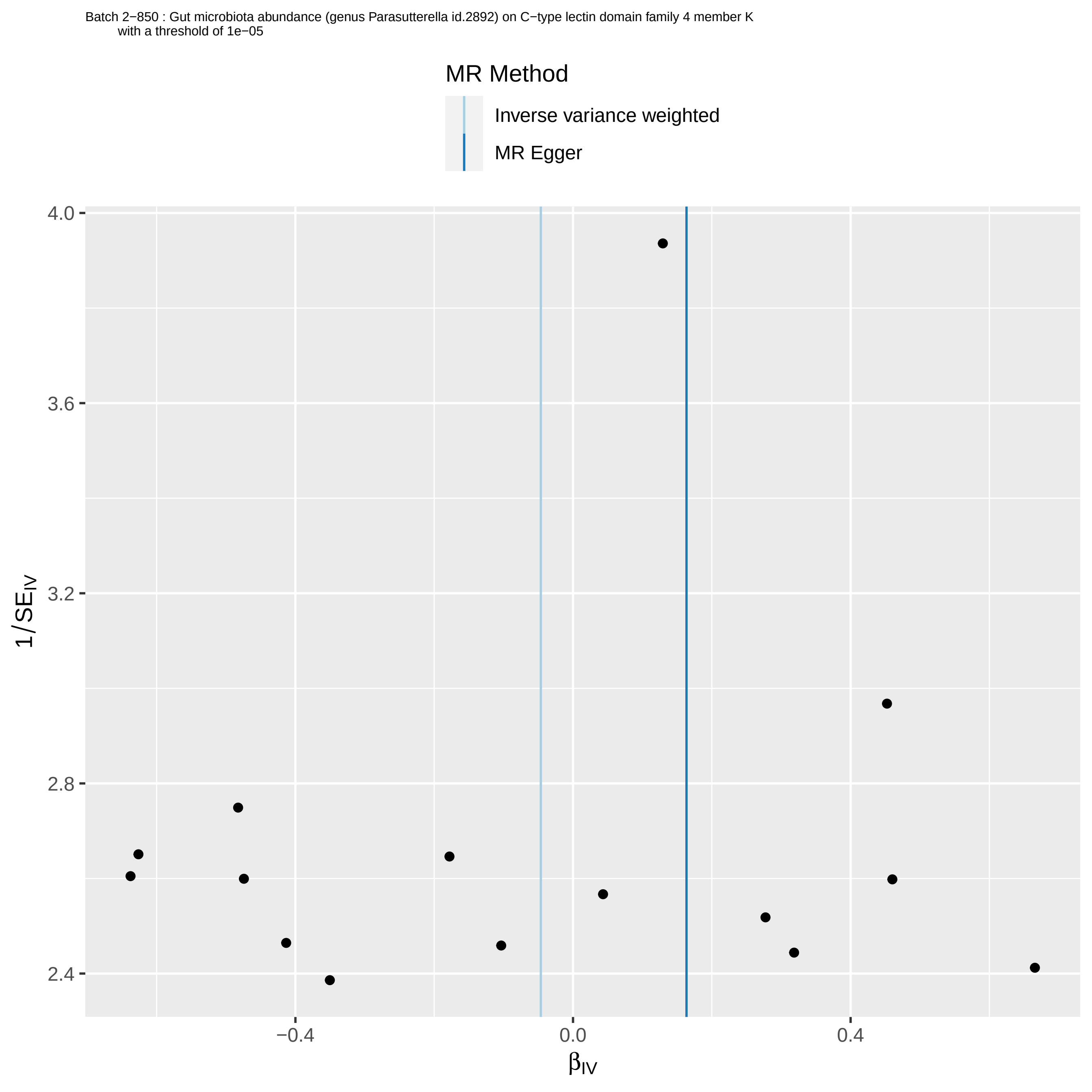
Batch 2−850 : Gut microbiota abundance (genus Parasutterella id.2892) on C−type lectin domain family 4 member K
with a threshold of 1e−05
MR Method
Inverse variance weighted
MR Egger
4.0
3.6
3.2
2.8
2.4
1/SE$_{IV}$
−0.4
0.0
0.4
β$_{IV}$

Batch 2−851 : Gut microbiota abundance (genus Catenibacterium id.2153) on CD209 antigen
with a threshold of 1e−05

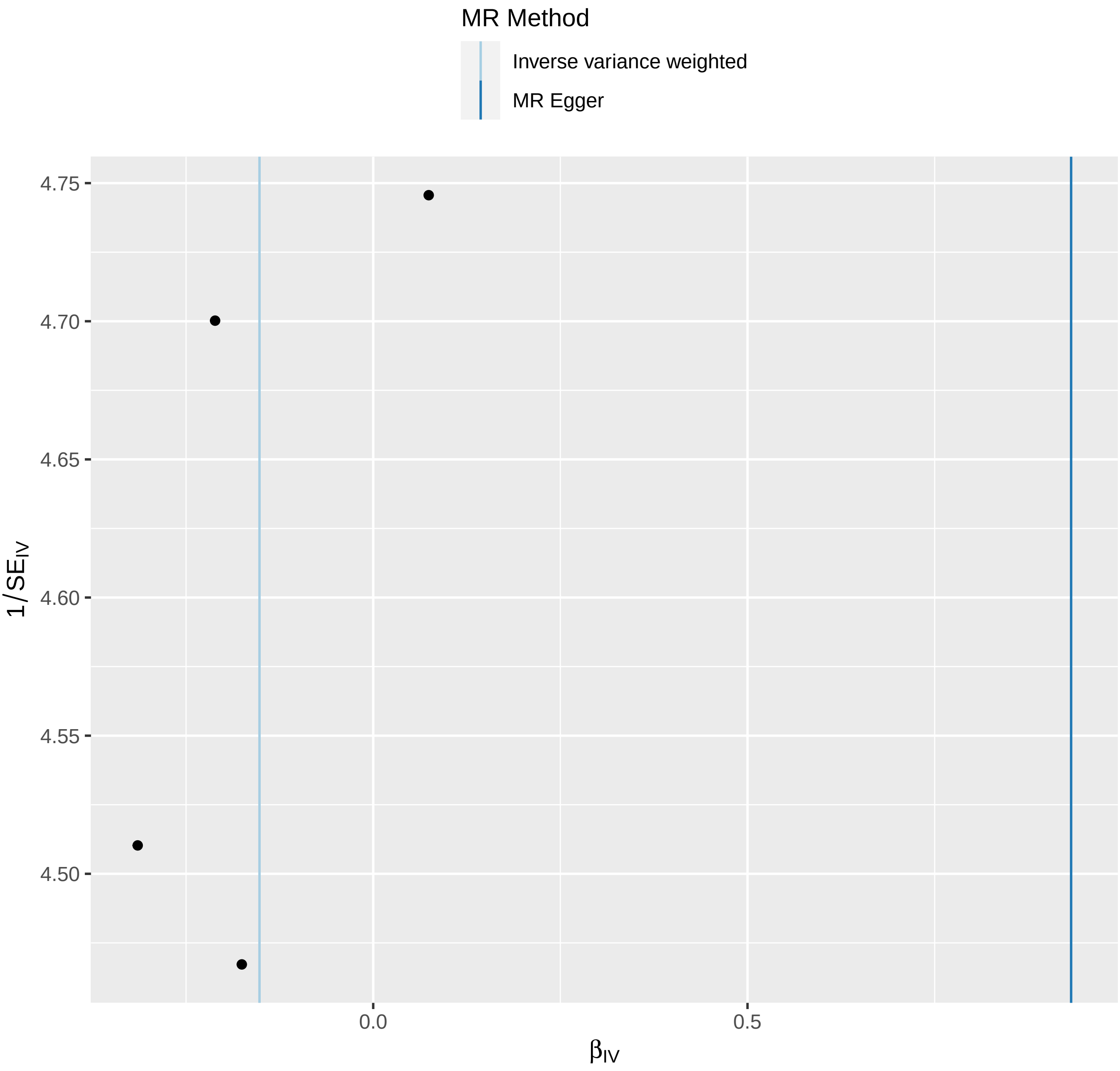

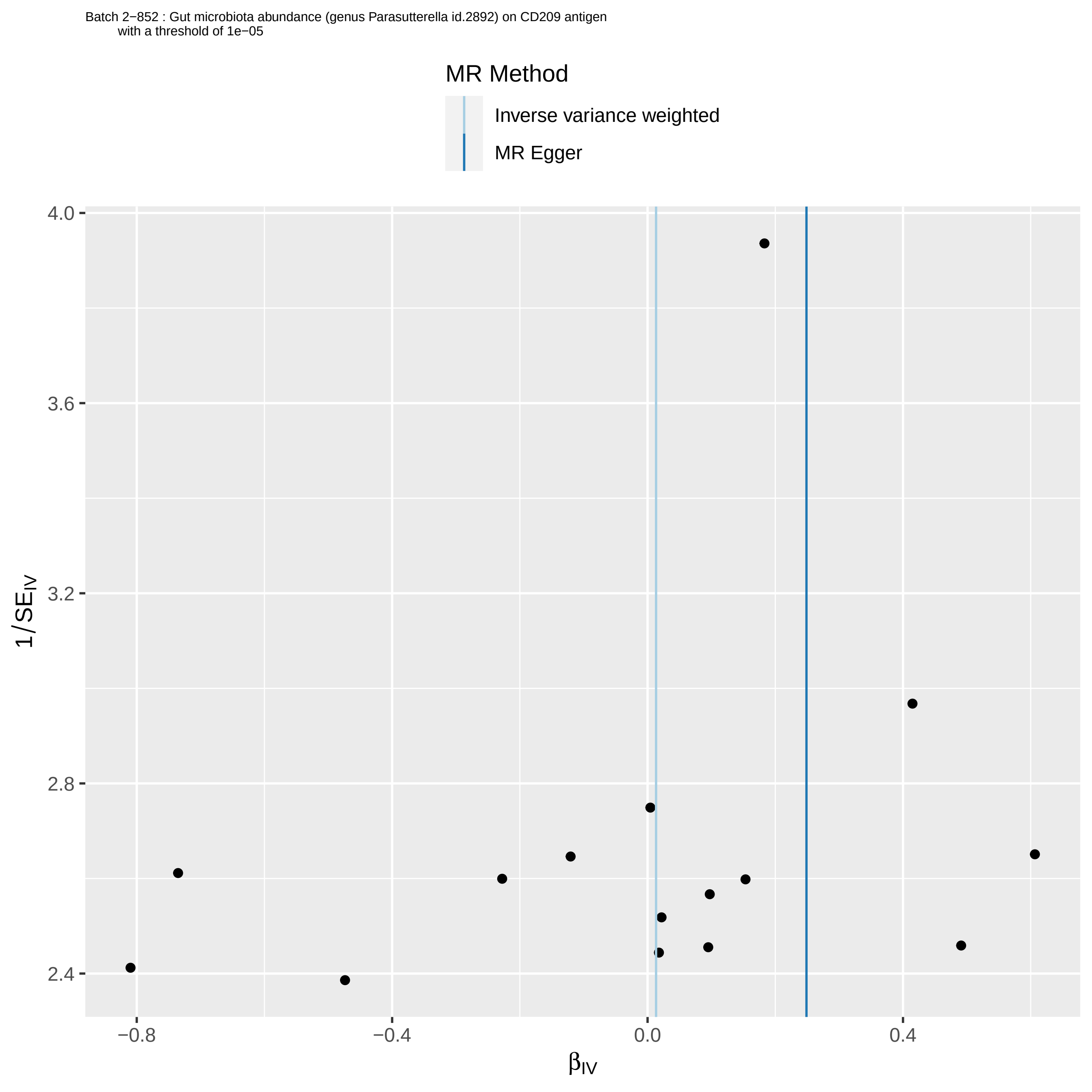

Batch 2−852 : Gut microbiota abundance (genus Parasutterella id.2892) on CD209 antigen
with a threshold of 1e−05
MR Method
Inverse variance weighted
MR Egger
4.0
3.6
3.2
2.8
2.4
1/SE_IV
−0.8
−0.4
0.0
0.4
β_IV

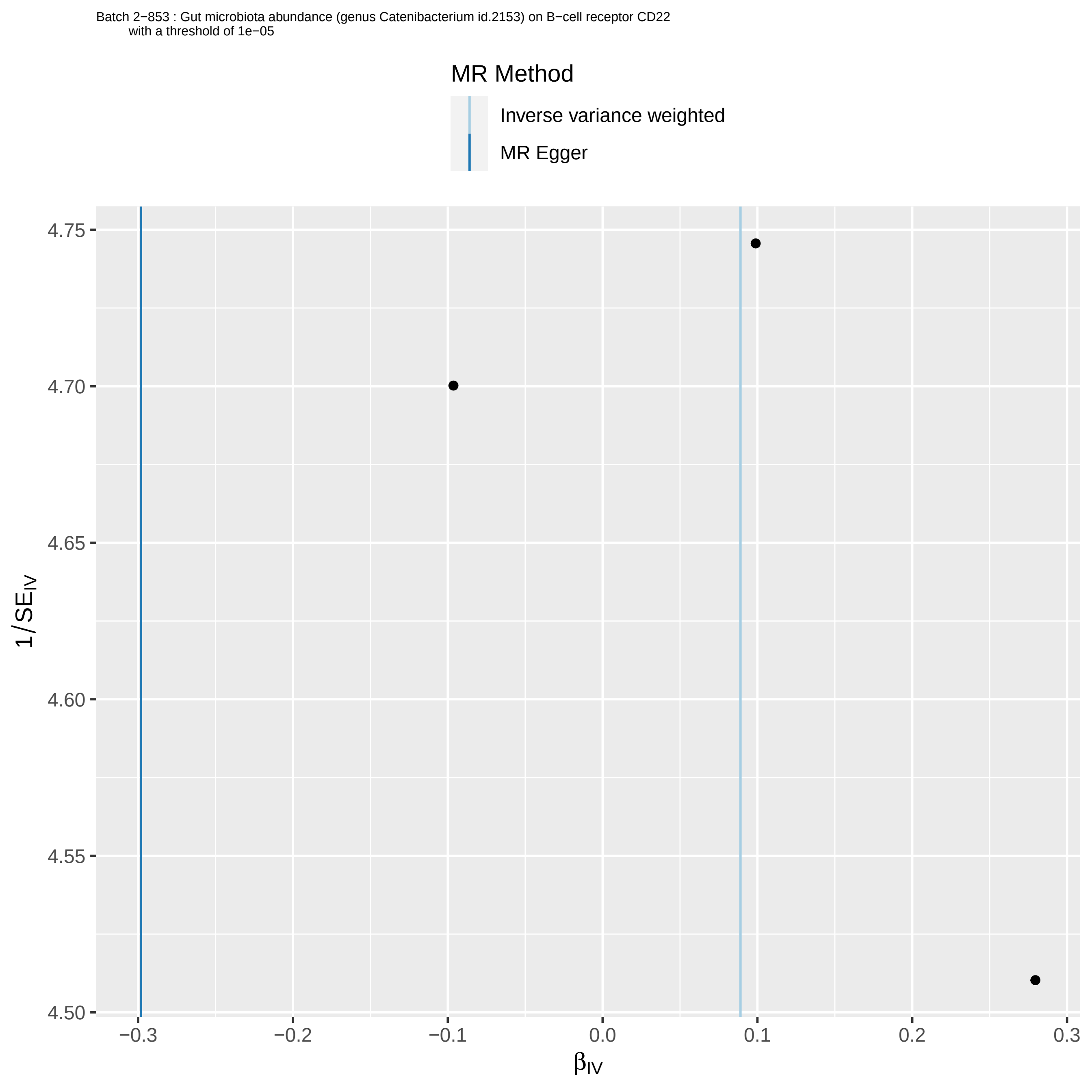
Batch 2−853 : Gut microbiota abundance (genus Catenibacterium id.2153) on B−cell receptor CD22
with a threshold of 1e−05
MR Method
Inverse variance weighted
MR Egger
4.75
4.70
4.65
4.60
4.55
4.50
1/SE_IV
−0.3
−0.2
−0.1
0.0
0.1
0.2
0.3
β_IV

Batch 2−854 : Gut microbiota abundance (genus Parasutterella id.2892) on B−cell receptor CD22
with a threshold of 1e−05

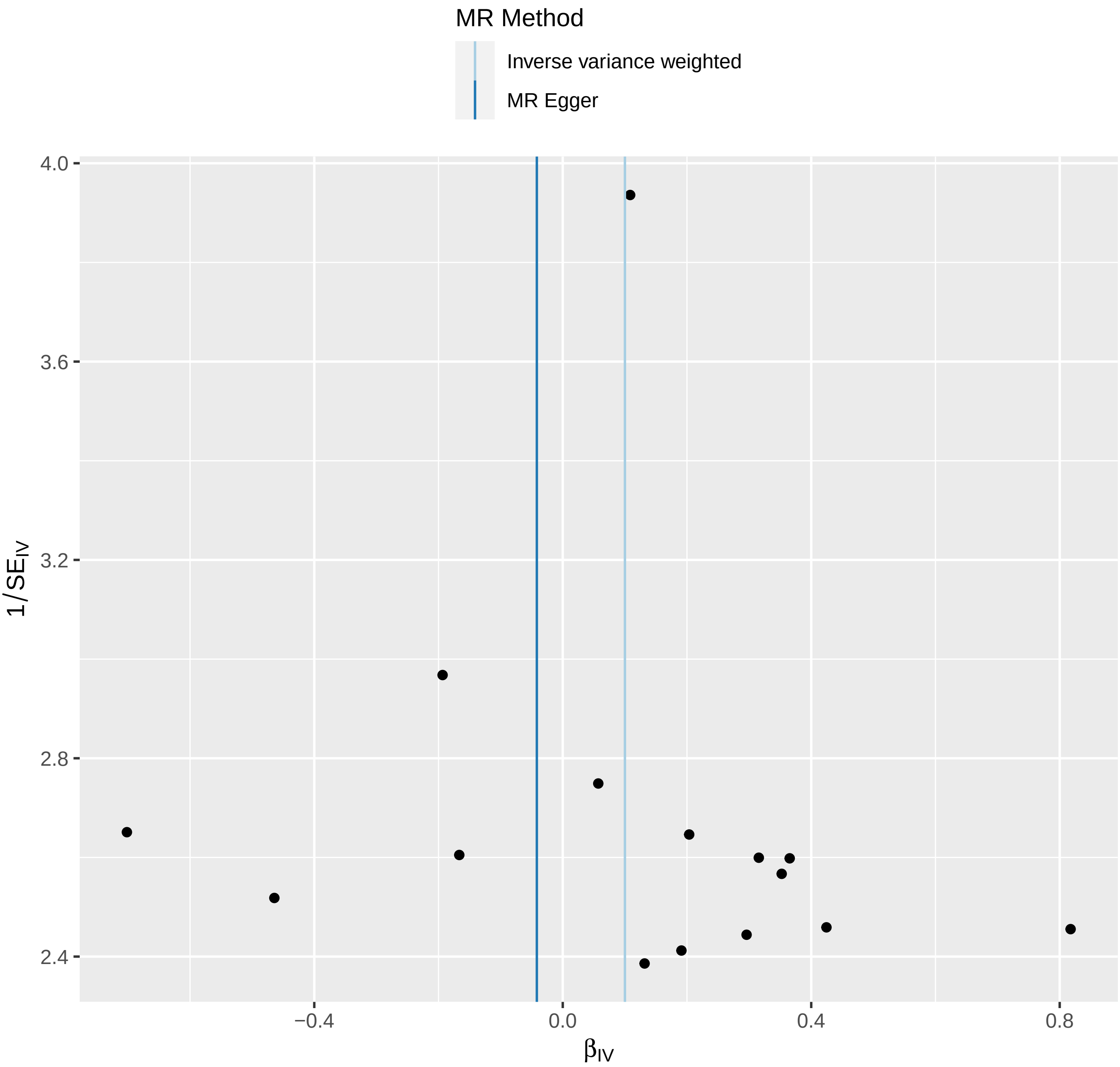

Batch 2−855 : Gut microbiota abundance (genus Catenibacterium id.2153) on CD226 antigen
with a threshold of 1e−05

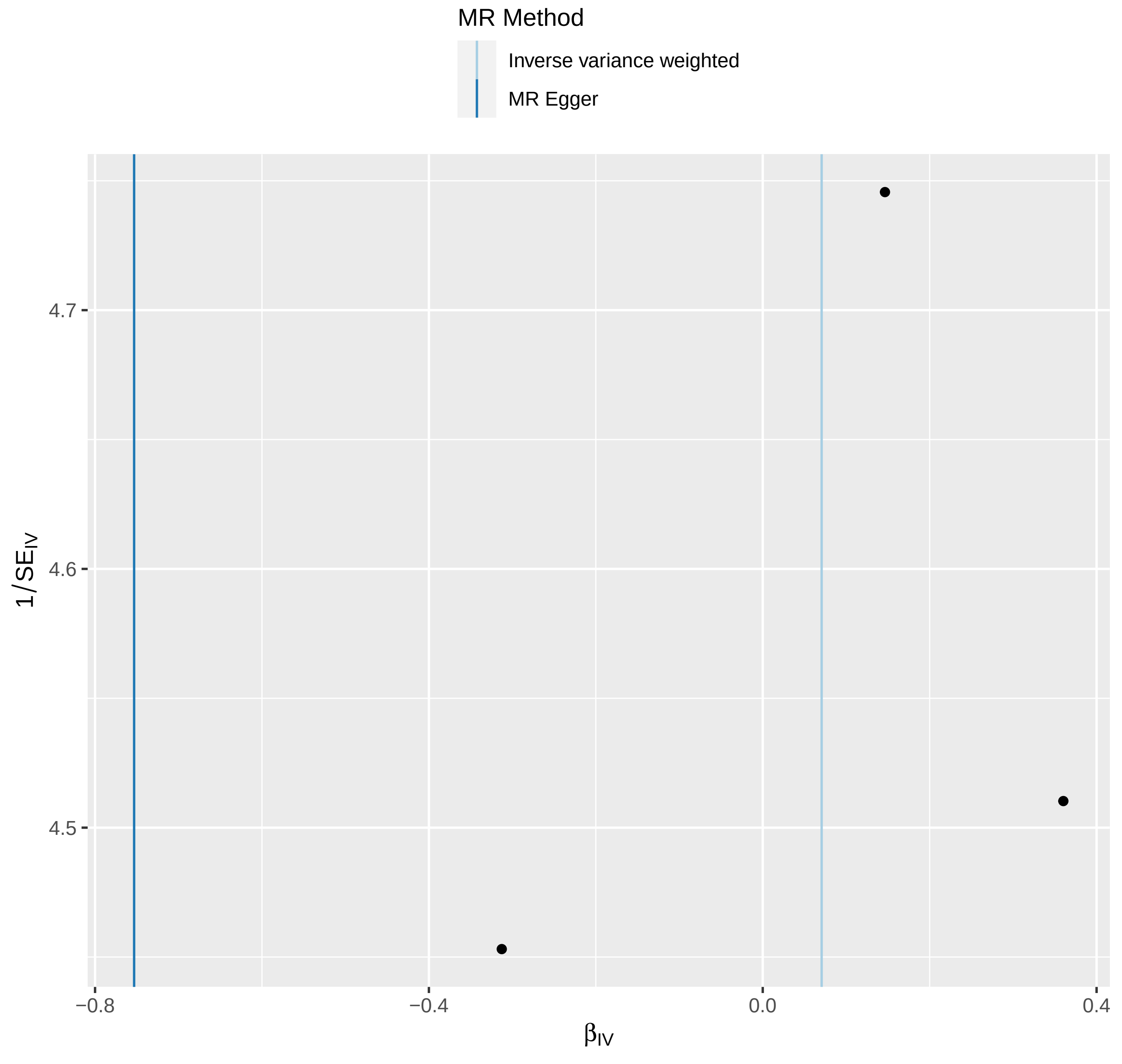

Batch 2−856 : Gut microbiota abundance (genus Parasutterella id.2892) on CD226 antigen
with a threshold of 1e−05

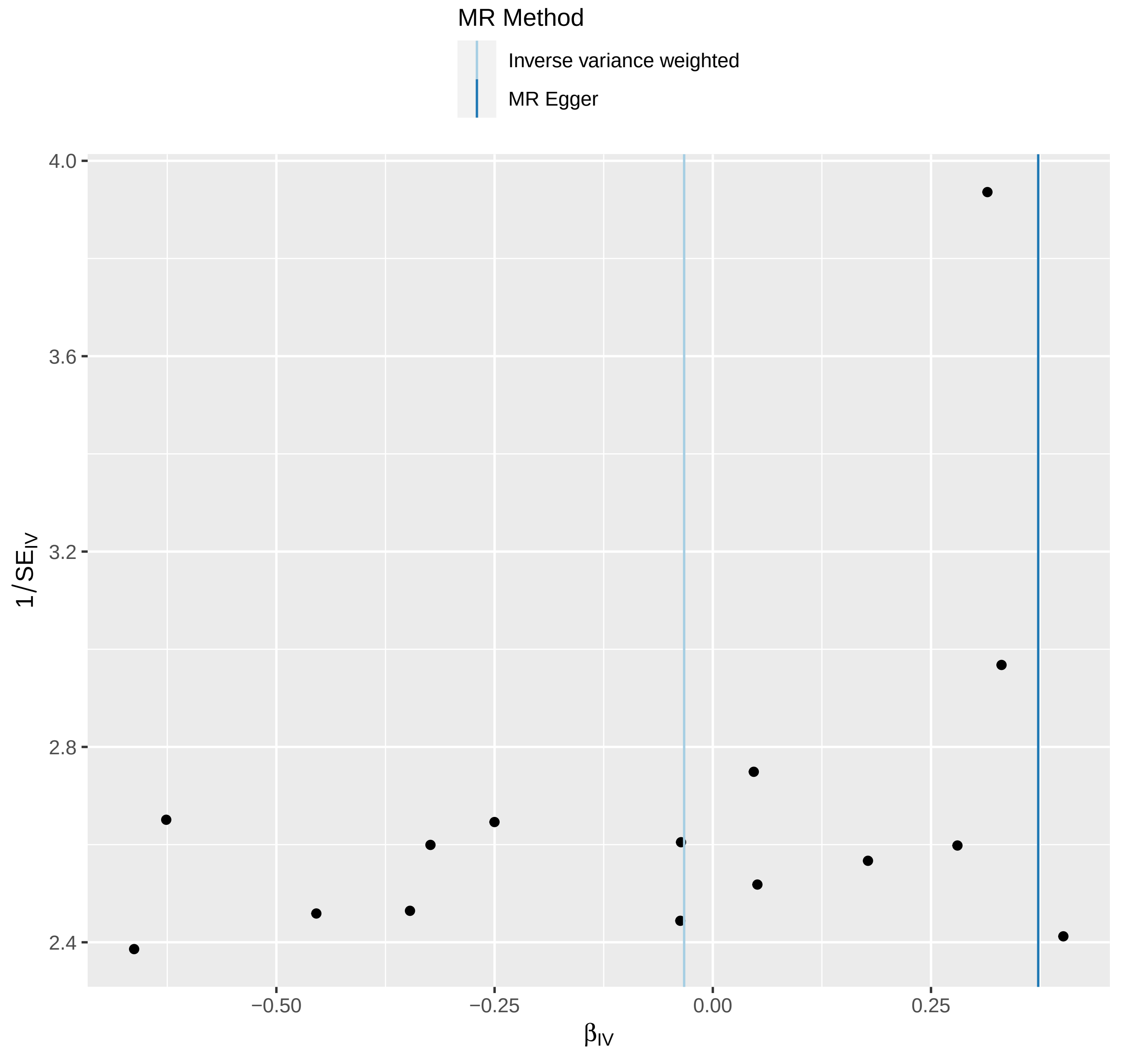

Batch 2−857 : Gut microbiota abundance (genus Catenibacterium id.2153) on CD226 antigen
with a threshold of 1e−05

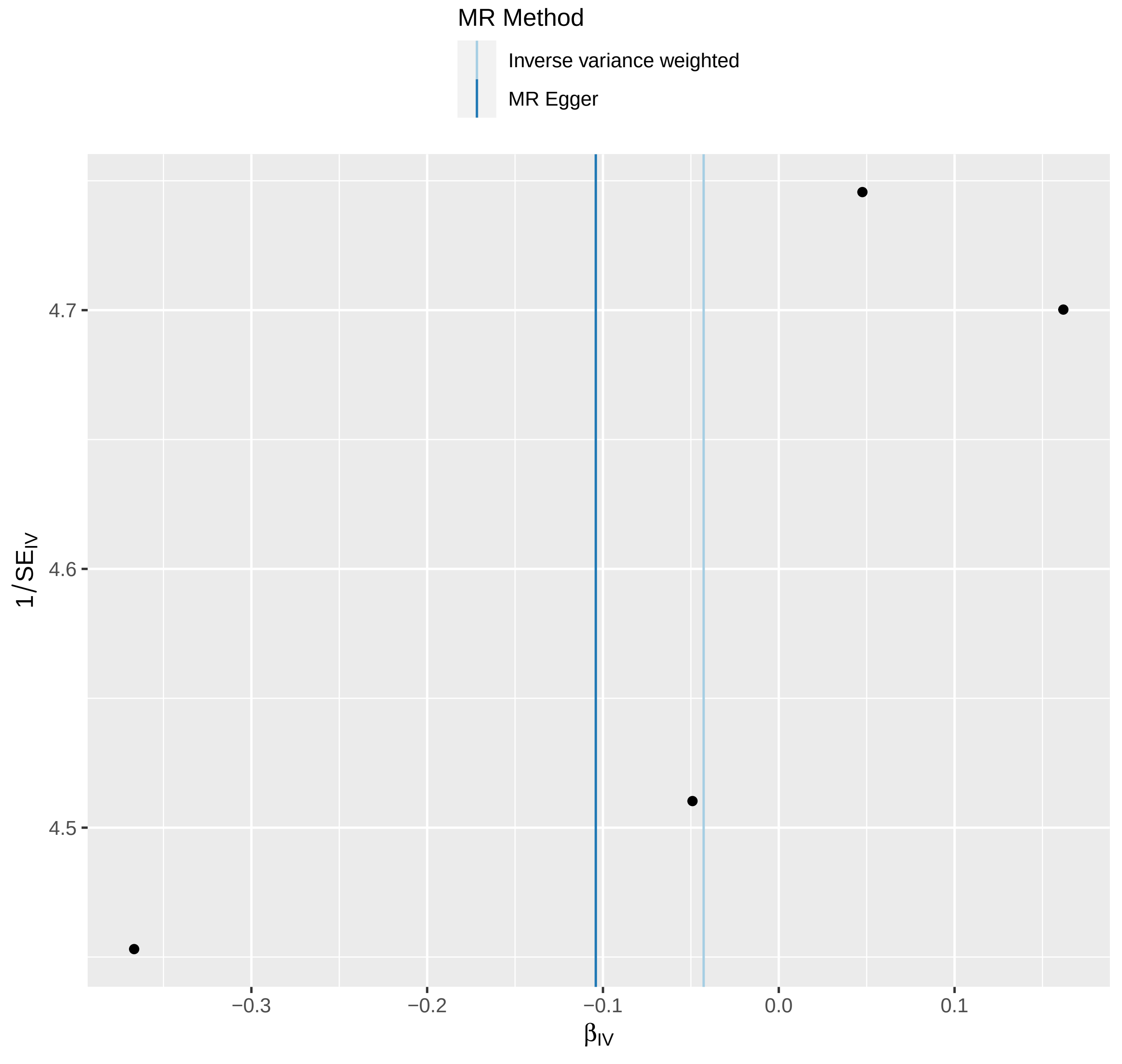

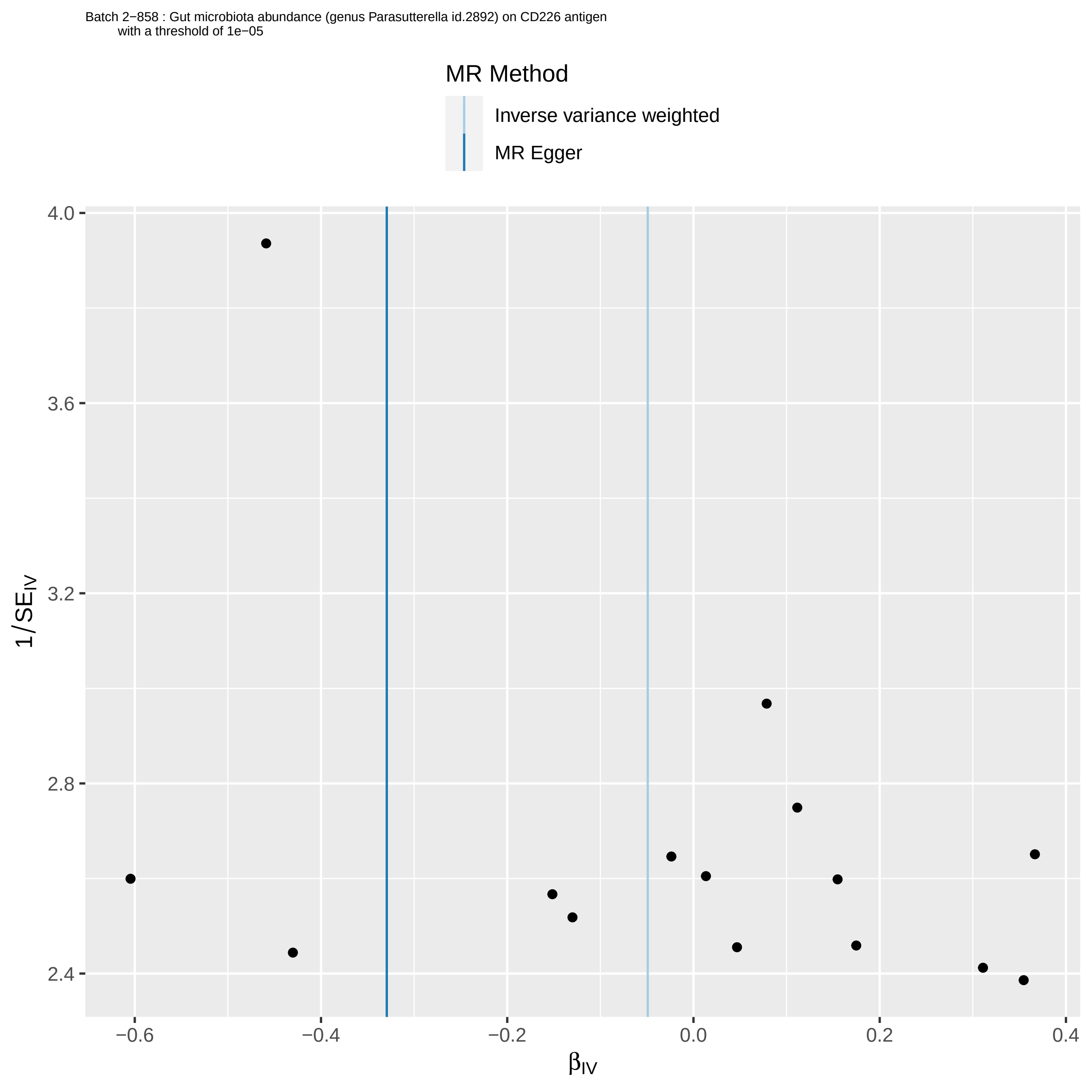

Batch 2−858 : Gut microbiota abundance (genus Parasutterella id.2892) on CD226 antigen
with a threshold of 1e−05
MR Method
Inverse variance weighted
MR Egger
4.0
3.6
3.2
2.8
2.4
1/SE_IV
−0.6
−0.4
−0.2
0.0
0.2
0.4
β_IV

Batch 2−859 : Gut microbiota abundance (genus Catenibacterium id.2153) on T−cell surface antigen CD2
with a threshold of 1e−05

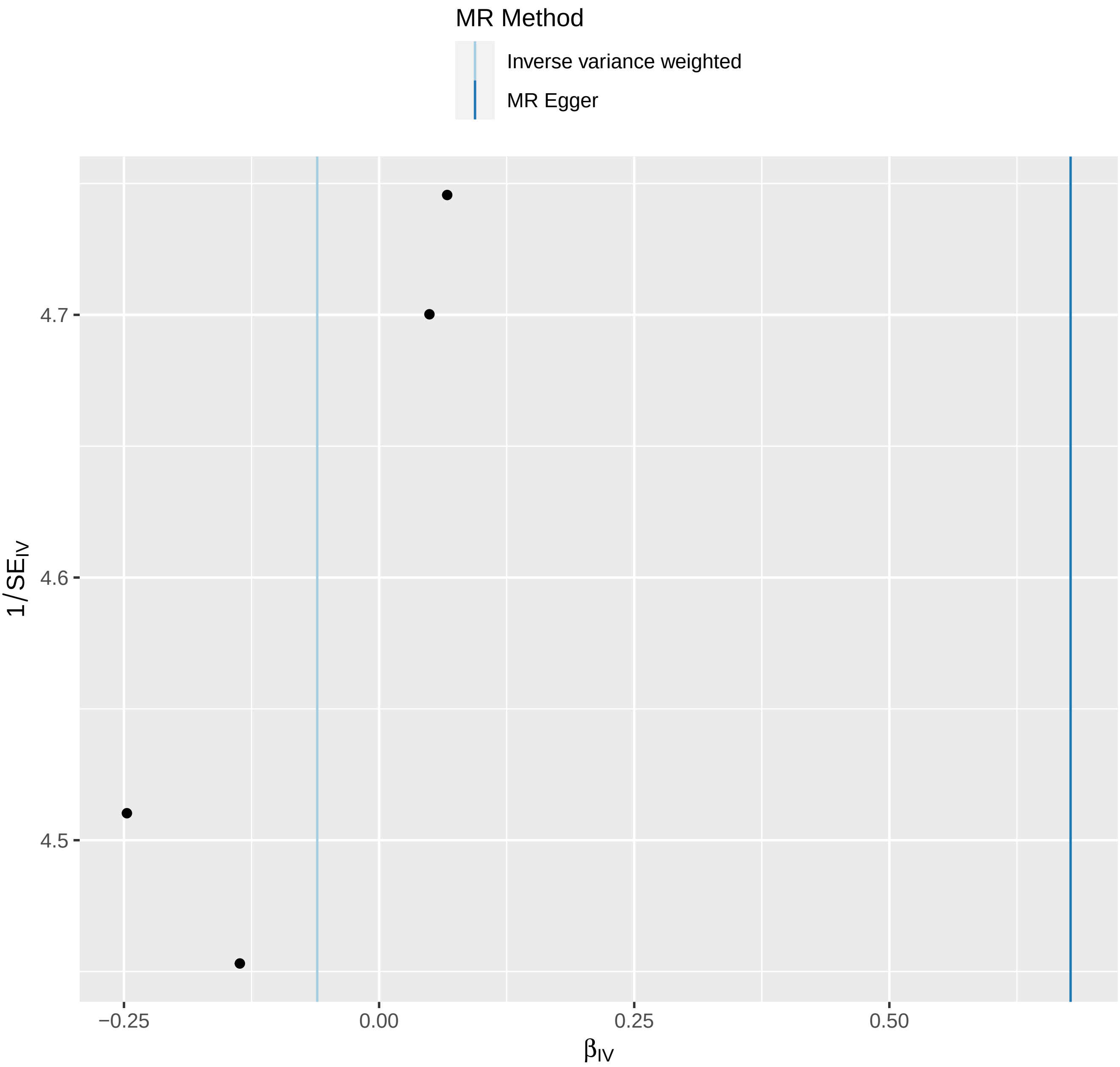

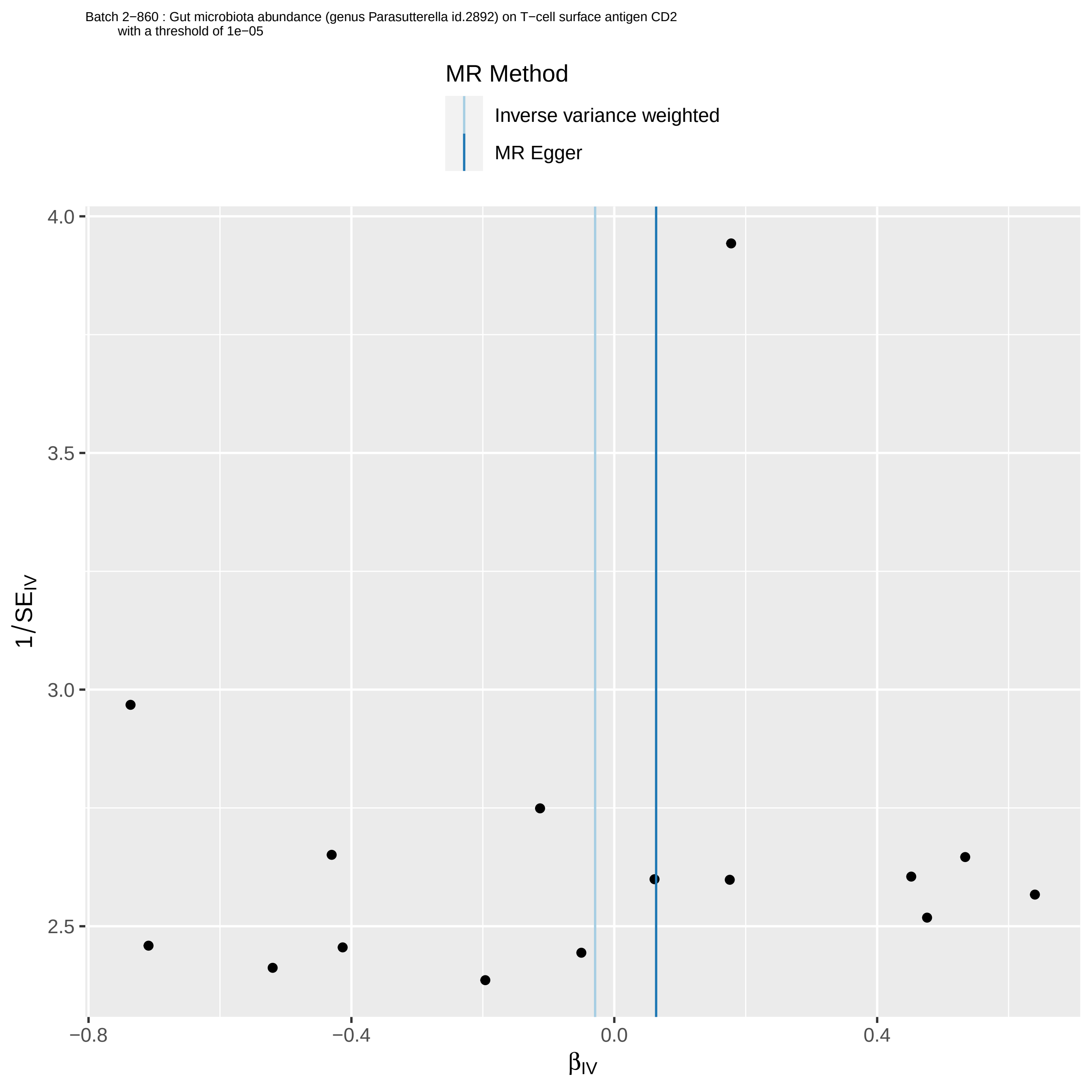

Batch 2−860 : Gut microbiota abundance (genus Parasutterella id.2892) on T−cell surface antigen CD2
with a threshold of 1e−05
MR Method
Inverse variance weighted
MR Egger
4.0
3.5
3.0
2.5
$1/SE_{IV}$
−0.8
−0.4
0.0
0.4
β_{IV}

Batch 2−861 : Gut microbiota abundance (genus Catenibacterium id.2153) on Programmed cell death 1 ligand 1
with a threshold of 1e−05

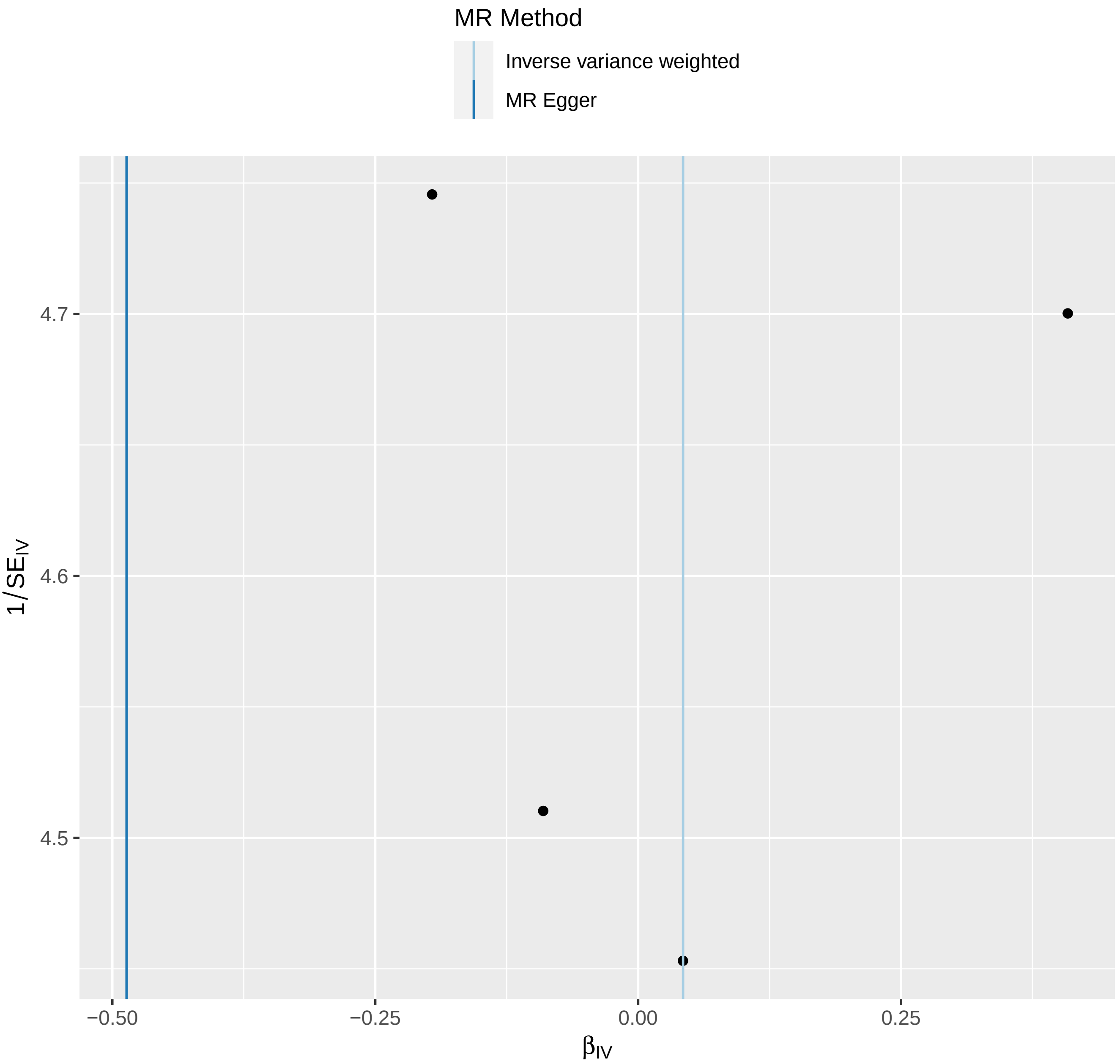

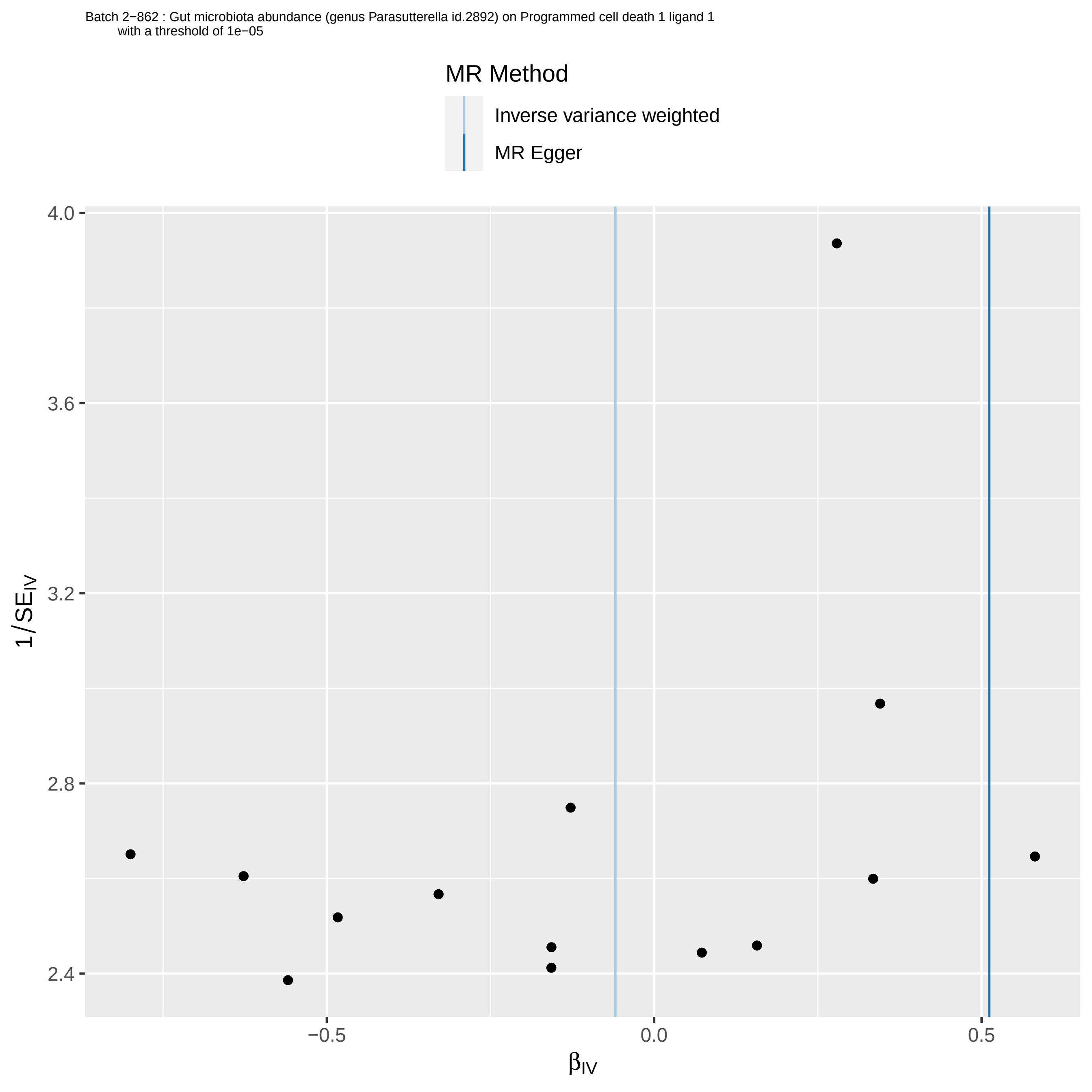
Batch 2−862 : Gut microbiota abundance (genus Parasutterella id.2892) on Programmed cell death 1 ligand 1
with a threshold of 1e−05
MR Method
Inverse variance weighted
MR Egger
4.0
3.6
3.2
2.8
2.4
1/SE_IV
−0.5
0.0
0.5
β_IV

Batch 2−863 : Gut microbiota abundance (genus Catenibacterium id.2153) on CMRF35−like molecule 8
with a threshold of 1e−05

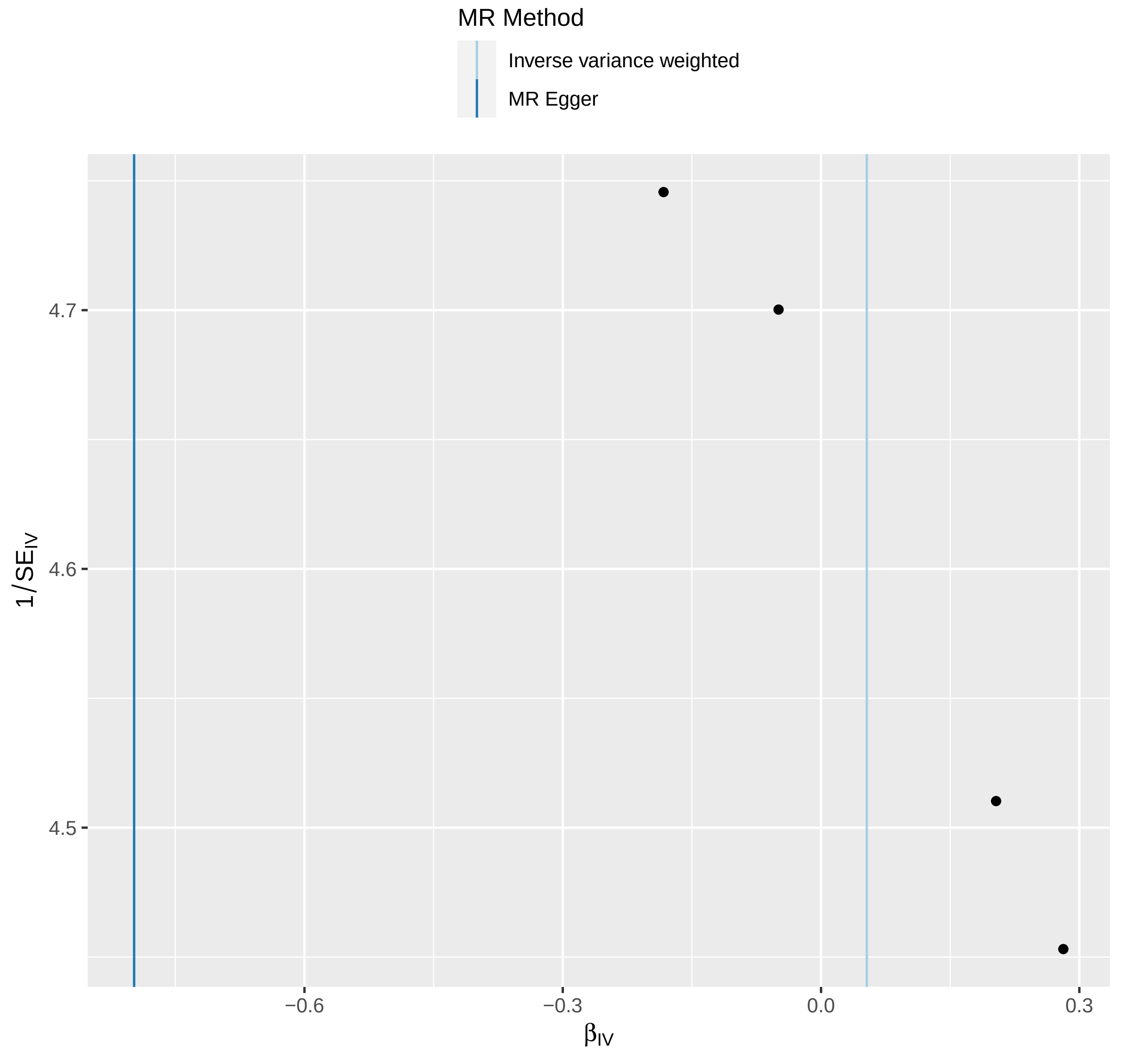

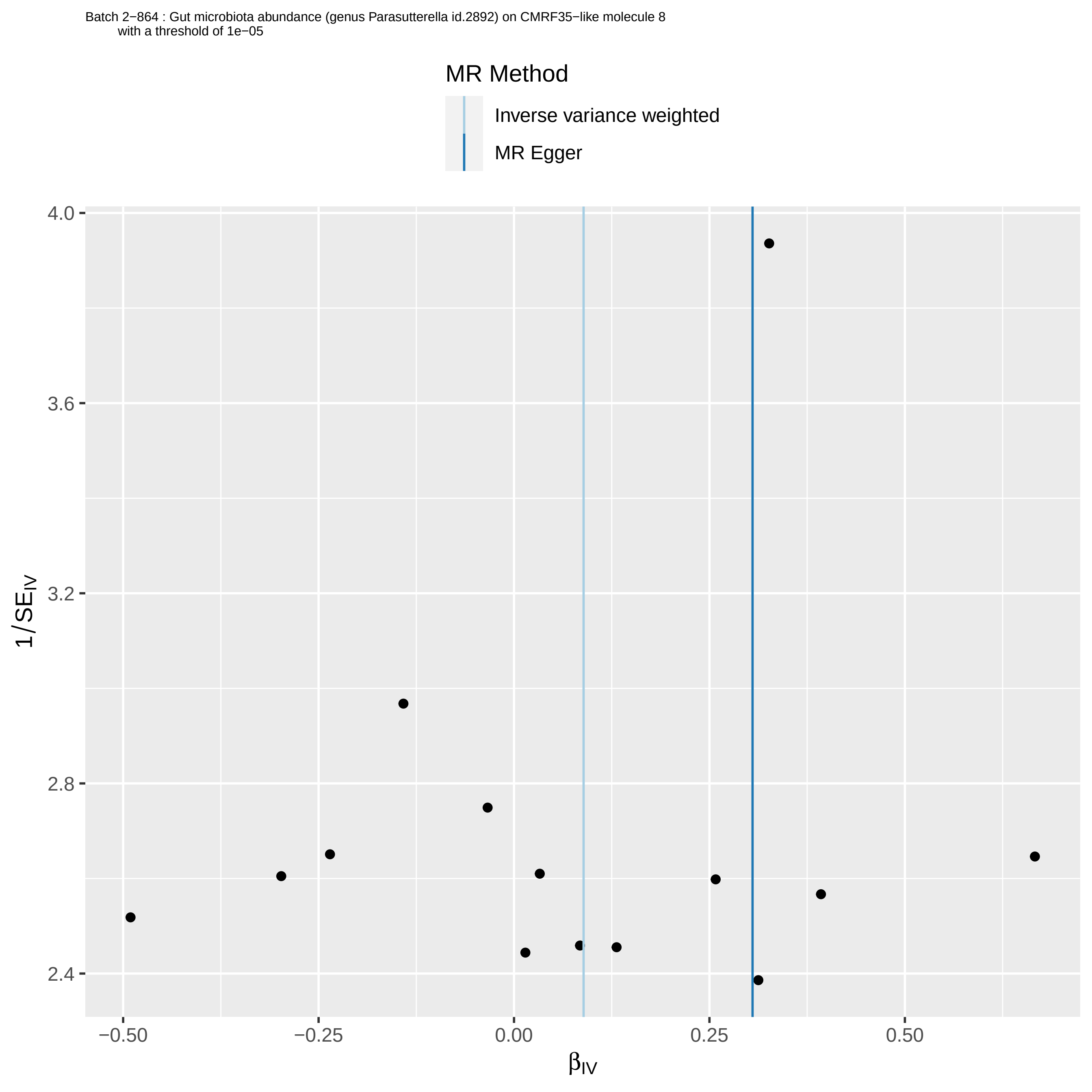

Batch 2−864 : Gut microbiota abundance (genus Parasutterella id.2892) on CMRF35−like molecule 8
with a threshold of 1e−05
MR Method
Inverse variance weighted
MR Egger
4.0
3.6
3.2
2.8
2.4
1/SE_IV
−0.50
−0.25
0.00
0.25
0.50
β_IV

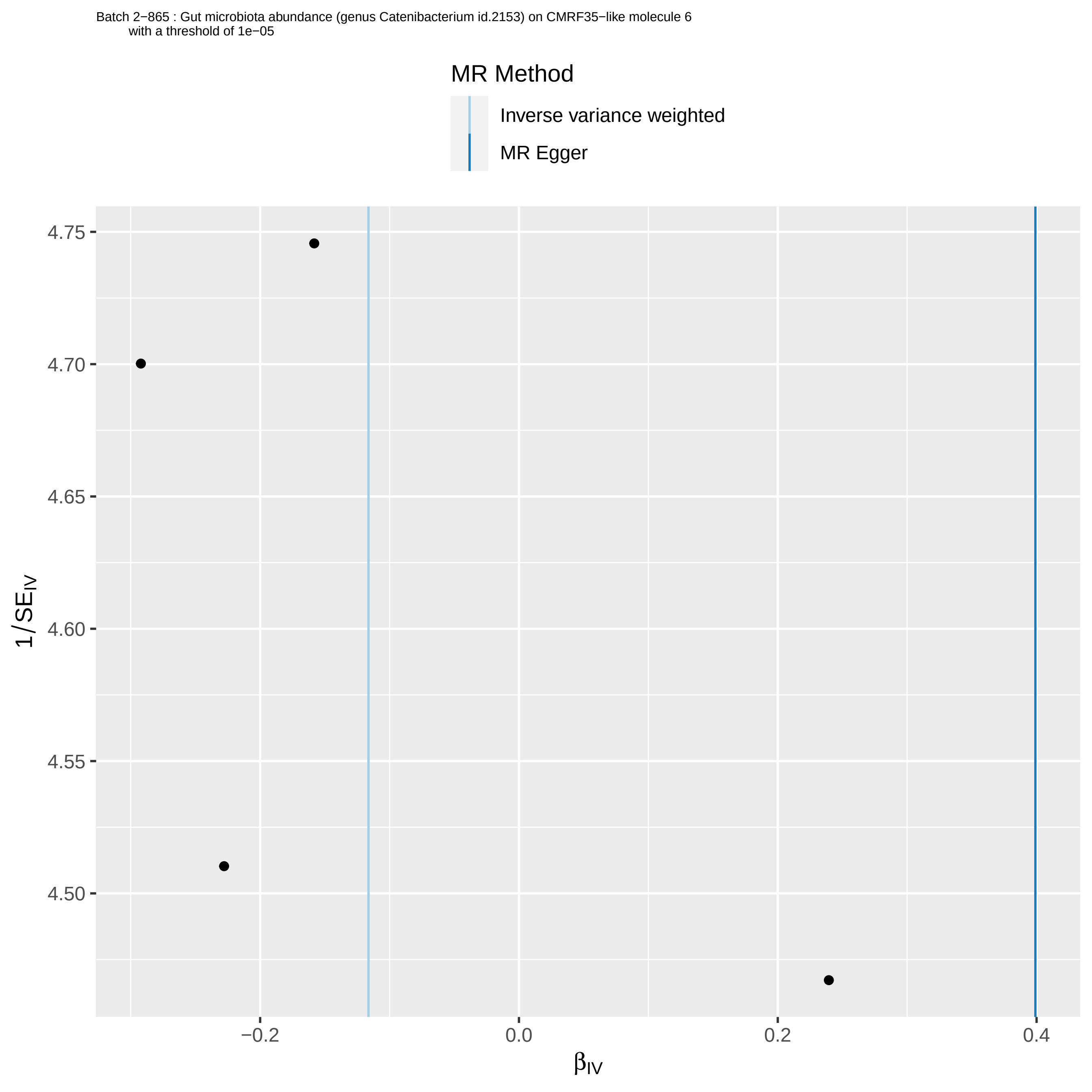

Batch 2−865 : Gut microbiota abundance (genus Catenibacterium id.2153) on CMRF35−like molecule 6
with a threshold of 1e−05
MR Method
Inverse variance weighted
MR Egger
4.75
4.70
4.65
4.60
4.55
4.50
1/SE_IV
−0.2
0.0
0.2
0.4
β_IV

Batch 2−866 : Gut microbiota abundance (genus Parasutterella id.2892) on CMRF35−like molecule 6
with a threshold of 1e−05

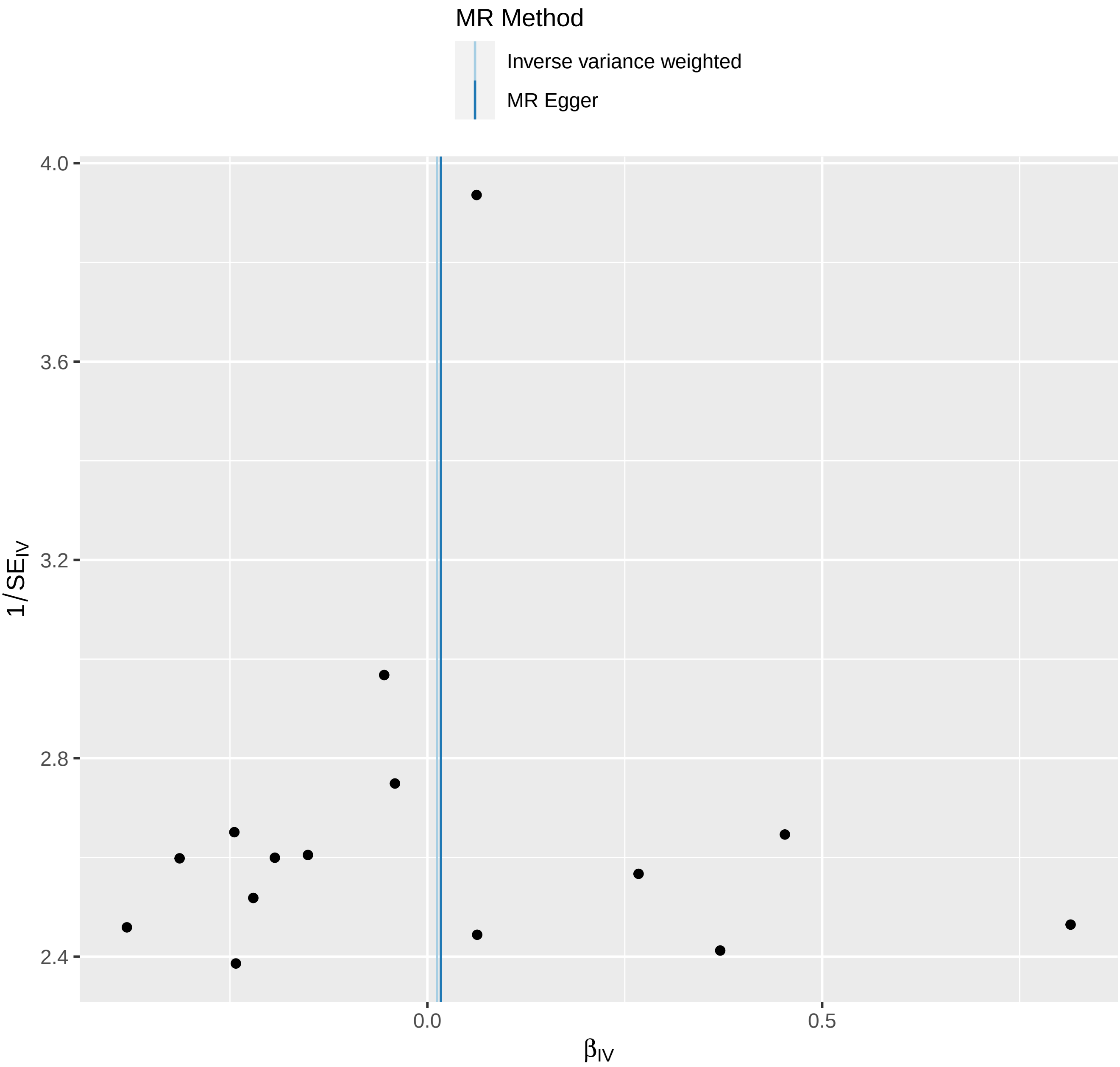

Batch 2−867 : Gut microbiota abundance (genus Catenibacterium id.2153) on CMRF35−like molecule 6
with a threshold of 1e−05

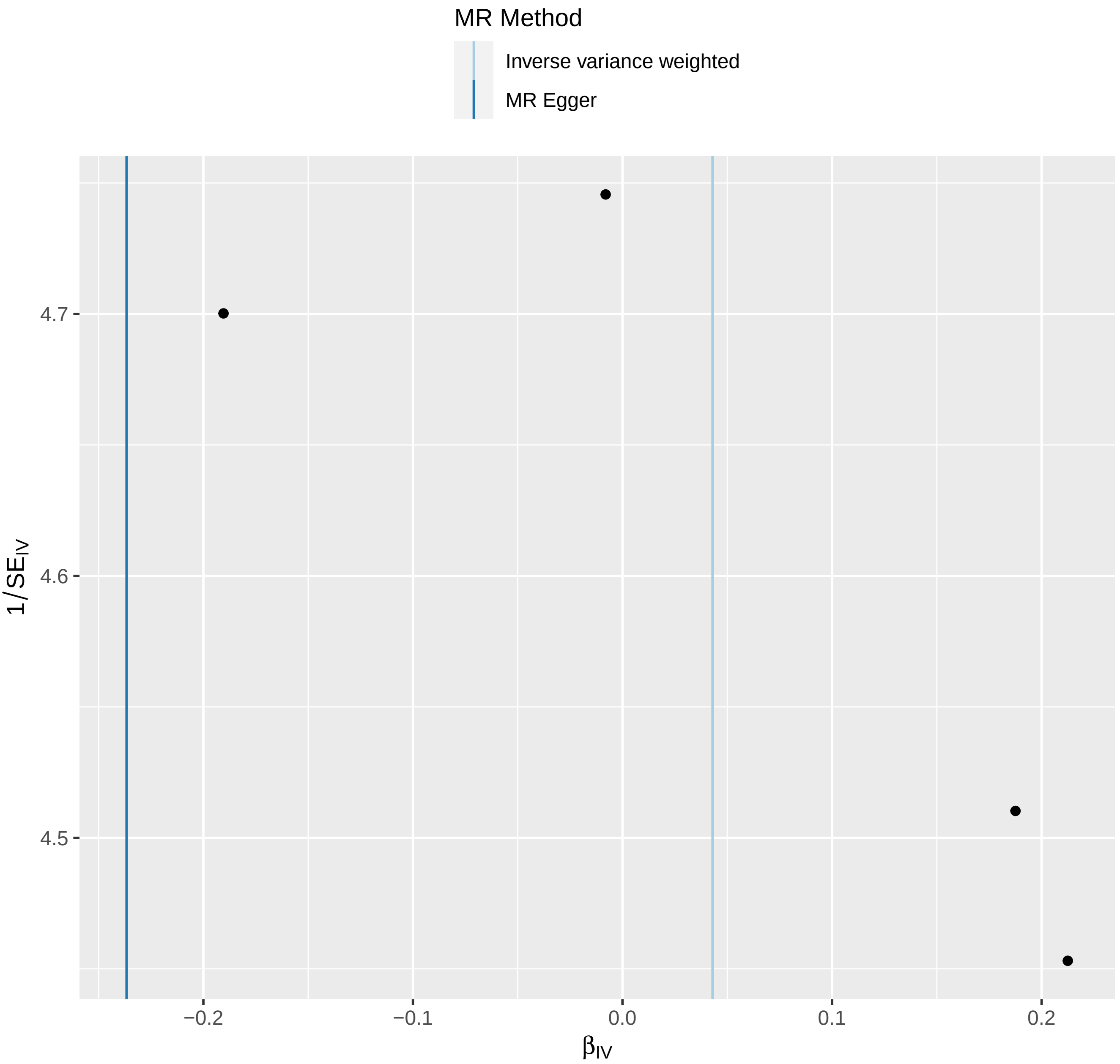

Batch 2−868 : Gut microbiota abundance (genus Parasutterella id.2892) on CMRF35−like molecule 6
with a threshold of 1e−05

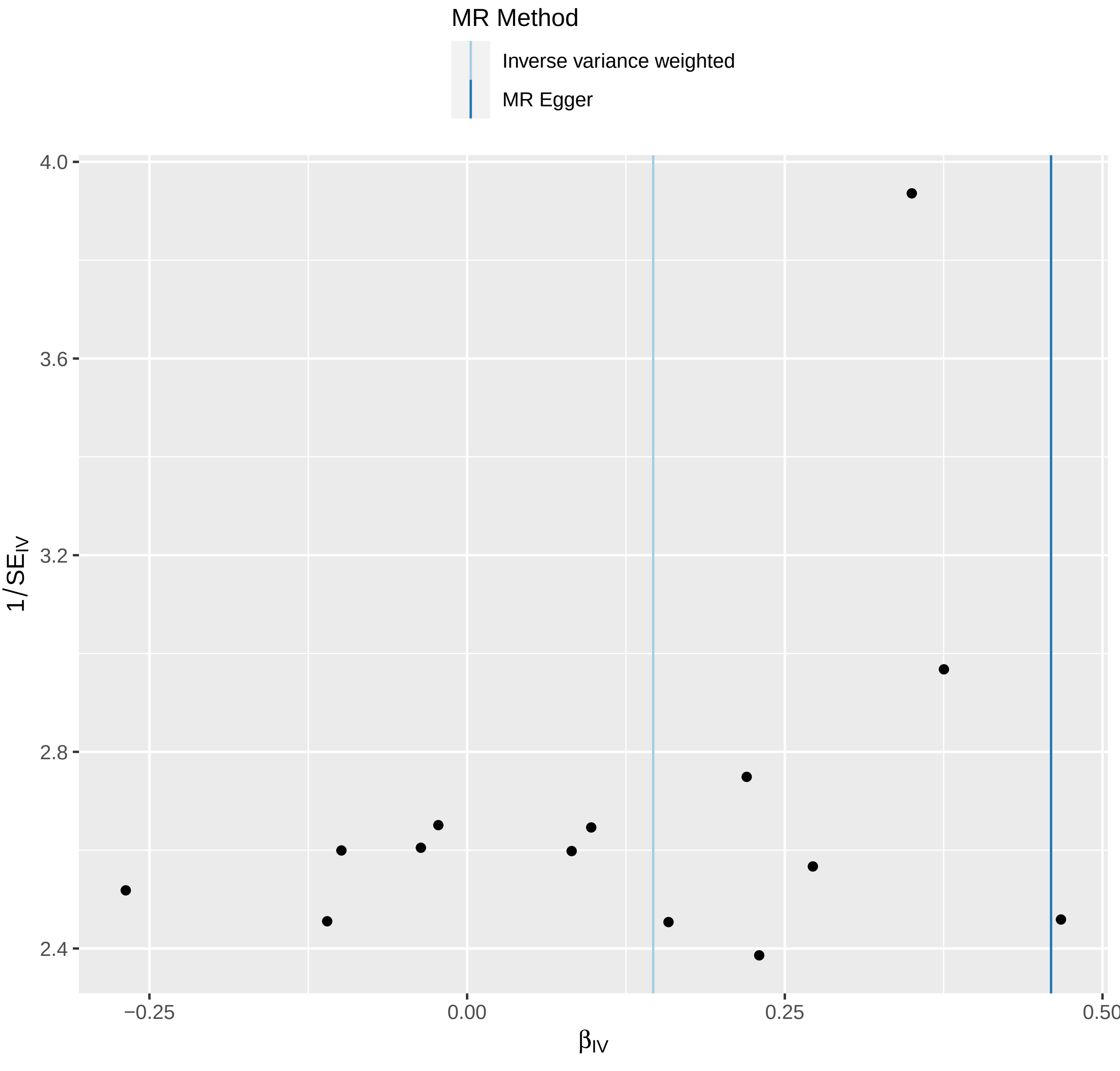

Batch 2−869 : Gut microbiota abundance (genus Catenibacterium id.2153) on CMRF35−like molecule 2
with a threshold of 1e−05

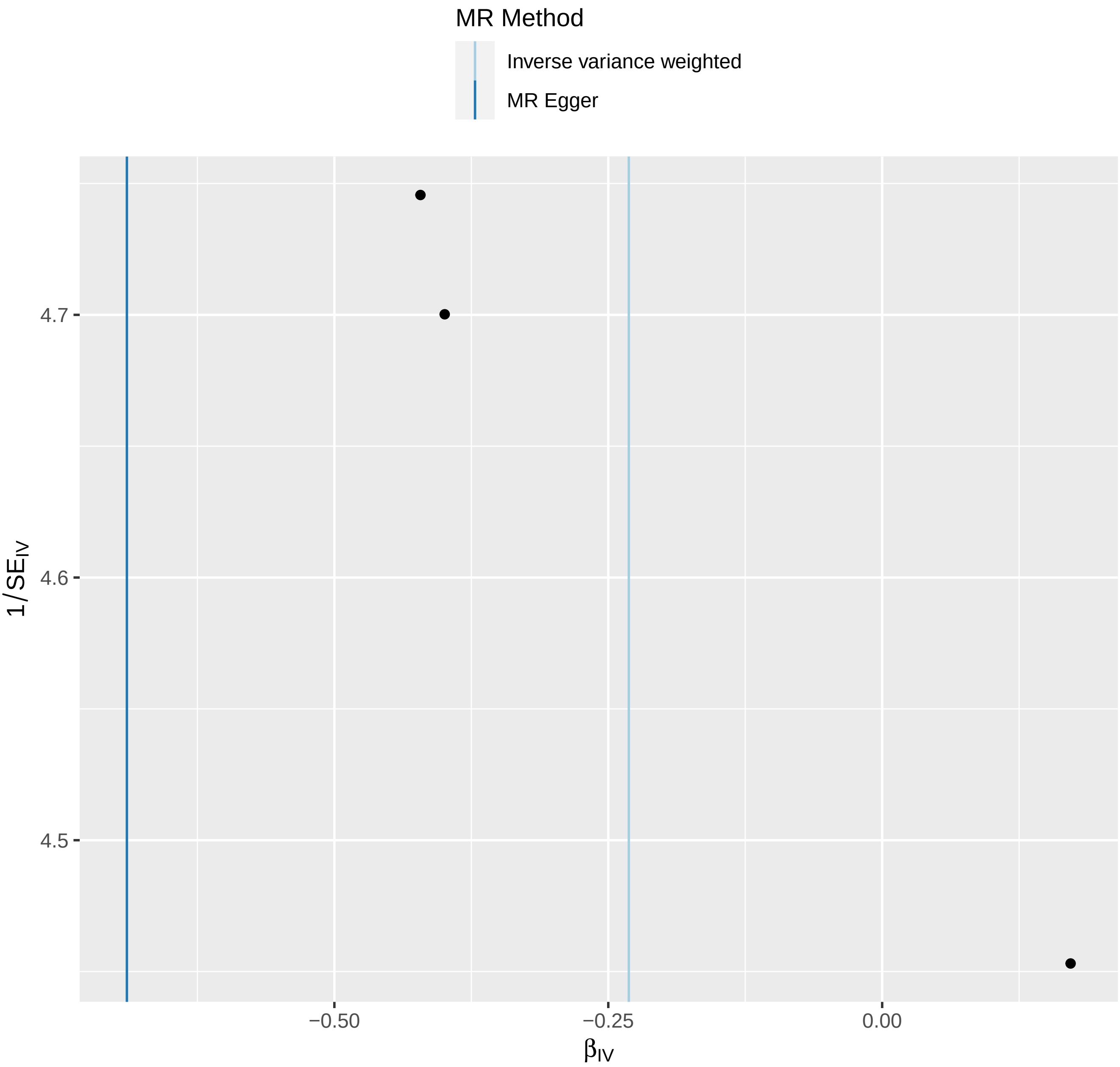

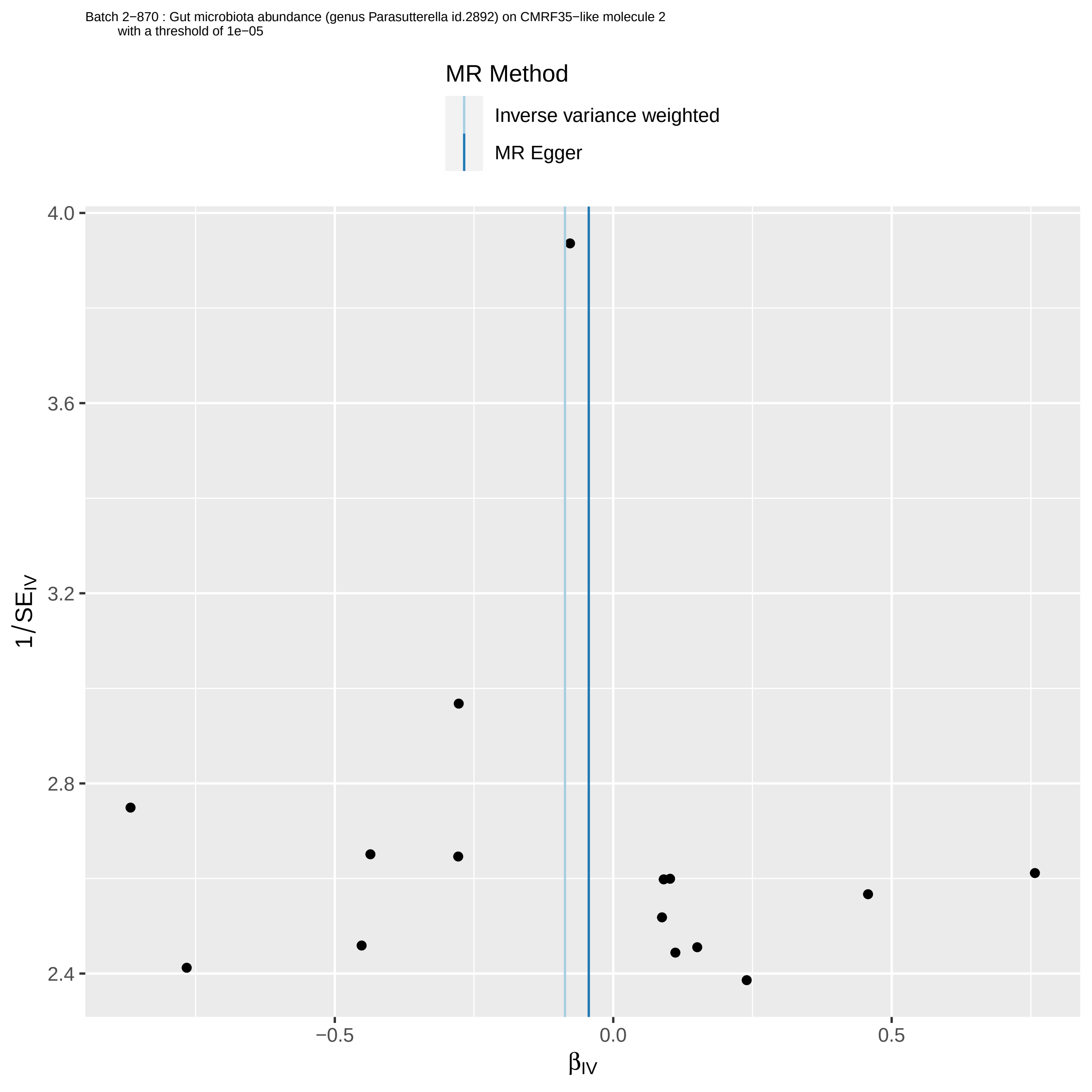

Batch 2−870 : Gut microbiota abundance (genus Parasutterella id.2892) on CMRF35−like molecule 2
with a threshold of 1e−05
MR Method
Inverse variance weighted
MR Egger
4.0
3.6
3.2
2.8
2.4
1/SE_IV
−0.5
0.0
0.5
β_IV

Batch 2−871 : Gut microbiota abundance (genus Catenibacterium id.2153) on CMRF35−like molecule 7
with a threshold of 1e−05

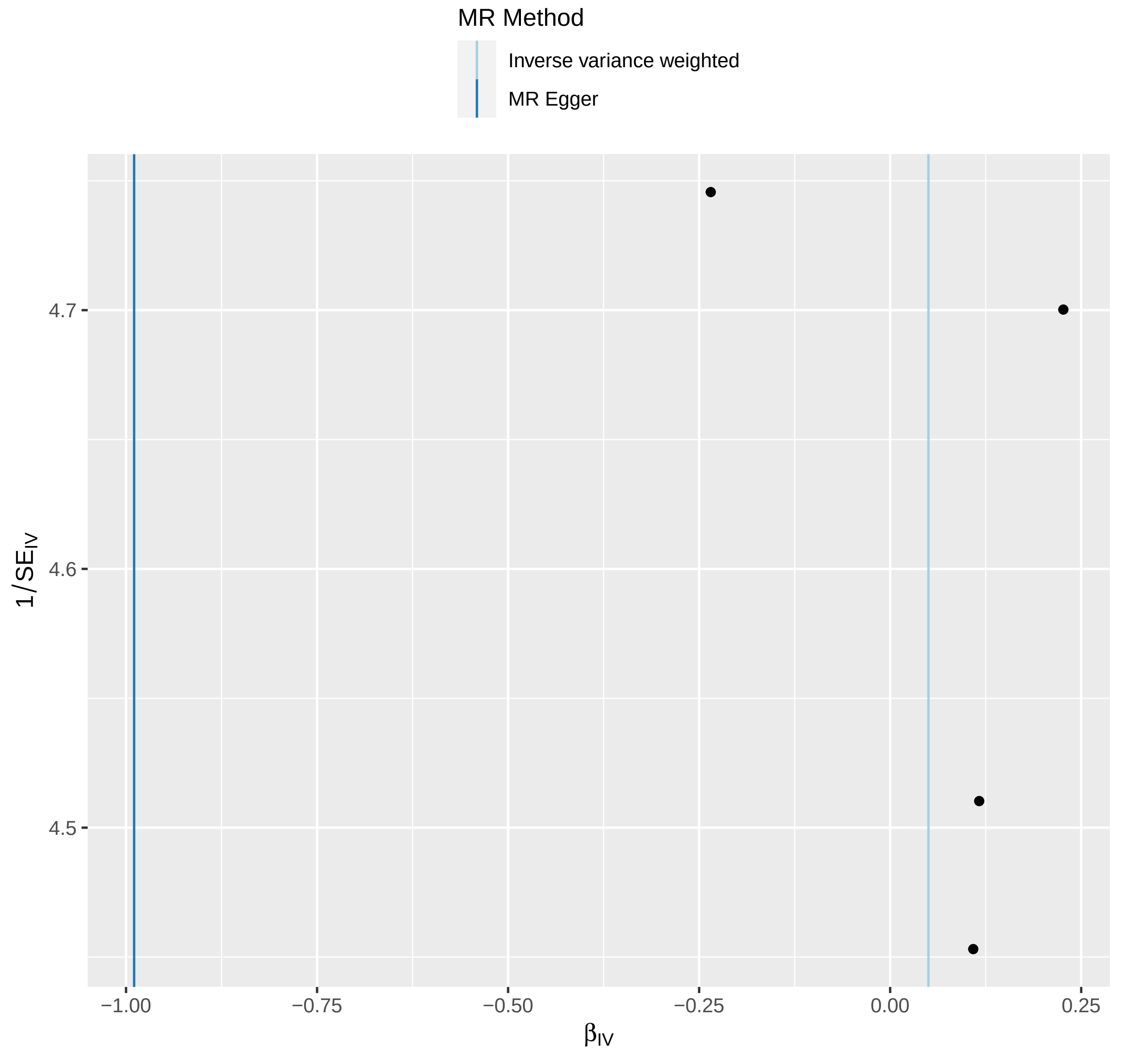

Batch 2−872 : Gut microbiota abundance (genus Parasutterella id.2892) on CMRF35−like molecule 7
with a threshold of 1e−05

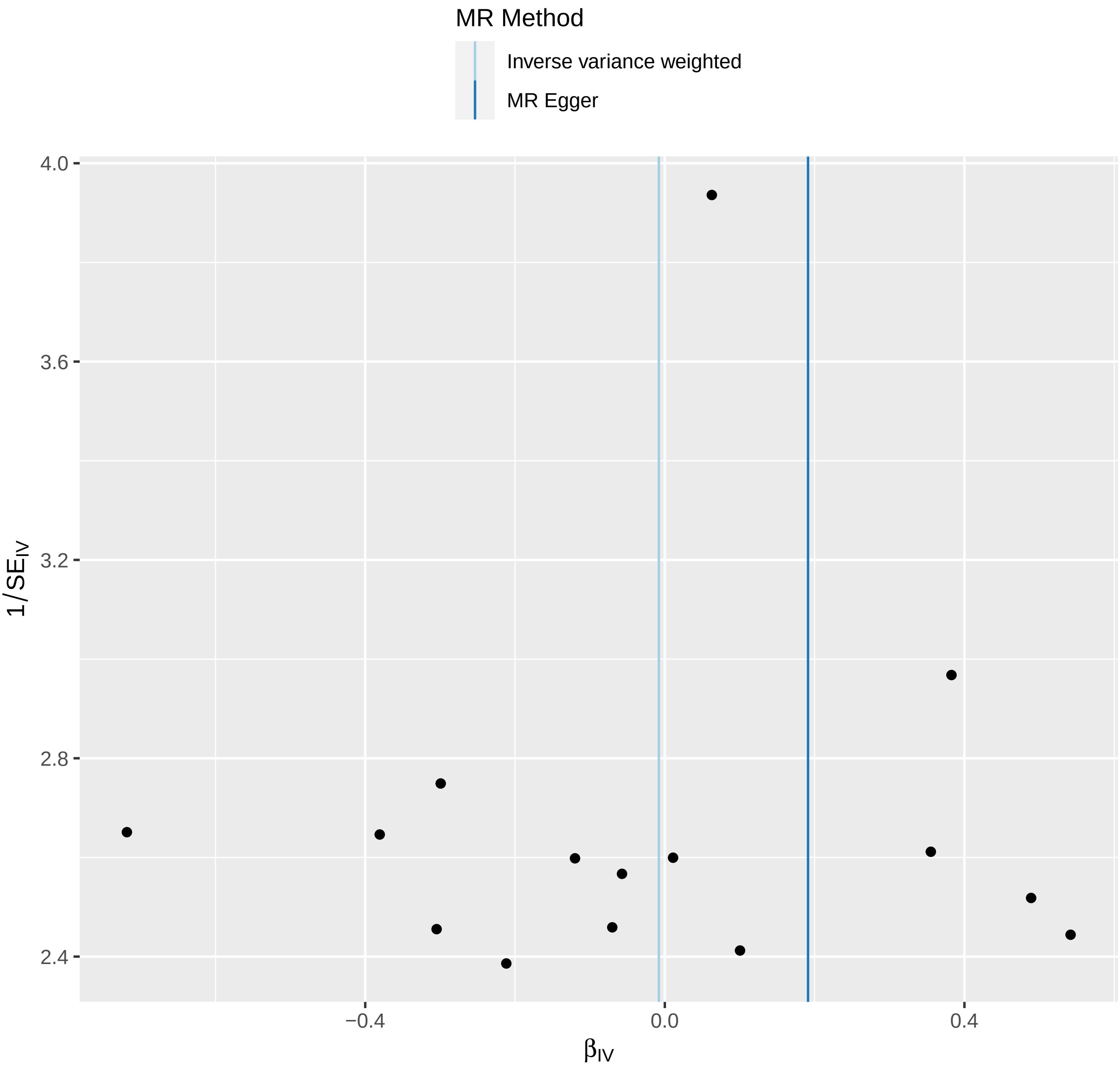

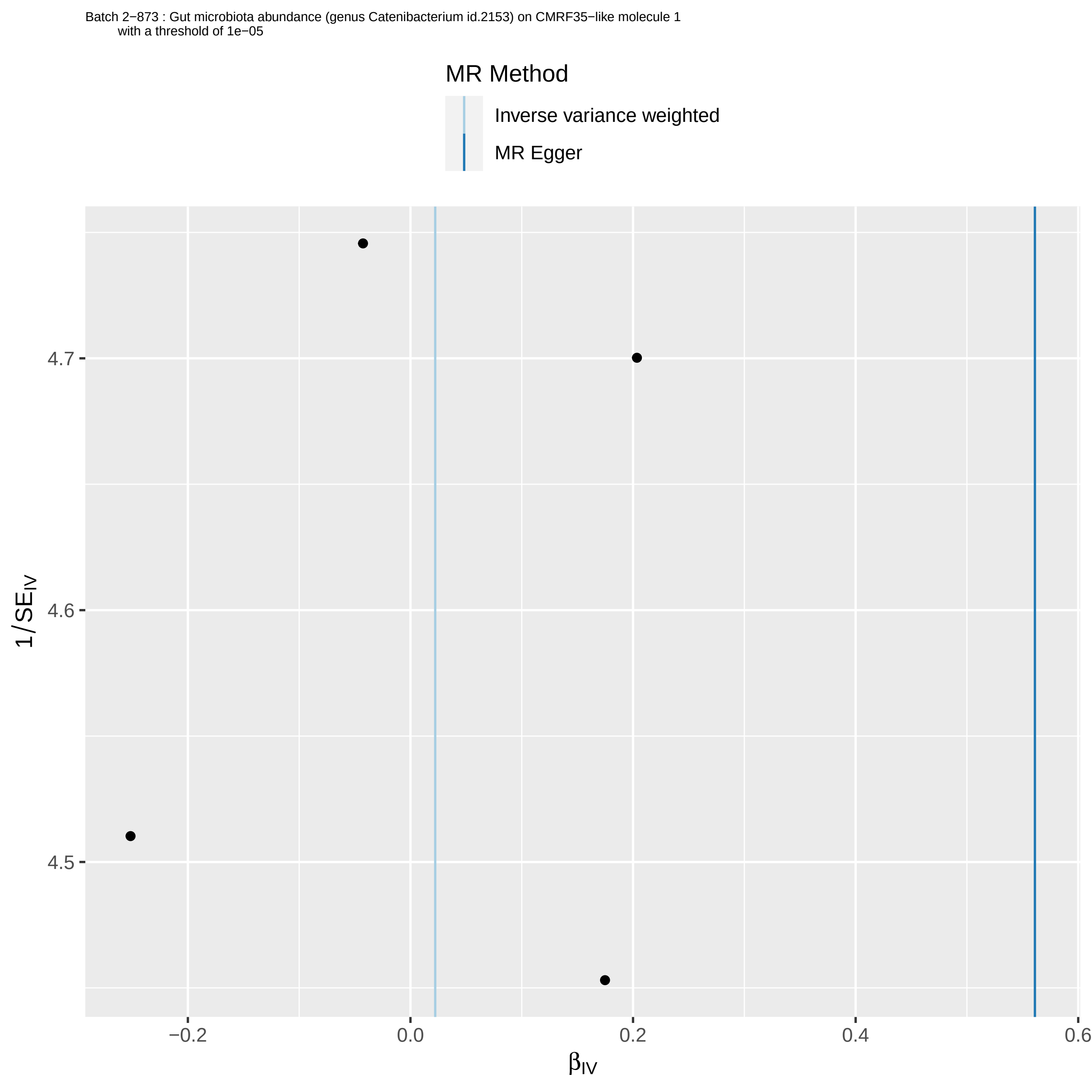

Batch 2−873 : Gut microbiota abundance (genus Catenibacterium id.2153) on CMRF35−like molecule 1
with a threshold of 1e−05
MR Method
Inverse variance weighted
MR Egger
4.7
4.6
4.5
1/SE_IV
−0.2
0.0
0.2
0.4
0.6
β_IV

Batch 2−874 : Gut microbiota abundance (genus Parasutterella id.2892) on CMRF35−like molecule 1
with a threshold of 1e−05

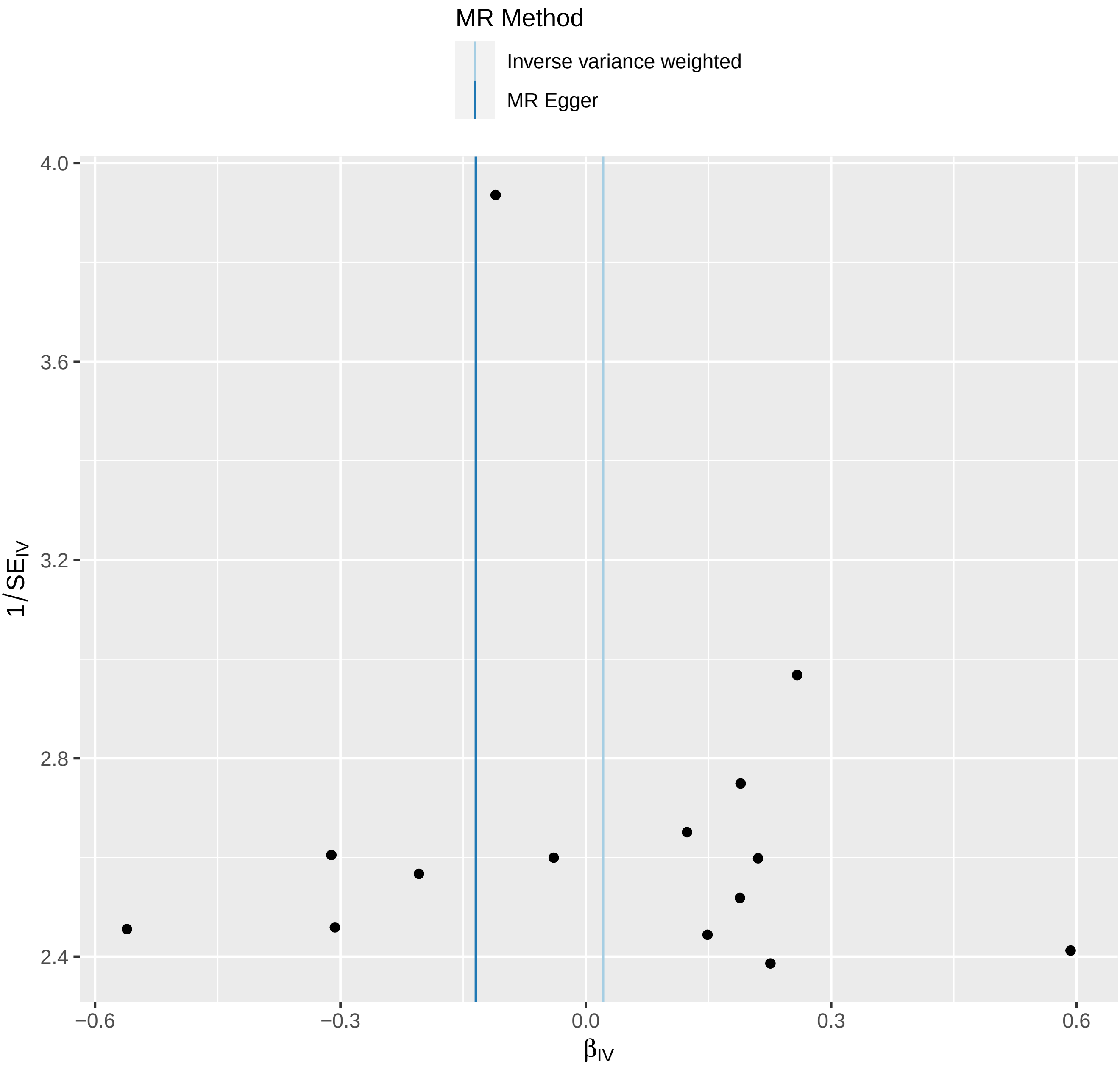

Batch 2−875 : Gut microbiota abundance (genus Catenibacterium id.2153) on CMRF35−like molecule 9
with a threshold of 1e−05

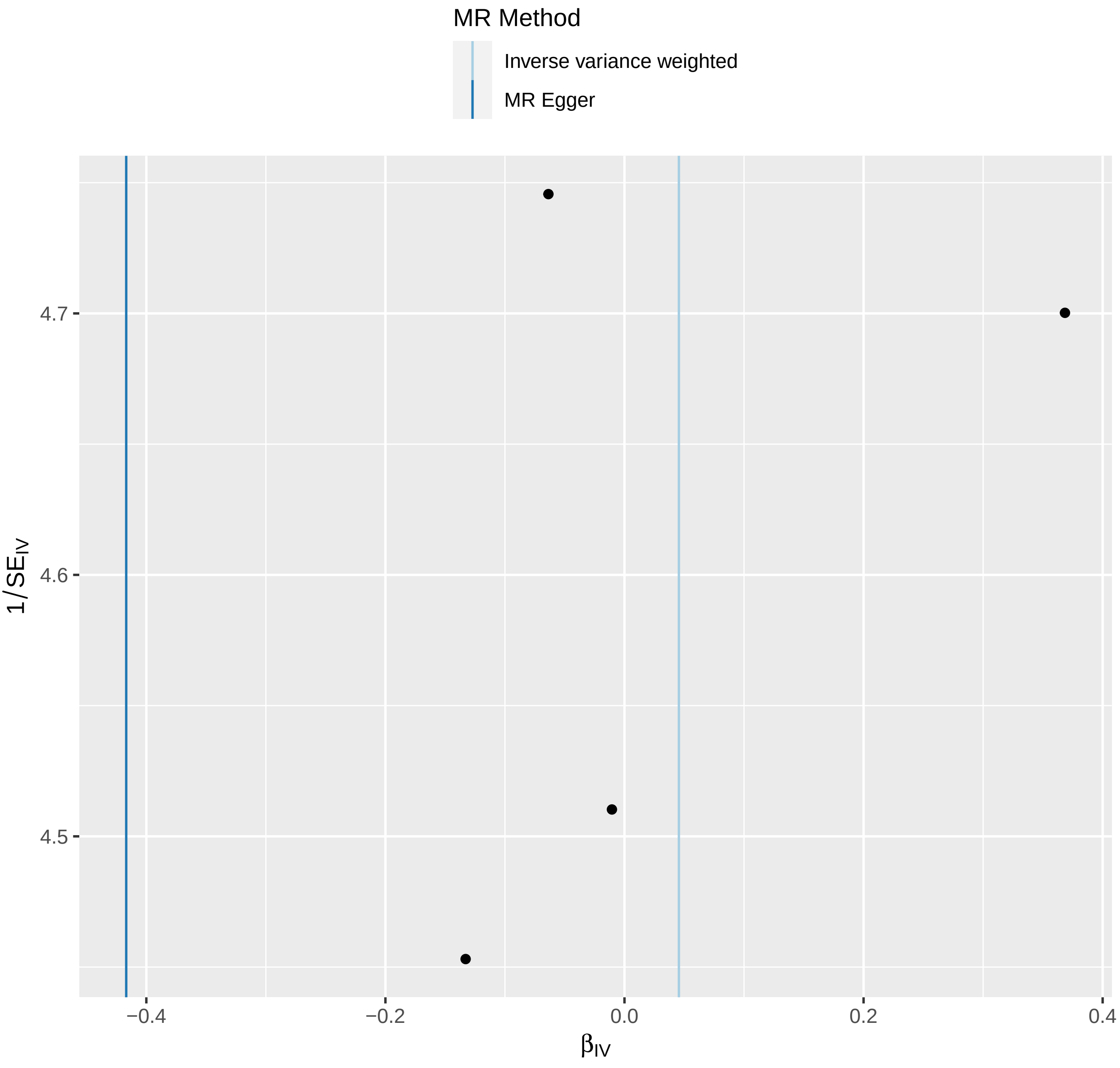

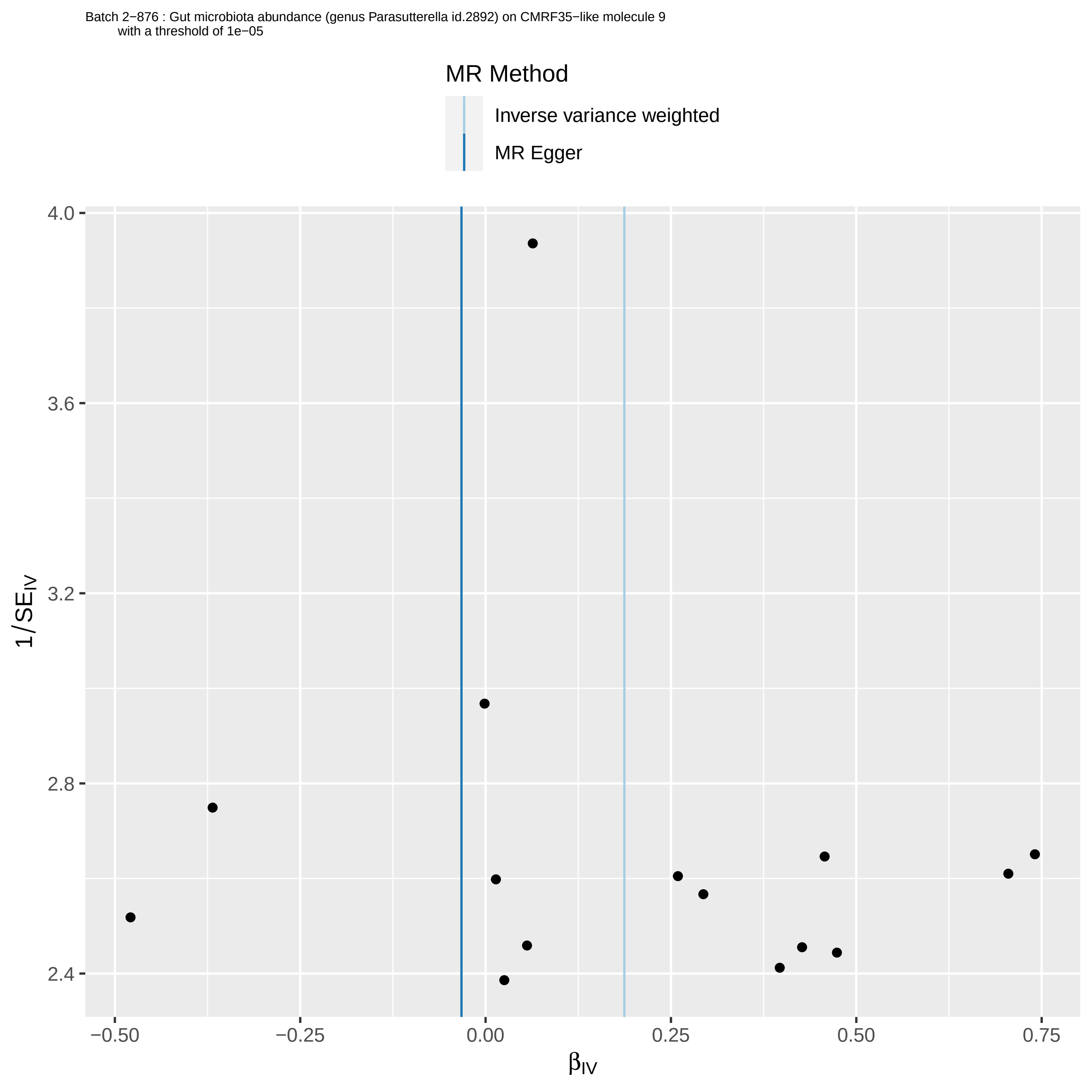

Batch 2−876 : Gut microbiota abundance (genus Parasutterella id.2892) on CMRF35−like molecule 9
with a threshold of 1e−05
MR Method
Inverse variance weighted
MR Egger
4.0
3.6
3.2
2.8
2.4
1/SE_IV
−0.50
−0.25
0.00
0.25
0.50
0.75
β_IV

Batch 2−877 : Gut microbiota abundance (genus Catenibacterium id.2153) on Myeloid cell surface antigen CD33
with a threshold of 1e−05

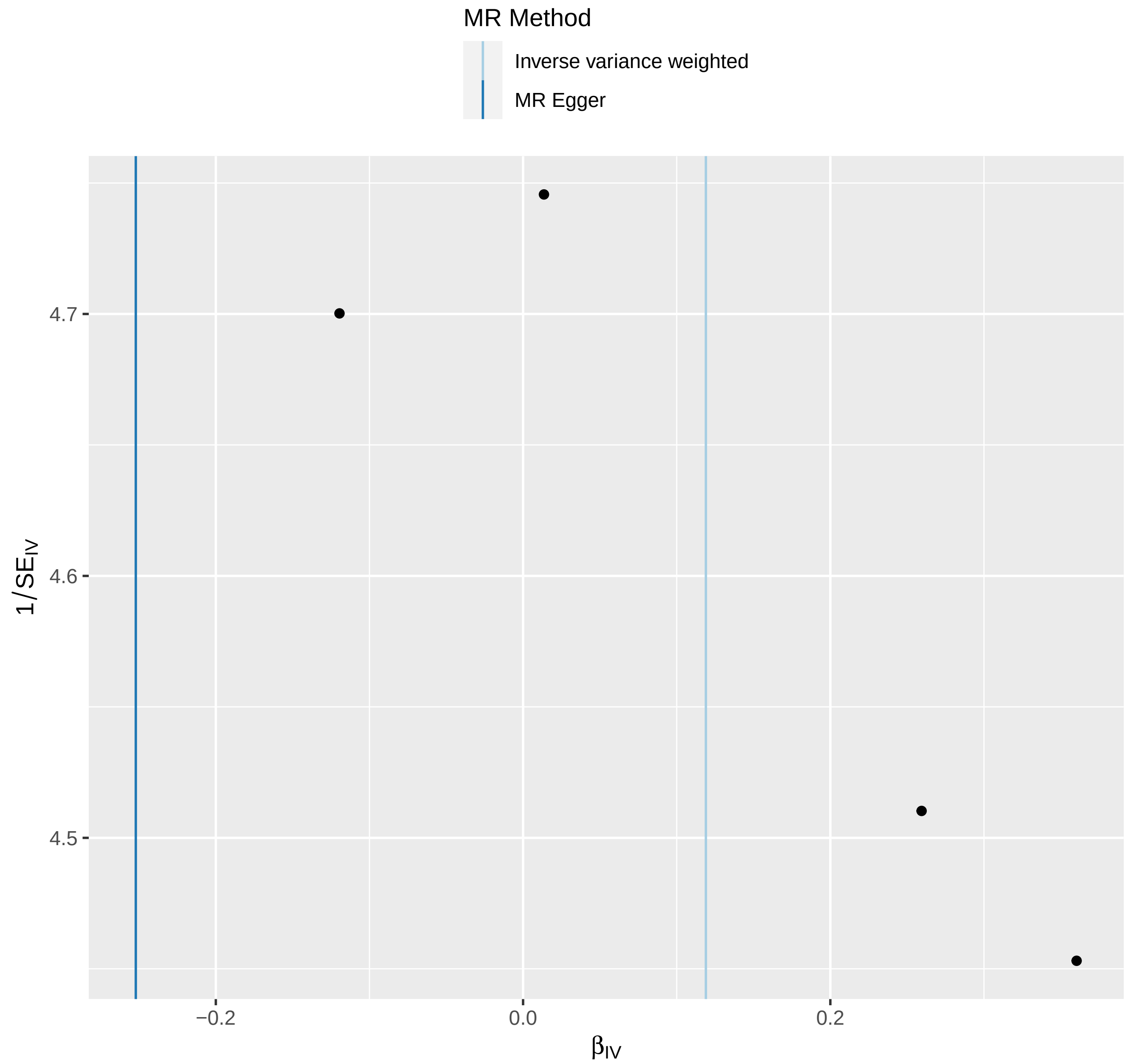

Batch 2−878 : Gut microbiota abundance (genus Parasutterella id.2892) on Myeloid cell surface antigen CD33
with a threshold of 1e−05

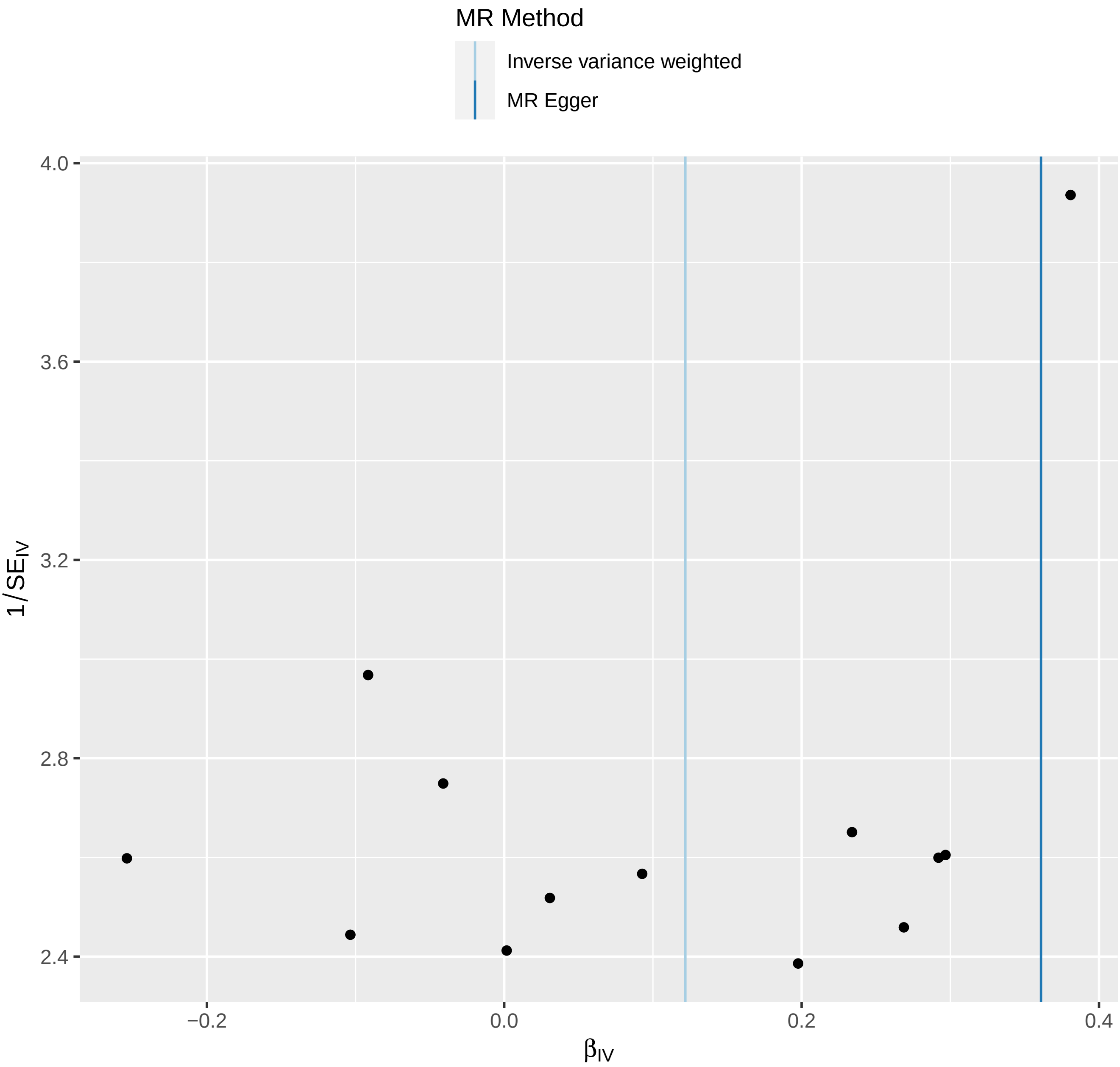

Batch 2−879 : Gut microbiota abundance (genus Catenibacterium id.2153) on Hematopoietic progenitor cell antigen CD34
with a threshold of 1e−05

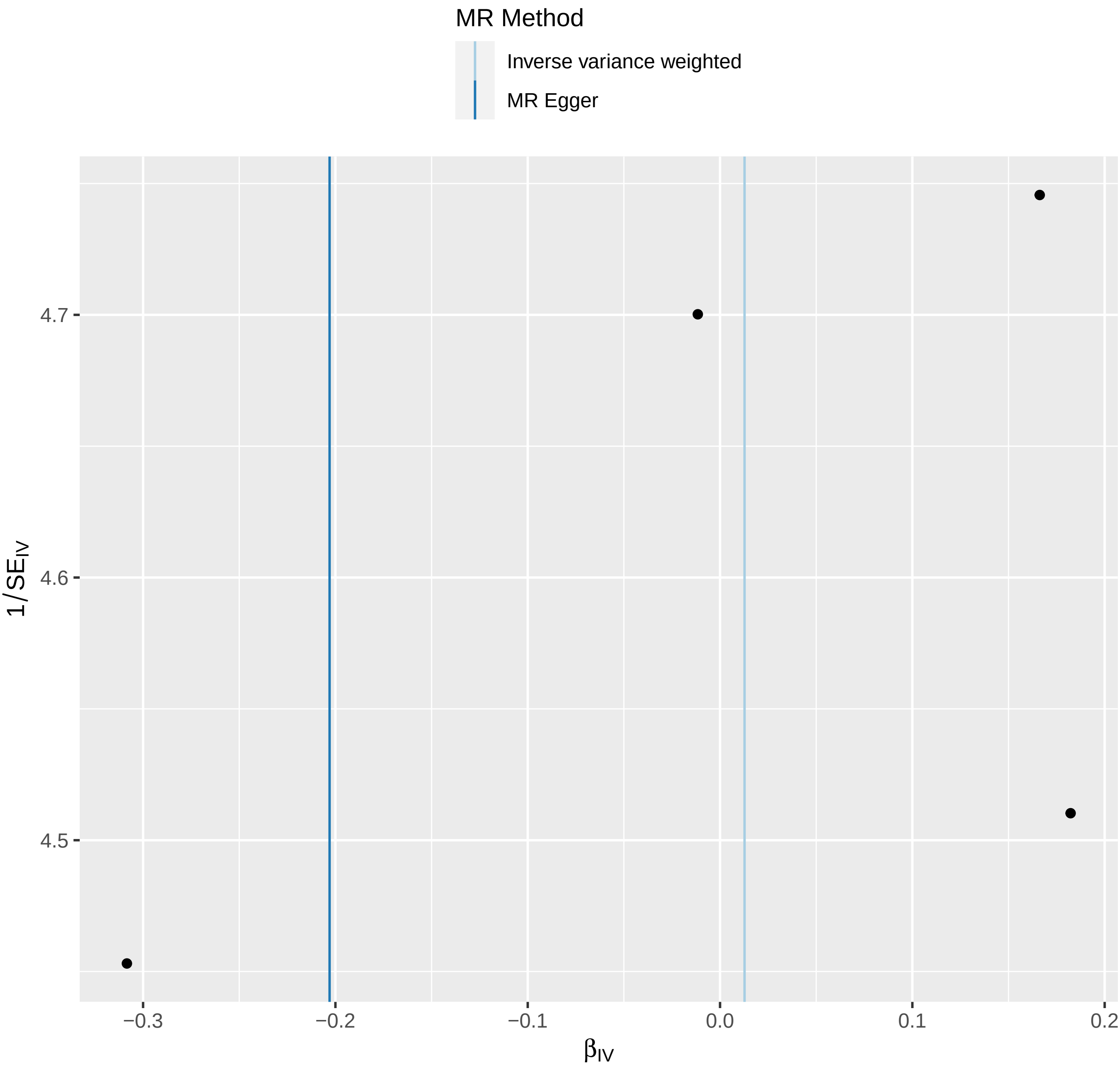

Batch 2−880 : Gut microbiota abundance (genus Parasutterella id.2892) on Hematopoietic progenitor cell antigen CD34
with a threshold of 1e−05

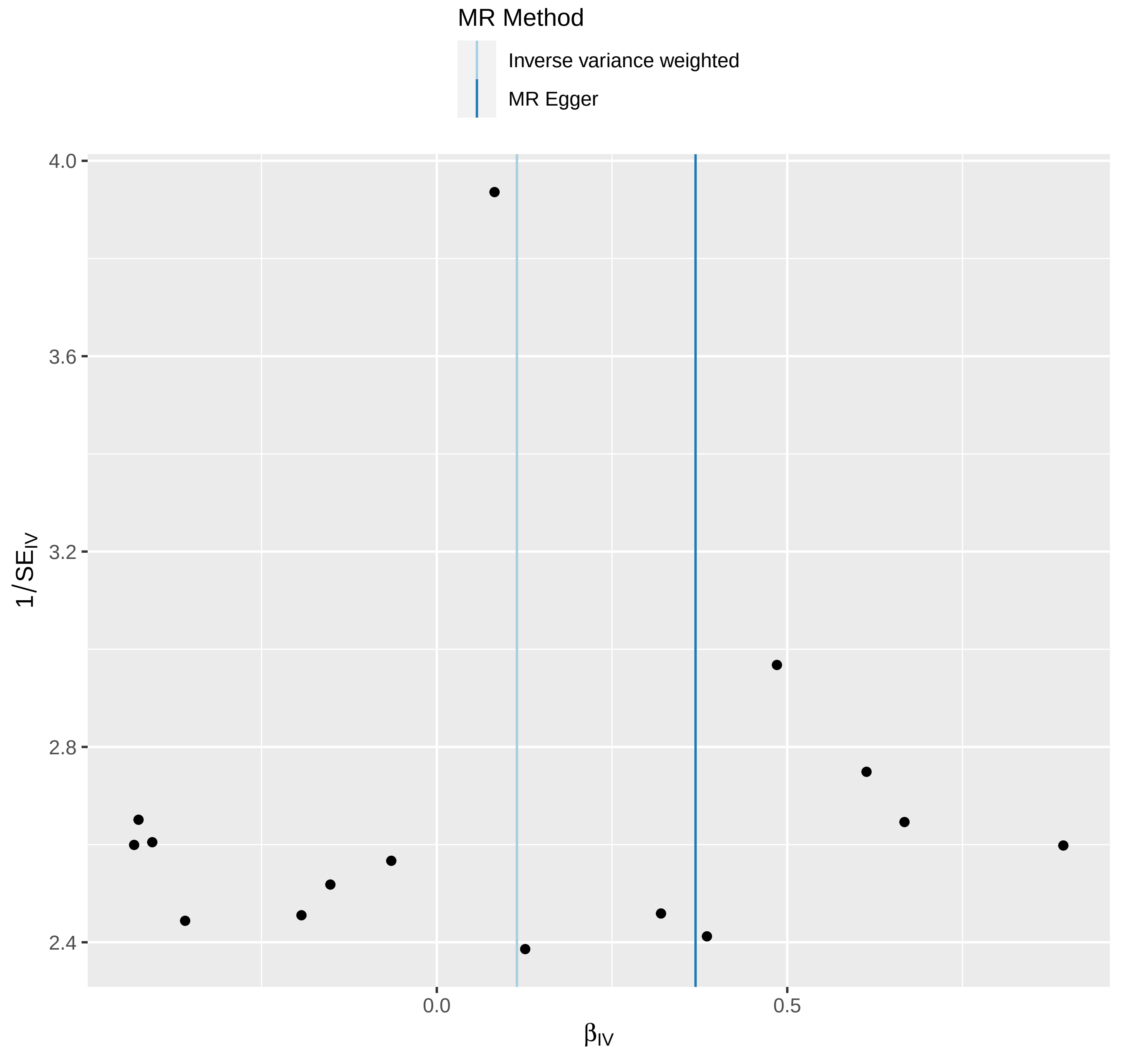

Batch 2−881 : Gut microbiota abundance (genus Catenibacterium id.2153) on Hematopoietic progenitor cell antigen CD34
with a threshold of 1e−05

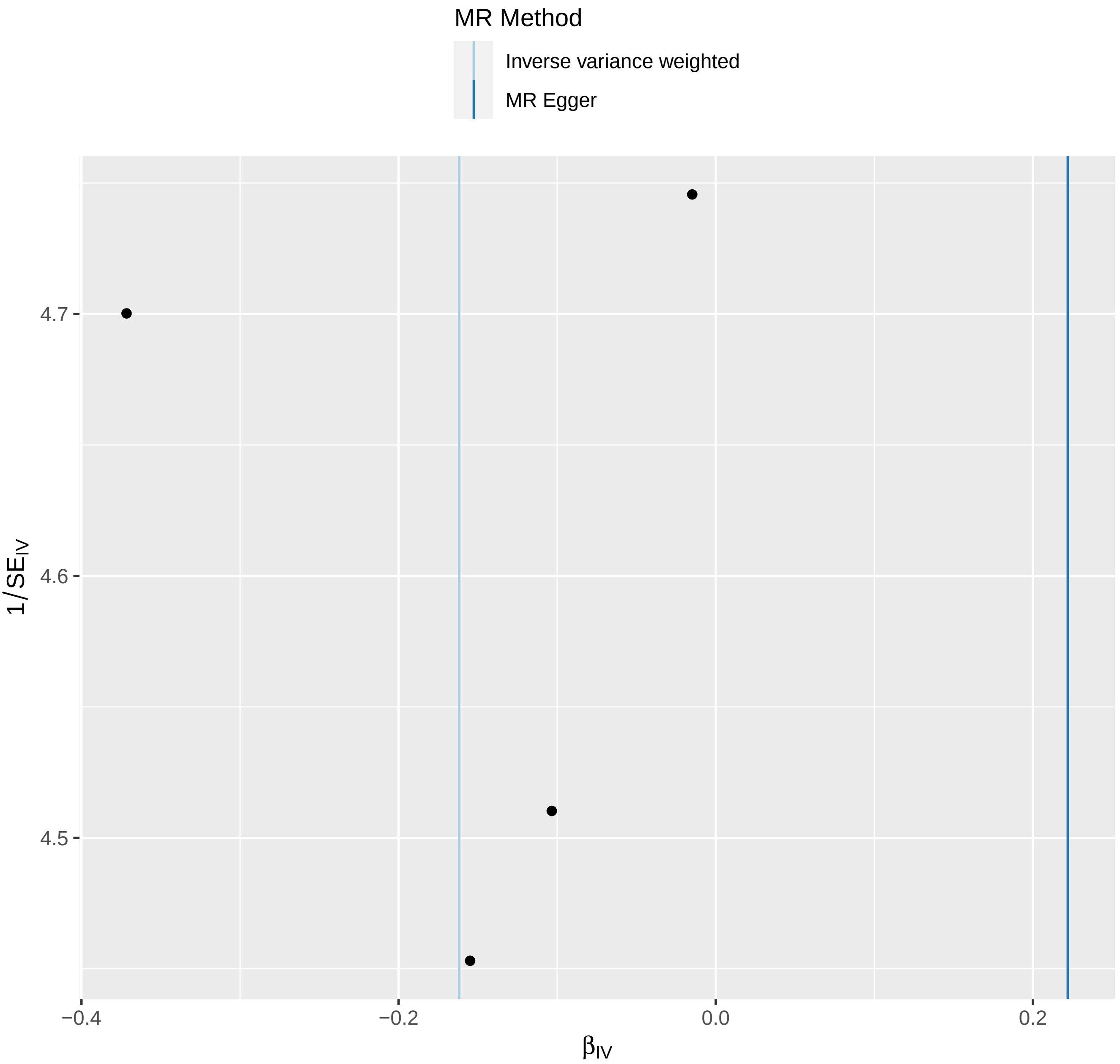

Batch 2−882 : Gut microbiota abundance (genus Parasutterella id.2892) on Hematopoietic progenitor cell antigen CD34
with a threshold of 1e−05

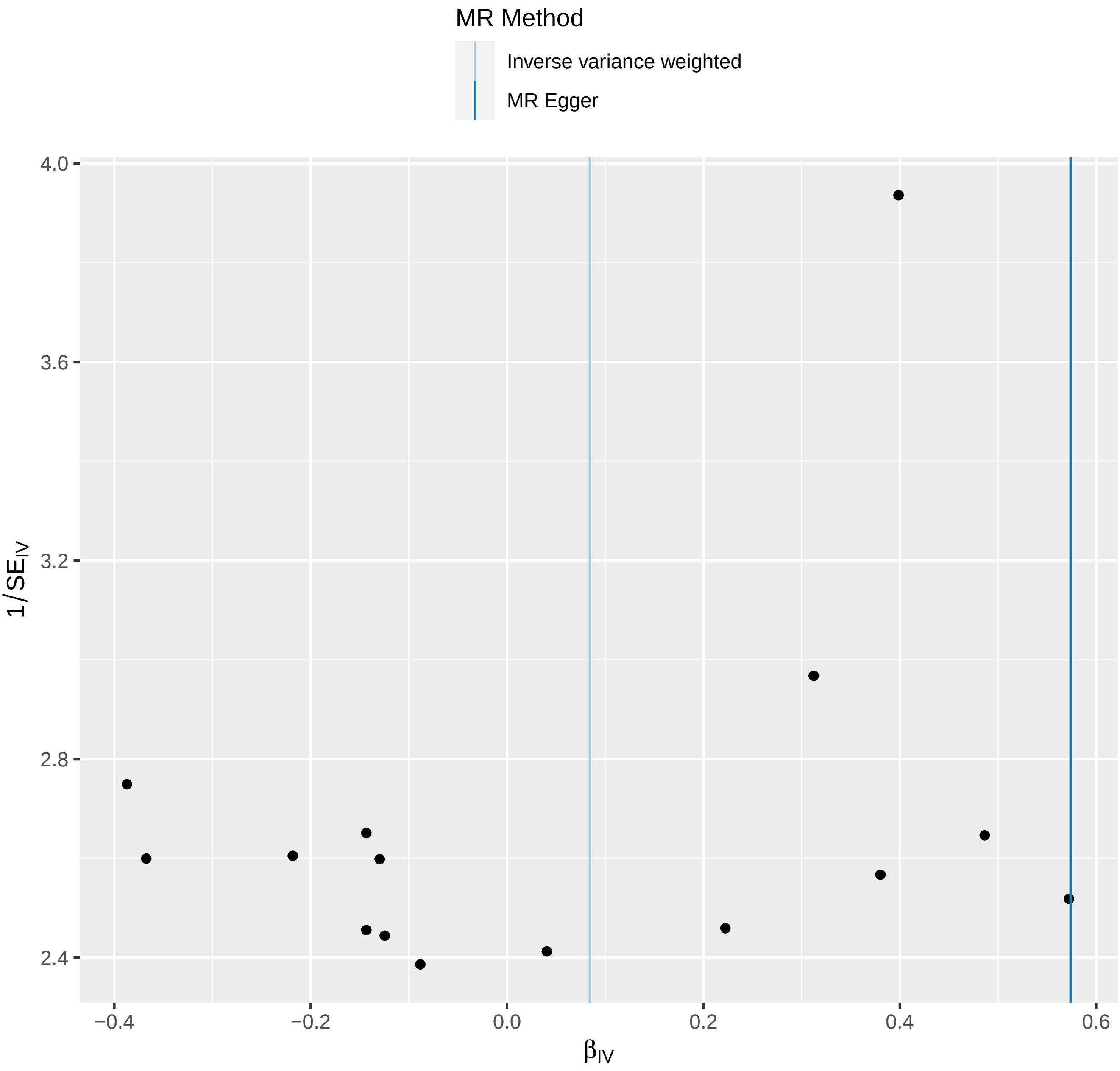

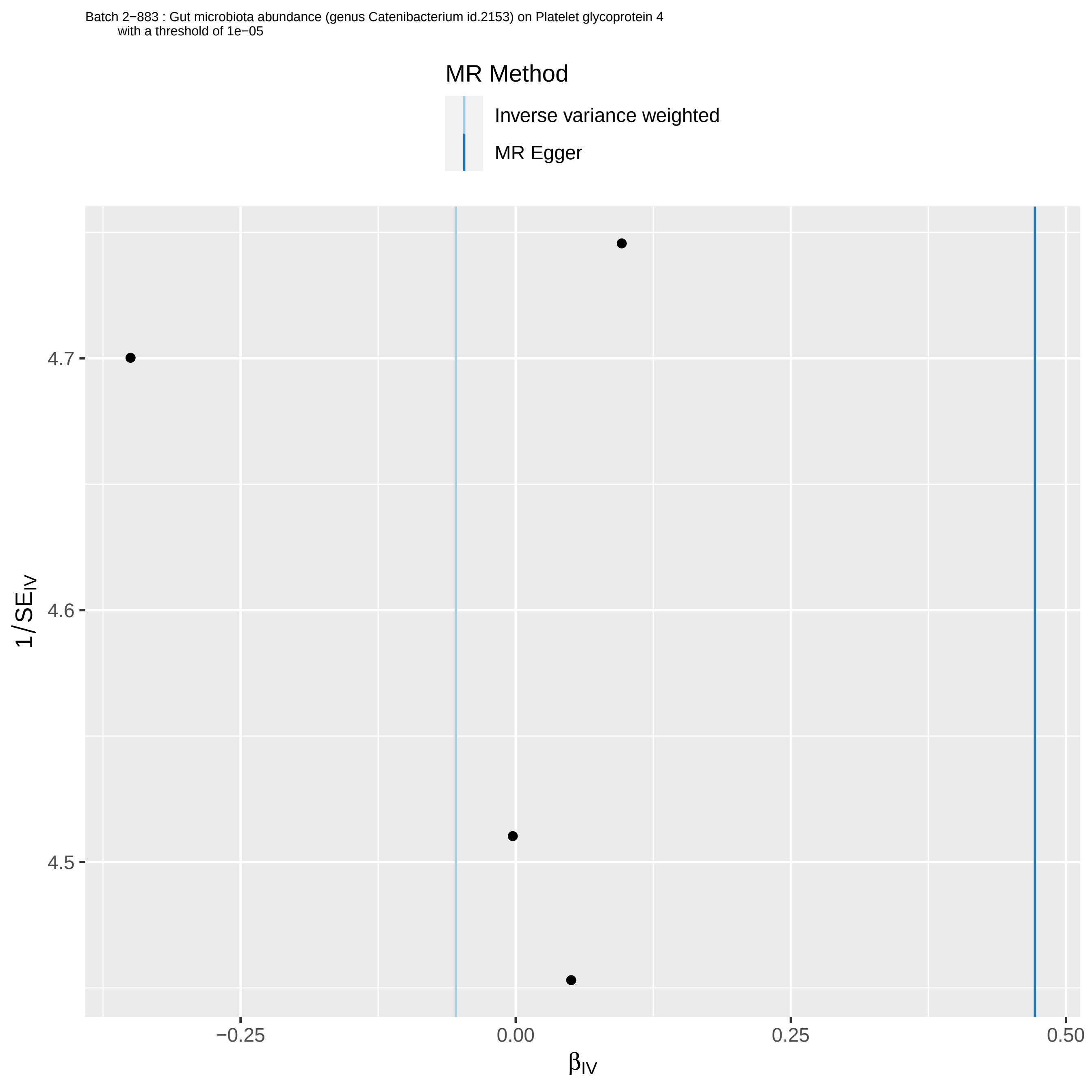
Batch 2−883 : Gut microbiota abundance (genus Catenibacterium id.2153) on Platelet glycoprotein 4
with a threshold of 1e−05
MR Method
Inverse variance weighted
MR Egger
4.7
4.6
4.5
1/SE_IV
−0.25
0.00
0.25
0.50
β_IV

Batch 2−884 : Gut microbiota abundance (genus Parasutterella id.2892) on Platelet glycoprotein 4
with a threshold of 1e−05

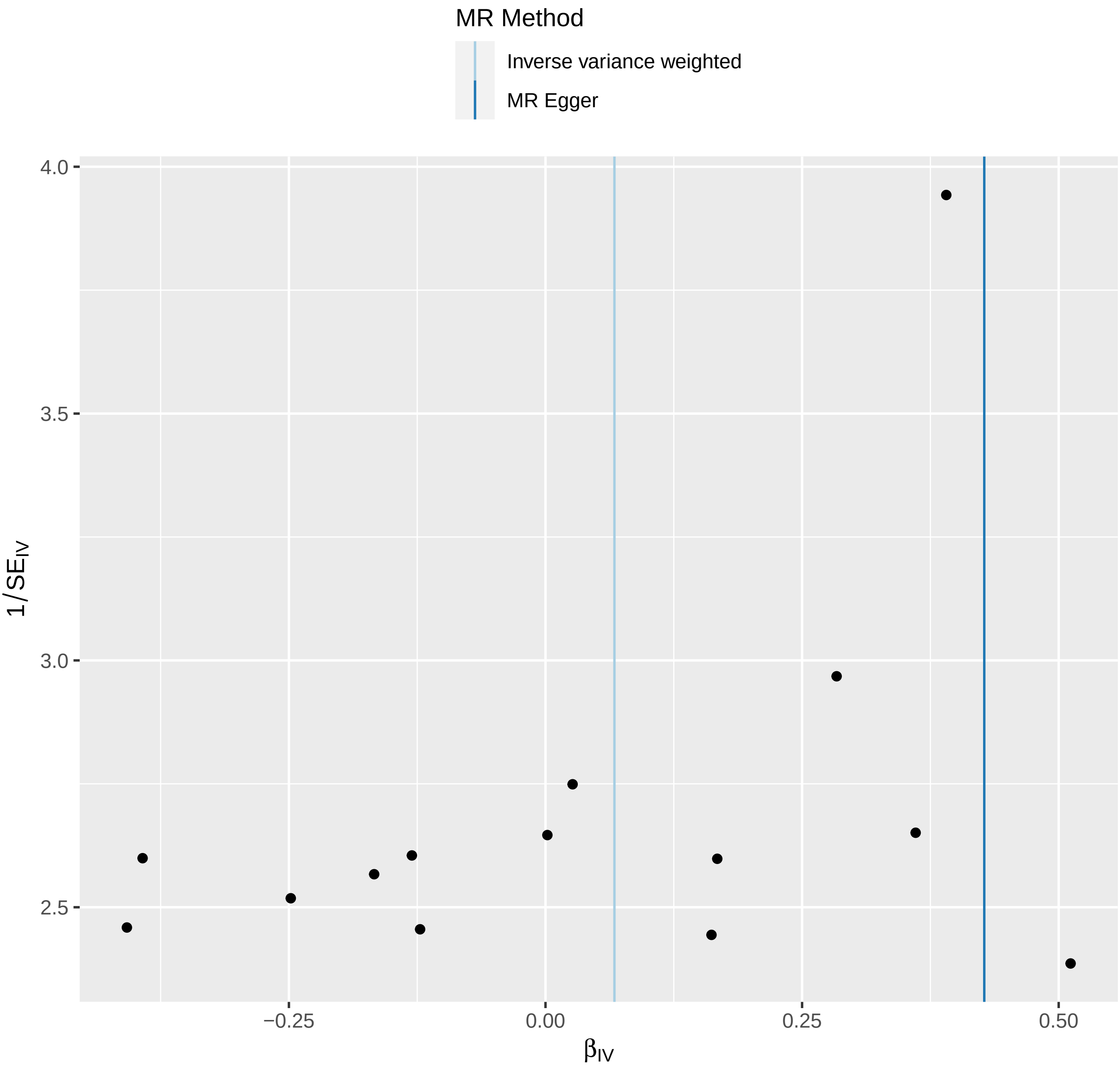

Batch 2−885 : Gut microbiota abundance (genus Catenibacterium id.2153) on ADP−ribosyl cyclase/cyclic ADP−ribose hydrolase 1
with a threshold of 1e−05

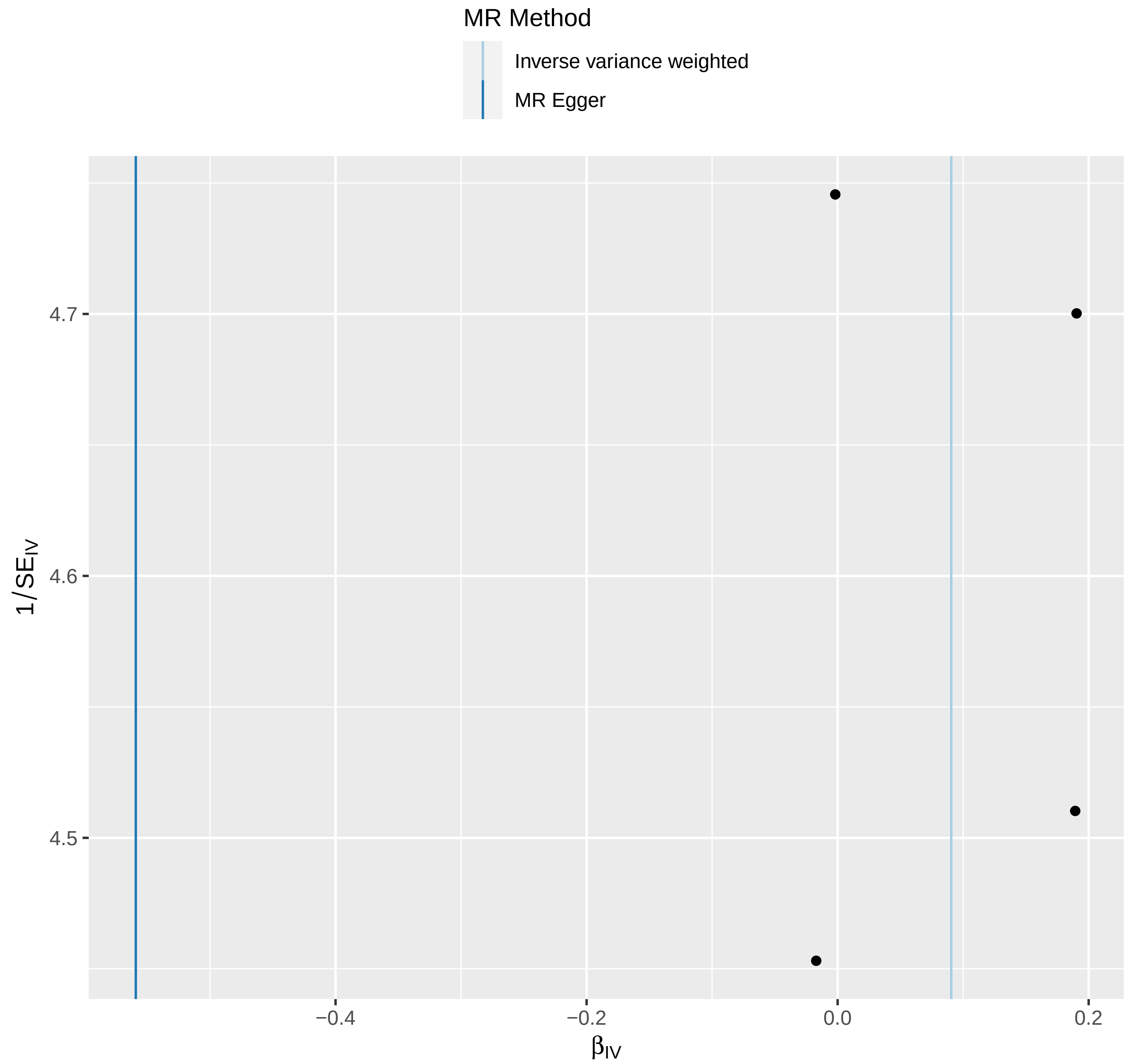

Batch 2−886 : Gut microbiota abundance (genus Parasutterella id.2892) on ADP−ribosyl cyclase/cyclic ADP−ribose hydrolase 1
with a threshold of 1e−05

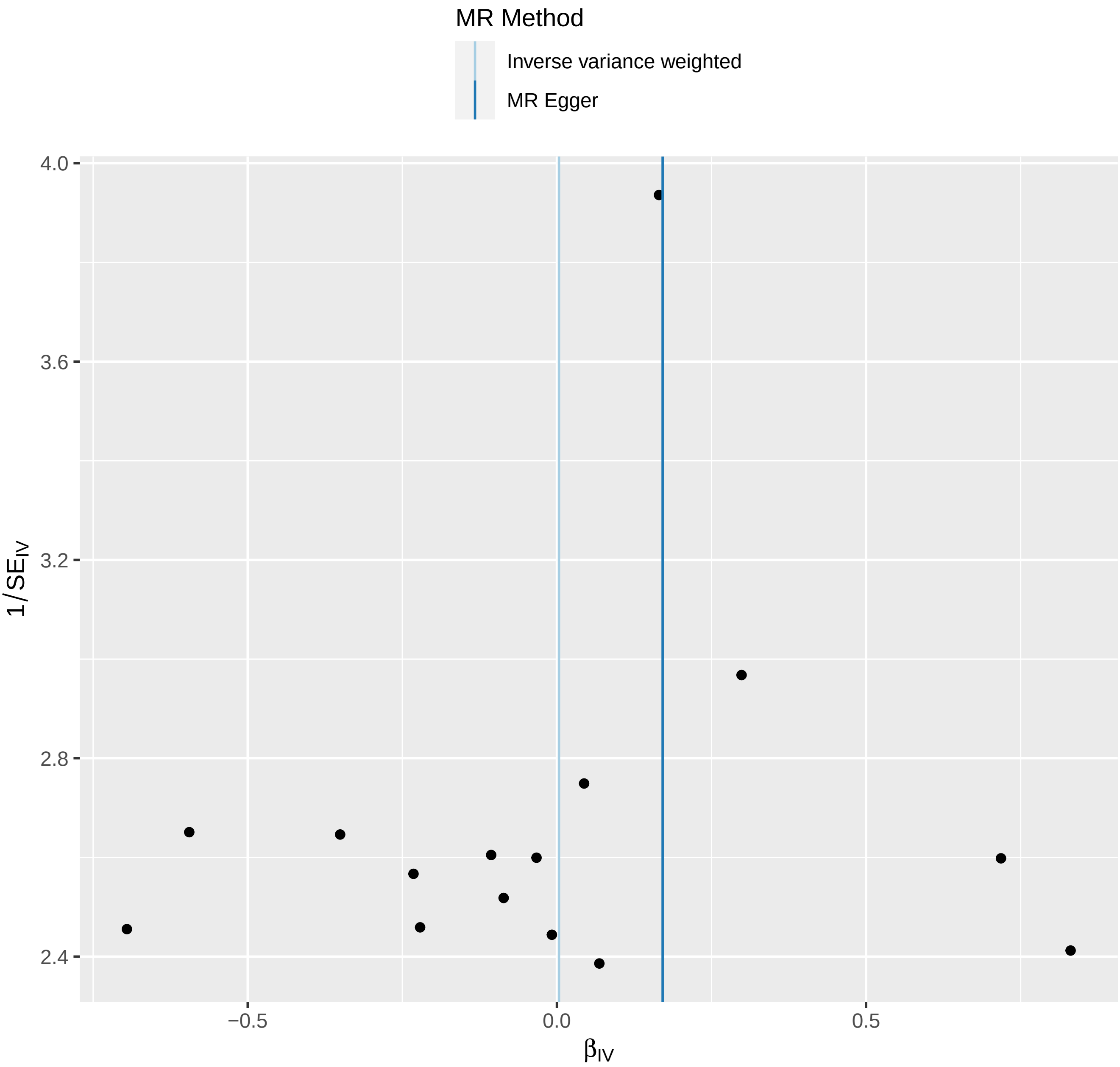

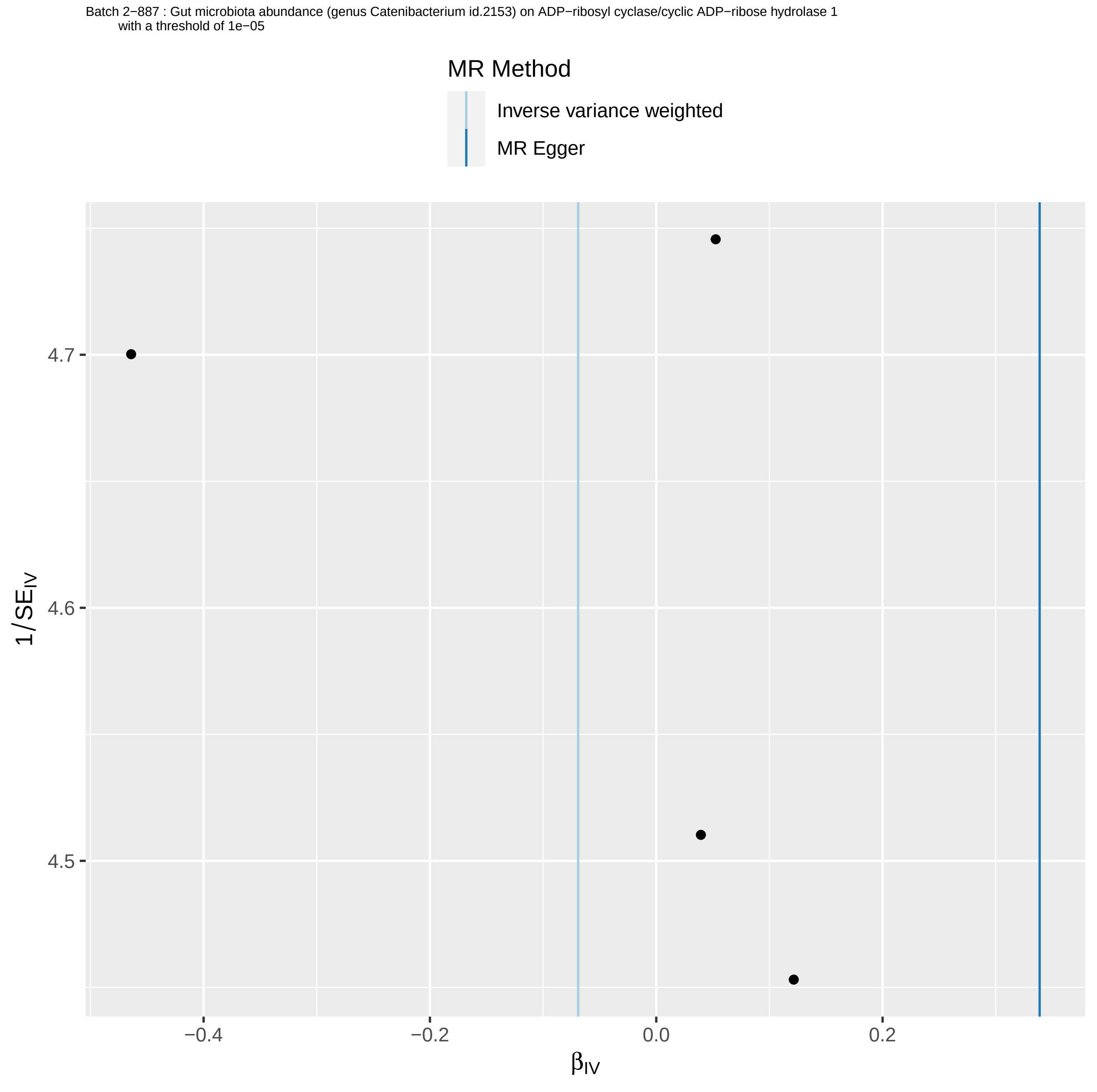

Batch 2−887 : Gut microbiota abundance (genus Catenibacterium id.2153) on ADP−ribosyl cyclase/cyclic ADP−ribose hydrolase 1
with a threshold of 1e−05
MR Method
Inverse variance weighted
MR Egger
4.7
4.6
4.5
1/SE_IV
−0.4
−0.2
0.0
0.2
β_IV

Batch 2−888 : Gut microbiota abundance (genus Parasutterella id.2892) on ADP−ribosyl cyclase/cyclic ADP−ribose hydrolase 1
with a threshold of 1e−05

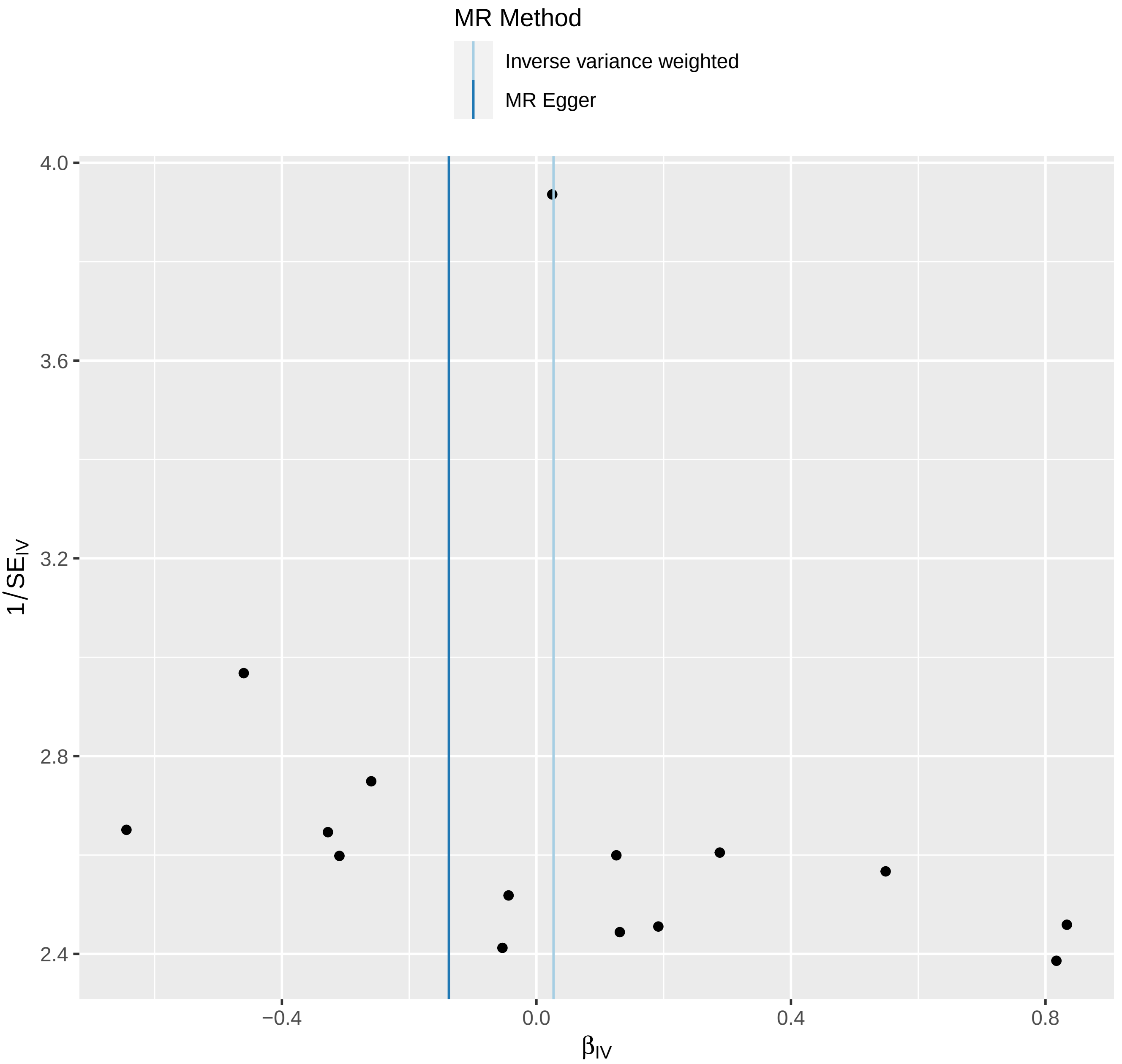

Batch 2−889 : Gut microbiota abundance (genus Catenibacterium id.2153) on T−cell surface glycoprotein CD3 epsilon chain with a threshold of 1e−05

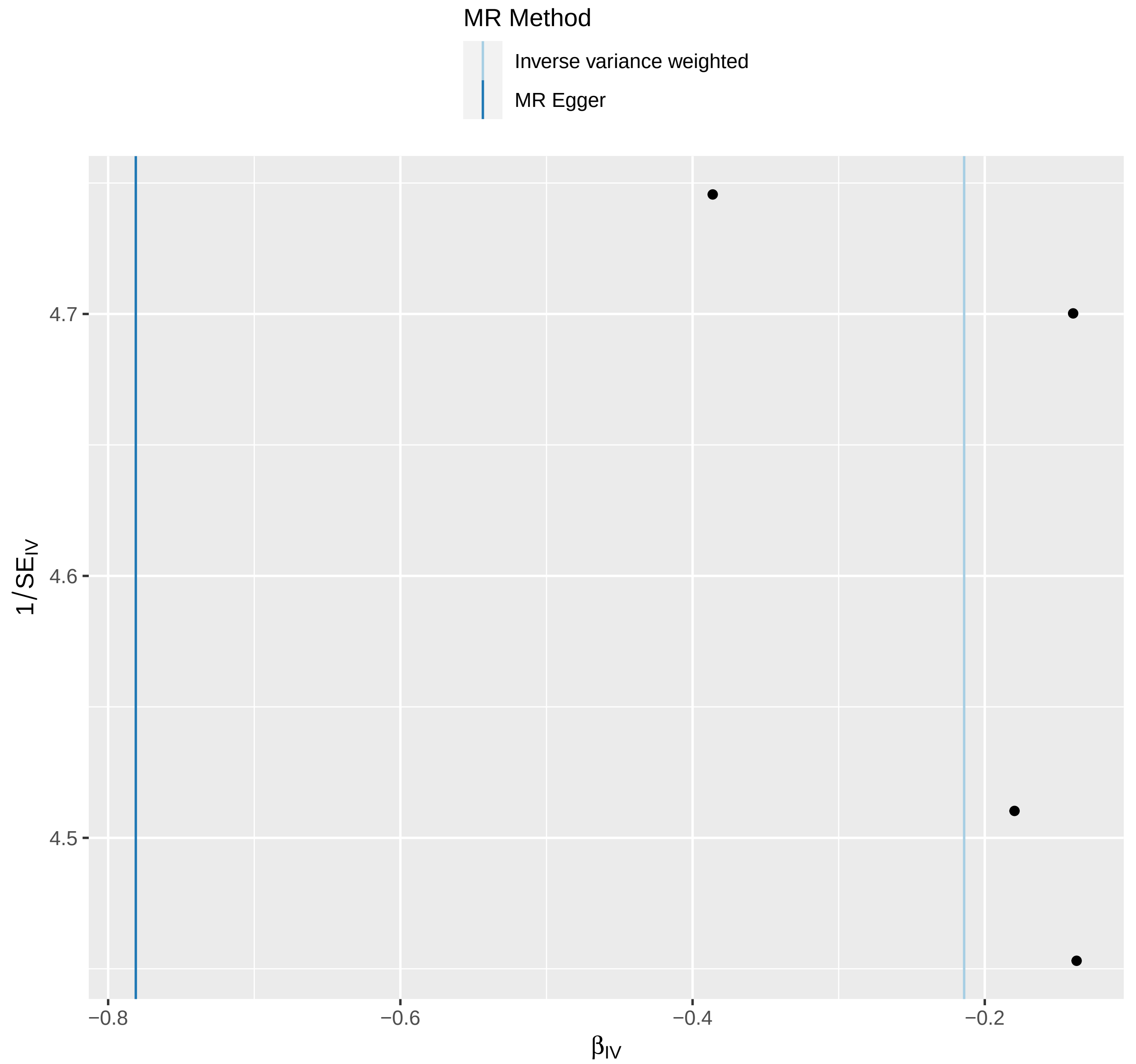

Batch 2−890 : Gut microbiota abundance (genus Parasutterella id.2892) on T−cell surface glycoprotein CD3 epsilon chain
with a threshold of 1e−05

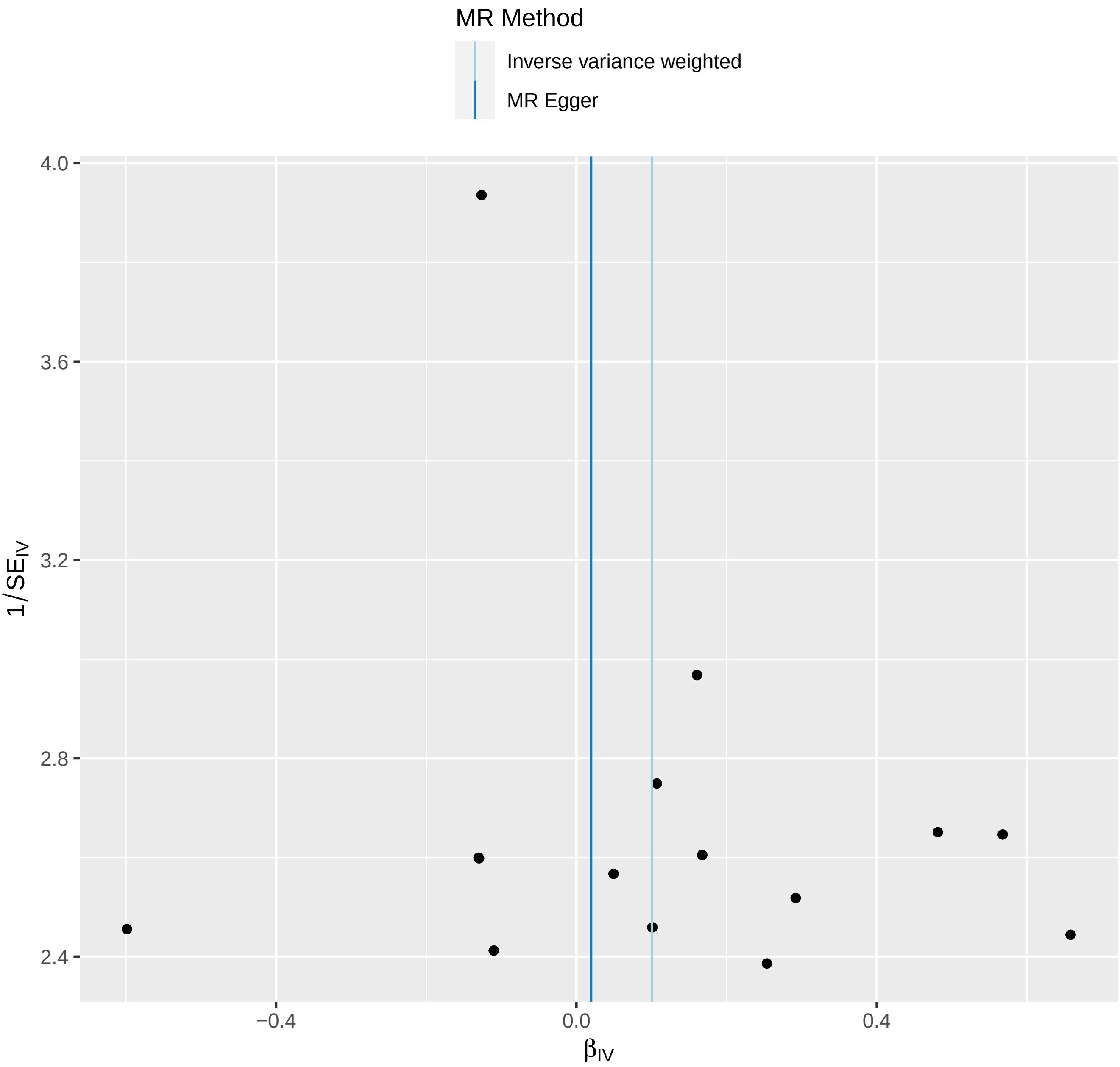

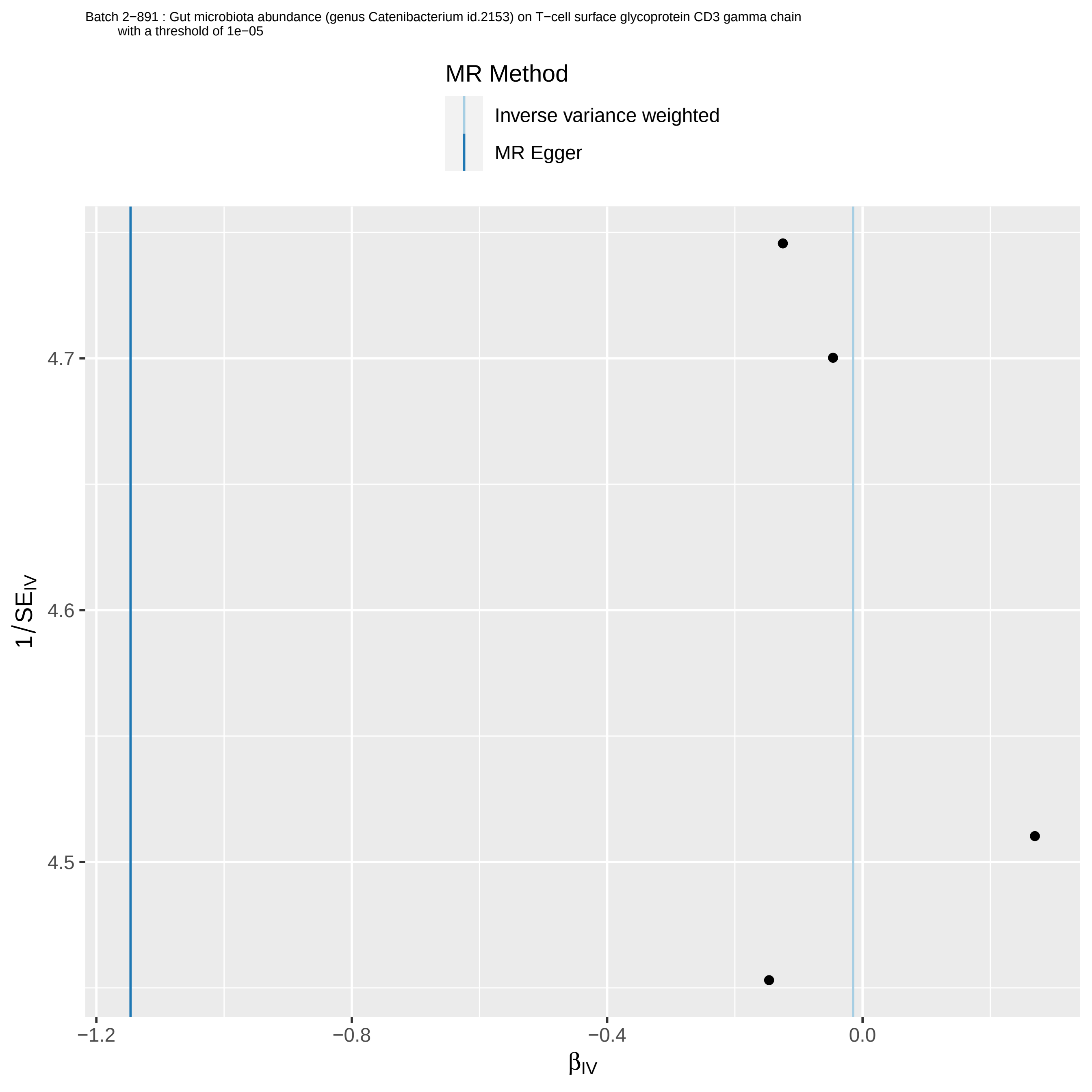

Batch 2−891 : Gut microbiota abundance (genus Catenibacterium id.2153) on T−cell surface glycoprotein CD3 gamma chain
with a threshold of 1e−05
MR Method
Inverse variance weighted
MR Egger
4.7
4.6
4.5
$1/SE_{IV}$
−1.2
−0.8
−0.4
0.0
β_{IV}

Batch 2−892 : Gut microbiota abundance (genus Parasutterella id.2892) on T−cell surface glycoprotein CD3 gamma chain
with a threshold of 1e−05

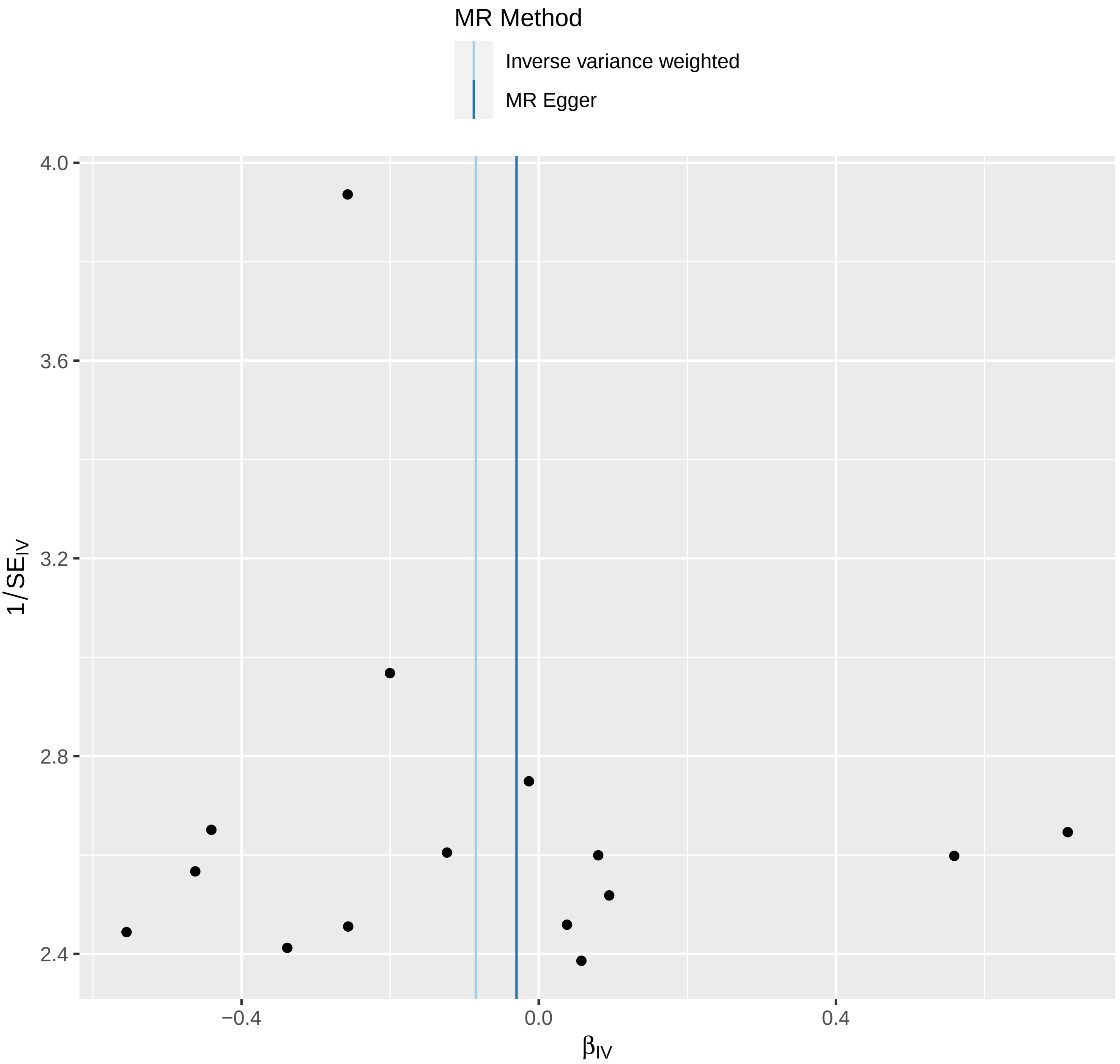

Batch 2−893 : Gut microbiota abundance (genus Catenibacterium id.2153) on CD40 ligand
with a threshold of 1e−05

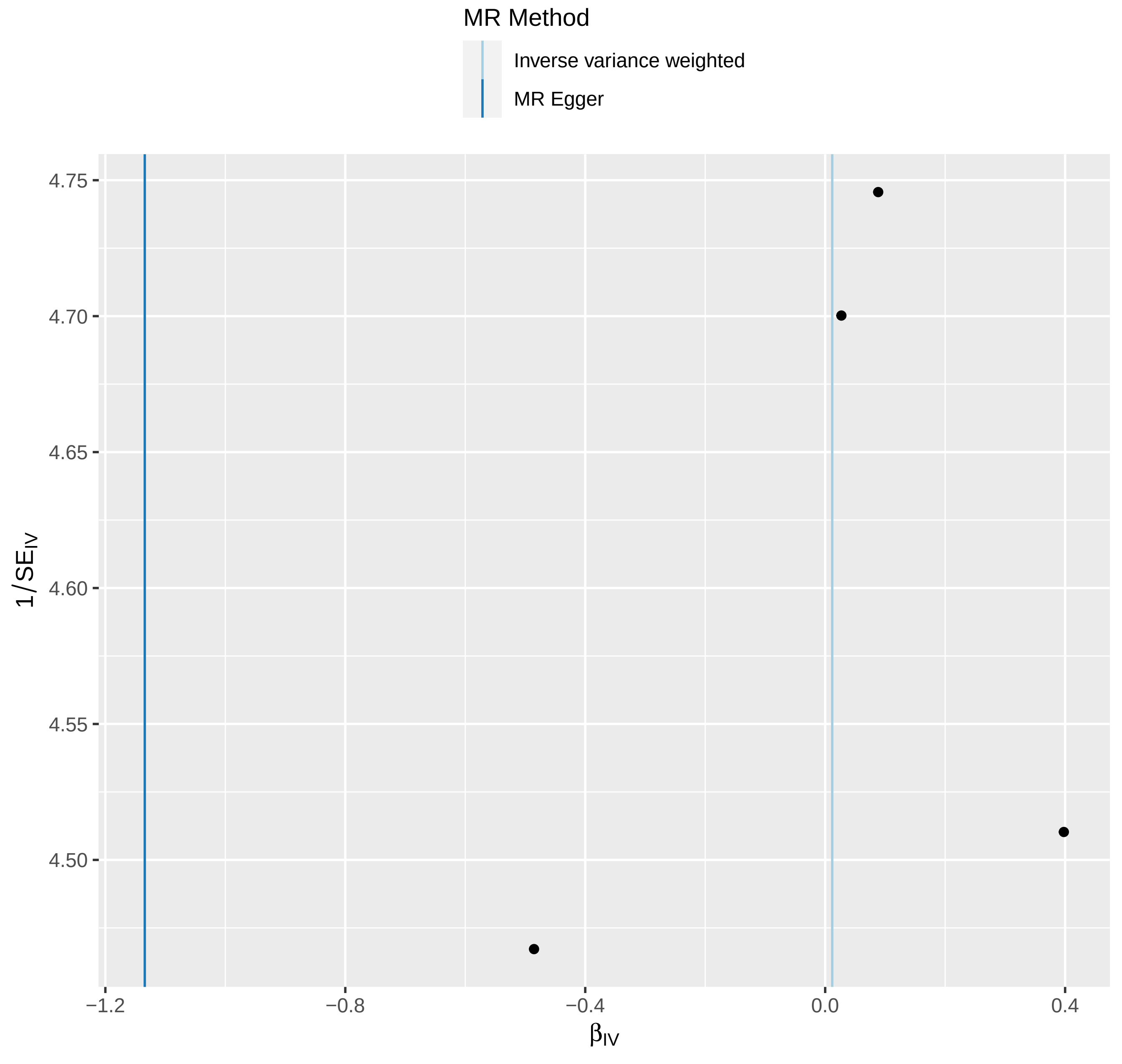

Batch 2−894 : Gut microbiota abundance (genus Parasutterella id.2892) on CD40 ligand
with a threshold of 1e−05

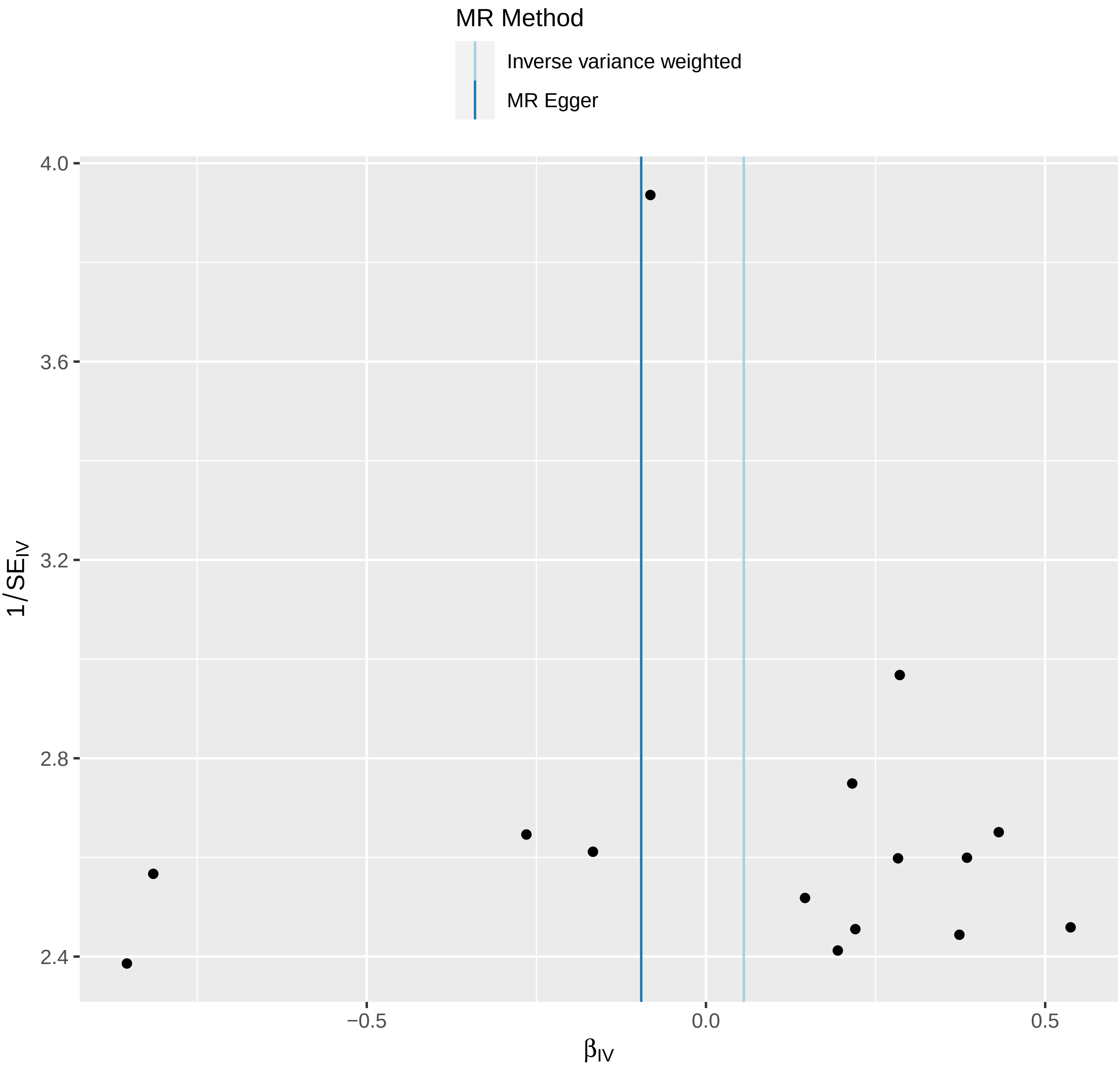

Batch 2−895 : Gut microbiota abundance (genus Catenibacterium id.2153) on T−cell surface glycoprotein CD4
with a threshold of 1e−05

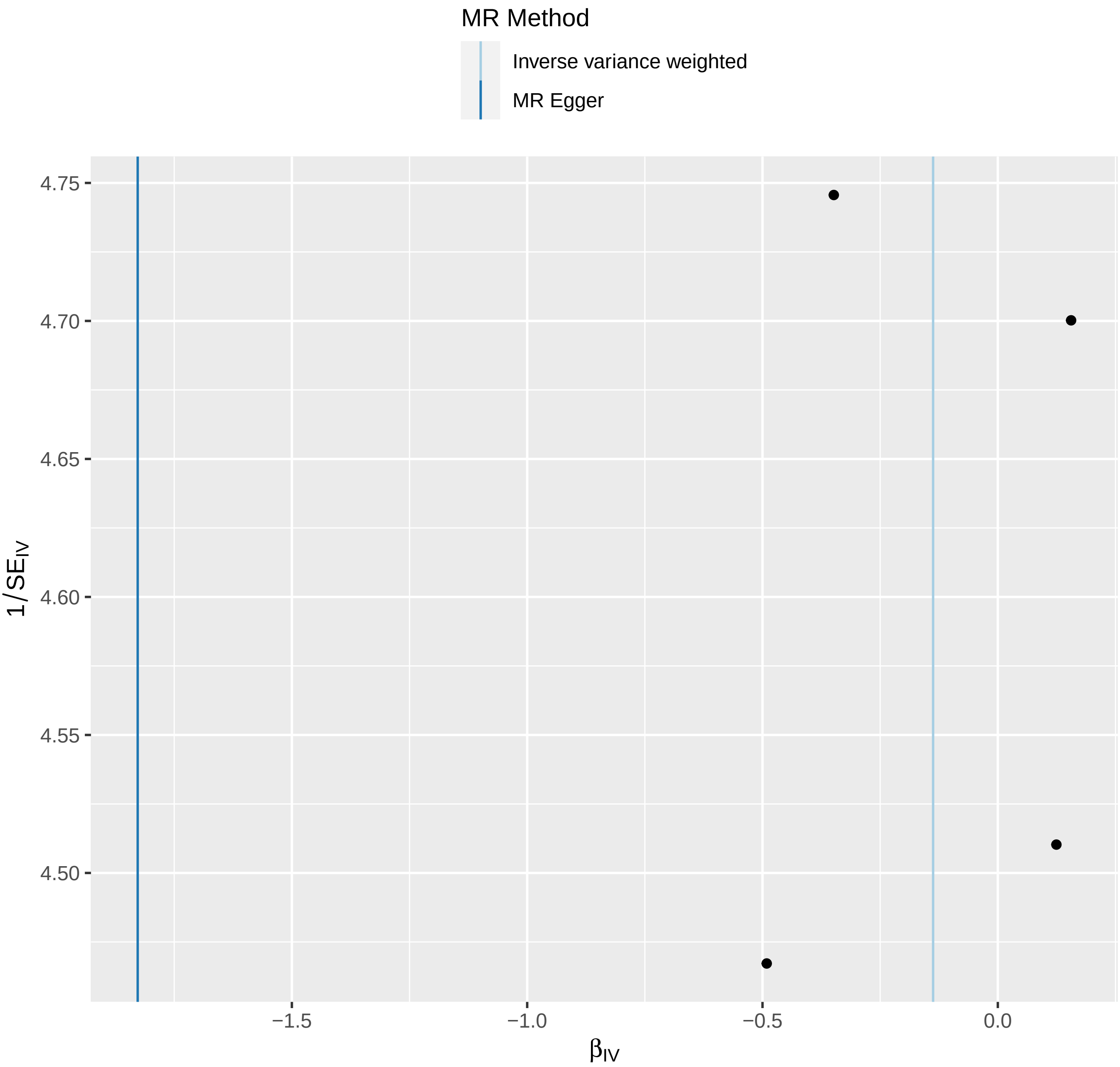

Batch 2−896 : Gut microbiota abundance (genus Parasutterella id.2892) on T−cell surface glycoprotein CD4
with a threshold of 1e−05

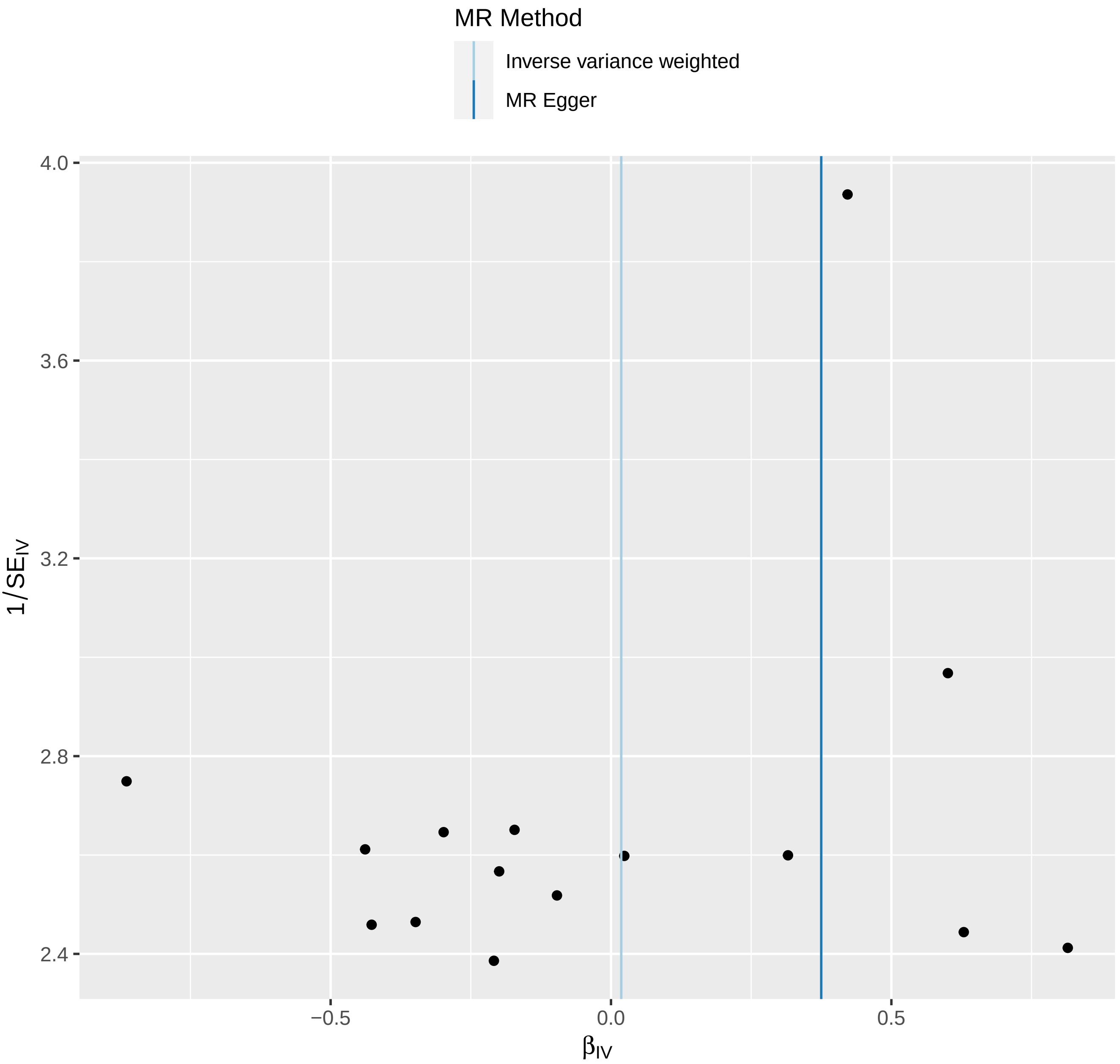

Batch 2−897 : Gut microbiota abundance (genus Catenibacterium id.2153) on CD44 antigen
with a threshold of 1e−05

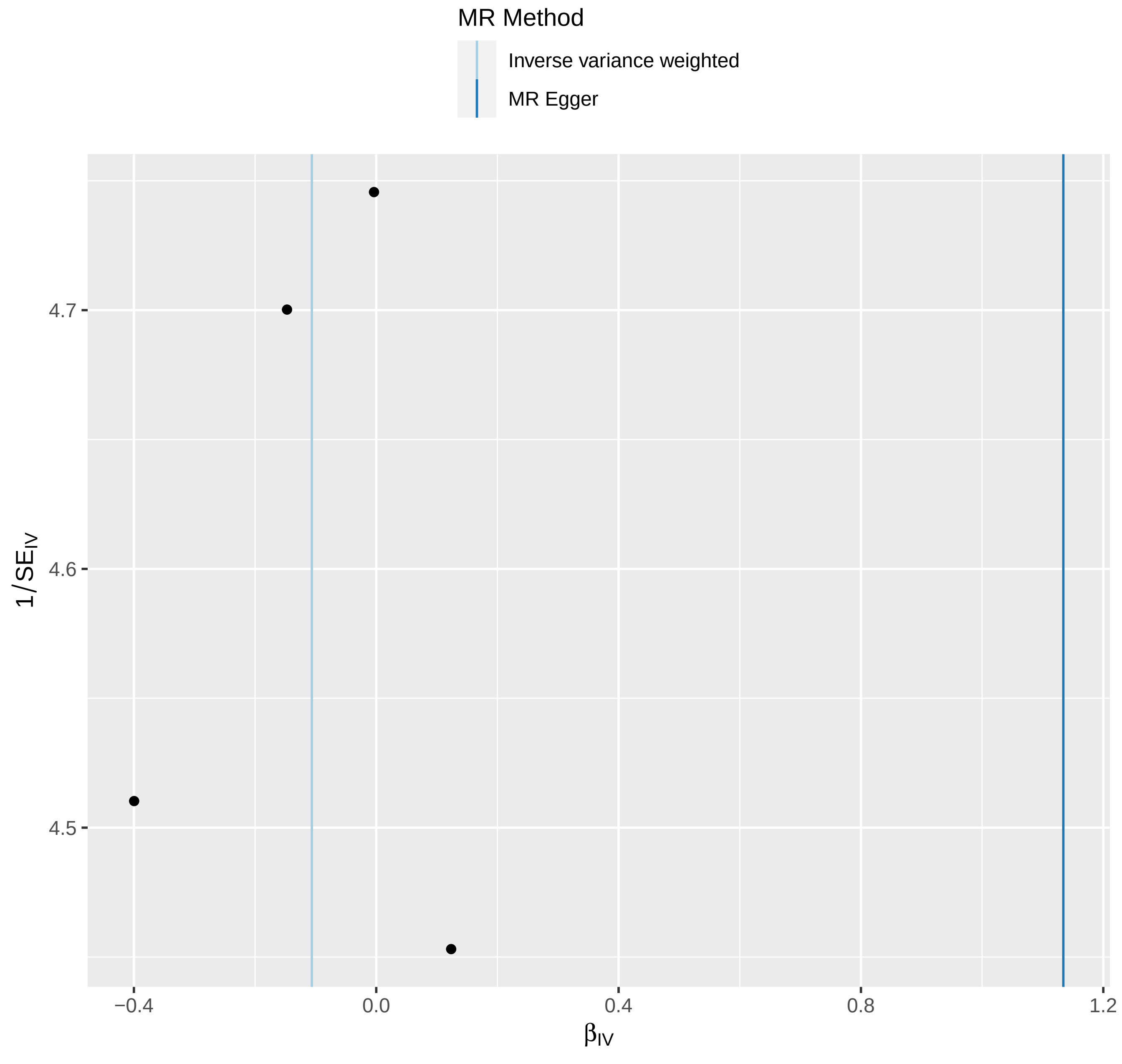

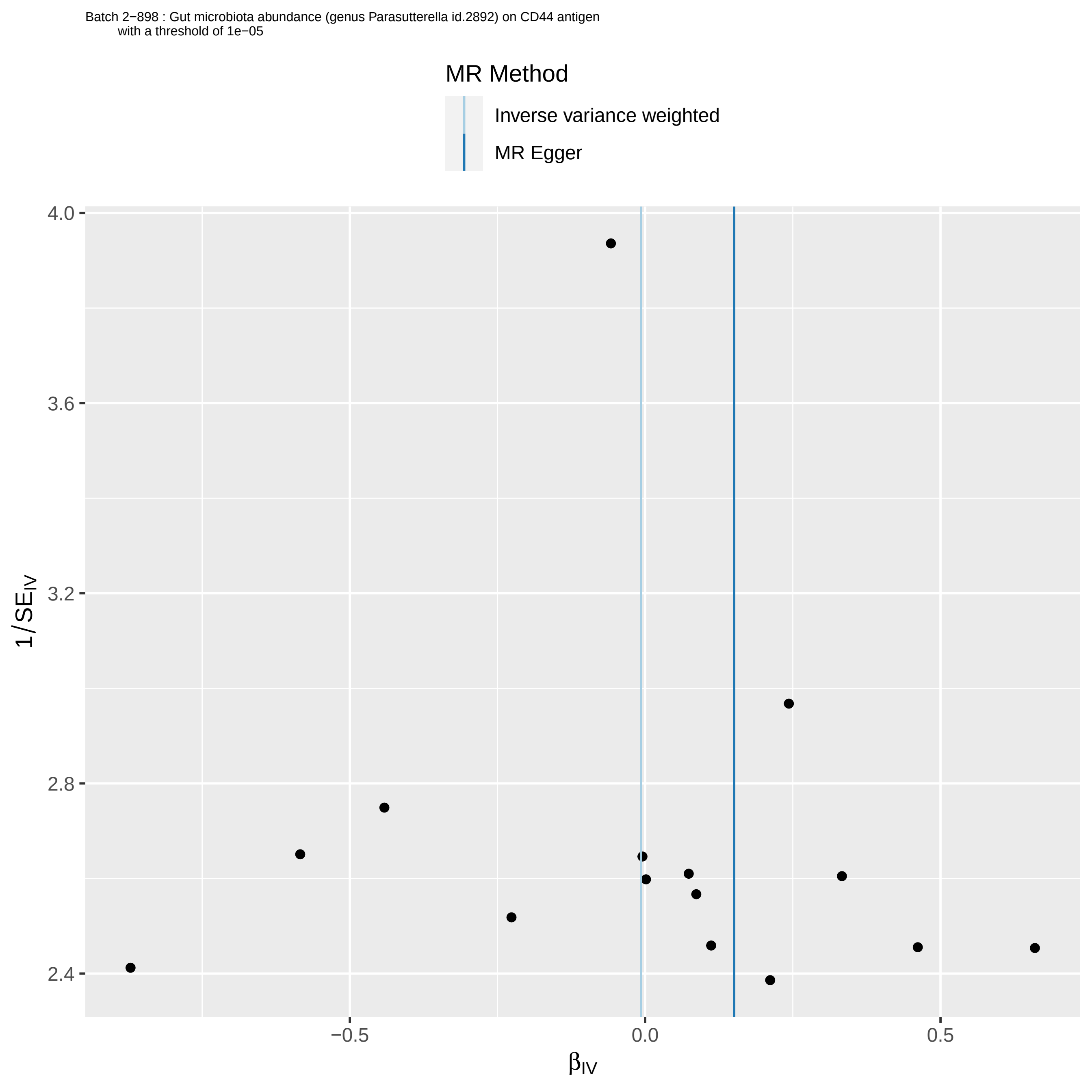
Batch 2−898 : Gut microbiota abundance (genus Parasutterella id.2892) on CD44 antigen
with a threshold of 1e−05
MR Method
Inverse variance weighted
MR Egger
4.0
3.6
3.2
2.8
2.4
1/SE_IV
−0.5
0.0
0.5
β_IV

Batch 2−899 : Gut microbiota abundance (genus Catenibacterium id.2153) on Leukocyte surface antigen CD47
with a threshold of 1e−05

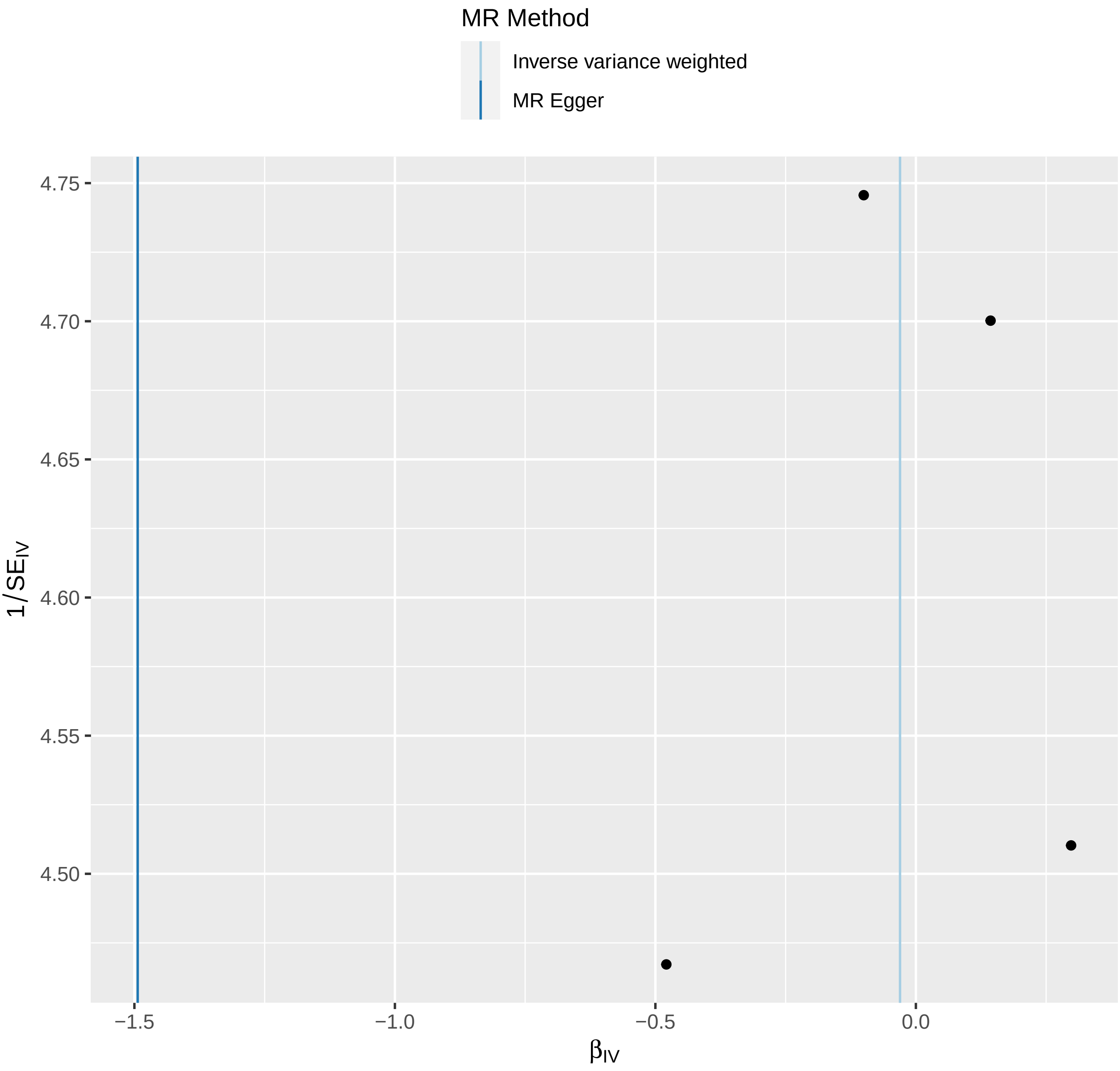

Batch 2−900 : Gut microbiota abundance (genus Parasutterella id.2892) on Leukocyte surface antigen CD47
with a threshold of 1e−05

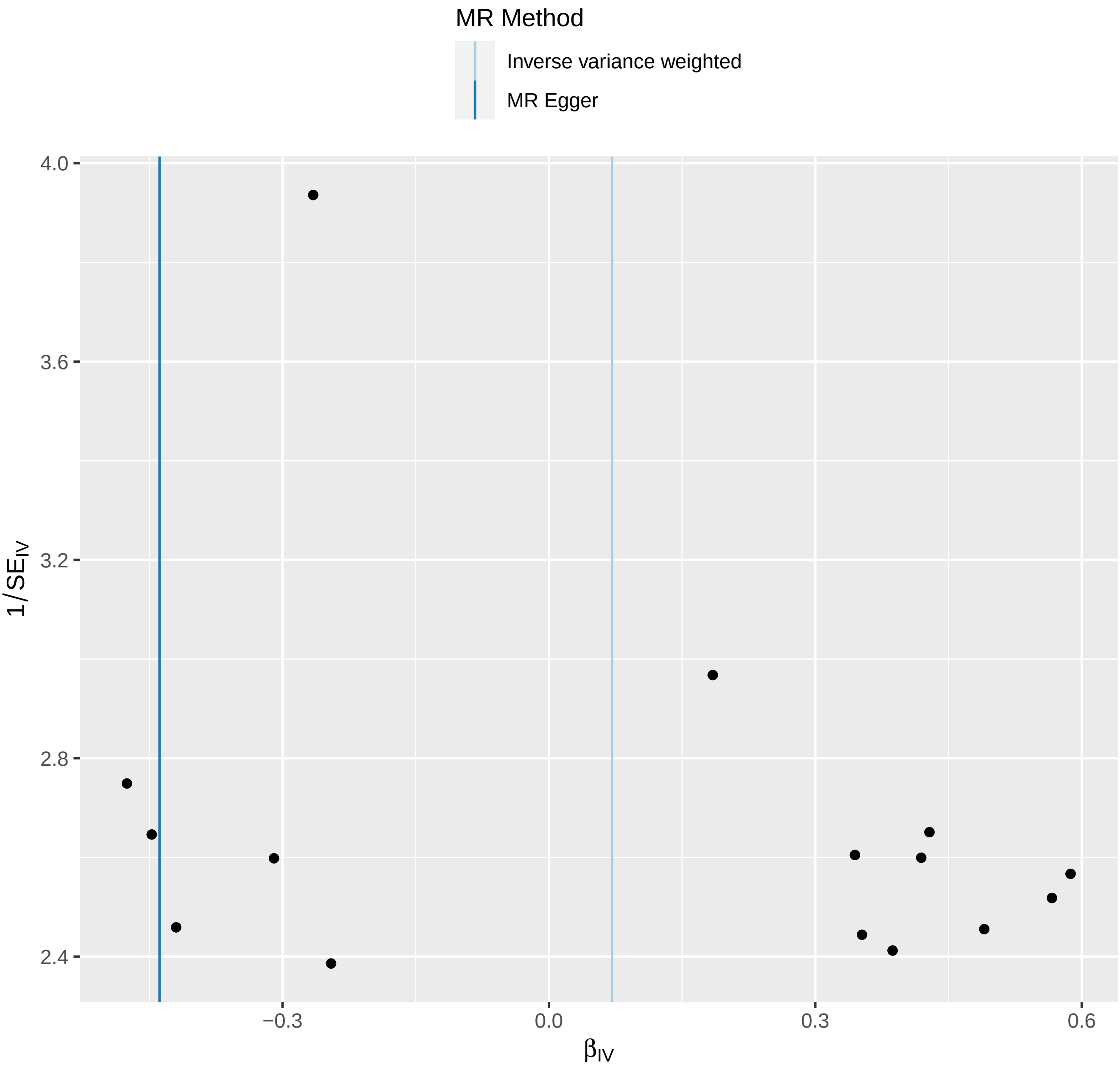

Batch 2−901 : Gut microbiota abundance (genus Catenibacterium id.2153) on CD48 antigen
with a threshold of 1e−05

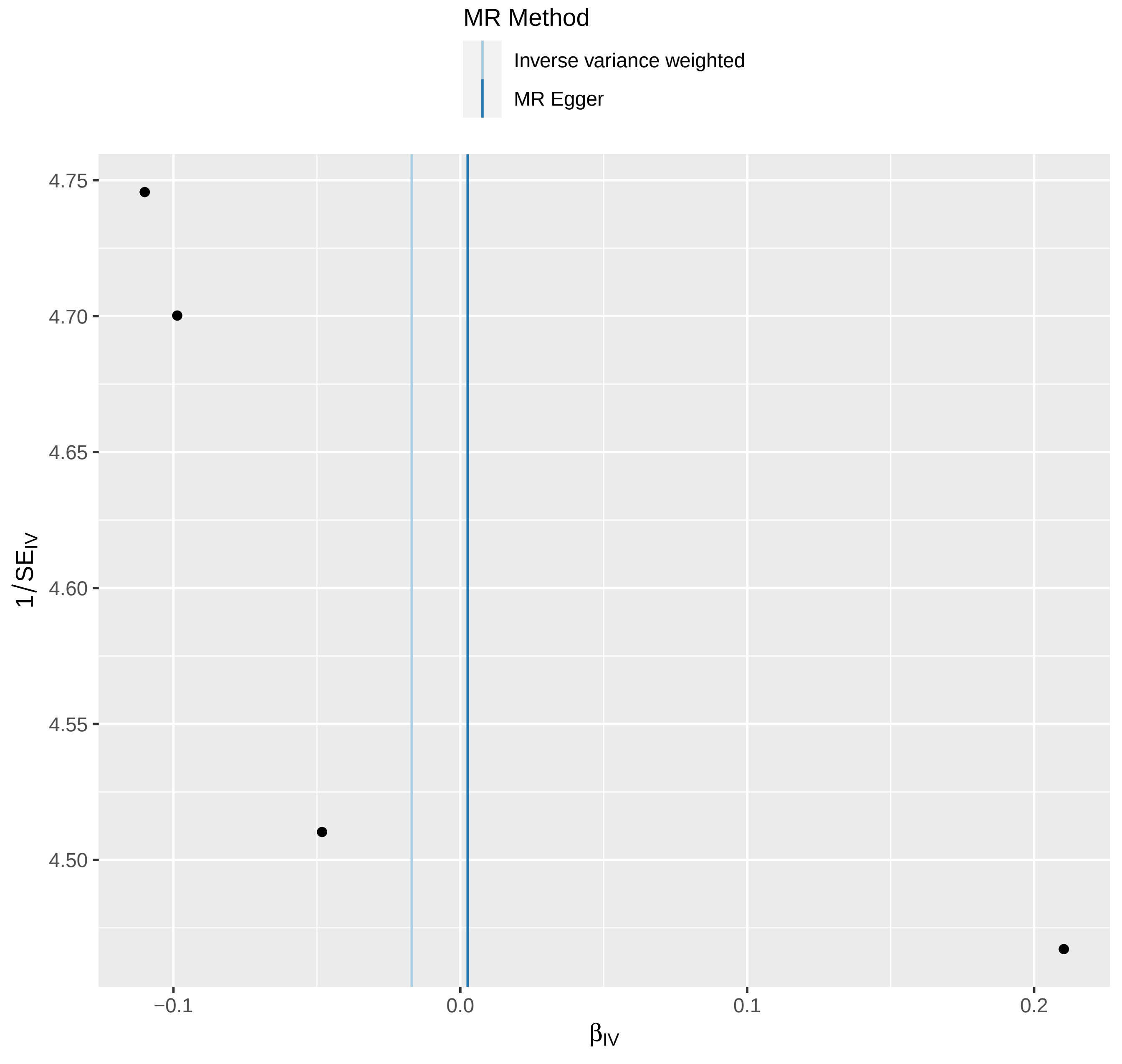

Batch 2−902 : Gut microbiota abundance (genus Parasutterella id.2892) on CD48 antigen
with a threshold of 1e−05

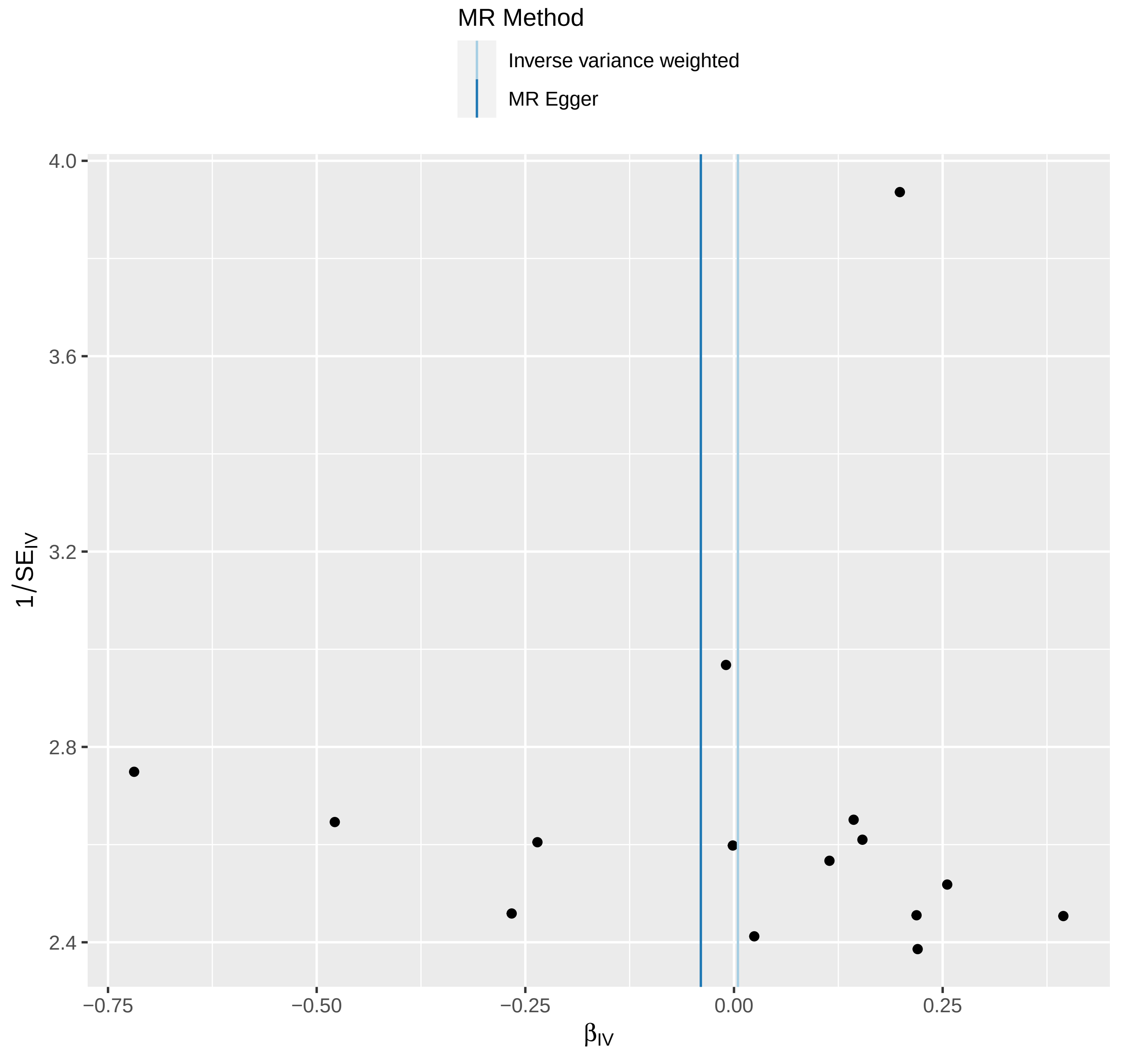

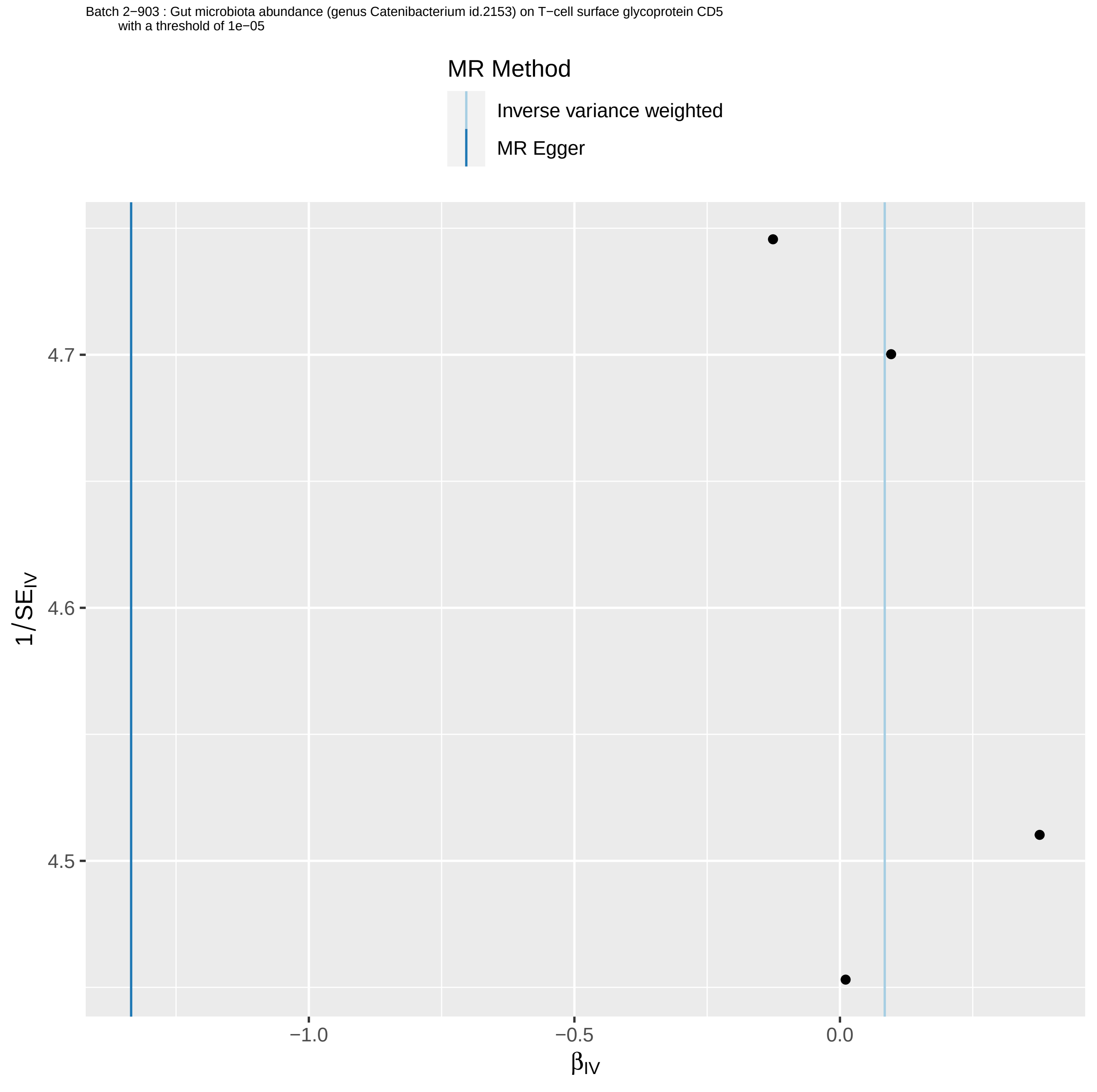
Batch 2−903 : Gut microbiota abundance (genus Catenibacterium id.2153) on T−cell surface glycoprotein CD5
with a threshold of 1e−05
MR Method
Inverse variance weighted
MR Egger
4.7
4.6
4.5
1/SE_IV
−1.0
−0.5
0.0
β_IV

Batch 2−904 : Gut microbiota abundance (genus Parasutterella id.2892) on T−cell surface glycoprotein CD5
with a threshold of 1e−05

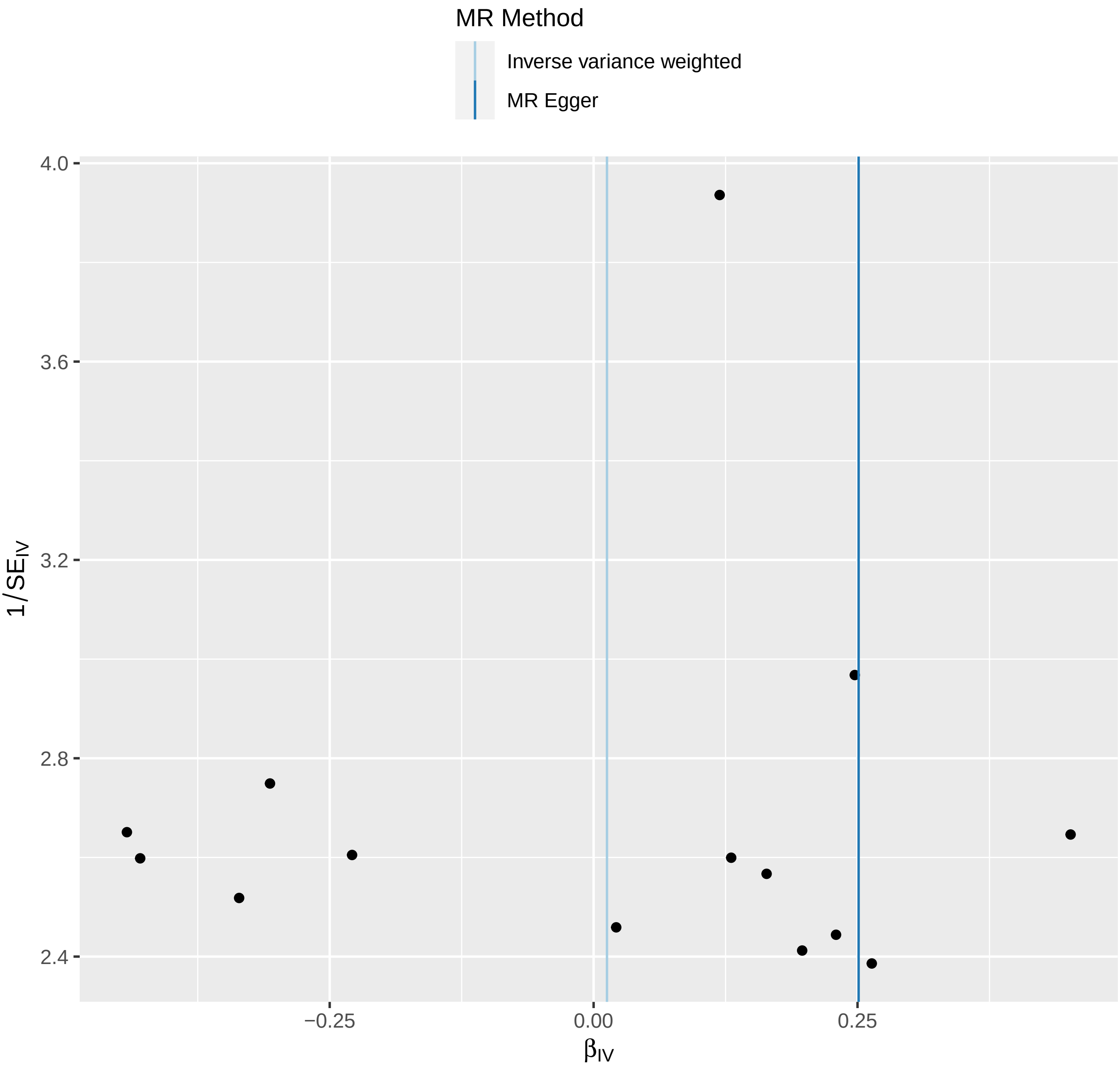

Batch 2−905 : Gut microbiota abundance (genus Catenibacterium id.2153) on Complement decay−accelerating factor
with a threshold of 1e−05

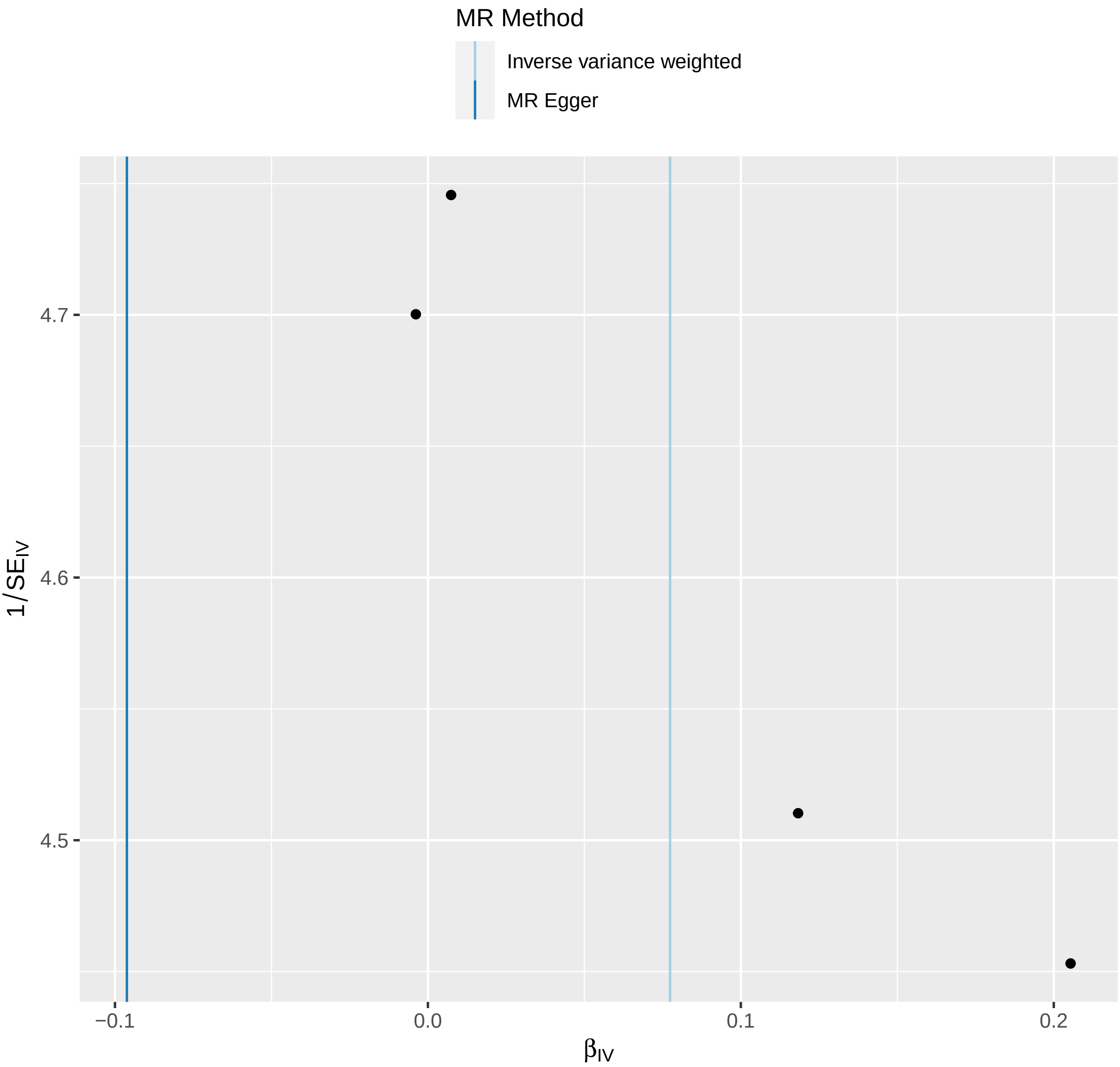

Batch 2−906 : Gut microbiota abundance (genus Parasutterella id.2892) on Complement decay−accelerating factor
with a threshold of 1e−05

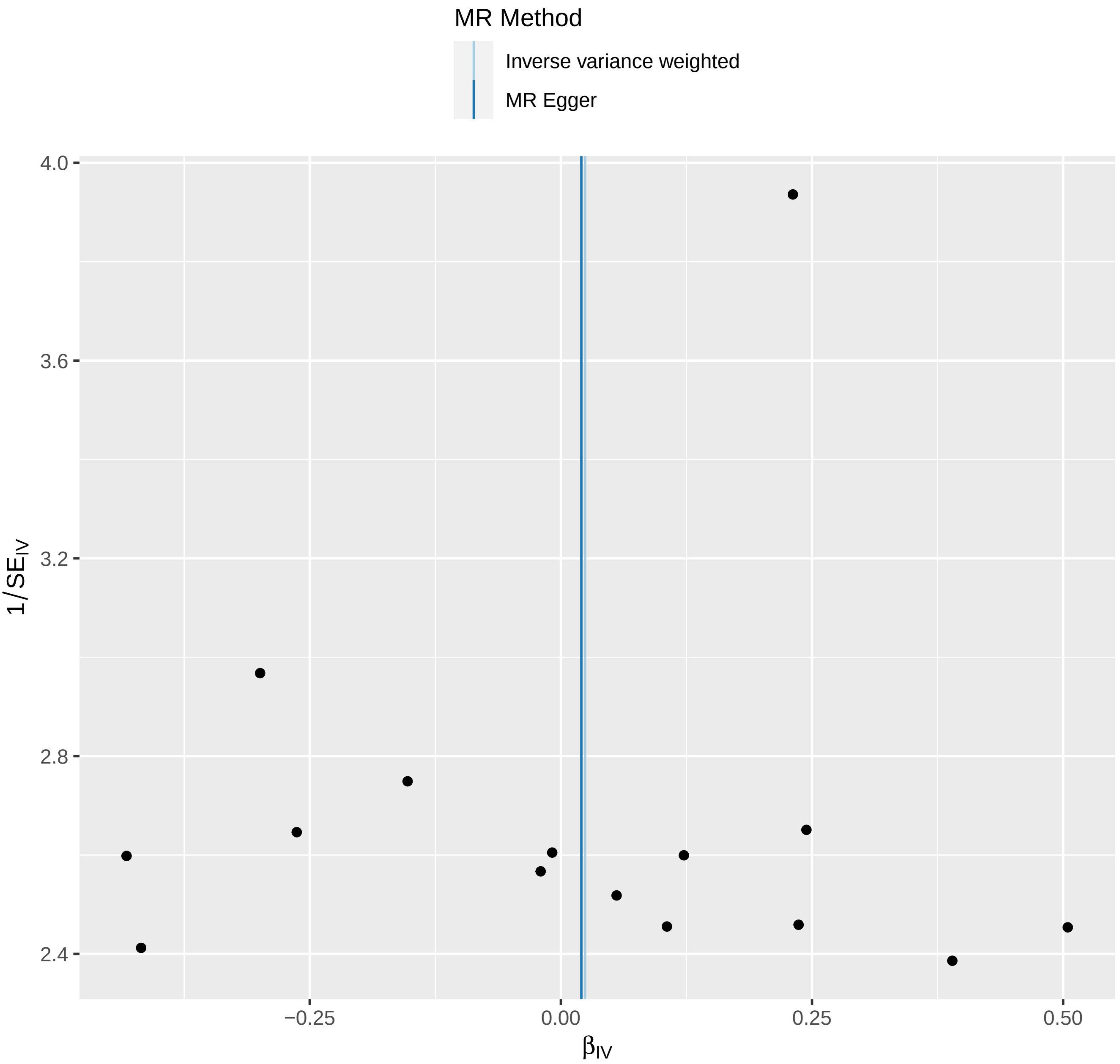

Batch 2−907 : Gut microbiota abundance (genus Catenibacterium id.2153) on Lymphocyte function−associated antigen 3 with a threshold of 1e−05

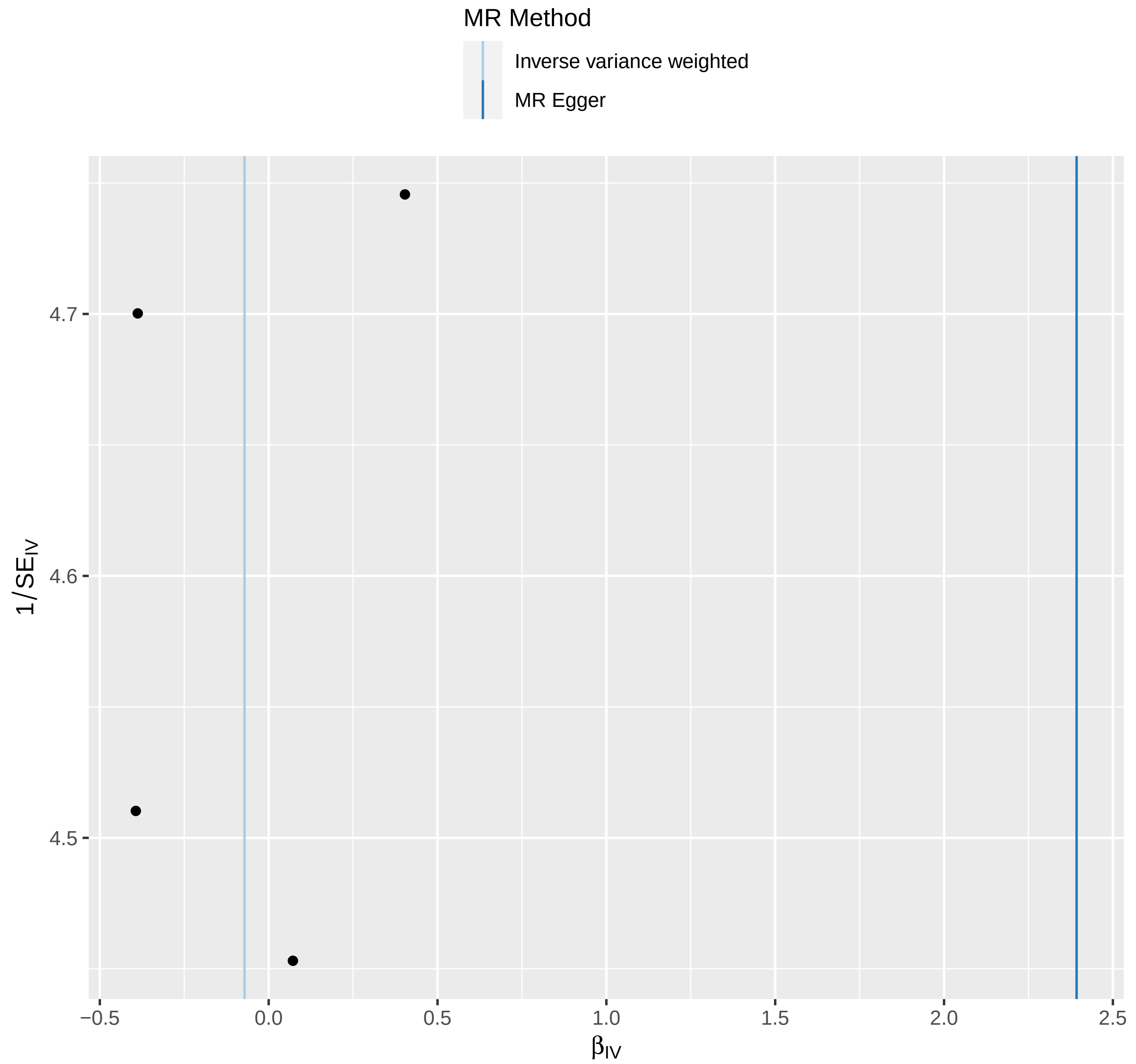

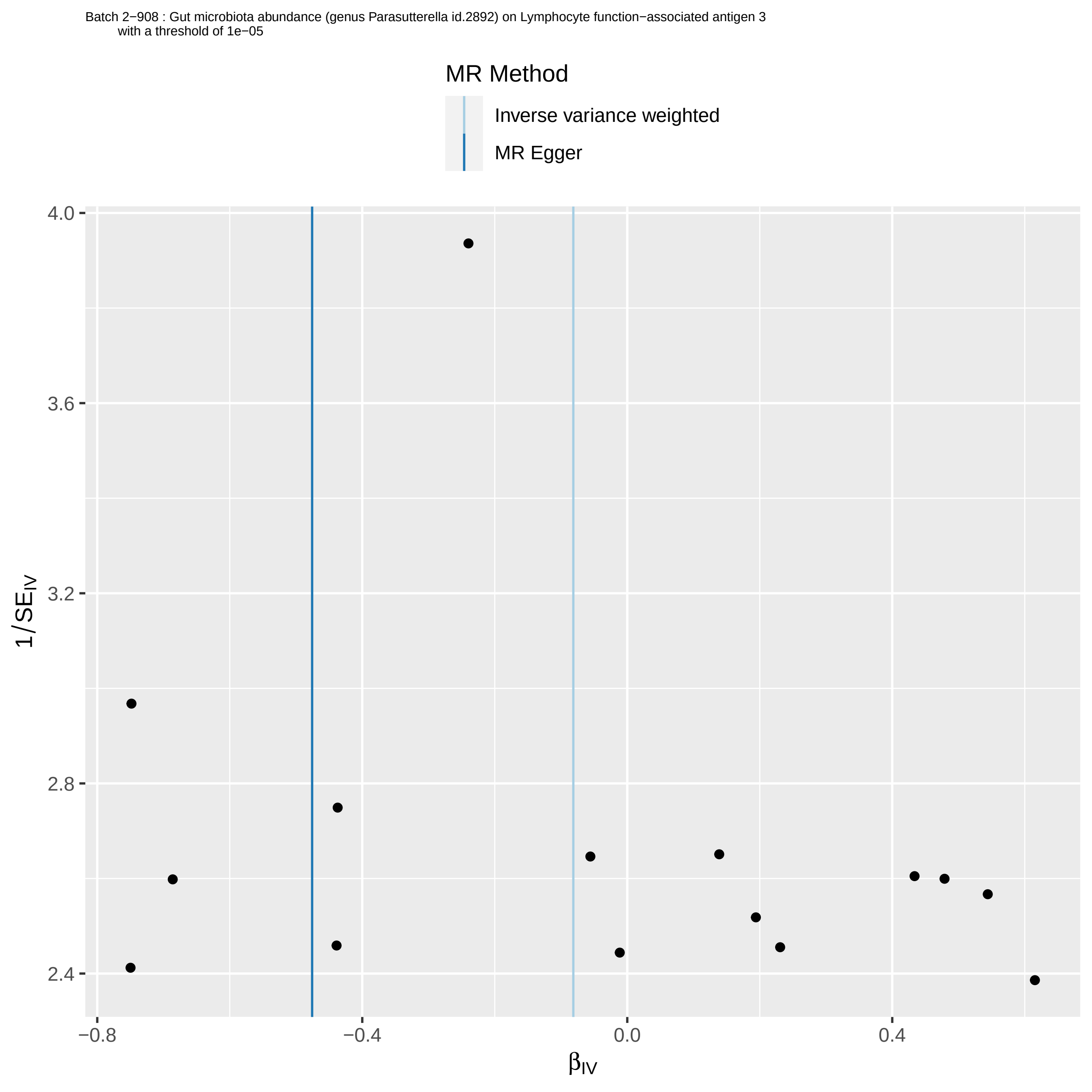

Batch 2−908 : Gut microbiota abundance (genus Parasutterella id.2892) on Lymphocyte function−associated antigen 3
with a threshold of 1e−05
MR Method
Inverse variance weighted
MR Egger
4.0
3.6
3.2
2.8
2.4
1/SE$_{IV}$
−0.8
−0.4
0.0
0.4
β$_{IV}$

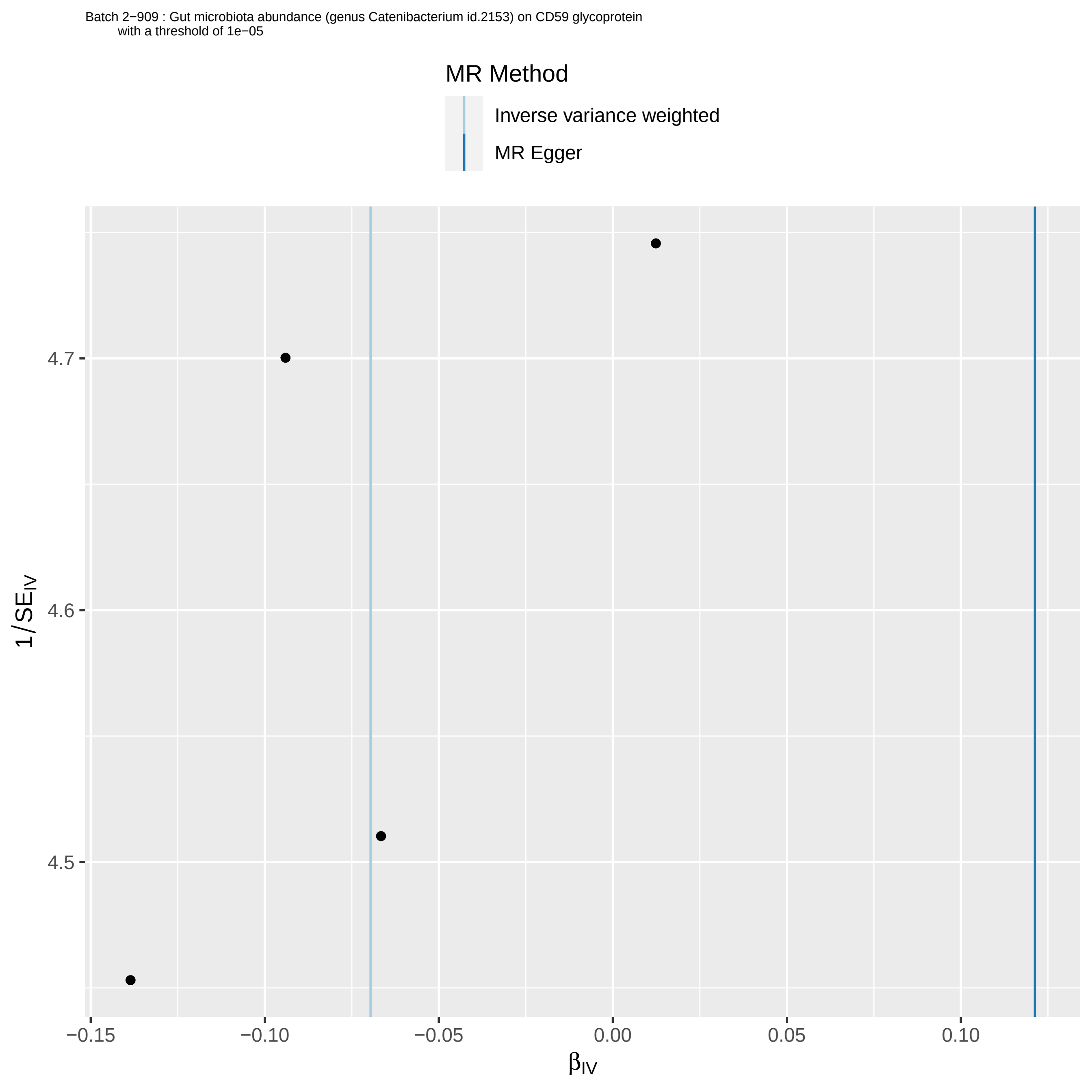

Batch 2−909 : Gut microbiota abundance (genus Catenibacterium id.2153) on CD59 glycoprotein
with a threshold of 1e−05
MR Method
Inverse variance weighted
MR Egger
4.7
4.6
4.5
1/SE$_{IV}$
−0.15
−0.10
−0.05
0.00
0.05
0.10
β$_{IV}$

Batch 2−910 : Gut microbiota abundance (genus Parasutterella id.2892) on CD59 glycoprotein
with a threshold of 1e−05

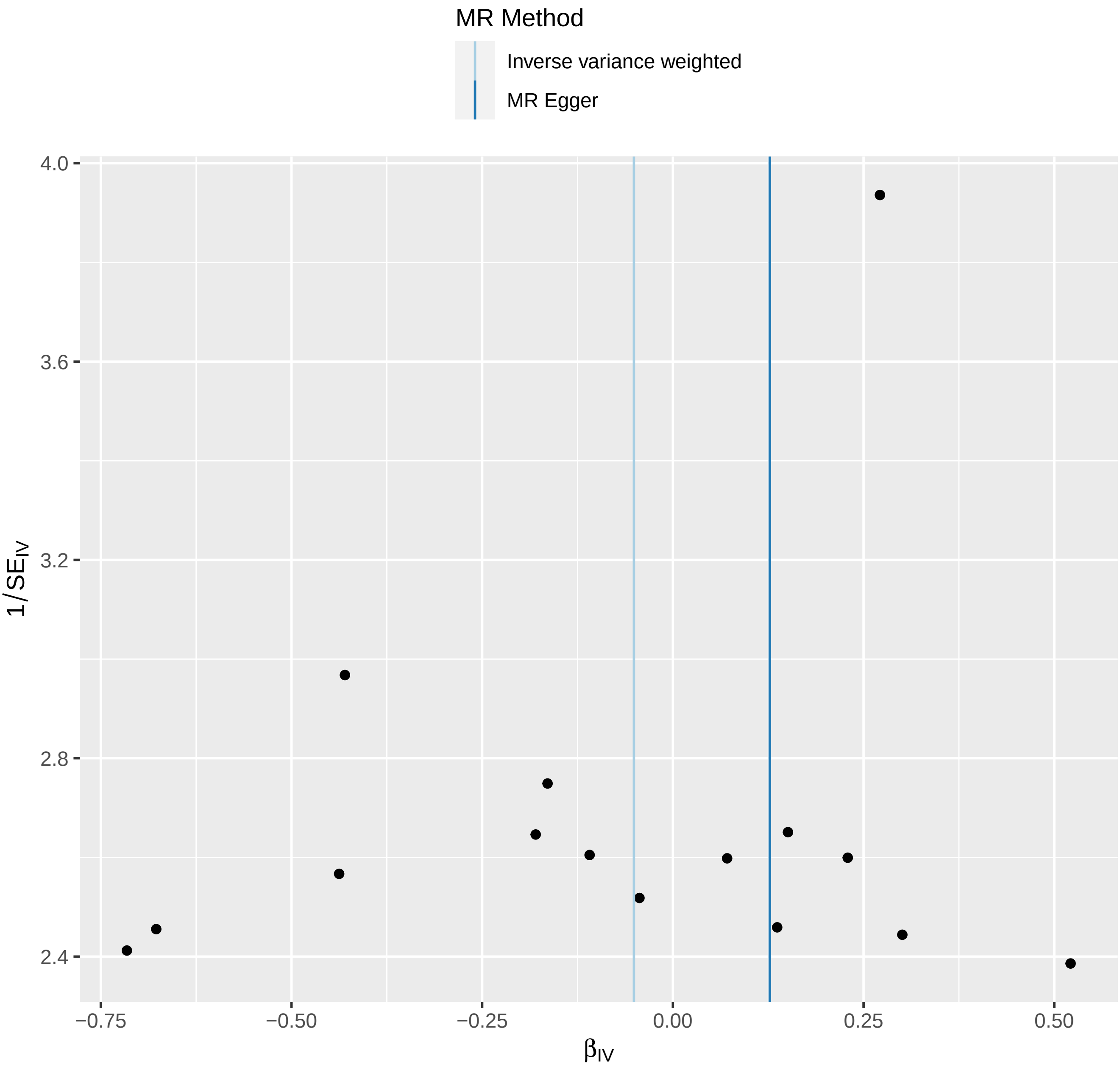

Batch 2−911 : Gut microbiota abundance (genus Catenibacterium id.2153) on CD63 antigen
with a threshold of 1e−05

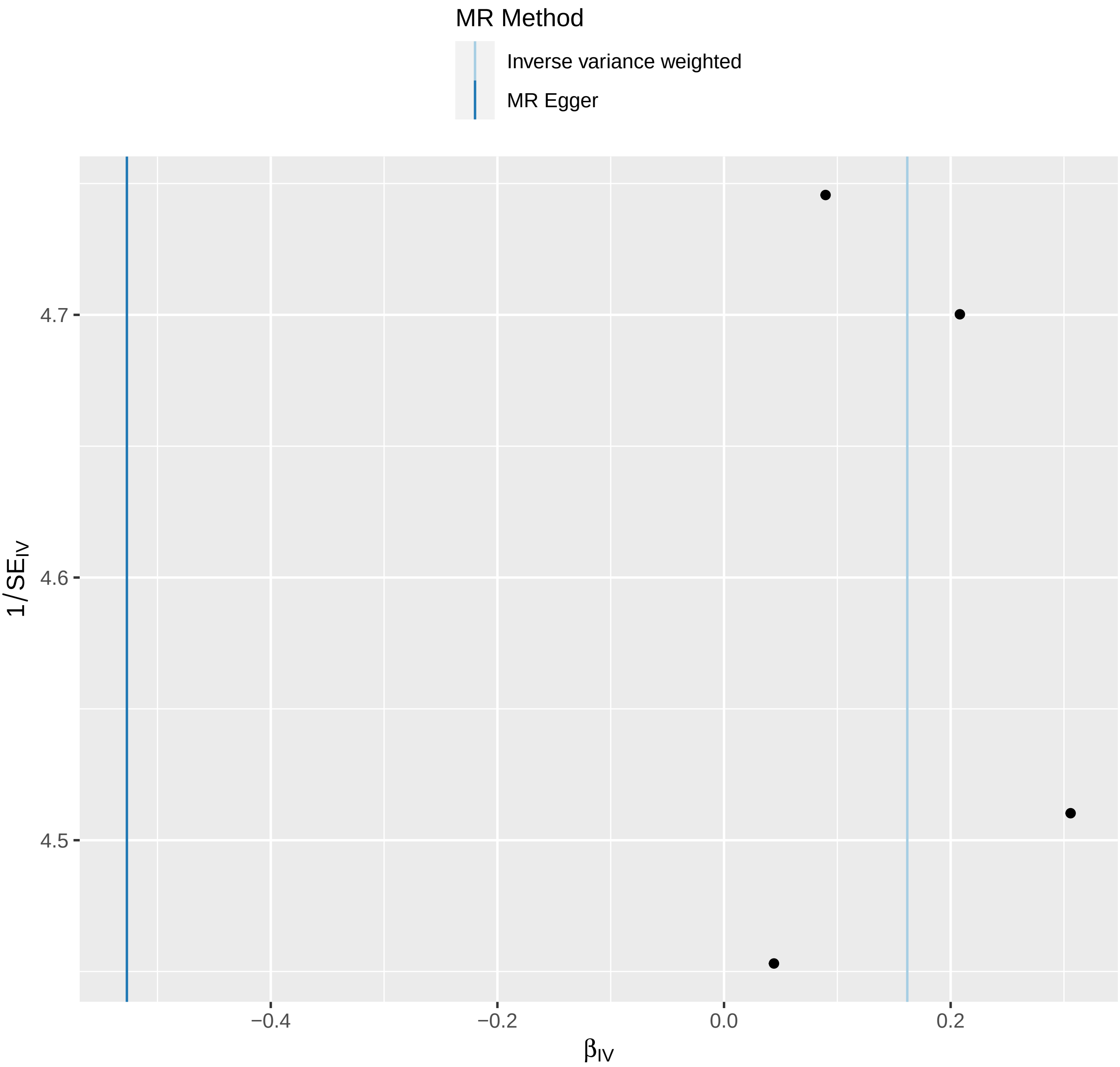

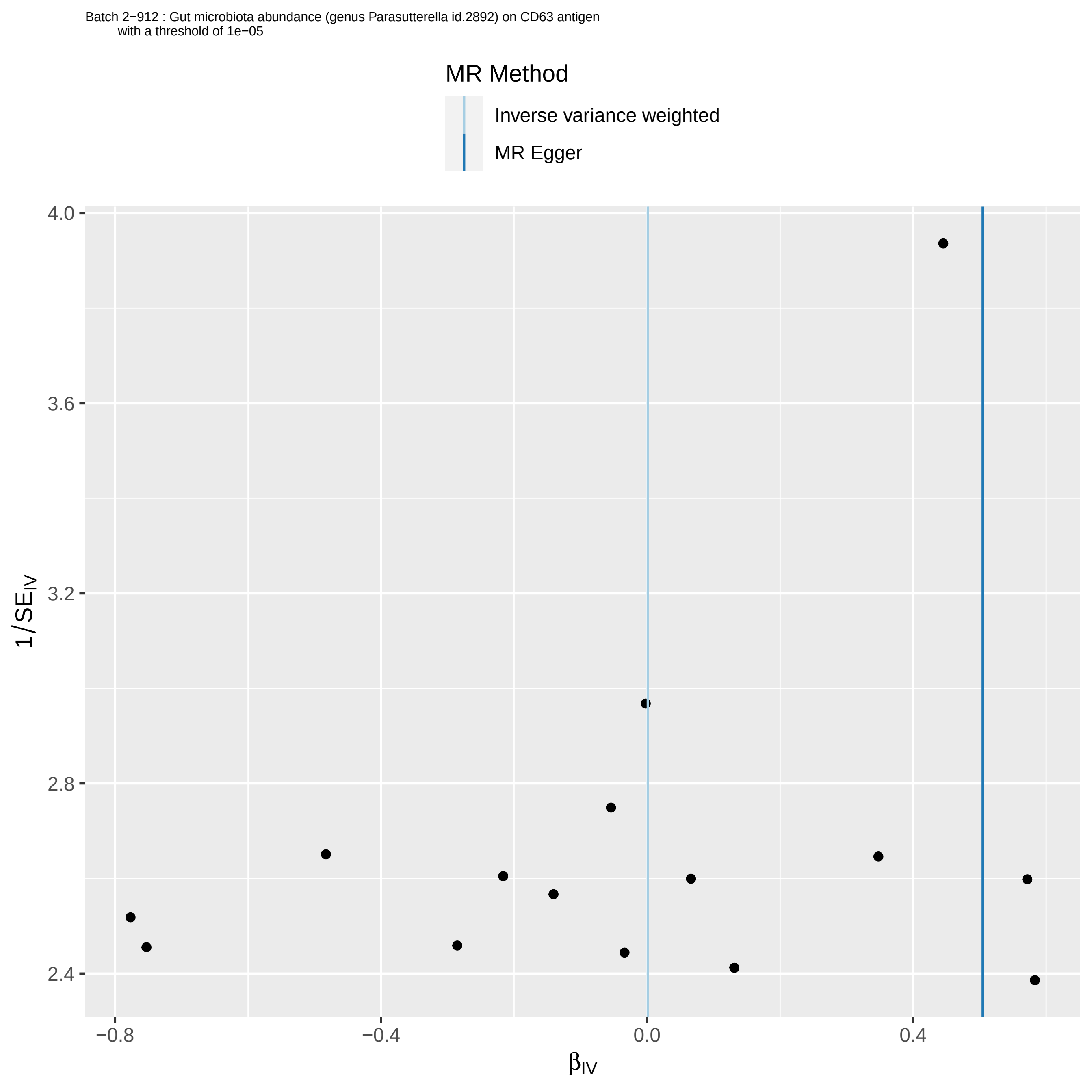
Batch 2−912 : Gut microbiota abundance (genus Parasutterella id.2892) on CD63 antigen
with a threshold of 1e−05
MR Method
Inverse variance weighted
MR Egger
4.0
3.6
3.2
2.8
2.4
1/SE_IV
−0.8
−0.4
0.0
0.4
β_IV

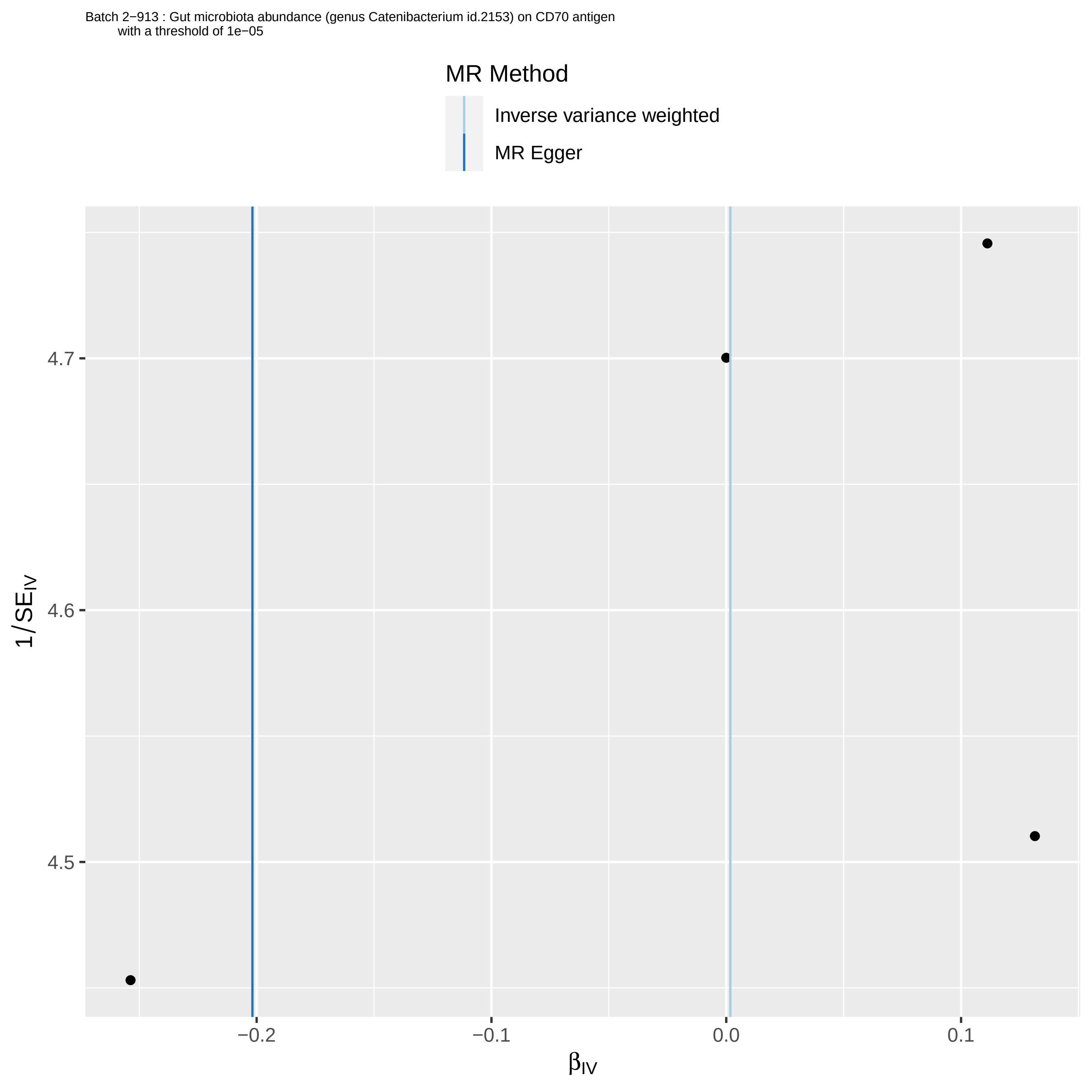

Batch 2−913 : Gut microbiota abundance (genus Catenibacterium id.2153) on CD70 antigen
with a threshold of 1e−05
MR Method
Inverse variance weighted
MR Egger
4.7
4.6
4.5
$1/SE_{IV}$
−0.2
−0.1
0.0
0.1
β_{IV}

Batch 2−914 : Gut microbiota abundance (genus Parasutterella id.2892) on CD70 antigen
with a threshold of 1e−05

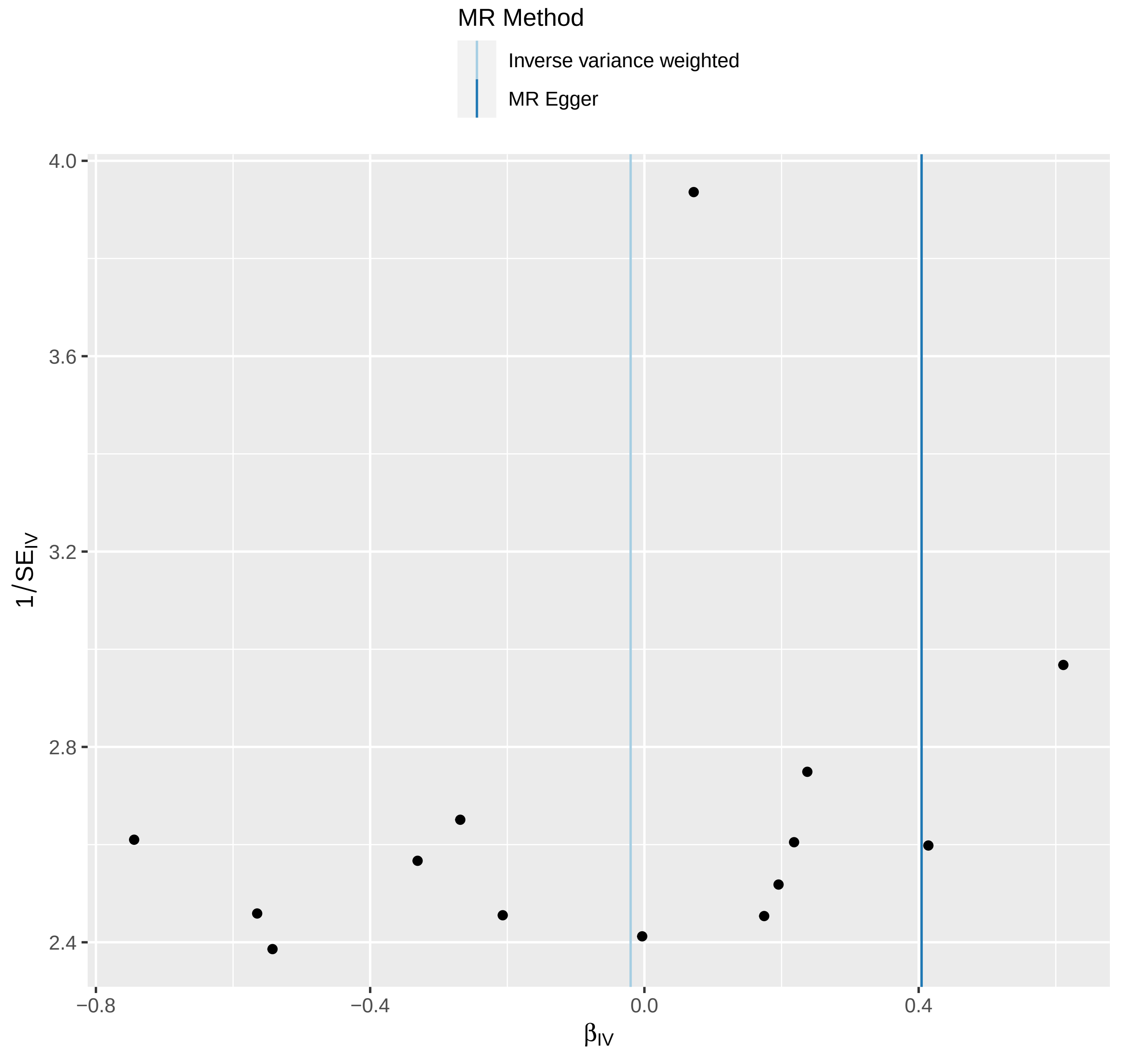

Batch 2−915 : Gut microbiota abundance (genus Catenibacterium id.2153) on CD70 antigen
with a threshold of 1e−05

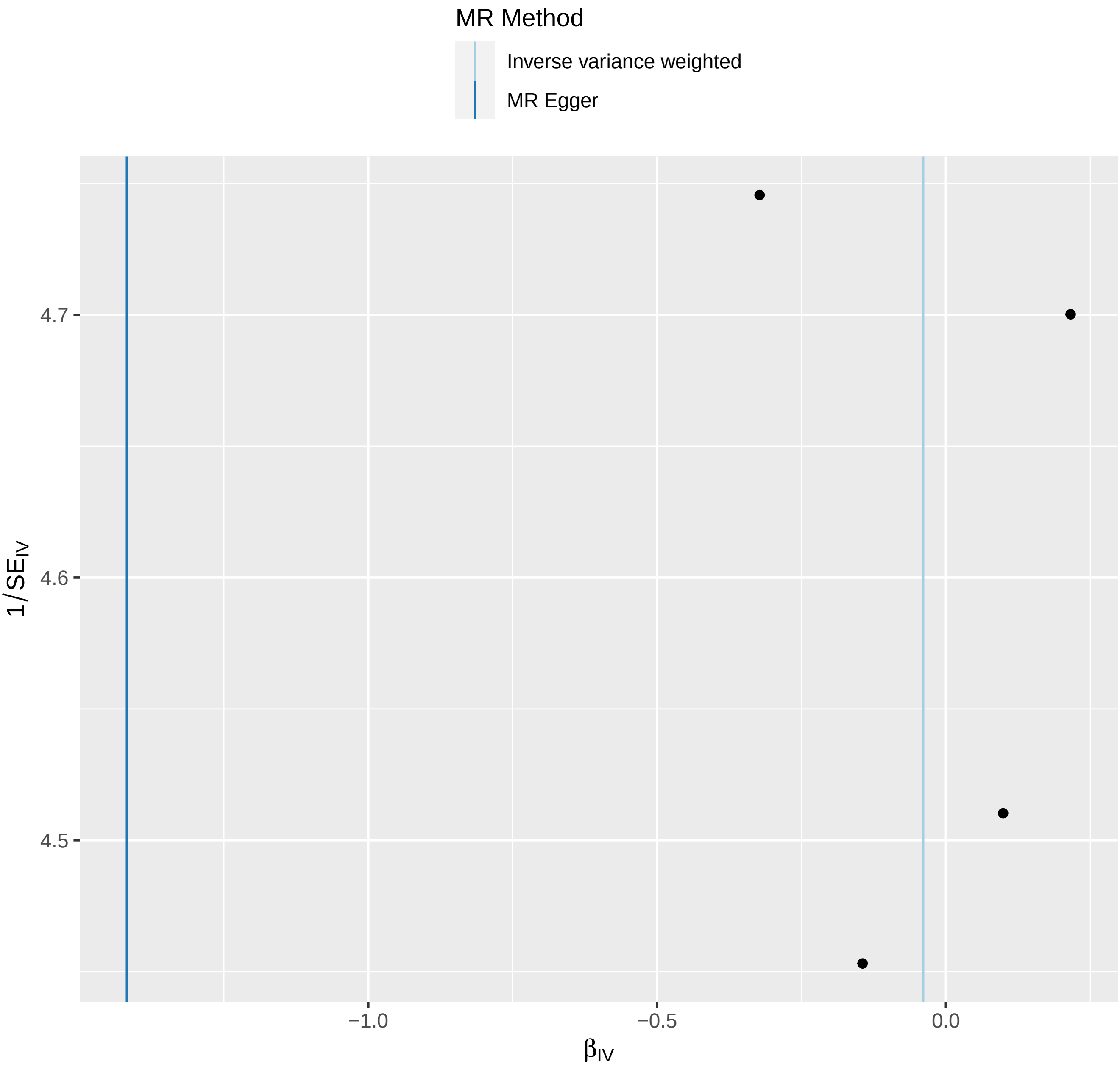

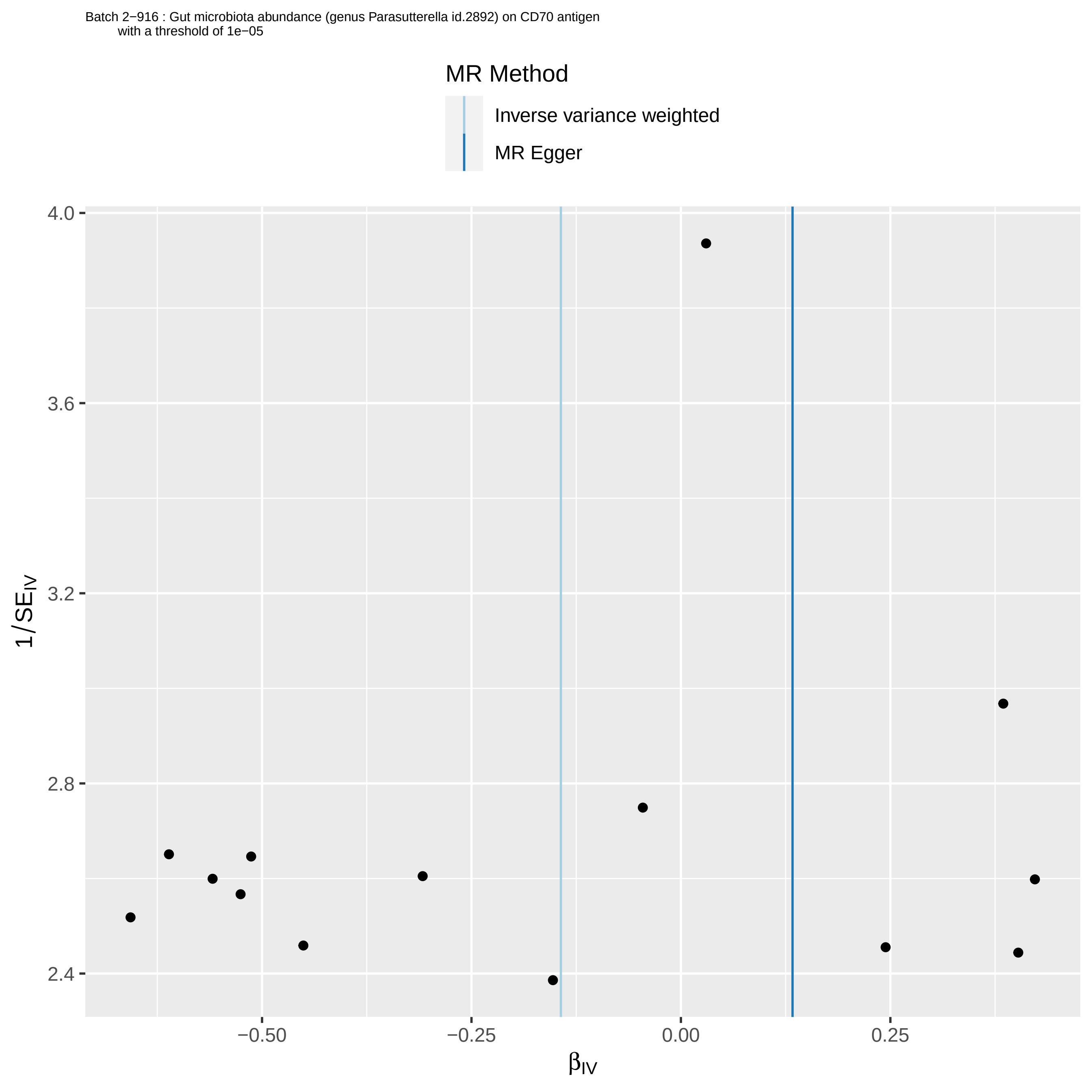
Batch 2−916 : Gut microbiota abundance (genus Parasutterella id.2892) on CD70 antigen
with a threshold of 1e−05
MR Method
Inverse variance weighted
MR Egger
4.0
3.6
3.2
2.8
2.4
1/SE$_{IV}$
−0.50
−0.25
0.00
0.25
β_{IV}

Batch 2−917 : Gut microbiota abundance (genus Catenibacterium id.2153) on HLA class II histocompatibility antigen gamma chain with a threshold of 1e−05

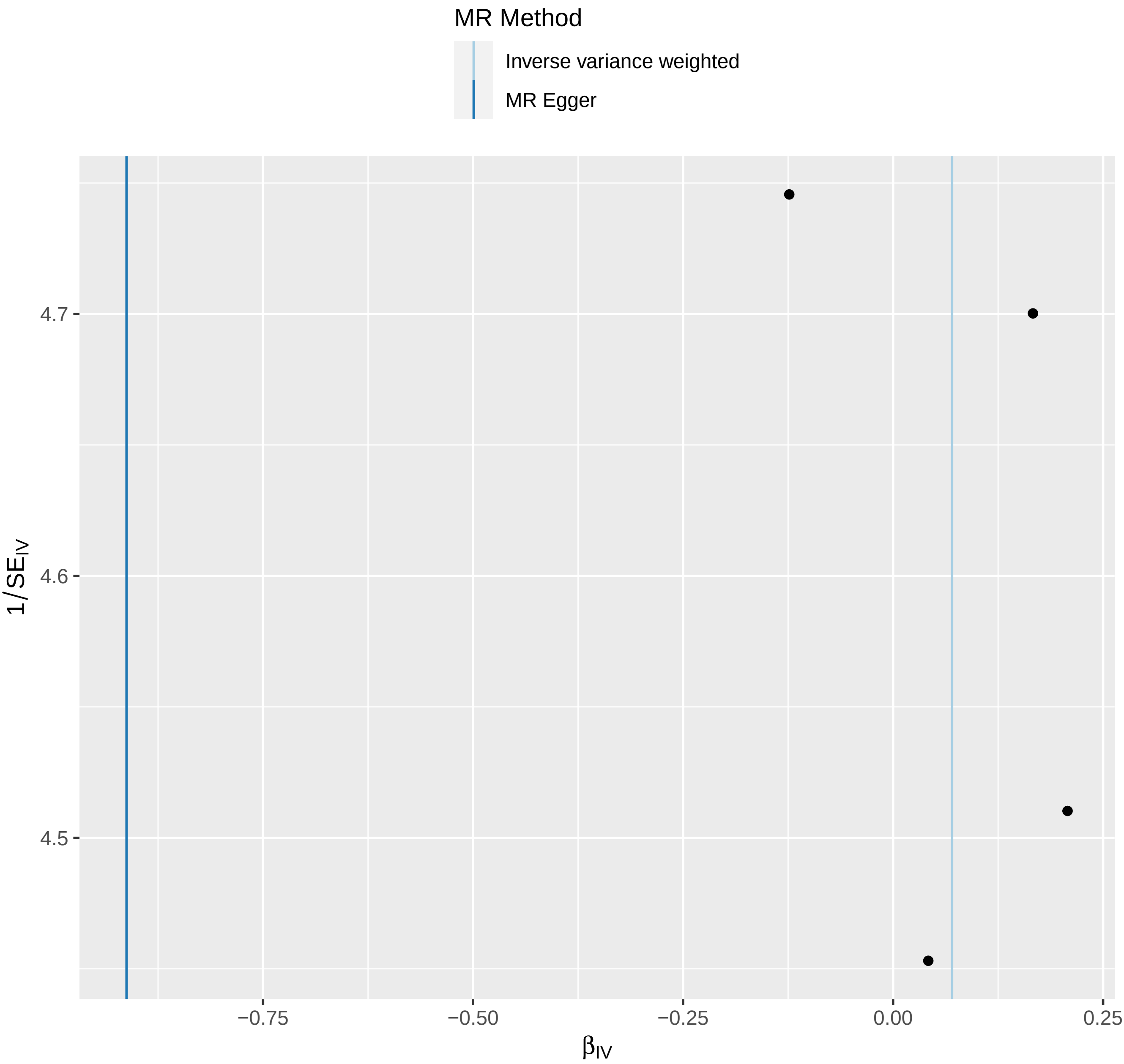

Batch 2−918 : Gut microbiota abundance (genus Parasutterella id.2892) on HLA class II histocompatibility antigen gamma chain with a threshold of 1e−05

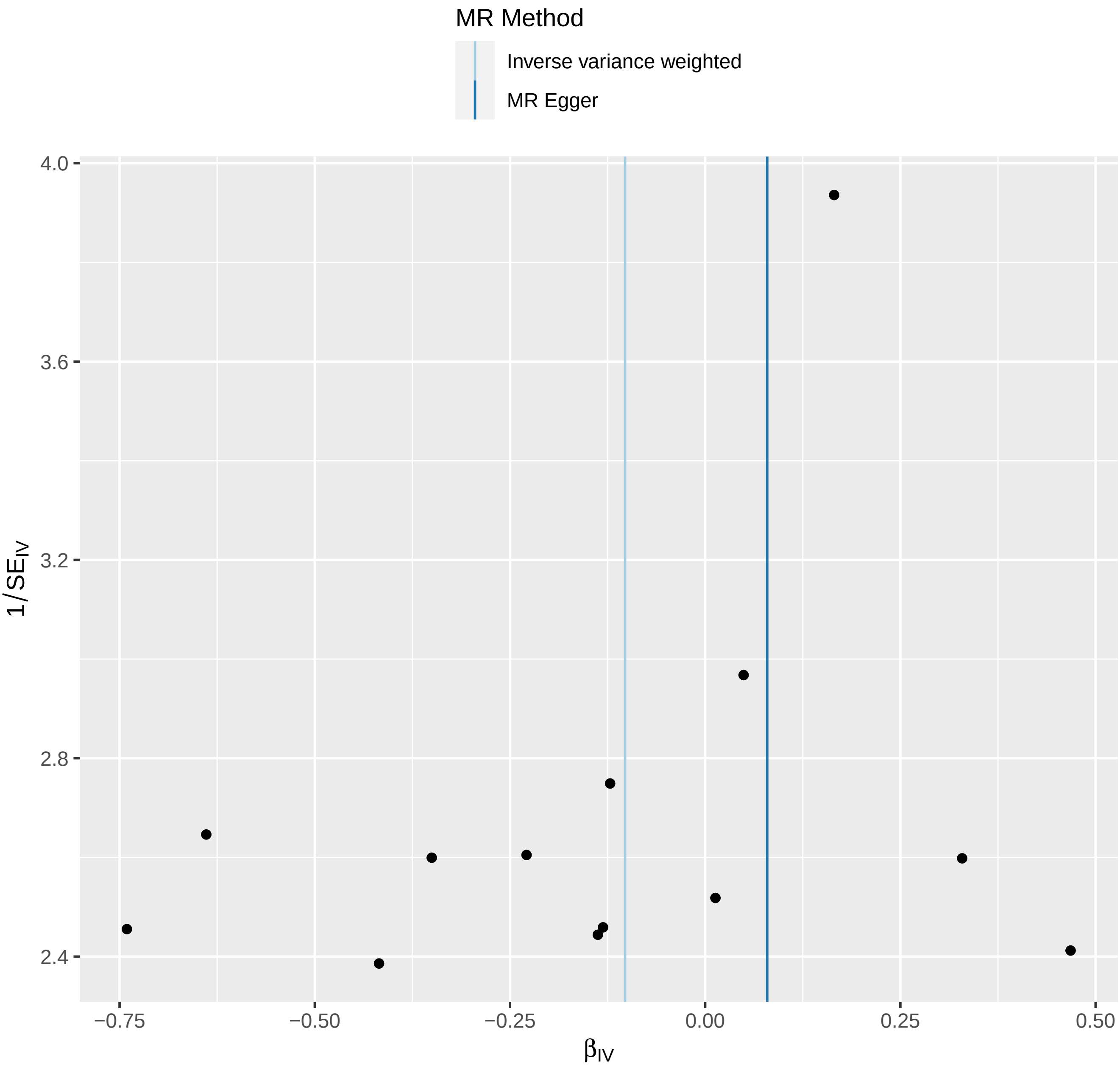

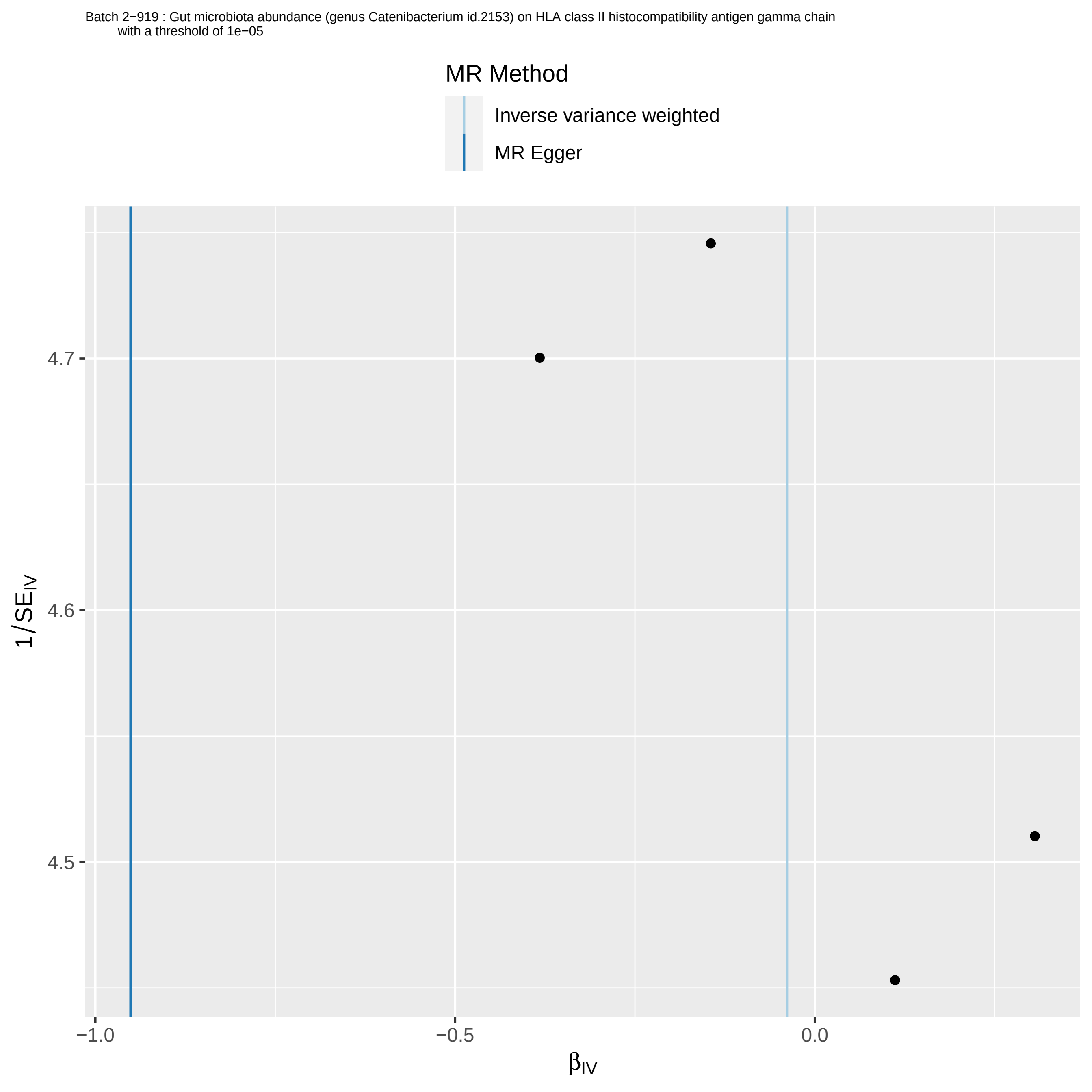

Batch 2−919 : Gut microbiota abundance (genus Catenibacterium id.2153) on HLA class II histocompatibility antigen gamma chain
with a threshold of 1e−05
MR Method
Inverse variance weighted
MR Egger
4.7
4.6
4.5
1/SE_IV
−1.0
−0.5
0.0
β_IV

Batch 2−920 : Gut microbiota abundance (genus Parasutterella id.2892) on HLA class II histocompatibility antigen gamma chain
with a threshold of 1e−05

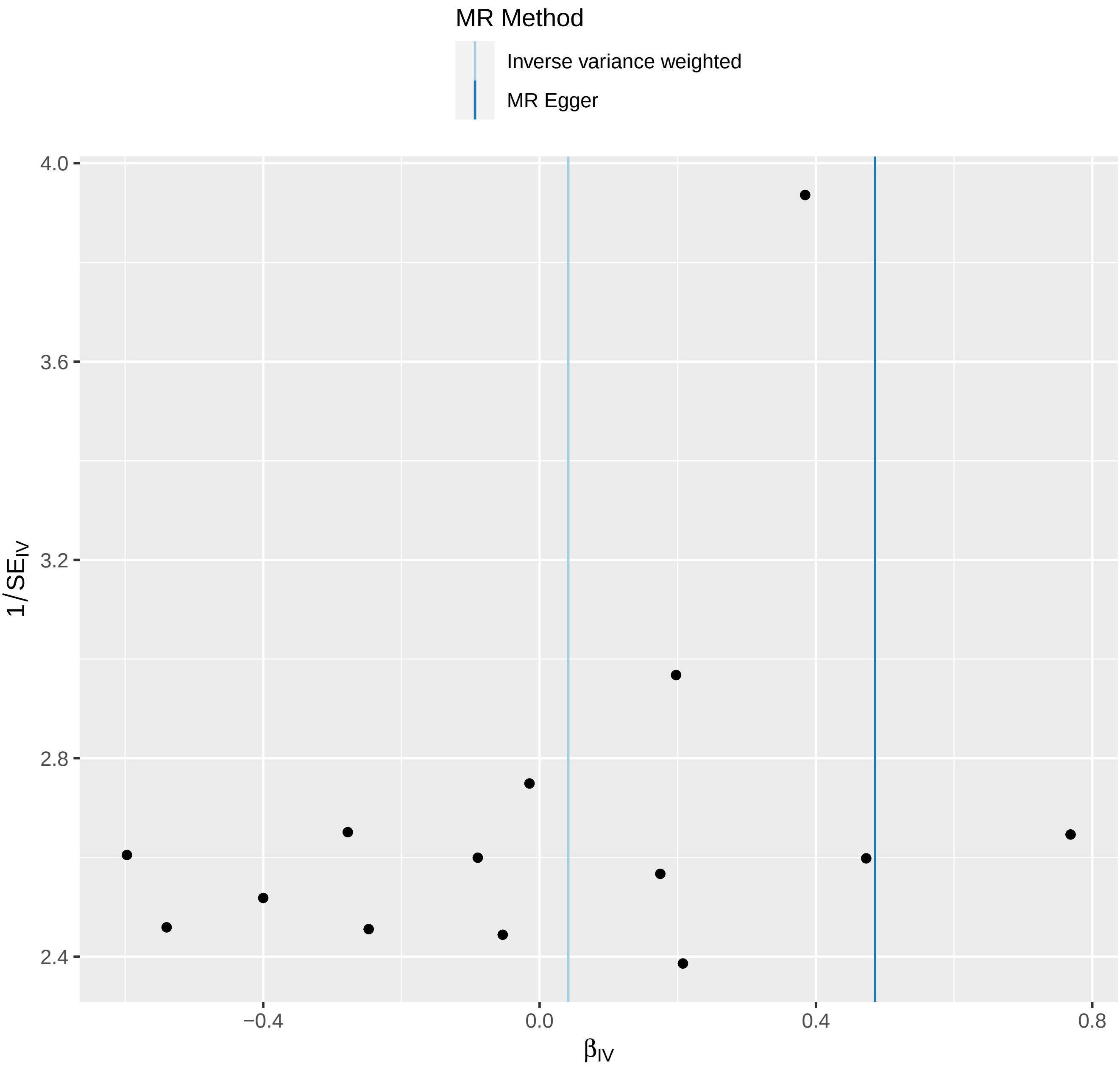

Batch 2−921 : Gut microbiota abundance (genus Catenibacterium id.2153) on B−cell antigen receptor complex−associated protein alpha chain with a threshold of 1e−05

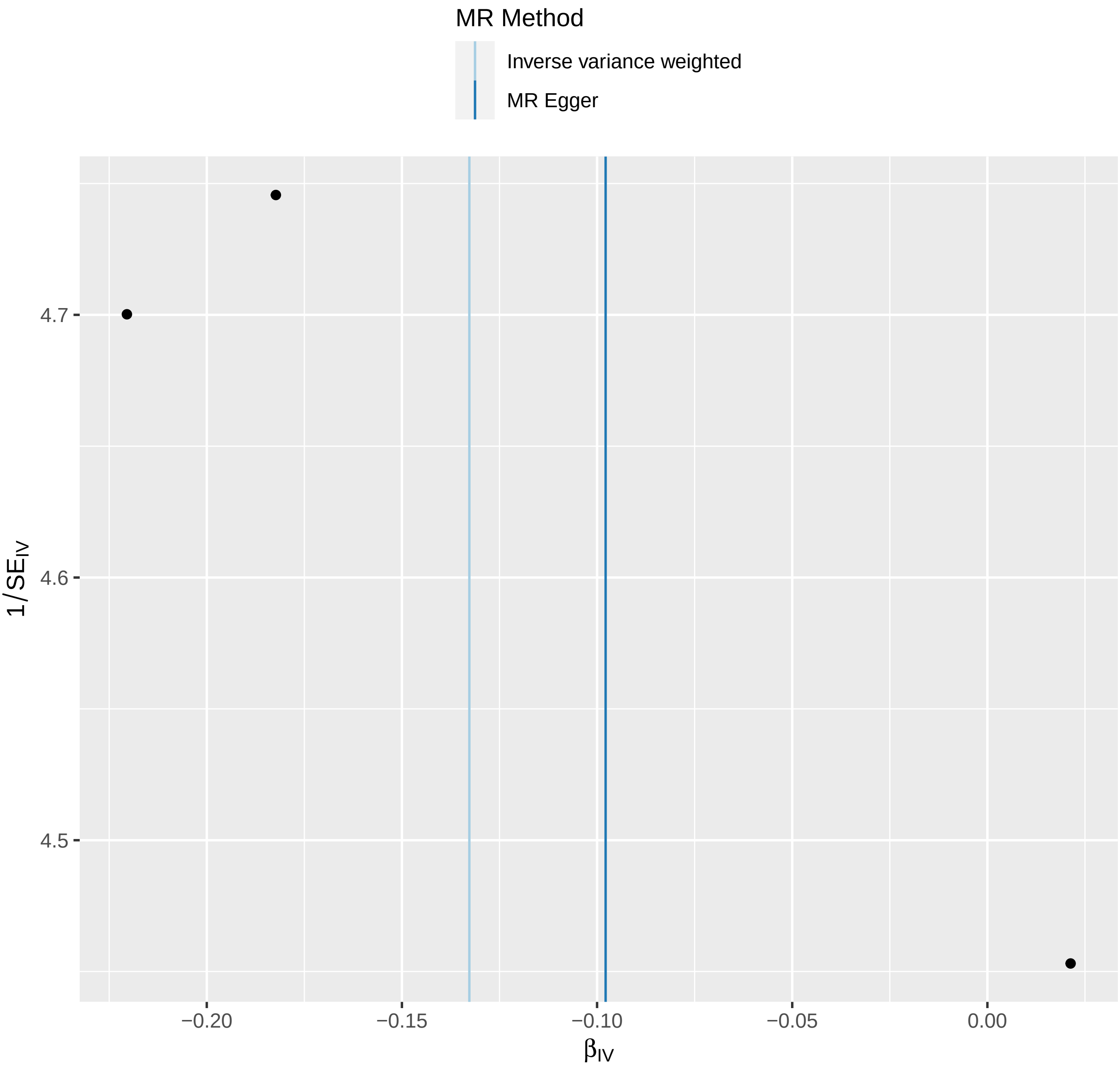

Batch 2−922 : Gut microbiota abundance (genus Parasutterella id.2892) on B−cell antigen receptor complex−associated protein alpha chain
with a threshold of 1e−05

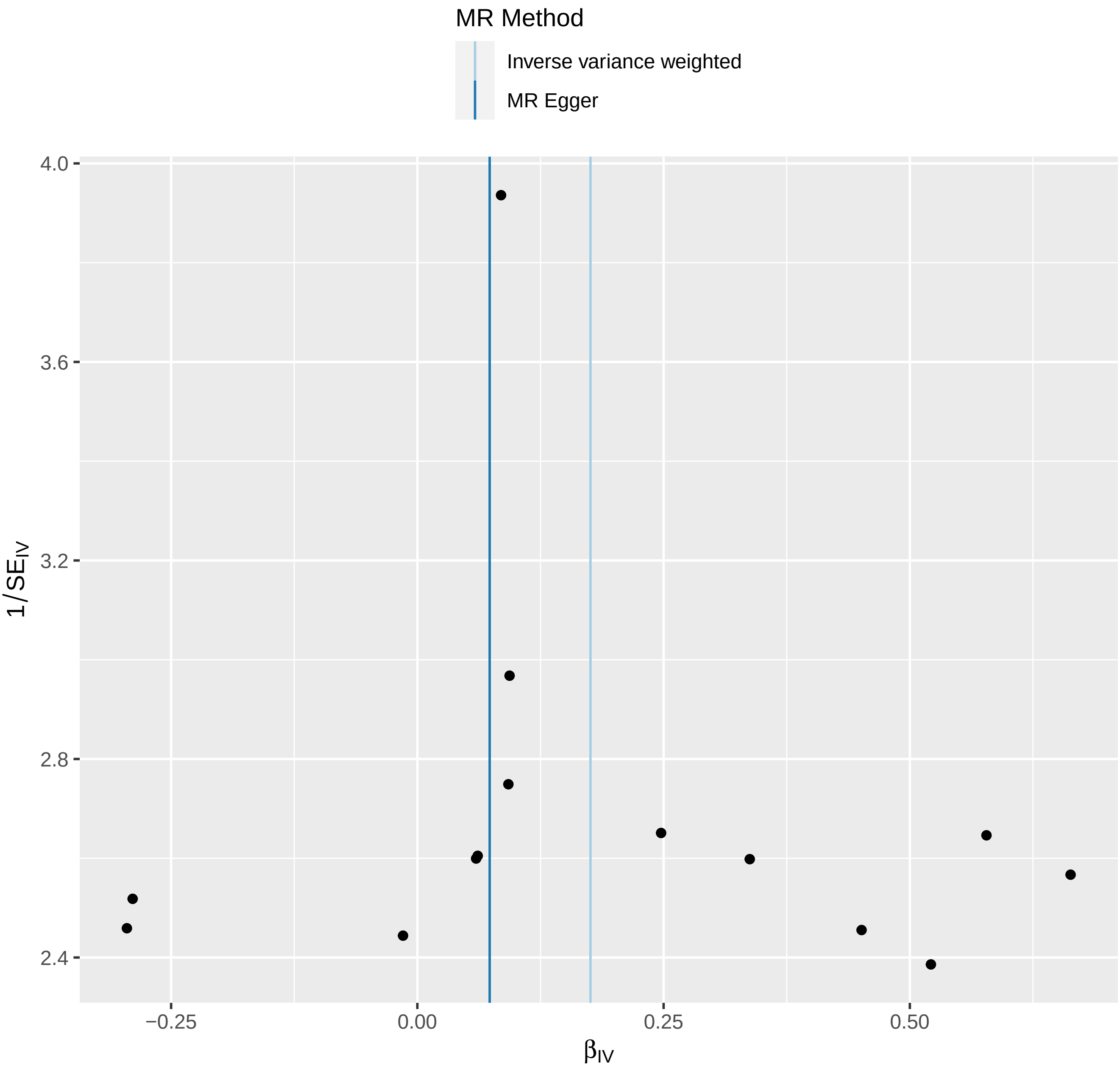

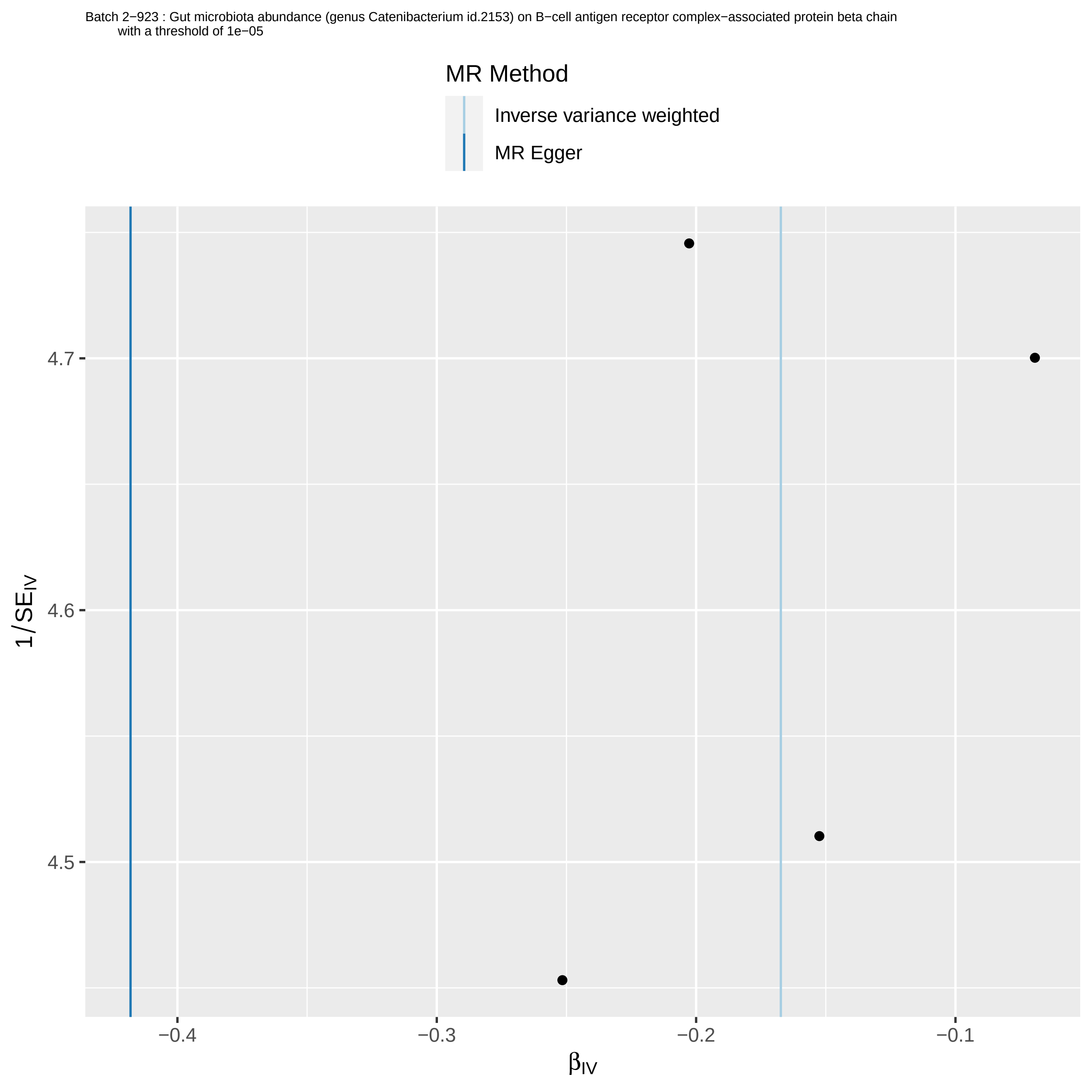

Batch 2−923 : Gut microbiota abundance (genus Catenibacterium id.2153) on B−cell antigen receptor complex−associated protein beta chain
with a threshold of 1e−05
MR Method
Inverse variance weighted
MR Egger
4.7
4.6
4.5
1/SE_IV
−0.4
−0.3
−0.2
−0.1
β_IV

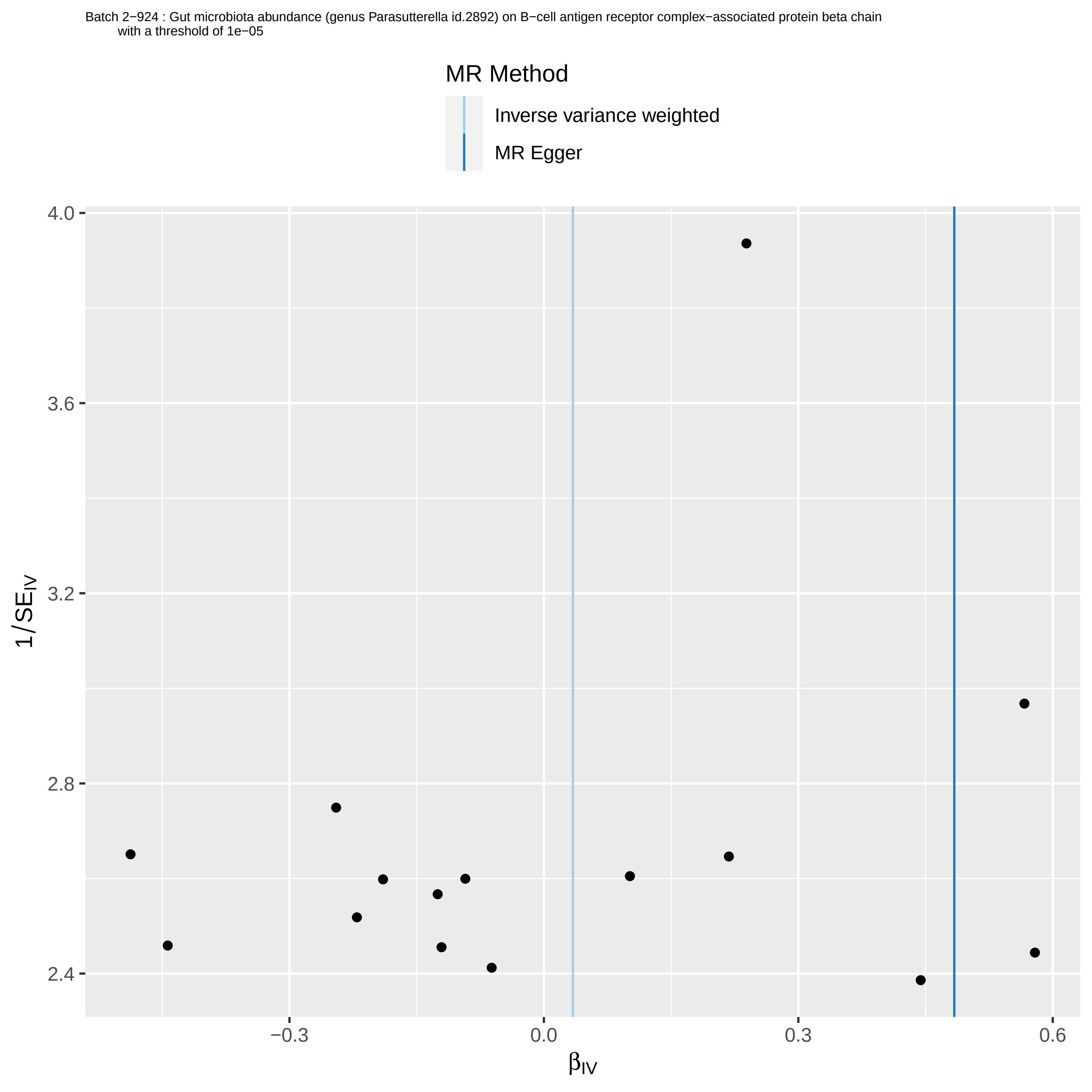
Batch 2−924 : Gut microbiota abundance (genus Parasutterella id.2892) on B−cell antigen receptor complex−associated protein beta chain
with a threshold of 1e−05
MR Method
Inverse variance weighted
MR Egger
4.0
3.6
3.2
2.8
2.4
1/SE_IV
−0.3
0.0
0.3
0.6
β_IV

Batch 2−925 : Gut microbiota abundance (genus Catenibacterium id.2153) on T−lymphocyte activation antigen CD80
with a threshold of 1e−05

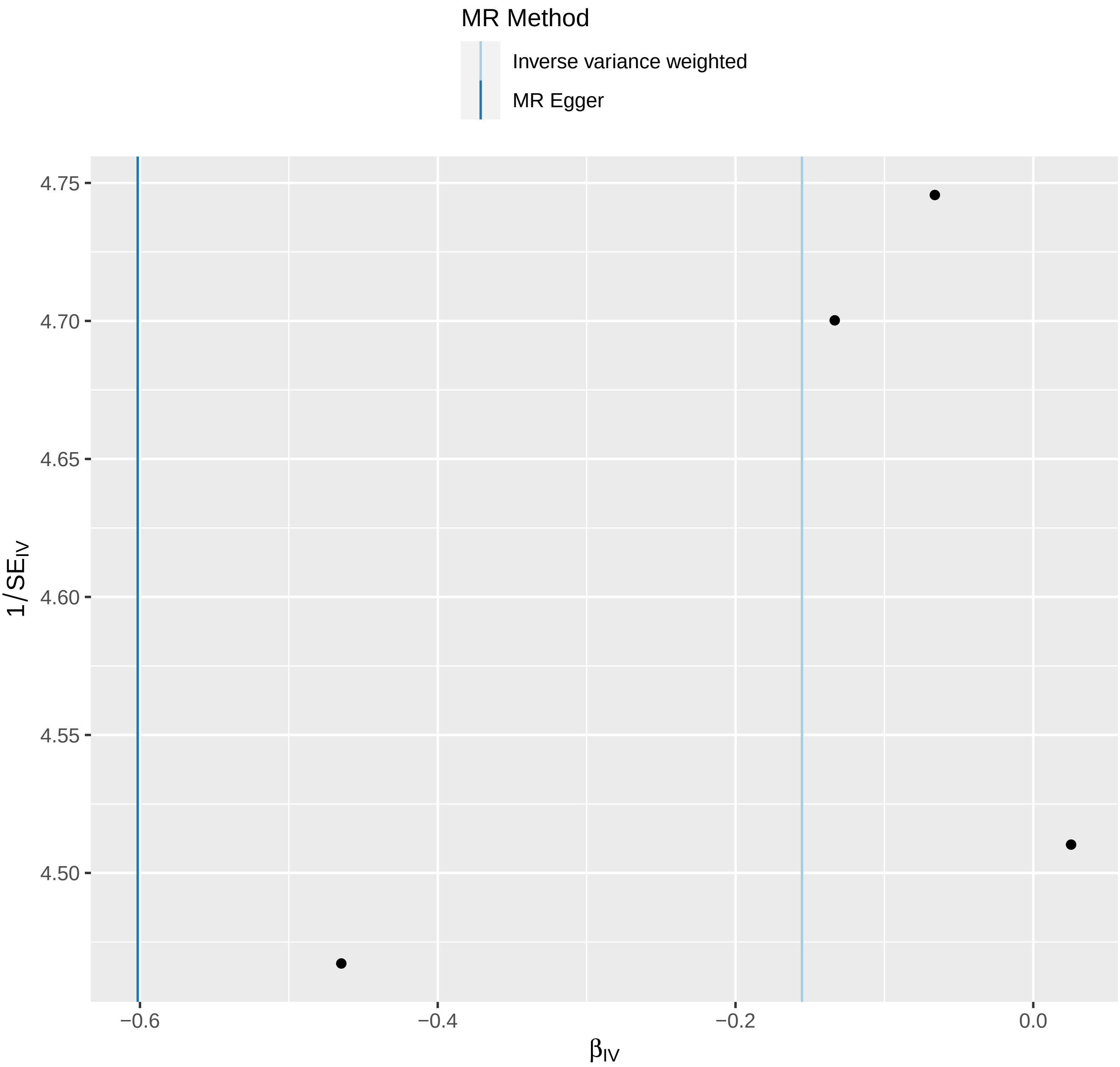

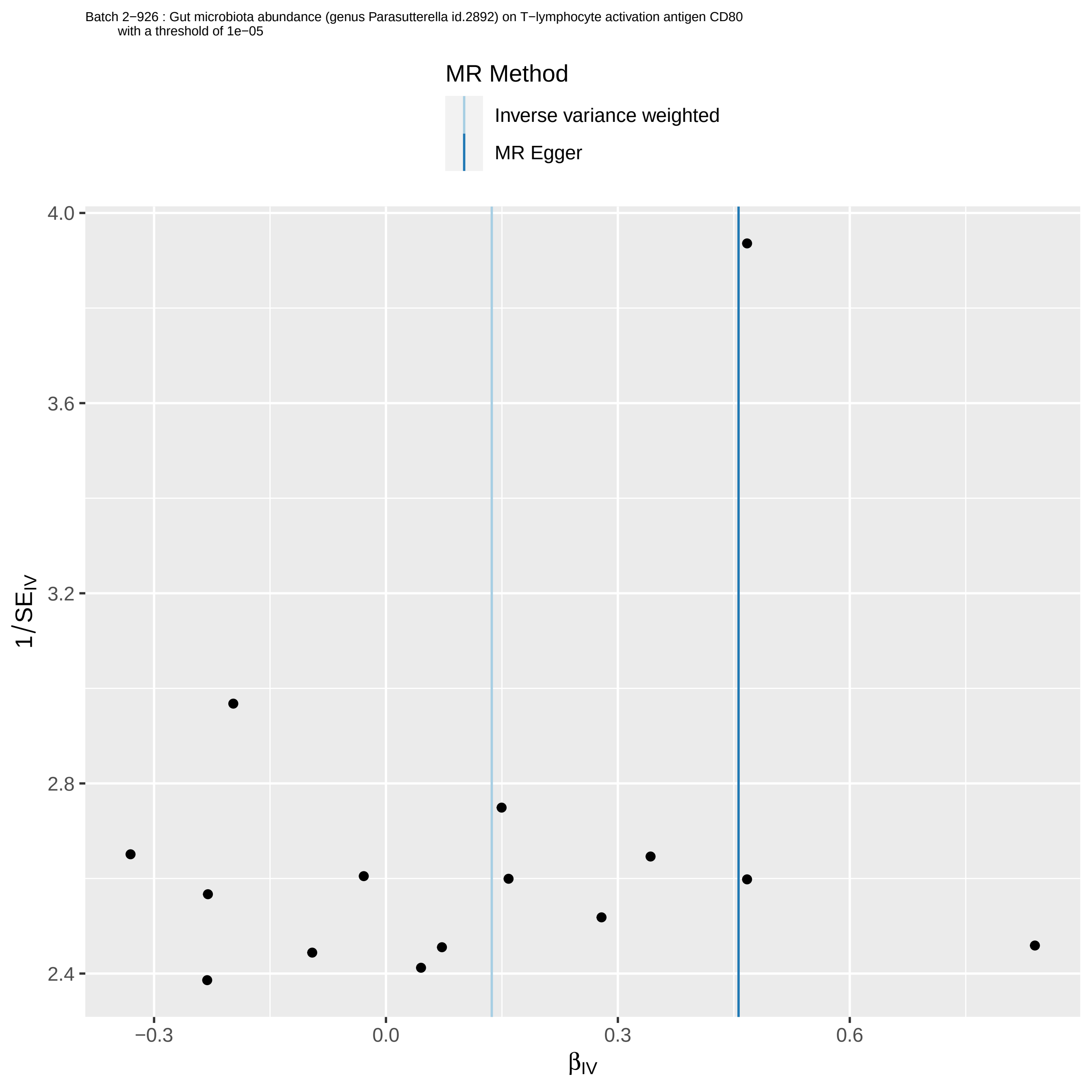

Batch 2−926 : Gut microbiota abundance (genus Parasutterella id.2892) on T−lymphocyte activation antigen CD80
with a threshold of 1e−05
MR Method
Inverse variance weighted
MR Egger
4.0
3.6
3.2
2.8
2.4
$1/SE_{IV}$
−0.3
0.0
0.3
0.6
β_{IV}

Batch 2−927 : Gut microbiota abundance (genus Catenibacterium id.2153) on SLAM family member 5
with a threshold of 1e−05

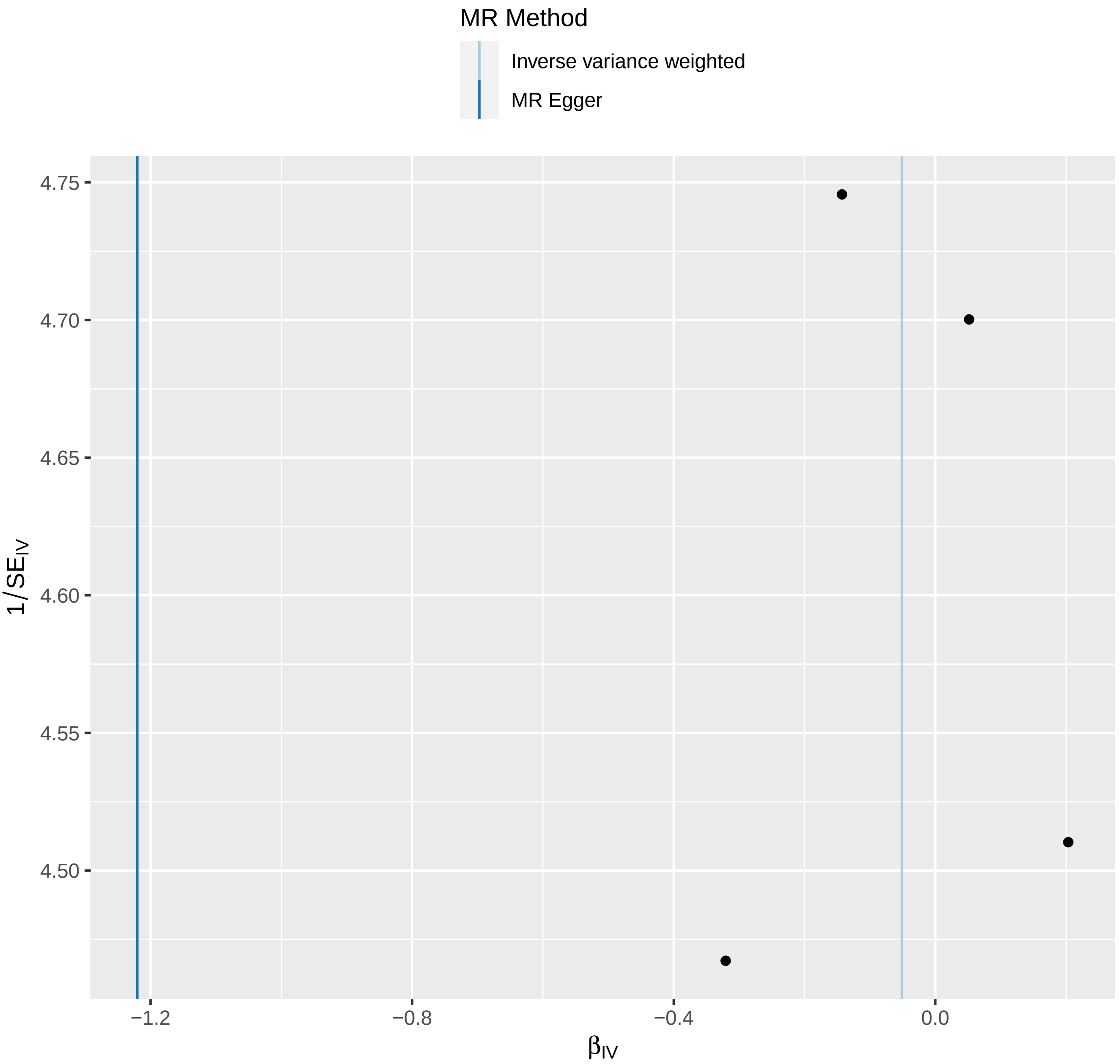

Batch 2−928 : Gut microbiota abundance (genus Parasutterella id.2892) on SLAM family member 5
with a threshold of 1e−05

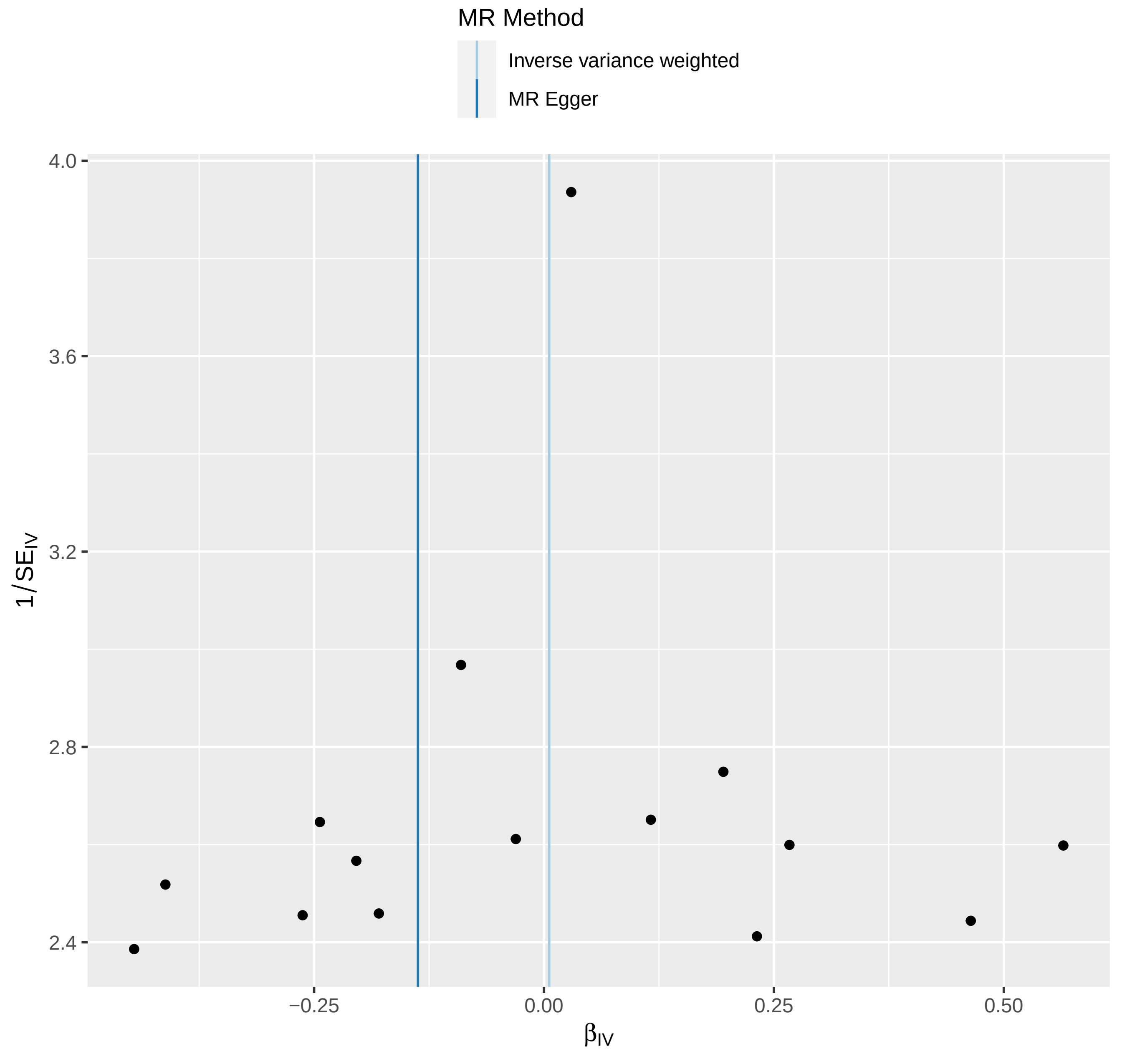

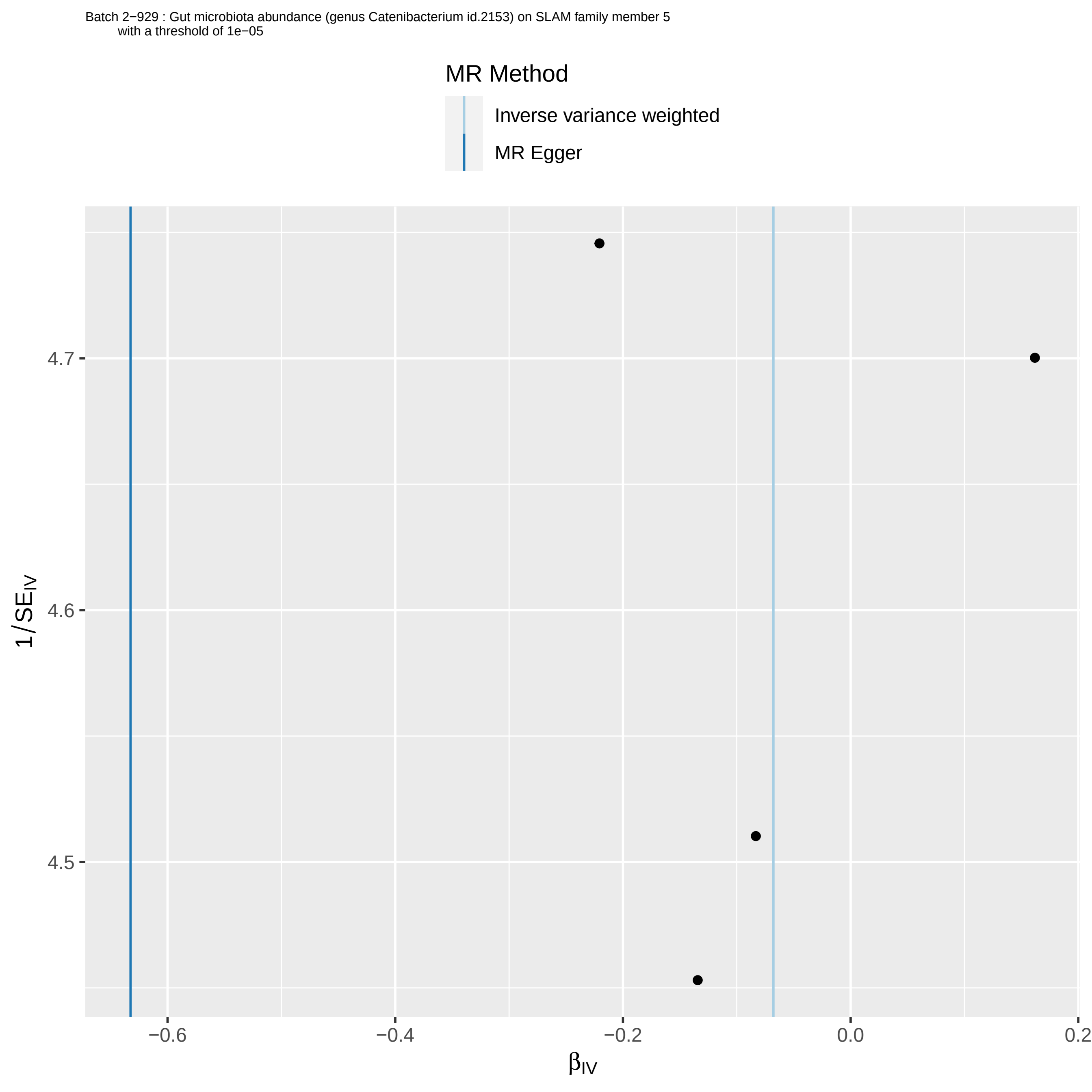

Batch 2−929 : Gut microbiota abundance (genus Catenibacterium id.2153) on SLAM family member 5
with a threshold of 1e−05
MR Method
Inverse variance weighted
MR Egger
4.7
4.6
4.5
1/SE_IV
−0.6
−0.4
−0.2
0.0
0.2
β_IV

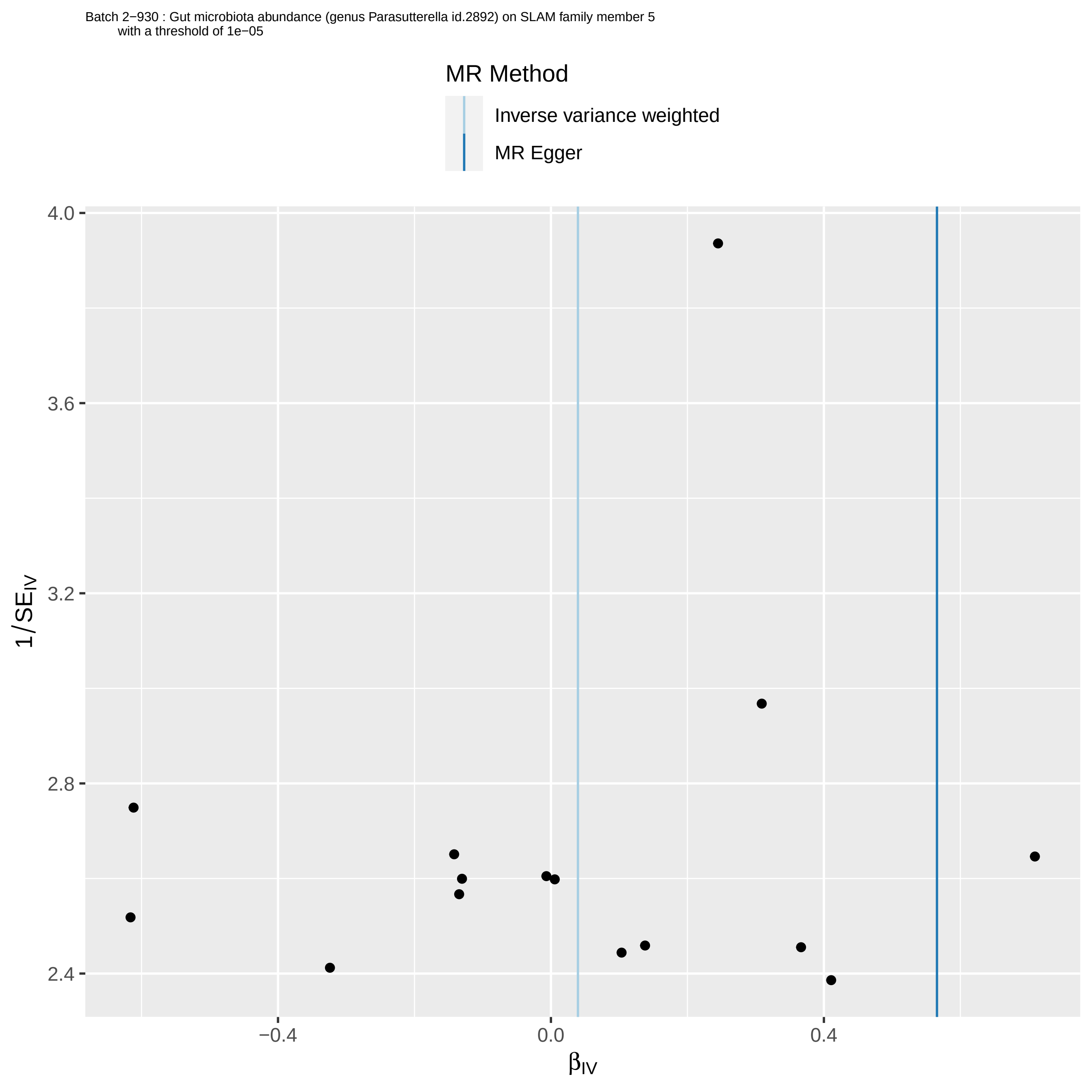
Batch 2−930 : Gut microbiota abundance (genus Parasutterella id.2892) on SLAM family member 5
with a threshold of 1e−05
MR Method
Inverse variance weighted
MR Egger
4.0
3.6
3.2
2.8
2.4
1/SE$_{IV}$
−0.4
0.0
0.4
β$_{IV}$

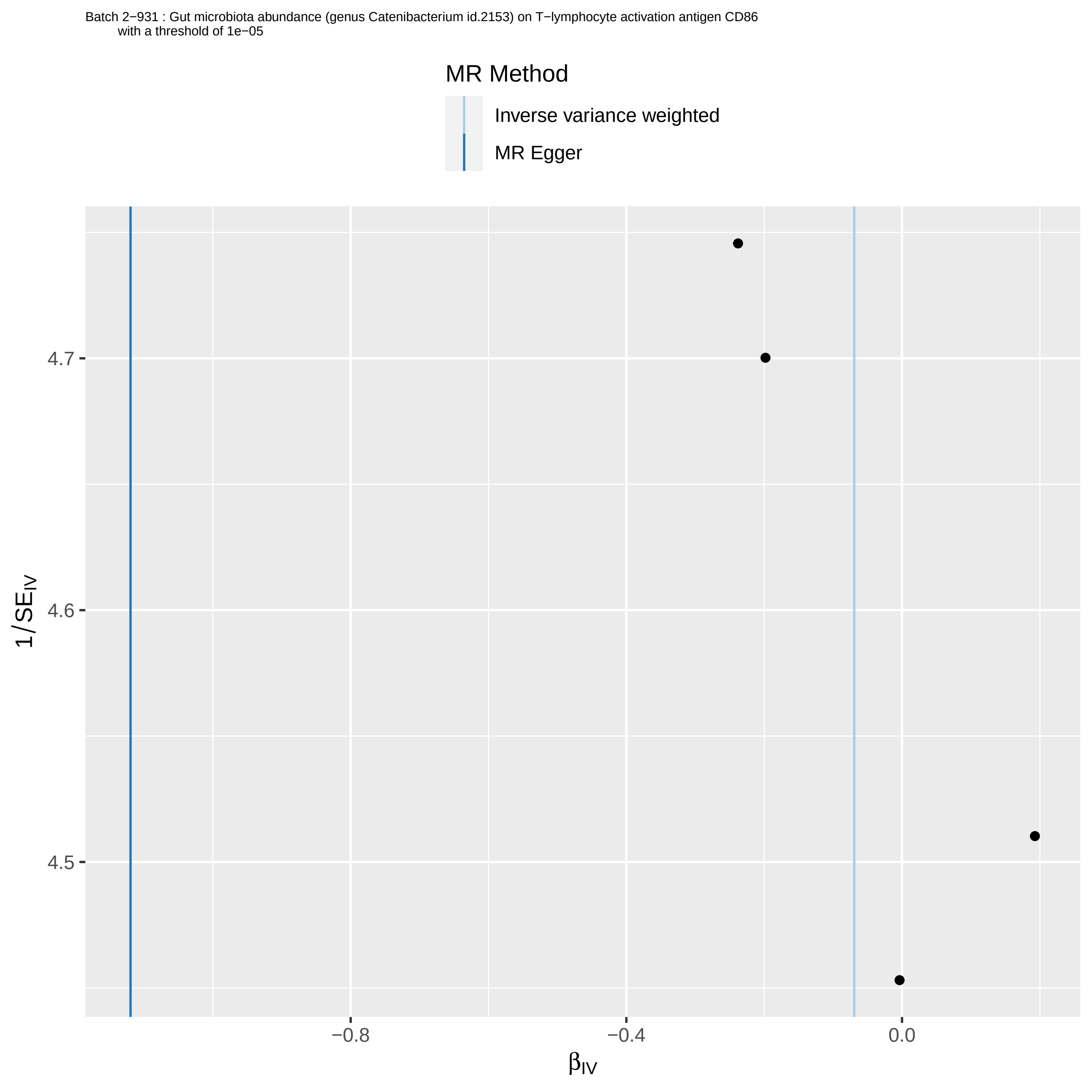
Batch 2−931 : Gut microbiota abundance (genus Catenibacterium id.2153) on T−lymphocyte activation antigen CD86
with a threshold of 1e−05
MR Method
Inverse variance weighted
MR Egger
4.7
4.6
4.5
1/SE_IV
−0.8
−0.4
0.0
β_IV

Batch 2−932 : Gut microbiota abundance (genus Parasutterella id.2892) on T−lymphocyte activation antigen CD86
with a threshold of 1e−05

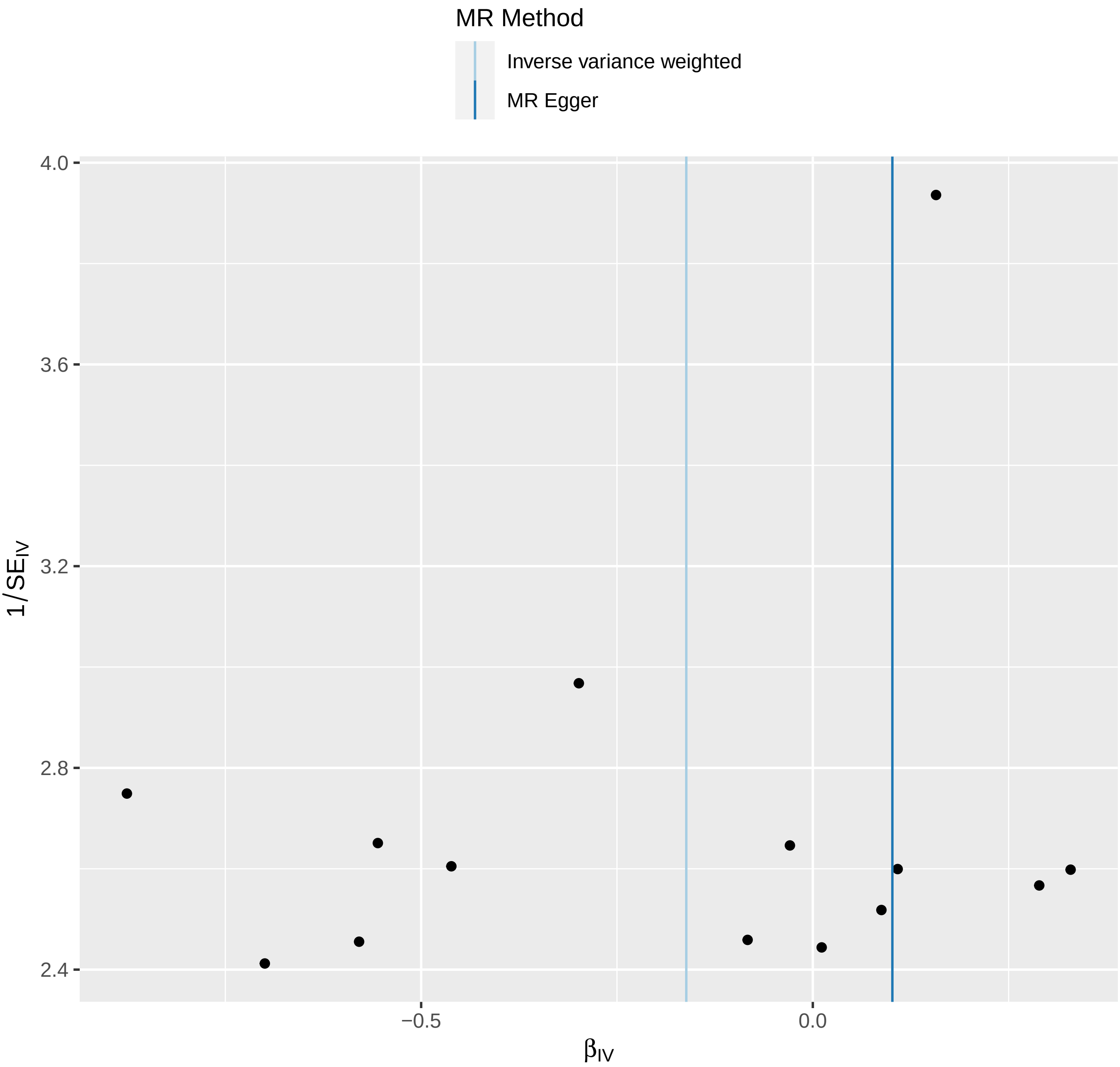

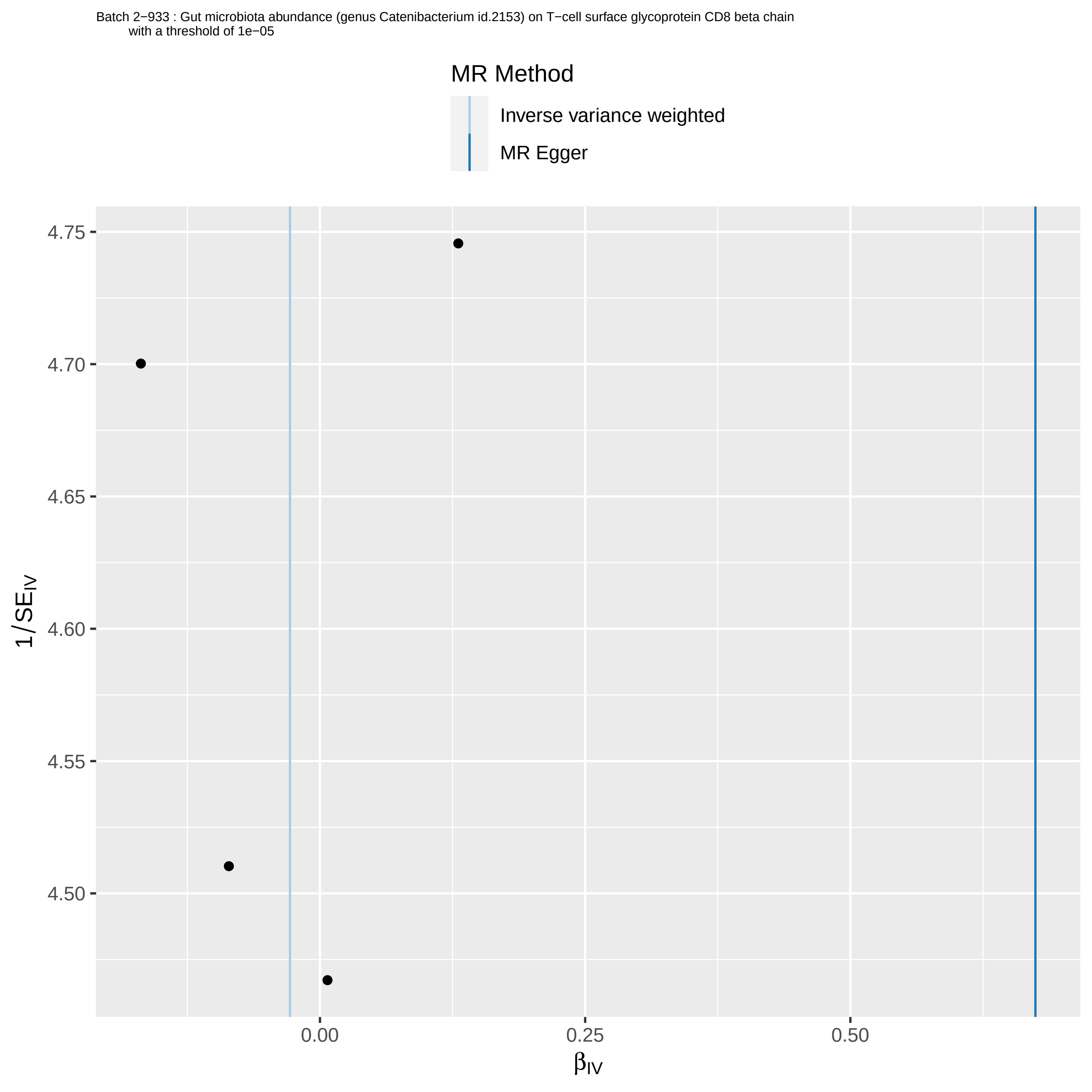

Batch 2−933 : Gut microbiota abundance (genus Catenibacterium id.2153) on T−cell surface glycoprotein CD8 beta chain
with a threshold of 1e−05
MR Method
Inverse variance weighted
MR Egger
4.75
4.70
4.65
4.60
4.55
4.50
1/SE_IV
0.00
0.25
0.50
β_IV

Batch 2−934 : Gut microbiota abundance (genus Parasutterella id.2892) on T−cell surface glycoprotein CD8 beta chain
with a threshold of 1e−05

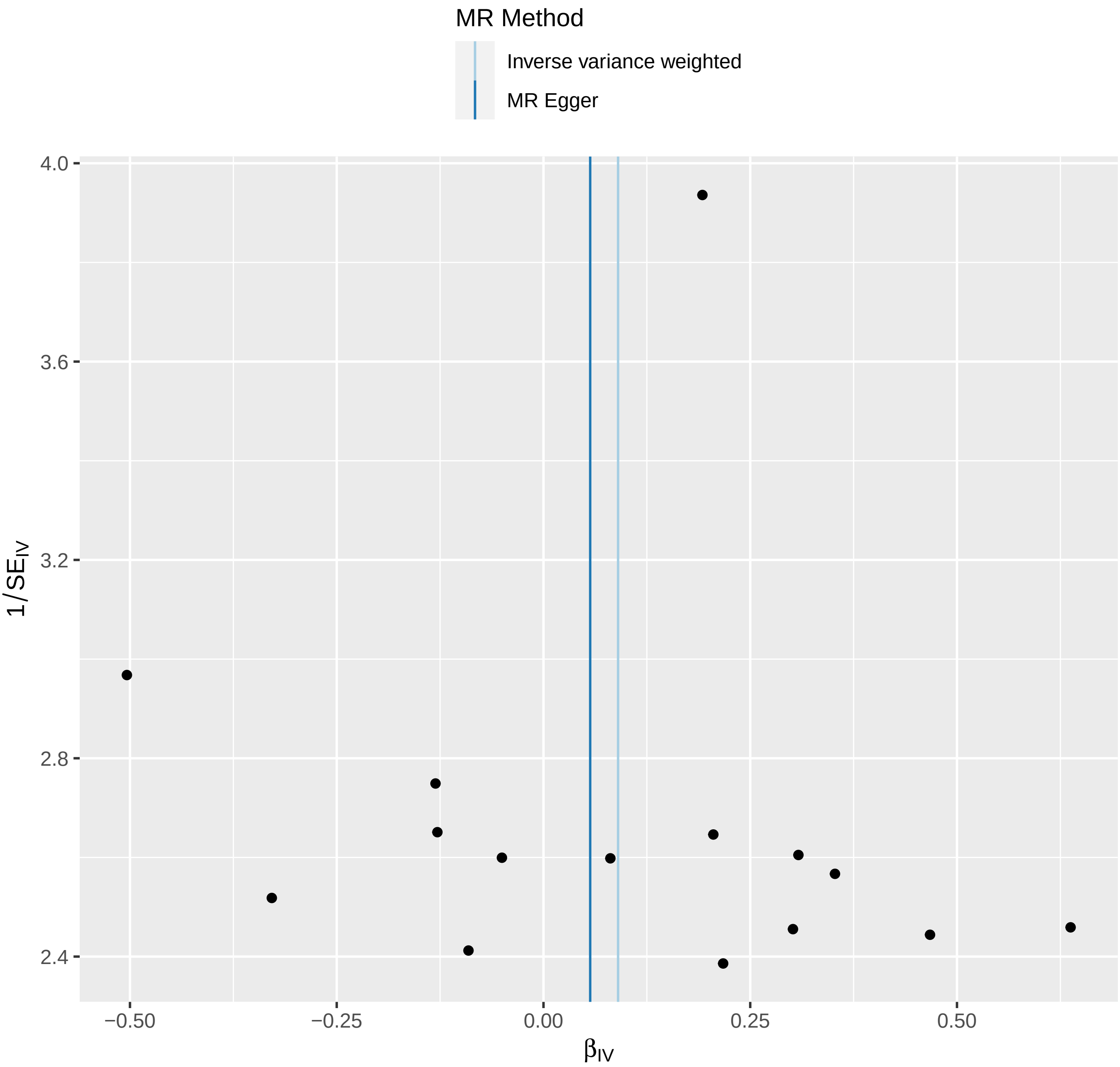

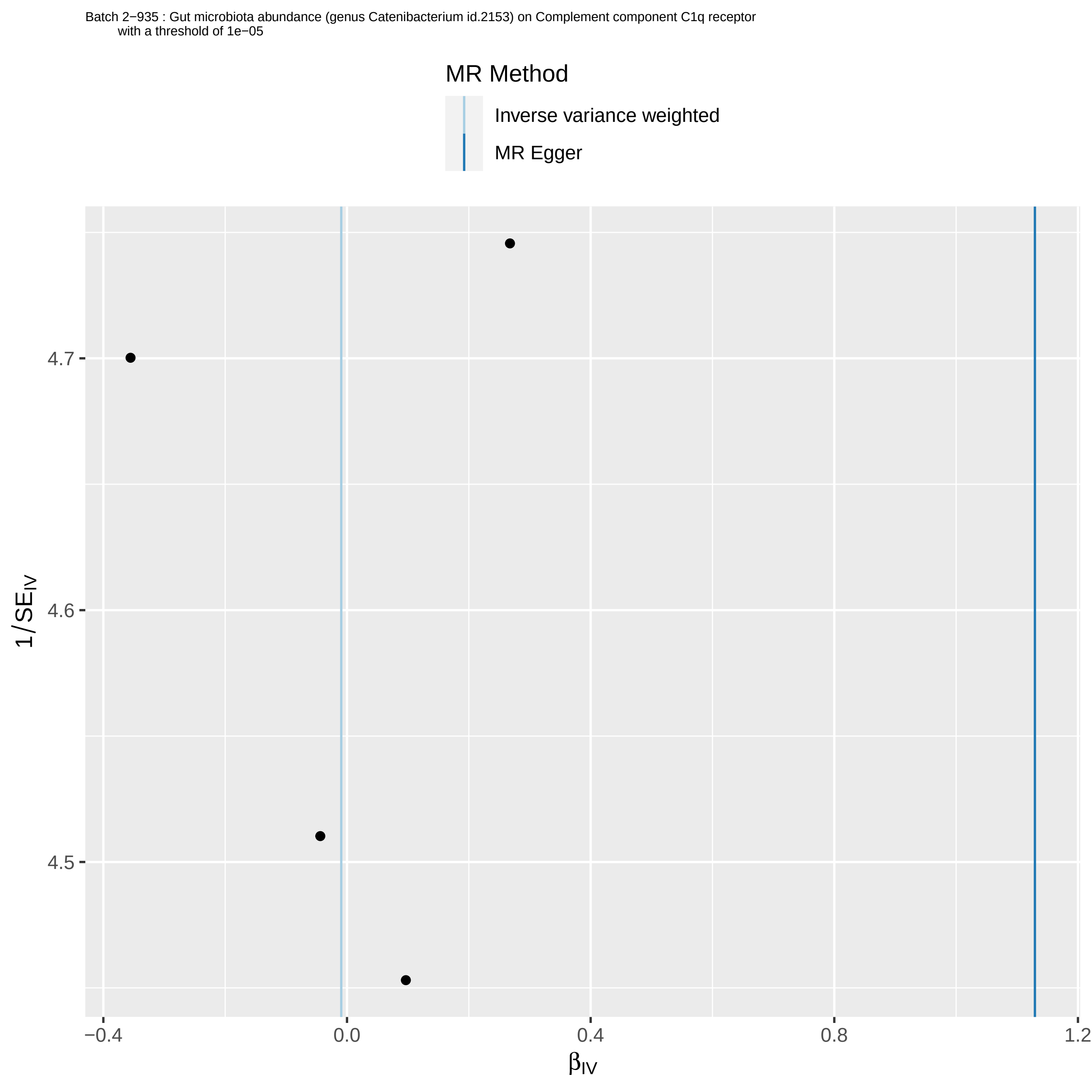

Batch 2−935 : Gut microbiota abundance (genus Catenibacterium id.2153) on Complement component C1q receptor
with a threshold of 1e−05
MR Method
Inverse variance weighted
MR Egger
4.7
4.6
4.5
$1/SE_{IV}$
−0.4
0.0
0.4
0.8
1.2
β_{IV}

Batch 2−936 : Gut microbiota abundance (genus Parasutterella id.2892) on Complement component C1q receptor
with a threshold of 1e−05

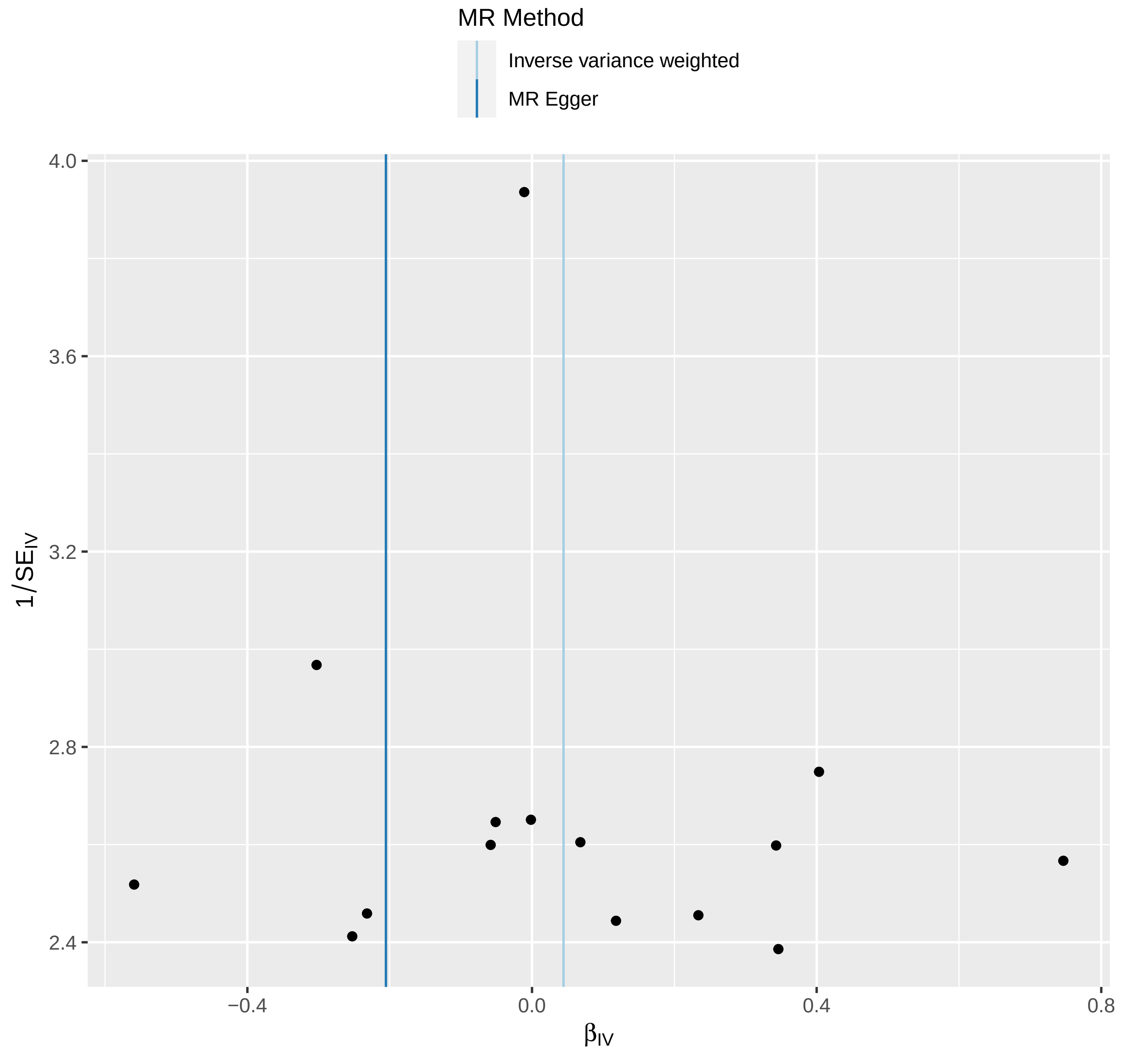

Batch 2−937 : Gut microbiota abundance (genus Catenibacterium id.2153) on Complement component C1q receptor with a threshold of 1e−05

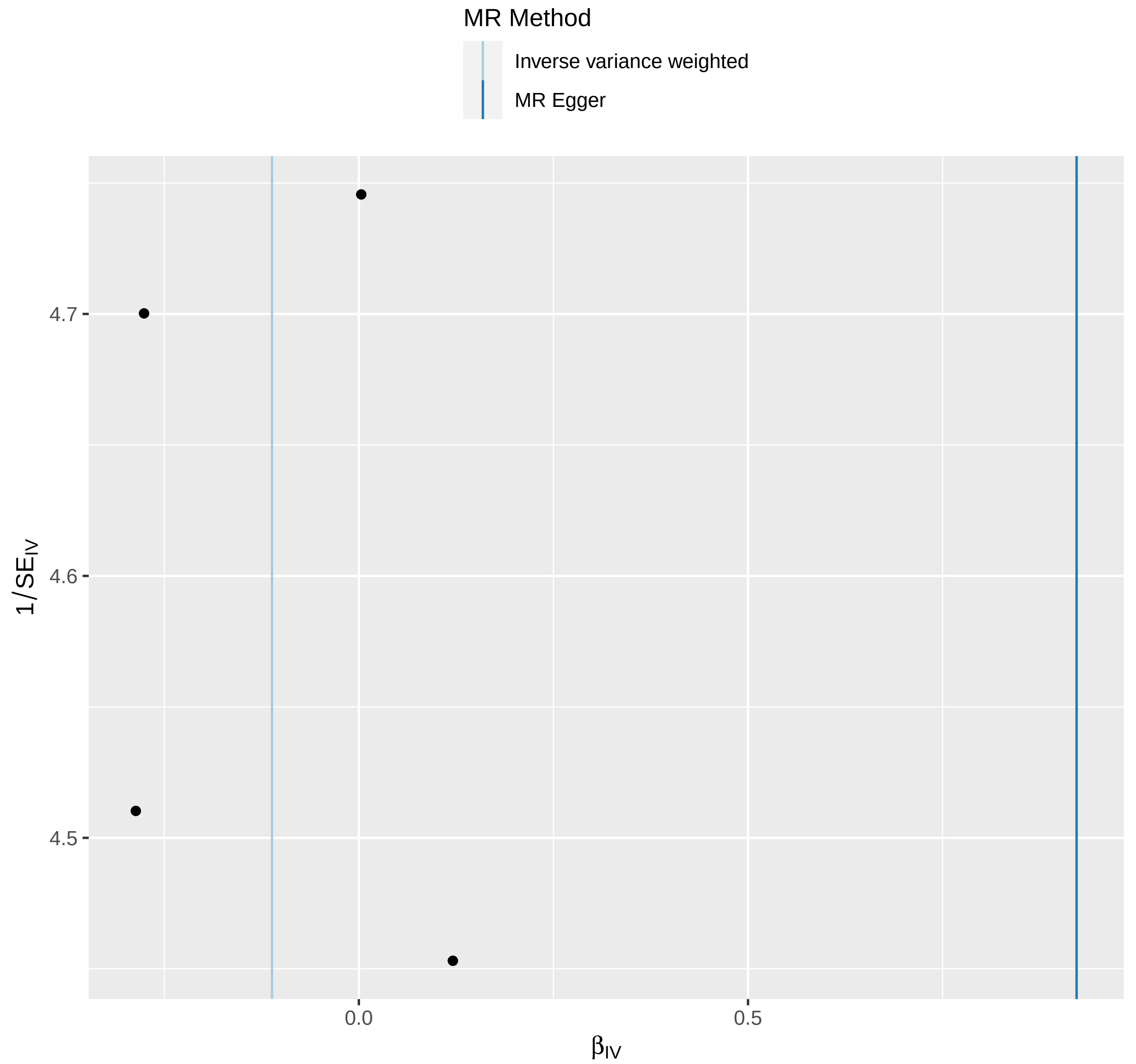

Batch 2−938 : Gut microbiota abundance (genus Parasutterella id.2892) on Complement component C1q receptor
with a threshold of 1e−05

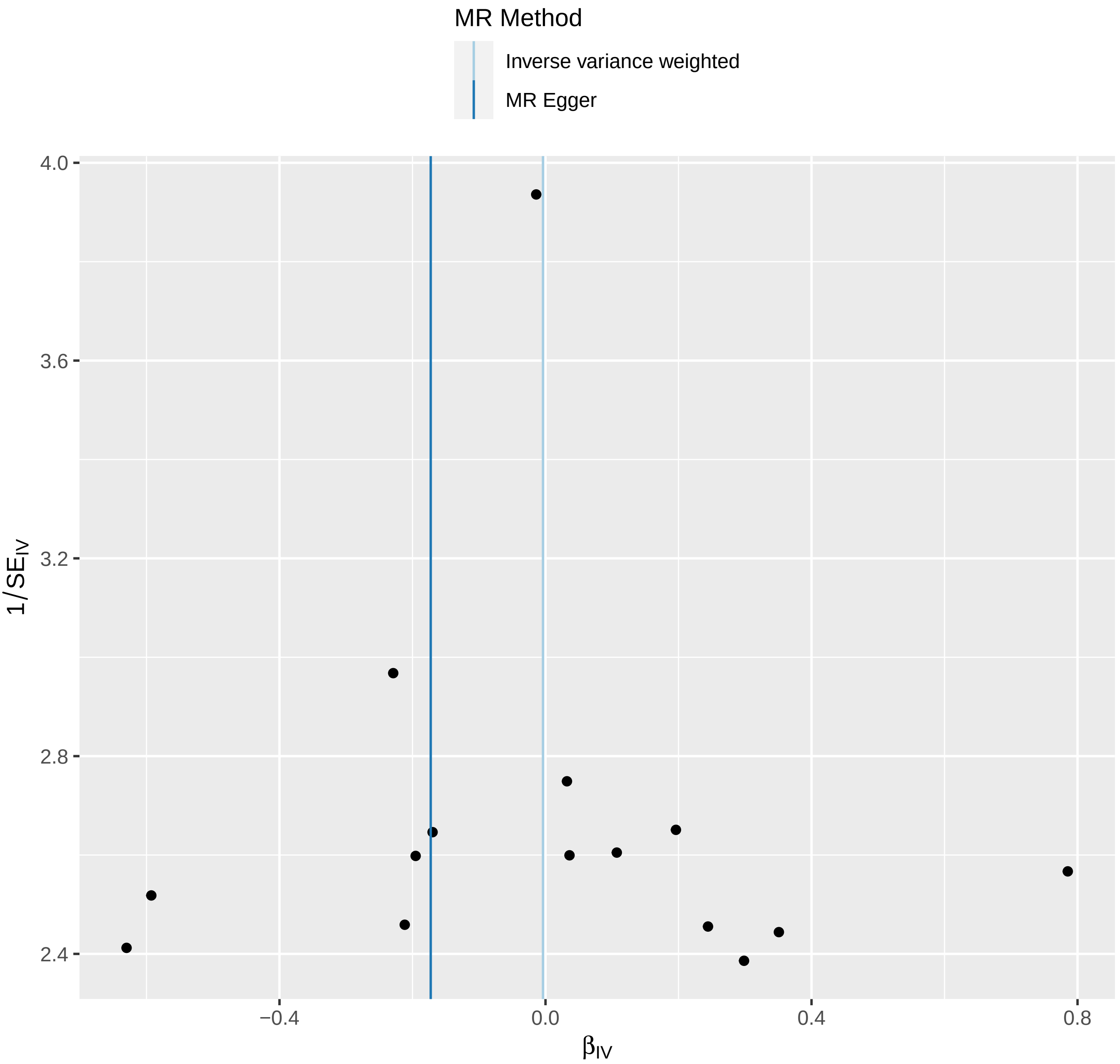

Batch 2−939 : Gut microbiota abundance (genus Catenibacterium id.2153) on T−cell surface protein tactile
with a threshold of 1e−05

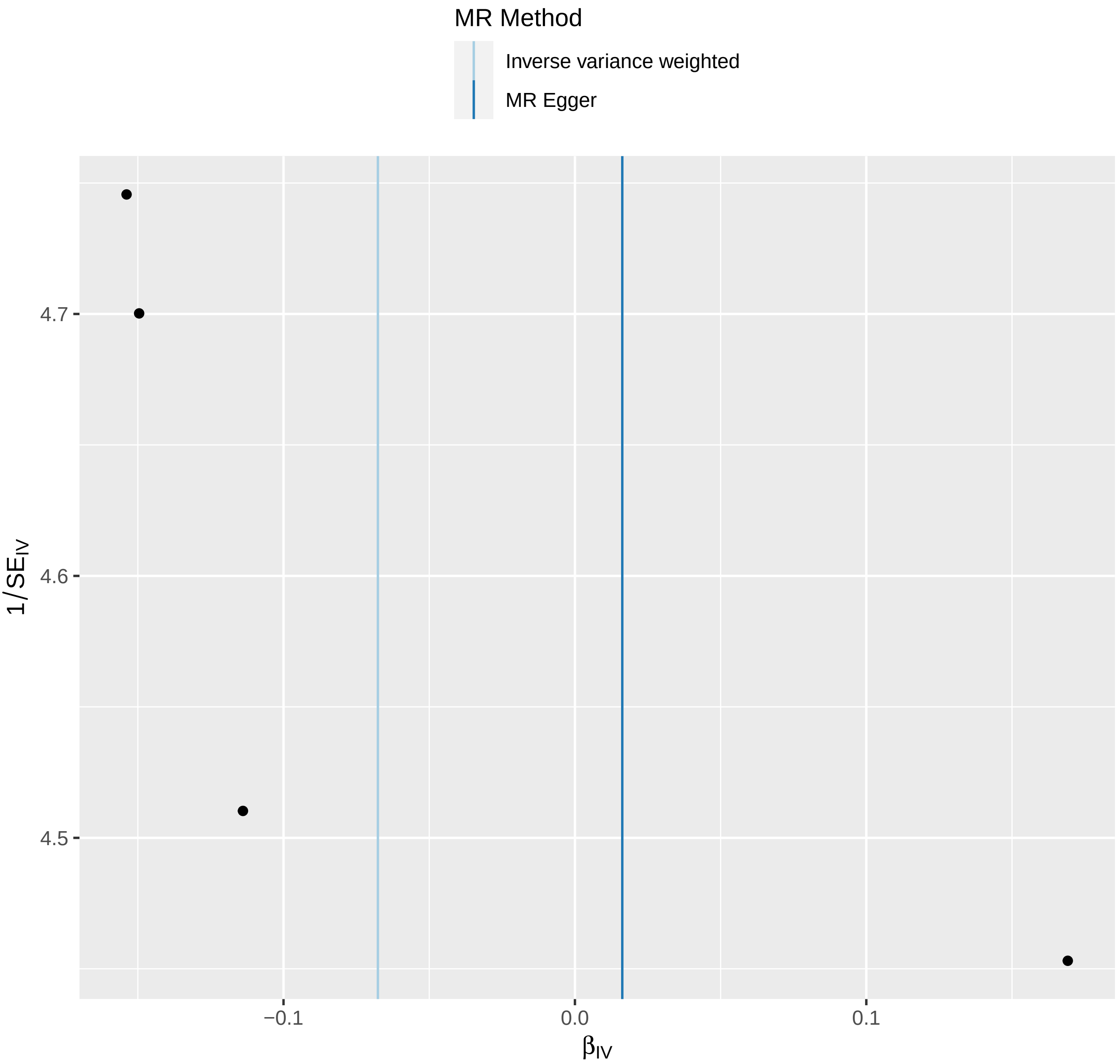

Batch 2−940 : Gut microbiota abundance (genus Parasutterella id.2892) on T−cell surface protein tactile
with a threshold of 1e−05

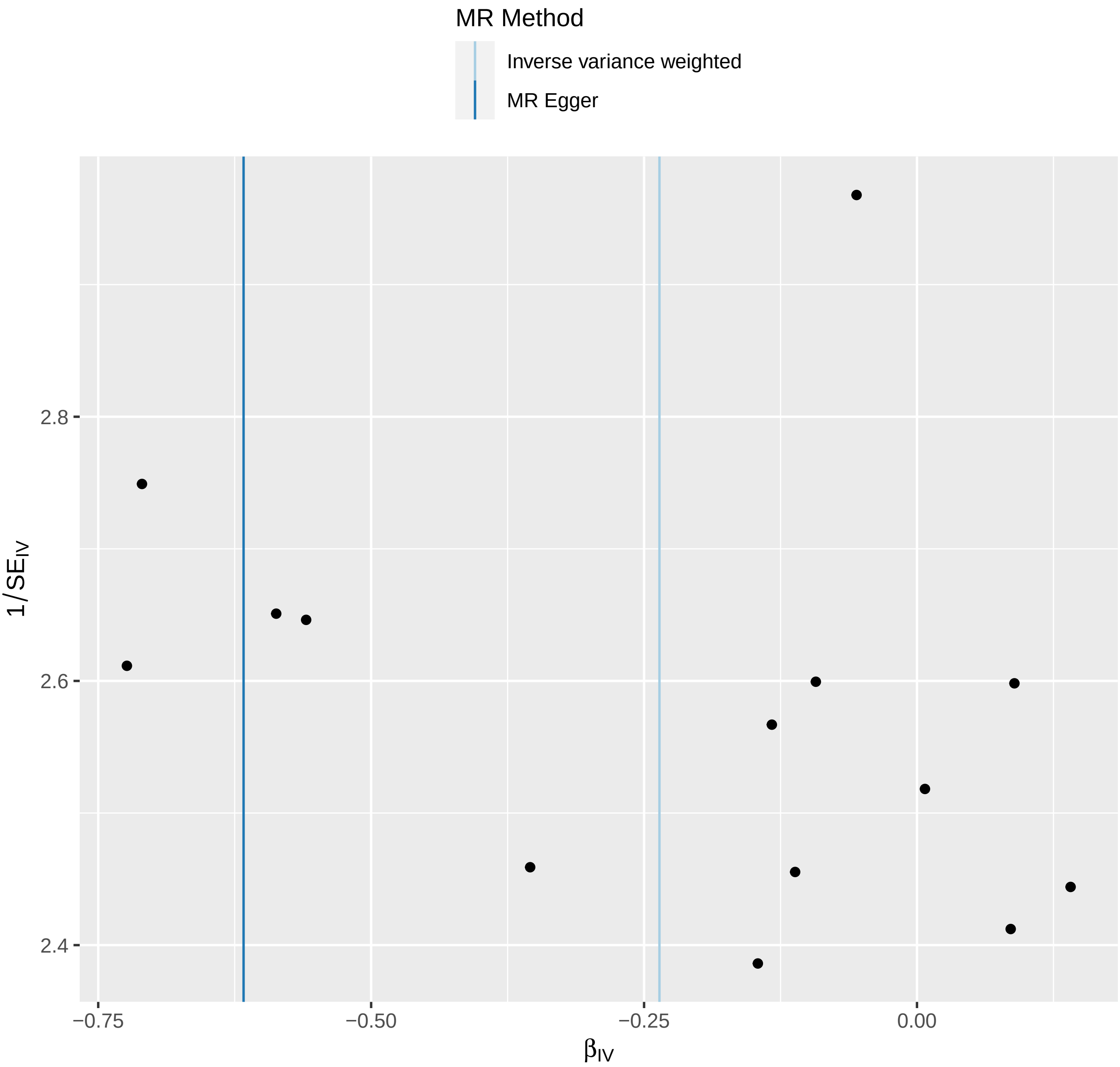

Batch 2−941 : Gut microbiota abundance (genus Catenibacterium id.2153) on CD97 antigen
with a threshold of 1e−05

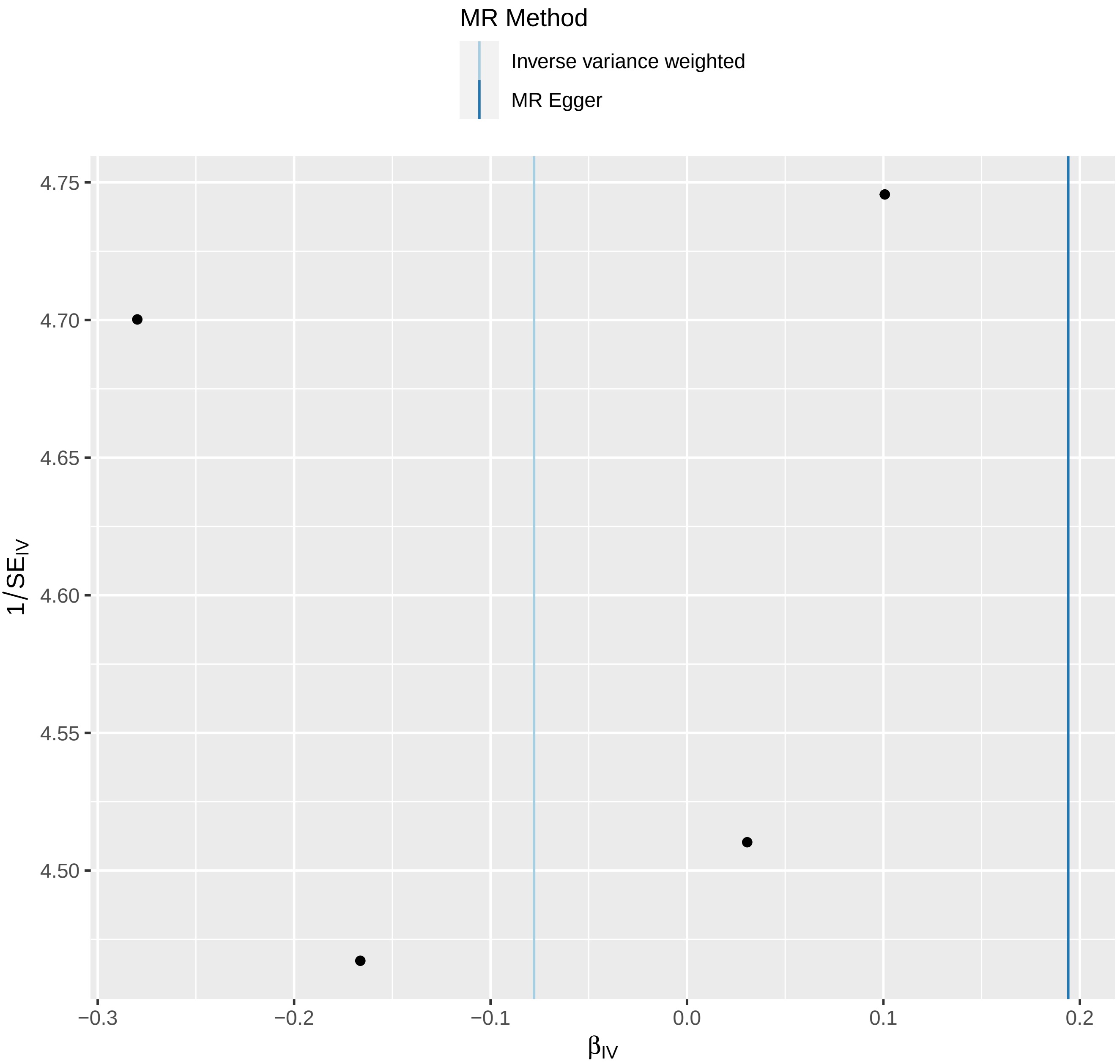

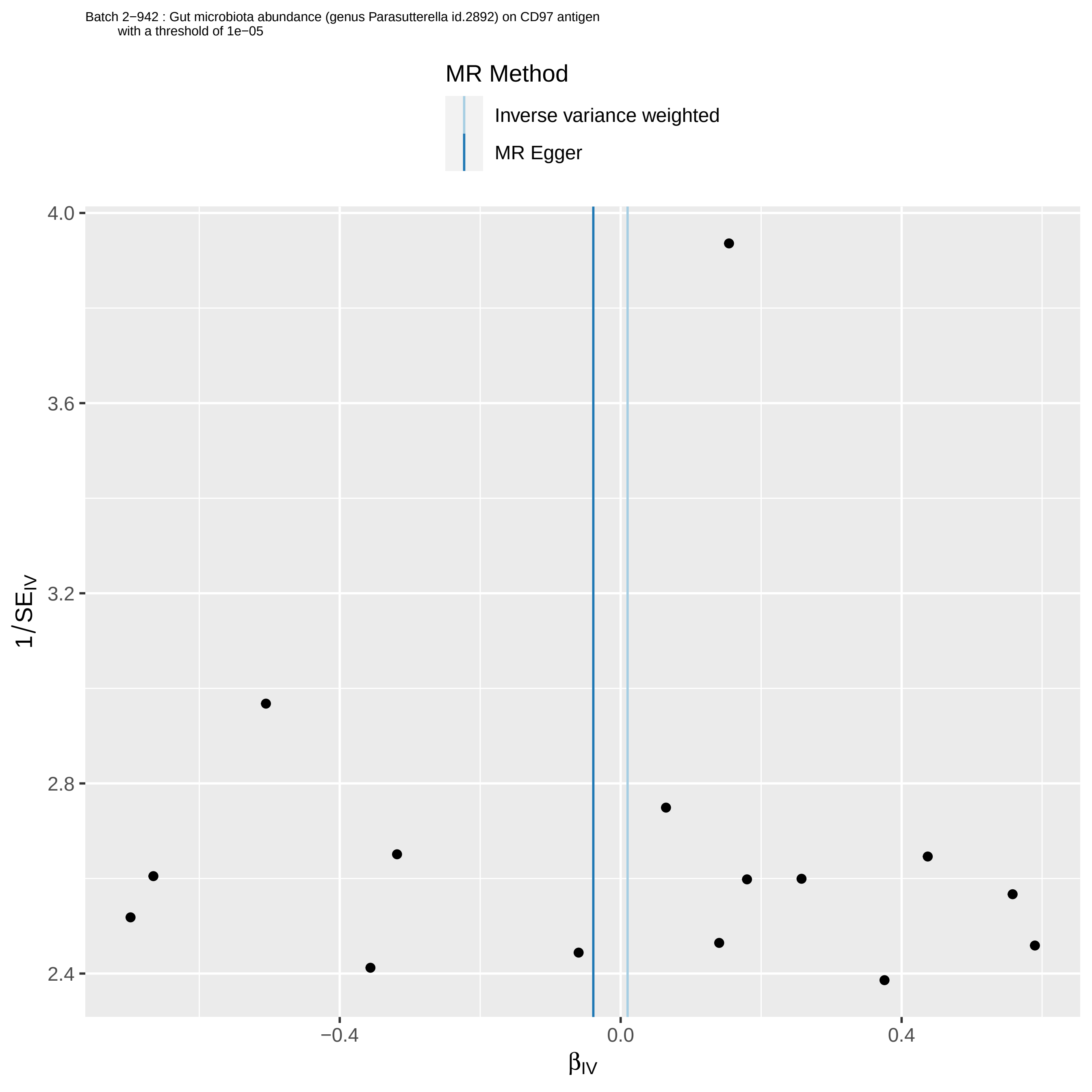
Batch 2−942 : Gut microbiota abundance (genus Parasutterella id.2892) on CD97 antigen
with a threshold of 1e−05
MR Method
Inverse variance weighted
MR Egger
4.0
3.6
3.2
2.8
2.4
1/SE_IV
−0.4
0.0
0.4
β_IV

Batch 2−943 : Gut microbiota abundance (genus Catenibacterium id.2153) on M−phase inducer phosphatase 2
with a threshold of 1e−05

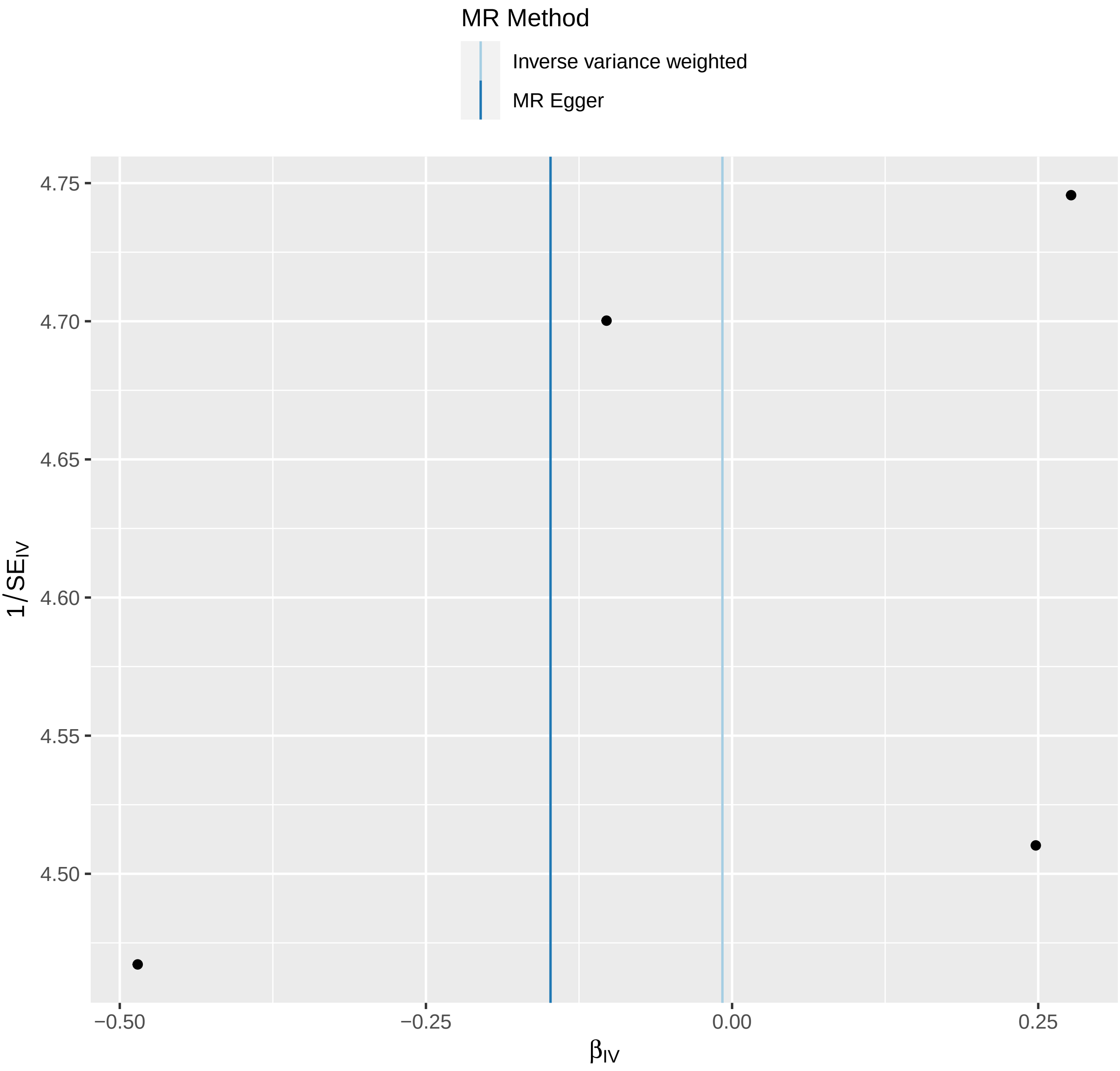

Batch 2−944 : Gut microbiota abundance (genus Parasutterella id.2892) on M−phase inducer phosphatase 2
with a threshold of 1e−05

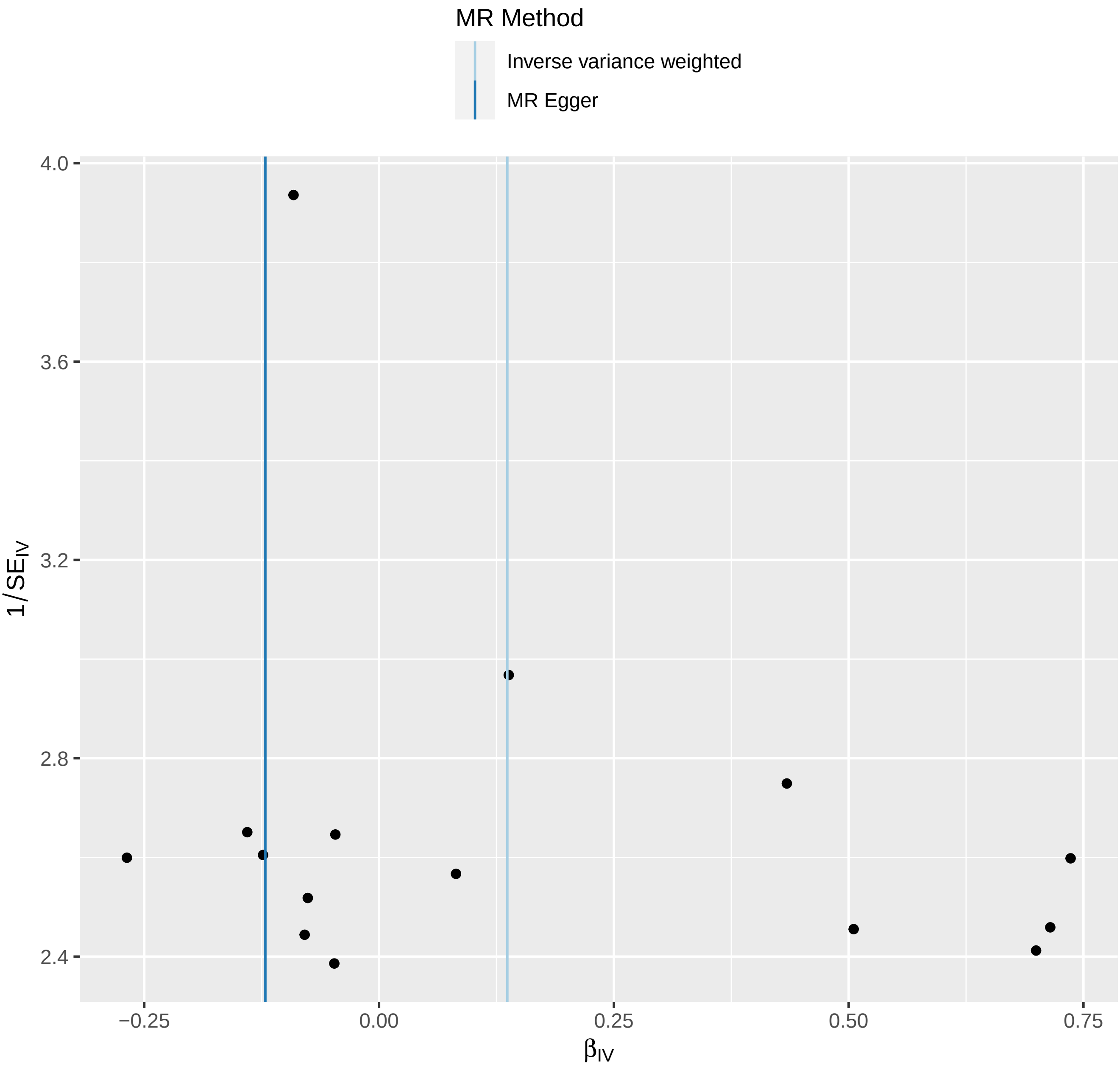

Batch 2−945 : Gut microbiota abundance (genus Catenibacterium id.2153) on Cyclin−dependent kinase 1:G2/mitotic−specific cyclin−B1 complex with a threshold of 1e−05

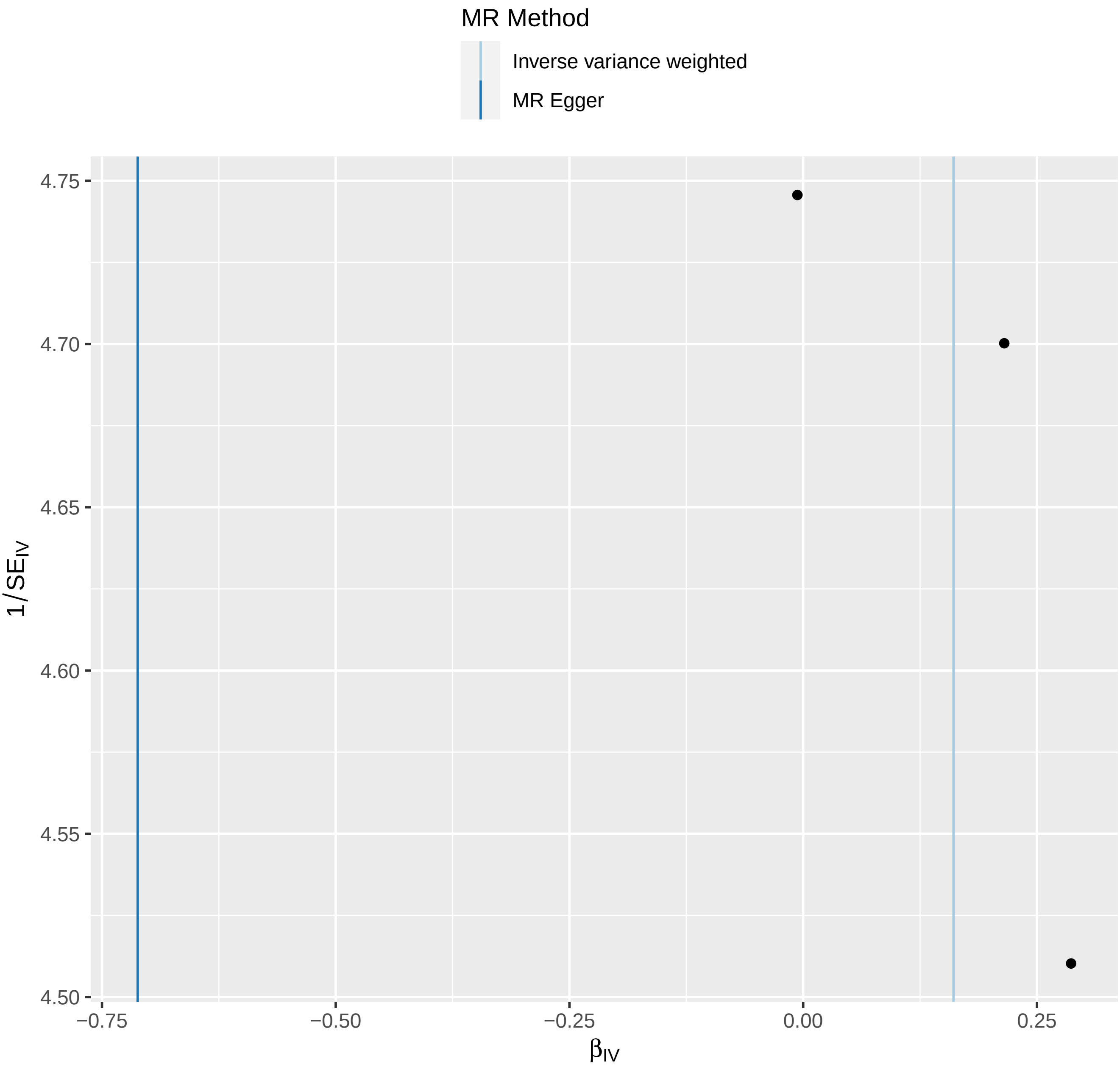

Batch 2−946 : Gut microbiota abundance (genus Parasutterella id.2892) on Cyclin−dependent kinase 1:G2/mitotic−specific cyclin−B1 complex
with a threshold of 1e−05

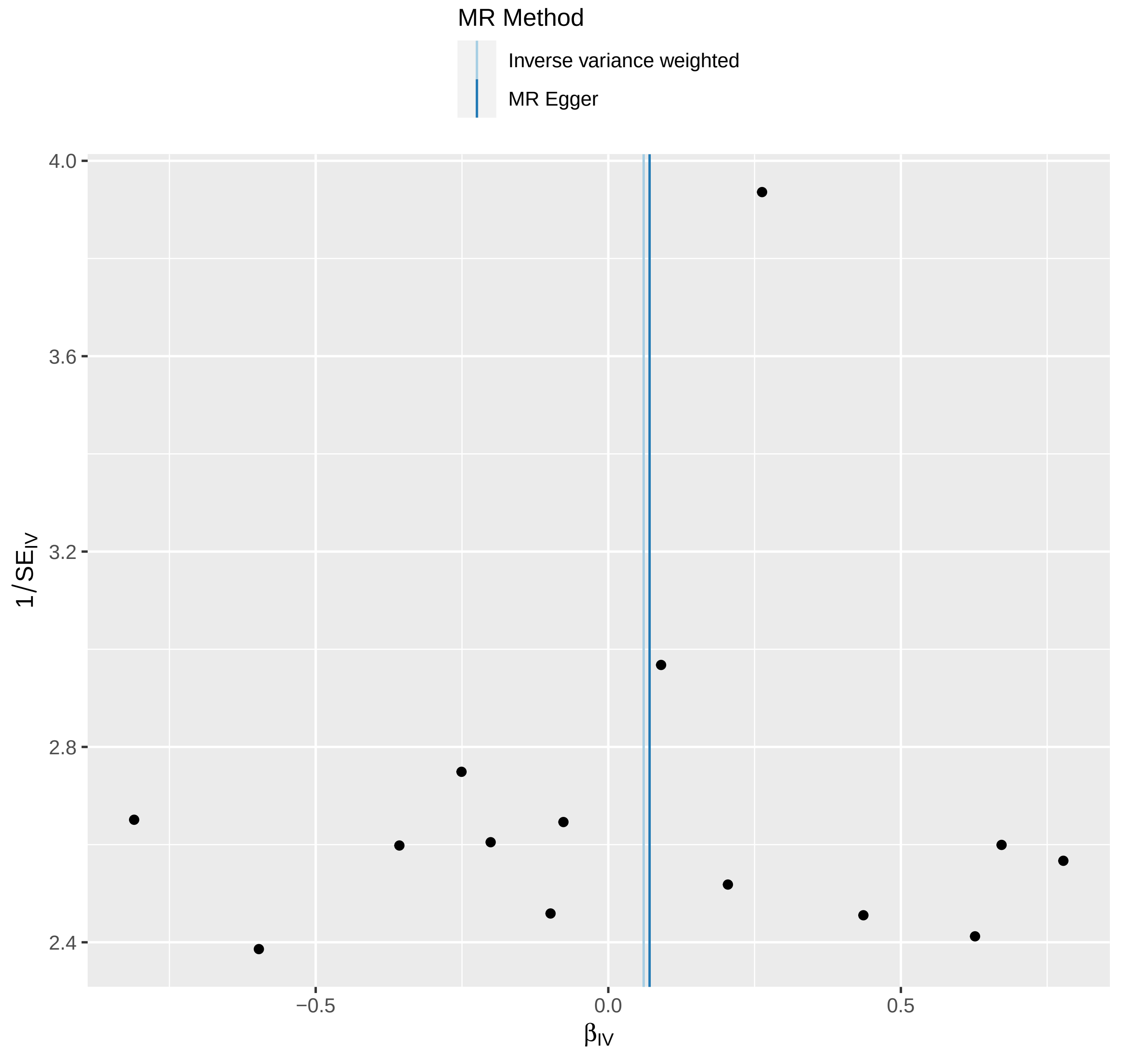

Batch 2−947 : Gut microbiota abundance (genus Catenibacterium id.2153) on Cell division control protein 42 homolog
with a threshold of 1e−05

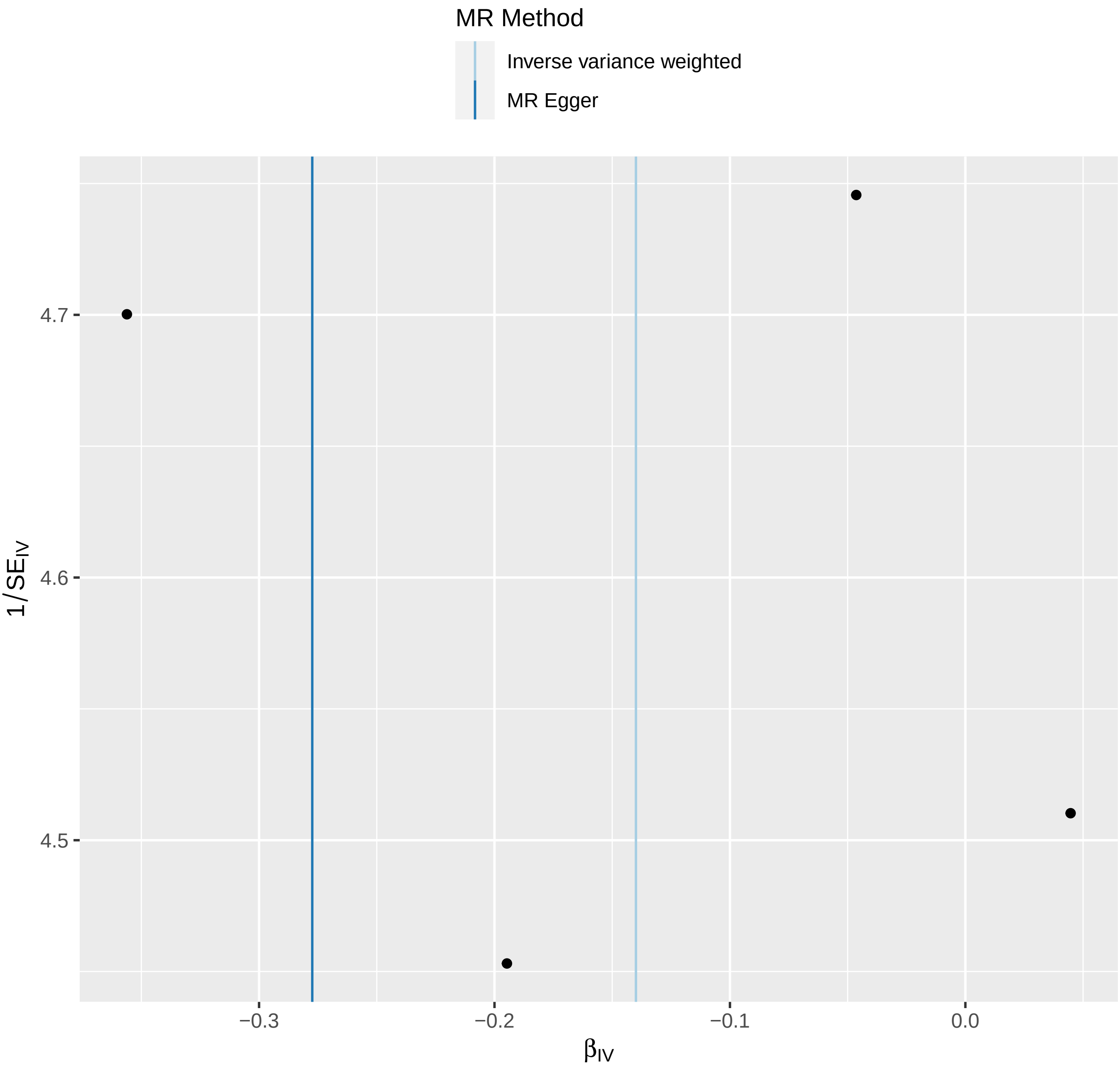

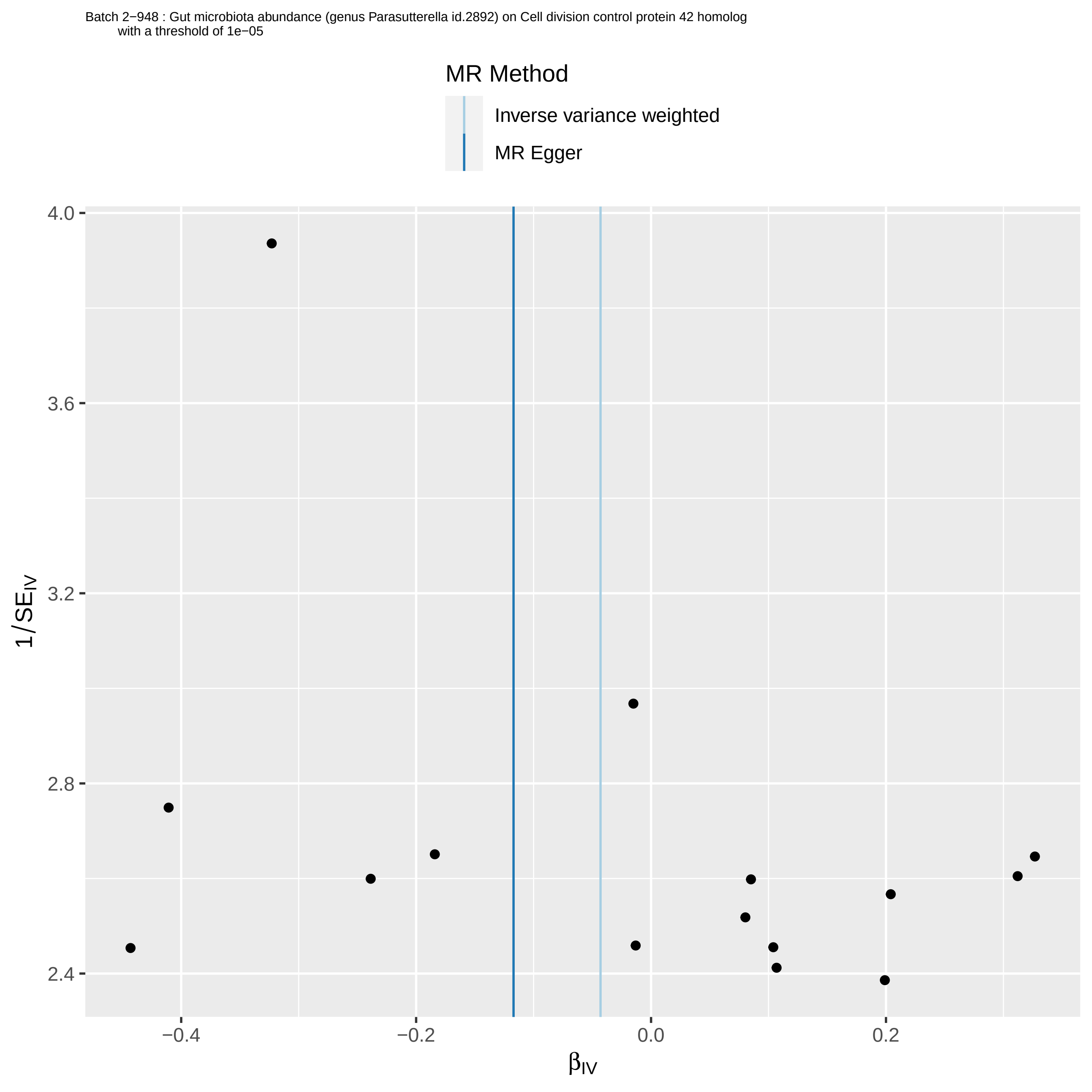
Batch 2−948 : Gut microbiota abundance (genus Parasutterella id.2892) on Cell division control protein 42 homolog
with a threshold of 1e−05
MR Method
Inverse variance weighted
MR Egger
4.0
3.6
3.2
2.8
2.4
1/SE_IV
−0.4
−0.2
0.0
0.2
β_IV

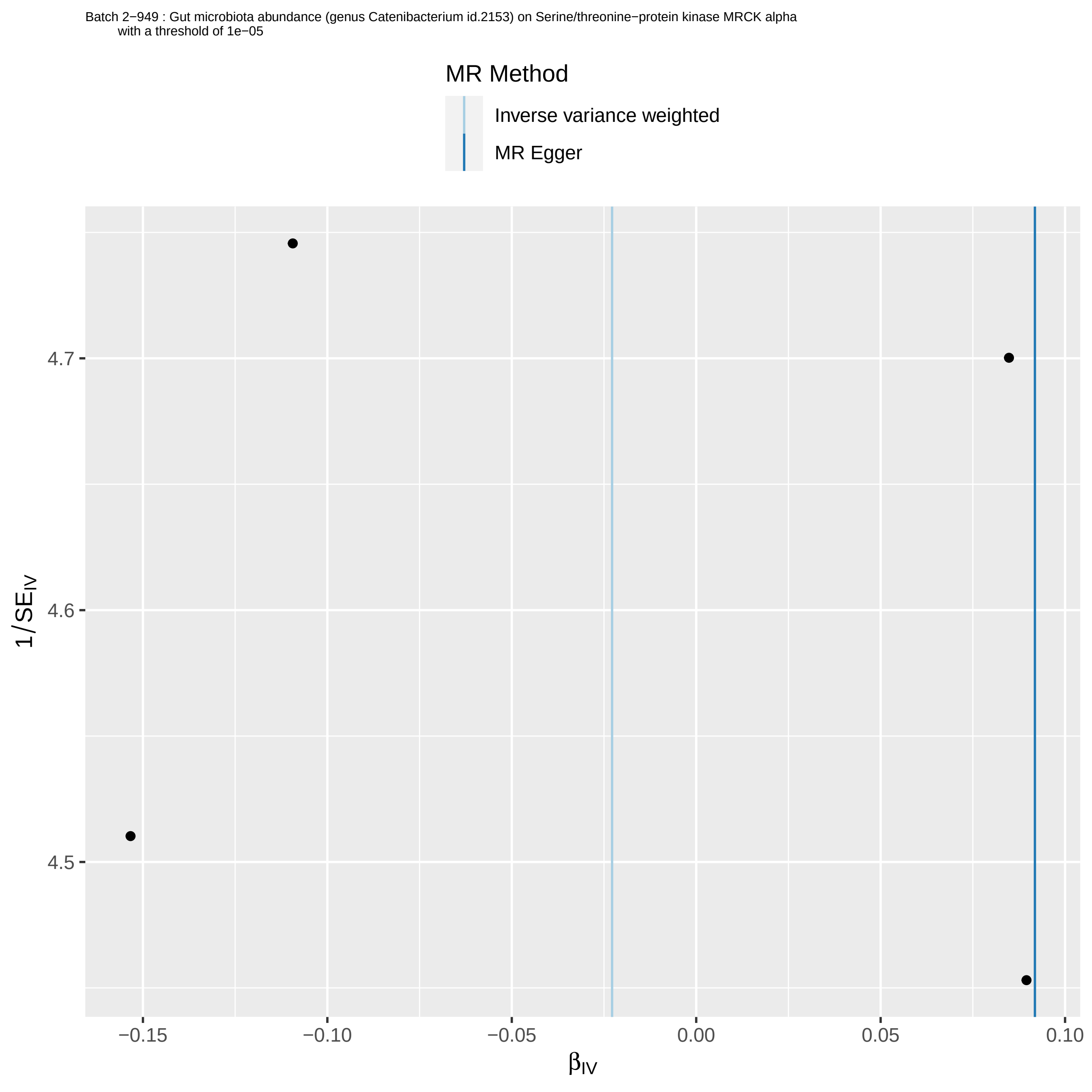
Batch 2−949 : Gut microbiota abundance (genus Catenibacterium id.2153) on Serine/threonine−protein kinase MRCK alpha
with a threshold of 1e−05
MR Method
Inverse variance weighted
MR Egger
4.7
4.6
4.5
1/SE_IV
−0.15
−0.10
−0.05
0.00
0.05
0.10
β_IV

Batch 2−950 : Gut microbiota abundance (genus Parasutterella id.2892) on Serine/threonine−protein kinase MRCK alpha with a threshold of 1e−05

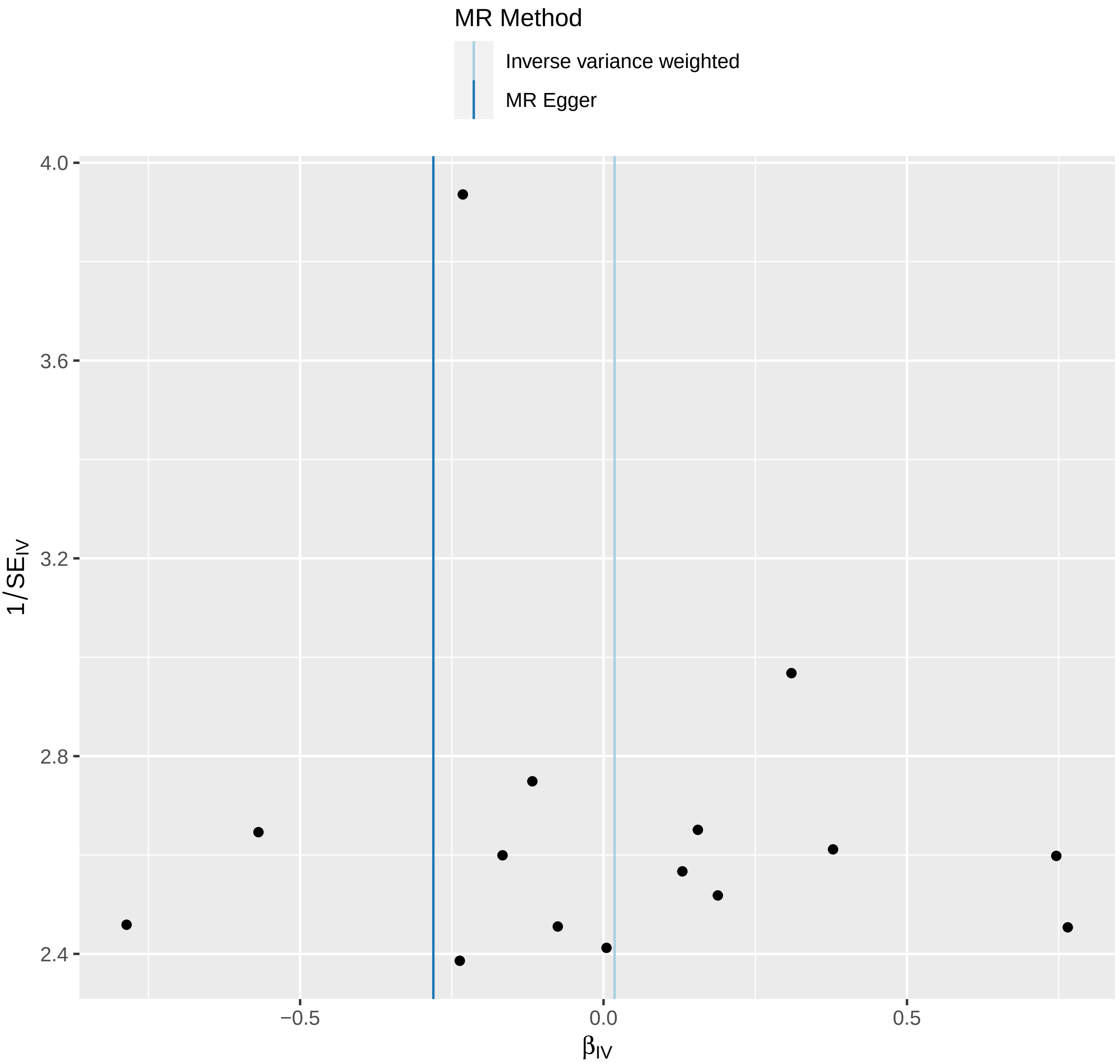

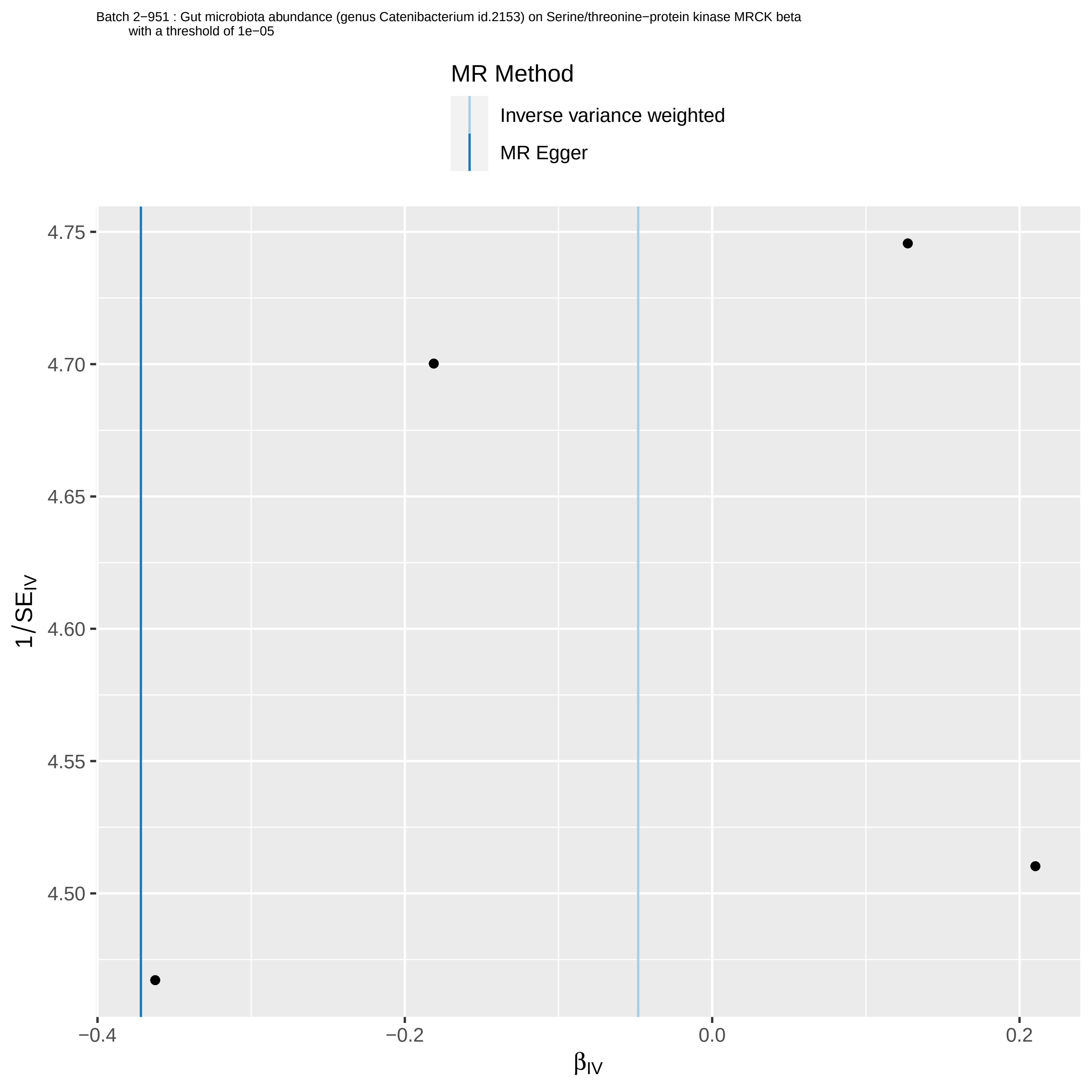
Batch 2−951 : Gut microbiota abundance (genus Catenibacterium id.2153) on Serine/threonine−protein kinase MRCK beta
with a threshold of 1e−05
MR Method
Inverse variance weighted
MR Egger
4.75
4.70
4.65
4.60
4.55
4.50
1/SE_IV
−0.4
−0.2
0.0
0.2
β_IV

Batch 2−952 : Gut microbiota abundance (genus Parasutterella id.2892) on Serine/threonine−protein kinase MRCK beta
with a threshold of 1e−05

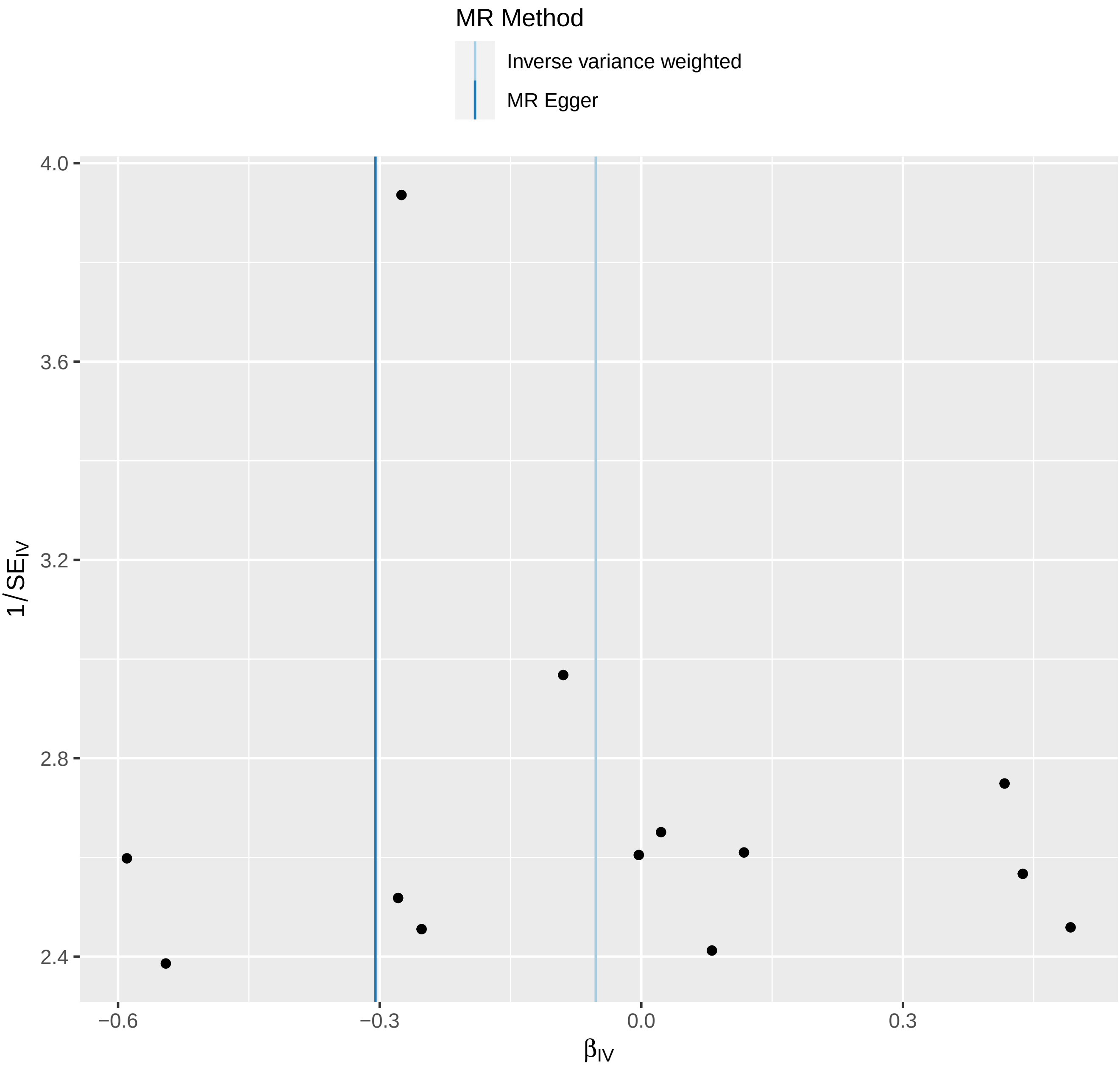

Batch 2−953 : Gut microbiota abundance (genus Catenibacterium id.2153) on CUB domain−containing protein 1
with a threshold of 1e−05

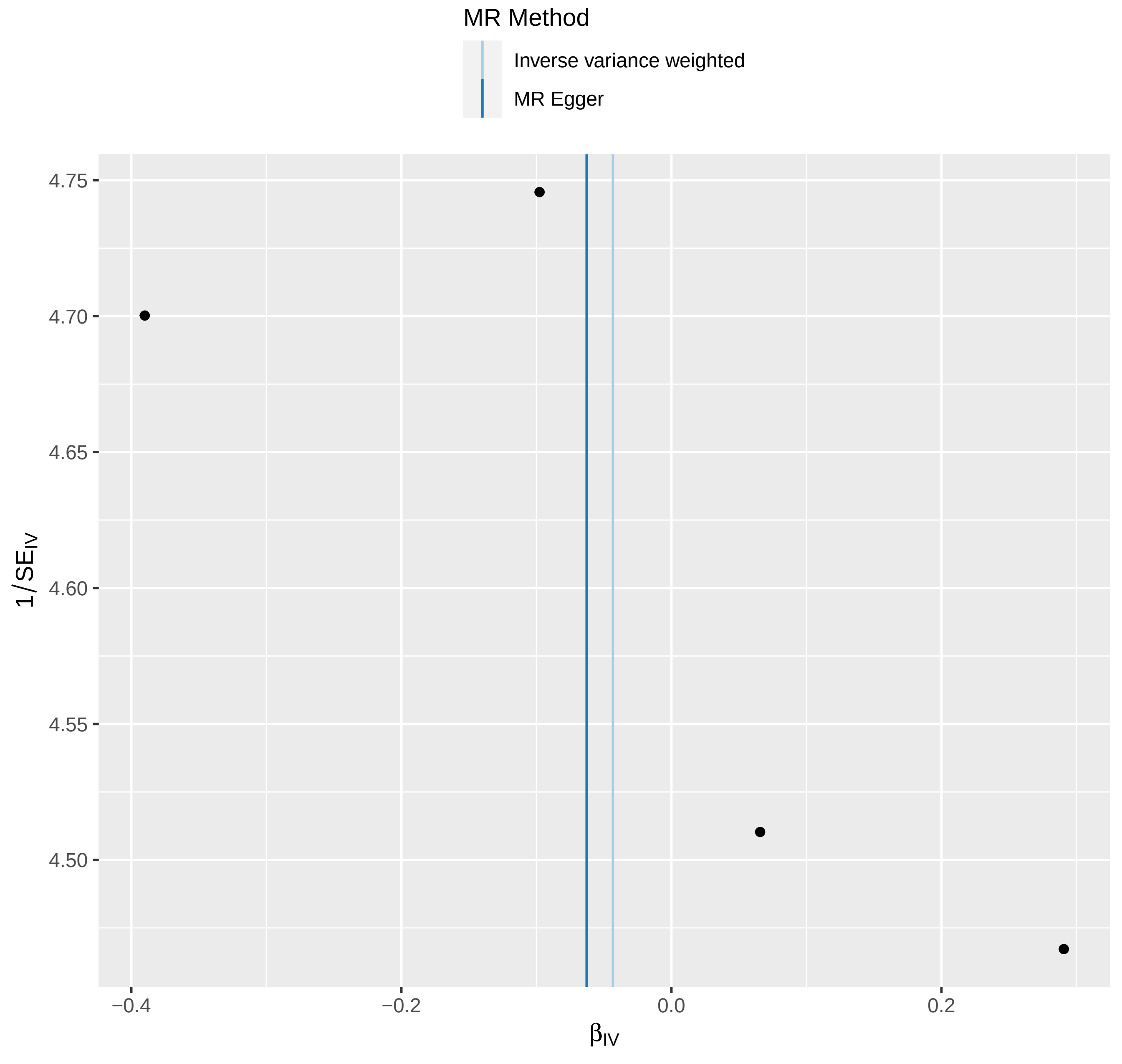

Batch 2−954 : Gut microbiota abundance (genus Parasutterella id.2892) on CUB domain−containing protein 1
with a threshold of 1e−05

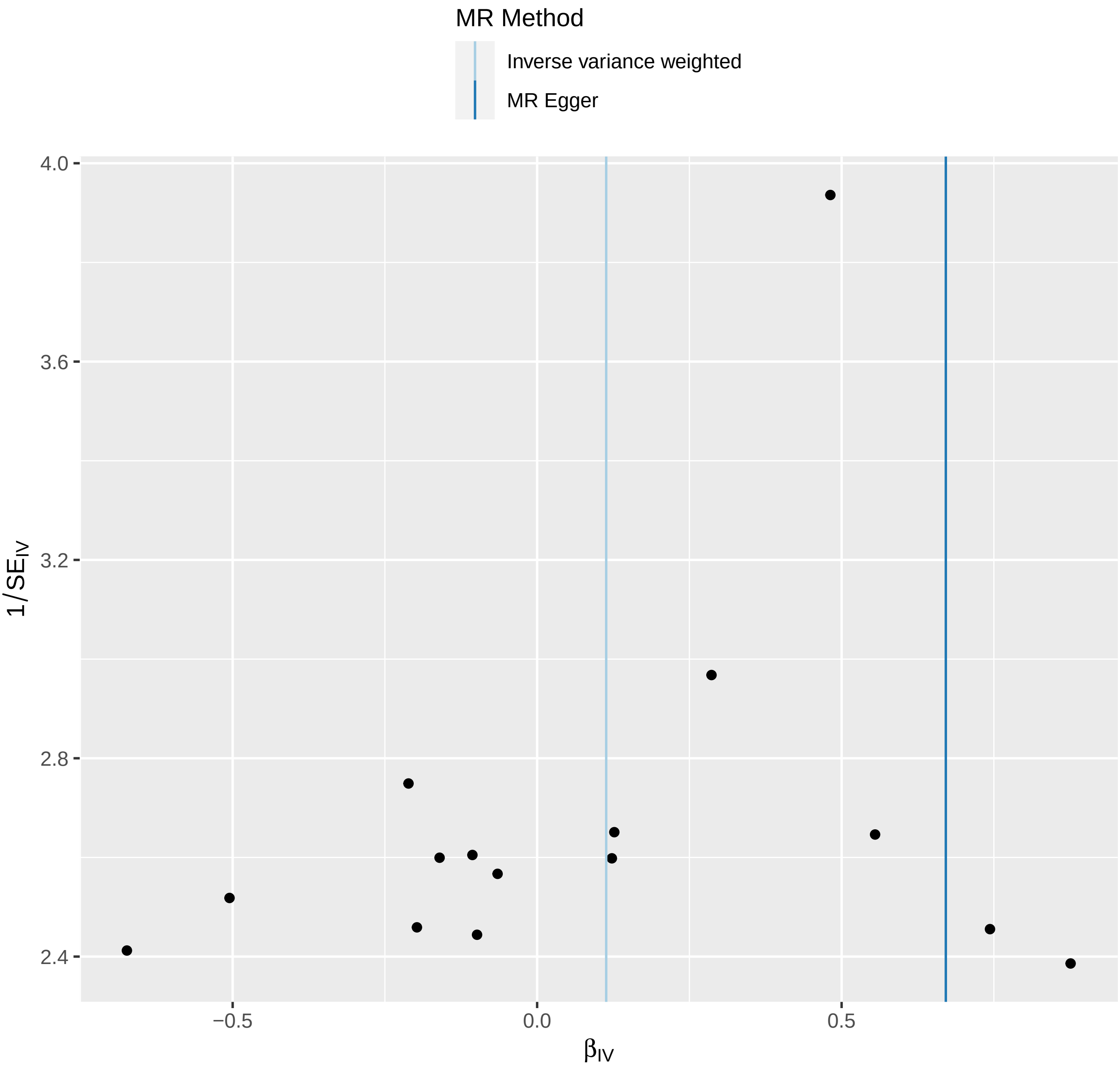

Batch 2−955 : Gut microbiota abundance (genus Catenibacterium id.2153) on Cadherin−12
with a threshold of 1e−05

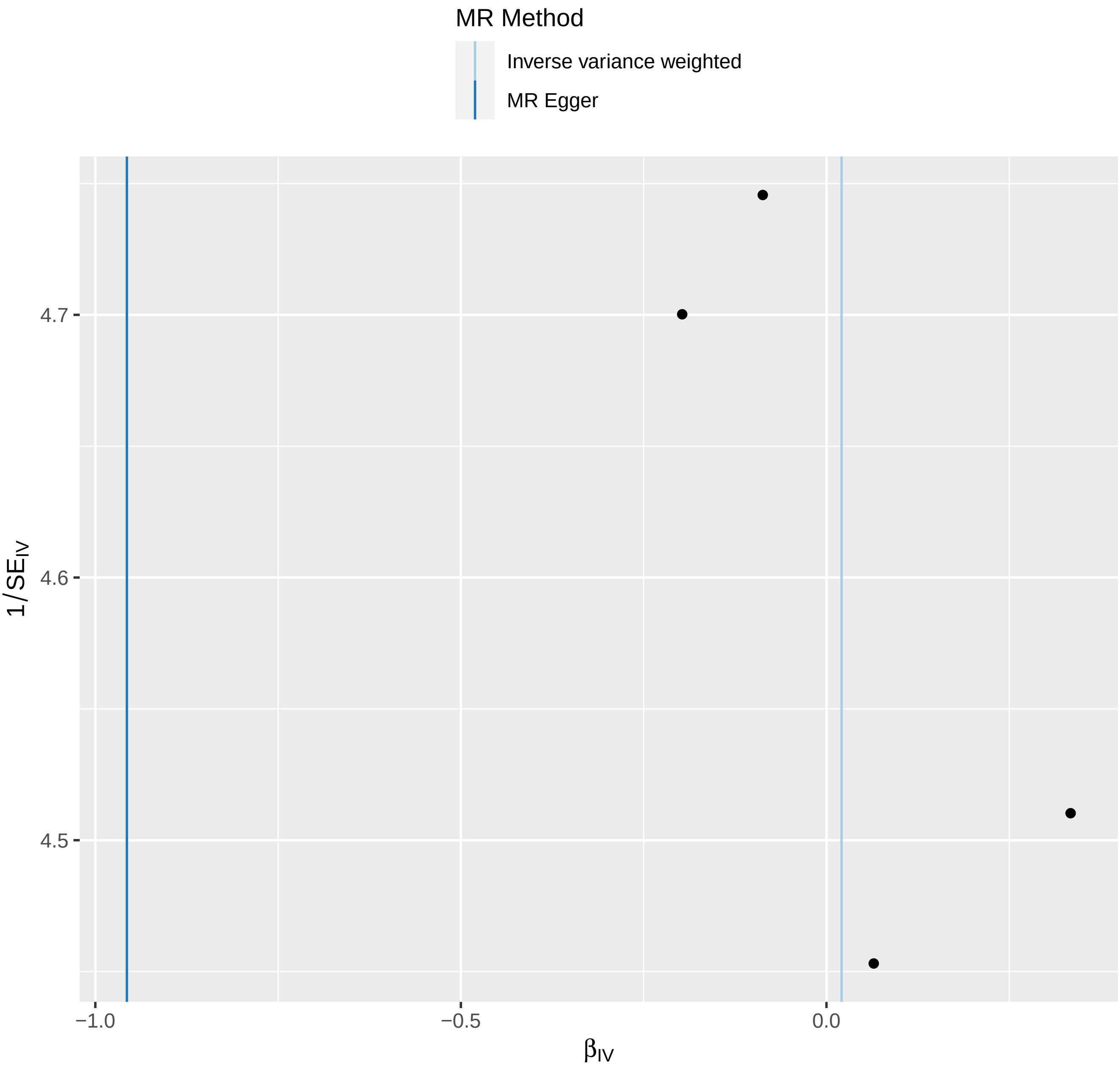

Batch 2−956 : Gut microbiota abundance (genus Parasutterella id.2892) on Cadherin−12
with a threshold of 1e−05

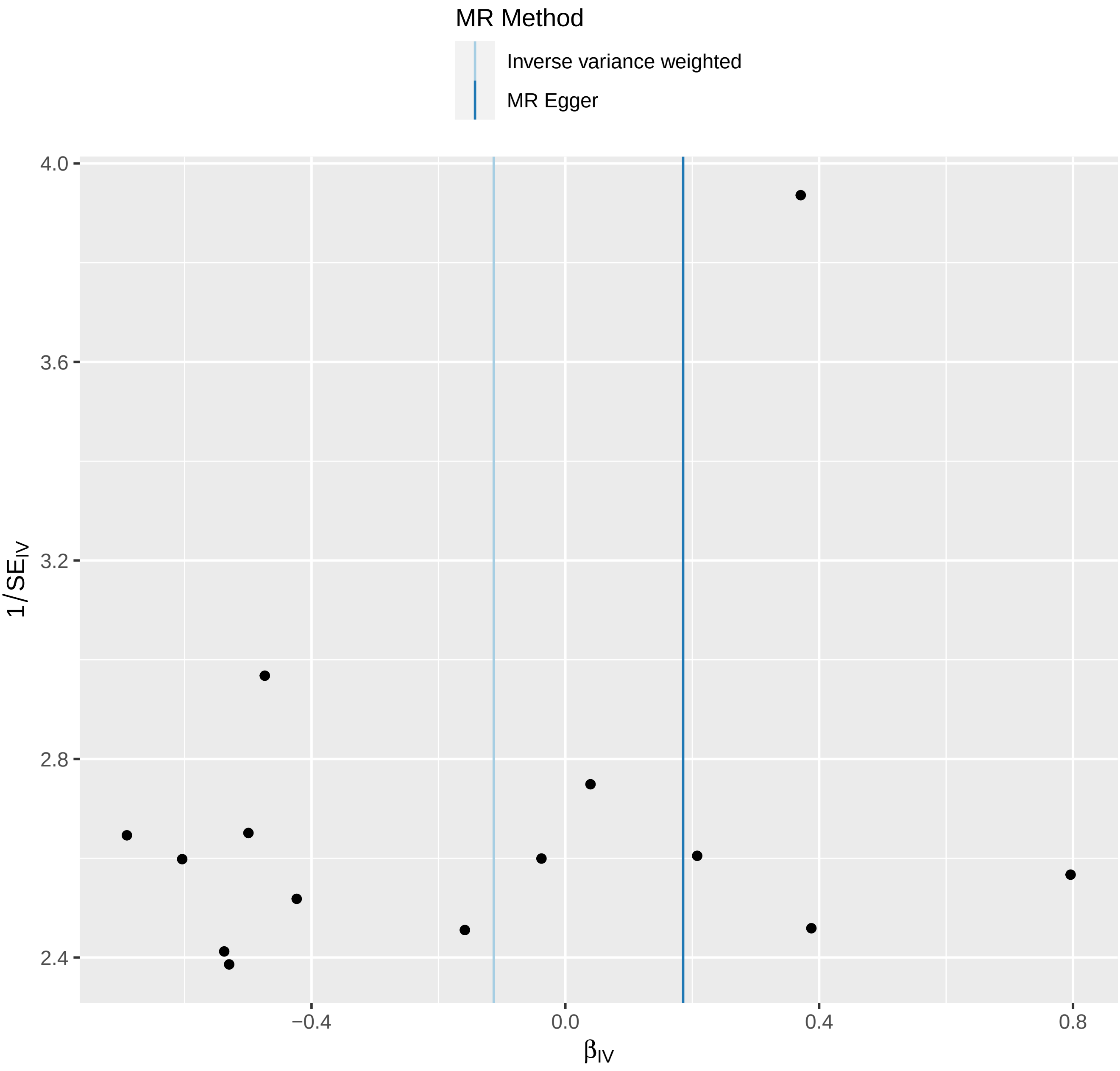

Batch 2−957 : Gut microbiota abundance (genus Catenibacterium id.2153) on Cadherin−12
with a threshold of 1e−05

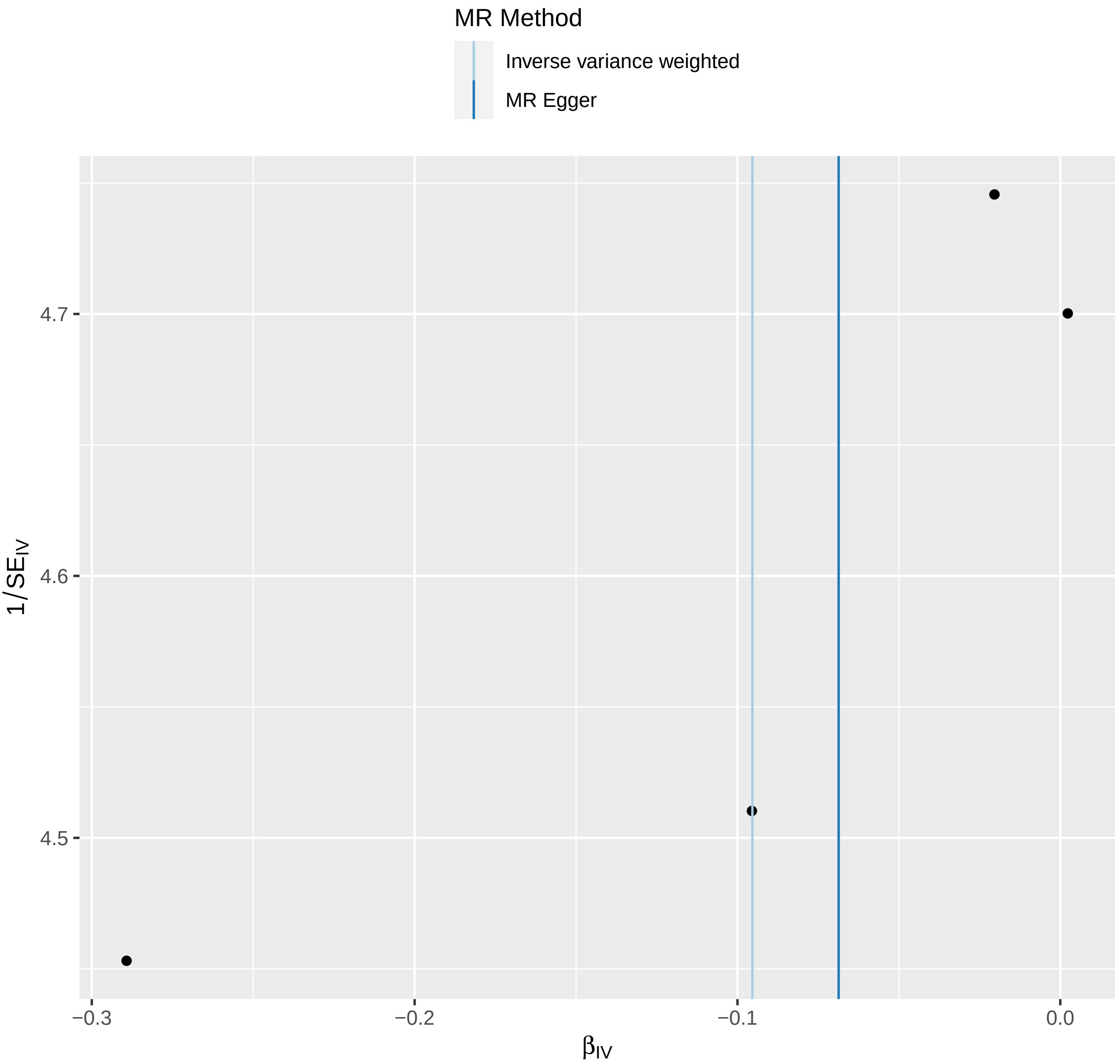

Batch 2−958 : Gut microbiota abundance (genus Parasutterella id.2892) on Cadherin−12
with a threshold of 1e−05

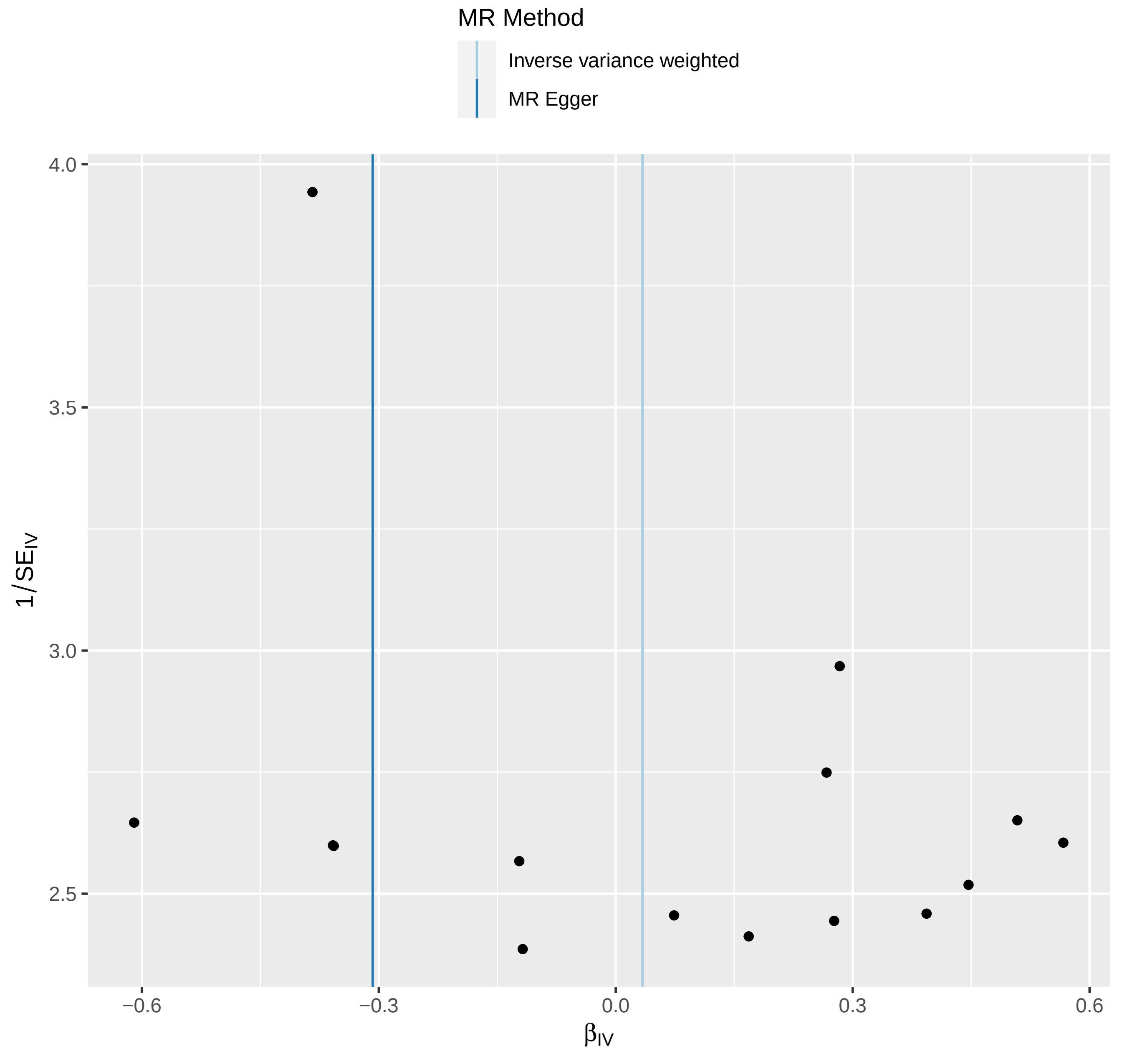

Batch 2−959 : Gut microbiota abundance (genus Catenibacterium id.2153) on Cadherin−1
with a threshold of 1e−05

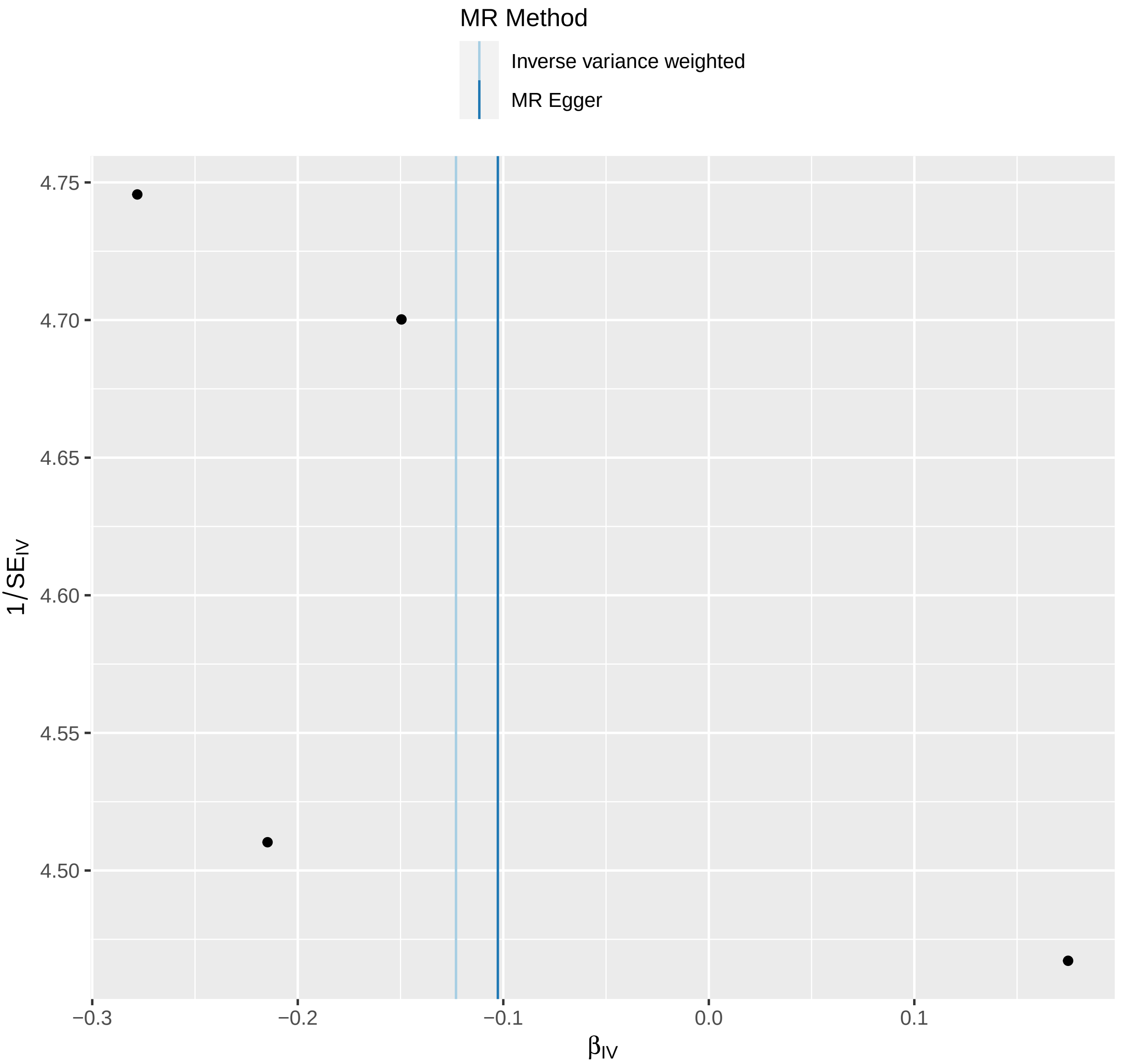

Batch 2−960 : Gut microbiota abundance (genus Parasutterella id.2892) on Cadherin−1
with a threshold of 1e−05

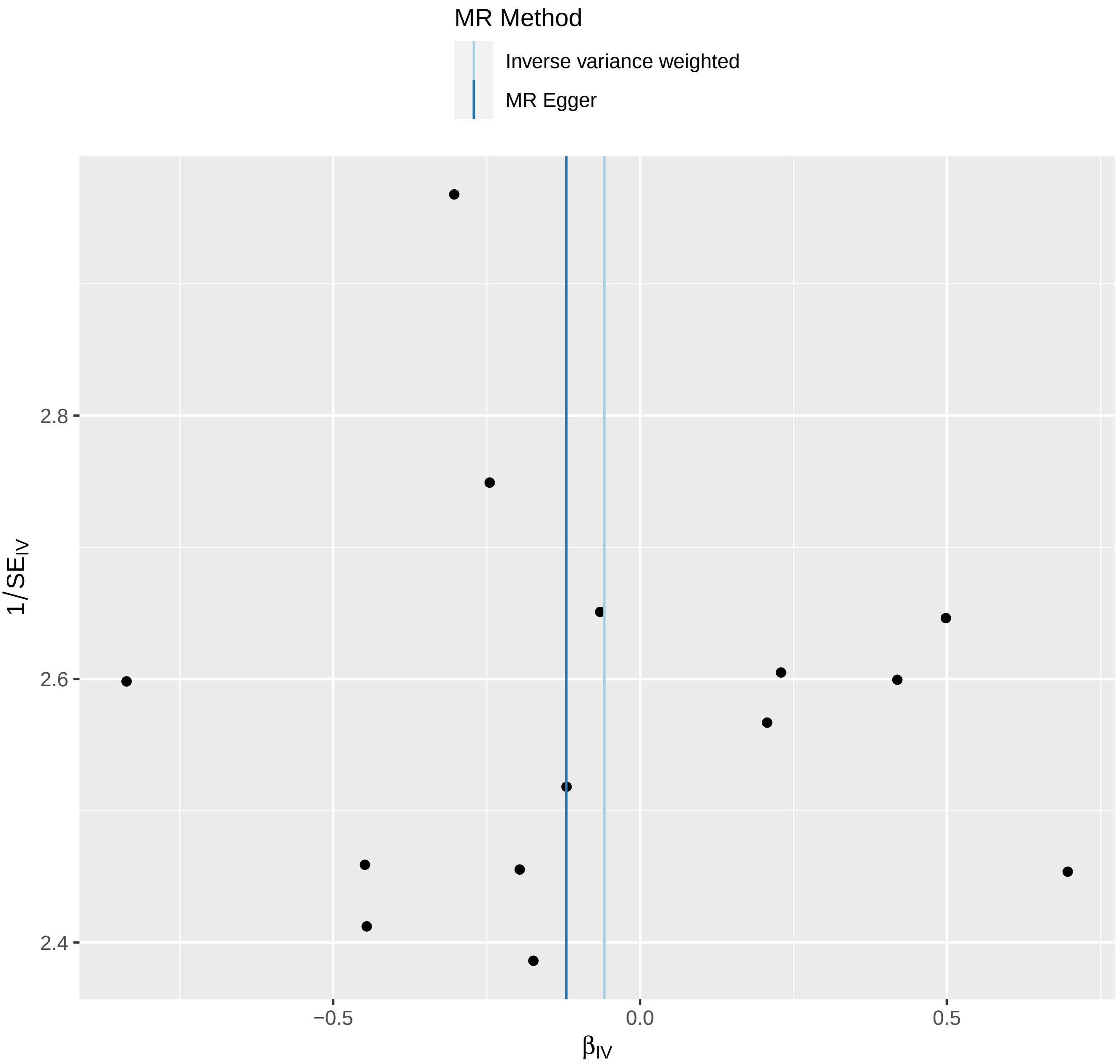

Batch 2−961 : Gut microbiota abundance (genus Catenibacterium id.2153) on Cadherin−15
with a threshold of 1e−05

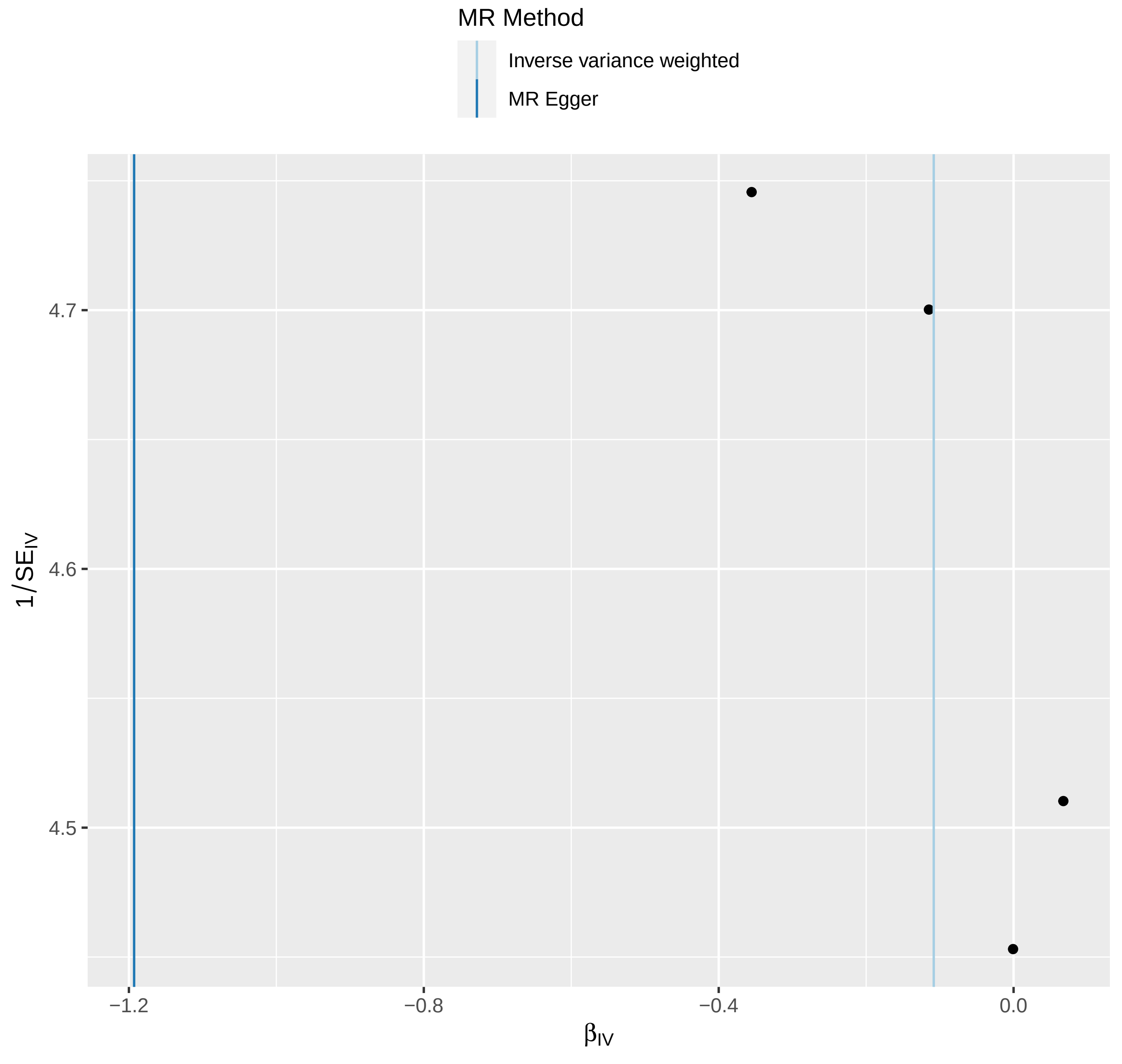

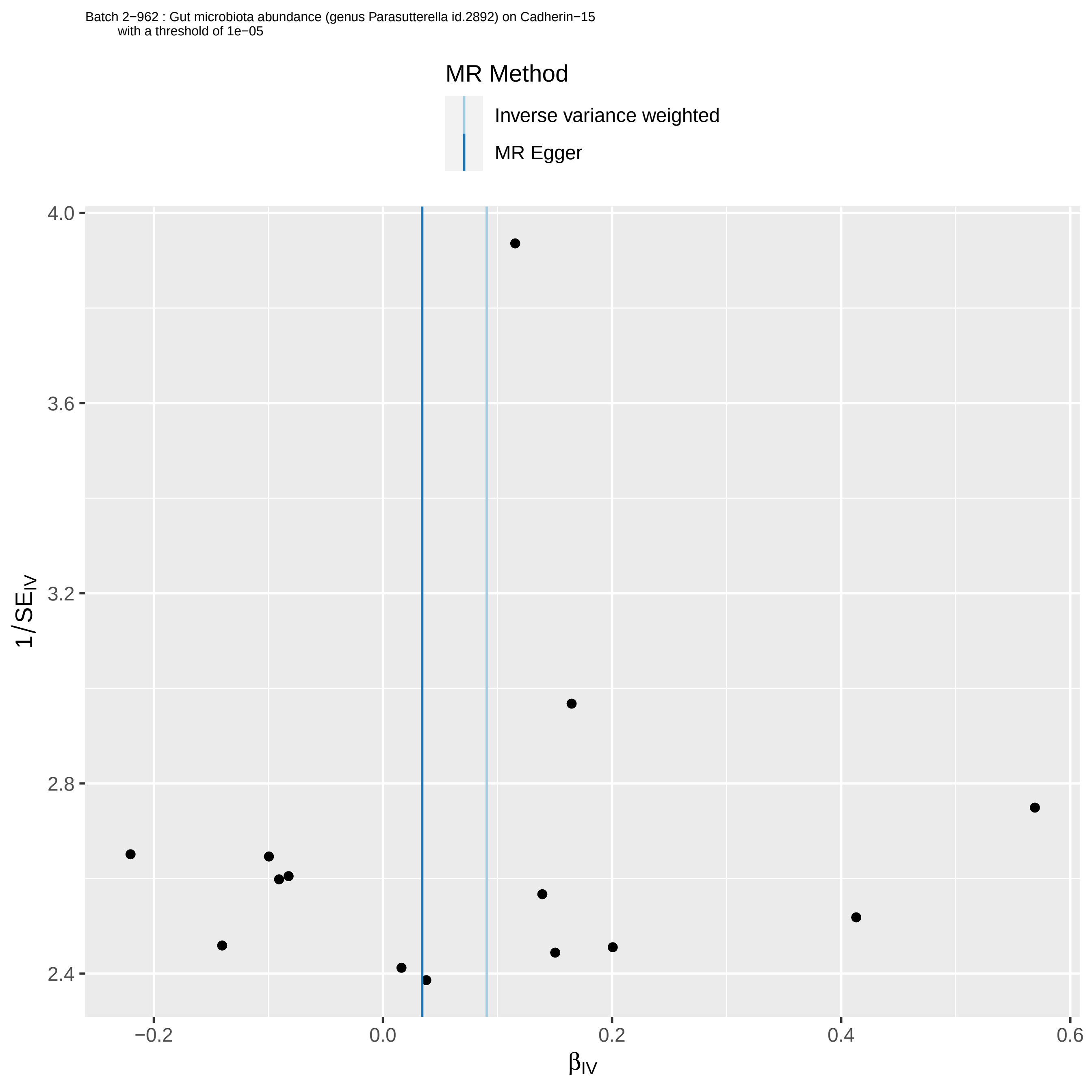
Batch 2−962 : Gut microbiota abundance (genus Parasutterella id.2892) on Cadherin−15
with a threshold of 1e−05
MR Method
Inverse variance weighted
MR Egger
4.0
3.6
3.2
2.8
2.4
1/SE_IV
−0.2
0.0
0.2
0.4
0.6
β_IV

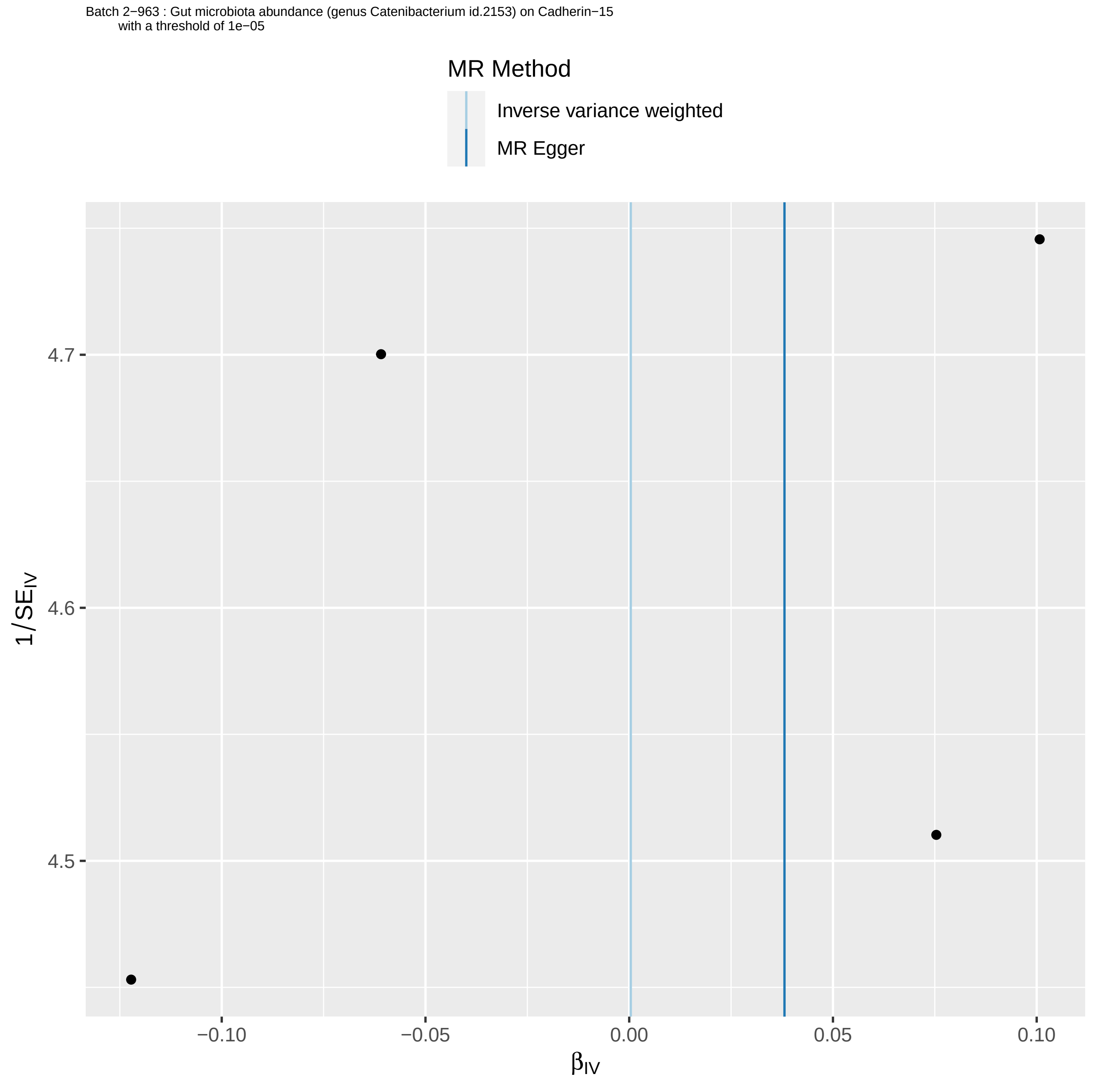
Batch 2−963 : Gut microbiota abundance (genus Catenibacterium id.2153) on Cadherin−15
with a threshold of 1e−05
MR Method
Inverse variance weighted
MR Egger
4.7
4.6
4.5
1/SE_IV
−0.10
−0.05
0.00
0.05
0.10
β_IV

Batch 2−964 : Gut microbiota abundance (genus Parasutterella id.2892) on Cadherin−15
with a threshold of 1e−05

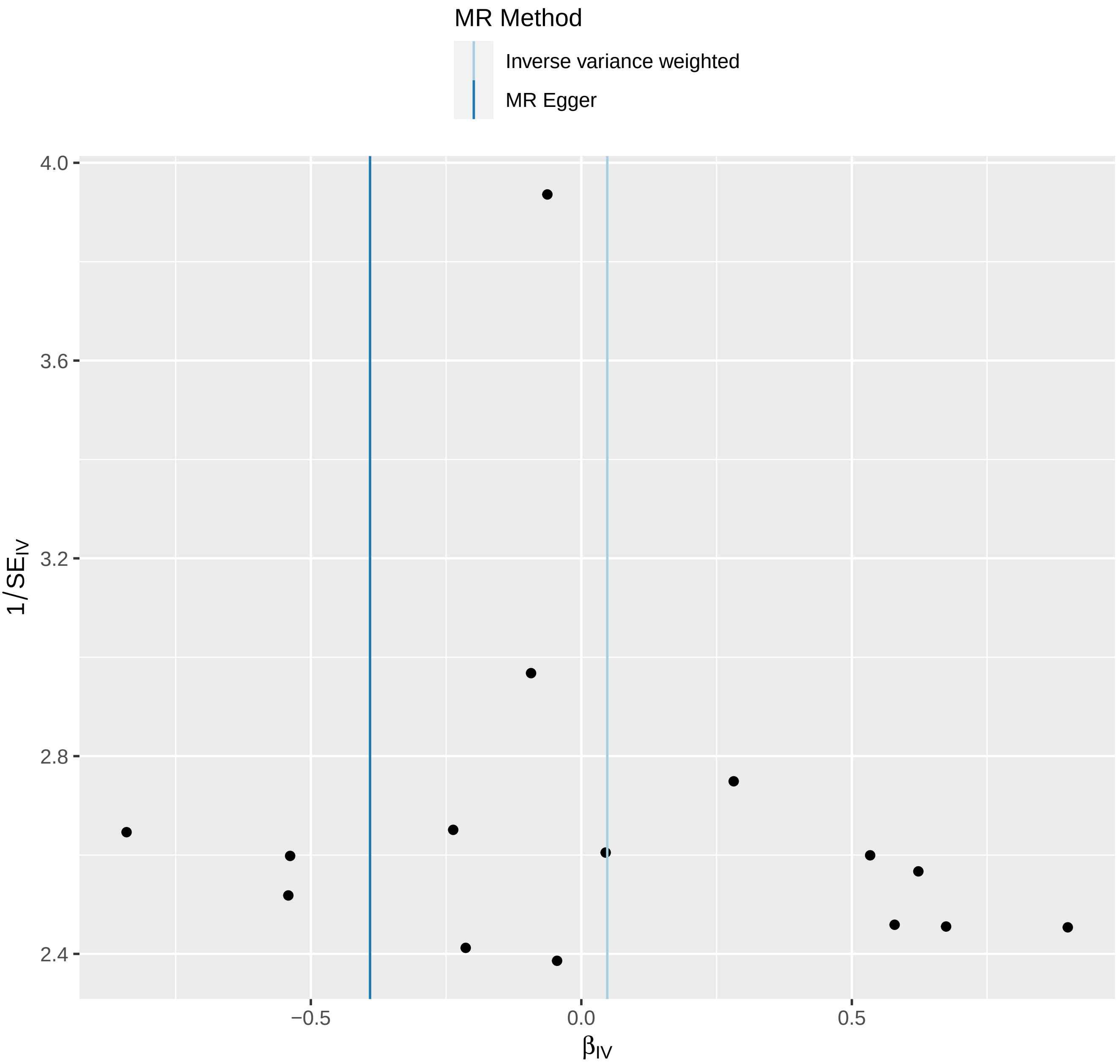

Batch 2−965 : Gut microbiota abundance (genus Catenibacterium id.2153) on Cadherin−2
with a threshold of 1e−05

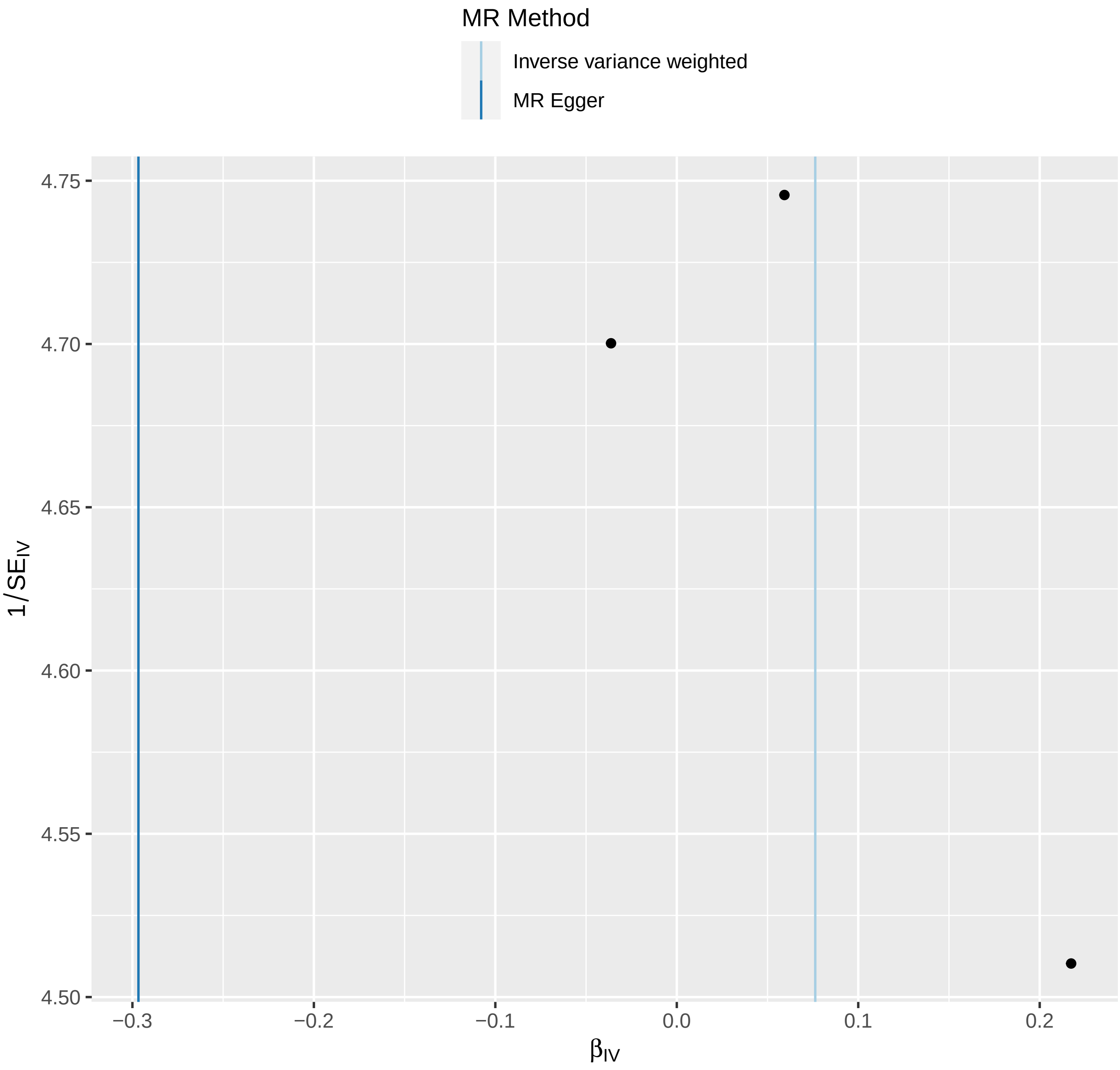

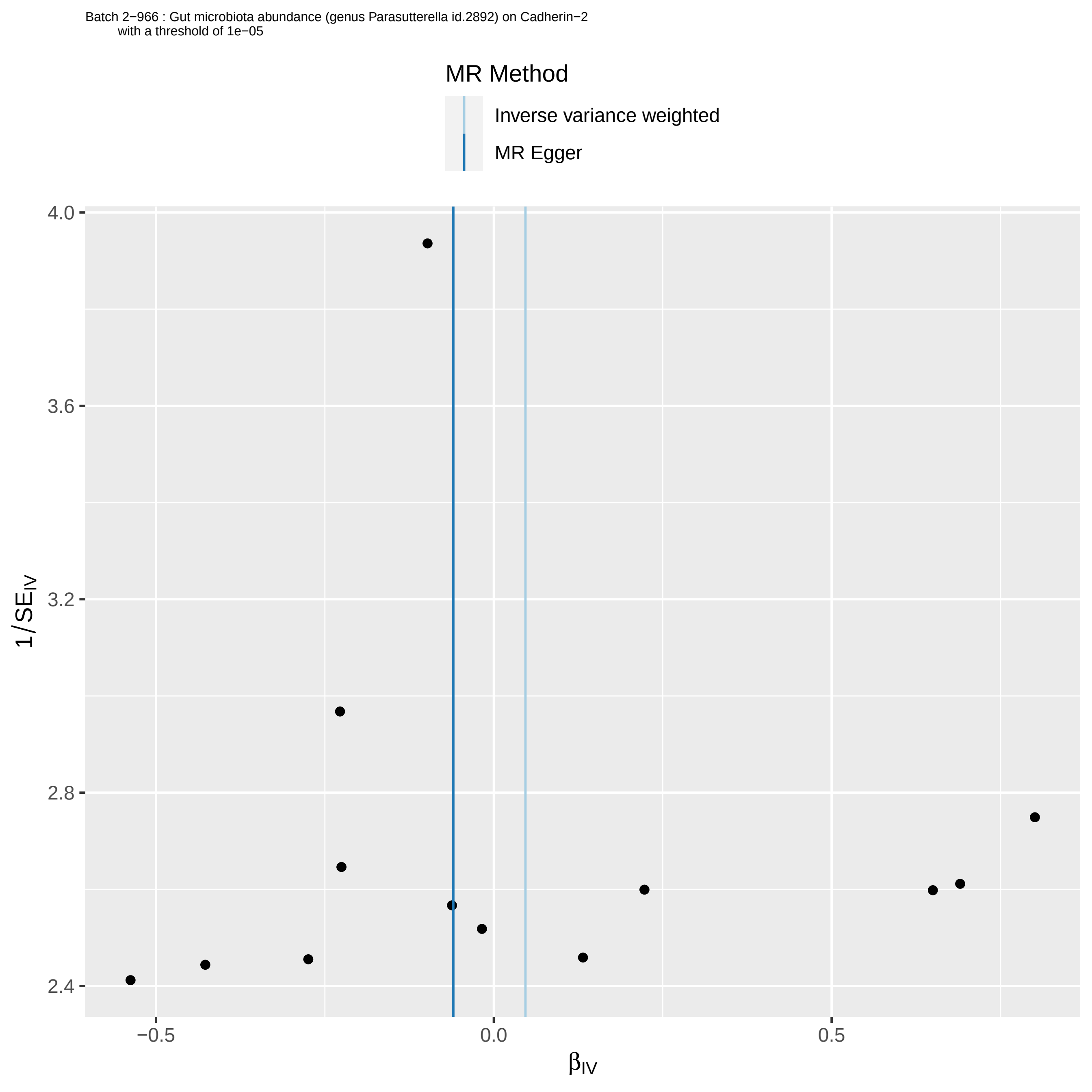
Batch 2−966 : Gut microbiota abundance (genus Parasutterella id.2892) on Cadherin−2
with a threshold of 1e−05
MR Method
Inverse variance weighted
MR Egger
4.0
3.6
3.2
2.8
2.4
$1/SE_{IV}$
−0.5
0.0
0.5
β_{IV}

Batch 2−967 : Gut microbiota abundance (genus Catenibacterium id.2153) on Cadherin−3
with a threshold of 1e−05

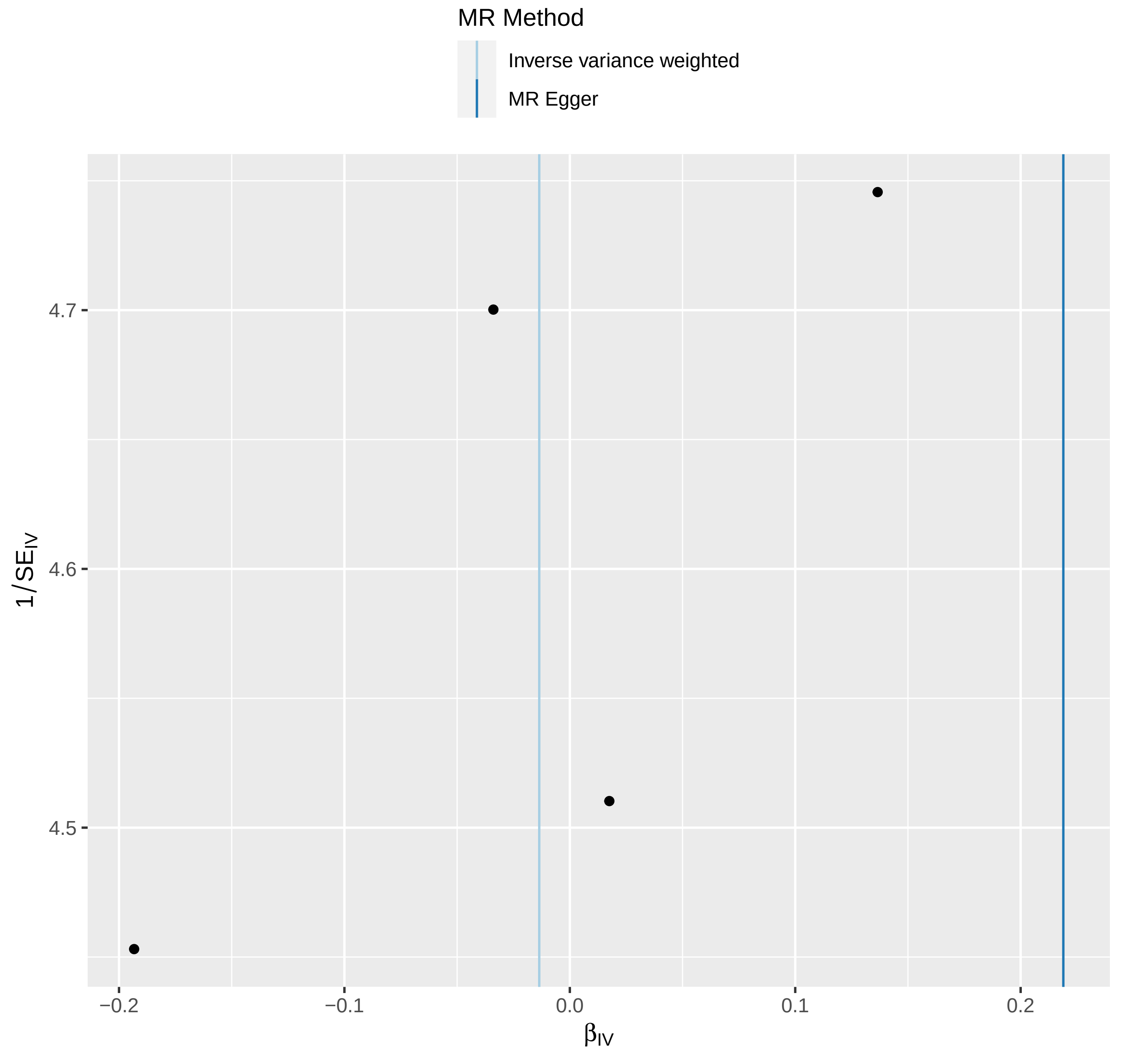

Batch 2−968 : Gut microbiota abundance (genus Parasutterella id.2892) on Cadherin−3
with a threshold of 1e−05

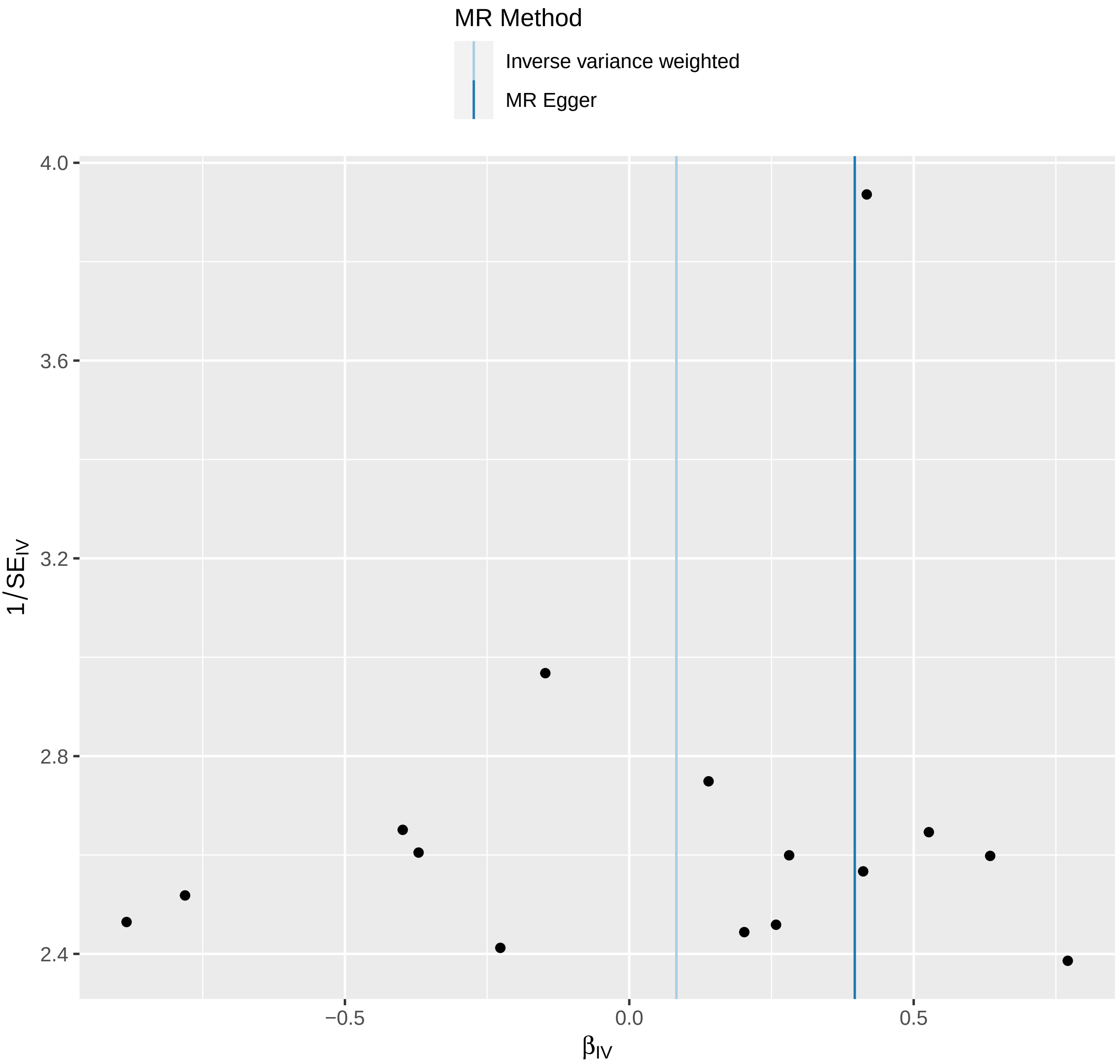

Batch 2−969 : Gut microbiota abundance (genus Catenibacterium id.2153) on Cadherin−5
with a threshold of 1e−05

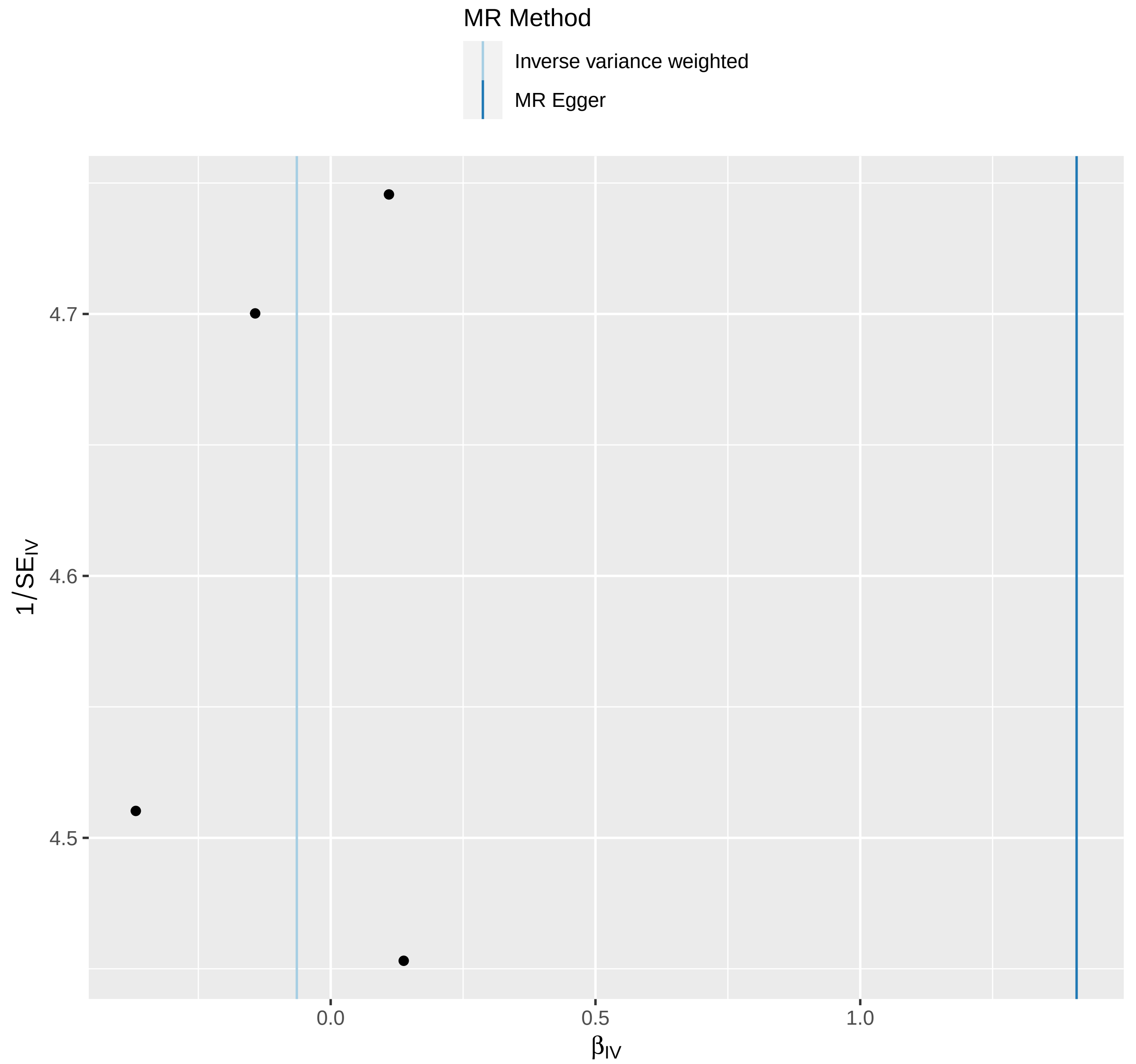

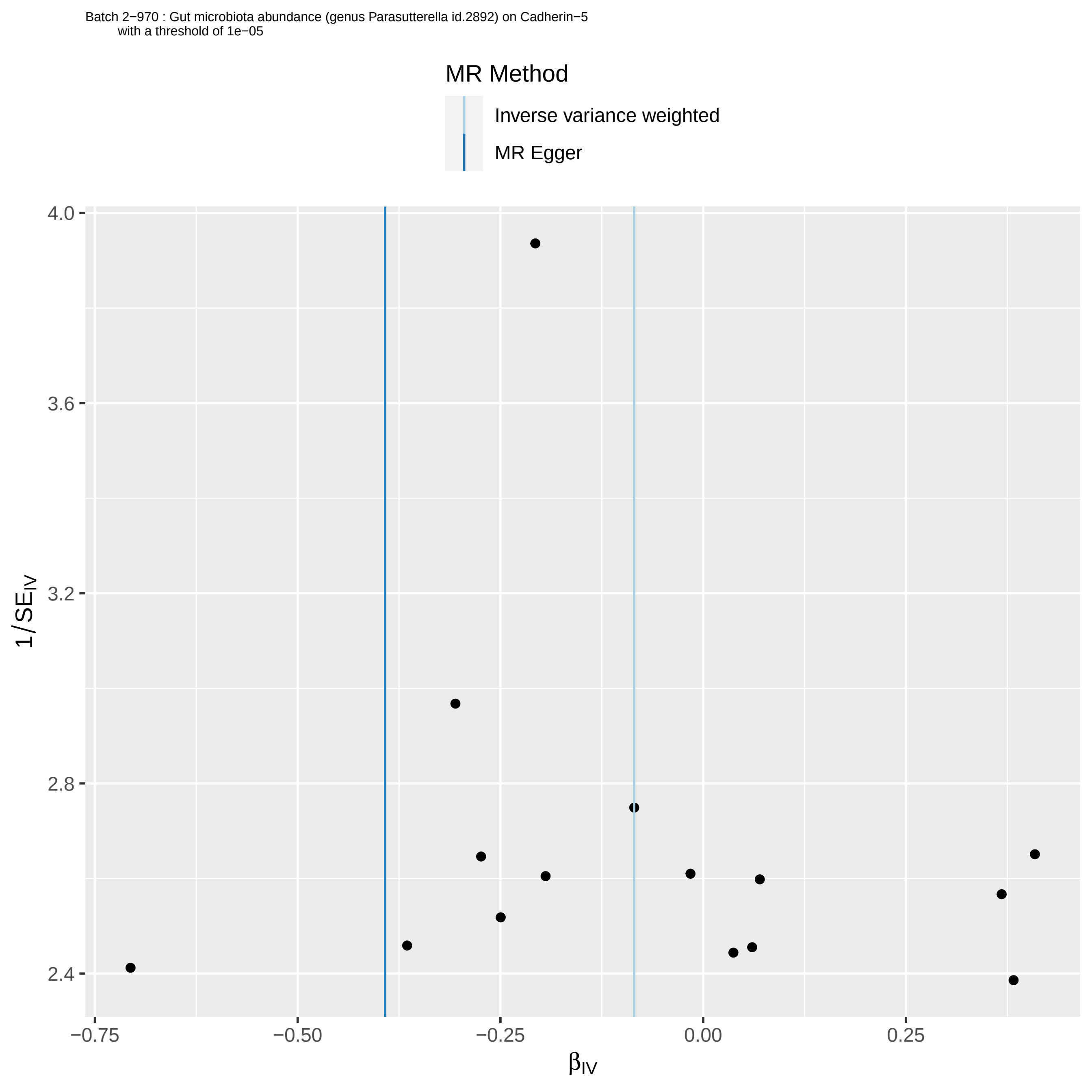
Batch 2−970 : Gut microbiota abundance (genus Parasutterella id.2892) on Cadherin−5
with a threshold of 1e−05
MR Method
Inverse variance weighted
MR Egger
4.0
3.6
3.2
2.8
2.4
1/SE_IV
−0.75
−0.50
−0.25
0.00
0.25
β_IV

Batch 2−971 : Gut microbiota abundance (genus Catenibacterium id.2153) on Cadherin−7
with a threshold of 1e−05

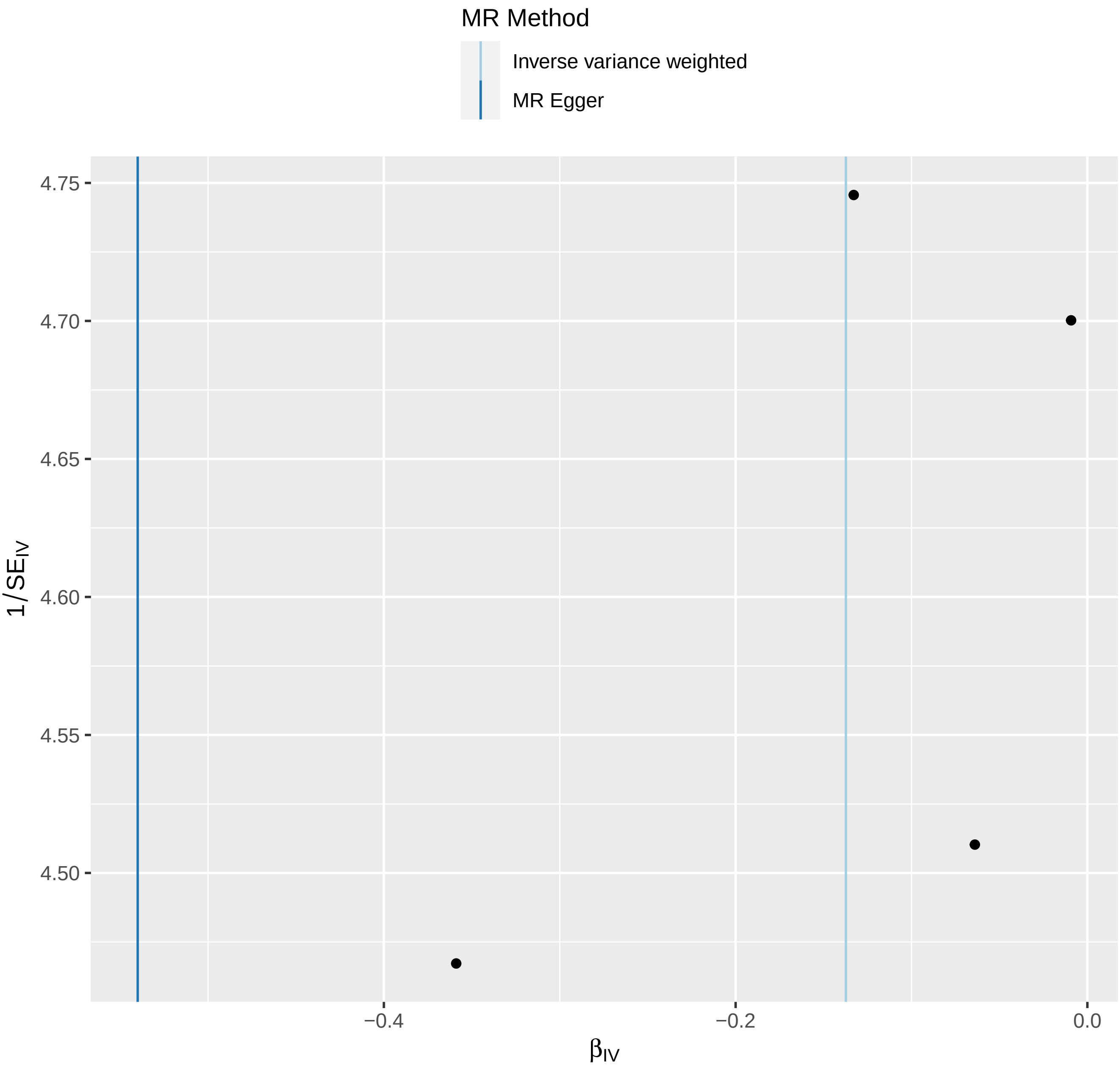

Batch 2−972 : Gut microbiota abundance (genus Parasutterella id.2892) on Cadherin−7
with a threshold of 1e−05

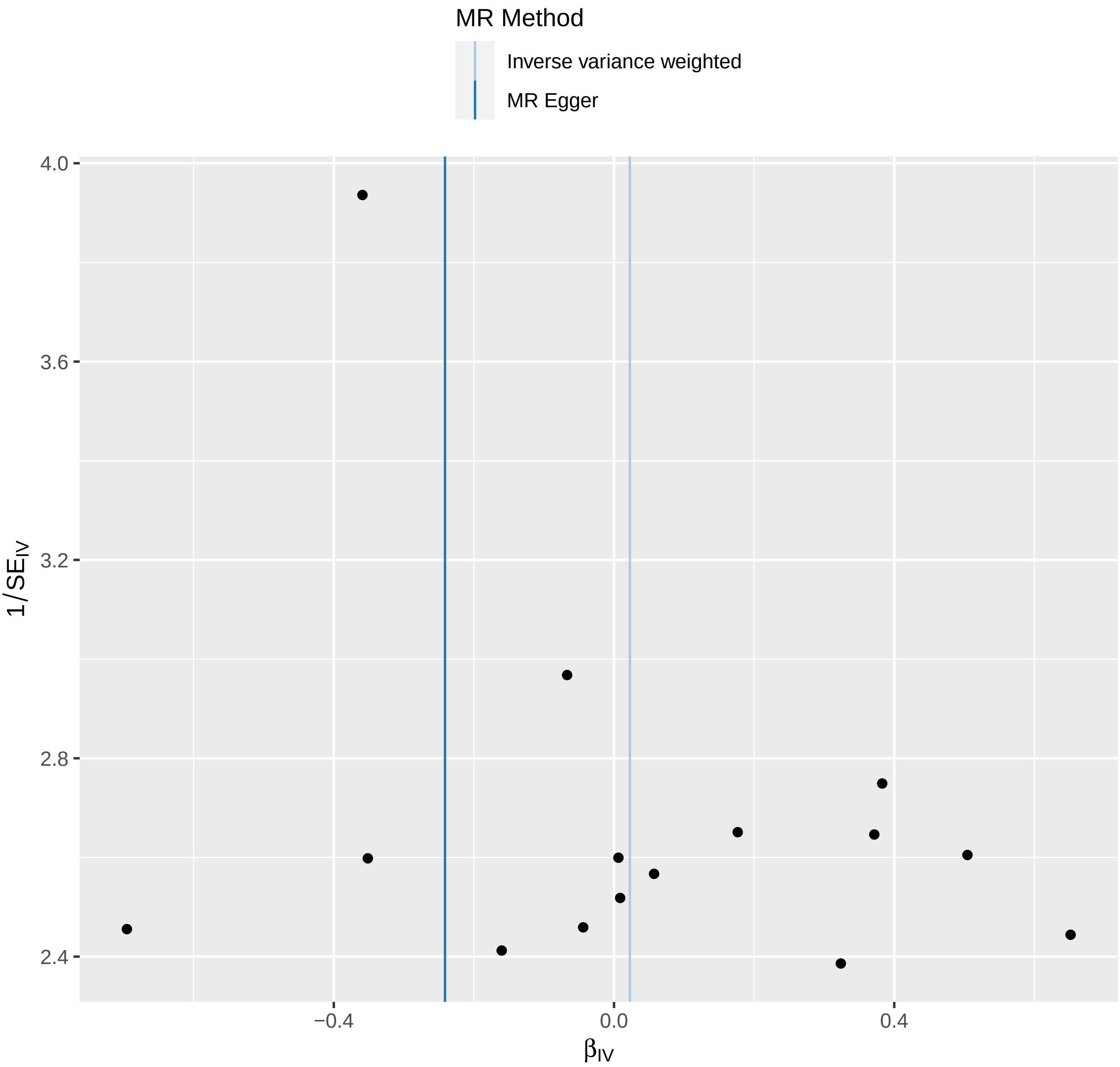

Batch 2−973 : Gut microbiota abundance (genus Catenibacterium id.2153) on Cadherin−related family member 1
with a threshold of 1e−05

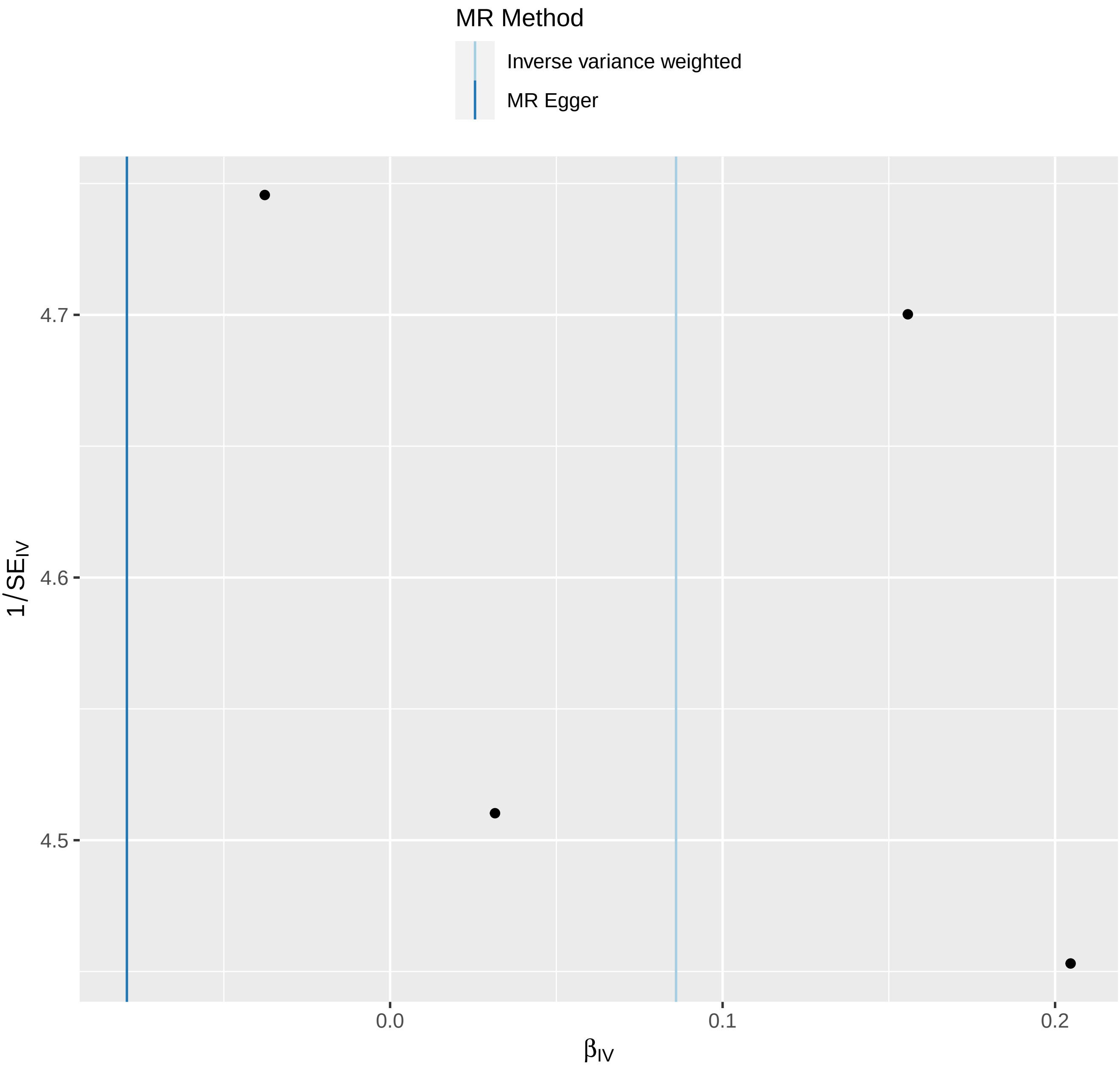

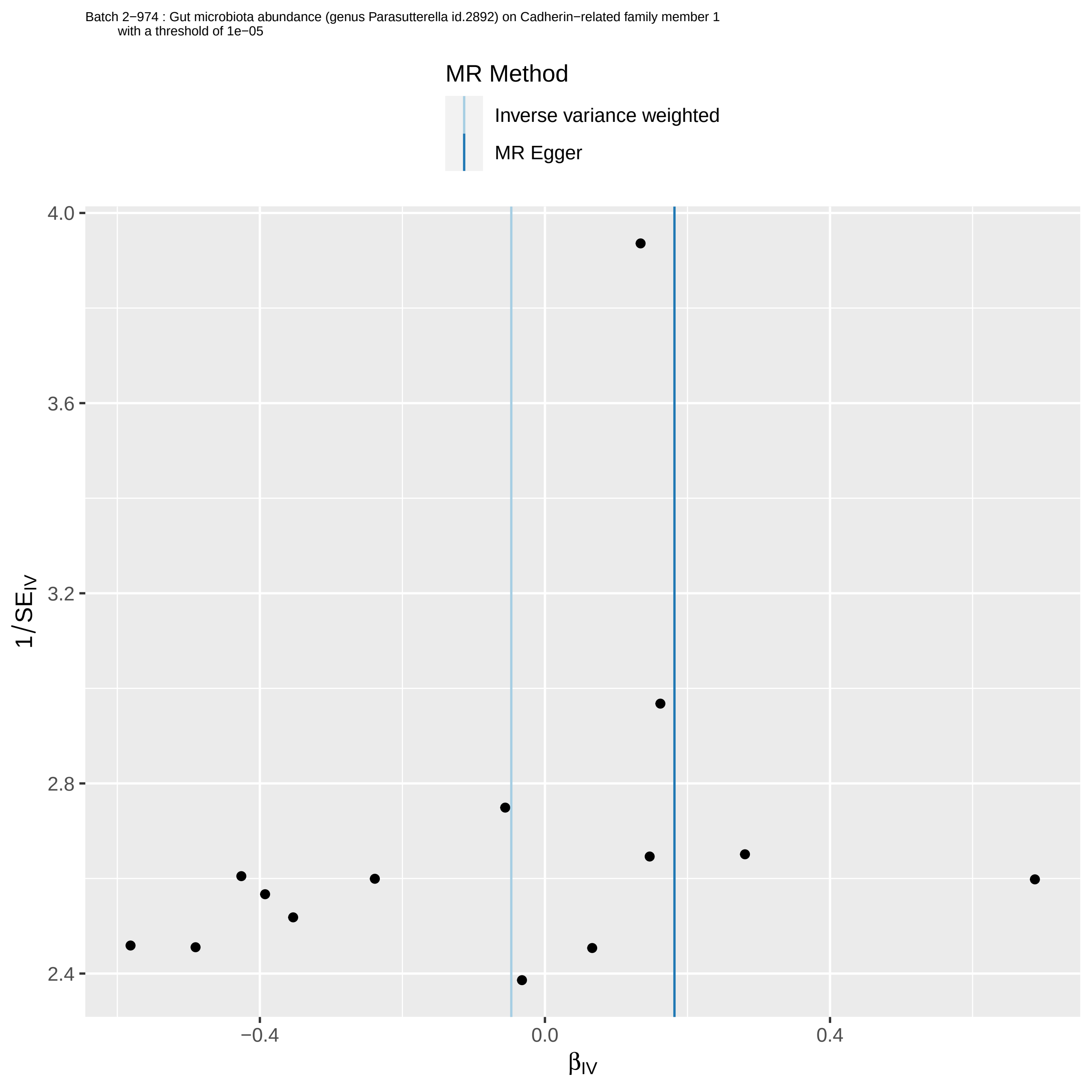

Batch 2−974 : Gut microbiota abundance (genus Parasutterella id.2892) on Cadherin−related family member 1
with a threshold of 1e−05
MR Method
Inverse variance weighted
MR Egger
4.0
3.6
3.2
2.8
2.4
1/SE_IV
−0.4
0.0
0.4
β_IV

Batch 2−975 : Gut microbiota abundance (genus Catenibacterium id.2153) on Cadherin−related family member 3
with a threshold of 1e−05

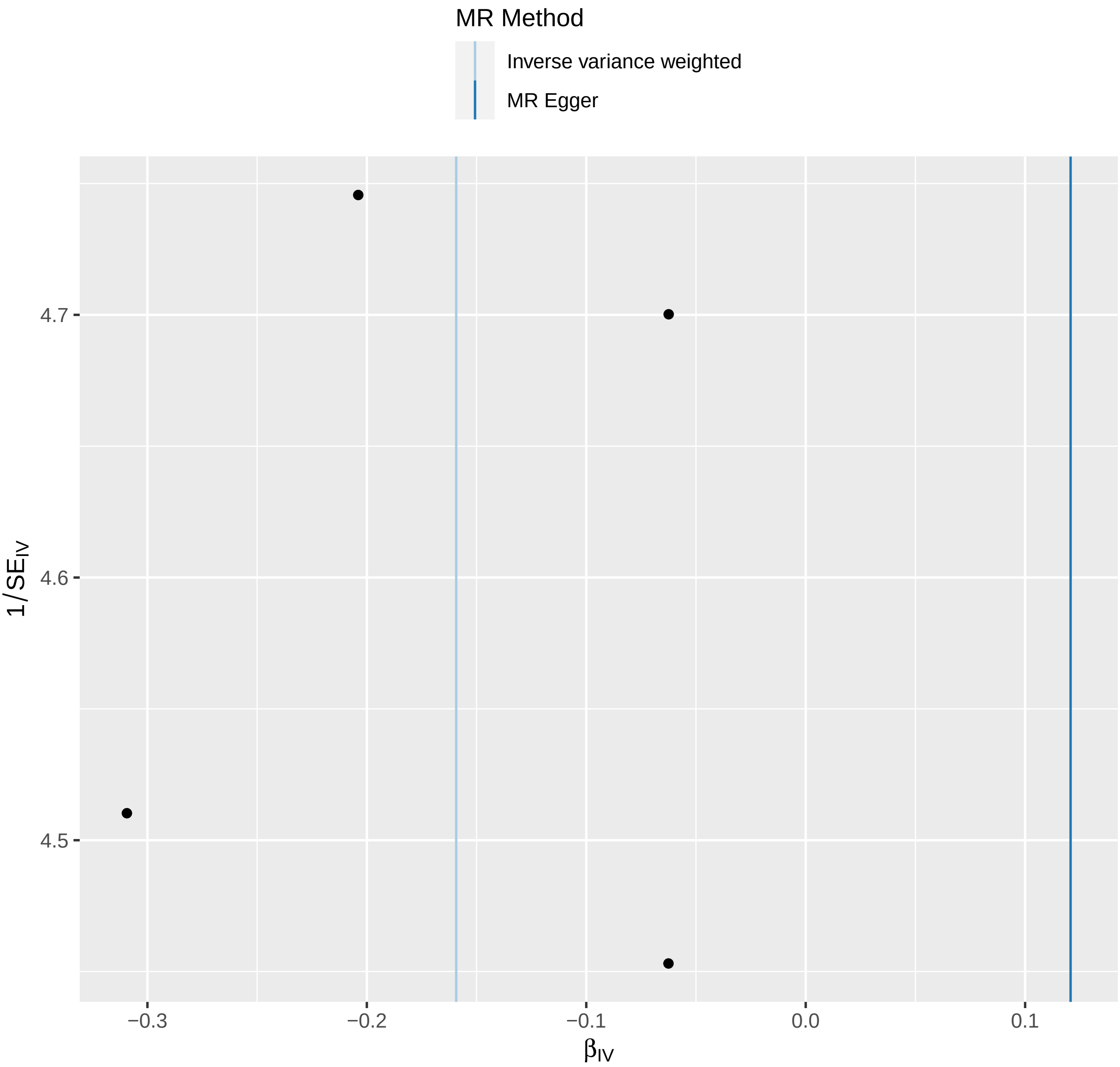

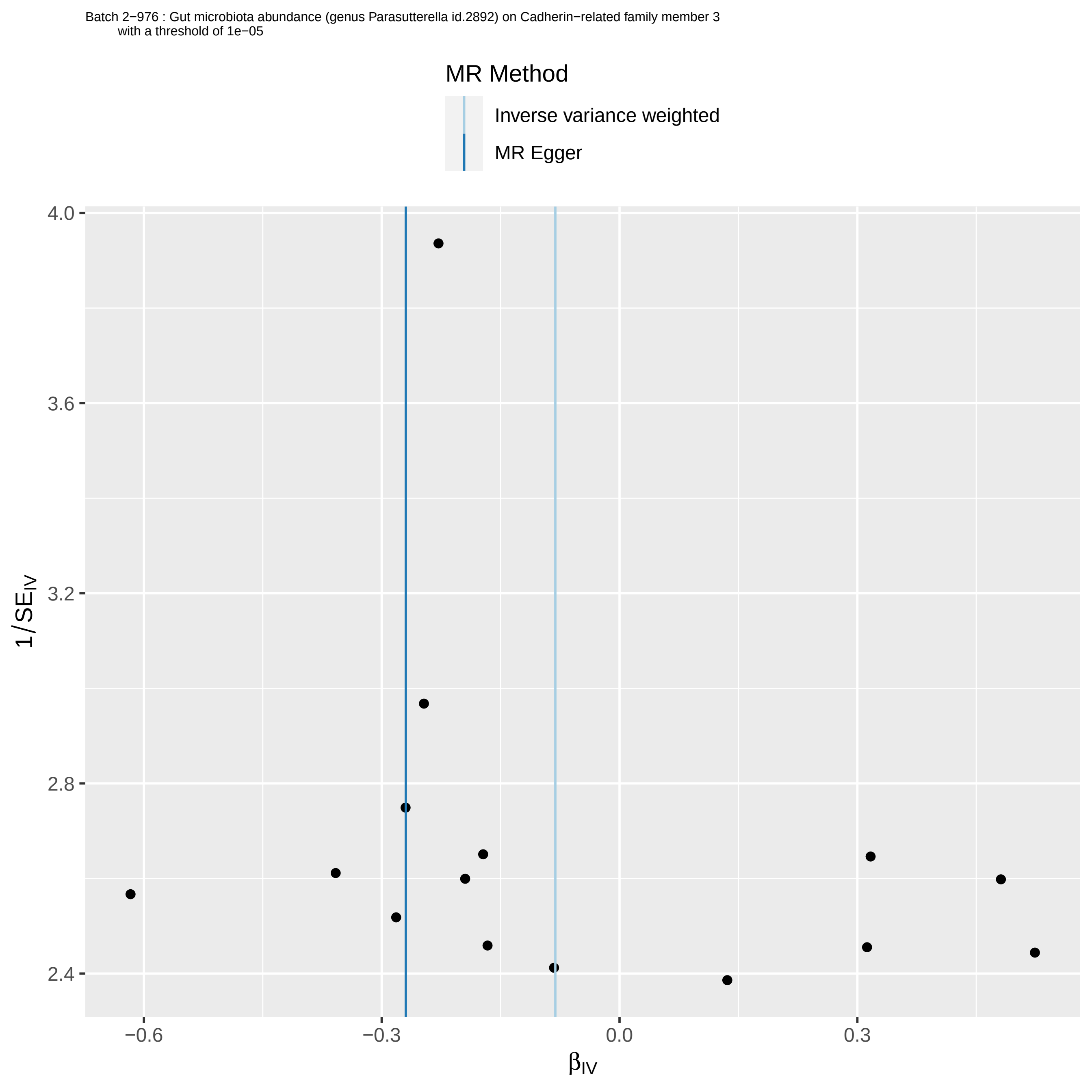
Batch 2−976 : Gut microbiota abundance (genus Parasutterella id.2892) on Cadherin−related family member 3
with a threshold of 1e−05
MR Method
Inverse variance weighted
MR Egger
4.0
3.6
3.2
2.8
2.4
1/SE_IV
−0.6
−0.3
0.0
0.3
β_IV

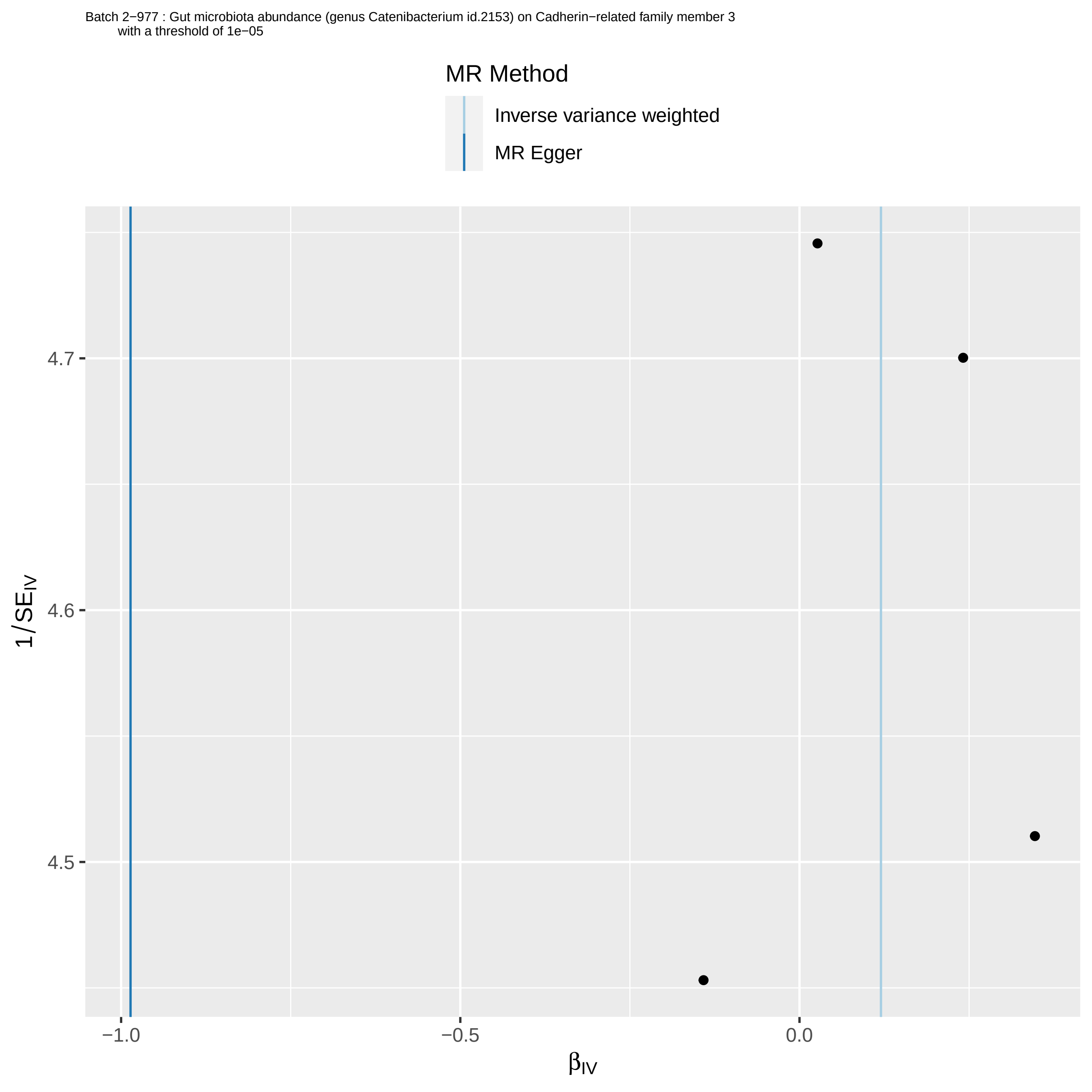
Batch 2−977 : Gut microbiota abundance (genus Catenibacterium id.2153) on Cadherin−related family member 3
with a threshold of 1e−05
MR Method
Inverse variance weighted
MR Egger
4.7
4.6
4.5
1/SE_IV
−1.0
−0.5
0.0
β_IV

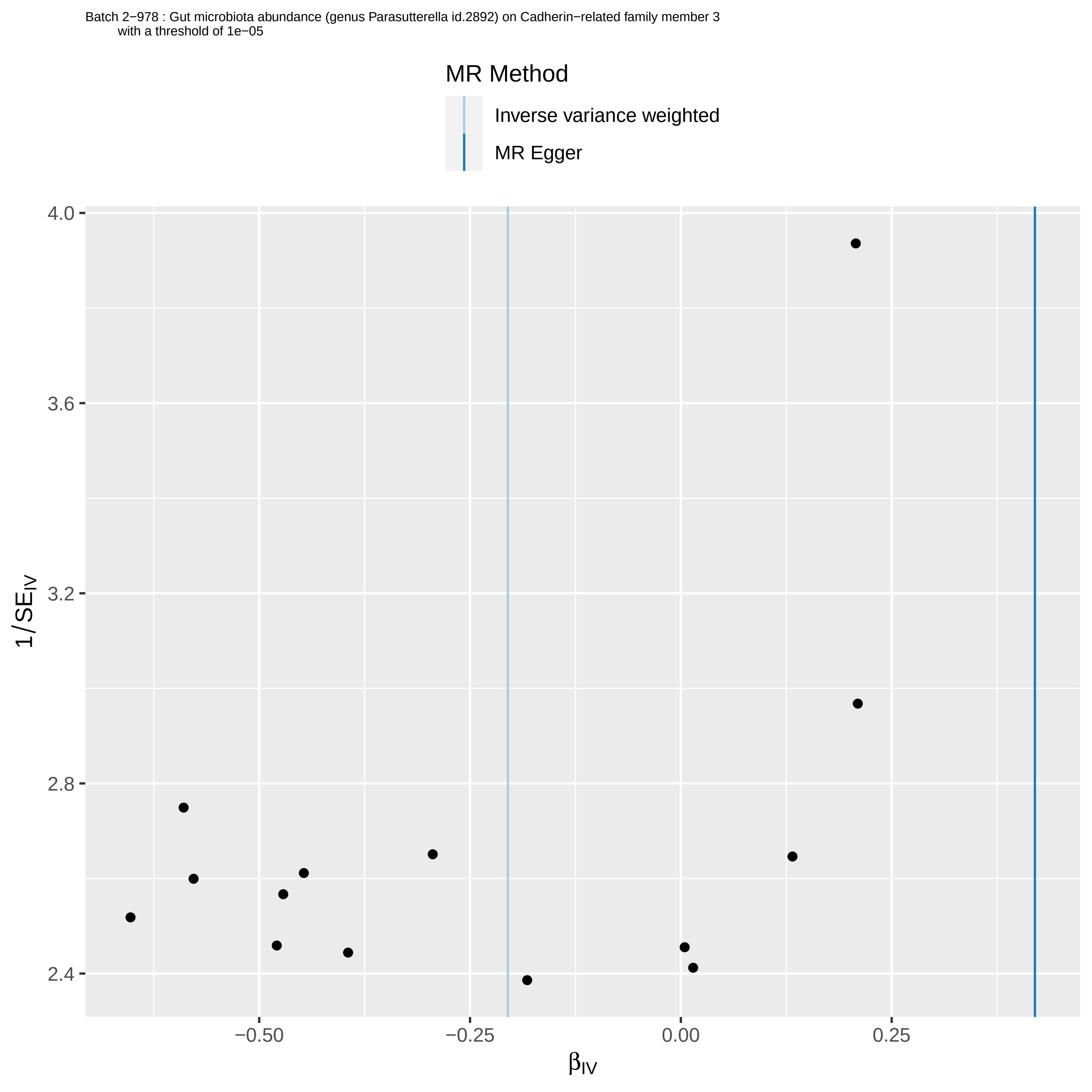

Batch 2−978 : Gut microbiota abundance (genus Parasutterella id.2892) on Cadherin−related family member 3
with a threshold of 1e−05
MR Method
Inverse variance weighted
MR Egger
4.0
3.6
3.2
2.8
2.4
1/SE_IV
−0.50
−0.25
0.00
0.25
β_IV

Batch 2−979 : Gut microbiota abundance (genus Catenibacterium id.2153) on Cadherin−related family member 5
with a threshold of 1e−05

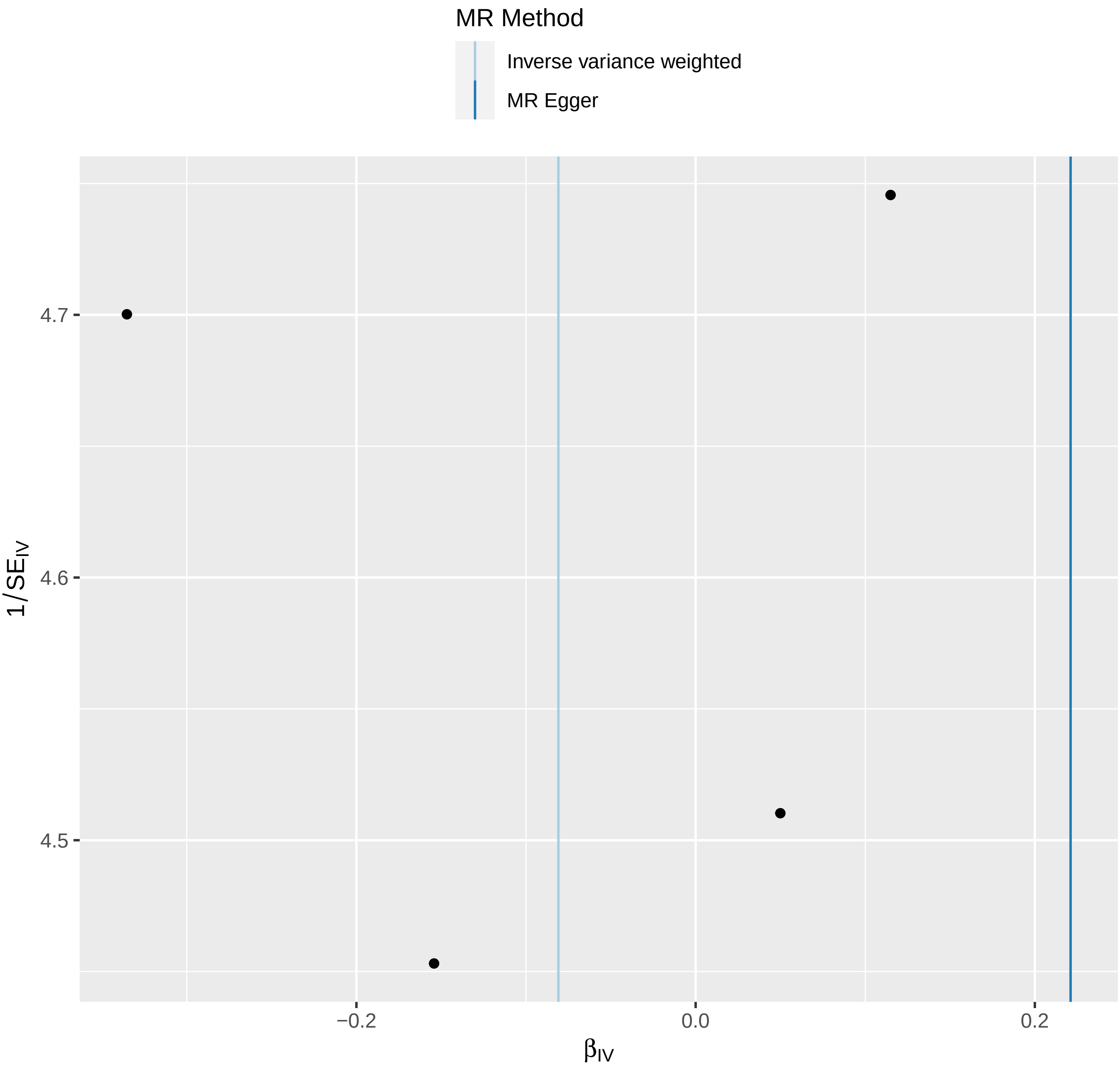

Batch 2−980 : Gut microbiota abundance (genus Parasutterella id.2892) on Cadherin−related family member 5
with a threshold of 1e−05

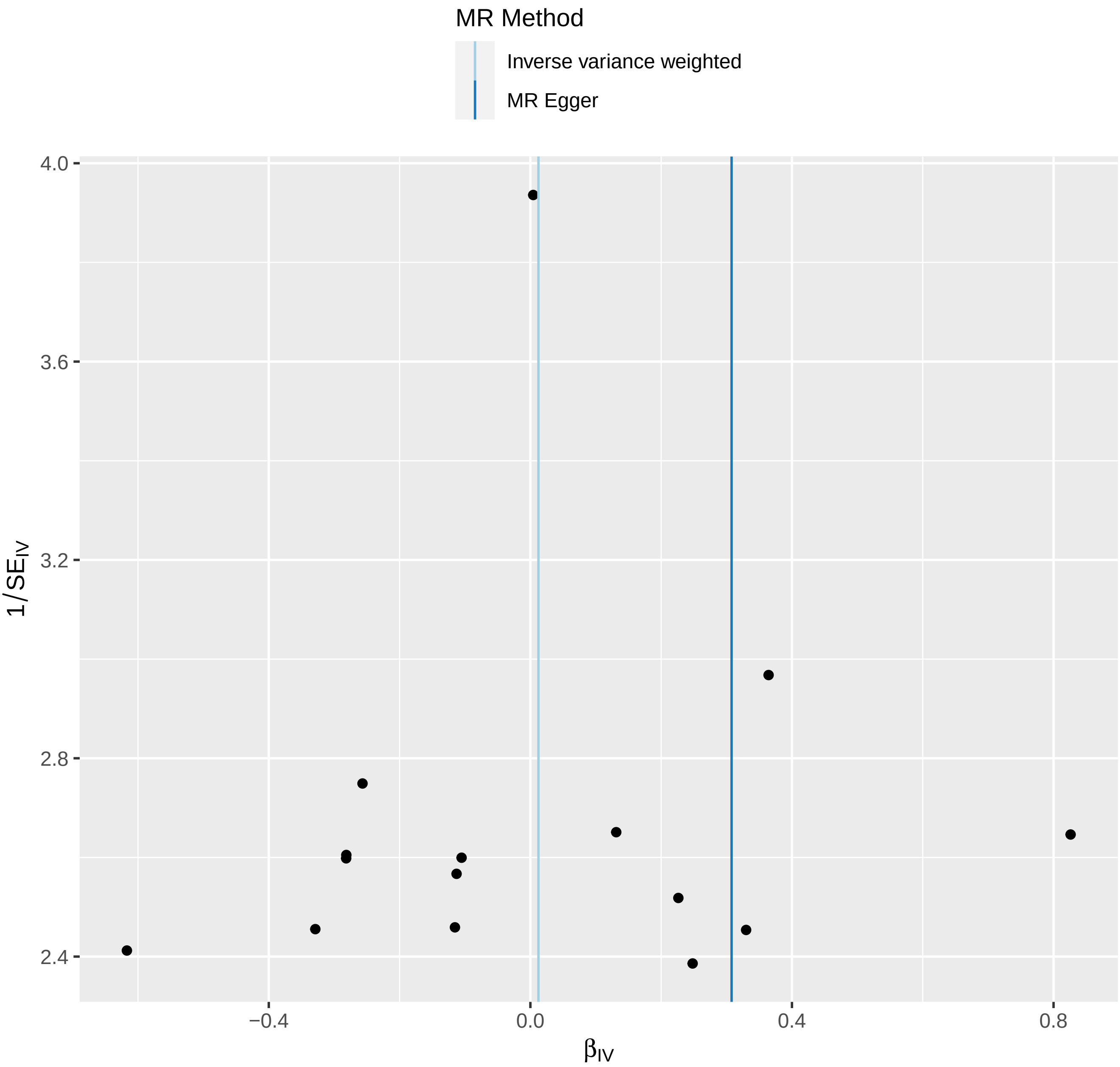

Batch 2−981 : Gut microbiota abundance (genus Catenibacterium id.2153) on Cyclin−dependent kinase 2−associated protein 1
with a threshold of 1e−05

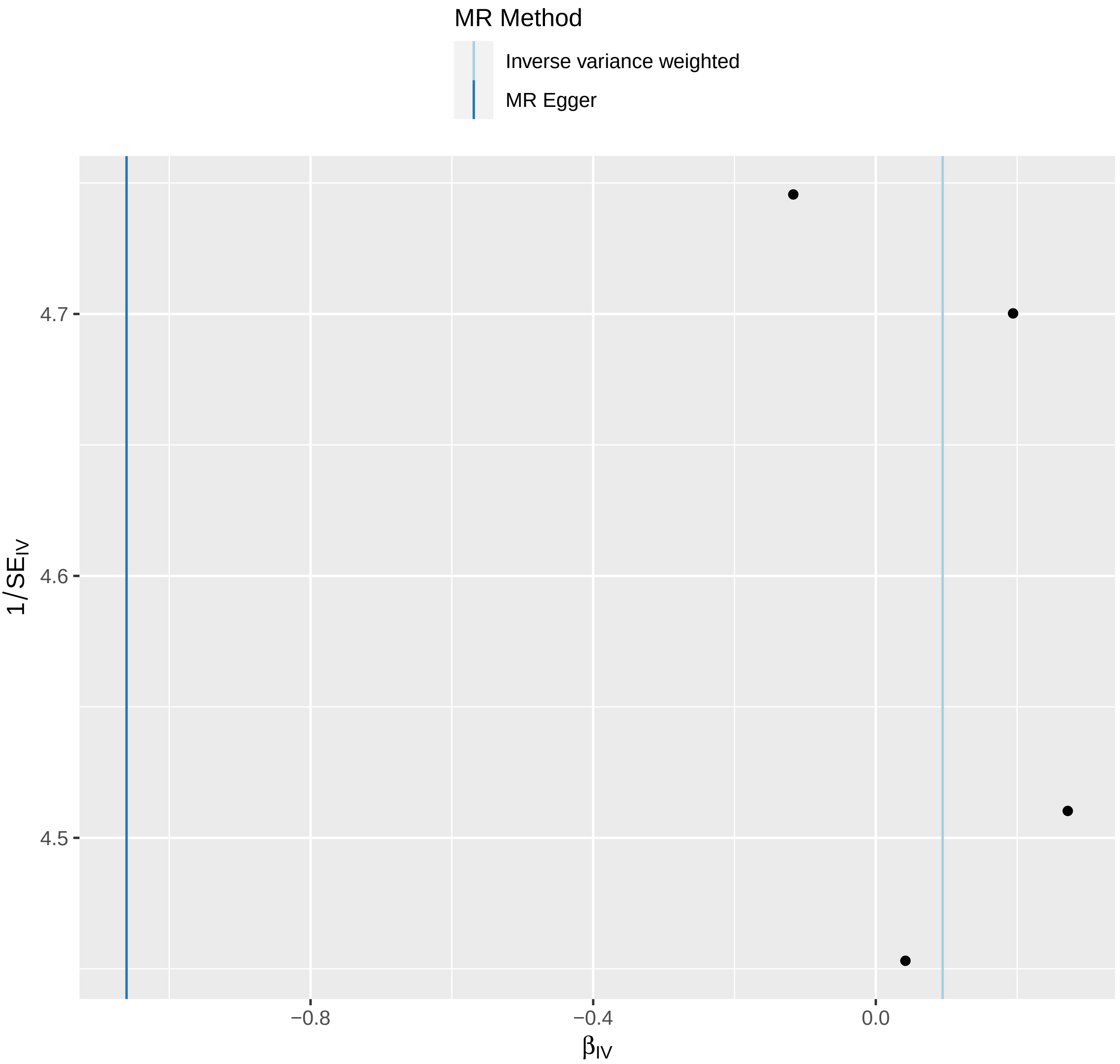

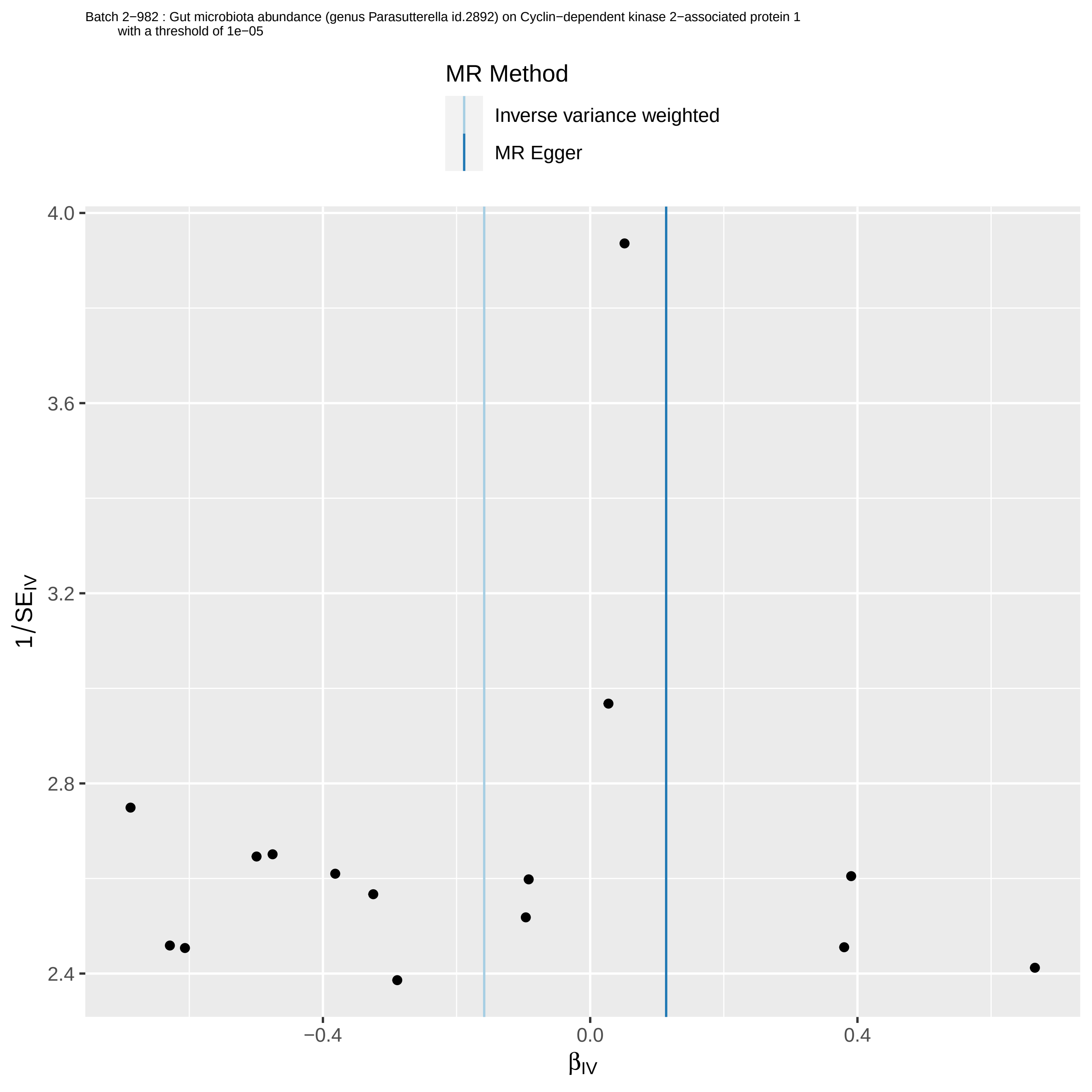

Batch 2−982 : Gut microbiota abundance (genus Parasutterella id.2892) on Cyclin−dependent kinase 2−associated protein 1
with a threshold of 1e−05
MR Method
Inverse variance weighted
MR Egger
4.0
3.6
3.2
2.8
2.4
1/SE_IV
−0.4
0.0
0.4
β_IV

Batch 2−983 : Gut microbiota abundance (genus Catenibacterium id.2153) on Cyclin−dependent kinase 2:Cyclin−A2 complex with a threshold of 1e−05

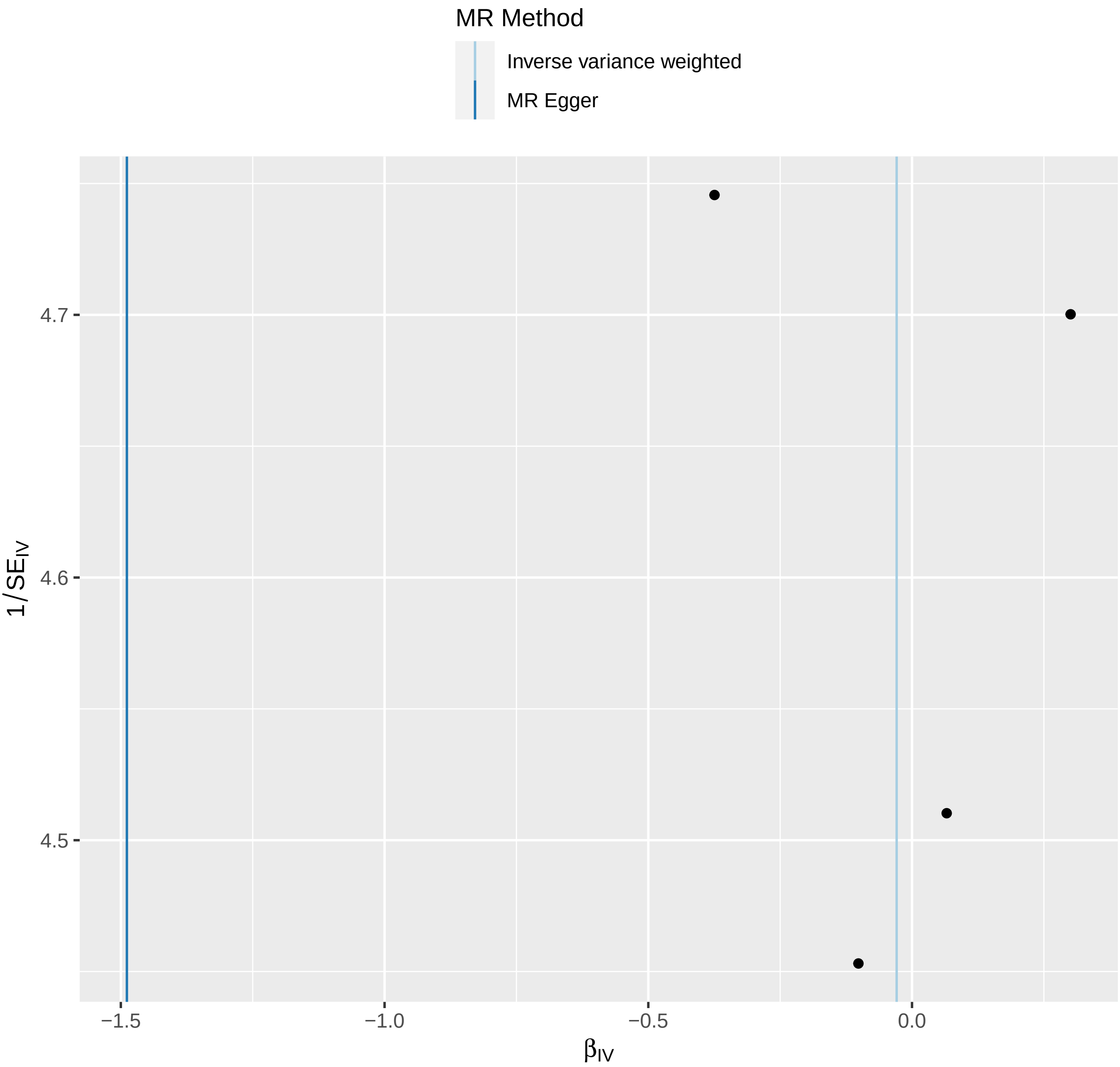

Batch 2−984 : Gut microbiota abundance (genus Parasutterella id.2892) on Cyclin−dependent kinase 2:Cyclin−A2 complex
with a threshold of 1e−05

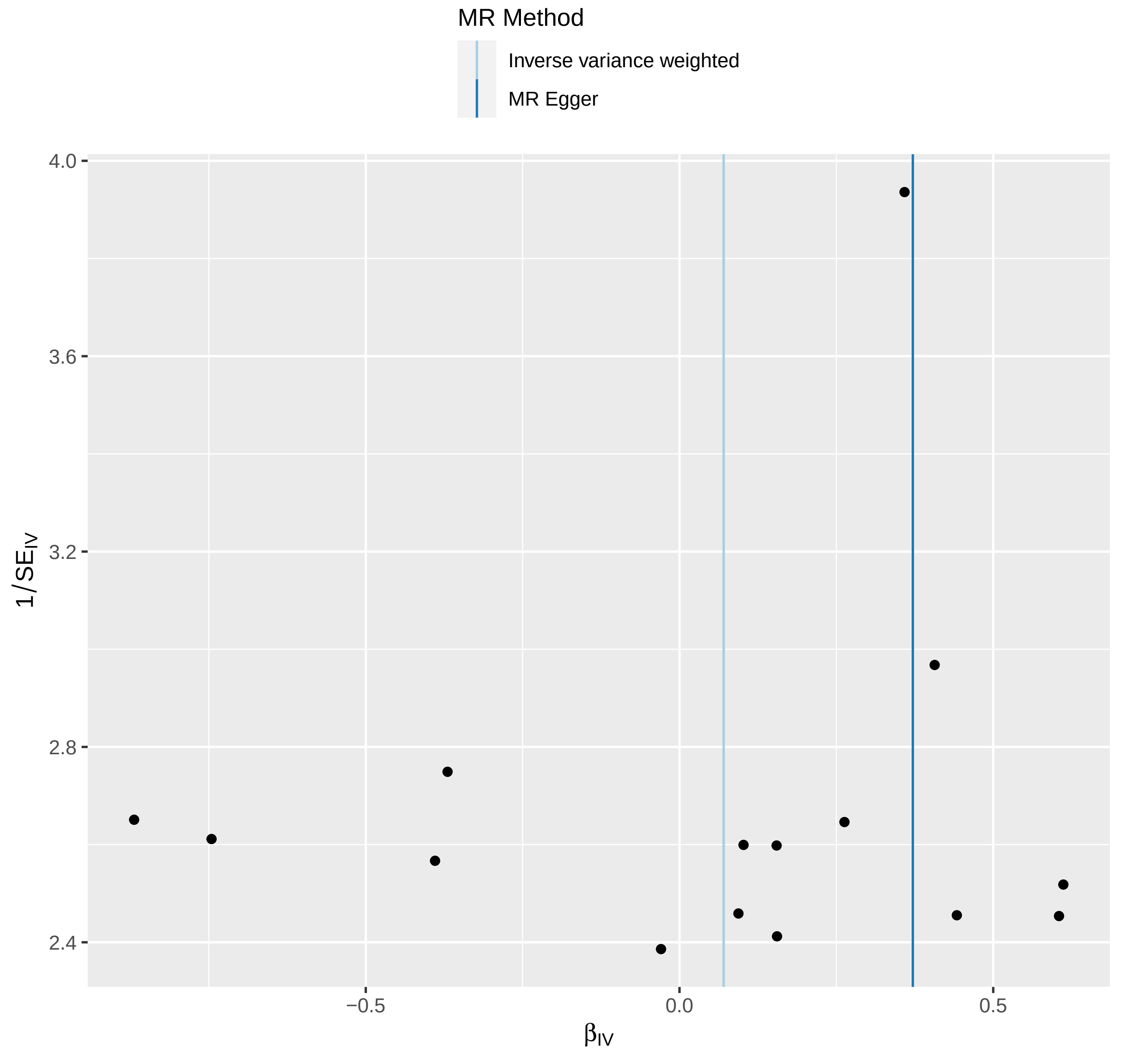

Batch 2−985 : Gut microbiota abundance (genus Catenibacterium id.2153) on Cyclin−dependent kinase 5:Cyclin−dependent kinase 5 activator 1 complex with a threshold of 1e−05

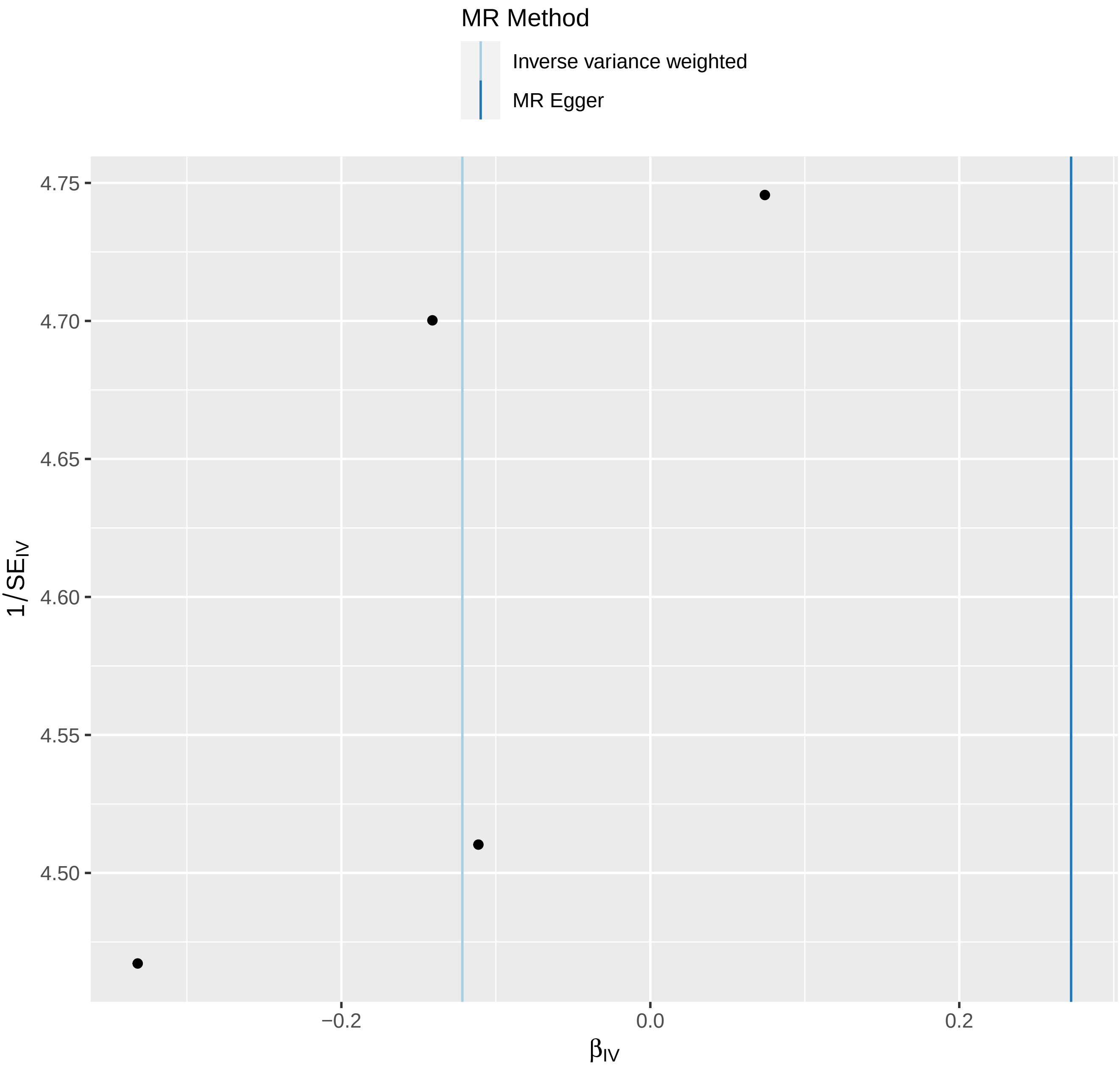

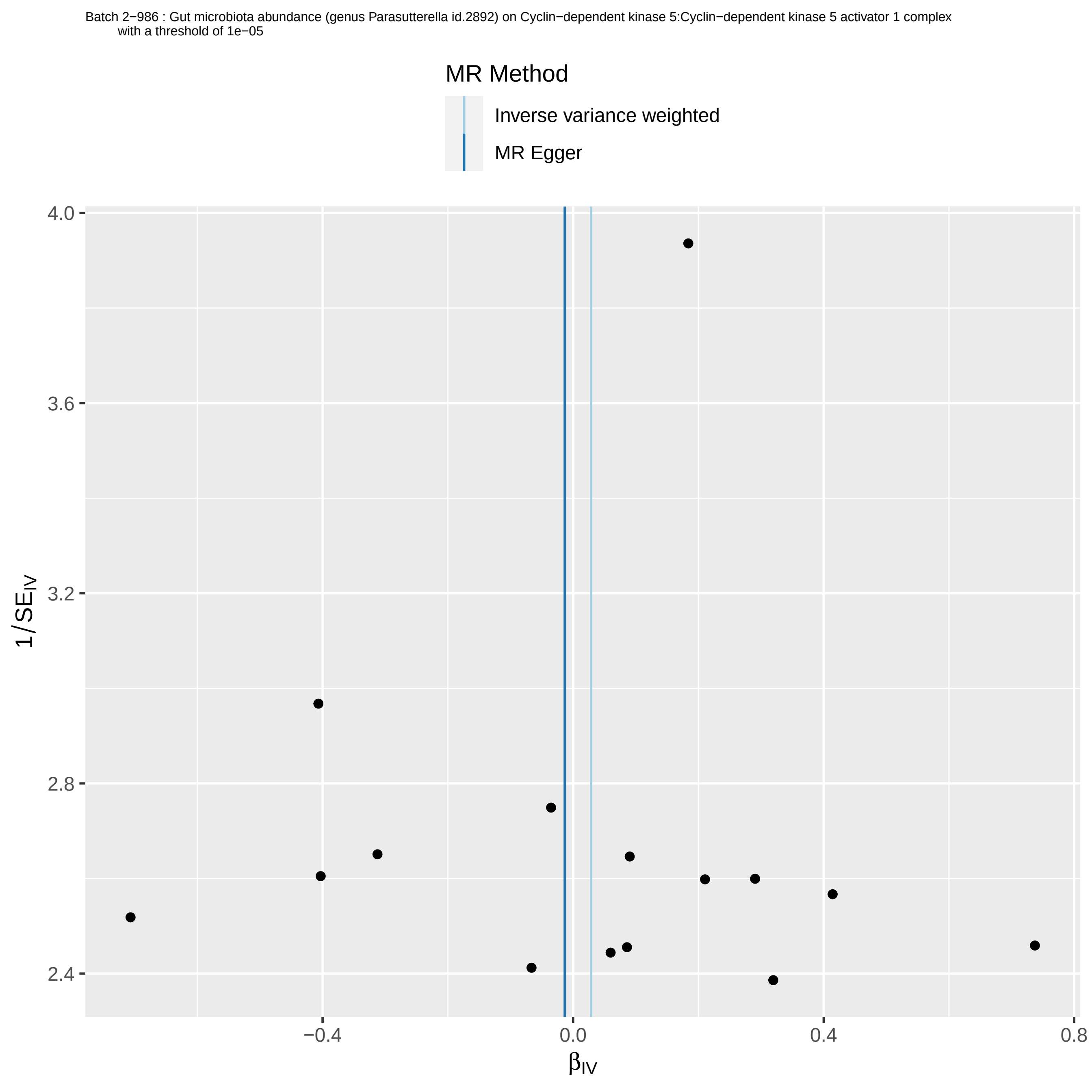

Batch 2−986 : Gut microbiota abundance (genus Parasutterella id.2892) on Cyclin−dependent kinase 5:Cyclin−dependent kinase 5 activator 1 complex with a threshold of 1e−05
MR Method
Inverse variance weighted
MR Egger
4.0
3.6
3.2
2.8
2.4
1/SE_IV
−0.4
0.0
0.4
0.8
β_IV

Batch 2−987 : Gut microbiota abundance (genus Catenibacterium id.2153) on Cyclin−dependent kinase 8:Cyclin−C complex
with a threshold of 1e−05

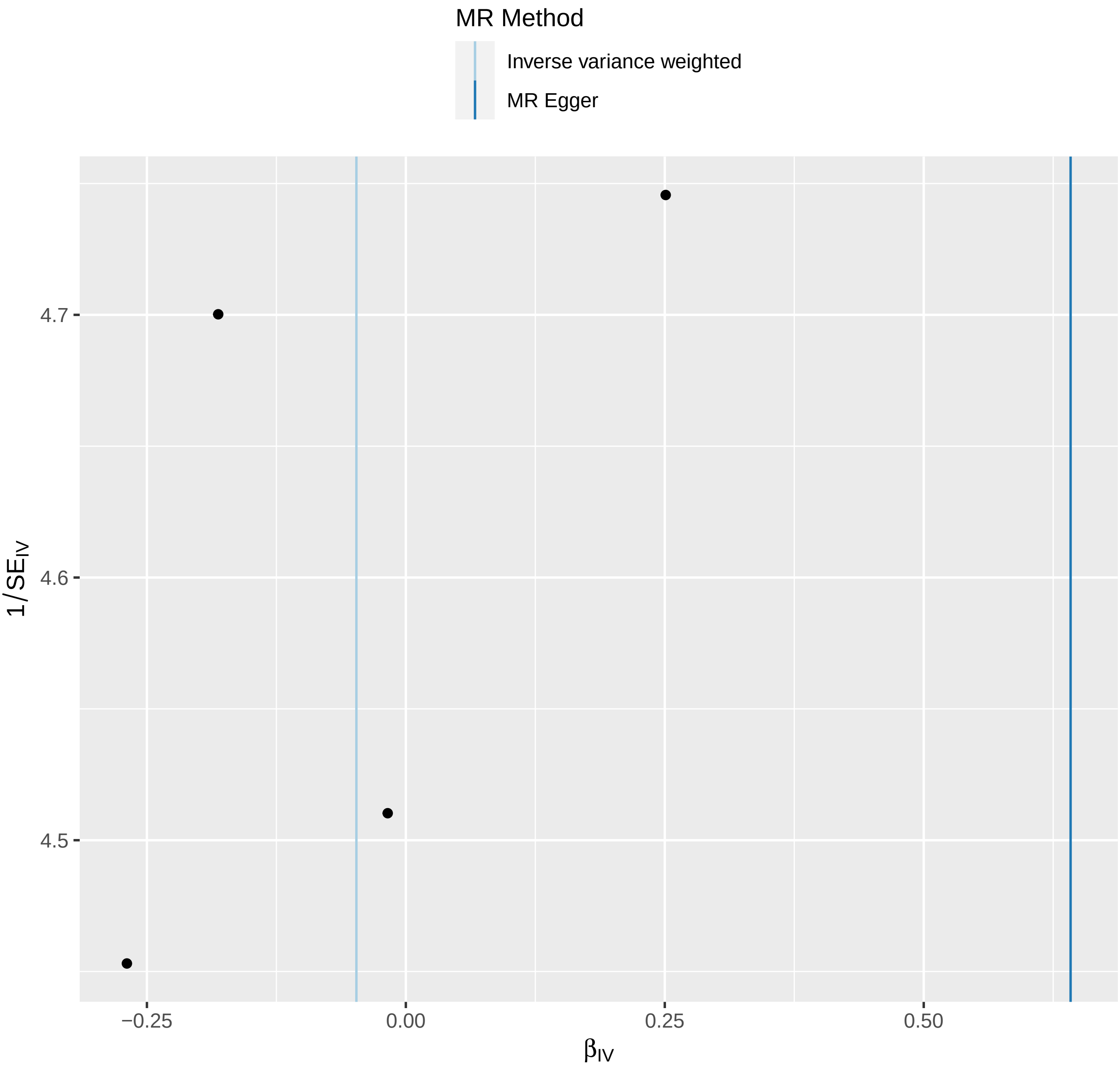

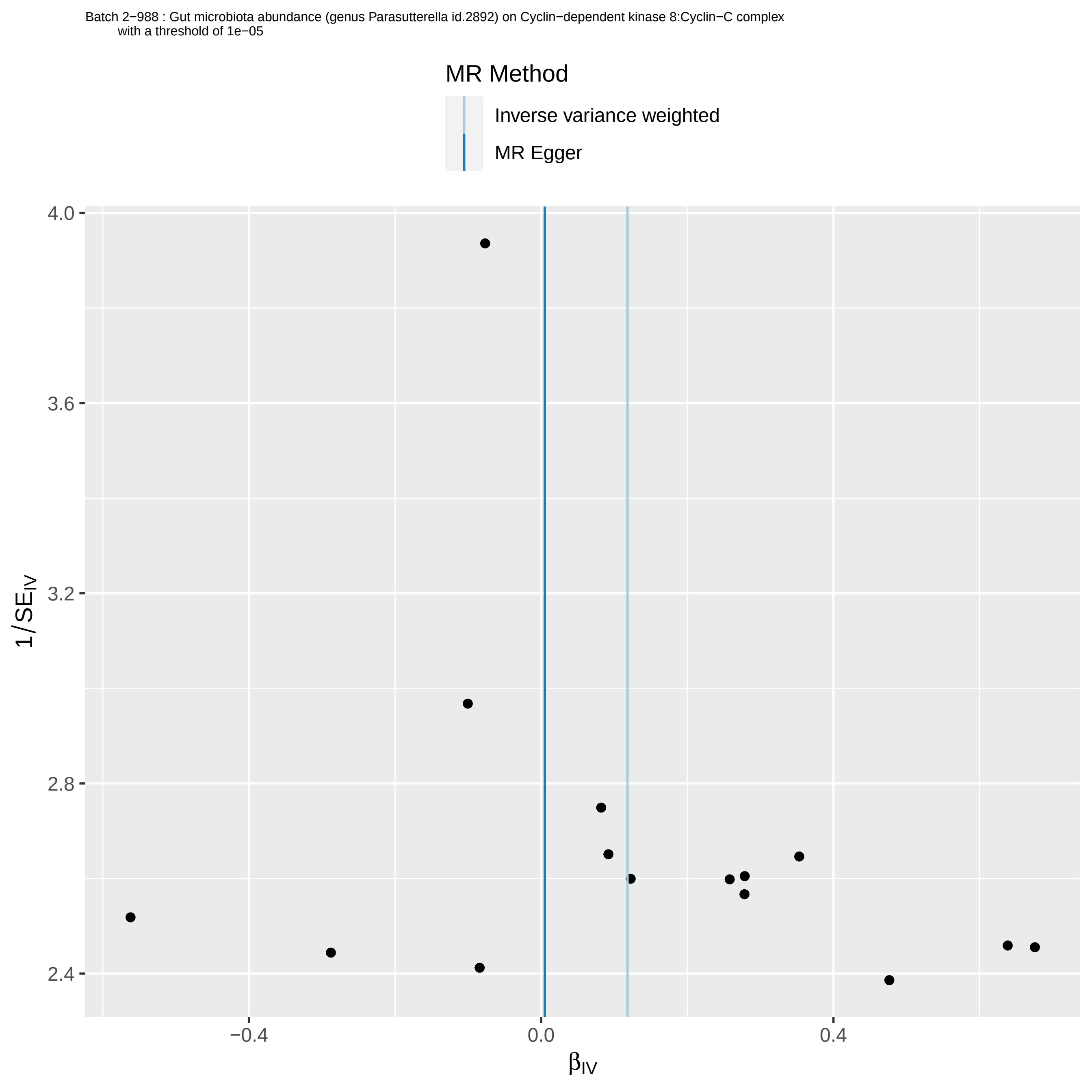

Batch 2−988 : Gut microbiota abundance (genus Parasutterella id.2892) on Cyclin−dependent kinase 8:Cyclin−C complex
with a threshold of 1e−05
MR Method
Inverse variance weighted
MR Egger
4.0
3.6
3.2
2.8
2.4
1/SE_IV
−0.4
0.0
0.4
β_IV

Batch 2−989 : Gut microbiota abundance (genus Catenibacterium id.2153) on Cyclin−dependent kinase inhibitor 1B
with a threshold of 1e−05

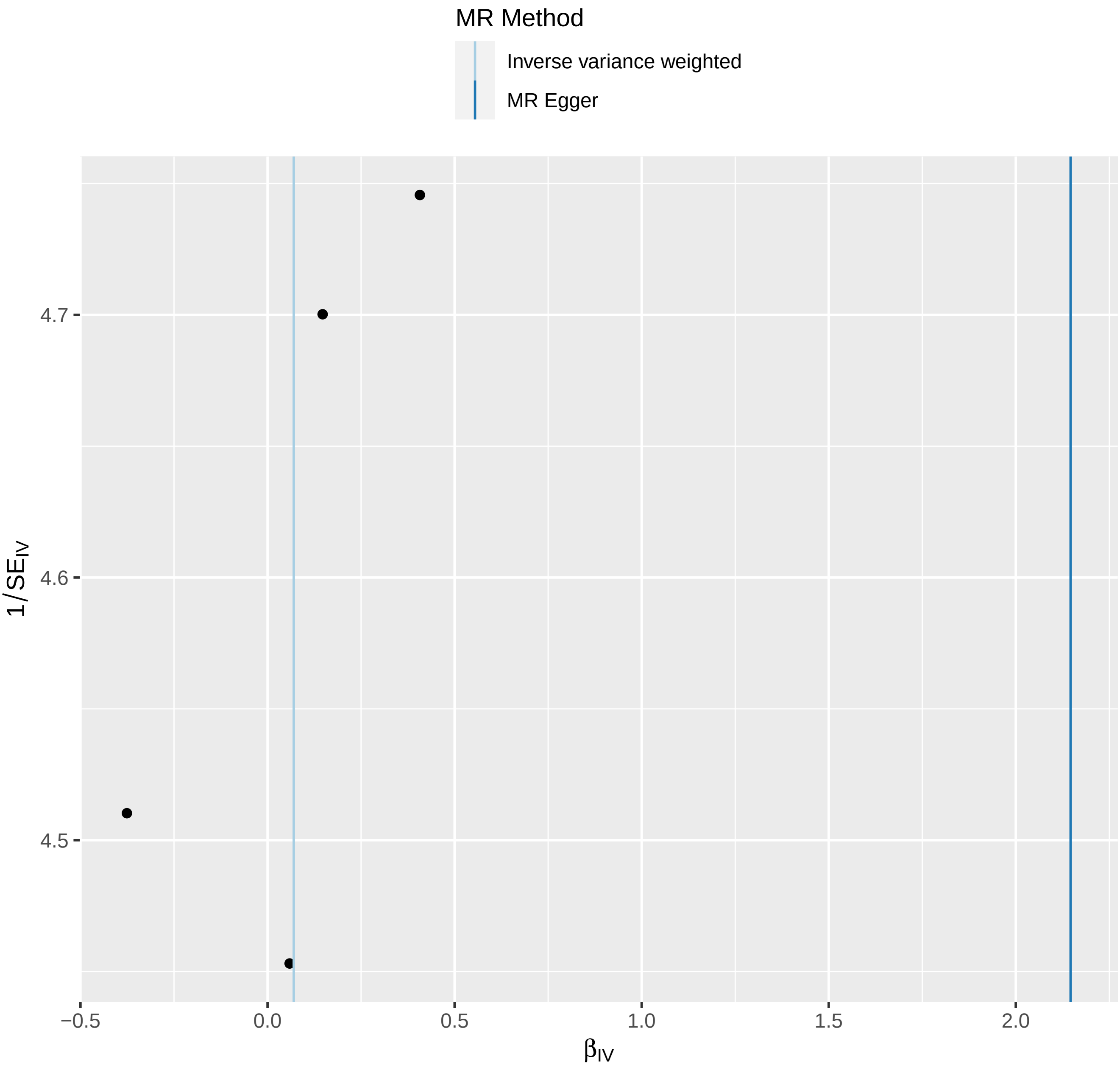

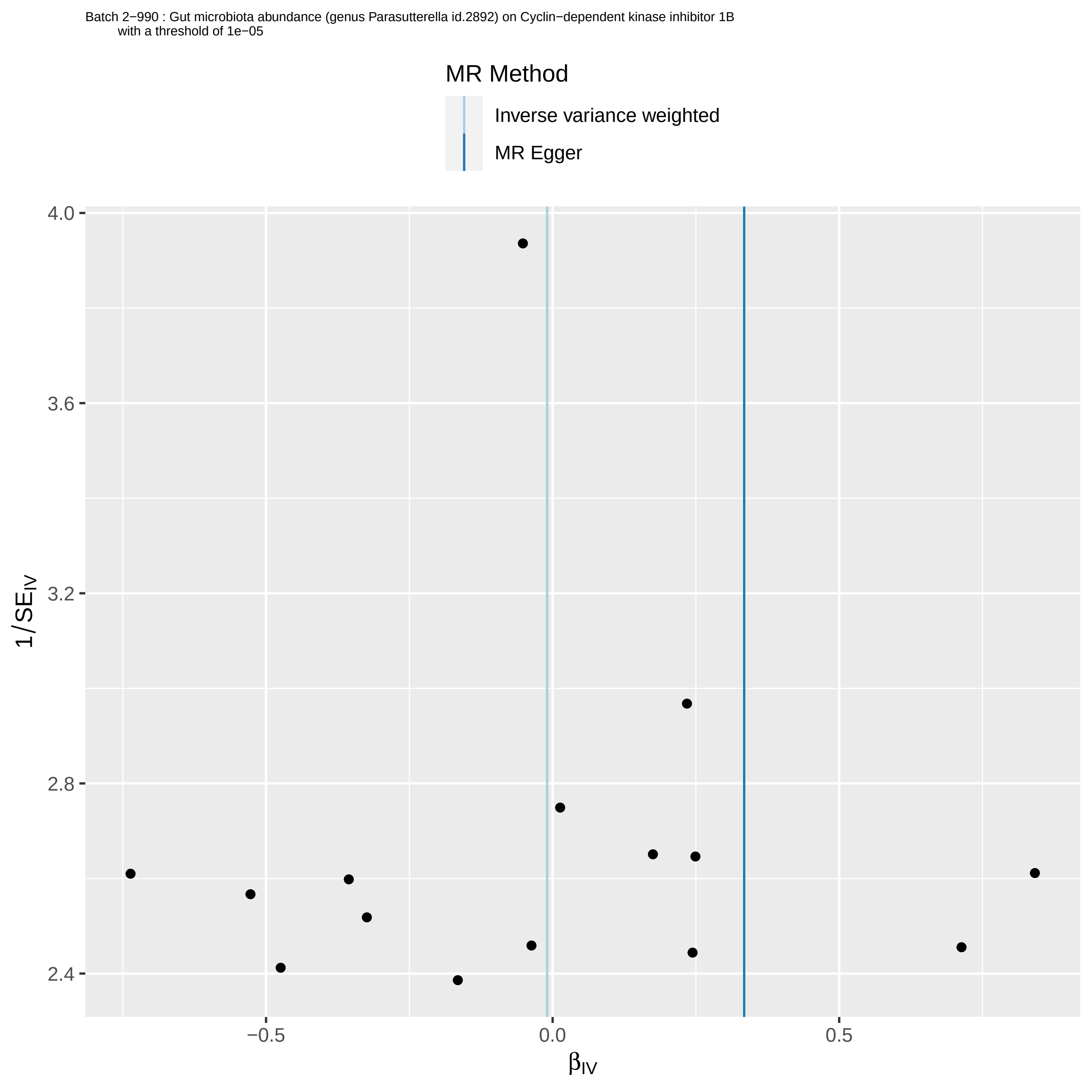

Batch 2−990 : Gut microbiota abundance (genus Parasutterella id.2892) on Cyclin−dependent kinase inhibitor 1B
with a threshold of 1e−05
MR Method
Inverse variance weighted
MR Egger
4.0
3.6
3.2
2.8
2.4
1/SEIV
−0.5
0.0
0.5
βIV

Batch 2−991 : Gut microbiota abundance (genus Catenibacterium id.2153) on Cyclin−dependent kinase 4 inhibitor B
with a threshold of 1e−05

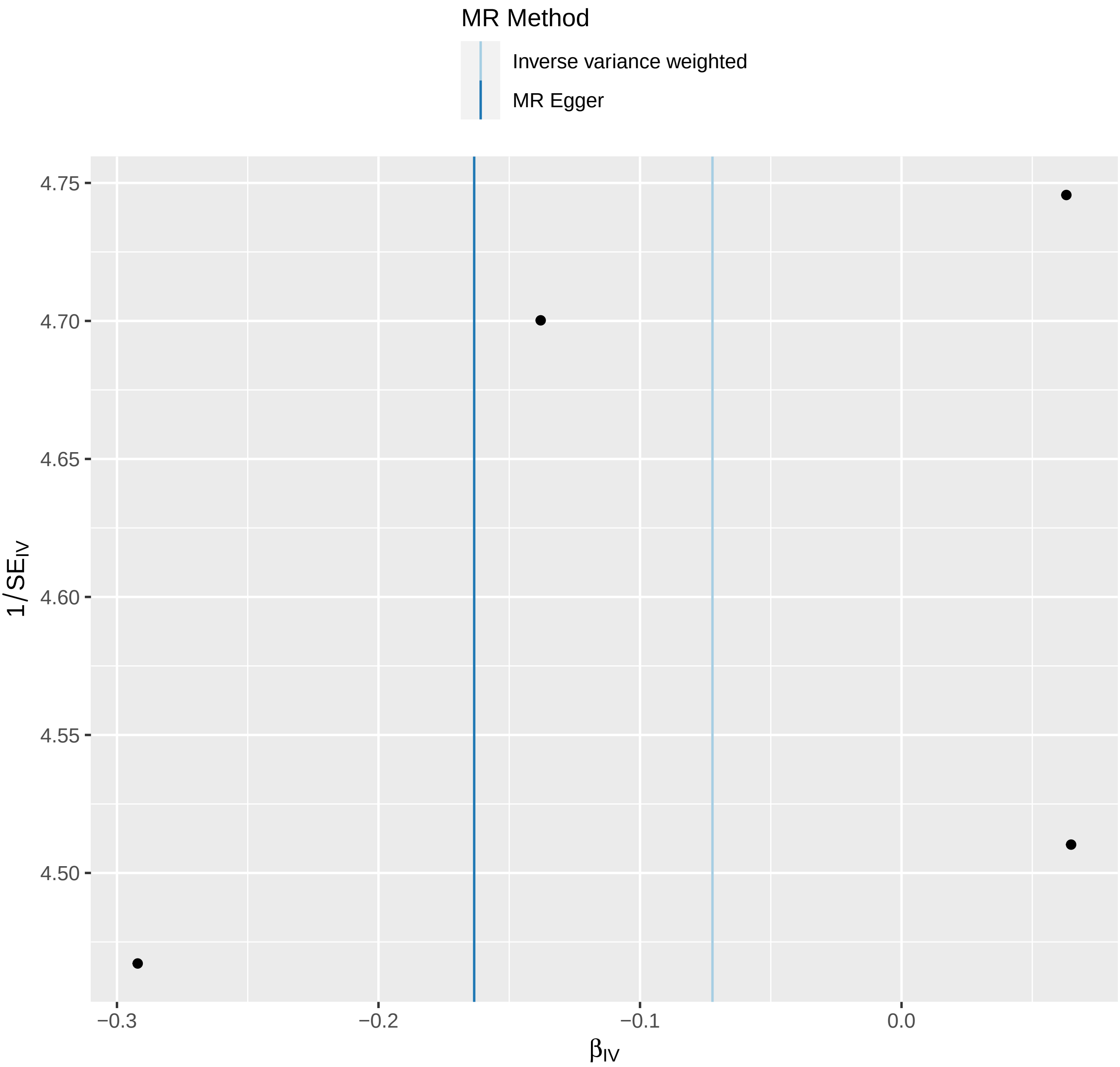

Batch 2−992 : Gut microbiota abundance (genus Parasutterella id.2892) on Cyclin−dependent kinase 4 inhibitor B
with a threshold of 1e−05

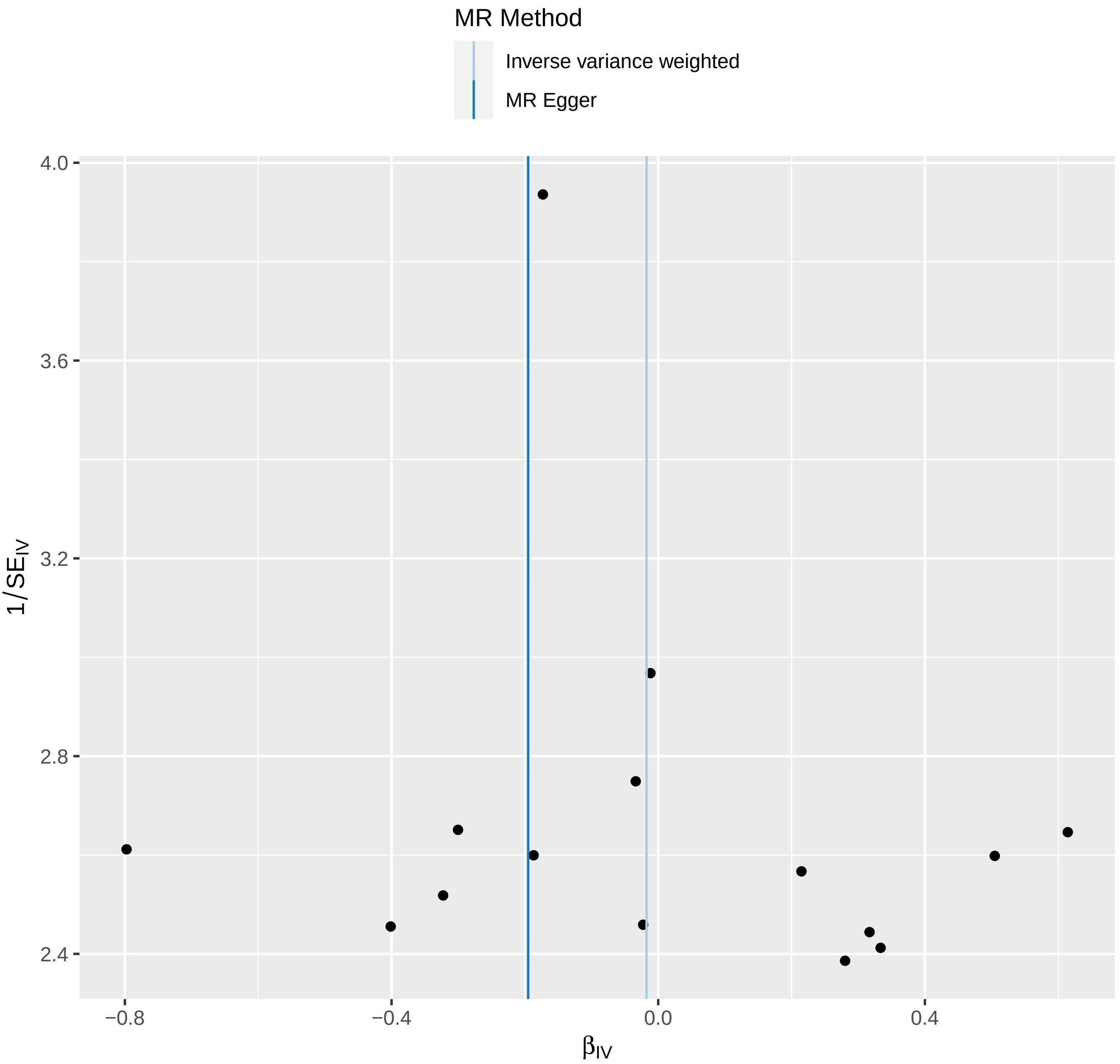

Batch 2−993 : Gut microbiota abundance (genus Catenibacterium id.2153) on Cyclin−dependent kinase inhibitor 3
with a threshold of 1e−05

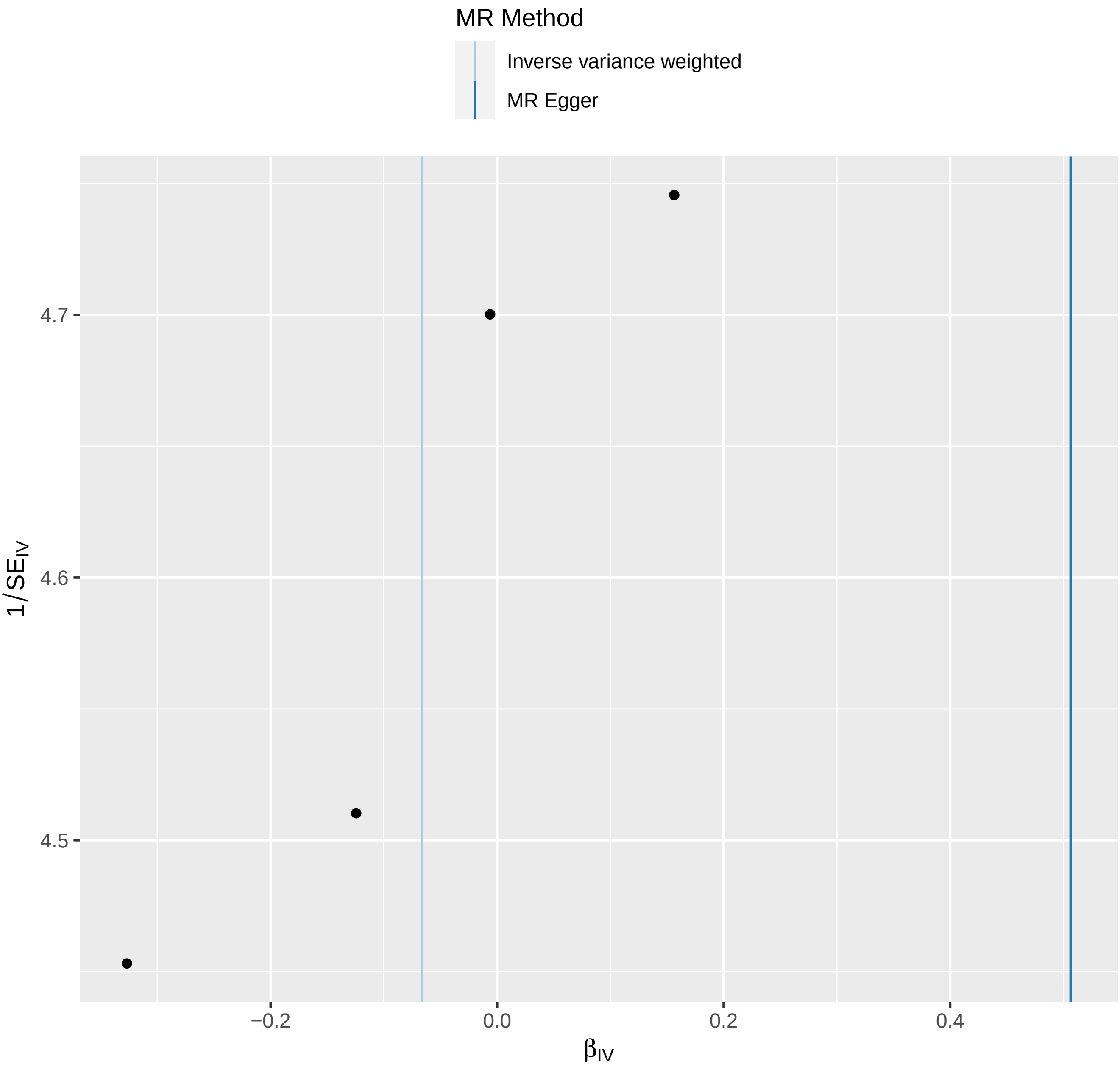

Batch 2−994 : Gut microbiota abundance (genus Parasutterella id.2892) on Cyclin−dependent kinase inhibitor 3
with a threshold of 1e−05

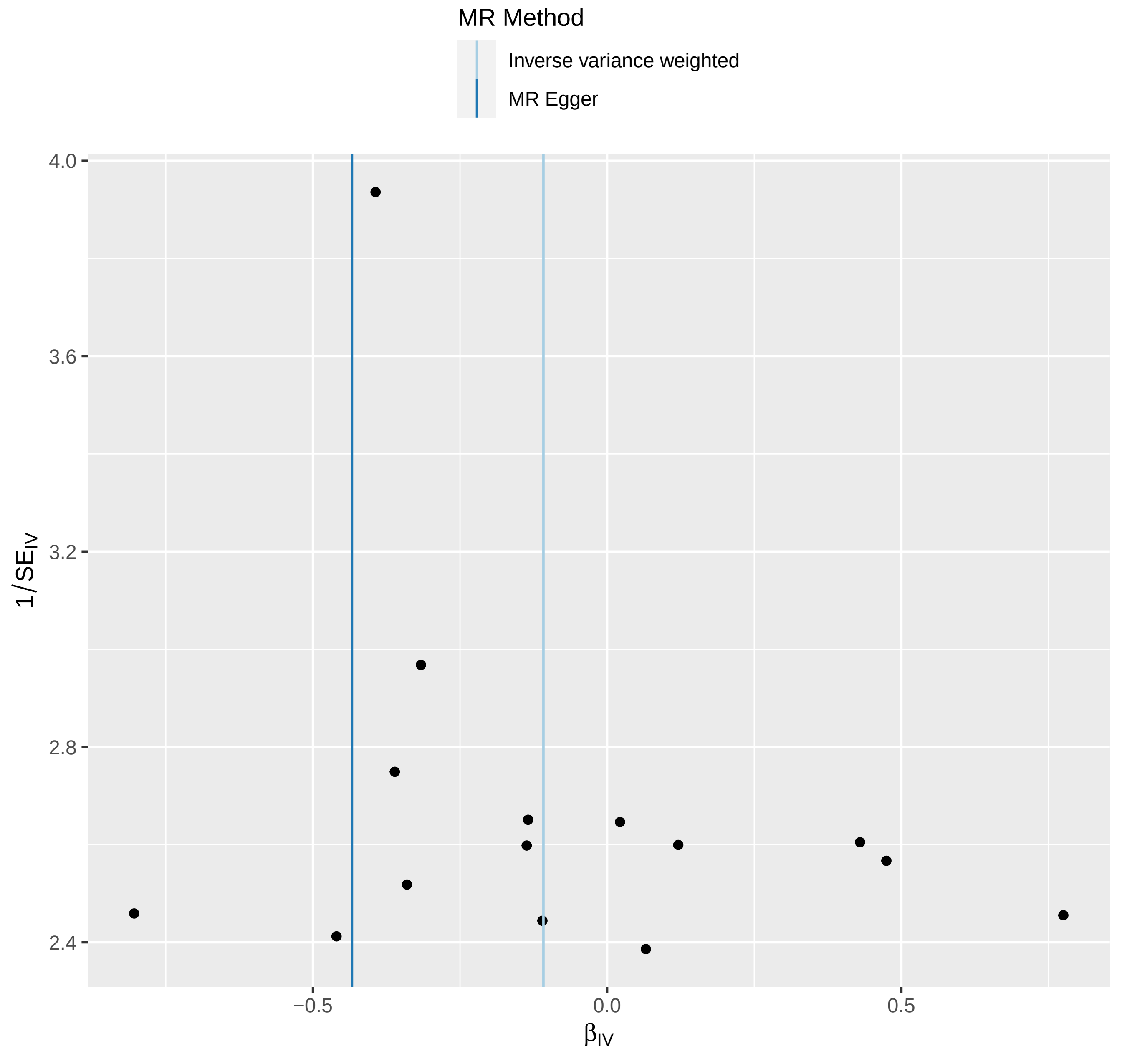

Batch 2−995 : Gut microbiota abundance (genus Catenibacterium id.2153) on Cerebral dopamine neurotrophic factor
with a threshold of 1e−05

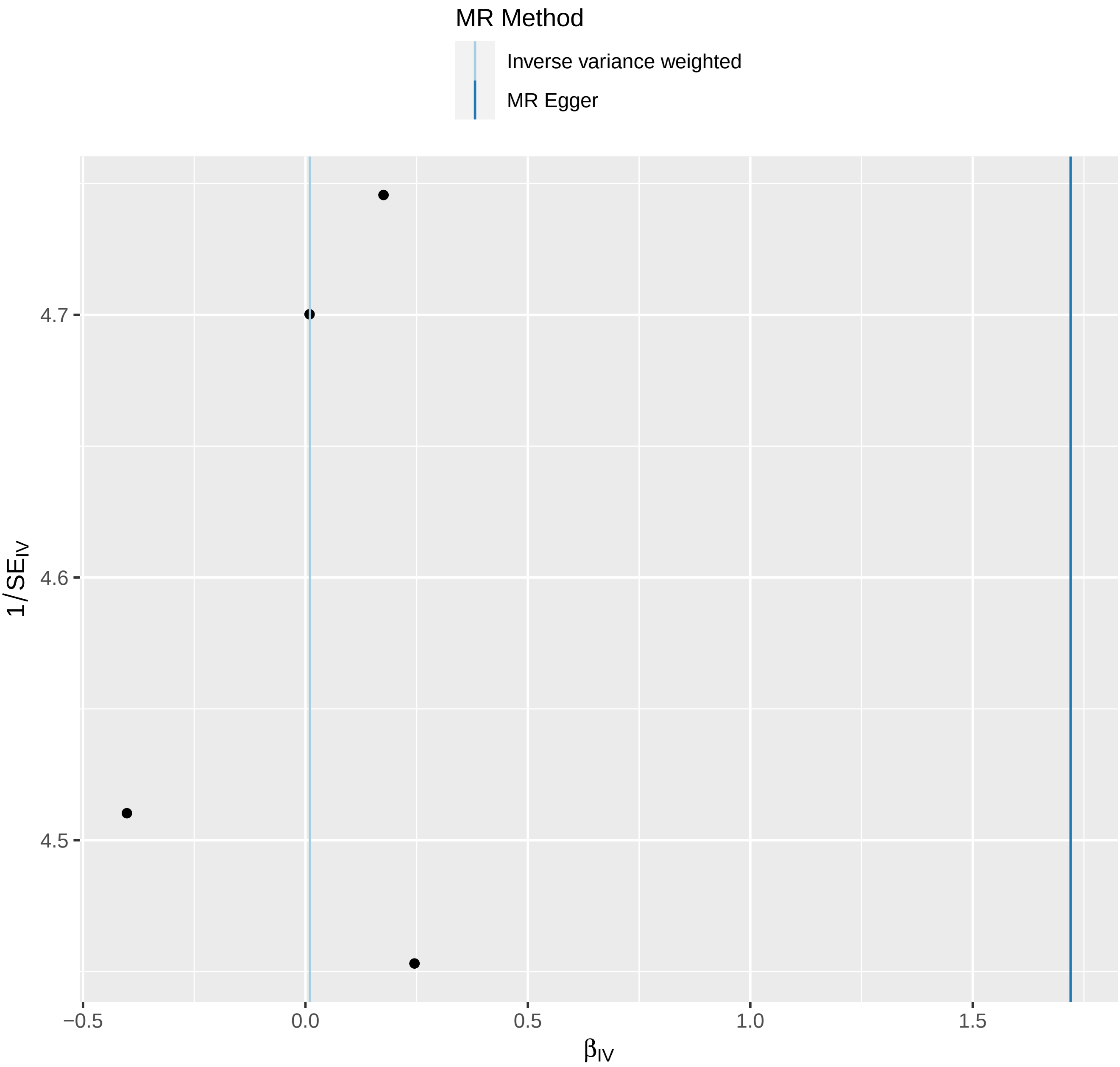

Batch 2−996 : Gut microbiota abundance (genus Parasutterella id.2892) on Cerebral dopamine neurotrophic factor
with a threshold of 1e−05

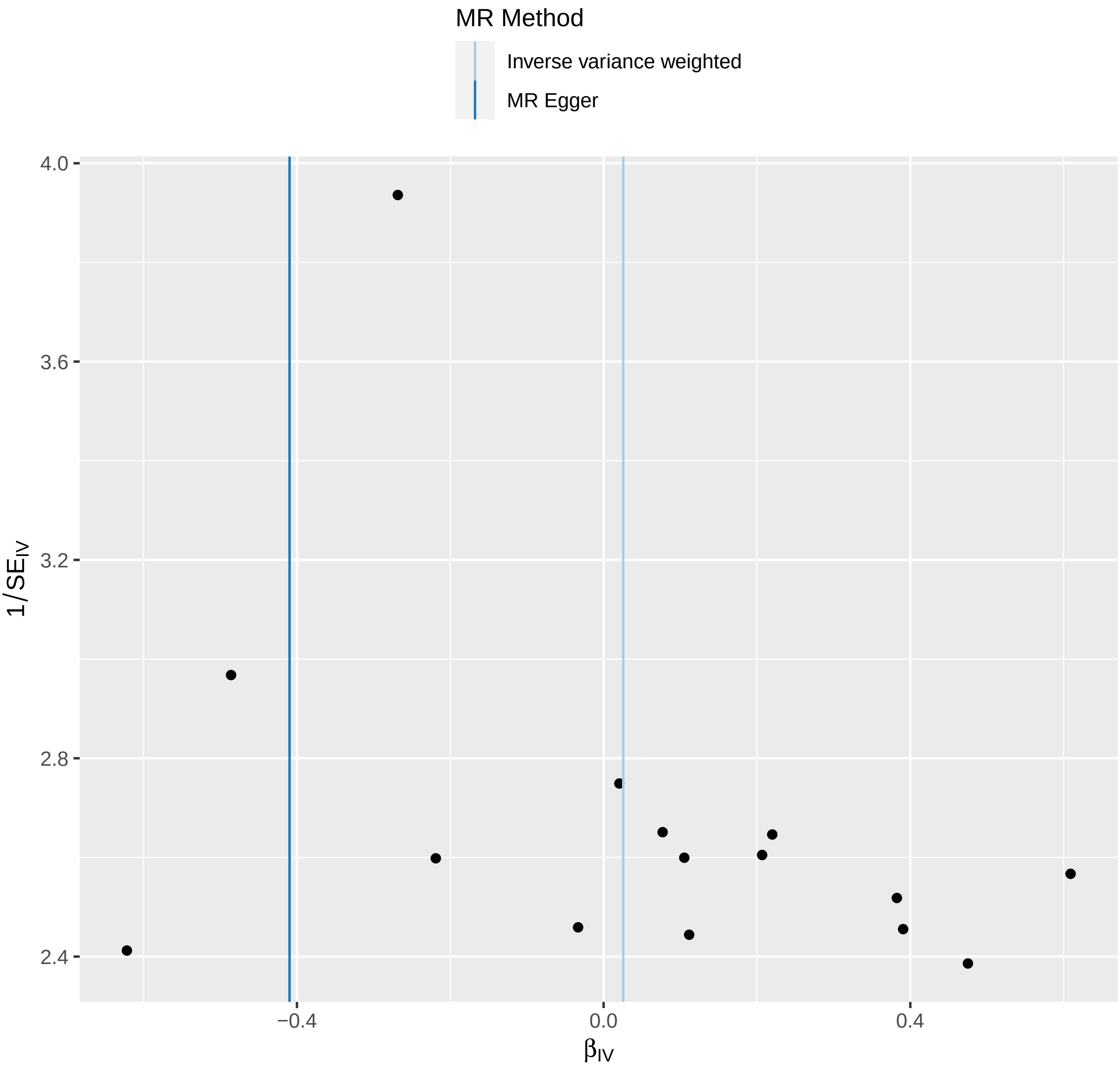

Batch 2−997 : Gut microbiota abundance (genus Catenibacterium id.2153) on Cell adhesion molecule−related/down−regulated by oncogenes with a threshold of 1e−05

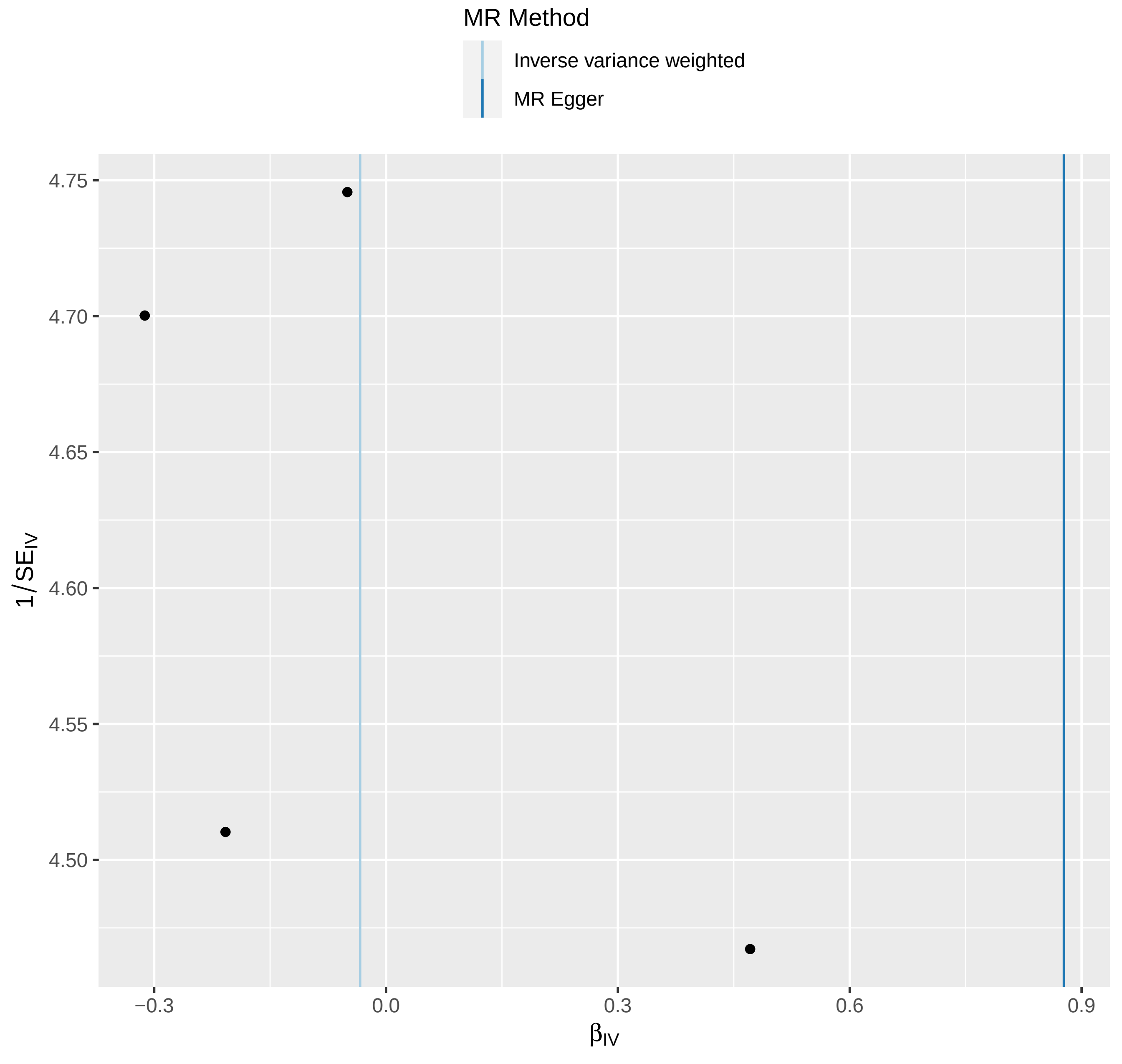

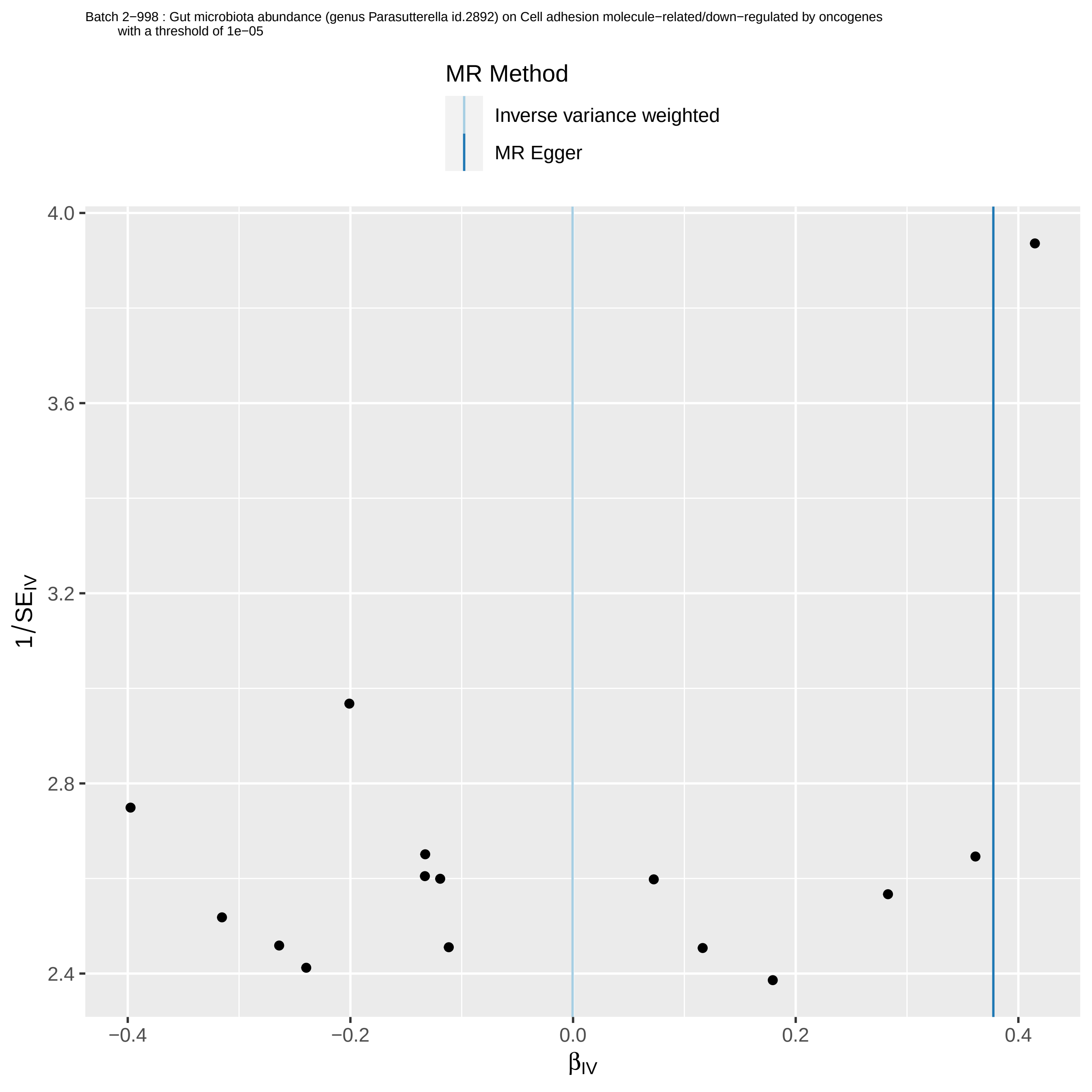
Batch 2−998 : Gut microbiota abundance (genus Parasutterella id.2892) on Cell adhesion molecule−related/down−regulated by oncogenes
with a threshold of 1e−05
MR Method
Inverse variance weighted
MR Egger
4.0
3.6
3.2
2.8
2.4
1/SE_IV
−0.4
−0.2
0.0
0.2
0.4
β_IV

Batch 2−999 : Gut microbiota abundance (genus Catenibacterium id.2153) on Corneodesmosin
with a threshold of 1e−05

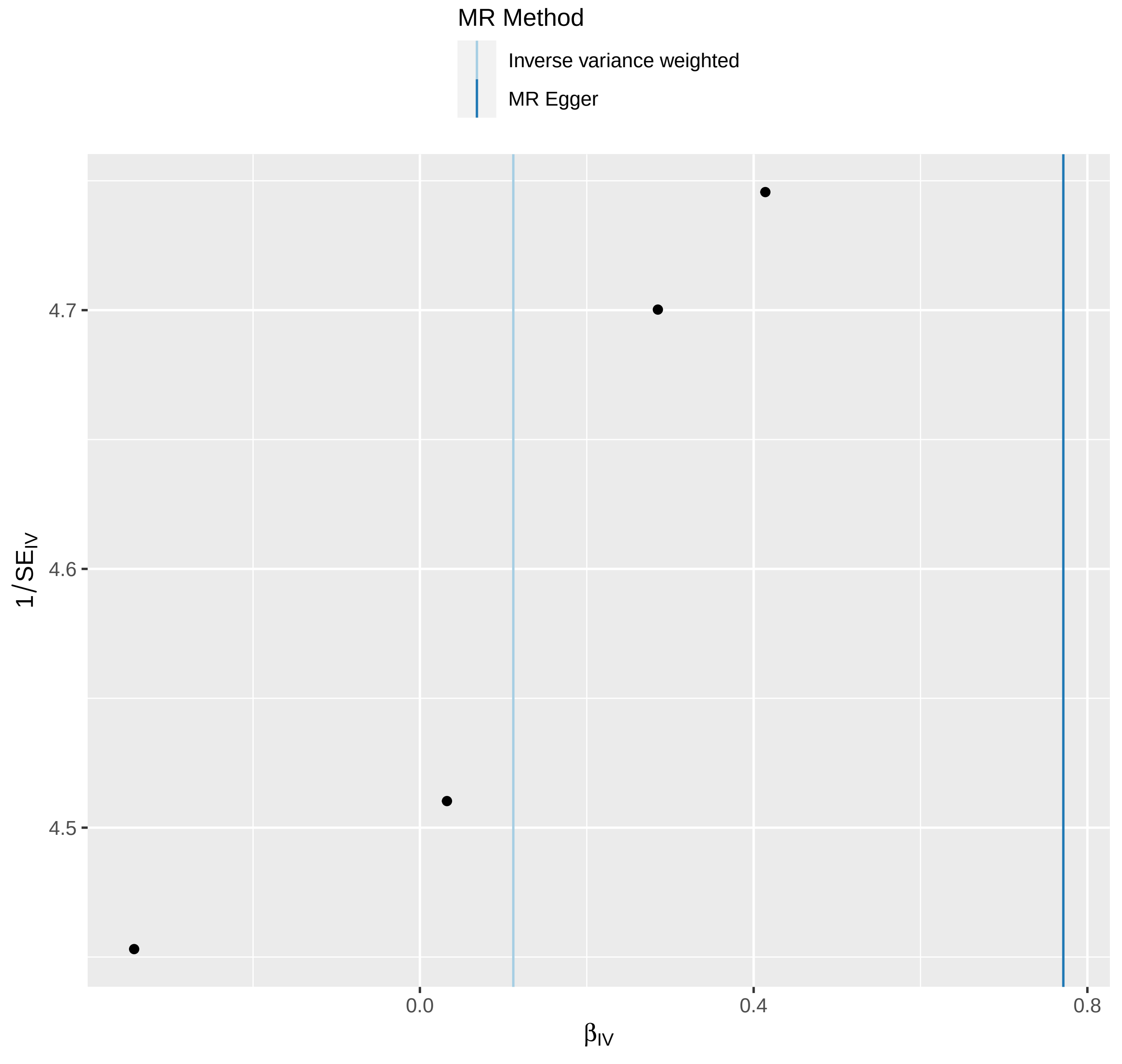

Batch 2−1000 : Gut microbiota abundance (genus Parasutterella id.2892) on Corneodesmosin
with a threshold of 1e−05

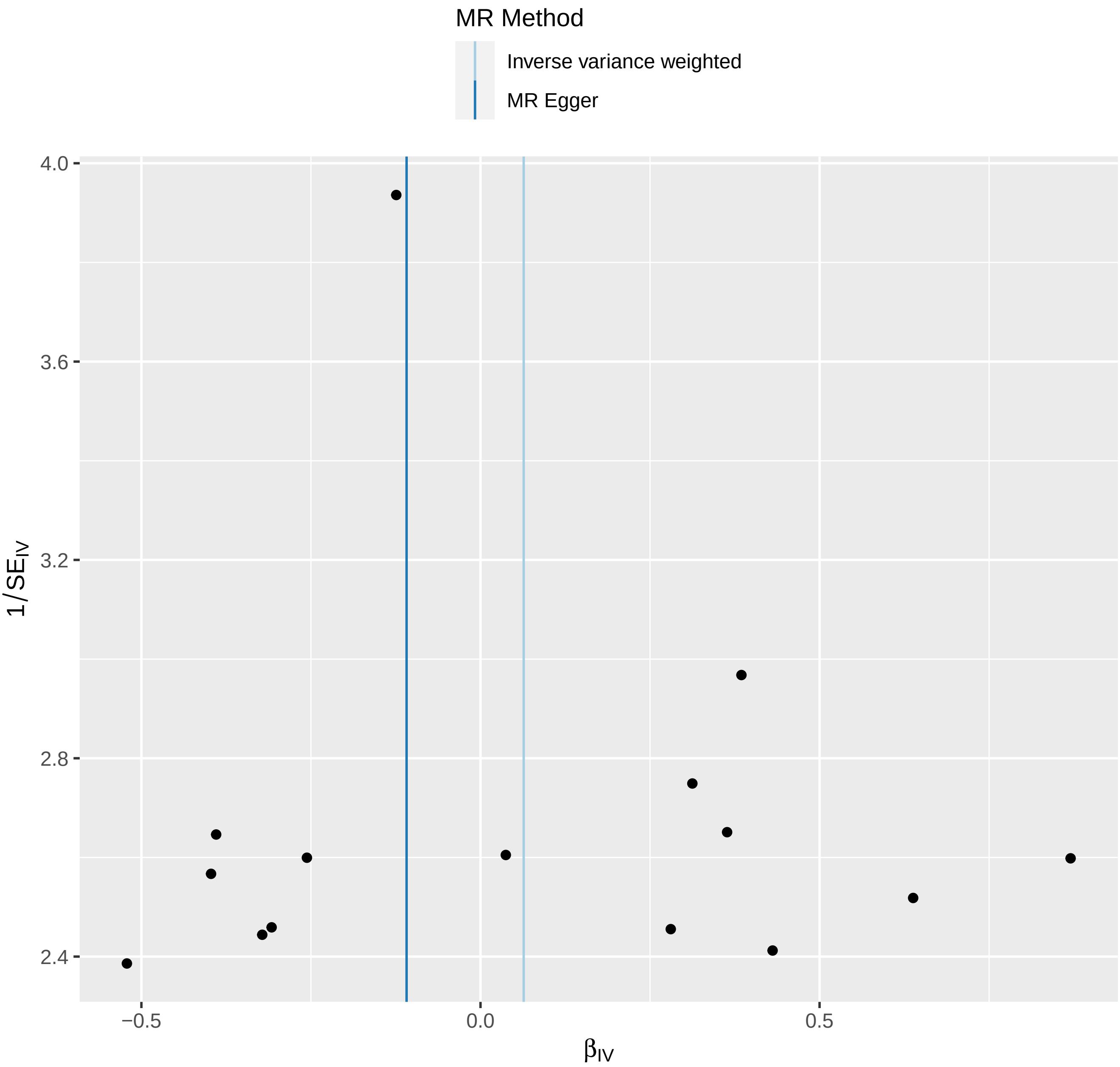

Batch 2−1001 : Gut microbiota abundance (genus Catenibacterium id.2153) on Testis−specific chromodomain protein Y 1
with a threshold of 1e−05

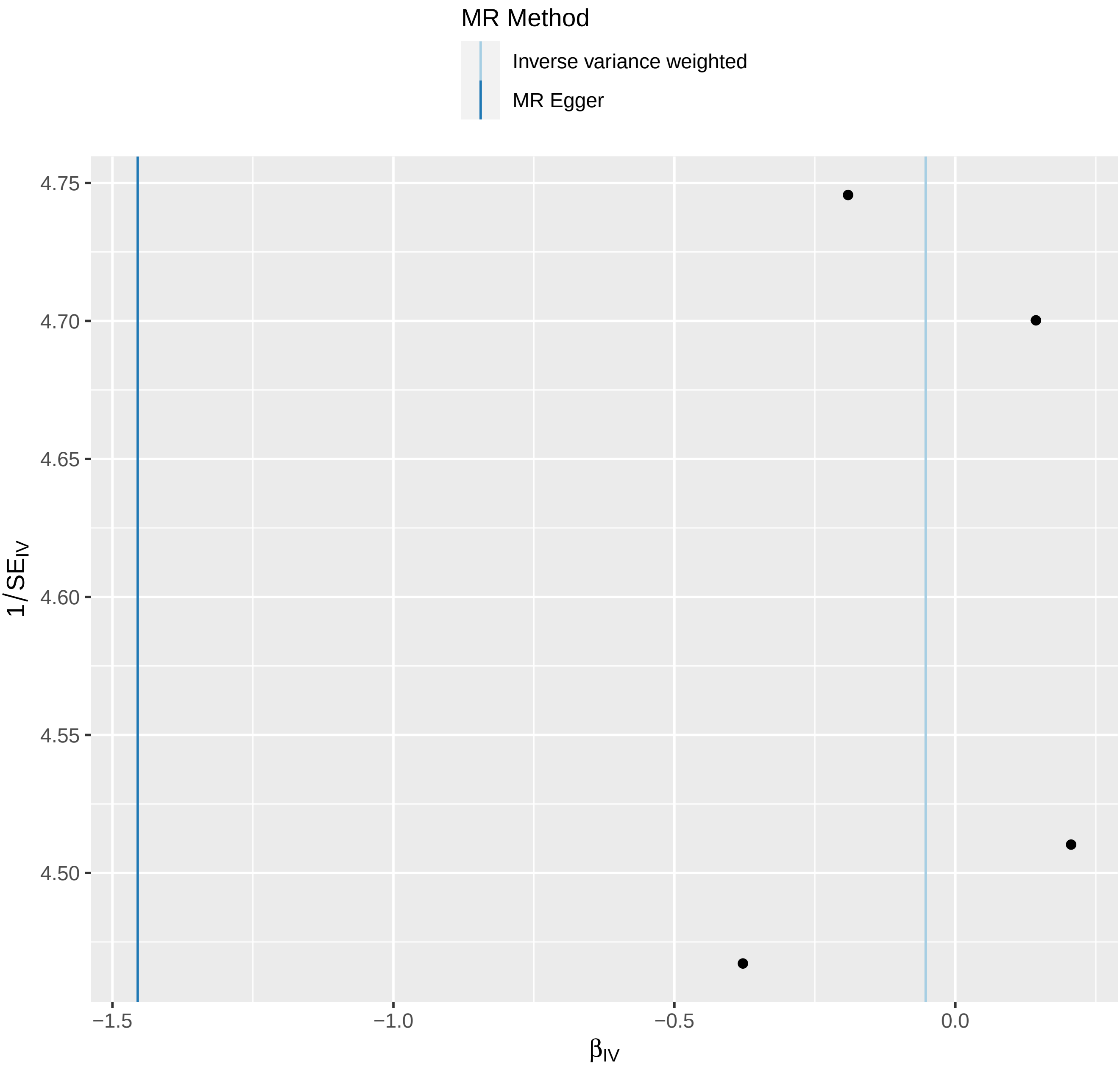

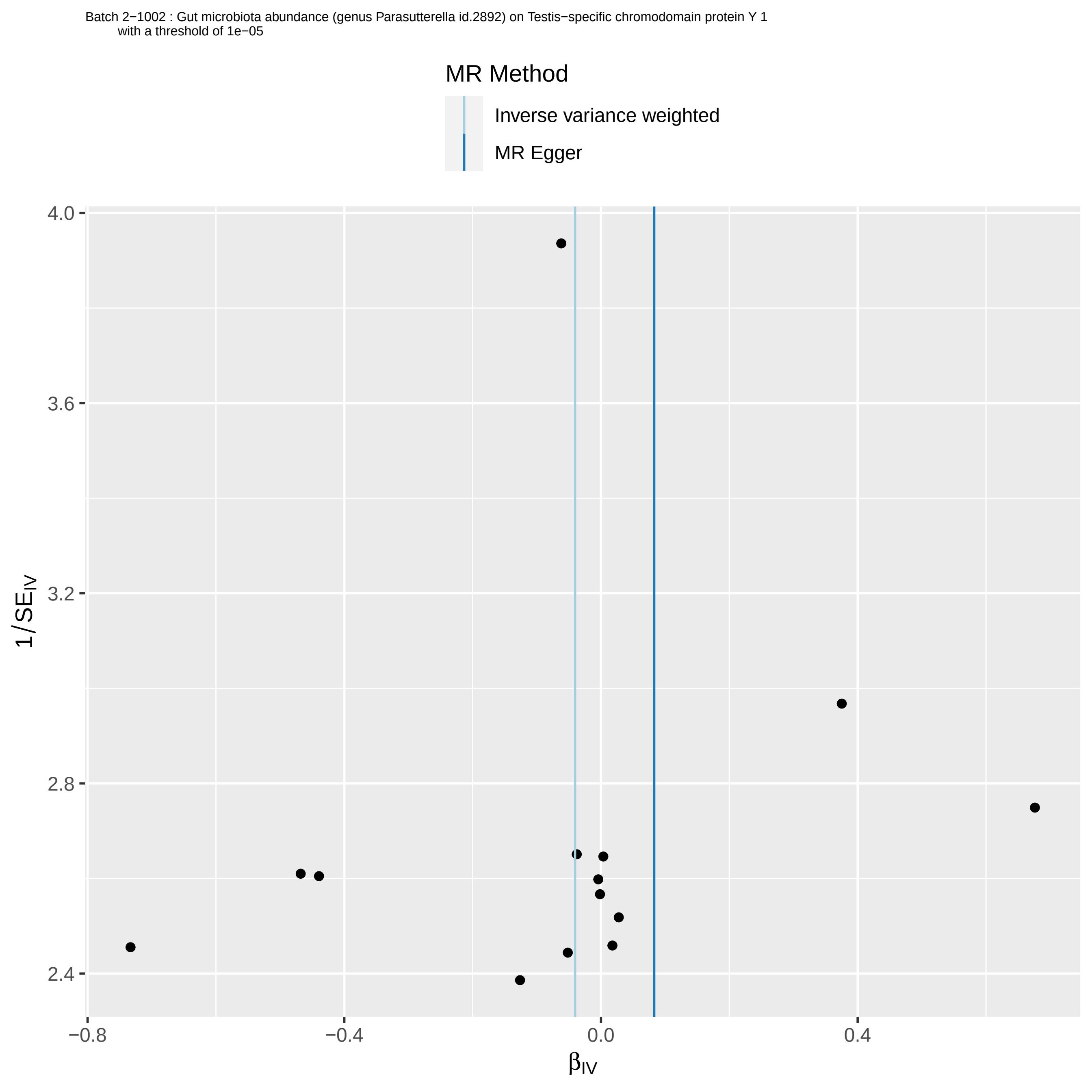
Batch 2−1002 : Gut microbiota abundance (genus Parasutterella id.2892) on Testis−specific chromodomain protein Y 1
with a threshold of 1e−05
MR Method
Inverse variance weighted
MR Egger
4.0
3.6
3.2
2.8
2.4
1/SE_IV
−0.8
−0.4
0.0
0.4
β_IV

Batch 2−1003 : Gut microbiota abundance (genus Catenibacterium id.2153) on Carcinoembryonic antigen−related cell adhesion molecule 1
with a threshold of 1e−05

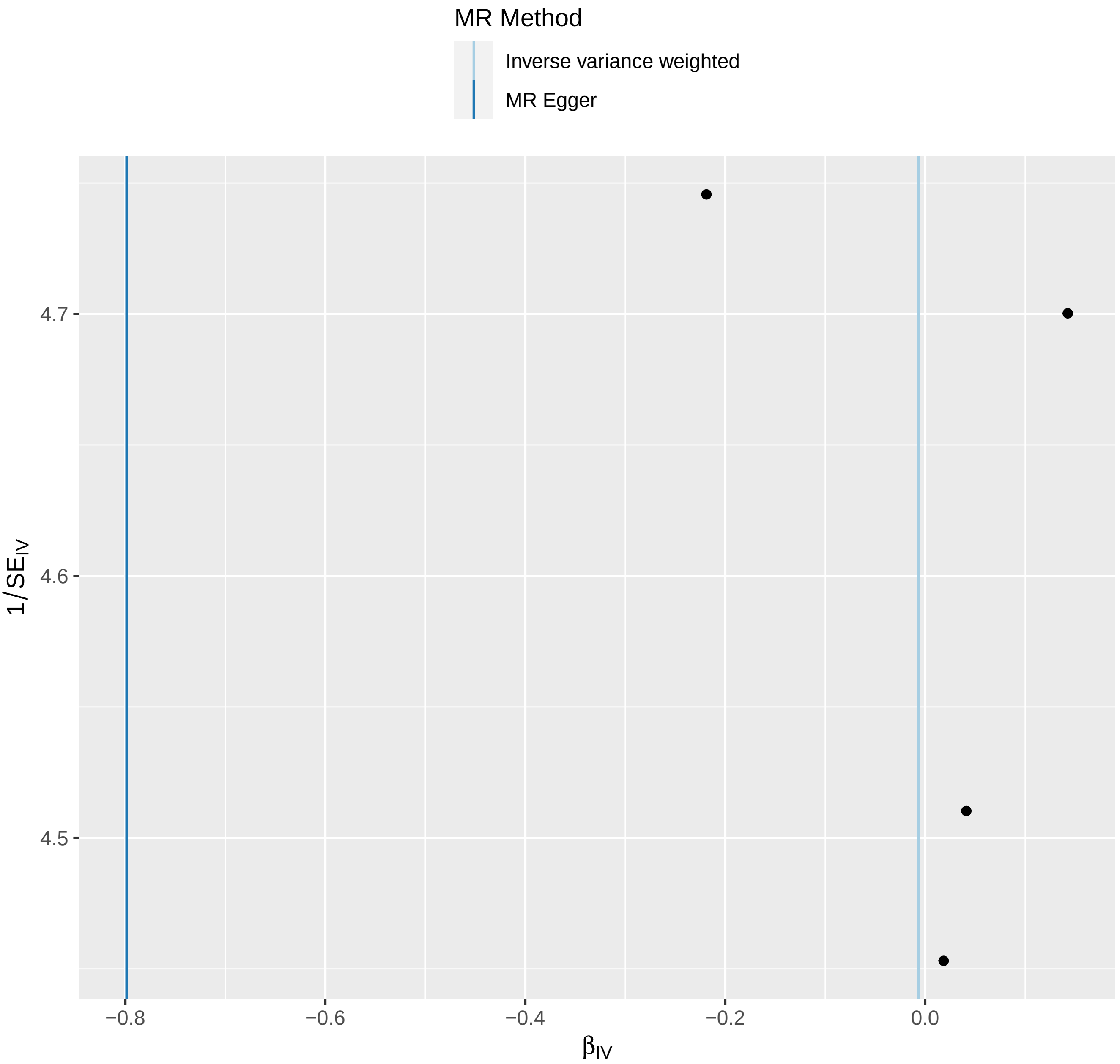

Batch 2−1004 : Gut microbiota abundance (genus Parasutterella id.2892) on Carcinoembryonic antigen−related cell adhesion molecule 1 with a threshold of 1e−05

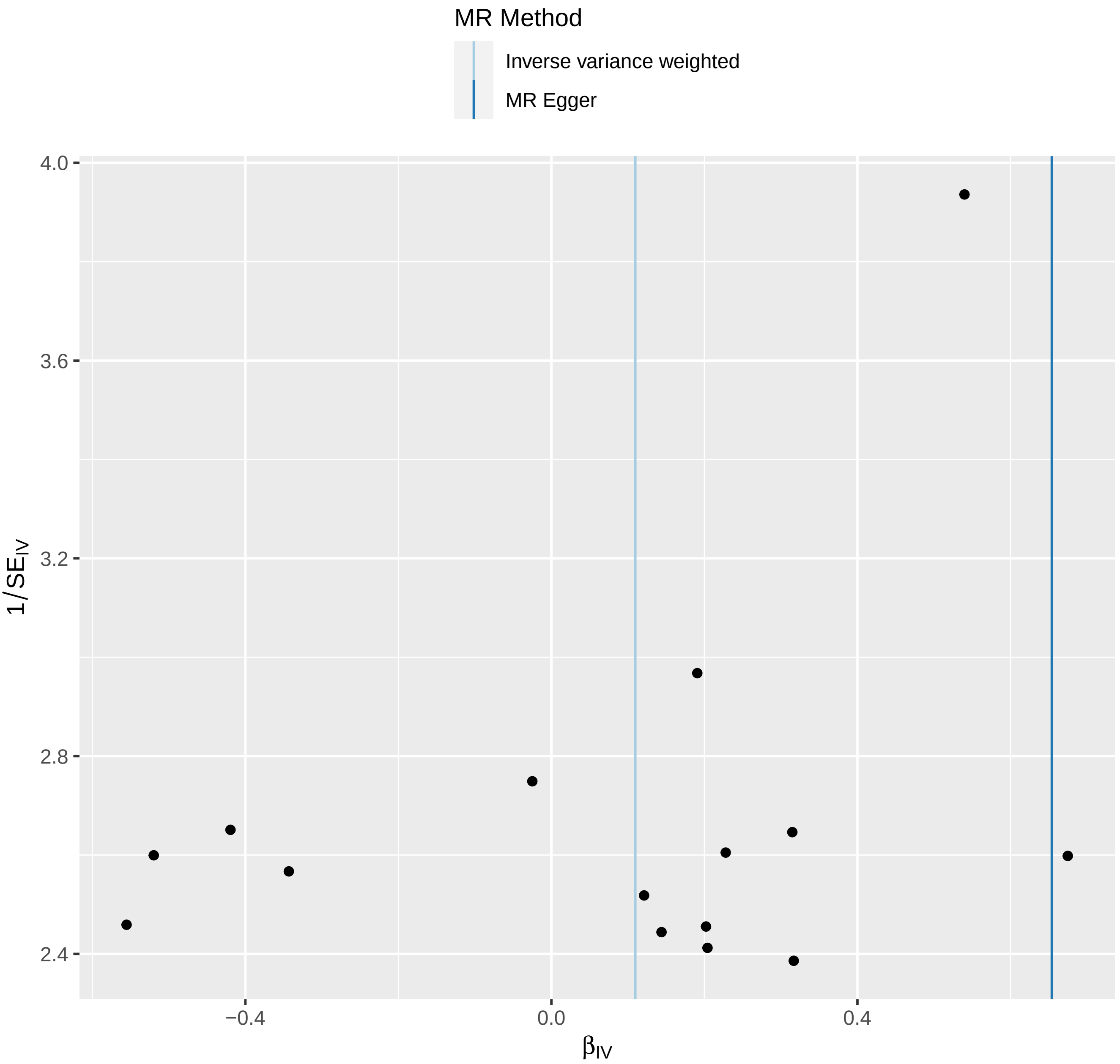

Batch 2−1005 : Gut microbiota abundance (genus Catenibacterium id.2153) on Carcinoembryonic antigen−related cell adhesion molecule 21
with a threshold of 1e−05

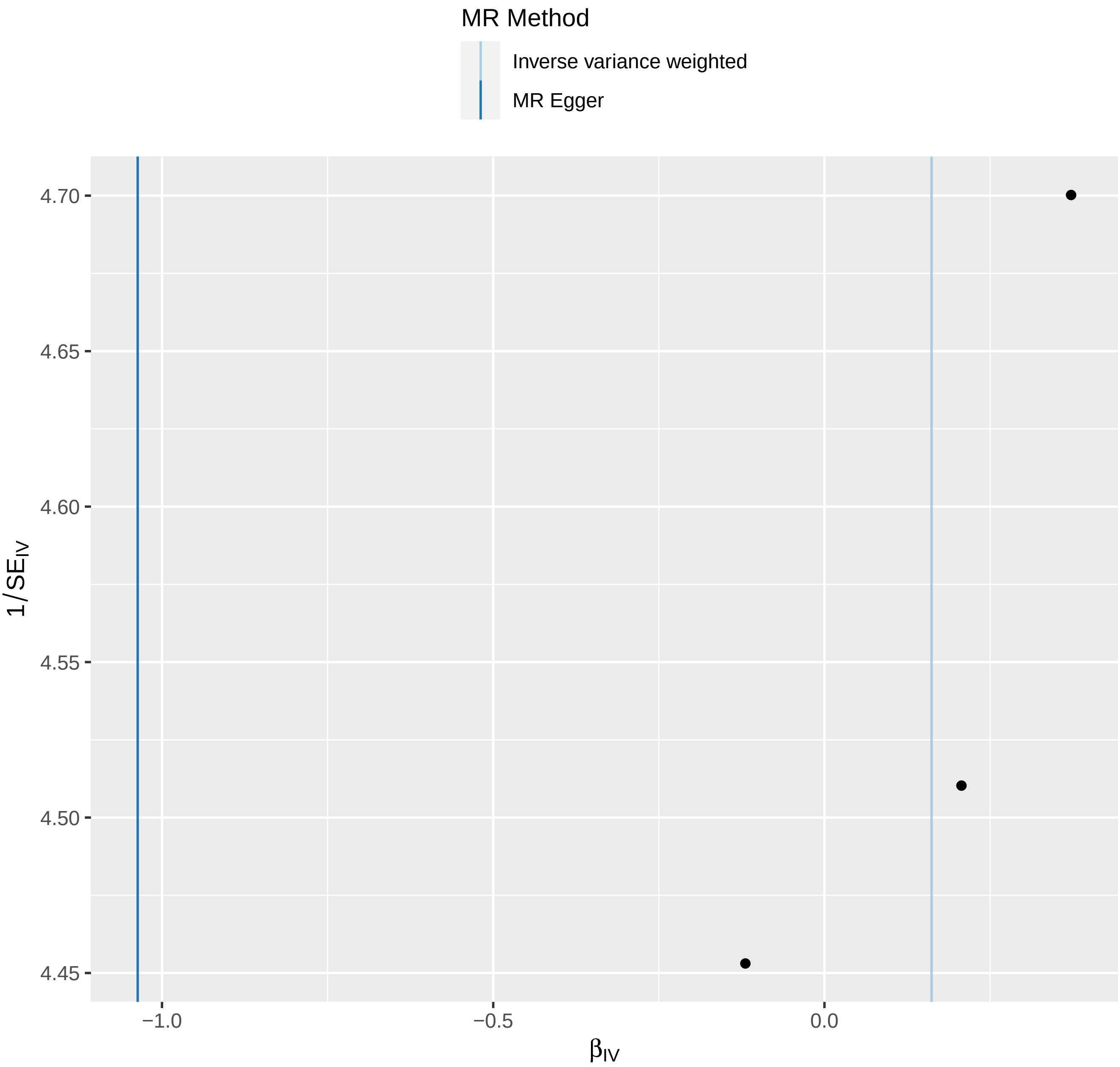

Batch 2−1006 : Gut microbiota abundance (genus Parasutterella id.2892) on Carcinoembryonic antigen−related cell adhesion molecule 21 with a threshold of 1e−05

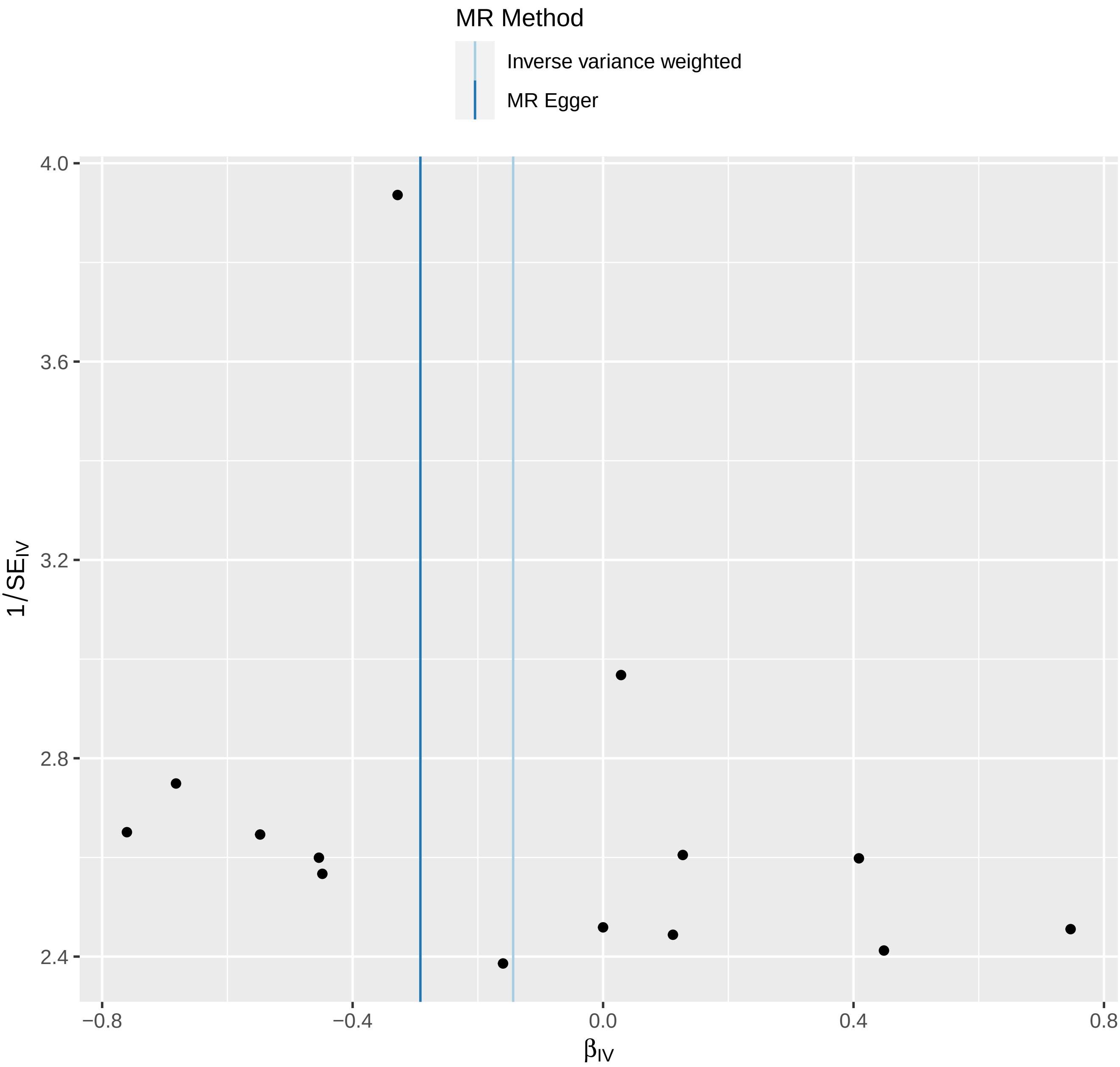

Batch 2−1007 : Gut microbiota abundance (genus Catenibacterium id.2153) on Carcinoembryonic antigen−related cell adhesion molecule 4
with a threshold of 1e−05

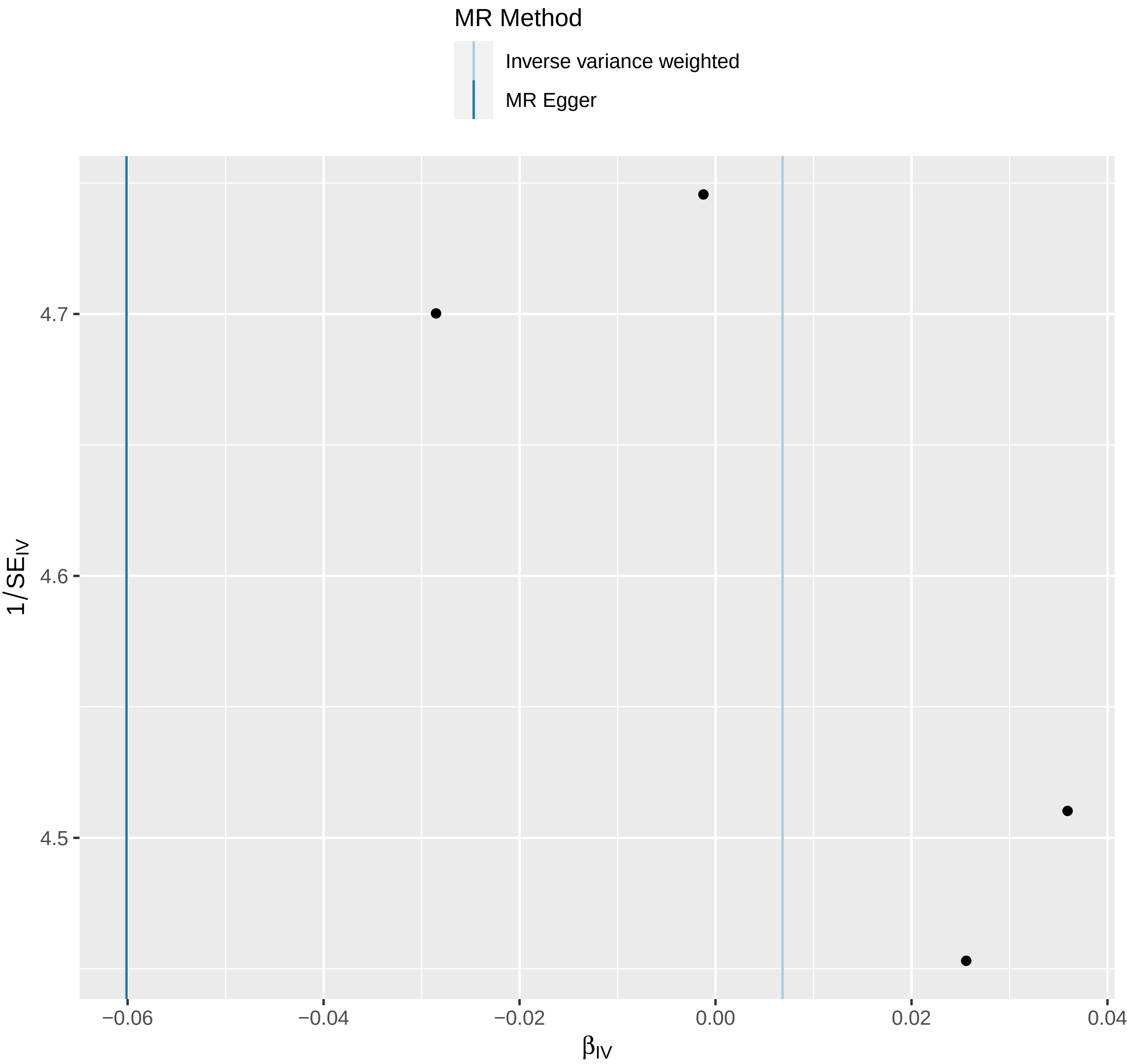

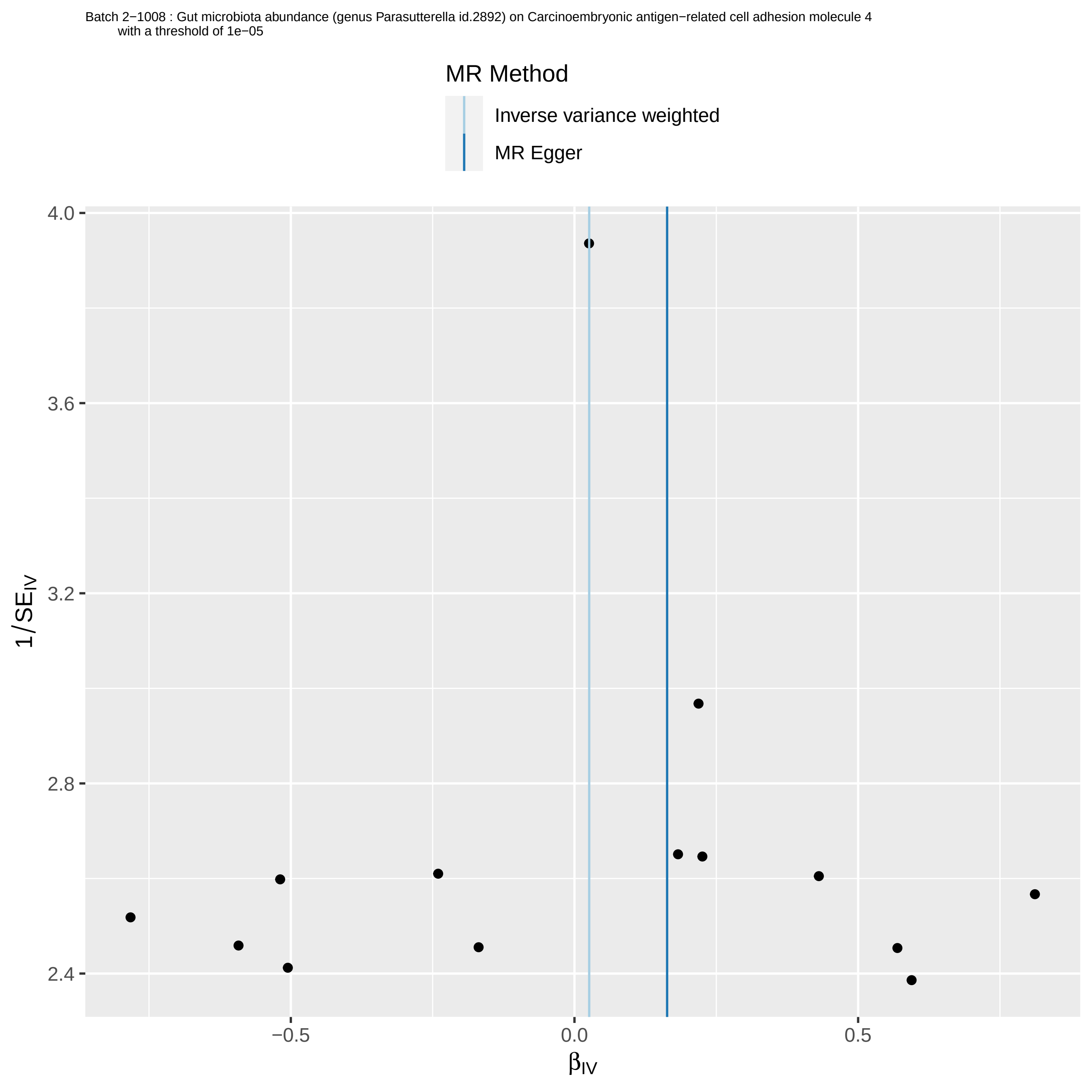

Batch 2−1008 : Gut microbiota abundance (genus Parasutterella id.2892) on Carcinoembryonic antigen−related cell adhesion molecule 4
with a threshold of 1e−05
MR Method
Inverse variance weighted
MR Egger
4.0
3.6
3.2
2.8
2.4
1/SE_IV
−0.5
0.0
0.5
β_IV

Batch 2−1009 : Gut microbiota abundance (genus Catenibacterium id.2153) on CCAAT/enhancer−binding protein beta
with a threshold of 1e−05

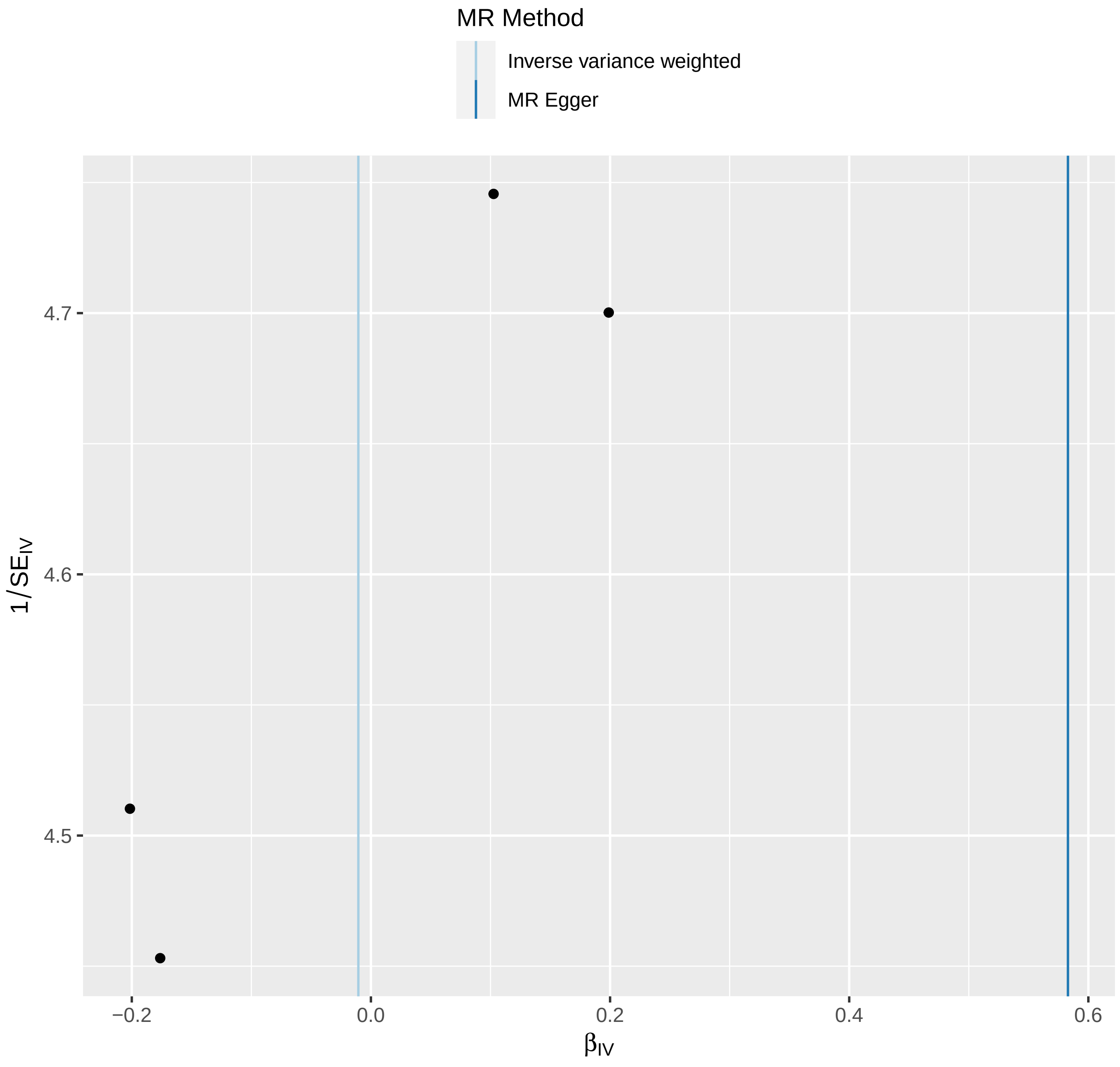

Batch 2−1010 : Gut microbiota abundance (genus Parasutterella id.2892) on CCAAT/enhancer−binding protein beta
with a threshold of 1e−05

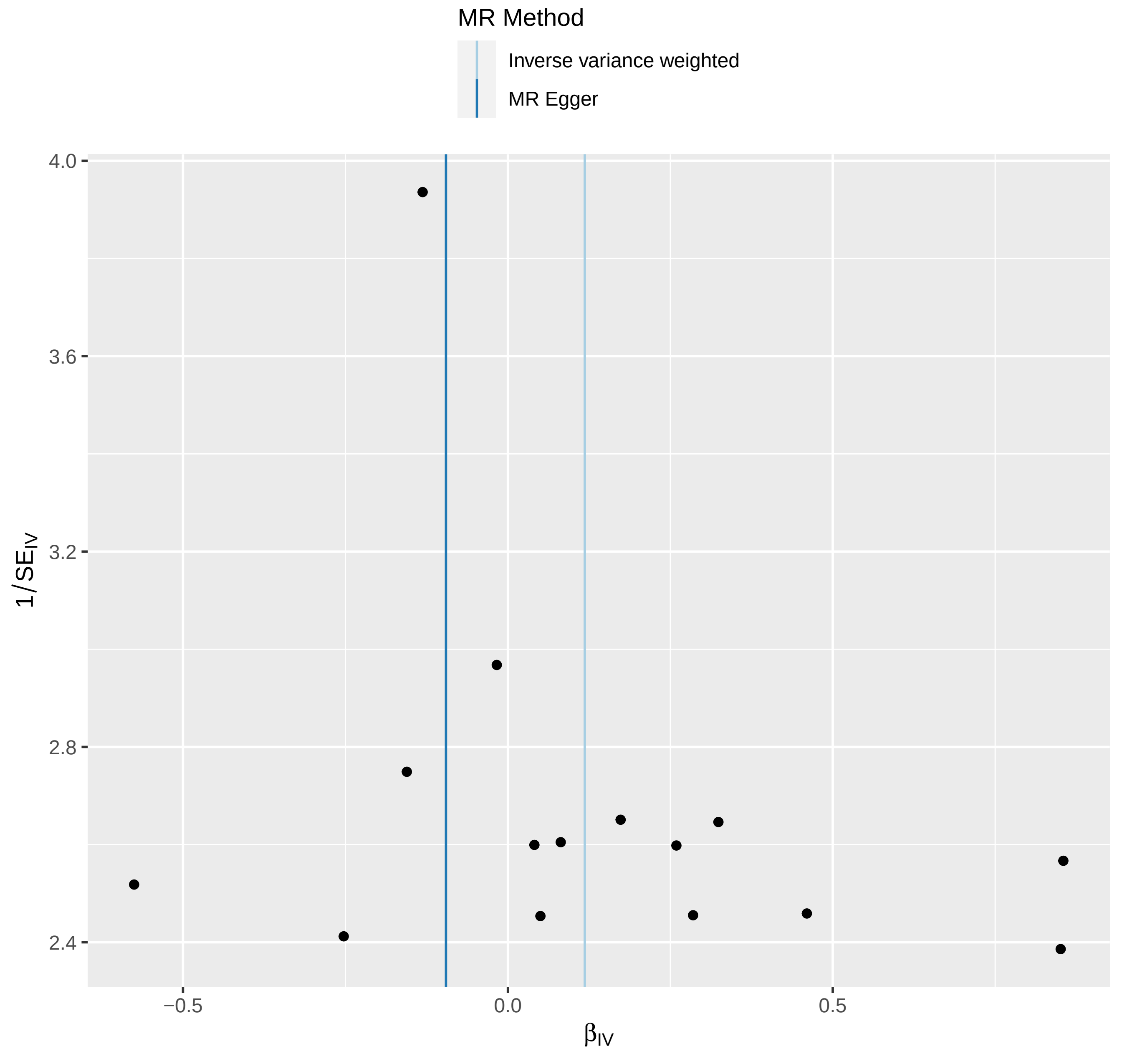

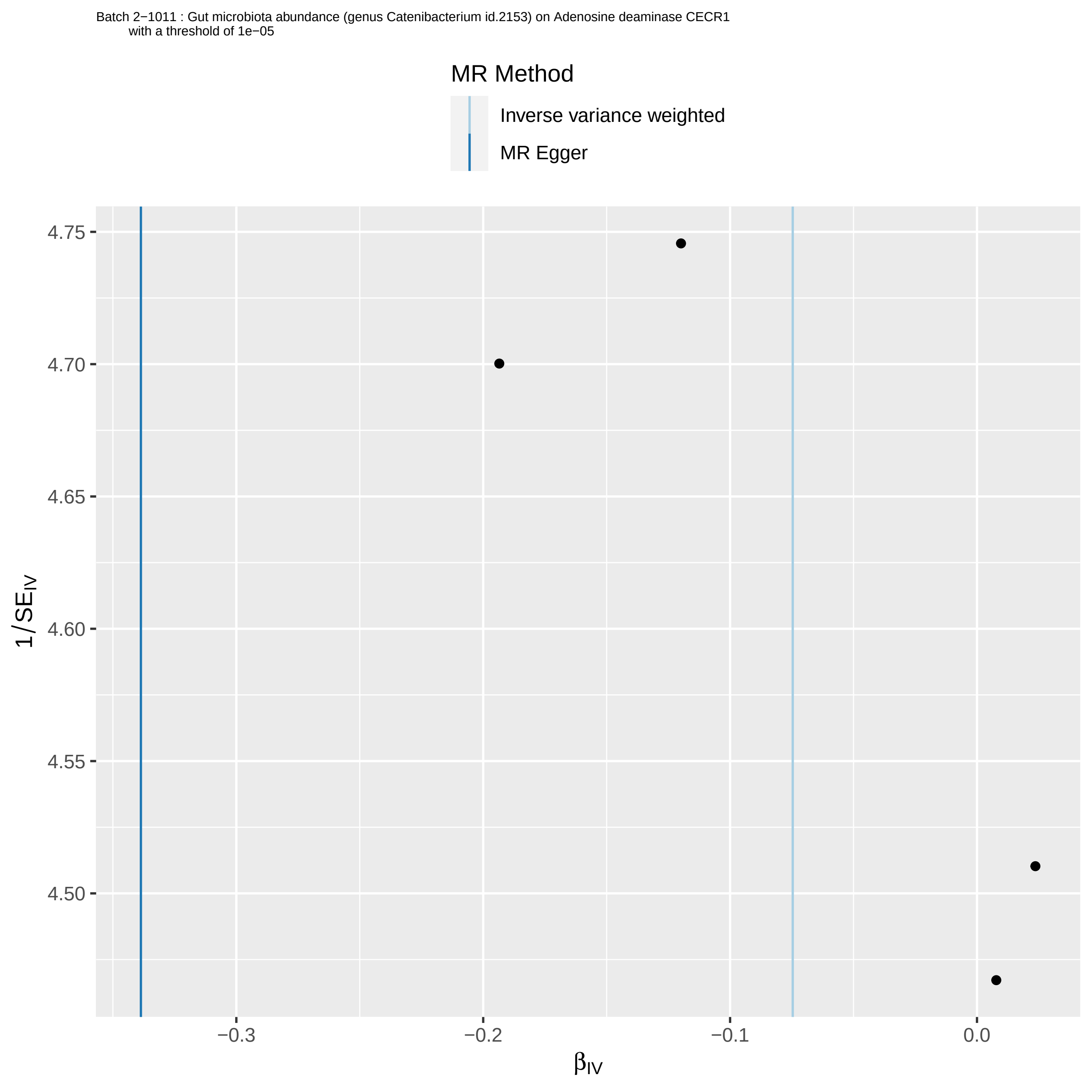

Batch 2−1011 : Gut microbiota abundance (genus Catenibacterium id.2153) on Adenosine deaminase CECR1
with a threshold of 1e−05
MR Method
Inverse variance weighted
MR Egger
4.75
4.70
4.65
4.60
4.55
4.50
1/SE_IV
−0.3
−0.2
−0.1
0.0
β_IV

Batch 2−1012 : Gut microbiota abundance (genus Parasutterella id.2892) on Adenosine deaminase CECR1
with a threshold of 1e−05

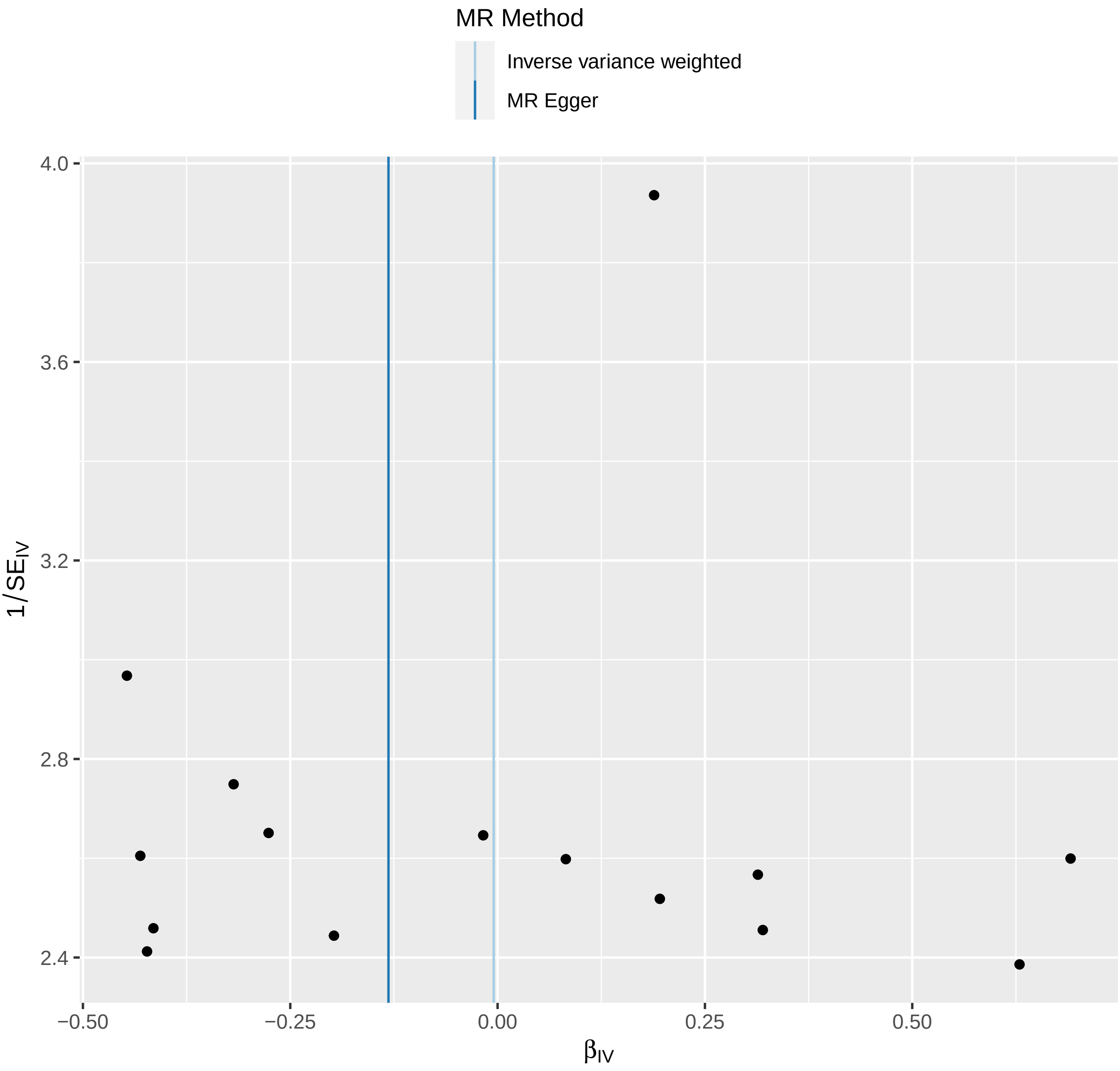

Batch 2−1013 : Gut microbiota abundance (genus Catenibacterium id.2153) on Bile salt−activated lipase
with a threshold of 1e−05

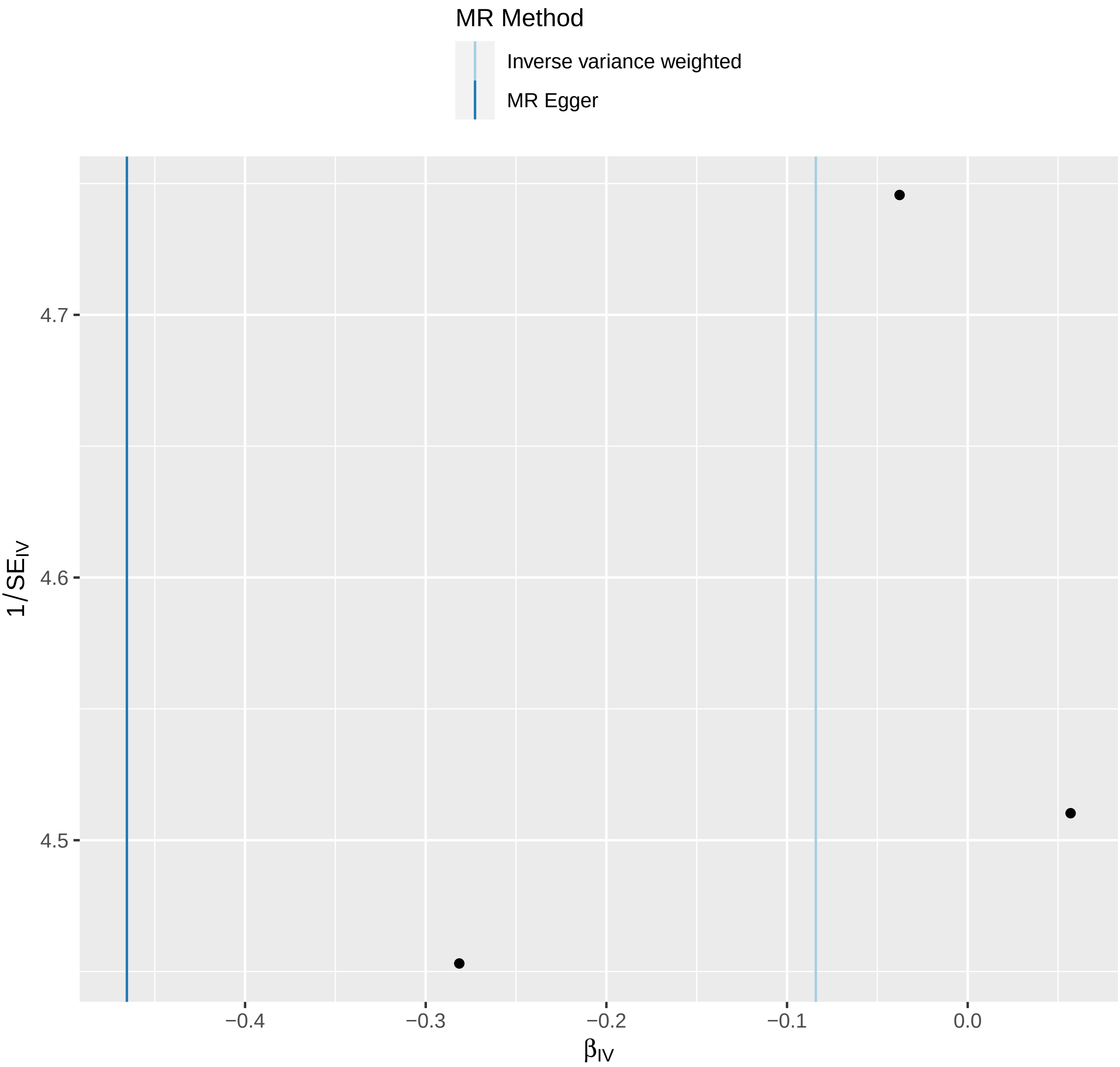

Batch 2−1014 : Gut microbiota abundance (genus Parasutterella id.2892) on Bile salt−activated lipase
with a threshold of 1e−05

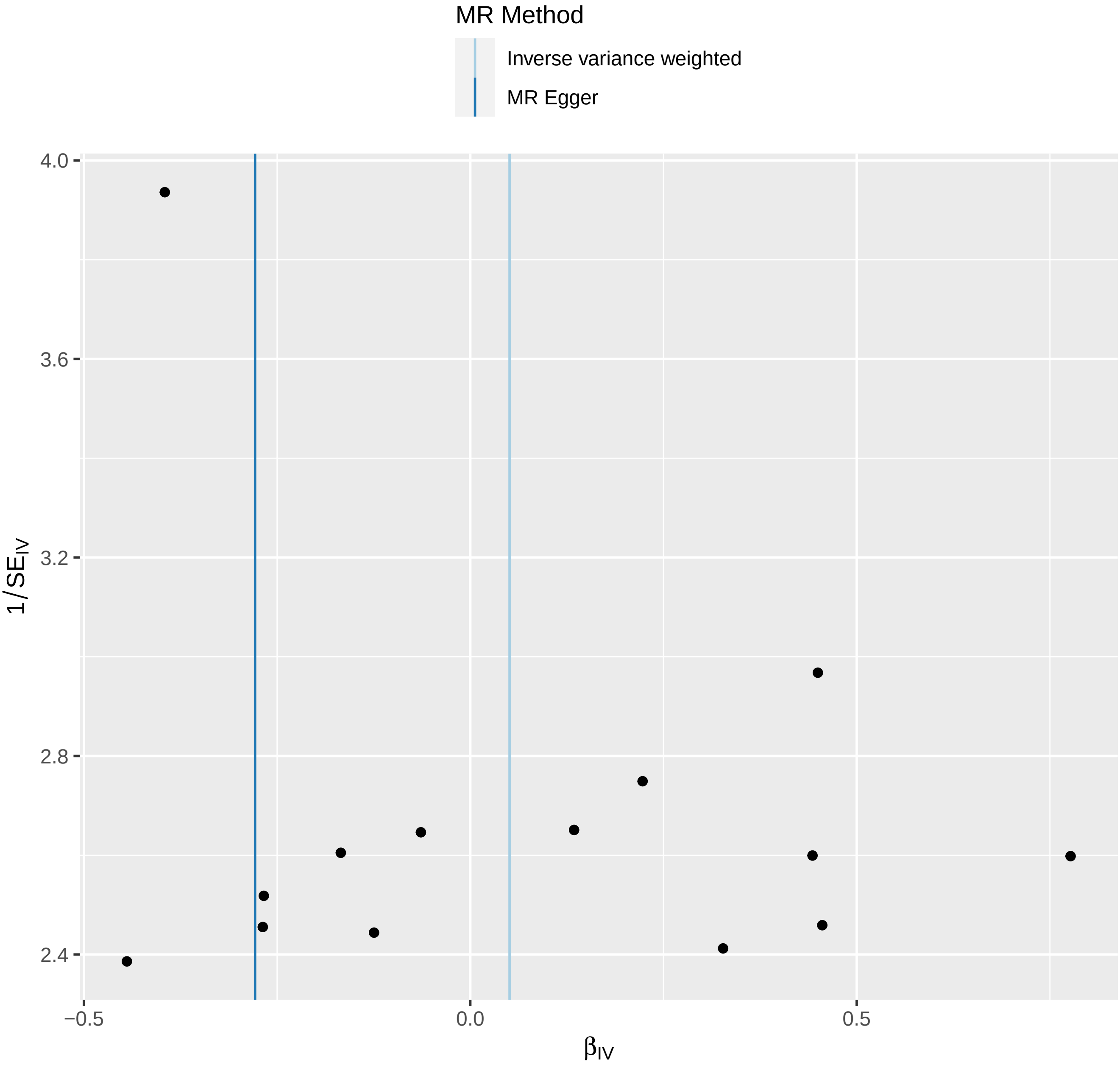

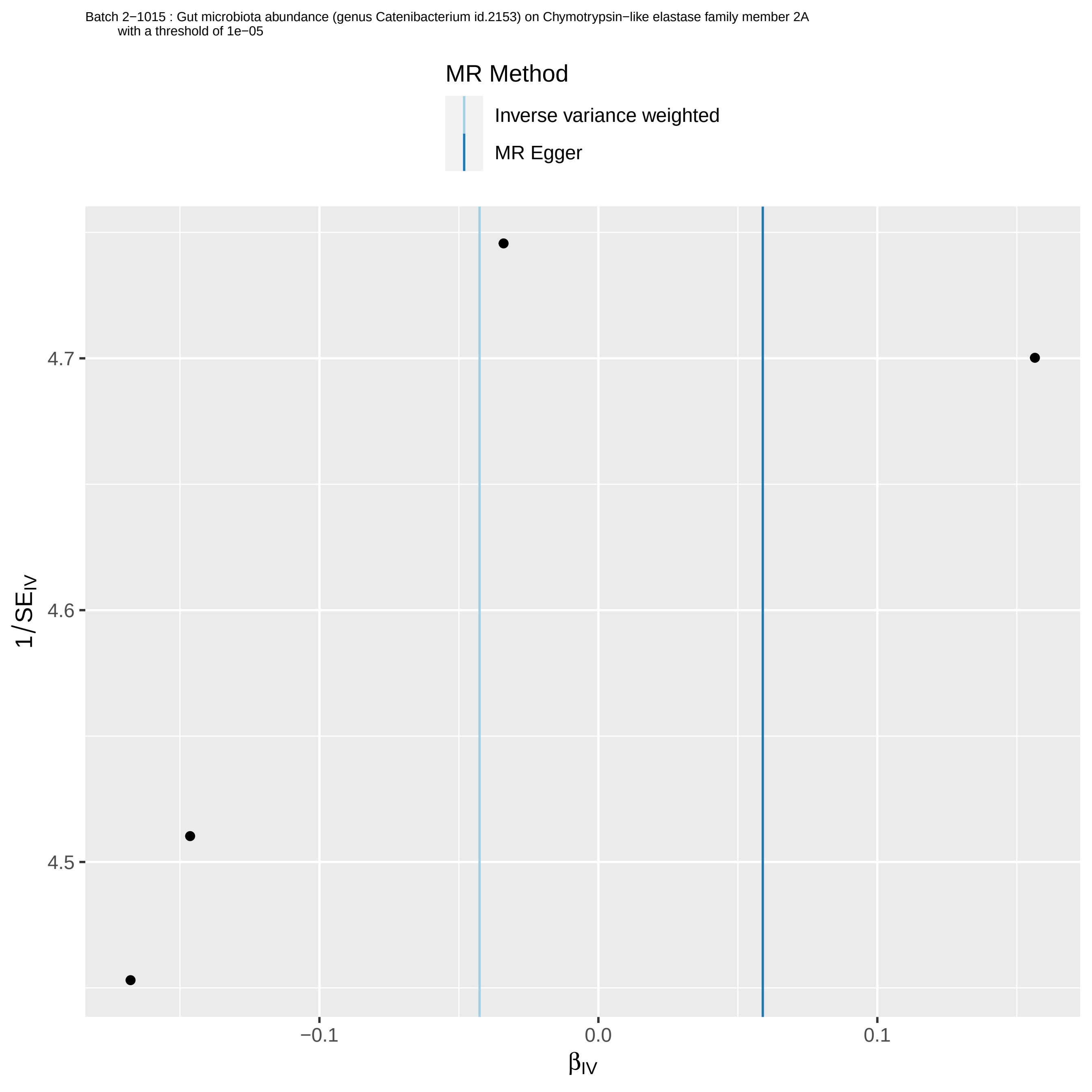
Batch 2−1015 : Gut microbiota abundance (genus Catenibacterium id.2153) on Chymotrypsin−like elastase family member 2A
with a threshold of 1e−05
MR Method
Inverse variance weighted
MR Egger
4.7
4.6
4.5
1/SE_IV
−0.1
0.0
0.1
β_IV

Batch 2−1016 : Gut microbiota abundance (genus Parasutterella id.2892) on Chymotrypsin−like elastase family member 2A
with a threshold of 1e−05

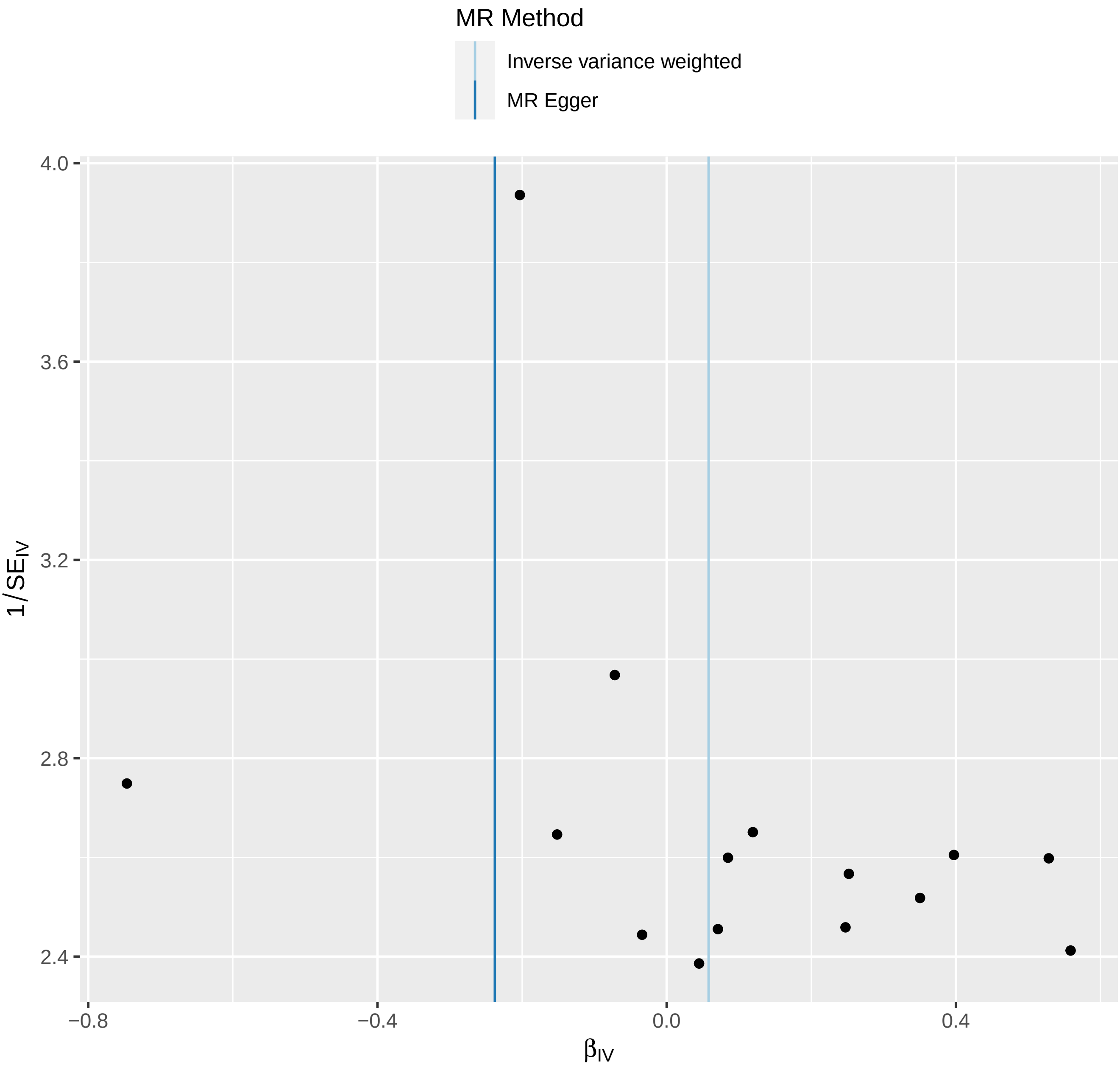

Batch 2−1017 : Gut microbiota abundance (genus Catenibacterium id.2153) on Chymotrypsin−like elastase family member 3B
with a threshold of 1e−05

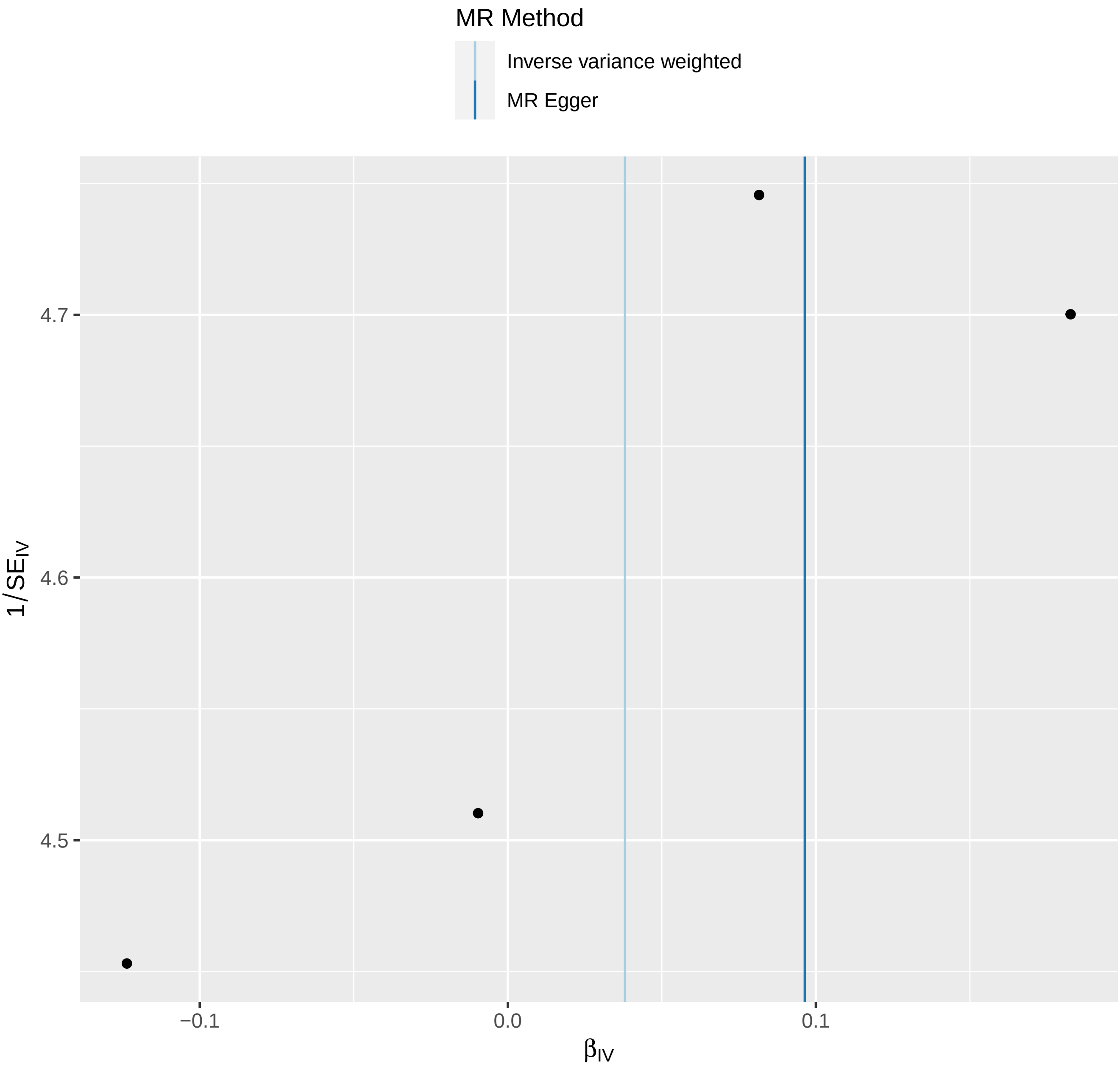

Batch 2−1018 : Gut microbiota abundance (genus Parasutterella id.2892) on Chymotrypsin−like elastase family member 3B
with a threshold of 1e−05

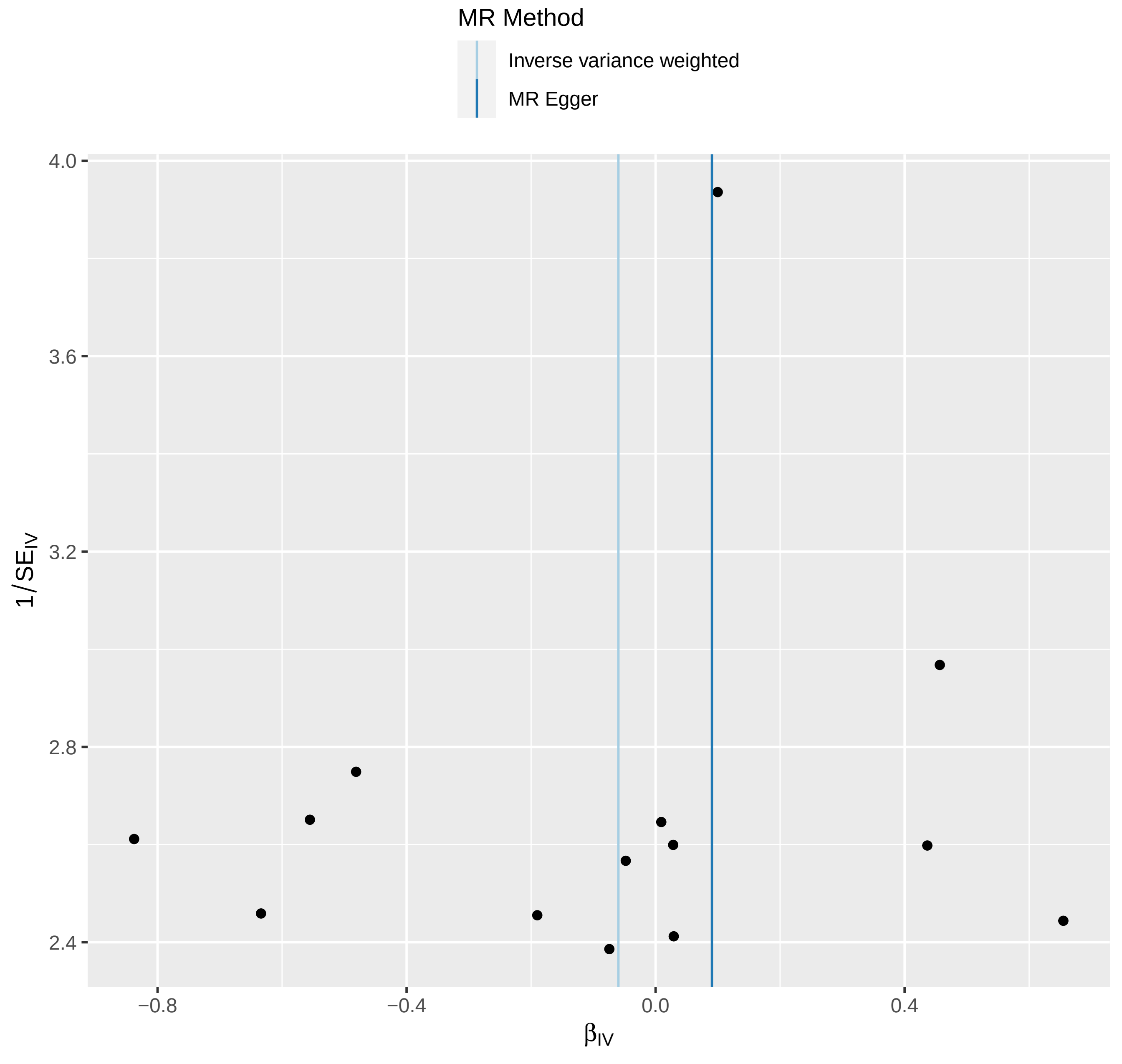

Batch 2−1019 : Gut microbiota abundance (genus Catenibacterium id.2153) on CUGBP Elav−like family member 2
with a threshold of 1e−05

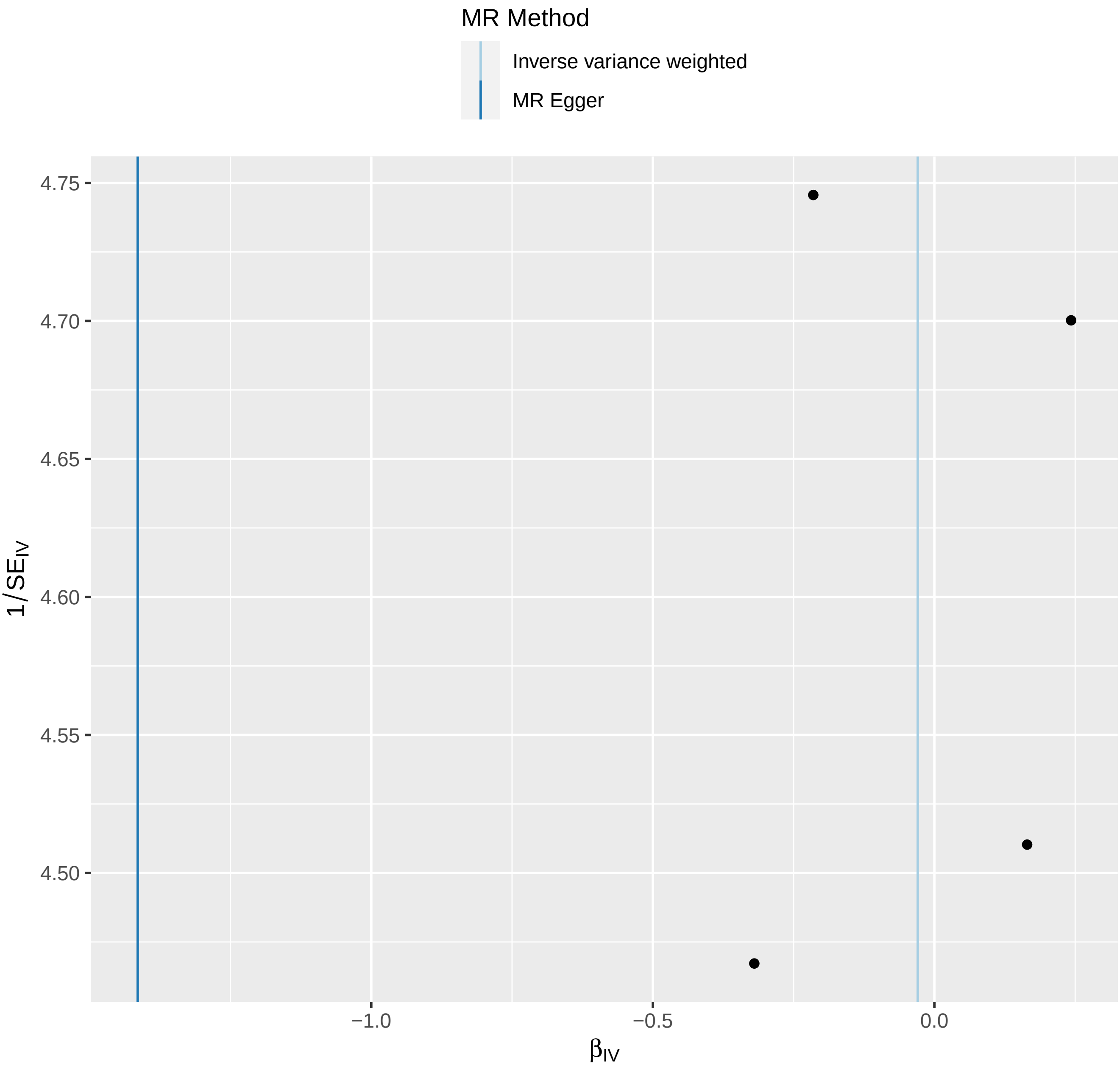

Batch 2−1020 : Gut microbiota abundance (genus Parasutterella id.2892) on CUGBP Elav−like family member 2
with a threshold of 1e−05

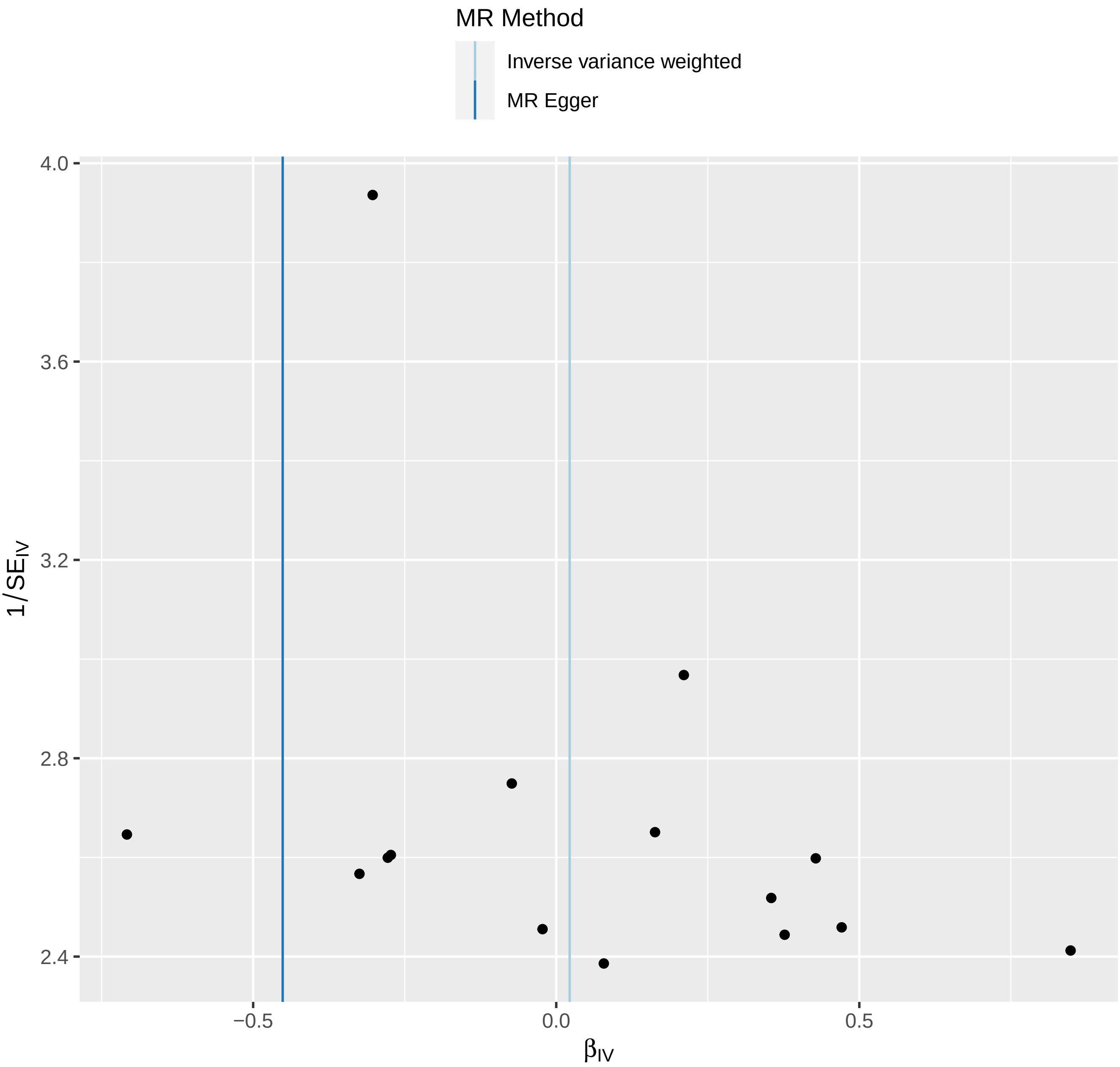

Batch 2−1021 : Gut microbiota abundance (genus Catenibacterium id.2153) on Centromere protein W
with a threshold of 1e−05

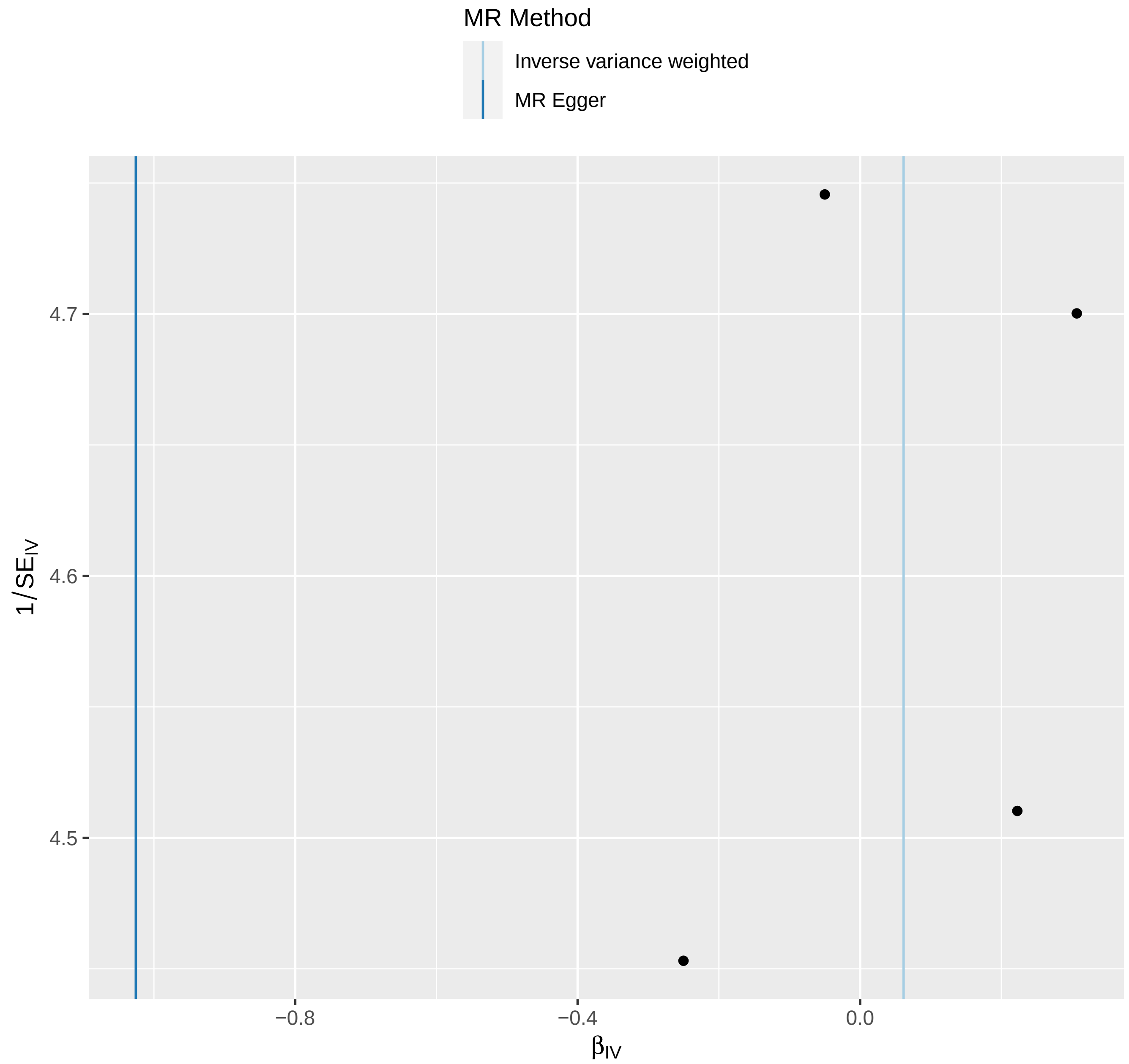

Batch 2−1022 : Gut microbiota abundance (genus Parasutterella id.2892) on Centromere protein W with a threshold of 1e−05

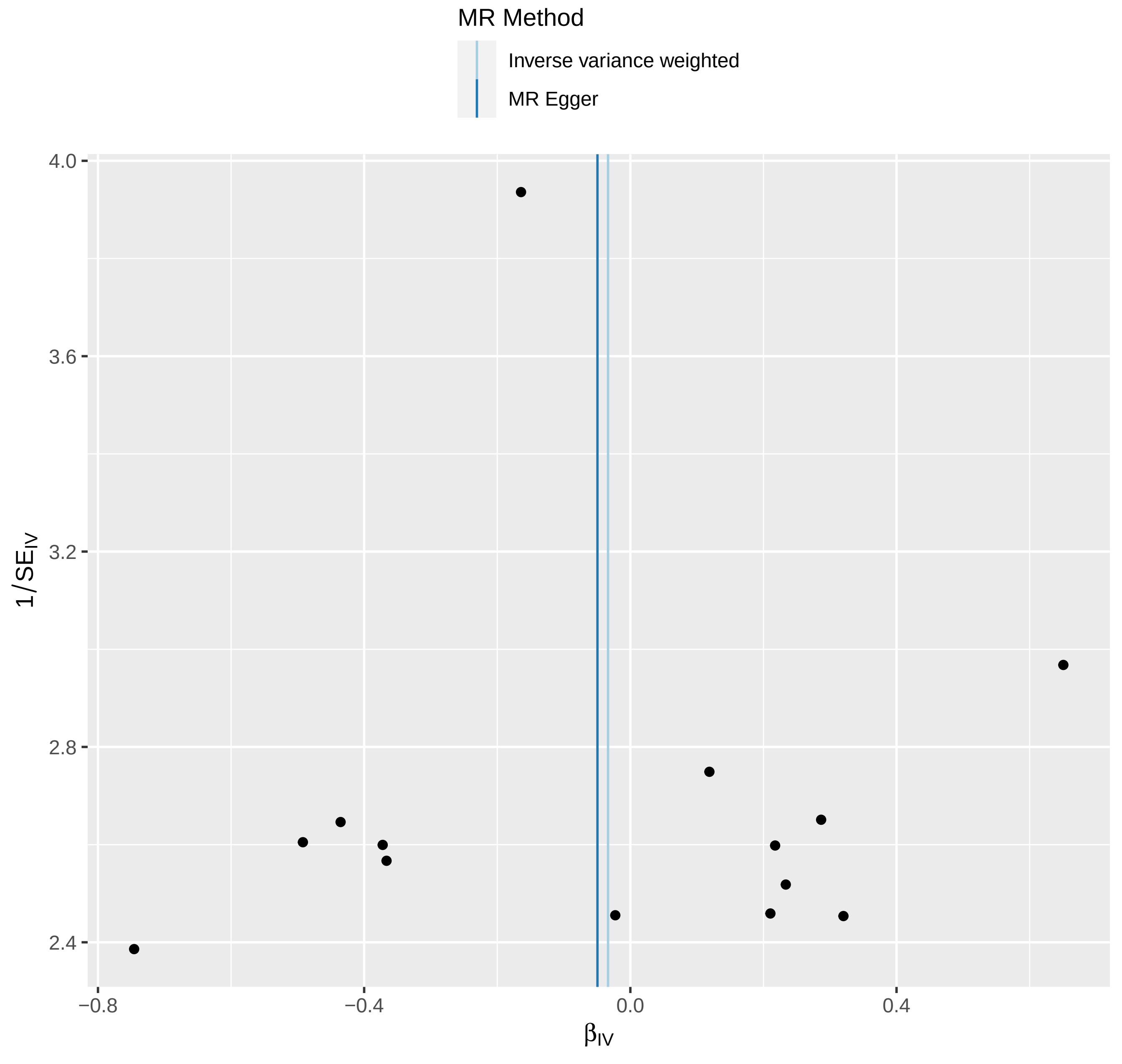

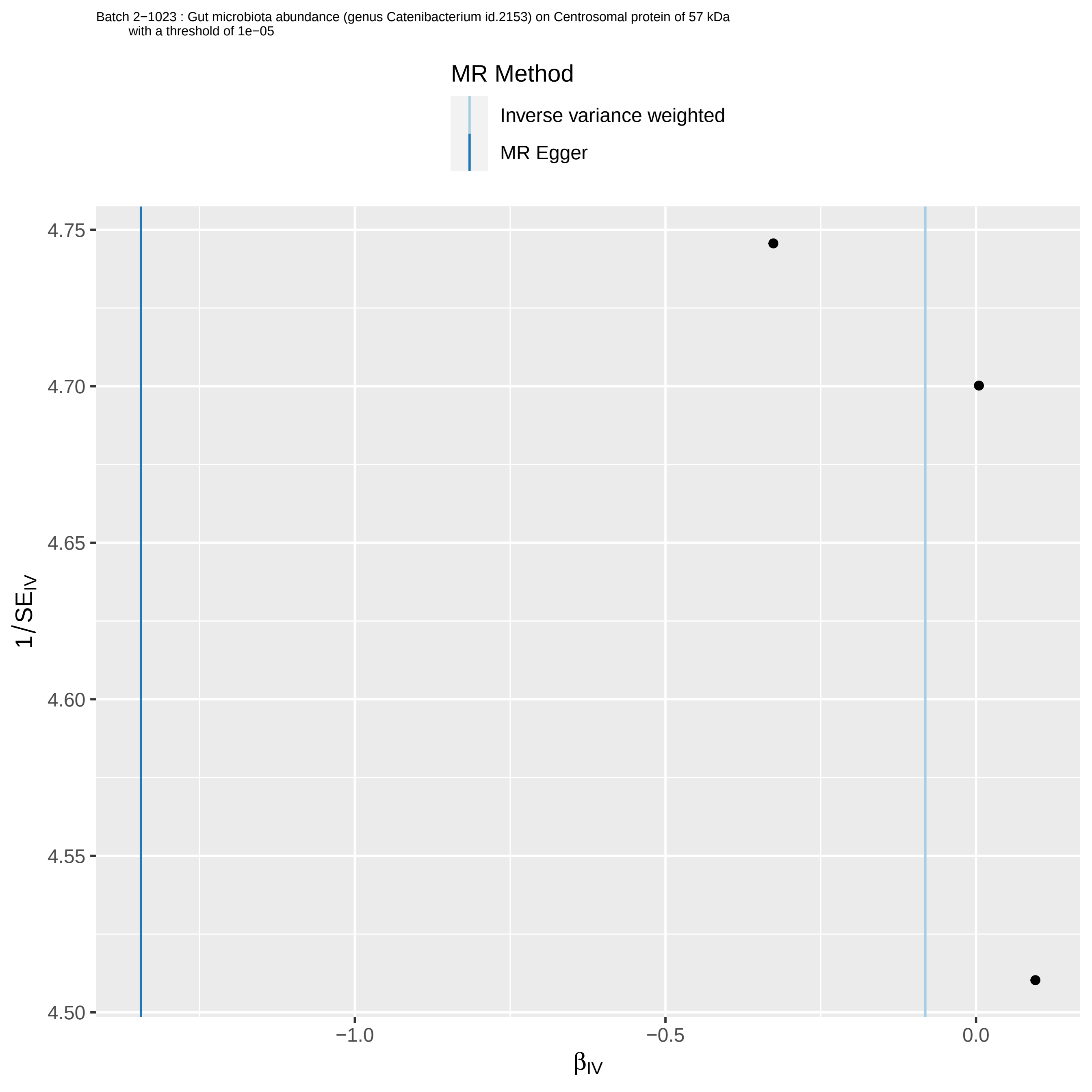

Batch 2−1023 : Gut microbiota abundance (genus Catenibacterium id.2153) on Centrosomal protein of 57 kDa
with a threshold of 1e−05
MR Method
Inverse variance weighted
MR Egger
4.75
4.70
4.65
4.60
4.55
4.50
1/SE_IV
−1.0
−0.5
0.0
β_IV

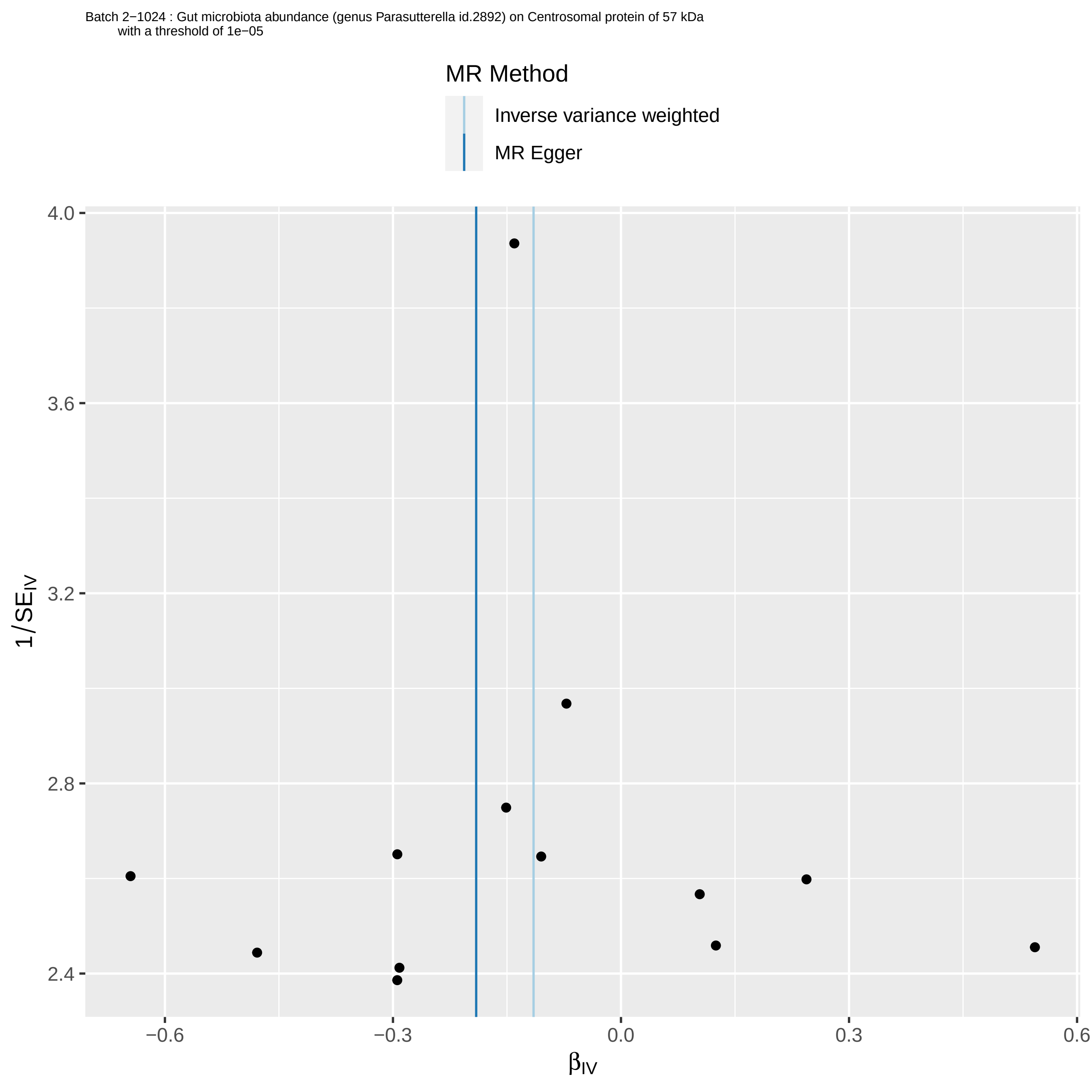
Batch 2−1024 : Gut microbiota abundance (genus Parasutterella id.2892) on Centrosomal protein of 57 kDa
with a threshold of 1e−05
MR Method
Inverse variance weighted
MR Egger
4.0
3.6
3.2
2.8
2.4
1/SE_IV
−0.6
−0.3
0.0
0.3
0.6
β_IV

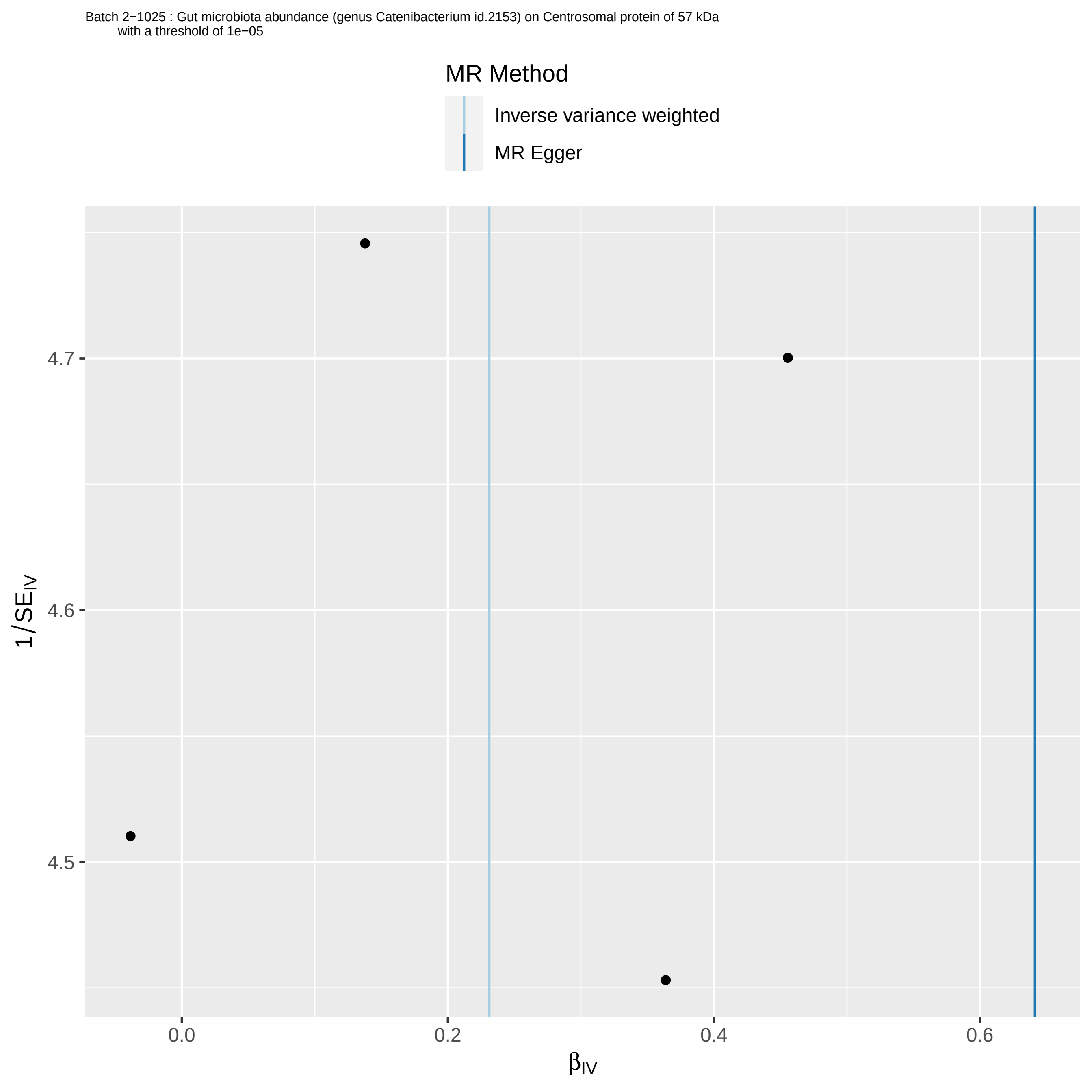
Batch 2−1025 : Gut microbiota abundance (genus Catenibacterium id.2153) on Centrosomal protein of 57 kDa
with a threshold of 1e−05
MR Method
Inverse variance weighted
MR Egger
4.7
4.6
4.5
1/SE_IV
0.0
0.2
0.4
0.6
β_IV

Batch 2−1026 : Gut microbiota abundance (genus Parasutterella id.2892) on Centrosomal protein of 57 kDa
with a threshold of 1e−05

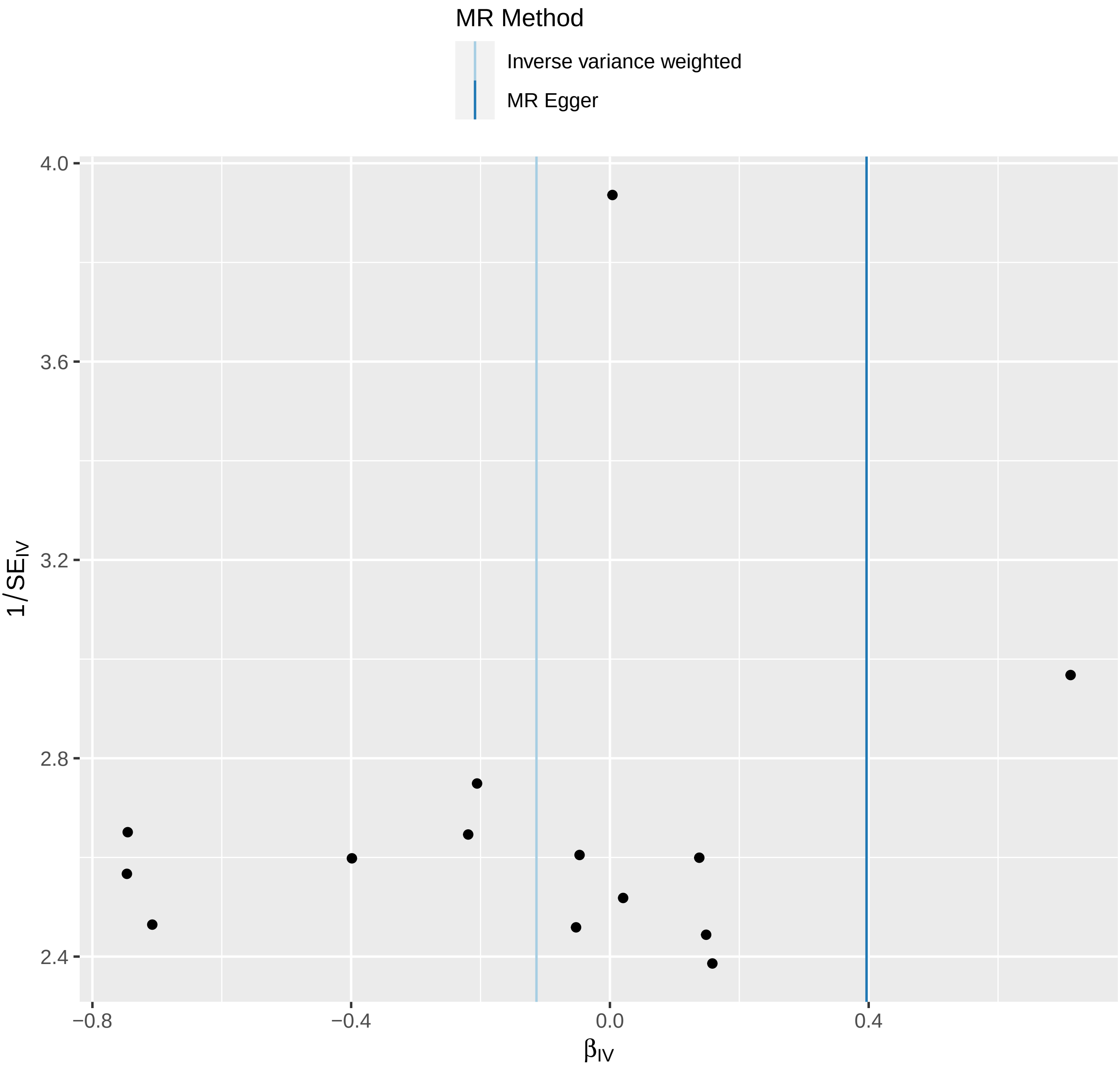

Batch 2−1027 : Gut microbiota abundance (genus Catenibacterium id.2153) on Centrosomal protein of 57 kDa
with a threshold of 1e−05

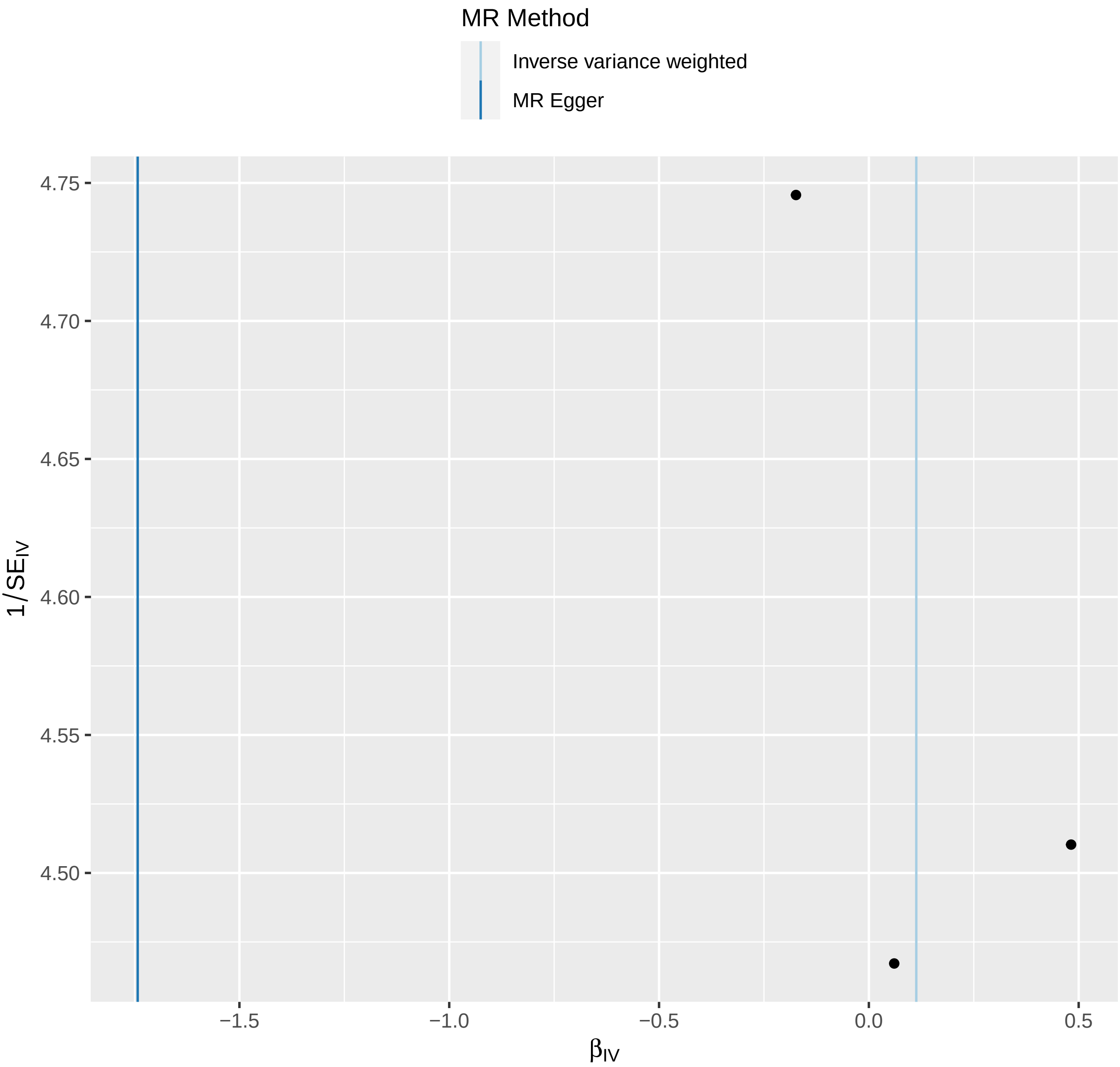

Batch 2−1028 : Gut microbiota abundance (genus Parasutterella id.2892) on Centrosomal protein of 57 kDa
with a threshold of 1e−05

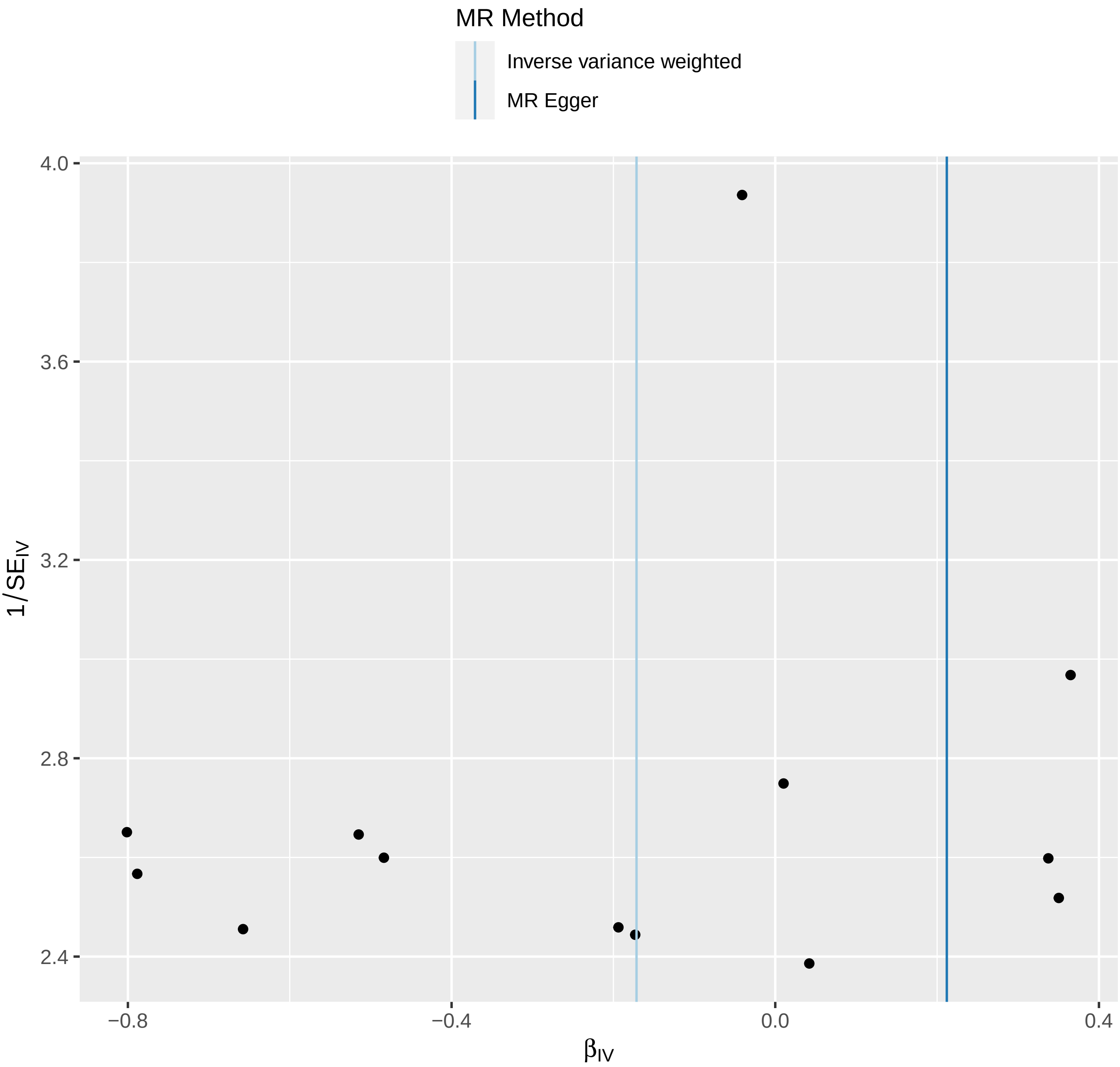

Batch 2−1029 : Gut microbiota abundance (genus Catenibacterium id.2153) on Ceramide synthase 5
with a threshold of 1e−05

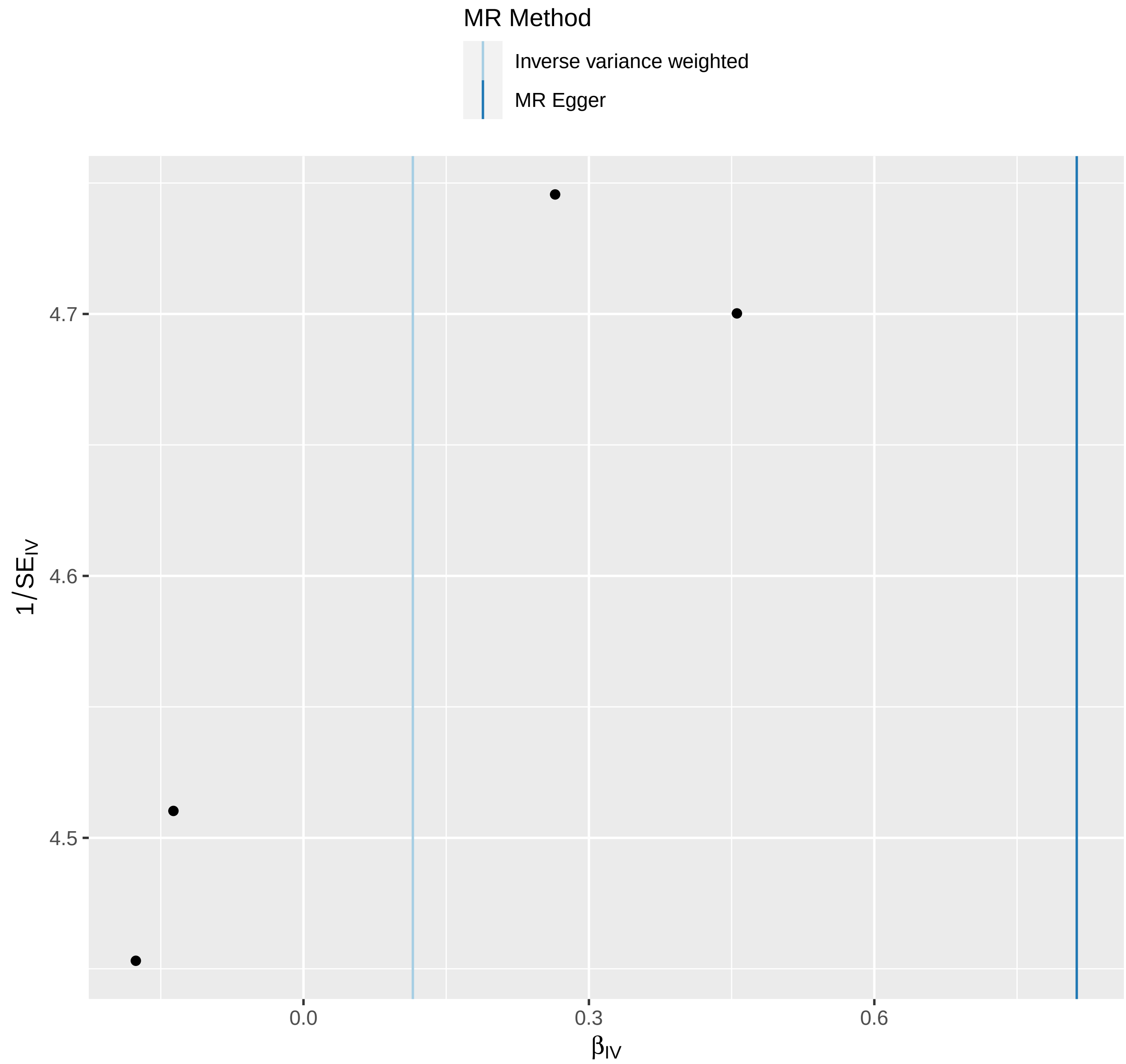

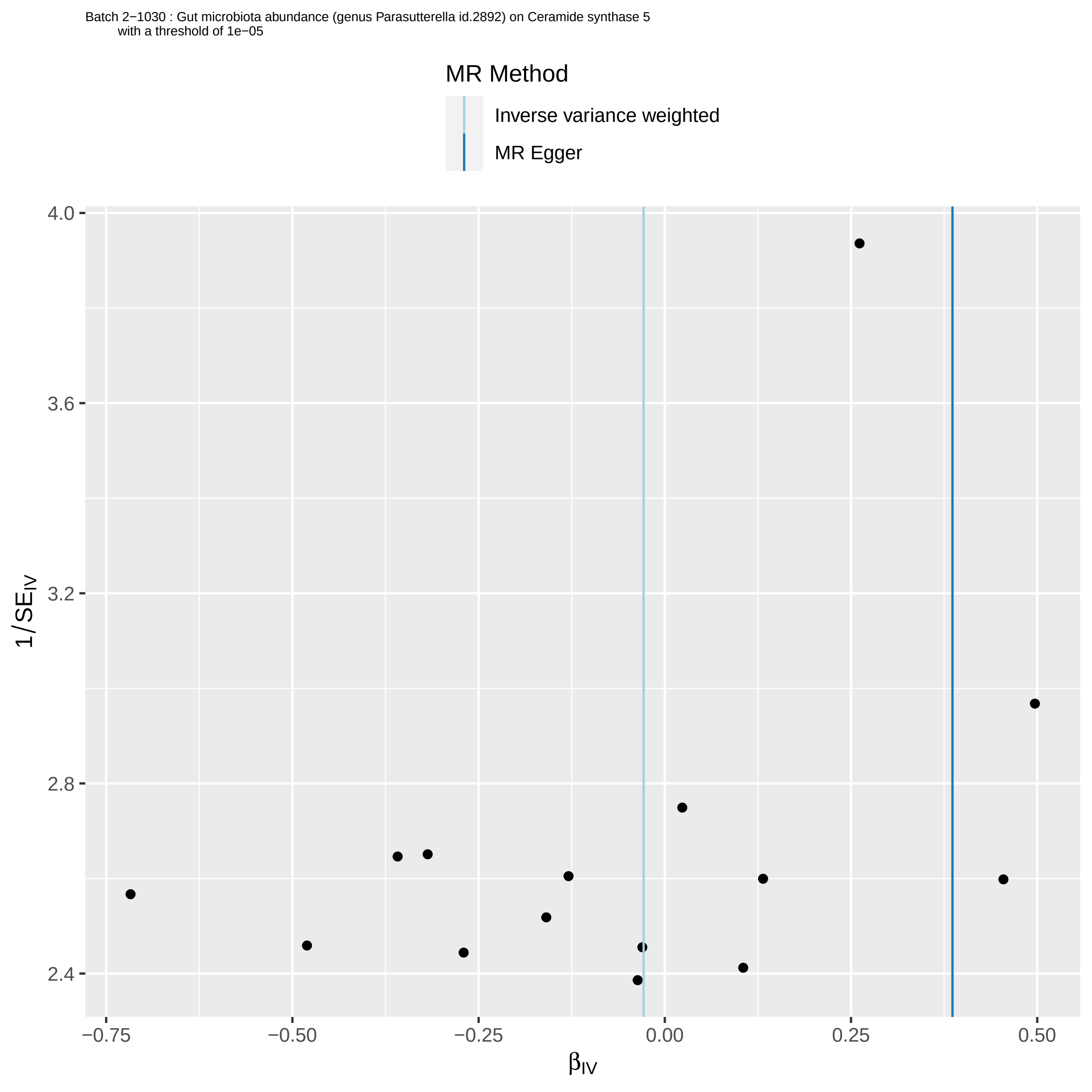
Batch 2−1030 : Gut microbiota abundance (genus Parasutterella id.2892) on Ceramide synthase 5
with a threshold of 1e−05
MR Method
Inverse variance weighted
MR Egger
4.0
3.6
3.2
2.8
2.4
1/SE_IV
−0.75
−0.50
−0.25
0.00
0.25
0.50
β_IV

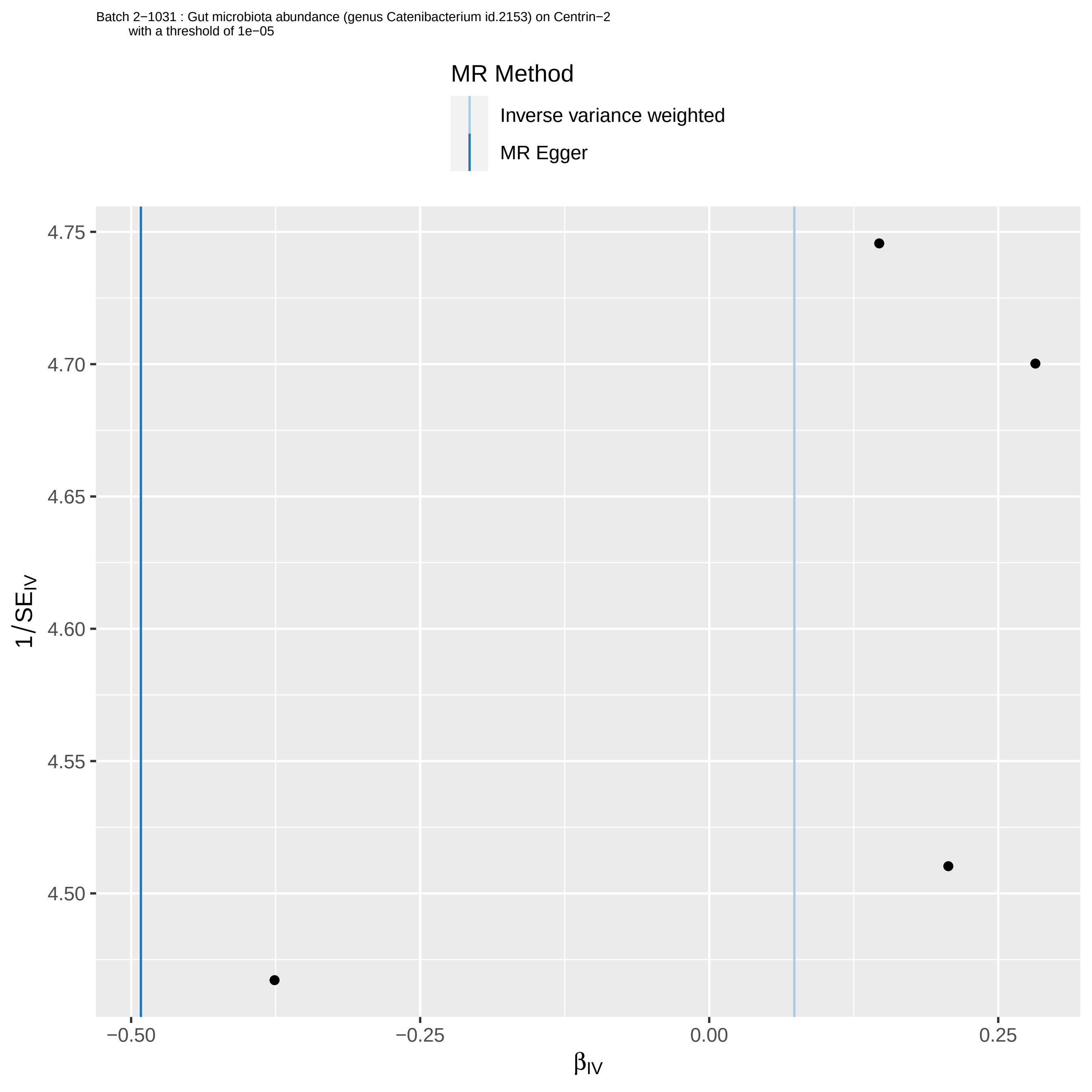
Batch 2−1031 : Gut microbiota abundance (genus Catenibacterium id.2153) on Centrin−2
with a threshold of 1e−05
MR Method
Inverse variance weighted
MR Egger
4.75
4.70
4.65
4.60
4.55
4.50
1/SE_IV
−0.50
−0.25
0.00
0.25
β_IV

Batch 2−1032 : Gut microbiota abundance (genus Parasutterella id.2892) on Centrin−2
with a threshold of 1e−05

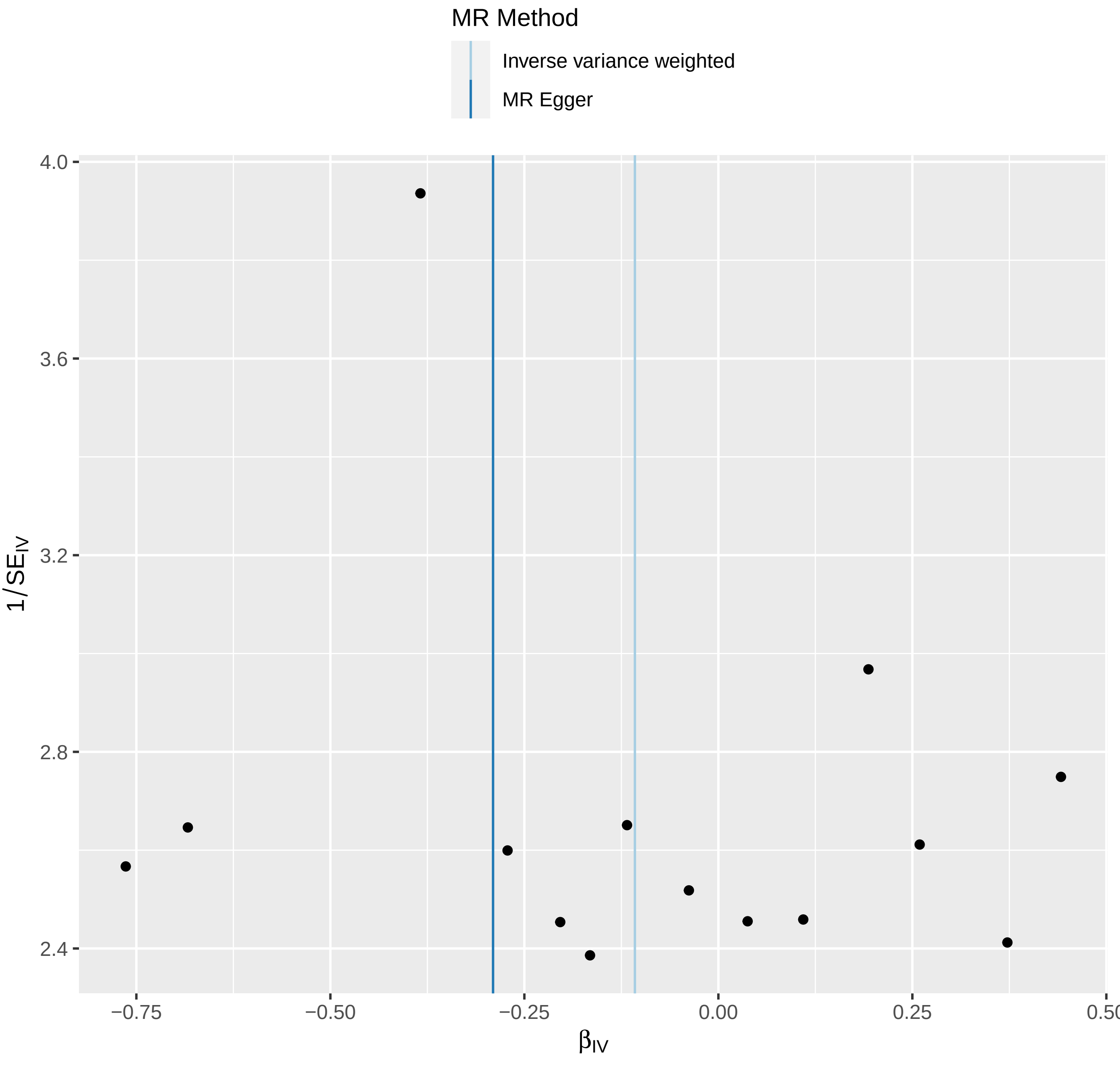

Batch 2−1033 : Gut microbiota abundance (genus Catenibacterium id.2153) on Cilia− and flagella−associated protein 45
with a threshold of 1e−05

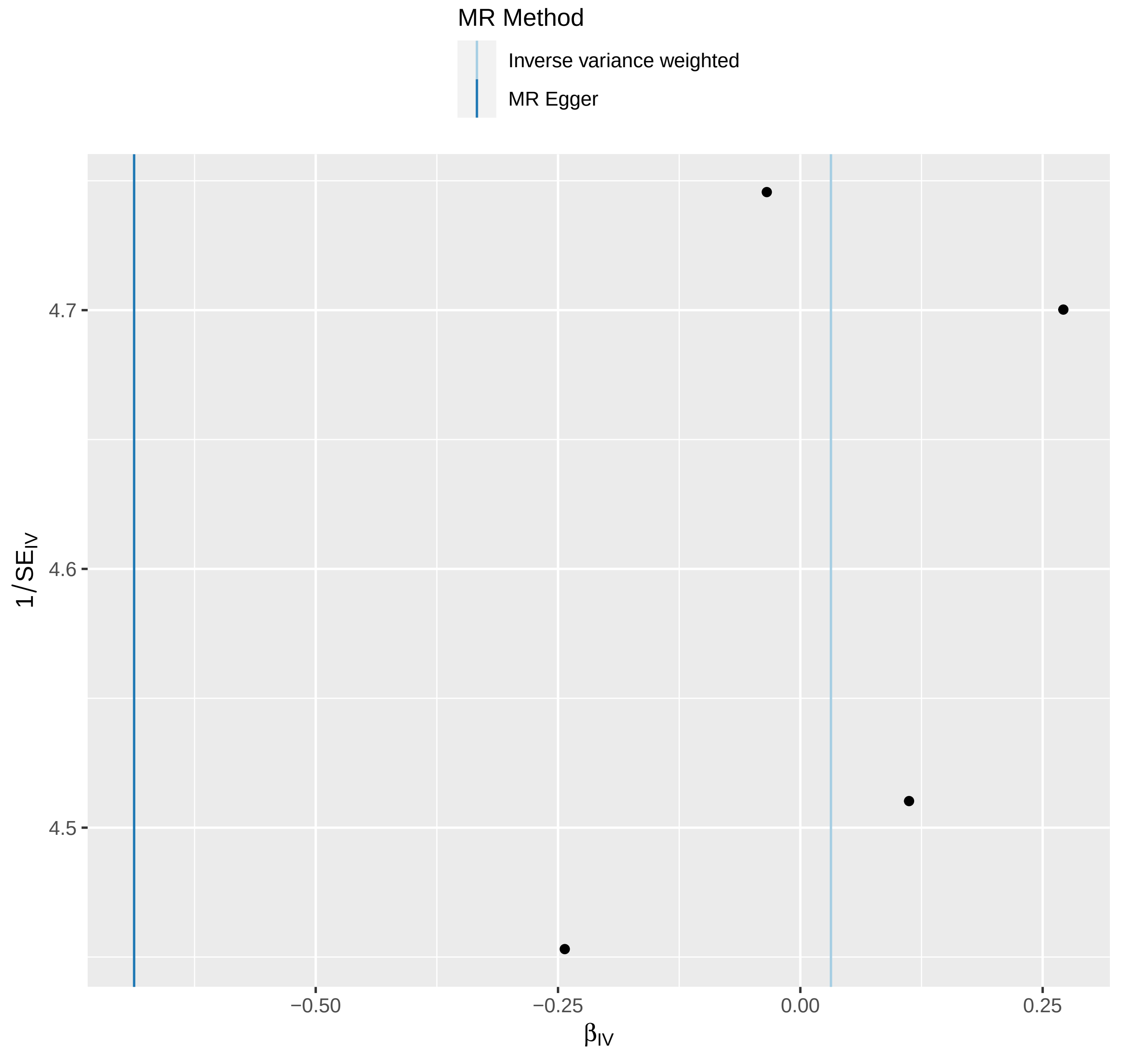

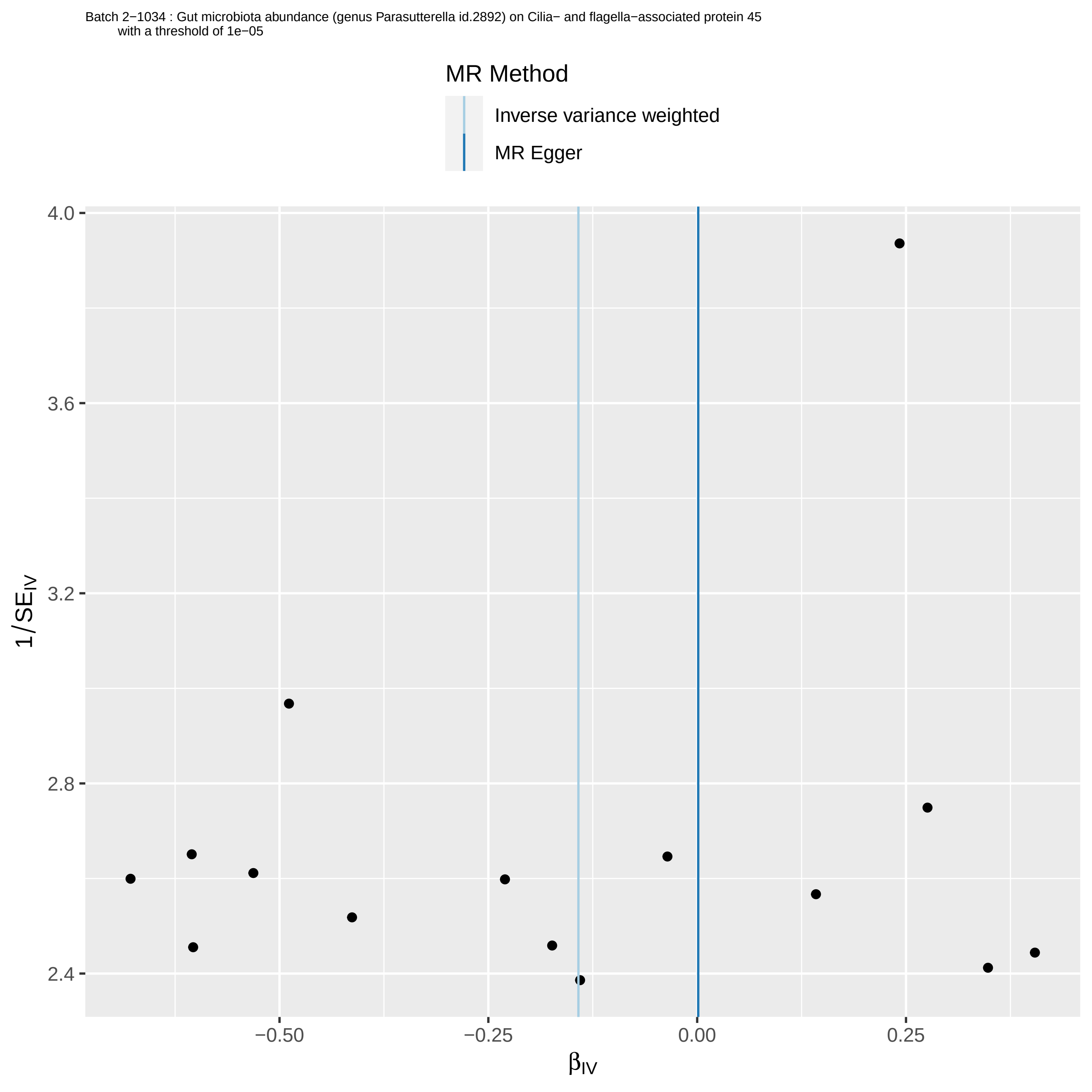

Batch 2−1034 : Gut microbiota abundance (genus Parasutterella id.2892) on Cilia− and flagella−associated protein 45
with a threshold of 1e−05
MR Method
Inverse variance weighted
MR Egger
4.0
3.6
3.2
2.8
2.4
$1/SE_{IV}$
−0.50
−0.25
0.00
0.25
β_{IV}

Batch 2−1035 : Gut microbiota abundance (genus Catenibacterium id.2153) on Complement factor B
with a threshold of 1e−05

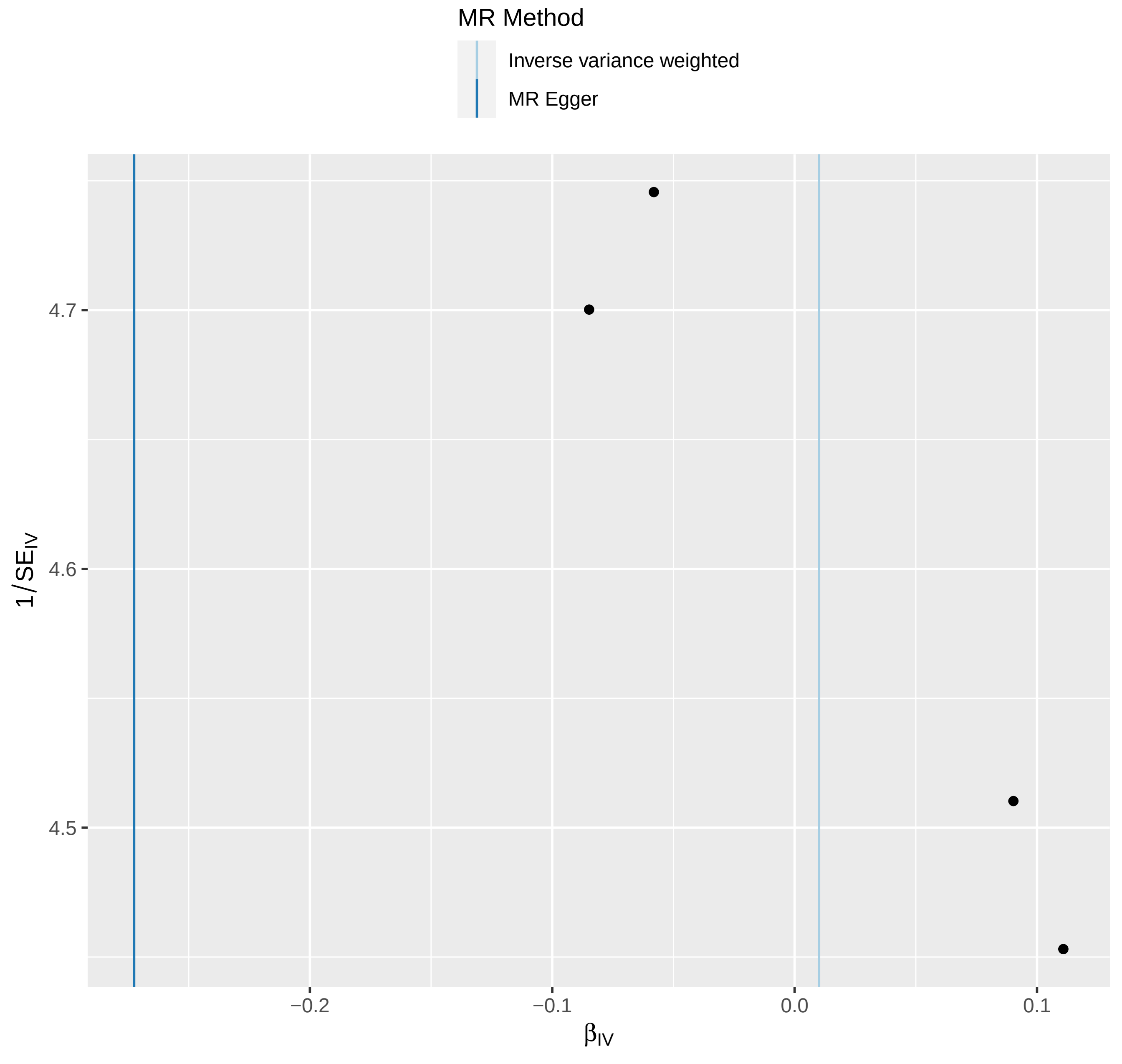

Batch 2−1036 : Gut microbiota abundance (genus Parasutterella id.2892) on Complement factor B
with a threshold of 1e−05

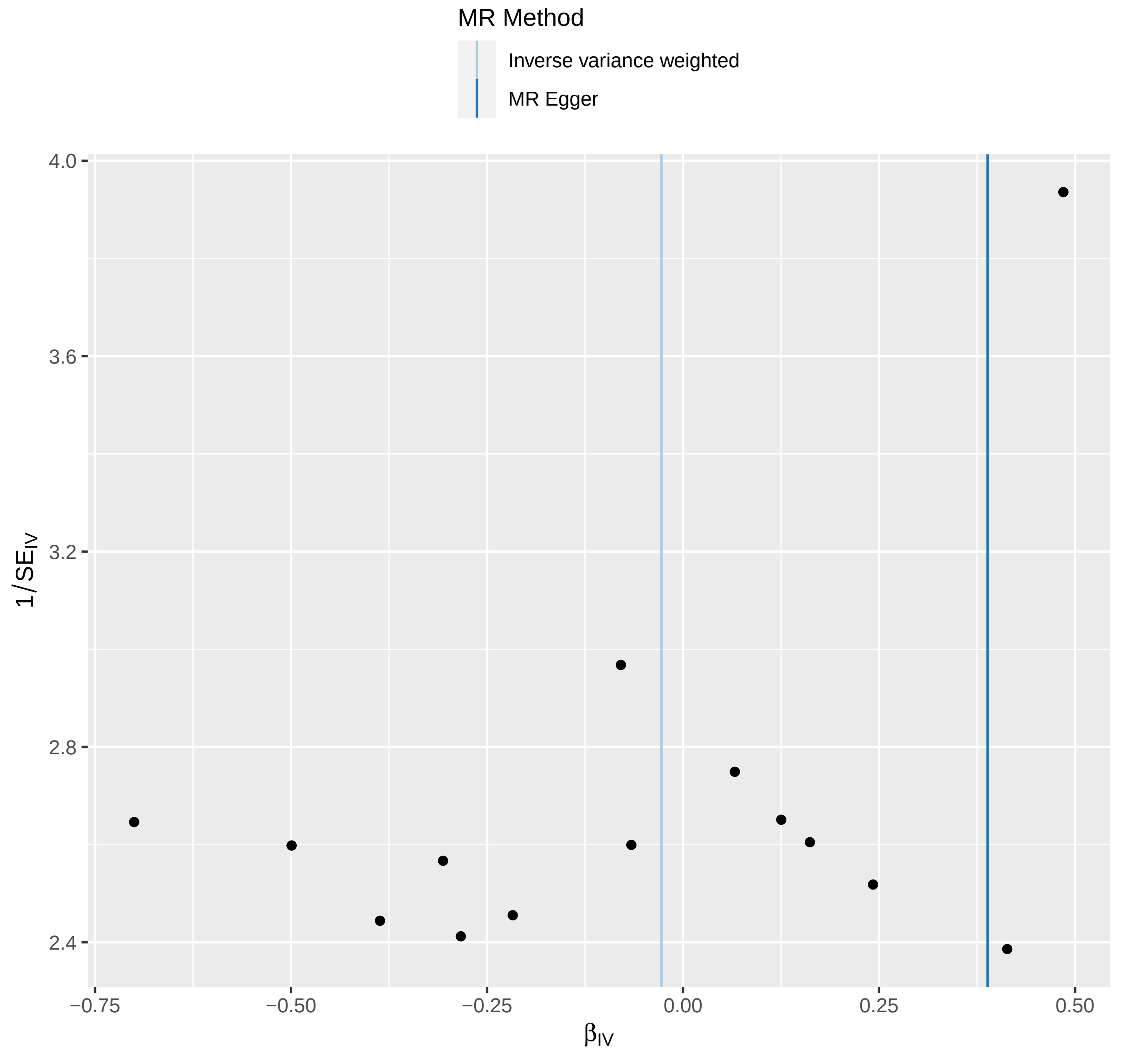

Batch 2−1037 : Gut microbiota abundance (genus Catenibacterium id.2153) on Cryptic protein
with a threshold of 1e−05

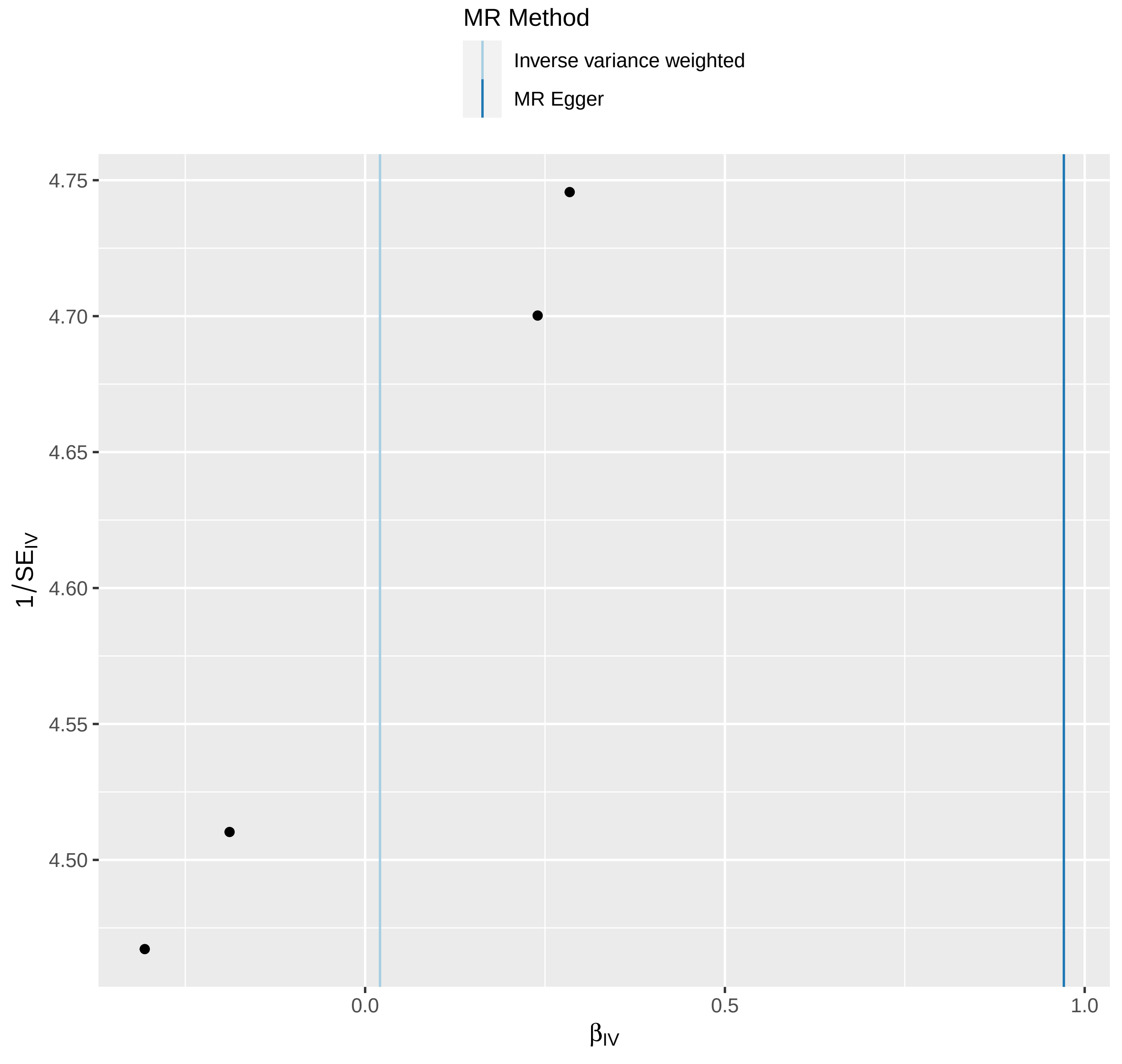

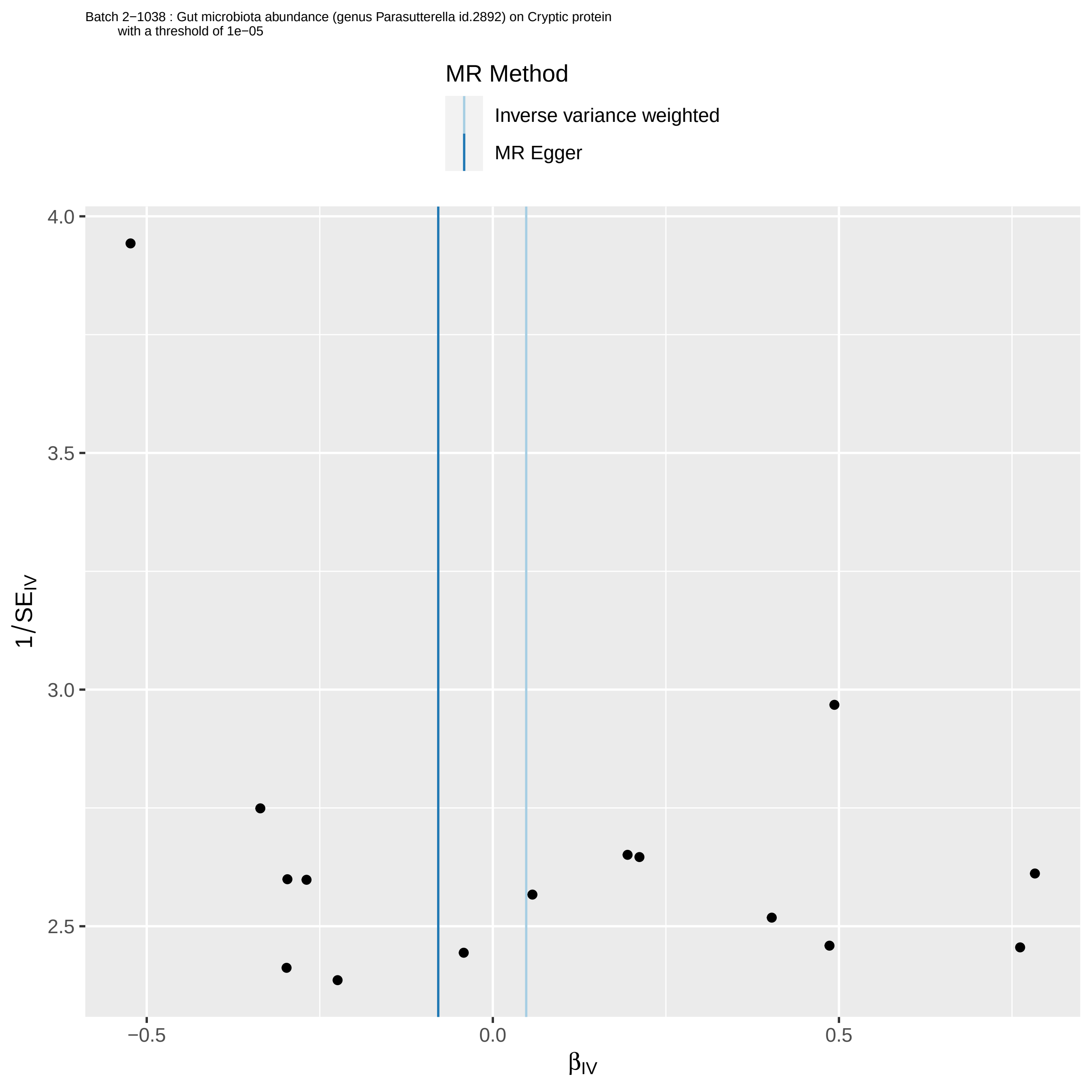
Batch 2−1038 : Gut microbiota abundance (genus Parasutterella id.2892) on Cryptic protein
with a threshold of 1e−05
MR Method
Inverse variance weighted
MR Egger
4.0
3.5
3.0
2.5
1/SE_IV
−0.5
0.0
0.5
β_IV

Batch 2−1039 : Gut microbiota abundance (genus Catenibacterium id.2153) on Complement factor H
with a threshold of 1e−05

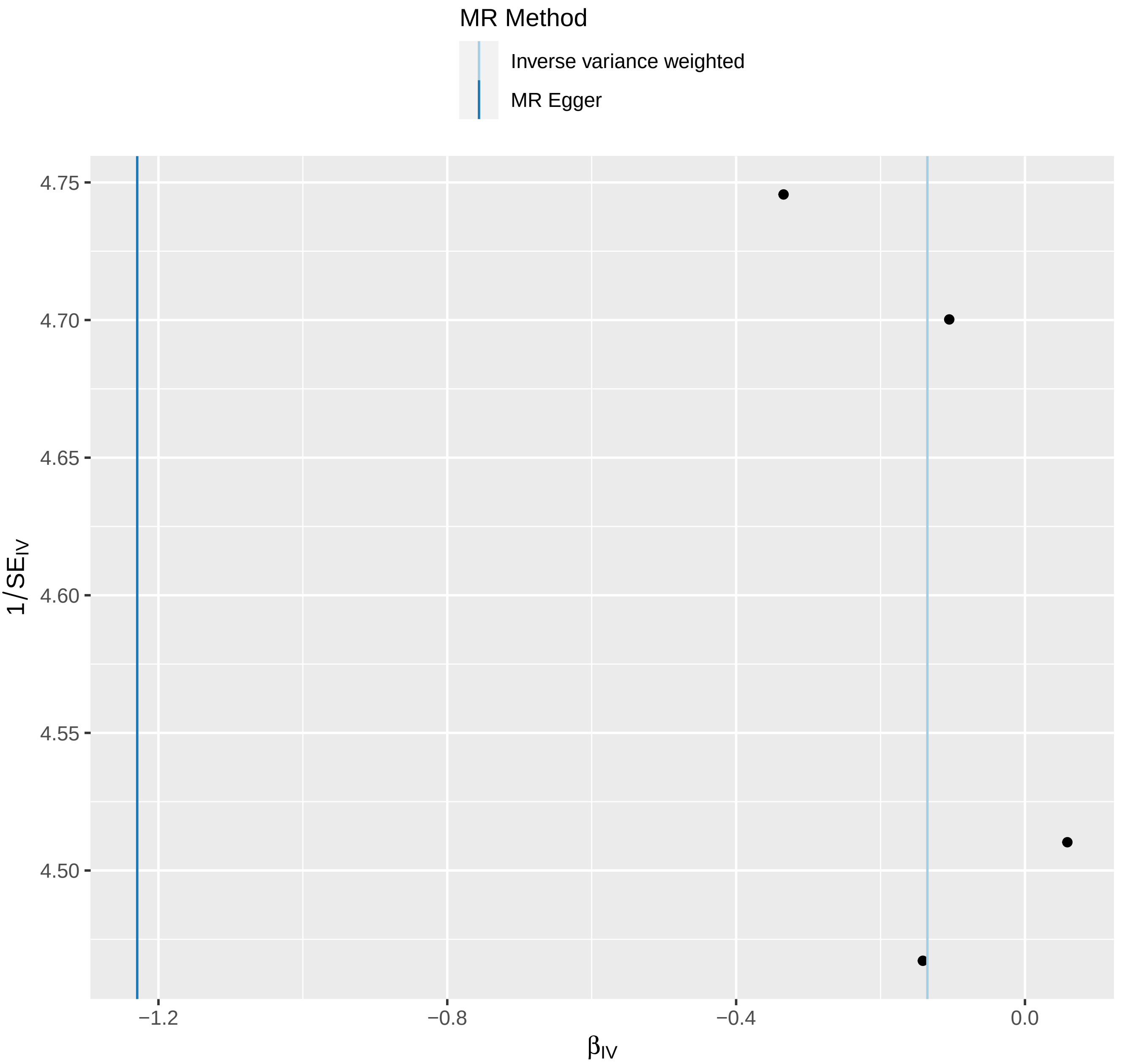

Batch 2−1040 : Gut microbiota abundance (genus Parasutterella id.2892) on Complement factor H
with a threshold of 1e−05

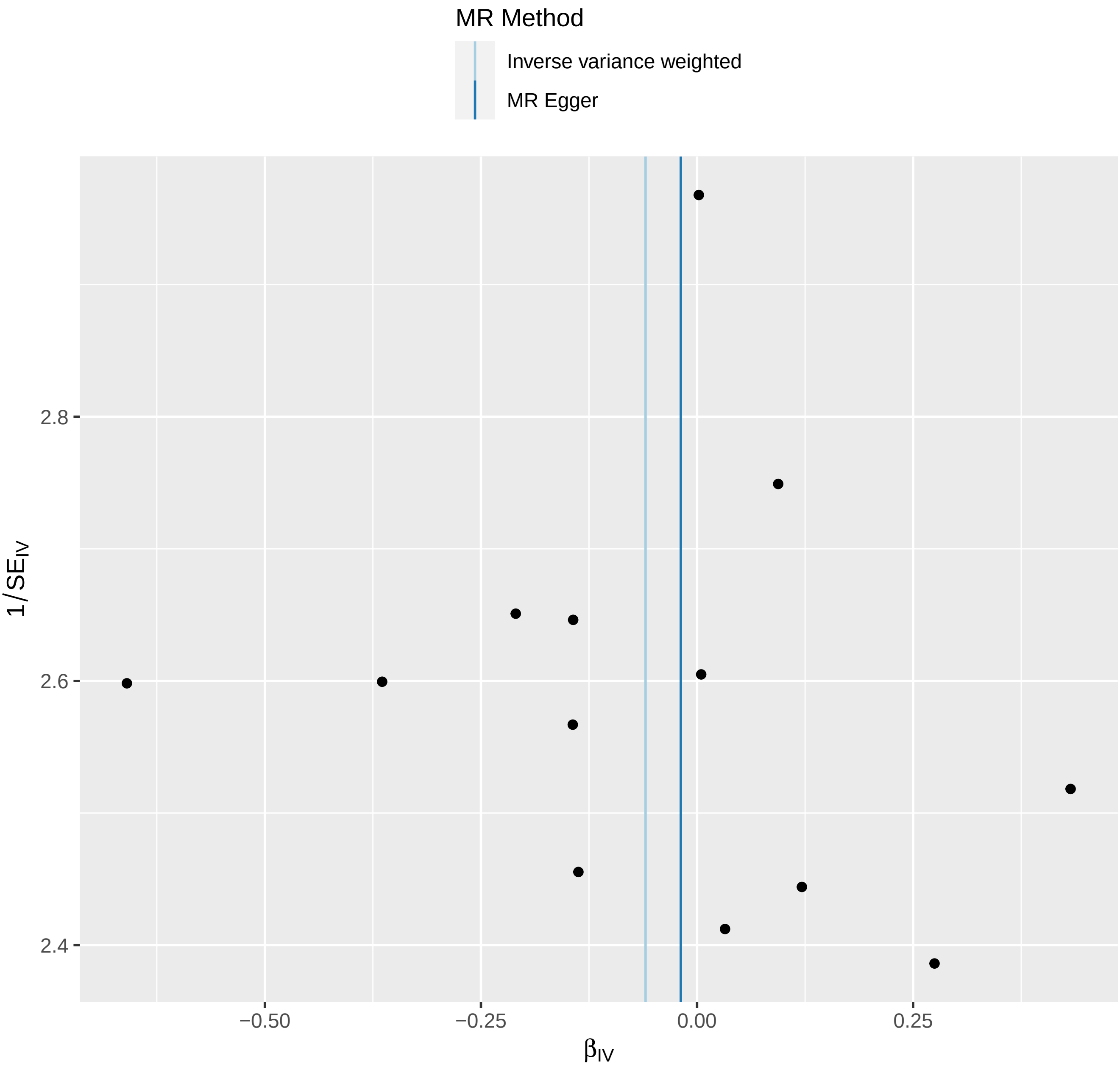

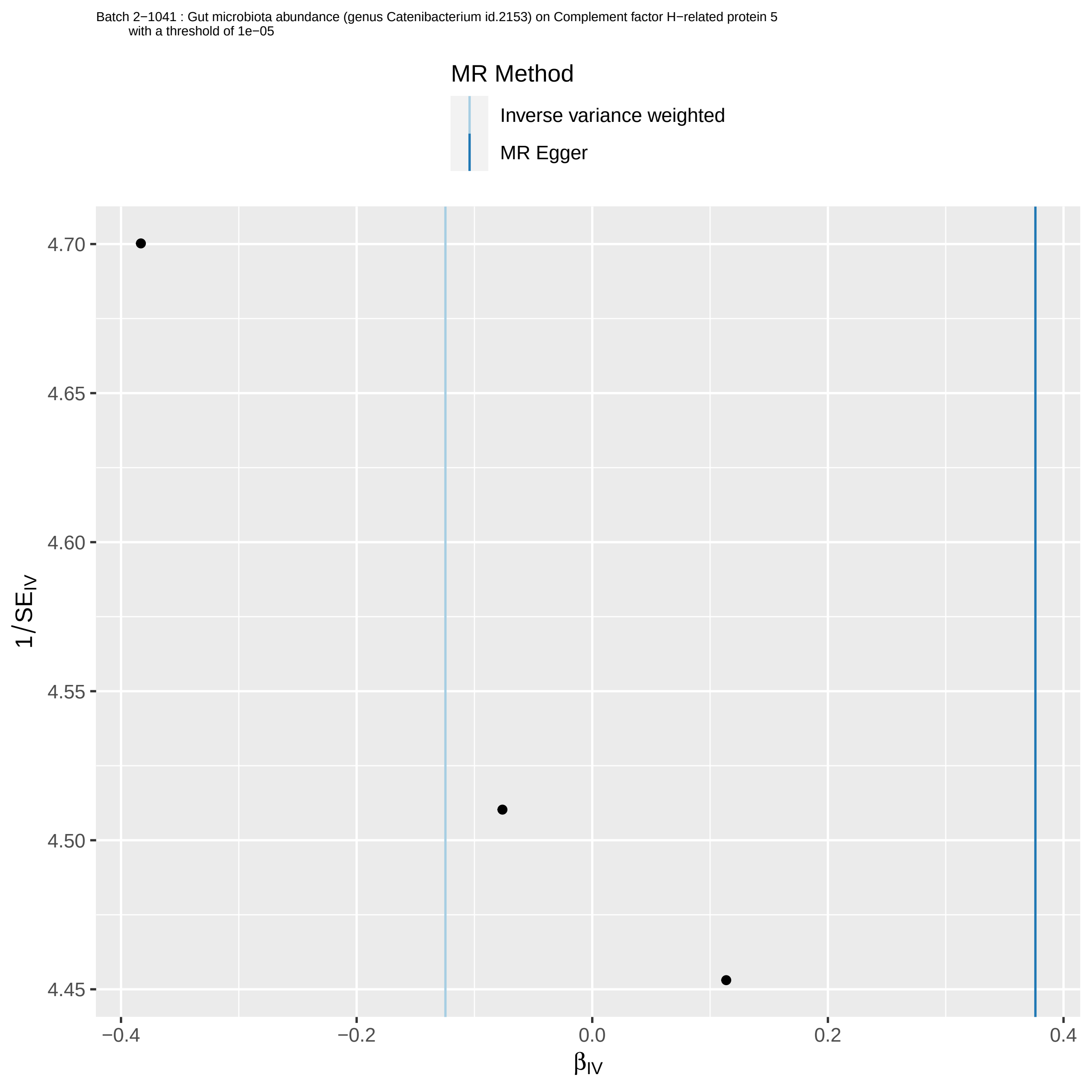
Batch 2−1041 : Gut microbiota abundance (genus Catenibacterium id.2153) on Complement factor H−related protein 5
with a threshold of 1e−05
MR Method
Inverse variance weighted
MR Egger
4.70
4.65
4.60
4.55
4.50
4.45
1/SE_IV
−0.4
−0.2
0.0
0.2
0.4
β_IV

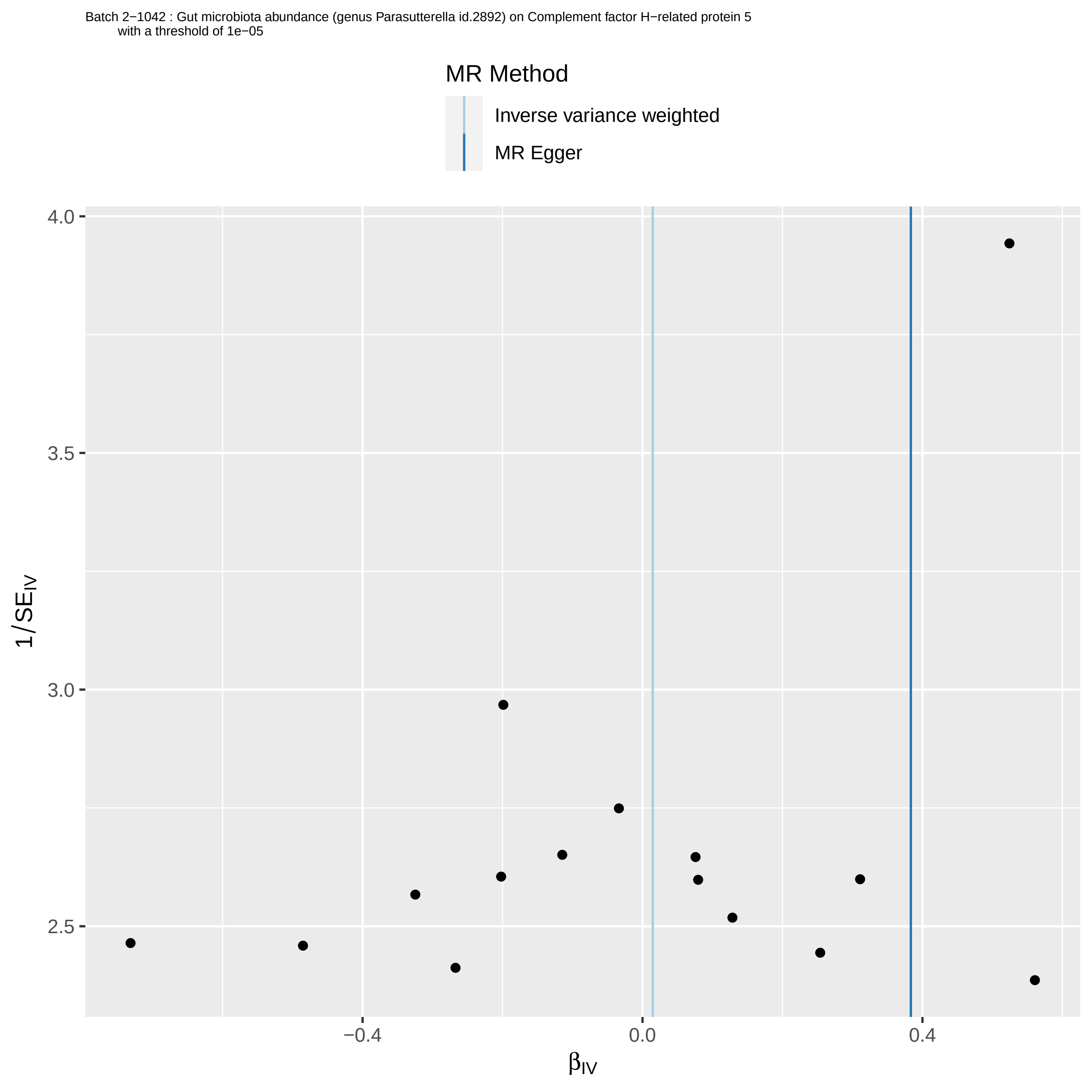

Batch 2−1042 : Gut microbiota abundance (genus Parasutterella id.2892) on Complement factor H−related protein 5
with a threshold of 1e−05
MR Method
Inverse variance weighted
MR Egger
4.0
3.5
3.0
2.5
1/SE_IV
−0.4
0.0
0.4
β_IV

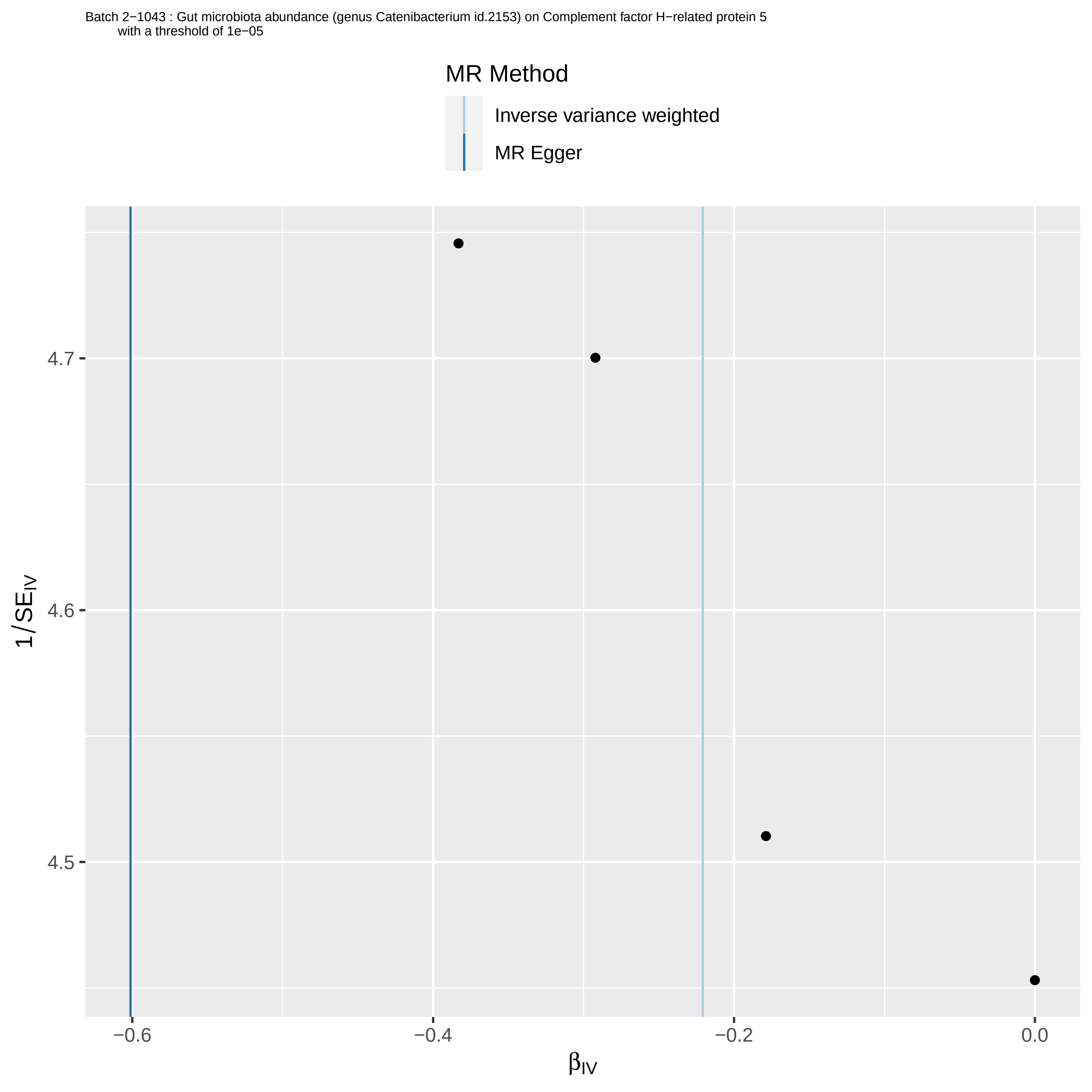

Batch 2−1043 : Gut microbiota abundance (genus Catenibacterium id.2153) on Complement factor H−related protein 5
with a threshold of 1e−05
MR Method
Inverse variance weighted
MR Egger
4.7
4.6
4.5
1/SE_IV
−0.6
−0.4
−0.2
0.0
β_IV

Batch 2−1044 : Gut microbiota abundance (genus Parasutterella id.2892) on Complement factor H−related protein 5
with a threshold of 1e−05

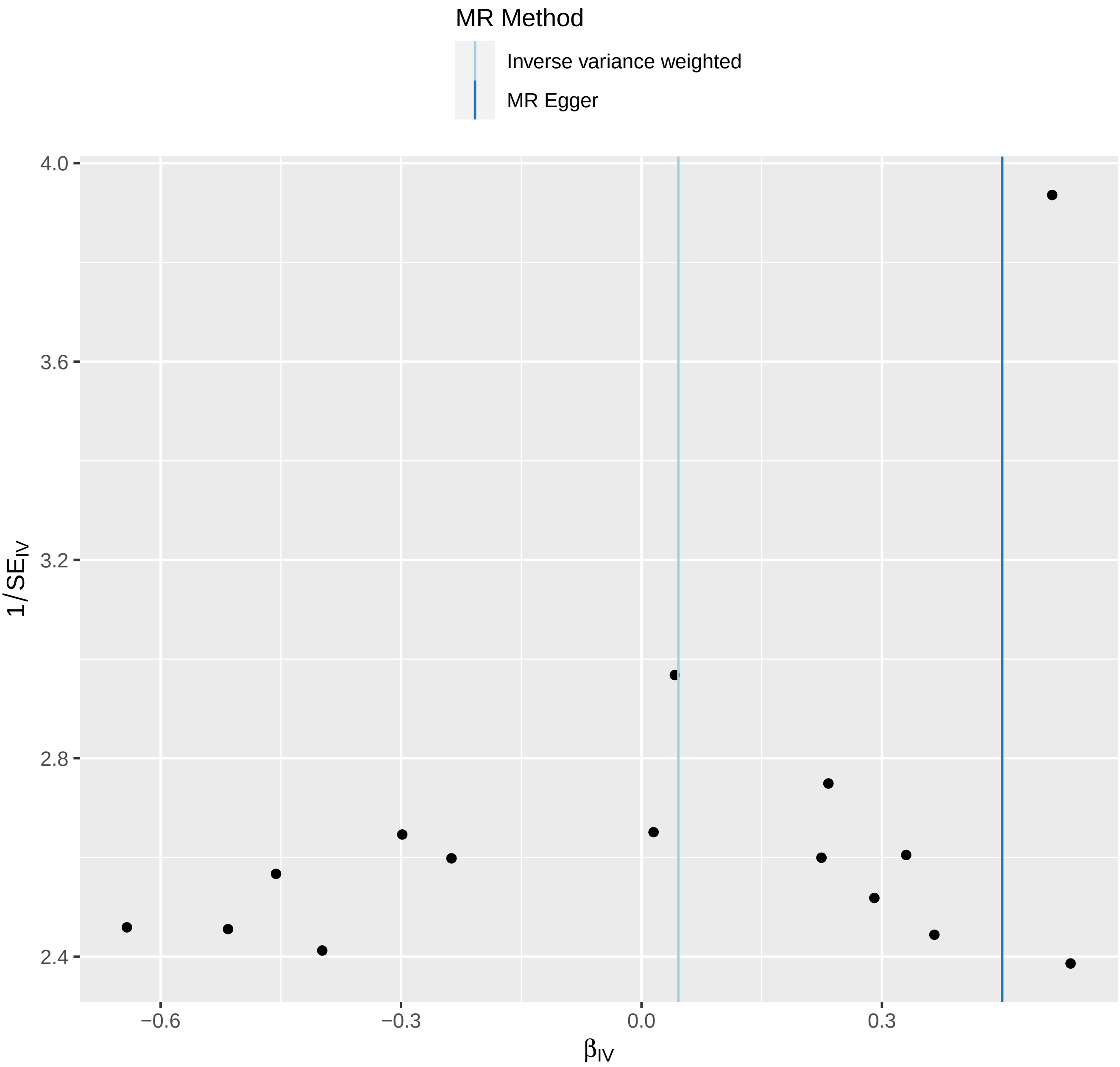

Batch 2−1045 : Gut microbiota abundance (genus Catenibacterium id.2153) on Complement factor I
with a threshold of 1e−05

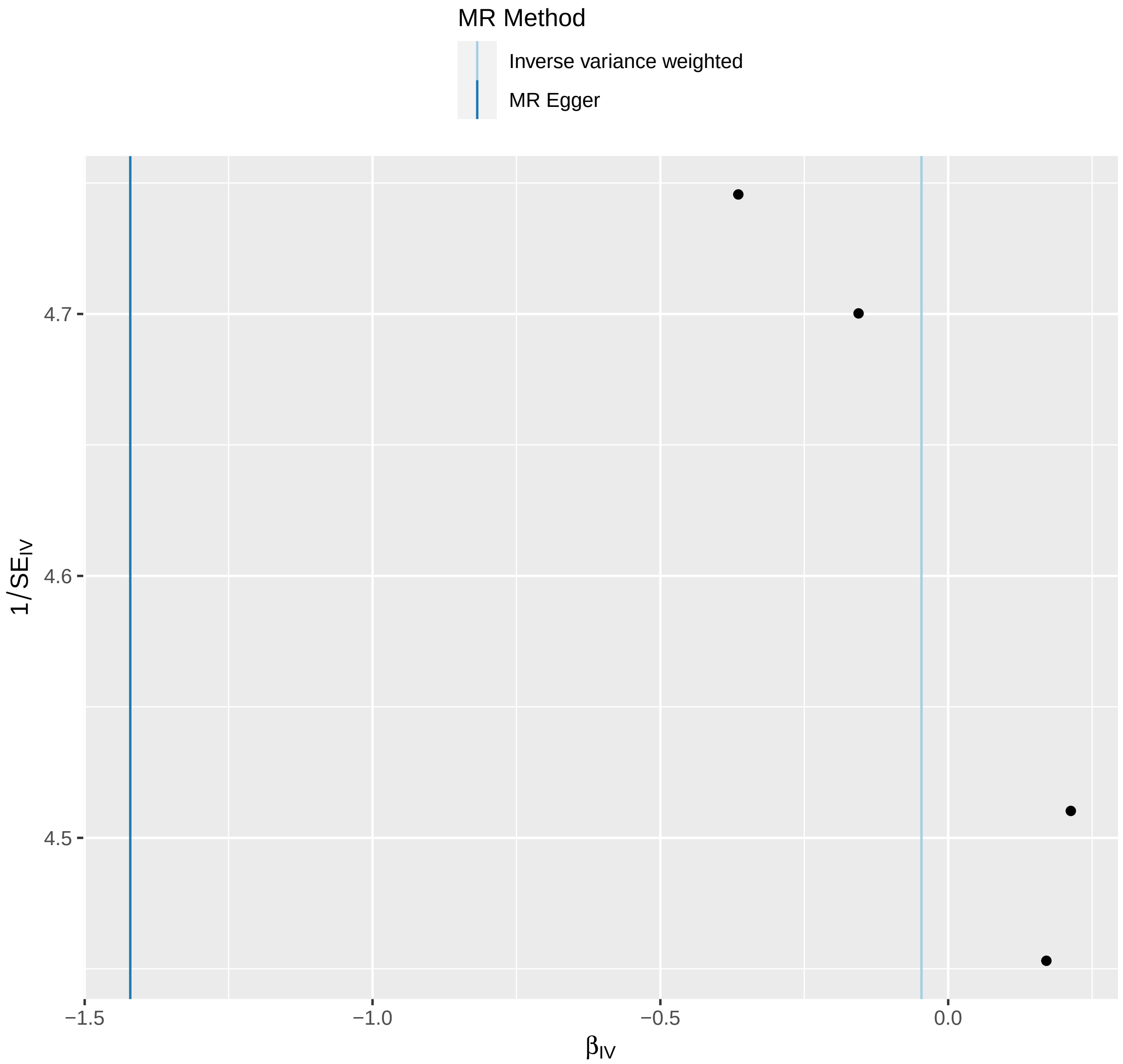

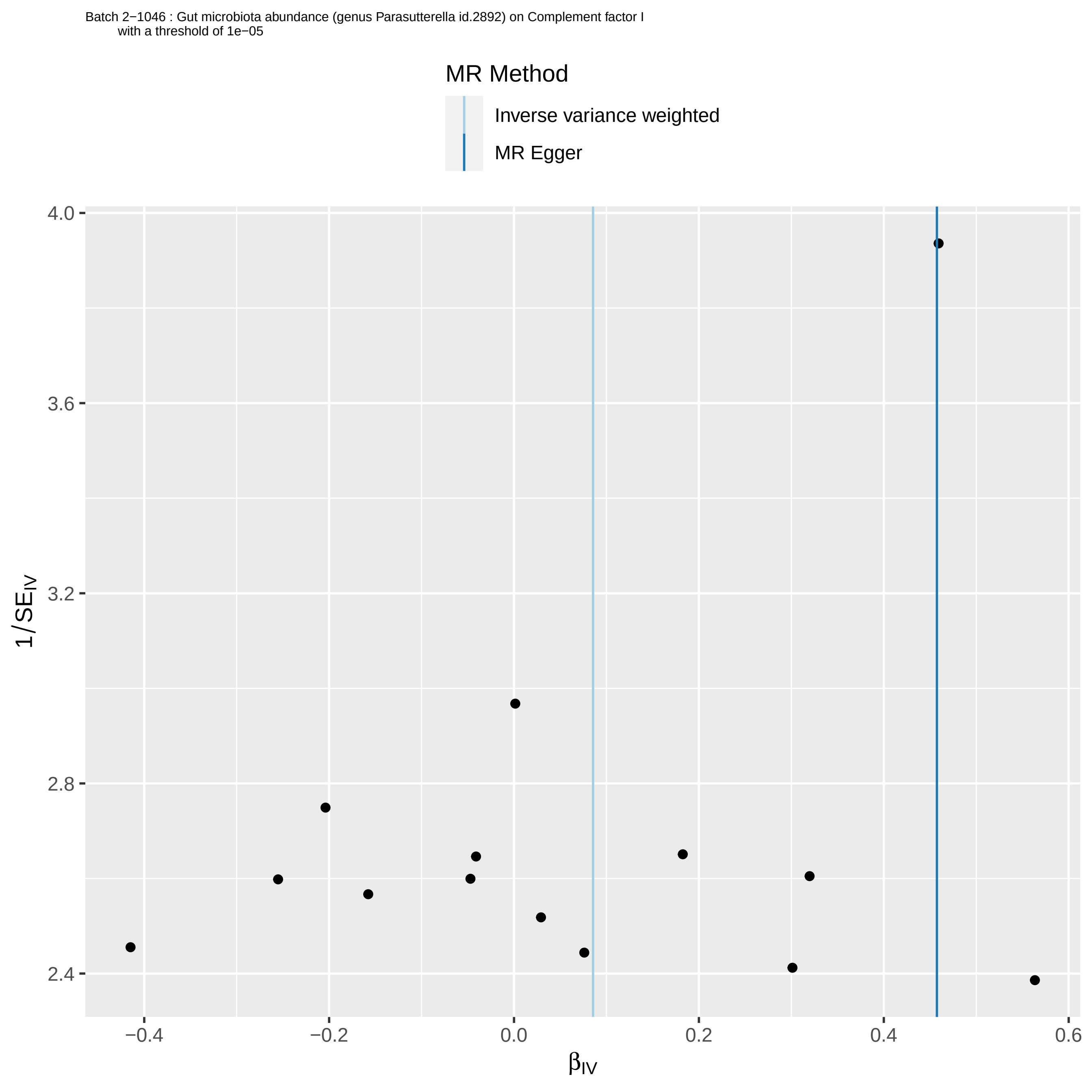
Batch 2−1046 : Gut microbiota abundance (genus Parasutterella id.2892) on Complement factor I
with a threshold of 1e−05
MR Method
Inverse variance weighted
MR Egger
4.0
3.6
3.2
2.8
2.4
1/SE_IV
−0.4
−0.2
0.0
0.2
0.4
0.6
β_IV

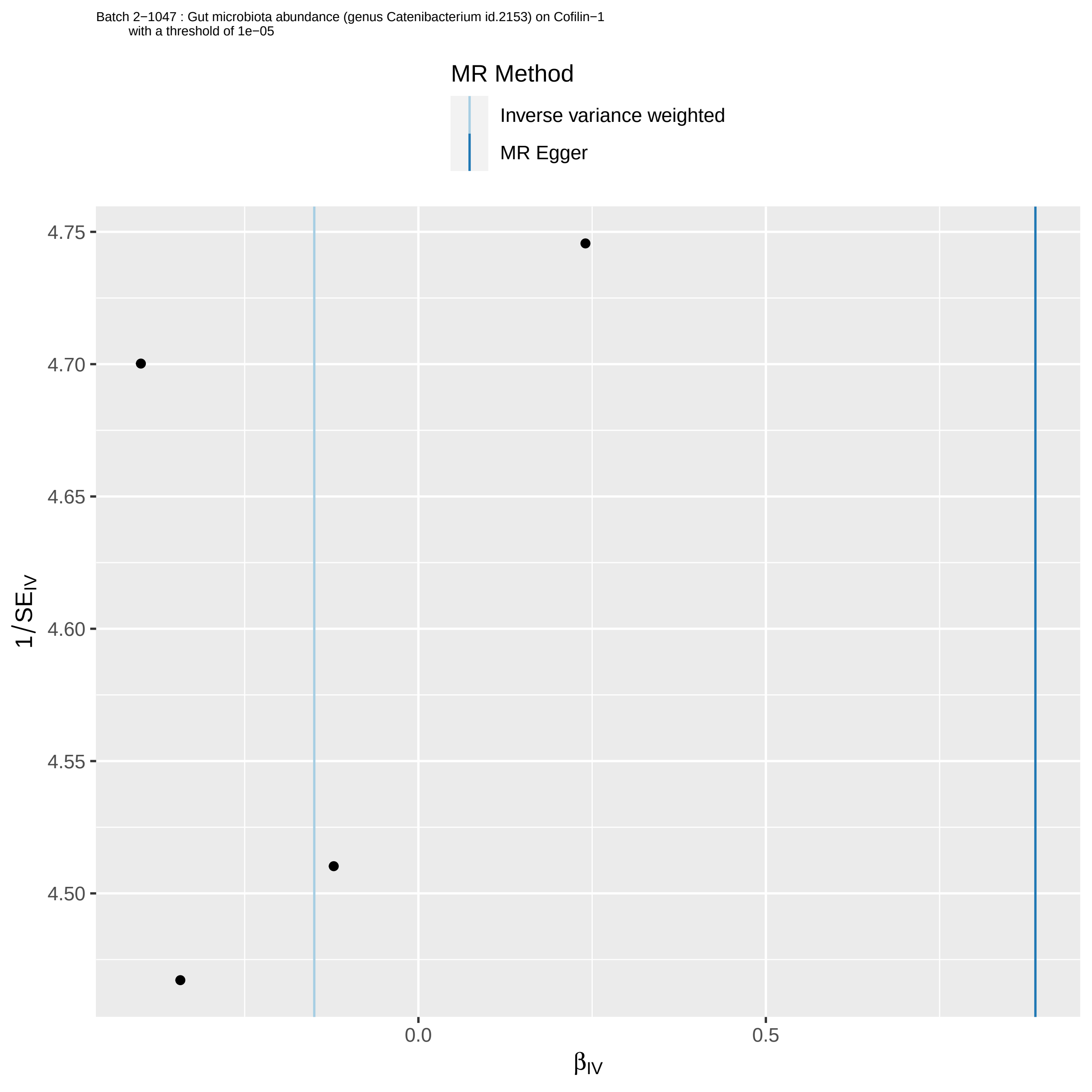
Batch 2−1047 : Gut microbiota abundance (genus Catenibacterium id.2153) on Cofilin−1
with a threshold of 1e−05
MR Method
Inverse variance weighted
MR Egger
4.75
4.70
4.65
4.60
4.55
4.50
$1/SE_{IV}$
0.0
0.5
β_{IV}

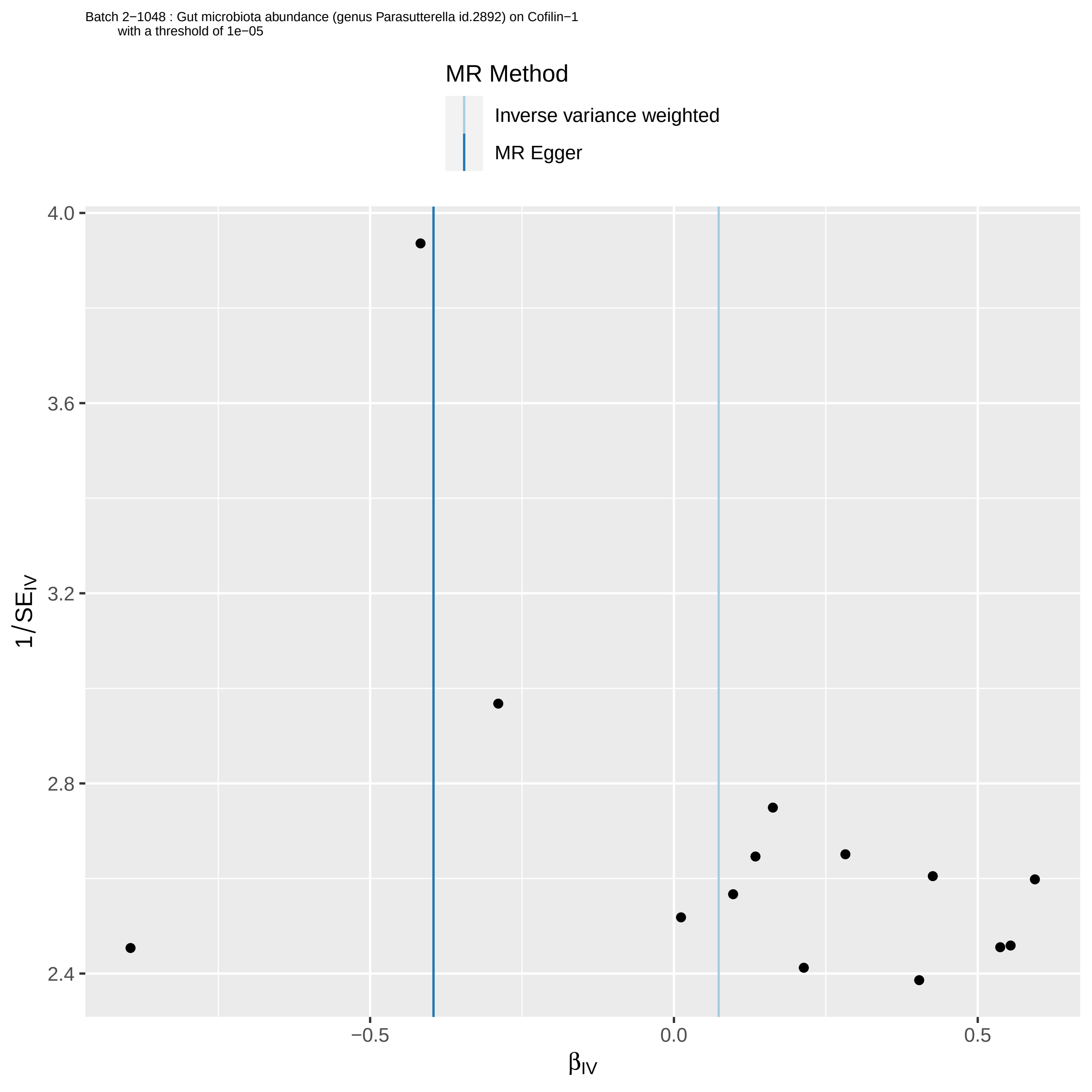
Batch 2−1048 : Gut microbiota abundance (genus Parasutterella id.2892) on Cofilin−1
with a threshold of 1e−05
MR Method
Inverse variance weighted
MR Egger
4.0
3.6
3.2
2.8
2.4
1/SE_IV
−0.5
0.0
0.5
β_IV

Batch 2−1049 : Gut microbiota abundance (genus Catenibacterium id.2153) on Properdin
with a threshold of 1e−05

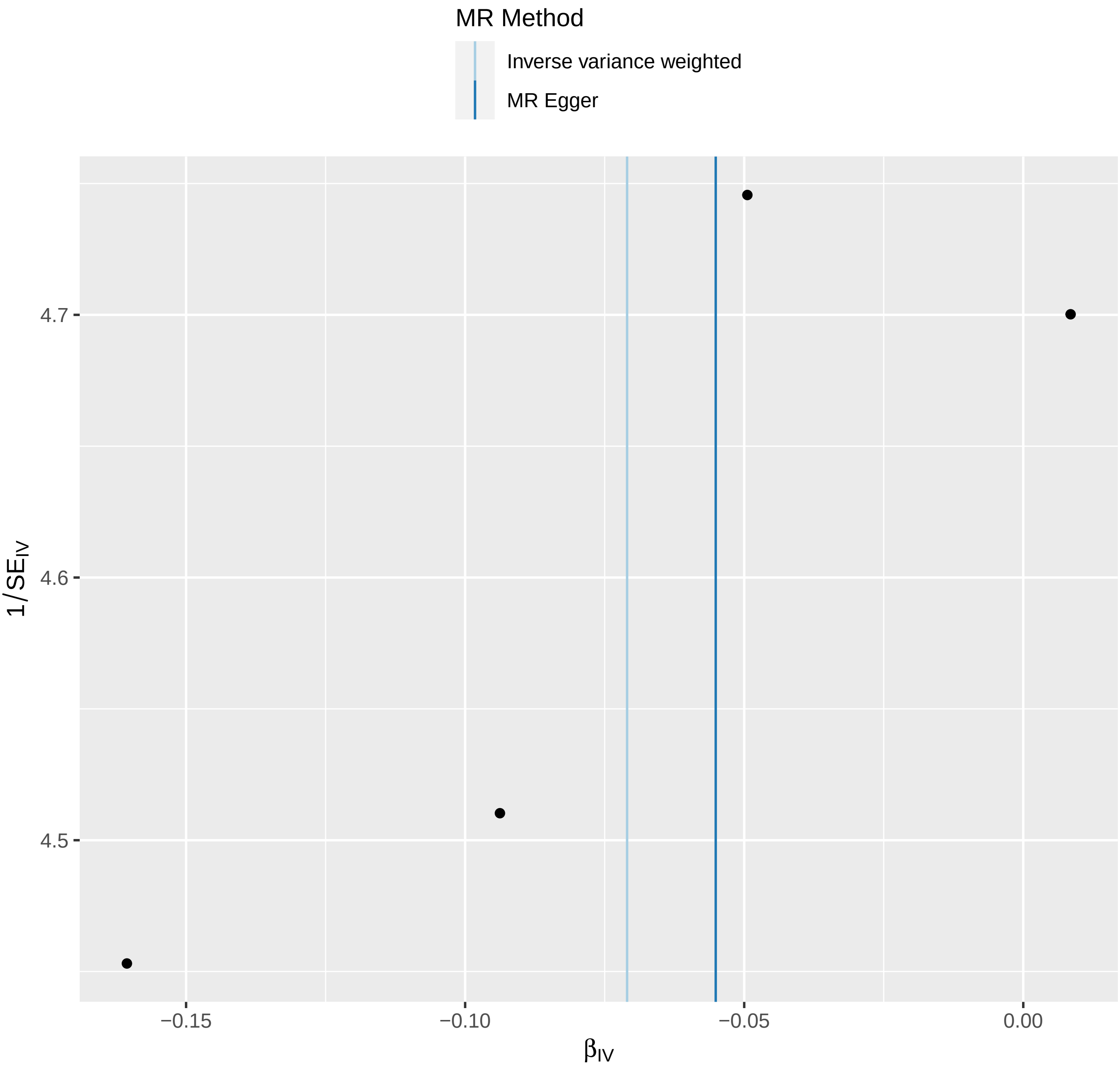

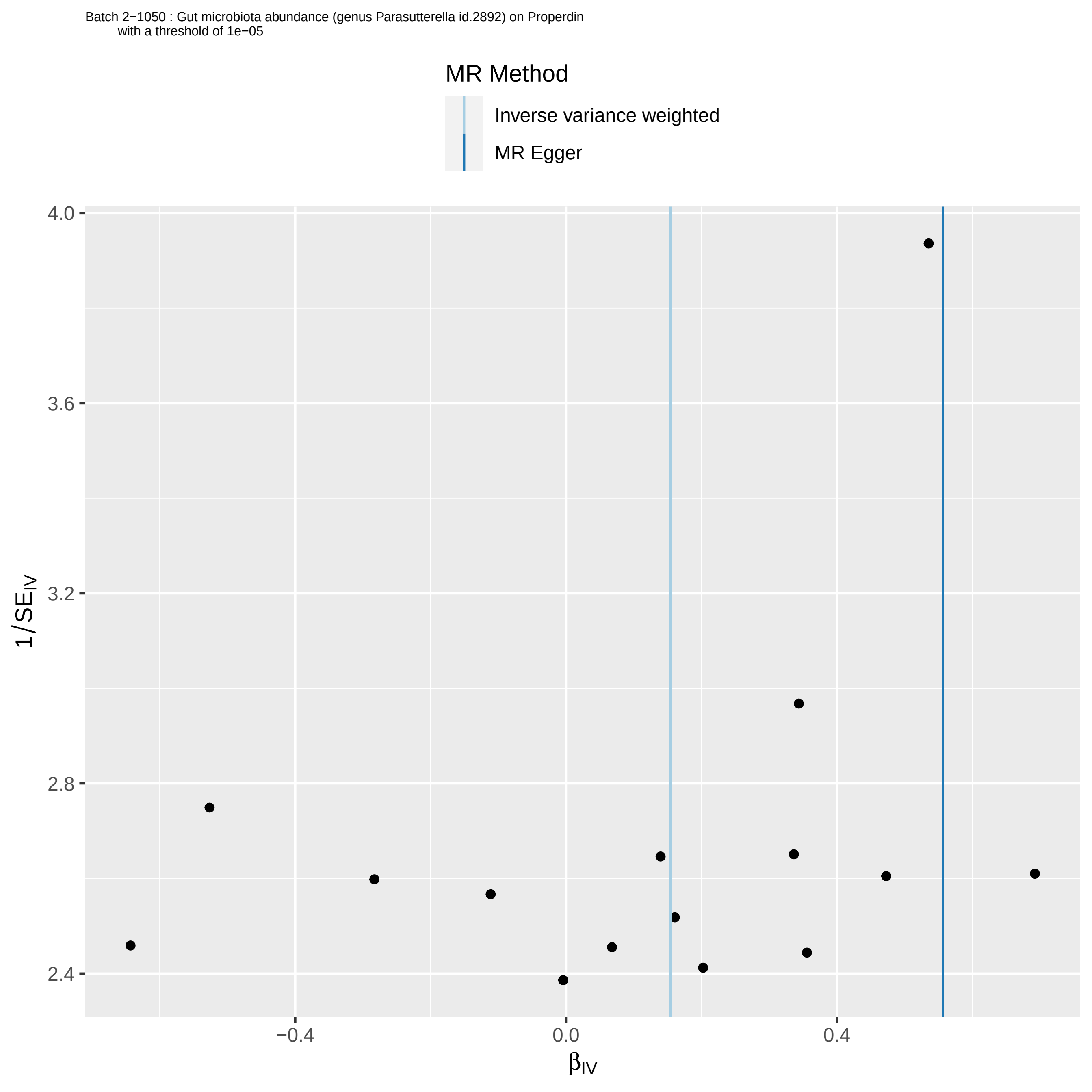

Batch 2−1050 : Gut microbiota abundance (genus Parasutterella id.2892) on Properdin
with a threshold of 1e−05
MR Method
Inverse variance weighted
MR Egger
4.0
3.6
3.2
2.8
2.4
1/SE_IV
−0.4
0.0
0.4
β_IV

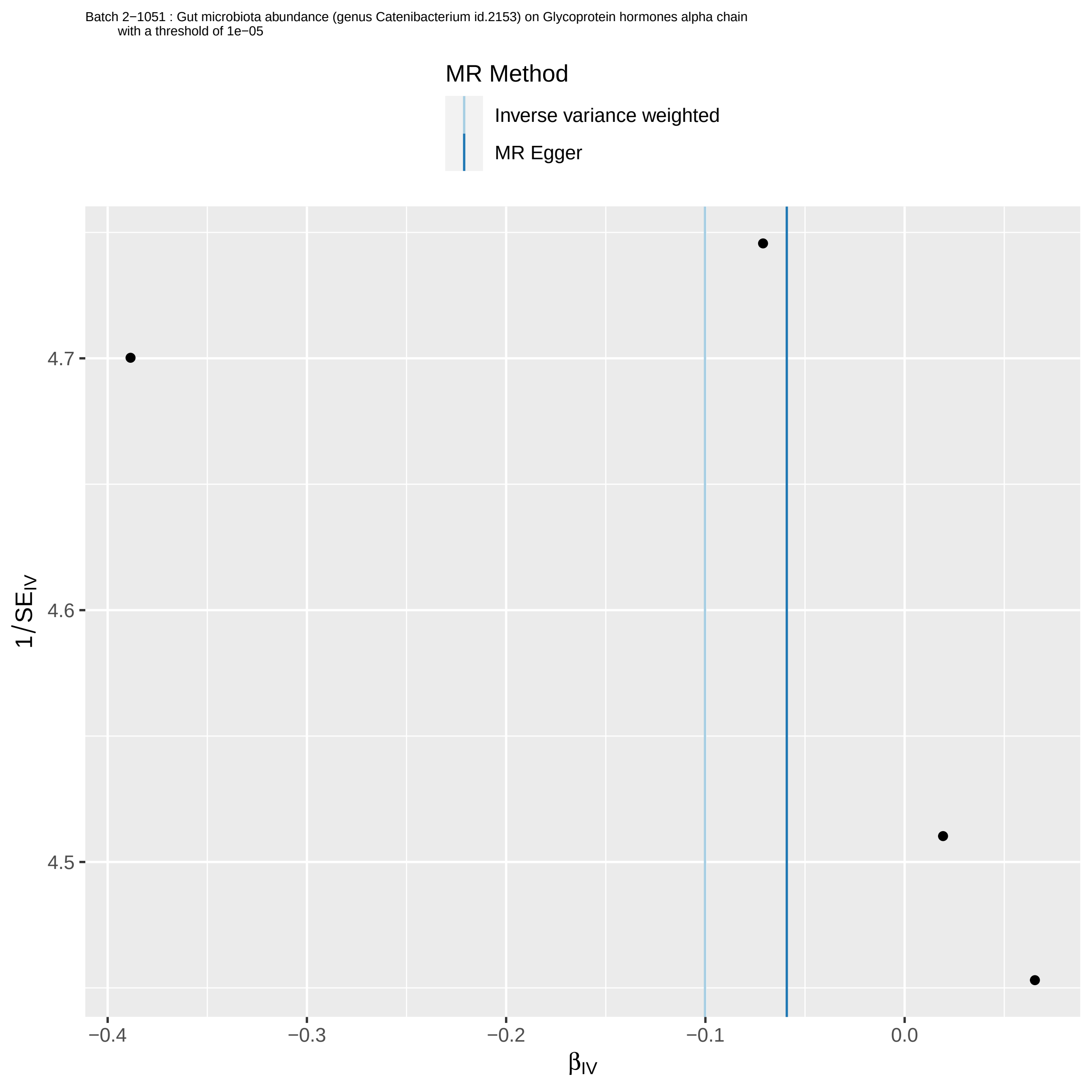
Batch 2−1051 : Gut microbiota abundance (genus Catenibacterium id.2153) on Glycoprotein hormones alpha chain
with a threshold of 1e−05
MR Method
Inverse variance weighted
MR Egger
4.7
4.6
4.5
1/SE_IV
−0.4
−0.3
−0.2
−0.1
0.0
β_IV

Batch 2−1052 : Gut microbiota abundance (genus Parasutterella id.2892) on Glycoprotein hormones alpha chain
with a threshold of 1e−05

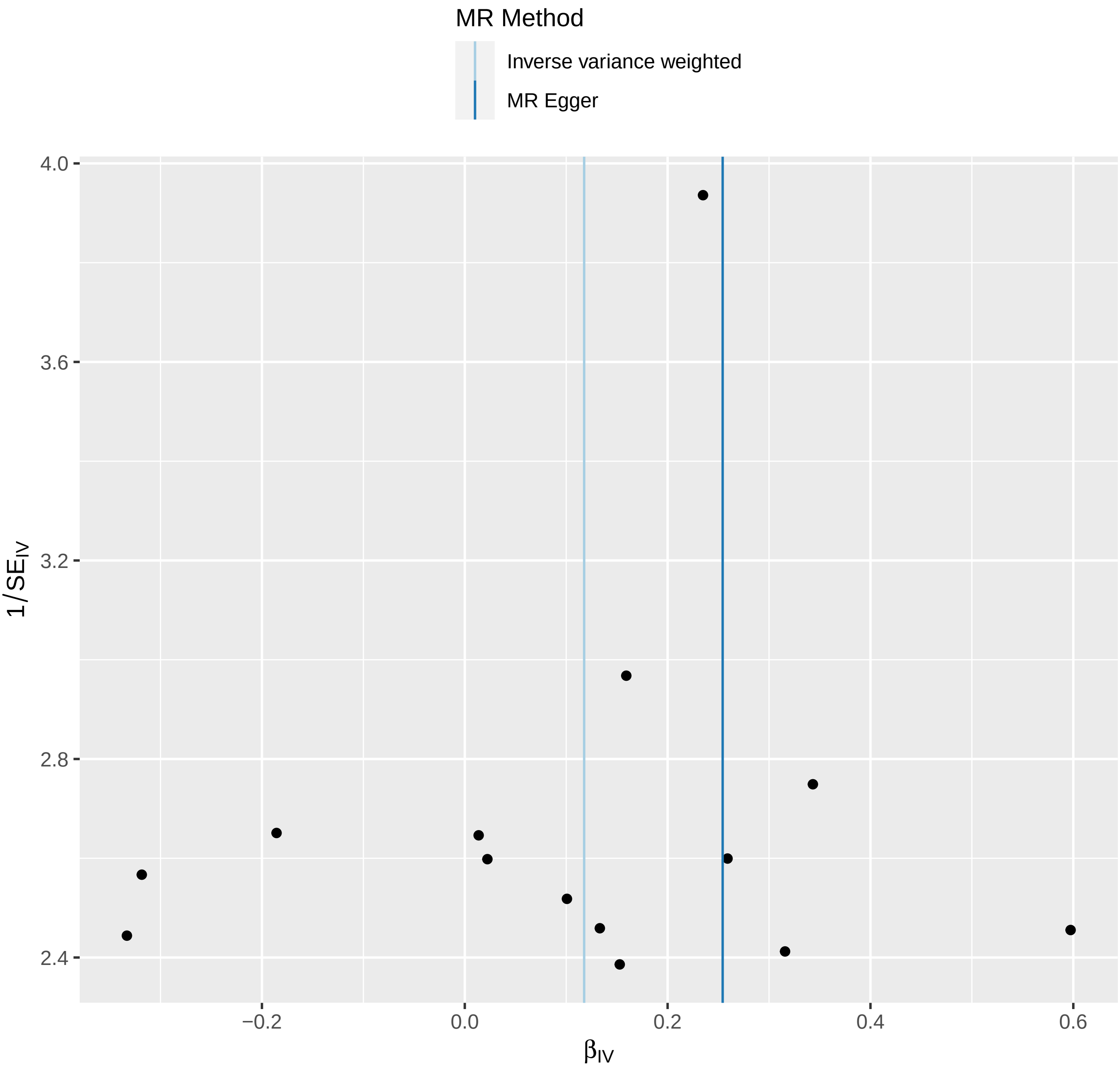

Batch 2−1053 : Gut microbiota abundance (genus Catenibacterium id.2153) on Human Chorionic Gonadotropin
with a threshold of 1e−05

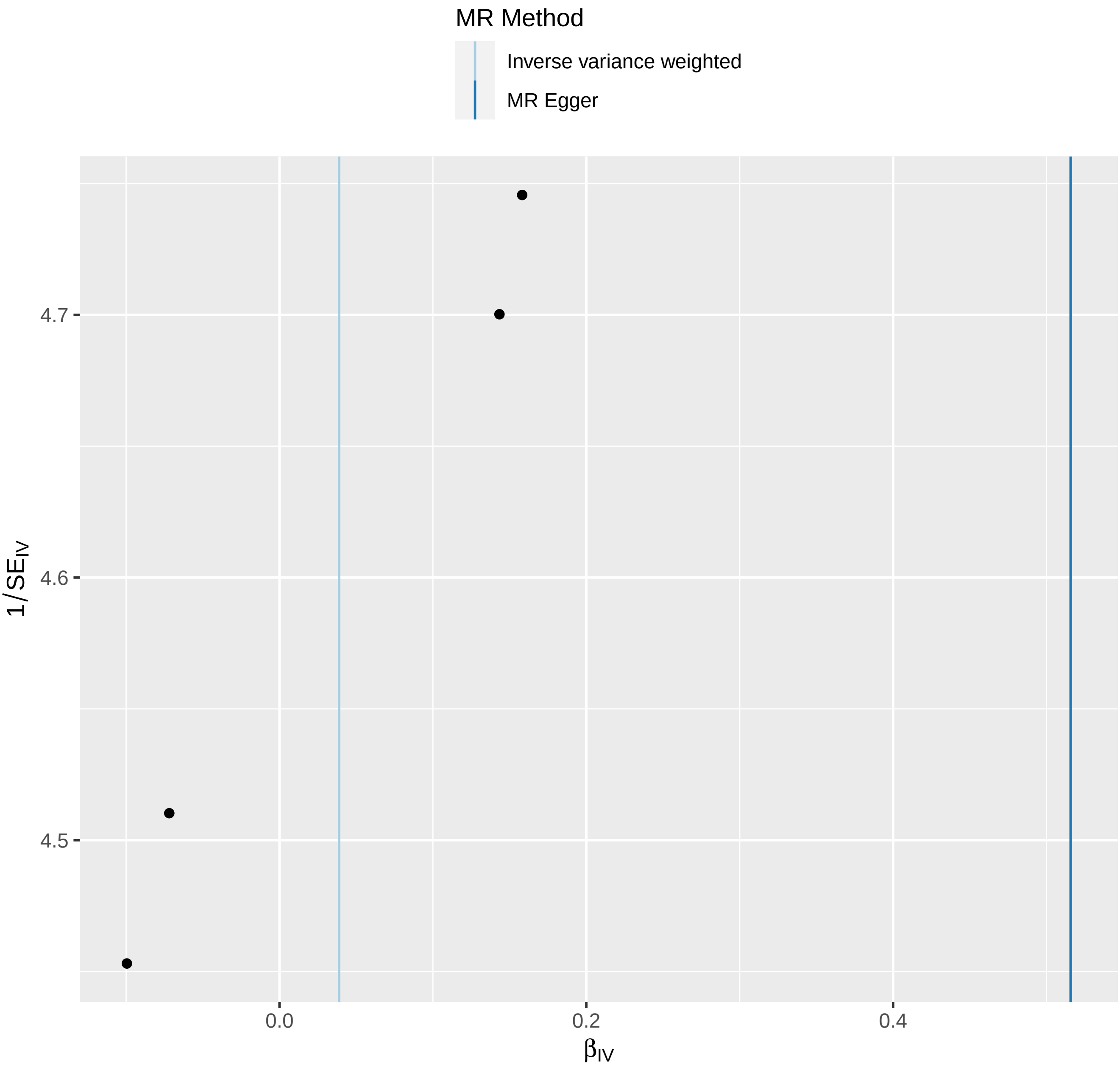

Batch 2−1054 : Gut microbiota abundance (genus Parasutterella id.2892) on Human Chorionic Gonadotropin
with a threshold of 1e−05

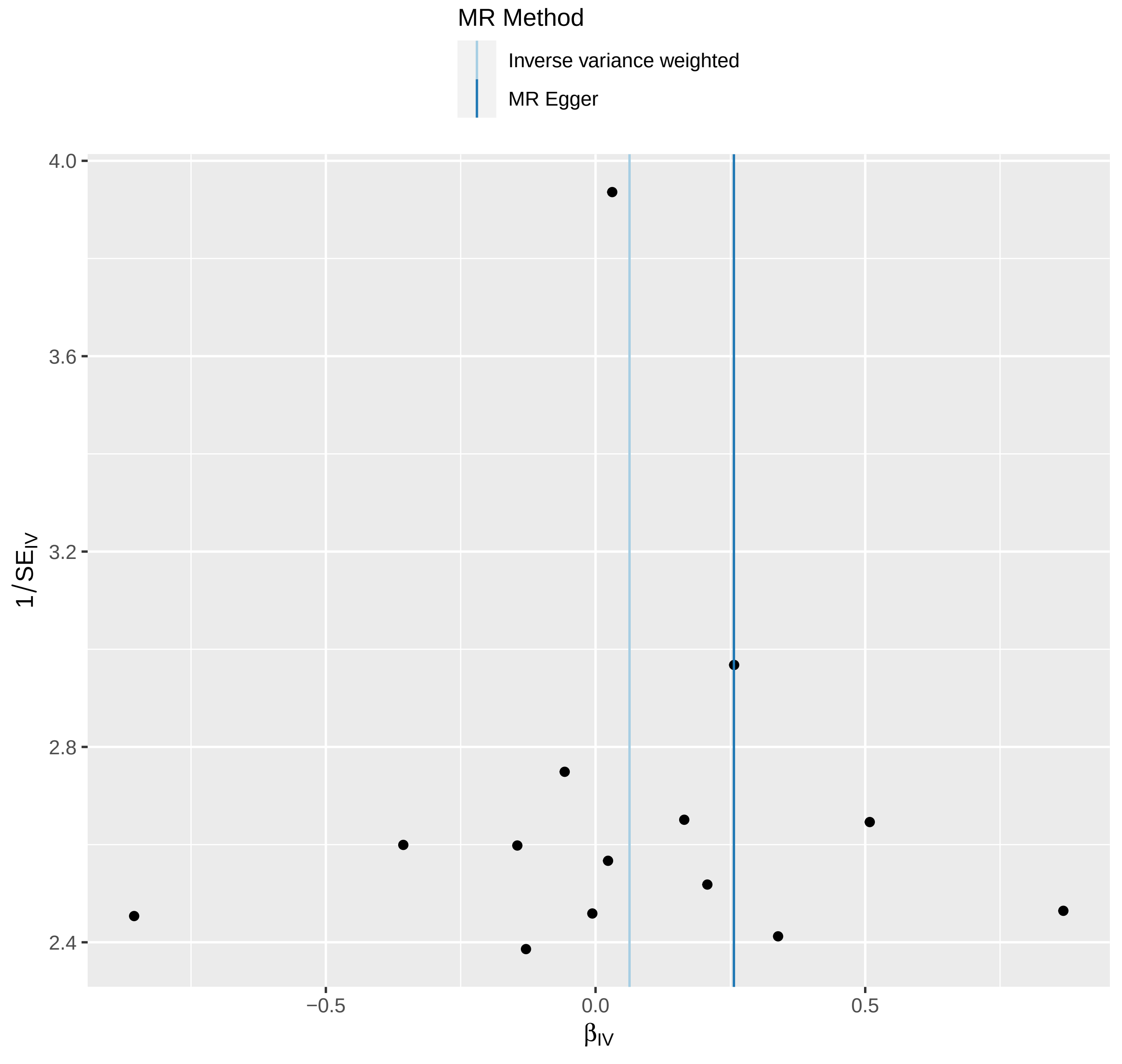

Batch 2−1055 : Gut microbiota abundance (genus Catenibacterium id.2153) on Follicle stimulating hormone
with a threshold of 1e−05

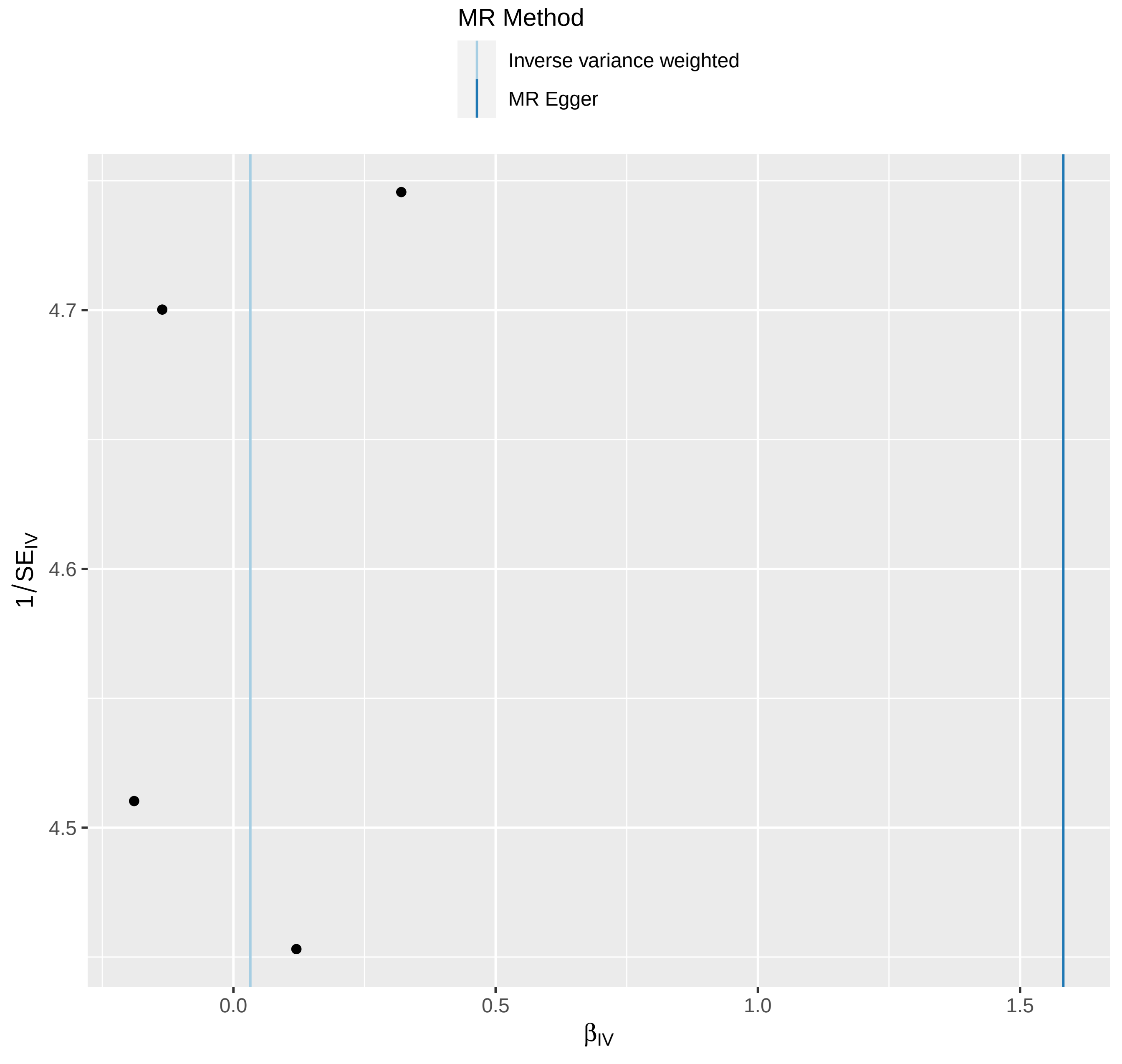

Batch 2−1056 : Gut microbiota abundance (genus Parasutterella id.2892) on Follicle stimulating hormone
with a threshold of 1e−05

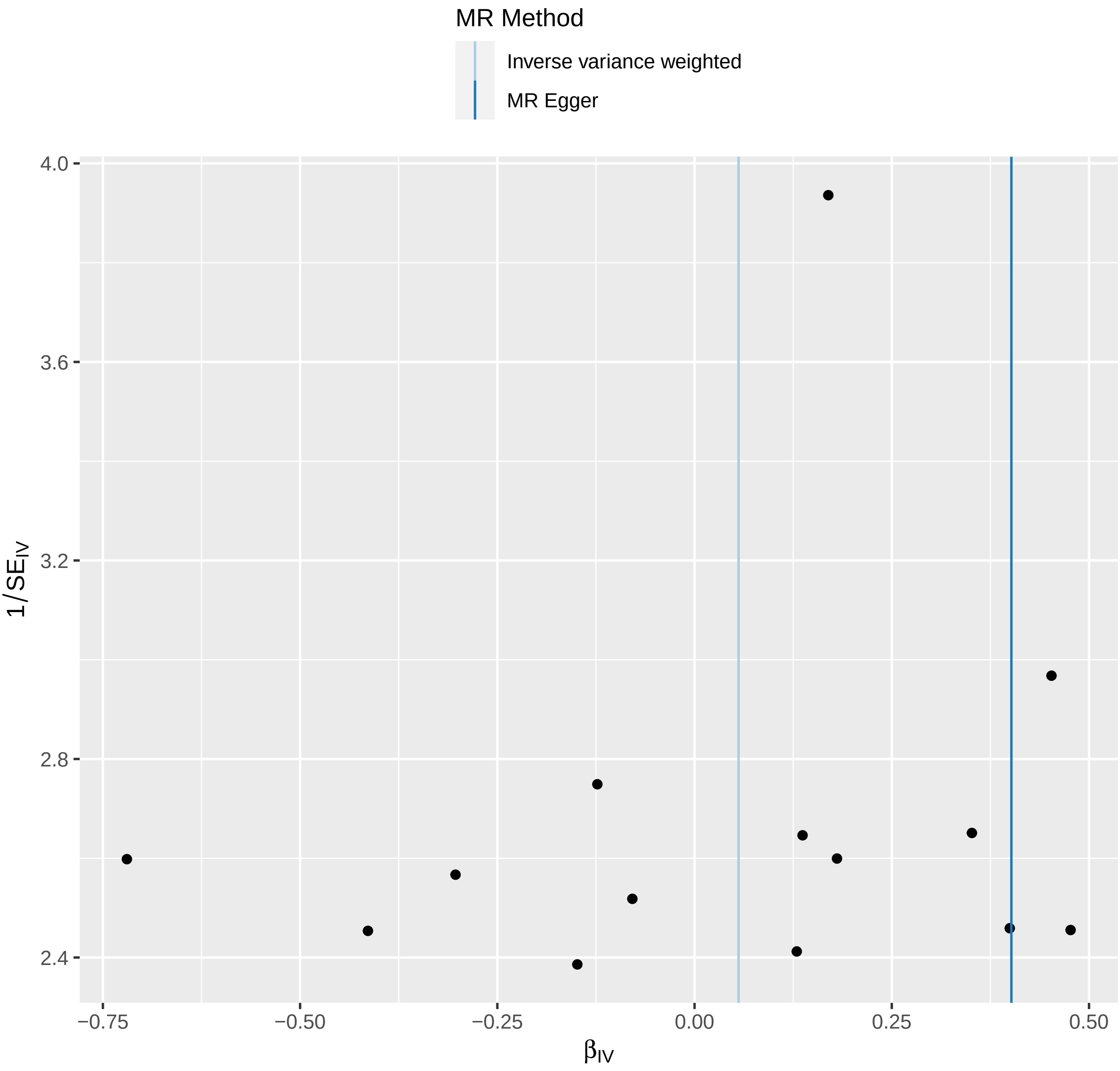

Batch 2−1057 : Gut microbiota abundance (genus Catenibacterium id.2153) on Luteinizing hormone
with a threshold of 1e−05

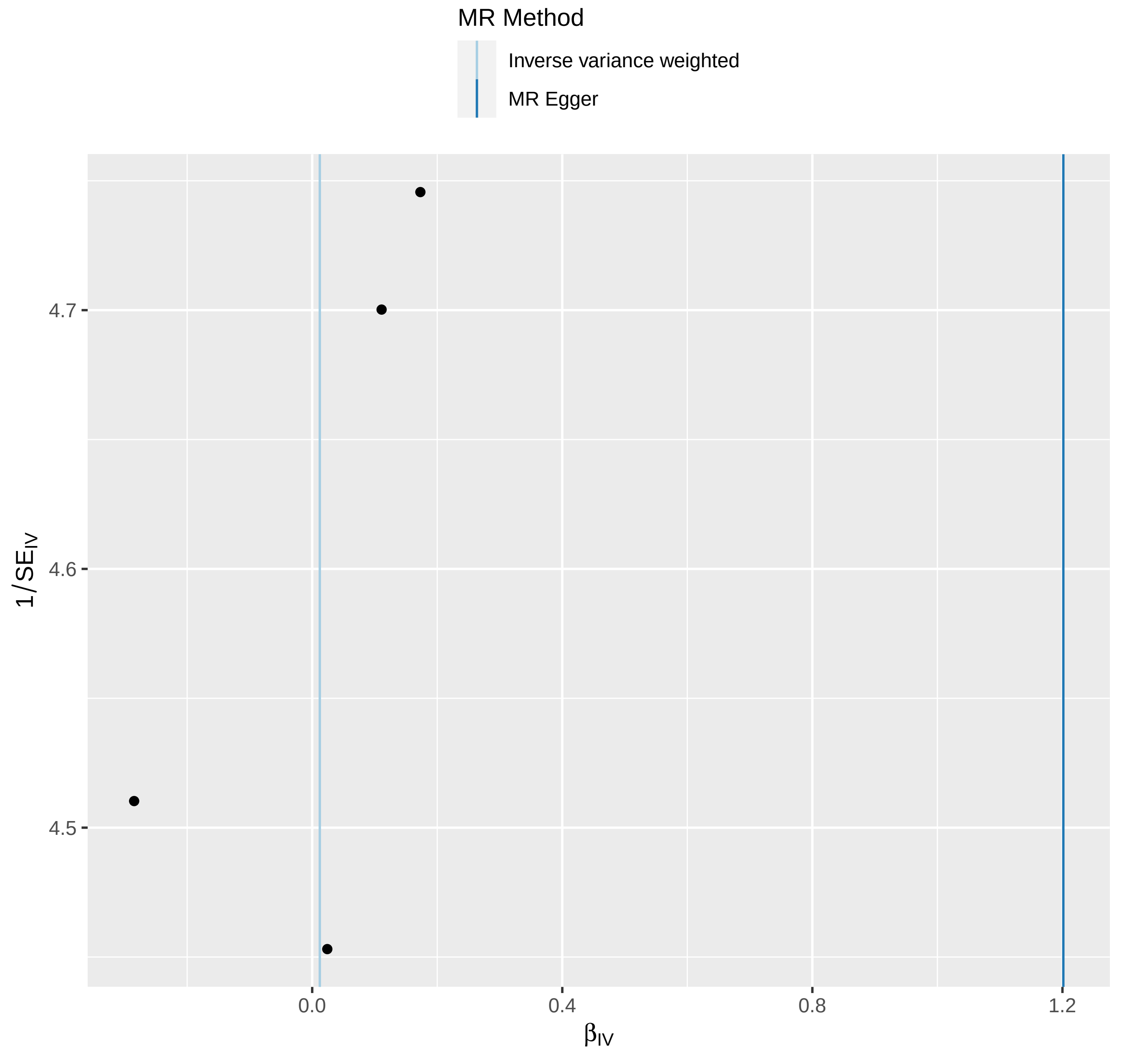

Batch 2−1058 : Gut microbiota abundance (genus Parasutterella id.2892) on Luteinizing hormone
with a threshold of 1e−05

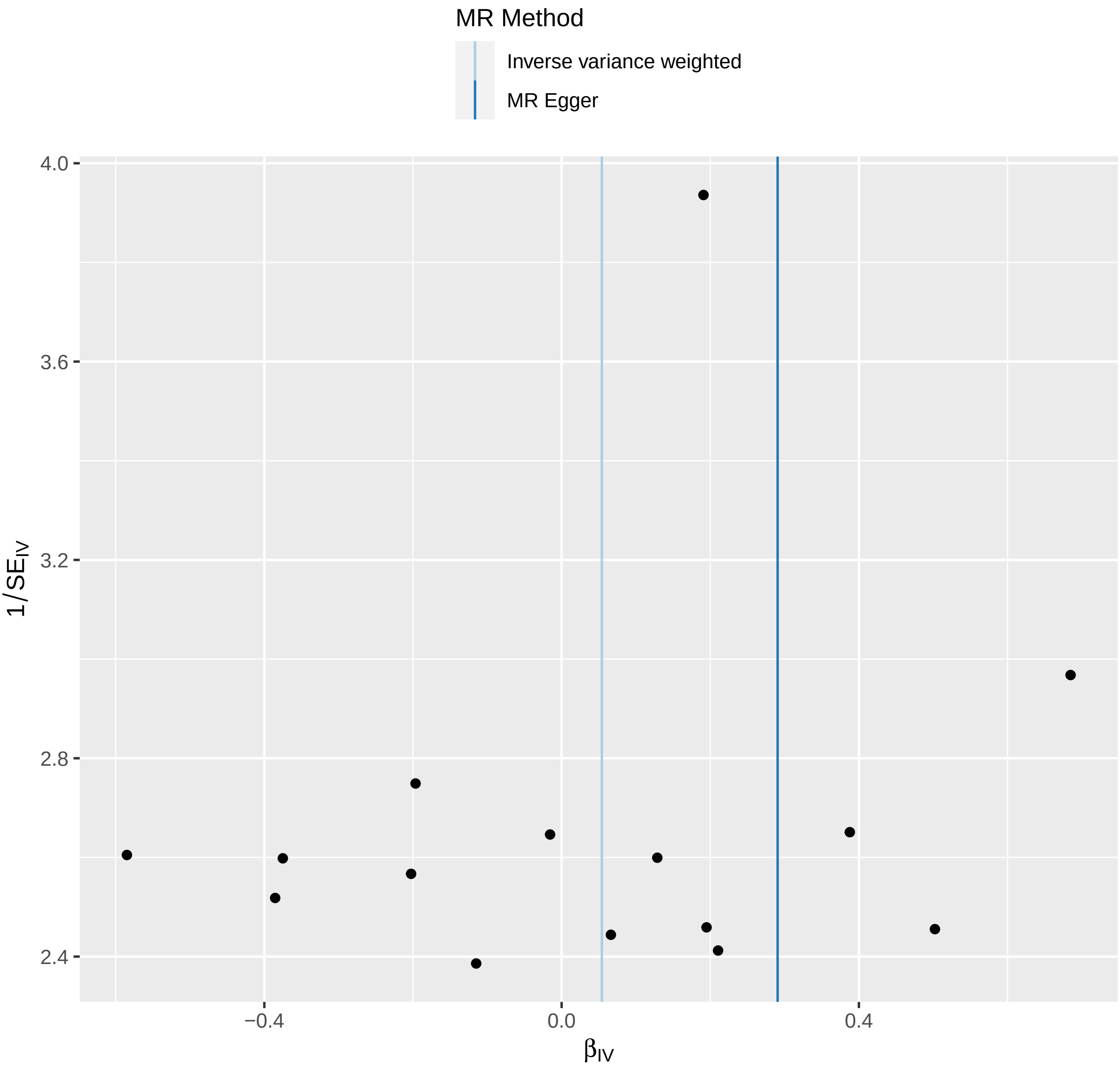

Batch 2−1059 : Gut microbiota abundance (genus Catenibacterium id.2153) on Thyroid Stimulating Hormone
with a threshold of 1e−05

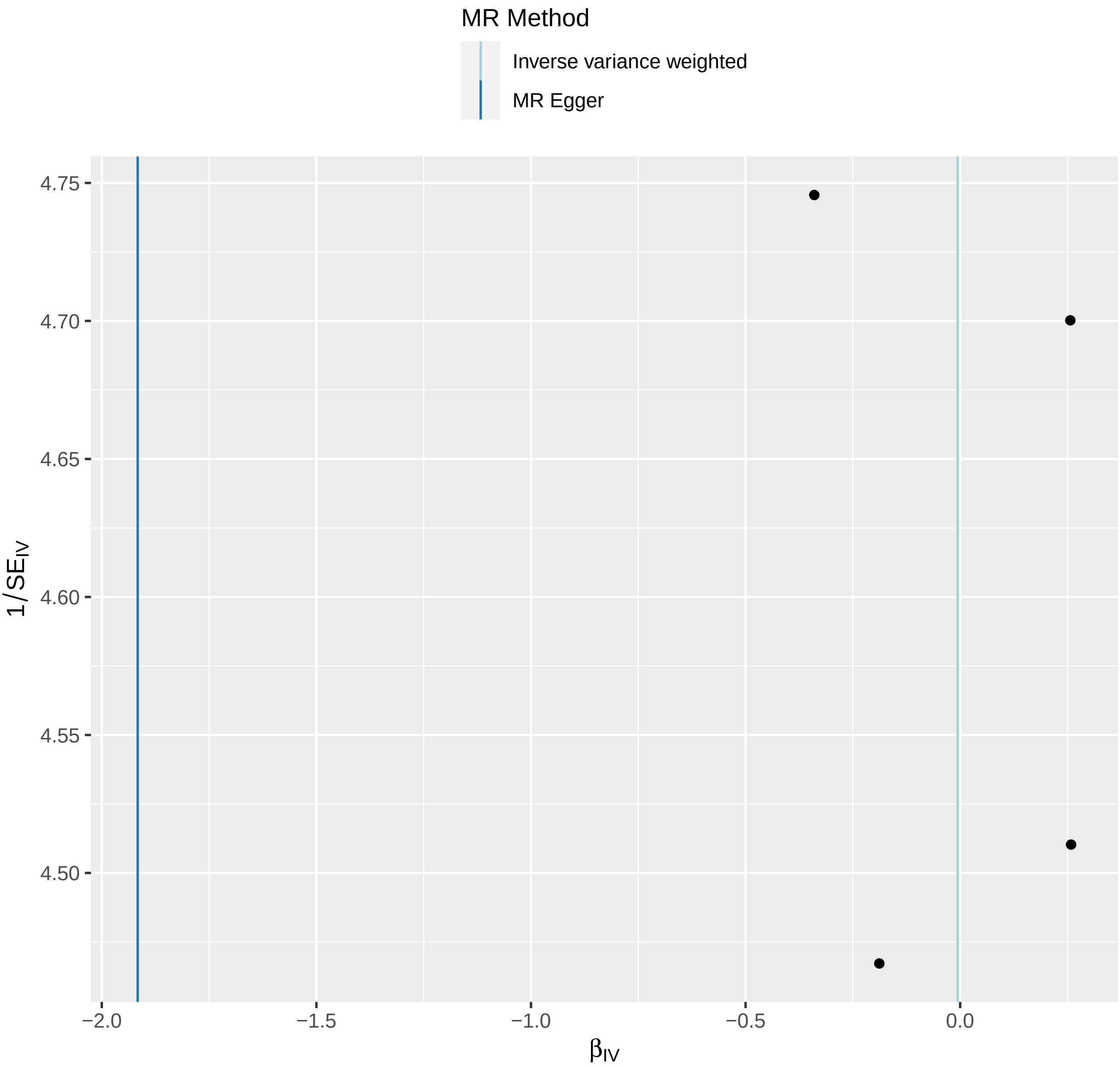

Batch 2−1060 : Gut microbiota abundance (genus Parasutterella id.2892) on Thyroid Stimulating Hormone
with a threshold of 1e−05

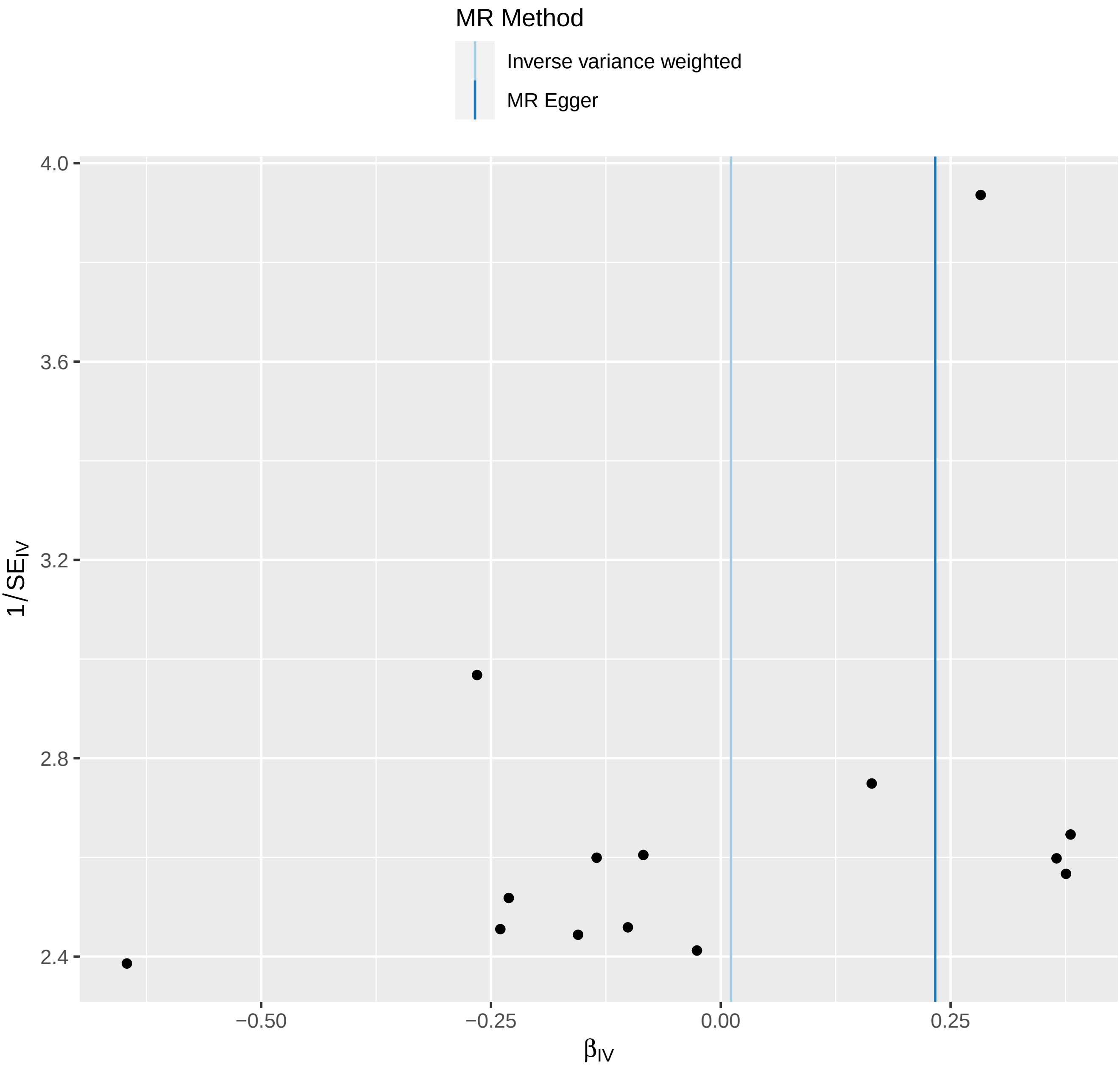

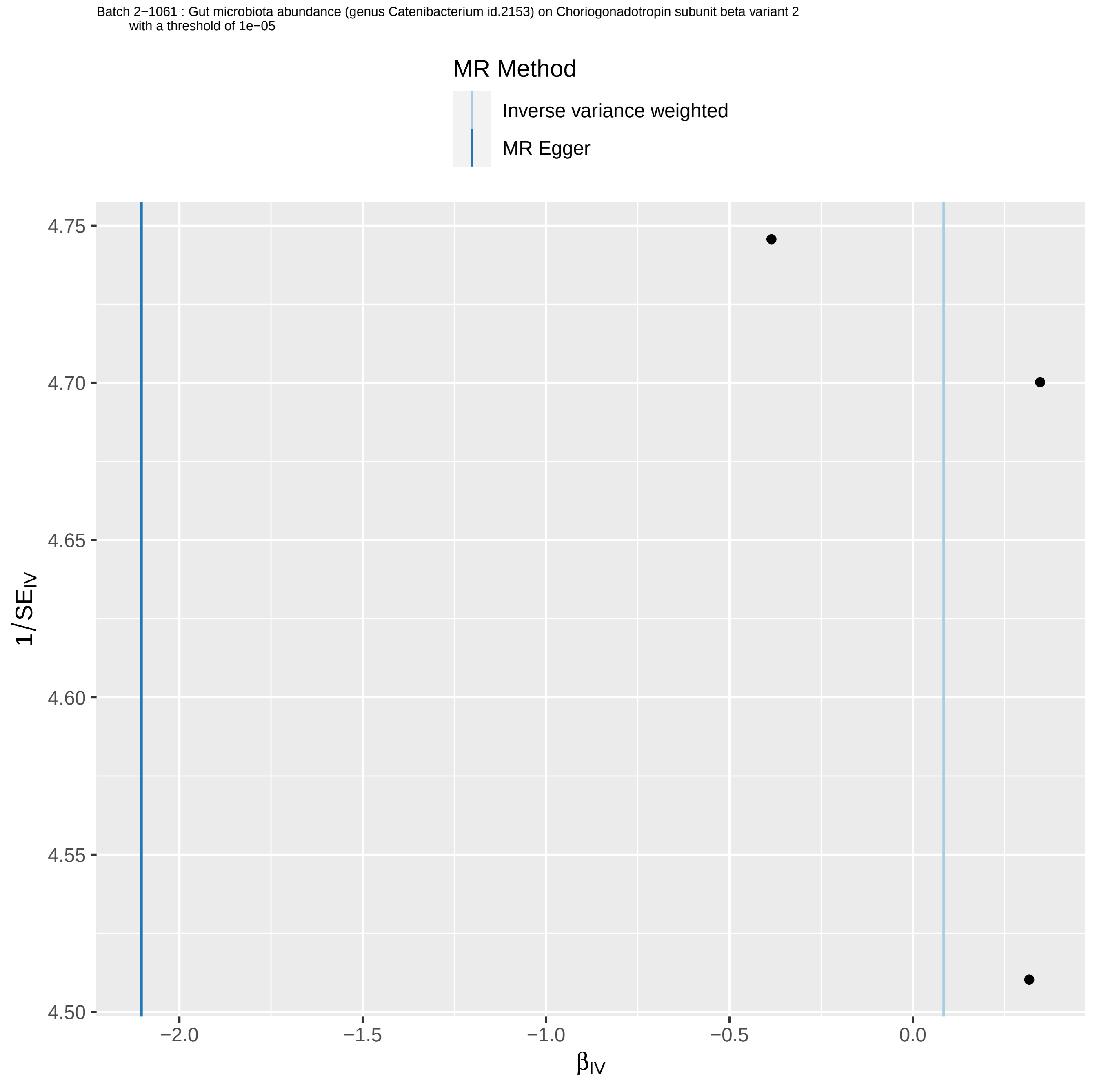
Batch 2−1061 : Gut microbiota abundance (genus Catenibacterium id.2153) on Choriogonadotropin subunit beta variant 2
with a threshold of 1e−05
MR Method
Inverse variance weighted
MR Egger
1/SE_IV
4.75
4.70
4.65
4.60
4.55
4.50
−2.0
−1.5
−1.0
−0.5
0.0
β_IV

Batch 2−1062 : Gut microbiota abundance (genus Parasutterella id.2892) on Choriogonadotropin subunit beta variant 2 with a threshold of 1e−05

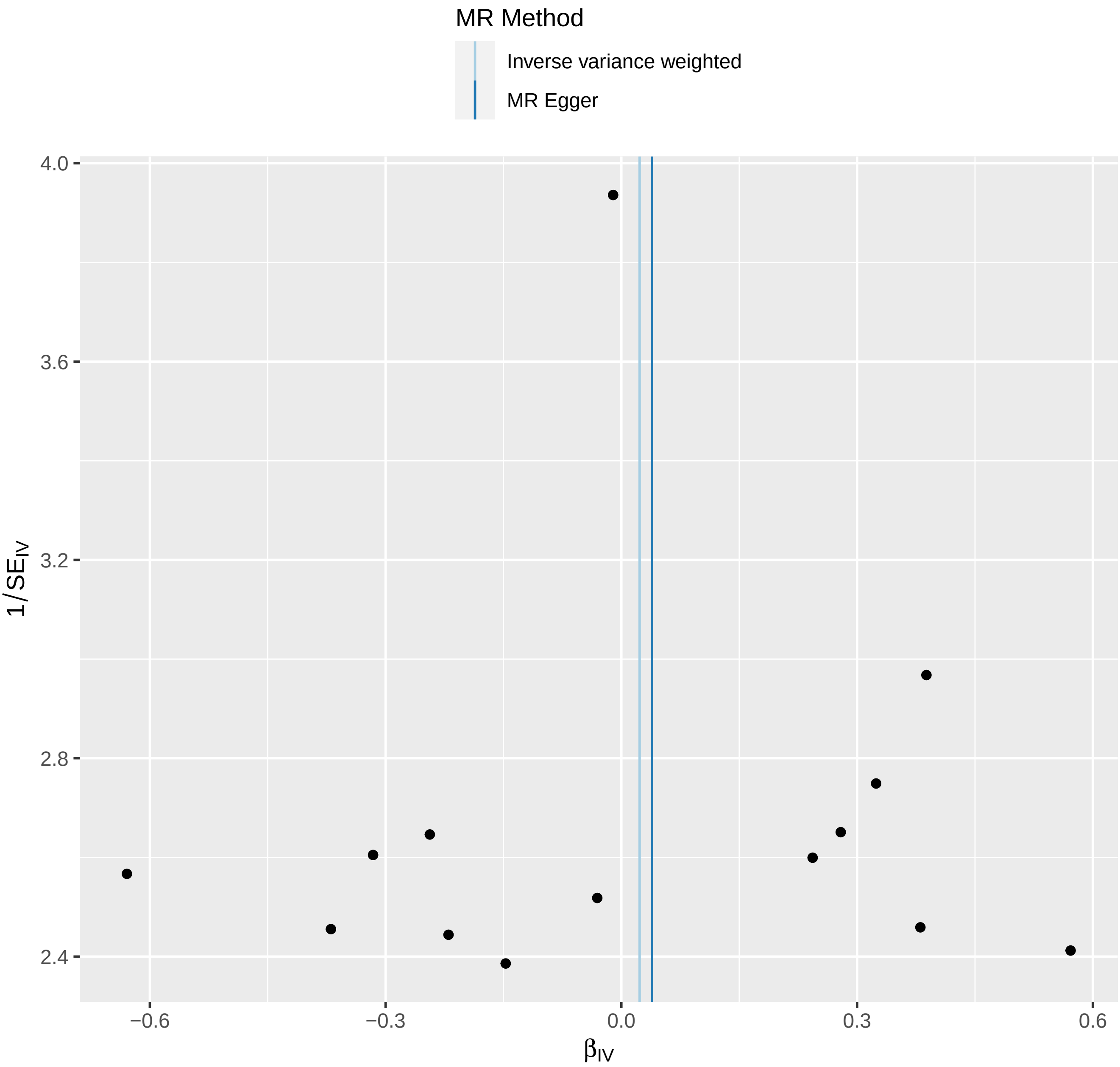

Batch 2−1063 : Gut microbiota abundance (genus Catenibacterium id.2153) on Cell growth regulator with EF hand domain protein 1 with a threshold of 1e−05

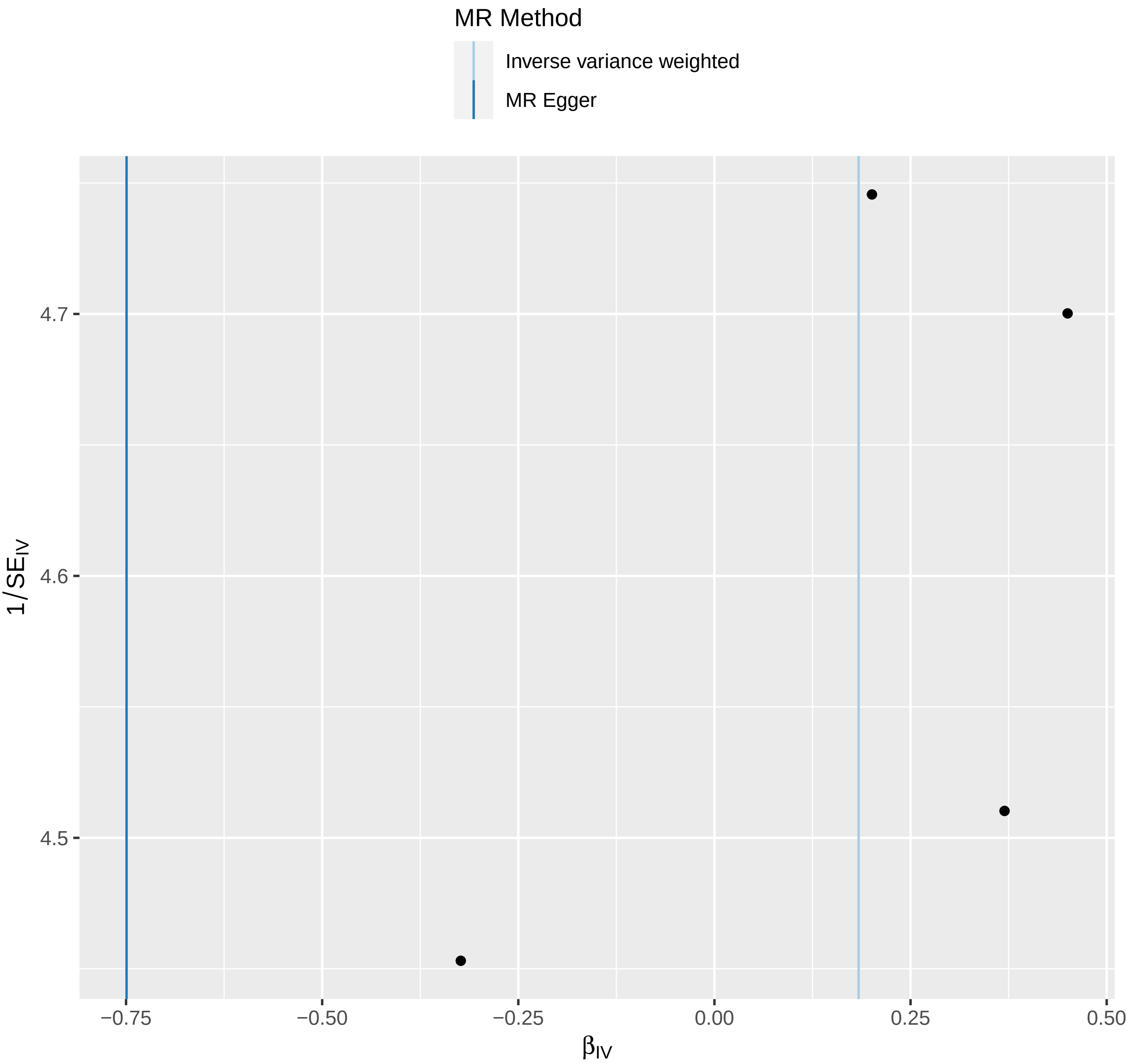

Batch 2−1064 : Gut microbiota abundance (genus Parasutterella id.2892) on Cell growth regulator with EF hand domain protein 1 with a threshold of 1e−05

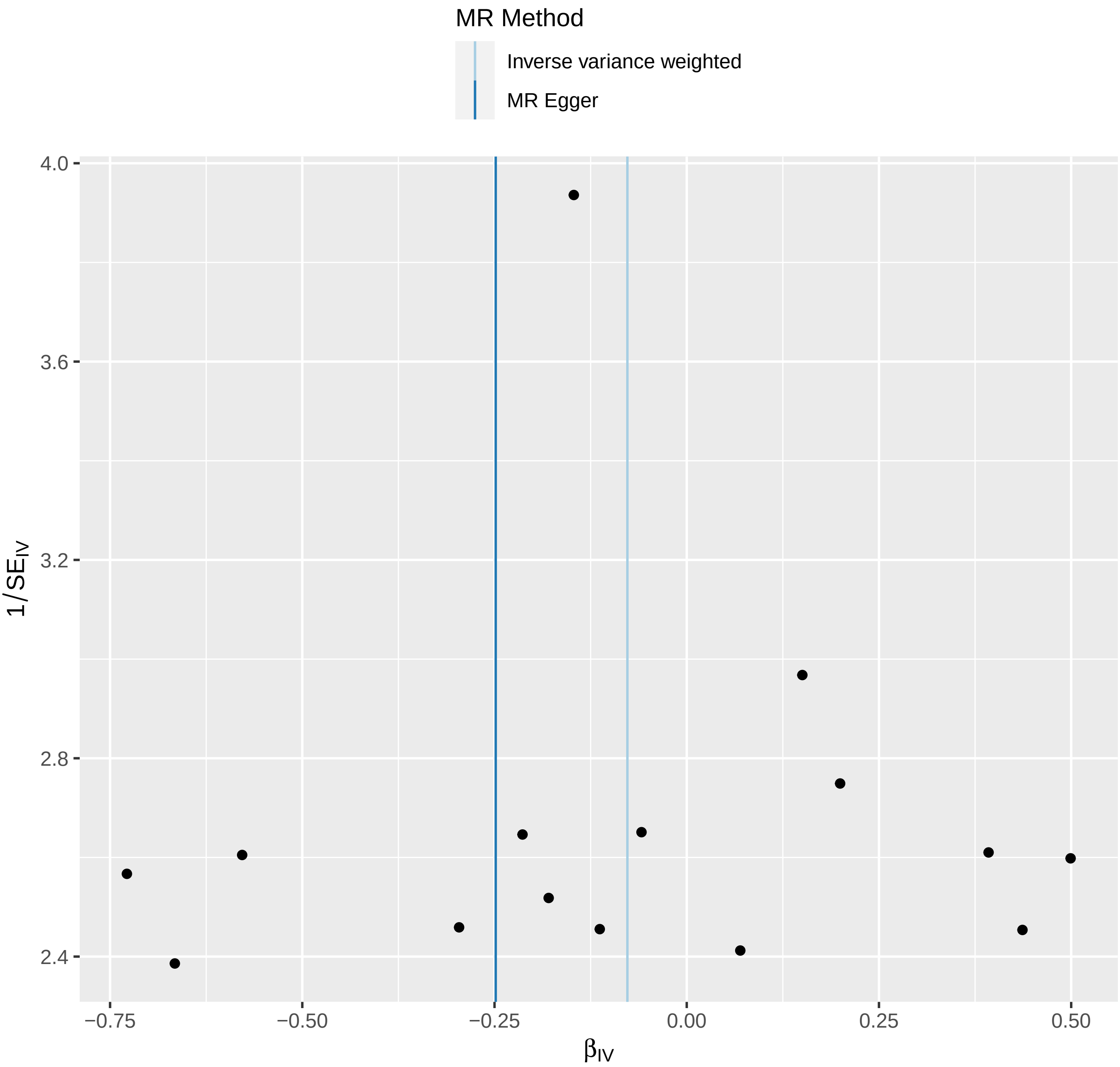

Batch 2−1065 : Gut microbiota abundance (genus Catenibacterium id.2153) on Chondroadherin
with a threshold of 1e−05

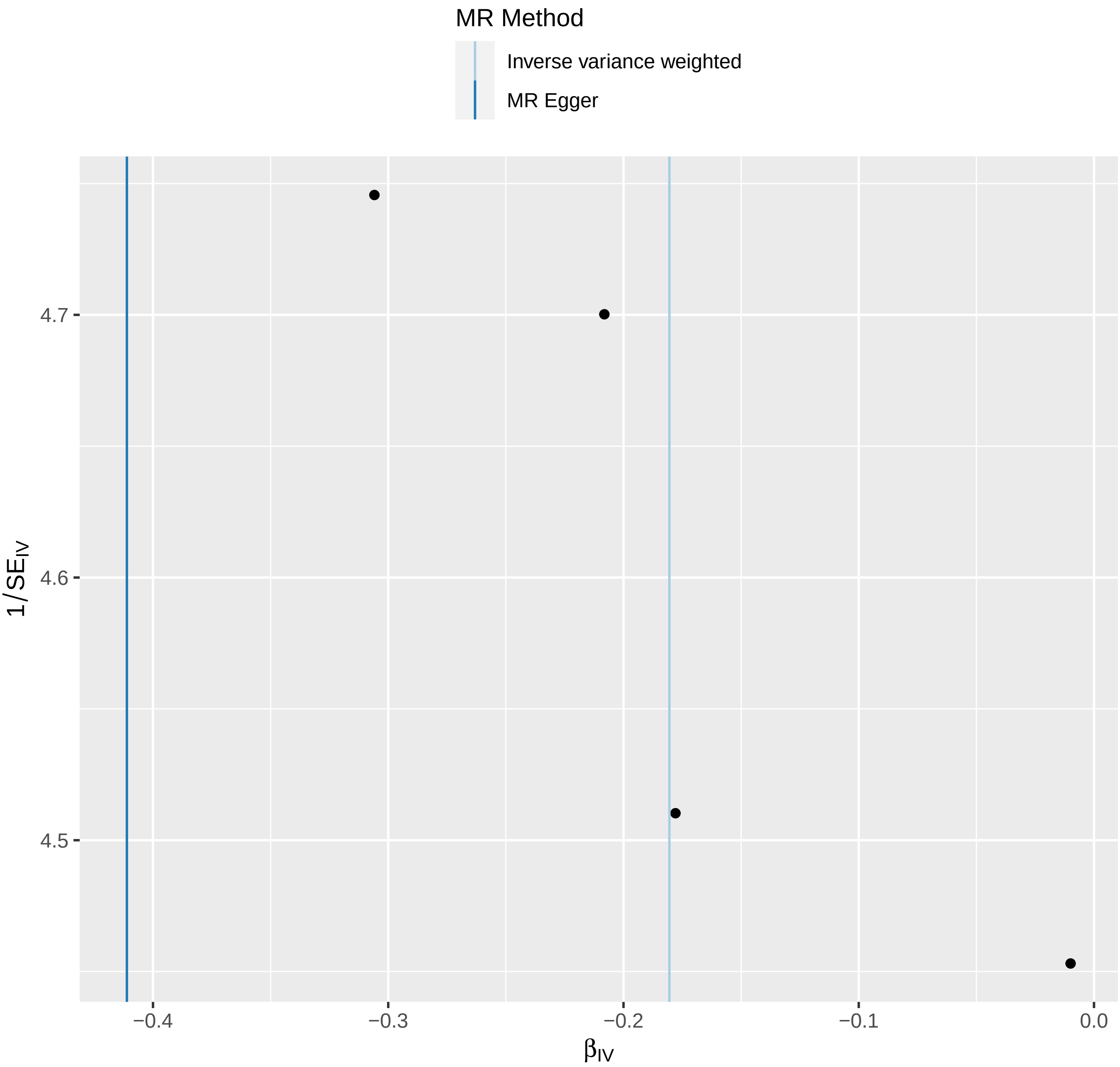

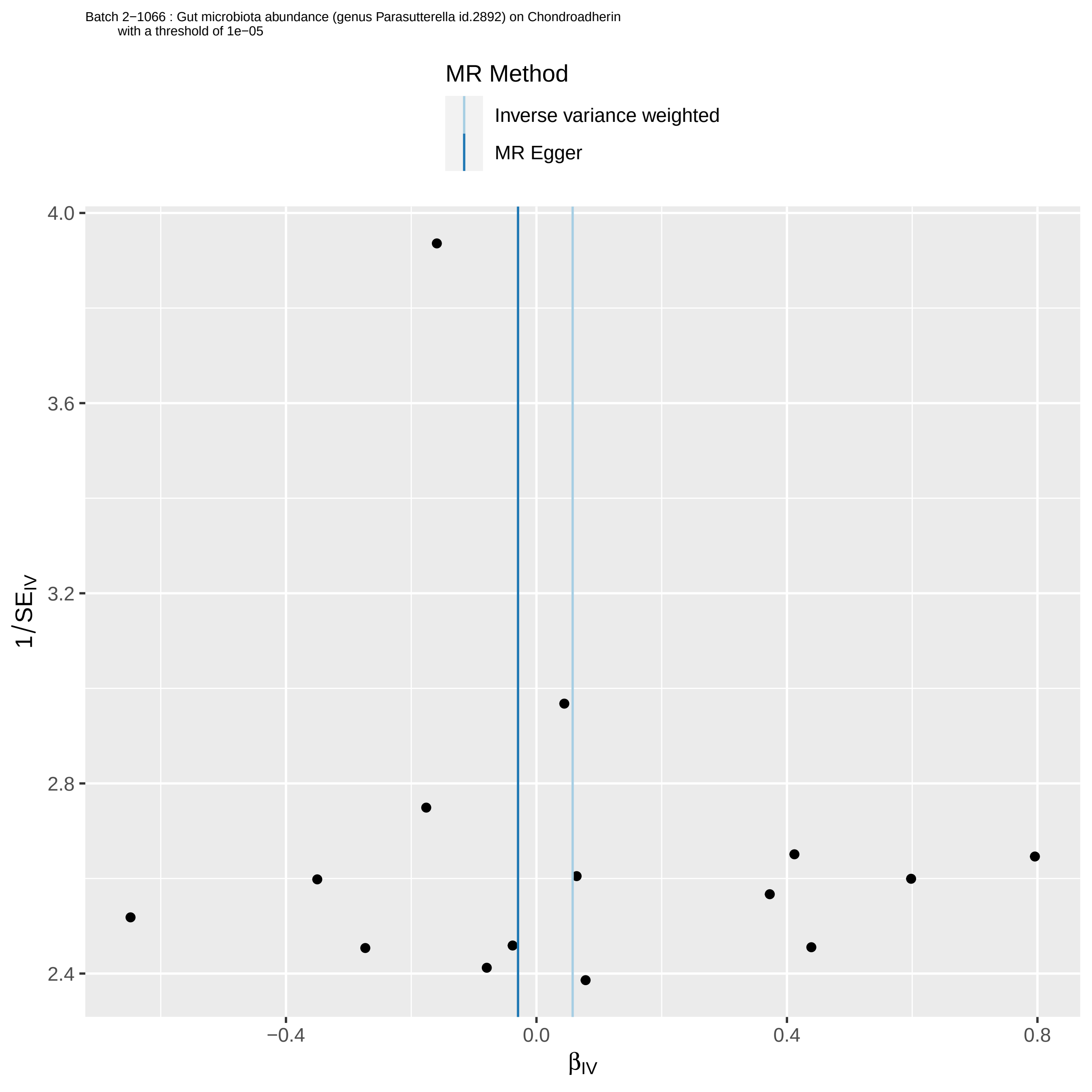

Batch 2−1066 : Gut microbiota abundance (genus Parasutterella id.2892) on Chondroadherin
with a threshold of 1e−05
MR Method
Inverse variance weighted
MR Egger
4.0
3.6
3.2
2.8
2.4
$1/SE_{IV}$
−0.4
0.0
0.4
0.8
β_{IV}

Batch 2−1067 : Gut microbiota abundance (genus Catenibacterium id.2153) on Coiled−coil−helix−coiled−coil−helix domain−containing protein 10, mitochondrial with a threshold of 1e−05

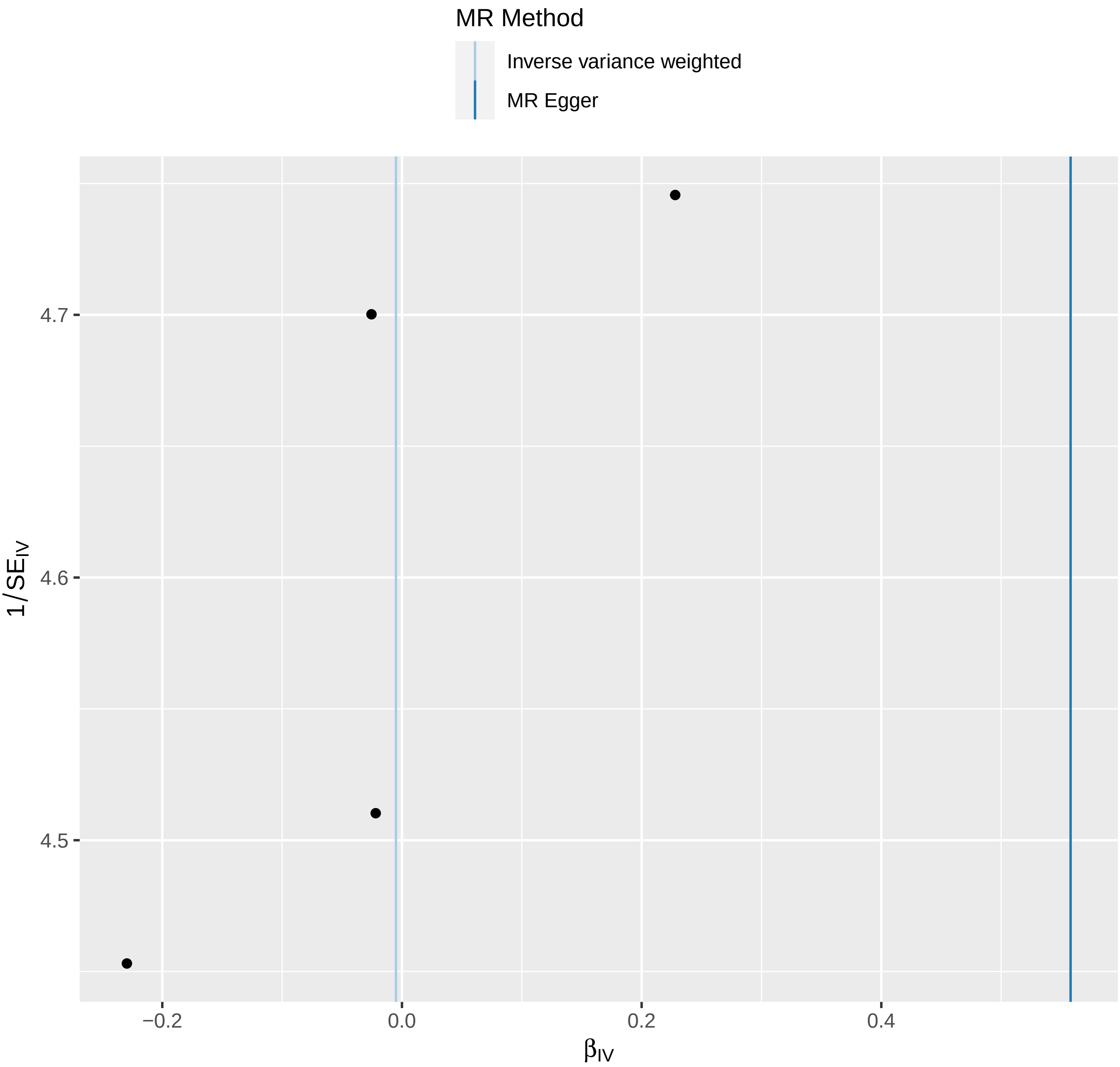

Batch 2−1068 : Gut microbiota abundance (genus Parasutterella id.2892) on Coiled−coil−helix−coiled−coil−helix domain−containing protein 10, mitochondrial
with a threshold of 1e−05

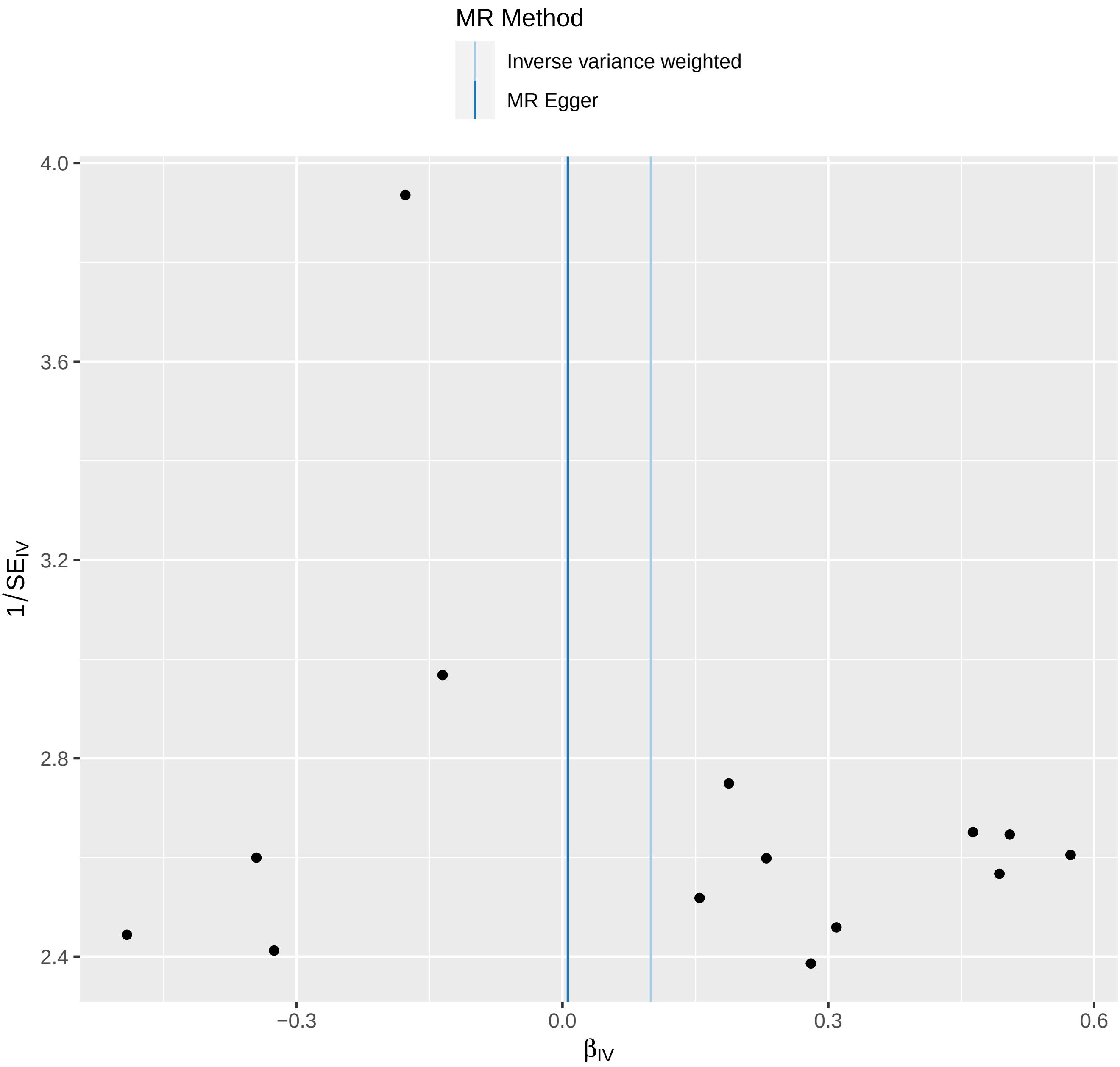

Batch 2−1069 : Gut microbiota abundance (genus Catenibacterium id.2153) on Coiled−coil−helix−coiled−coil−helix domain−containing protein 2 with a threshold of 1e−05

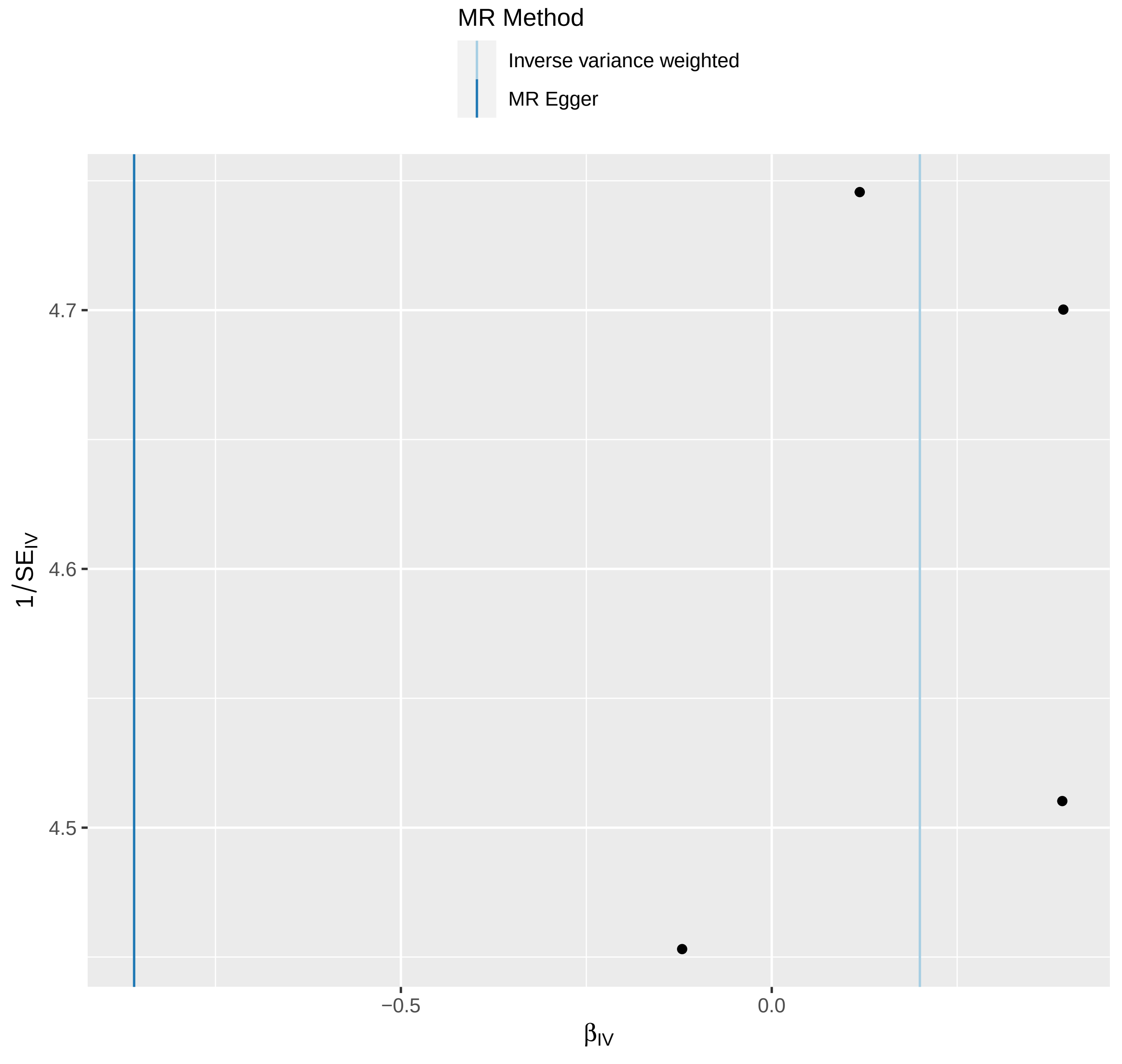

Batch 2−1070 : Gut microbiota abundance (genus Parasutterella id.2892) on Coiled−coil−helix−coiled−coil−helix domain−containing protein 2 with a threshold of 1e−05

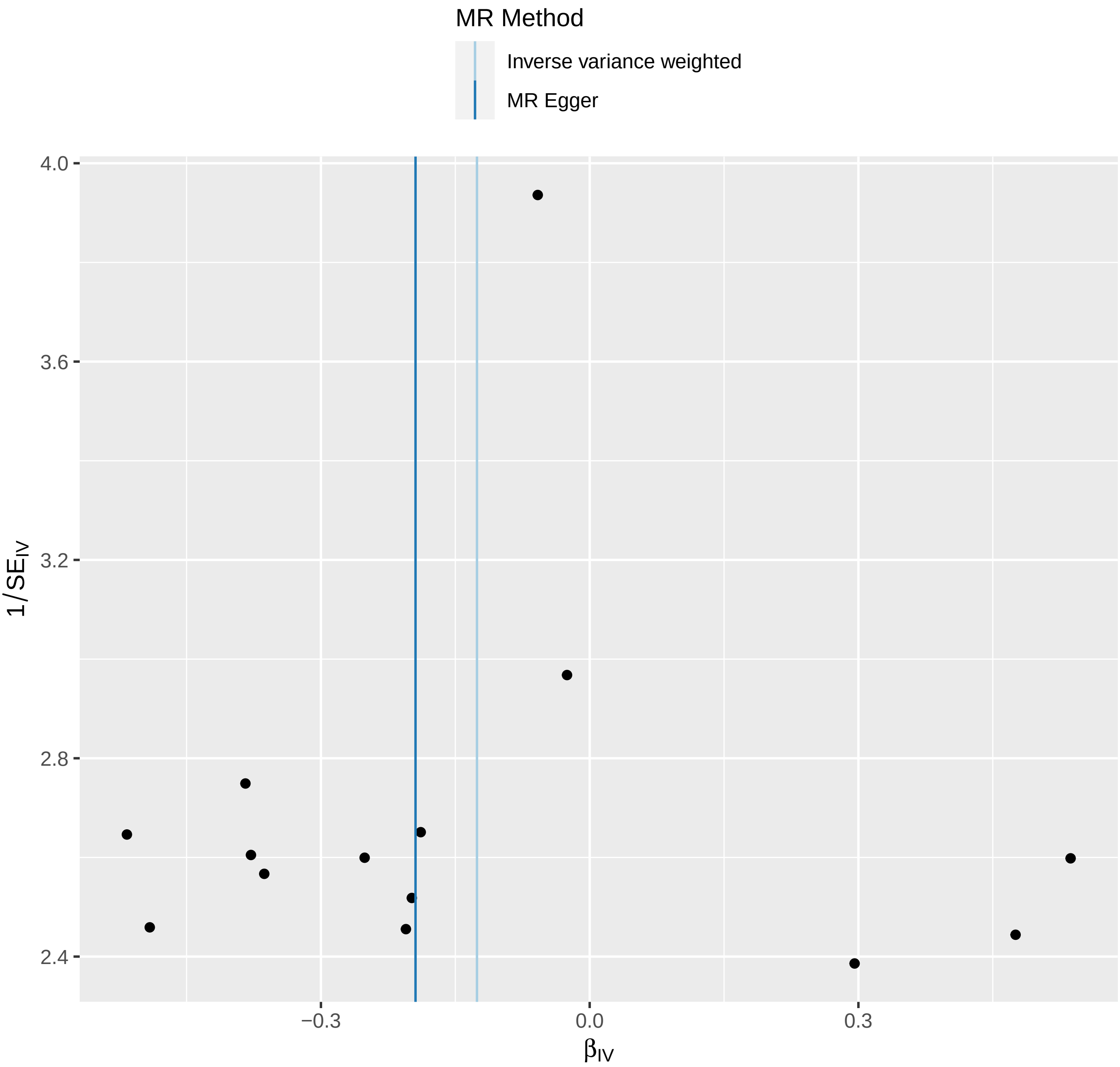

Batch 2−1071 : Gut microbiota abundance (genus Catenibacterium id.2153) on Chromodomain−helicase−DNA−binding protein 7
with a threshold of 1e−05

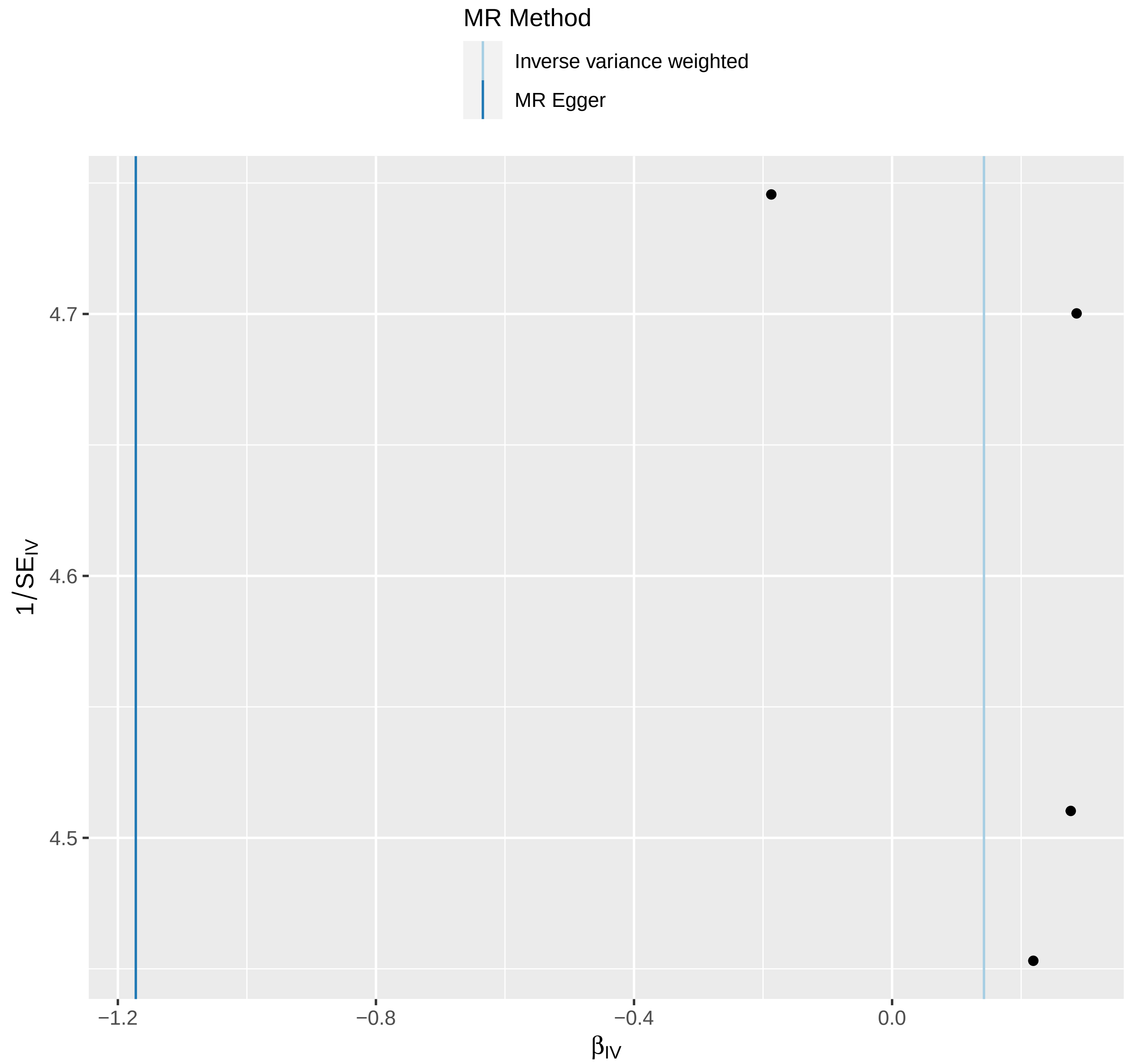

Batch 2−1072 : Gut microbiota abundance (genus Parasutterella id.2892) on Chromodomain−helicase−DNA−binding protein 7
with a threshold of 1e−05

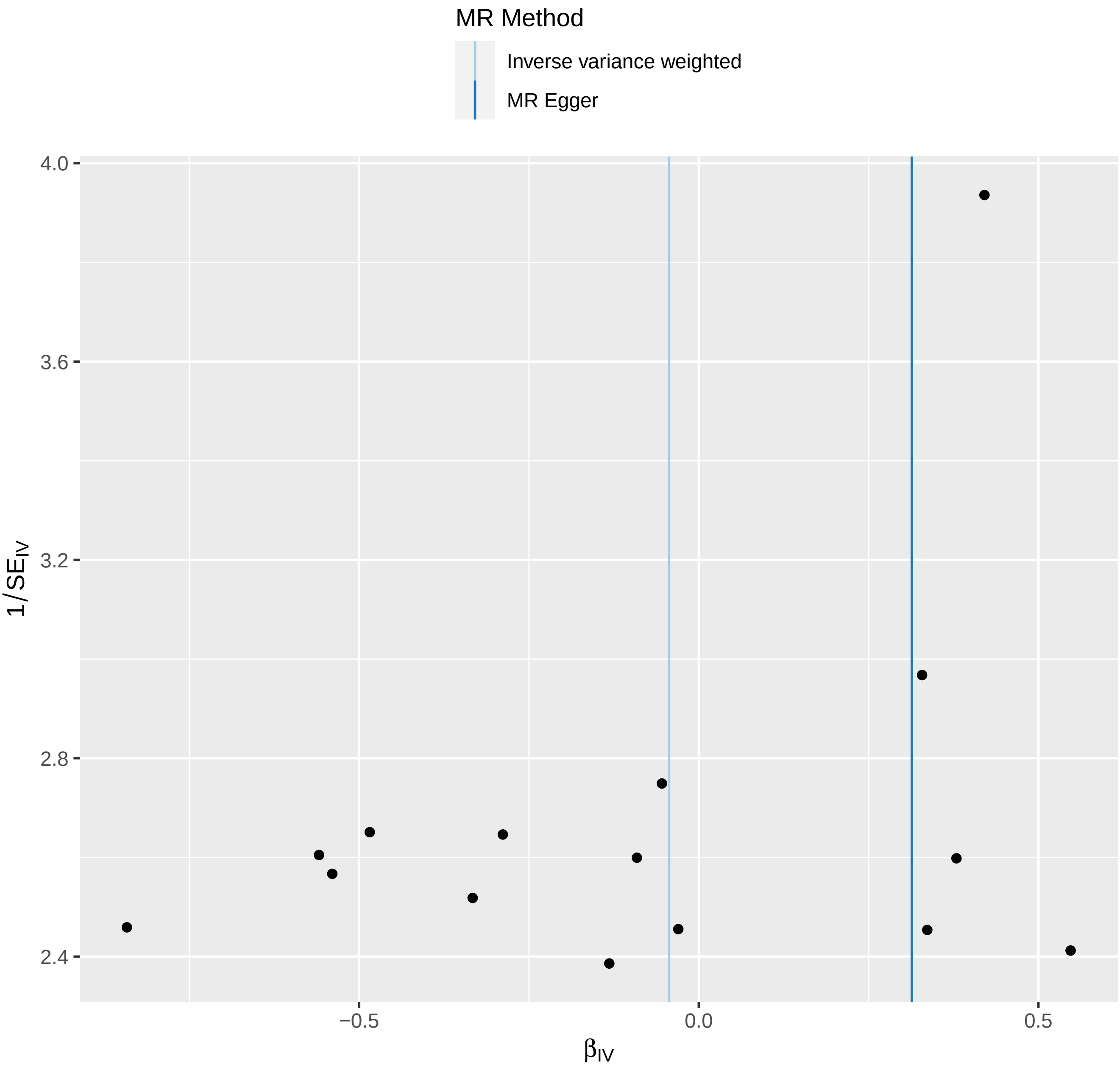

Batch 2−1073 : Gut microbiota abundance (genus Catenibacterium id.2153) on Serine/threonine−protein kinase Chk1
with a threshold of 1e−05

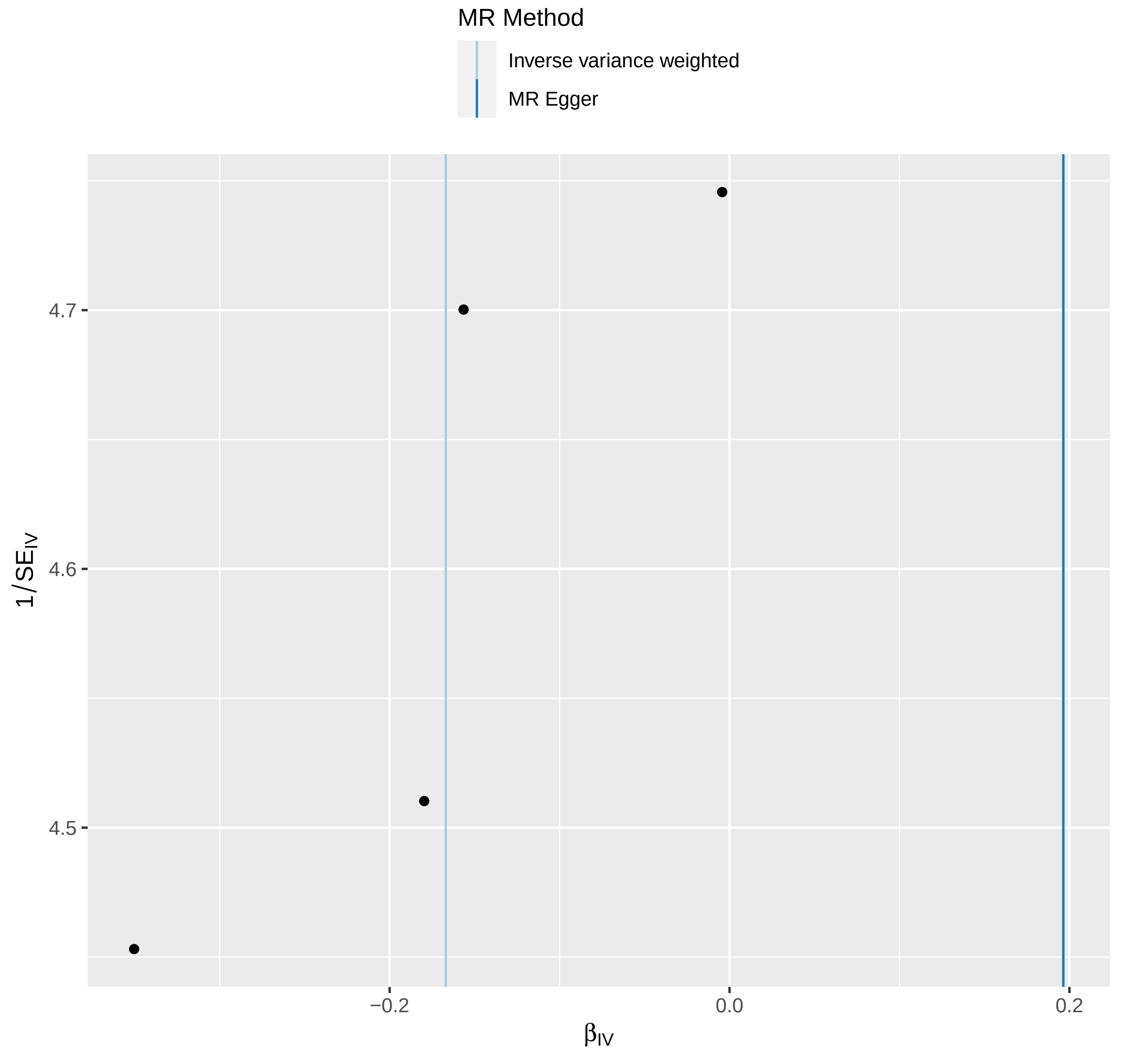

Batch 2−1074 : Gut microbiota abundance (genus Parasutterella id.2892) on Serine/threonine−protein kinase Chk1
with a threshold of 1e−05

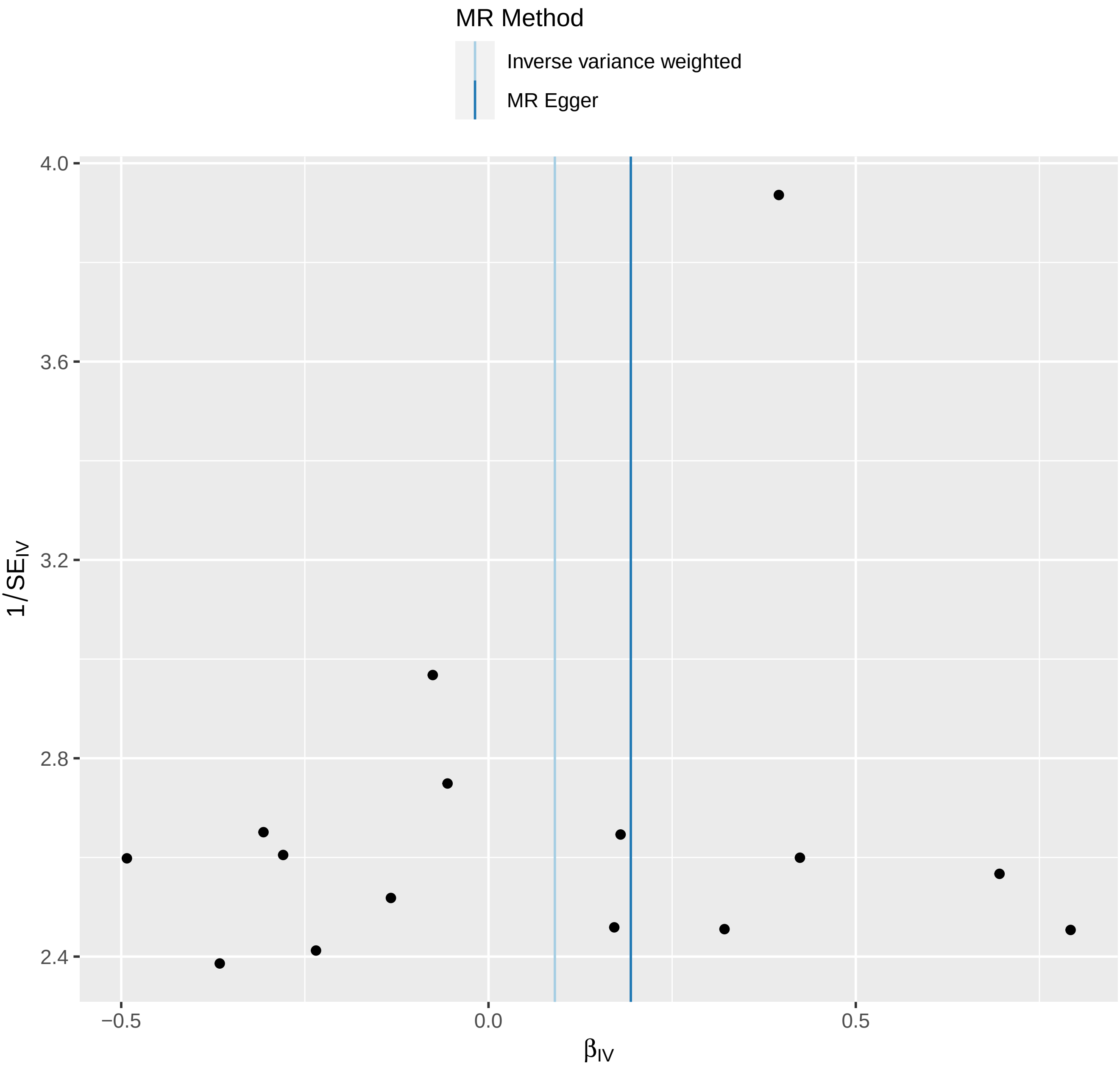

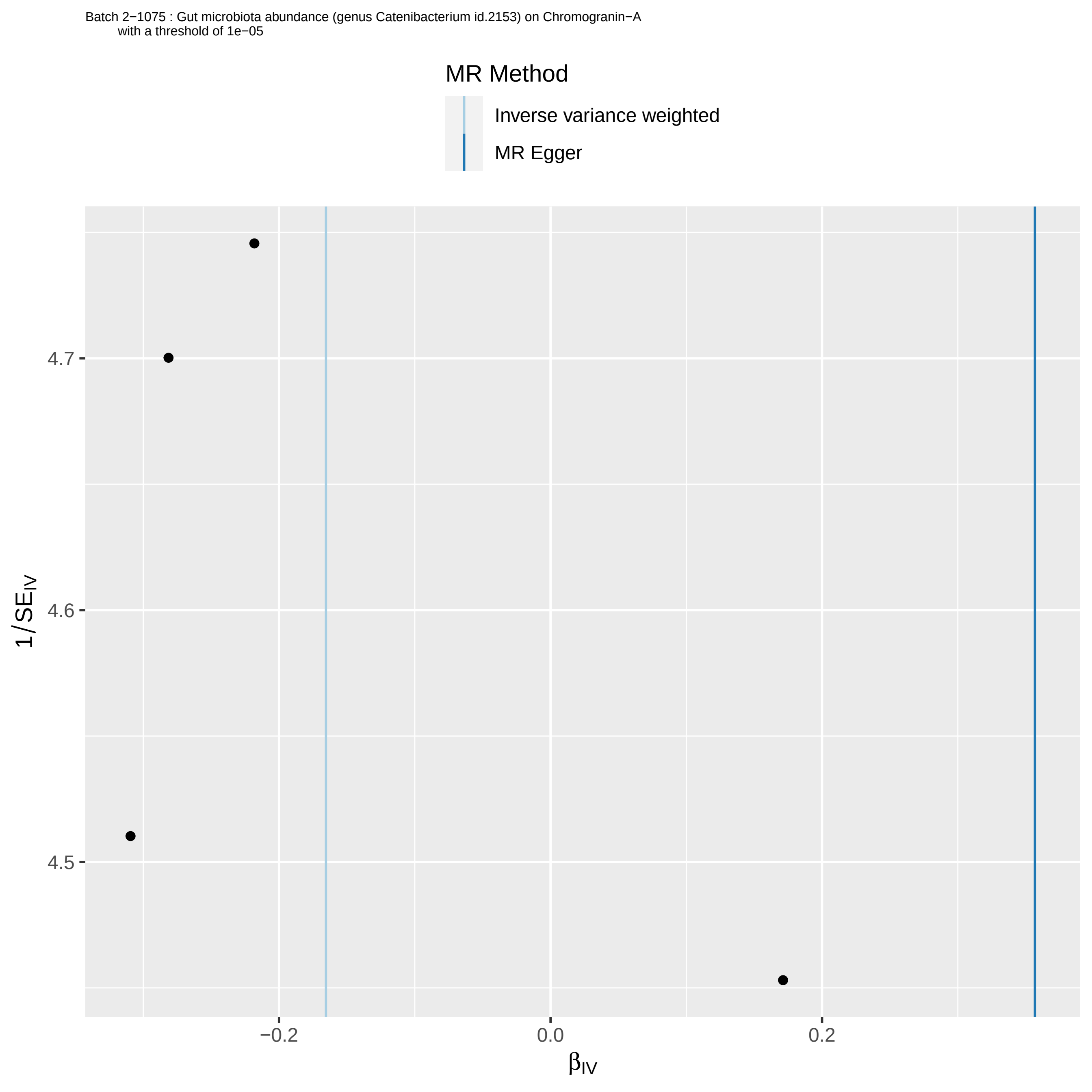
Batch 2−1075 : Gut microbiota abundance (genus Catenibacterium id.2153) on Chromogranin−A
with a threshold of 1e−05
MR Method
Inverse variance weighted
MR Egger
4.7
4.6
4.5
1/SE_IV
−0.2
0.0
0.2
β_IV

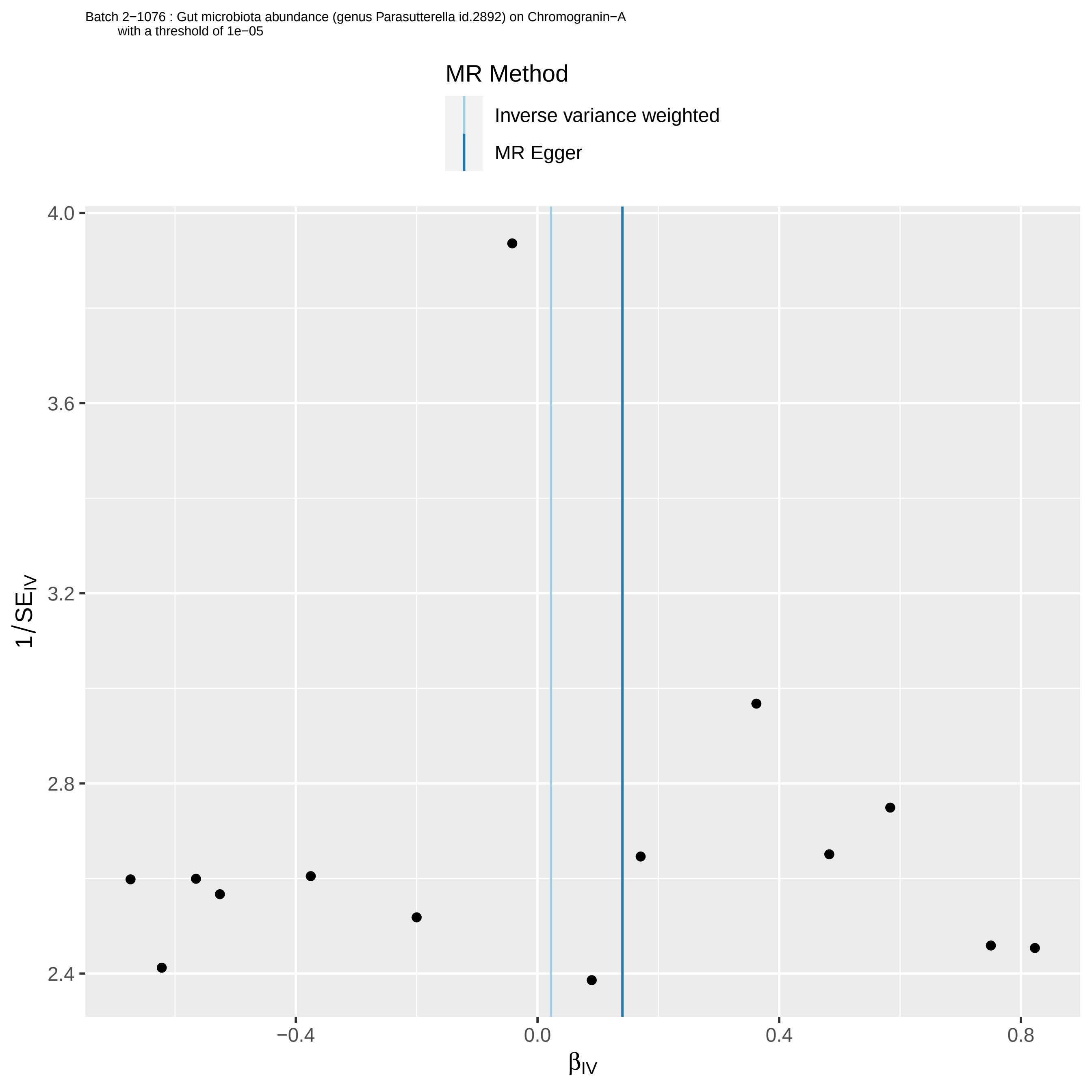
Batch 2−1076 : Gut microbiota abundance (genus Parasutterella id.2892) on Chromogranin−A
with a threshold of 1e−05
MR Method
Inverse variance weighted
MR Egger
4.0
3.6
3.2
2.8
2.4
1/SE$_{IV}$
−0.4
0.0
0.4
0.8
β$_{IV}$

Batch 2−1077 : Gut microbiota abundance (genus Catenibacterium id.2153) on Secretogranin−1
with a threshold of 1e−05

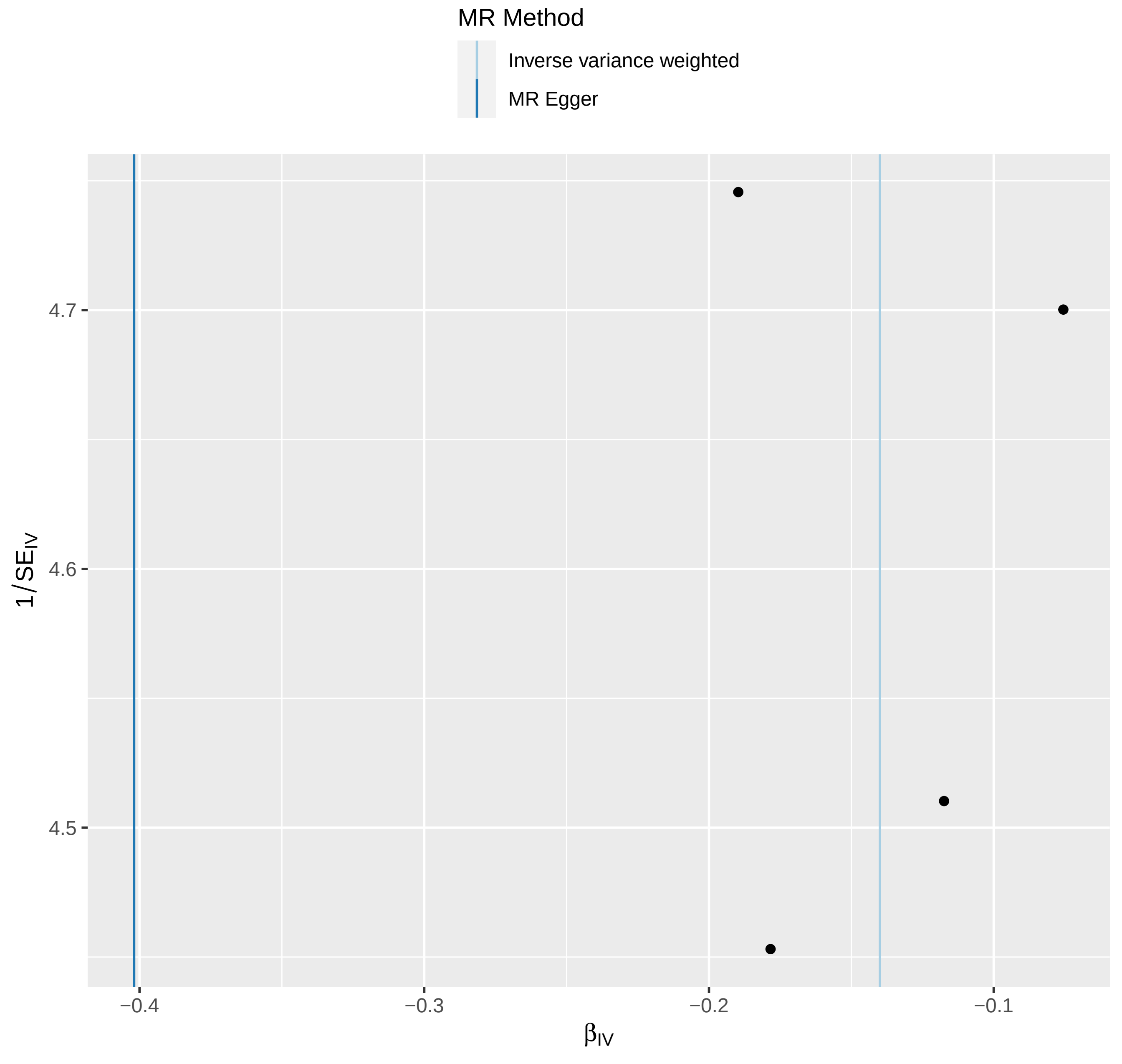

Batch 2−1078 : Gut microbiota abundance (genus Parasutterella id.2892) on Secretogranin−1
with a threshold of 1e−05

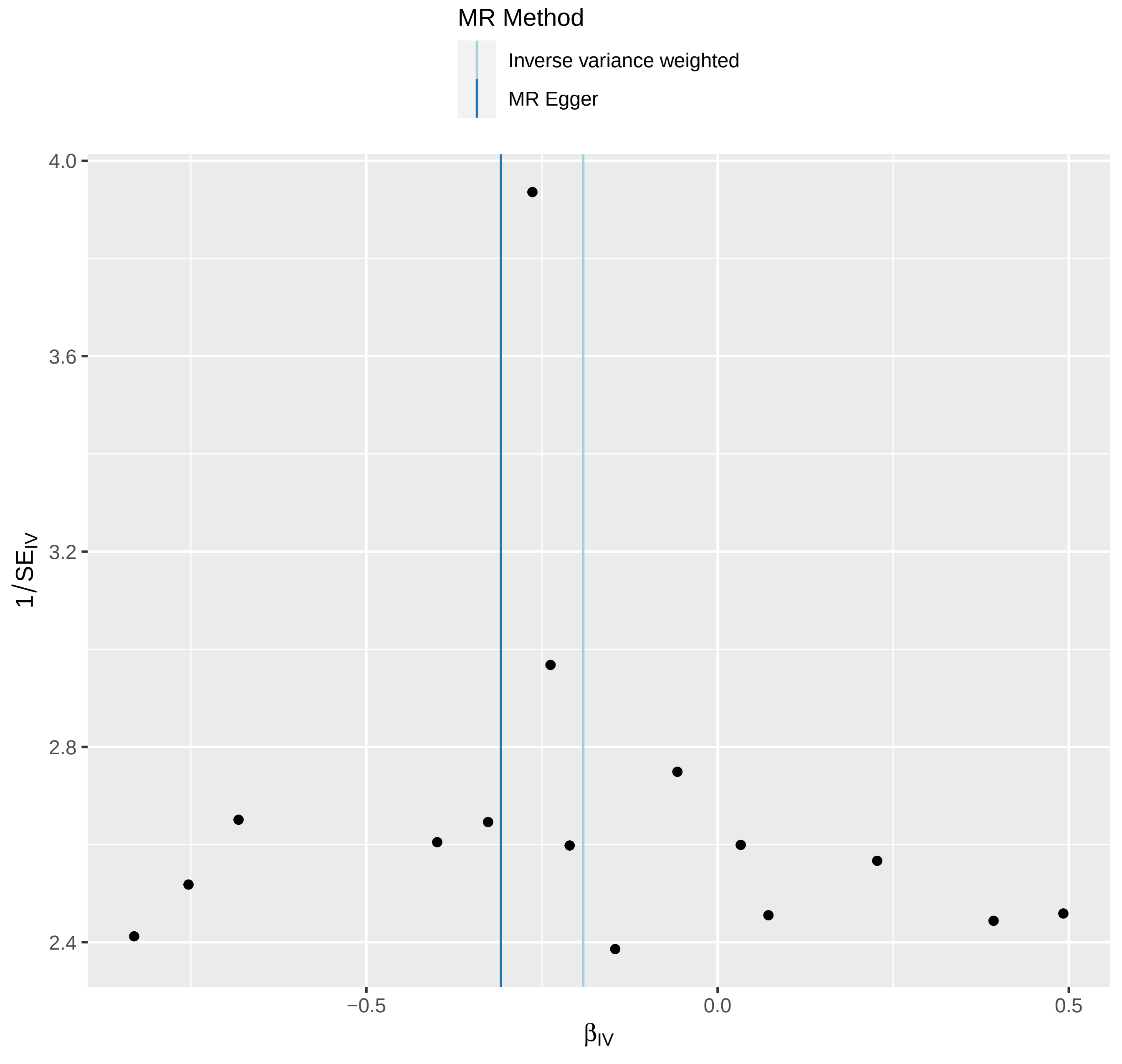

Batch 2−1079 : Gut microbiota abundance (genus Catenibacterium id.2153) on Chitinase−3−like protein 1
with a threshold of 1e−05

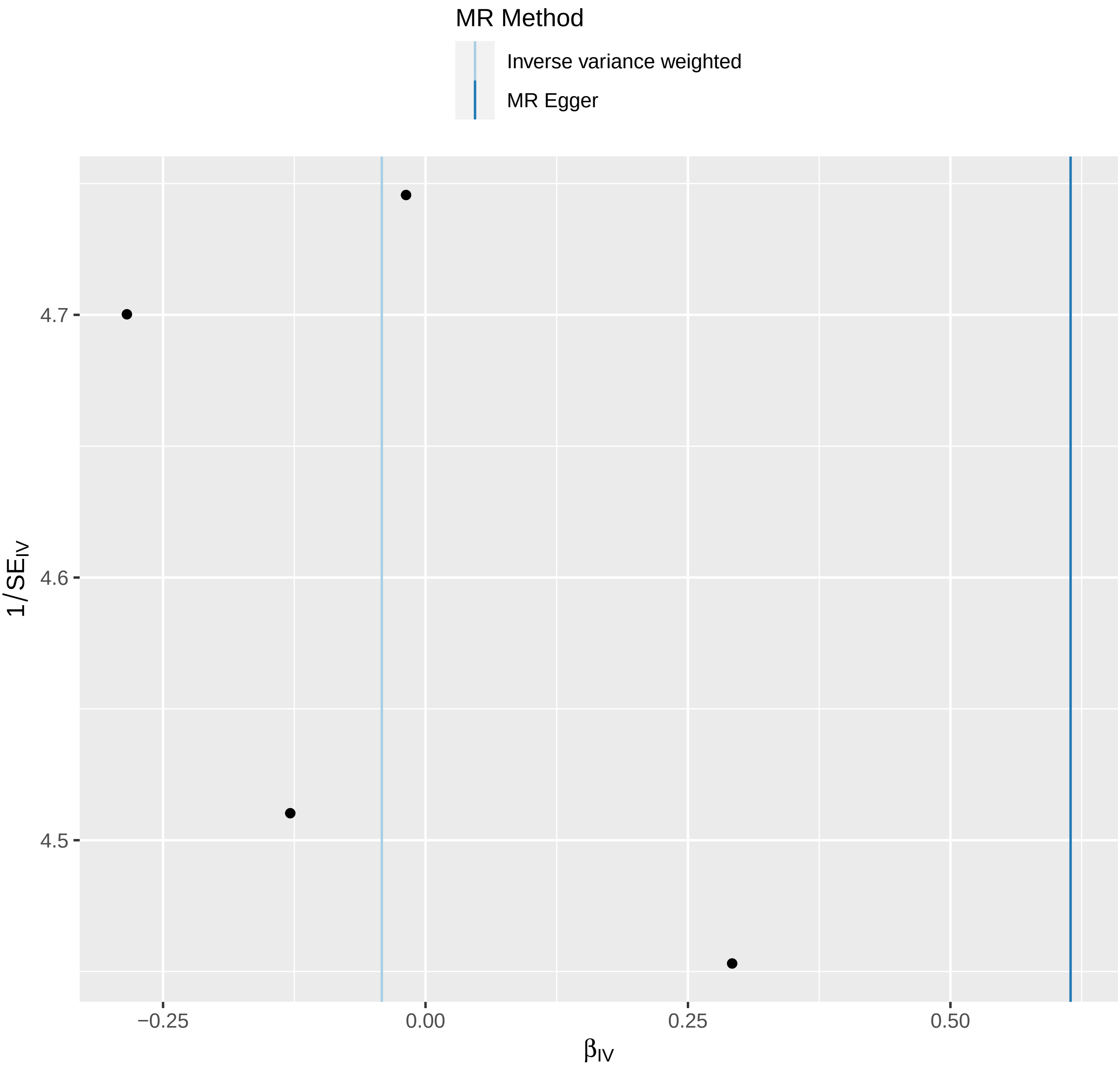

Batch 2−1080 : Gut microbiota abundance (genus Parasutterella id.2892) on Chitinase−3−like protein 1
with a threshold of 1e−05

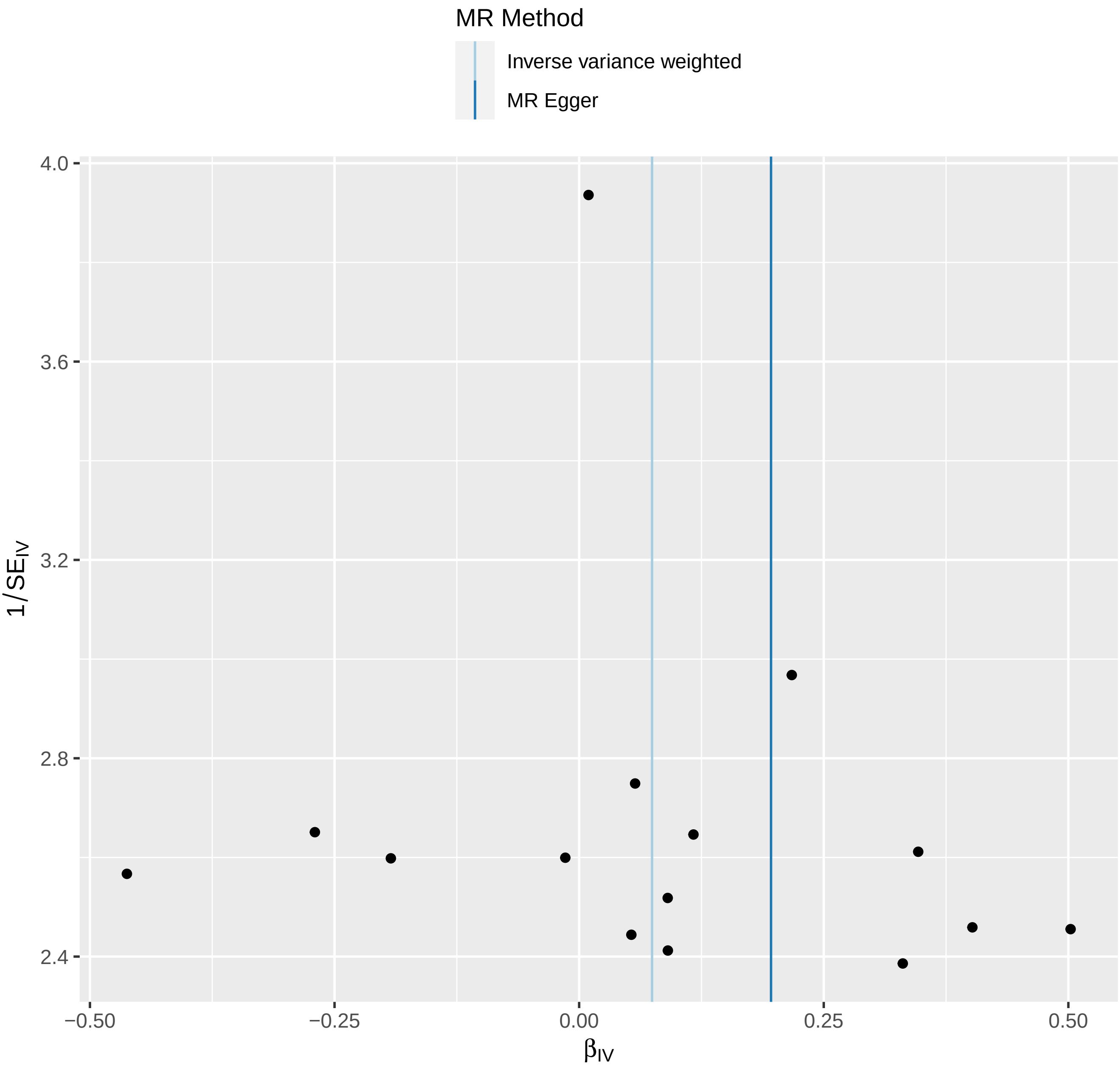

Batch 2−1081 : Gut microbiota abundance (genus Catenibacterium id.2153) on Chitinase−3−like protein 2
with a threshold of 1e−05

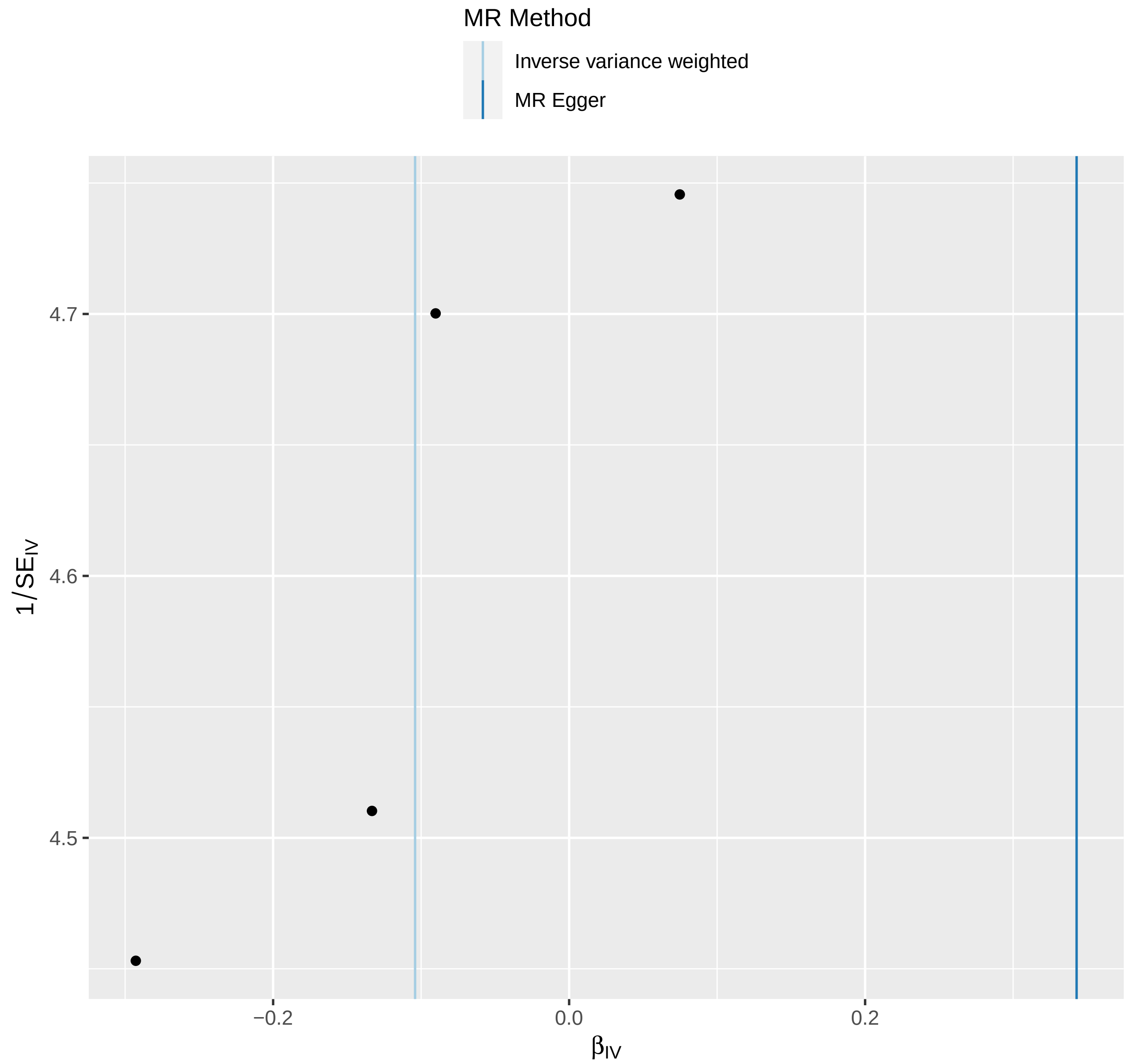

Batch 2−1082 : Gut microbiota abundance (genus Parasutterella id.2892) on Chitinase−3−like protein 2
with a threshold of 1e−05

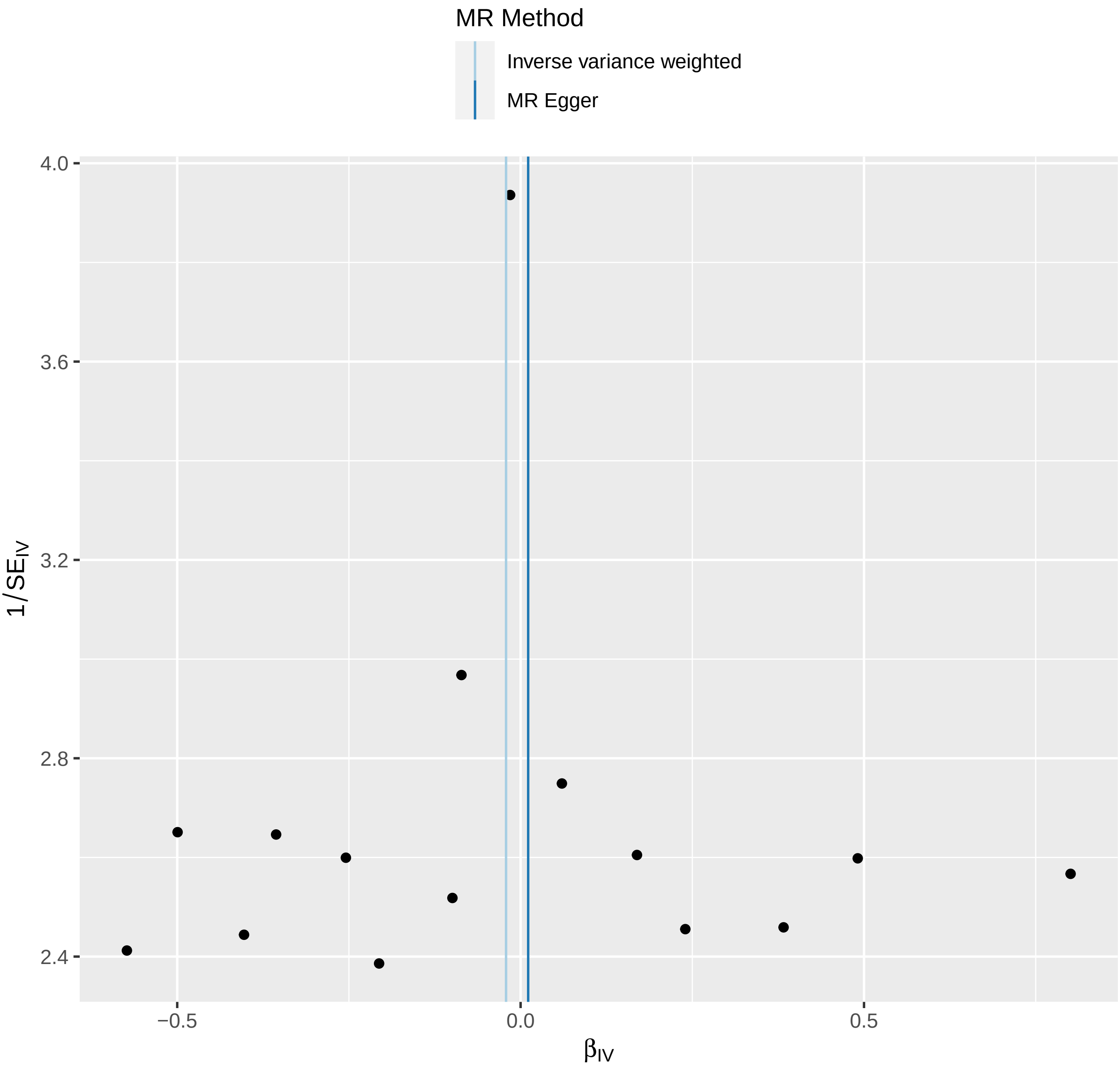

Batch 2−1083 : Gut microbiota abundance (genus Catenibacterium id.2153) on Cysteine−rich hydrophobic domain−containing protein 2
with a threshold of 1e−05

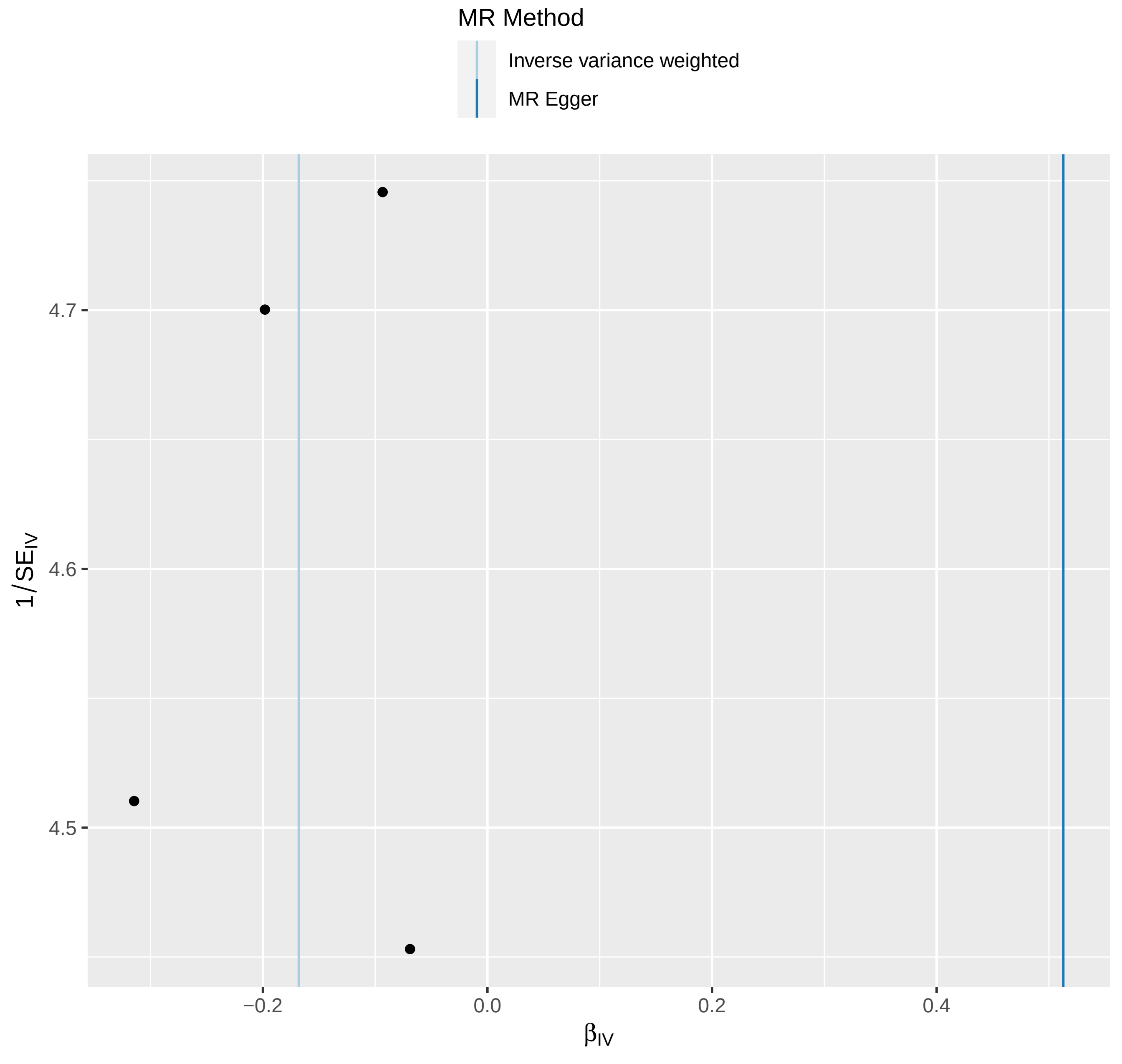

Batch 2−1084 : Gut microbiota abundance (genus Parasutterella id.2892) on Cysteine−rich hydrophobic domain−containing protein 2
with a threshold of 1e−05

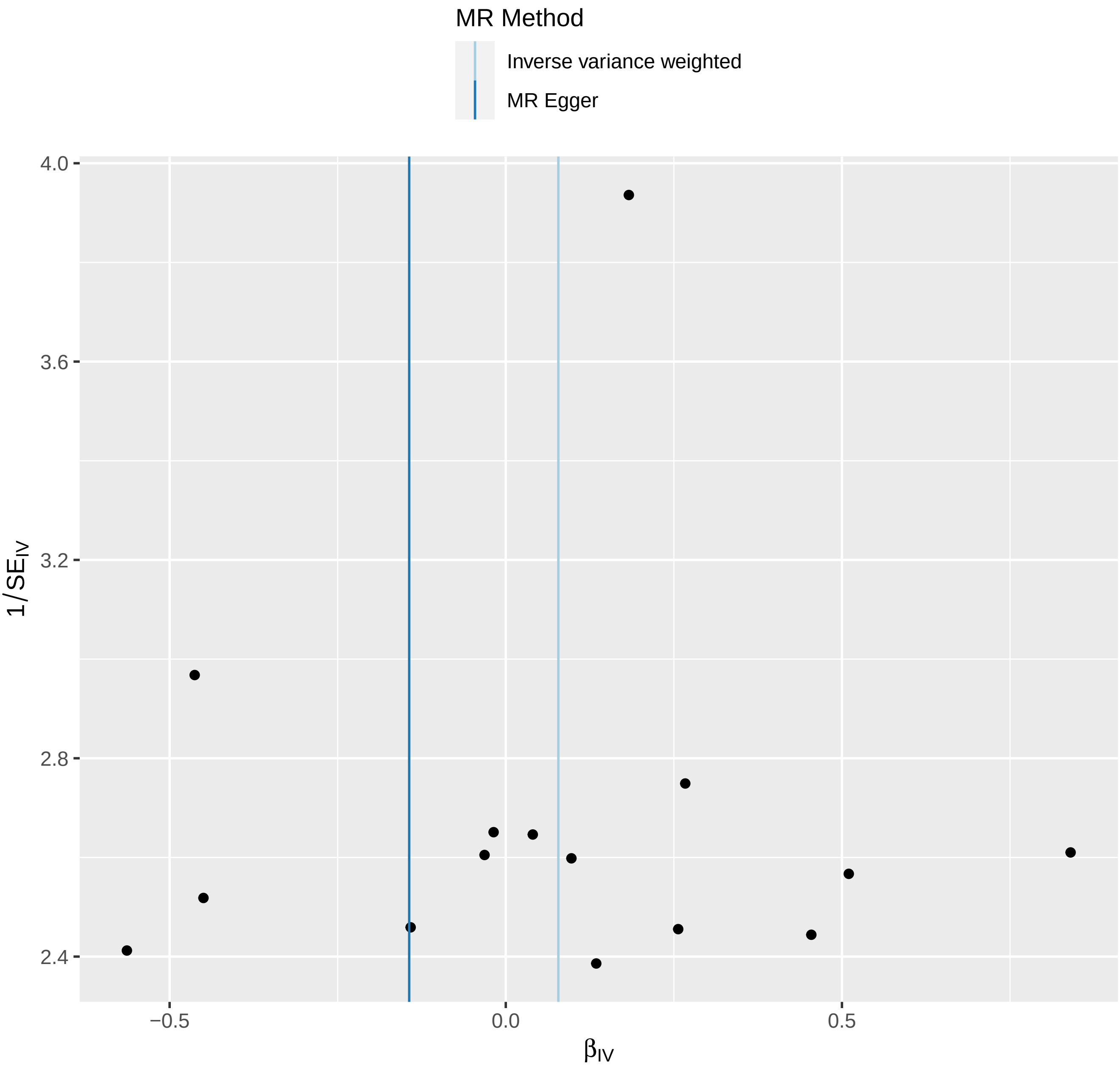

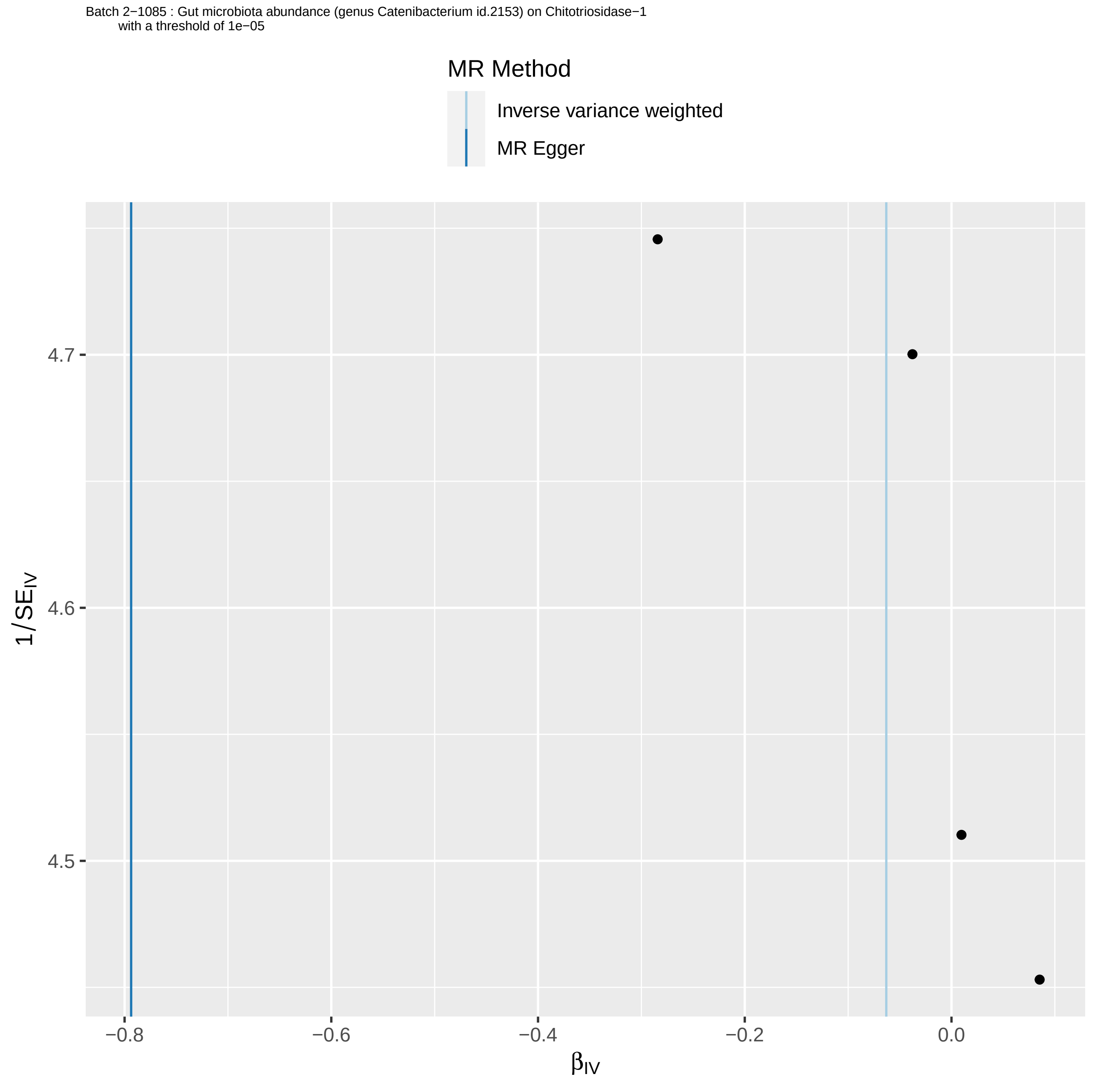

Batch 2−1085 : Gut microbiota abundance (genus Catenibacterium id.2153) on Chitotriosidase−1
with a threshold of 1e−05
MR Method
Inverse variance weighted
MR Egger
4.7
4.6
4.5
1/SE_IV
−0.8
−0.6
−0.4
−0.2
0.0
β_IV

Batch 2−1086 : Gut microbiota abundance (genus Parasutterella id.2892) on Chitotriosidase−1
with a threshold of 1e−05

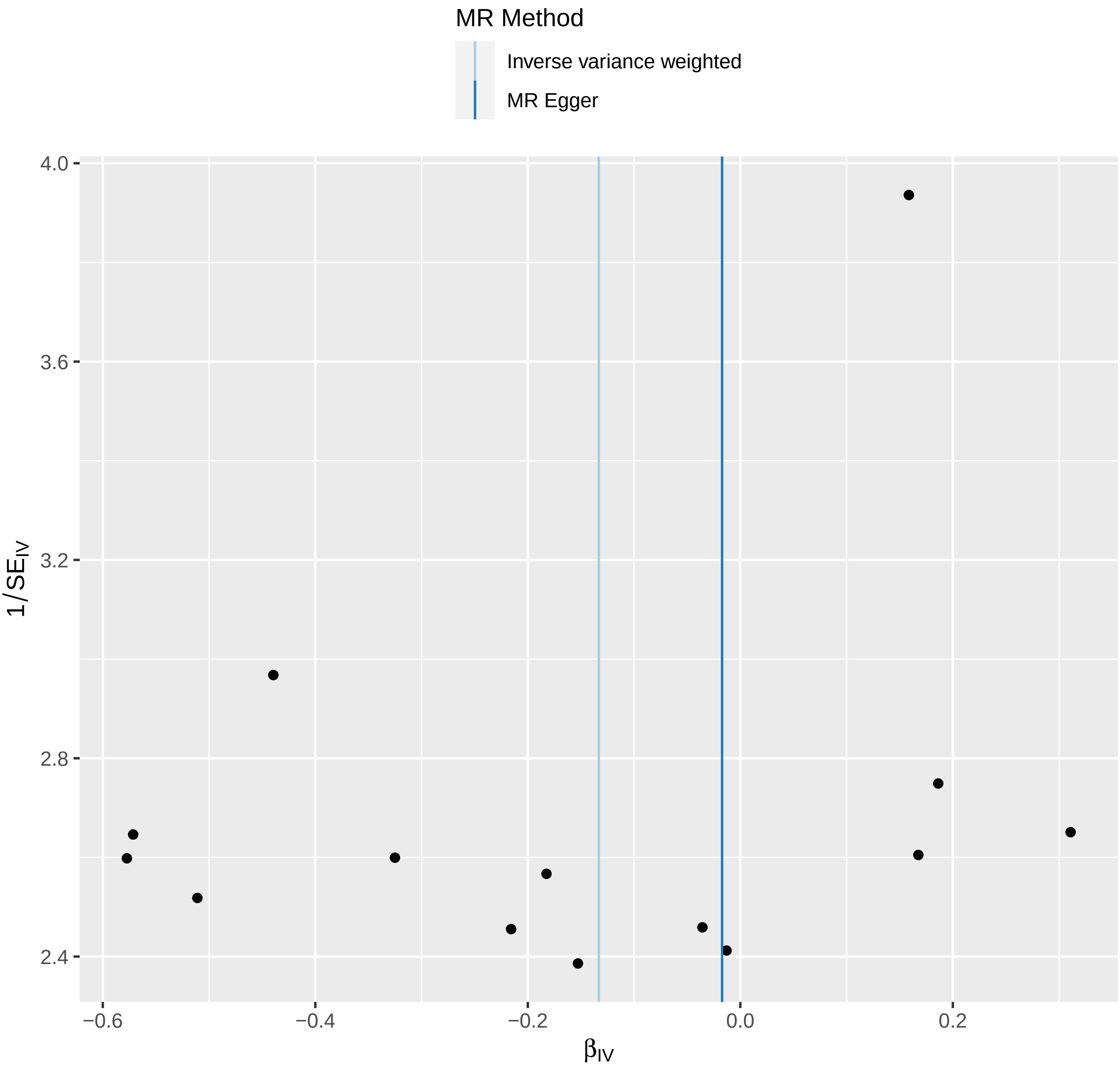

Batch 2−1087 : Gut microbiota abundance (genus Catenibacterium id.2153) on Choline/ethanolamine kinase
with a threshold of 1e−05

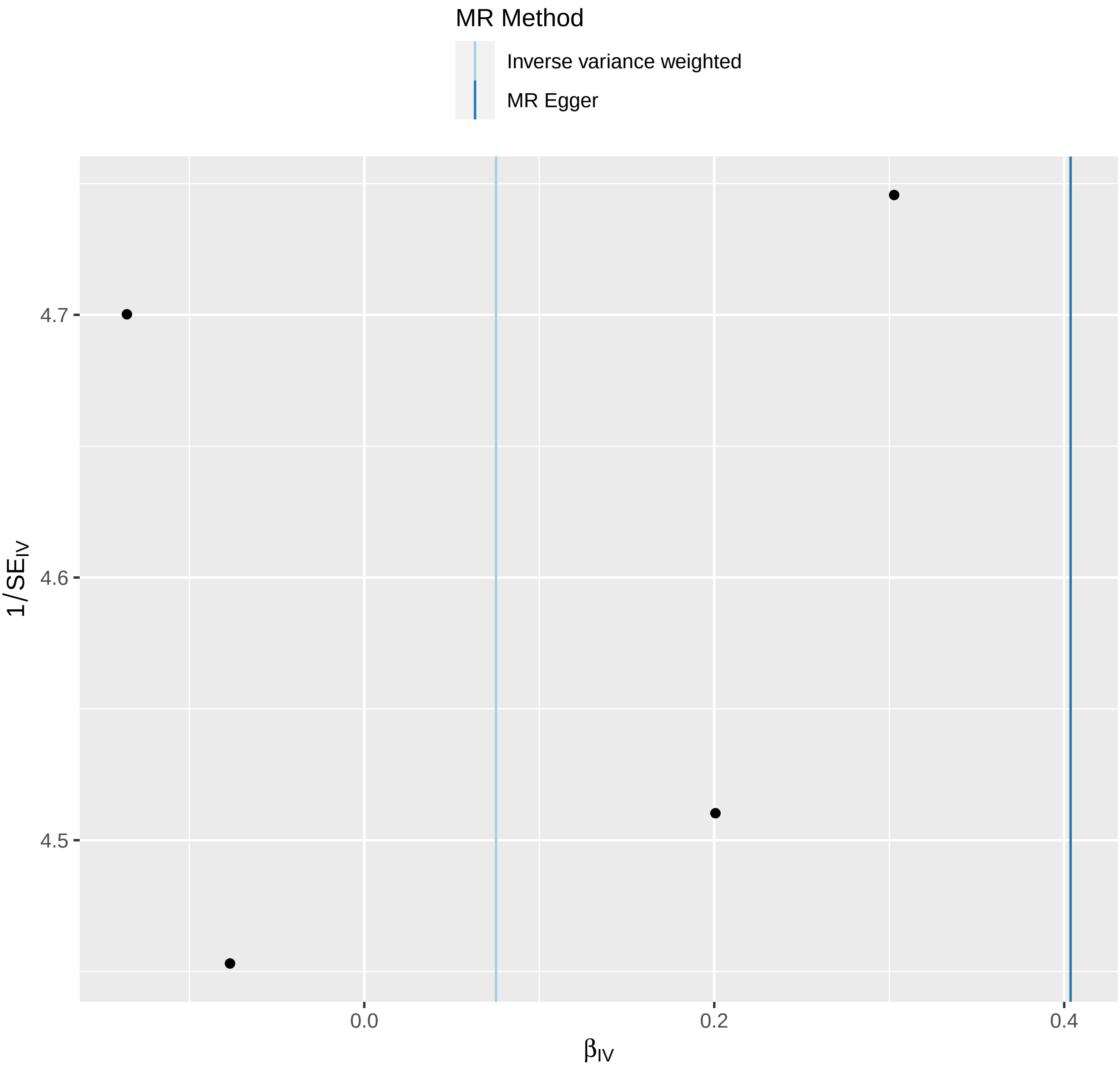

Batch 2−1088 : Gut microbiota abundance (genus Parasutterella id.2892) on Choline/ethanolamine kinase
with a threshold of 1e−05

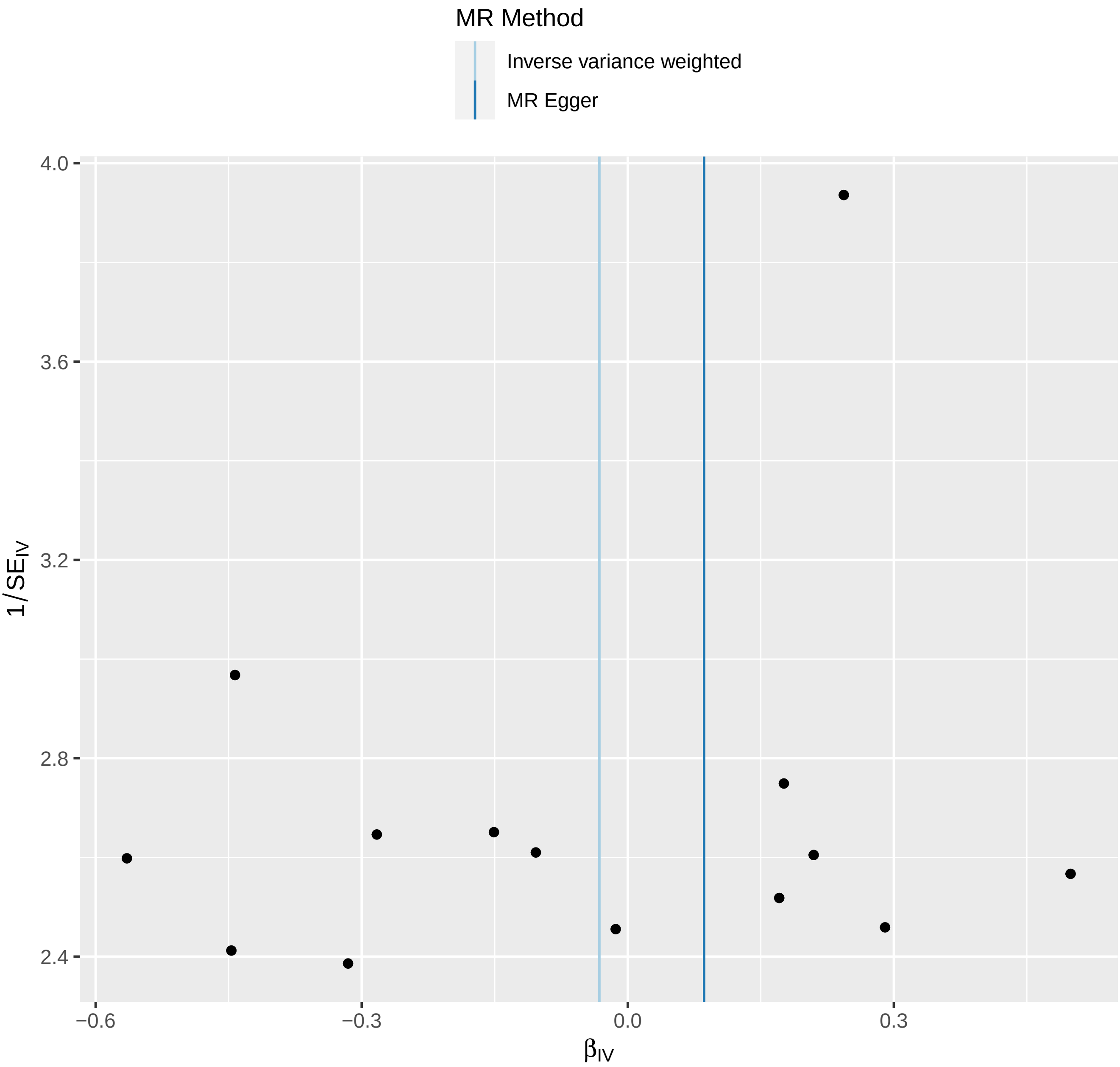

Batch 2−1089 : Gut microbiota abundance (genus Catenibacterium id.2153) on Neural cell adhesion molecule L1−like protein
with a threshold of 1e−05

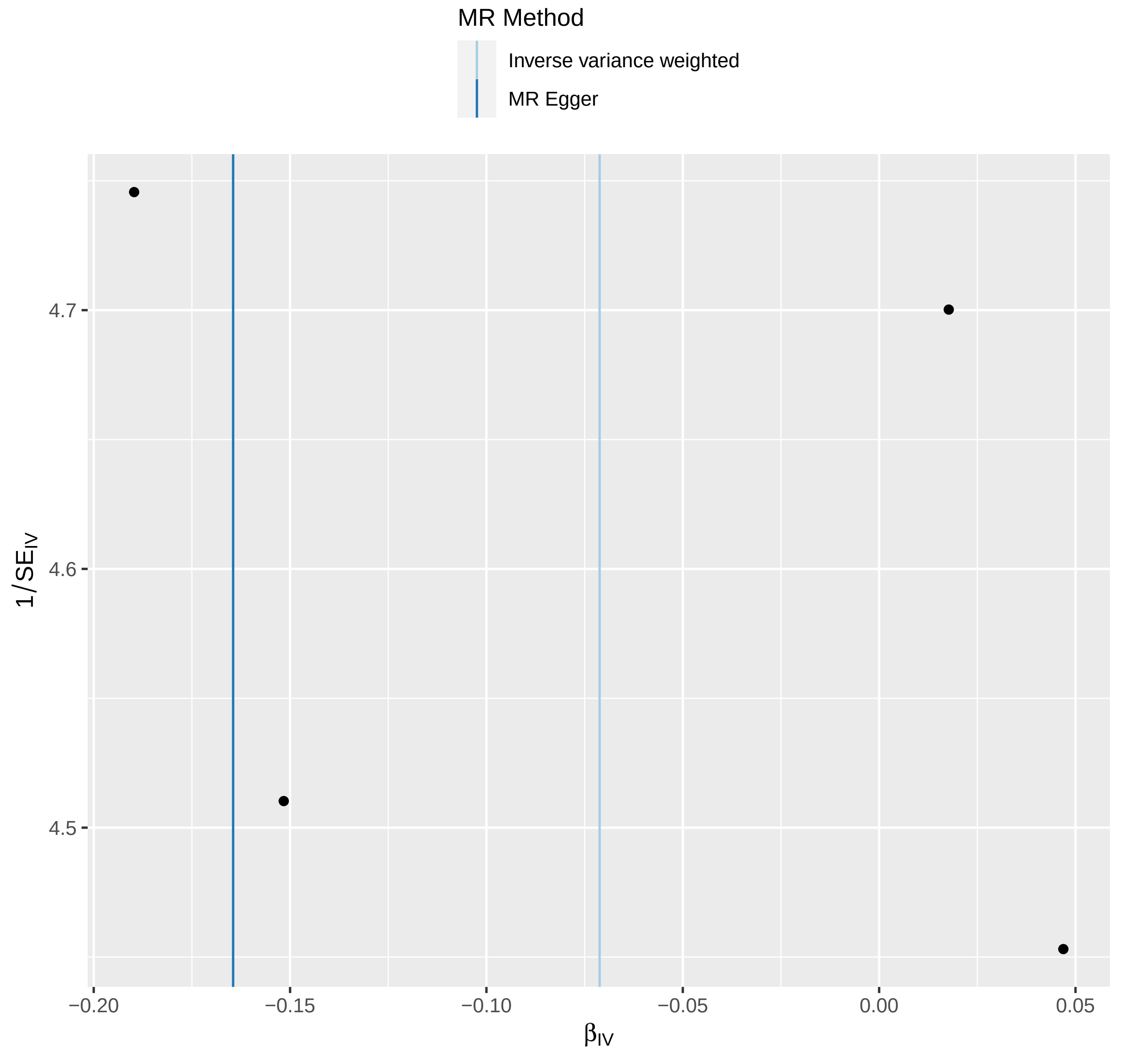

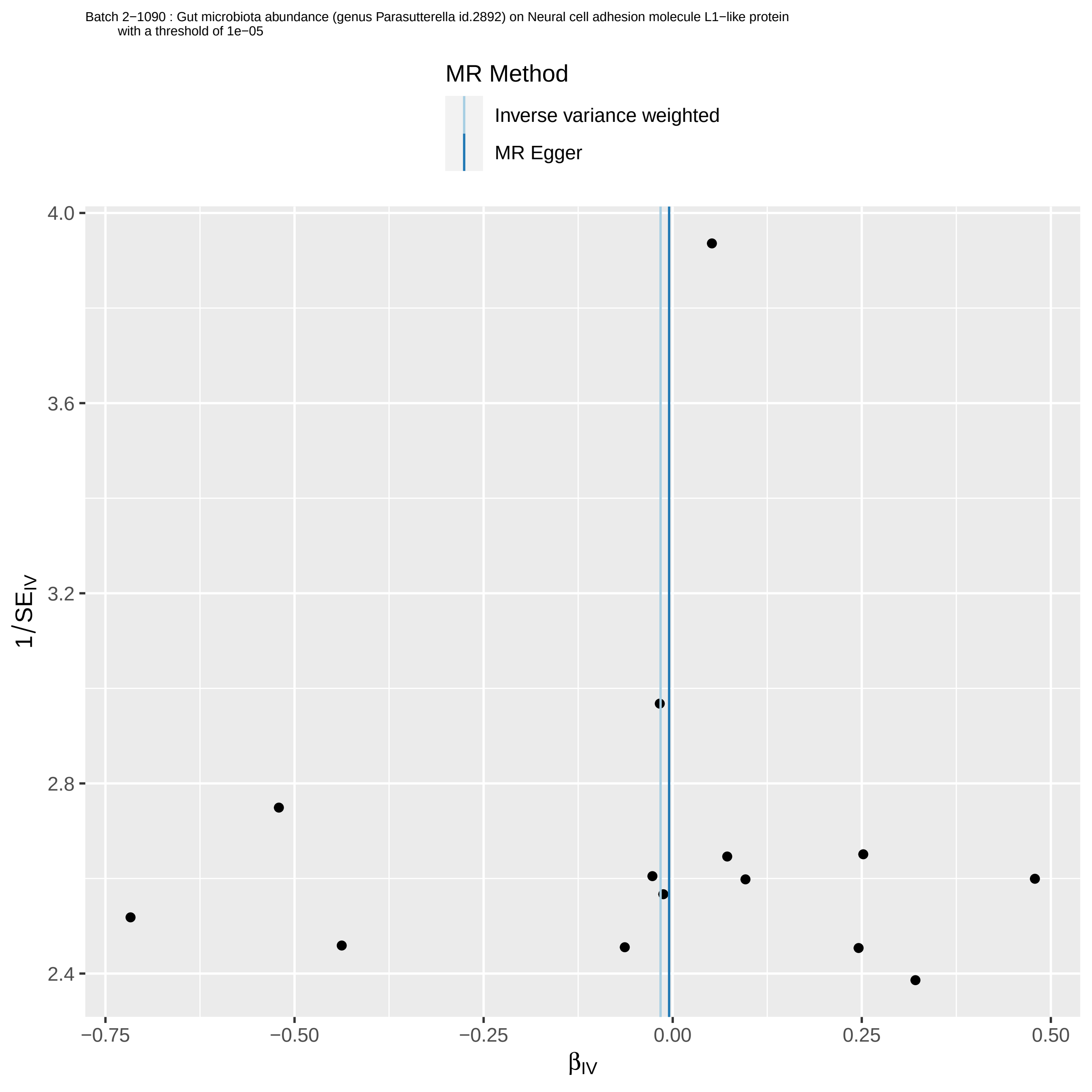
Batch 2−1090 : Gut microbiota abundance (genus Parasutterella id.2892) on Neural cell adhesion molecule L1−like protein
with a threshold of 1e−05
MR Method
Inverse variance weighted
MR Egger
4.0
3.6
3.2
2.8
2.4
1/SE_IV
−0.75
−0.50
−0.25
0.00
0.25
0.50
β_IV

Batch 2−1091 : Gut microbiota abundance (genus Catenibacterium id.2153) on Calcineurin B homologous protein 1
with a threshold of 1e−05

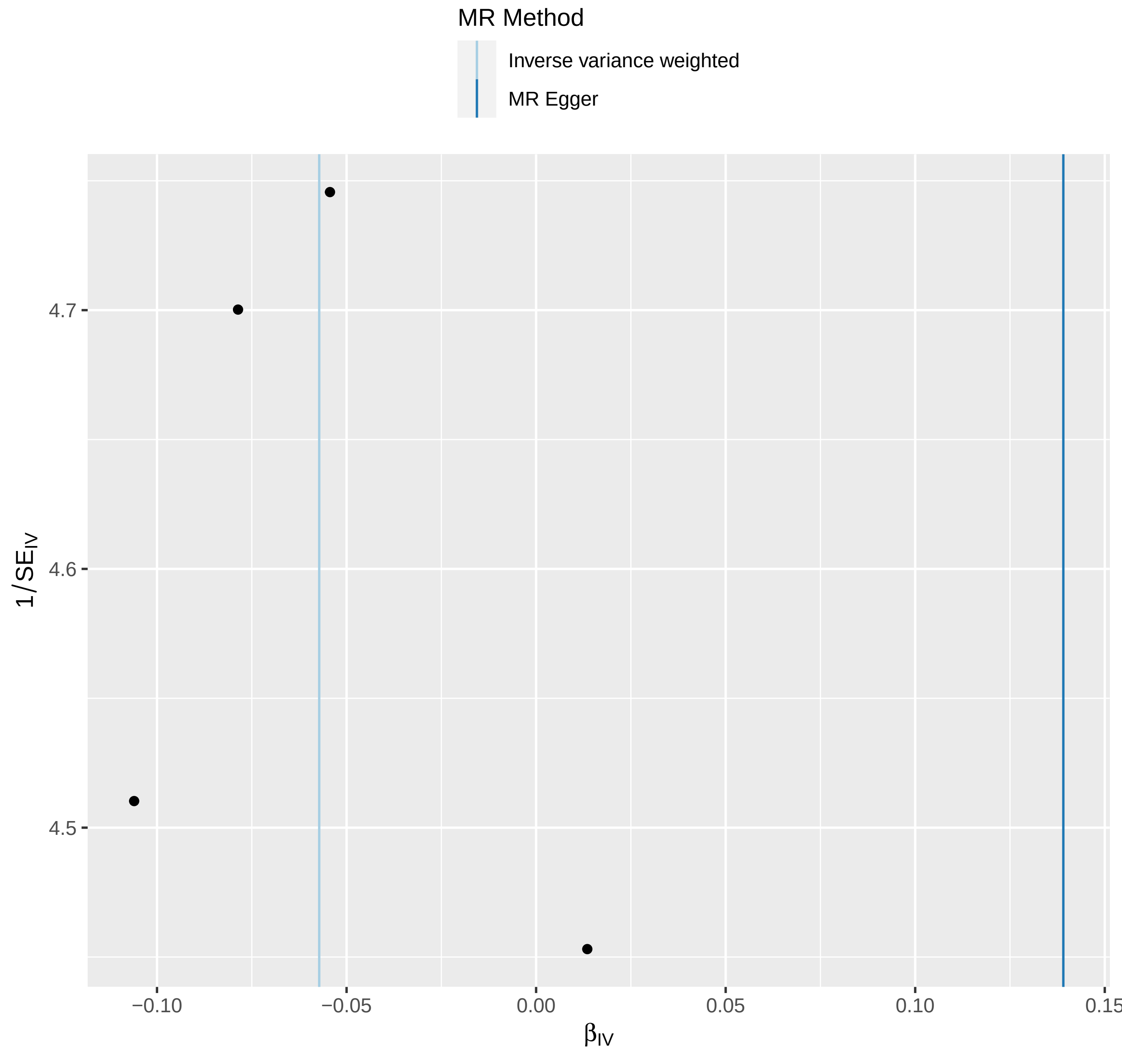

Batch 2−1092 : Gut microbiota abundance (genus Parasutterella id.2892) on Calcineurin B homologous protein 1
with a threshold of 1e−05

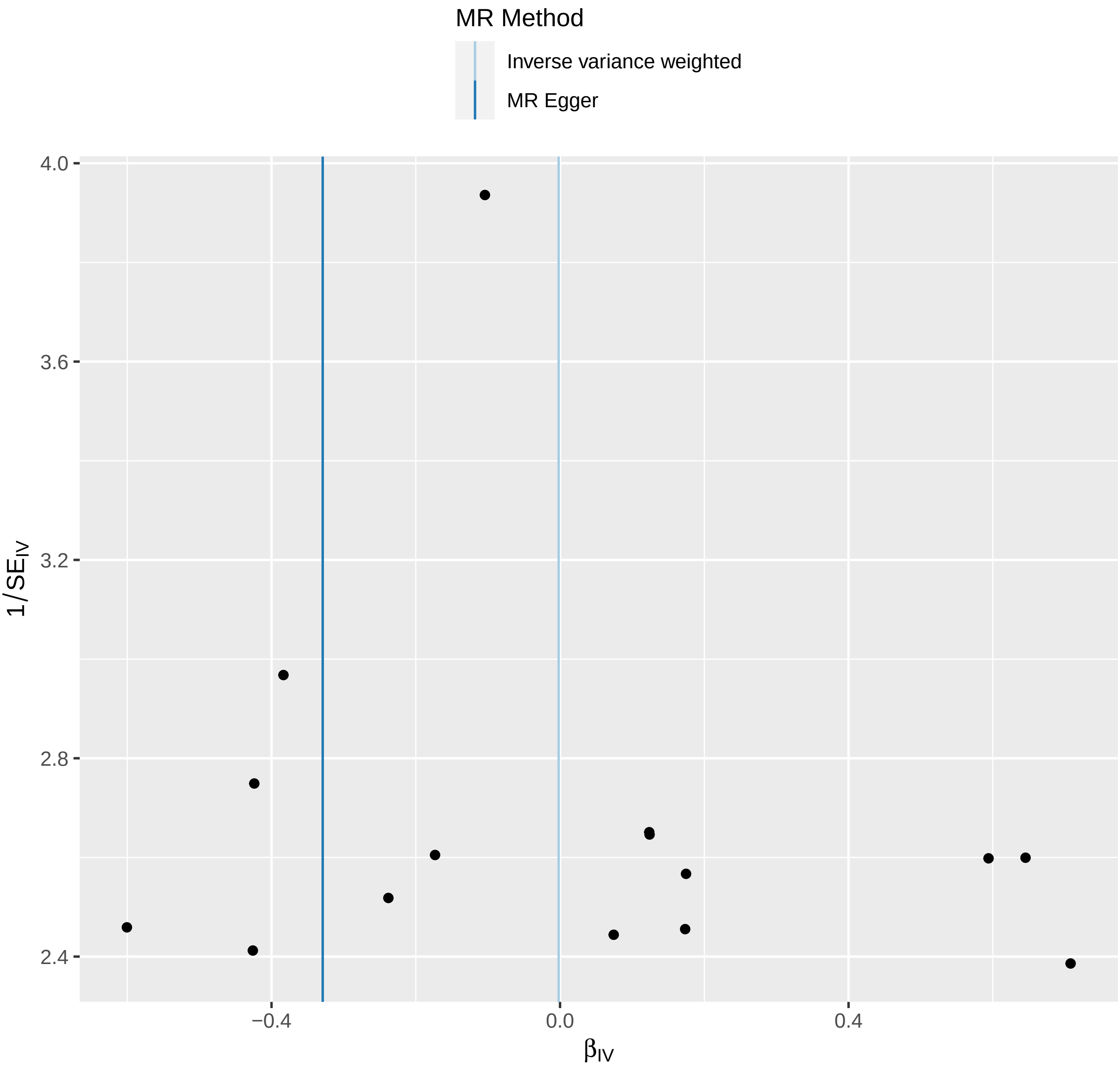

Batch 2−1093 : Gut microbiota abundance (genus Catenibacterium id.2153) on Chordin
with a threshold of 1e−05

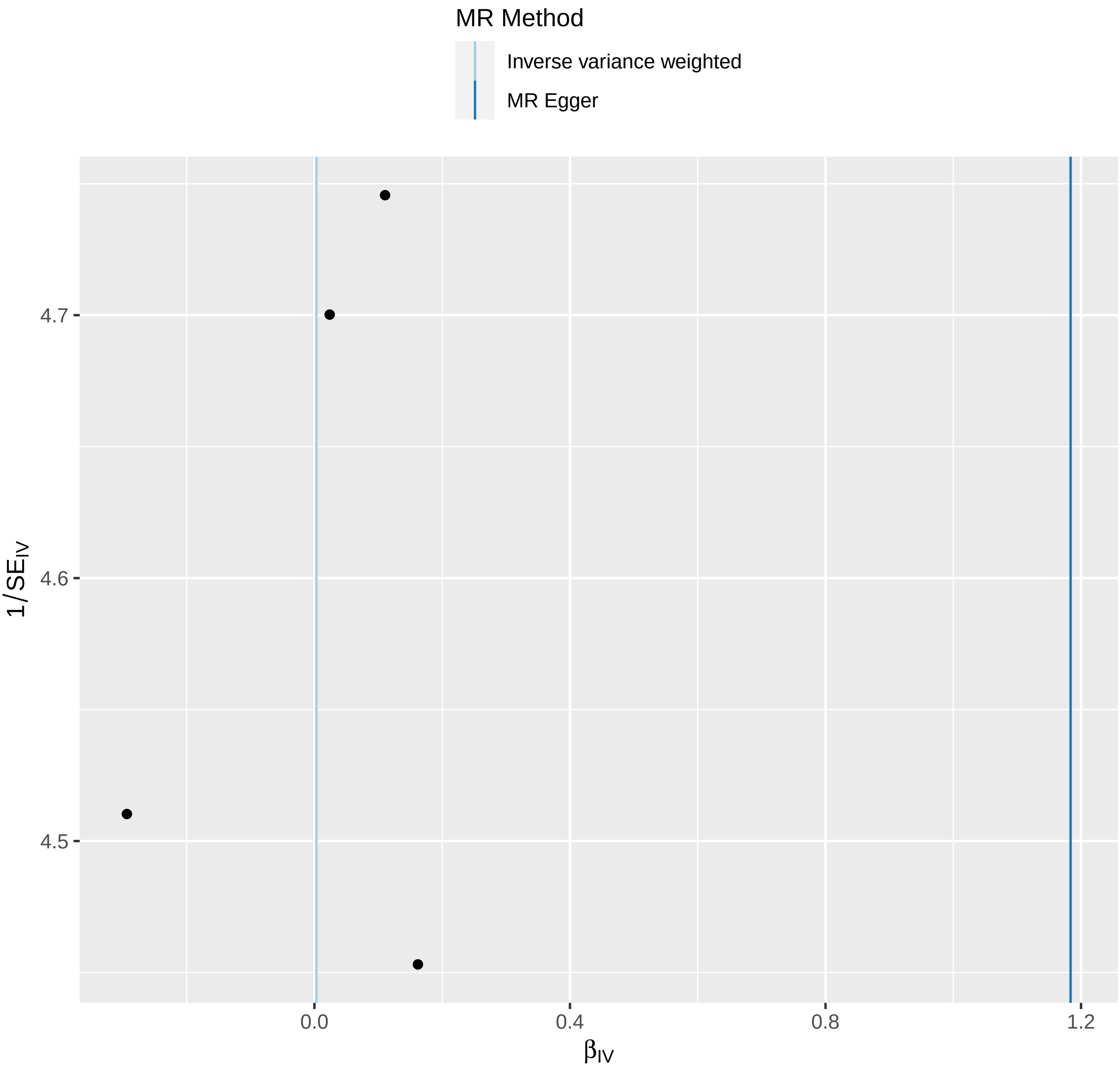

Batch 2−1094 : Gut microbiota abundance (genus Parasutterella id.2892) on Chordin
with a threshold of 1e−05

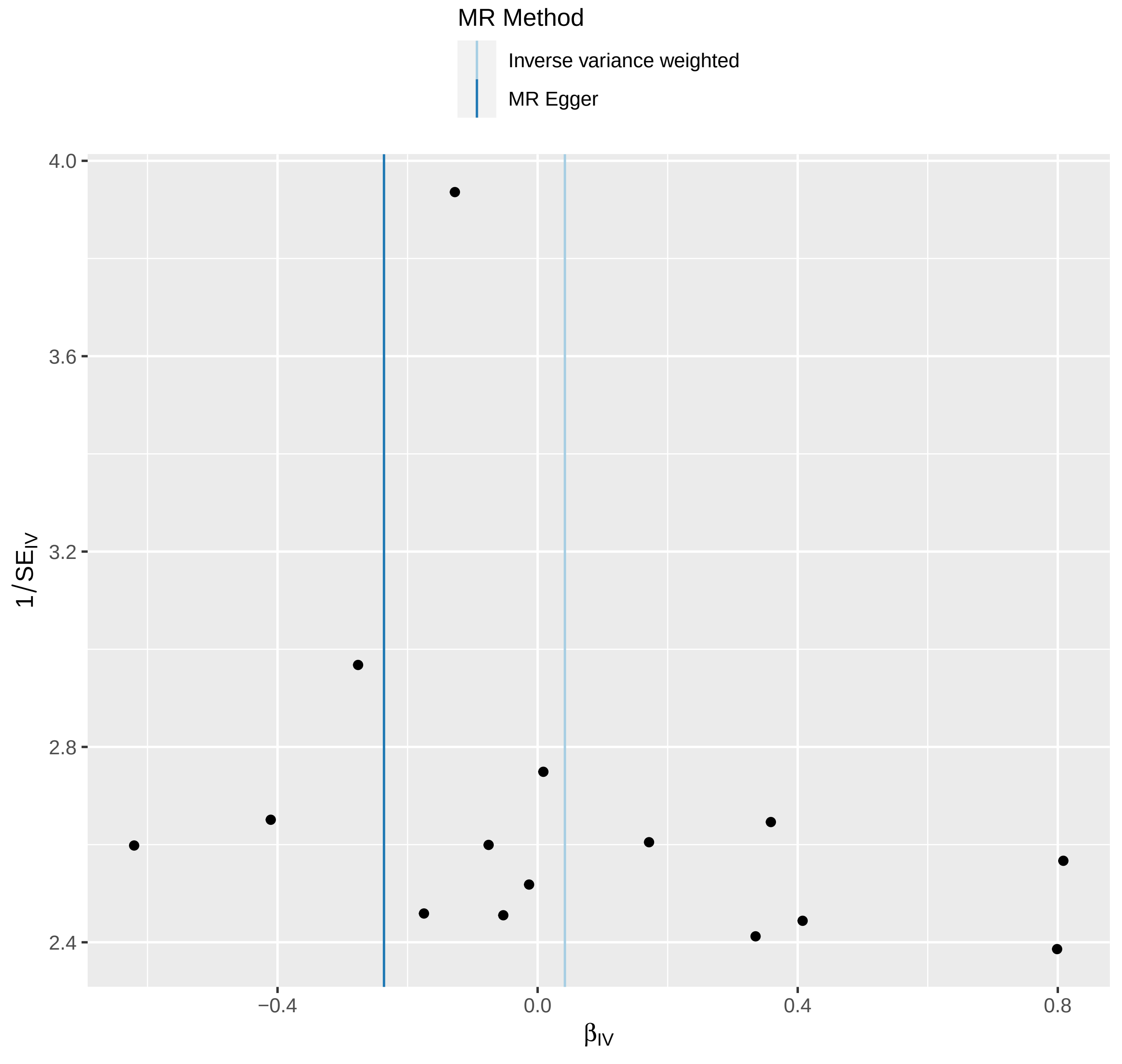

Batch 2−1095 : Gut microbiota abundance (genus Catenibacterium id.2153) on Chordin−like protein 1
with a threshold of 1e−05

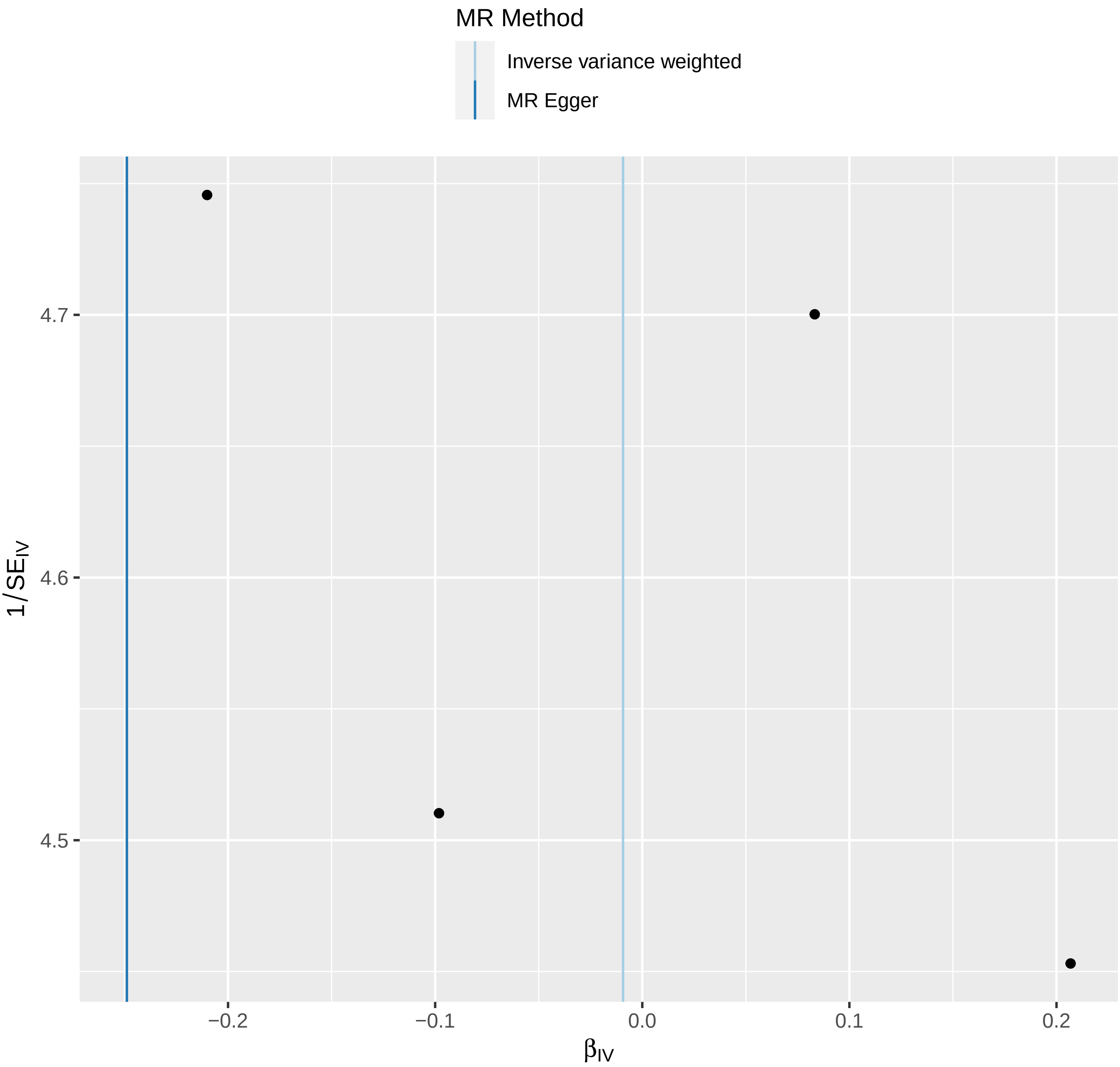

Batch 2−1096 : Gut microbiota abundance (genus Parasutterella id.2892) on Chordin−like protein 1
with a threshold of 1e−05

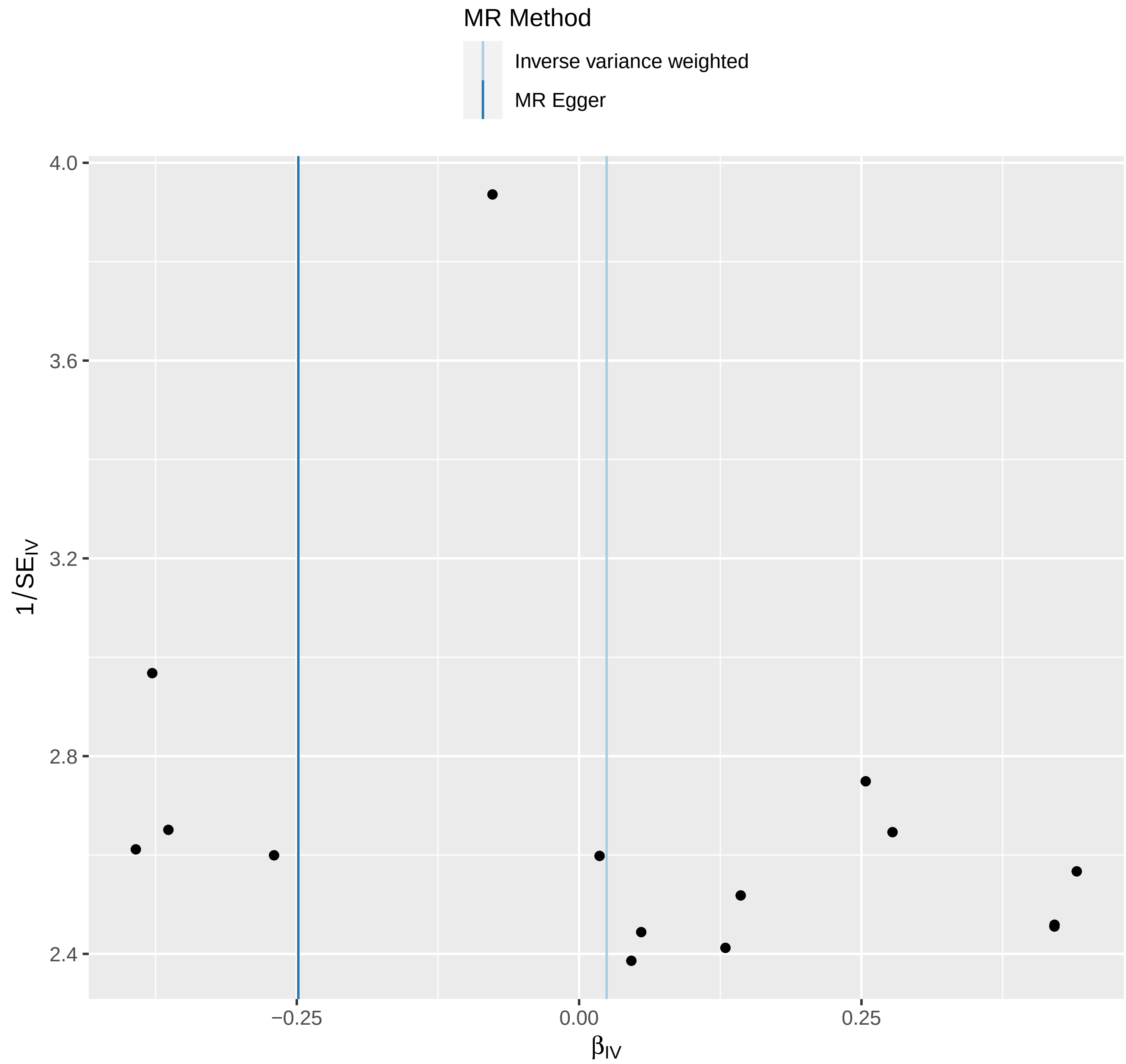

Batch 2−1097 : Gut microbiota abundance (genus Catenibacterium id.2153) on Chordin−like protein 2
with a threshold of 1e−05

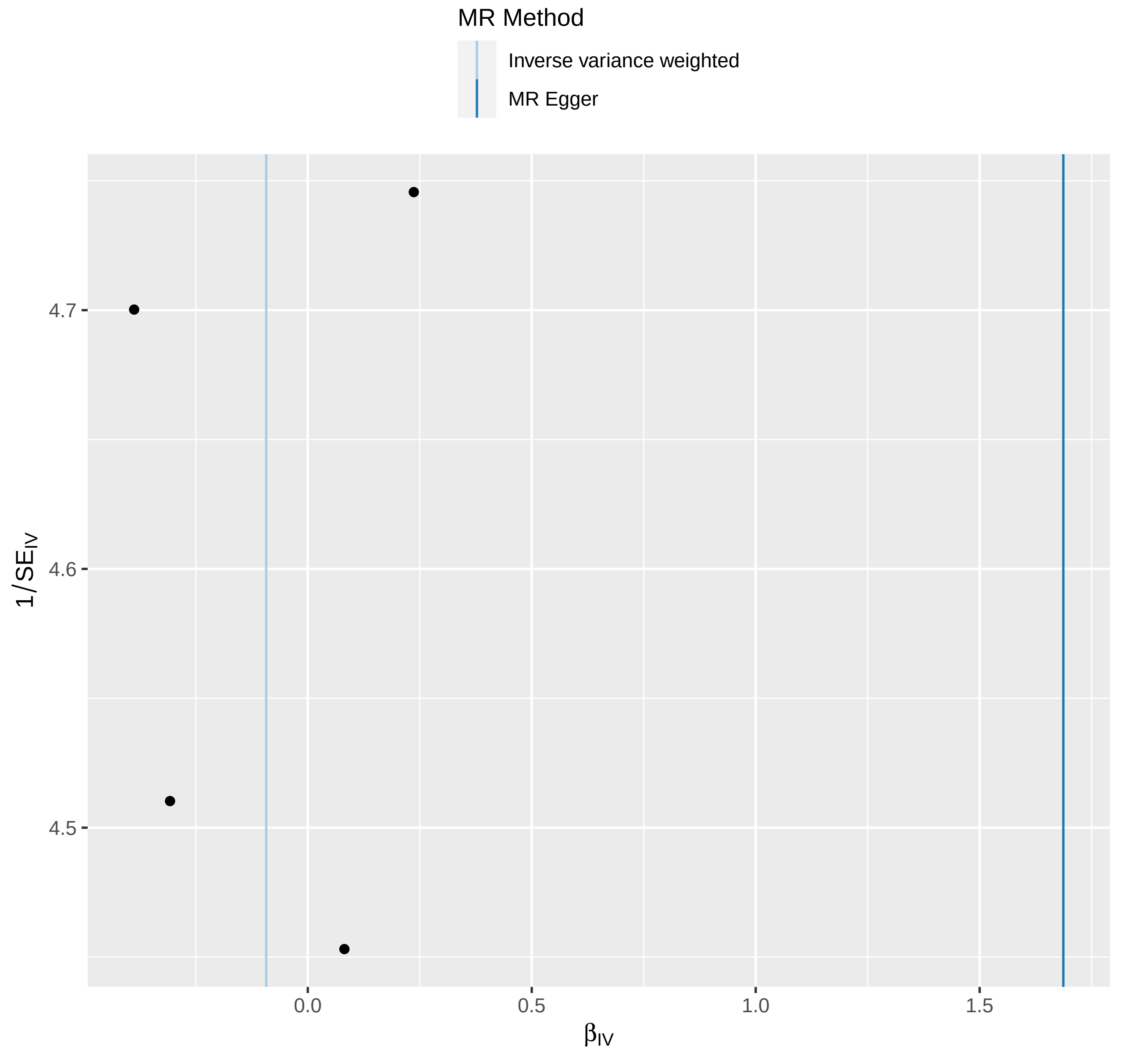

Batch 2−1098 : Gut microbiota abundance (genus Parasutterella id.2892) on Chordin−like protein 2
with a threshold of 1e−05

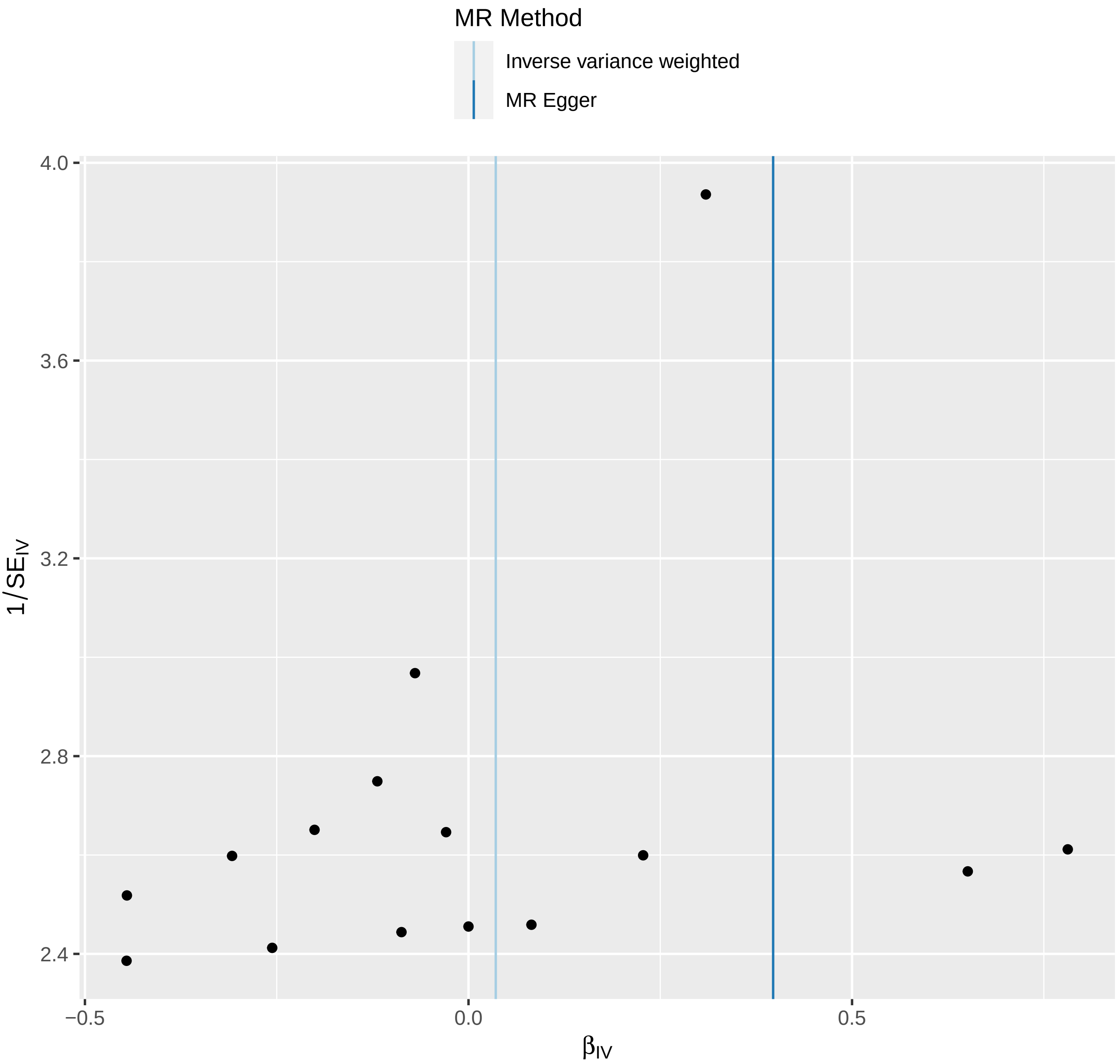

Batch 2−1099 : Gut microbiota abundance (genus Catenibacterium id.2153) on Carbohydrate sulfotransferase 11
with a threshold of 1e−05

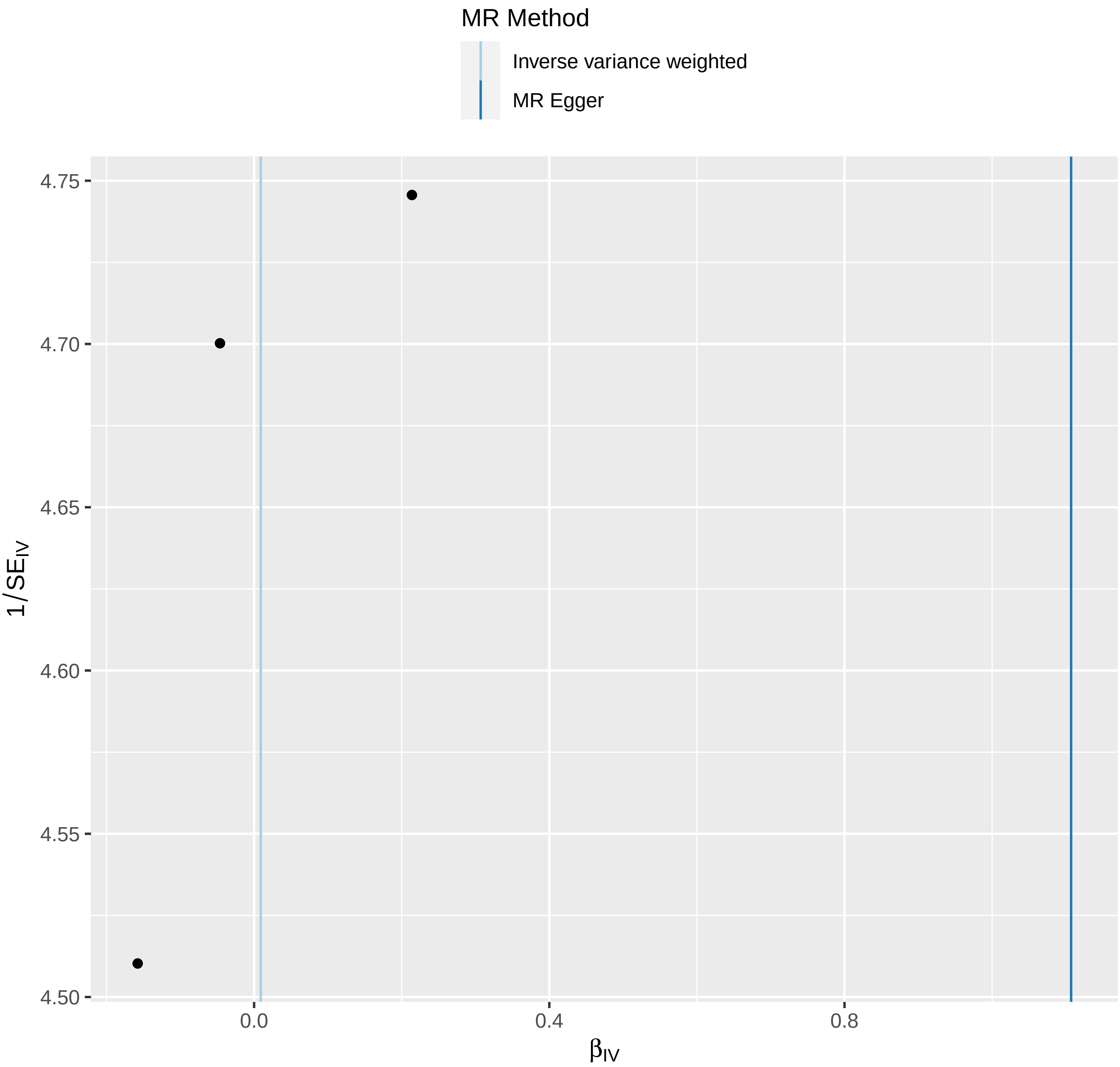

Batch 2−1100 : Gut microbiota abundance (genus Parasutterella id.2892) on Carbohydrate sulfotransferase 11
with a threshold of 1e−05

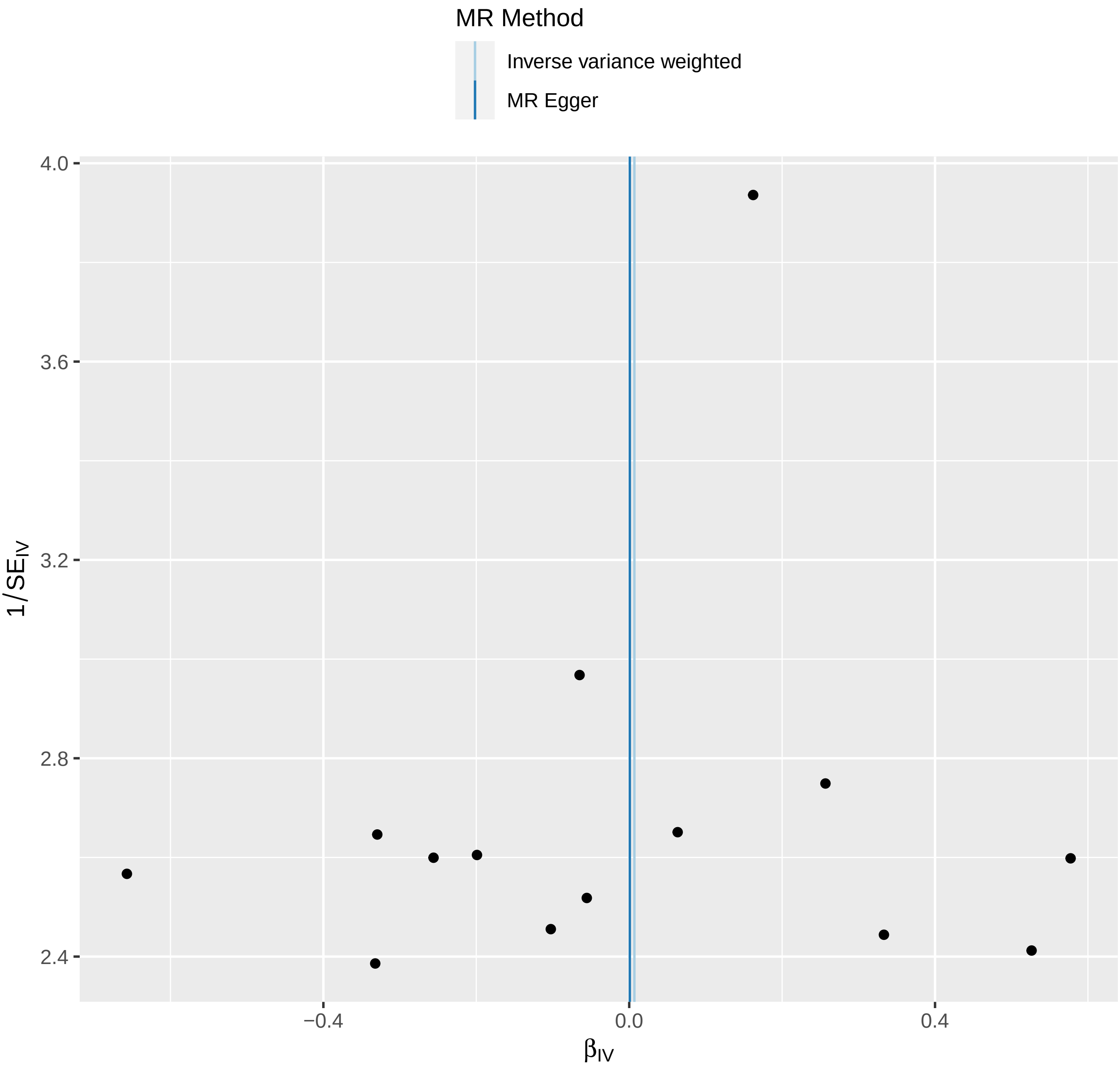

Batch 2−1101 : Gut microbiota abundance (genus Catenibacterium id.2153) on Carbohydrate sulfotransferase 14
with a threshold of 1e−05

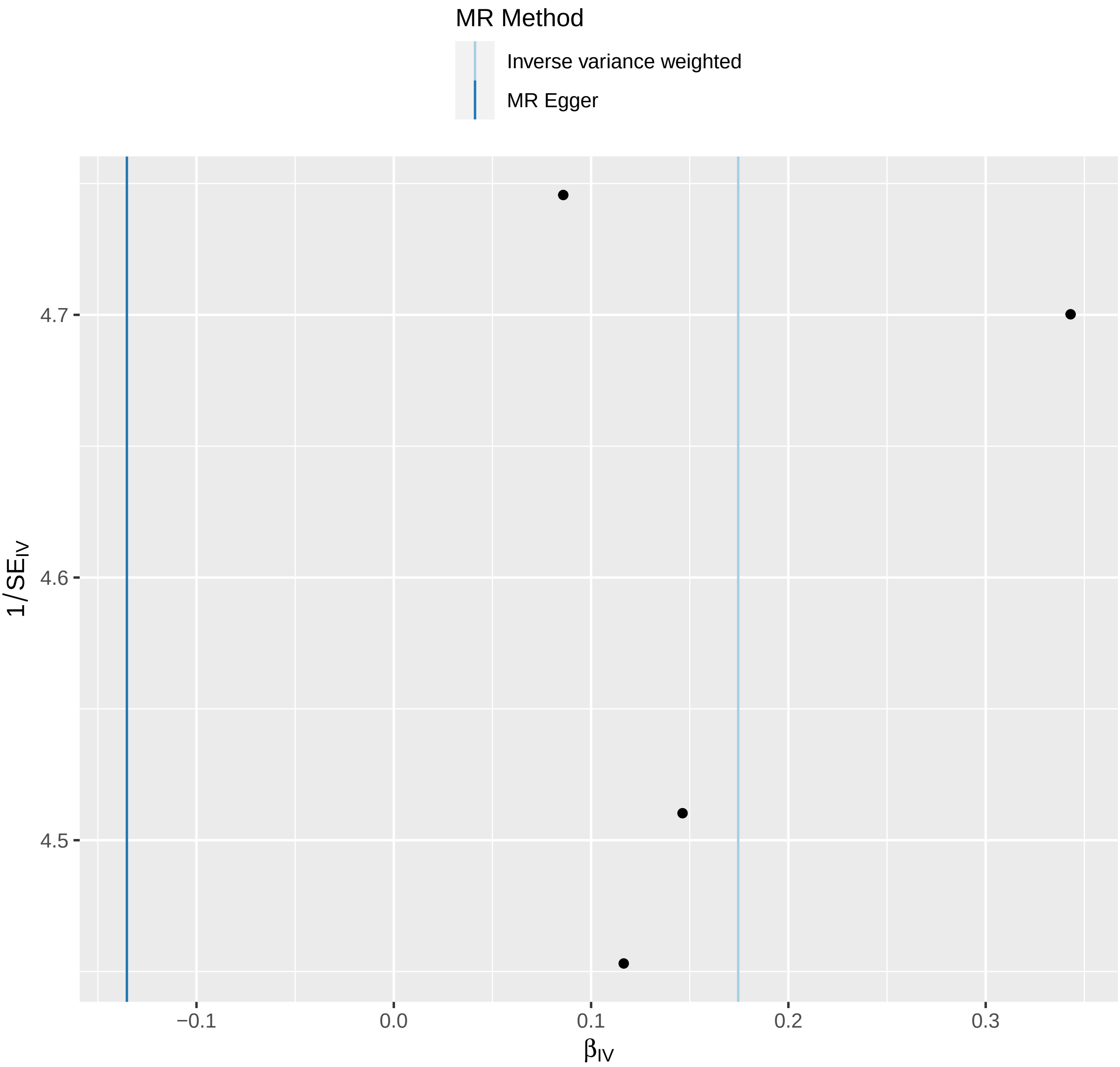

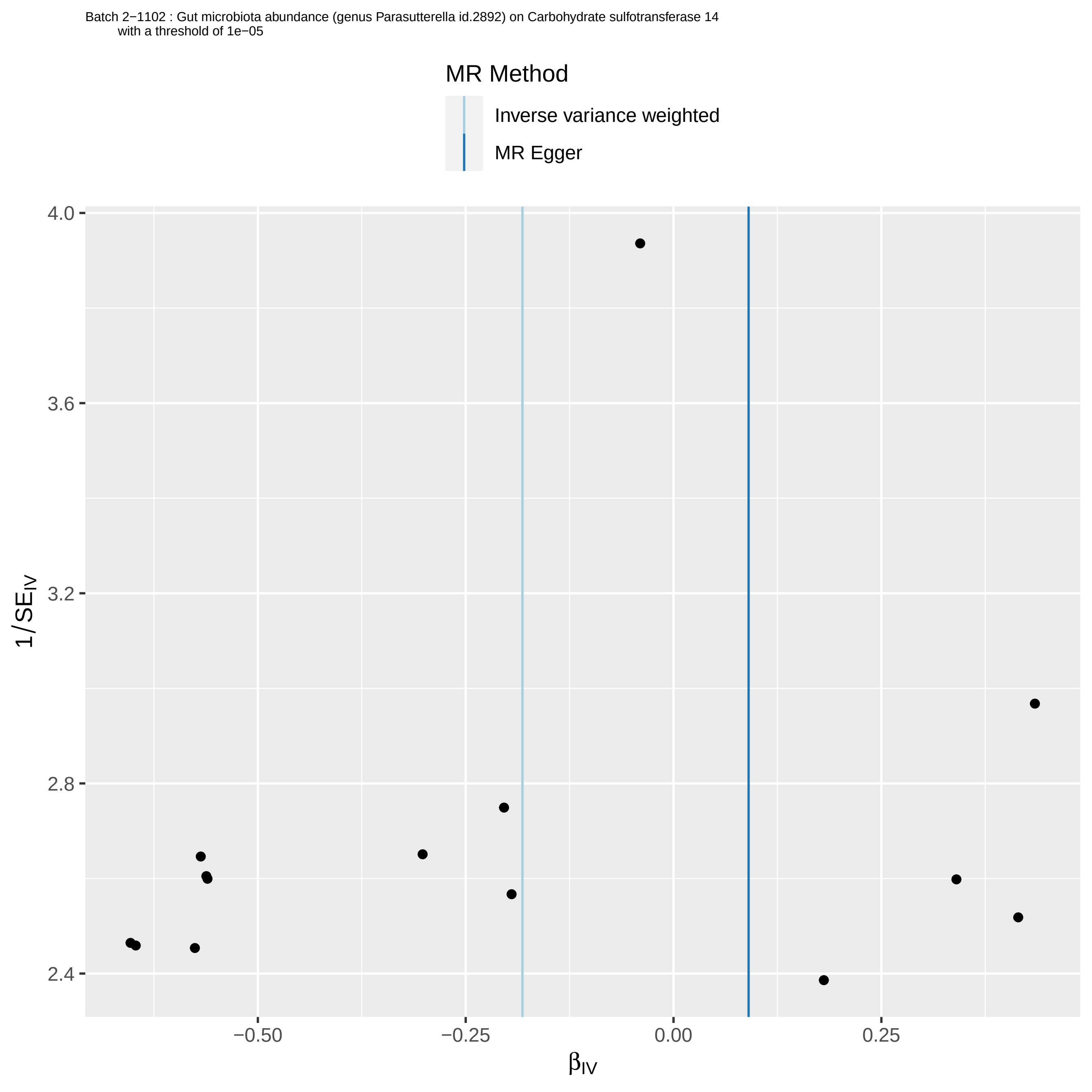
Batch 2−1102 : Gut microbiota abundance (genus Parasutterella id.2892) on Carbohydrate sulfotransferase 14
with a threshold of 1e−05
MR Method
Inverse variance weighted
MR Egger
4.0
3.6
3.2
2.8
2.4
1/SE_IV
−0.50
−0.25
0.00
0.25
β_IV

Batch 2−1103 : Gut microbiota abundance (genus Catenibacterium id.2153) on Carbohydrate sulfotransferase 15
with a threshold of 1e−05

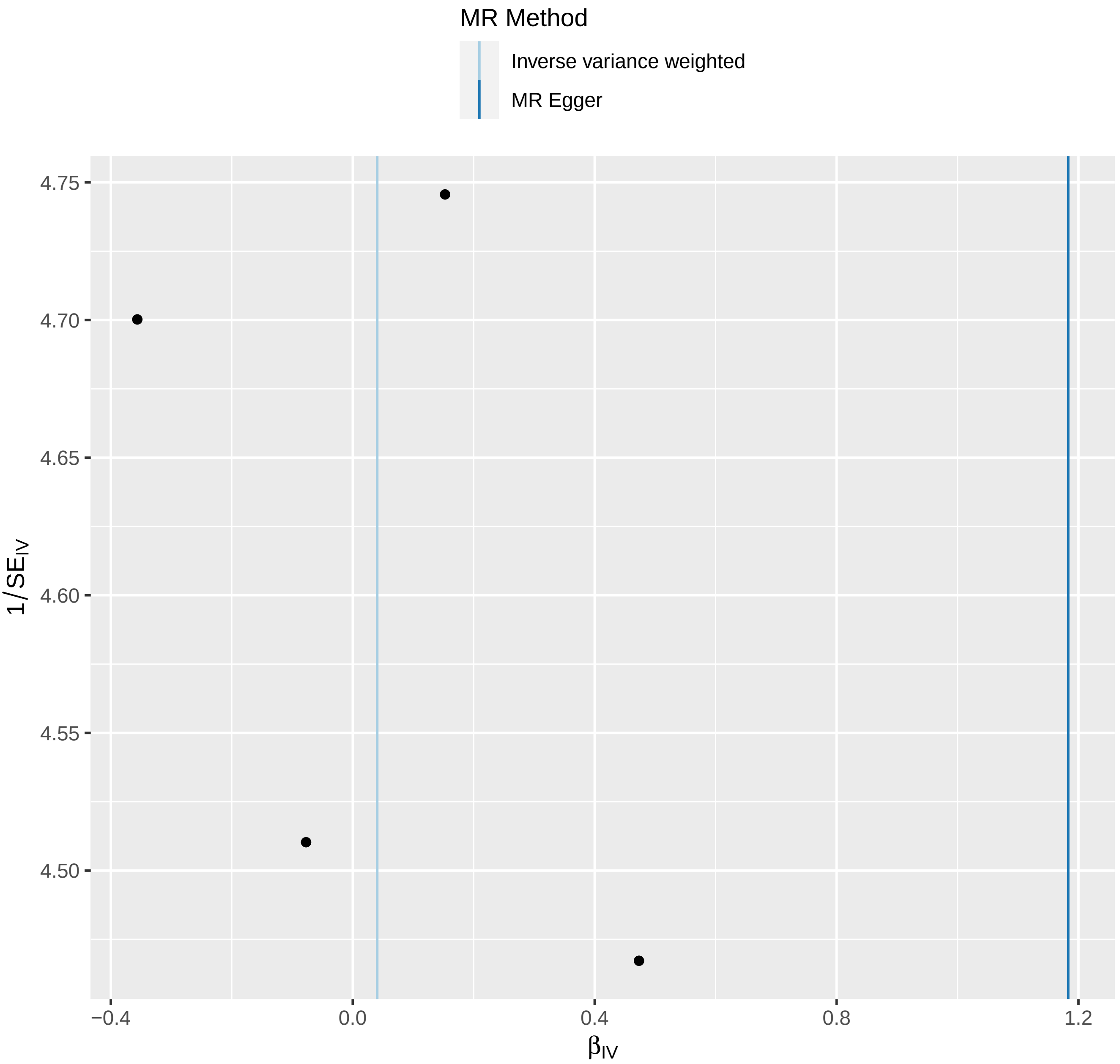

Batch 2−1104 : Gut microbiota abundance (genus Parasutterella id.2892) on Carbohydrate sulfotransferase 15
with a threshold of 1e−05

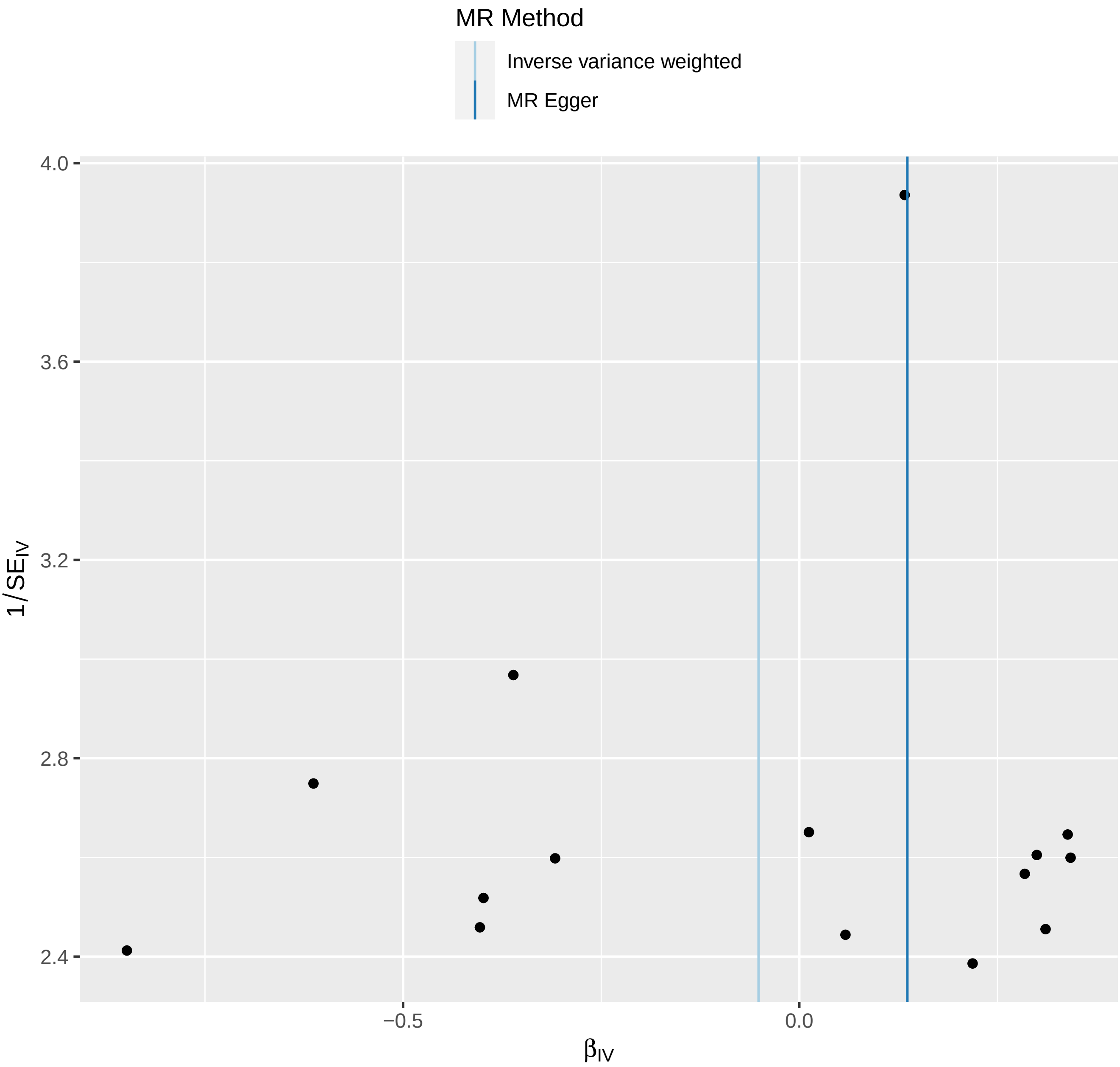

Batch 2−1105 : Gut microbiota abundance (genus Catenibacterium id.2153) on Carbohydrate sulfotransferase 15
with a threshold of 1e−05

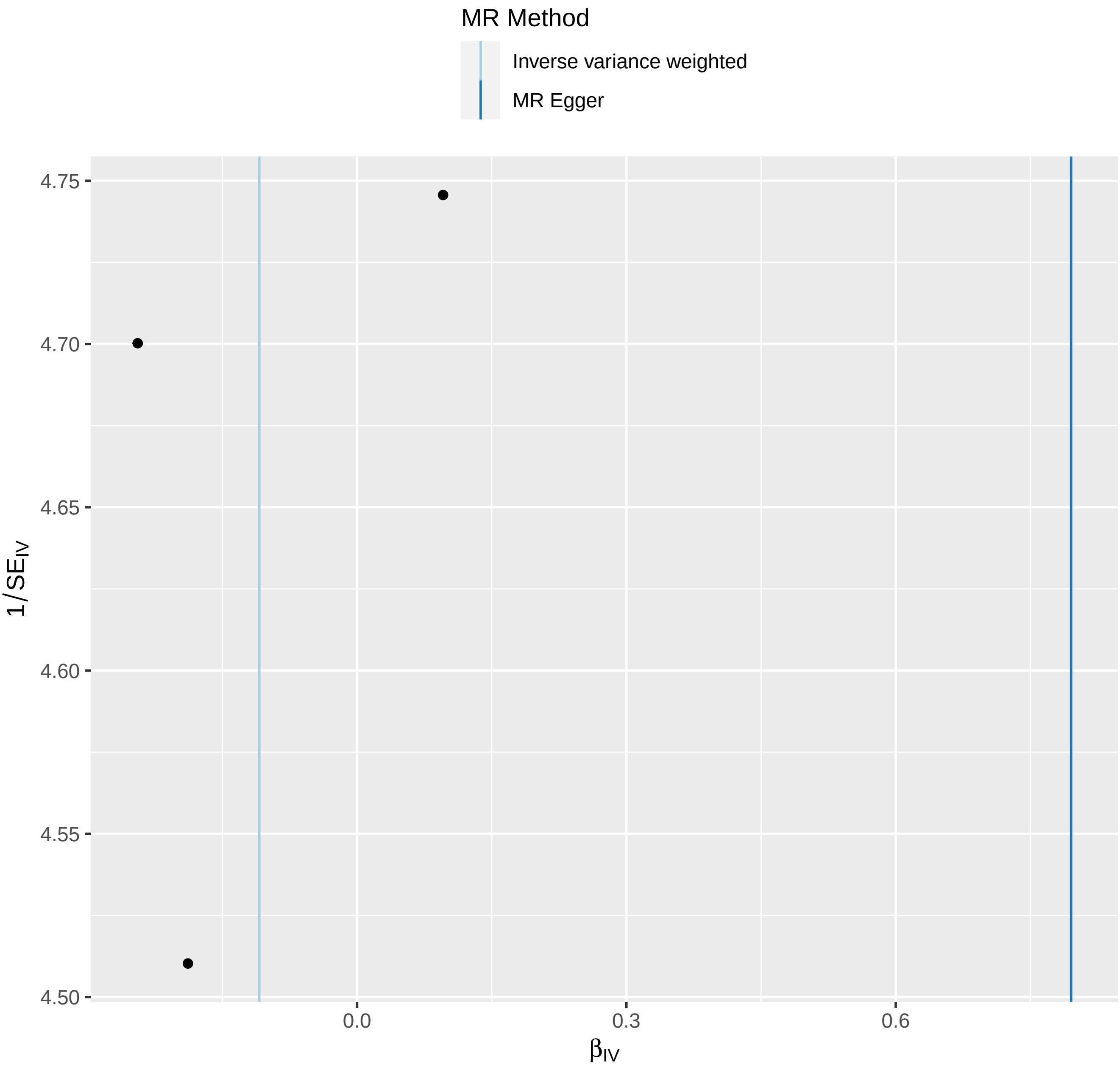

Batch 2−1106 : Gut microbiota abundance (genus Parasutterella id.2892) on Carbohydrate sulfotransferase 15
with a threshold of 1e−05

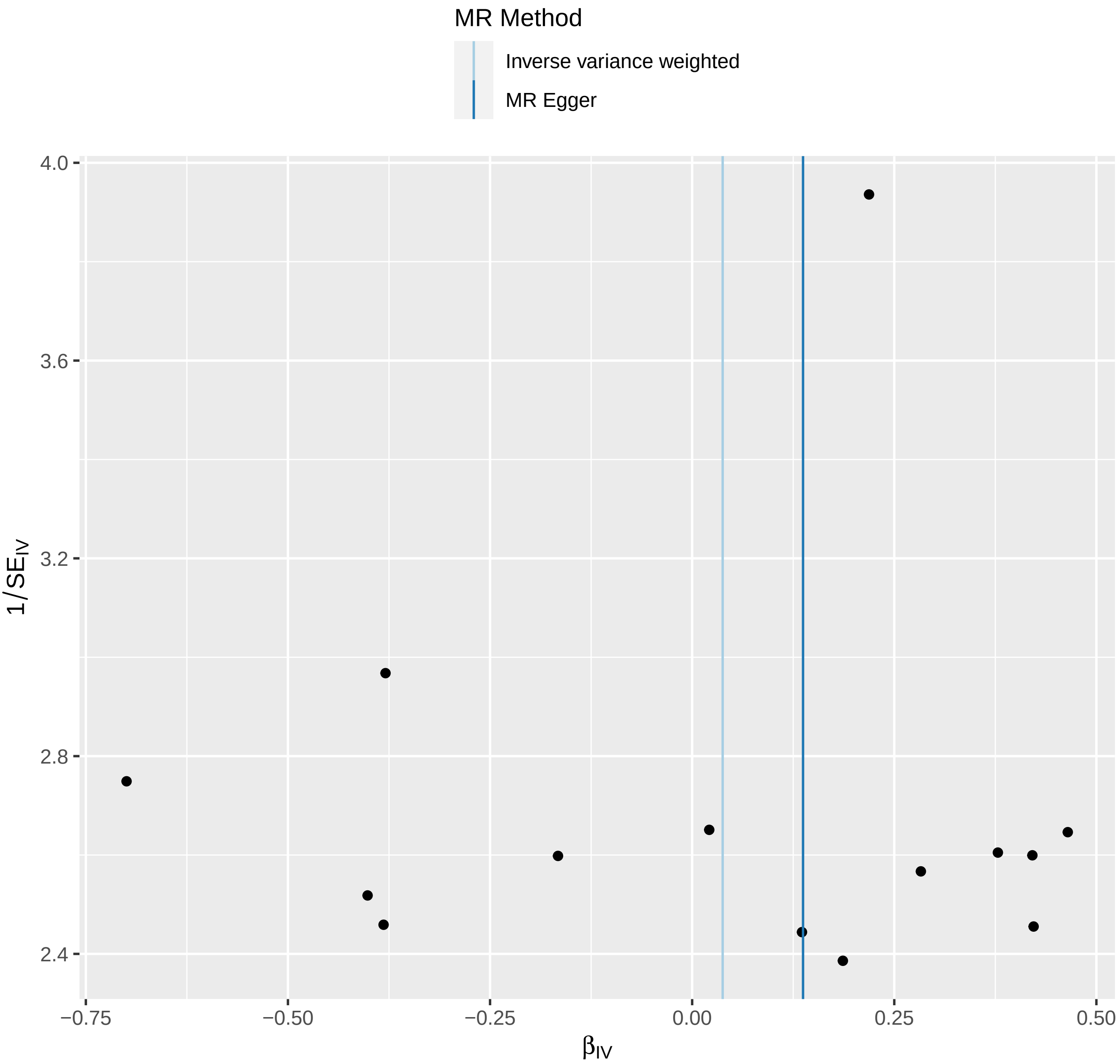

Batch 2−1107 : Gut microbiota abundance (genus Catenibacterium id.2153) on Carbohydrate sulfotransferase 1
with a threshold of 1e−05

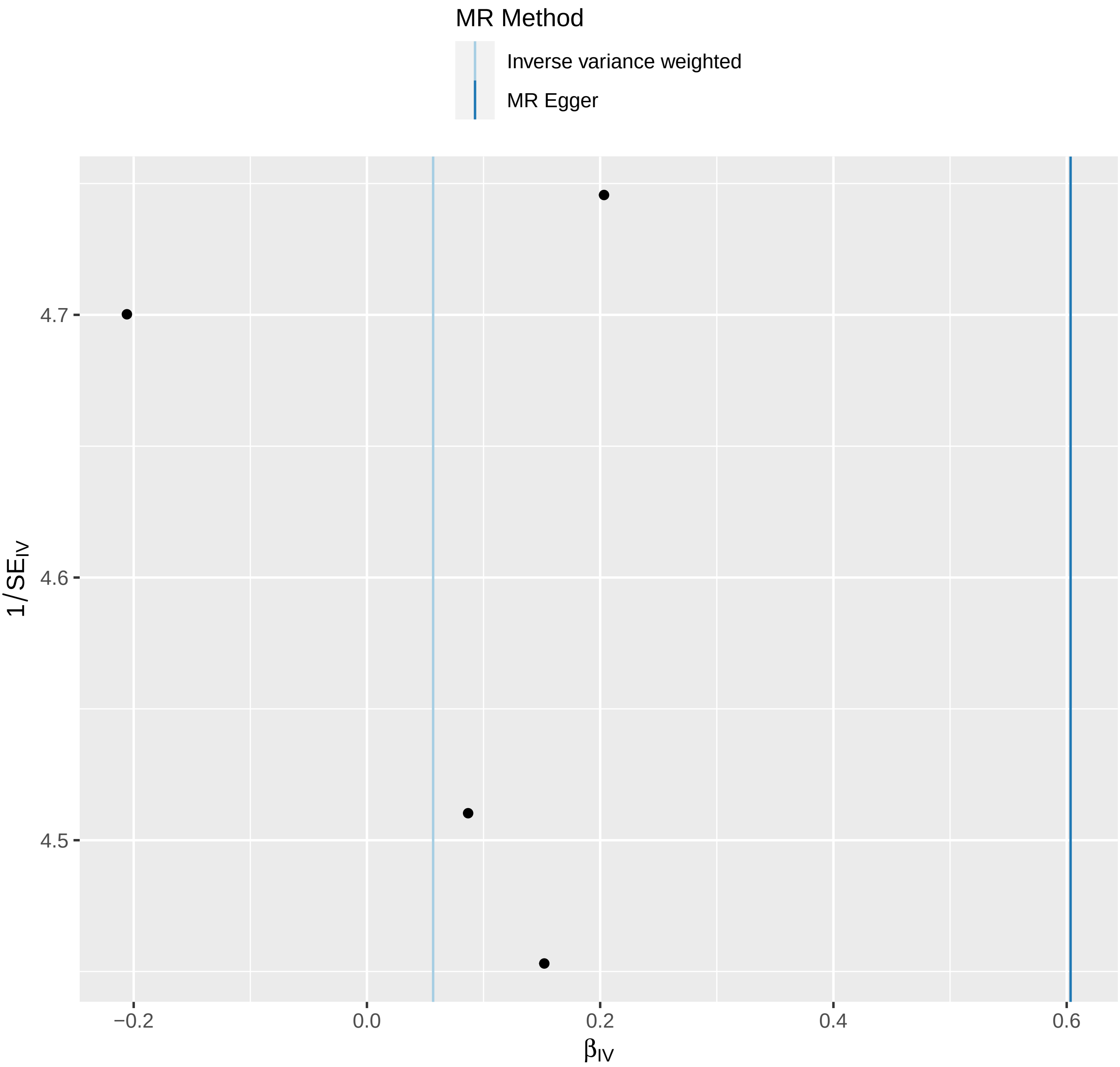

Batch 2−1108 : Gut microbiota abundance (genus Parasutterella id.2892) on Carbohydrate sulfotransferase 1
with a threshold of 1e−05

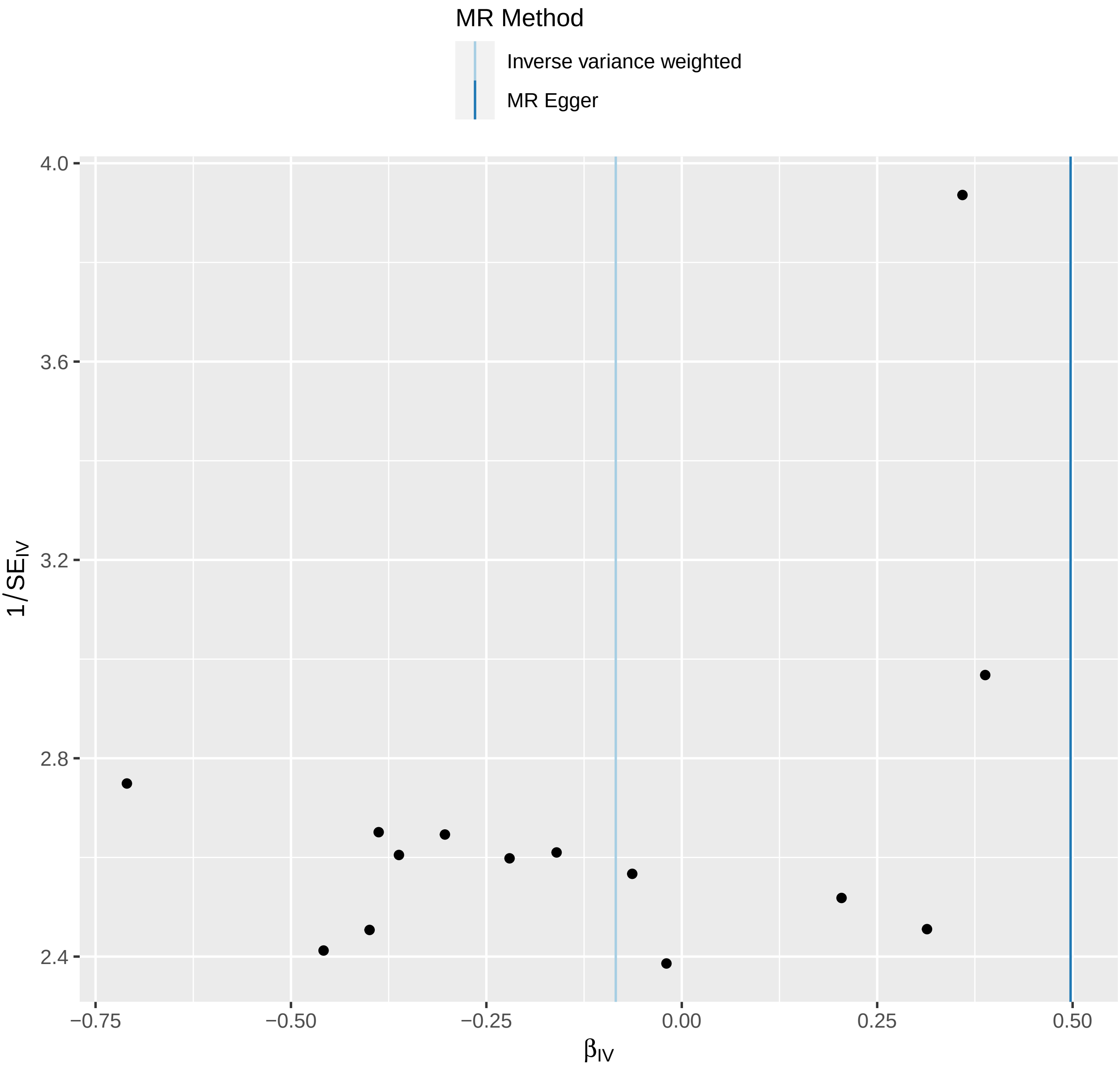

Batch 2−1109 : Gut microbiota abundance (genus Catenibacterium id.2153) on Carbohydrate sulfotransferase 2
with a threshold of 1e−05

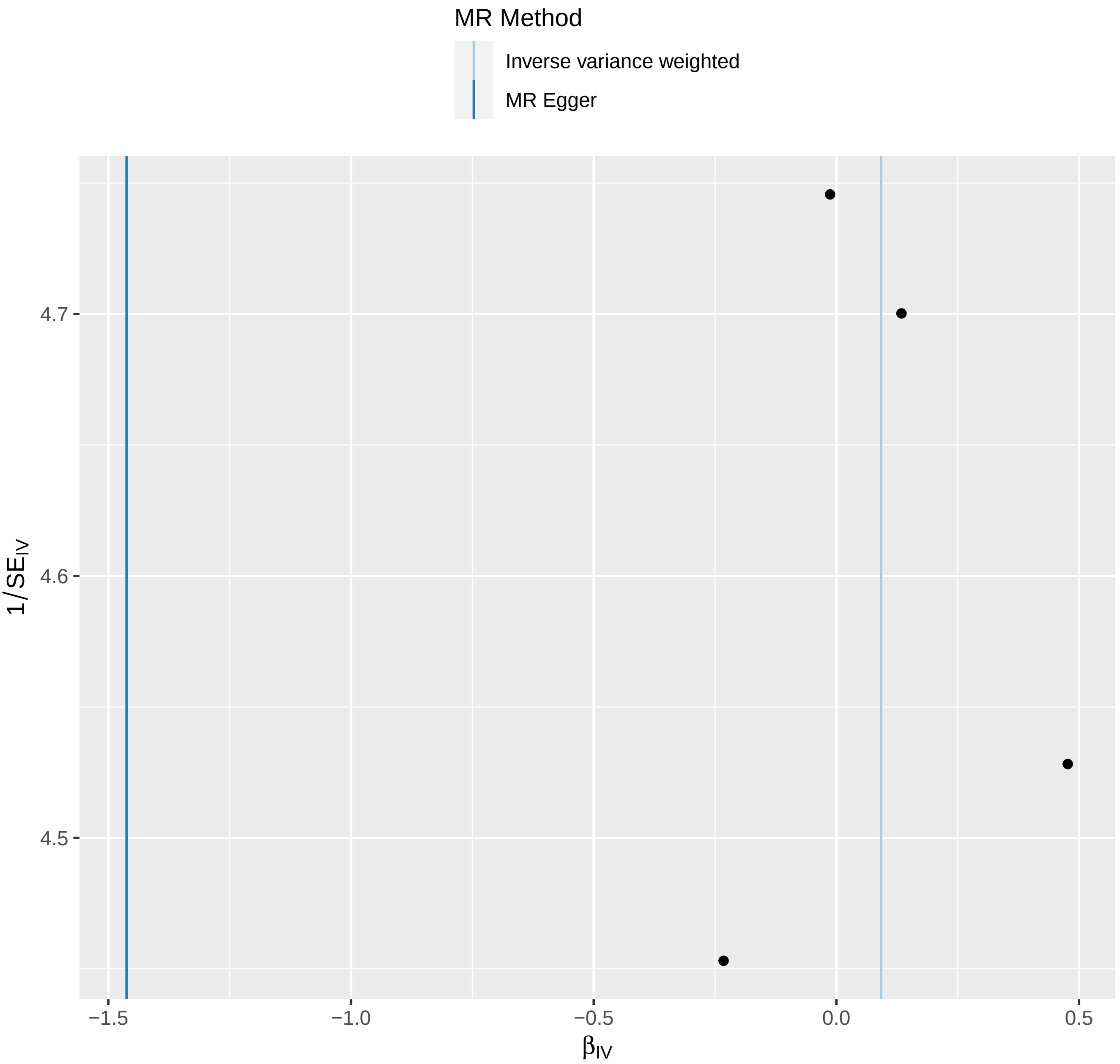

Batch 2−1110 : Gut microbiota abundance (genus Parasutterella id.2892) on Carbohydrate sulfotransferase 2
with a threshold of 1e−05

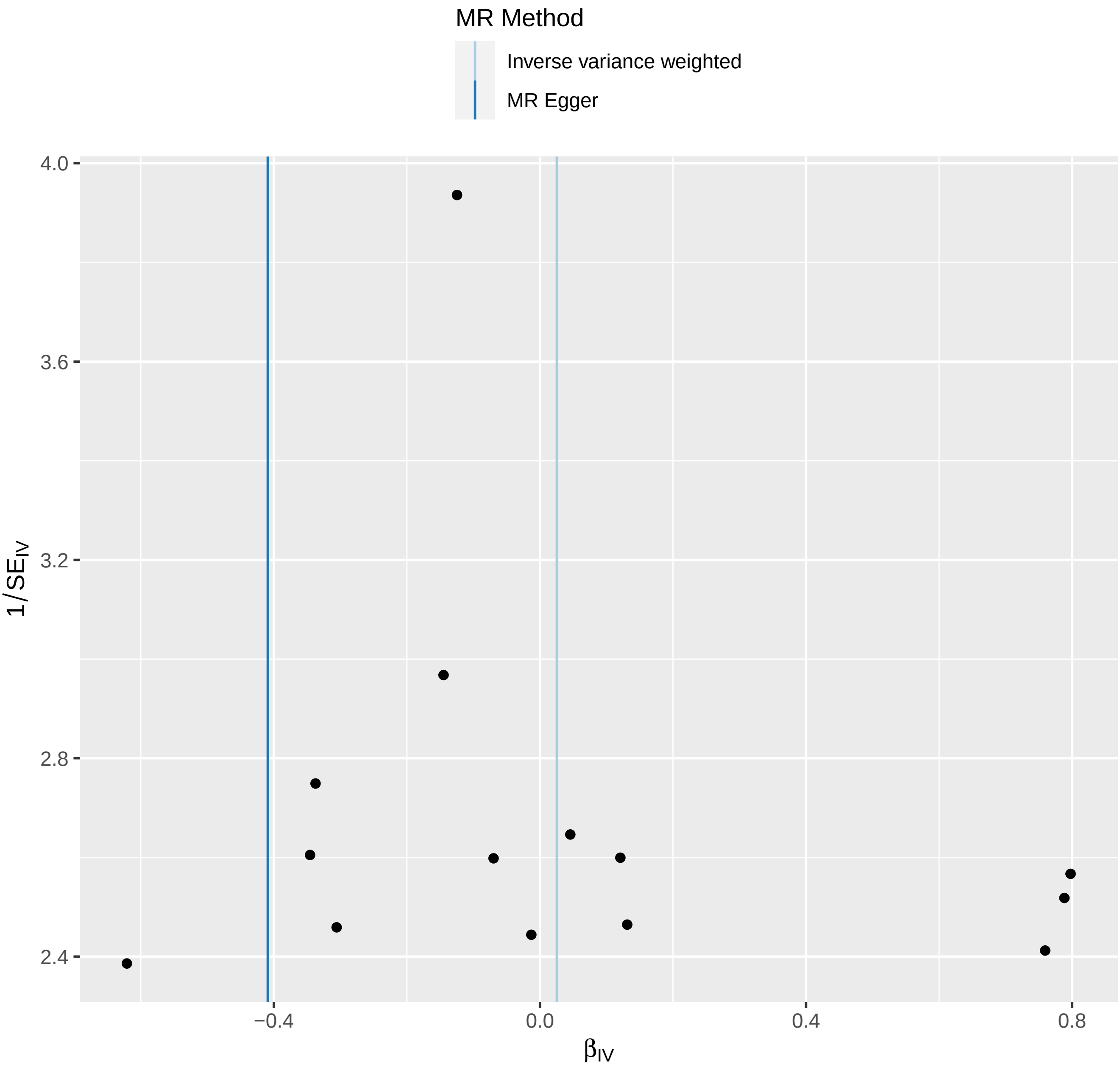

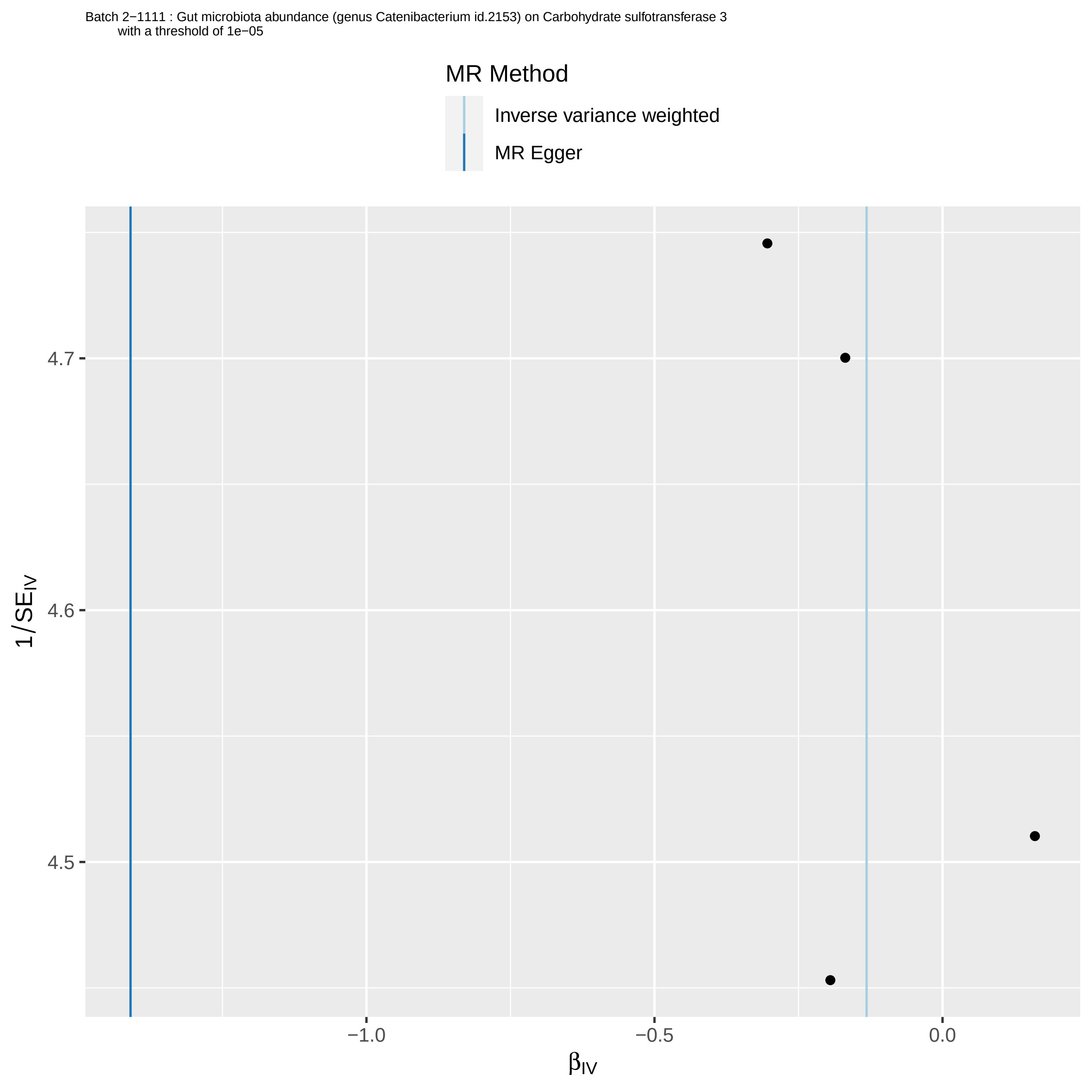
Batch 2−1111 : Gut microbiota abundance (genus Catenibacterium id.2153) on Carbohydrate sulfotransferase 3
with a threshold of 1e−05
MR Method
Inverse variance weighted
MR Egger
4.7
4.6
4.5
1/SE_IV
−1.0
−0.5
0.0
β_IV

Batch 2−1112 : Gut microbiota abundance (genus Parasutterella id.2892) on Carbohydrate sulfotransferase 3
with a threshold of 1e−05

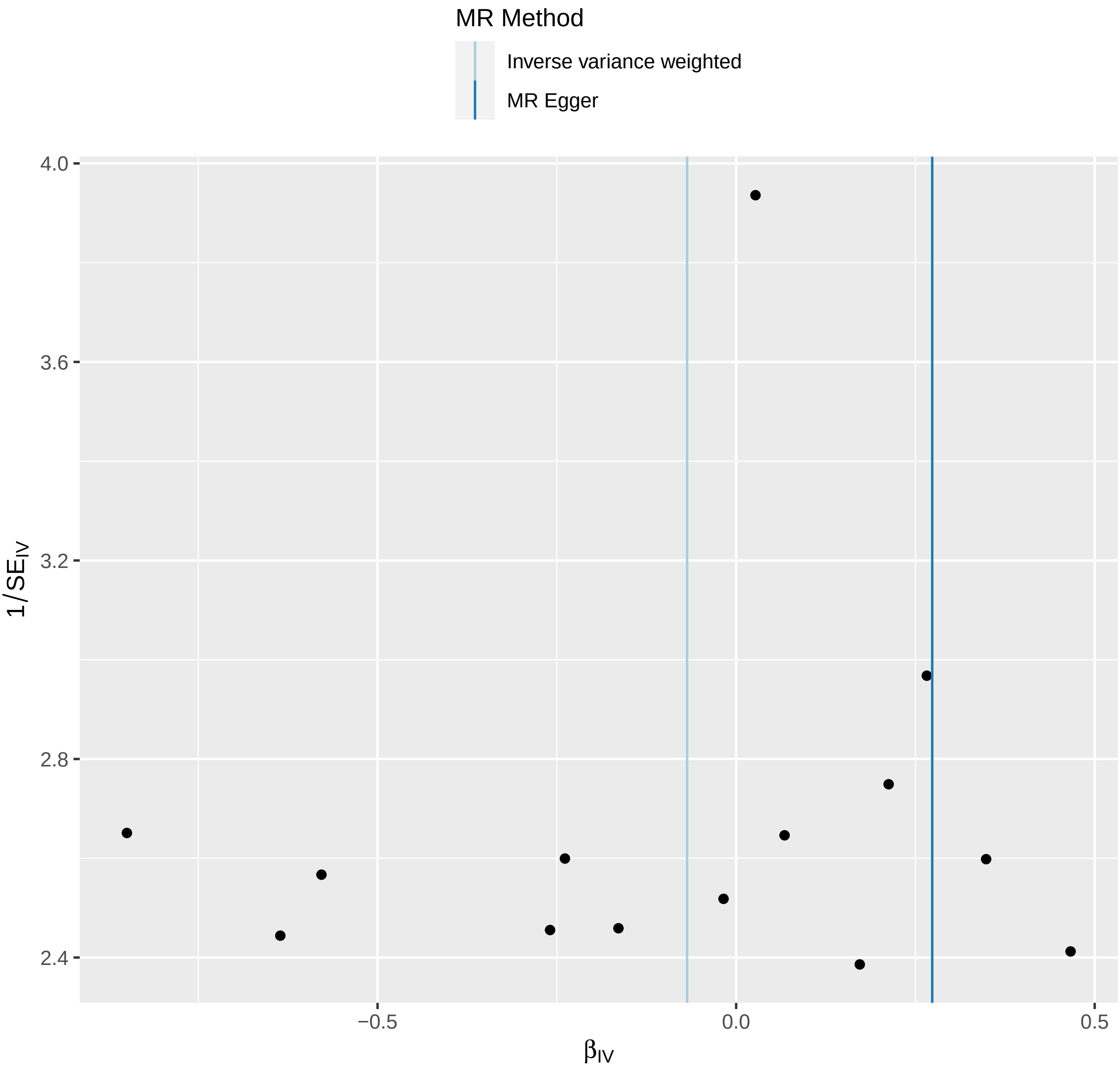

Batch 2−1113 : Gut microbiota abundance (genus Catenibacterium id.2153) on Carbohydrate sulfotransferase 5
with a threshold of 1e−05

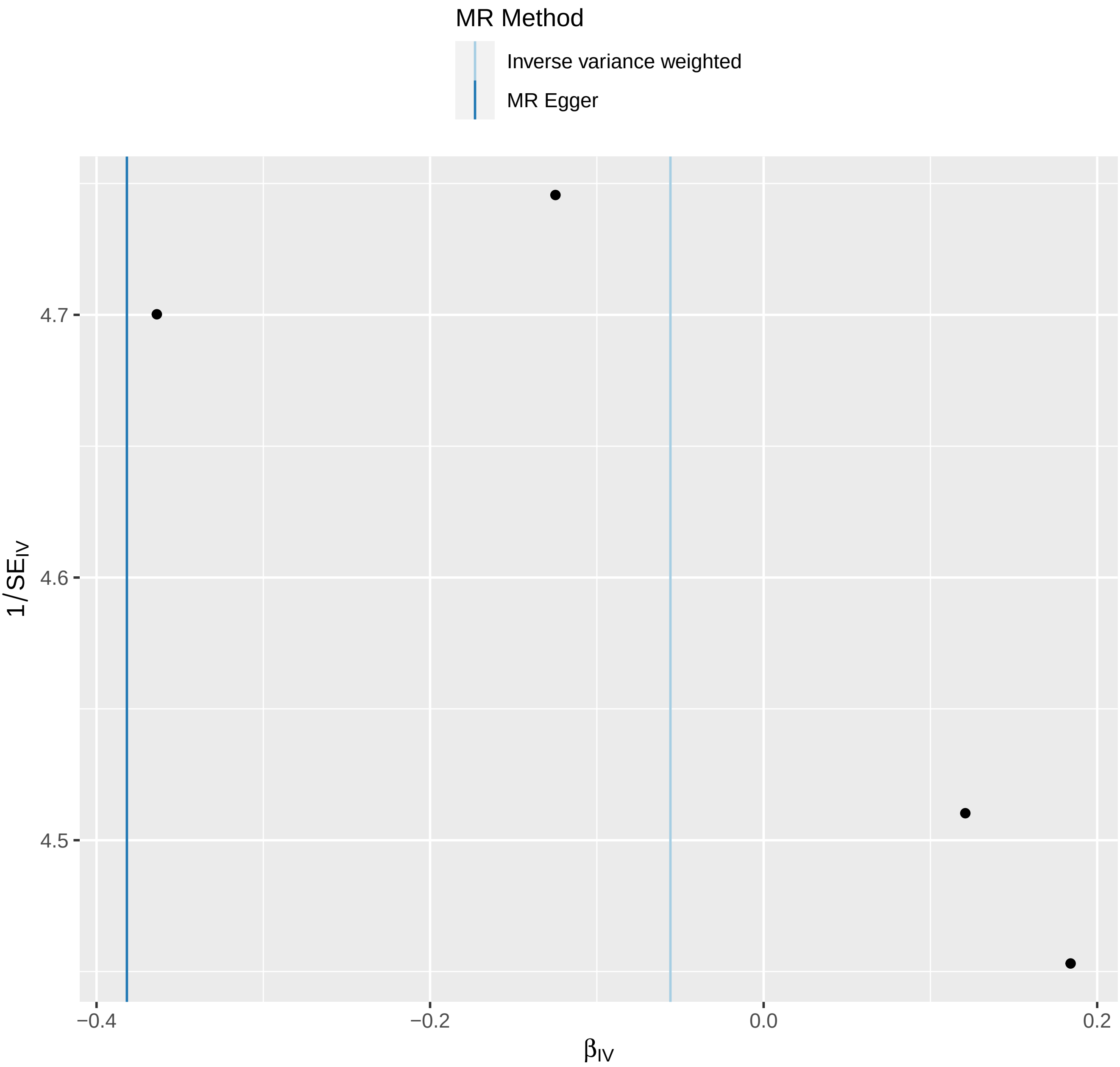

Batch 2−1114 : Gut microbiota abundance (genus Parasutterella id.2892) on Carbohydrate sulfotransferase 5
with a threshold of 1e−05

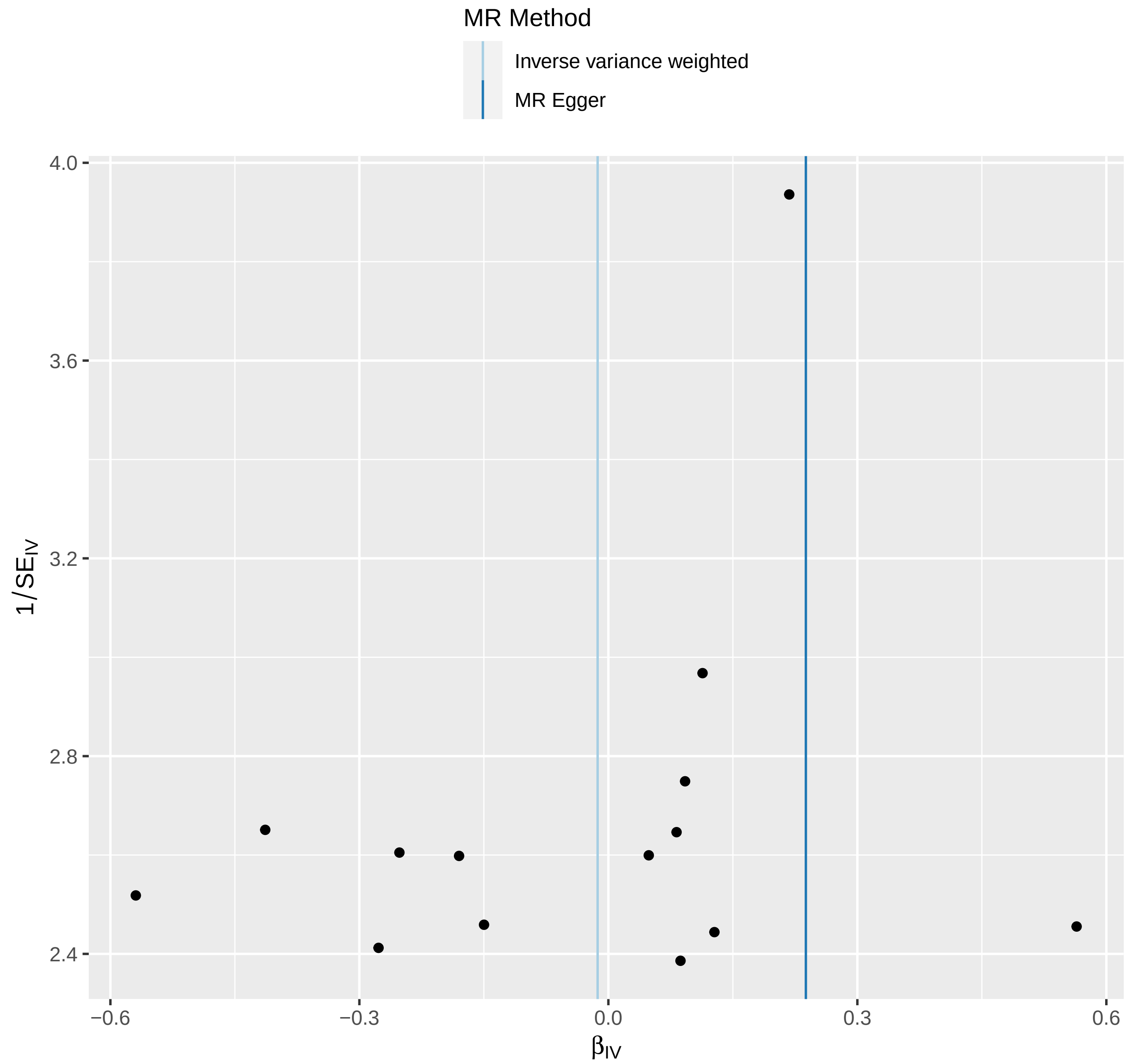

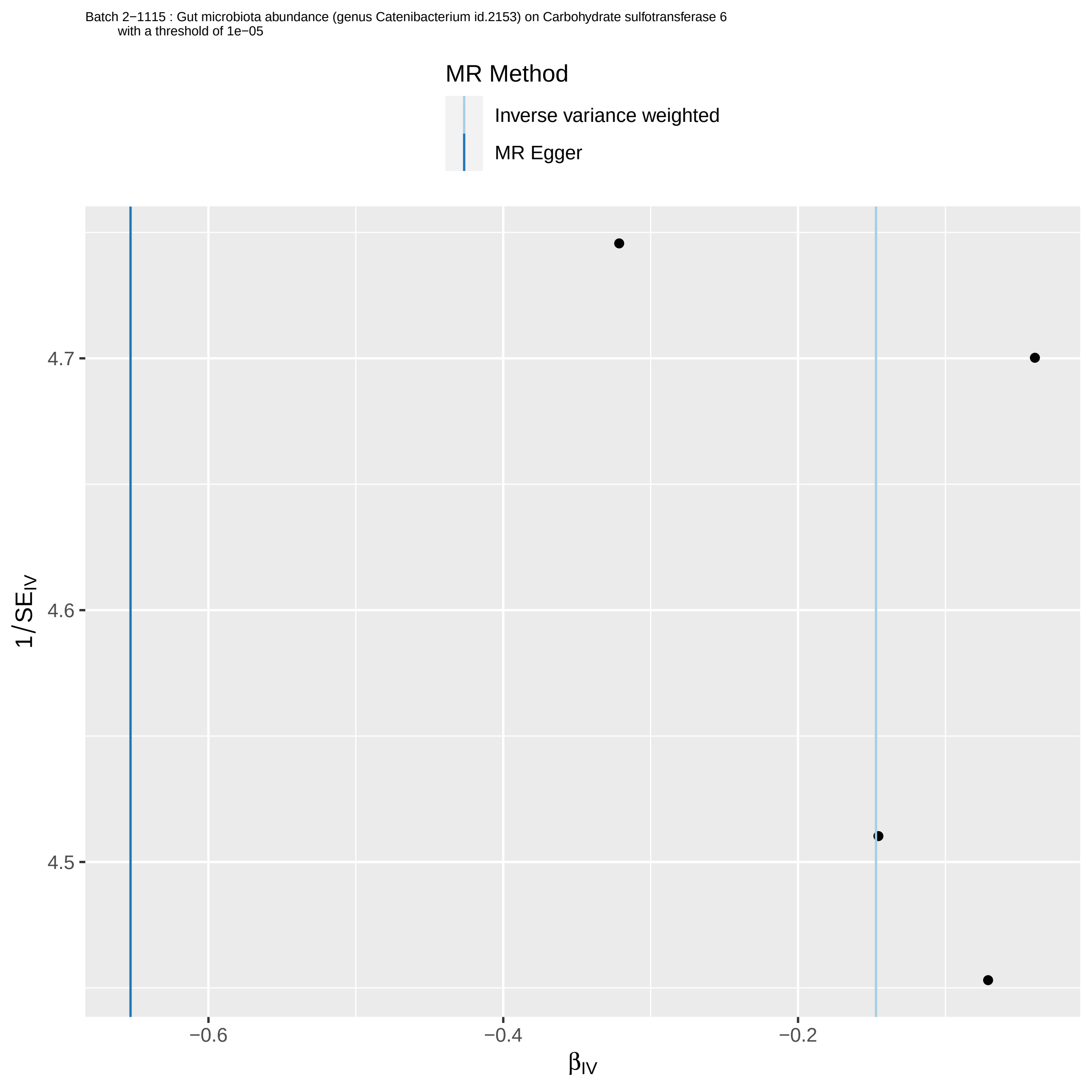
Batch 2−1115 : Gut microbiota abundance (genus Catenibacterium id.2153) on Carbohydrate sulfotransferase 6
with a threshold of 1e−05
MR Method
Inverse variance weighted
MR Egger
4.7
4.6
4.5
1/SE_IV
−0.6
−0.4
−0.2
β_IV

Batch 2−1116 : Gut microbiota abundance (genus Parasutterella id.2892) on Carbohydrate sulfotransferase 6
with a threshold of 1e−05

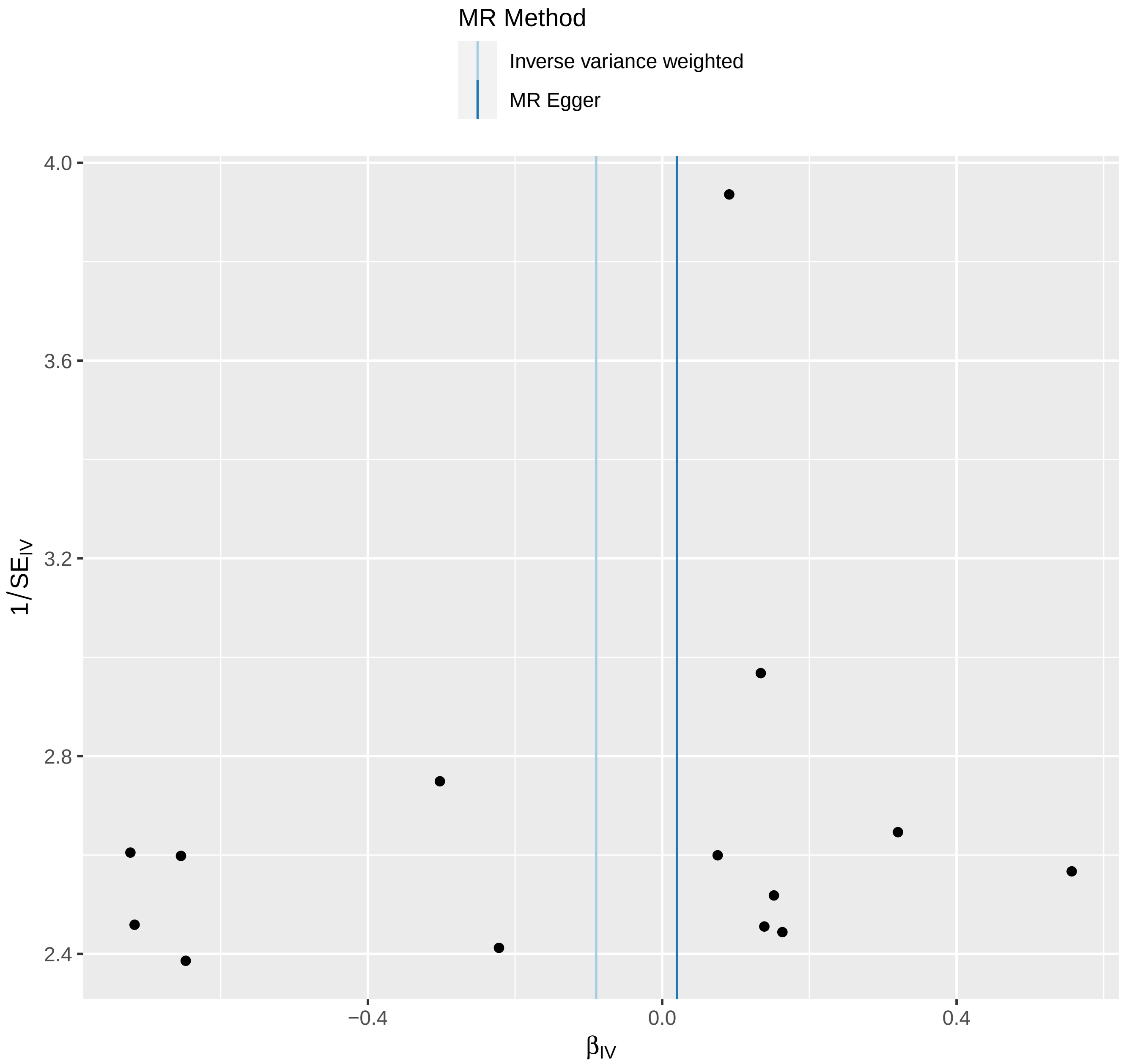

Batch 2−1117 : Gut microbiota abundance (genus Catenibacterium id.2153) on Carbohydrate sulfotransferase 9
with a threshold of 1e−05

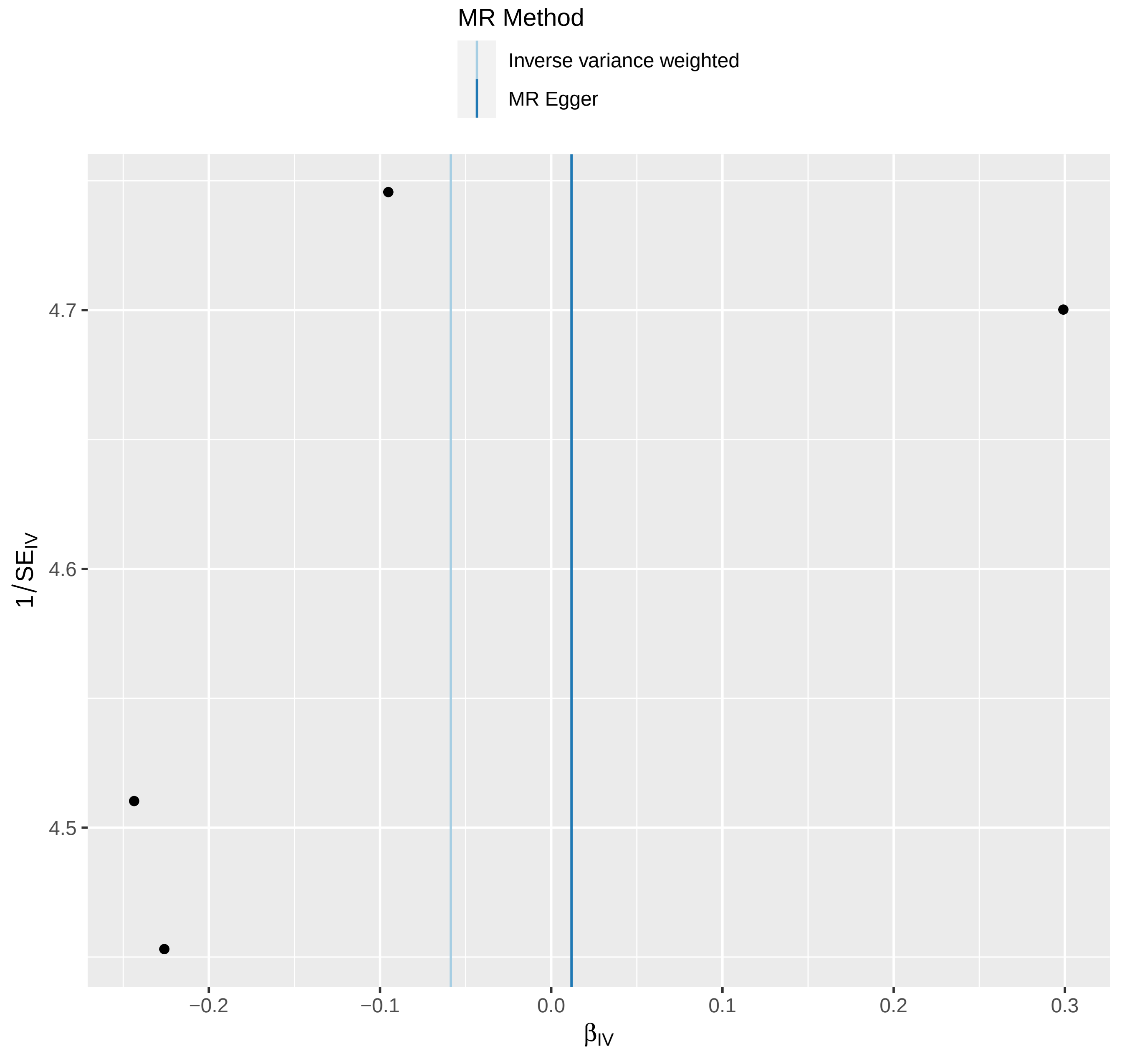

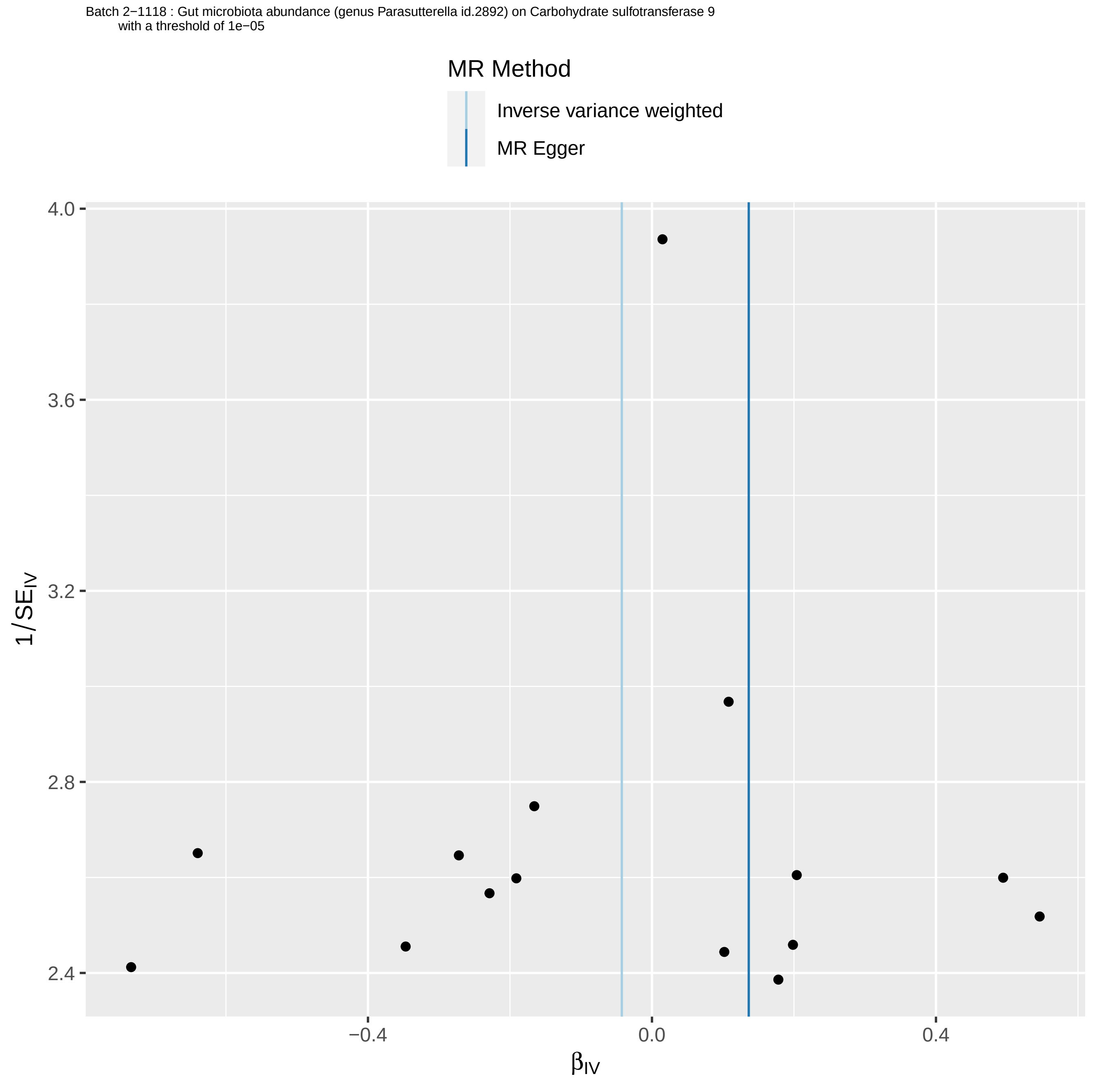
Batch 2−1118 : Gut microbiota abundance (genus Parasutterella id.2892) on Carbohydrate sulfotransferase 9
with a threshold of 1e−05
MR Method
Inverse variance weighted
MR Egger
4.0
3.6
3.2
2.8
2.4
1/SE_IV
−0.4
0.0
0.4
β_IV

Batch 2−1119 : Gut microbiota abundance (genus Catenibacterium id.2153) on Cartilage intermediate layer protein 1 with a threshold of 1e−05

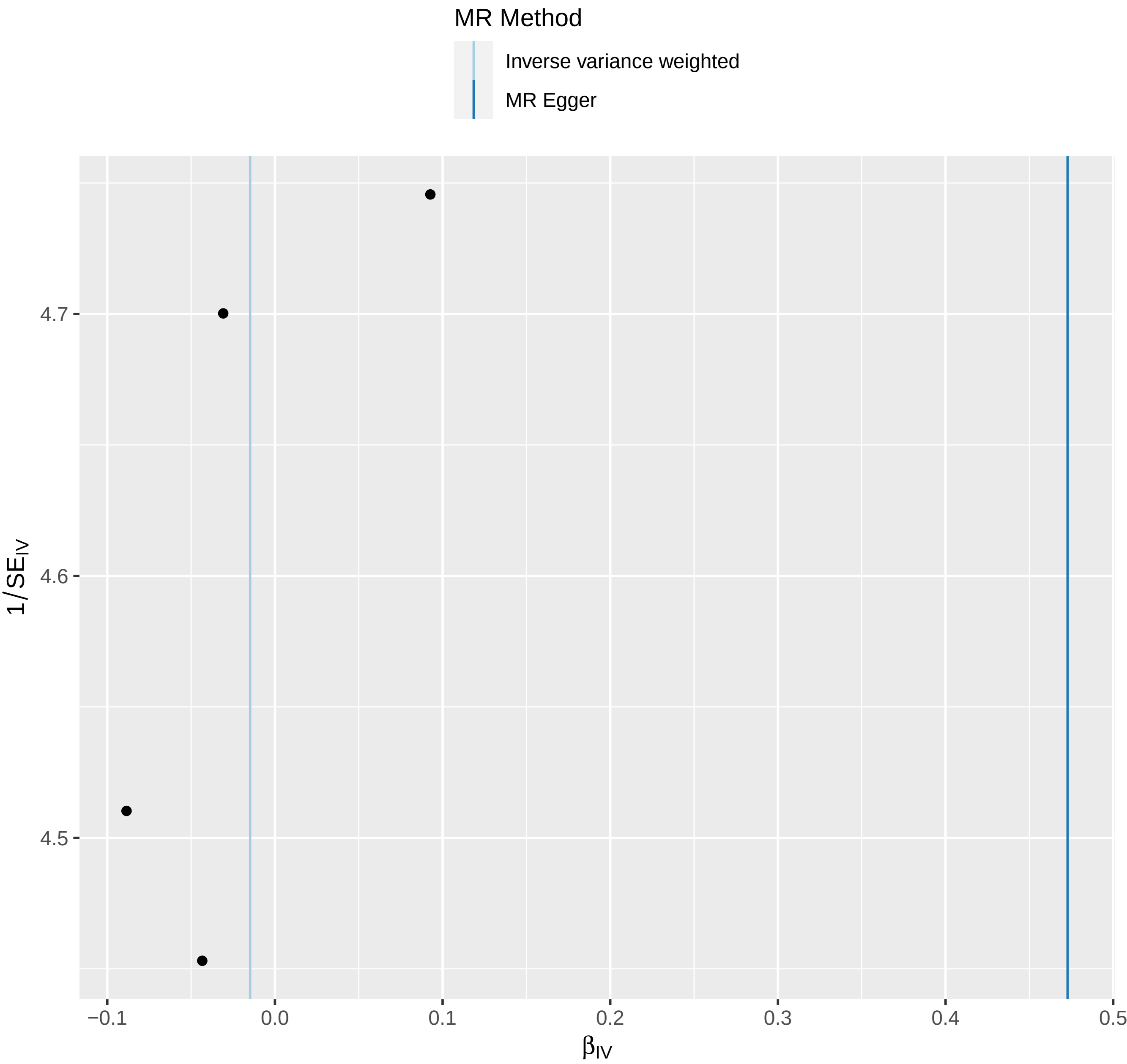

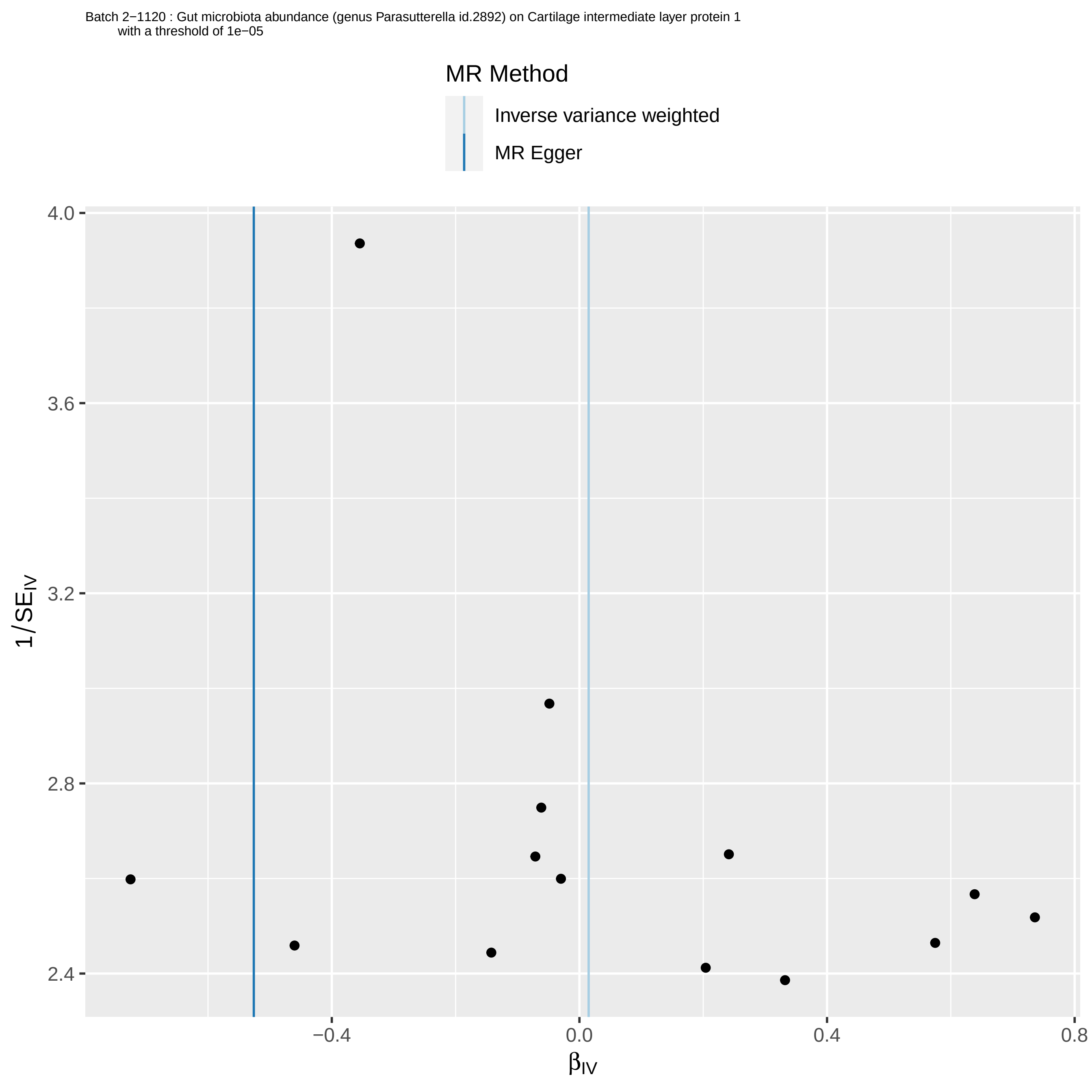
Batch 2−1120 : Gut microbiota abundance (genus Parasutterella id.2892) on Cartilage intermediate layer protein 1
with a threshold of 1e−05
MR Method
Inverse variance weighted
MR Egger
4.0
3.6
3.2
2.8
2.4
1/SE$_{IV}$
−0.4
0.0
0.4
0.8
β_{IV}

Batch 2−1121 : Gut microbiota abundance (genus Catenibacterium id.2153) on Cold−inducible RNA−binding protein
with a threshold of 1e−05

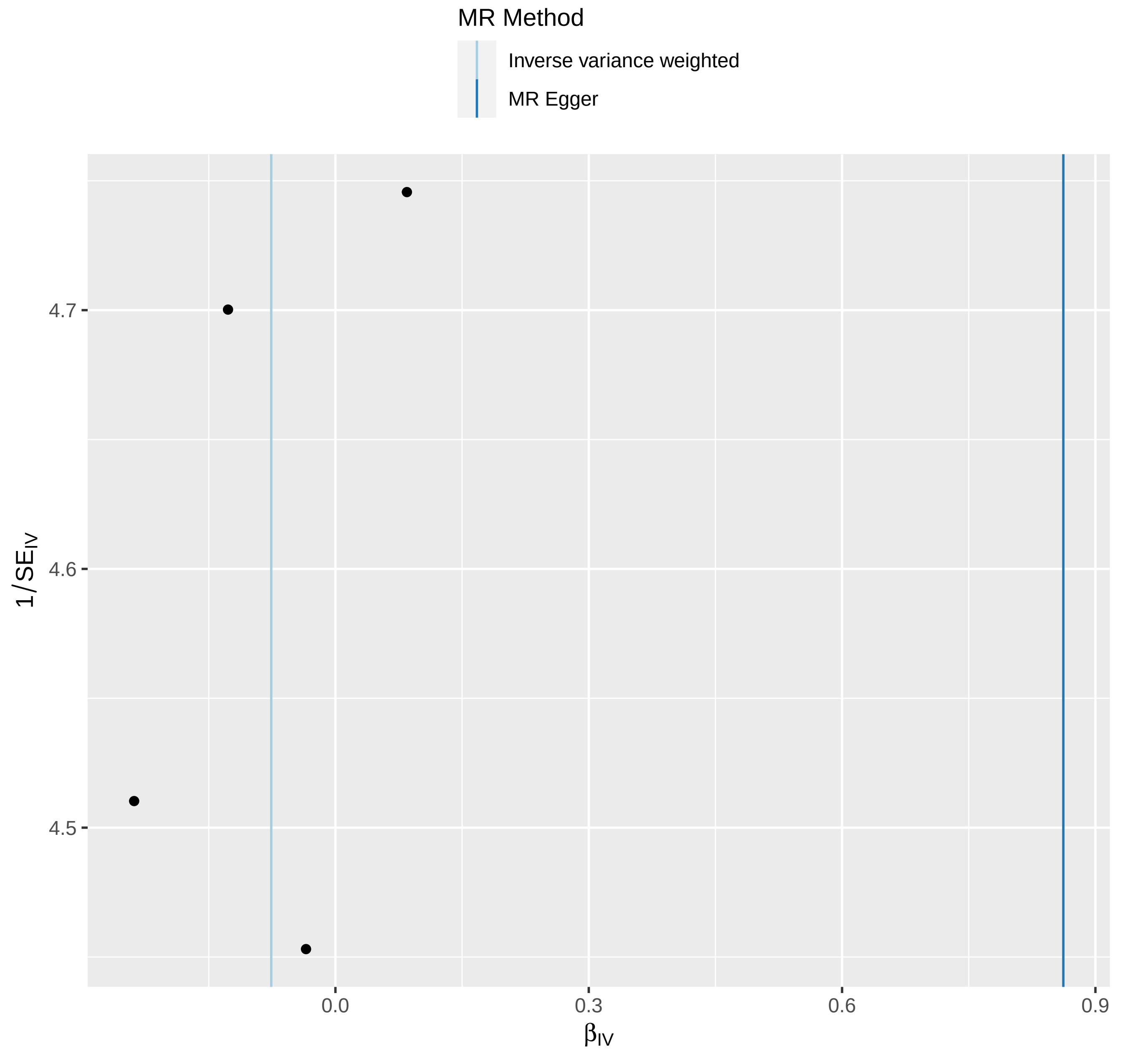

Batch 2−1122 : Gut microbiota abundance (genus Parasutterella id.2892) on Cold−inducible RNA−binding protein
with a threshold of 1e−05

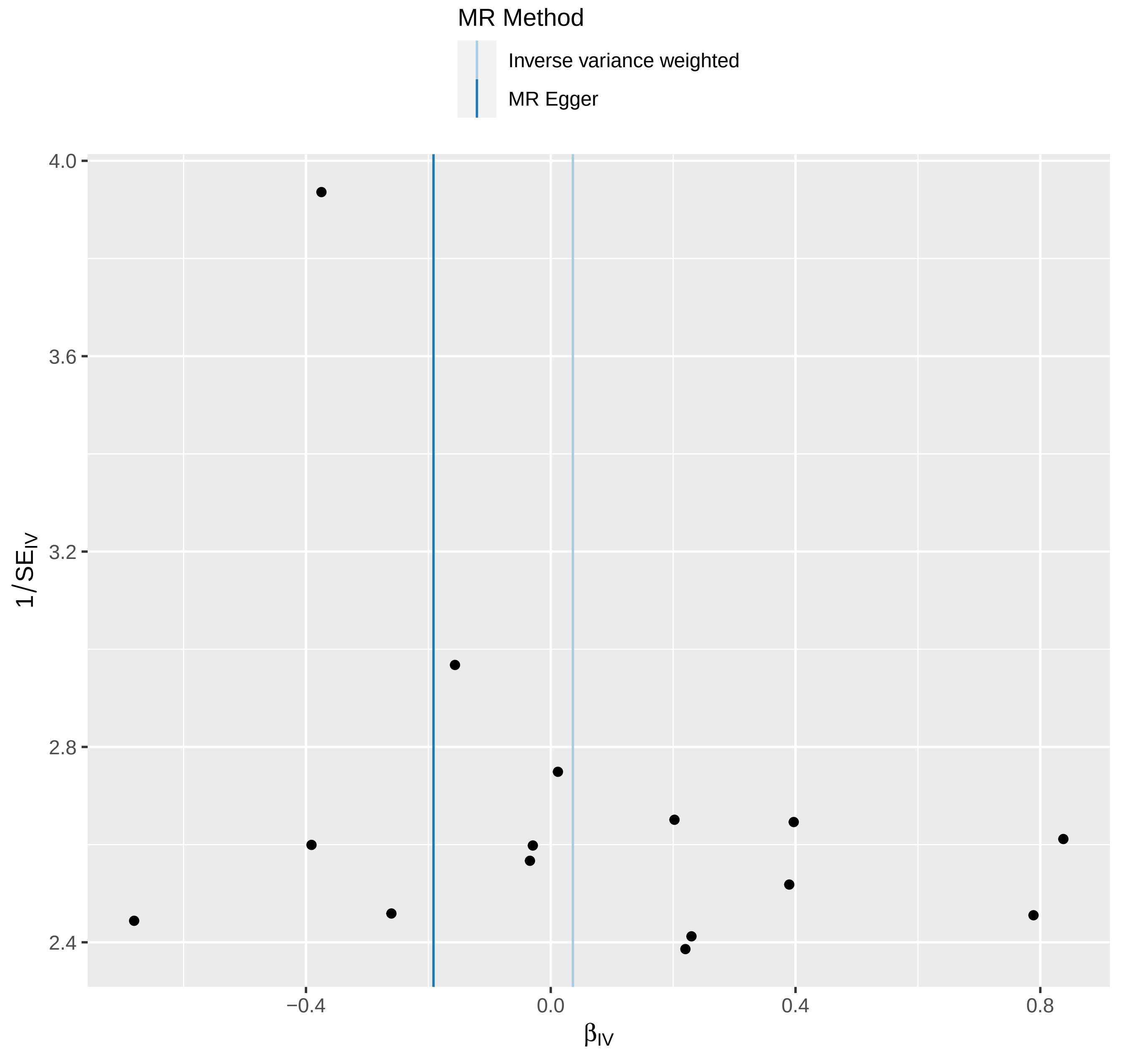

Batch 2−1123 : Gut microbiota abundance (genus Catenibacterium id.2153) on CDGSH iron−sulfur domain−containing protein 1
with a threshold of 1e−05

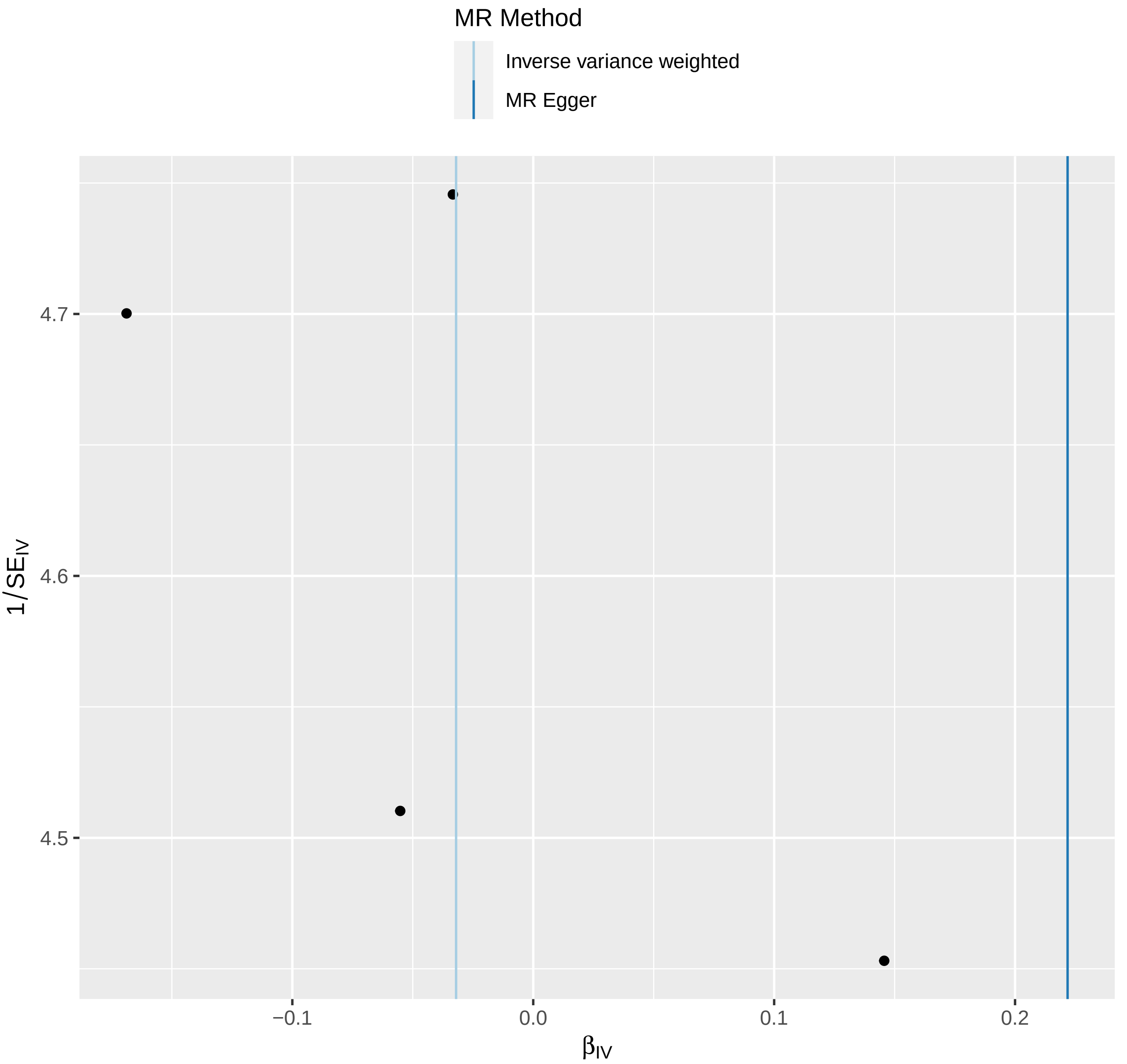

Batch 2−1124 : Gut microbiota abundance (genus Parasutterella id.2892) on CDGSH iron−sulfur domain−containing protein 1 with a threshold of 1e−05

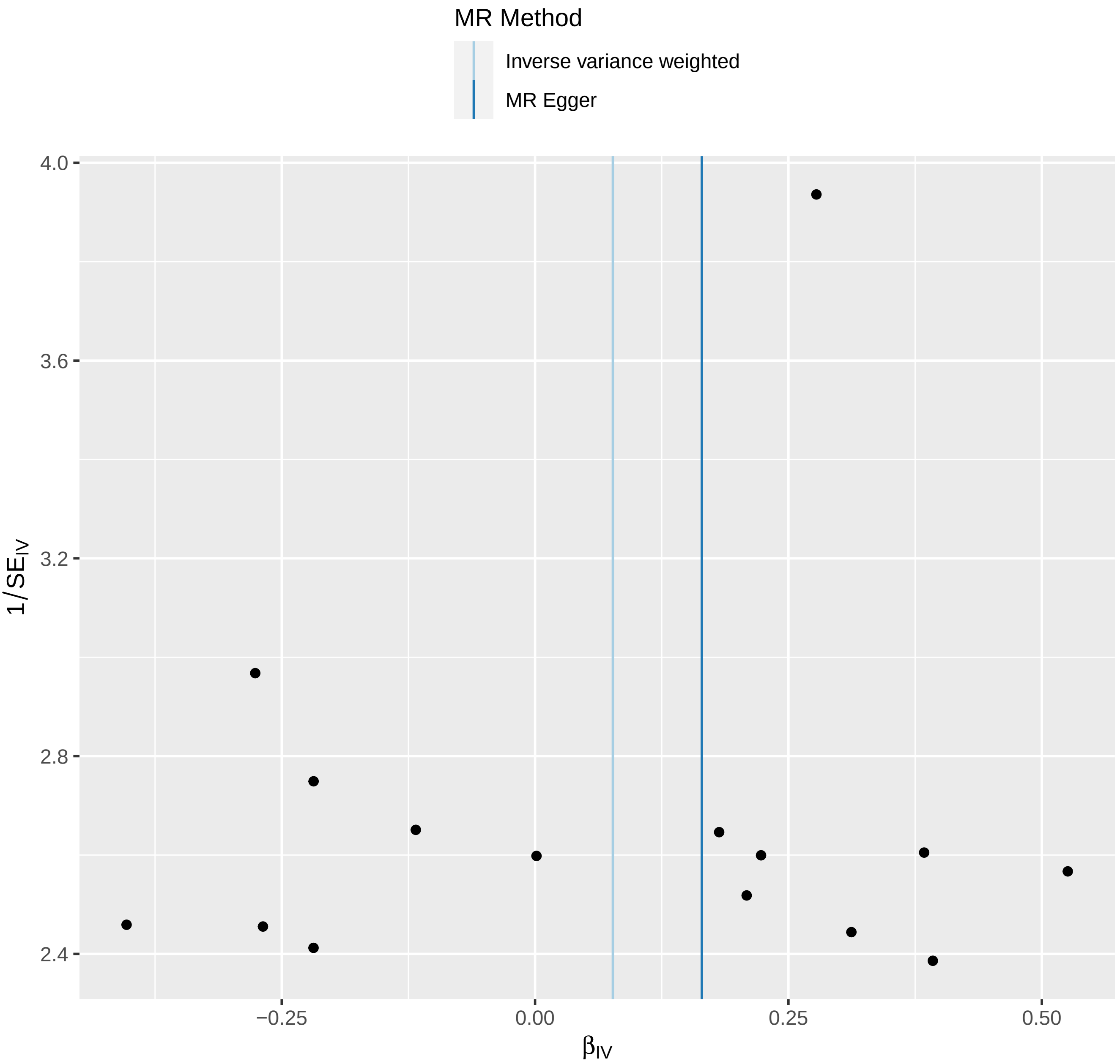

Batch 2−1125 : Gut microbiota abundance (genus Catenibacterium id.2153) on CDGSH iron−sulfur domain−containing protein 2 with a threshold of 1e−05

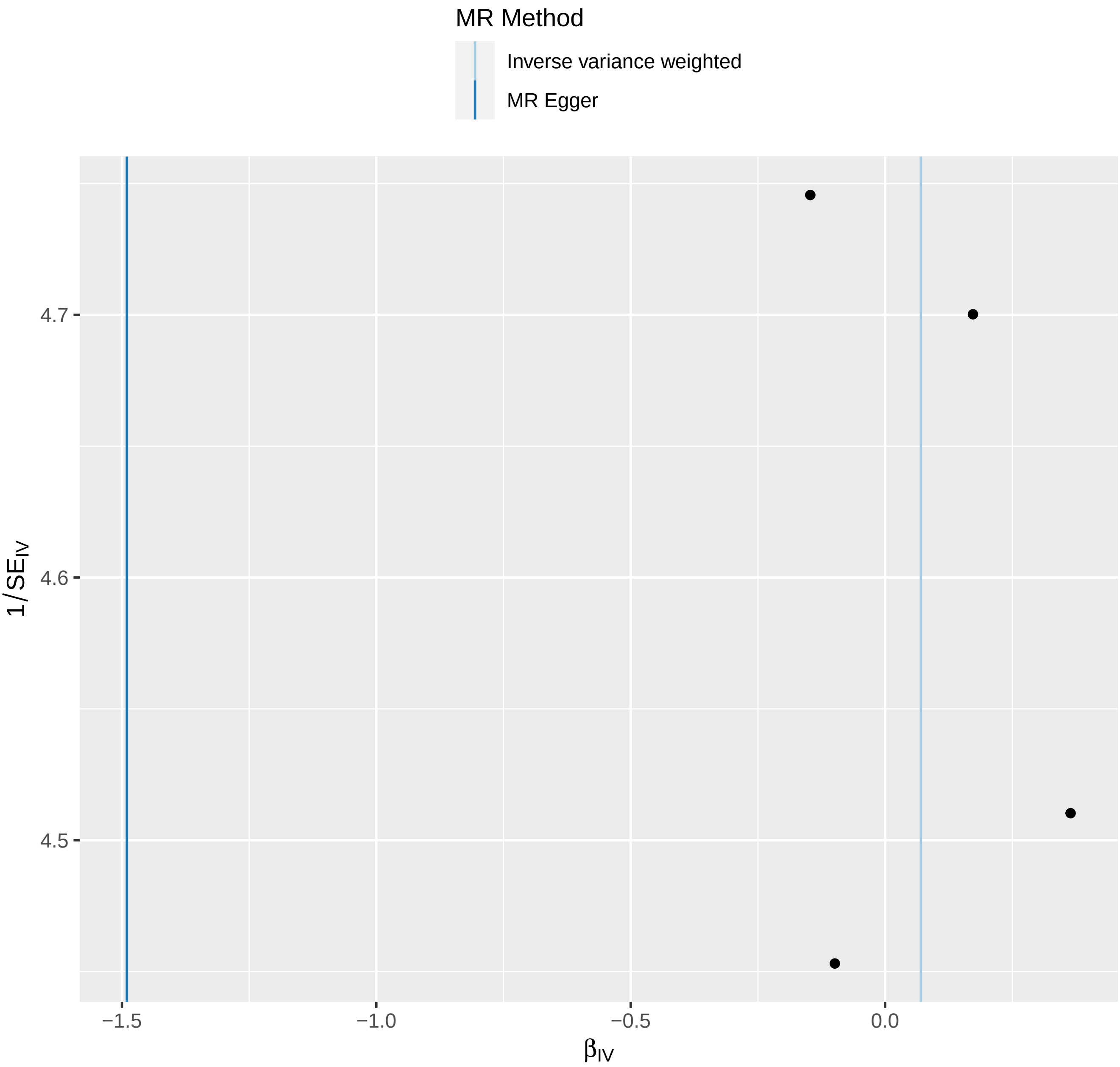

Batch 2−1126 : Gut microbiota abundance (genus Parasutterella id.2892) on CDGSH iron−sulfur domain−containing protein 2
with a threshold of 1e−05

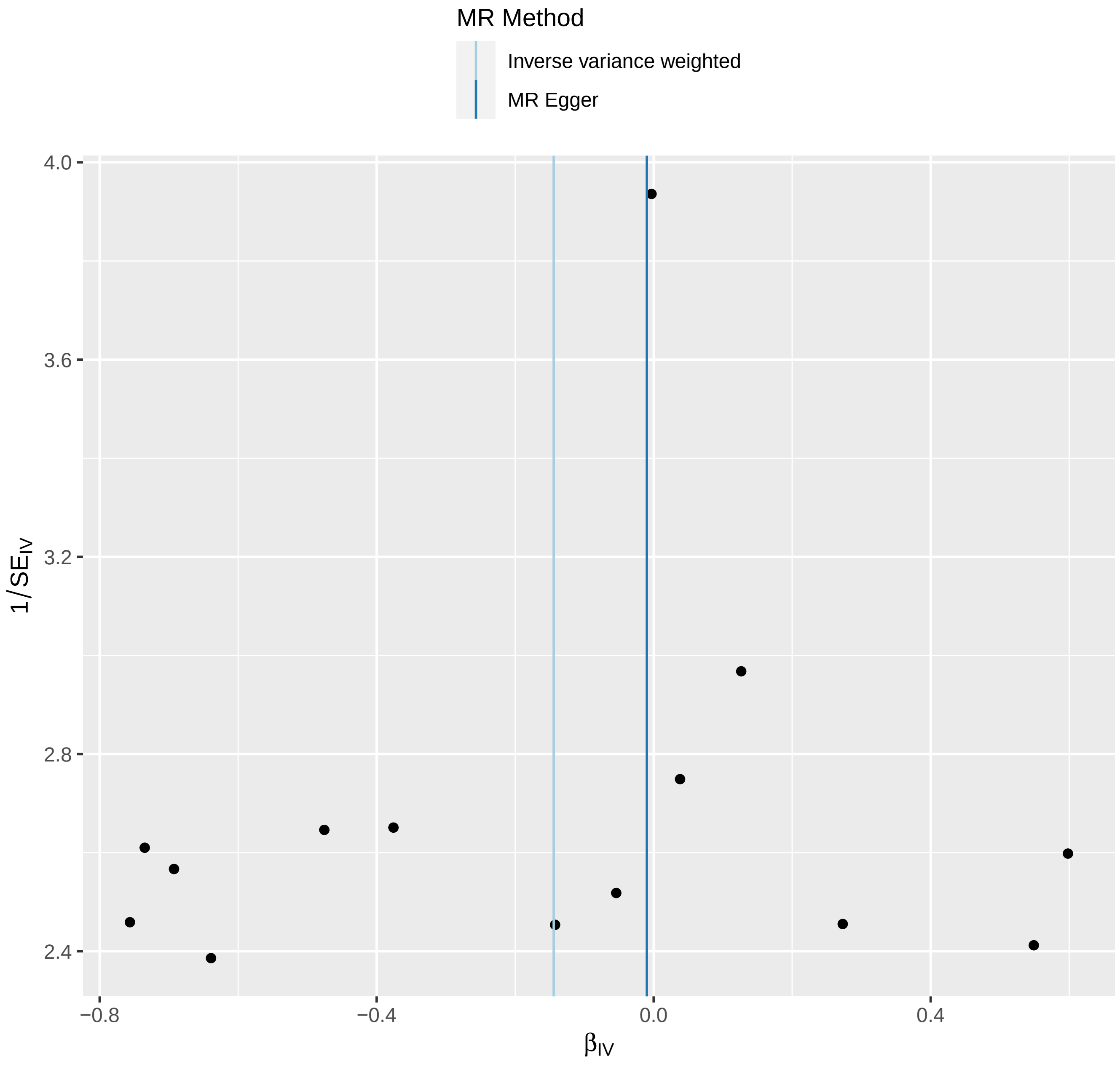

Batch 2−1127 : Gut microbiota abundance (genus Catenibacterium id.2153) on Cytoskeleton−associated protein 2
with a threshold of 1e−05

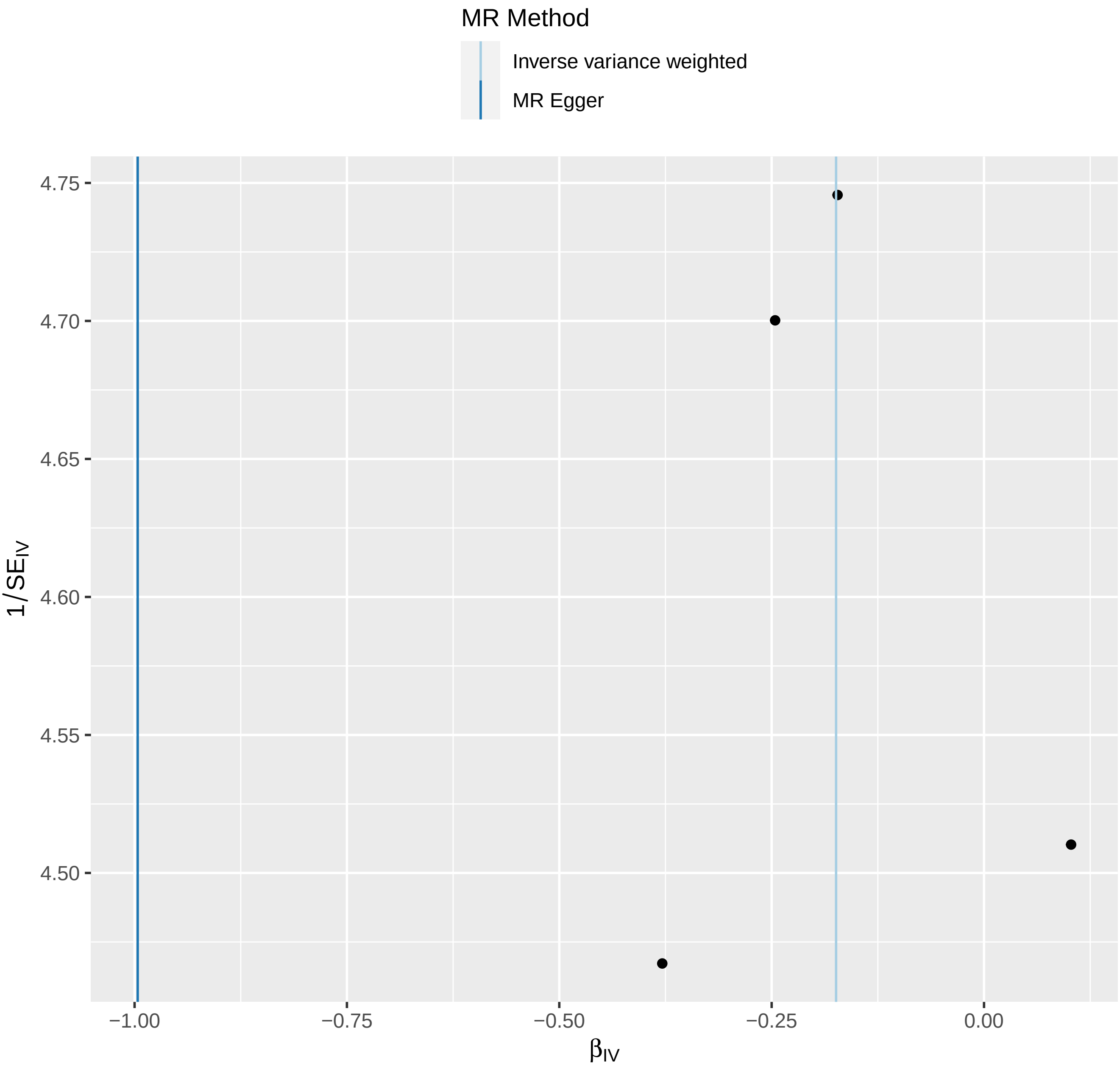

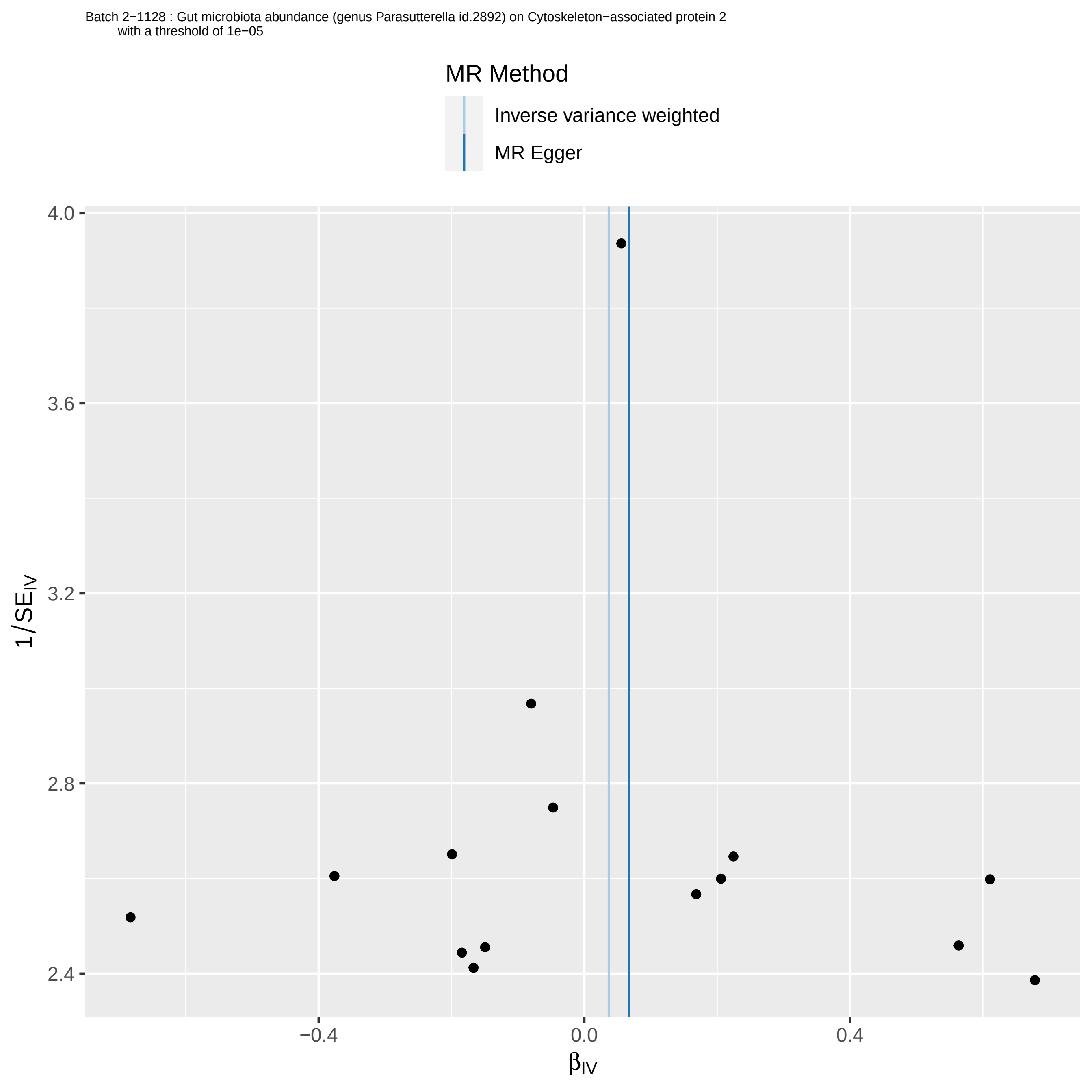
Batch 2−1128 : Gut microbiota abundance (genus Parasutterella id.2892) on Cytoskeleton−associated protein 2
with a threshold of 1e−05
MR Method
Inverse variance weighted
MR Egger
4.0
3.6
3.2
2.8
2.4
1/SE_IV
−0.4
0.0
0.4
β_IV

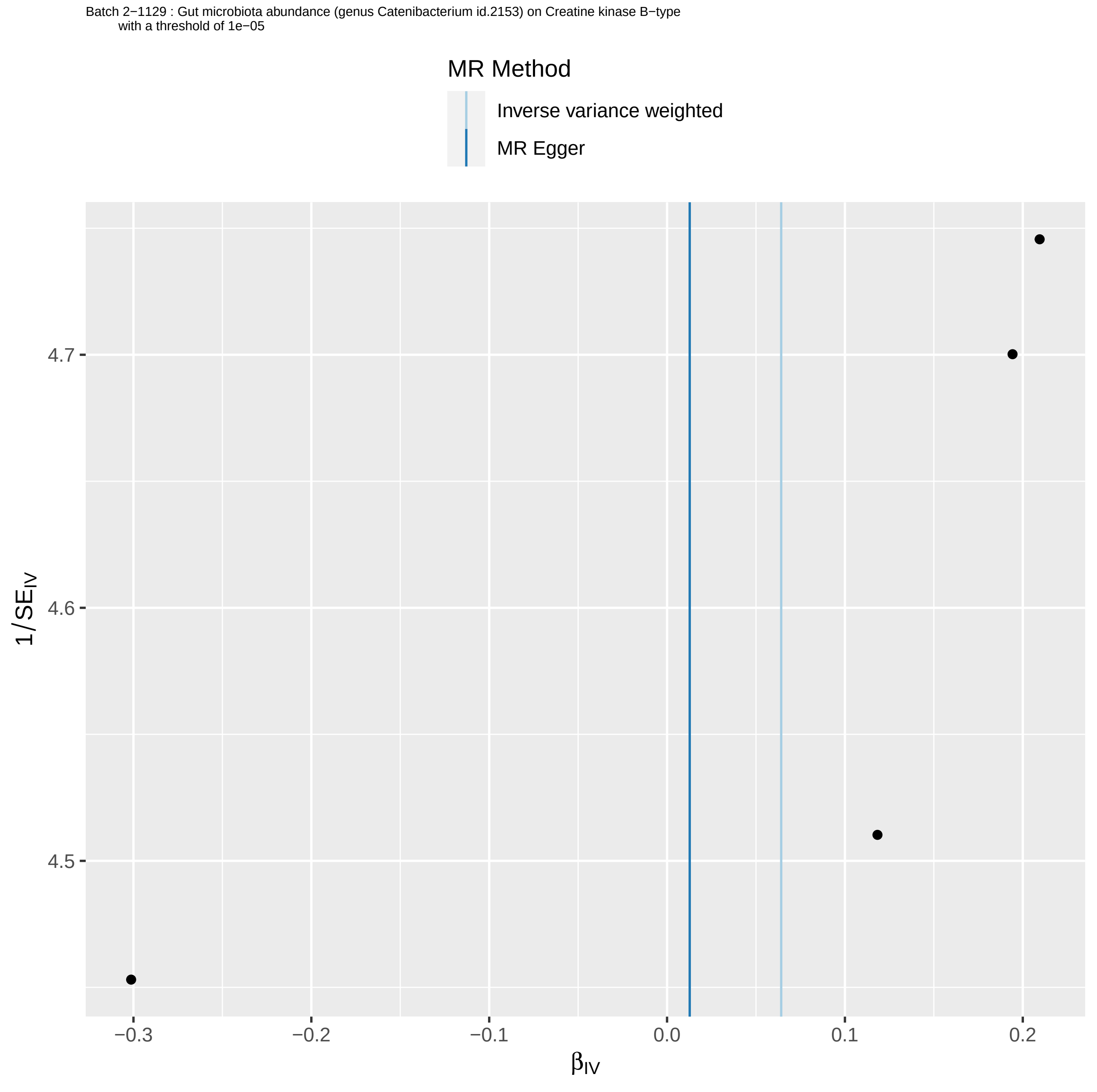
Batch 2−1129 : Gut microbiota abundance (genus Catenibacterium id.2153) on Creatine kinase B−type
with a threshold of 1e−05
MR Method
Inverse variance weighted
MR Egger
4.7
4.6
4.5
1/SE_IV
−0.3
−0.2
−0.1
0.0
0.1
0.2
β_IV

Batch 2−1130 : Gut microbiota abundance (genus Parasutterella id.2892) on Creatine kinase B−type
with a threshold of 1e−05

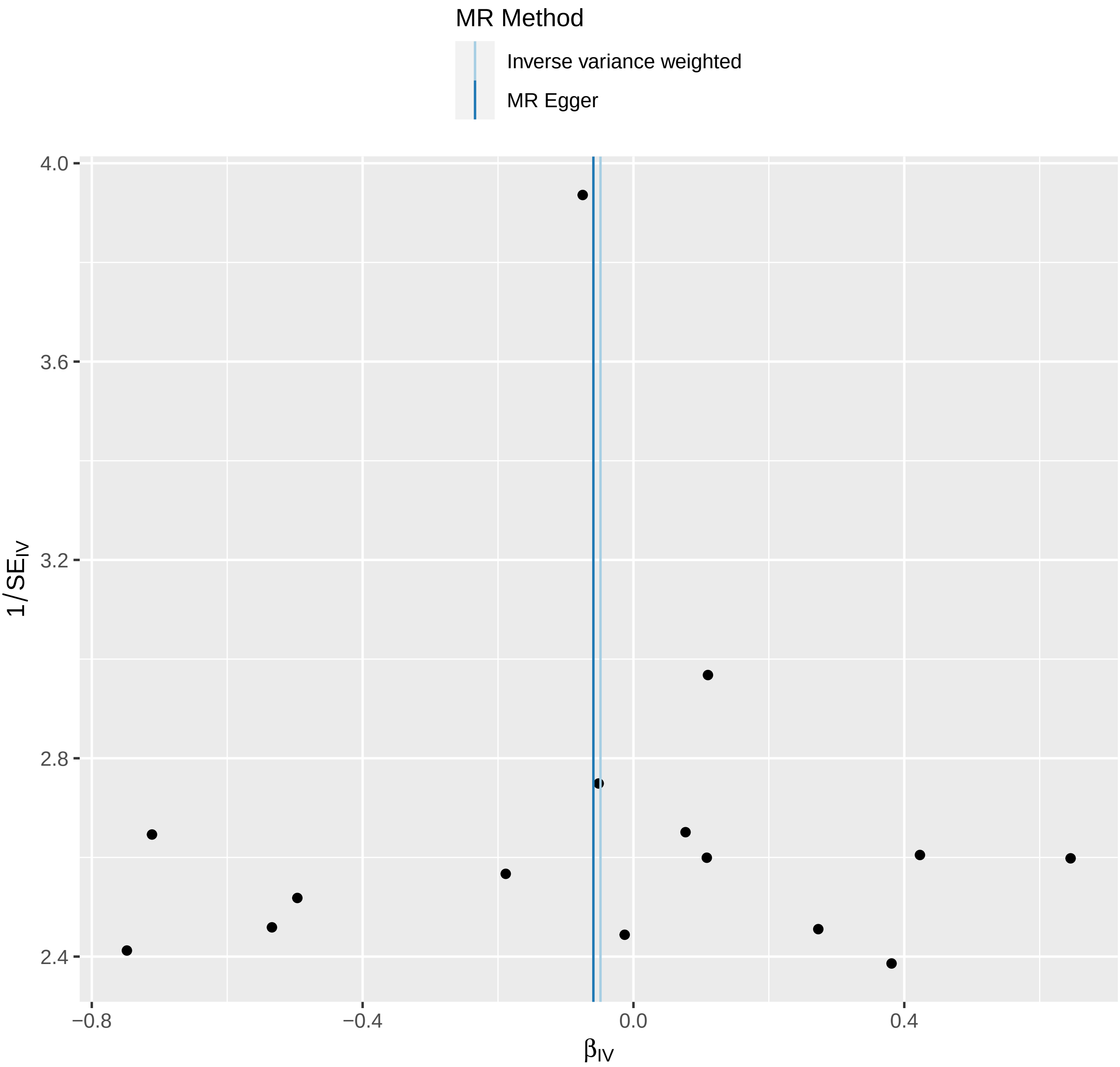

Batch 2−1131 : Gut microbiota abundance (genus Catenibacterium id.2153) on Creatine kinase M−type
with a threshold of 1e−05

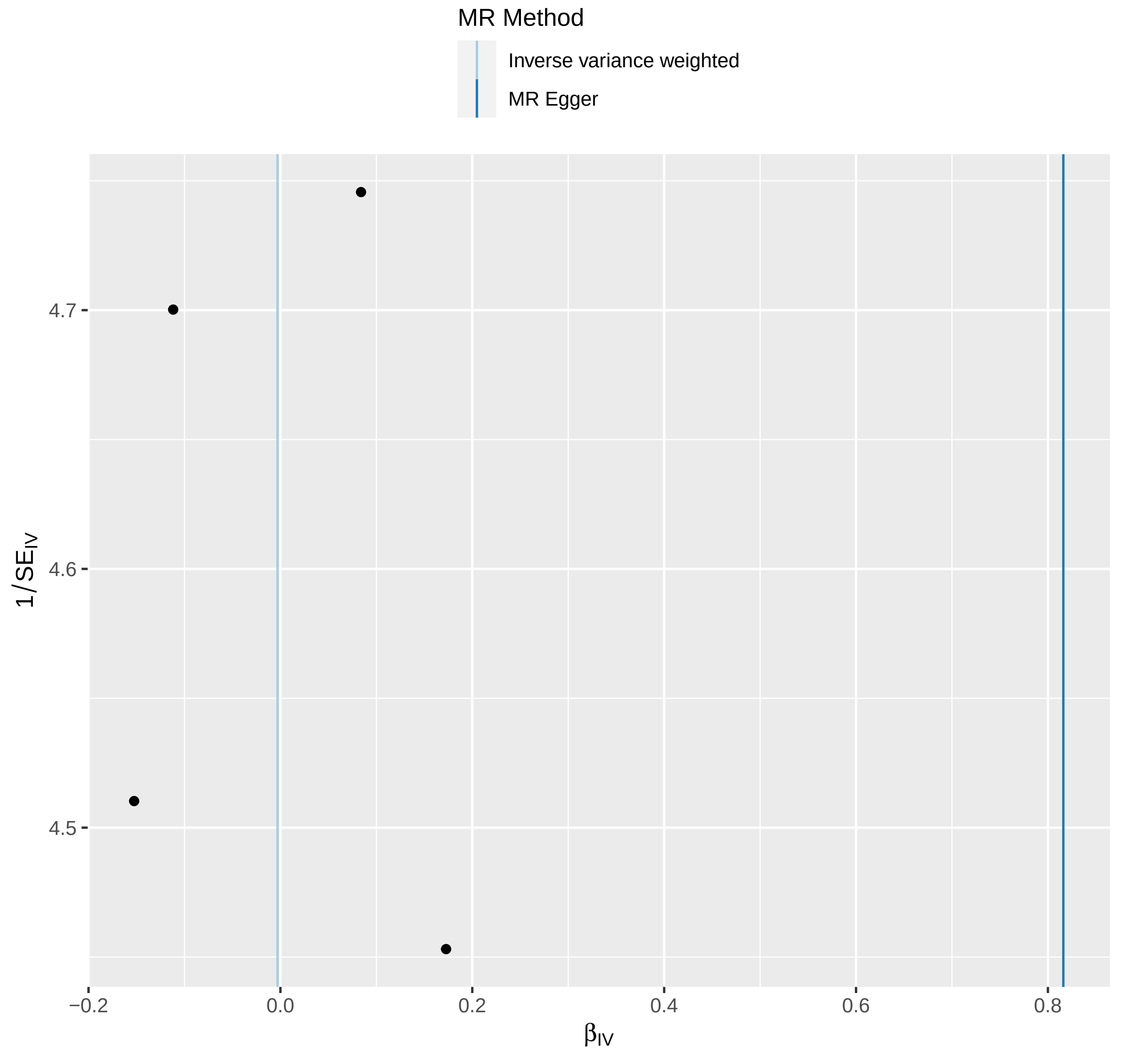

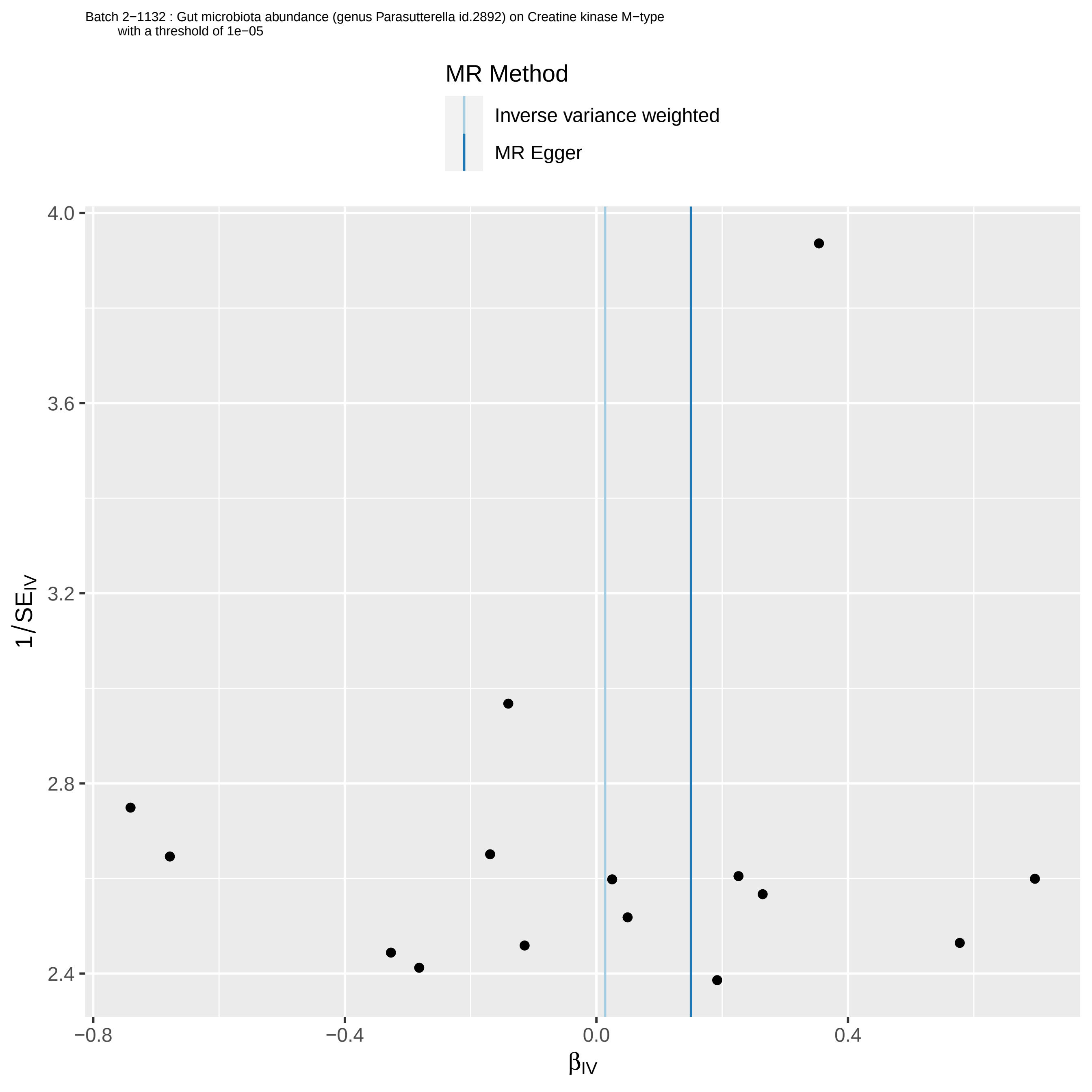

Batch 2−1132 : Gut microbiota abundance (genus Parasutterella id.2892) on Creatine kinase M−type
with a threshold of 1e−05
MR Method
Inverse variance weighted
MR Egger
4.0
3.6
3.2
2.8
2.4
1/SE_IV
−0.8
−0.4
0.0
0.4
β_IV

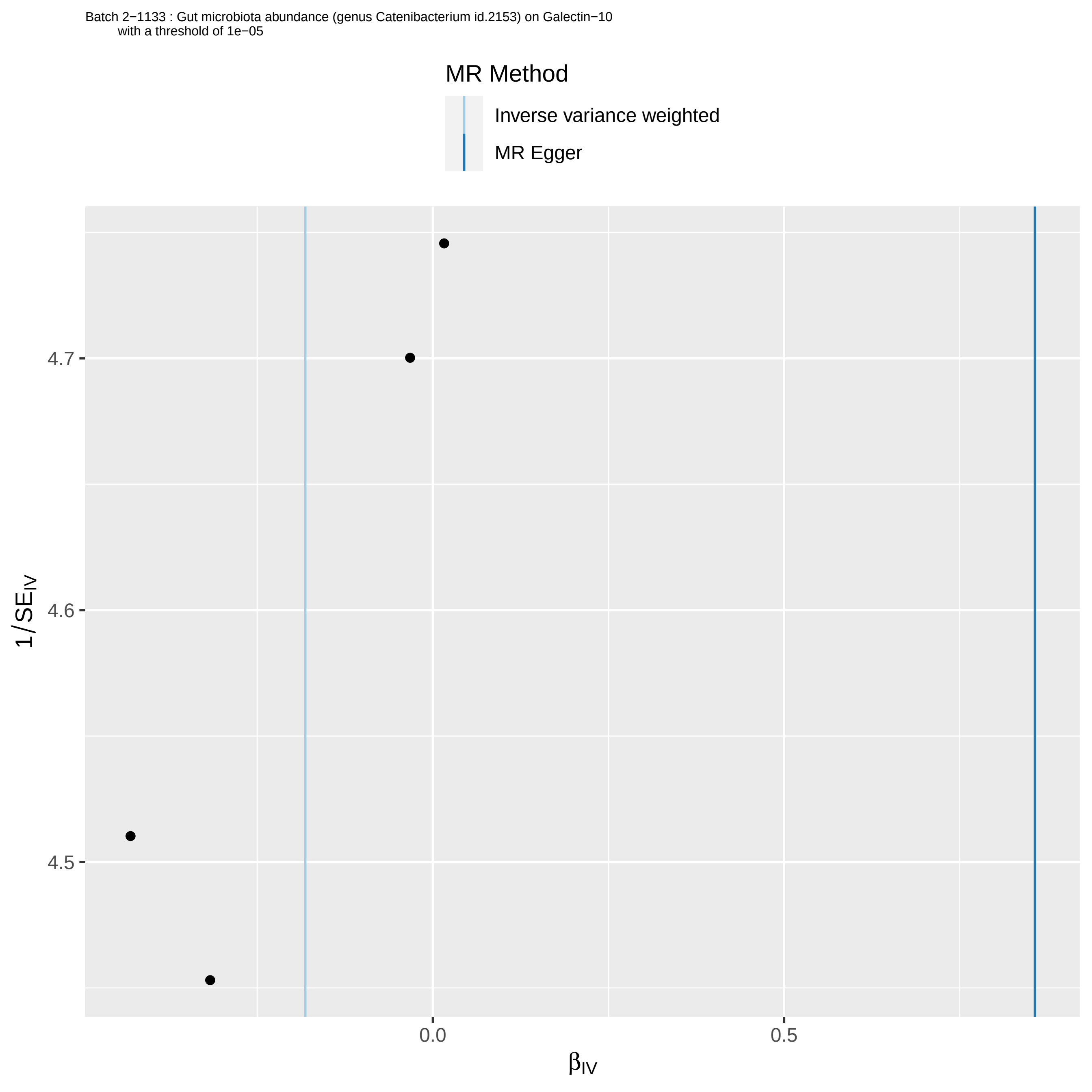
Batch 2−1133 : Gut microbiota abundance (genus Catenibacterium id.2153) on Galectin−10
with a threshold of 1e−05
MR Method
Inverse variance weighted
MR Egger
4.7
4.6
4.5
1/SE_IV
0.0
0.5
β_IV

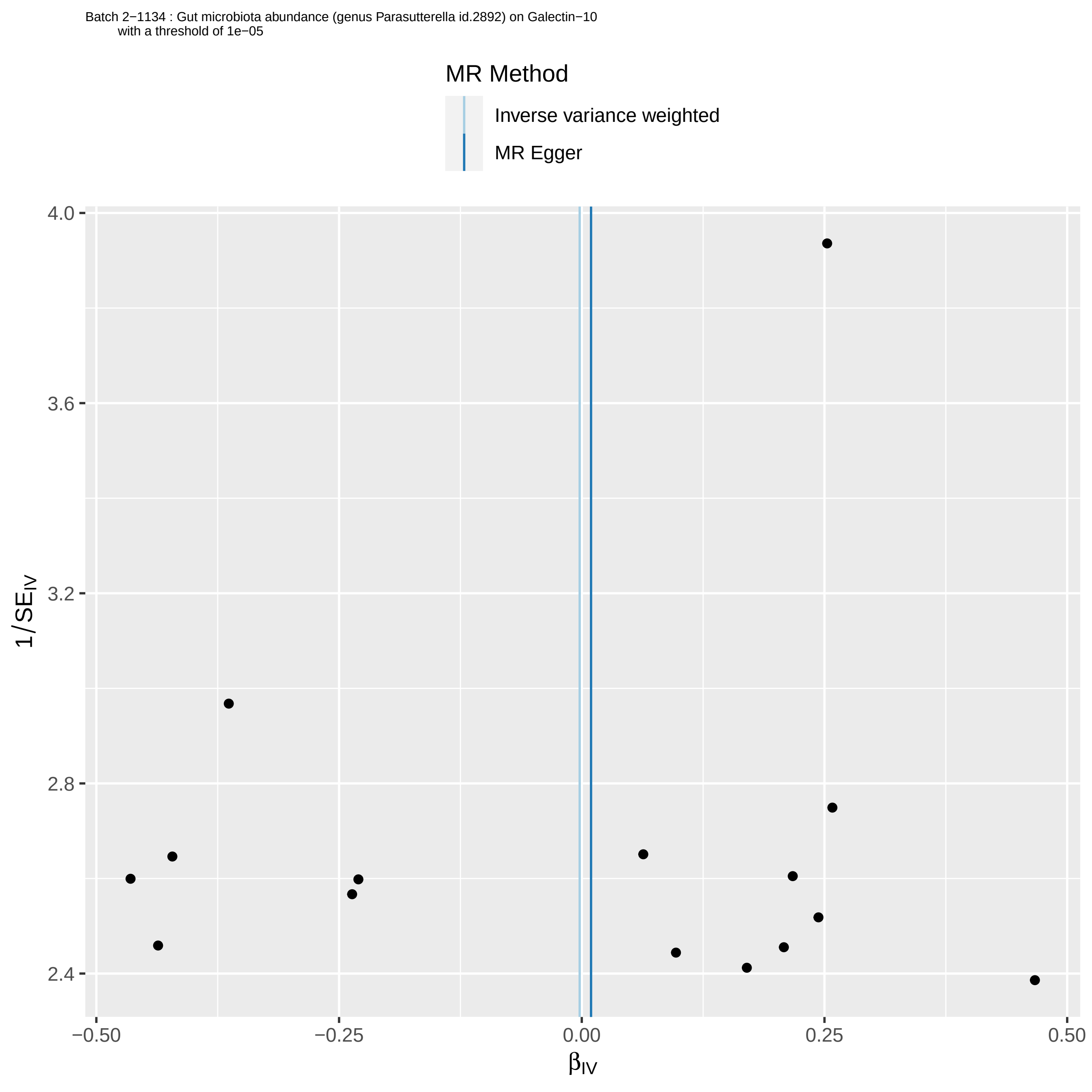
Batch 2−1134 : Gut microbiota abundance (genus Parasutterella id.2892) on Galectin−10
with a threshold of 1e−05
MR Method
Inverse variance weighted
MR Egger
4.0
3.6
3.2
2.8
2.4
1/SE_IV
−0.50
−0.25
0.00
0.25
0.50
β_IV

Batch 2−1135 : Gut microbiota abundance (genus Catenibacterium id.2153) on C−type lectin domain family 10 member A
with a threshold of 1e−05

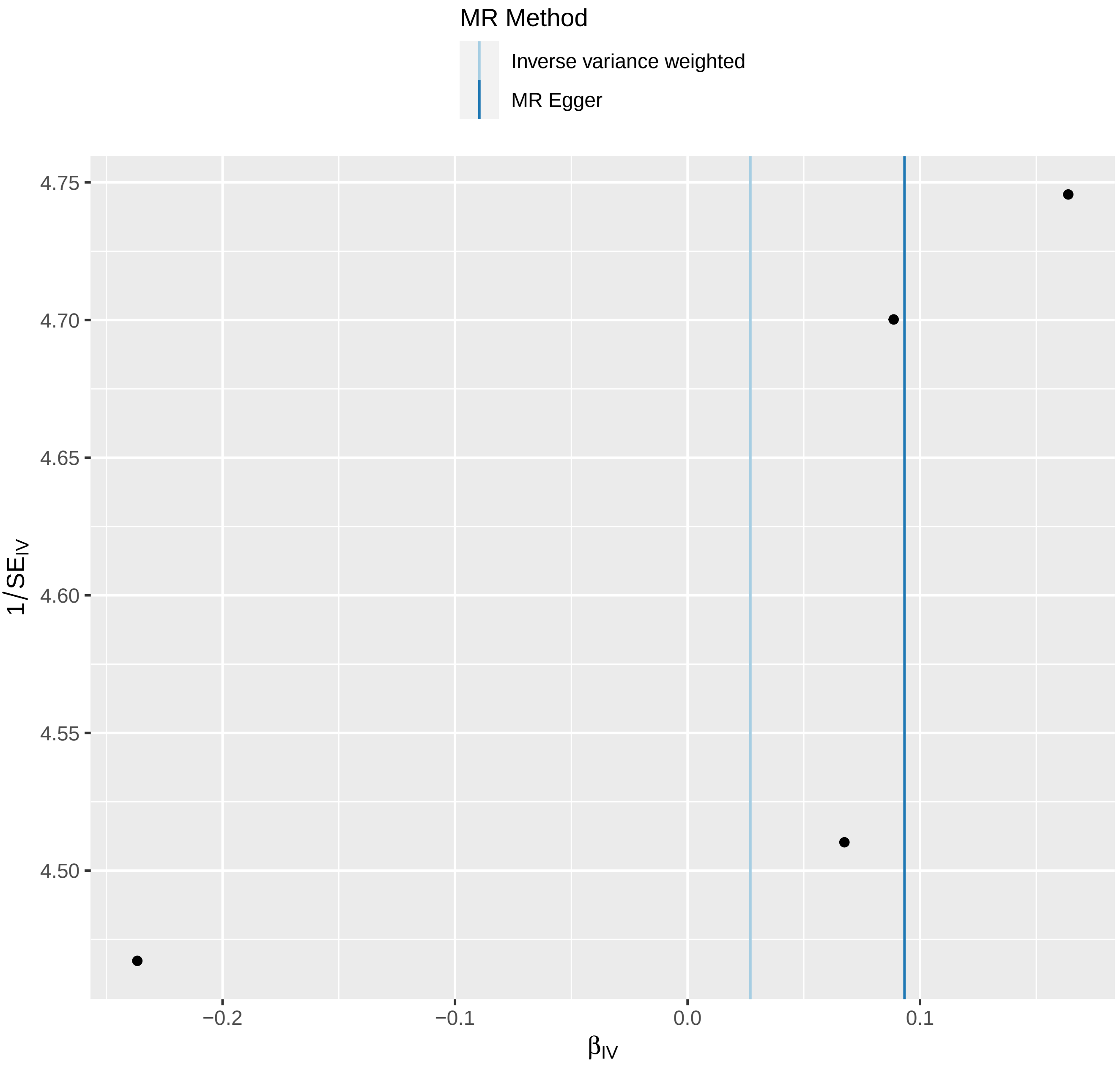

Batch 2−1136 : Gut microbiota abundance (genus Parasutterella id.2892) on C−type lectin domain family 10 member A
with a threshold of 1e−05

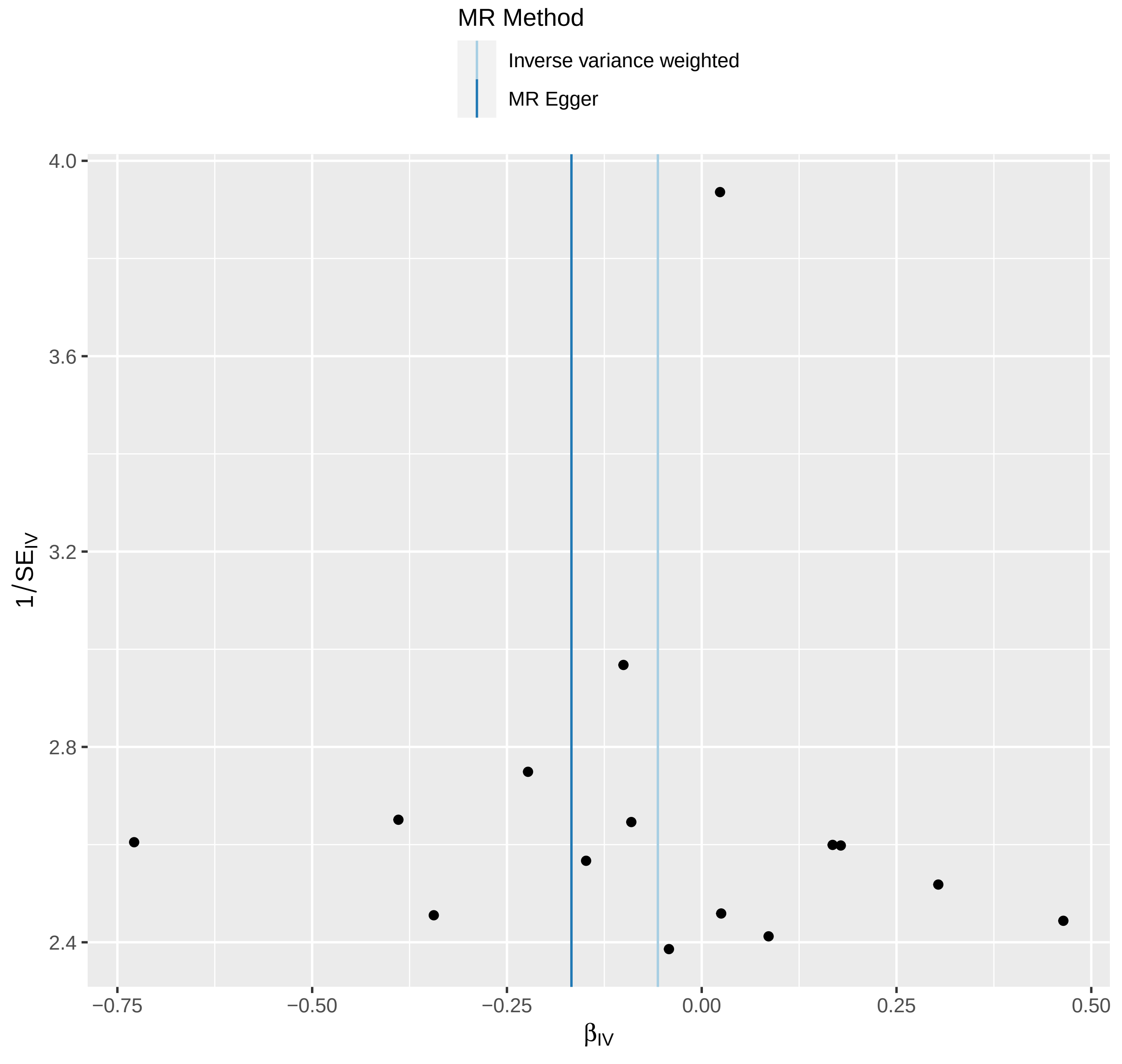

Batch 2−1137 : Gut microbiota abundance (genus Catenibacterium id.2153) on Stem Cell Growth Factor−beta
with a threshold of 1e−05

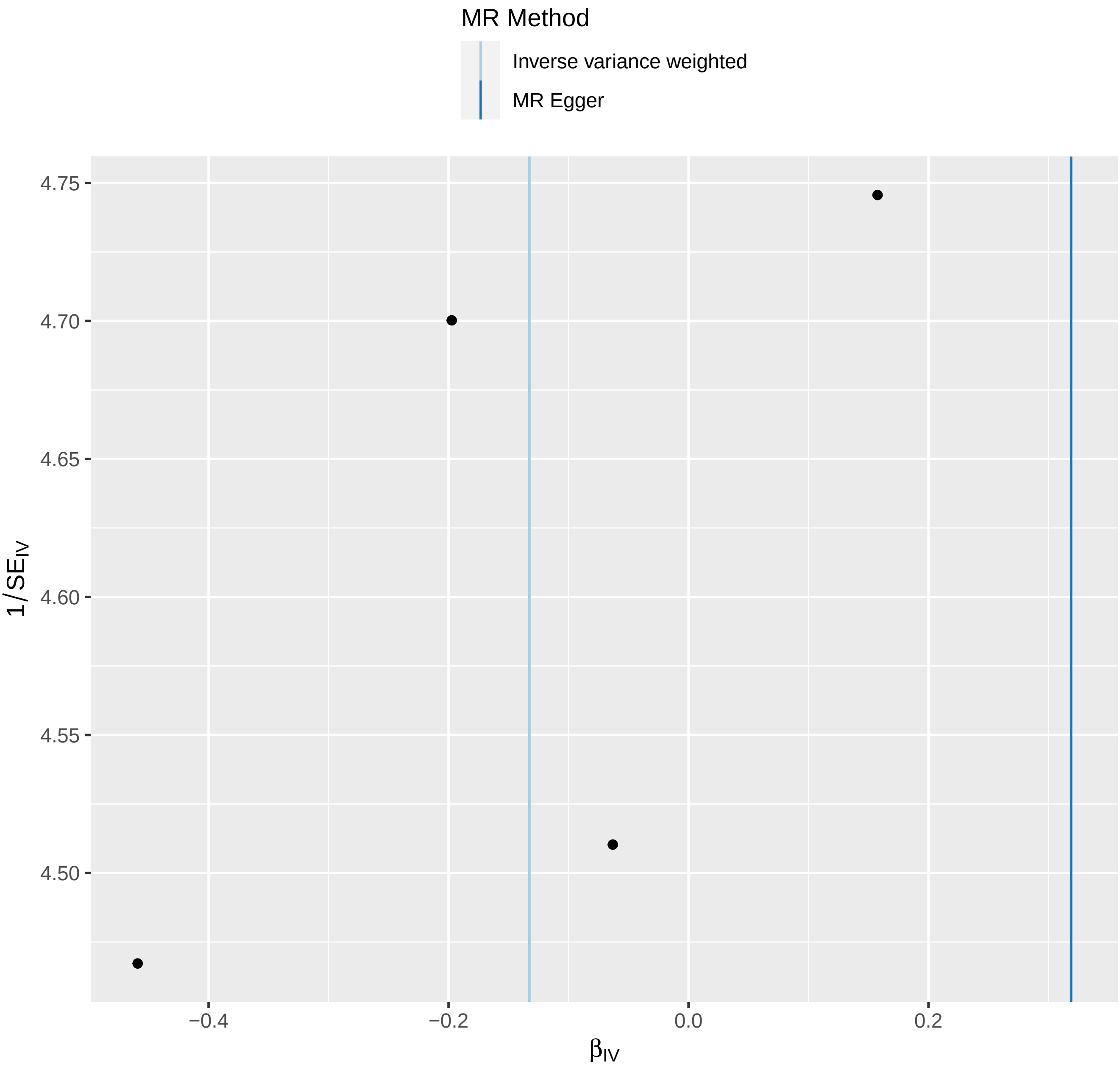

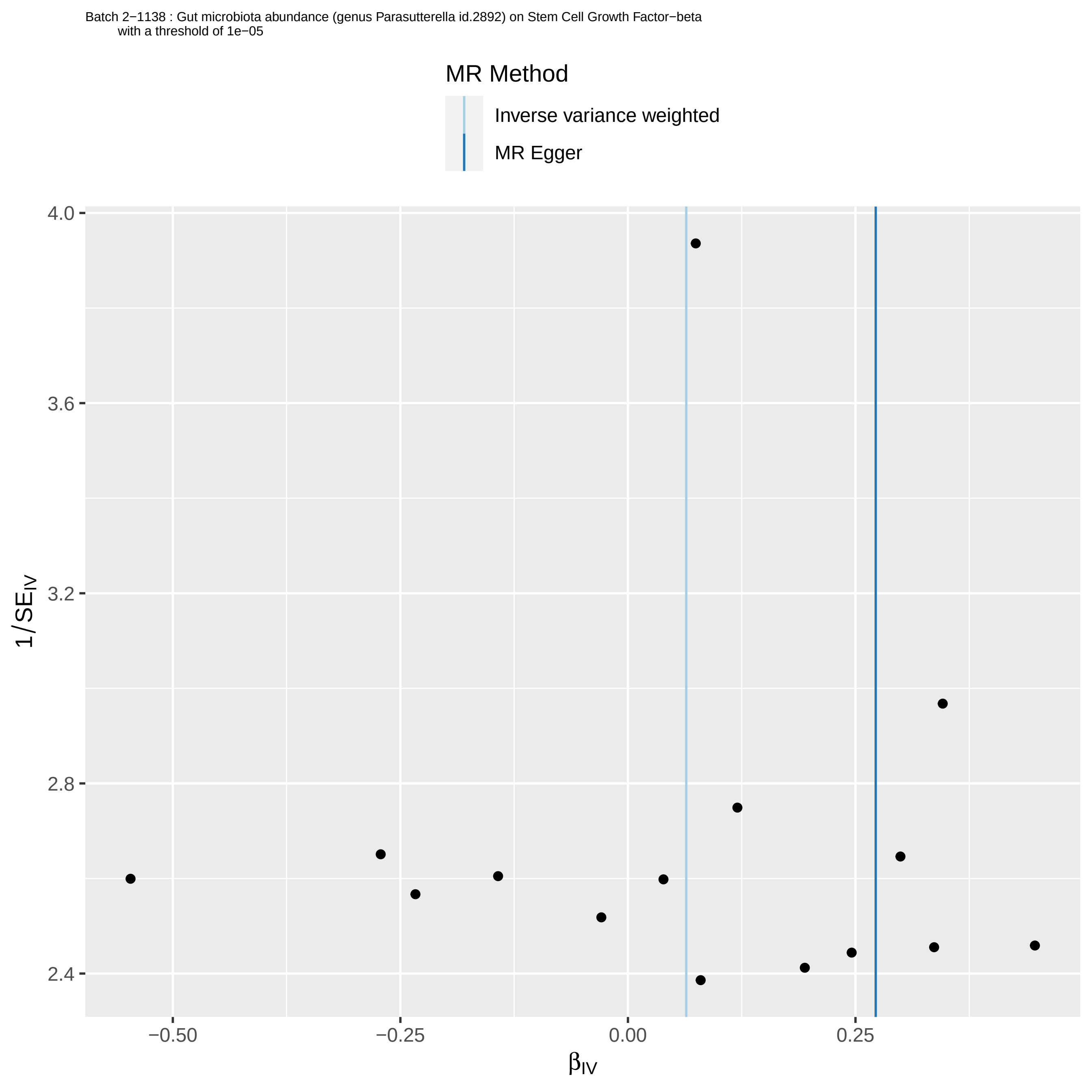

Batch 2−1138 : Gut microbiota abundance (genus Parasutterella id.2892) on Stem Cell Growth Factor−beta
with a threshold of 1e−05
MR Method
Inverse variance weighted
MR Egger
4.0
3.6
3.2
2.8
2.4
1/SE_IV
−0.50
−0.25
0.00
0.25
β_IV

Batch 2−1139 : Gut microbiota abundance (genus Catenibacterium id.2153) on C−type lectin domain family 12 member A
with a threshold of 1e−05

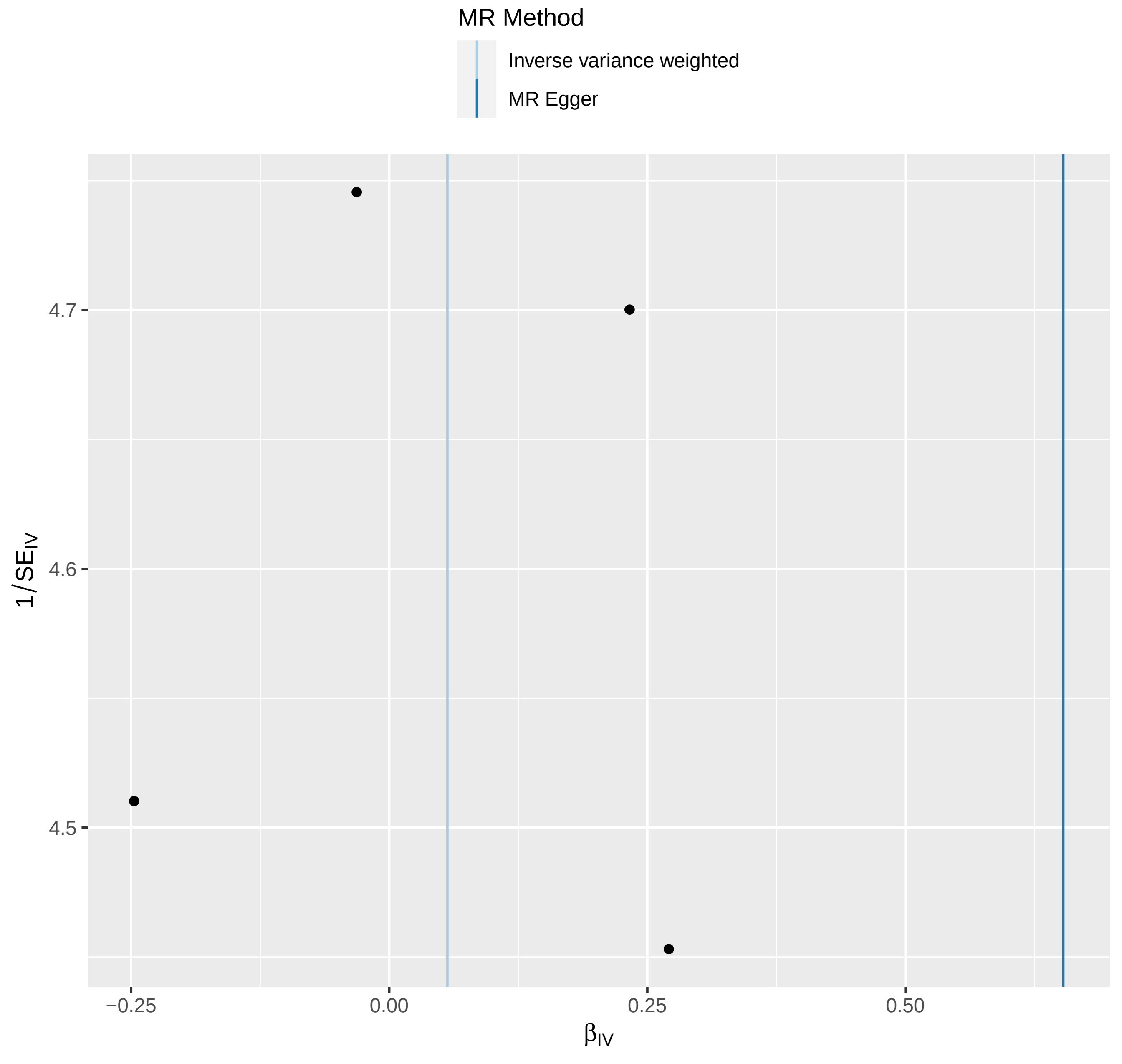

Batch 2−1140 : Gut microbiota abundance (genus Parasutterella id.2892) on C−type lectin domain family 12 member A
with a threshold of 1e−05

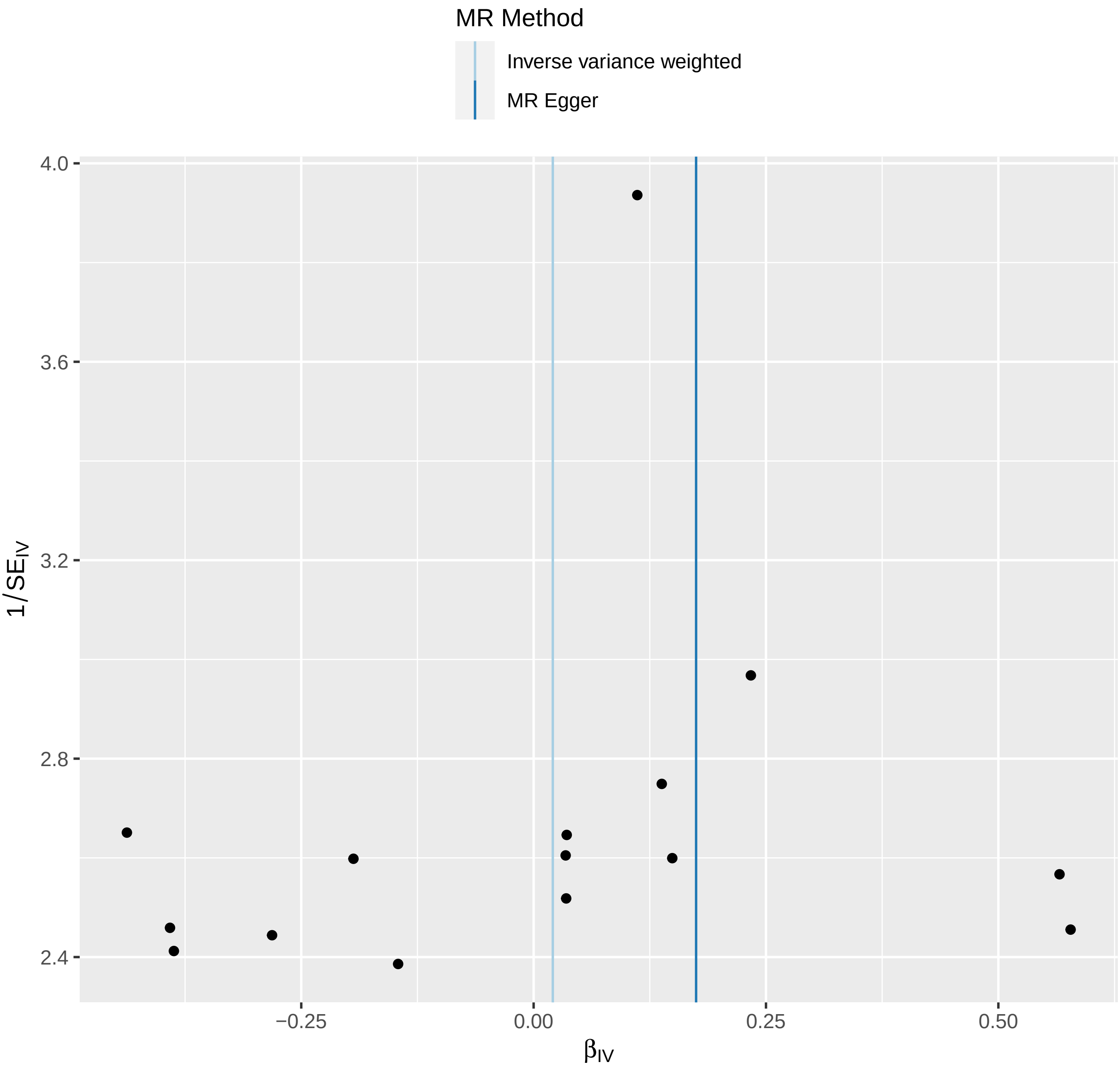

Batch 2−1141 : Gut microbiota abundance (genus Catenibacterium id.2153) on C−type lectin domain family 14 member A
with a threshold of 1e−05

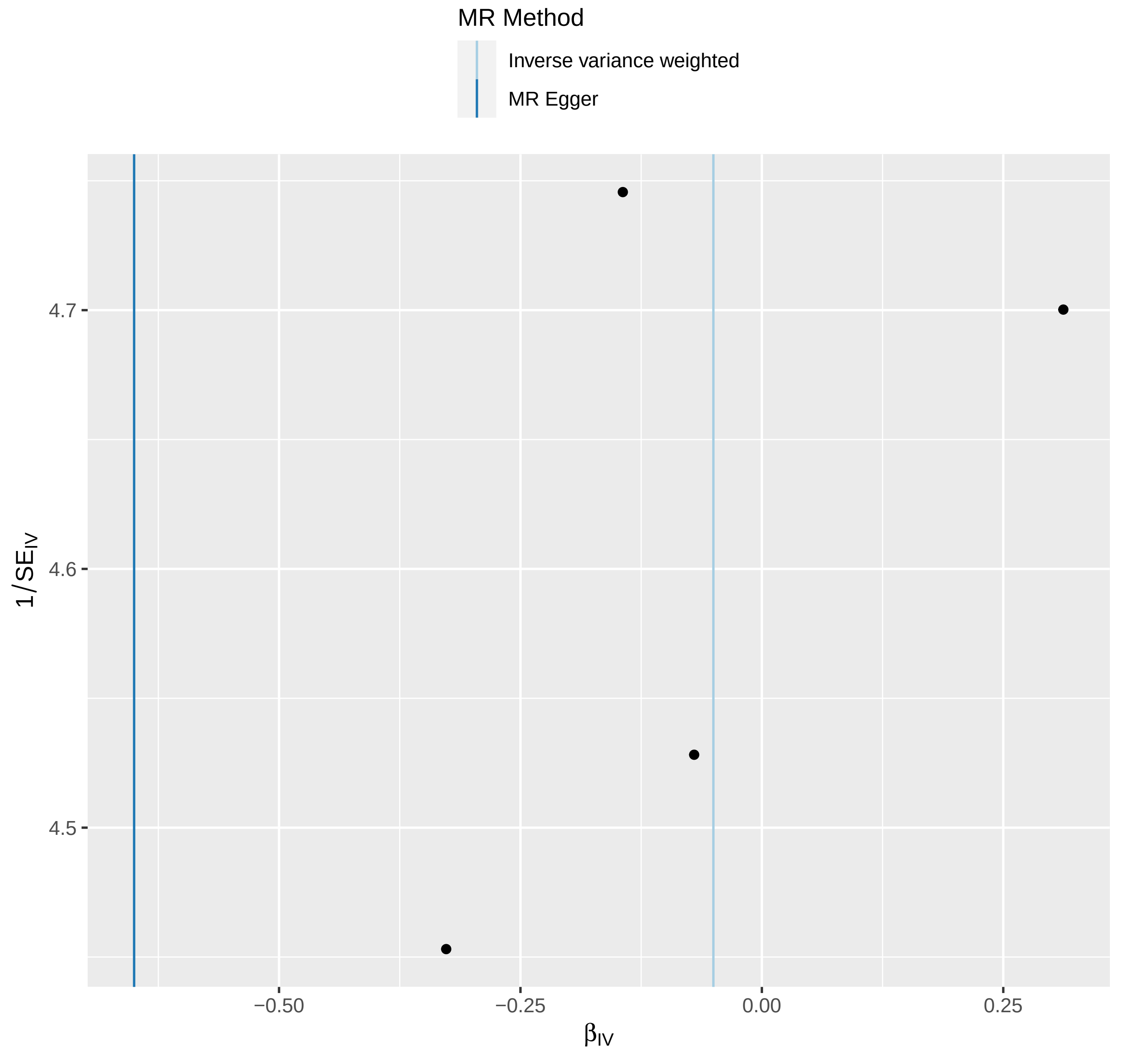

Batch 2−1142 : Gut microbiota abundance (genus Parasutterella id.2892) on C−type lectin domain family 14 member A
with a threshold of 1e−05

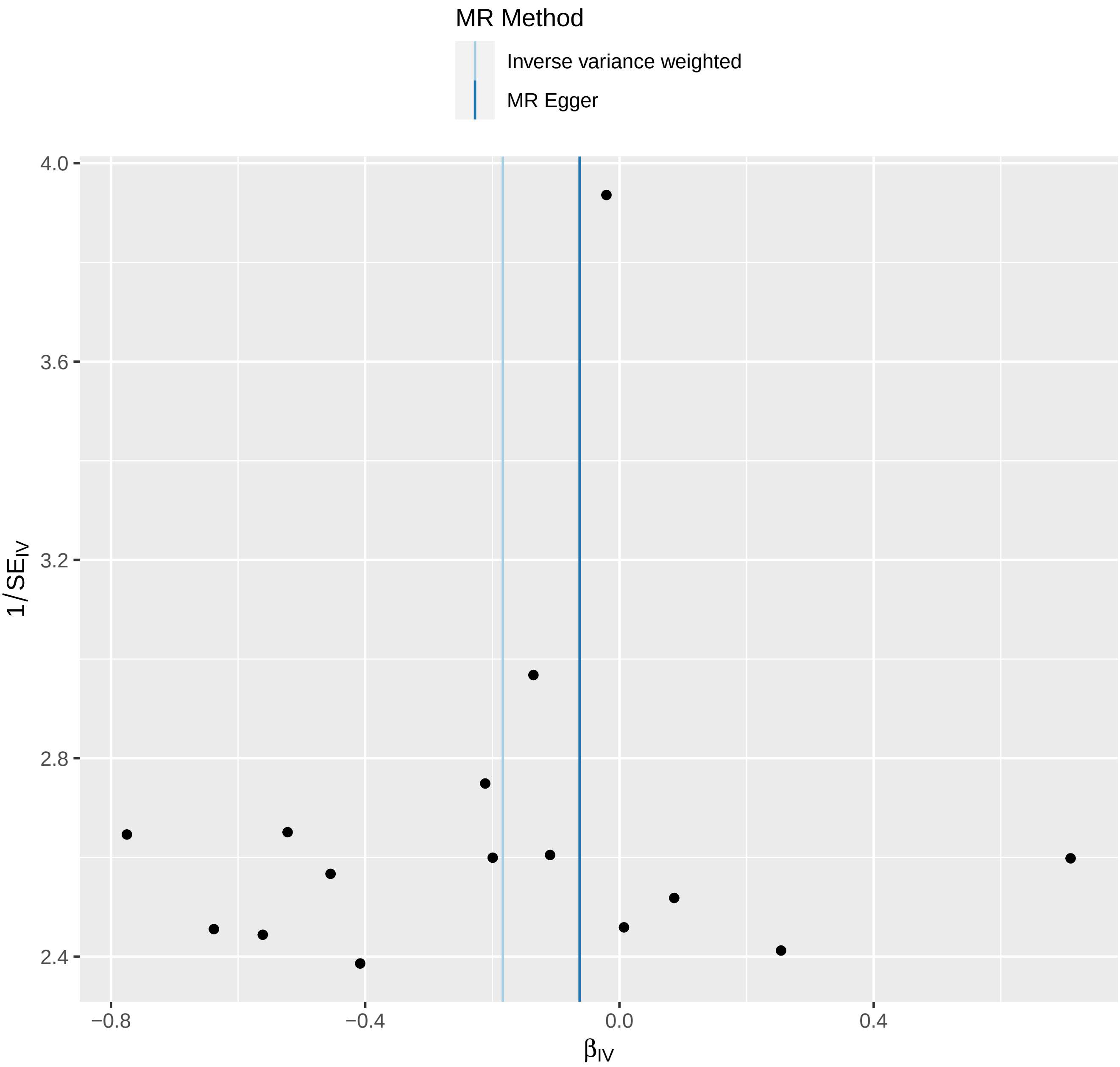

Batch 2−1143 : Gut microbiota abundance (genus Catenibacterium id.2153) on C−type lectin domain family 2 member A
with a threshold of 1e−05

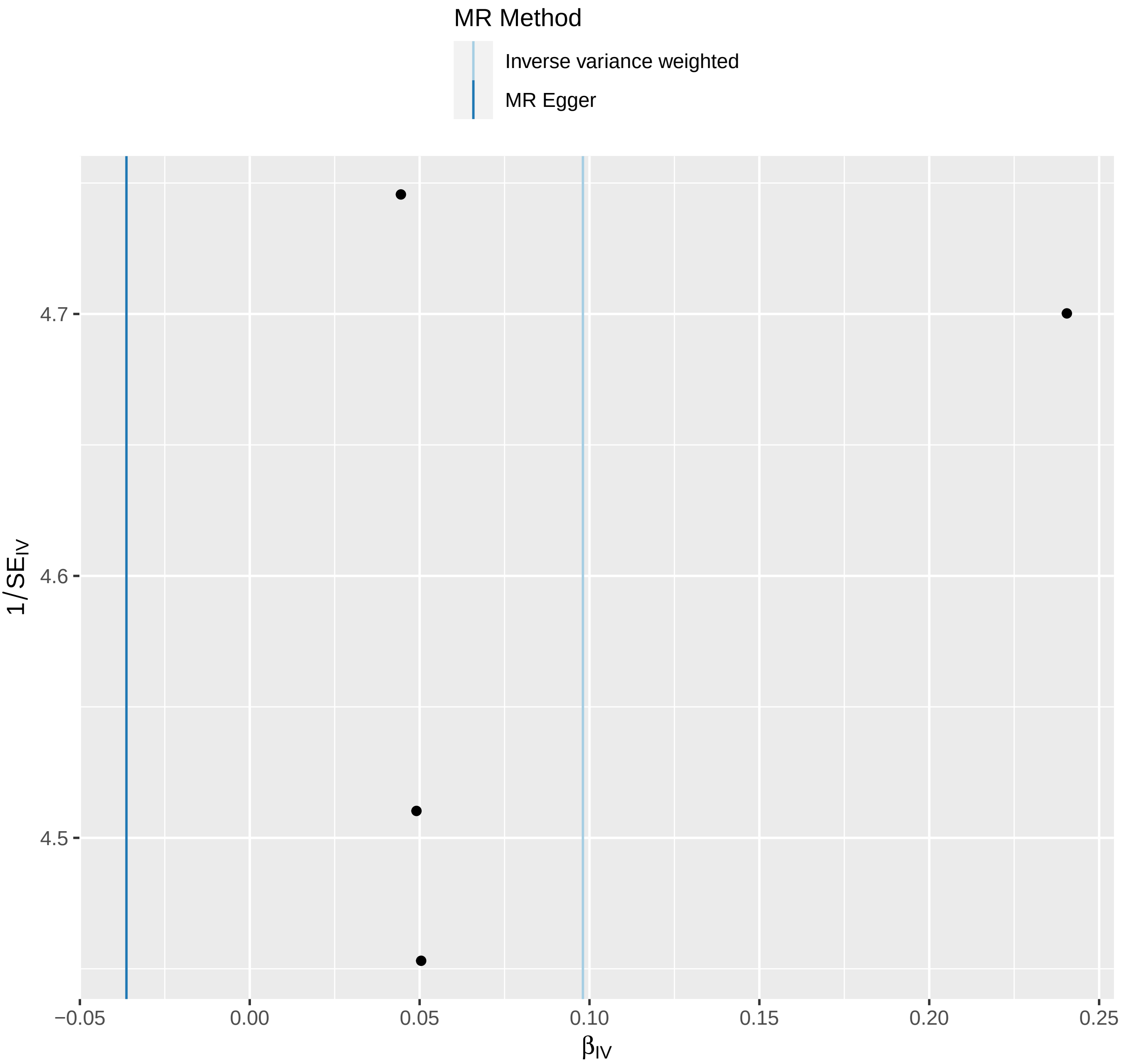

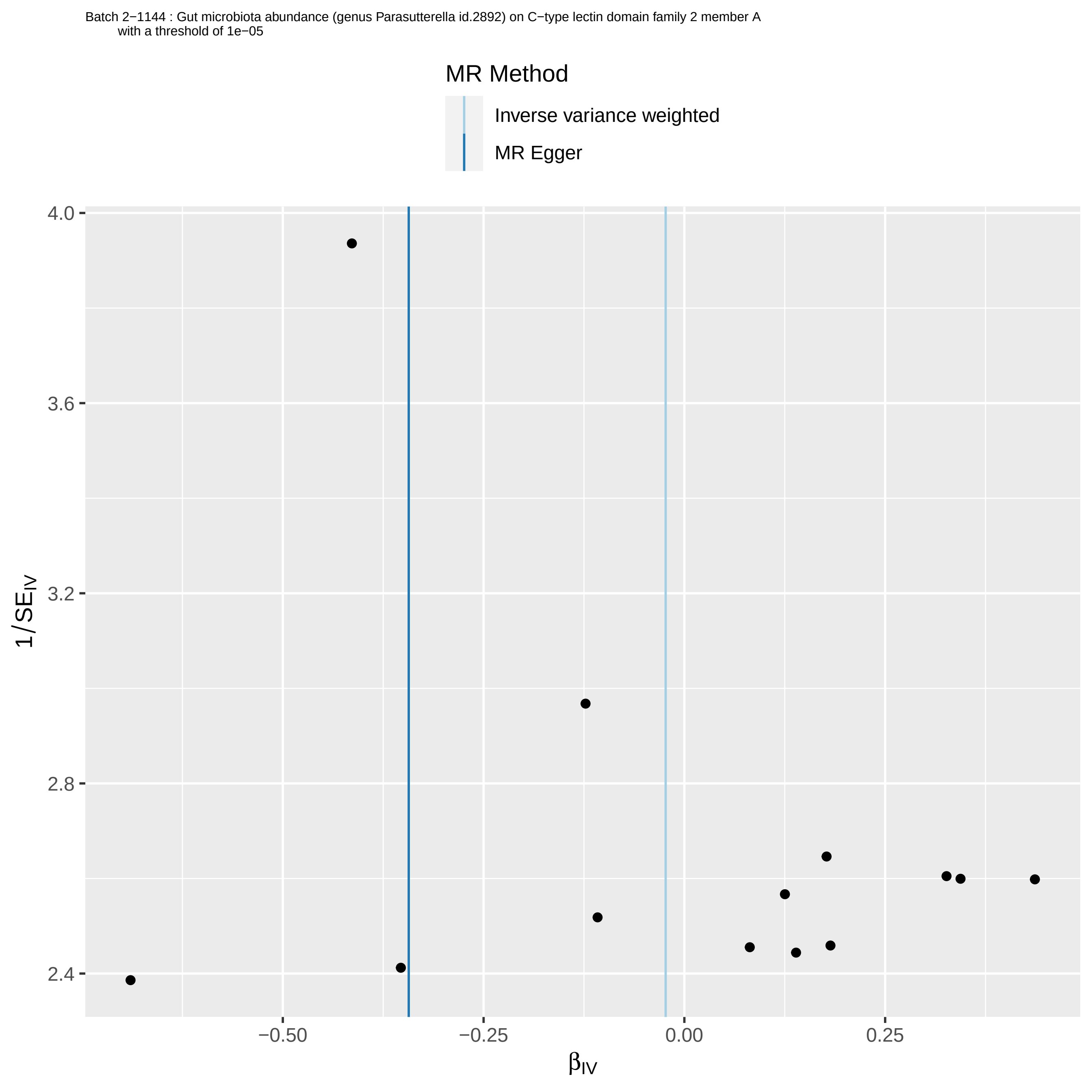
Batch 2−1144 : Gut microbiota abundance (genus Parasutterella id.2892) on C−type lectin domain family 2 member A
with a threshold of 1e−05
MR Method
Inverse variance weighted
MR Egger
4.0
3.6
3.2
2.8
2.4
$1/SE_{IV}$
−0.50
−0.25
0.00
0.25
β_{IV}

Batch 2−1145 : Gut microbiota abundance (genus Catenibacterium id.2153) on C−type lectin domain family 2 member B
with a threshold of 1e−05

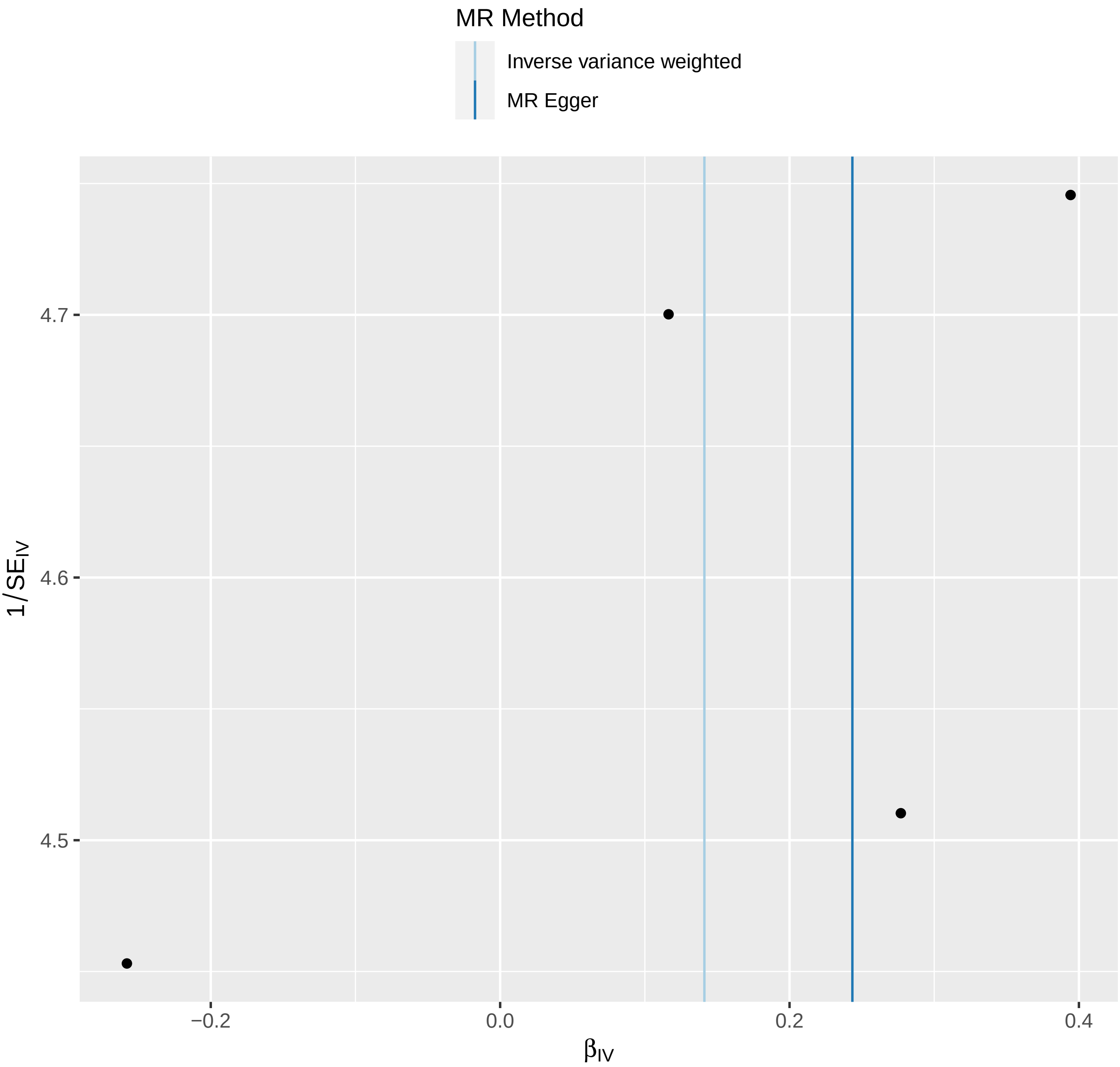

Batch 2−1146 : Gut microbiota abundance (genus Parasutterella id.2892) on C−type lectin domain family 2 member B
with a threshold of 1e−05

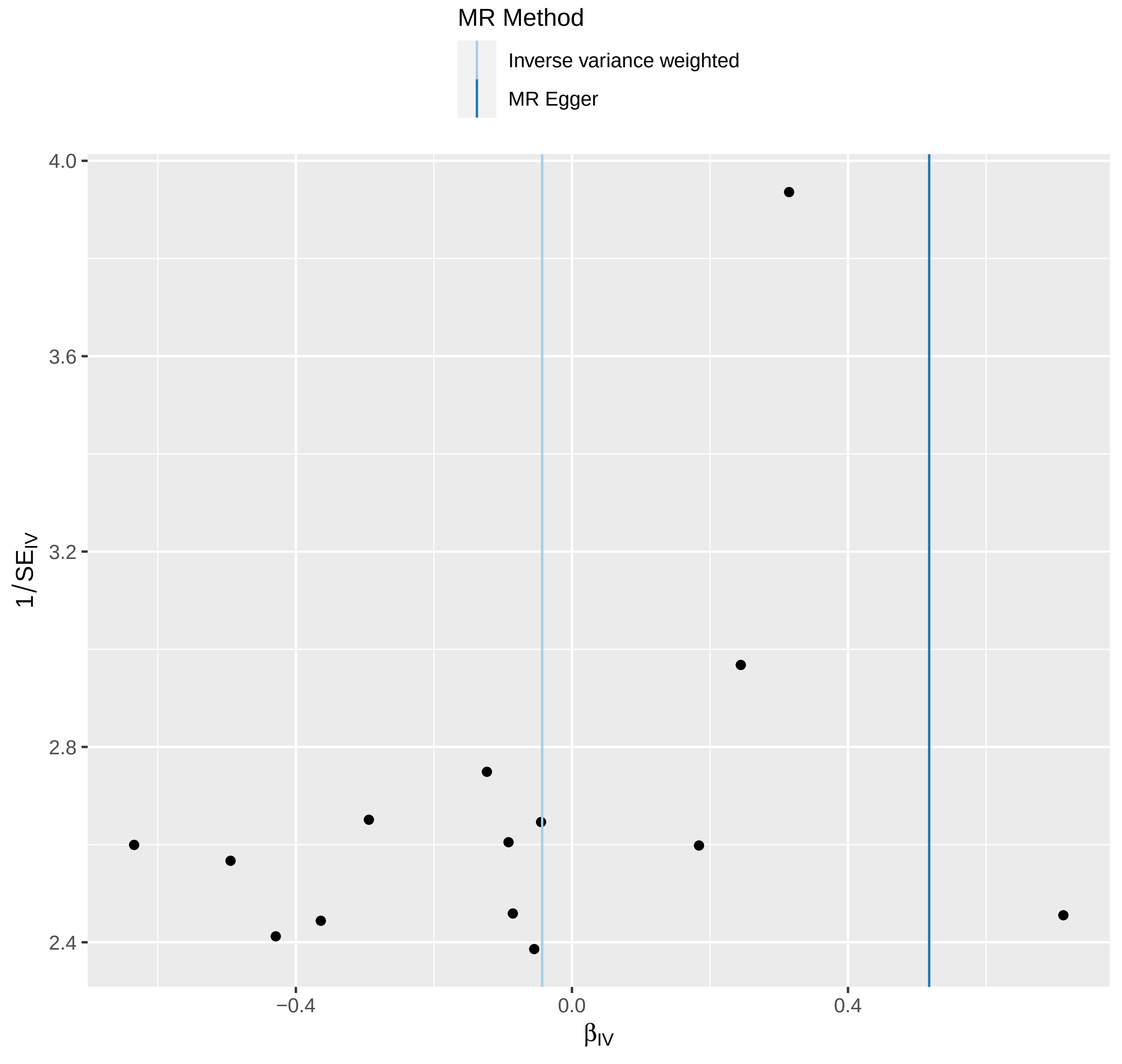

Batch 2−1147 : Gut microbiota abundance (genus Catenibacterium id.2153) on C−type lectin domain family 2 member D
with a threshold of 1e−05

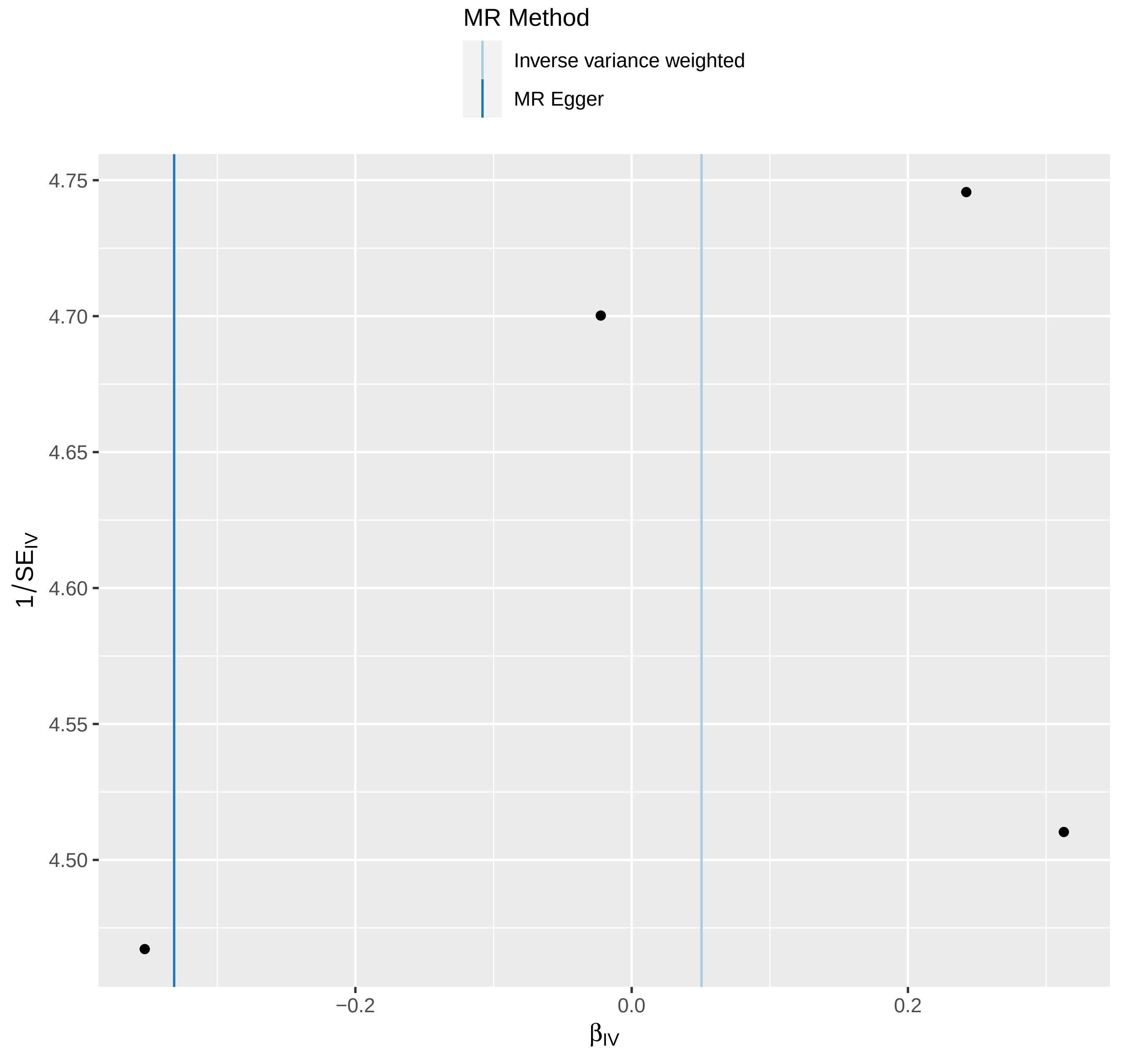

Batch 2−1148 : Gut microbiota abundance (genus Parasutterella id.2892) on C−type lectin domain family 2 member D
with a threshold of 1e−05

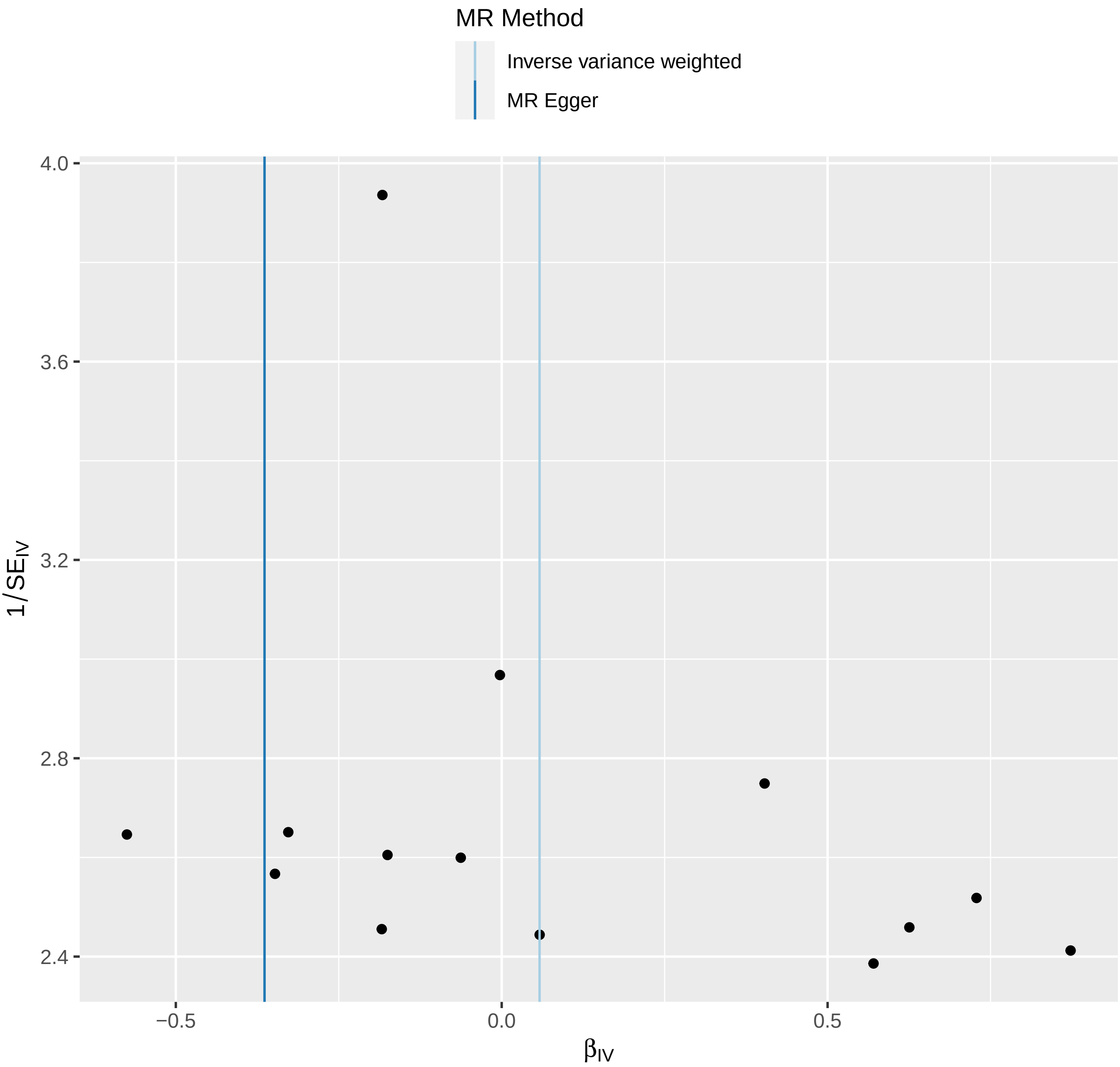

Batch 2−1149 : Gut microbiota abundance (genus Catenibacterium id.2153) on C−type lectin domain family 2 member L
with a threshold of 1e−05

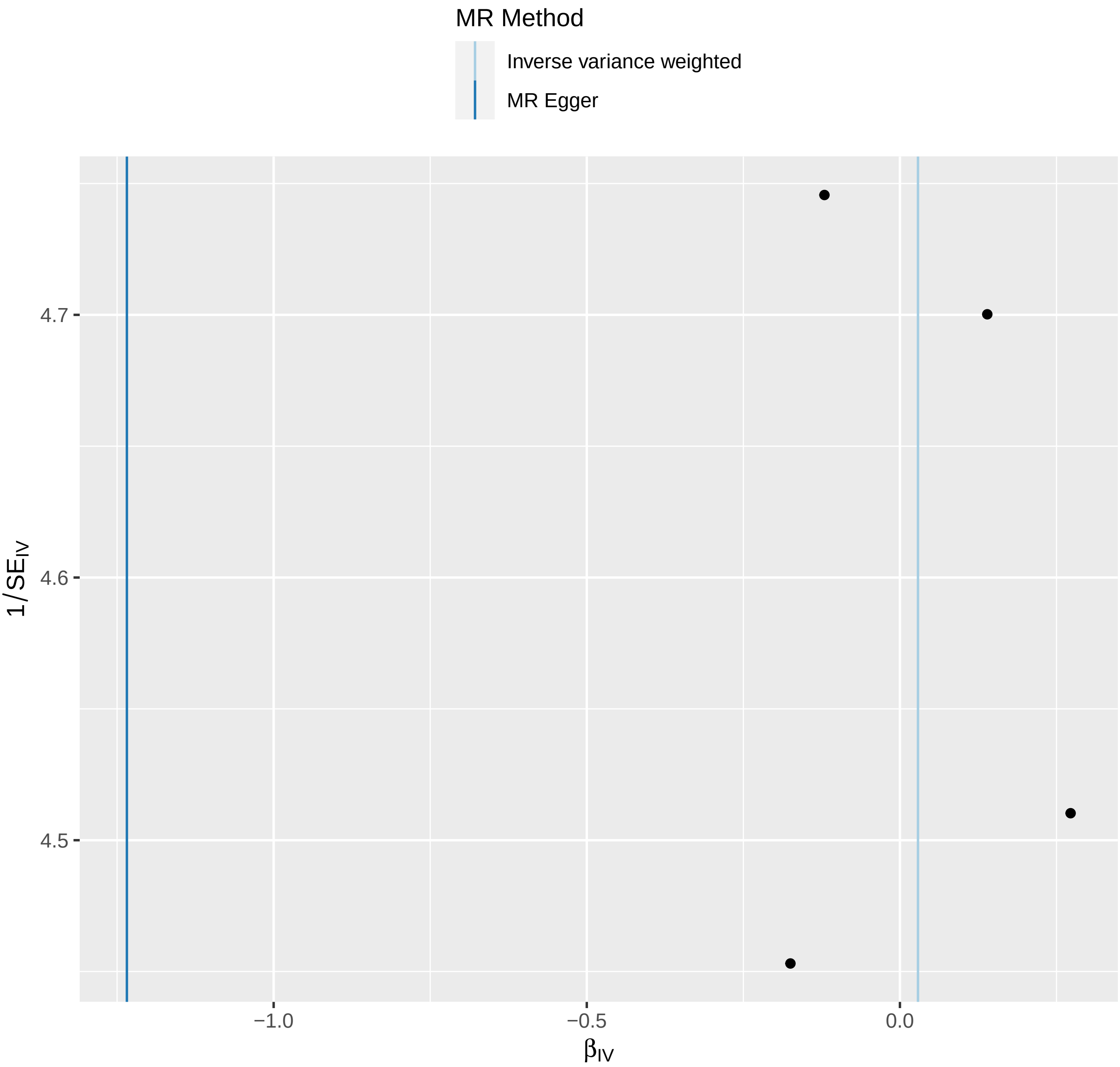

Batch 2−1150 : Gut microbiota abundance (genus Parasutterella id.2892) on C−type lectin domain family 2 member L
with a threshold of 1e−05

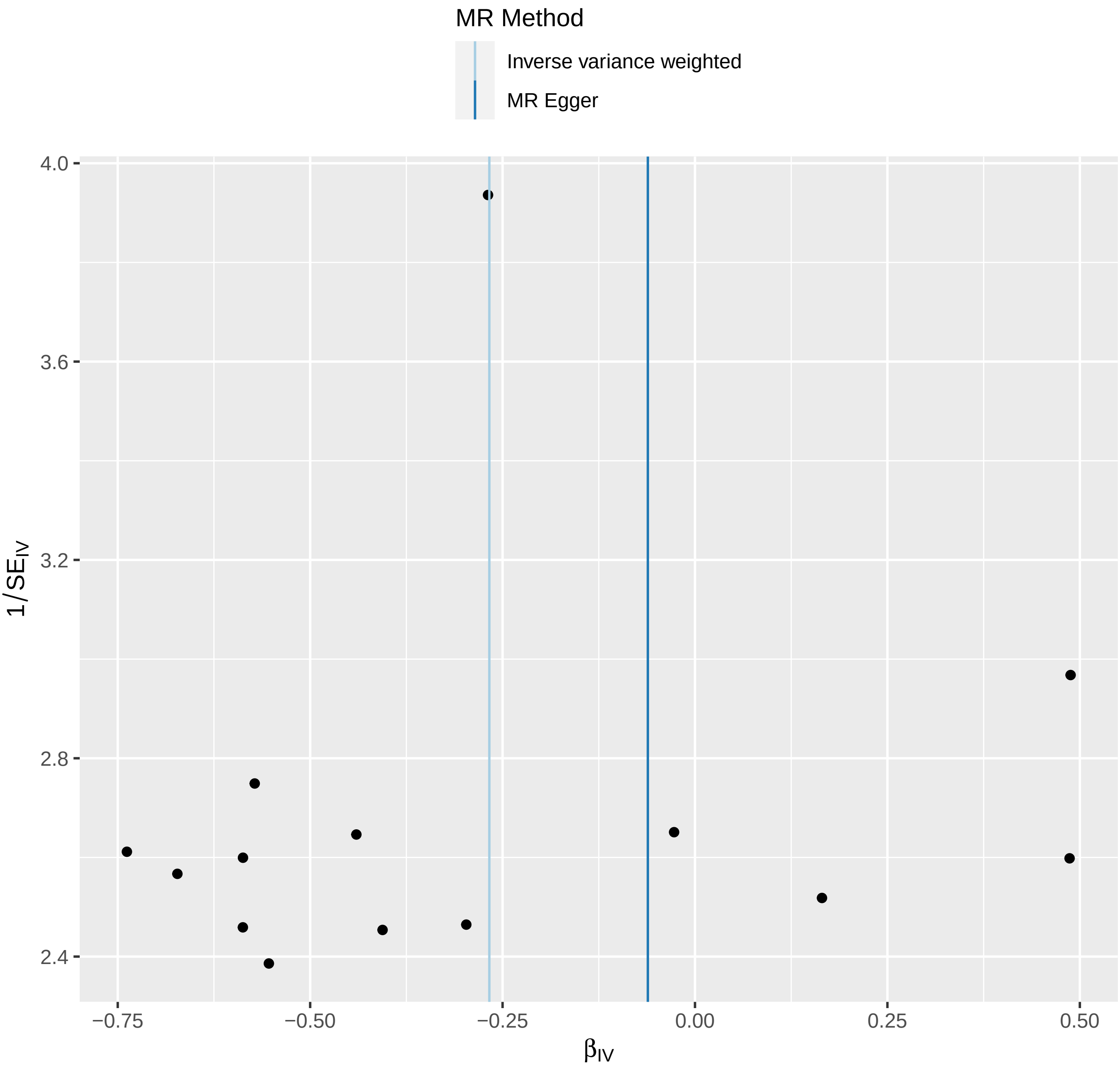

Batch 2−1151 : Gut microbiota abundance (genus Catenibacterium id.2153) on C−type lectin domain family 4 member D
with a threshold of 1e−05

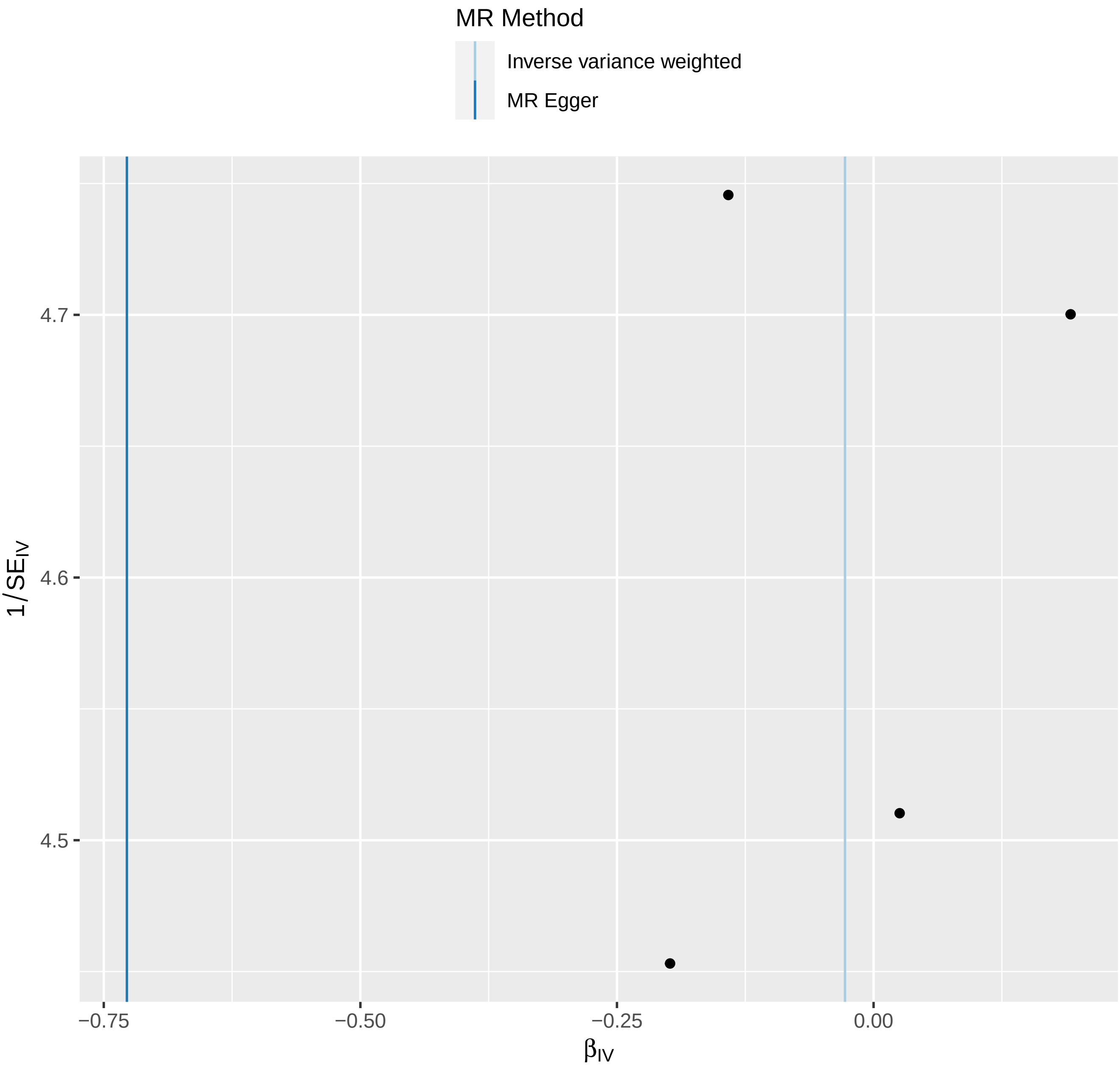

Batch 2−1152 : Gut microbiota abundance (genus Parasutterella id.2892) on C−type lectin domain family 4 member D
with a threshold of 1e−05

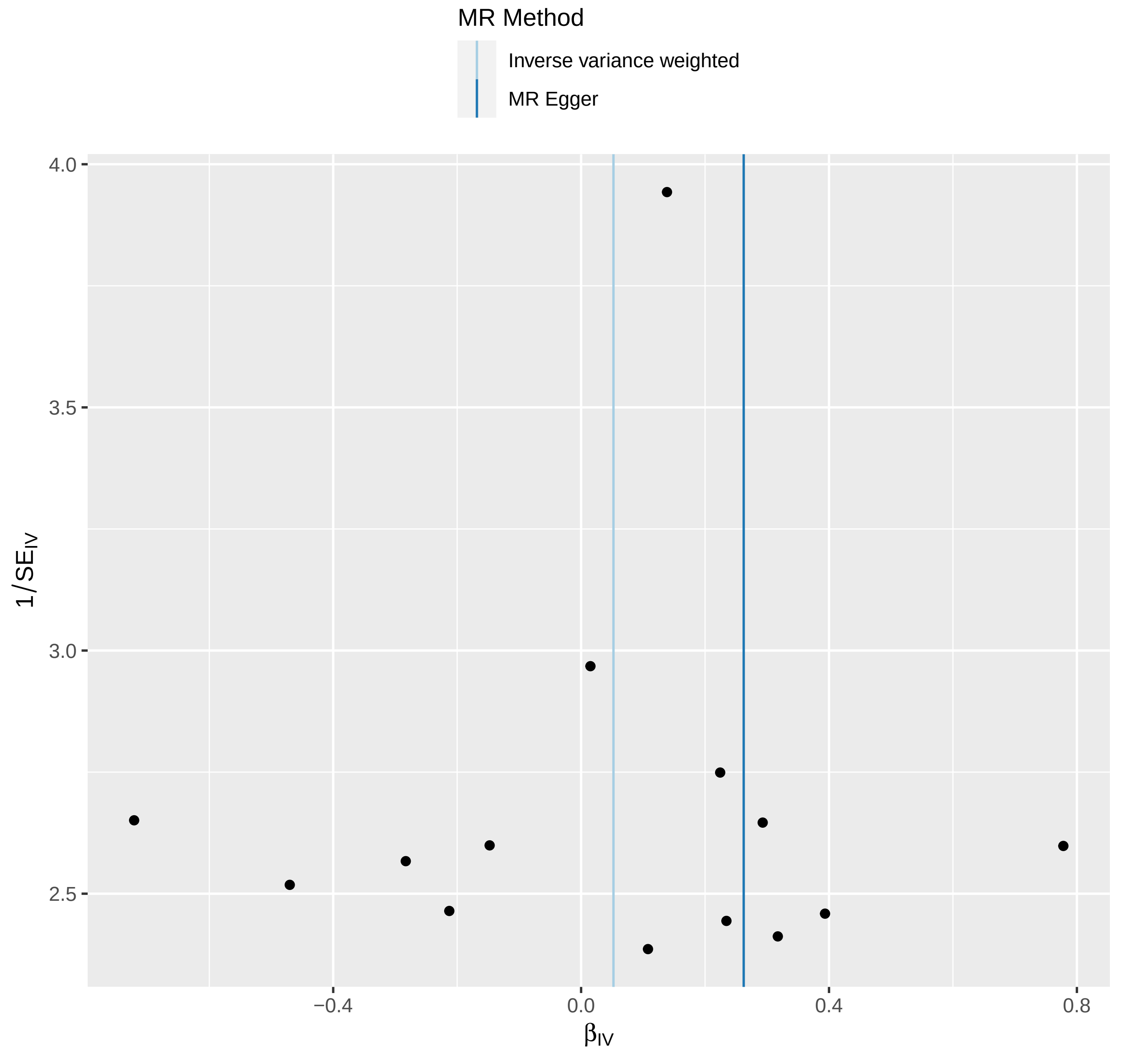

Batch 2−1153 : Gut microbiota abundance (genus Catenibacterium id.2153) on C−type lectin domain family 4 member E
with a threshold of 1e−05

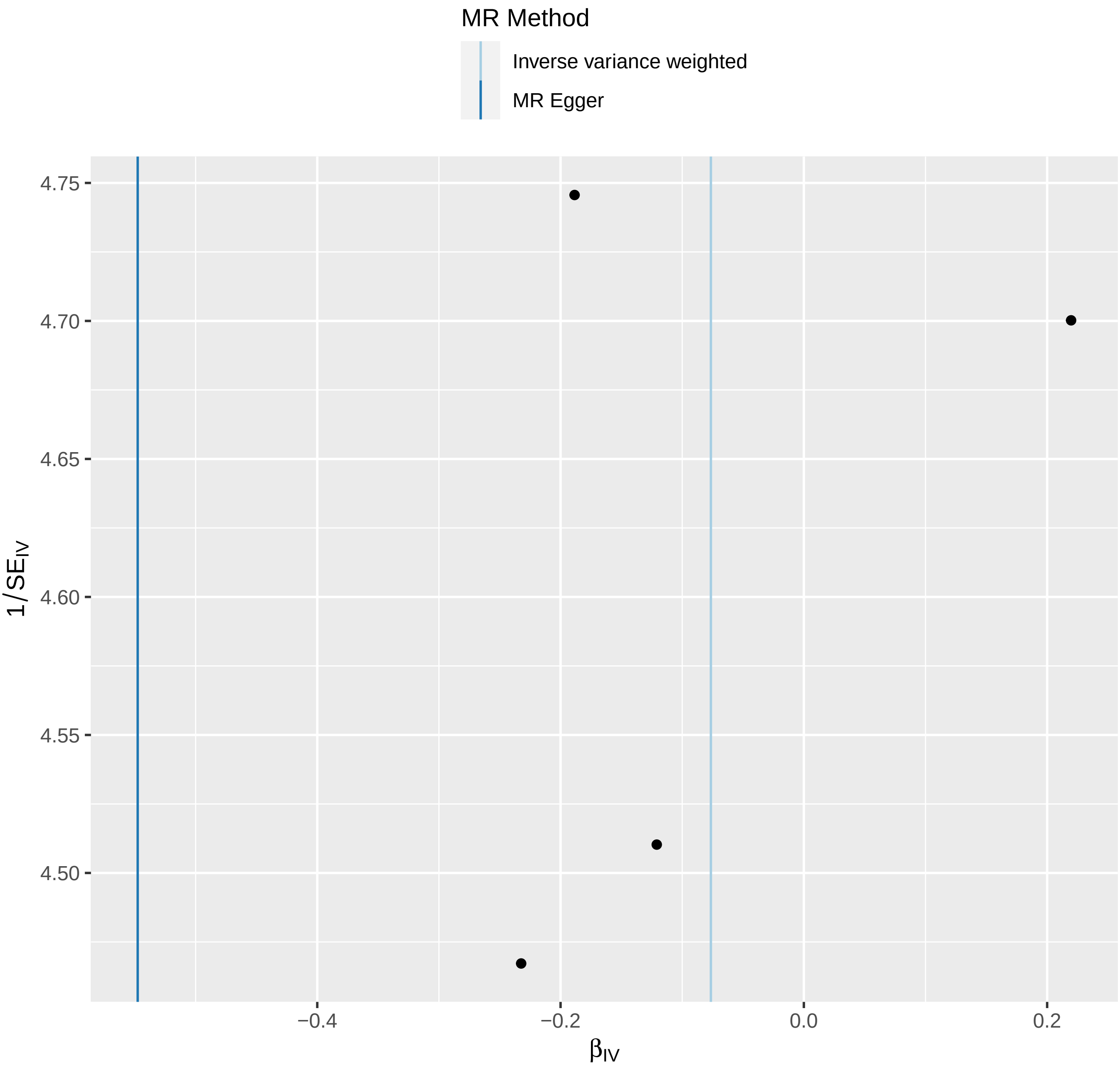

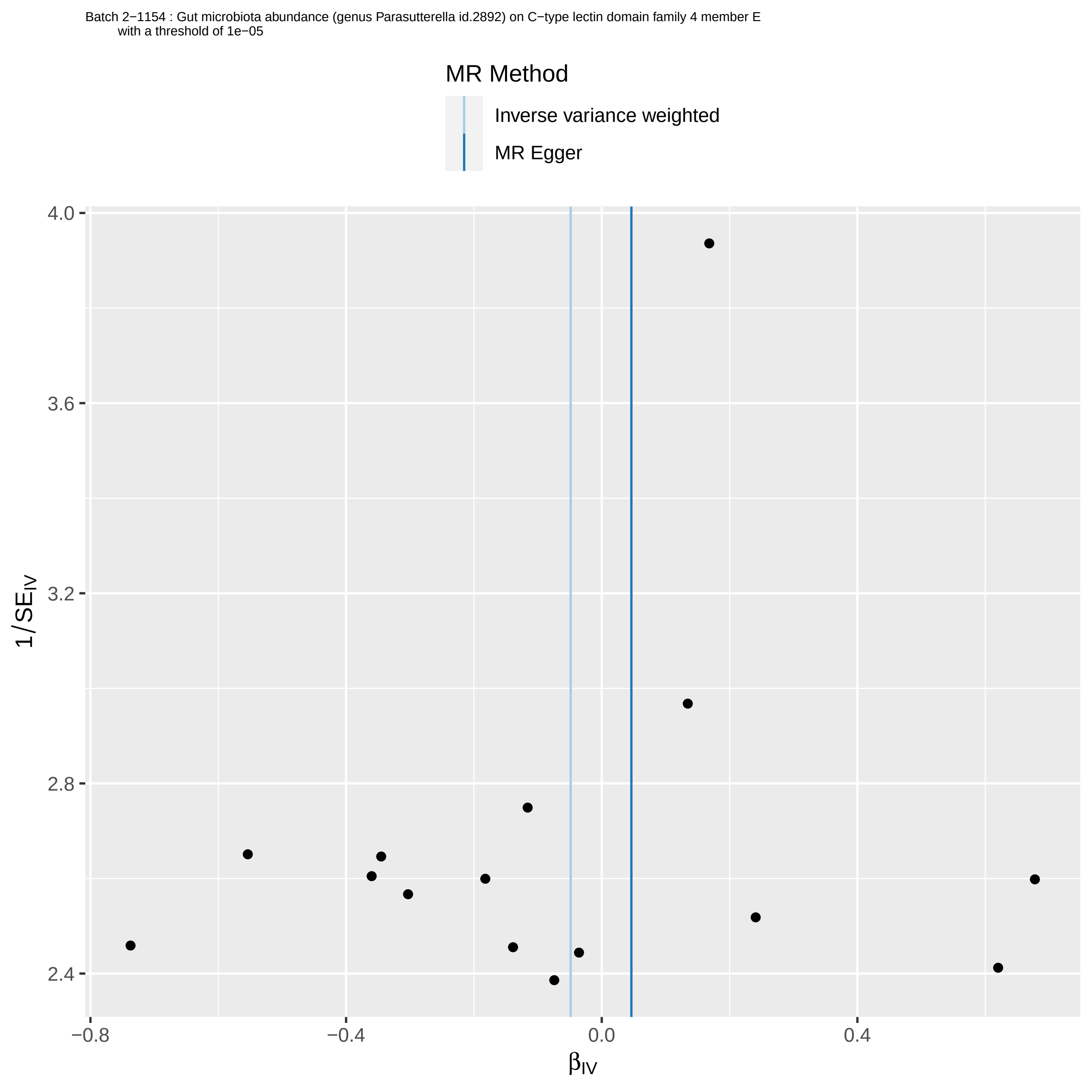

Batch 2−1154 : Gut microbiota abundance (genus Parasutterella id.2892) on C−type lectin domain family 4 member E
with a threshold of 1e−05
MR Method
Inverse variance weighted
MR Egger
4.0
3.6
3.2
2.8
2.4
1/SE_IV
−0.8
−0.4
0.0
0.4
β_IV

Batch 2−1155 : Gut microbiota abundance (genus Catenibacterium id.2153) on C−type lectin domain family 4 member M
with a threshold of 1e−05

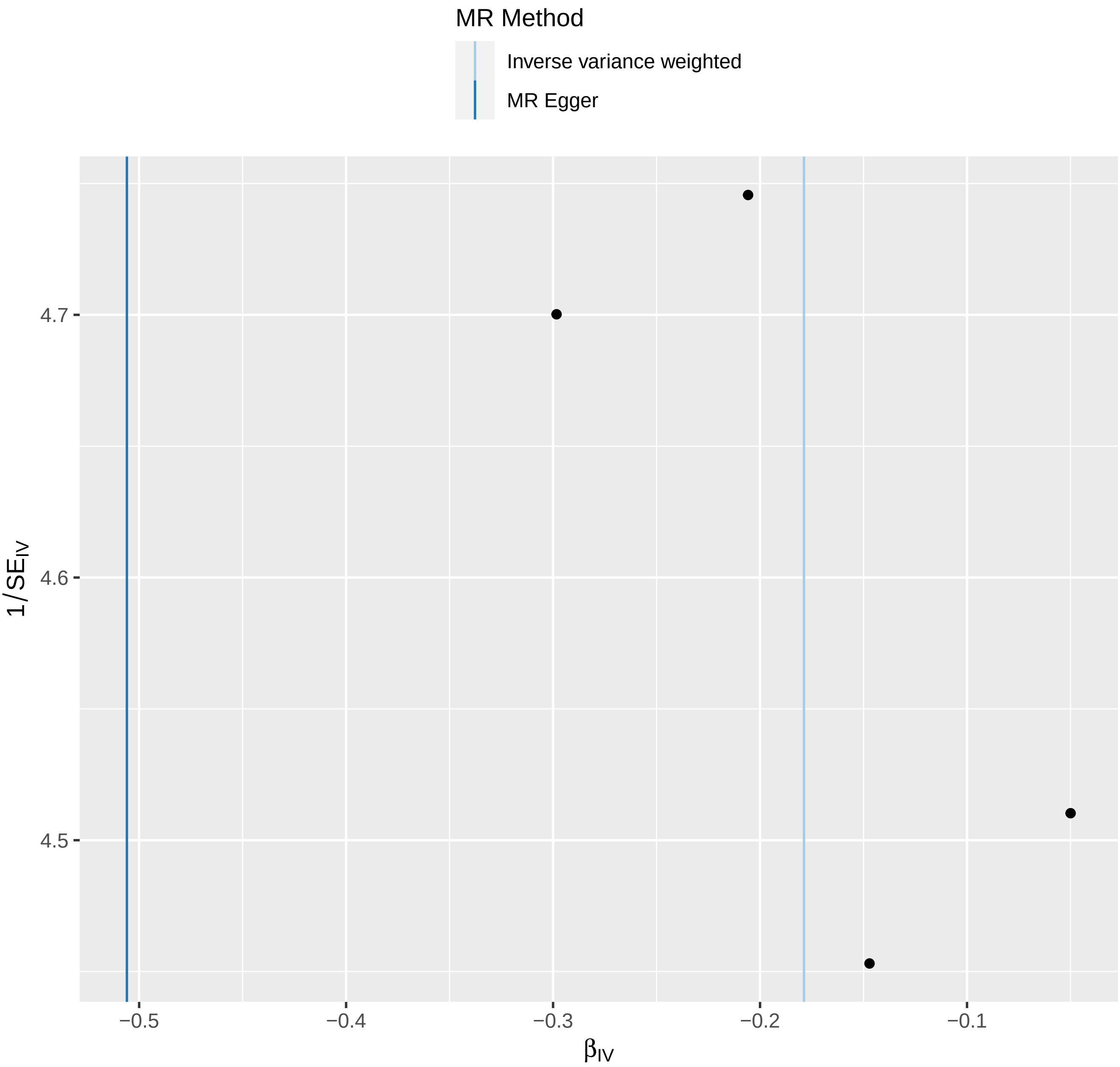

Batch 2−1156 : Gut microbiota abundance (genus Parasutterella id.2892) on C−type lectin domain family 4 member M
with a threshold of 1e−05

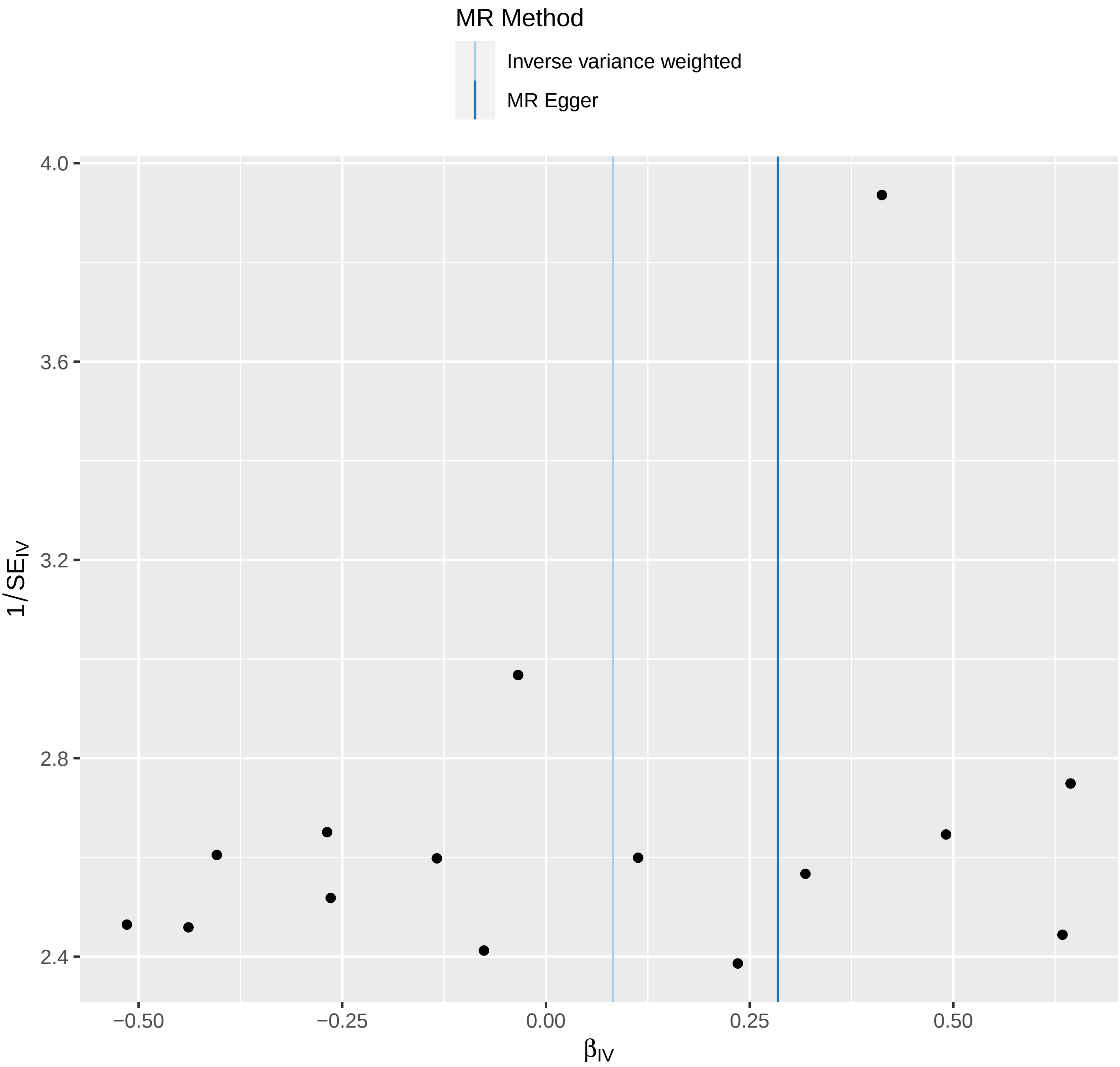

Batch 2−1157 : Gut microbiota abundance (genus Catenibacterium id.2153) on C−type lectin domain family 5 member A
with a threshold of 1e−05

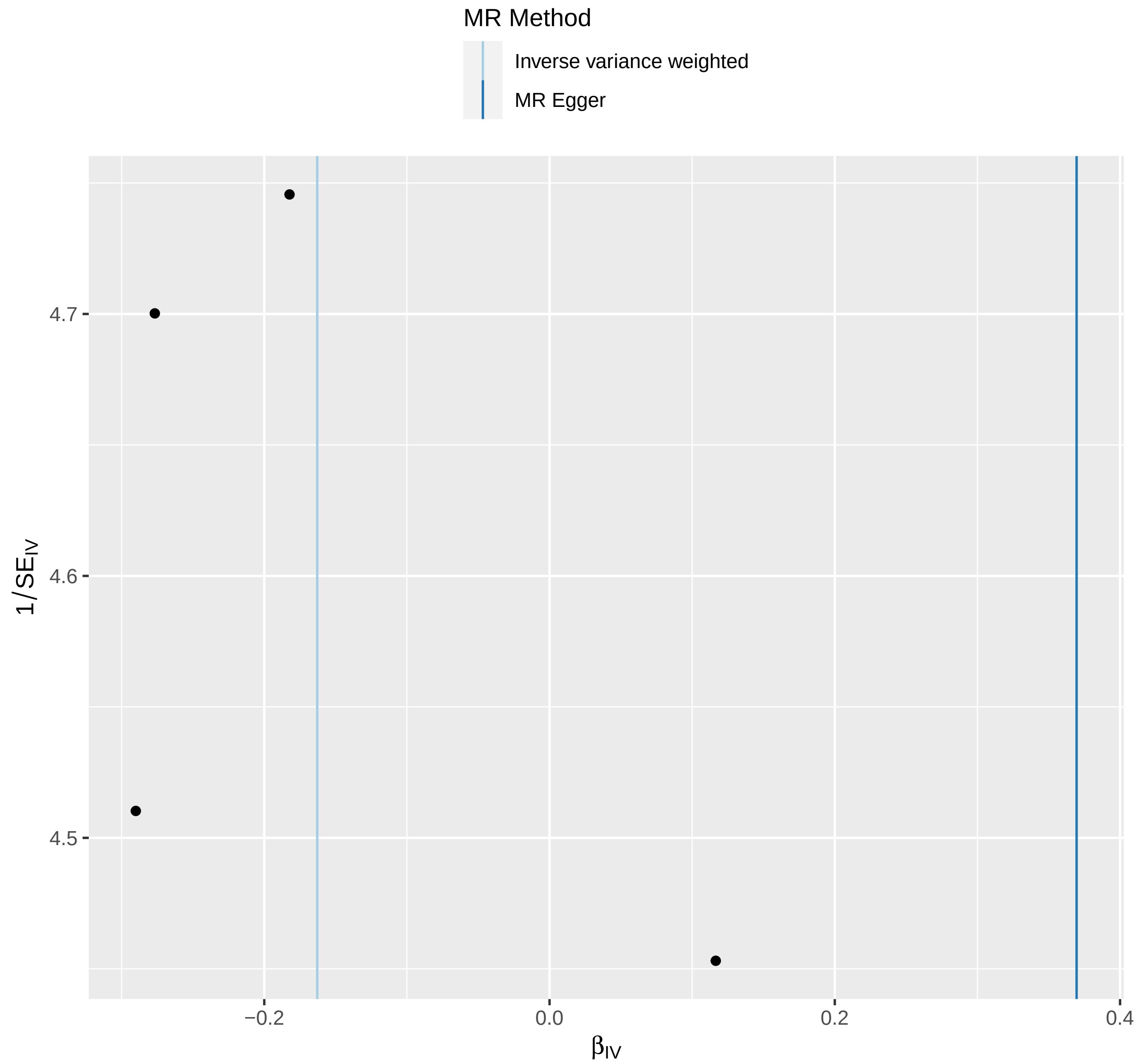

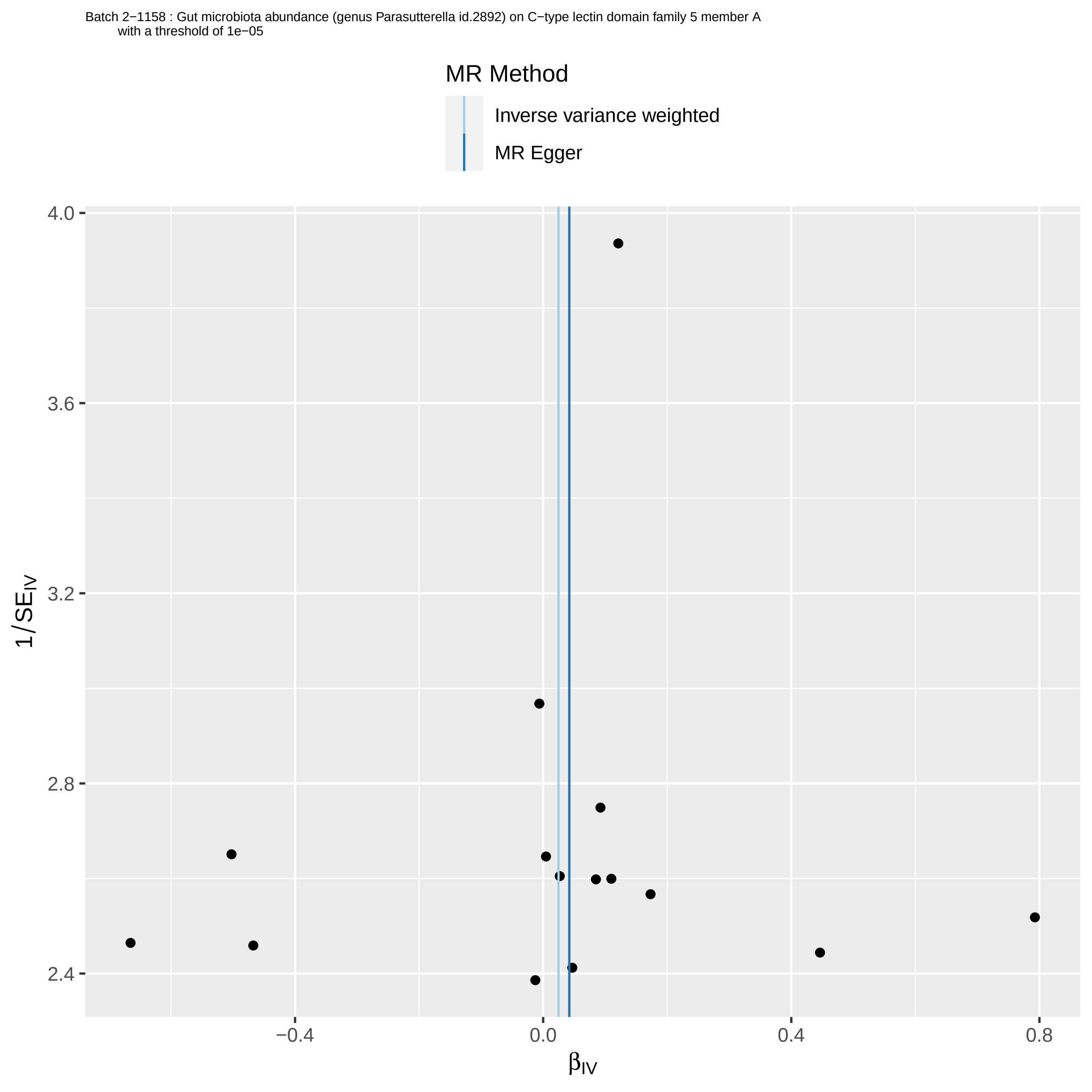
Batch 2−1158 : Gut microbiota abundance (genus Parasutterella id.2892) on C−type lectin domain family 5 member A
with a threshold of 1e−05
MR Method
Inverse variance weighted
MR Egger
4.0
3.6
3.2
2.8
2.4
$1/SE_{IV}$
−0.4
0.0
0.4
0.8
β_{IV}

Batch 2−1159 : Gut microbiota abundance (genus Catenibacterium id.2153) on C−type lectin domain family 6 member A
with a threshold of 1e−05

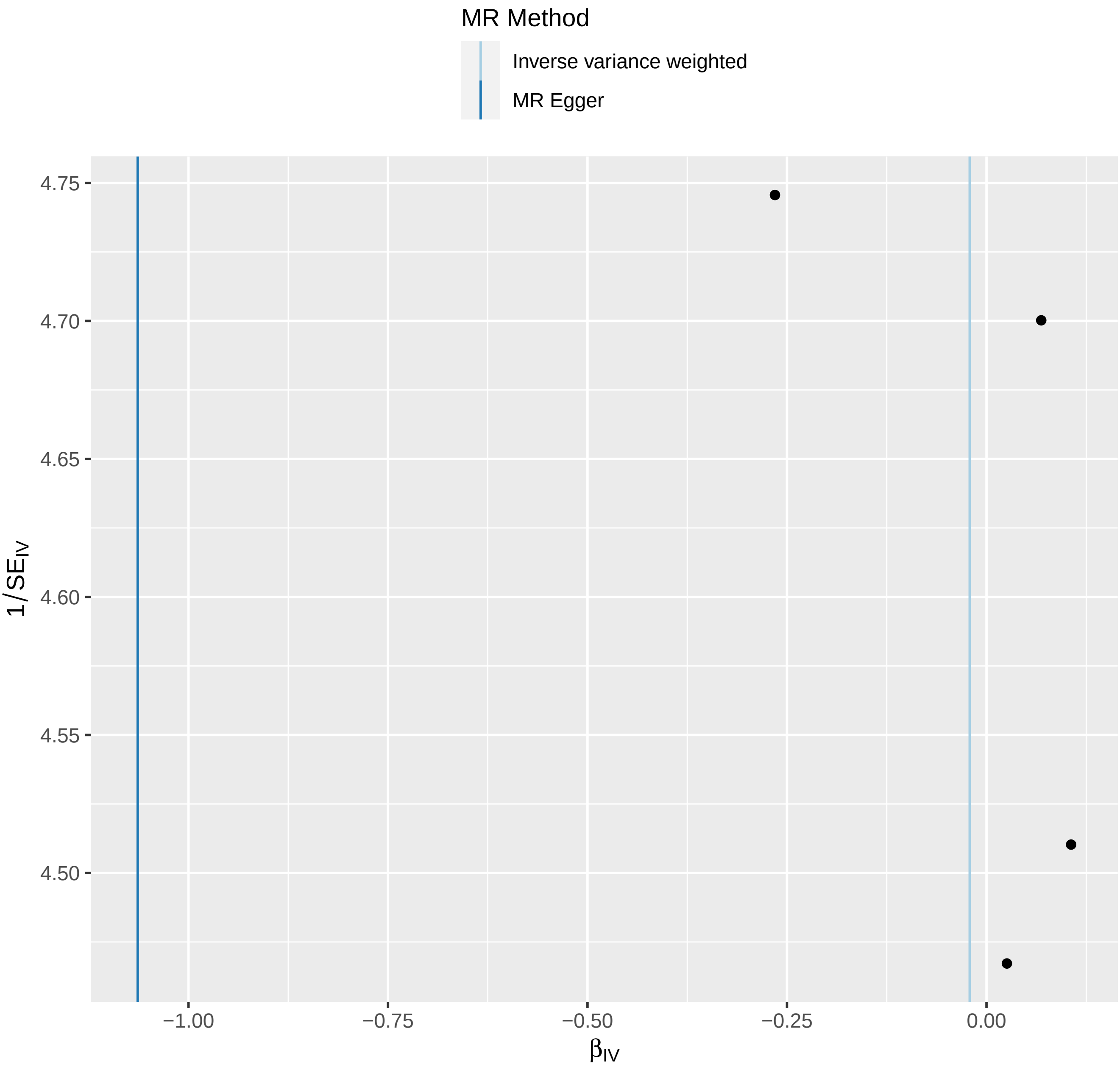

Batch 2−1160 : Gut microbiota abundance (genus Parasutterella id.2892) on C−type lectin domain family 6 member A with a threshold of 1e−05

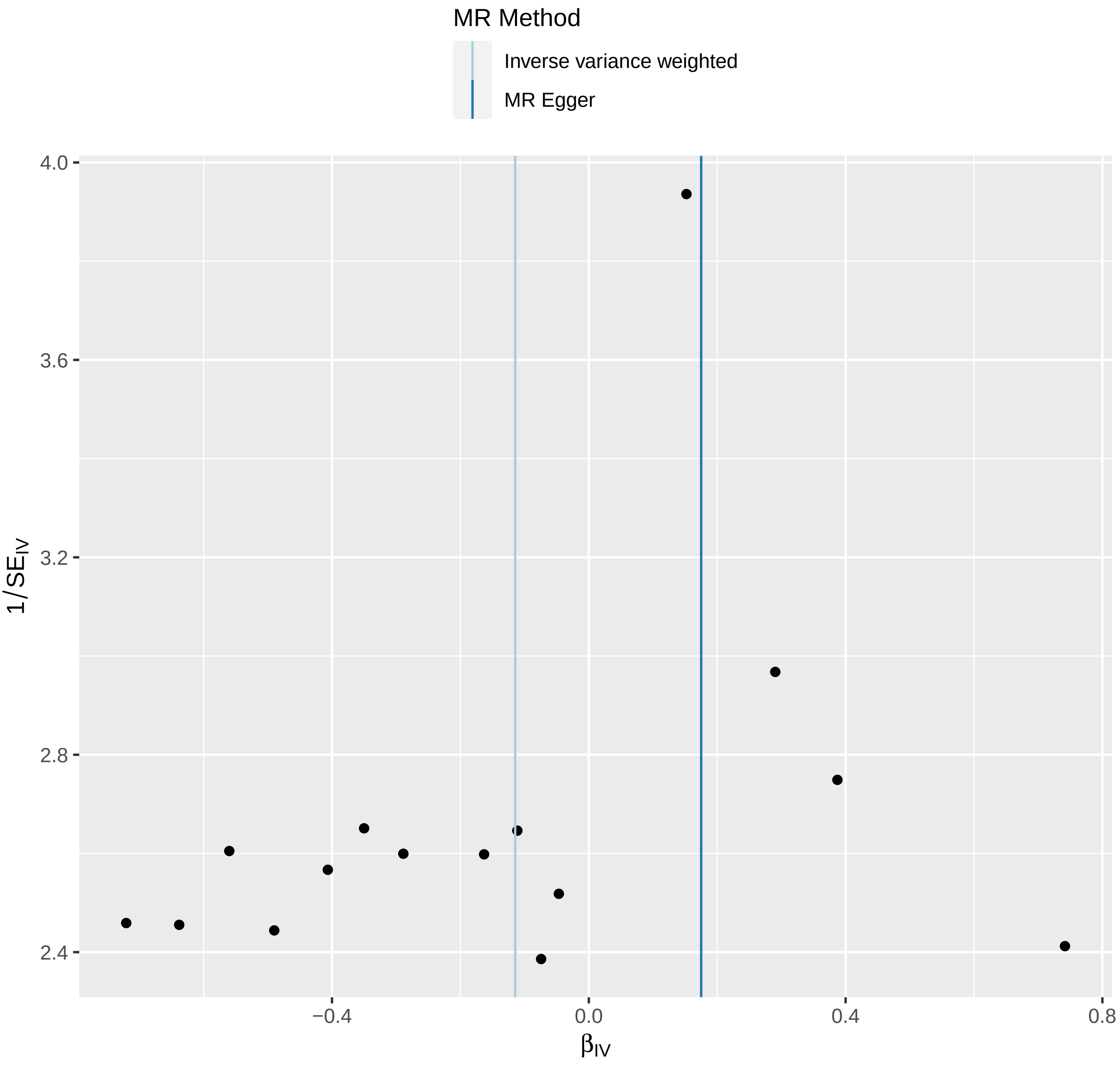

Batch 2−1161 : Gut microbiota abundance (genus Catenibacterium id.2153) on C−type lectin domain family 7 member A
with a threshold of 1e−05

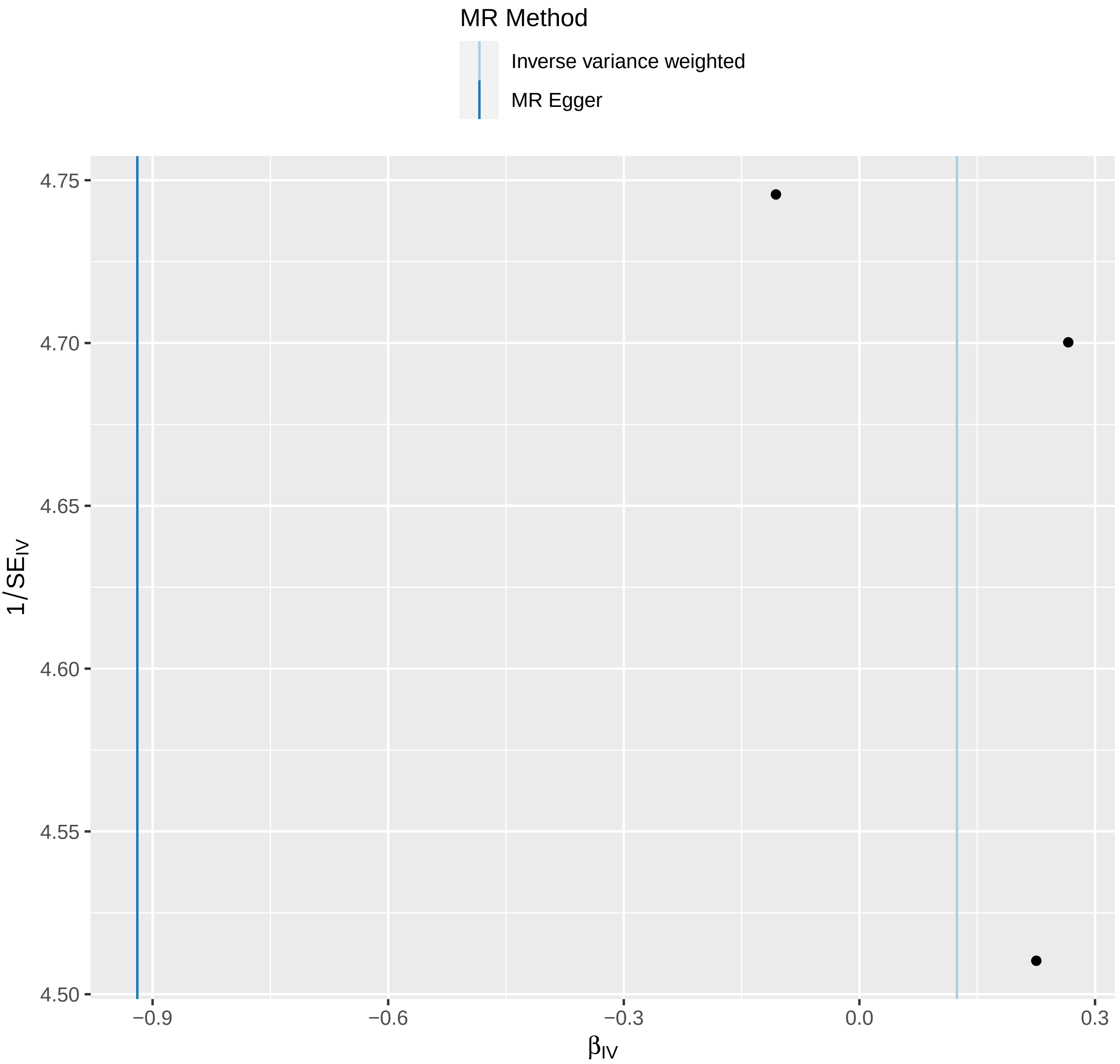

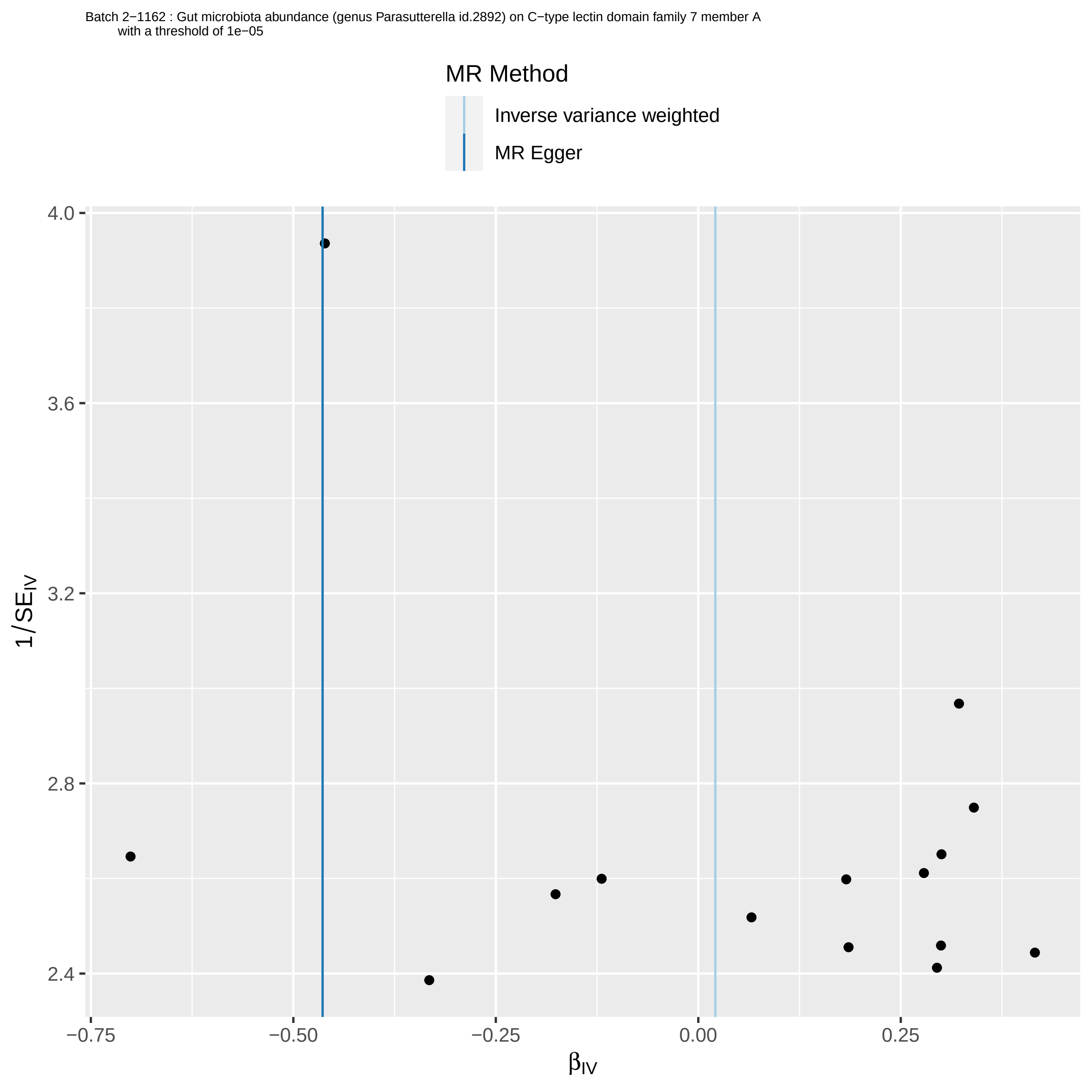

Batch 2−1162 : Gut microbiota abundance (genus Parasutterella id.2892) on C−type lectin domain family 7 member A
with a threshold of 1e−05
MR Method
Inverse variance weighted
MR Egger
4.0
3.6
3.2
2.8
2.4
1/SE_IV
−0.75
−0.50
−0.25
0.00
0.25
β_IV

Batch 2−1163 : Gut microbiota abundance (genus Catenibacterium id.2153) on C−type lectin−like domain family 1
with a threshold of 1e−05

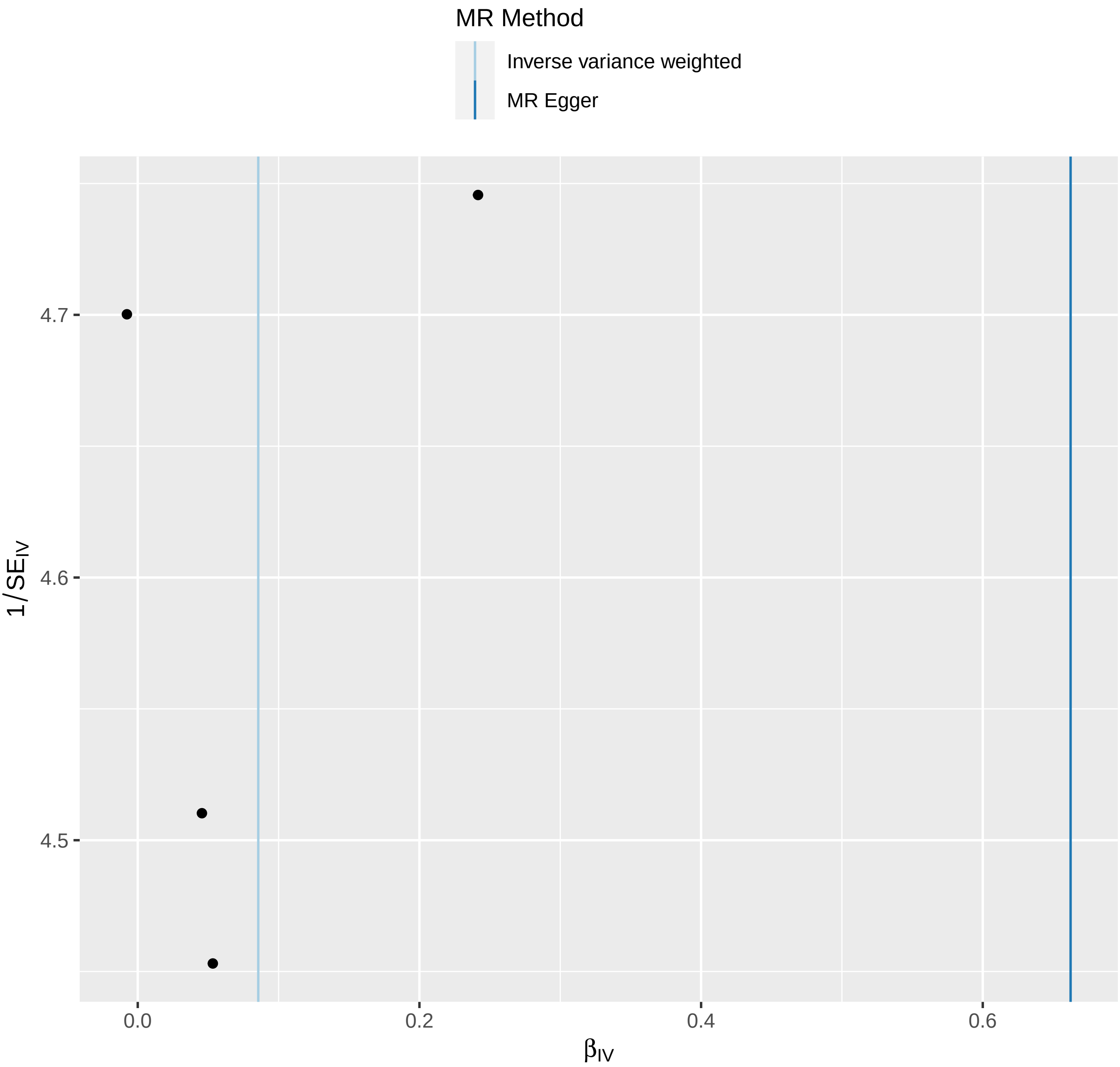

Batch 2−1164 : Gut microbiota abundance (genus Parasutterella id.2892) on C−type lectin−like domain family 1
with a threshold of 1e−05

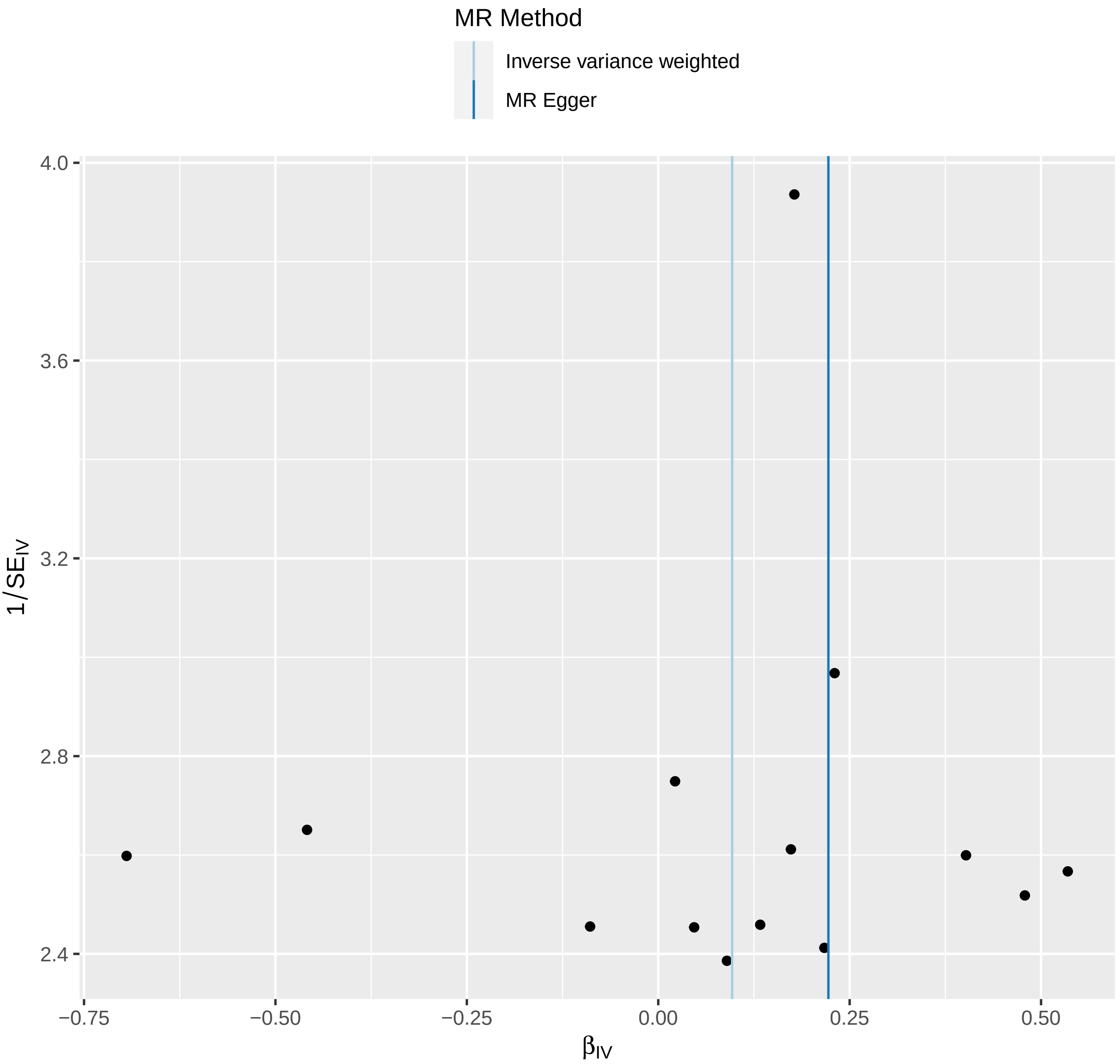

Batch 2−1165 : Gut microbiota abundance (genus Catenibacterium id.2153) on Chloride intracellular channel protein 4 with a threshold of 1e−05

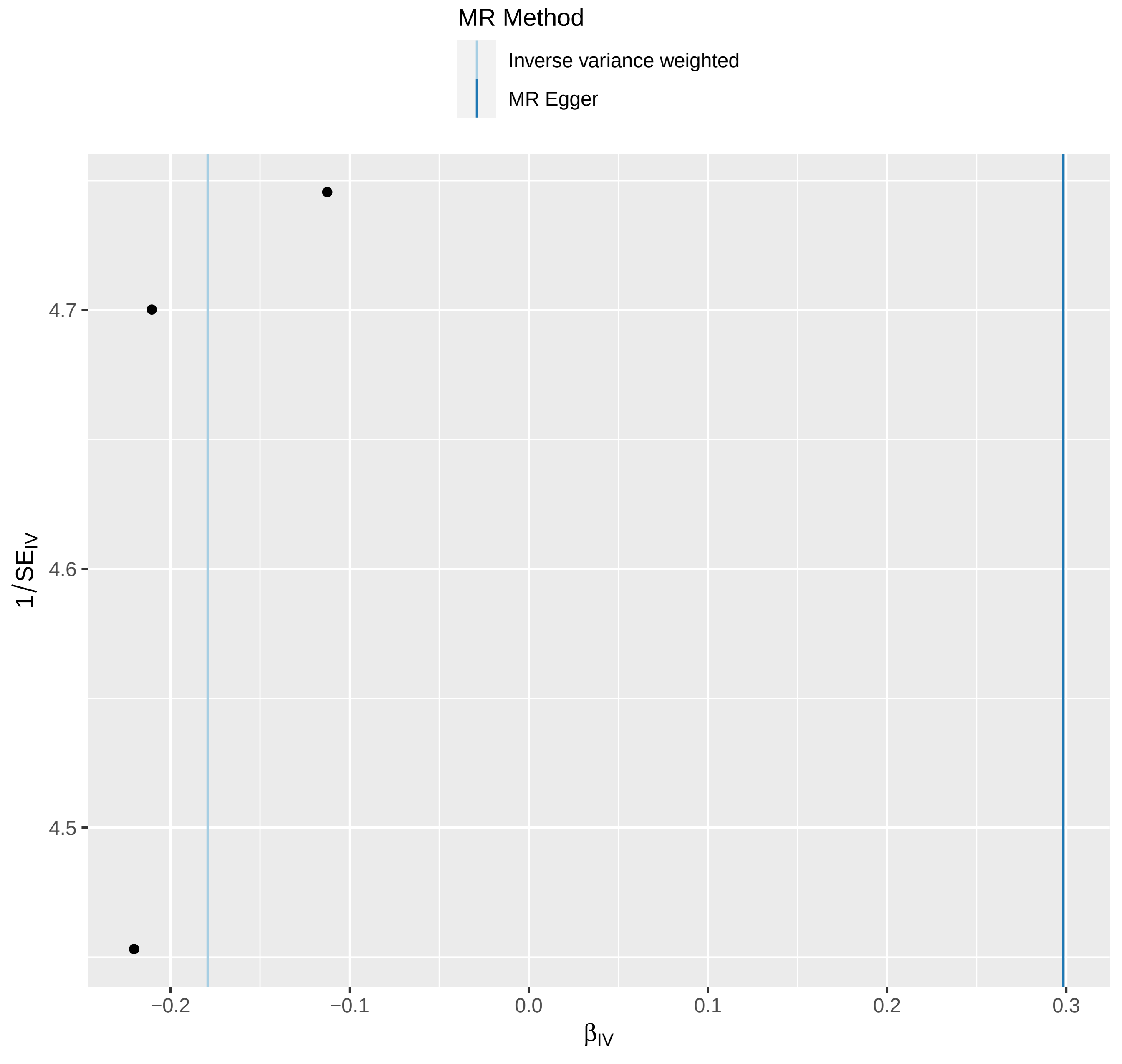

Batch 2−1166 : Gut microbiota abundance (genus Parasutterella id.2892) on Chloride intracellular channel protein 4
with a threshold of 1e−05

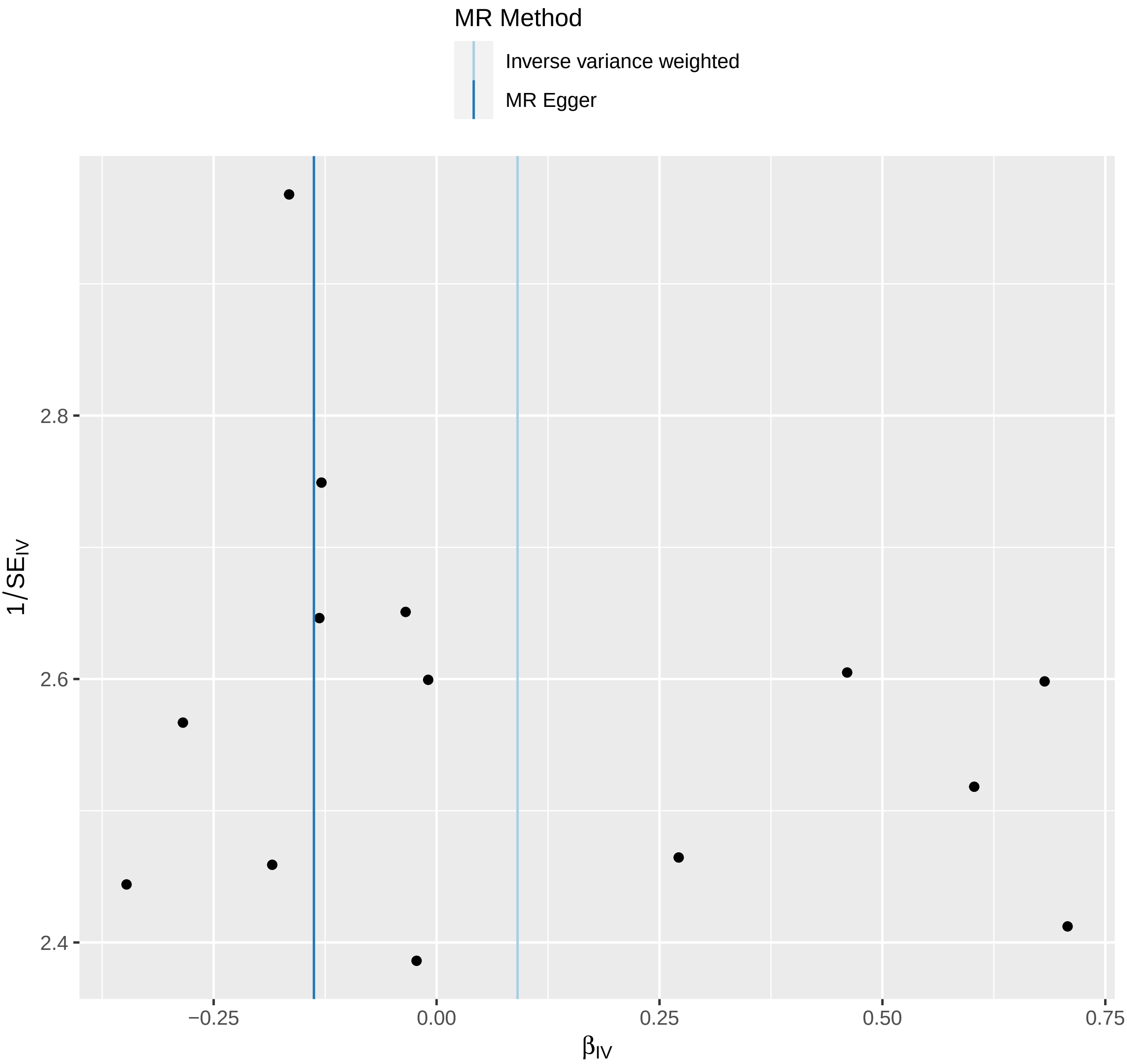

Batch 2−1167 : Gut microbiota abundance (genus Catenibacterium id.2153) on Chloride intracellular channel protein 5
with a threshold of 1e−05

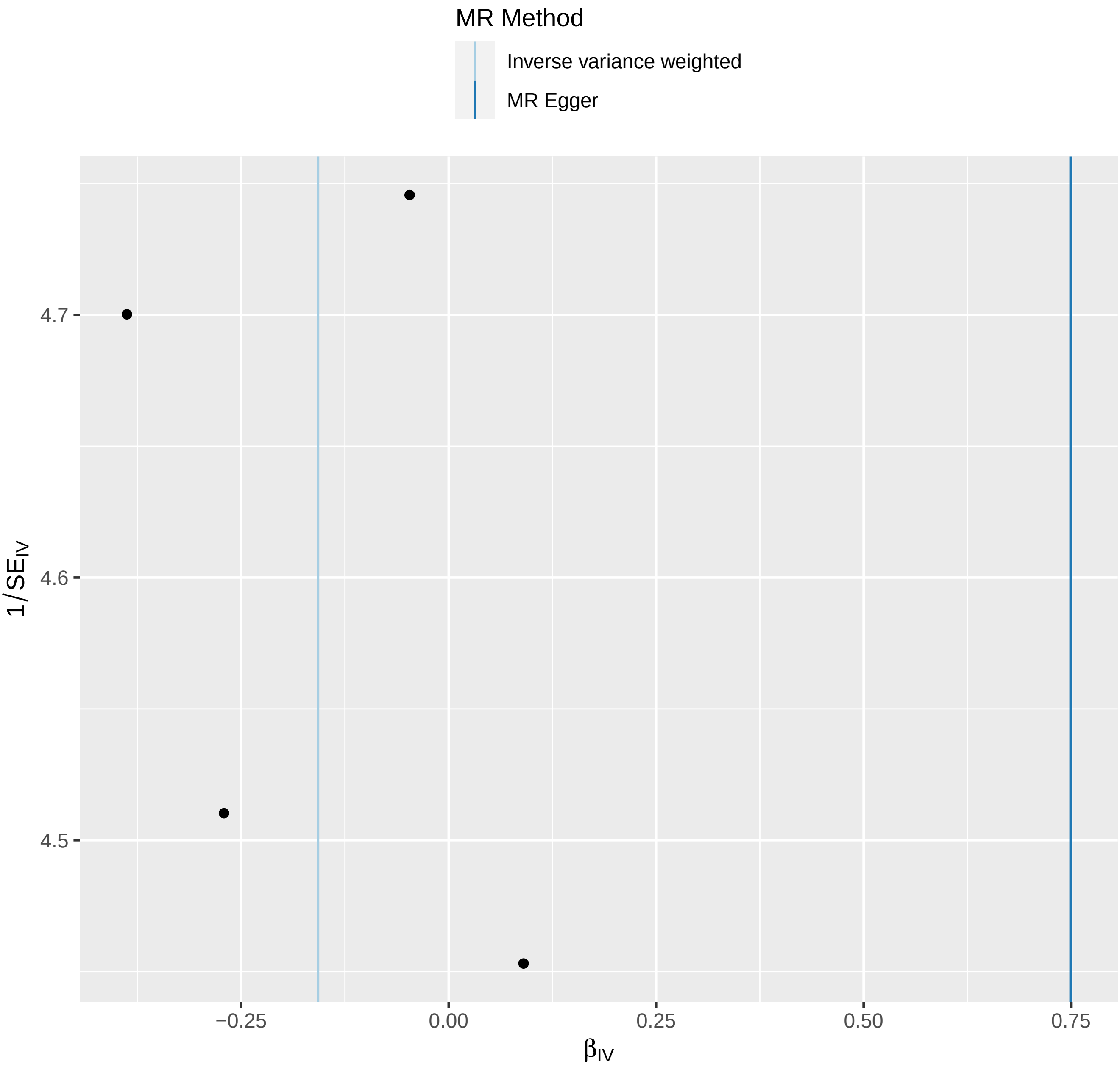

Batch 2−1168 : Gut microbiota abundance (genus Parasutterella id.2892) on Chloride intracellular channel protein 5 with a threshold of 1e−05

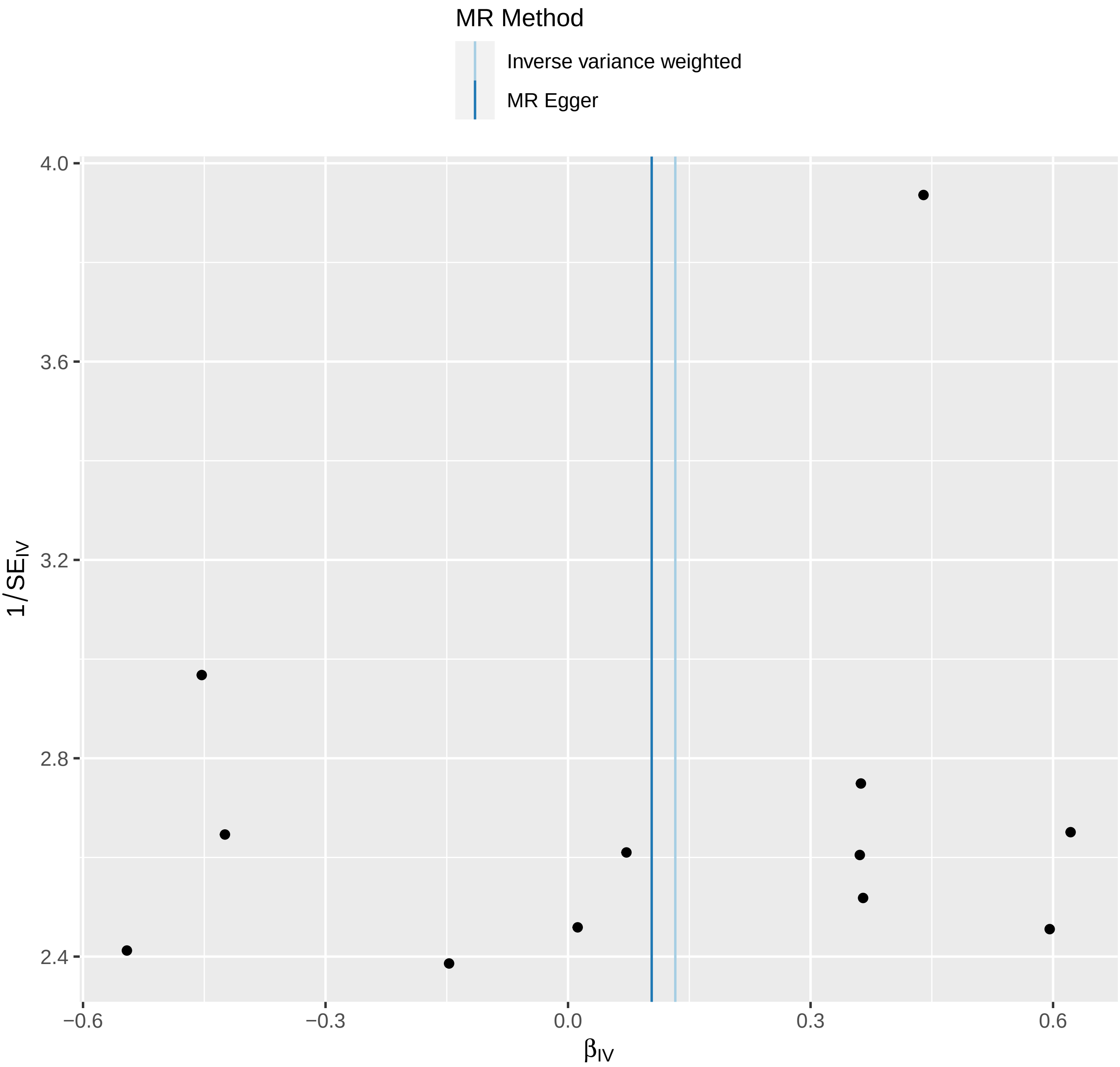

Batch 2−1169 : Gut microbiota abundance (genus Catenibacterium id.2153) on Clathrin interactor 1
with a threshold of 1e−05

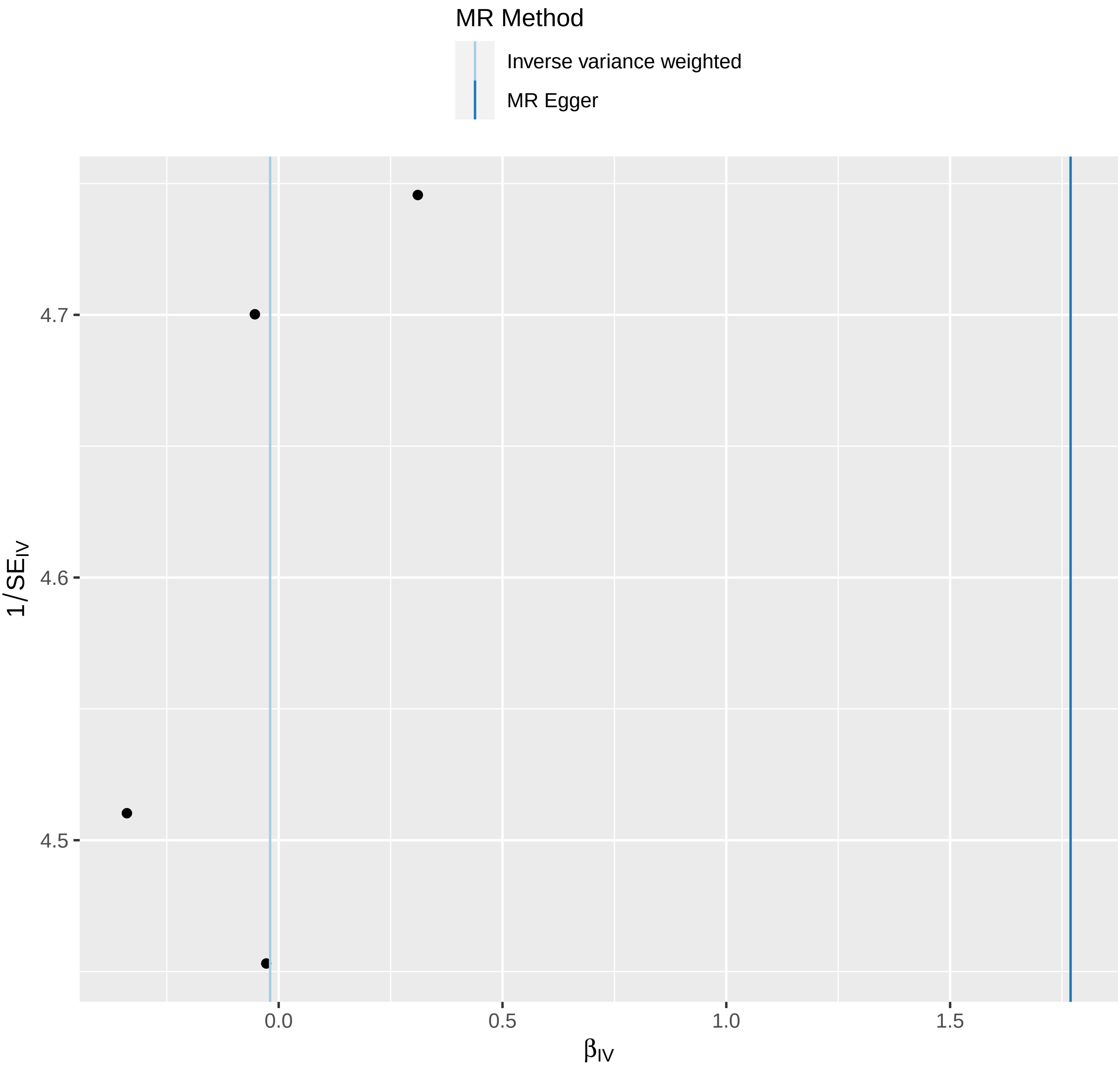

Batch 2−1170 : Gut microbiota abundance (genus Parasutterella id.2892) on Clathrin interactor 1
with a threshold of 1e−05

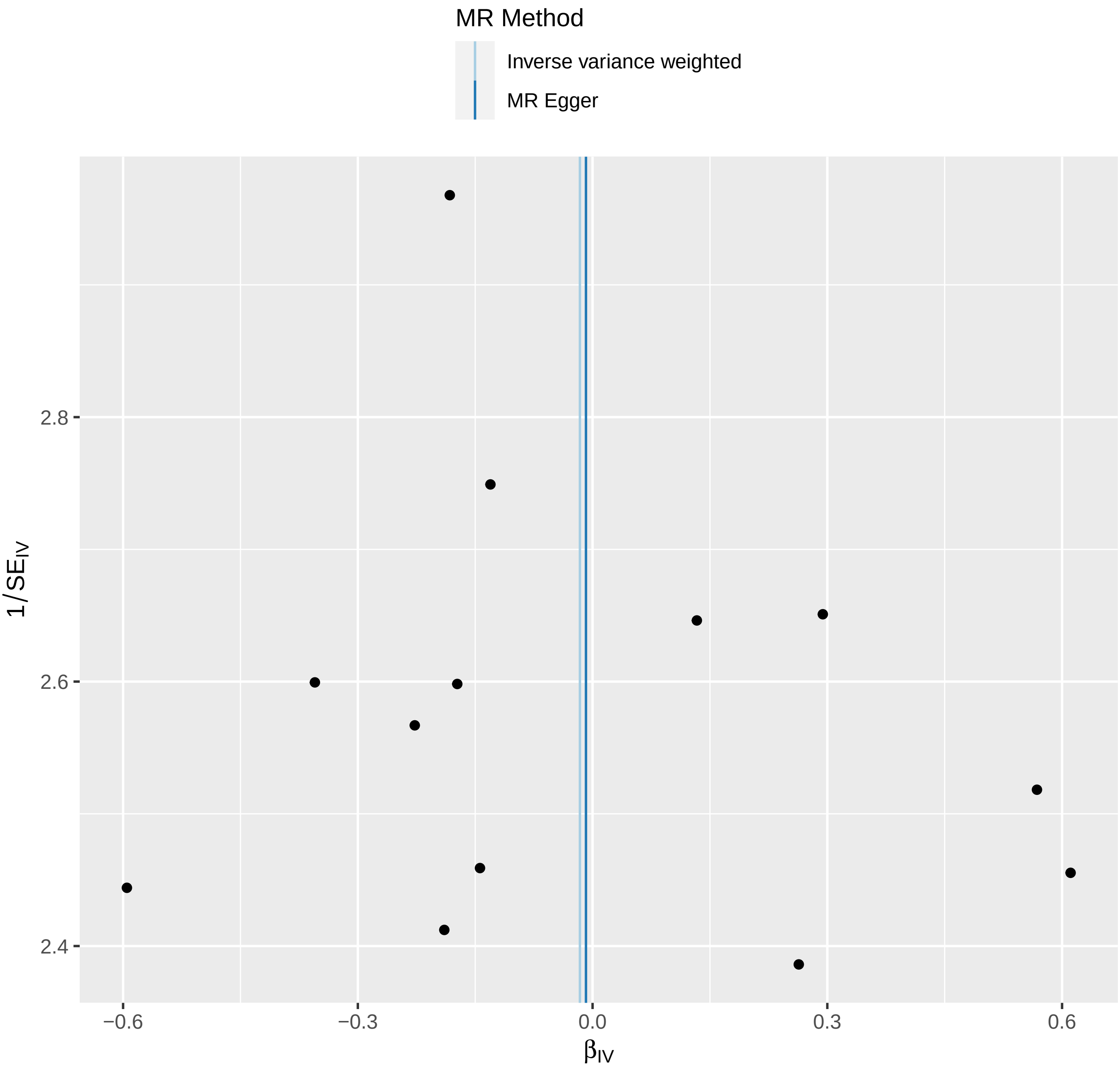

Batch 2−1171 : Gut microbiota abundance (genus Catenibacterium id.2153) on Dual specificity protein kinase CLK2
with a threshold of 1e−05

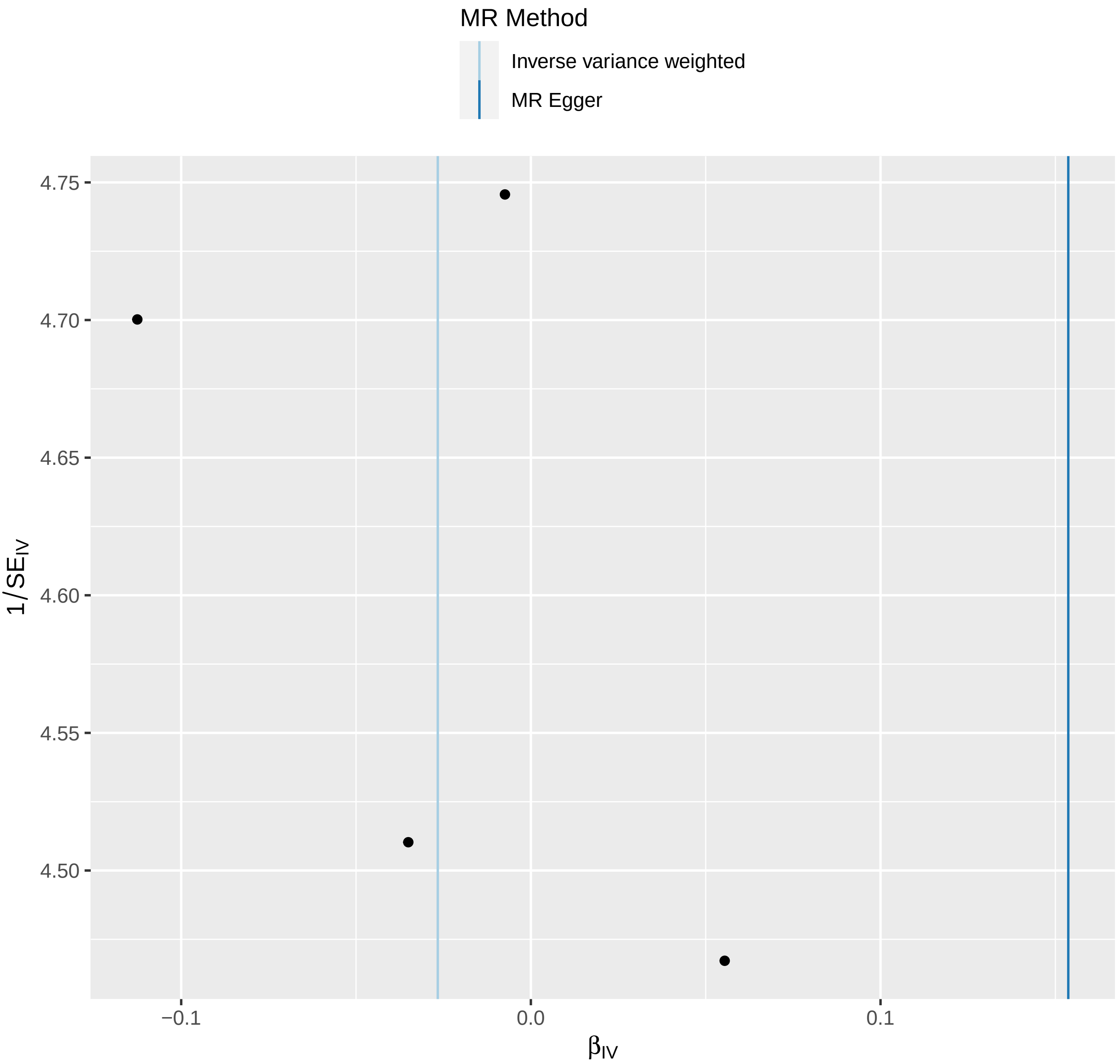

Batch 2−1172 : Gut microbiota abundance (genus Parasutterella id.2892) on Dual specificity protein kinase CLK2
with a threshold of 1e−05

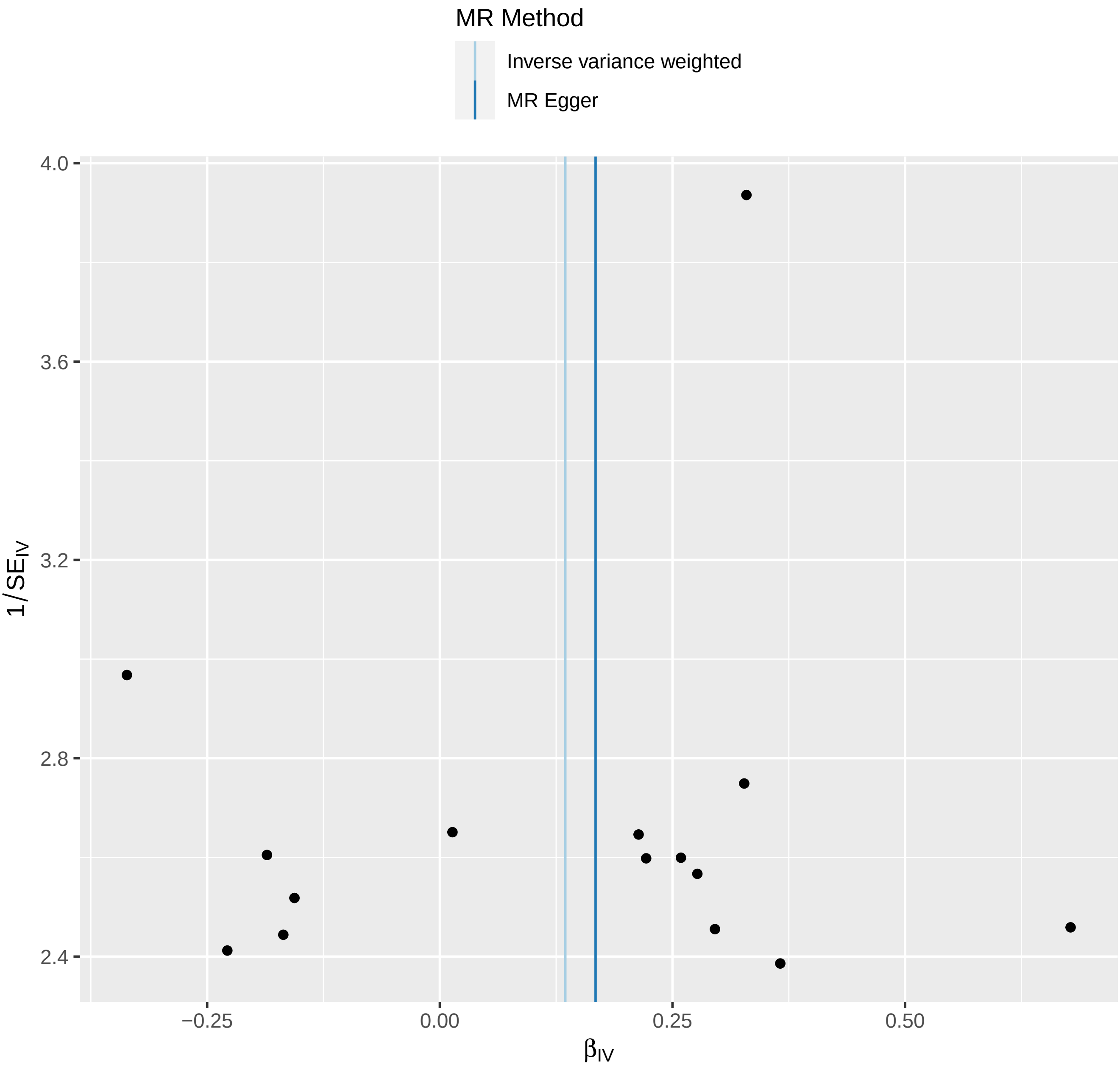

Batch 2−1173 : Gut microbiota abundance (genus Catenibacterium id.2153) on CXADR−like membrane protein
with a threshold of 1e−05

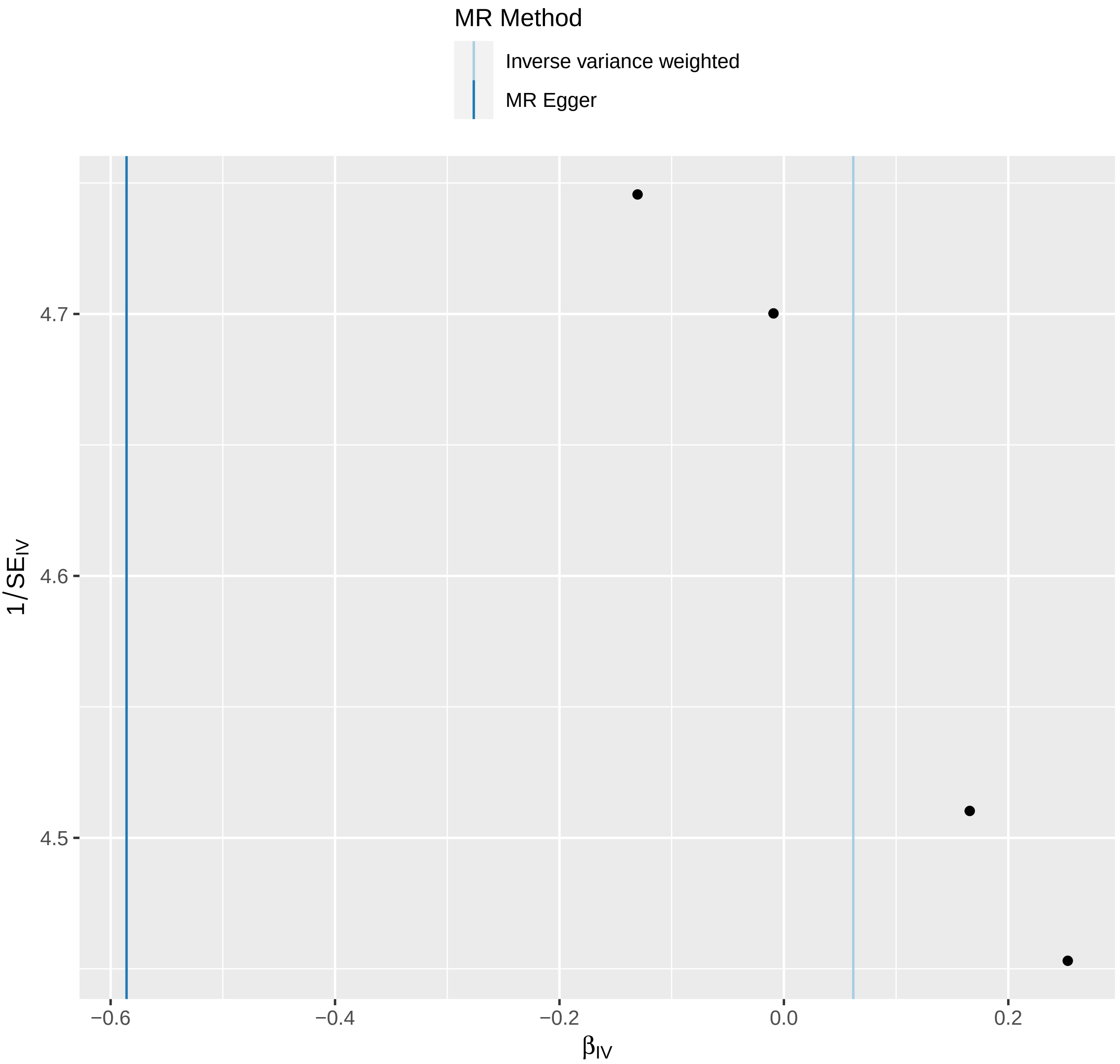

Batch 2−1174 : Gut microbiota abundance (genus Parasutterella id.2892) on CXADR−like membrane protein
with a threshold of 1e−05

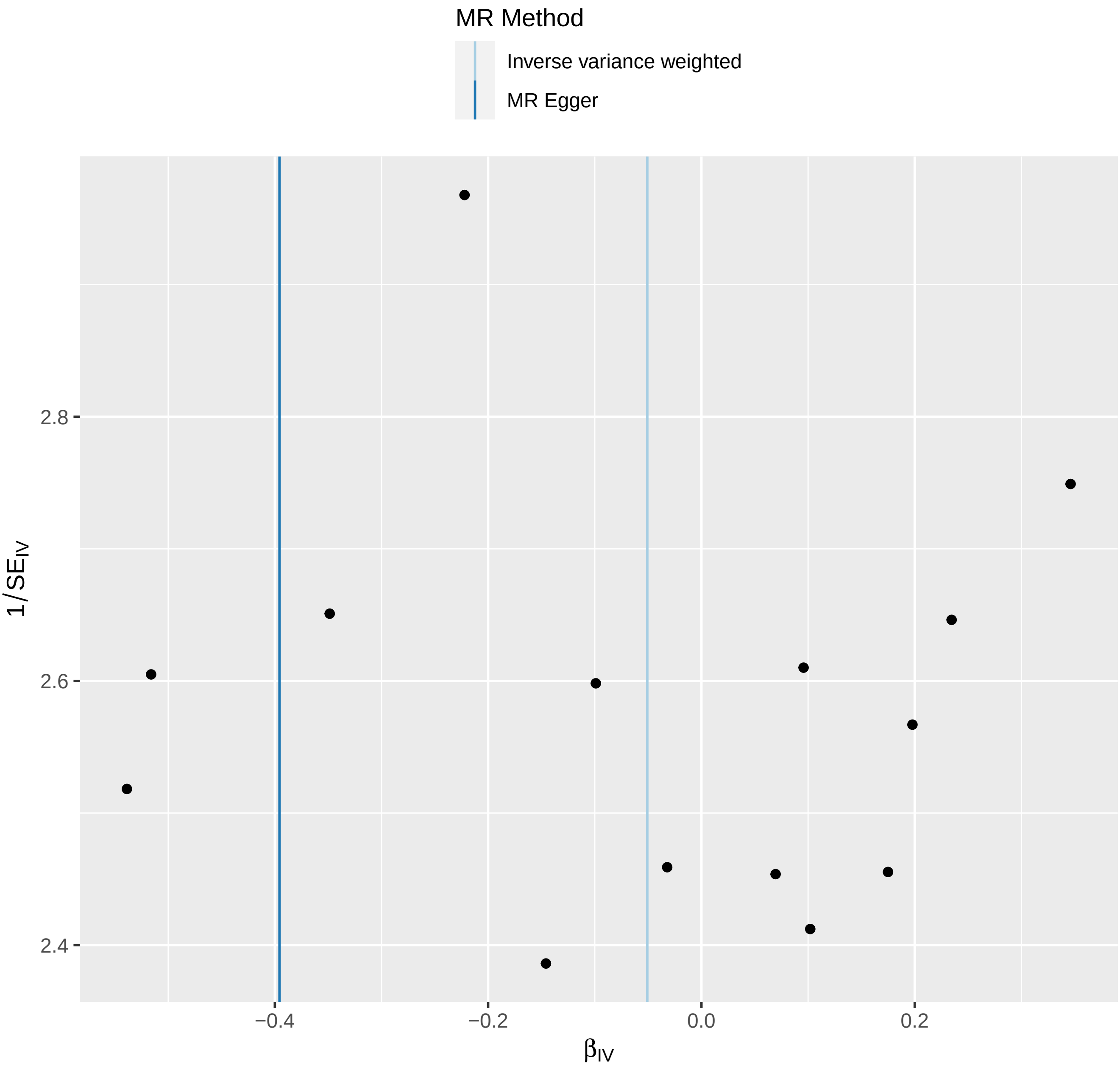

Batch 2−1175 : Gut microbiota abundance (genus Catenibacterium id.2153) on CXADR−like membrane protein
with a threshold of 1e−05

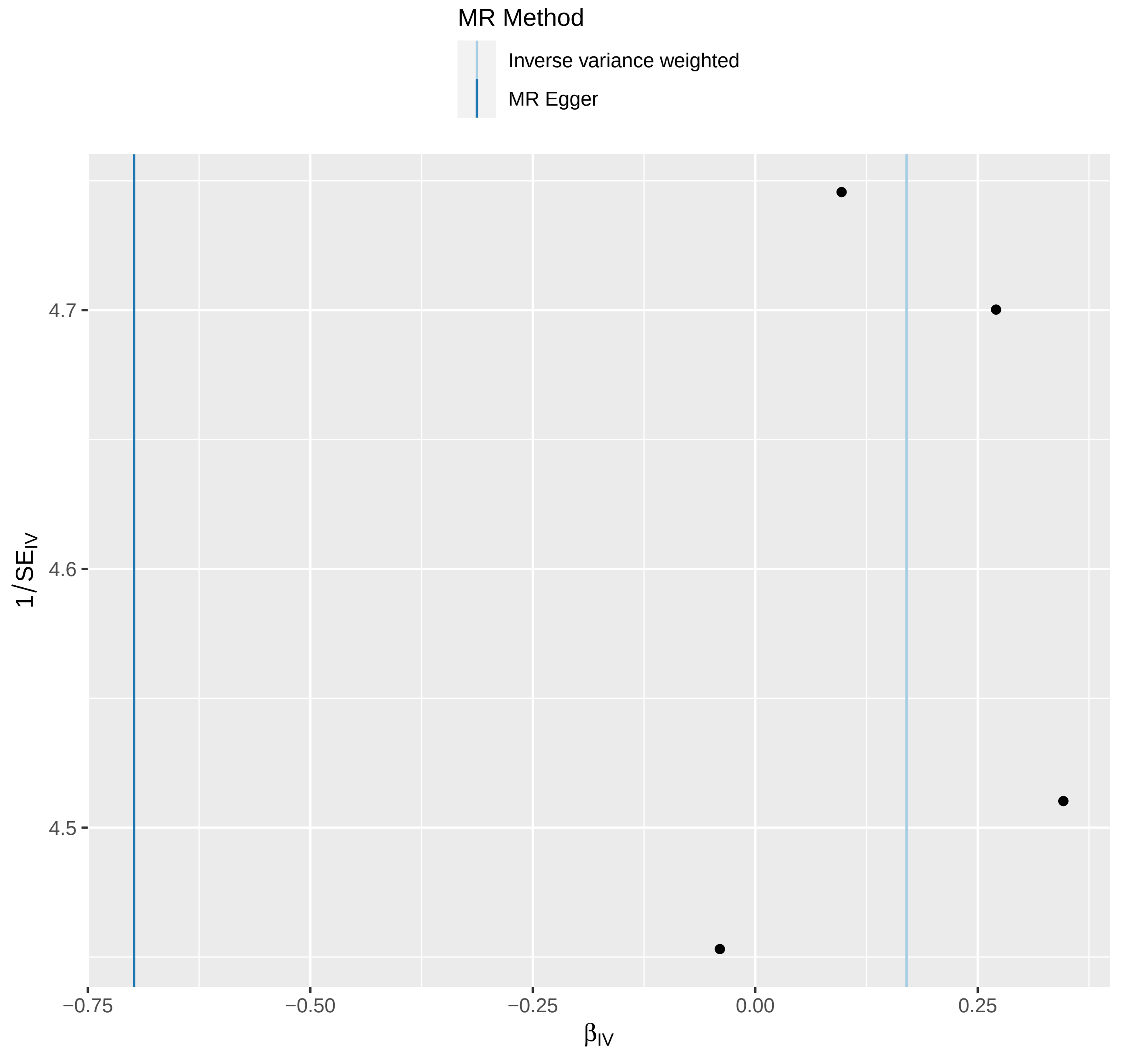

Batch 2−1176 : Gut microbiota abundance (genus Parasutterella id.2892) on CXADR−like membrane protein
with a threshold of 1e−05

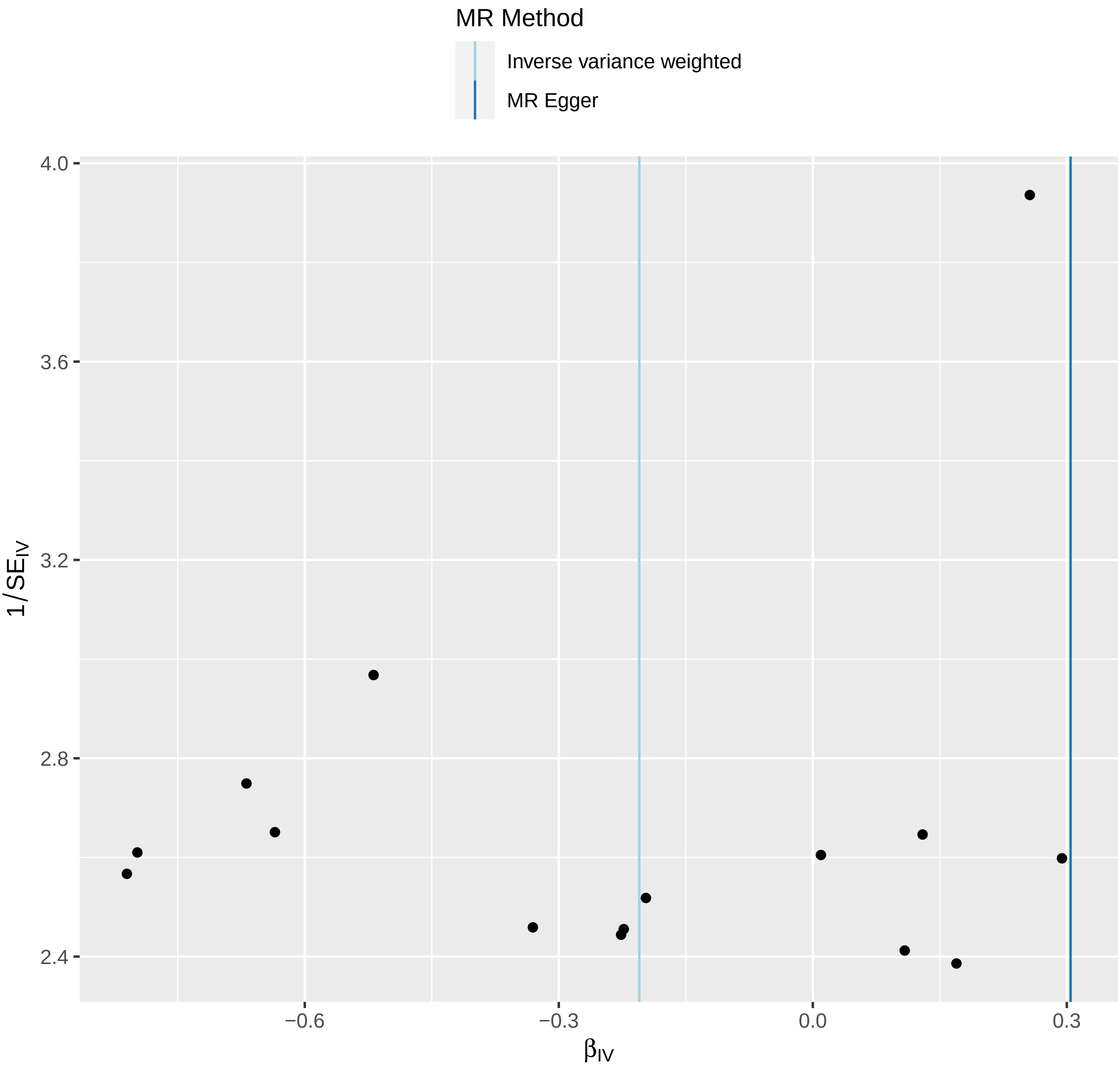

Batch 2−1177 : Gut microbiota abundance (genus Catenibacterium id.2153) on Ceroid−lipofuscinosis neuronal protein 5
with a threshold of 1e−05

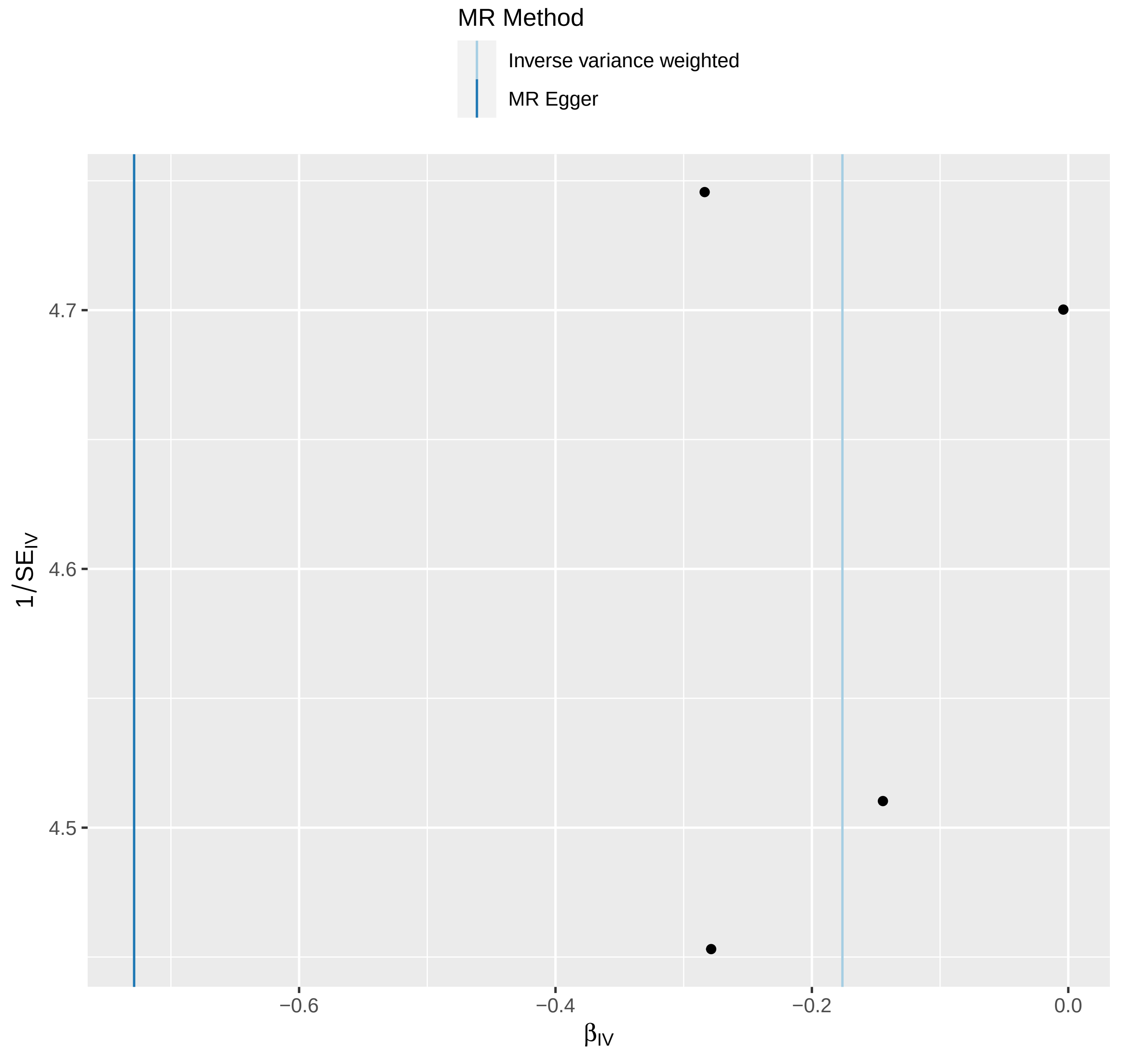

Batch 2−1178 : Gut microbiota abundance (genus Parasutterella id.2892) on Ceroid−lipofuscinosis neuronal protein 5
with a threshold of 1e−05

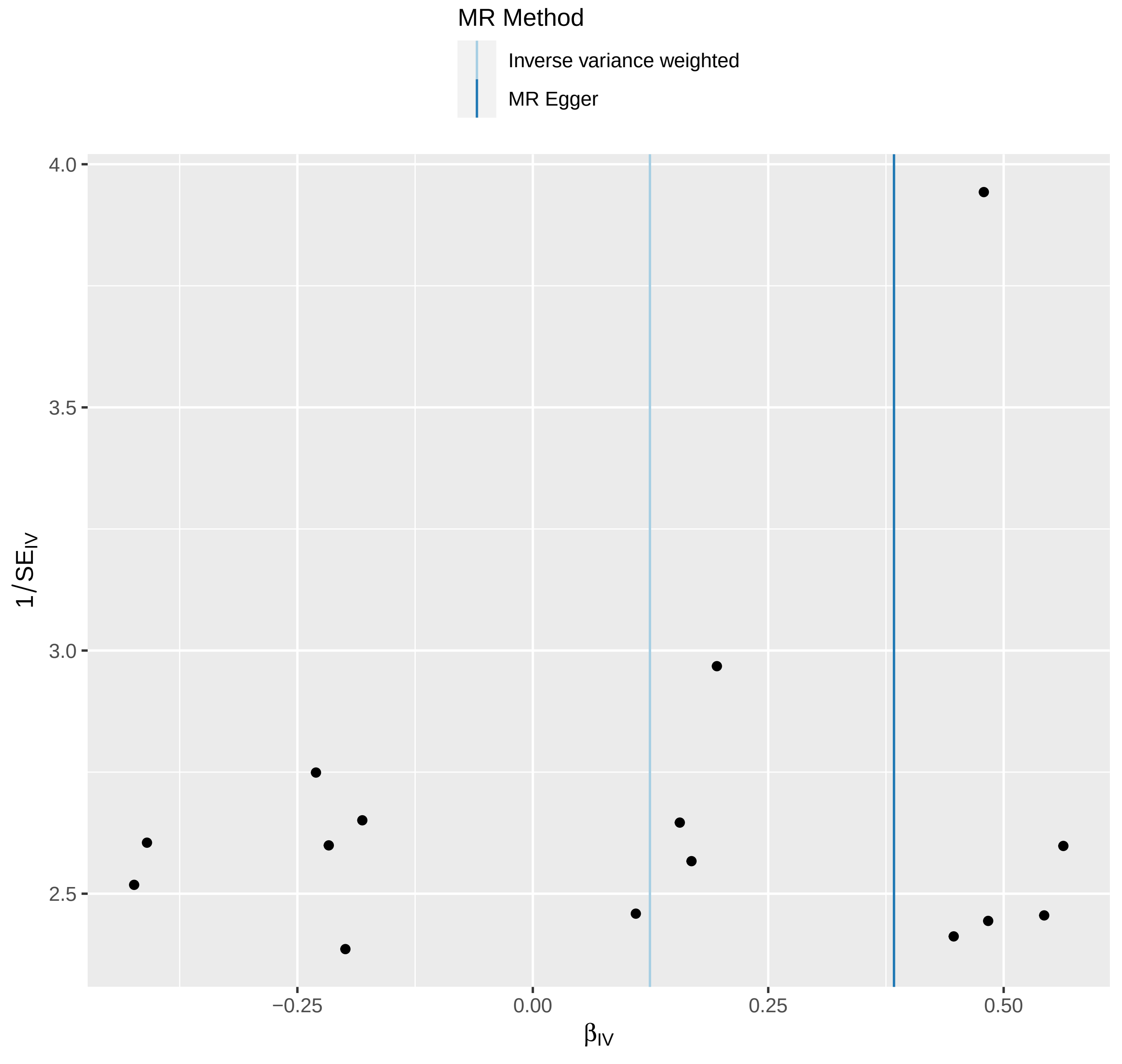

Batch 2−1179 : Gut microbiota abundance (genus Catenibacterium id.2153) on Ceroid−lipofuscinosis neuronal protein 5 with a threshold of 1e−05

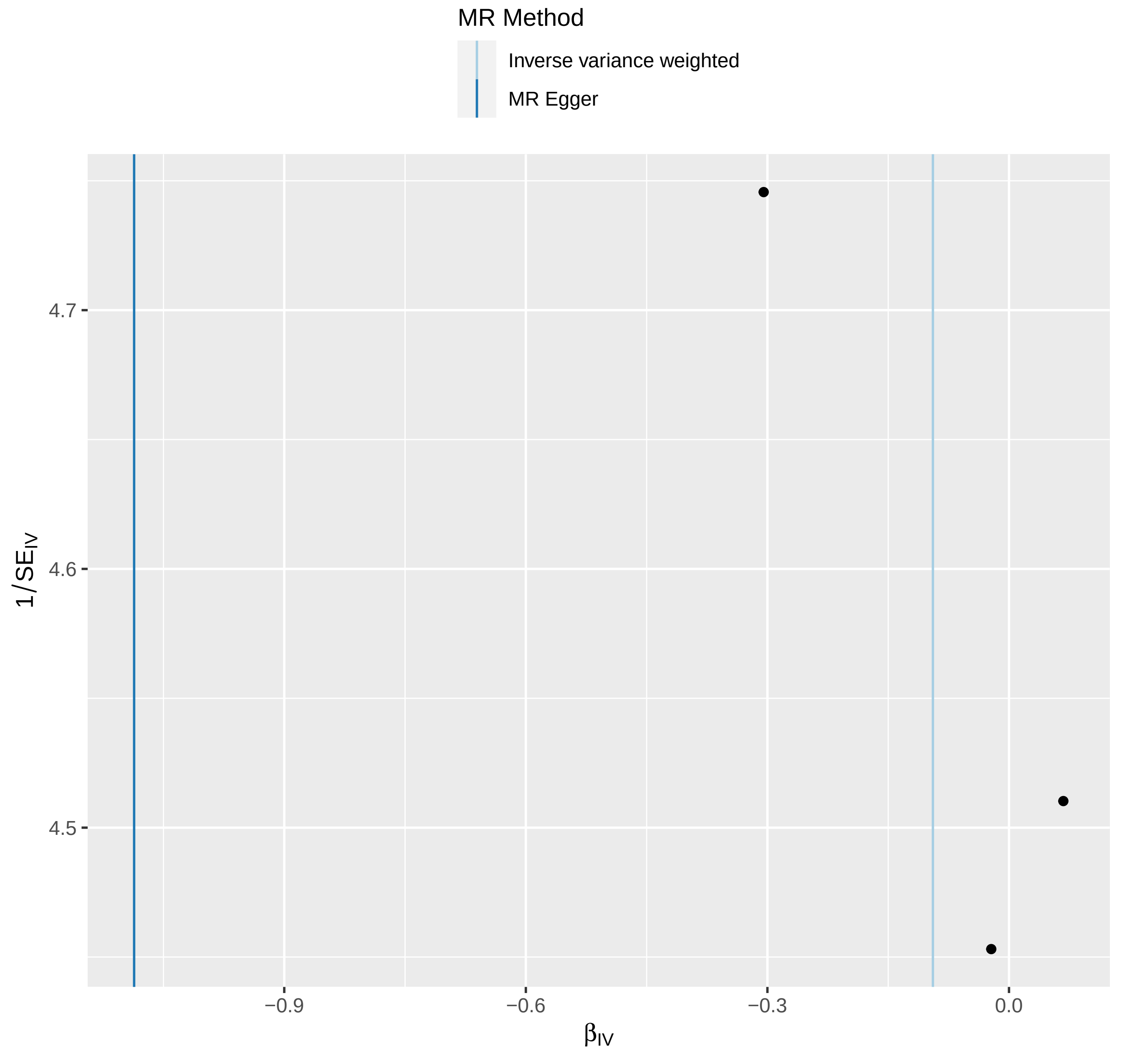

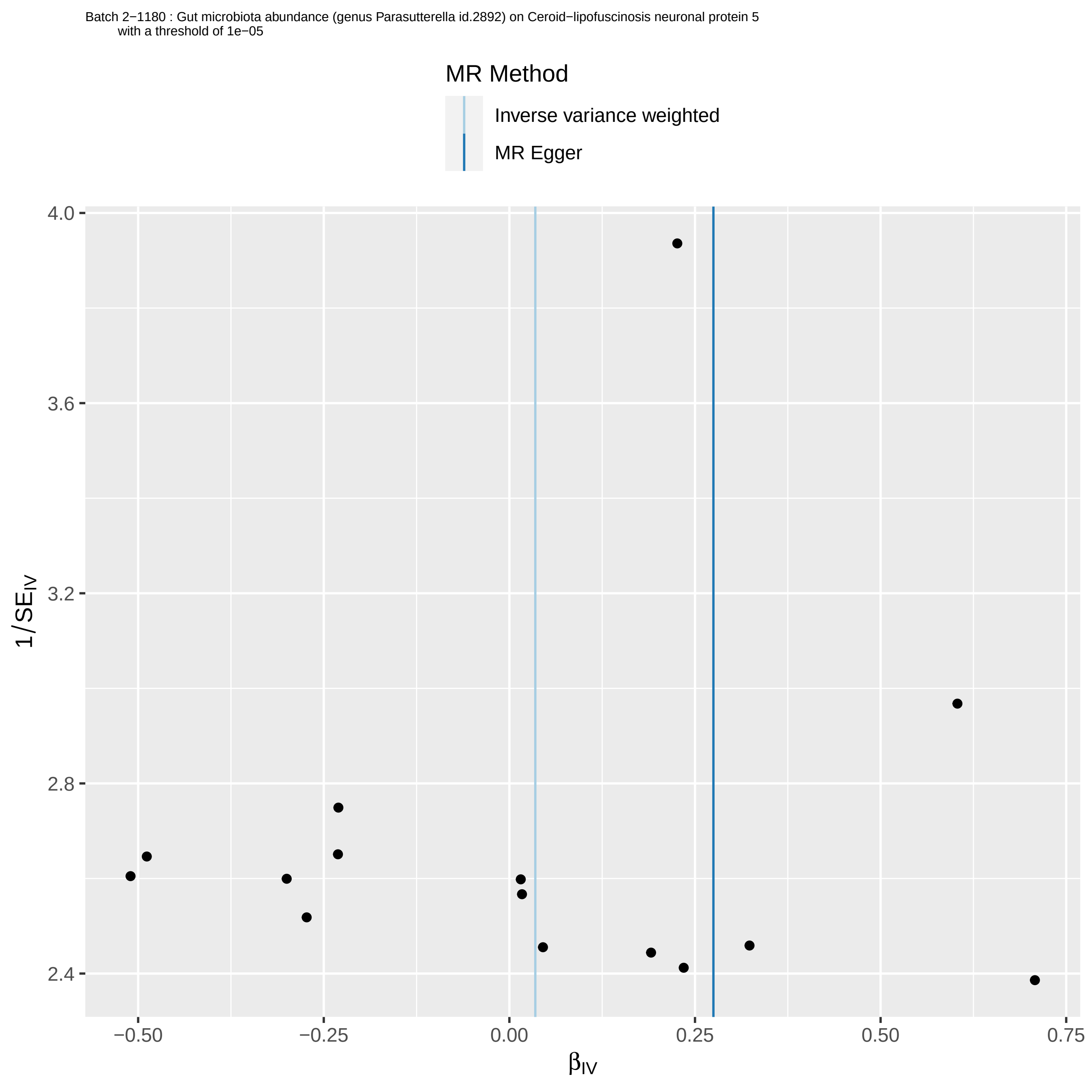

Batch 2−1180 : Gut microbiota abundance (genus Parasutterella id.2892) on Ceroid−lipofuscinosis neuronal protein 5
with a threshold of 1e−05
MR Method
Inverse variance weighted
MR Egger
4.0
3.6
3.2
2.8
2.4
1/SE_IV
−0.50
−0.25
0.00
0.25
0.50
0.75
β_IV

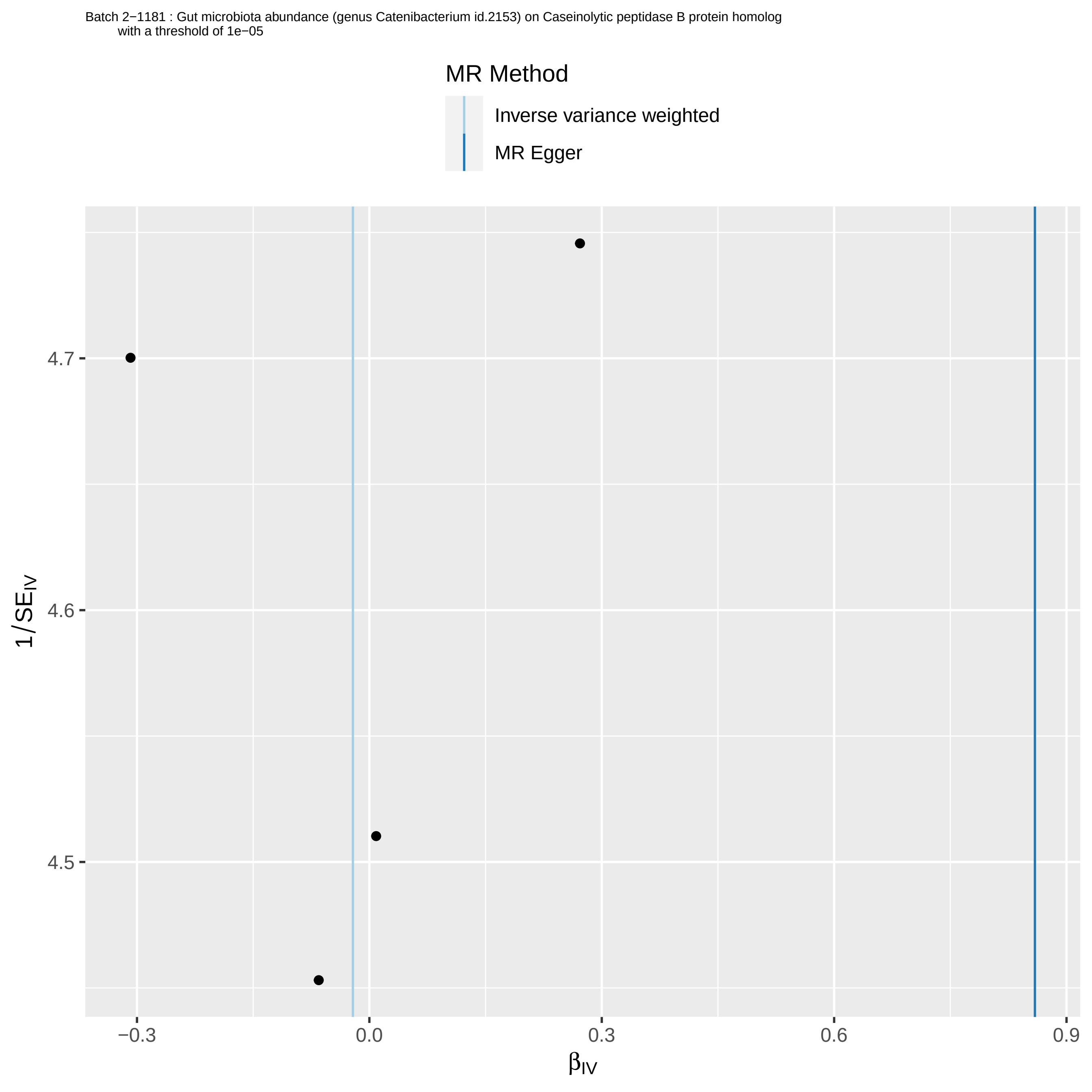
Batch 2−1181 : Gut microbiota abundance (genus Catenibacterium id.2153) on Caseinolytic peptidase B protein homolog
with a threshold of 1e−05
MR Method
Inverse variance weighted
MR Egger
4.7
4.6
4.5
1/SE_IV
−0.3
0.0
0.3
0.6
0.9
β_IV

Batch 2−1182 : Gut microbiota abundance (genus Parasutterella id.2892) on Caseinolytic peptidase B protein homolog with a threshold of 1e−05

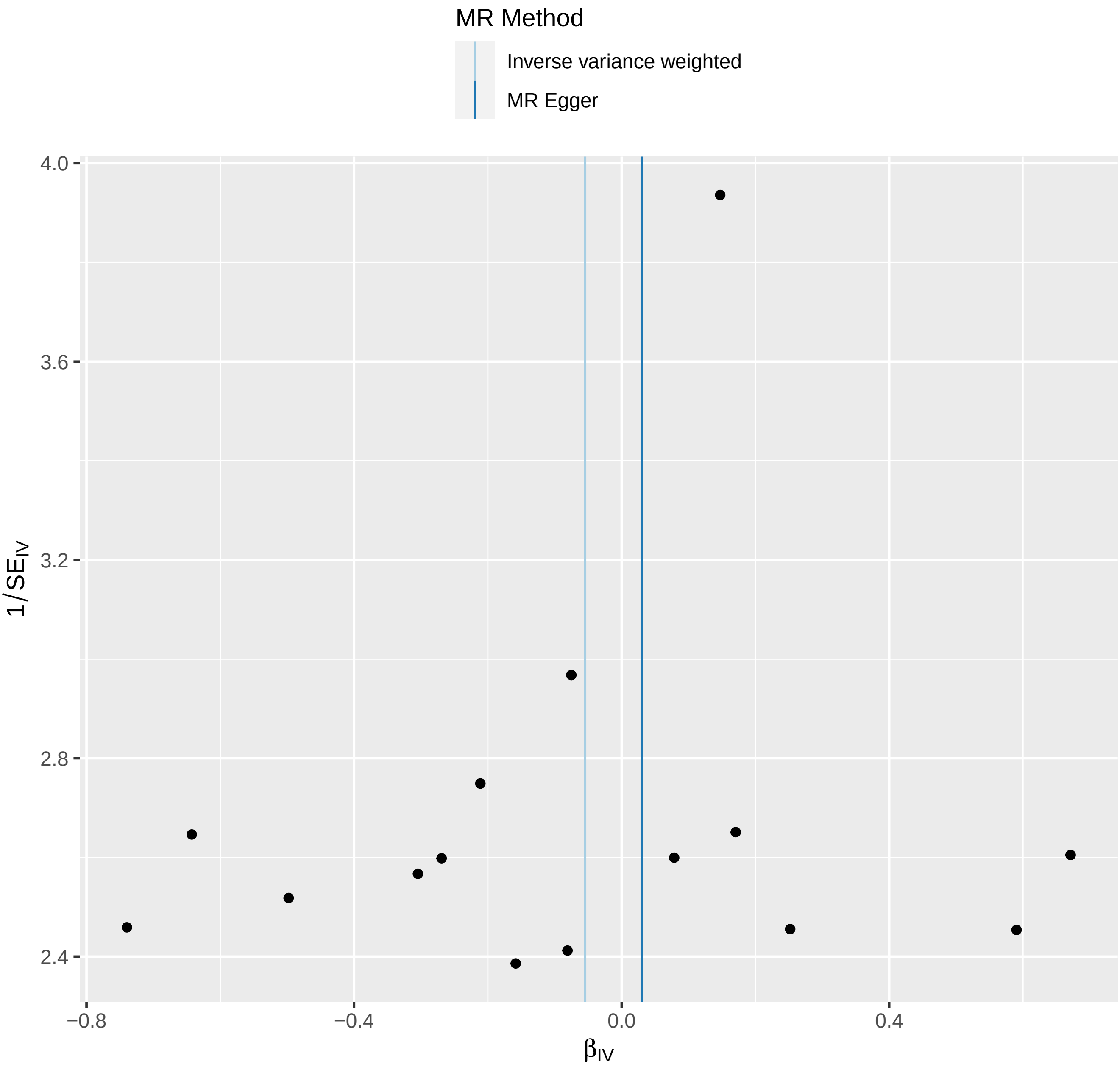

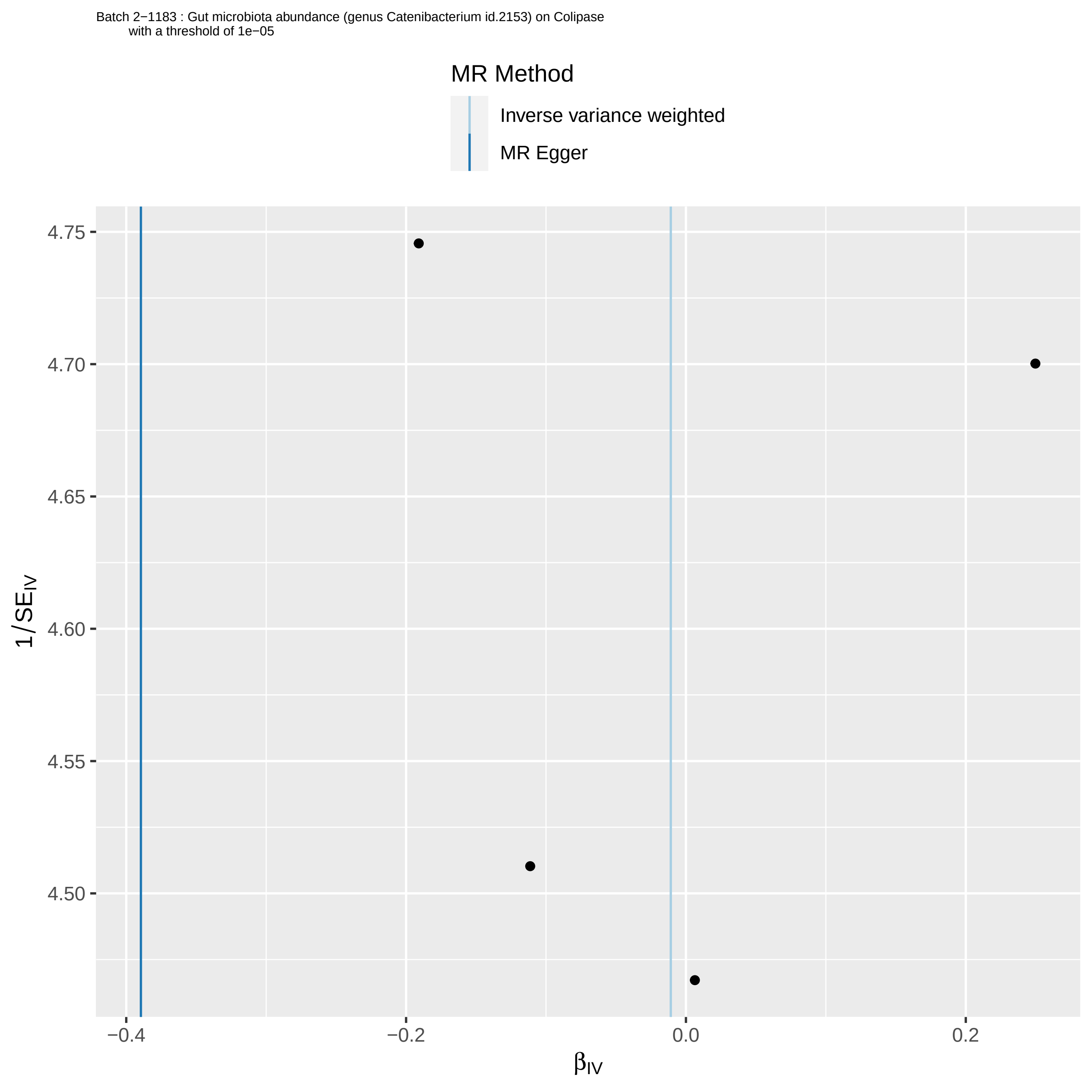
Batch 2−1183 : Gut microbiota abundance (genus Catenibacterium id.2153) on Colipase
with a threshold of 1e−05
MR Method
Inverse variance weighted
MR Egger
4.75
4.70
4.65
4.60
4.55
4.50
$1/SE_{IV}$
−0.4
−0.2
0.0
0.2
β_{IV}

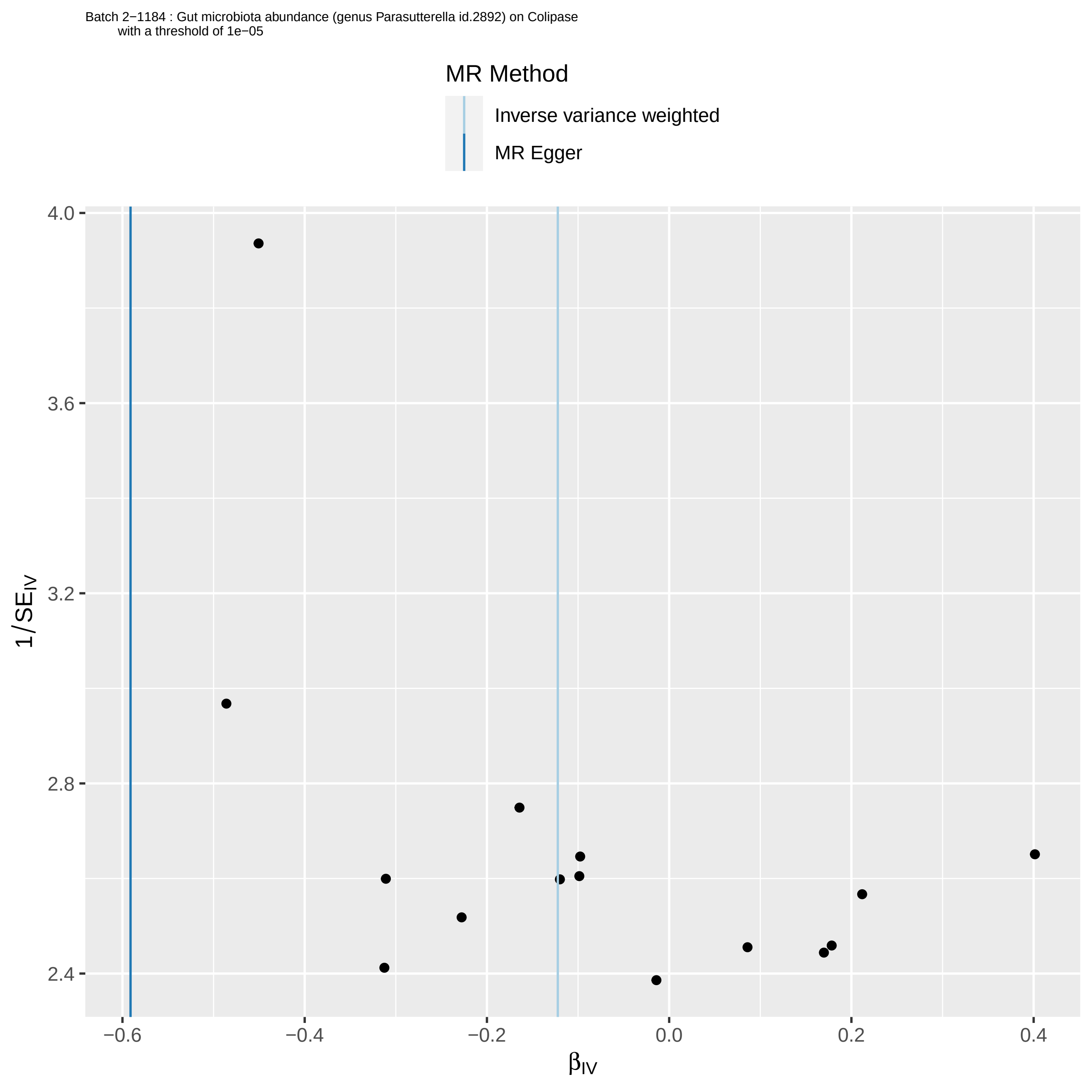

Batch 2−1184 : Gut microbiota abundance (genus Parasutterella id.2892) on Colipase
with a threshold of 1e−05
MR Method
Inverse variance weighted
MR Egger
4.0
3.6
3.2
2.8
2.4
1/SE$_{IV}$
−0.6
−0.4
−0.2
0.0
0.2
0.4
β$_{IV}$

Batch 2−1185 : Gut microbiota abundance (genus Catenibacterium id.2153) on Colipase−like protein 2
with a threshold of 1e−05

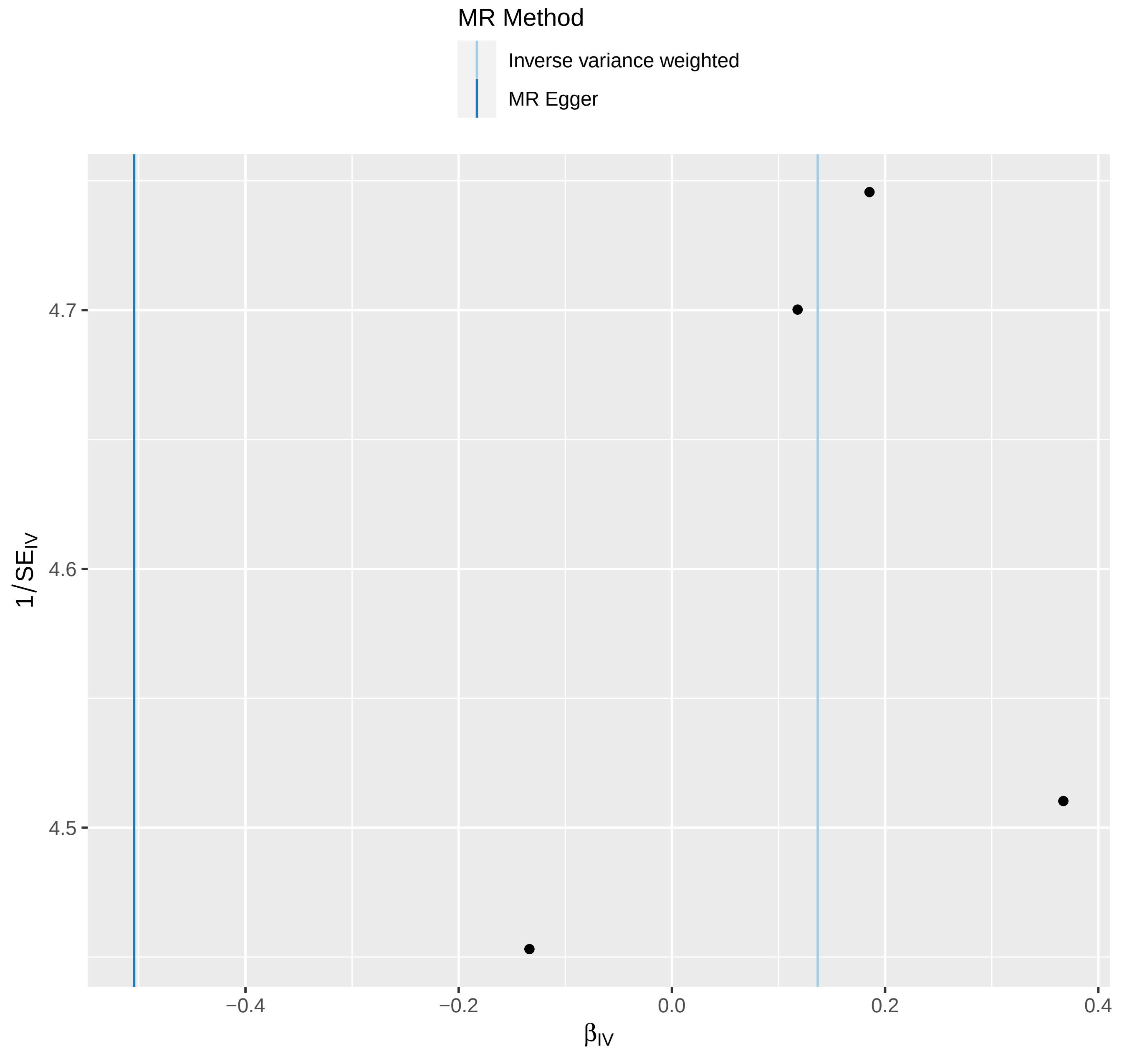

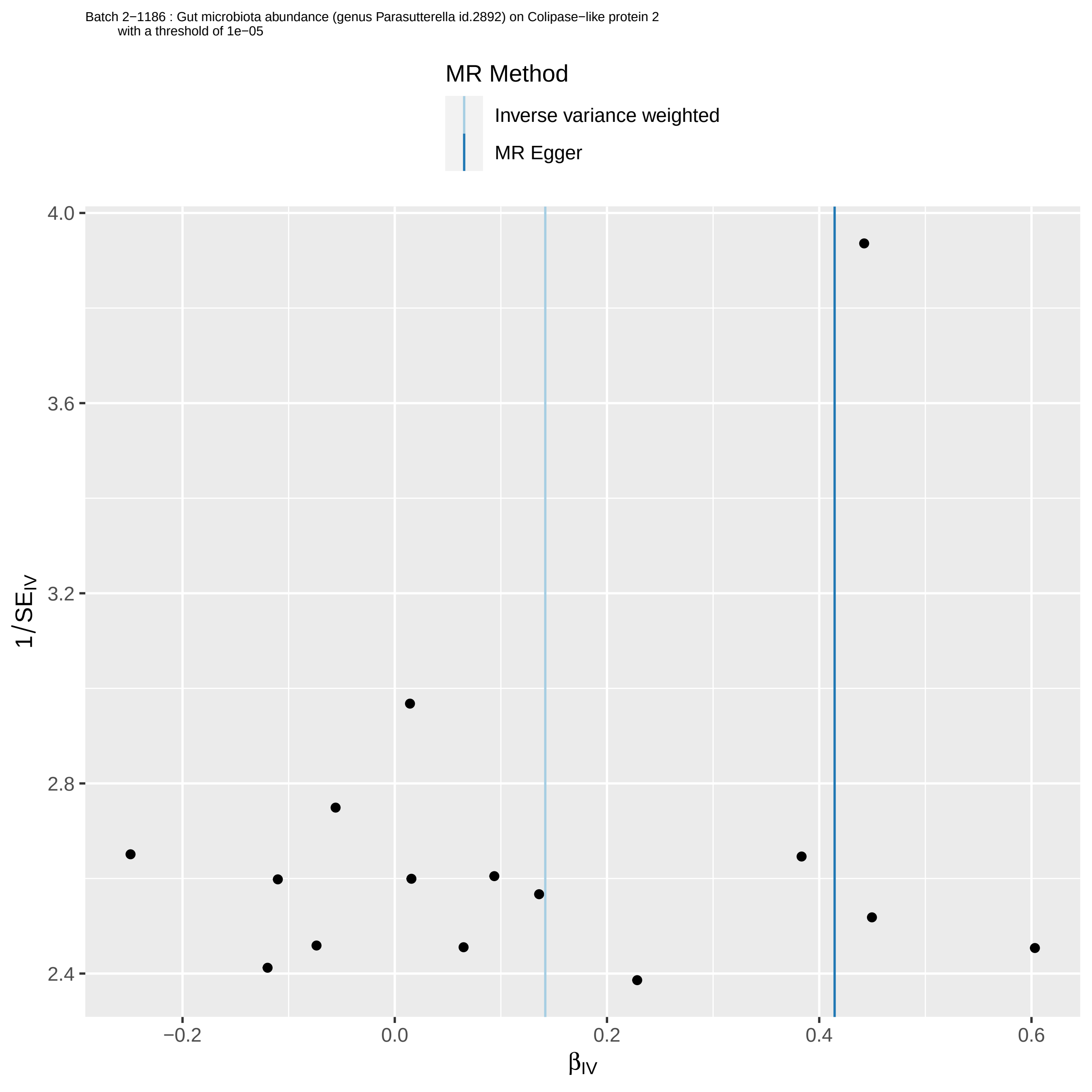
Batch 2−1186 : Gut microbiota abundance (genus Parasutterella id.2892) on Colipase−like protein 2
with a threshold of 1e−05
MR Method
Inverse variance weighted
MR Egger
4.0
3.6
3.2
2.8
2.4
1/SE_IV
−0.2
0.0
0.2
0.4
0.6
β_IV

Batch 2−1187 : Gut microbiota abundance (genus Catenibacterium id.2153) on Calsyntenin−3
with a threshold of 1e−05

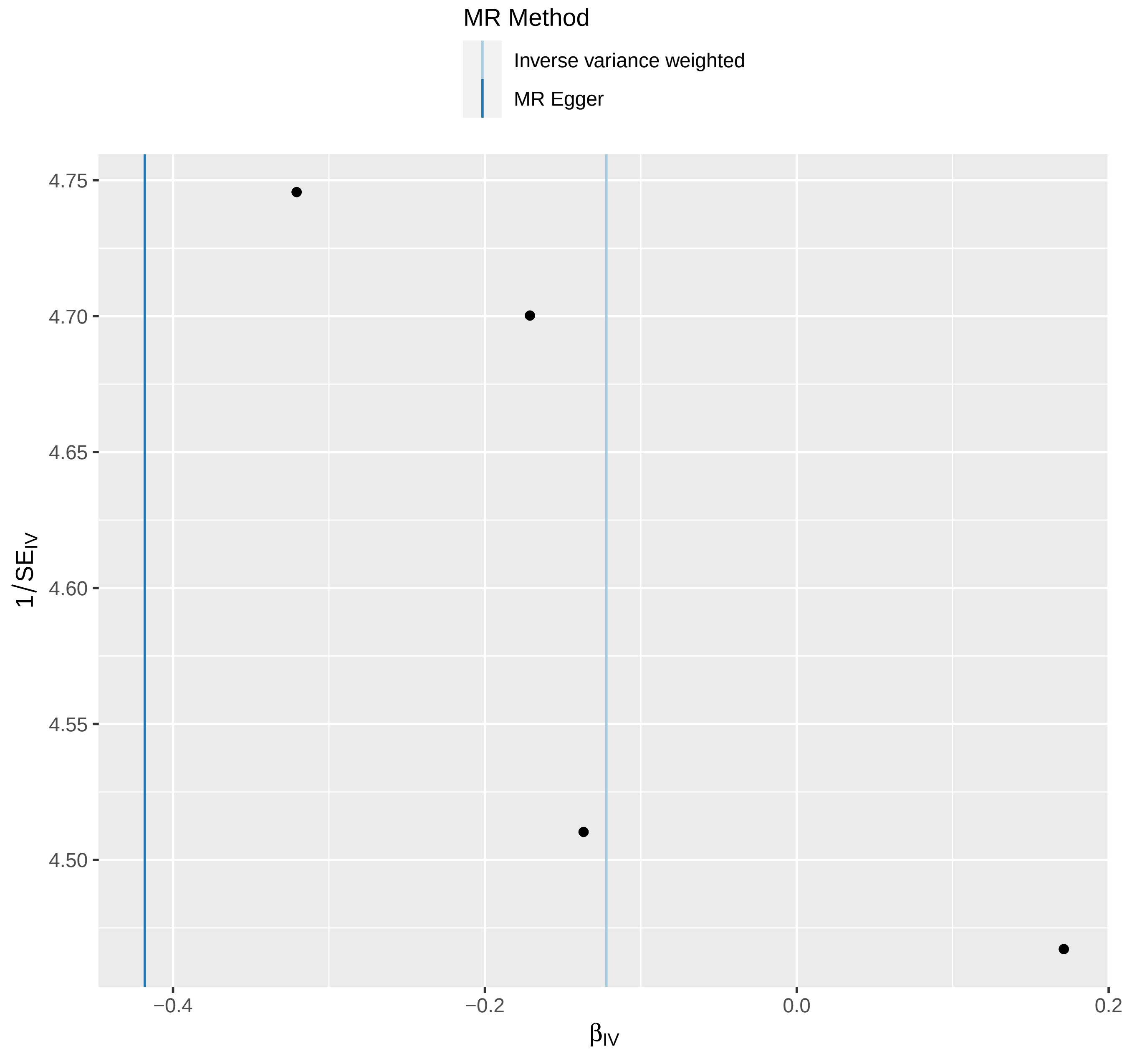

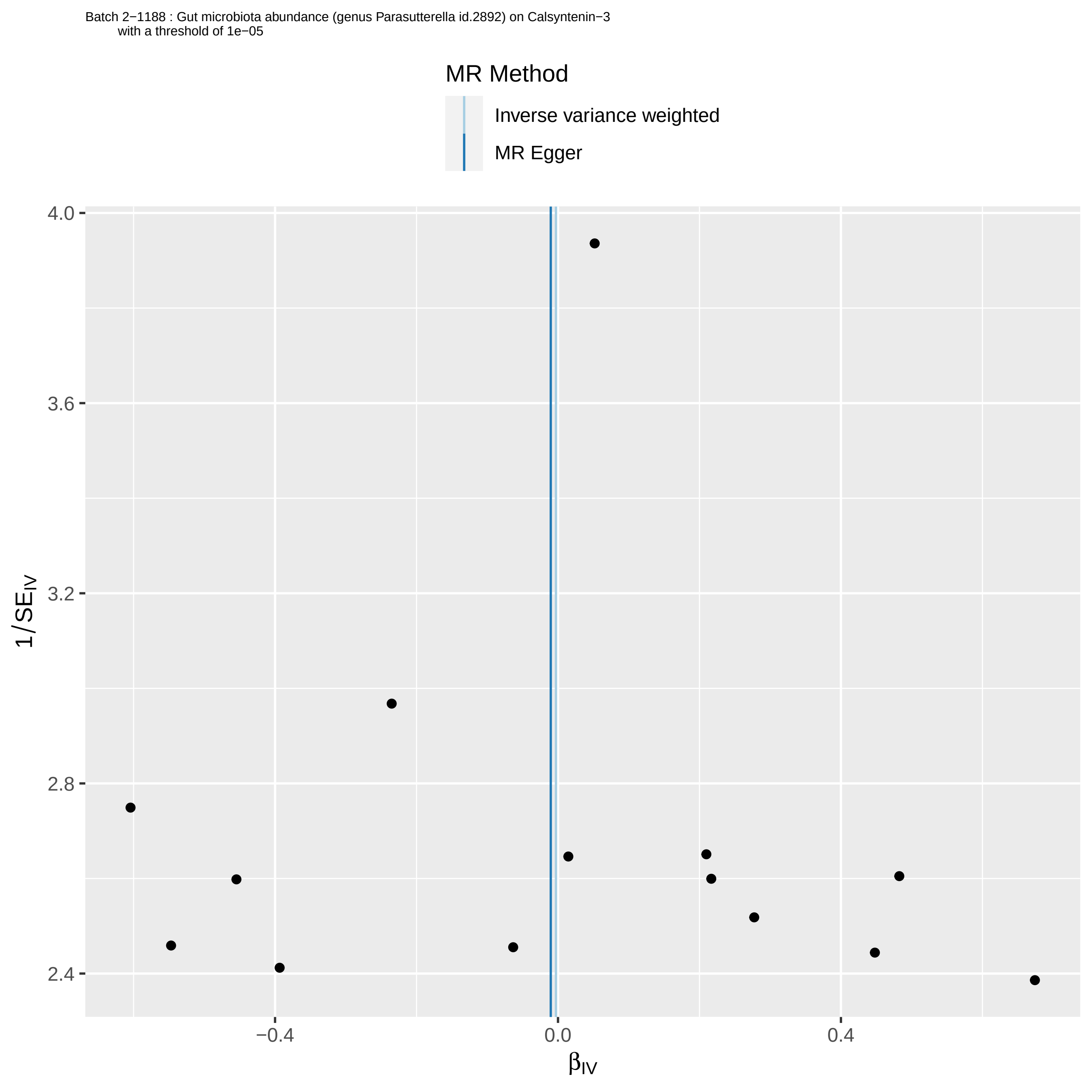
Batch 2−1188 : Gut microbiota abundance (genus Parasutterella id.2892) on Calsyntenin−3
with a threshold of 1e−05
MR Method
Inverse variance weighted
MR Egger
4.0
3.6
3.2
2.8
2.4
1/SE_IV
−0.4
0.0
0.4
β_IV

Batch 2−1189 : Gut microbiota abundance (genus Catenibacterium id.2153) on Clathrin heavy chain 1
with a threshold of 1e−05

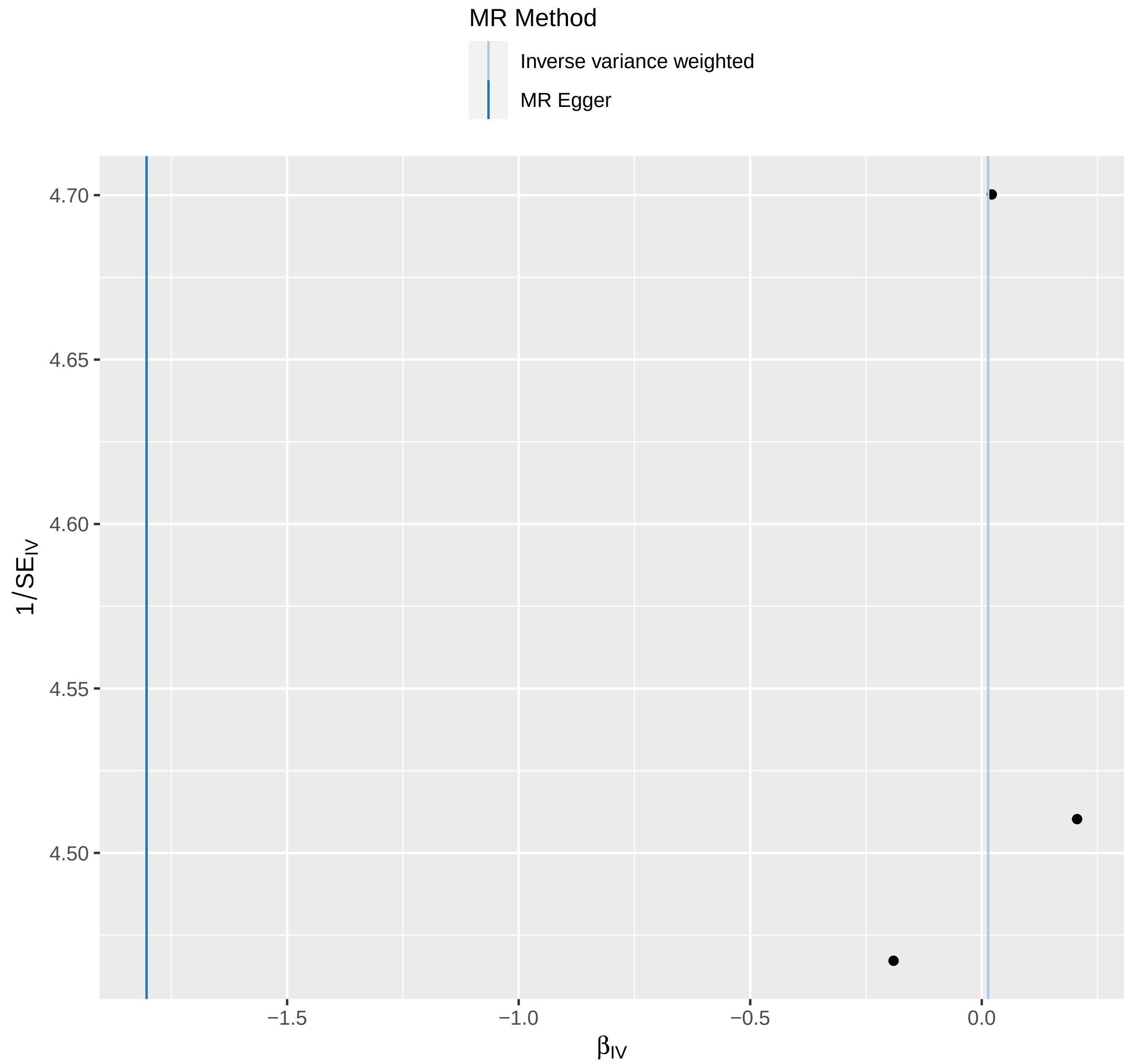

Batch 2−1190 : Gut microbiota abundance (genus Parasutterella id.2892) on Clathrin heavy chain 1
with a threshold of 1e−05

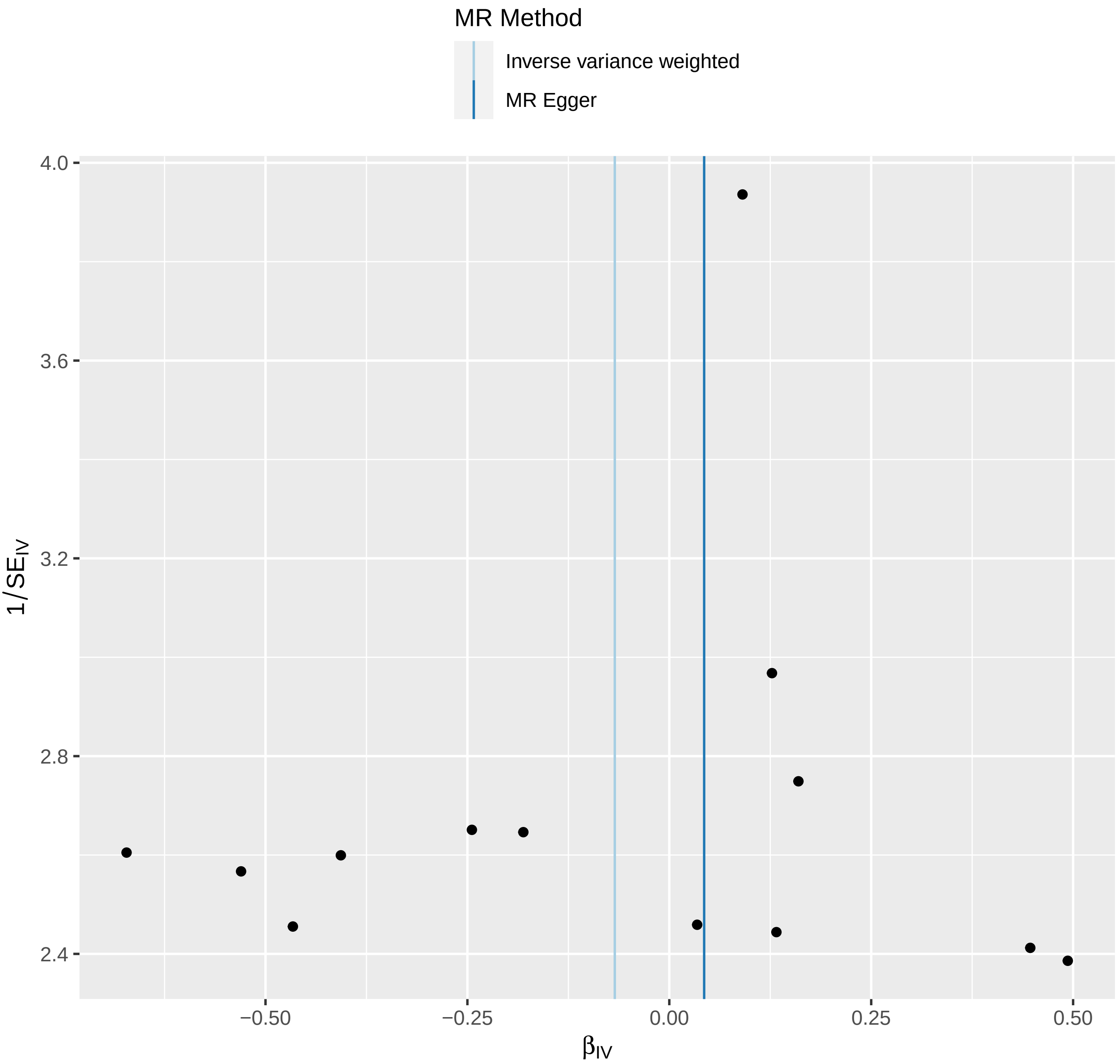

Batch 2−1191 : Gut microbiota abundance (genus Catenibacterium id.2153) on Clusterin
with a threshold of 1e−05

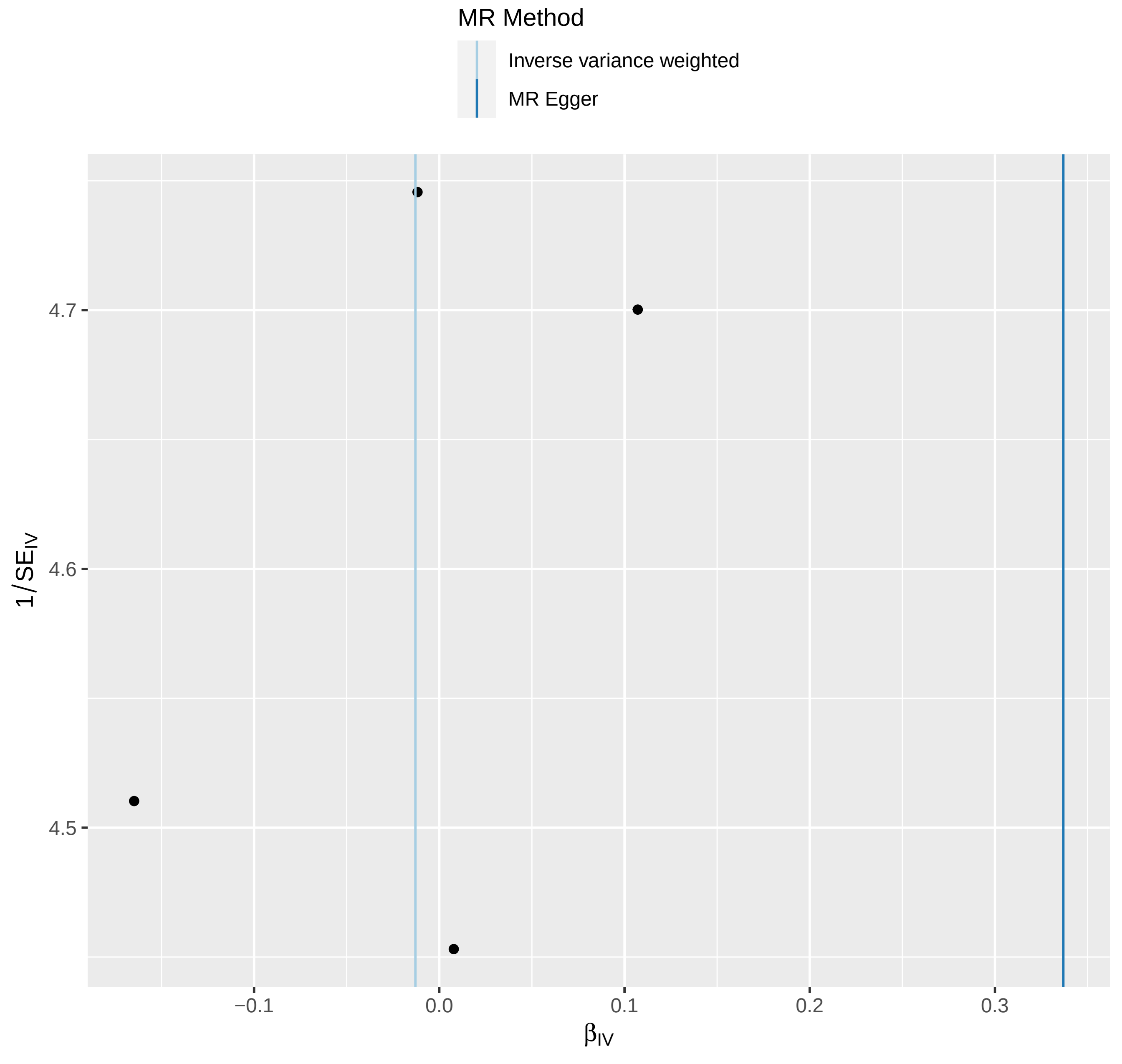

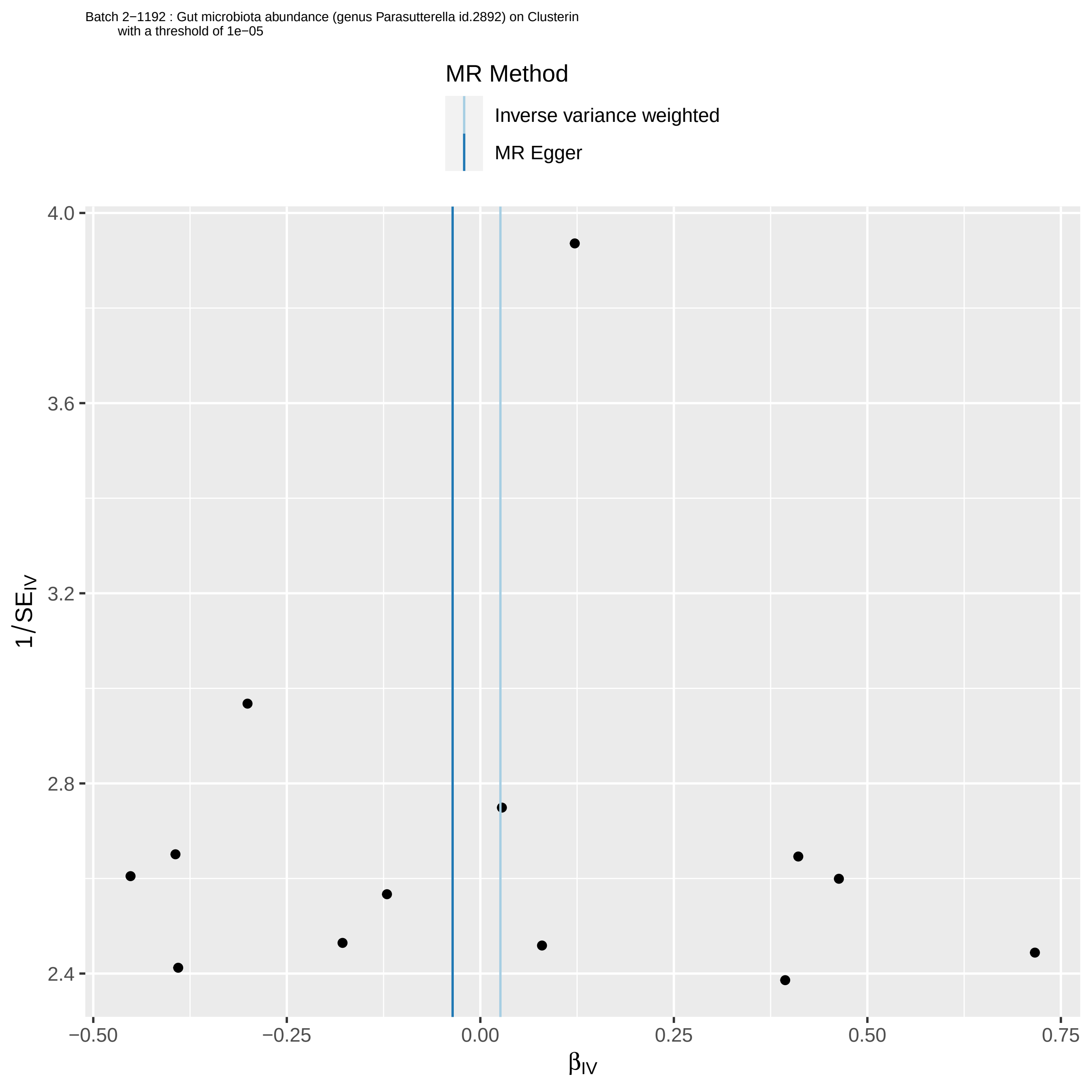

Batch 2−1192 : Gut microbiota abundance (genus Parasutterella id.2892) on Clusterin
with a threshold of 1e−05
MR Method
Inverse variance weighted
MR Egger
4.0
3.6
3.2
2.8
2.4
1/SE_IV
−0.50
−0.25
0.00
0.25
0.50
0.75
β_IV

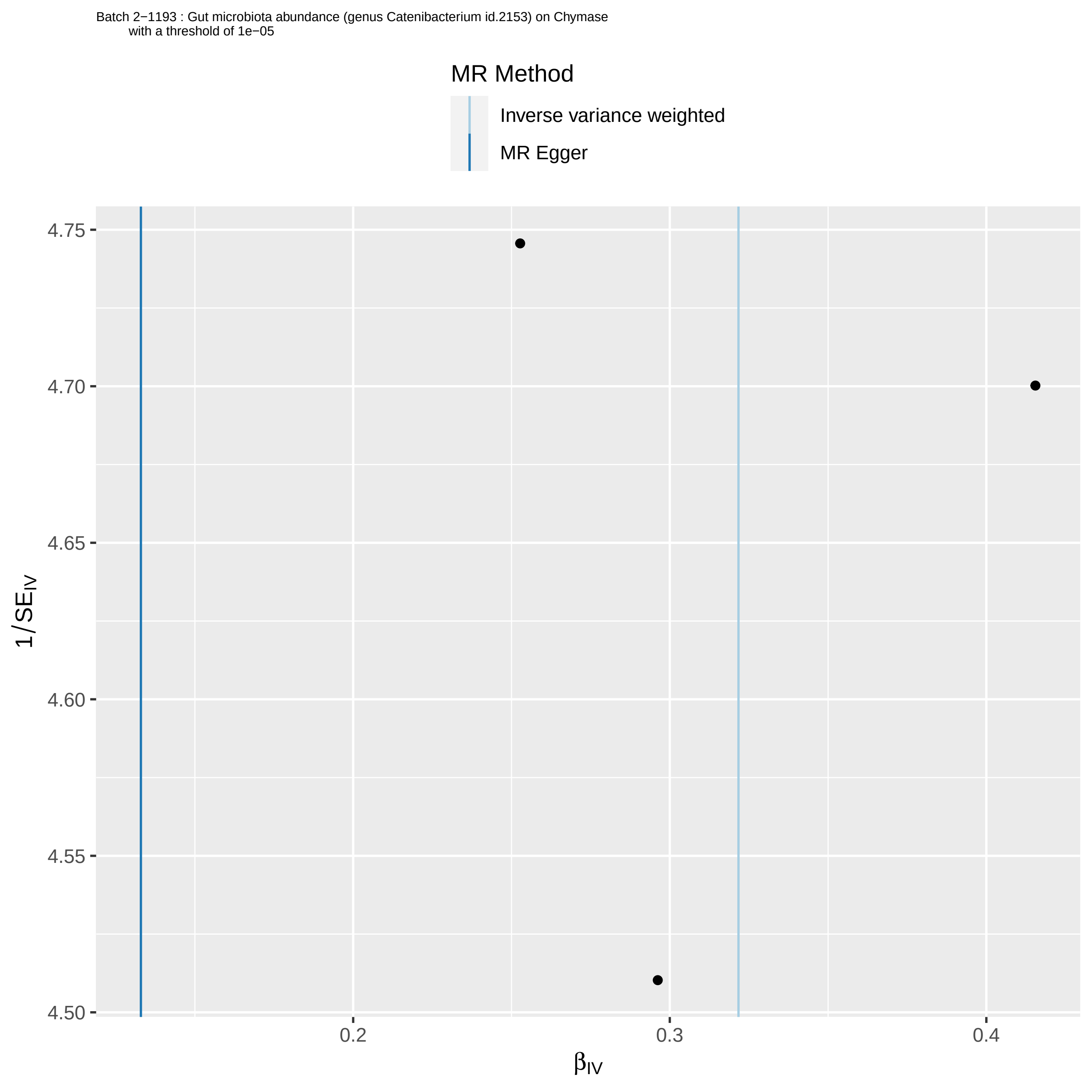
Batch 2−1193 : Gut microbiota abundance (genus Catenibacterium id.2153) on Chymase
with a threshold of 1e−05
MR Method
Inverse variance weighted
MR Egger
4.75
4.70
4.65
4.60
4.55
4.50
1/SE_IV
0.2
0.3
0.4
β_IV

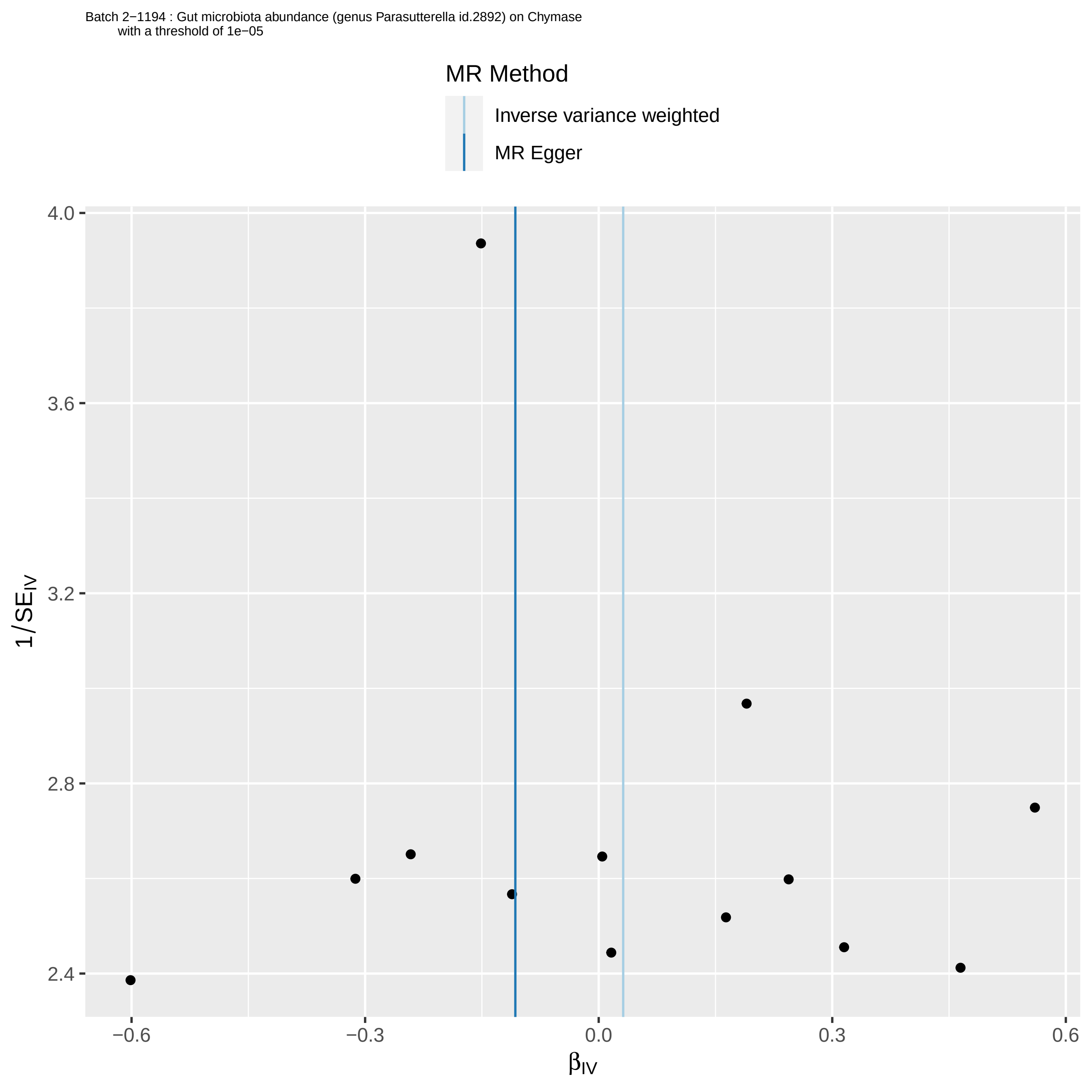
Batch 2−1194 : Gut microbiota abundance (genus Parasutterella id.2892) on Chymase
with a threshold of 1e−05
MR Method
Inverse variance weighted
MR Egger
4.0
3.6
3.2
2.8
2.4
1/SE_IV
−0.6
−0.3
0.0
0.3
0.6
β_IV

Batch 2−1195 : Gut microbiota abundance (genus Catenibacterium id.2153) on UMP−CMP kinase with a threshold of 1e−05

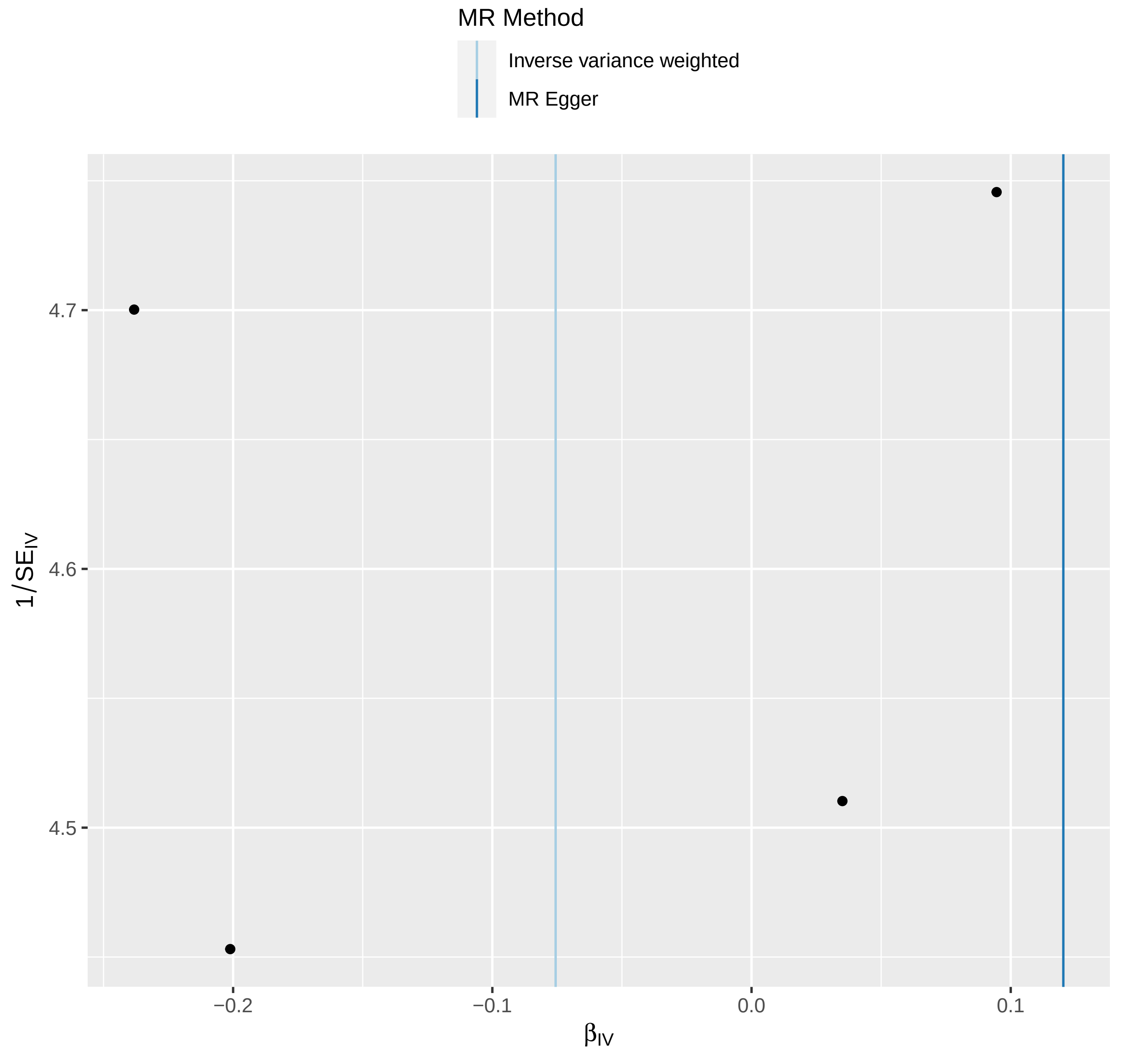

Batch 2−1196 : Gut microbiota abundance (genus Parasutterella id.2892) on UMP−CMP kinase
with a threshold of 1e−05

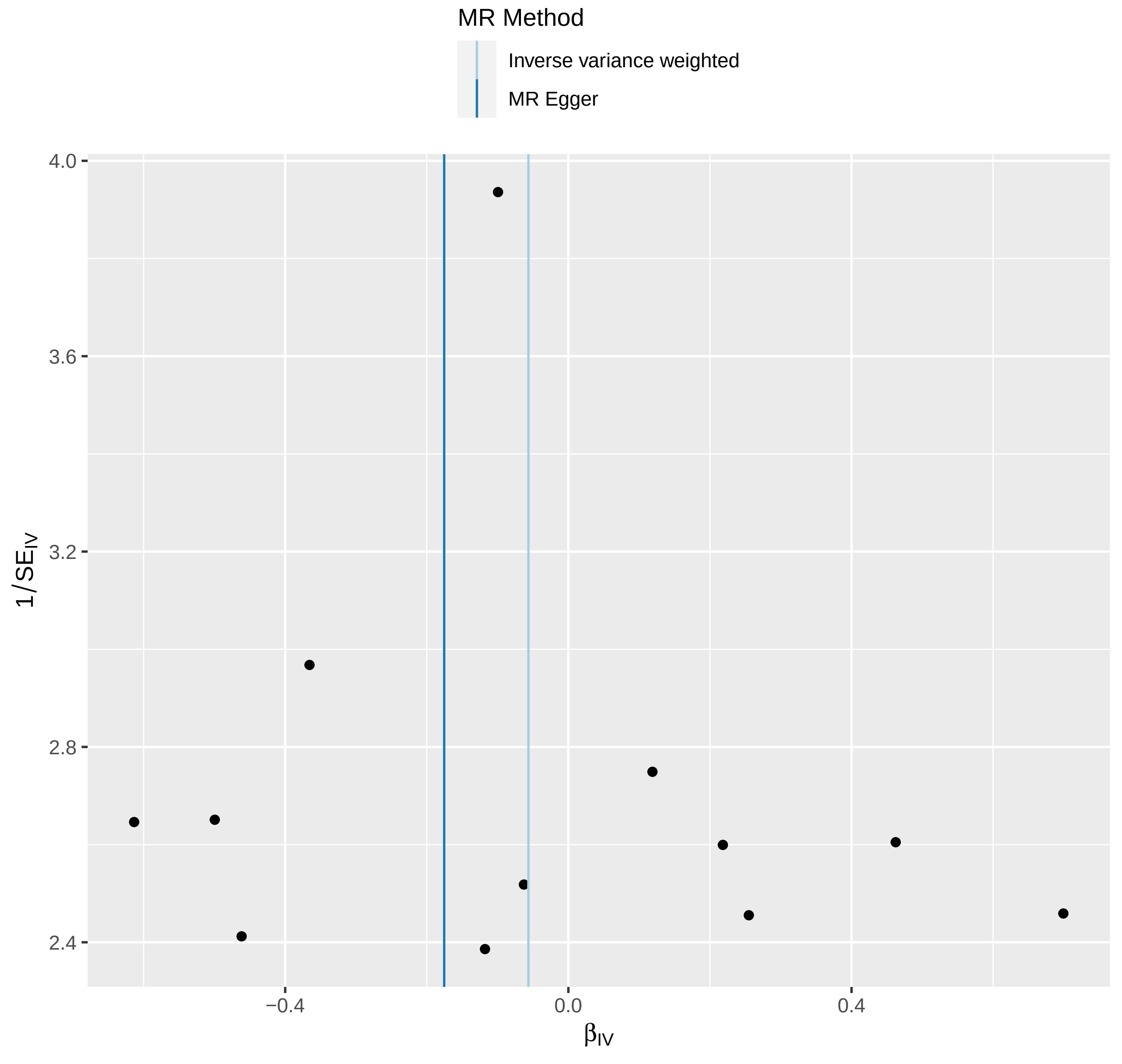

Batch 2−1197 : Gut microbiota abundance (genus Catenibacterium id.2153) on UMP−CMP kinase
with a threshold of 1e−05

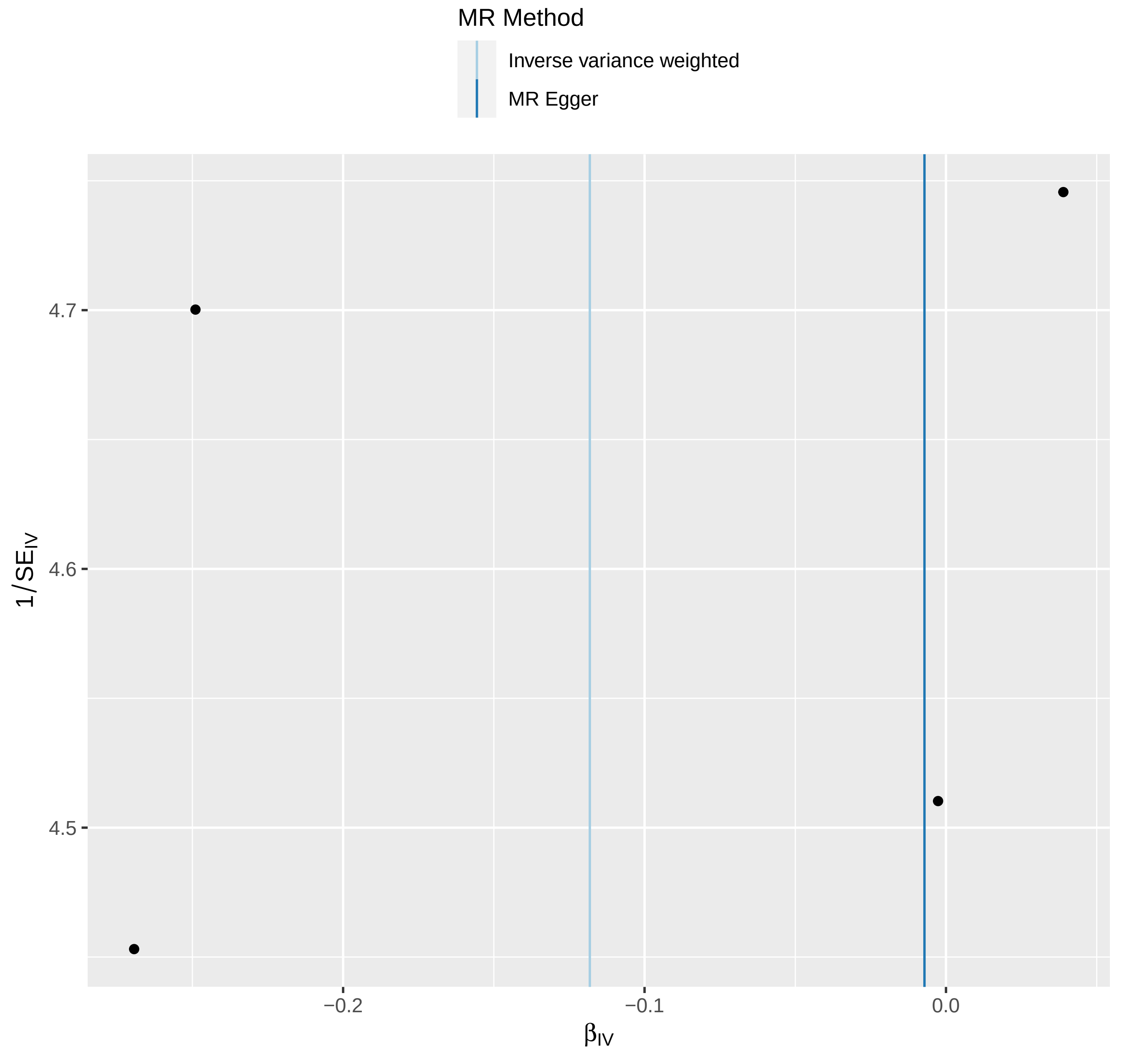

Batch 2−1198 : Gut microbiota abundance (genus Parasutterella id.2892) on UMP−CMP kinase
with a threshold of 1e−05

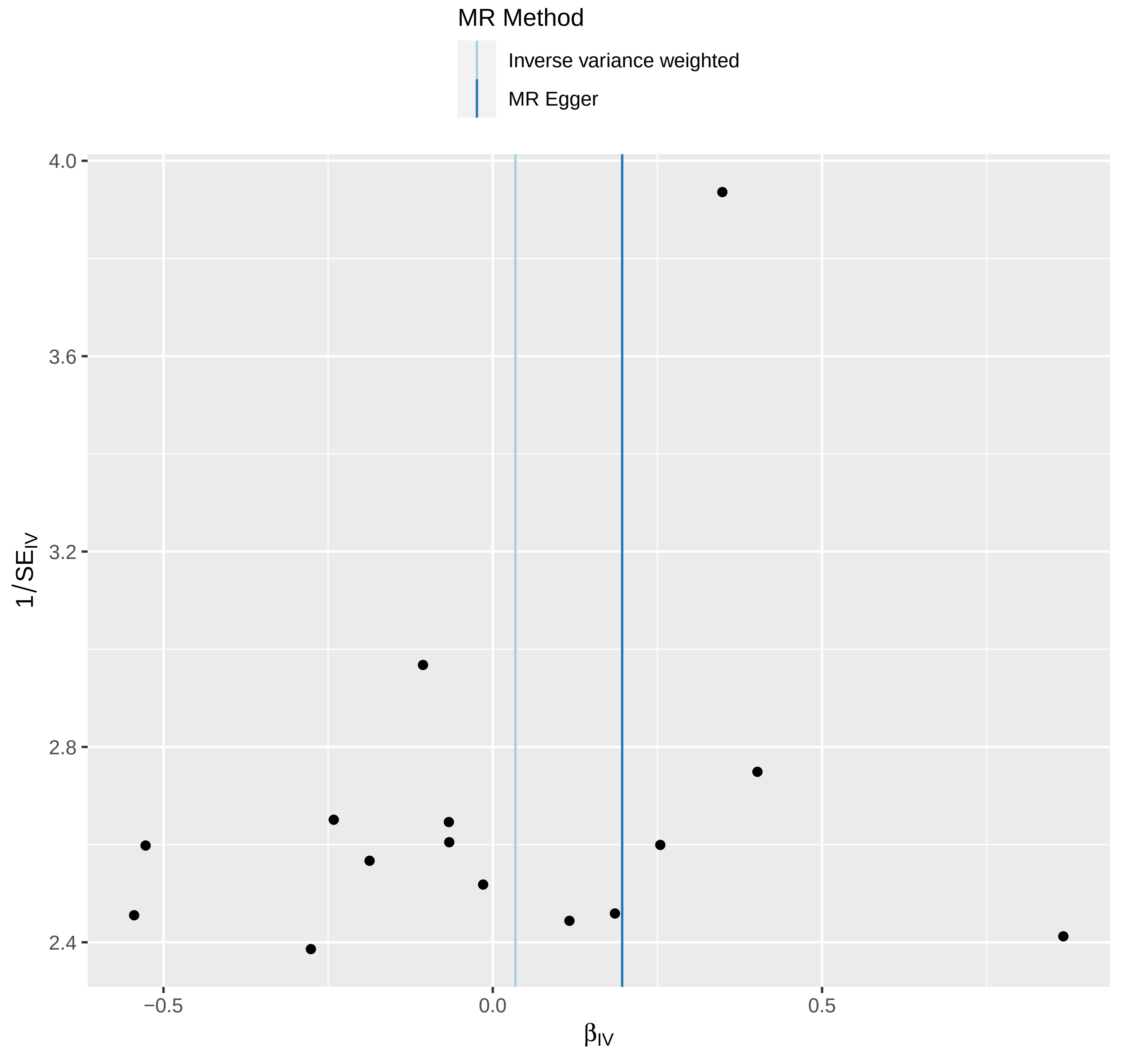

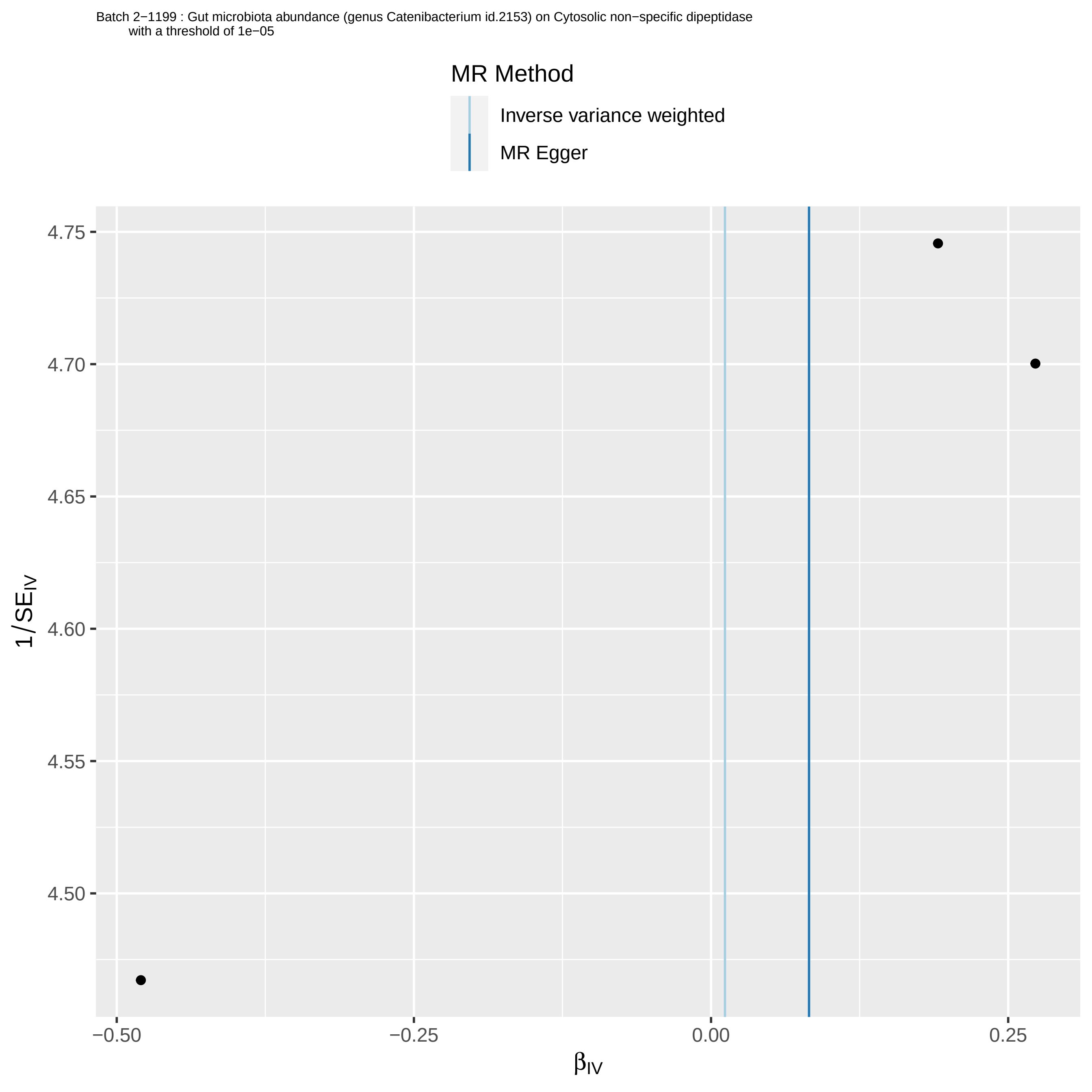
Batch 2−1199 : Gut microbiota abundance (genus Catenibacterium id.2153) on Cytosolic non−specific dipeptidase
with a threshold of 1e−05
MR Method
Inverse variance weighted
MR Egger
4.75
4.70
4.65
4.60
4.55
4.50
1/SE_IV
−0.50
−0.25
0.00
0.25
β_IV

Batch 2−1200 : Gut microbiota abundance (genus Parasutterella id.2892) on Cytosolic non−specific dipeptidase
with a threshold of 1e−05

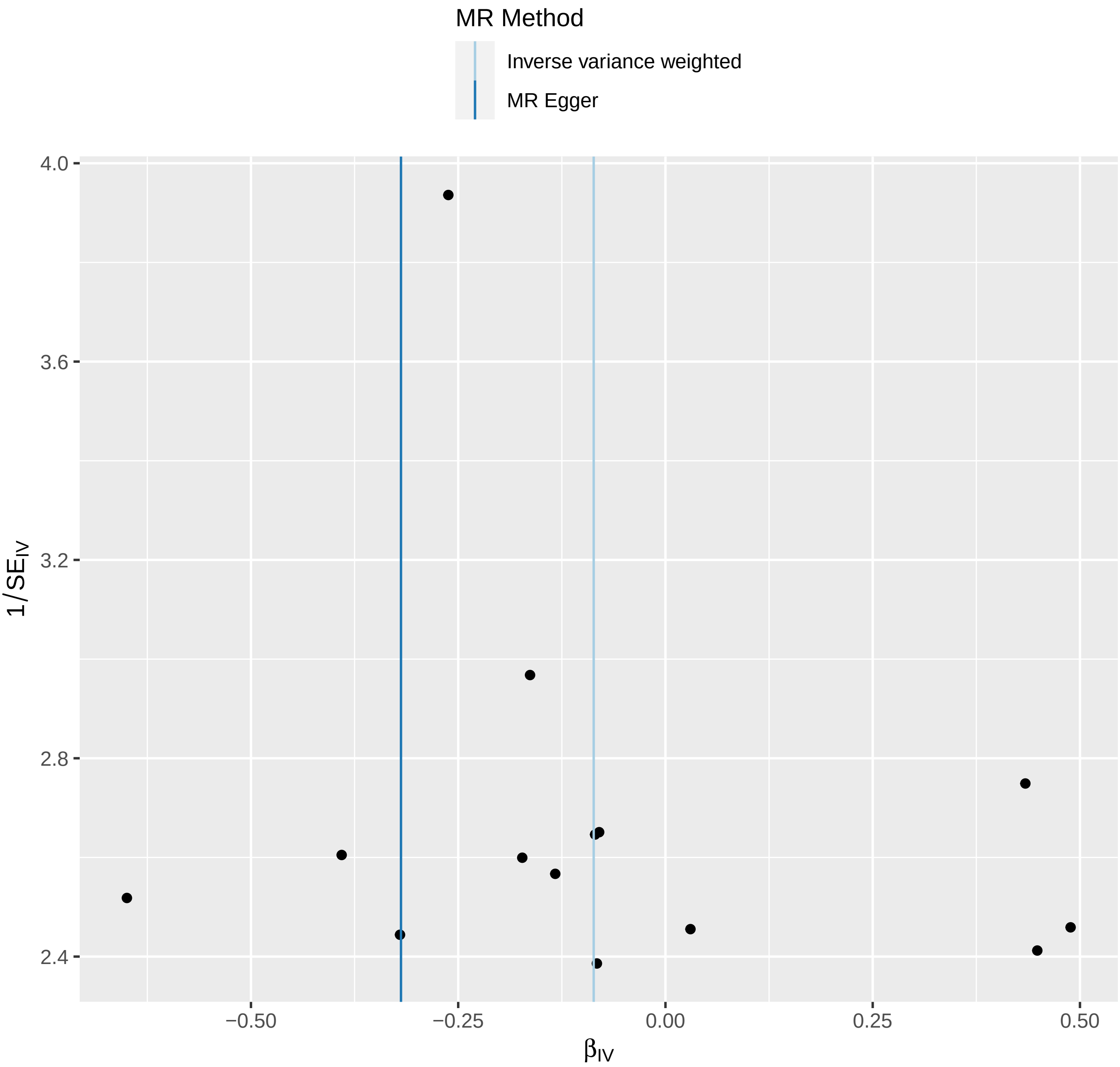

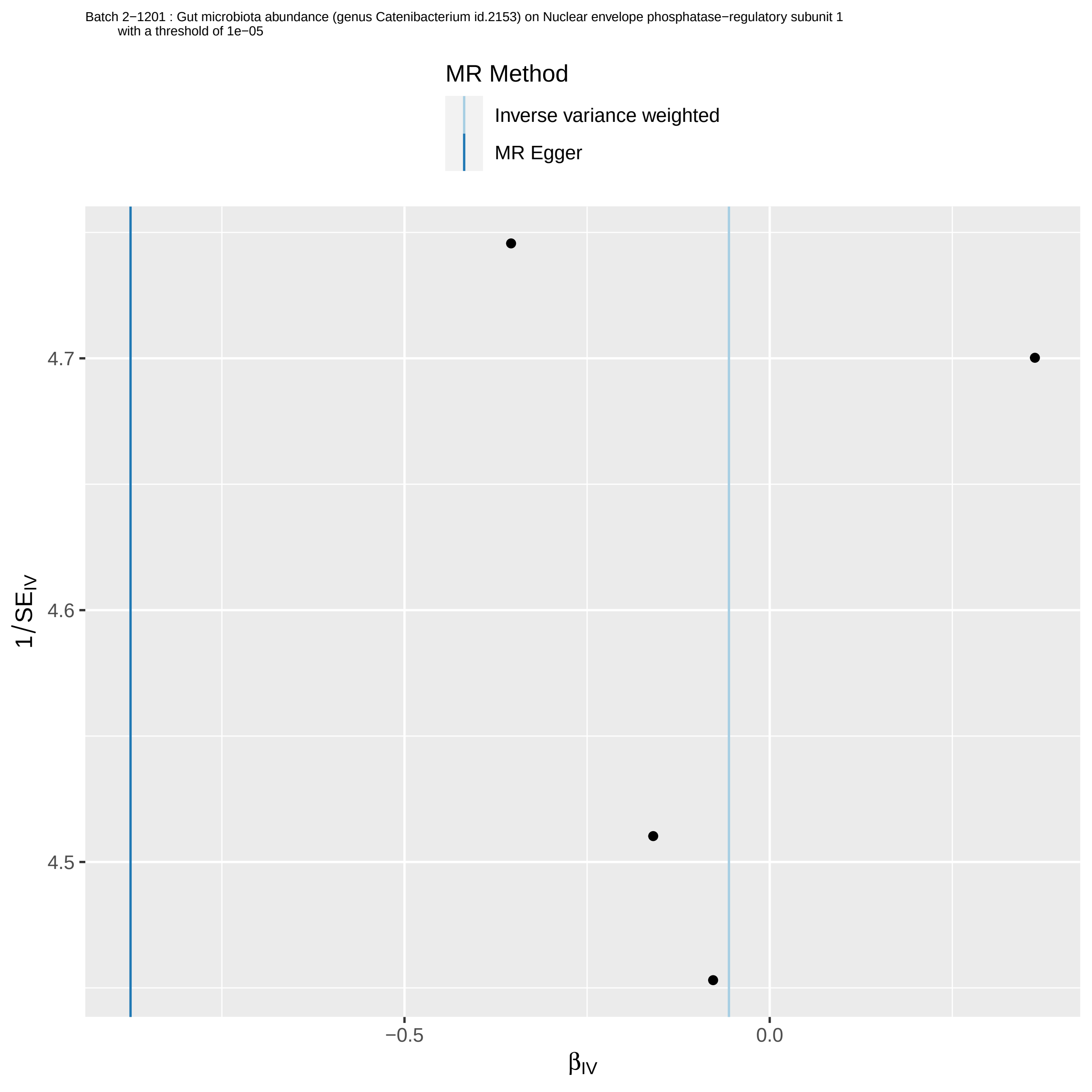
Batch 2−1201 : Gut microbiota abundance (genus Catenibacterium id.2153) on Nuclear envelope phosphatase−regulatory subunit 1
with a threshold of 1e−05
MR Method
Inverse variance weighted
MR Egger
4.7
4.6
4.5
1/SE_IV
−0.5
0.0
β_IV

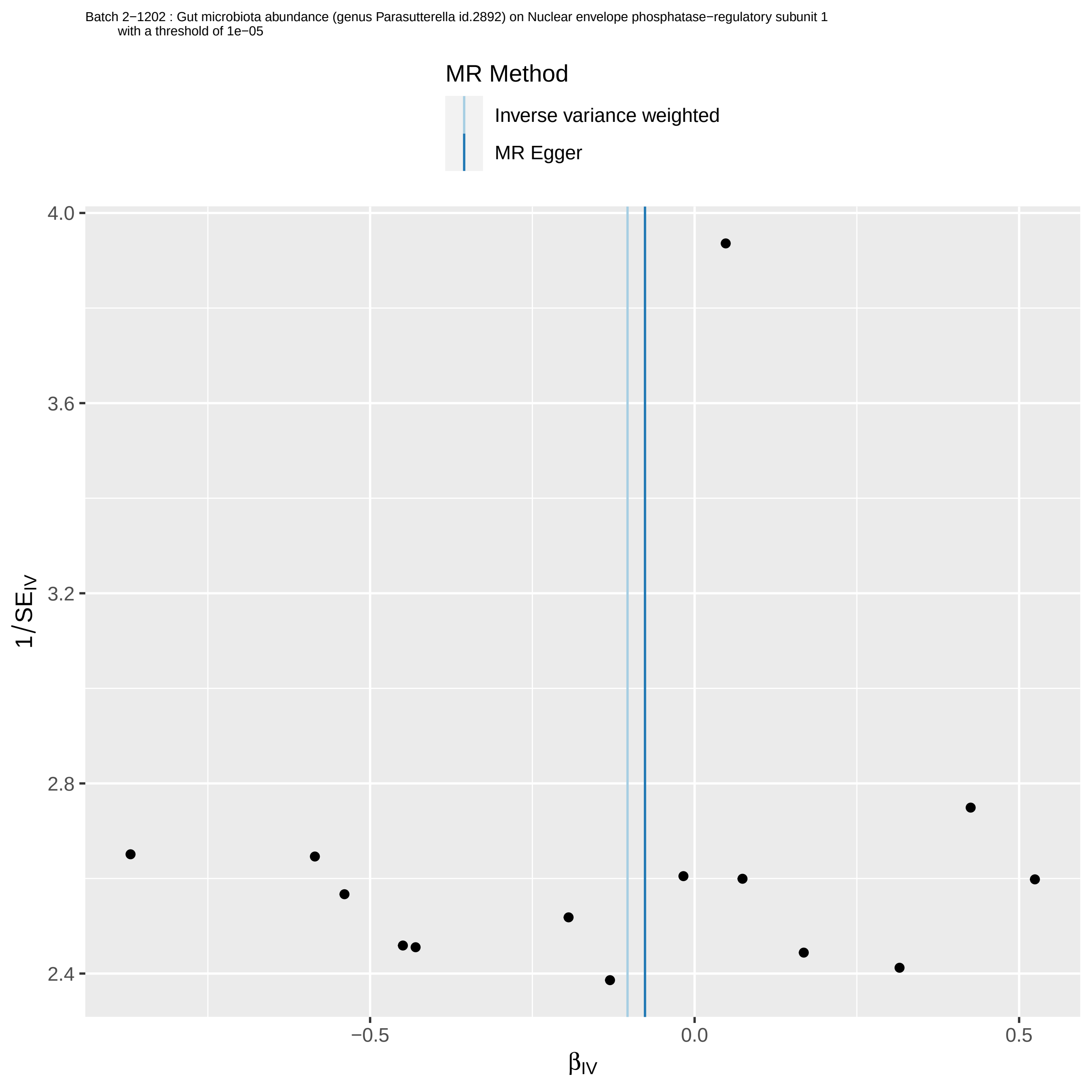

Batch 2−1202 : Gut microbiota abundance (genus Parasutterella id.2892) on Nuclear envelope phosphatase−regulatory subunit 1
with a threshold of 1e−05
MR Method
Inverse variance weighted
MR Egger
4.0
3.6
3.2
2.8
2.4
1/SE_IV
−0.5
0.0
0.5
β_IV

Batch 2−1203 : Gut microbiota abundance (genus Catenibacterium id.2153) on CCR4−NOT transcription complex subunit 1 with a threshold of 1e−05

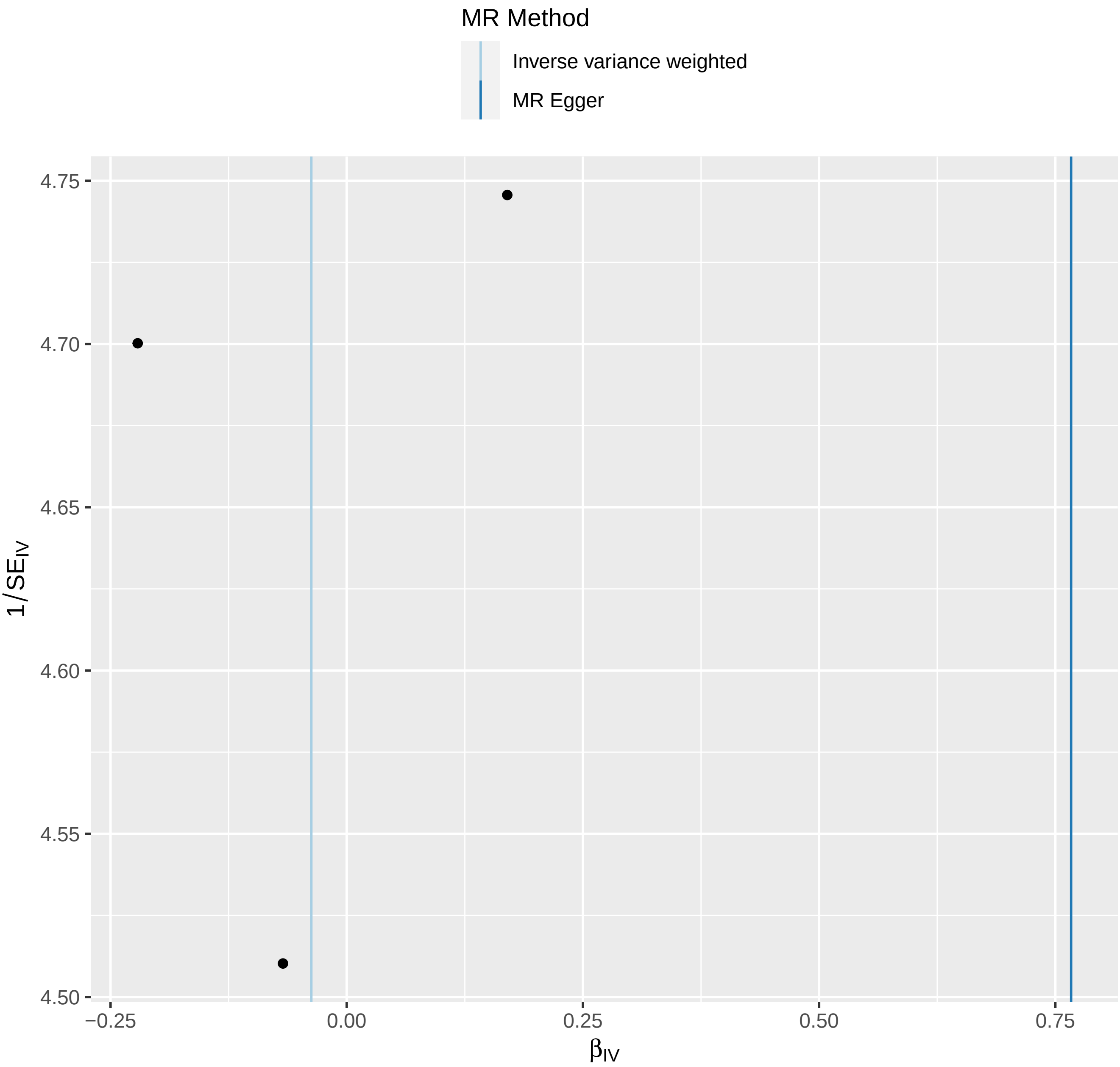

Batch 2−1204 : Gut microbiota abundance (genus Parasutterella id.2892) on CCR4−NOT transcription complex subunit 1
with a threshold of 1e−05

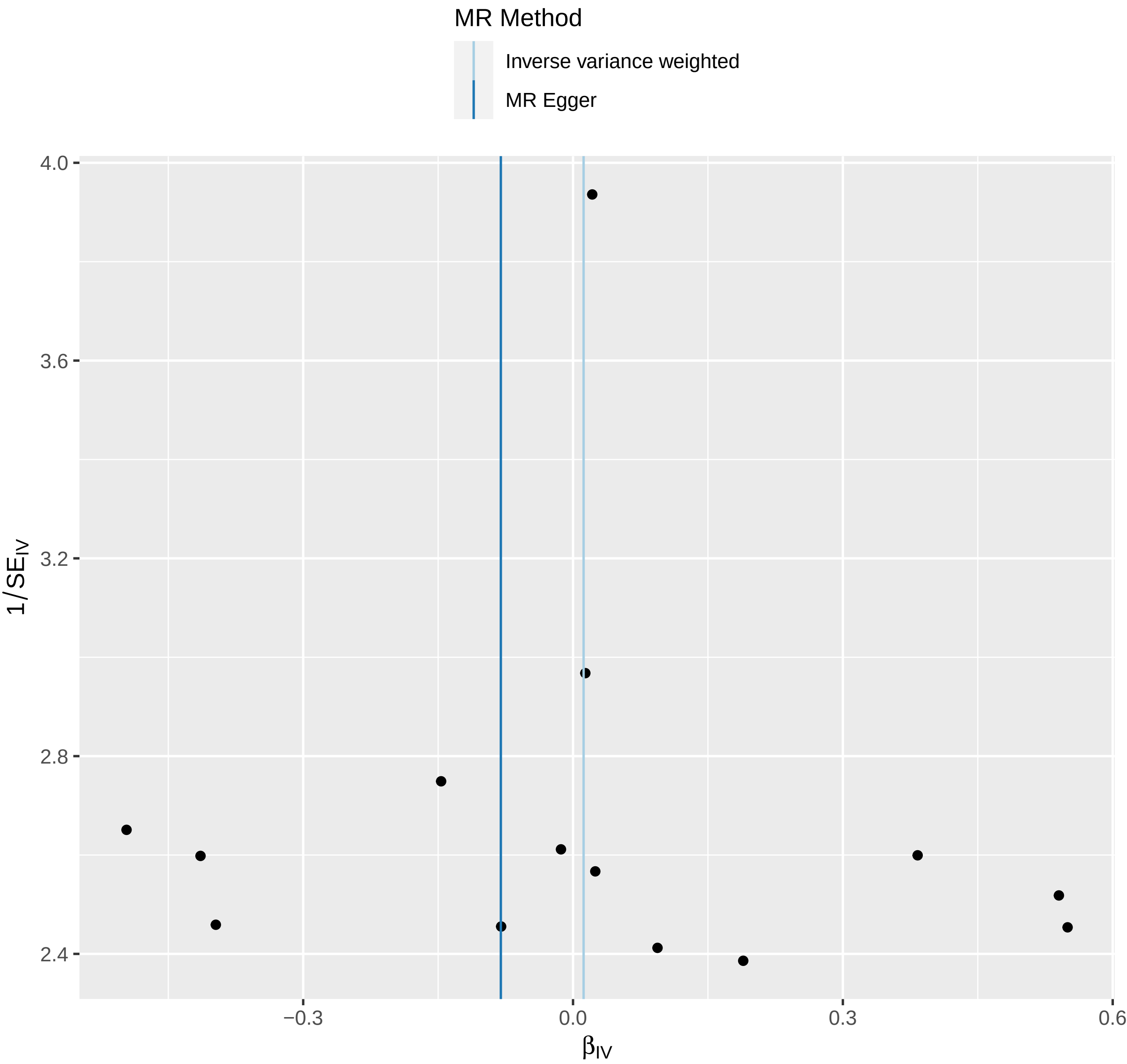

Batch 2−1205 : Gut microbiota abundance (genus Catenibacterium id.2153) on 2',3'−cyclic−nucleotide 3'−phosphodiesterase with a threshold of 1e−05

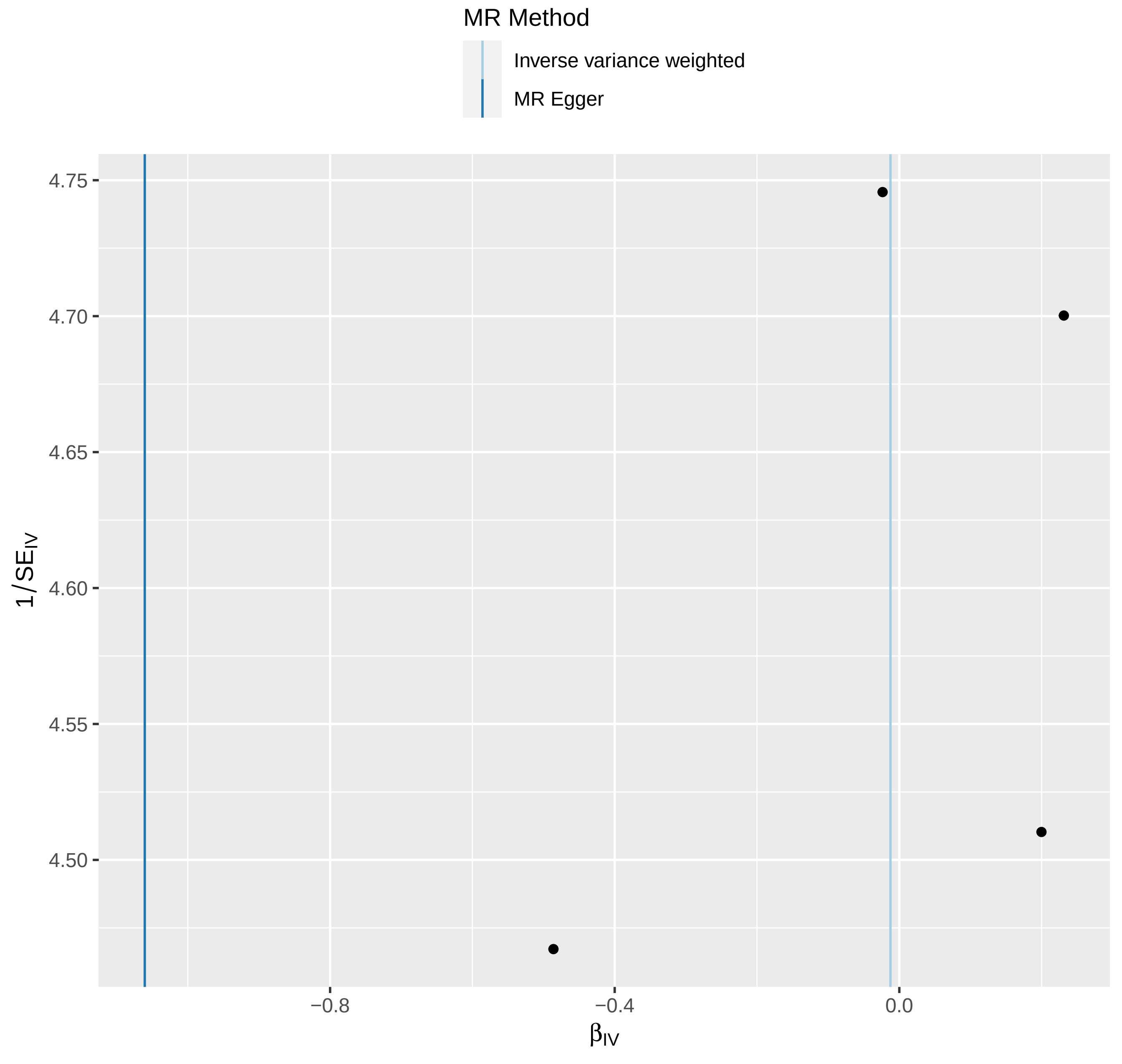

Batch 2−1206 : Gut microbiota abundance (genus Parasutterella id.2892) on 2',3'−cyclic−nucleotide 3'−phosphodiesterase with a threshold of 1e−05

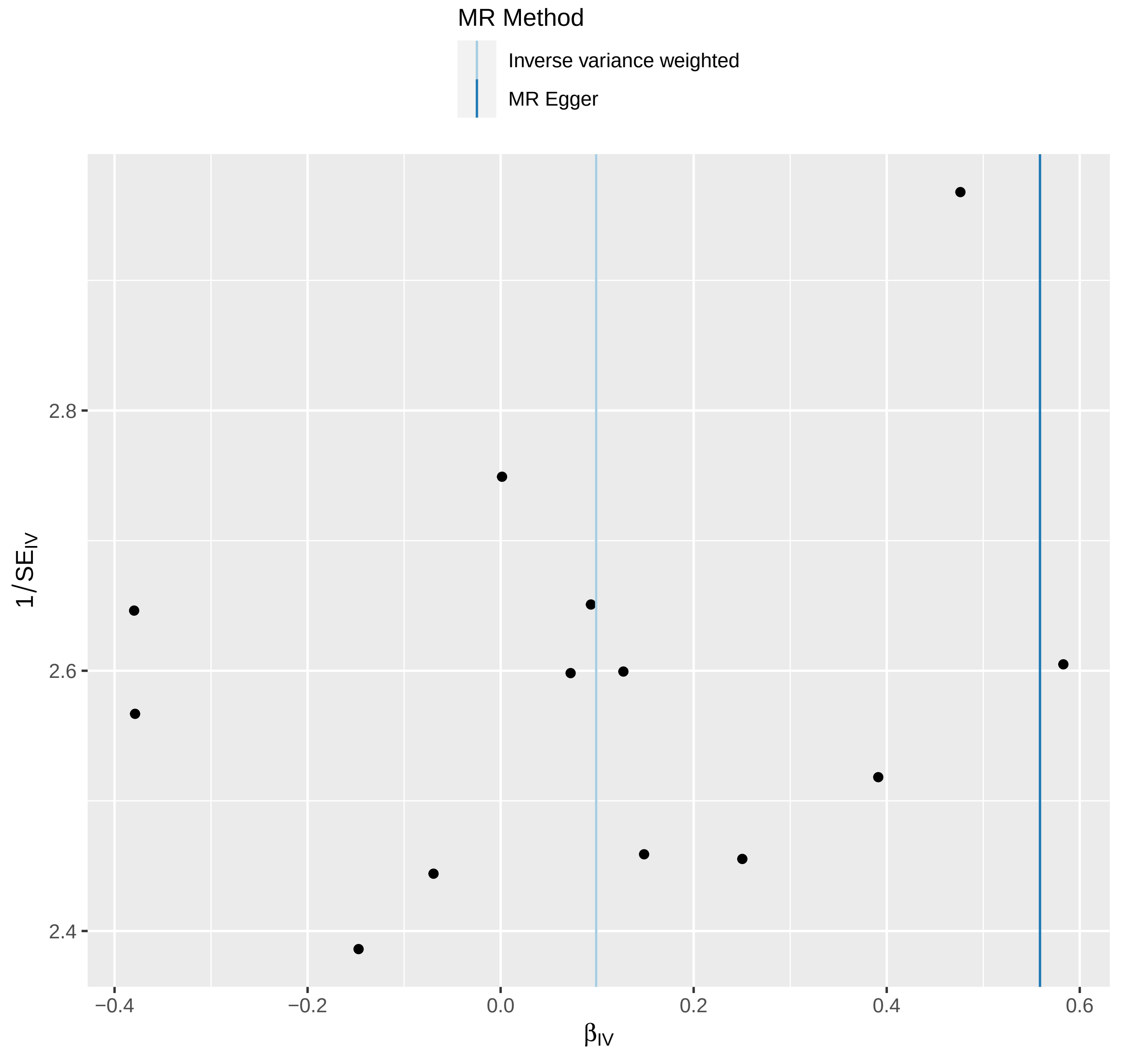

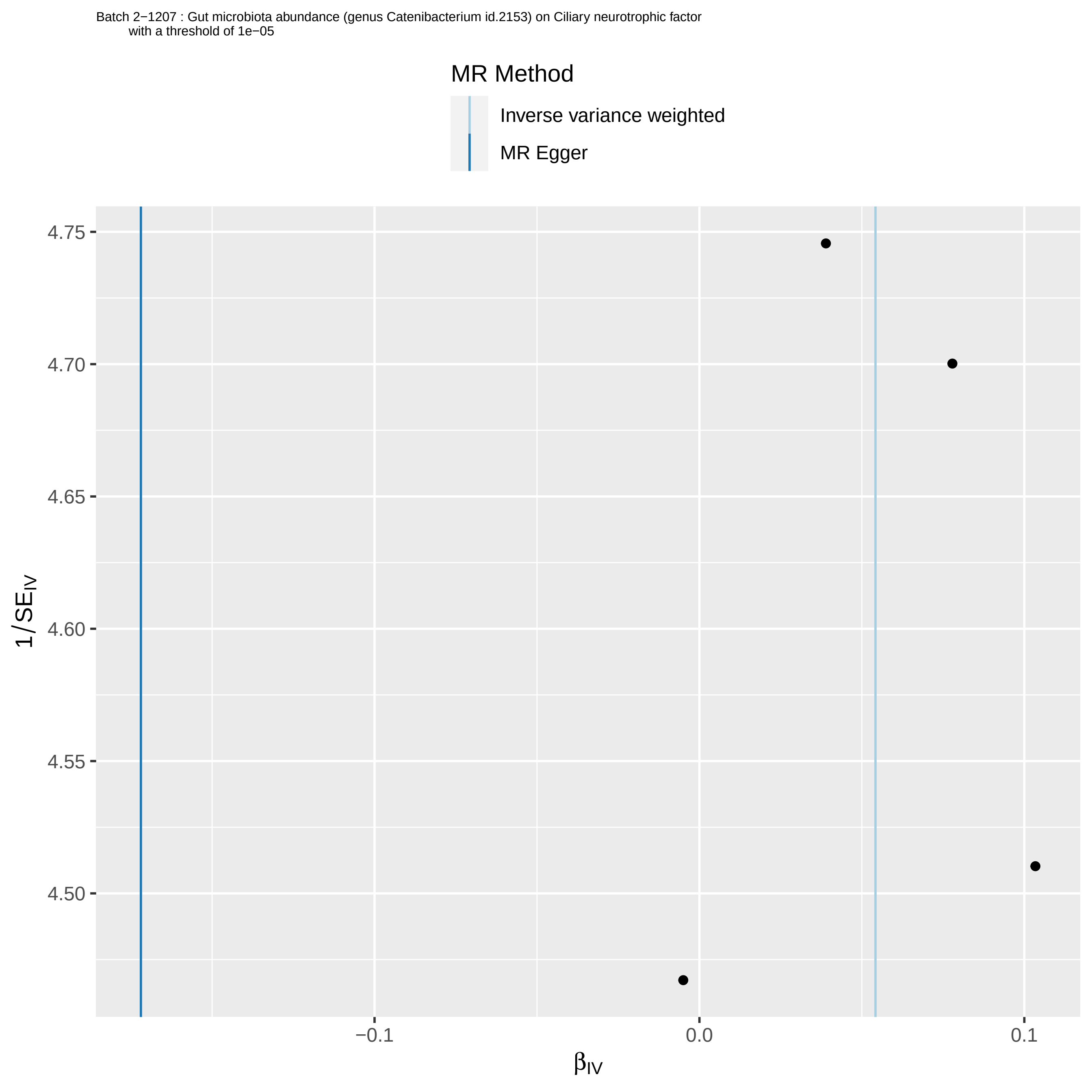

Batch 2−1207 : Gut microbiota abundance (genus Catenibacterium id.2153) on Ciliary neurotrophic factor
with a threshold of 1e−05
MR Method
Inverse variance weighted
MR Egger
4.75
4.70
4.65
4.60
4.55
4.50
1/SE$_{IV}$
−0.1
0.0
0.1
β_{IV}

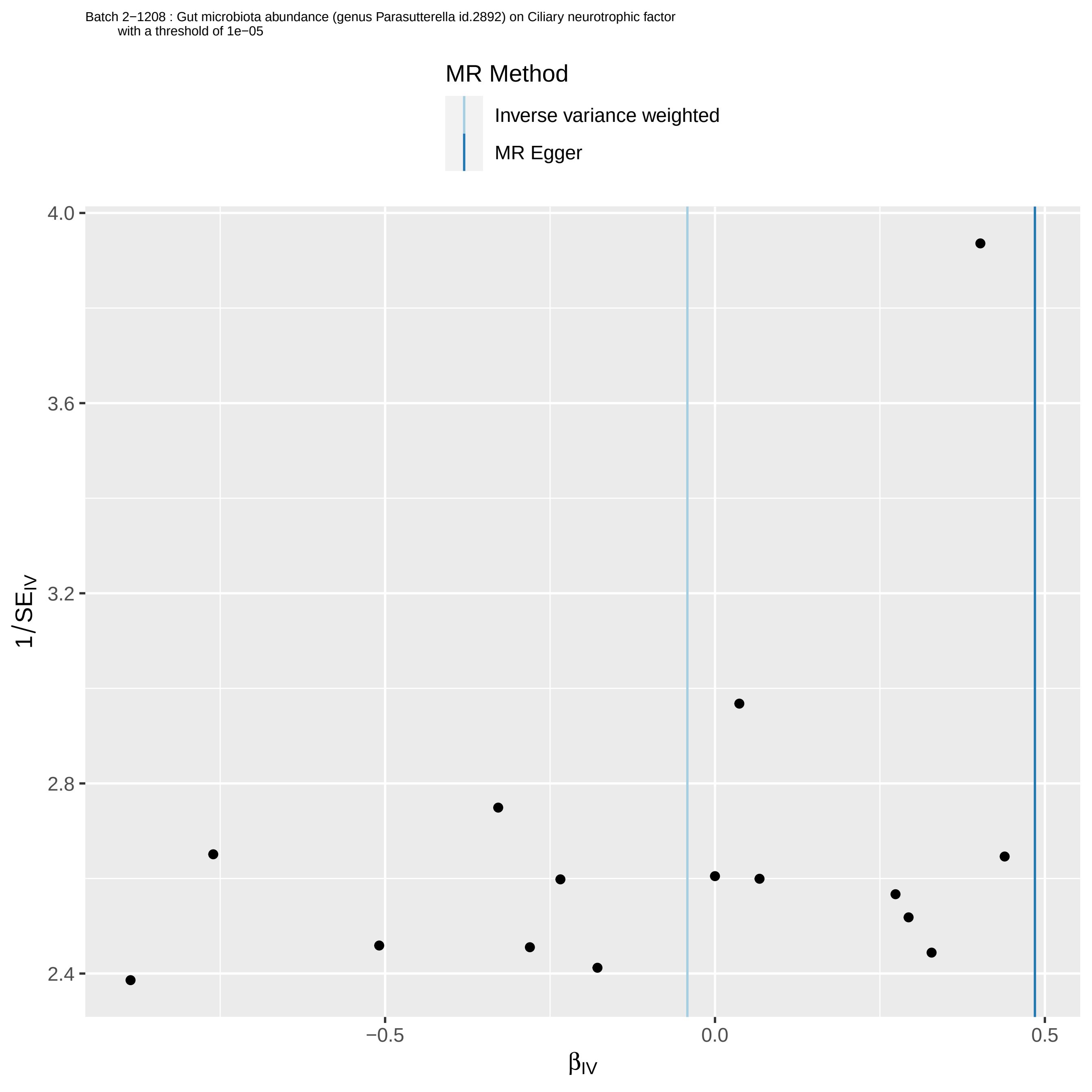
Batch 2−1208 : Gut microbiota abundance (genus Parasutterella id.2892) on Ciliary neurotrophic factor
with a threshold of 1e−05
MR Method
Inverse variance weighted
MR Egger
4.0
3.6
3.2
2.8
2.4
1/SE_IV
−0.5
0.0
0.5
β_IV

Batch 2−1209 : Gut microbiota abundance (genus Catenibacterium id.2153) on Ciliary neurotrophic factor receptor subunit alpha with a threshold of 1e−05

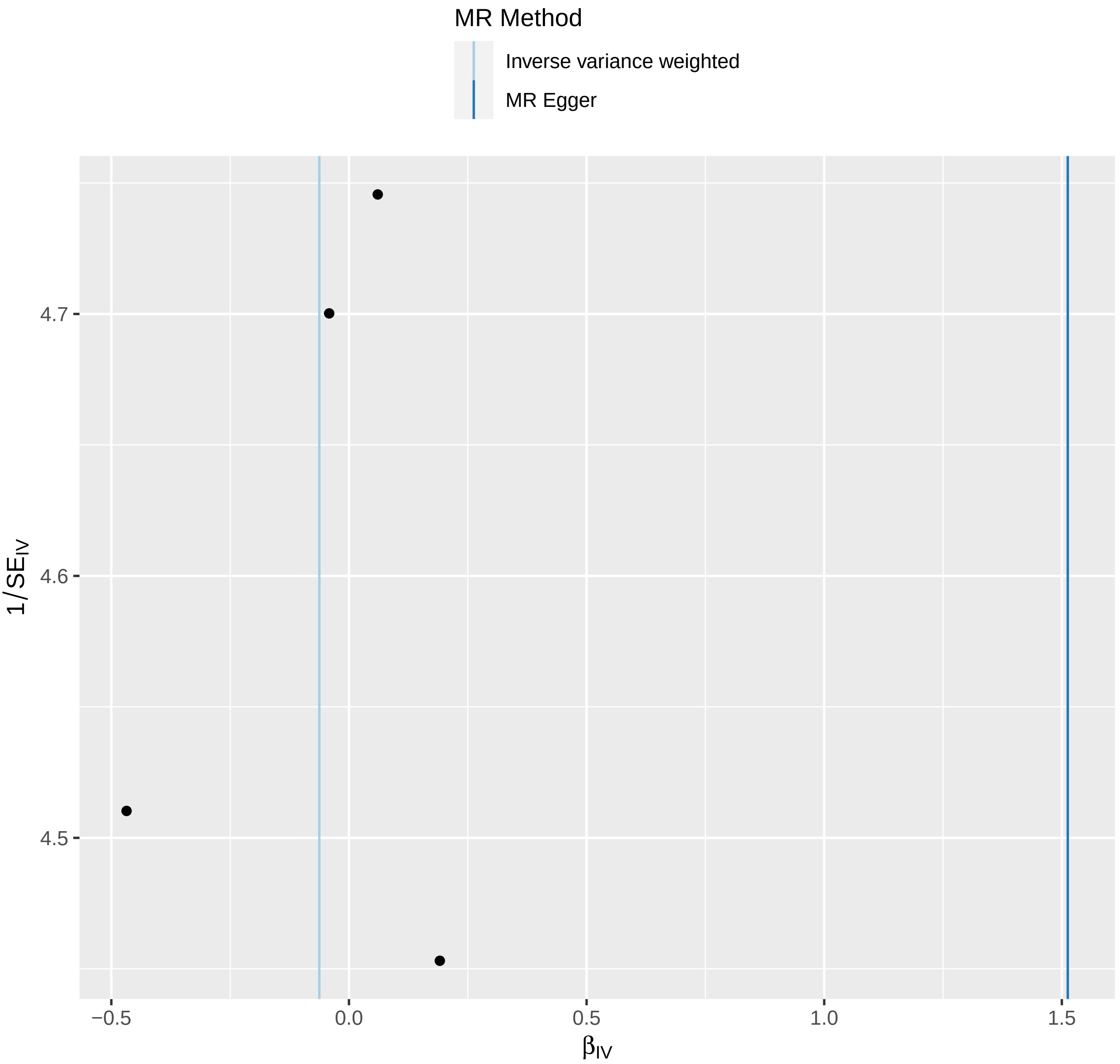

Batch 2−1210 : Gut microbiota abundance (genus Parasutterella id.2892) on Ciliary neurotrophic factor receptor subunit alpha
with a threshold of 1e−05

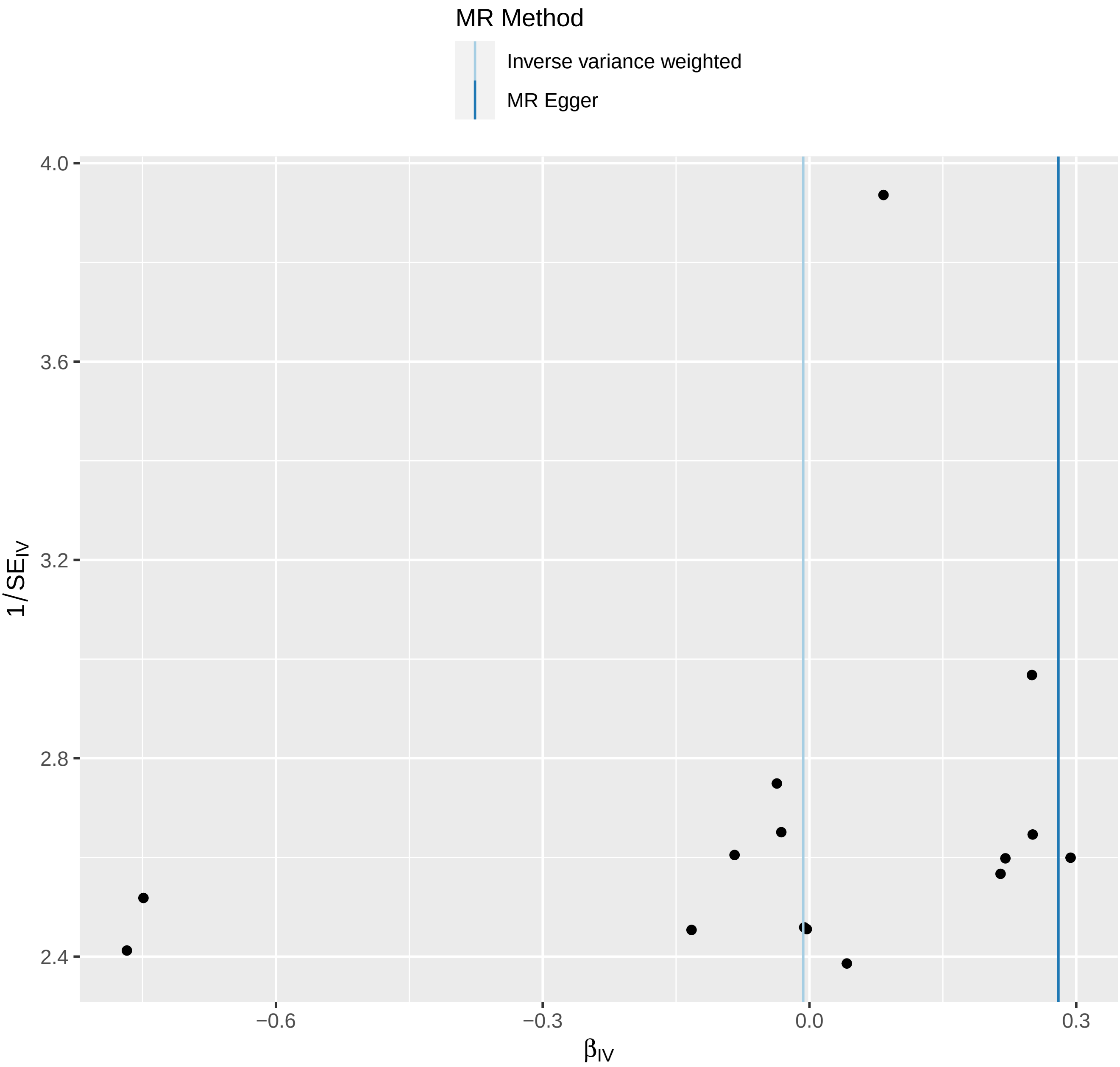

Batch 2−1211 : Gut microbiota abundance (genus Catenibacterium id.2153) on Ciliary neurotrophic factor receptor subunit alpha
with a threshold of 1e−05

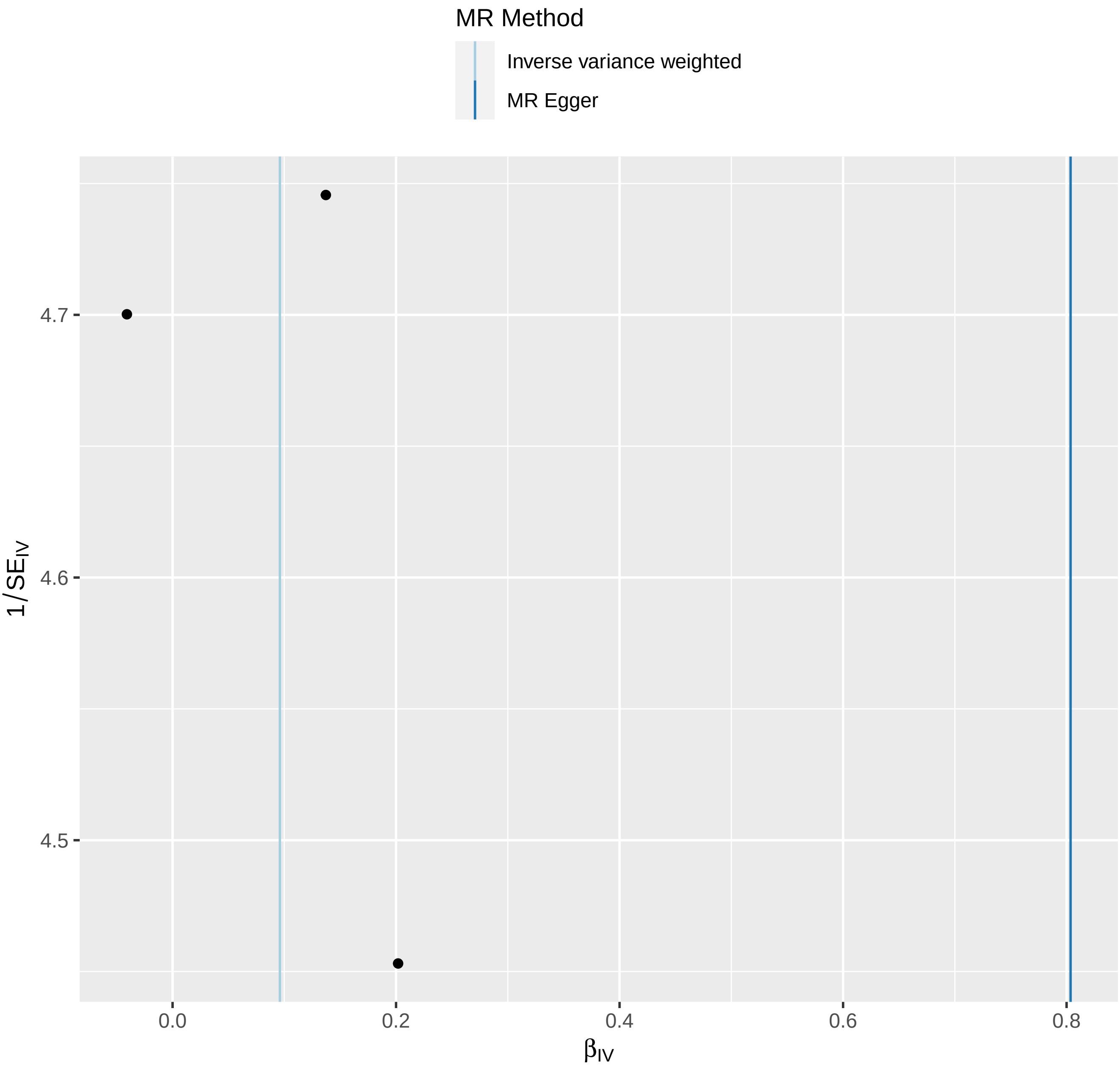

Batch 2−1212 : Gut microbiota abundance (genus Parasutterella id.2892) on Ciliary neurotrophic factor receptor subunit alpha
with a threshold of 1e−05

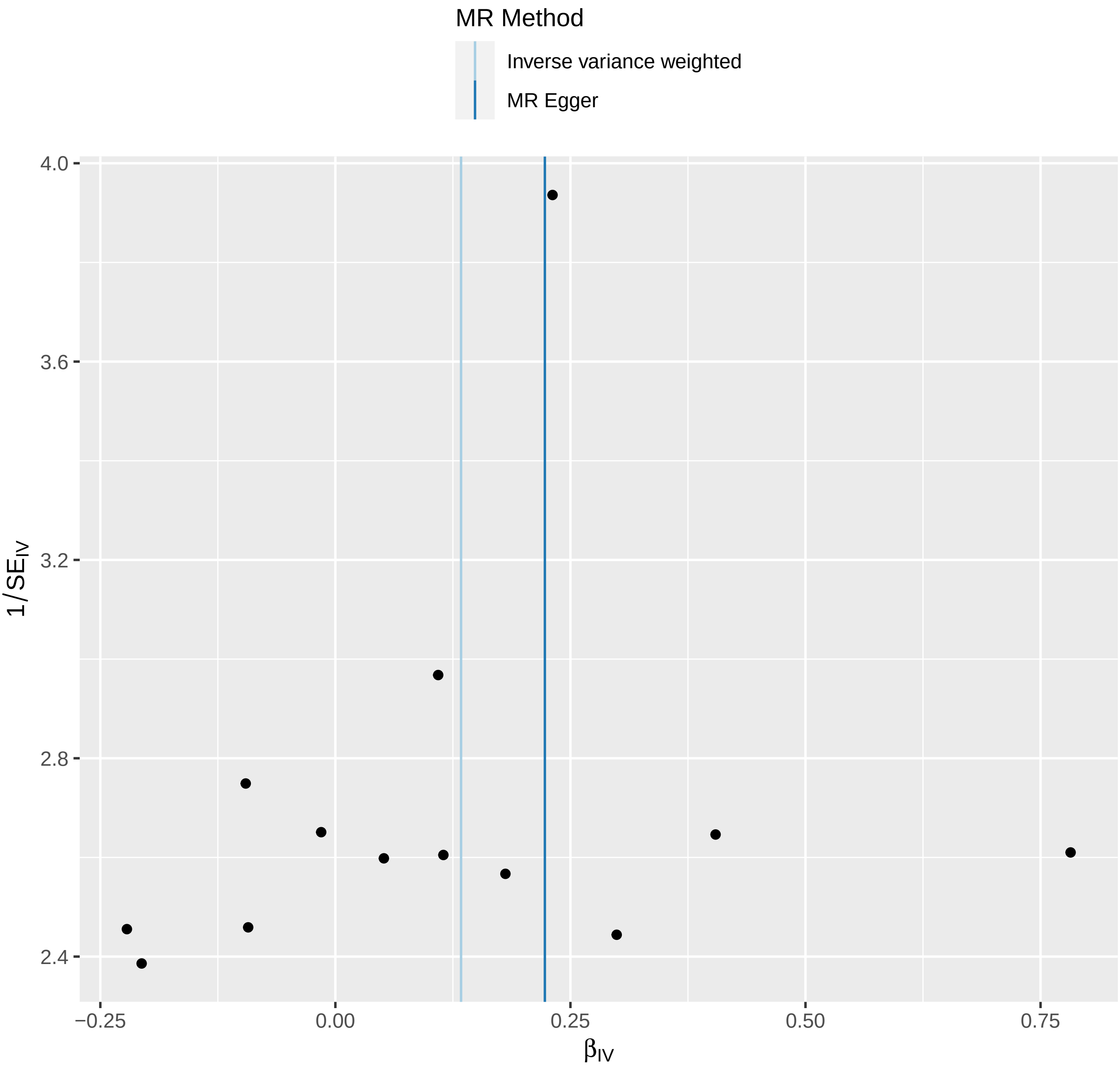

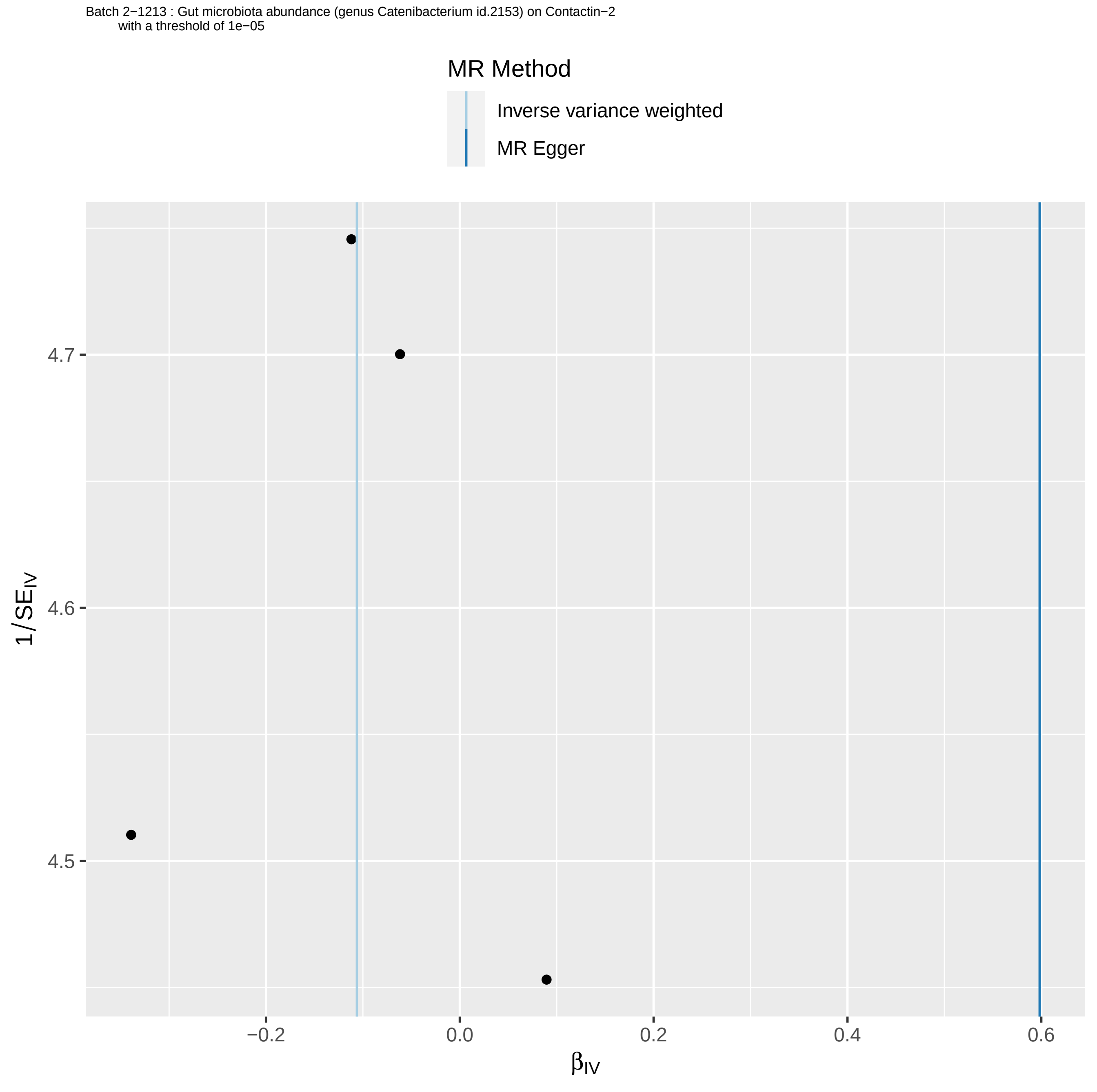
Batch 2−1213 : Gut microbiota abundance (genus Catenibacterium id.2153) on Contactin−2
with a threshold of 1e−05
MR Method
Inverse variance weighted
MR Egger
4.7
4.6
4.5
1/SE$_{IV}$
−0.2
0.0
0.2
0.4
0.6
β$_{IV}$

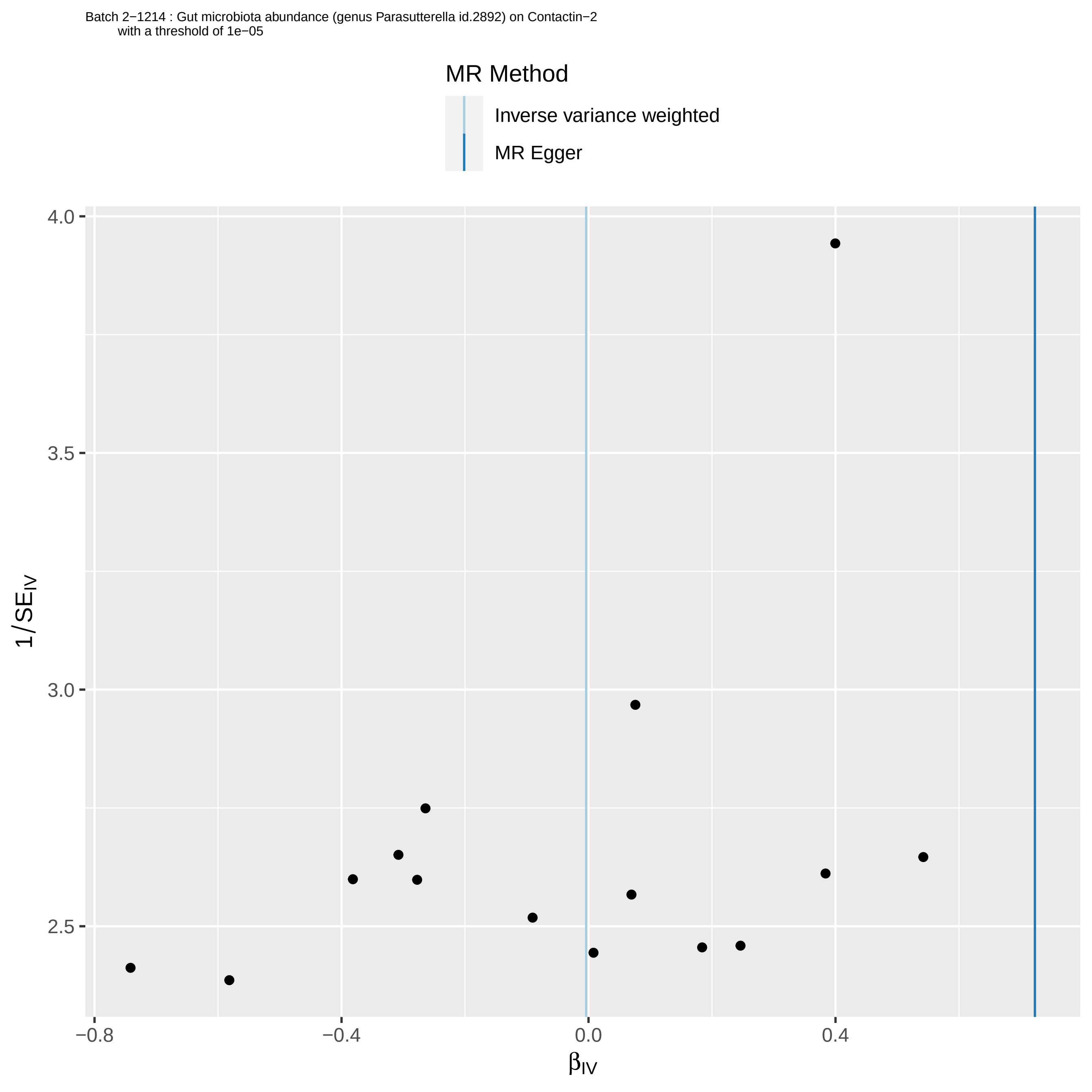
Batch 2−1214 : Gut microbiota abundance (genus Parasutterella id.2892) on Contactin−2
with a threshold of 1e−05
MR Method
Inverse variance weighted
MR Egger
4.0
3.5
3.0
2.5
1/SE_IV
−0.8
−0.4
0.0
0.4
β_IV

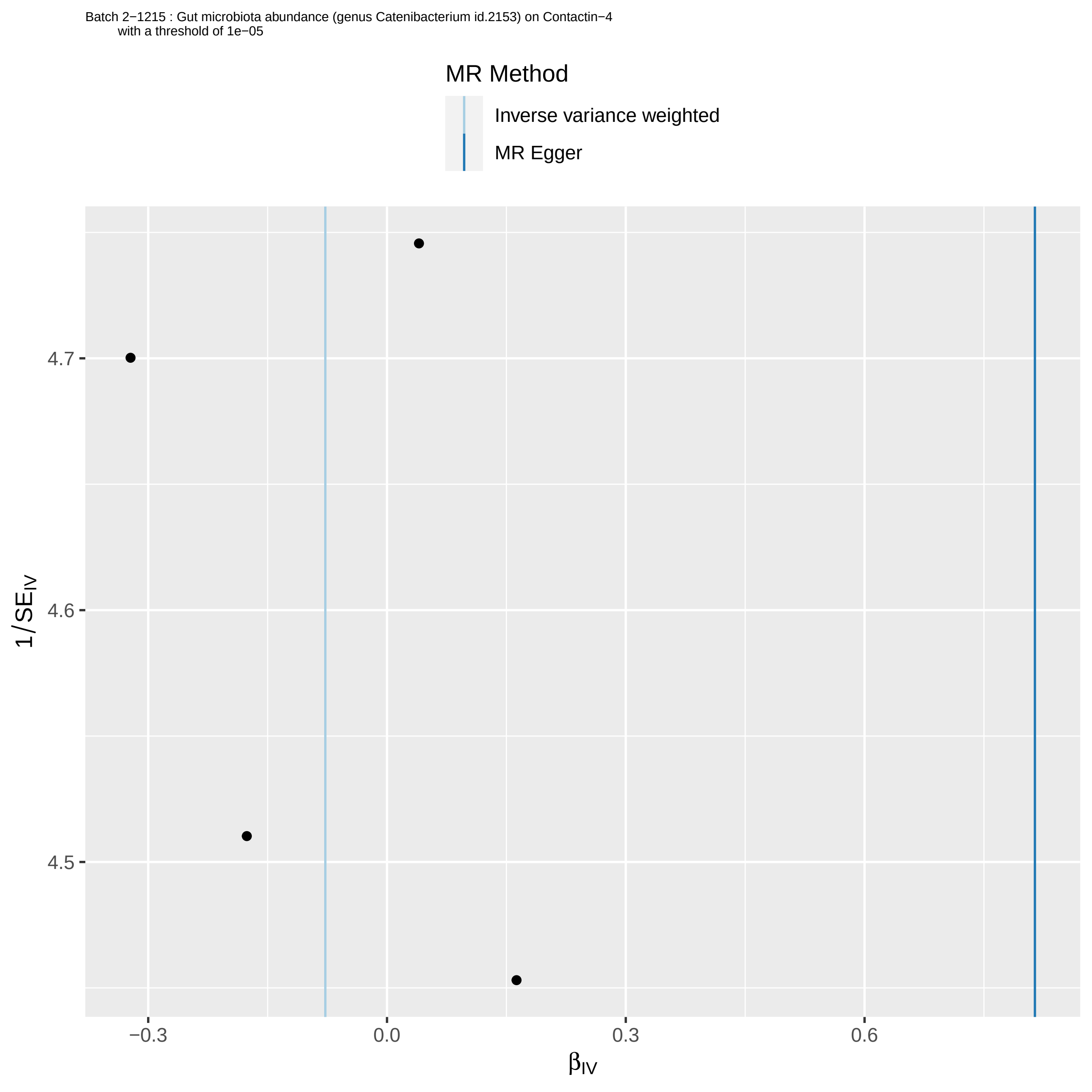

Batch 2−1215 : Gut microbiota abundance (genus Catenibacterium id.2153) on Contactin−4
with a threshold of 1e−05
MR Method
Inverse variance weighted
MR Egger
4.7
4.6
4.5
$1/SE_{IV}$
−0.3
0.0
0.3
0.6
β_{IV}

Batch 2−1216 : Gut microbiota abundance (genus Parasutterella id.2892) on Contactin−4
with a threshold of 1e−05

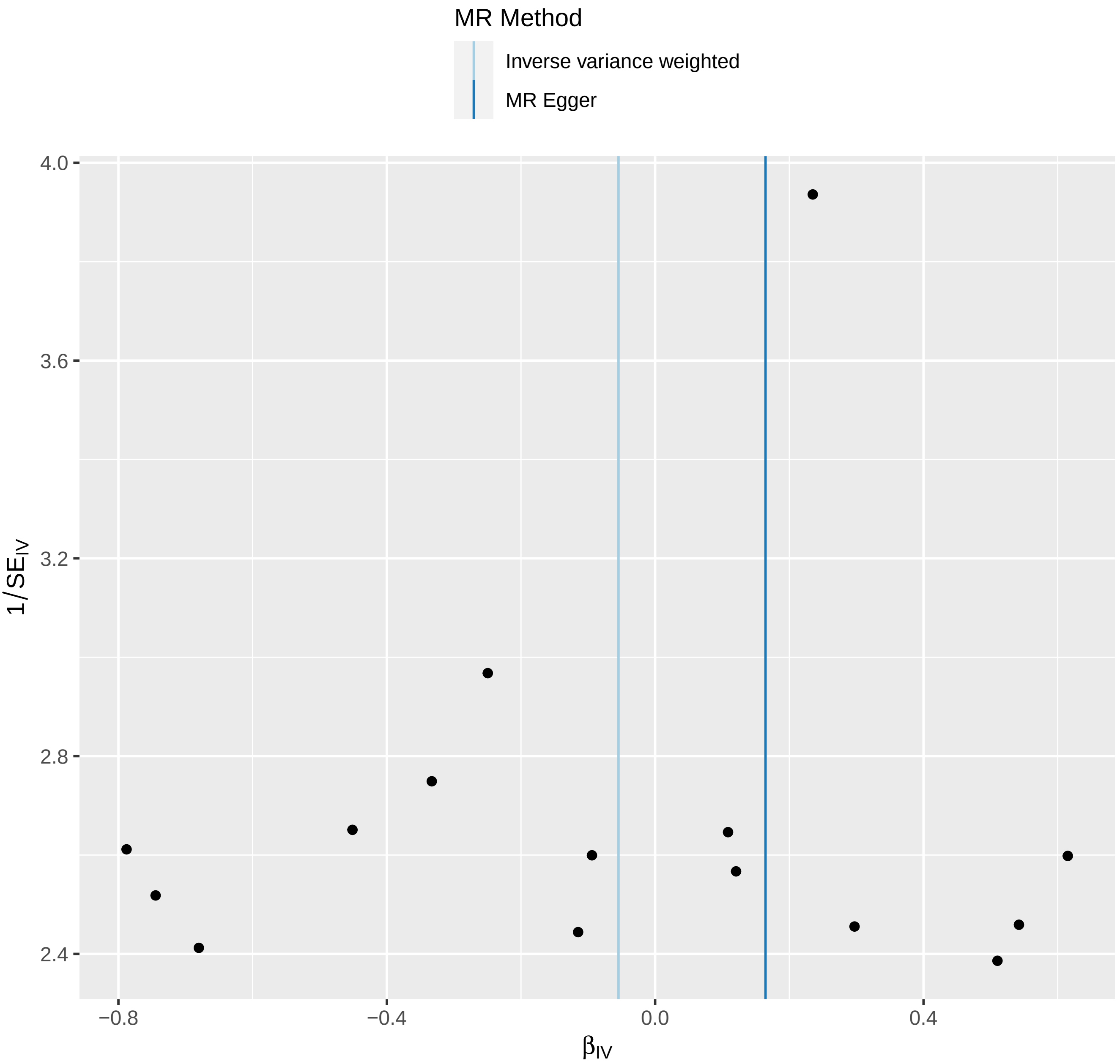

Batch 2−1217 : Gut microbiota abundance (genus Catenibacterium id.2153) on Contactin−5
with a threshold of 1e−05

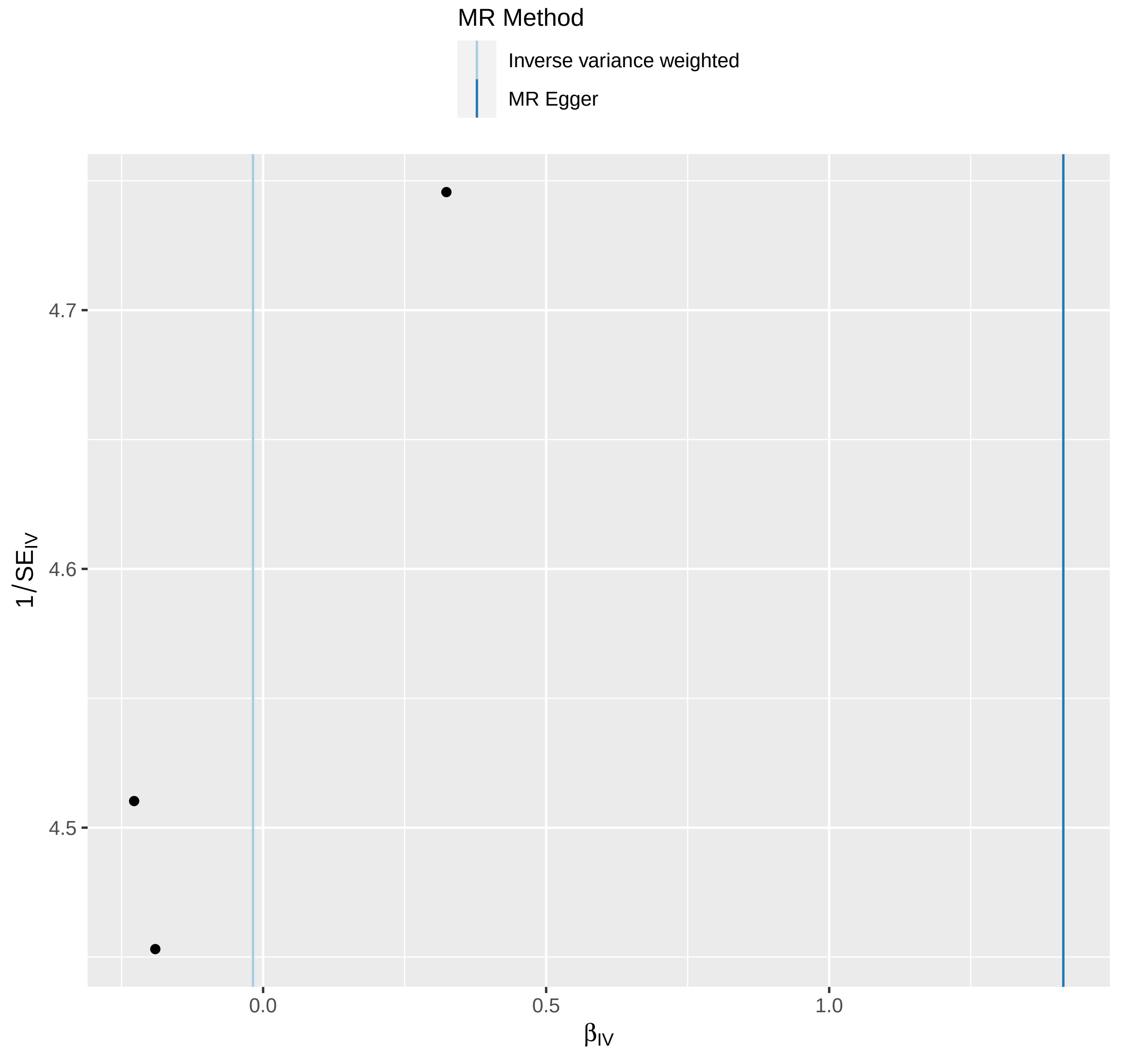

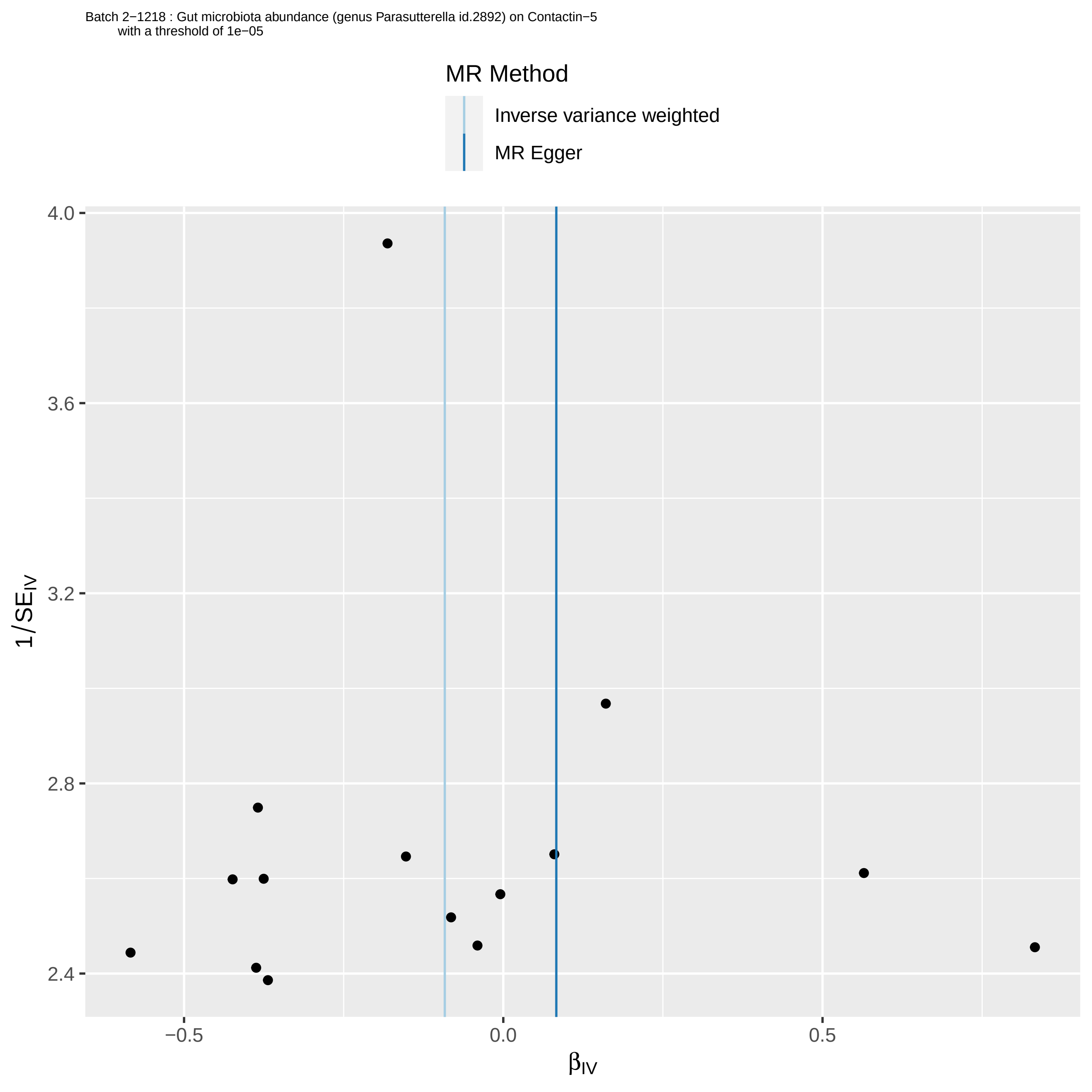

Batch 2−1218 : Gut microbiota abundance (genus Parasutterella id.2892) on Contactin−5
with a threshold of 1e−05
MR Method
Inverse variance weighted
MR Egger
4.0
3.6
3.2
2.8
2.4
1/SE_IV
−0.5
0.0
0.5
β_IV

Batch 2−1219 : Gut microbiota abundance (genus Catenibacterium id.2153) on Contactin−associated protein 1
with a threshold of 1e−05

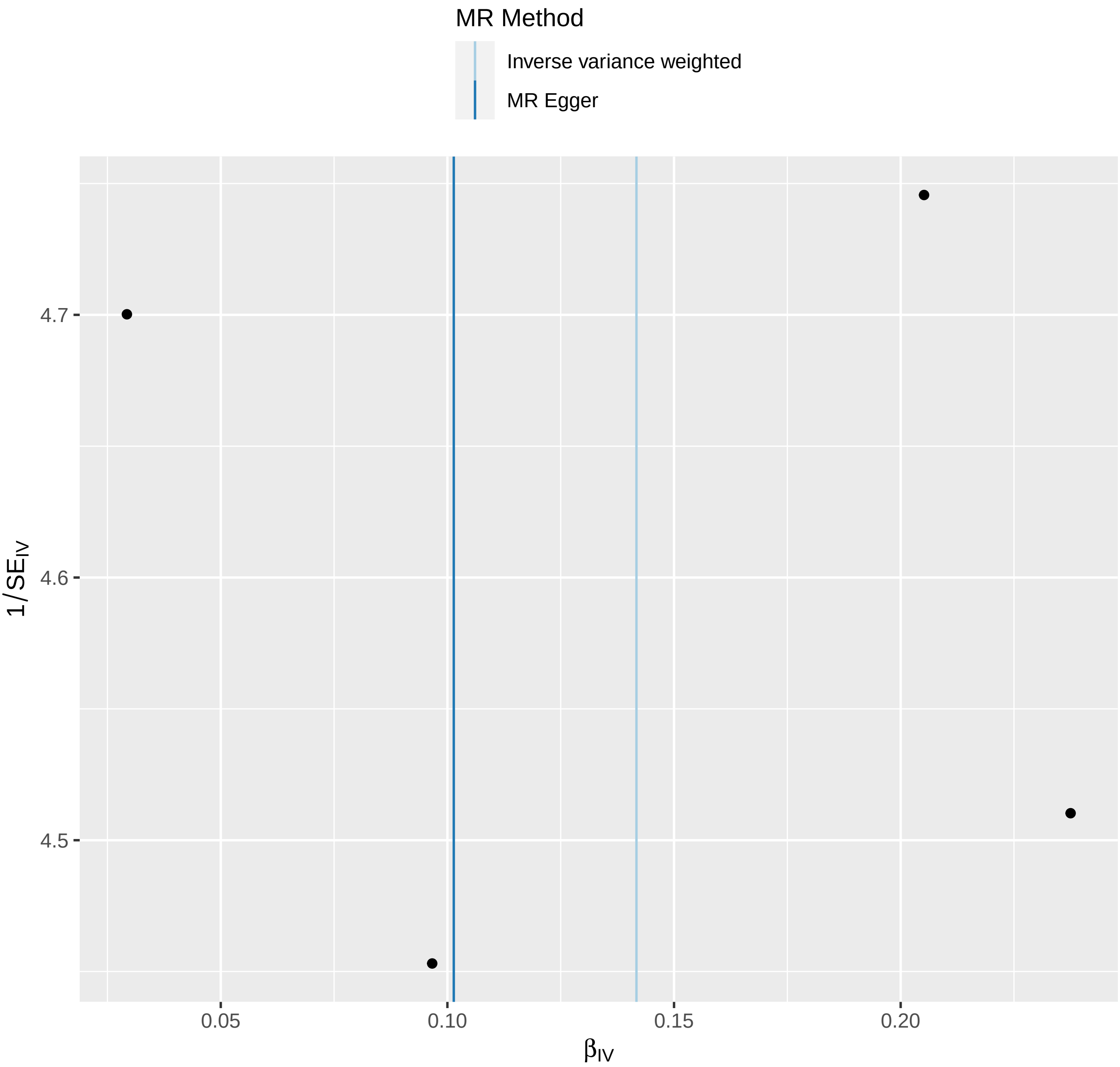

Batch 2−1220 : Gut microbiota abundance (genus Parasutterella id.2892) on Contactin−associated protein 1
with a threshold of 1e−05

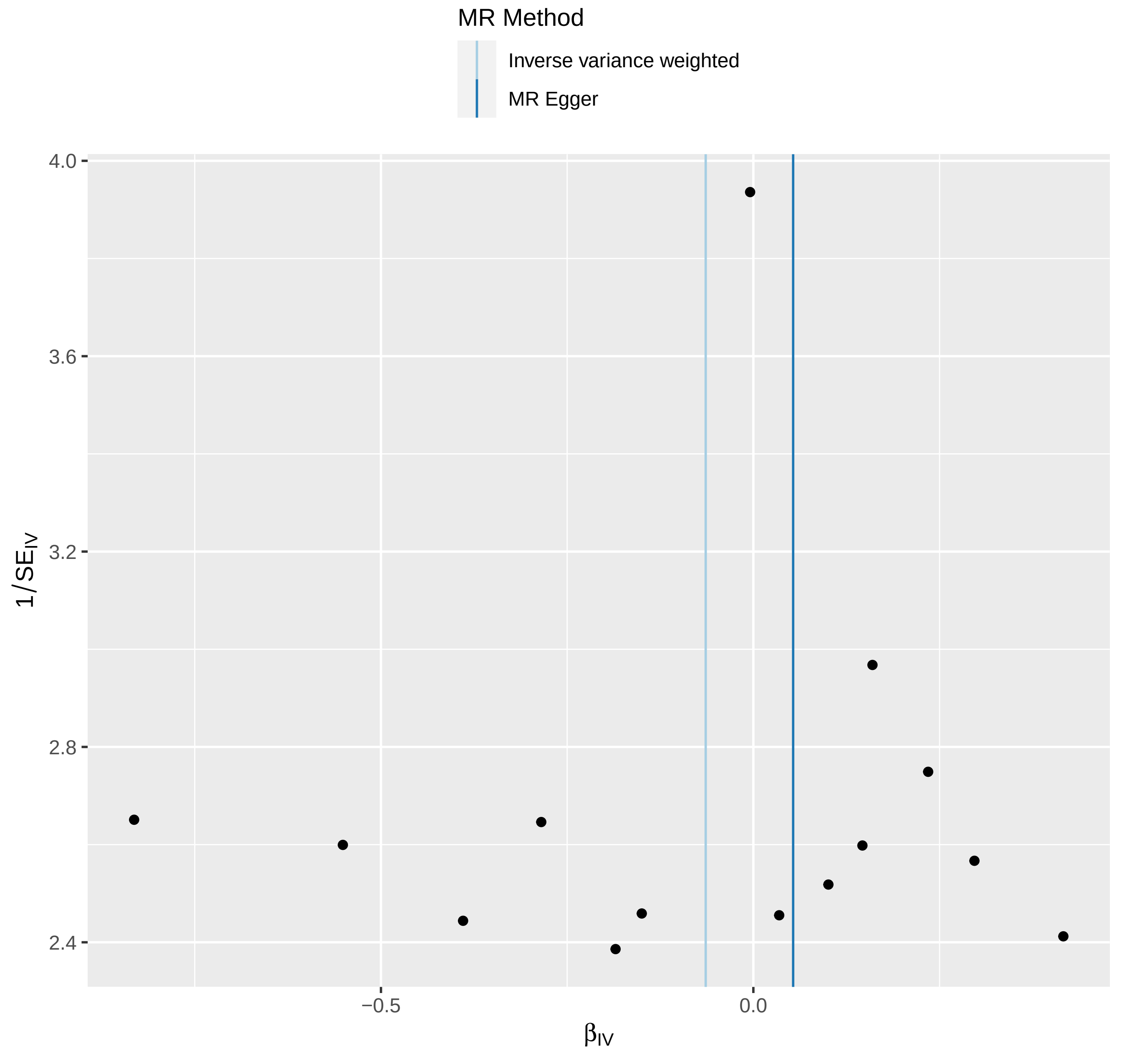

Batch 2−1221 : Gut microbiota abundance (genus Catenibacterium id.2153) on Contactin−associated protein−like 2
with a threshold of 1e−05

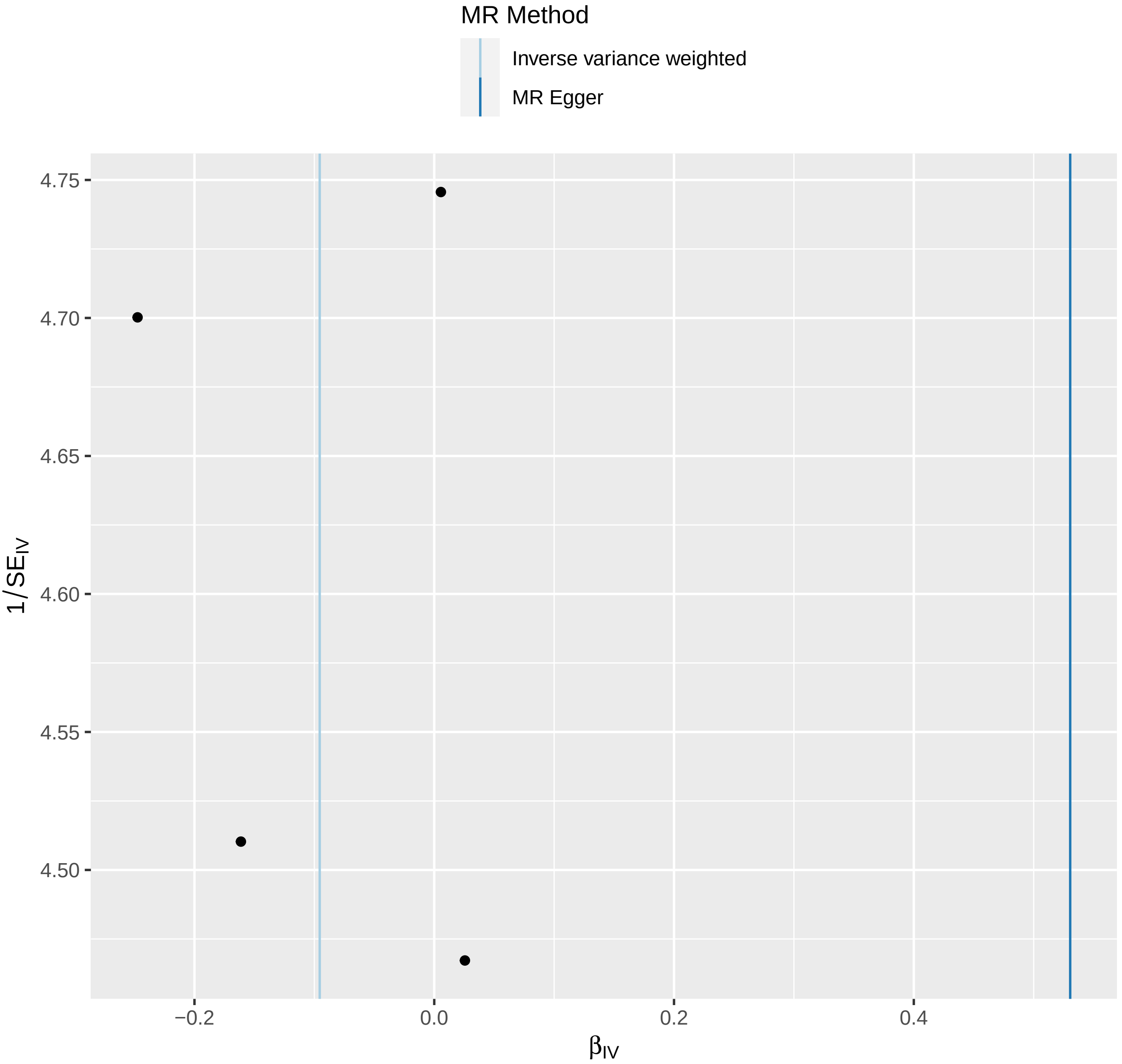

Batch 2−1222 : Gut microbiota abundance (genus Parasutterella id.2892) on Contactin−associated protein−like 2
with a threshold of 1e−05

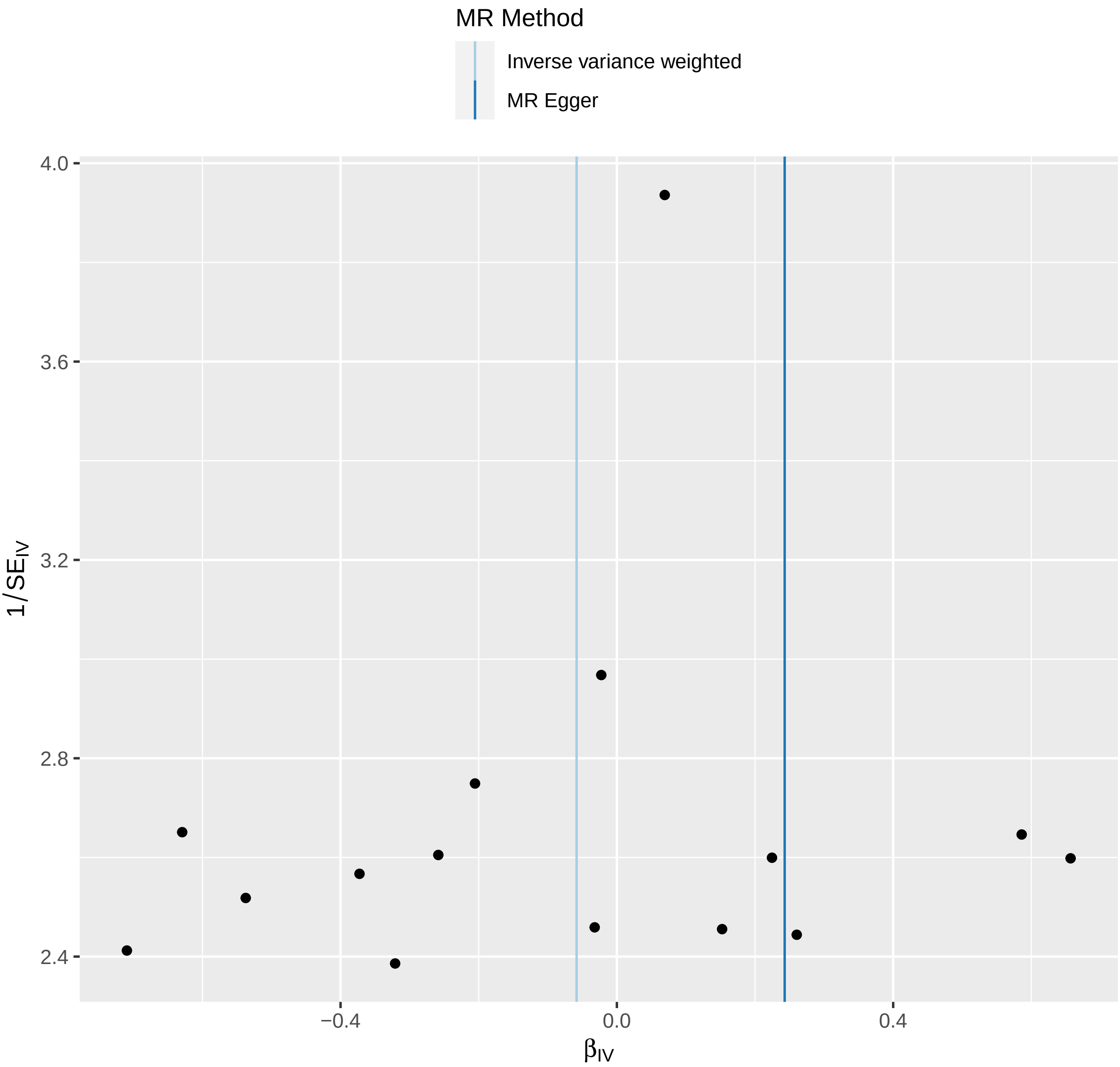

Batch 2−1223 : Gut microbiota abundance (genus Catenibacterium id.2153) on Cytochrome c oxidase assembly factor 3 homolog, mitochondrial
with a threshold of 1e−05

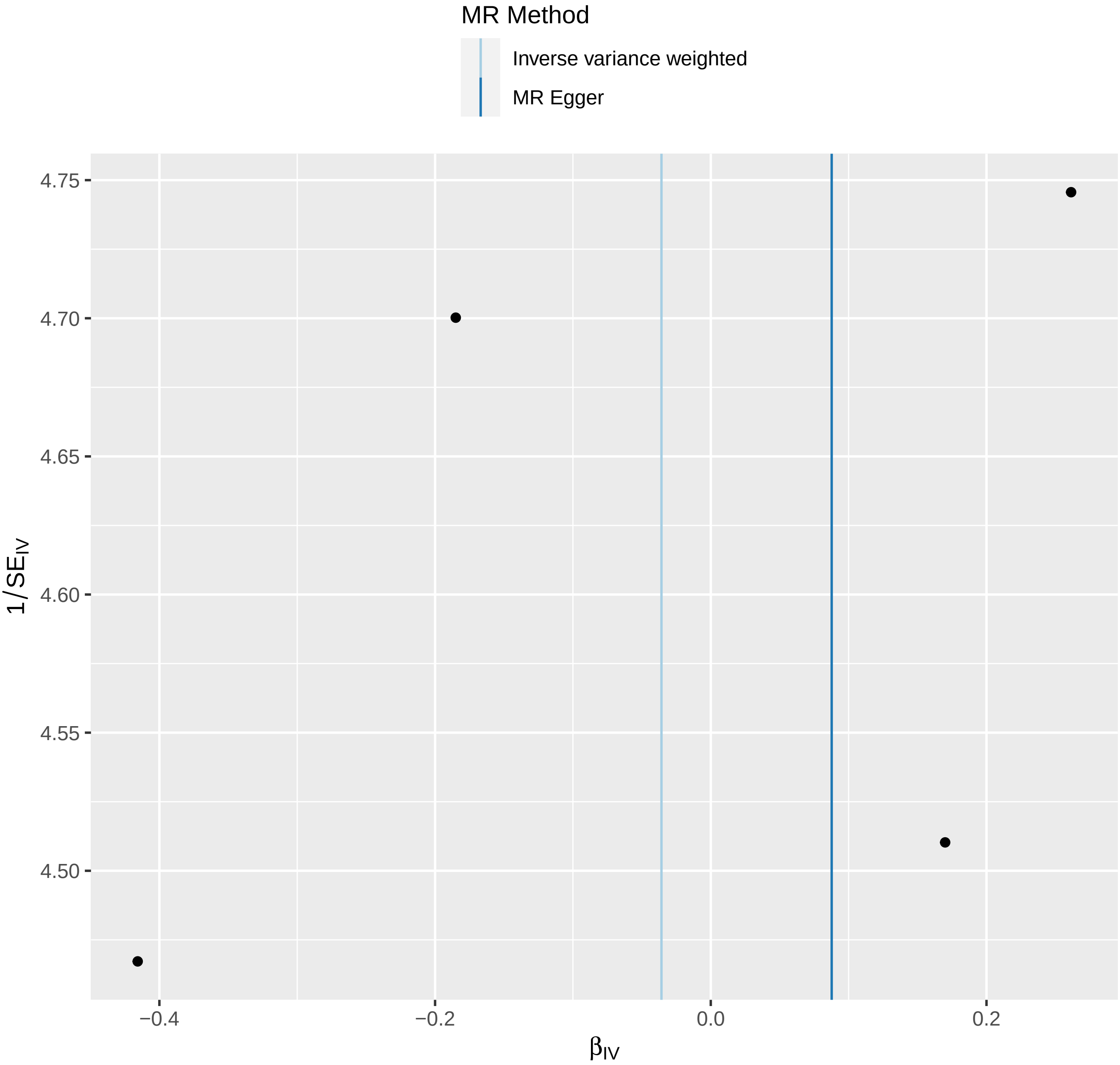

Batch 2−1224 : Gut microbiota abundance (genus Parasutterella id.2892) on Cytochrome c oxidase assembly factor 3 homolog, mitochondrial
with a threshold of 1e−05

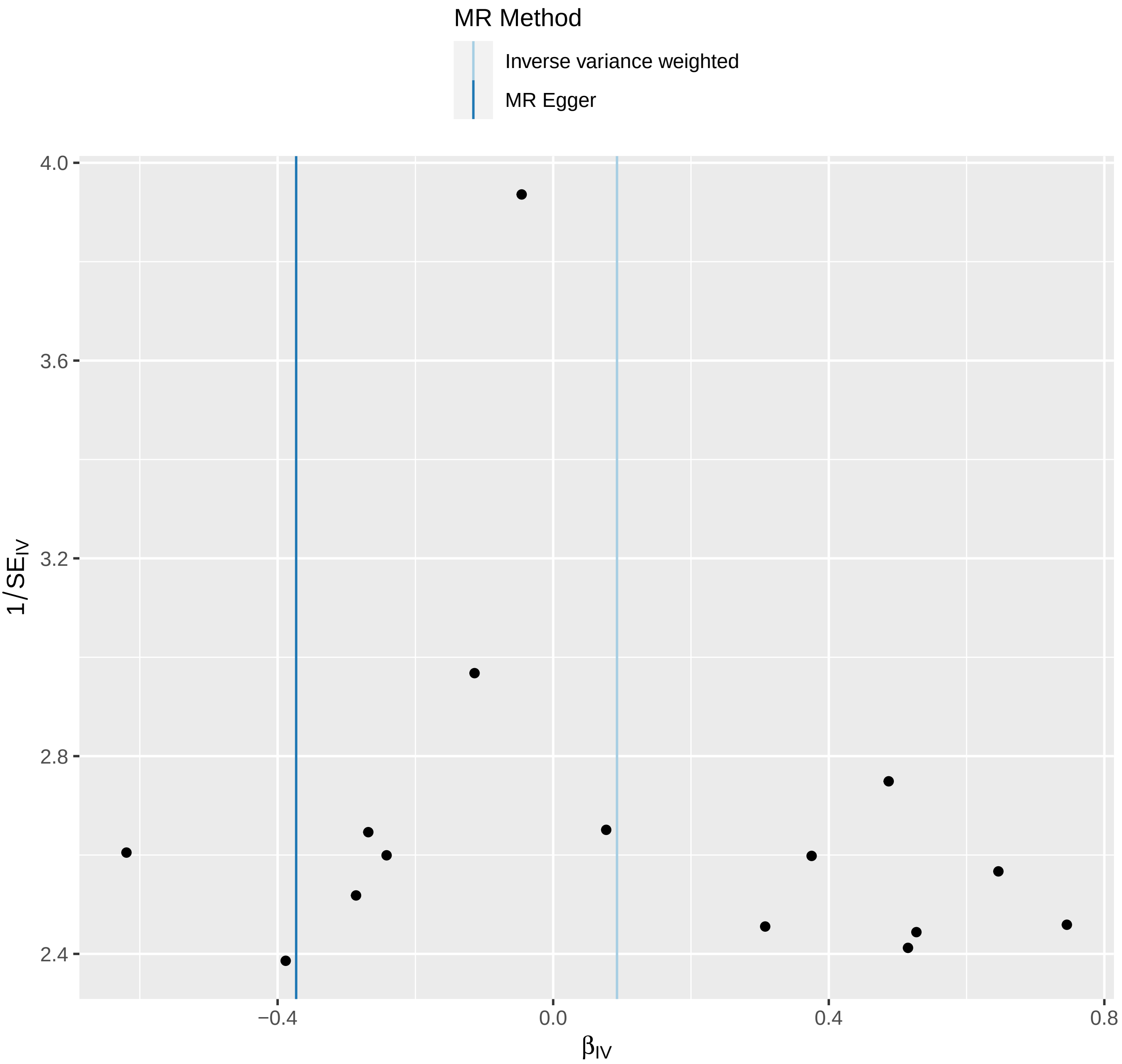

Batch 2−1225 : Gut microbiota abundance (genus Catenibacterium id.2153) on Cochlin
with a threshold of 1e−05

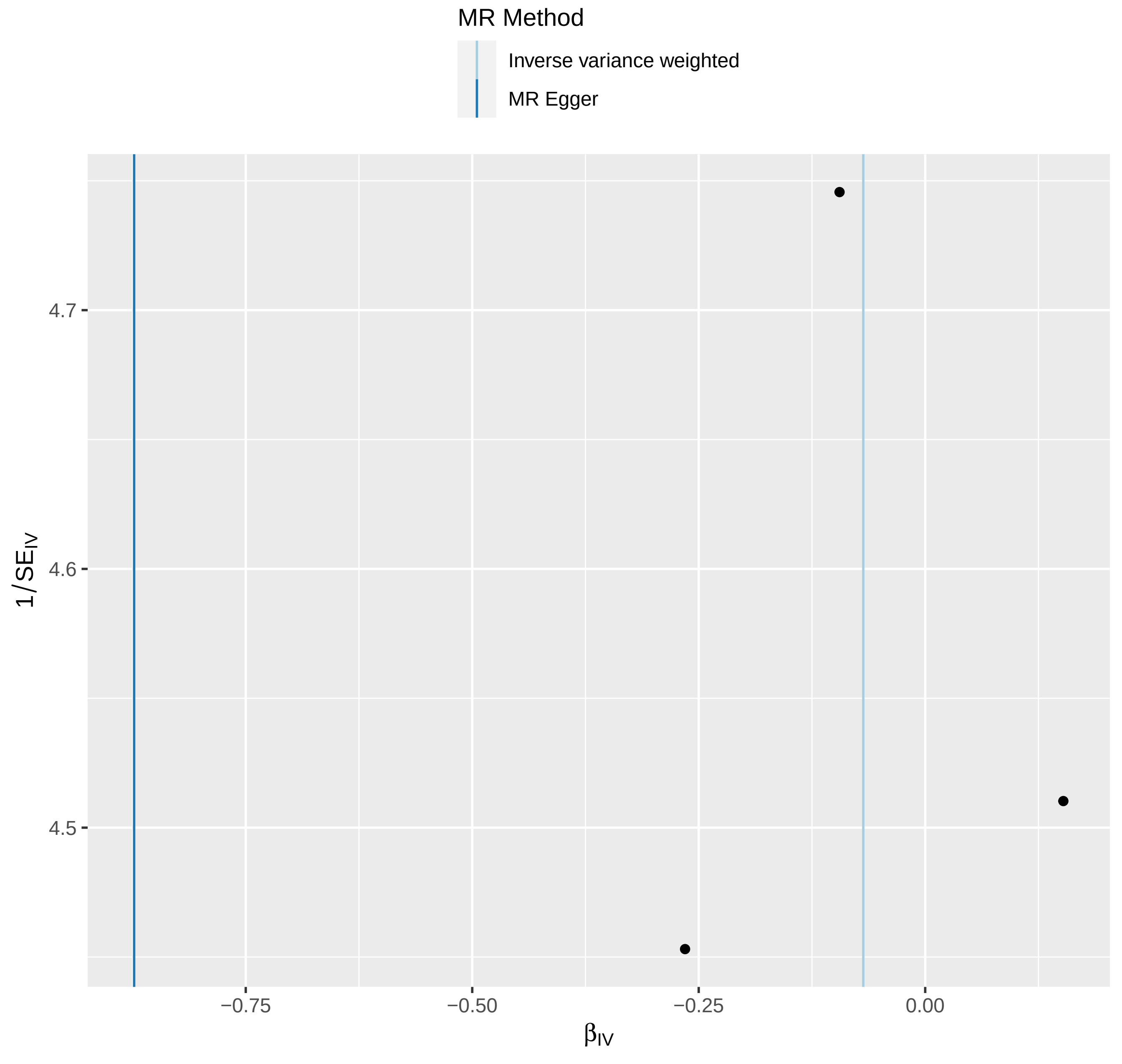

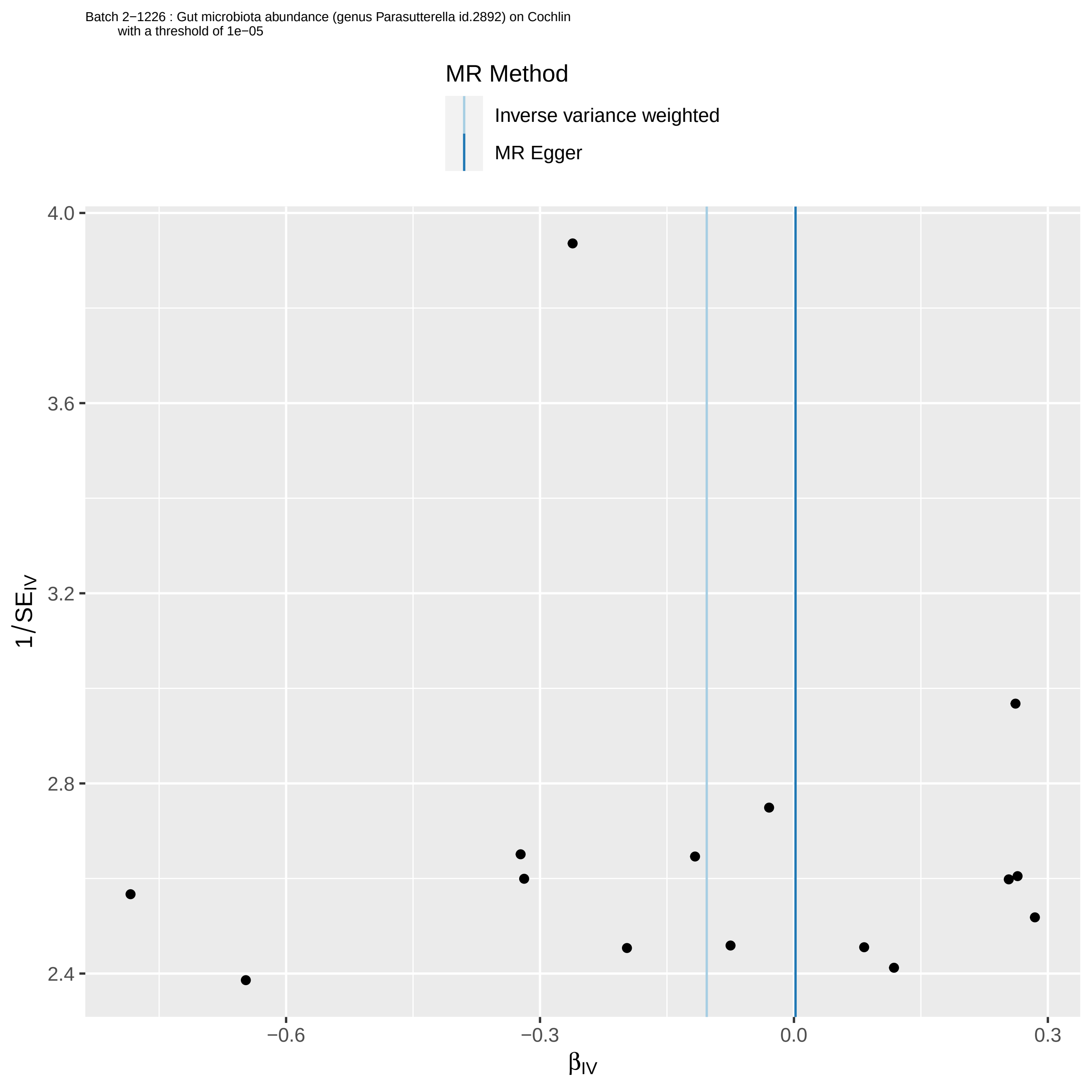

Batch 2−1226 : Gut microbiota abundance (genus Parasutterella id.2892) on Cochlin
with a threshold of 1e−05
MR Method
Inverse variance weighted
MR Egger
4.0
3.6
3.2
2.8
2.4
1/SE_IV
−0.6
−0.3
0.0
0.3
β_IV

Batch 2−1227 : Gut microbiota abundance (genus Catenibacterium id.2153) on Conserved oligomeric Golgi complex subunit 8 with a threshold of 1e−05

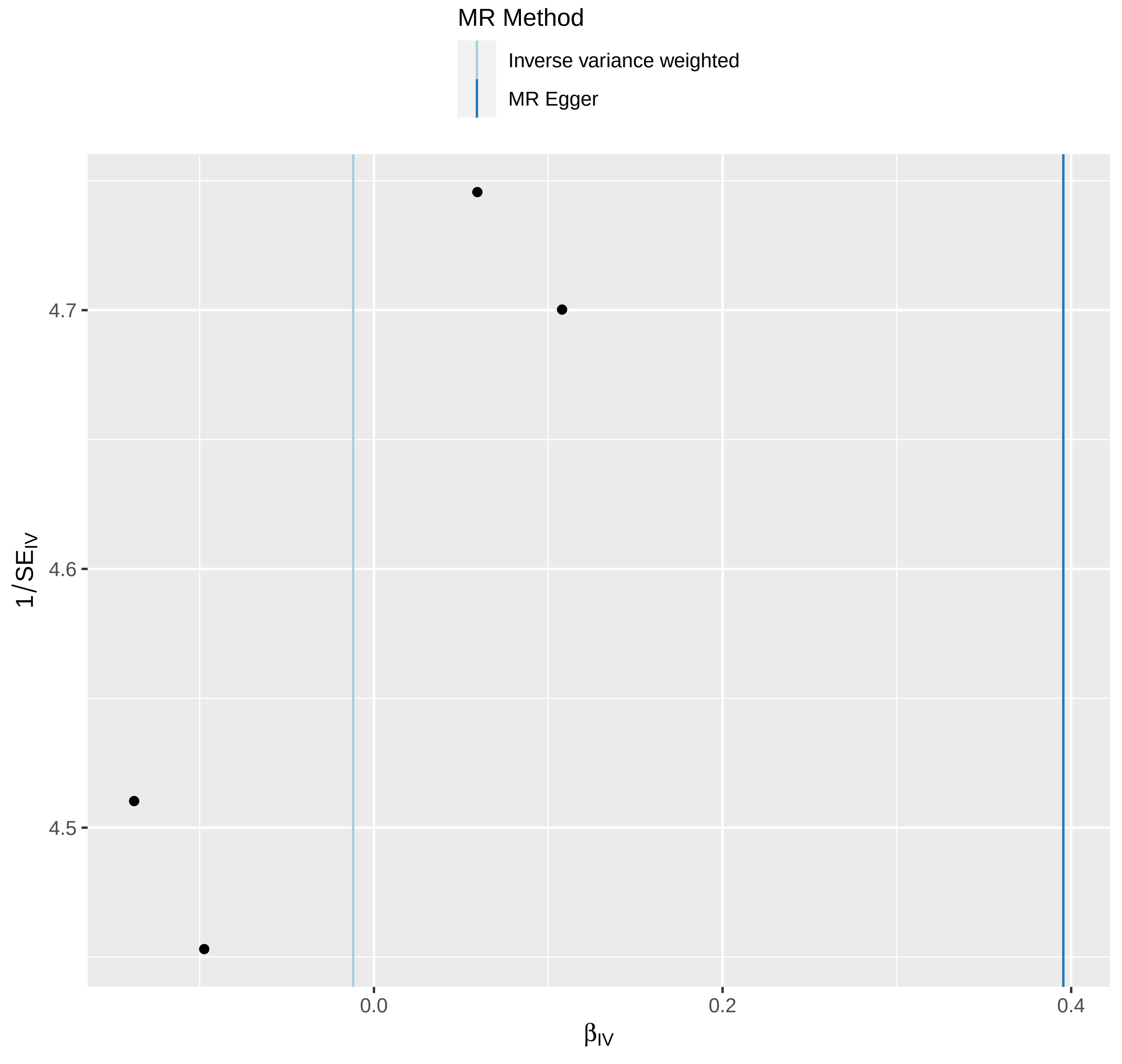

Batch 2−1228 : Gut microbiota abundance (genus Parasutterella id.2892) on Conserved oligomeric Golgi complex subunit 8
with a threshold of 1e−05

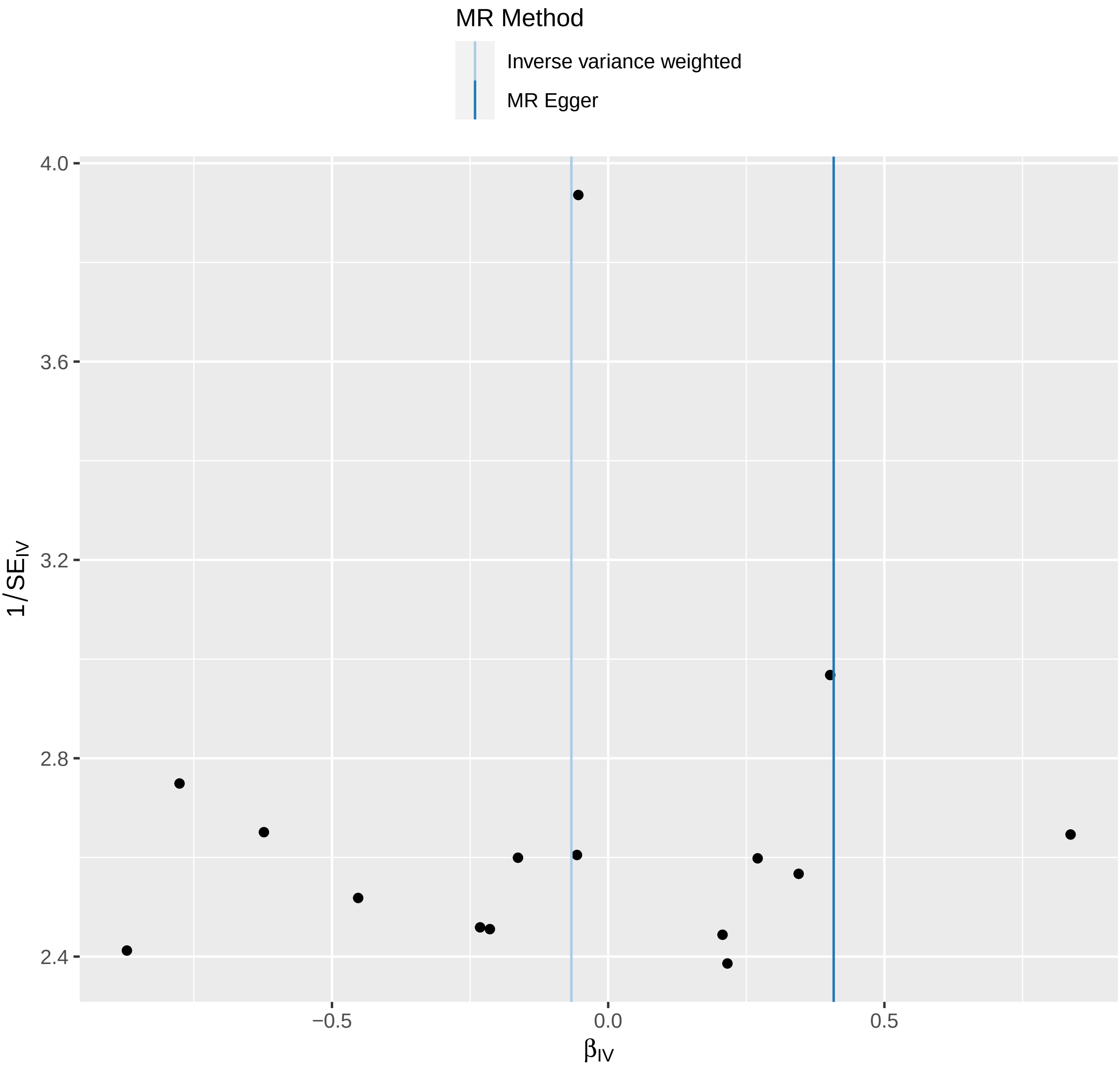

Batch 2−1229 : Gut microbiota abundance (genus Catenibacterium id.2153) on Collagen alpha−2(XI) chain
with a threshold of 1e−05

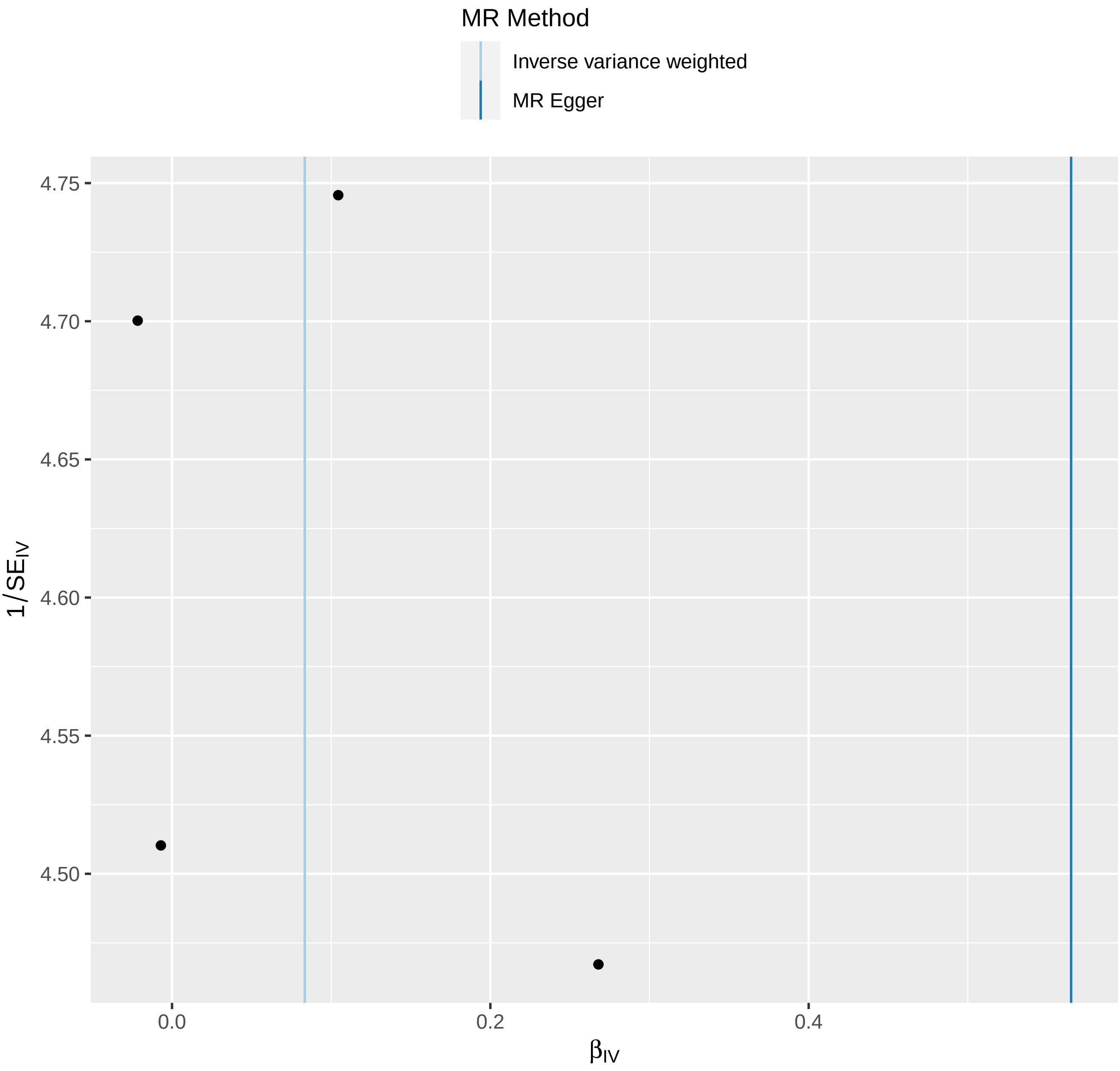

Batch 2−1230 : Gut microbiota abundance (genus Parasutterella id.2892) on Collagen alpha−2(XI) chain
with a threshold of 1e−05

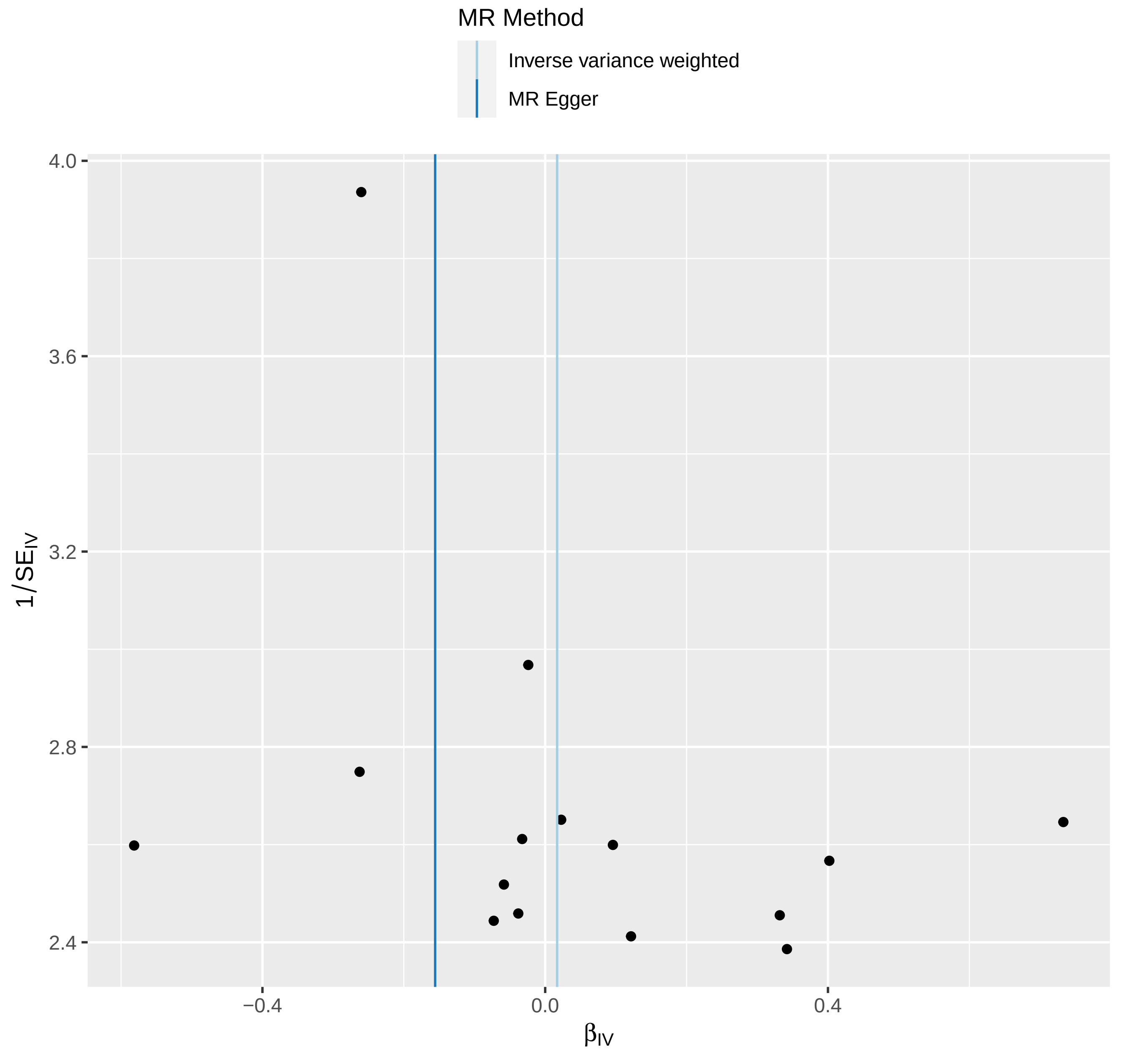

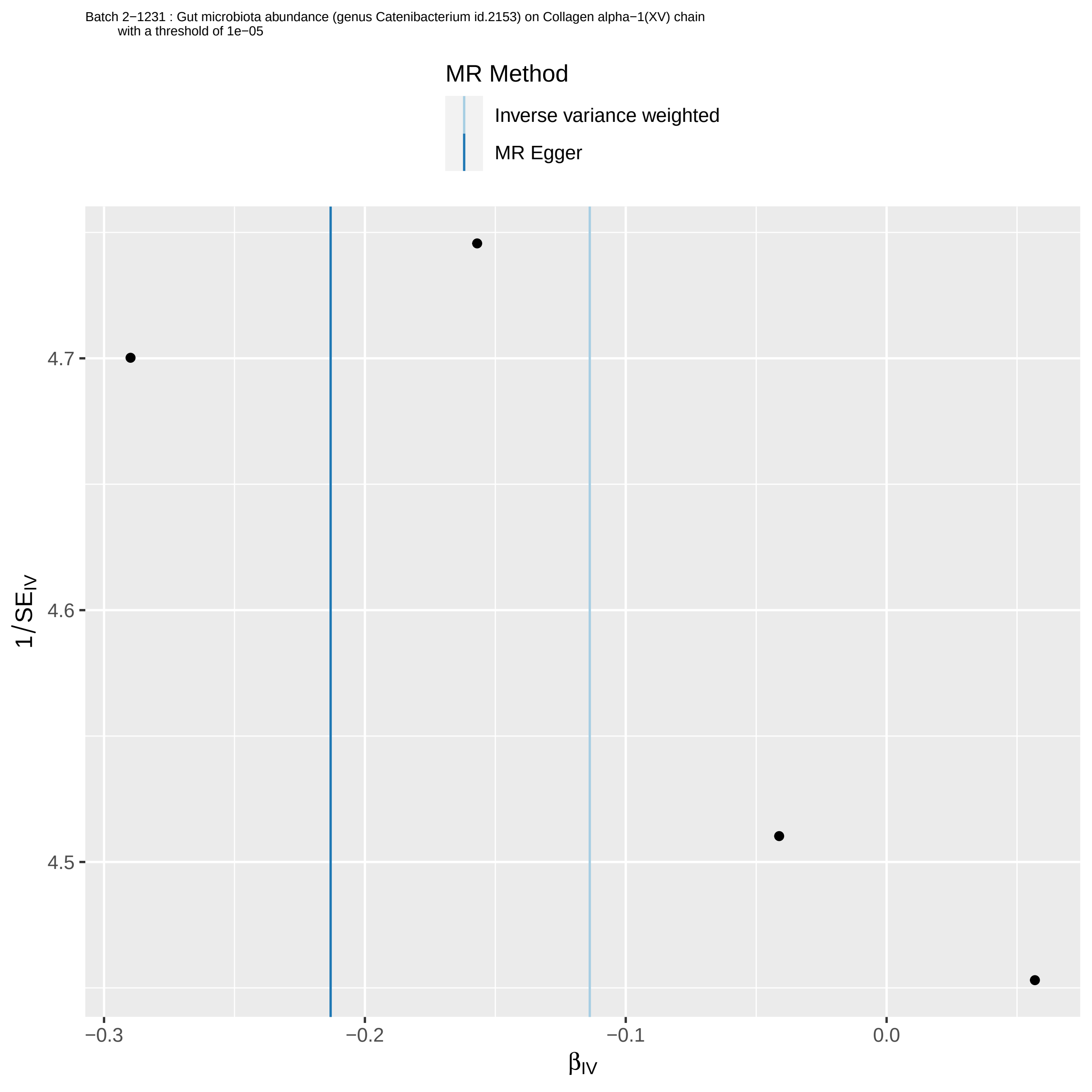
Batch 2−1231 : Gut microbiota abundance (genus Catenibacterium id.2153) on Collagen alpha−1(XV) chain
with a threshold of 1e−05
MR Method
Inverse variance weighted
MR Egger
4.7
4.6
4.5
1/SE_IV
−0.3
−0.2
−0.1
0.0
β_IV

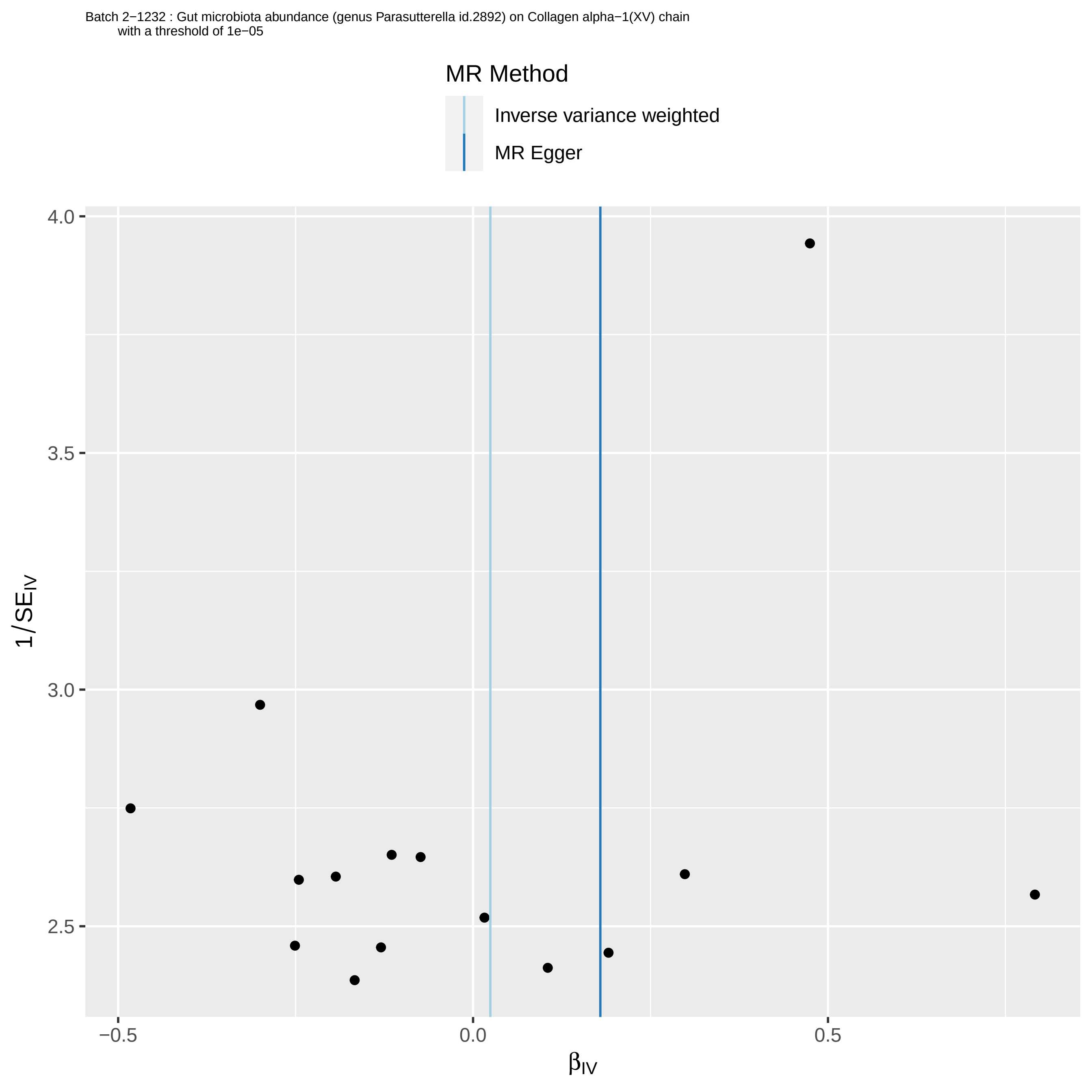

Batch 2−1232 : Gut microbiota abundance (genus Parasutterella id.2892) on Collagen alpha−1(XV) chain
with a threshold of 1e−05
MR Method
Inverse variance weighted
MR Egger
4.0
3.5
3.0
2.5
1/SE_IV
−0.5
0.0
0.5
β_IV

Batch 2−1233 : Gut microbiota abundance (genus Catenibacterium id.2153) on Endostatin
with a threshold of 1e−05

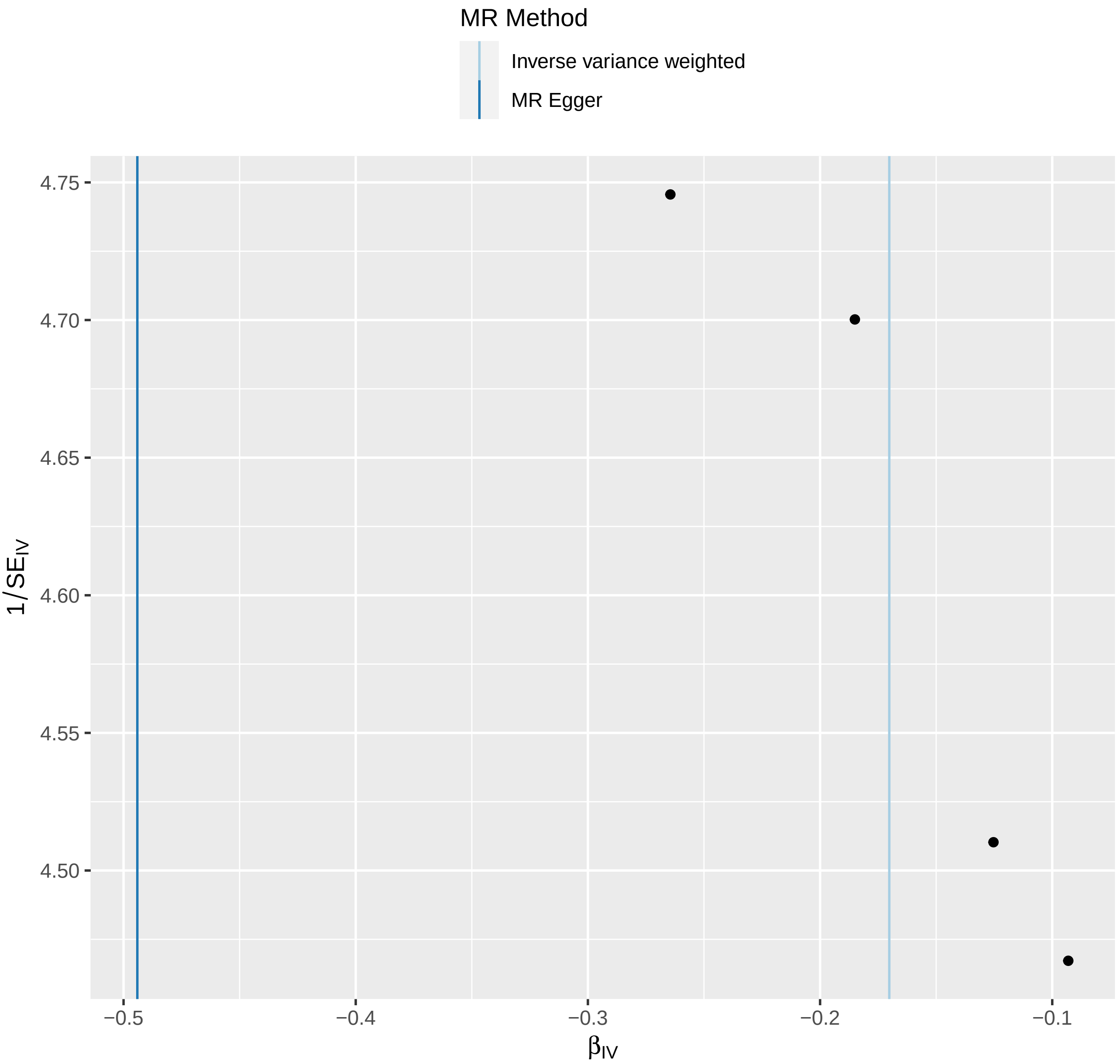

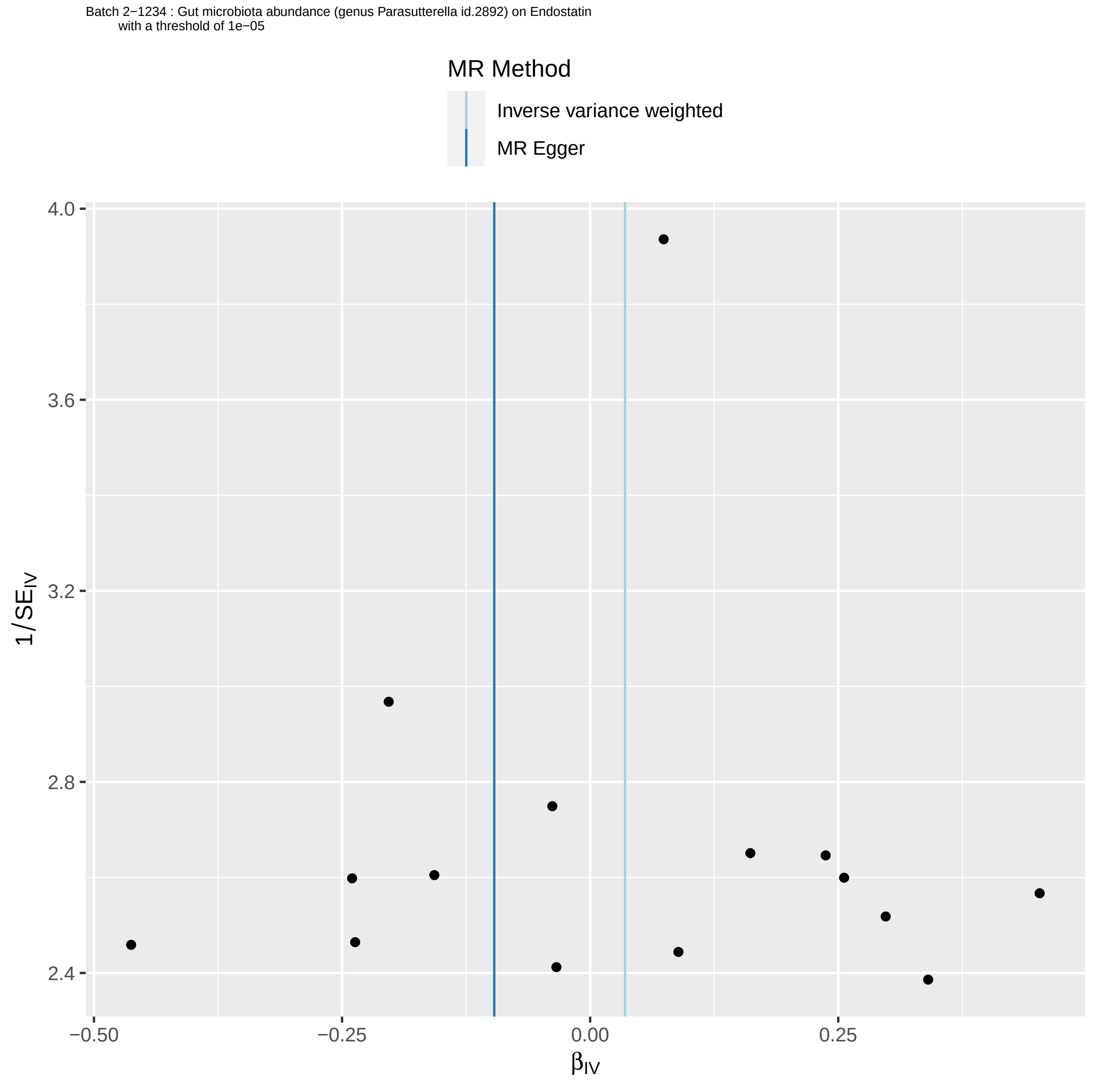
Batch 2−1234 : Gut microbiota abundance (genus Parasutterella id.2892) on Endostatin
with a threshold of 1e−05
MR Method
Inverse variance weighted
MR Egger
4.0
3.6
3.2
2.8
2.4
1/SE$_{IV}$
−0.50
−0.25
0.00
0.25
β$_{IV}$

Batch 2−1235 : Gut microbiota abundance (genus Catenibacterium id.2153) on Collagen alpha−1(I) chain
with a threshold of 1e−05

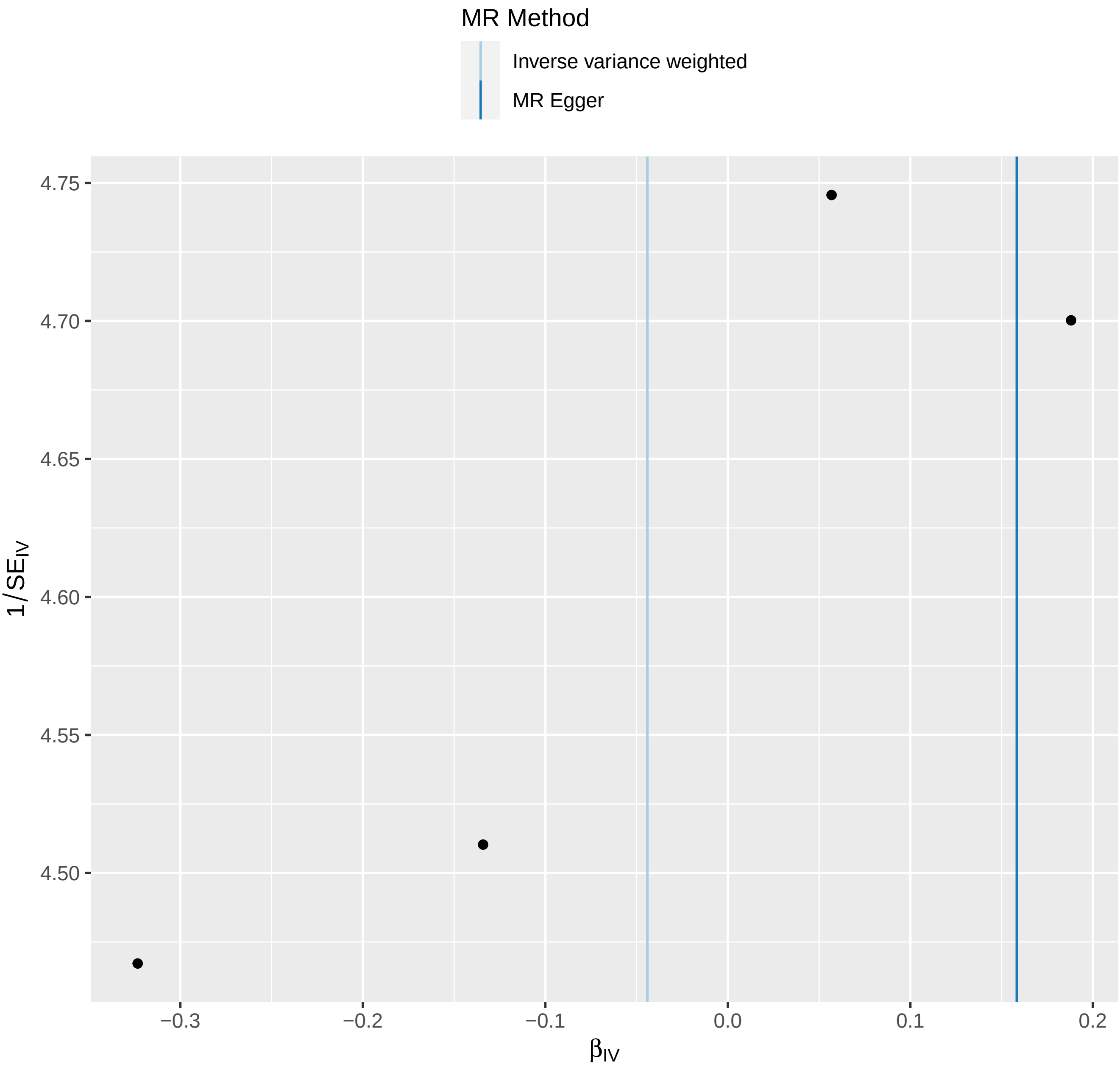

Batch 2−1236 : Gut microbiota abundance (genus Parasutterella id.2892) on Collagen alpha−1(I) chain
with a threshold of 1e−05

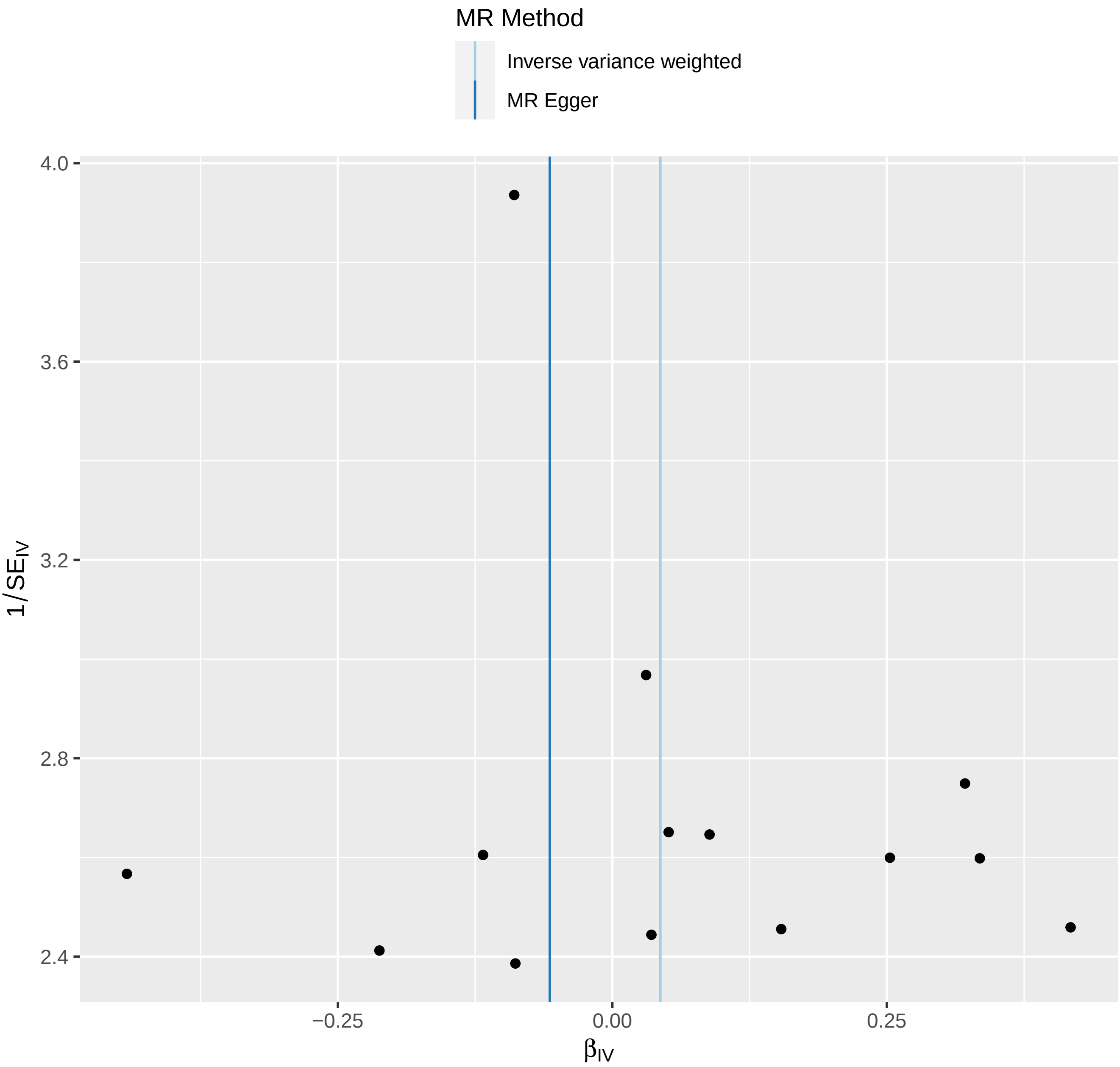

Batch 2−1237 : Gut microbiota abundance (genus Catenibacterium id.2153) on Collagen alpha−1(XX) chain
with a threshold of 1e−05

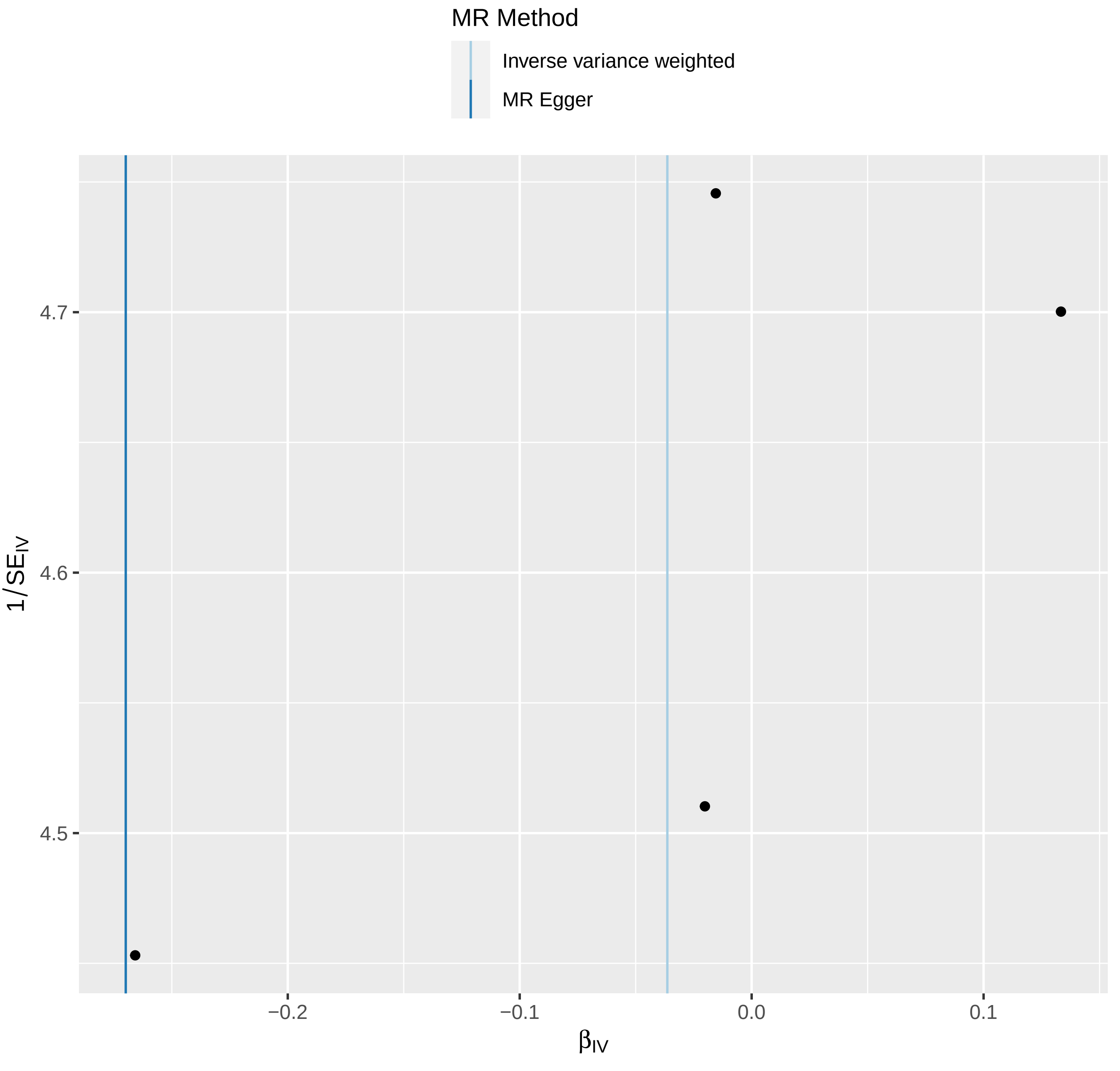

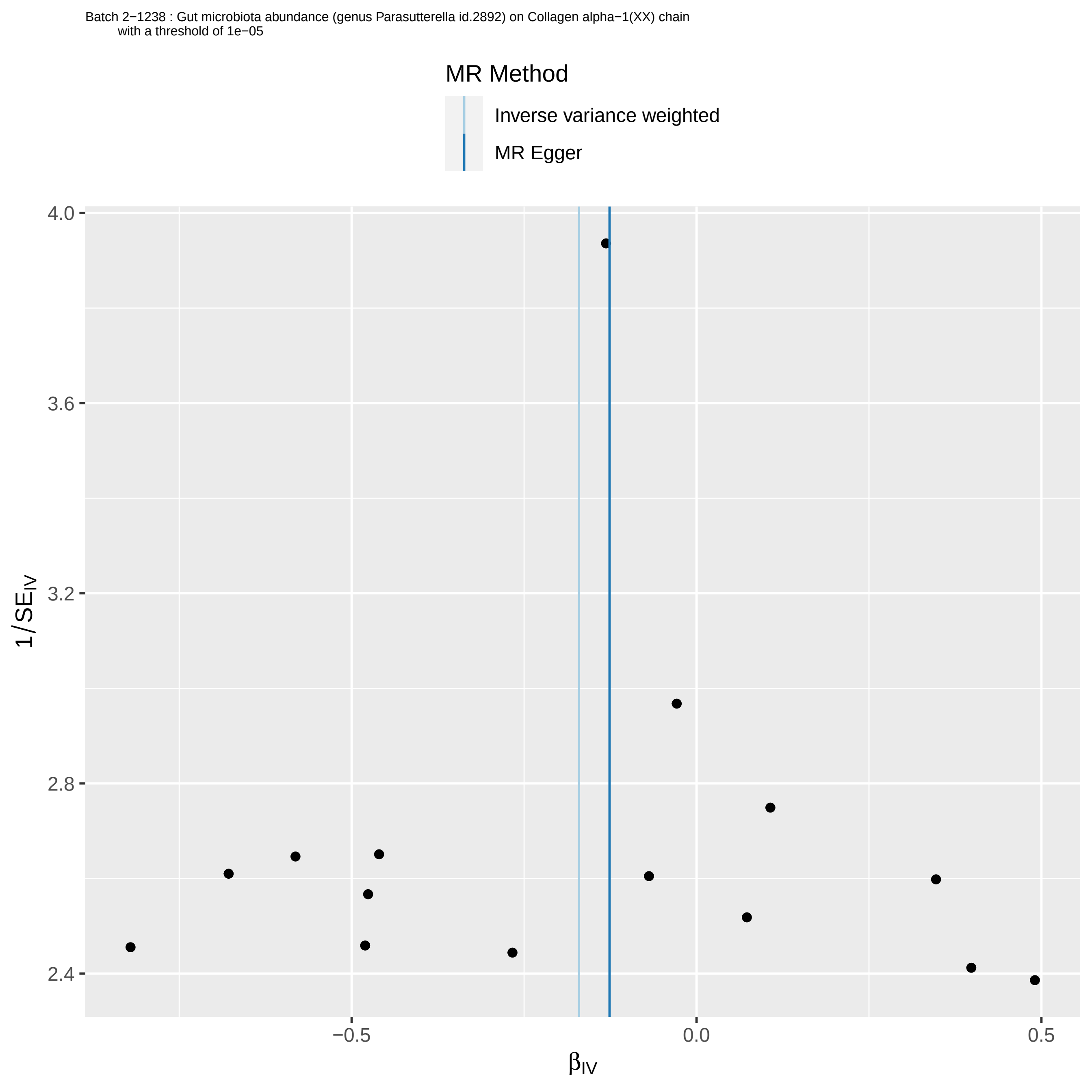
Batch 2−1238 : Gut microbiota abundance (genus Parasutterella id.2892) on Collagen alpha−1(XX) chain
with a threshold of 1e−05
MR Method
Inverse variance weighted
MR Egger
1/SE_IV
4.0
3.6
3.2
2.8
2.4
−0.5
0.0
0.5
β_IV

Batch 2−1239 : Gut microbiota abundance (genus Catenibacterium id.2153) on Collagen alpha−1(XXVI) chain
with a threshold of 1e−05

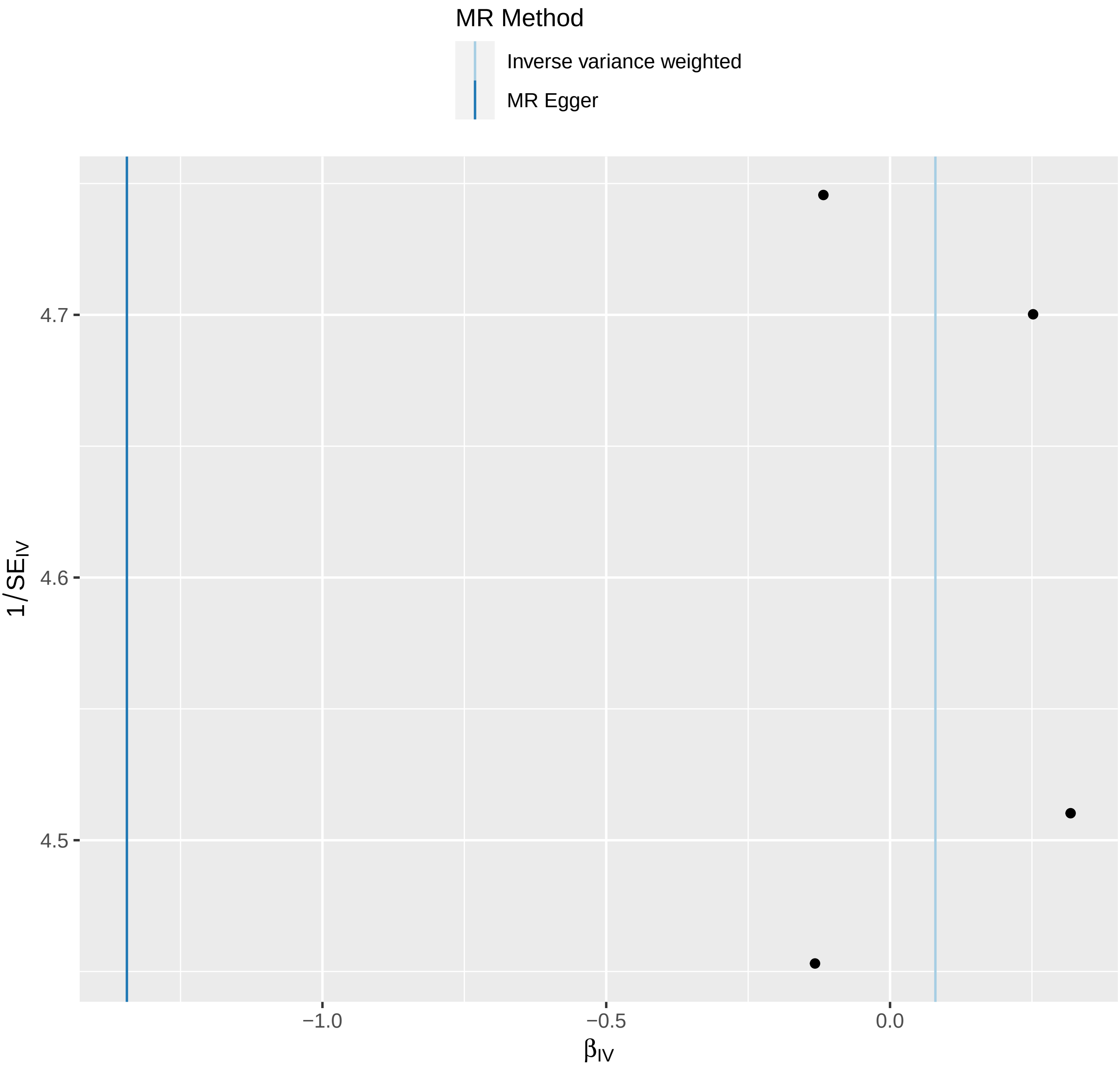

Batch 2−1240 : Gut microbiota abundance (genus Parasutterella id.2892) on Collagen alpha−1(XXVI) chain
with a threshold of 1e−05

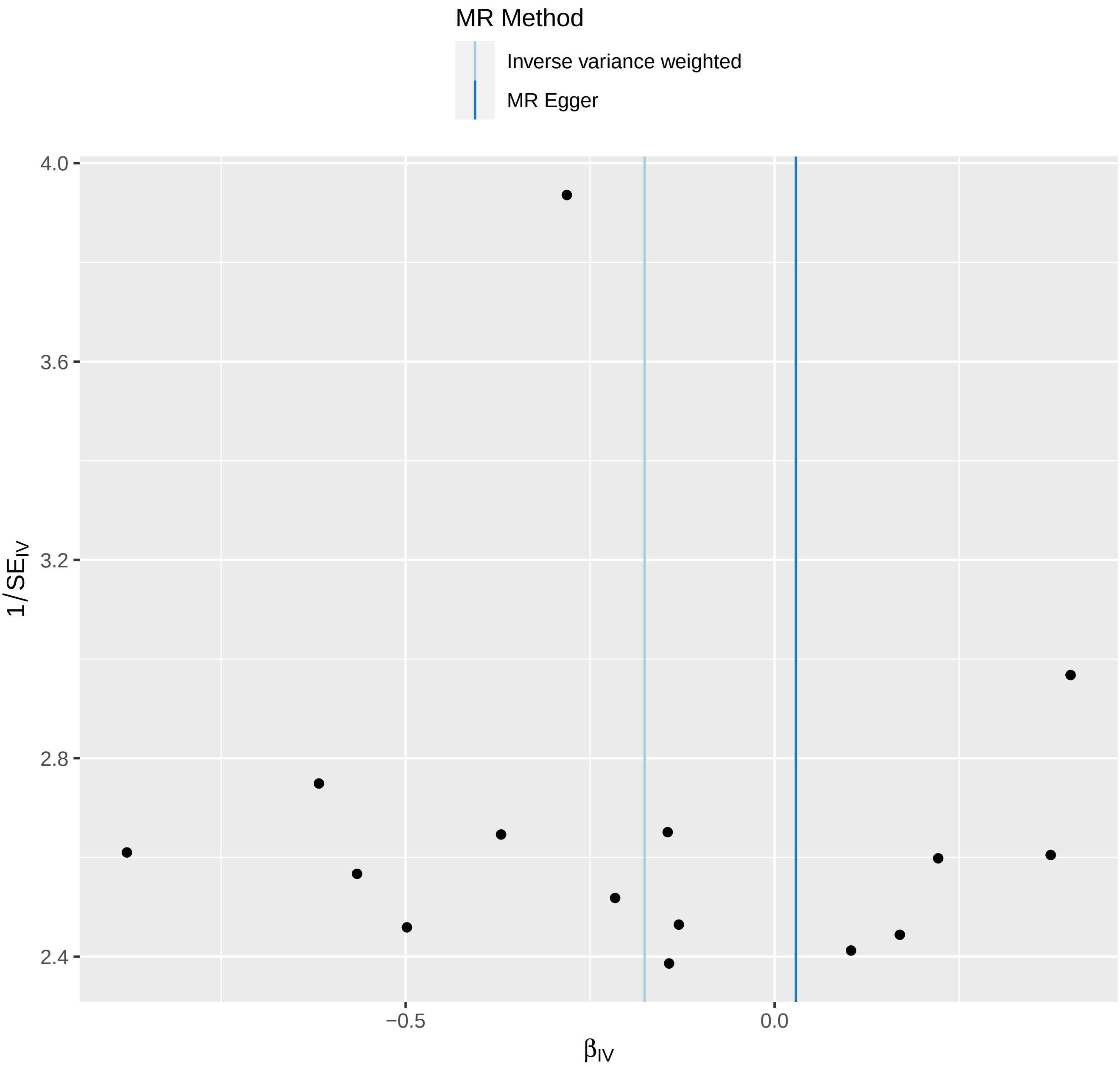

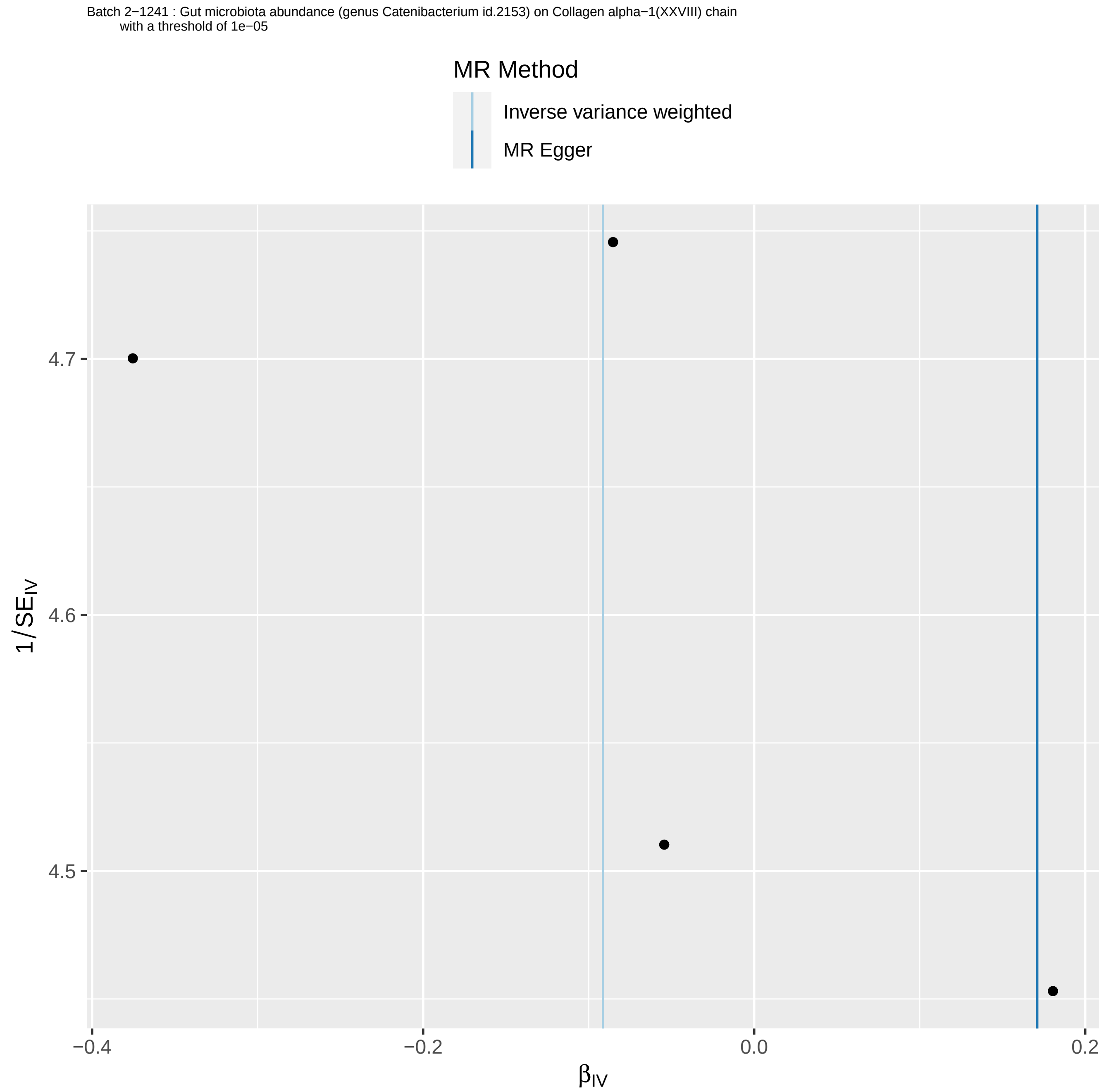
Batch 2−1241 : Gut microbiota abundance (genus Catenibacterium id.2153) on Collagen alpha−1(XXVIII) chain
with a threshold of 1e−05
MR Method
Inverse variance weighted
MR Egger
4.7
4.6
4.5
1/SE_IV
−0.4
−0.2
0.0
0.2
β_IV

Batch 2−1242 : Gut microbiota abundance (genus Parasutterella id.2892) on Collagen alpha−1(XXVIII) chain with a threshold of 1e−05

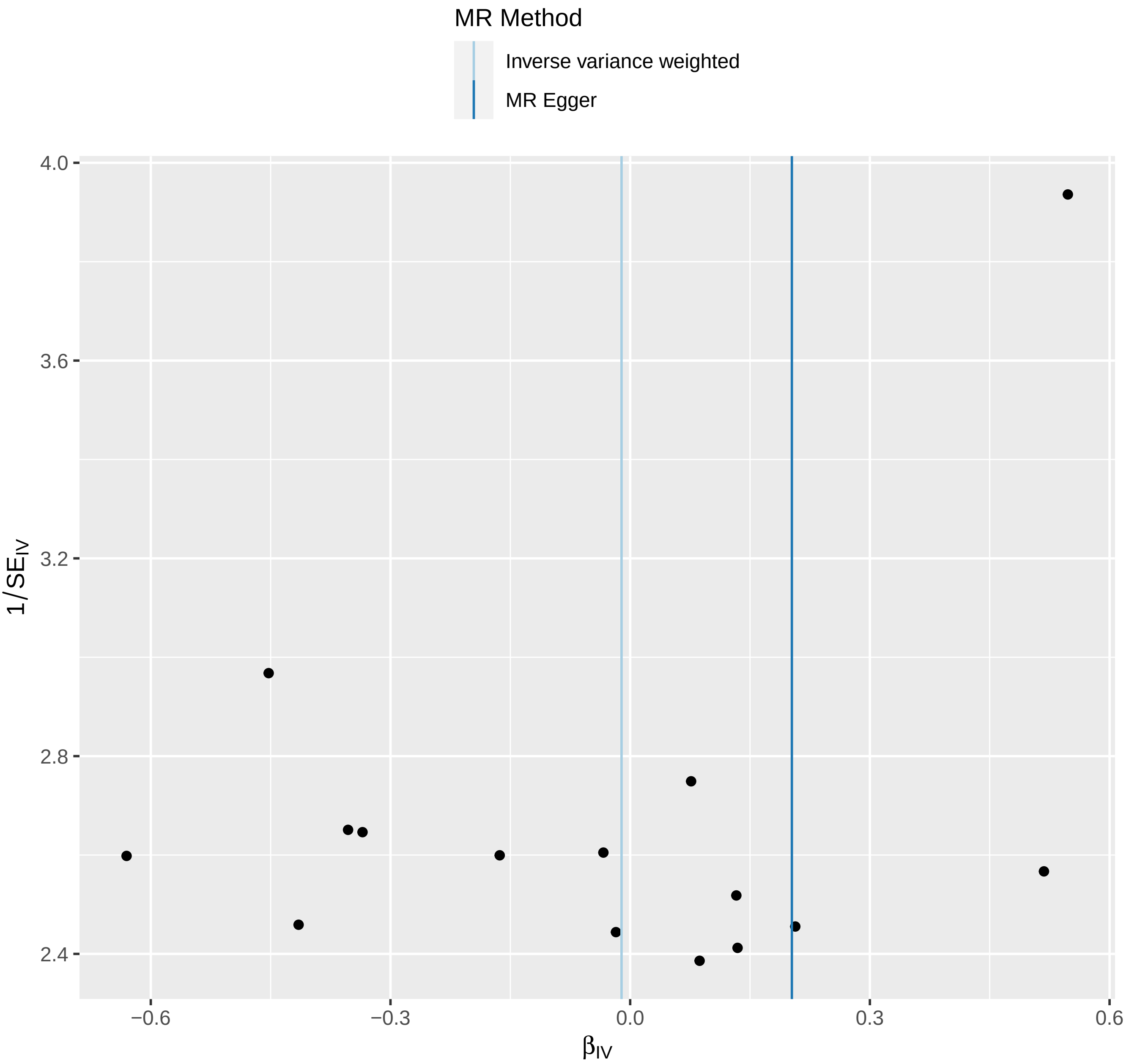

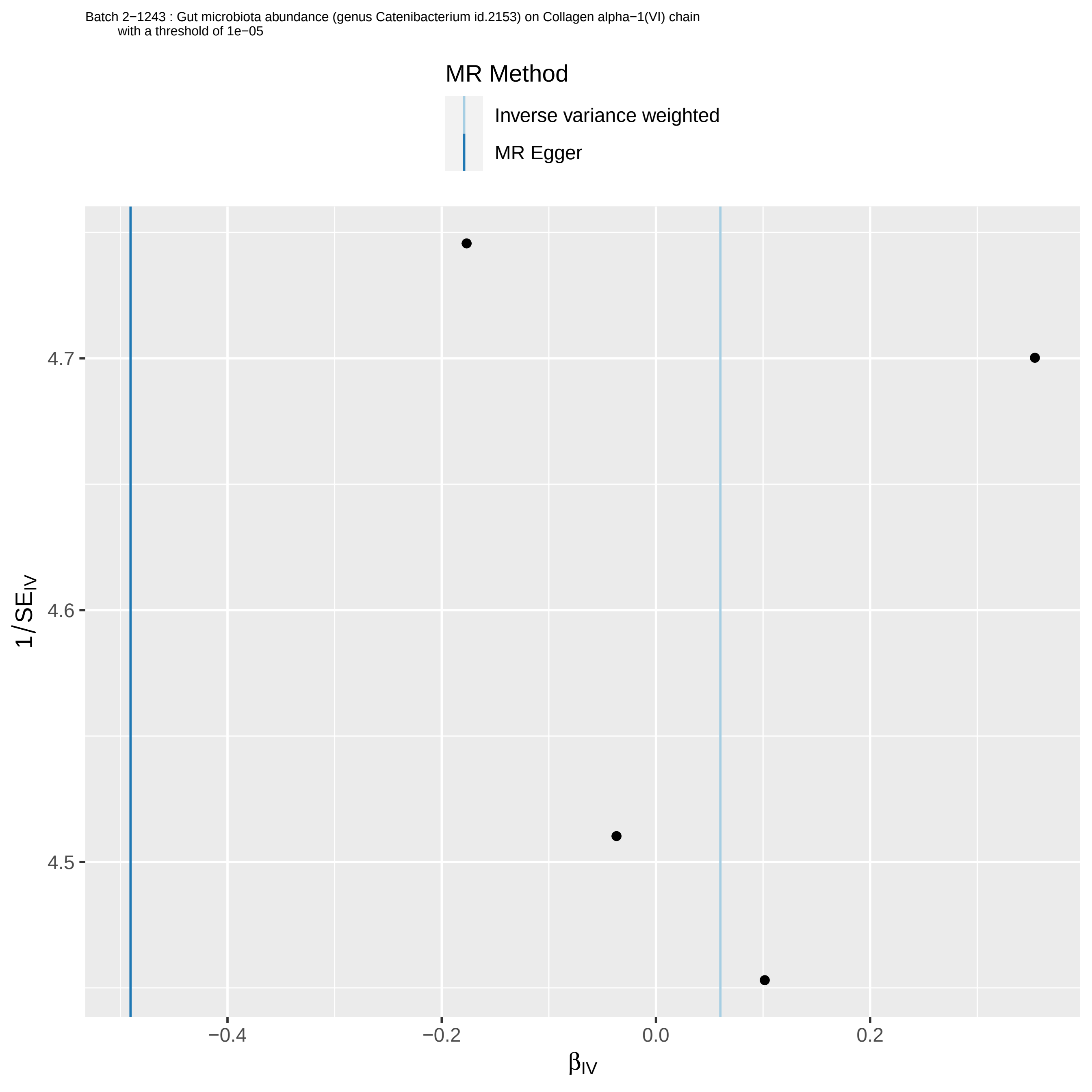

Batch 2−1243 : Gut microbiota abundance (genus Catenibacterium id.2153) on Collagen alpha−1(VI) chain
with a threshold of 1e−05
MR Method
Inverse variance weighted
MR Egger
4.7
4.6
4.5
1/SE_IV
−0.4
−0.2
0.0
0.2
β_IV

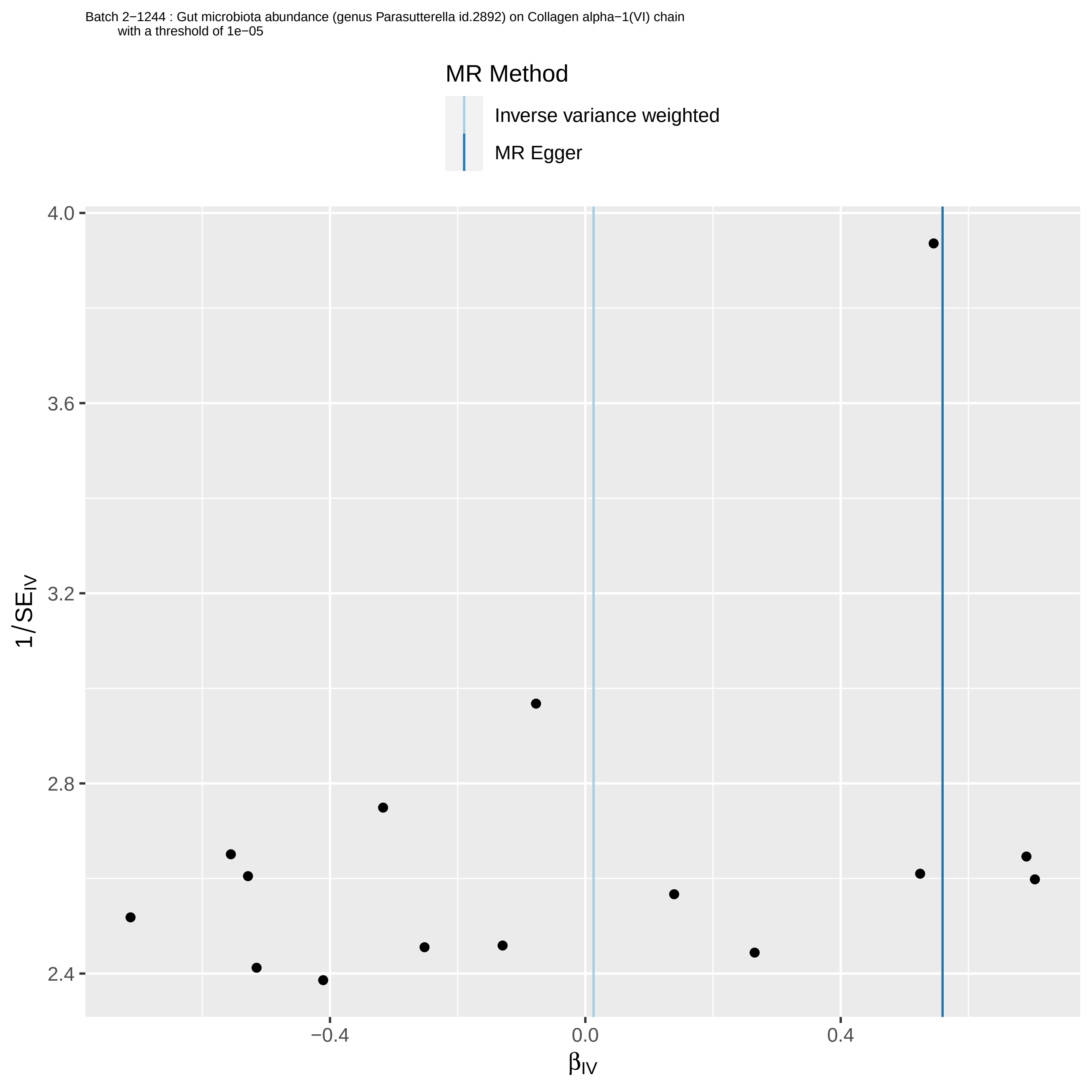

Batch 2−1244 : Gut microbiota abundance (genus Parasutterella id.2892) on Collagen alpha−1(VI) chain
with a threshold of 1e−05
MR Method
Inverse variance weighted
MR Egger
4.0
3.6
3.2
2.8
2.4
1/SE_IV
−0.4
0.0
0.4
β_IV

Batch 2−1245 : Gut microbiota abundance (genus Catenibacterium id.2153) on Collagen alpha−3(VI) chain
with a threshold of 1e−05

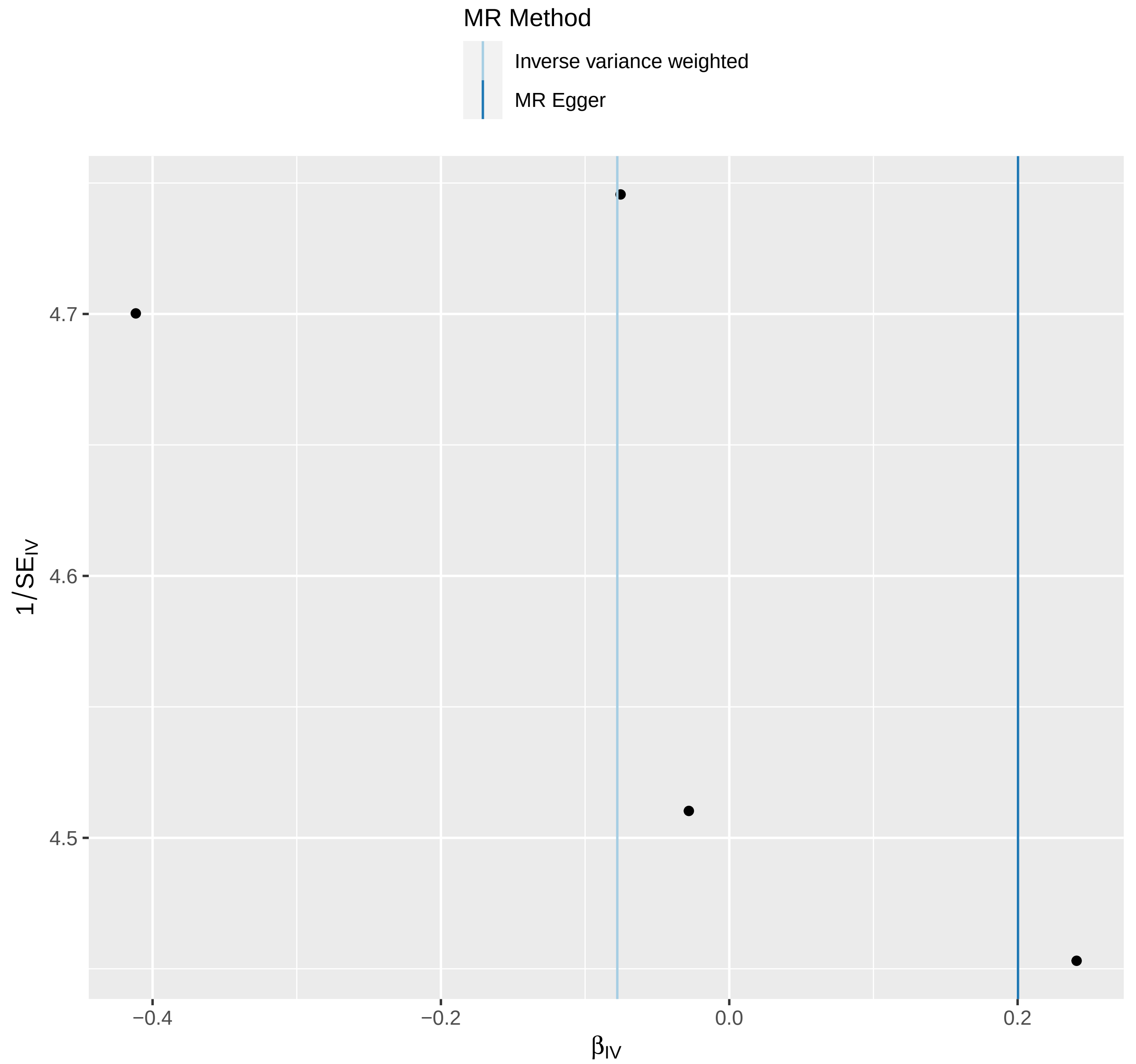

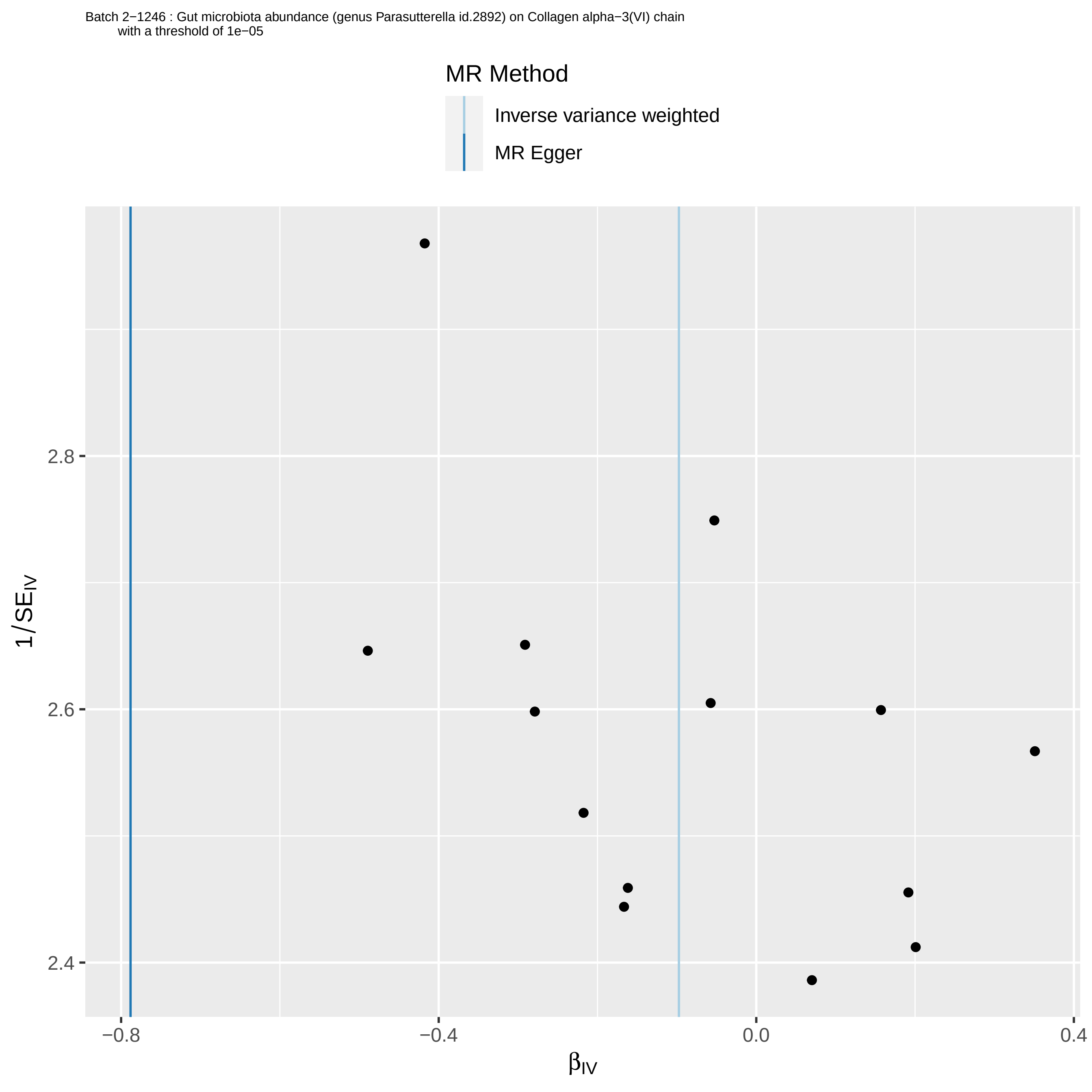

Batch 2−1246 : Gut microbiota abundance (genus Parasutterella id.2892) on Collagen alpha−3(VI) chain
with a threshold of 1e−05
MR Method
Inverse variance weighted
MR Egger
2.8
2.6
2.4
1/SE_IV
−0.8
−0.4
0.0
0.4
β_IV

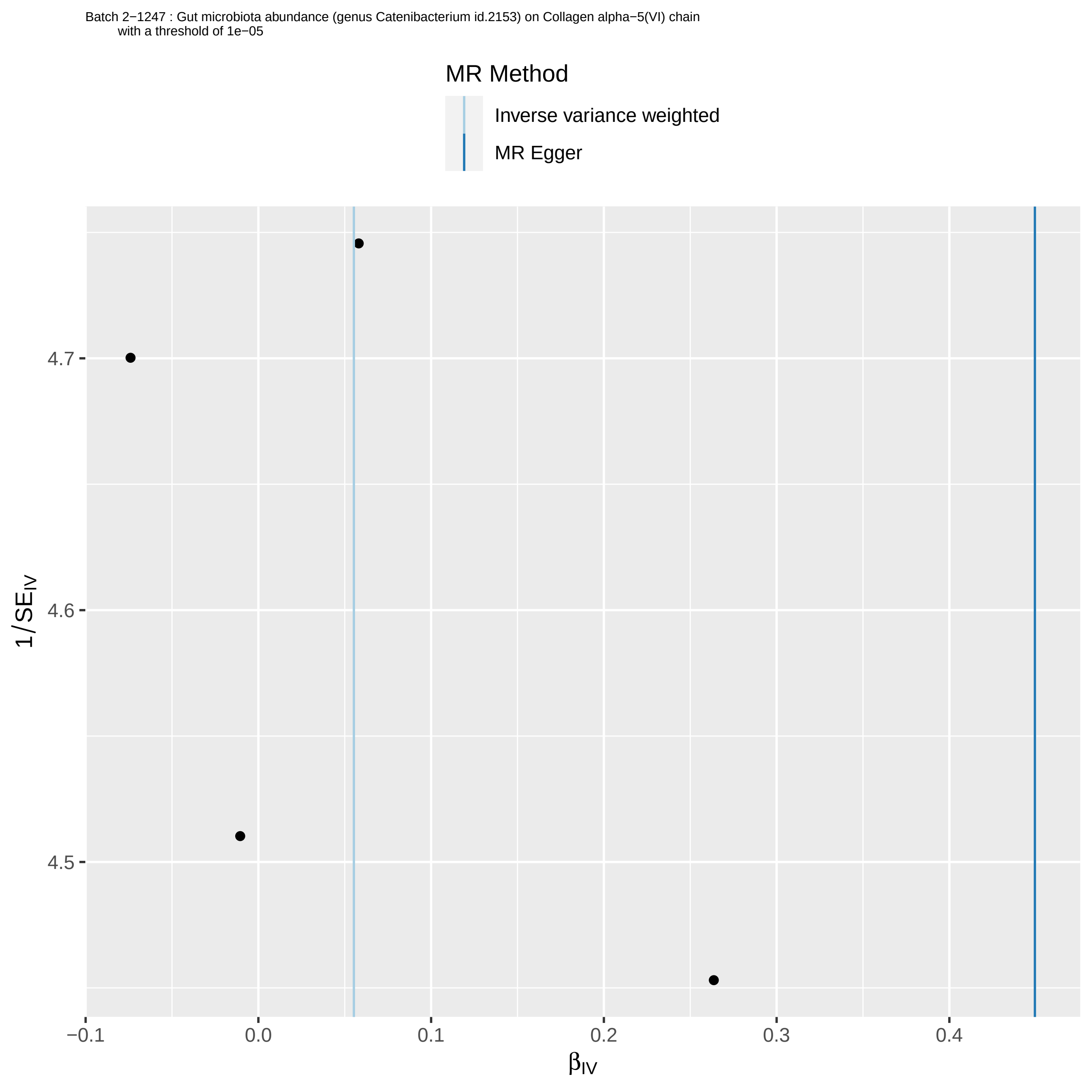

Batch 2−1247 : Gut microbiota abundance (genus Catenibacterium id.2153) on Collagen alpha−5(VI) chain
with a threshold of 1e−05
MR Method
Inverse variance weighted
MR Egger
4.7
4.6
4.5
1/SE_IV
−0.1
0.0
0.1
0.2
0.3
0.4
β_IV

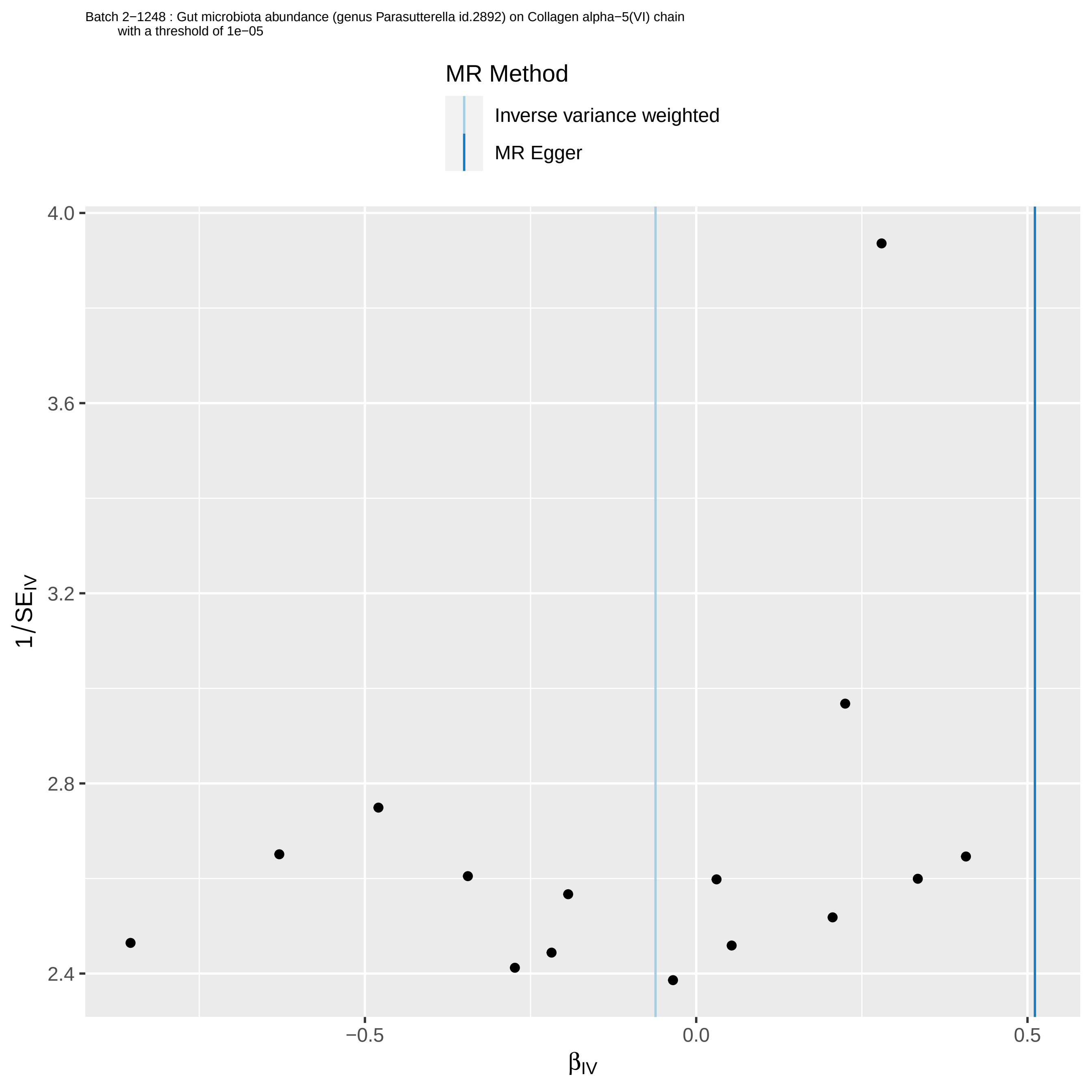
Batch 2−1248 : Gut microbiota abundance (genus Parasutterella id.2892) on Collagen alpha−5(VI) chain
with a threshold of 1e−05
MR Method
Inverse variance weighted
MR Egger
4.0
3.6
3.2
2.8
2.4
1/SE_IV
−0.5
0.0
0.5
β_IV

Batch 2−1249 : Gut microbiota abundance (genus Catenibacterium id.2153) on Collagen alpha−1(VIII) chain
with a threshold of 1e−05

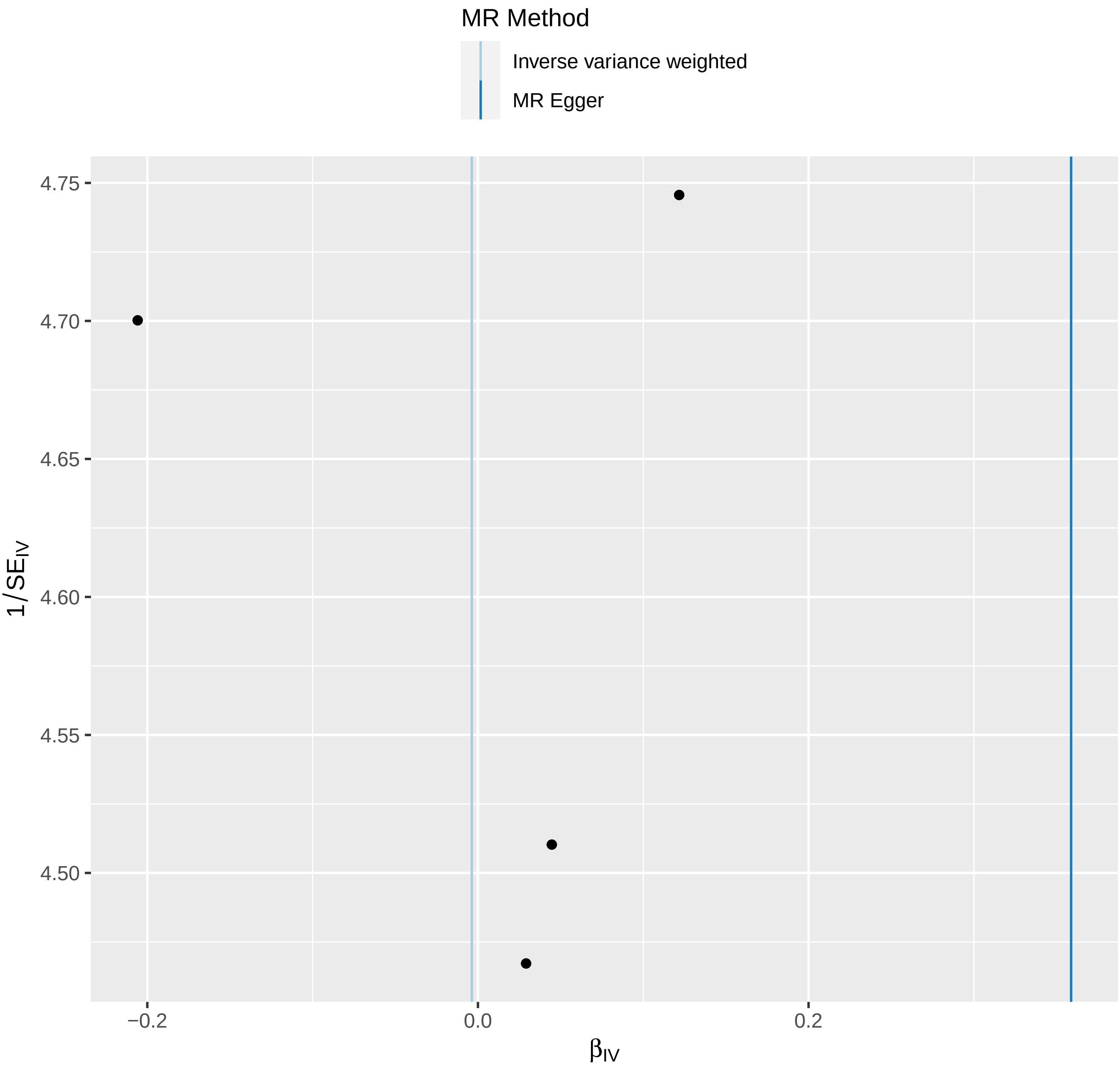

Batch 2−1250 : Gut microbiota abundance (genus Parasutterella id.2892) on Collagen alpha−1(VIII) chain
with a threshold of 1e−05

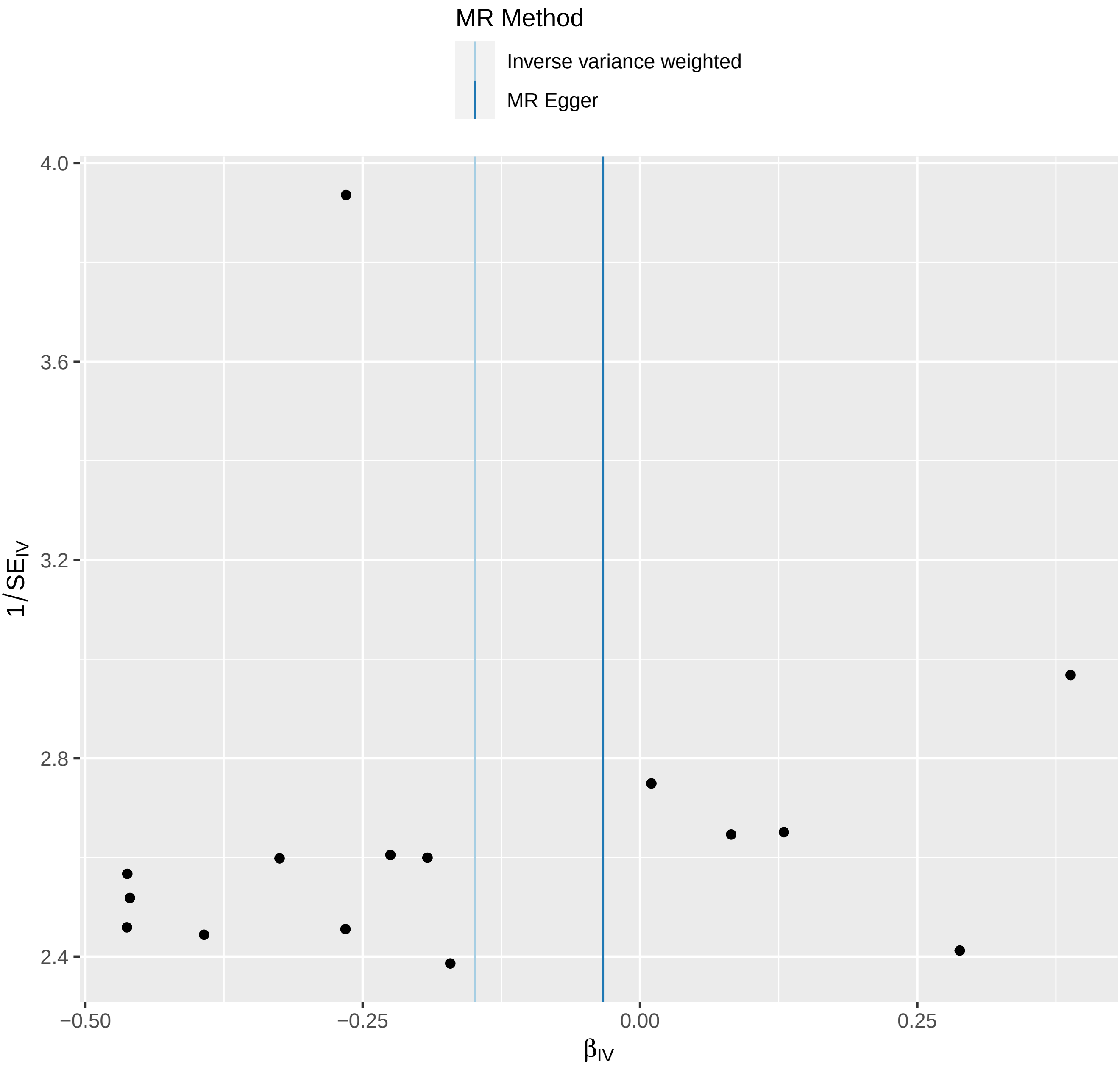

Batch 2−1251 : Gut microbiota abundance (genus Catenibacterium id.2153) on Collagen alpha−2(IX) chain
with a threshold of 1e−05

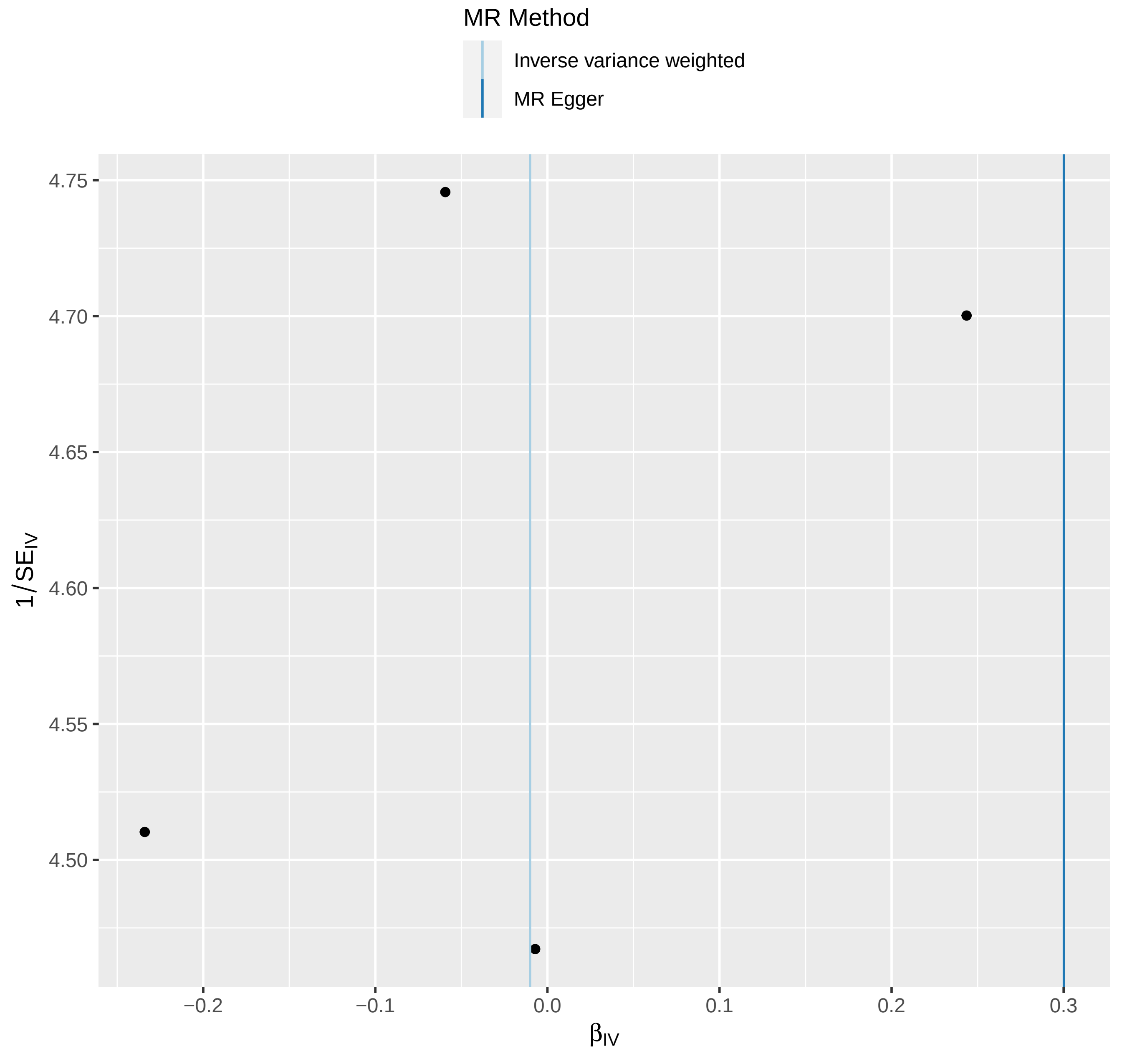

Batch 2−1252 : Gut microbiota abundance (genus Parasutterella id.2892) on Collagen alpha−2(IX) chain
with a threshold of 1e−05

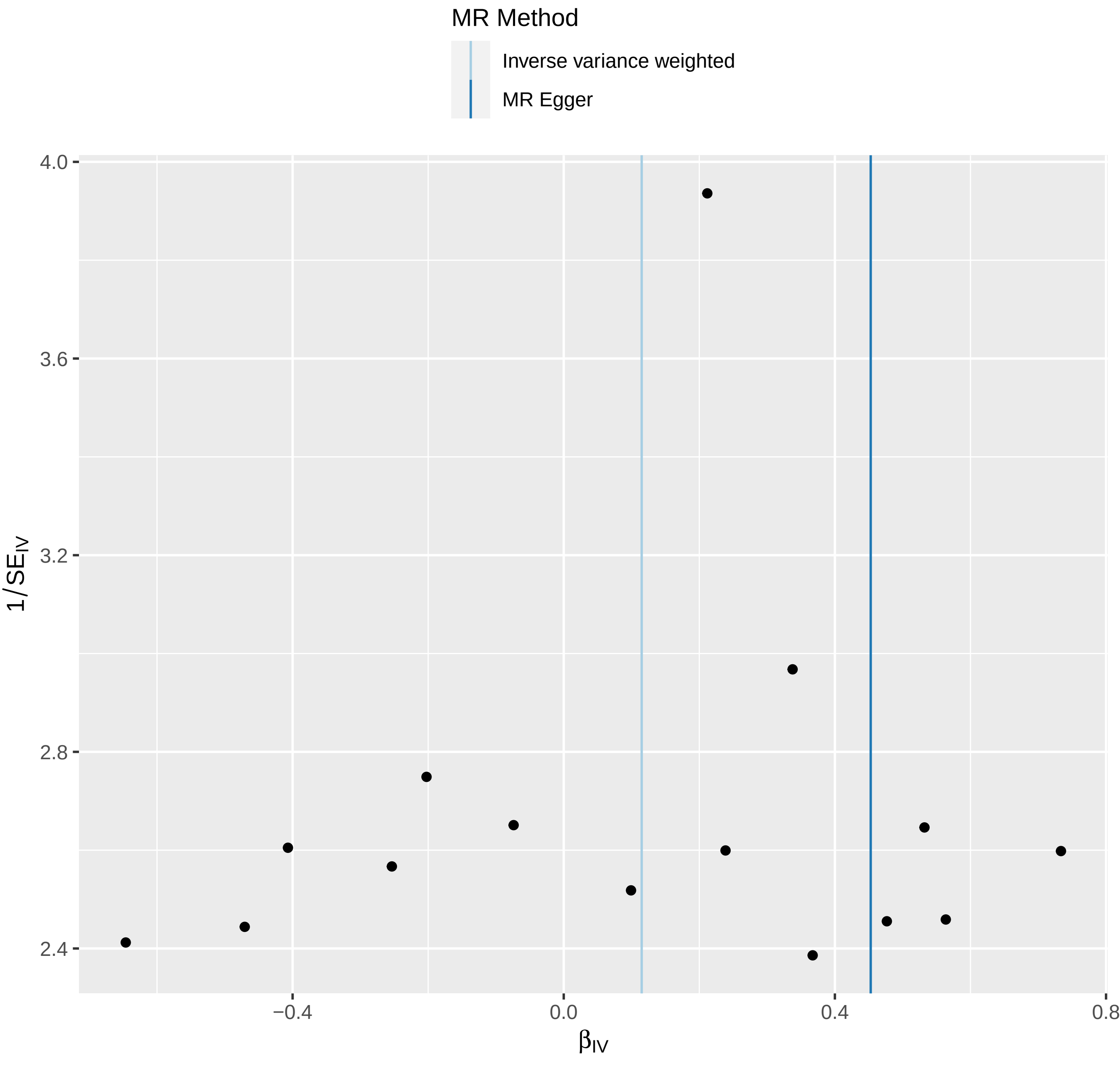

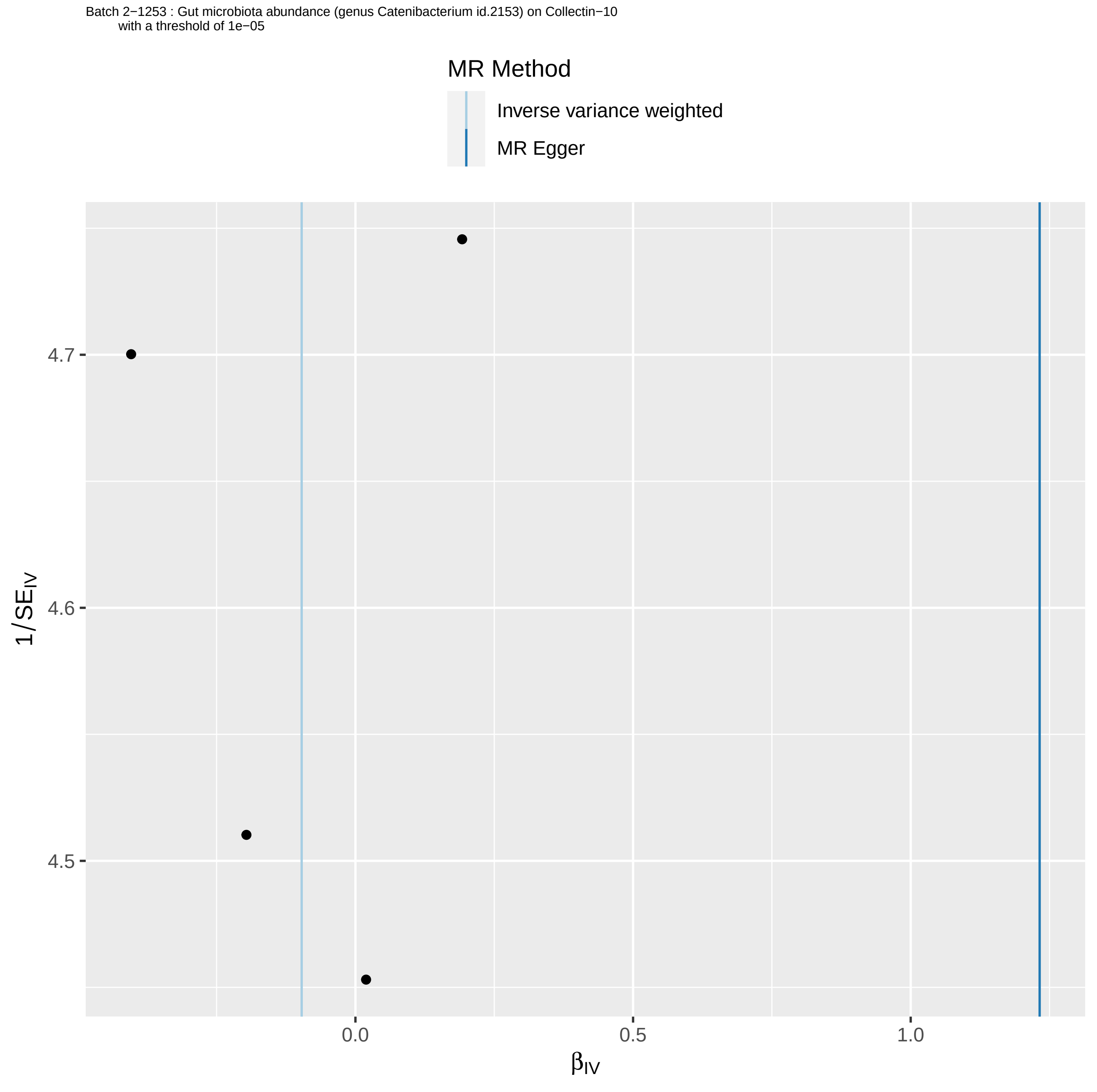
Batch 2−1253 : Gut microbiota abundance (genus Catenibacterium id.2153) on Collectin−10
with a threshold of 1e−05
MR Method
Inverse variance weighted
MR Egger
4.7
4.6
4.5
1/SE_IV
0.0
0.5
1.0
β_IV

Batch 2−1254 : Gut microbiota abundance (genus Parasutterella id.2892) on Collectin−10
with a threshold of 1e−05

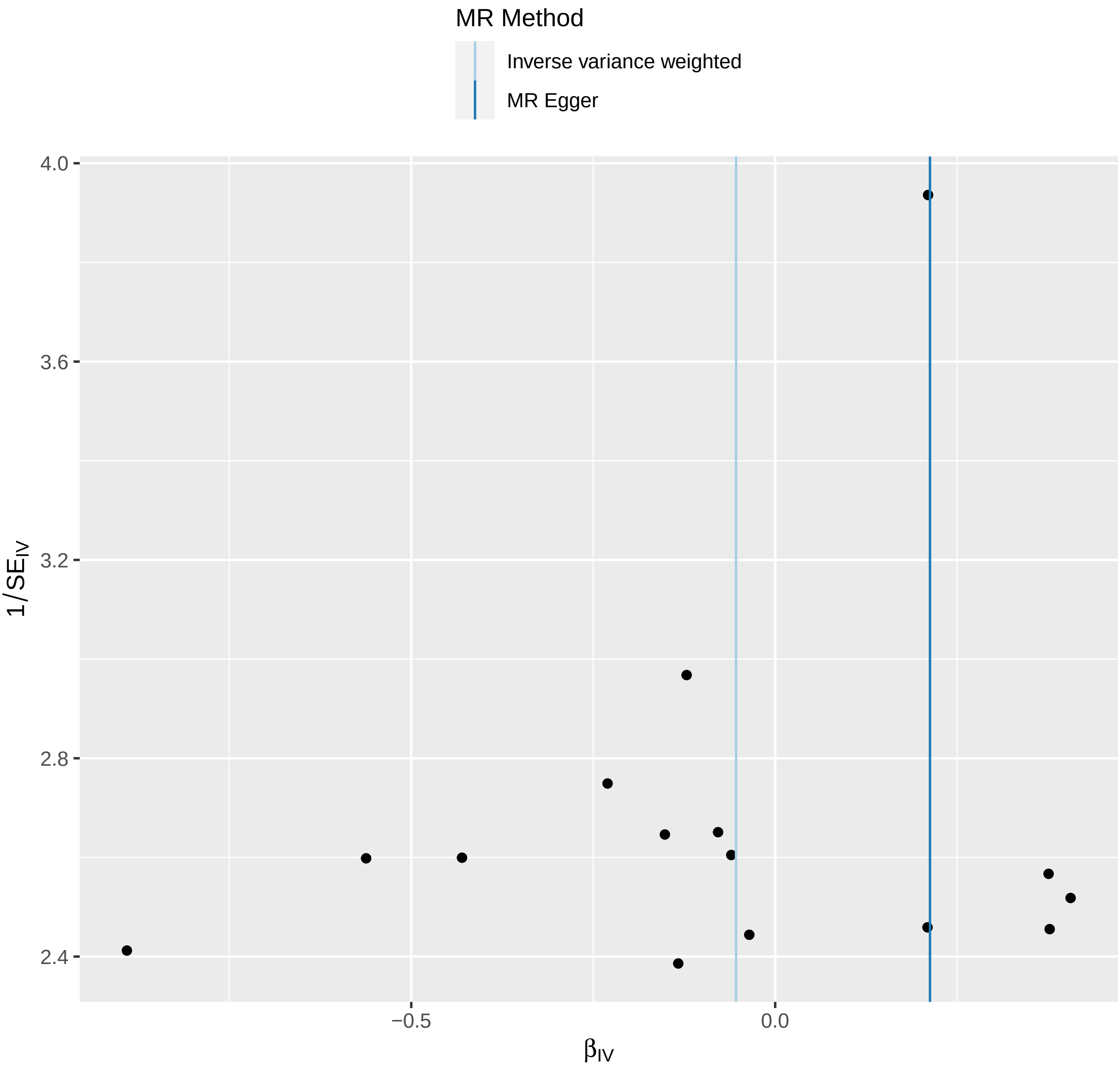

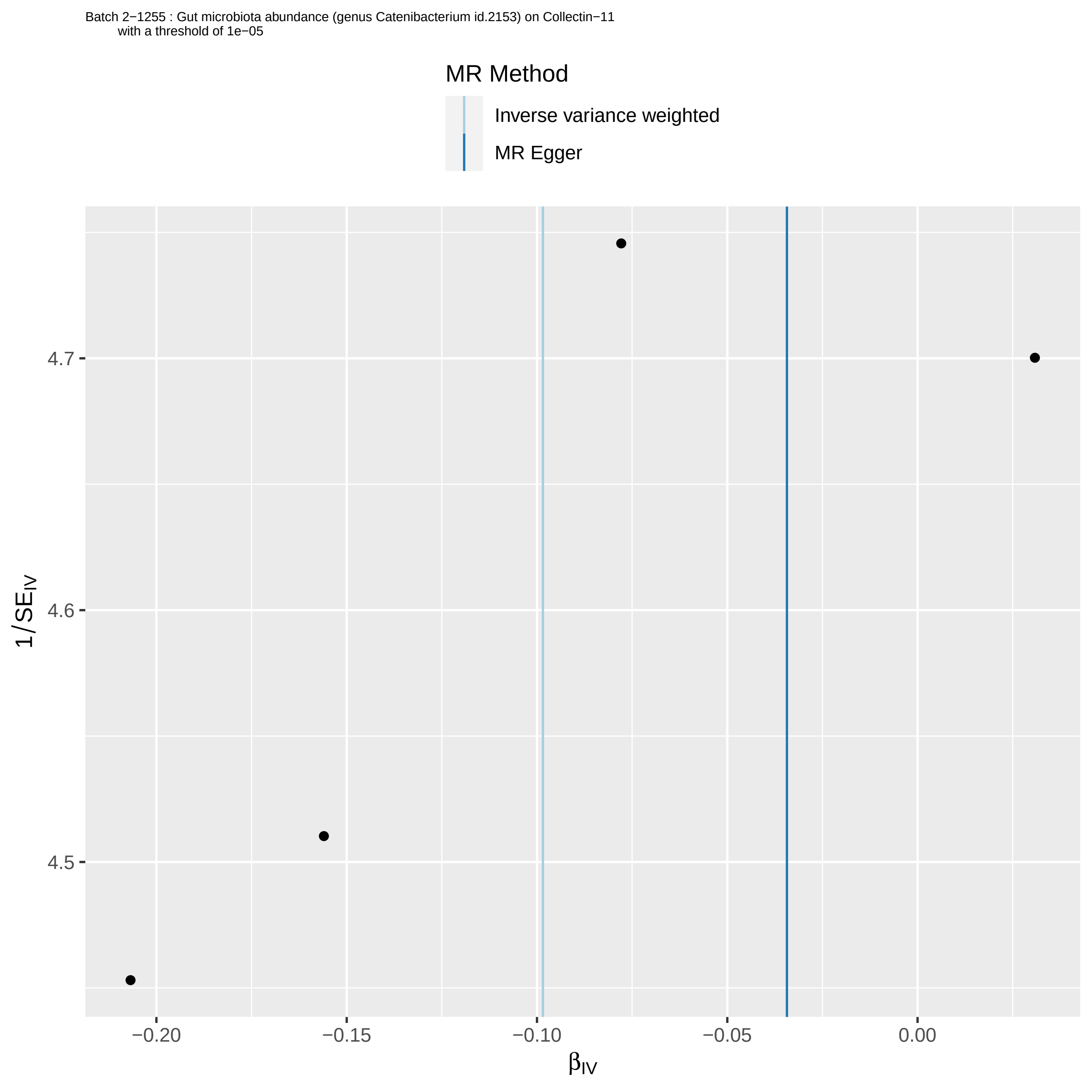
Batch 2−1255 : Gut microbiota abundance (genus Catenibacterium id.2153) on Collectin−11
with a threshold of 1e−05
MR Method
Inverse variance weighted
MR Egger
4.7
4.6
4.5
1/SE_IV
−0.20
−0.15
−0.10
−0.05
0.00
β_IV

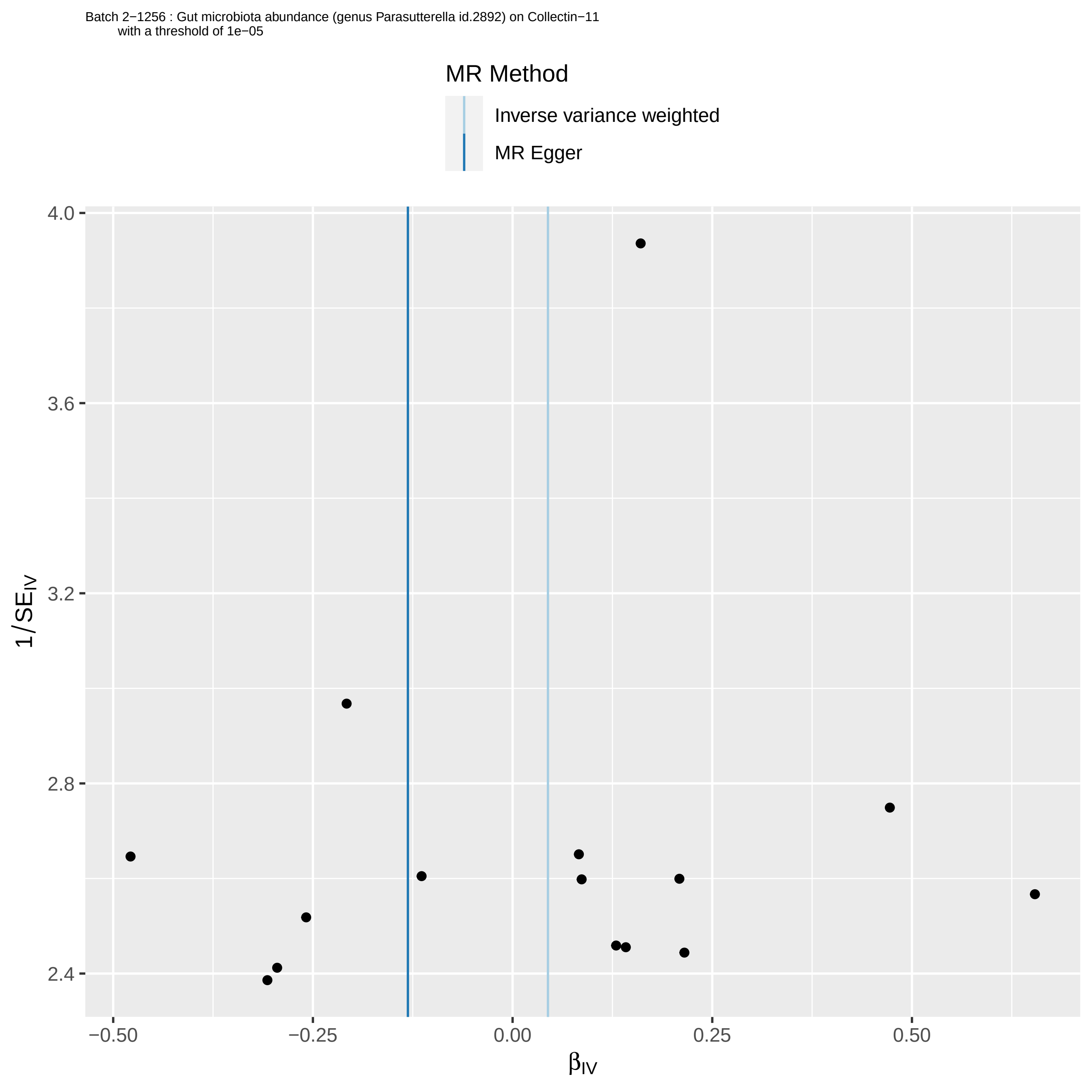
Batch 2−1256 : Gut microbiota abundance (genus Parasutterella id.2892) on Collectin−11
with a threshold of 1e−05
MR Method
Inverse variance weighted
MR Egger
4.0
3.6
3.2
2.8
2.4
1/SE_IV
−0.50
−0.25
0.00
0.25
0.50
β_IV

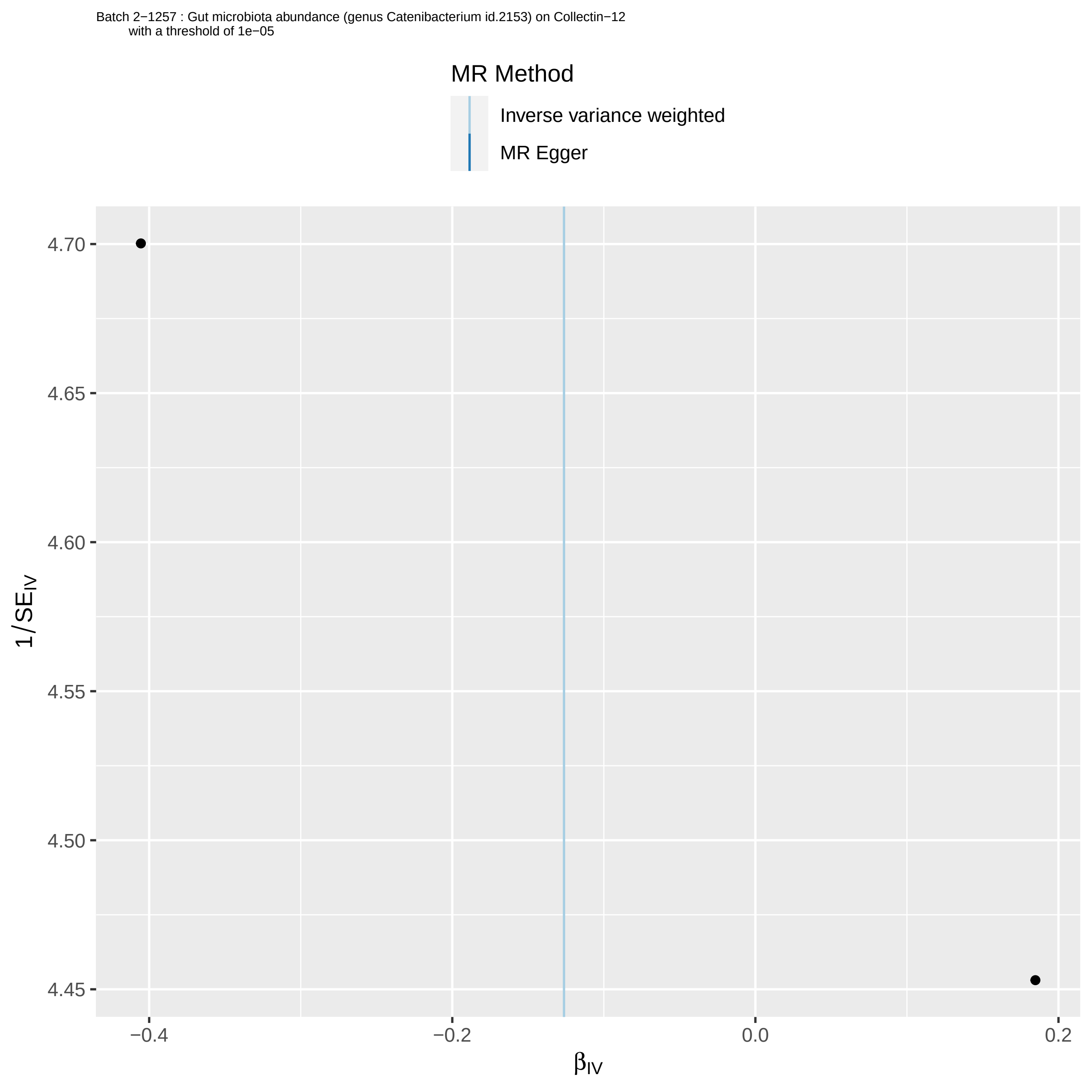

Batch 2−1257 : Gut microbiota abundance (genus Catenibacterium id.2153) on Collectin−12
with a threshold of 1e−05
MR Method
Inverse variance weighted
MR Egger
4.70
4.65
4.60
4.55
4.50
4.45
1/SE_IV
−0.4
−0.2
0.0
0.2
β_IV

Batch 2−1258 : Gut microbiota abundance (genus Parasutterella id.2892) on Collectin−12
with a threshold of 1e−05

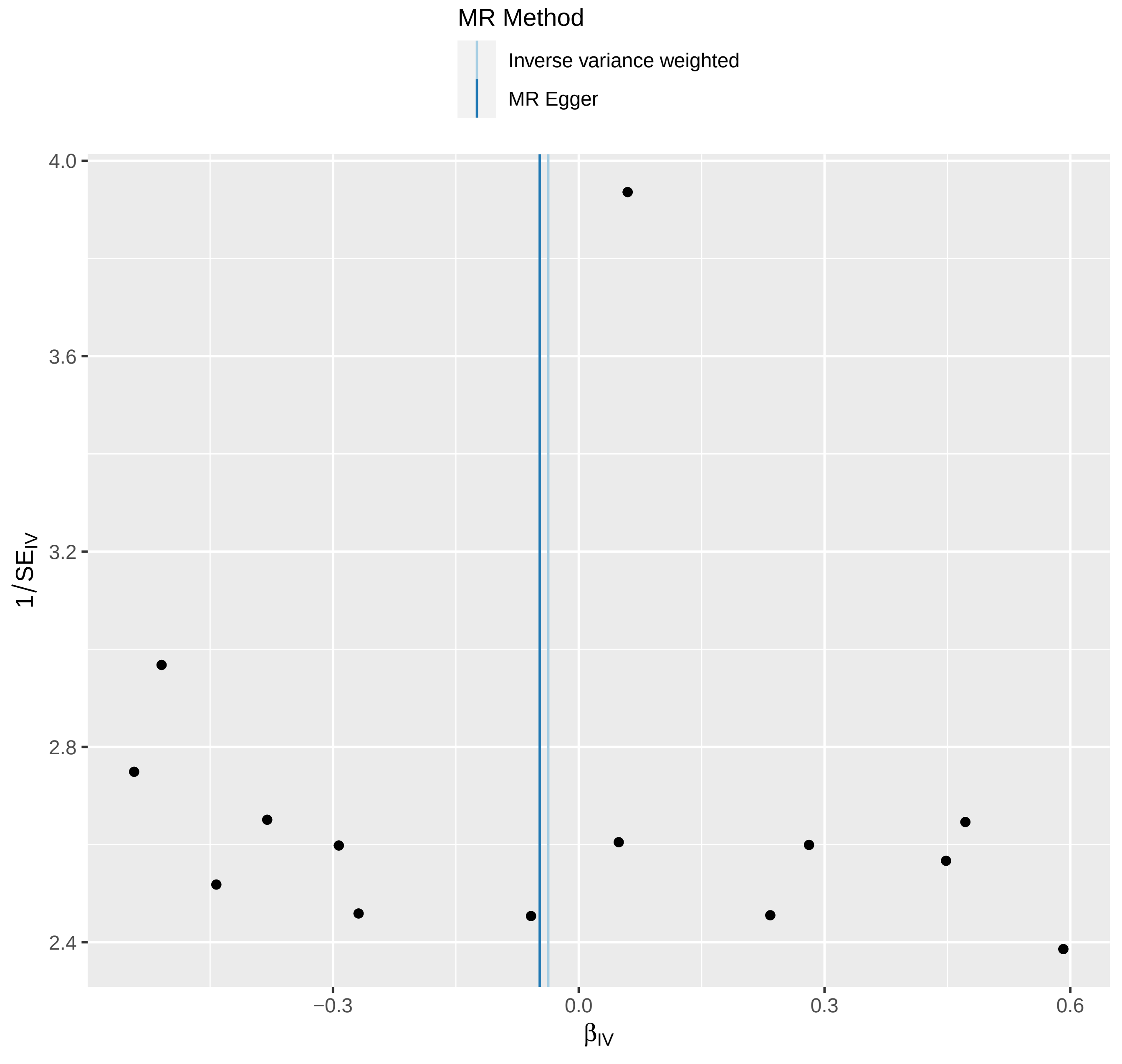

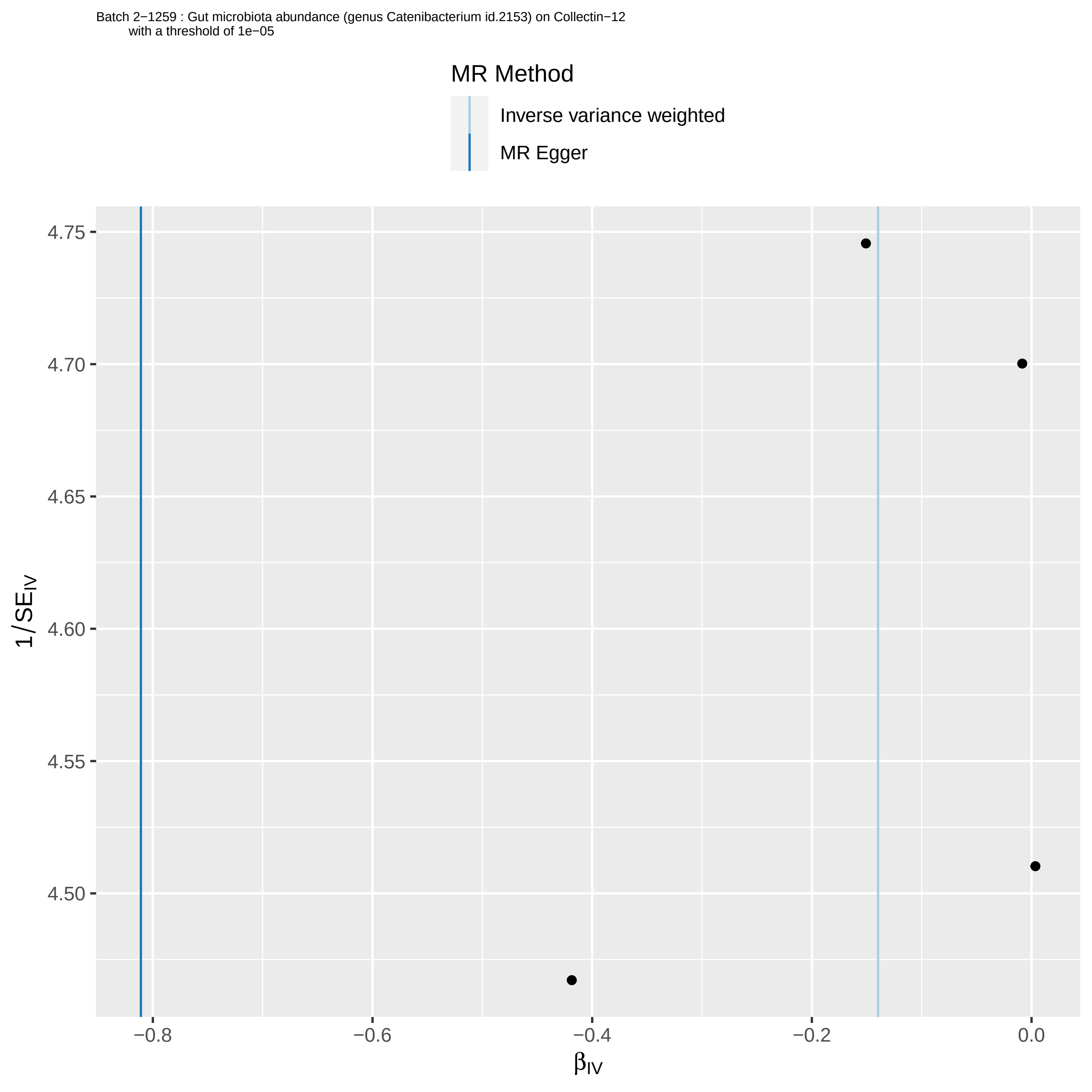

Batch 2−1259 : Gut microbiota abundance (genus Catenibacterium id.2153) on Collectin−12
with a threshold of 1e−05
MR Method
Inverse variance weighted
MR Egger
4.75
4.70
4.65
4.60
4.55
4.50
1/SE_IV
−0.8
−0.6
−0.4
−0.2
0.0
β_IV

Batch 2−1260 : Gut microbiota abundance (genus Parasutterella id.2892) on Collectin−12
with a threshold of 1e−05

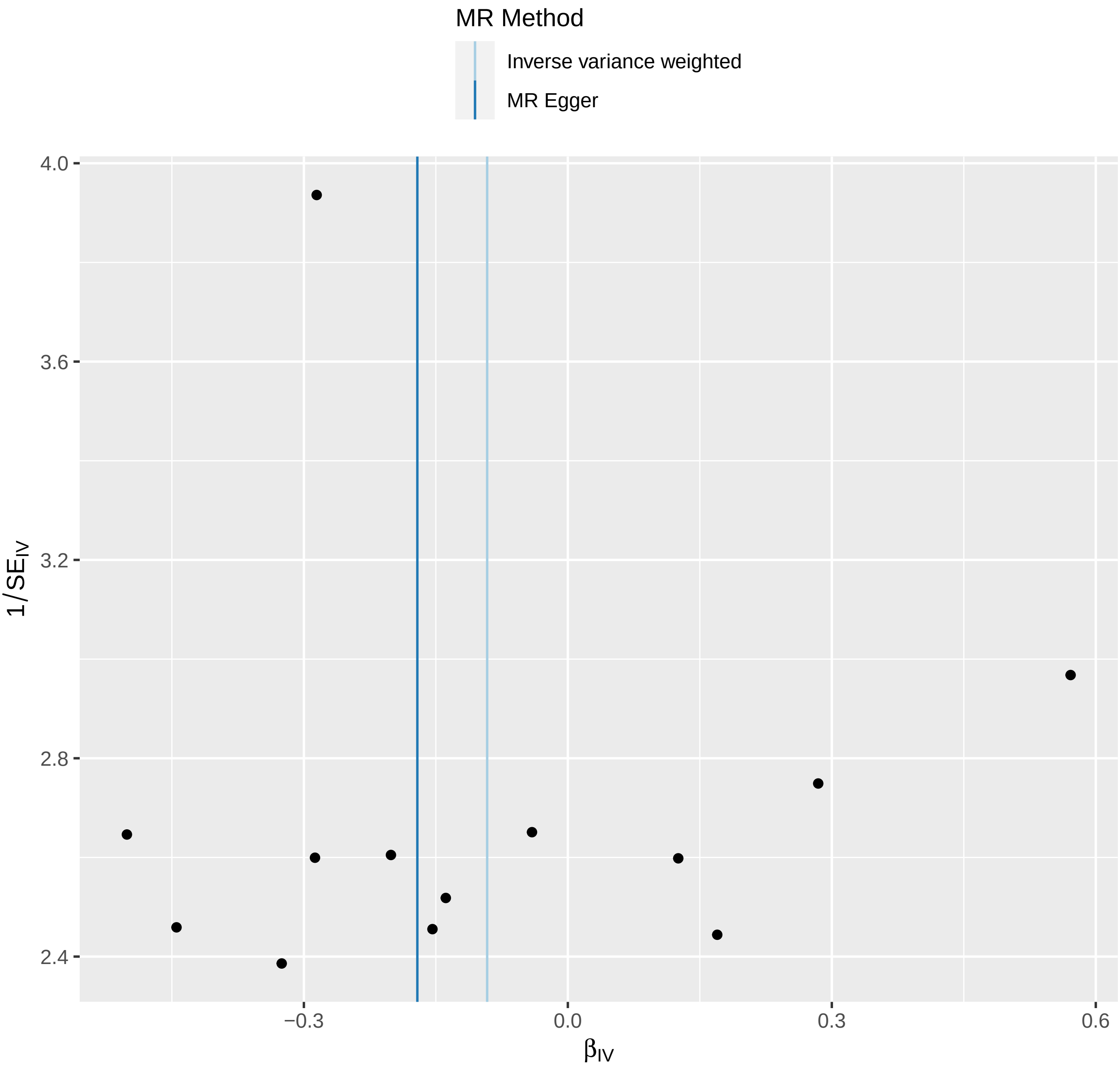

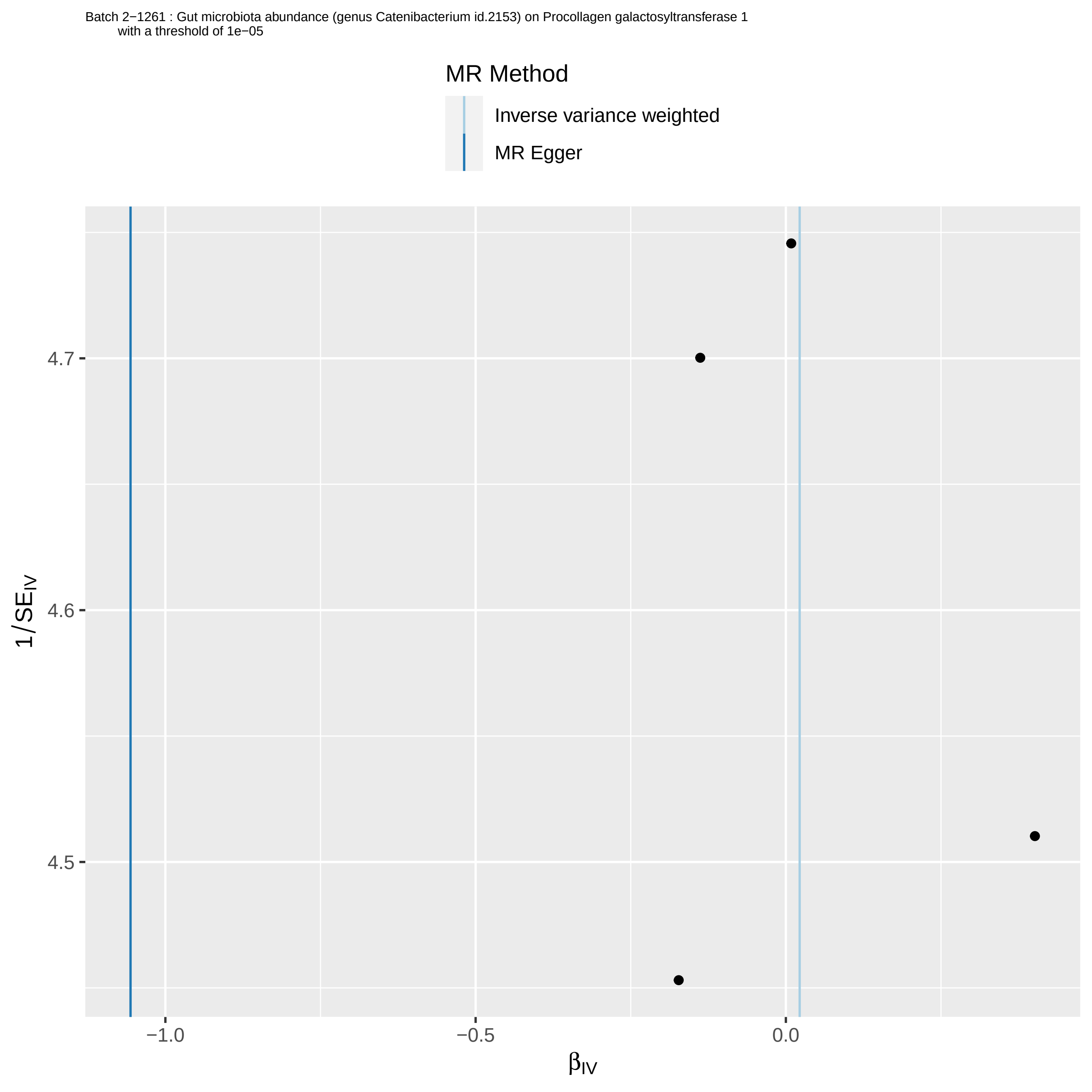
Batch 2−1261 : Gut microbiota abundance (genus Catenibacterium id.2153) on Procollagen galactosyltransferase 1
with a threshold of 1e−05
MR Method
Inverse variance weighted
MR Egger
4.7
4.6
4.5
1/SE$_{IV}$
−1.0
−0.5
0.0
β_{IV}

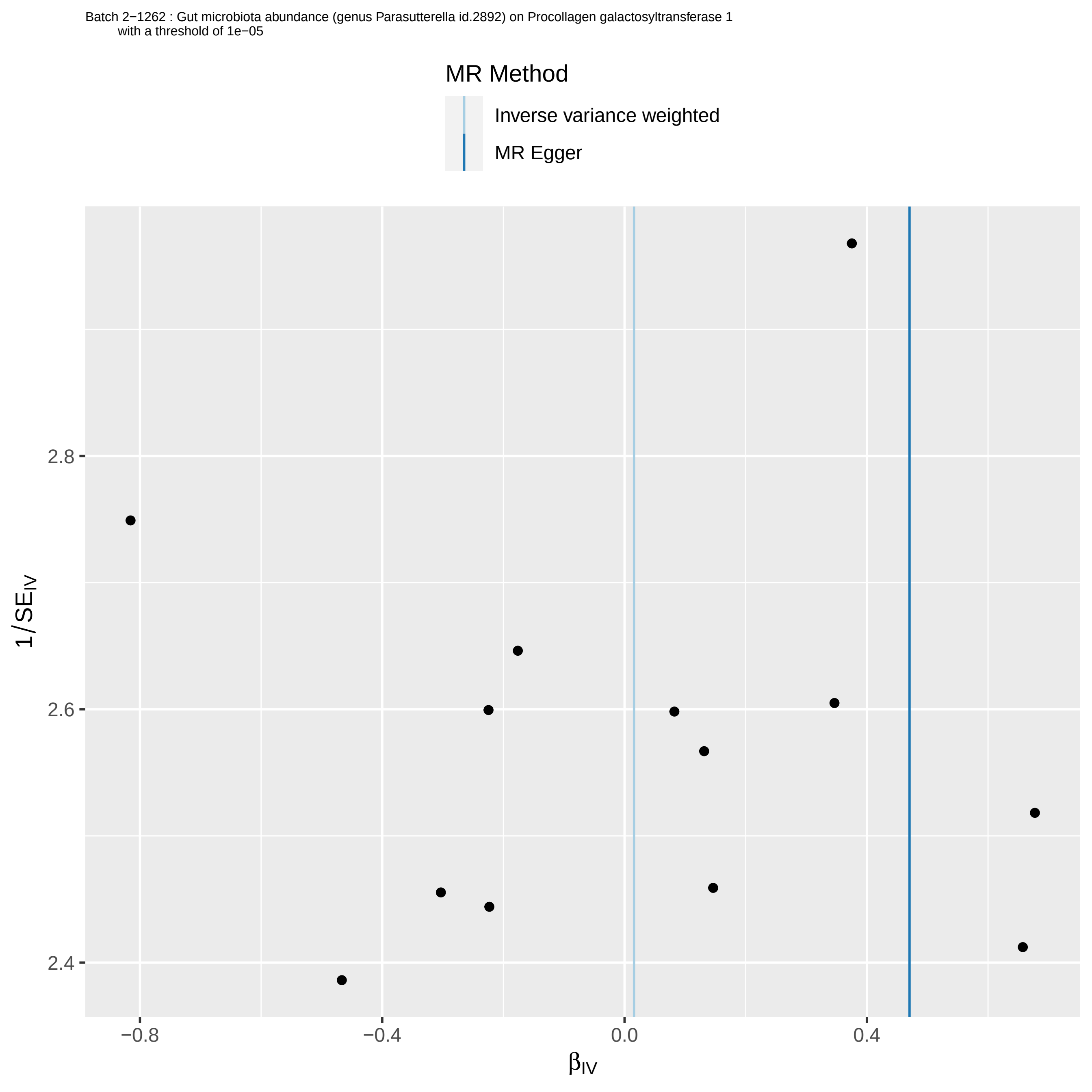
Batch 2−1262 : Gut microbiota abundance (genus Parasutterella id.2892) on Procollagen galactosyltransferase 1
with a threshold of 1e−05
MR Method
Inverse variance weighted
MR Egger
2.8
2.6
2.4
1/SE_IV
−0.8
−0.4
0.0
0.4
β_IV

Batch 2−1263 : Gut microbiota abundance (genus Catenibacterium id.2153) on Procollagen galactosyltransferase 2
with a threshold of 1e−05

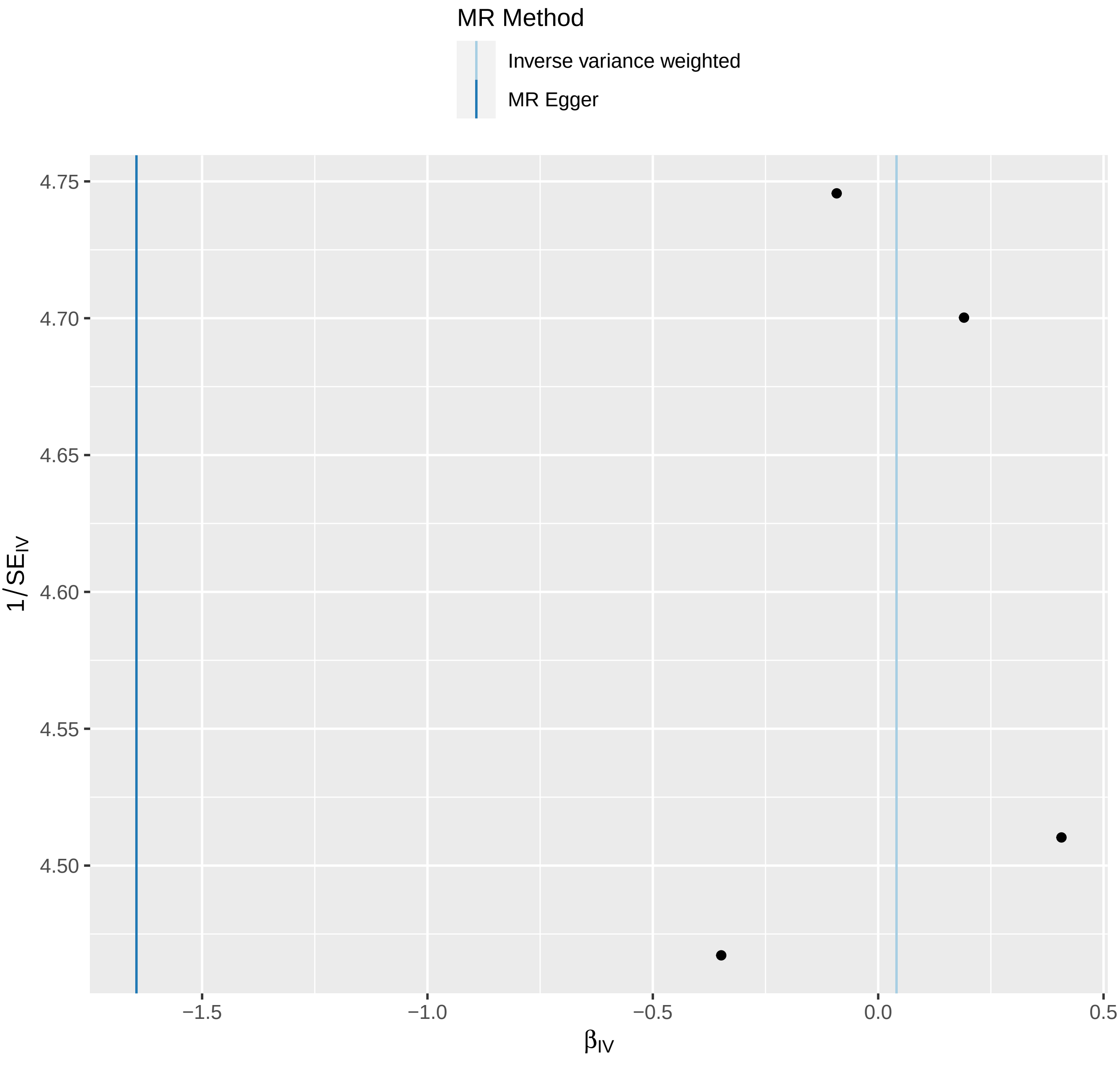

Batch 2−1264 : Gut microbiota abundance (genus Parasutterella id.2892) on Procollagen galactosyltransferase 2
with a threshold of 1e−05

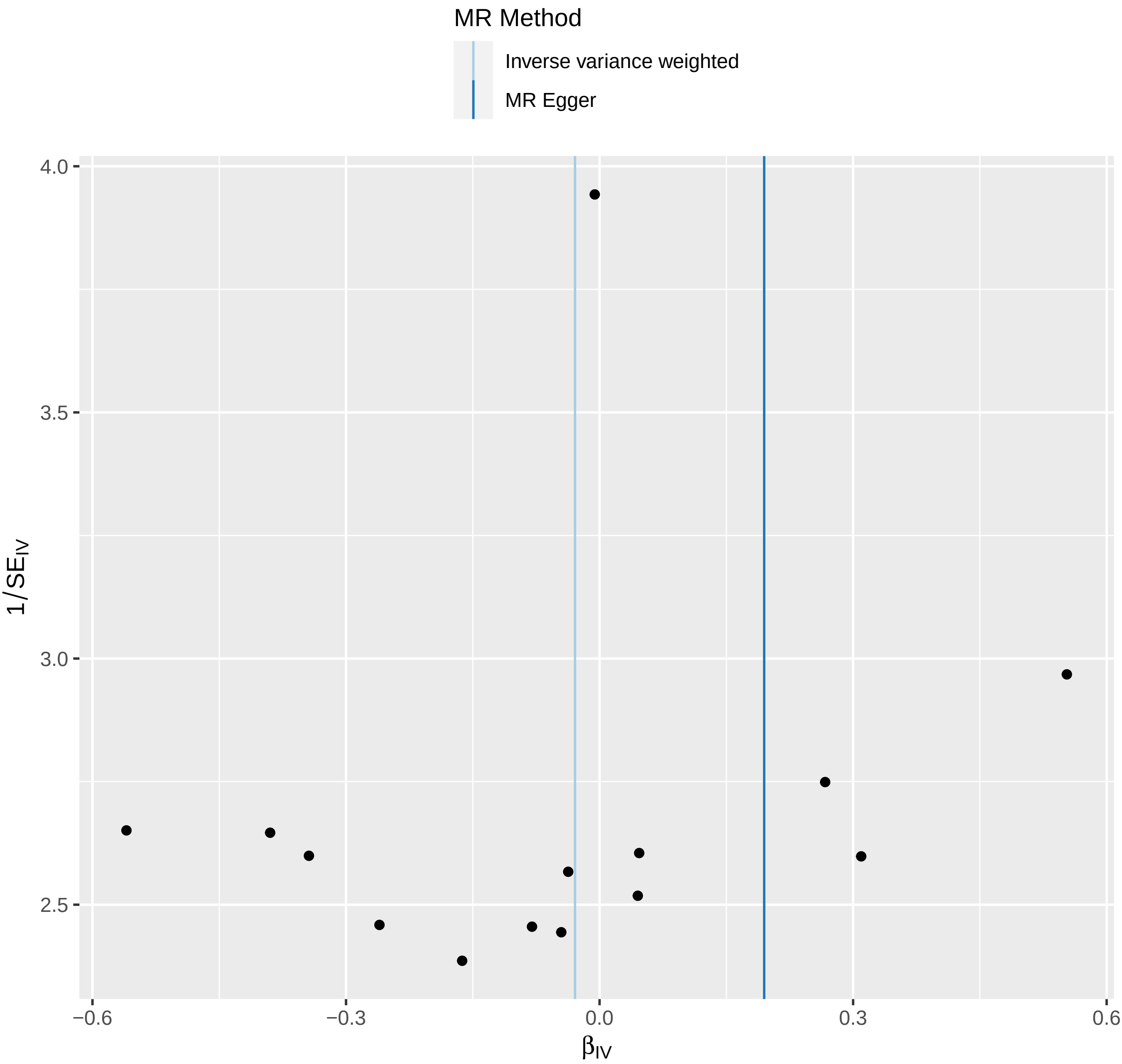

Batch 2−1265 : Gut microbiota abundance (genus Catenibacterium id.2153) on COMM domain−containing protein 1
with a threshold of 1e−05

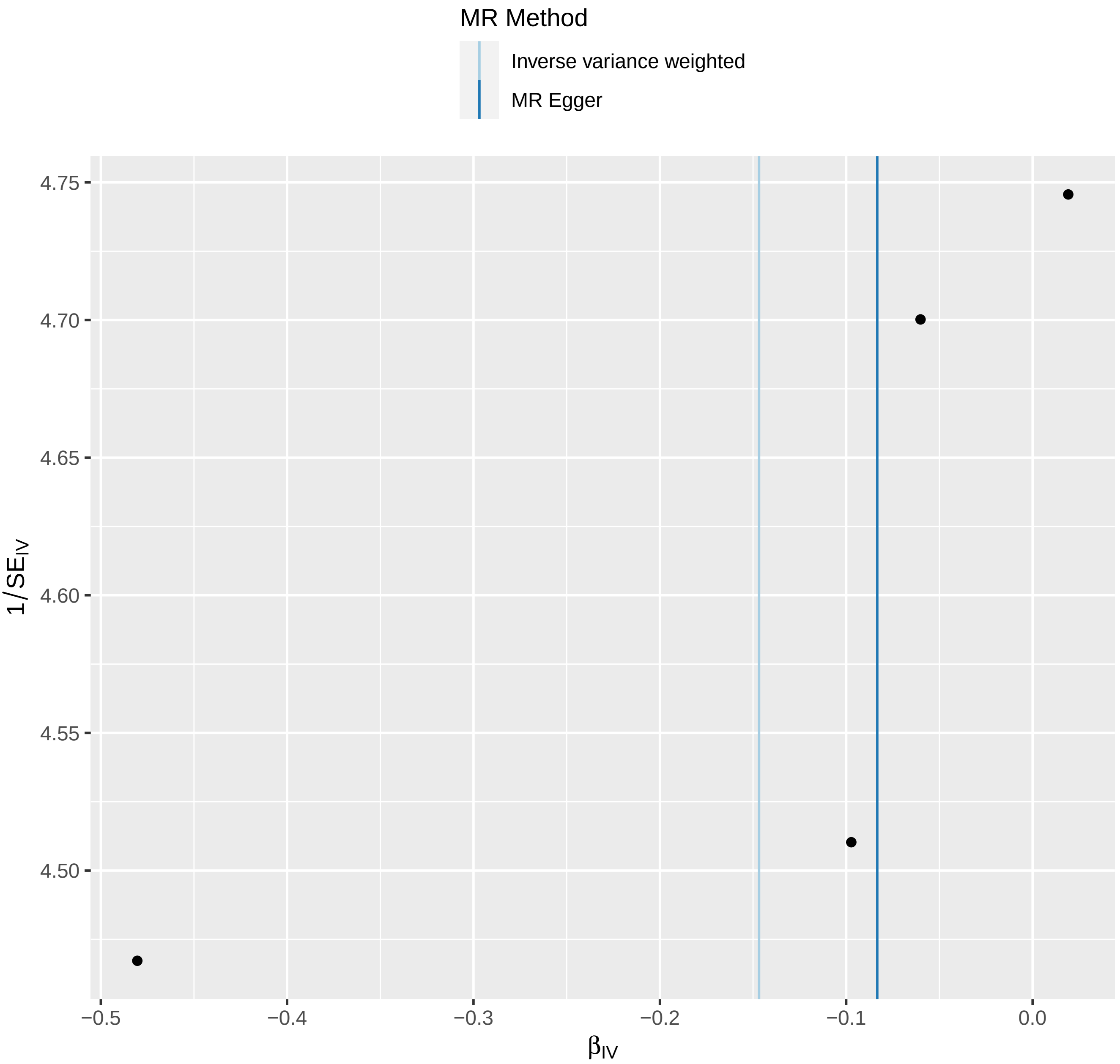

Batch 2−1266 : Gut microbiota abundance (genus Parasutterella id.2892) on COMM domain−containing protein 1
with a threshold of 1e−05

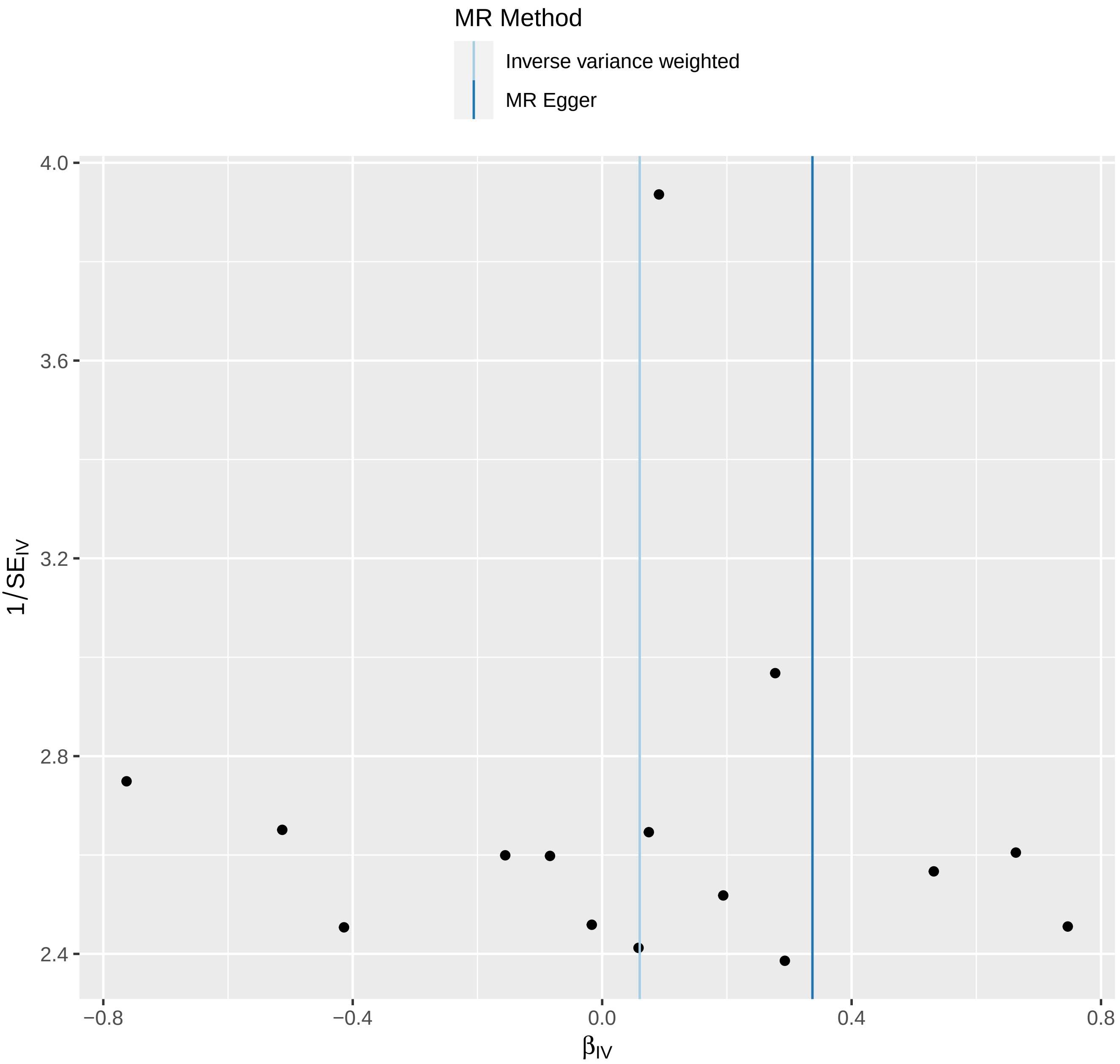

Batch 2−1267 : Gut microbiota abundance (genus Catenibacterium id.2153) on COMM domain−containing protein 7
with a threshold of 1e−05

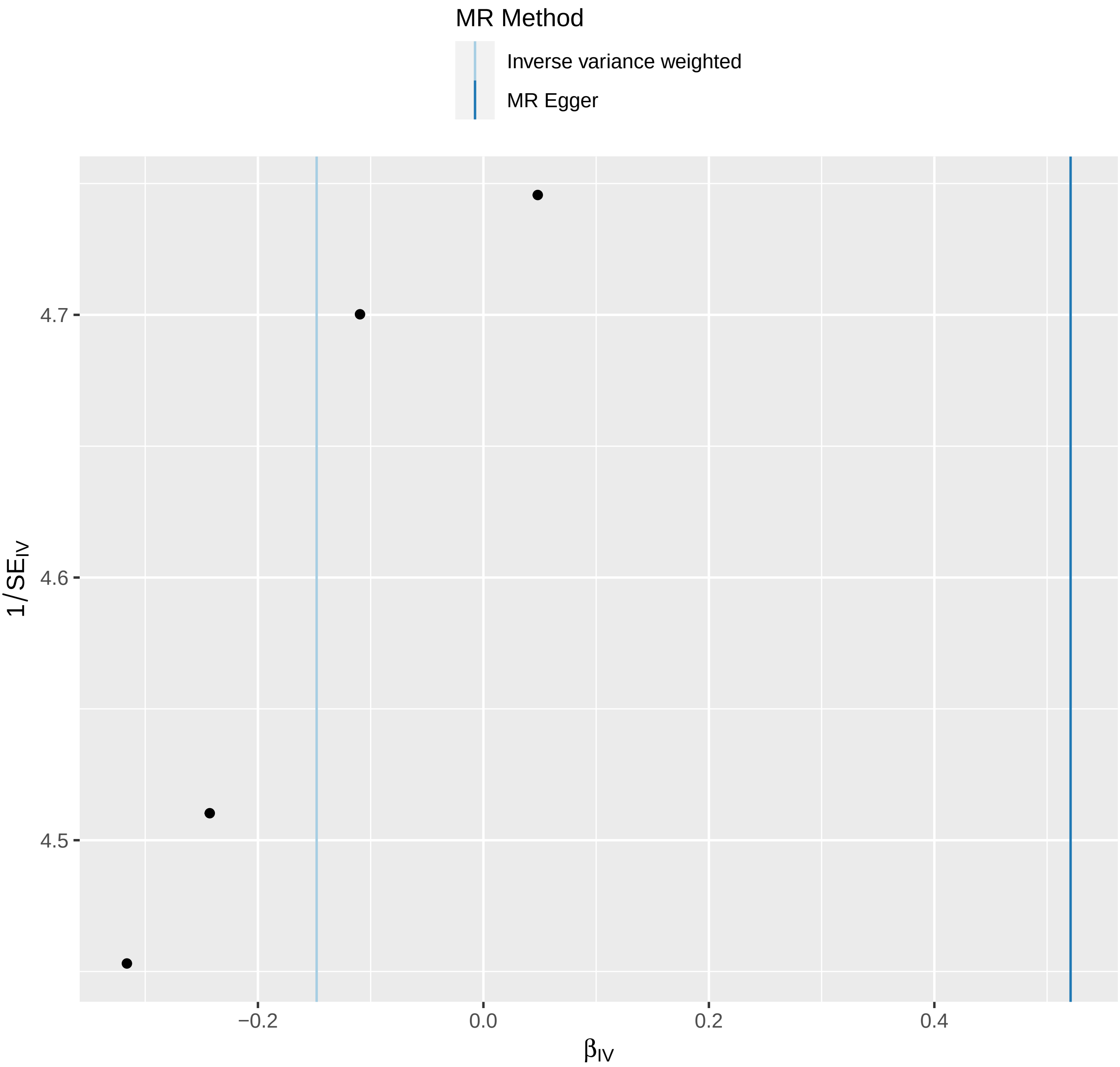

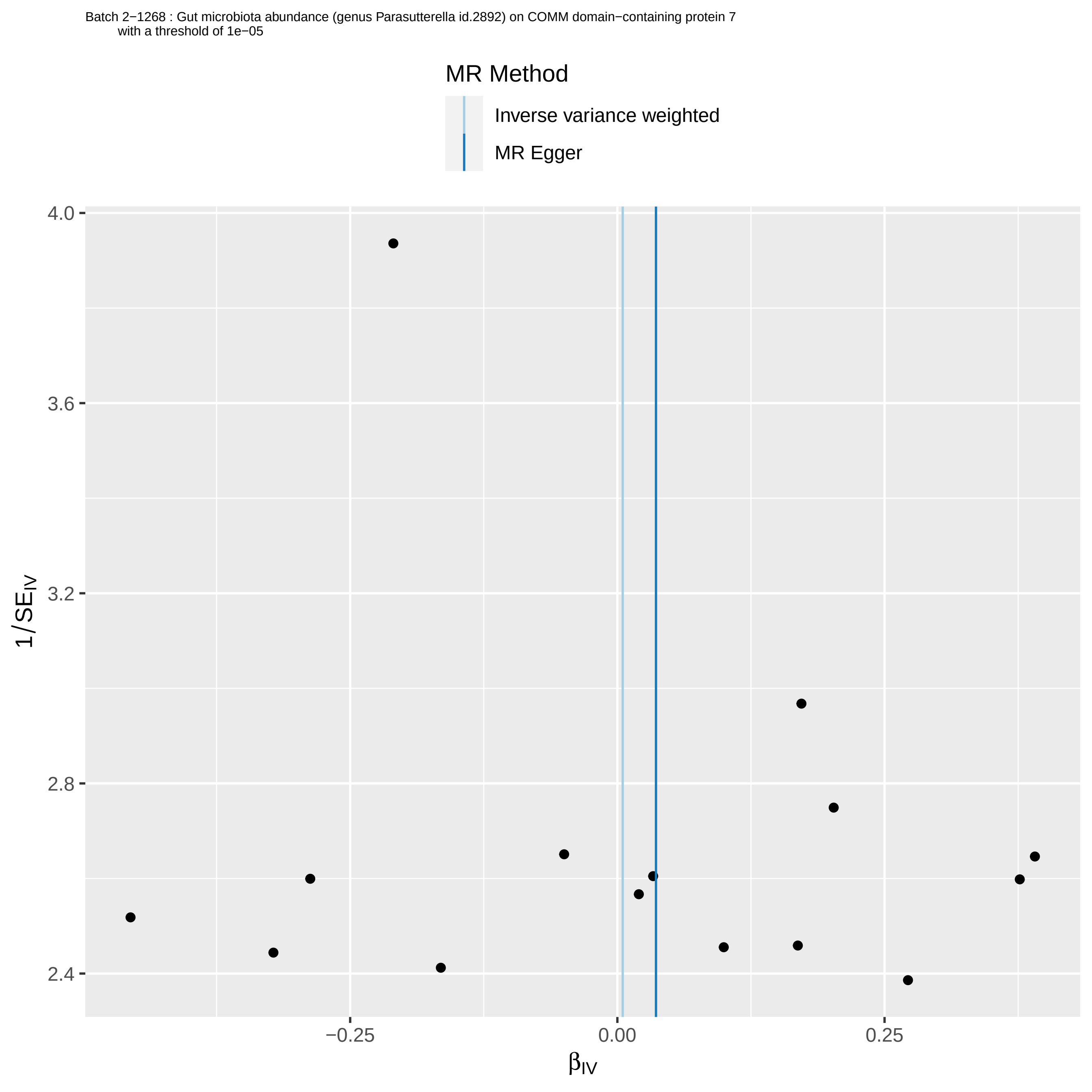
Batch 2−1268 : Gut microbiota abundance (genus Parasutterella id.2892) on COMM domain−containing protein 7
with a threshold of 1e−05
MR Method
Inverse variance weighted
MR Egger
4.0
3.6
3.2
2.8
2.4
1/SE$_{IV}$
−0.25
0.00
0.25
β$_{IV}$

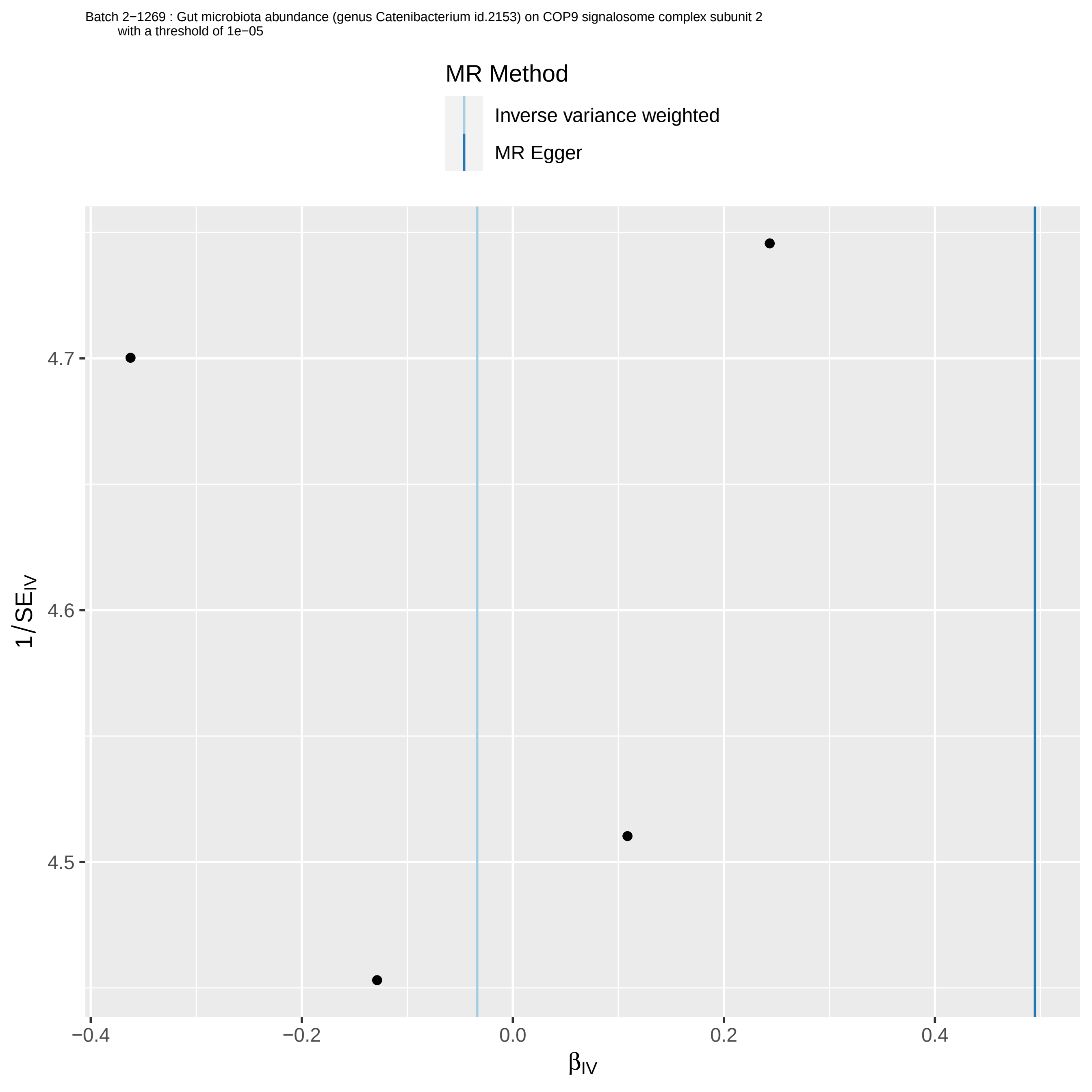

Batch 2−1269 : Gut microbiota abundance (genus Catenibacterium id.2153) on COP9 signalosome complex subunit 2
with a threshold of 1e−05
MR Method
Inverse variance weighted
MR Egger
4.7
4.6
4.5
1/SE_IV
−0.4
−0.2
0.0
0.2
0.4
β_IV

Batch 2−1270 : Gut microbiota abundance (genus Parasutterella id.2892) on COP9 signalosome complex subunit 2
with a threshold of 1e−05

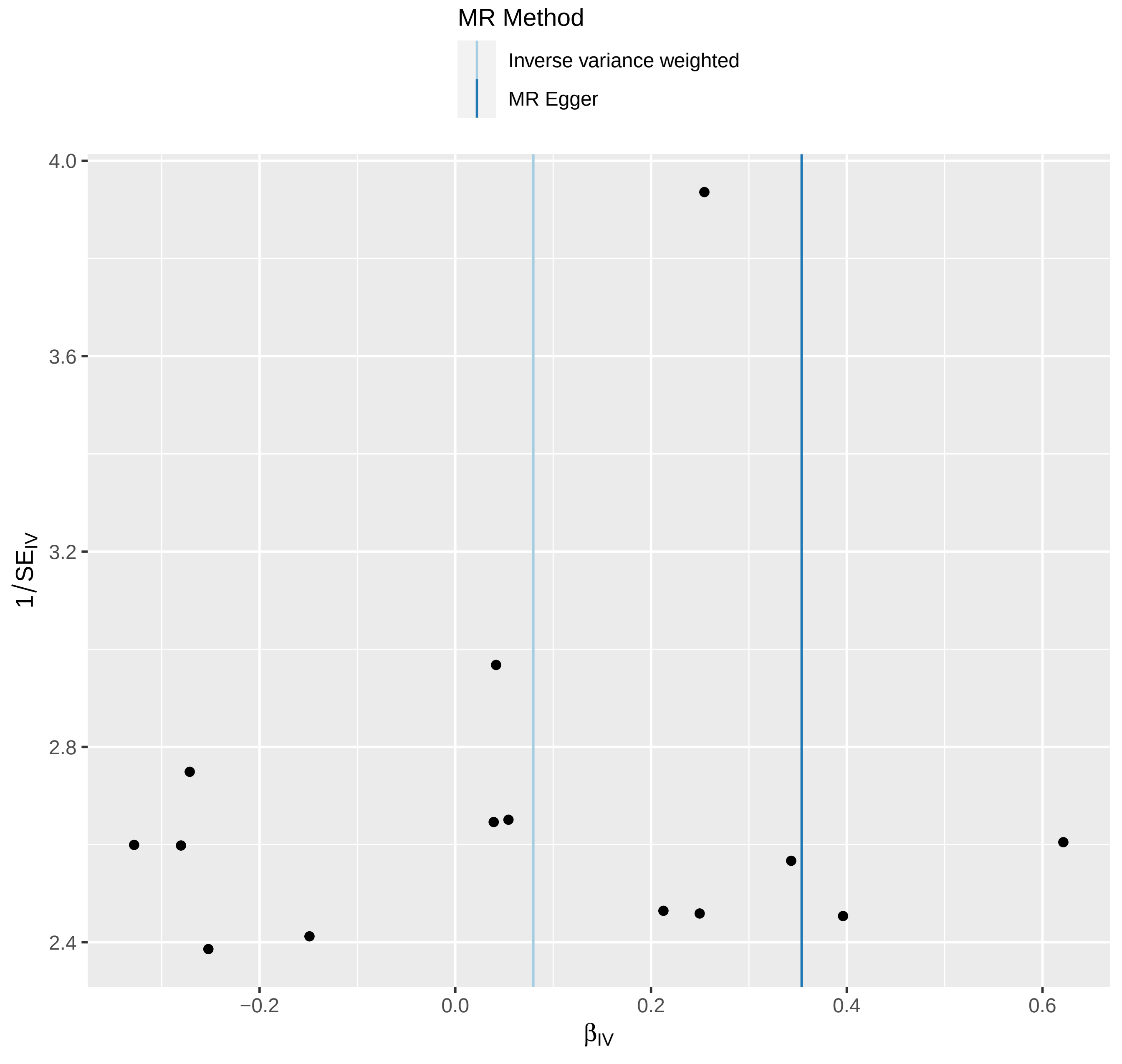

Batch 2−1271 : Gut microbiota abundance (genus Catenibacterium id.2153) on Coactosin−like protein
with a threshold of 1e−05

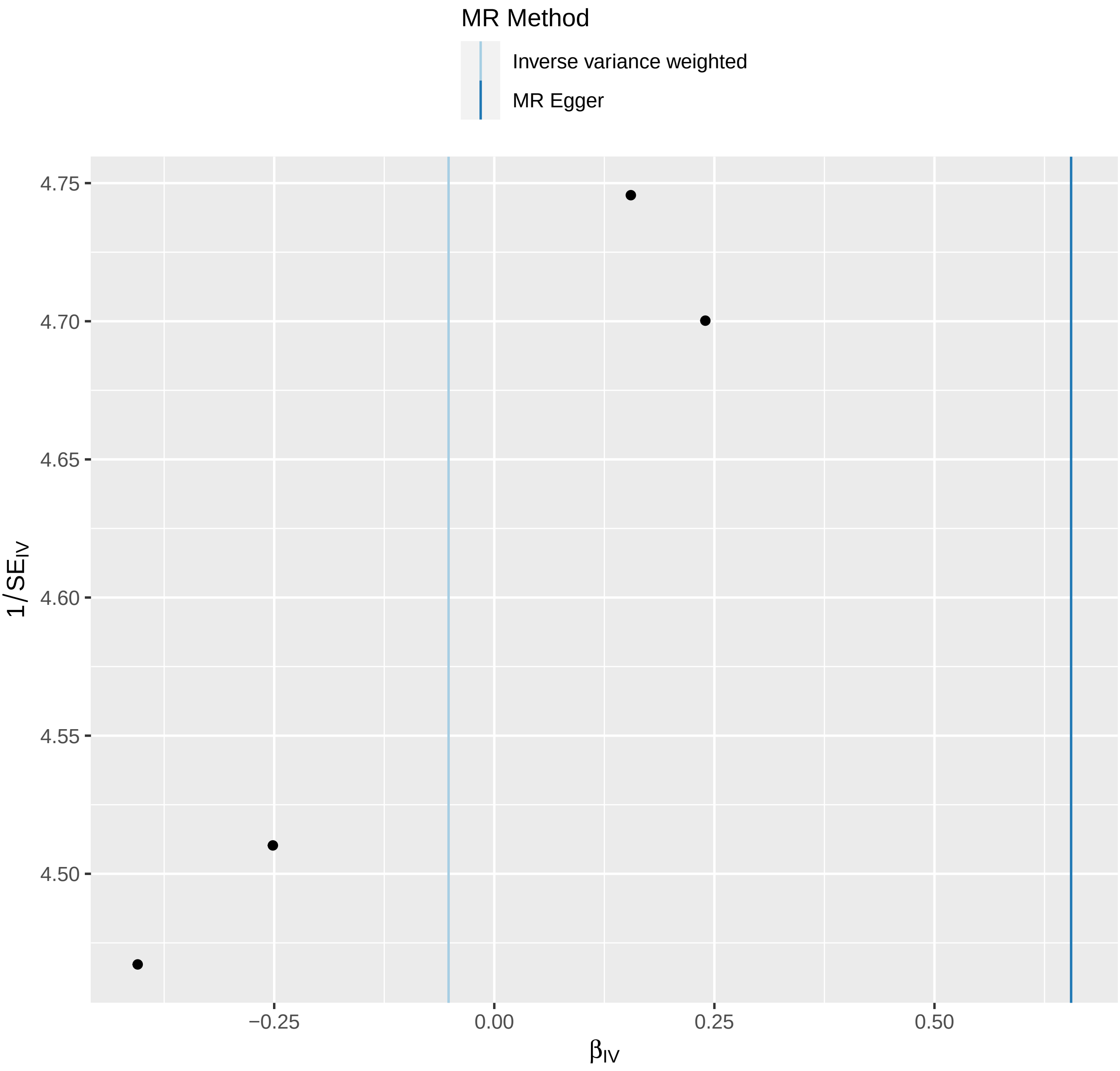

Batch 2−1272 : Gut microbiota abundance (genus Parasutterella id.2892) on Coactosin−like protein
with a threshold of 1e−05

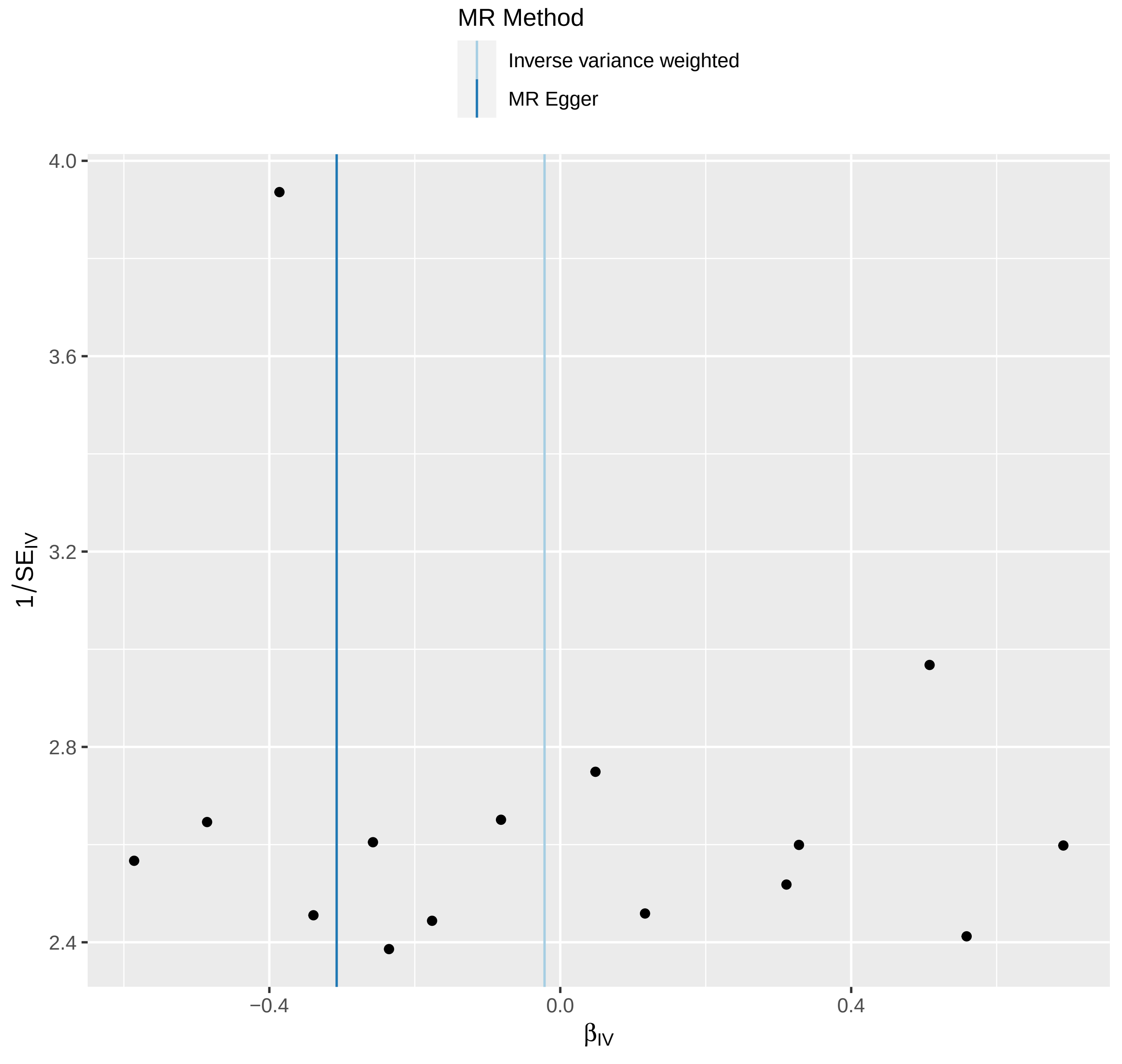

Batch 2−1273 : Gut microbiota abundance (genus Catenibacterium id.2153) on Cytochrome c oxidase subunit 4 isoform 2, mitochondrial
with a threshold of 1e−05

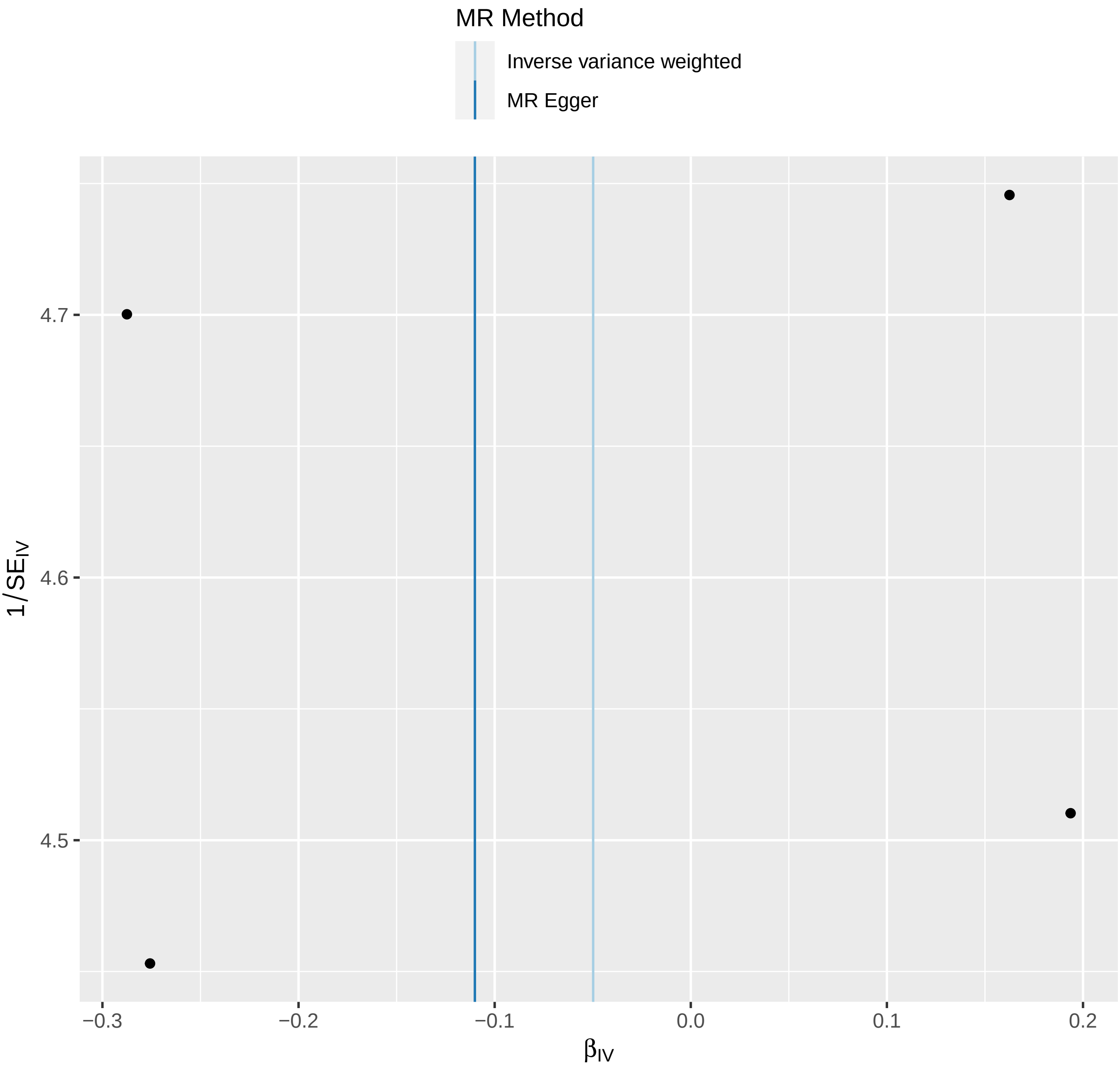

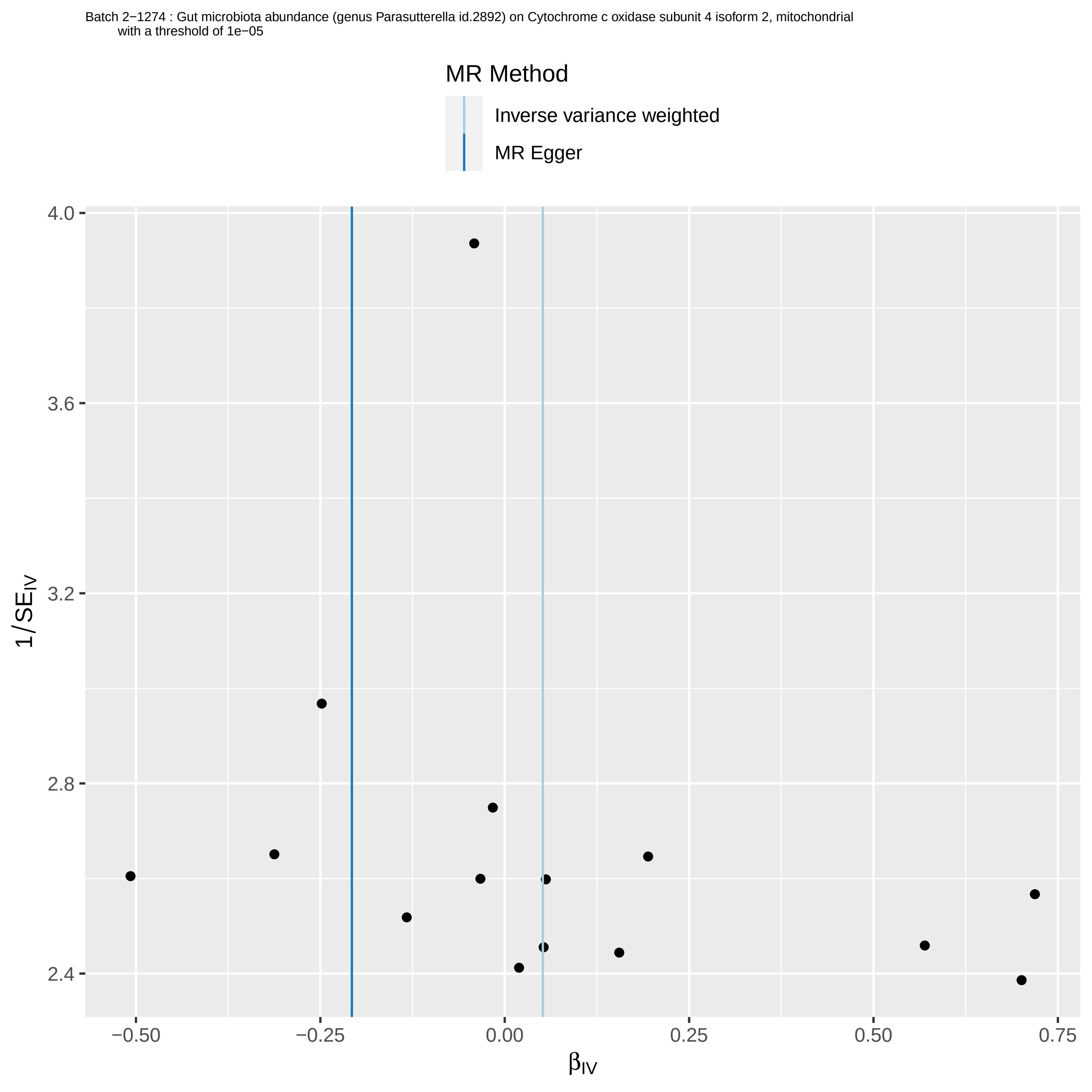
Batch 2−1274 : Gut microbiota abundance (genus Parasutterella id.2892) on Cytochrome c oxidase subunit 4 isoform 2, mitochondrial
with a threshold of 1e−05
MR Method
Inverse variance weighted
MR Egger
4.0
3.6
3.2
2.8
2.4
1/SE$_{IV}$
−0.50
−0.25
0.00
0.25
0.50
0.75
β_{IV}

Batch 2−1275 : Gut microbiota abundance (genus Catenibacterium id.2153) on Cytochrome c oxidase subunit 5B, mitochondrial
with a threshold of 1e−05

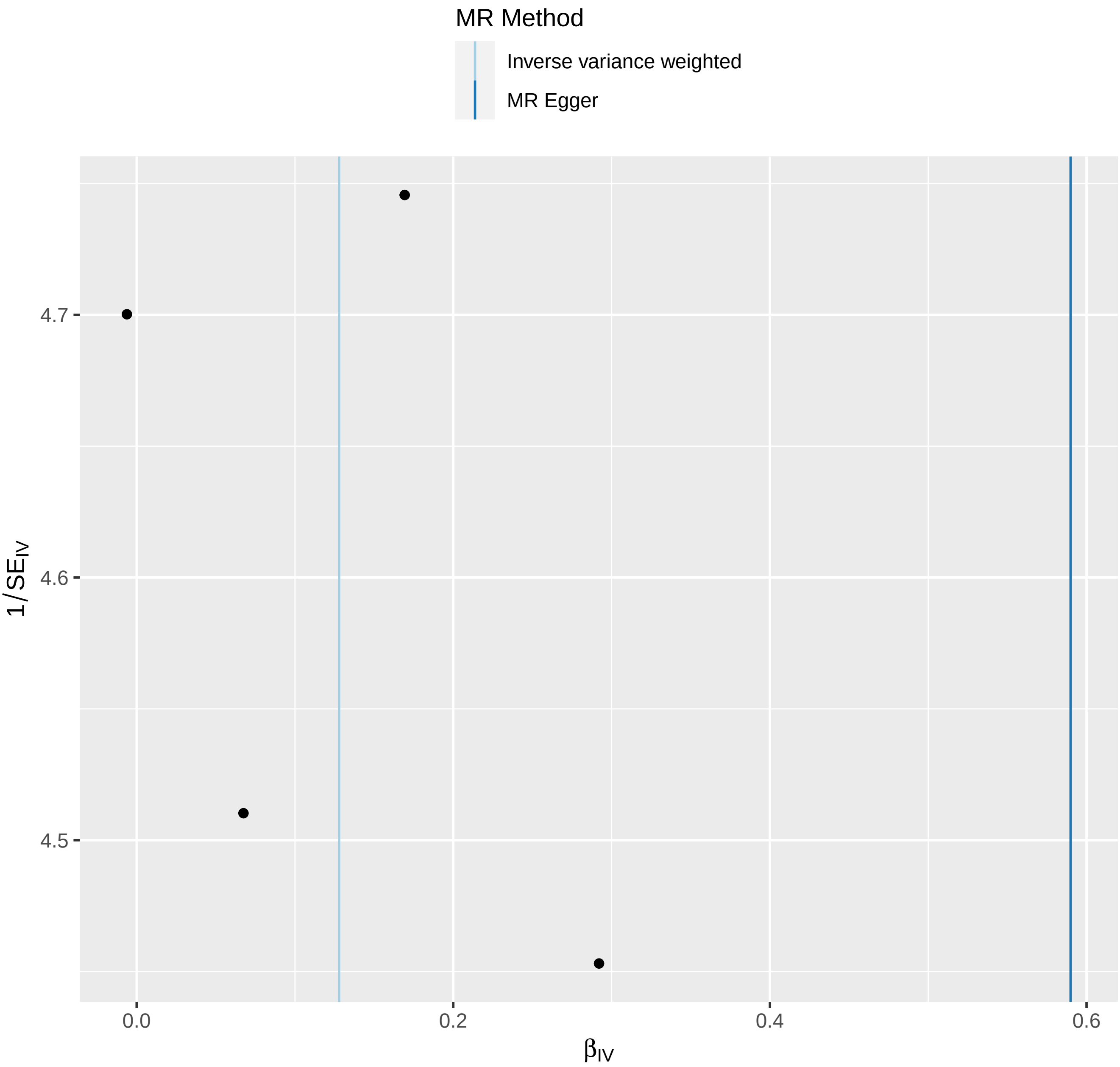

Batch 2−1276 : Gut microbiota abundance (genus Parasutterella id.2892) on Cytochrome c oxidase subunit 5B, mitochondrial
with a threshold of 1e−05

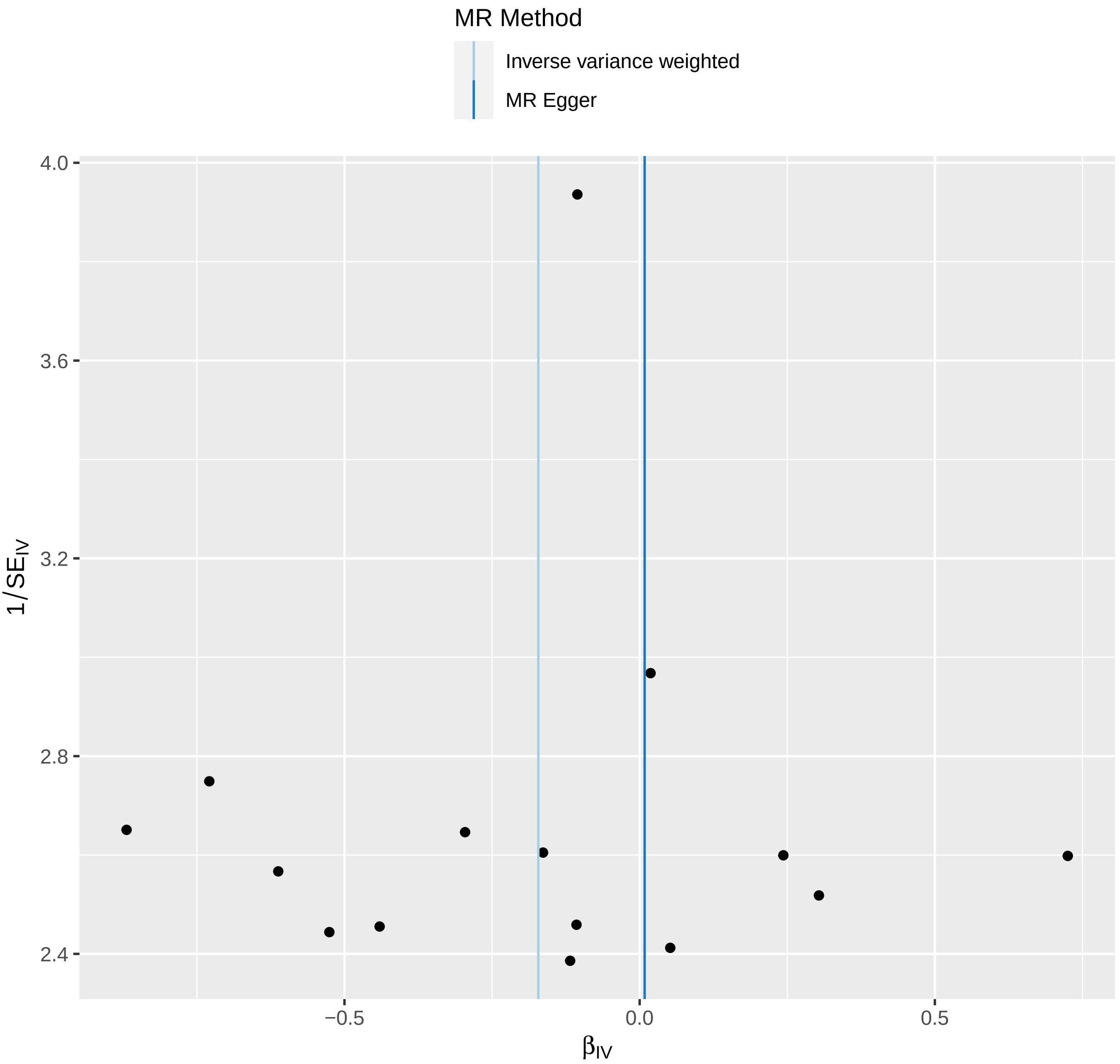

Batch 2−1277 : Gut microbiota abundance (genus Catenibacterium id.2153) on Cytochrome c oxidase subunit 6C
with a threshold of 1e−05

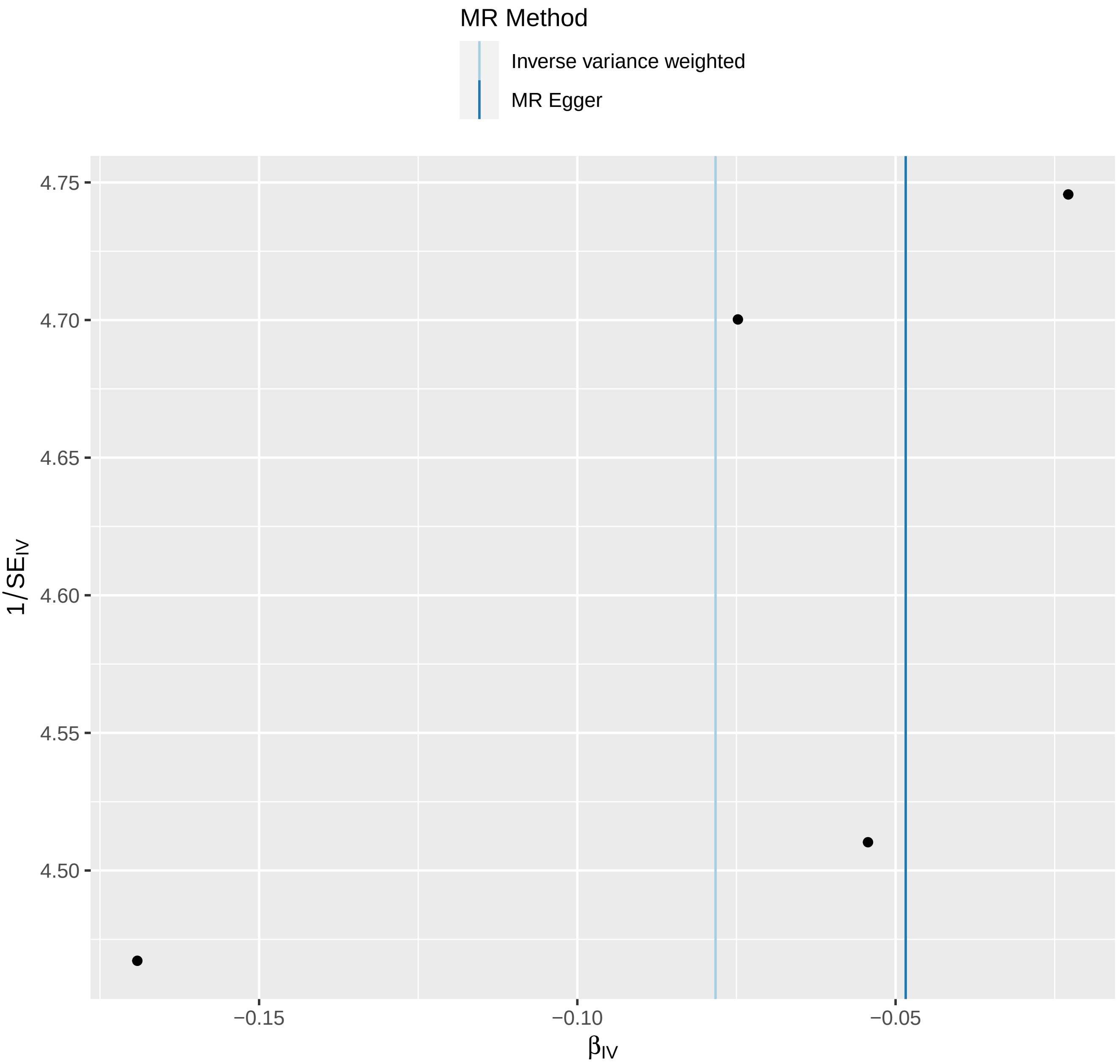

Batch 2−1278 : Gut microbiota abundance (genus Parasutterella id.2892) on Cytochrome c oxidase subunit 6C
with a threshold of 1e−05

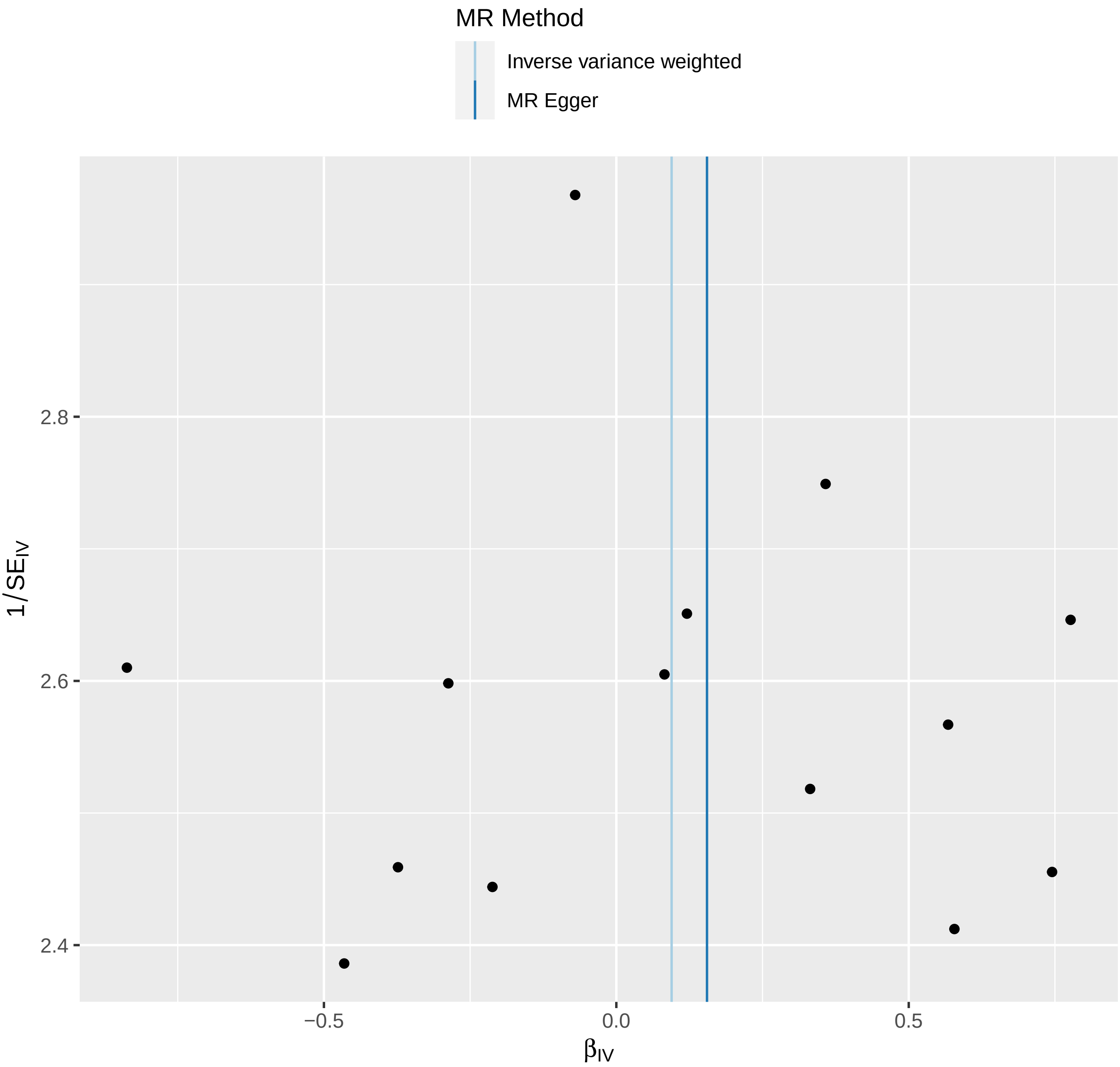

Batch 2−1279 : Gut microbiota abundance (genus Catenibacterium id.2153) on Cytochrome c oxidase subunit 7A1, mitochondrial
with a threshold of 1e−05

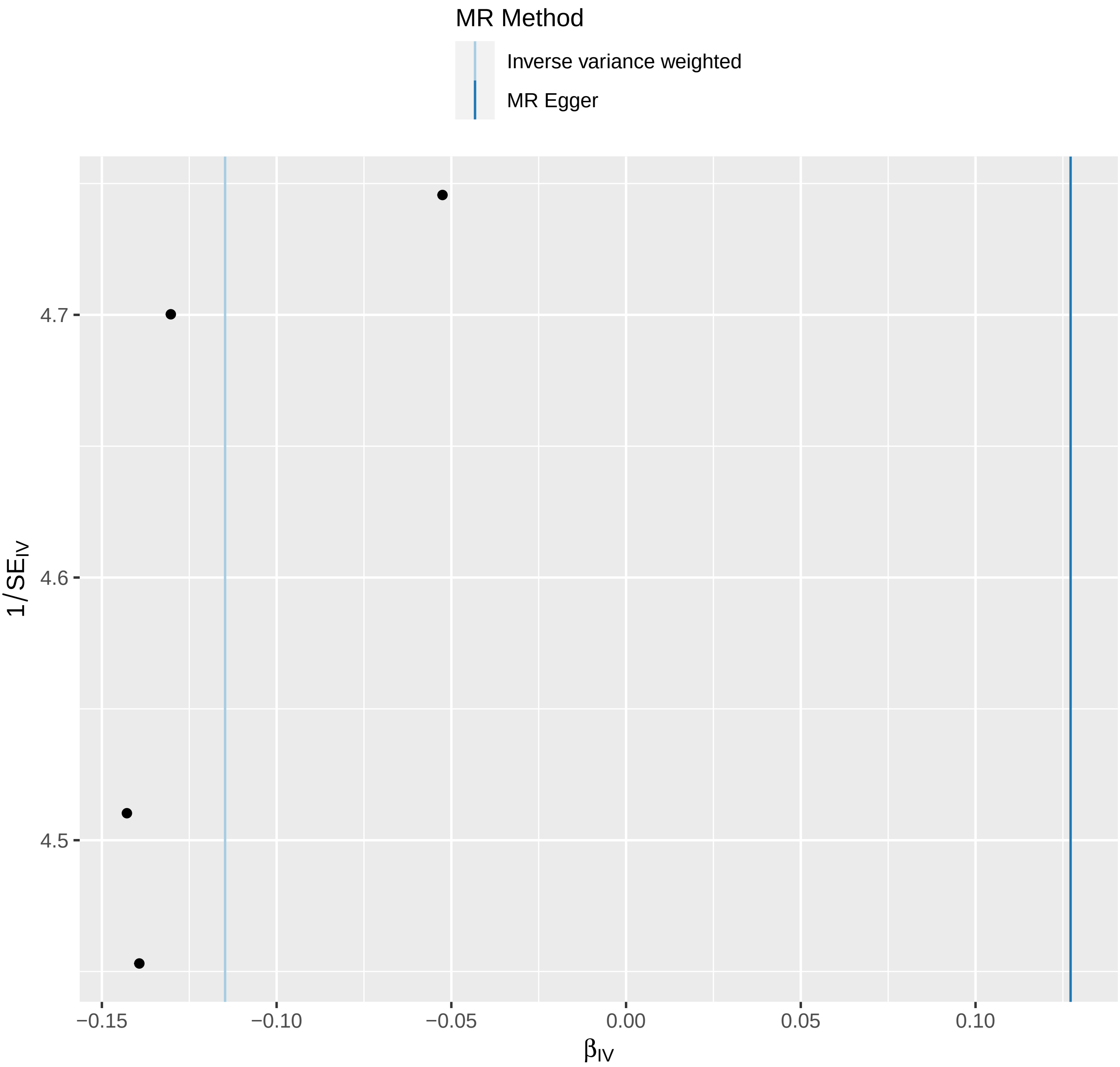

Batch 2−1280 : Gut microbiota abundance (genus Parasutterella id.2892) on Cytochrome c oxidase subunit 7A1, mitochondrial
with a threshold of 1e−05

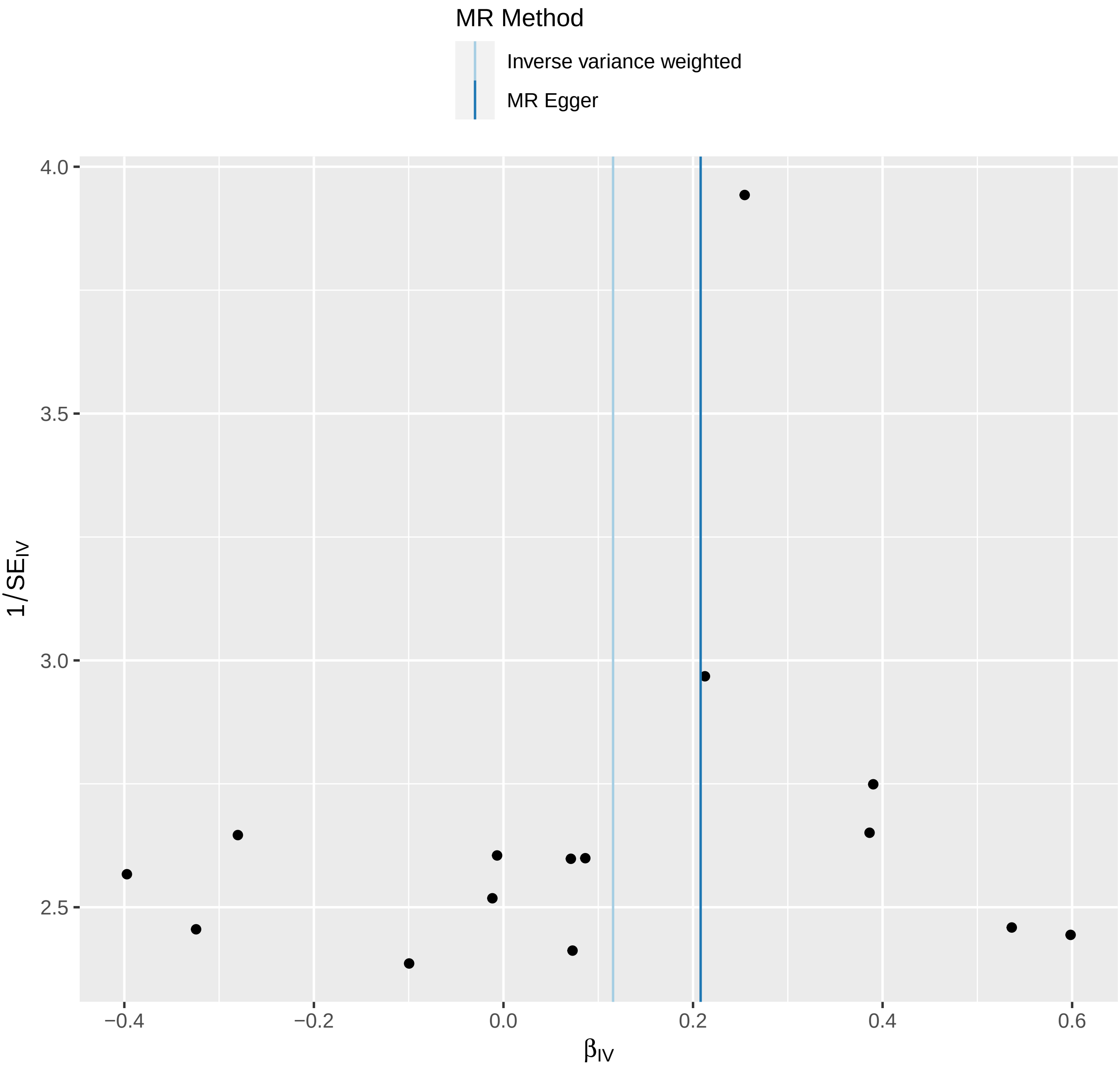

Batch 2−1281 : Gut microbiota abundance (genus Catenibacterium id.2153) on Cytochrome c oxidase subunit 8A, mitochondrial
with a threshold of 1e−05

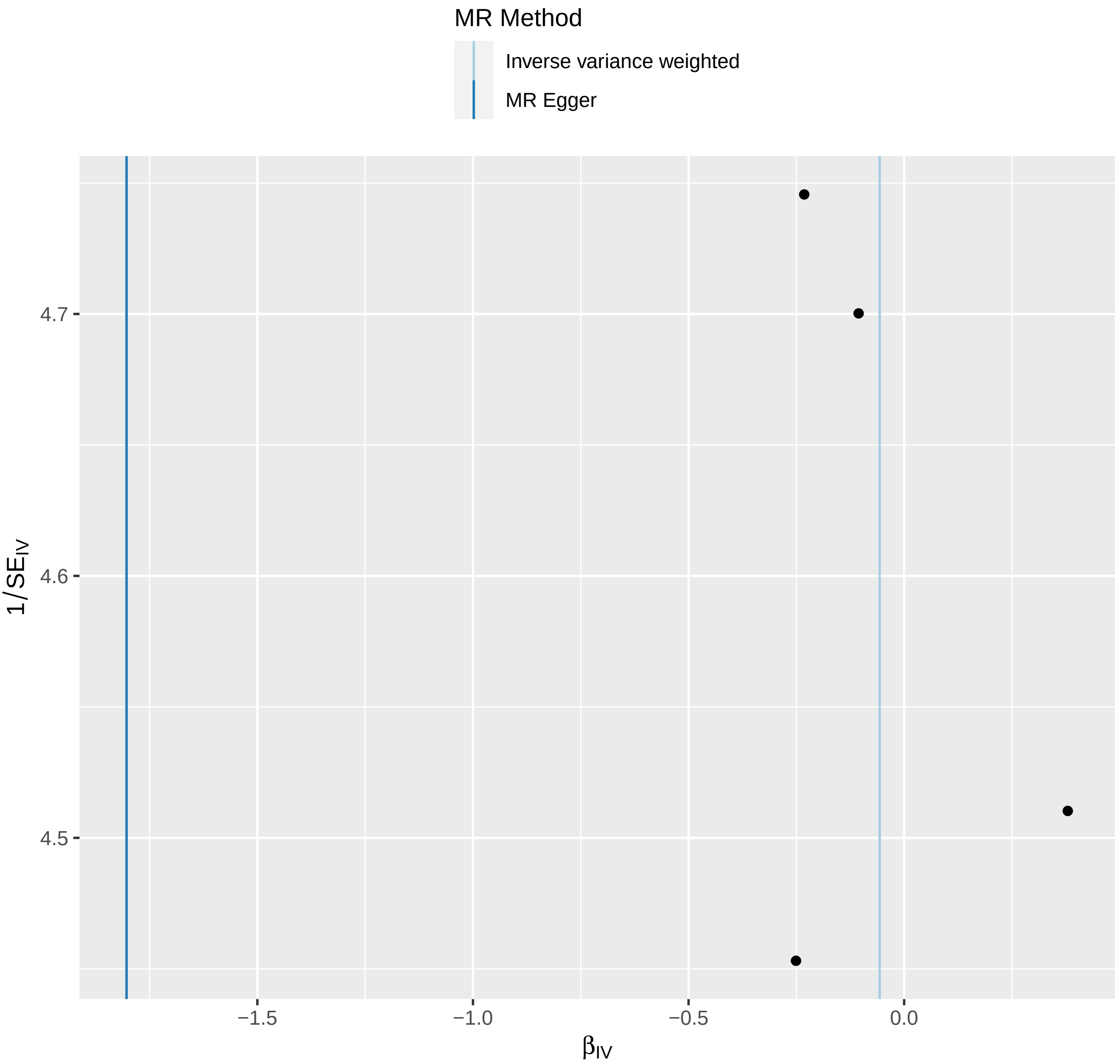

Batch 2−1282 : Gut microbiota abundance (genus Parasutterella id.2892) on Cytochrome c oxidase subunit 8A, mitochondrial
with a threshold of 1e−05

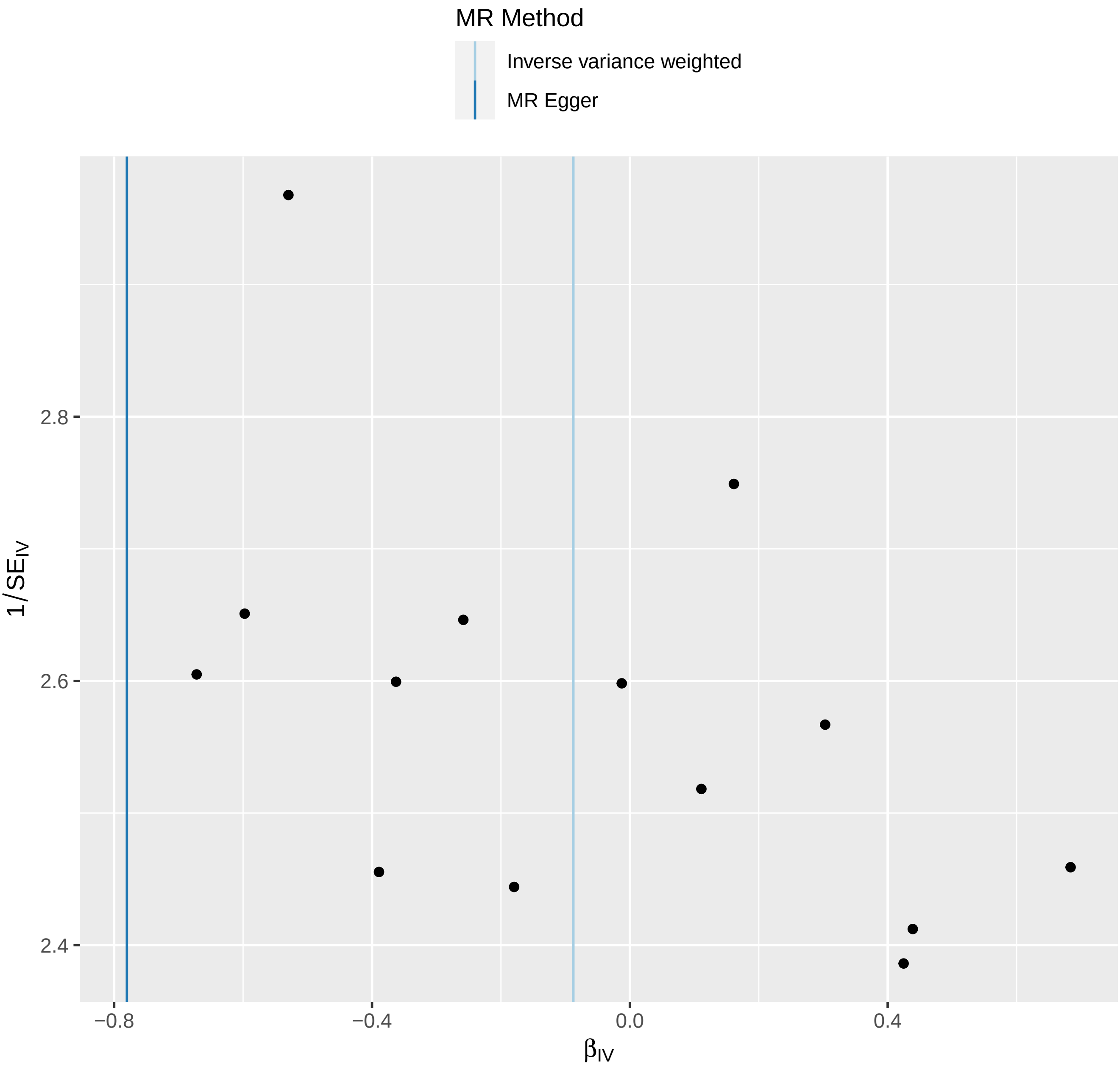

Batch 2−1283 : Gut microbiota abundance (genus Catenibacterium id.2153) on Carboxypeptidase A2
with a threshold of 1e−05

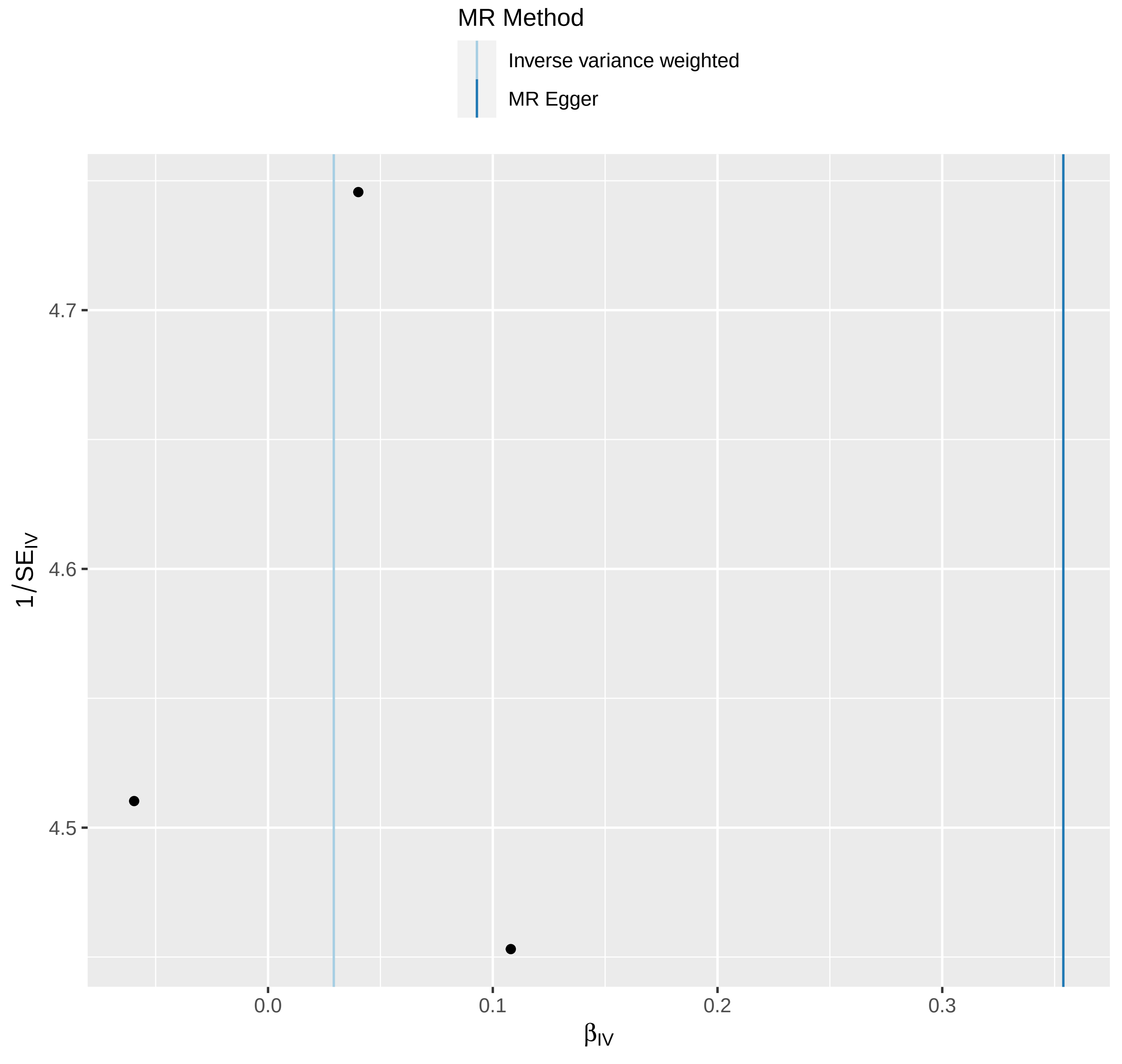

Batch 2−1284 : Gut microbiota abundance (genus Parasutterella id.2892) on Carboxypeptidase A2
with a threshold of 1e−05

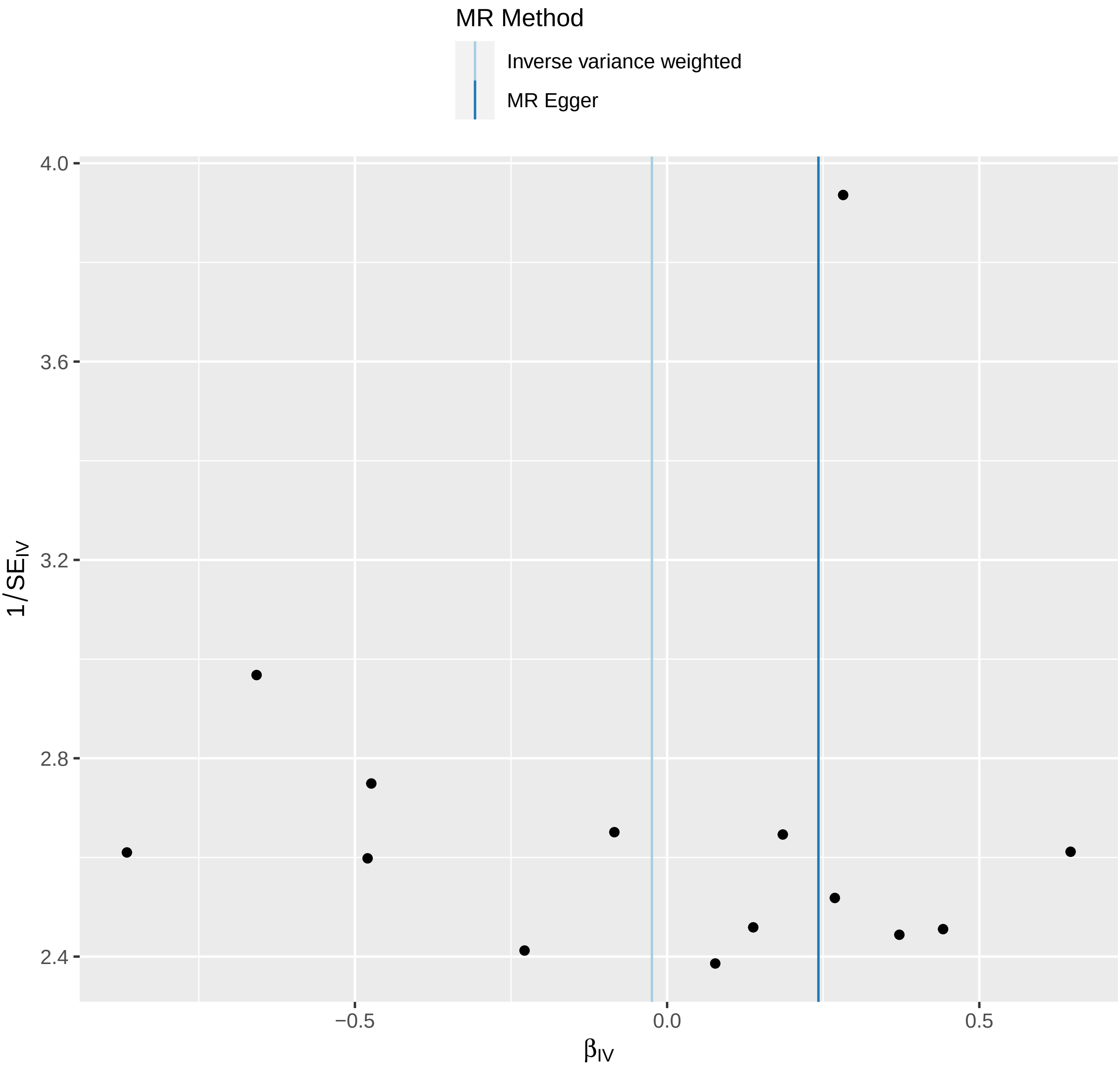

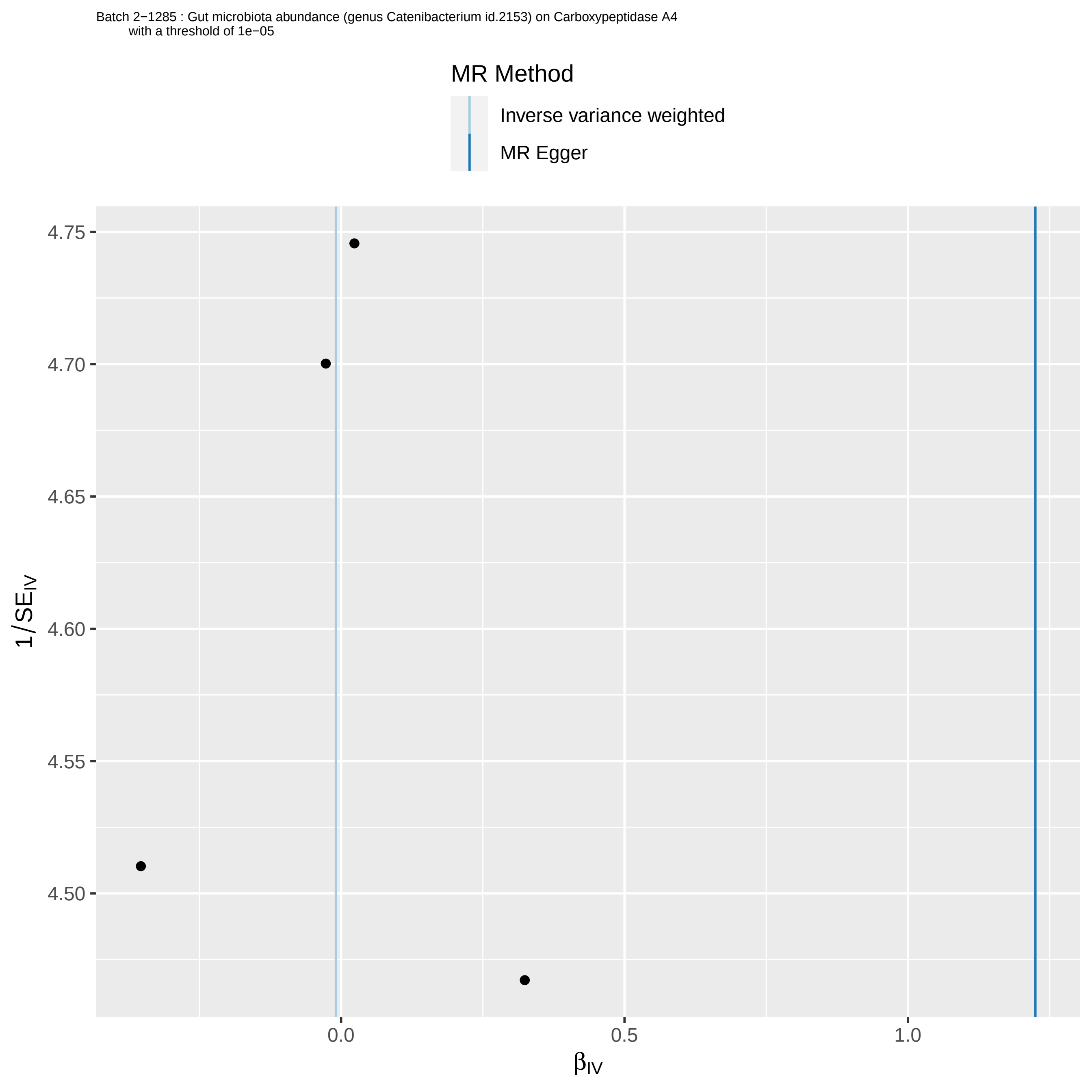

Batch 2−1285 : Gut microbiota abundance (genus Catenibacterium id.2153) on Carboxypeptidase A4
with a threshold of 1e−05
MR Method
Inverse variance weighted
MR Egger
4.75
4.70
4.65
4.60
4.55
4.50
1/SE_IV
0.0
0.5
1.0
β_IV

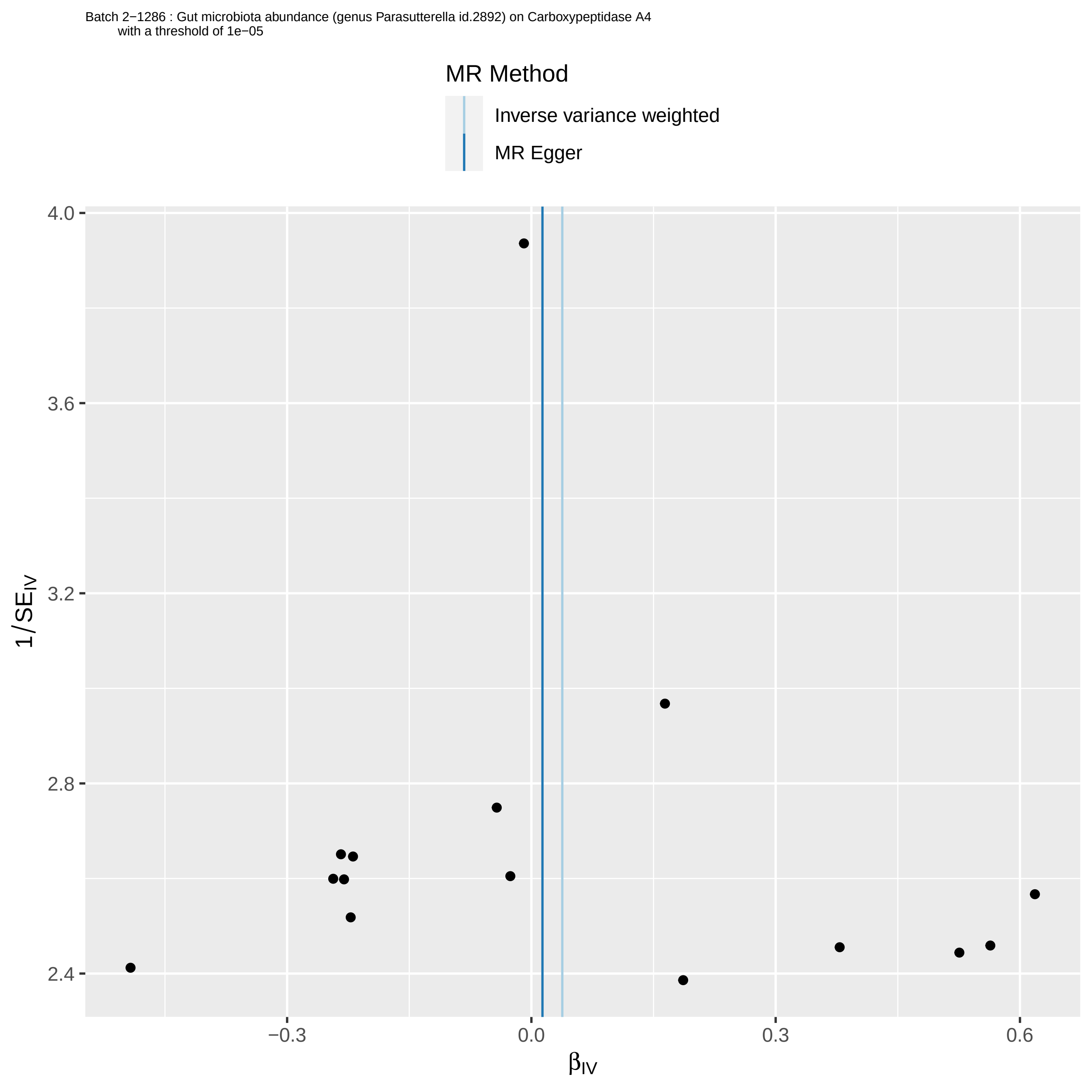

Batch 2−1286 : Gut microbiota abundance (genus Parasutterella id.2892) on Carboxypeptidase A4
with a threshold of 1e−05
MR Method
Inverse variance weighted
MR Egger
4.0
3.6
3.2
2.8
2.4
−0.3
0.0
0.3
0.6
$1/SE_{IV}$
β_{IV}

Batch 2−1287 : Gut microbiota abundance (genus Catenibacterium id.2153) on Carboxypeptidase B
with a threshold of 1e−05

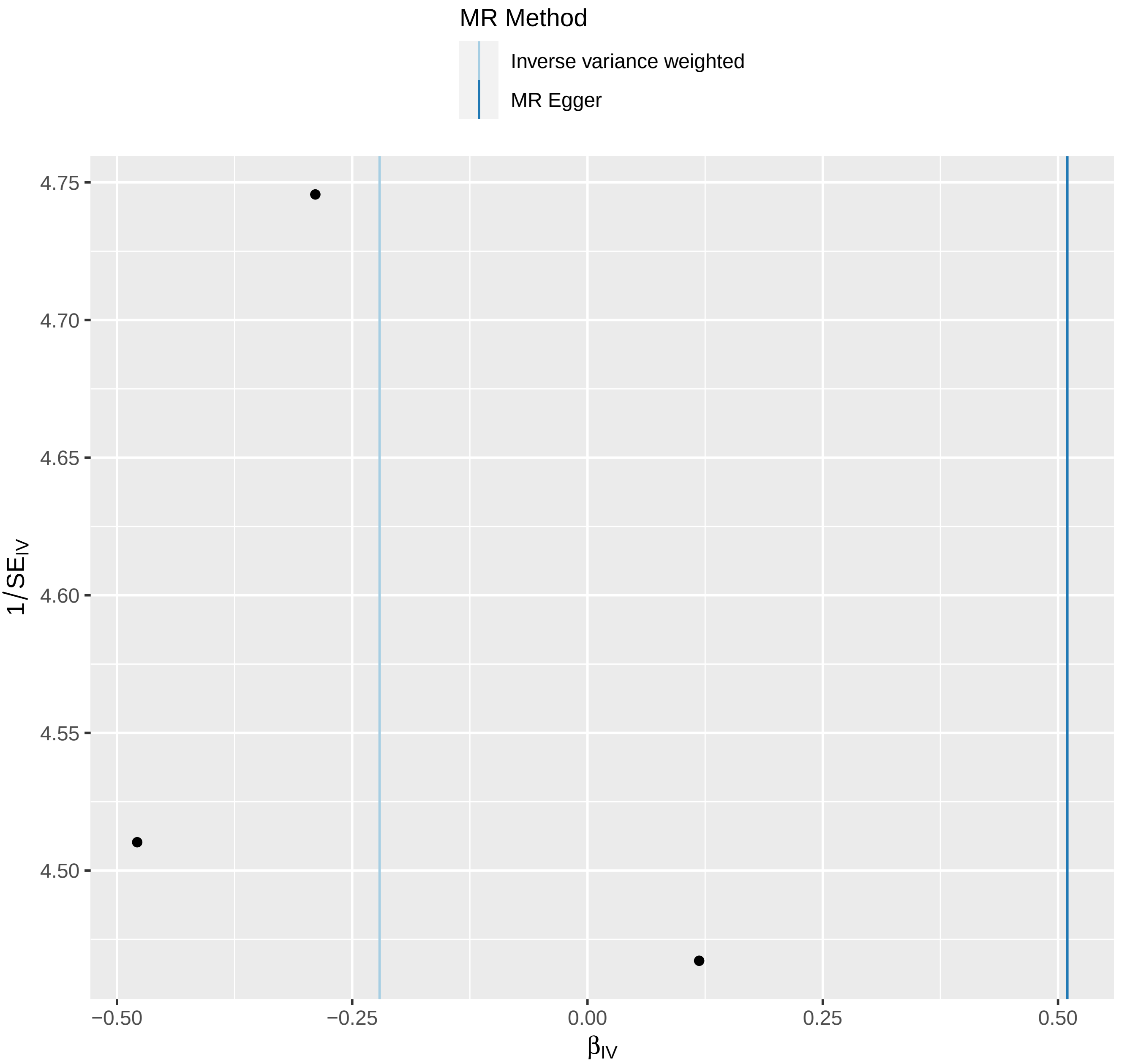

Batch 2−1288 : Gut microbiota abundance (genus Parasutterella id.2892) on Carboxypeptidase B
with a threshold of 1e−05

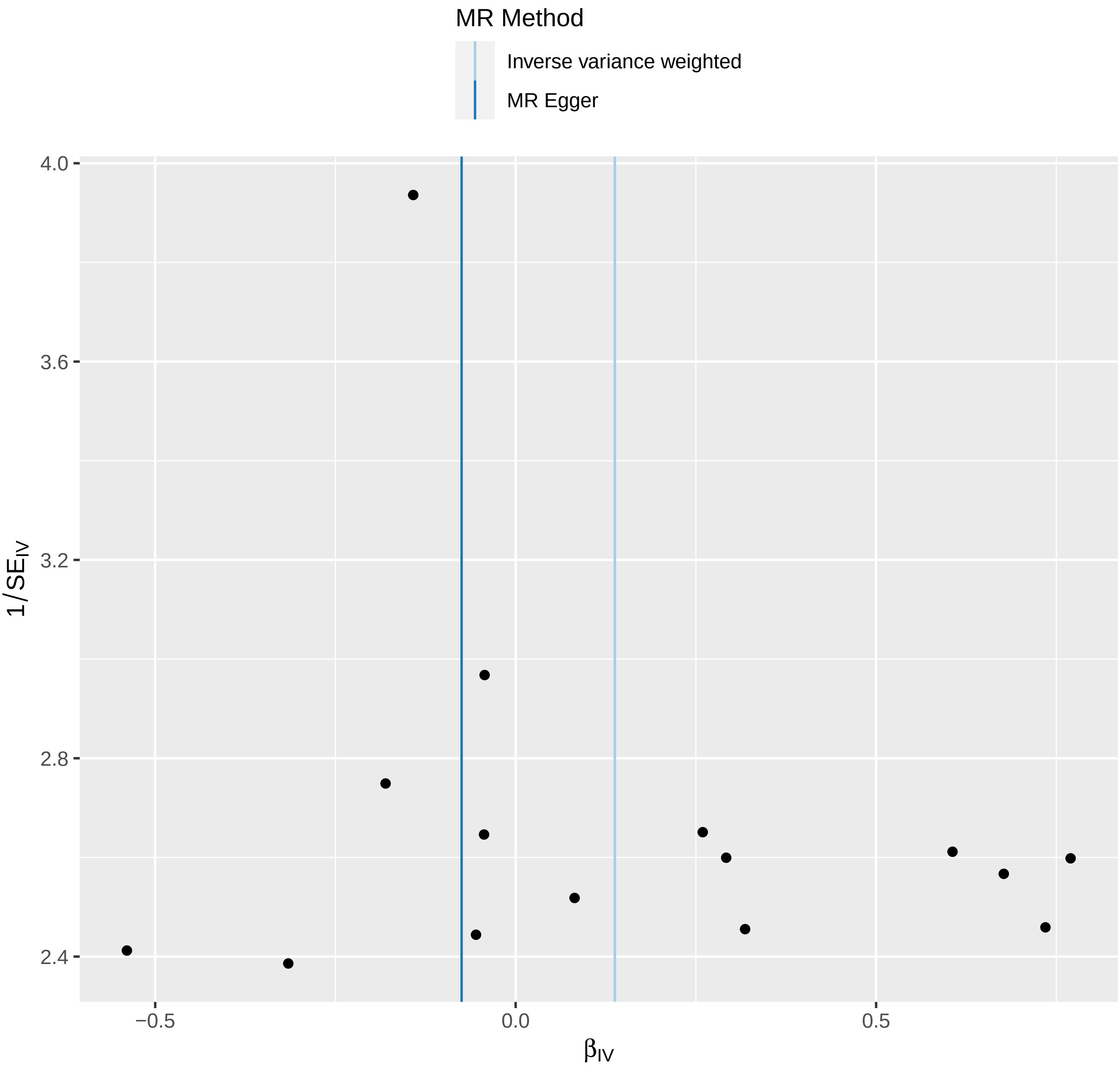

Batch 2−1289 : Gut microbiota abundance (genus Catenibacterium id.2153) on Carboxypeptidase M
with a threshold of 1e−05

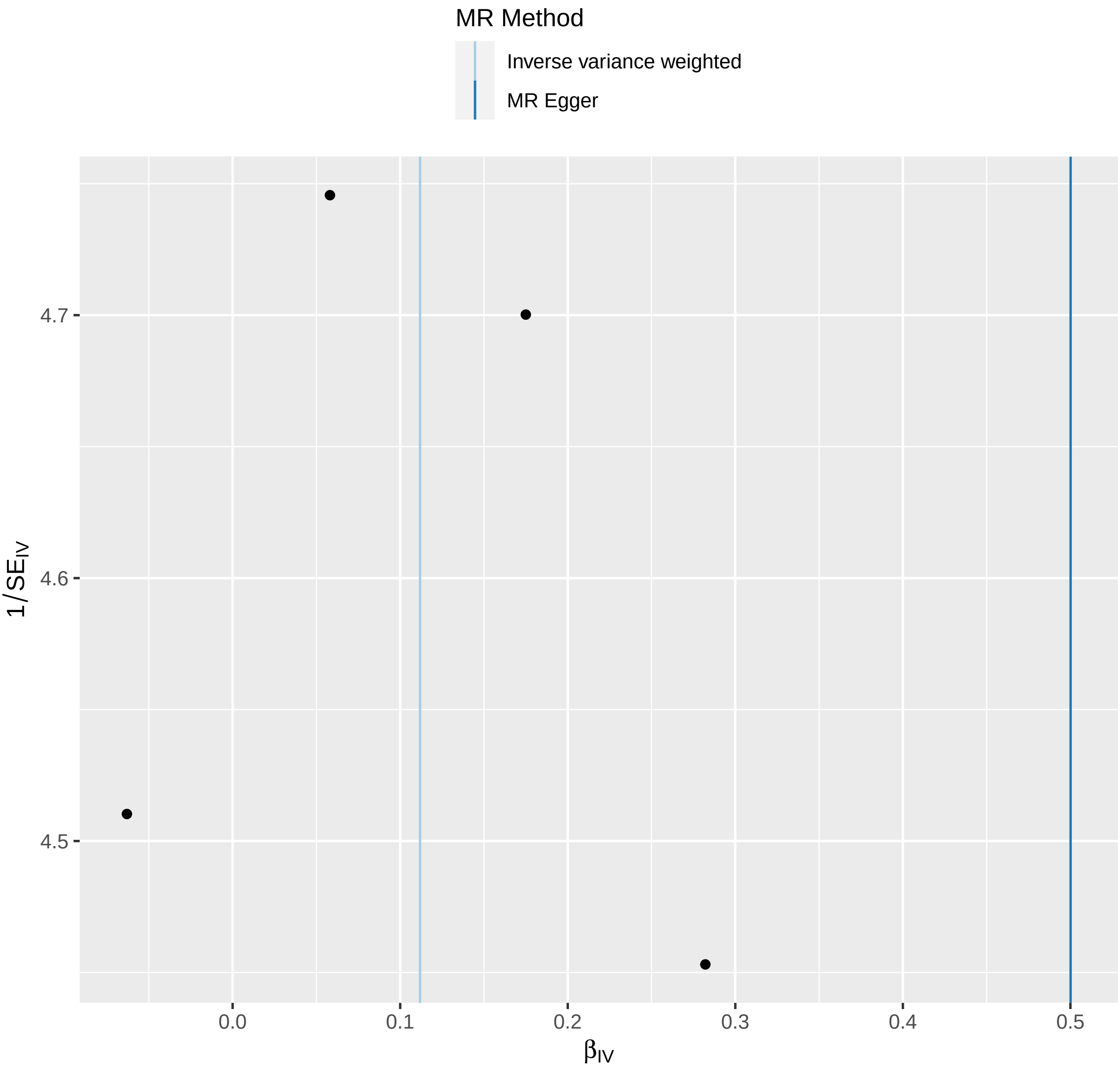

Batch 2−1290 : Gut microbiota abundance (genus Parasutterella id.2892) on Carboxypeptidase M
with a threshold of 1e−05

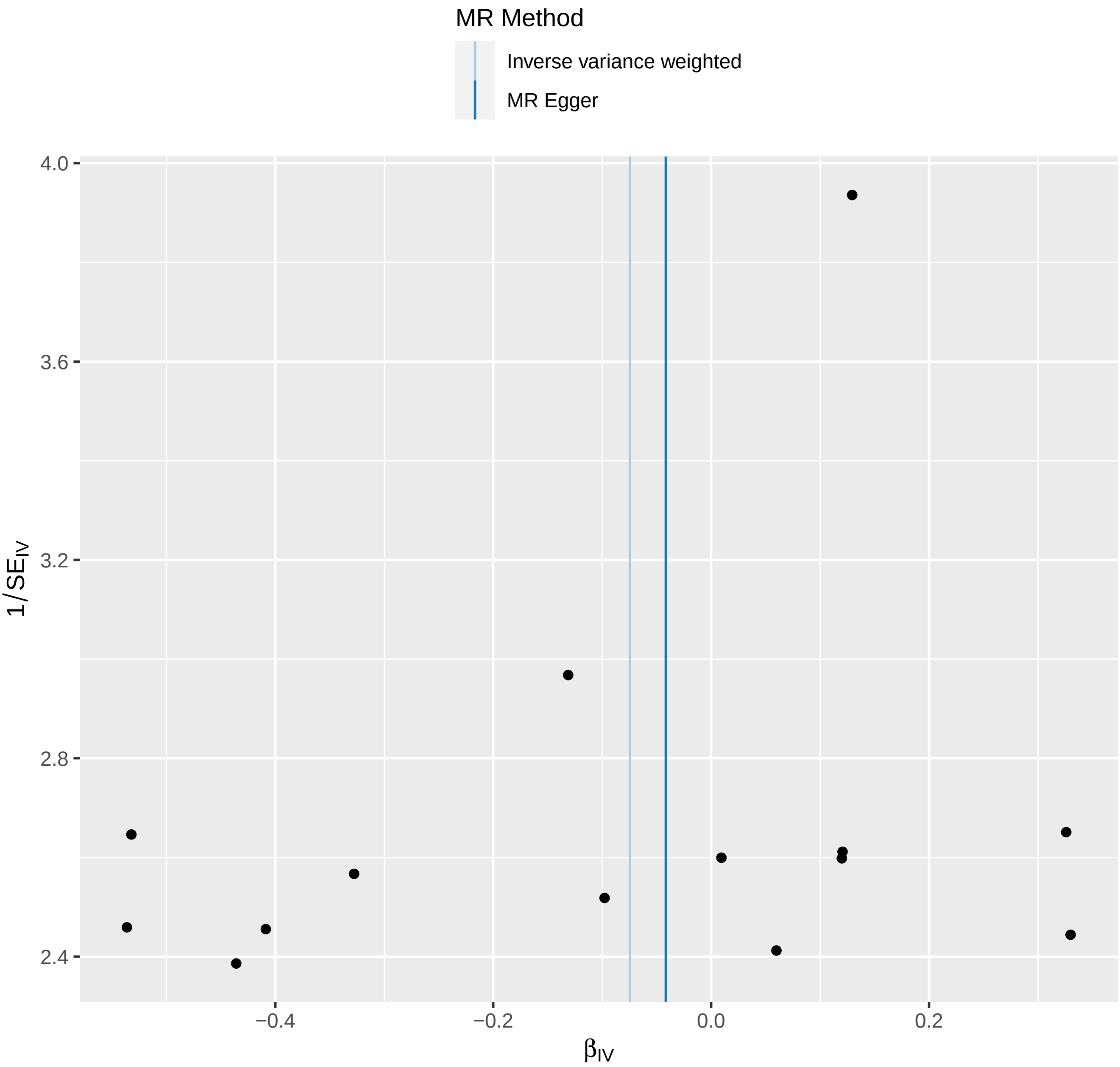

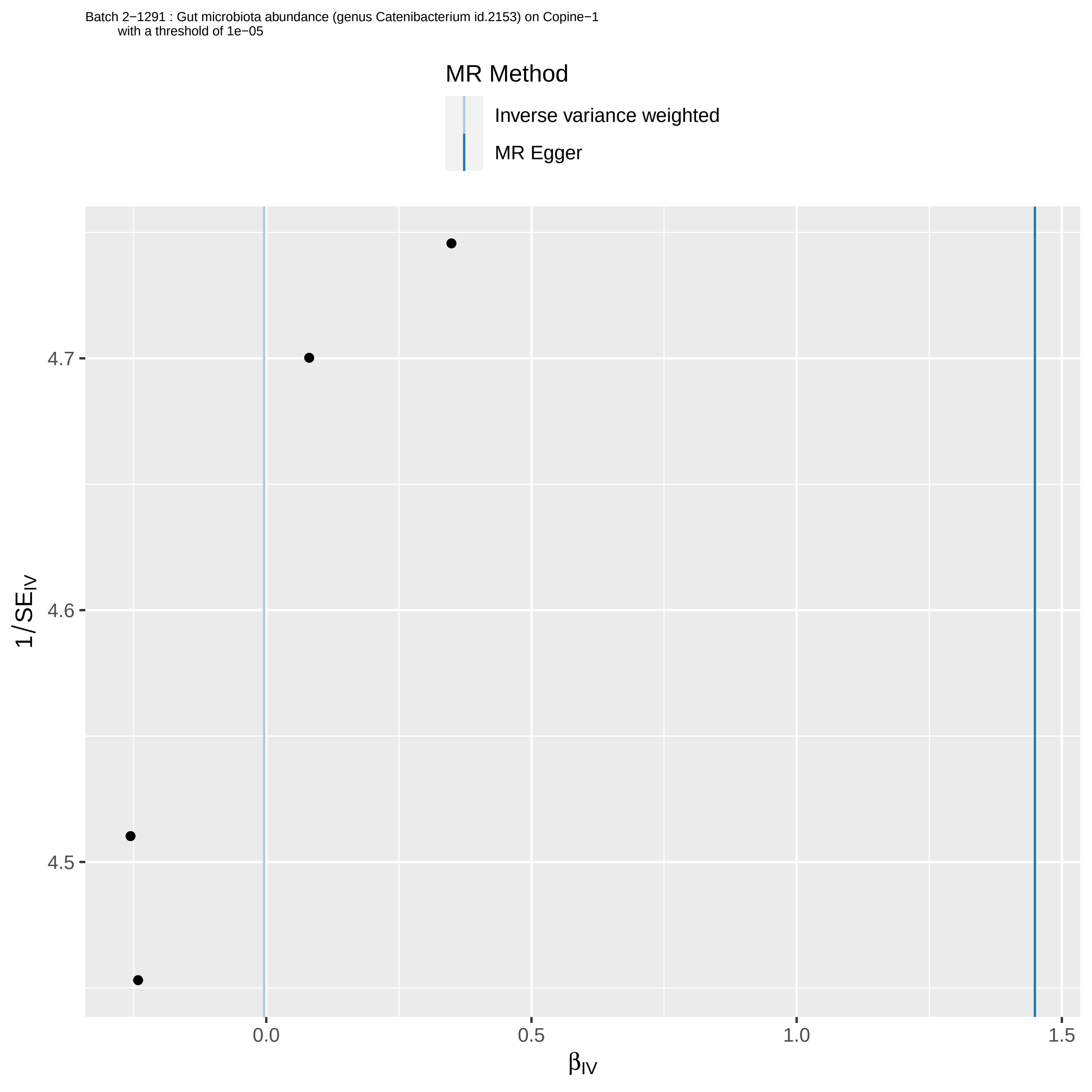

Batch 2−1291 : Gut microbiota abundance (genus Catenibacterium id.2153) on Copine−1
with a threshold of 1e−05
MR Method
Inverse variance weighted
MR Egger
4.7
4.6
4.5
1/SE$_{IV}$
0.0
0.5
1.0
1.5
β$_{IV}$

Batch 2−1292 : Gut microbiota abundance (genus Parasutterella id.2892) on Copine−1
with a threshold of 1e−05

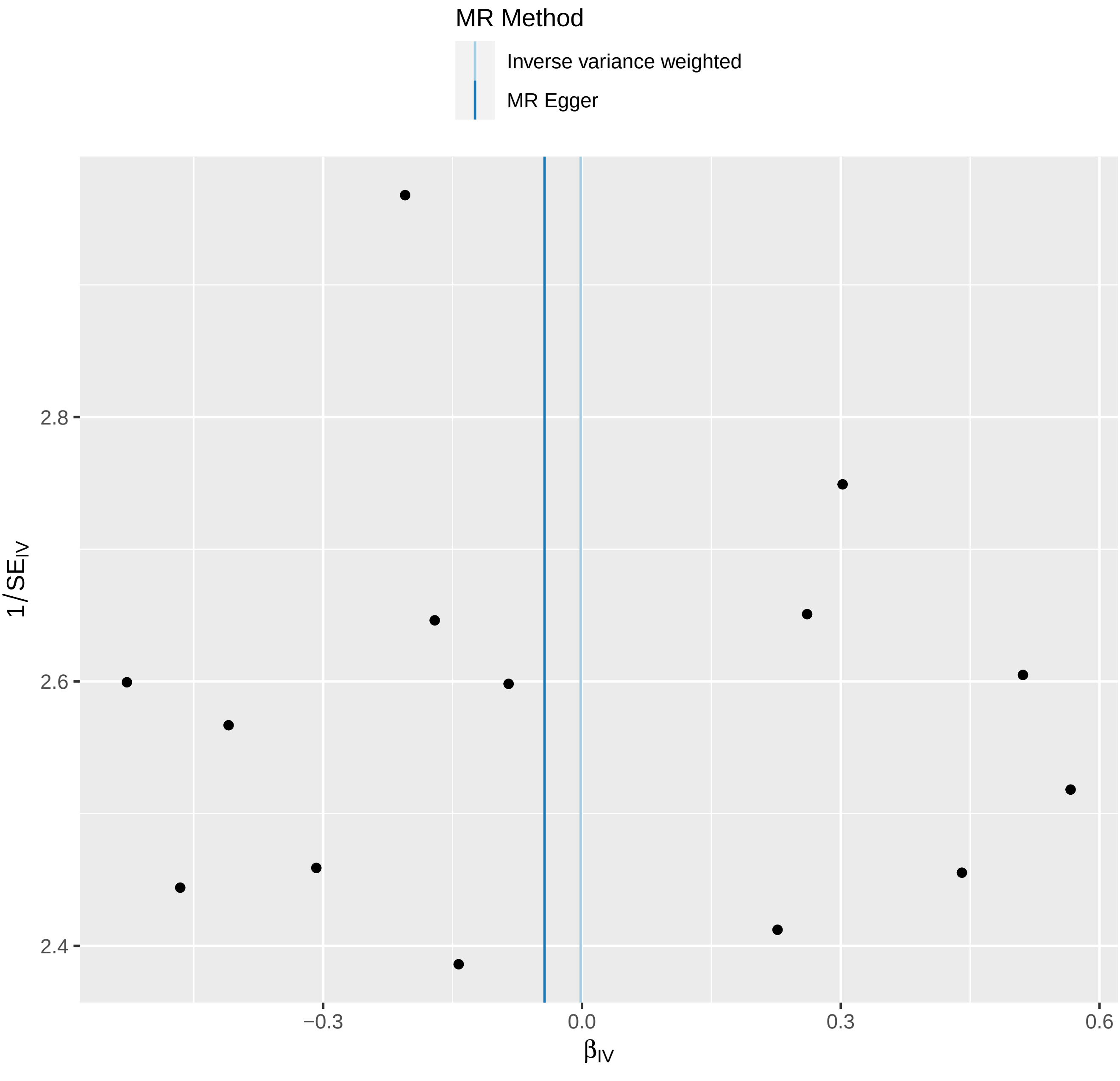

Batch 2−1293 : Gut microbiota abundance (genus Catenibacterium id.2153) on Probable serine carboxypeptidase CPVL
with a threshold of 1e−05

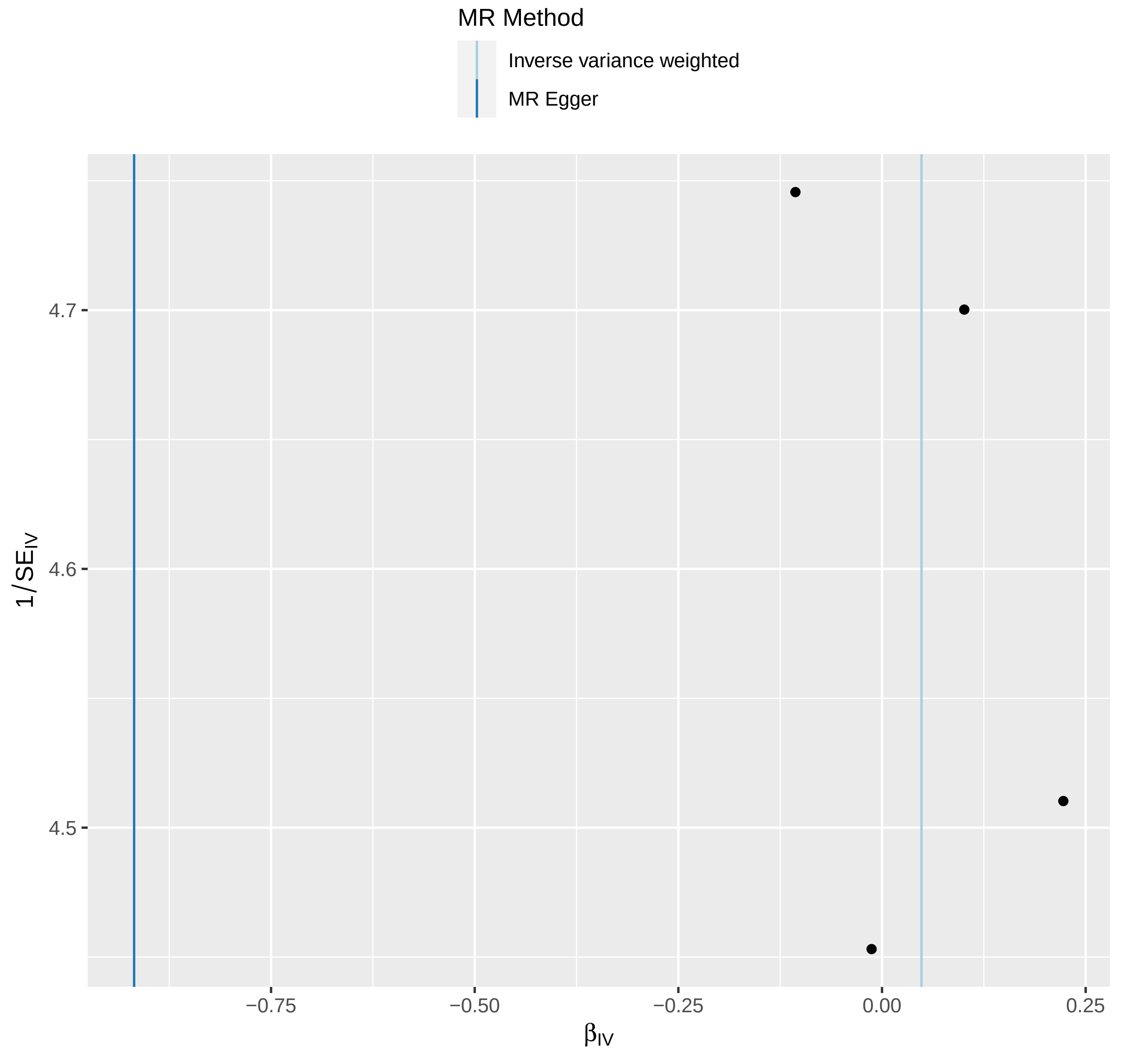

Batch 2−1294 : Gut microbiota abundance (genus Parasutterella id.2892) on Probable serine carboxypeptidase CPVL
with a threshold of 1e−05

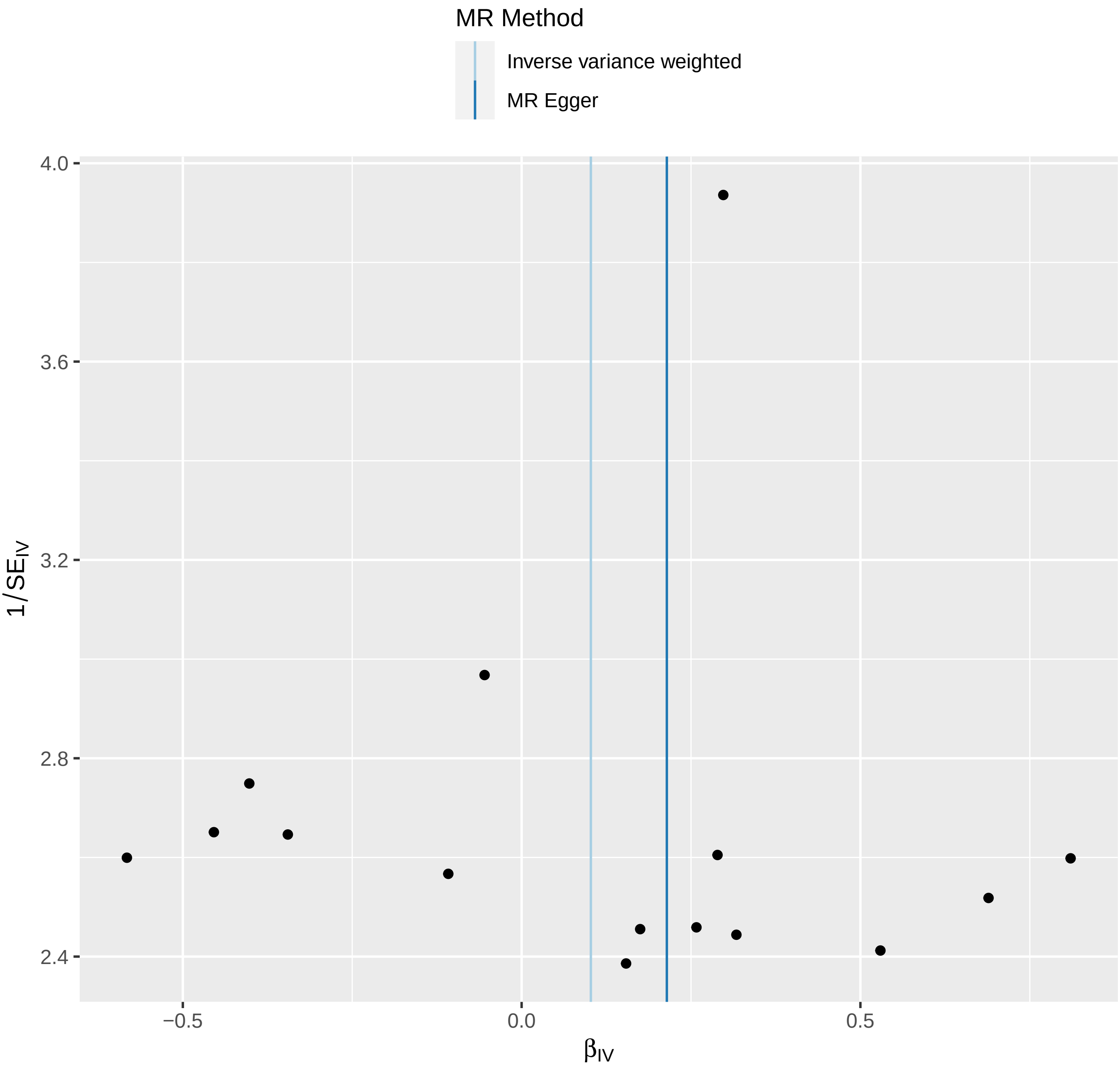

Batch 2−1295 : Gut microbiota abundance (genus Catenibacterium id.2153) on Probable carboxypeptidase X1
with a threshold of 1e−05

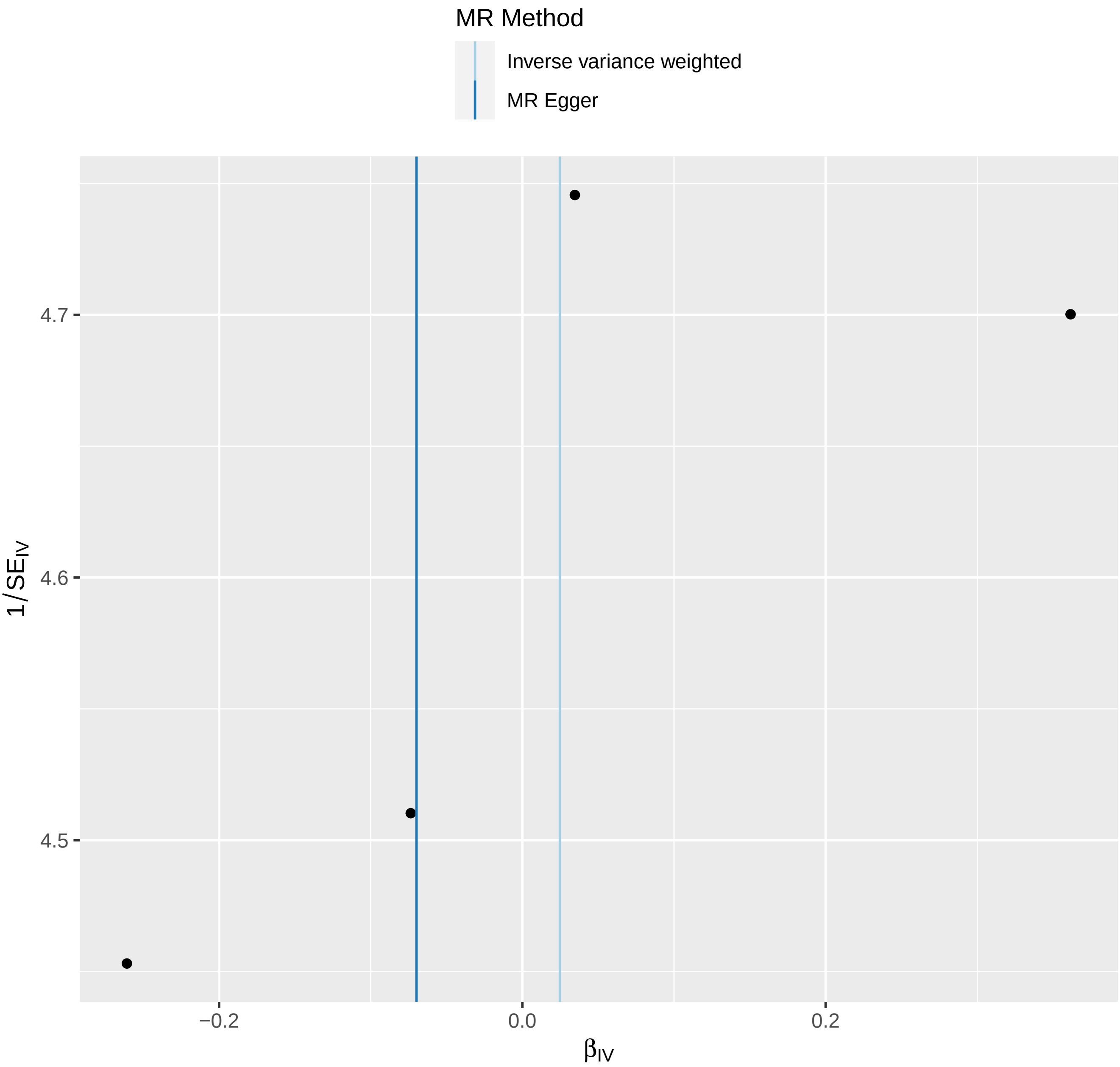

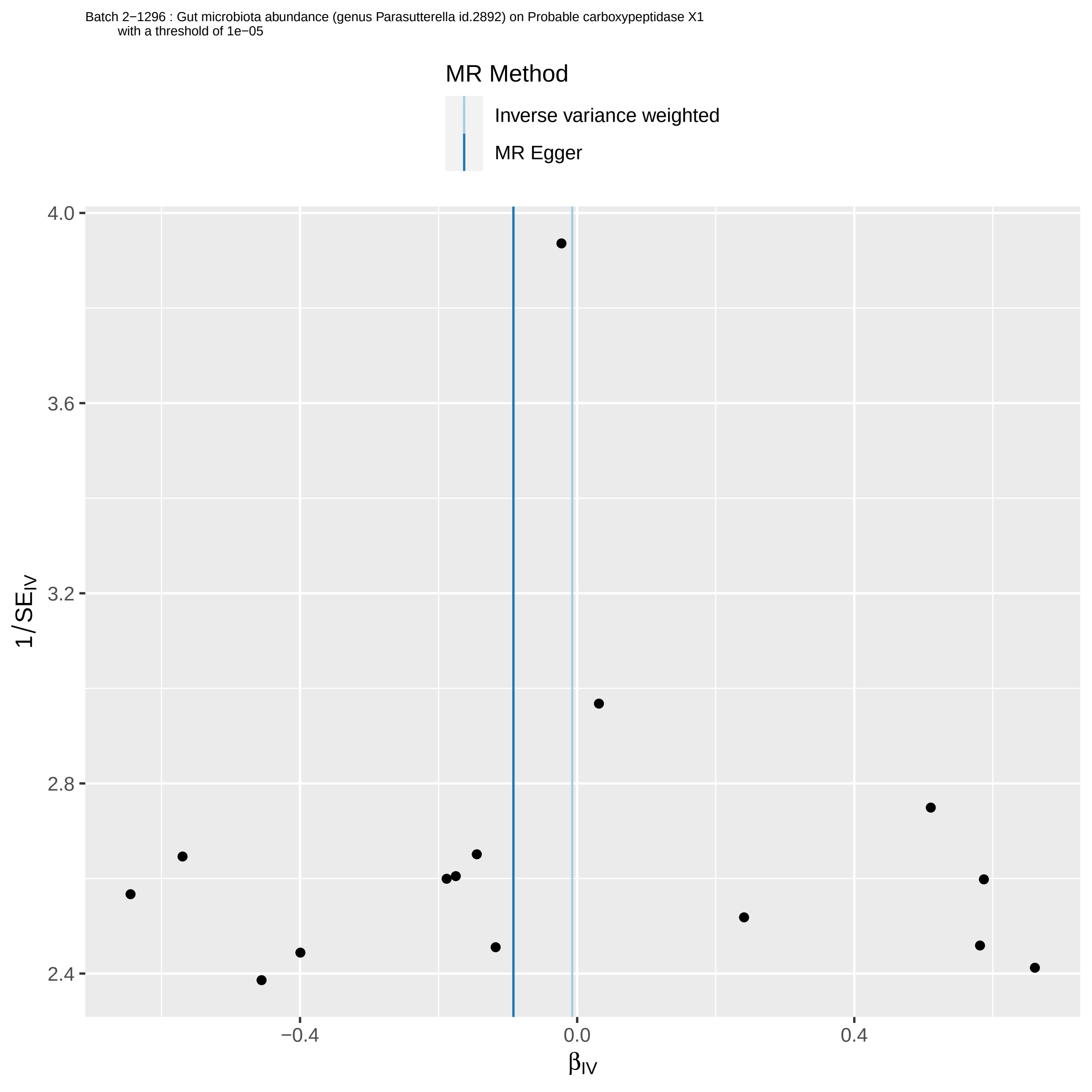

Batch 2−1296 : Gut microbiota abundance (genus Parasutterella id.2892) on Probable carboxypeptidase X1
with a threshold of 1e−05
MR Method
Inverse variance weighted
MR Egger
4.0
3.6
3.2
2.8
2.4
1/SE_IV
−0.4
0.0
0.4
β_IV

Batch 2−1297 : Gut microbiota abundance (genus Catenibacterium id.2153) on Carboxypeptidase Z
with a threshold of 1e−05

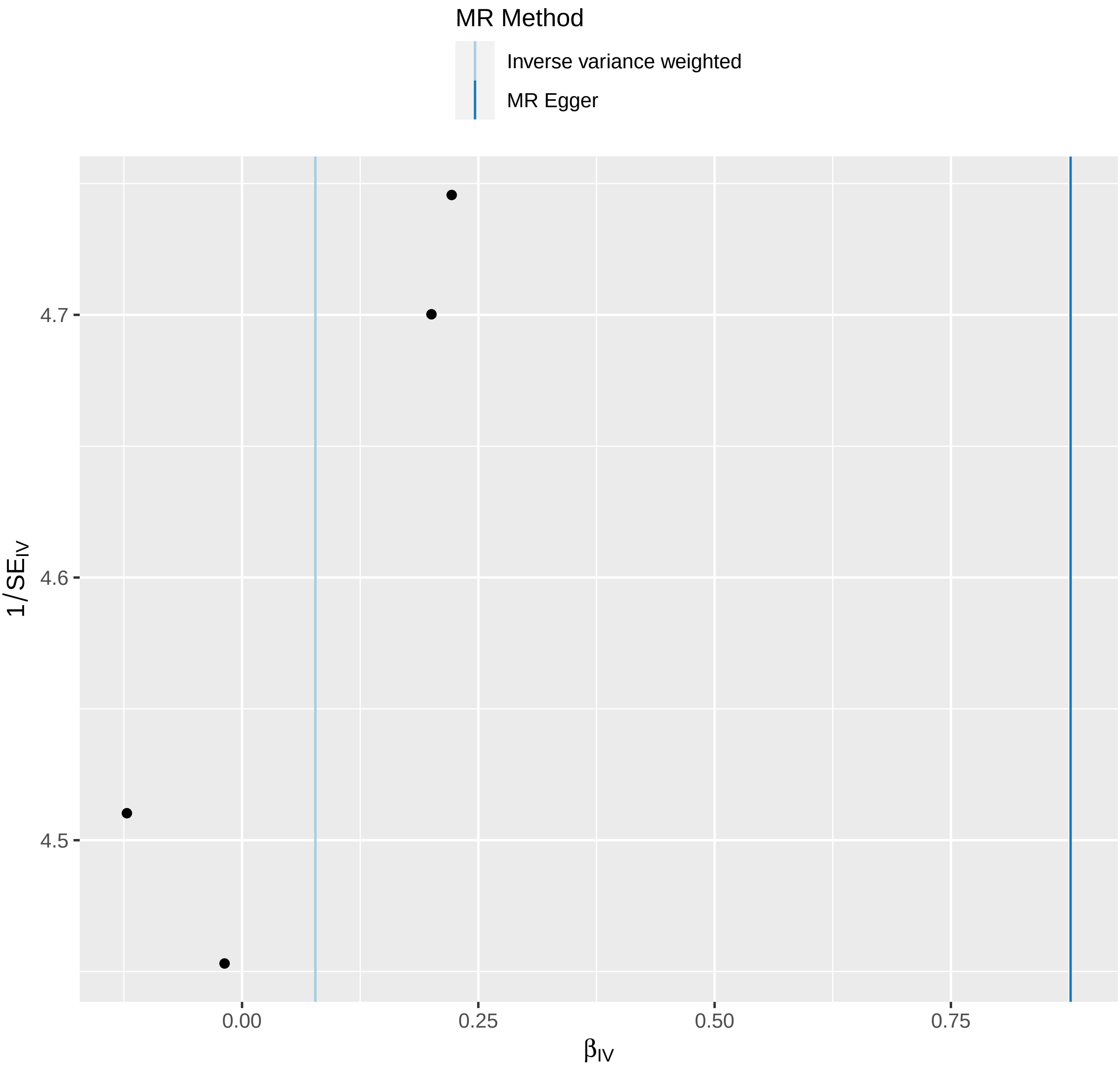

Batch 2−1298 : Gut microbiota abundance (genus Parasutterella id.2892) on Carboxypeptidase Z
with a threshold of 1e−05

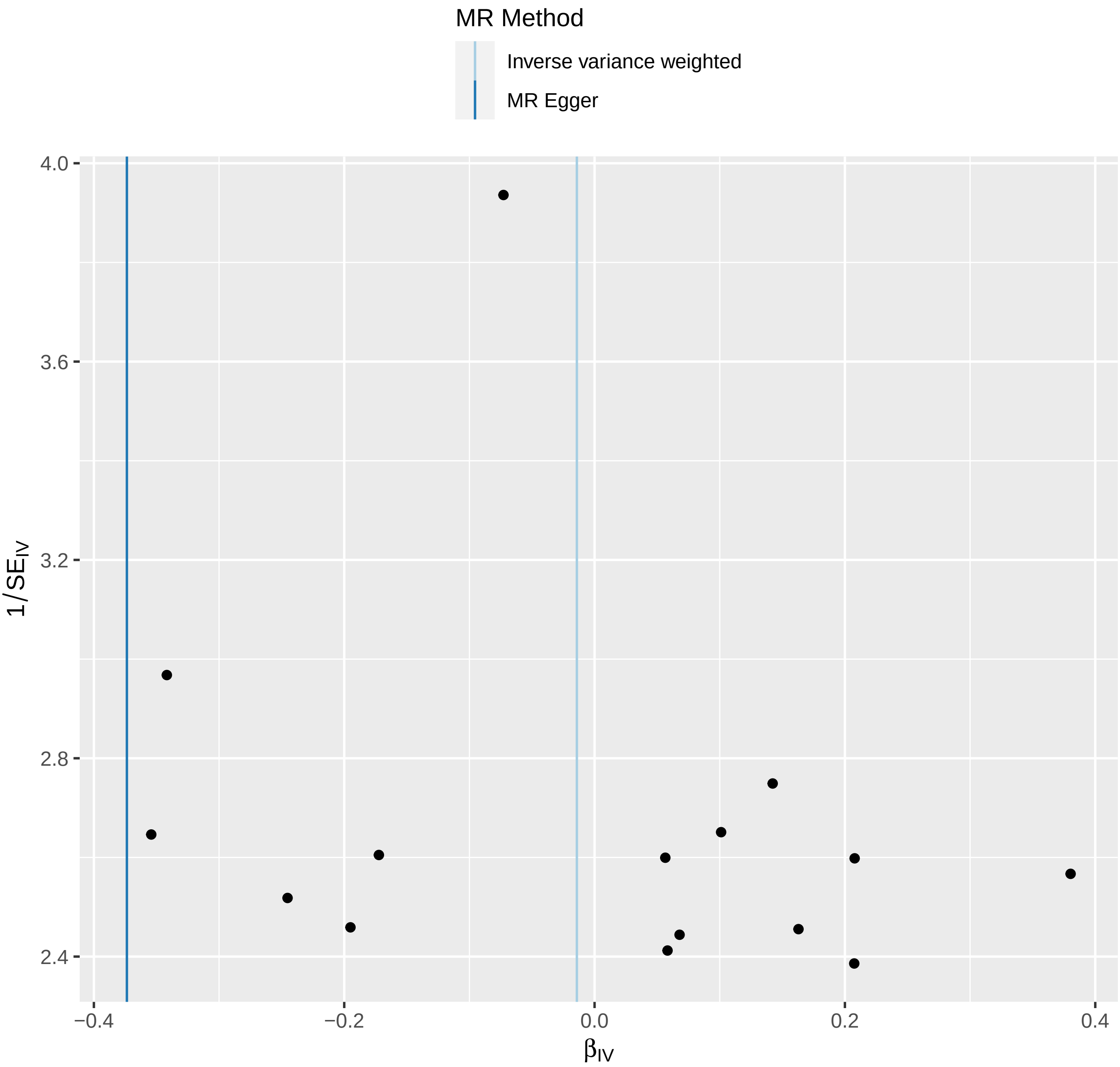

Batch 2−1299 : Gut microbiota abundance (genus Catenibacterium id.2153) on Cellular retinoic acid−binding protein 1
with a threshold of 1e−05

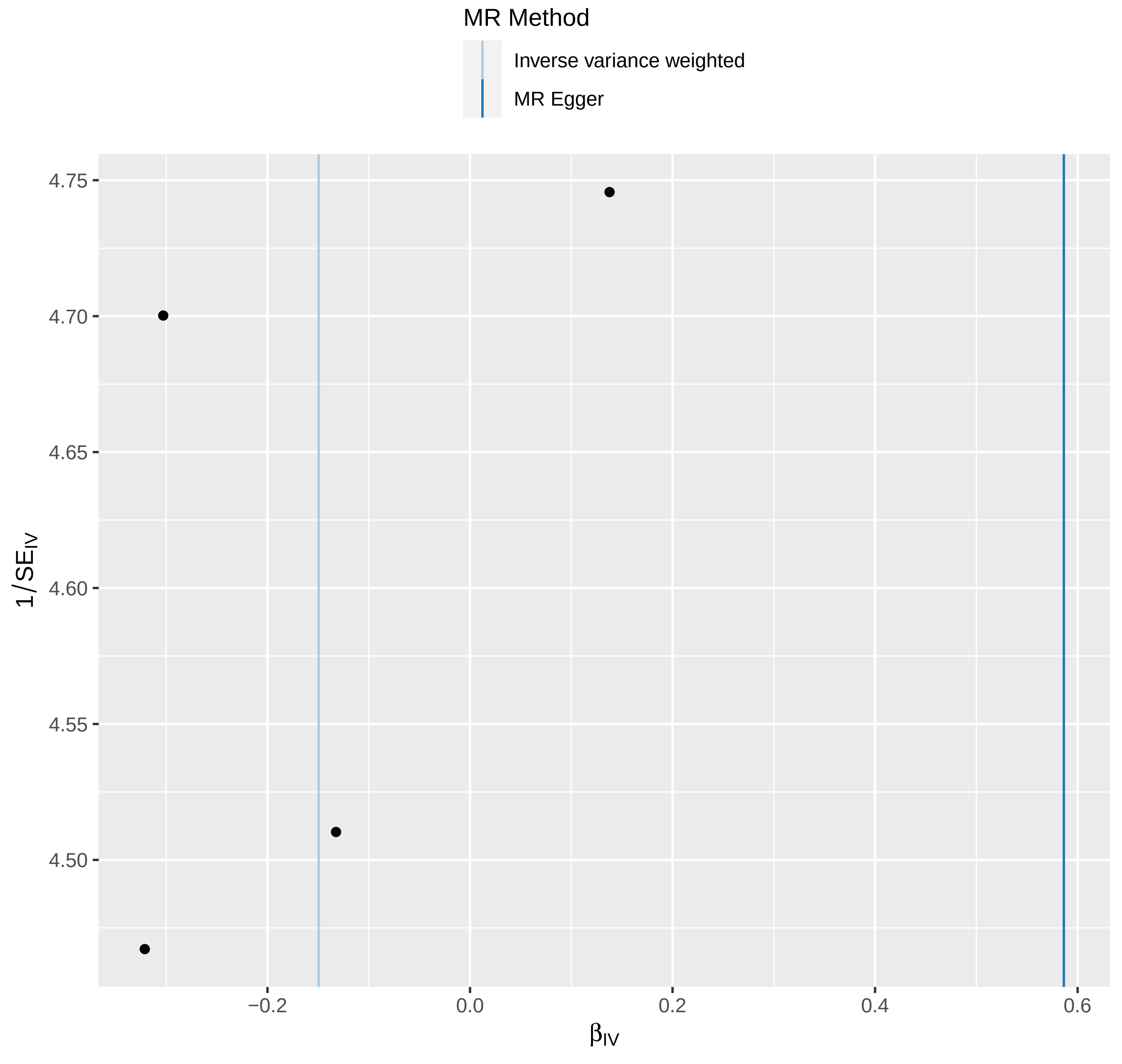

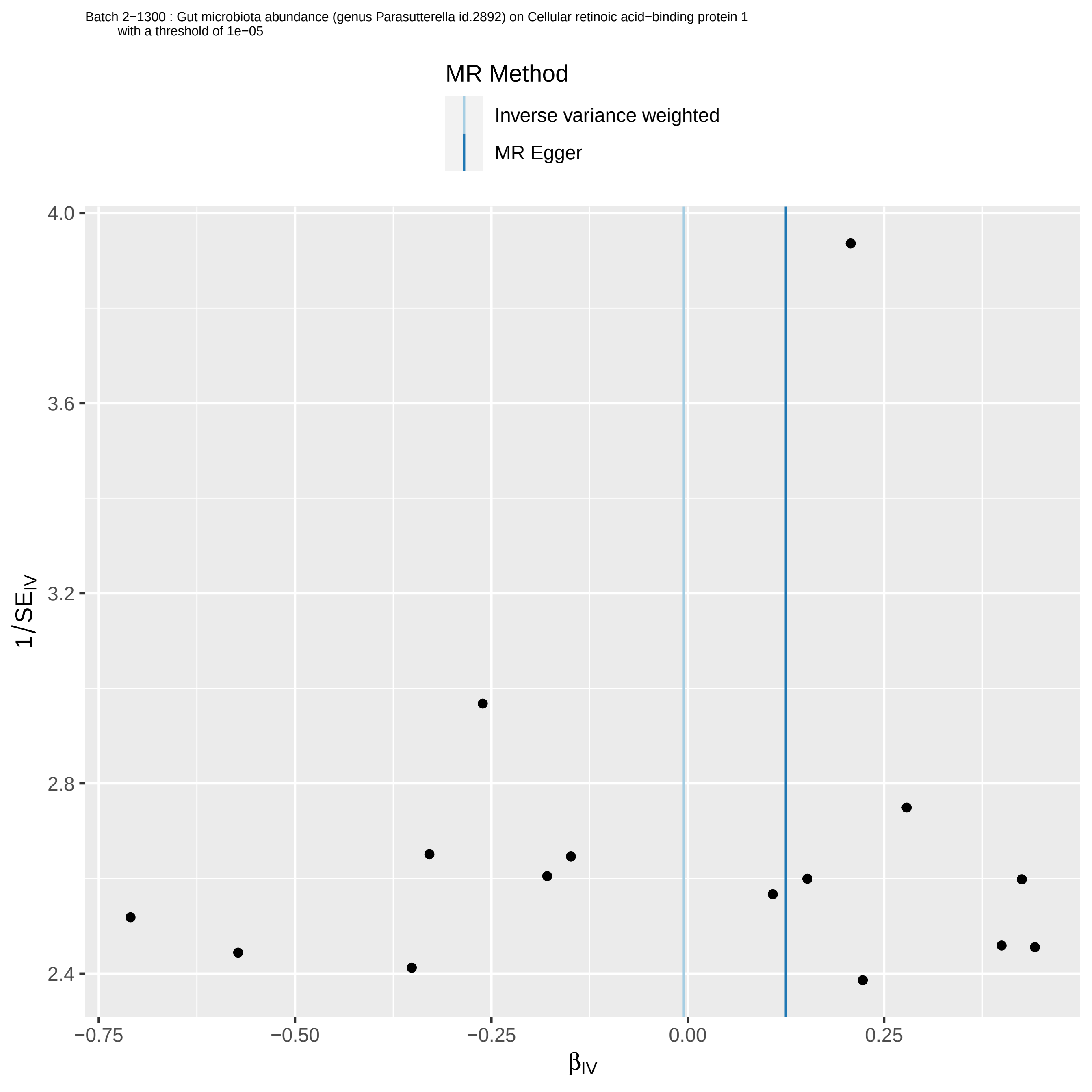

Batch 2−1300 : Gut microbiota abundance (genus Parasutterella id.2892) on Cellular retinoic acid−binding protein 1
with a threshold of 1e−05
MR Method
Inverse variance weighted
MR Egger
4.0
3.6
3.2
2.8
2.4
1/SE_IV
−0.75
−0.50
−0.25
0.00
0.25
β_IV

Batch 2−1301 : Gut microbiota abundance (genus Catenibacterium id.2153) on Cellular retinoic acid−binding protein 2
with a threshold of 1e−05

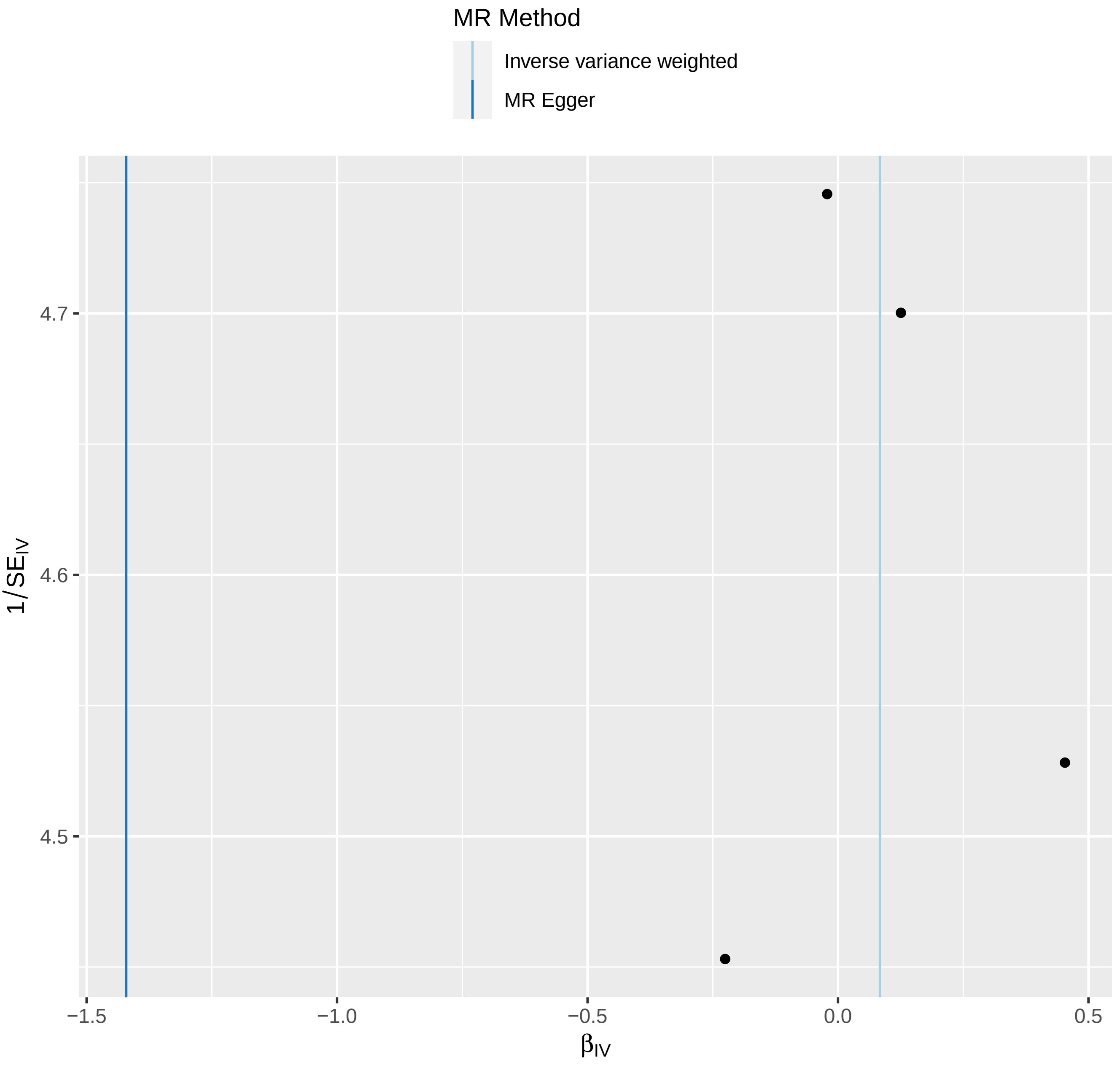

Batch 2−1302 : Gut microbiota abundance (genus Parasutterella id.2892) on Cellular retinoic acid−binding protein 2
with a threshold of 1e−05

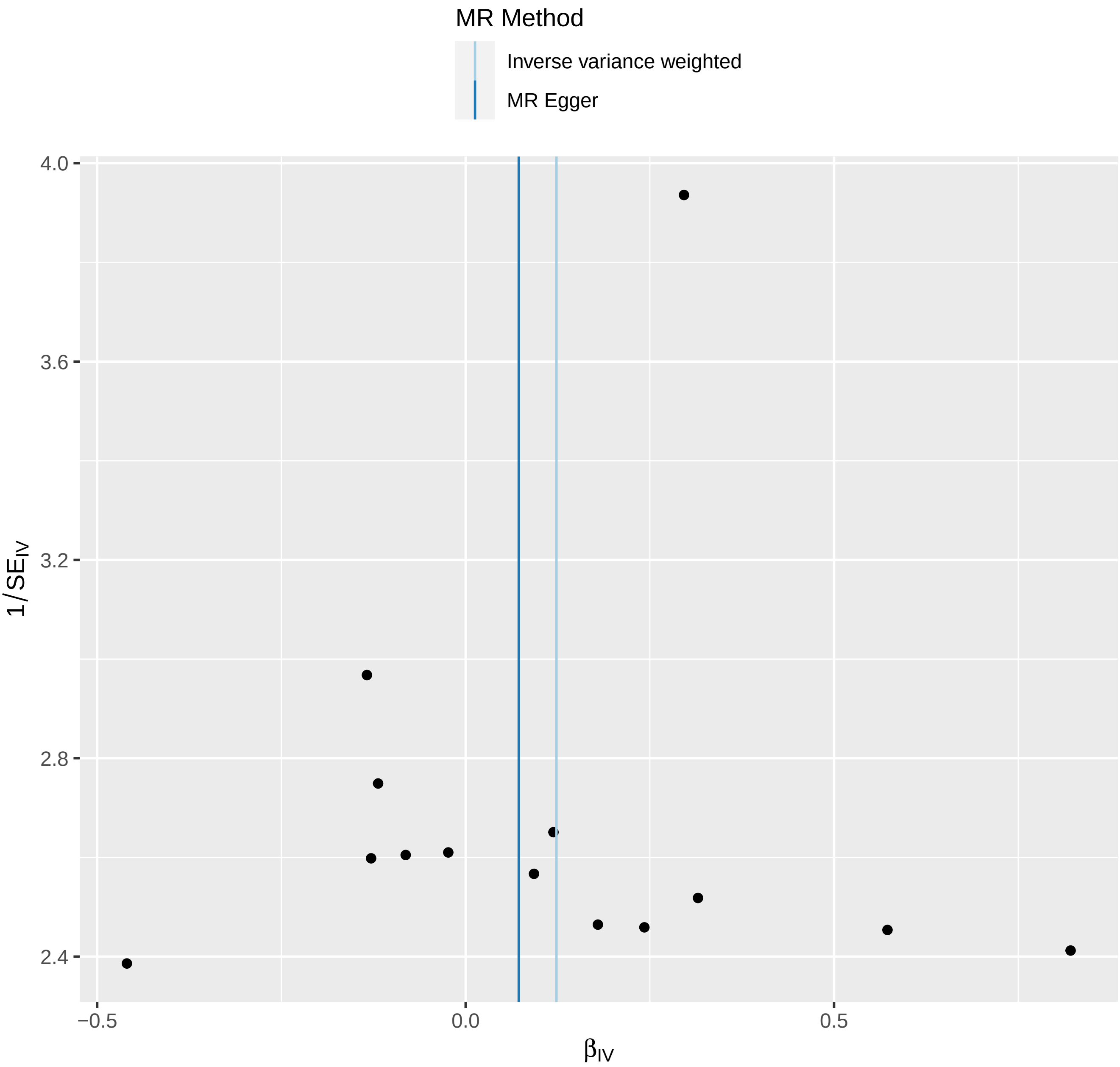

Batch 2−1303 : Gut microbiota abundance (genus Catenibacterium id.2153) on Protein crumbs homolog 1 with a threshold of 1e−05

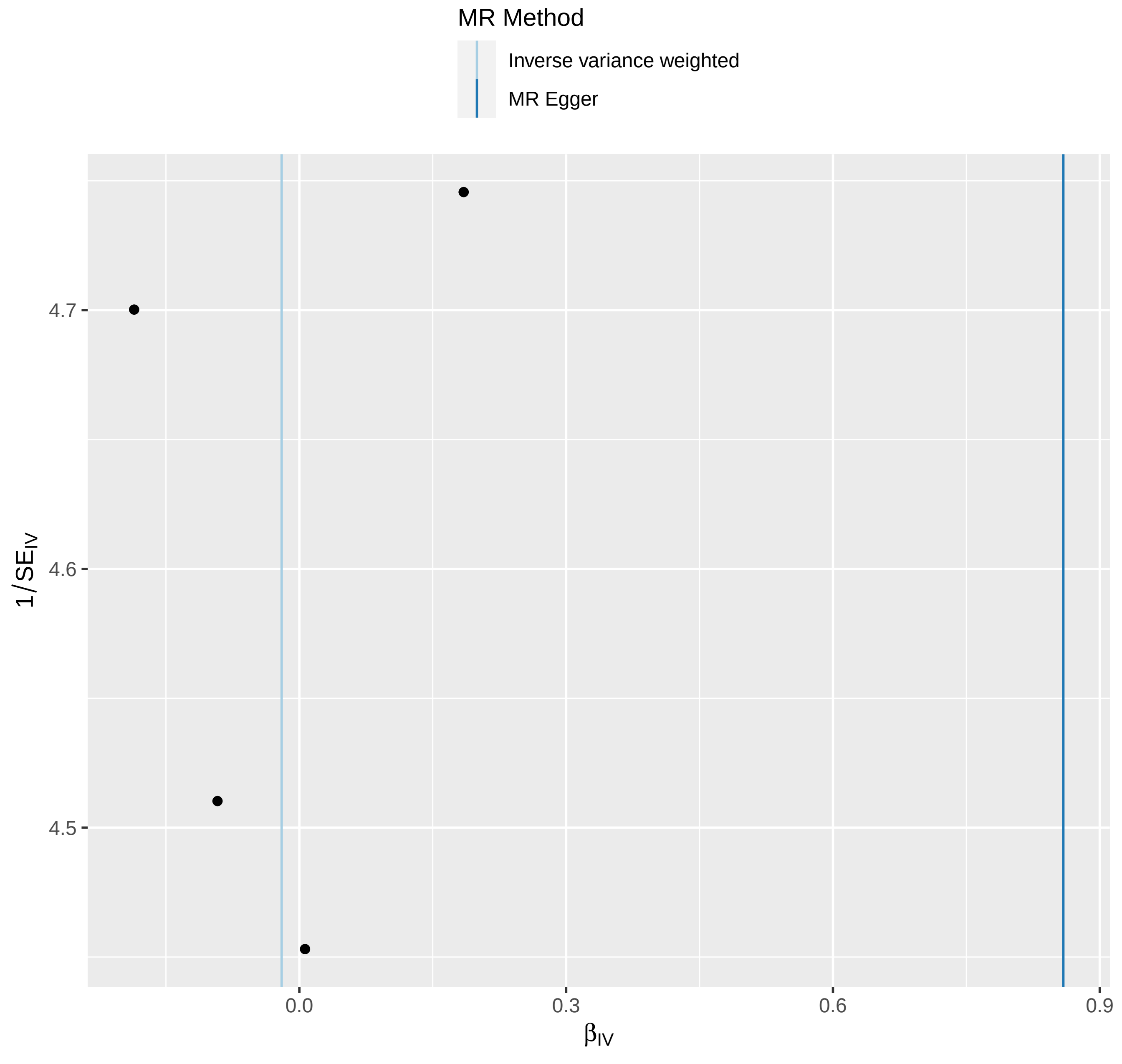

Batch 2−1304 : Gut microbiota abundance (genus Parasutterella id.2892) on Protein crumbs homolog 1
with a threshold of 1e−05

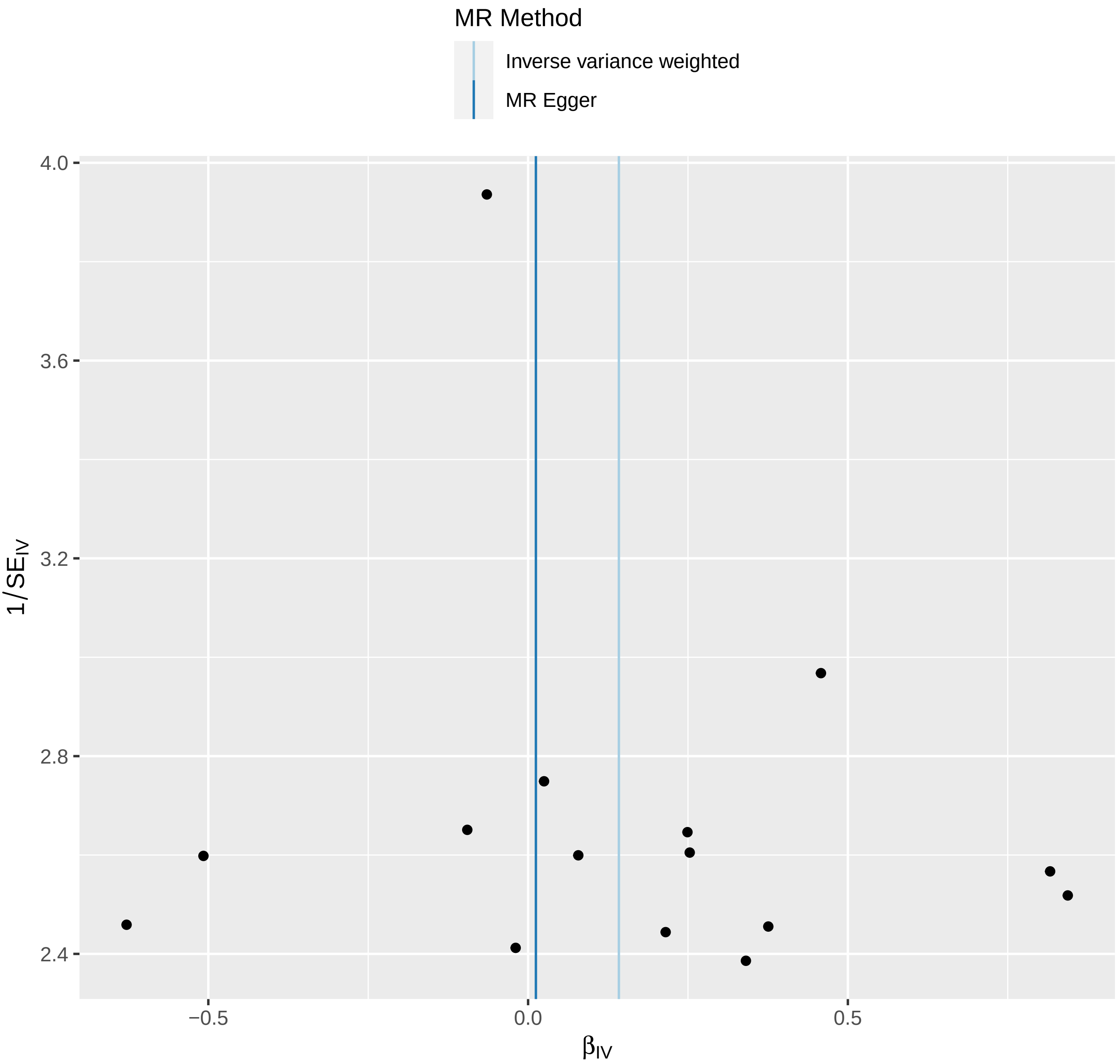

Batch 2−1305 : Gut microbiota abundance (genus Catenibacterium id.2153) on Cyclic AMP−responsive element−binding protein 3−like protein 1 with a threshold of 1e−05

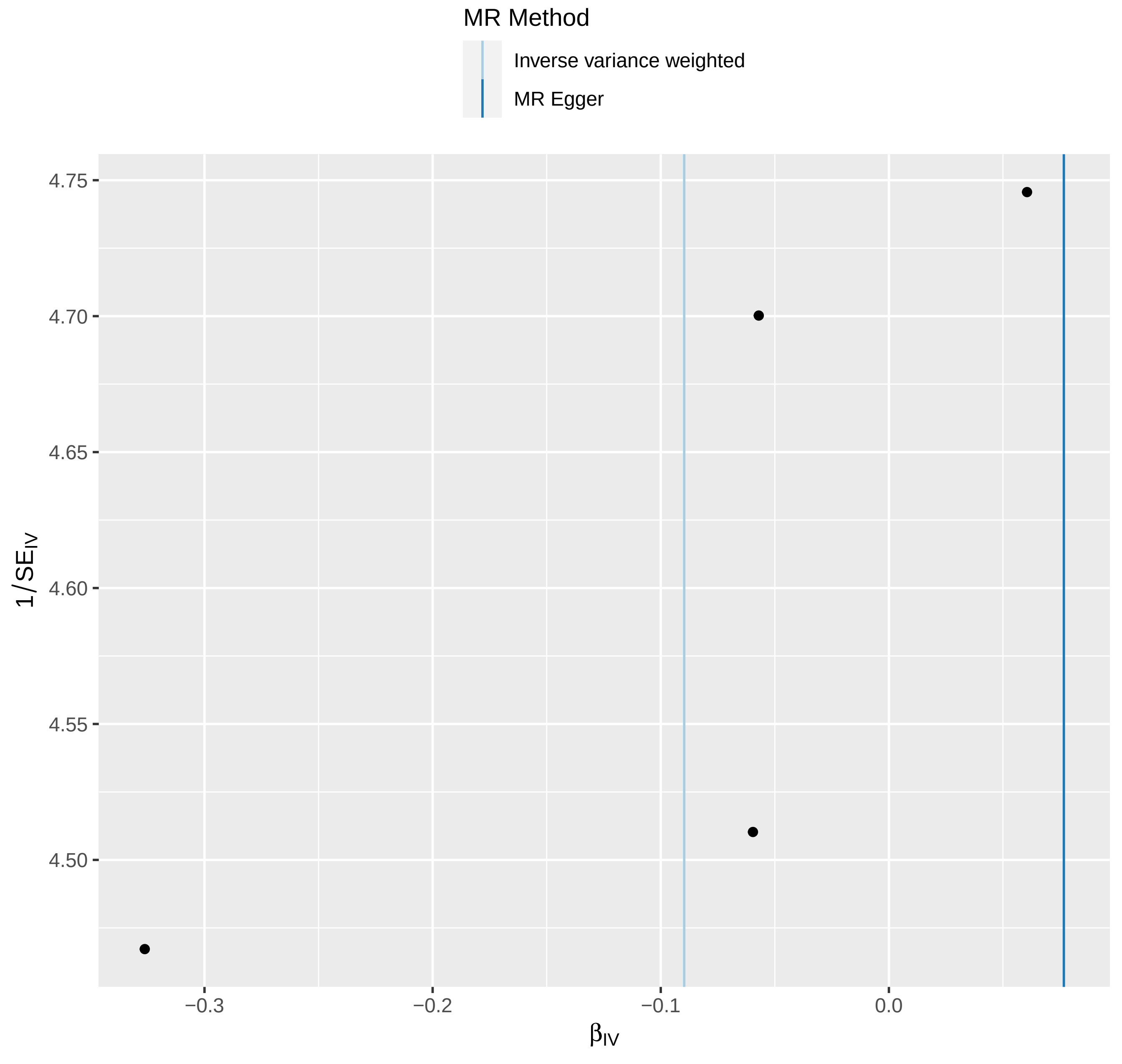

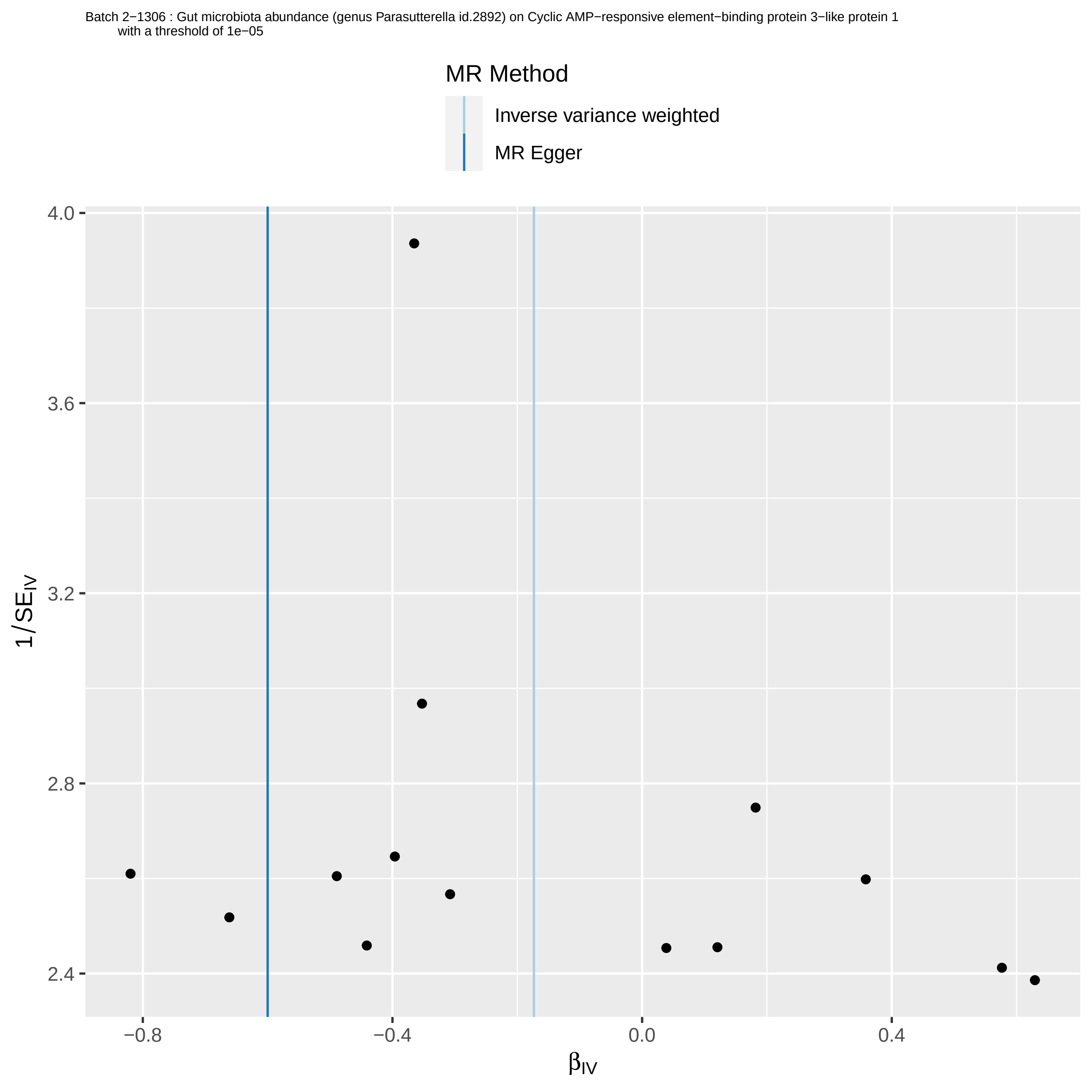
Batch 2−1306 : Gut microbiota abundance (genus Parasutterella id.2892) on Cyclic AMP−responsive element−binding protein 3−like protein 1
with a threshold of 1e−05
MR Method
Inverse variance weighted
MR Egger
4.0
3.6
3.2
2.8
2.4
1/SE_IV
−0.8
−0.4
0.0
0.4
β_IV

Batch 2−1307 : Gut microbiota abundance (genus Catenibacterium id.2153) on Cyclic AMP−responsive element−binding protein 3−like protein 4 with a threshold of 1e−05

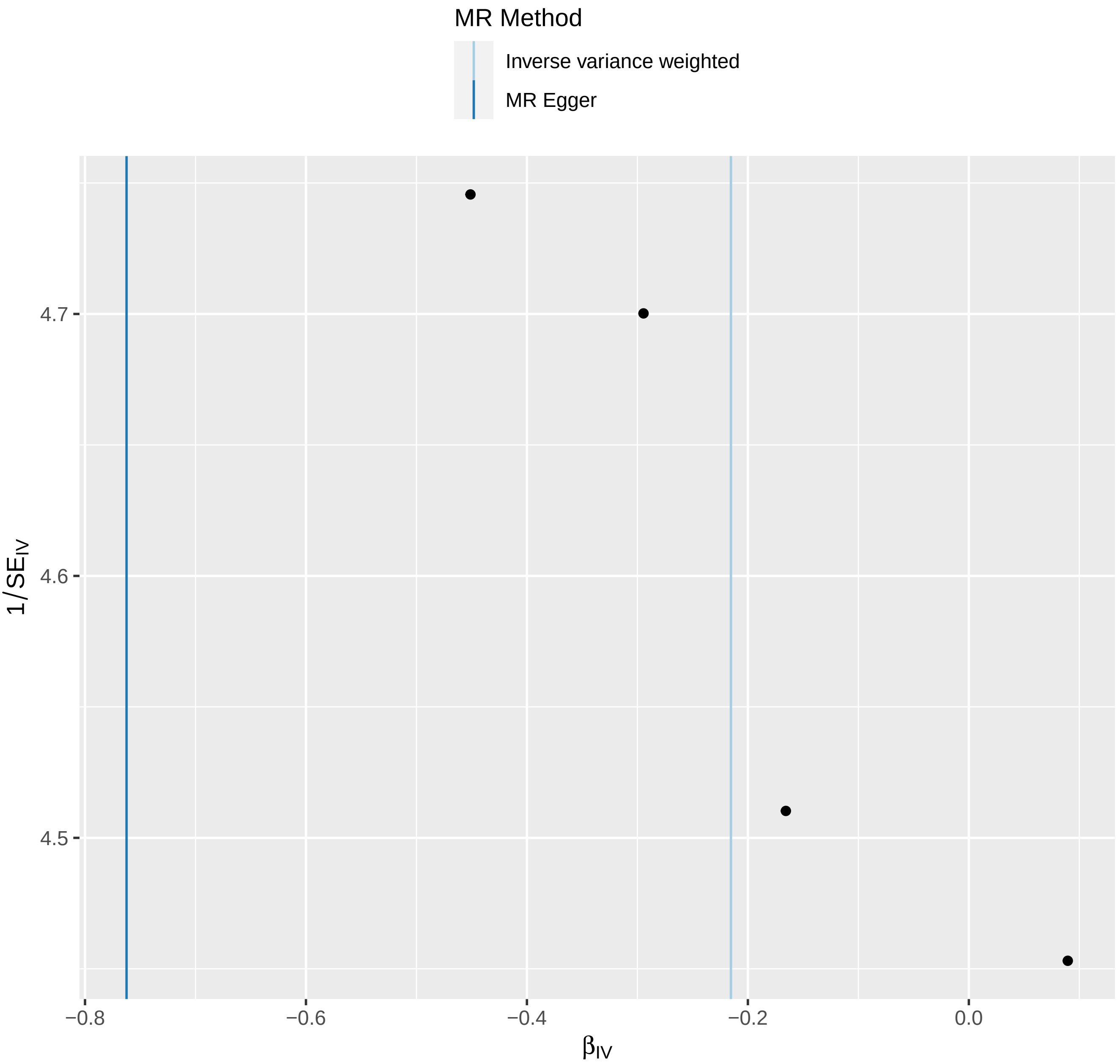

Batch 2−1308 : Gut microbiota abundance (genus Parasutterella id.2892) on Cyclic AMP−responsive element−binding protein 3−like protein 4 with a threshold of 1e−05

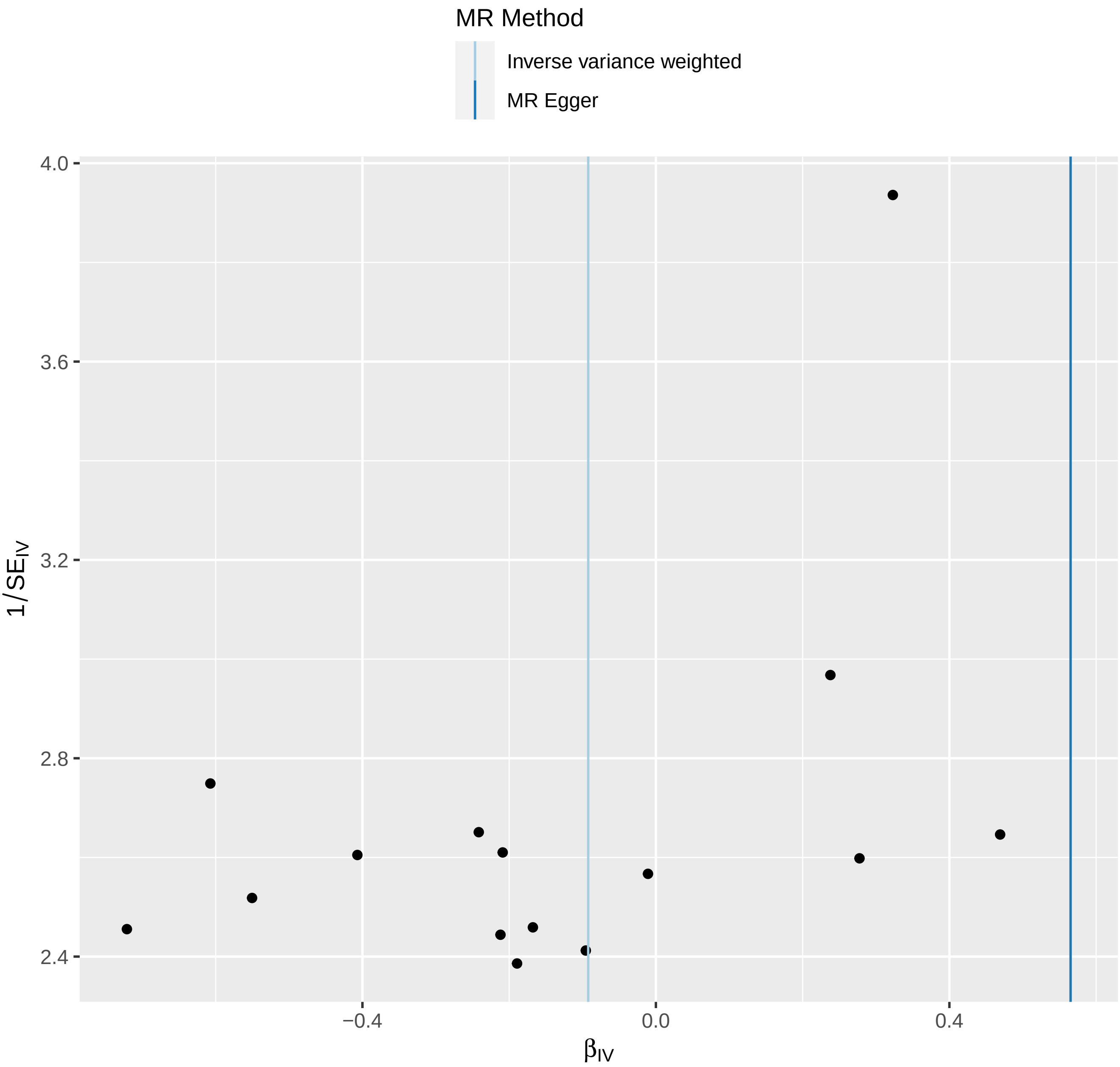

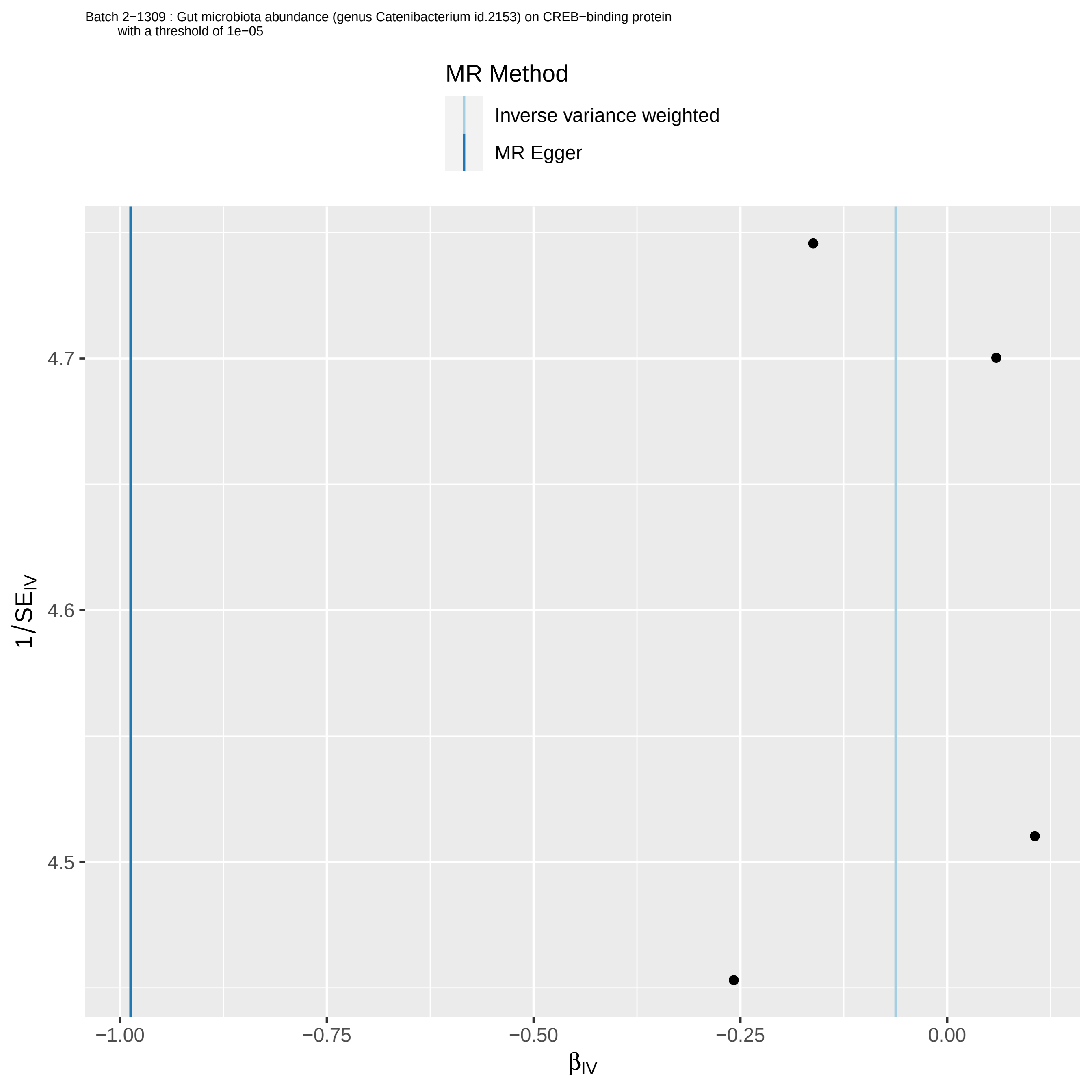

Batch 2−1309 : Gut microbiota abundance (genus Catenibacterium id.2153) on CREB−binding protein
with a threshold of 1e−05
MR Method
Inverse variance weighted
MR Egger
4.7
4.6
4.5
1/SE_IV
−1.00
−0.75
−0.50
−0.25
0.00
β_IV

Batch 2−1310 : Gut microbiota abundance (genus Parasutterella id.2892) on CREB−binding protein
with a threshold of 1e−05

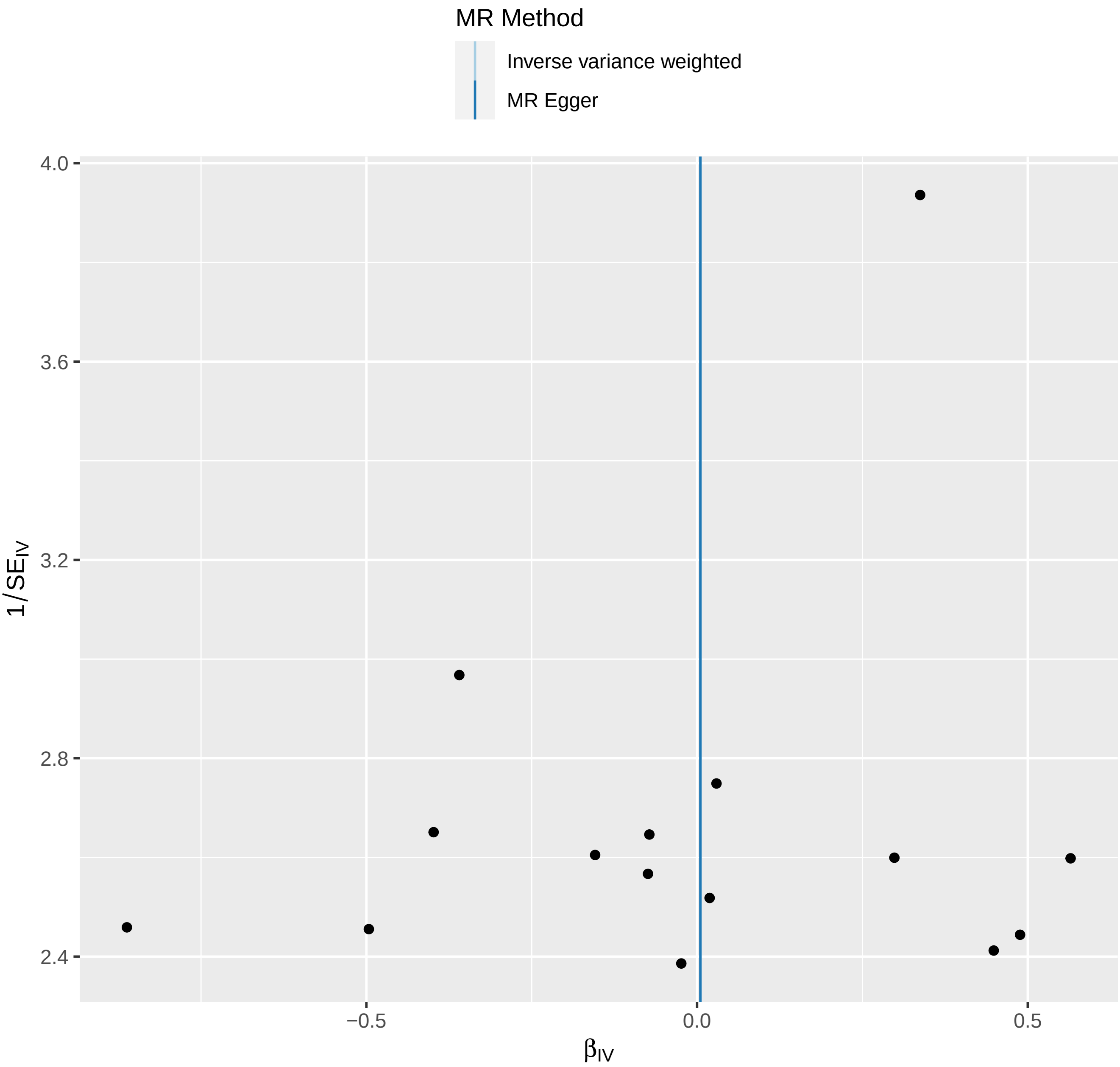

Batch 2−1311 : Gut microbiota abundance (genus Catenibacterium id.2153) on Protein CREG1
with a threshold of 1e−05

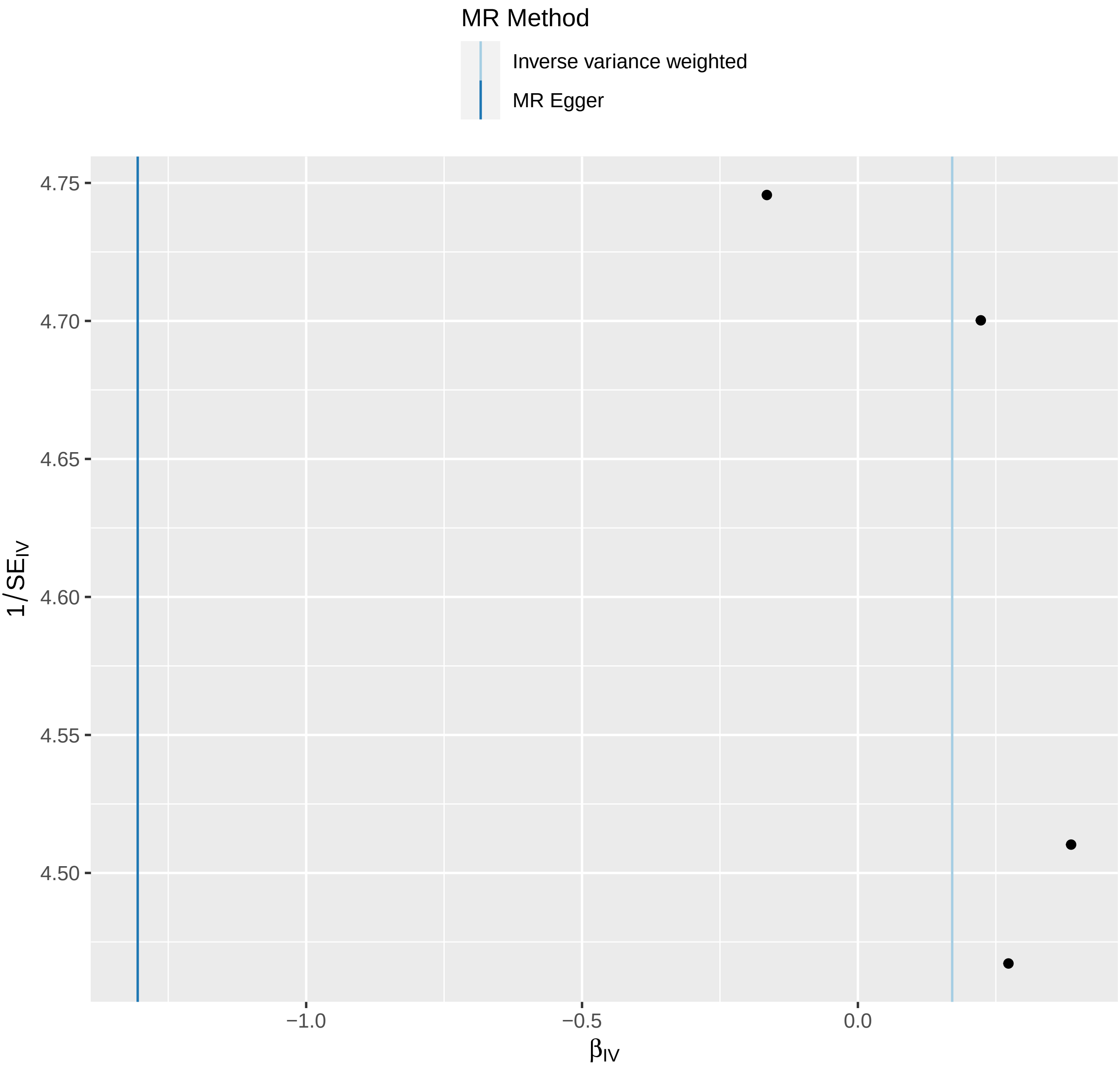

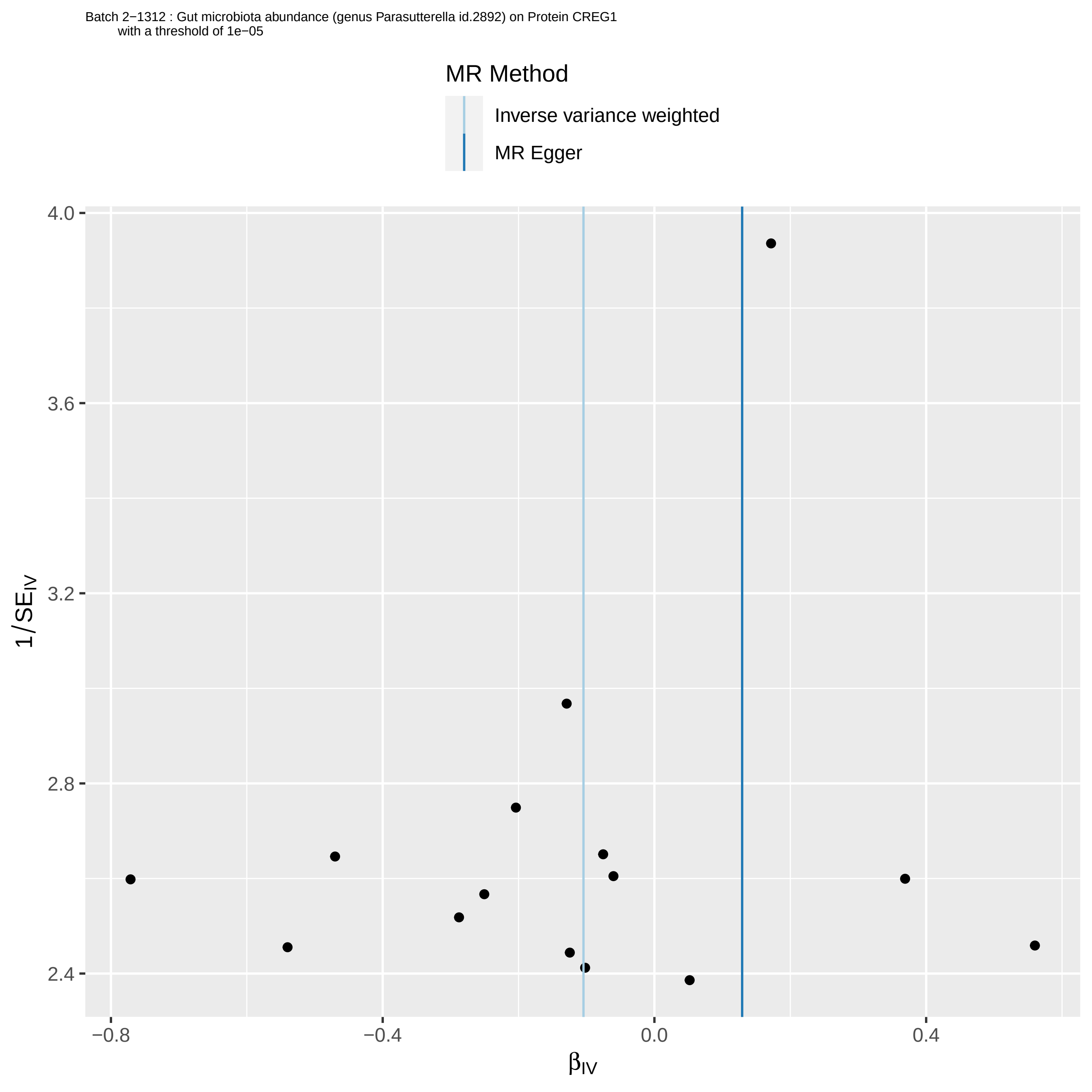
Batch 2−1312 : Gut microbiota abundance (genus Parasutterella id.2892) on Protein CREG1
with a threshold of 1e−05
MR Method
Inverse variance weighted
MR Egger
4.0
3.6
3.2
2.8
2.4
1/SE_IV
−0.8
−0.4
0.0
0.4
β_IV

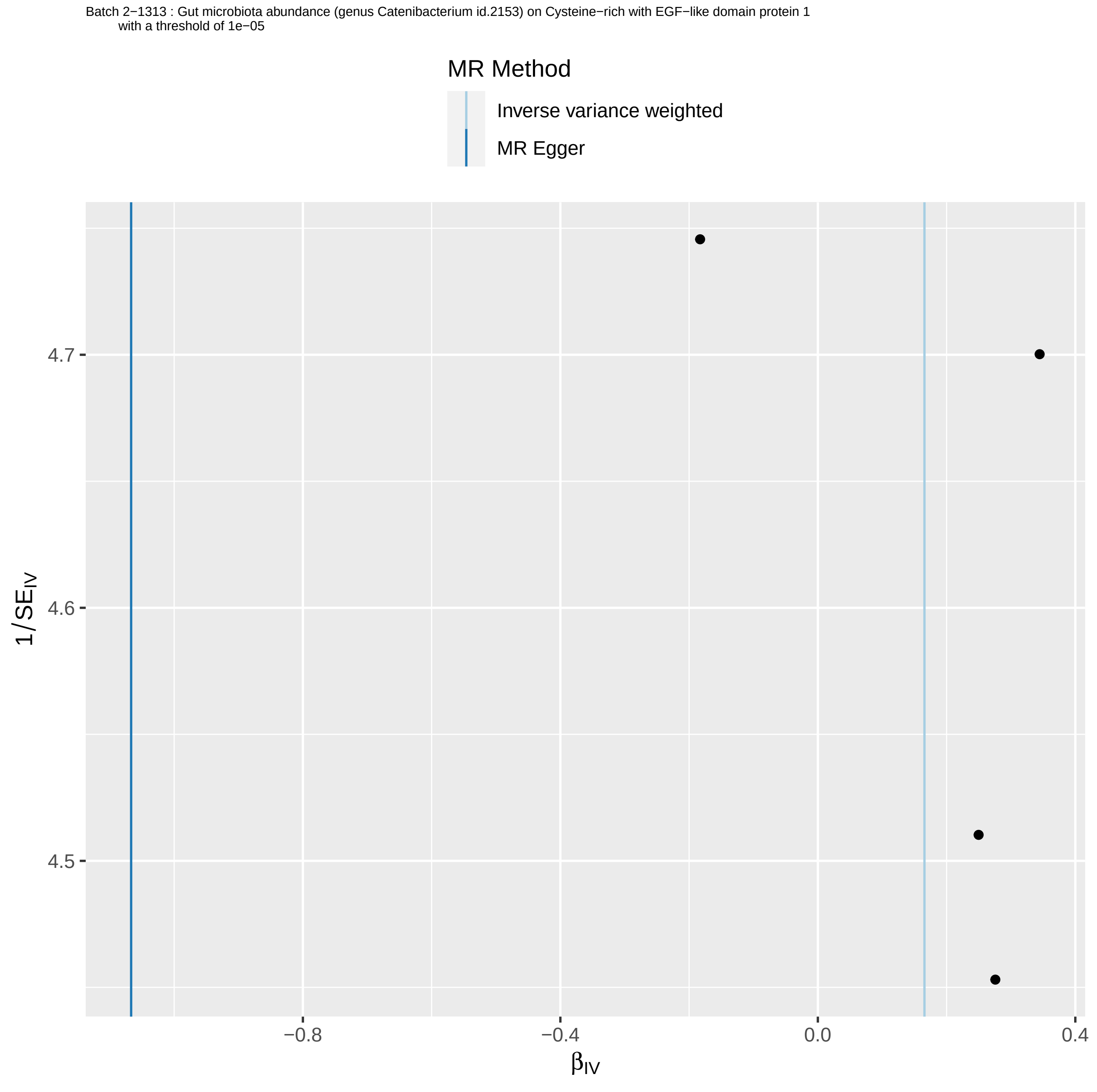
Batch 2−1313 : Gut microbiota abundance (genus Catenibacterium id.2153) on Cysteine−rich with EGF−like domain protein 1
with a threshold of 1e−05
MR Method
Inverse variance weighted
MR Egger
4.7
4.6
4.5
1/SE_IV
−0.8
−0.4
0.0
0.4
β_IV

Batch 2−1314 : Gut microbiota abundance (genus Parasutterella id.2892) on Cysteine−rich with EGF−like domain protein 1 with a threshold of 1e−05

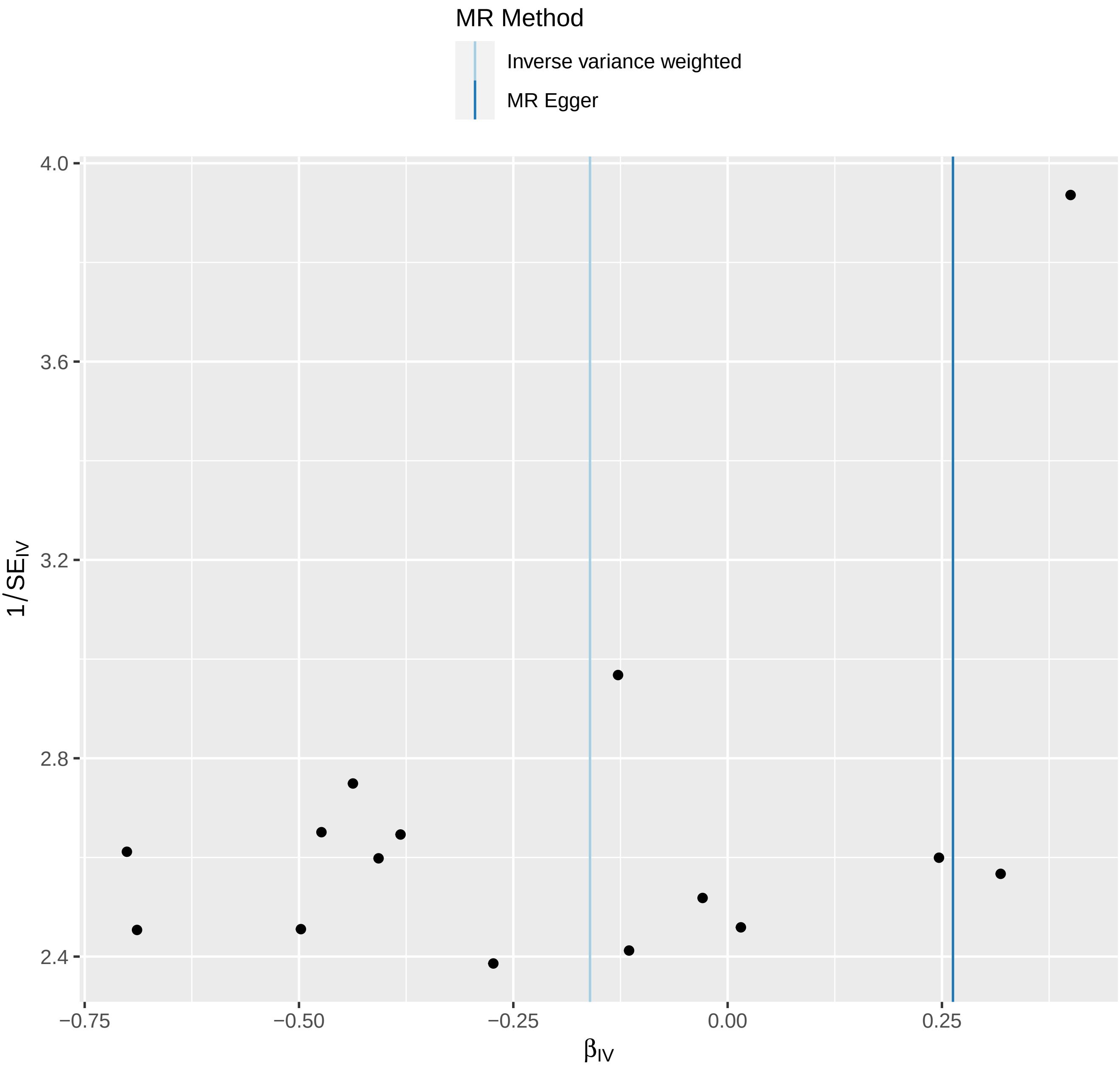

Batch 2−1315 : Gut microbiota abundance (genus Catenibacterium id.2153) on Corticoliberin
with a threshold of 1e−05

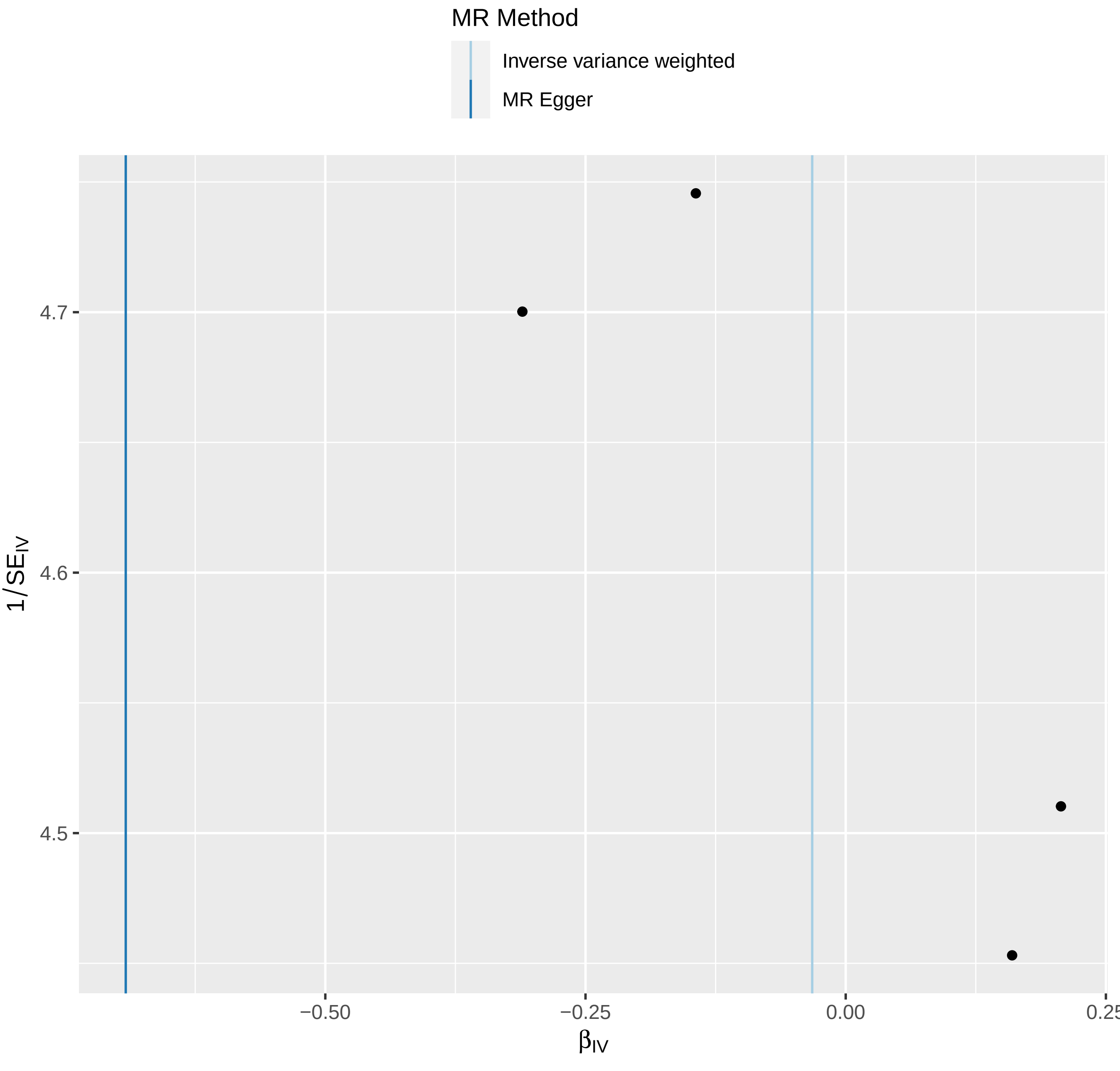

Batch 2−1316 : Gut microbiota abundance (genus Parasutterella id.2892) on Corticoliberin
with a threshold of 1e−05

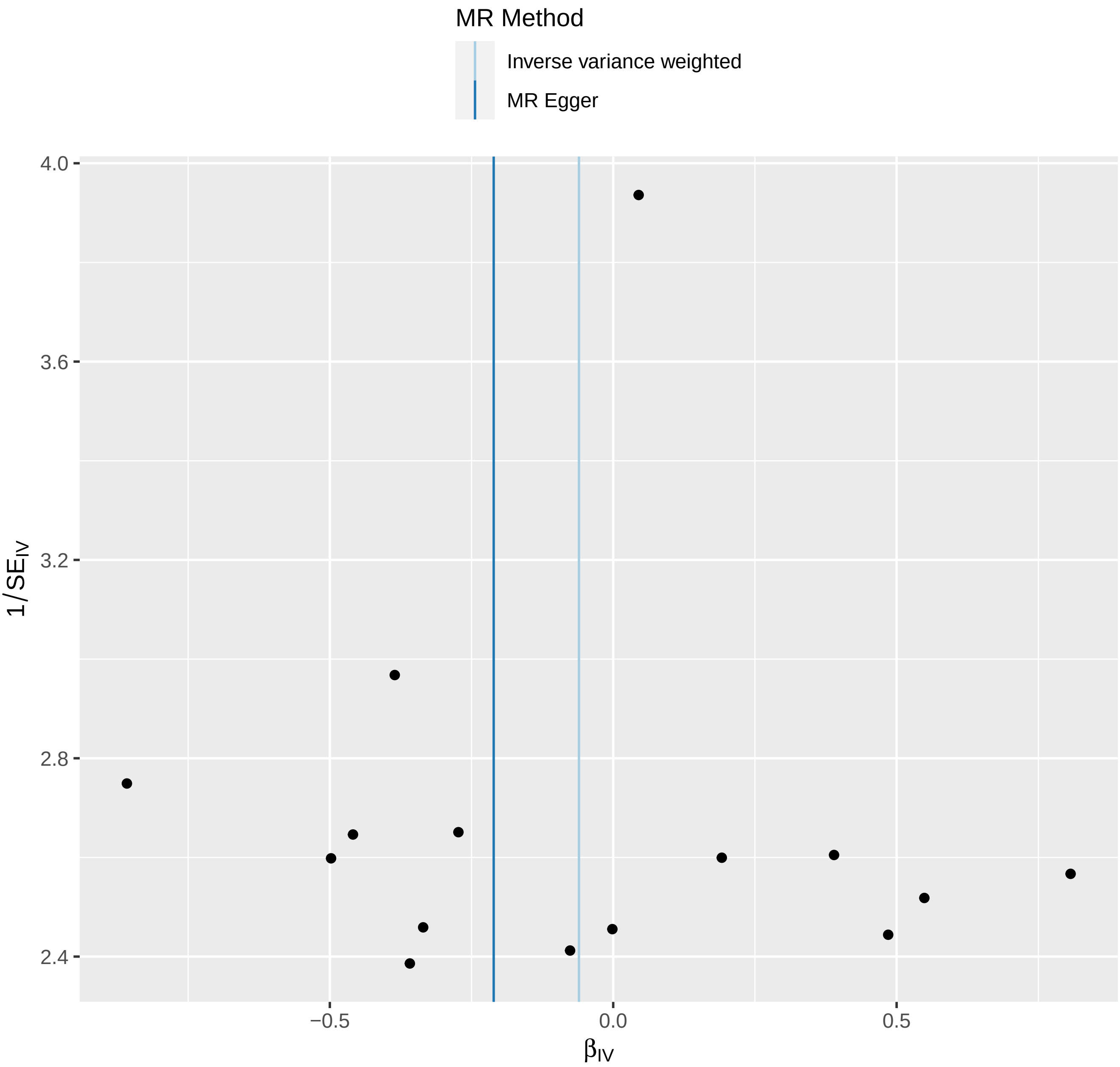

Batch 2−1317 : Gut microbiota abundance (genus Catenibacterium id.2153) on Corticotropin−releasing factor−binding protein
with a threshold of 1e−05

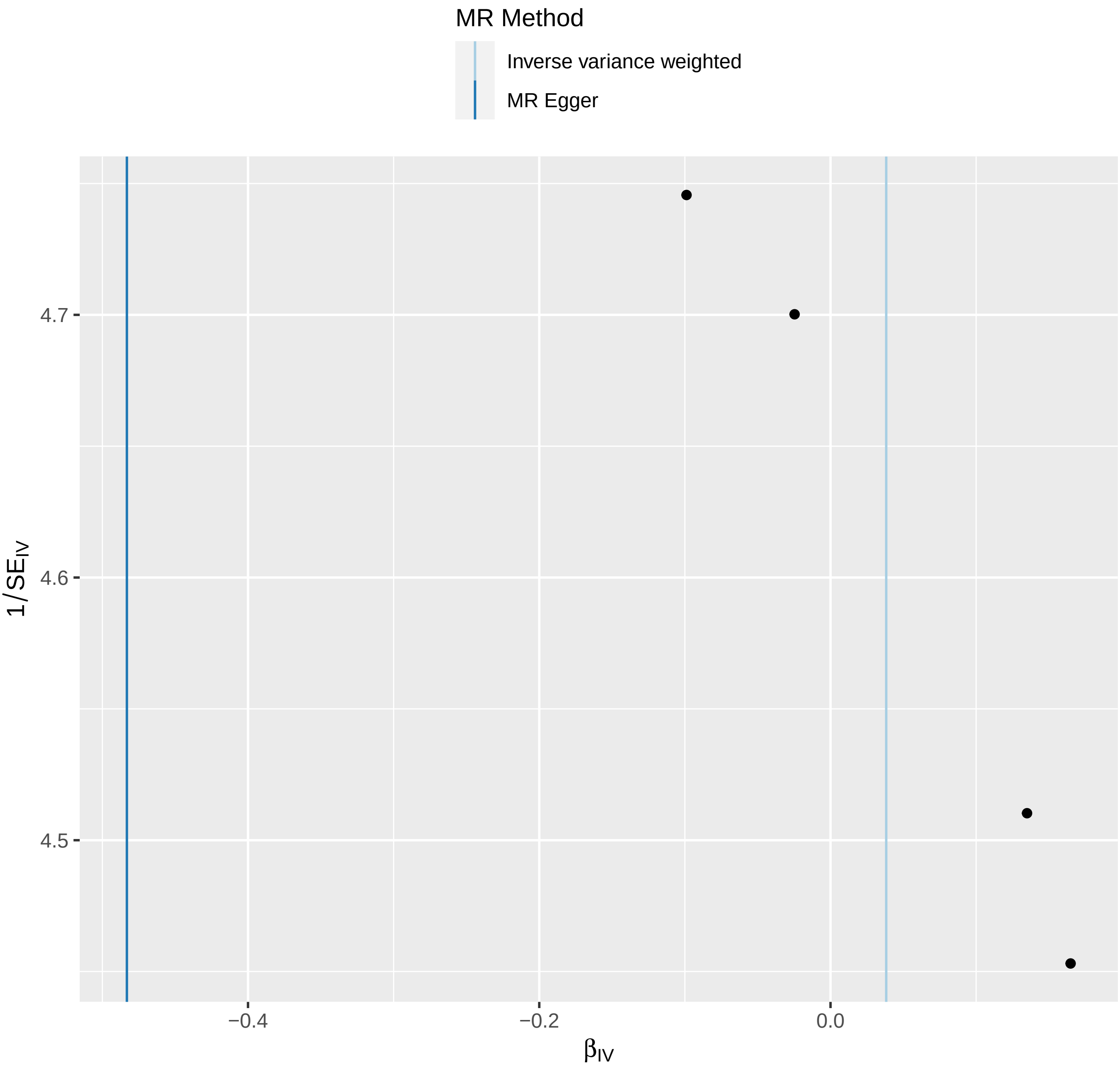

Batch 2−1318 : Gut microbiota abundance (genus Parasutterella id.2892) on Corticotropin−releasing factor−binding protein
with a threshold of 1e−05

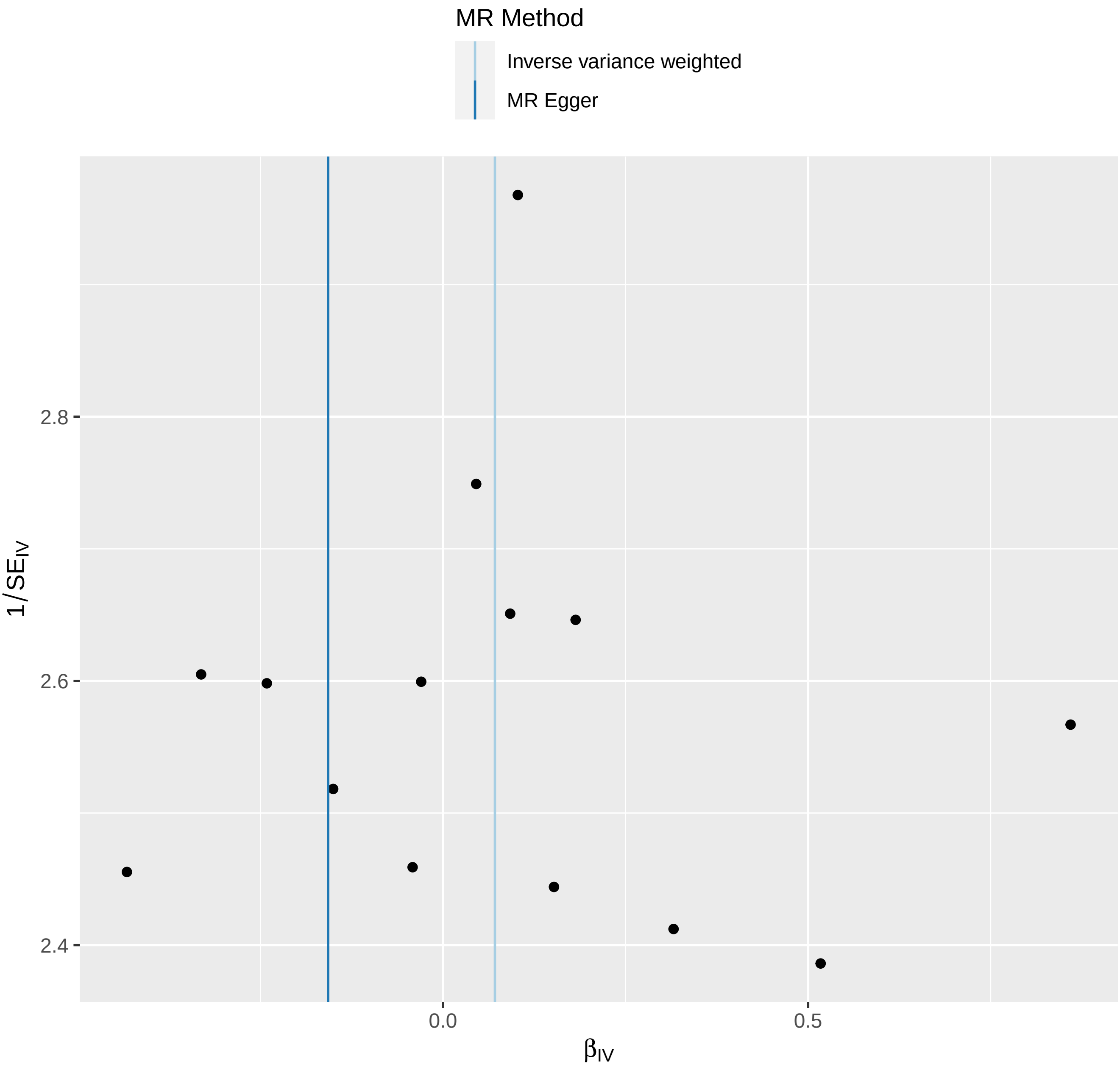

Batch 2−1319 : Gut microbiota abundance (genus Catenibacterium id.2153) on Cysteine−rich motor neuron 1 protein
with a threshold of 1e−05

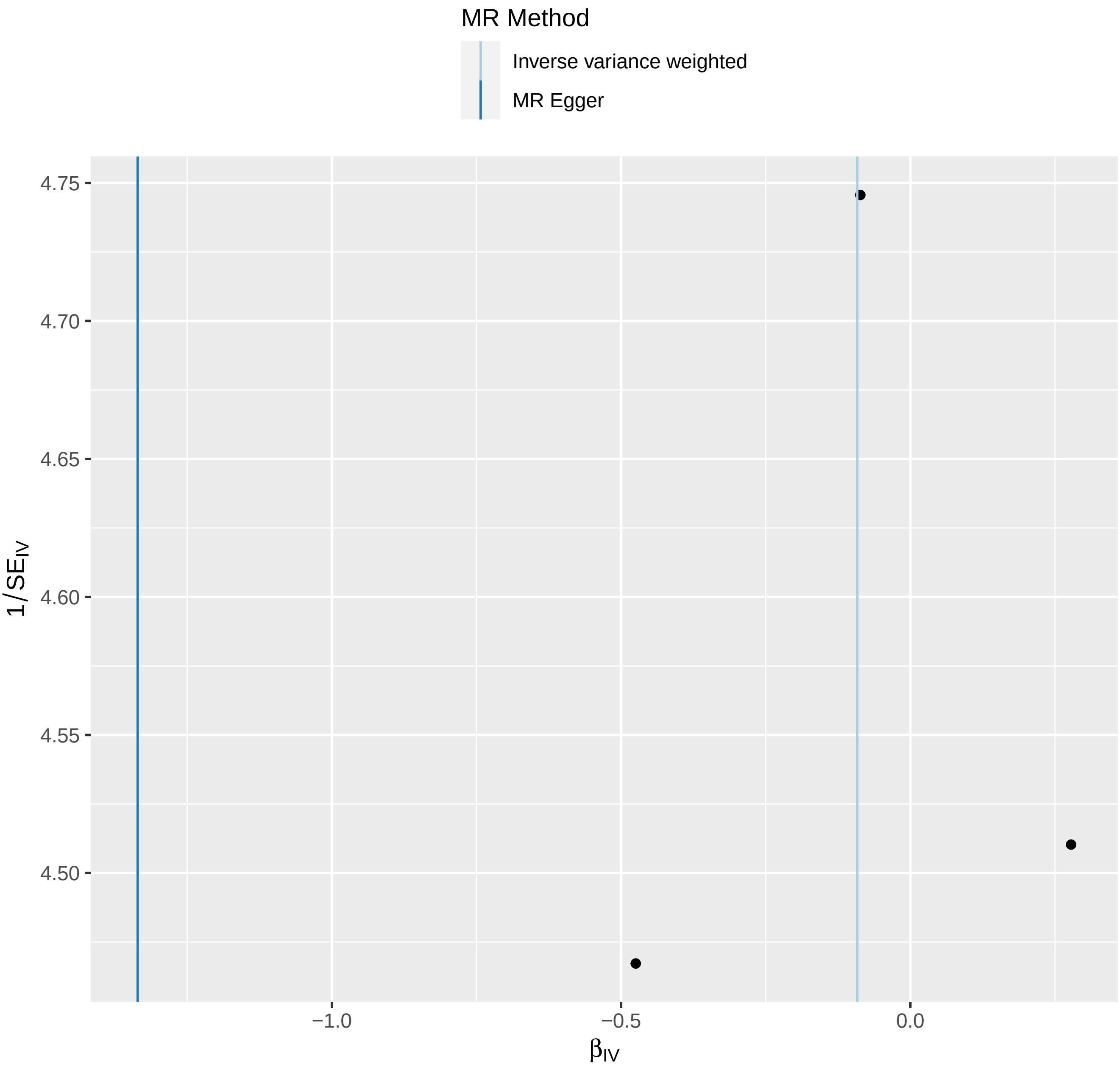

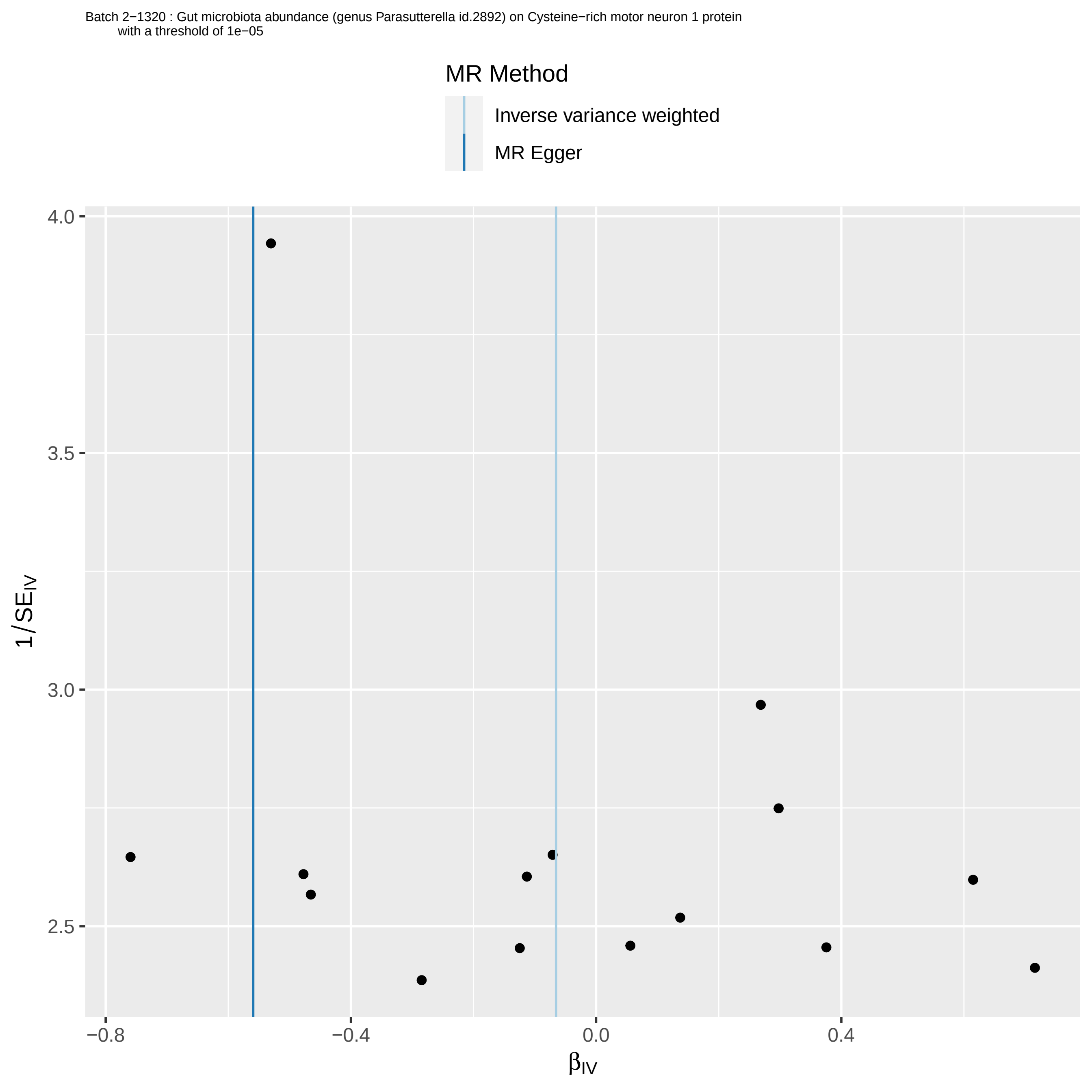

Batch 2−1320 : Gut microbiota abundance (genus Parasutterella id.2892) on Cysteine−rich motor neuron 1 protein
with a threshold of 1e−05
MR Method
Inverse variance weighted
MR Egger
4.0
3.5
3.0
2.5
1/SE_IV
−0.8
−0.4
0.0
0.4
β_IV

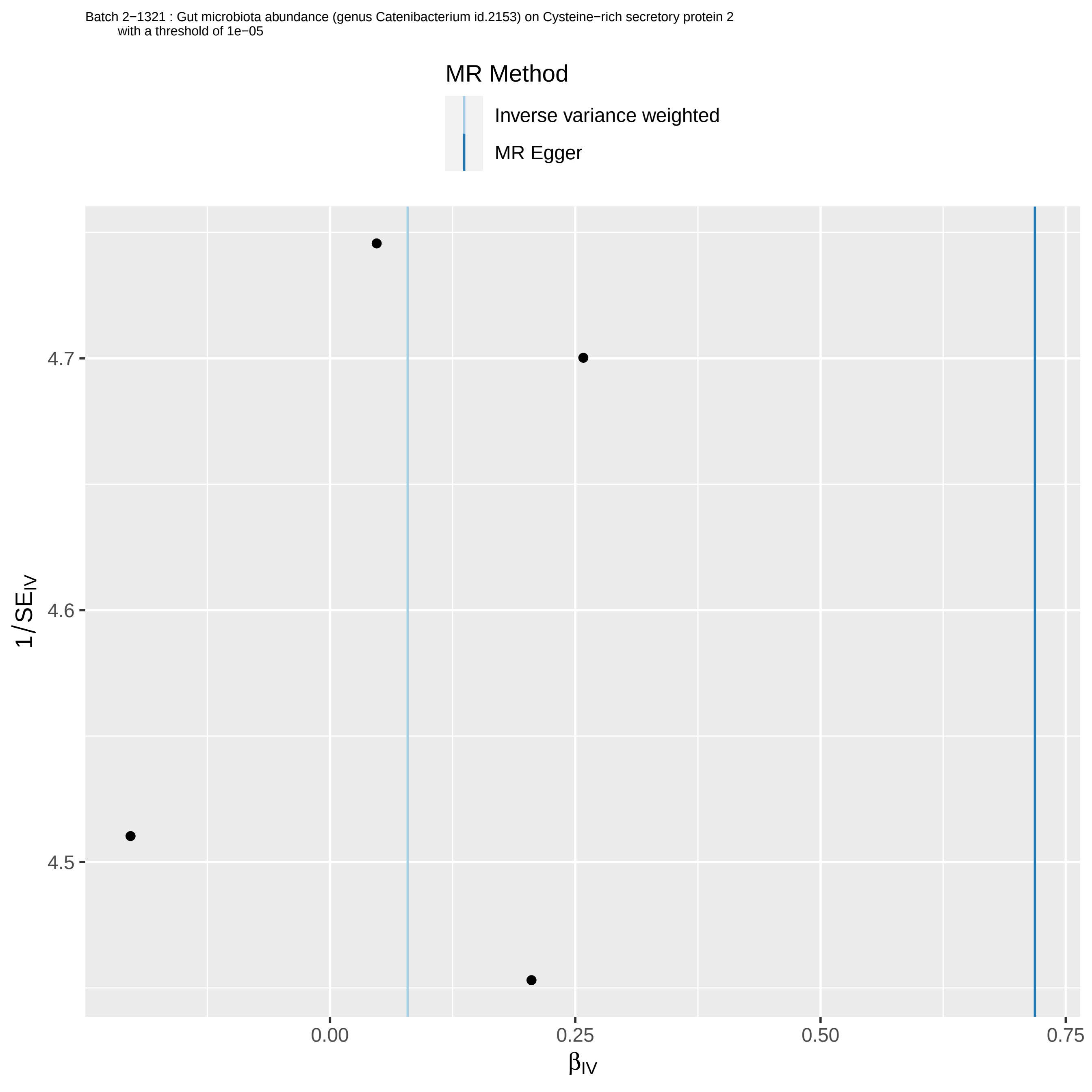
Batch 2−1321 : Gut microbiota abundance (genus Catenibacterium id.2153) on Cysteine−rich secretory protein 2
with a threshold of 1e−05
MR Method
Inverse variance weighted
MR Egger
4.7
4.6
4.5
1/SE_IV
0.00
0.25
0.50
0.75
β_IV

Batch 2−1322 : Gut microbiota abundance (genus Parasutterella id.2892) on Cysteine−rich secretory protein 2
with a threshold of 1e−05

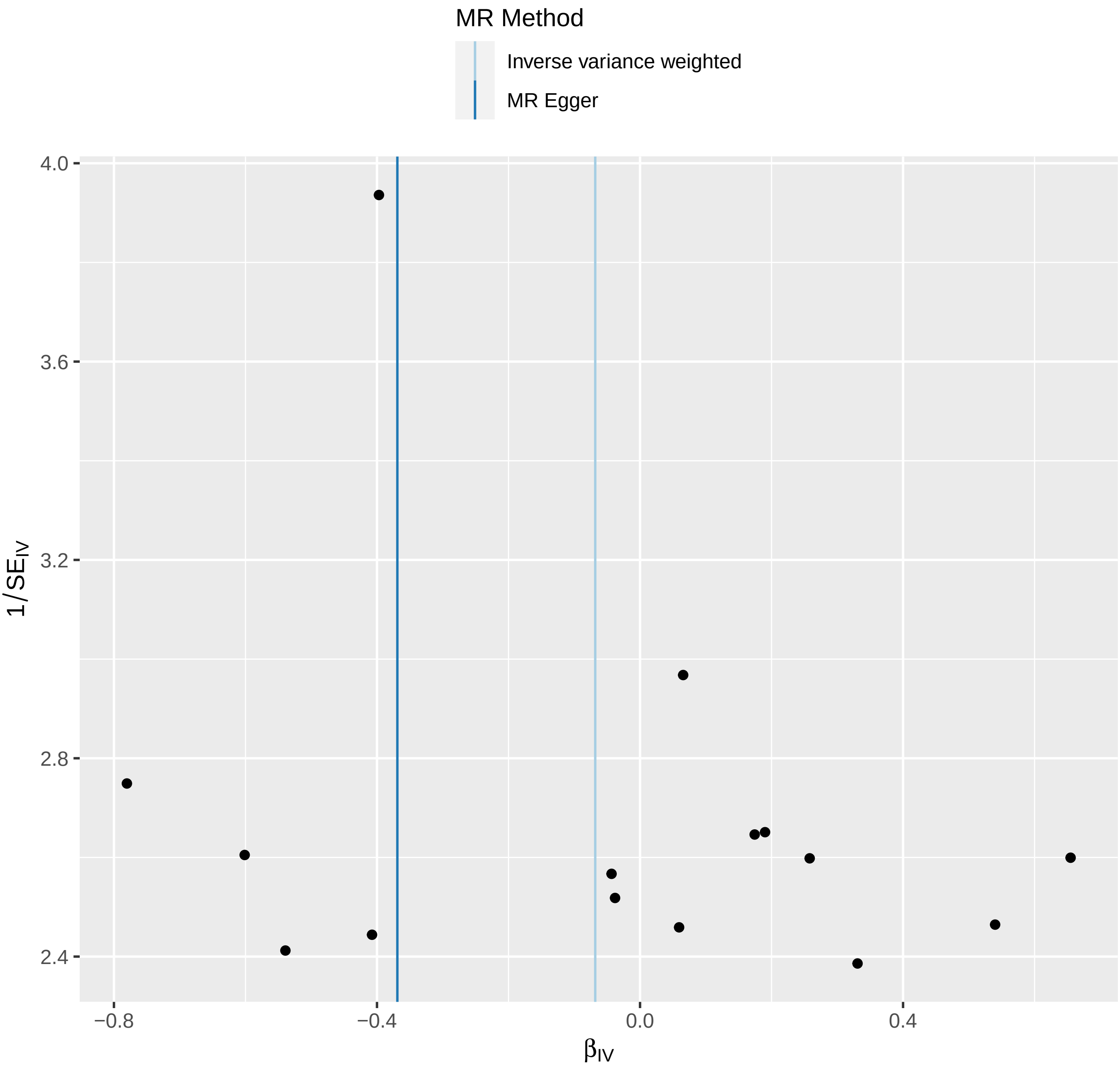

Batch 2−1323 : Gut microbiota abundance (genus Catenibacterium id.2153) on Cysteine−rich secretory protein 3 with a threshold of 1e−05

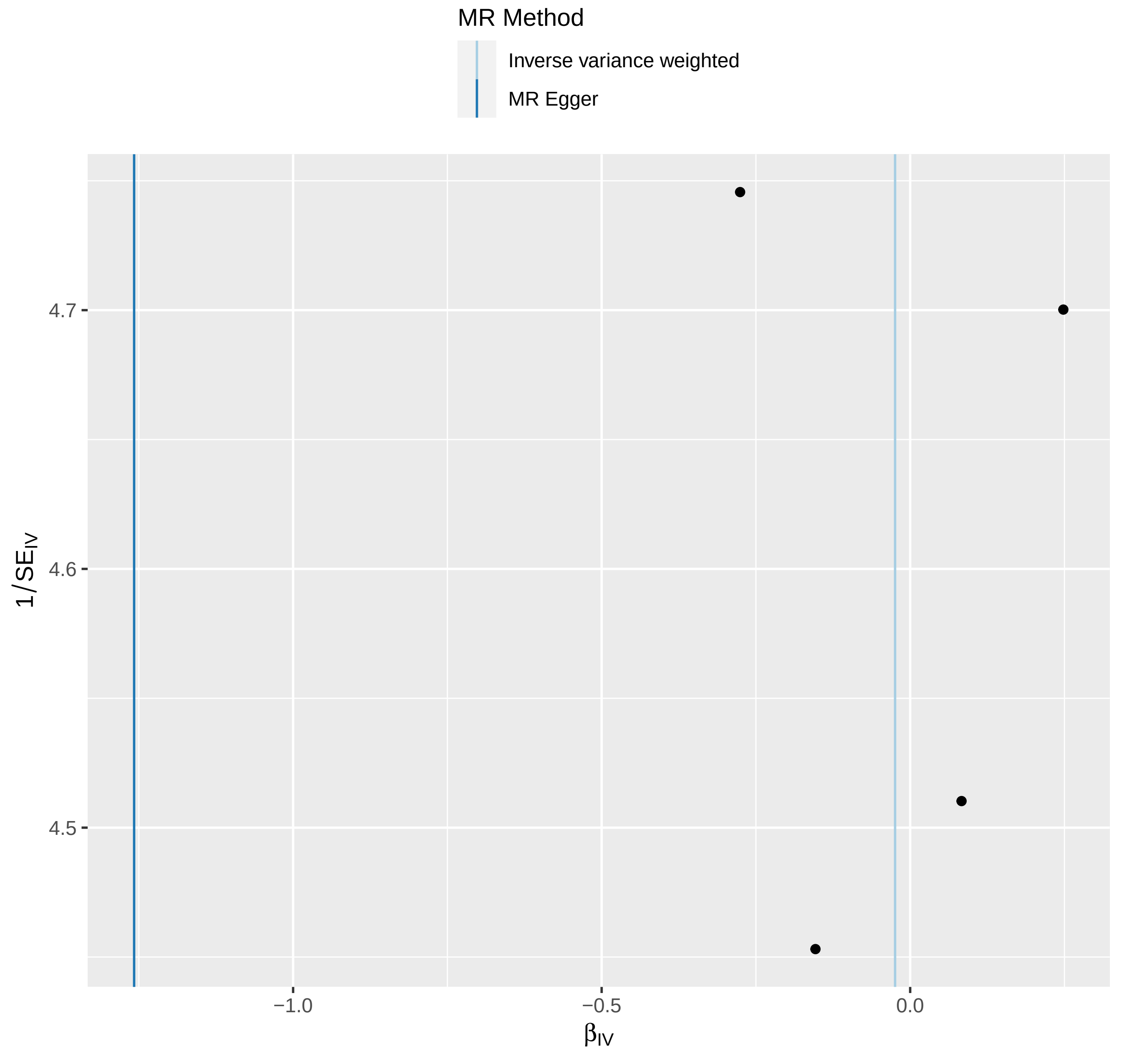

Batch 2−1324 : Gut microbiota abundance (genus Parasutterella id.2892) on Cysteine−rich secretory protein 3
with a threshold of 1e−05

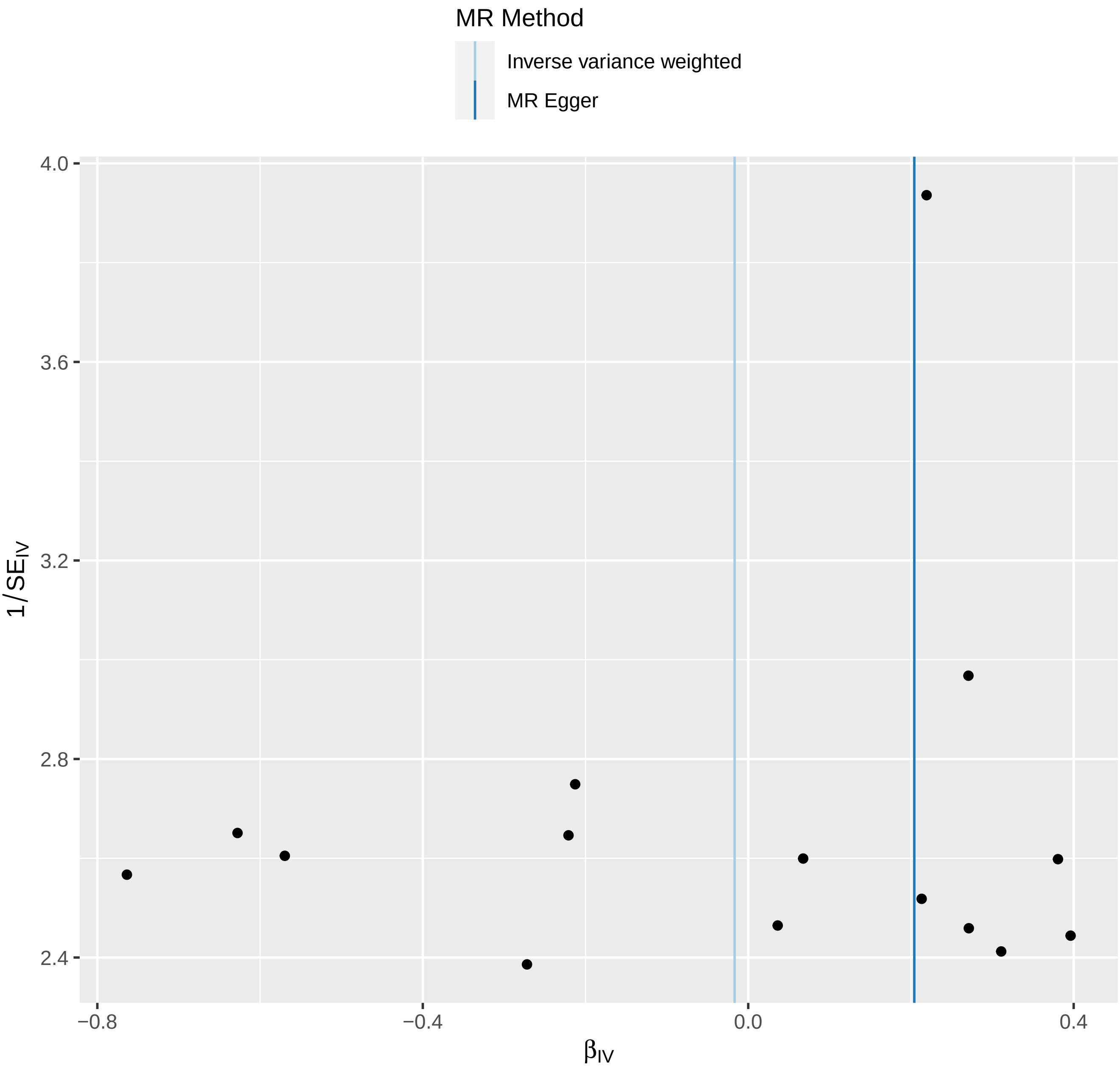

Batch 2−1325 : Gut microbiota abundance (genus Catenibacterium id.2153) on Cysteine−rich secretory protein LCCL domain−containing 2
with a threshold of 1e−05

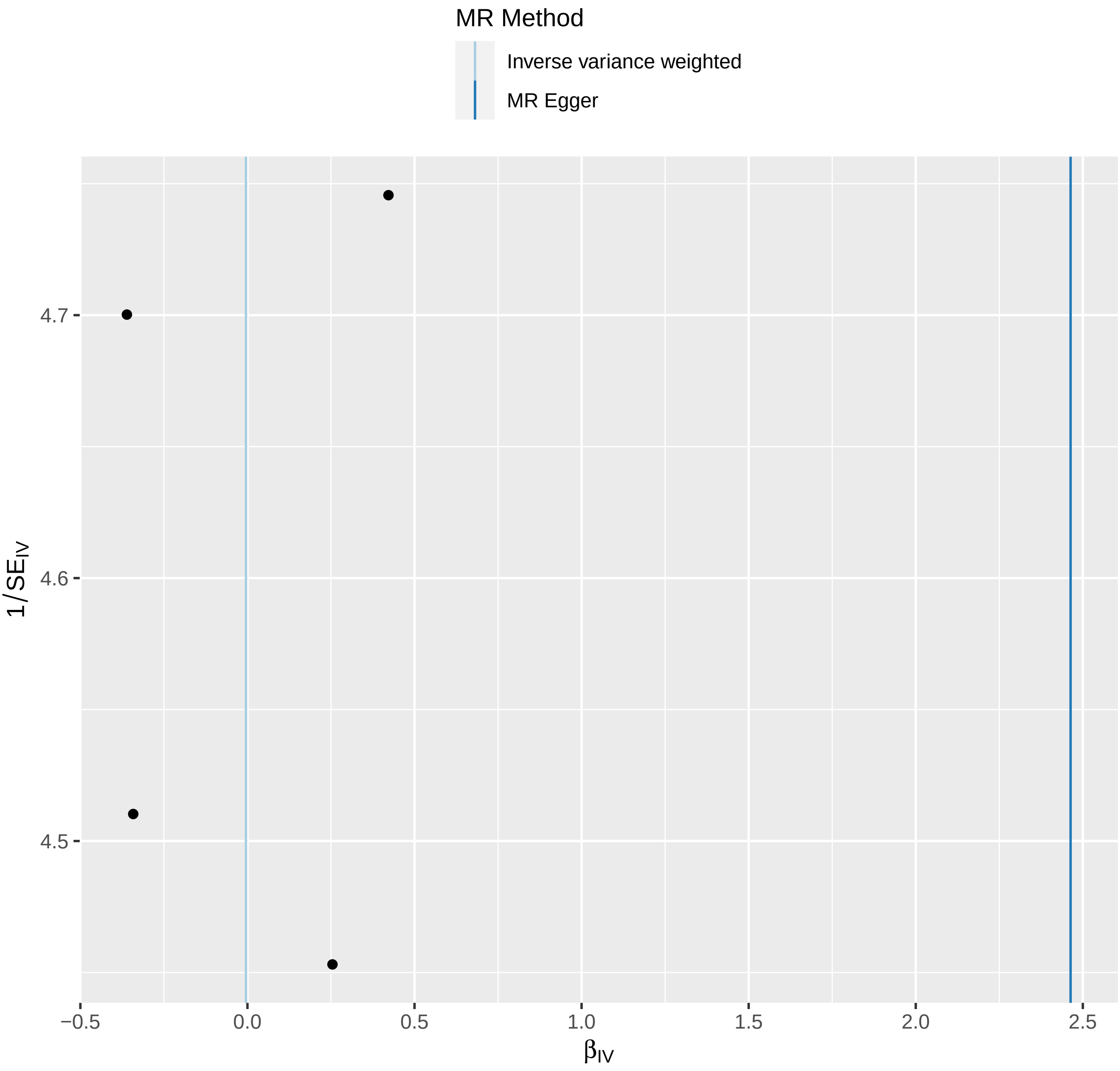

Batch 2−1326 : Gut microbiota abundance (genus Parasutterella id.2892) on Cysteine−rich secretory protein LCCL domain−containing 2
with a threshold of 1e−05

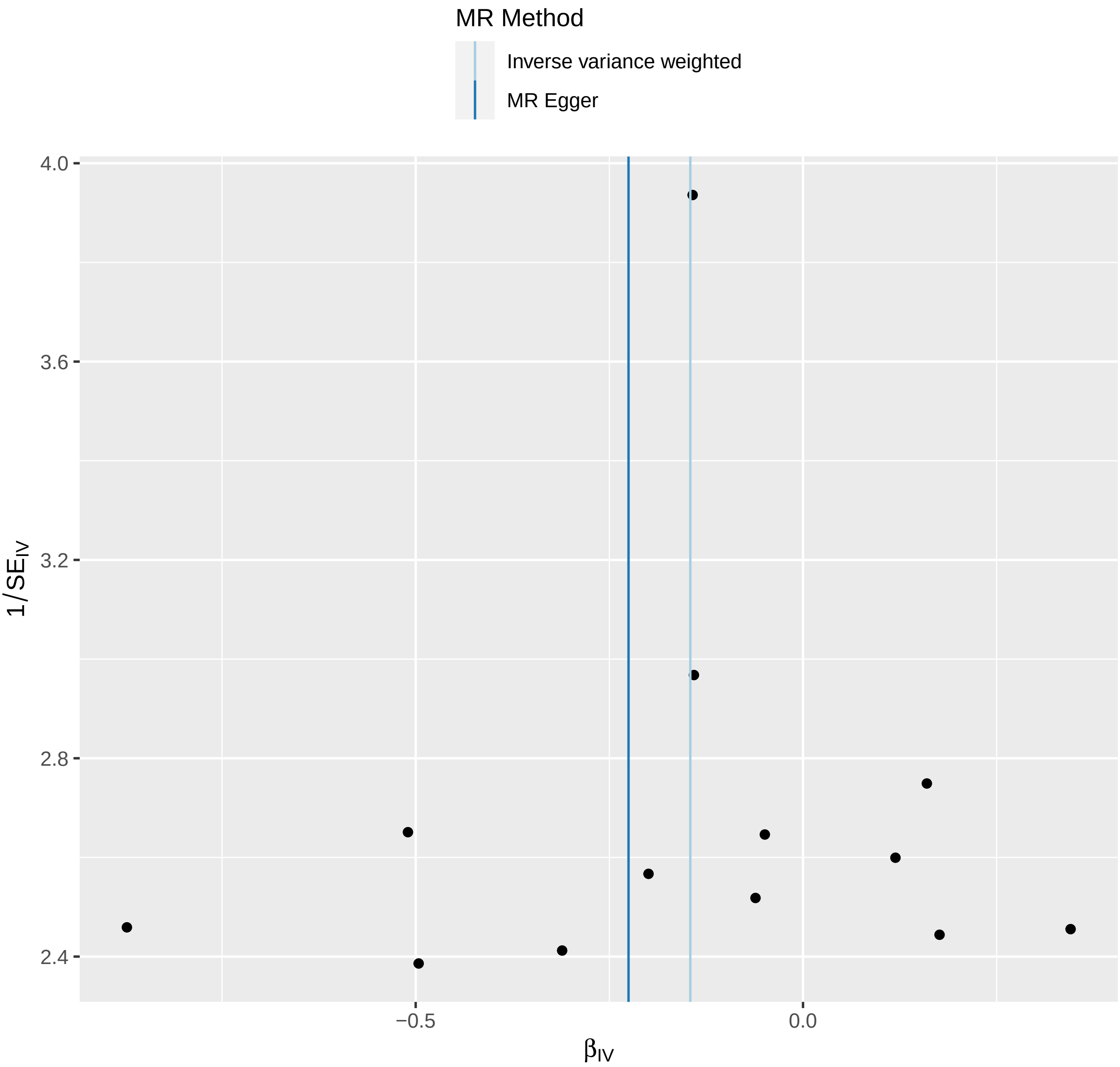

Batch 2−1327 : Gut microbiota abundance (genus Catenibacterium id.2153) on Adapter molecule crk
with a threshold of 1e−05

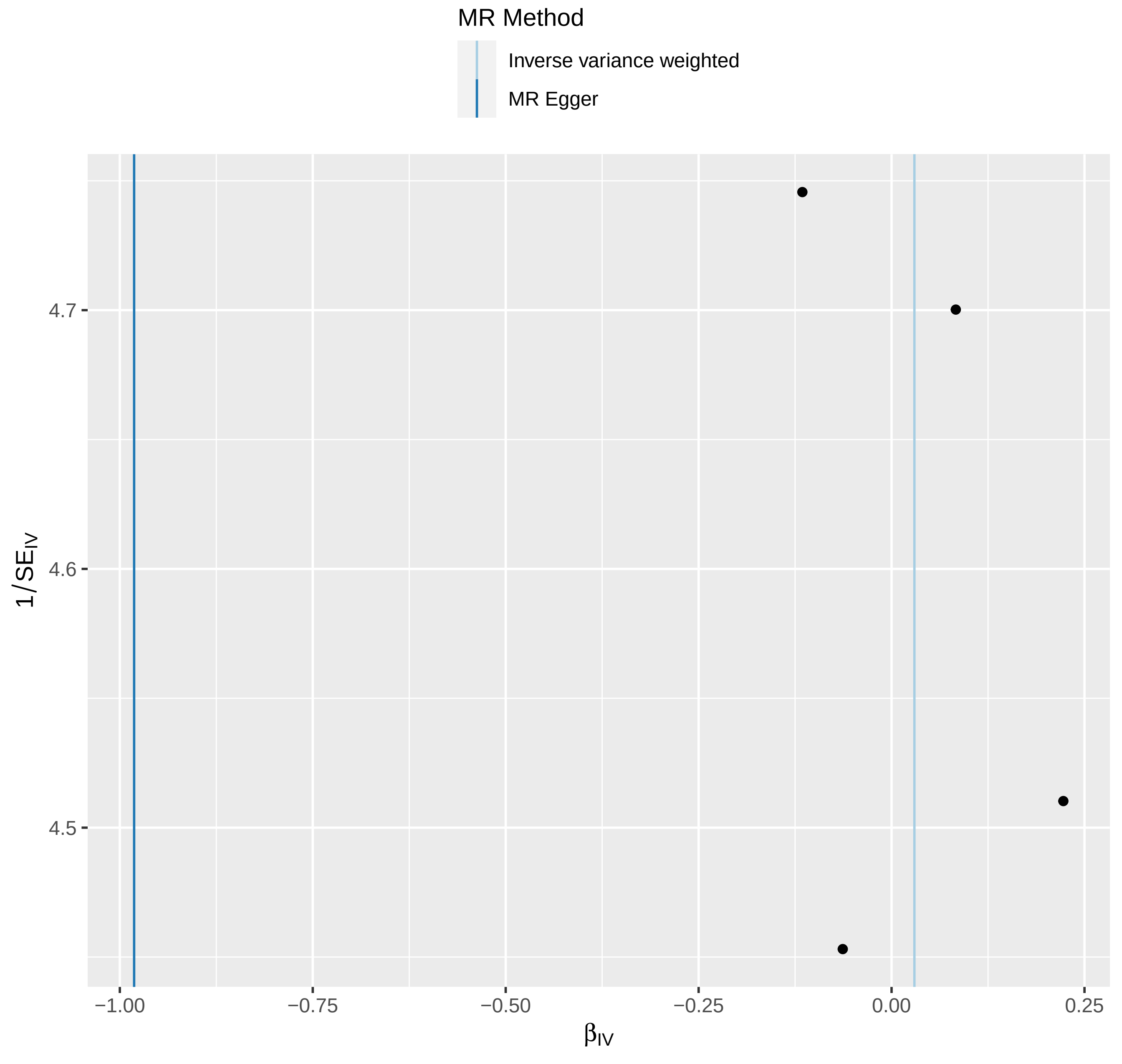

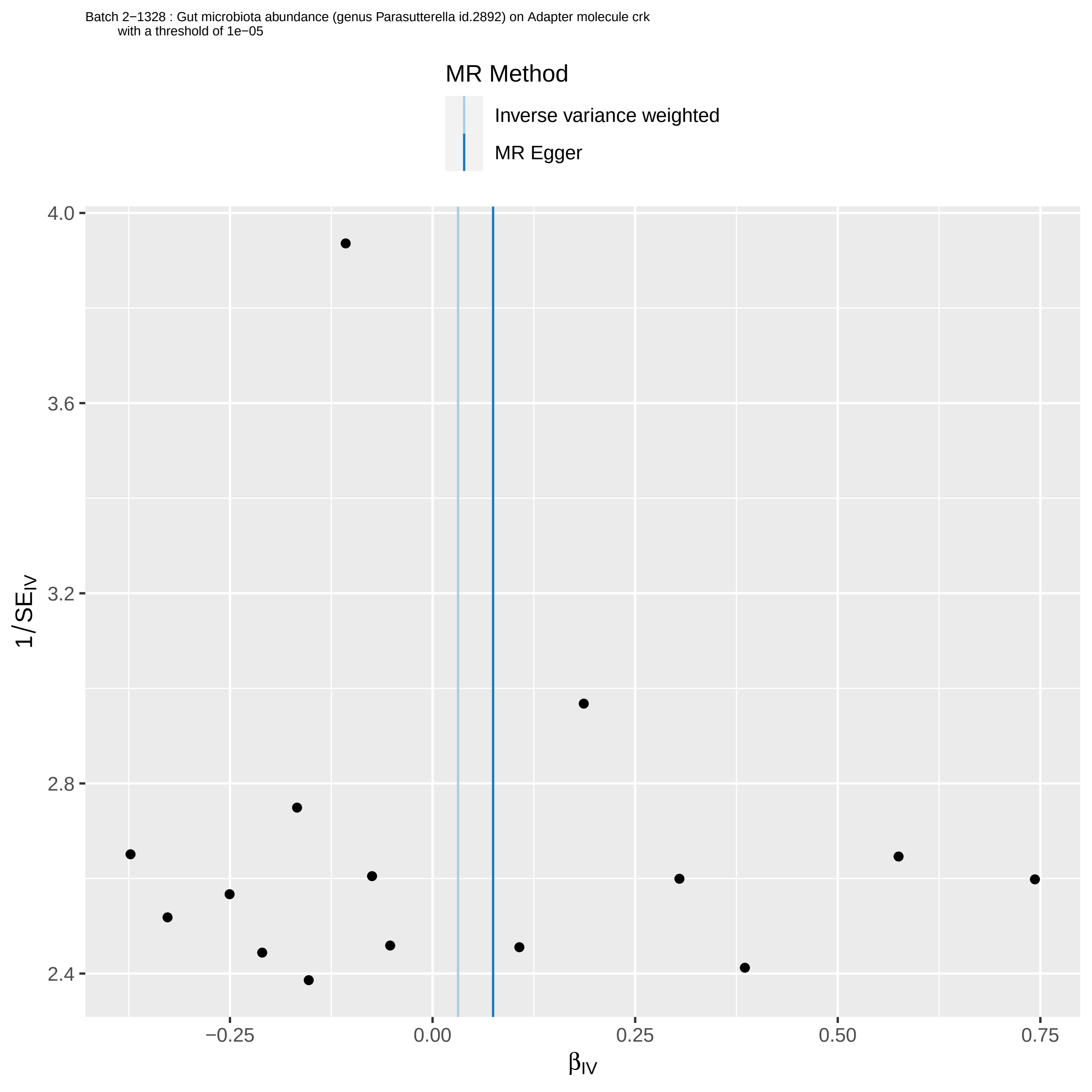

Batch 2−1328 : Gut microbiota abundance (genus Parasutterella id.2892) on Adapter molecule crk
with a threshold of 1e−05
MR Method
Inverse variance weighted
MR Egger
4.0
3.6
3.2
2.8
2.4
$1/SE_{IV}$
−0.25
0.00
0.25
0.50
0.75
β_{IV}

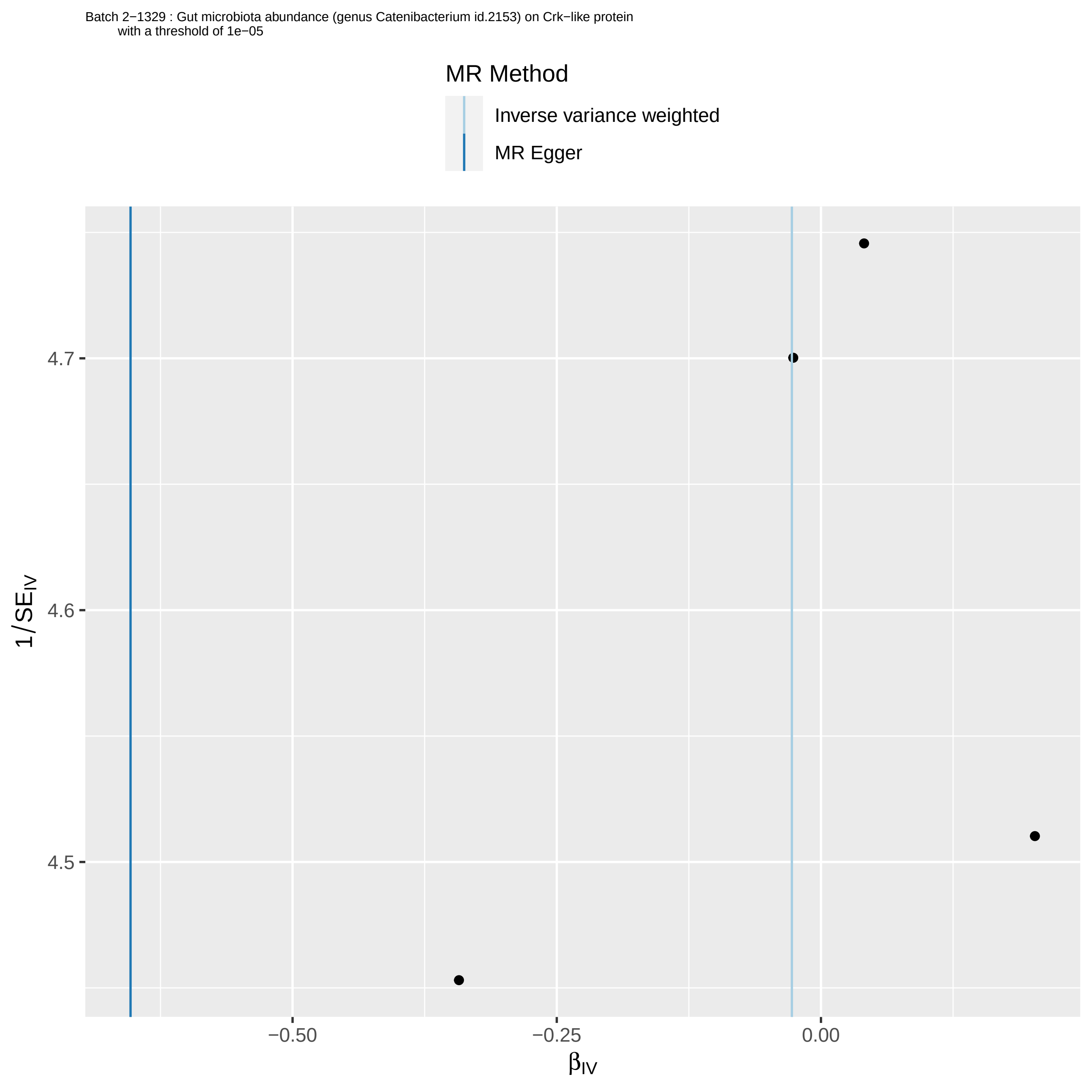
Batch 2−1329 : Gut microbiota abundance (genus Catenibacterium id.2153) on Crk−like protein
with a threshold of 1e−05
MR Method
Inverse variance weighted
MR Egger
4.7
4.6
4.5
1/SE$_{IV}$
−0.50
−0.25
0.00
β$_{IV}$

Batch 2−1330 : Gut microbiota abundance (genus Parasutterella id.2892) on Crk−like protein
with a threshold of 1e−05

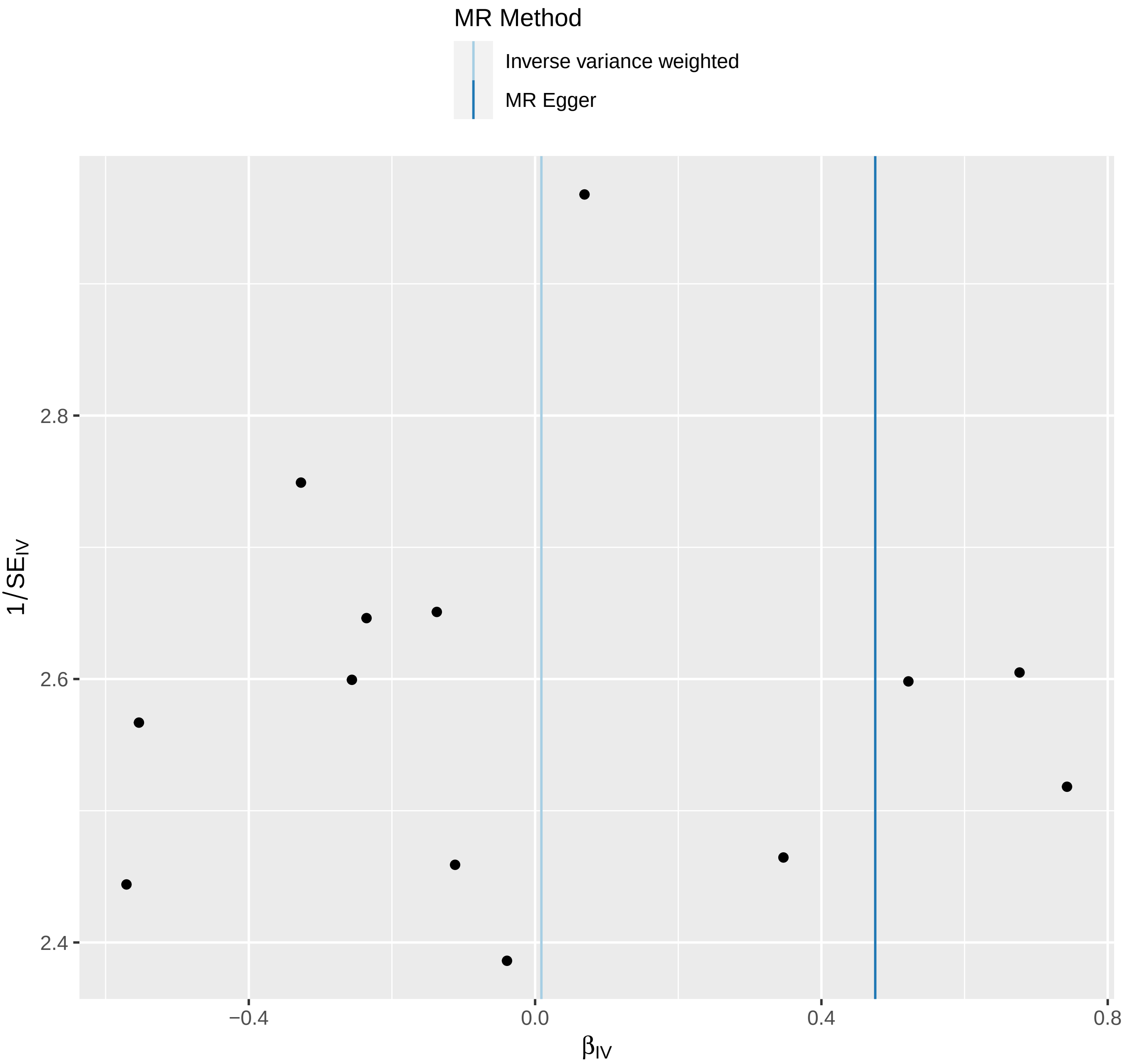

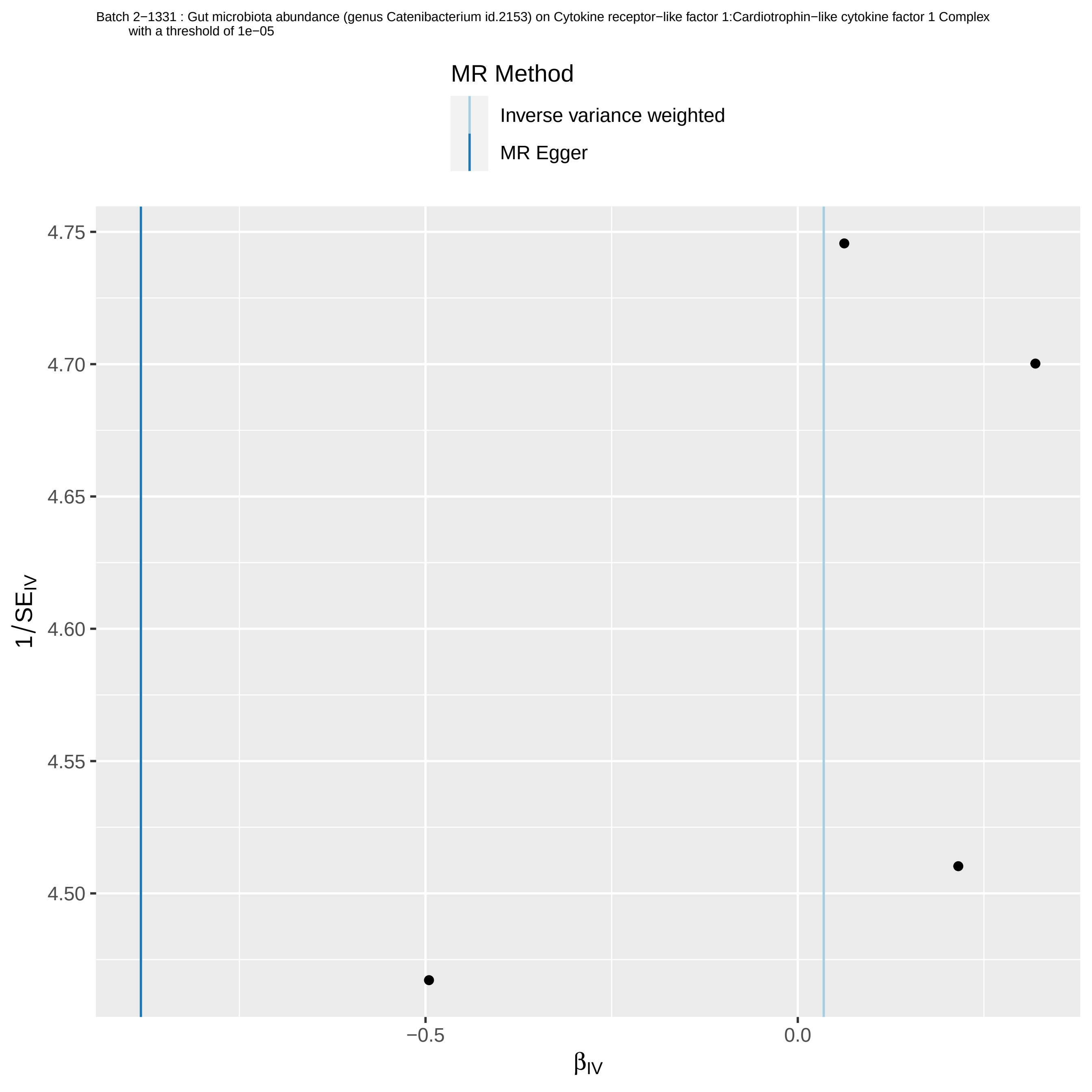
Batch 2−1331 : Gut microbiota abundance (genus Catenibacterium id.2153) on Cytokine receptor−like factor 1:Cardiotrophin−like cytokine factor 1 Complex with a threshold of 1e−05
MR Method
Inverse variance weighted
MR Egger
4.75
4.70
4.65
4.60
4.55
4.50
1/SE_IV
−0.5
0.0
β_IV

Batch 2−1332 : Gut microbiota abundance (genus Parasutterella id.2892) on Cytokine receptor−like factor 1:Cardiotrophin−like cytokine factor 1 Complex
with a threshold of 1e−05

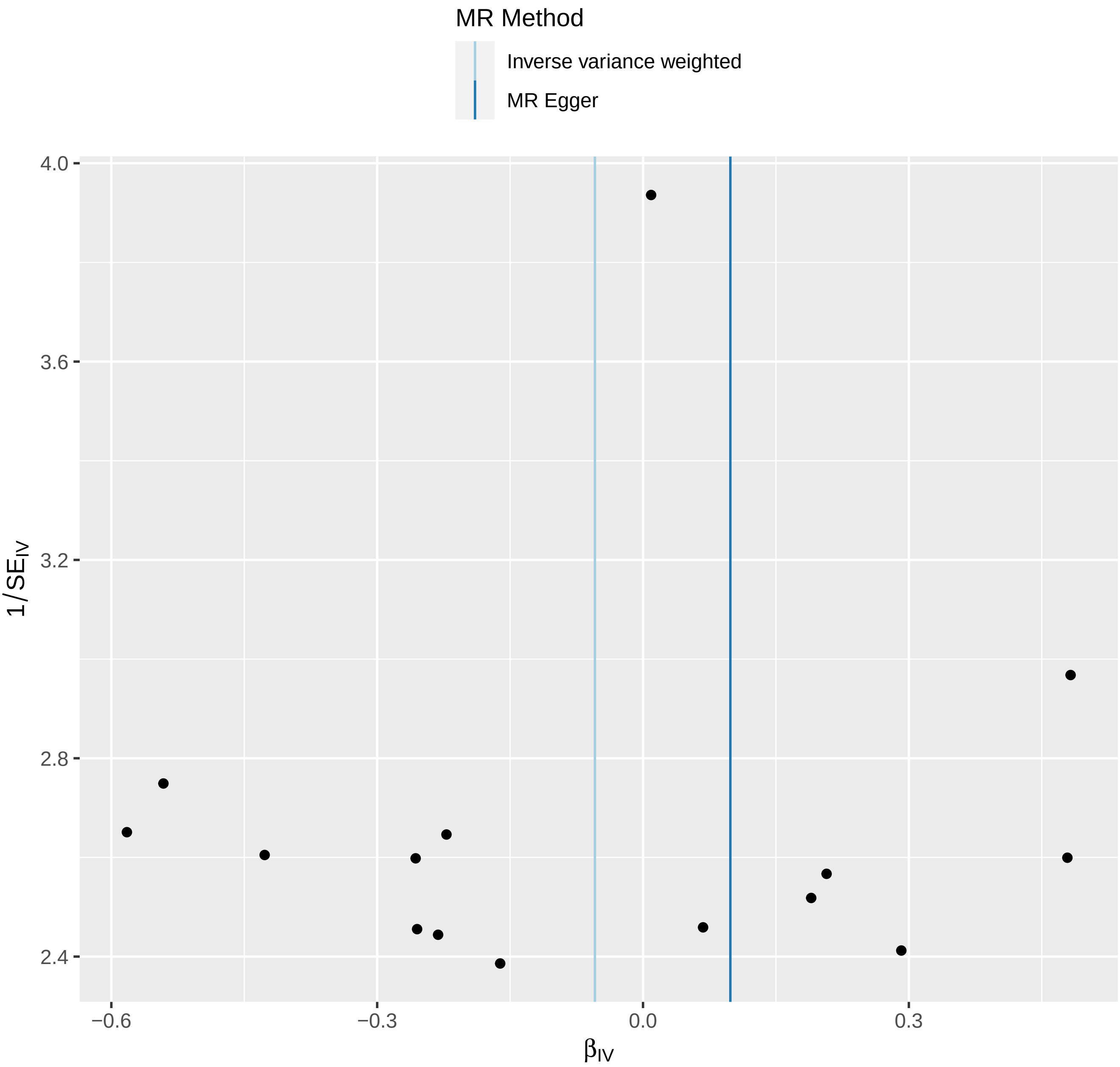

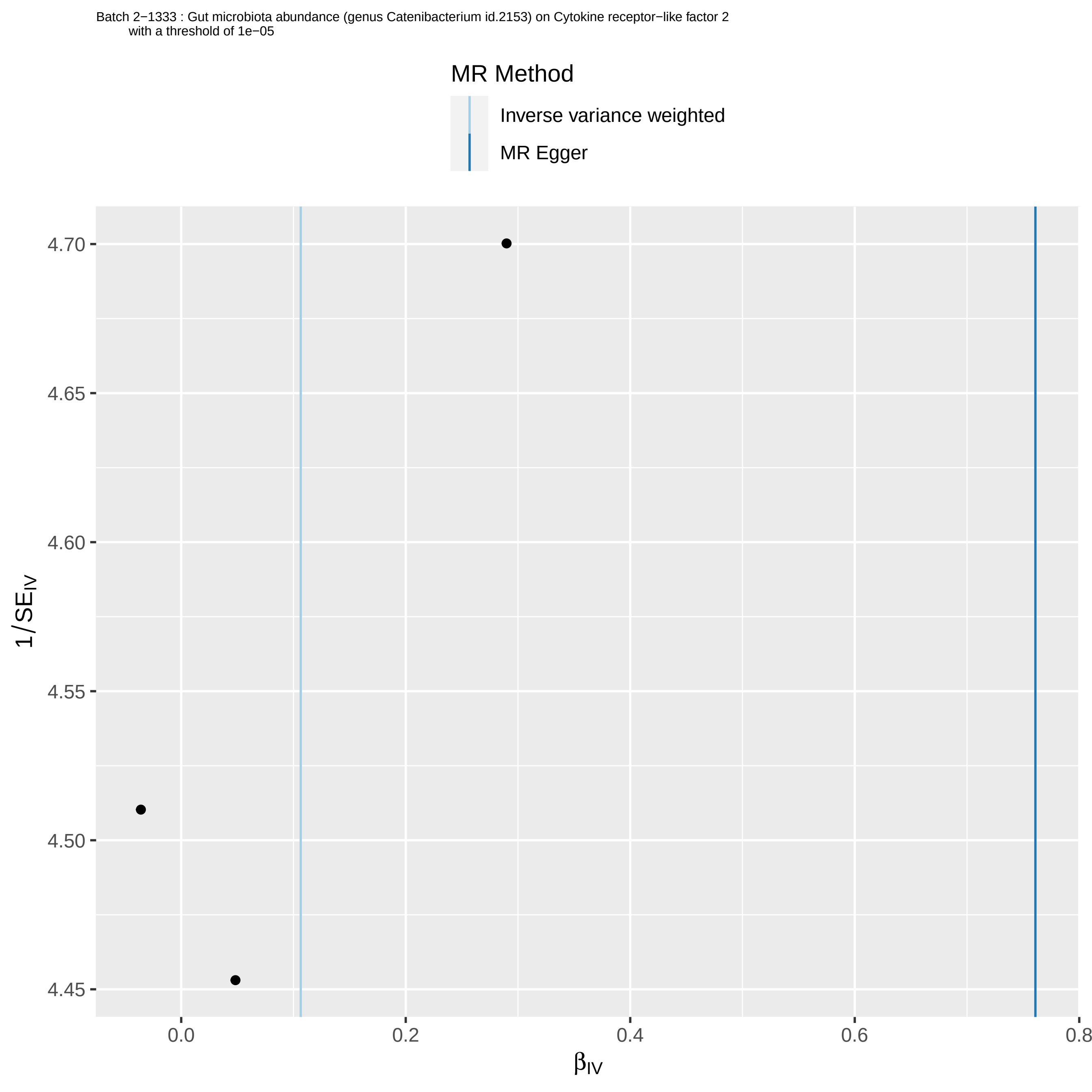

Batch 2−1333 : Gut microbiota abundance (genus Catenibacterium id.2153) on Cytokine receptor−like factor 2
with a threshold of 1e−05
MR Method
Inverse variance weighted
MR Egger
4.70
4.65
4.60
4.55
4.50
4.45
1/SE_IV
0.0
0.2
0.4
0.6
0.8
β_IV

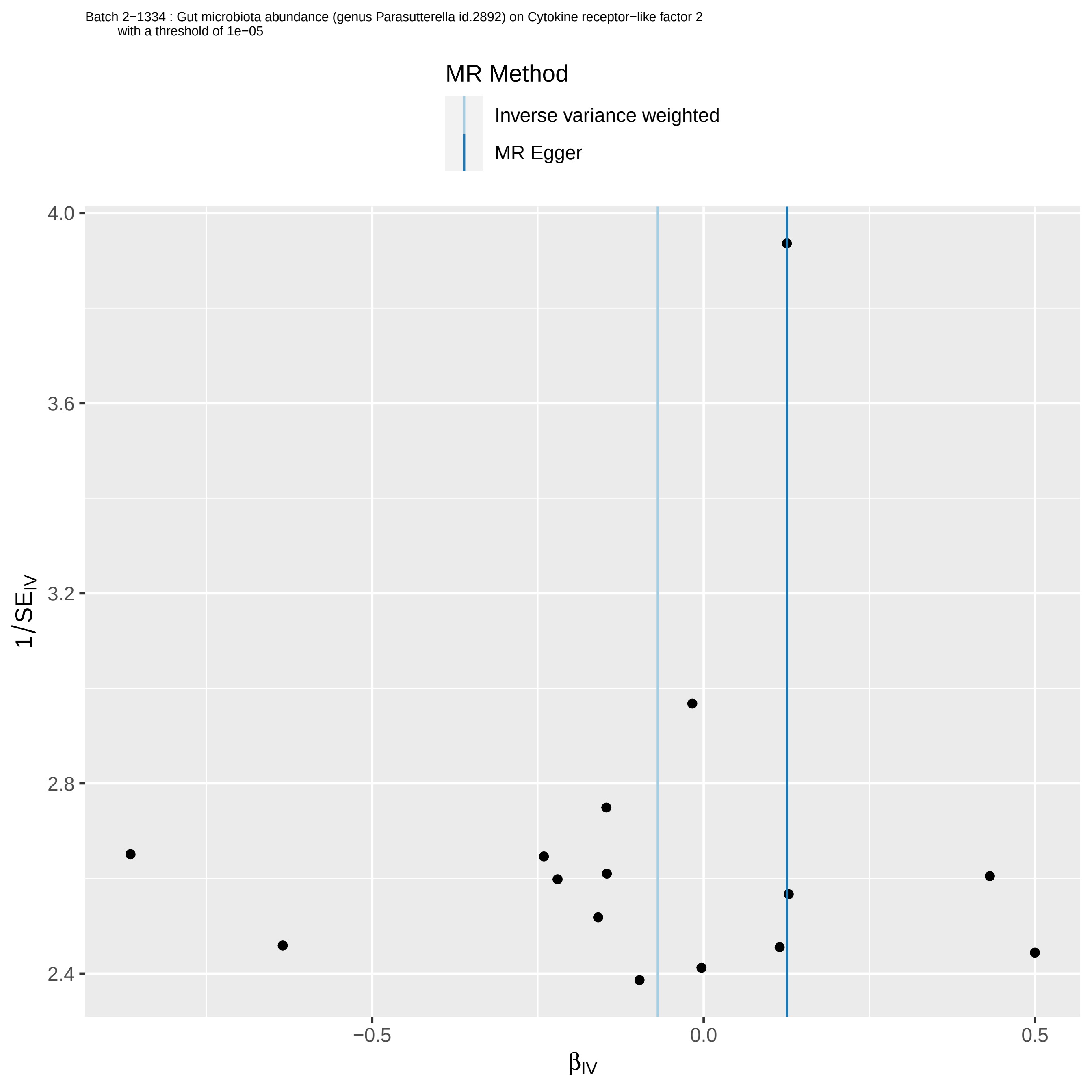

Batch 2−1334 : Gut microbiota abundance (genus Parasutterella id.2892) on Cytokine receptor−like factor 2
with a threshold of 1e−05
MR Method
Inverse variance weighted
MR Egger
4.0
3.6
3.2
2.8
2.4
1/SE_IV
−0.5
0.0
0.5
β_IV

Batch 2−1335 : Gut microbiota abundance (genus Catenibacterium id.2153) on Cytokine receptor−like factor 2
with a threshold of 1e−05

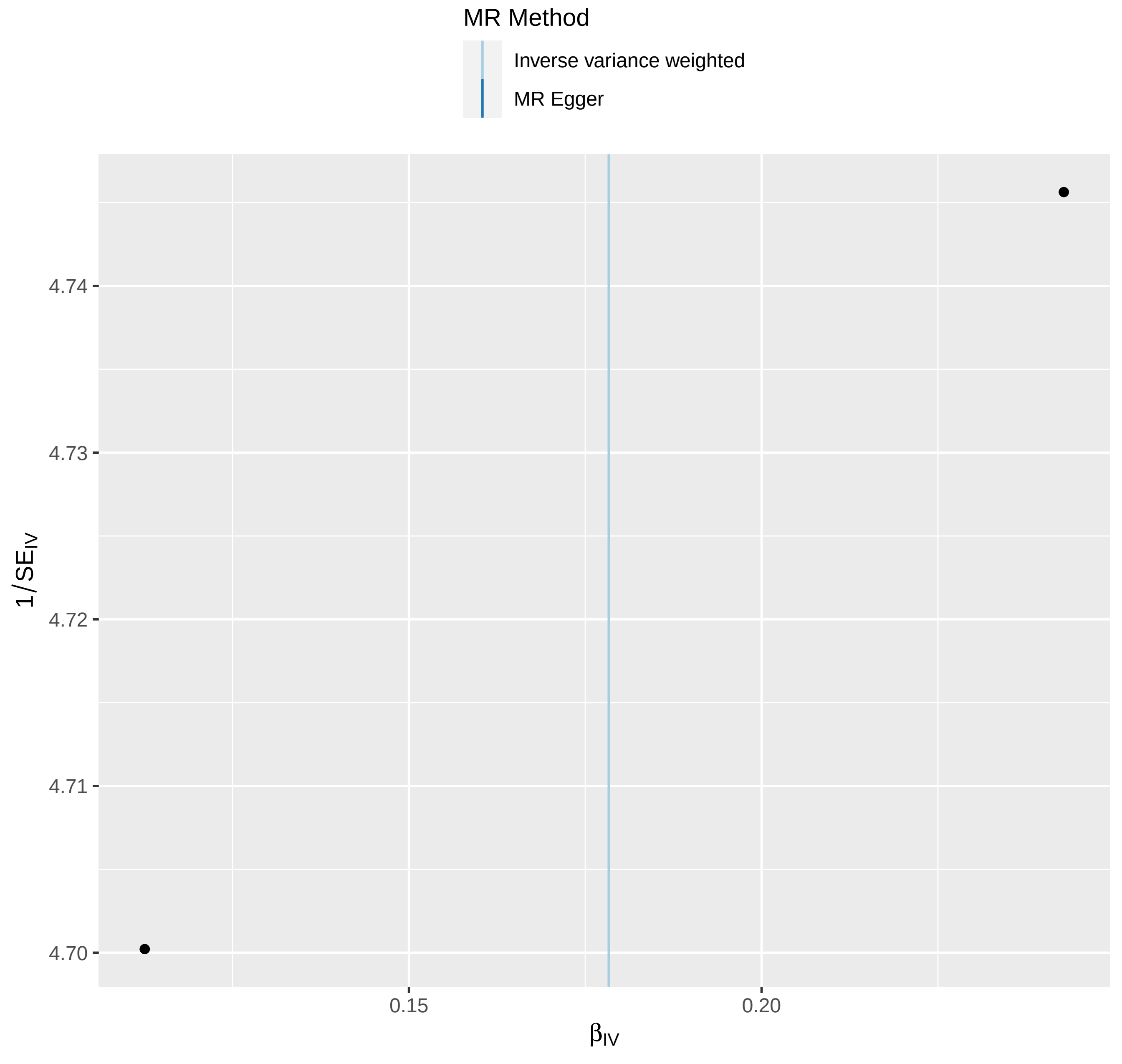

Batch 2−1336 : Gut microbiota abundance (genus Parasutterella id.2892) on Cytokine receptor−like factor 2
with a threshold of 1e−05

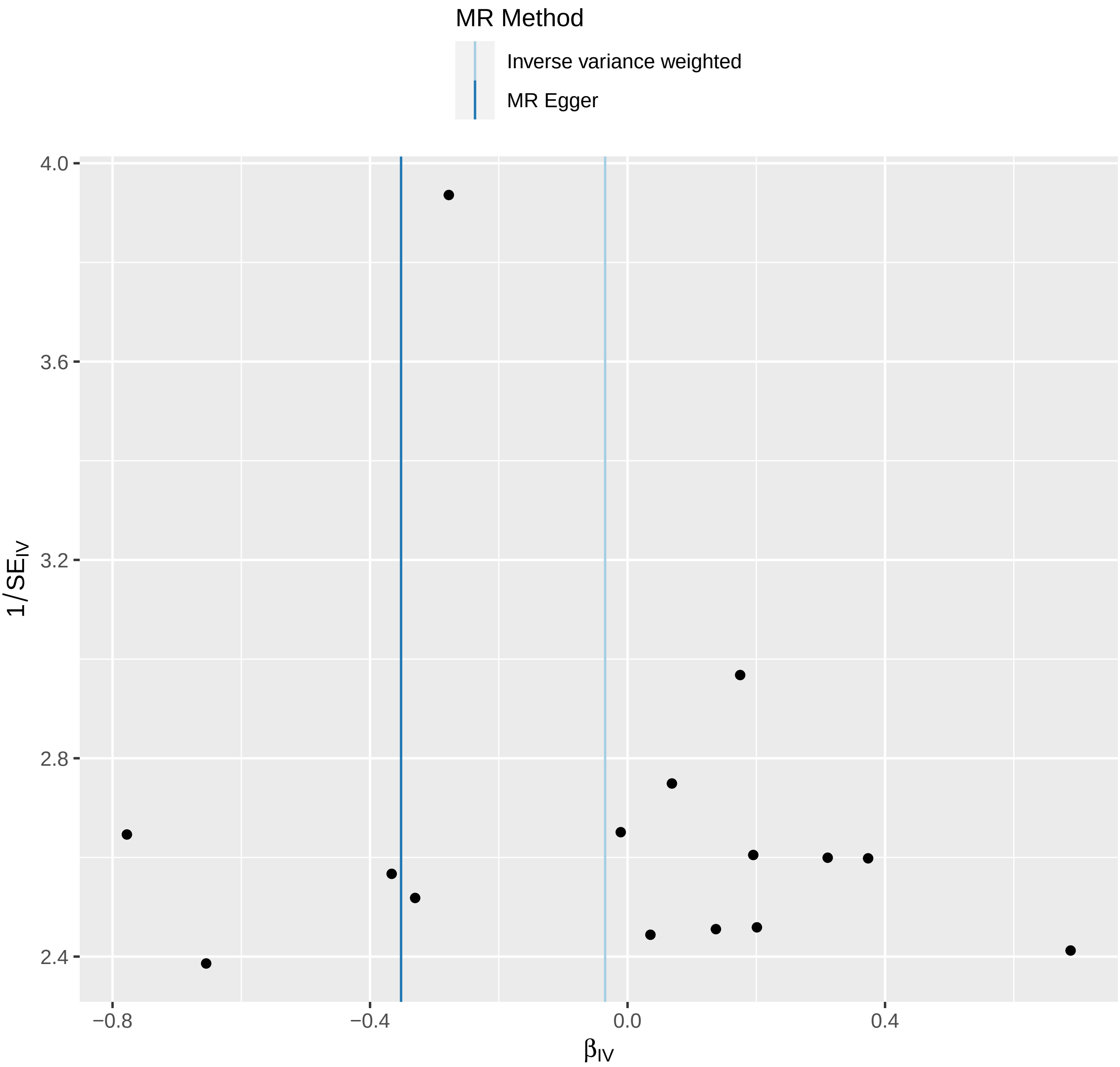

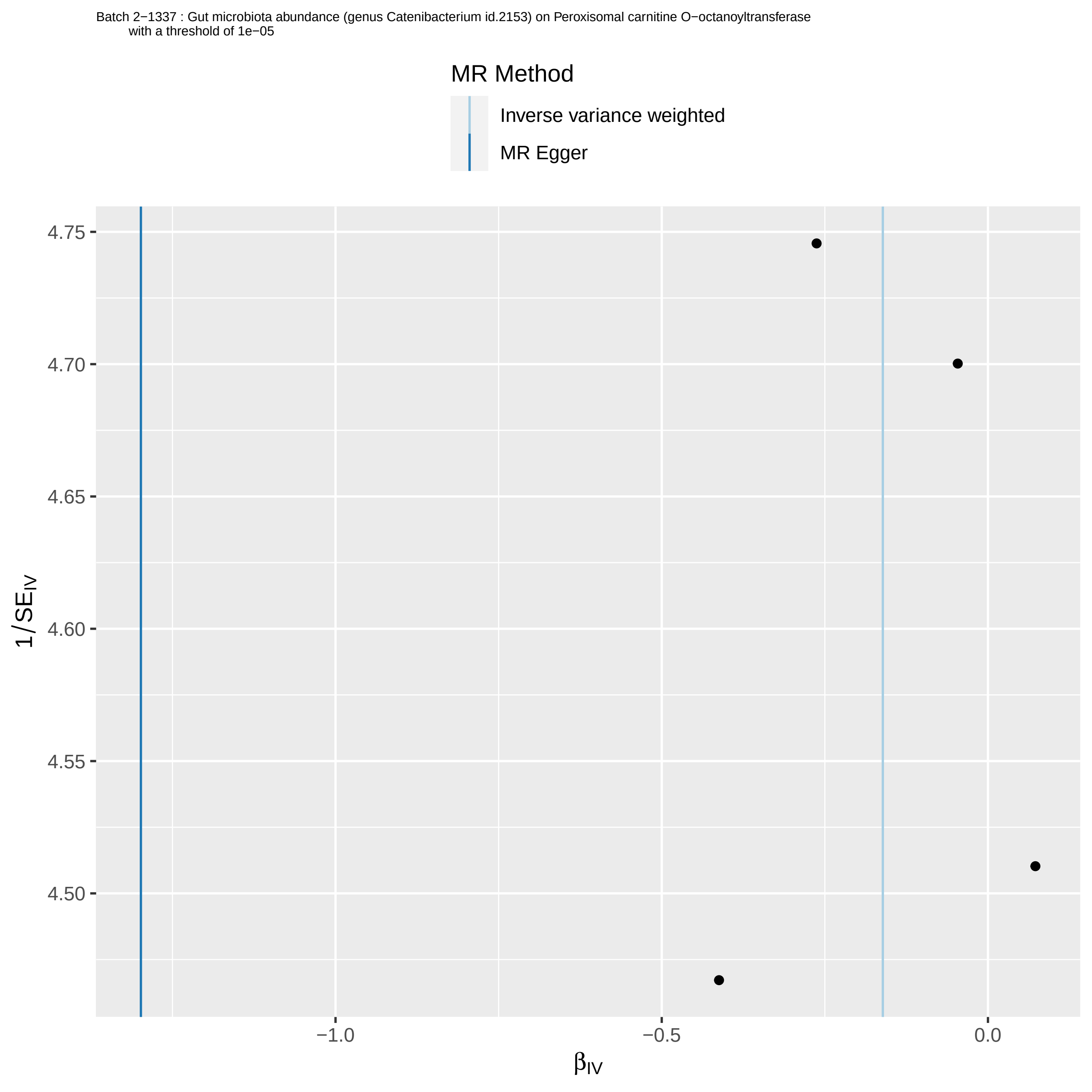
Batch 2−1337 : Gut microbiota abundance (genus Catenibacterium id.2153) on Peroxisomal carnitine O−octanoyltransferase
with a threshold of 1e−05
MR Method
Inverse variance weighted
MR Egger
4.75
4.70
4.65
4.60
4.55
4.50
1/SE_IV
−1.0
−0.5
0.0
β_IV

Batch 2−1338 : Gut microbiota abundance (genus Parasutterella id.2892) on Peroxisomal carnitine O−octanoyltransferase
with a threshold of 1e−05

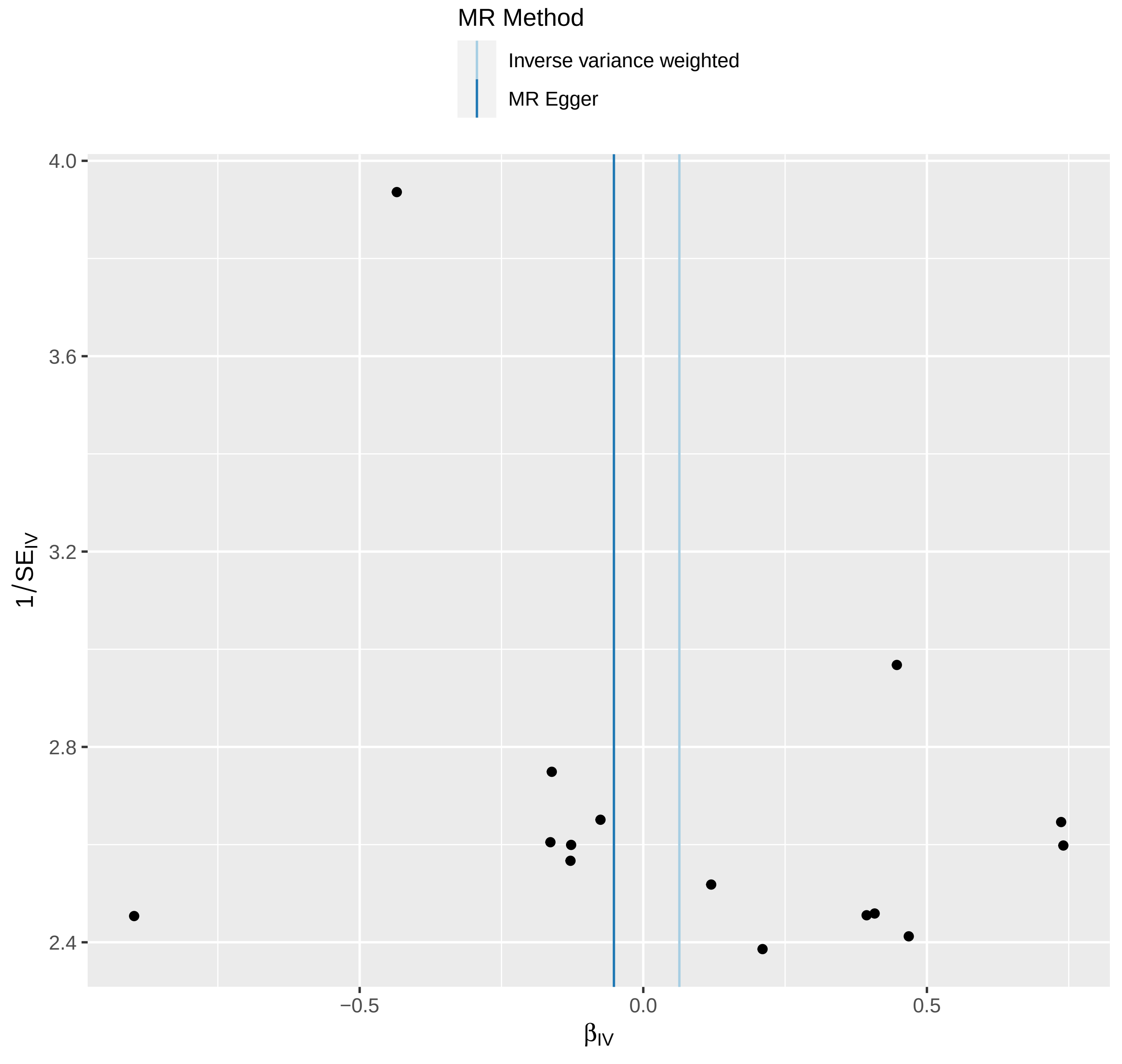

Batch 2−1339 : Gut microbiota abundance (genus Catenibacterium id.2153) on C−reactive protein
with a threshold of 1e−05

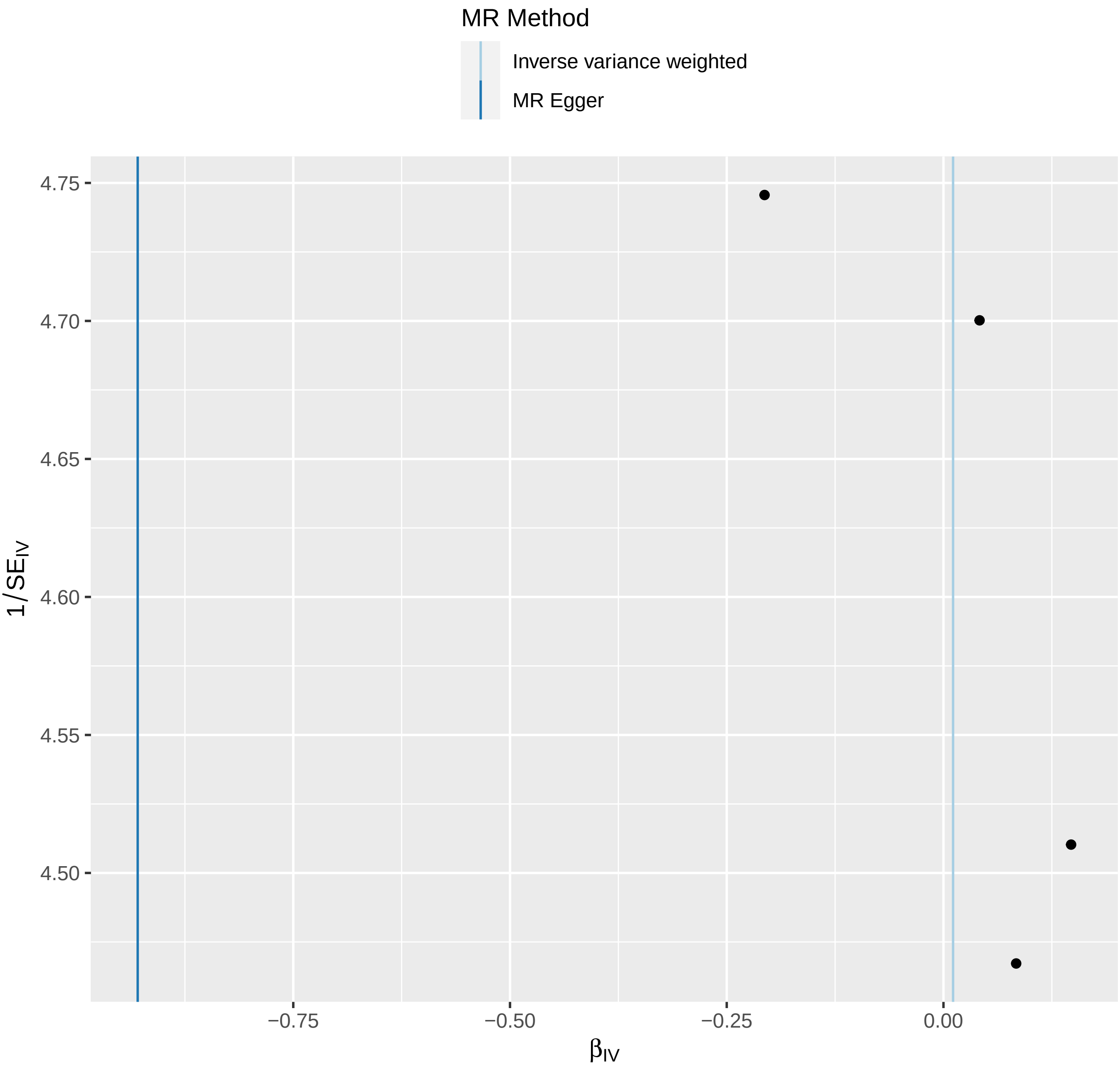

Batch 2−1340 : Gut microbiota abundance (genus Parasutterella id.2892) on C−reactive protein
with a threshold of 1e−05

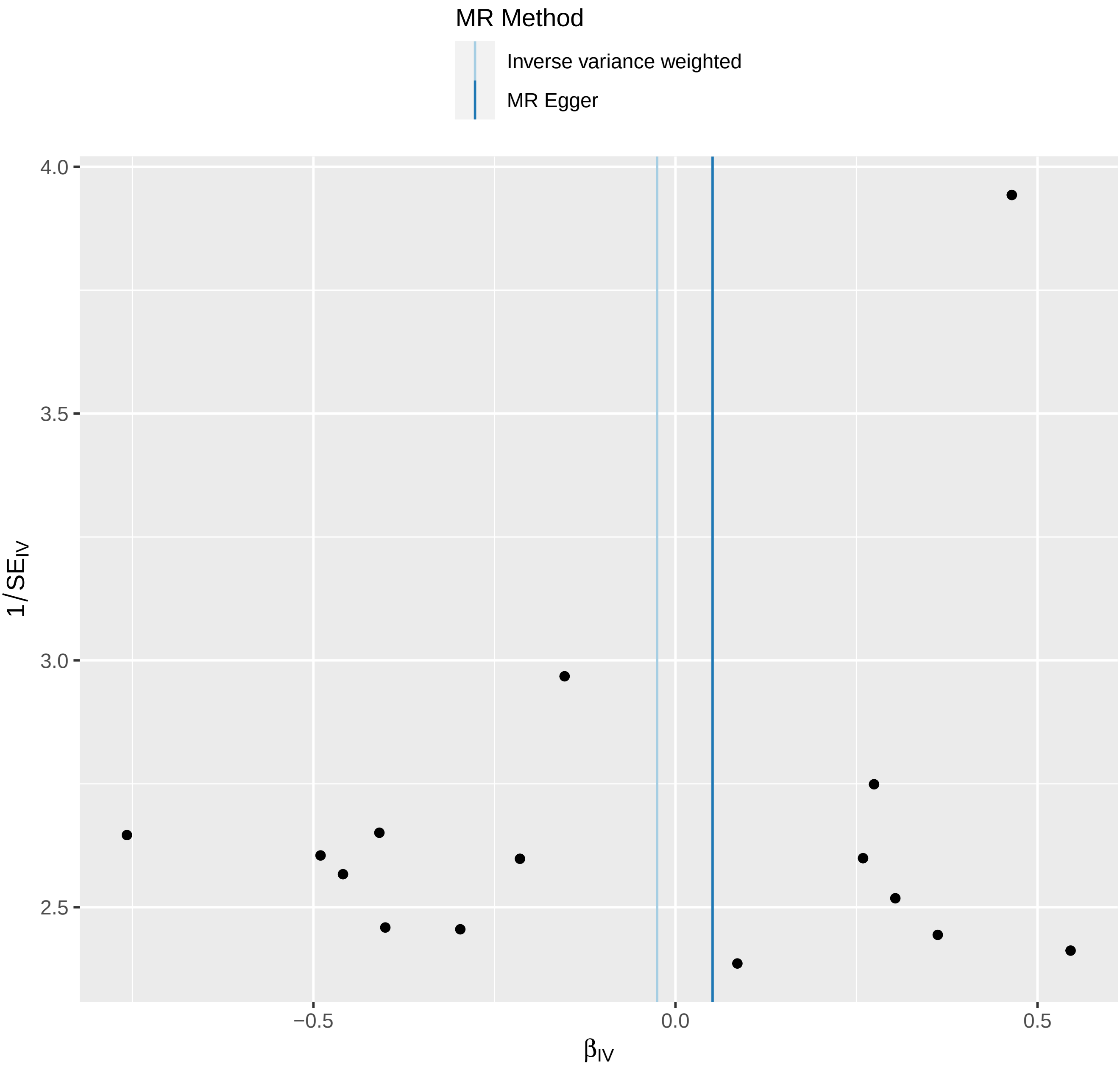

Batch 2−1341 : Gut microbiota abundance (genus Catenibacterium id.2153) on Cytotoxic and regulatory T−cell molecule
with a threshold of 1e−05

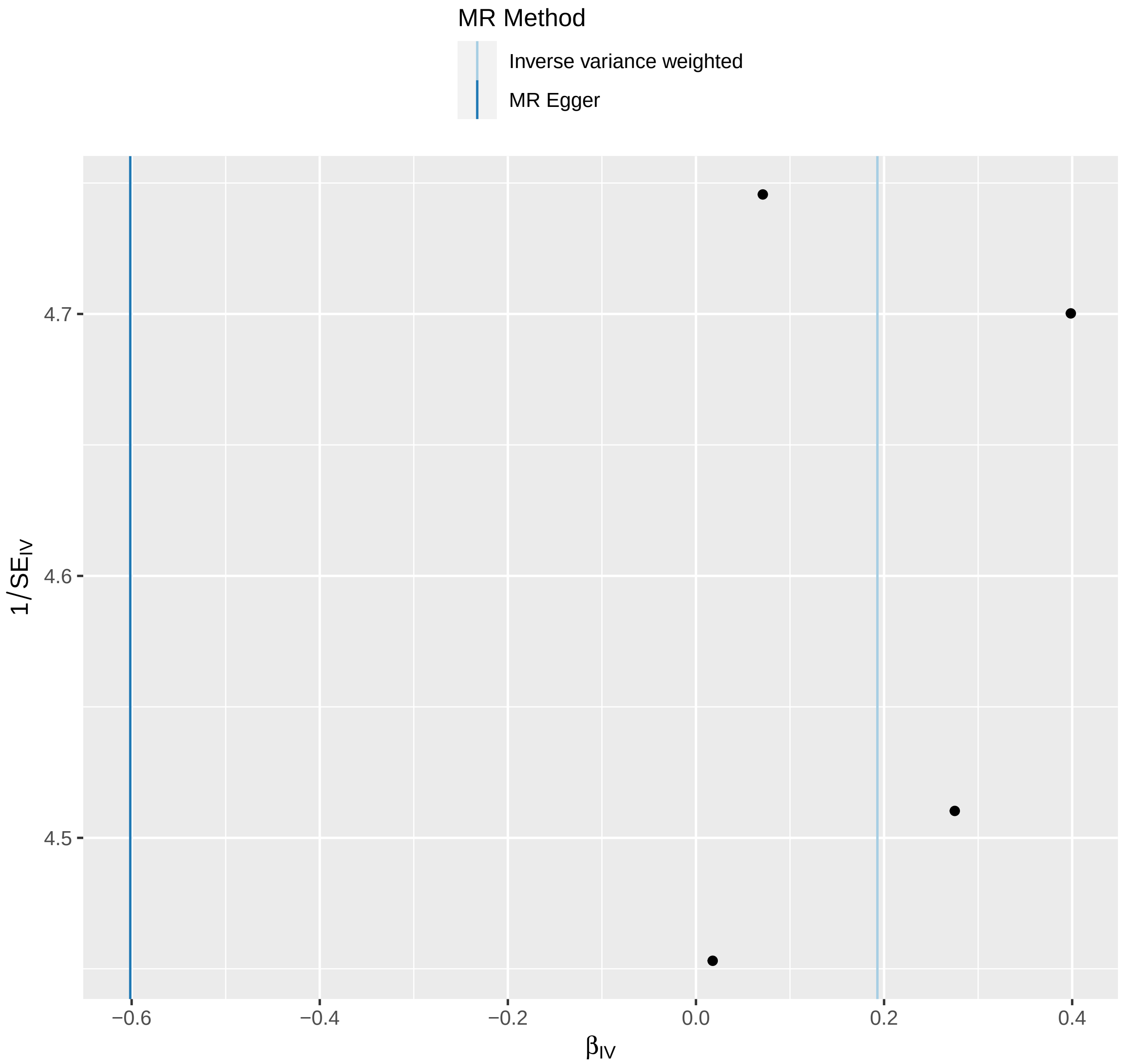

Batch 2−1342 : Gut microbiota abundance (genus Parasutterella id.2892) on Cytotoxic and regulatory T−cell molecule
with a threshold of 1e−05

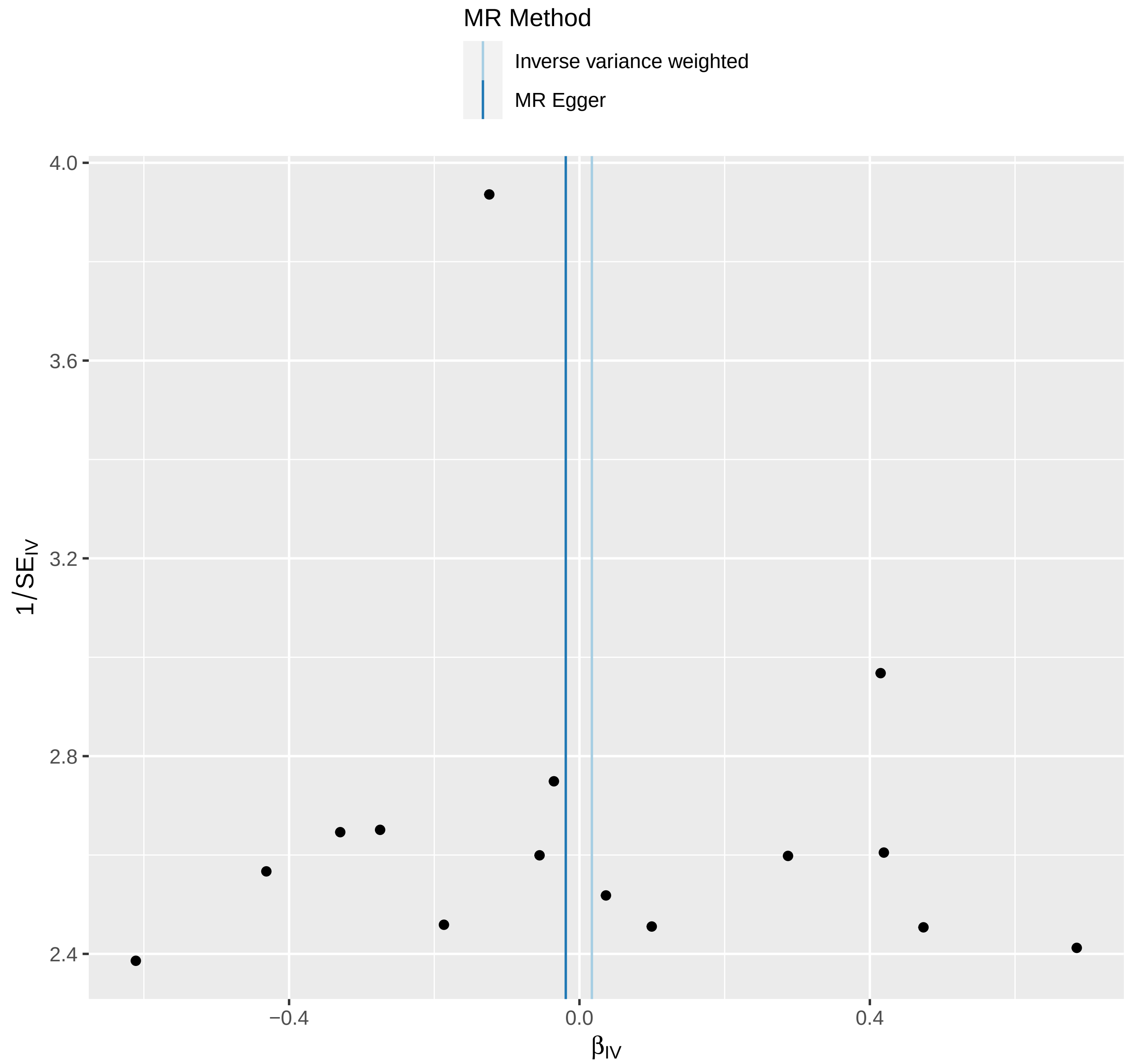

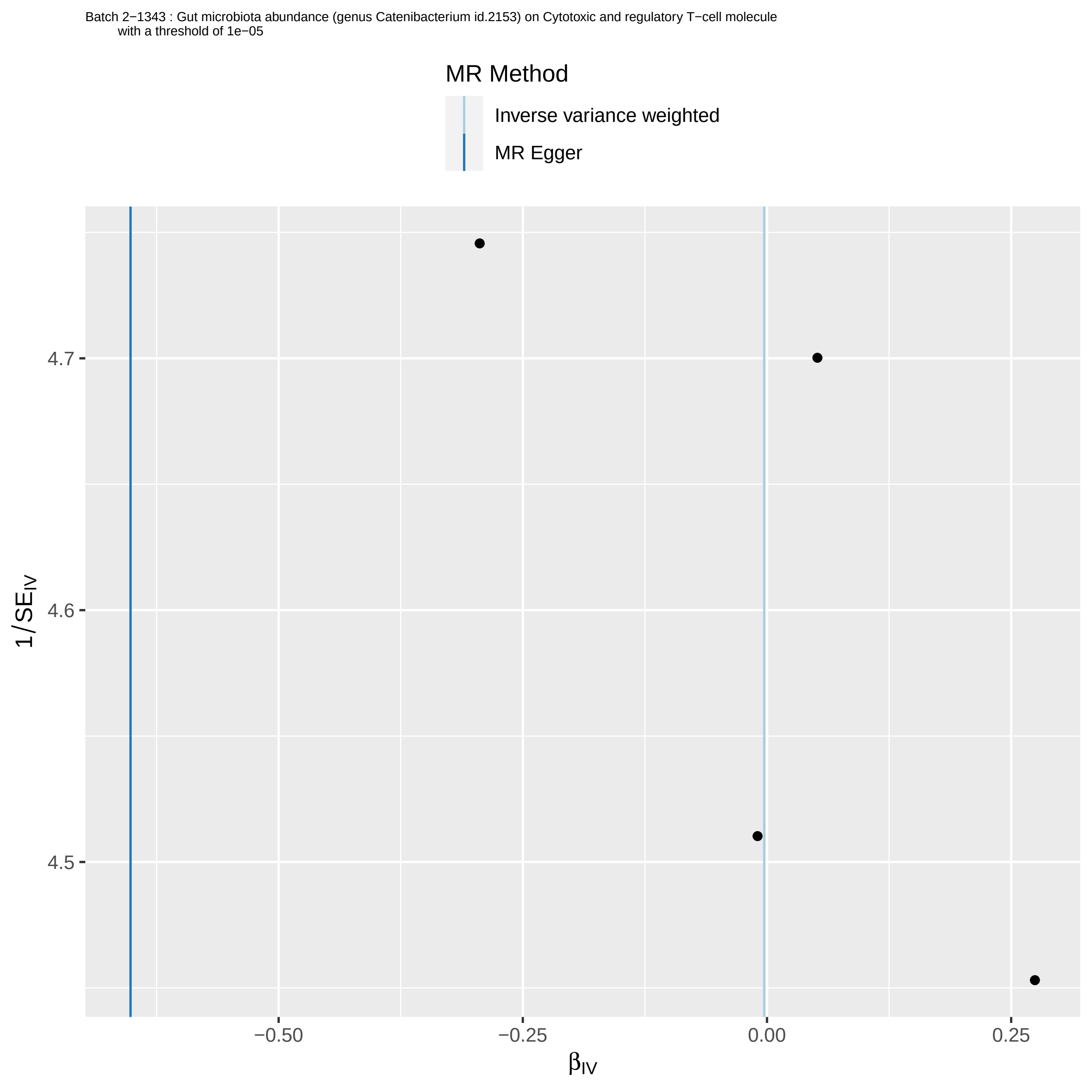

Batch 2−1343 : Gut microbiota abundance (genus Catenibacterium id.2153) on Cytotoxic and regulatory T−cell molecule
with a threshold of 1e−05
MR Method
Inverse variance weighted
MR Egger
4.7
4.6
4.5
1/SE_IV
−0.50
−0.25
0.00
0.25
β_IV

Batch 2−1344 : Gut microbiota abundance (genus Parasutterella id.2892) on Cytotoxic and regulatory T−cell molecule
with a threshold of 1e−05

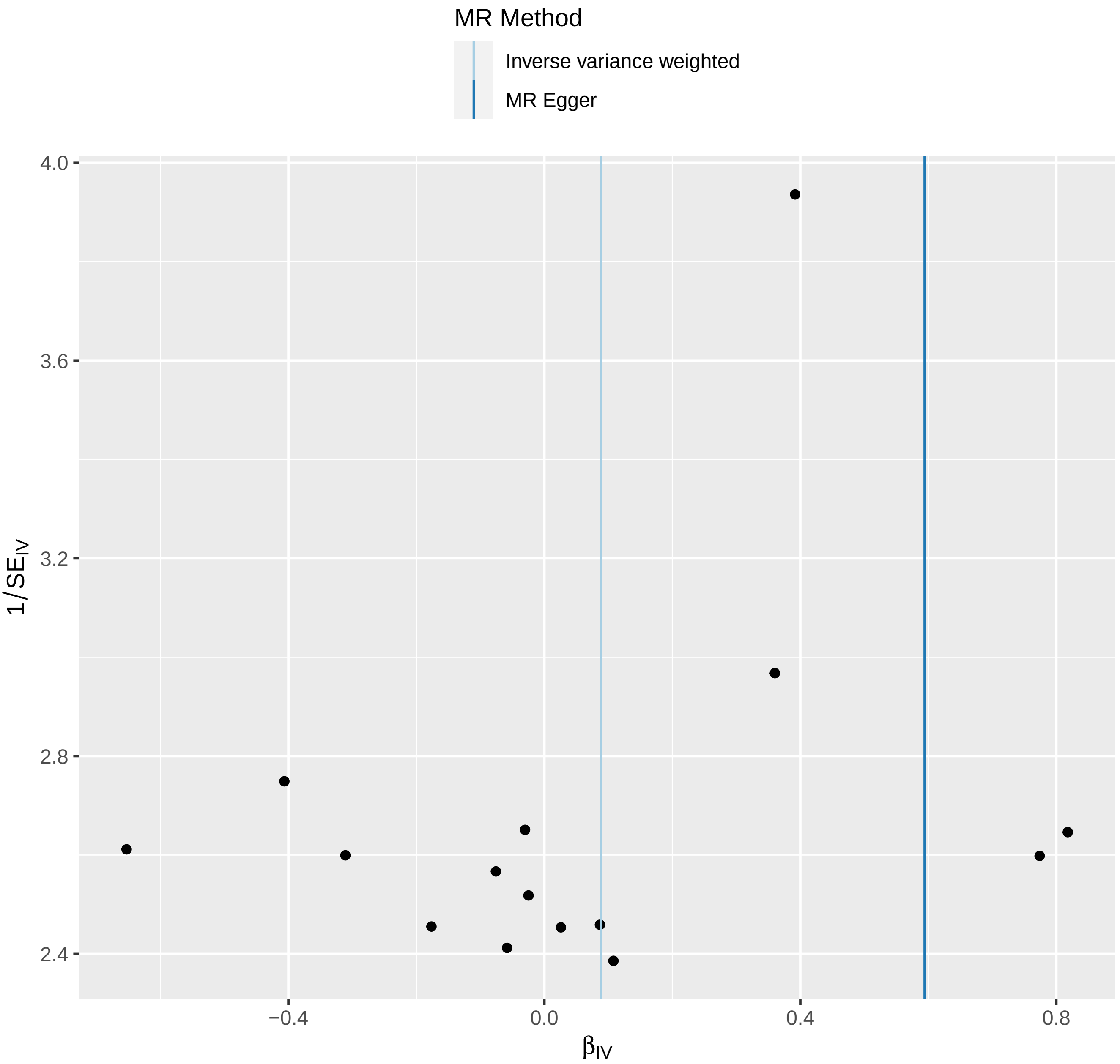

Batch 2−1345 : Gut microbiota abundance (genus Catenibacterium id.2153) on Alpha−crystallin A chain
with a threshold of 1e−05

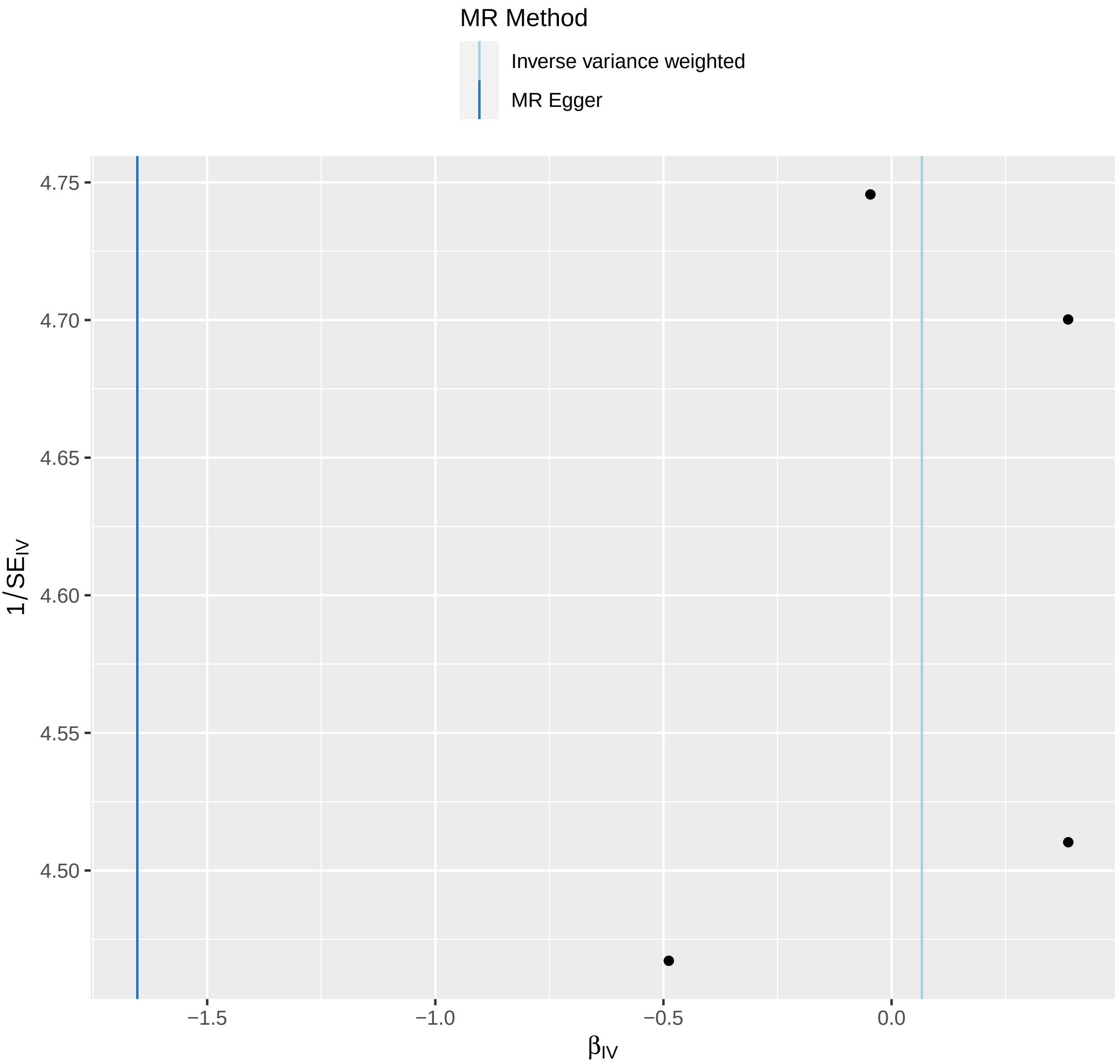

Batch 2−1346 : Gut microbiota abundance (genus Parasutterella id.2892) on Alpha−crystallin A chain
with a threshold of 1e−05

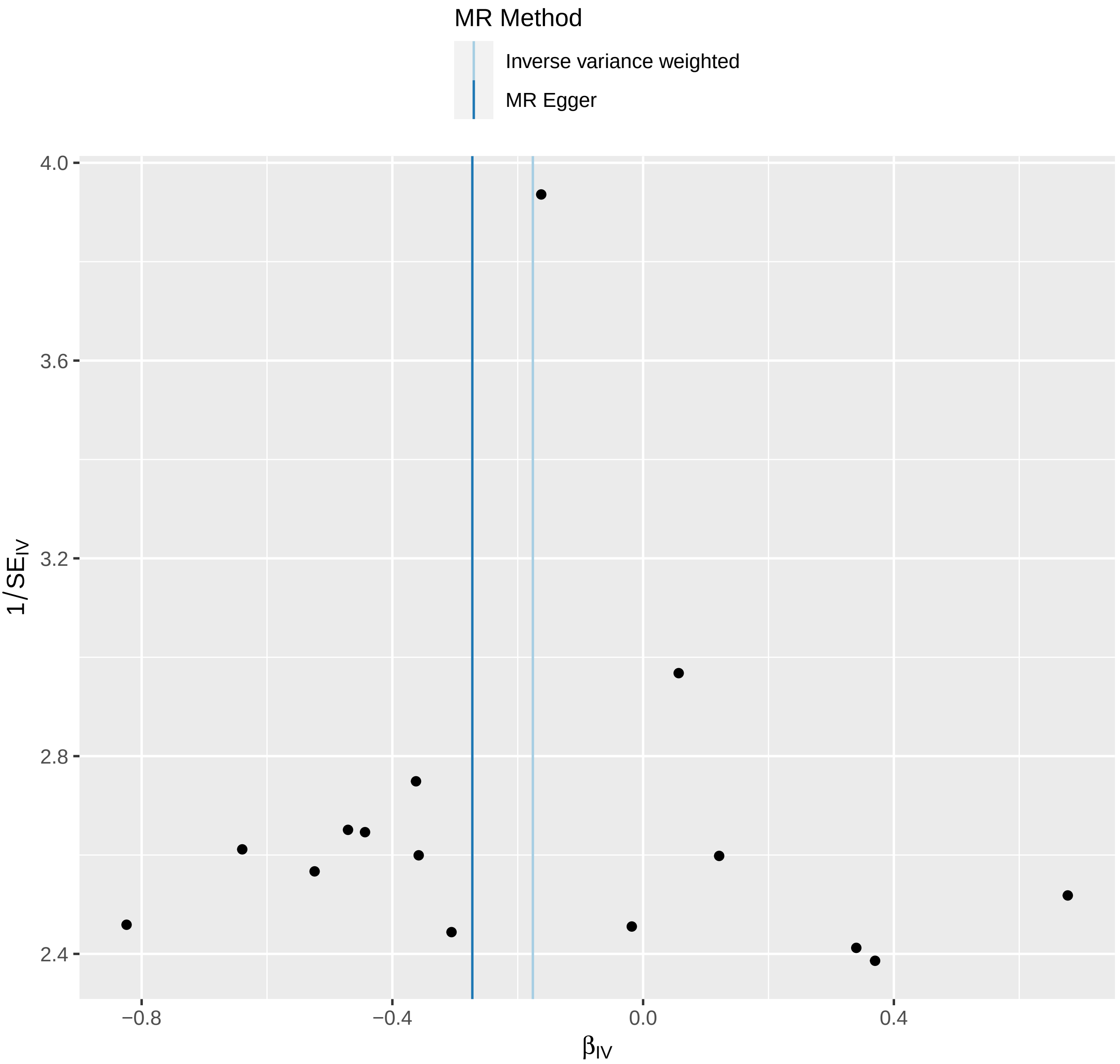

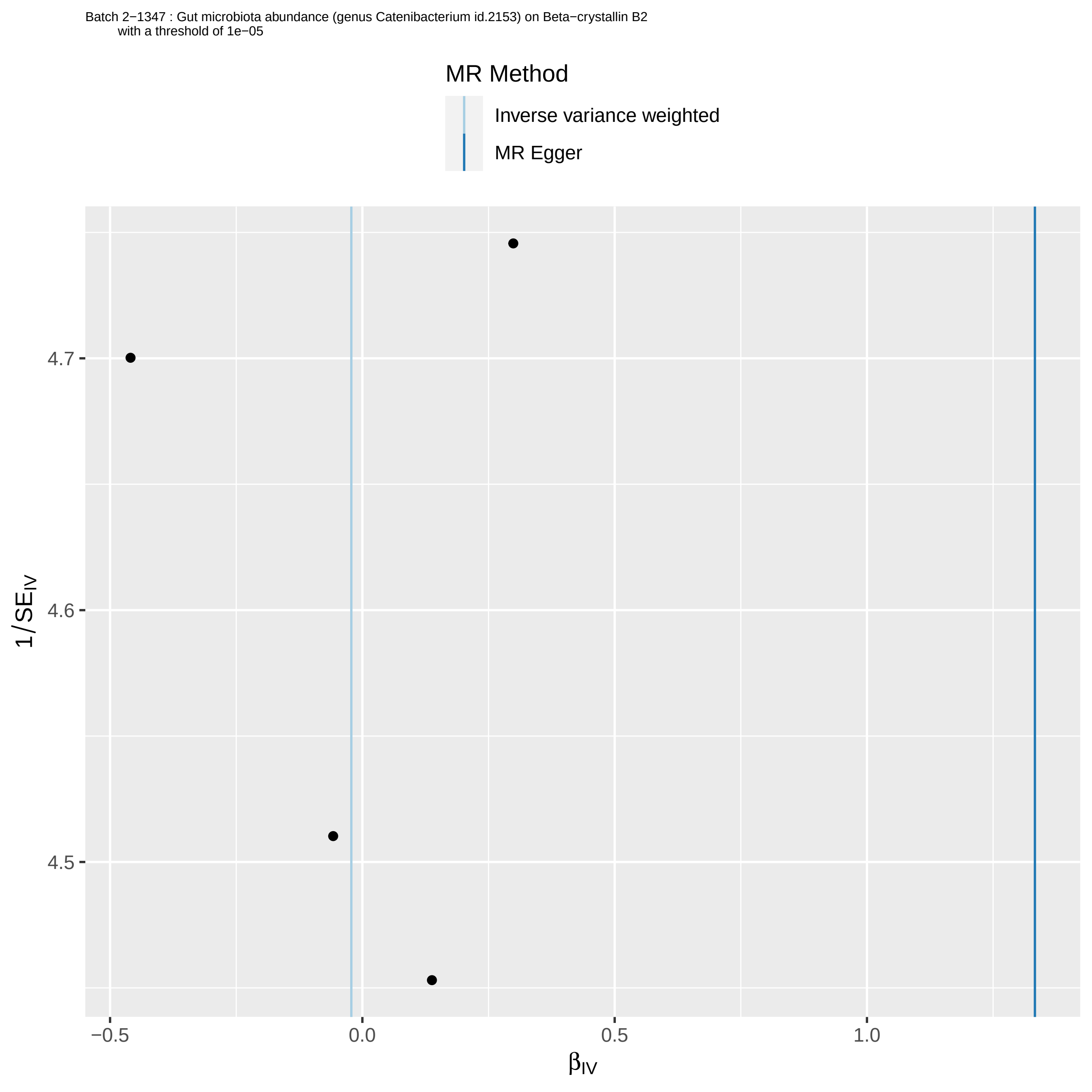

Batch 2−1347 : Gut microbiota abundance (genus Catenibacterium id.2153) on Beta−crystallin B2
with a threshold of 1e−05
MR Method
Inverse variance weighted
MR Egger
4.7
4.6
4.5
1/SE_IV
−0.5
0.0
0.5
1.0
β_IV

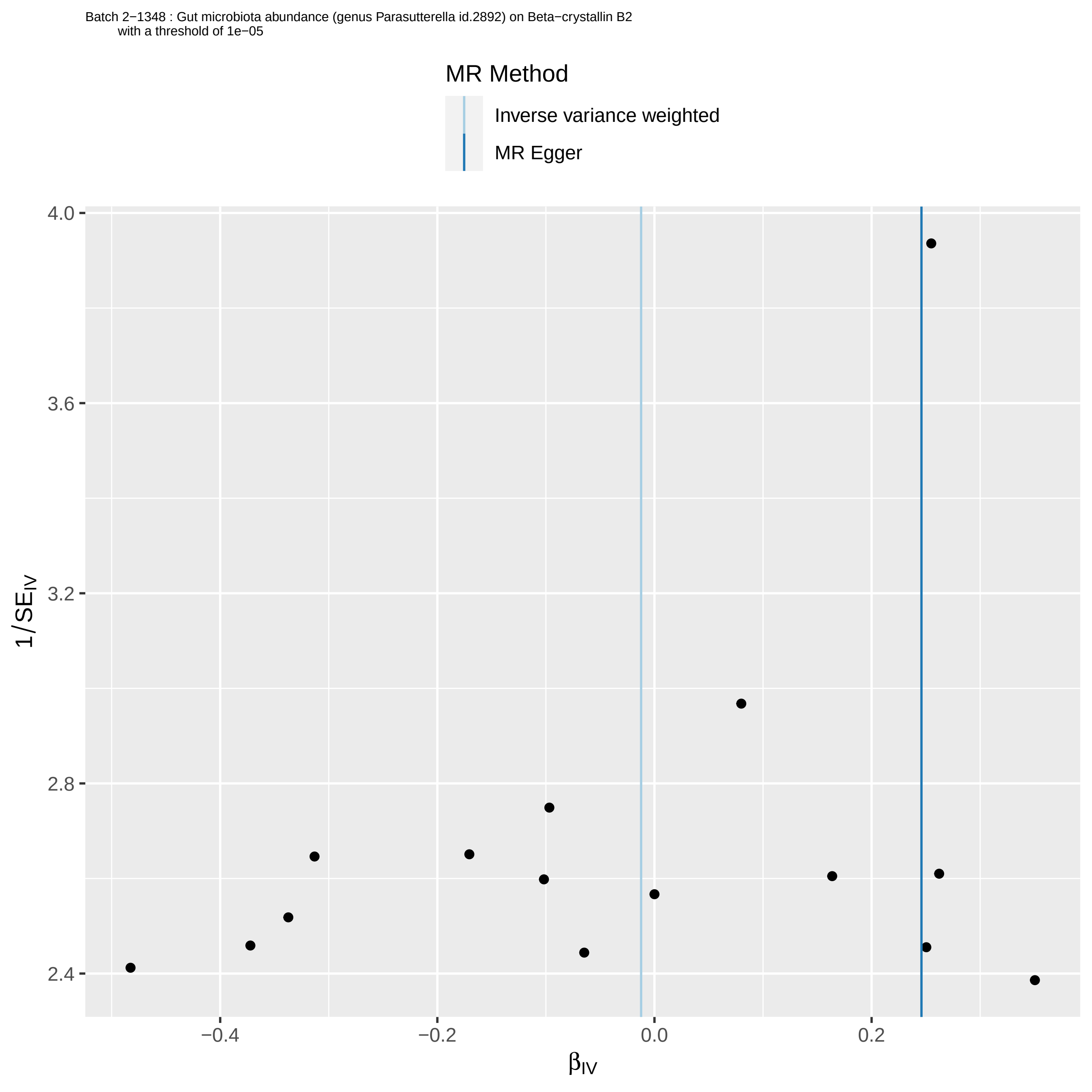

Batch 2−1348 : Gut microbiota abundance (genus Parasutterella id.2892) on Beta−crystallin B2
with a threshold of 1e−05
MR Method
Inverse variance weighted
MR Egger
4.0
3.6
3.2
2.8
2.4
1/SE_IV
−0.4
−0.2
0.0
0.2
β_IV

Batch 2−1349 : Gut microbiota abundance (genus Catenibacterium id.2153) on Gamma−crystallin D
with a threshold of 1e−05

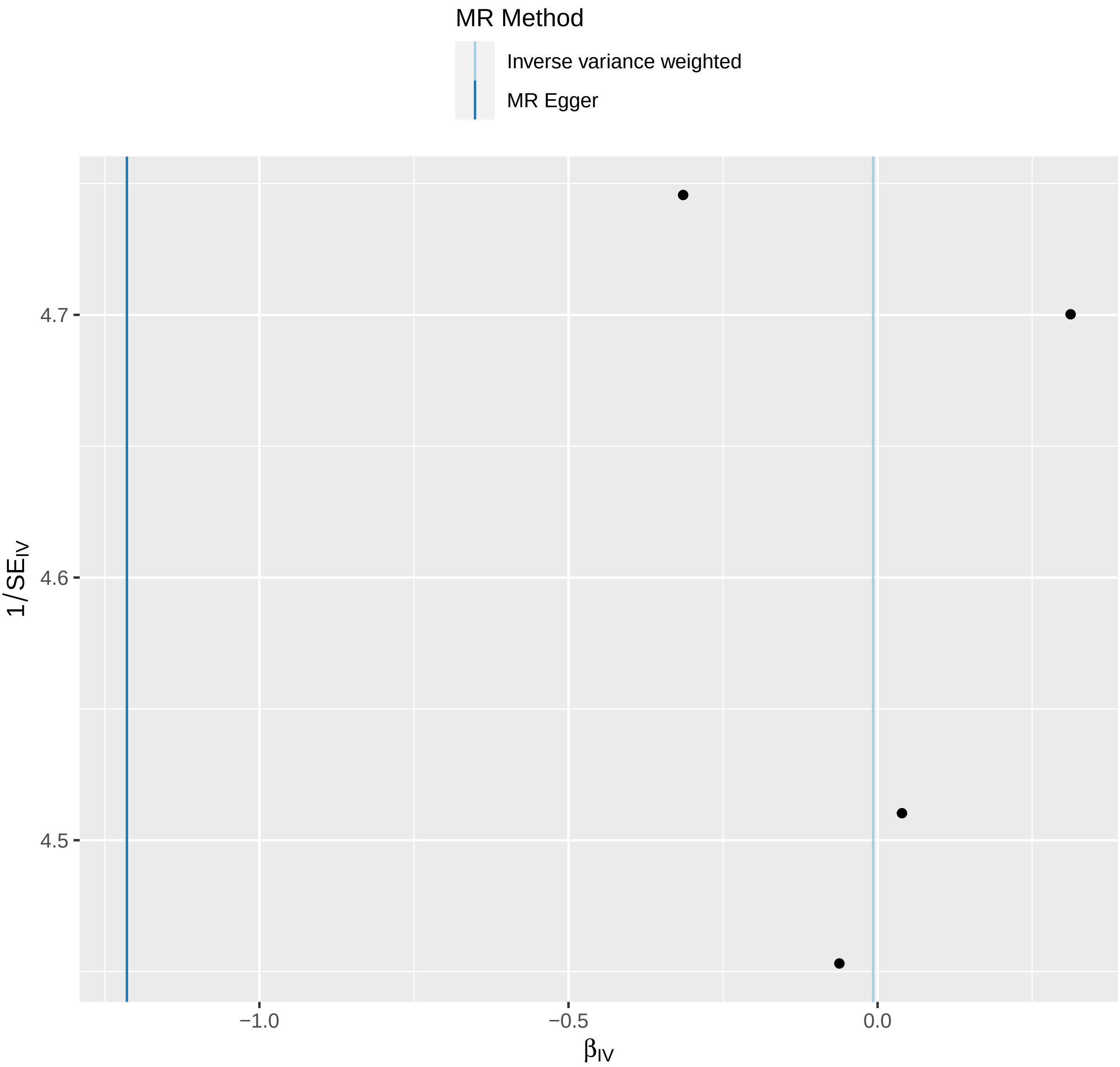

Batch 2−1350 : Gut microbiota abundance (genus Parasutterella id.2892) on Gamma−crystallin D
with a threshold of 1e−05

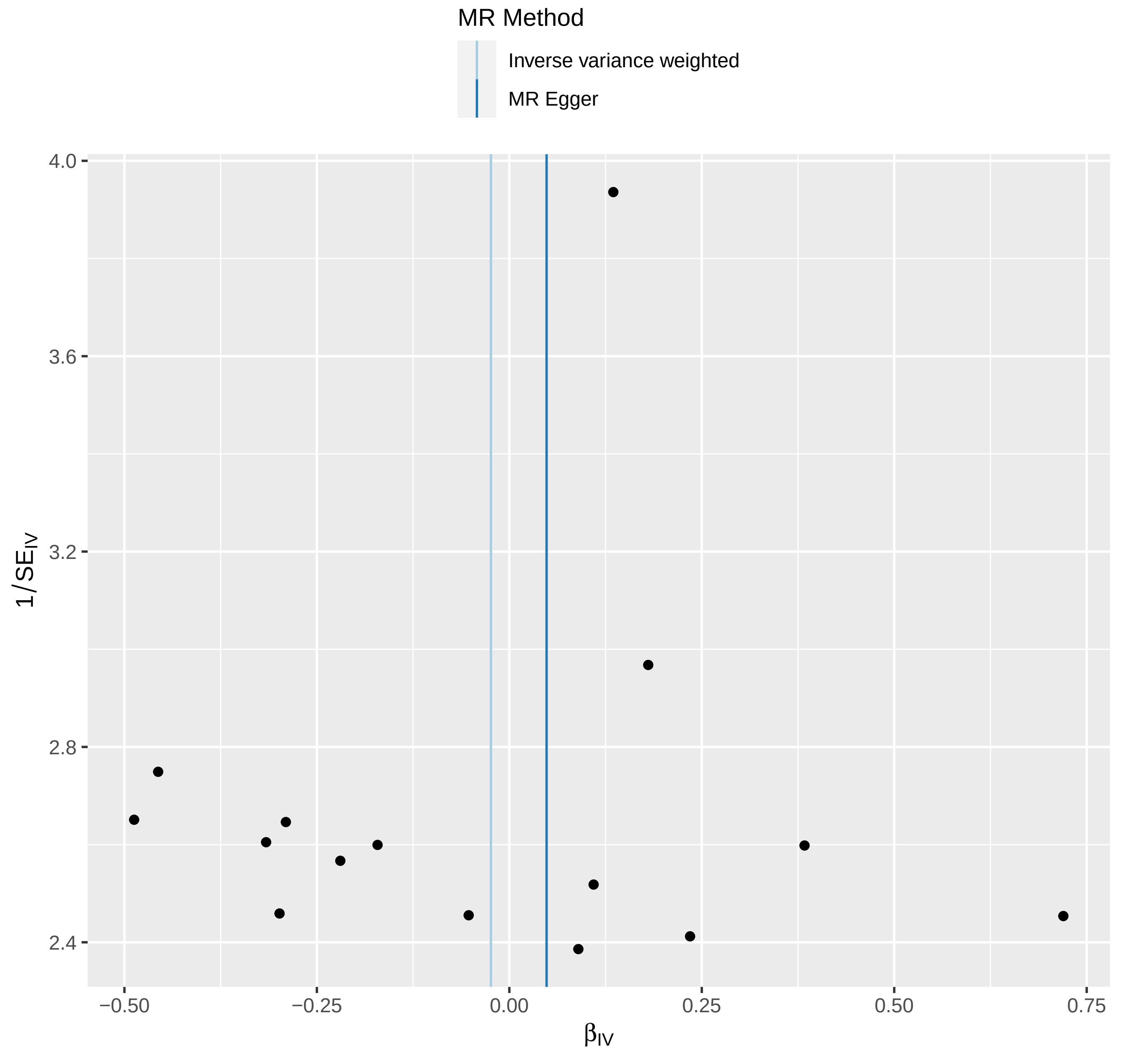

Batch 2−1351 : Gut microbiota abundance (genus Catenibacterium id.2153) on Quinone oxidoreductase−like protein 1
with a threshold of 1e−05

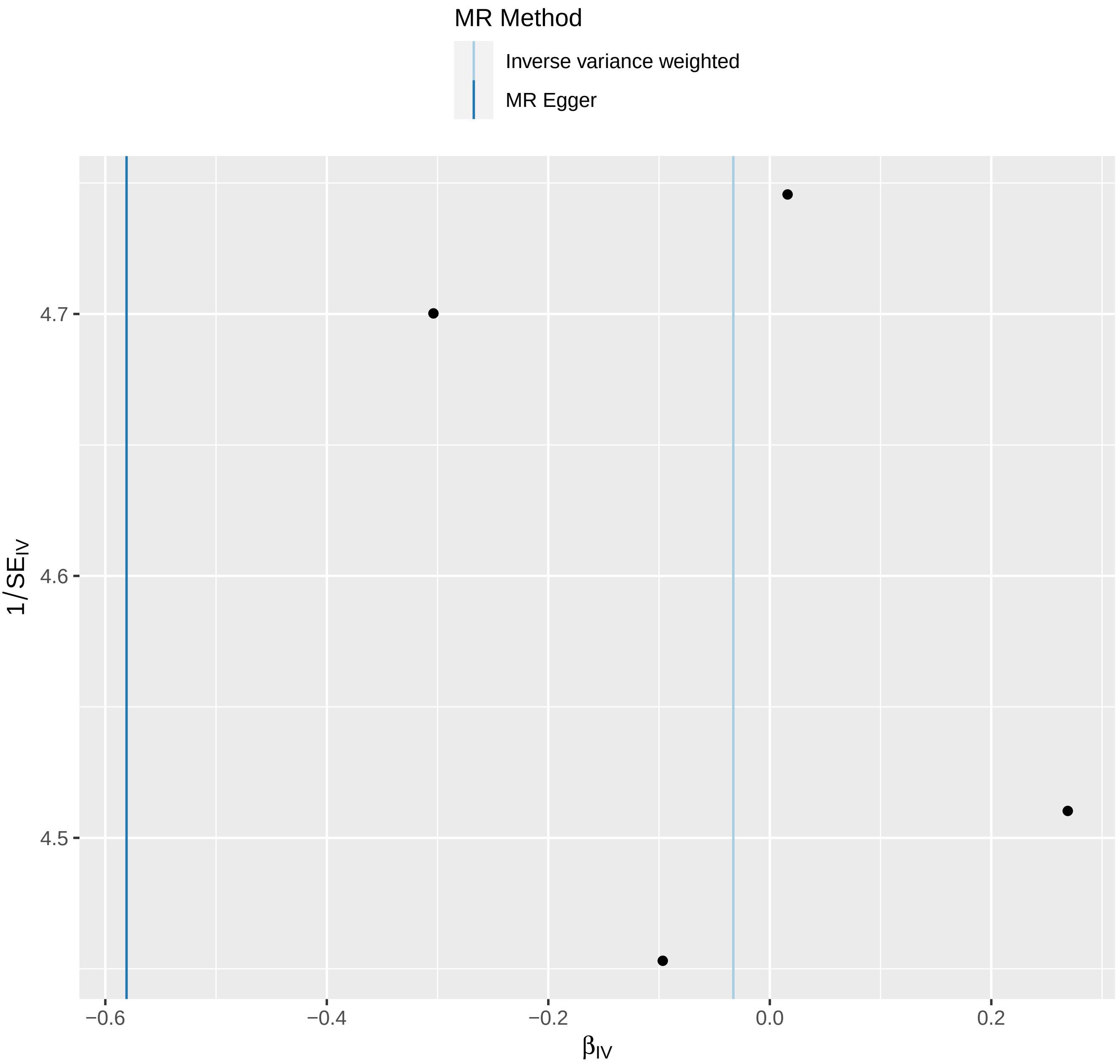

Batch 2−1352 : Gut microbiota abundance (genus Parasutterella id.2892) on Quinone oxidoreductase−like protein 1
with a threshold of 1e−05

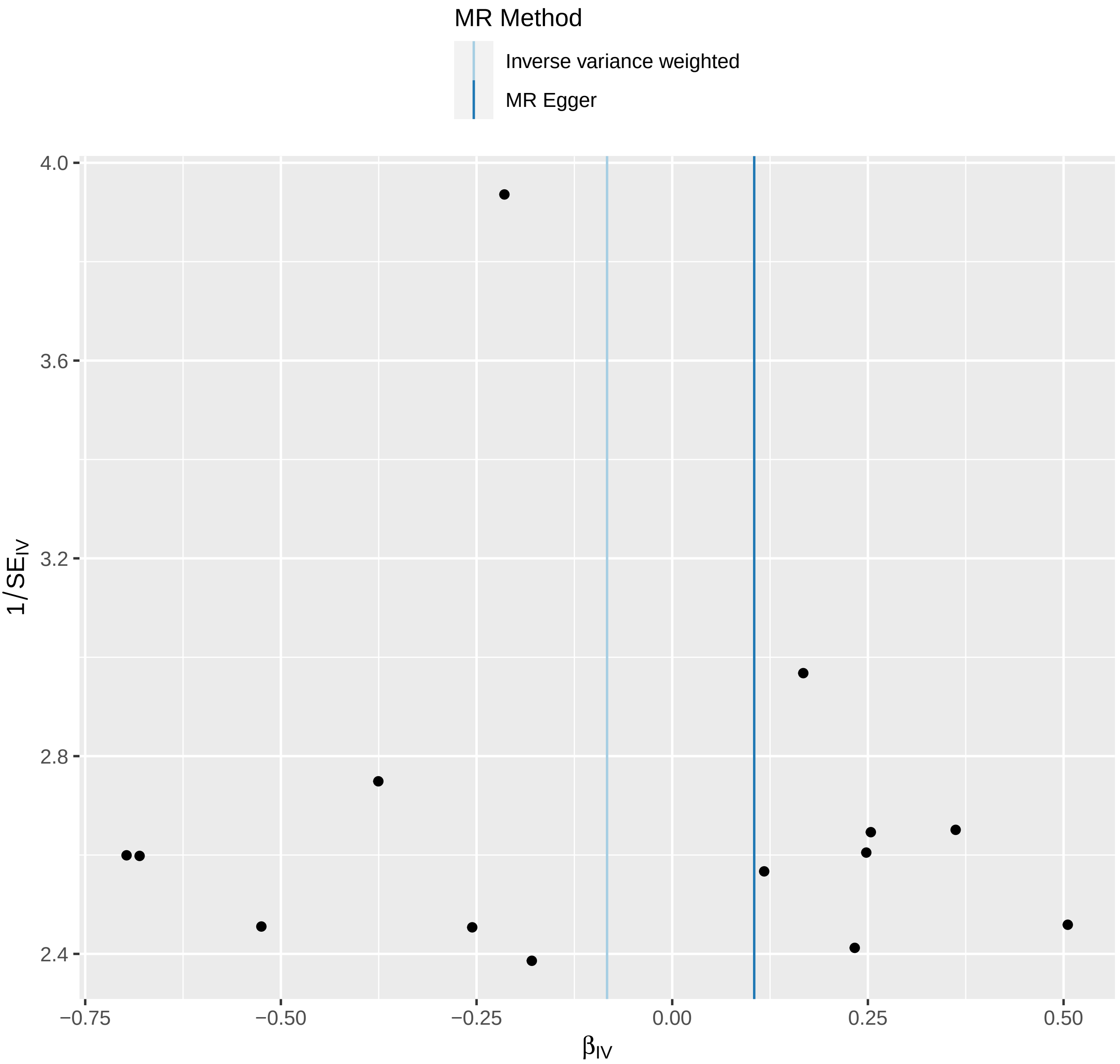

Batch 2−1353 : Gut microbiota abundance (genus Catenibacterium id.2153) on Putative chondrosarcoma−associated gene 1 protein
with a threshold of 1e−05

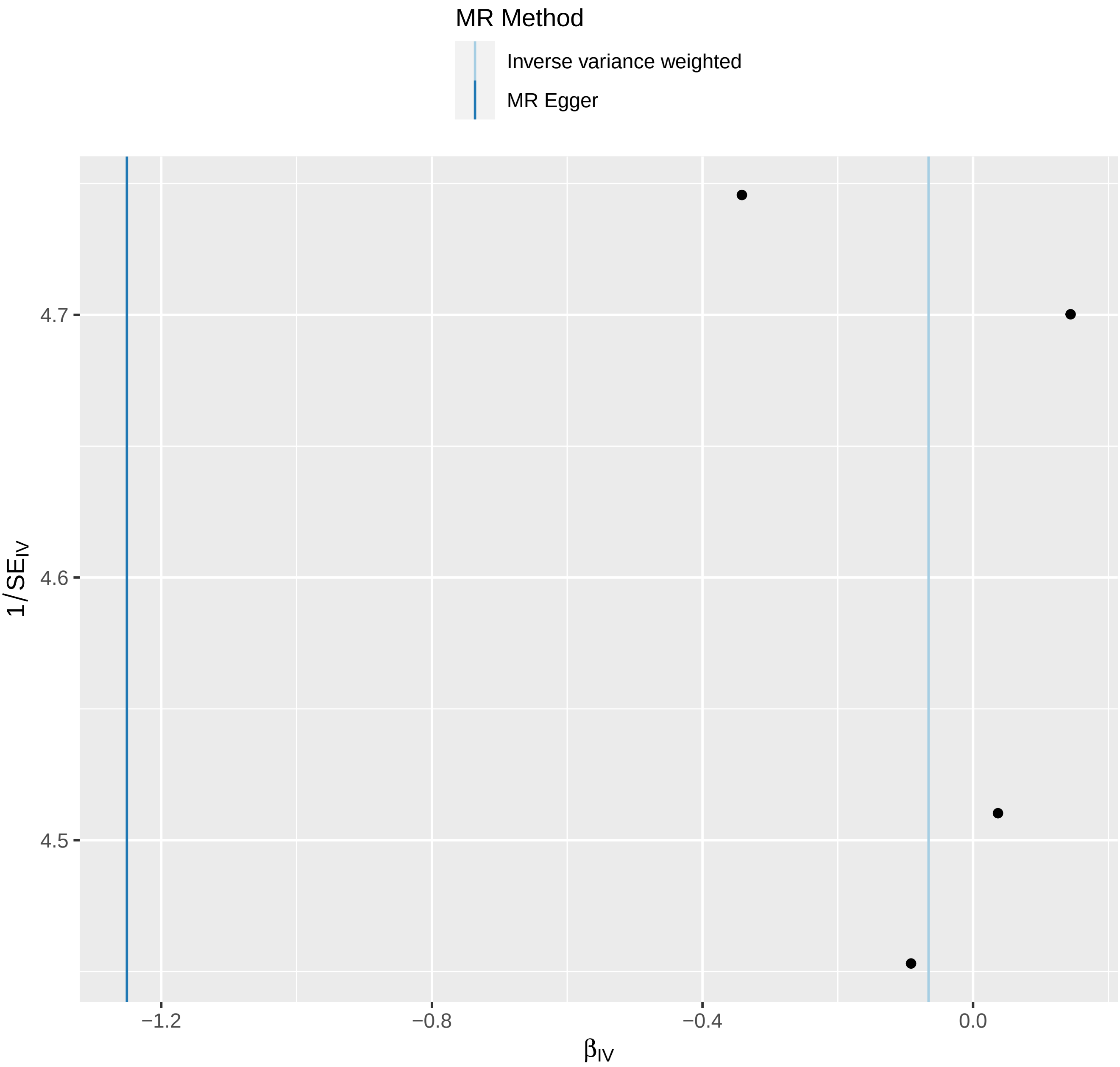

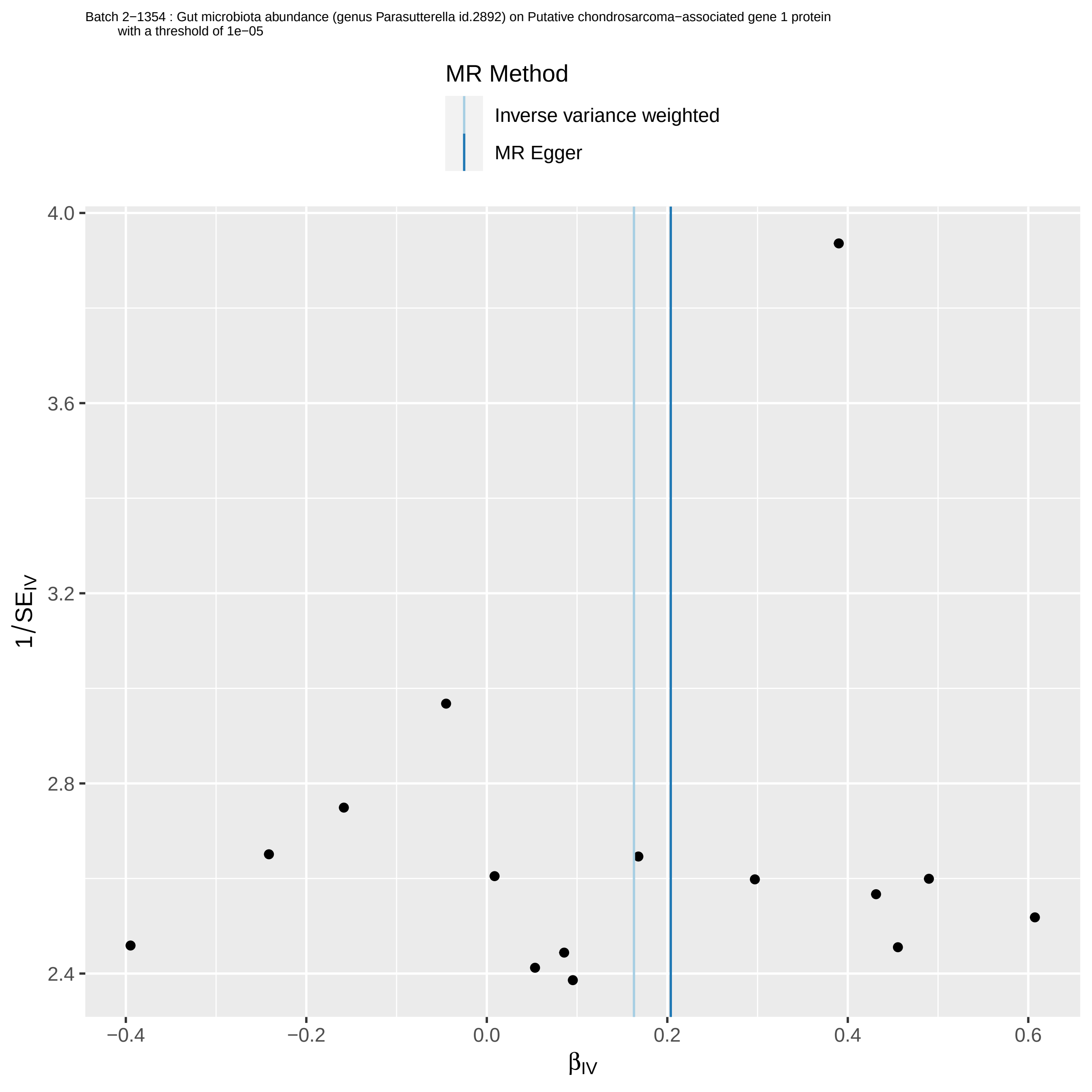
Batch 2−1354 : Gut microbiota abundance (genus Parasutterella id.2892) on Putative chondrosarcoma−associated gene 1 protein
with a threshold of 1e−05
MR Method
Inverse variance weighted
MR Egger
4.0
3.6
3.2
2.8
2.4
1/SE_IV
−0.4
−0.2
0.0
0.2
0.4
0.6
β_IV

Batch 2−1355 : Gut microbiota abundance (genus Catenibacterium id.2153) on Cold shock domain−containing protein C2
with a threshold of 1e−05

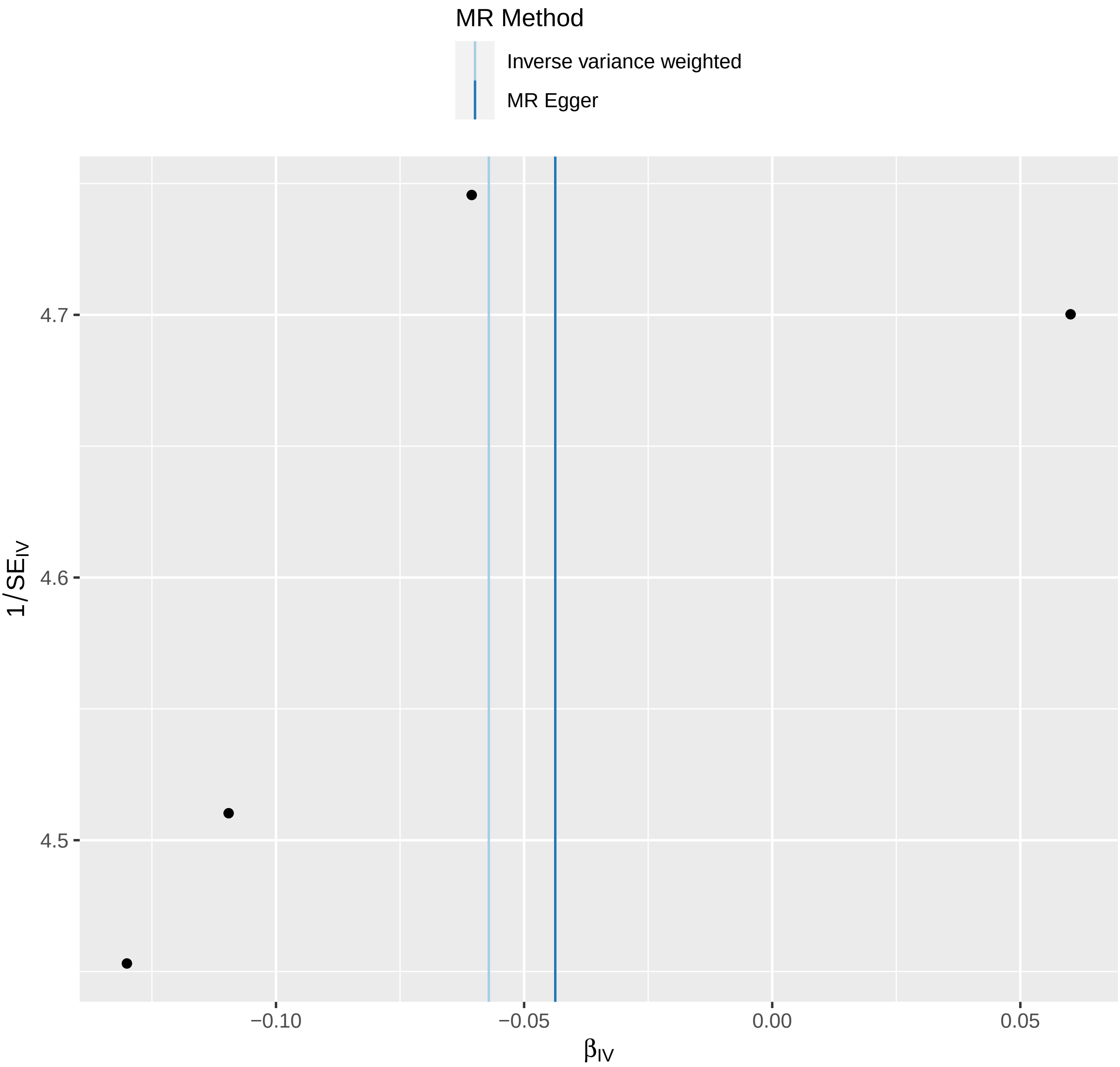

Batch 2−1356 : Gut microbiota abundance (genus Parasutterella id.2892) on Cold shock domain−containing protein C2
with a threshold of 1e−05

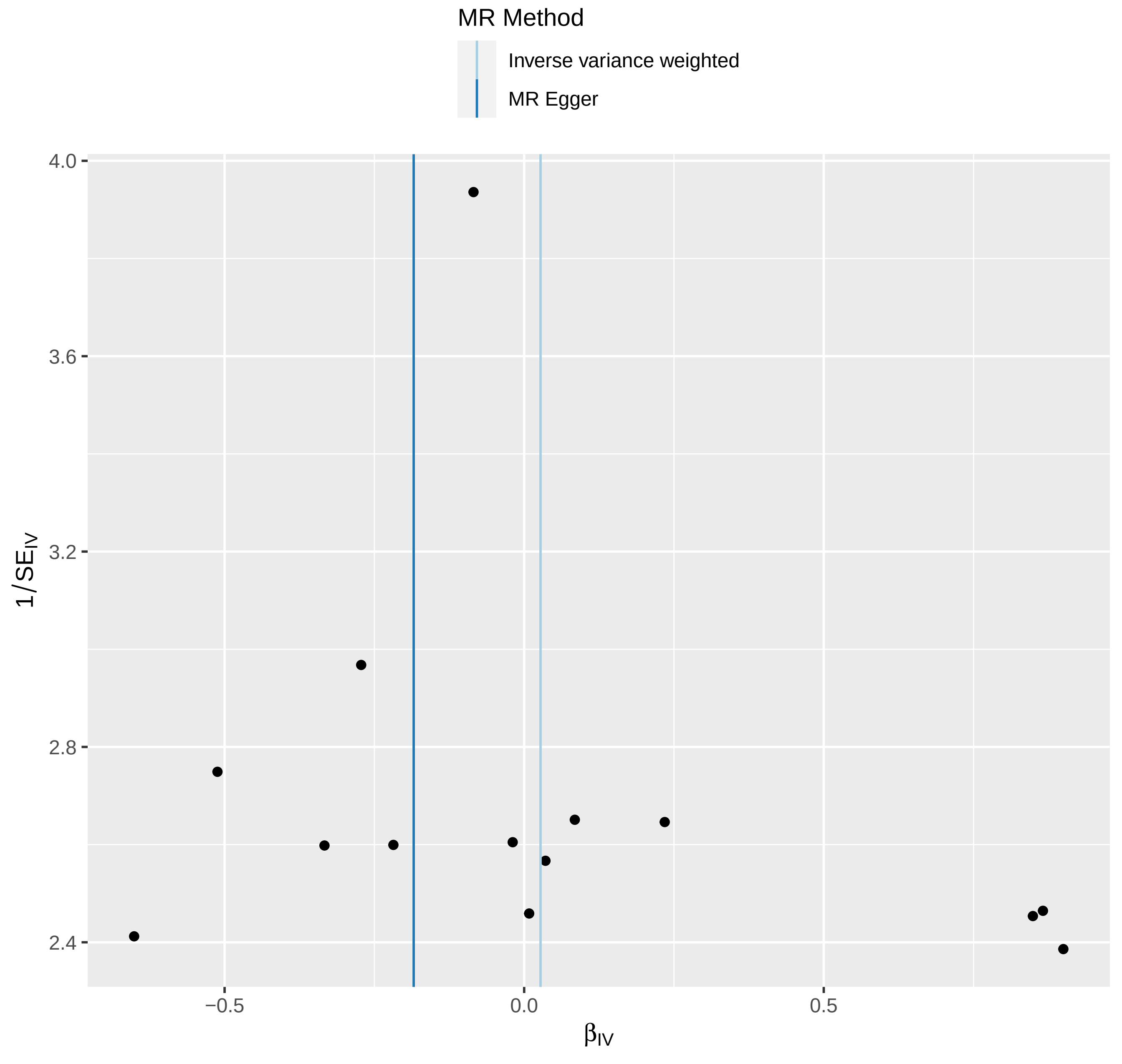

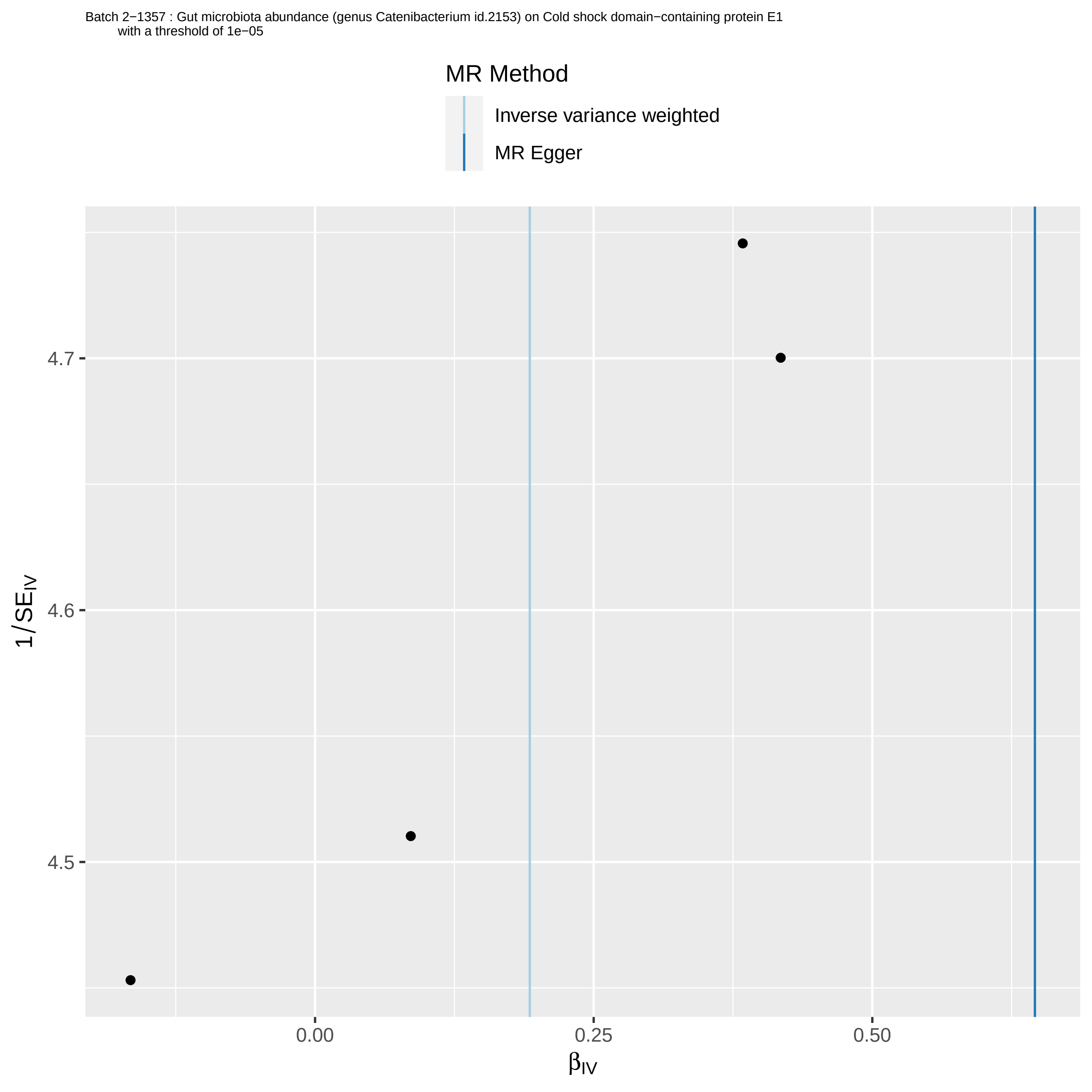
Batch 2−1357 : Gut microbiota abundance (genus Catenibacterium id.2153) on Cold shock domain−containing protein E1
with a threshold of 1e−05
MR Method
Inverse variance weighted
MR Egger
4.7
4.6
4.5
1/SE$_{IV}$
0.00
0.25
0.50
β$_{IV}$

Batch 2−1358 : Gut microbiota abundance (genus Parasutterella id.2892) on Cold shock domain−containing protein E1 with a threshold of 1e−05

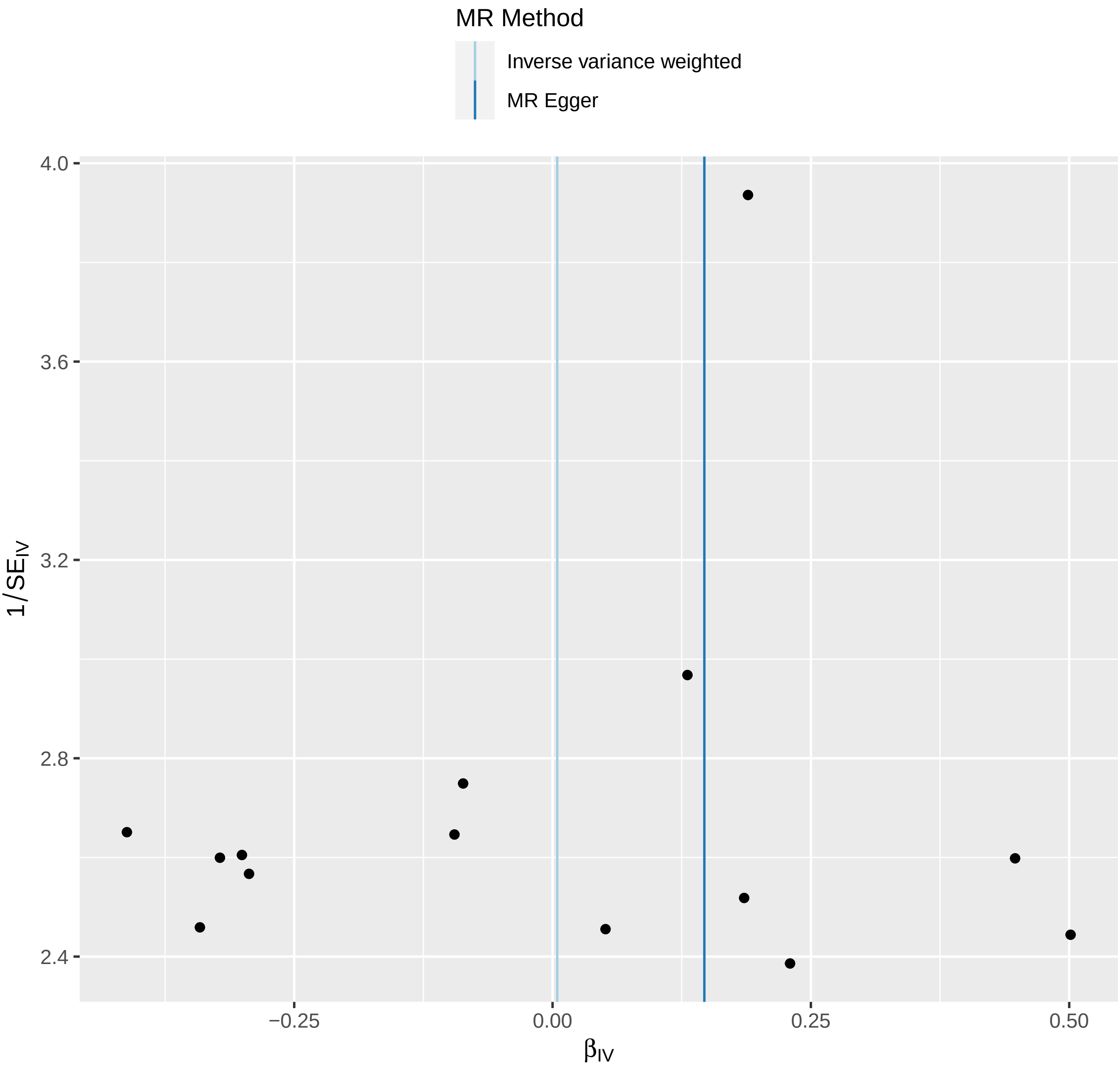

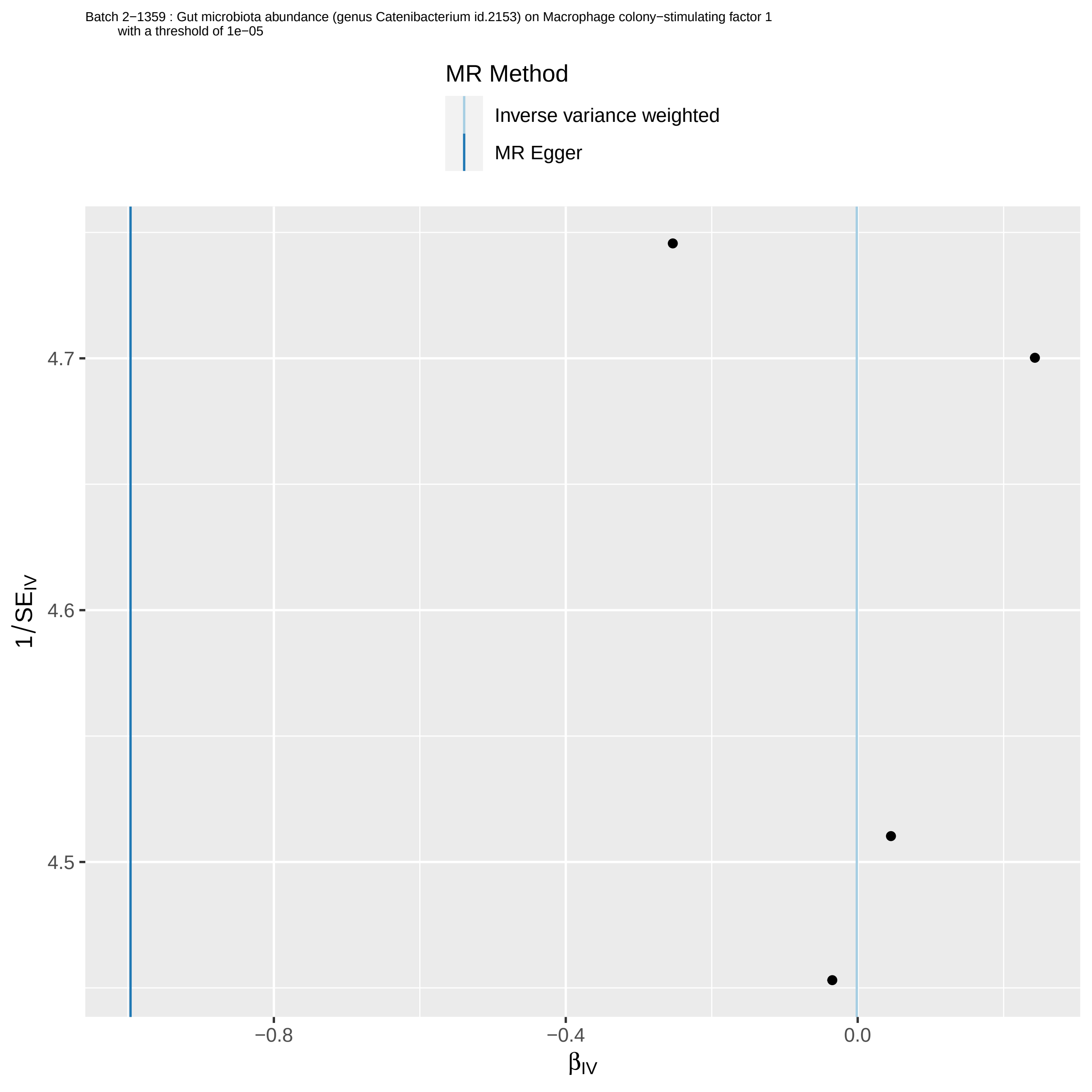

Batch 2−1359 : Gut microbiota abundance (genus Catenibacterium id.2153) on Macrophage colony−stimulating factor 1
with a threshold of 1e−05
MR Method
Inverse variance weighted
MR Egger
4.7
4.6
4.5
1/SE_IV
−0.8
−0.4
0.0
β_IV

Batch 2−1360 : Gut microbiota abundance (genus Parasutterella id.2892) on Macrophage colony−stimulating factor 1
with a threshold of 1e−05

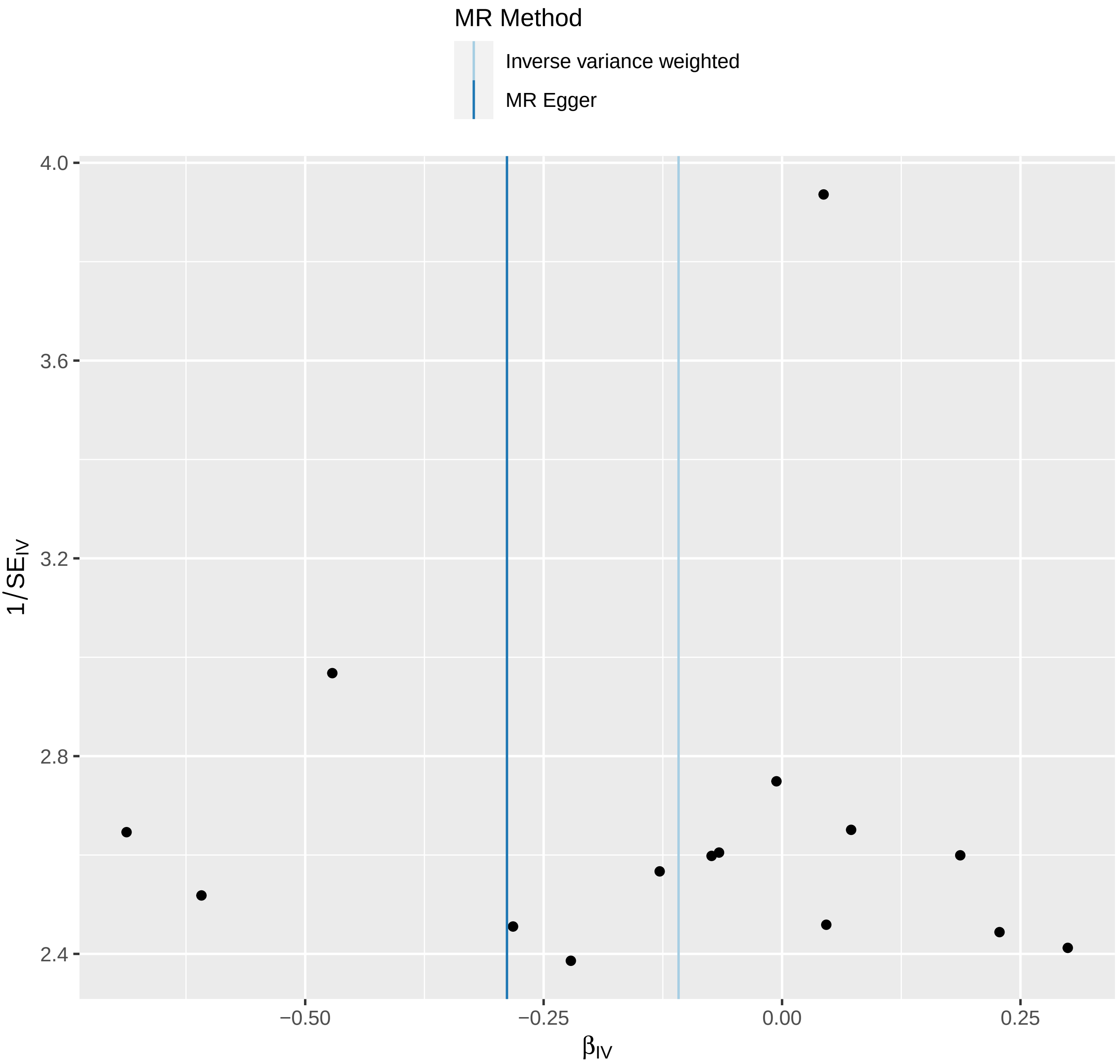

Batch 2−1361 : Gut microbiota abundance (genus Catenibacterium id.2153) on Granulocyte−macrophage colony−stimulating factor
with a threshold of 1e−05

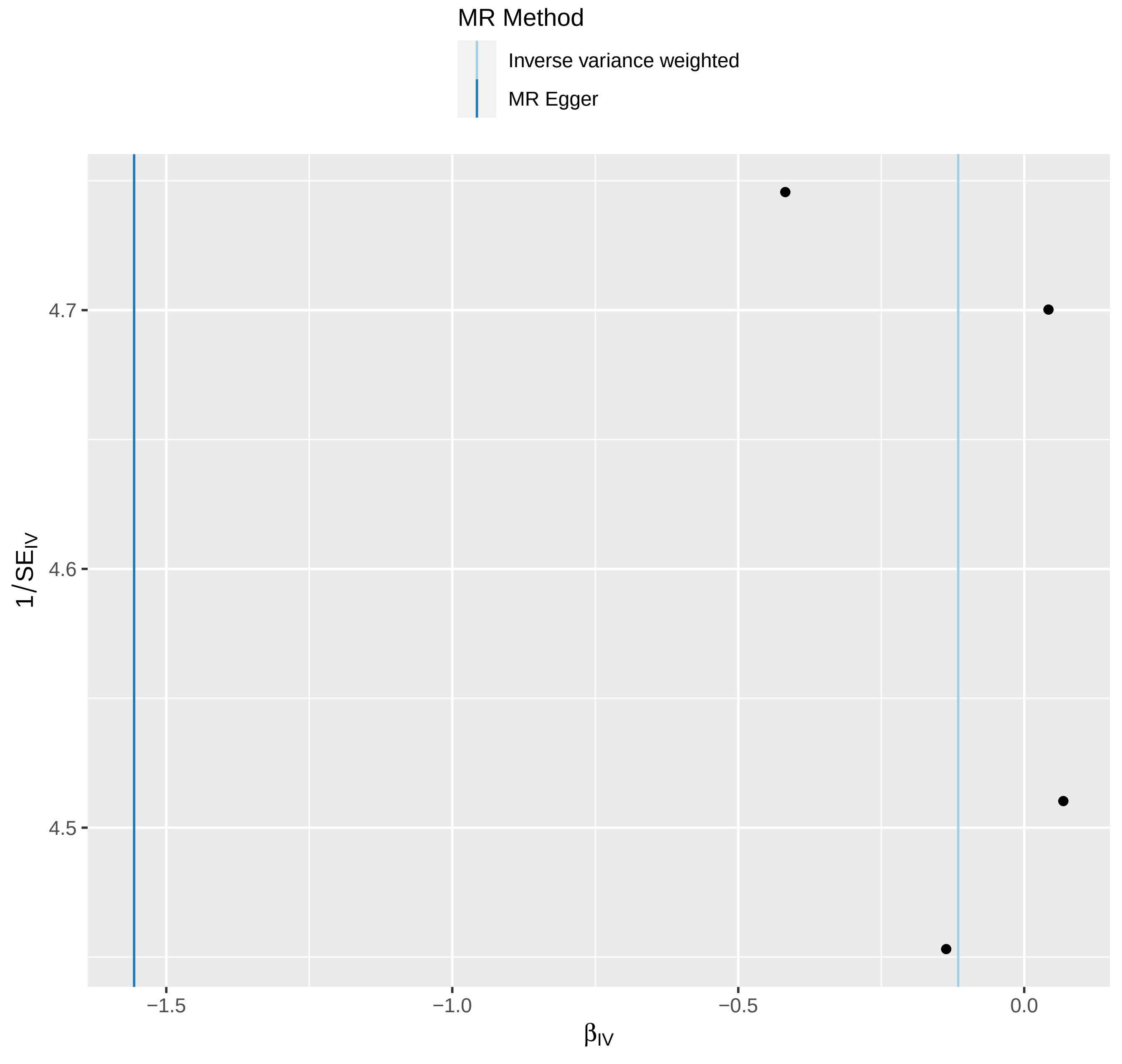

Batch 2−1362 : Gut microbiota abundance (genus Parasutterella id.2892) on Granulocyte−macrophage colony−stimulating factor
with a threshold of 1e−05

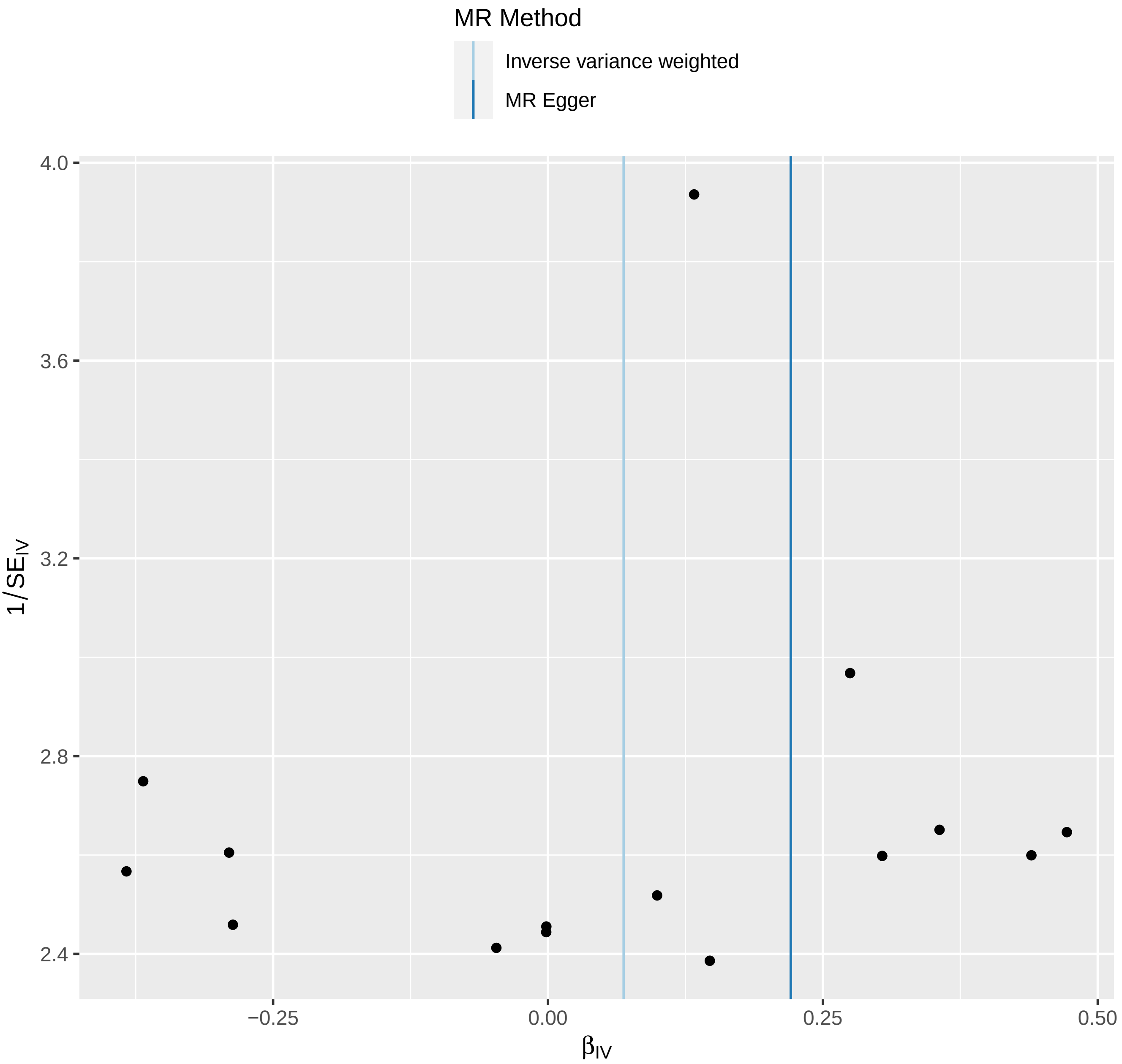

Batch 2−1363 : Gut microbiota abundance (genus Catenibacterium id.2153) on Granulocyte−macrophage colony−stimulating factor receptor subunit alpha with a threshold of 1e−05

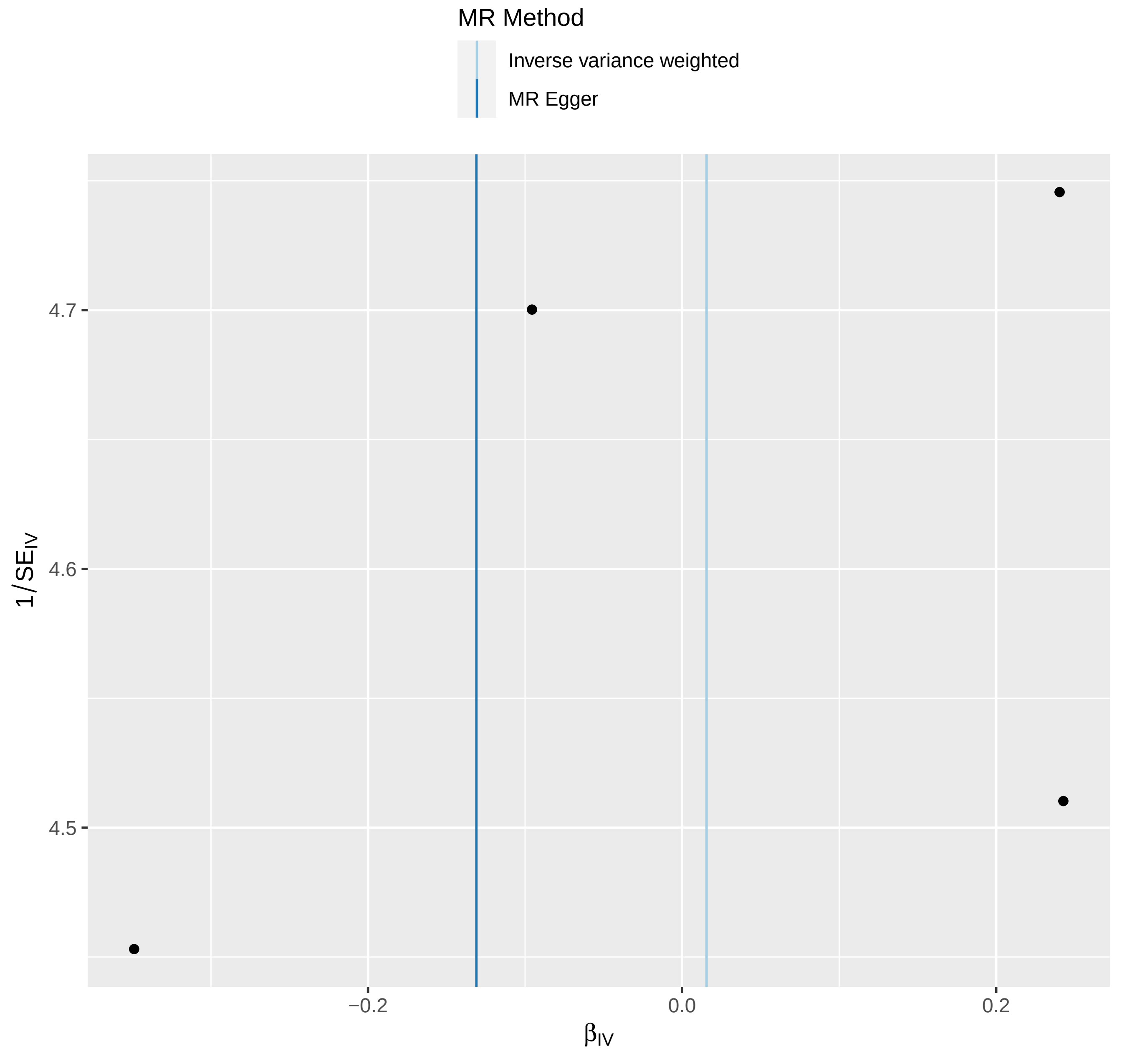

Batch 2−1364 : Gut microbiota abundance (genus Parasutterella id.2892) on Granulocyte−macrophage colony−stimulating factor receptor subunit alpha with a threshold of 1e−05

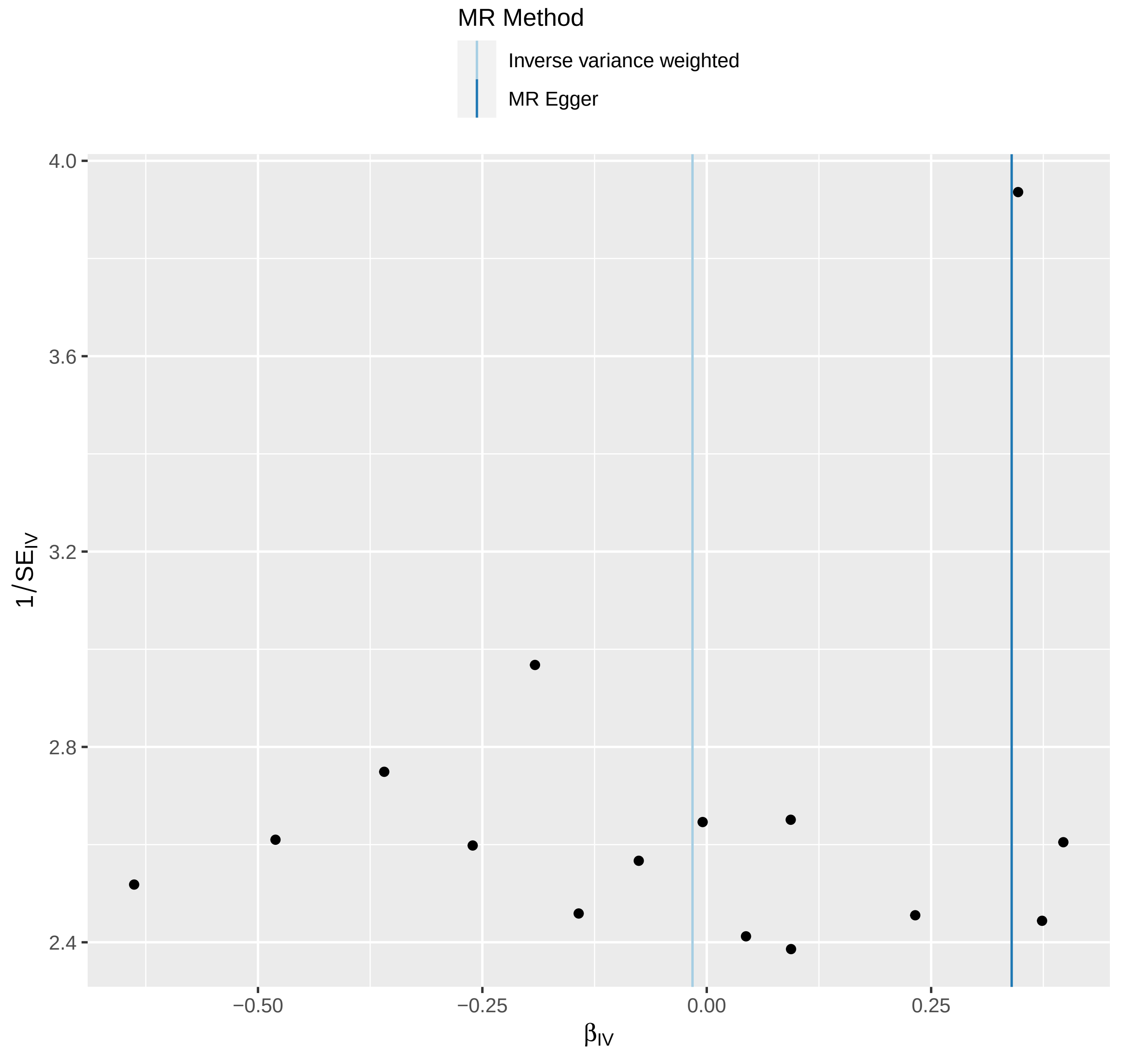

Batch 2−1365 : Gut microbiota abundance (genus Catenibacterium id.2153) on Cytokine receptor common subunit beta with a threshold of 1e−05

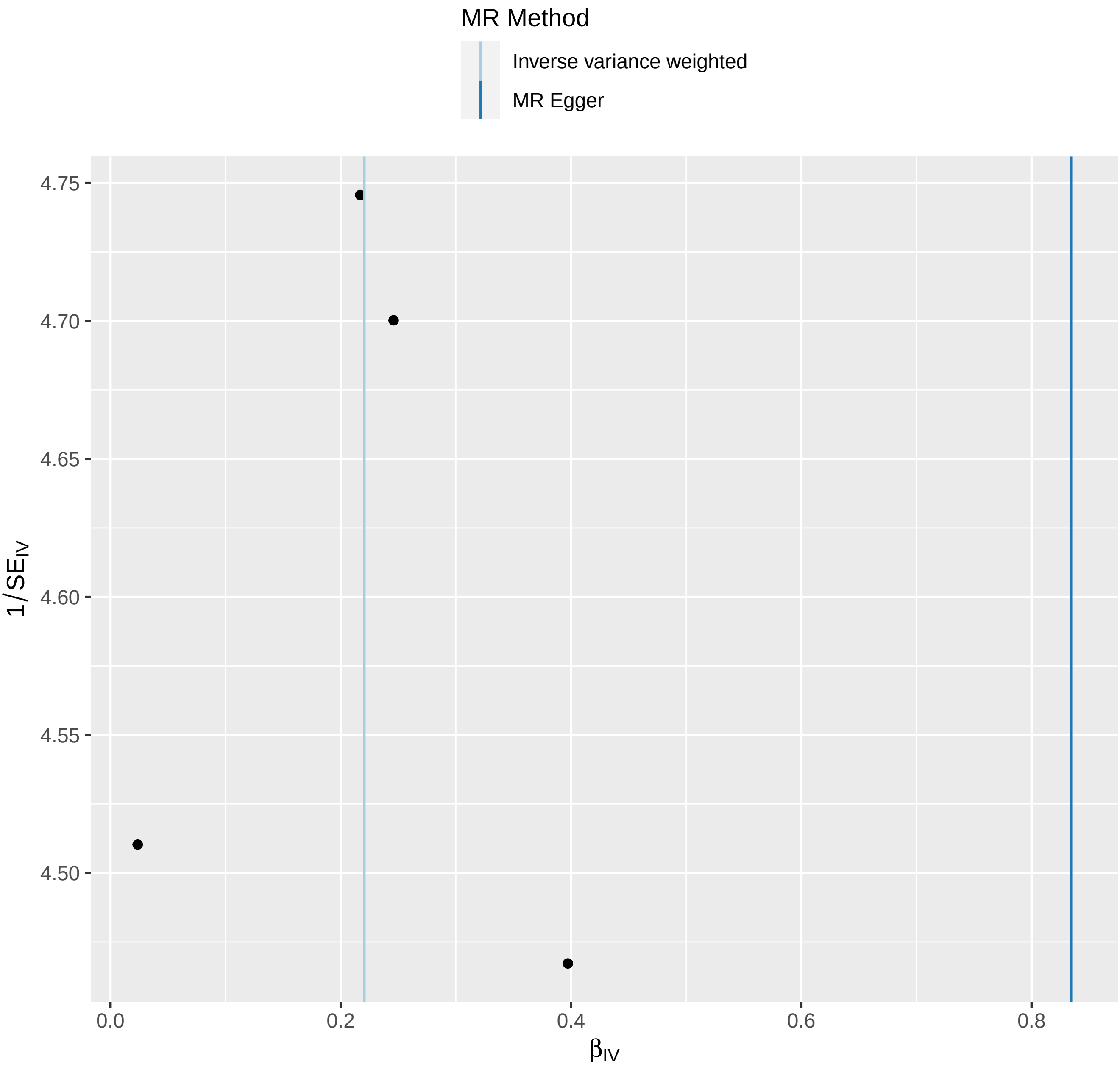

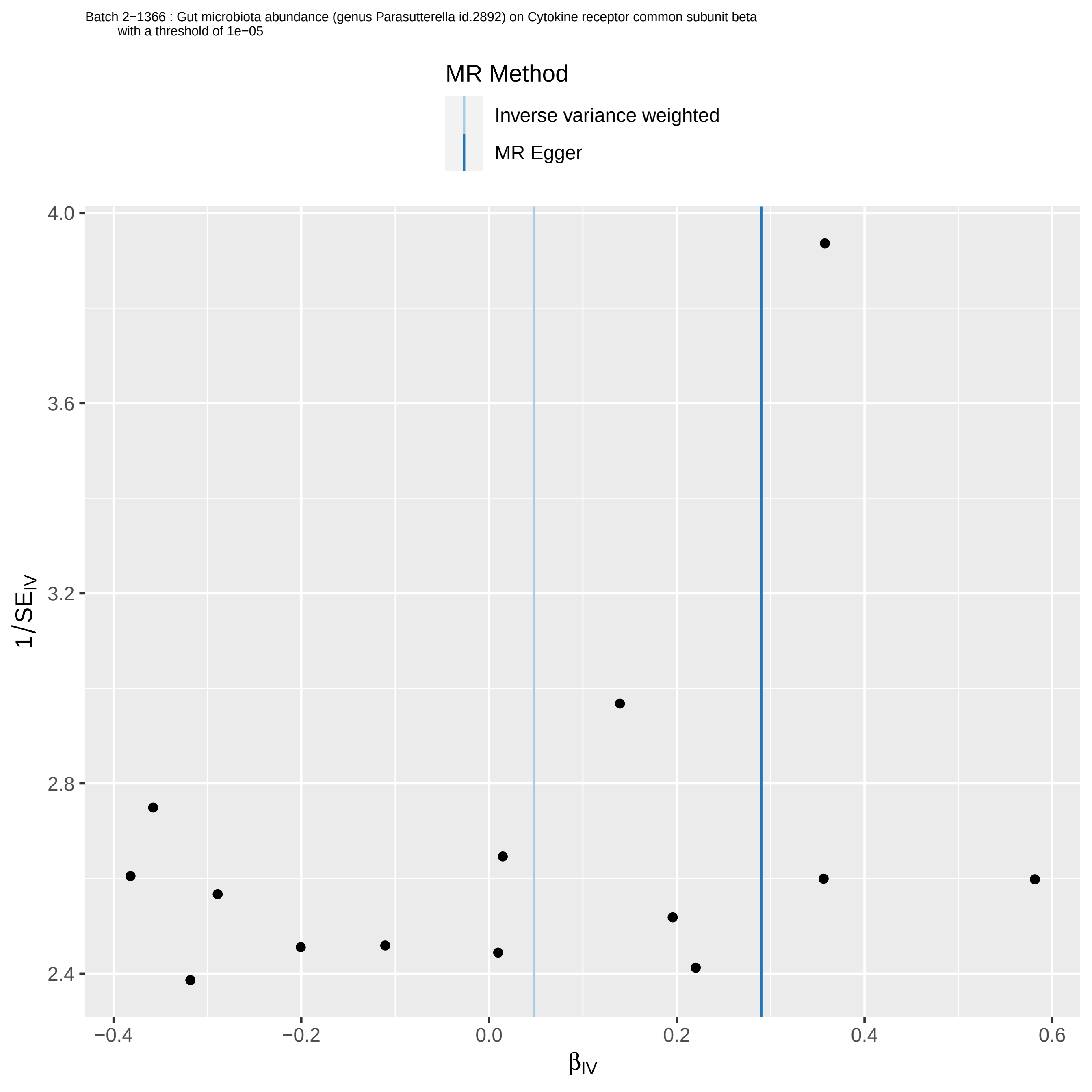

Batch 2−1366 : Gut microbiota abundance (genus Parasutterella id.2892) on Cytokine receptor common subunit beta
with a threshold of 1e−05
MR Method
Inverse variance weighted
MR Egger
4.0
3.6
3.2
2.8
2.4
1/SE_IV
−0.4
−0.2
0.0
0.2
0.4
0.6
β_IV

Batch 2−1367 : Gut microbiota abundance (genus Catenibacterium id.2153) on Cytokine receptor common subunit beta
with a threshold of 1e−05

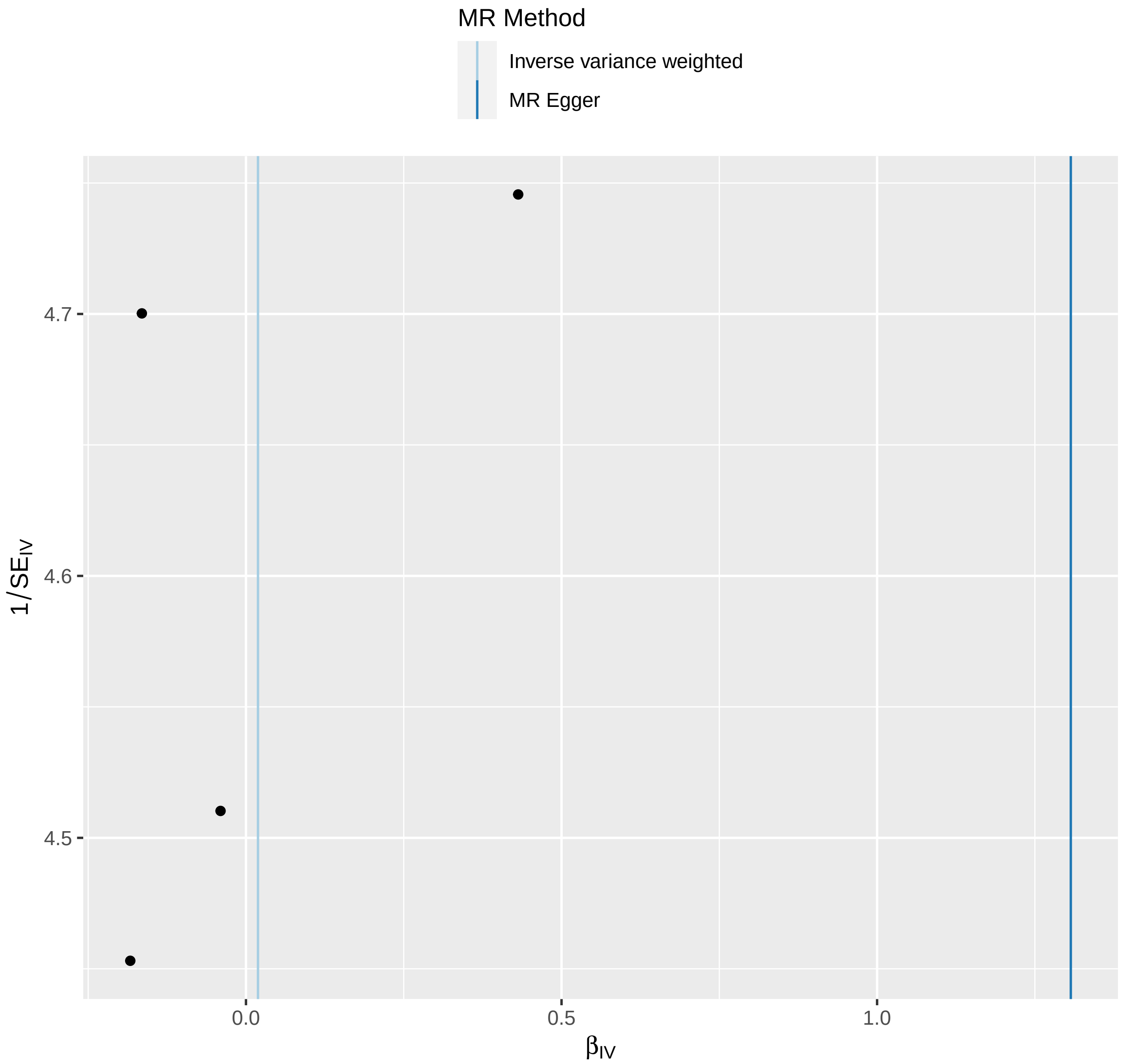

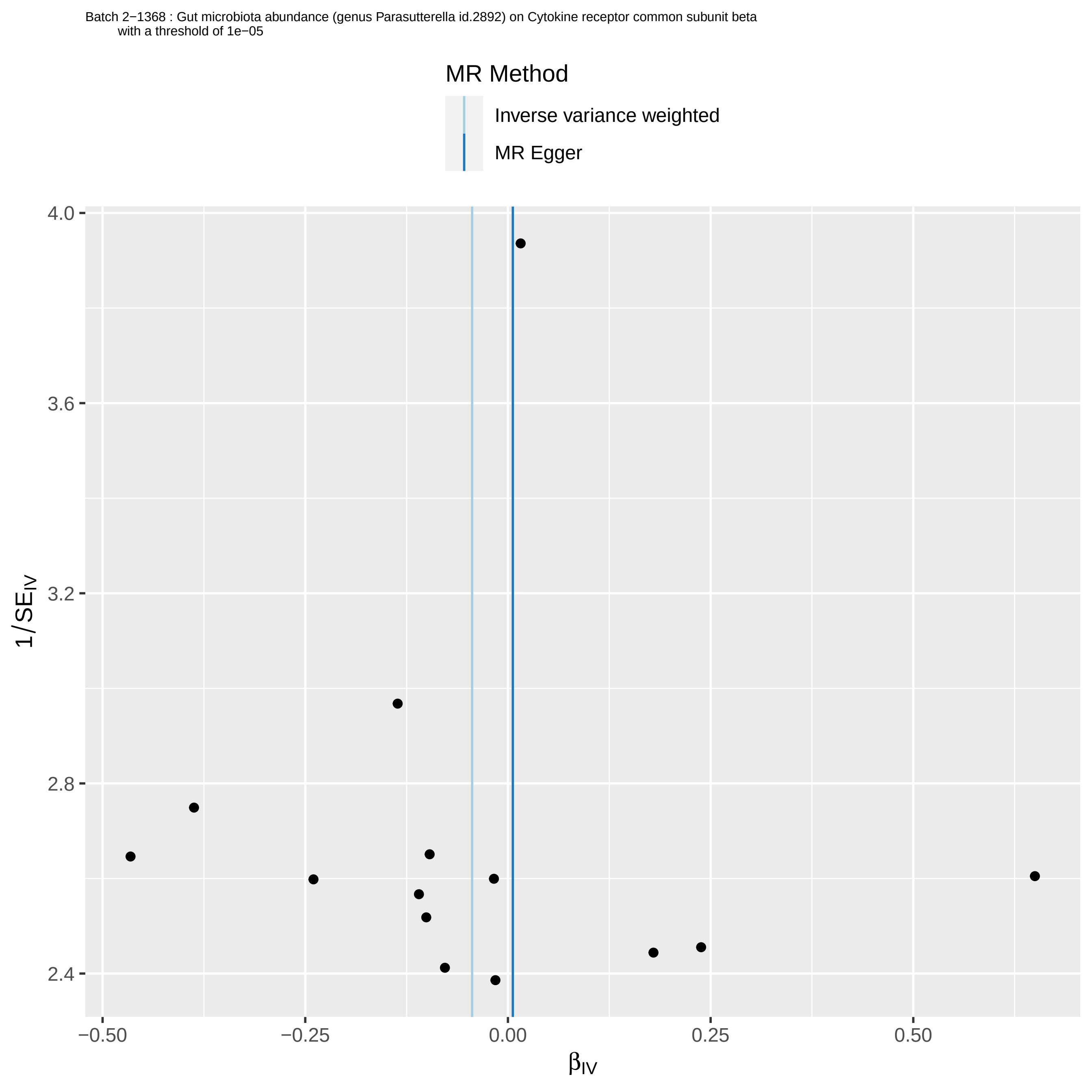

Batch 2−1368 : Gut microbiota abundance (genus Parasutterella id.2892) on Cytokine receptor common subunit beta
with a threshold of 1e−05
MR Method
Inverse variance weighted
MR Egger
4.0
3.6
3.2
2.8
2.4
1/SE_IV
−0.50
−0.25
0.00
0.25
0.50
β_IV

Batch 2−1369 : Gut microbiota abundance (genus Catenibacterium id.2153) on Granulocyte colony−stimulating factor with a threshold of 1e−05

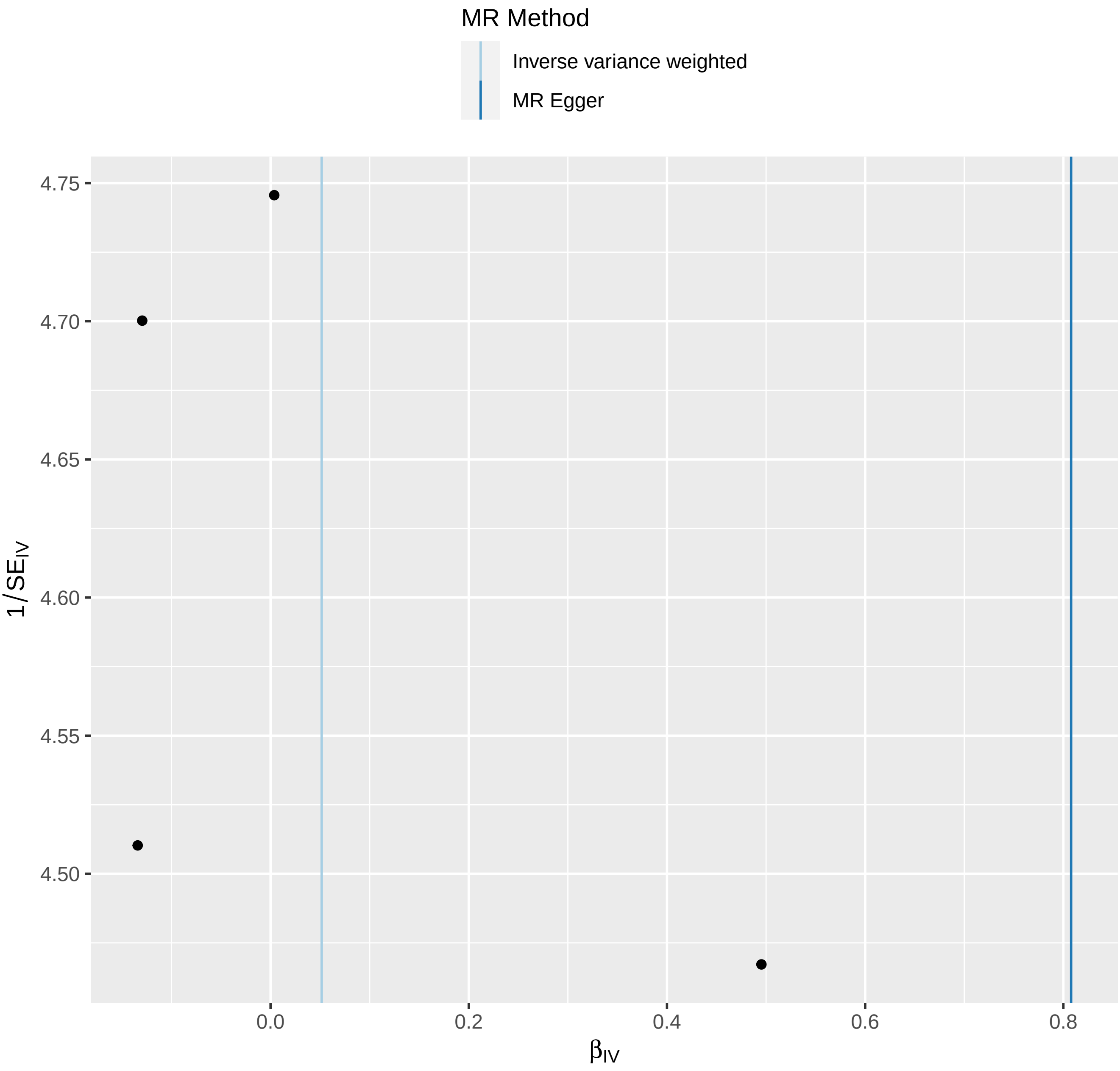

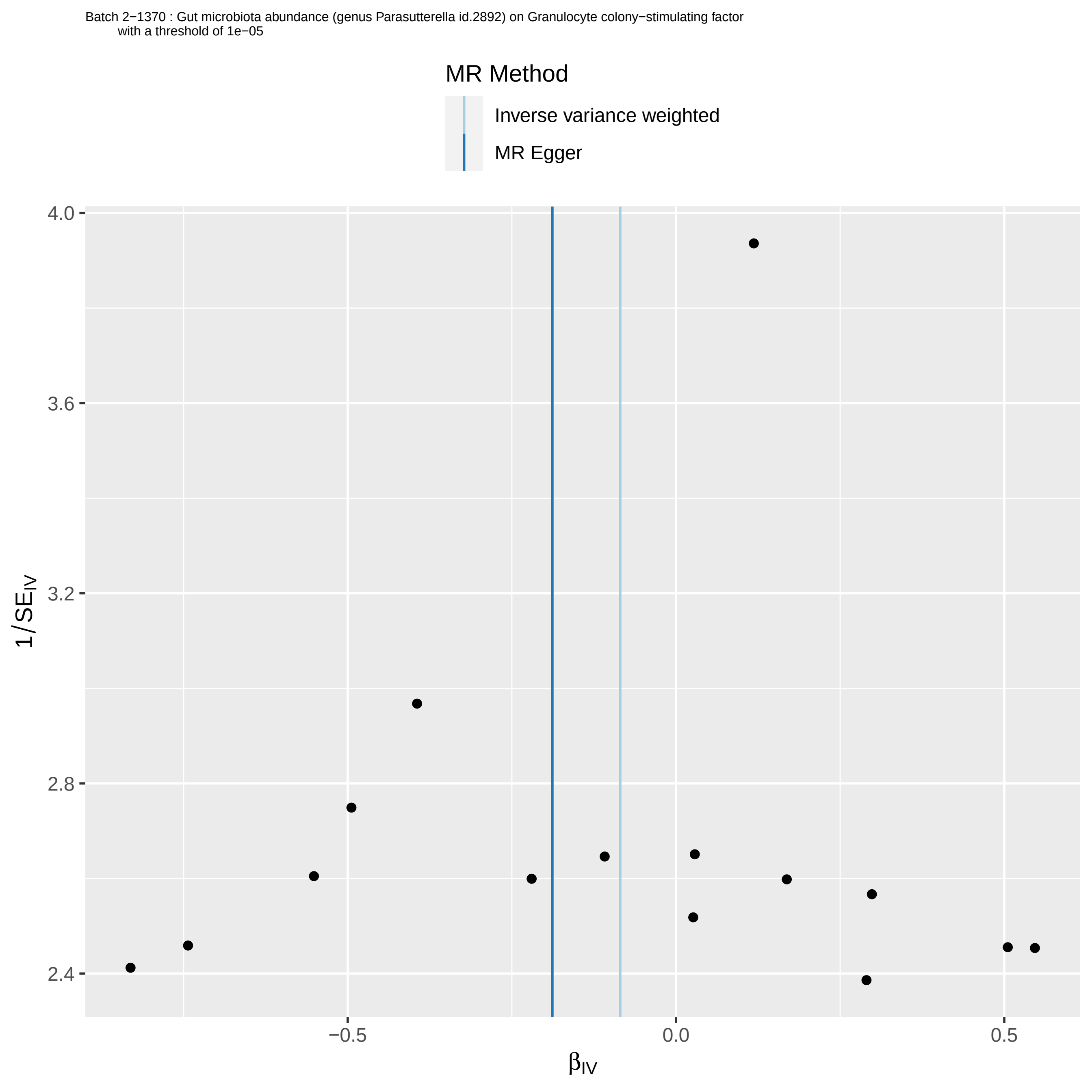

Batch 2−1370 : Gut microbiota abundance (genus Parasutterella id.2892) on Granulocyte colony−stimulating factor
with a threshold of 1e−05
MR Method
Inverse variance weighted
MR Egger
4.0
3.6
3.2
2.8
2.4
1/SE_IV
−0.5
0.0
0.5
β_IV

Batch 2−1371 : Gut microbiota abundance (genus Catenibacterium id.2153) on Granulocyte colony−stimulating factor receptor
with a threshold of 1e−05

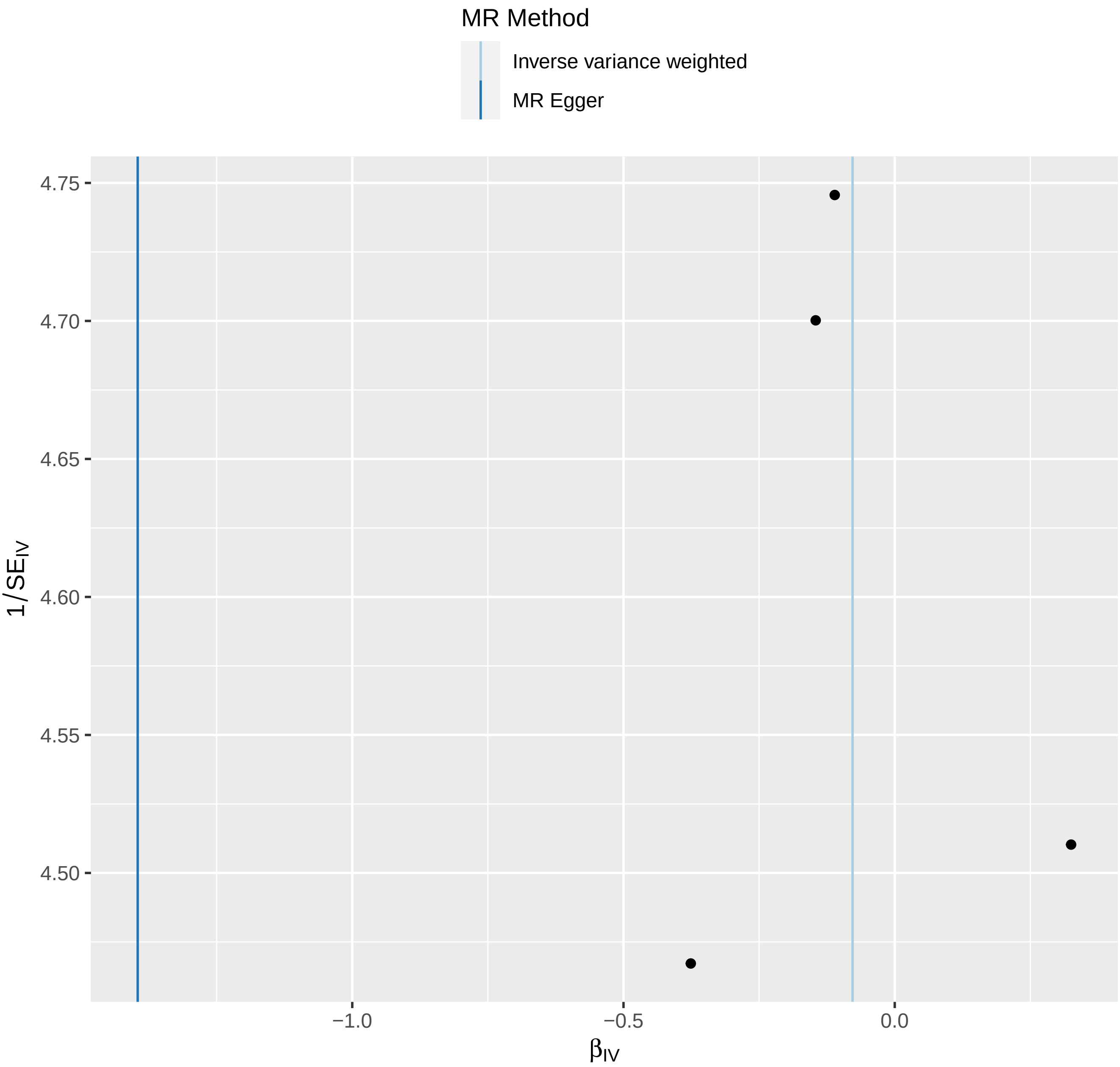

Batch 2−1372 : Gut microbiota abundance (genus Parasutterella id.2892) on Granulocyte colony−stimulating factor receptor with a threshold of 1e−05

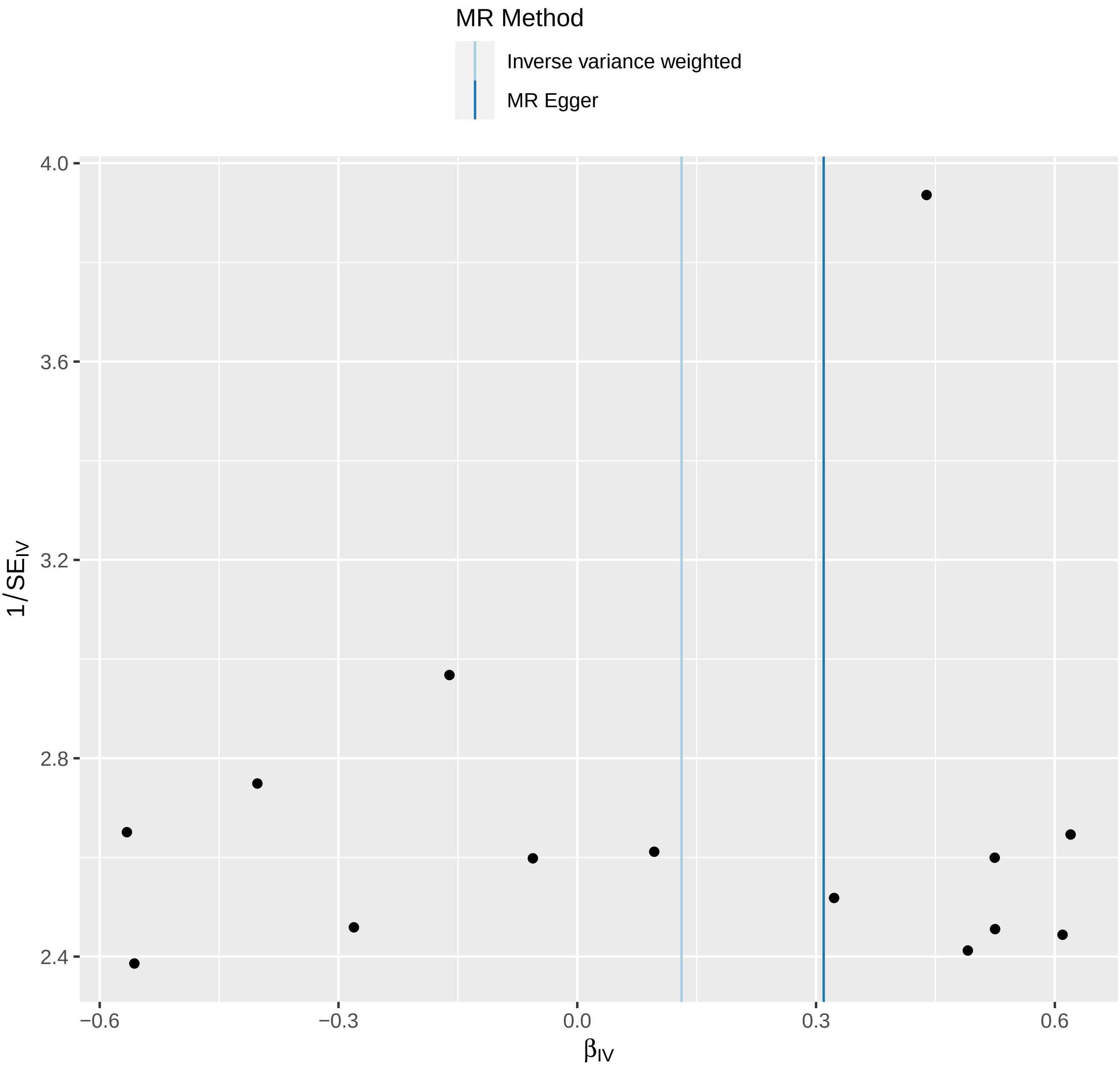

Batch 2−1373 : Gut microbiota abundance (genus Catenibacterium id.2153) on Chondroitin sulfate N−acetylgalactosaminyltransferase 2 with a threshold of 1e−05

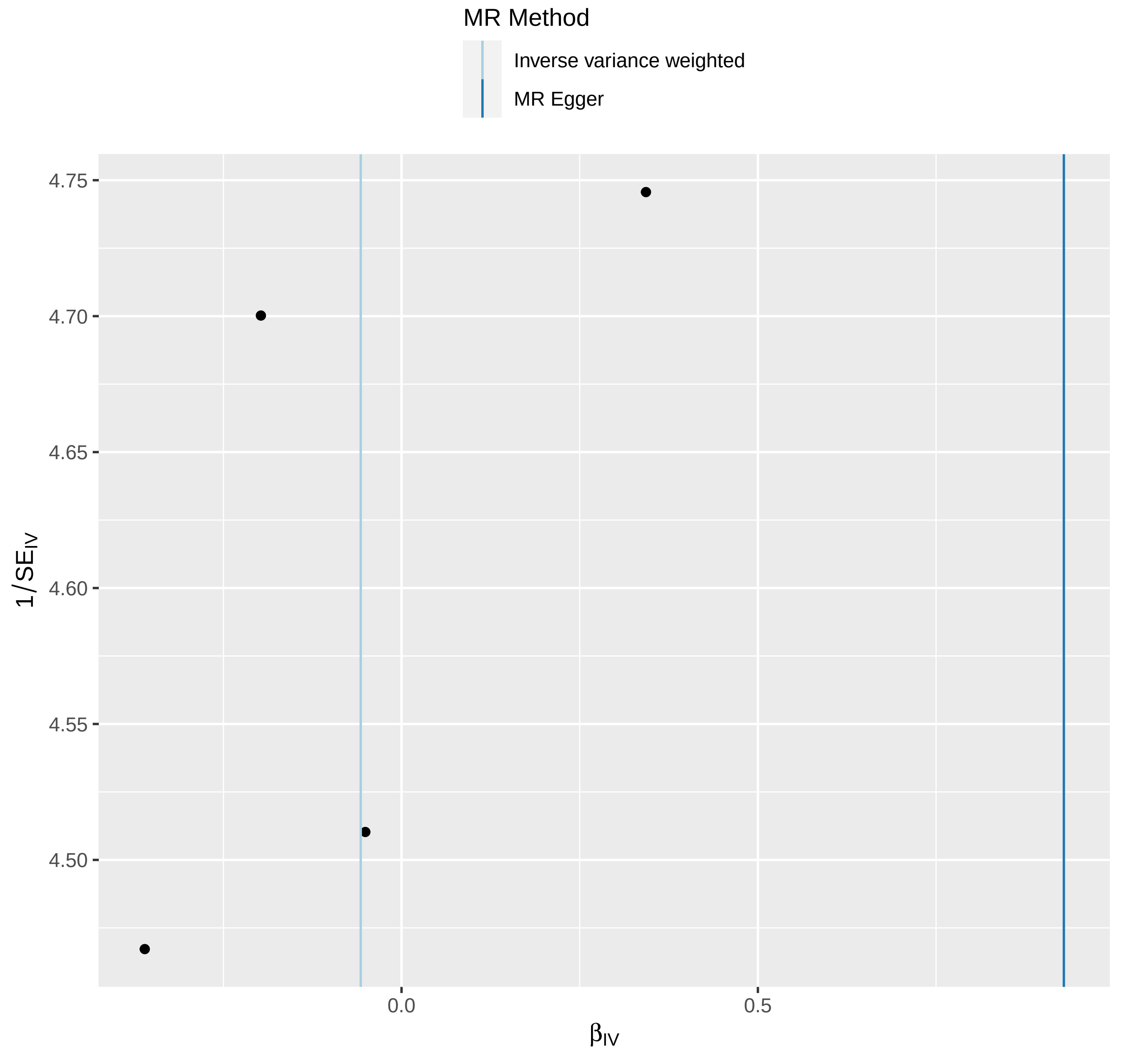

Batch 2−1374 : Gut microbiota abundance (genus Parasutterella id.2892) on Chondroitin sulfate N−acetylgalactosaminyltransferase 2
with a threshold of 1e−05

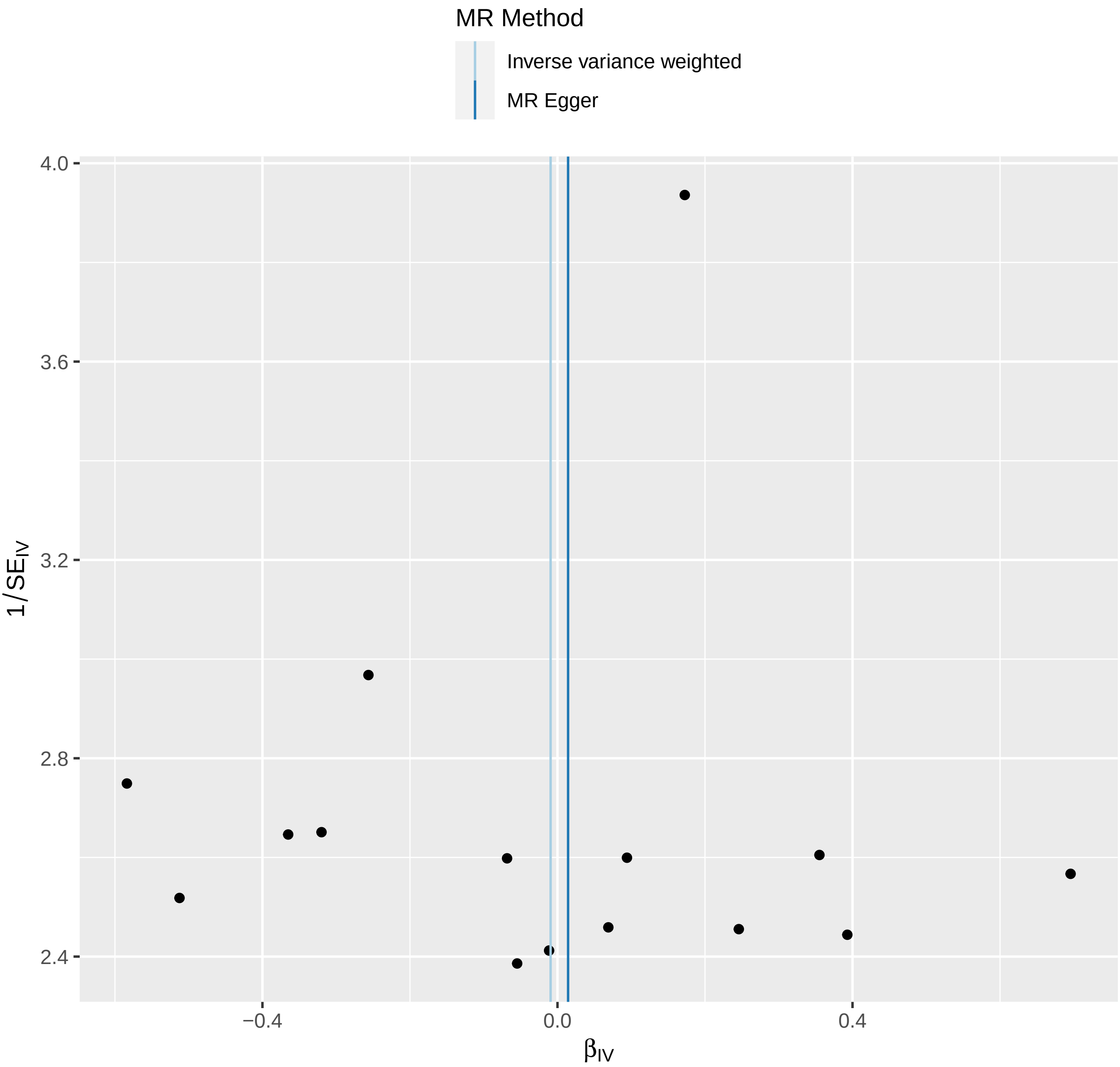

Batch 2−1375 : Gut microbiota abundance (genus Catenibacterium id.2153) on Chorionic somatomammotropin hormone
with a threshold of 1e−05

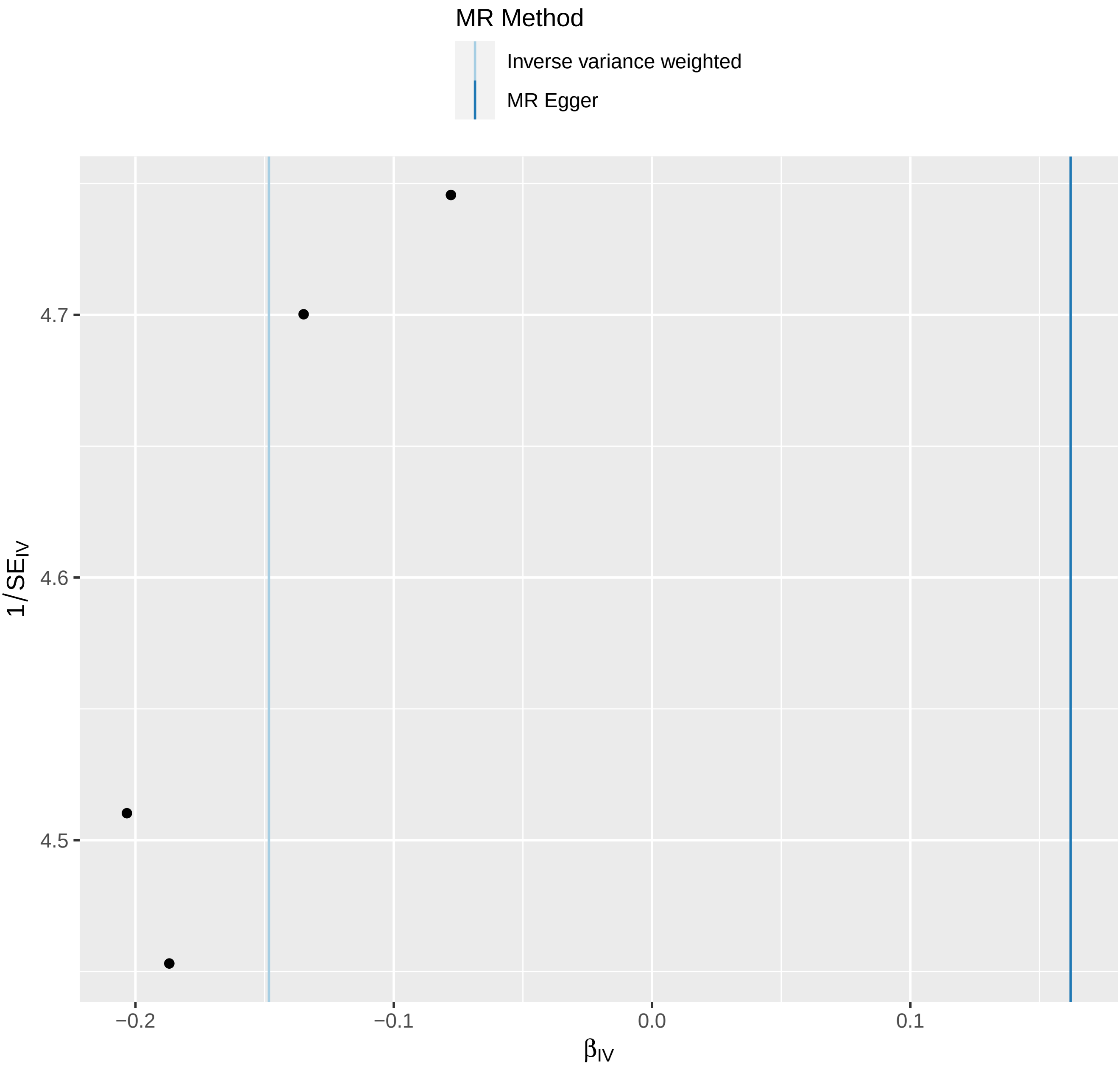

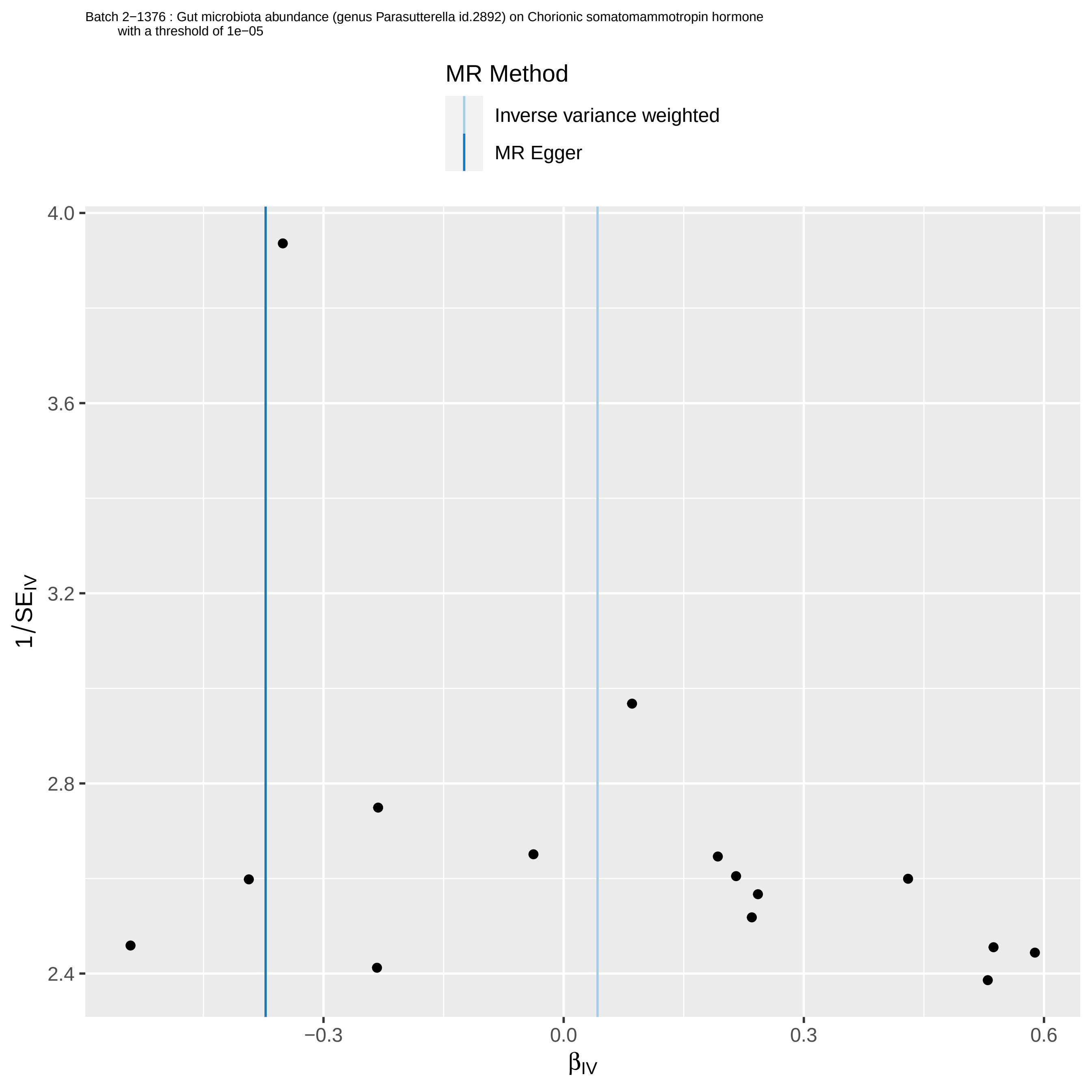
Batch 2−1376 : Gut microbiota abundance (genus Parasutterella id.2892) on Chorionic somatomammotropin hormone
with a threshold of 1e−05
MR Method
Inverse variance weighted
MR Egger
4.0
3.6
3.2
2.8
2.4
1/SE$_{IV}$
−0.3
0.0
0.3
0.6
β_{IV}

Batch 2−1377 : Gut microbiota abundance (genus Catenibacterium id.2153) on Tyrosine−protein kinase CSK
with a threshold of 1e−05

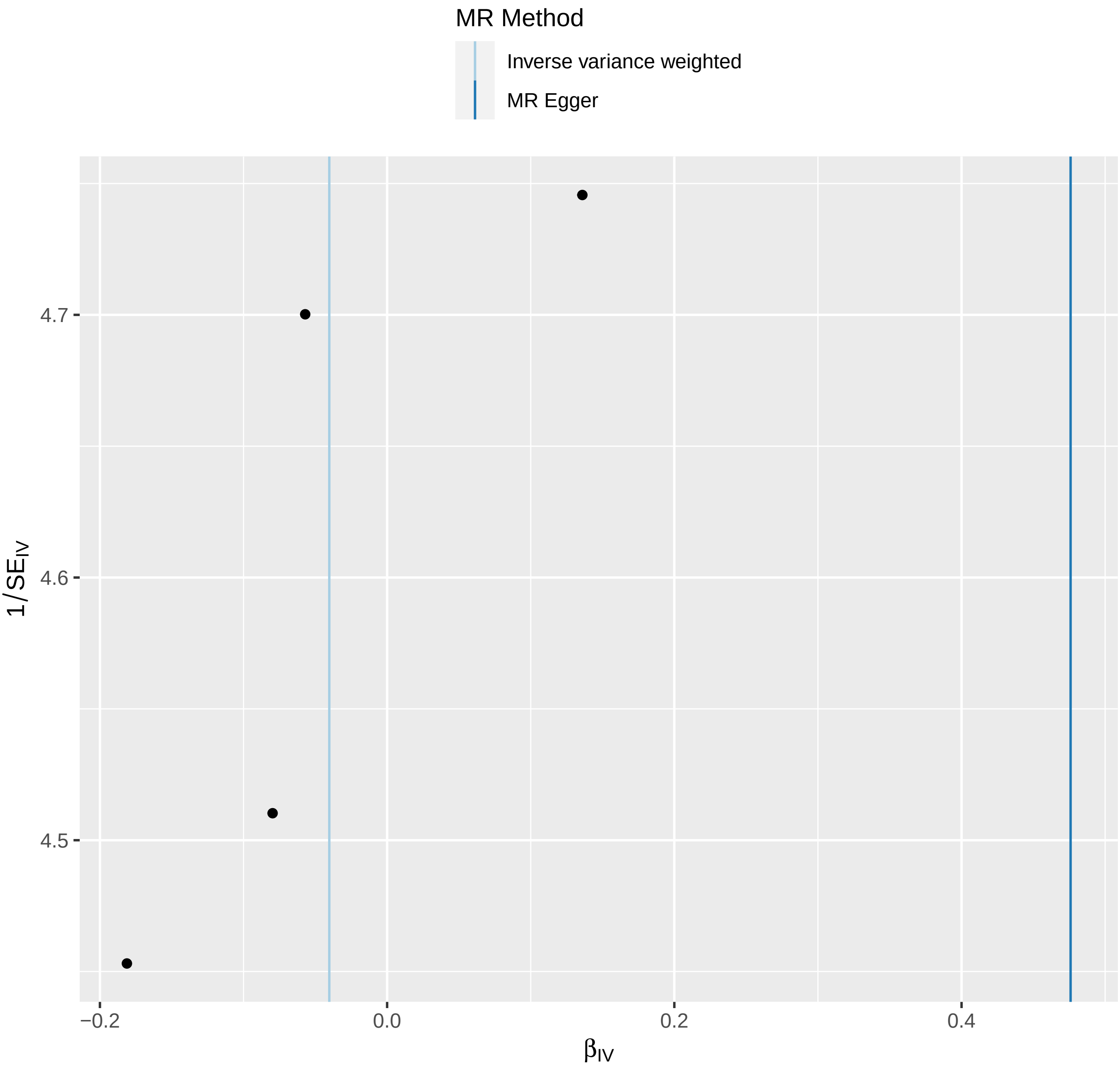

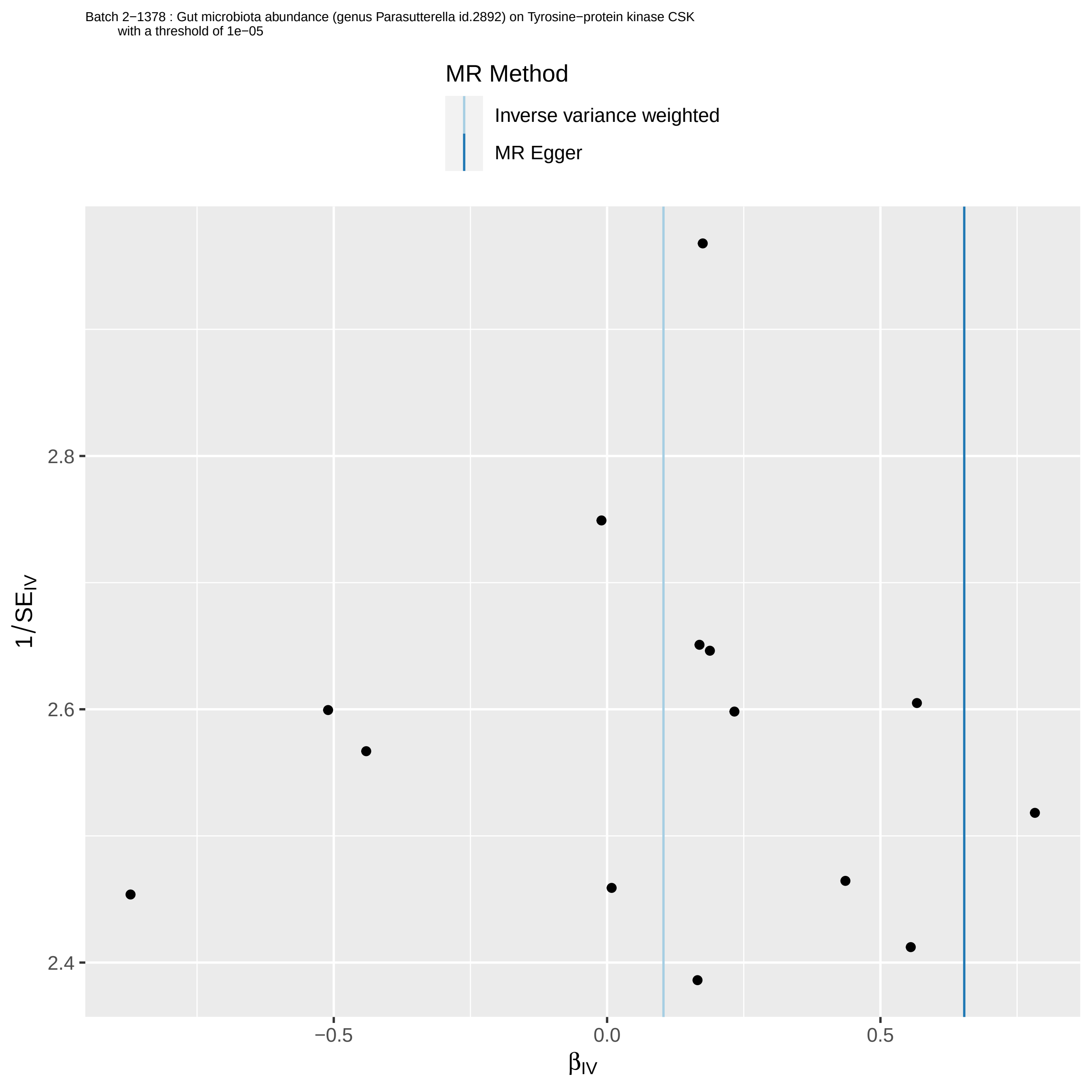
Batch 2−1378 : Gut microbiota abundance (genus Parasutterella id.2892) on Tyrosine−protein kinase CSK
with a threshold of 1e−05
MR Method
Inverse variance weighted
MR Egger
2.8
2.6
2.4
1/SE_IV
−0.5
0.0
0.5
β_IV

Batch 2−1379 : Gut microbiota abundance (genus Catenibacterium id.2153) on CUB and sushi domain−containing protein 1
with a threshold of 1e−05

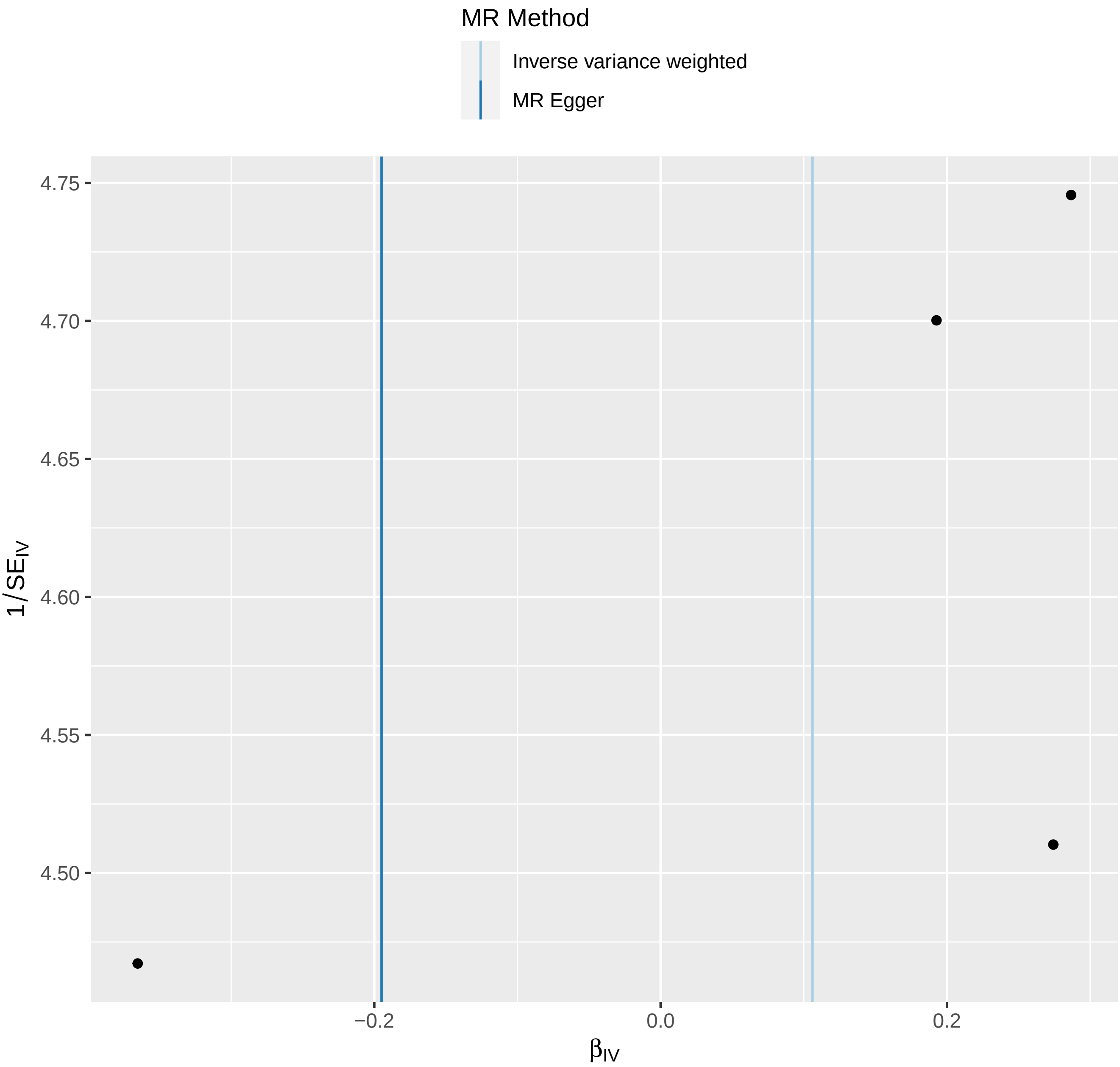

Batch 2−1380 : Gut microbiota abundance (genus Parasutterella id.2892) on CUB and sushi domain−containing protein 1
with a threshold of 1e−05

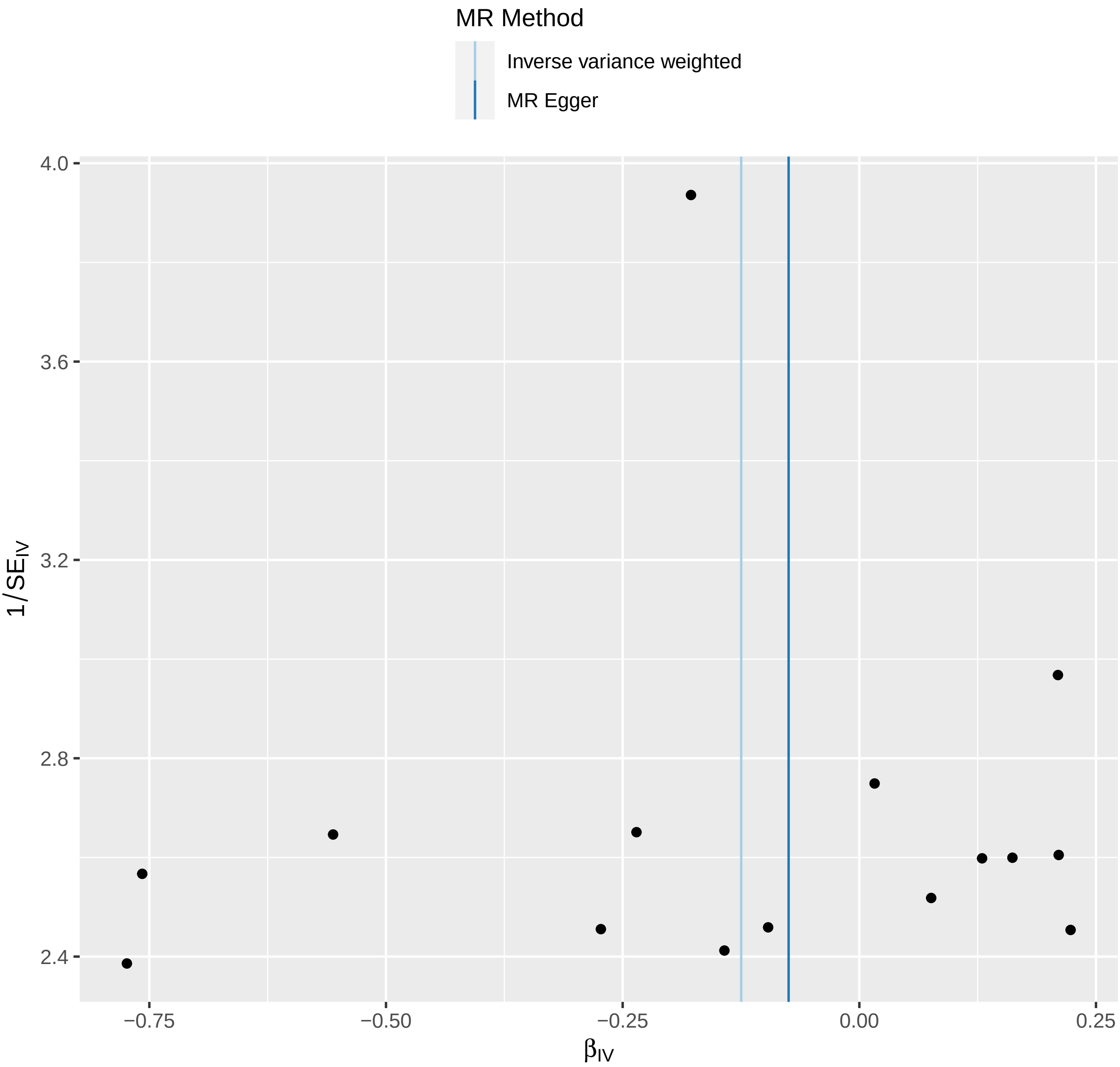

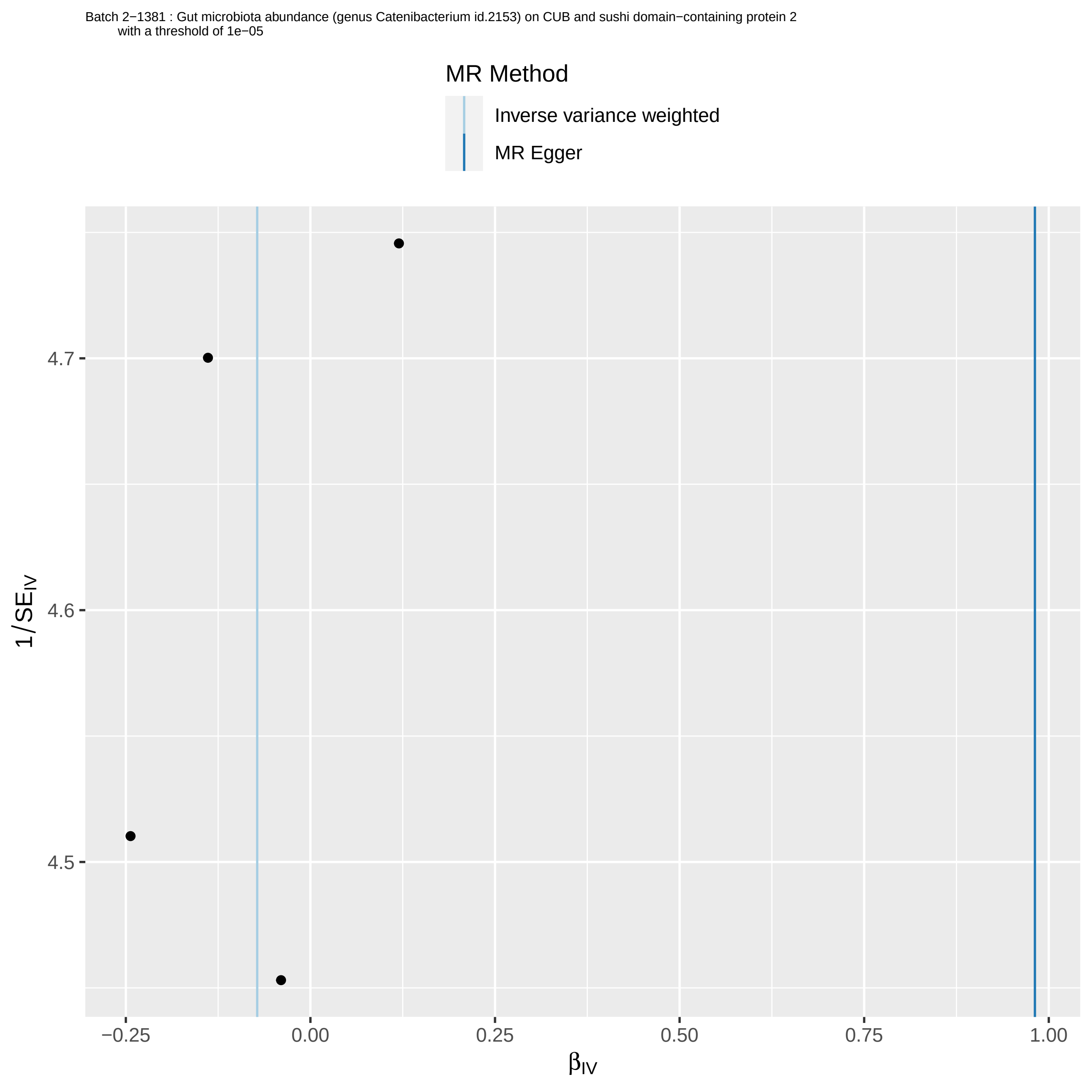

Batch 2−1381 : Gut microbiota abundance (genus Catenibacterium id.2153) on CUB and sushi domain−containing protein 2
with a threshold of 1e−05
MR Method
Inverse variance weighted
MR Egger
4.7
4.6
4.5
1/SE_IV
−0.25
0.00
0.25
0.50
0.75
1.00
β_IV

Batch 2−1382 : Gut microbiota abundance (genus Parasutterella id.2892) on CUB and sushi domain−containing protein 2
with a threshold of 1e−05

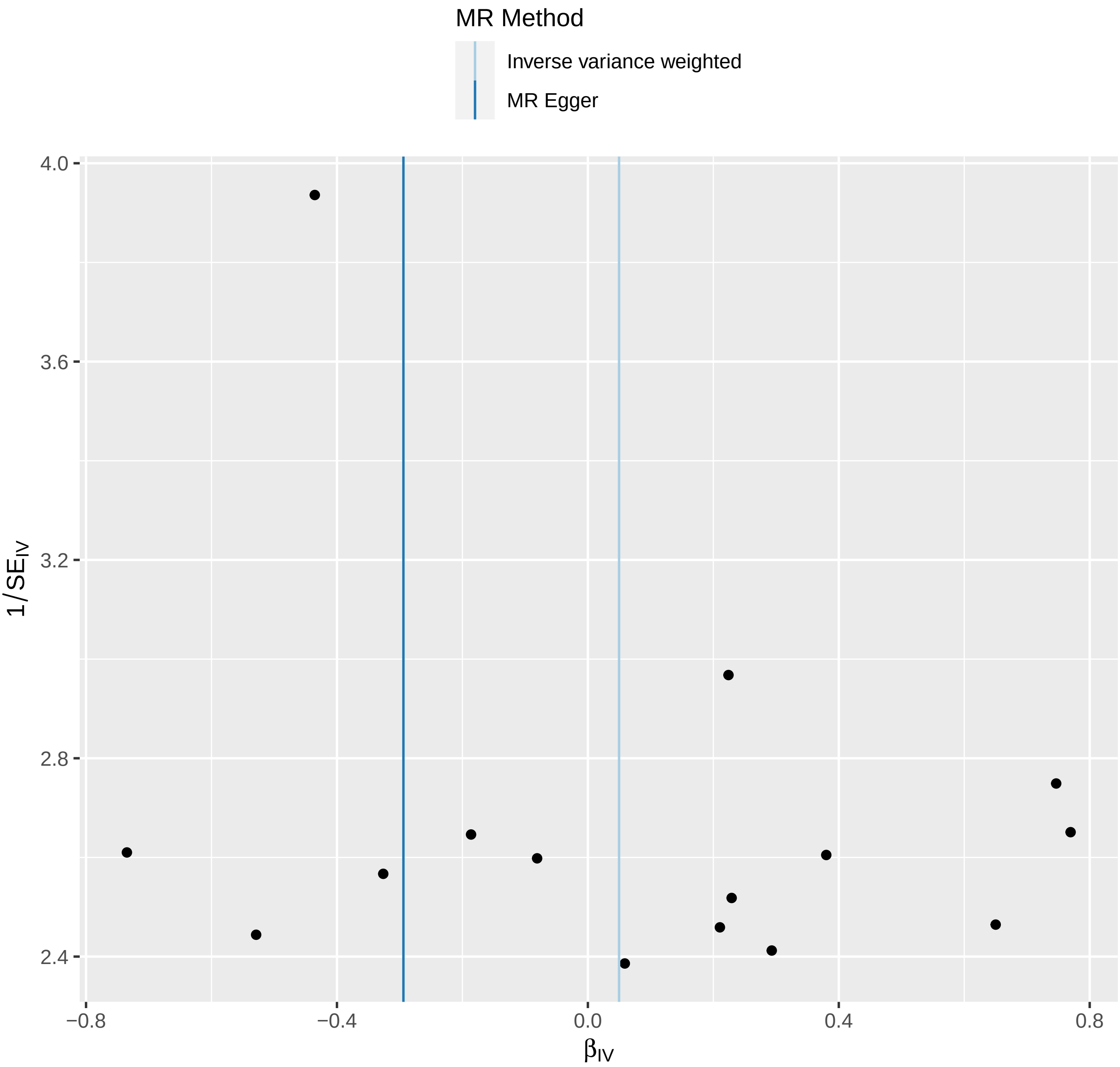

Batch 2−1383 : Gut microbiota abundance (genus Catenibacterium id.2153) on alpha−S1−casein
with a threshold of 1e−05

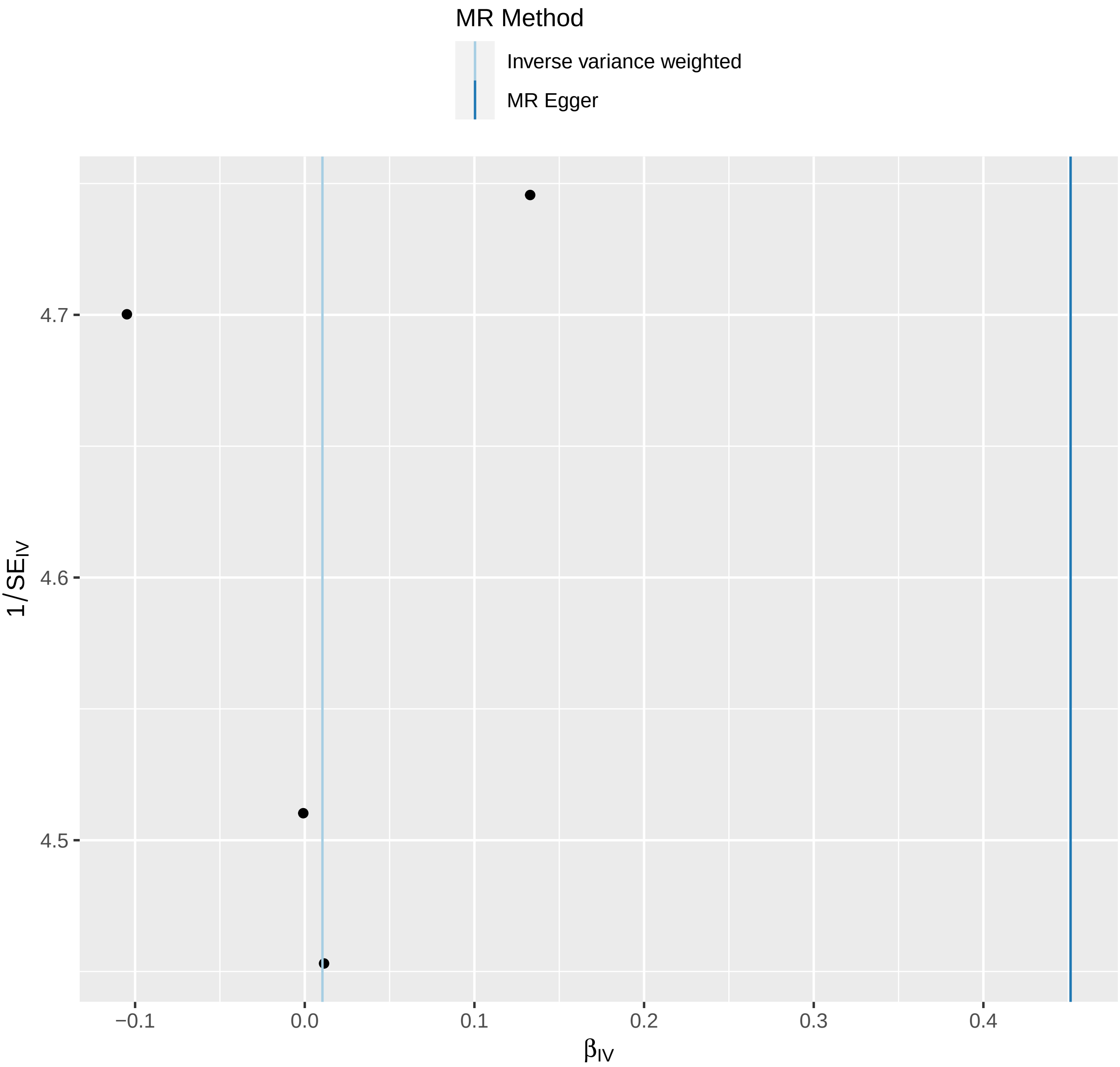

Batch 2−1384 : Gut microbiota abundance (genus Parasutterella id.2892) on alpha−S1−casein
with a threshold of 1e−05

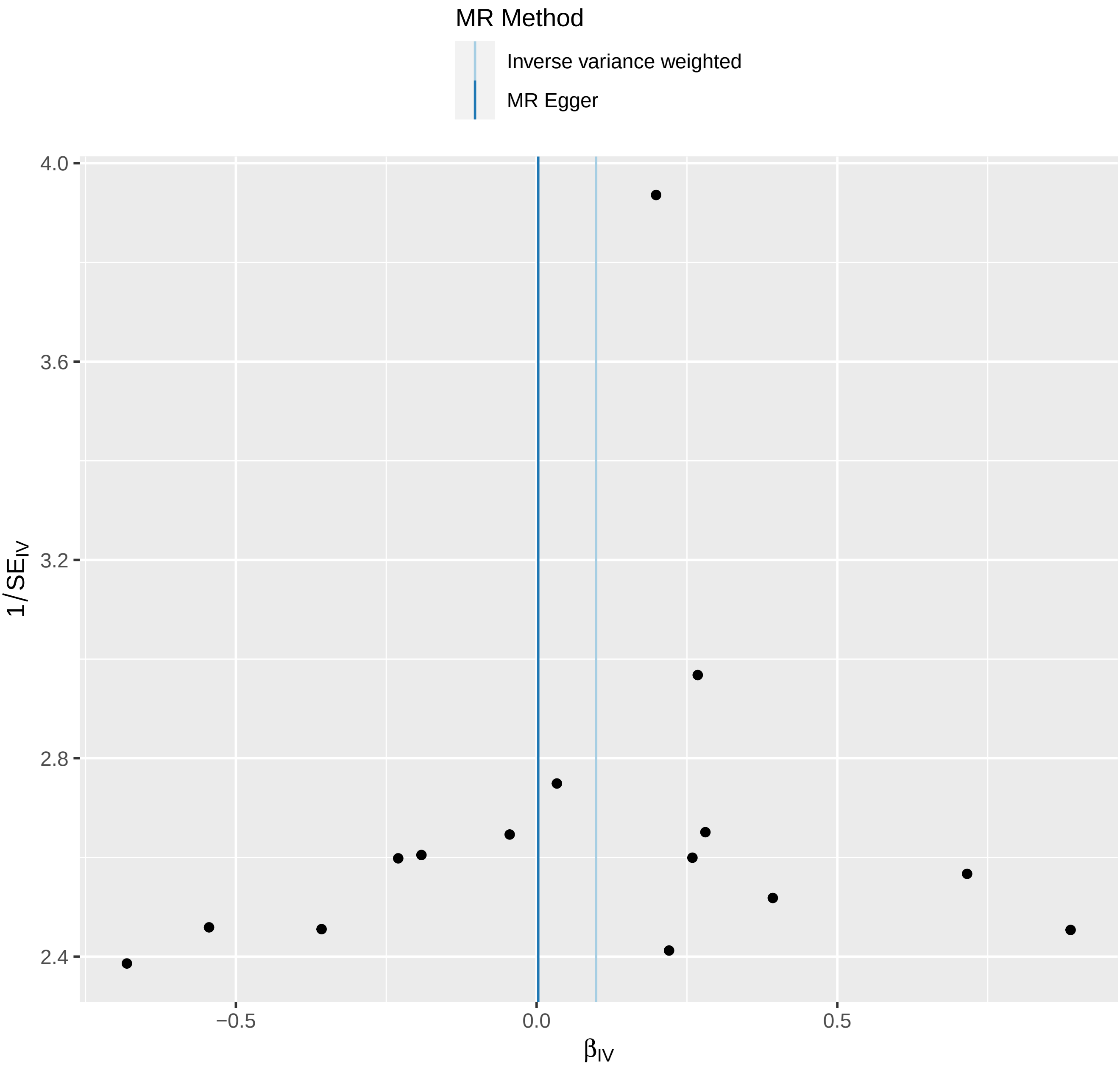

Batch 2−1385 : Gut microbiota abundance (genus Catenibacterium id.2153) on Casein kinase I isoform delta
with a threshold of 1e−05

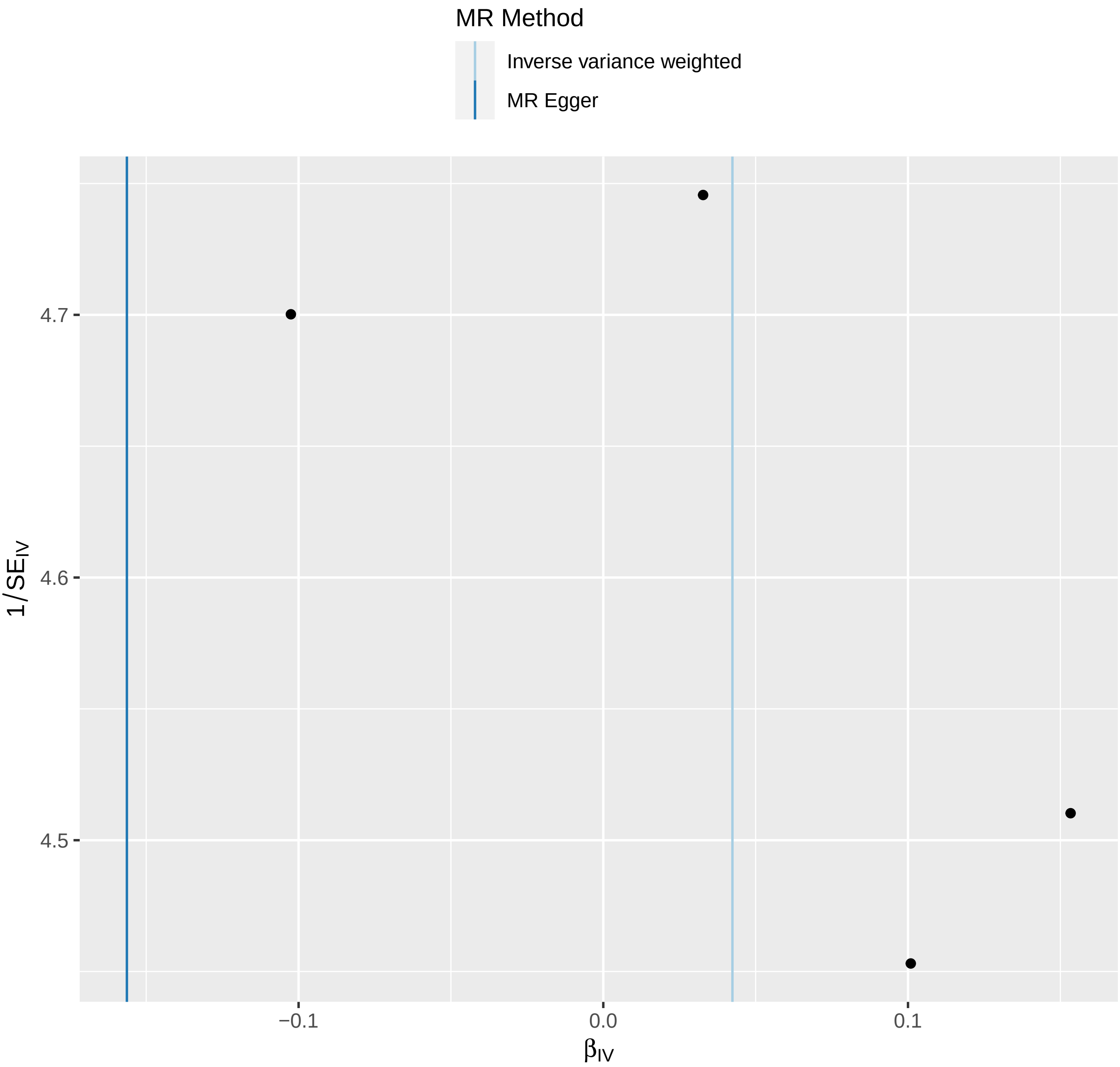

Batch 2−1386 : Gut microbiota abundance (genus Parasutterella id.2892) on Casein kinase I isoform delta
with a threshold of 1e−05

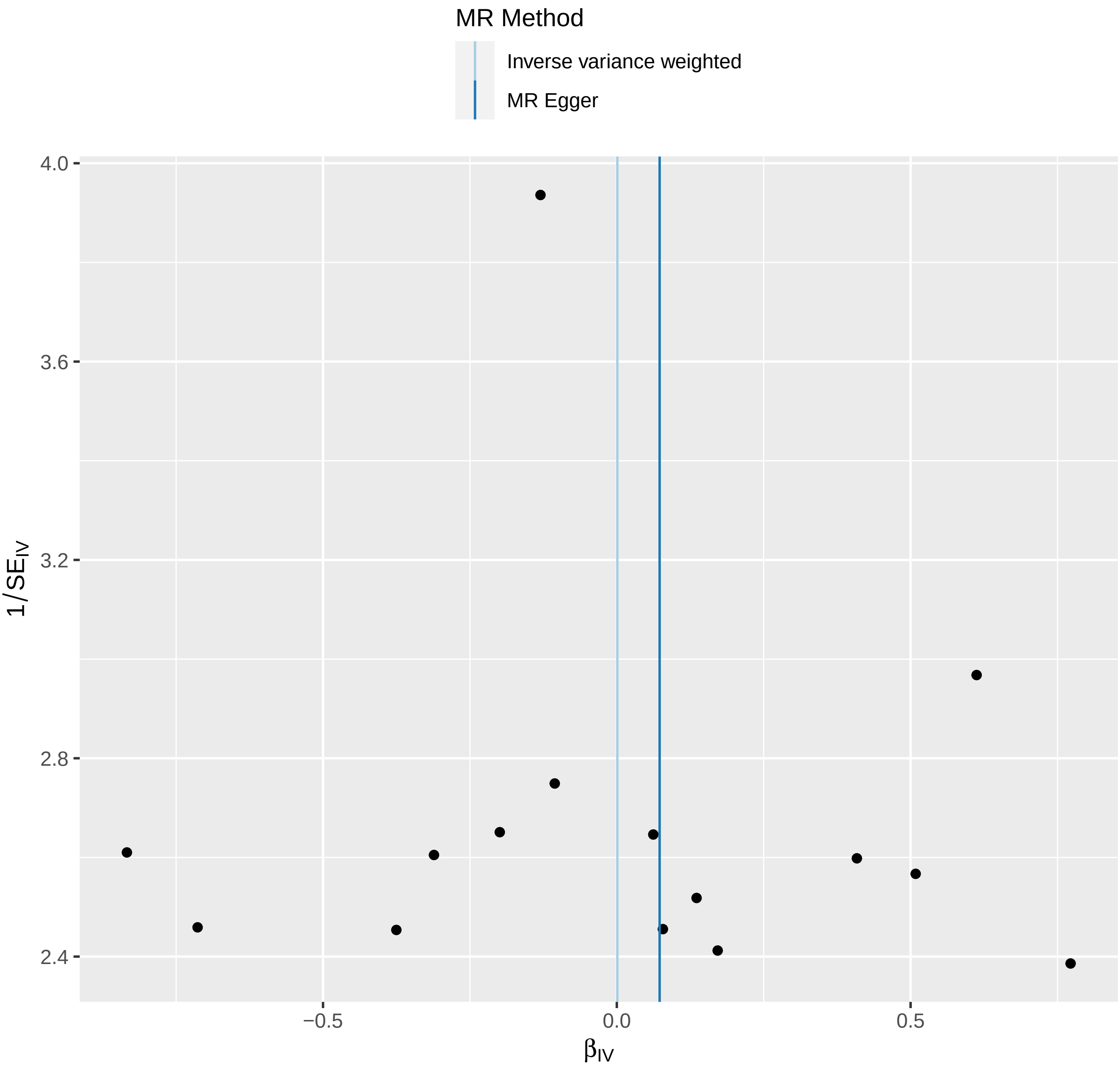

Batch 2−1387 : Gut microbiota abundance (genus Catenibacterium id.2153) on Casein kinase I isoform gamma−2
with a threshold of 1e−05

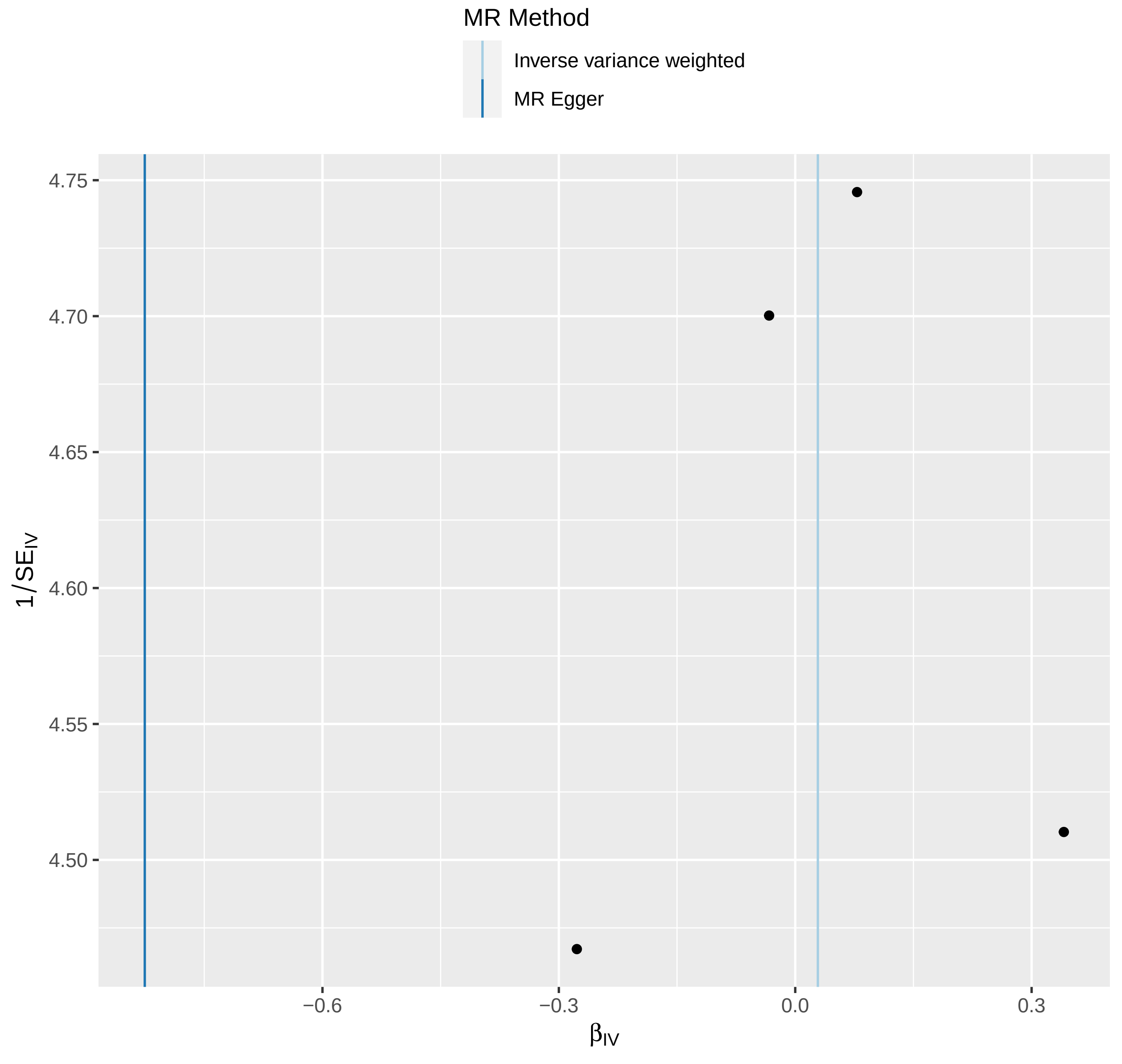

Batch 2−1388 : Gut microbiota abundance (genus Parasutterella id.2892) on Casein kinase I isoform gamma−2 with a threshold of 1e−05

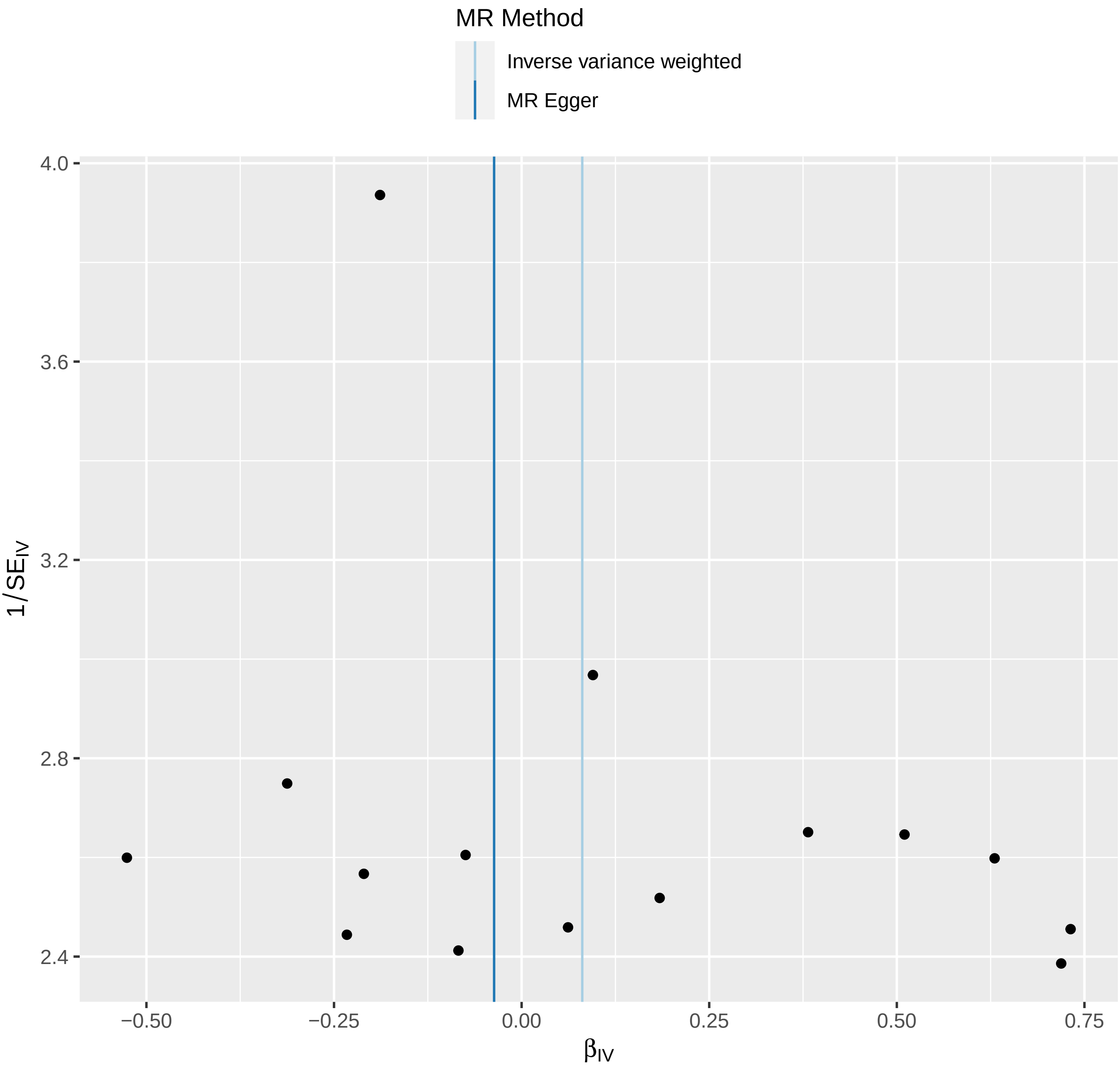

Batch 2−1389 : Gut microbiota abundance (genus Catenibacterium id.2153) on Casein kinase II subunit alpha
with a threshold of 1e−05

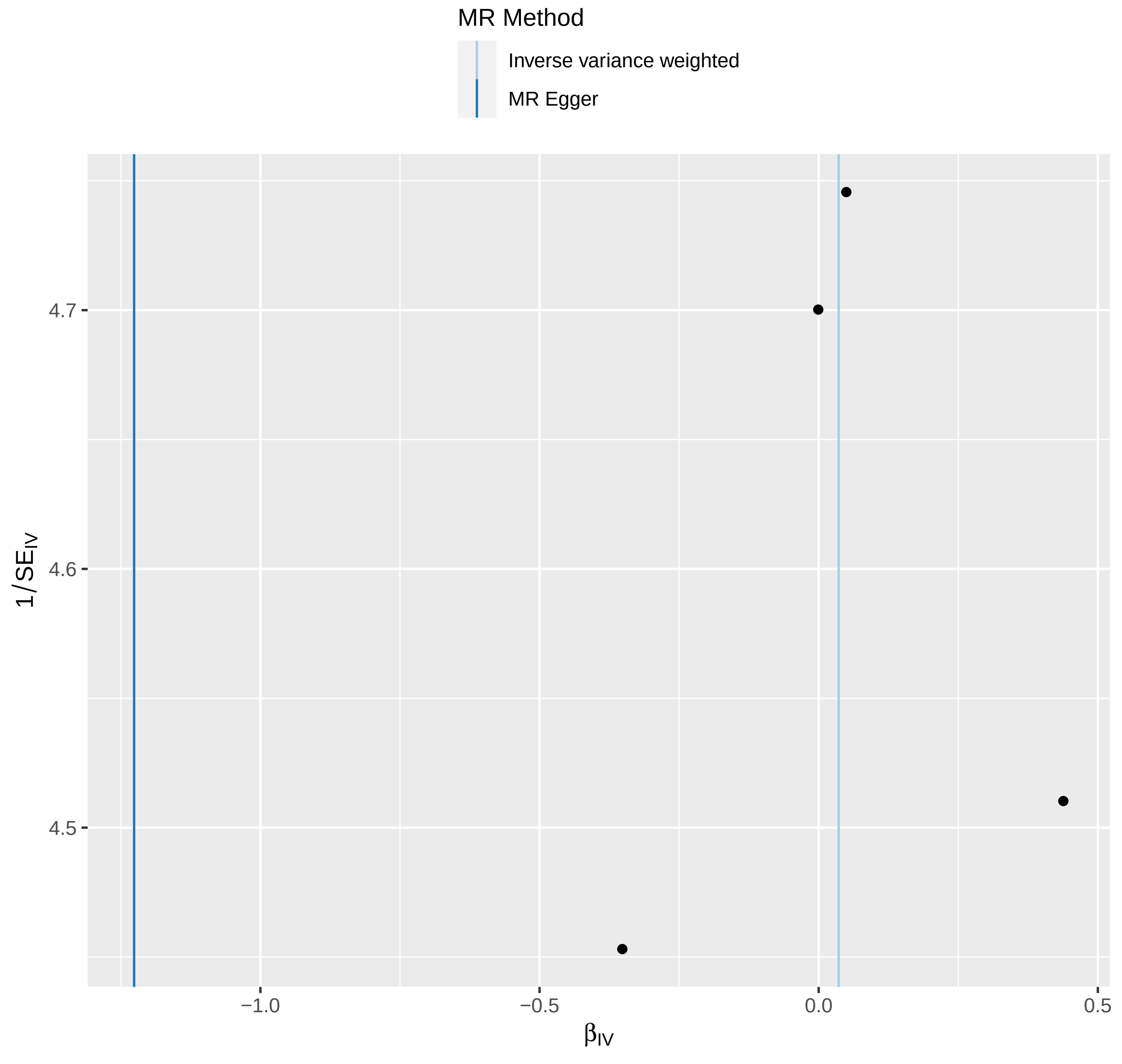

Batch 2−1390 : Gut microbiota abundance (genus Parasutterella id.2892) on Casein kinase II subunit alpha
with a threshold of 1e−05

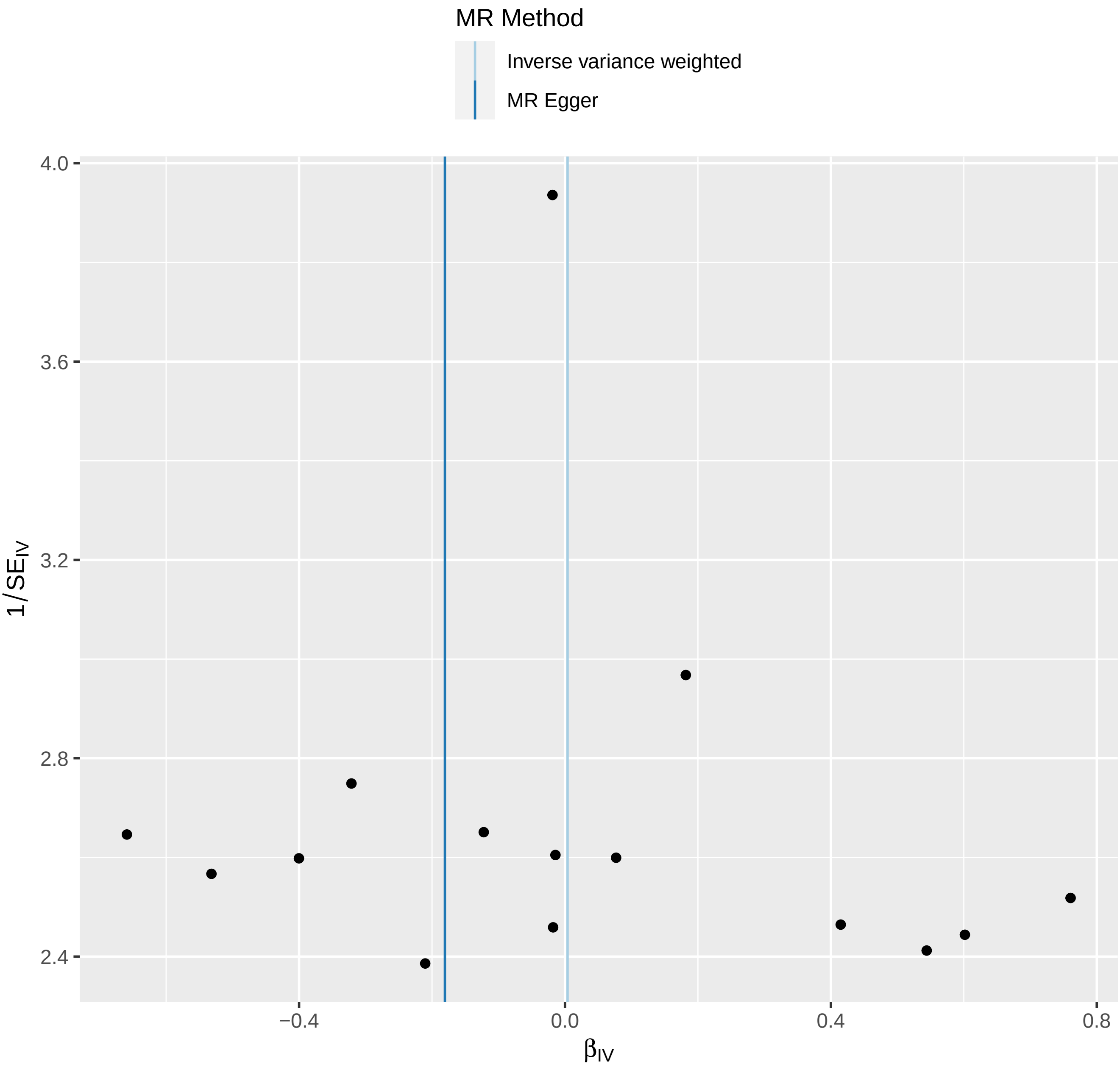

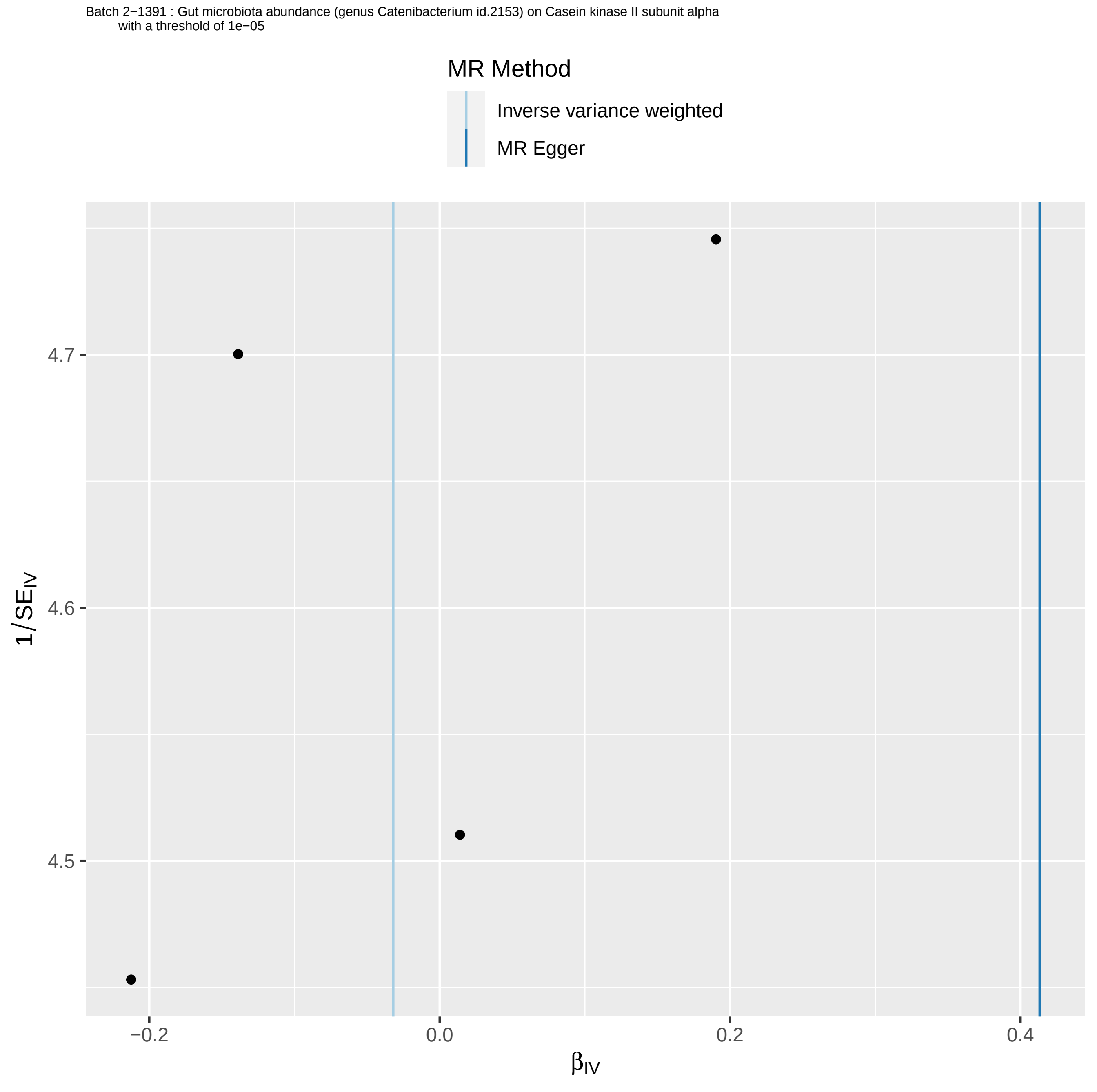
Batch 2−1391 : Gut microbiota abundance (genus Catenibacterium id.2153) on Casein kinase II subunit alpha
with a threshold of 1e−05
MR Method
Inverse variance weighted
MR Egger
4.7
4.6
4.5
1/SE_IV
−0.2
0.0
0.2
0.4
β_IV

Batch 2−1392 : Gut microbiota abundance (genus Parasutterella id.2892) on Casein kinase II subunit alpha
with a threshold of 1e−05

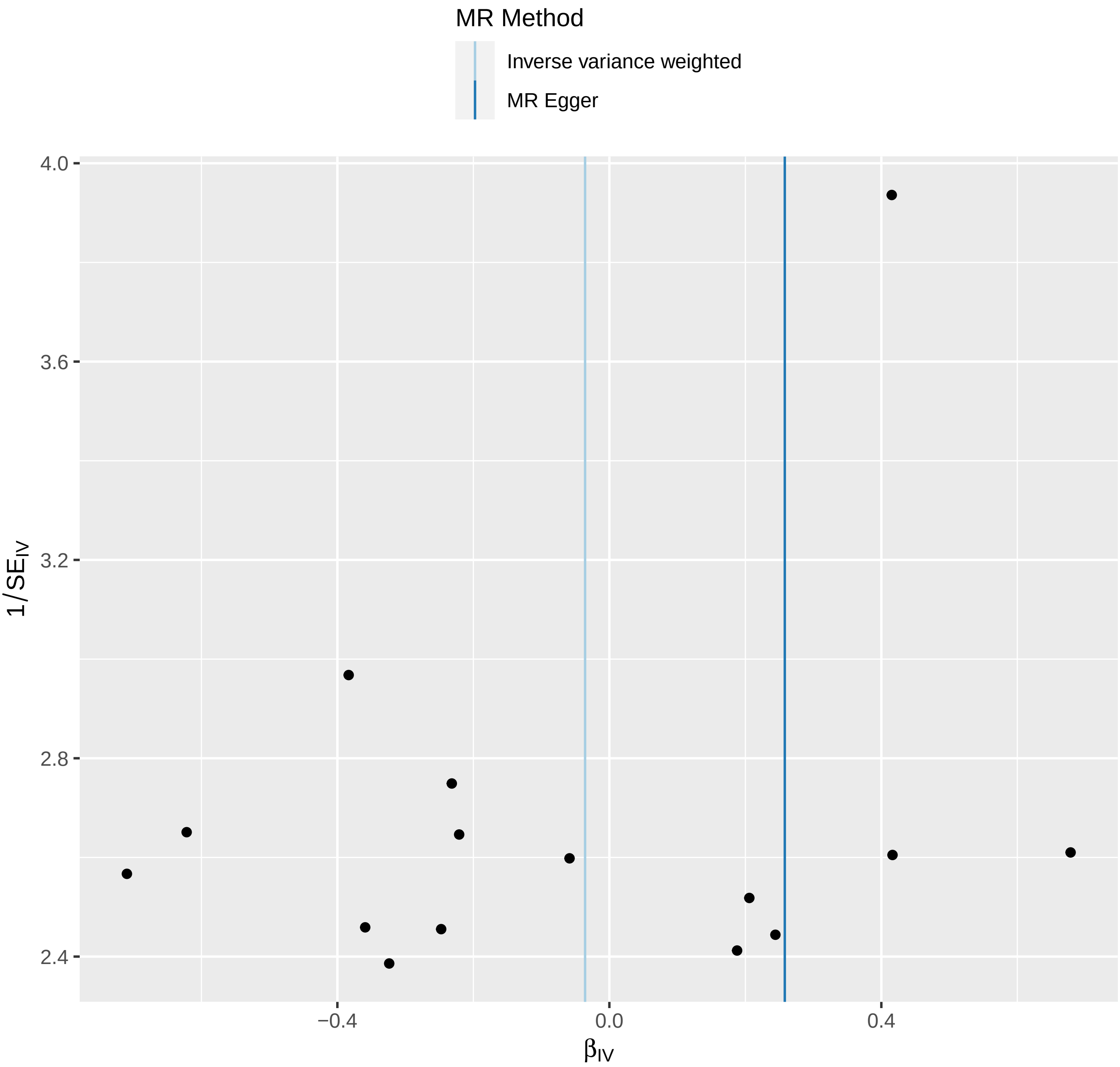

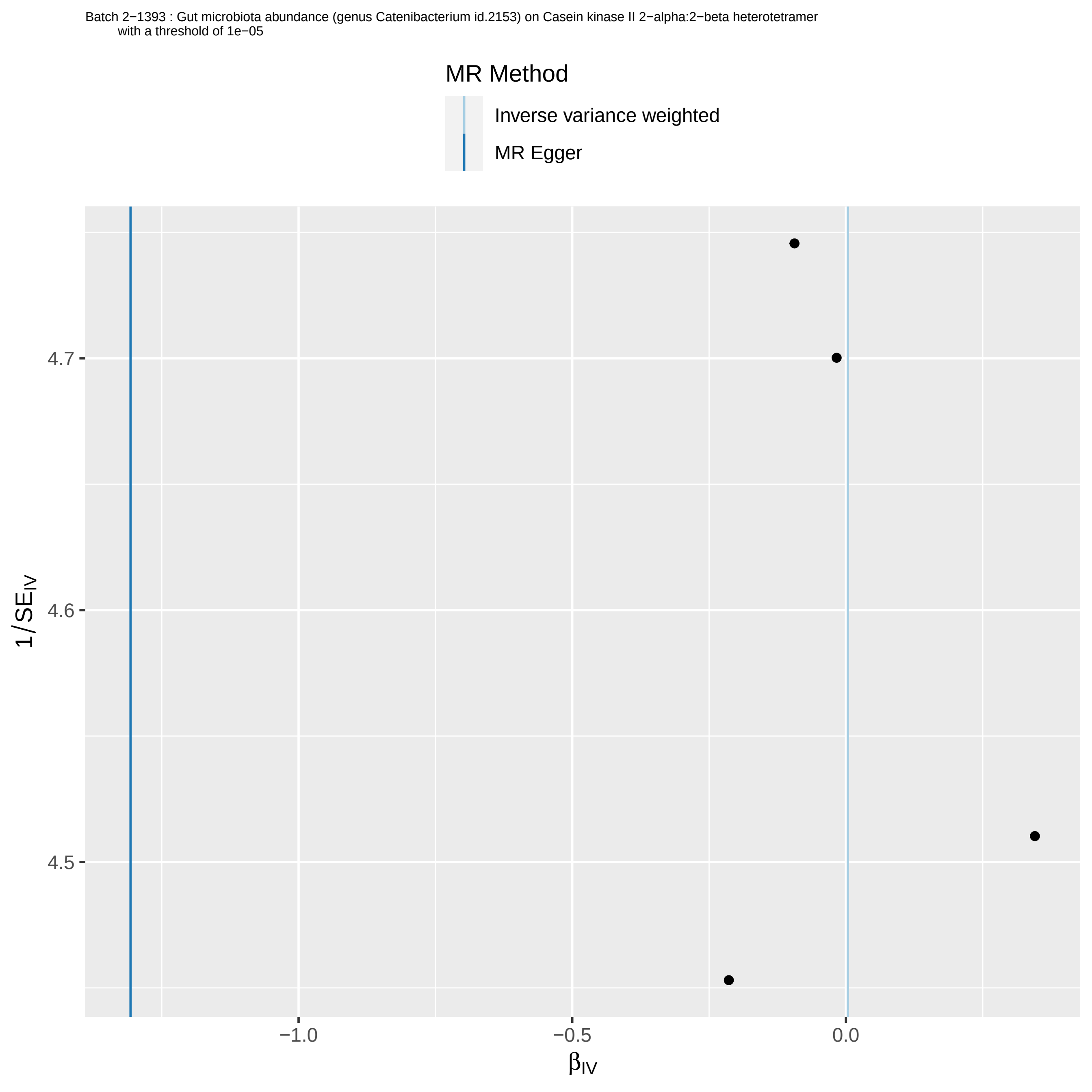

Batch 2−1393 : Gut microbiota abundance (genus Catenibacterium id.2153) on Casein kinase II 2−alpha:2−beta heterotetramer
with a threshold of 1e−05
MR Method
Inverse variance weighted
MR Egger
4.7
4.6
4.5
1/SE_IV
−1.0
−0.5
0.0
β_IV

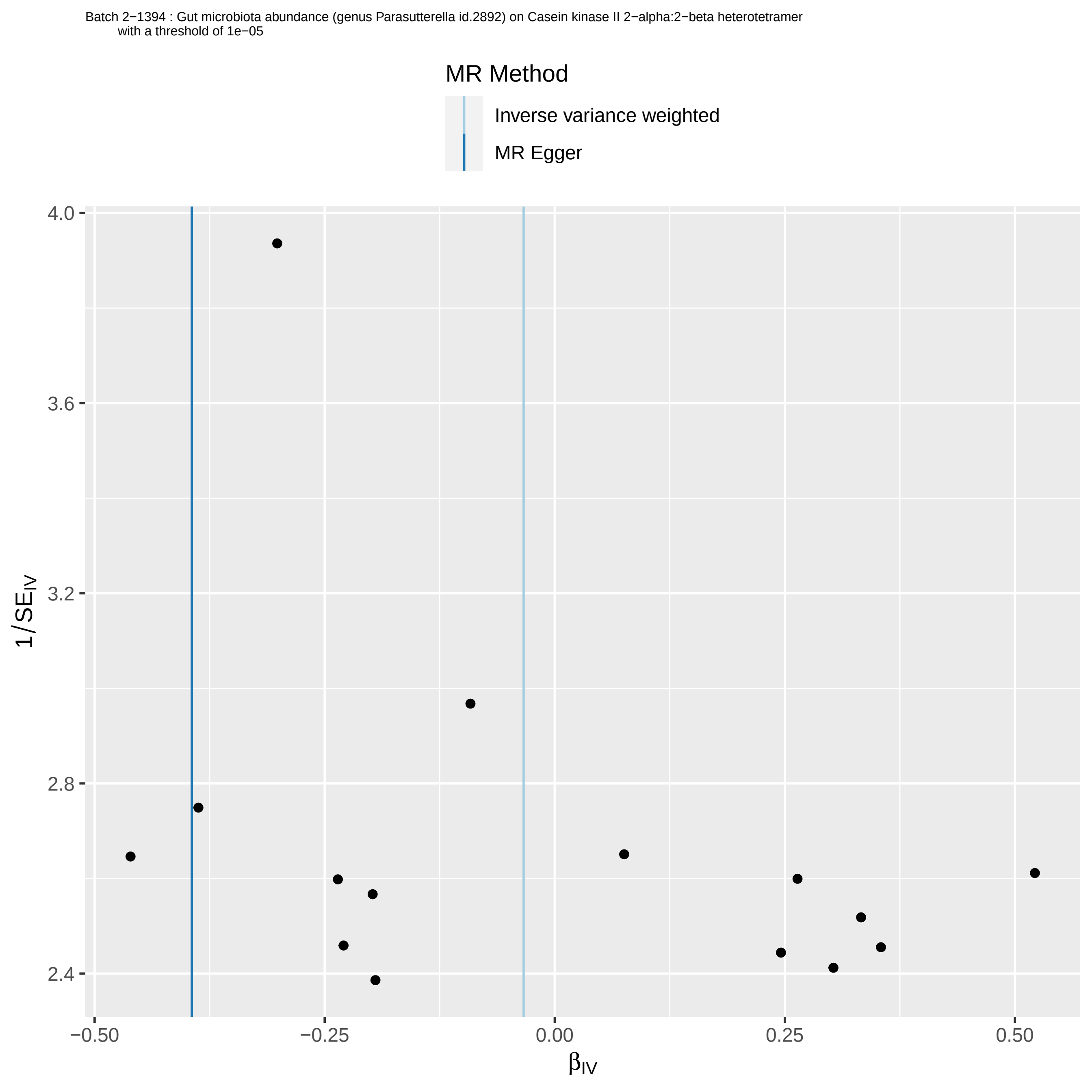

Batch 2−1394 : Gut microbiota abundance (genus Parasutterella id.2892) on Casein kinase II 2−alpha:2−beta heterotetramer
with a threshold of 1e−05
MR Method
Inverse variance weighted
MR Egger
4.0
3.6
3.2
2.8
2.4
1/SE_IV
−0.50
−0.25
0.00
0.25
0.50
β_IV

Batch 2−1395 : Gut microbiota abundance (genus Catenibacterium id.2153) on Casein kinase II 2−alpha':2−beta heterotetramer with a threshold of 1e−05

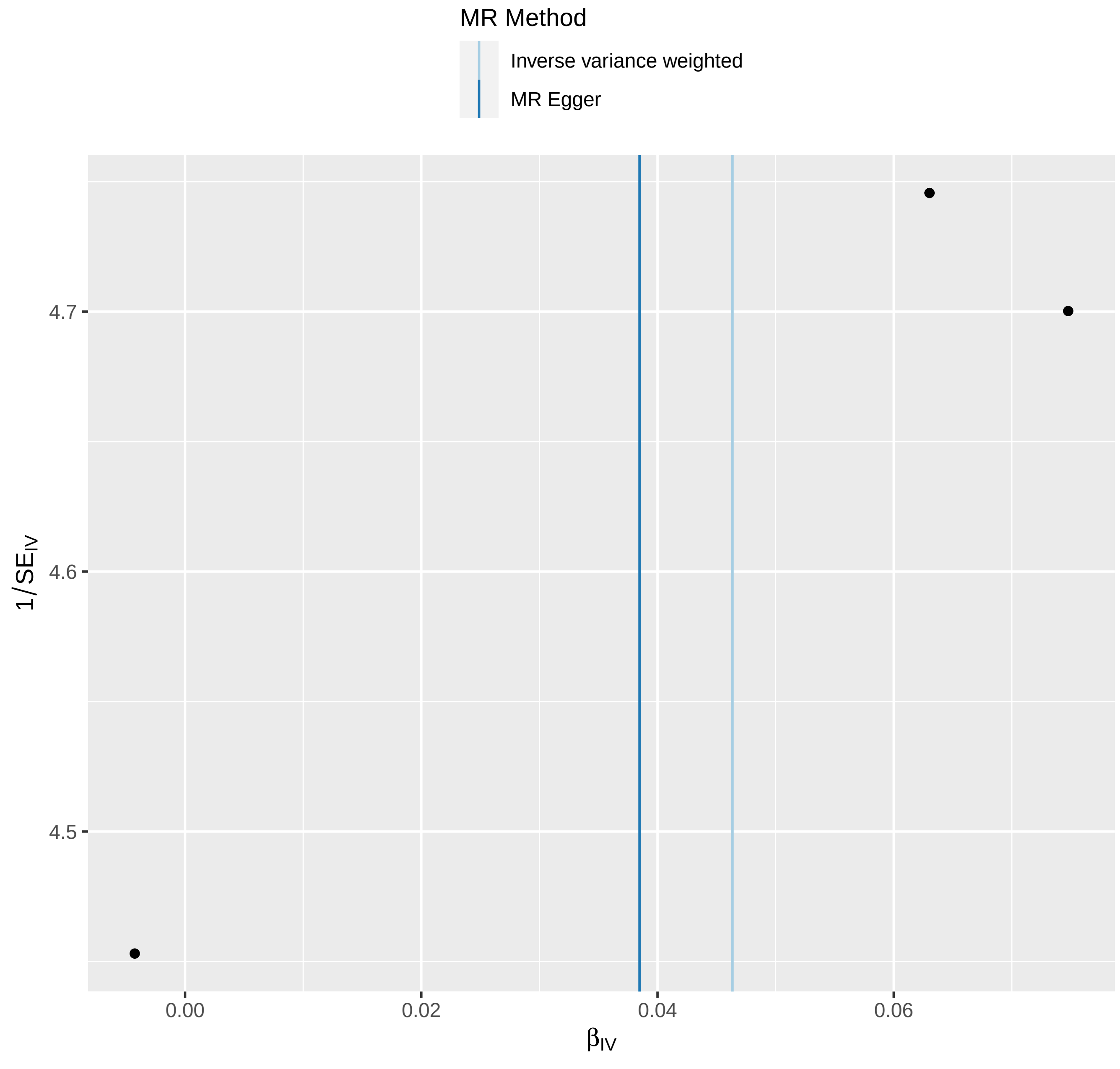

Batch 2−1396 : Gut microbiota abundance (genus Parasutterella id.2892) on Casein kinase II 2−alpha':2−beta heterotetramer with a threshold of 1e−05

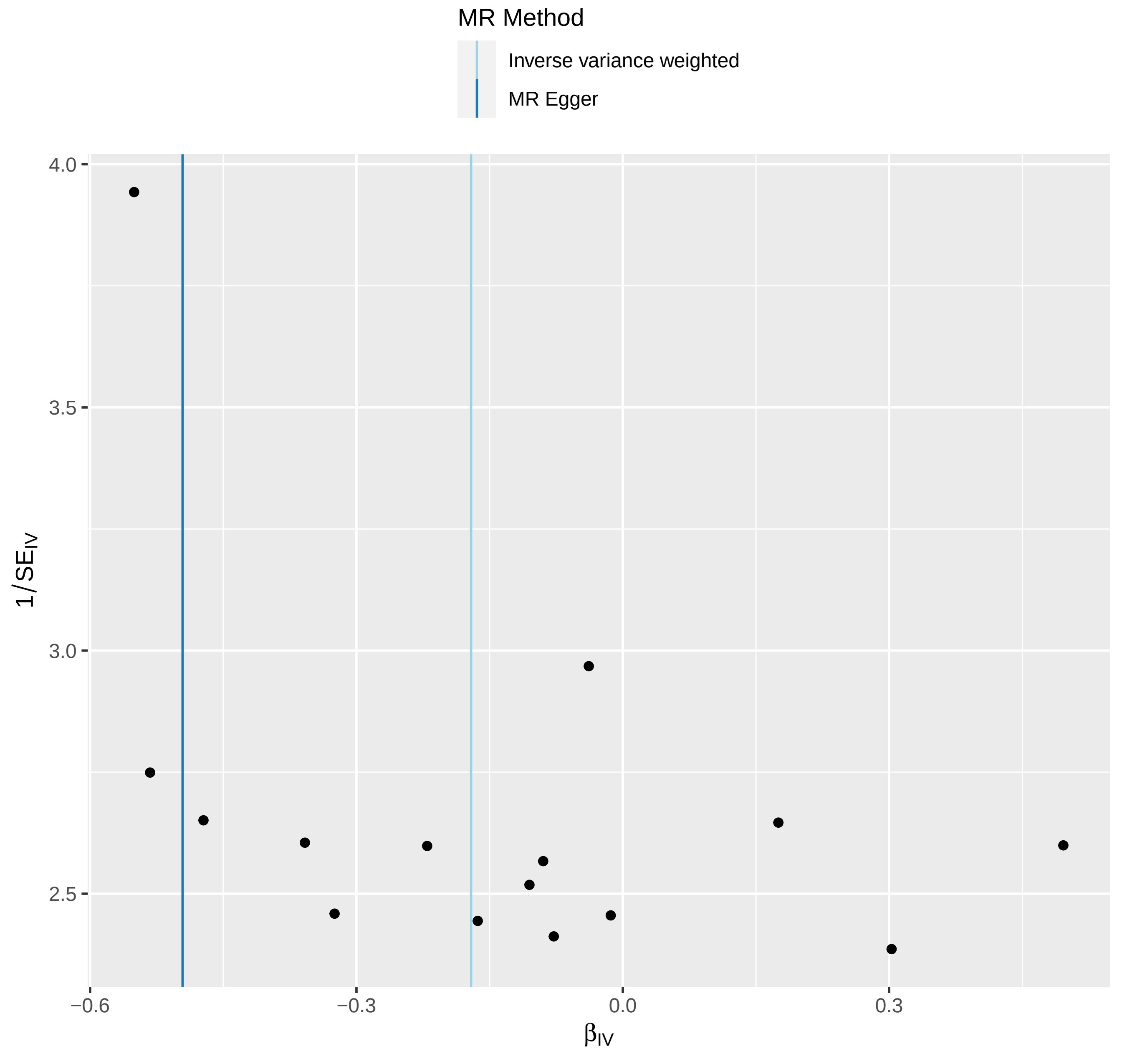

Batch 2−1397 : Gut microbiota abundance (genus Catenibacterium id.2153) on Chondroitin sulfate proteoglycan 4
with a threshold of 1e−05

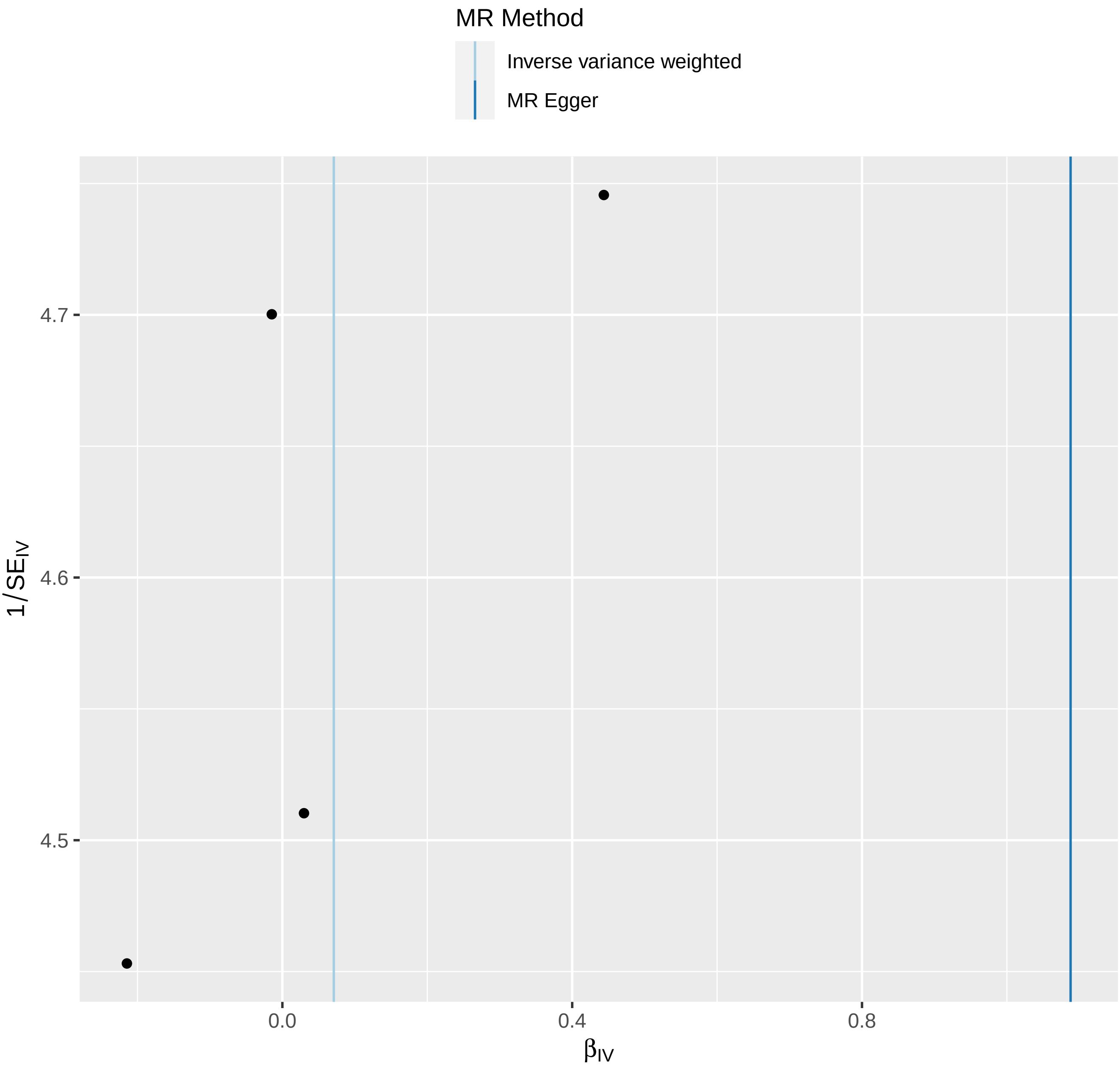

Batch 2−1398 : Gut microbiota abundance (genus Parasutterella id.2892) on Chondroitin sulfate proteoglycan 4
with a threshold of 1e−05

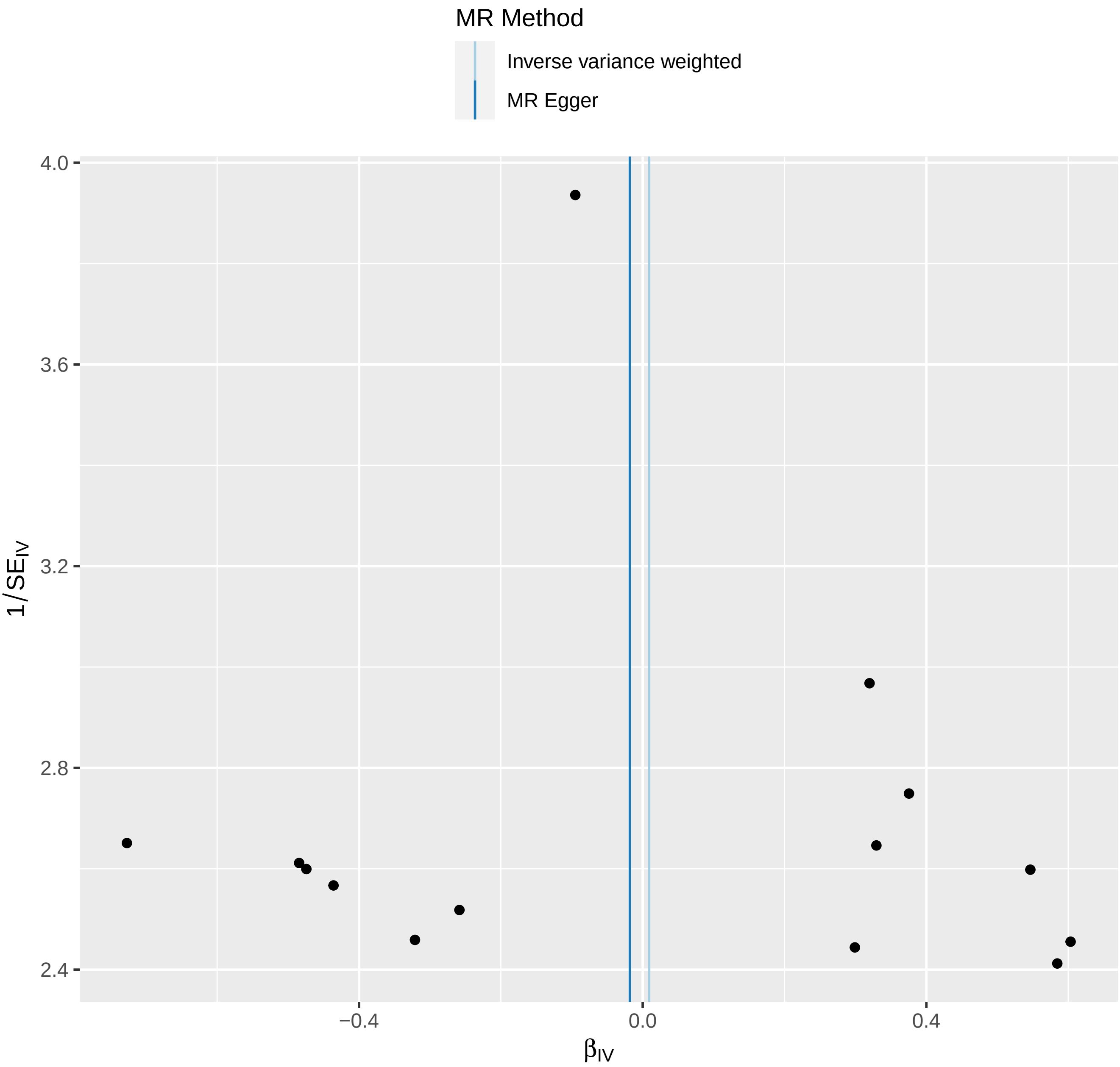

Batch 2−1399 : Gut microbiota abundance (genus Catenibacterium id.2153) on Cysteine and glycine−rich protein 2
with a threshold of 1e−05

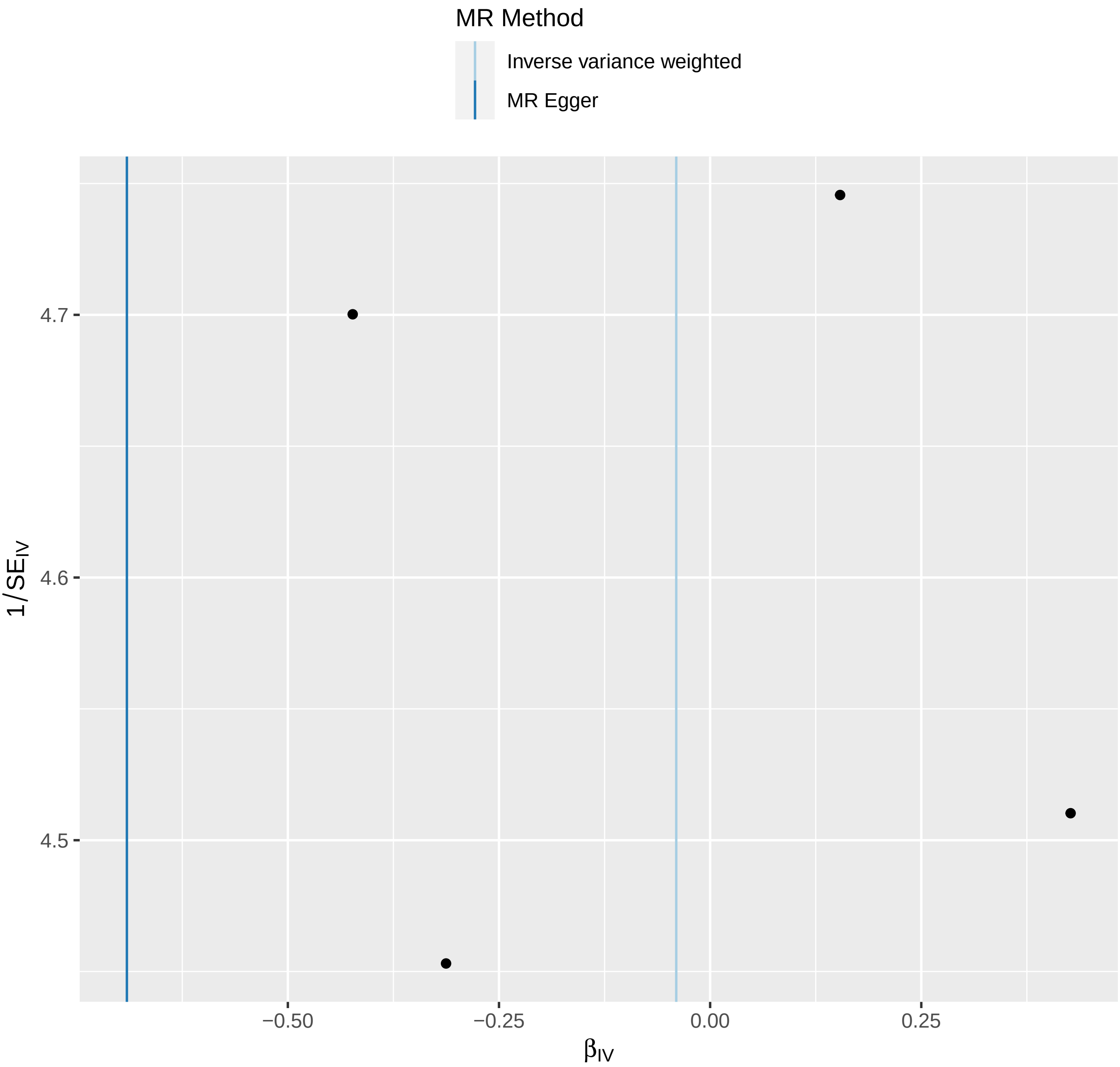

Batch 2−1400 : Gut microbiota abundance (genus Parasutterella id.2892) on Cysteine and glycine−rich protein 2
with a threshold of 1e−05

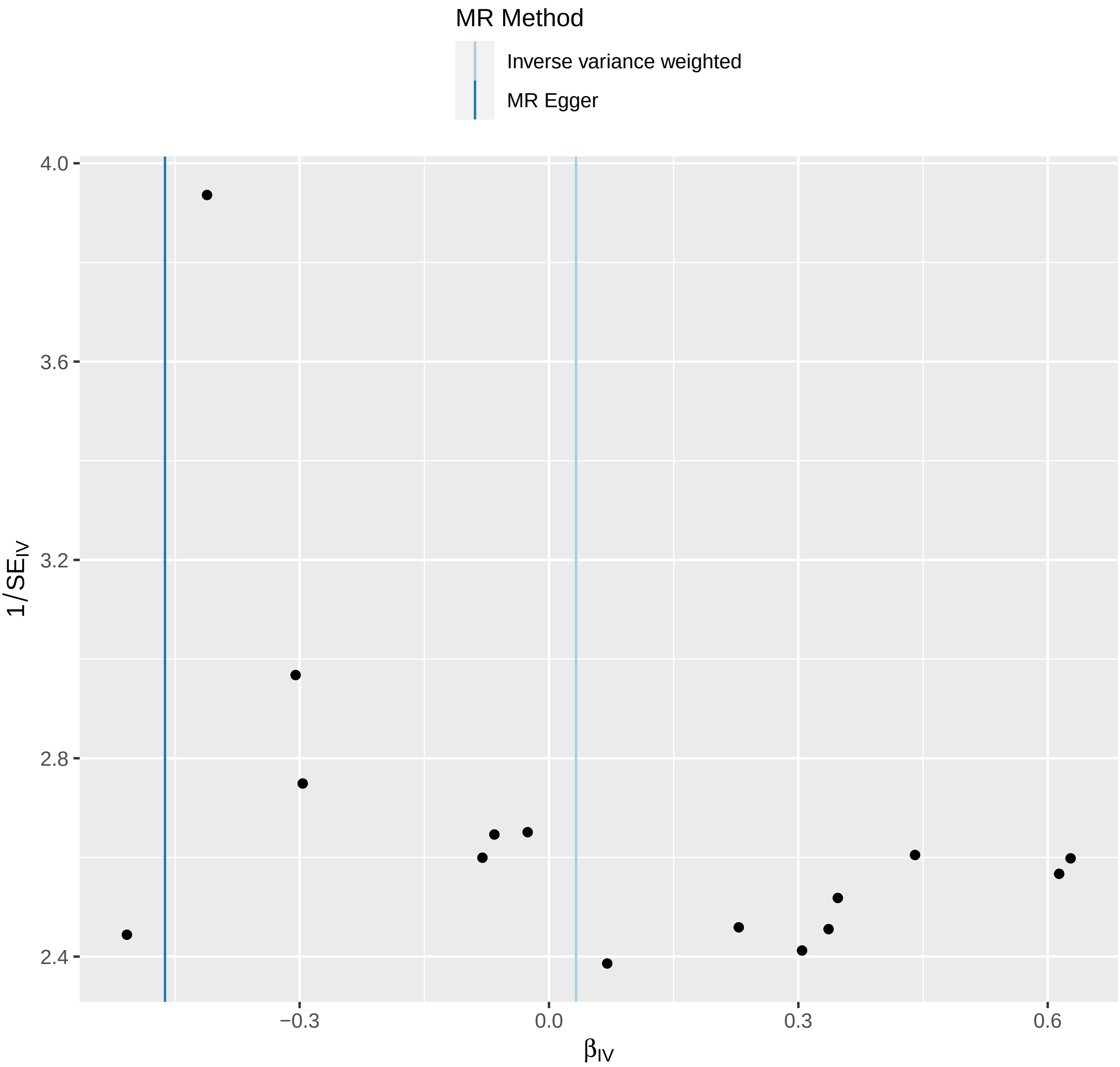

Batch 2−1401 : Gut microbiota abundance (genus Catenibacterium id.2153) on Cysteine and glycine−rich protein 3
with a threshold of 1e−05

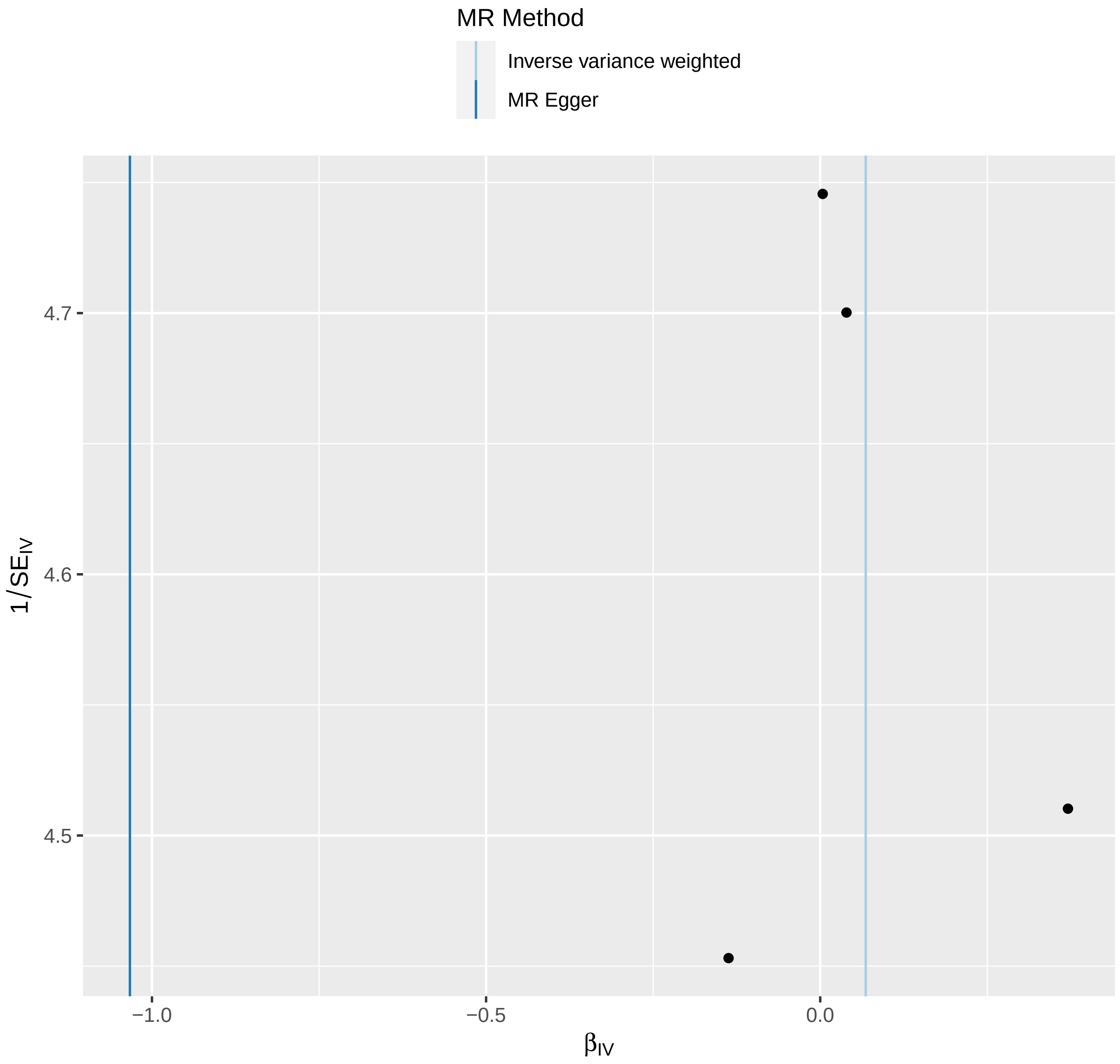

Batch 2−1402 : Gut microbiota abundance (genus Parasutterella id.2892) on Cysteine and glycine−rich protein 3
with a threshold of 1e−05

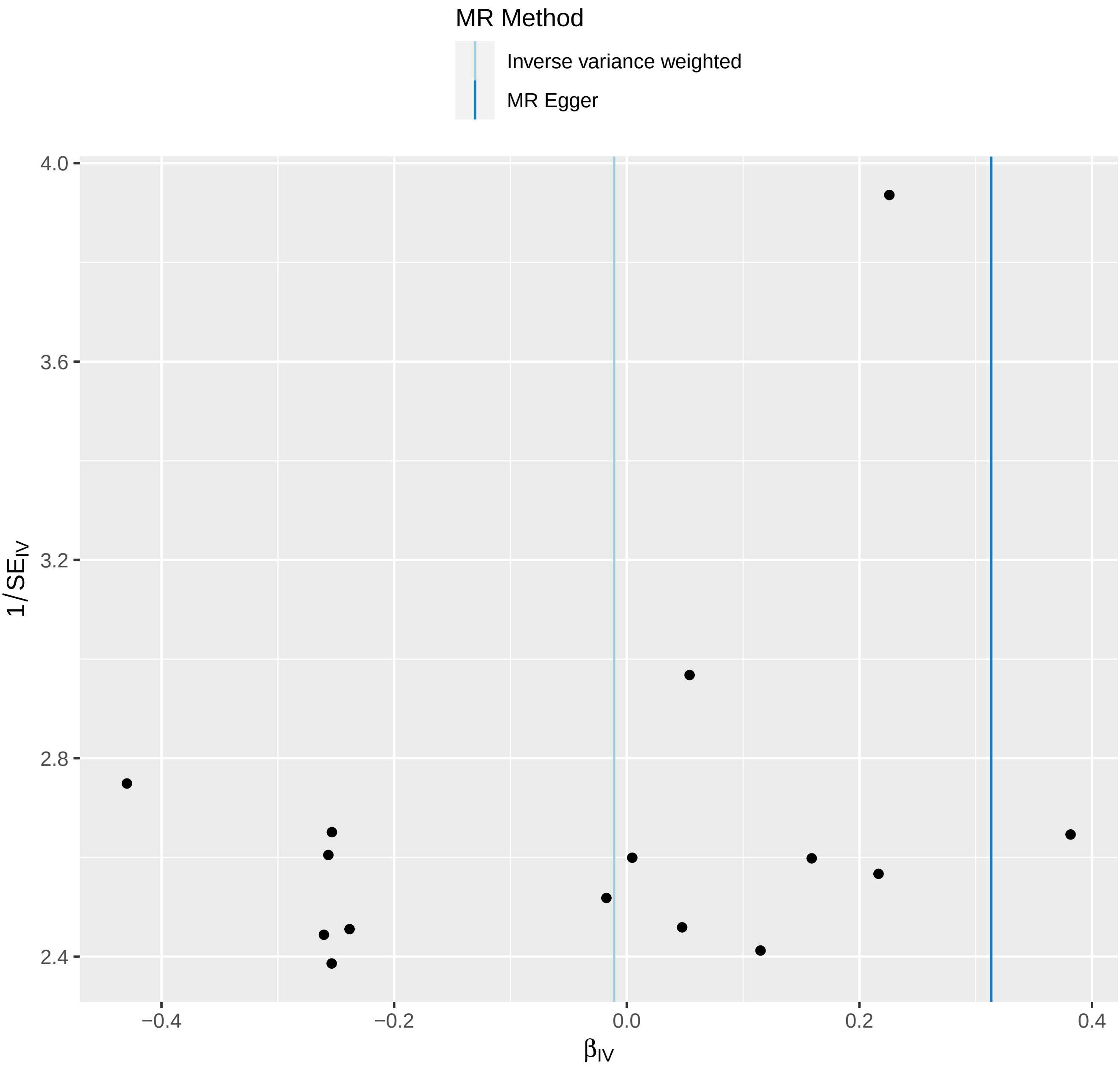

Batch 2−1403 : Gut microbiota abundance (genus Catenibacterium id.2153) on Cystatin−D
with a threshold of 1e−05

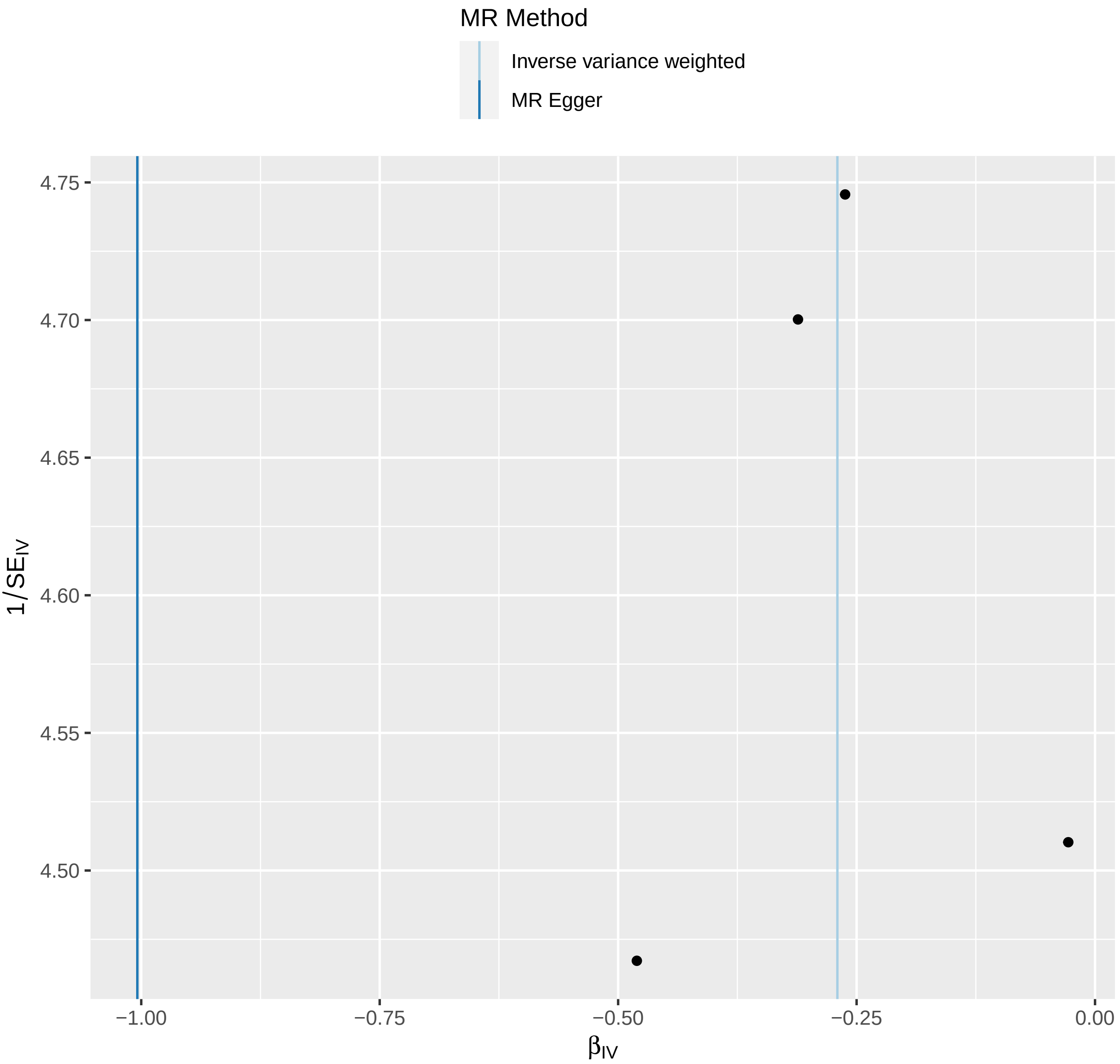

Batch 2−1404 : Gut microbiota abundance (genus Parasutterella id.2892) on Cystatin−D
with a threshold of 1e−05

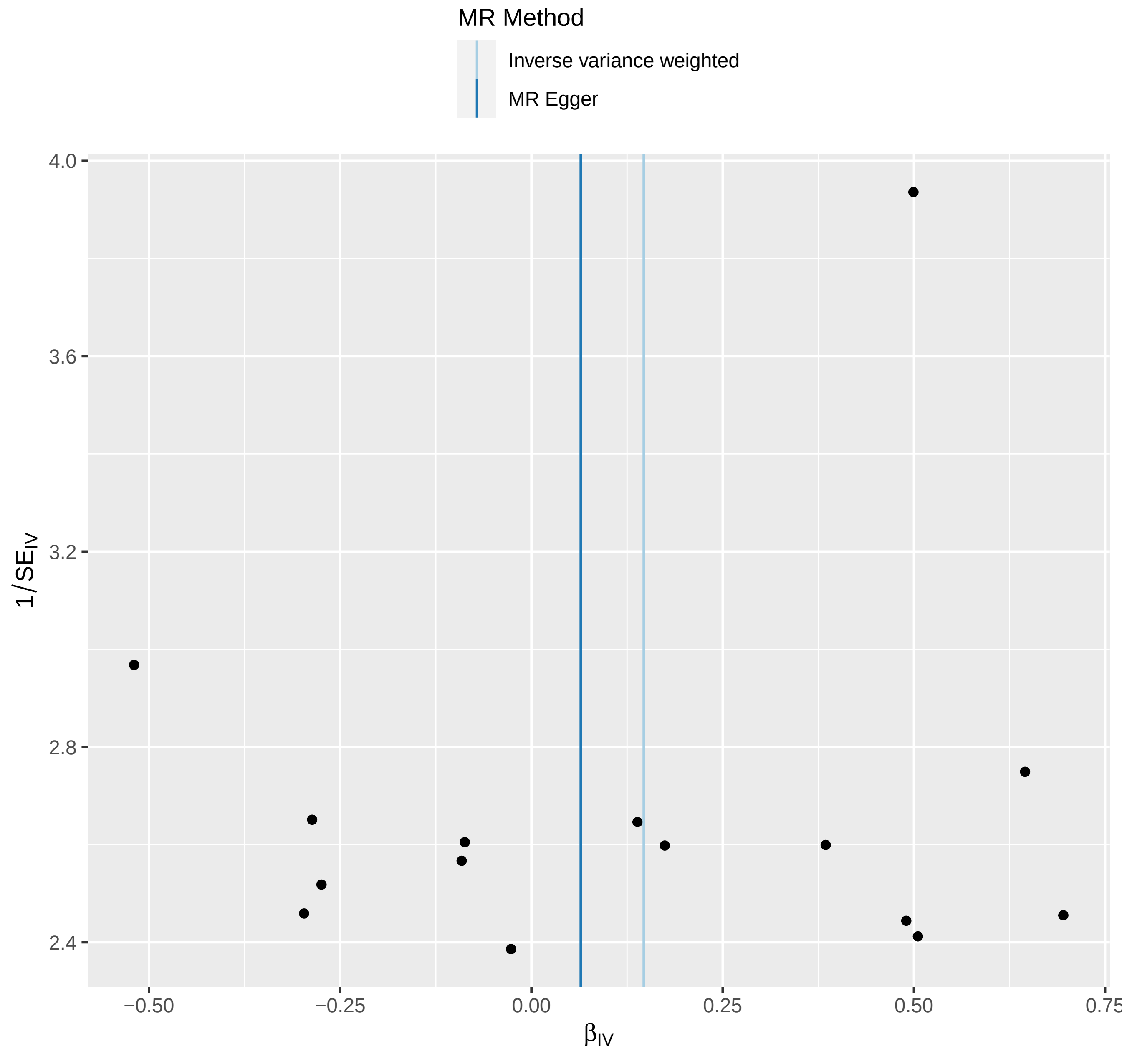

Batch 2−1405 : Gut microbiota abundance (genus Catenibacterium id.2153) on Cystatin−M
with a threshold of 1e−05

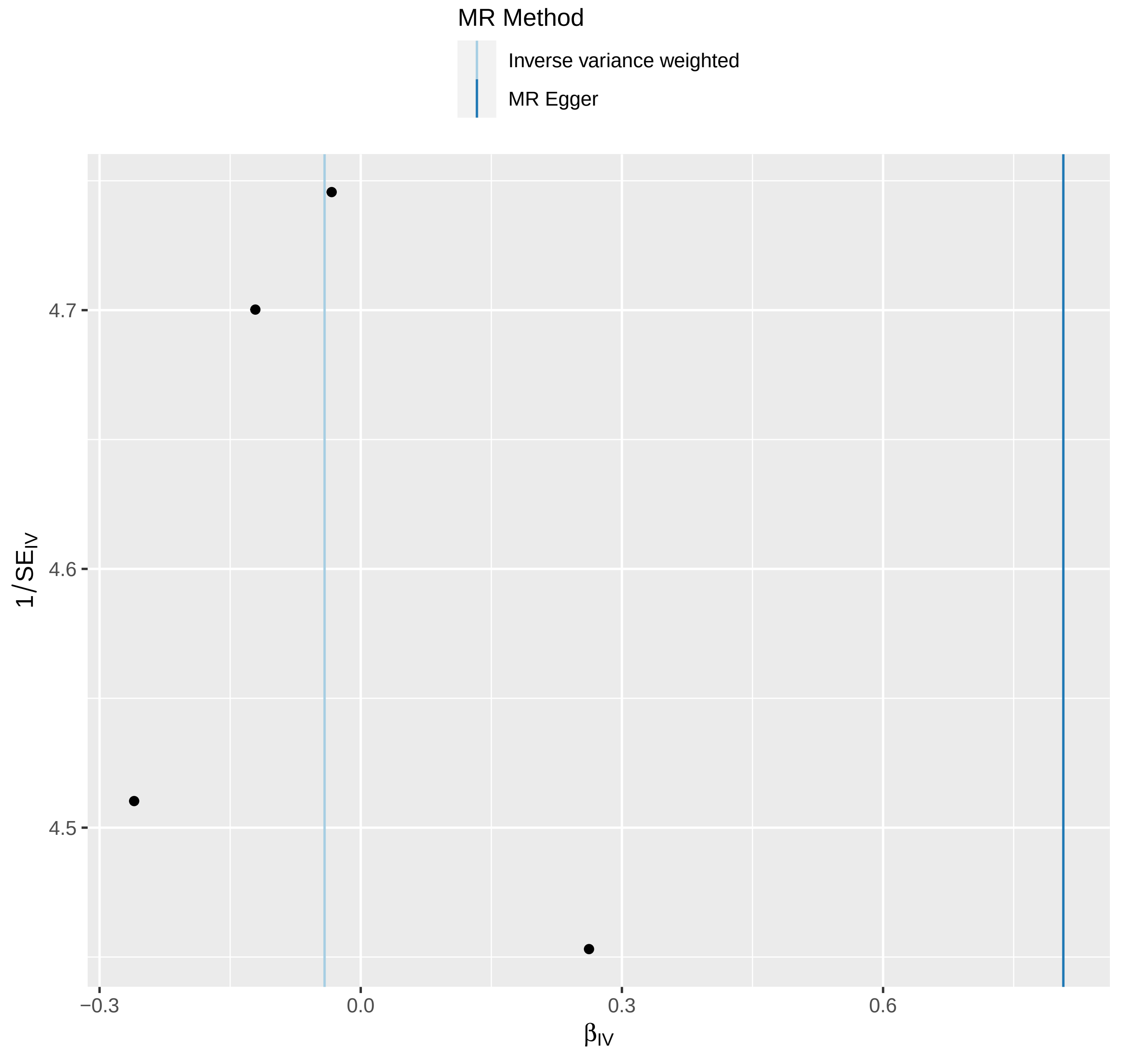

Batch 2−1406 : Gut microbiota abundance (genus Parasutterella id.2892) on Cystatin−M
with a threshold of 1e−05

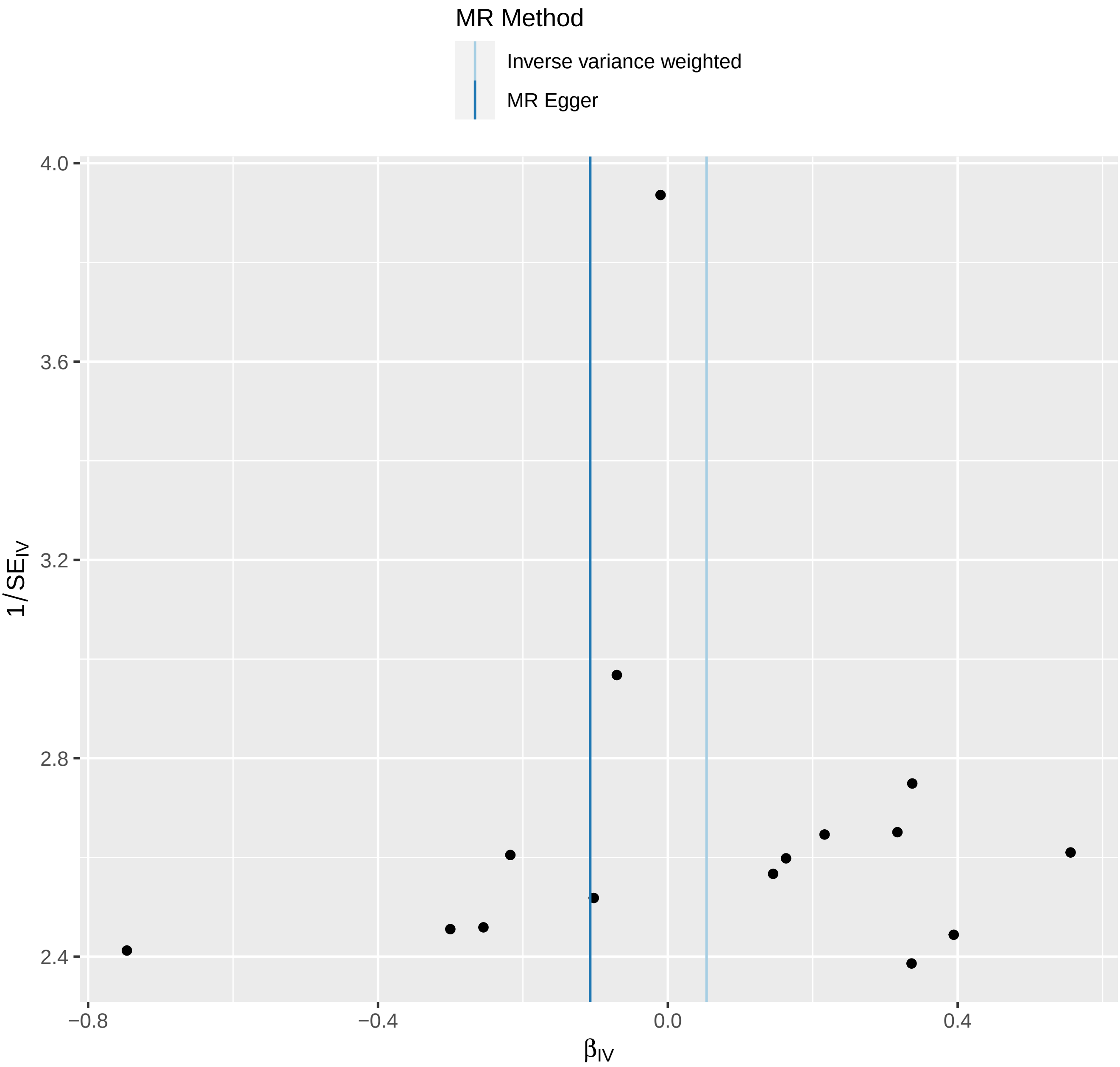

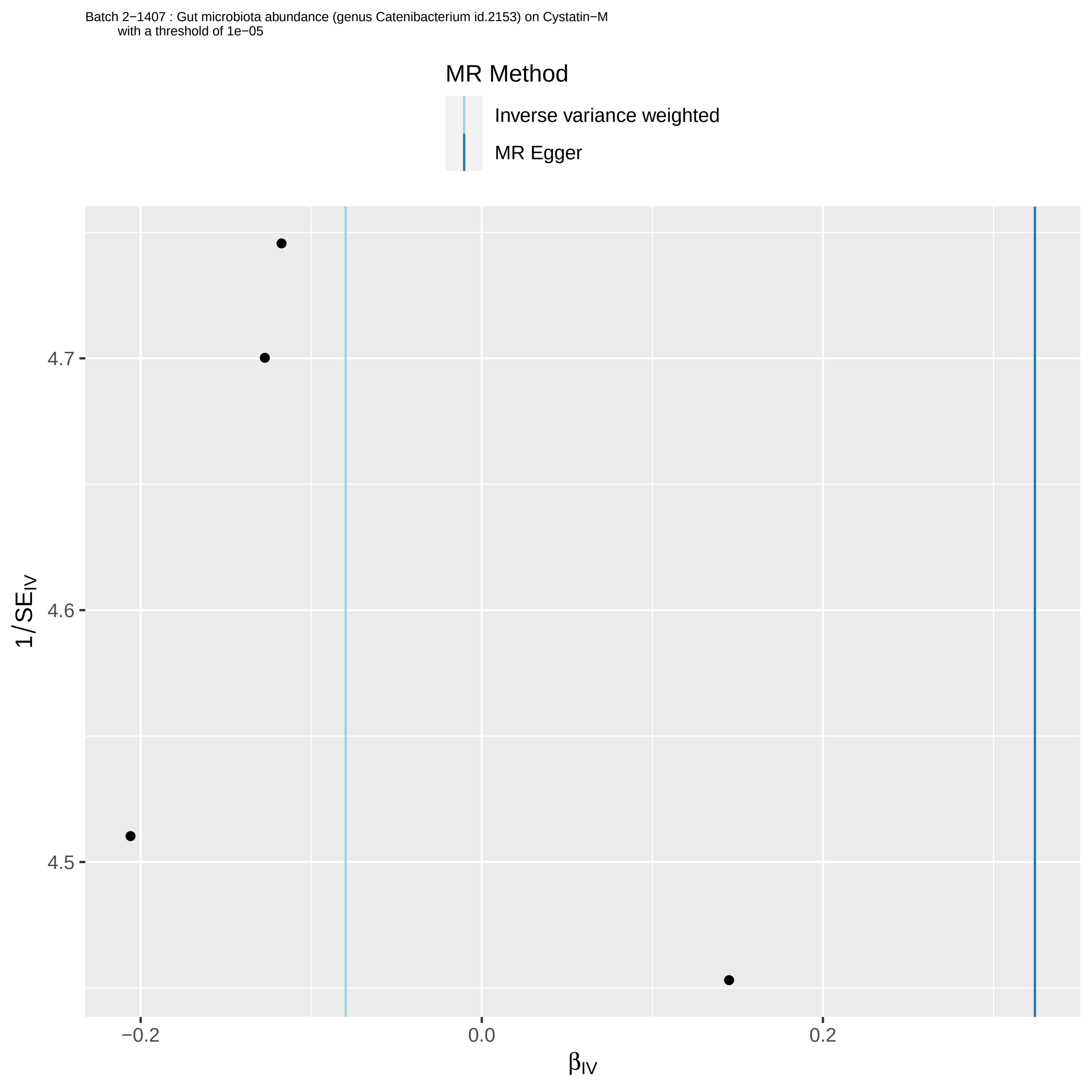

Batch 2−1407 : Gut microbiota abundance (genus Catenibacterium id.2153) on Cystatin−M
with a threshold of 1e−05
MR Method
Inverse variance weighted
MR Egger
4.7
4.6
4.5
1/SEIV
−0.2
0.0
0.2
βIV

Batch 2−1408 : Gut microbiota abundance (genus Parasutterella id.2892) on Cystatin−M
with a threshold of 1e−05

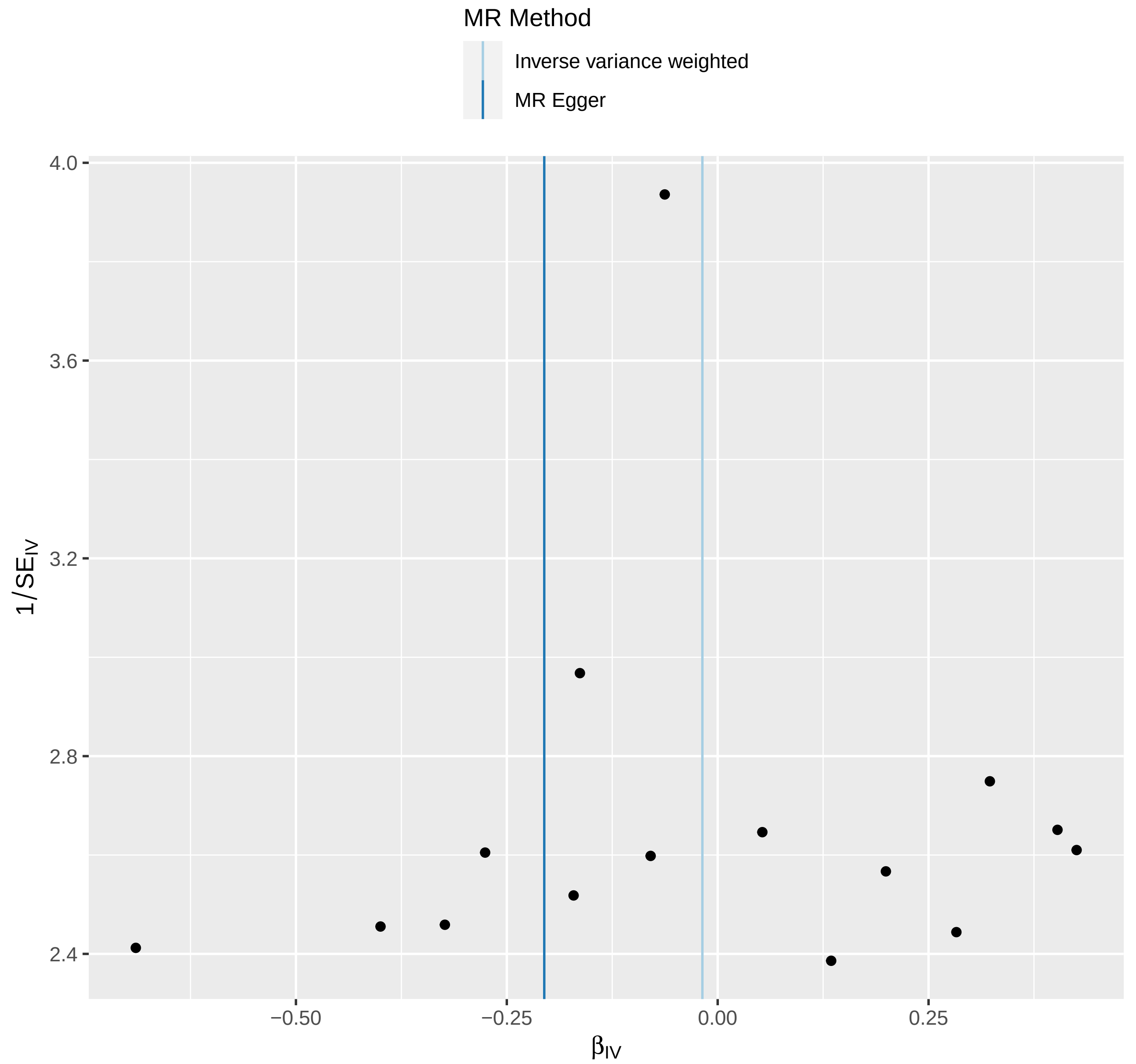

Batch 2−1409 : Gut microbiota abundance (genus Catenibacterium id.2153) on Cystatin−F
with a threshold of 1e−05

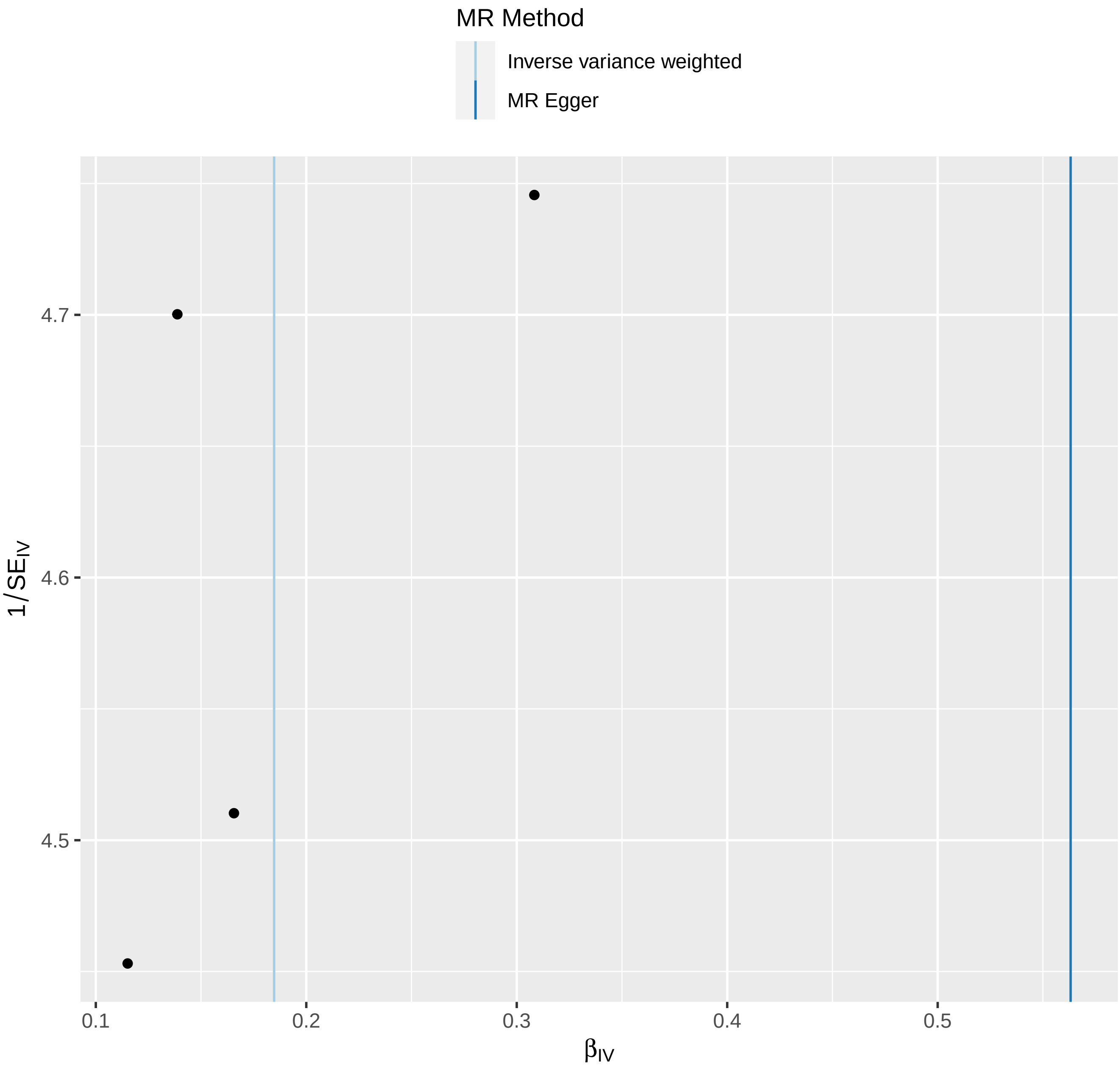

Batch 2−1410 : Gut microbiota abundance (genus Parasutterella id.2892) on Cystatin−F
with a threshold of 1e−05

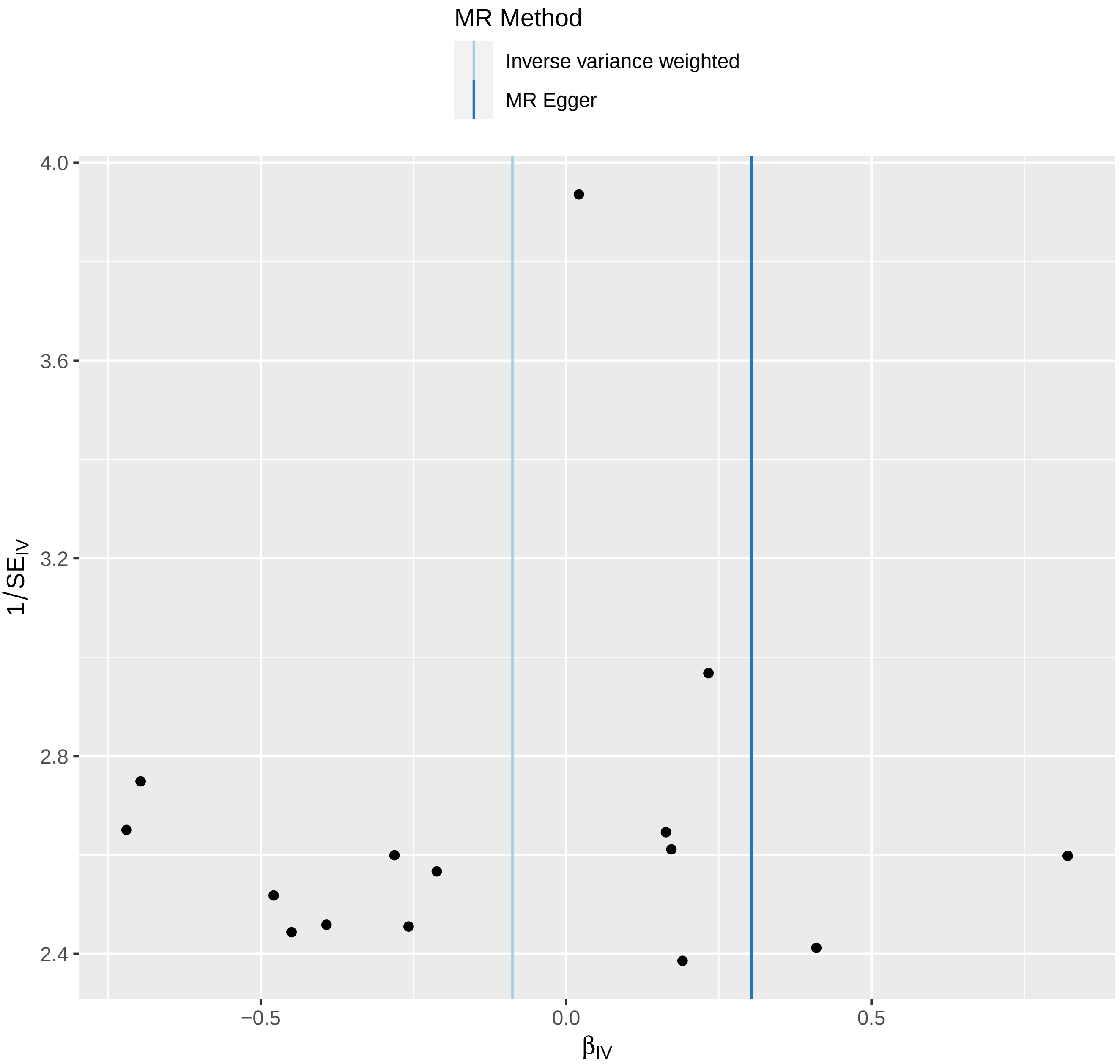

Batch 2−1411 : Gut microbiota abundance (genus Catenibacterium id.2153) on Cystatin−8
with a threshold of 1e−05

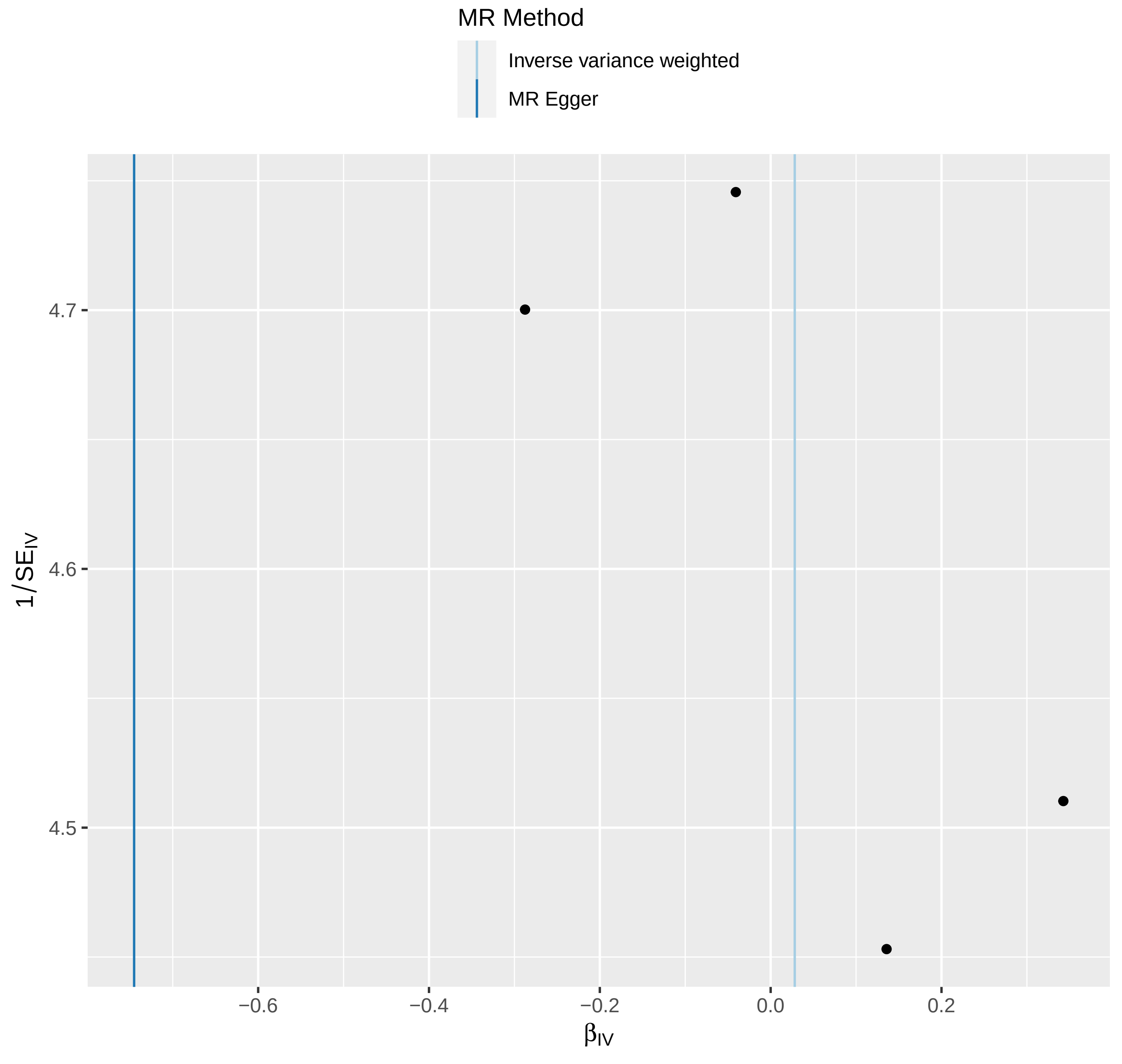

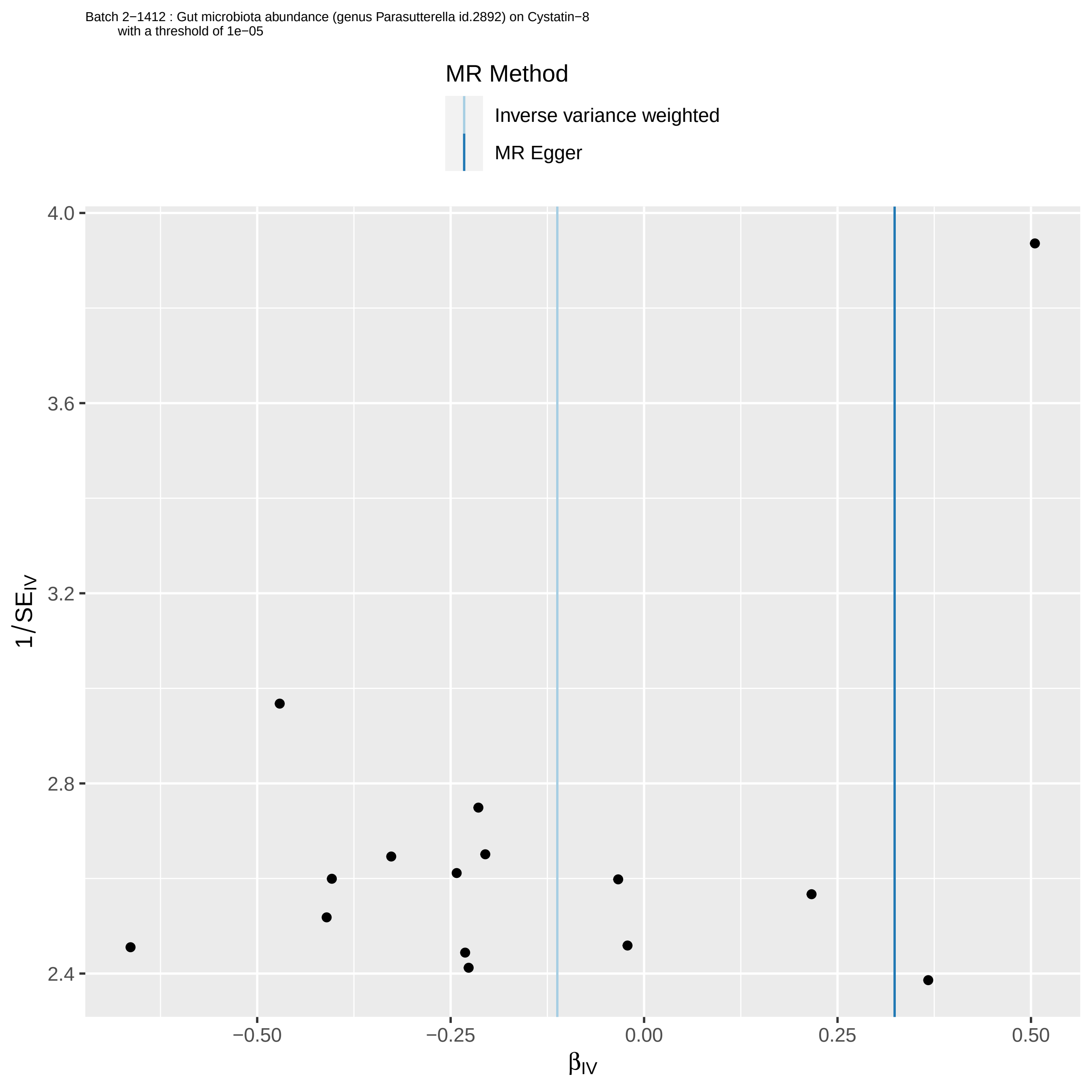

Batch 2−1412 : Gut microbiota abundance (genus Parasutterella id.2892) on Cystatin−8
with a threshold of 1e−05
MR Method
Inverse variance weighted
MR Egger
4.0
3.6
3.2
2.8
2.4
1/SE$_{IV}$
−0.50
−0.25
0.00
0.25
0.50
β$_{IV}$

Batch 2−1413 : Gut microbiota abundance (genus Catenibacterium id.2153) on Cancer/testis antigen 55
with a threshold of 1e−05

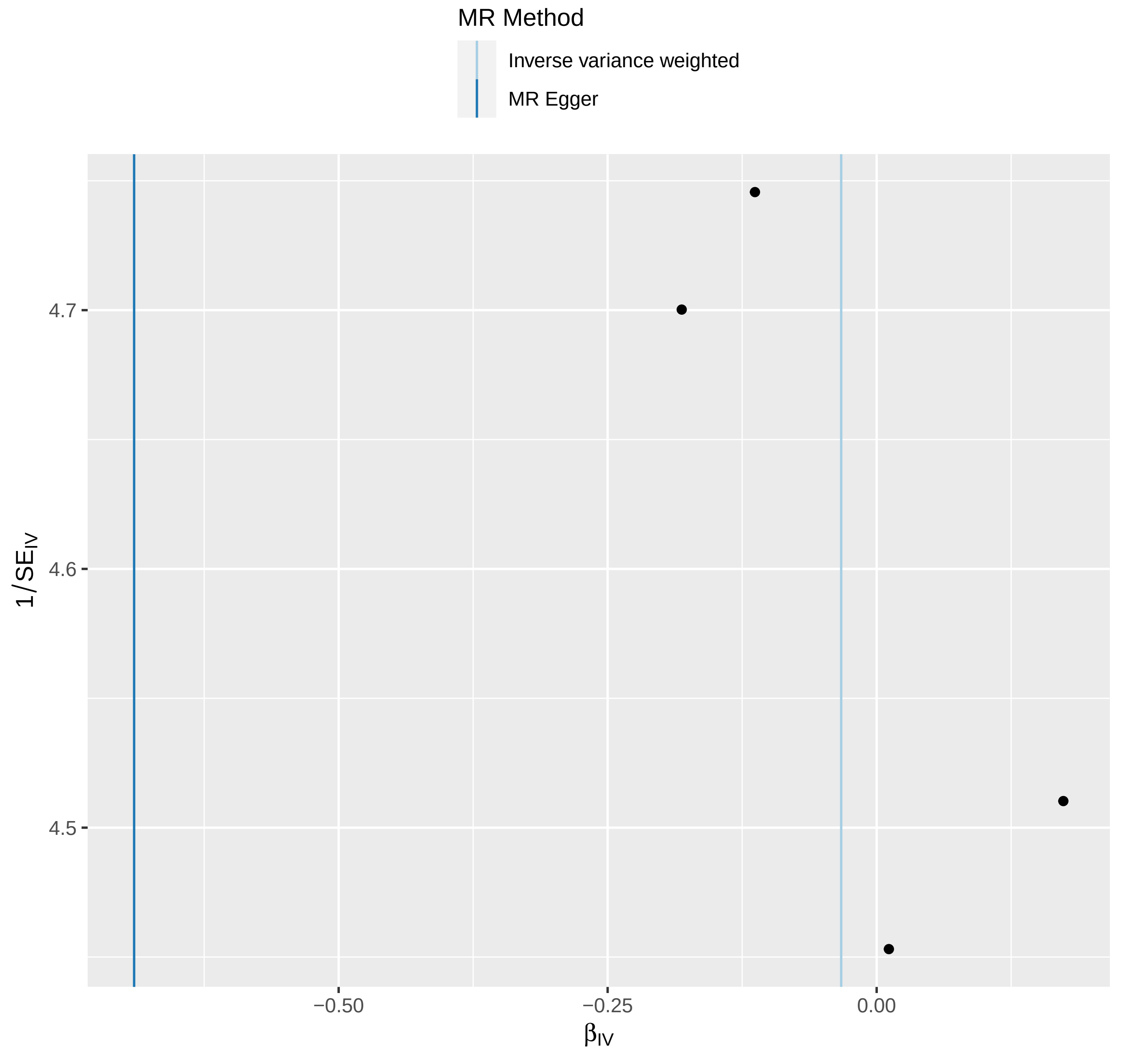

Batch 2−1414 : Gut microbiota abundance (genus Parasutterella id.2892) on Cancer/testis antigen 55
with a threshold of 1e−05

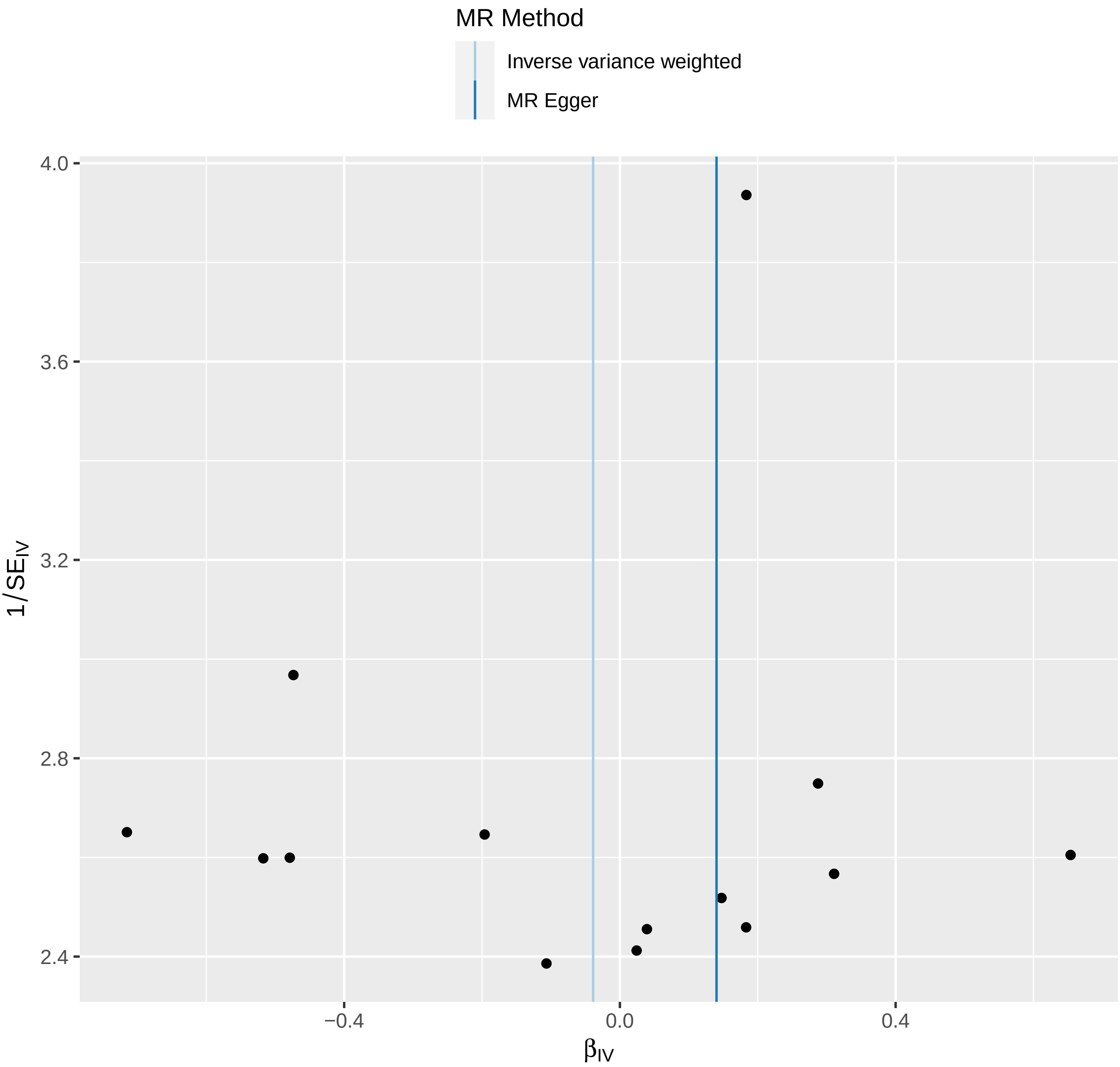

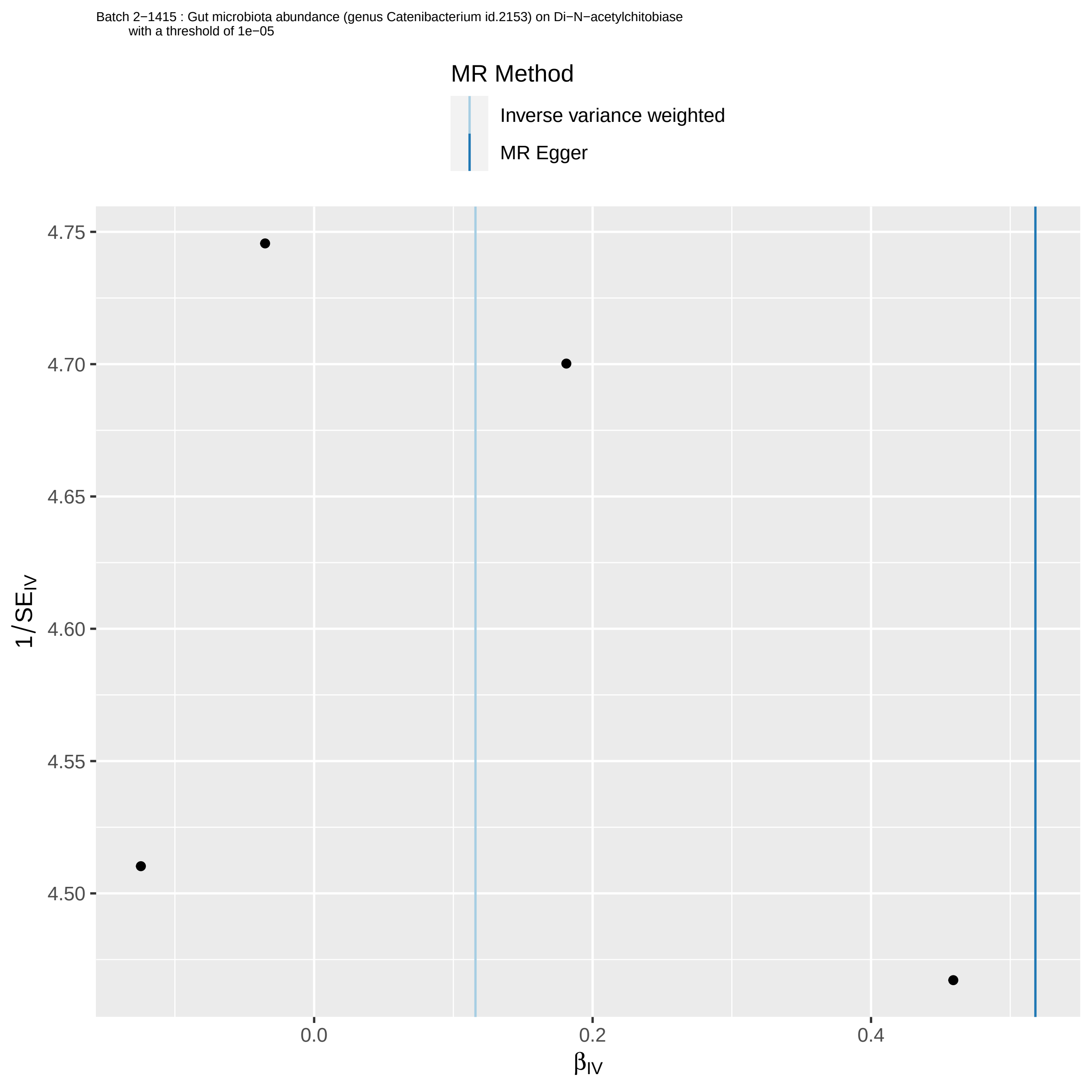

Batch 2−1415 : Gut microbiota abundance (genus Catenibacterium id.2153) on Di−N−acetylchitobiase
with a threshold of 1e−05
MR Method
Inverse variance weighted
MR Egger
4.75
4.70
4.65
4.60
4.55
4.50
1/SE_IV
0.0
0.2
0.4
β_IV

Batch 2−1416 : Gut microbiota abundance (genus Parasutterella id.2892) on Di−N−acetylchitobiase
with a threshold of 1e−05

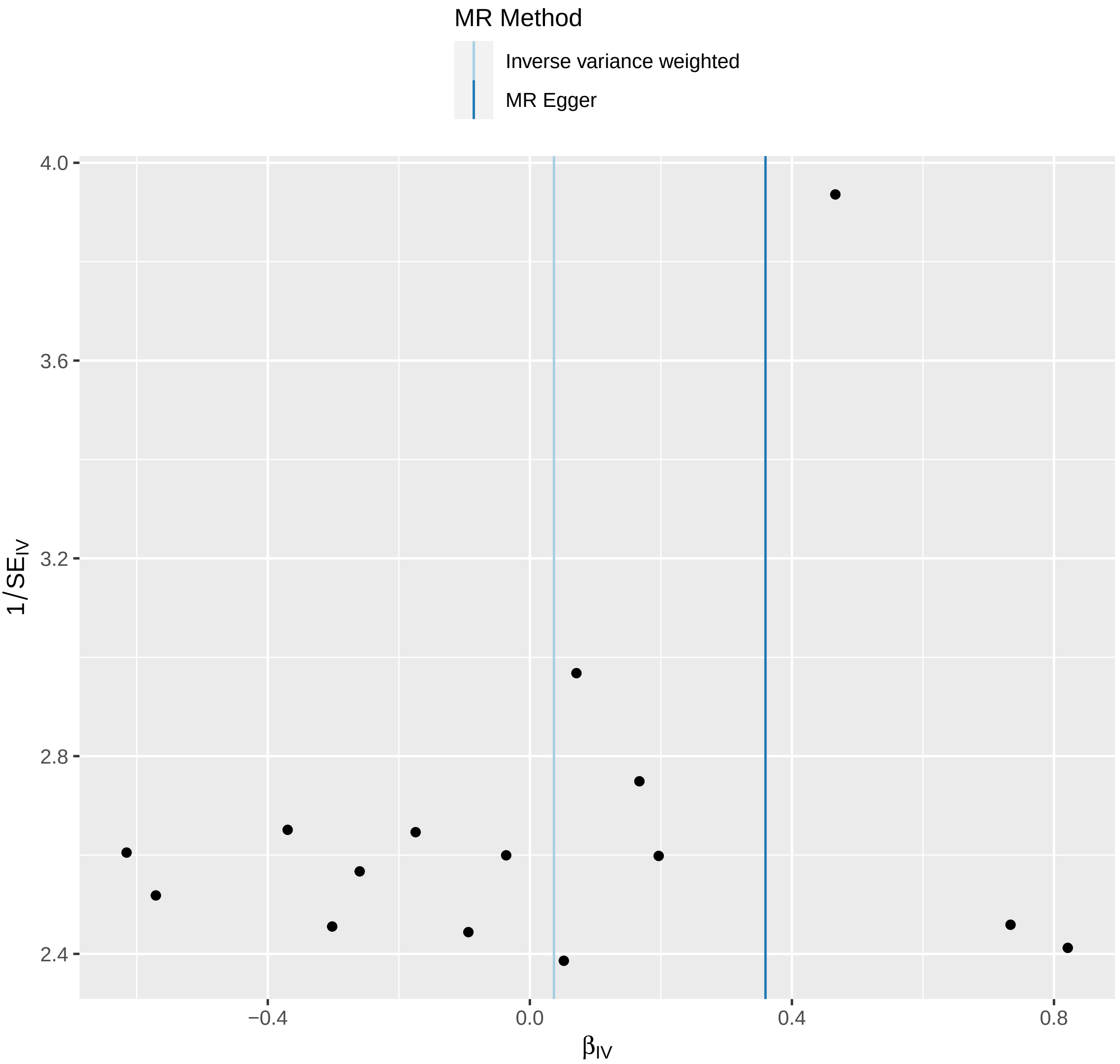

Batch 2−1417 : Gut microbiota abundance (genus Catenibacterium id.2153) on Transcriptional repressor CTCF
with a threshold of 1e−05

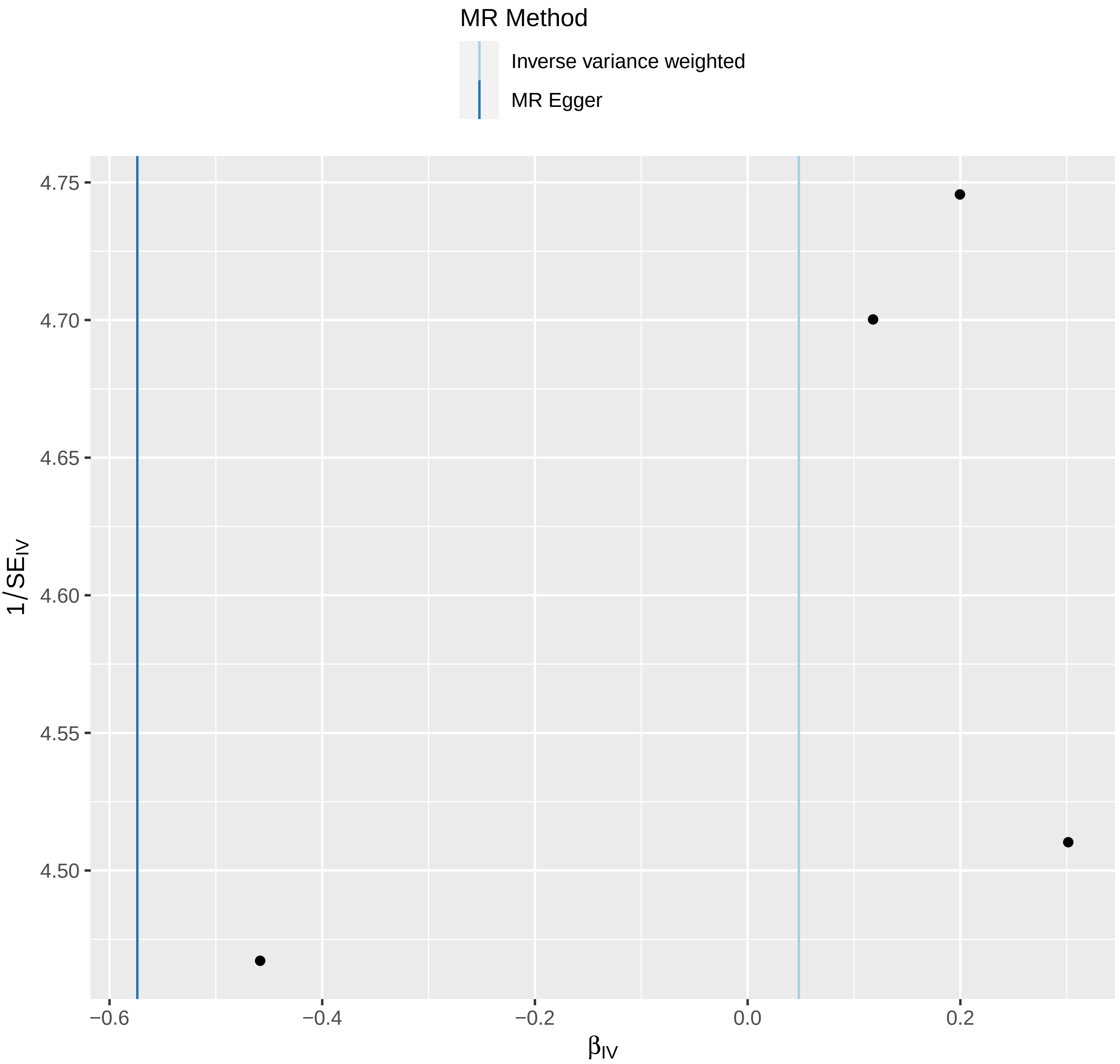

Batch 2−1418 : Gut microbiota abundance (genus Parasutterella id.2892) on Transcriptional repressor CTCF with a threshold of 1e−05

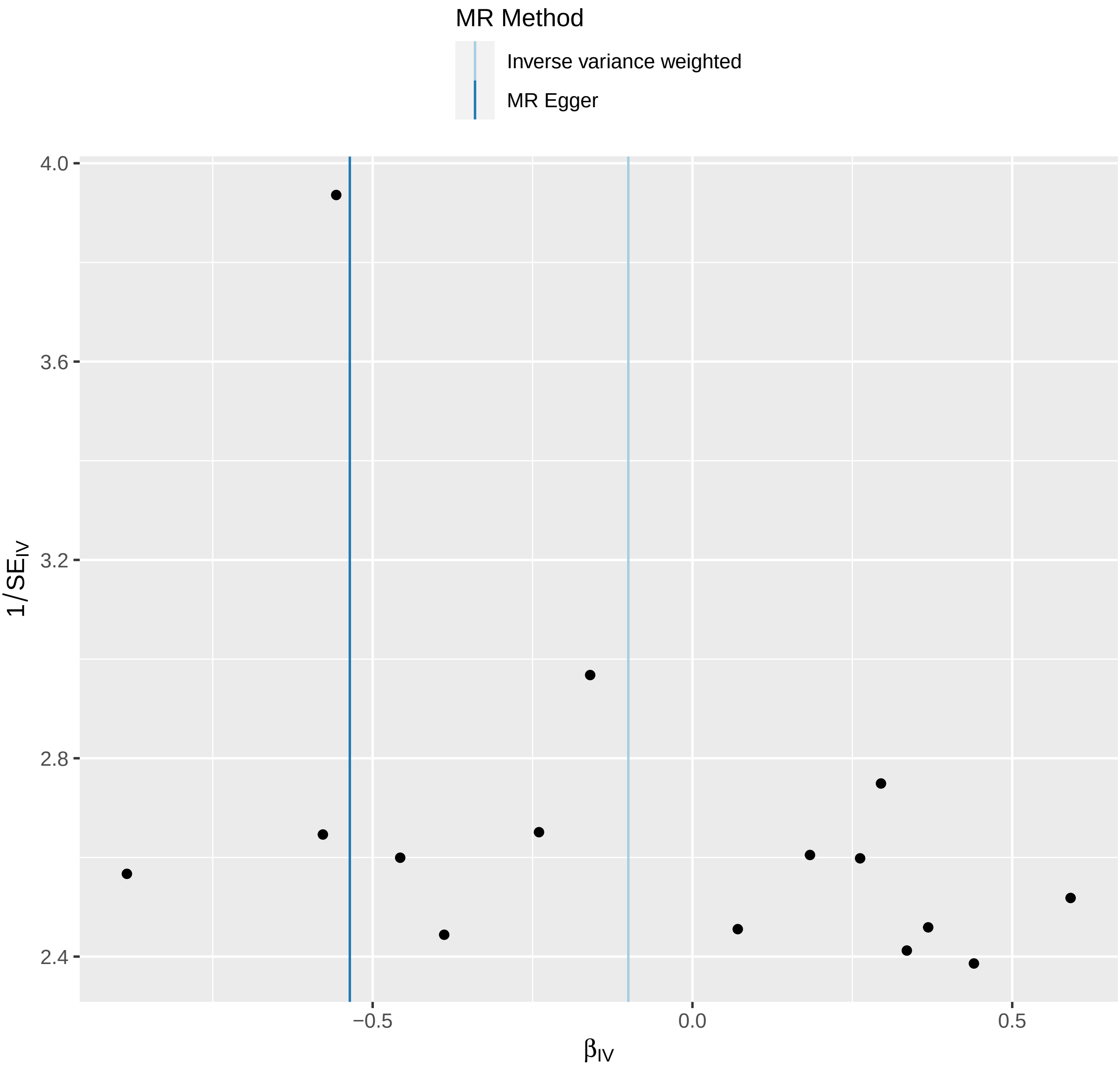

Batch 2−1419 : Gut microbiota abundance (genus Catenibacterium id.2153) on Cardiotrophin−1
with a threshold of 1e−05

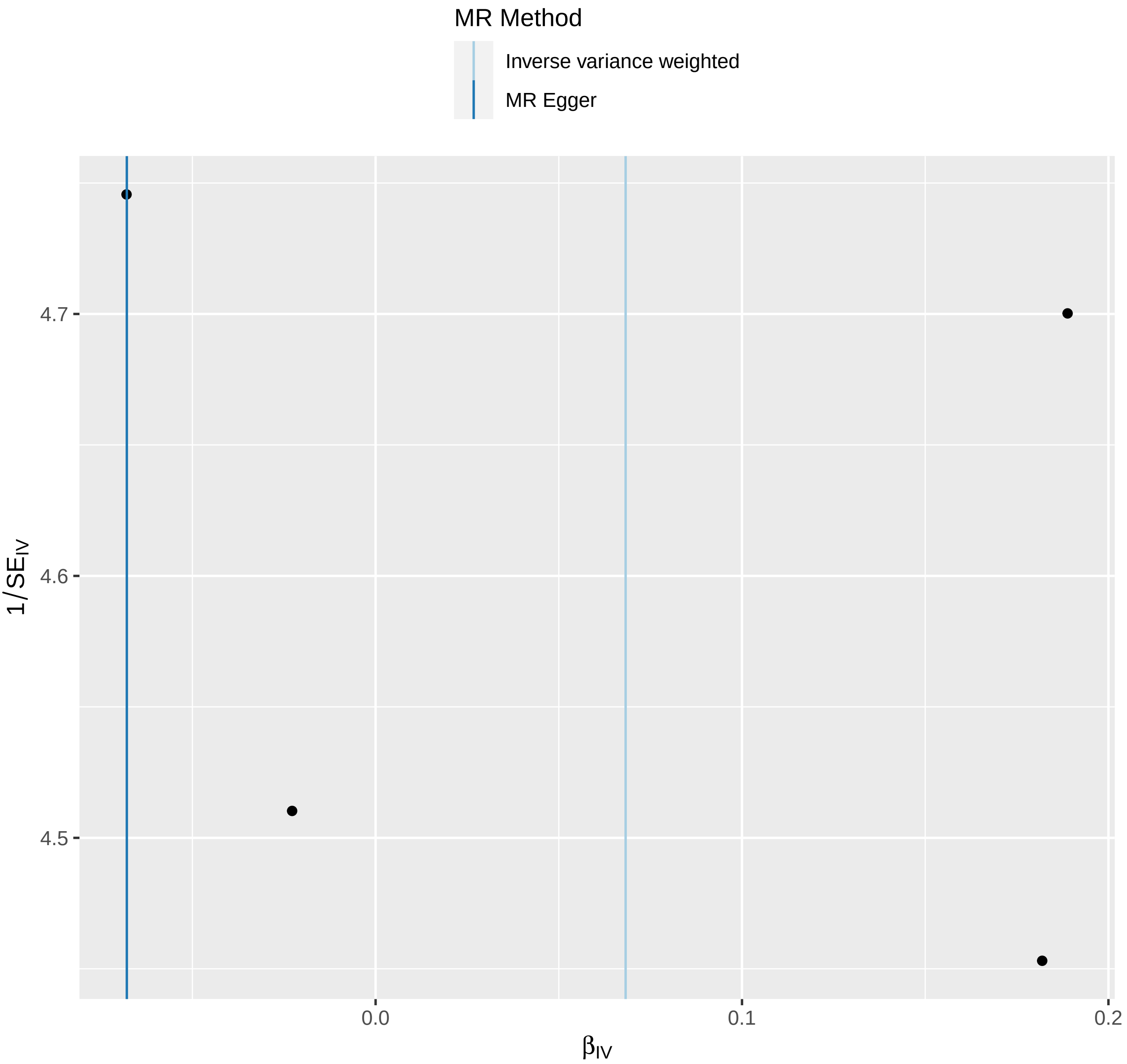

Batch 2−1420 : Gut microbiota abundance (genus Parasutterella id.2892) on Cardiotrophin−1
with a threshold of 1e−05

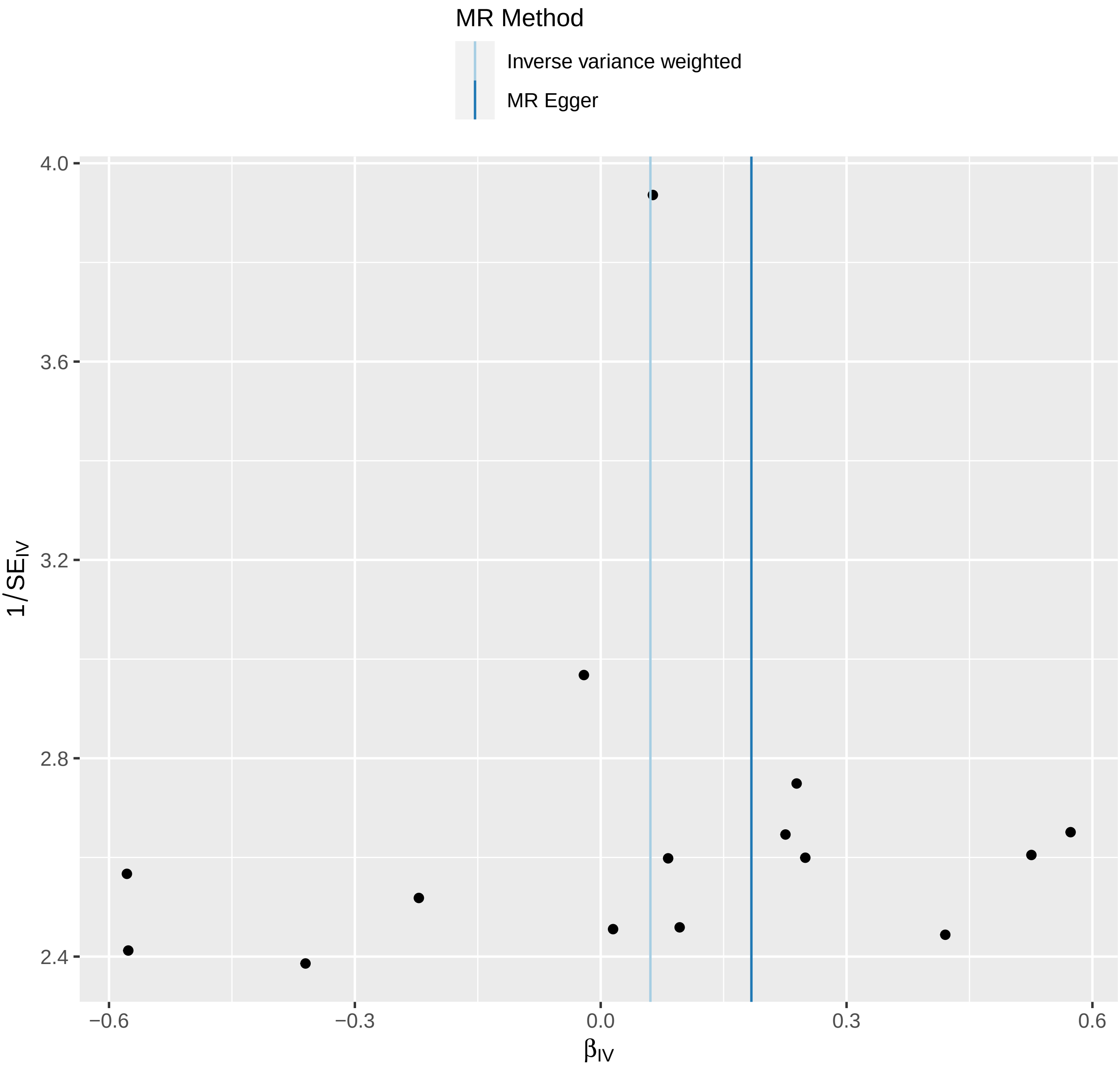

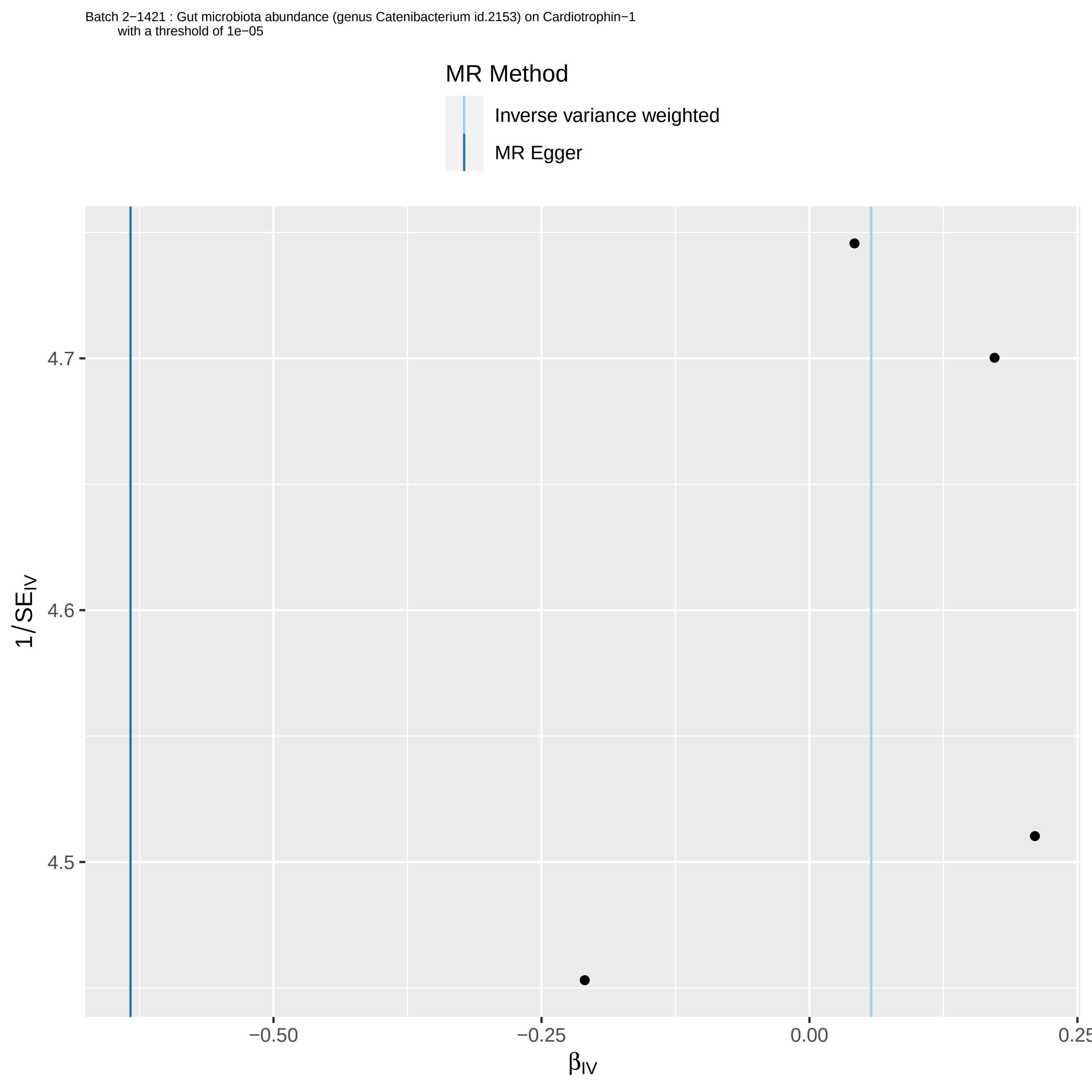

Batch 2−1421 : Gut microbiota abundance (genus Catenibacterium id.2153) on Cardiotrophin−1
with a threshold of 1e−05
MR Method
Inverse variance weighted
MR Egger
4.7
4.6
4.5
1/SE_IV
−0.50
−0.25
0.00
0.25
β_IV

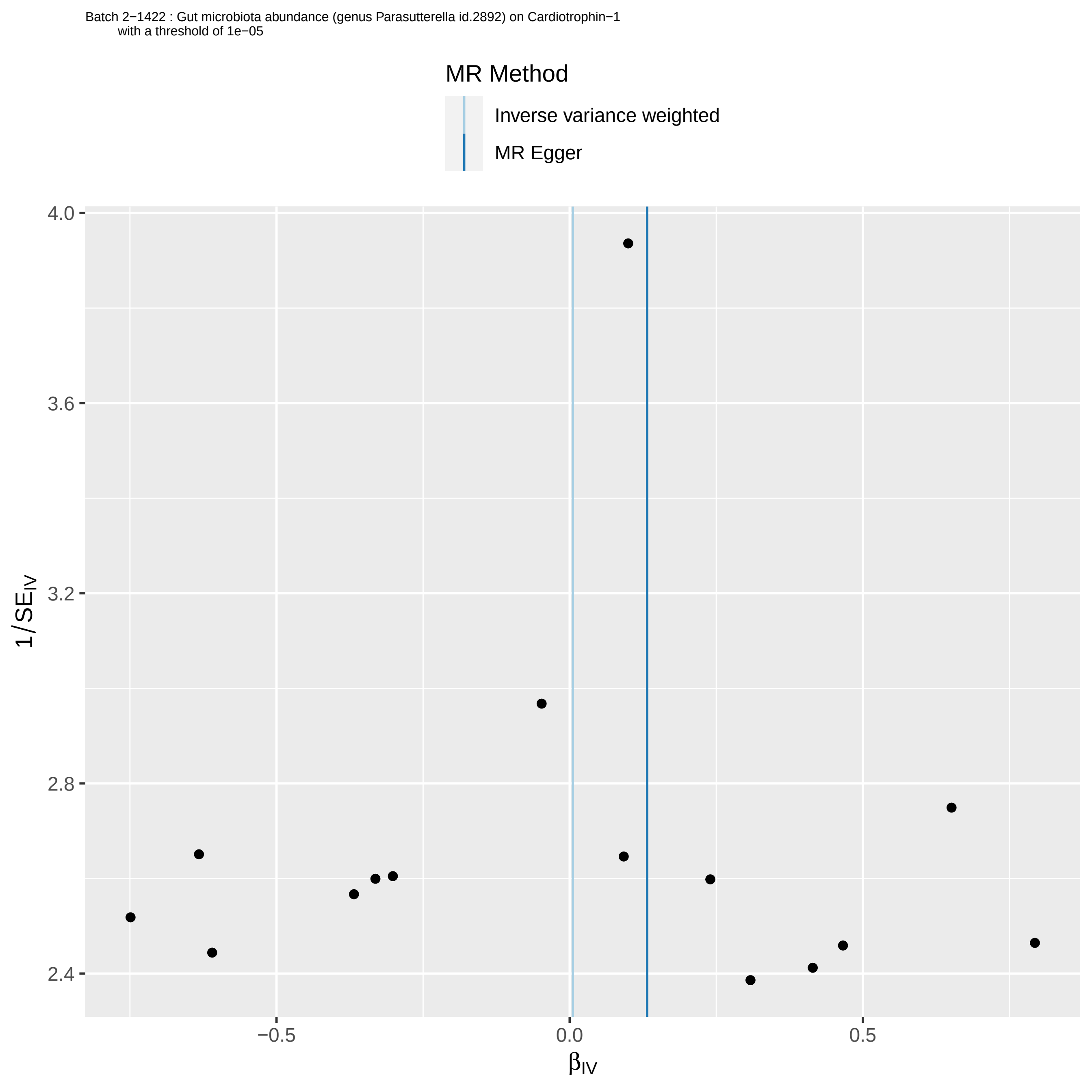
Batch 2−1422 : Gut microbiota abundance (genus Parasutterella id.2892) on Cardiotrophin−1
with a threshold of 1e−05
MR Method
Inverse variance weighted
MR Egger
4.0
3.6
3.2
2.8
2.4
1/SE_IV
−0.5
0.0
0.5
β_IV

Batch 2−1423 : Gut microbiota abundance (genus Catenibacterium id.2153) on Connective tissue growth factor
with a threshold of 1e−05

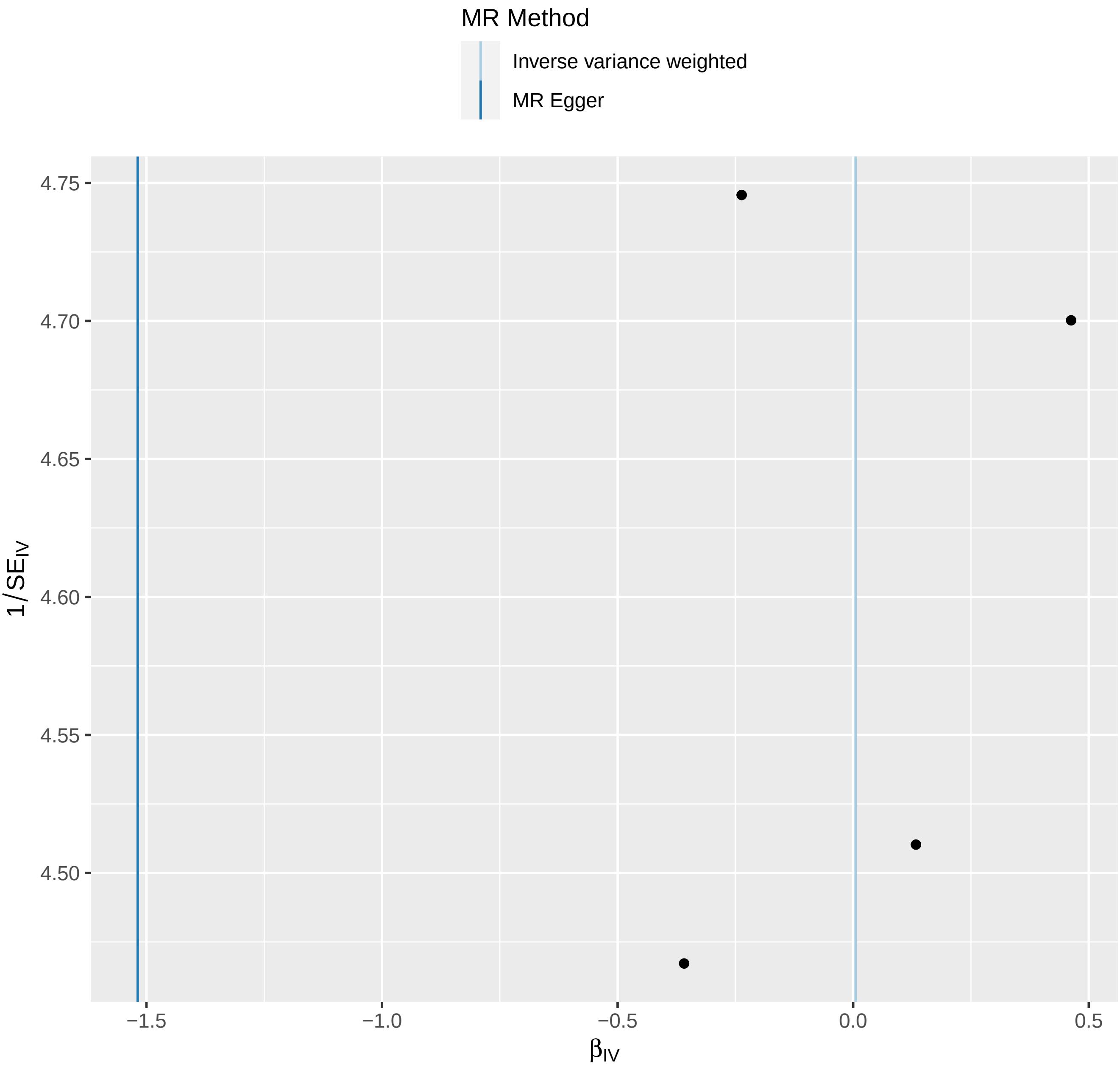

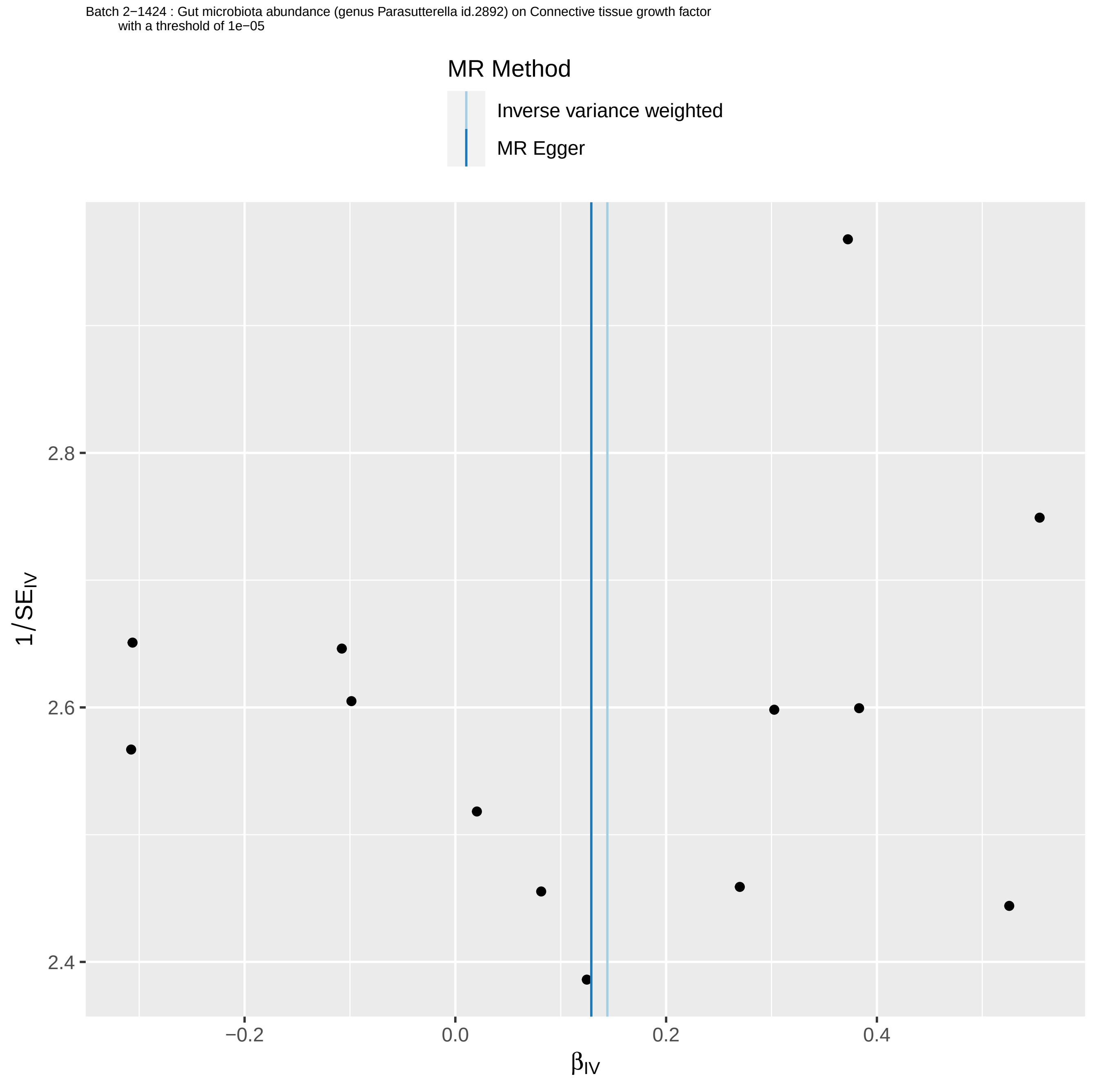
Batch 2−1424 : Gut microbiota abundance (genus Parasutterella id.2892) on Connective tissue growth factor
with a threshold of 1e−05
MR Method
Inverse variance weighted
MR Egger
2.8
2.6
2.4
1/SE_IV
−0.2
0.0
0.2
0.4
β_IV

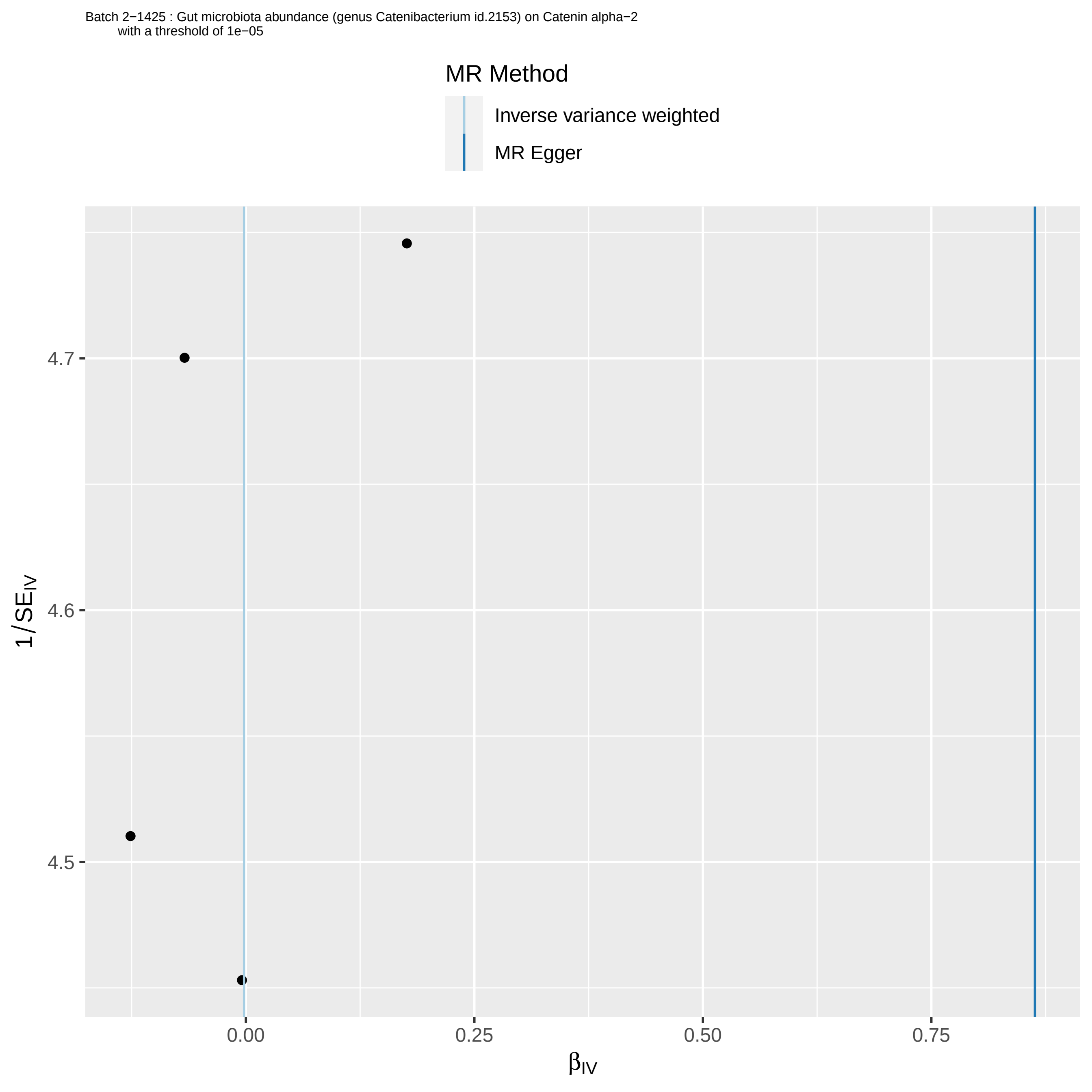

Batch 2−1425 : Gut microbiota abundance (genus Catenibacterium id.2153) on Catenin alpha−2
with a threshold of 1e−05
MR Method
Inverse variance weighted
MR Egger
4.7
4.6
4.5
1/SE_IV
0.00
0.25
0.50
0.75
β_IV

Batch 2−1426 : Gut microbiota abundance (genus Parasutterella id.2892) on Catenin alpha−2
with a threshold of 1e−05

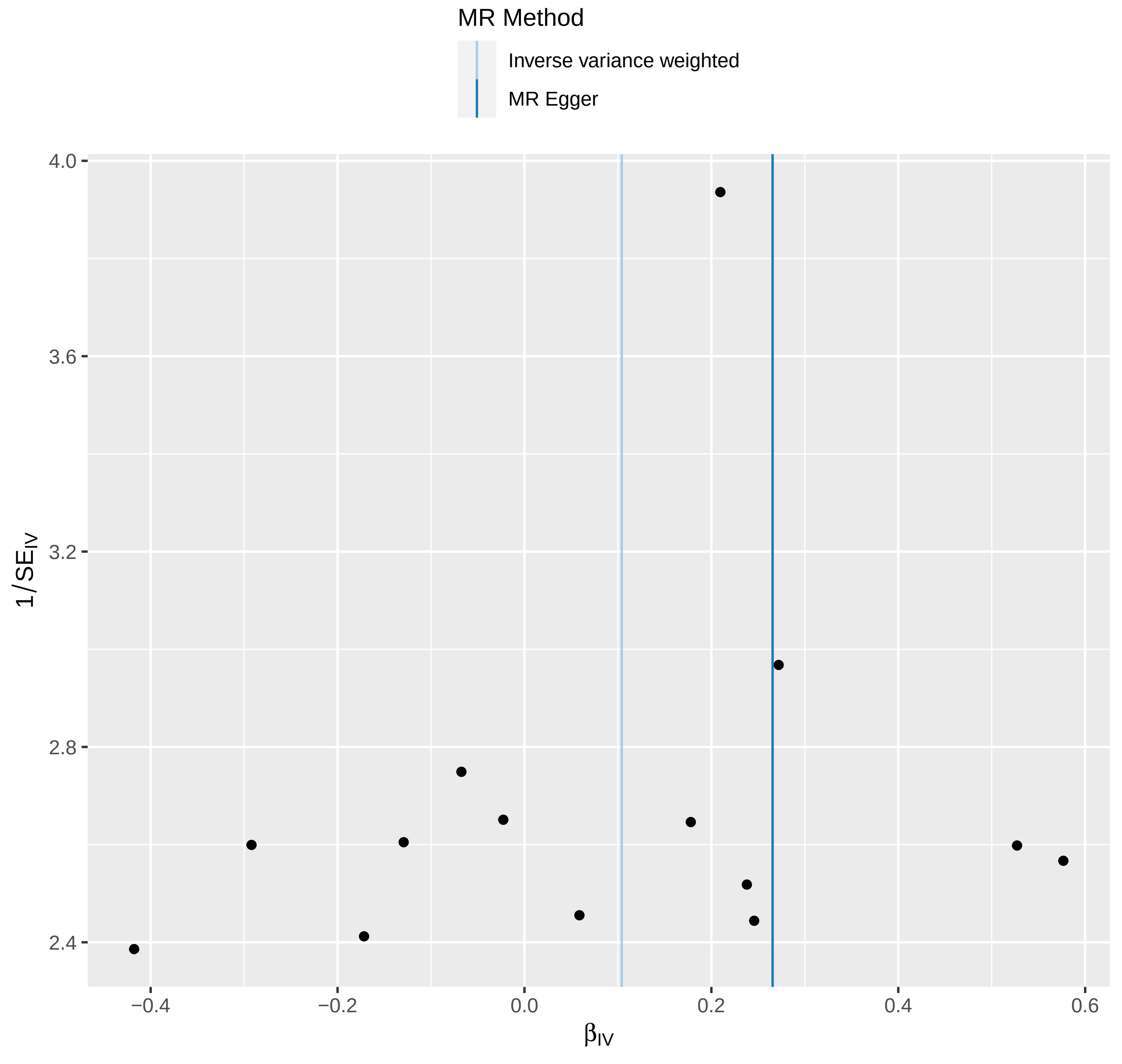

Batch 2−1427 : Gut microbiota abundance (genus Catenibacterium id.2153) on Chymotrypsinogen B
with a threshold of 1e−05

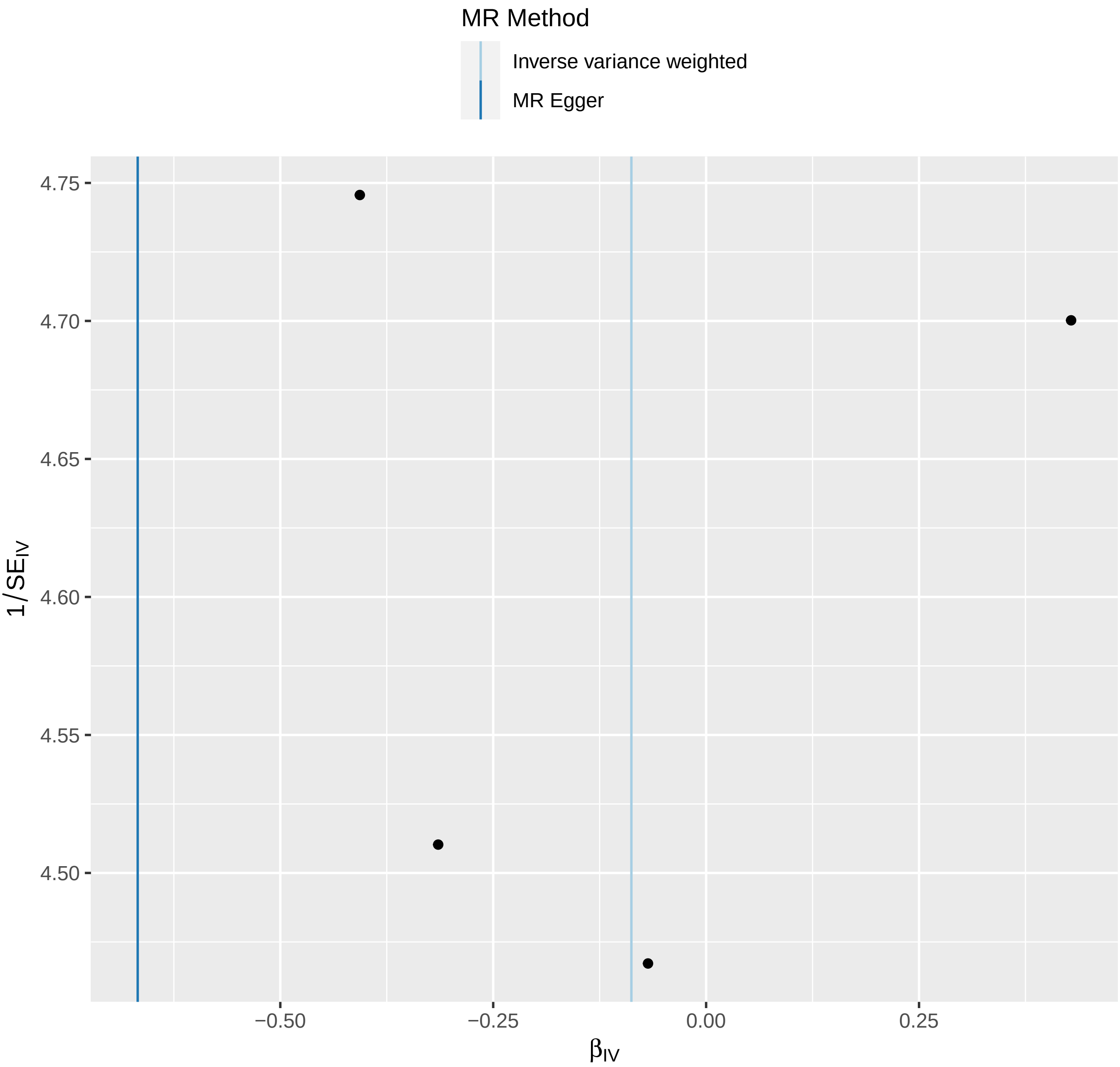

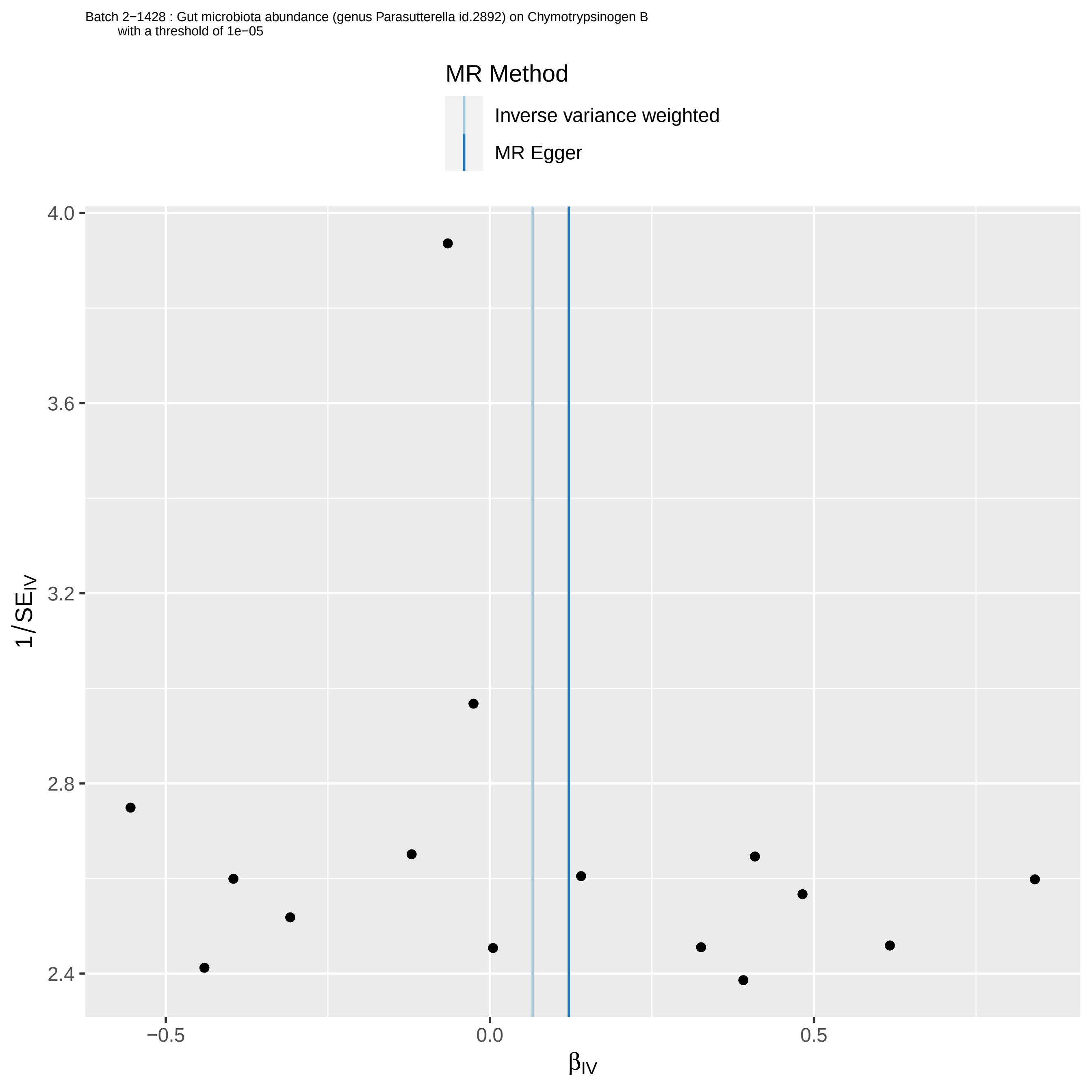
Batch 2−1428 : Gut microbiota abundance (genus Parasutterella id.2892) on Chymotrypsinogen B
with a threshold of 1e−05
MR Method
Inverse variance weighted
MR Egger
4.0
3.6
3.2
2.8
2.4
1/SE_IV
−0.5
0.0
0.5
β_IV

Batch 2−1429 : Gut microbiota abundance (genus Catenibacterium id.2153) on Chymotrypsin−C
with a threshold of 1e−05

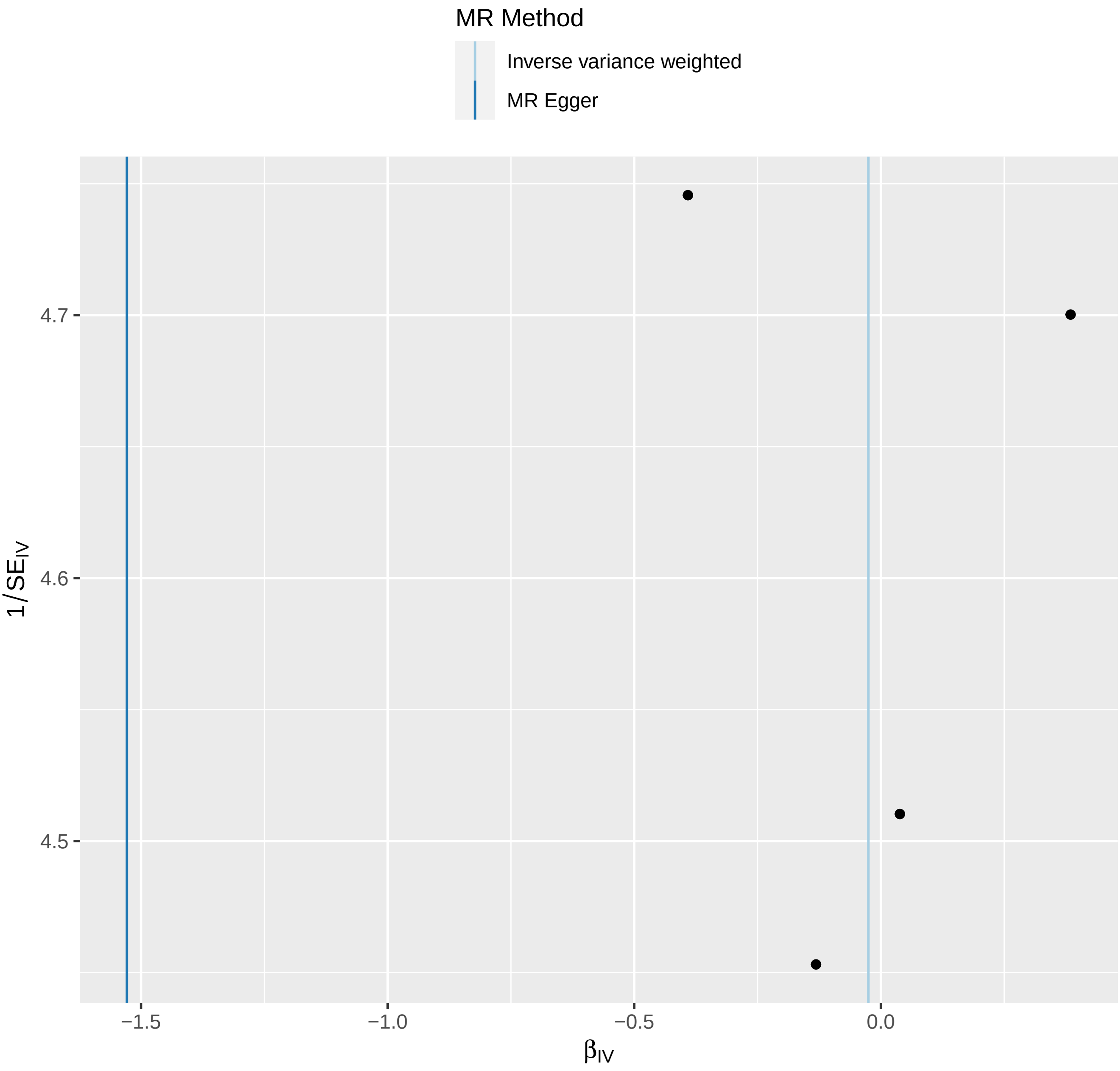

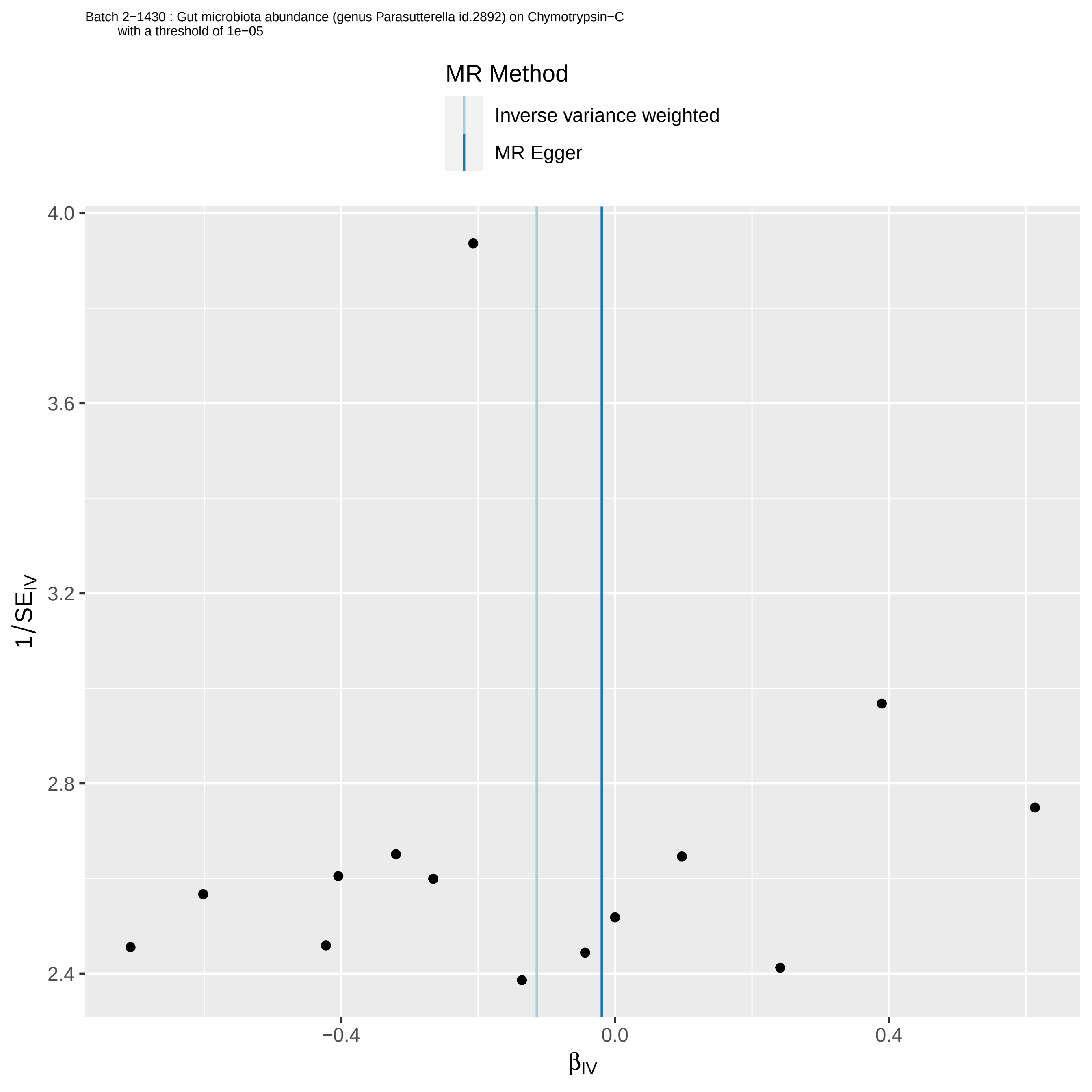
Batch 2−1430 : Gut microbiota abundance (genus Parasutterella id.2892) on Chymotrypsin−C
with a threshold of 1e−05
MR Method
Inverse variance weighted
MR Egger
4.0
3.6
3.2
2.8
2.4
1/SE_IV
−0.4
0.0
0.4
β_IV

Batch 2−1431 : Gut microbiota abundance (genus Catenibacterium id.2153) on Chymotrypsin−like protease CTRL−1
with a threshold of 1e−05

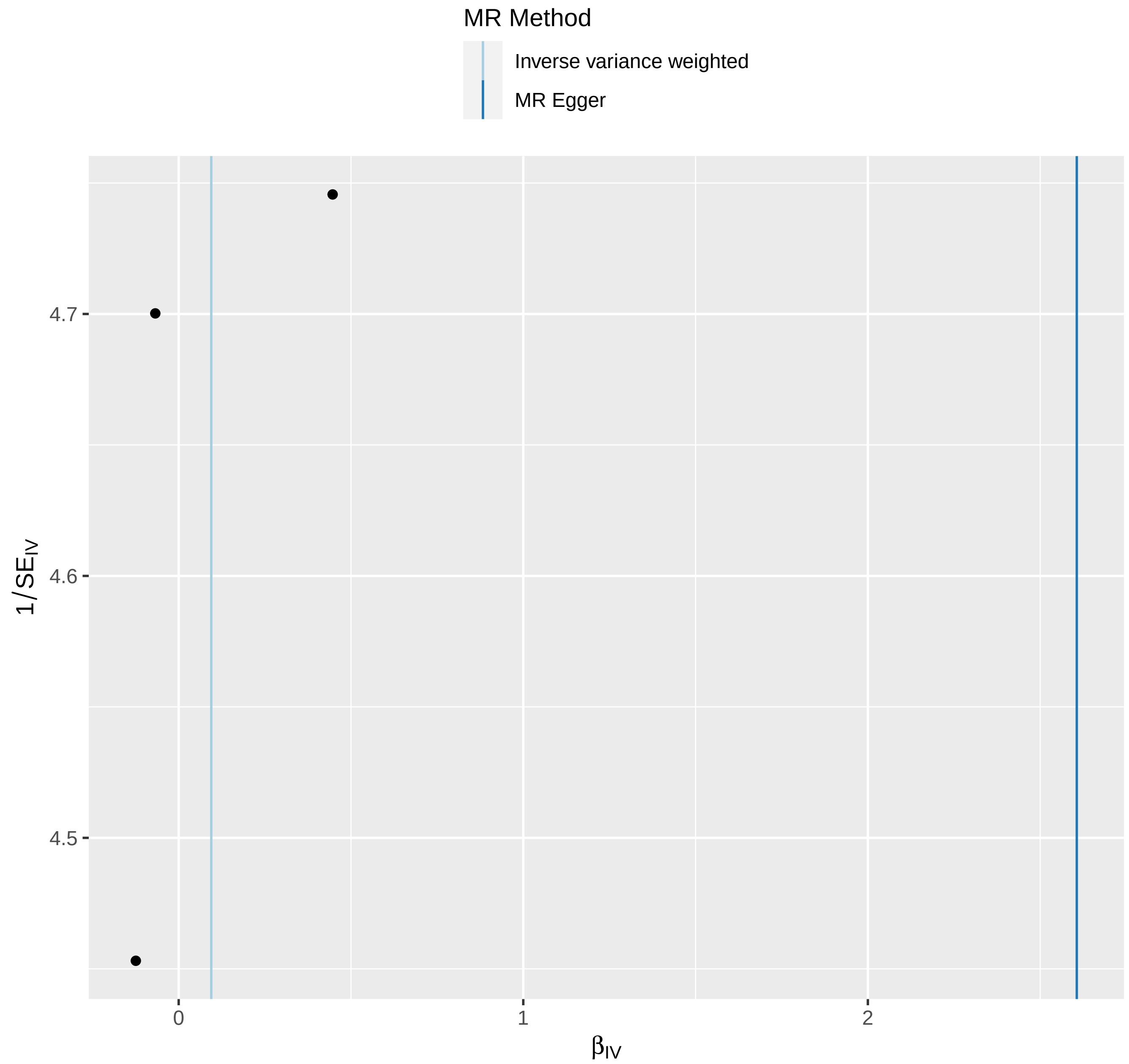

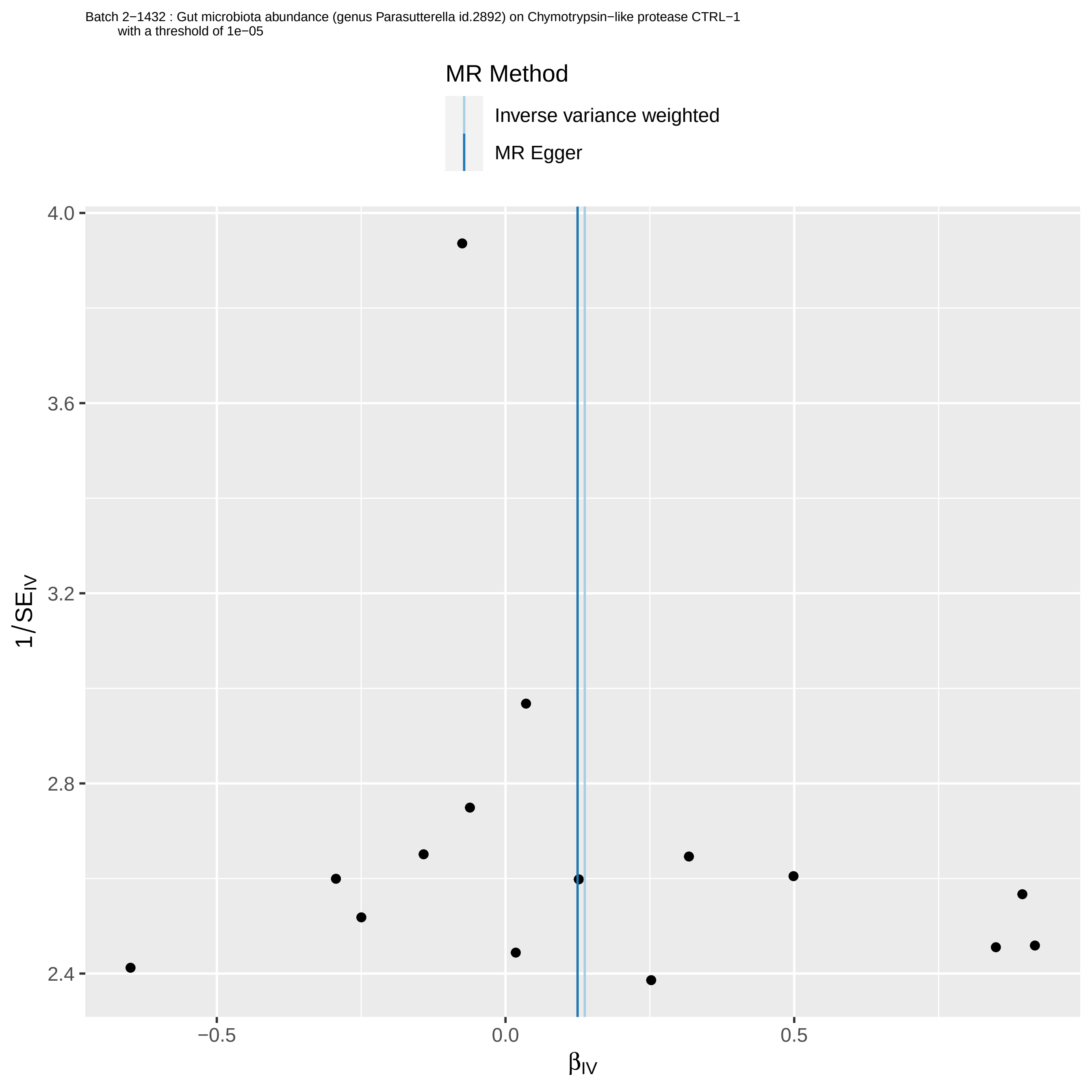
Batch 2−1432 : Gut microbiota abundance (genus Parasutterella id.2892) on Chymotrypsin−like protease CTRL−1
with a threshold of 1e−05
MR Method
Inverse variance weighted
MR Egger
4.0
3.6
3.2
2.8
2.4
1/SE_IV
−0.5
0.0
0.5
β_IV

Batch 2−1433 : Gut microbiota abundance (genus Catenibacterium id.2153) on Lysosomal protective protein
with a threshold of 1e−05

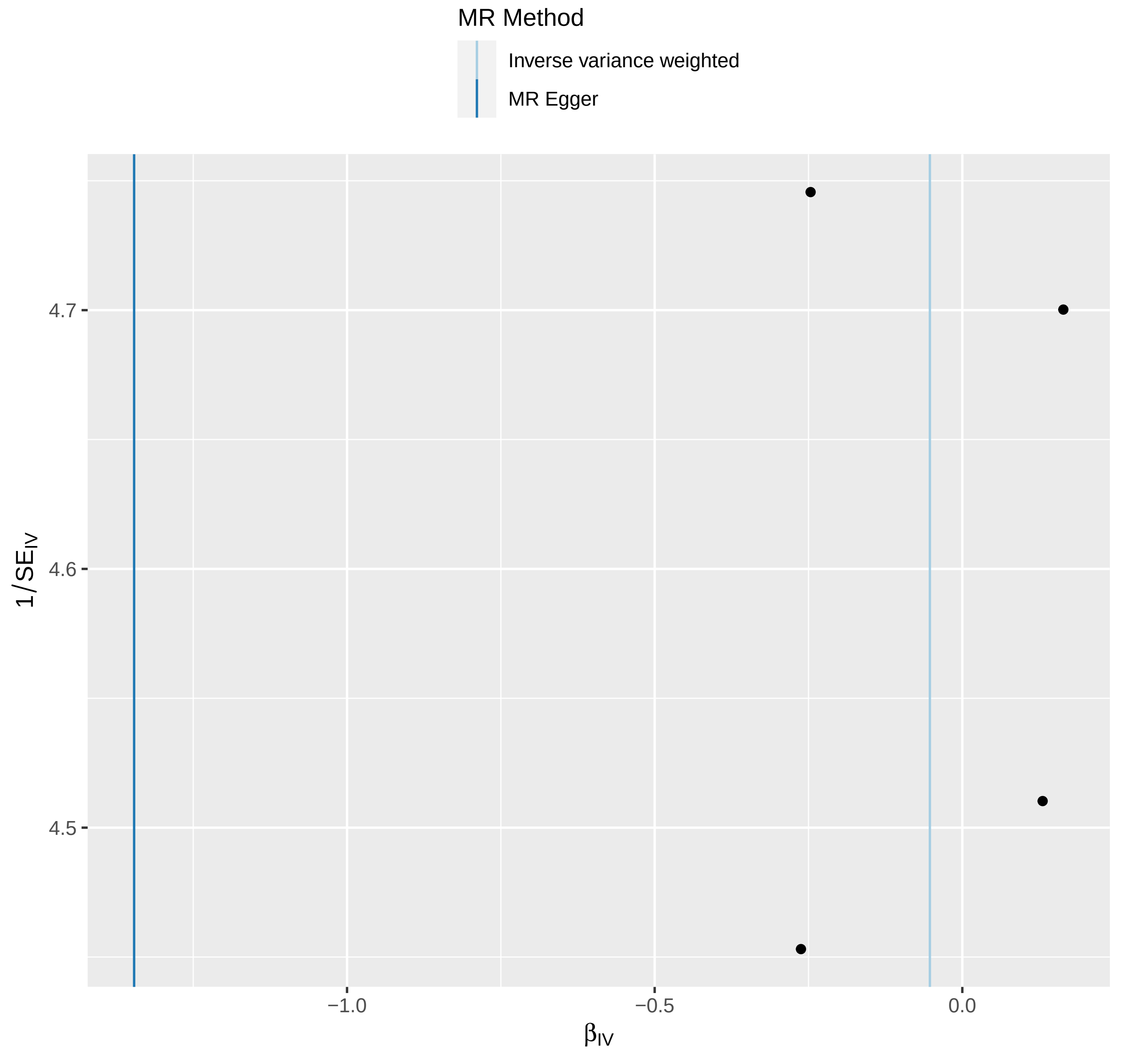

Batch 2−1434 : Gut microbiota abundance (genus Parasutterella id.2892) on Lysosomal protective protein
with a threshold of 1e−05

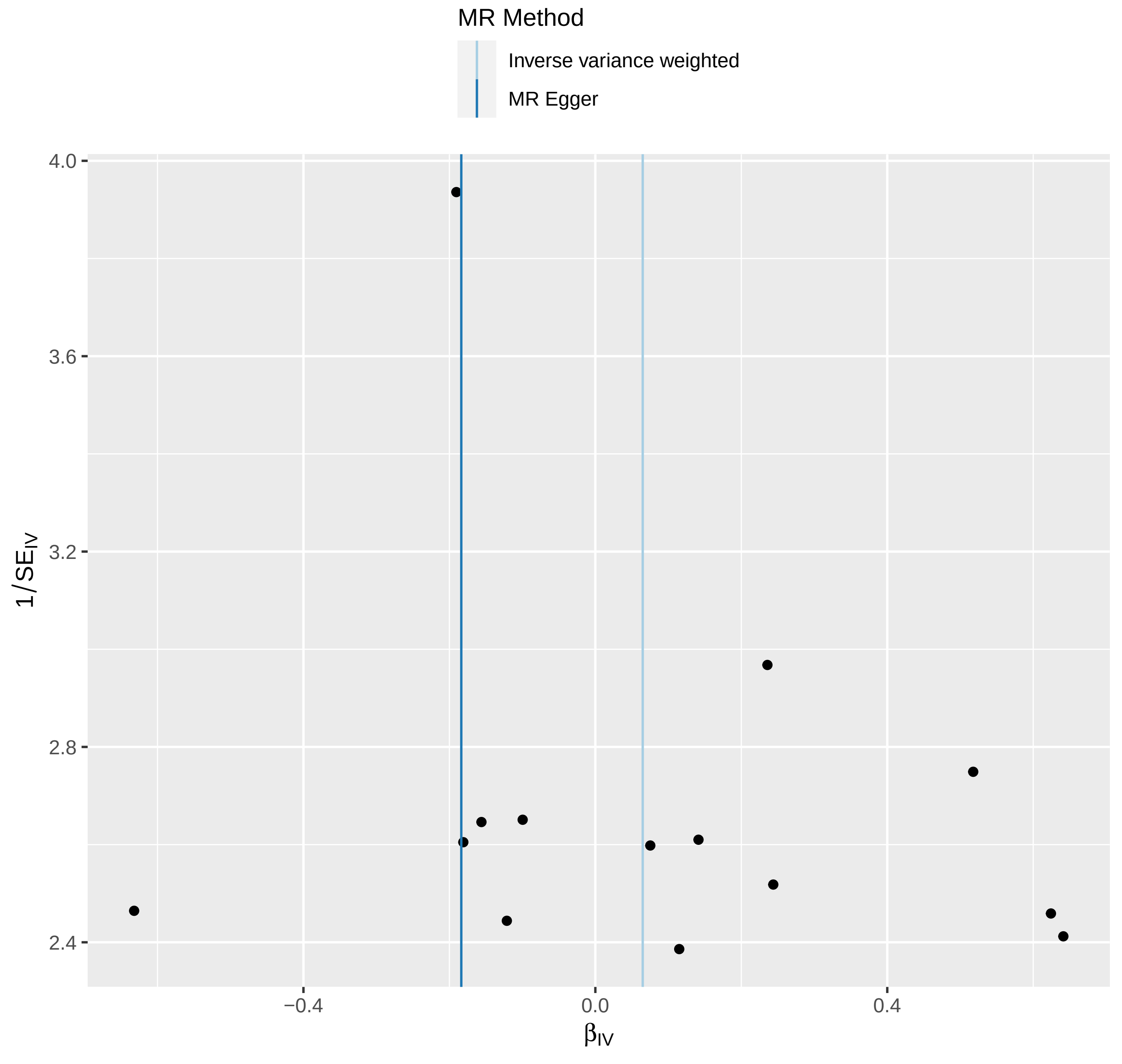

Batch 2−1435 : Gut microbiota abundance (genus Catenibacterium id.2153) on Cathepsin B
with a threshold of 1e−05

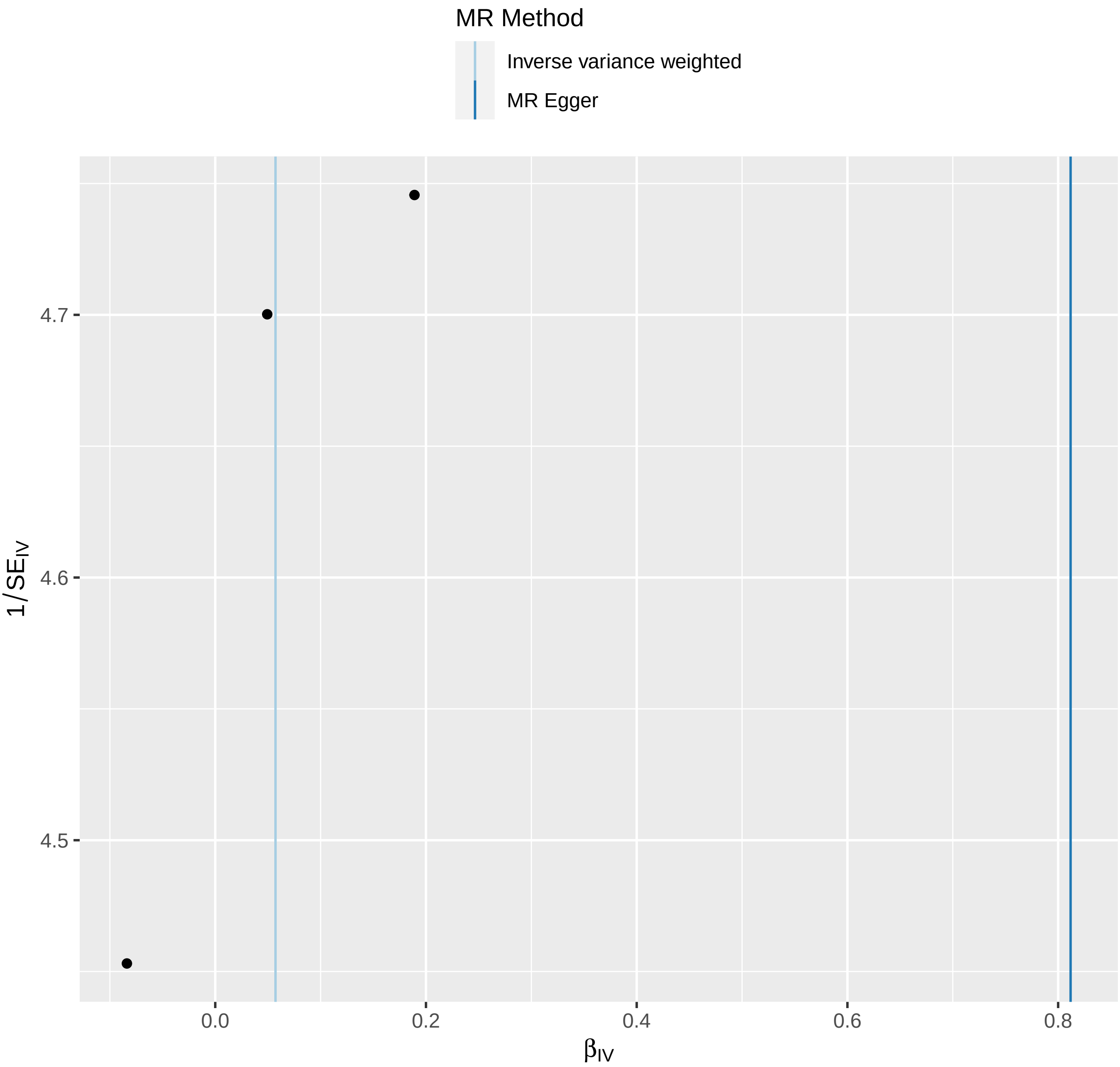

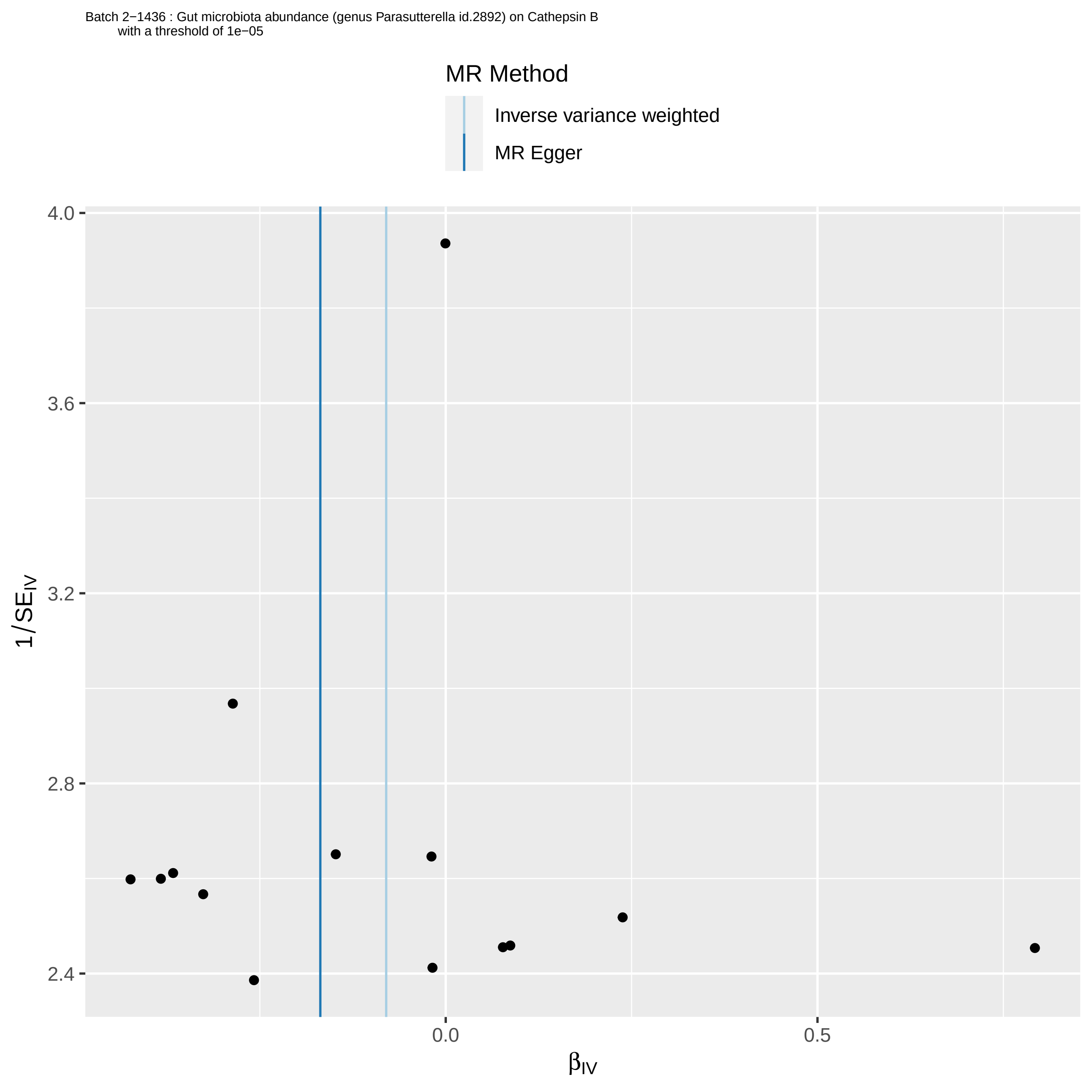
Batch 2−1436 : Gut microbiota abundance (genus Parasutterella id.2892) on Cathepsin B
with a threshold of 1e−05
MR Method
Inverse variance weighted
MR Egger
4.0
3.6
3.2
2.8
2.4
1/SE$_{IV}$
0.0
0.5
β_{IV}

Batch 2−1437 : Gut microbiota abundance (genus Catenibacterium id.2153) on Dipeptidyl peptidase 1
with a threshold of 1e−05

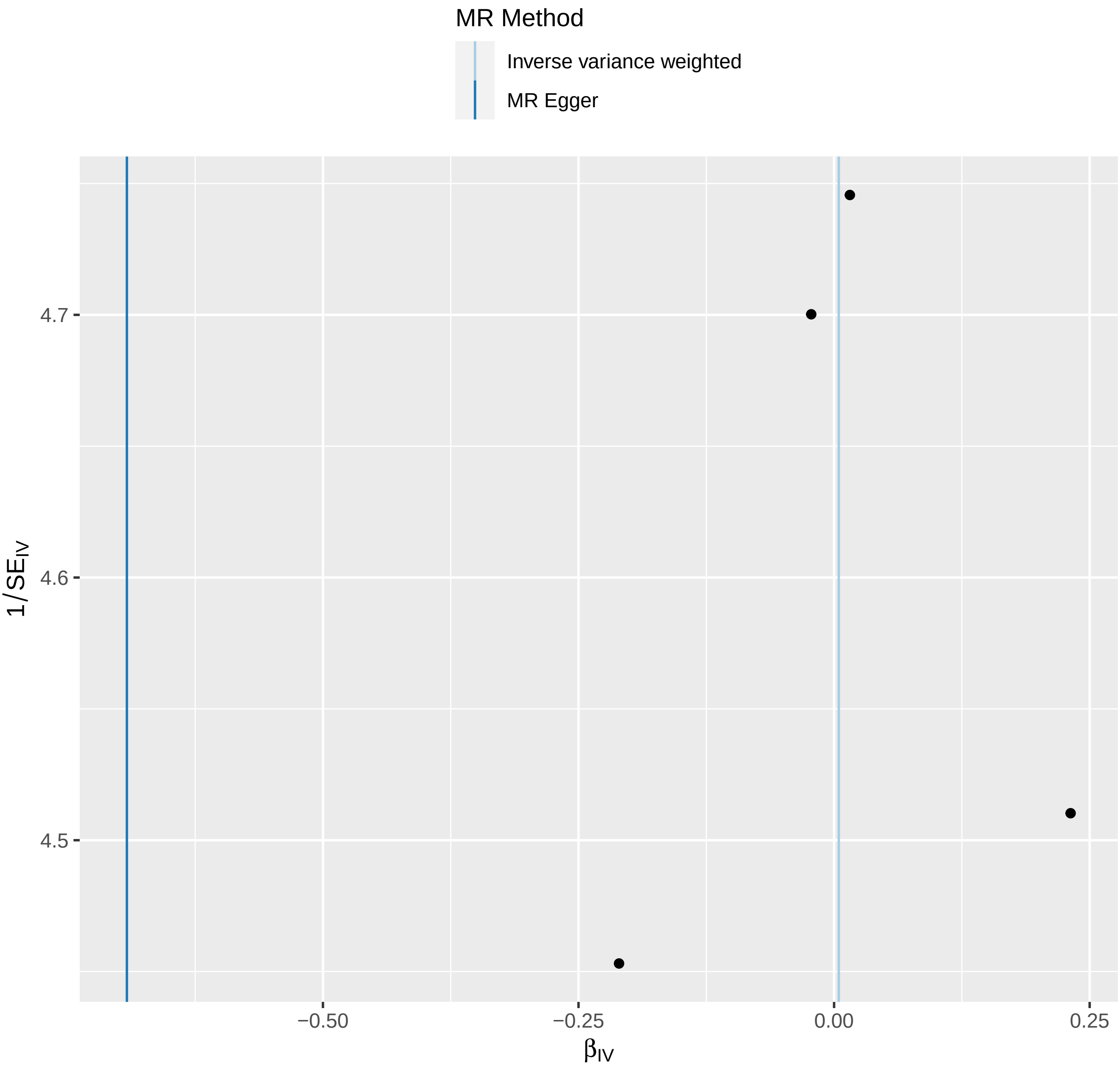

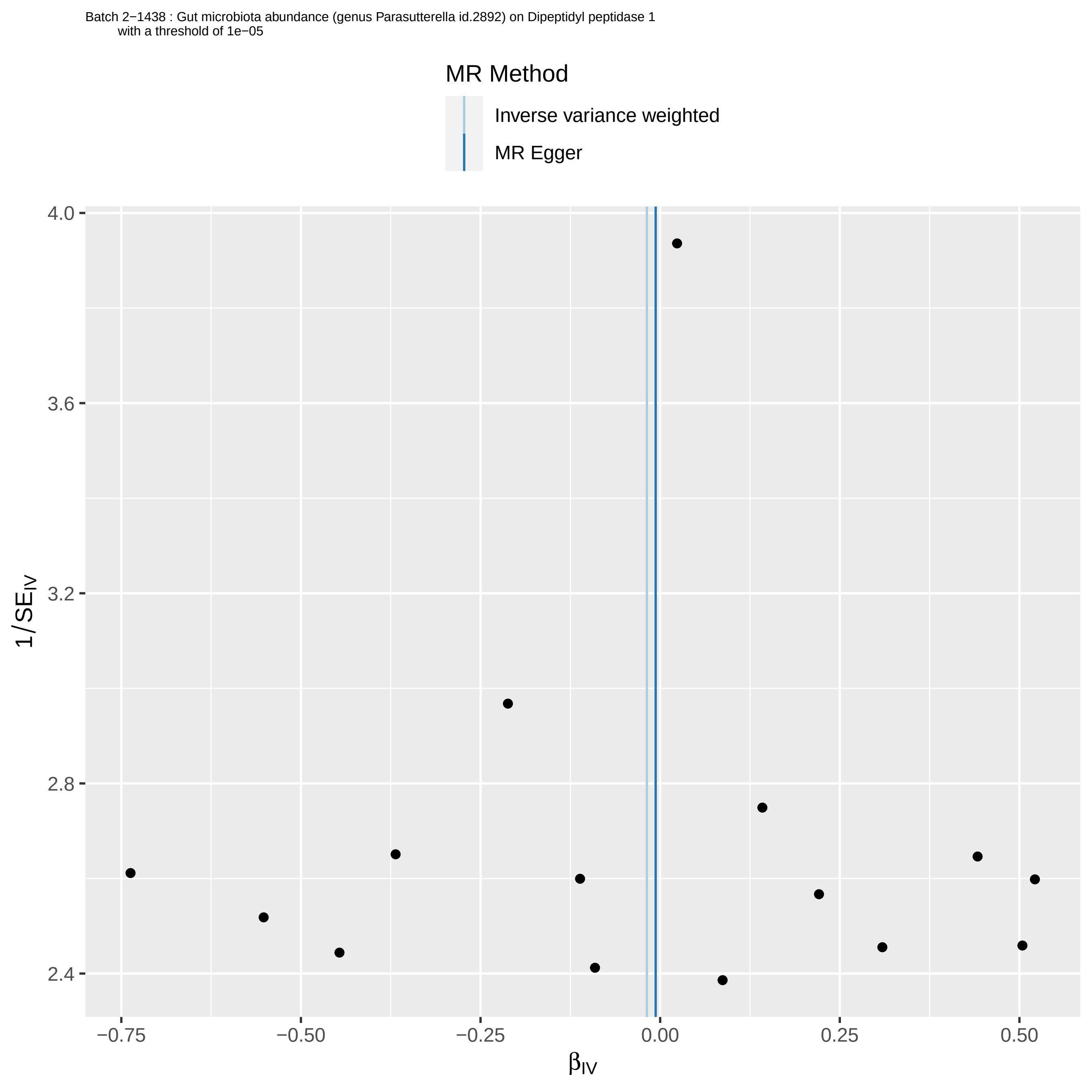
Batch 2−1438 : Gut microbiota abundance (genus Parasutterella id.2892) on Dipeptidyl peptidase 1
with a threshold of 1e−05
MR Method
Inverse variance weighted
MR Egger
4.0
3.6
3.2
2.8
2.4
1/SE_IV
−0.75
−0.50
−0.25
0.00
0.25
0.50
β_IV

Batch 2−1439 : Gut microbiota abundance (genus Catenibacterium id.2153) on Cathepsin E
with a threshold of 1e−05

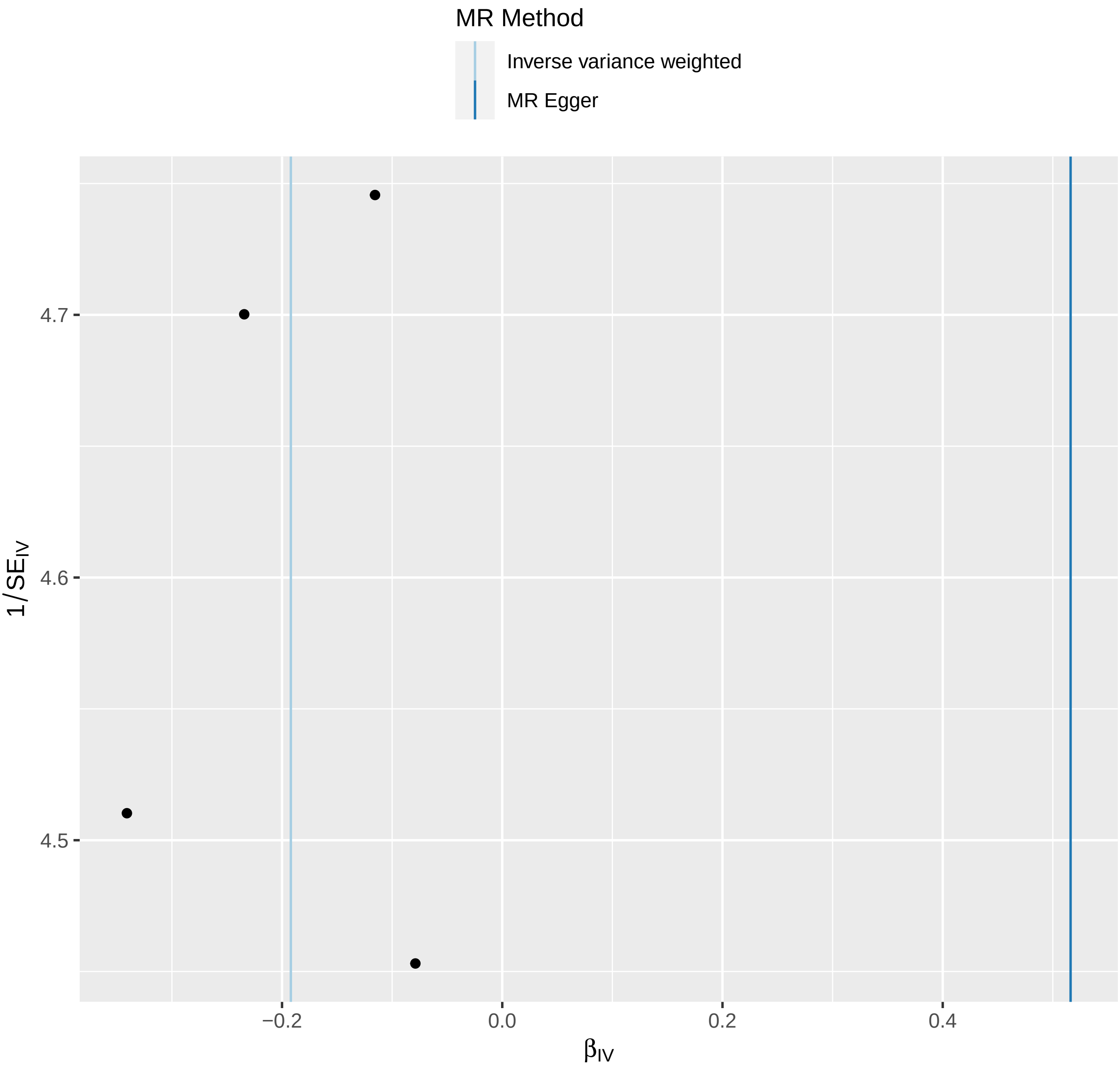

Batch 2−1440 : Gut microbiota abundance (genus Parasutterella id.2892) on Cathepsin E
with a threshold of 1e−05

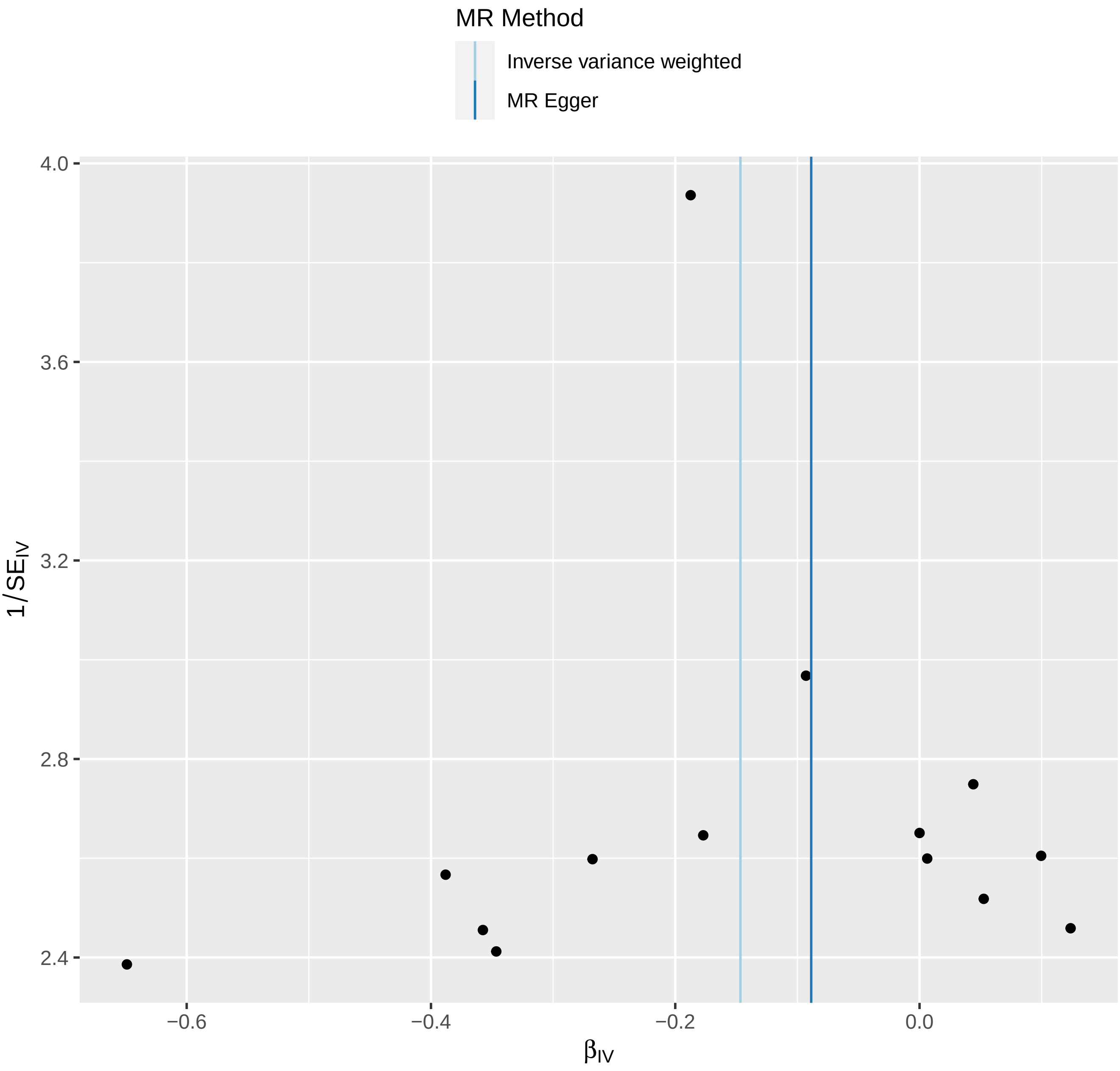

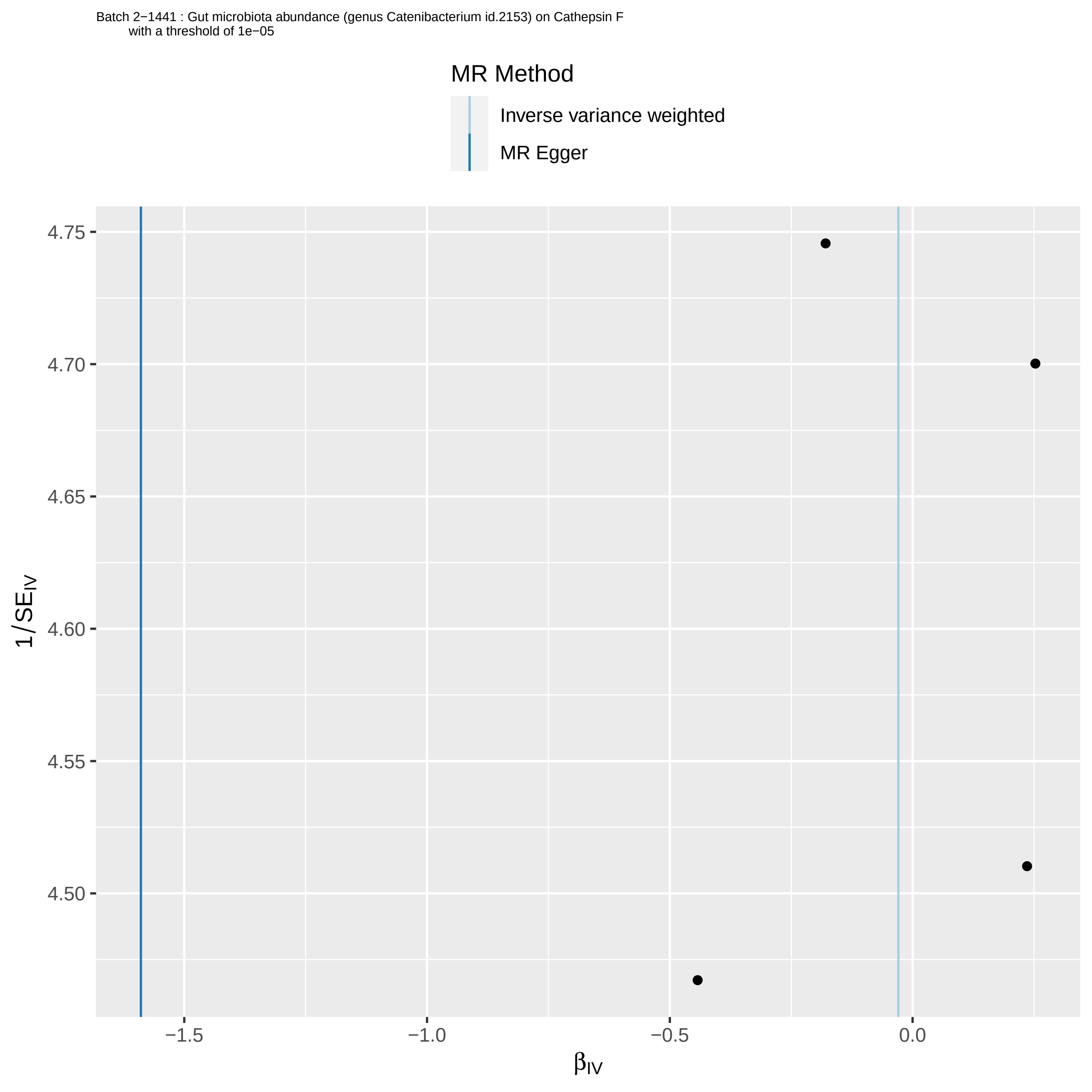

Batch 2−1441 : Gut microbiota abundance (genus Catenibacterium id.2153) on Cathepsin F
with a threshold of 1e−05
MR Method
Inverse variance weighted
MR Egger
4.75
4.70
4.65
4.60
4.55
4.50
1/SE_IV
−1.5
−1.0
−0.5
0.0
β_IV

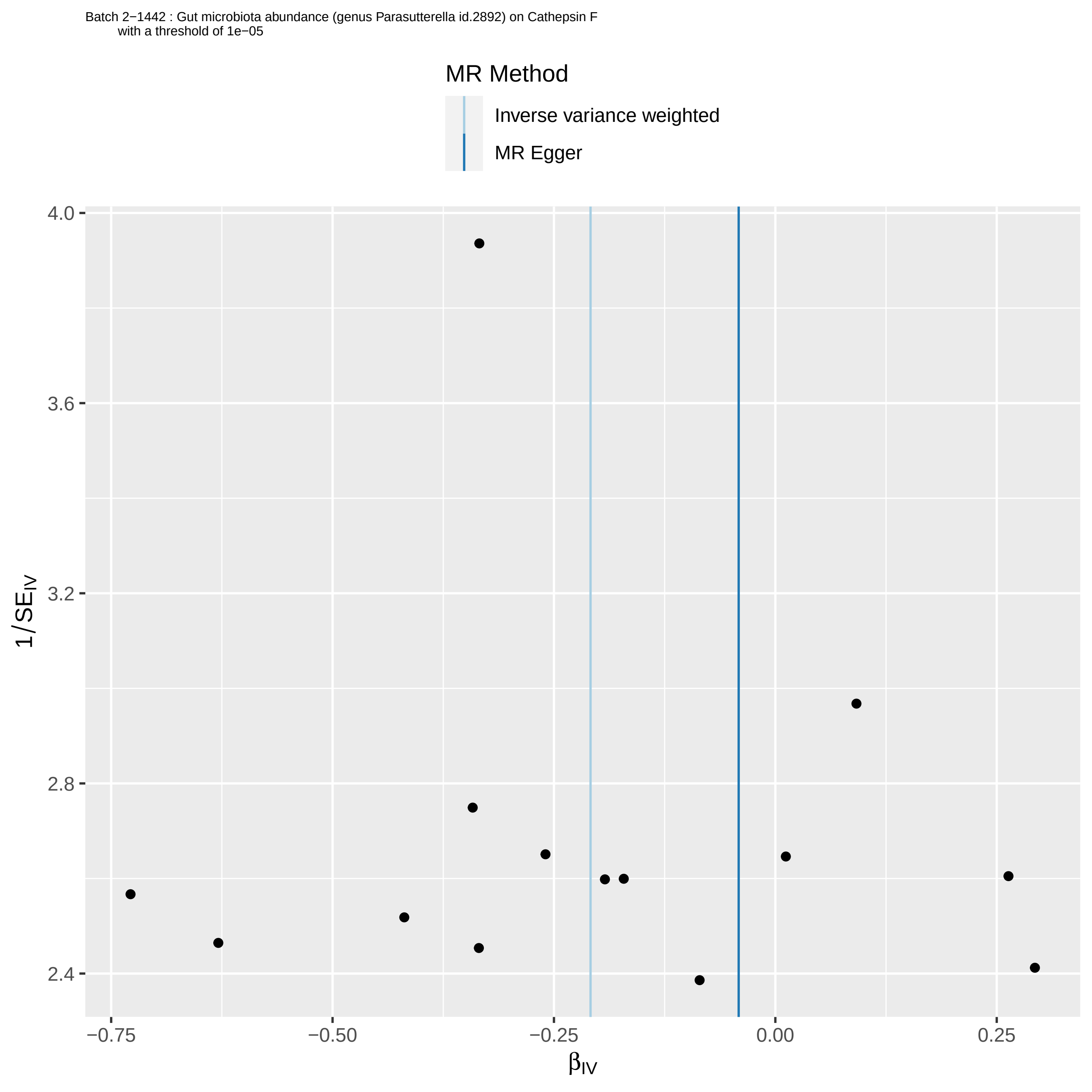

Batch 2−1442 : Gut microbiota abundance (genus Parasutterella id.2892) on Cathepsin F
with a threshold of 1e−05
MR Method
Inverse variance weighted
MR Egger
4.0
3.6
3.2
2.8
2.4
1/SE_IV
−0.75
−0.50
−0.25
0.00
0.25
β_IV

Batch 2−1443 : Gut microbiota abundance (genus Catenibacterium id.2153) on Cathepsin F
with a threshold of 1e−05

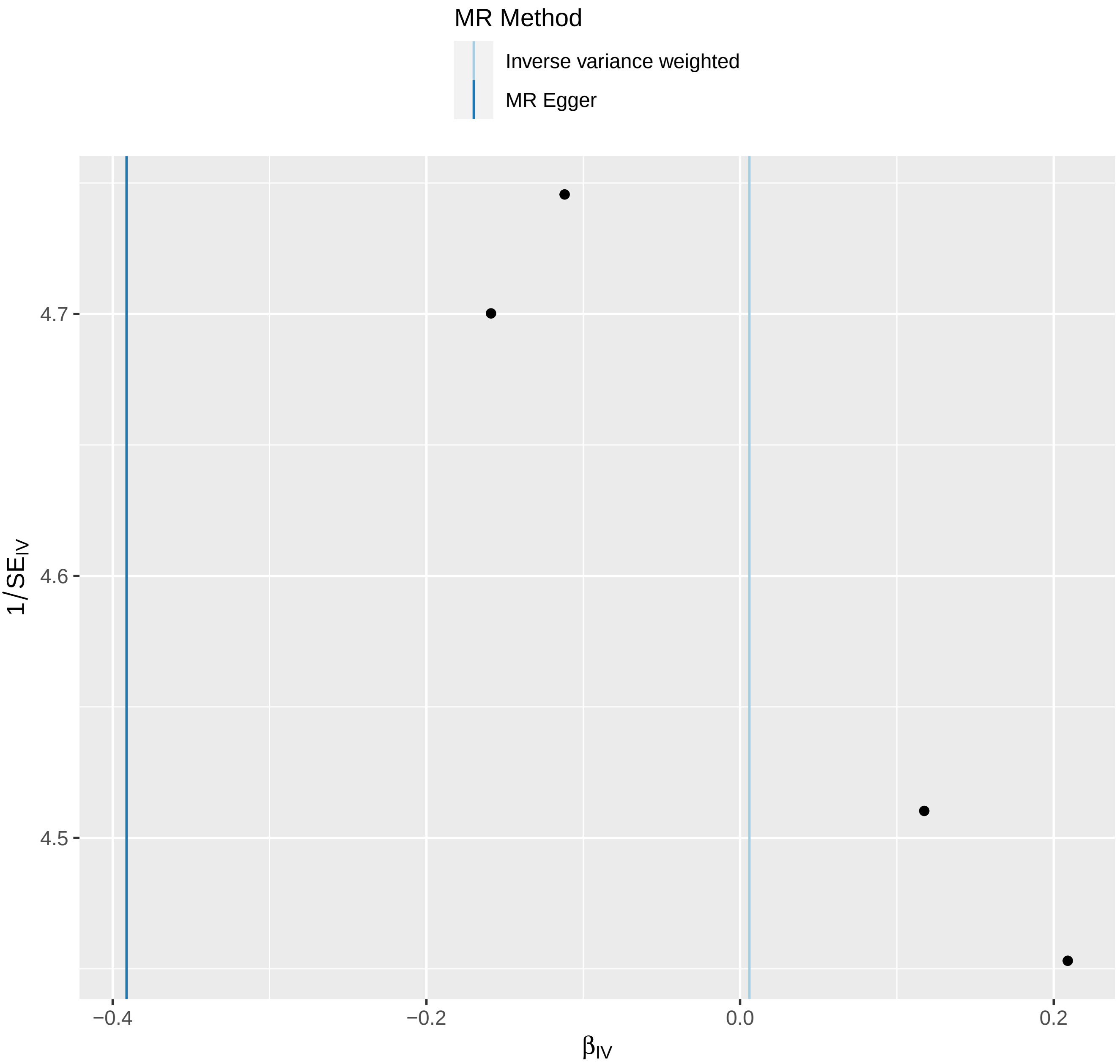

Batch 2−1444 : Gut microbiota abundance (genus Parasutterella id.2892) on Cathepsin F
with a threshold of 1e−05

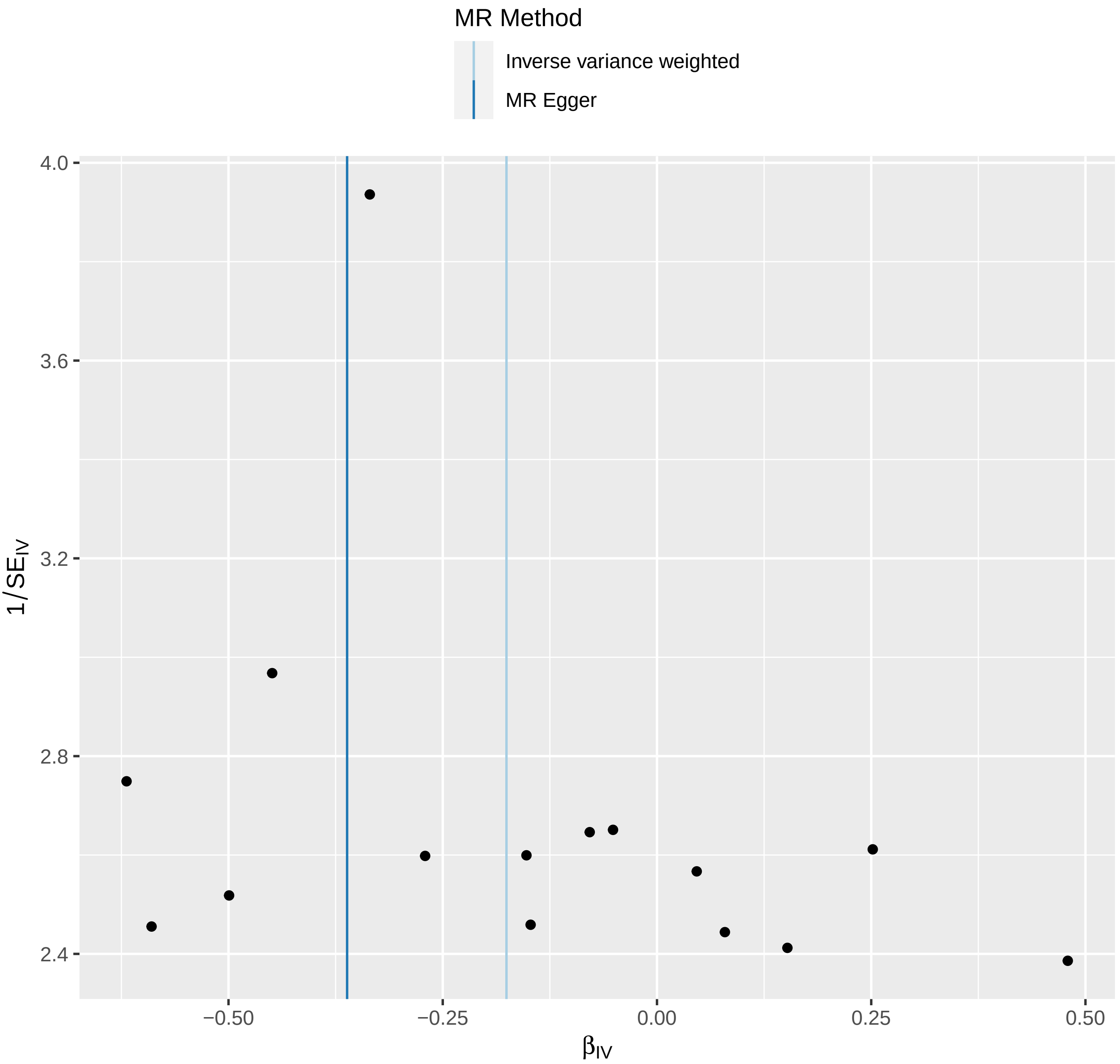

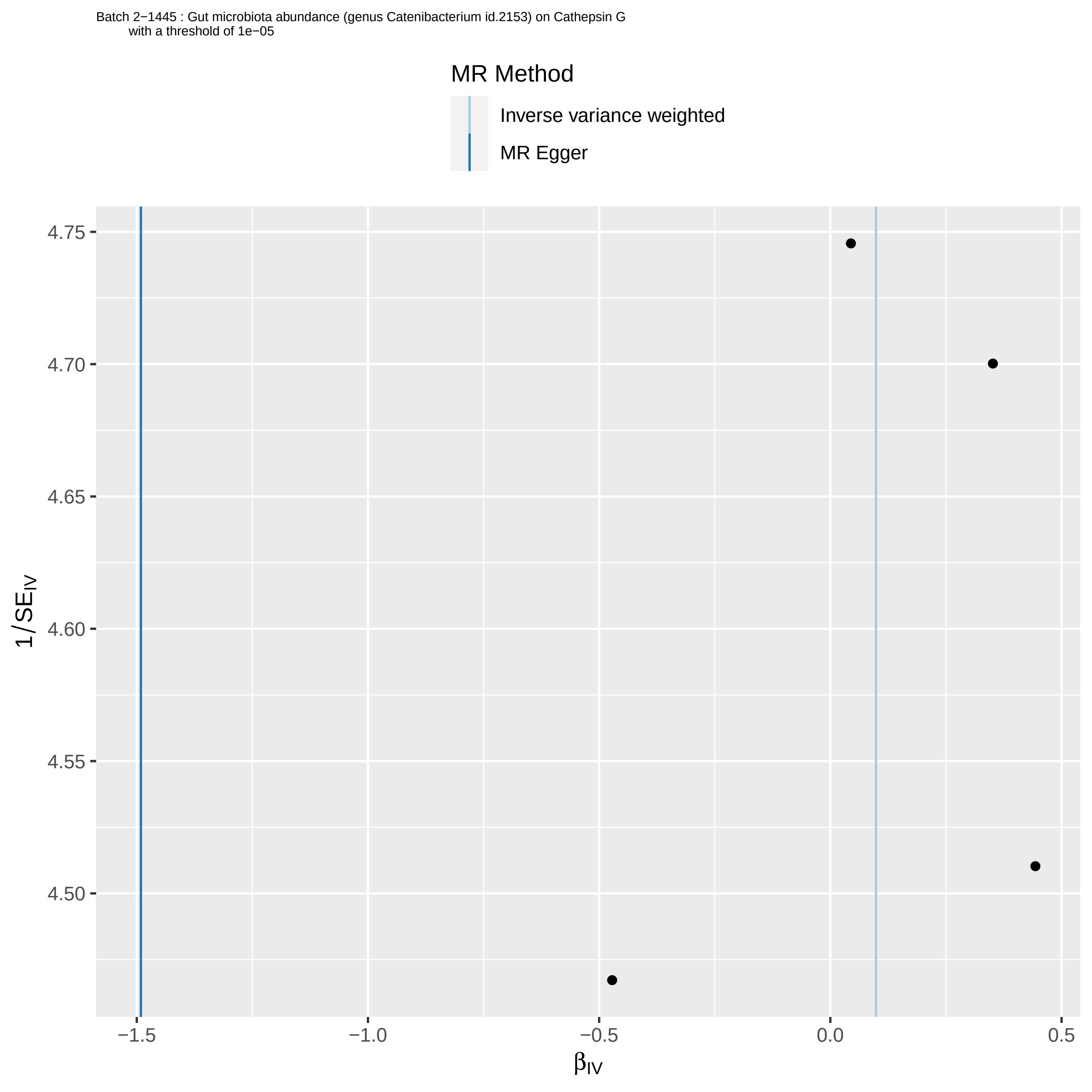
Batch 2−1445 : Gut microbiota abundance (genus Catenibacterium id.2153) on Cathepsin G
with a threshold of 1e−05
MR Method
Inverse variance weighted
MR Egger
4.75
4.70
4.65
4.60
4.55
4.50
1/SE_IV
−1.5
−1.0
−0.5
0.0
0.5
β_IV

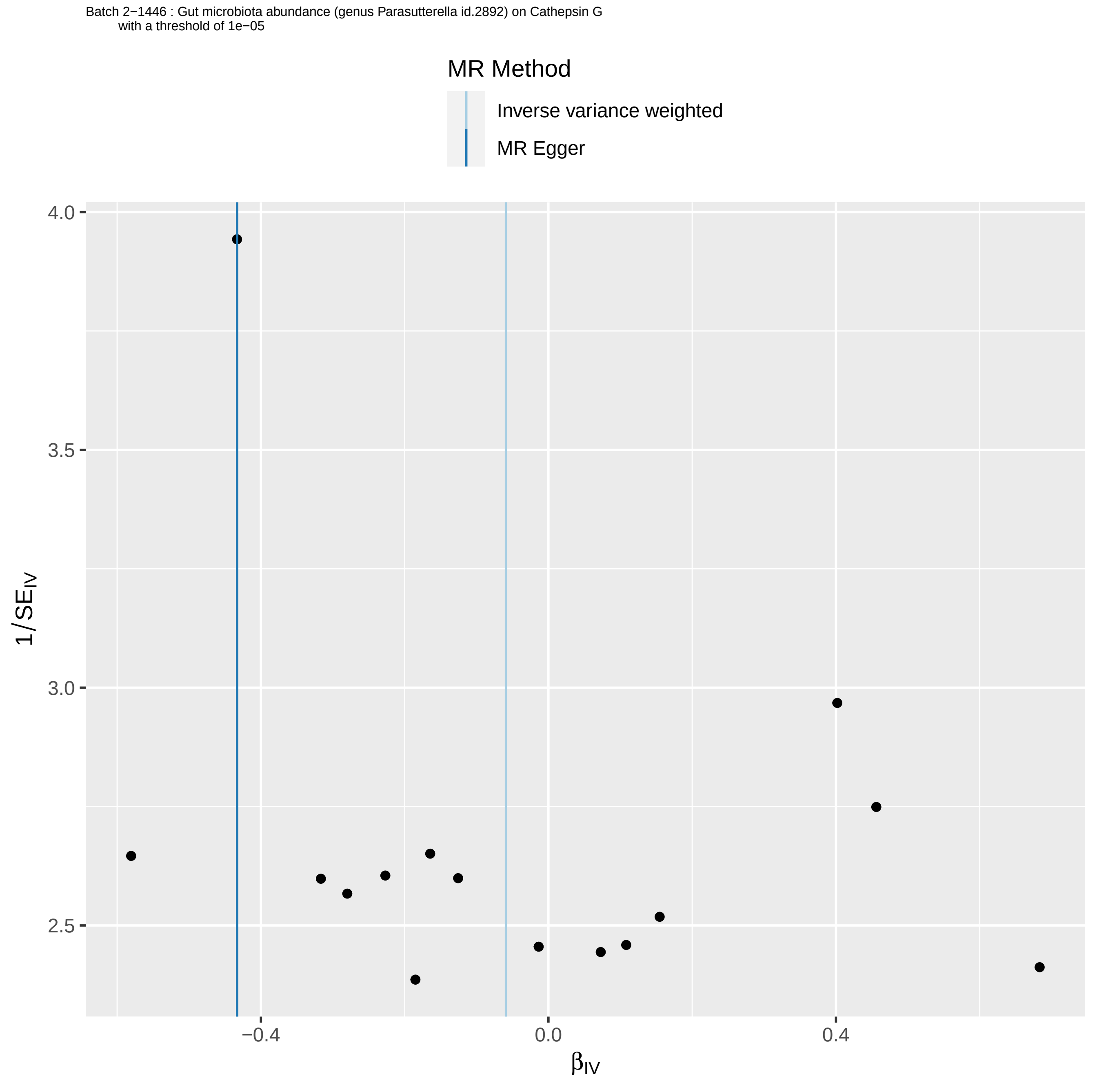

Batch 2−1446 : Gut microbiota abundance (genus Parasutterella id.2892) on Cathepsin G
with a threshold of 1e−05
MR Method
Inverse variance weighted
MR Egger
4.0
3.5
3.0
2.5
$1/SE_{IV}$
−0.4
0.0
0.4
β_{IV}

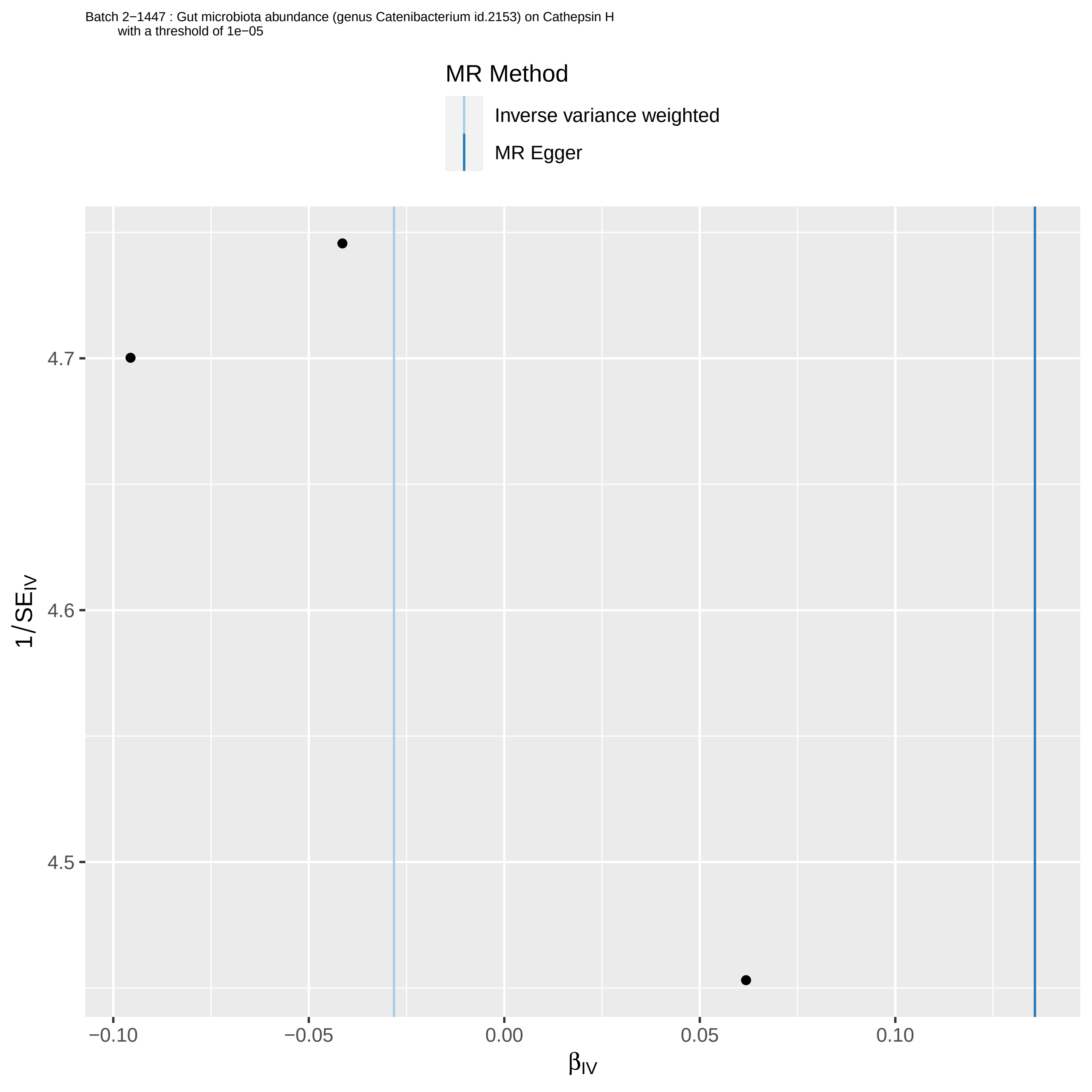

Batch 2−1447 : Gut microbiota abundance (genus Catenibacterium id.2153) on Cathepsin H
with a threshold of 1e−05
MR Method
Inverse variance weighted
MR Egger
4.7
4.6
4.5
1/SE_IV
−0.10
−0.05
0.00
0.05
0.10
β_IV

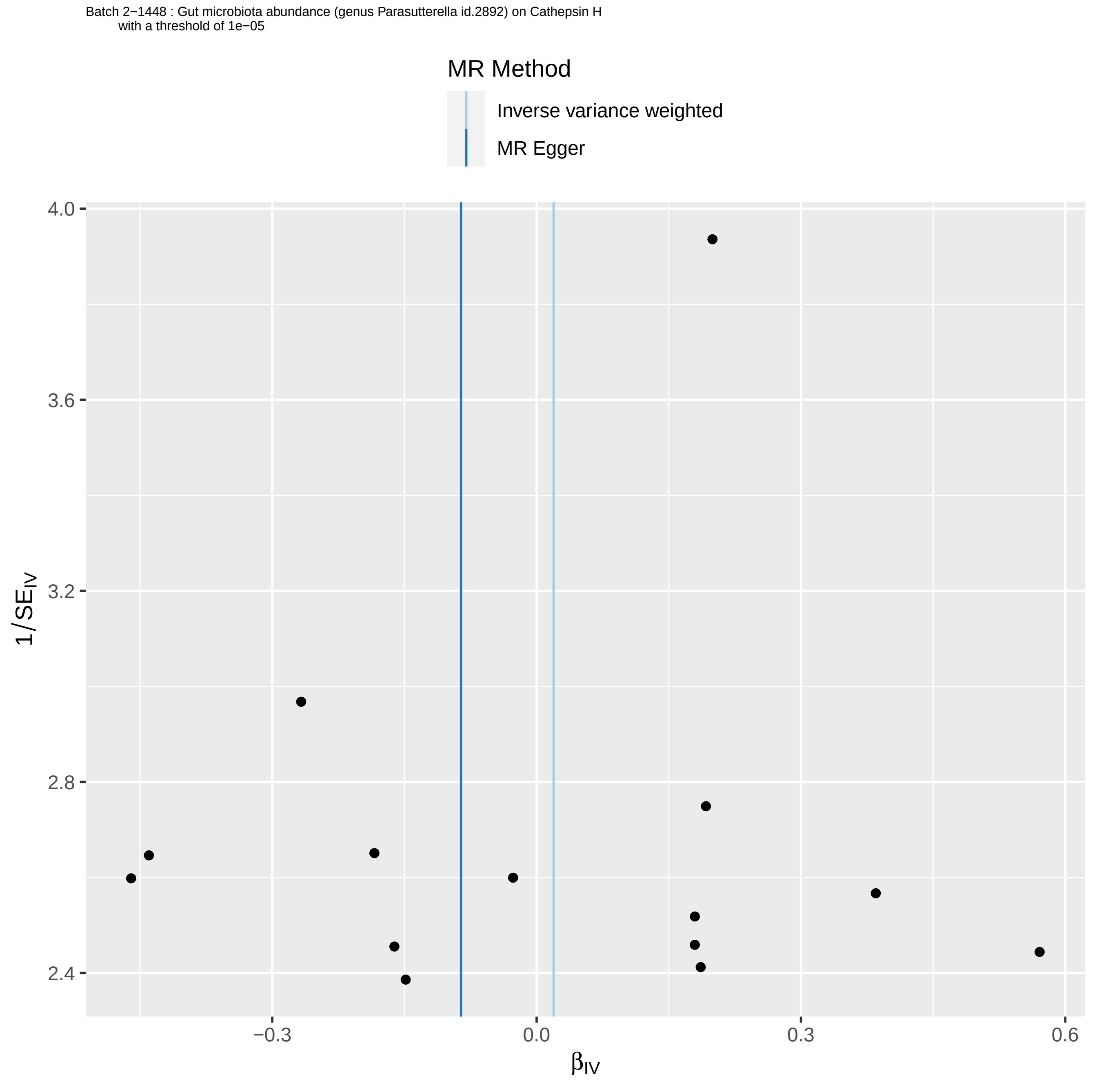
Batch 2−1448 : Gut microbiota abundance (genus Parasutterella id.2892) on Cathepsin H
with a threshold of 1e−05
MR Method
Inverse variance weighted
MR Egger
4.0
3.6
3.2
2.8
2.4
1/SE_IV
−0.3
0.0
0.3
0.6
β_IV

Batch 2−1449 : Gut microbiota abundance (genus Catenibacterium id.2153) on Cathepsin H
with a threshold of 1e−05

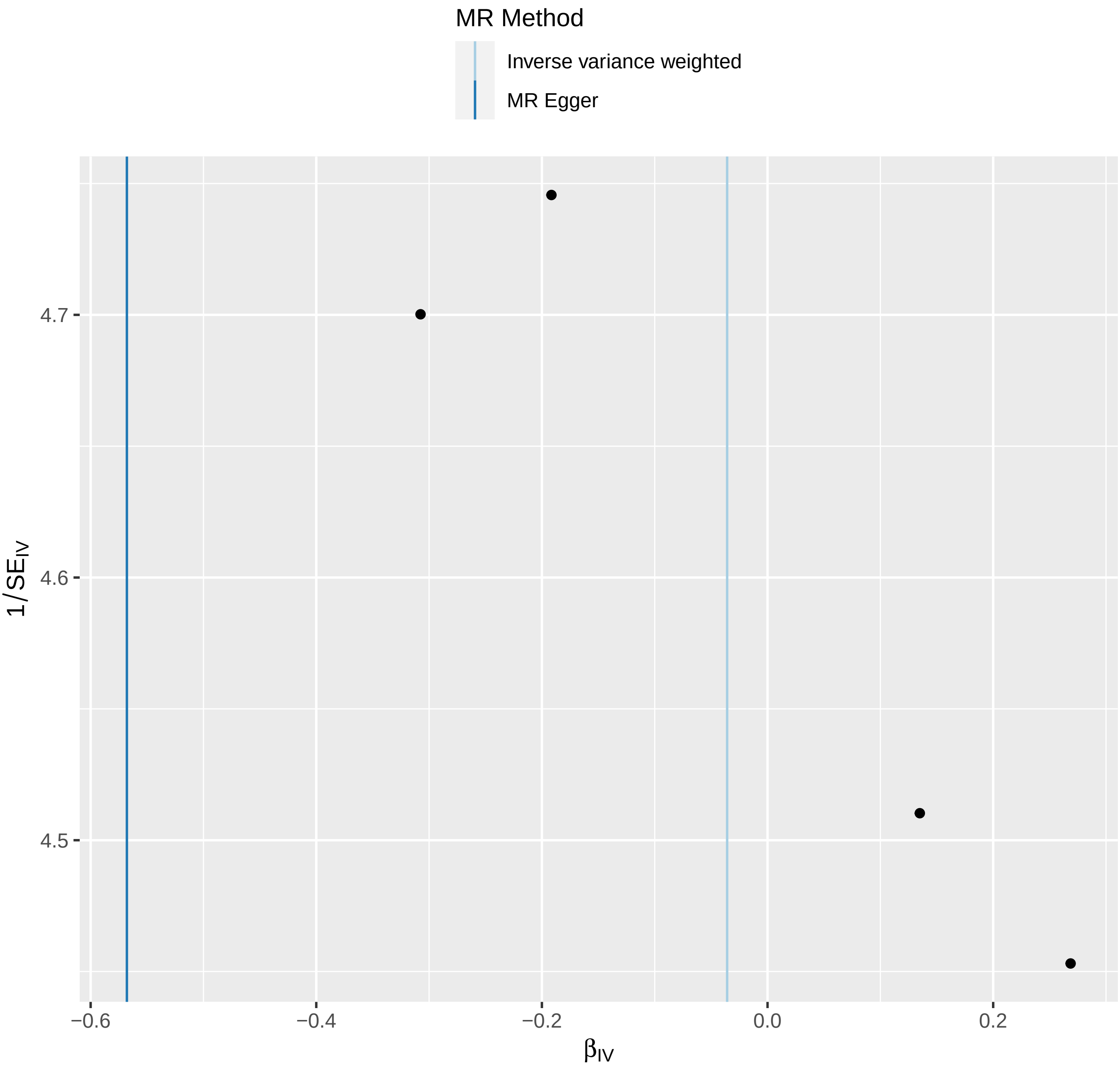

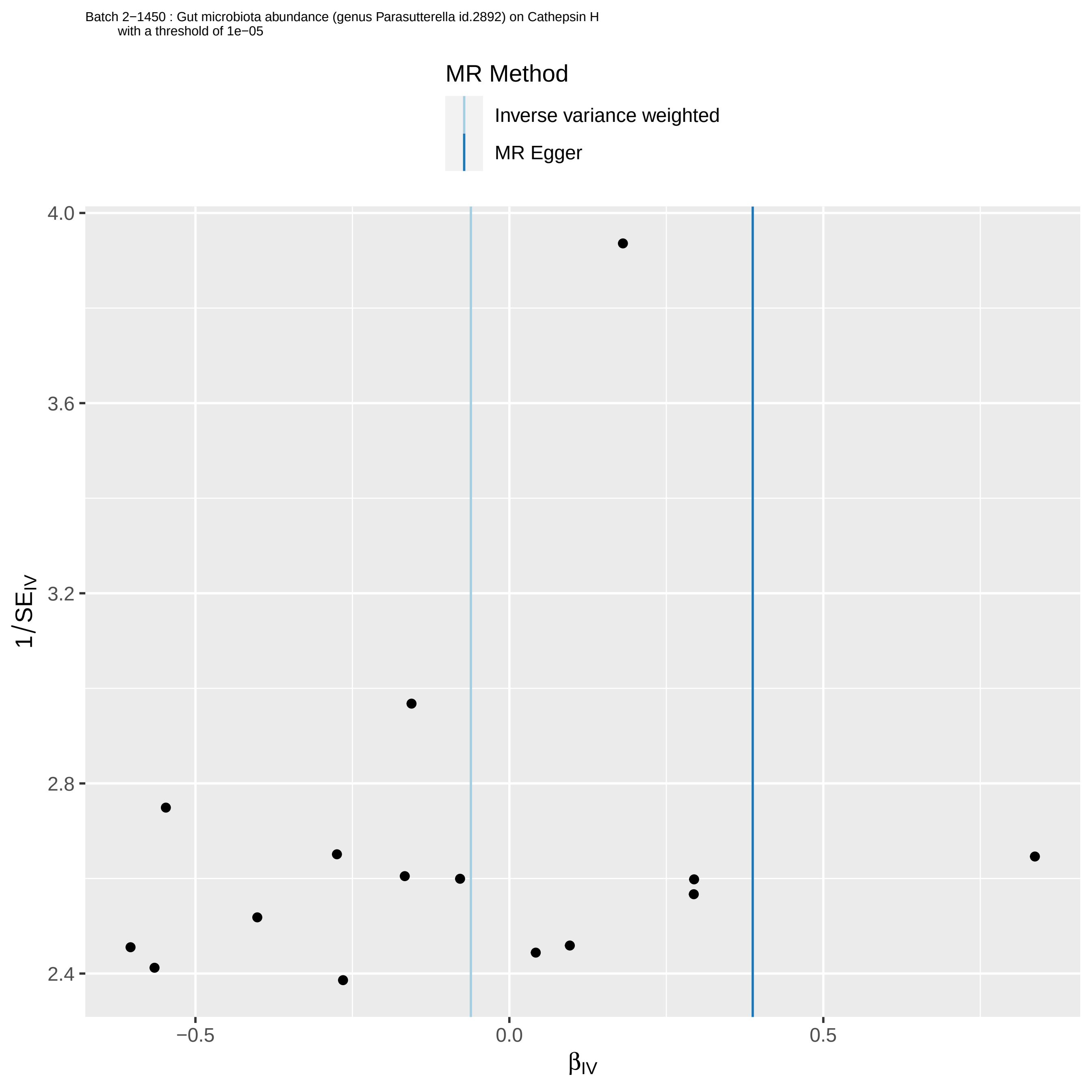

Batch 2−1450 : Gut microbiota abundance (genus Parasutterella id.2892) on Cathepsin H
with a threshold of 1e−05
MR Method
Inverse variance weighted
MR Egger
4.0
3.6
3.2
2.8
2.4
1/SE_IV
−0.5
0.0
0.5
β_IV

Batch 2−1451 : Gut microbiota abundance (genus Catenibacterium id.2153) on Cathepsin O
with a threshold of 1e−05

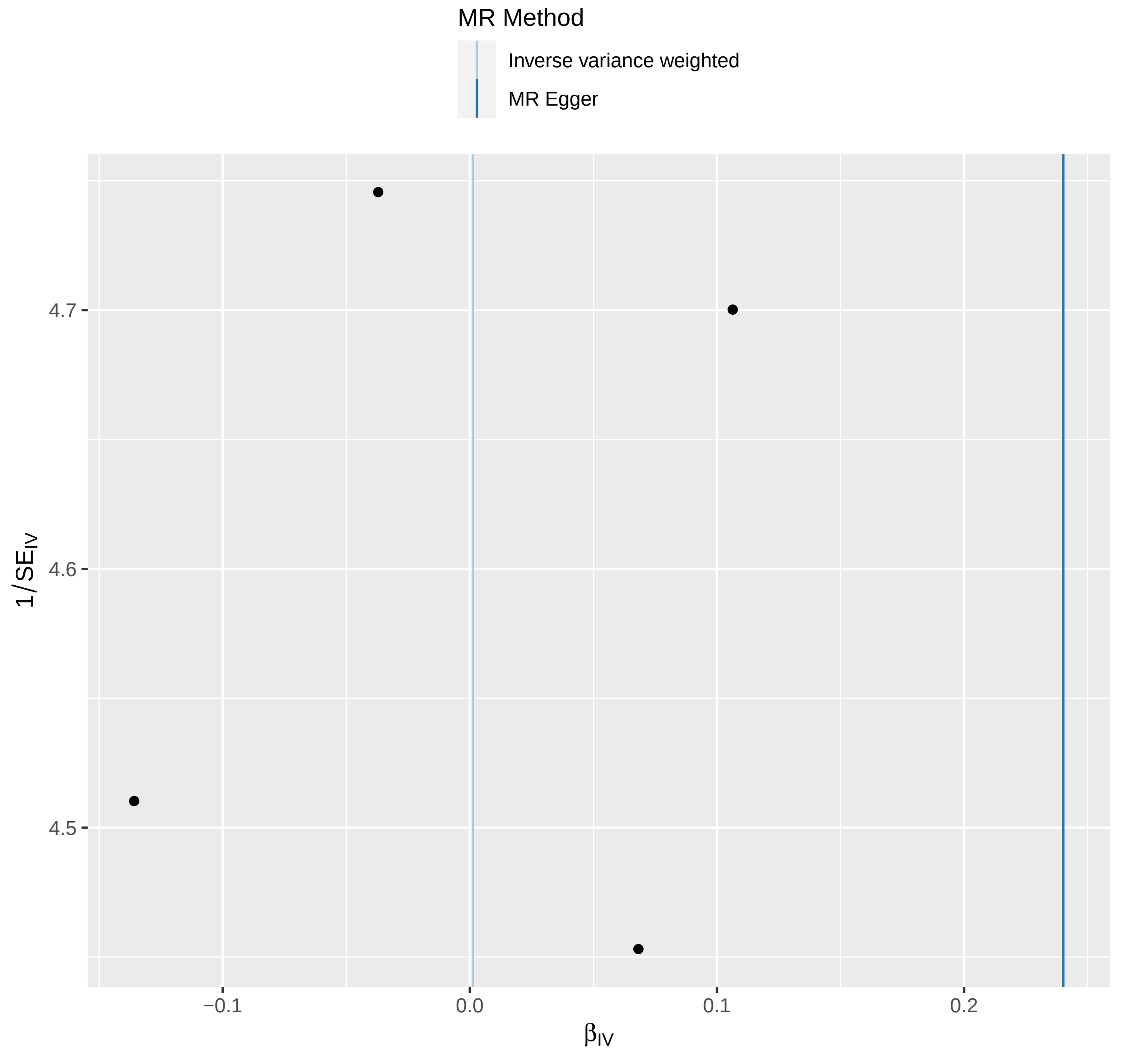

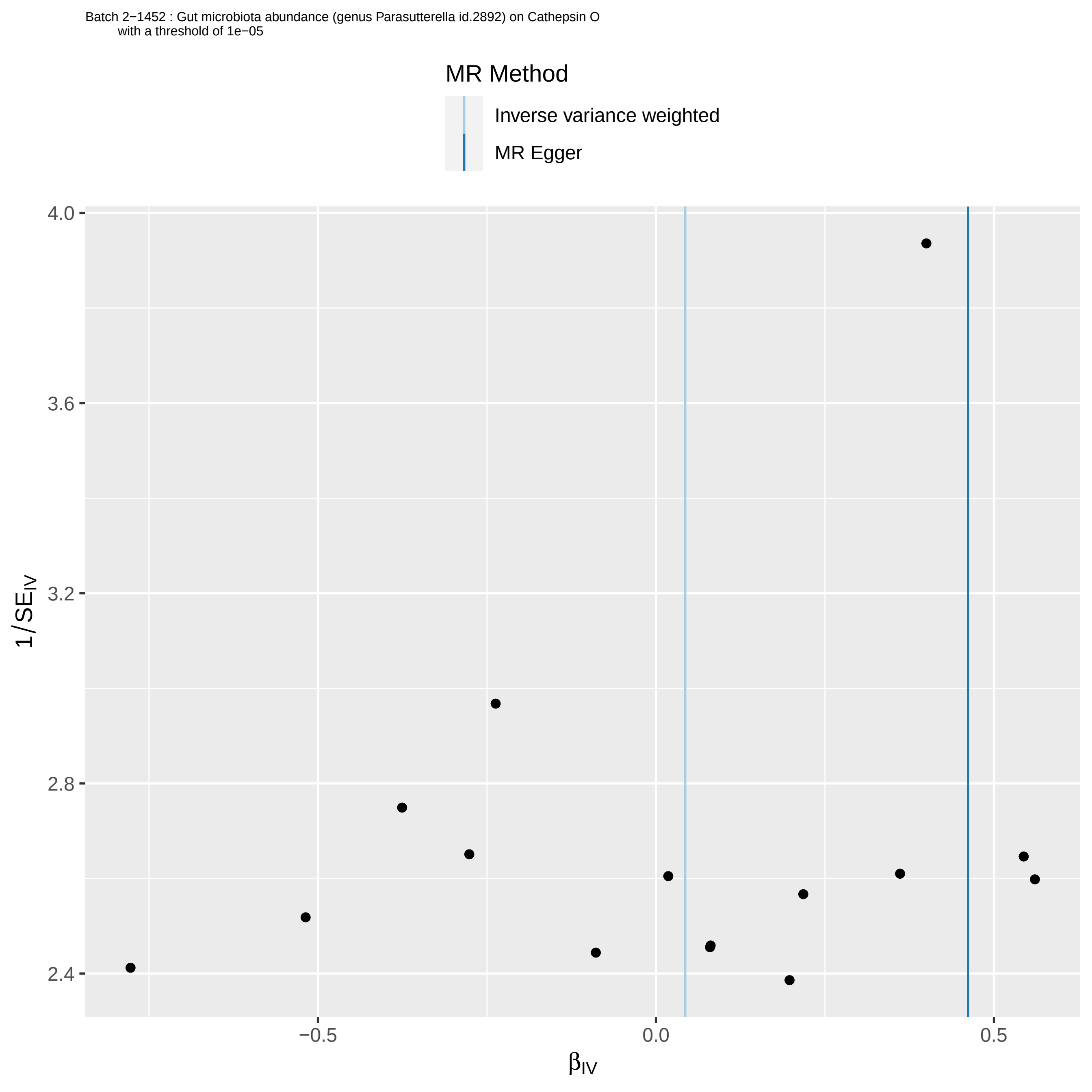
Batch 2−1452 : Gut microbiota abundance (genus Parasutterella id.2892) on Cathepsin O
with a threshold of 1e−05
MR Method
Inverse variance weighted
MR Egger
4.0
3.6
3.2
2.8
2.4
1/SE_IV
−0.5
0.0
0.5
β_IV

Batch 2−1453 : Gut microbiota abundance (genus Catenibacterium id.2153) on Cathepsin S
with a threshold of 1e−05

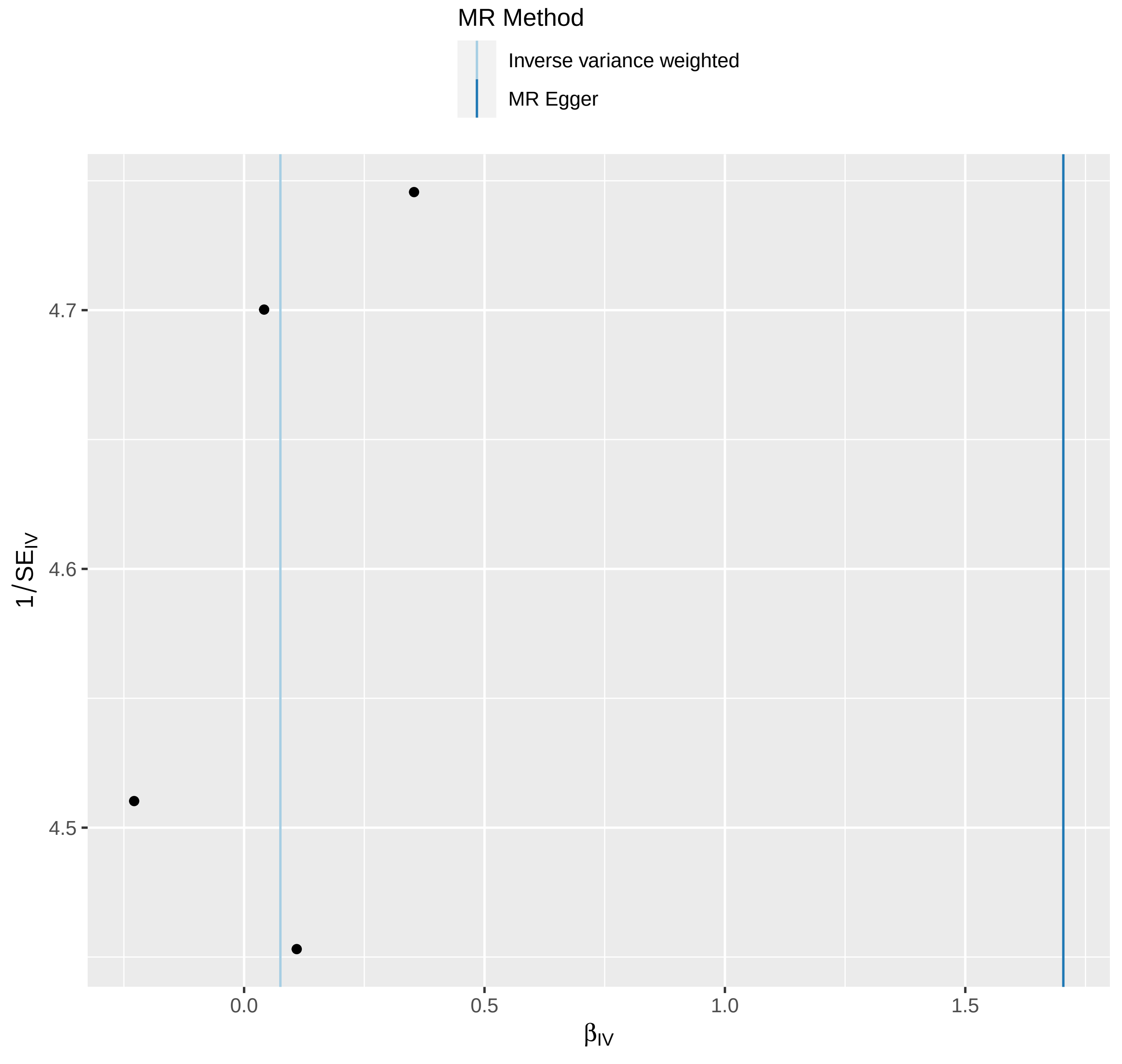

Batch 2−1454 : Gut microbiota abundance (genus Parasutterella id.2892) on Cathepsin S
with a threshold of 1e−05

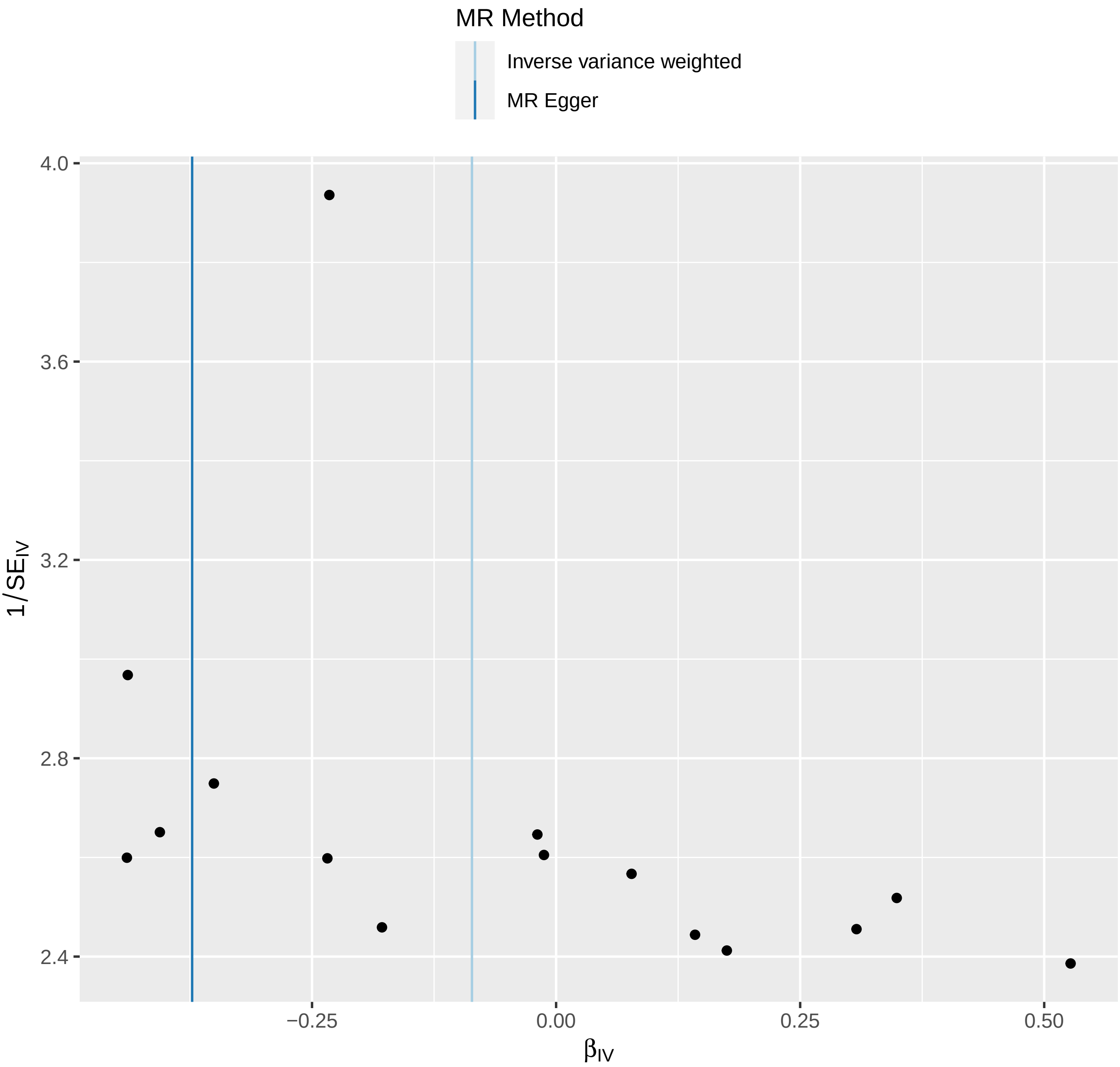

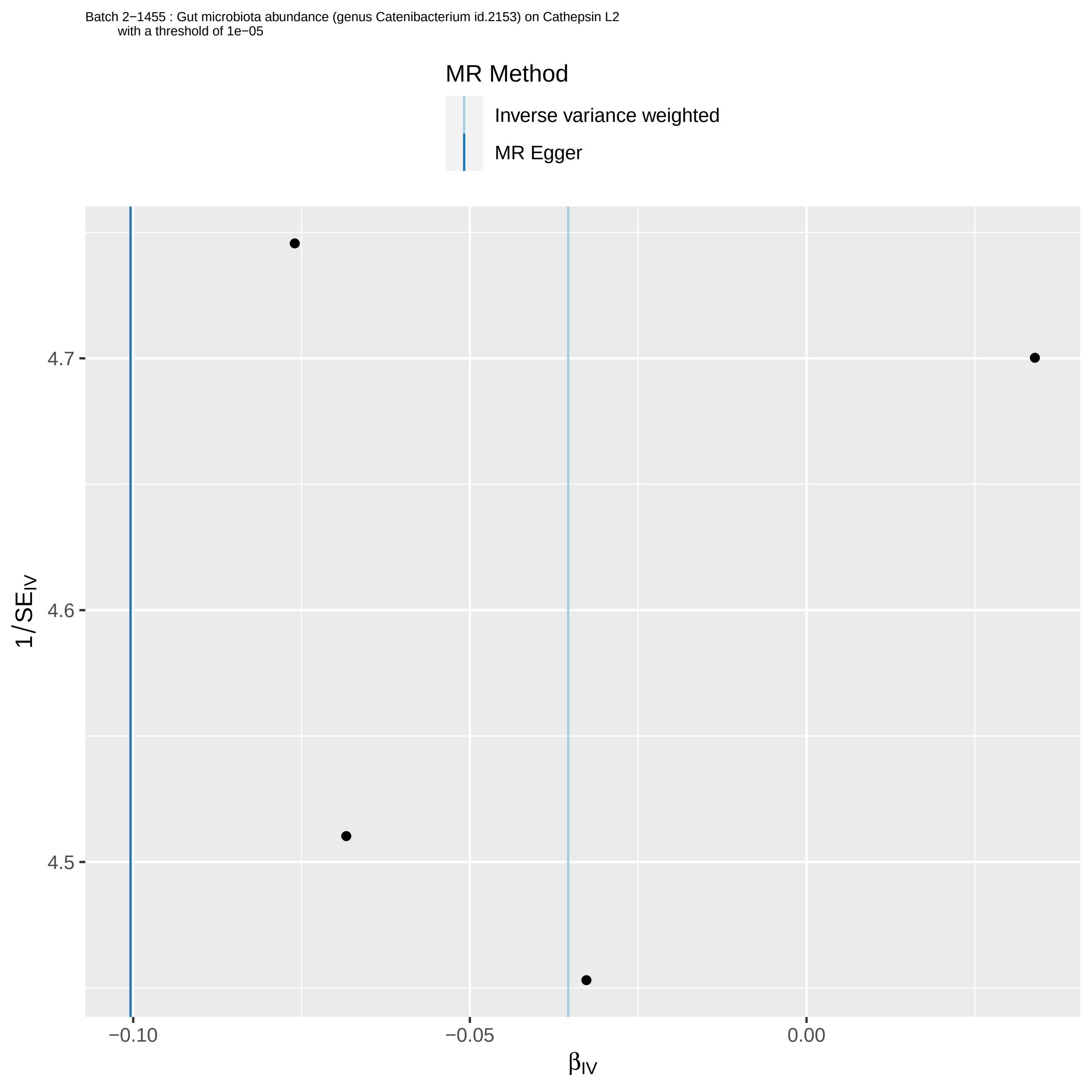
Batch 2−1455 : Gut microbiota abundance (genus Catenibacterium id.2153) on Cathepsin L2
with a threshold of 1e−05
MR Method
Inverse variance weighted
MR Egger
4.7
4.6
4.5
1/SE$_{IV}$
−0.10
−0.05
0.00
β$_{IV}$

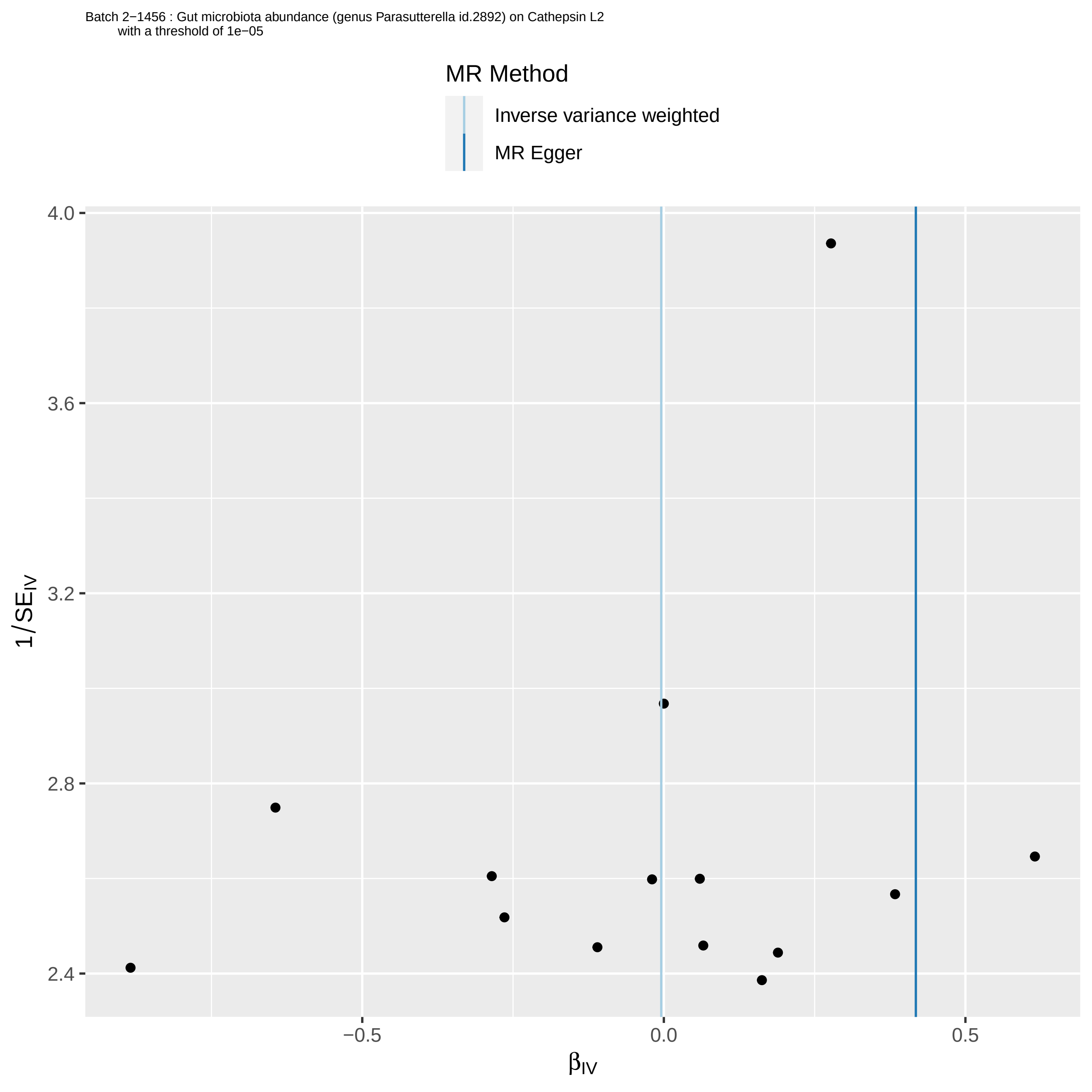
Batch 2−1456 : Gut microbiota abundance (genus Parasutterella id.2892) on Cathepsin L2
with a threshold of 1e−05
MR Method
Inverse variance weighted
MR Egger
4.0
3.6
3.2
2.8
2.4
1/SE_IV
−0.5
0.0
0.5
β_IV

Batch 2−1457 : Gut microbiota abundance (genus Catenibacterium id.2153) on Cathepsin Z
with a threshold of 1e−05

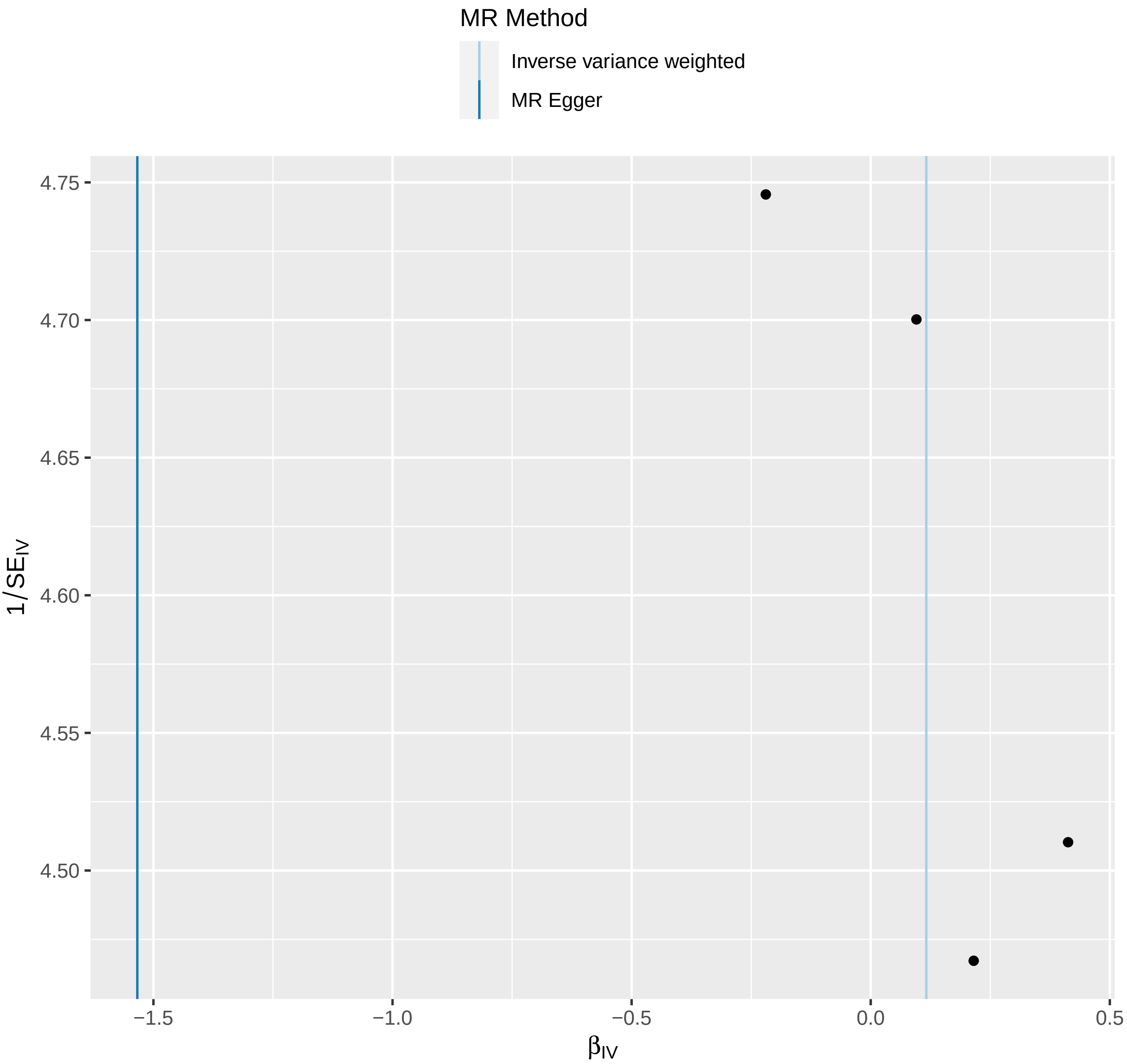

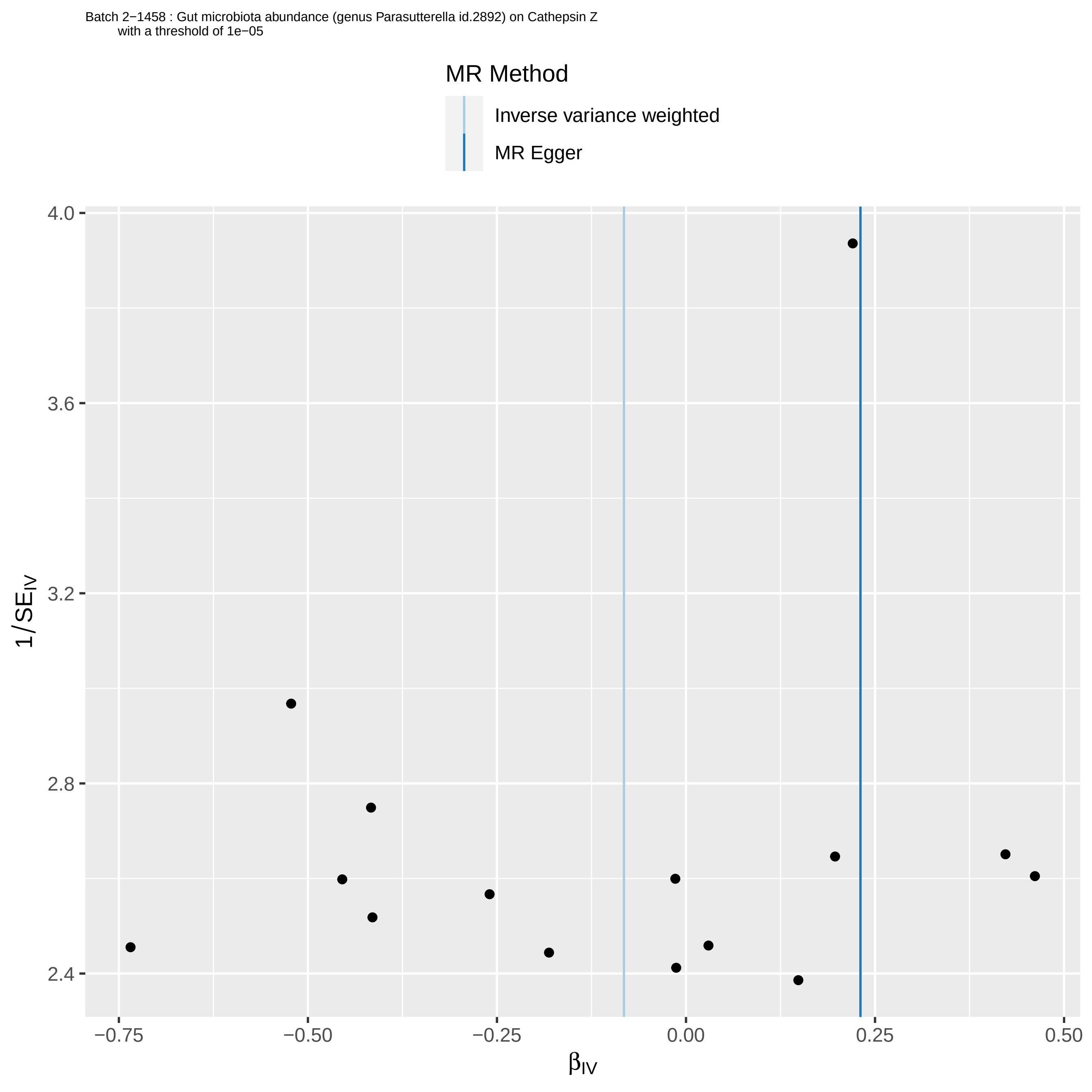
Batch 2−1458 : Gut microbiota abundance (genus Parasutterella id.2892) on Cathepsin Z
with a threshold of 1e−05
MR Method
Inverse variance weighted
MR Egger
1/SE_IV
4.0
3.6
3.2
2.8
2.4
−0.75
−0.50
−0.25
0.00
0.25
0.50
β_IV

Batch 2−1459 : Gut microbiota abundance (genus Catenibacterium id.2153) on Cortexin−3
with a threshold of 1e−05

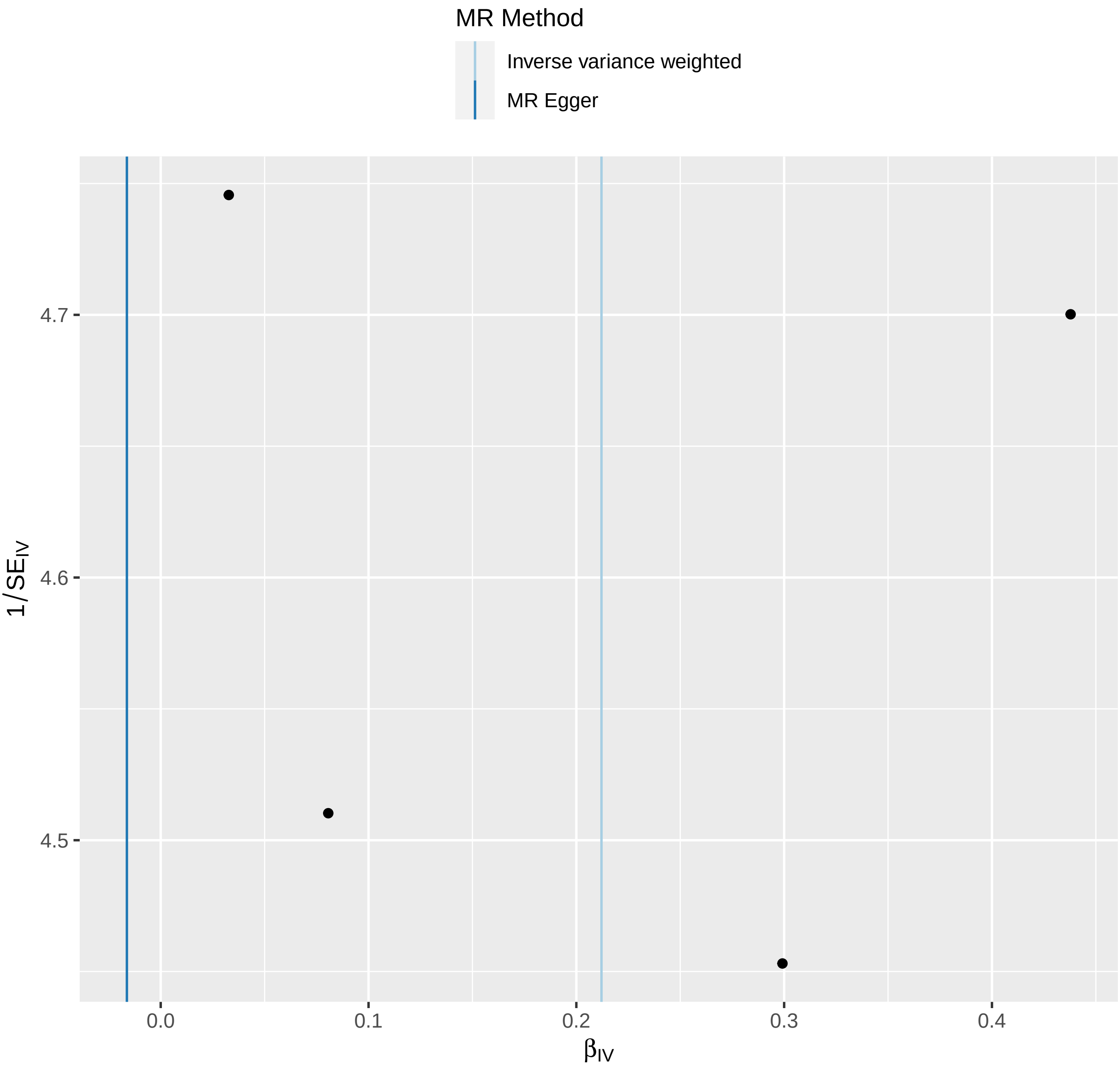

Batch 2−1460 : Gut microbiota abundance (genus Parasutterella id.2892) on Cortexin−3
with a threshold of 1e−05

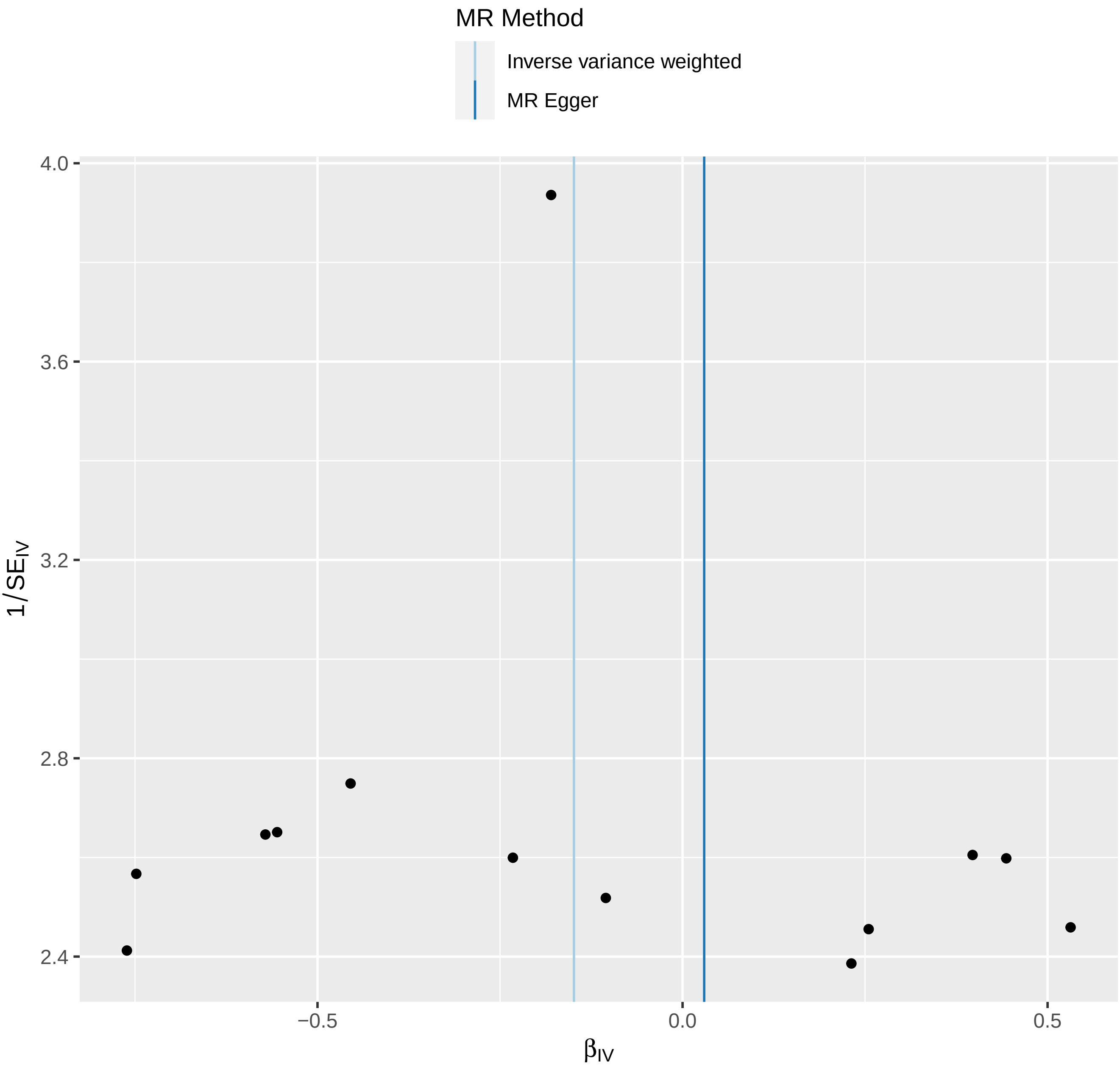

Batch 2−1461 : Gut microbiota abundance (genus Catenibacterium id.2153) on cubilin
with a threshold of 1e−05

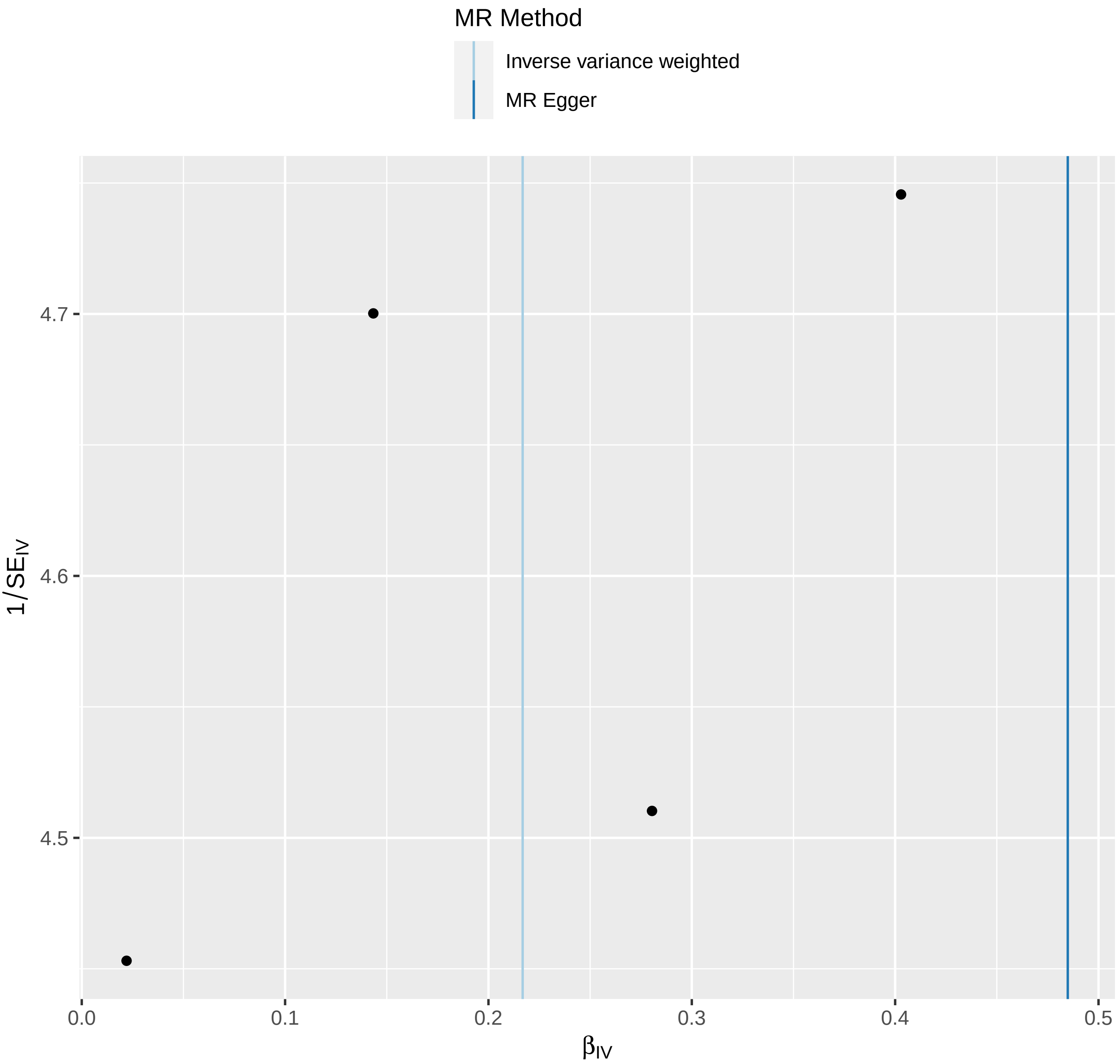

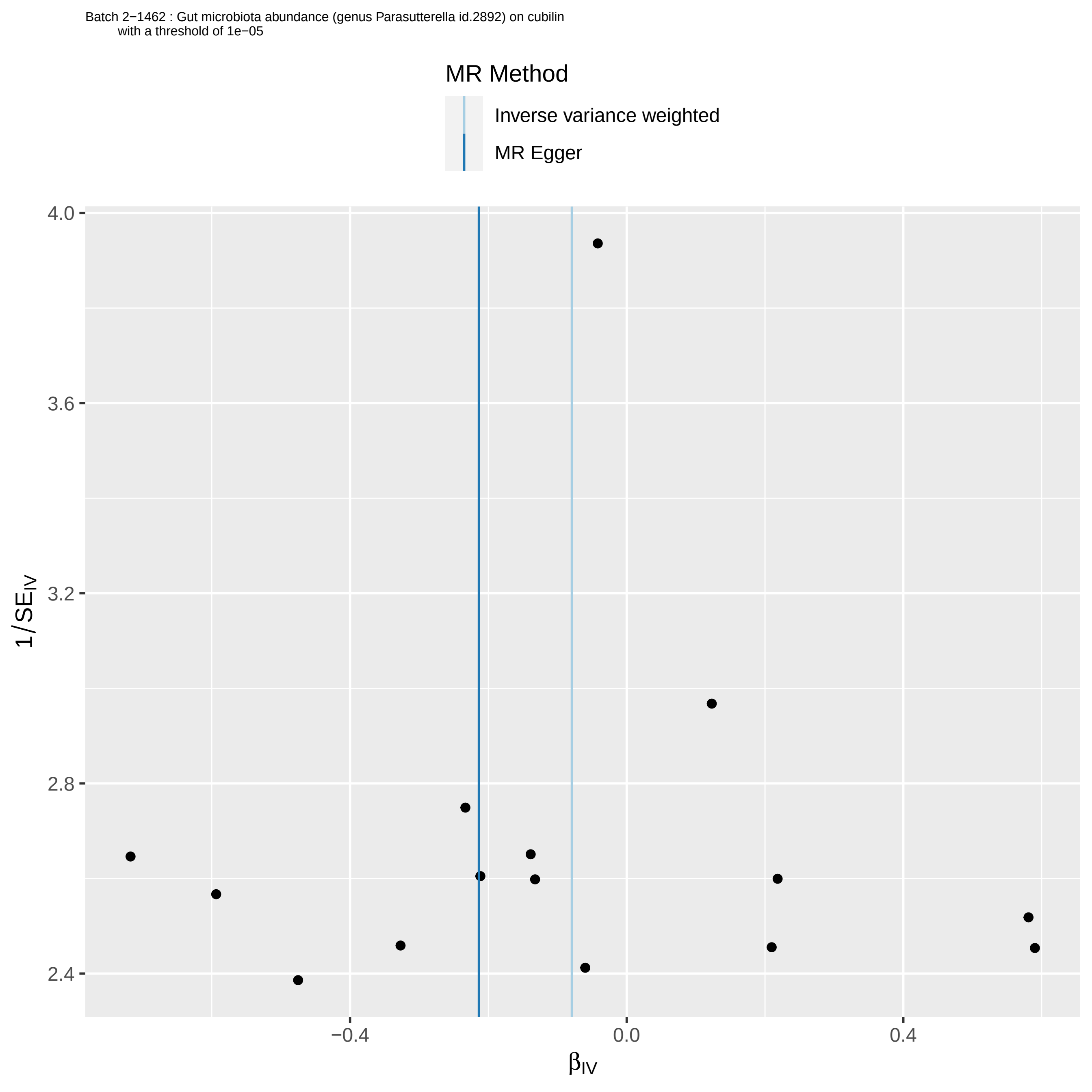
Batch 2−1462 : Gut microbiota abundance (genus Parasutterella id.2892) on cubilin
with a threshold of 1e−05
MR Method
Inverse variance weighted
MR Egger
4.0
3.6
3.2
2.8
2.4
1/SE$_{IV}$
−0.4
0.0
0.4
β$_{IV}$

Batch 2−1463 : Gut microbiota abundance (genus Catenibacterium id.2153) on Cullin−3
with a threshold of 1e−05

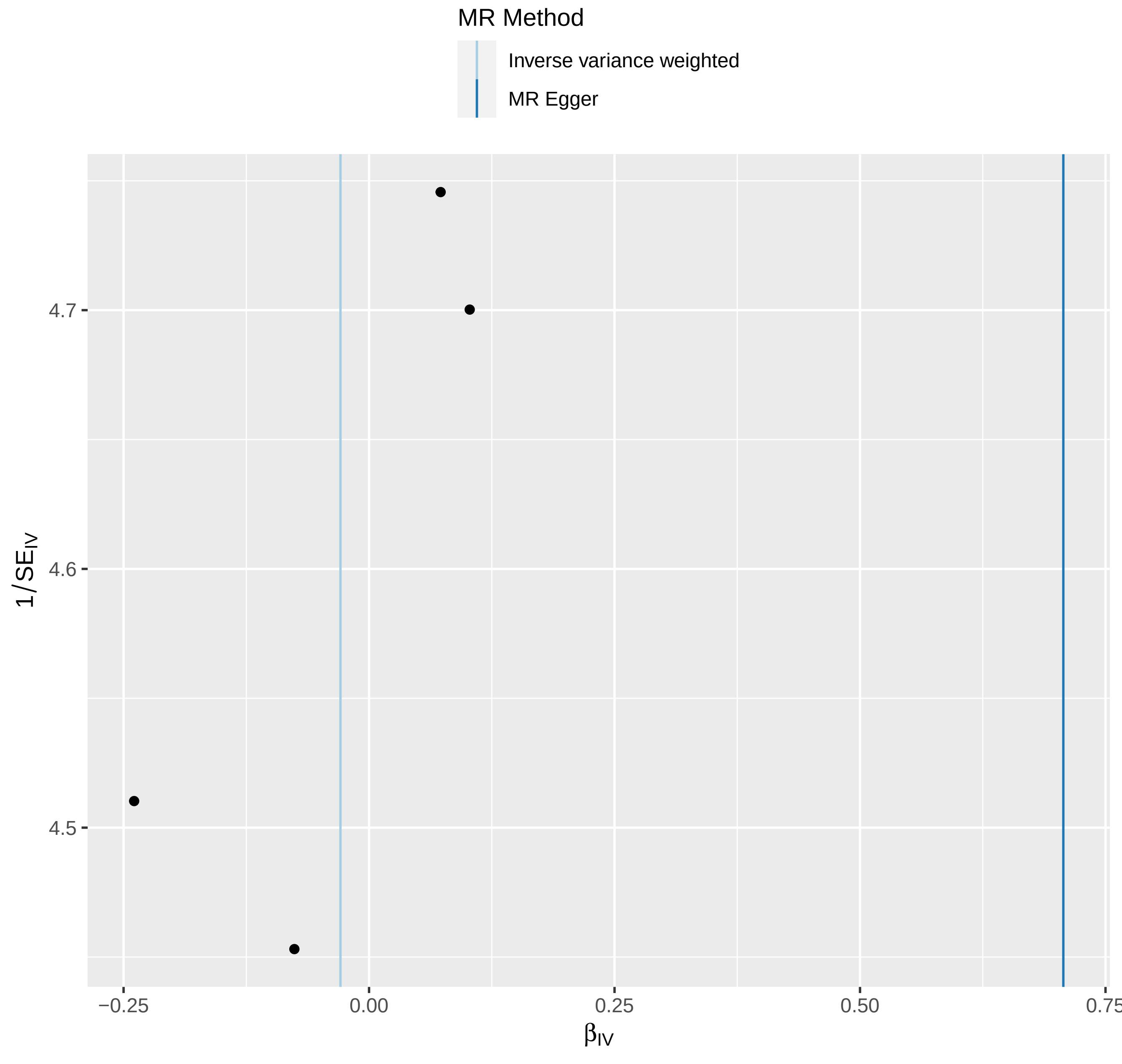

Batch 2−1464 : Gut microbiota abundance (genus Parasutterella id.2892) on Cullin−3
with a threshold of 1e−05

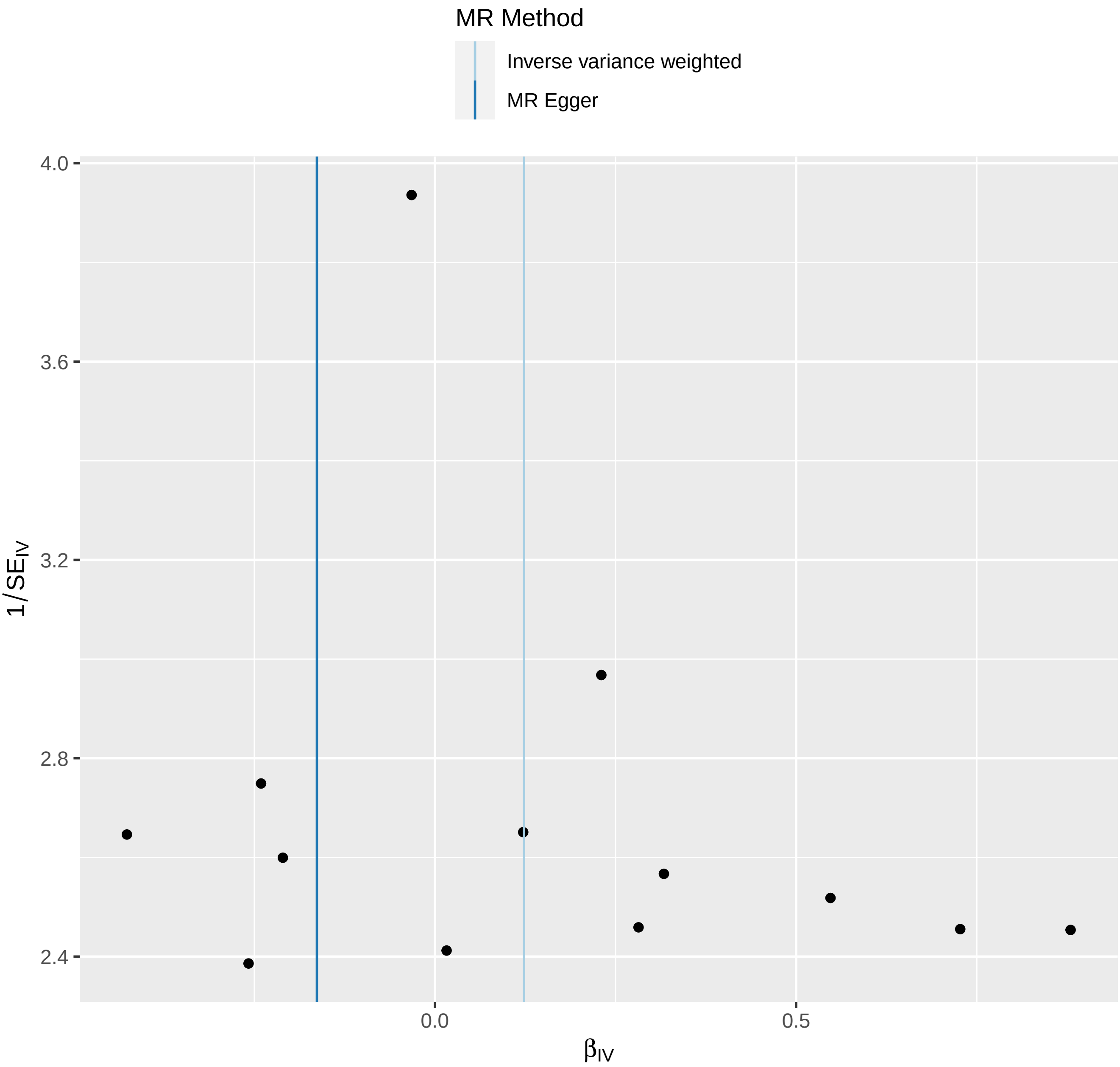

Batch 2−1465 : Gut microbiota abundance (genus Catenibacterium id.2153) on Cullin−4B
with a threshold of 1e−05

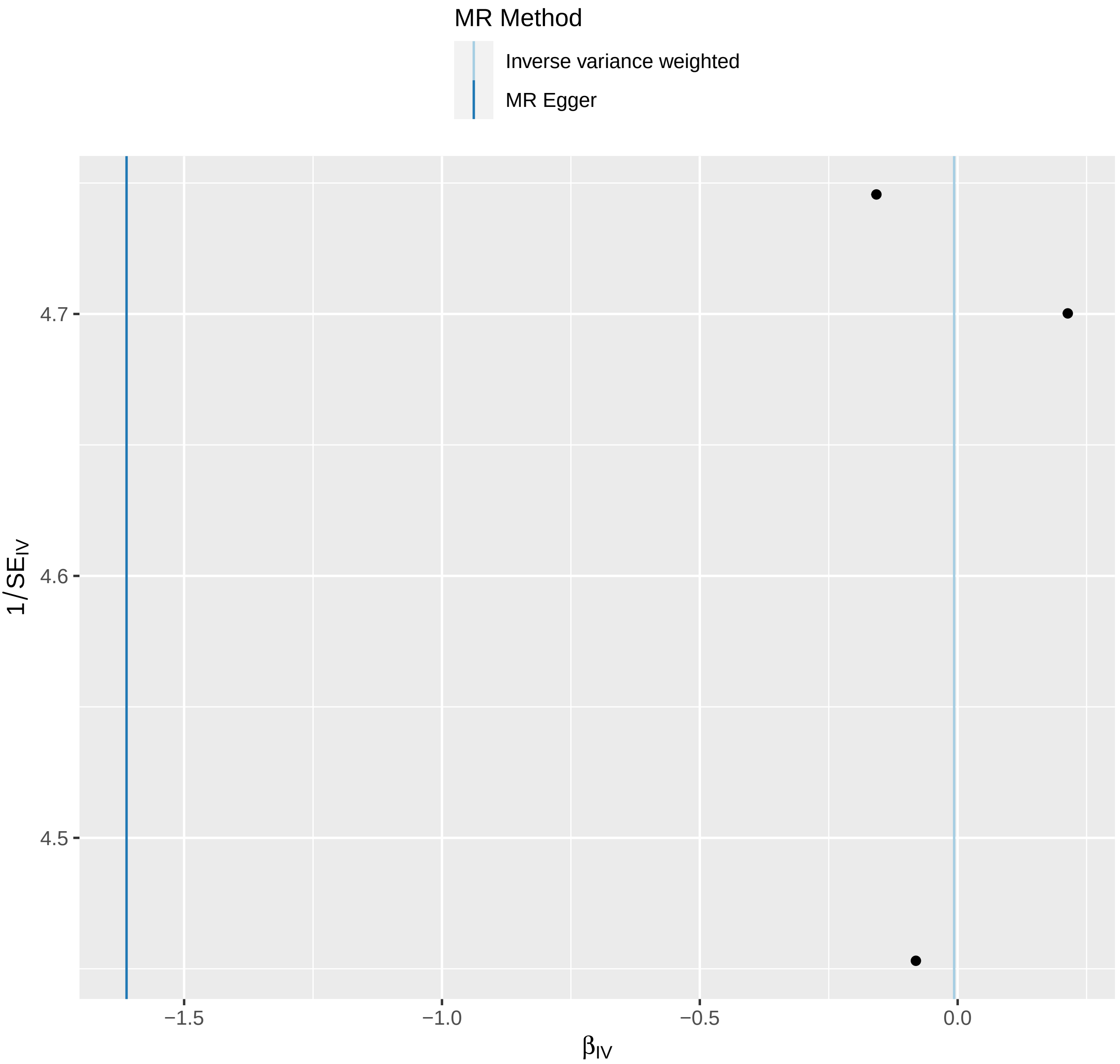

Batch 2−1466 : Gut microbiota abundance (genus Parasutterella id.2892) on Cullin−4B
with a threshold of 1e−05

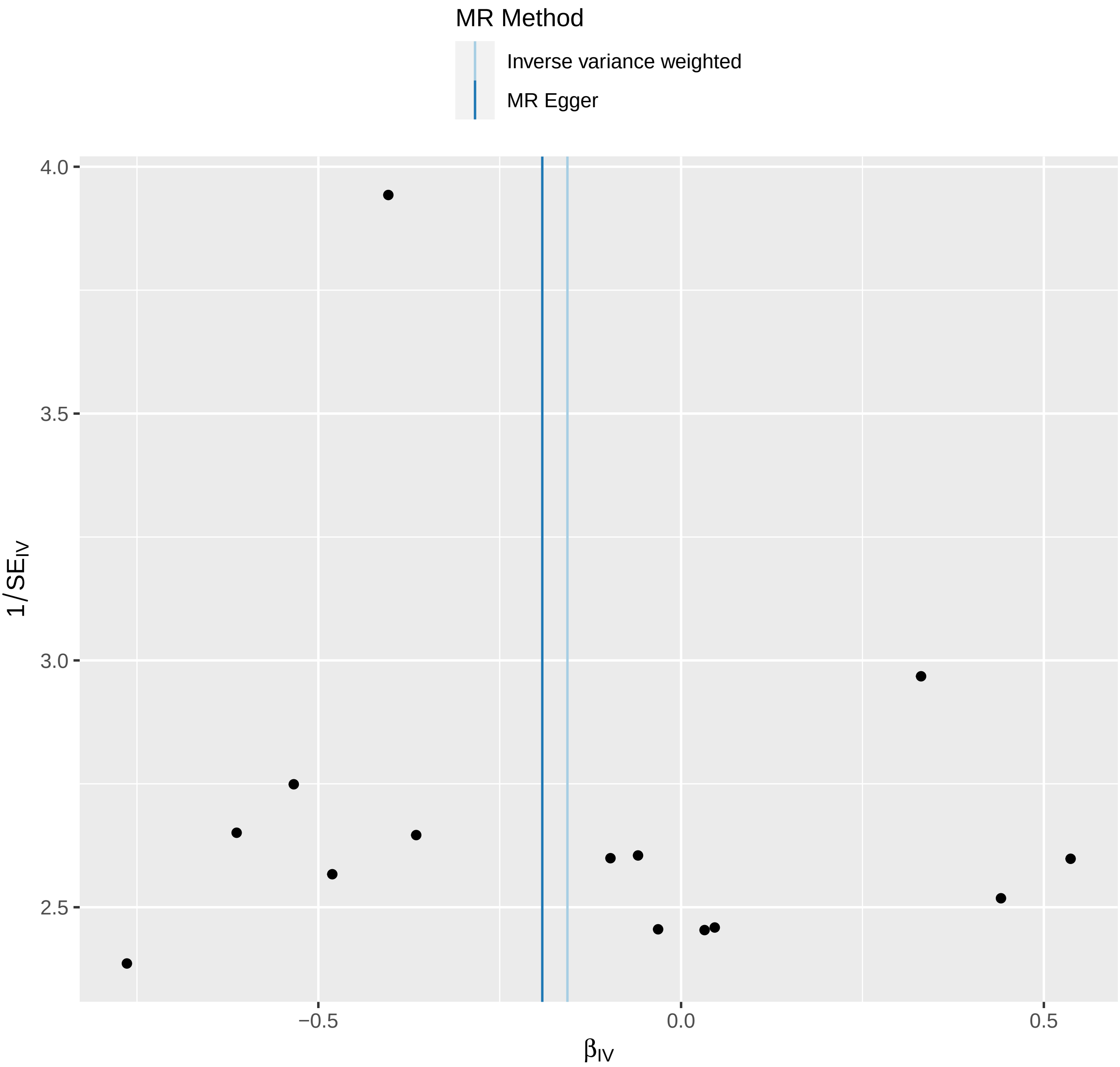

Batch 2−1467 : Gut microbiota abundance (genus Catenibacterium id.2153) on Cullin−9
with a threshold of 1e−05

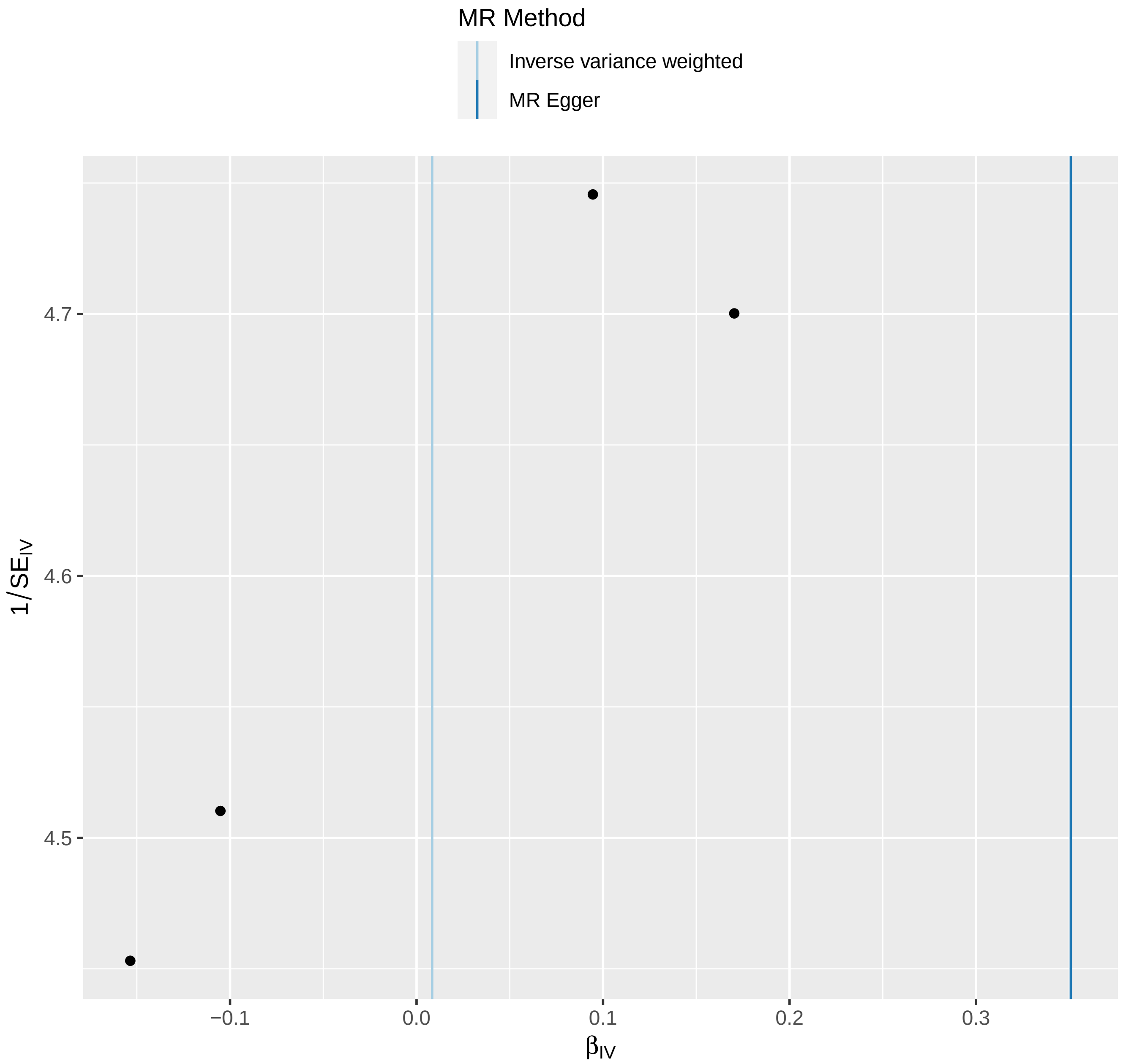

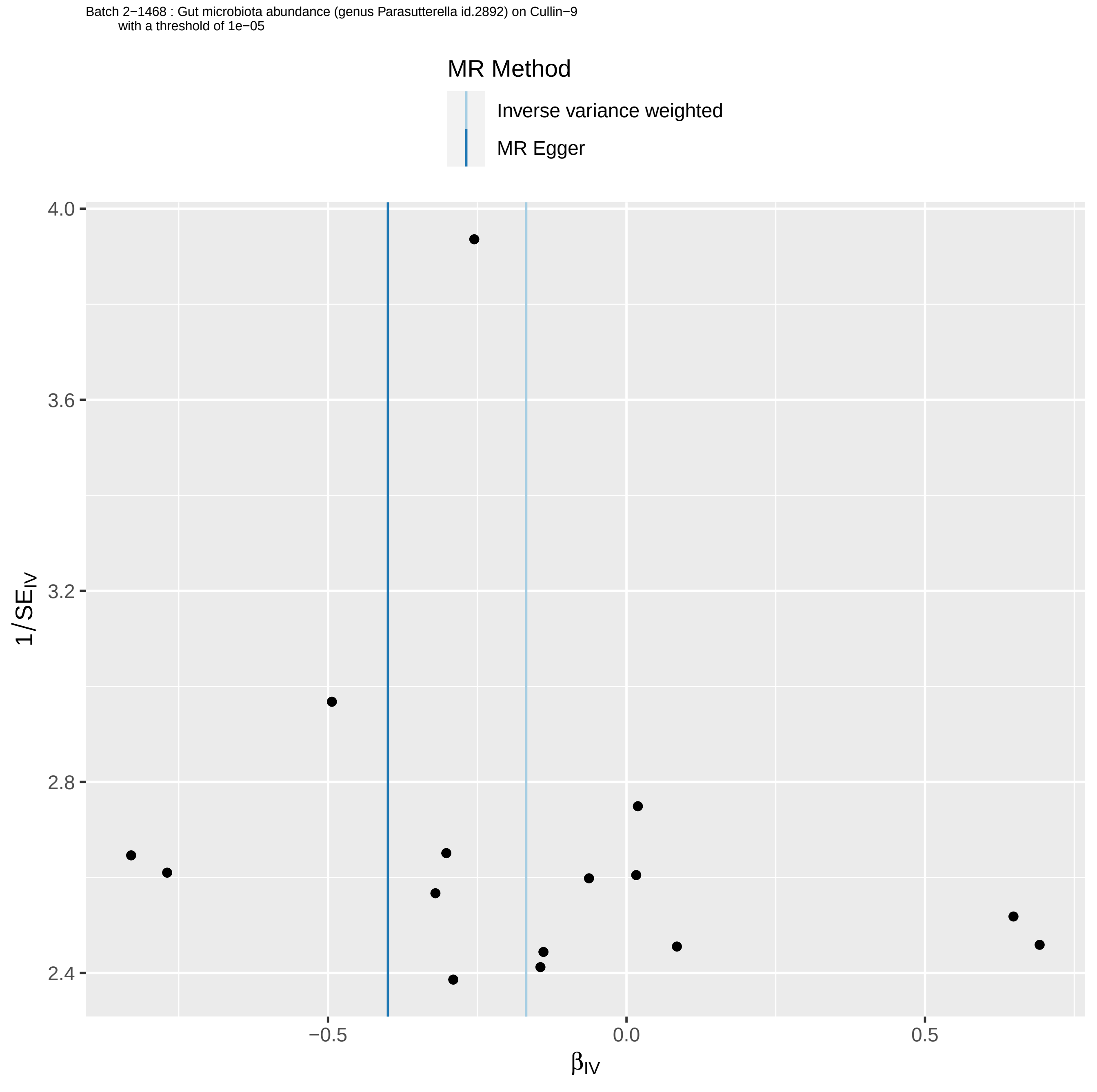

Batch 2−1468 : Gut microbiota abundance (genus Parasutterella id.2892) on Cullin−9
with a threshold of 1e−05
MR Method
Inverse variance weighted
MR Egger
4.0
3.6
3.2
2.8
2.4
1/SE$_{IV}$
−0.5
0.0
0.5
β$_{IV}$

Batch 2−1469 : Gut microbiota abundance (genus Catenibacterium id.2153) on CUB and zona pellucida−like domain−containing protein 1 with a threshold of 1e−05

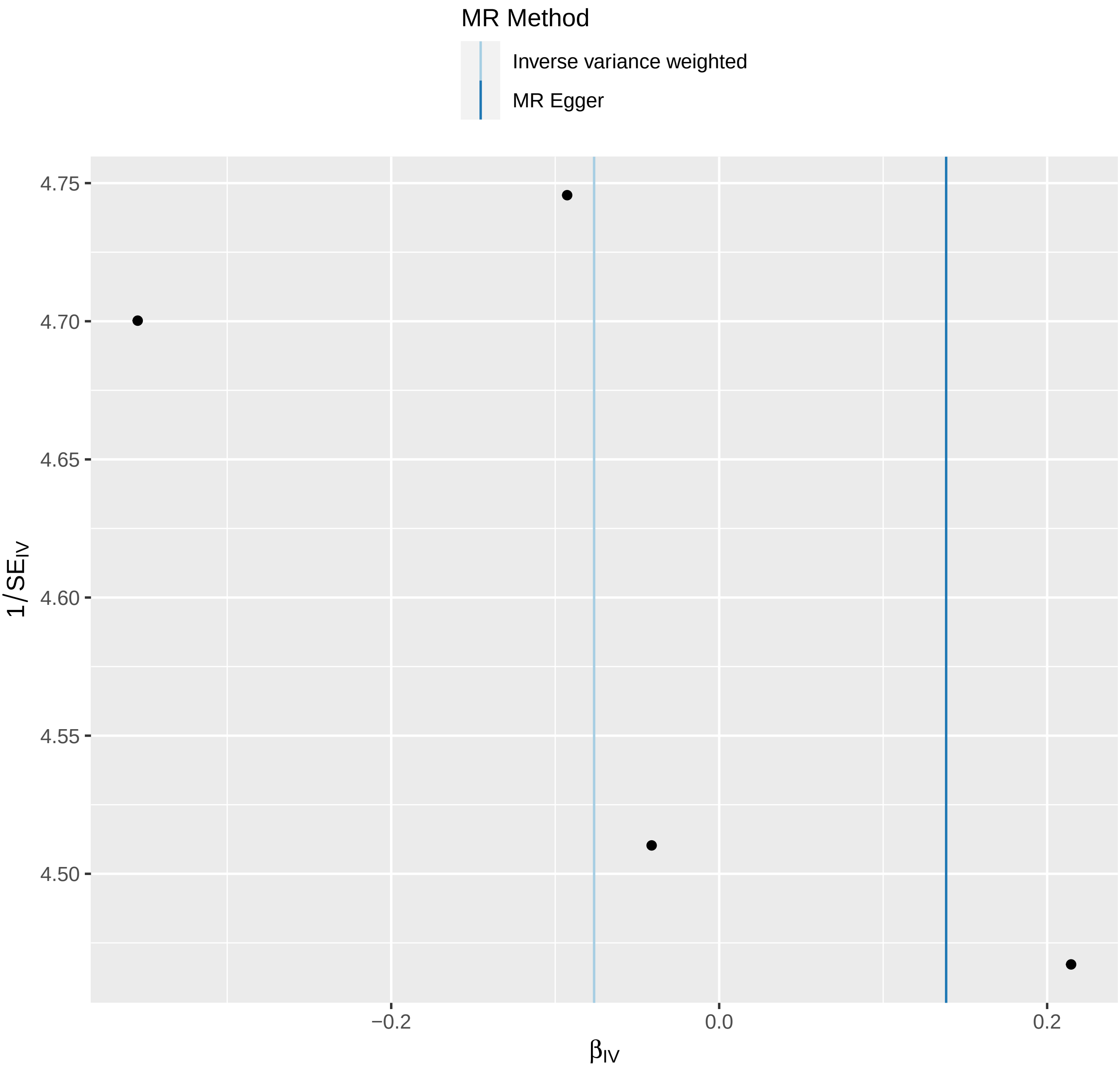

Batch 2−1470 : Gut microbiota abundance (genus Parasutterella id.2892) on CUB and zona pellucida−like domain−containing protein 1
with a threshold of 1e−05

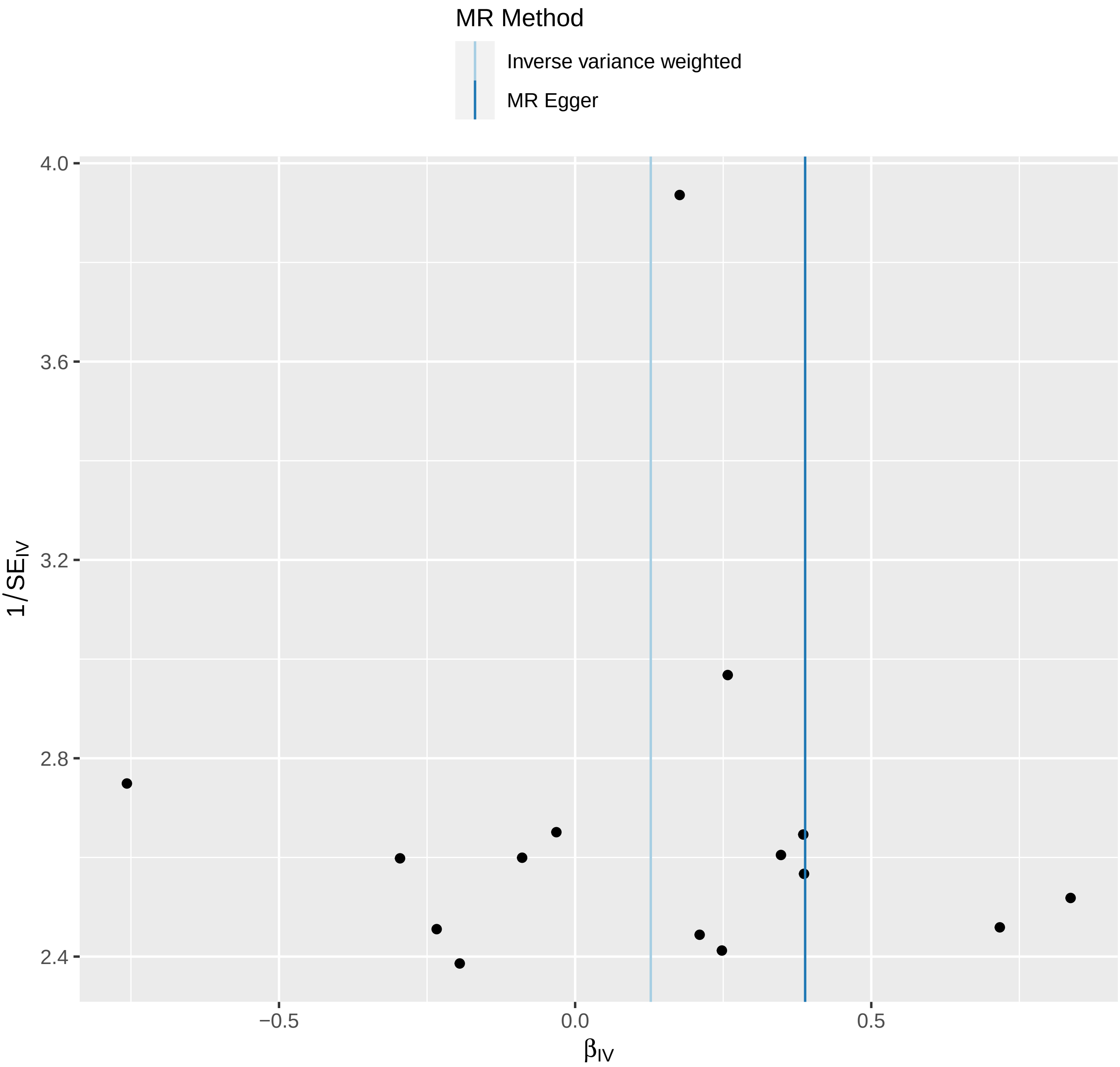

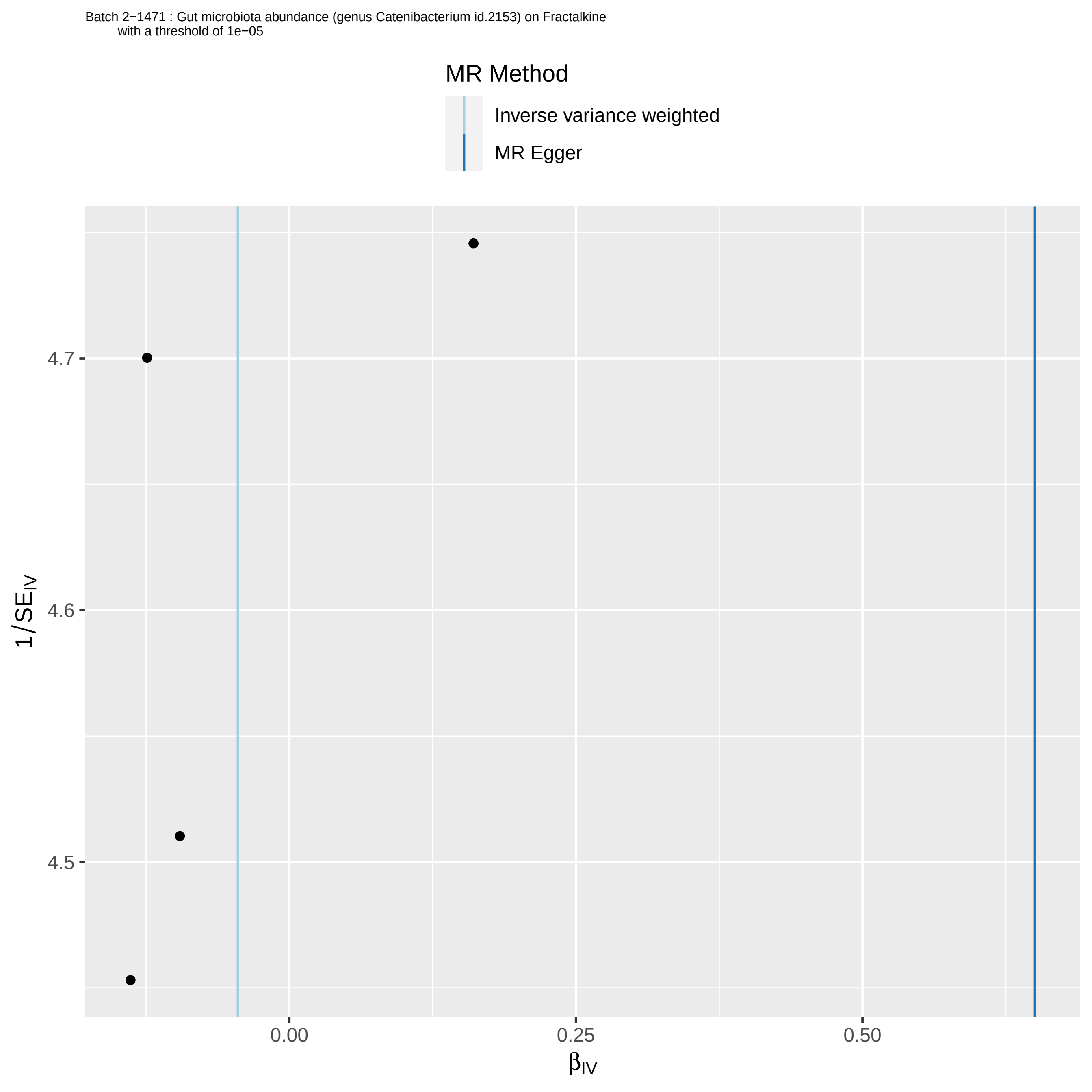
Batch 2−1471 : Gut microbiota abundance (genus Catenibacterium id.2153) on Fractalkine
with a threshold of 1e−05
MR Method
Inverse variance weighted
MR Egger
4.7
4.6
4.5
$1/SE_{IV}$
0.00
0.25
0.50
β_{IV}

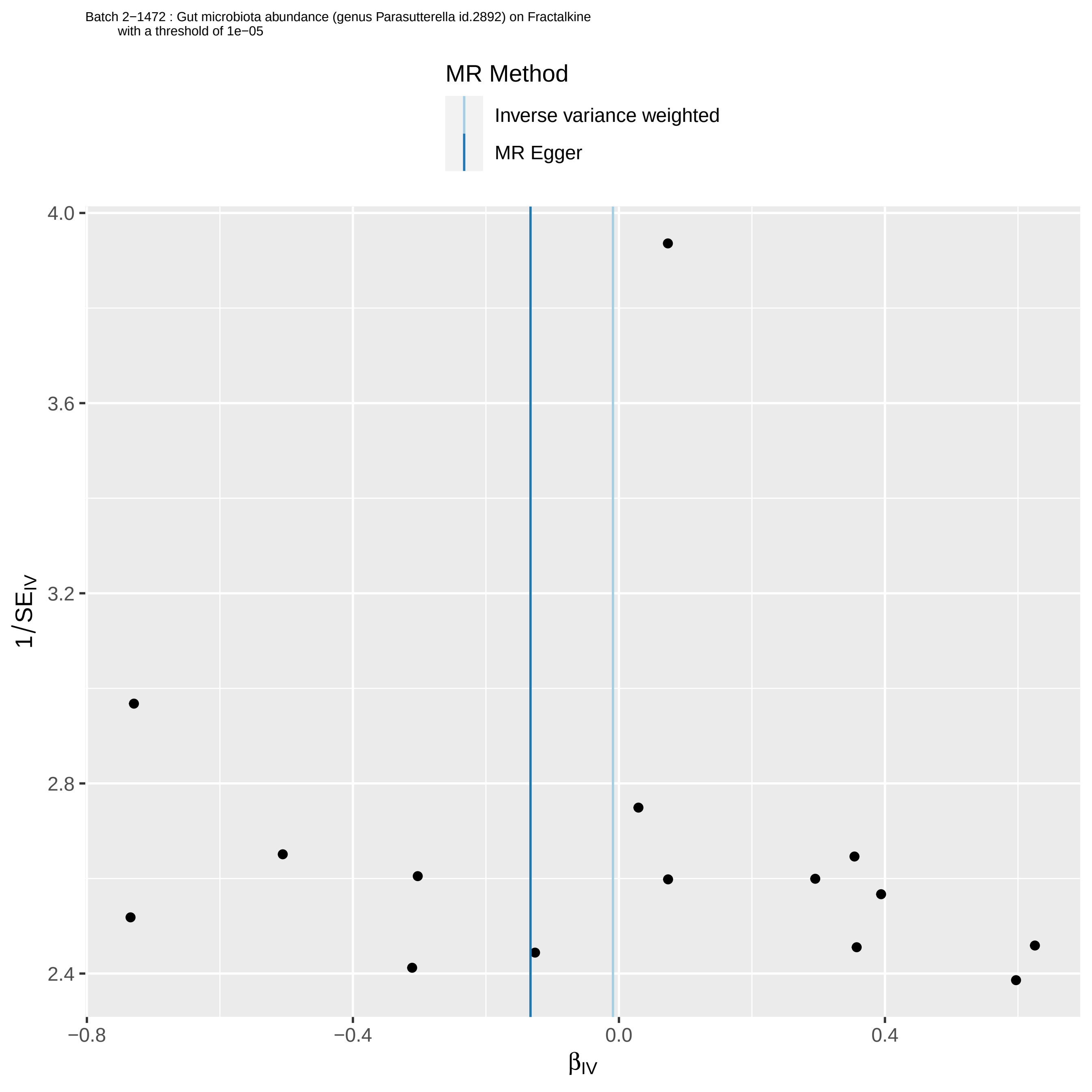
Batch 2−1472 : Gut microbiota abundance (genus Parasutterella id.2892) on Fractalkine
with a threshold of 1e−05
MR Method
Inverse variance weighted
MR Egger
4.0
3.6
3.2
2.8
2.4
1/SE_IV
−0.8
−0.4
0.0
0.4
β_IV

Batch 2−1473 : Gut microbiota abundance (genus Catenibacterium id.2153) on Coxsackievirus and adenovirus receptor
with a threshold of 1e−05

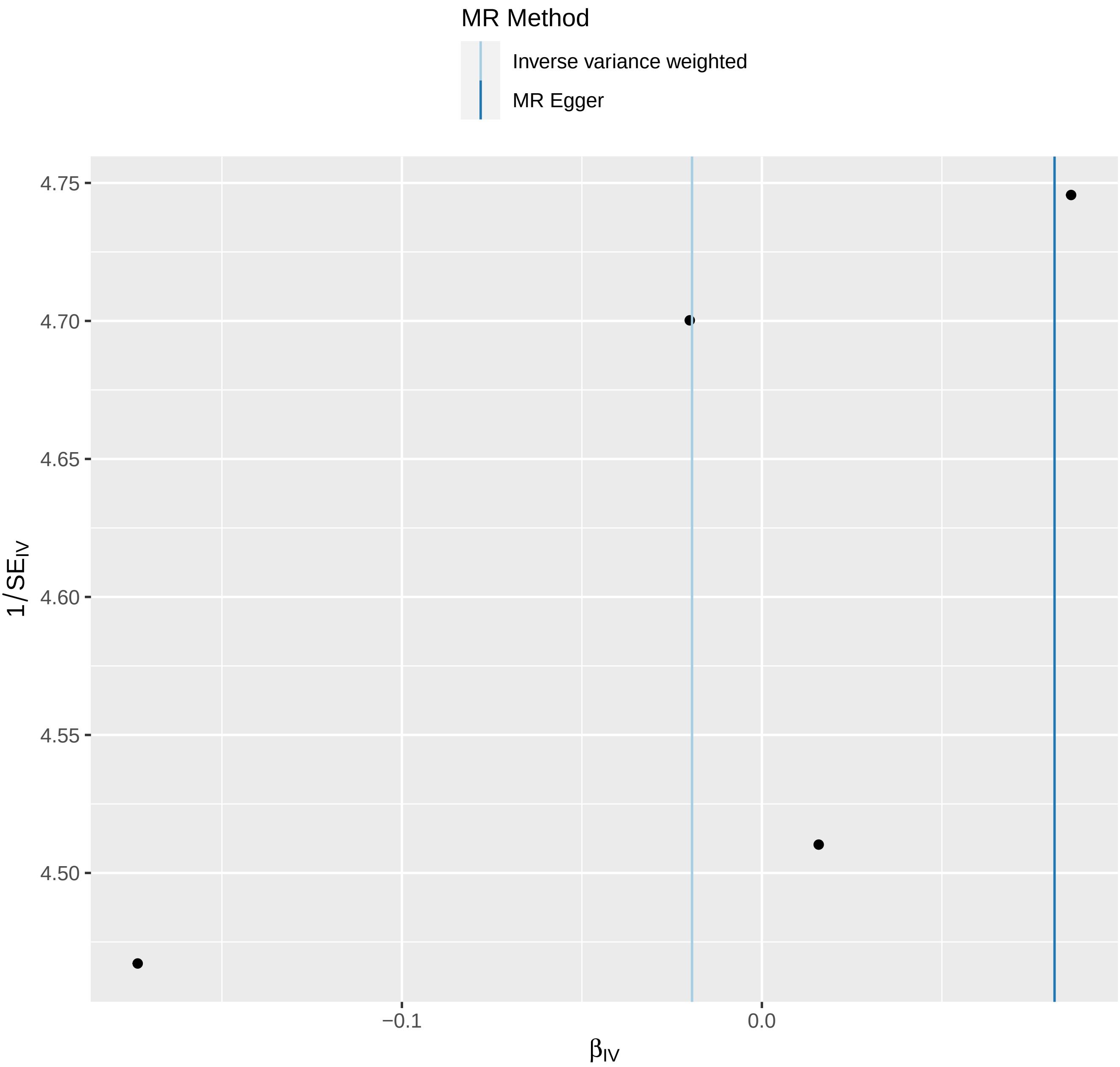

Batch 2−1474 : Gut microbiota abundance (genus Parasutterella id.2892) on Coxsackievirus and adenovirus receptor with a threshold of 1e−05

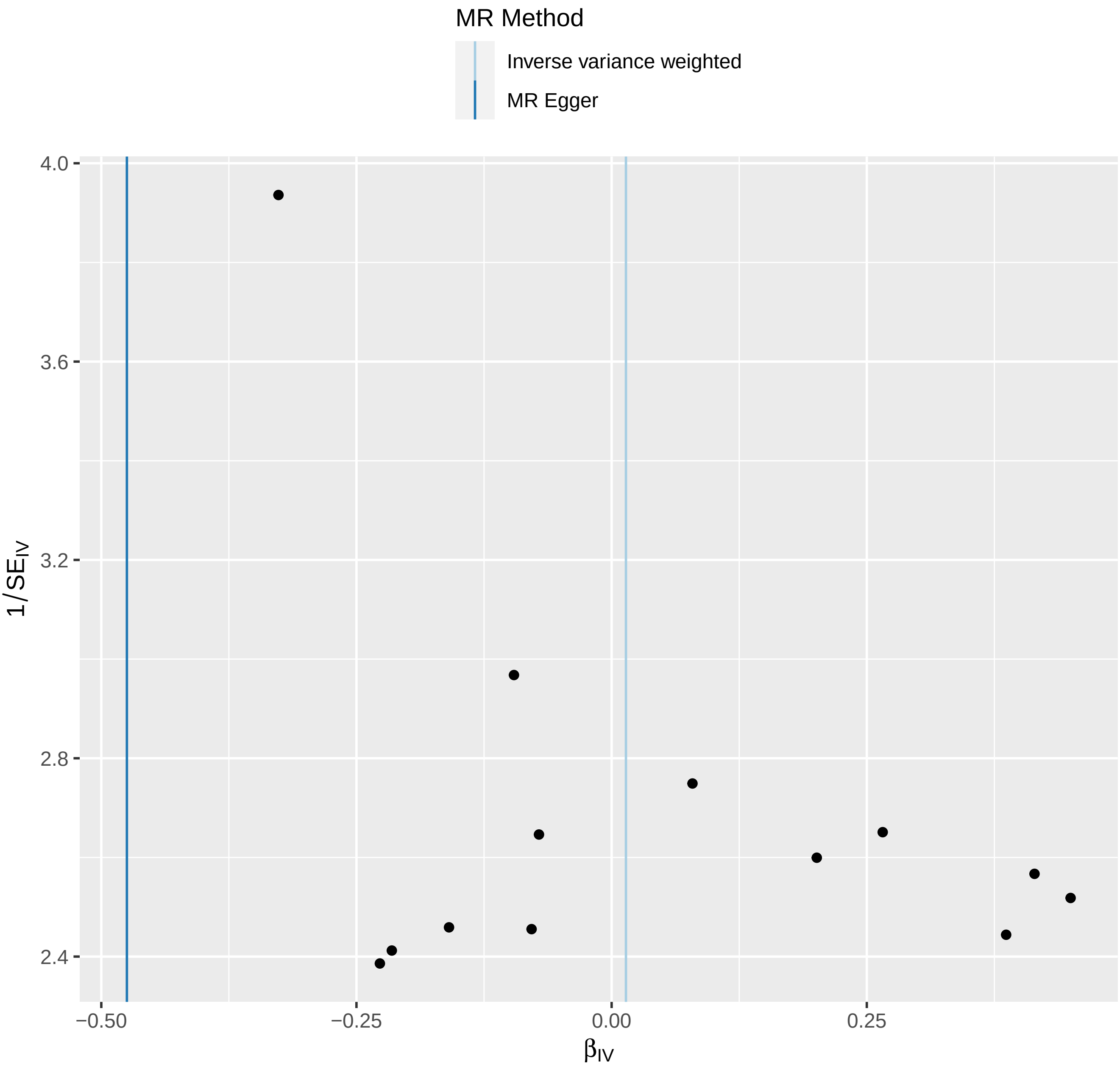

Batch 2−1475 : Gut microbiota abundance (genus Catenibacterium id.2153) on C−X−C motif chemokine 10
with a threshold of 1e−05

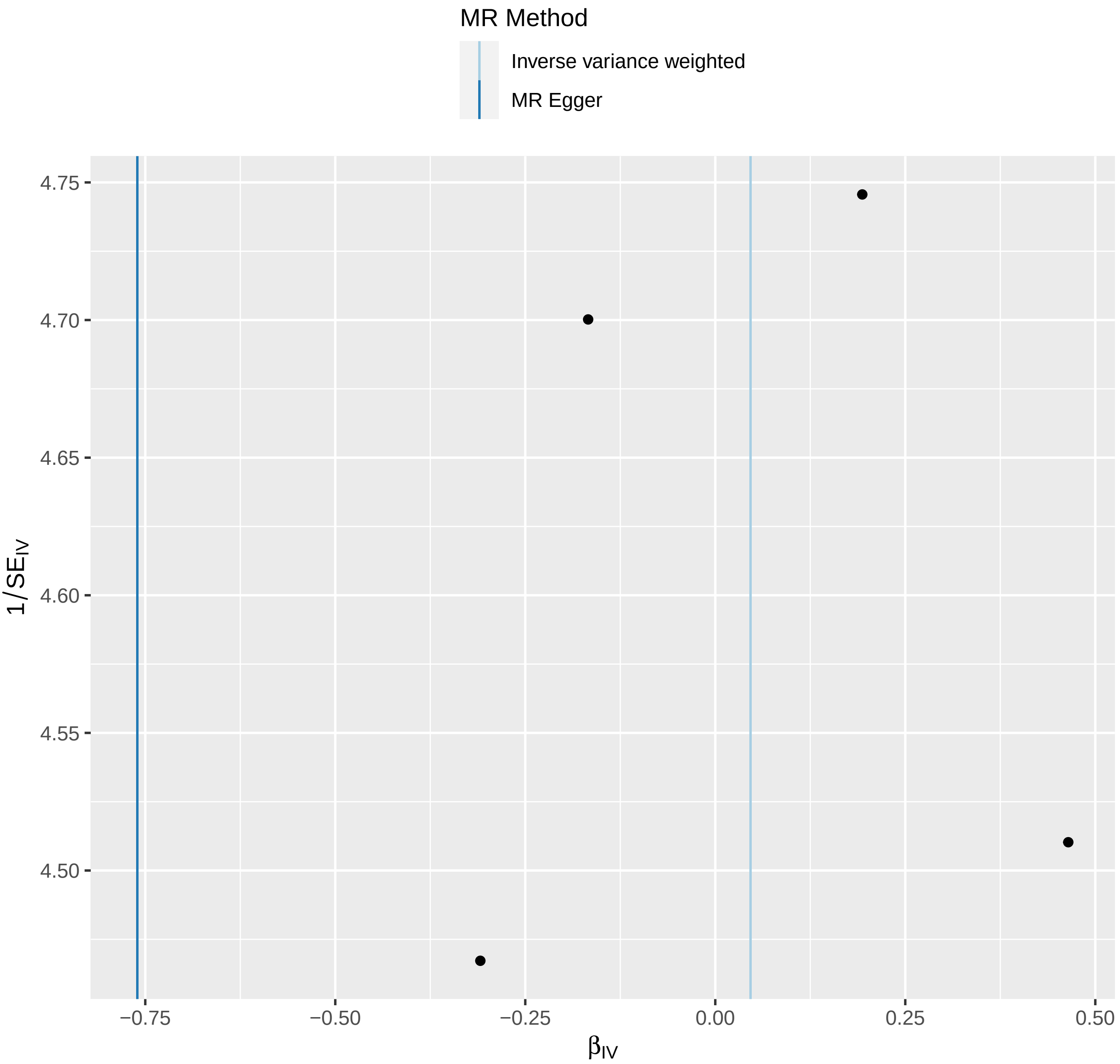

Batch 2−1476 : Gut microbiota abundance (genus Parasutterella id.2892) on C−X−C motif chemokine 10
with a threshold of 1e−05

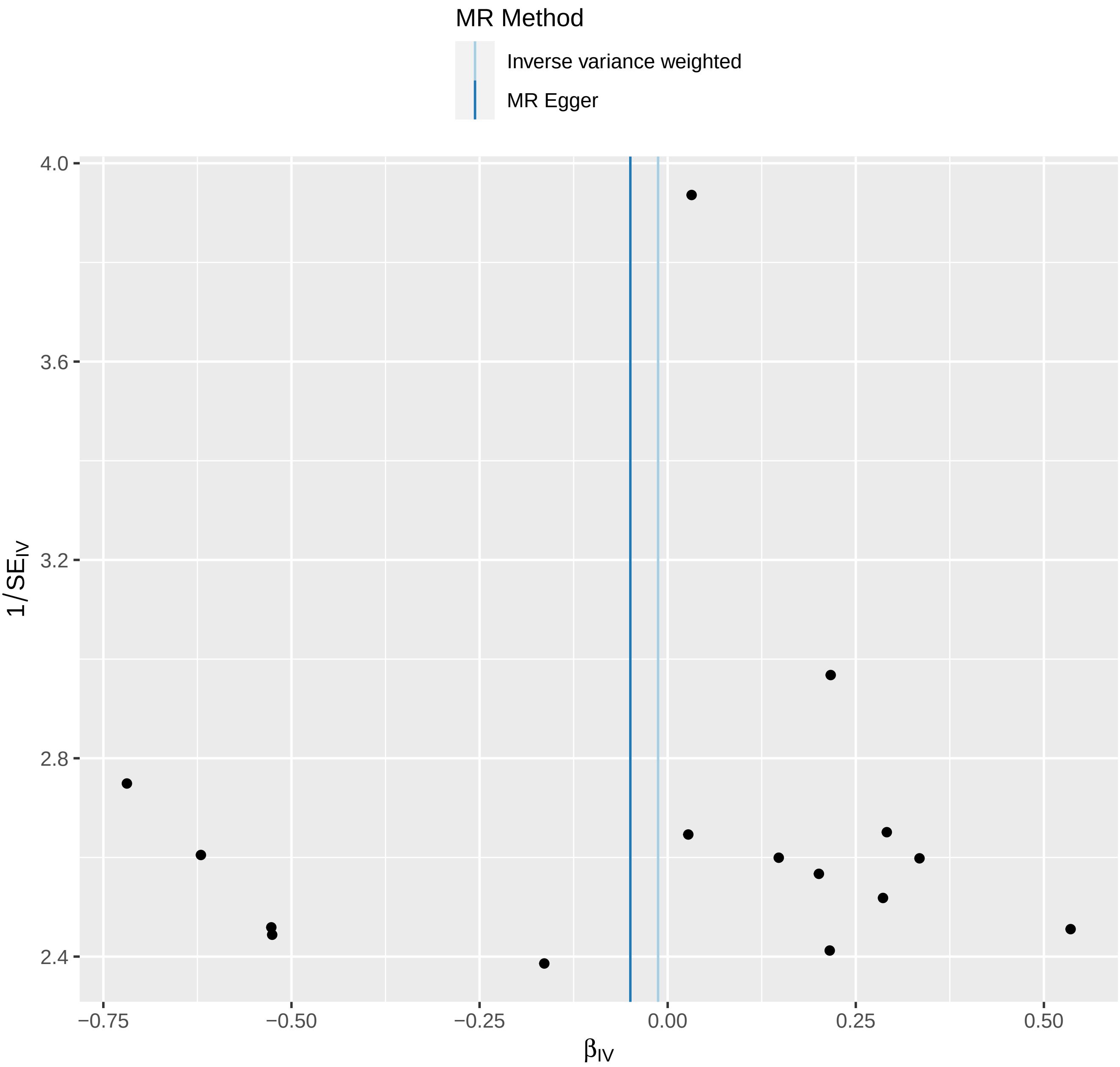

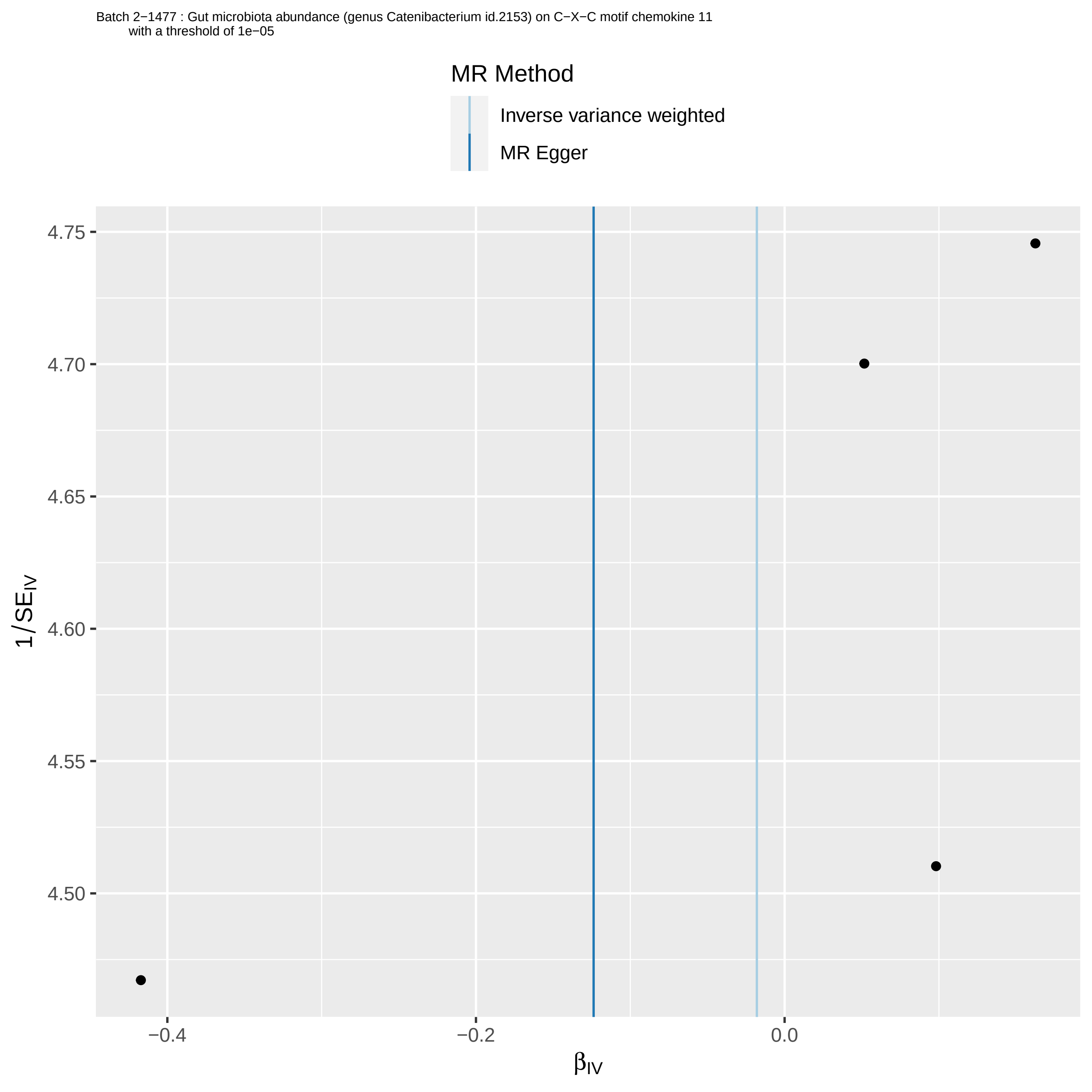
Batch 2−1477 : Gut microbiota abundance (genus Catenibacterium id.2153) on C−X−C motif chemokine 11
with a threshold of 1e−05
MR Method
Inverse variance weighted
MR Egger
4.75
4.70
4.65
4.60
4.55
4.50
1/SE$_{IV}$
−0.4
−0.2
0.0
β$_{IV}$

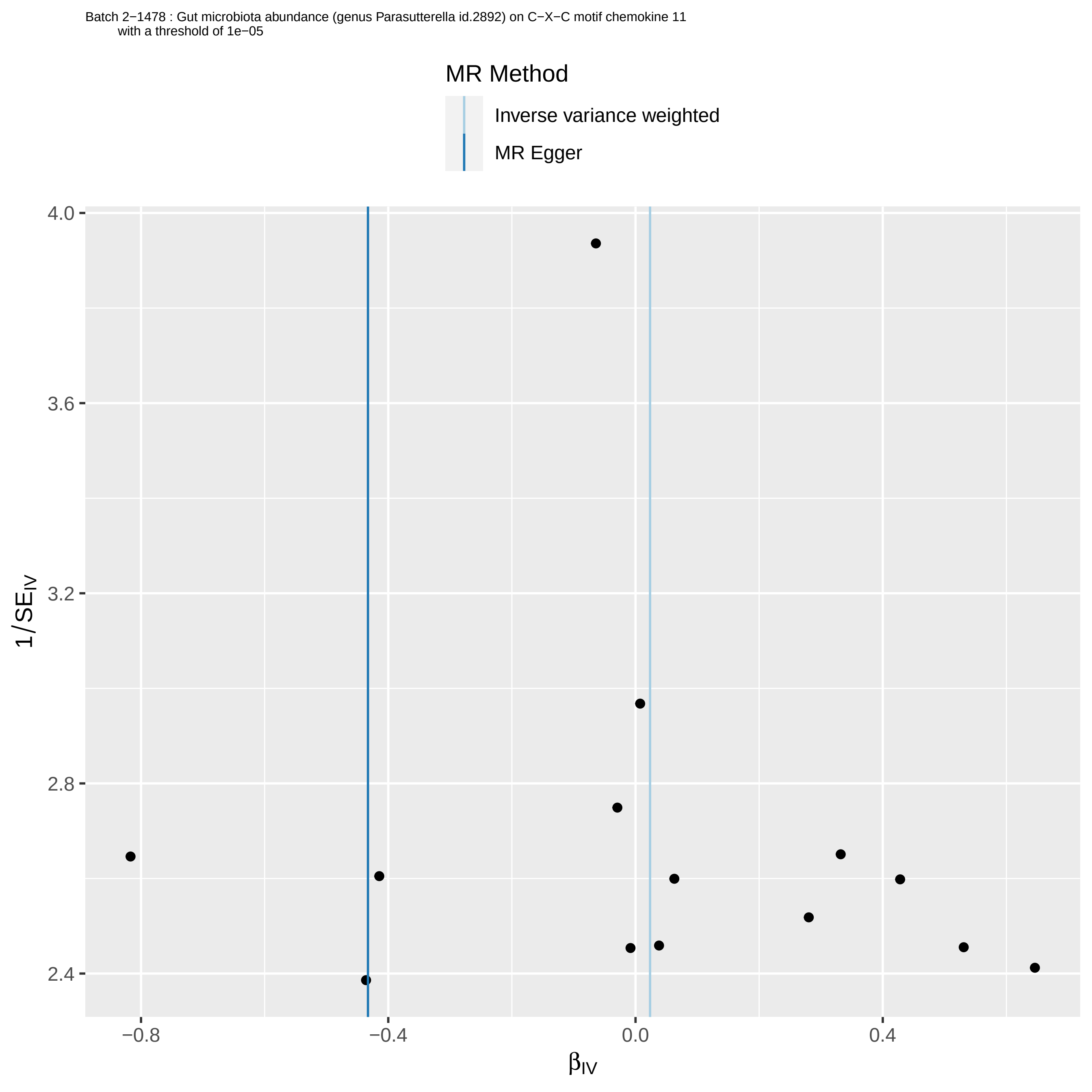

Batch 2−1478 : Gut microbiota abundance (genus Parasutterella id.2892) on C−X−C motif chemokine 11
with a threshold of 1e−05
MR Method
Inverse variance weighted
MR Egger
4.0
3.6
3.2
2.8
2.4
1/SE$_{IV}$
−0.8
−0.4
0.0
0.4
β_{IV}

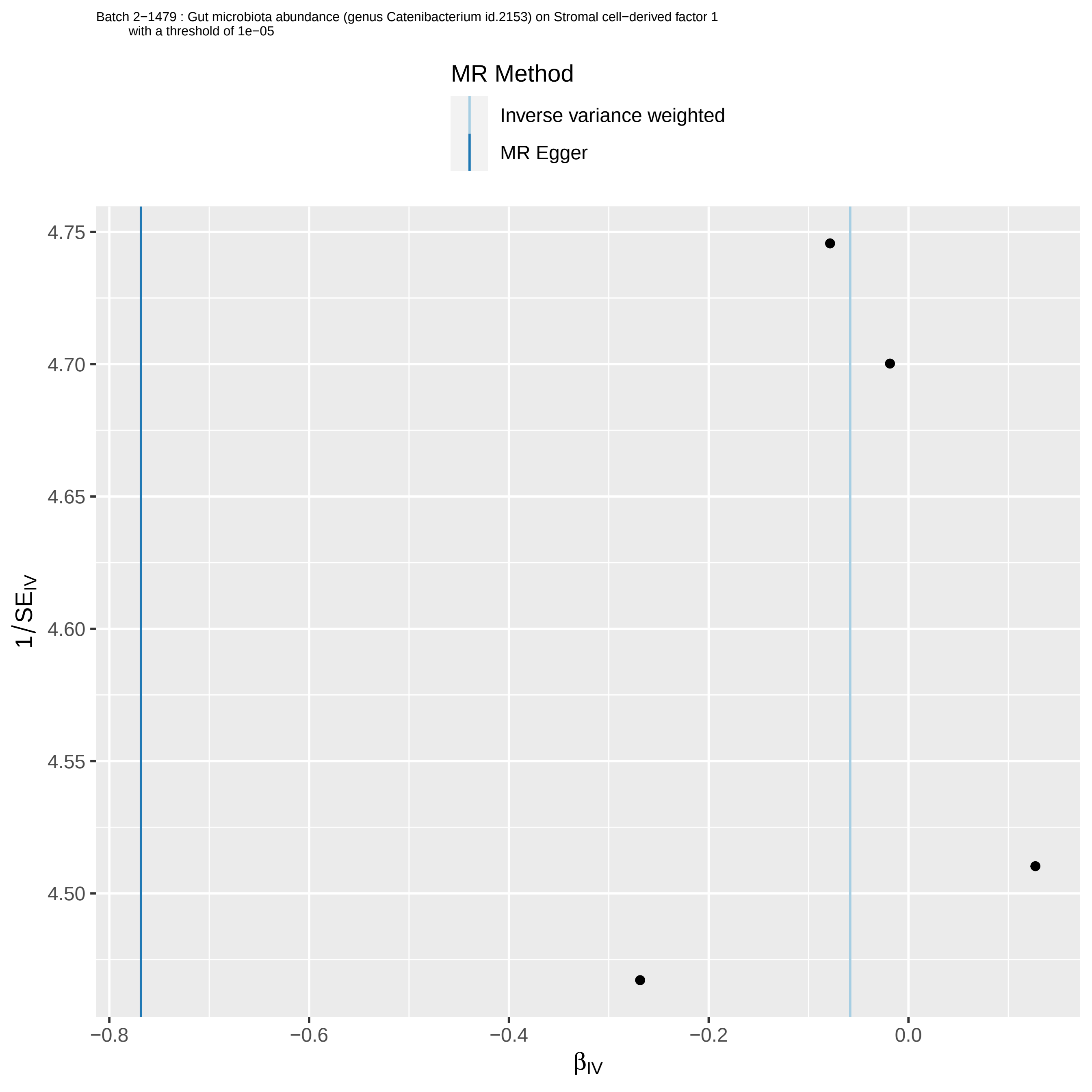
Batch 2−1479 : Gut microbiota abundance (genus Catenibacterium id.2153) on Stromal cell−derived factor 1
with a threshold of 1e−05
MR Method
Inverse variance weighted
MR Egger
4.75
4.70
4.65
4.60
4.55
4.50
1/SE_IV
−0.8
−0.6
−0.4
−0.2
0.0
β_IV

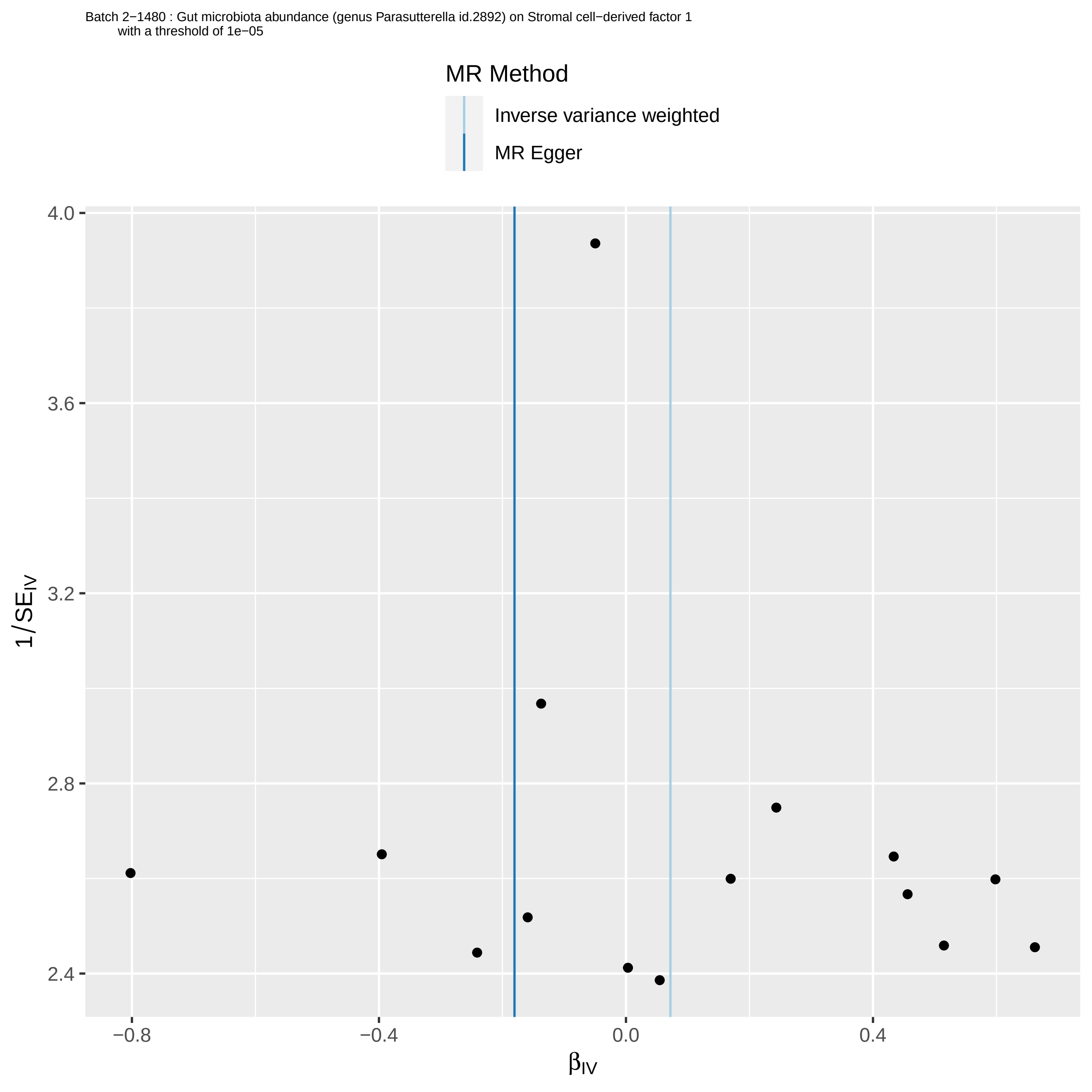
Batch 2−1480 : Gut microbiota abundance (genus Parasutterella id.2892) on Stromal cell−derived factor 1
with a threshold of 1e−05
MR Method
Inverse variance weighted
MR Egger
4.0
3.6
3.2
2.8
2.4
1/SE_IV
−0.8
−0.4
0.0
0.4
β_IV

Batch 2−1481 : Gut microbiota abundance (genus Catenibacterium id.2153) on Stromal cell−derived factor 1
with a threshold of 1e−05

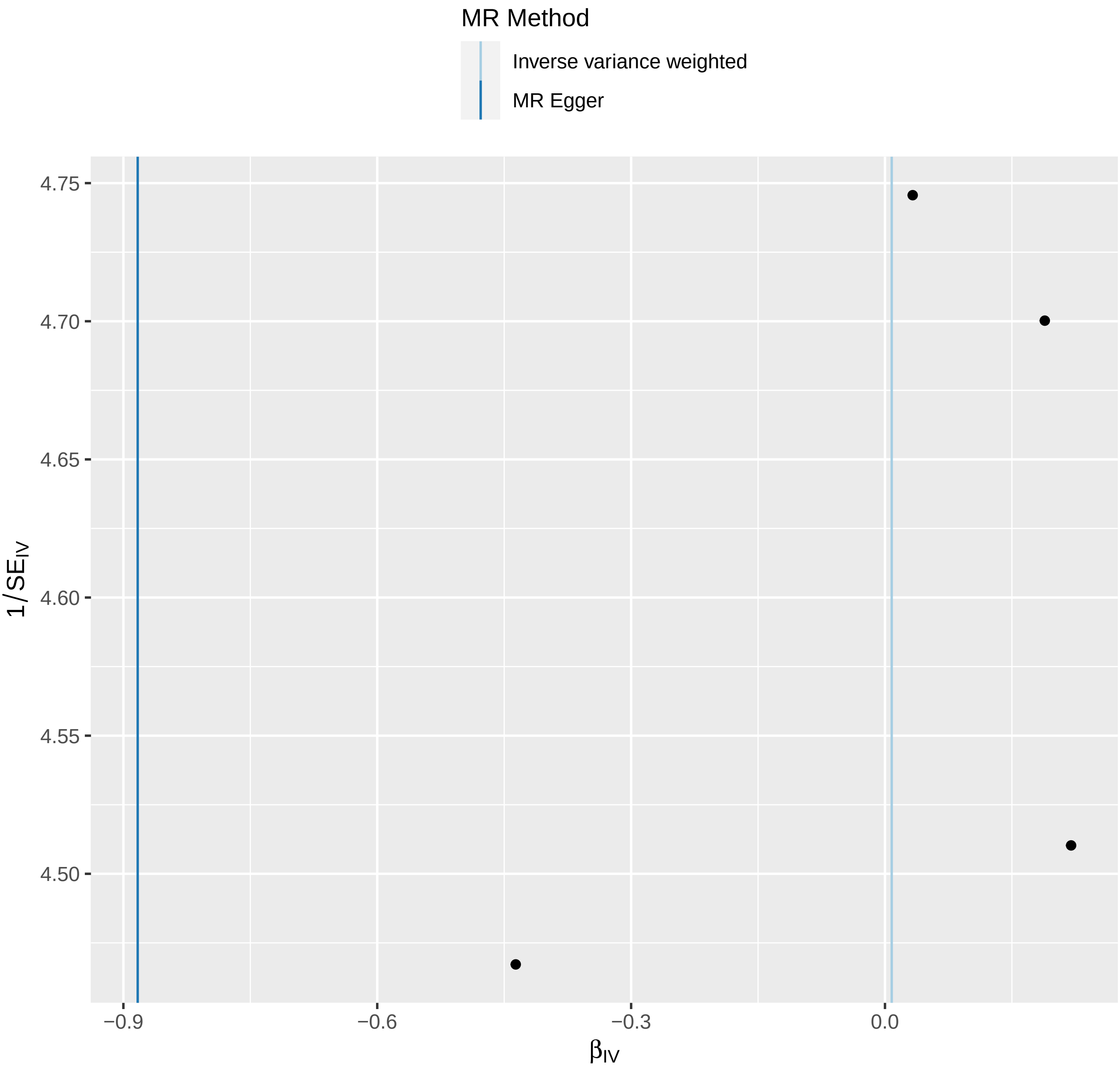

Batch 2−1482 : Gut microbiota abundance (genus Parasutterella id.2892) on Stromal cell−derived factor 1
with a threshold of 1e−05

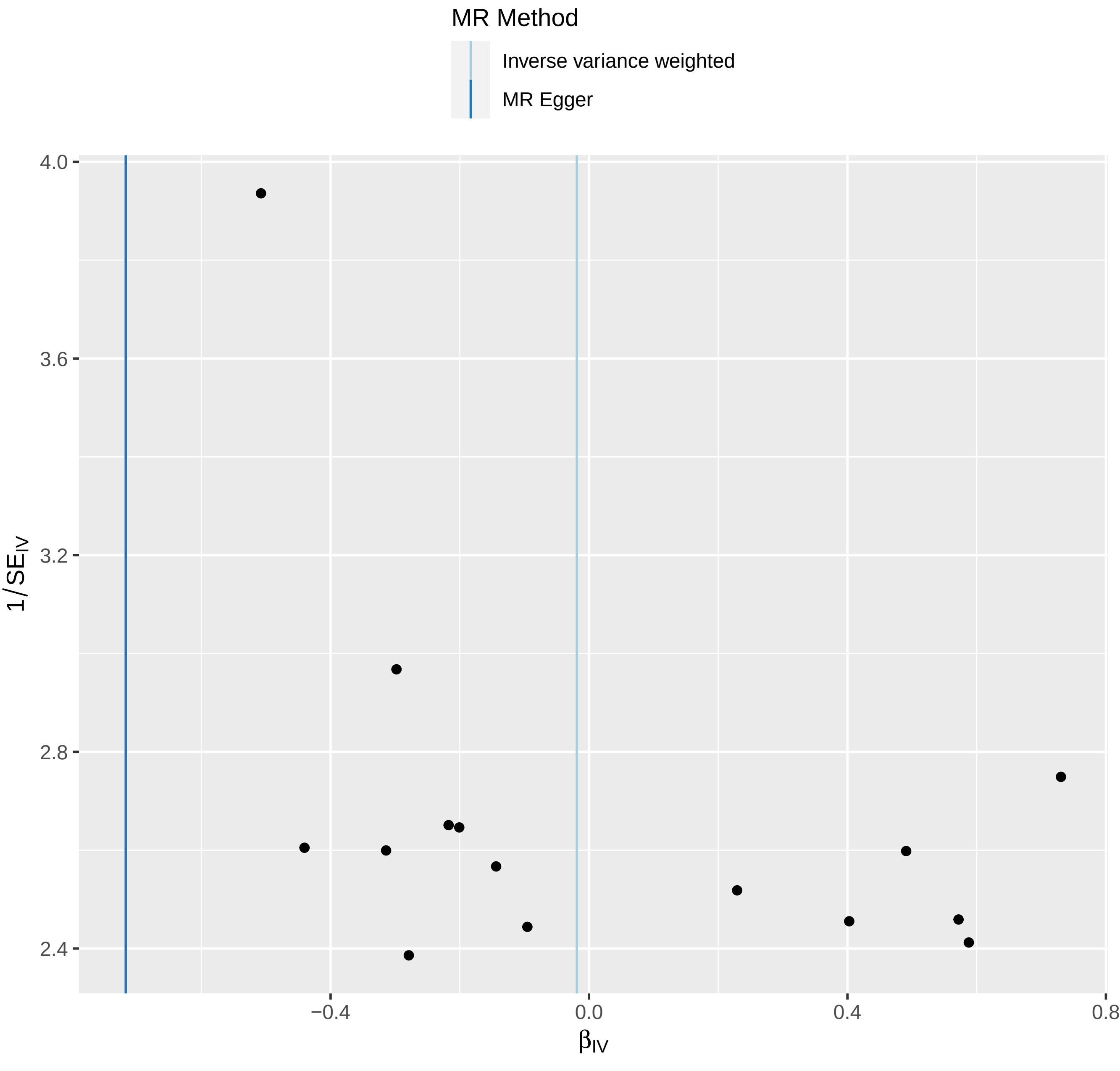

Batch 2−1483 : Gut microbiota abundance (genus Catenibacterium id.2153) on Growth−regulated alpha protein
with a threshold of 1e−05

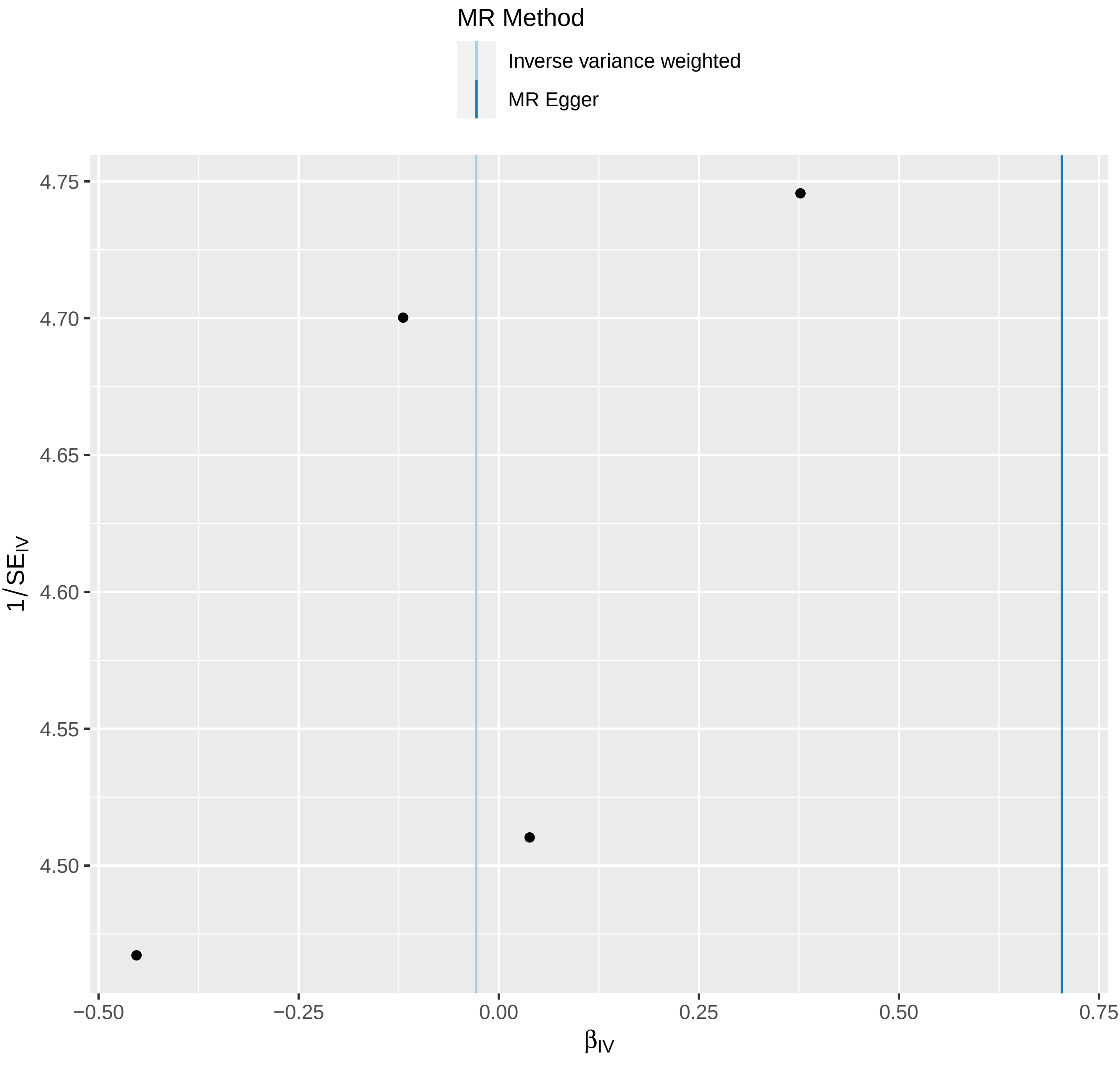

Batch 2−1484 : Gut microbiota abundance (genus Parasutterella id.2892) on Growth−regulated alpha protein
with a threshold of 1e−05

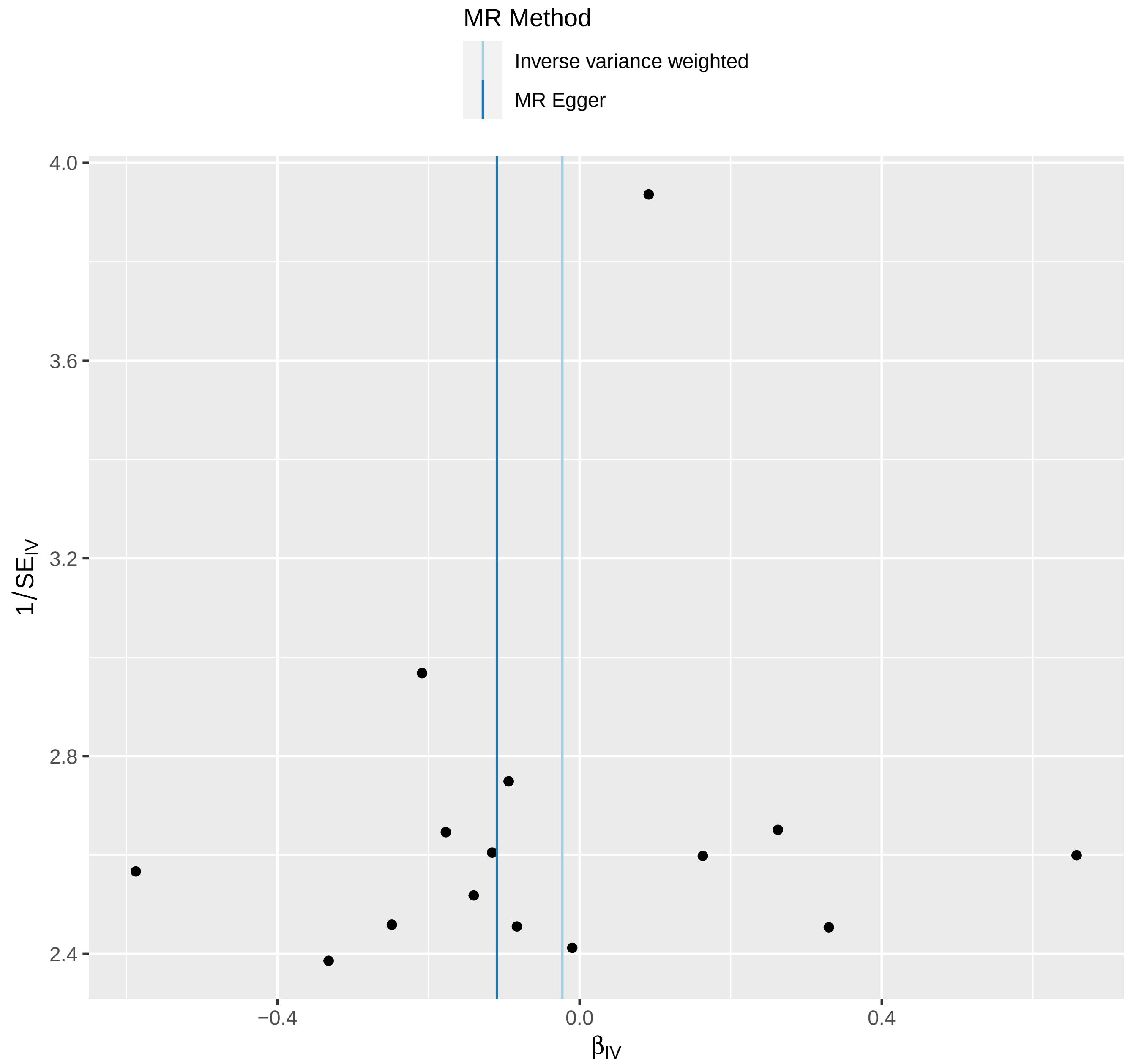

Batch 2−1485 : Gut microbiota abundance (genus Catenibacterium id.2153) on C−X−C motif chemokine 13 with a threshold of 1e−05

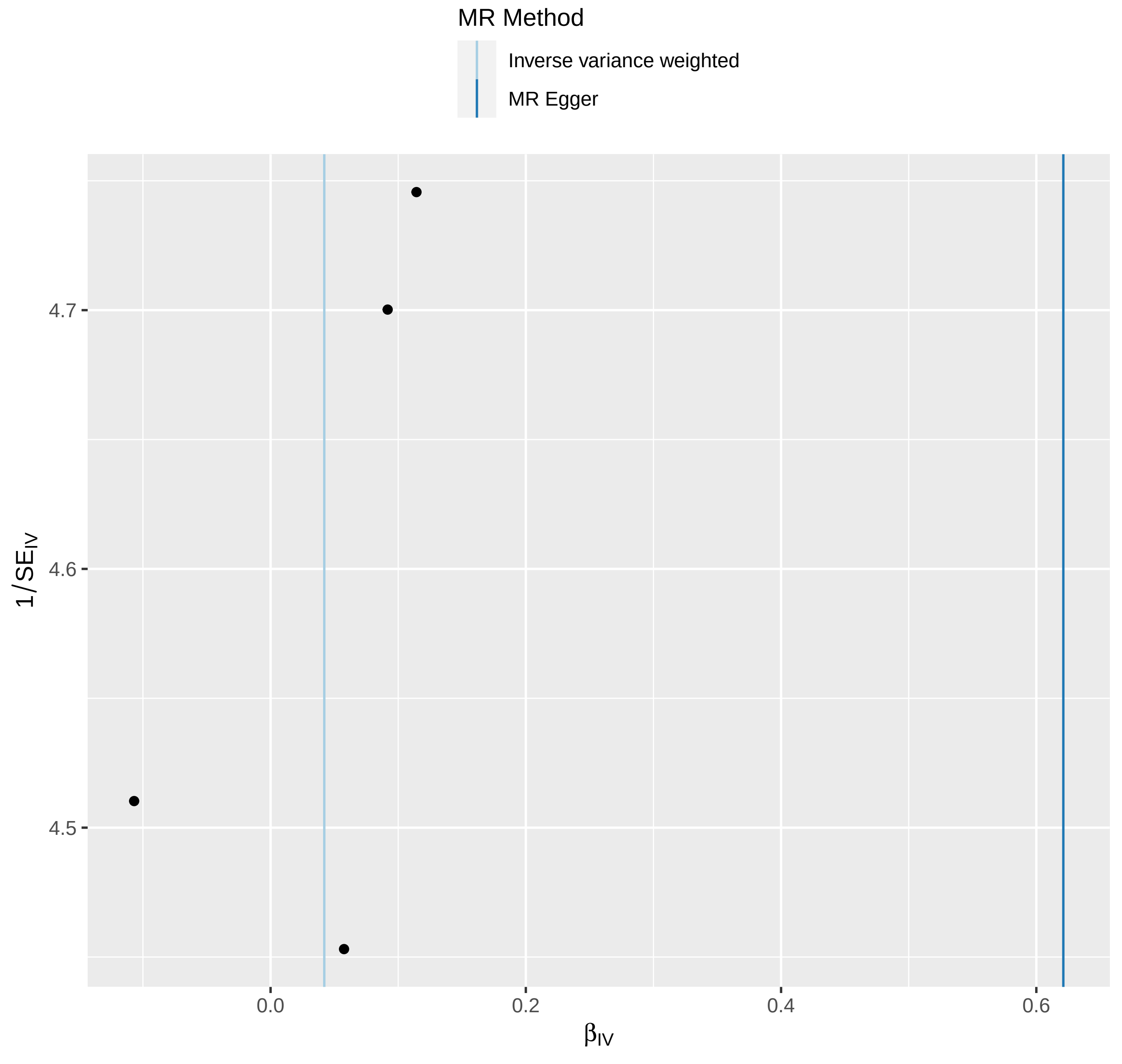

Batch 2−1486 : Gut microbiota abundance (genus Parasutterella id.2892) on C−X−C motif chemokine 13
with a threshold of 1e−05

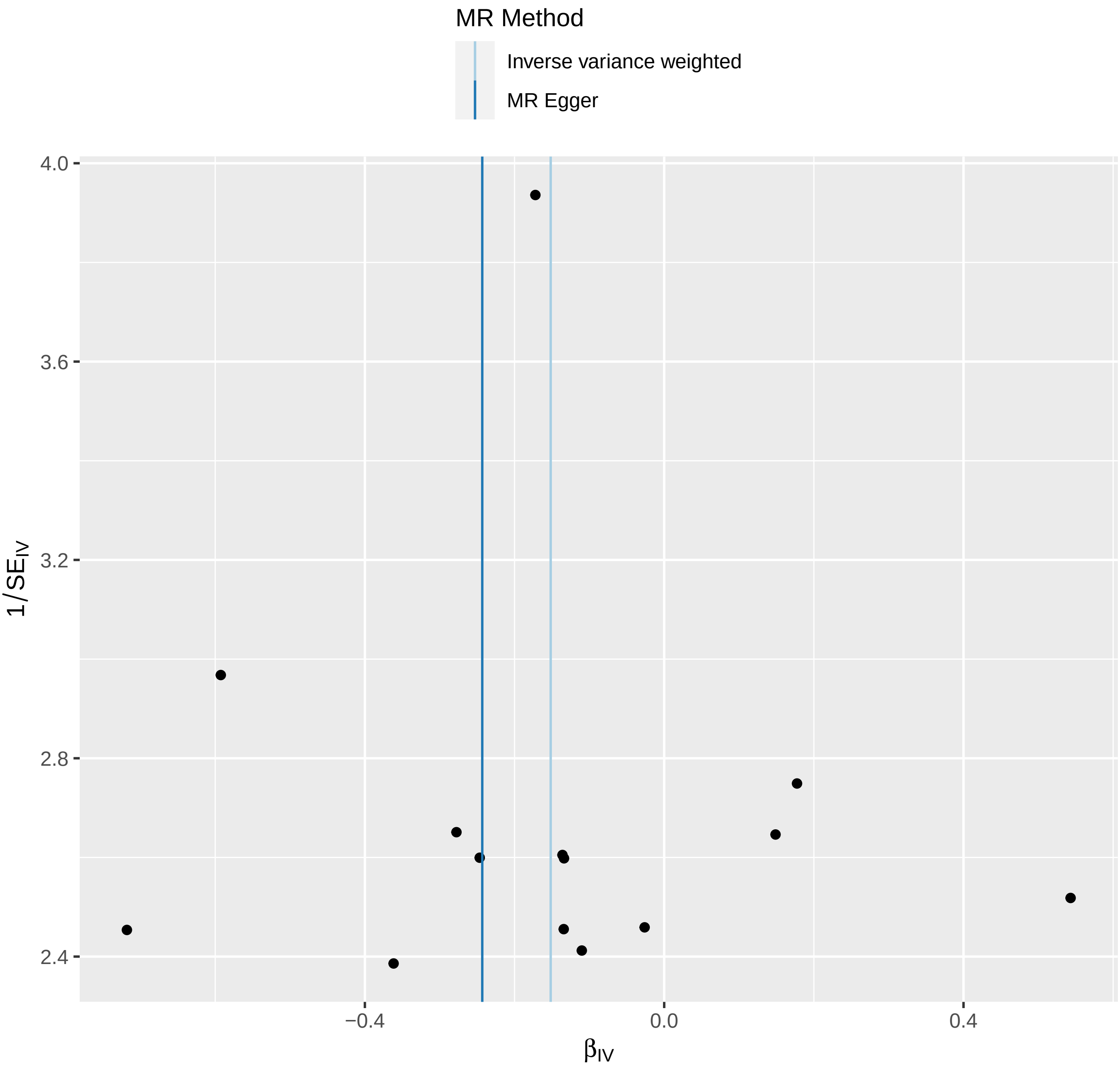

Batch 2−1487 : Gut microbiota abundance (genus Catenibacterium id.2153) on C−X−C motif chemokine 14
with a threshold of 1e−05

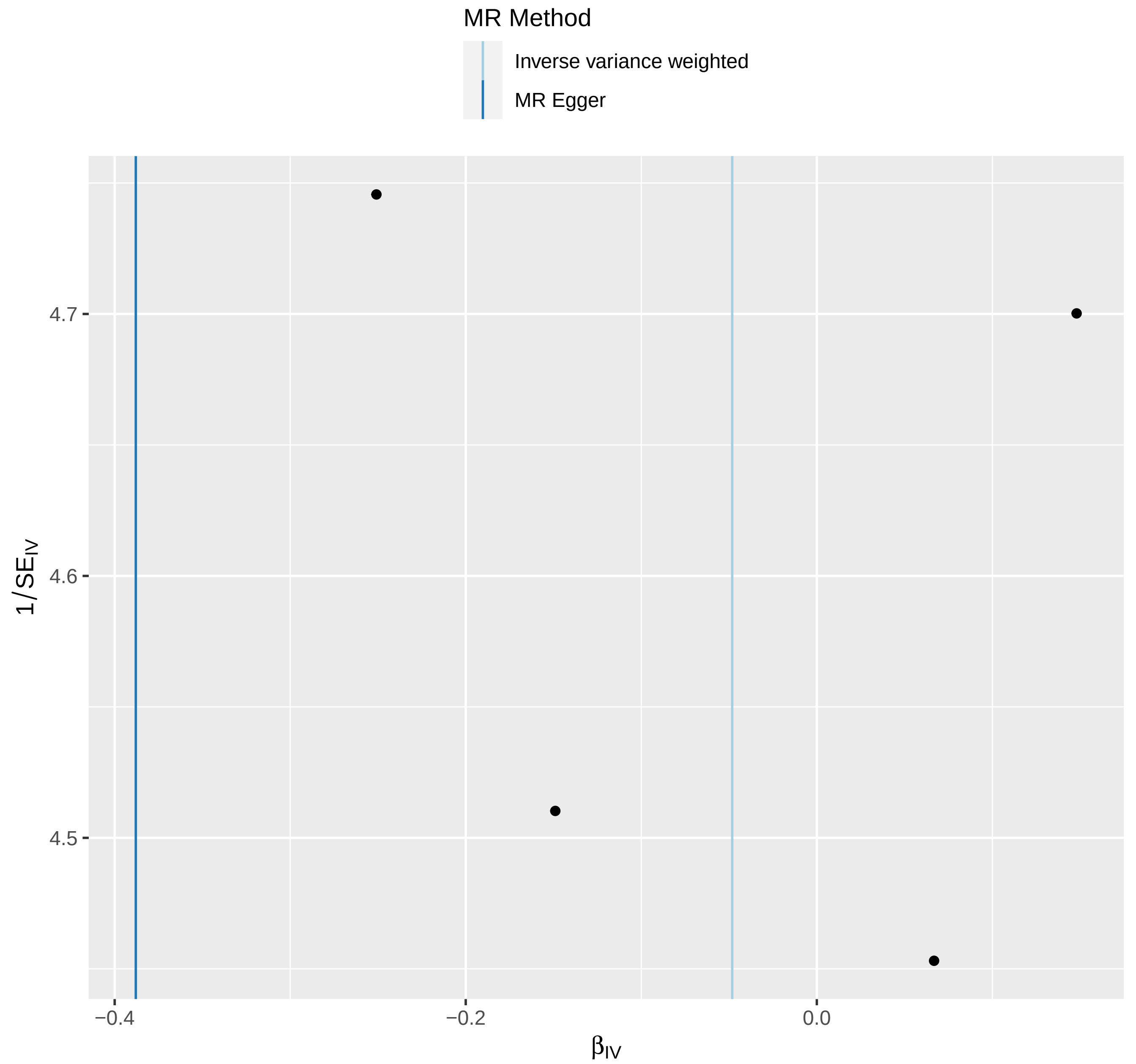

Batch 2−1488 : Gut microbiota abundance (genus Parasutterella id.2892) on C−X−C motif chemokine 14
with a threshold of 1e−05

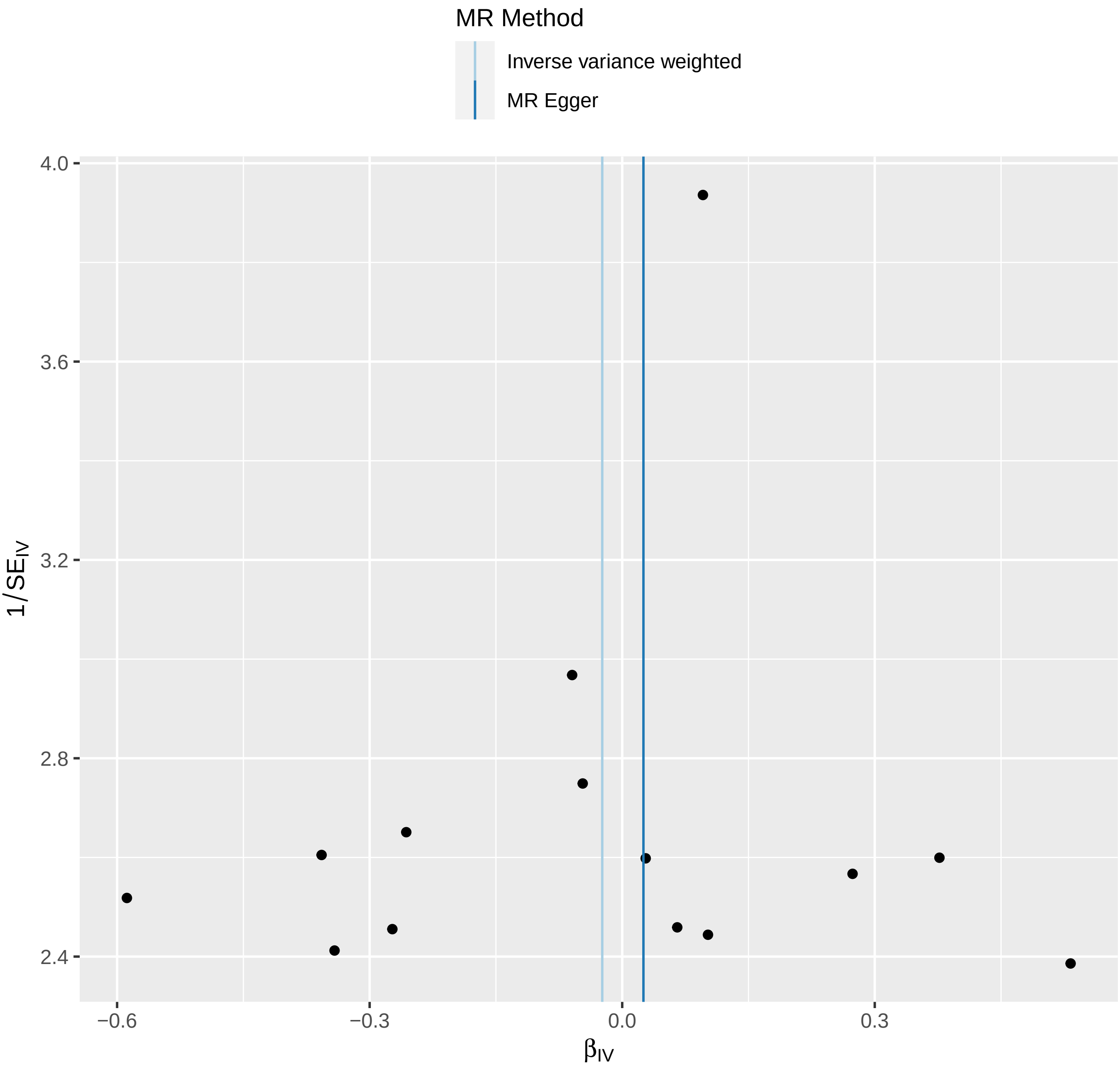

Batch 2−1489 : Gut microbiota abundance (genus Catenibacterium id.2153) on C−X−C motif chemokine 16
with a threshold of 1e−05

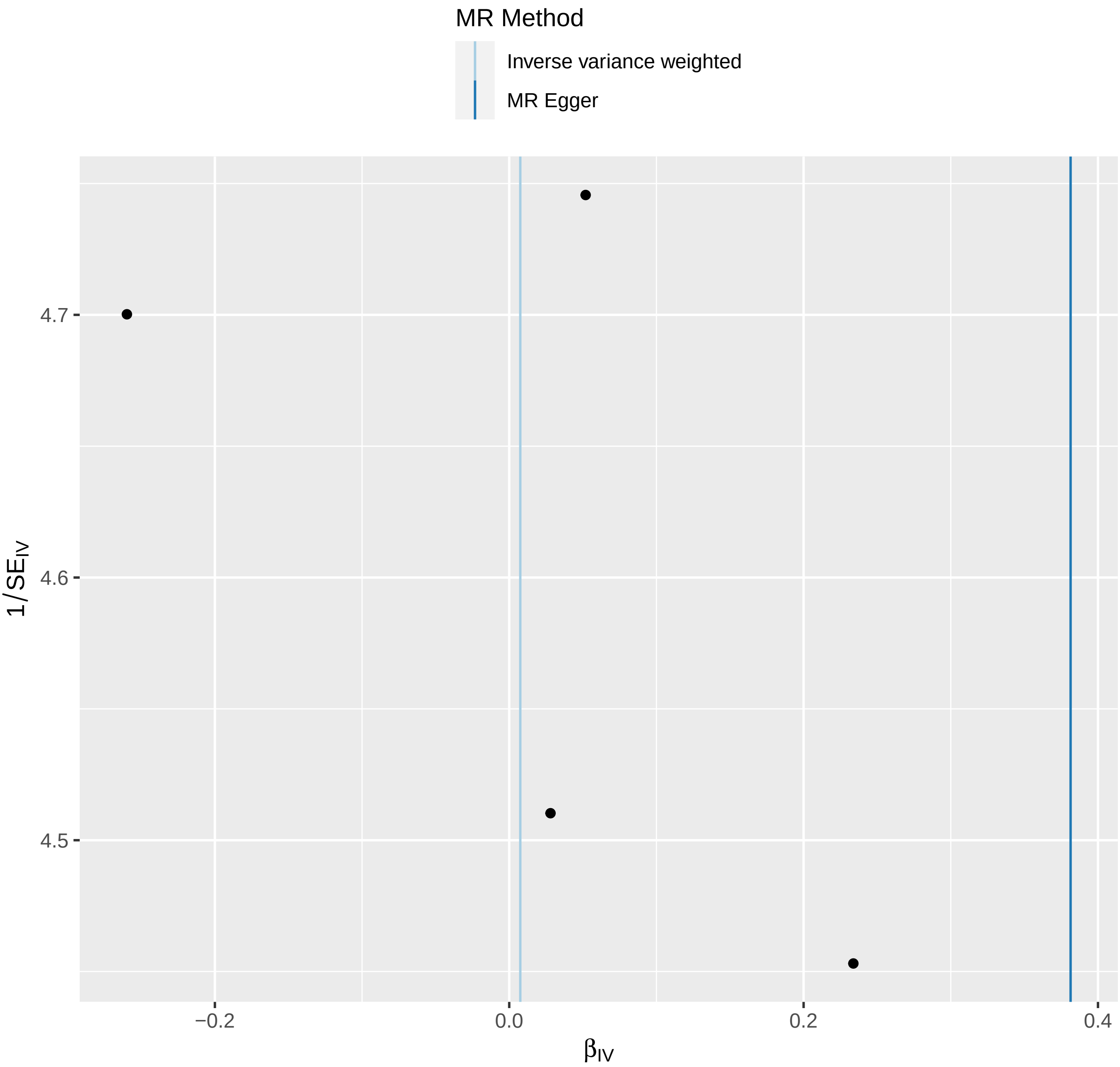

Batch 2−1490 : Gut microbiota abundance (genus Parasutterella id.2892) on C−X−C motif chemokine 16
with a threshold of 1e−05

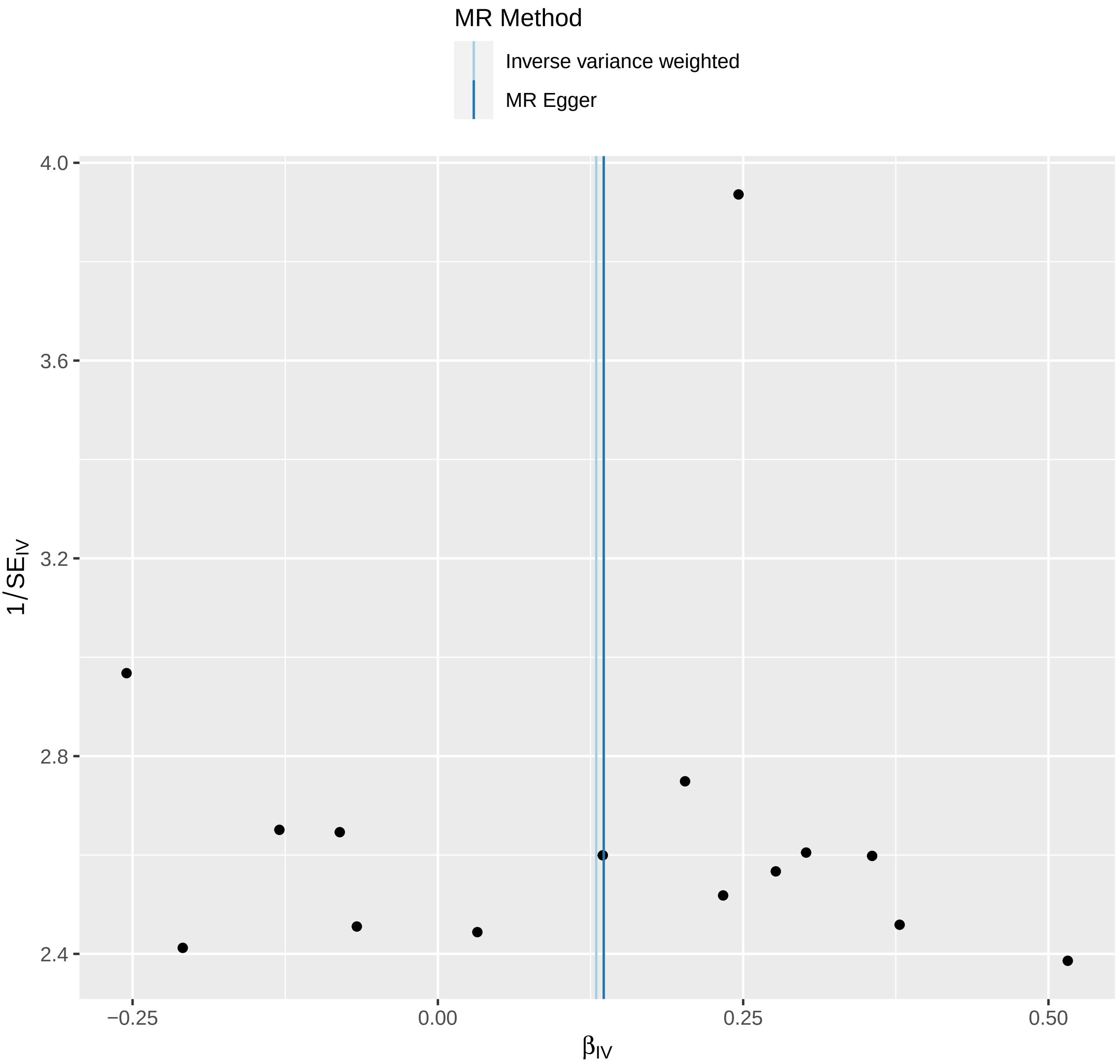

Batch 2−1491 : Gut microbiota abundance (genus Catenibacterium id.2153) on Gro−beta/gamma
with a threshold of 1e−05

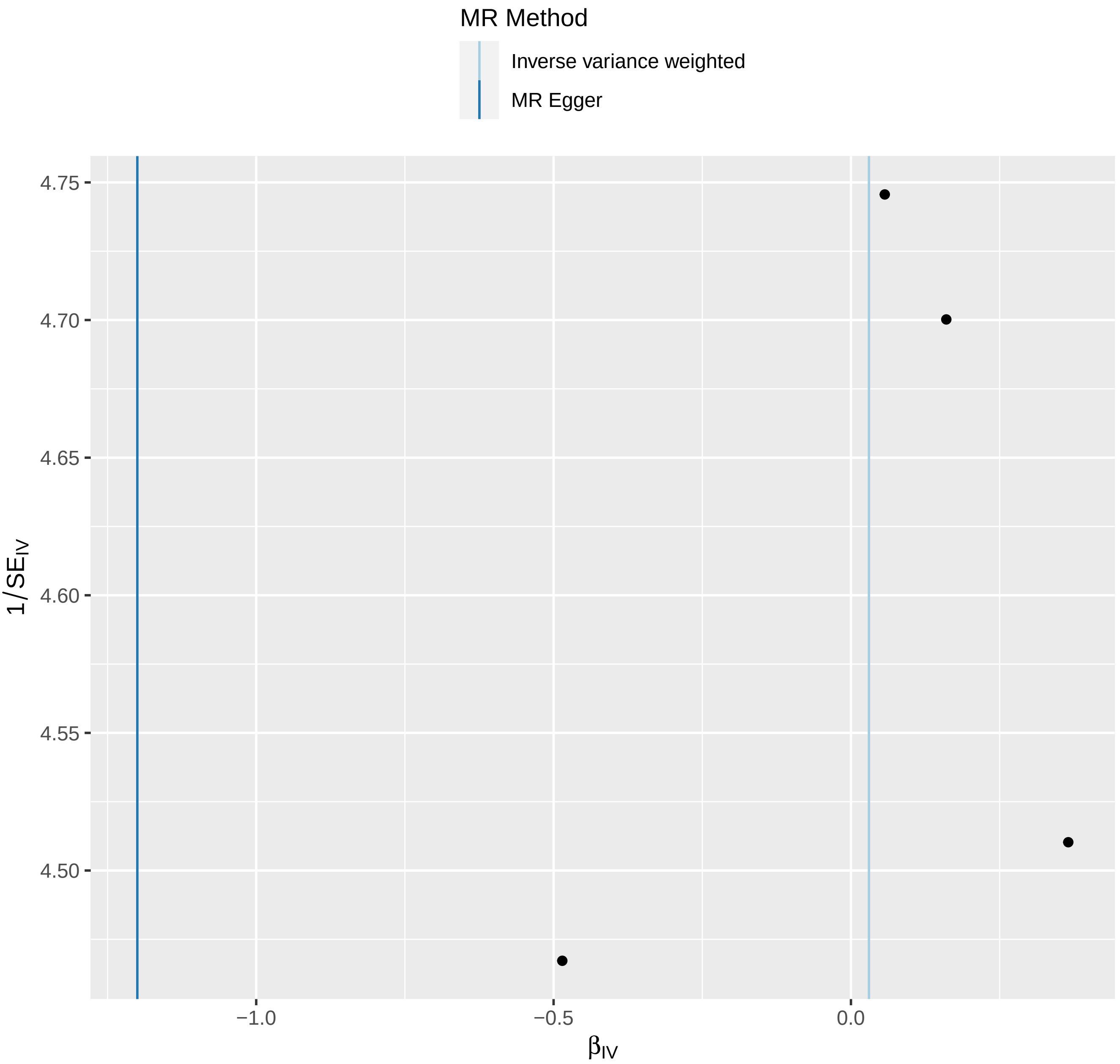

Batch 2−1492 : Gut microbiota abundance (genus Parasutterella id.2892) on Gro−beta/gamma
with a threshold of 1e−05

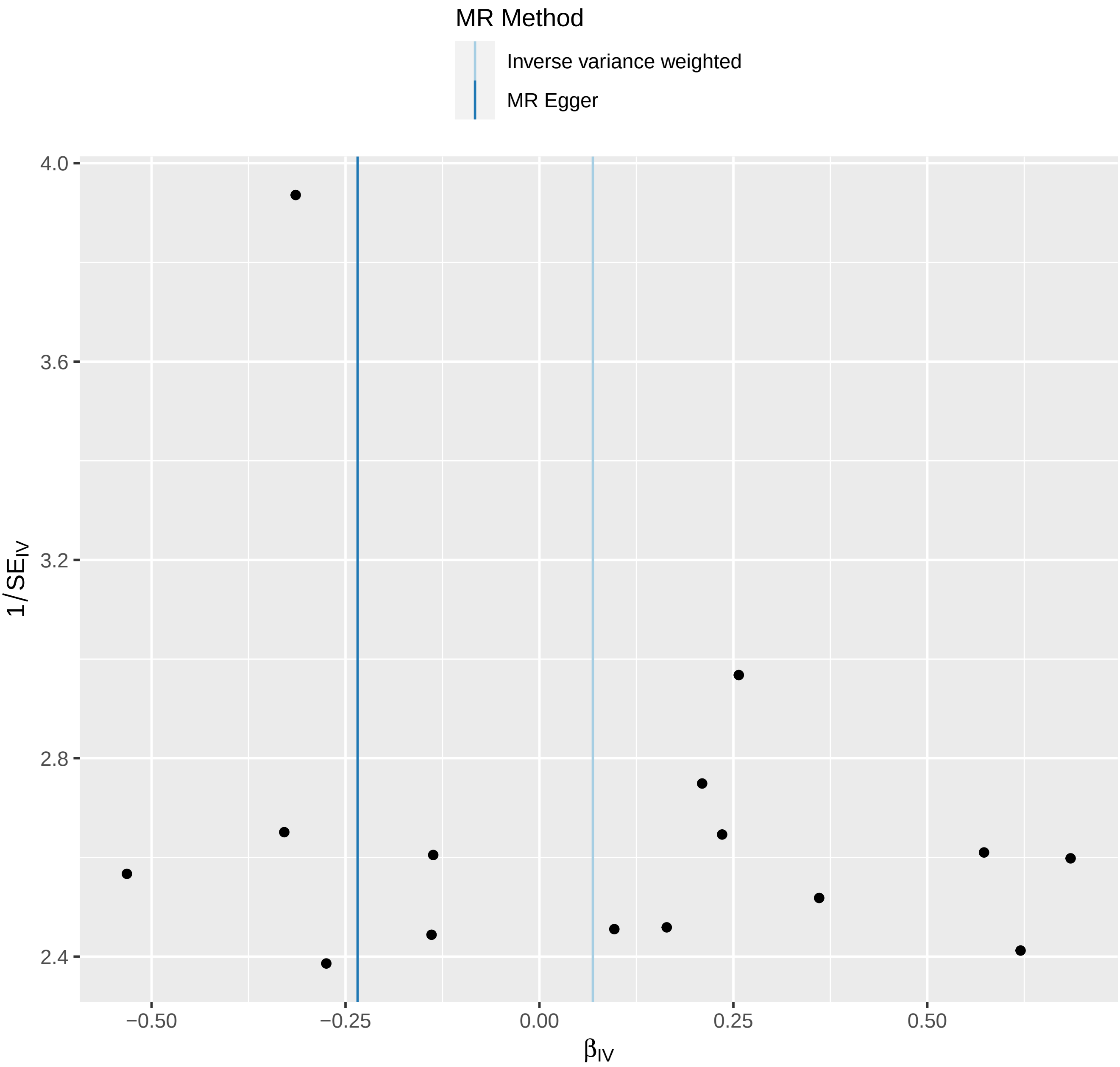

Batch 2−1493 : Gut microbiota abundance (genus Catenibacterium id.2153) on Gro−beta/gamma
with a threshold of 1e−05

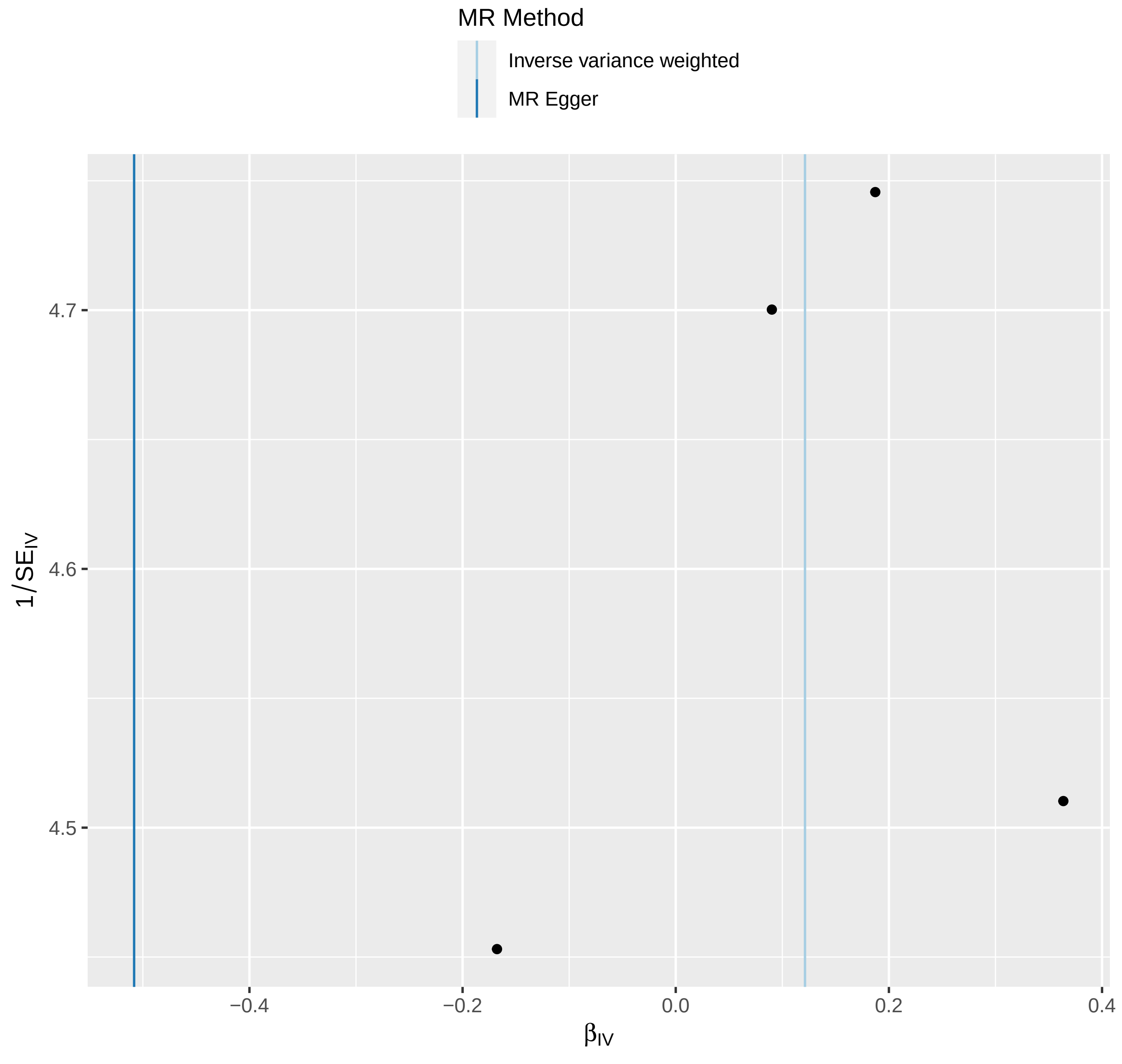

Batch 2−1494 : Gut microbiota abundance (genus Parasutterella id.2892) on Gro−beta/gamma
with a threshold of 1e−05

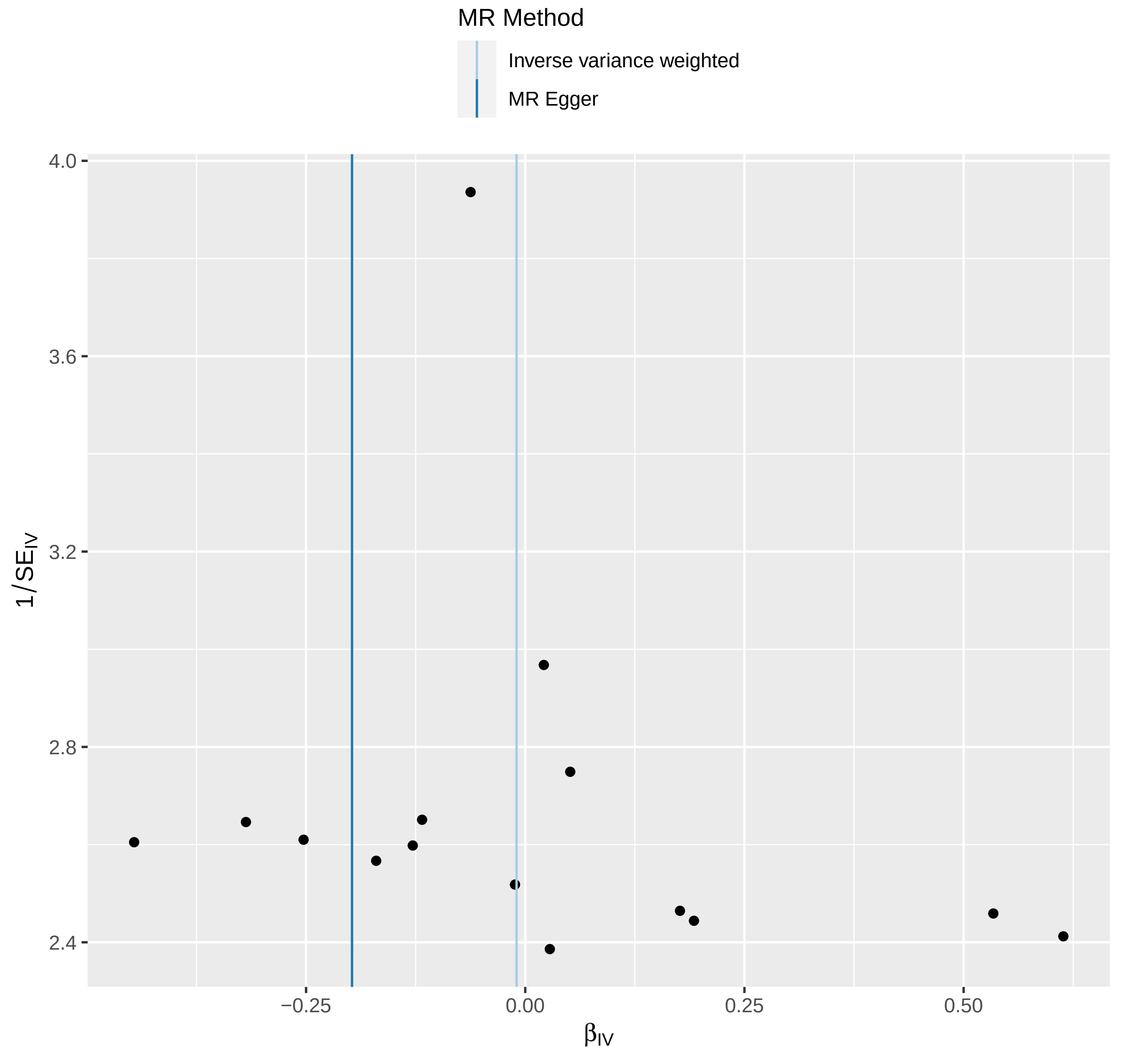

Batch 2−1495 : Gut microbiota abundance (genus Catenibacterium id.2153) on C−X−C motif chemokine 5
with a threshold of 1e−05

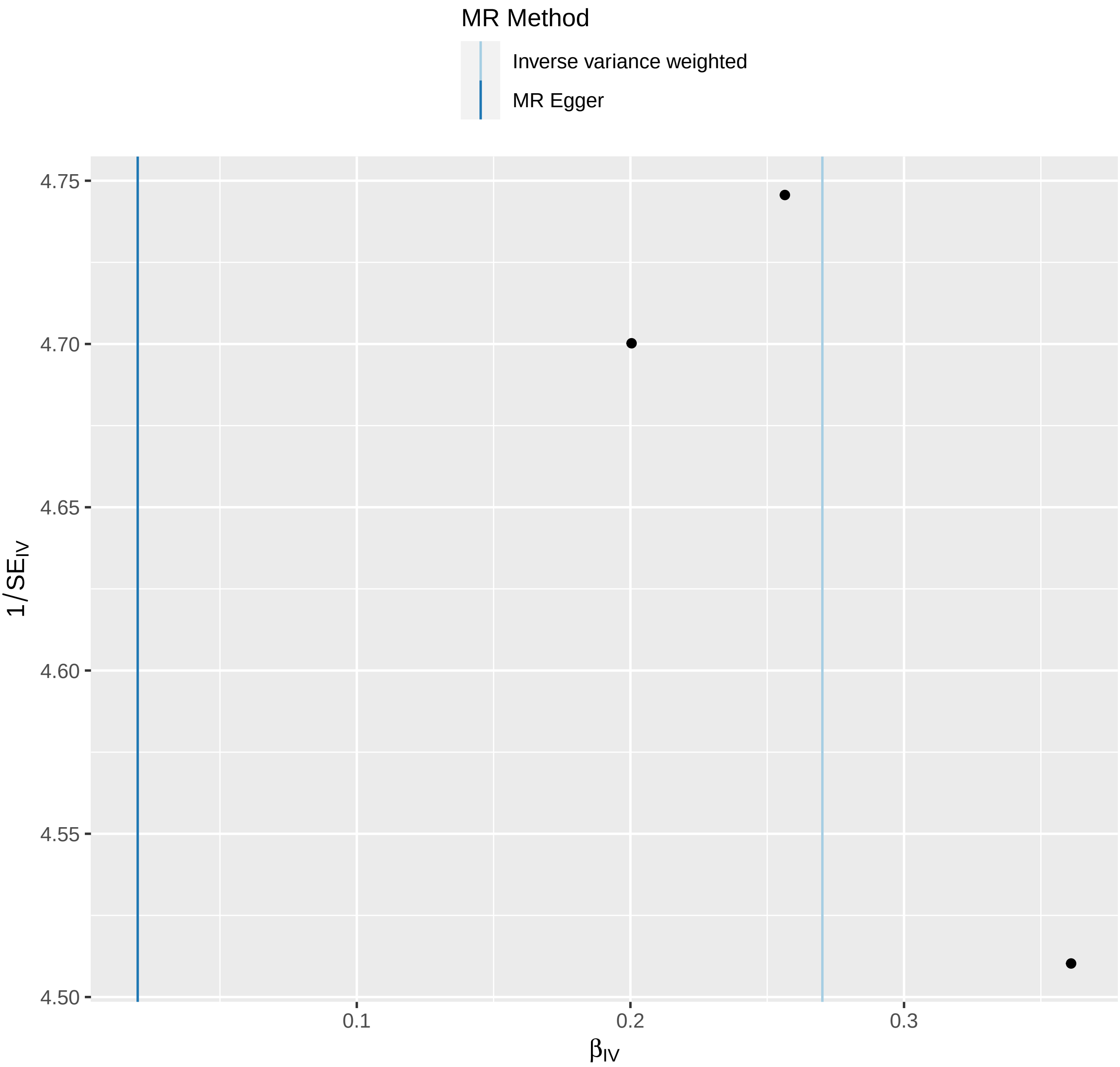

Batch 2−1496 : Gut microbiota abundance (genus Parasutterella id.2892) on C−X−C motif chemokine 5
with a threshold of 1e−05

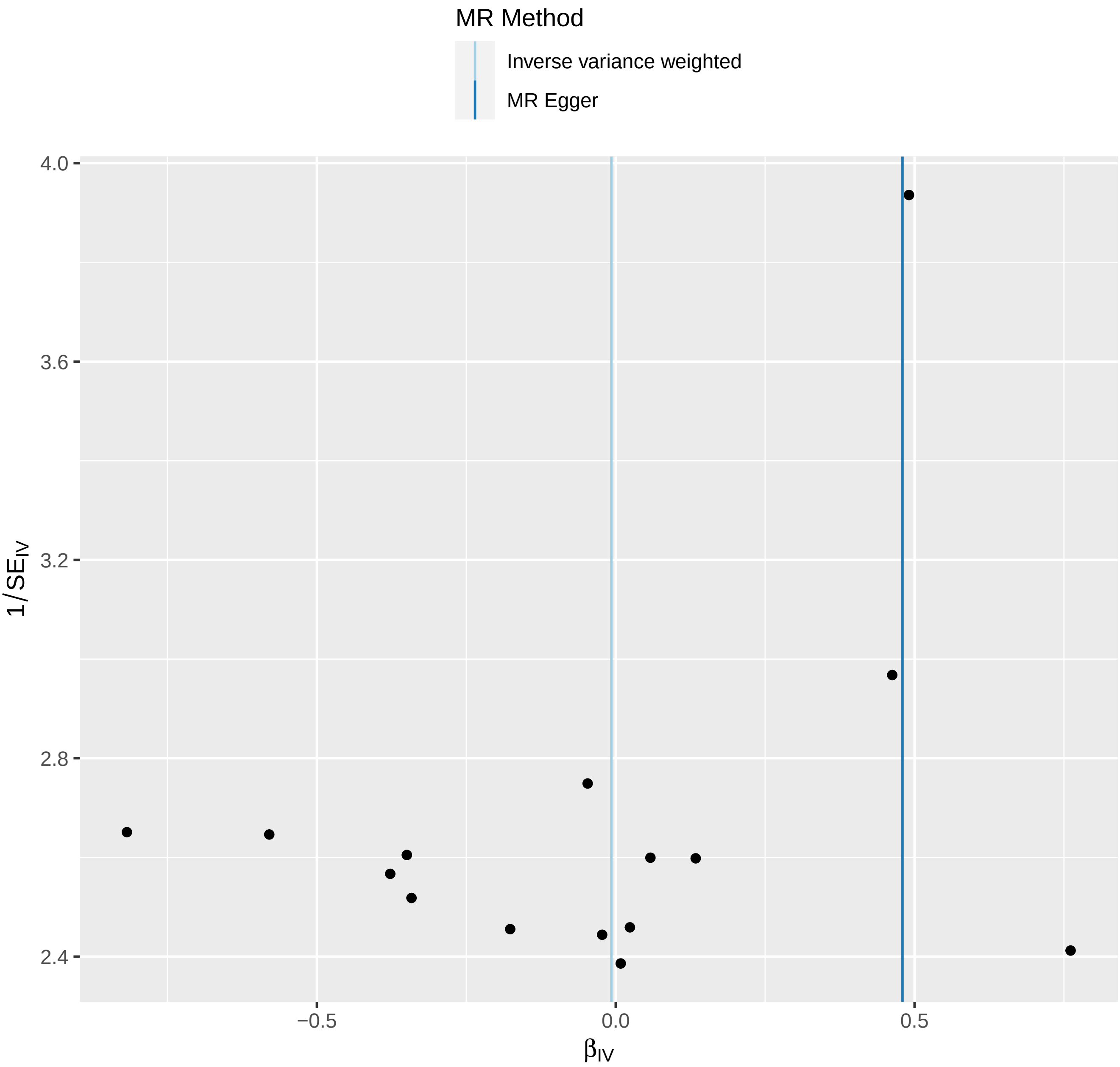

Batch 2−1497 : Gut microbiota abundance (genus Catenibacterium id.2153) on Interleukin−8
with a threshold of 1e−05

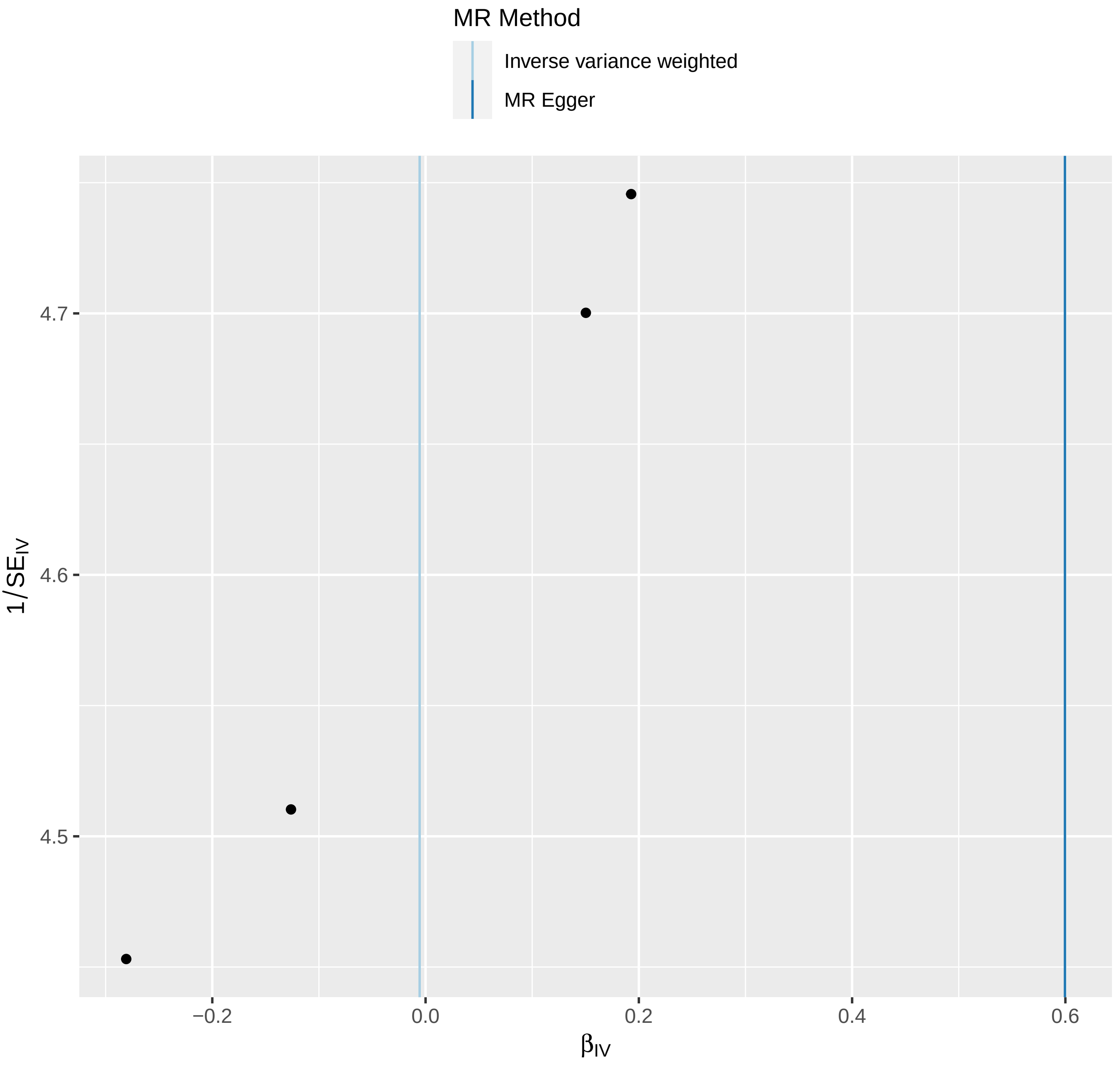

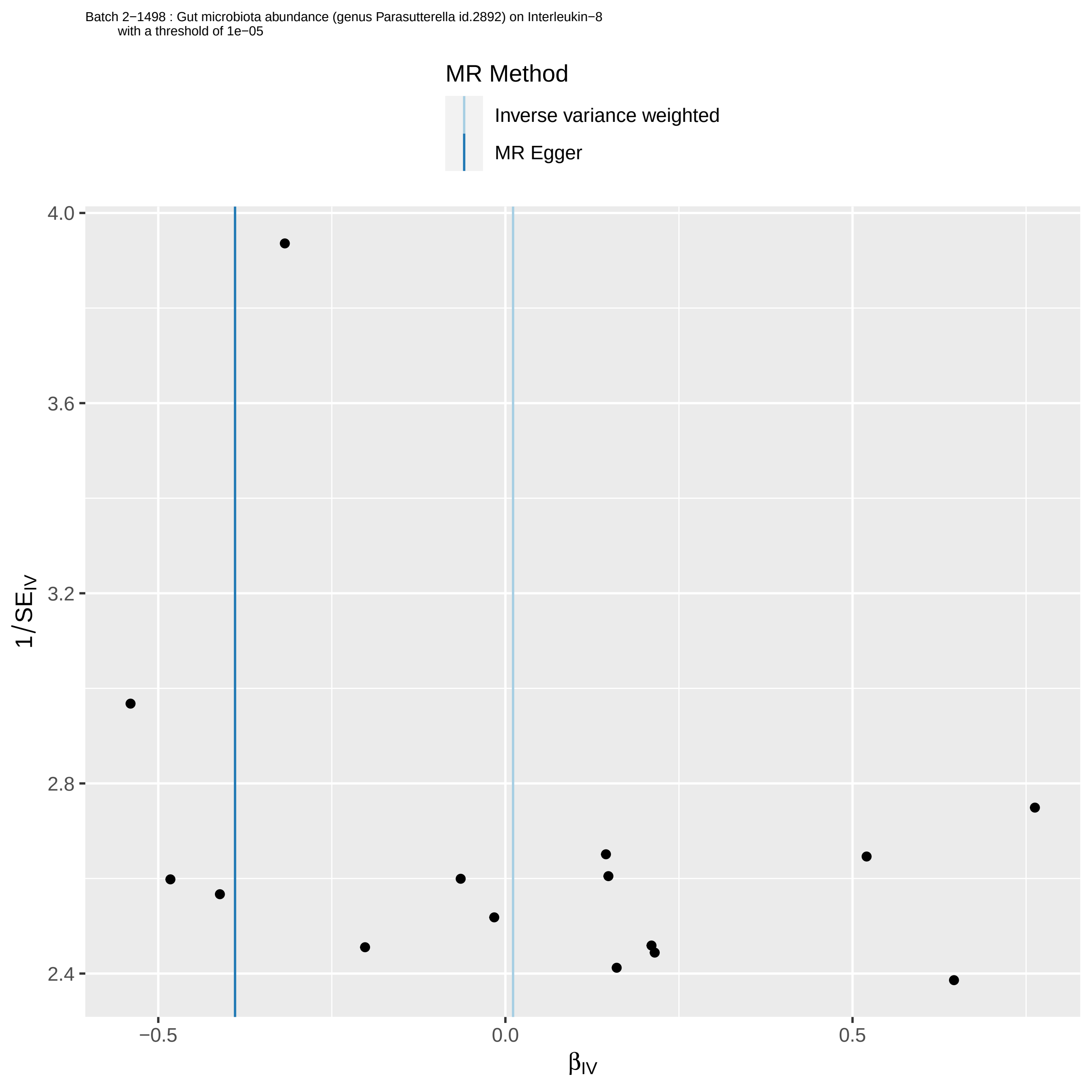

Batch 2−1498 : Gut microbiota abundance (genus Parasutterella id.2892) on Interleukin−8
with a threshold of 1e−05
MR Method
Inverse variance weighted
MR Egger
4.0
3.6
3.2
2.8
2.4
1/SE_IV
−0.5
0.0
0.5
β_IV

Batch 2−1499 : Gut microbiota abundance (genus Catenibacterium id.2153) on C−X−C motif chemokine 9 with a threshold of 1e−05

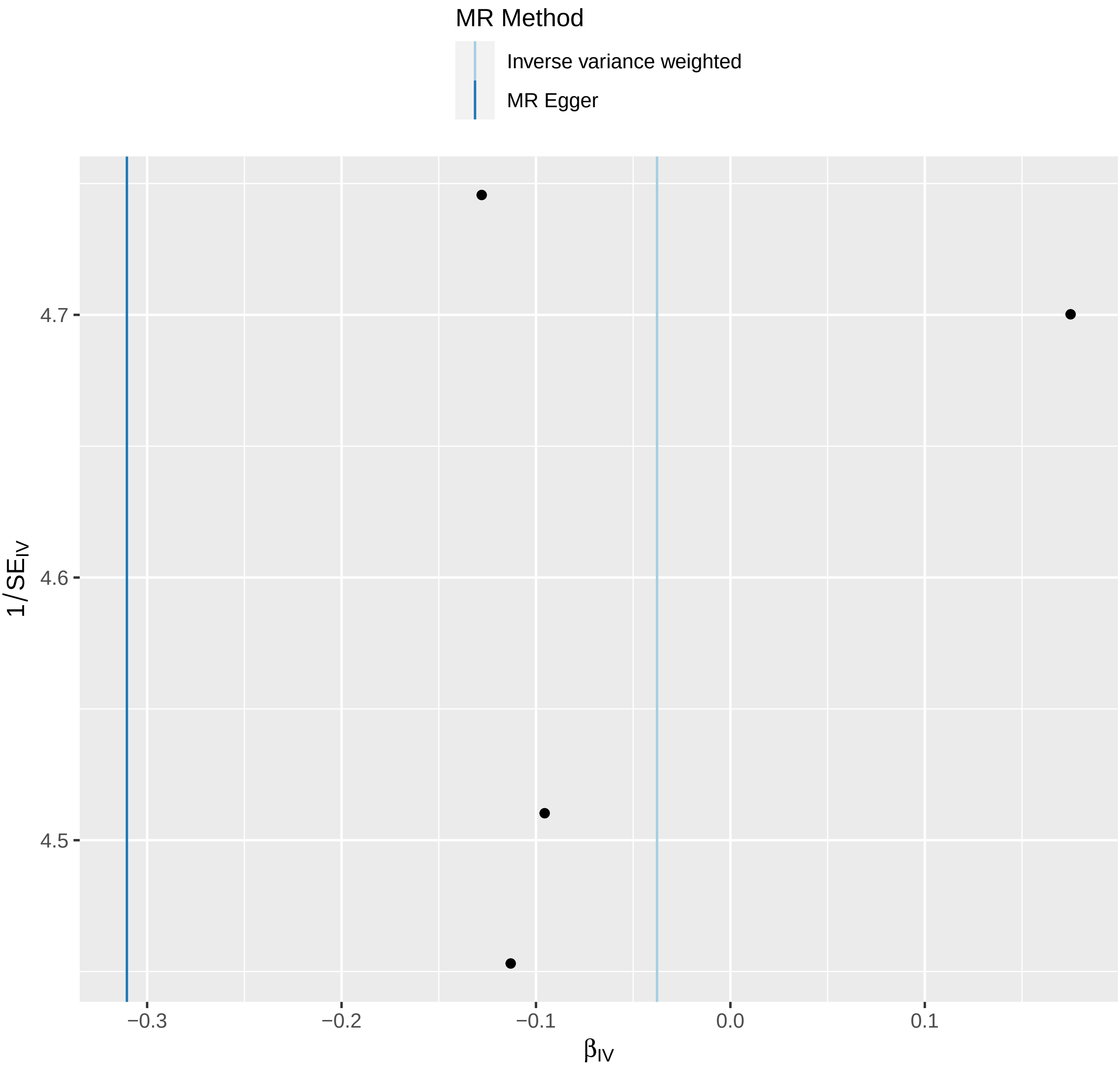

Batch 2−1500 : Gut microbiota abundance (genus Parasutterella id.2892) on C−X−C motif chemokine 9
with a threshold of 1e−05

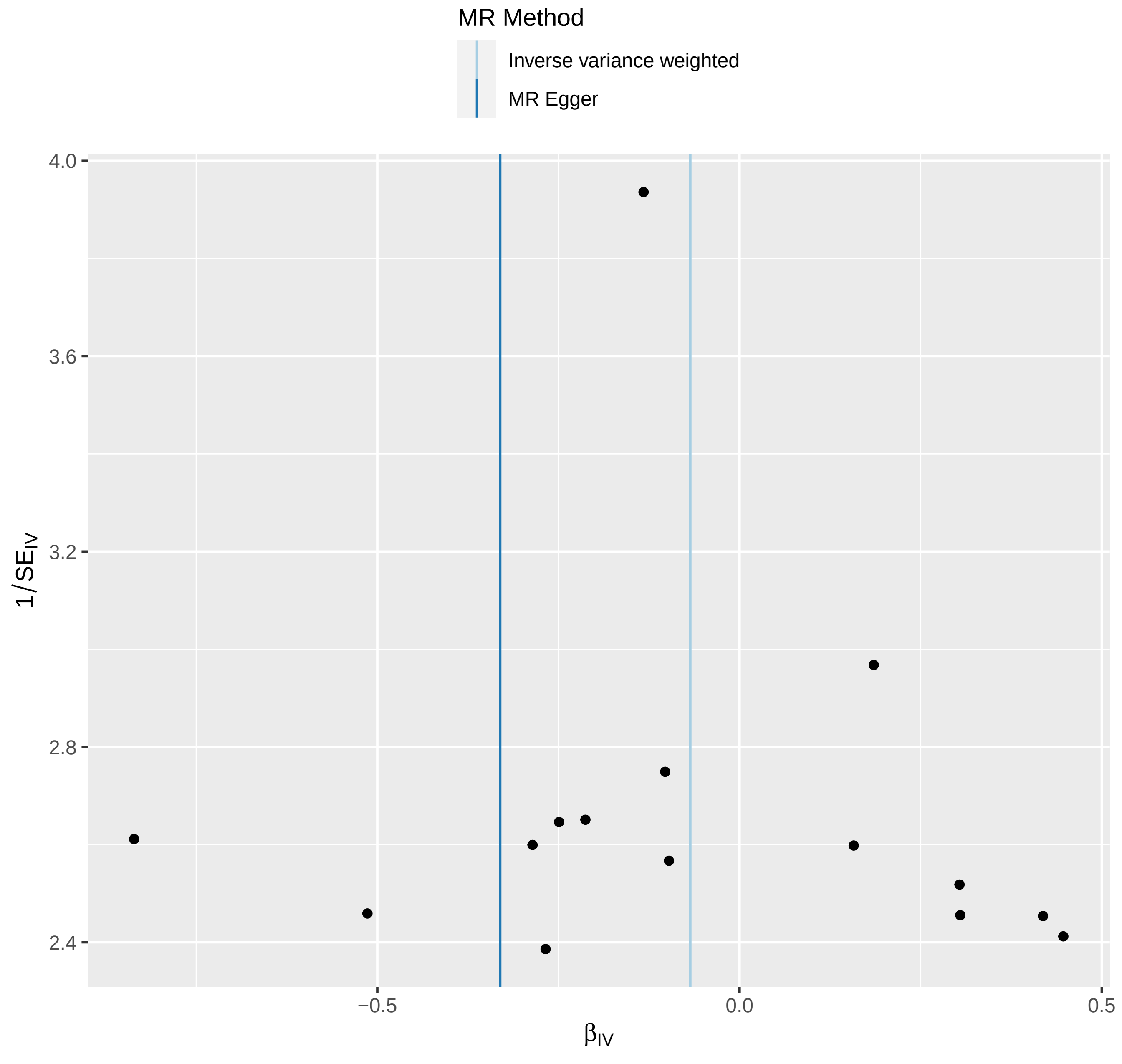

Batch 2−1501 : Gut microbiota abundance (genus Catenibacterium id.2153) on C−X−C motif chemokine 9
with a threshold of 1e−05

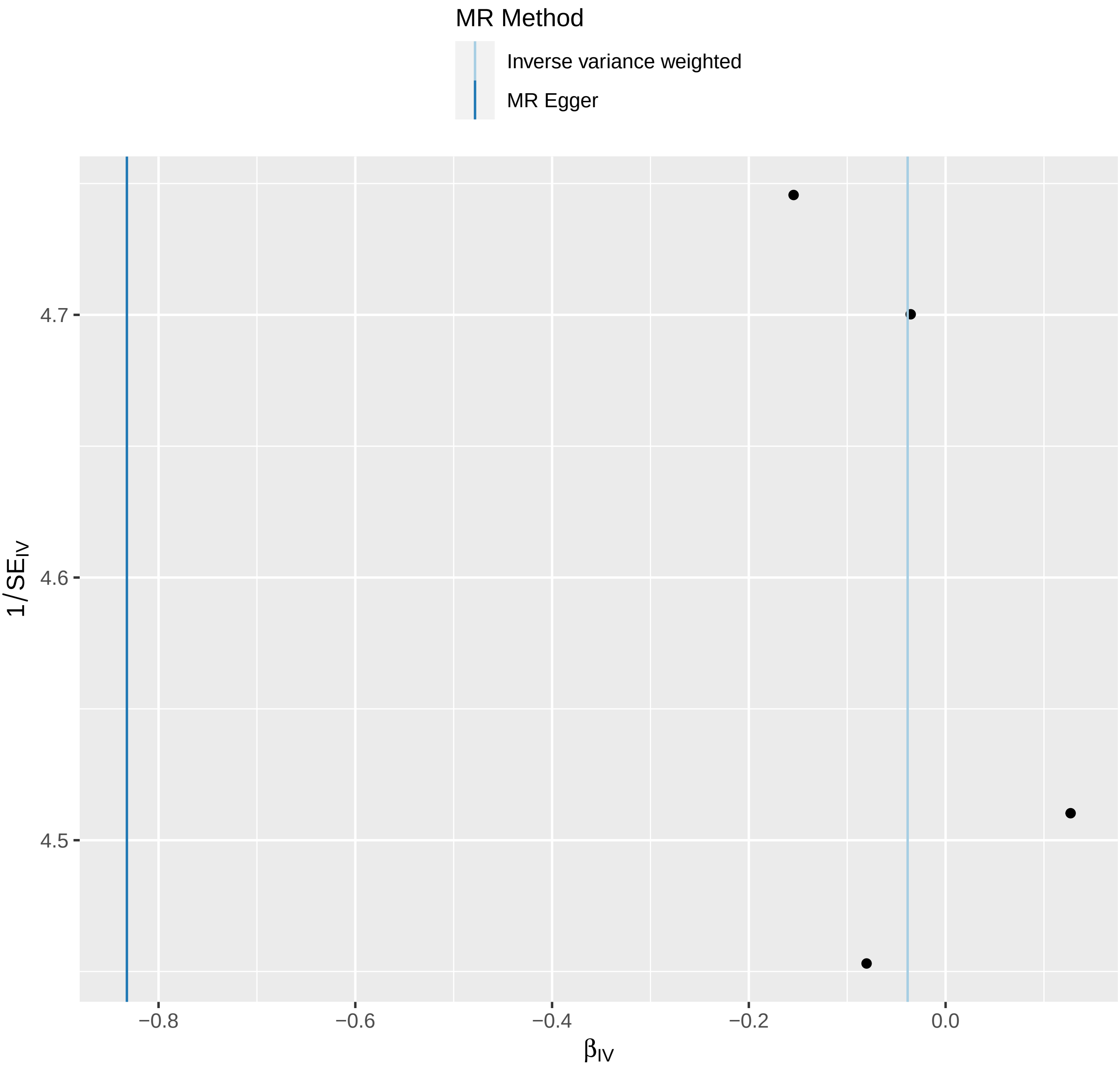

Batch 2−1502 : Gut microbiota abundance (genus Parasutterella id.2892) on C−X−C motif chemokine 9
with a threshold of 1e−05

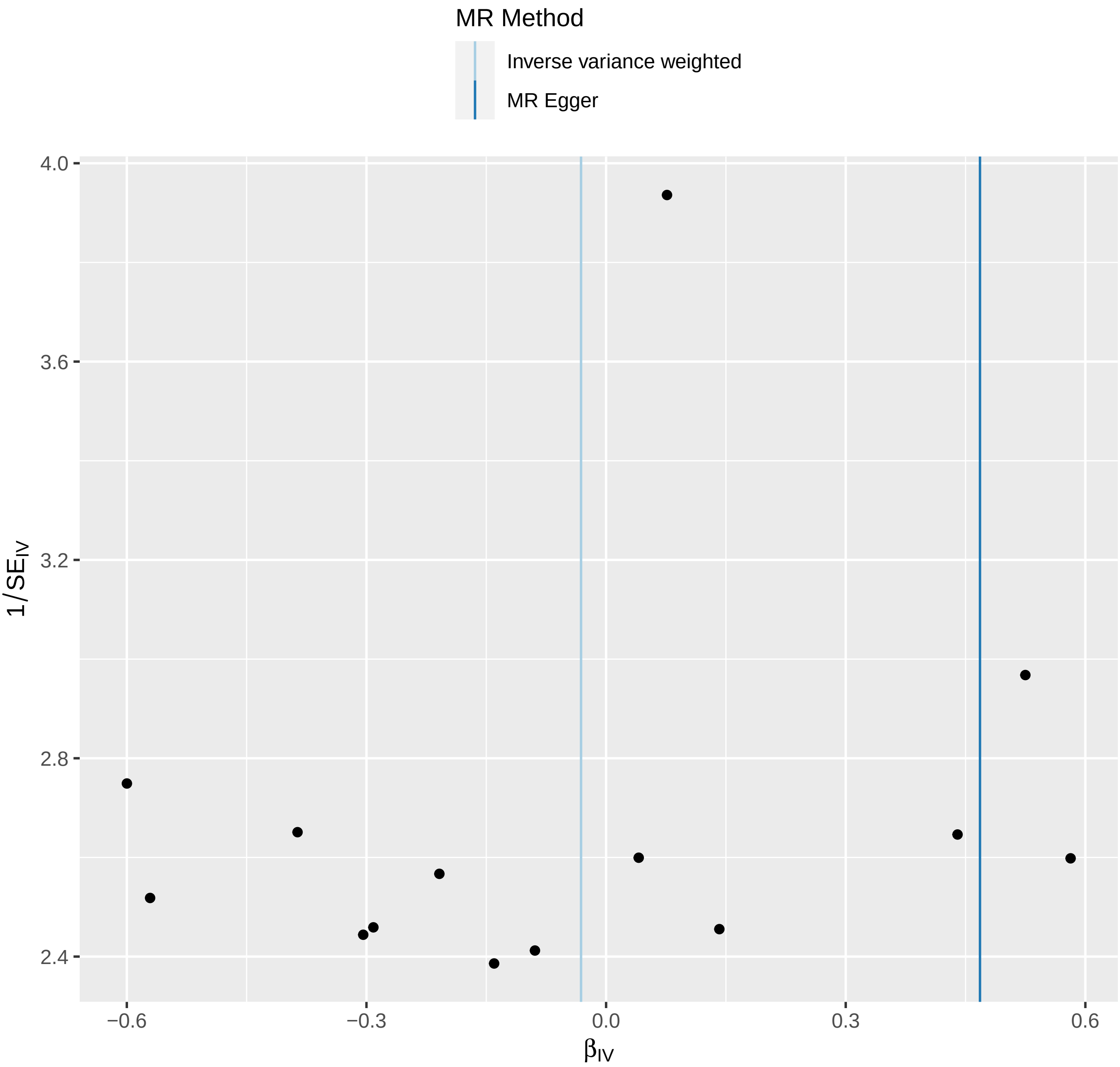

Batch 2−1503 : Gut microbiota abundance (genus Catenibacterium id.2153) on Cytochrome b5
with a threshold of 1e−05

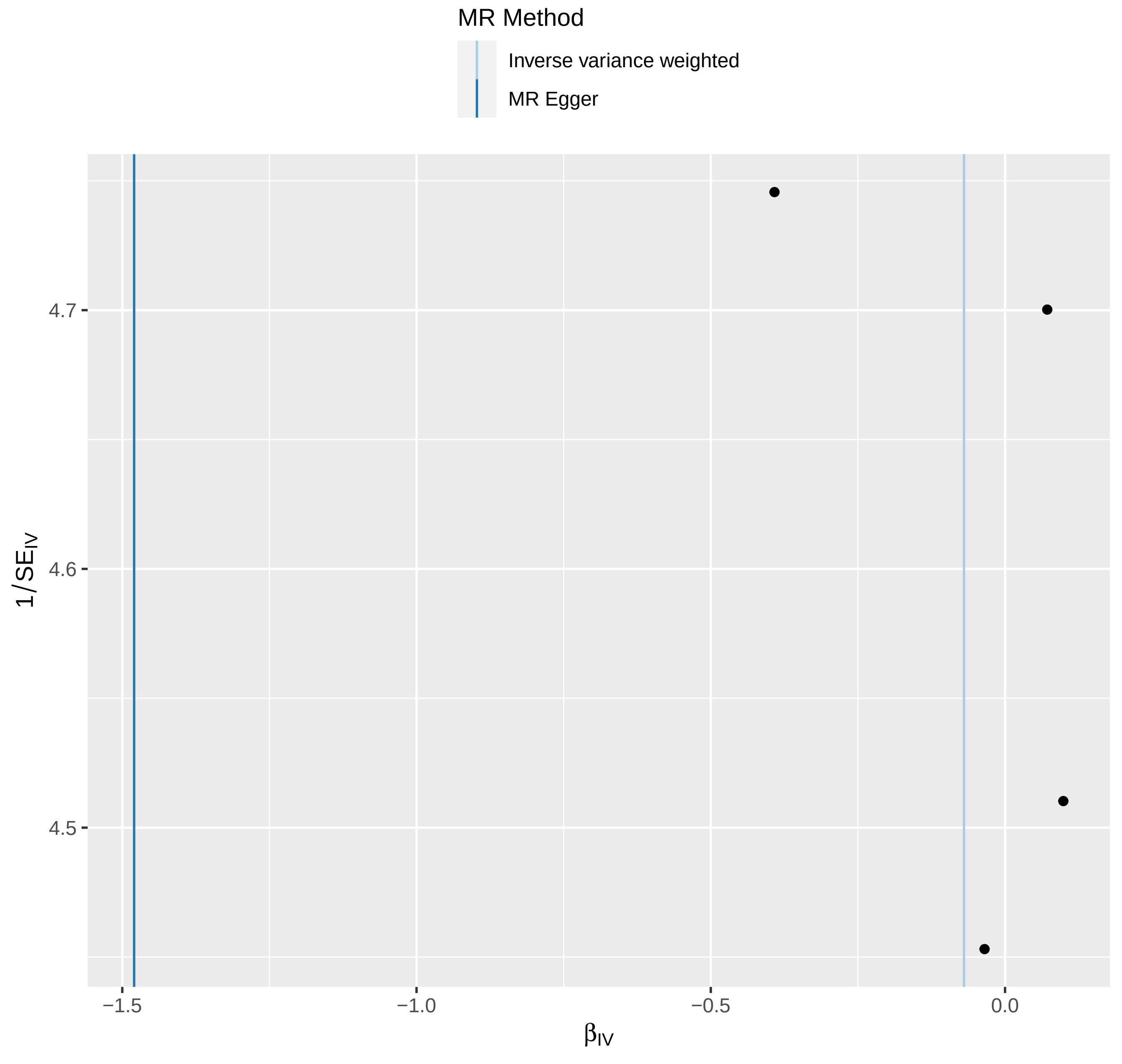

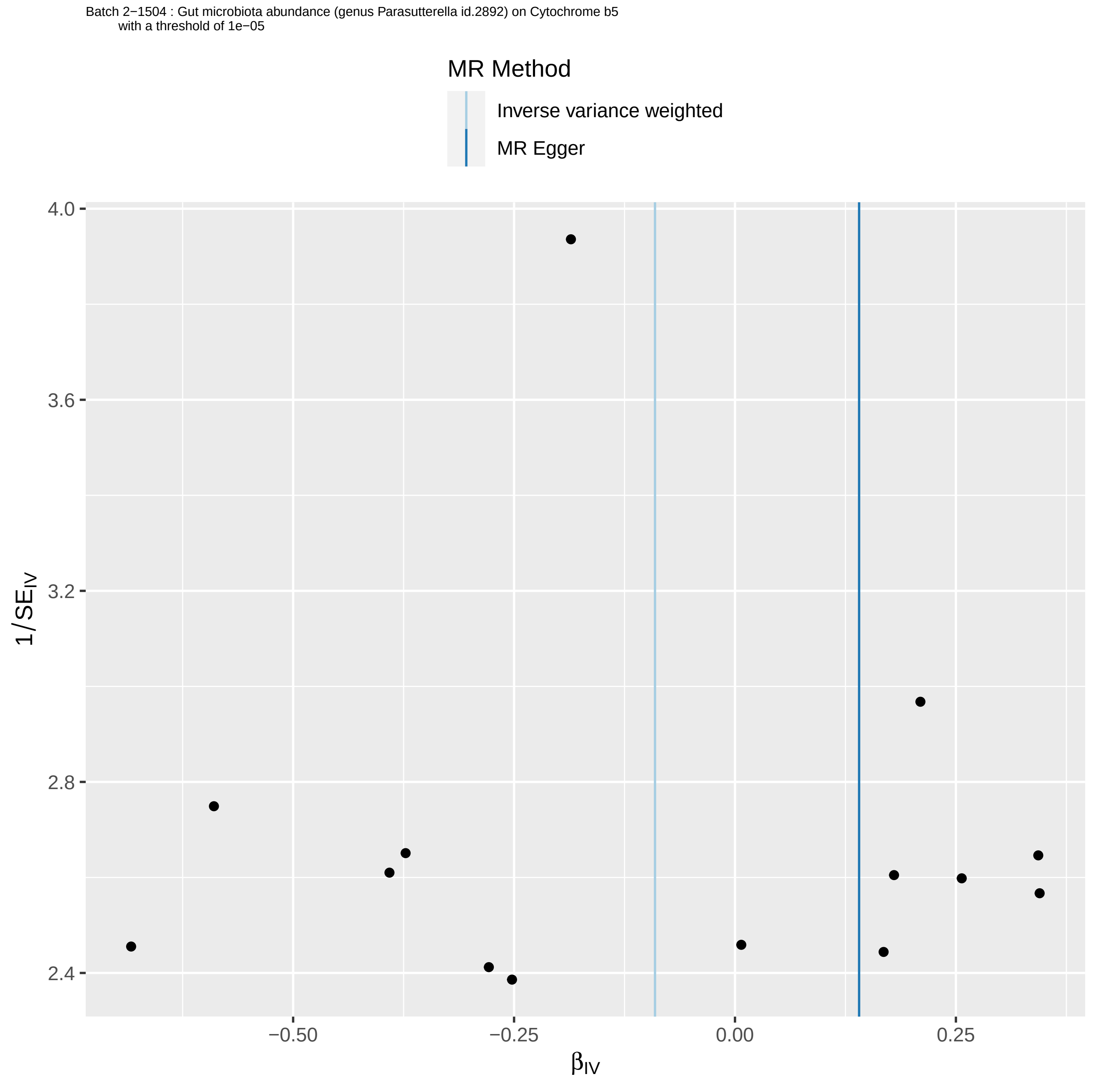

Batch 2−1504 : Gut microbiota abundance (genus Parasutterella id.2892) on Cytochrome b5
with a threshold of 1e−05
MR Method
Inverse variance weighted
MR Egger
4.0
3.6
3.2
2.8
2.4
1/SE$_{IV}$
−0.50
−0.25
0.00
0.25
β_{IV}

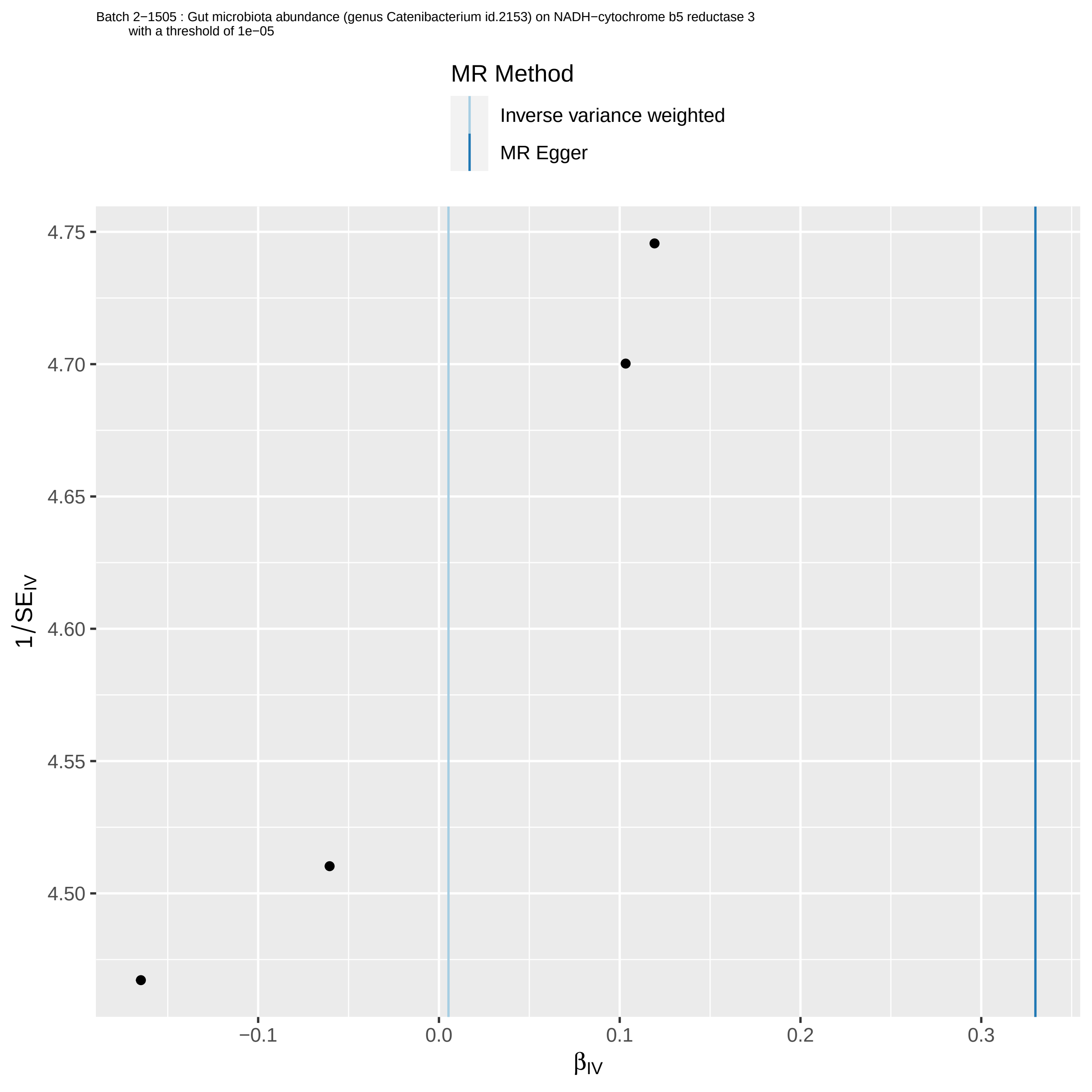
Batch 2−1505 : Gut microbiota abundance (genus Catenibacterium id.2153) on NADH−cytochrome b5 reductase 3
with a threshold of 1e−05
MR Method
Inverse variance weighted
MR Egger
4.75
4.70
4.65
4.60
4.55
4.50
−0.1
0.0
0.1
0.2
0.3
$1/SE_{IV}$
β_{IV}

Batch 2−1506 : Gut microbiota abundance (genus Parasutterella id.2892) on NADH−cytochrome b5 reductase 3
with a threshold of 1e−05

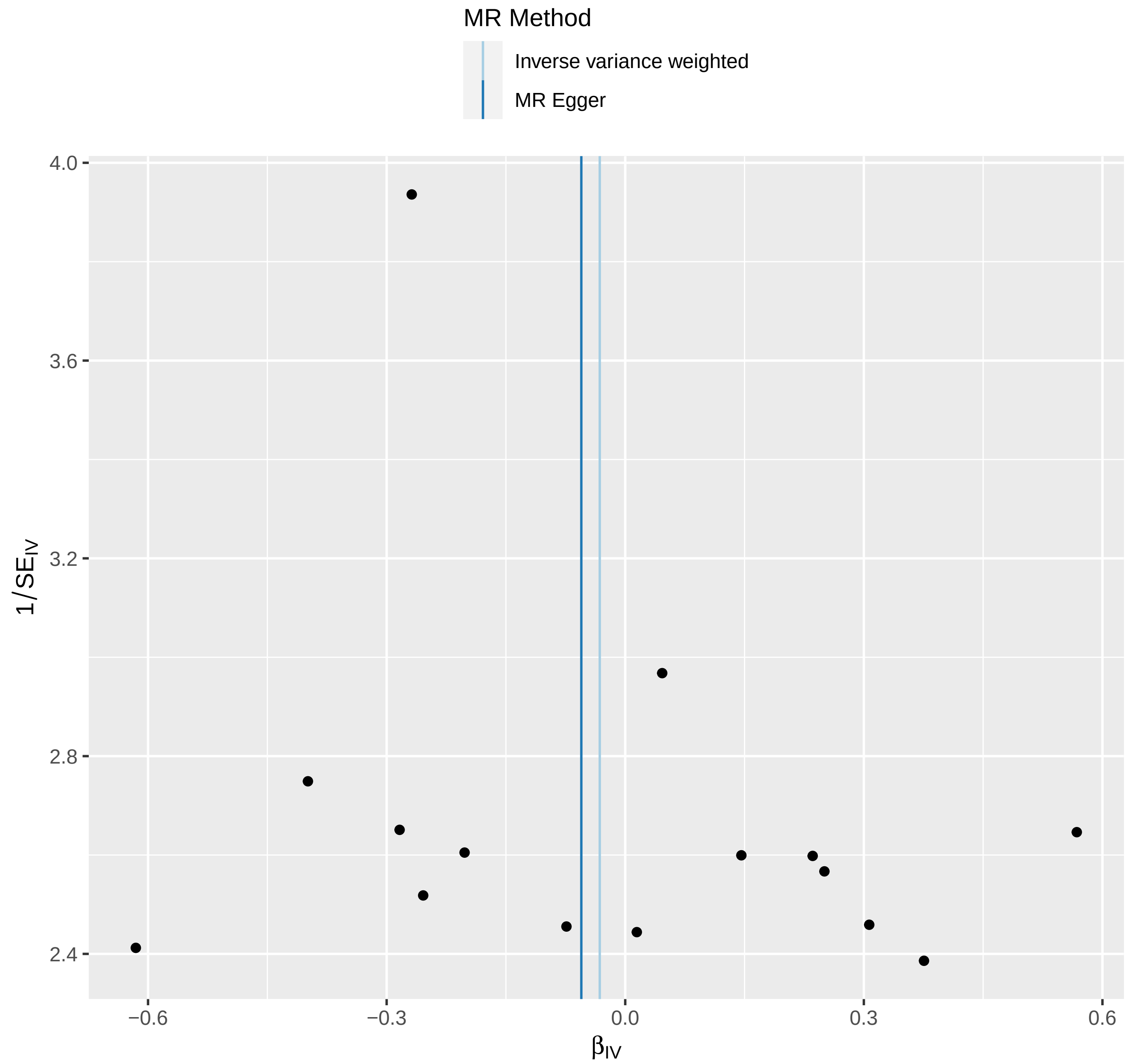

Batch 2−1507 : Gut microbiota abundance (genus Catenibacterium id.2153) on Cytochrome c
with a threshold of 1e−05

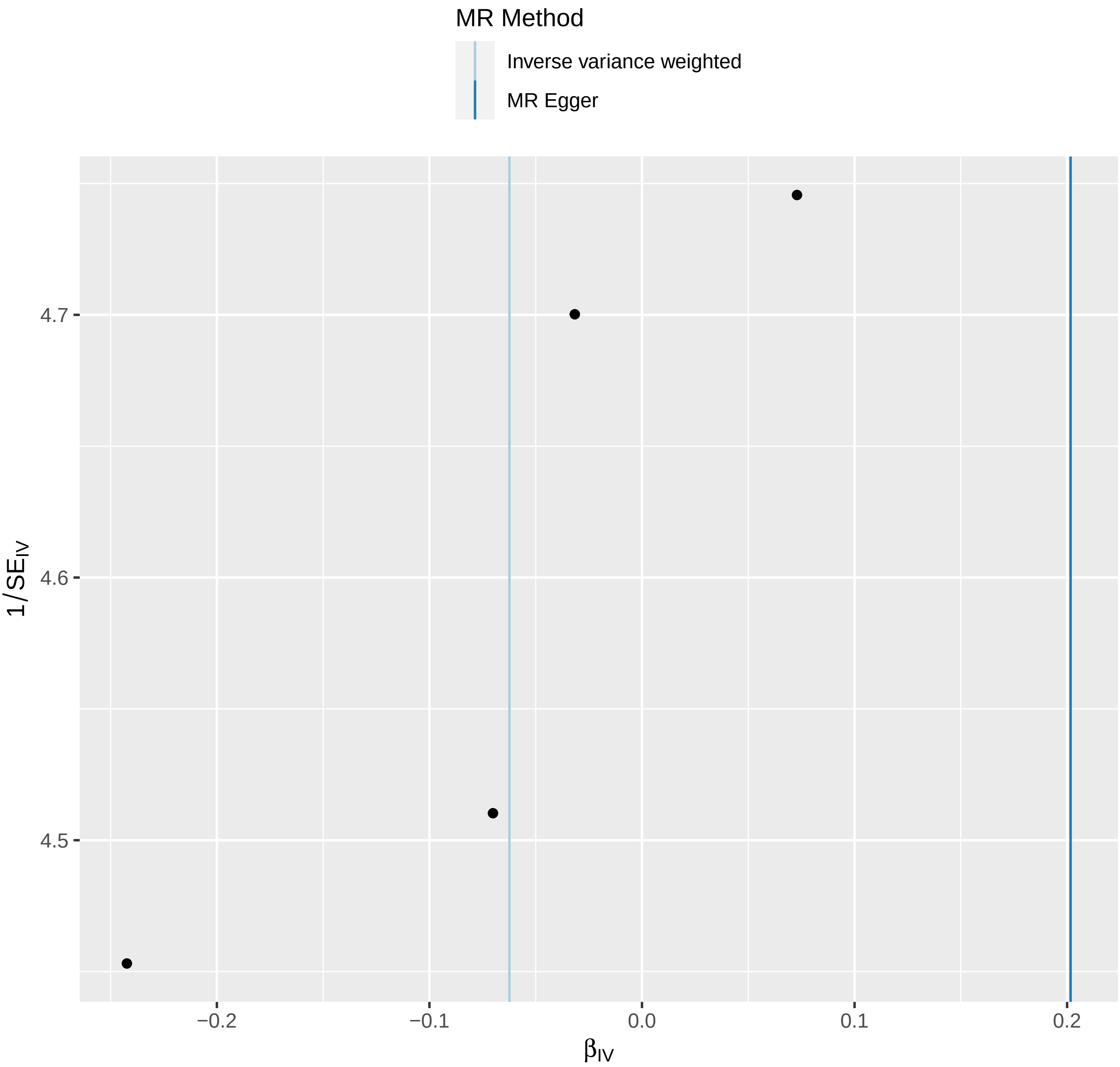

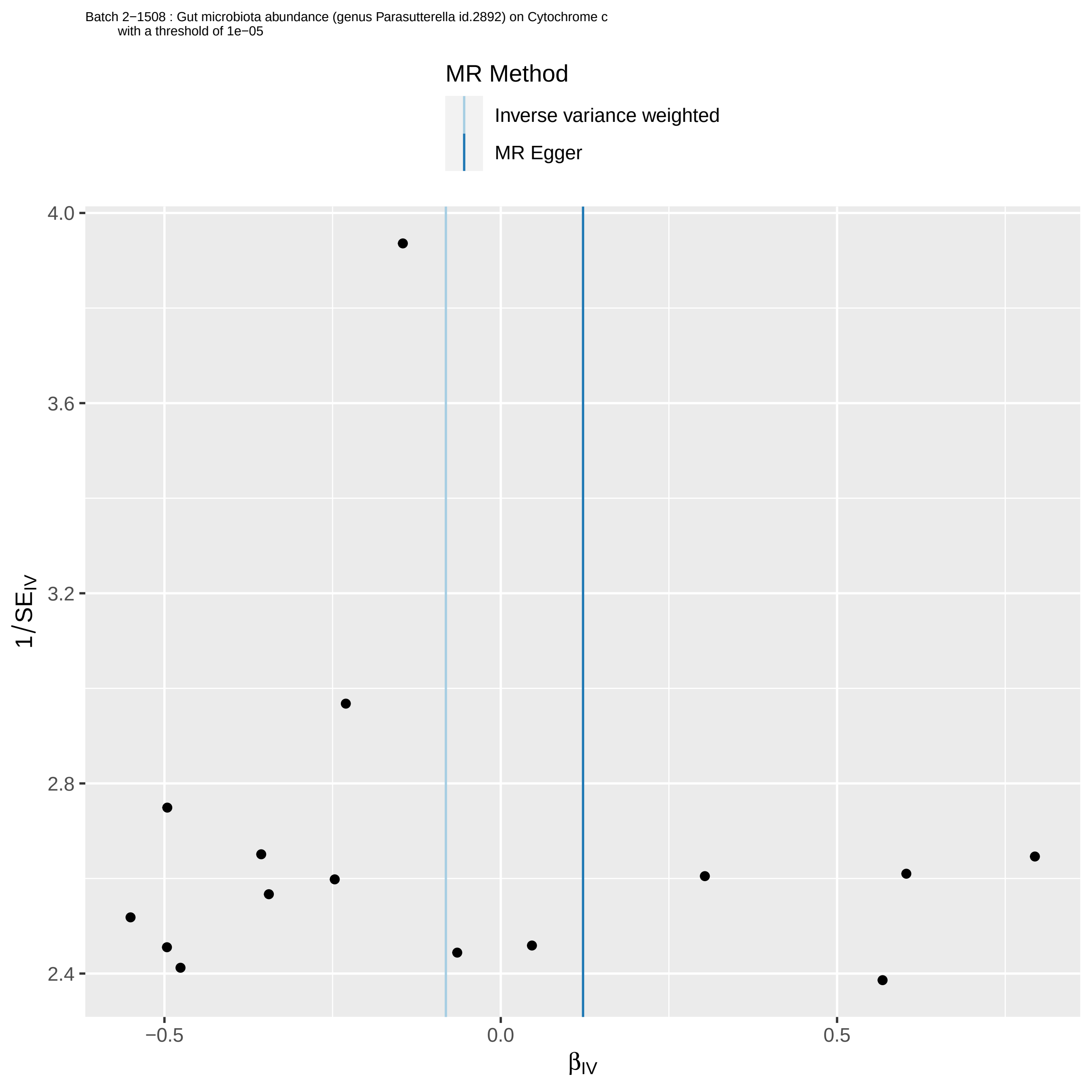

Batch 2−1508 : Gut microbiota abundance (genus Parasutterella id.2892) on Cytochrome c
with a threshold of 1e−05
MR Method
Inverse variance weighted
MR Egger
4.0
3.6
3.2
2.8
2.4
1/SE_IV
−0.5
0.0
0.5
β_IV

Batch 2−1509 : Gut microbiota abundance (genus Catenibacterium id.2153) on Cytoglobin
with a threshold of 1e−05

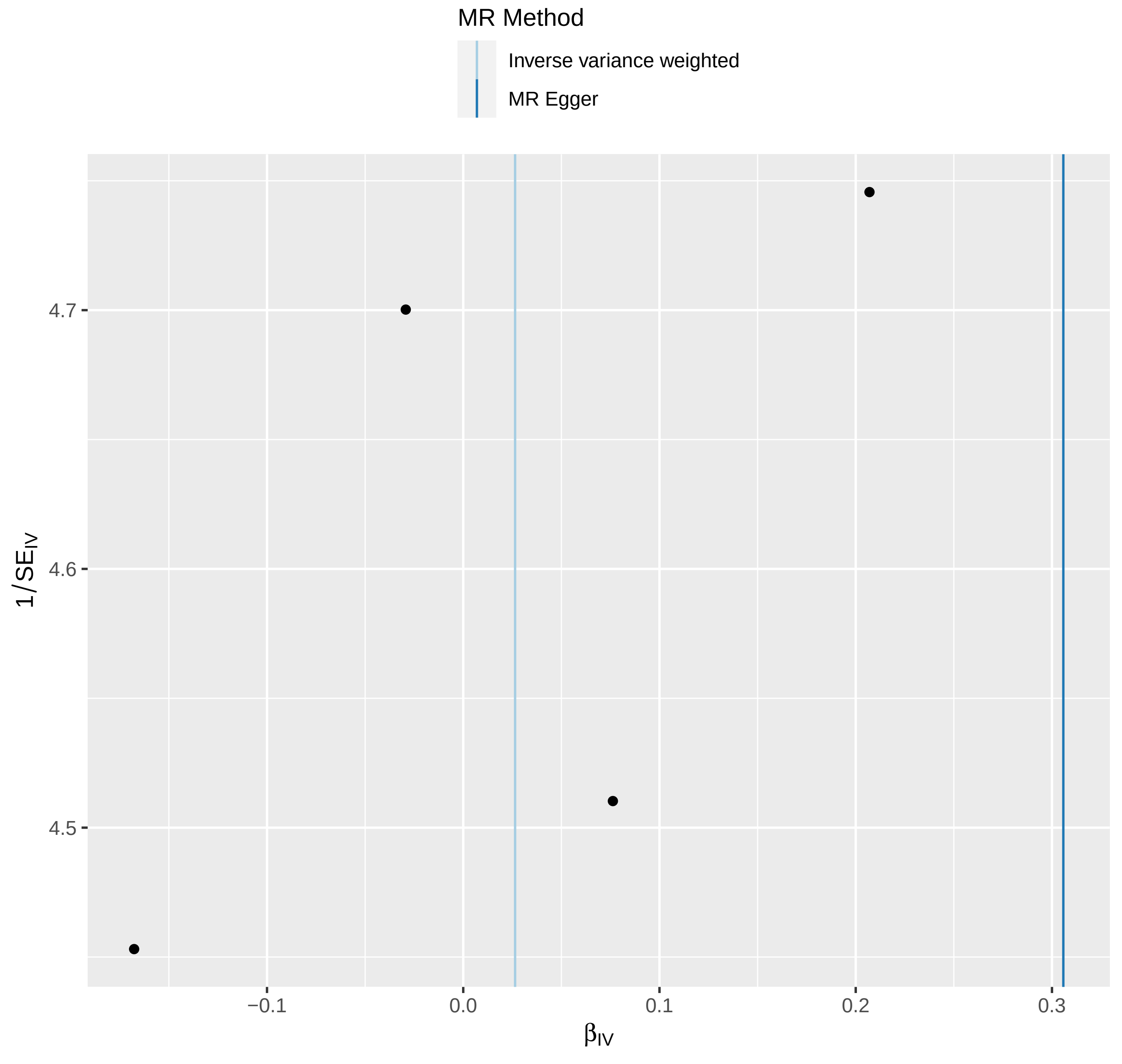

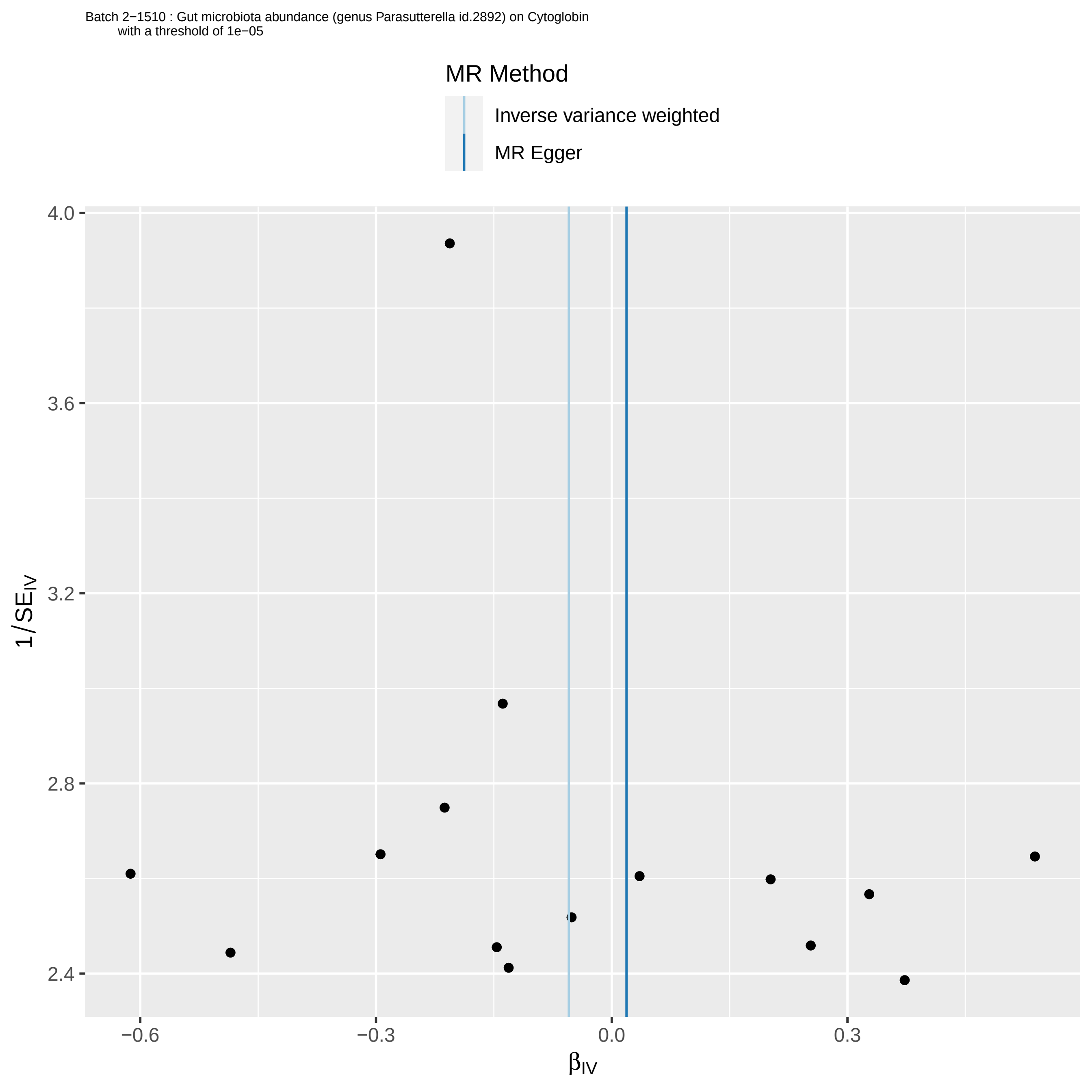

Batch 2−1510 : Gut microbiota abundance (genus Parasutterella id.2892) on Cytoglobin
with a threshold of 1e−05
MR Method
Inverse variance weighted
MR Egger
4.0
3.6
3.2
2.8
2.4
1/SE_IV
−0.6
−0.3
0.0
0.3
β_IV

Batch 2−1511 : Gut microbiota abundance (genus Catenibacterium id.2153) on Cytochrome P450 3A4
with a threshold of 1e−05

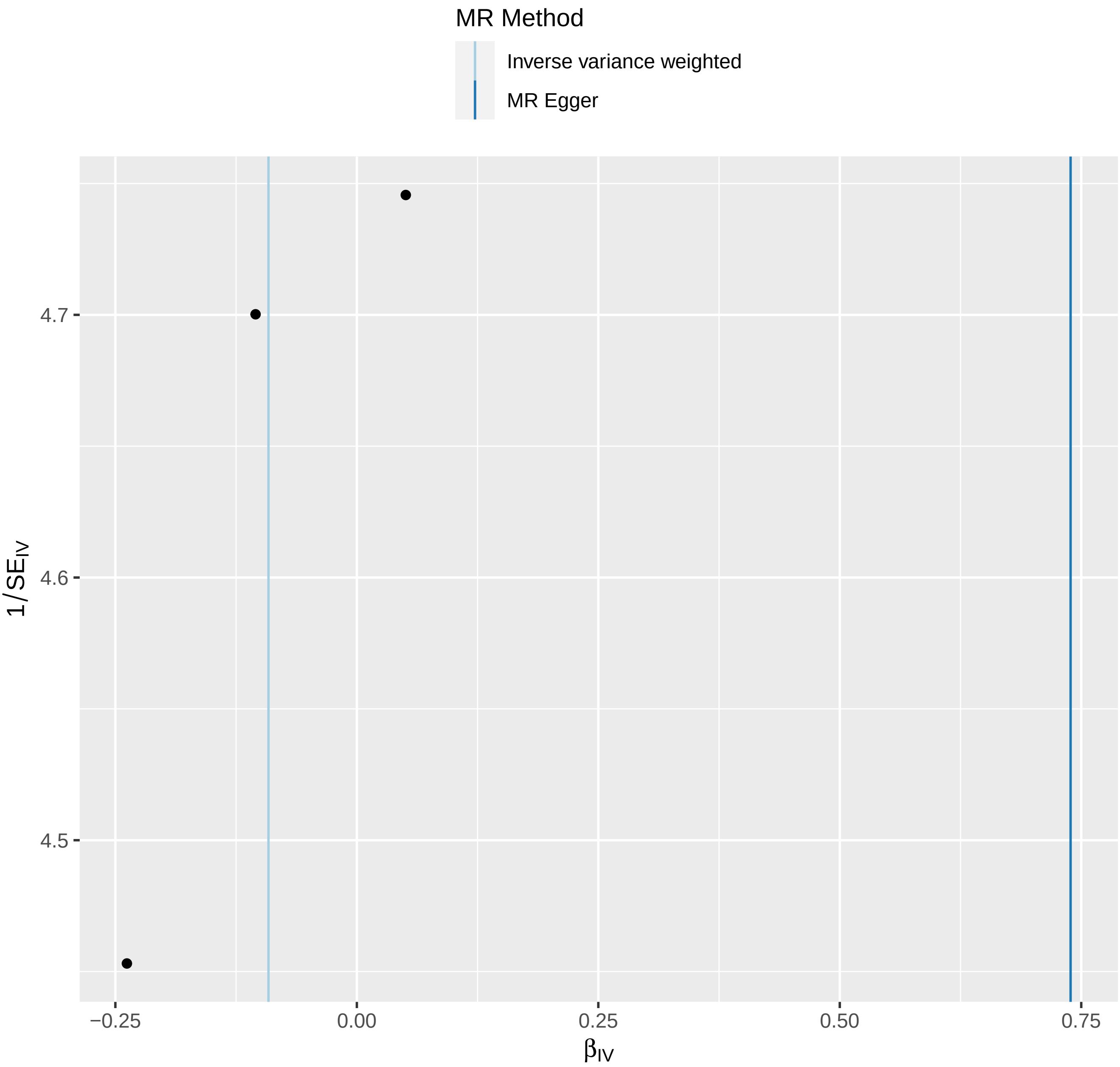

Batch 2−1512 : Gut microbiota abundance (genus Parasutterella id.2892) on Cytochrome P450 3A4
with a threshold of 1e−05

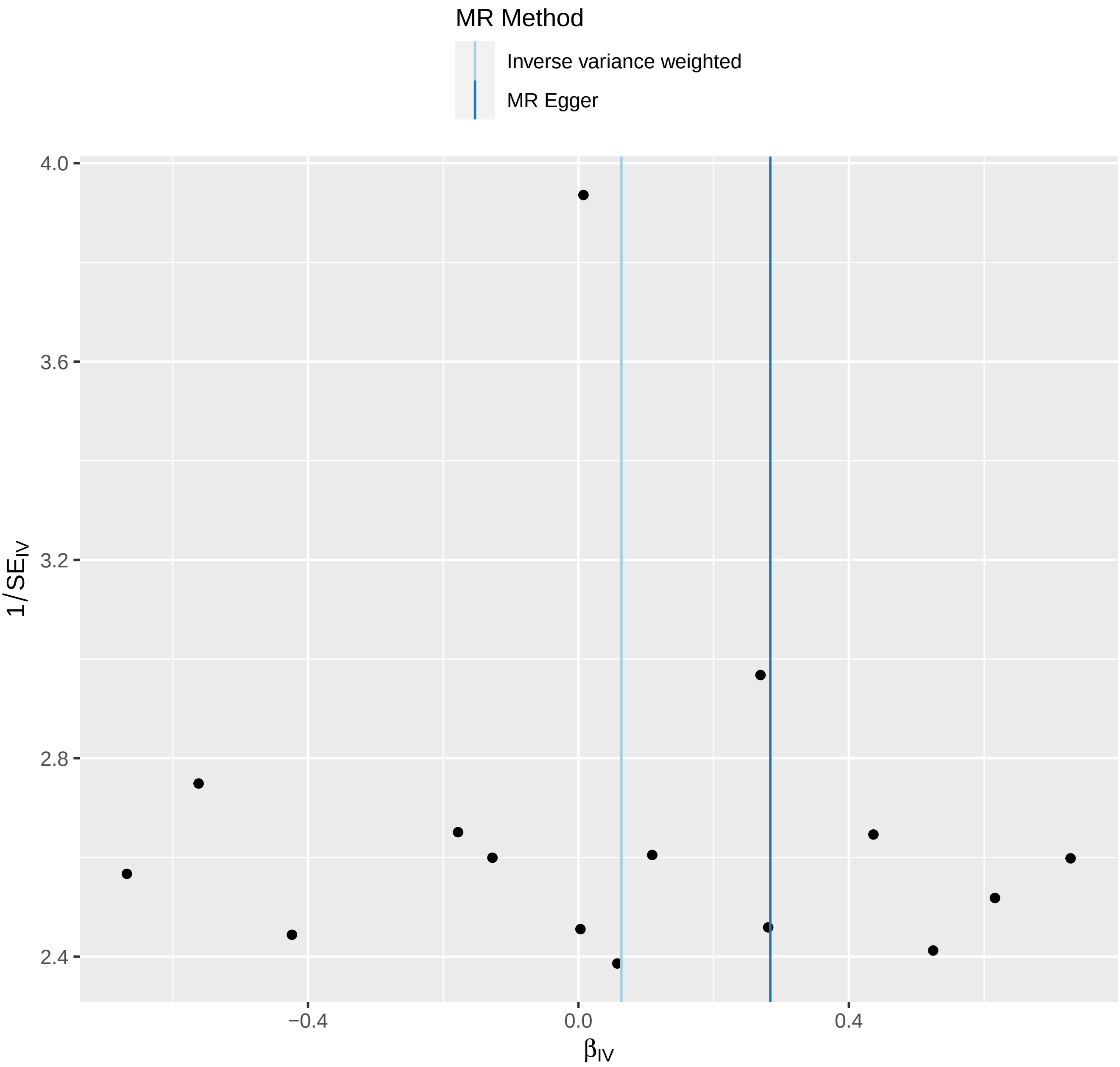

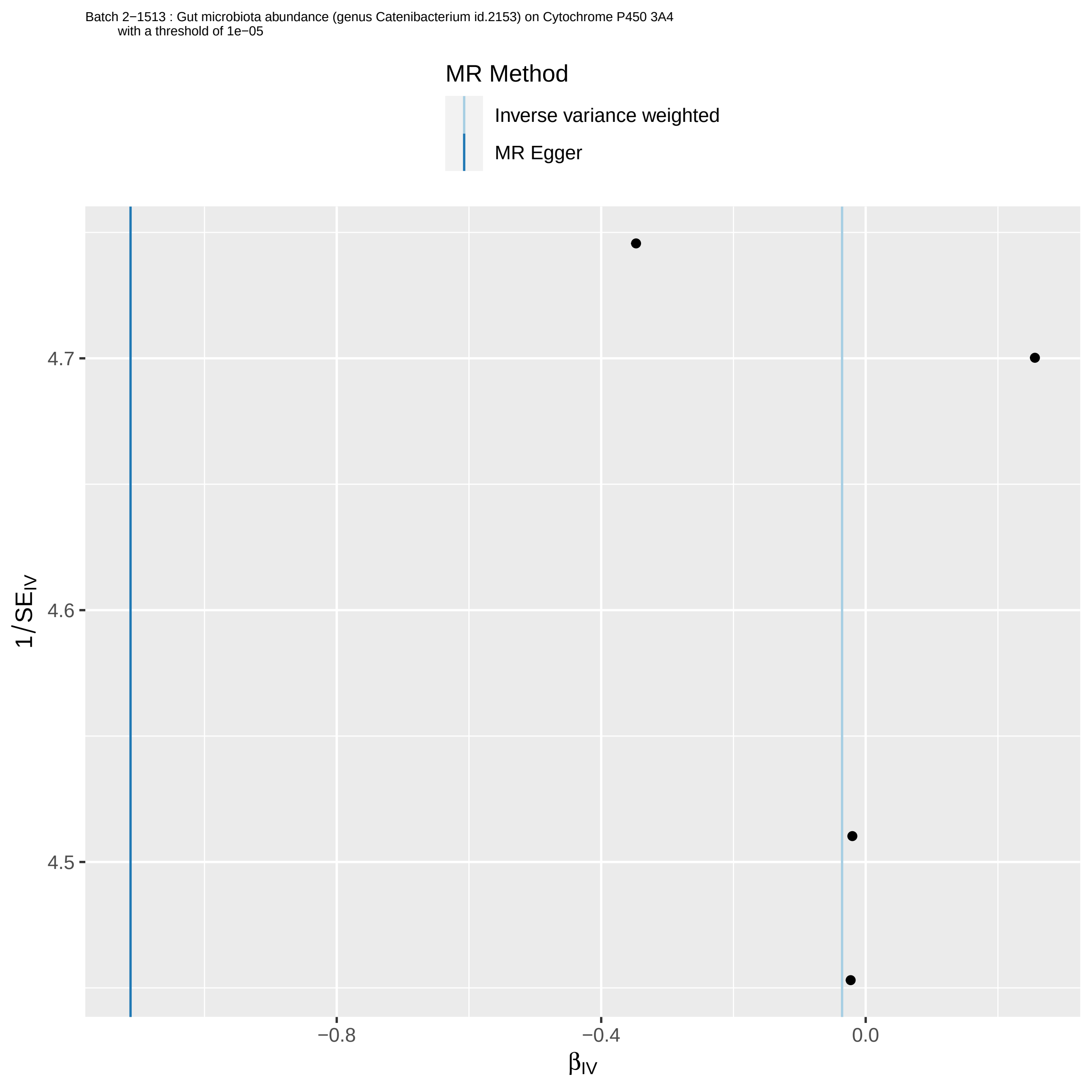

Batch 2−1513 : Gut microbiota abundance (genus Catenibacterium id.2153) on Cytochrome P450 3A4
with a threshold of 1e−05
MR Method
Inverse variance weighted
MR Egger
4.7
4.6
4.5
1/SE_IV
−0.8
−0.4
0.0
β_IV

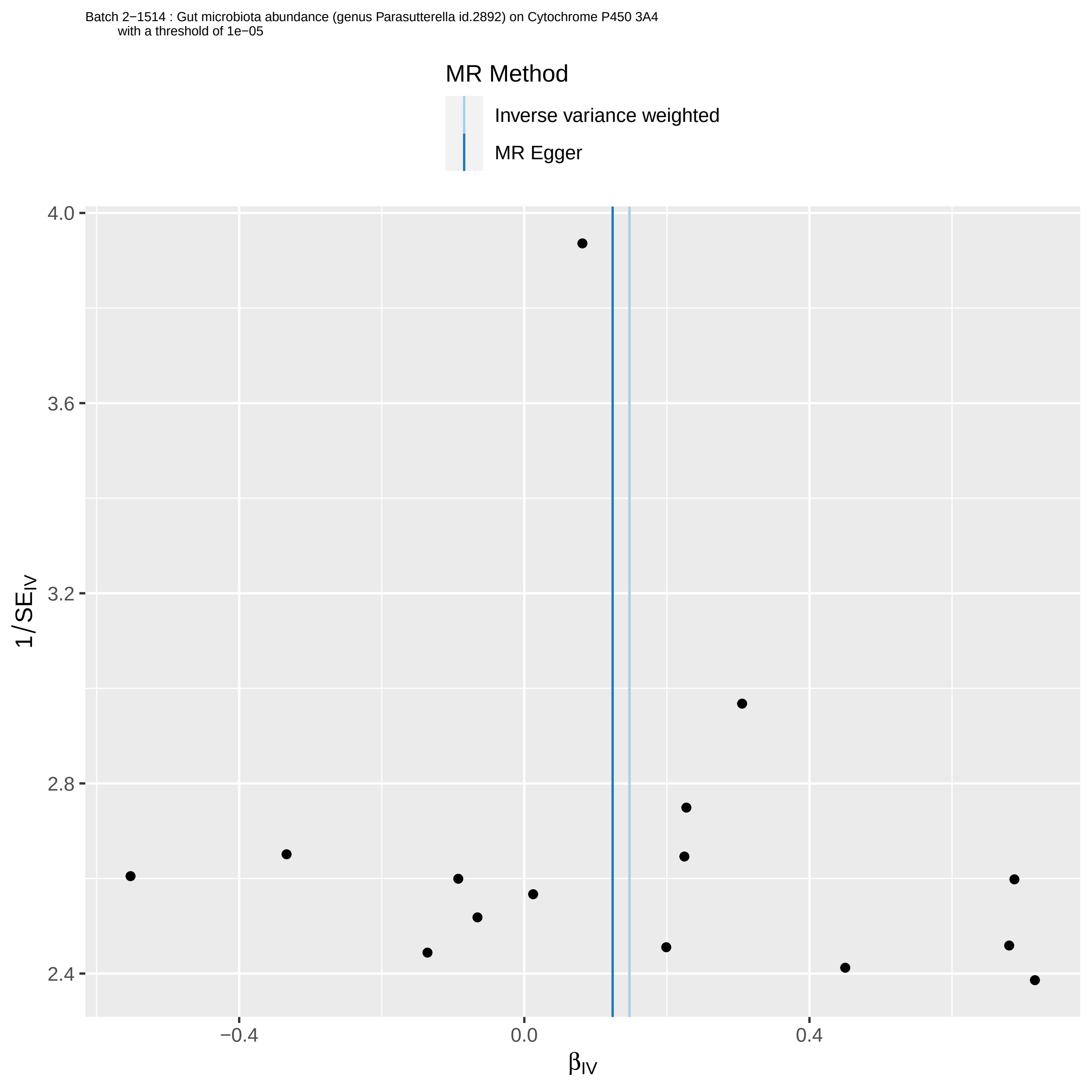

Batch 2−1514 : Gut microbiota abundance (genus Parasutterella id.2892) on Cytochrome P450 3A4
with a threshold of 1e−05
MR Method
Inverse variance weighted
MR Egger
4.0
3.6
3.2
2.8
2.4
1/SEIV
−0.4
0.0
0.4
βIV

Batch 2−1515 : Gut microbiota abundance (genus Catenibacterium id.2153) on Protein CYR61
with a threshold of 1e−05

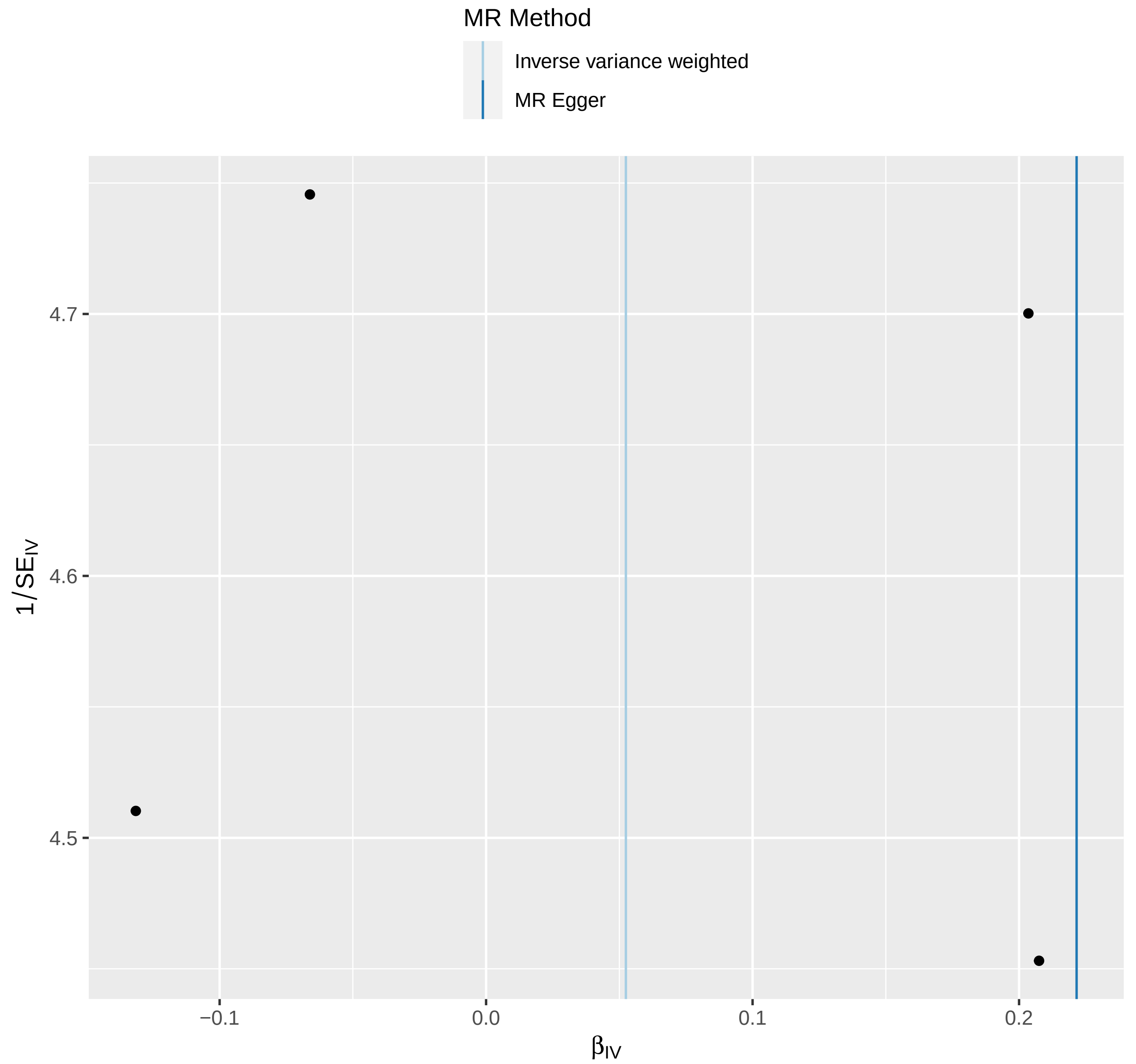

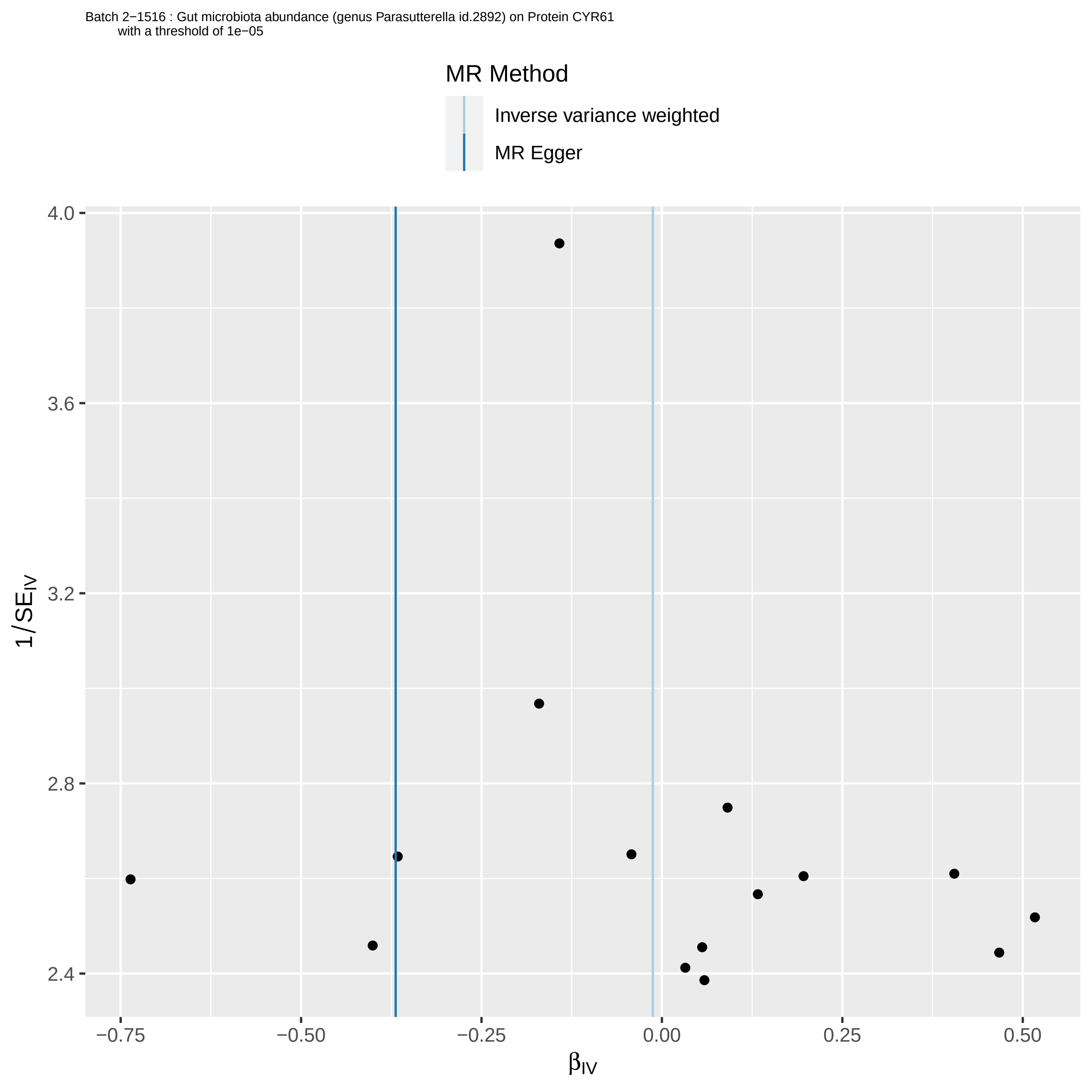
Batch 2−1516 : Gut microbiota abundance (genus Parasutterella id.2892) on Protein CYR61
with a threshold of 1e−05
MR Method
Inverse variance weighted
MR Egger
4.0
3.6
3.2
2.8
2.4
1/SE$_{IV}$
−0.75
−0.50
−0.25
0.00
0.25
0.50
β_{IV}

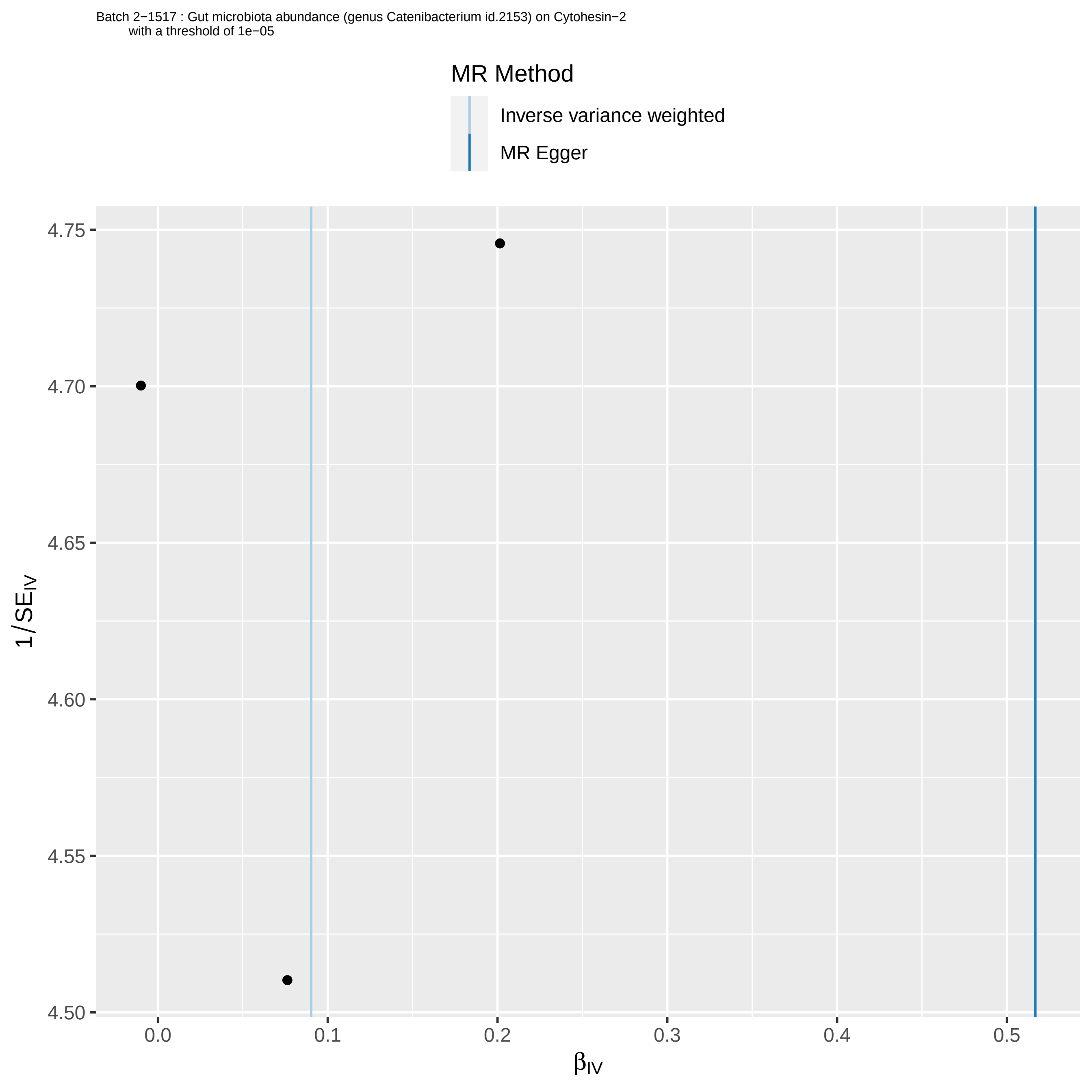

Batch 2−1517 : Gut microbiota abundance (genus Catenibacterium id.2153) on Cytohesin−2
with a threshold of 1e−05
MR Method
Inverse variance weighted
MR Egger
4.75
4.70
4.65
4.60
4.55
4.50
1/SE_IV
0.0
0.1
0.2
0.3
0.4
0.5
β_IV

Batch 2−1518 : Gut microbiota abundance (genus Parasutterella id.2892) on Cytohesin−2
with a threshold of 1e−05

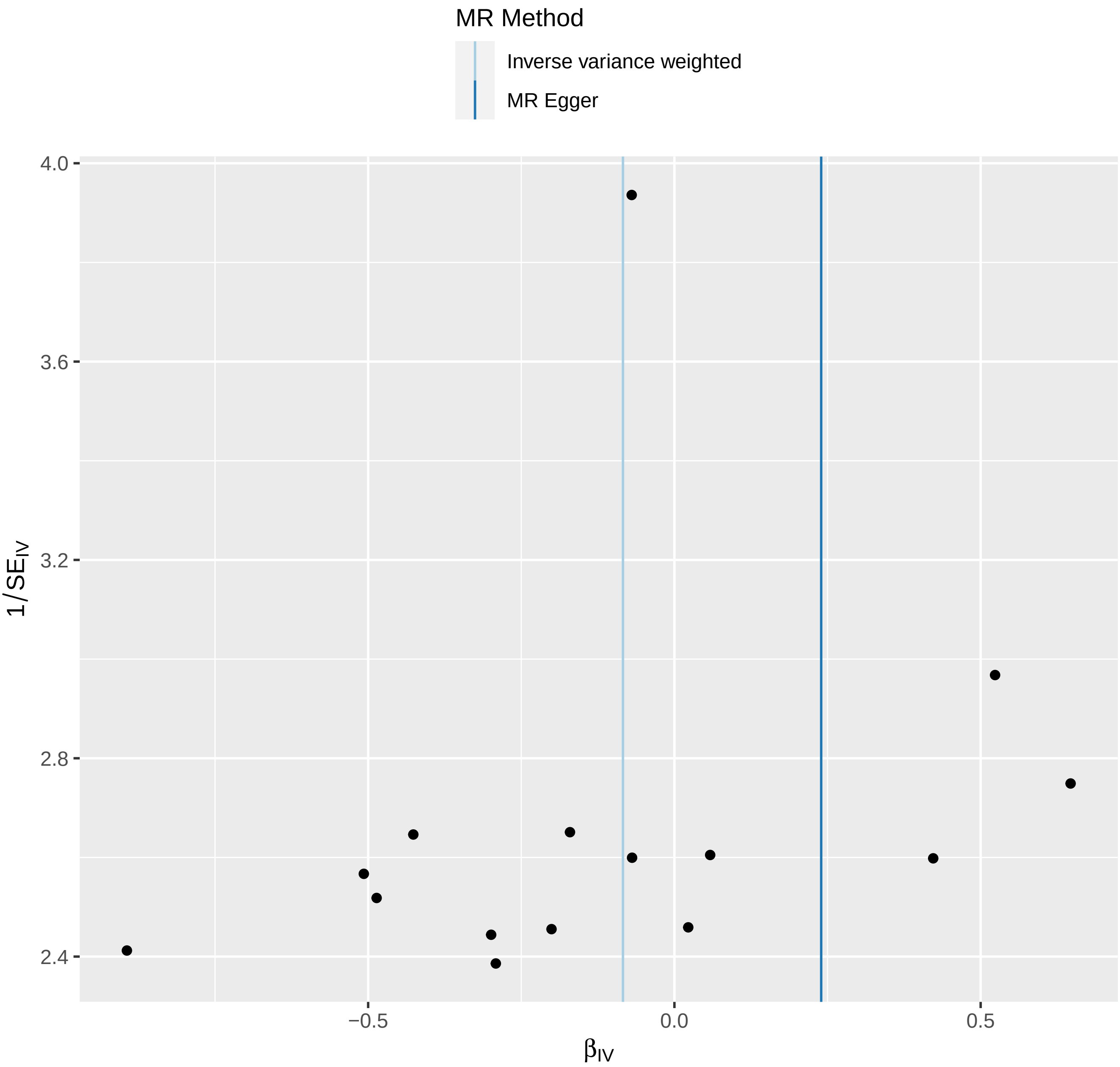

Batch 2−1519 : Gut microbiota abundance (genus Catenibacterium id.2153) on Cytohesin−4
with a threshold of 1e−05

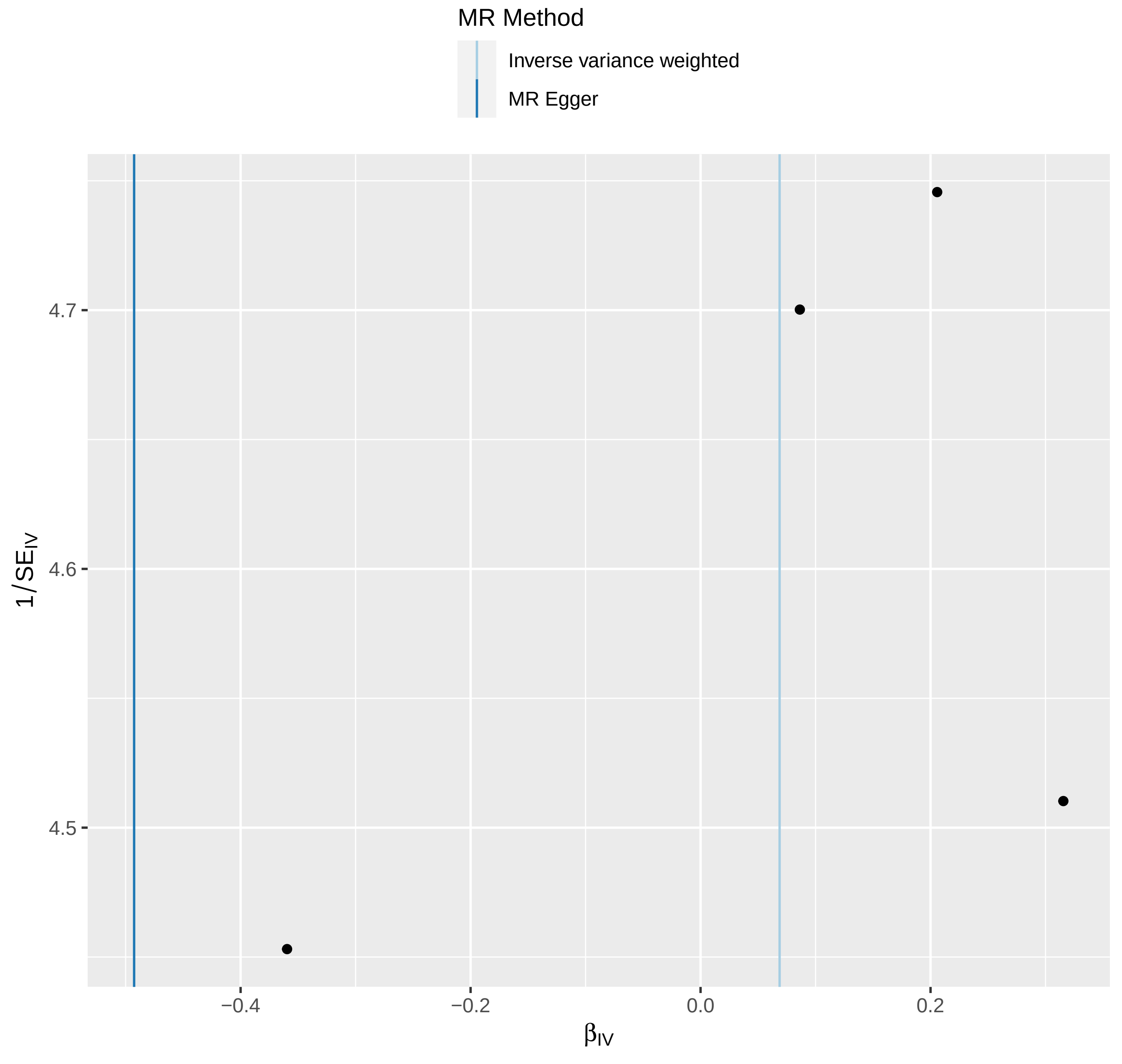

Batch 2−1520 : Gut microbiota abundance (genus Parasutterella id.2892) on Cytohesin−4
with a threshold of 1e−05

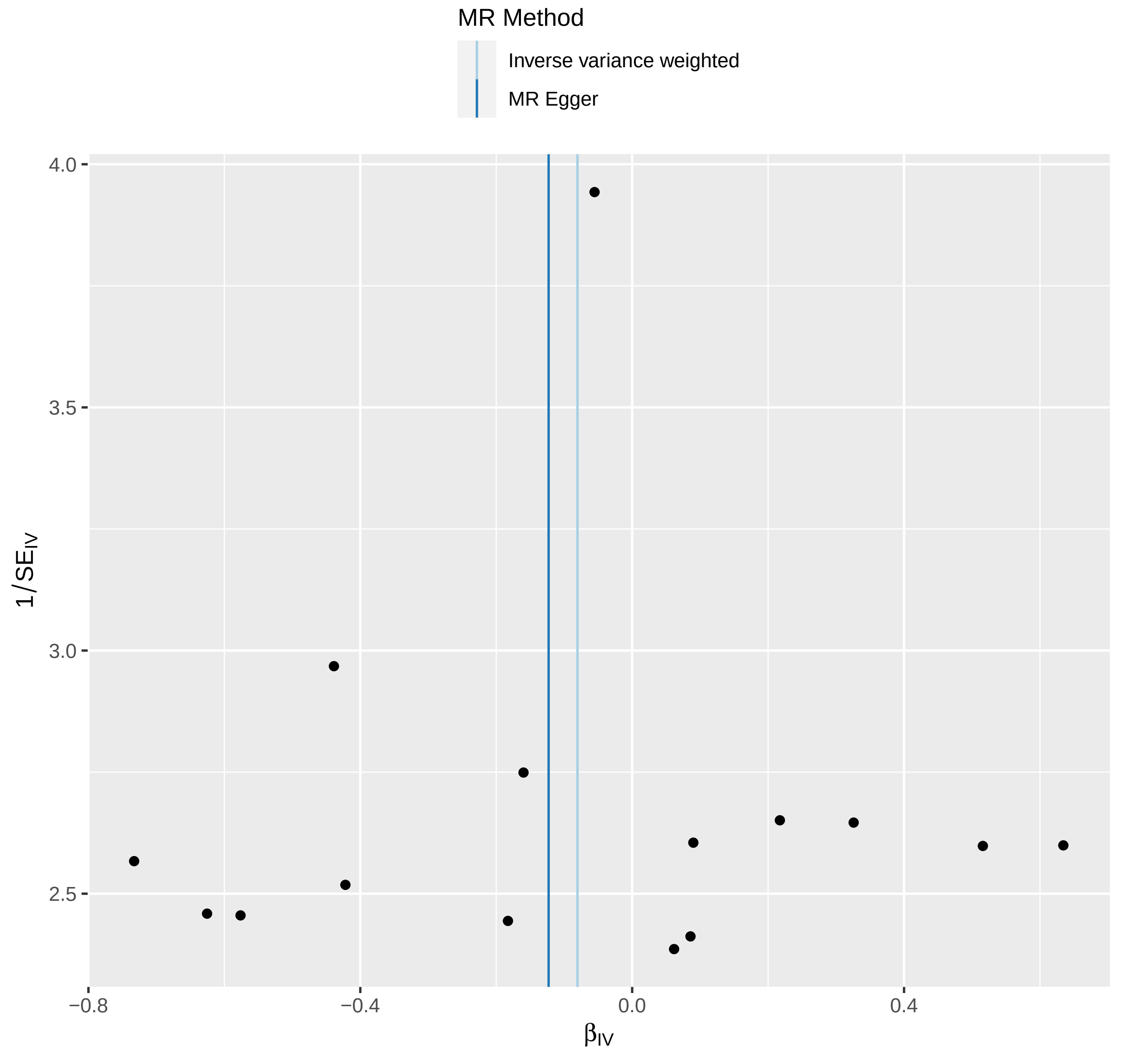

Batch 2−1521 : Gut microbiota abundance (genus Catenibacterium id.2153) on Cytohesin−interacting protein
with a threshold of 1e−05

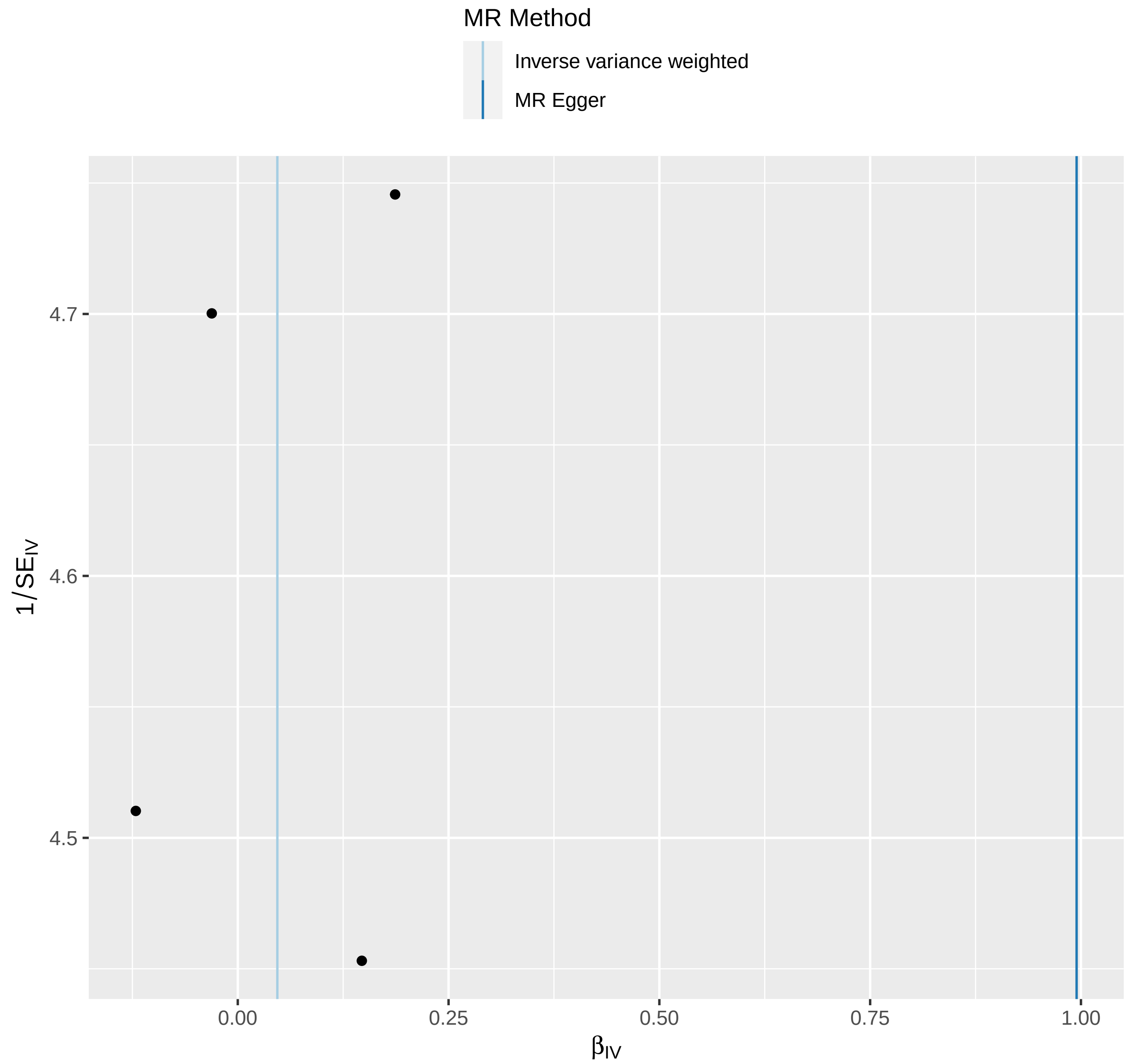

Batch 2−1522 : Gut microbiota abundance (genus Parasutterella id.2892) on Cytohesin−interacting protein
with a threshold of 1e−05

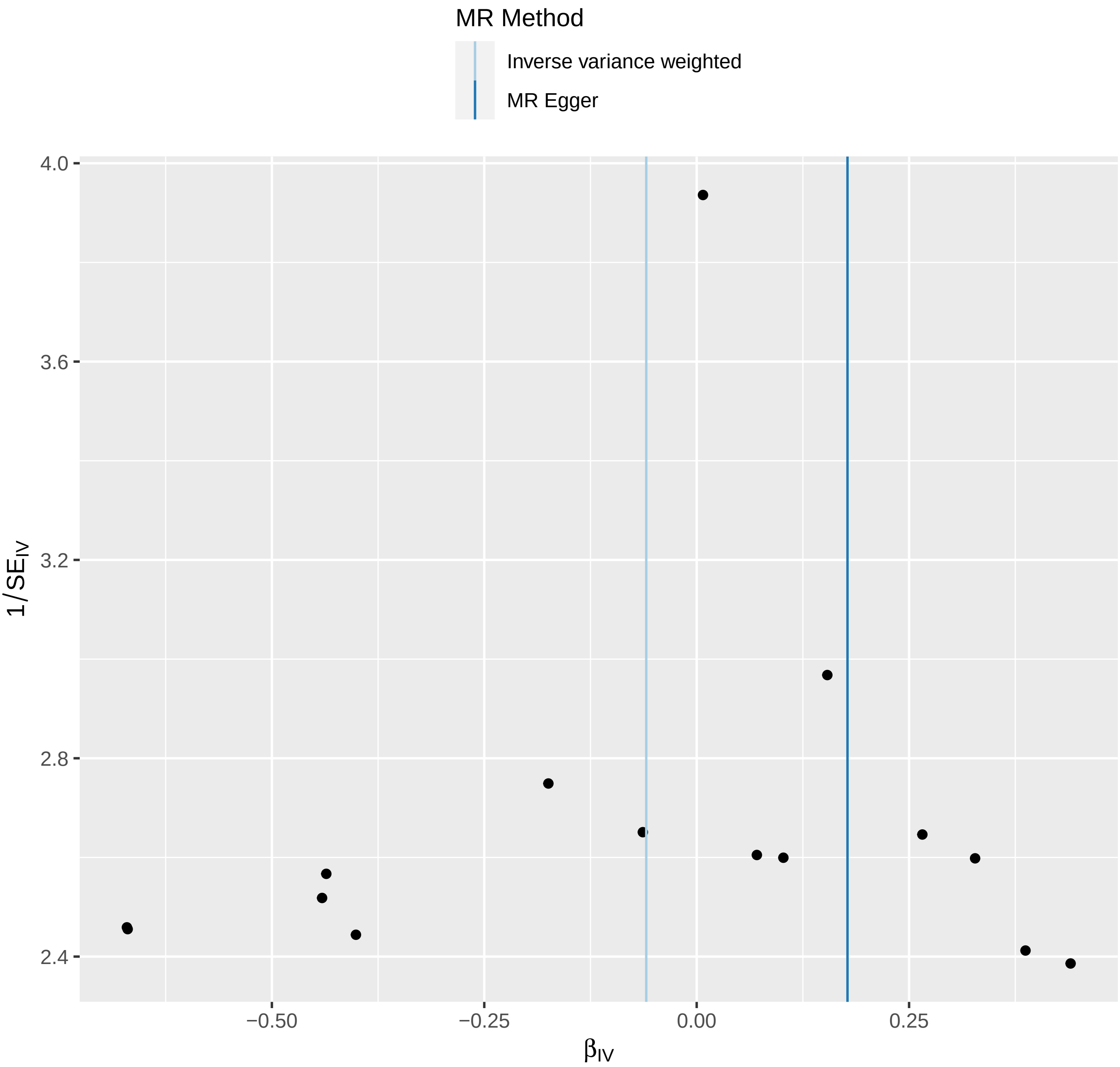

Batch 2−1523 : Gut microbiota abundance (genus Catenibacterium id.2153) on Dystroglycan
with a threshold of 1e−05

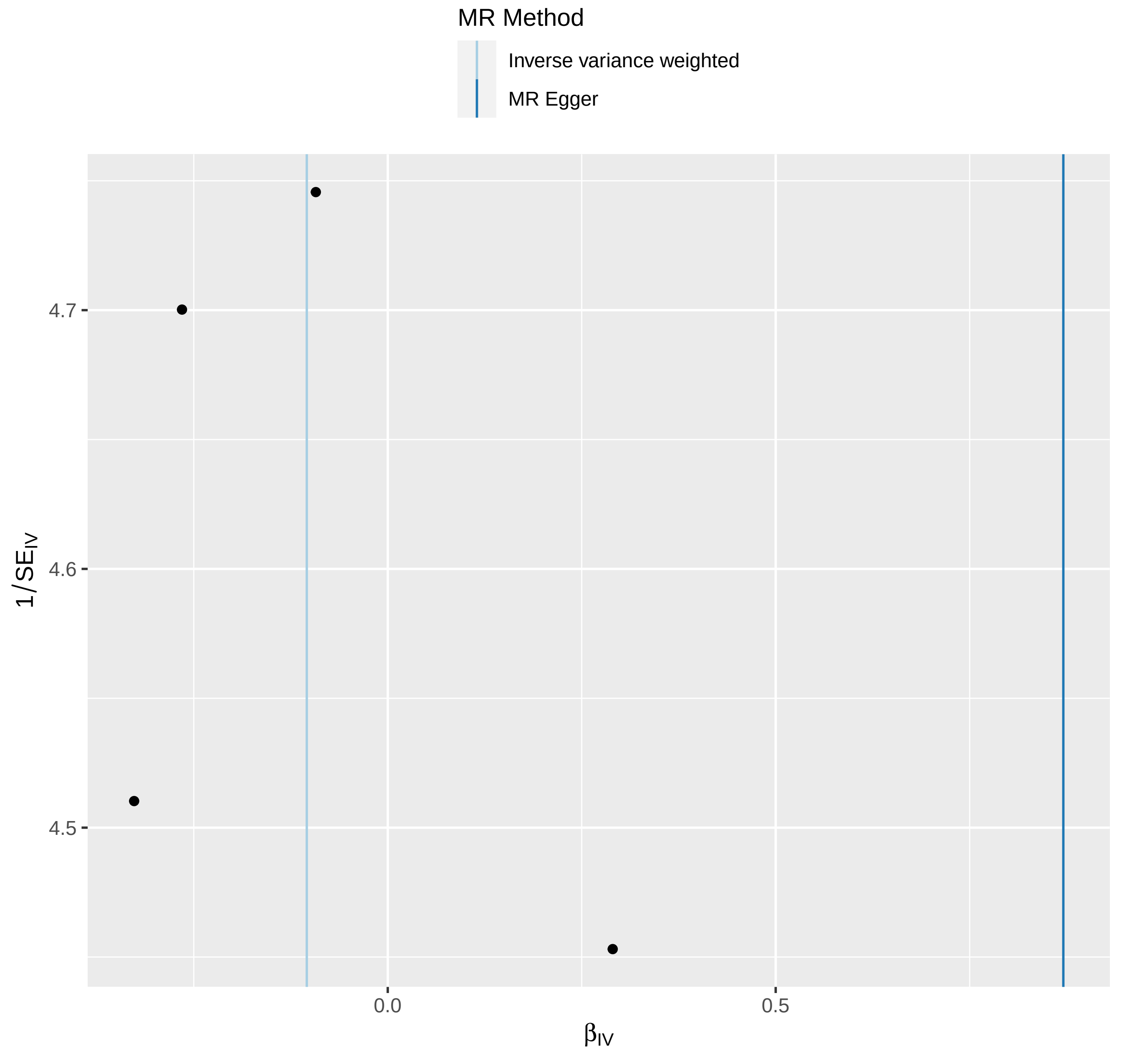

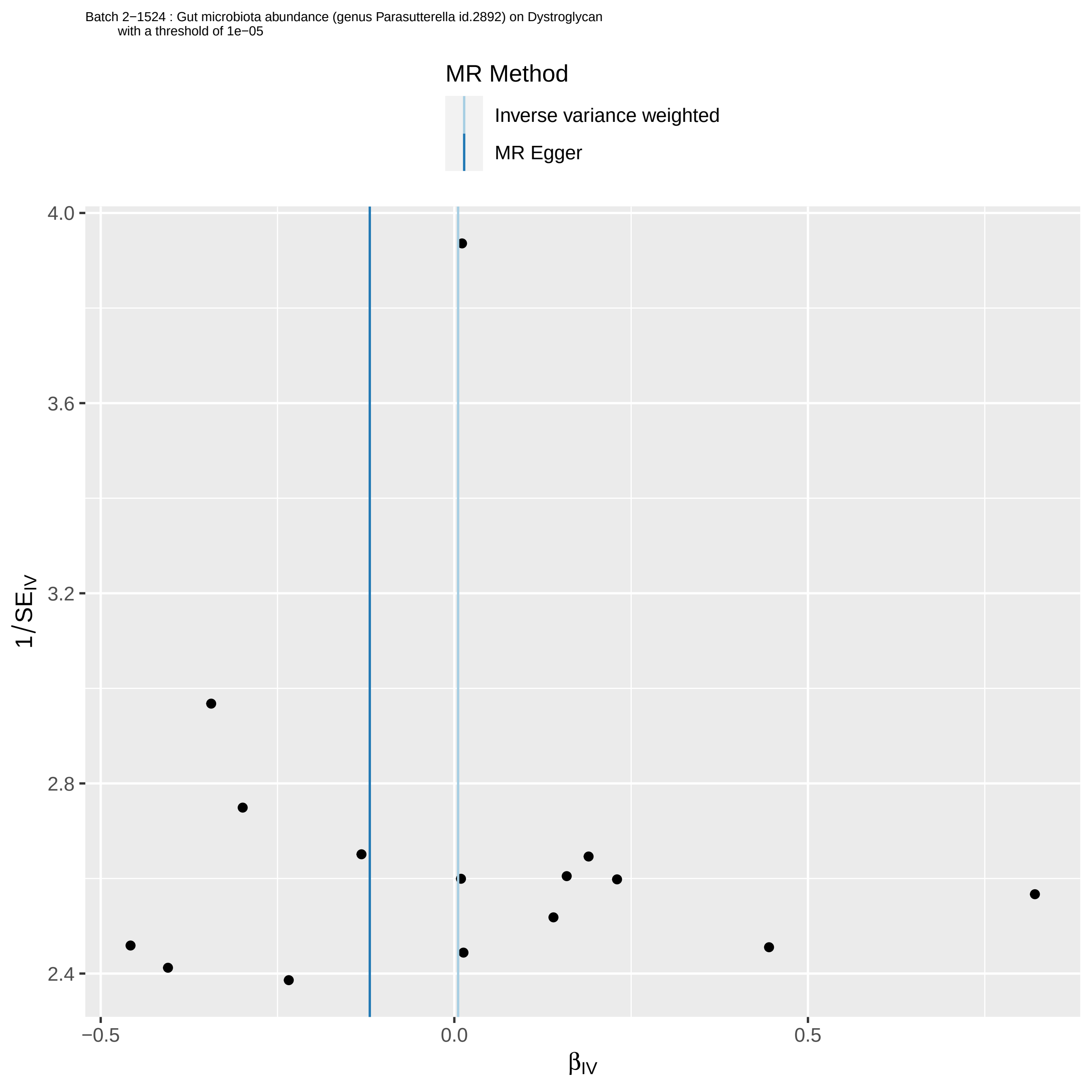
Batch 2−1524 : Gut microbiota abundance (genus Parasutterella id.2892) on Dystroglycan
with a threshold of 1e−05
MR Method
Inverse variance weighted
MR Egger
4.0
3.6
3.2
2.8
2.4
1/SE_IV
−0.5
0.0
0.5
β_IV

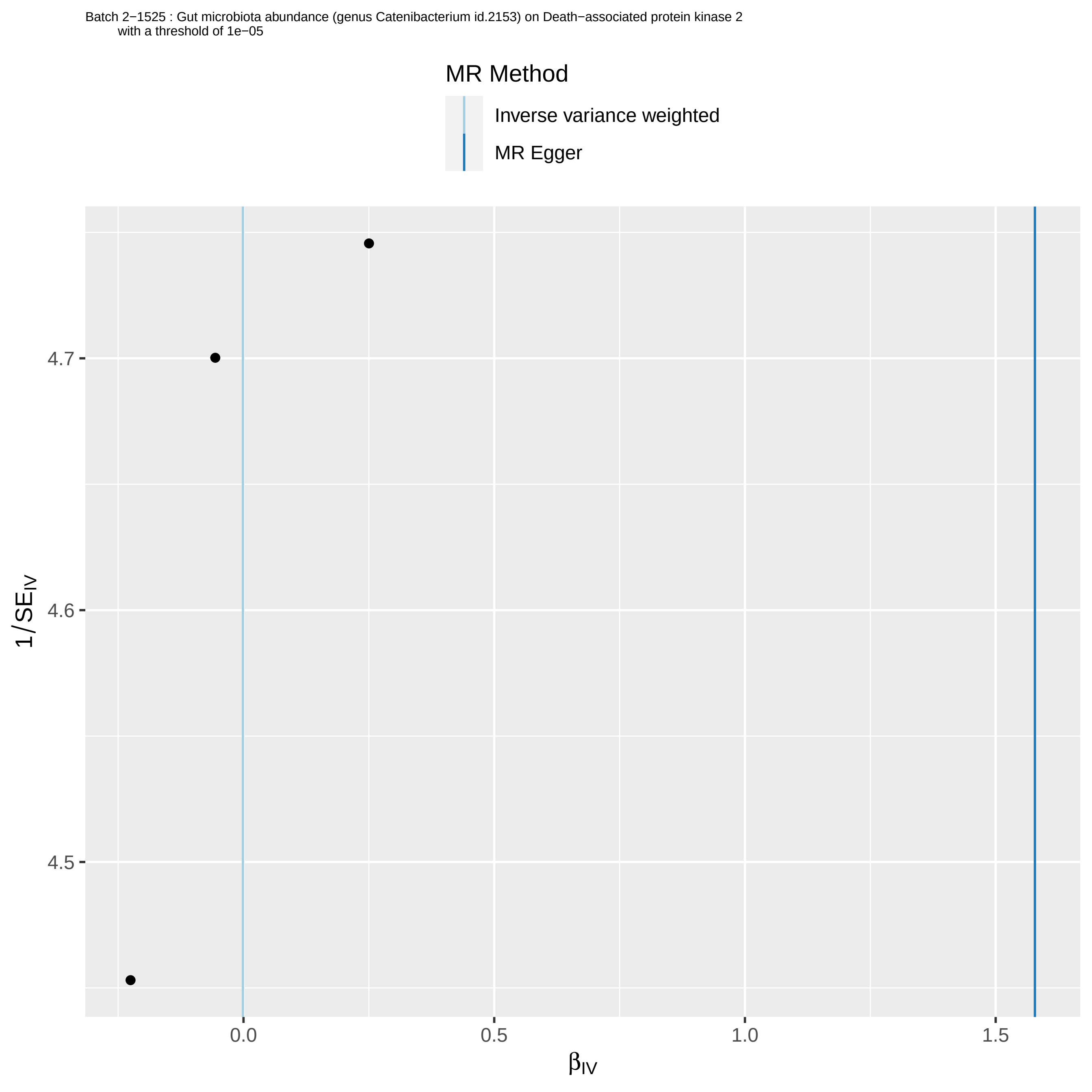

Batch 2−1525 : Gut microbiota abundance (genus Catenibacterium id.2153) on Death−associated protein kinase 2
with a threshold of 1e−05
MR Method
Inverse variance weighted
MR Egger
4.7
4.6
4.5
1/SE_IV
0.0
0.5
1.0
1.5
β_IV

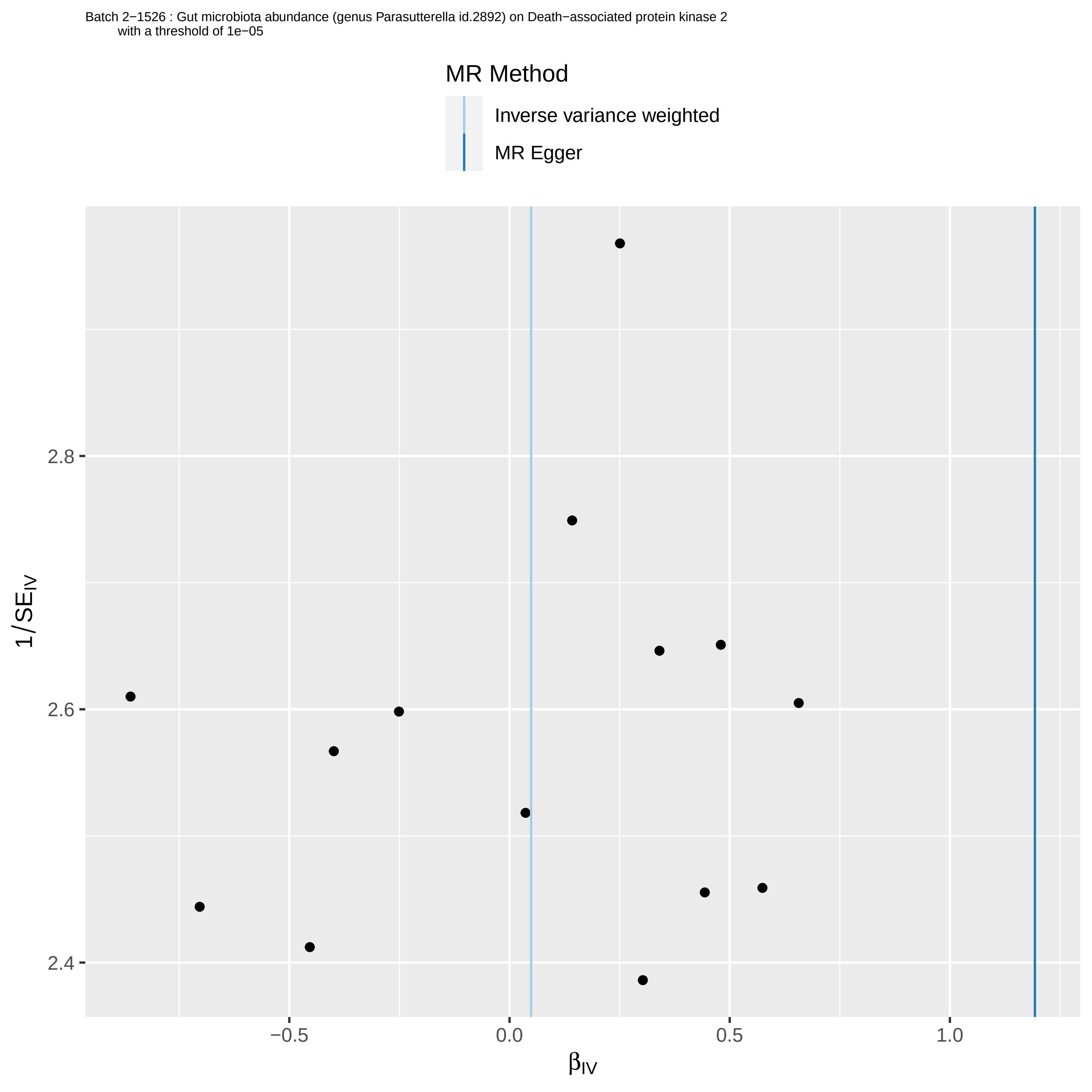

Batch 2−1526 : Gut microbiota abundance (genus Parasutterella id.2892) on Death−associated protein kinase 2
with a threshold of 1e−05
MR Method
Inverse variance weighted
MR Egger
2.8
2.6
2.4
1/SE_IV
−0.5
0.0
0.5
1.0
β_IV

Batch 2−1527 : Gut microbiota abundance (genus Catenibacterium id.2153) on Drebrin−like protein
with a threshold of 1e−05

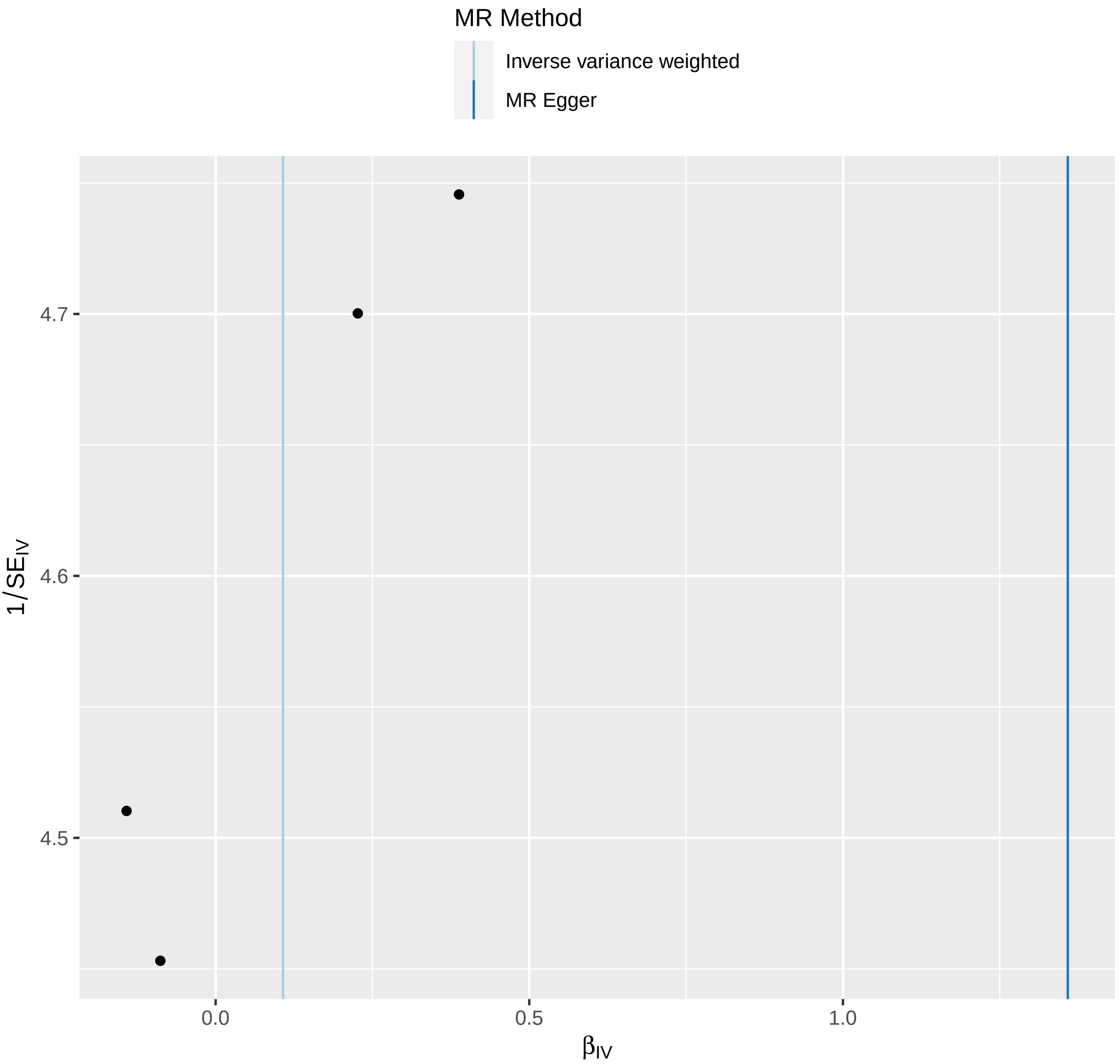

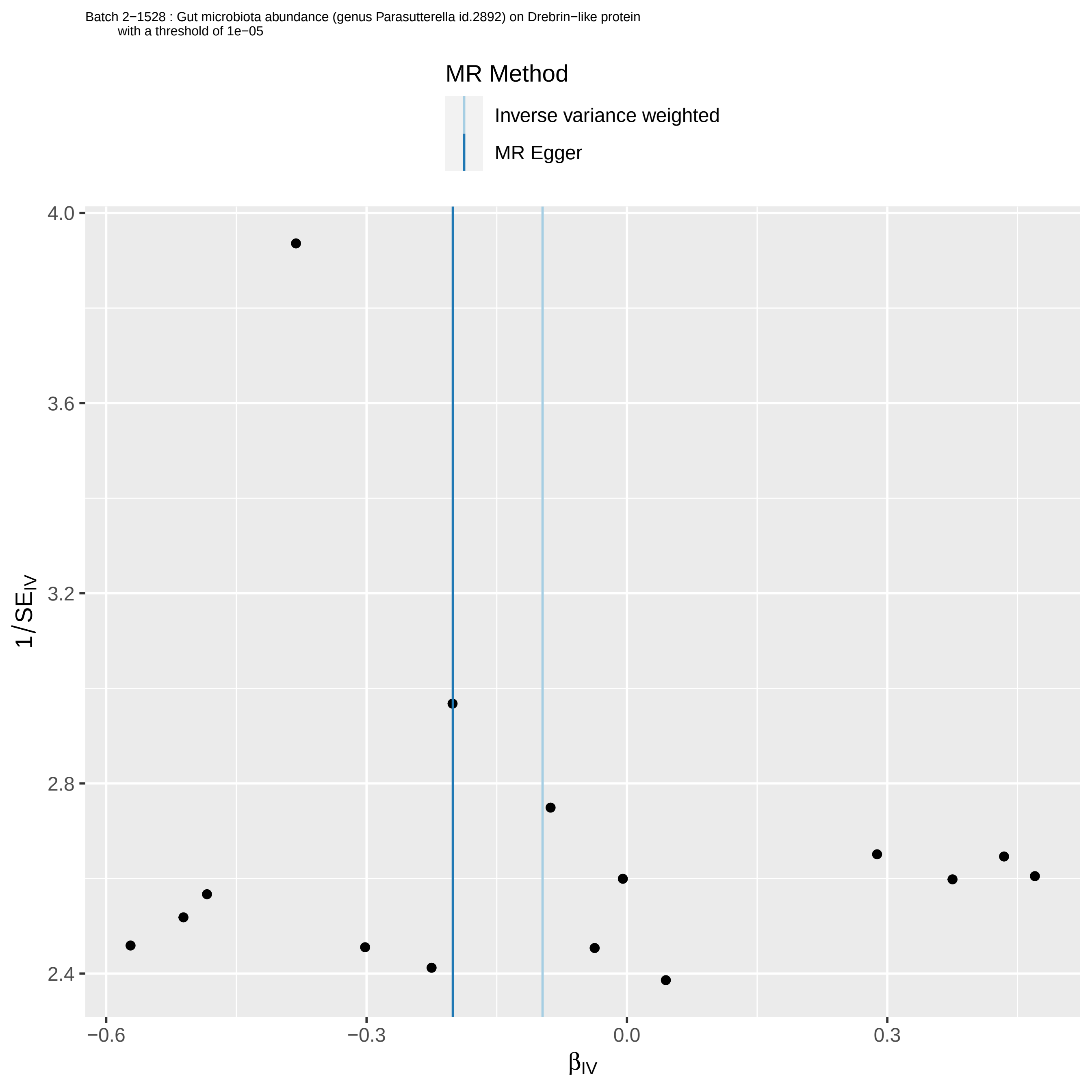

Batch 2−1528 : Gut microbiota abundance (genus Parasutterella id.2892) on Drebrin−like protein
with a threshold of 1e−05
MR Method
Inverse variance weighted
MR Egger
4.0
3.6
3.2
2.8
2.4
1/SE_IV
−0.6
−0.3
0.0
0.3
β_IV

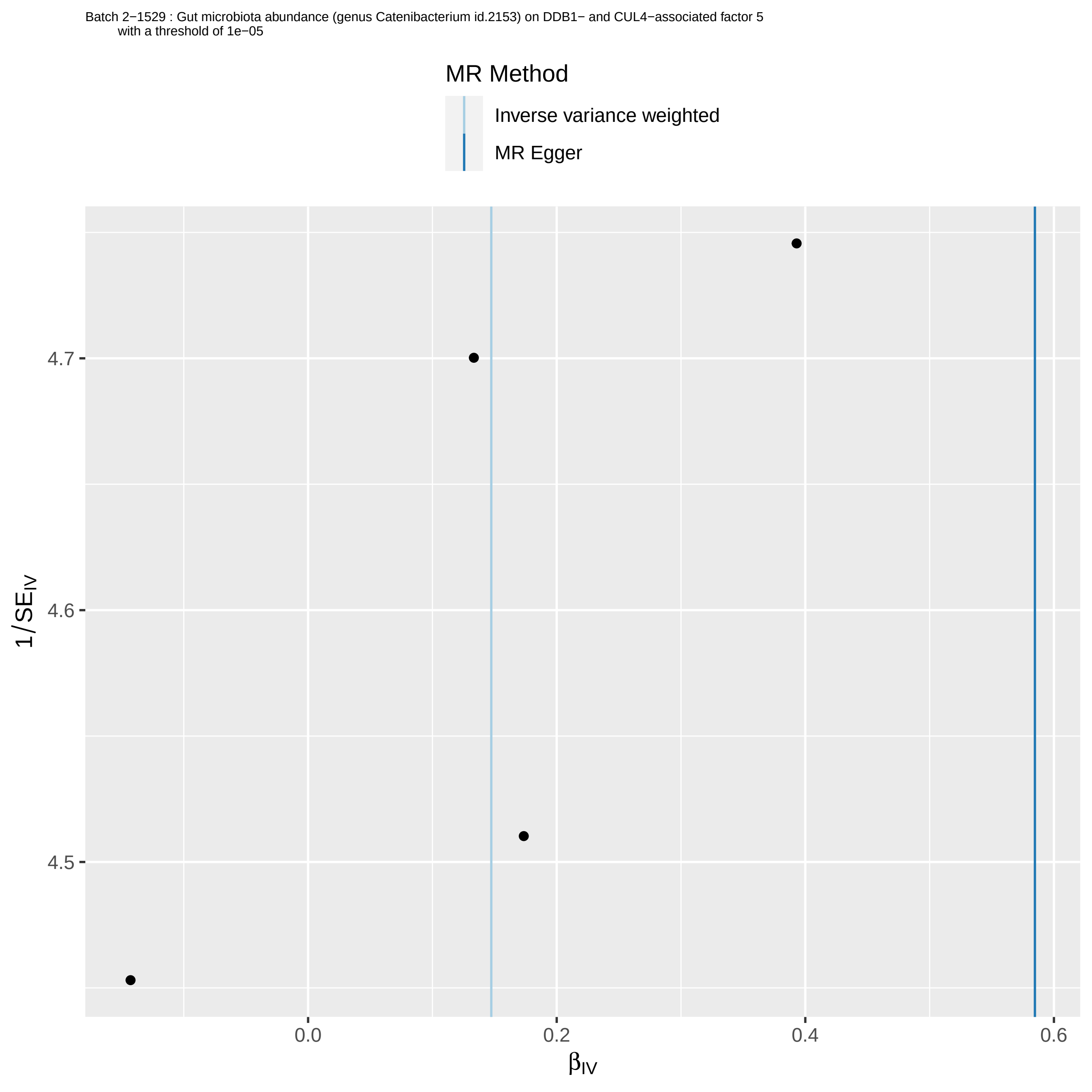

Batch 2−1529 : Gut microbiota abundance (genus Catenibacterium id.2153) on DDB1− and CUL4−associated factor 5 with a threshold of 1e−05
MR Method
Inverse variance weighted
MR Egger
4.7
4.6
4.5
1/SE_IV
0.0
0.2
0.4
0.6
β_IV

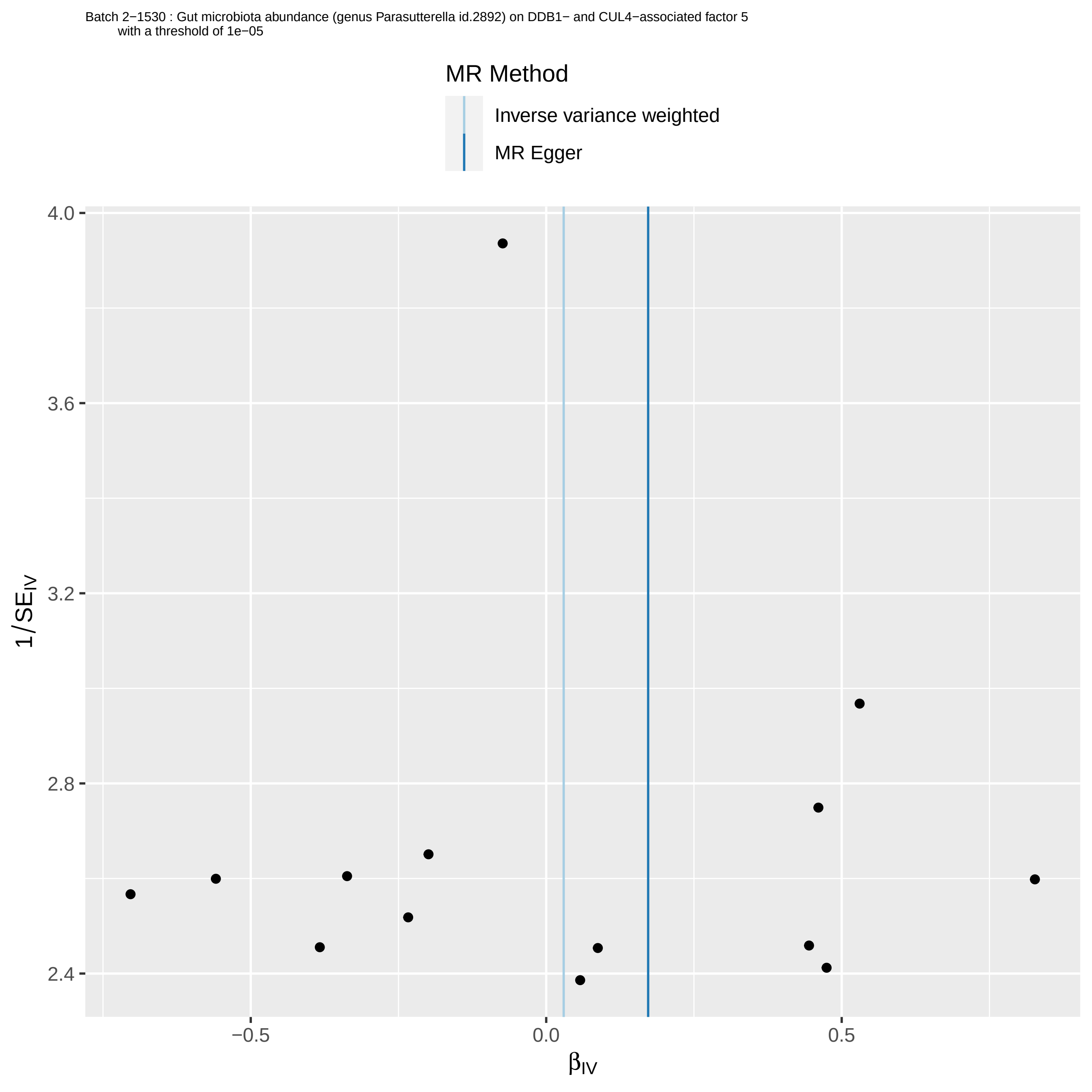

Batch 2−1530 : Gut microbiota abundance (genus Parasutterella id.2892) on DDB1− and CUL4−associated factor 5
with a threshold of 1e−05
MR Method
Inverse variance weighted
MR Egger
4.0
3.6
3.2
2.8
2.4
1/SE_IV
−0.5
0.0
0.5
β_IV

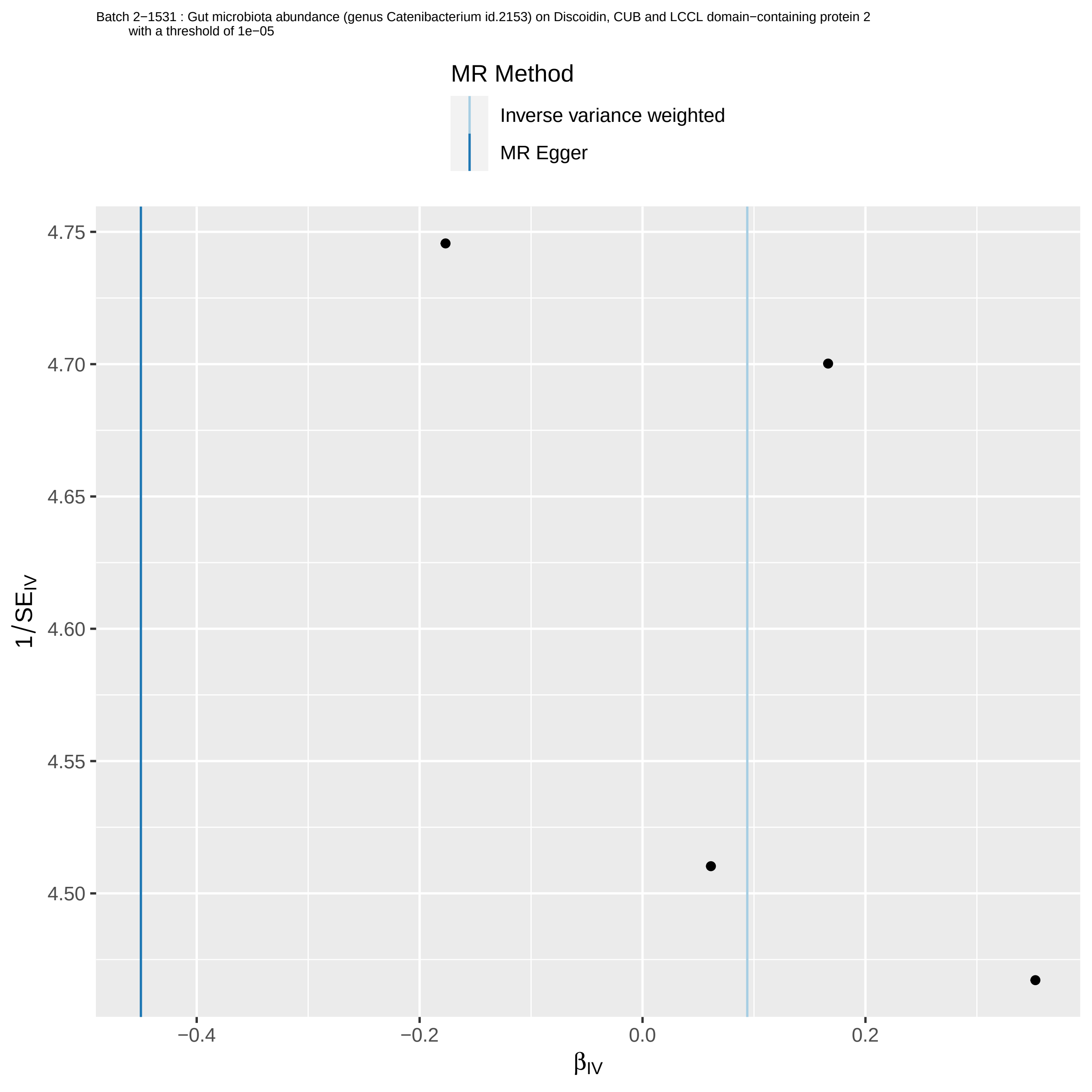
Batch 2−1531 : Gut microbiota abundance (genus Catenibacterium id.2153) on Discoidin, CUB and LCCL domain−containing protein 2
with a threshold of 1e−05
MR Method
Inverse variance weighted
MR Egger
4.75
4.70
4.65
4.60
4.55
4.50
1/SE_IV
−0.4
−0.2
0.0
0.2
β_IV

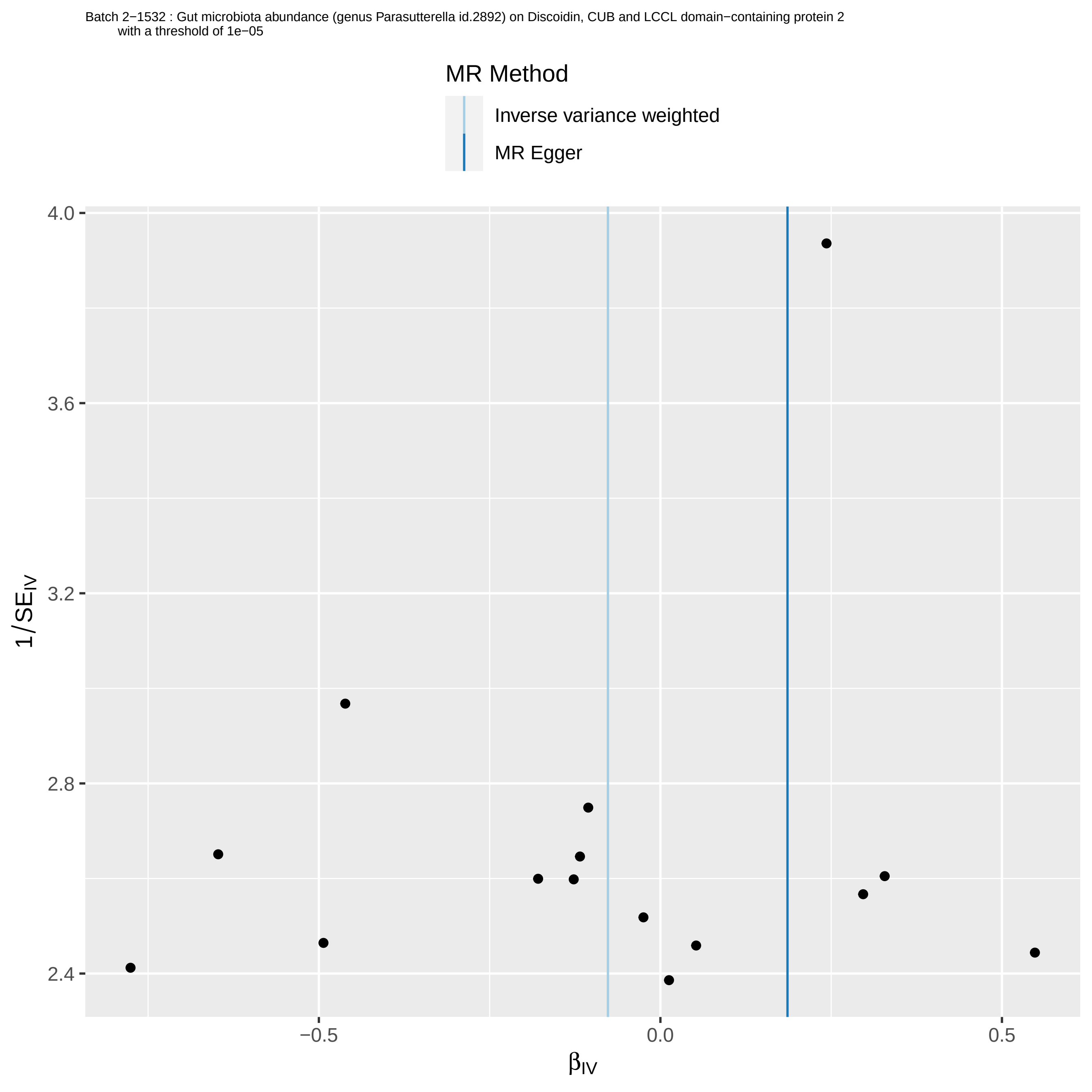

Batch 2−1532 : Gut microbiota abundance (genus Parasutterella id.2892) on Discoidin, CUB and LCCL domain−containing protein 2
with a threshold of 1e−05
MR Method
Inverse variance weighted
MR Egger
4.0
3.6
3.2
2.8
2.4
$1/SE_{IV}$
−0.5
0.0
0.5
β_{IV}

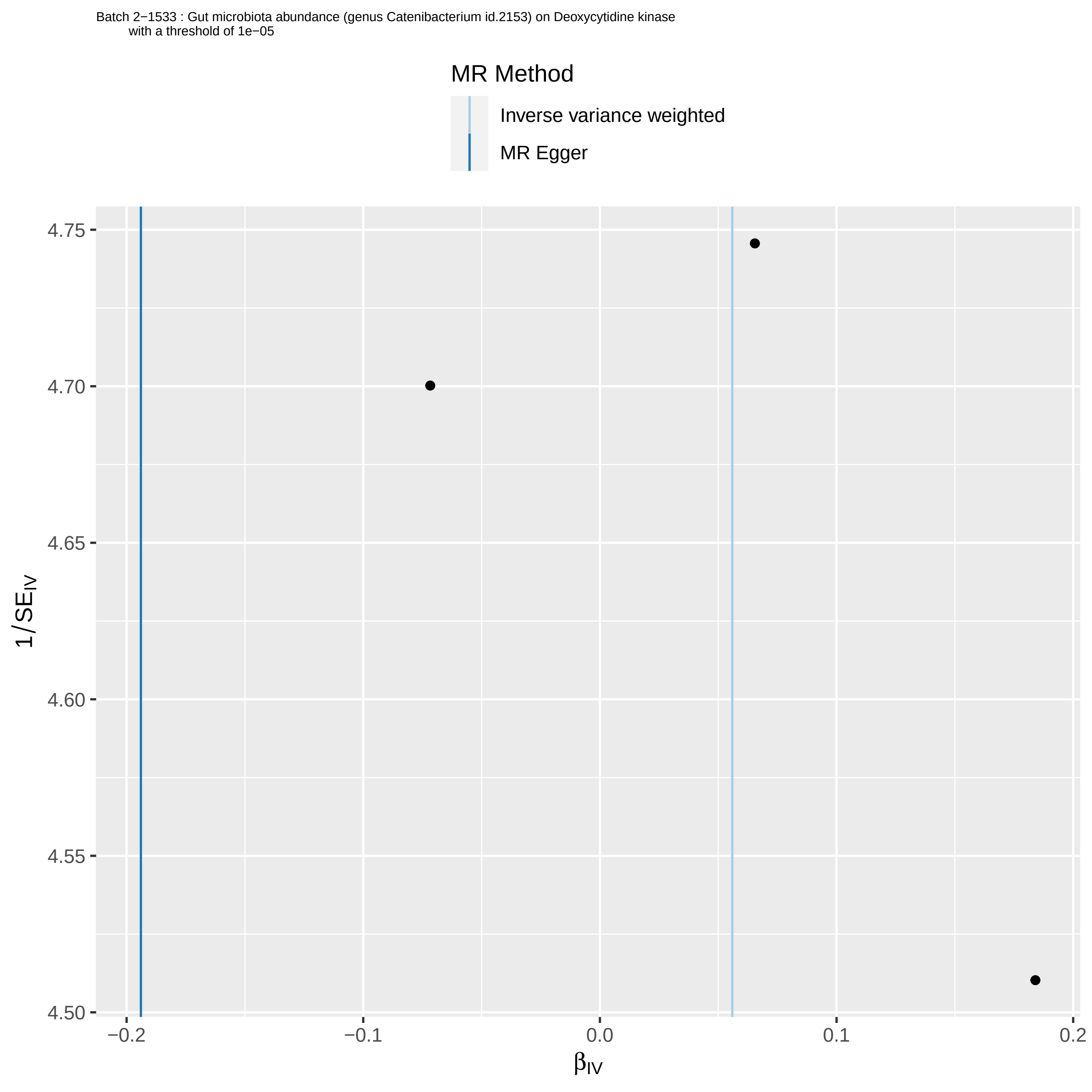

Batch 2−1533 : Gut microbiota abundance (genus Catenibacterium id.2153) on Deoxycytidine kinase
with a threshold of 1e−05
MR Method
Inverse variance weighted
MR Egger
4.75
4.70
4.65
4.60
4.55
4.50
1/SE_IV
−0.2
−0.1
0.0
0.1
0.2
β_IV

Batch 2−1534 : Gut microbiota abundance (genus Parasutterella id.2892) on Deoxycytidine kinase
with a threshold of 1e−05

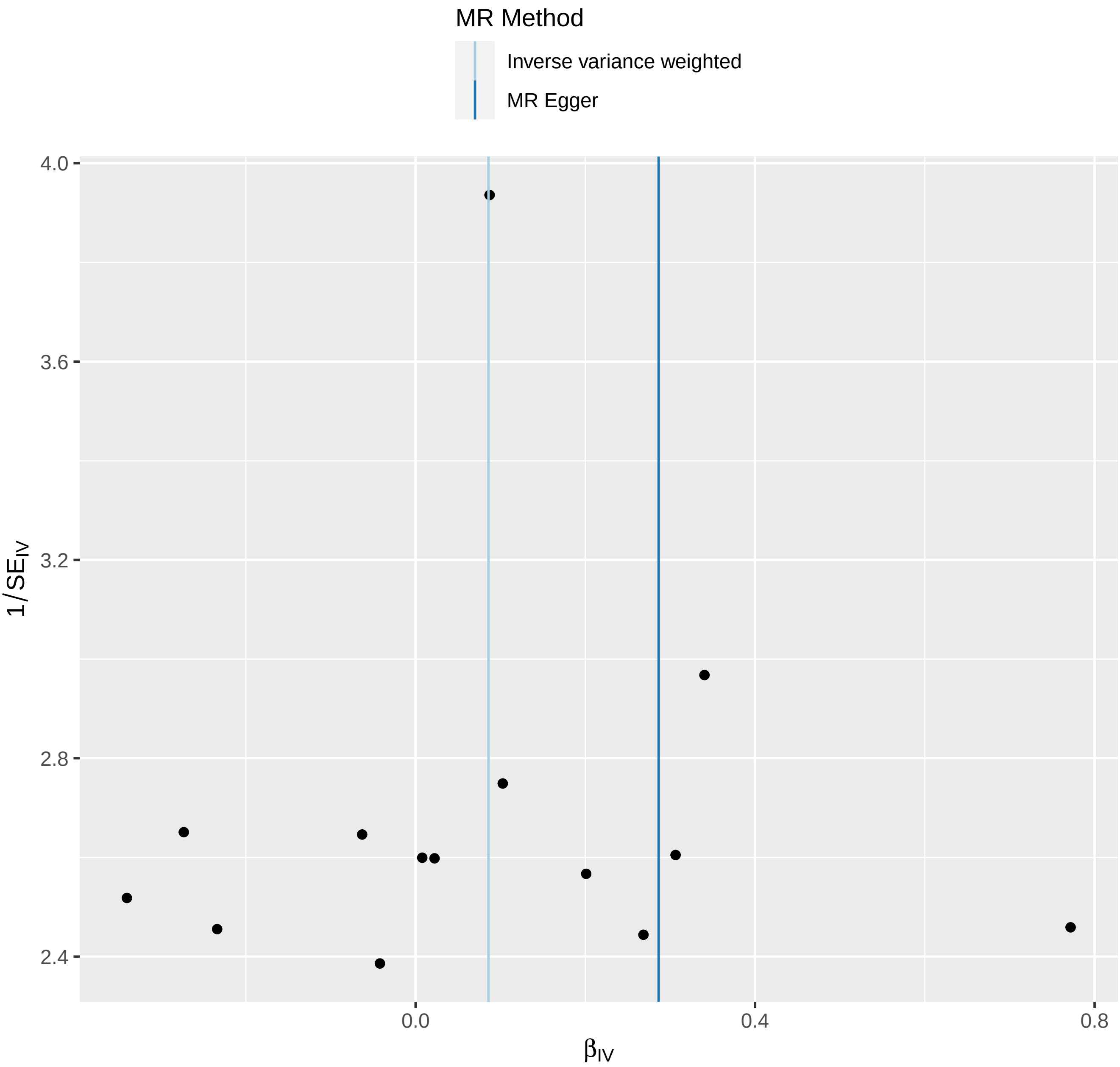

Batch 2−1535 : Gut microbiota abundance (genus Catenibacterium id.2153) on Serine/threonine−protein kinase DCLK3
with a threshold of 1e−05

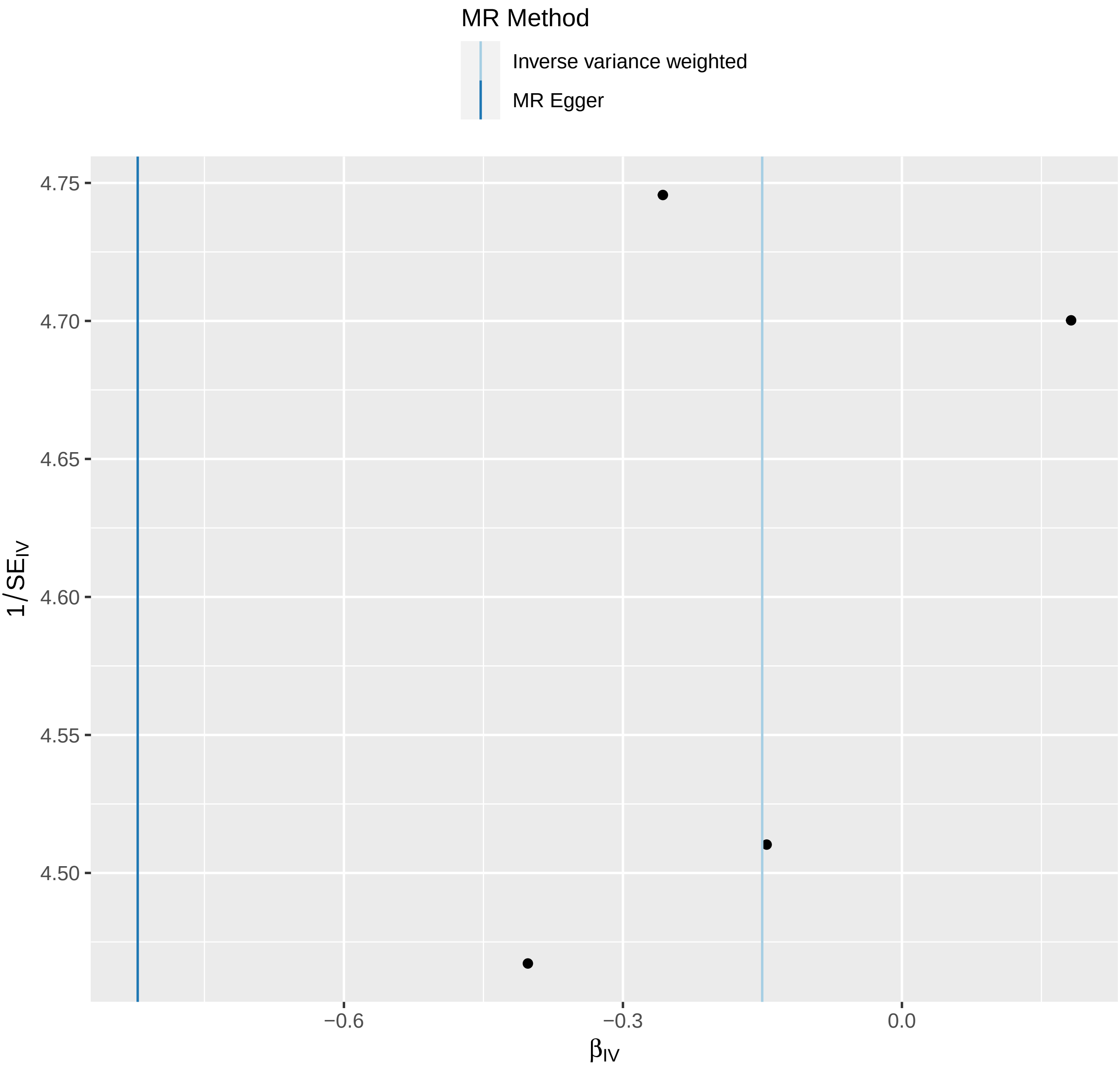

Batch 2−1536 : Gut microbiota abundance (genus Parasutterella id.2892) on Serine/threonine−protein kinase DCLK3
with a threshold of 1e−05

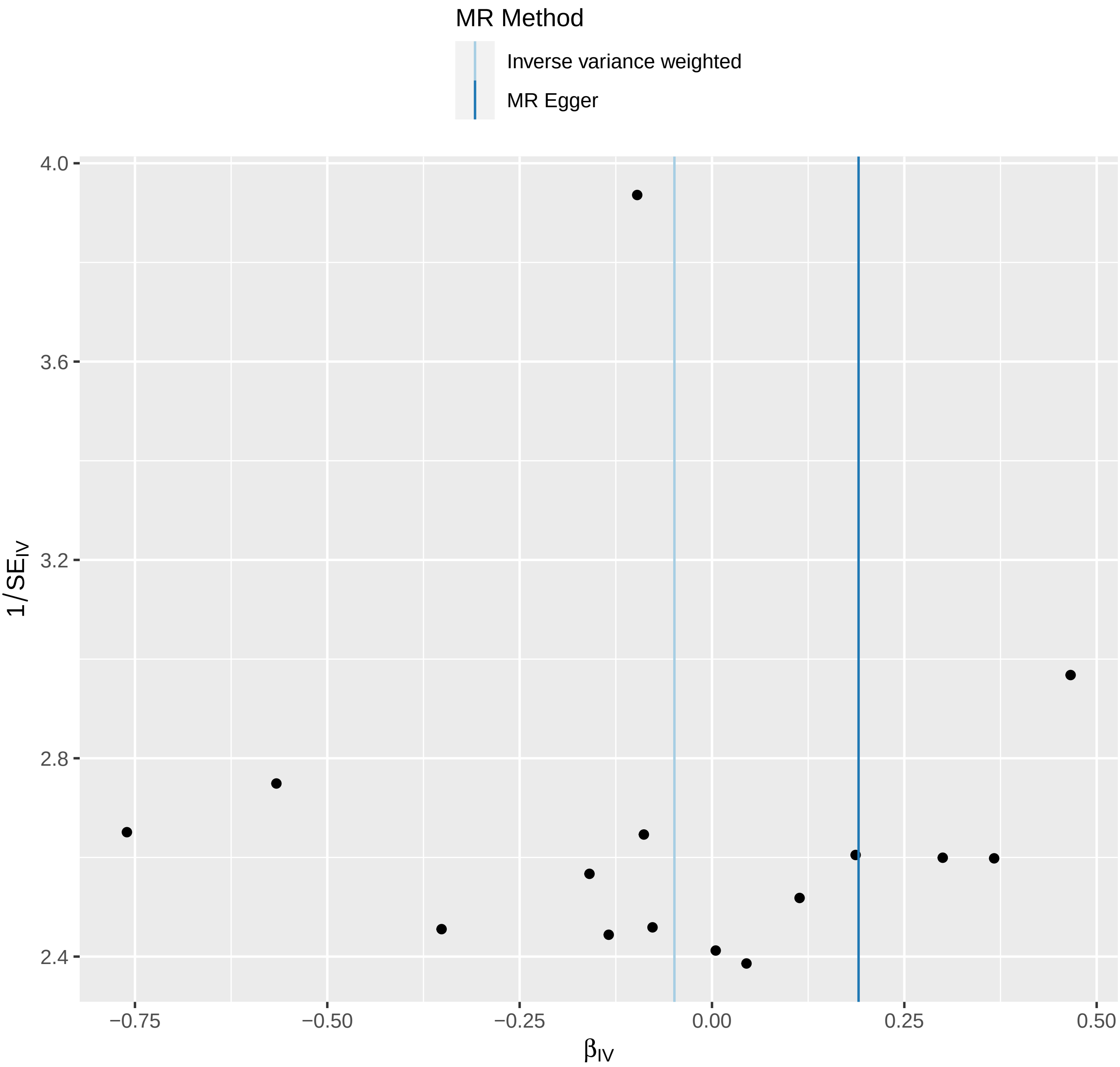

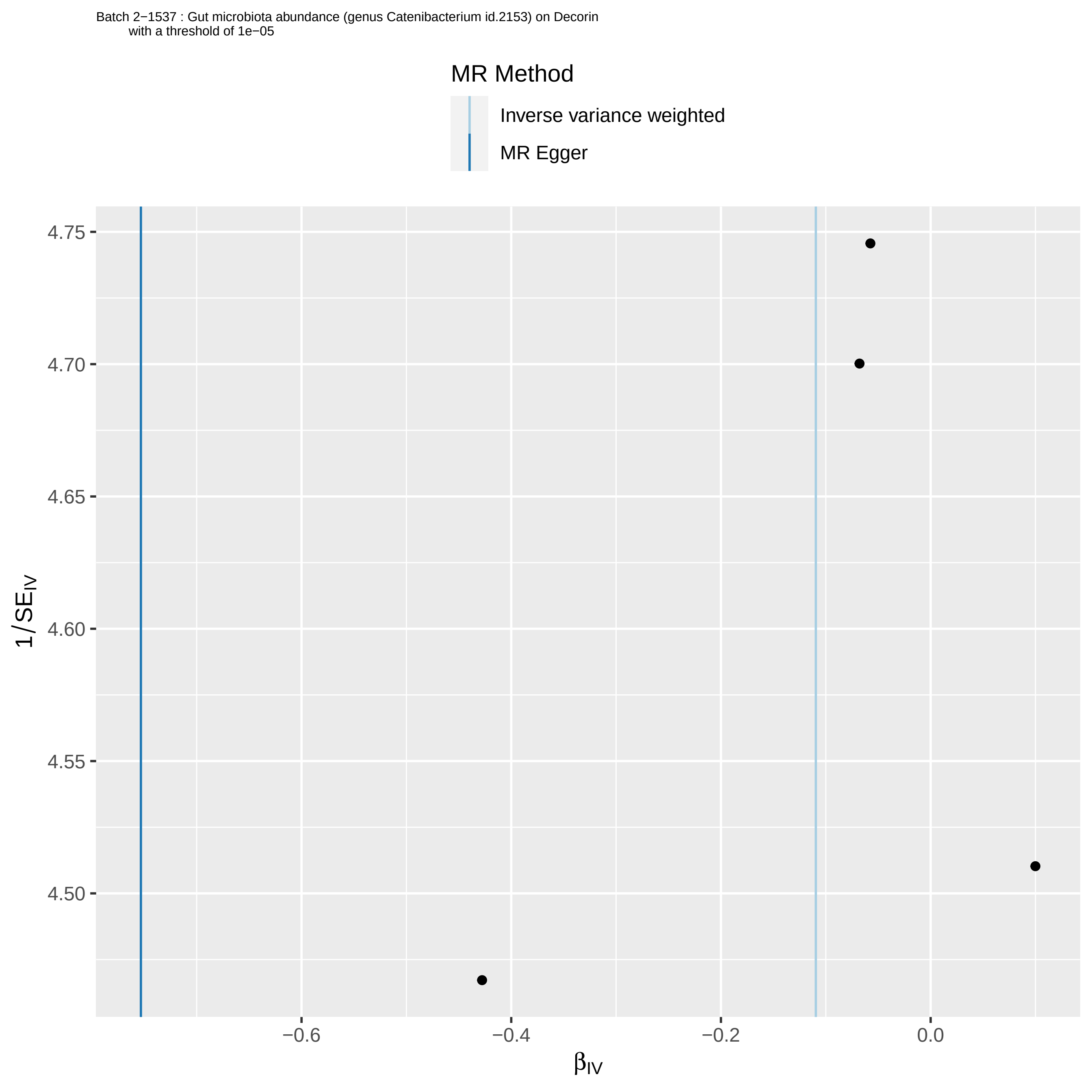
Batch 2−1537 : Gut microbiota abundance (genus Catenibacterium id.2153) on Decorin
with a threshold of 1e−05
MR Method
Inverse variance weighted
MR Egger
4.75
4.70
4.65
4.60
4.55
4.50
1/SE$_{IV}$
−0.6
−0.4
−0.2
0.0
β$_{IV}$

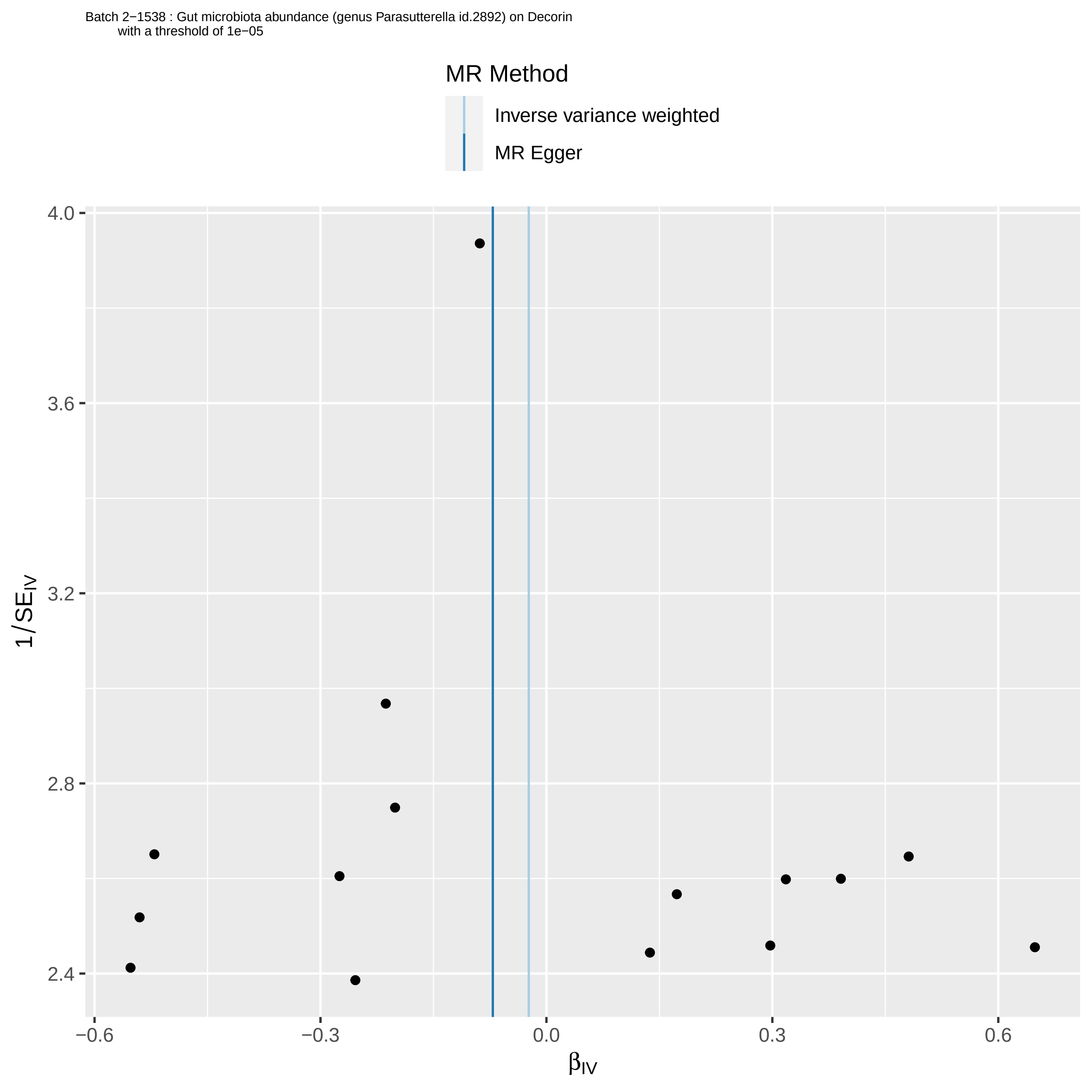
Batch 2−1538 : Gut microbiota abundance (genus Parasutterella id.2892) on Decorin
with a threshold of 1e−05
MR Method
Inverse variance weighted
MR Egger
4.0
3.6
3.2
2.8
2.4
1/SE_IV
−0.6
−0.3
0.0
0.3
0.6
β_IV

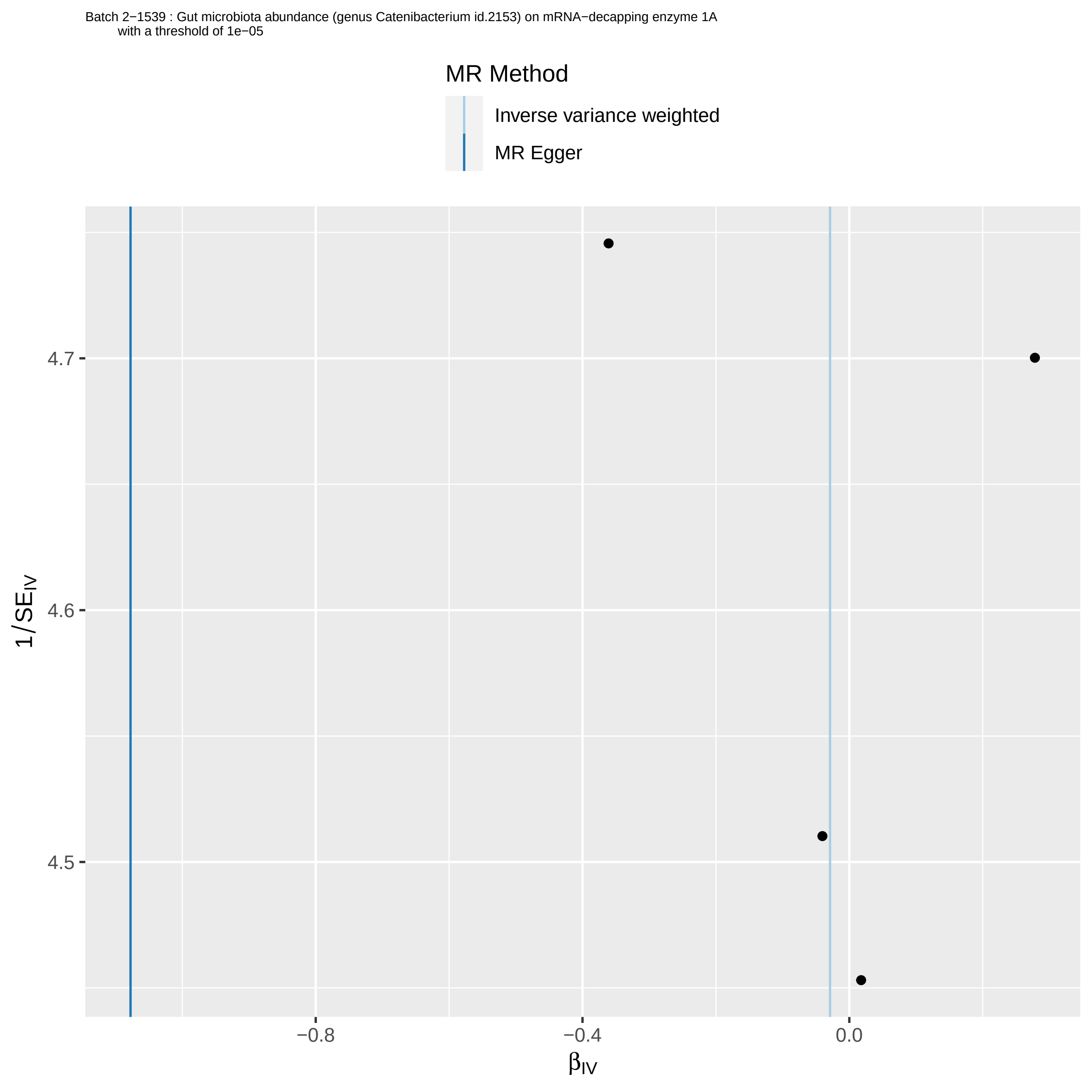

Batch 2−1539 : Gut microbiota abundance (genus Catenibacterium id.2153) on mRNA−decapping enzyme 1A
with a threshold of 1e−05
MR Method
Inverse variance weighted
MR Egger
4.7
4.6
4.5
1/SE_IV
−0.8
−0.4
0.0
β_IV

Batch 2−1540 : Gut microbiota abundance (genus Parasutterella id.2892) on mRNA−decapping enzyme 1A
with a threshold of 1e−05

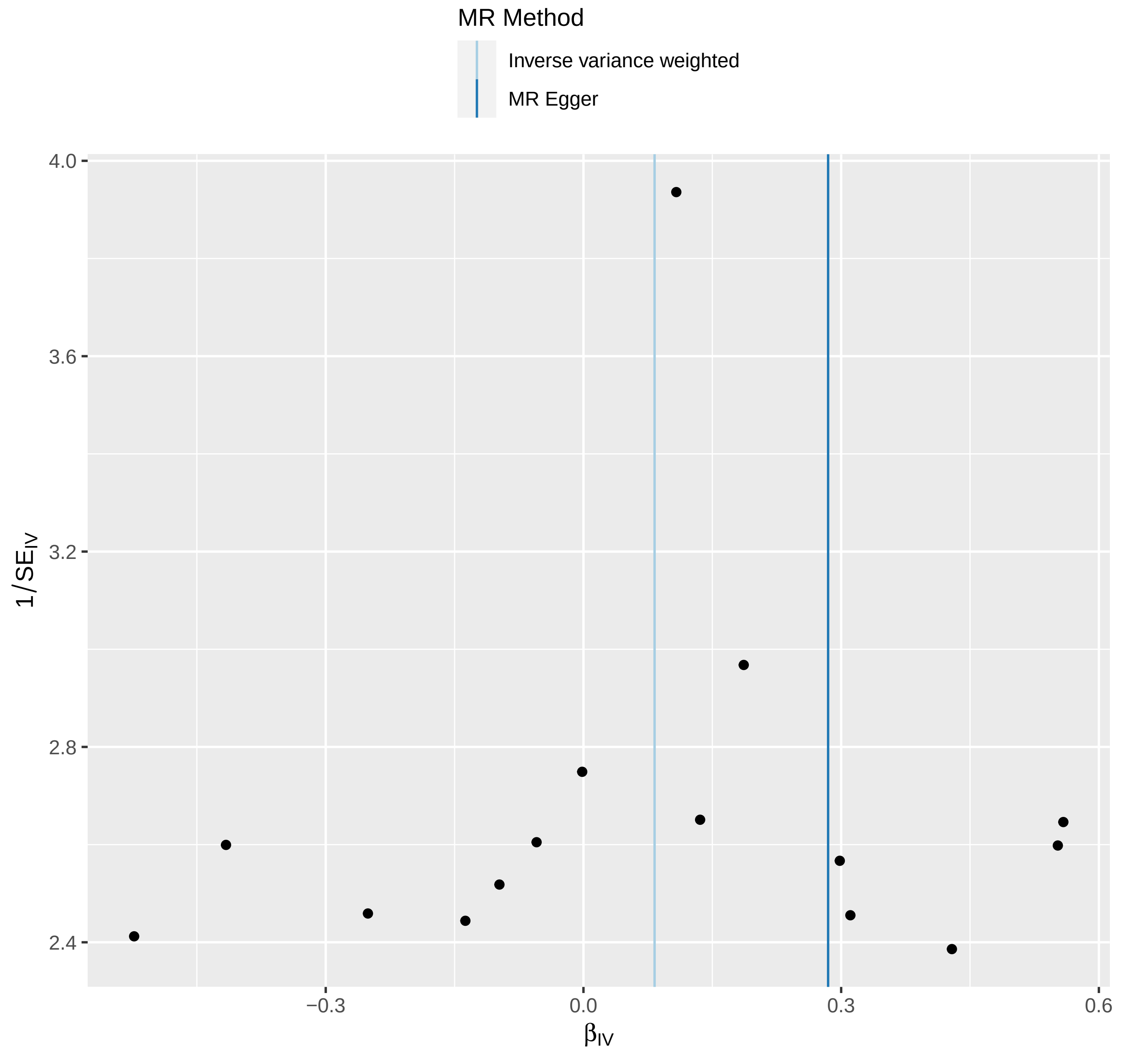

Batch 2−1541 : Gut microbiota abundance (genus Catenibacterium id.2153) on Dynactin subunit 2
with a threshold of 1e−05

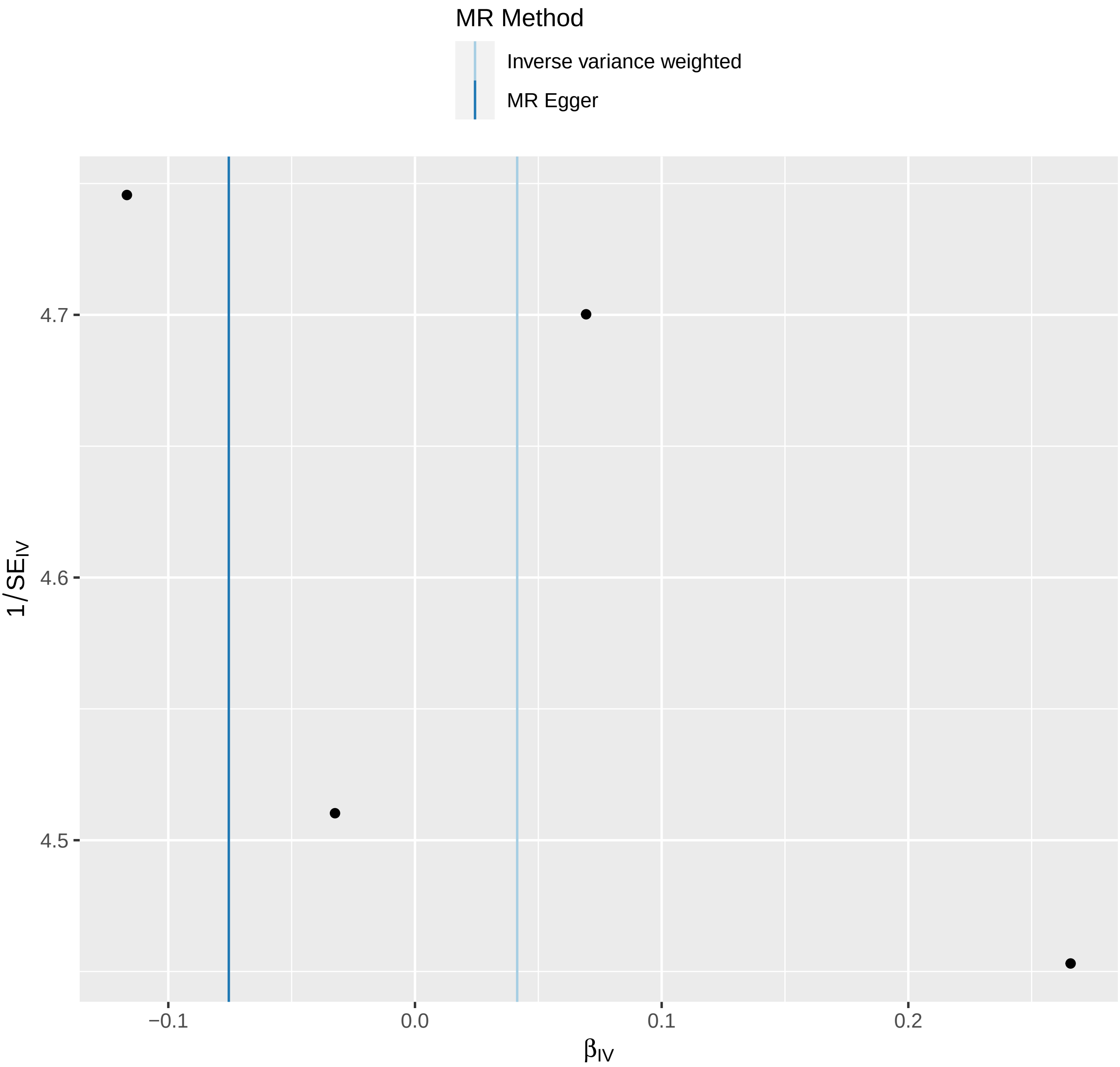

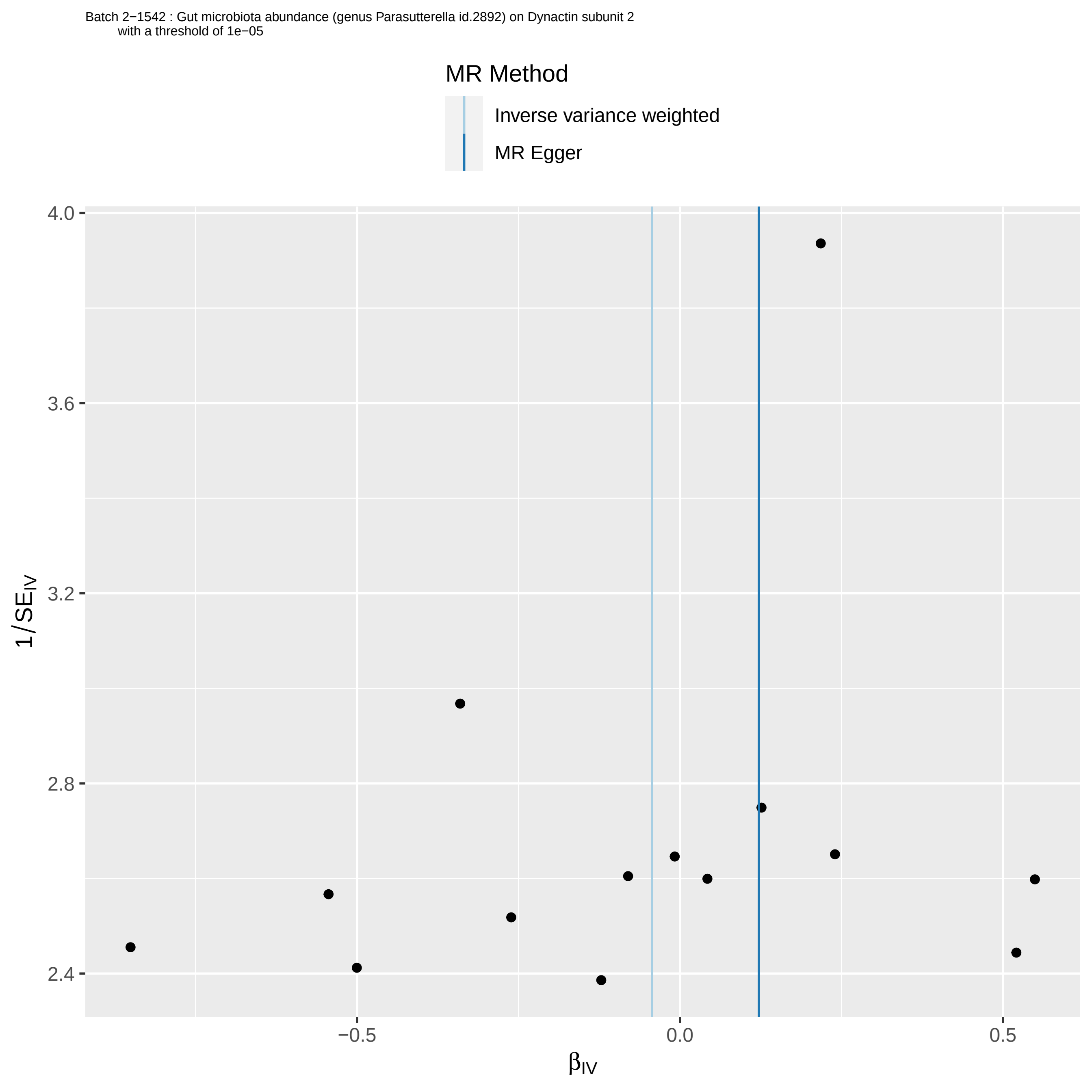
Batch 2−1542 : Gut microbiota abundance (genus Parasutterella id.2892) on Dynactin subunit 2
with a threshold of 1e−05
MR Method
Inverse variance weighted
MR Egger
4.0
3.6
3.2
2.8
2.4
1/SE$_{IV}$
−0.5
0.0
0.5
β_{IV}

Batch 2−1543 : Gut microbiota abundance (genus Catenibacterium id.2153) on dCTP pyrophosphatase 1
with a threshold of 1e−05

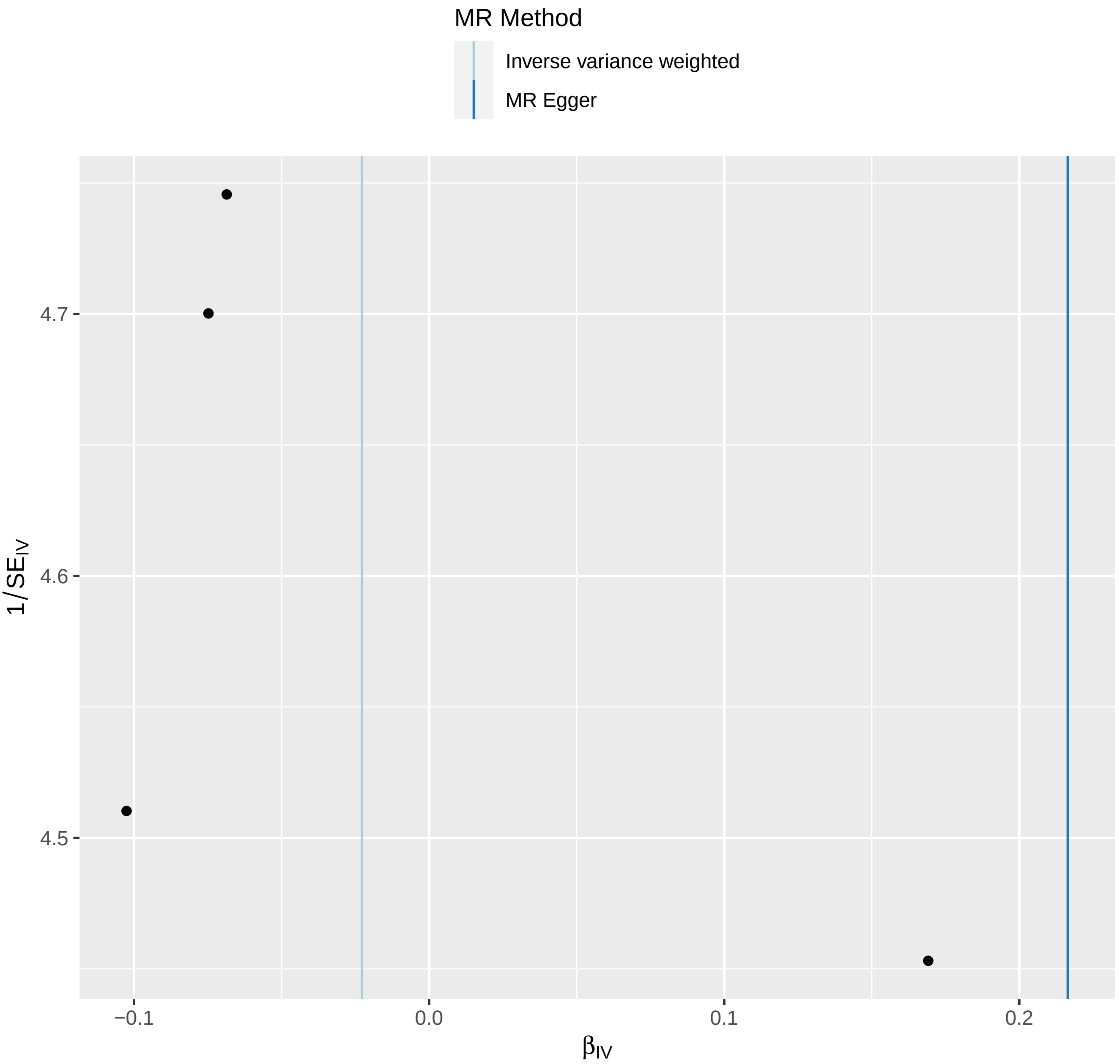

Batch 2−1544 : Gut microbiota abundance (genus Parasutterella id.2892) on dCTP pyrophosphatase 1
with a threshold of 1e−05

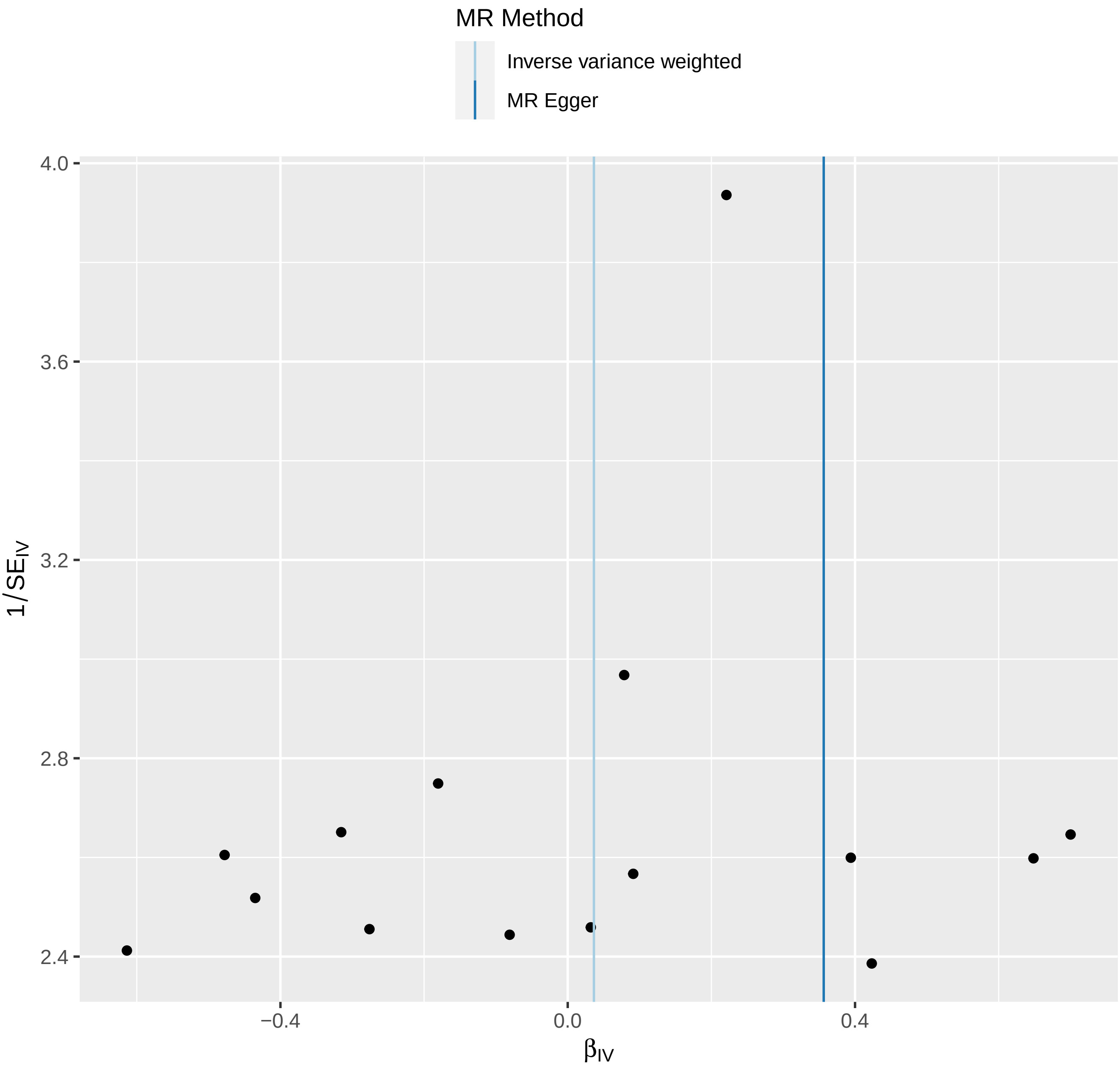

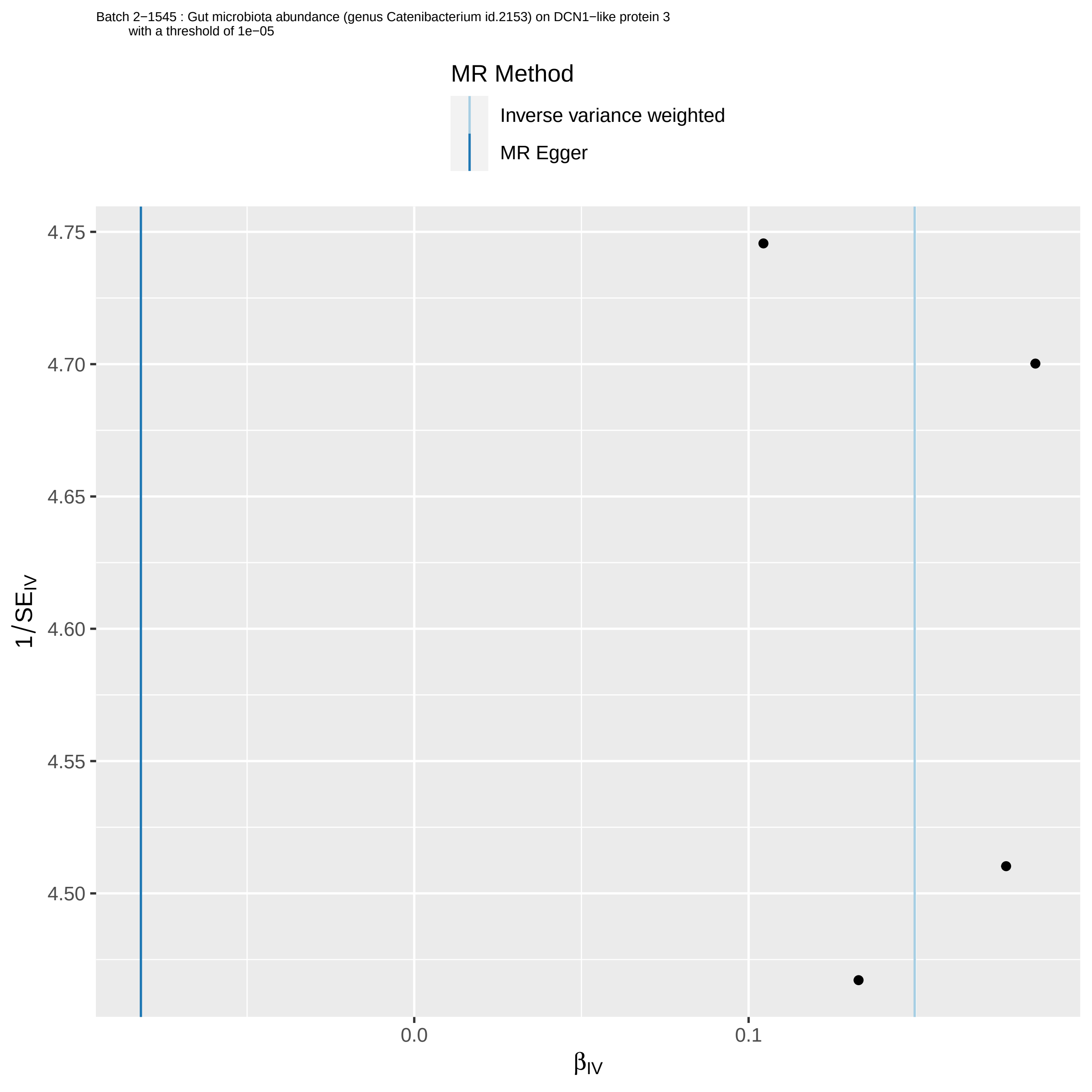
Batch 2−1545 : Gut microbiota abundance (genus Catenibacterium id.2153) on DCN1−like protein 3
with a threshold of 1e−05
MR Method
Inverse variance weighted
MR Egger
4.75
4.70
4.65
4.60
4.55
4.50
1/SE$_{IV}$
0.0
0.1
β$_{IV}$

Batch 2−1546 : Gut microbiota abundance (genus Parasutterella id.2892) on DCN1−like protein 3
with a threshold of 1e−05

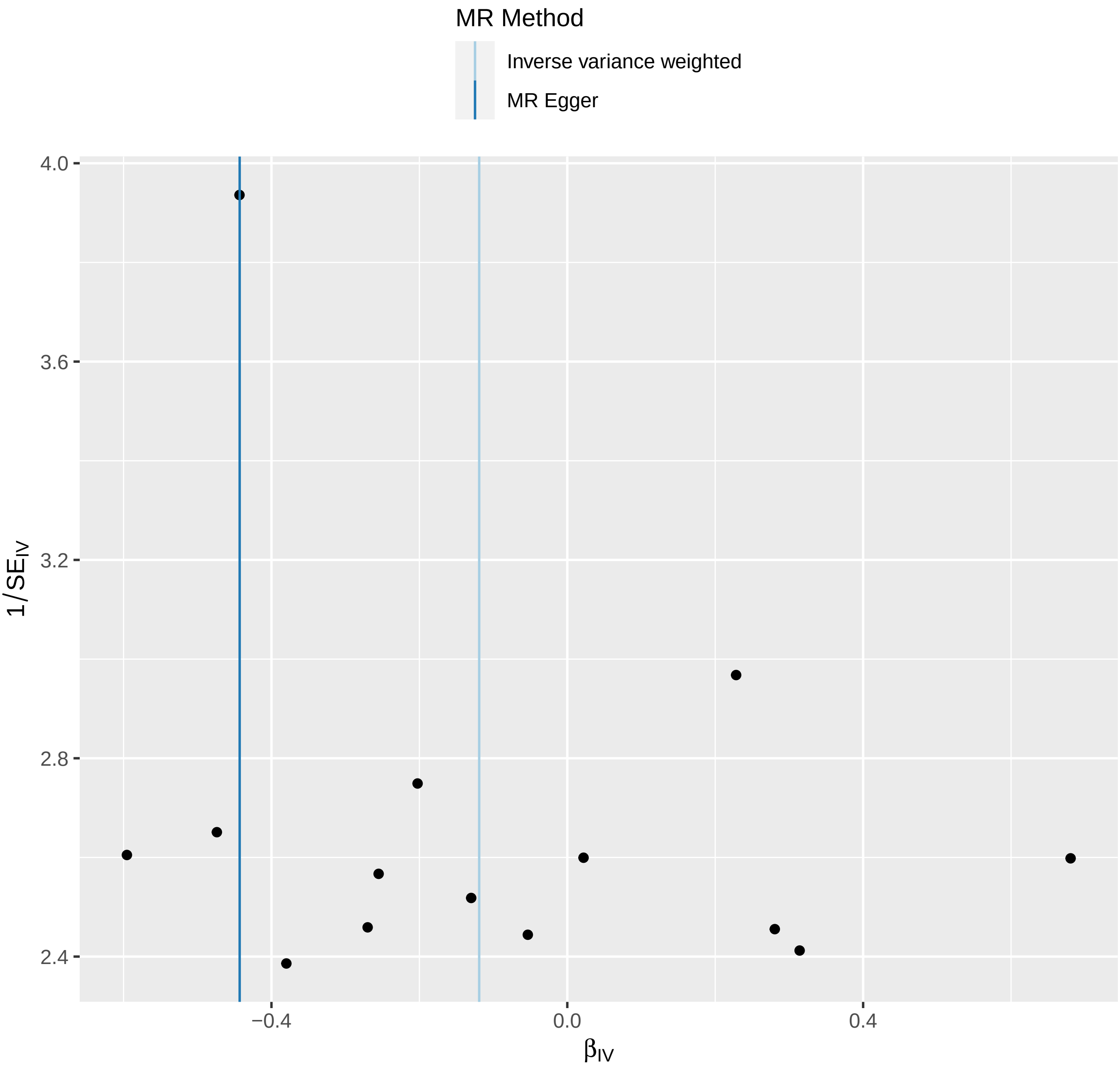

Batch 2−1547 : Gut microbiota abundance (genus Catenibacterium id.2153) on DCN1−like protein 5
with a threshold of 1e−05

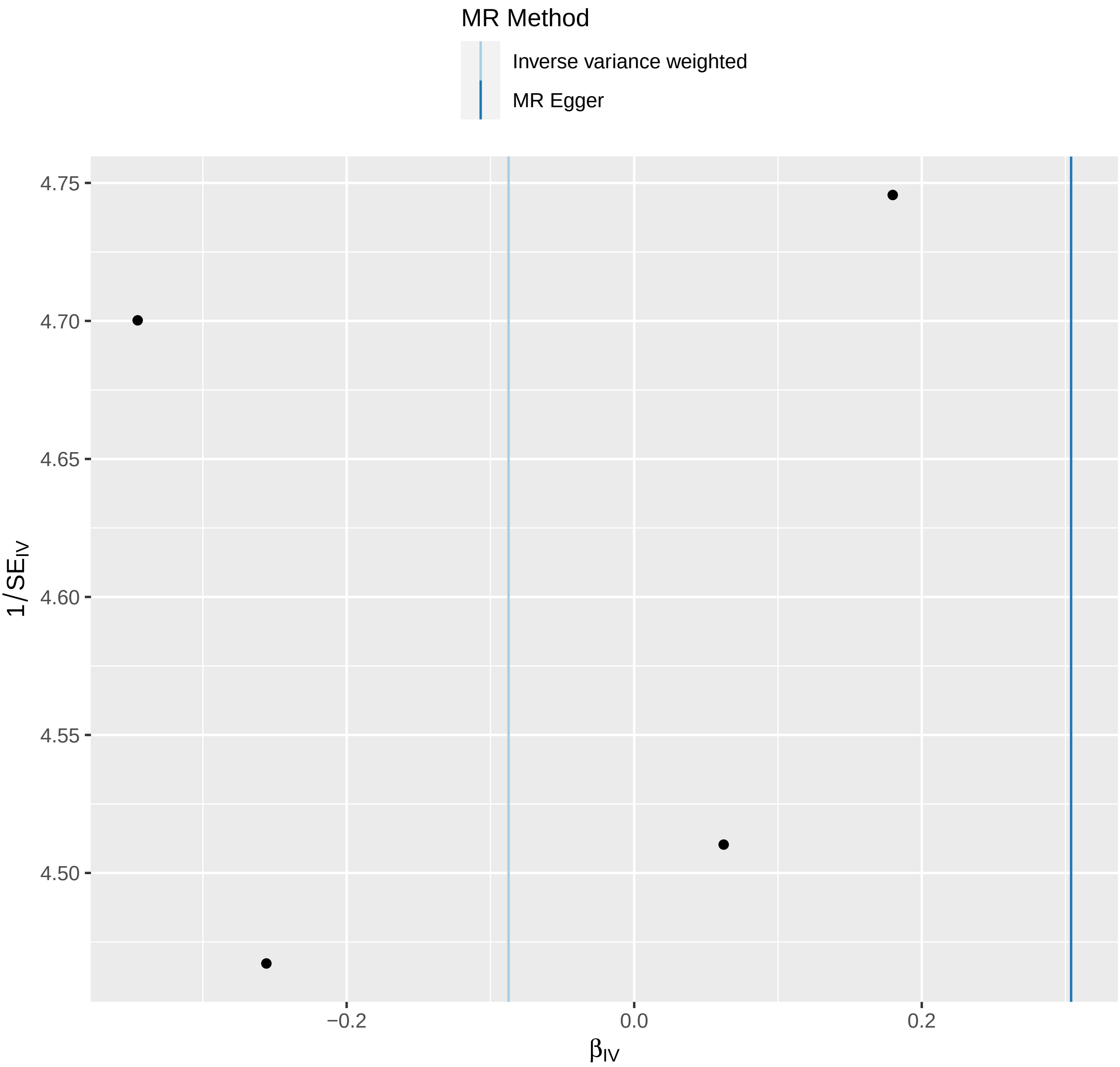

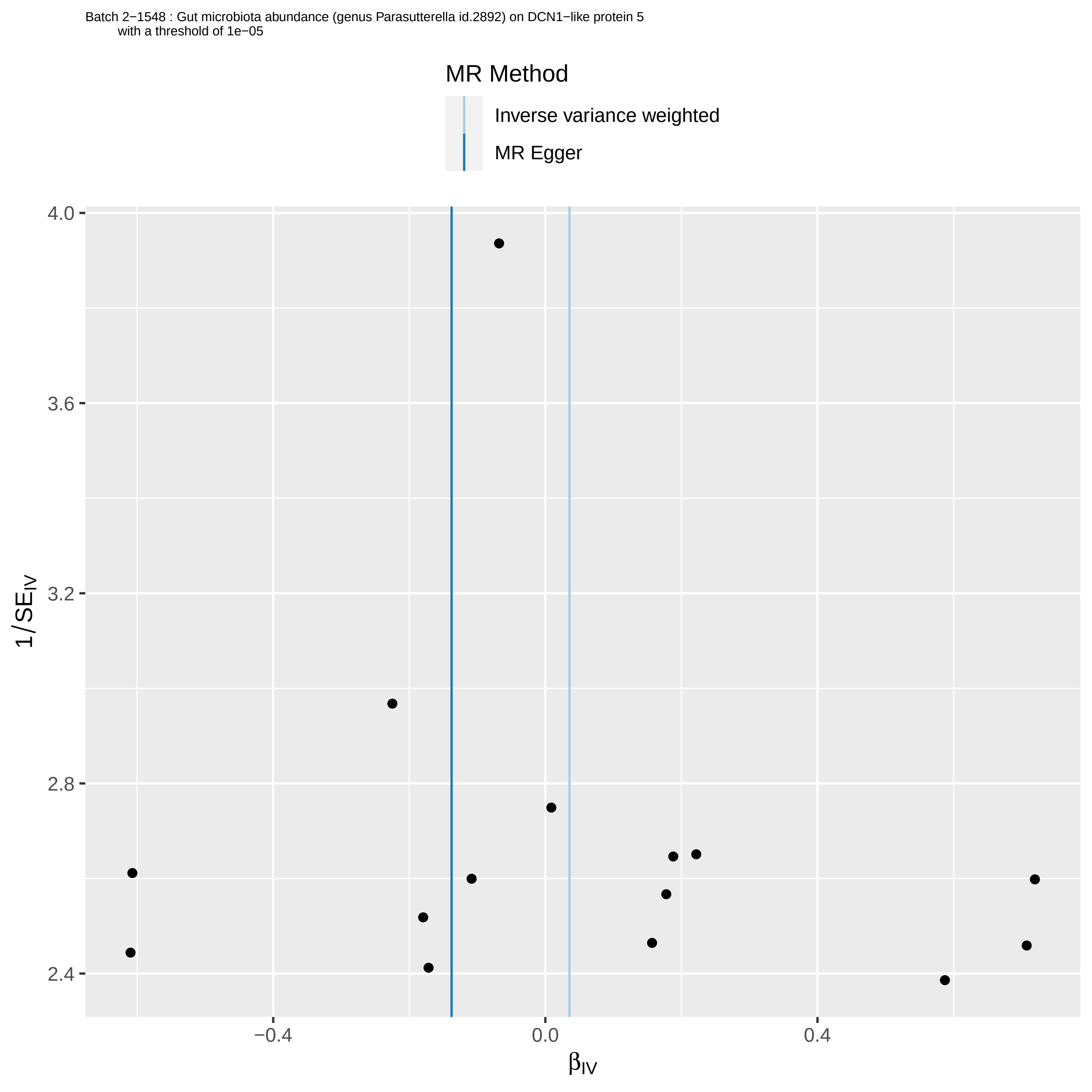

Batch 2−1548 : Gut microbiota abundance (genus Parasutterella id.2892) on DCN1−like protein 5
with a threshold of 1e−05
MR Method
Inverse variance weighted
MR Egger
4.0
3.6
3.2
2.8
2.4
1/SE_IV
−0.4
0.0
0.4
β_IV

Batch 2−1549 : Gut microbiota abundance (genus Catenibacterium id.2153) on Aromatic−L−amino−acid decarboxylase with a threshold of 1e−05

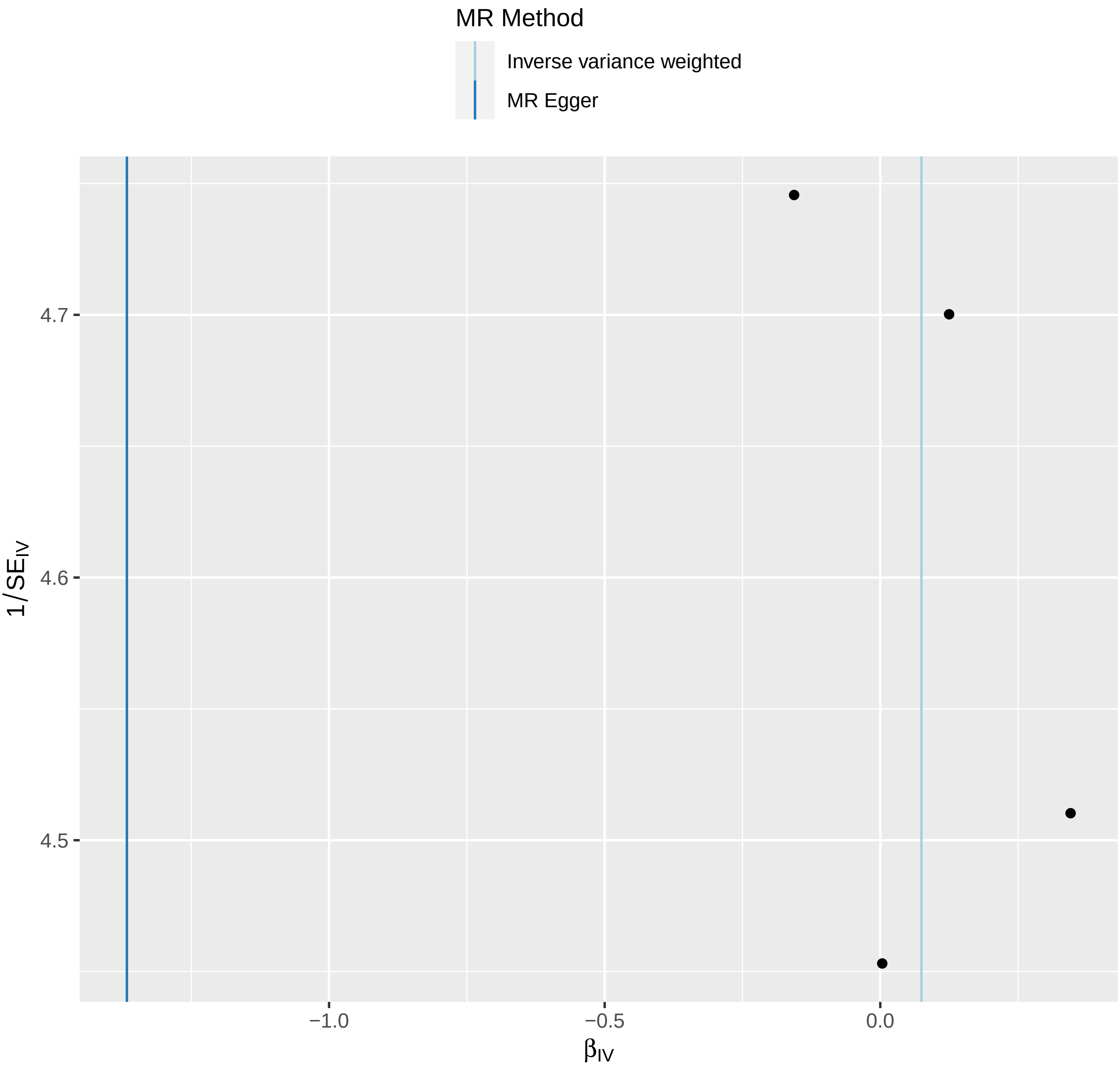

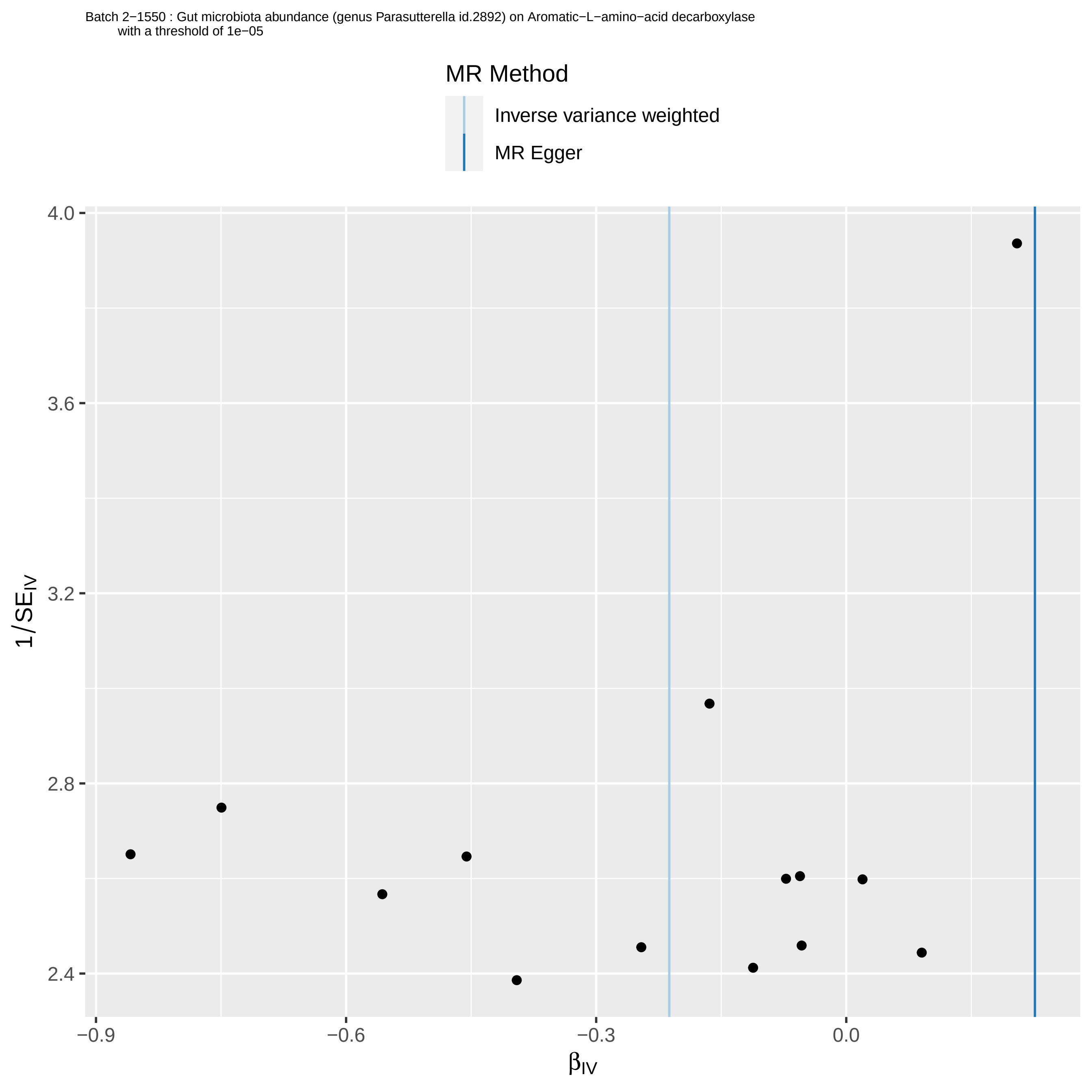
Batch 2−1550 : Gut microbiota abundance (genus Parasutterella id.2892) on Aromatic−L−amino−acid decarboxylase
with a threshold of 1e−05
MR Method
Inverse variance weighted
MR Egger
4.0
3.6
3.2
2.8
2.4
1/SE_IV
−0.9
−0.6
−0.3
0.0
β_IV

Batch 2−1551 : Gut microbiota abundance (genus Catenibacterium id.2153) on Discoidin domain−containing receptor 2
with a threshold of 1e−05

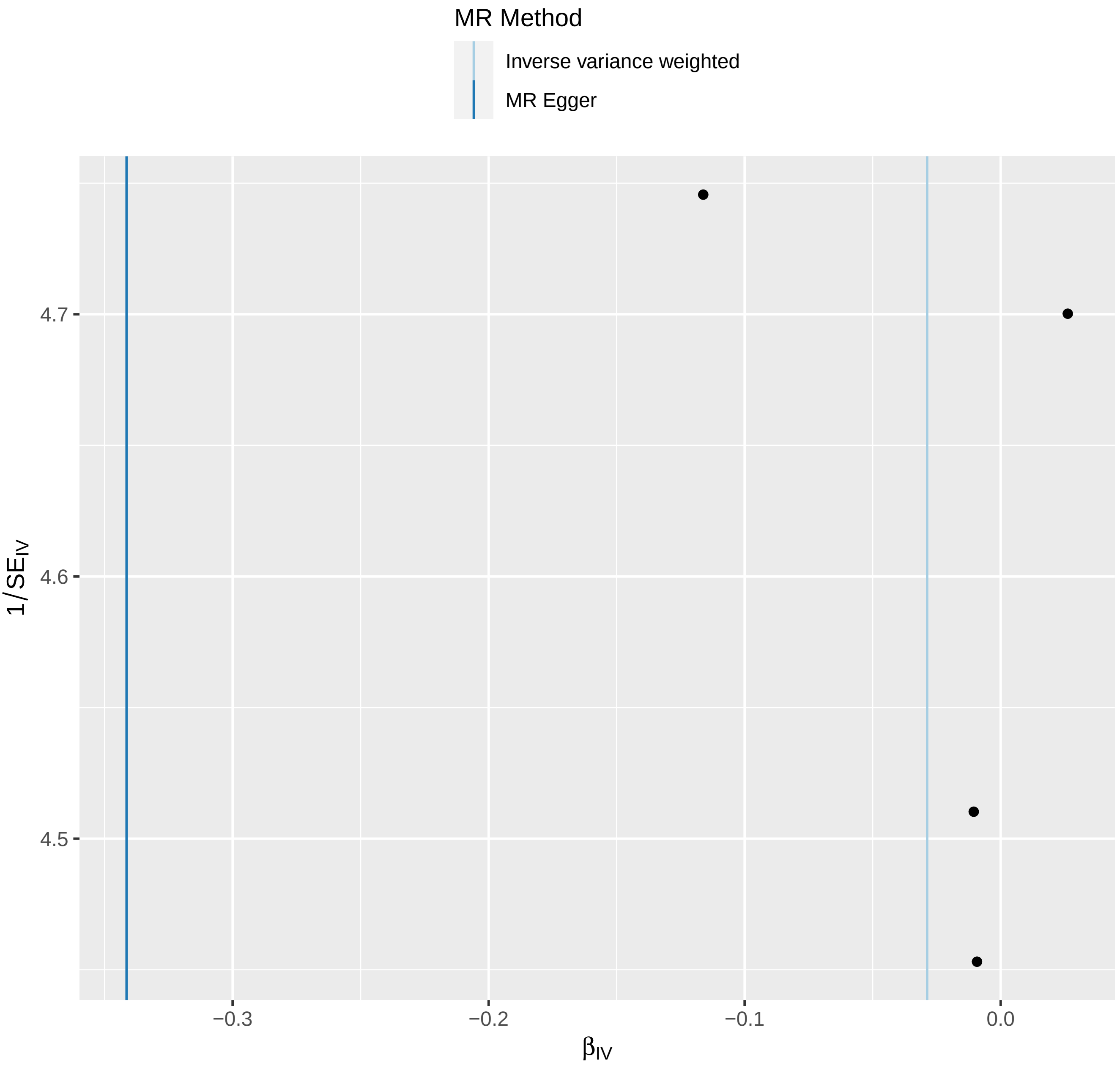

Batch 2−1552 : Gut microbiota abundance (genus Parasutterella id.2892) on Discoidin domain−containing receptor 2 with a threshold of 1e−05

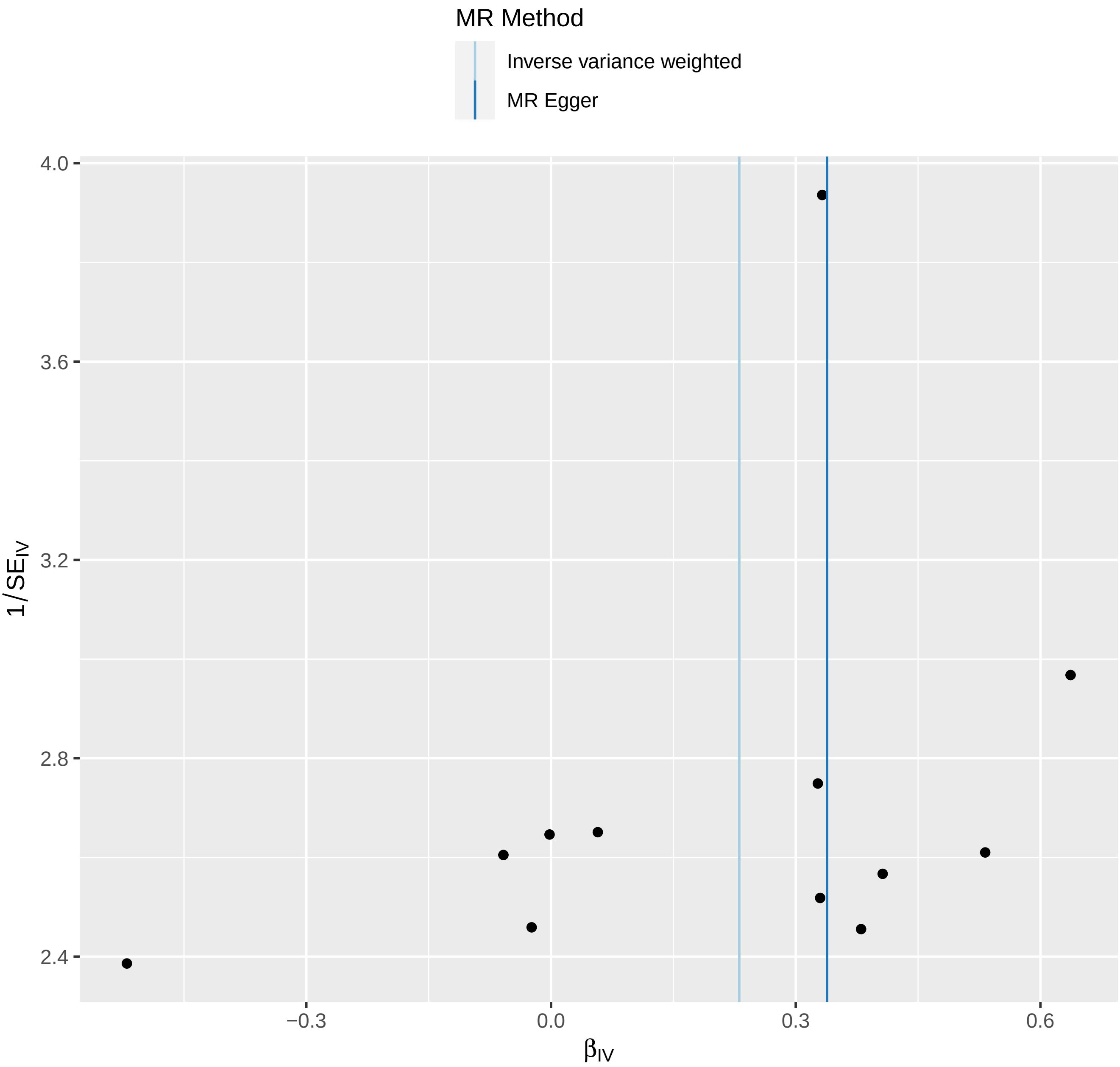

Batch 2−1553 : Gut microbiota abundance (genus Catenibacterium id.2153) on ATP−dependent RNA helicase DDX25
with a threshold of 1e−05

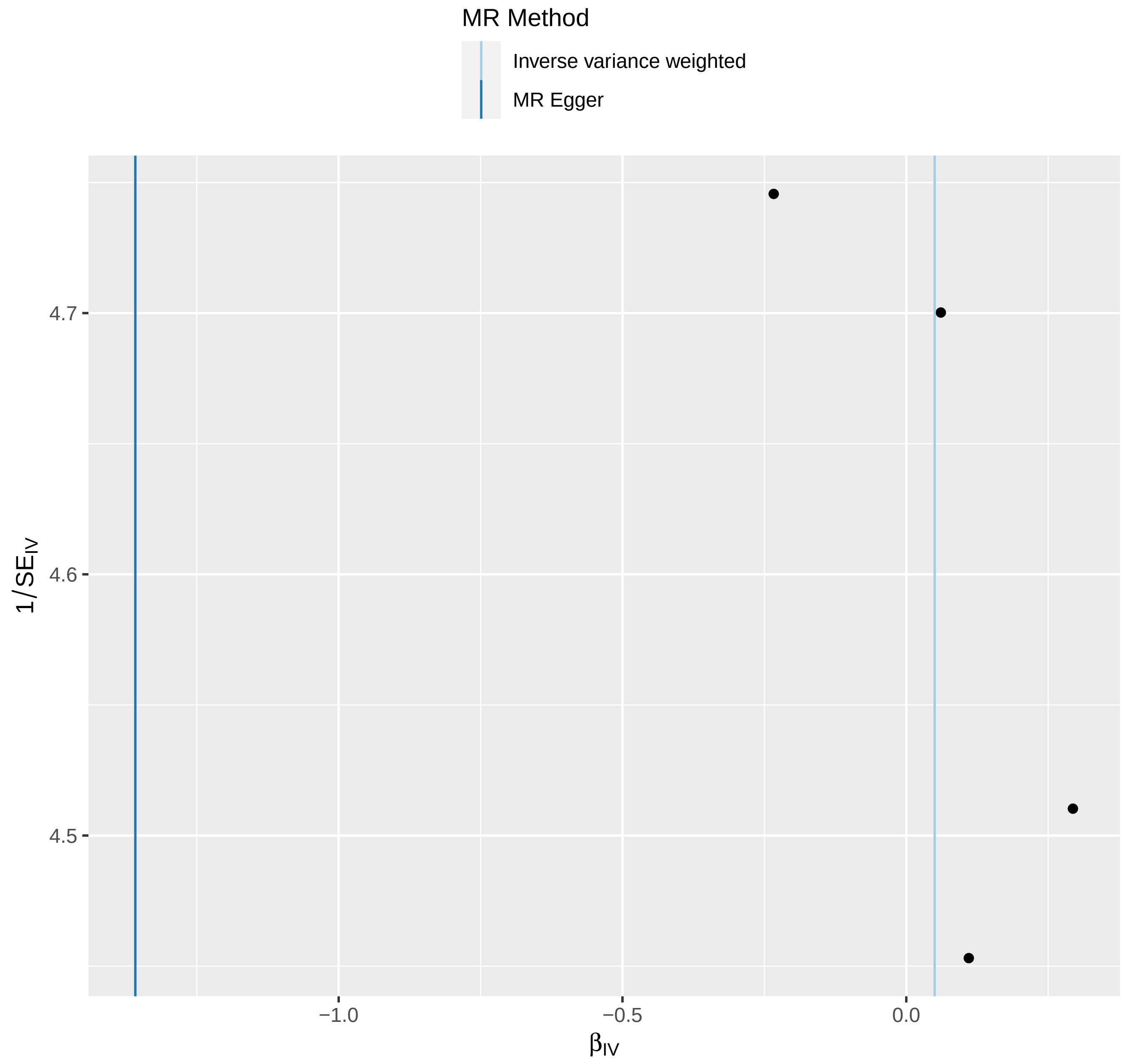

Batch 2−1554 : Gut microbiota abundance (genus Parasutterella id.2892) on ATP−dependent RNA helicase DDX25
with a threshold of 1e−05

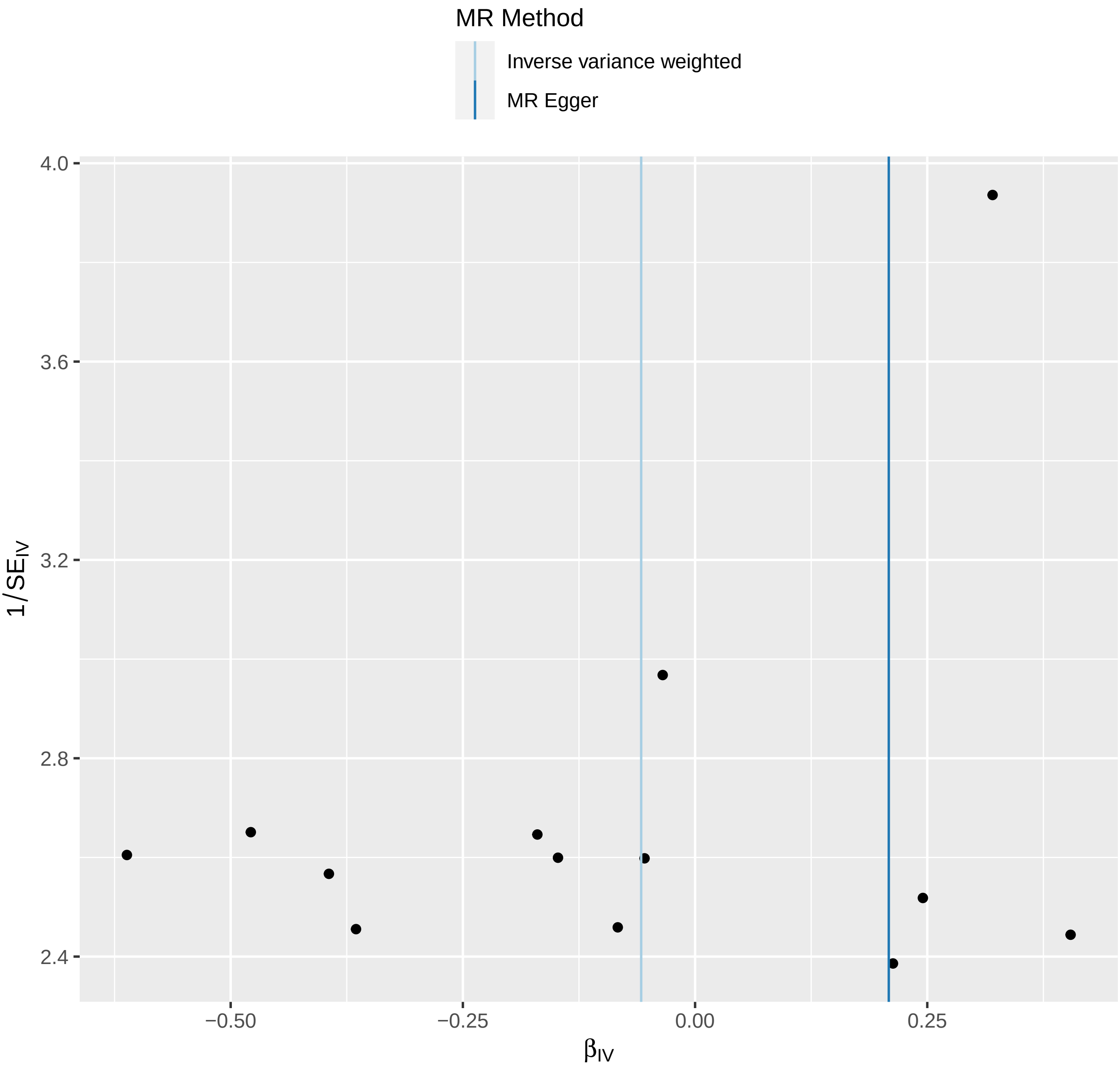

Batch 2−1555 : Gut microbiota abundance (genus Catenibacterium id.2153) on Spliceosome RNA helicase DDX39B
with a threshold of 1e−05

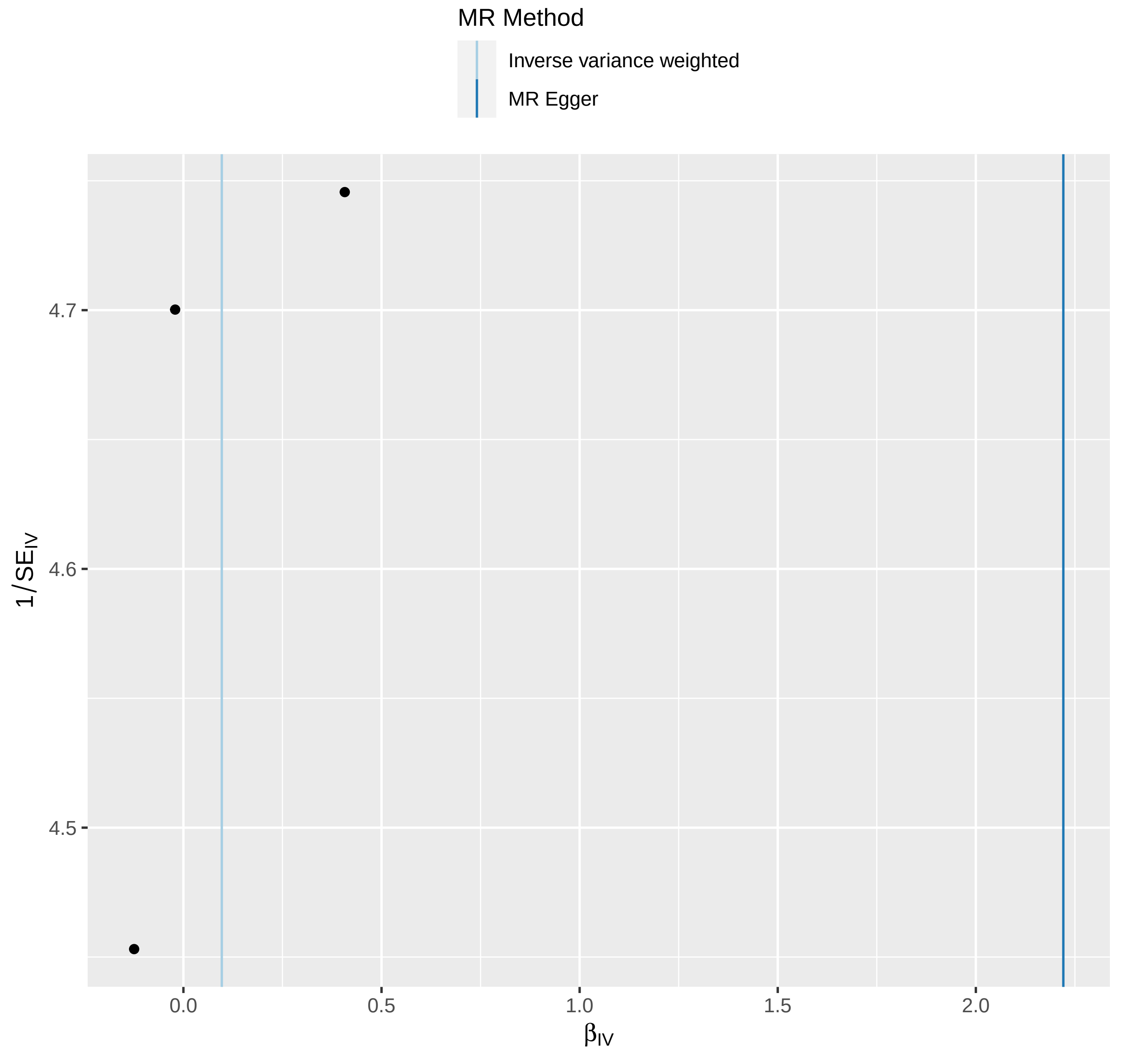

Batch 2−1556 : Gut microbiota abundance (genus Parasutterella id.2892) on Spliceosome RNA helicase DDX39B
with a threshold of 1e−05

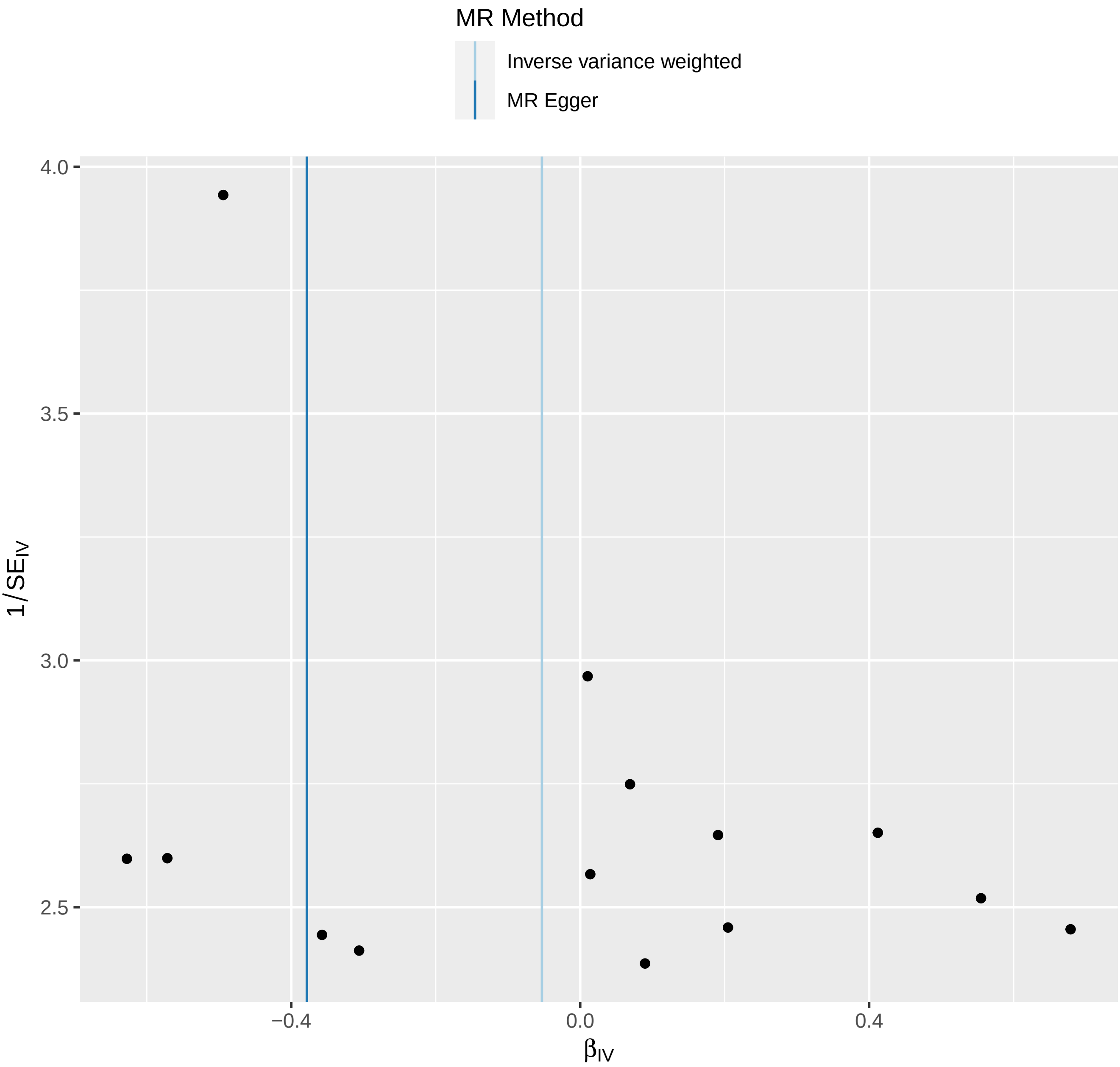

Batch 2−1557 : Gut microbiota abundance (genus Catenibacterium id.2153) on Probable ATP−dependent RNA helicase DDX58
with a threshold of 1e−05

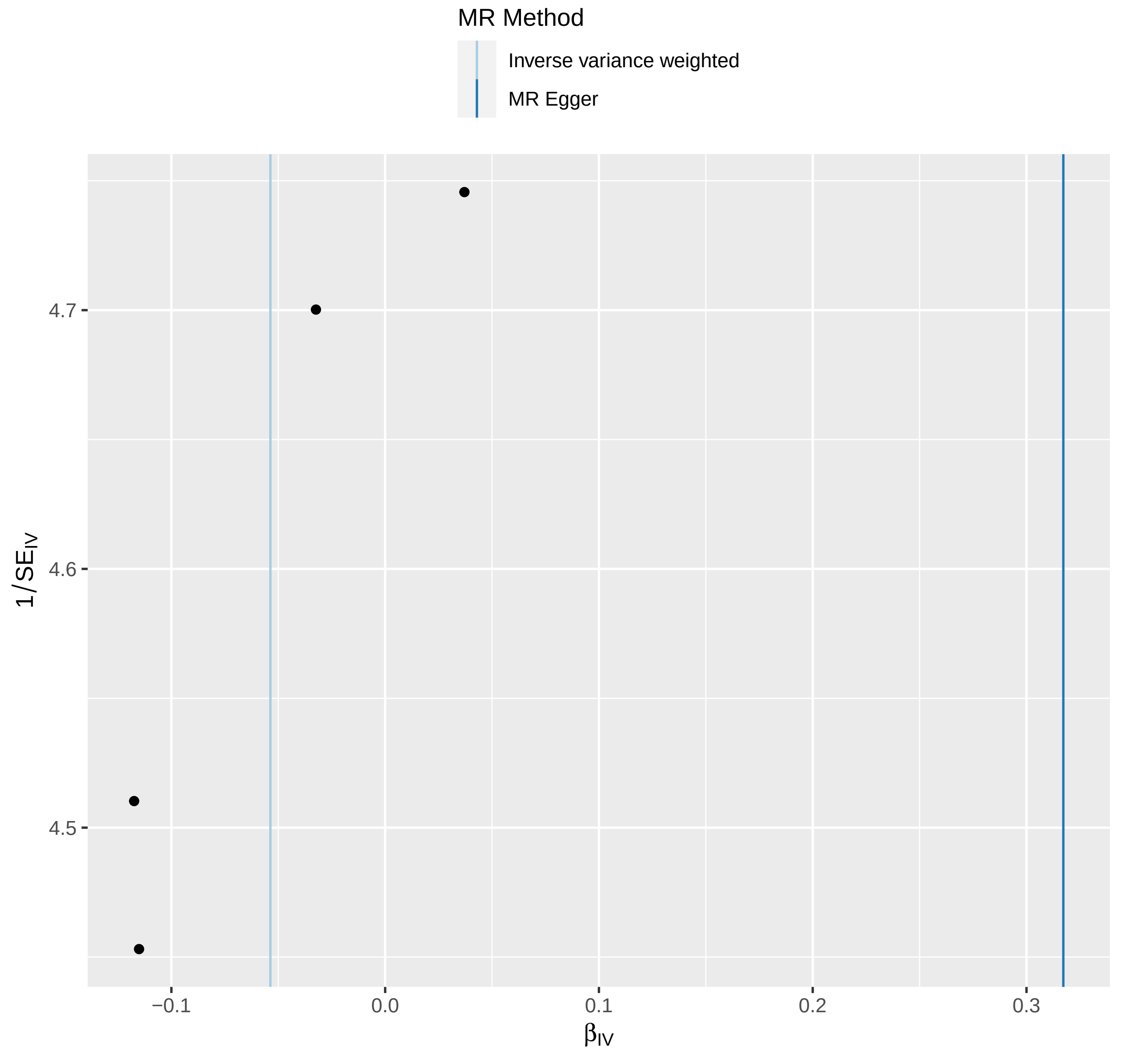

Batch 2−1558 : Gut microbiota abundance (genus Parasutterella id.2892) on Probable ATP−dependent RNA helicase DDX58
with a threshold of 1e−05

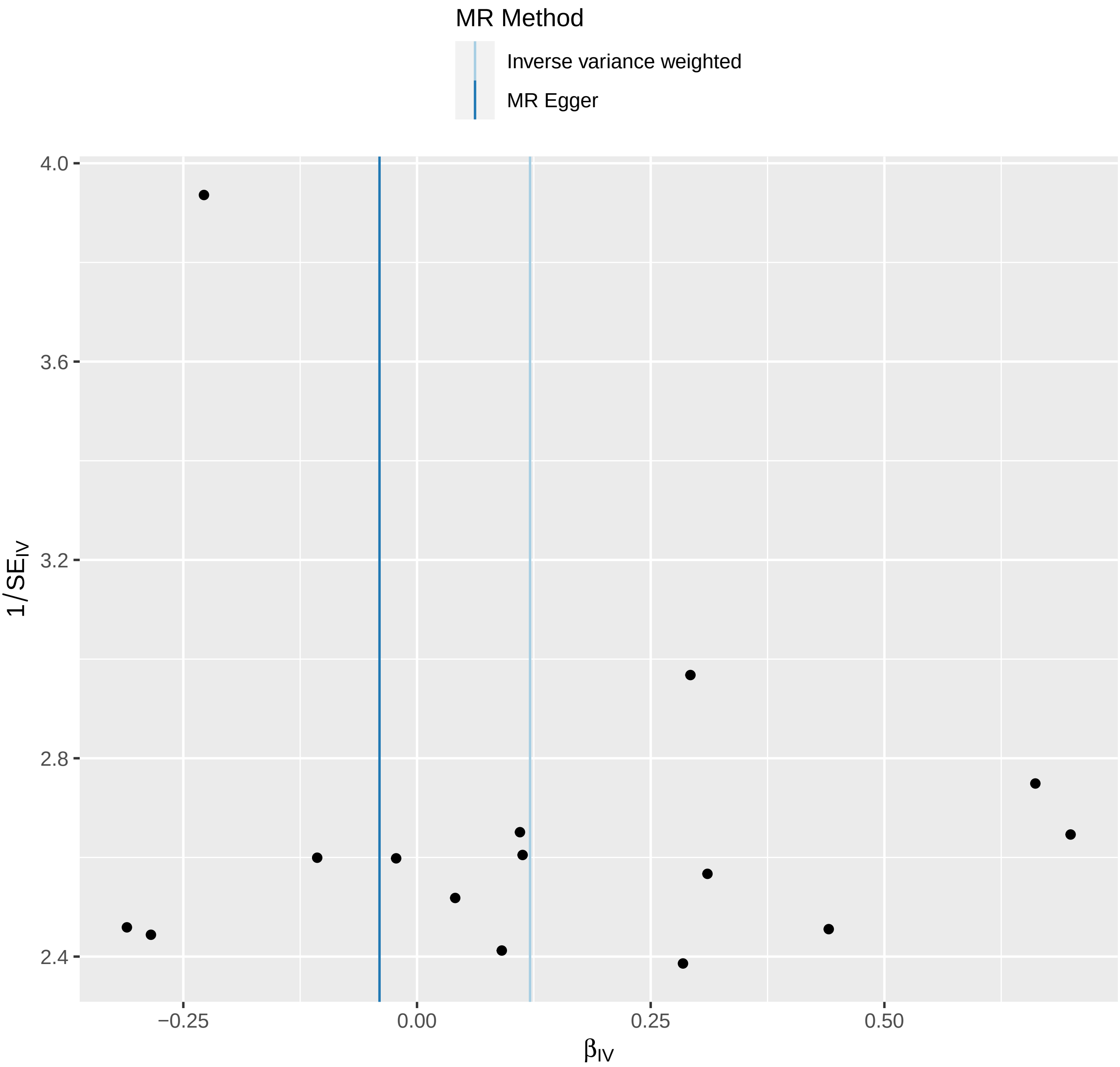

Batch 2−1559 : Gut microbiota abundance (genus Catenibacterium id.2153) on Deformed epidermal autoregulatory factor 1 homolog
with a threshold of 1e−05

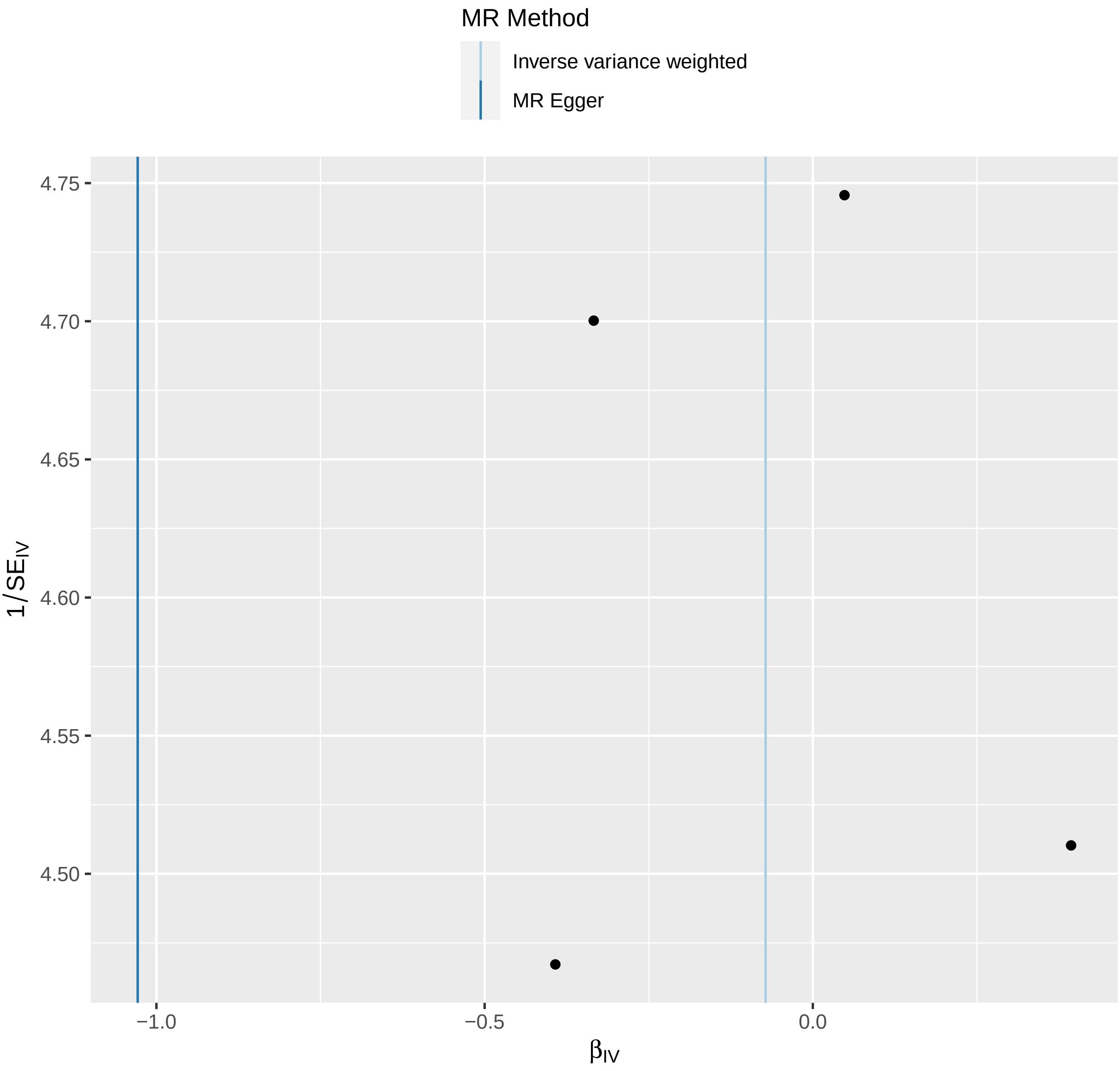

Batch 2−1560 : Gut microbiota abundance (genus Parasutterella id.2892) on Deformed epidermal autoregulatory factor 1 homolog
with a threshold of 1e−05

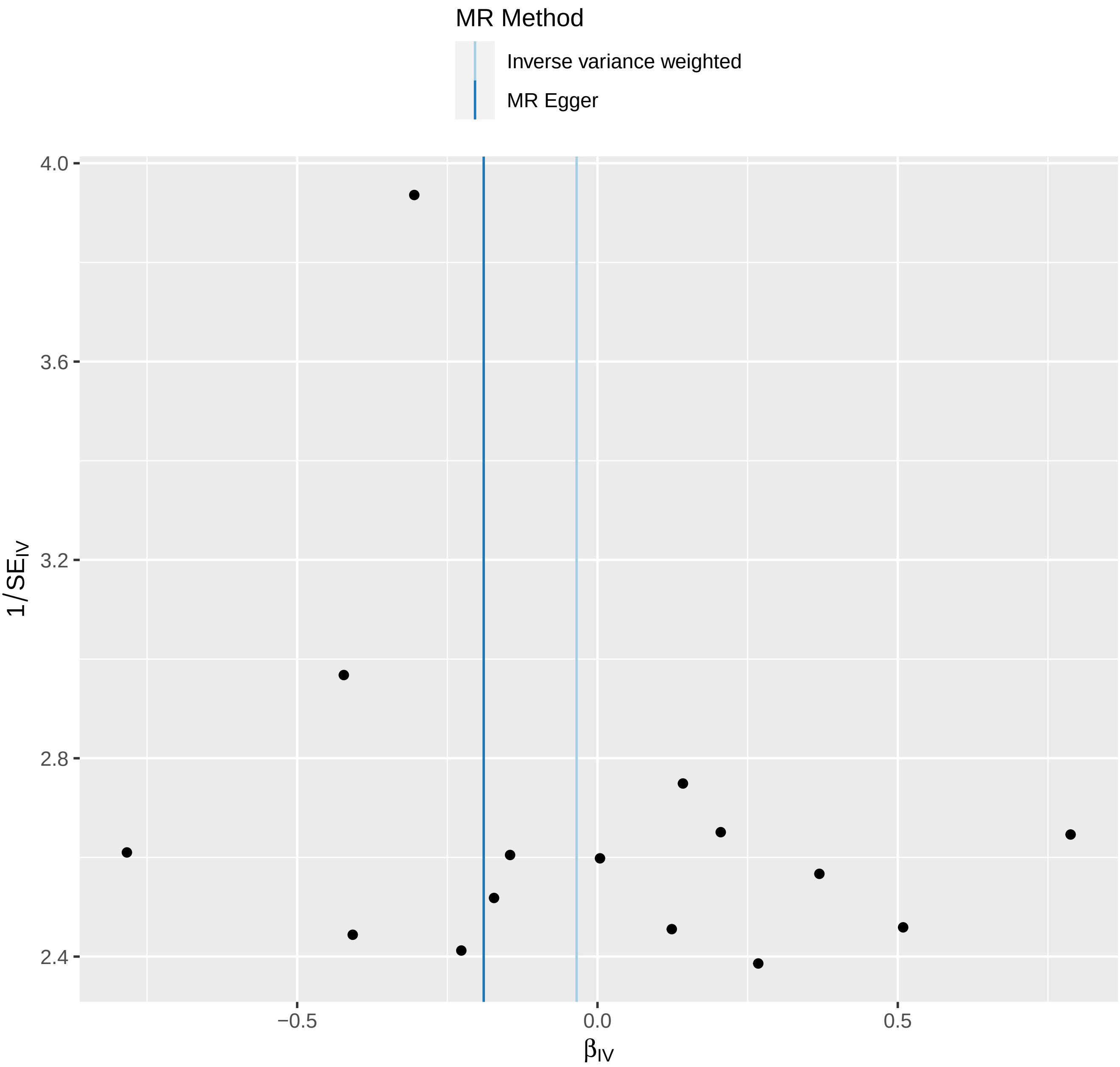

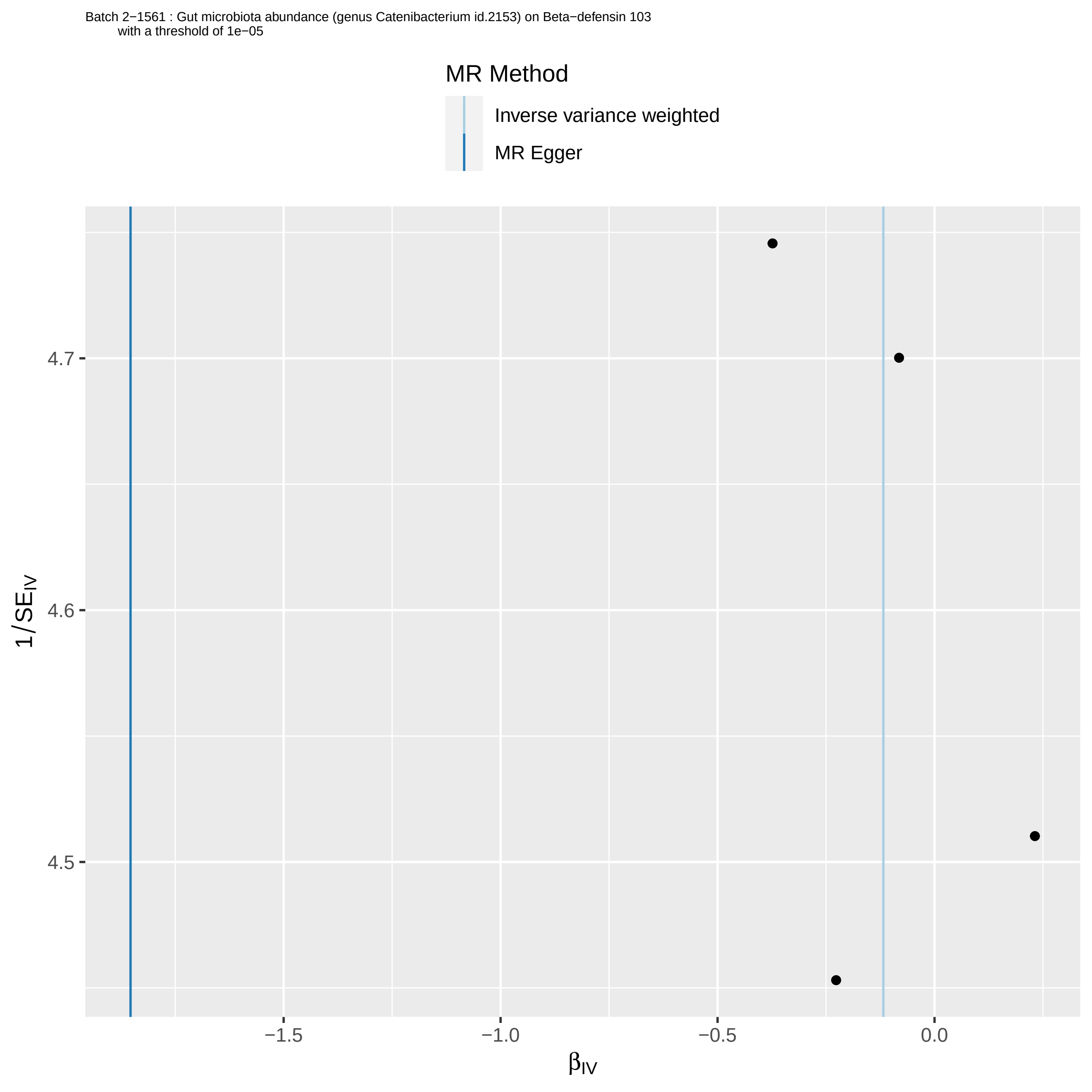
Batch 2−1561 : Gut microbiota abundance (genus Catenibacterium id.2153) on Beta−defensin 103
with a threshold of 1e−05
MR Method
Inverse variance weighted
MR Egger
4.7
4.6
4.5
1/SE_IV
−1.5
−1.0
−0.5
0.0
β_IV

Batch 2−1562 : Gut microbiota abundance (genus Parasutterella id.2892) on Beta−defensin 103
with a threshold of 1e−05

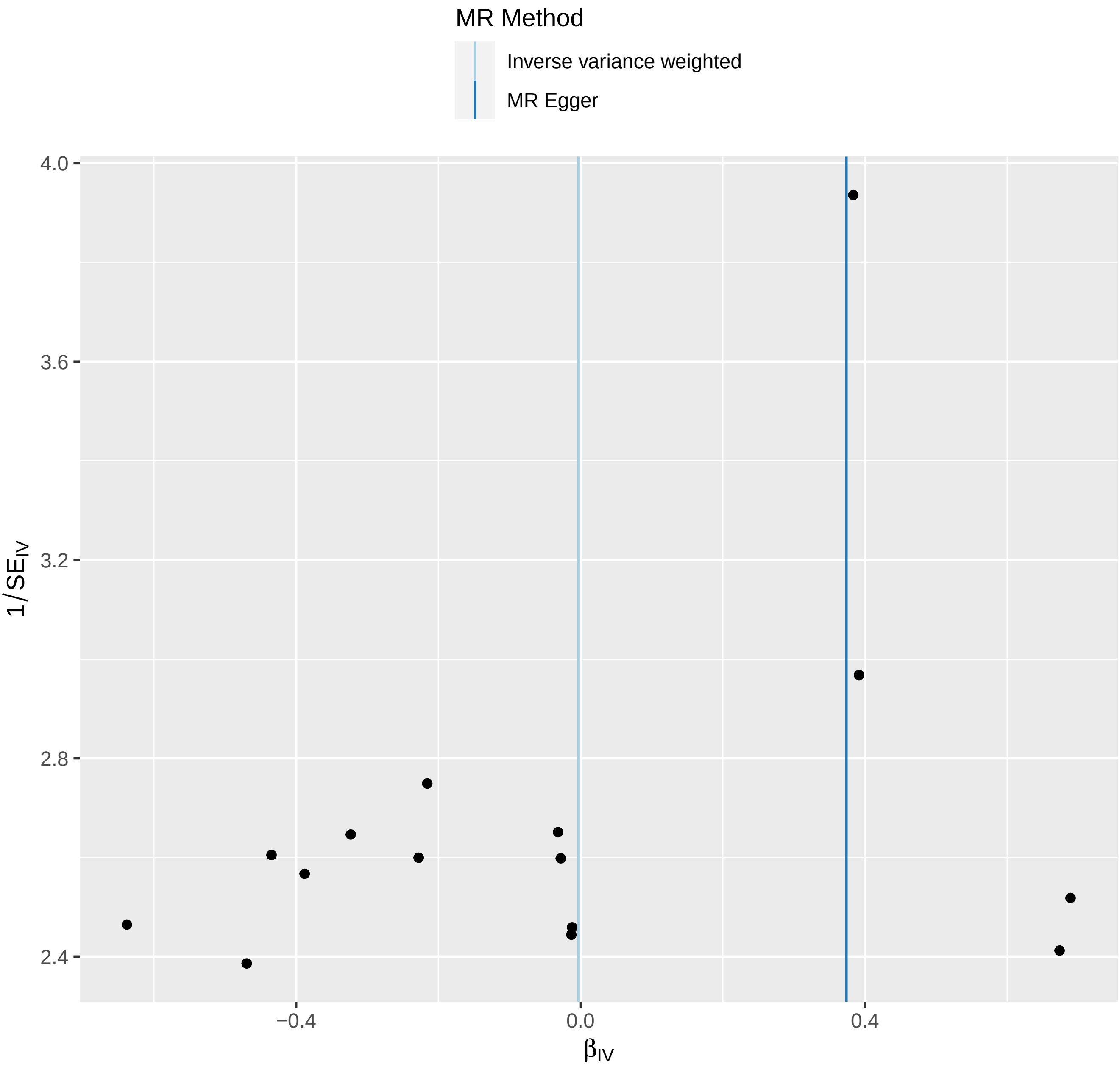

Batch 2−1563 : Gut microbiota abundance (genus Catenibacterium id.2153) on Beta−defensin 104
with a threshold of 1e−05

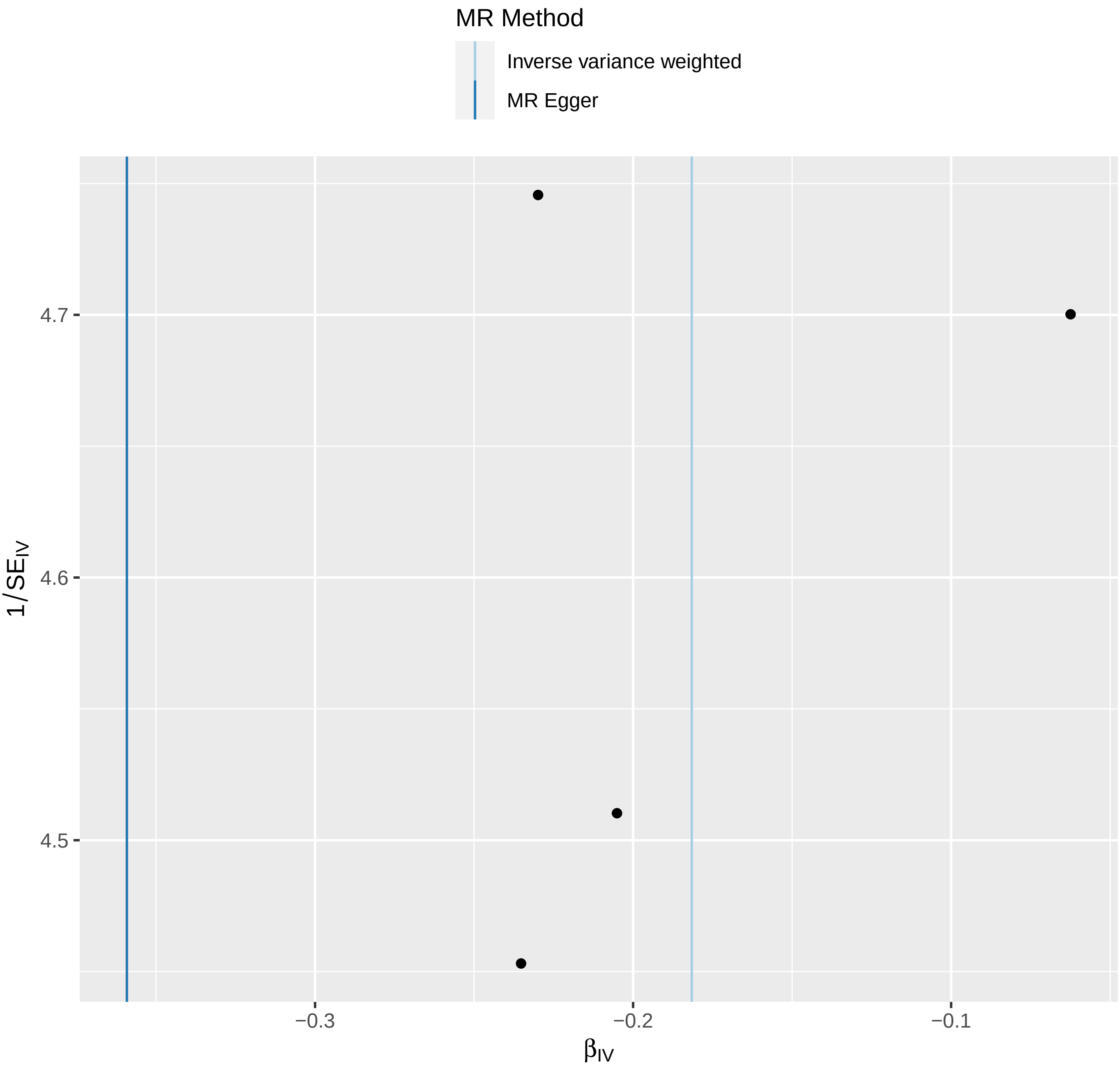

Batch 2−1564 : Gut microbiota abundance (genus Parasutterella id.2892) on Beta−defensin 104
with a threshold of 1e−05

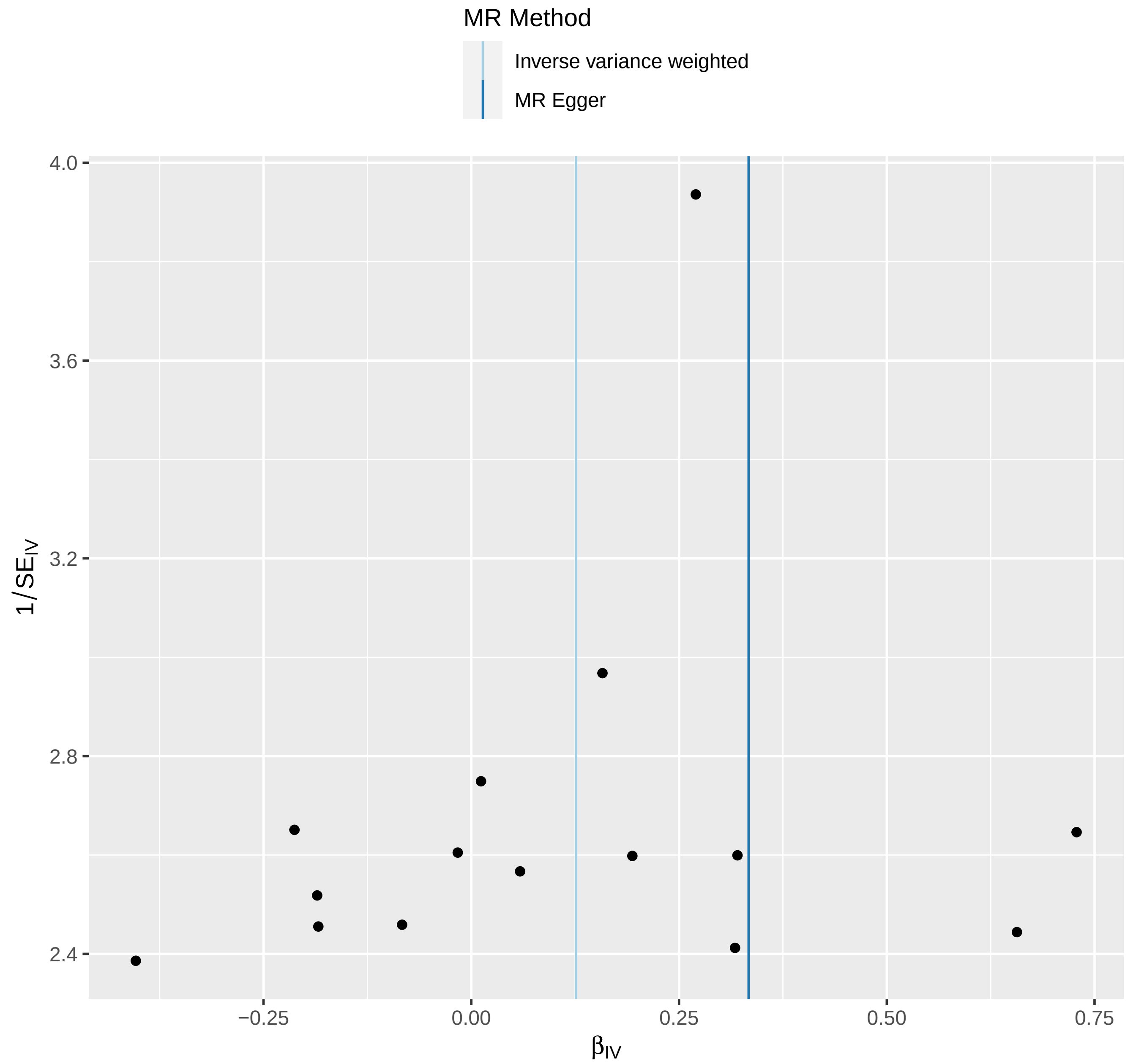

Batch 2−1565 : Gut microbiota abundance (genus Catenibacterium id.2153) on Beta−defensin 105
with a threshold of 1e−05

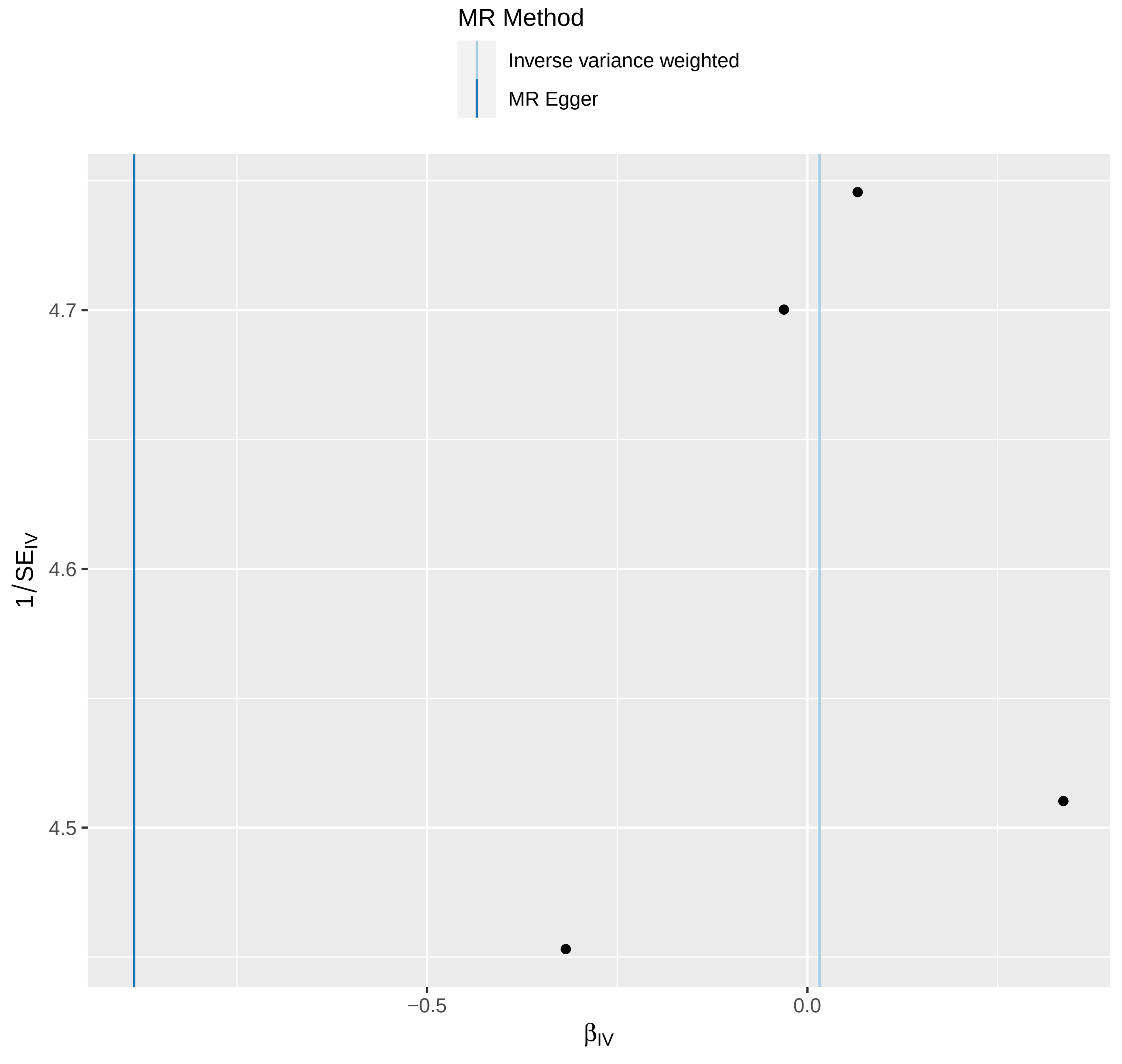

Batch 2−1566 : Gut microbiota abundance (genus Parasutterella id.2892) on Beta−defensin 105
with a threshold of 1e−05

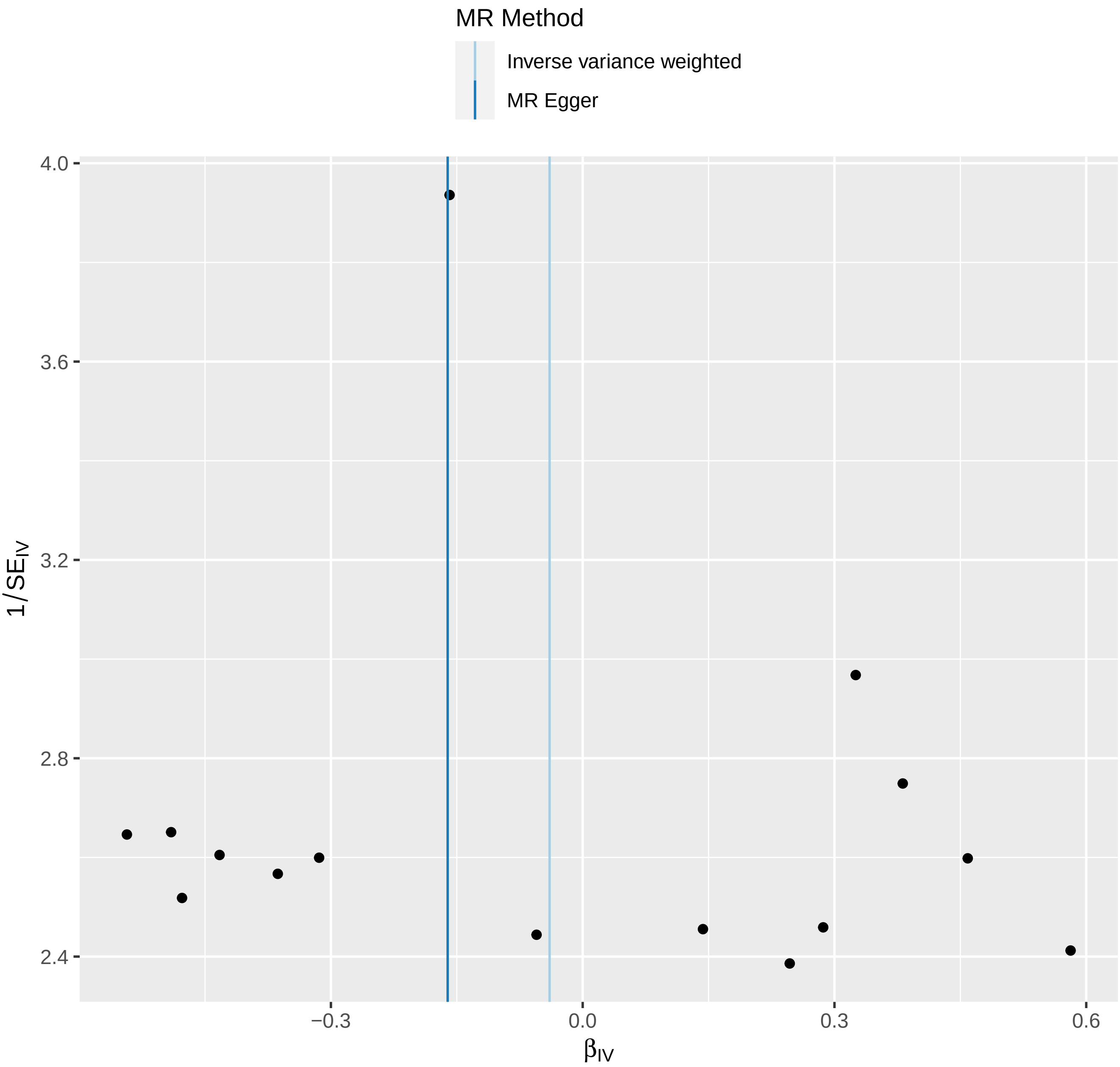

Batch 2−1567 : Gut microbiota abundance (genus Catenibacterium id.2153) on Beta−defensin 106
with a threshold of 1e−05

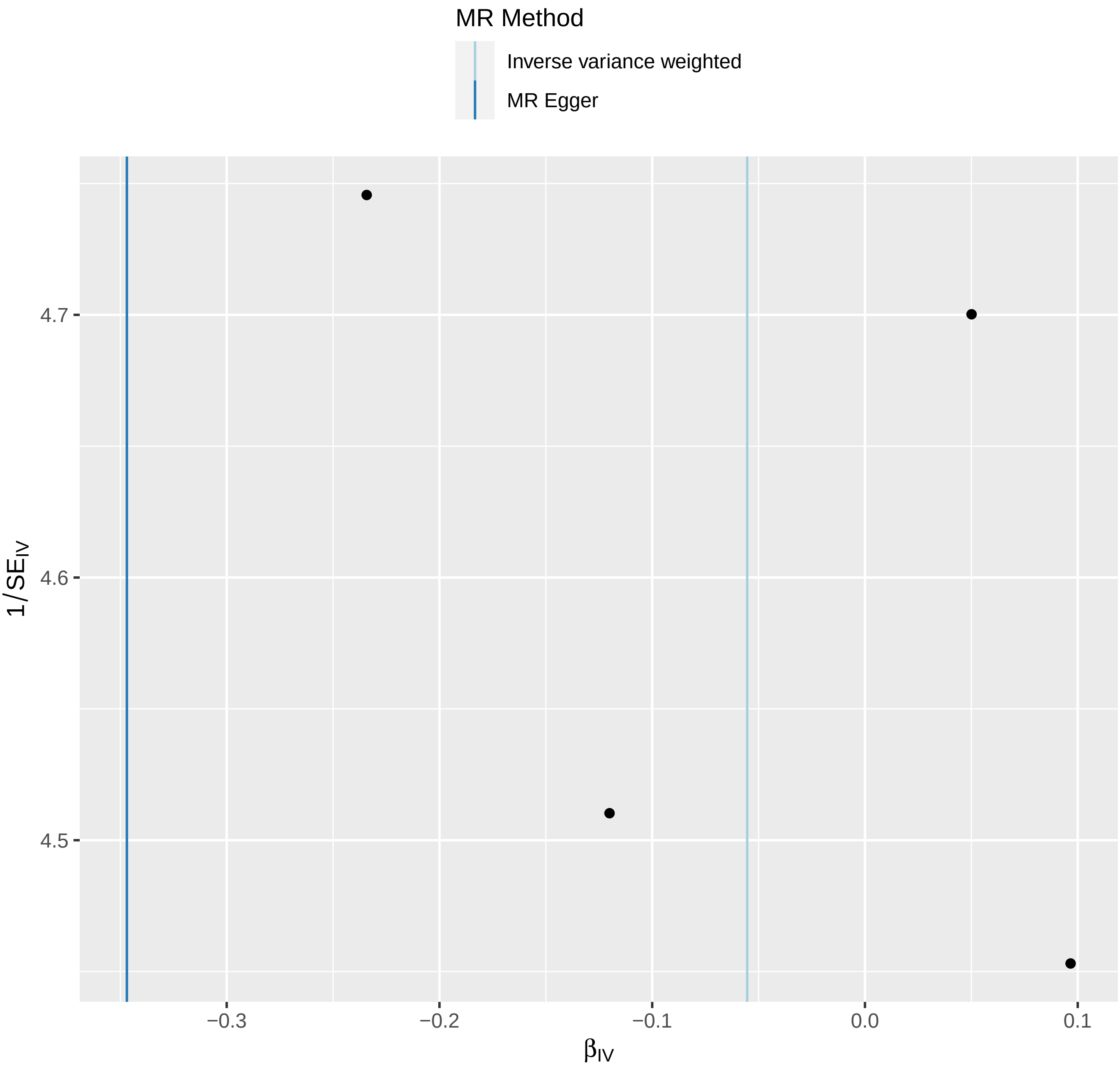

Batch 2−1568 : Gut microbiota abundance (genus Parasutterella id.2892) on Beta−defensin 106
with a threshold of 1e−05

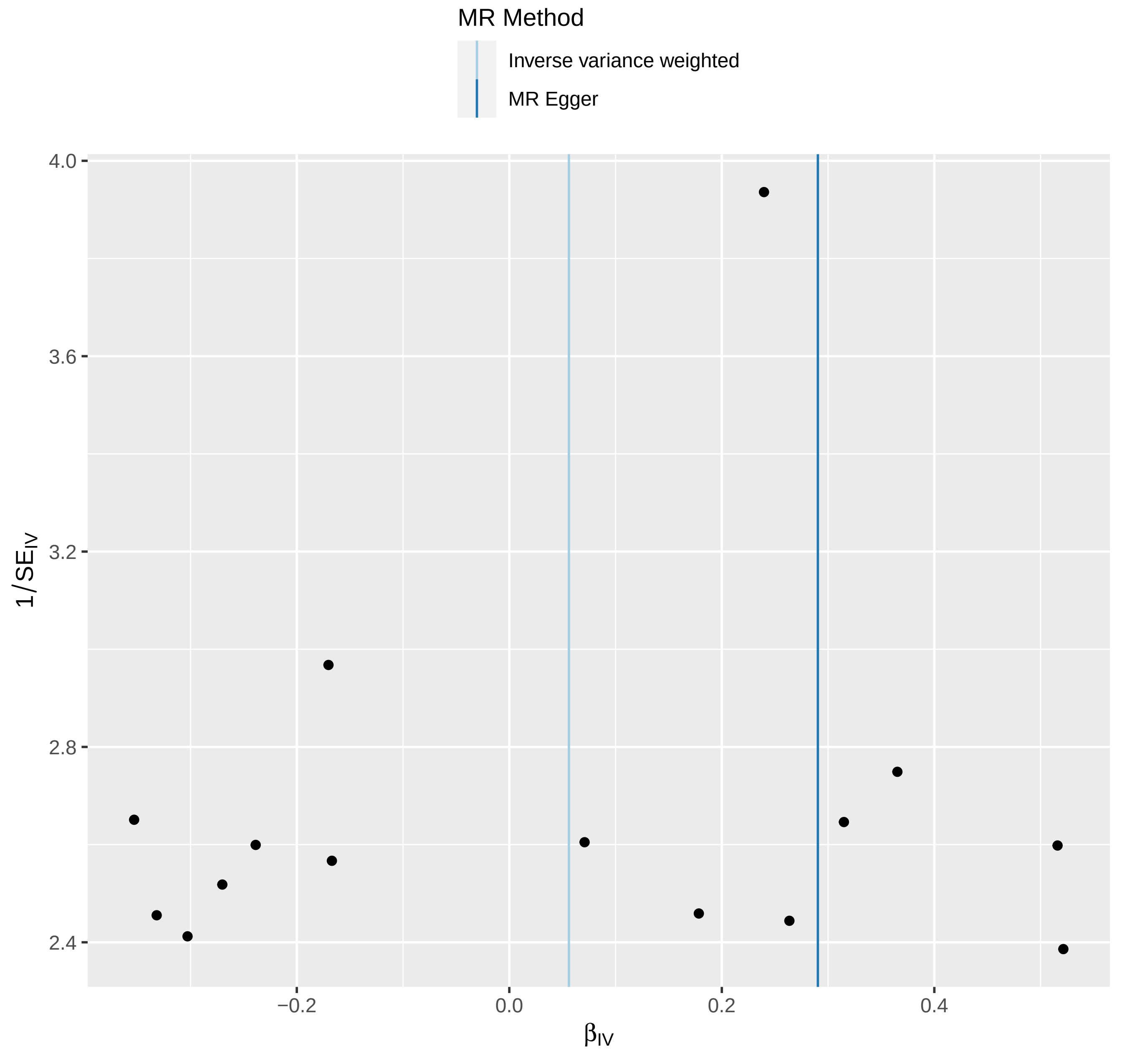

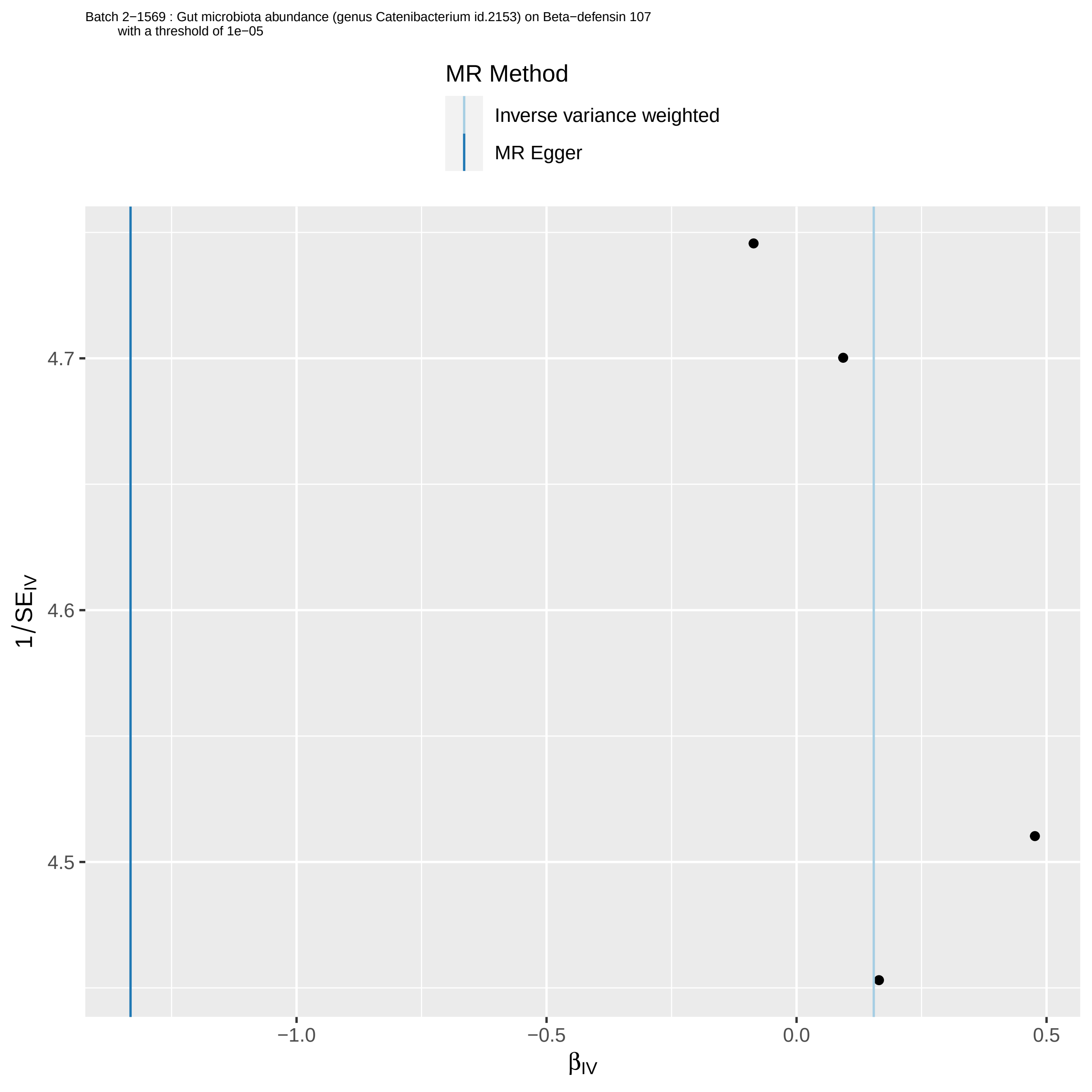

Batch 2−1569 : Gut microbiota abundance (genus Catenibacterium id.2153) on Beta−defensin 107
with a threshold of 1e−05
MR Method
Inverse variance weighted
MR Egger
4.7
4.6
4.5
1/SE_IV
−1.0
−0.5
0.0
0.5
β_IV

Batch 2−1570 : Gut microbiota abundance (genus Parasutterella id.2892) on Beta−defensin 107
with a threshold of 1e−05

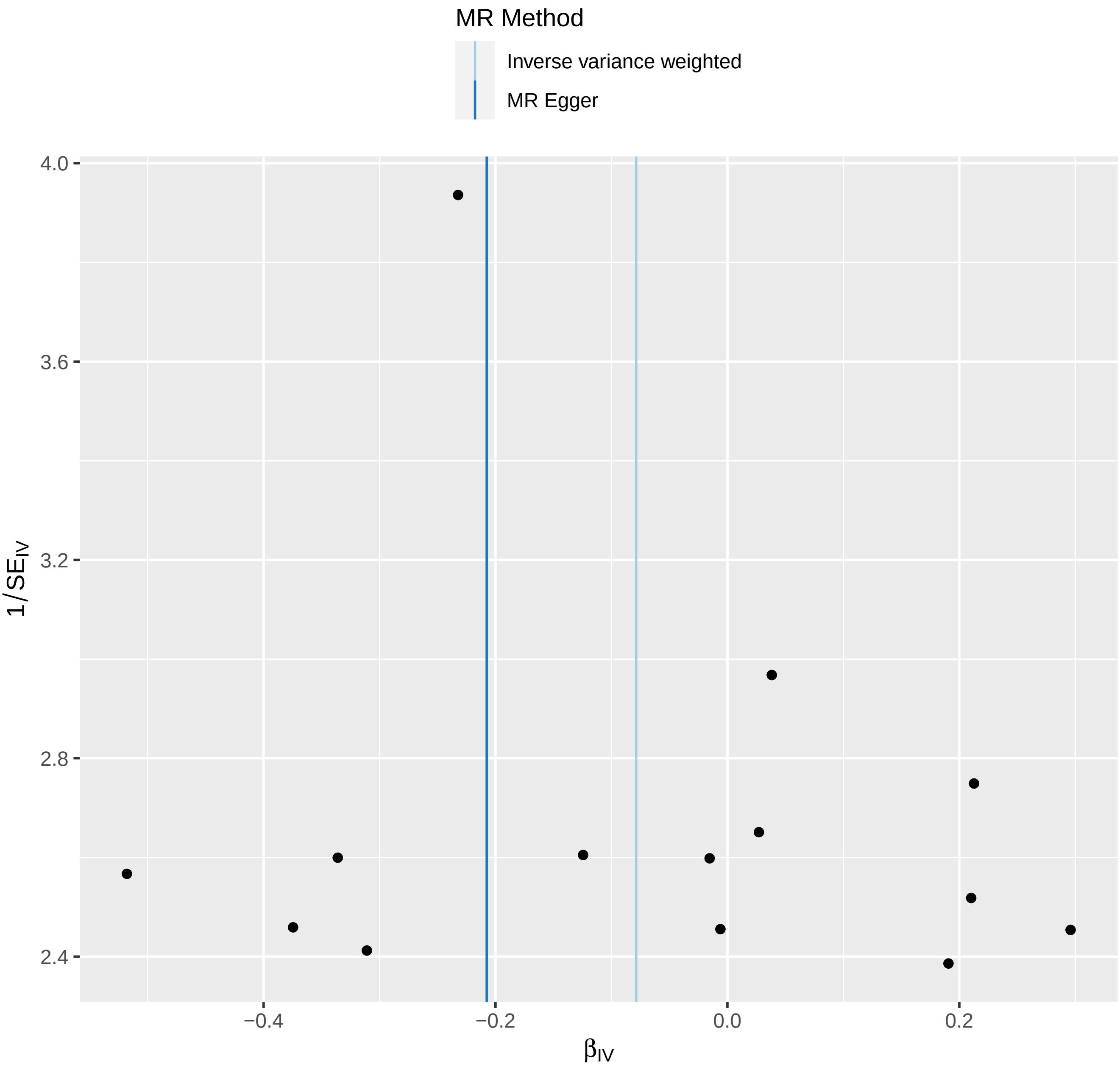

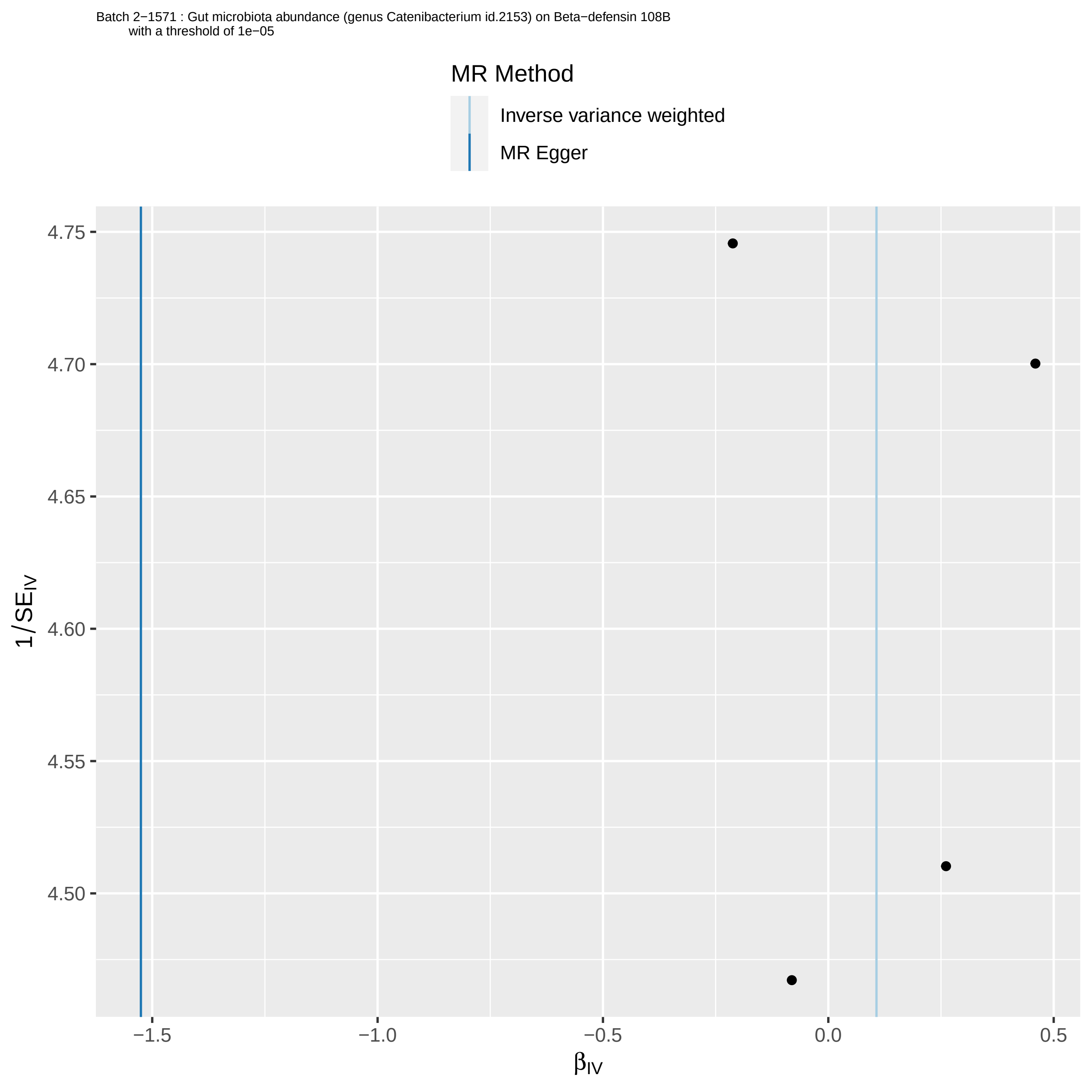
Batch 2−1571 : Gut microbiota abundance (genus Catenibacterium id.2153) on Beta−defensin 108B
with a threshold of 1e−05
MR Method
Inverse variance weighted
MR Egger
4.75
4.70
4.65
4.60
4.55
4.50
1/SEIV
−1.5
−1.0
−0.5
0.0
0.5
βIV

Batch 2−1572 : Gut microbiota abundance (genus Parasutterella id.2892) on Beta−defensin 108B
with a threshold of 1e−05

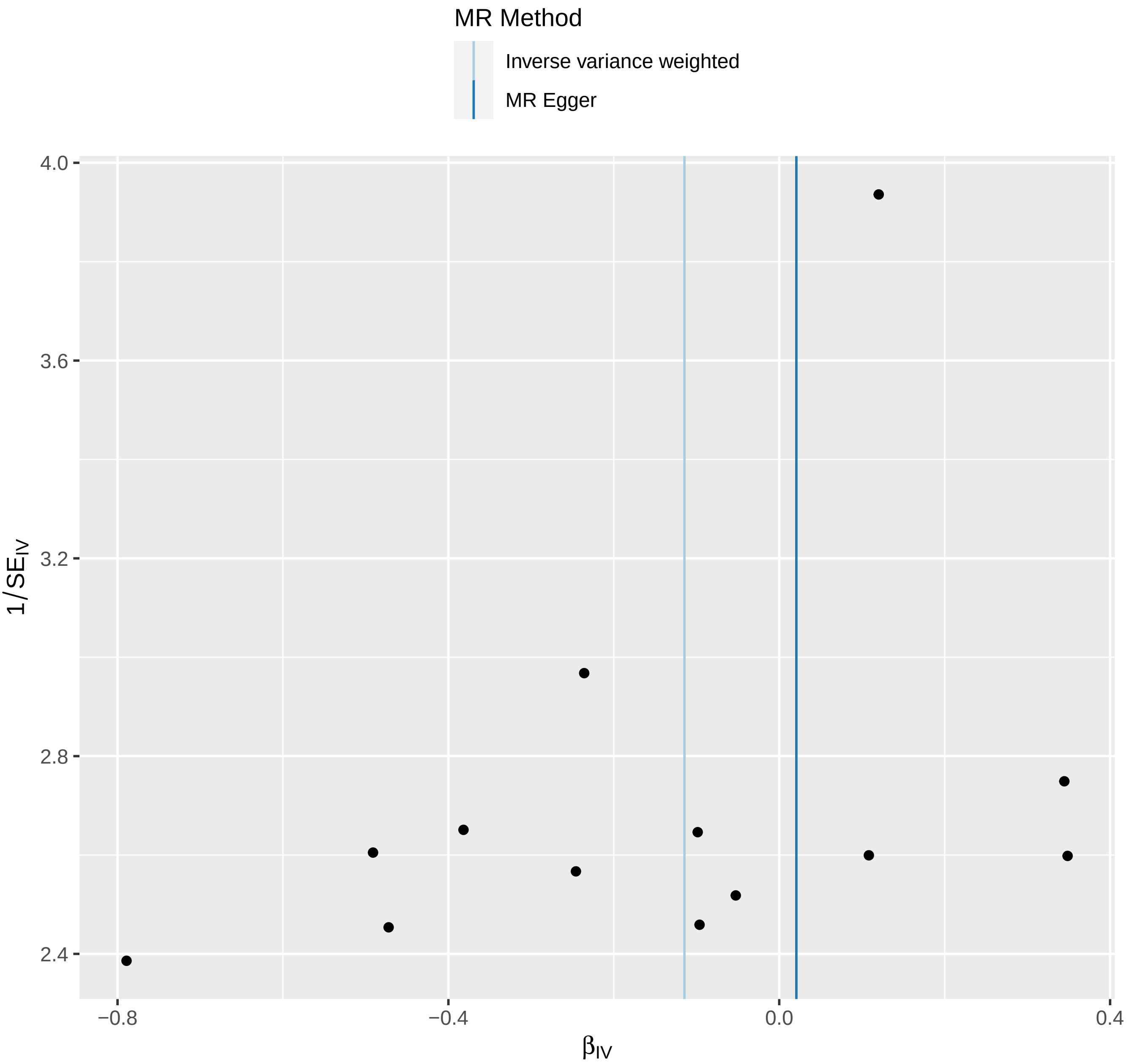

Batch 2−1573 : Gut microbiota abundance (genus Catenibacterium id.2153) on Beta−defensin 110
with a threshold of 1e−05

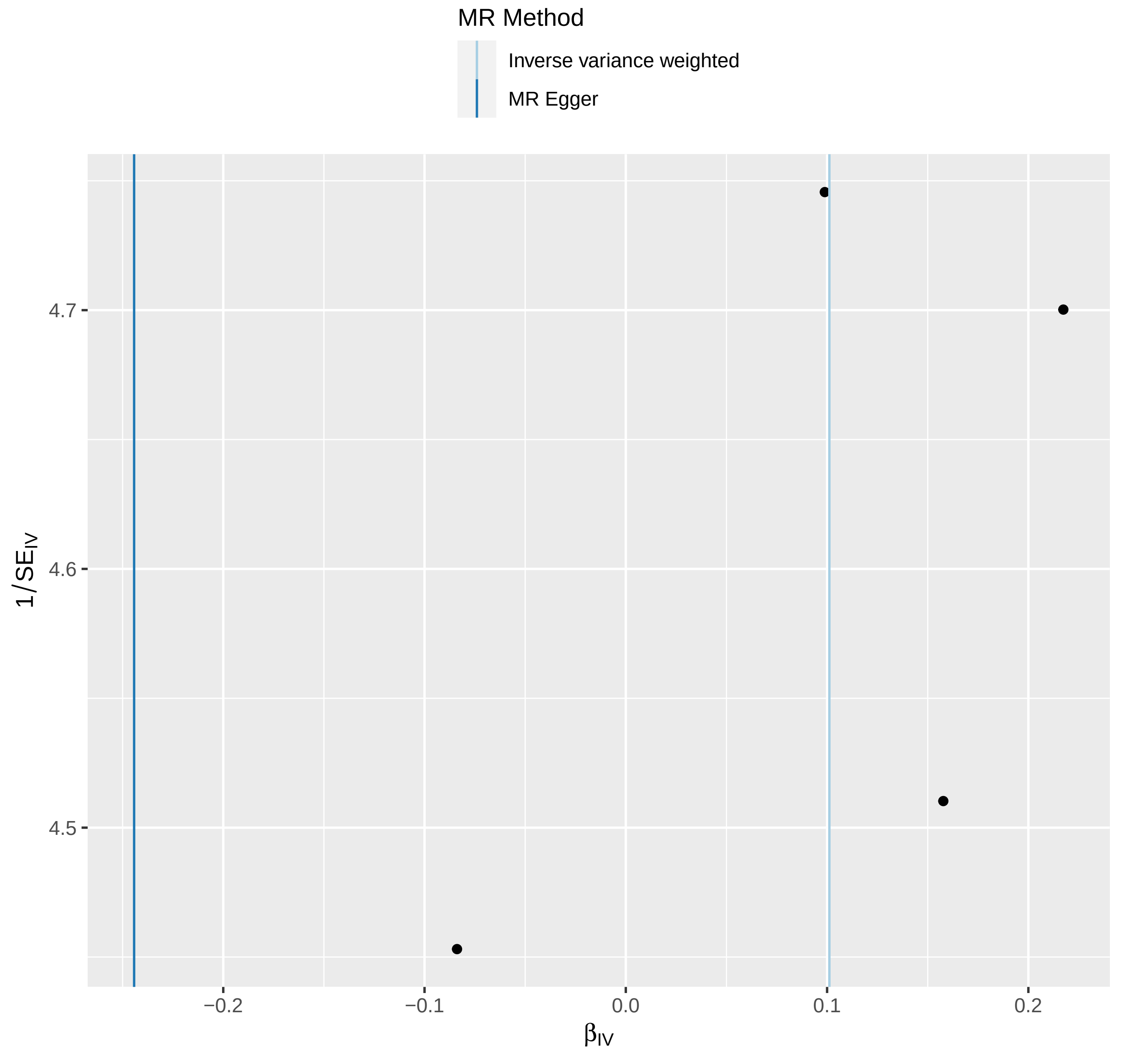

Batch 2−1574 : Gut microbiota abundance (genus Parasutterella id.2892) on Beta−defensin 110
with a threshold of 1e−05

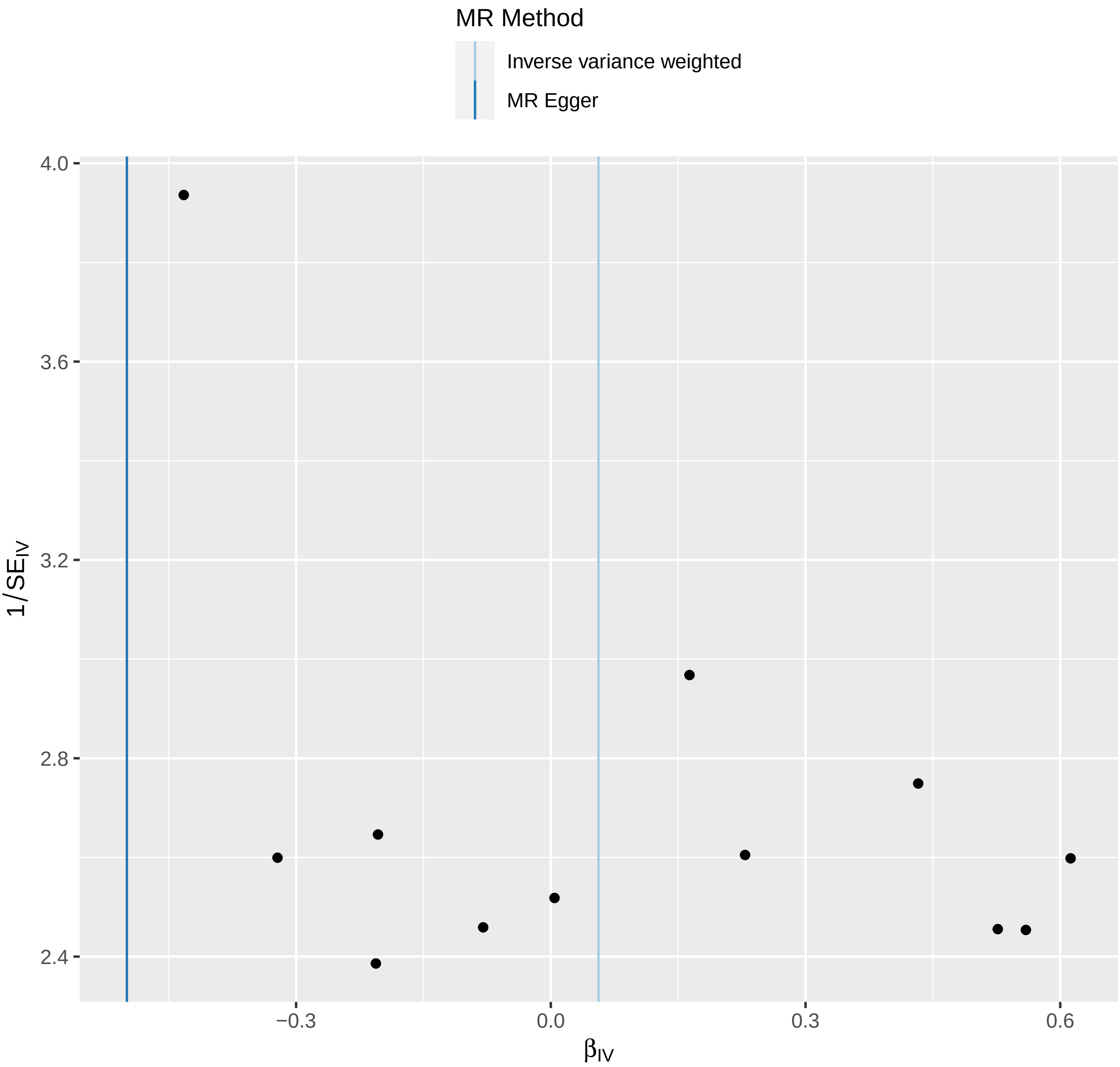

Batch 2−1575 : Gut microbiota abundance (genus Catenibacterium id.2153) on Beta−defensin 112
with a threshold of 1e−05

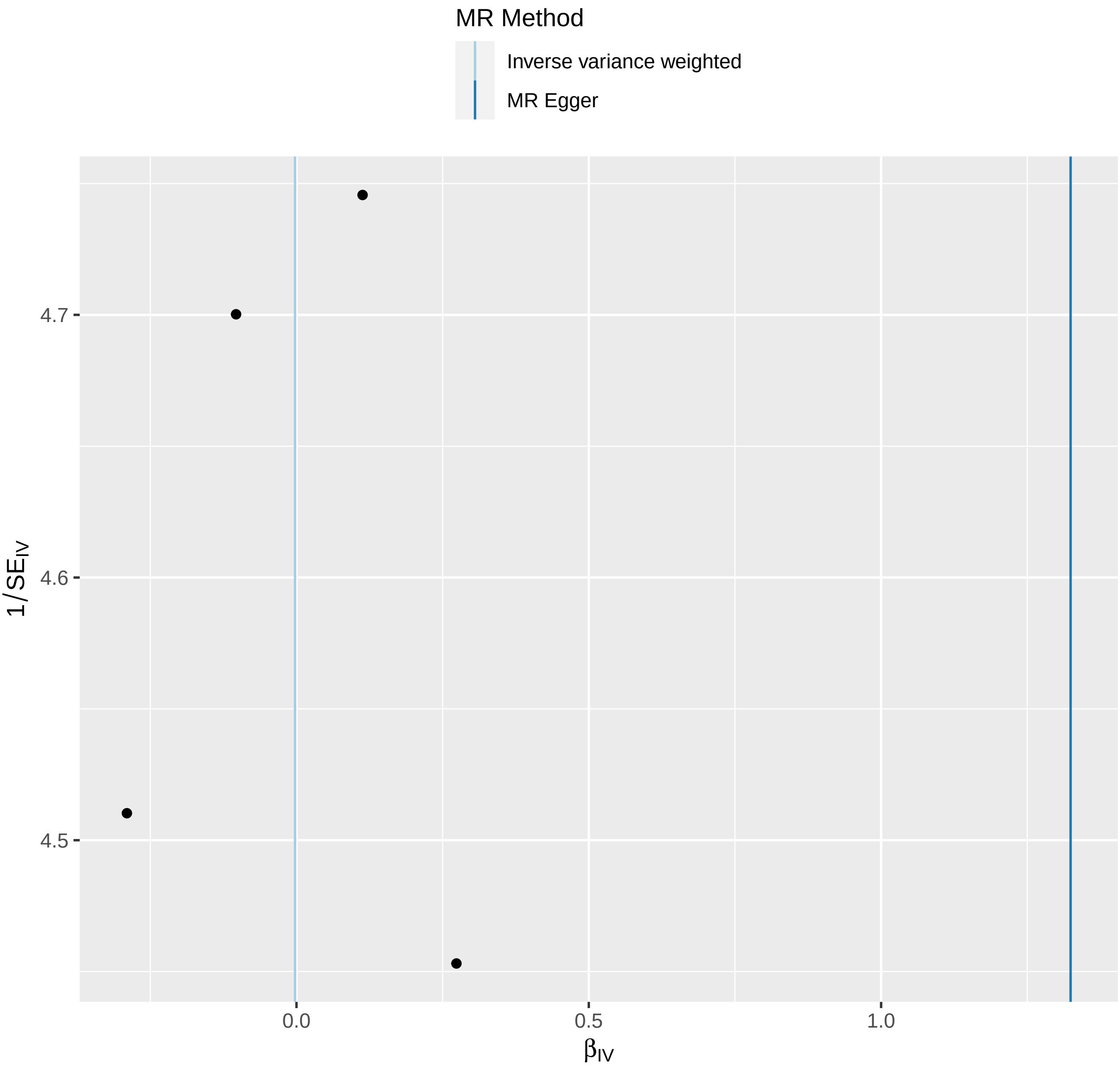

Batch 2−1576 : Gut microbiota abundance (genus Parasutterella id.2892) on Beta−defensin 112
with a threshold of 1e−05

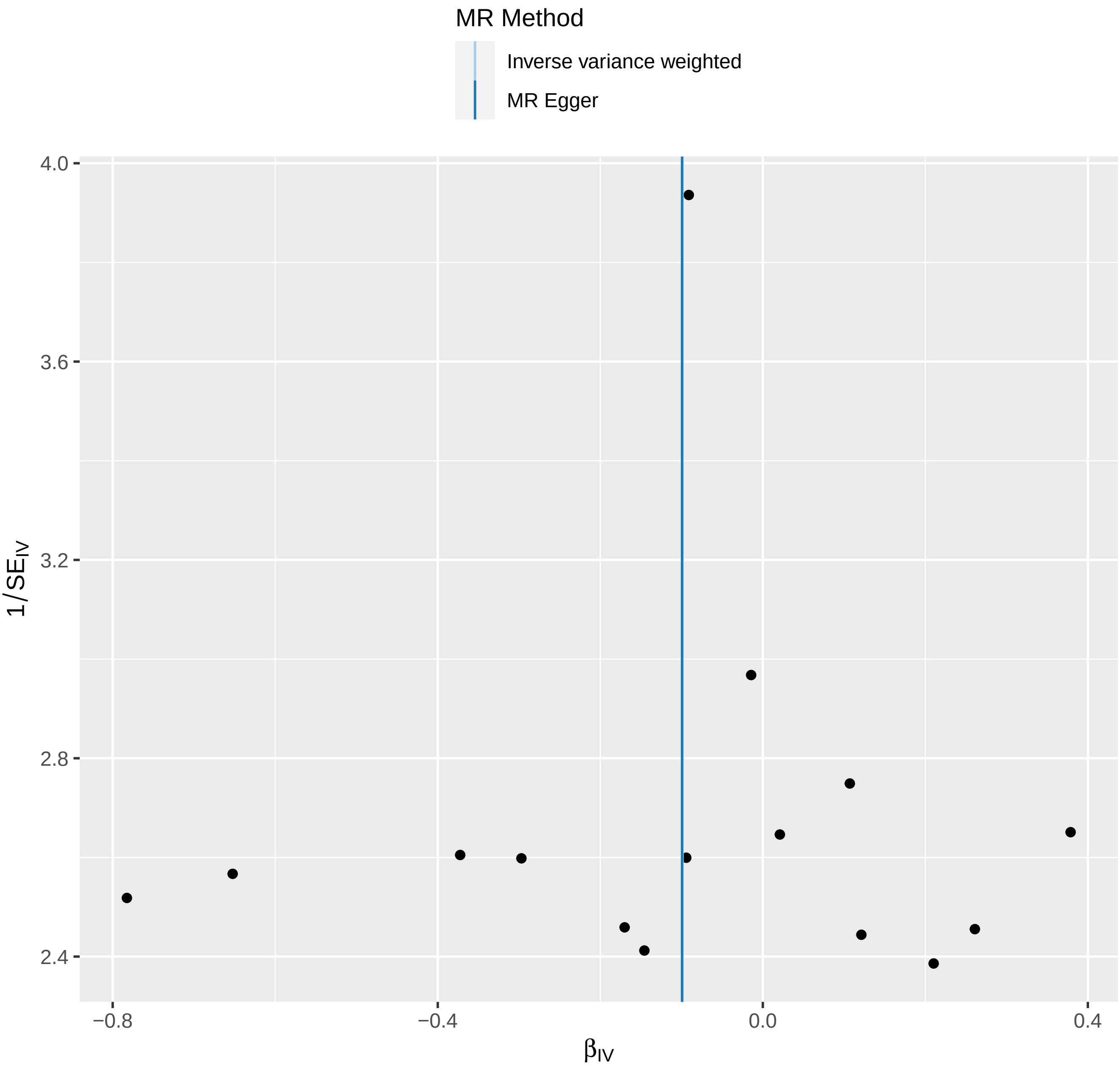

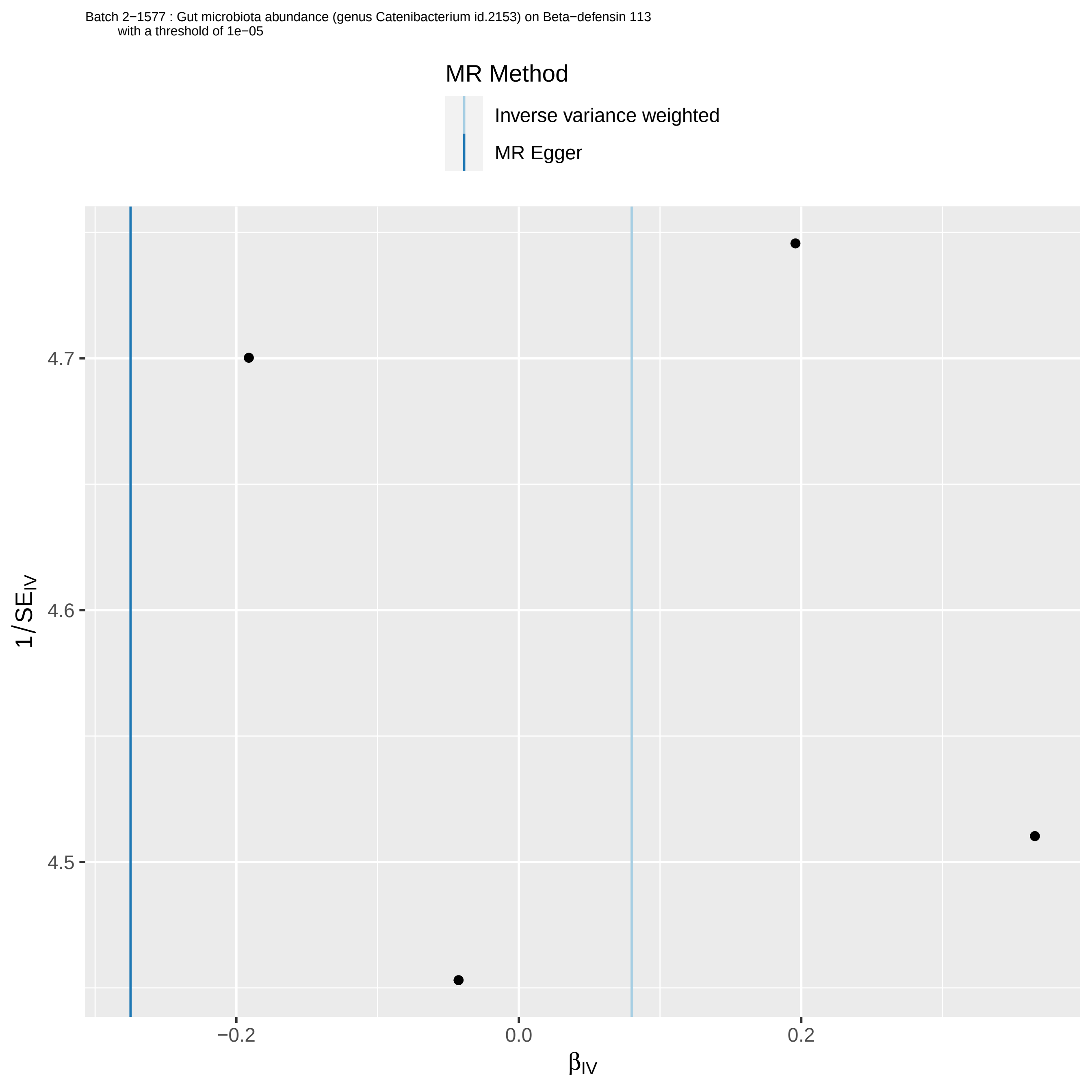

Batch 2−1577 : Gut microbiota abundance (genus Catenibacterium id.2153) on Beta−defensin 113
with a threshold of 1e−05
MR Method
Inverse variance weighted
MR Egger
4.7
4.6
4.5
1/SE_IV
−0.2
0.0
0.2
β_IV

Batch 2−1578 : Gut microbiota abundance (genus Parasutterella id.2892) on Beta−defensin 113
with a threshold of 1e−05

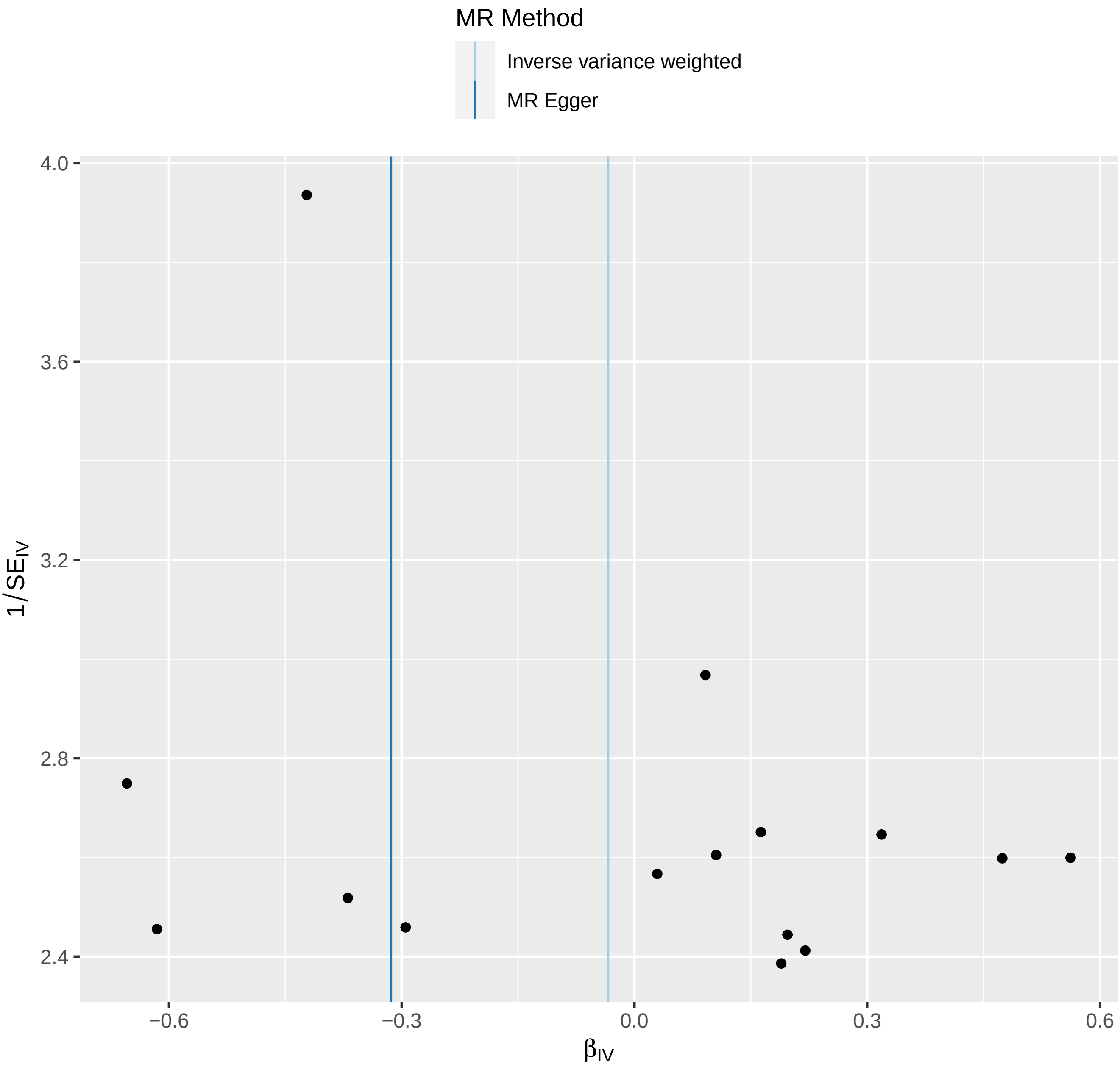

Batch 2−1579 : Gut microbiota abundance (genus Catenibacterium id.2153) on Beta−defensin 115
with a threshold of 1e−05

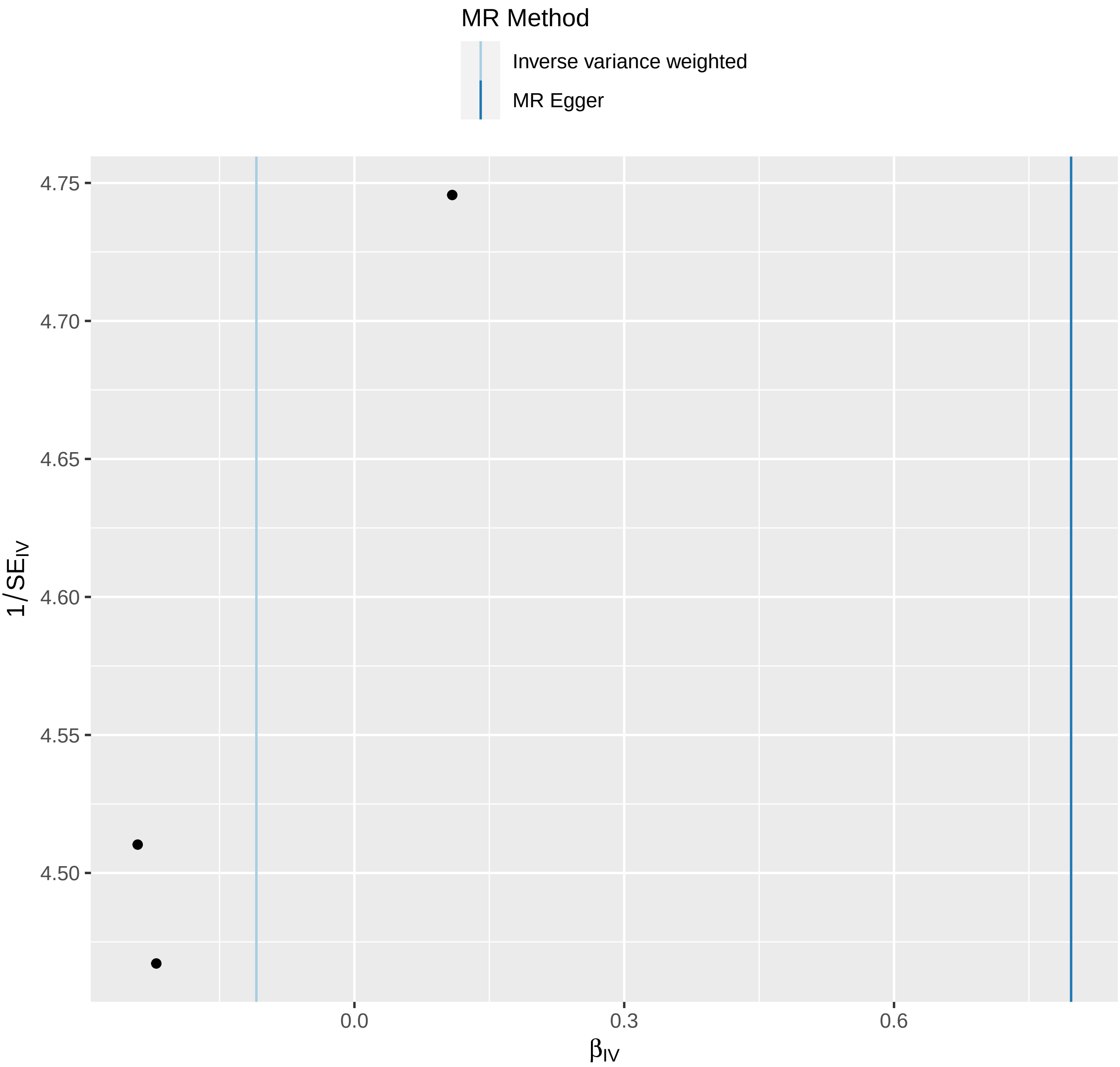

Batch 2−1580 : Gut microbiota abundance (genus Parasutterella id.2892) on Beta−defensin 115
with a threshold of 1e−05

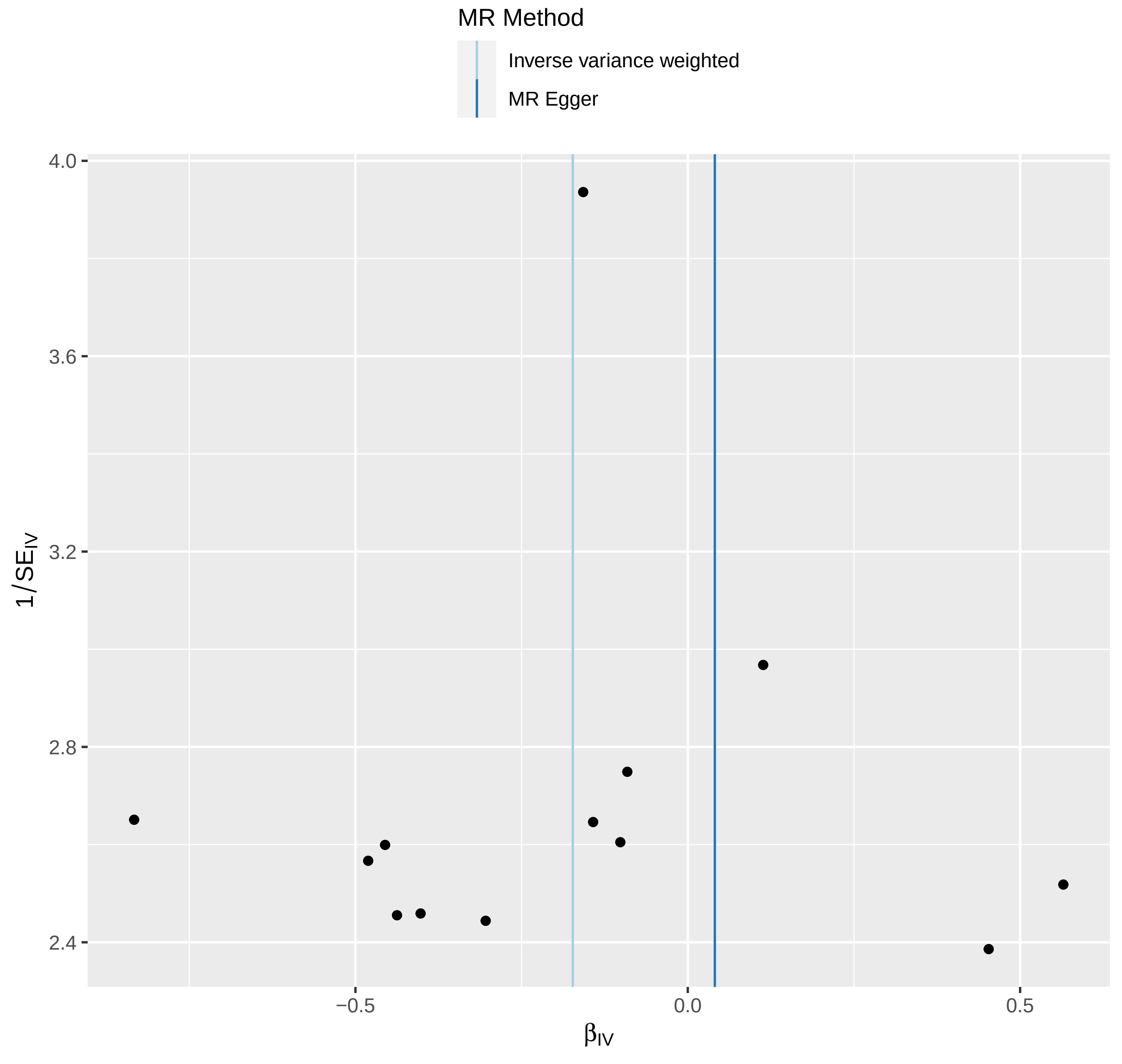

Batch 2−1581 : Gut microbiota abundance (genus Catenibacterium id.2153) on Beta−defensin 116
with a threshold of 1e−05

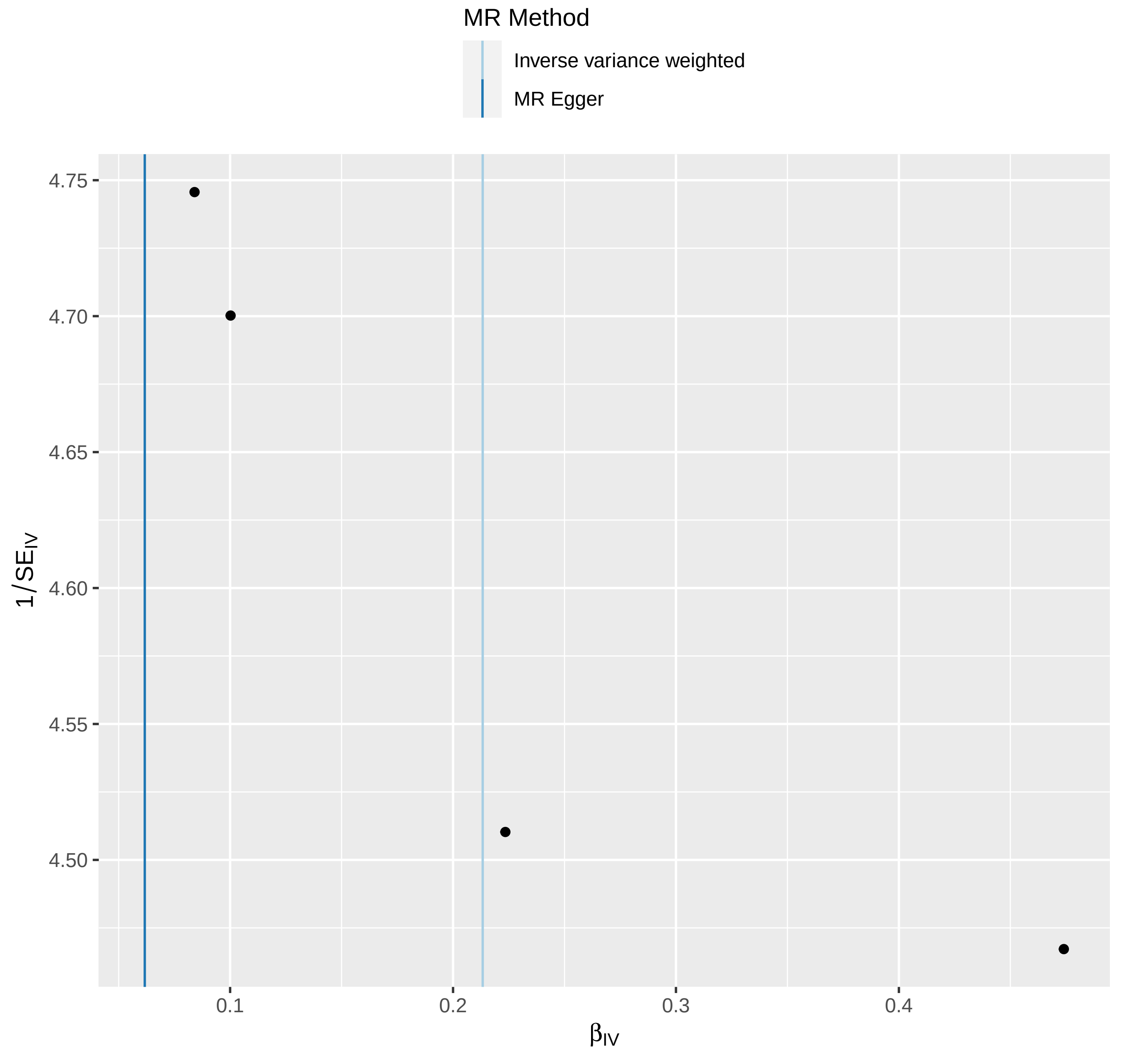

Batch 2−1582 : Gut microbiota abundance (genus Parasutterella id.2892) on Beta−defensin 116
with a threshold of 1e−05

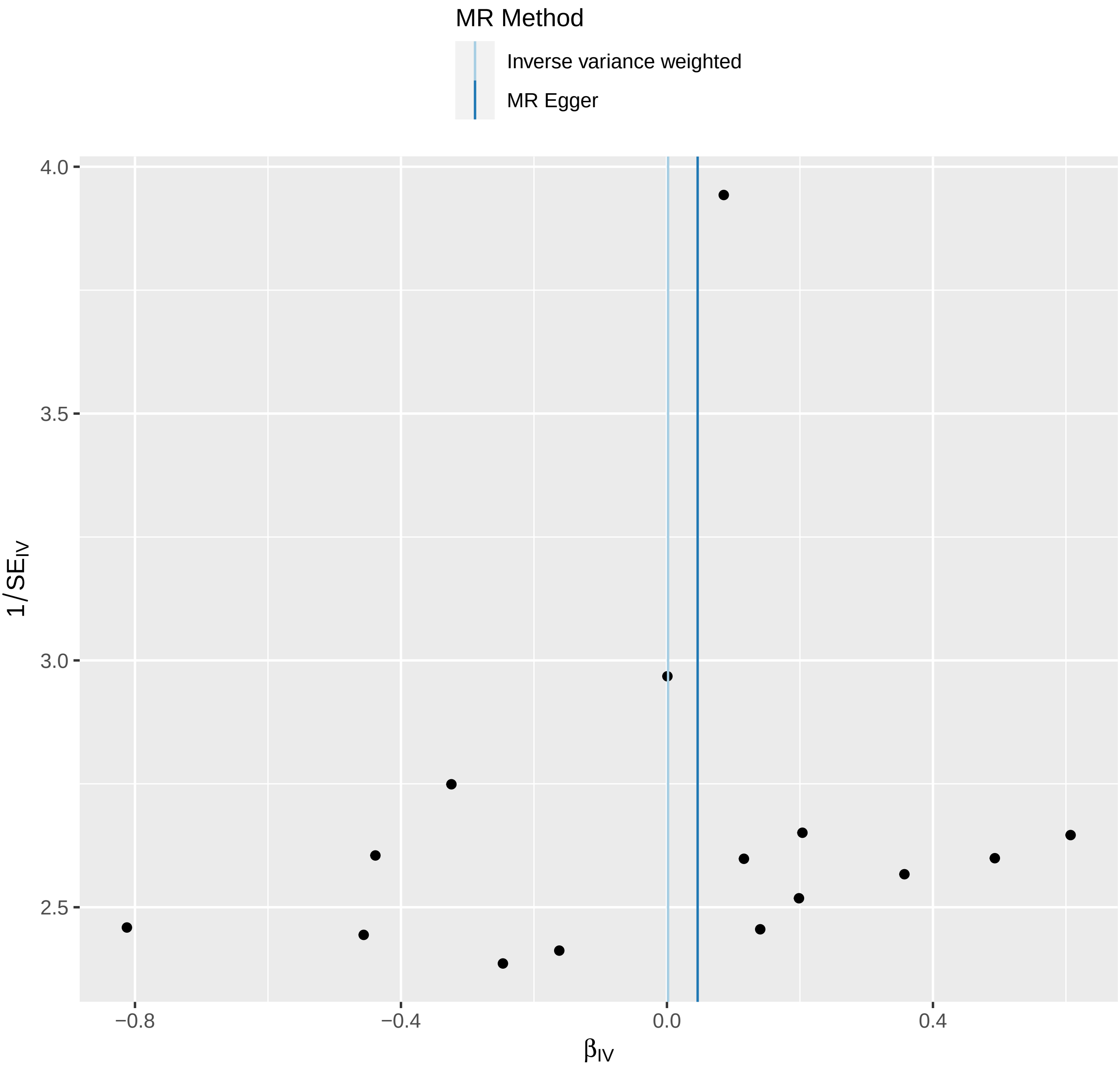

Batch 2−1583 : Gut microbiota abundance (genus Catenibacterium id.2153) on Beta−defensin 118
with a threshold of 1e−05

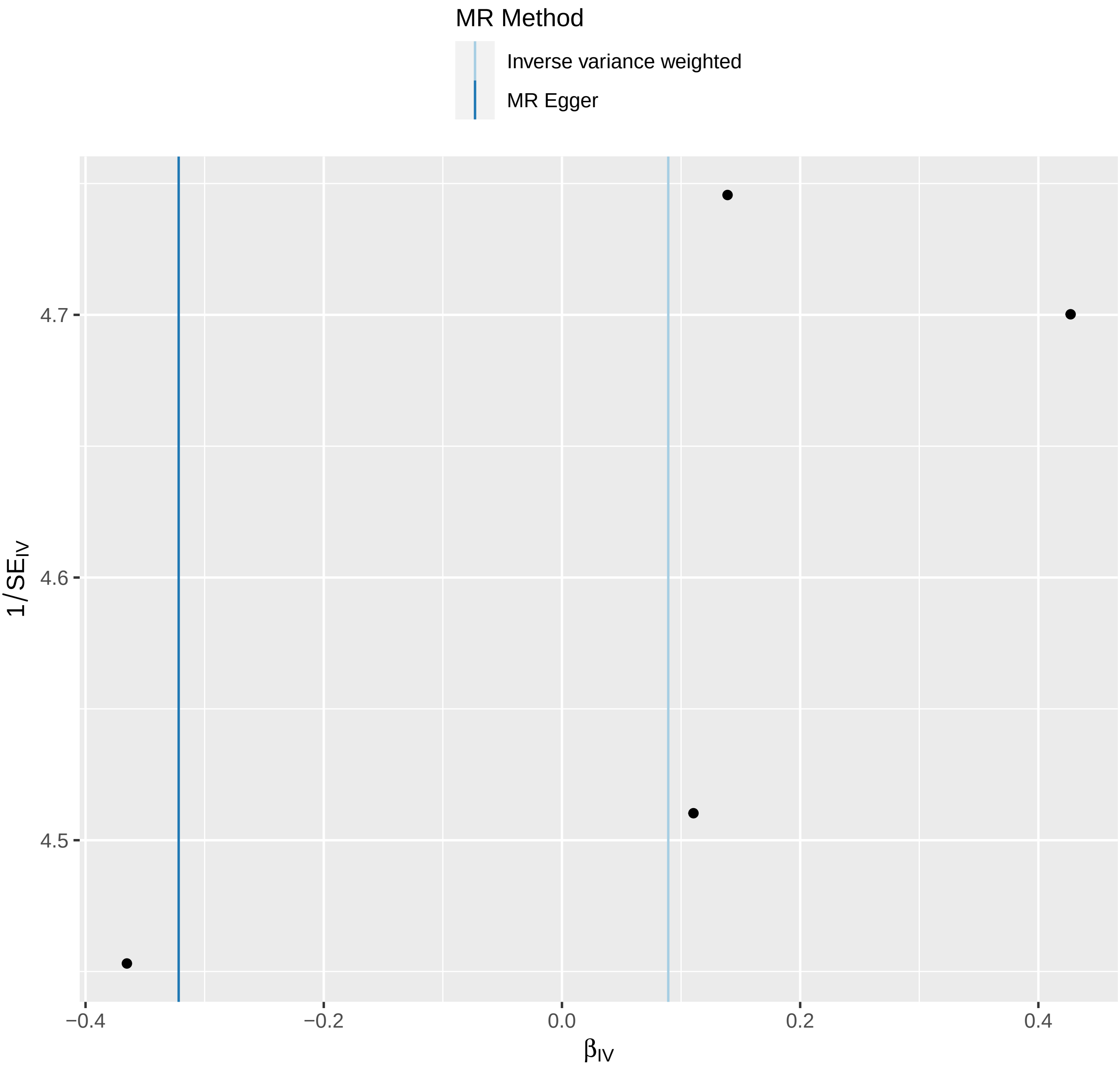

Batch 2−1584 : Gut microbiota abundance (genus Parasutterella id.2892) on Beta−defensin 118
with a threshold of 1e−05

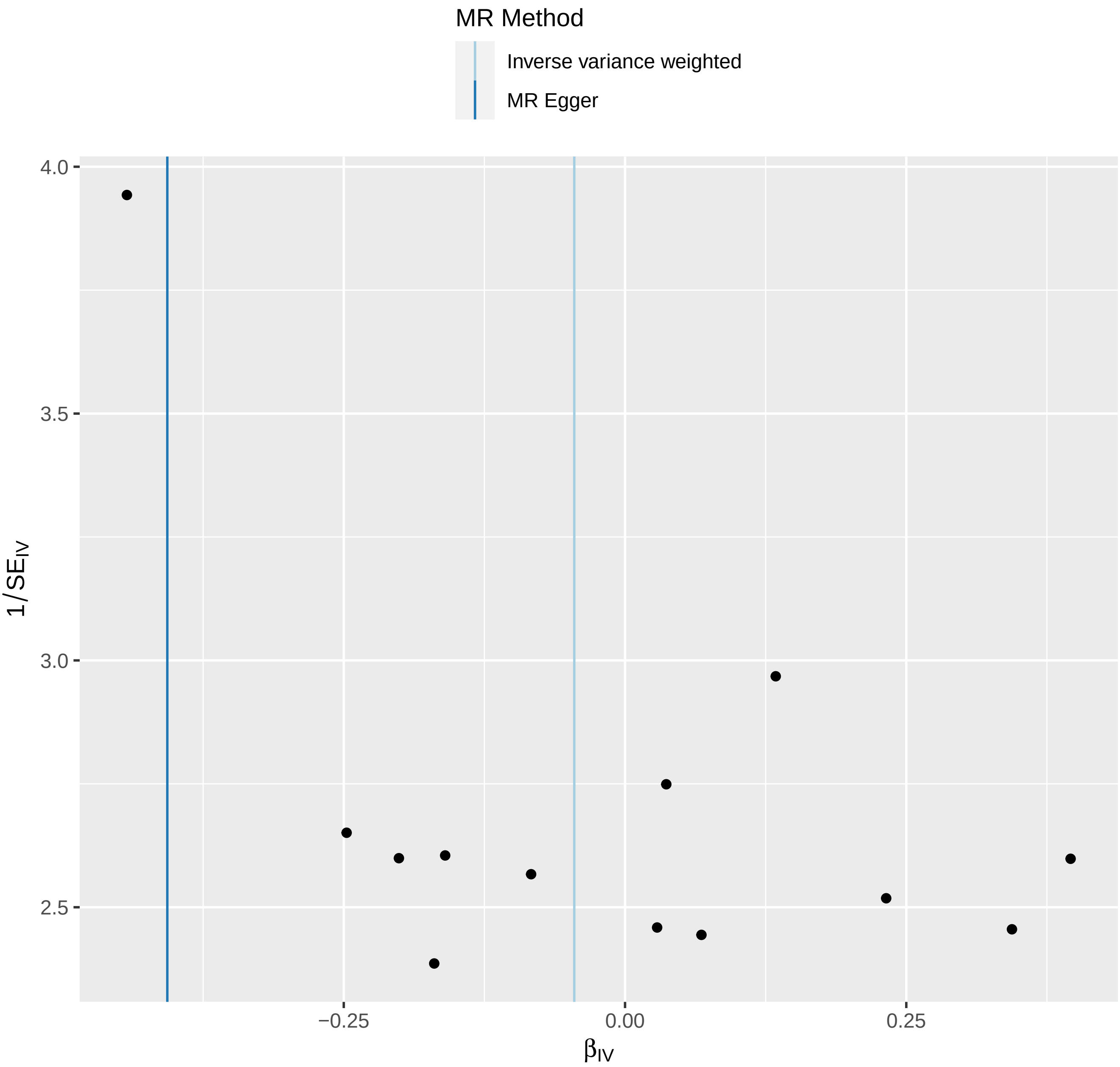

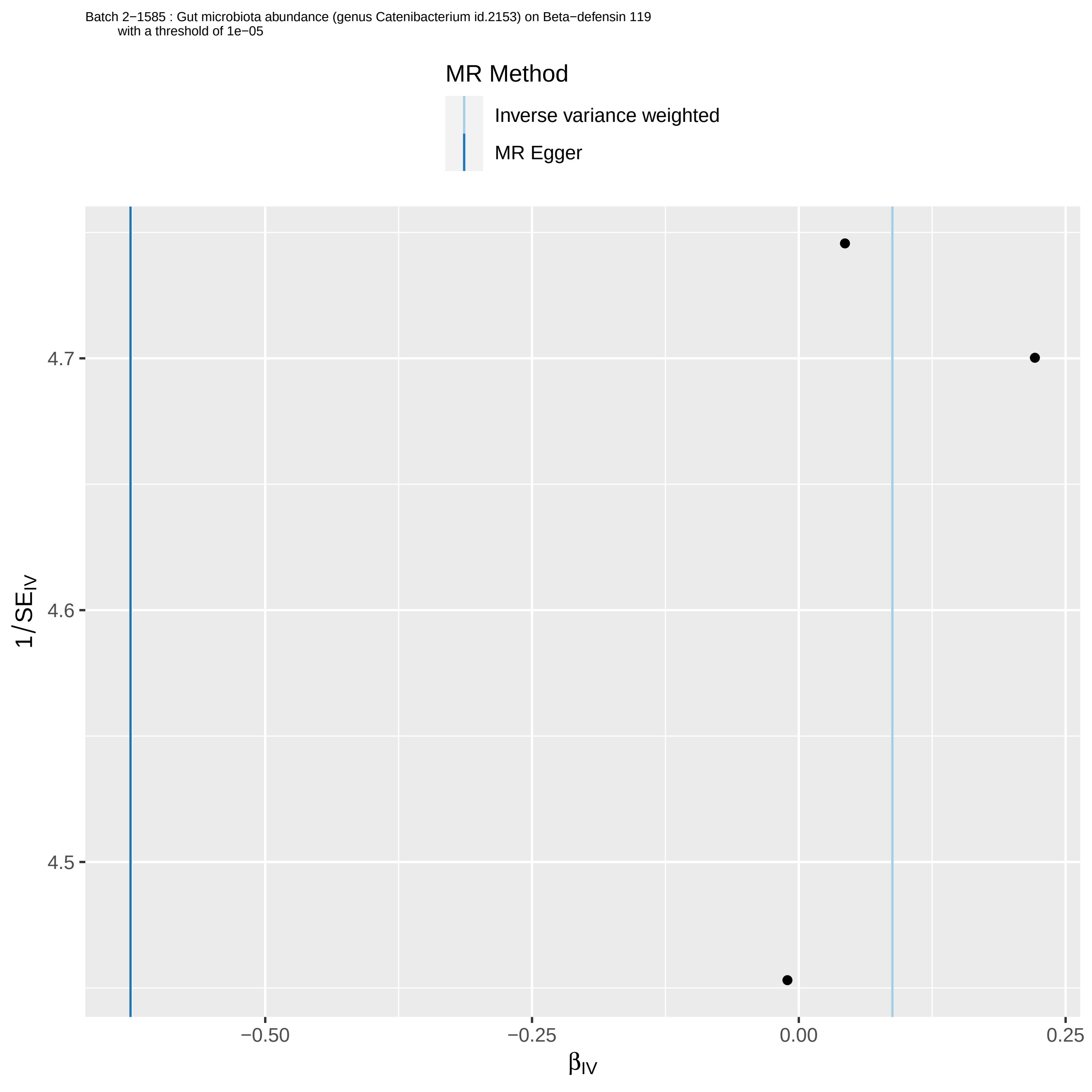

Batch 2−1585 : Gut microbiota abundance (genus Catenibacterium id.2153) on Beta−defensin 119
with a threshold of 1e−05
MR Method
Inverse variance weighted
MR Egger
4.7
4.6
4.5
1/SE_IV
−0.50
−0.25
0.00
0.25
β_IV

Batch 2−1586 : Gut microbiota abundance (genus Parasutterella id.2892) on Beta−defensin 119
with a threshold of 1e−05

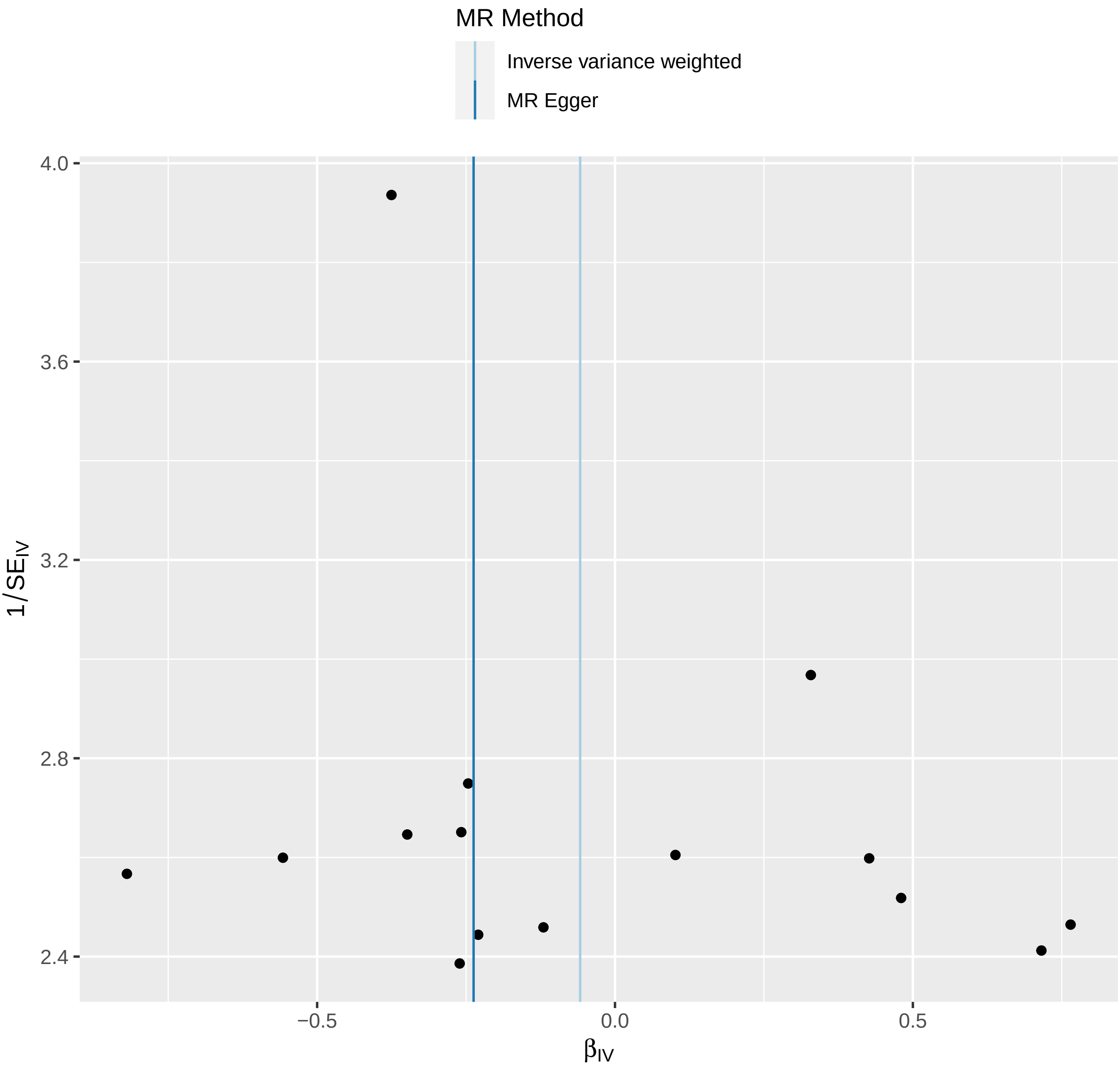

Batch 2−1587 : Gut microbiota abundance (genus Catenibacterium id.2153) on Beta−defensin 119
with a threshold of 1e−05

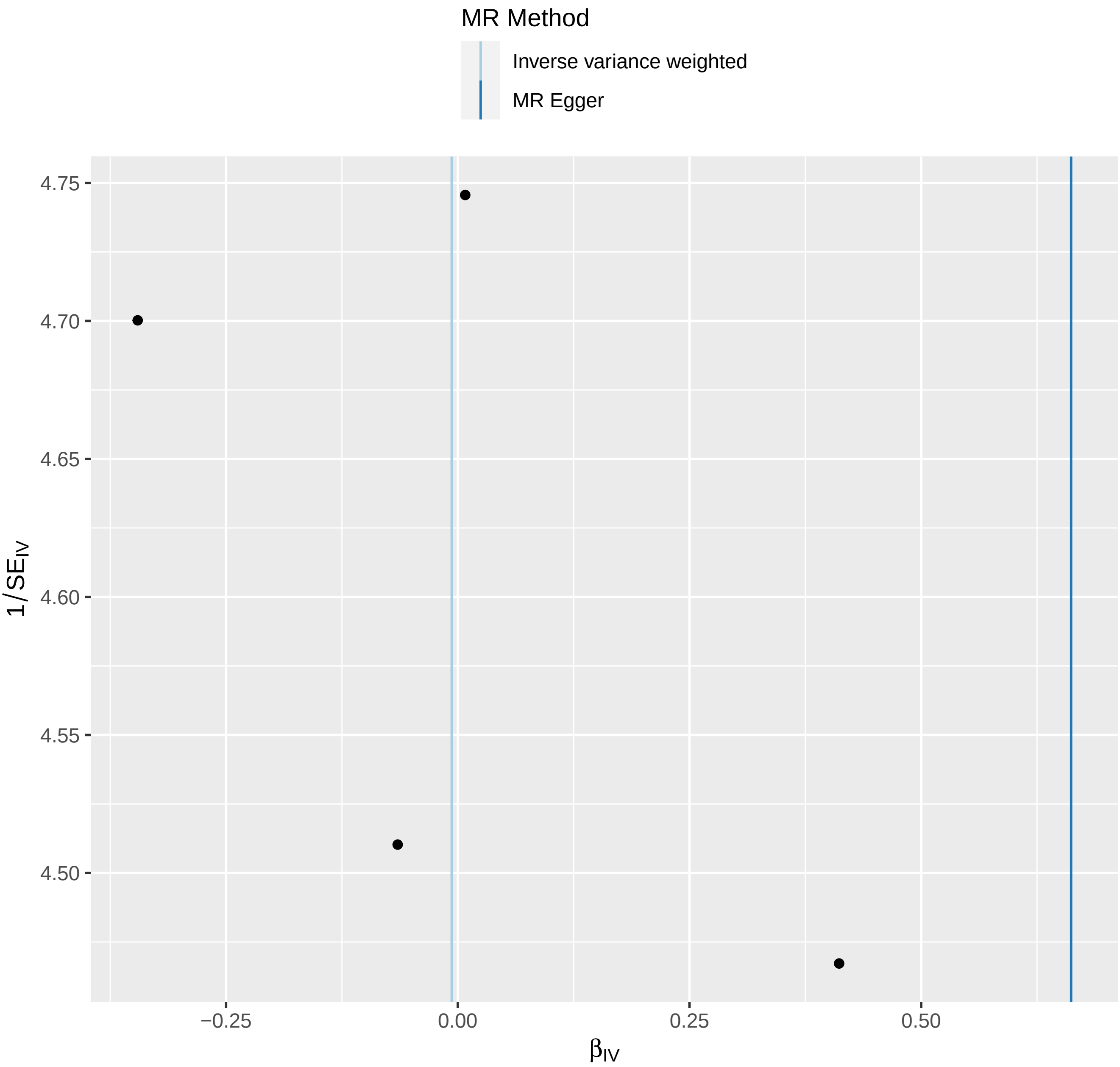

Batch 2−1588 : Gut microbiota abundance (genus Parasutterella id.2892) on Beta−defensin 119
with a threshold of 1e−05

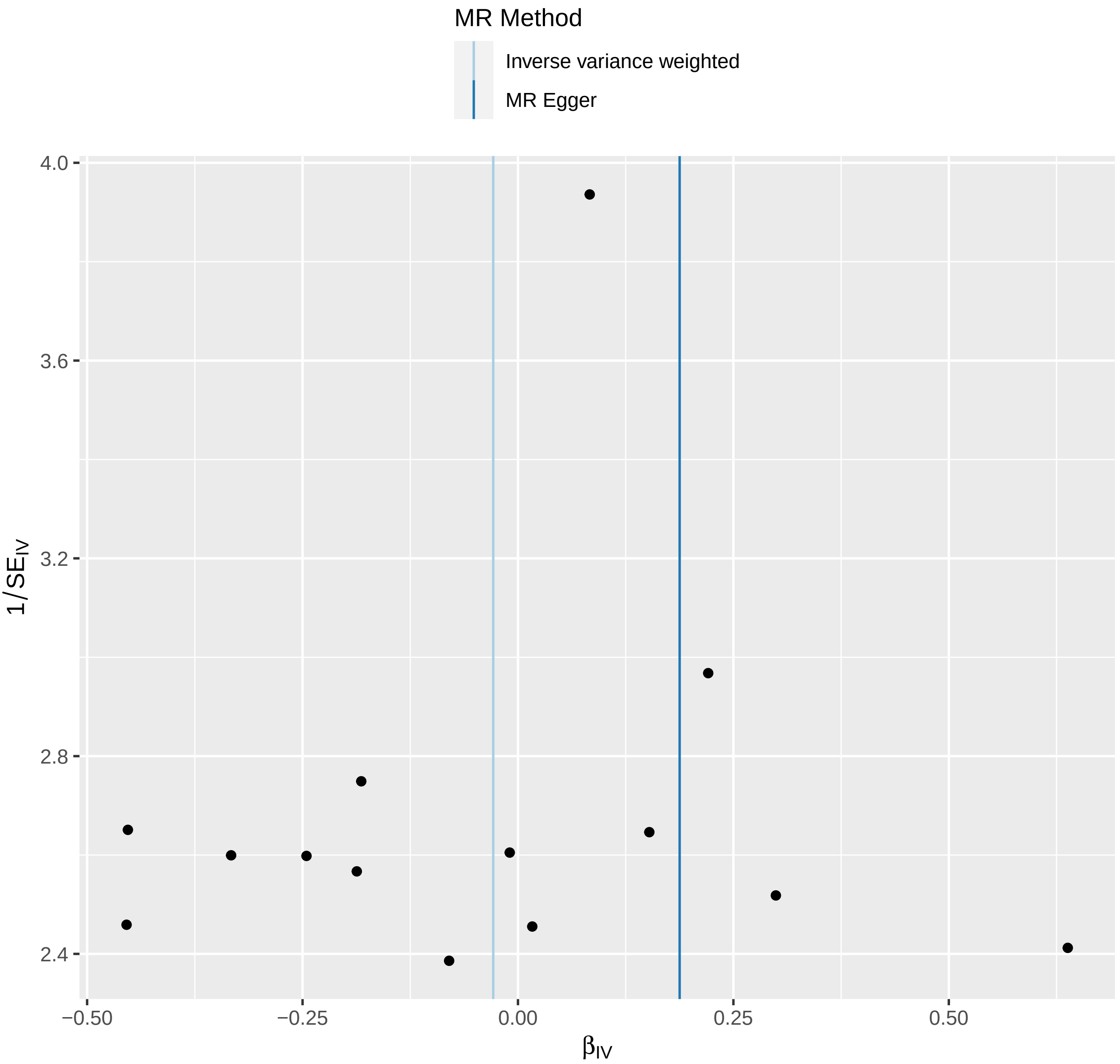

Batch 2−1589 : Gut microbiota abundance (genus Catenibacterium id.2153) on Beta−defensin 119
with a threshold of 1e−05

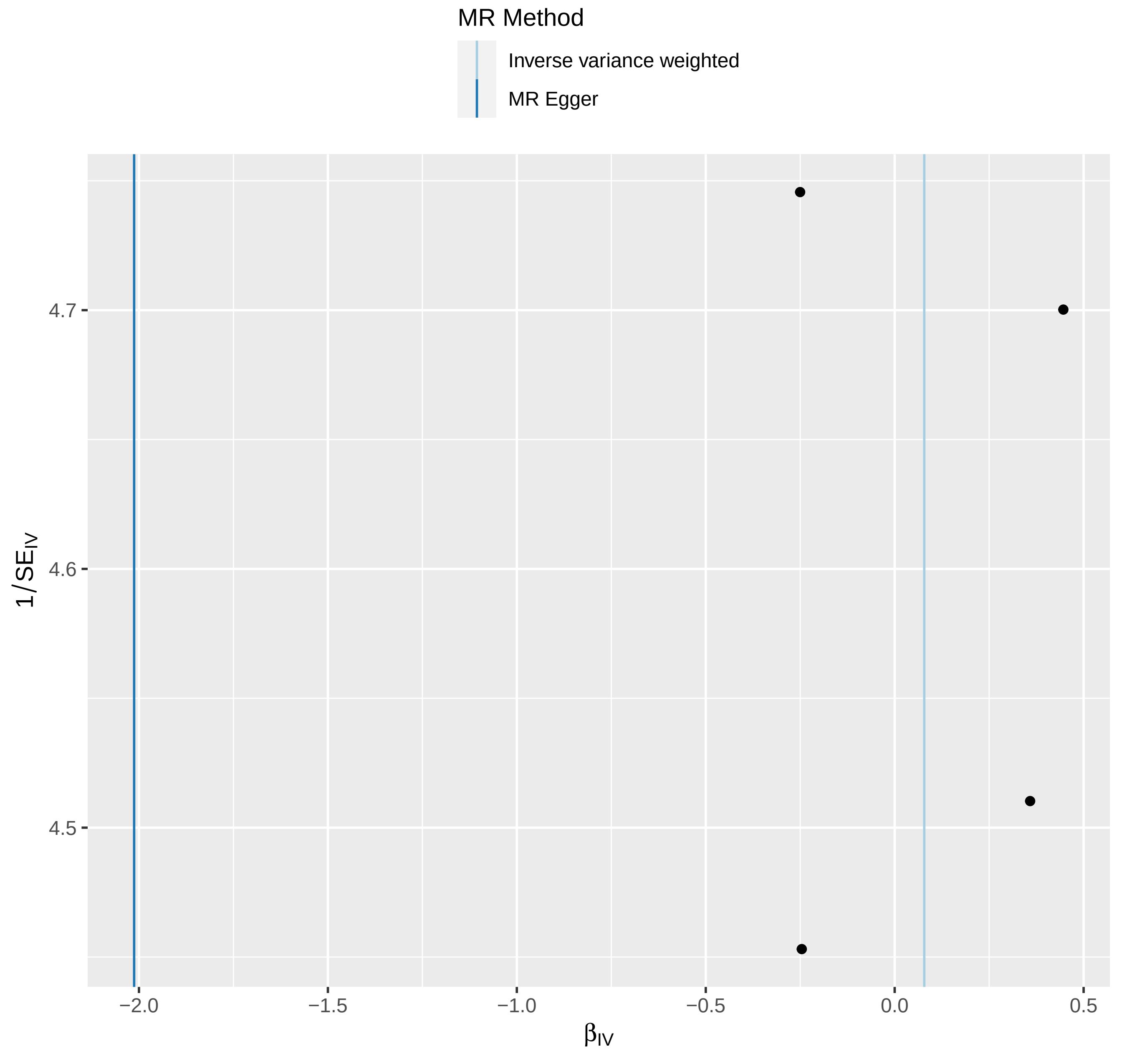

Batch 2−1590 : Gut microbiota abundance (genus Parasutterella id.2892) on Beta−defensin 119
with a threshold of 1e−05

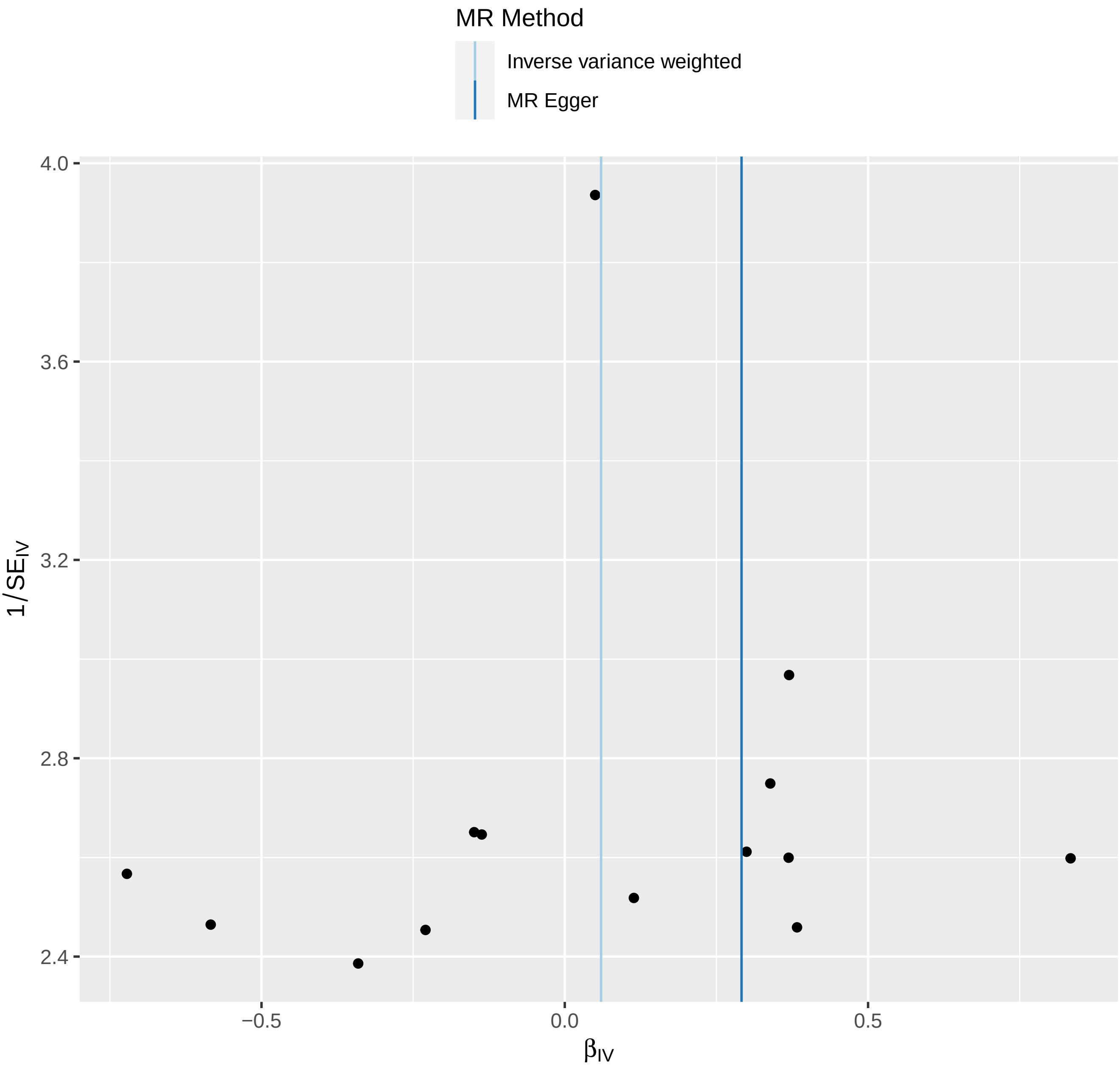

Batch 2−1591 : Gut microbiota abundance (genus Catenibacterium id.2153) on Beta−defensin 119
with a threshold of 1e−05

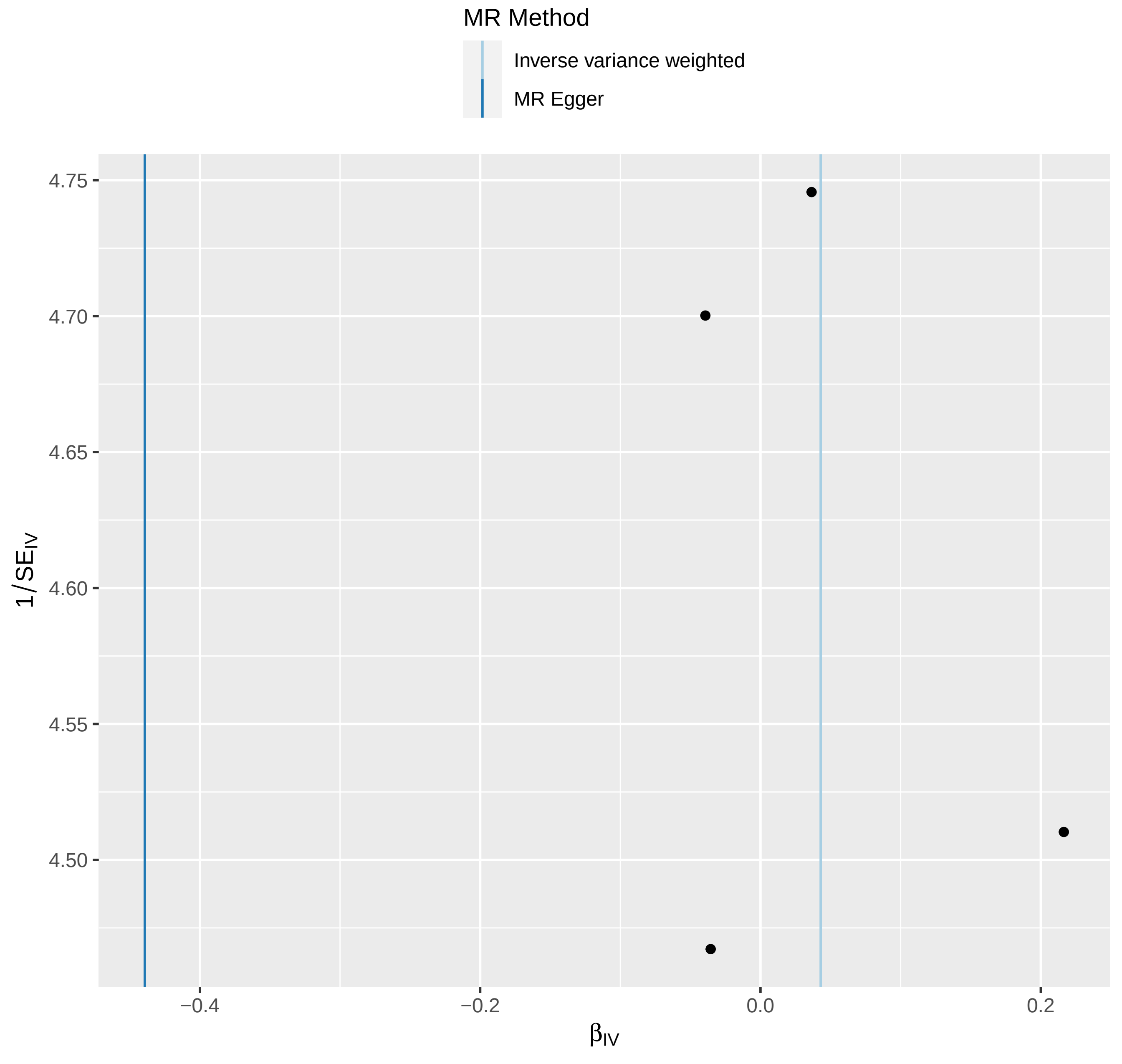

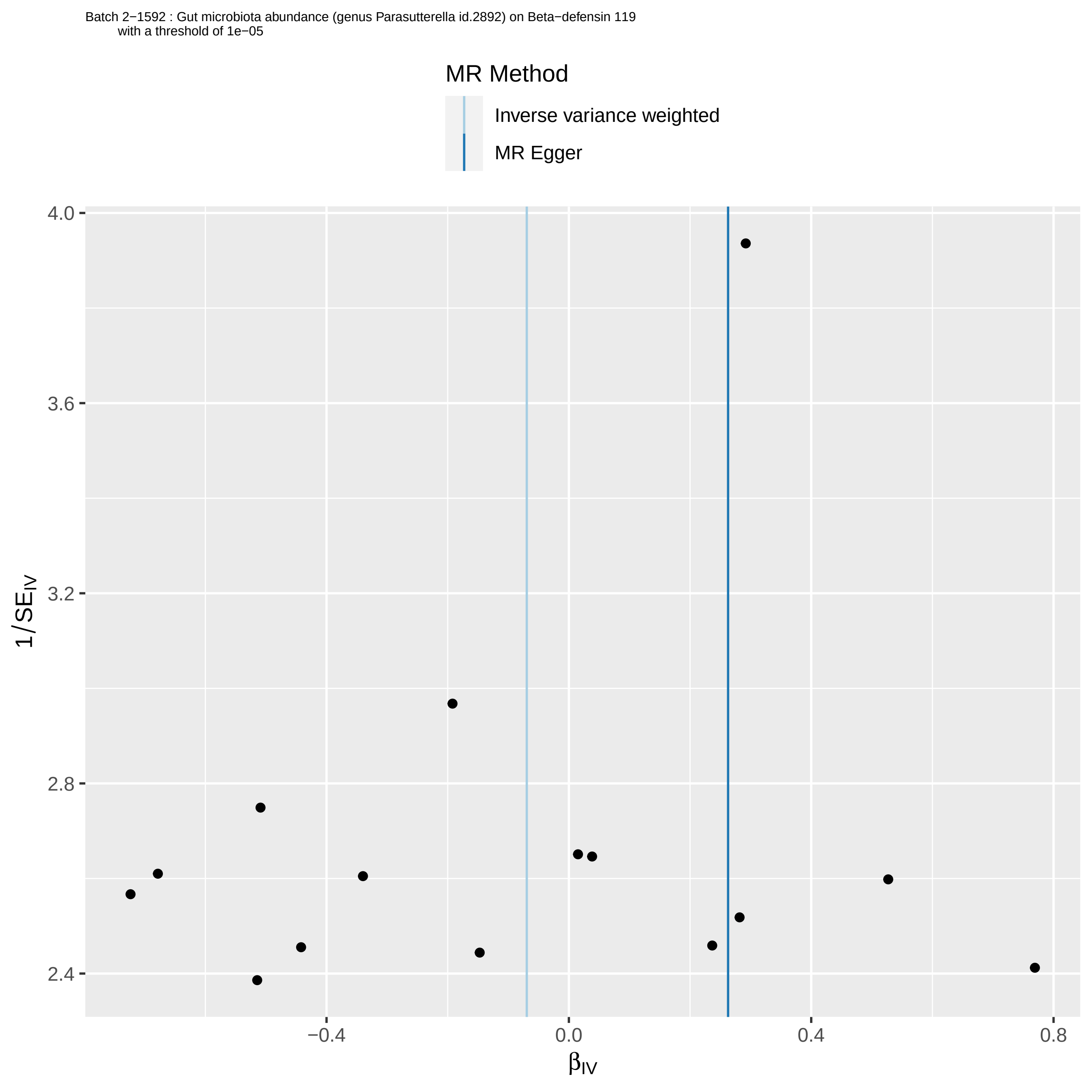

Batch 2−1592 : Gut microbiota abundance (genus Parasutterella id.2892) on Beta−defensin 119
with a threshold of 1e−05
MR Method
Inverse variance weighted
MR Egger
4.0
3.6
3.2
2.8
2.4
1/SE$_{IV}$
−0.4
0.0
0.4
0.8
β$_{IV}$

Batch 2−1593 : Gut microbiota abundance (genus Catenibacterium id.2153) on Beta−defensin 121
with a threshold of 1e−05

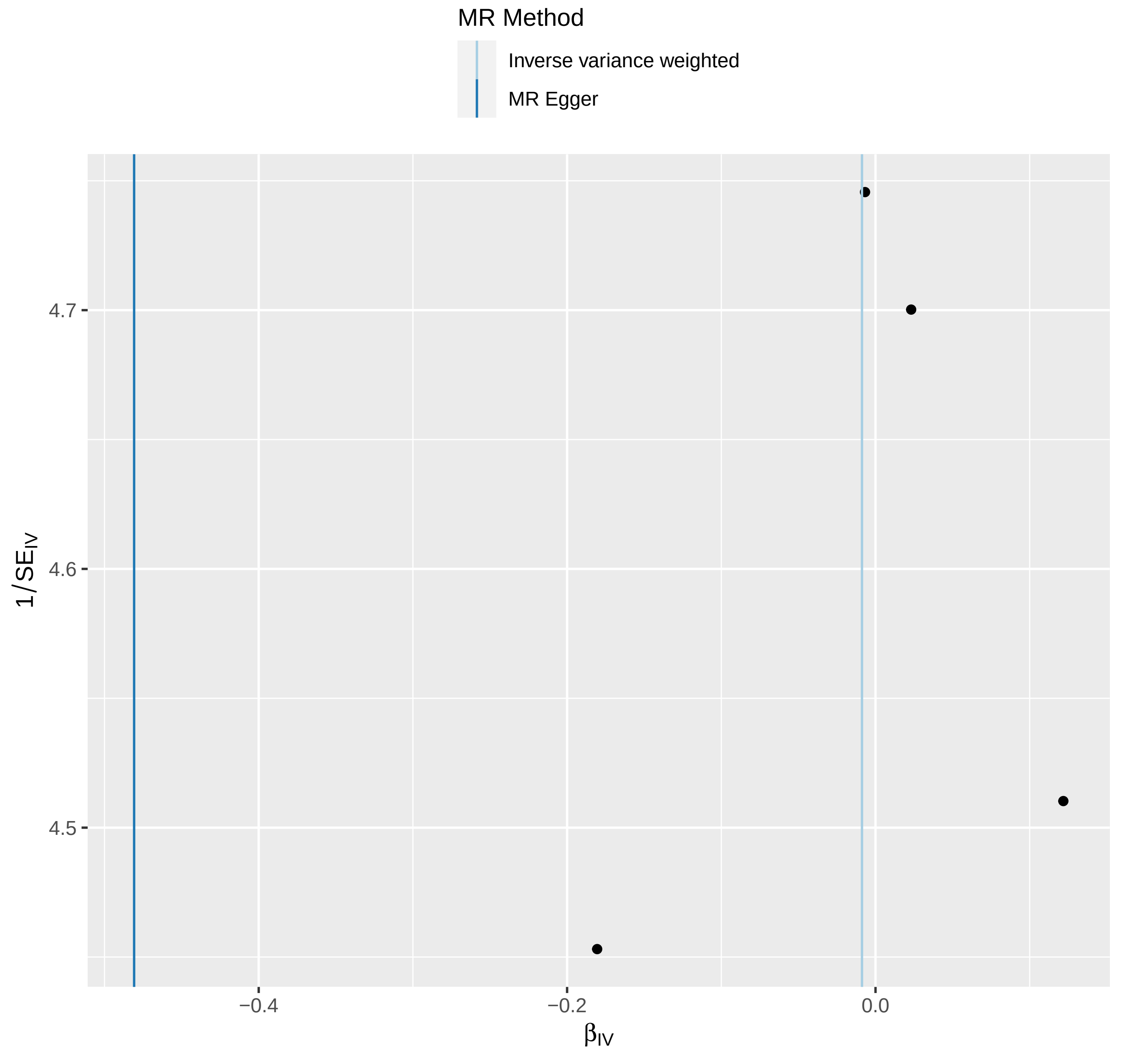

Batch 2−1594 : Gut microbiota abundance (genus Parasutterella id.2892) on Beta−defensin 121
with a threshold of 1e−05

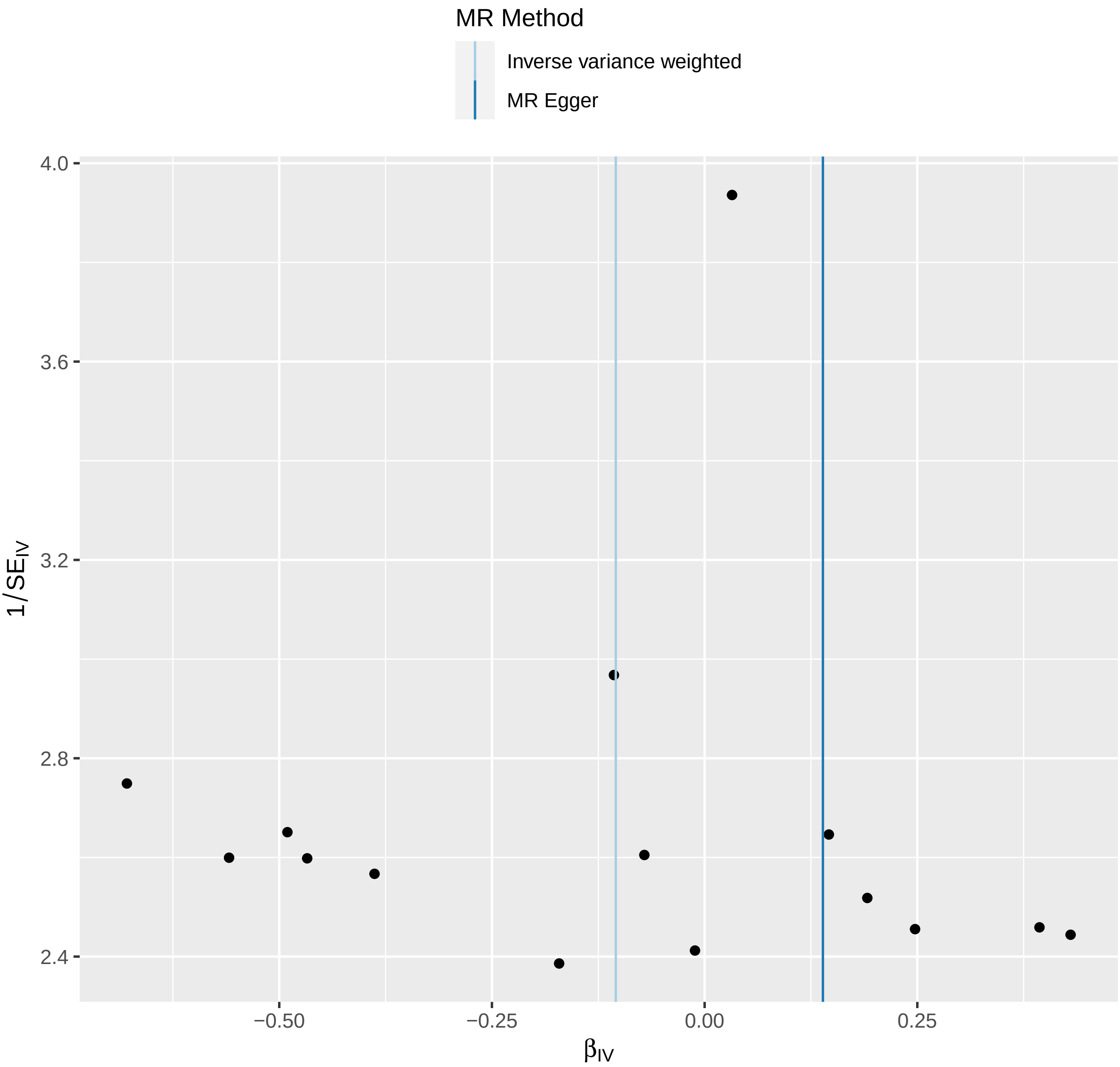

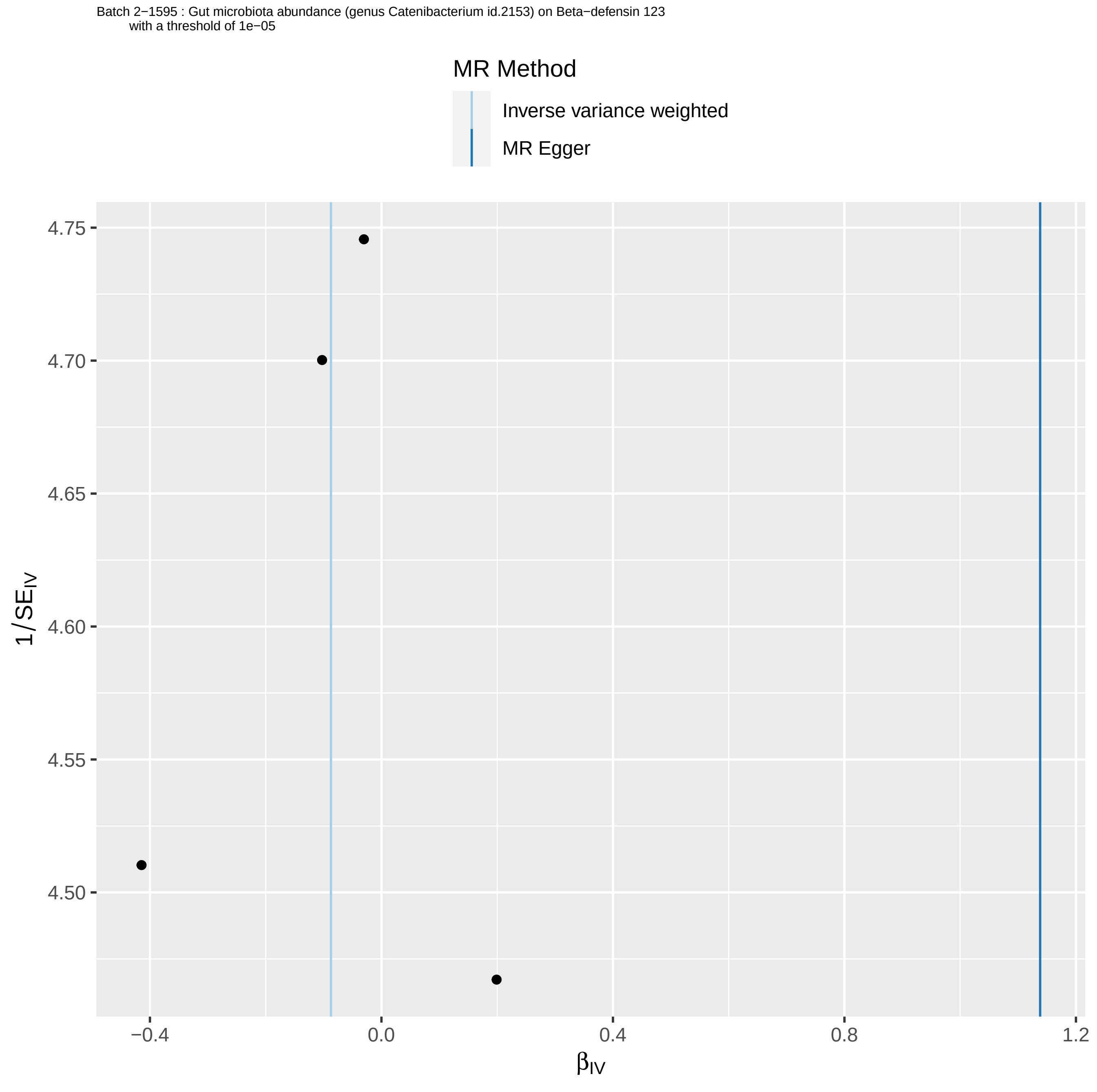
Batch 2−1595 : Gut microbiota abundance (genus Catenibacterium id.2153) on Beta−defensin 123
with a threshold of 1e−05
MR Method
Inverse variance weighted
MR Egger
4.75
4.70
4.65
4.60
4.55
4.50
1/SE_IV
−0.4
0.0
0.4
0.8
1.2
β_IV

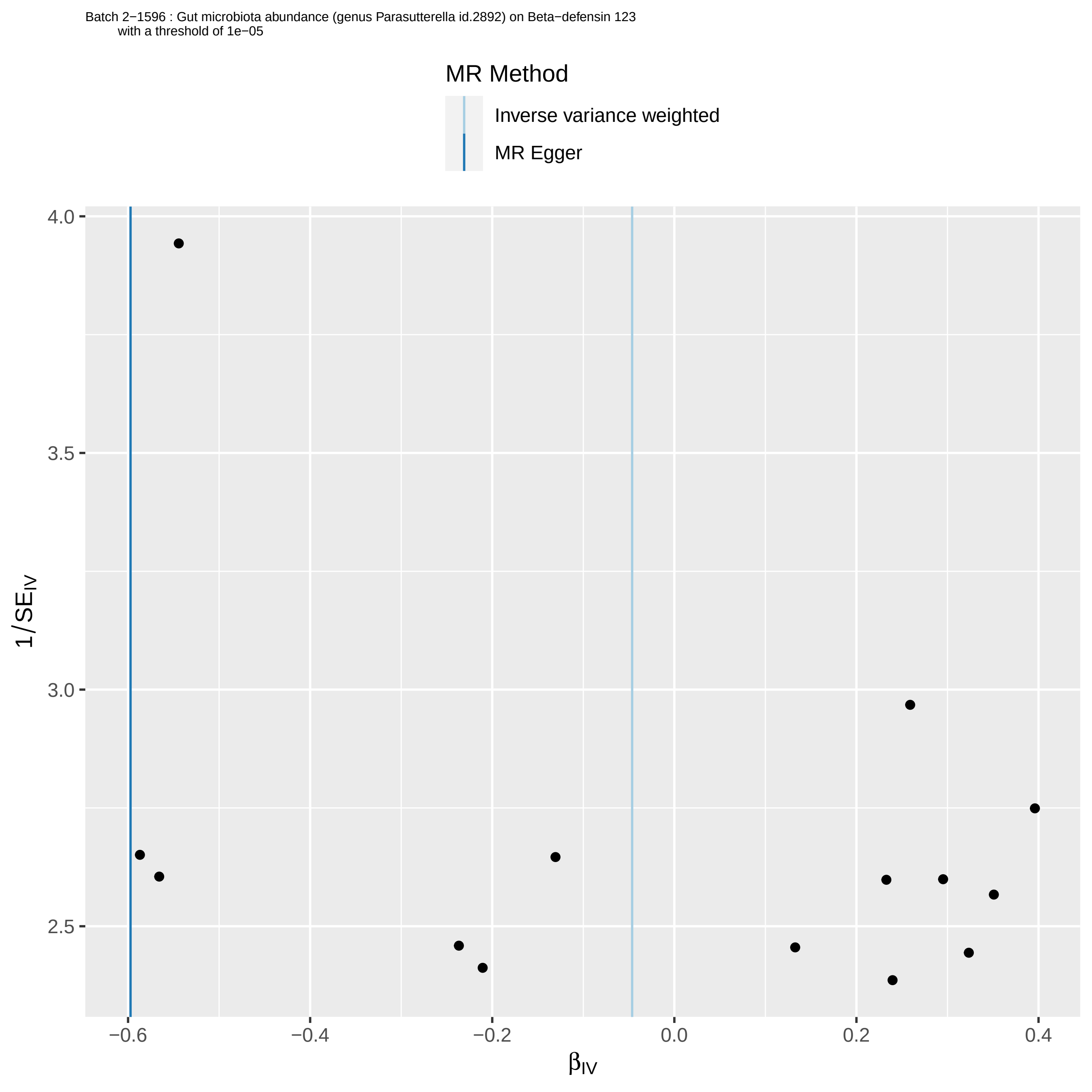

Batch 2−1596 : Gut microbiota abundance (genus Parasutterella id.2892) on Beta−defensin 123
with a threshold of 1e−05
MR Method
Inverse variance weighted
MR Egger
4.0
3.5
3.0
2.5
1/SE_IV
−0.6
−0.4
−0.2
0.0
0.2
0.4
β_IV

Batch 2−1597 : Gut microbiota abundance (genus Catenibacterium id.2153) on Beta−defensin 125
with a threshold of 1e−05

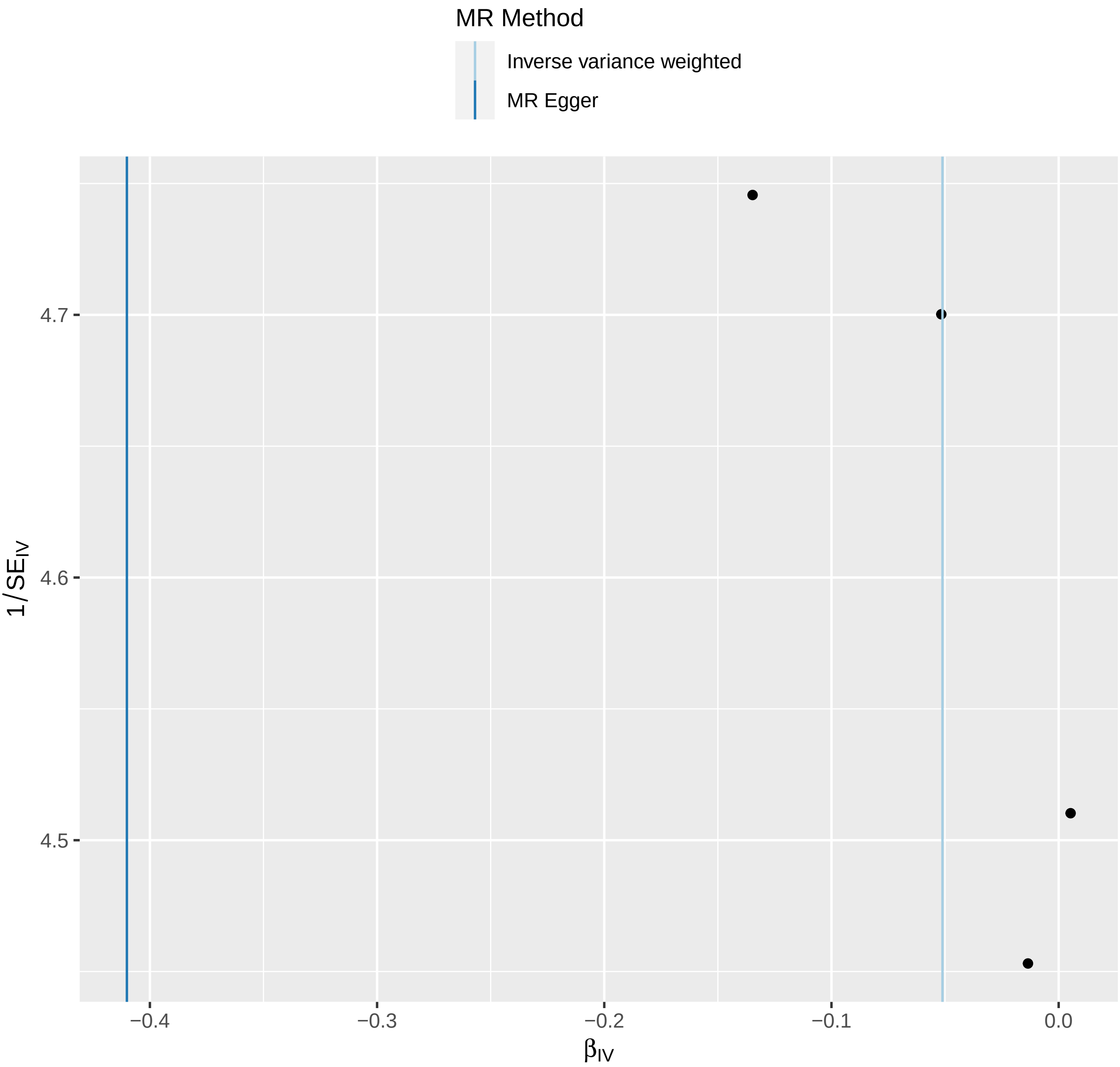

Batch 2−1598 : Gut microbiota abundance (genus Parasutterella id.2892) on Beta−defensin 125
with a threshold of 1e−05

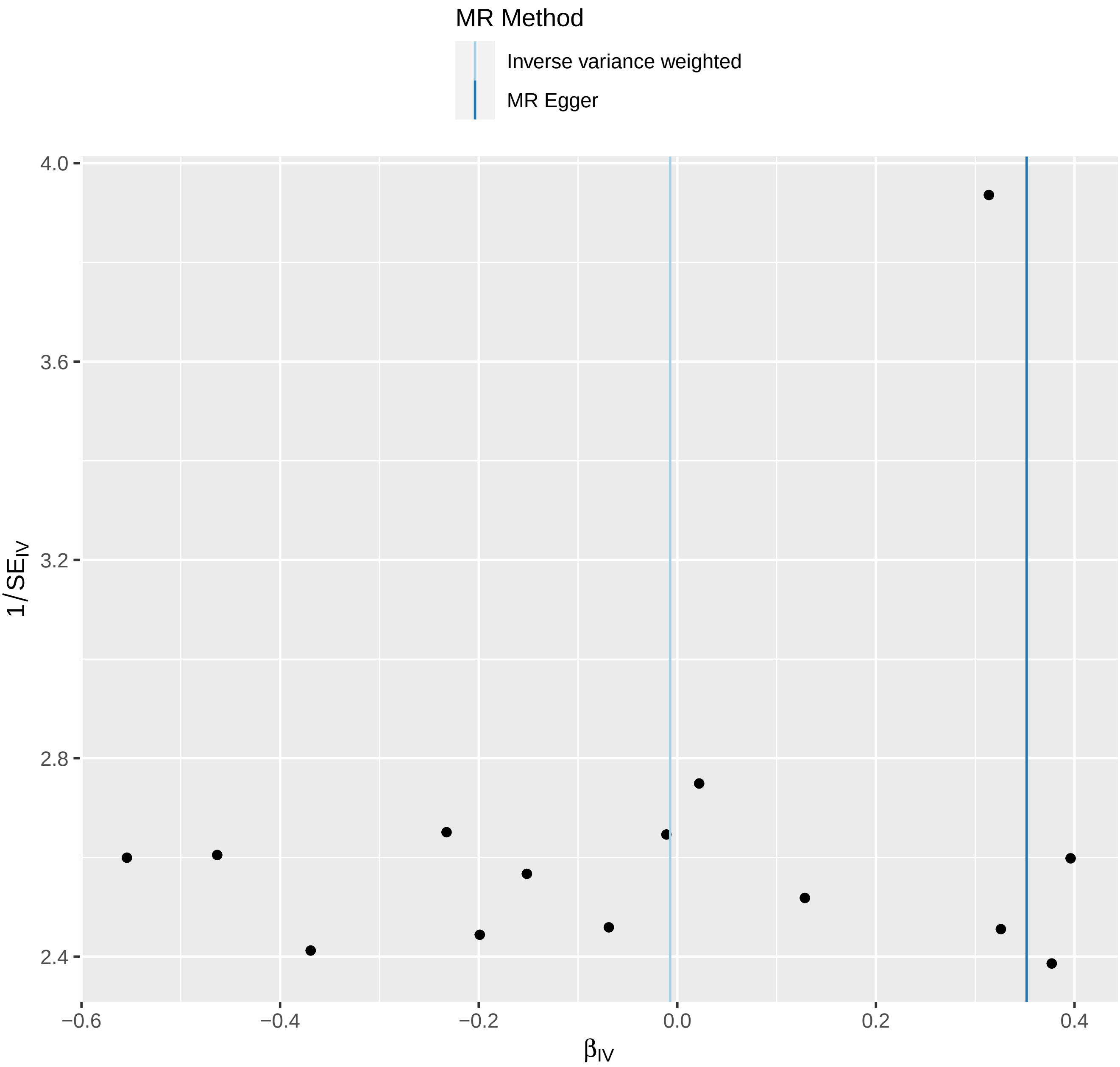

Batch 2−1599 : Gut microbiota abundance (genus Catenibacterium id.2153) on Beta−defensin 128
with a threshold of 1e−05

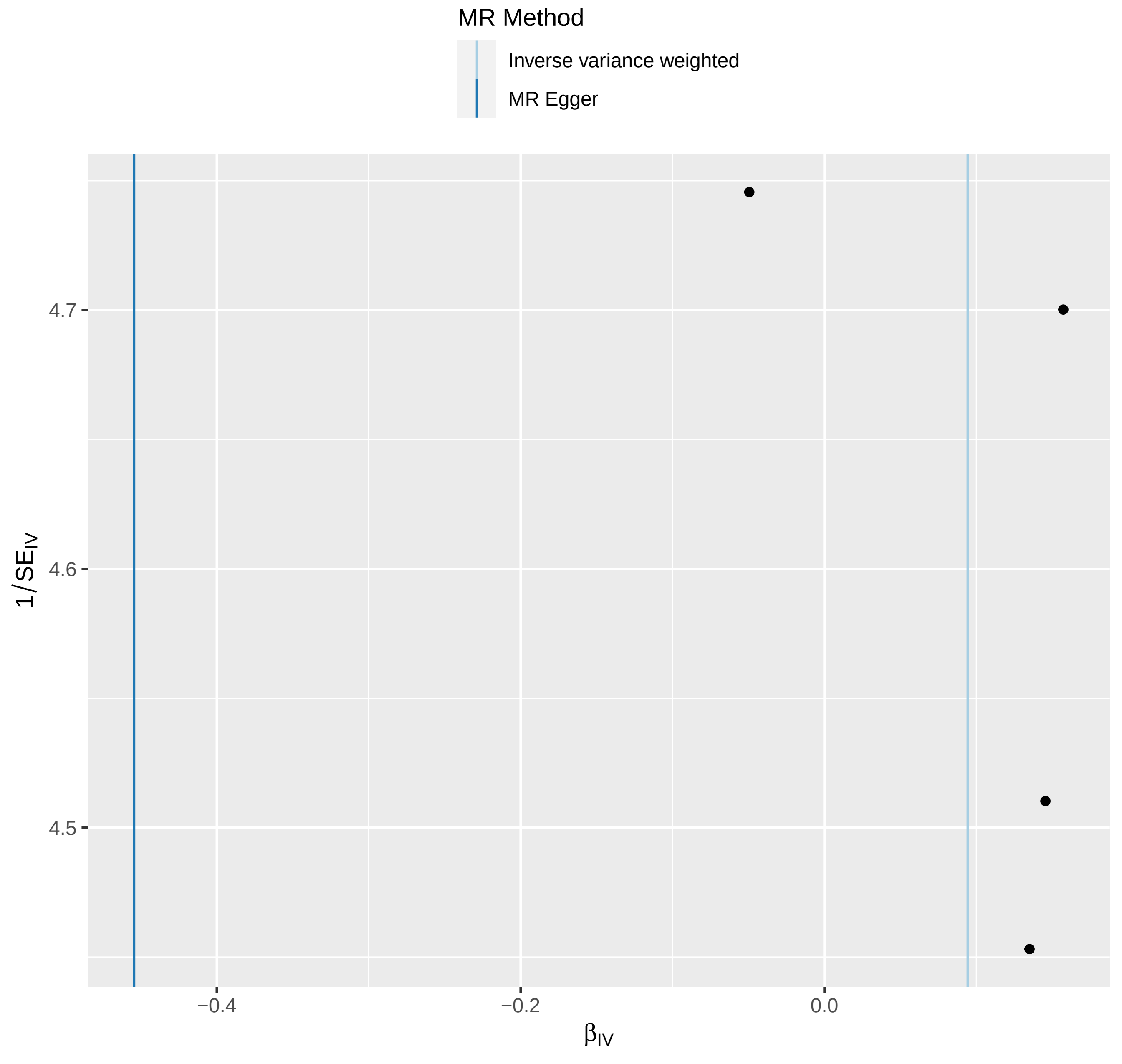

Batch 2−1600 : Gut microbiota abundance (genus Parasutterella id.2892) on Beta−defensin 128
with a threshold of 1e−05

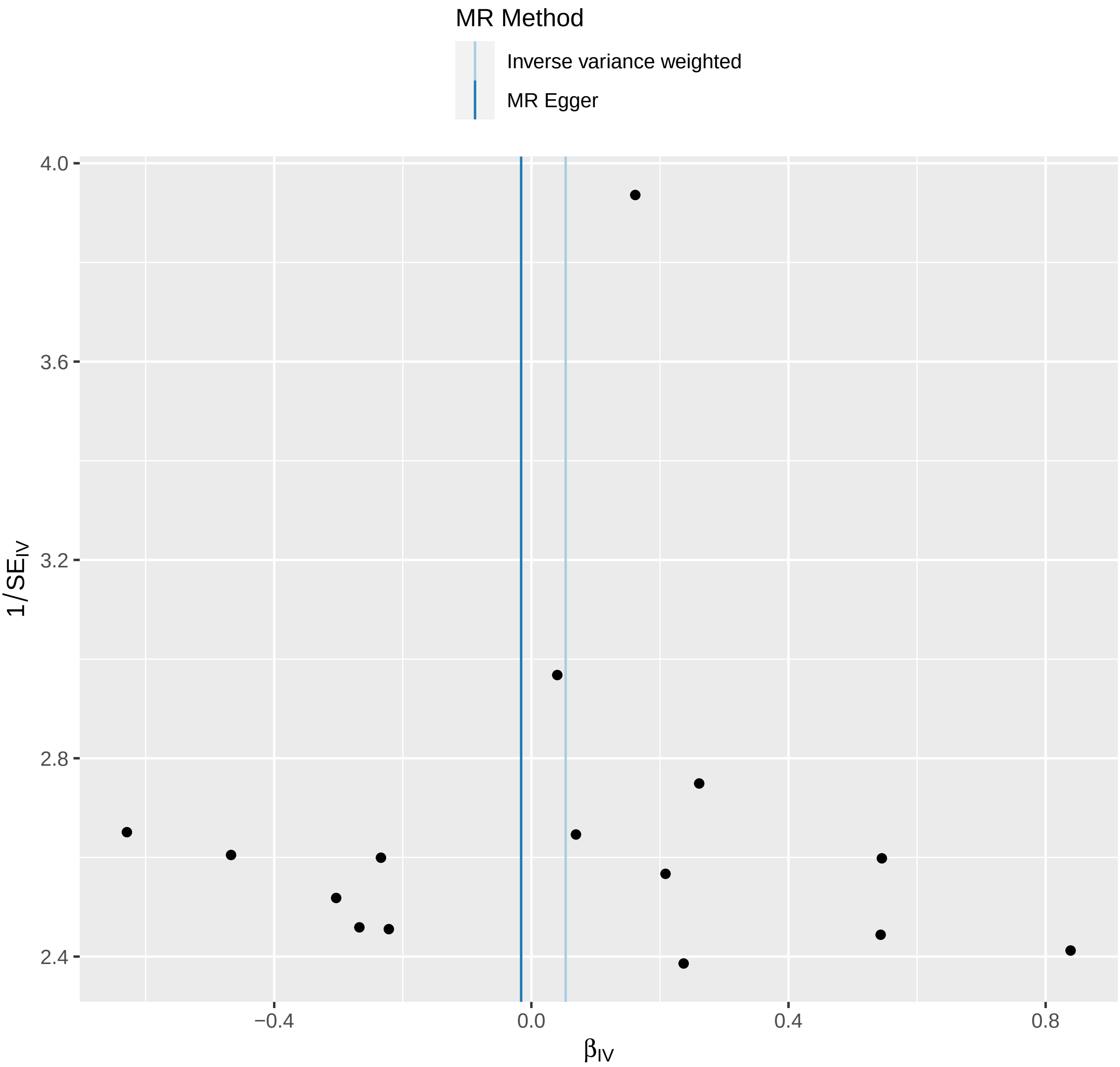

Batch 2−1601 : Gut microbiota abundance (genus Catenibacterium id.2153) on Beta−defensin 134
with a threshold of 1e−05

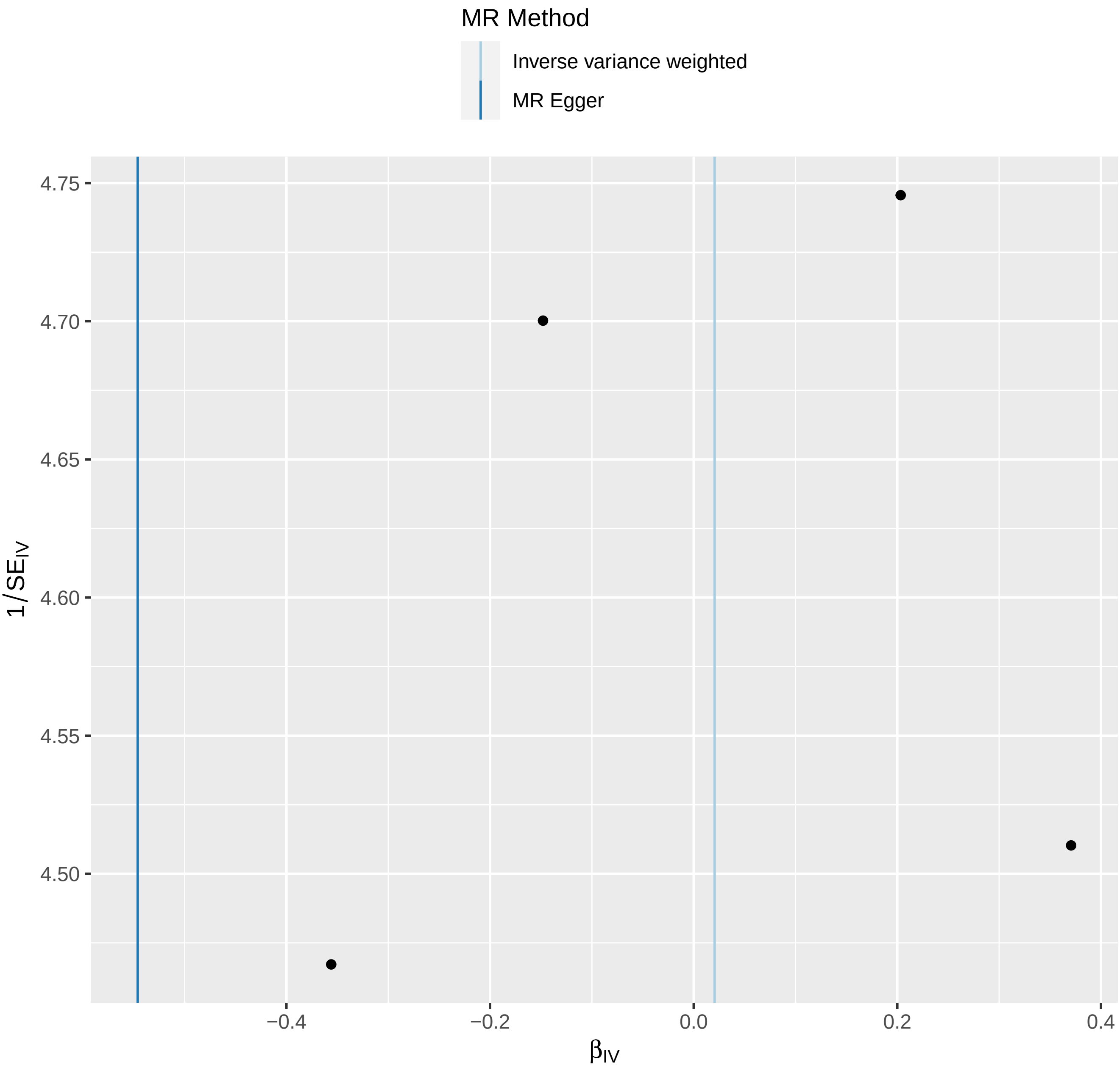

Batch 2−1602 : Gut microbiota abundance (genus Parasutterella id.2892) on Beta−defensin 134
with a threshold of 1e−05

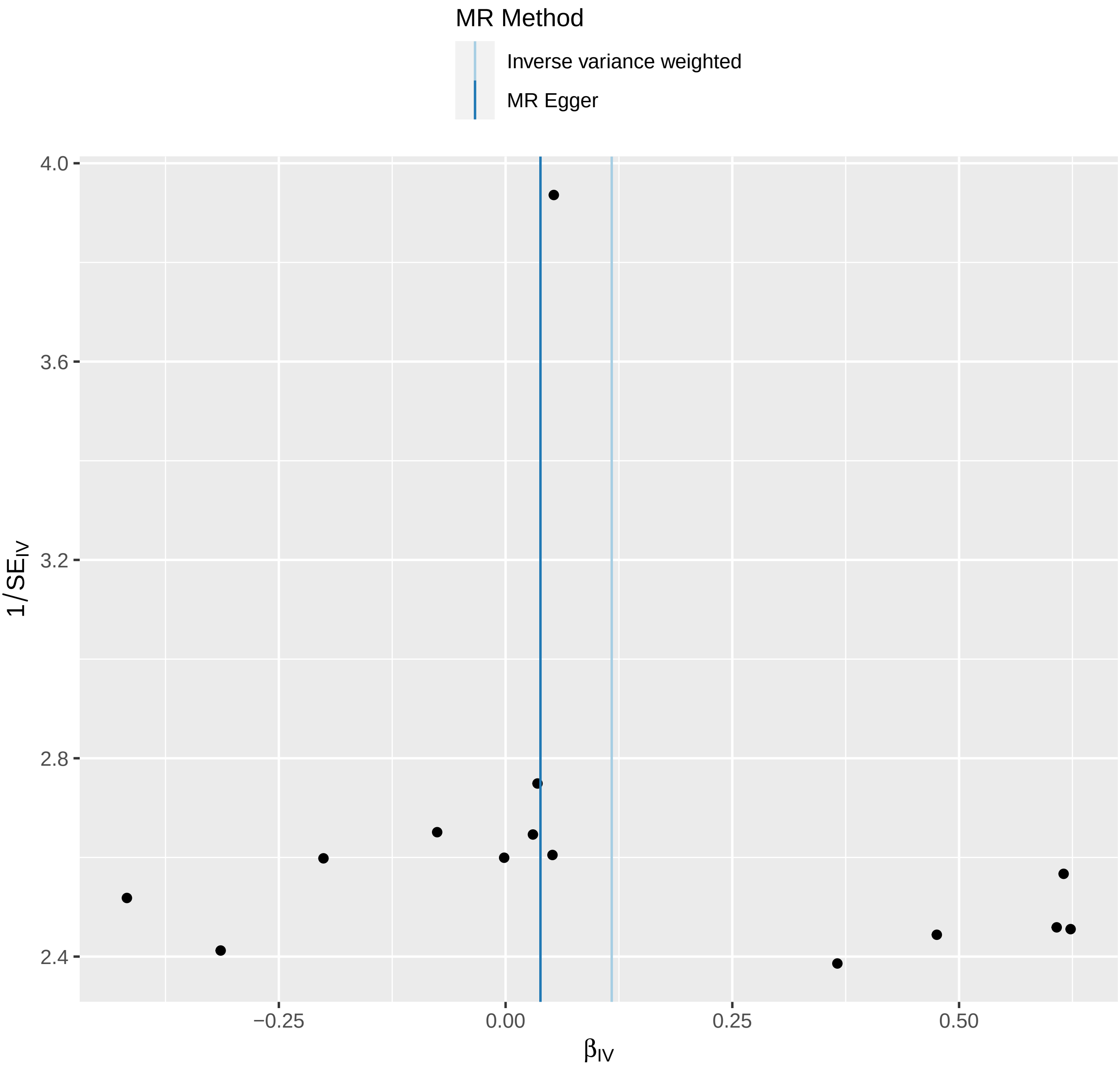

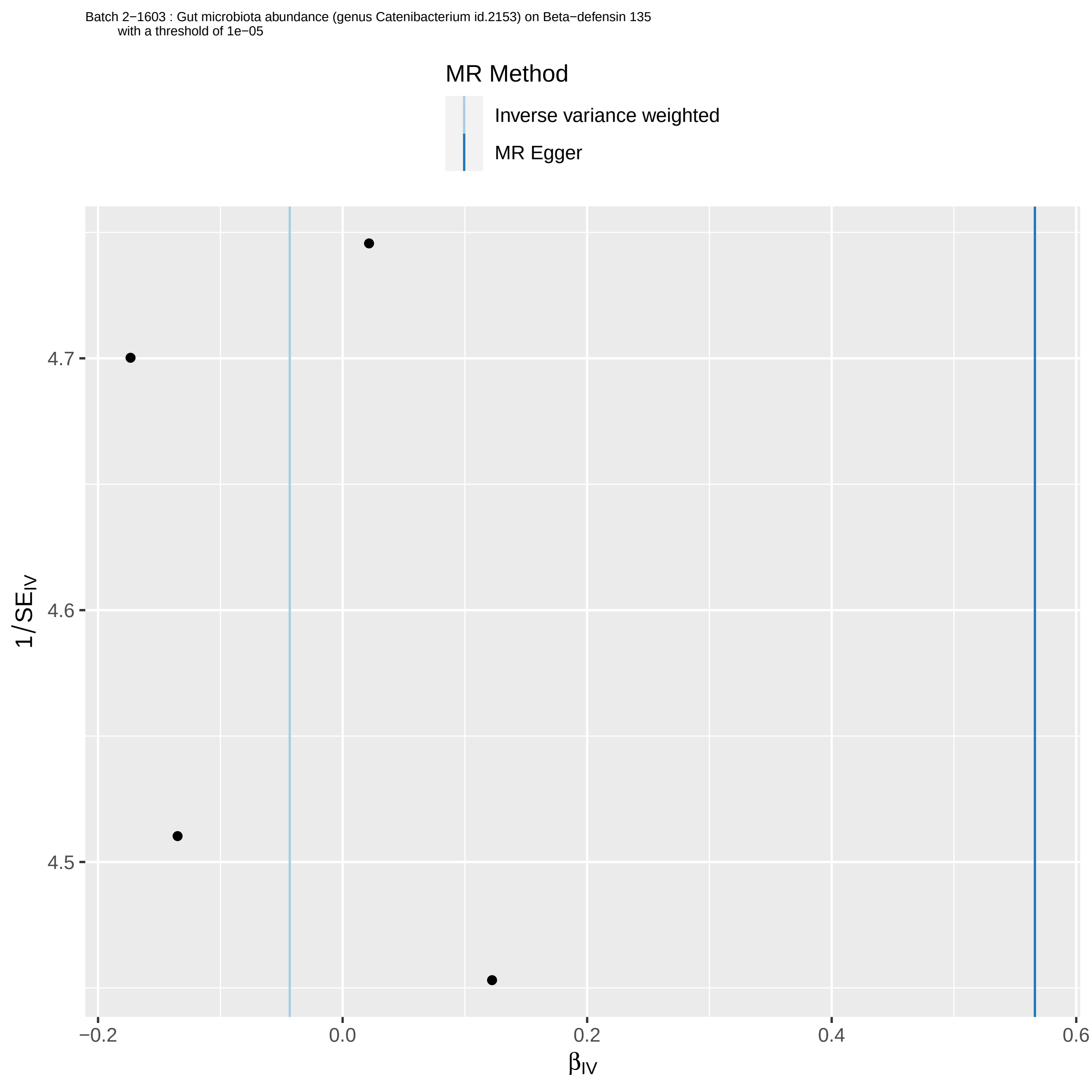

Batch 2−1603 : Gut microbiota abundance (genus Catenibacterium id.2153) on Beta−defensin 135
with a threshold of 1e−05
MR Method
Inverse variance weighted
MR Egger
4.7
4.6
4.5
1/SEIV
−0.2
0.0
0.2
0.4
0.6
βIV

Batch 2−1604 : Gut microbiota abundance (genus Parasutterella id.2892) on Beta−defensin 135
with a threshold of 1e−05

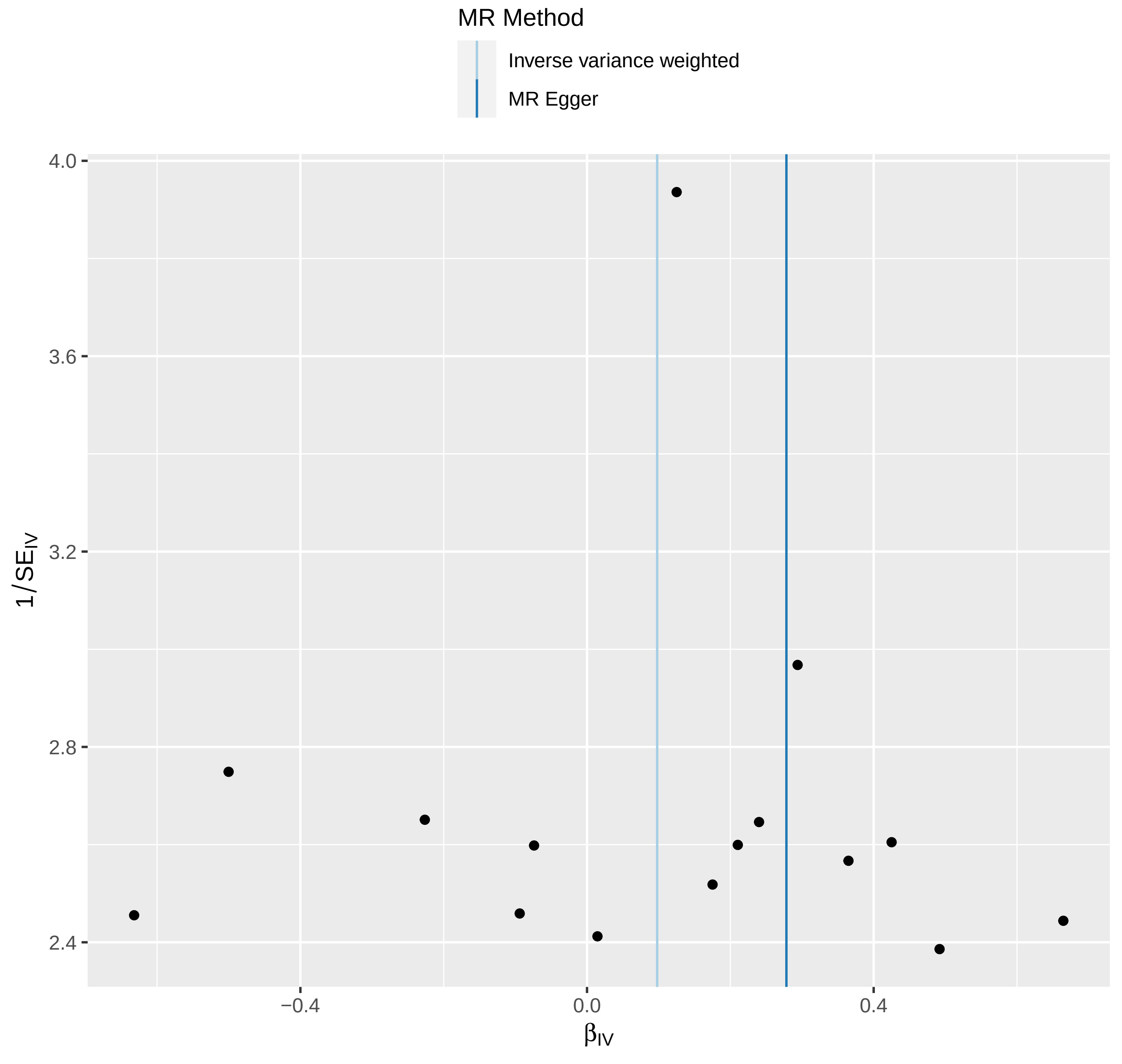

Batch 2−1605 : Gut microbiota abundance (genus Catenibacterium id.2153) on Beta−defensin 136
with a threshold of 1e−05

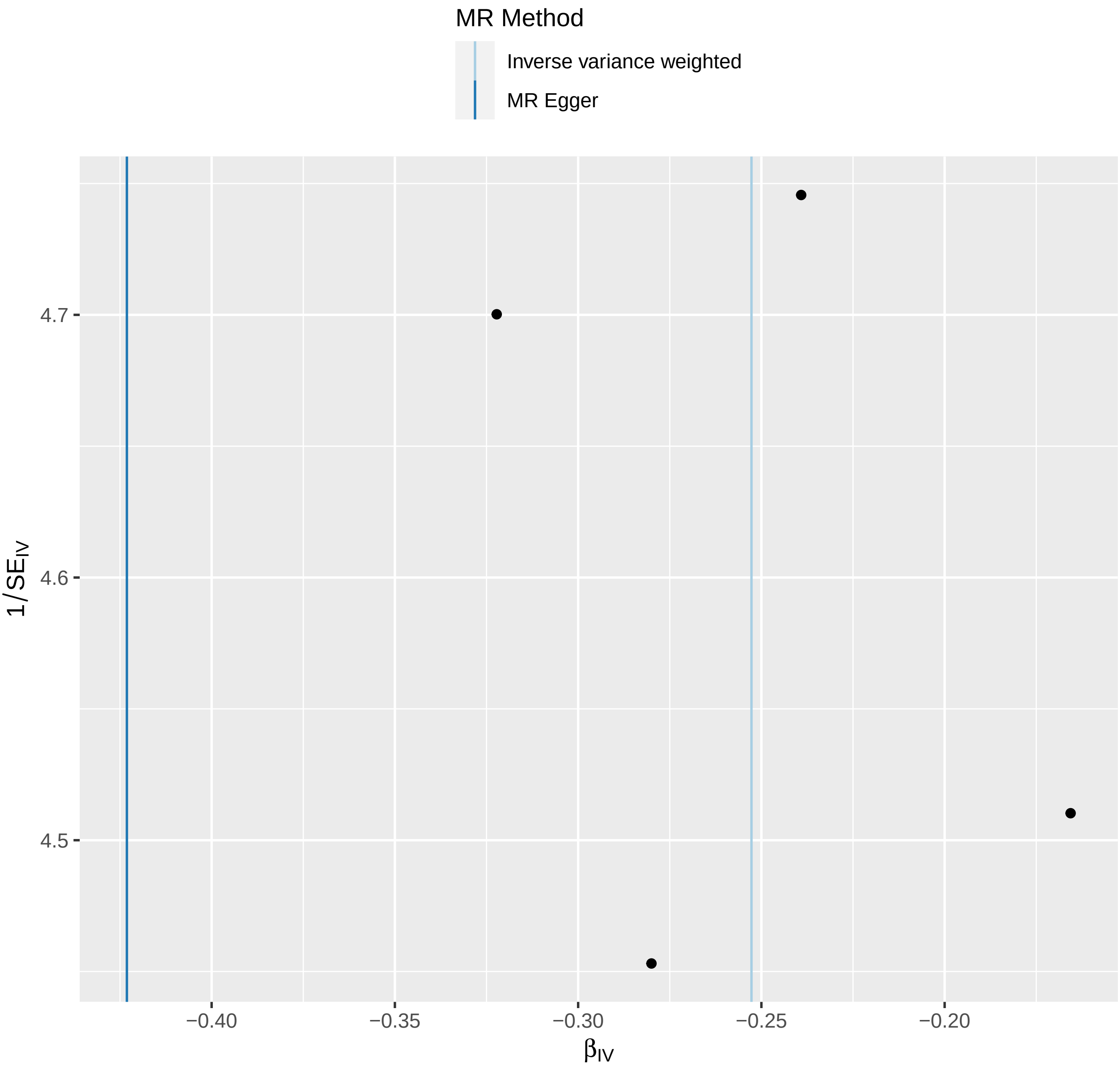

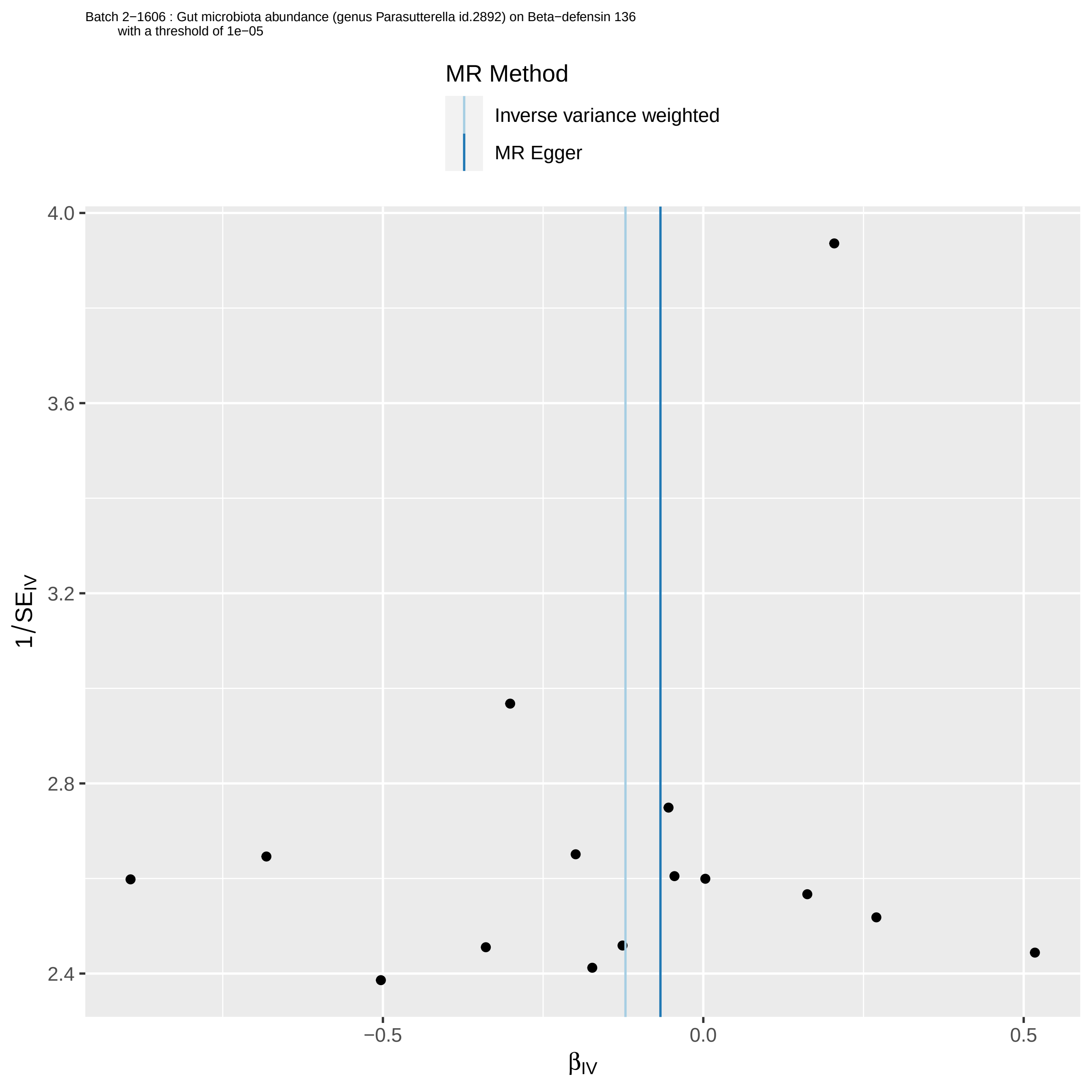
Batch 2−1606 : Gut microbiota abundance (genus Parasutterella id.2892) on Beta−defensin 136
with a threshold of 1e−05
MR Method
Inverse variance weighted
MR Egger
4.0
3.6
3.2
2.8
2.4
1/SE_IV
−0.5
0.0
0.5
β_IV

Batch 2−1607 : Gut microbiota abundance (genus Catenibacterium id.2153) on Beta−defensin 1
with a threshold of 1e−05

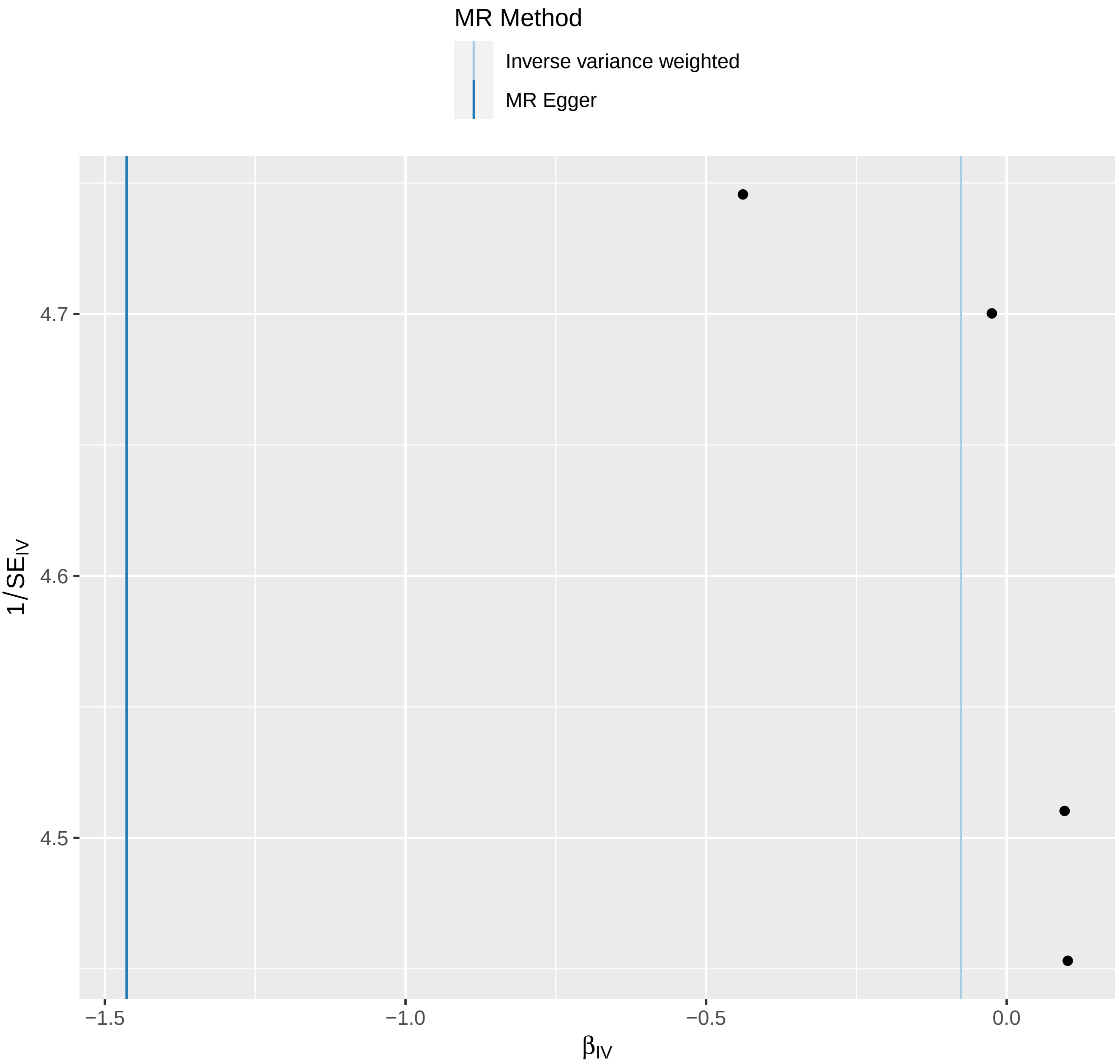

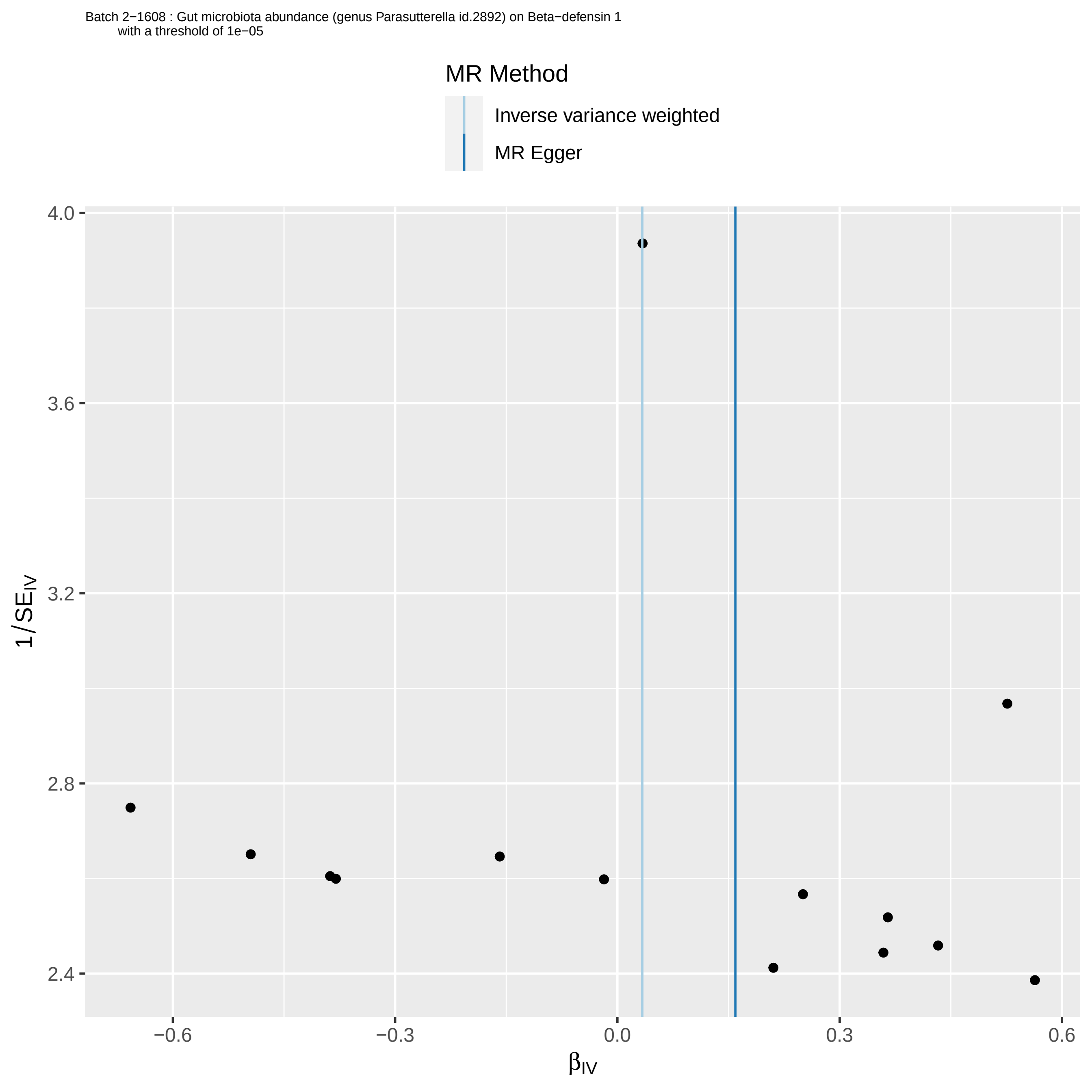

Batch 2−1608 : Gut microbiota abundance (genus Parasutterella id.2892) on Beta−defensin 1
with a threshold of 1e−05
MR Method
Inverse variance weighted
MR Egger
4.0
3.6
3.2
2.8
2.4
1/SE_IV
−0.6
−0.3
0.0
0.3
0.6
β_IV

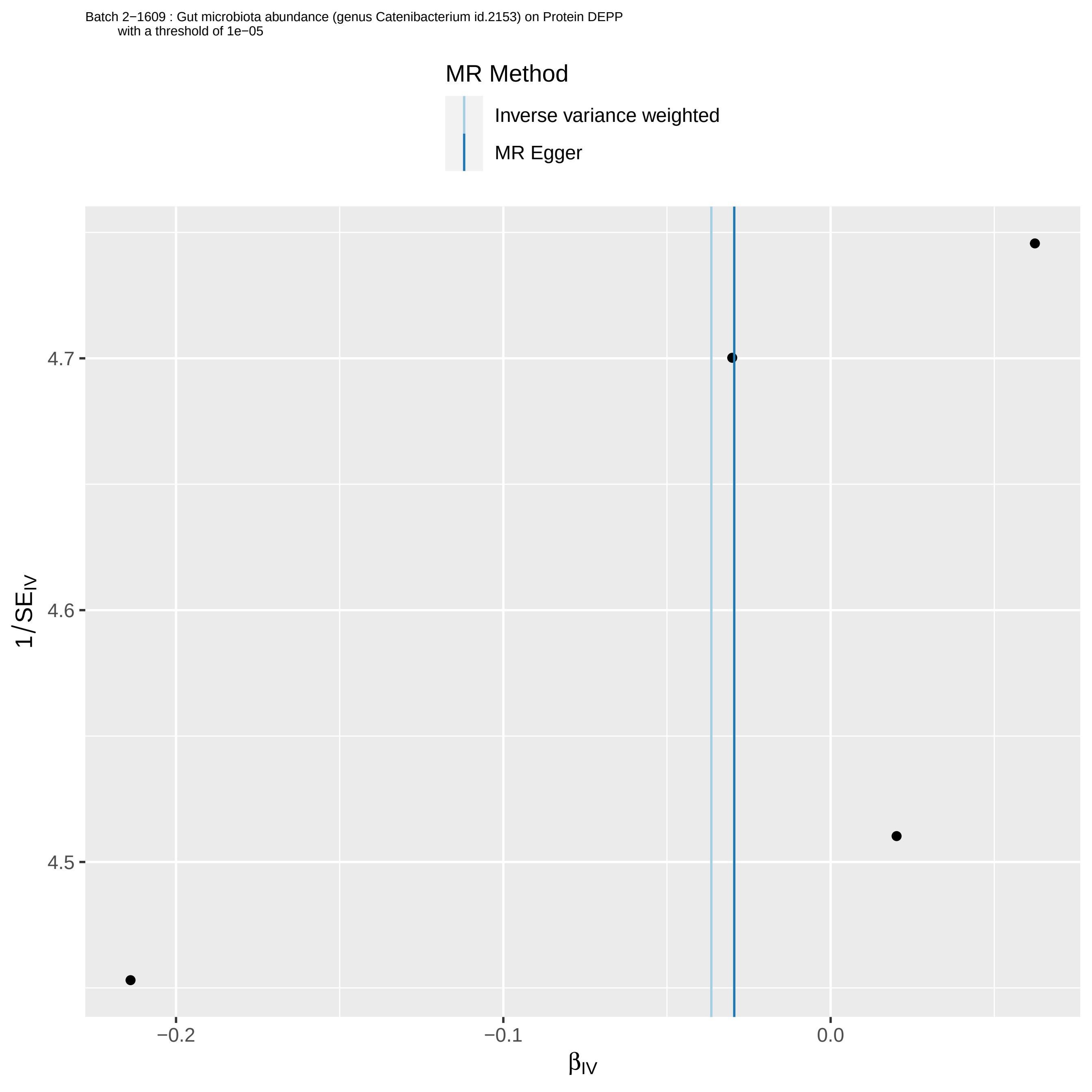

Batch 2−1609 : Gut microbiota abundance (genus Catenibacterium id.2153) on Protein DEPP
with a threshold of 1e−05
MR Method
Inverse variance weighted
MR Egger
4.7
4.6
4.5
1/SE_IV
−0.2
−0.1
0.0
β_IV

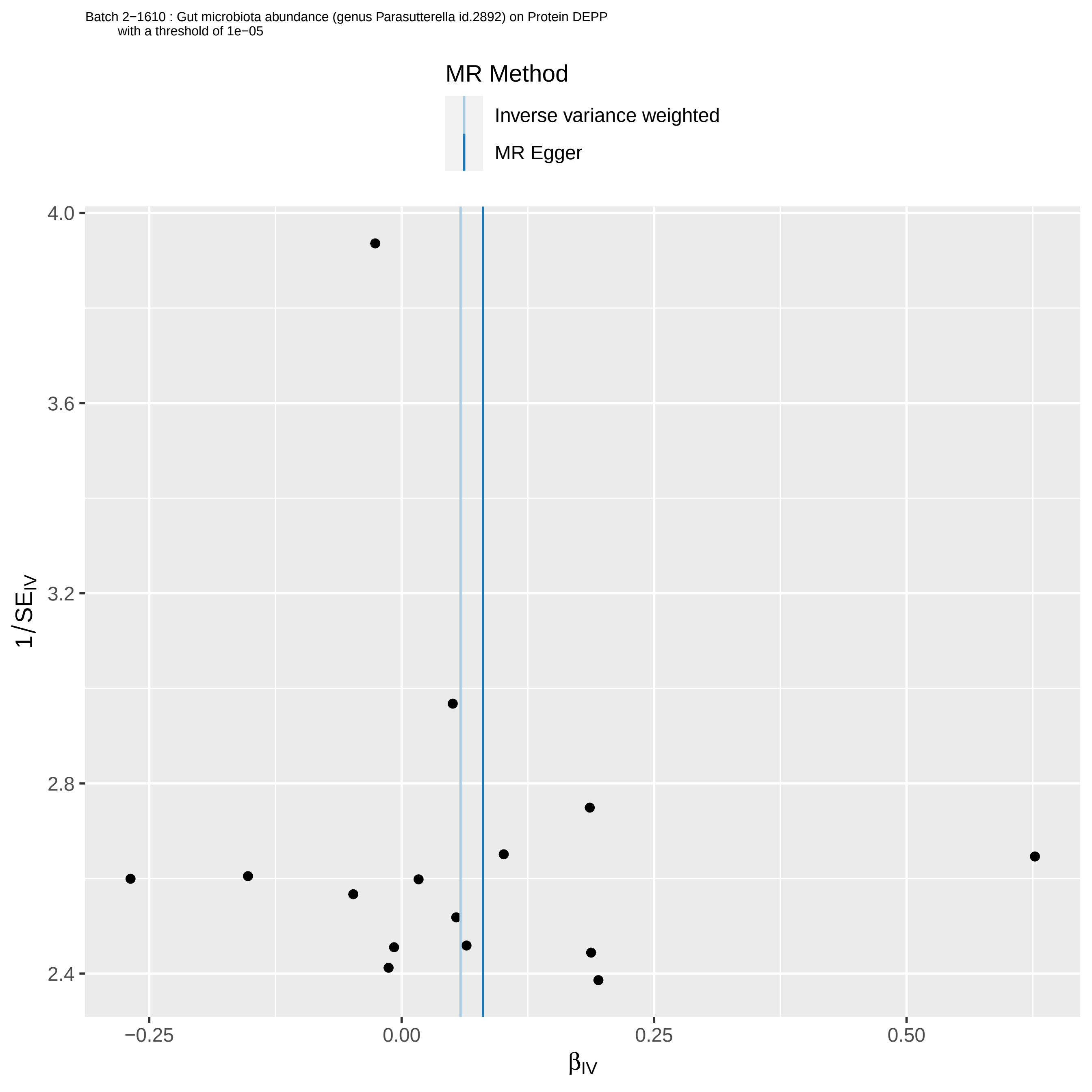

Batch 2−1610 : Gut microbiota abundance (genus Parasutterella id.2892) on Protein DEPP
with a threshold of 1e−05
MR Method
Inverse variance weighted
MR Egger
4.0
3.6
3.2
2.8
2.4
1/SE_IV
−0.25
0.00
0.25
0.50
β_IV

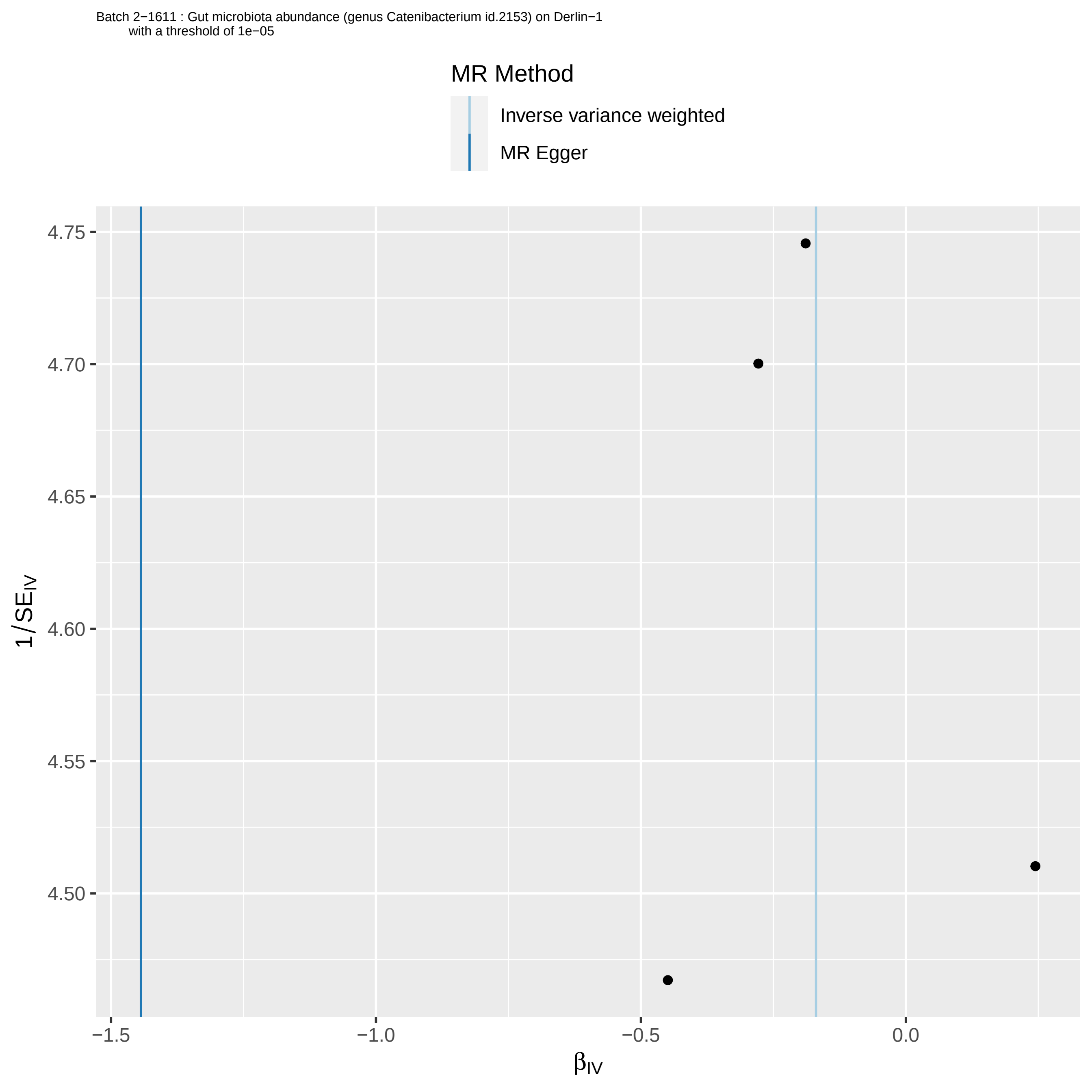

Batch 2−1611 : Gut microbiota abundance (genus Catenibacterium id.2153) on Derlin−1
with a threshold of 1e−05
MR Method
Inverse variance weighted
MR Egger
4.75
4.70
4.65
4.60
4.55
4.50
−1.5
−1.0
−0.5
0.0
$1/SE_{IV}$
β_{IV}

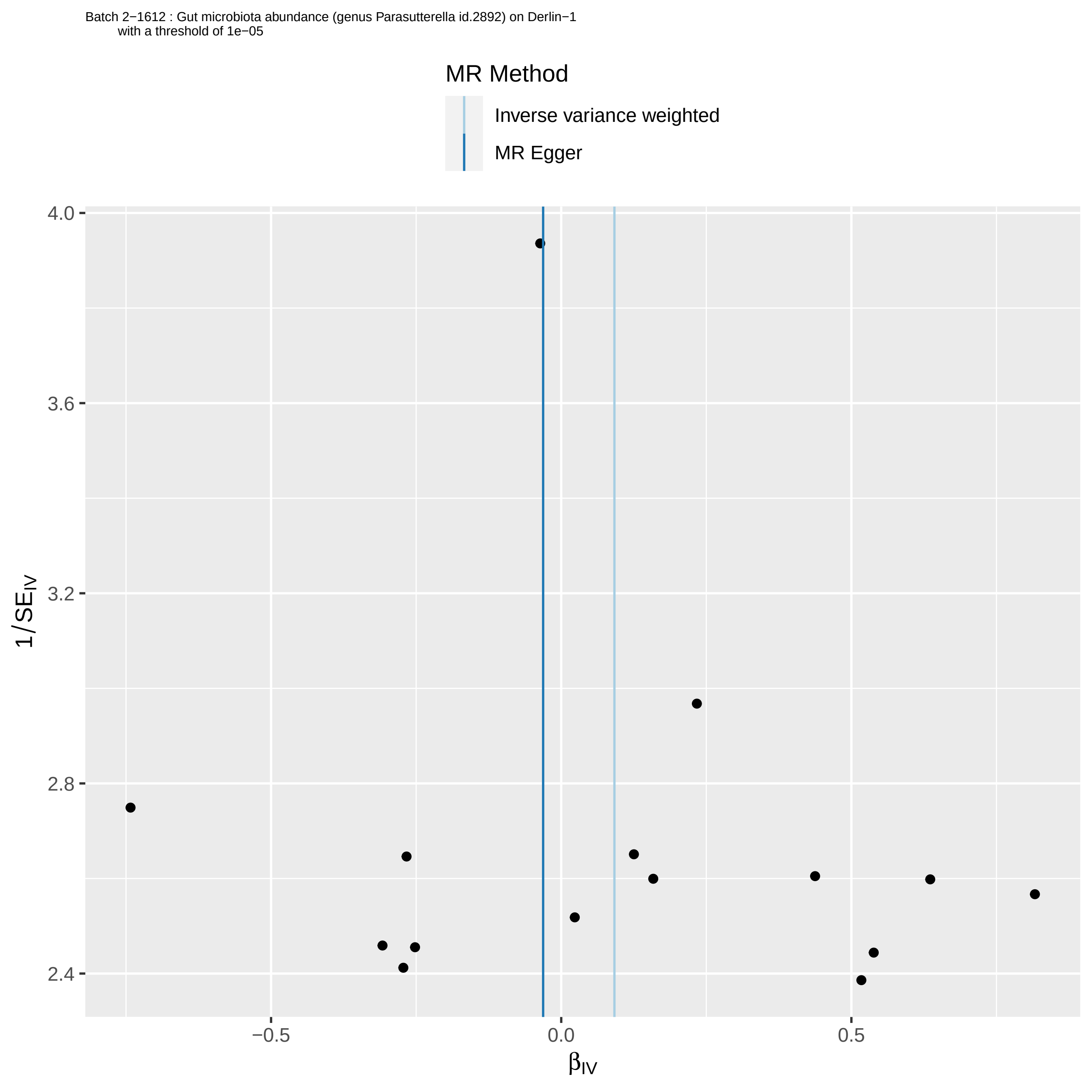
Batch 2−1612 : Gut microbiota abundance (genus Parasutterella id.2892) on Derlin−1
with a threshold of 1e−05
MR Method
Inverse variance weighted
MR Egger
4.0
3.6
3.2
2.8
2.4
1/SE_IV
−0.5
0.0
0.5
β_IV

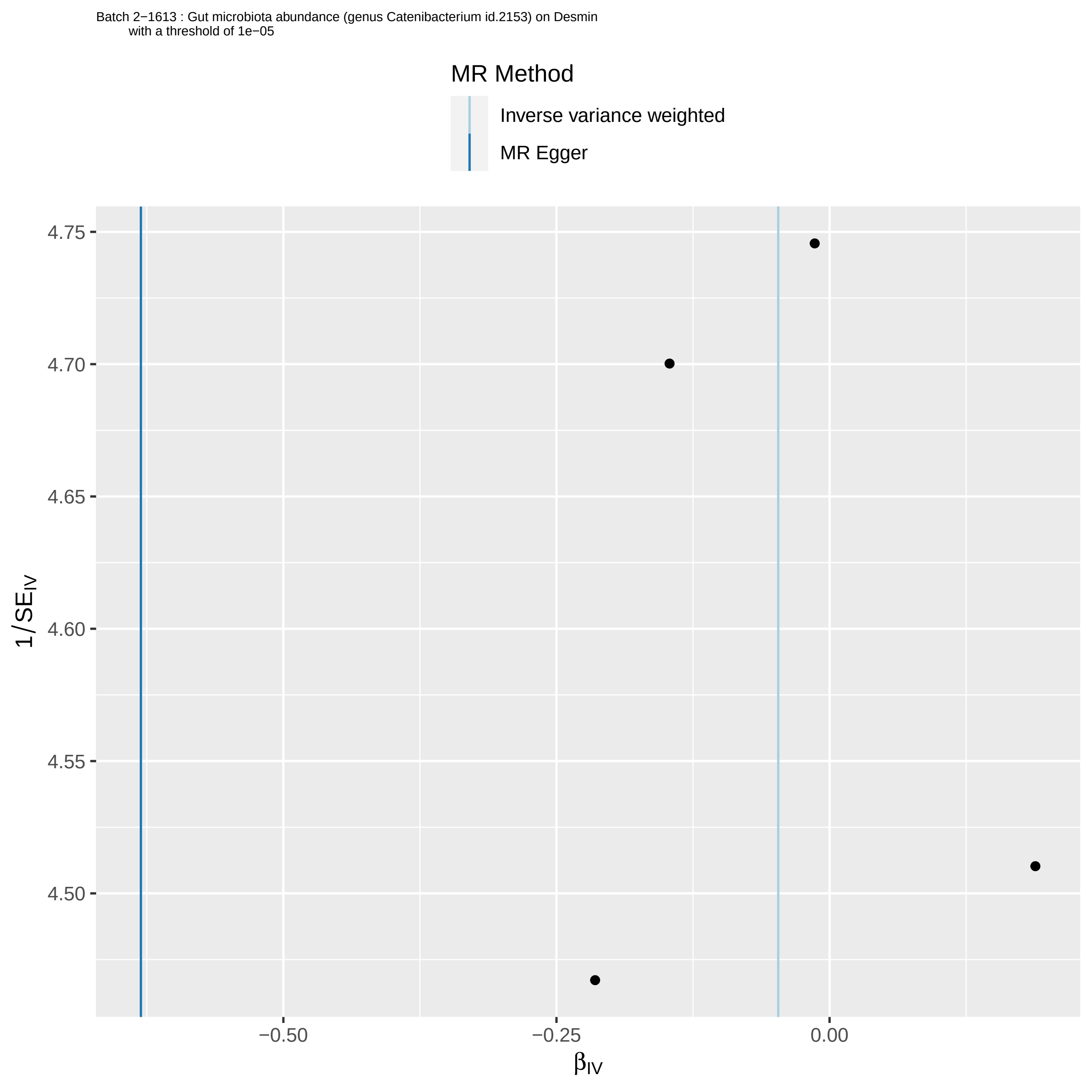
Batch 2−1613 : Gut microbiota abundance (genus Catenibacterium id.2153) on Desmin
with a threshold of 1e−05
MR Method
Inverse variance weighted
MR Egger
4.75
4.70
4.65
4.60
4.55
4.50
1/SE_IV
−0.50
−0.25
0.00
β_IV

Batch 2−1614 : Gut microbiota abundance (genus Parasutterella id.2892) on Desmin
with a threshold of 1e−05

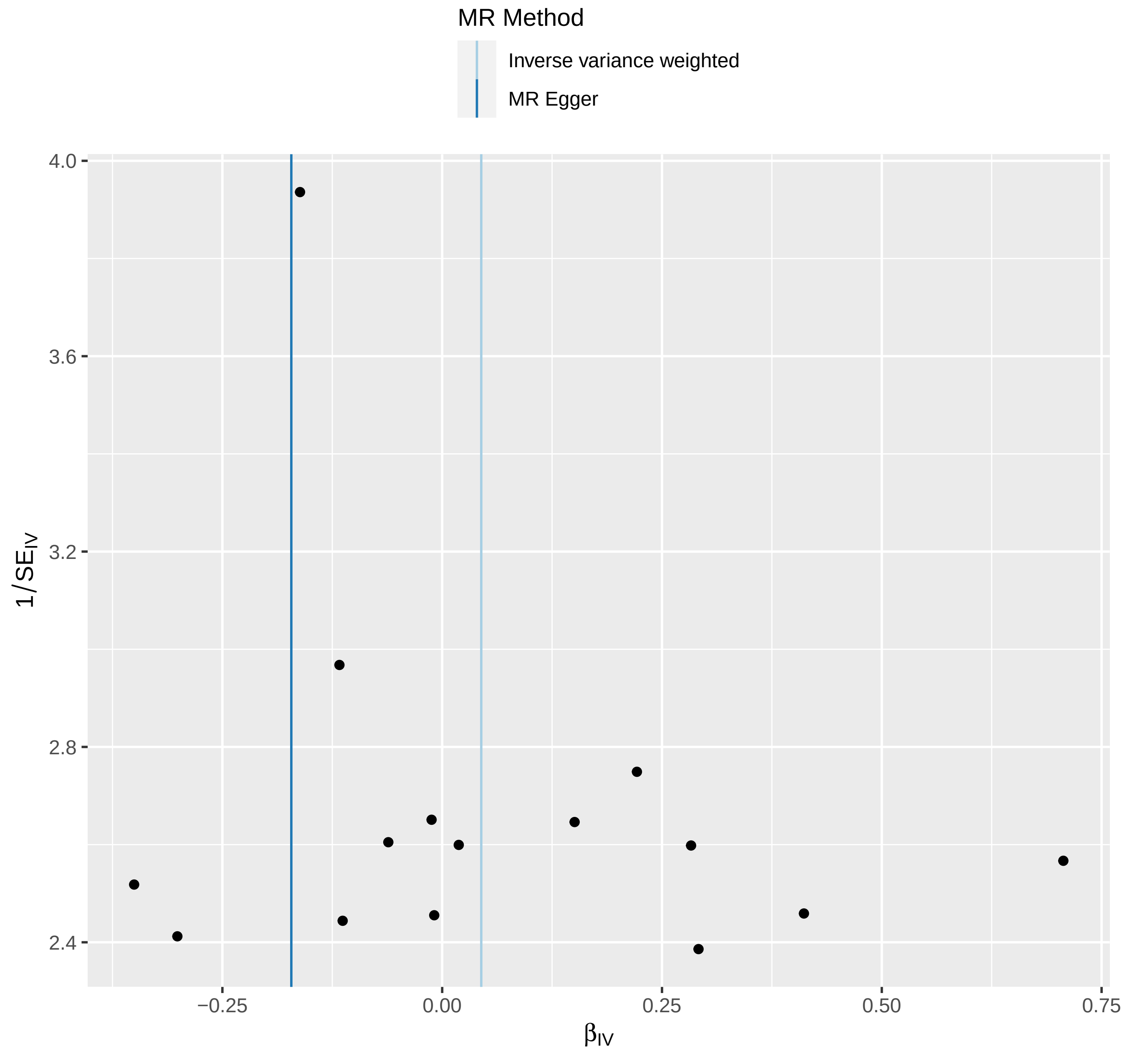

Batch 2−1615 : Gut microbiota abundance (genus Catenibacterium id.2153) on Protein DGCR14
with a threshold of 1e−05

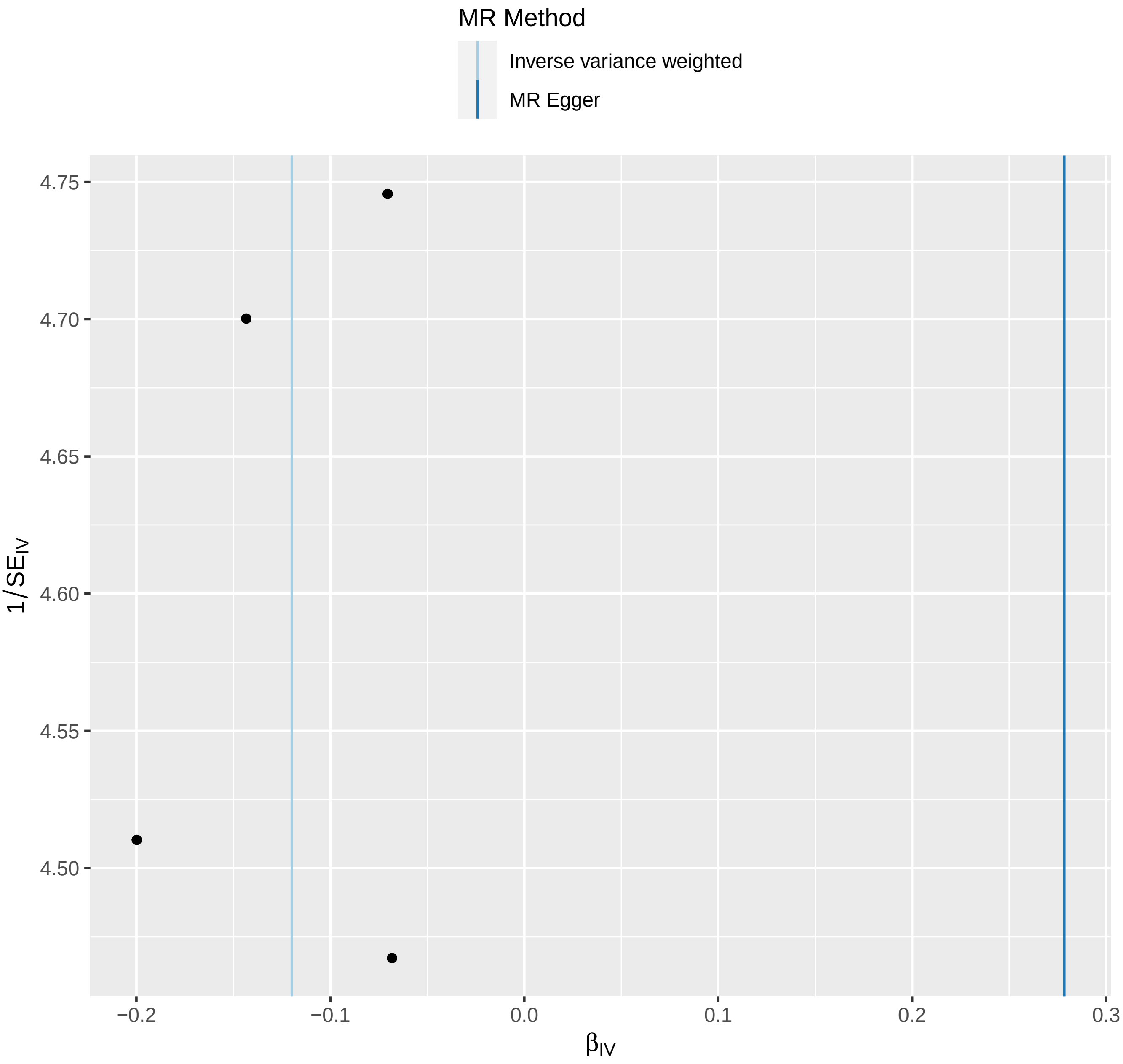

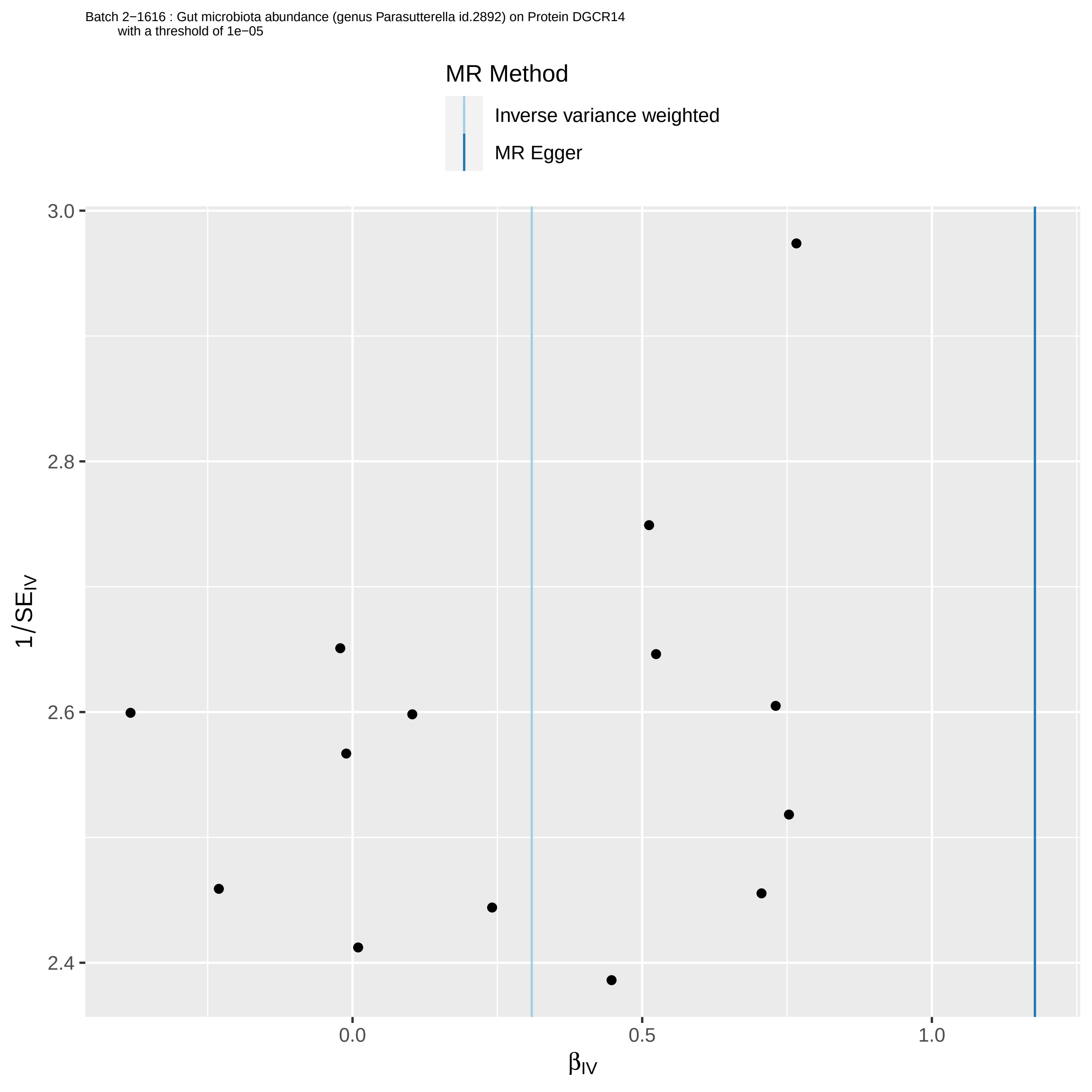
Batch 2−1616 : Gut microbiota abundance (genus Parasutterella id.2892) on Protein DGCR14
with a threshold of 1e−05
MR Method
Inverse variance weighted
MR Egger
3.0
2.8
2.6
2.4
1/SE_IV
0.0
0.5
1.0
β_IV

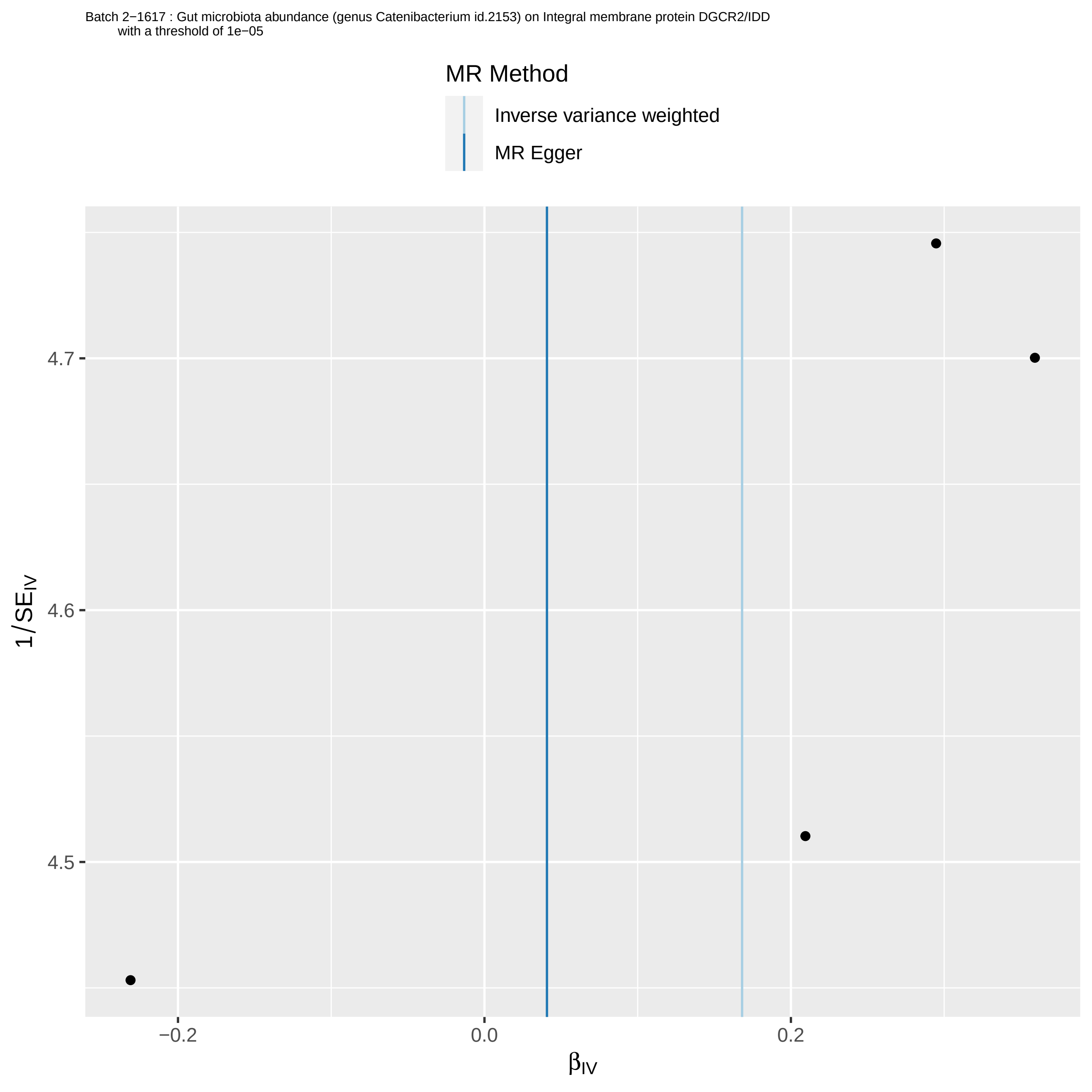

Batch 2−1617 : Gut microbiota abundance (genus Catenibacterium id.2153) on Integral membrane protein DGCR2/IDD
with a threshold of 1e−05
MR Method
Inverse variance weighted
MR Egger
4.7
4.6
4.5
1/SE_IV
−0.2
0.0
0.2
β_IV

Batch 2−1618 : Gut microbiota abundance (genus Parasutterella id.2892) on Integral membrane protein DGCR2/IDD
with a threshold of 1e−05

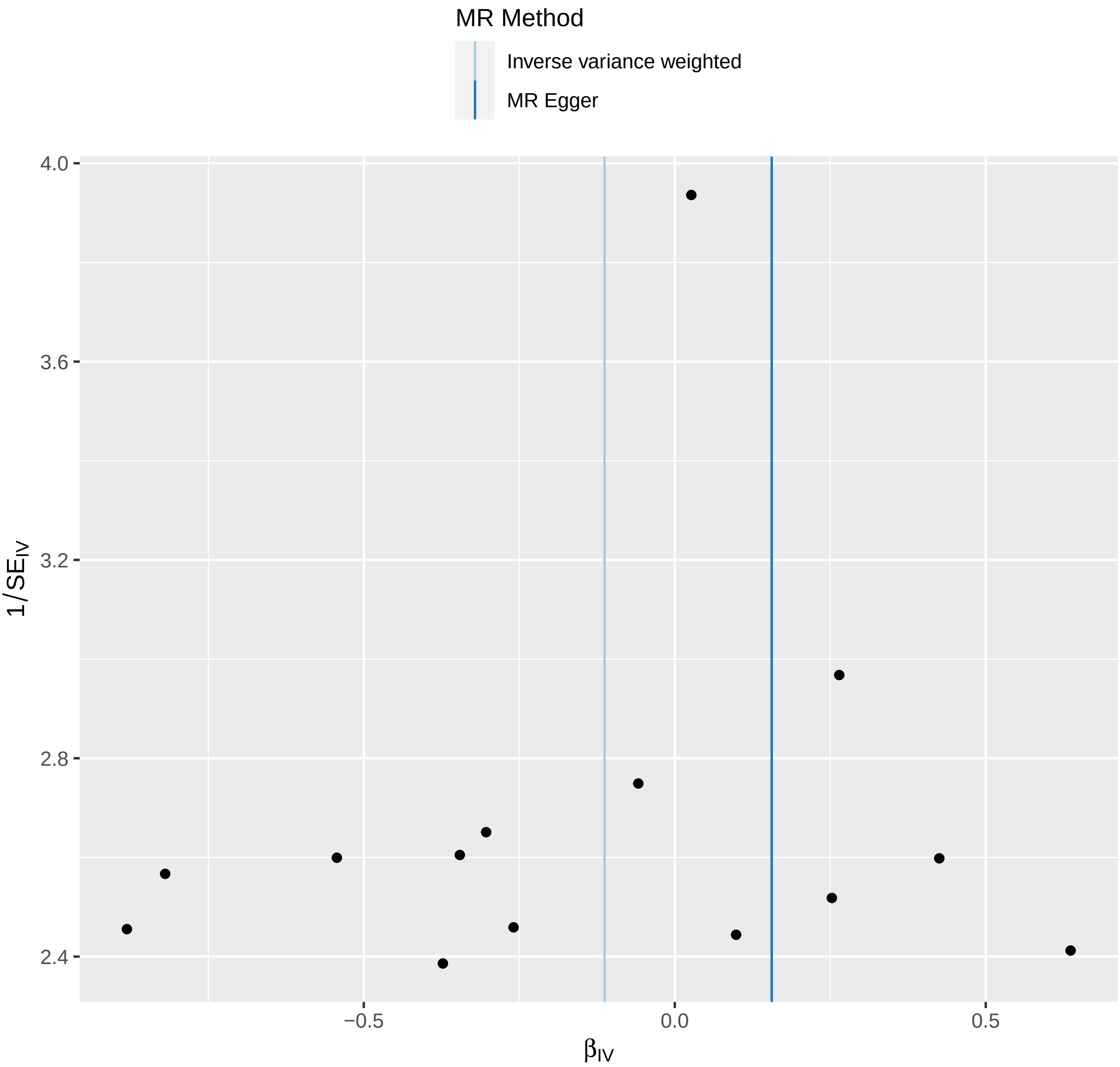

Batch 2−1619 : Gut microbiota abundance (genus Catenibacterium id.2153) on Protein DGCR6
with a threshold of 1e−05

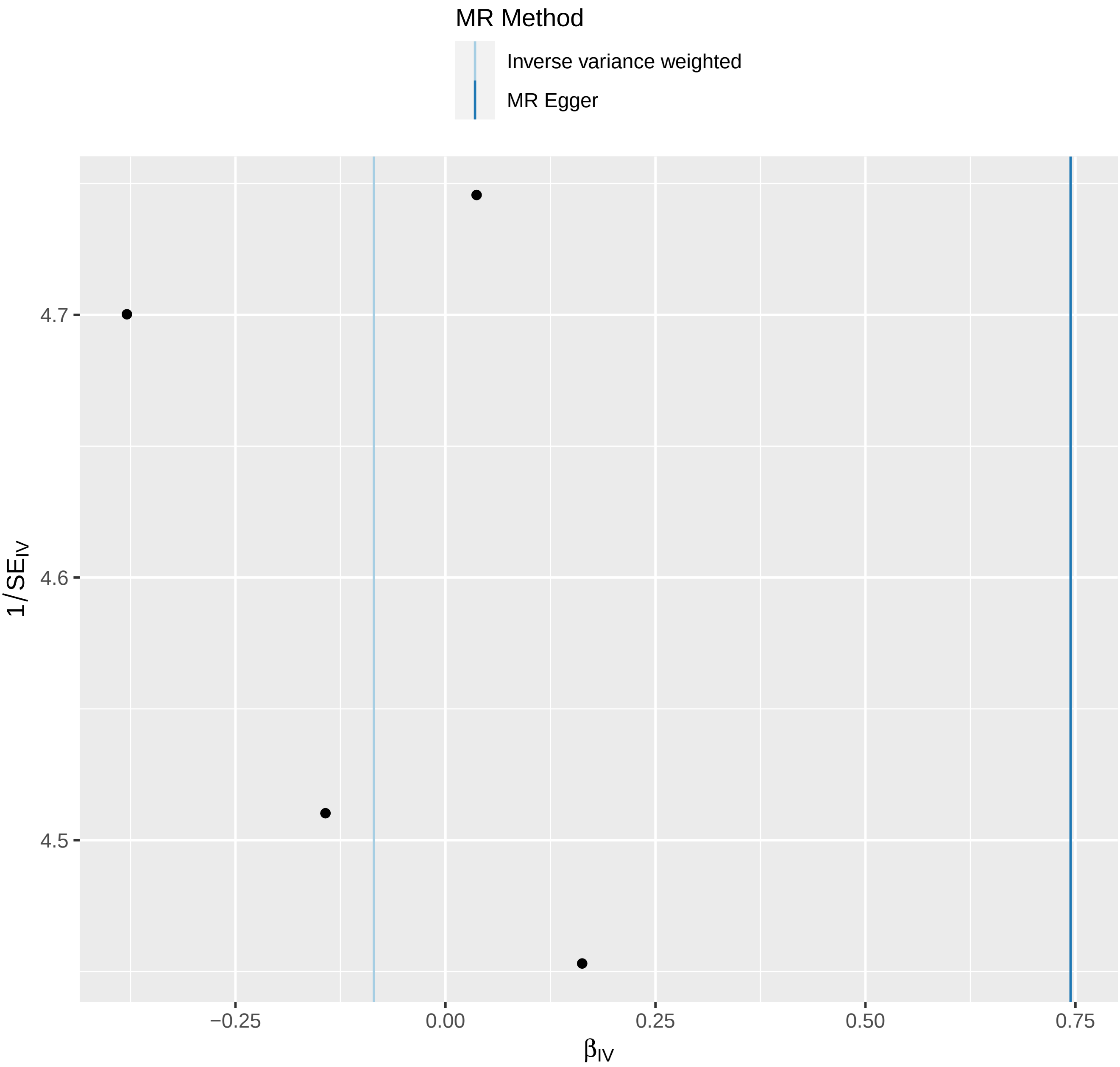

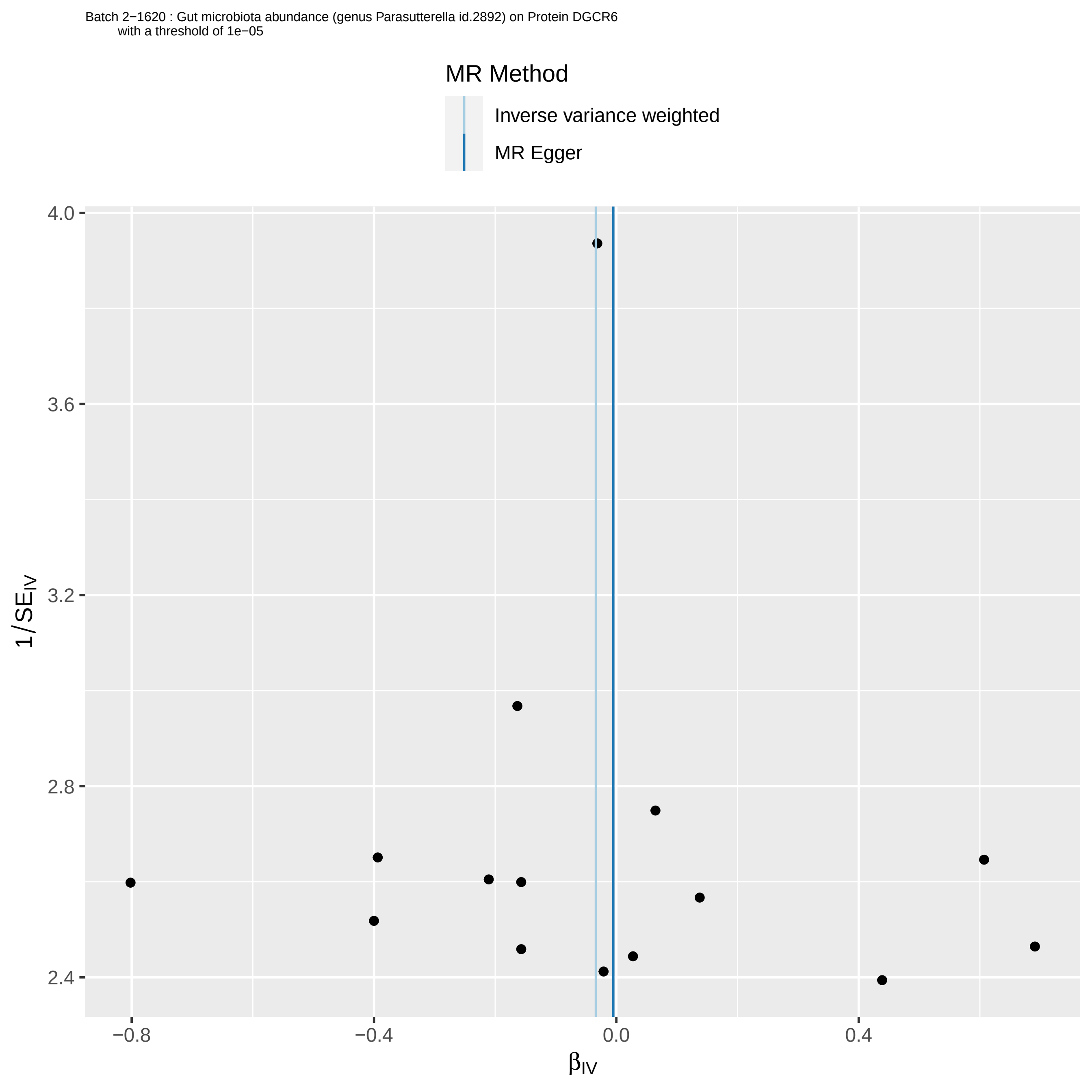
Batch 2−1620 : Gut microbiota abundance (genus Parasutterella id.2892) on Protein DGCR6
with a threshold of 1e−05
MR Method
Inverse variance weighted
MR Egger
4.0
3.6
3.2
2.8
2.4
1/SE_IV
−0.8
−0.4
0.0
0.4
β_IV

Batch 2−1621 : Gut microbiota abundance (genus Catenibacterium id.2153) on Diacylglycerol kinase beta
with a threshold of 1e−05

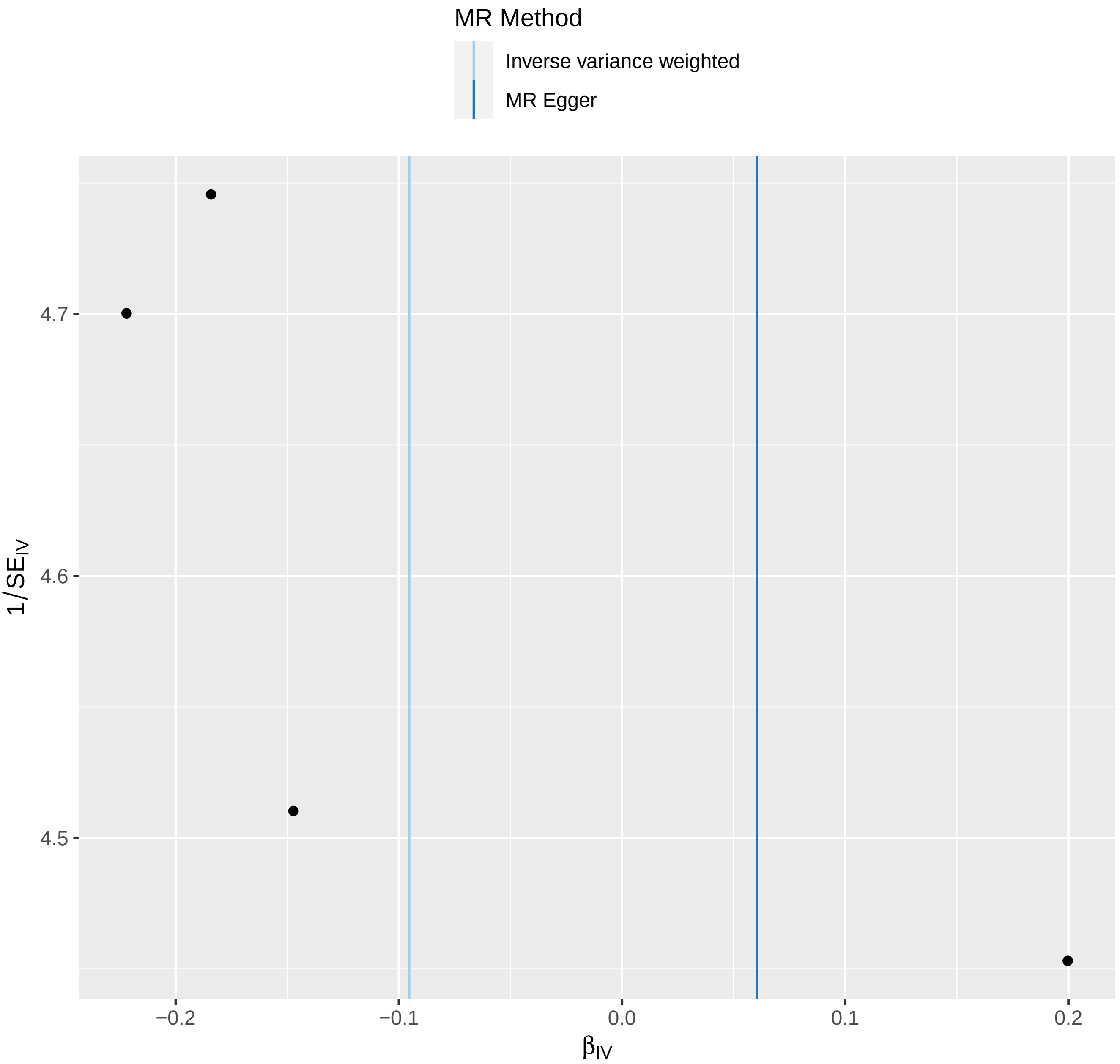

Batch 2−1622 : Gut microbiota abundance (genus Parasutterella id.2892) on Diacylglycerol kinase beta
with a threshold of 1e−05

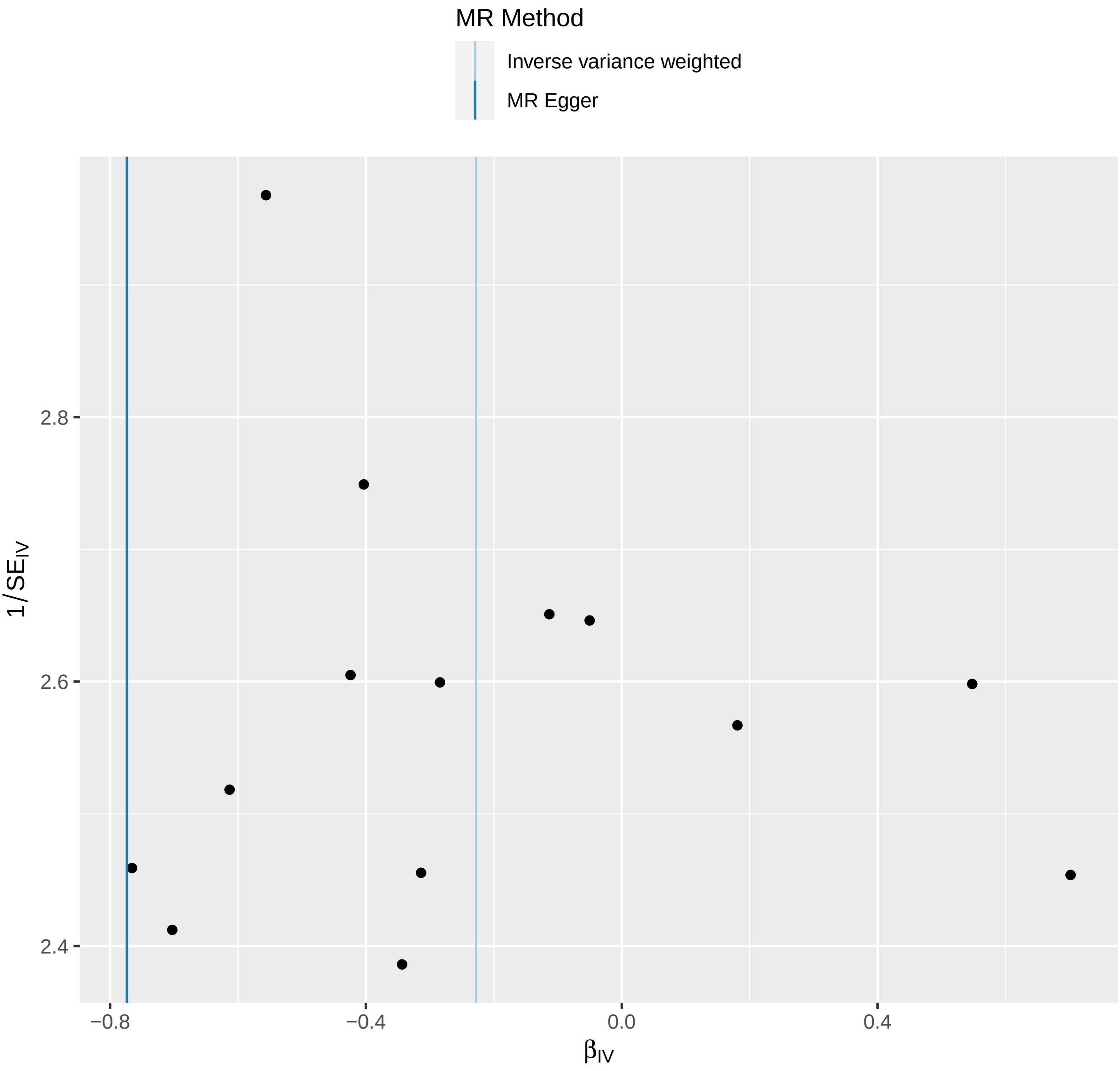

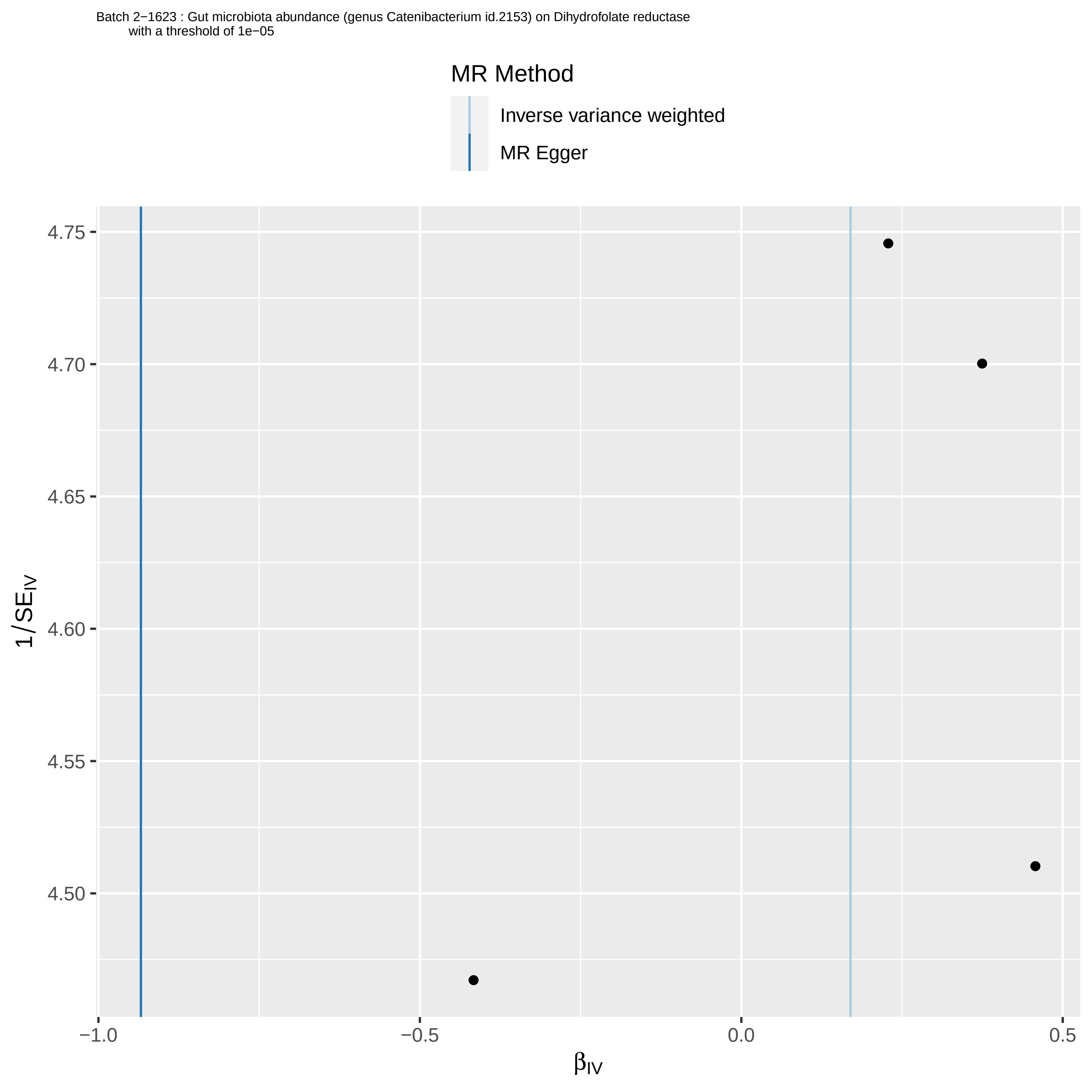

Batch 2−1623 : Gut microbiota abundance (genus Catenibacterium id.2153) on Dihydrofolate reductase
with a threshold of 1e−05
MR Method
Inverse variance weighted
MR Egger
4.75
4.70
4.65
4.60
4.55
4.50
1/SE_IV
−1.0
−0.5
0.0
0.5
β_IV

Batch 2−1624 : Gut microbiota abundance (genus Parasutterella id.2892) on Dihydrofolate reductase
with a threshold of 1e−05

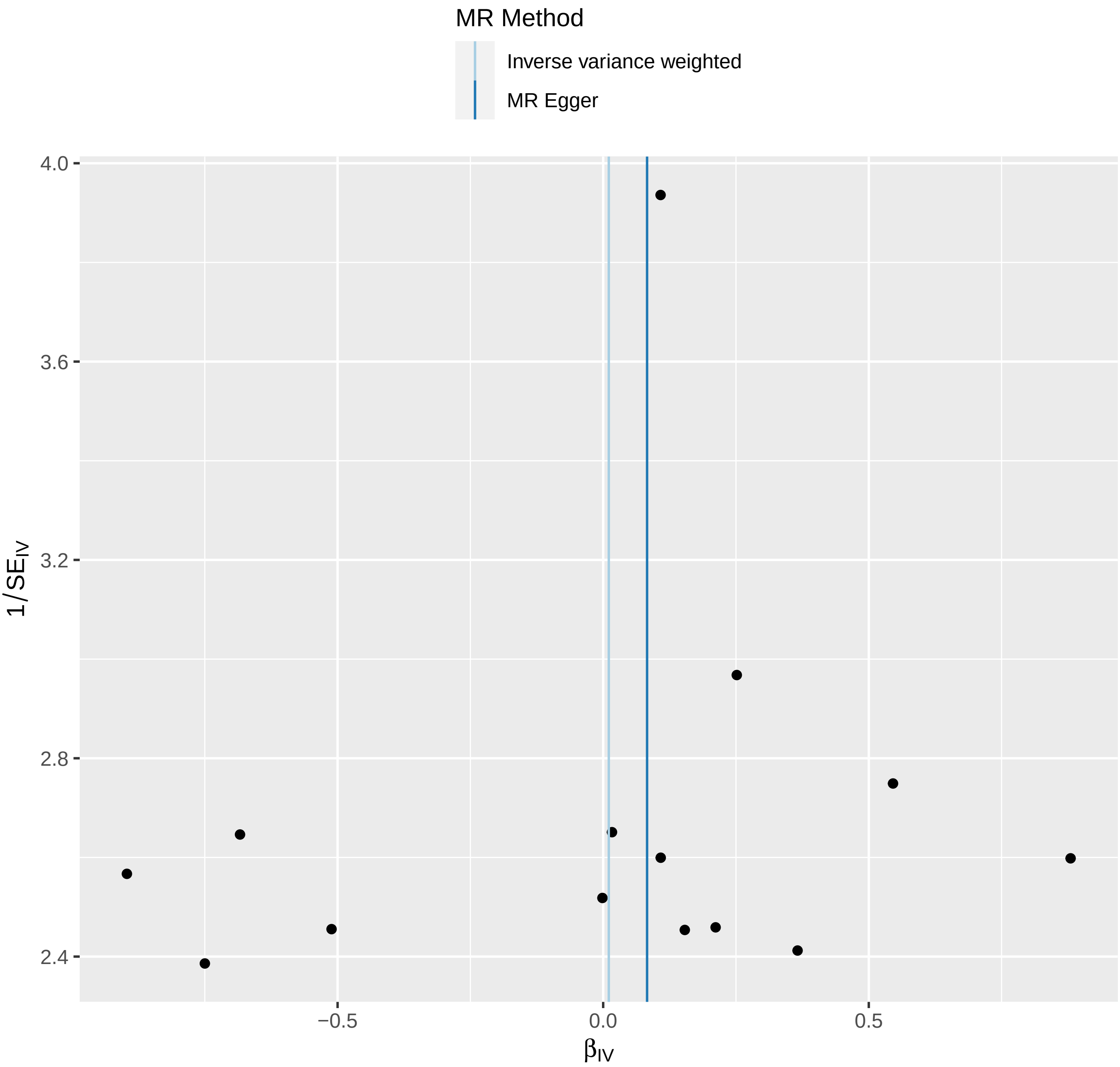

Batch 2−1625 : Gut microbiota abundance (genus Catenibacterium id.2153) on Desert hedgehog protein N−product
with a threshold of 1e−05

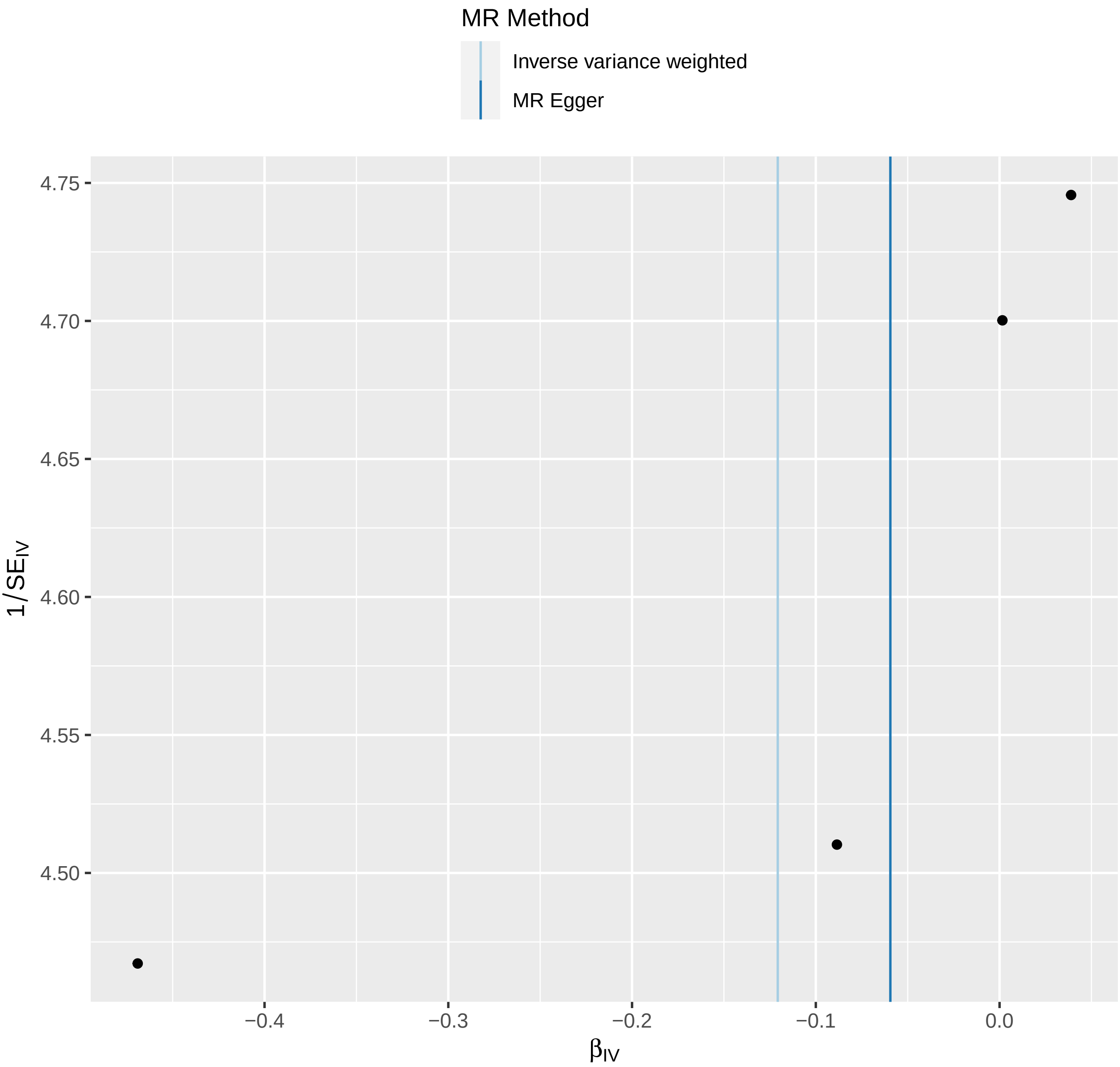

Batch 2−1626 : Gut microbiota abundance (genus Parasutterella id.2892) on Desert hedgehog protein N−product
with a threshold of 1e−05

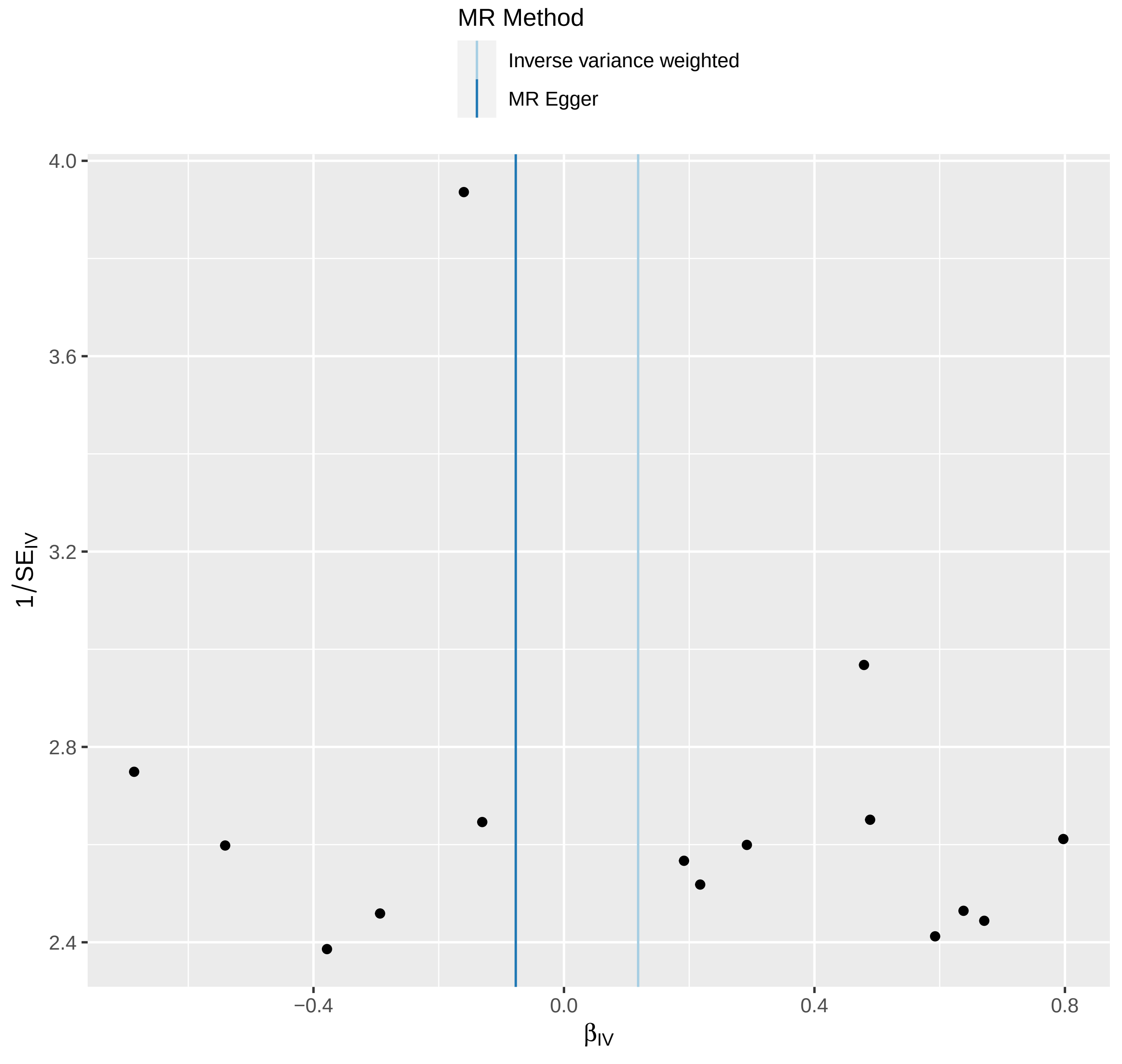

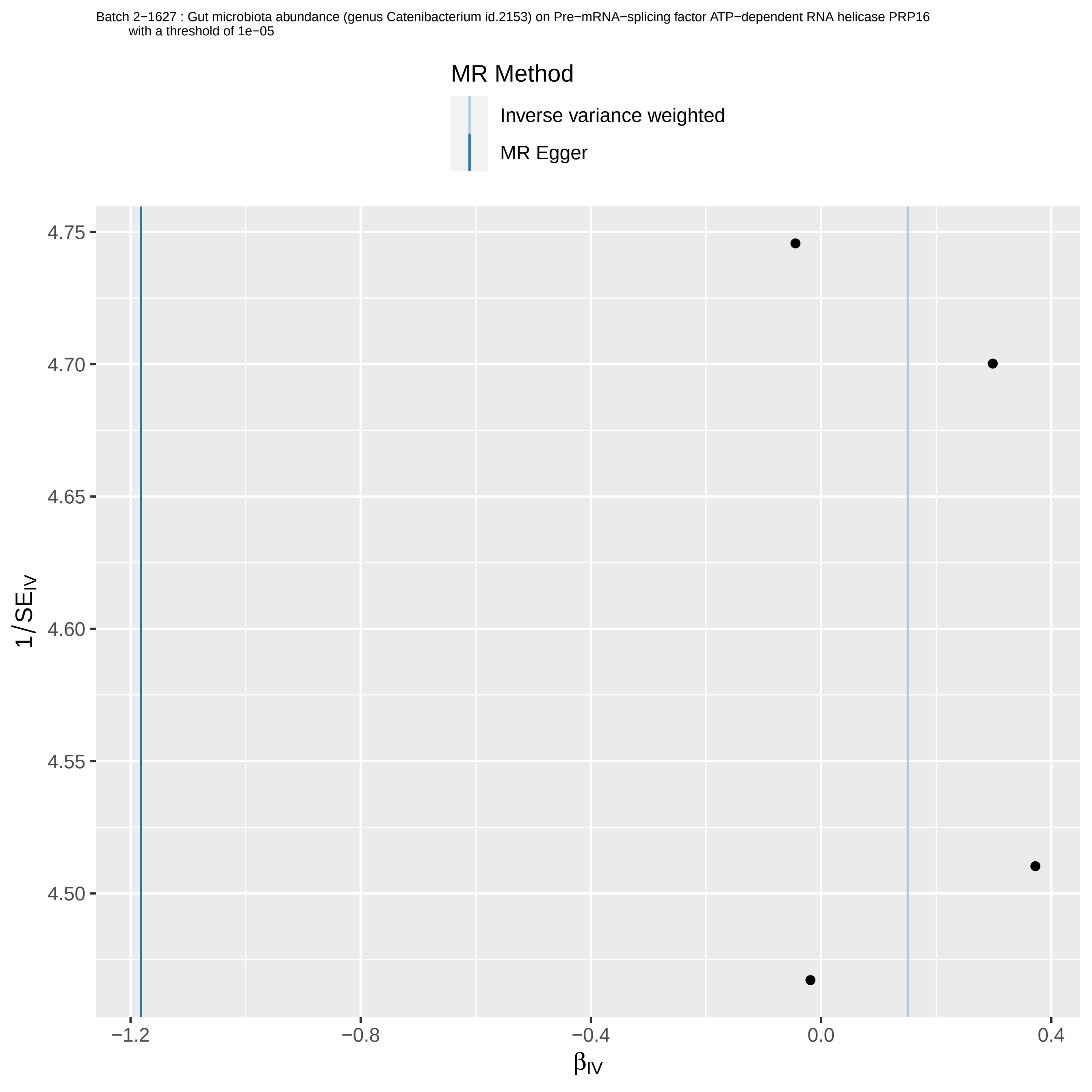
Batch 2−1627 : Gut microbiota abundance (genus Catenibacterium id.2153) on Pre−mRNA−splicing factor ATP−dependent RNA helicase PRP16
with a threshold of 1e−05
MR Method
Inverse variance weighted
MR Egger
4.75
4.70
4.65
4.60
4.55
4.50
1/SE_IV
−1.2
−0.8
−0.4
0.0
0.4
β_IV

Batch 2−1628 : Gut microbiota abundance (genus Parasutterella id.2892) on Pre−mRNA−splicing factor ATP−dependent RNA helicase PRP16
with a threshold of 1e−05

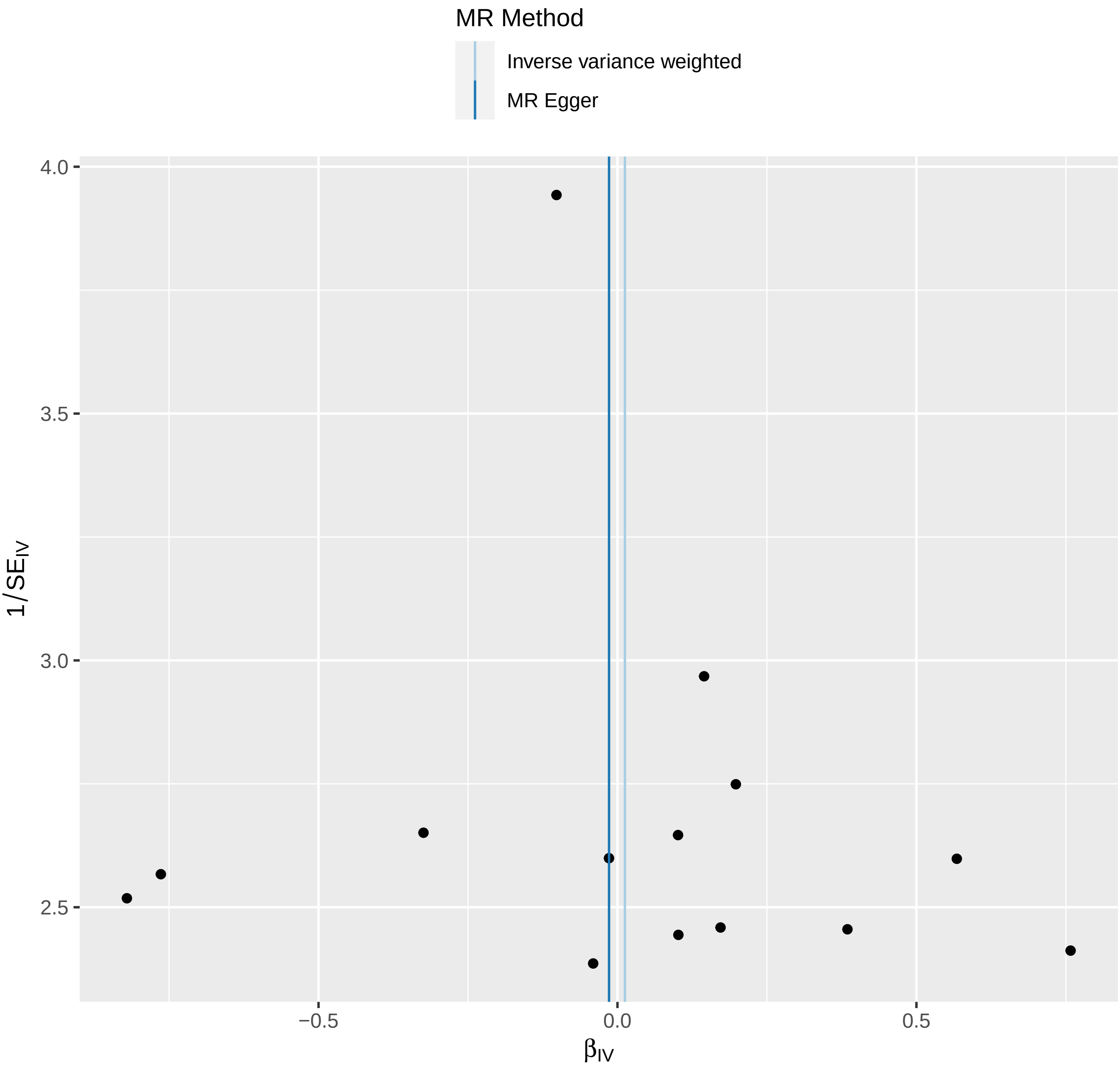

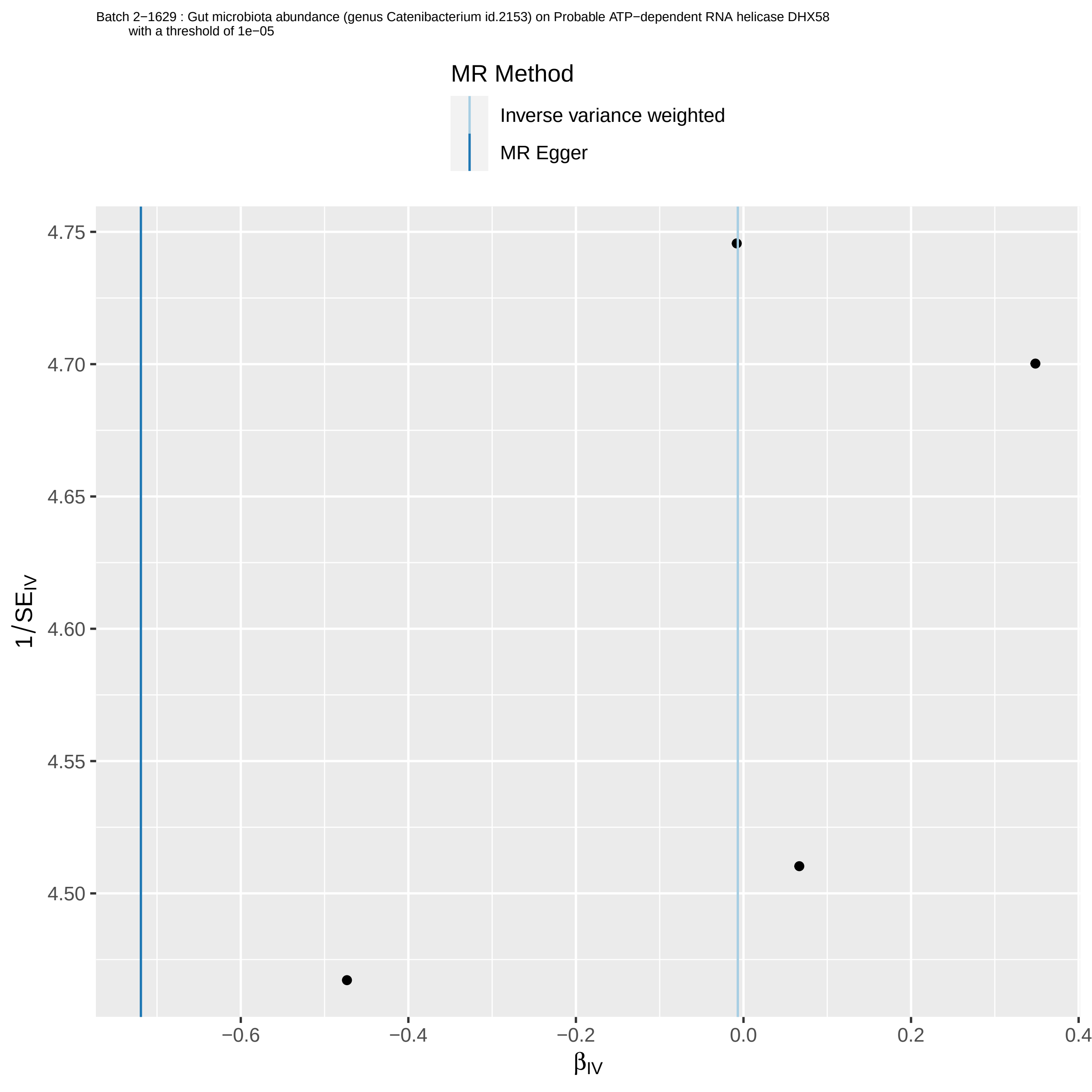

Batch 2−1629 : Gut microbiota abundance (genus Catenibacterium id.2153) on Probable ATP−dependent RNA helicase DHX58
with a threshold of 1e−05
MR Method
Inverse variance weighted
MR Egger
4.75
4.70
4.65
4.60
4.55
4.50
$1/SE_{IV}$
−0.6
−0.4
−0.2
0.0
0.2
0.4
β_{IV}

Batch 2−1630 : Gut microbiota abundance (genus Parasutterella id.2892) on Probable ATP−dependent RNA helicase DHX58
with a threshold of 1e−05

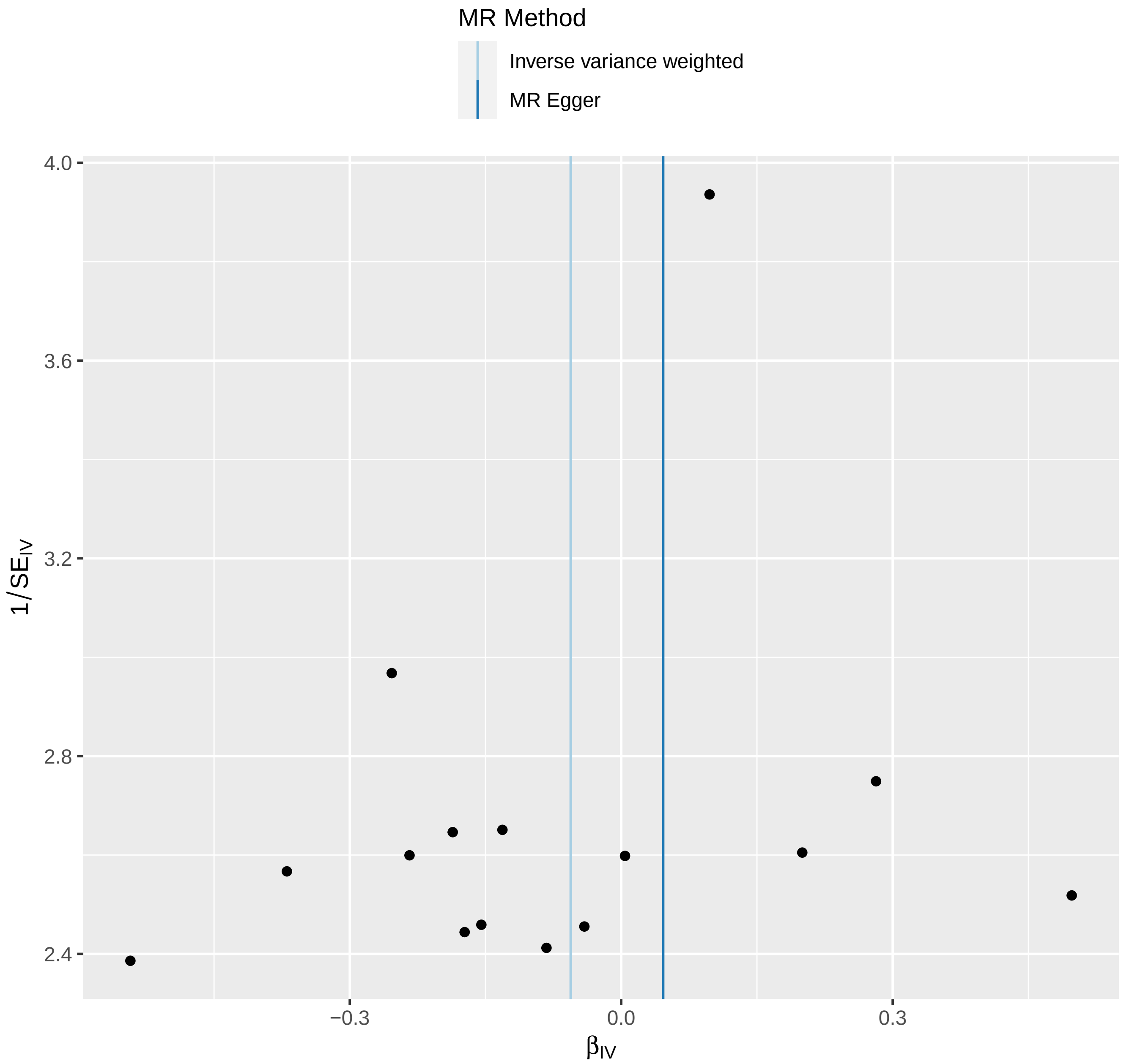

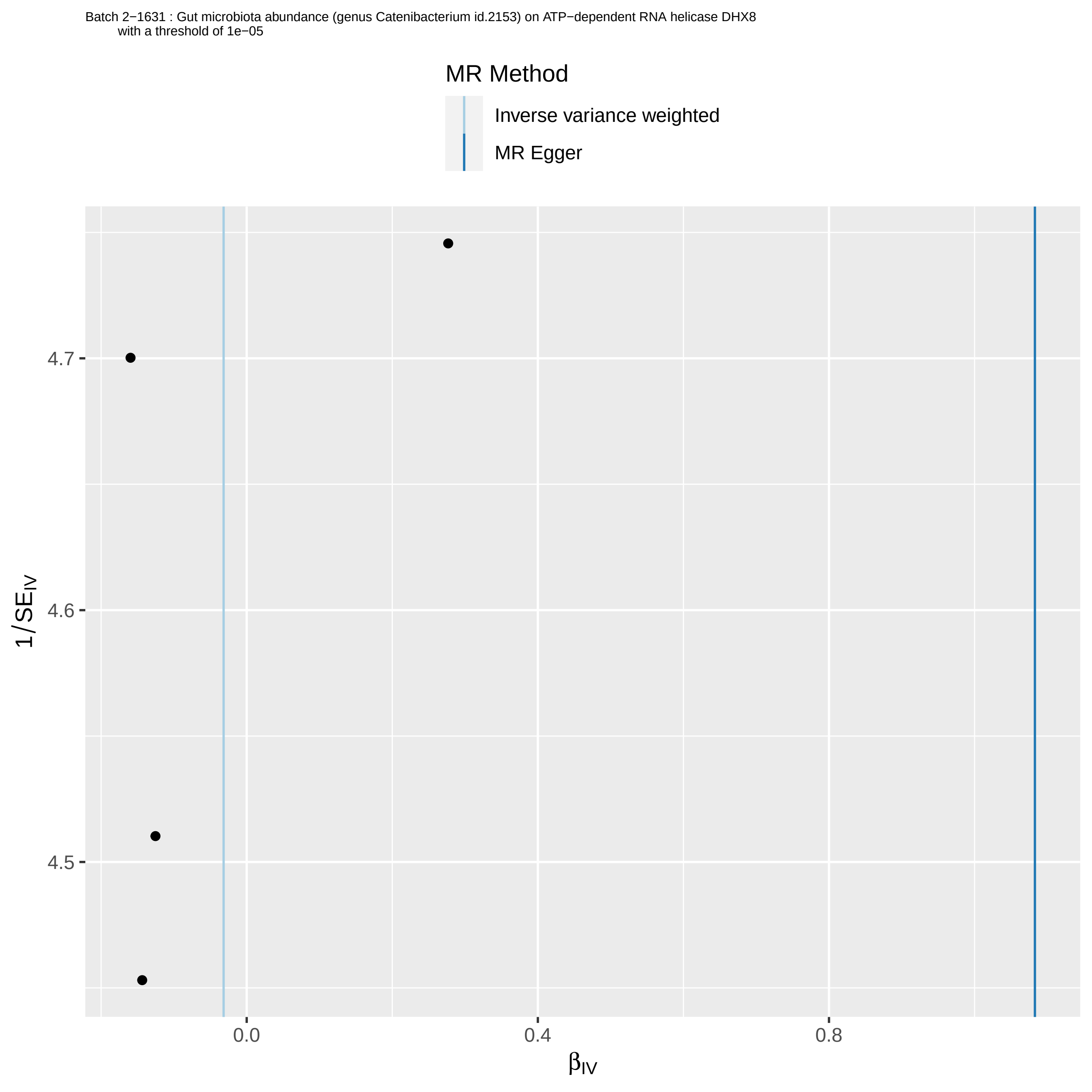
Batch 2−1631 : Gut microbiota abundance (genus Catenibacterium id.2153) on ATP−dependent RNA helicase DHX8
with a threshold of 1e−05
MR Method
Inverse variance weighted
MR Egger
4.7
4.6
4.5
1/SE_IV
0.0
0.4
0.8
β_IV

Batch 2−1632 : Gut microbiota abundance (genus Parasutterella id.2892) on ATP−dependent RNA helicase DHX8
with a threshold of 1e−05

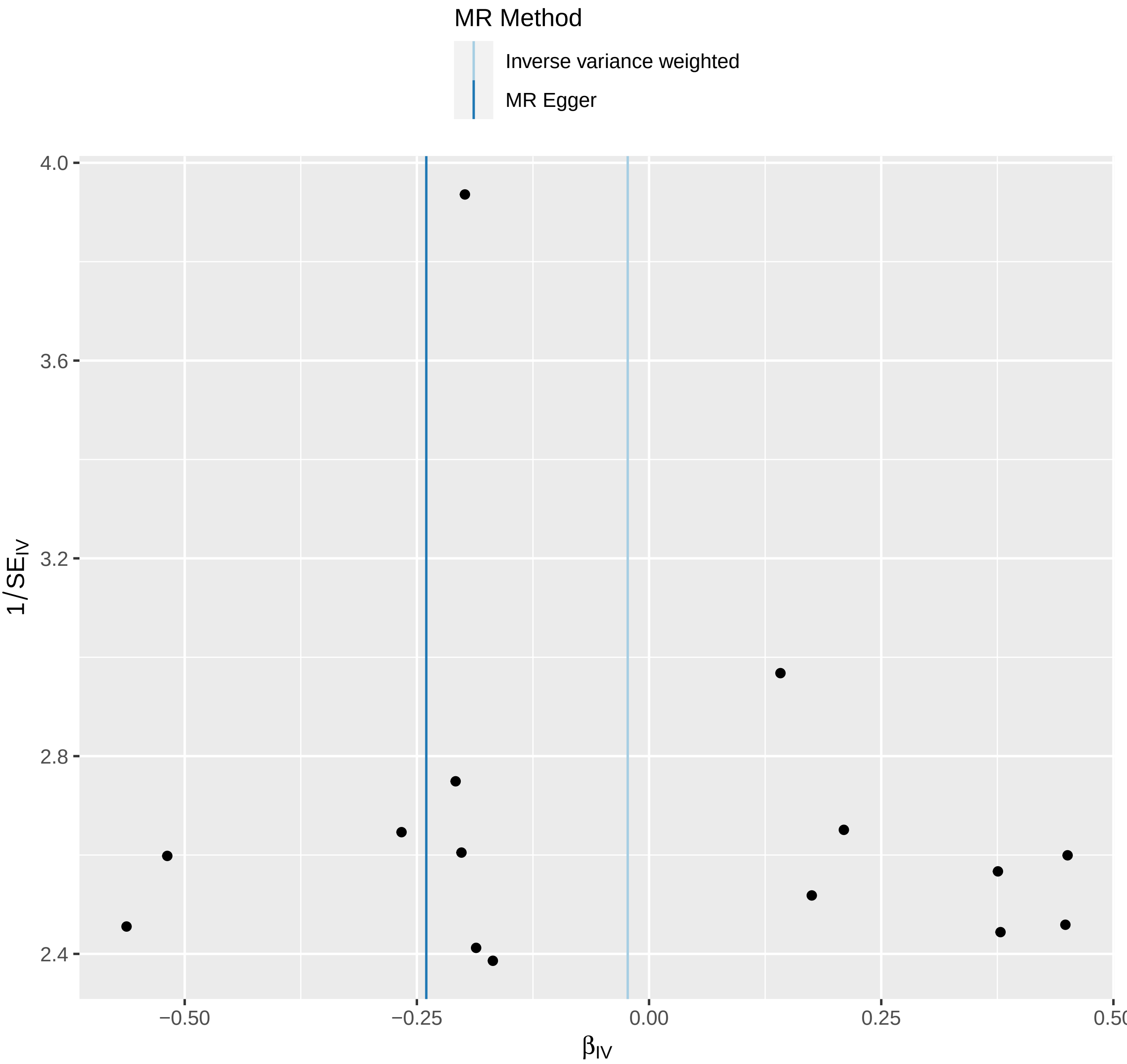

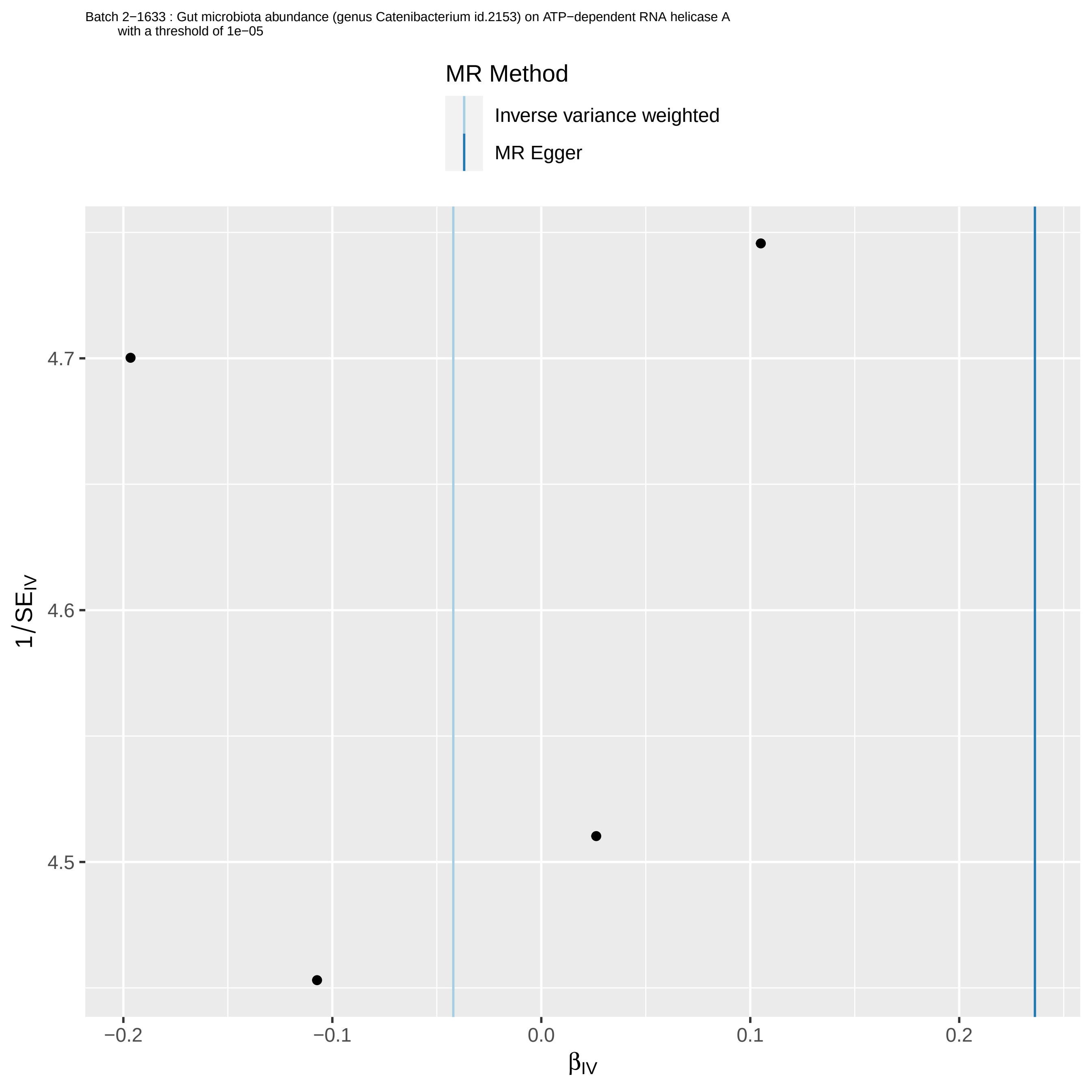

Batch 2−1633 : Gut microbiota abundance (genus Catenibacterium id.2153) on ATP−dependent RNA helicase A
with a threshold of 1e−05
MR Method
Inverse variance weighted
MR Egger
4.7
4.6
4.5
1/SE_IV
−0.2
−0.1
0.0
0.1
0.2
β_IV

Batch 2−1634 : Gut microbiota abundance (genus Parasutterella id.2892) on ATP−dependent RNA helicase A
with a threshold of 1e−05

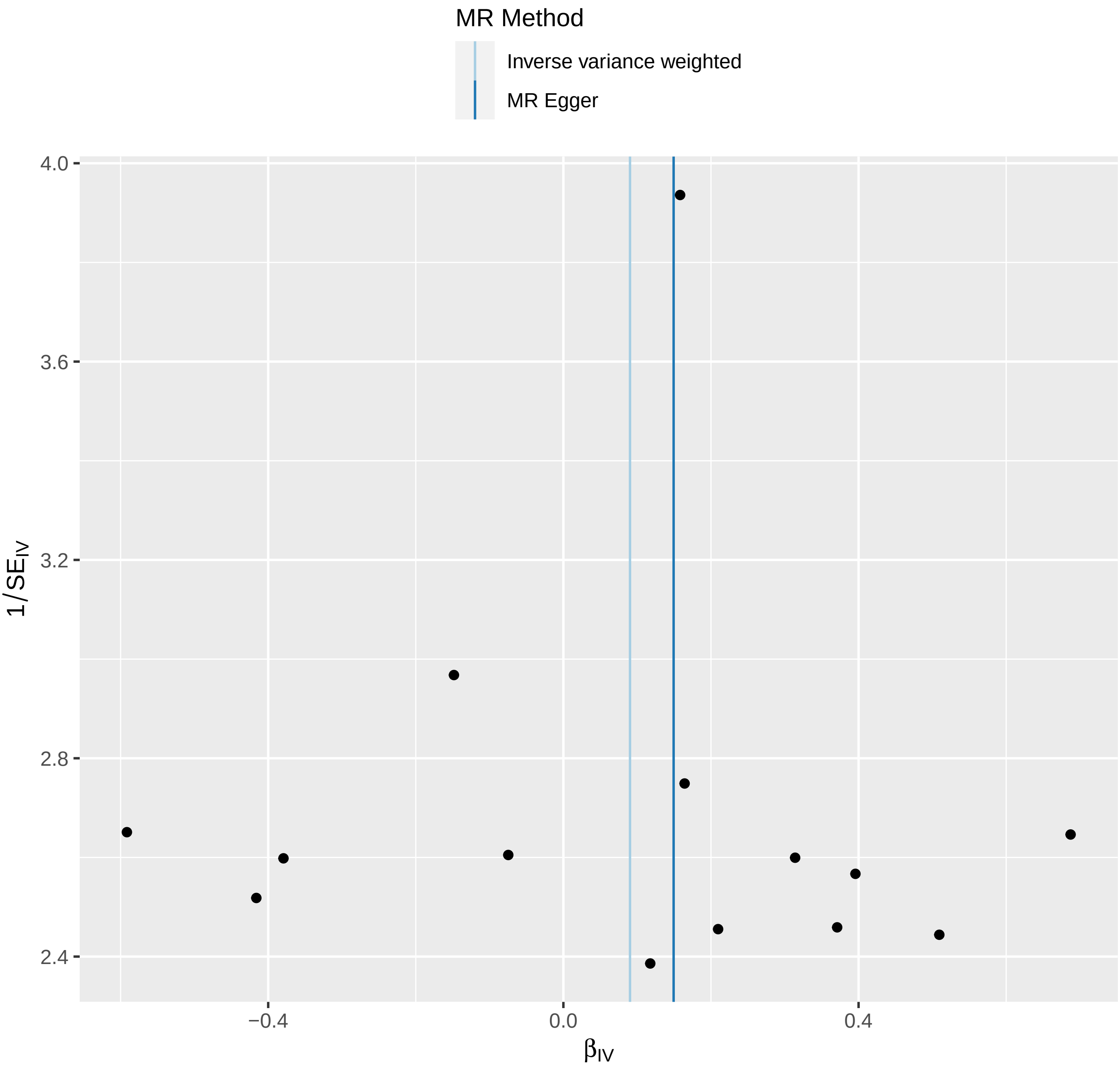

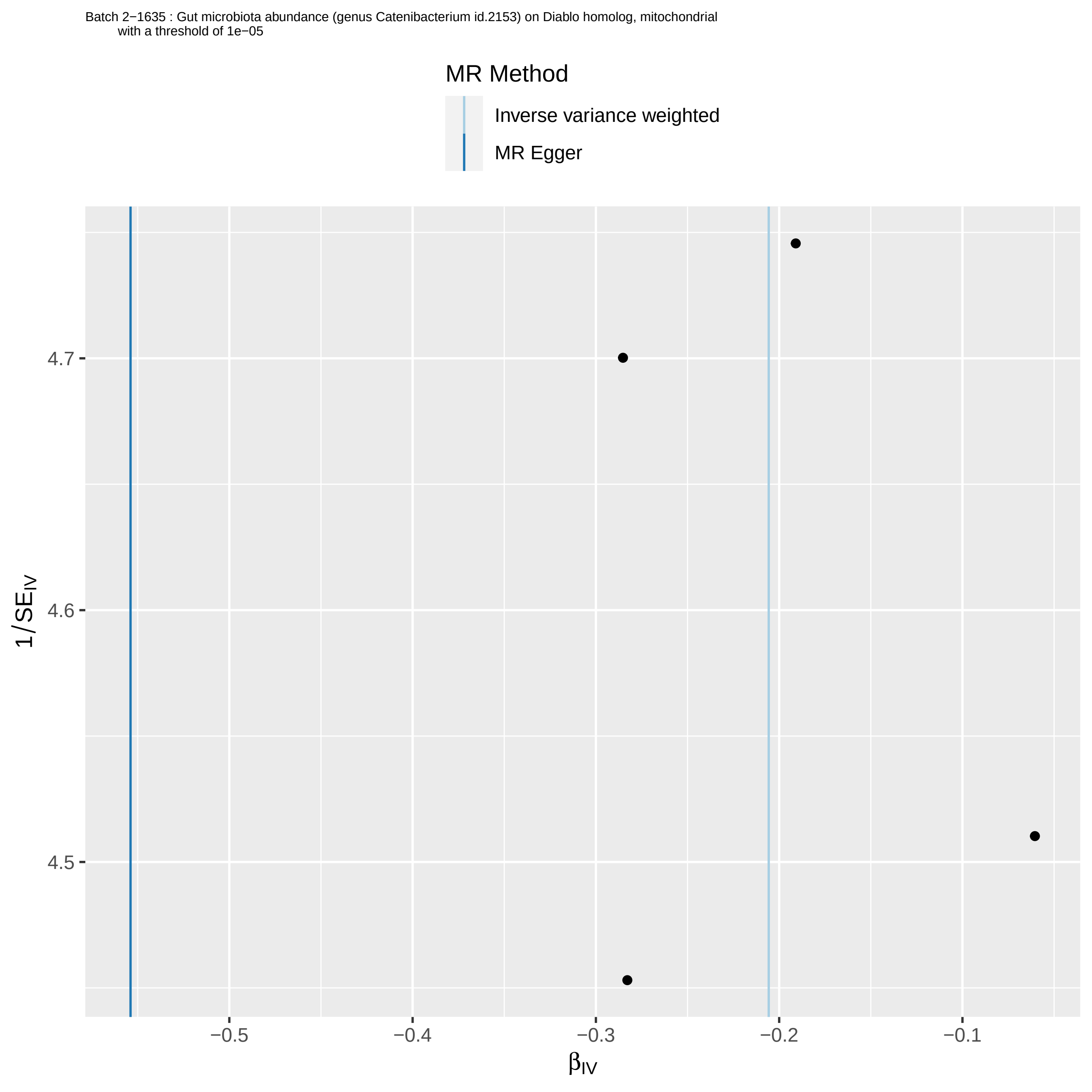

Batch 2−1635 : Gut microbiota abundance (genus Catenibacterium id.2153) on Diablo homolog, mitochondrial
with a threshold of 1e−05
MR Method
Inverse variance weighted
MR Egger
4.7
4.6
4.5
1/SE_IV
−0.5
−0.4
−0.3
−0.2
−0.1
β_IV

Batch 2−1636 : Gut microbiota abundance (genus Parasutterella id.2892) on Diablo homolog, mitochondrial
with a threshold of 1e−05

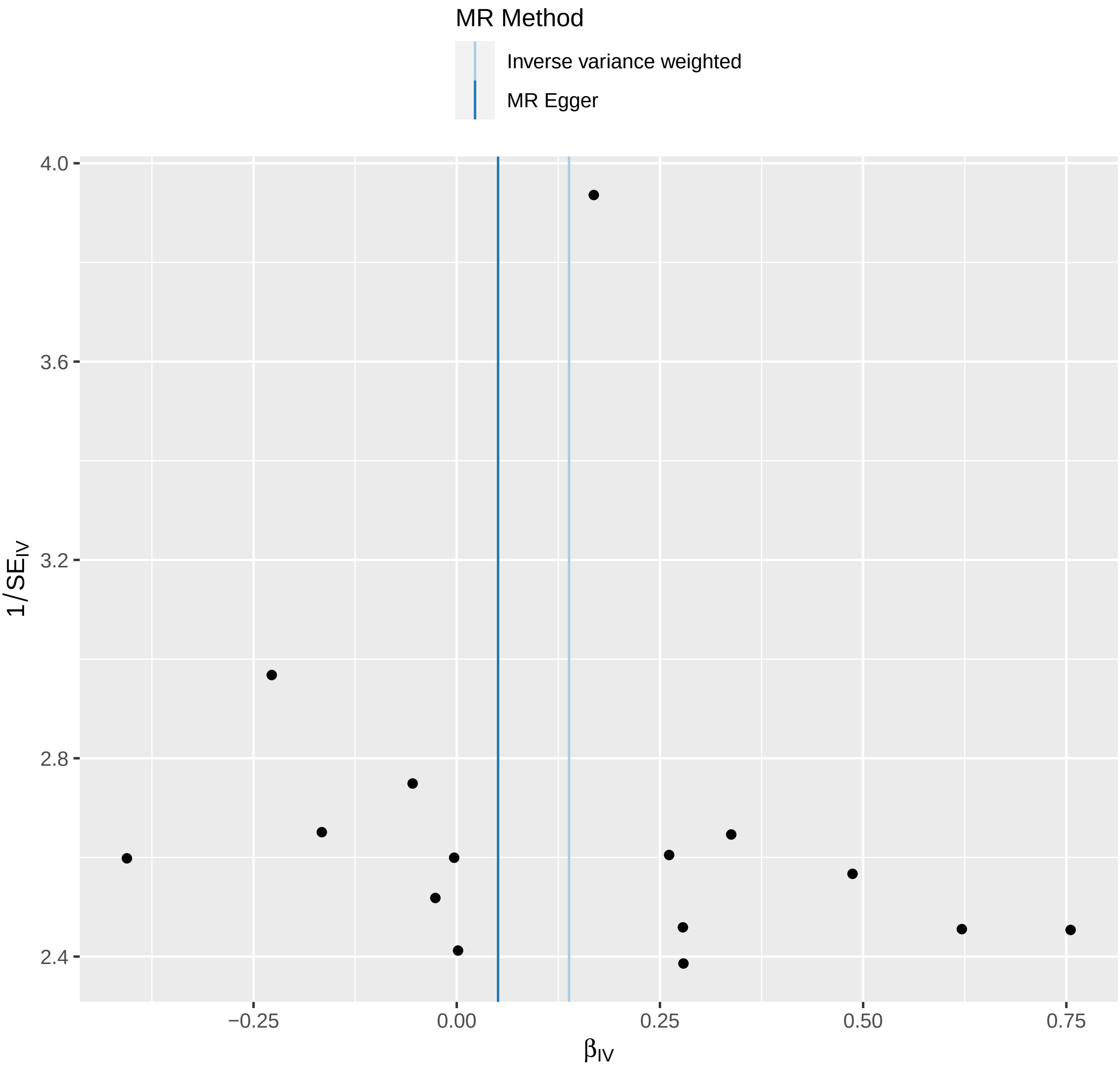

Batch 2−1637 : Gut microbiota abundance (genus Catenibacterium id.2153) on Probable dimethyladenosine transferase with a threshold of 1e−05

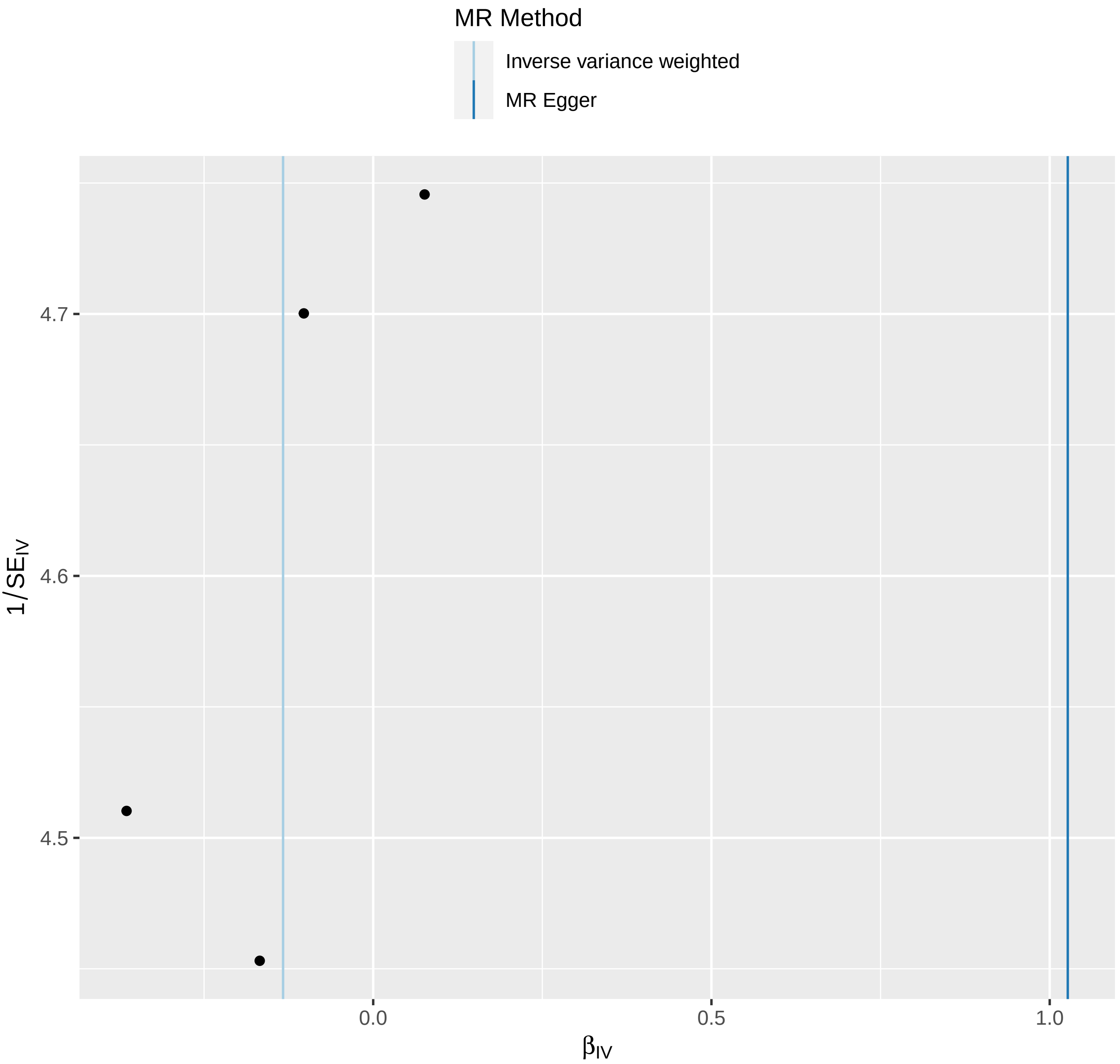

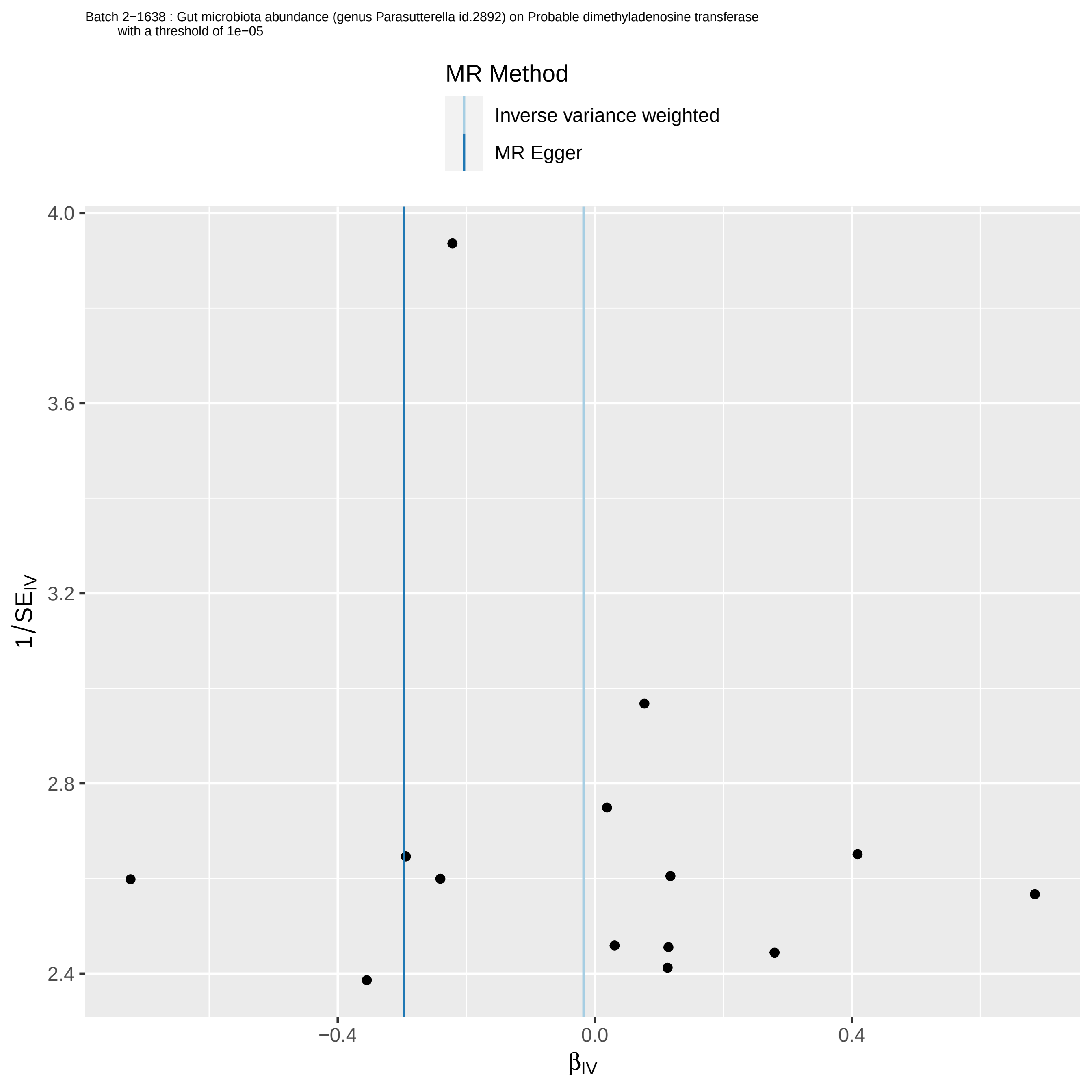

Batch 2−1638 : Gut microbiota abundance (genus Parasutterella id.2892) on Probable dimethyladenosine transferase
with a threshold of 1e−05
MR Method
Inverse variance weighted
MR Egger
4.0
3.6
3.2
2.8
2.4
1/SE_IV
−0.4
0.0
0.4
β_IV

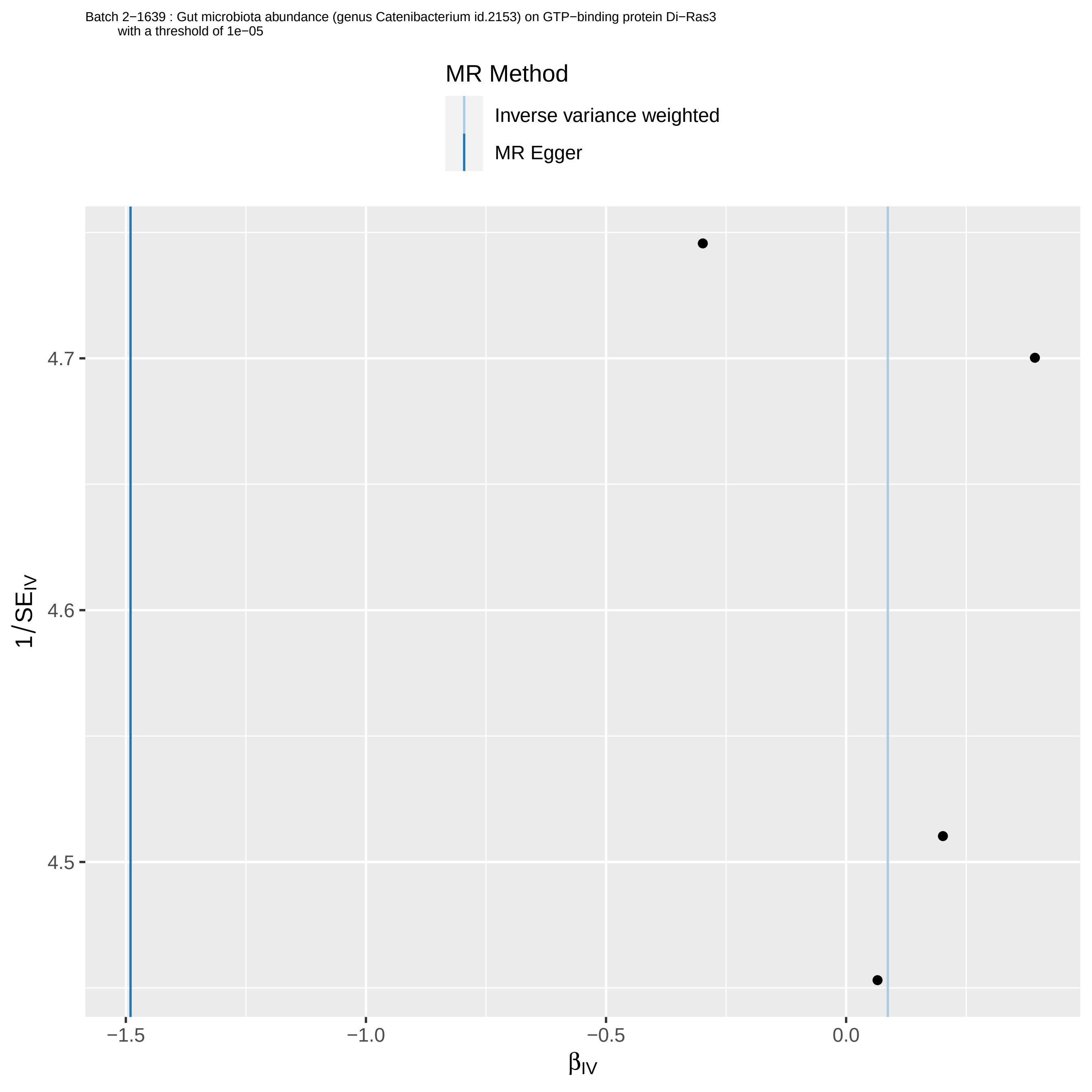
Batch 2−1639 : Gut microbiota abundance (genus Catenibacterium id.2153) on GTP−binding protein Di−Ras3
with a threshold of 1e−05
MR Method
Inverse variance weighted
MR Egger
4.7
4.6
4.5
1/SE_IV
−1.5
−1.0
−0.5
0.0
β_IV

Batch 2−1640 : Gut microbiota abundance (genus Parasutterella id.2892) on GTP−binding protein Di−Ras3
with a threshold of 1e−05

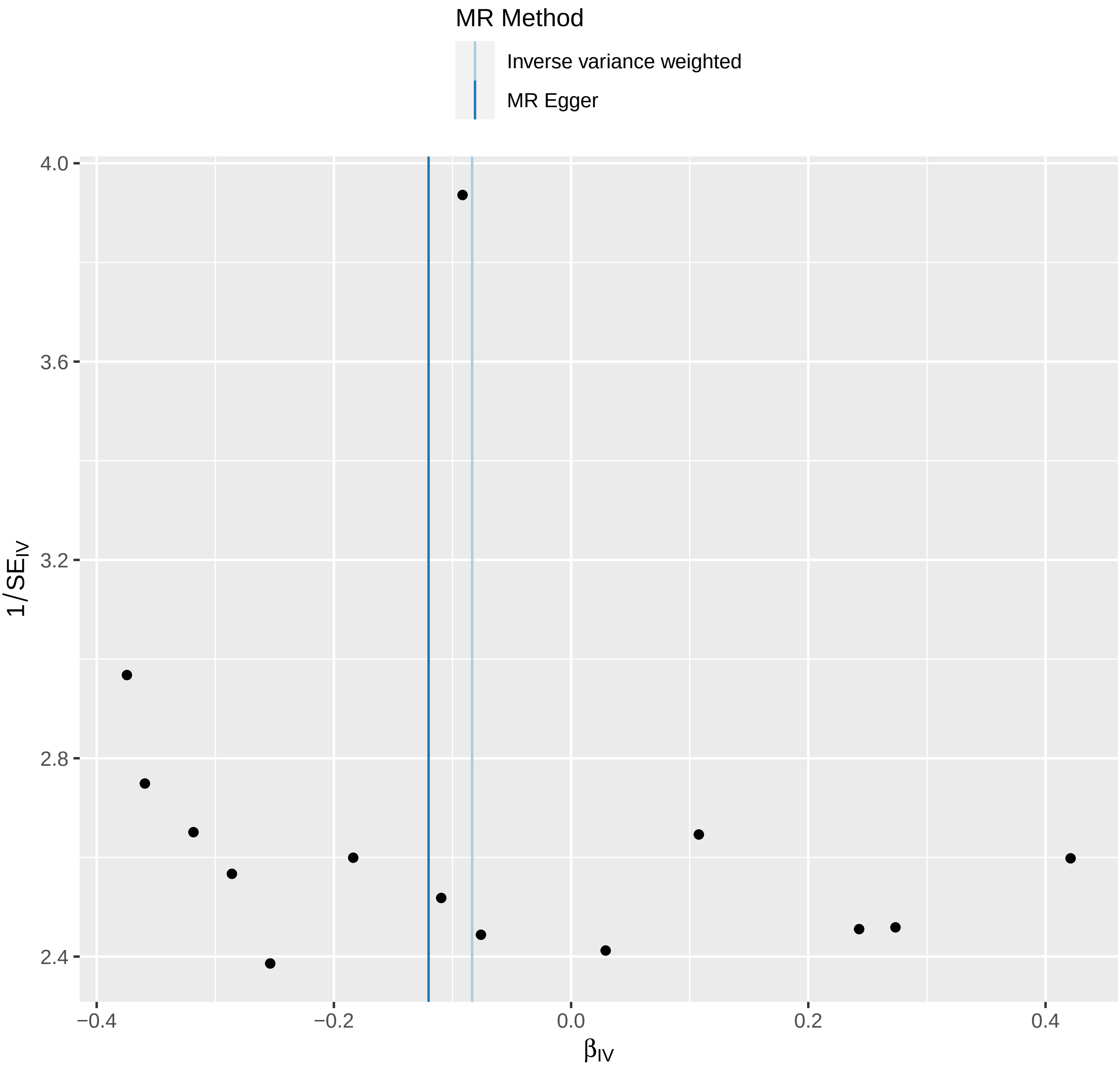

Batch 2−1641 : Gut microbiota abundance (genus Catenibacterium id.2153) on Dickkopf−related protein 1
with a threshold of 1e−05

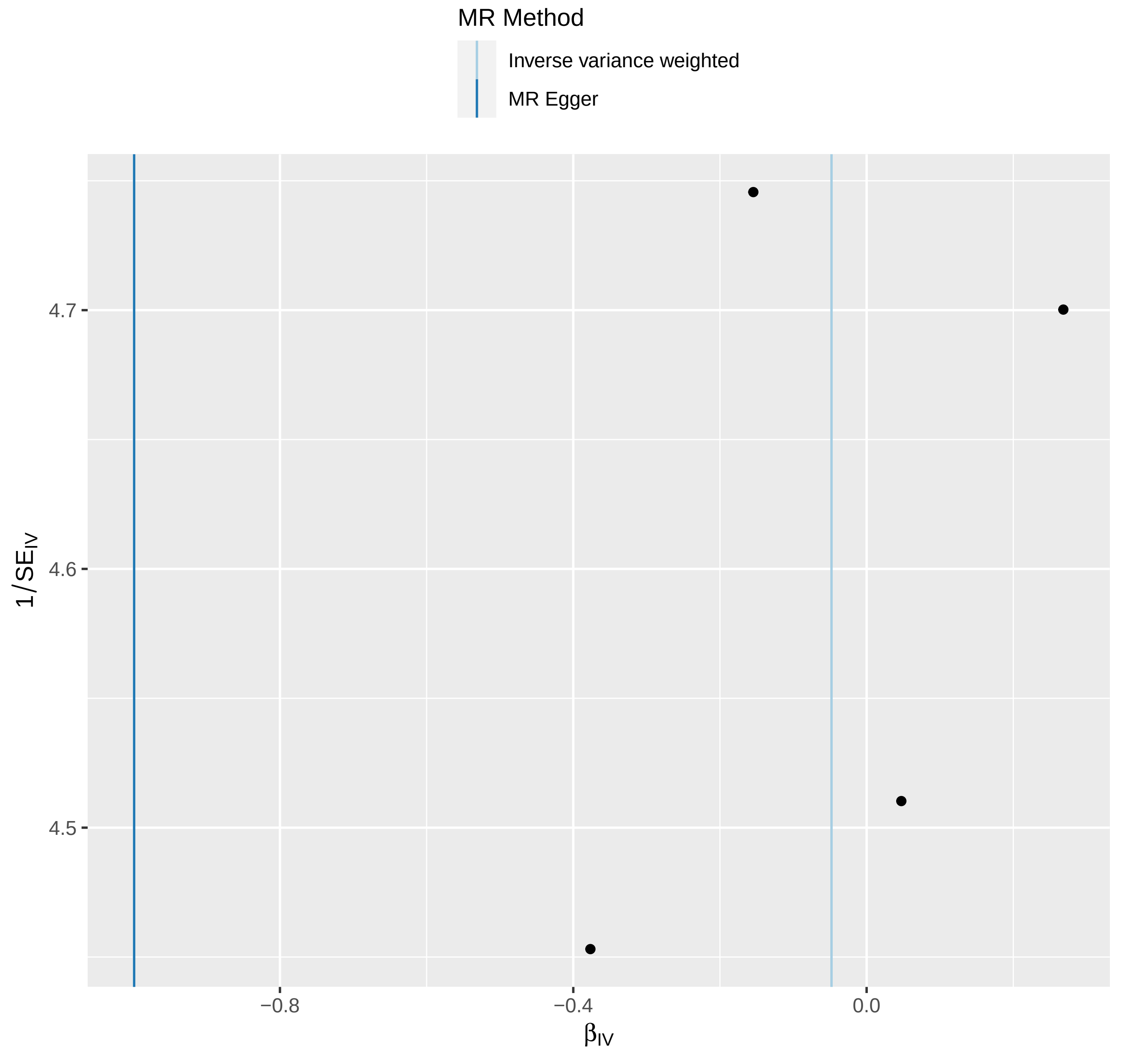

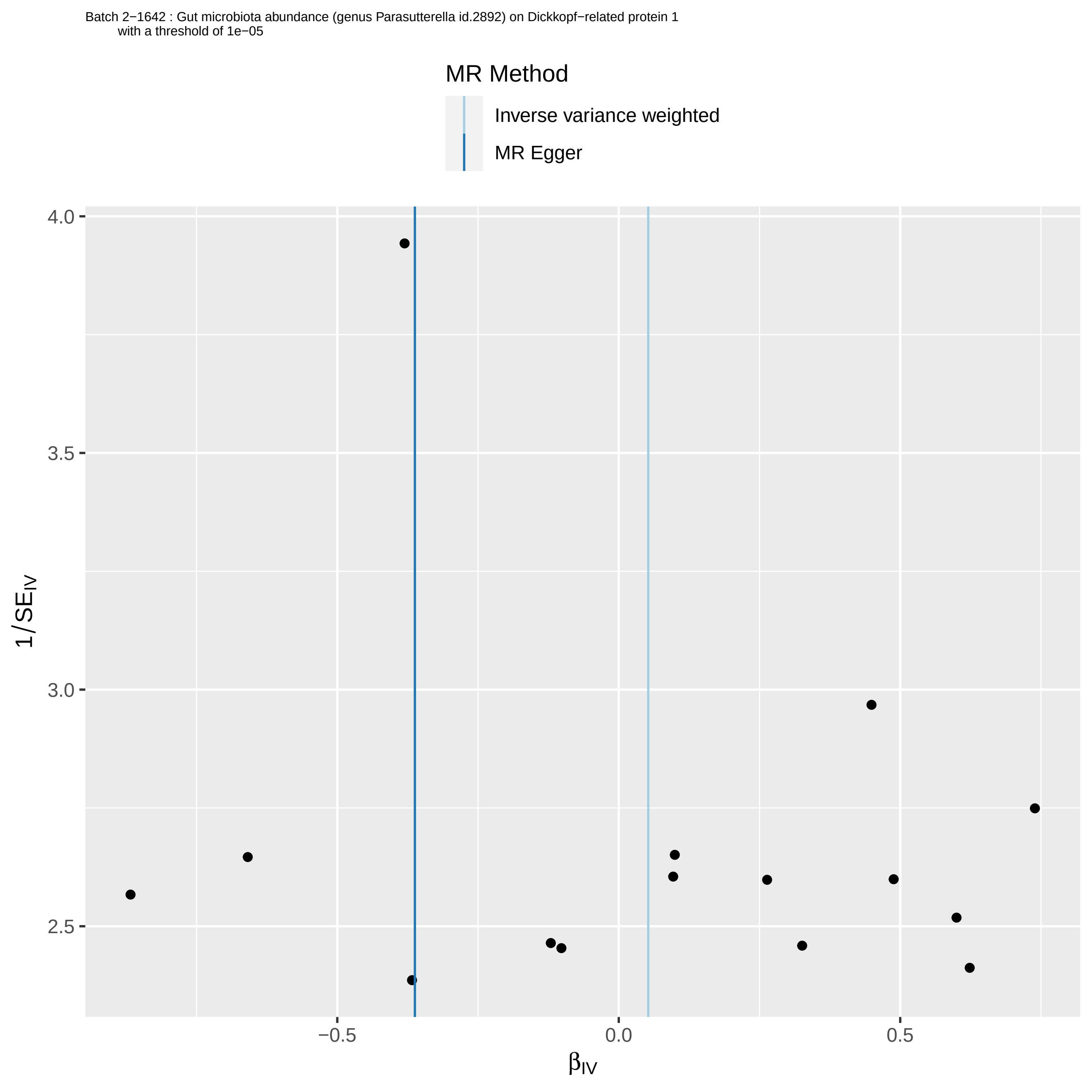

Batch 2−1642 : Gut microbiota abundance (genus Parasutterella id.2892) on Dickkopf−related protein 1
with a threshold of 1e−05
MR Method
Inverse variance weighted
MR Egger
4.0
3.5
3.0
2.5
1/SE_IV
−0.5
0.0
0.5
β_IV

Batch 2−1643 : Gut microbiota abundance (genus Catenibacterium id.2153) on Dickkopf−related protein 3
with a threshold of 1e−05

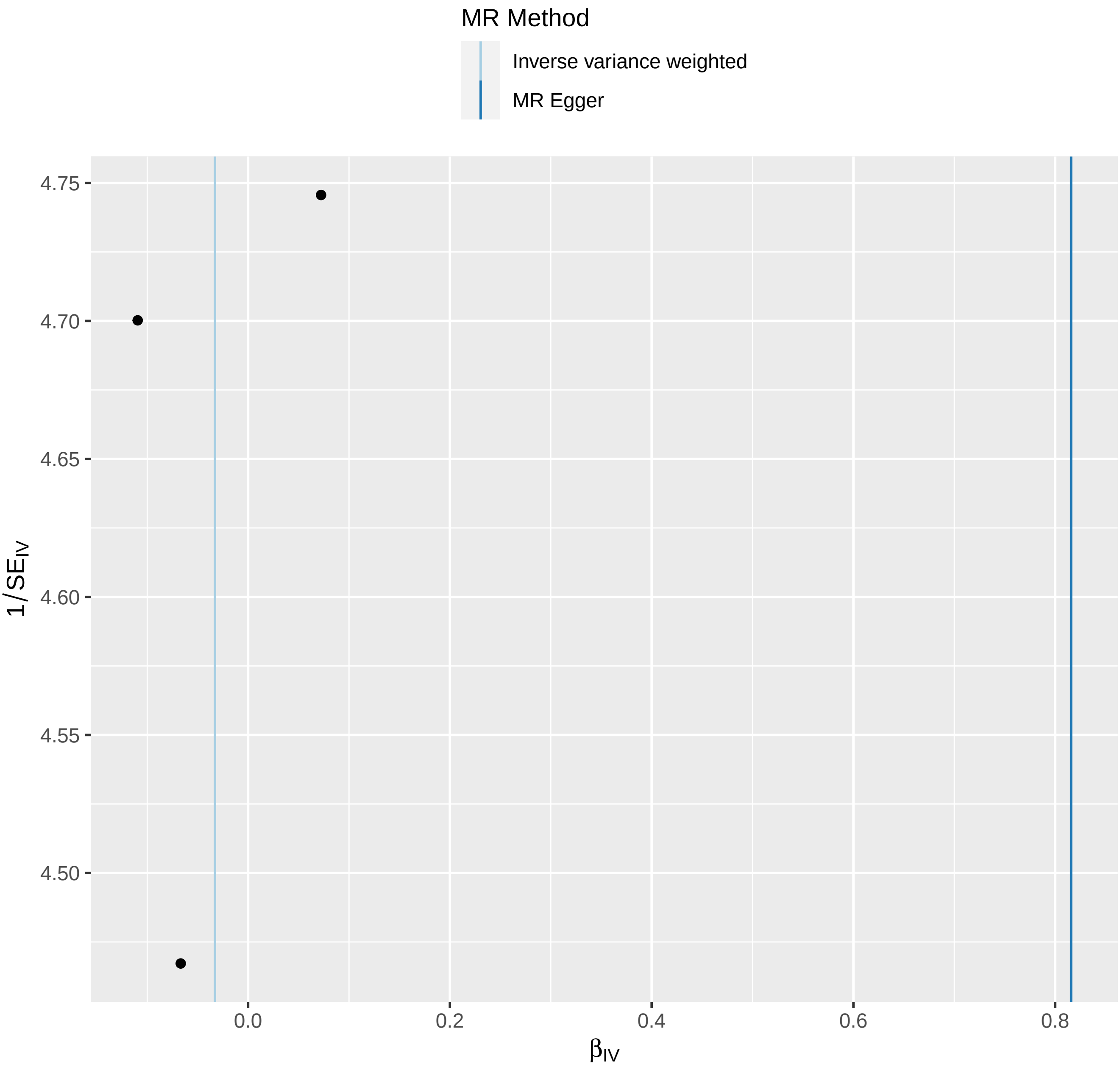

Batch 2−1644 : Gut microbiota abundance (genus Parasutterella id.2892) on Dickkopf−related protein 3
with a threshold of 1e−05

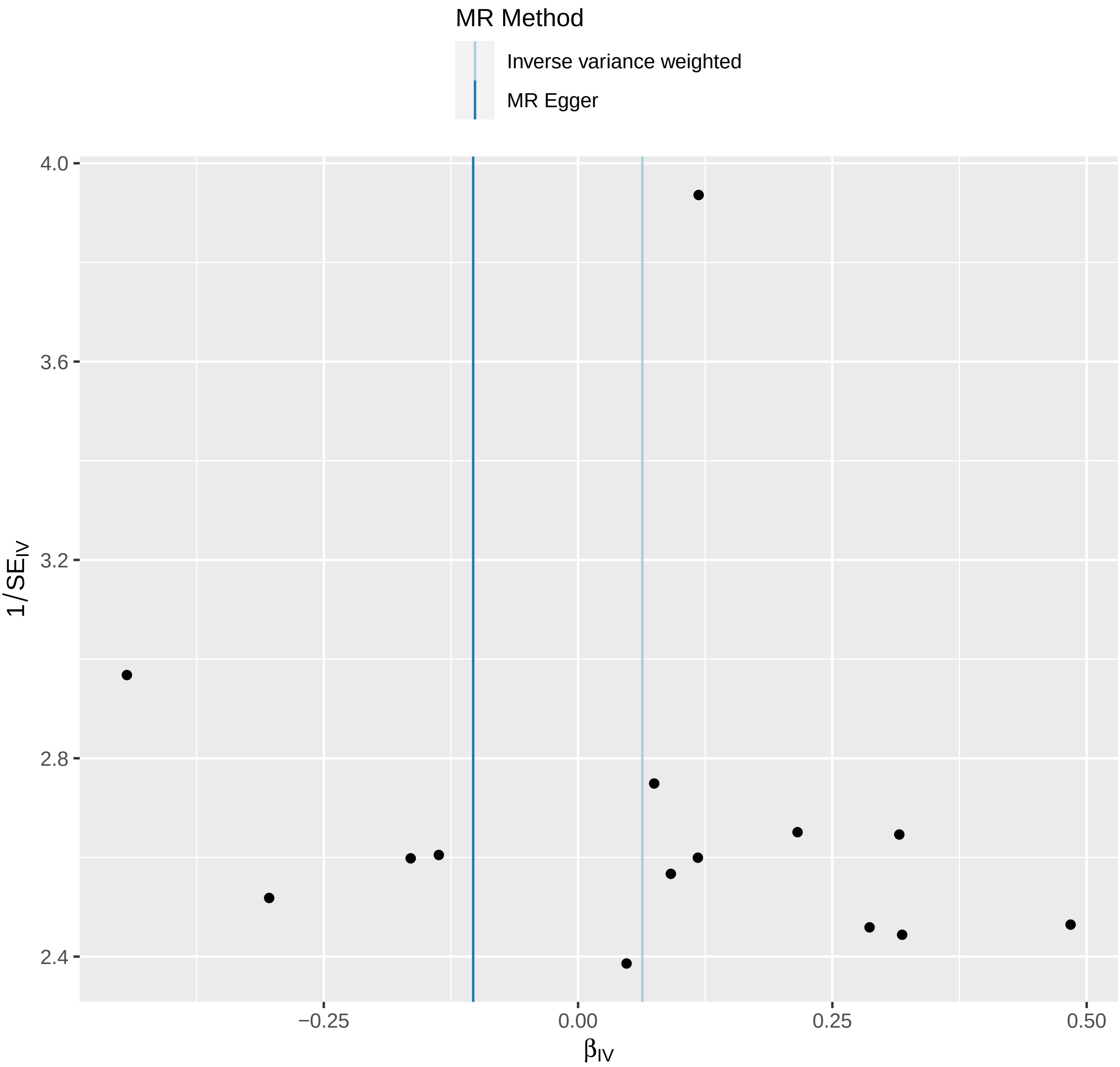

Batch 2−1645 : Gut microbiota abundance (genus Catenibacterium id.2153) on Dickkopf−related protein 4
with a threshold of 1e−05

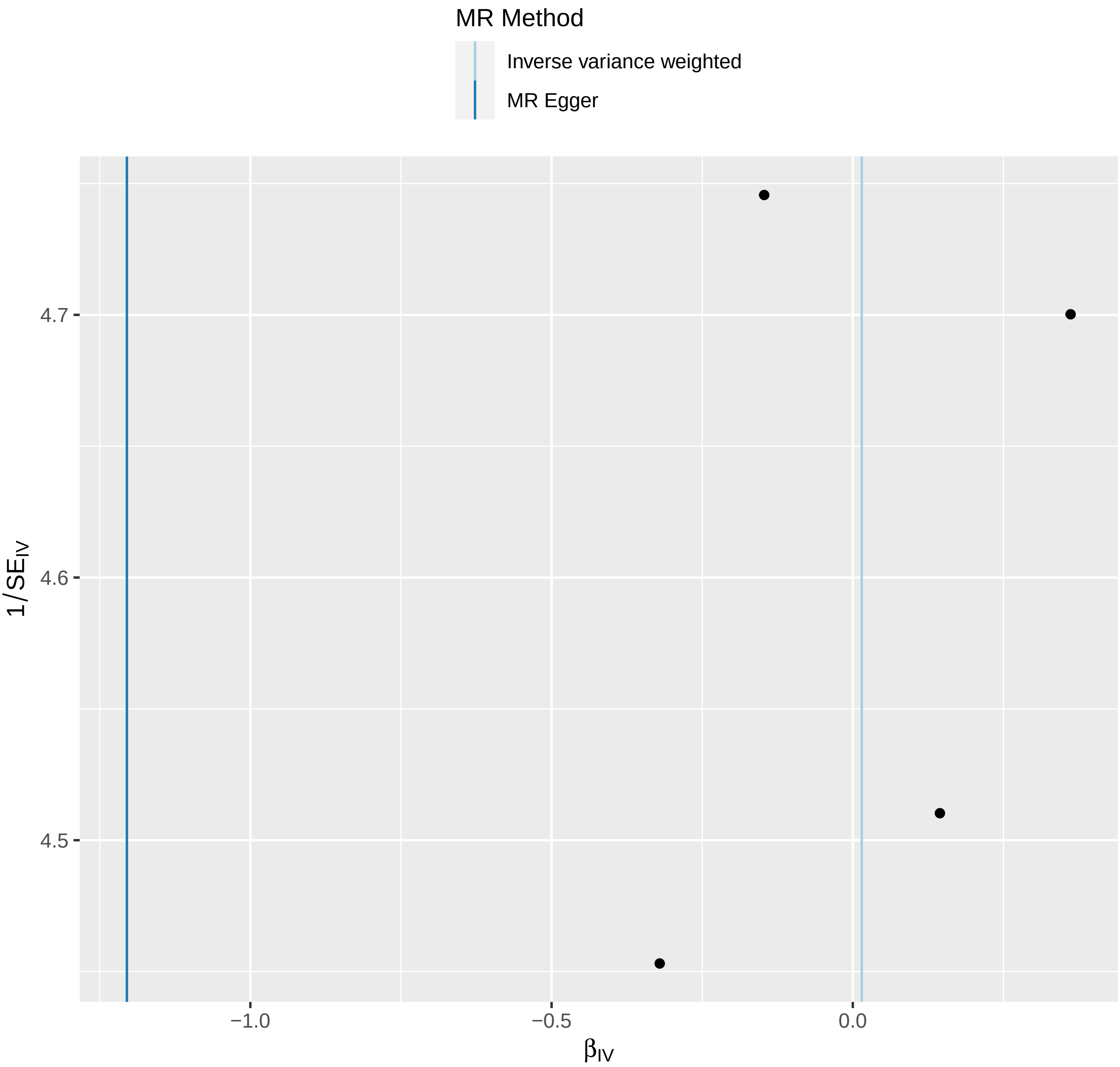

Batch 2−1646 : Gut microbiota abundance (genus Parasutterella id.2892) on Dickkopf−related protein 4
with a threshold of 1e−05

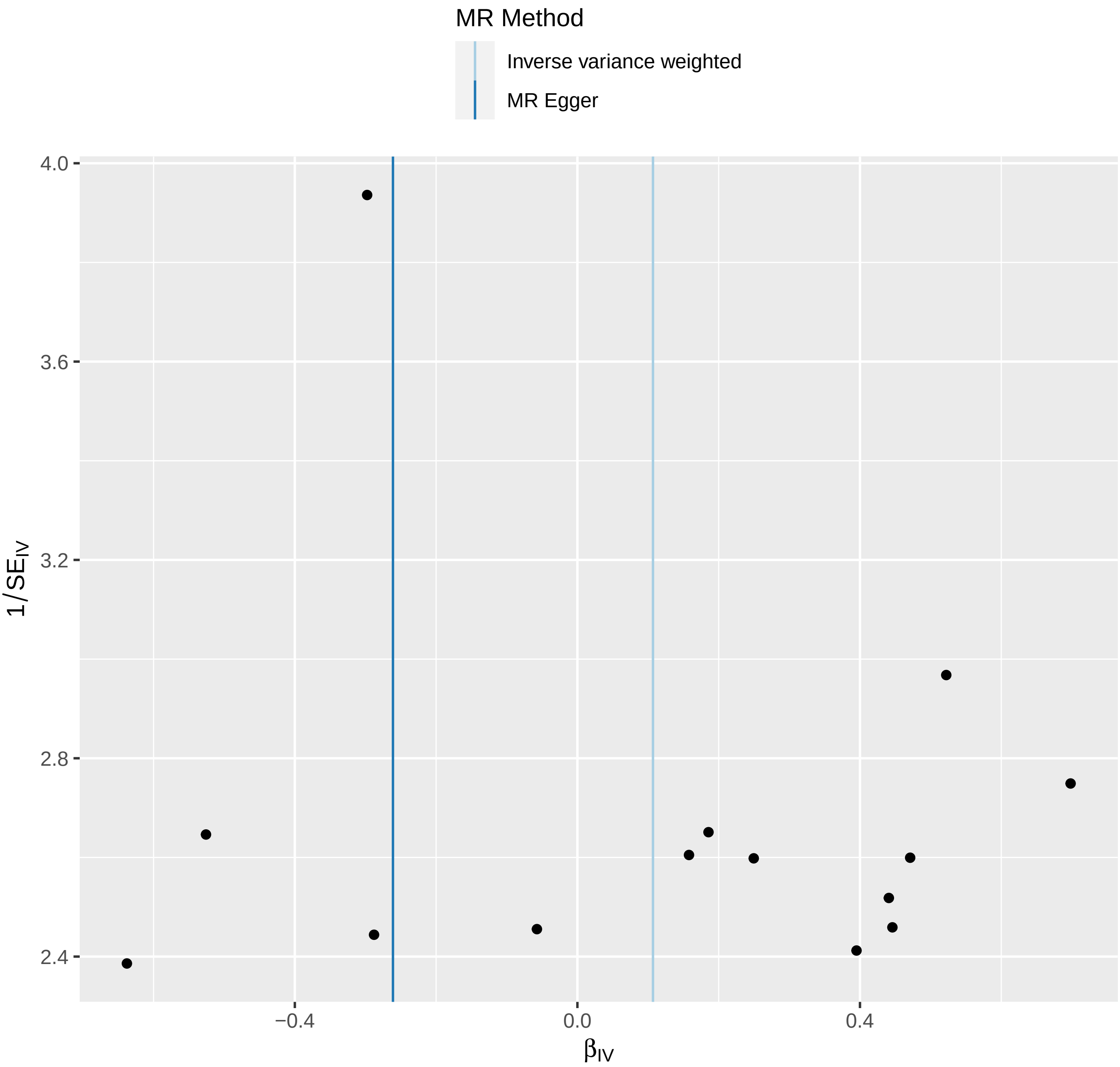

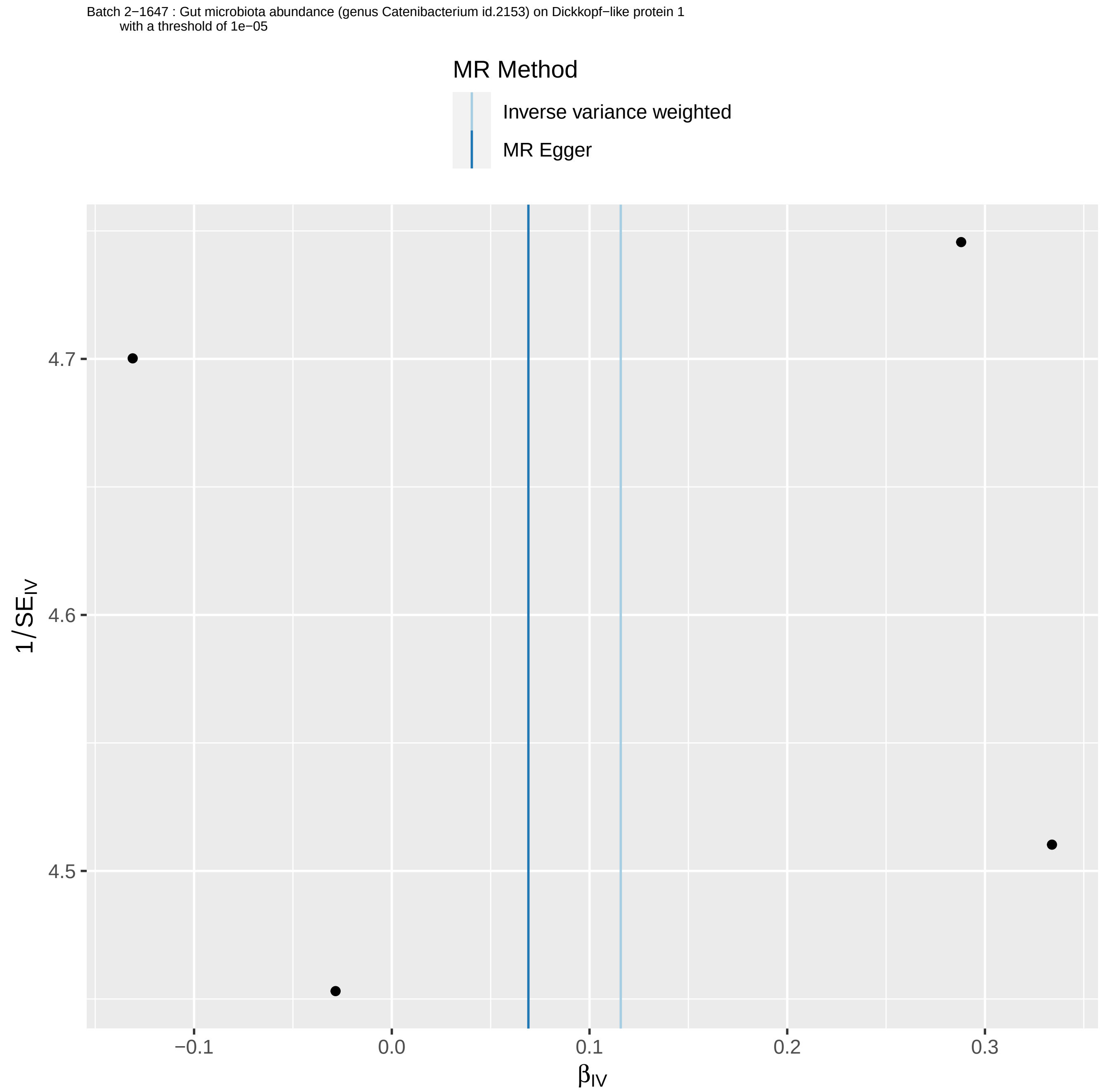
Batch 2−1647 : Gut microbiota abundance (genus Catenibacterium id.2153) on Dickkopf−like protein 1
with a threshold of 1e−05
MR Method
Inverse variance weighted
MR Egger
4.7
4.6
4.5
1/SE_IV
−0.1
0.0
0.1
0.2
0.3
β_IV

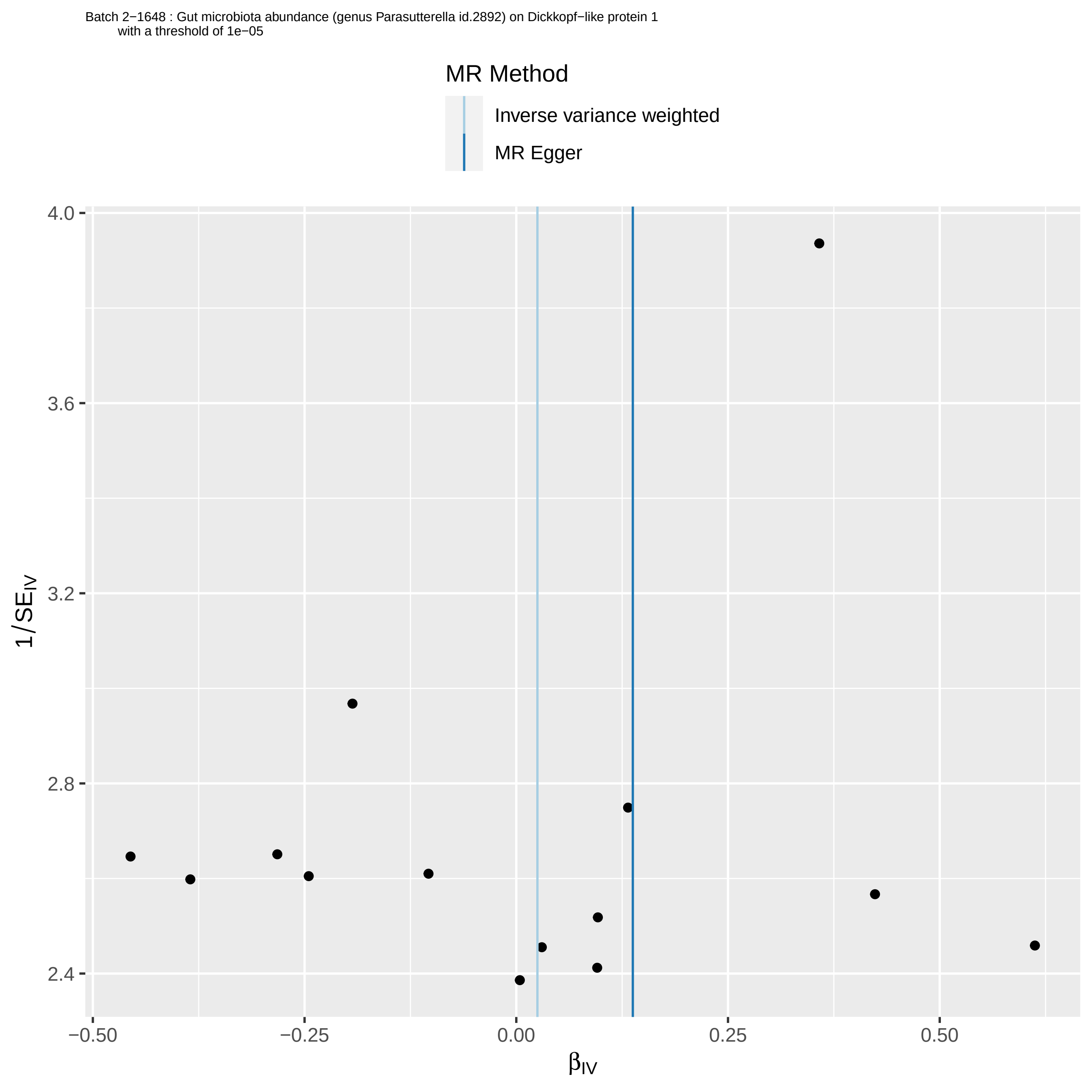
Batch 2−1648 : Gut microbiota abundance (genus Parasutterella id.2892) on Dickkopf−like protein 1
with a threshold of 1e−05
MR Method
Inverse variance weighted
MR Egger
4.0
3.6
3.2
2.8
2.4
1/SE_IV
−0.50
−0.25
0.00
0.25
0.50
β_IV

Batch 2−1649 : Gut microbiota abundance (genus Catenibacterium id.2153) on Dihydrolipoyl dehydrogenase, mitochondrial
with a threshold of 1e−05

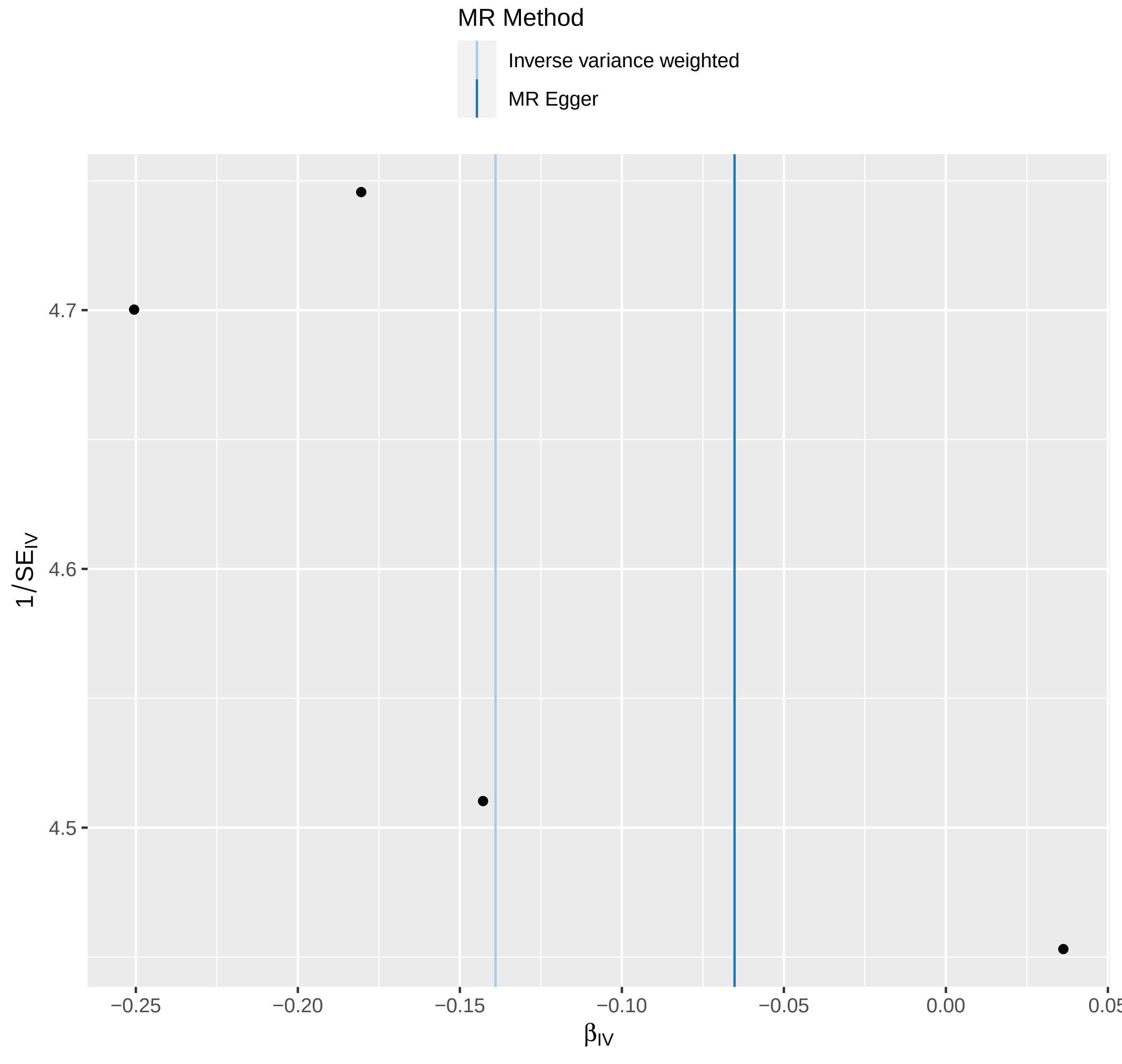

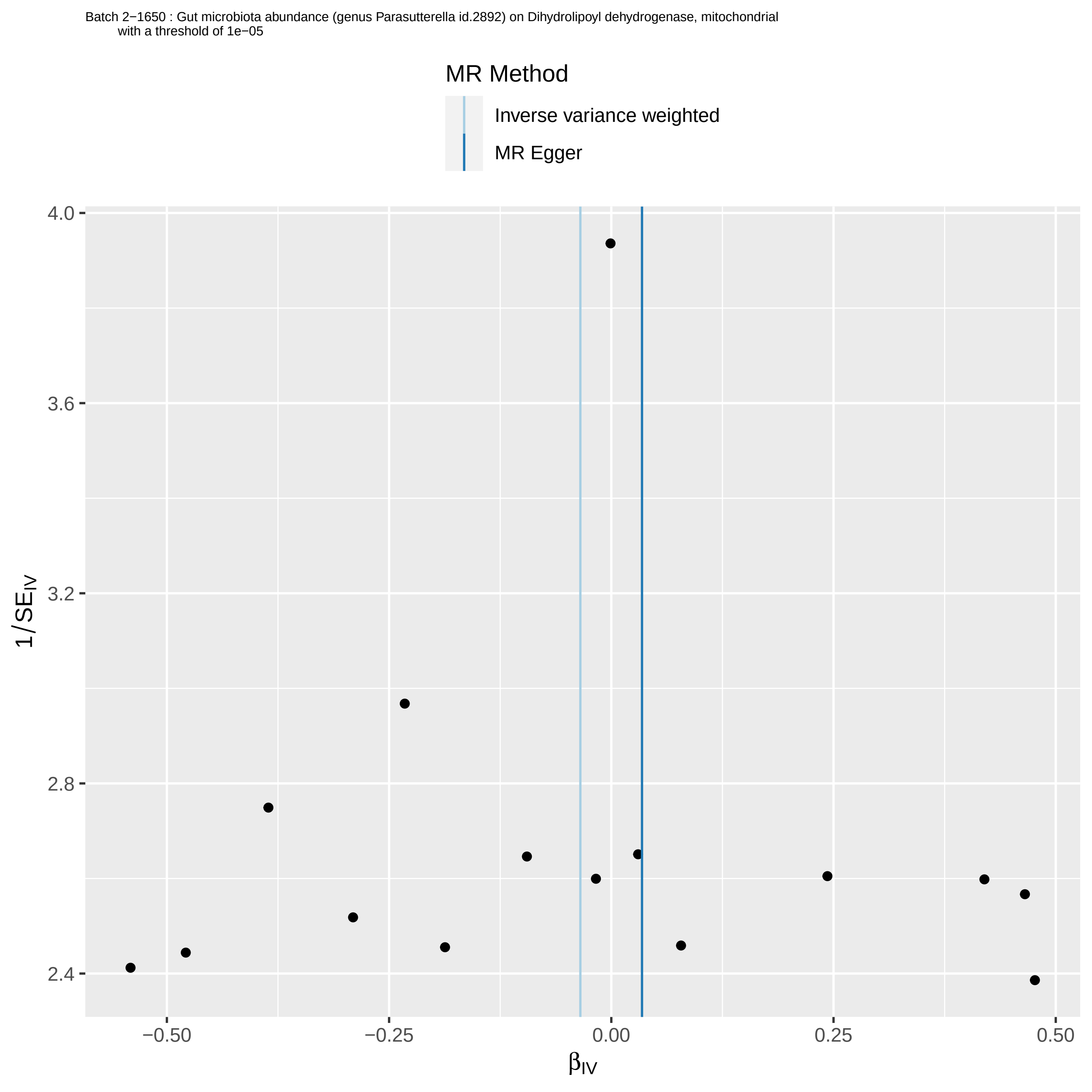

Batch 2−1650 : Gut microbiota abundance (genus Parasutterella id.2892) on Dihydrolipoyl dehydrogenase, mitochondrial
with a threshold of 1e−05
MR Method
Inverse variance weighted
MR Egger
4.0
3.6
3.2
2.8
2.4
1/SE_IV
−0.50
−0.25
0.00
0.25
0.50
β_IV

Batch 2−1651 : Gut microbiota abundance (genus Catenibacterium id.2153) on Disks large homolog 3
with a threshold of 1e−05

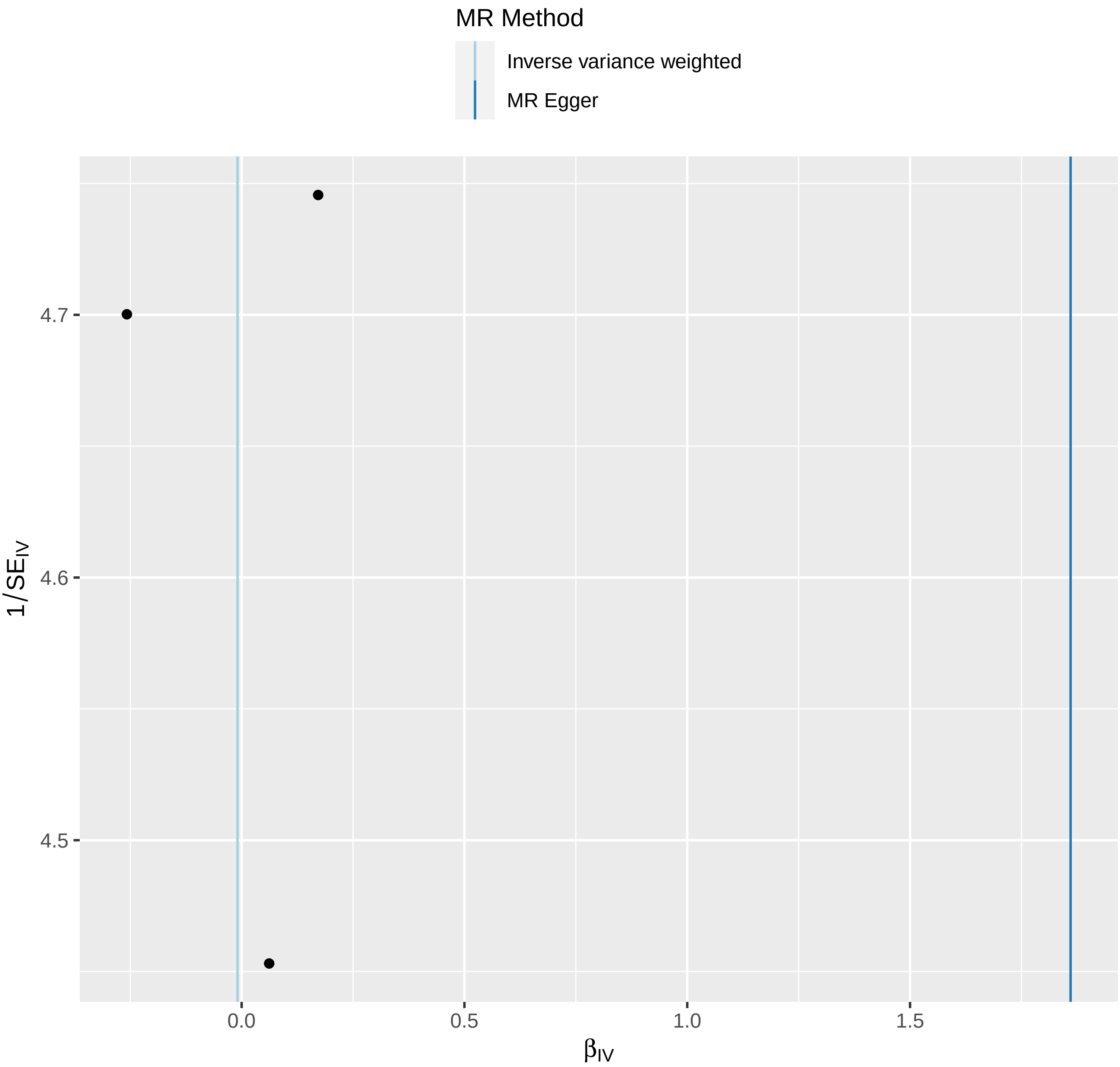

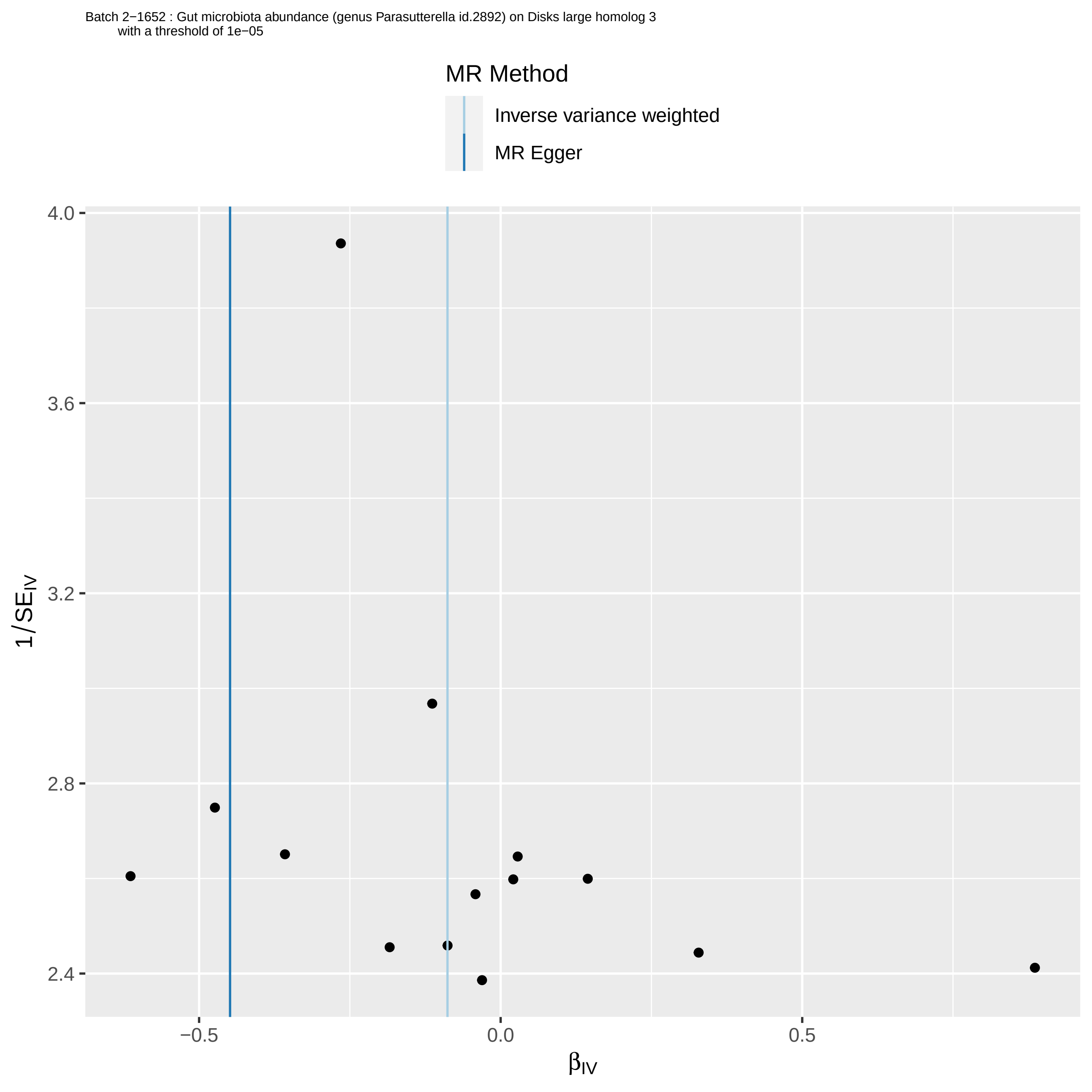

Batch 2−1652 : Gut microbiota abundance (genus Parasutterella id.2892) on Disks large homolog 3
with a threshold of 1e−05
MR Method
Inverse variance weighted
MR Egger
4.0
3.6
3.2
2.8
2.4
1/SE_IV
−0.5
0.0
0.5
β_IV

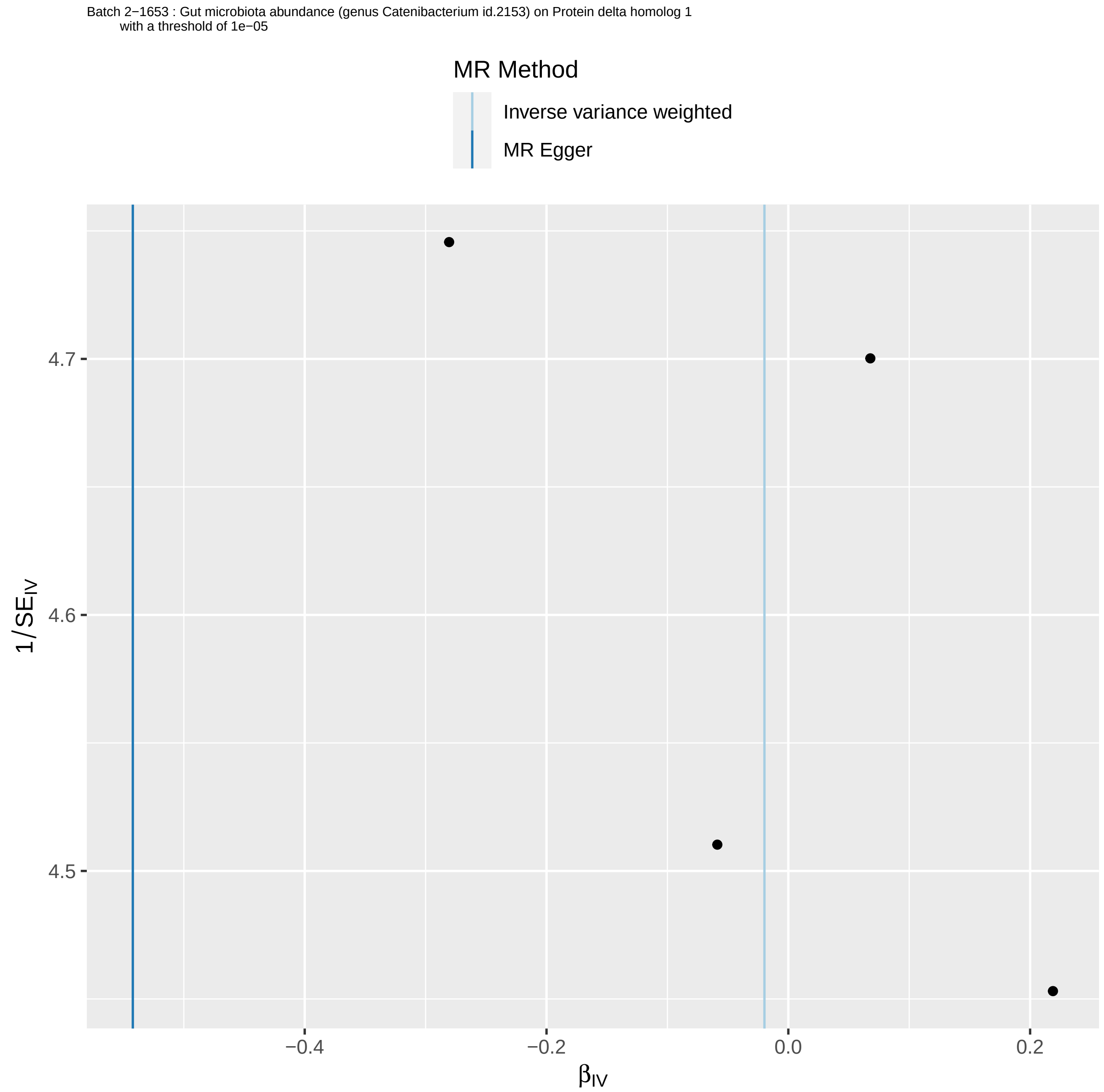

Batch 2−1653 : Gut microbiota abundance (genus Catenibacterium id.2153) on Protein delta homolog 1
with a threshold of 1e−05
MR Method
Inverse variance weighted
MR Egger
4.7
4.6
4.5
1/SE_IV
−0.4
−0.2
0.0
0.2
β_IV

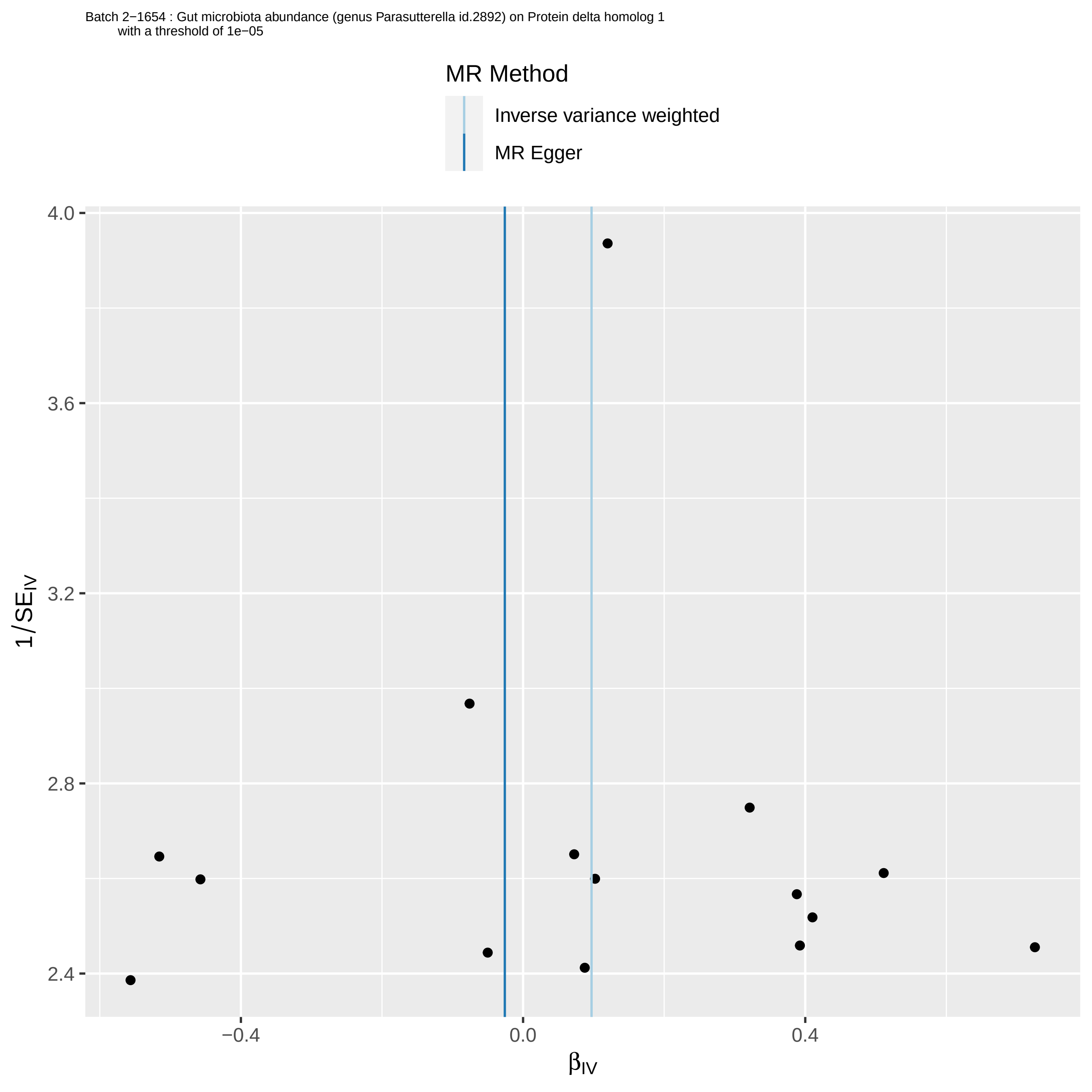
Batch 2−1654 : Gut microbiota abundance (genus Parasutterella id.2892) on Protein delta homolog 1
with a threshold of 1e−05
MR Method
Inverse variance weighted
MR Egger
4.0
3.6
3.2
2.8
2.4
1/SE_IV
−0.4
0.0
0.4
β_IV

Batch 2−1655 : Gut microbiota abundance (genus Catenibacterium id.2153) on Protein delta homolog 1
with a threshold of 1e−05

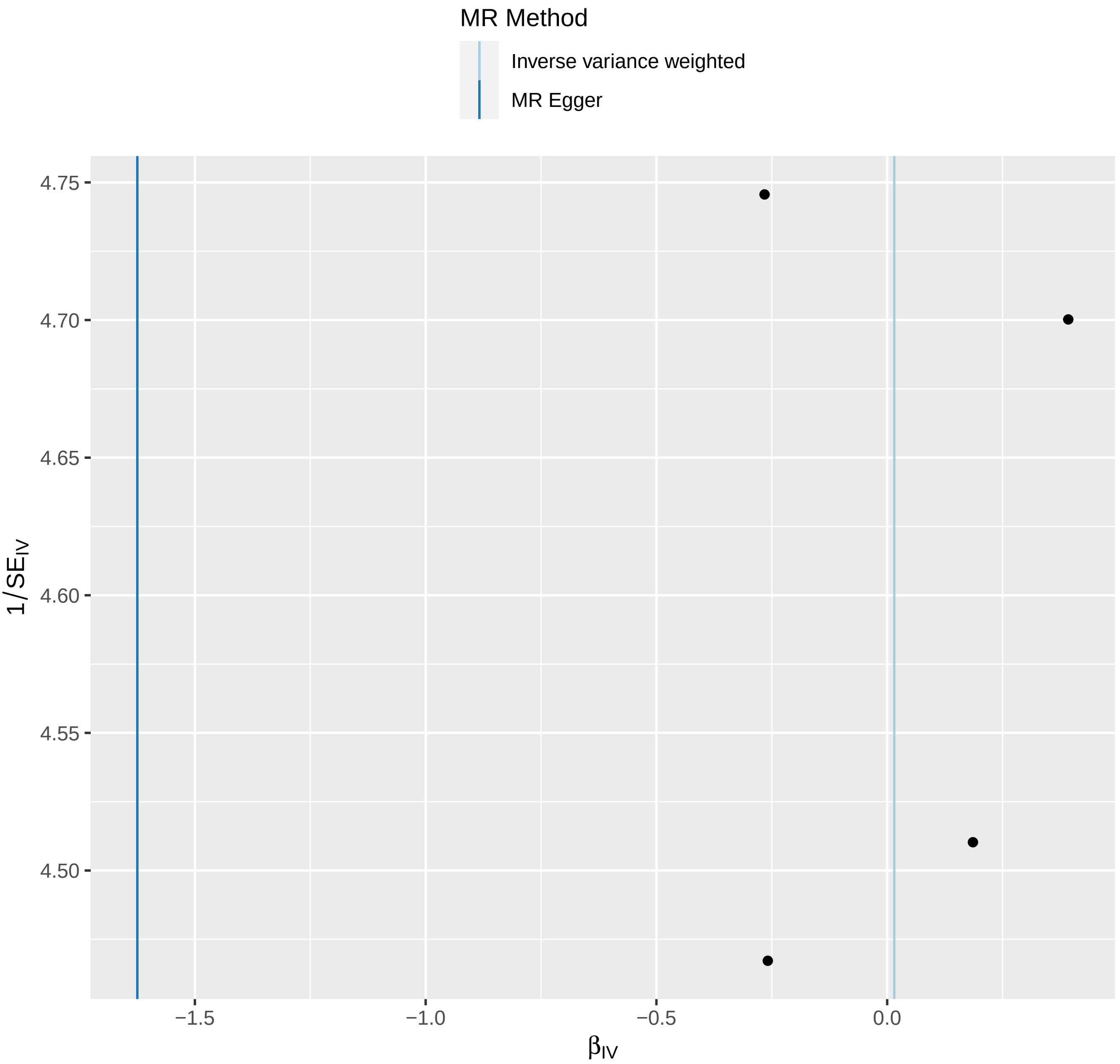

Batch 2−1656 : Gut microbiota abundance (genus Parasutterella id.2892) on Protein delta homolog 1
with a threshold of 1e−05

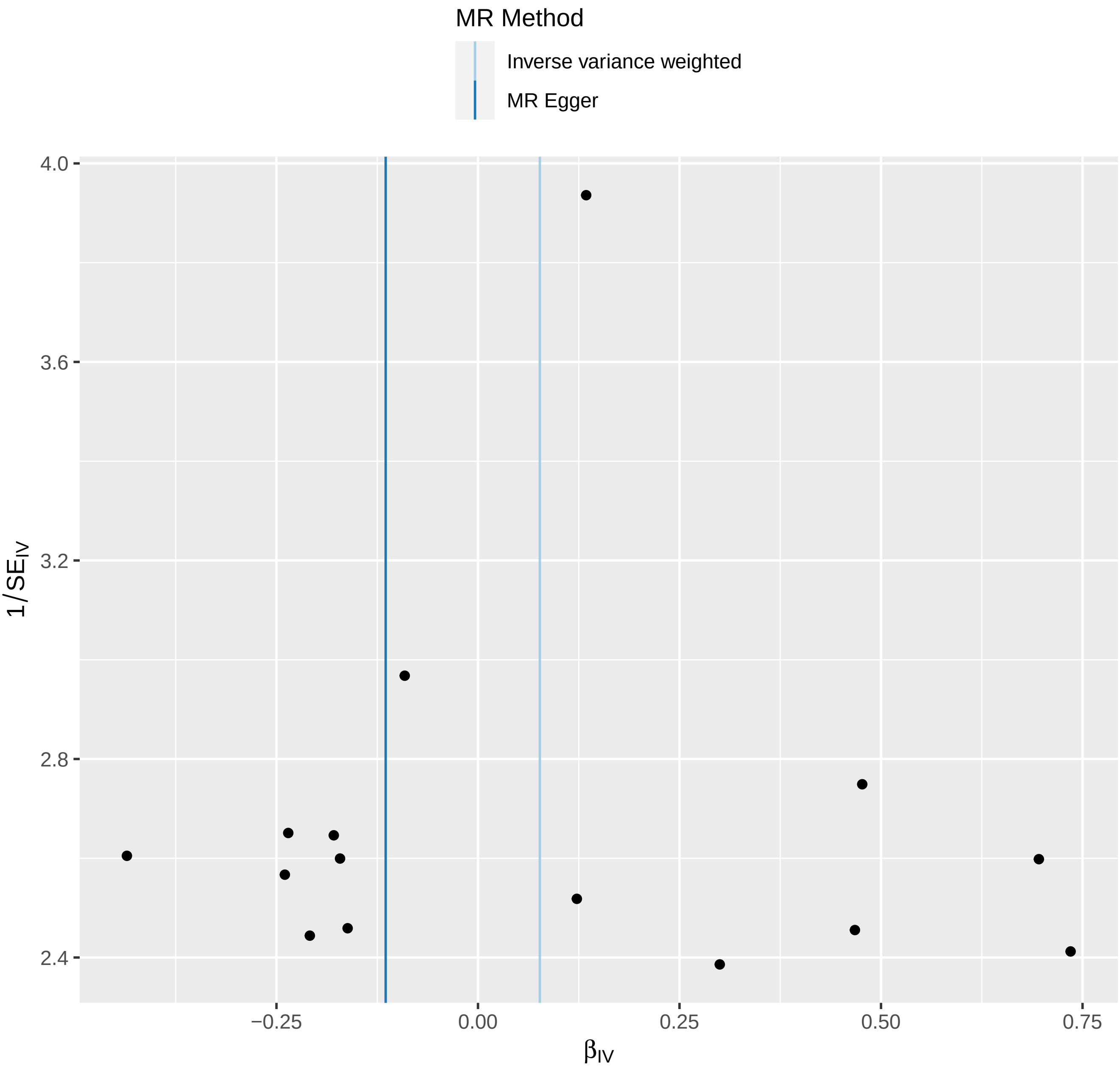

Batch 2−1657 : Gut microbiota abundance (genus Catenibacterium id.2153) on Protein delta homolog 2
with a threshold of 1e−05

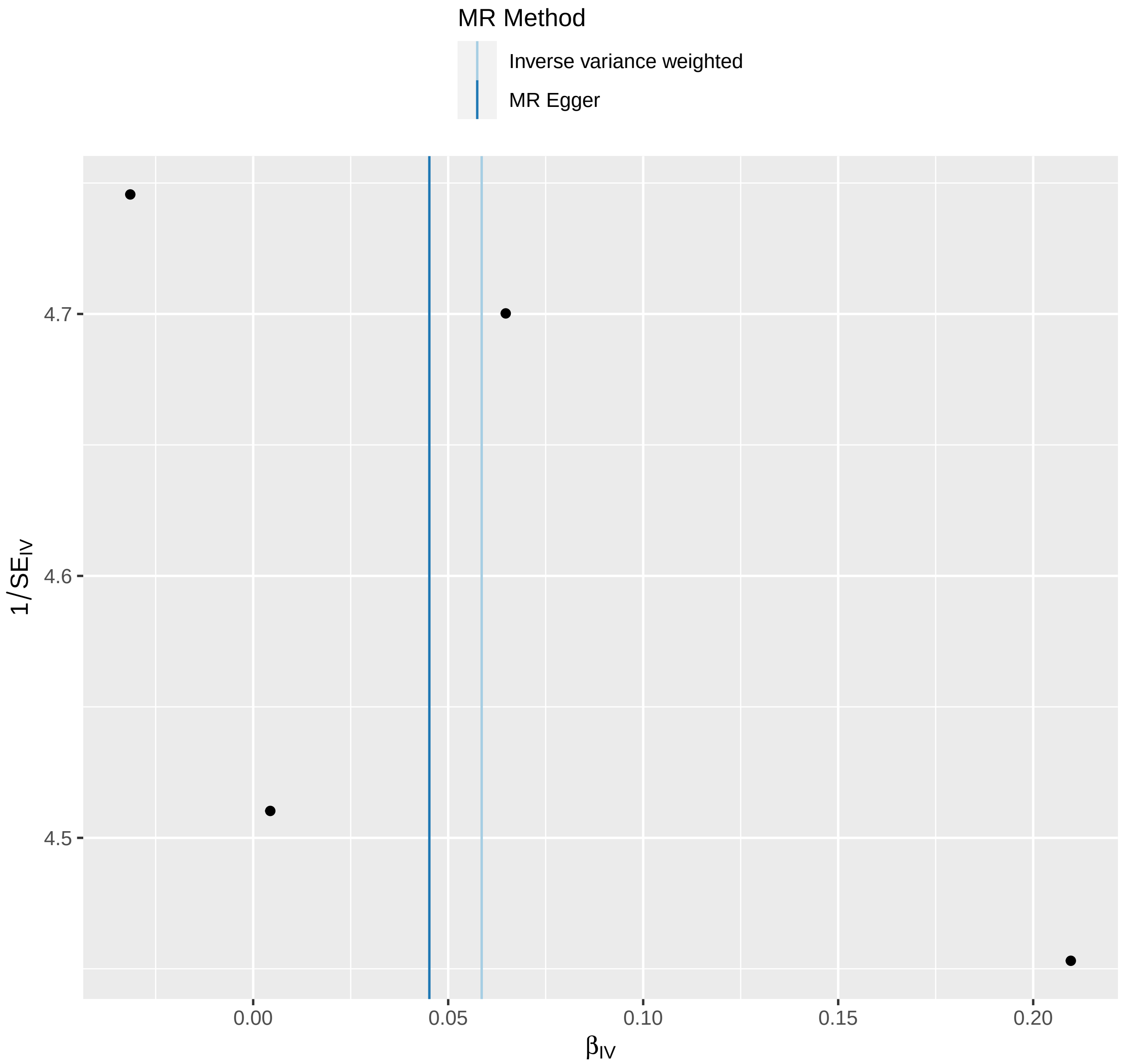

Batch 2−1658 : Gut microbiota abundance (genus Parasutterella id.2892) on Protein delta homolog 2
with a threshold of 1e−05

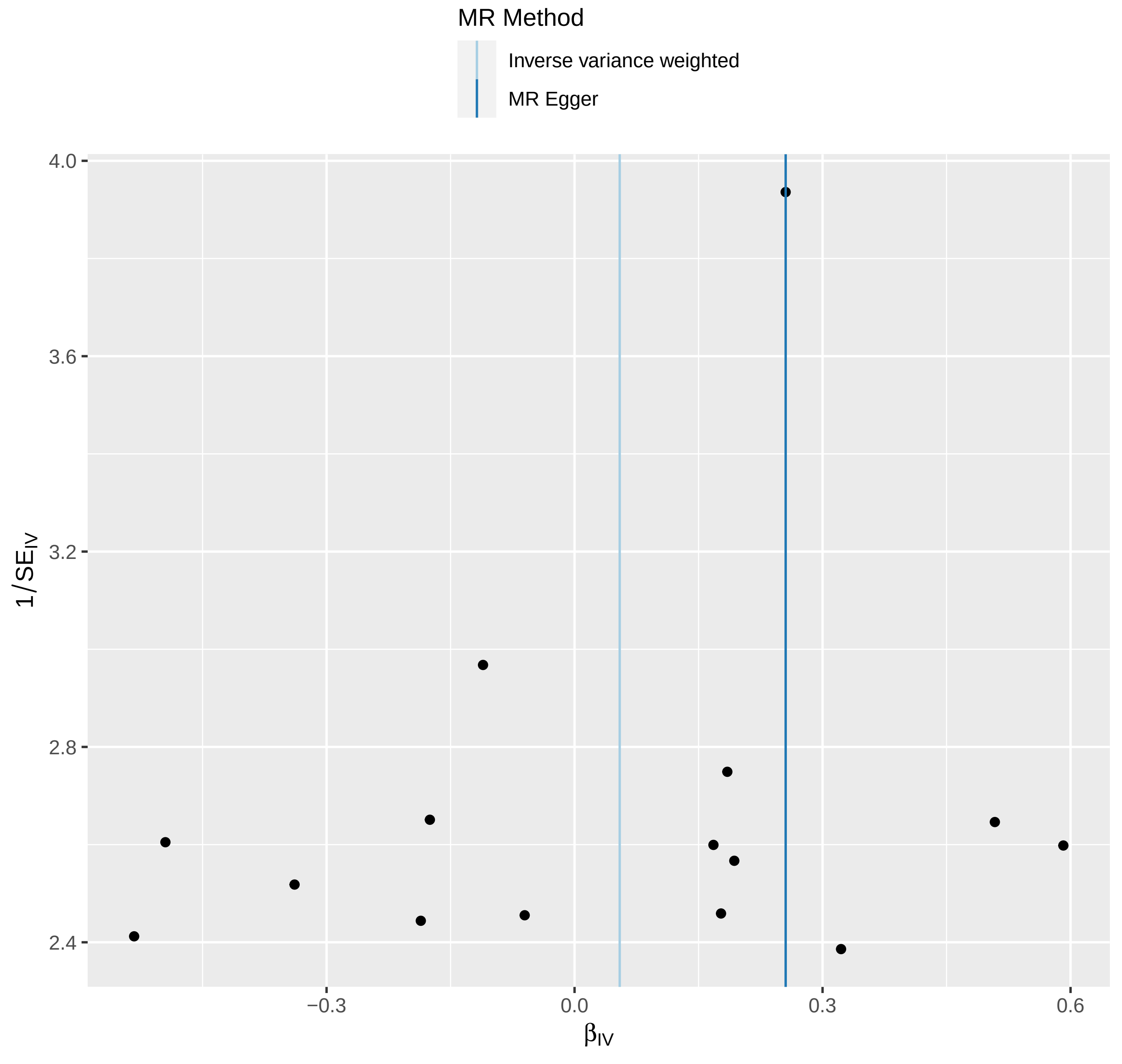

Batch 2−1659 : Gut microbiota abundance (genus Catenibacterium id.2153) on Delta−like protein 1
with a threshold of 1e−05

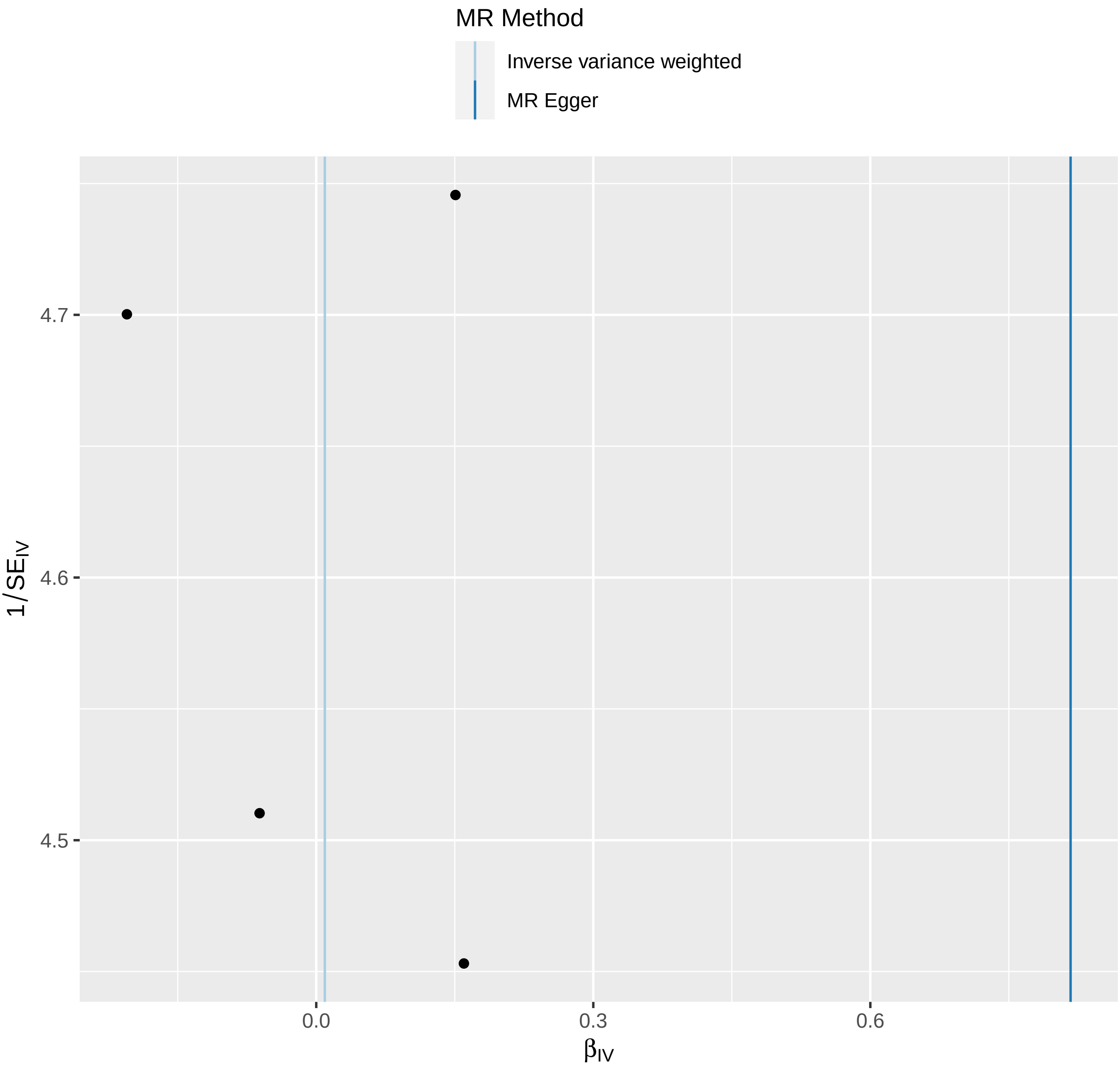

Batch 2−1660 : Gut microbiota abundance (genus Parasutterella id.2892) on Delta−like protein 1
with a threshold of 1e−05

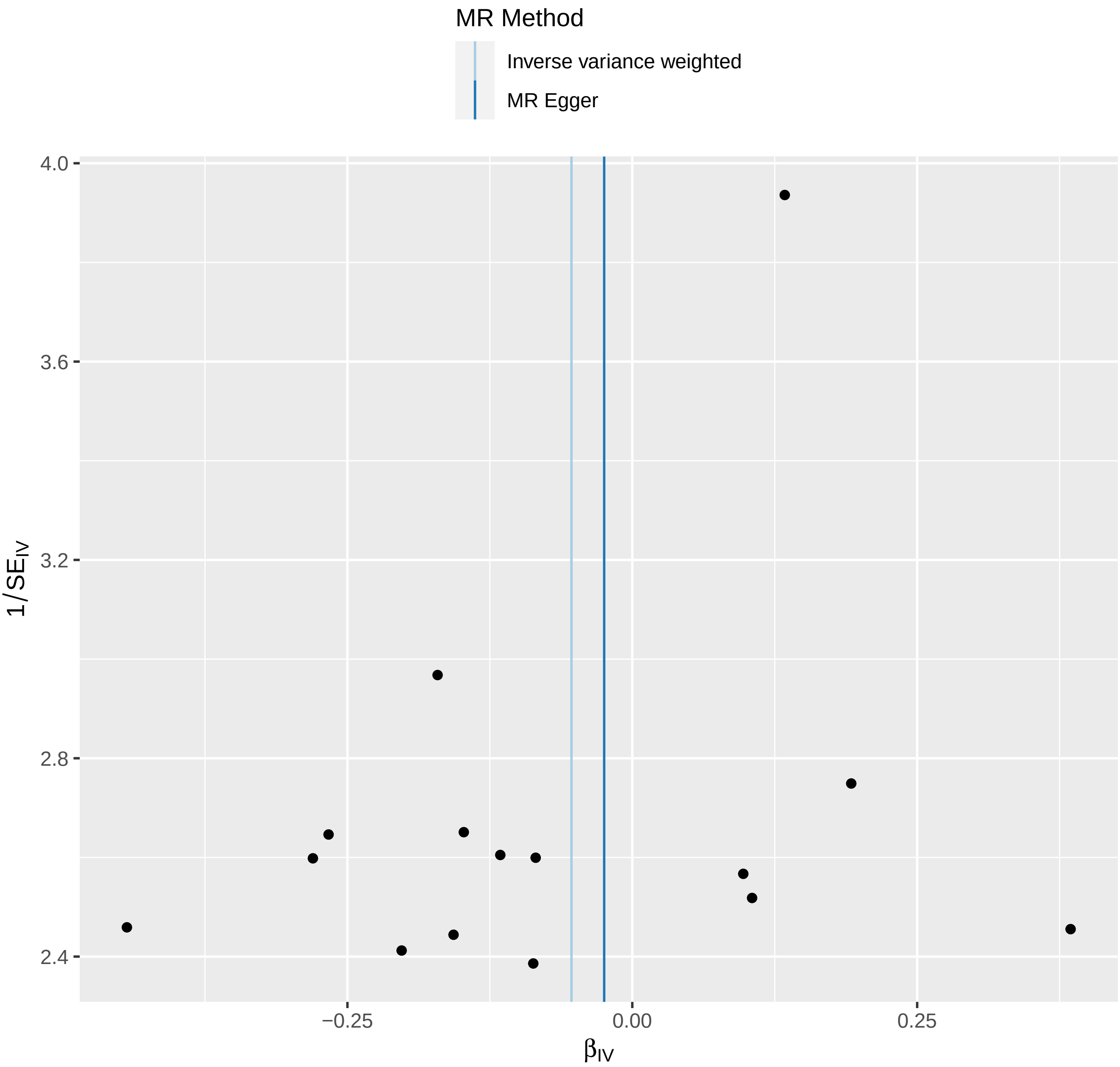

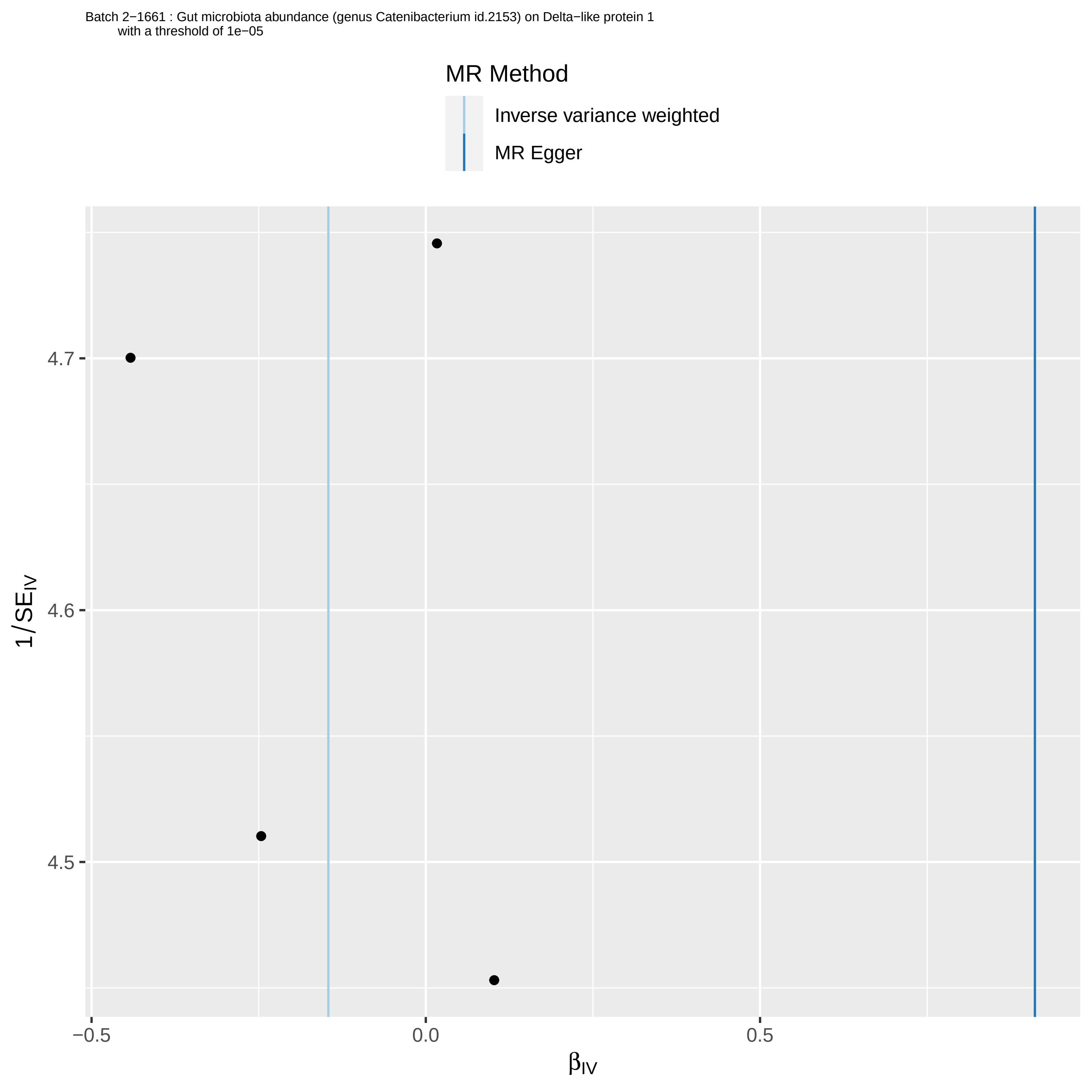
Batch 2−1661 : Gut microbiota abundance (genus Catenibacterium id.2153) on Delta−like protein 1
with a threshold of 1e−05
MR Method
Inverse variance weighted
MR Egger
4.7
4.6
4.5
1/SE$_{IV}$
−0.5
0.0
0.5
β_{IV}

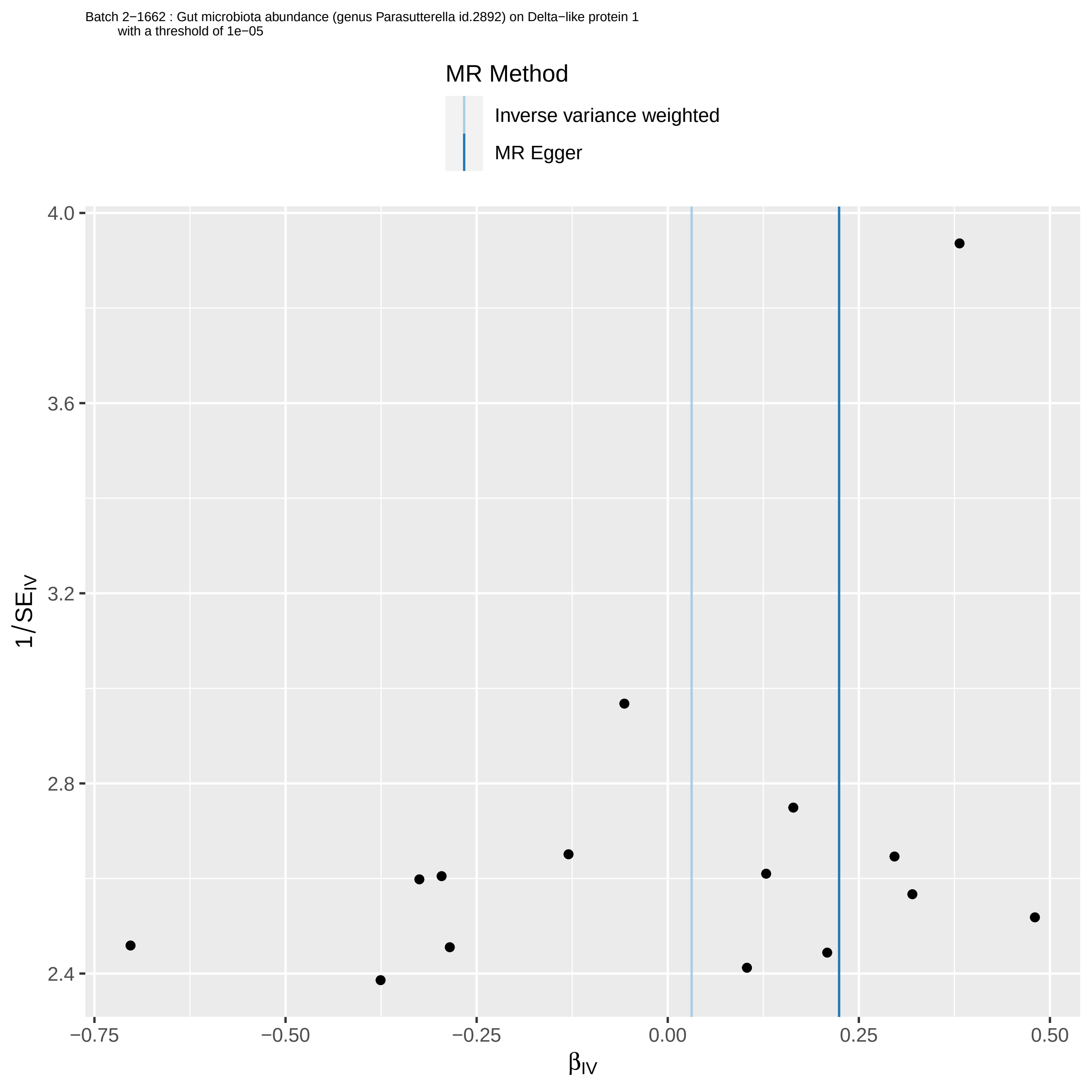

Batch 2−1662 : Gut microbiota abundance (genus Parasutterella id.2892) on Delta−like protein 1
with a threshold of 1e−05
MR Method
Inverse variance weighted
MR Egger
4.0
3.6
3.2
2.8
2.4
1/SE_IV
−0.75
−0.50
−0.25
0.00
0.25
0.50
β_IV

Batch 2−1663 : Gut microbiota abundance (genus Catenibacterium id.2153) on Delta−like protein 4
with a threshold of 1e−05

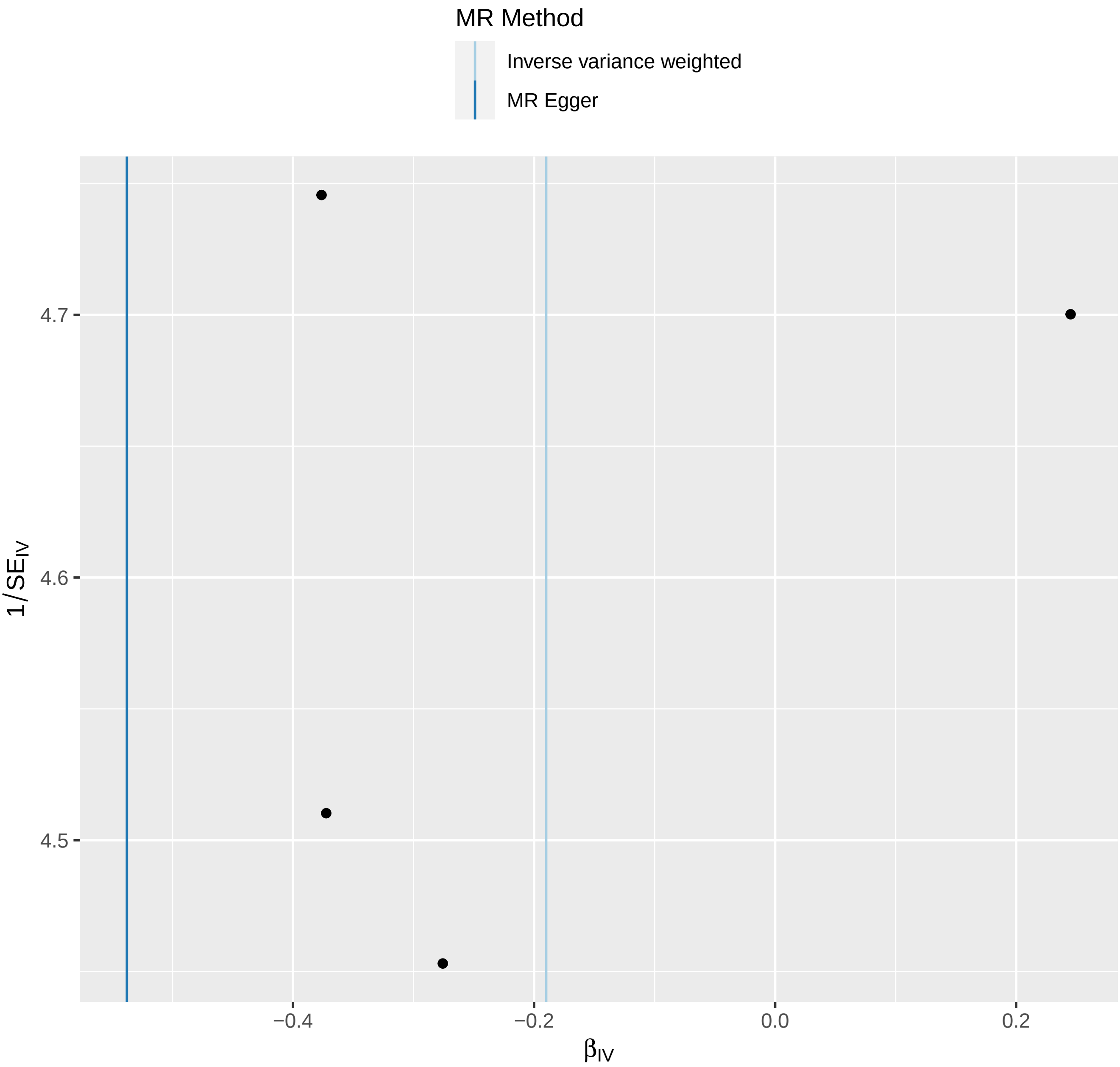

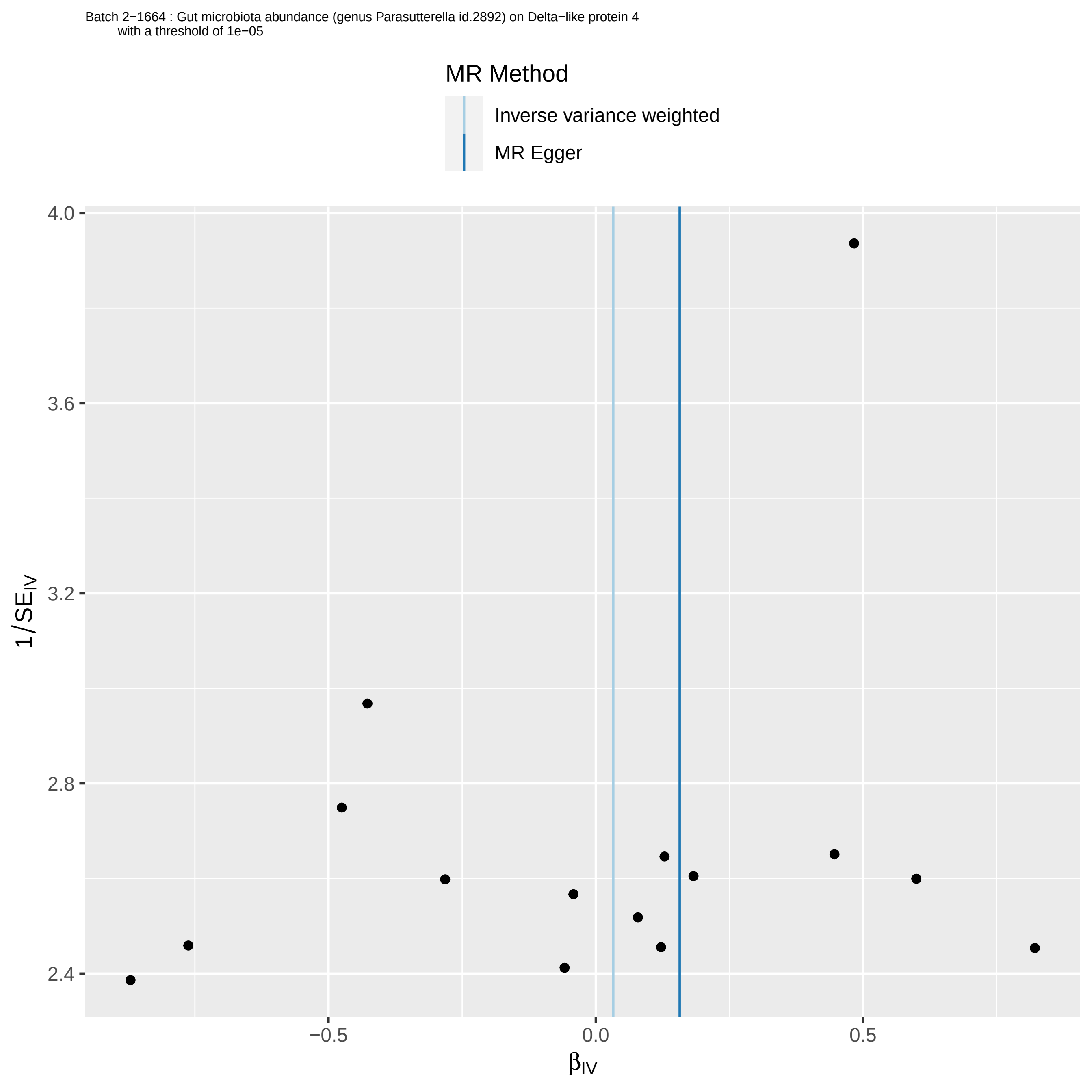
Batch 2−1664 : Gut microbiota abundance (genus Parasutterella id.2892) on Delta−like protein 4
with a threshold of 1e−05
MR Method
Inverse variance weighted
MR Egger
4.0
3.6
3.2
2.8
2.4
1/SE_IV
−0.5
0.0
0.5
β_IV

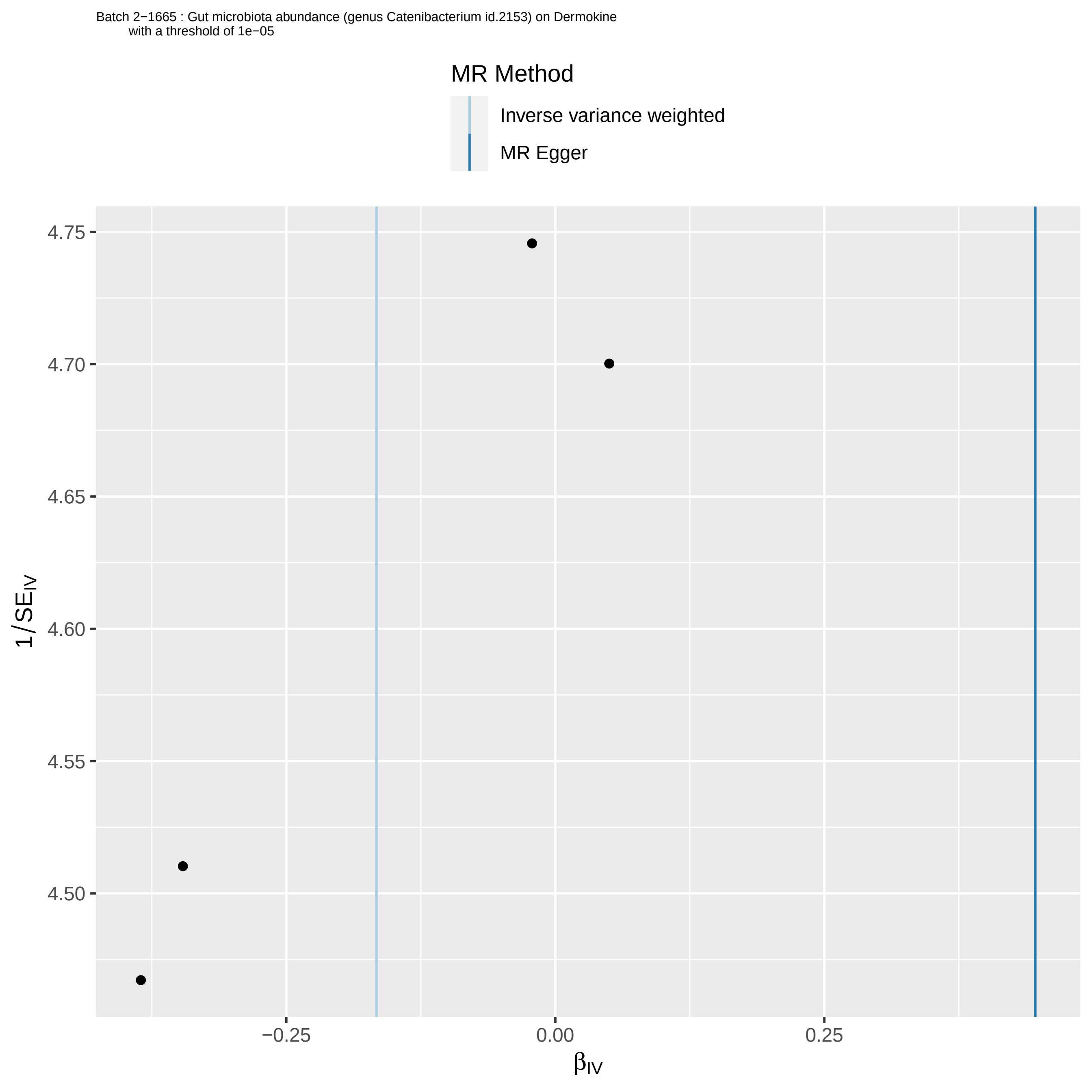
Batch 2−1665 : Gut microbiota abundance (genus Catenibacterium id.2153) on Dermokine
with a threshold of 1e−05
MR Method
Inverse variance weighted
MR Egger
4.75
4.70
4.65
4.60
4.55
4.50
1/SE$_{IV}$
−0.25
0.00
0.25
β$_{IV}$

Batch 2−1666 : Gut microbiota abundance (genus Parasutterella id.2892) on Dermokine
with a threshold of 1e−05

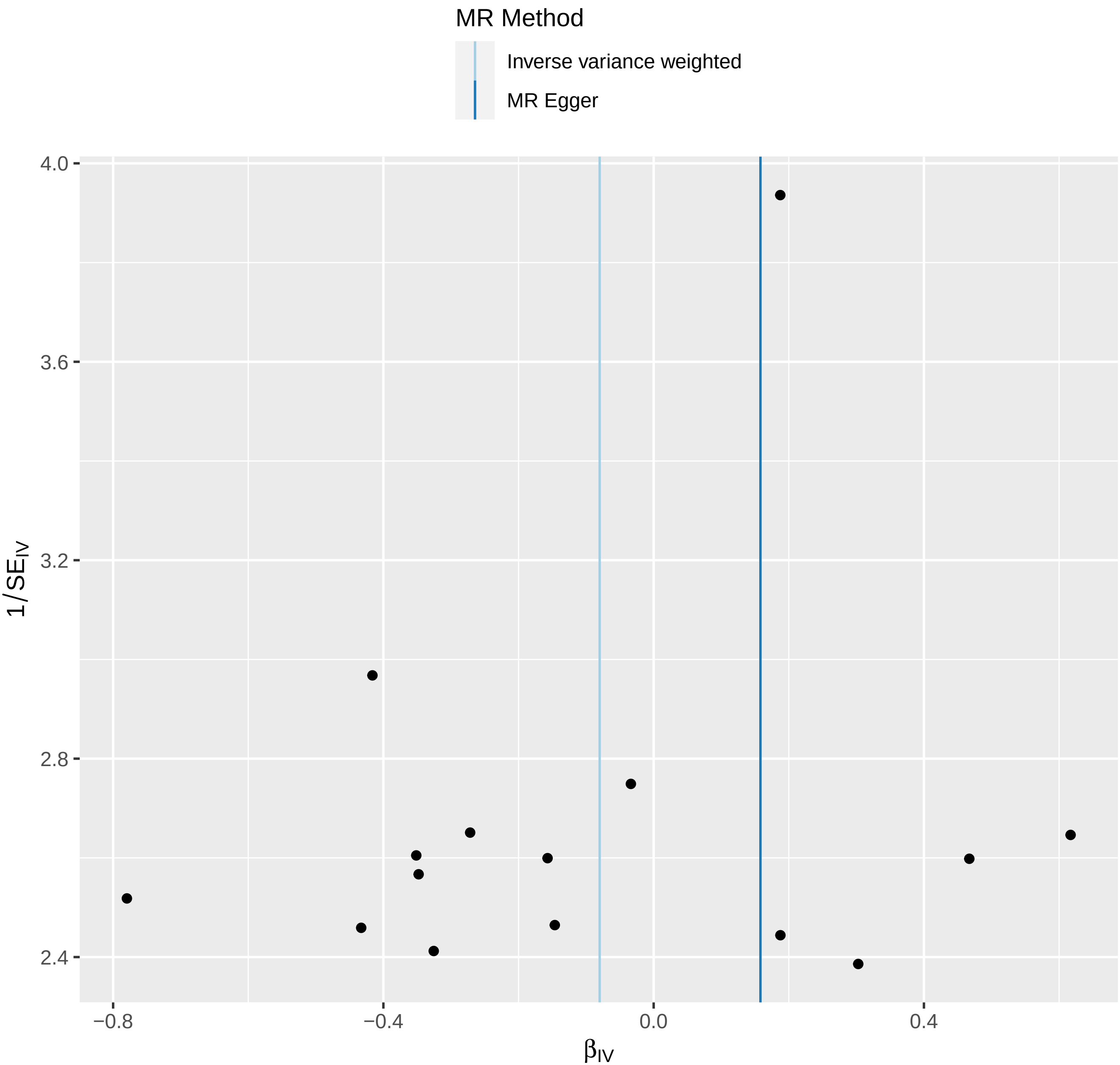

Batch 2−1667 : Gut microbiota abundance (genus Catenibacterium id.2153) on Myotonin−protein kinase
with a threshold of 1e−05

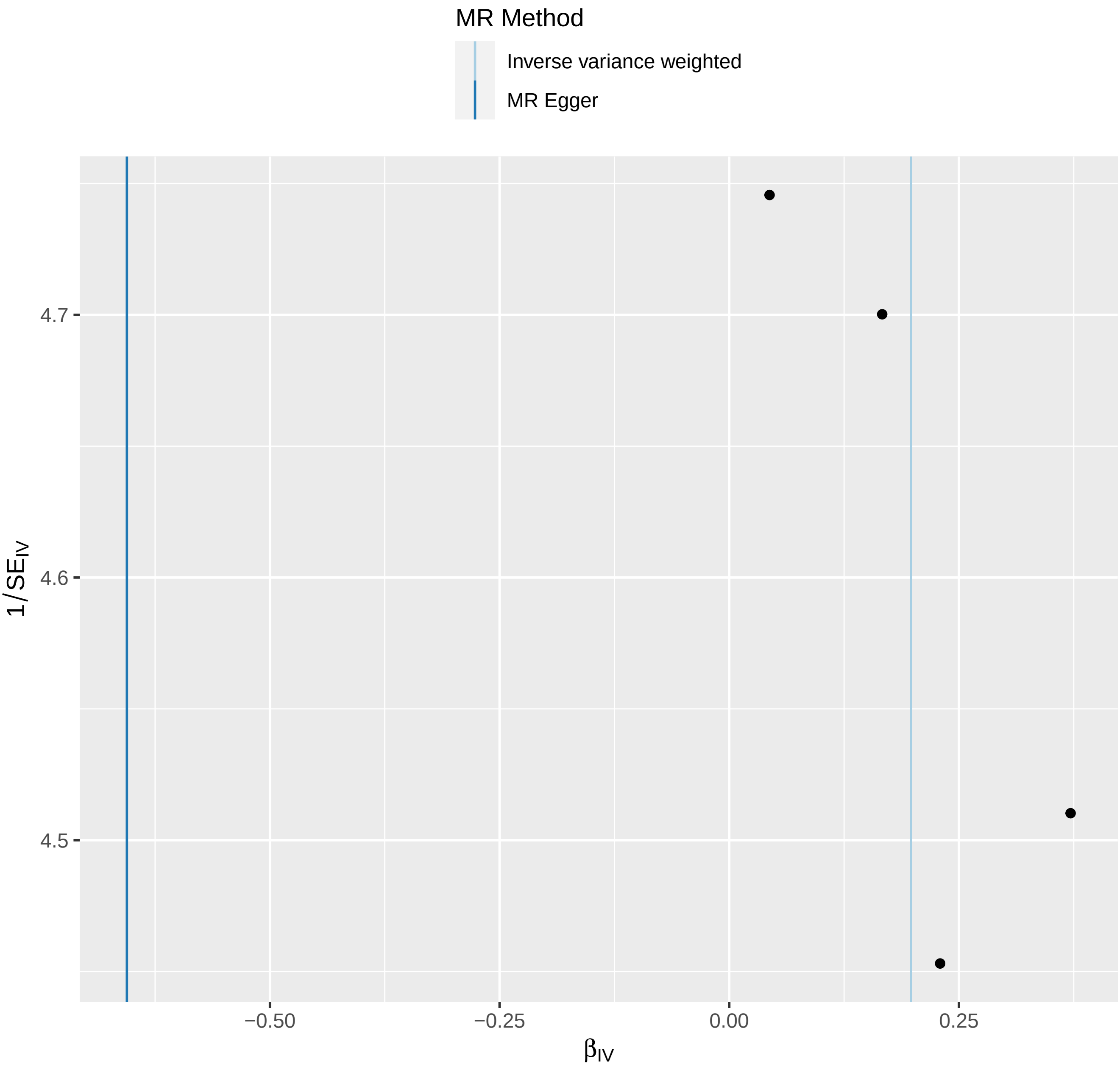

Batch 2−1668 : Gut microbiota abundance (genus Parasutterella id.2892) on Myotonin−protein kinase
with a threshold of 1e−05

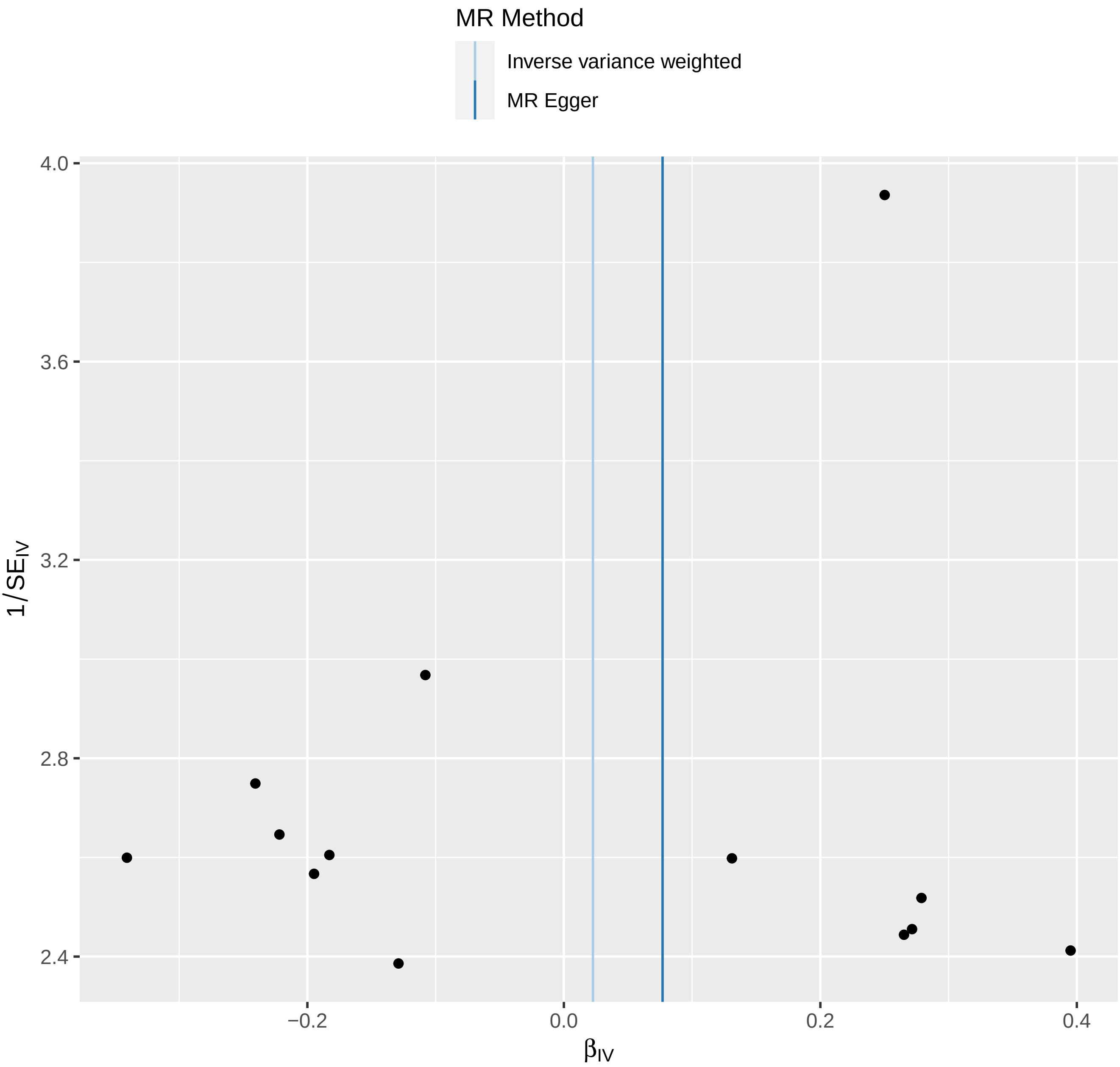

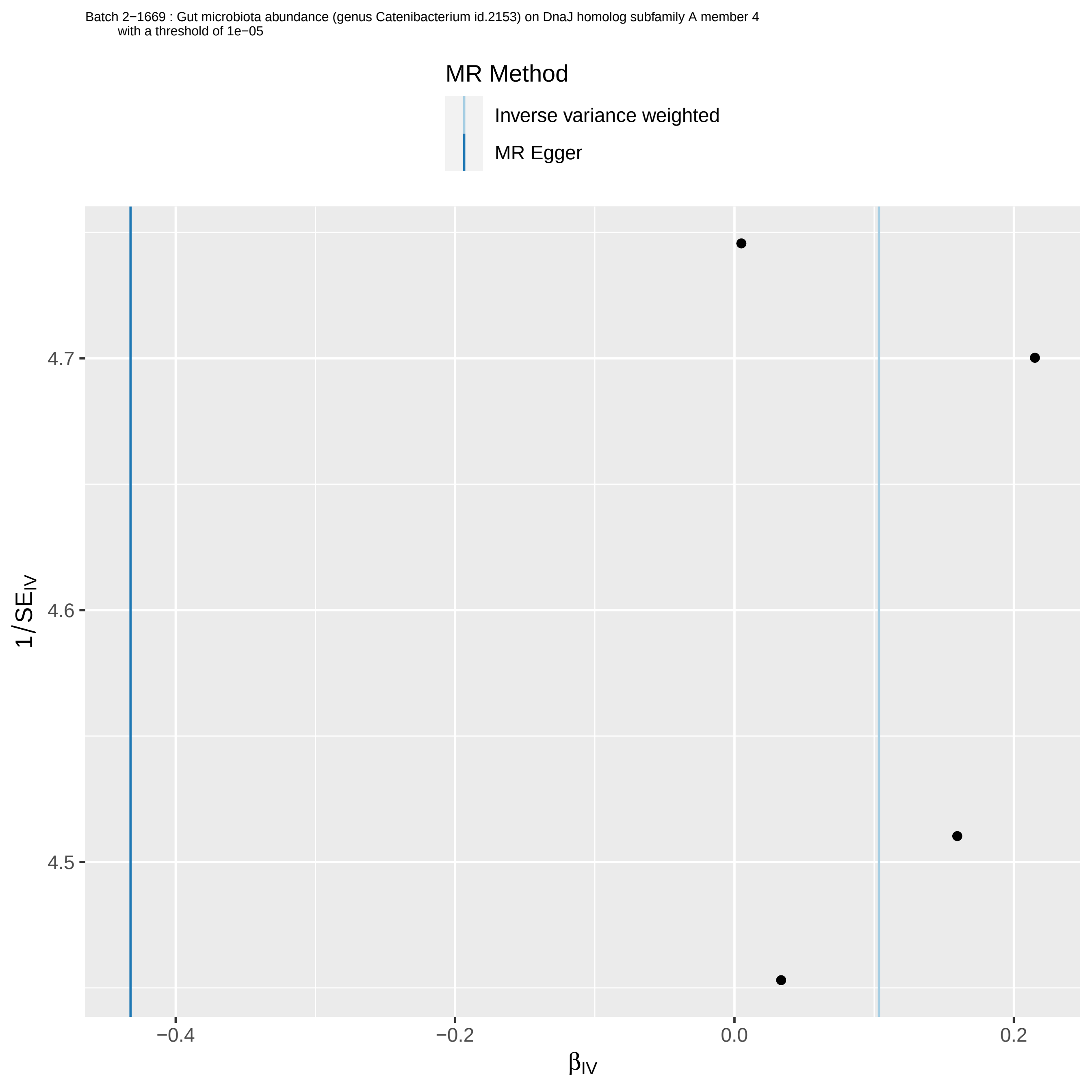
Batch 2−1669 : Gut microbiota abundance (genus Catenibacterium id.2153) on DnaJ homolog subfamily A member 4
with a threshold of 1e−05
MR Method
Inverse variance weighted
MR Egger
4.7
4.6
4.5
1/SE_IV
−0.4
−0.2
0.0
0.2
β_IV

Batch 2−1670 : Gut microbiota abundance (genus Parasutterella id.2892) on DnaJ homolog subfamily A member 4
with a threshold of 1e−05

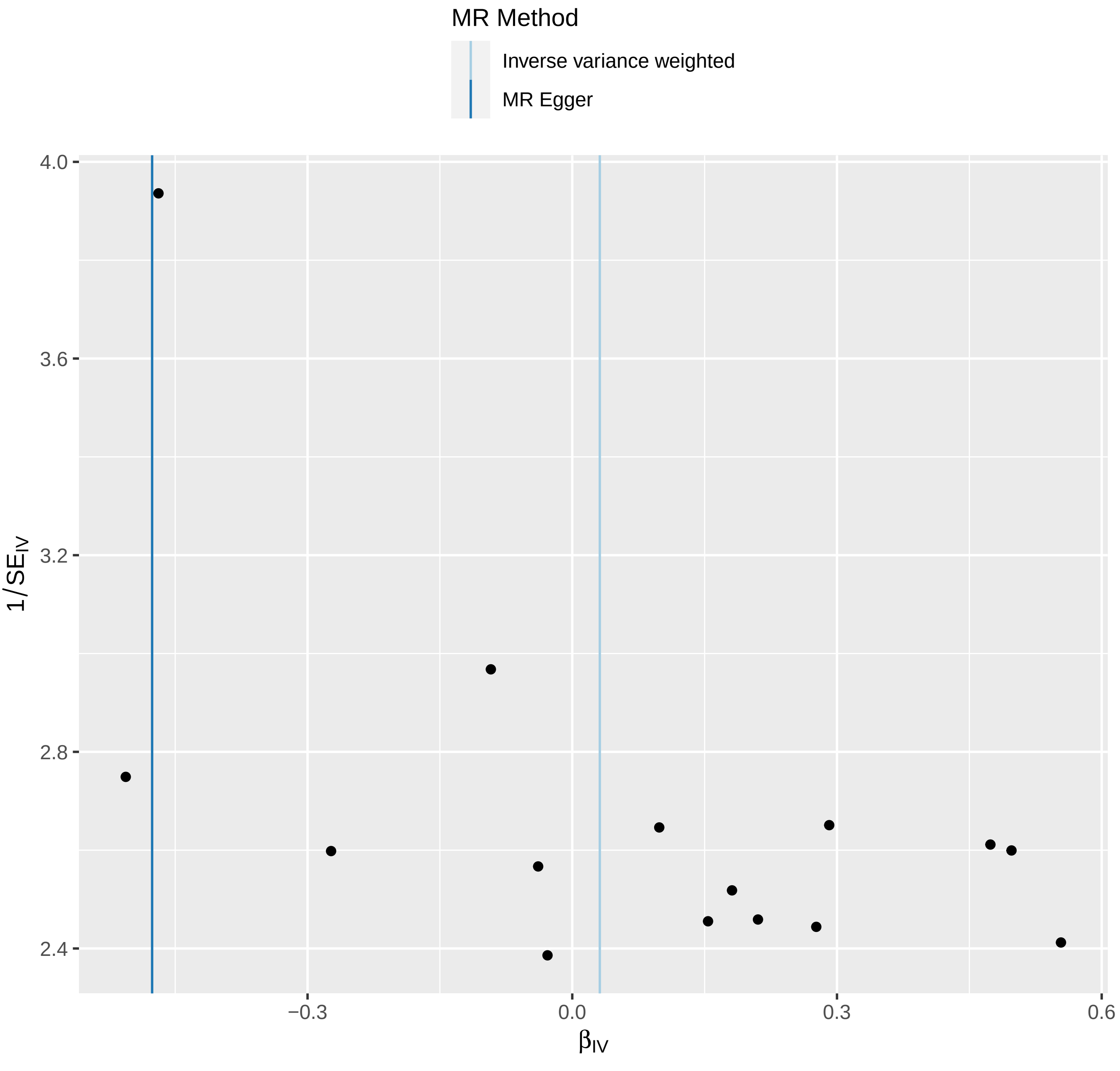

Batch 2−1671 : Gut microbiota abundance (genus Catenibacterium id.2153) on DnaJ homolog subfamily B member 11
with a threshold of 1e−05

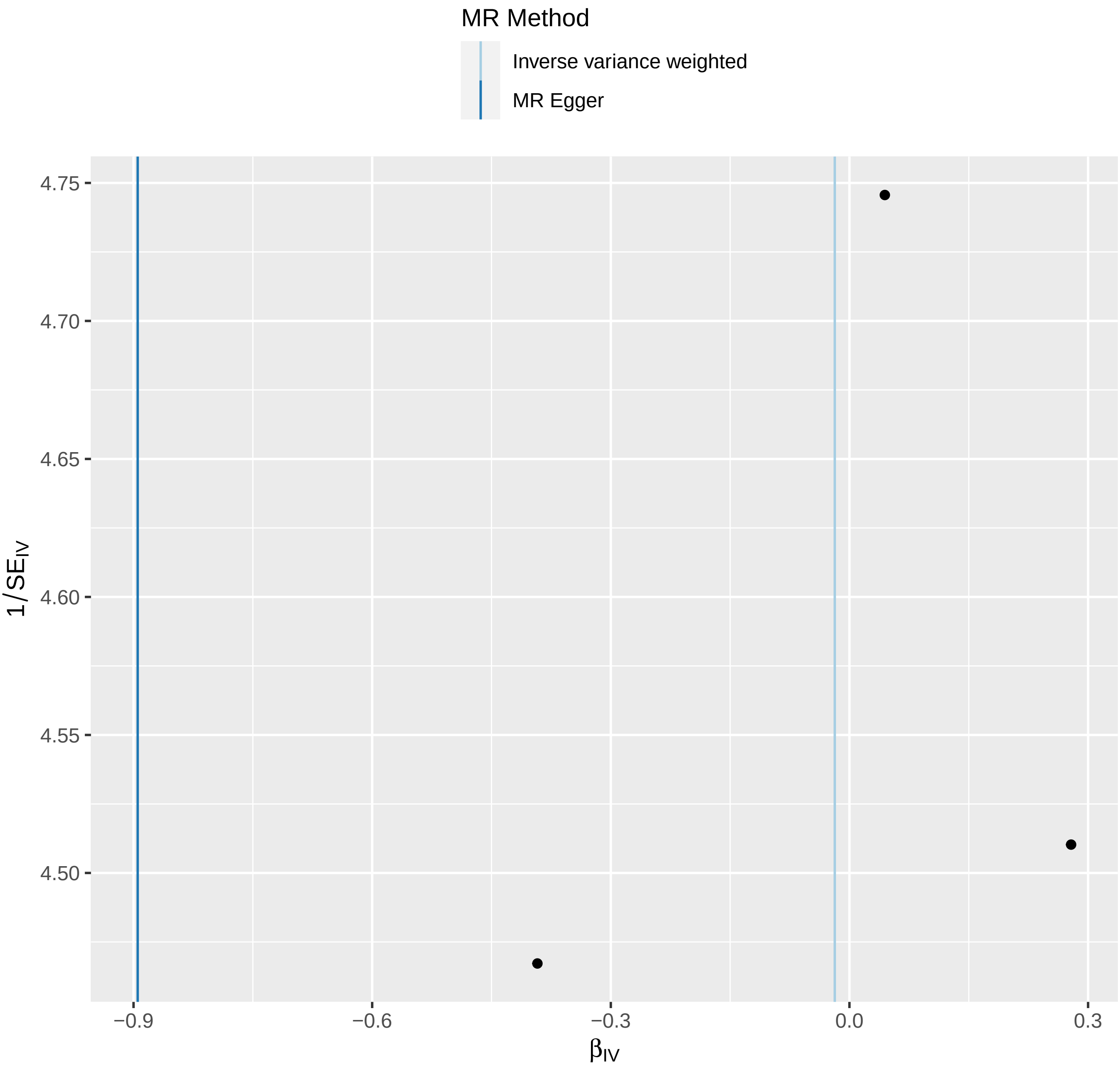

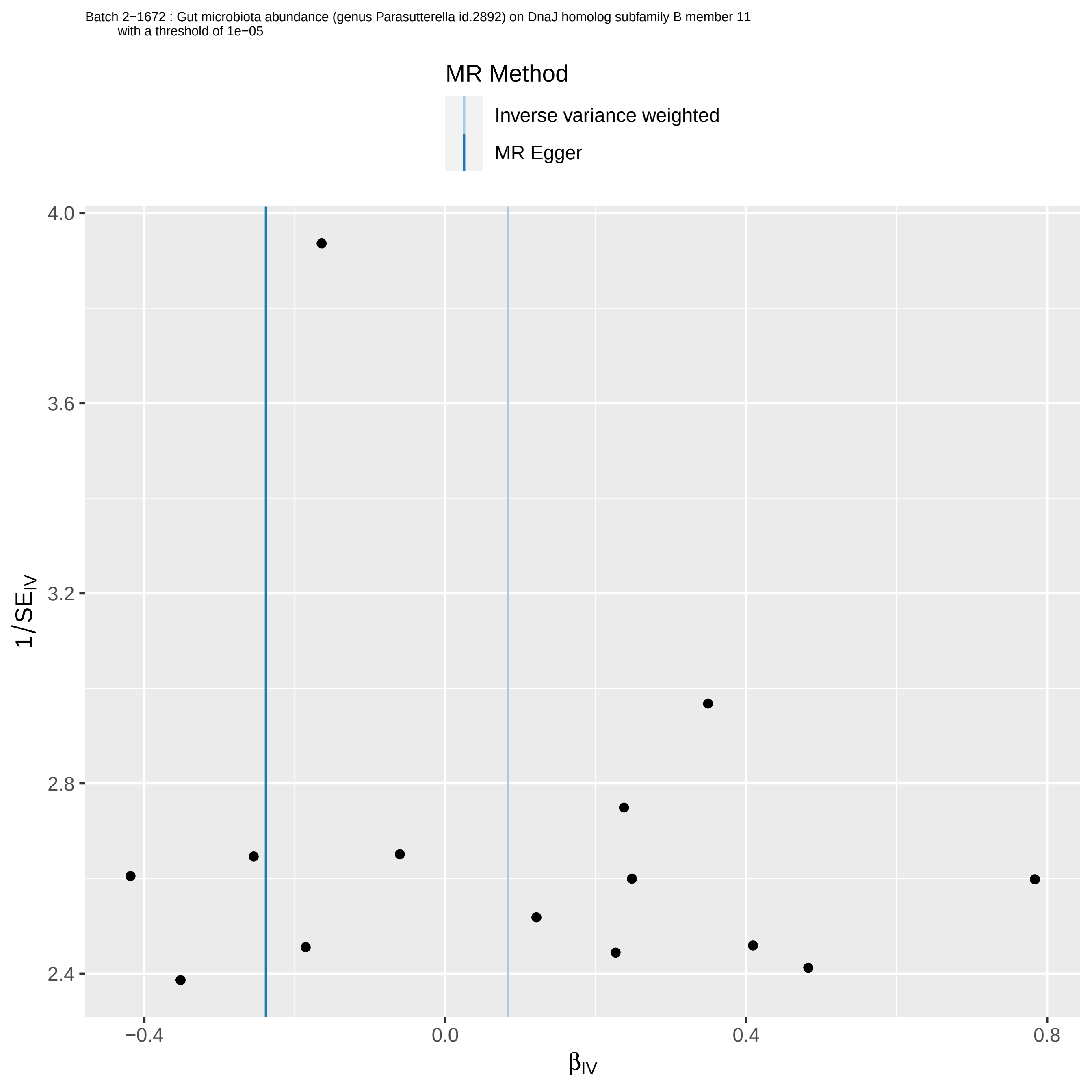

Batch 2−1672 : Gut microbiota abundance (genus Parasutterella id.2892) on DnaJ homolog subfamily B member 11
with a threshold of 1e−05
MR Method
Inverse variance weighted
MR Egger
4.0
3.6
3.2
2.8
2.4
1/SE_IV
−0.4
0.0
0.4
0.8
β_IV

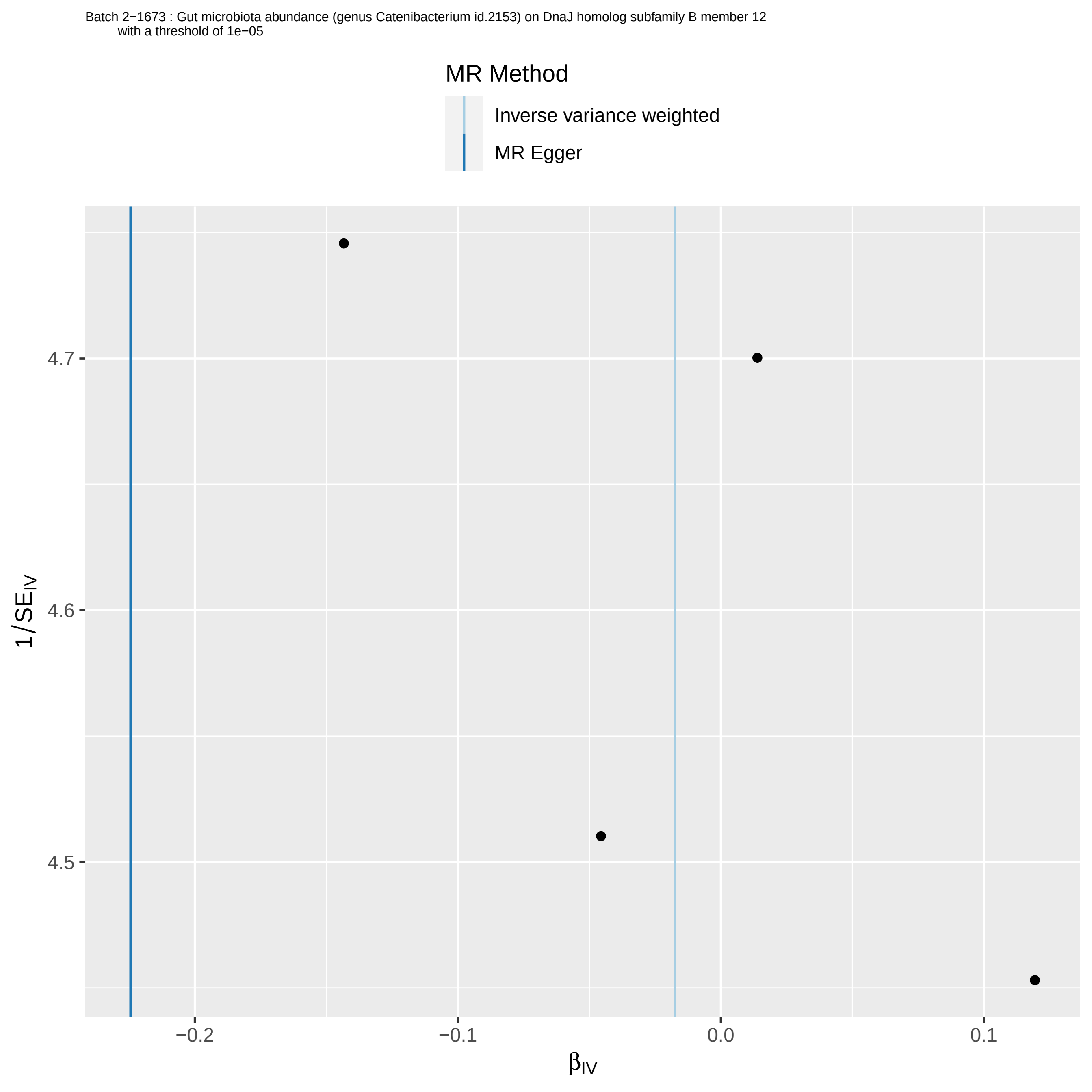

Batch 2−1673 : Gut microbiota abundance (genus Catenibacterium id.2153) on DnaJ homolog subfamily B member 12
with a threshold of 1e−05
MR Method
Inverse variance weighted
MR Egger
4.7
4.6
4.5
1/SE_IV
−0.2
−0.1
0.0
0.1
β_IV

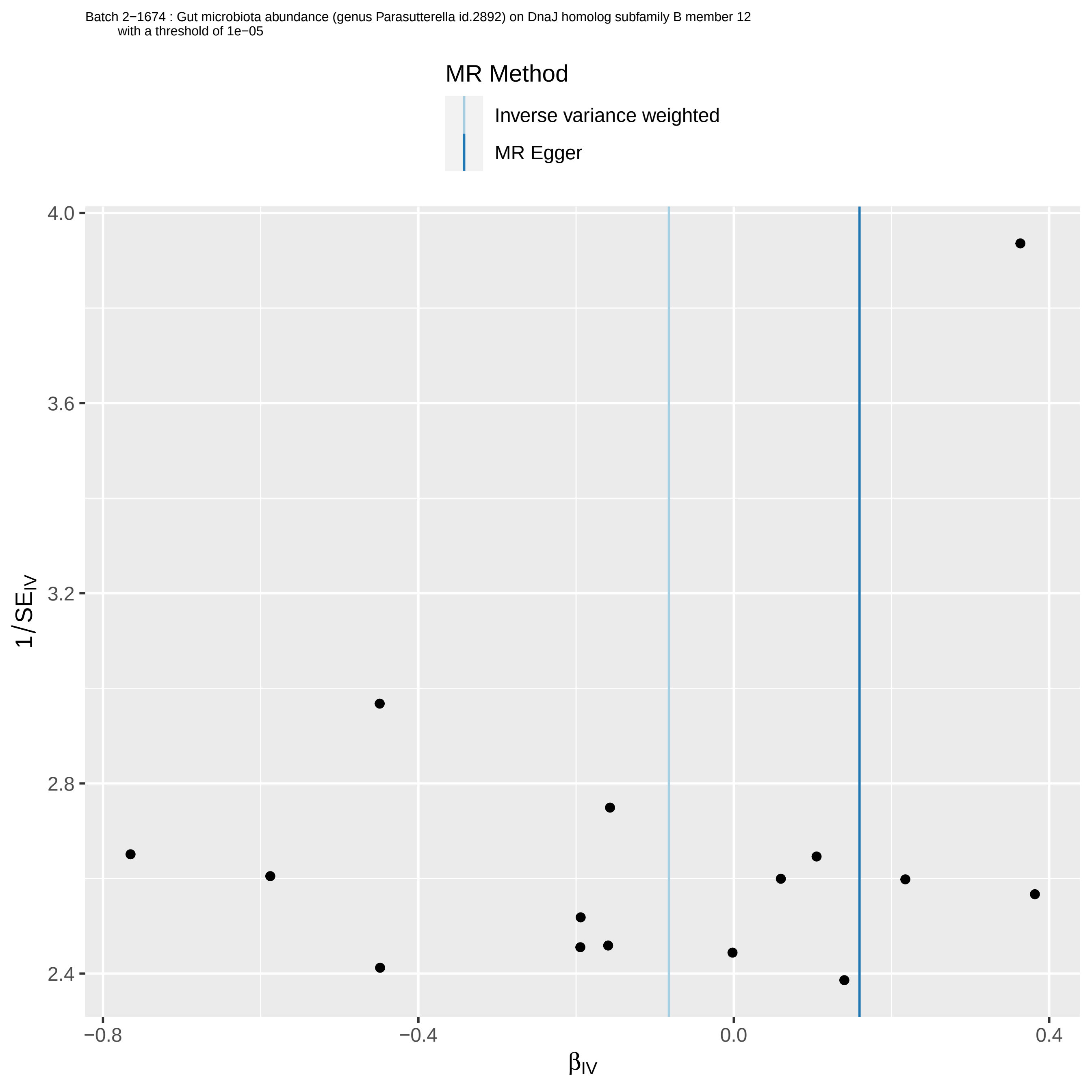
Batch 2−1674 : Gut microbiota abundance (genus Parasutterella id.2892) on DnaJ homolog subfamily B member 12
with a threshold of 1e−05
MR Method
Inverse variance weighted
MR Egger
4.0
3.6
3.2
2.8
2.4
1/SE_IV
−0.8
−0.4
0.0
0.4
β_IV

Batch 2−1675 : Gut microbiota abundance (genus Catenibacterium id.2153) on DnaJ homolog subfamily B member 1
with a threshold of 1e−05

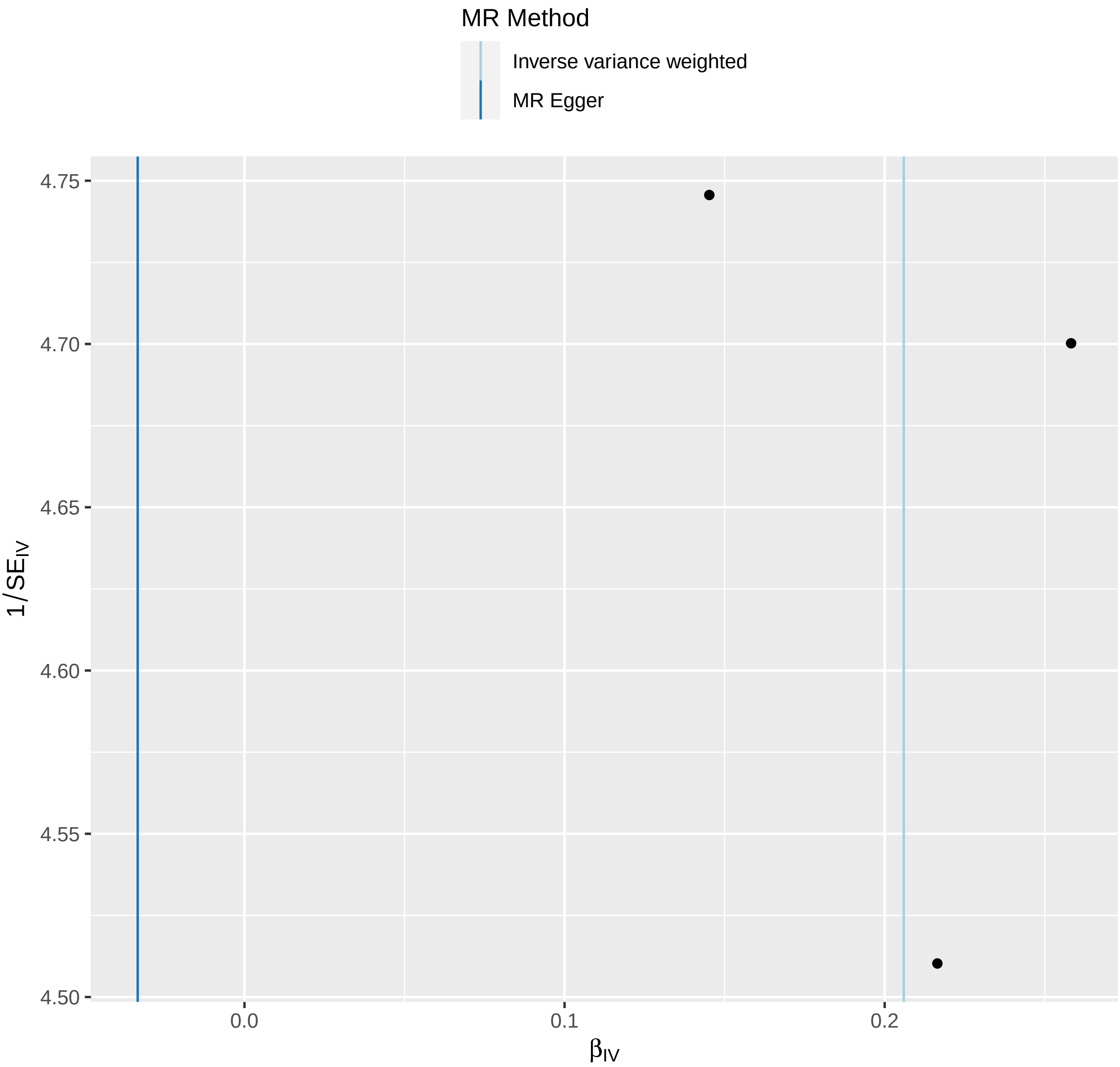

Batch 2−1676 : Gut microbiota abundance (genus Parasutterella id.2892) on DnaJ homolog subfamily B member 1
with a threshold of 1e−05

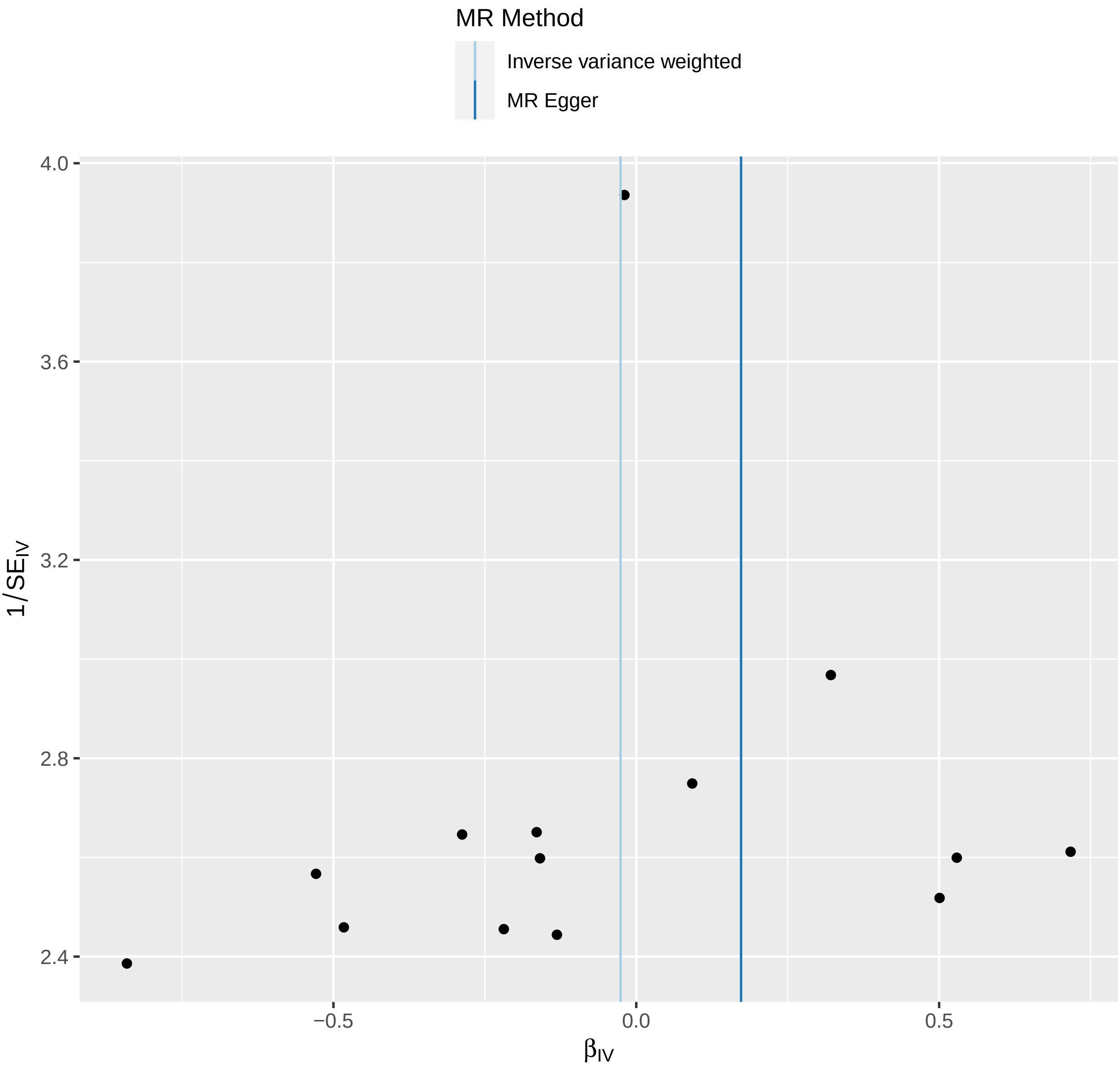

Batch 2−1677 : Gut microbiota abundance (genus Catenibacterium id.2153) on DnaJ homolog subfamily B member 14
with a threshold of 1e−05

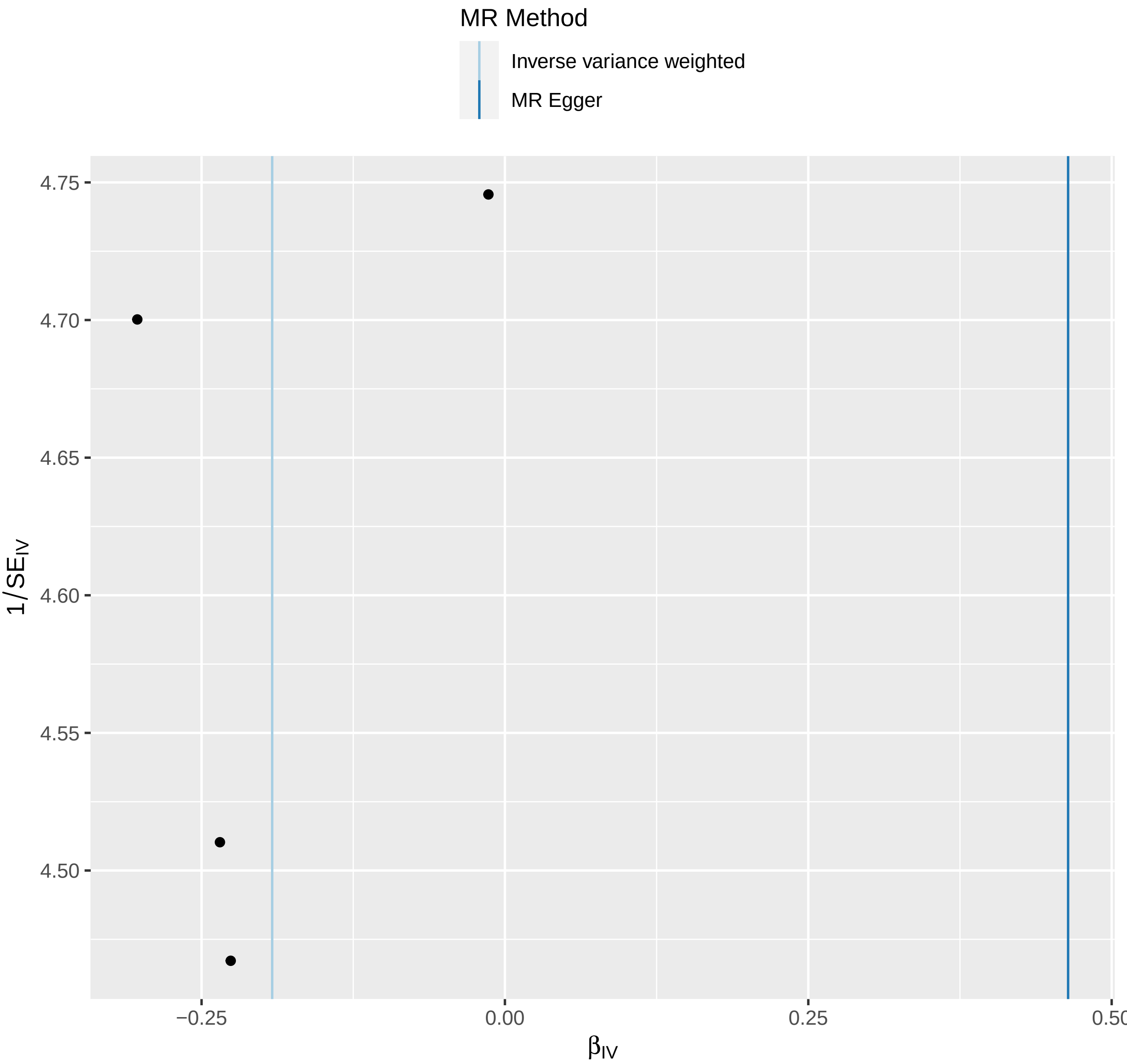

Batch 2−1678 : Gut microbiota abundance (genus Parasutterella id.2892) on DnaJ homolog subfamily B member 14
with a threshold of 1e−05

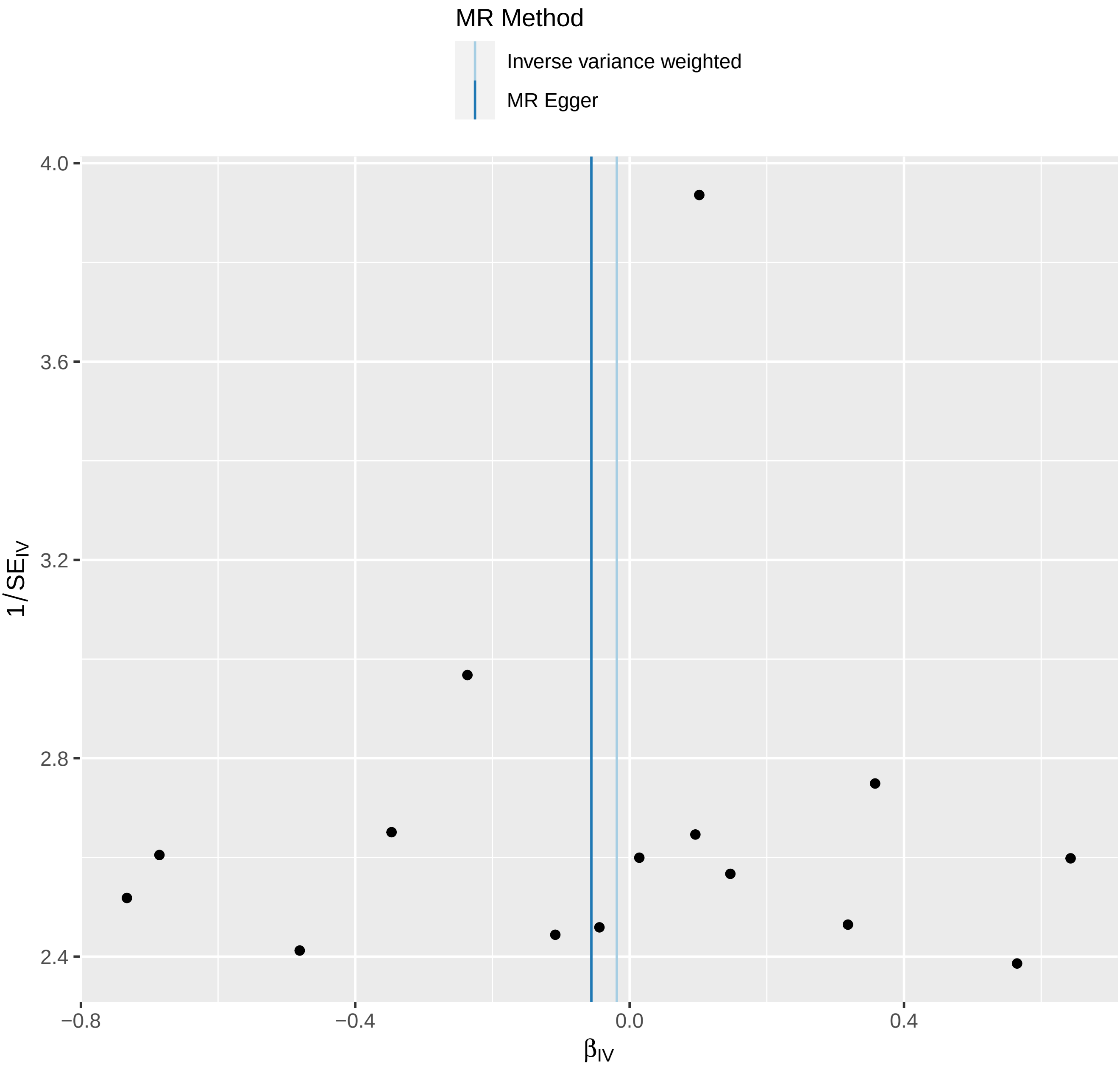

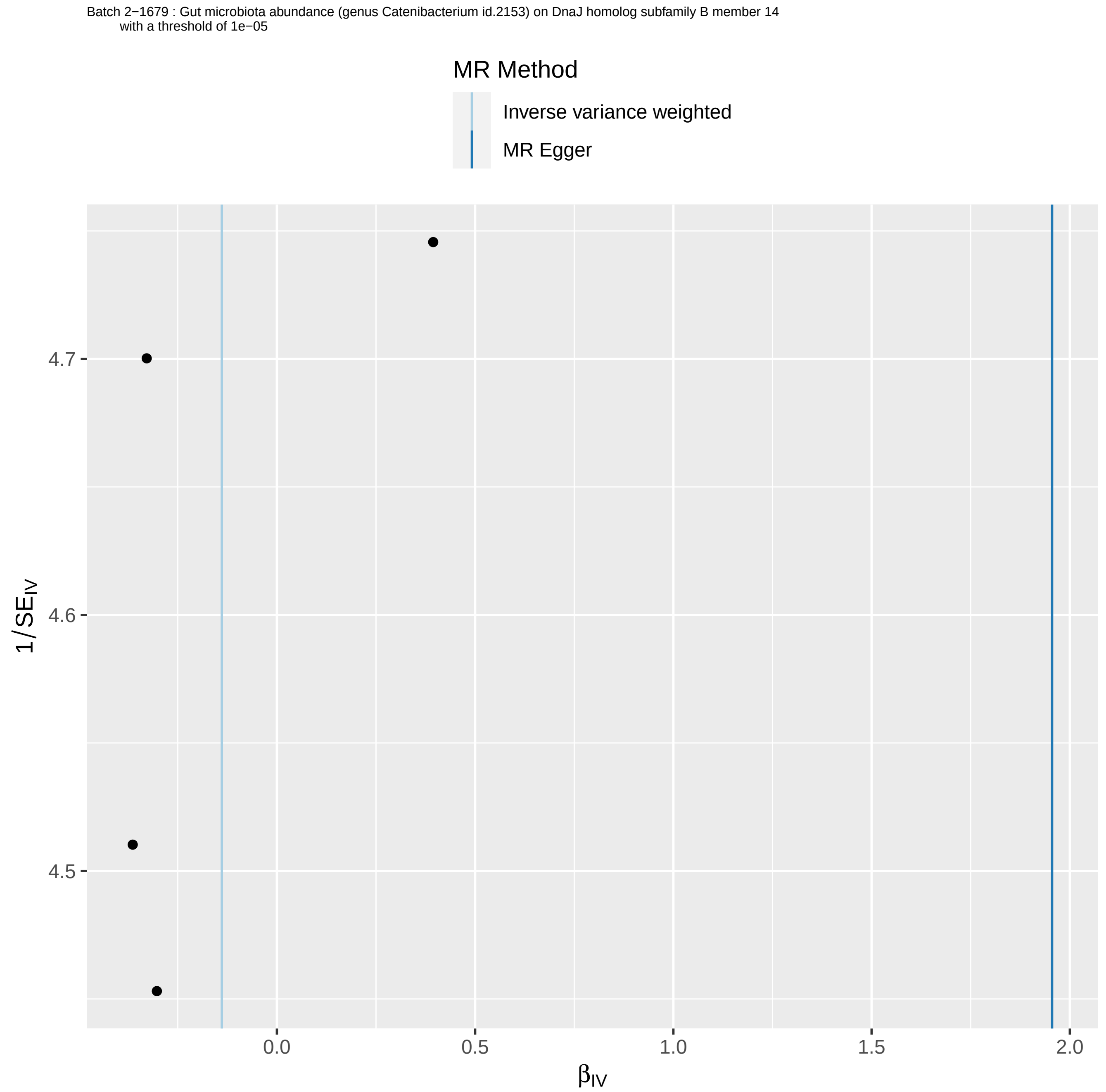
Batch 2−1679 : Gut microbiota abundance (genus Catenibacterium id.2153) on DnaJ homolog subfamily B member 14
with a threshold of 1e−05
MR Method
Inverse variance weighted
MR Egger
4.7
4.6
4.5
1/SE_IV
0.0
0.5
1.0
1.5
2.0
β_IV

Batch 2−1680 : Gut microbiota abundance (genus Parasutterella id.2892) on DnaJ homolog subfamily B member 14
with a threshold of 1e−05

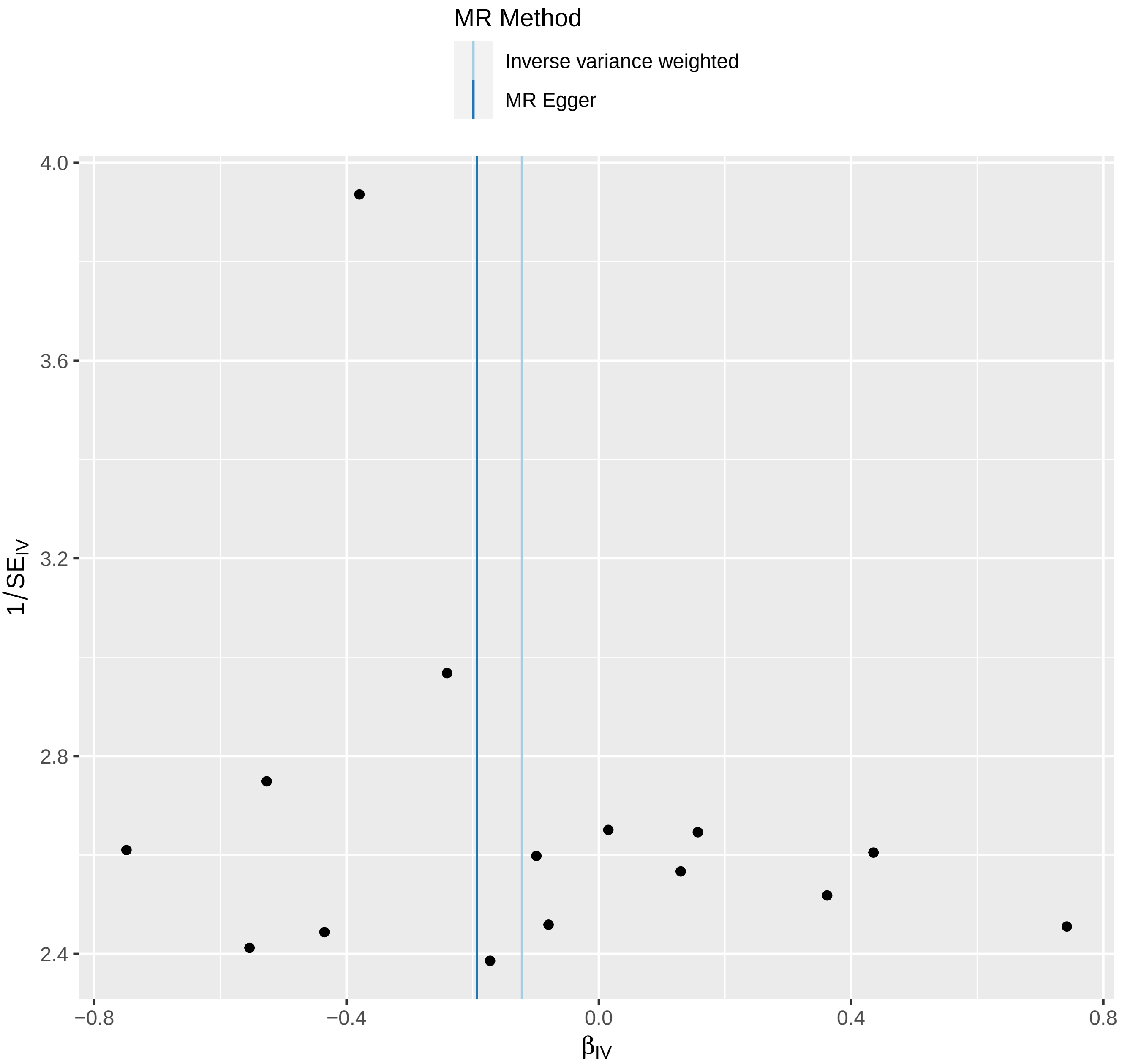

Batch 2−1681 : Gut microbiota abundance (genus Catenibacterium id.2153) on DnaJ homolog subfamily B member 2
with a threshold of 1e−05

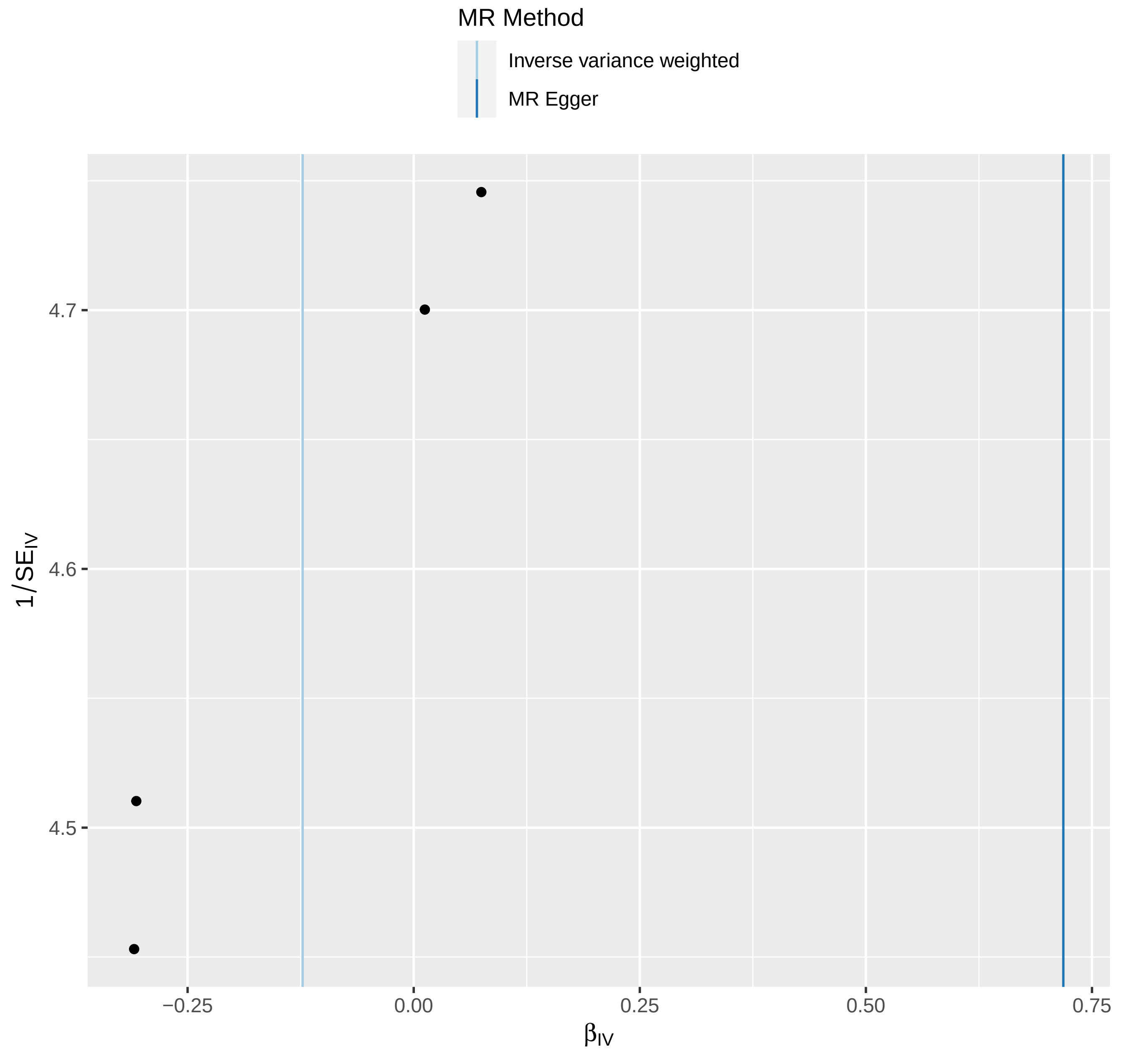

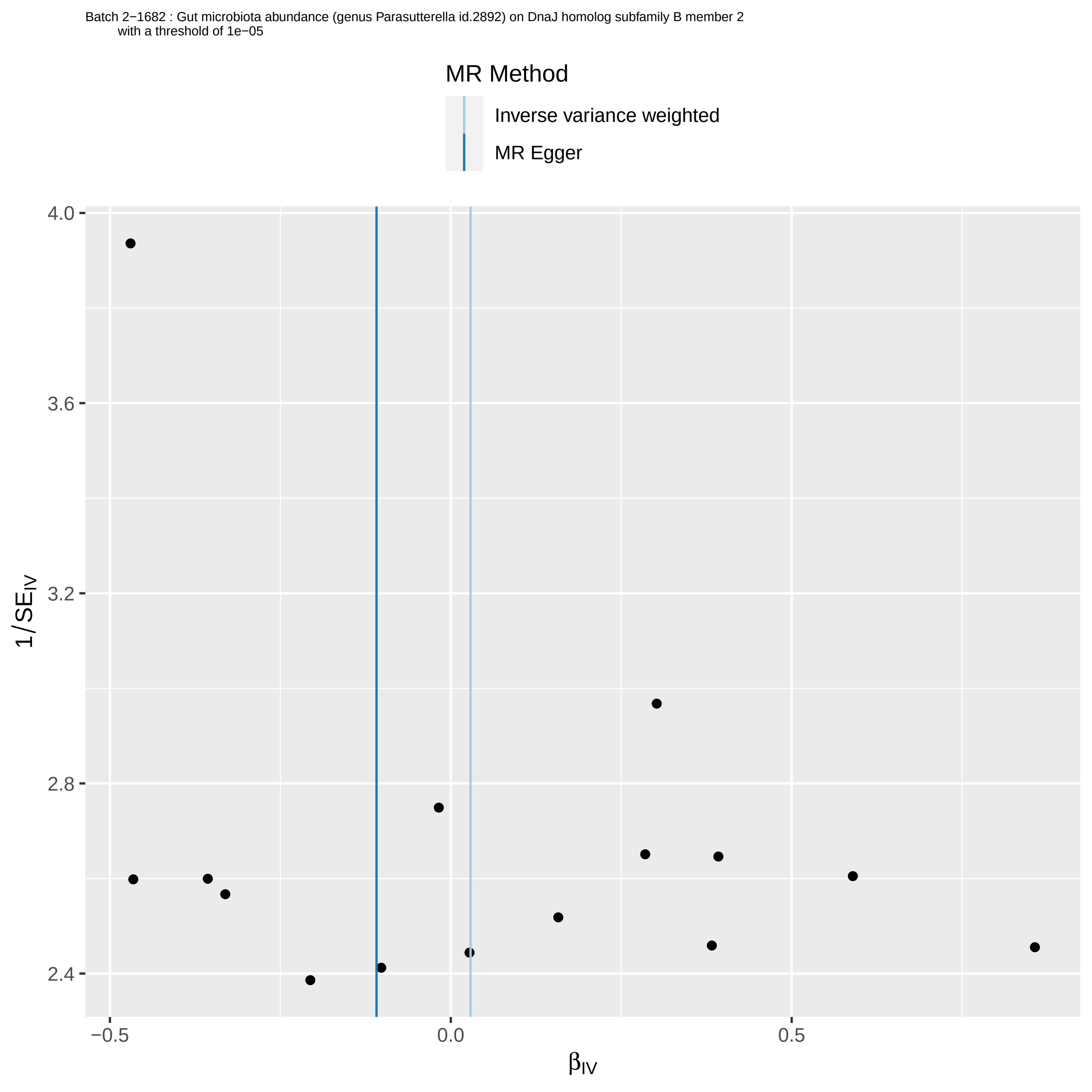

Batch 2−1682 : Gut microbiota abundance (genus Parasutterella id.2892) on DnaJ homolog subfamily B member 2
with a threshold of 1e−05
MR Method
Inverse variance weighted
MR Egger
4.0
3.6
3.2
2.8
2.4
1/SE_IV
−0.5
0.0
0.5
β_IV

Batch 2−1683 : Gut microbiota abundance (genus Catenibacterium id.2153) on DnaJ homolog subfamily B member 9 with a threshold of 1e−05

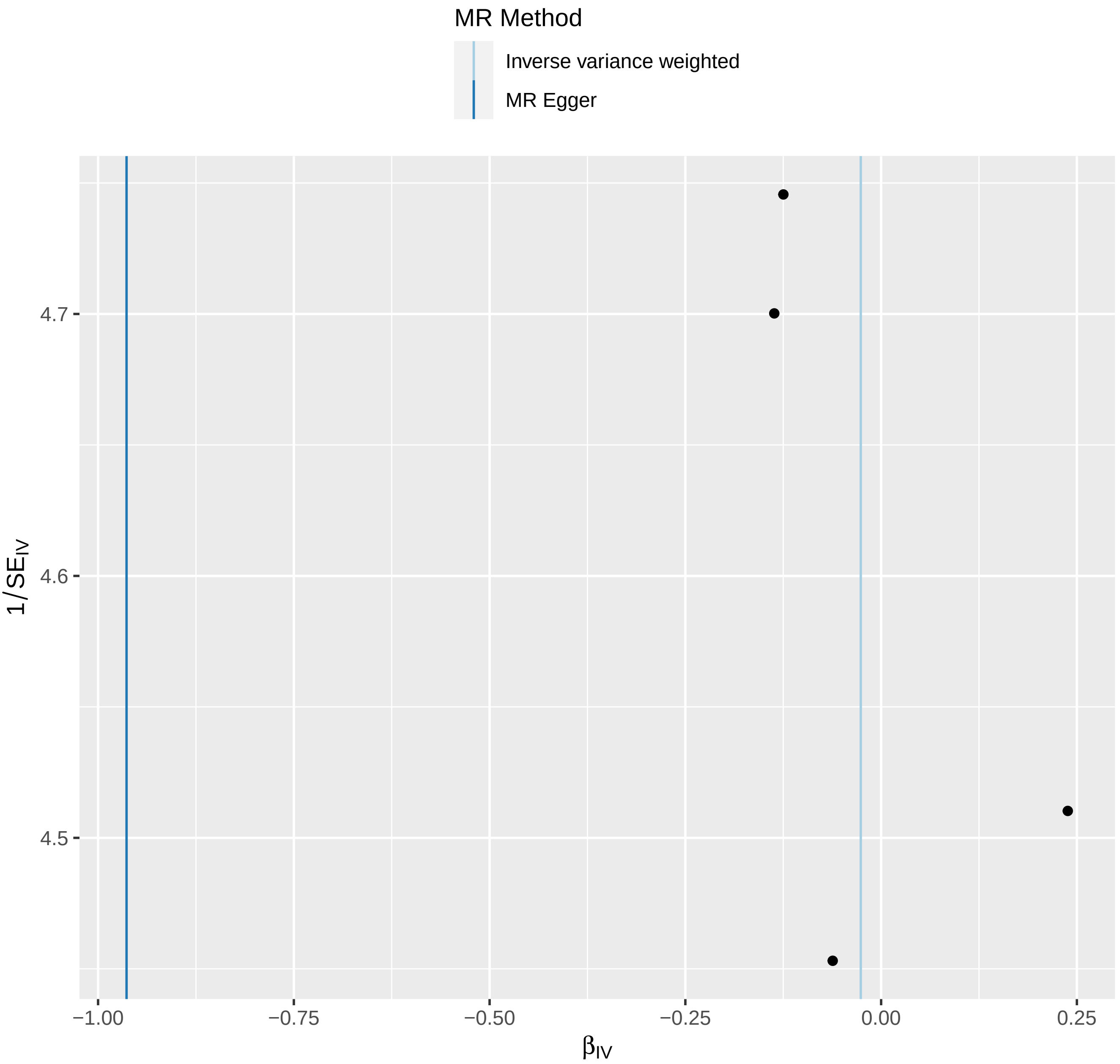

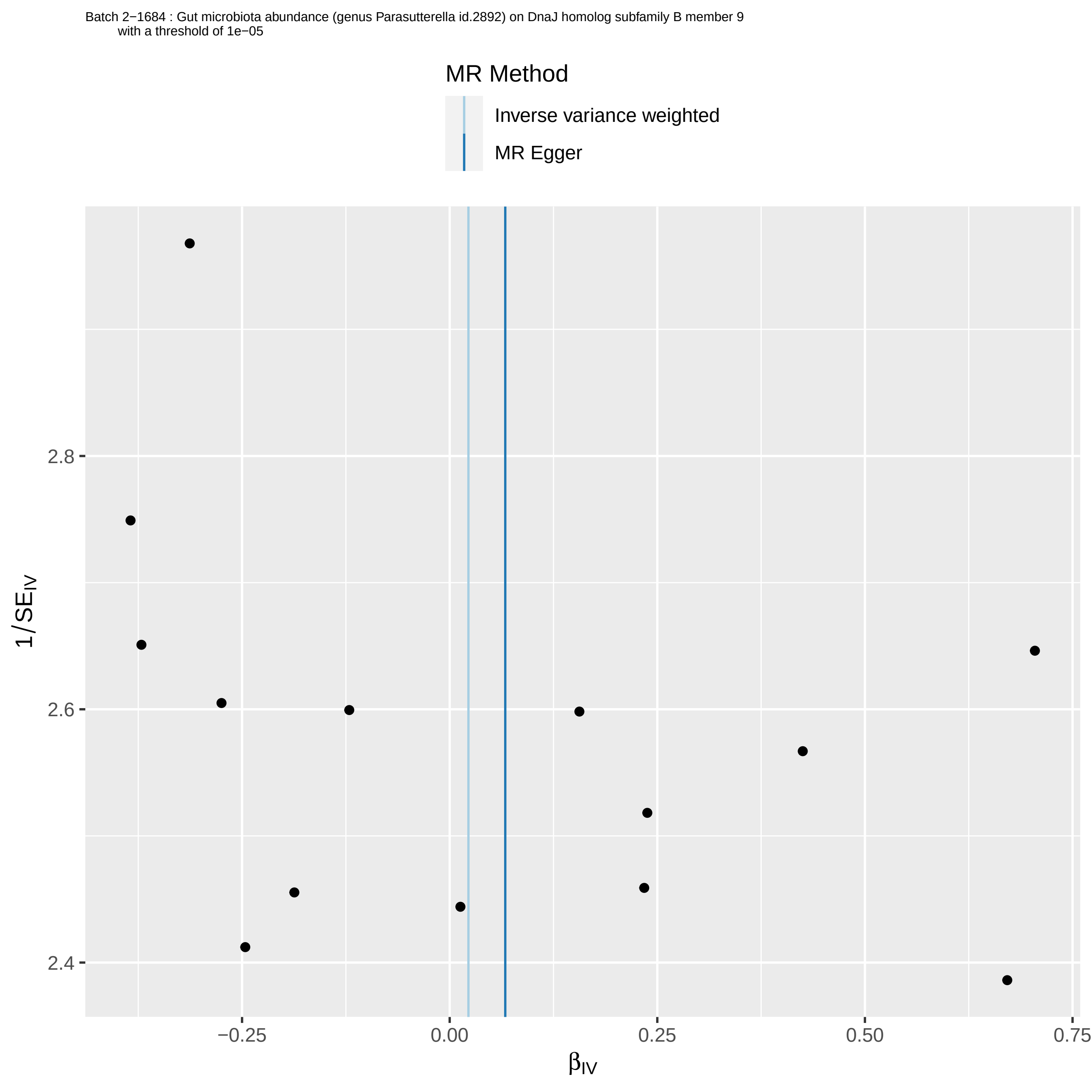
Batch 2−1684 : Gut microbiota abundance (genus Parasutterella id.2892) on DnaJ homolog subfamily B member 9
with a threshold of 1e−05
MR Method
Inverse variance weighted
MR Egger
2.8
2.6
2.4
1/SE_IV
−0.25
0.00
0.25
0.50
0.75
β_IV

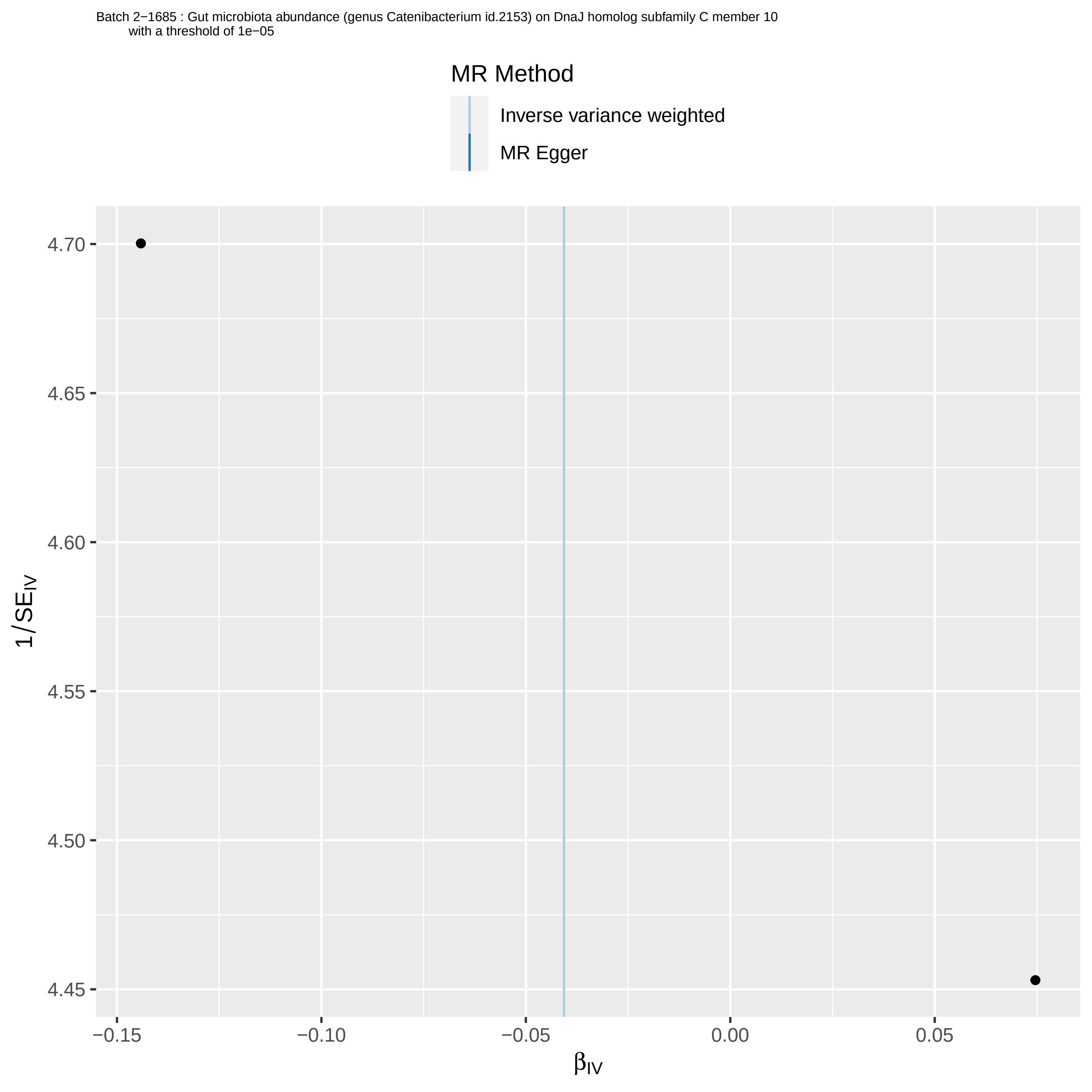
Batch 2−1685 : Gut microbiota abundance (genus Catenibacterium id.2153) on DnaJ homolog subfamily C member 10
with a threshold of 1e−05
MR Method
Inverse variance weighted
MR Egger
4.70
4.65
4.60
4.55
4.50
4.45
1/SE_IV
−0.15
−0.10
−0.05
0.00
0.05
β_IV

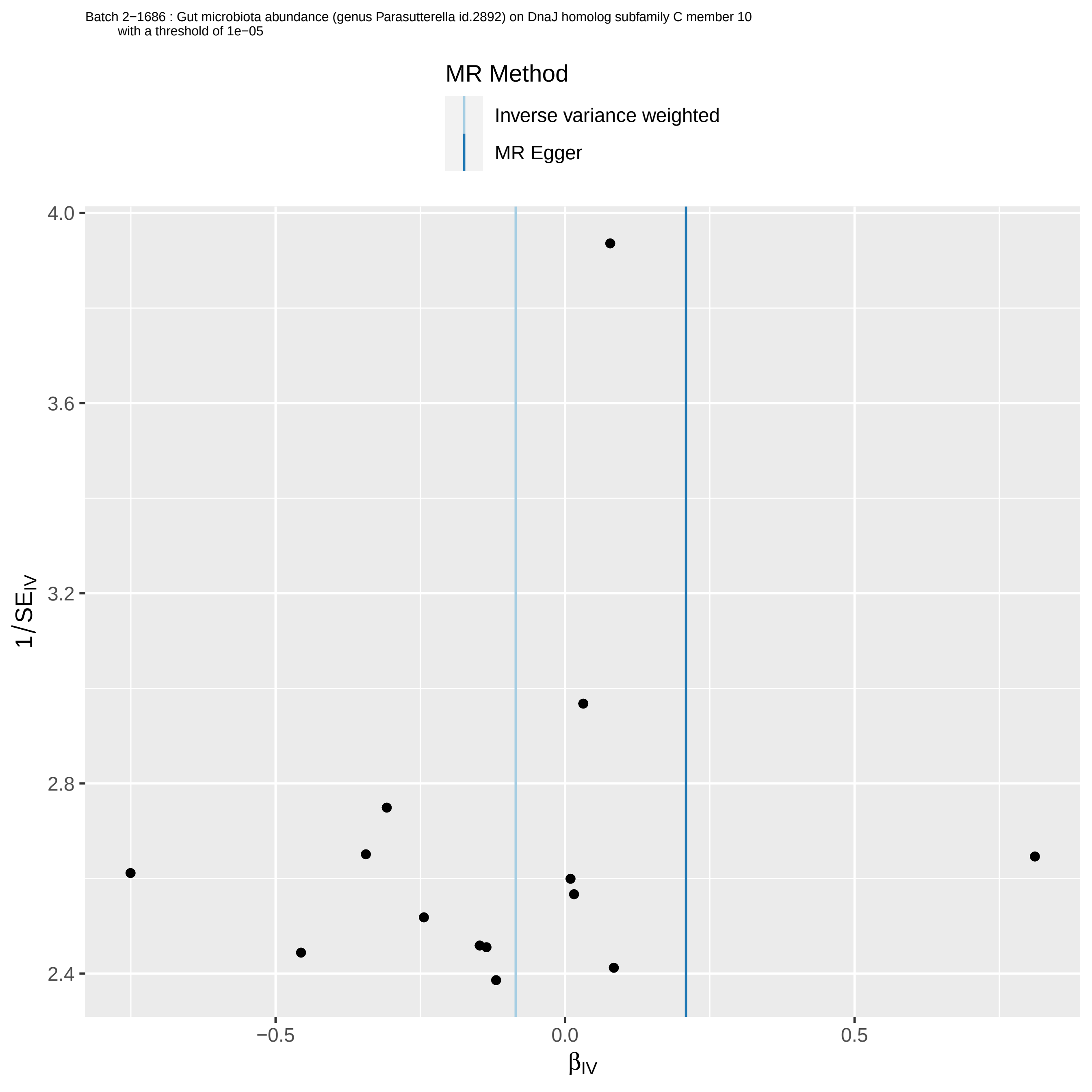

Batch 2−1686 : Gut microbiota abundance (genus Parasutterella id.2892) on DnaJ homolog subfamily C member 10
with a threshold of 1e−05
MR Method
Inverse variance weighted
MR Egger
4.0
3.6
3.2
2.8
2.4
1/SE$_{IV}$
−0.5
0.0
0.5
β_{IV}

Batch 2−1687 : Gut microbiota abundance (genus Catenibacterium id.2153) on DnaJ homolog subfamily C member 15
with a threshold of 1e−05

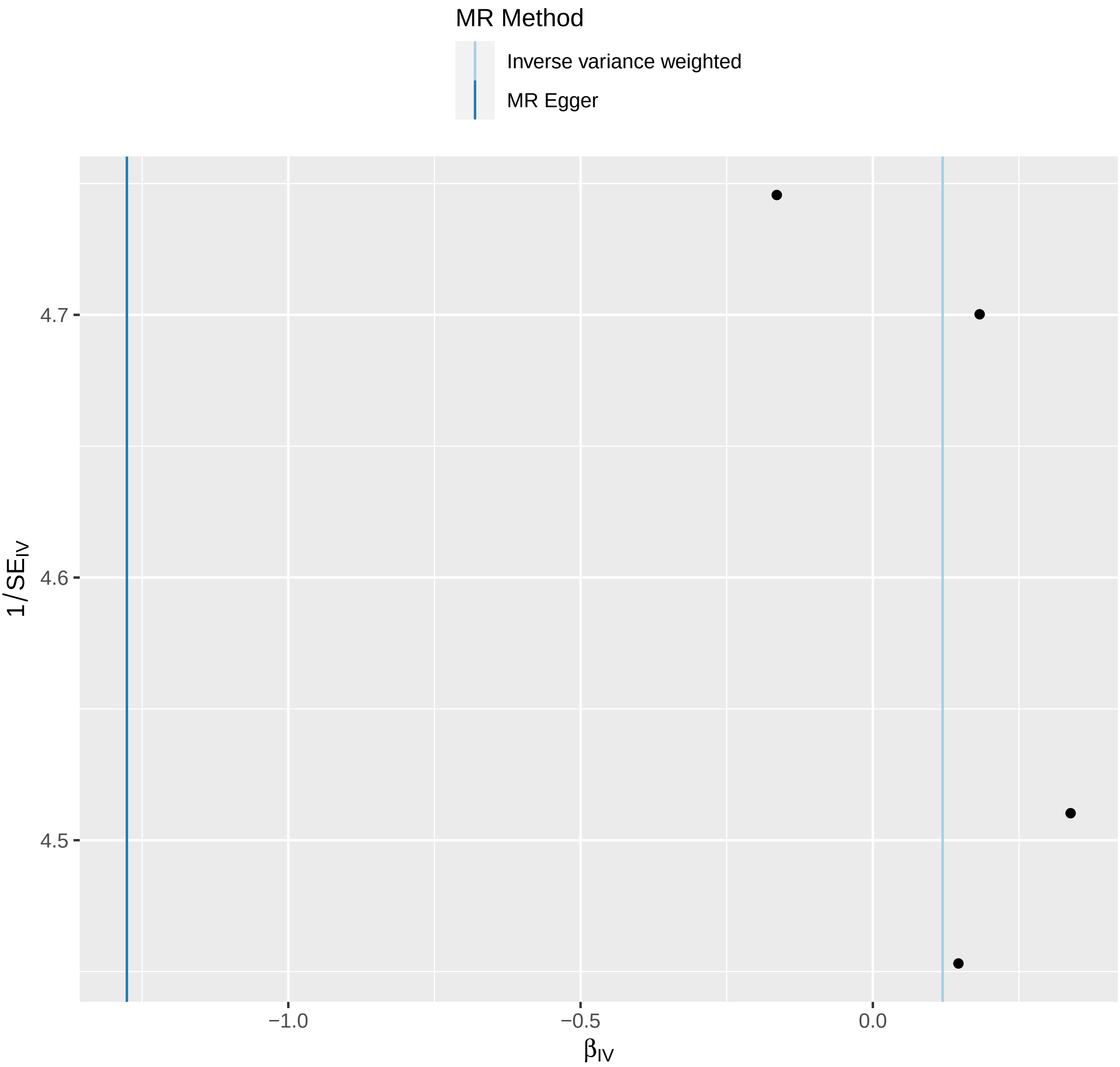

Batch 2−1688 : Gut microbiota abundance (genus Parasutterella id.2892) on DnaJ homolog subfamily C member 15
with a threshold of 1e−05

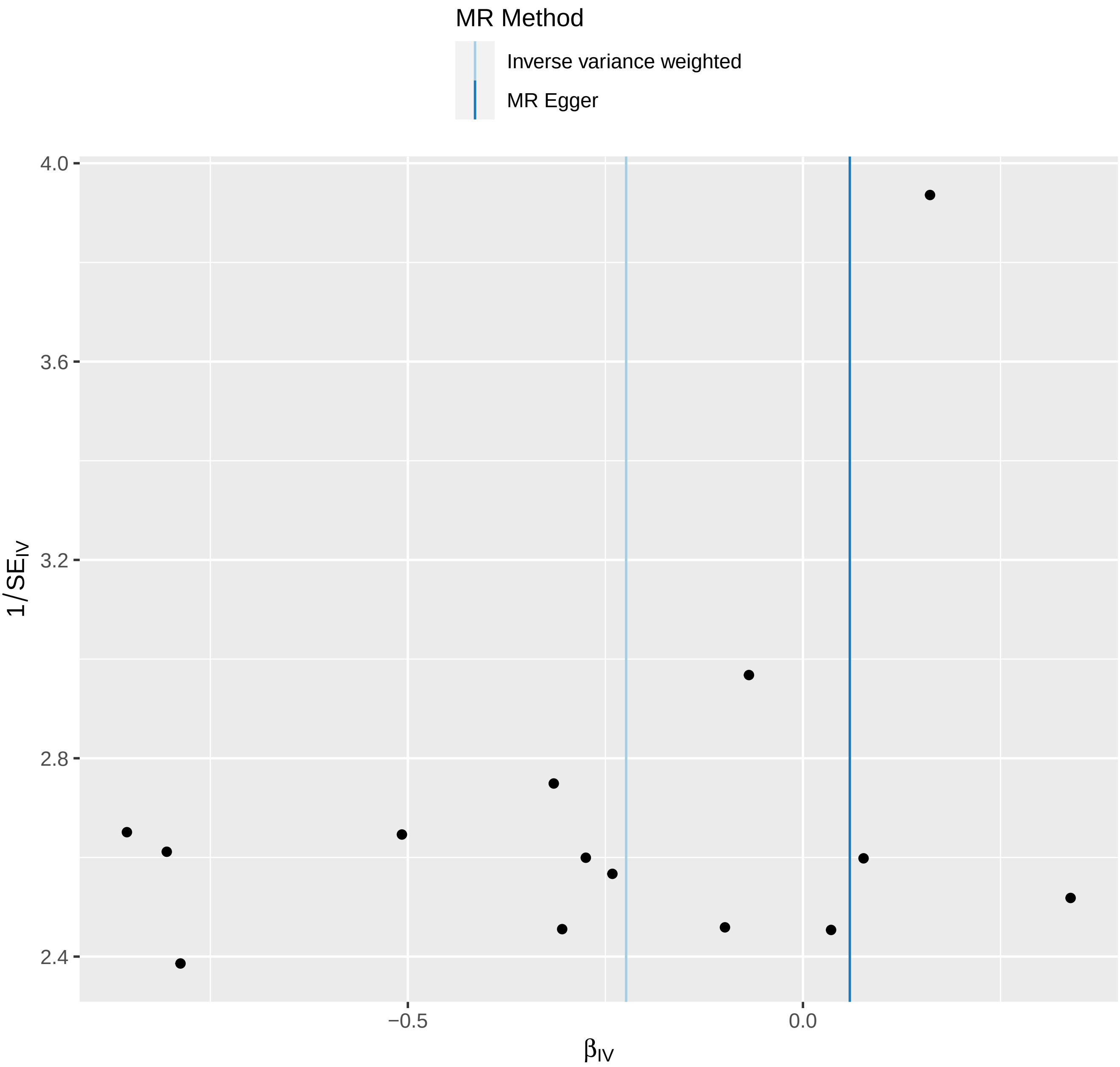

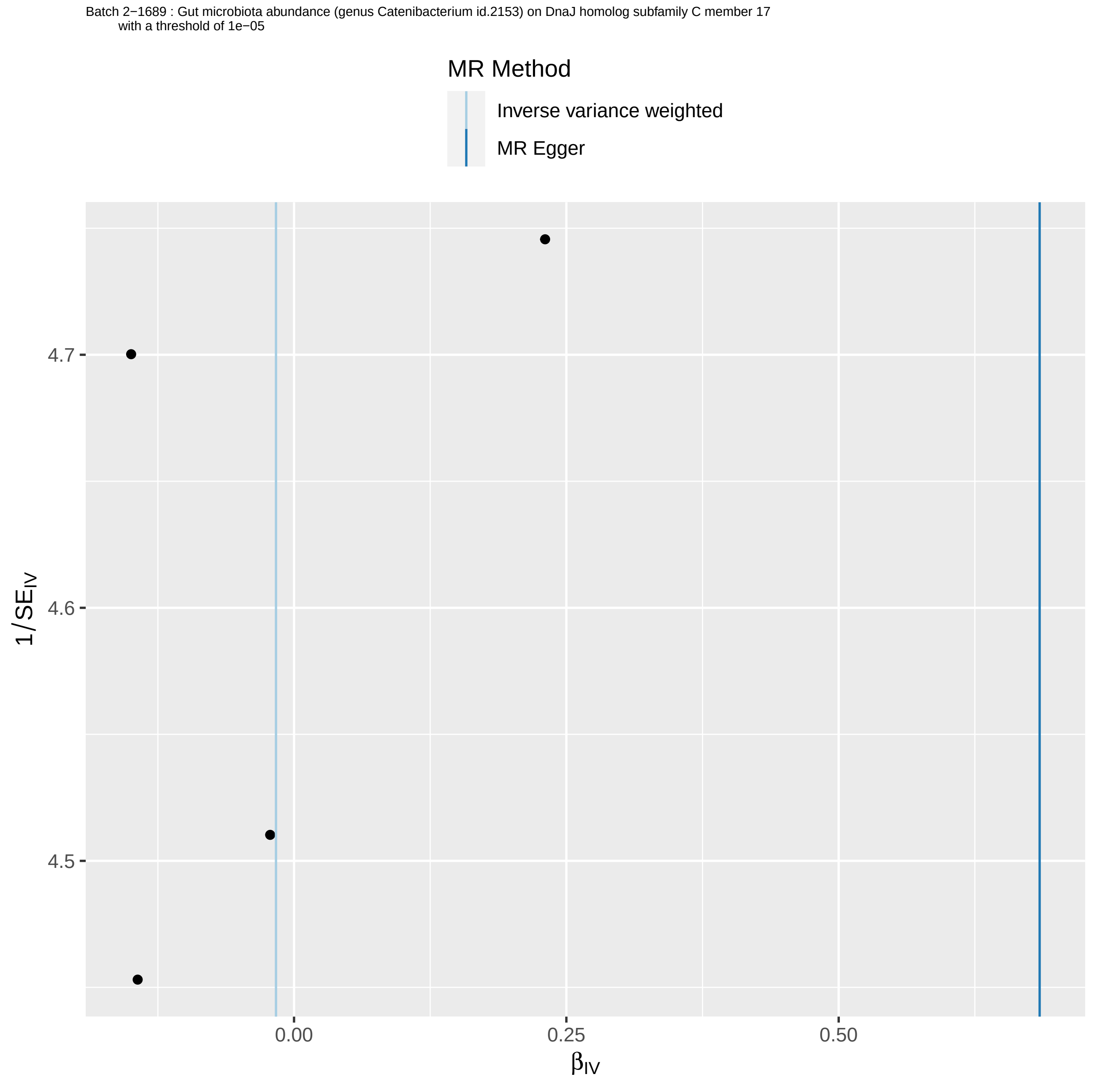

Batch 2−1689 : Gut microbiota abundance (genus Catenibacterium id.2153) on DnaJ homolog subfamily C member 17
with a threshold of 1e−05
MR Method
Inverse variance weighted
MR Egger
4.7
4.6
4.5
1/SE_IV
0.00
0.25
0.50
β_IV

Batch 2−1690 : Gut microbiota abundance (genus Parasutterella id.2892) on DnaJ homolog subfamily C member 17
with a threshold of 1e−05

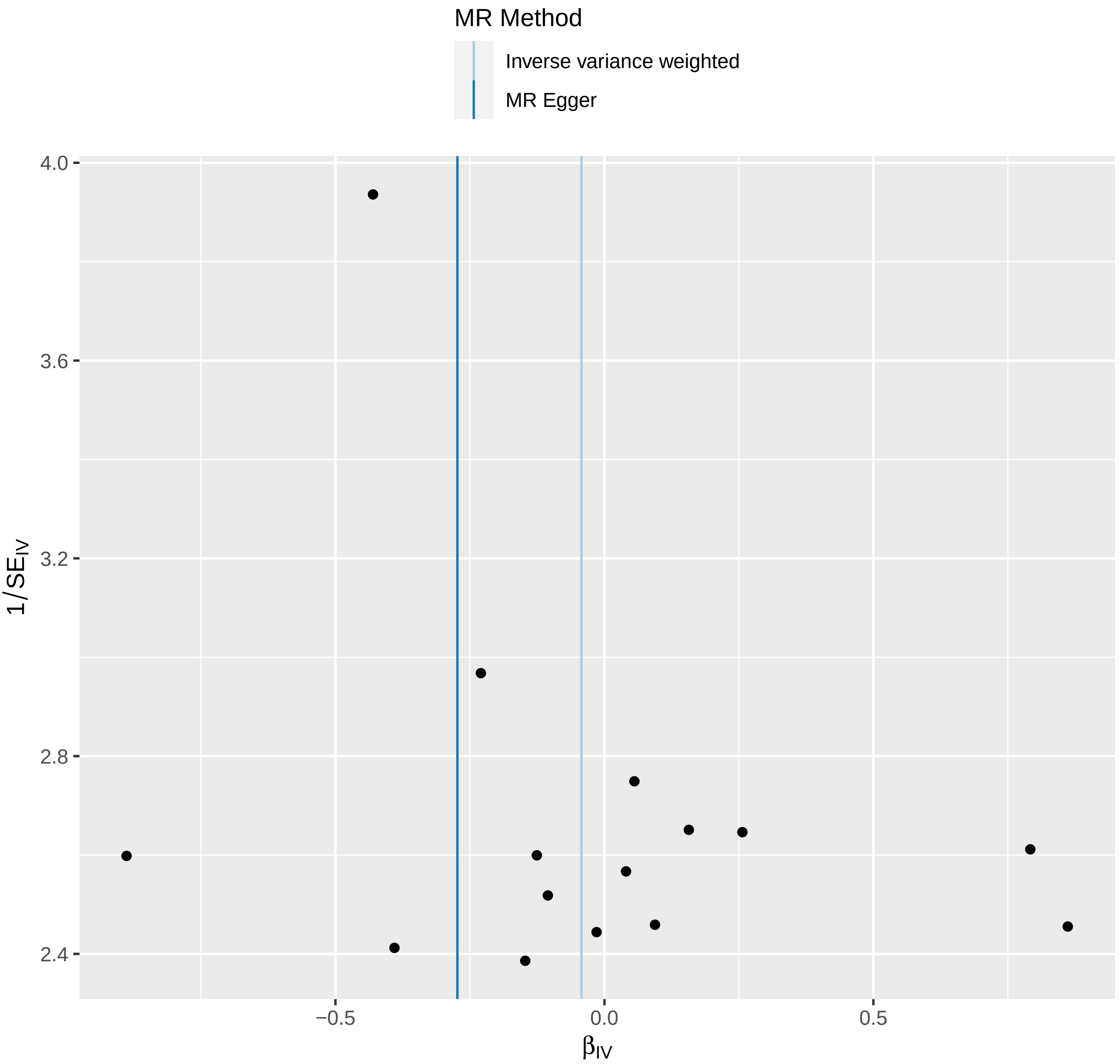

Batch 2−1691 : Gut microbiota abundance (genus Catenibacterium id.2153) on DnaJ homolog subfamily C member 18
with a threshold of 1e−05

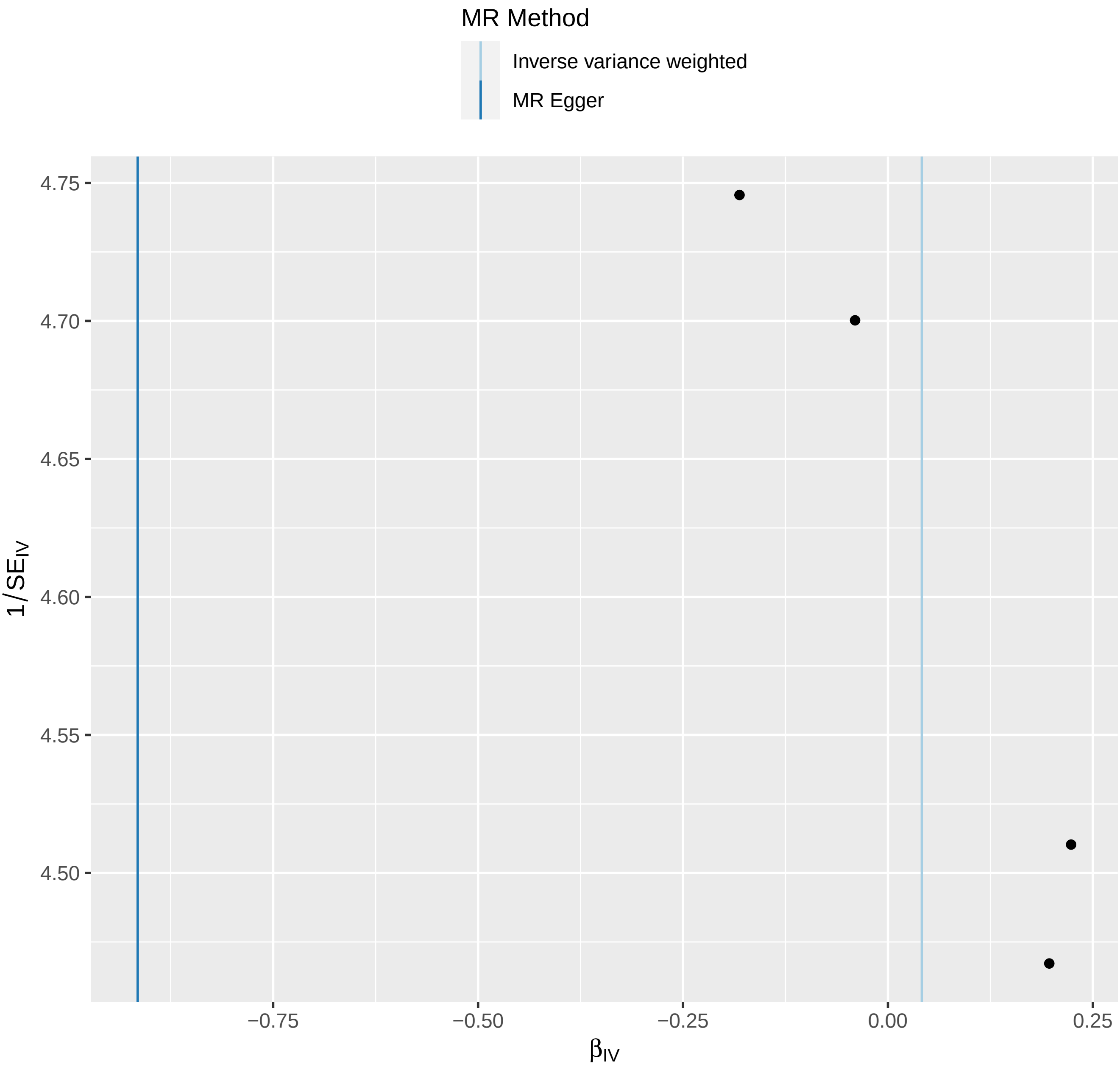

Batch 2−1692 : Gut microbiota abundance (genus Parasutterella id.2892) on DnaJ homolog subfamily C member 18
with a threshold of 1e−05

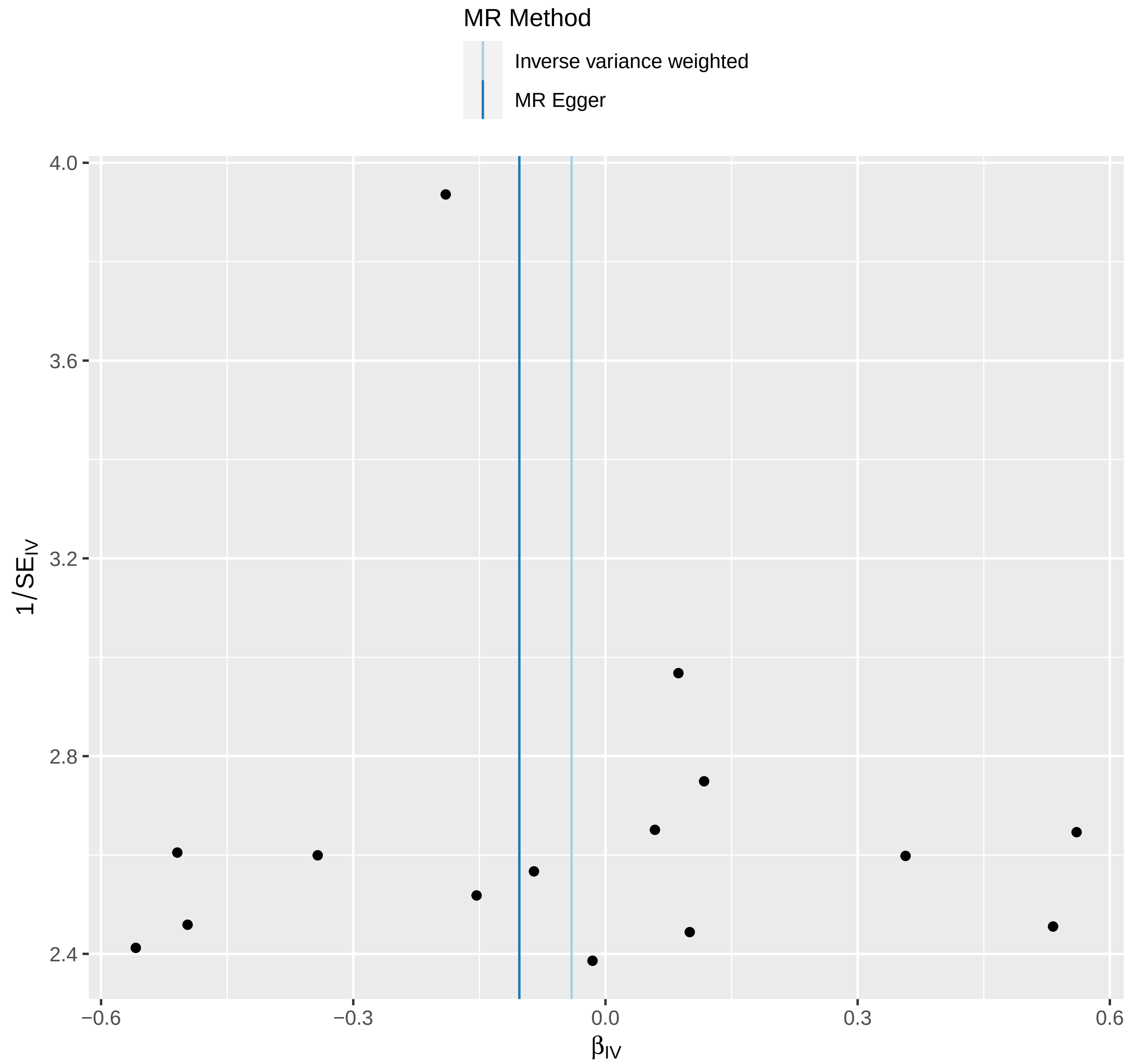

Batch 2−1693 : Gut microbiota abundance (genus Catenibacterium id.2153) on Mitochondrial import inner membrane translocase subunit TIM14 with a threshold of 1e−05

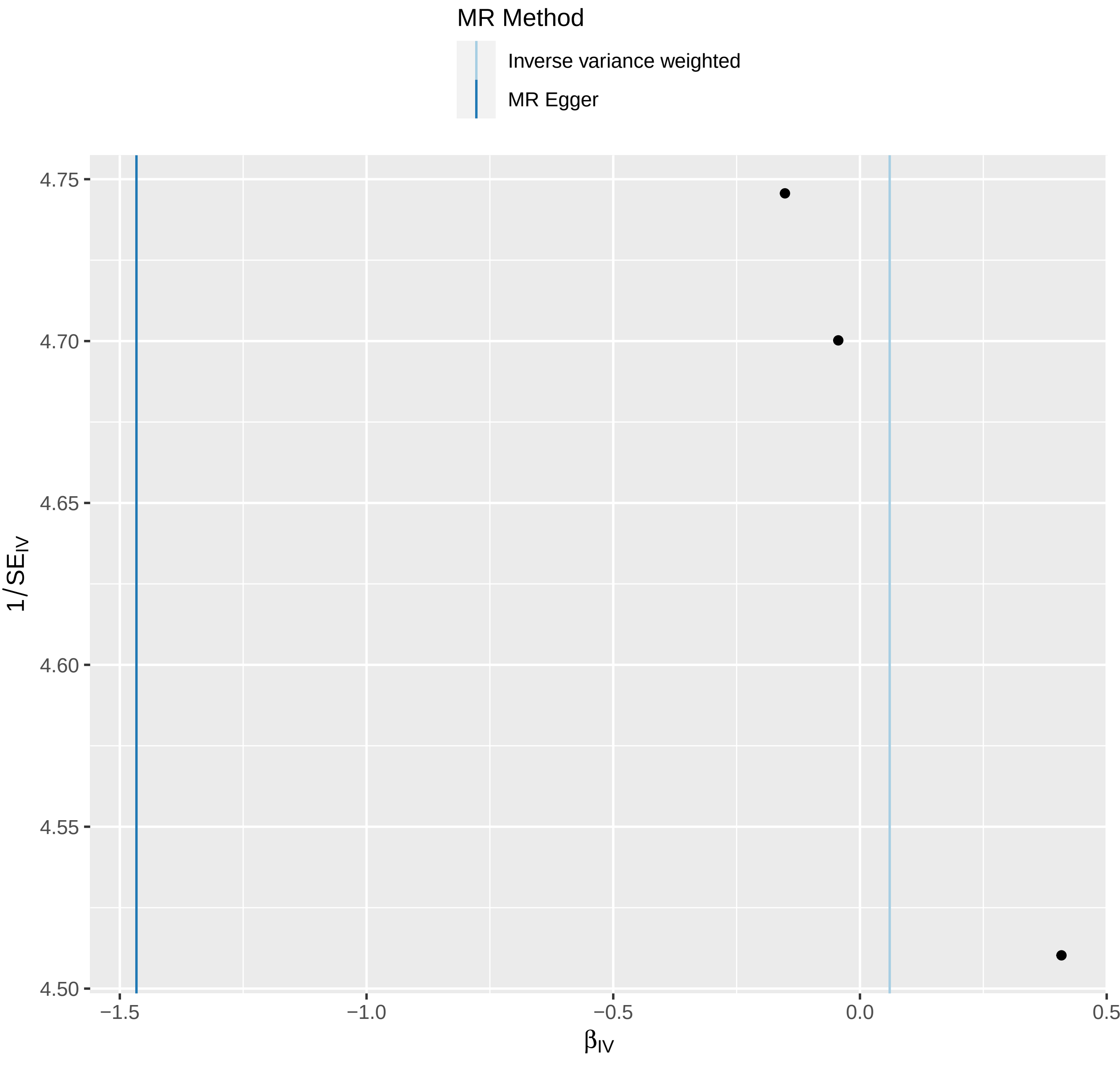

Batch 2−1694 : Gut microbiota abundance (genus Parasutterella id.2892) on Mitochondrial import inner membrane translocase subunit TIM14
with a threshold of 1e−05

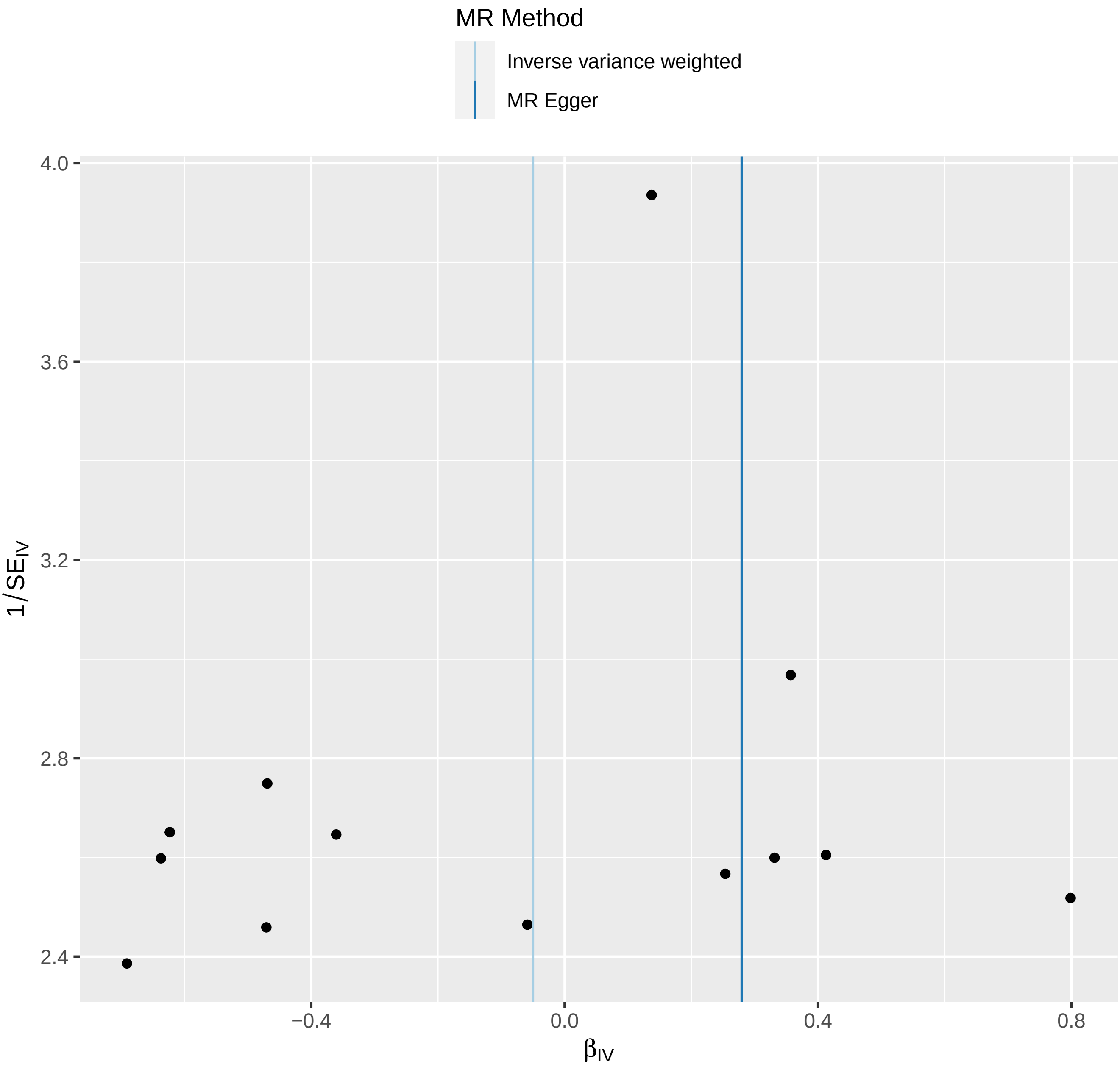

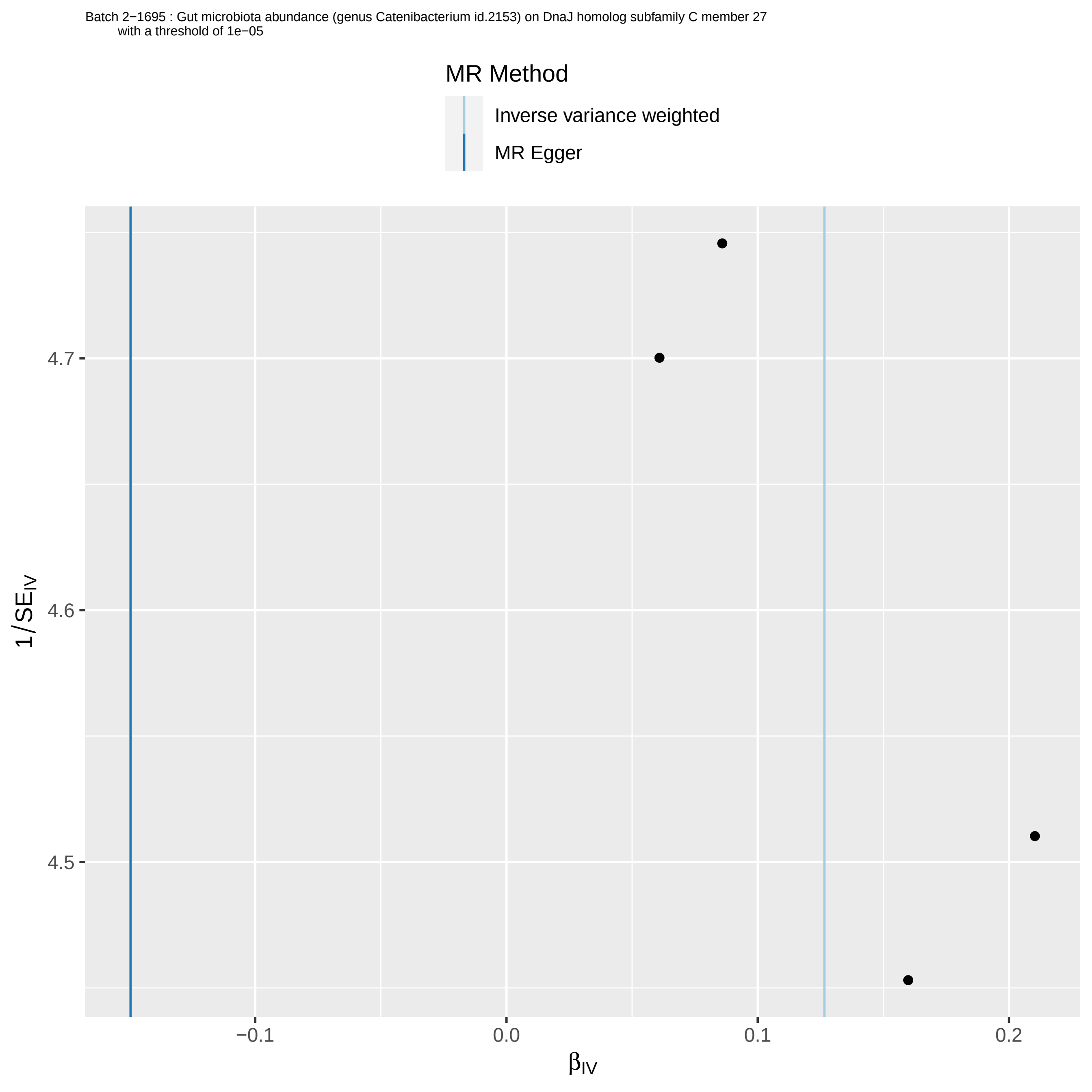
Batch 2−1695 : Gut microbiota abundance (genus Catenibacterium id.2153) on DnaJ homolog subfamily C member 27
with a threshold of 1e−05
MR Method
Inverse variance weighted
MR Egger
4.7
4.6
4.5
1/SE$_{IV}$
−0.1
0.0
0.1
0.2
β_{IV}

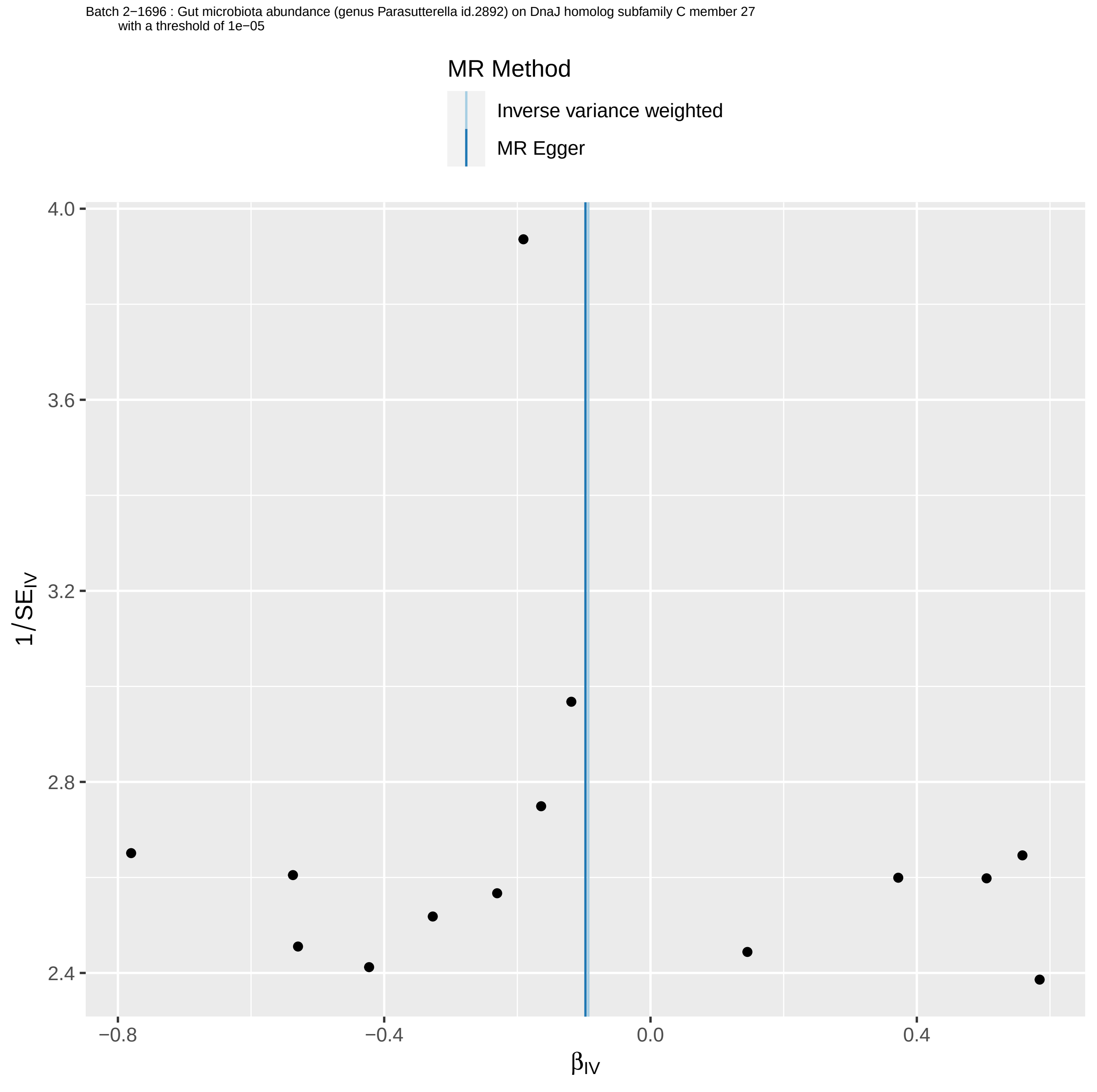
Batch 2−1696 : Gut microbiota abundance (genus Parasutterella id.2892) on DnaJ homolog subfamily C member 27
with a threshold of 1e−05
MR Method
Inverse variance weighted
MR Egger
4.0
3.6
3.2
2.8
2.4
$1/SE_{IV}$
−0.8
−0.4
0.0
0.4
β_{IV}

Batch 2−1697 : Gut microbiota abundance (genus Catenibacterium id.2153) on DnaJ homolog subfamily C member 30 with a threshold of 1e−05

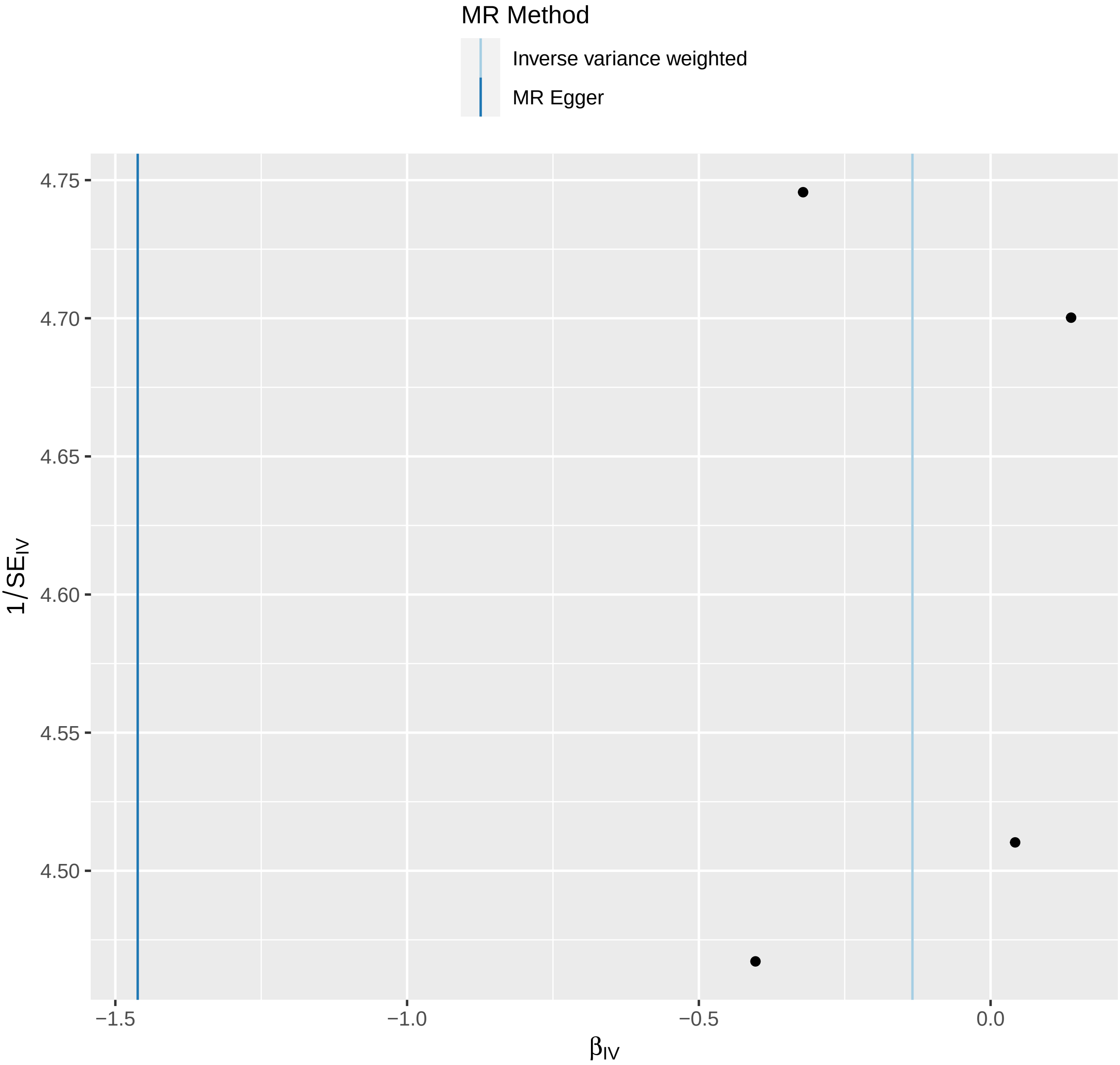

Batch 2−1698 : Gut microbiota abundance (genus Parasutterella id.2892) on DnaJ homolog subfamily C member 30
with a threshold of 1e−05

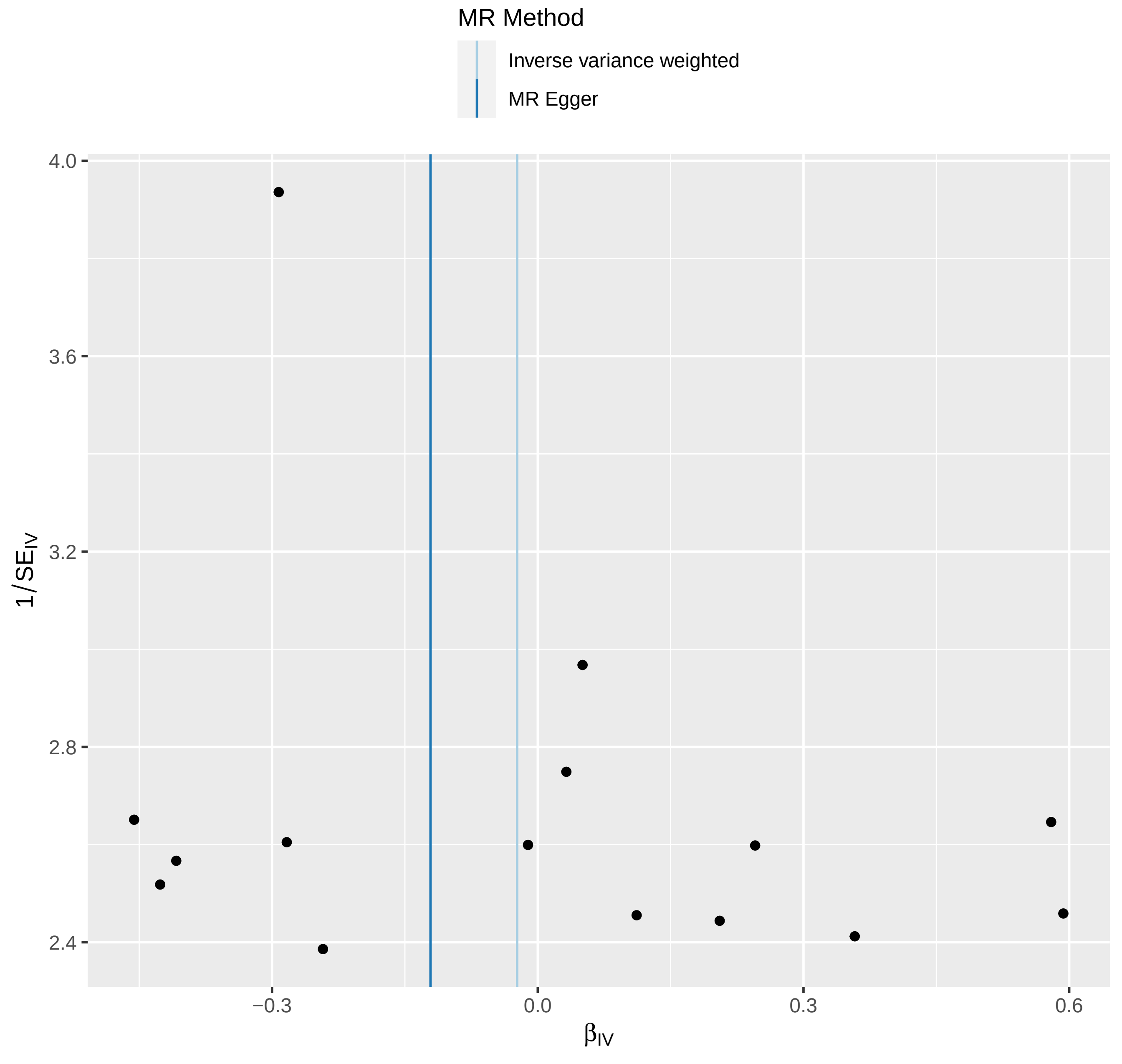

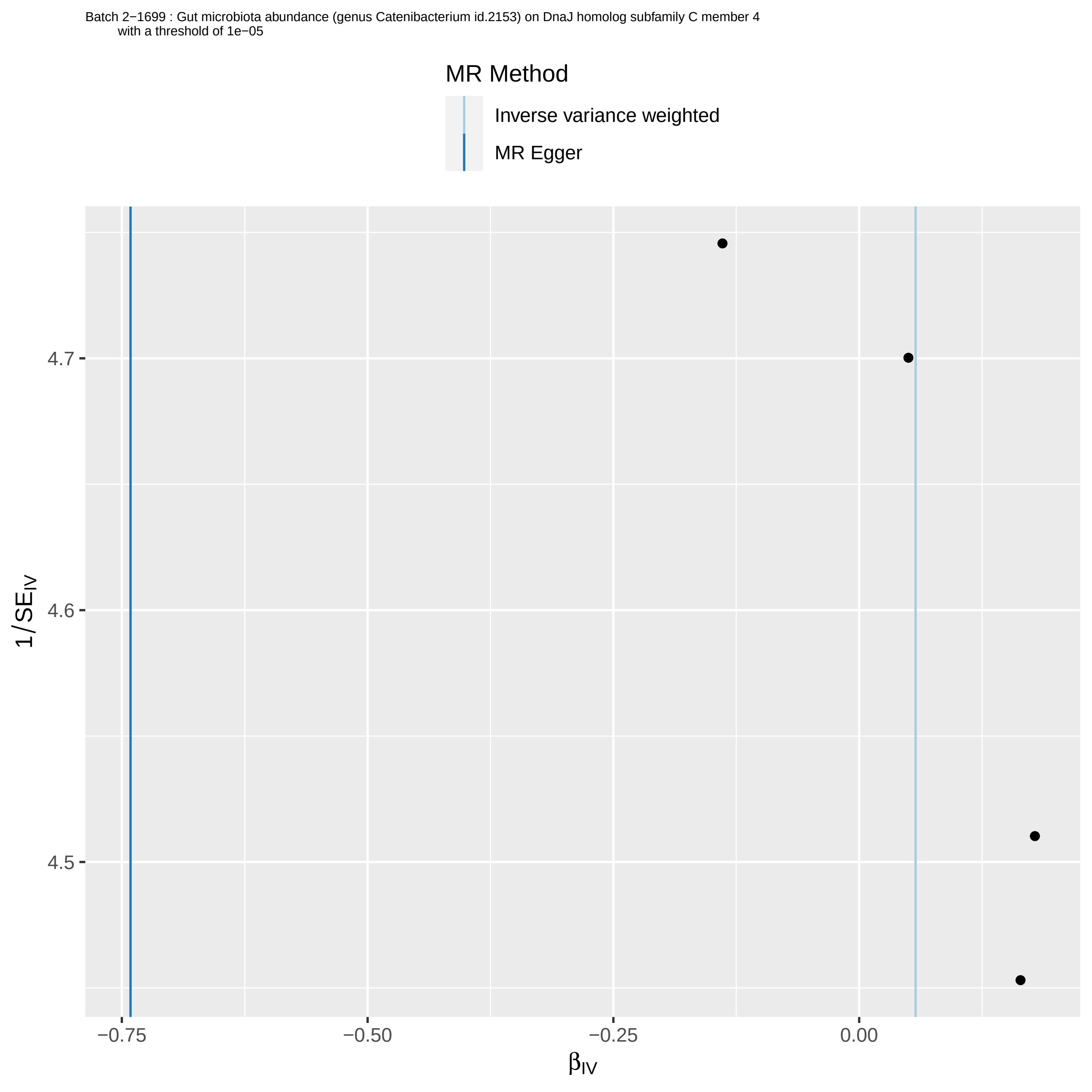

Batch 2−1699 : Gut microbiota abundance (genus Catenibacterium id.2153) on DnaJ homolog subfamily C member 4
with a threshold of 1e−05
MR Method
Inverse variance weighted
MR Egger
4.7
4.6
4.5
1/SE$_{IV}$
−0.75
−0.50
−0.25
0.00
β$_{IV}$

Batch 2−1700 : Gut microbiota abundance (genus Parasutterella id.2892) on DnaJ homolog subfamily C member 4
with a threshold of 1e−05

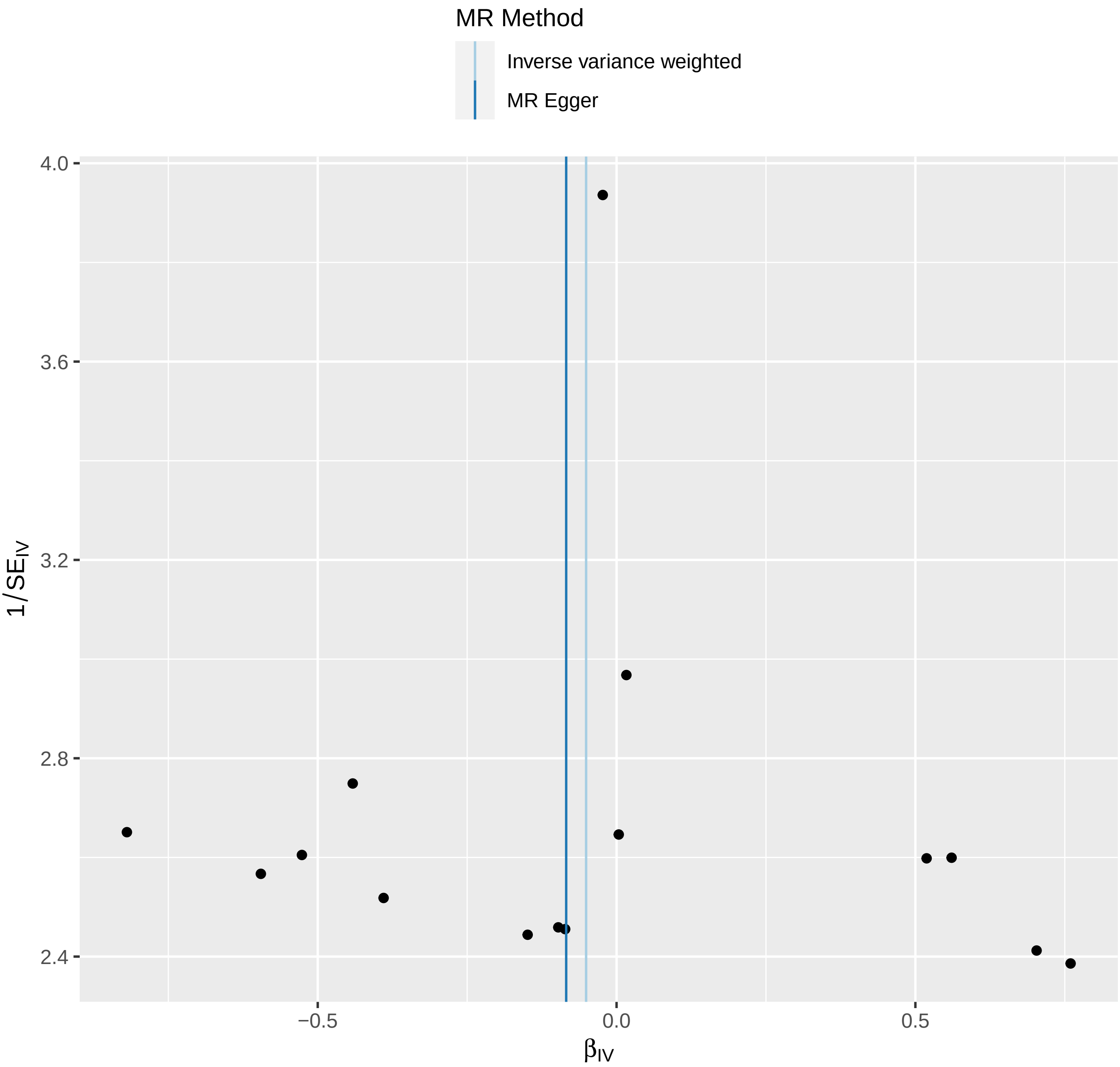

Batch 2−1701 : Gut microbiota abundance (genus Catenibacterium id.2153) on Deoxyribonuclease−2−beta
with a threshold of 1e−05

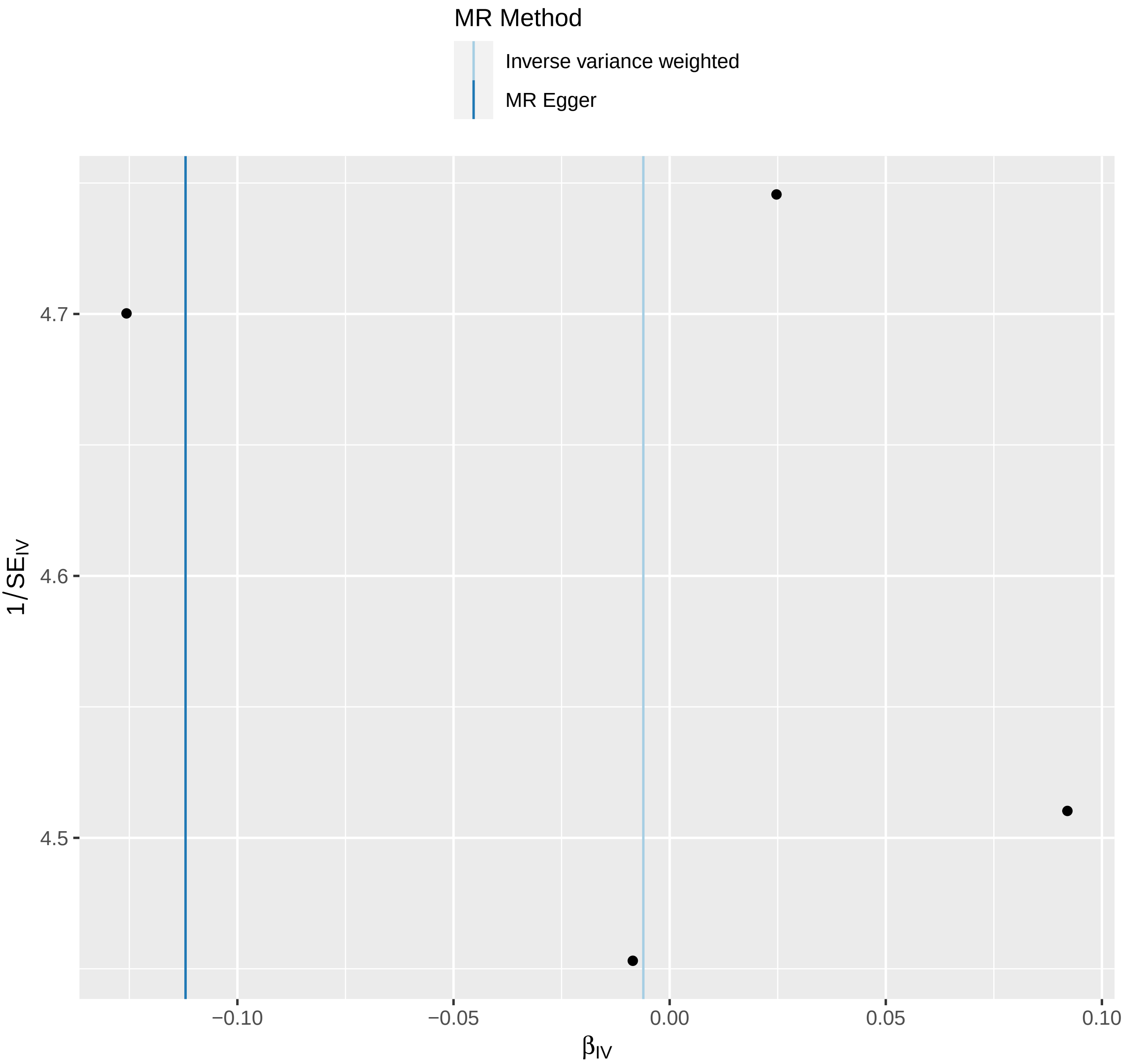

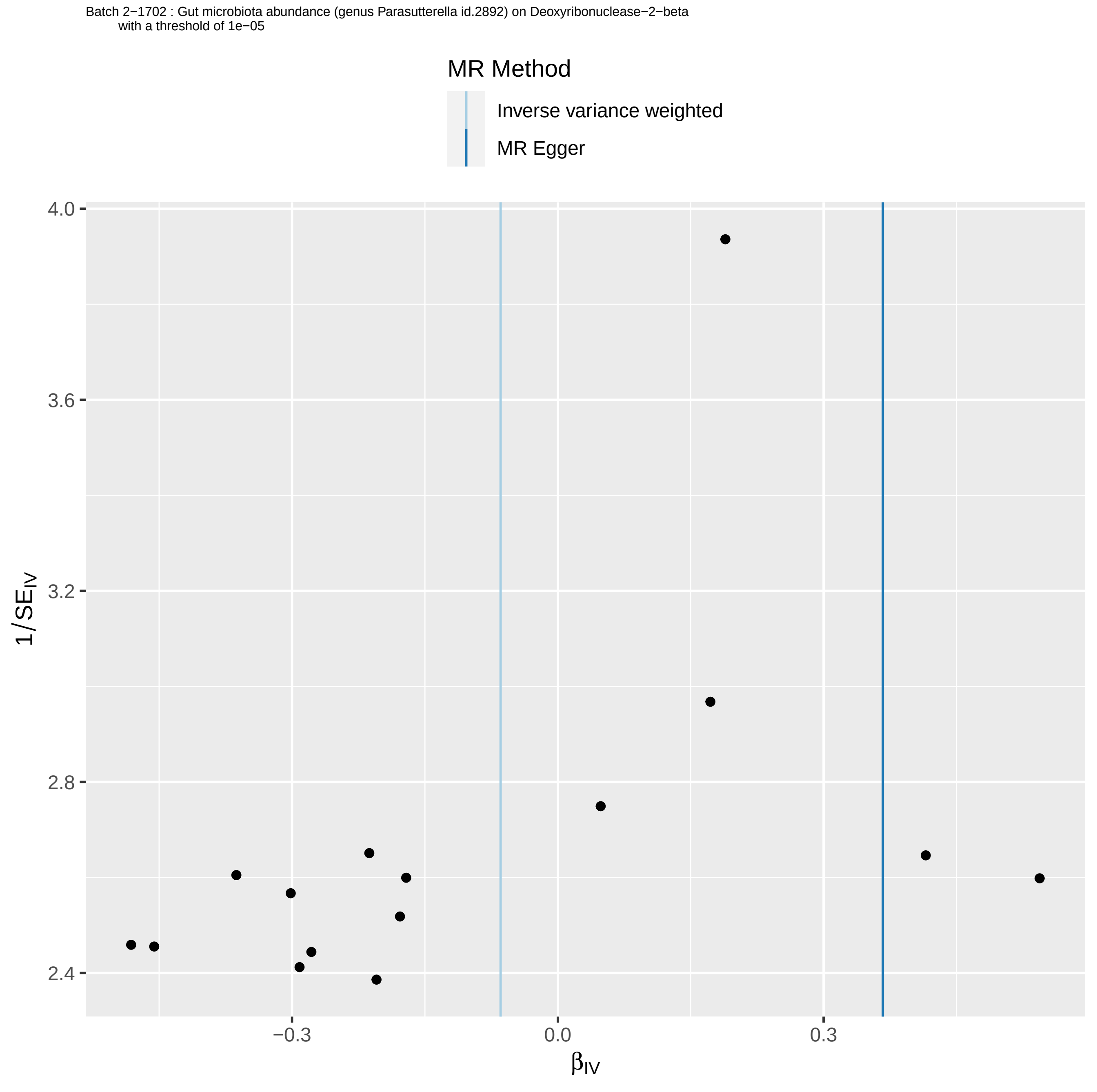

Batch 2−1702 : Gut microbiota abundance (genus Parasutterella id.2892) on Deoxyribonuclease−2−beta
with a threshold of 1e−05
MR Method
Inverse variance weighted
MR Egger
4.0
3.6
3.2
2.8
2.4
1/SE$_{IV}$
−0.3
0.0
0.3
β$_{IV}$

Batch 2−1703 : Gut microbiota abundance (genus Catenibacterium id.2153) on Delta and Notch−like epidermal growth factor−related receptor with a threshold of 1e−05

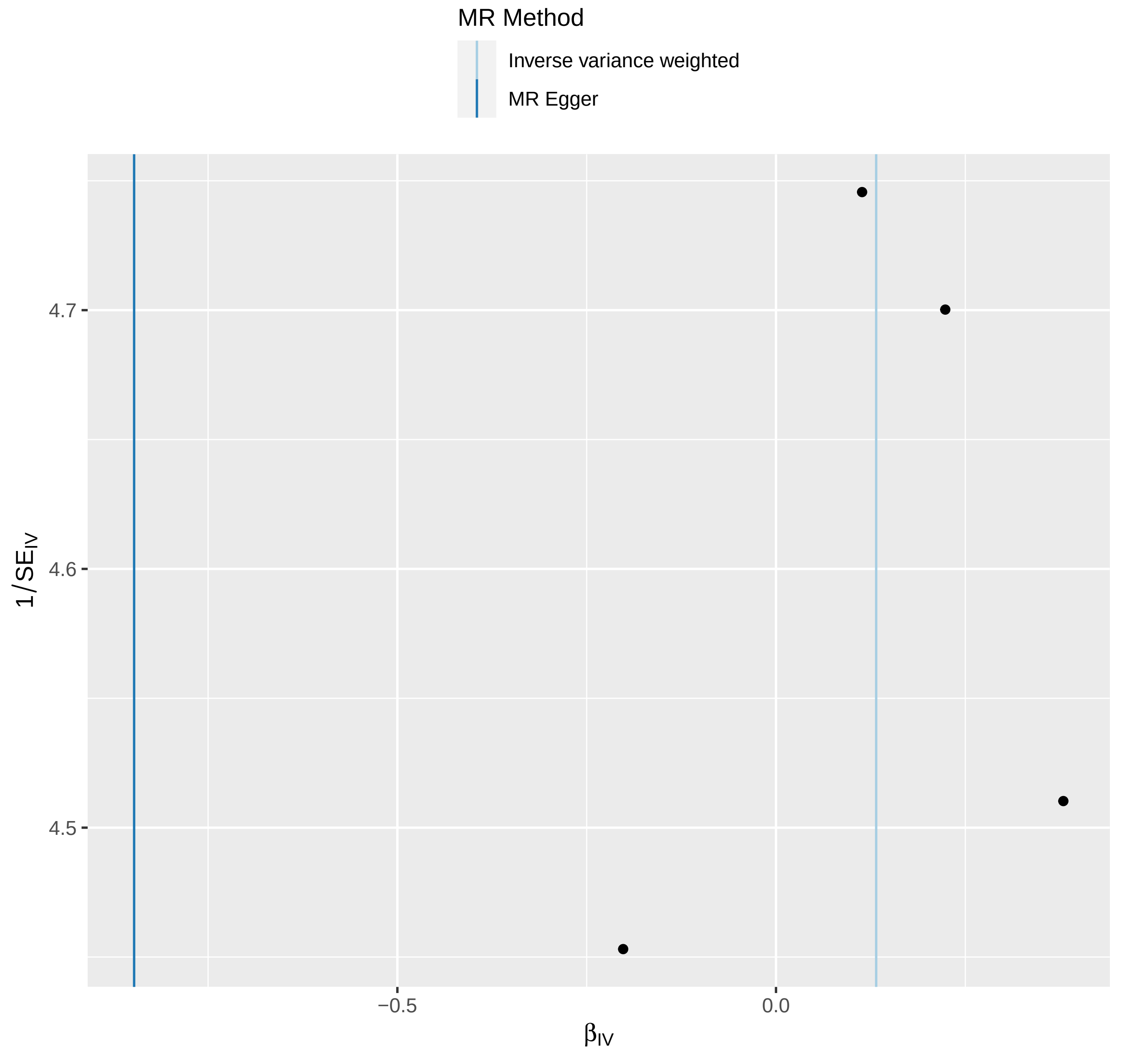

Batch 2−1704 : Gut microbiota abundance (genus Parasutterella id.2892) on Delta and Notch−like epidermal growth factor−related receptor with a threshold of 1e−05

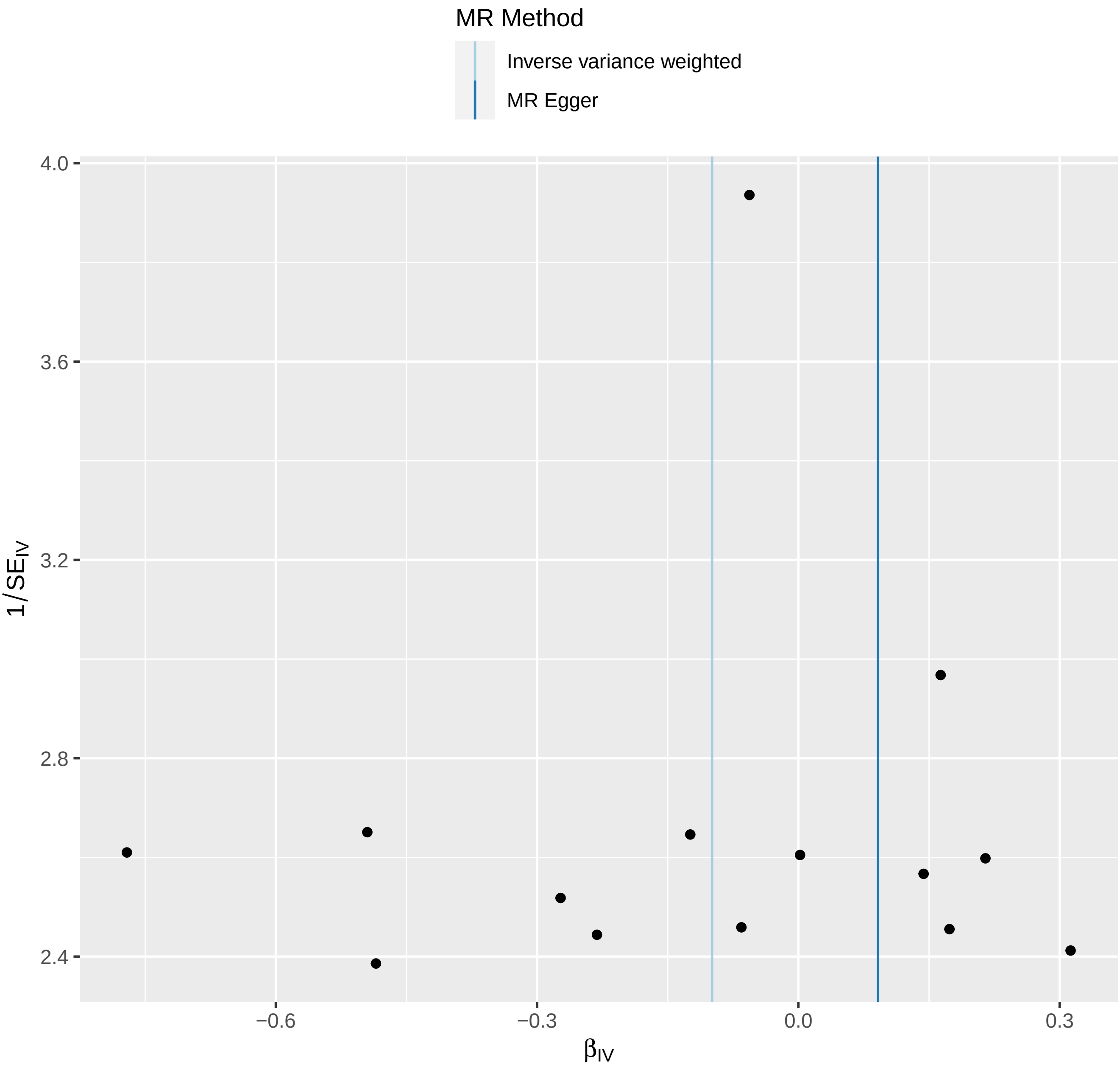

Batch 2−1705 : Gut microbiota abundance (genus Catenibacterium id.2153) on Delta and Notch−like epidermal growth factor−related receptor with a threshold of 1e−05

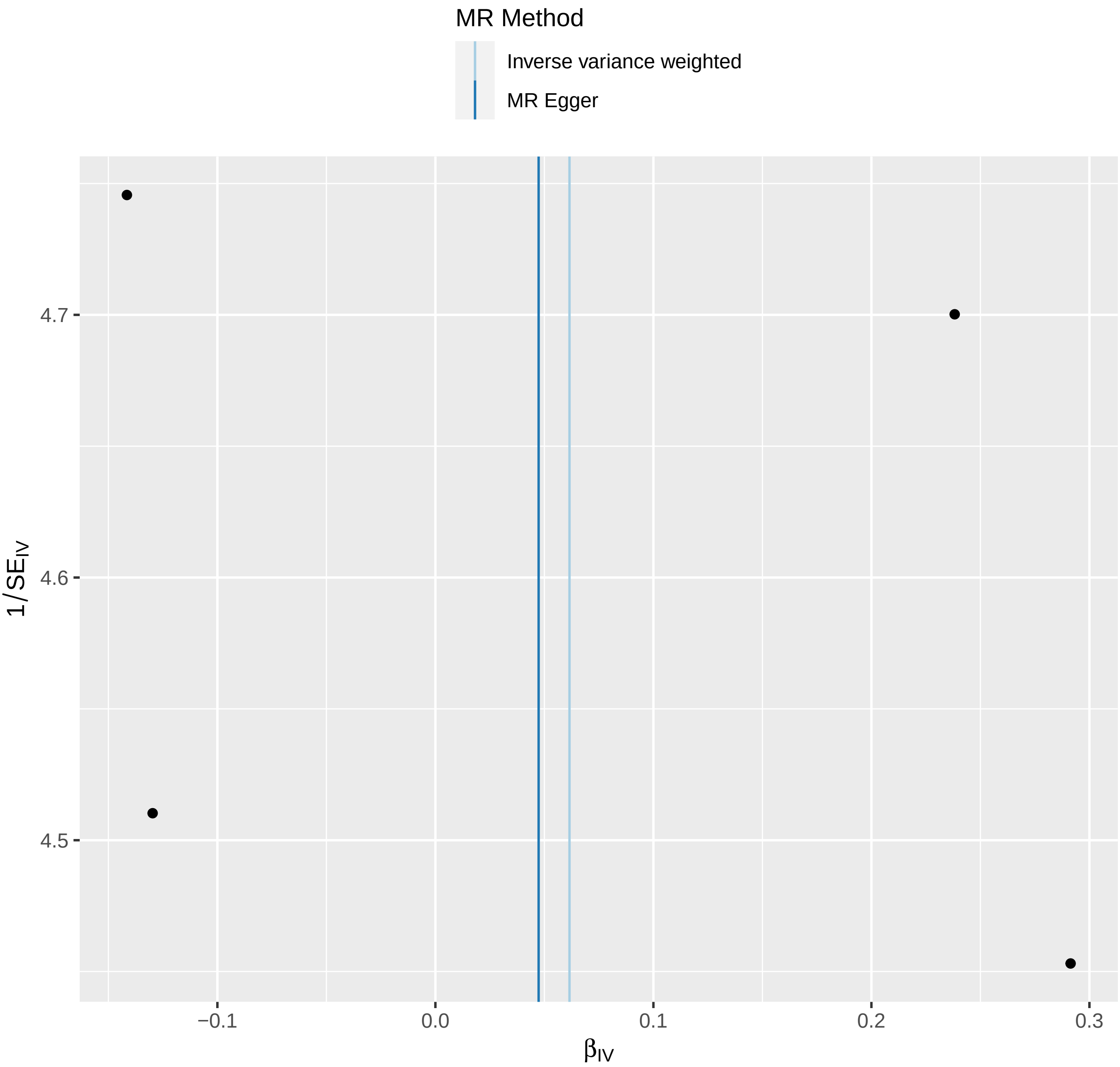

Batch 2−1706 : Gut microbiota abundance (genus Parasutterella id.2892) on Delta and Notch−like epidermal growth factor−related receptor
with a threshold of 1e−05

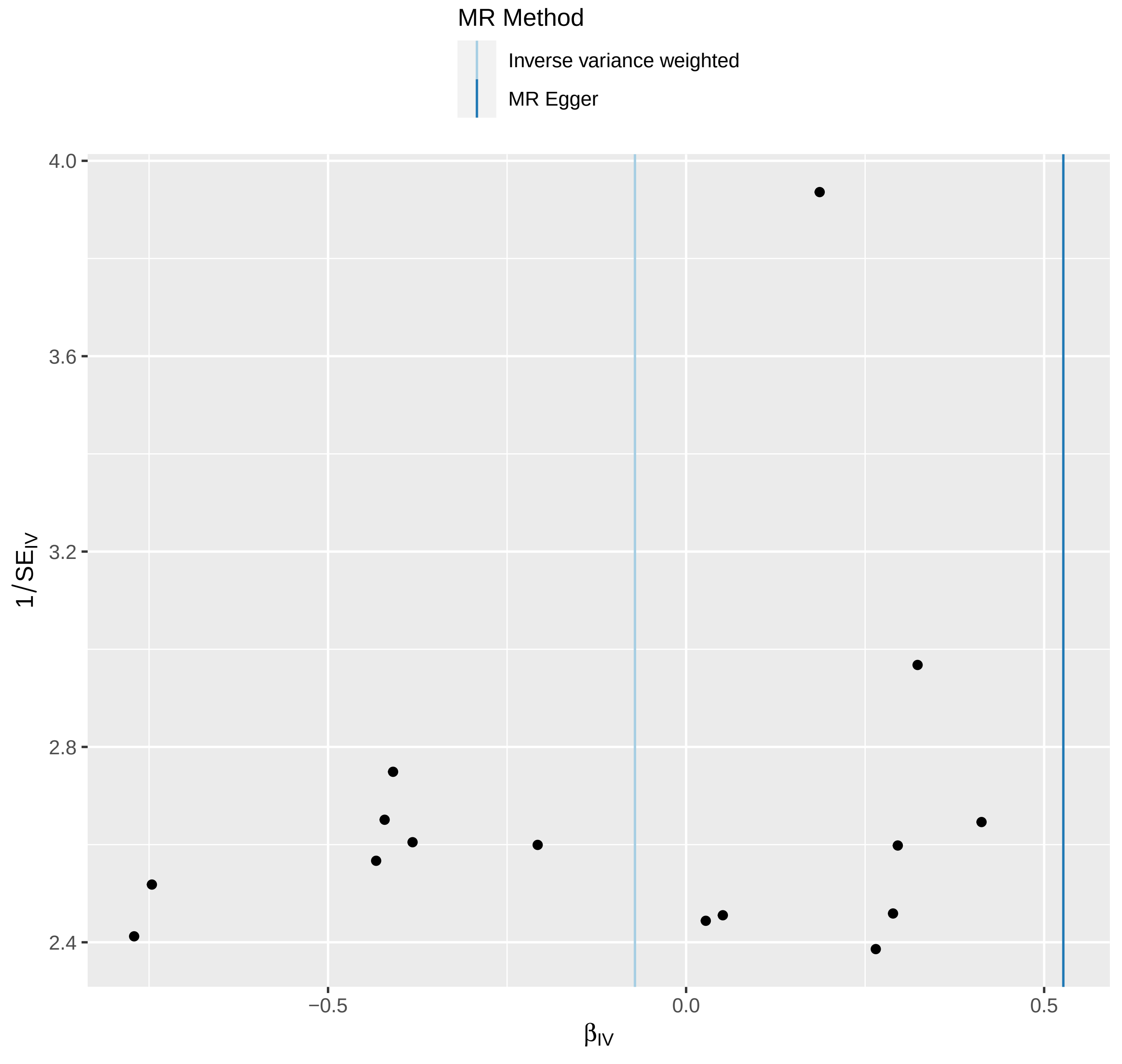

Batch 2−1707 : Gut microbiota abundance (genus Catenibacterium id.2153) on Dedicator of cytokinesis protein 9
with a threshold of 1e−05

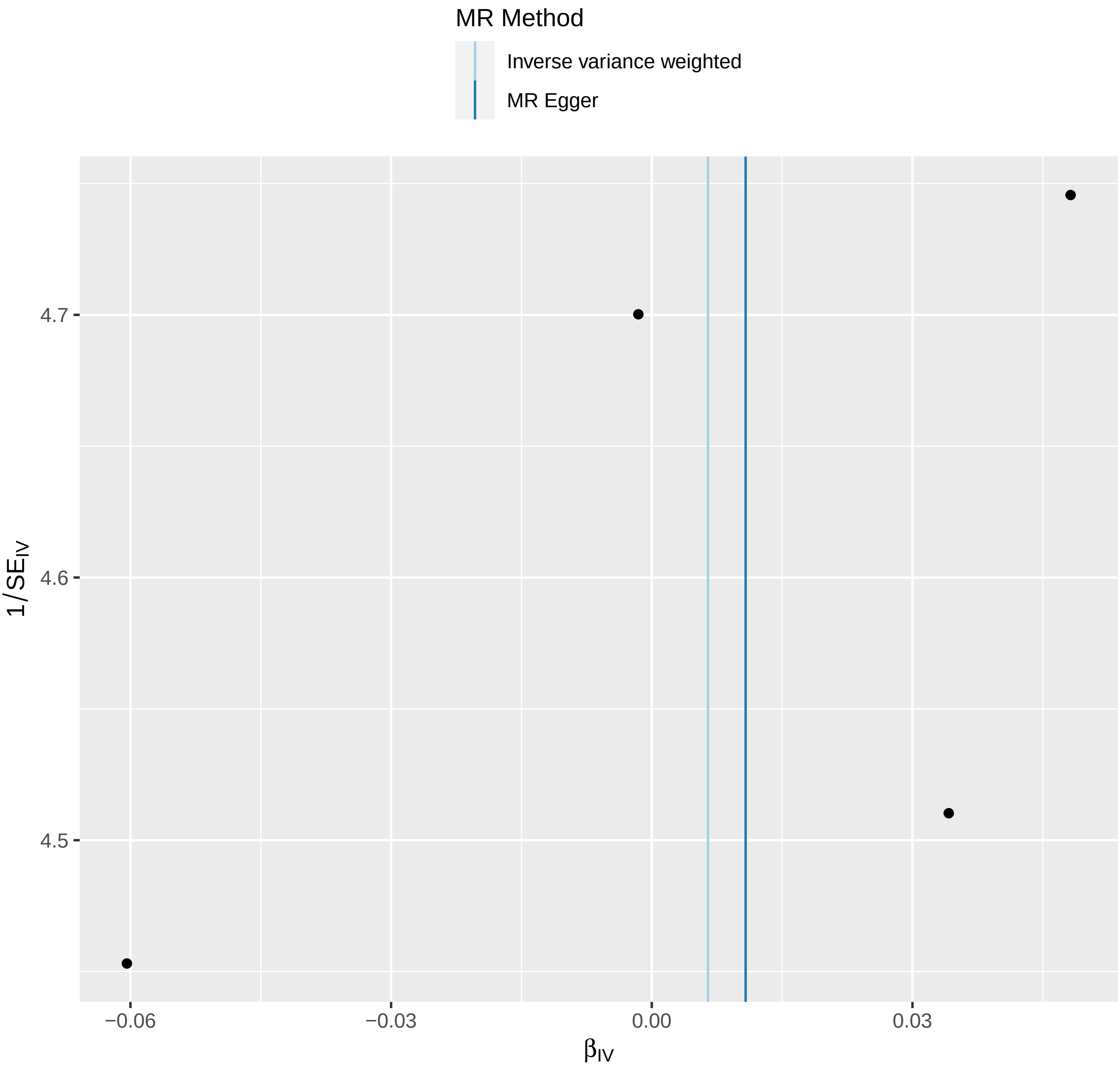

Batch 2−1708 : Gut microbiota abundance (genus Parasutterella id.2892) on Dedicator of cytokinesis protein 9 with a threshold of 1e−05

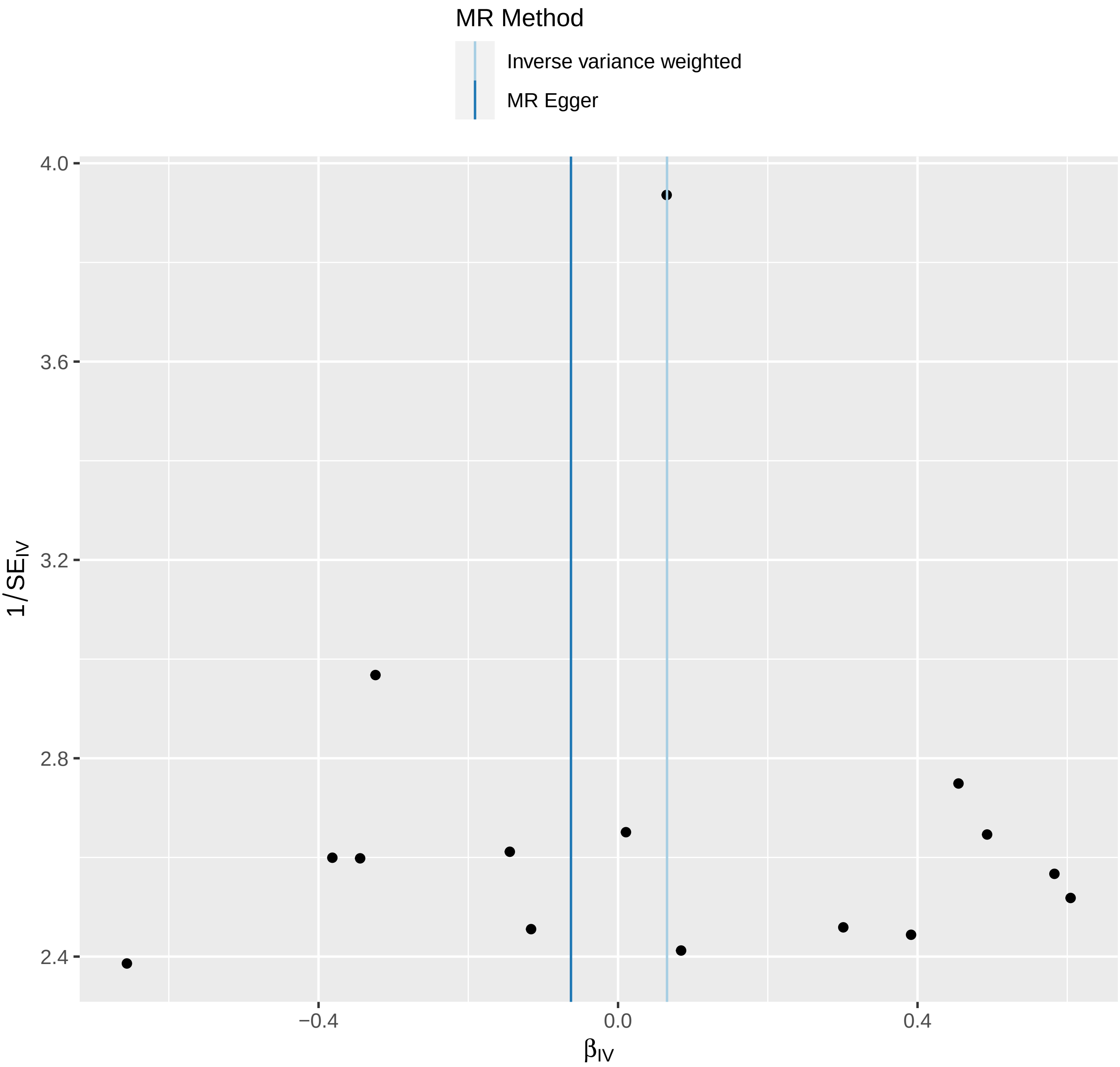

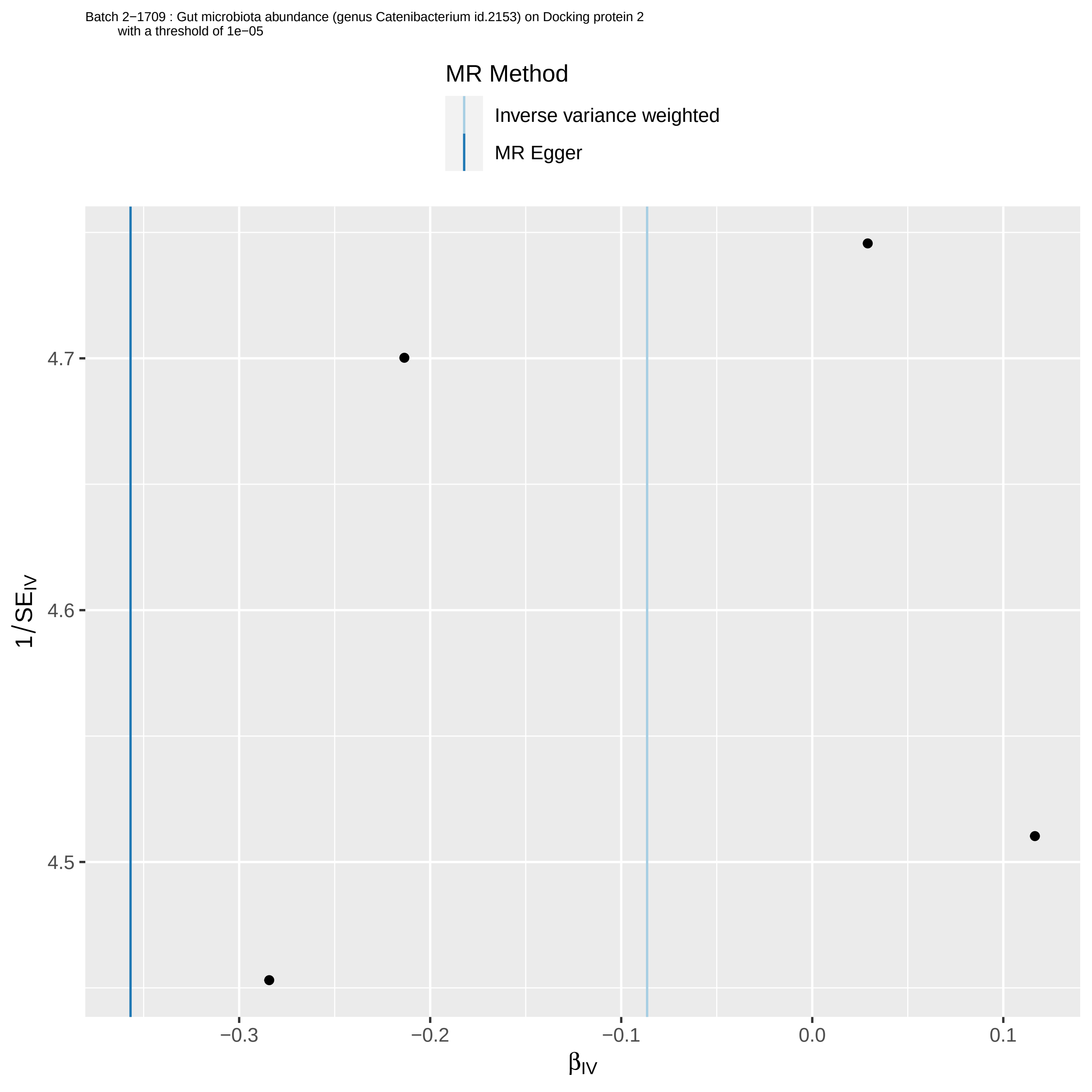
Batch 2−1709 : Gut microbiota abundance (genus Catenibacterium id.2153) on Docking protein 2
with a threshold of 1e−05
MR Method
Inverse variance weighted
MR Egger
4.7
4.6
4.5
1/SE_IV
−0.3
−0.2
−0.1
0.0
0.1
β_IV

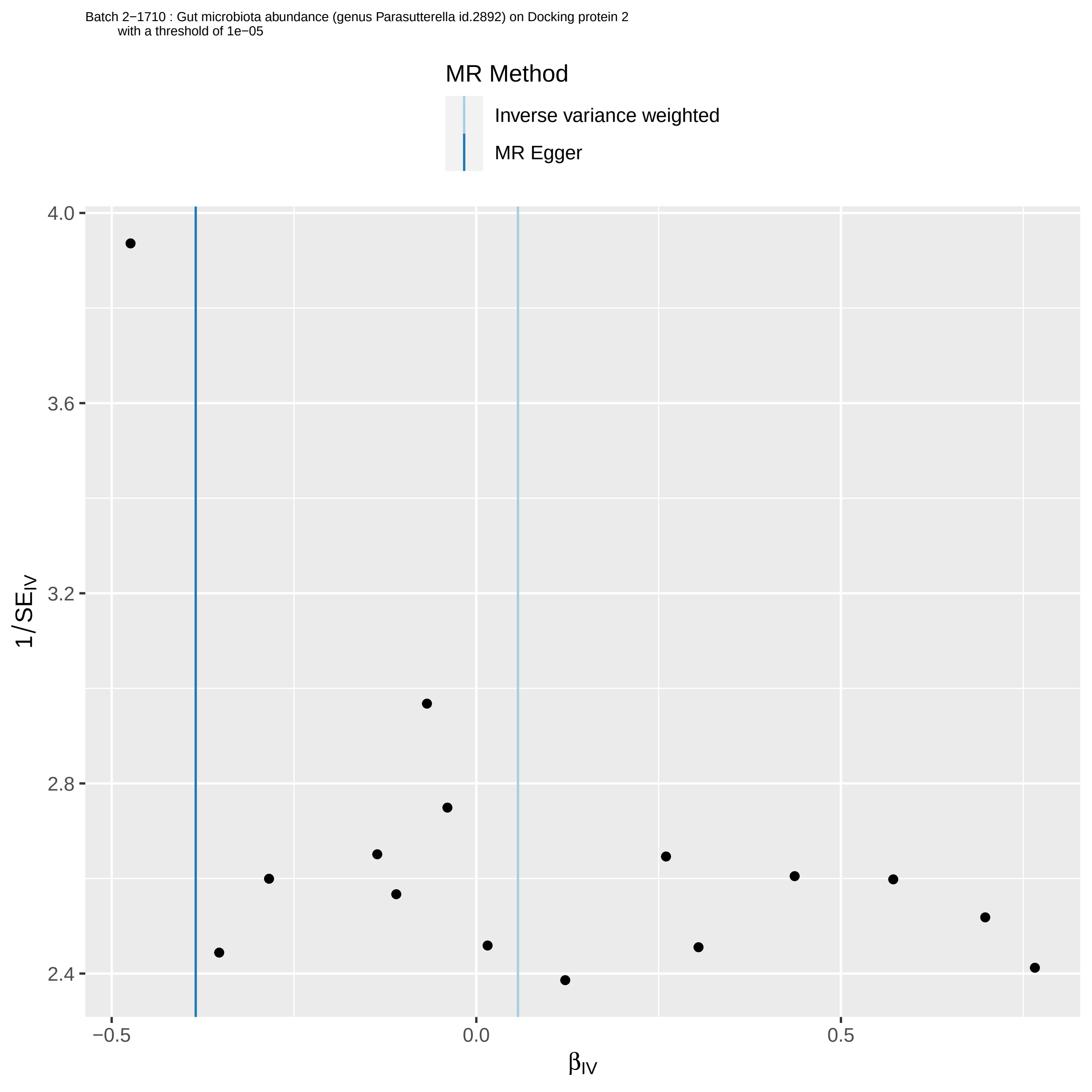

Batch 2−1710 : Gut microbiota abundance (genus Parasutterella id.2892) on Docking protein 2
with a threshold of 1e−05
MR Method
Inverse variance weighted
MR Egger
4.0
3.6
3.2
2.8
2.4
1/SE_IV
−0.5
0.0
0.5
β_IV

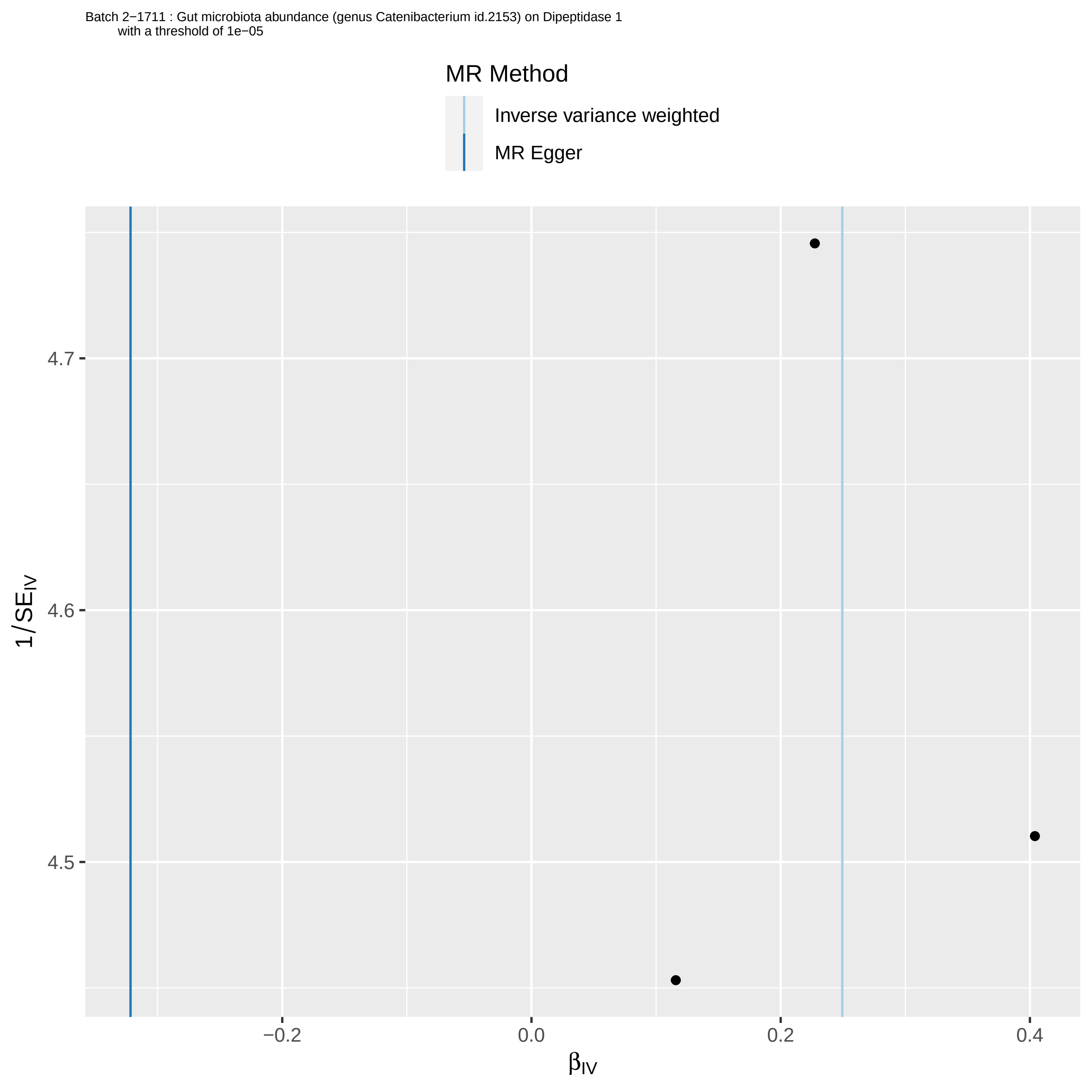

Batch 2−1711 : Gut microbiota abundance (genus Catenibacterium id.2153) on Dipeptidase 1
with a threshold of 1e−05
MR Method
Inverse variance weighted
MR Egger
4.7
4.6
4.5
1/SE$_{IV}$
−0.2
0.0
0.2
0.4
β$_{IV}$

Batch 2−1712 : Gut microbiota abundance (genus Parasutterella id.2892) on Dipeptidase 1
with a threshold of 1e−05

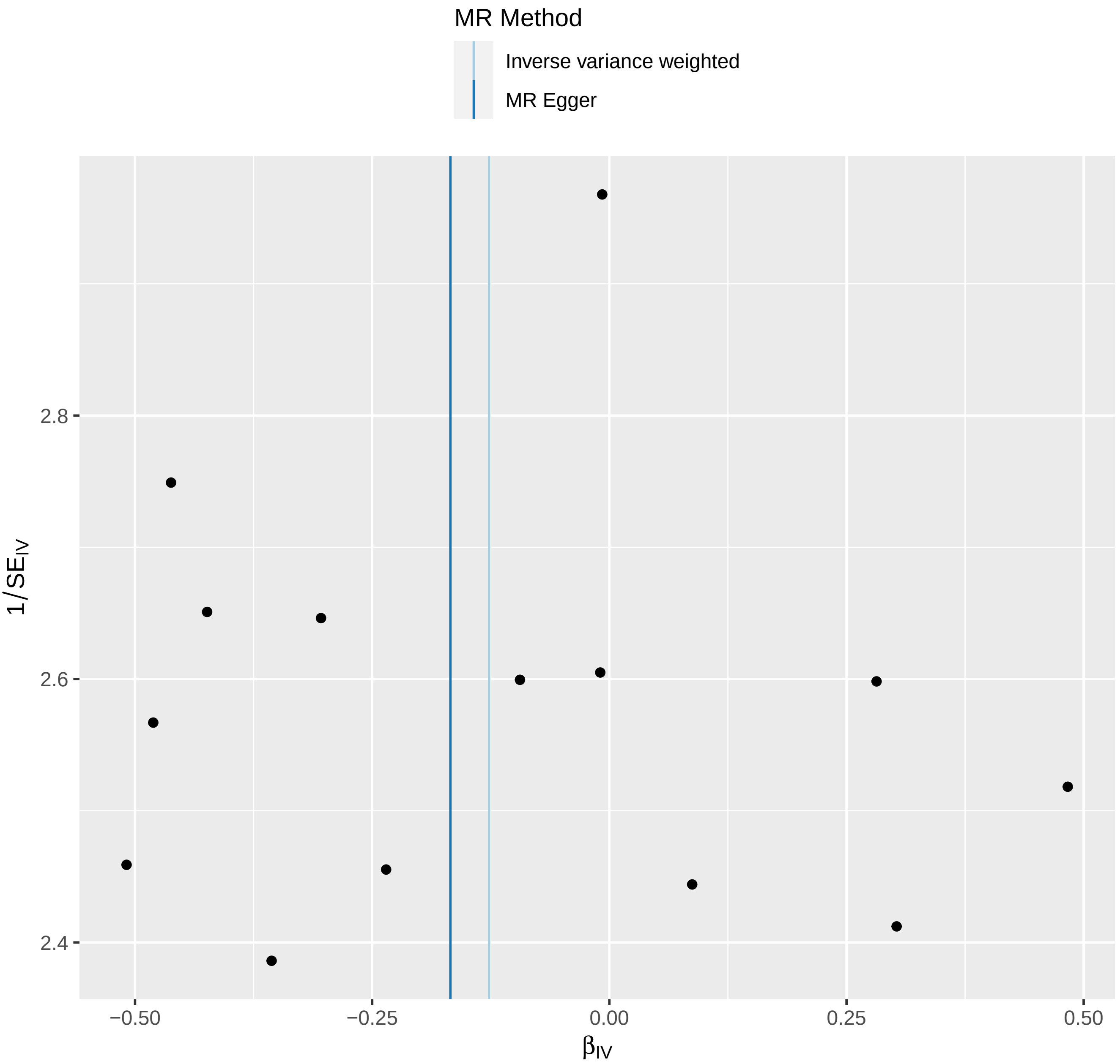

Batch 2−1713 : Gut microbiota abundance (genus Catenibacterium id.2153) on Dipeptidase 2
with a threshold of 1e−05

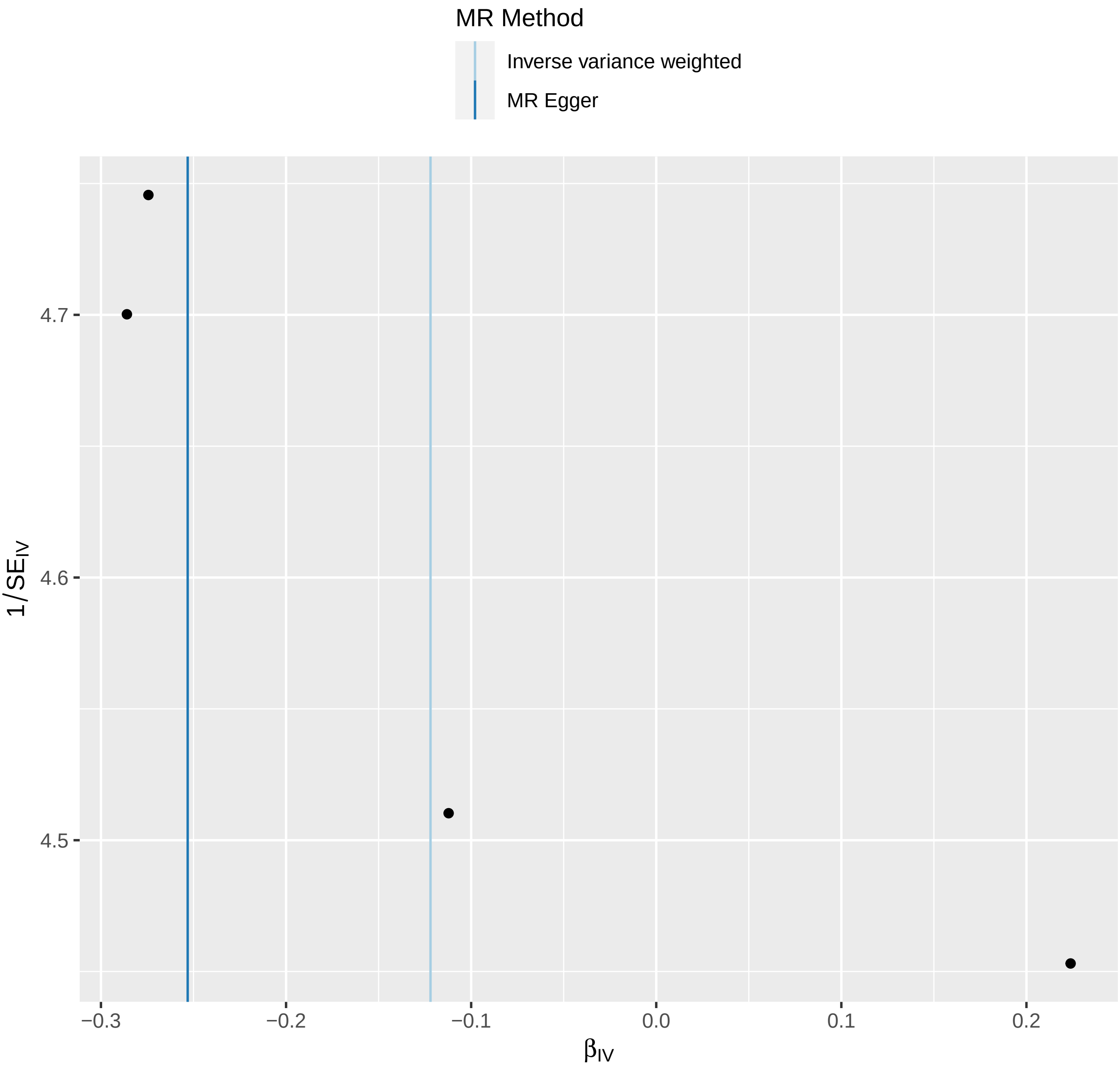

Batch 2−1714 : Gut microbiota abundance (genus Parasutterella id.2892) on Dipeptidase 2
with a threshold of 1e−05

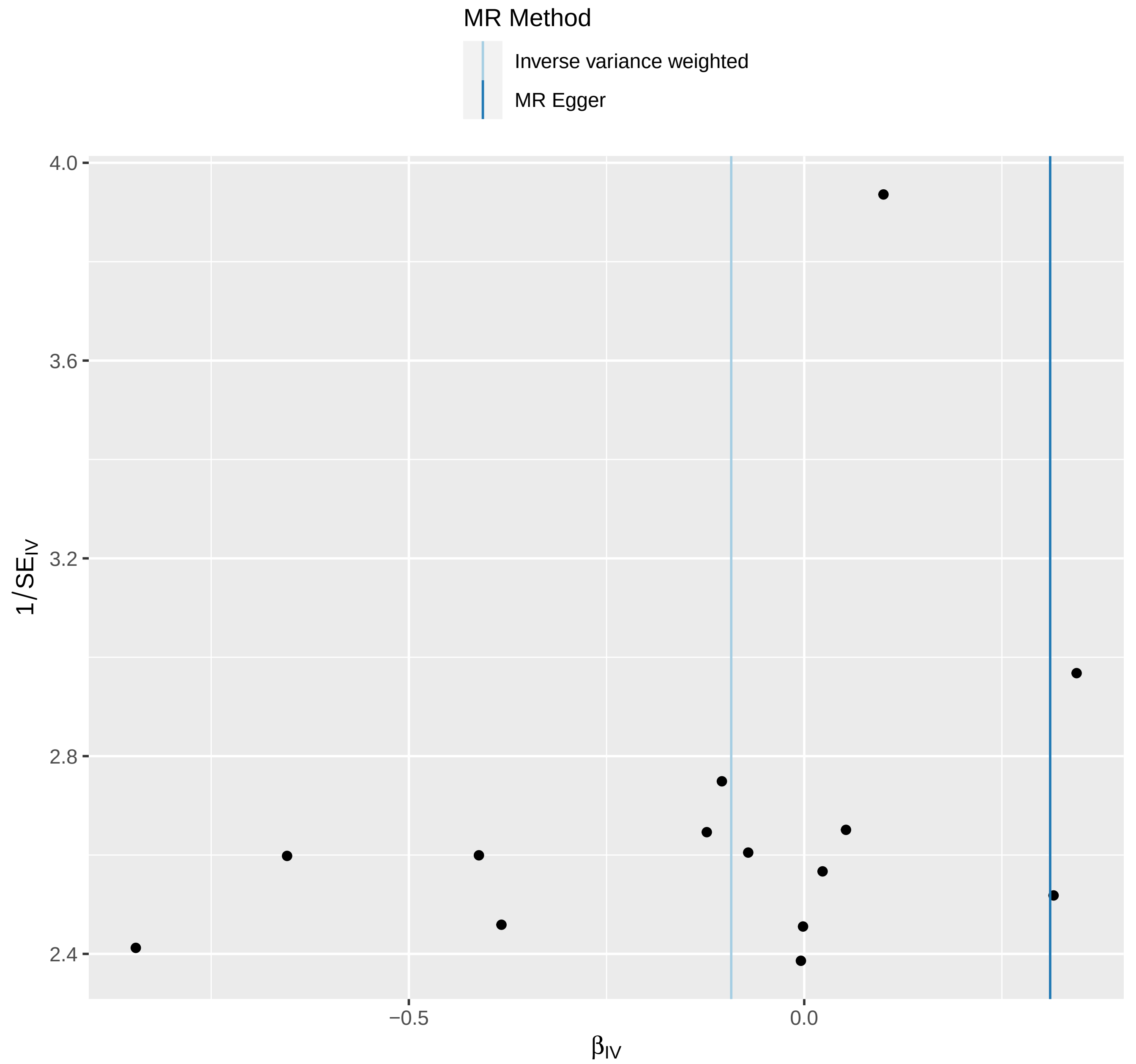

Batch 2−1715 : Gut microbiota abundance (genus Catenibacterium id.2153) on Inactive dipeptidyl peptidase 10
with a threshold of 1e−05

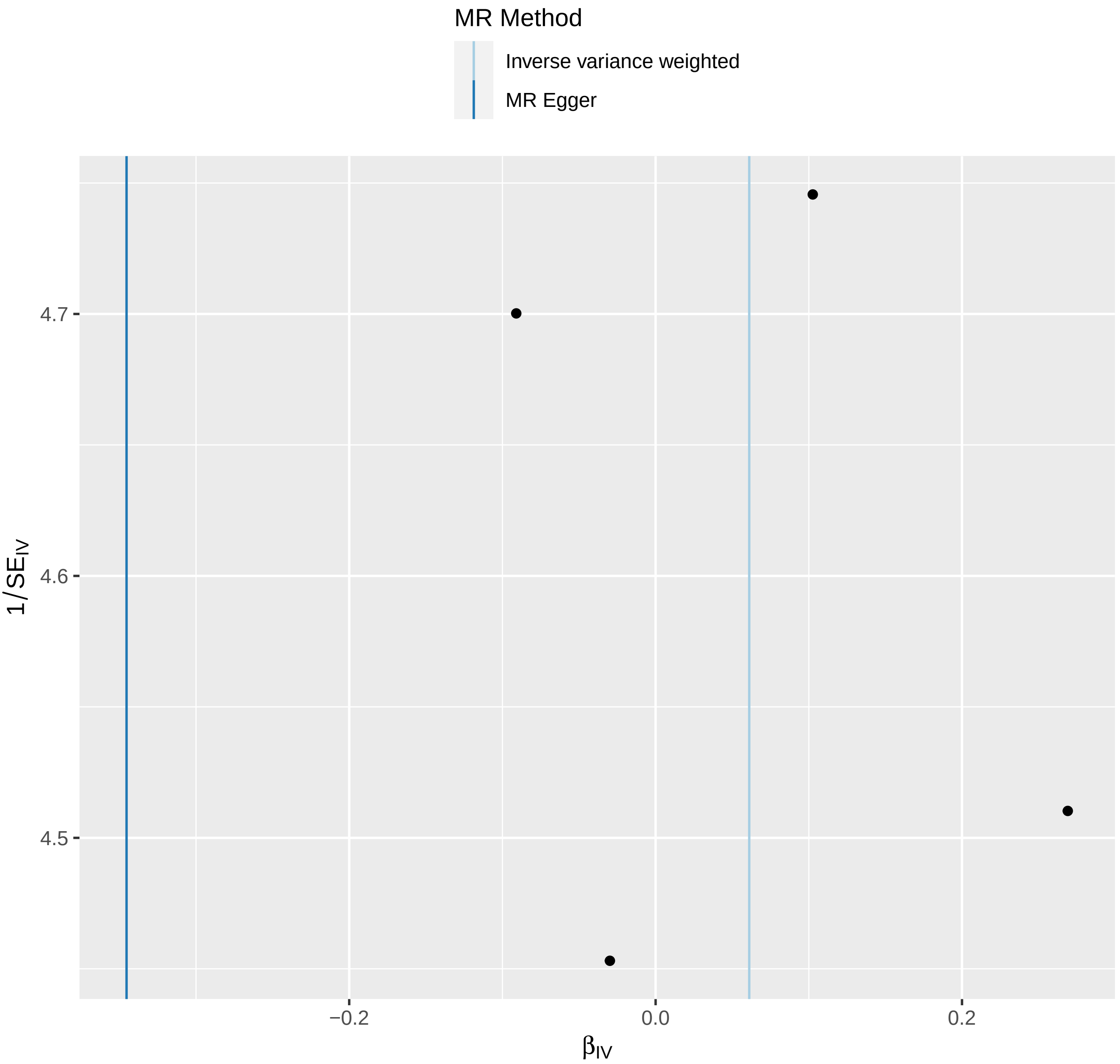

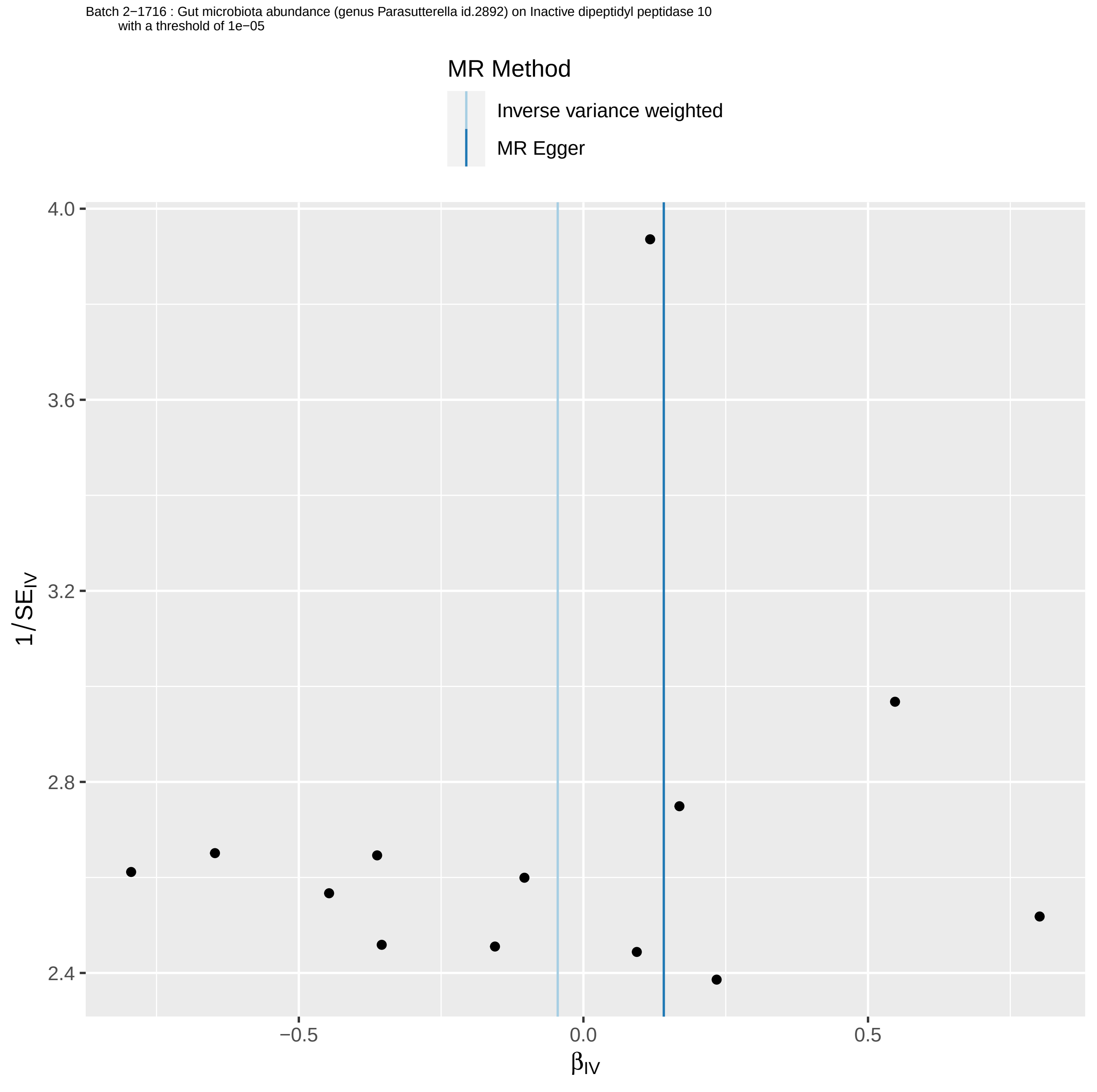

Batch 2−1716 : Gut microbiota abundance (genus Parasutterella id.2892) on Inactive dipeptidyl peptidase 10
with a threshold of 1e−05
MR Method
Inverse variance weighted
MR Egger
4.0
3.6
3.2
2.8
2.4
1/SE_IV
−0.5
0.0
0.5
β_IV

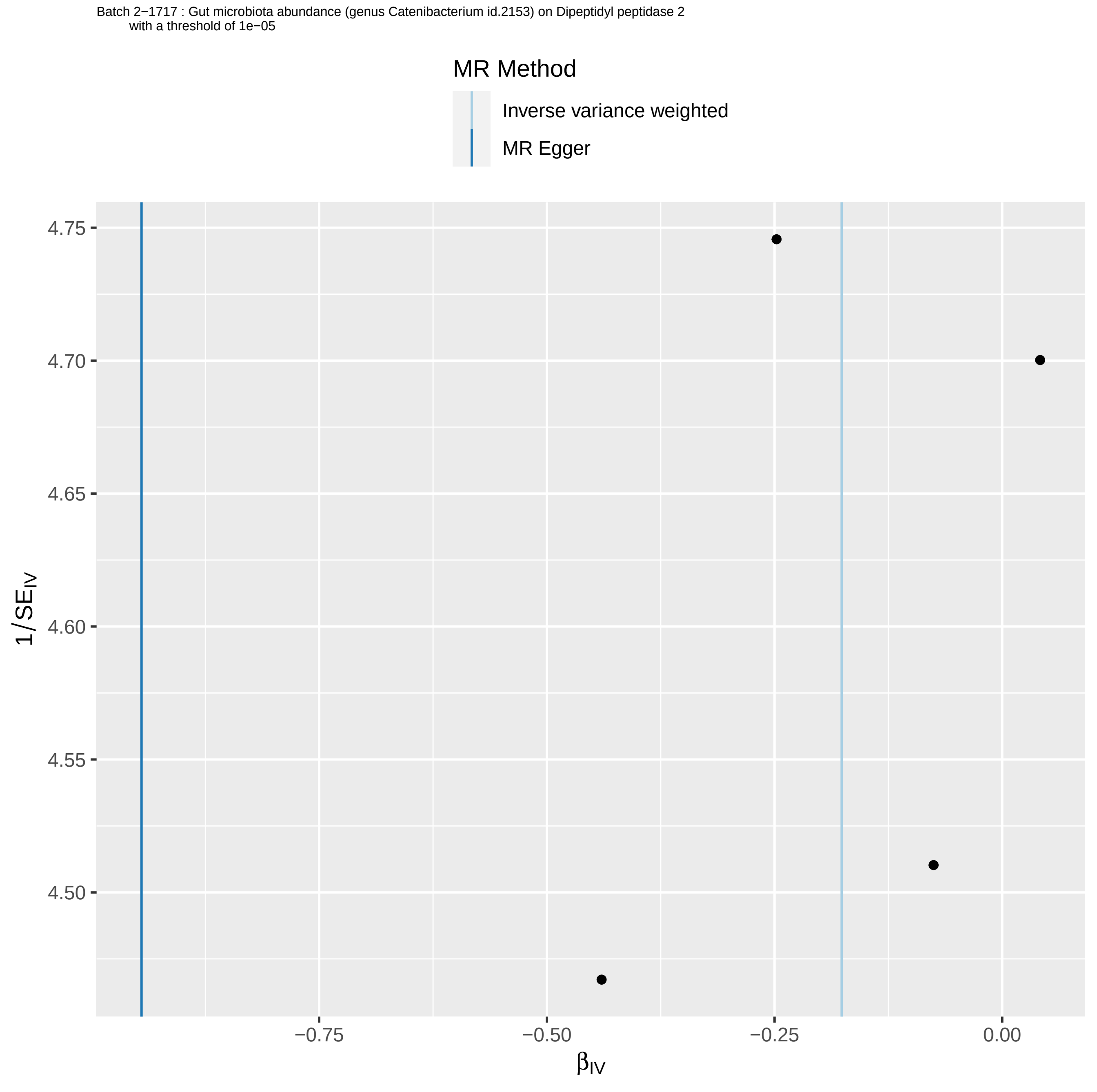
Batch 2−1717 : Gut microbiota abundance (genus Catenibacterium id.2153) on Dipeptidyl peptidase 2
with a threshold of 1e−05
MR Method
Inverse variance weighted
MR Egger
4.75
4.70
4.65
4.60
4.55
4.50
1/SE_IV
−0.75
−0.50
−0.25
0.00
β_IV

Batch 2−1718 : Gut microbiota abundance (genus Parasutterella id.2892) on Dipeptidyl peptidase 2
with a threshold of 1e−05

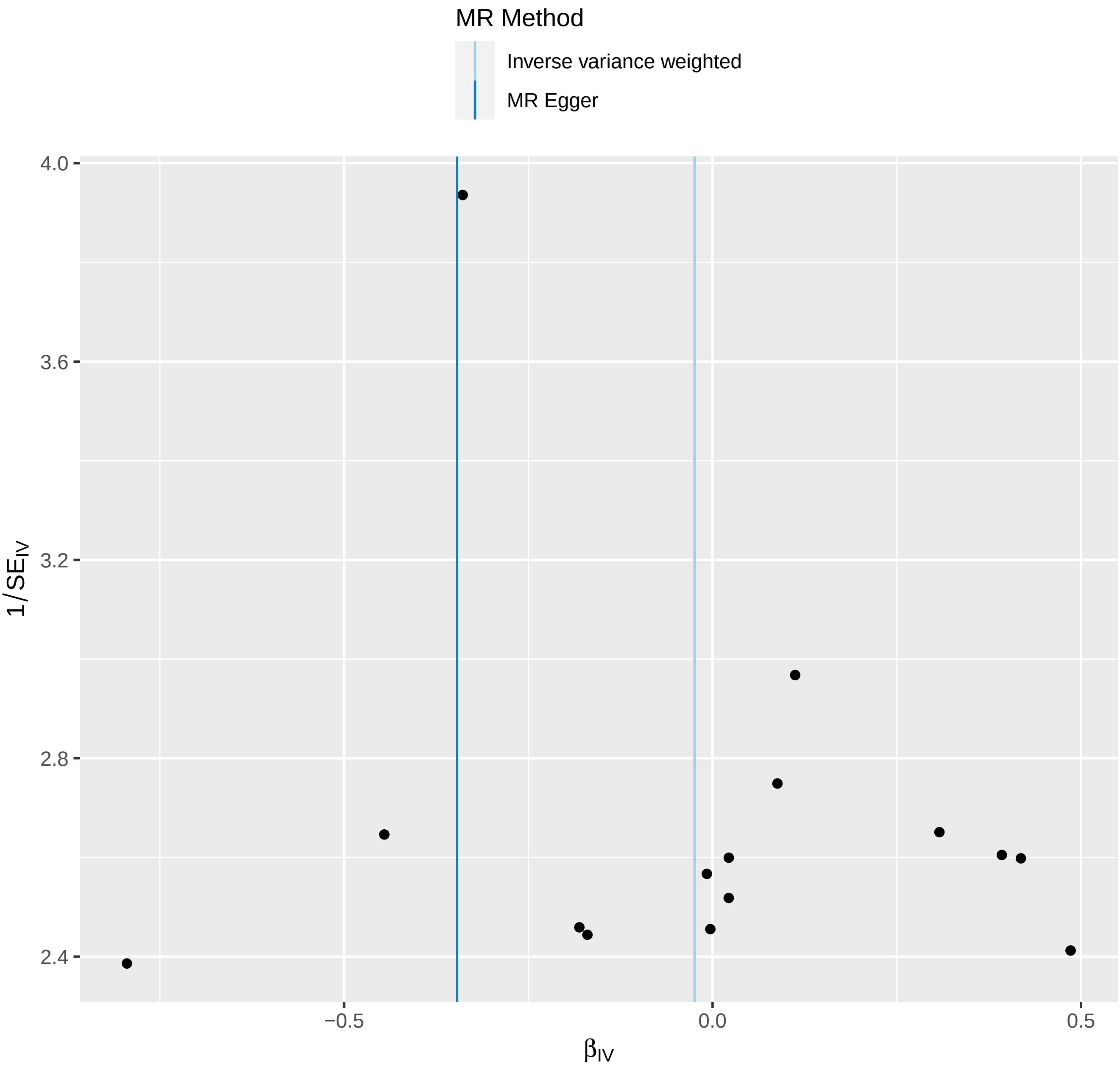

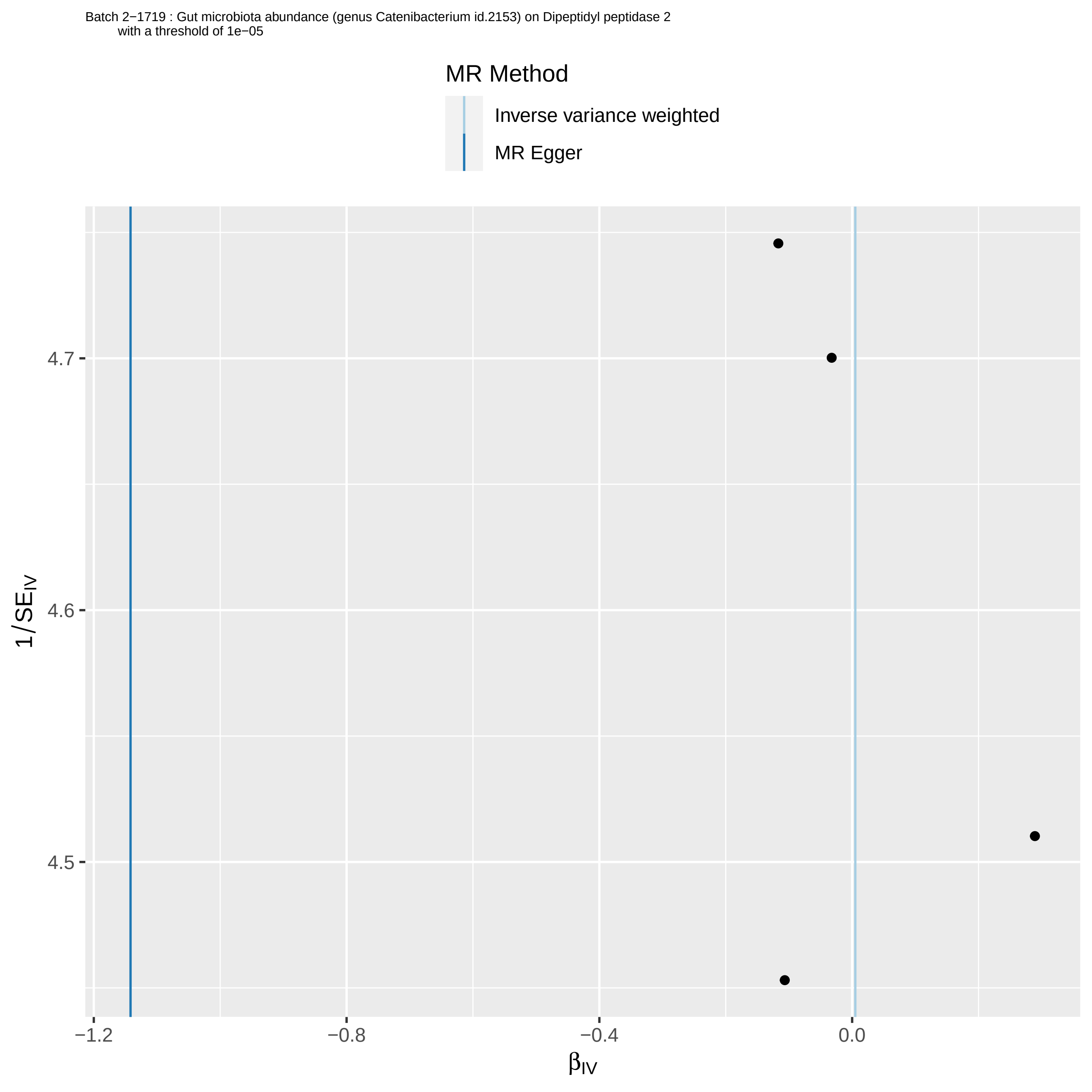
Batch 2−1719 : Gut microbiota abundance (genus Catenibacterium id.2153) on Dipeptidyl peptidase 2
with a threshold of 1e−05
MR Method
Inverse variance weighted
MR Egger
4.7
4.6
4.5
1/SE$_{IV}$
−1.2
−0.8
−0.4
0.0
β$_{IV}$

Batch 2−1720 : Gut microbiota abundance (genus Parasutterella id.2892) on Dipeptidyl peptidase 2
with a threshold of 1e−05

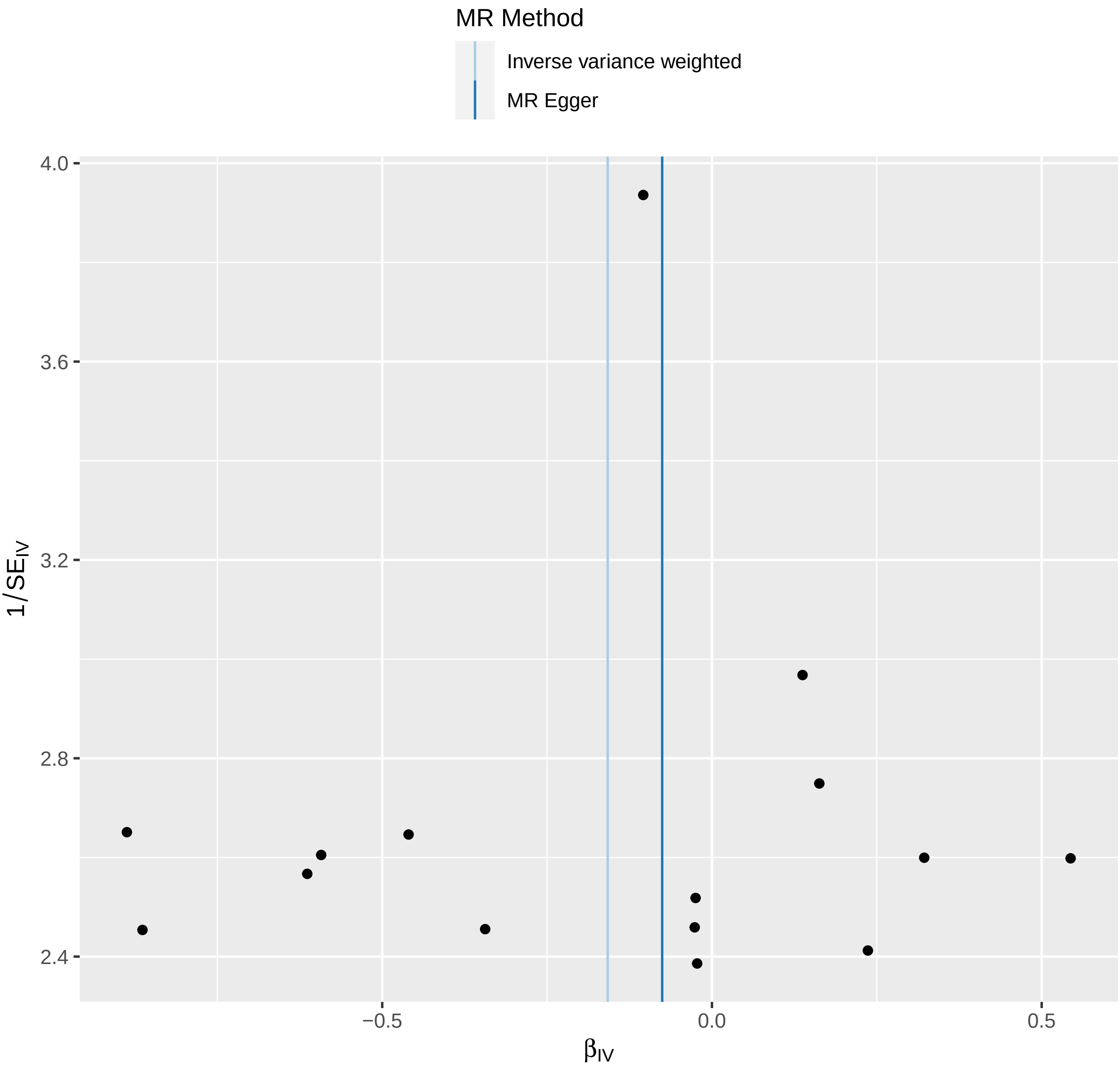

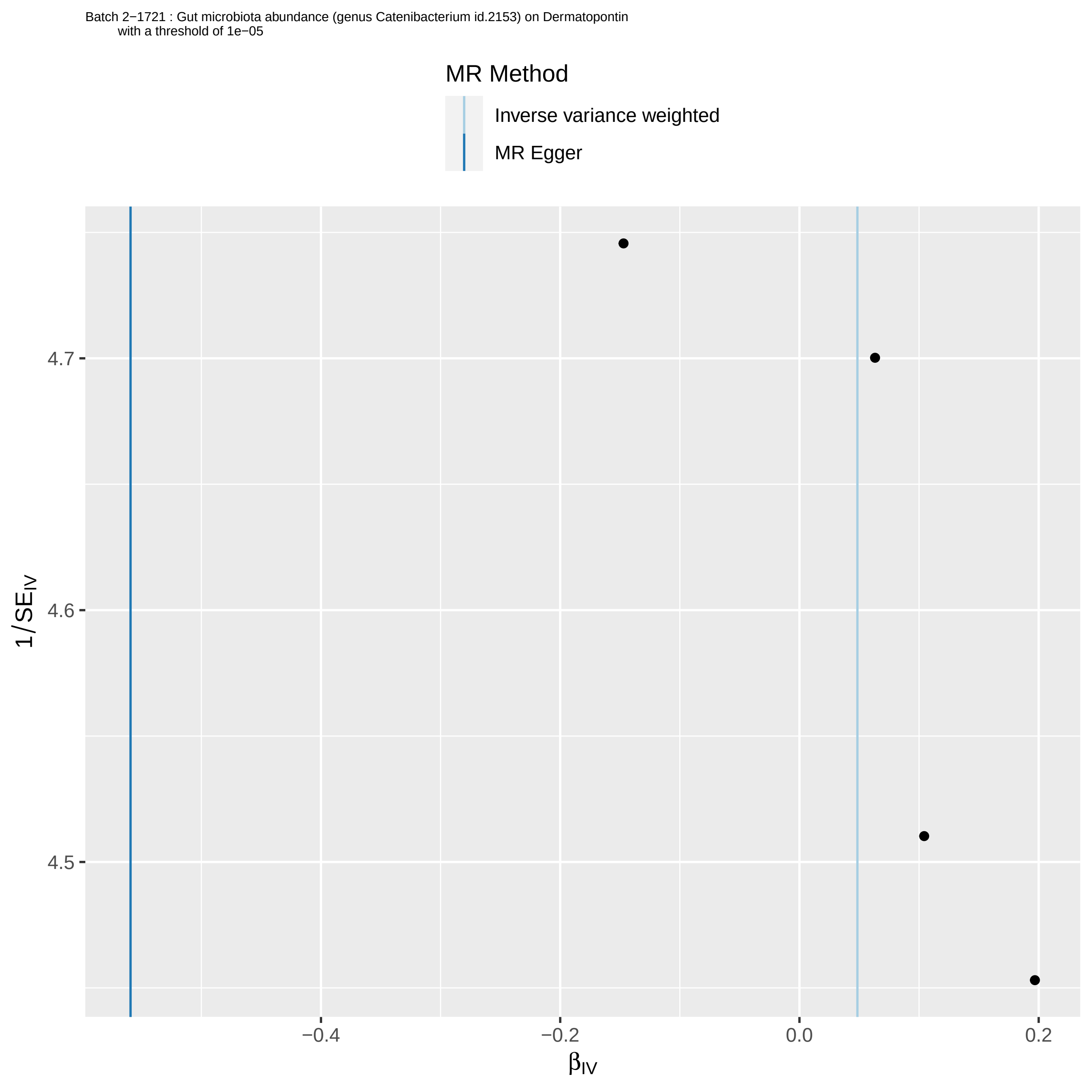
Batch 2−1721 : Gut microbiota abundance (genus Catenibacterium id.2153) on Dermatopontin
with a threshold of 1e−05
MR Method
Inverse variance weighted
MR Egger
4.7
4.6
4.5
1/SE_IV
−0.4
−0.2
0.0
0.2
β_IV

Batch 2−1722 : Gut microbiota abundance (genus Parasutterella id.2892) on Dermatopontin
with a threshold of 1e−05

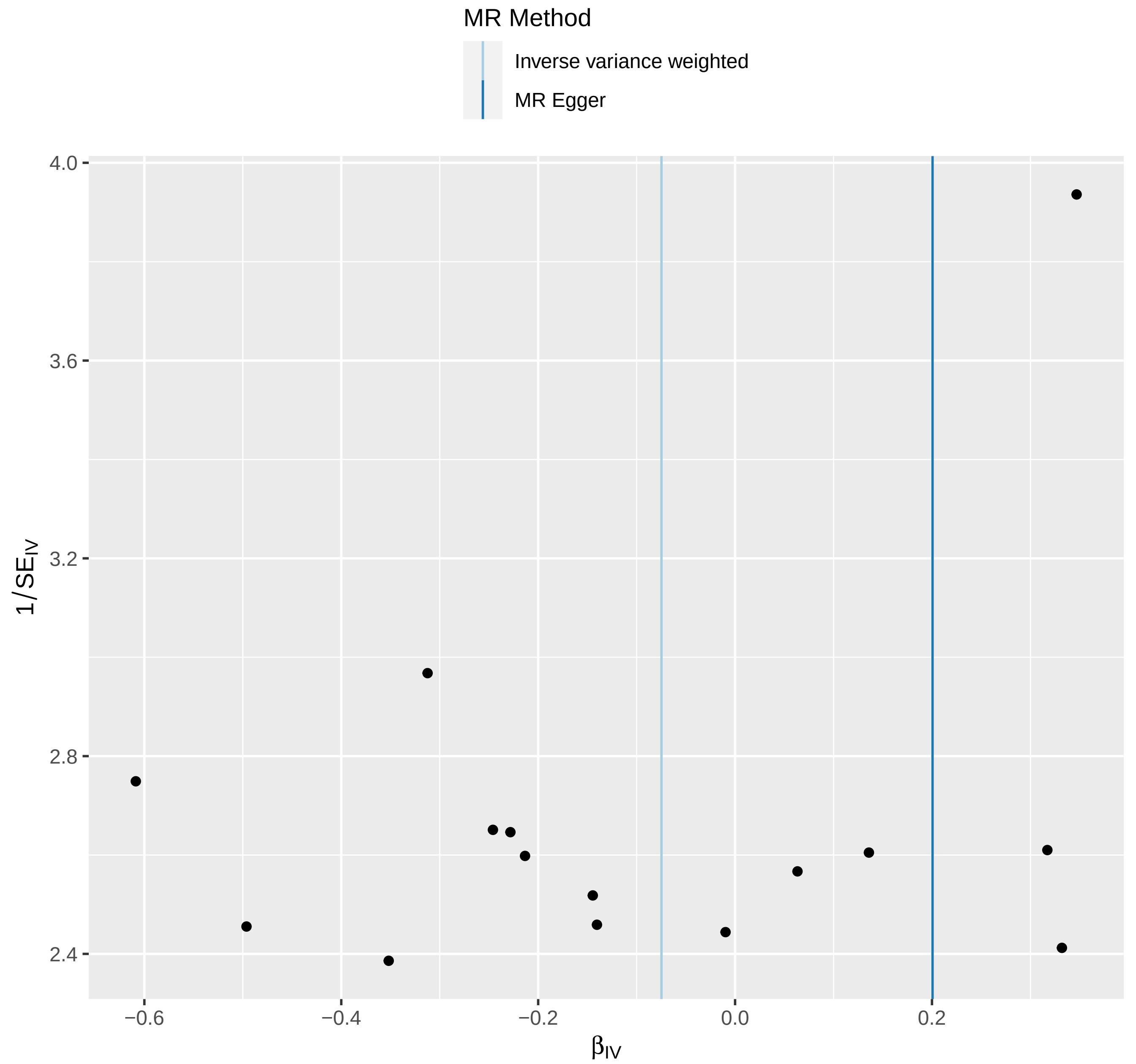

Batch 2−1723 : Gut microbiota abundance (genus Catenibacterium id.2153) on Protein dpy−30 homolog
with a threshold of 1e−05

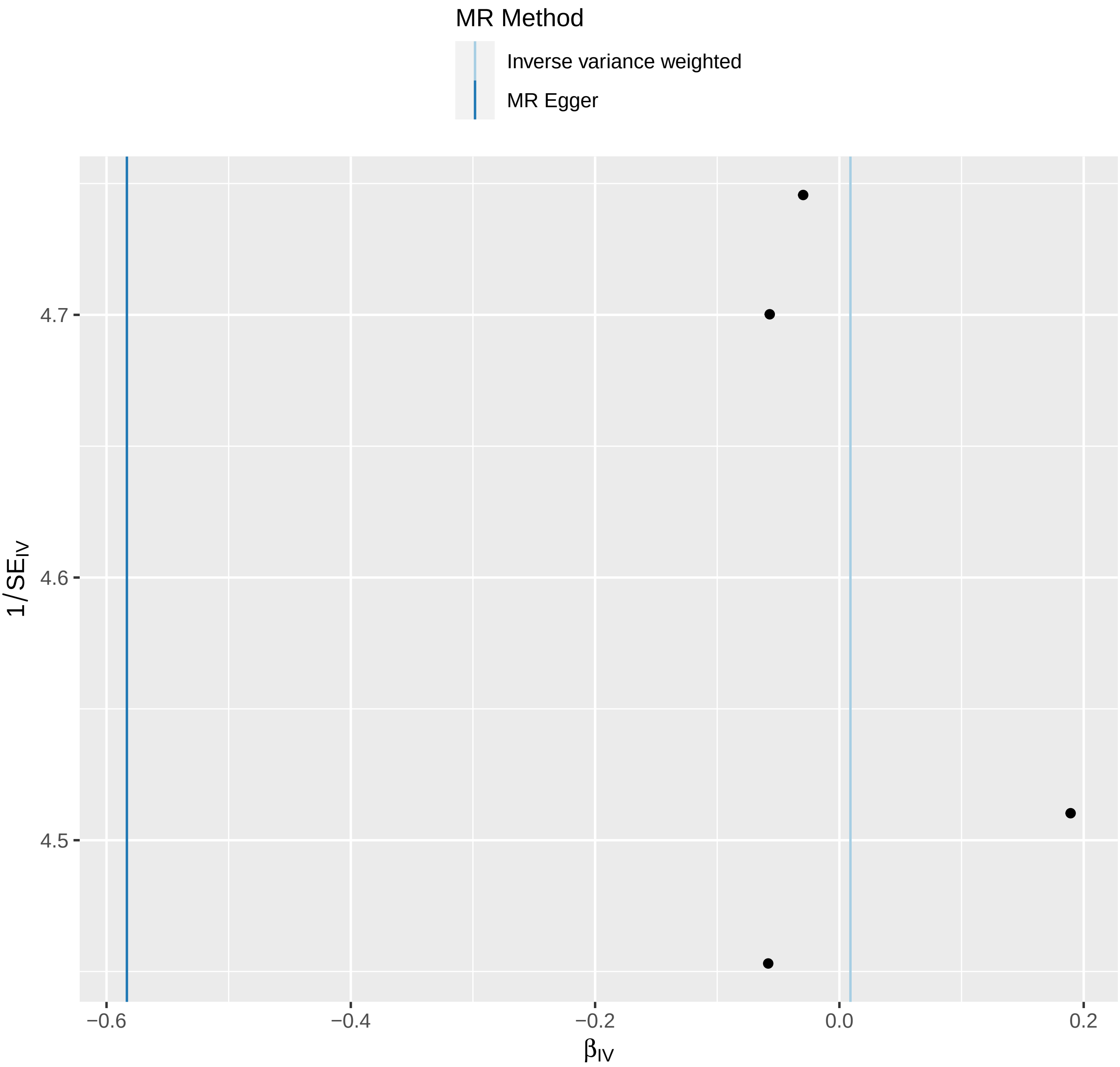

Batch 2−1724 : Gut microbiota abundance (genus Parasutterella id.2892) on Protein dpy−30 homolog
with a threshold of 1e−05

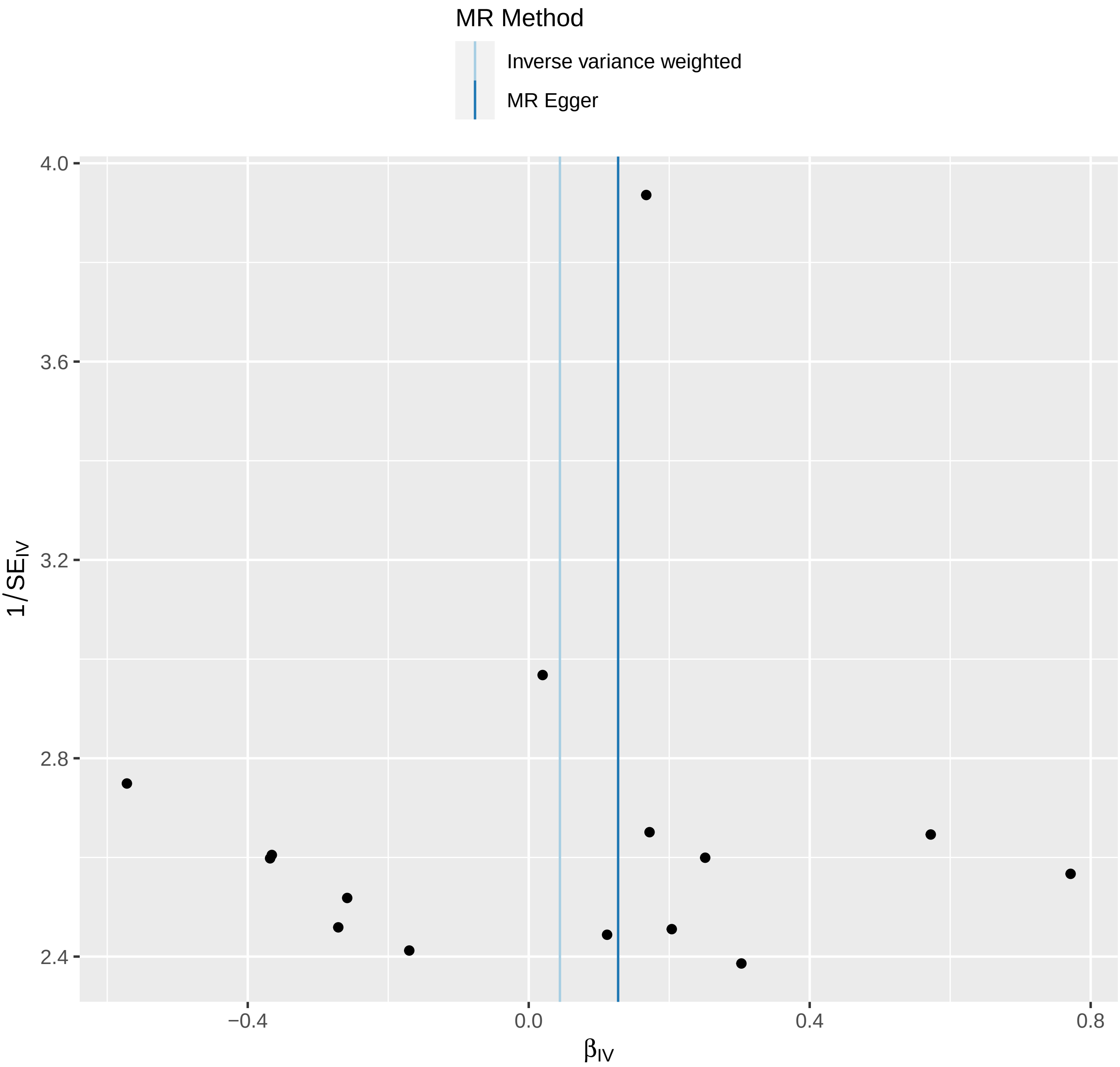

Batch 2−1725 : Gut microbiota abundance (genus Catenibacterium id.2153) on Dihydropyrimidinase−related protein 3
with a threshold of 1e−05

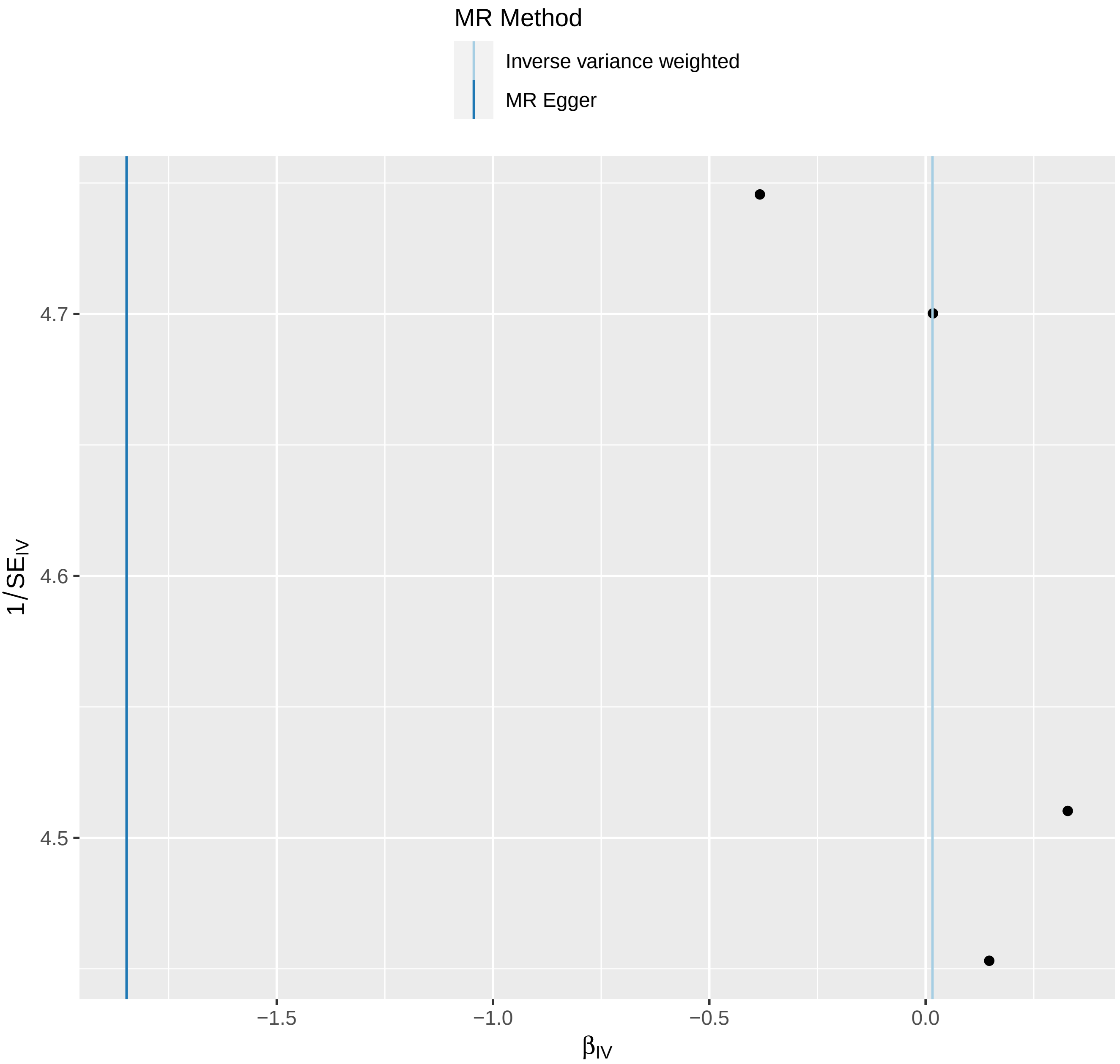

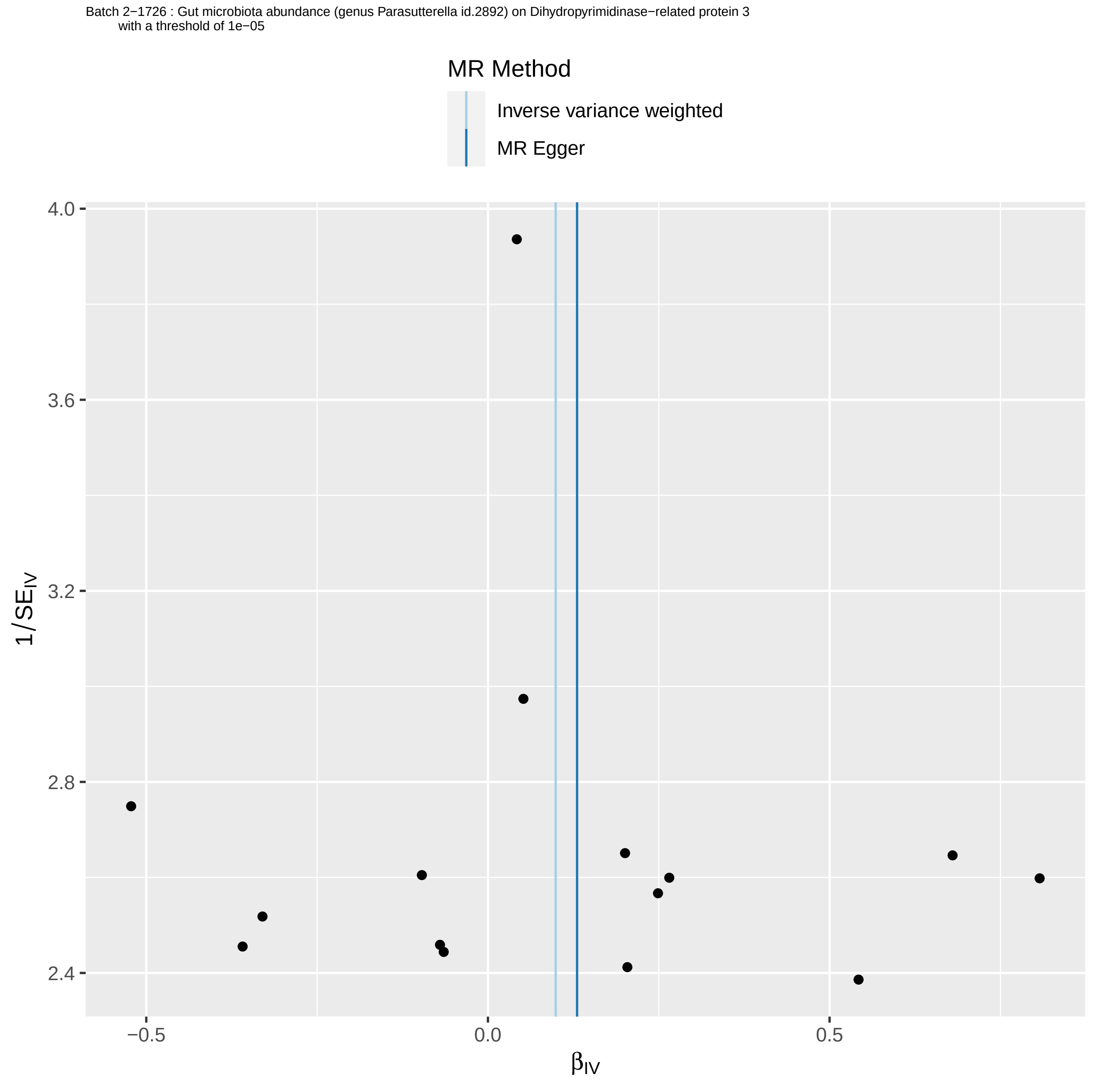
Batch 2−1726 : Gut microbiota abundance (genus Parasutterella id.2892) on Dihydropyrimidinase−related protein 3
with a threshold of 1e−05
MR Method
Inverse variance weighted
MR Egger
4.0
3.6
3.2
2.8
2.4
1/SE_IV
−0.5
0.0
0.5
β_IV

Batch 2−1727 : Gut microbiota abundance (genus Catenibacterium id.2153) on Dihydropyrimidinase−related protein 5
with a threshold of 1e−05

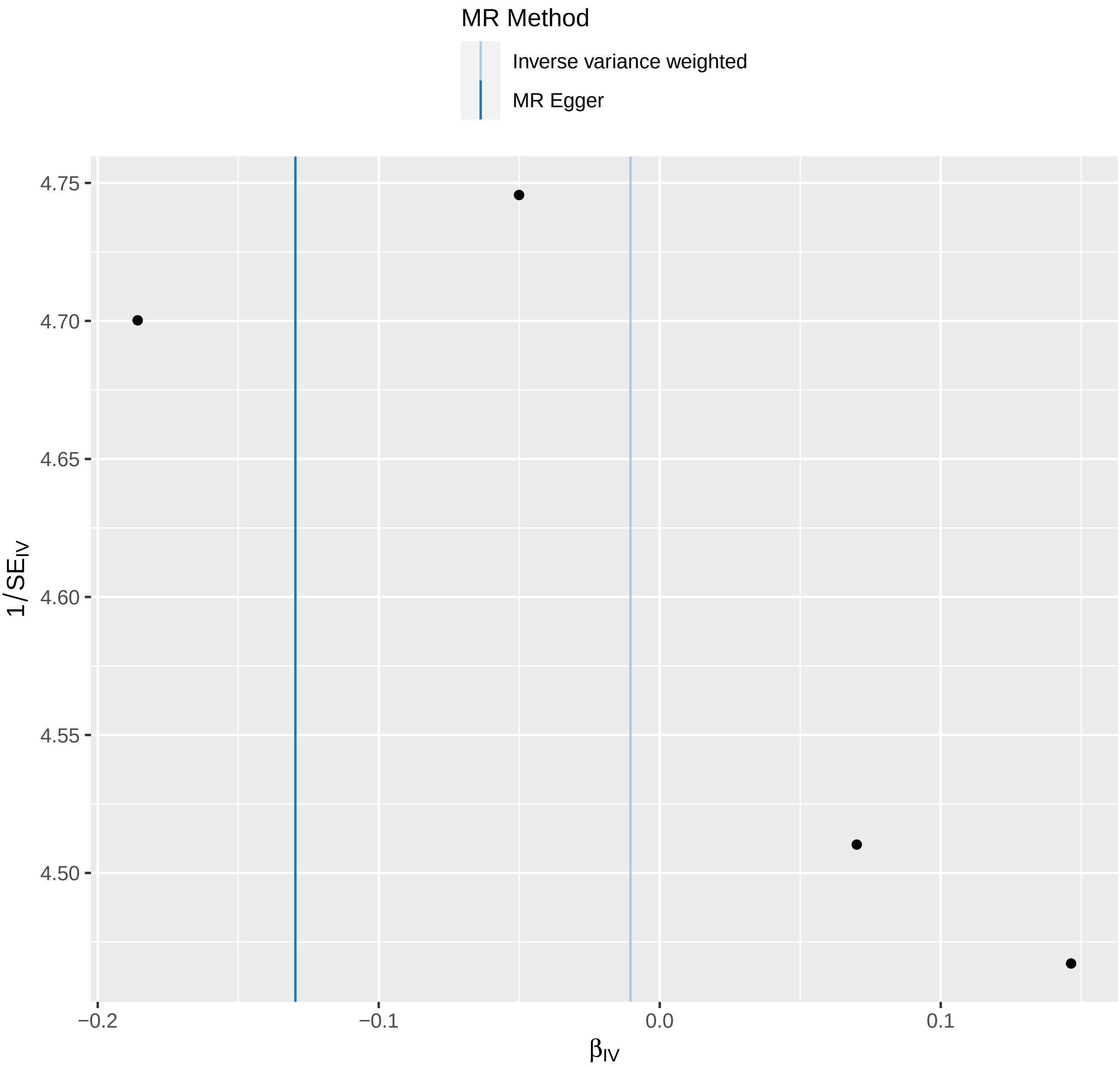

Batch 2−1728 : Gut microbiota abundance (genus Parasutterella id.2892) on Dihydropyrimidinase−related protein 5
with a threshold of 1e−05

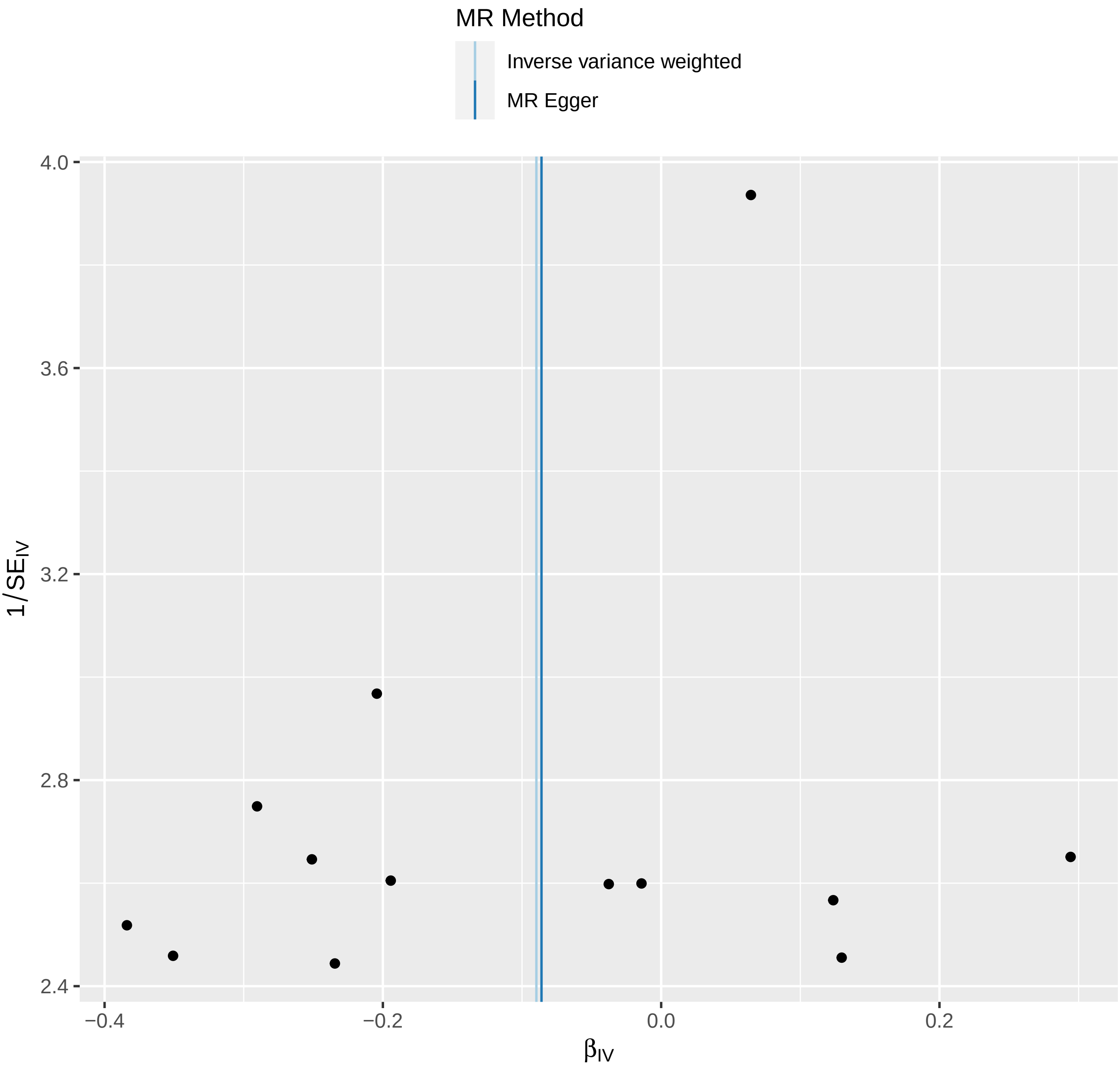

Batch 2−1729 : Gut microbiota abundance (genus Catenibacterium id.2153) on Dorsal root ganglia homeobox protein
with a threshold of 1e−05

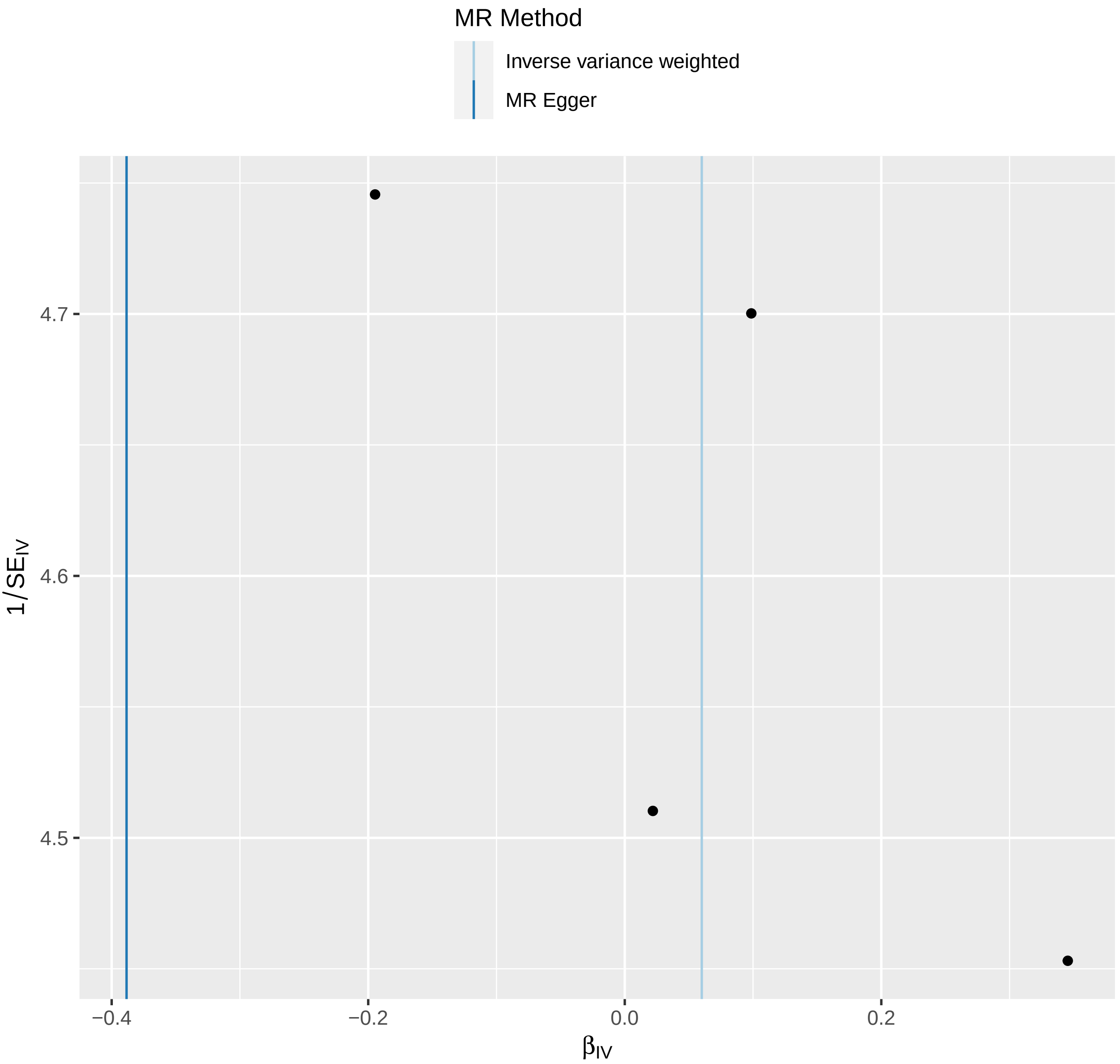

Batch 2−1730 : Gut microbiota abundance (genus Parasutterella id.2892) on Dorsal root ganglia homeobox protein
with a threshold of 1e−05

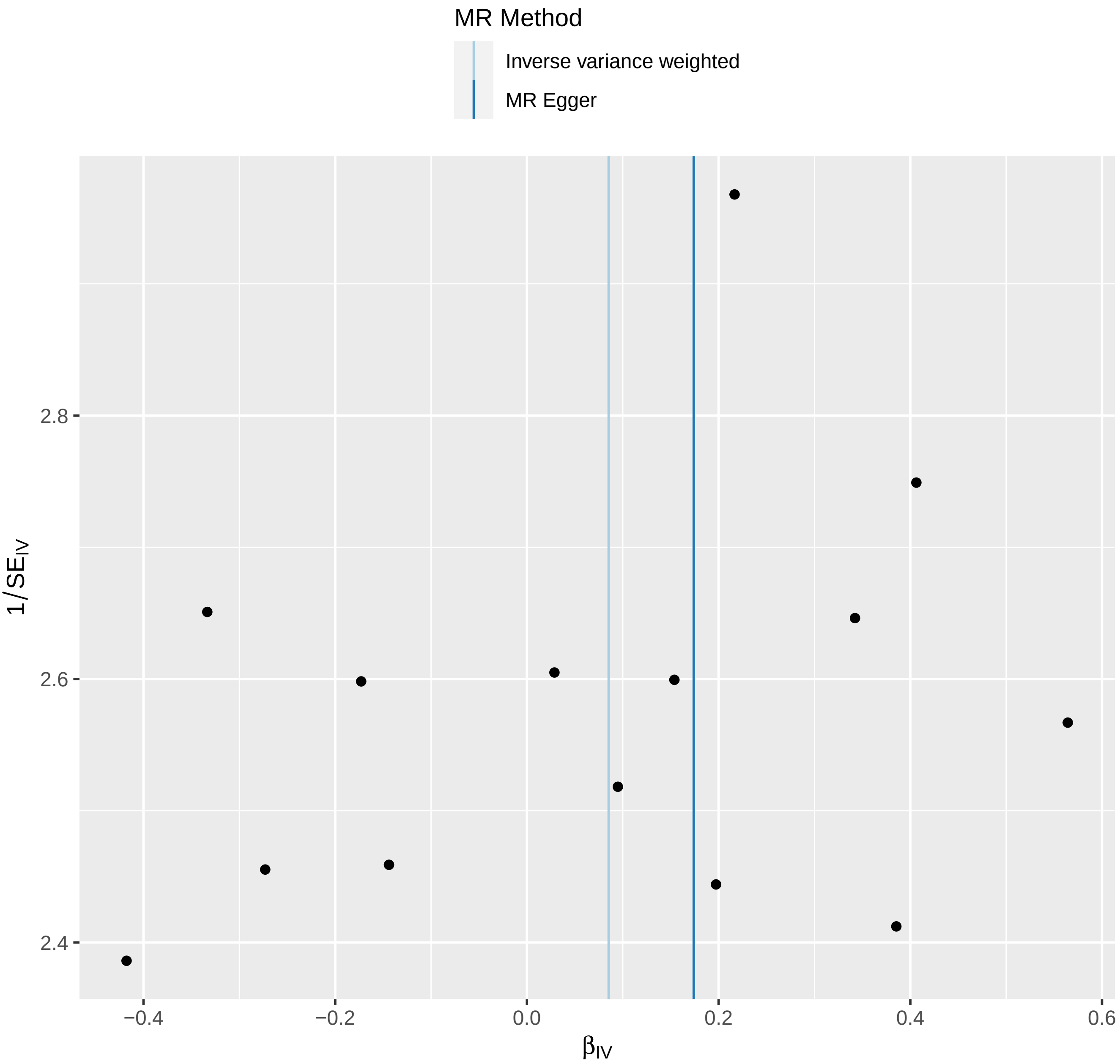

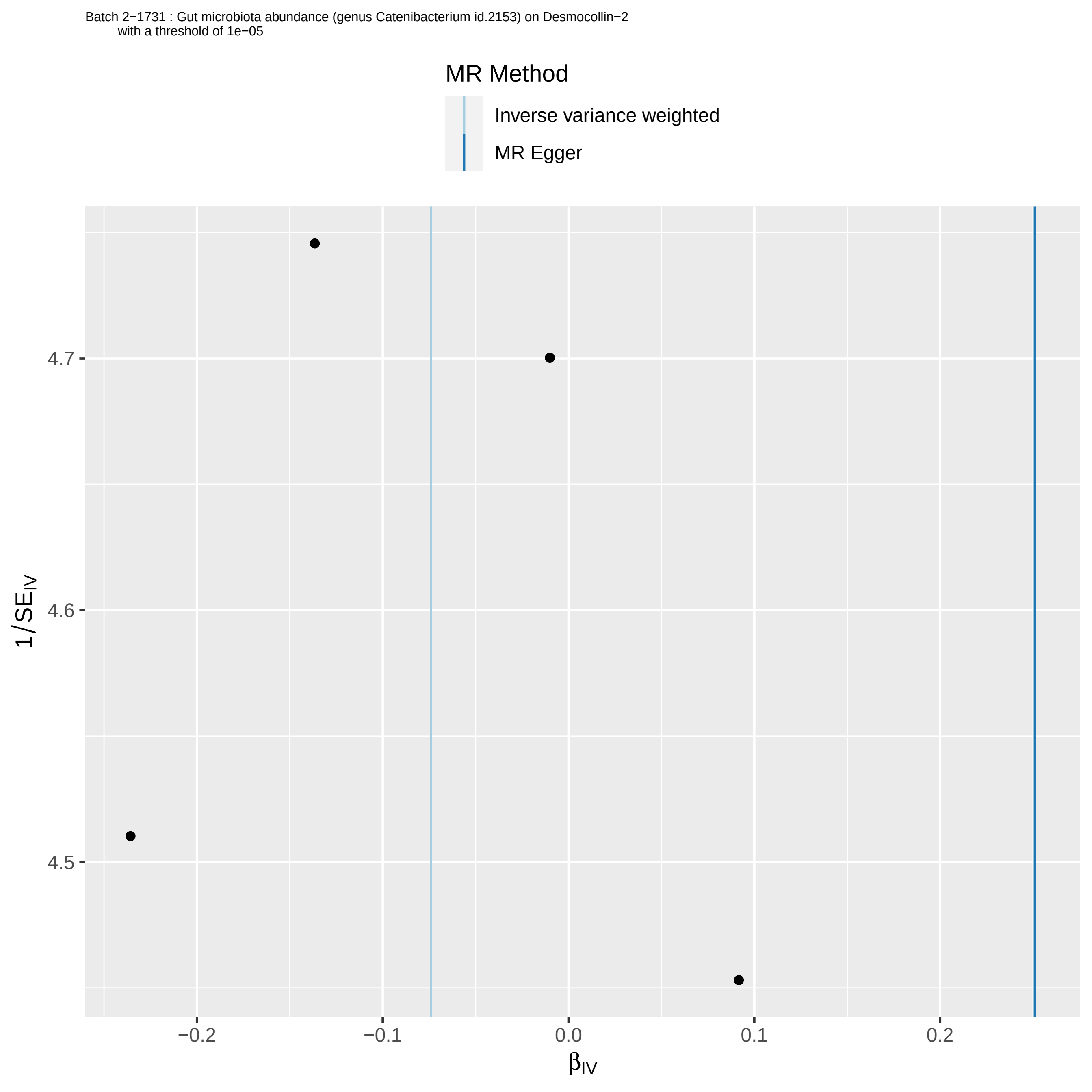
Batch 2−1731 : Gut microbiota abundance (genus Catenibacterium id.2153) on Desmocollin−2
with a threshold of 1e−05
MR Method
Inverse variance weighted
MR Egger
4.7
4.6
4.5
1/SE_IV
−0.2
−0.1
0.0
0.1
0.2
β_IV

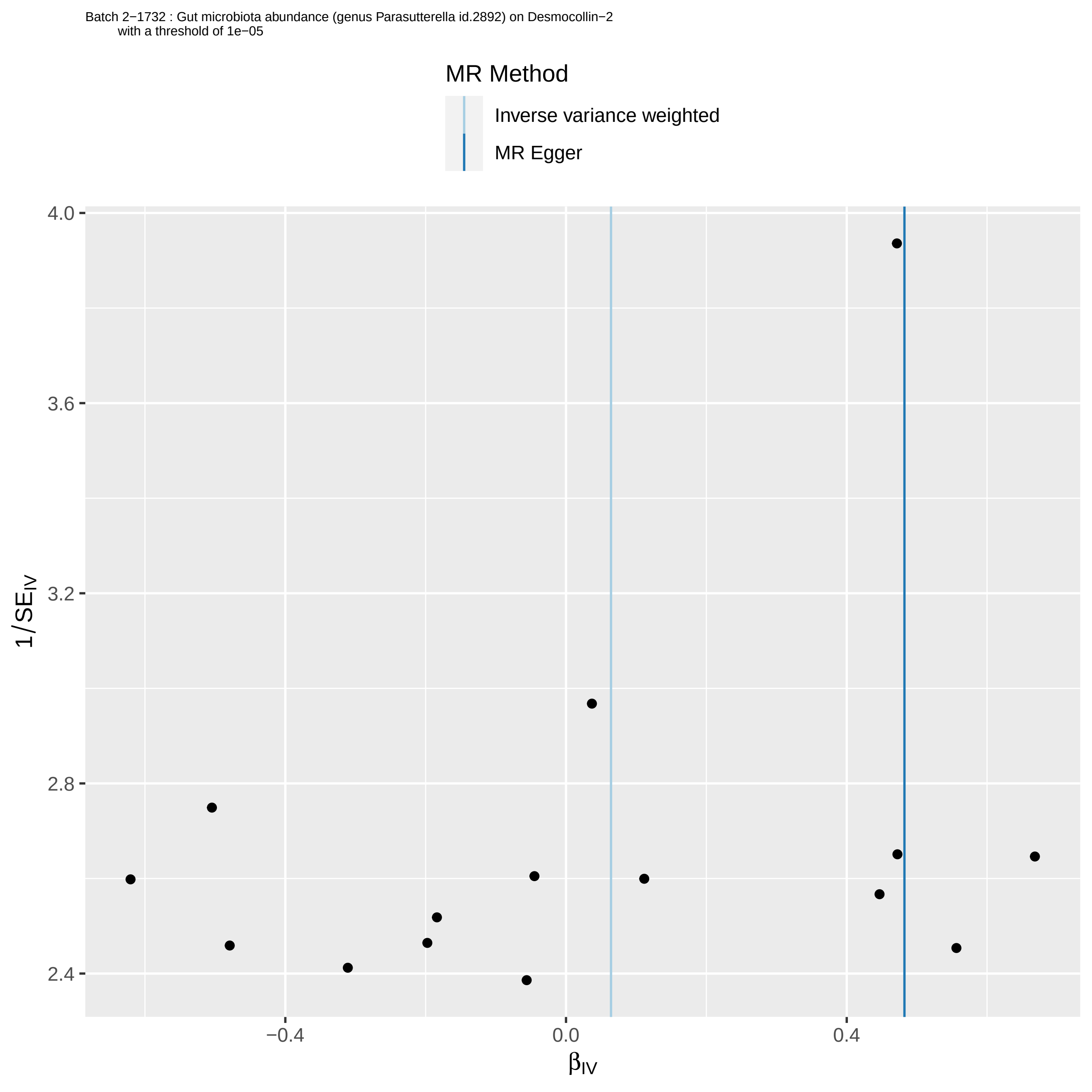
Batch 2−1732 : Gut microbiota abundance (genus Parasutterella id.2892) on Desmocollin−2
with a threshold of 1e−05
MR Method
Inverse variance weighted
MR Egger
4.0
3.6
3.2
2.8
2.4
1/SE_IV
−0.4
0.0
0.4
β_IV

Batch 2−1733 : Gut microbiota abundance (genus Catenibacterium id.2153) on Desmocollin−3
with a threshold of 1e−05

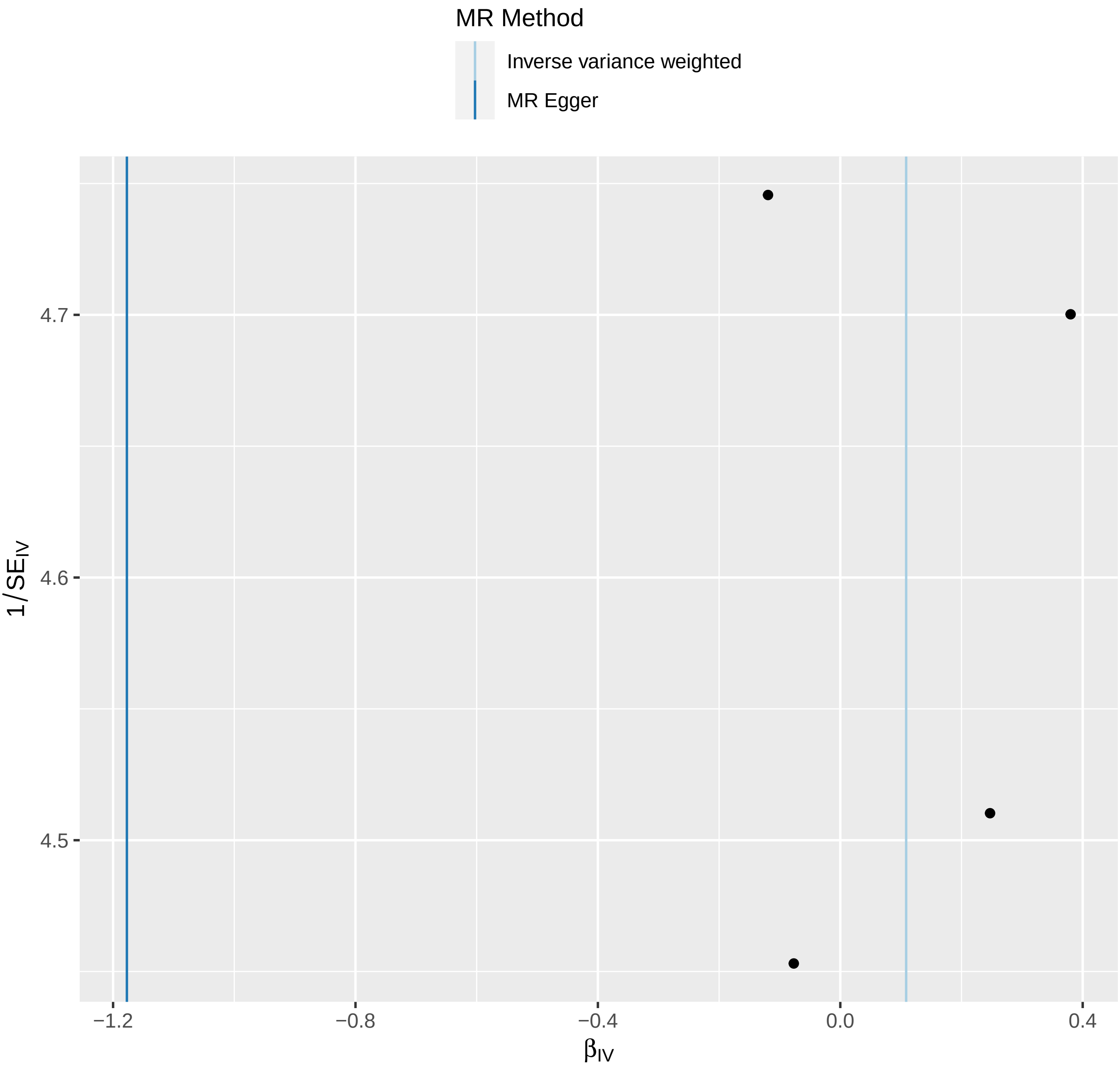

Batch 2−1734 : Gut microbiota abundance (genus Parasutterella id.2892) on Desmocollin−3
with a threshold of 1e−05

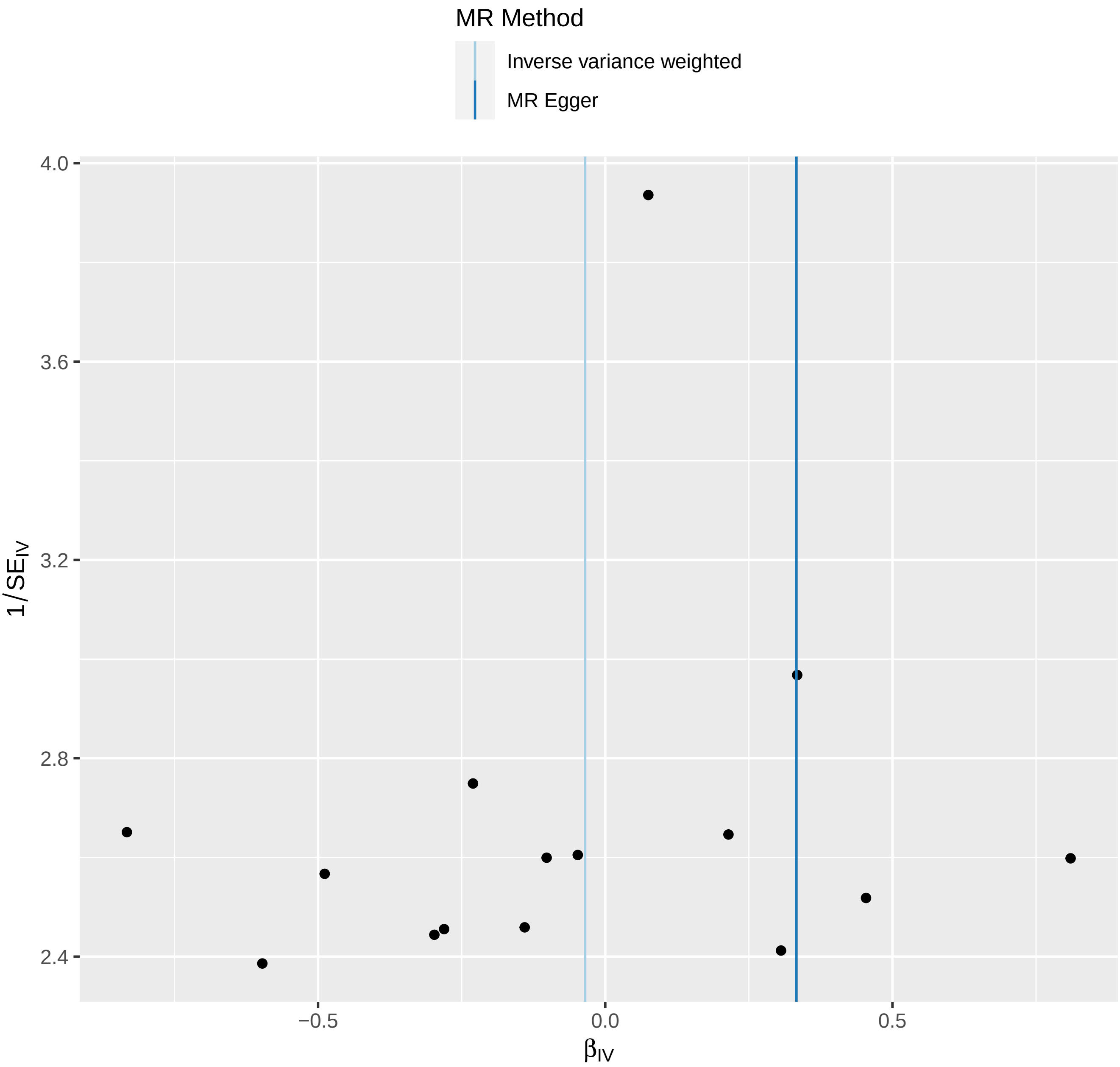

Batch 2−1735 : Gut microbiota abundance (genus Catenibacterium id.2153) on Down syndrome cell adhesion molecule
with a threshold of 1e−05

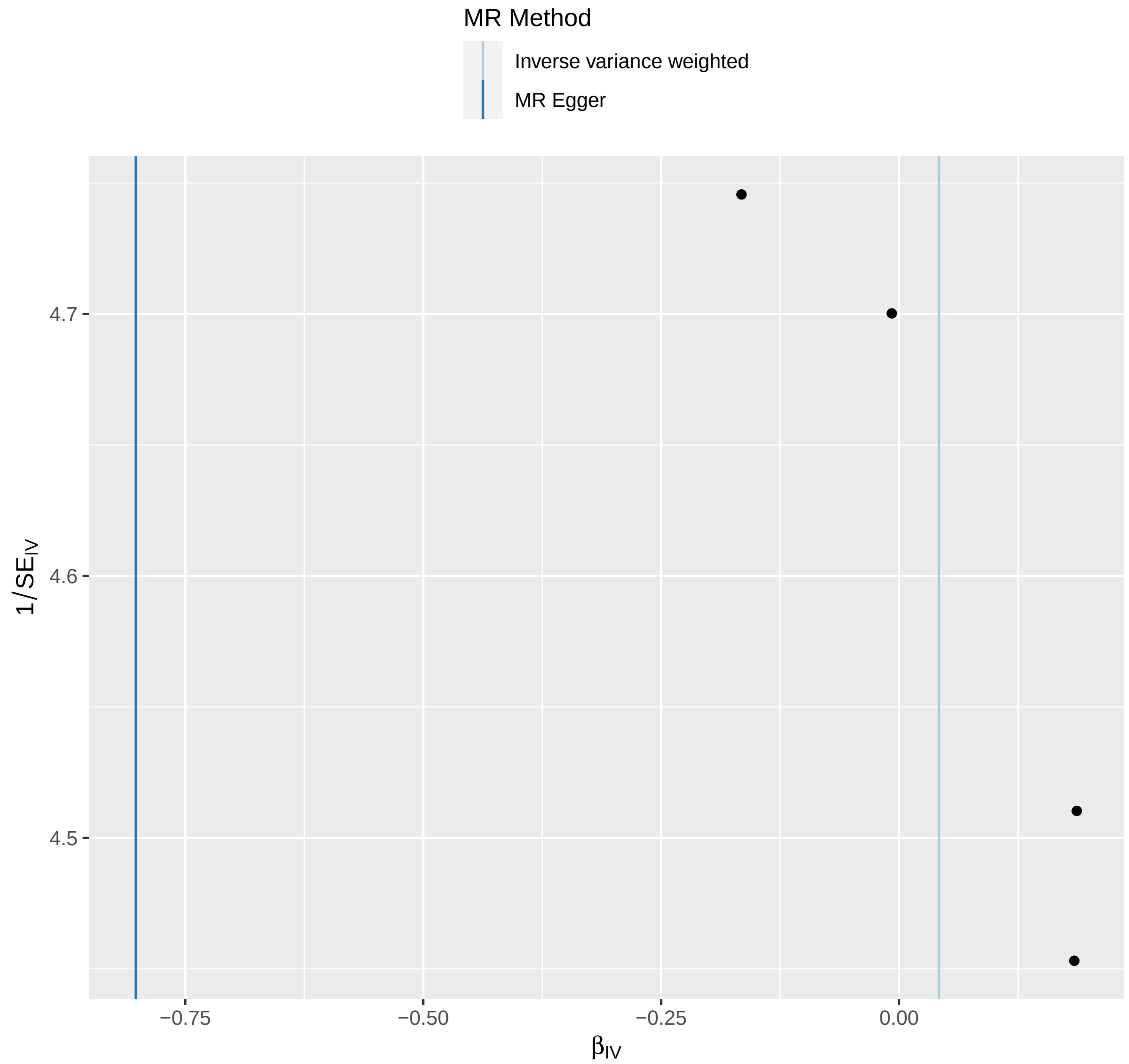

Batch 2−1736 : Gut microbiota abundance (genus Parasutterella id.2892) on Down syndrome cell adhesion molecule
with a threshold of 1e−05

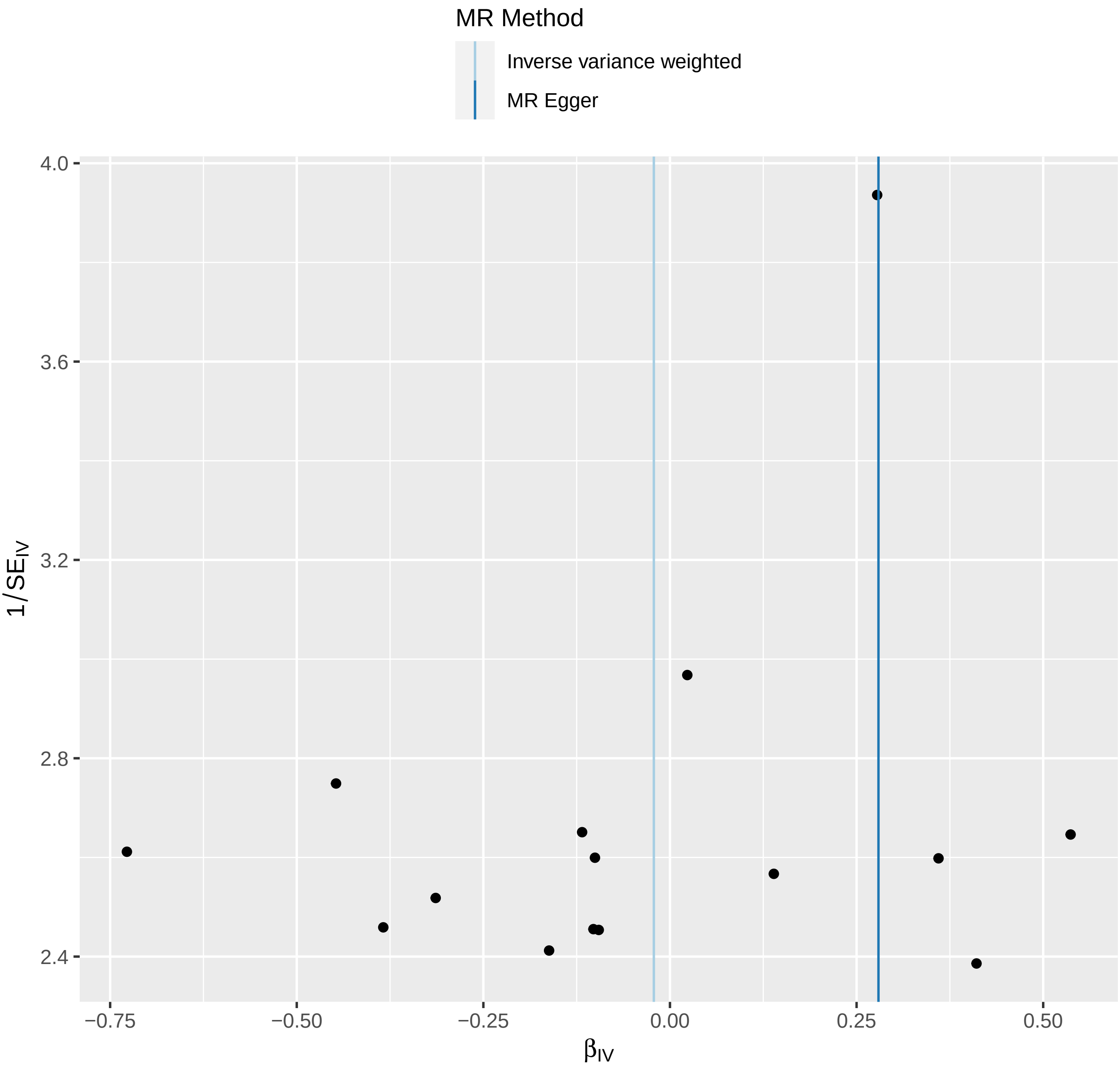

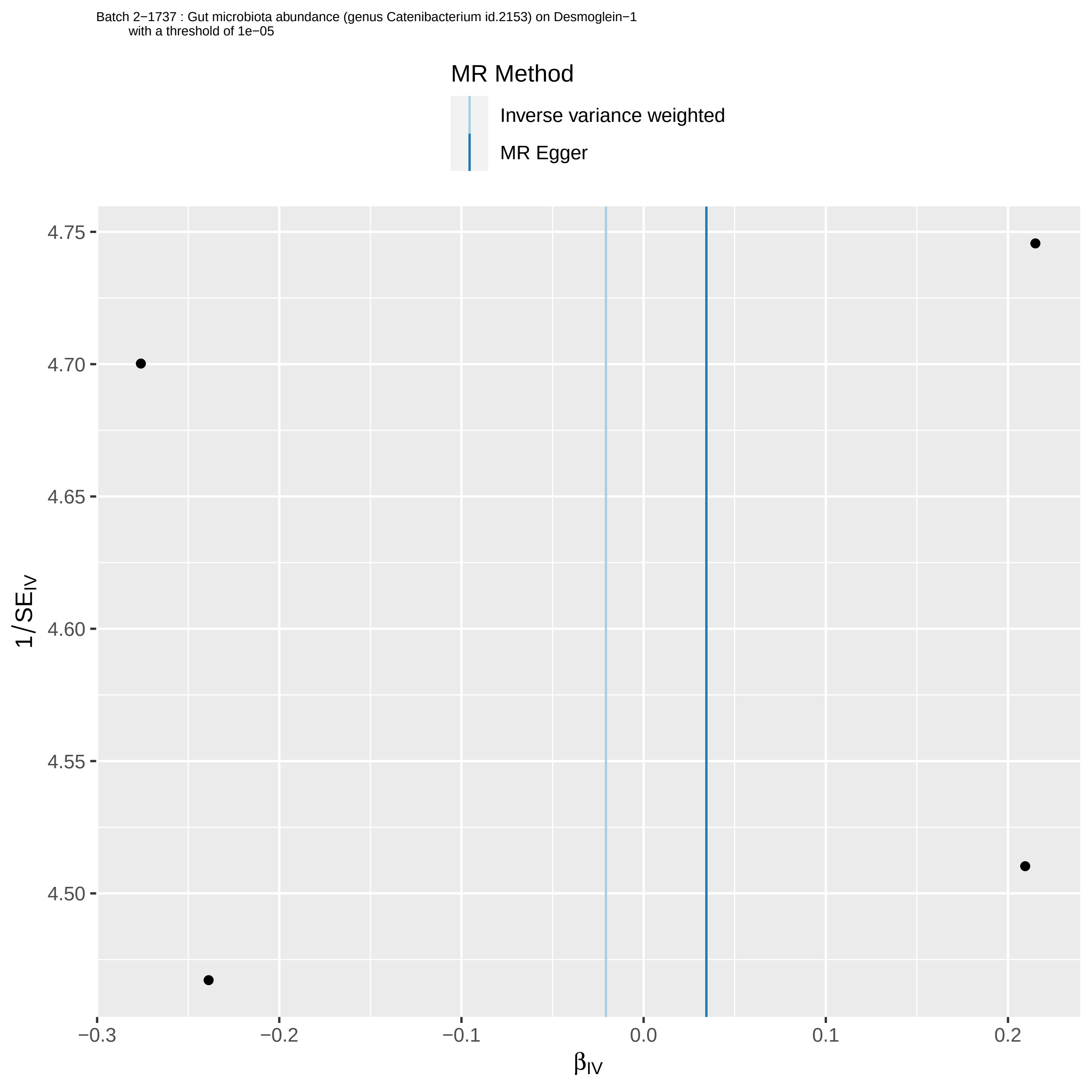

Batch 2−1737 : Gut microbiota abundance (genus Catenibacterium id.2153) on Desmoglein−1
with a threshold of 1e−05
MR Method
Inverse variance weighted
MR Egger
4.75
4.70
4.65
4.60
4.55
4.50
1/SE_IV
−0.3
−0.2
−0.1
0.0
0.1
0.2
β_IV

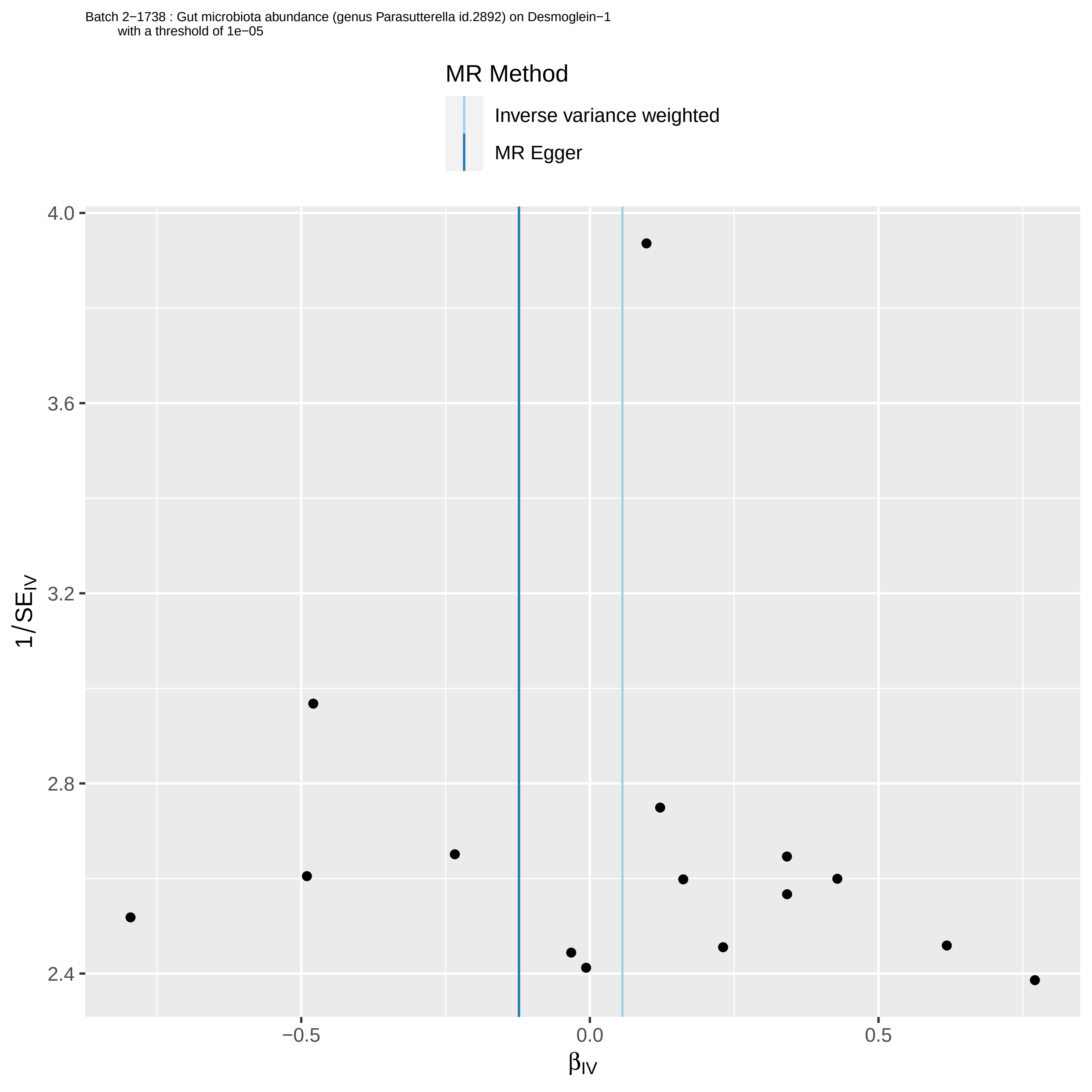
Batch 2−1738 : Gut microbiota abundance (genus Parasutterella id.2892) on Desmoglein−1
with a threshold of 1e−05
MR Method
Inverse variance weighted
MR Egger
4.0
3.6
3.2
2.8
2.4
1/SE_IV
−0.5
0.0
0.5
β_IV

Batch 2−1739 : Gut microbiota abundance (genus Catenibacterium id.2153) on Desmoglein−2
with a threshold of 1e−05

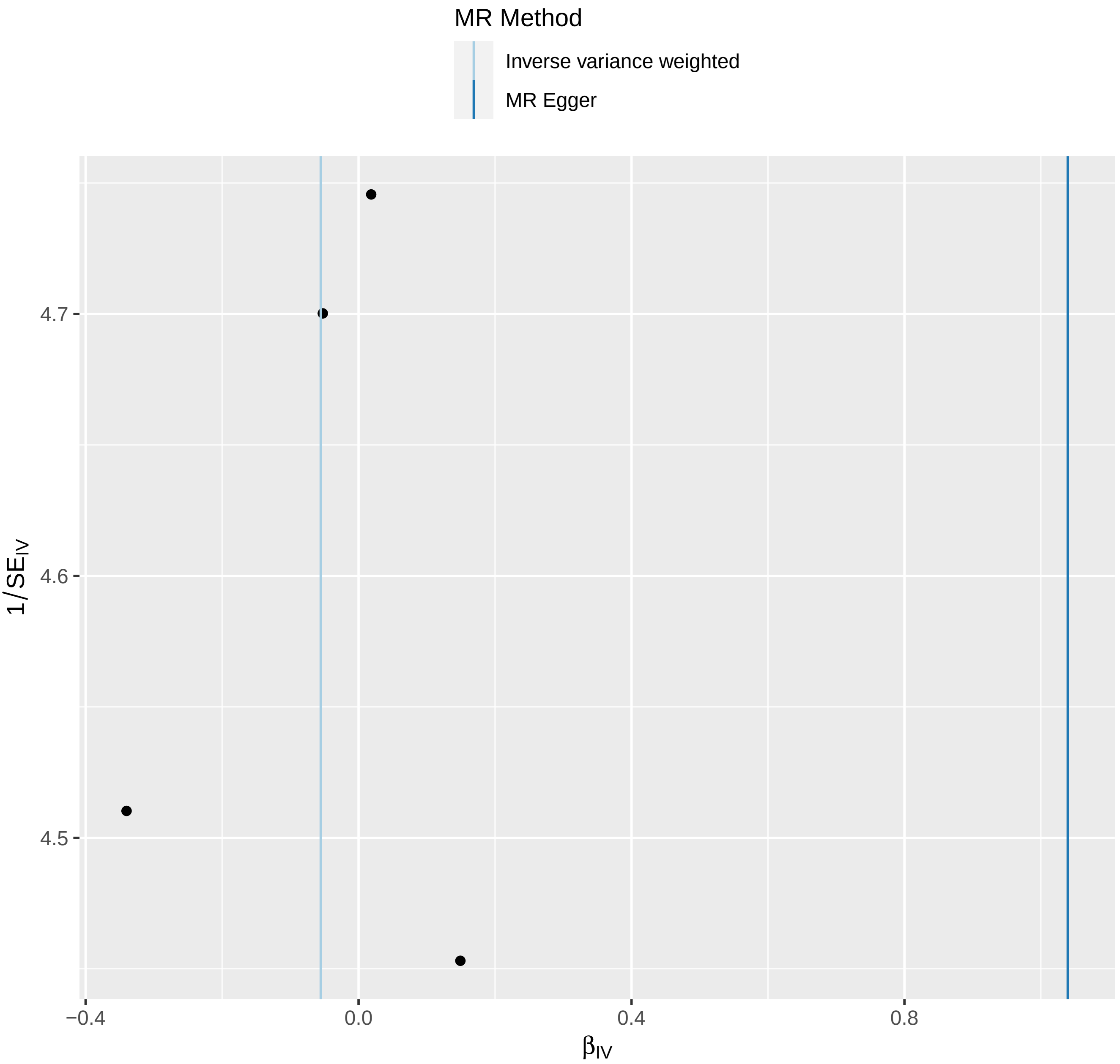

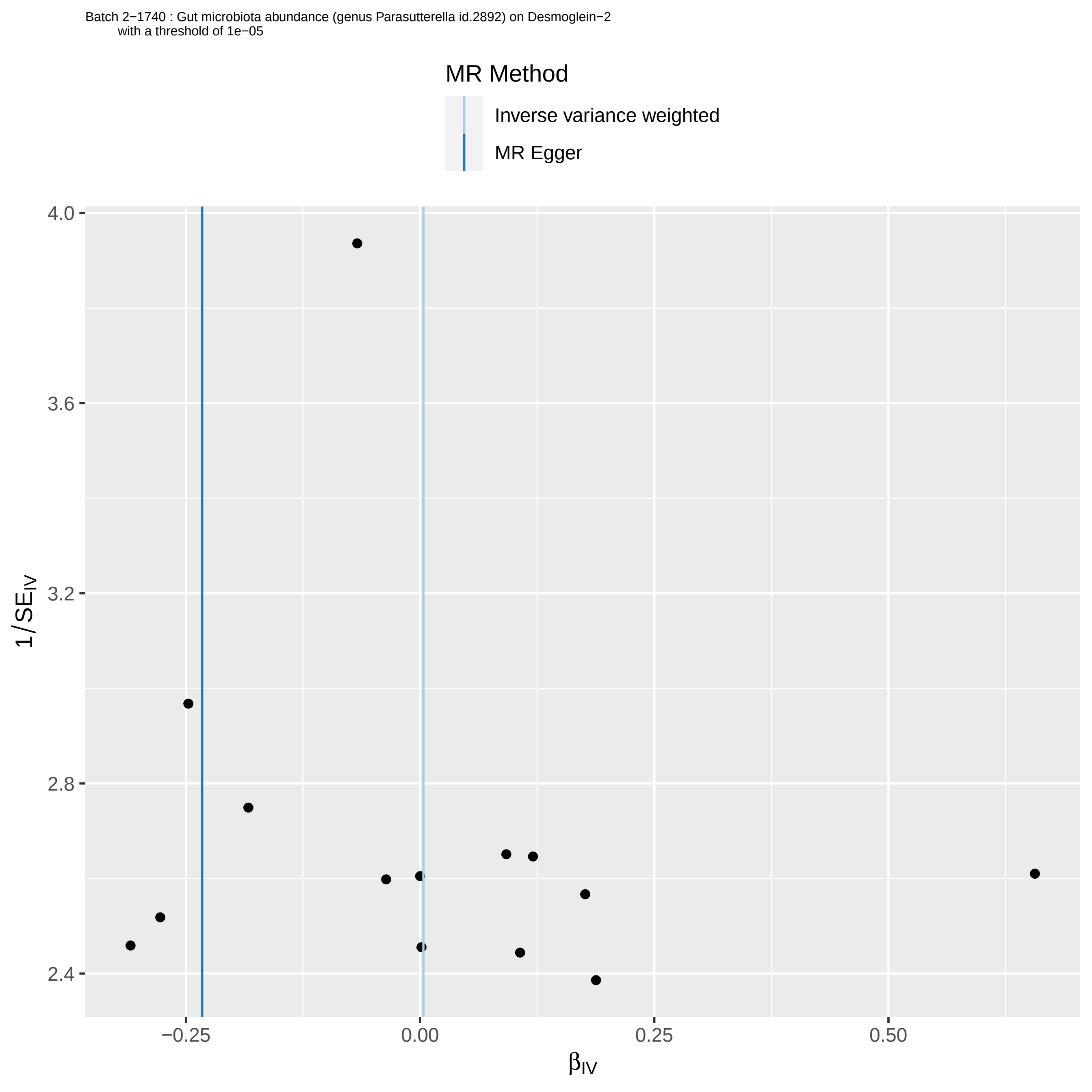
Batch 2−1740 : Gut microbiota abundance (genus Parasutterella id.2892) on Desmoglein−2
with a threshold of 1e−05
MR Method
Inverse variance weighted
MR Egger
4.0
3.6
3.2
2.8
2.4
1/SE_IV
−0.25
0.00
0.25
0.50
β_IV

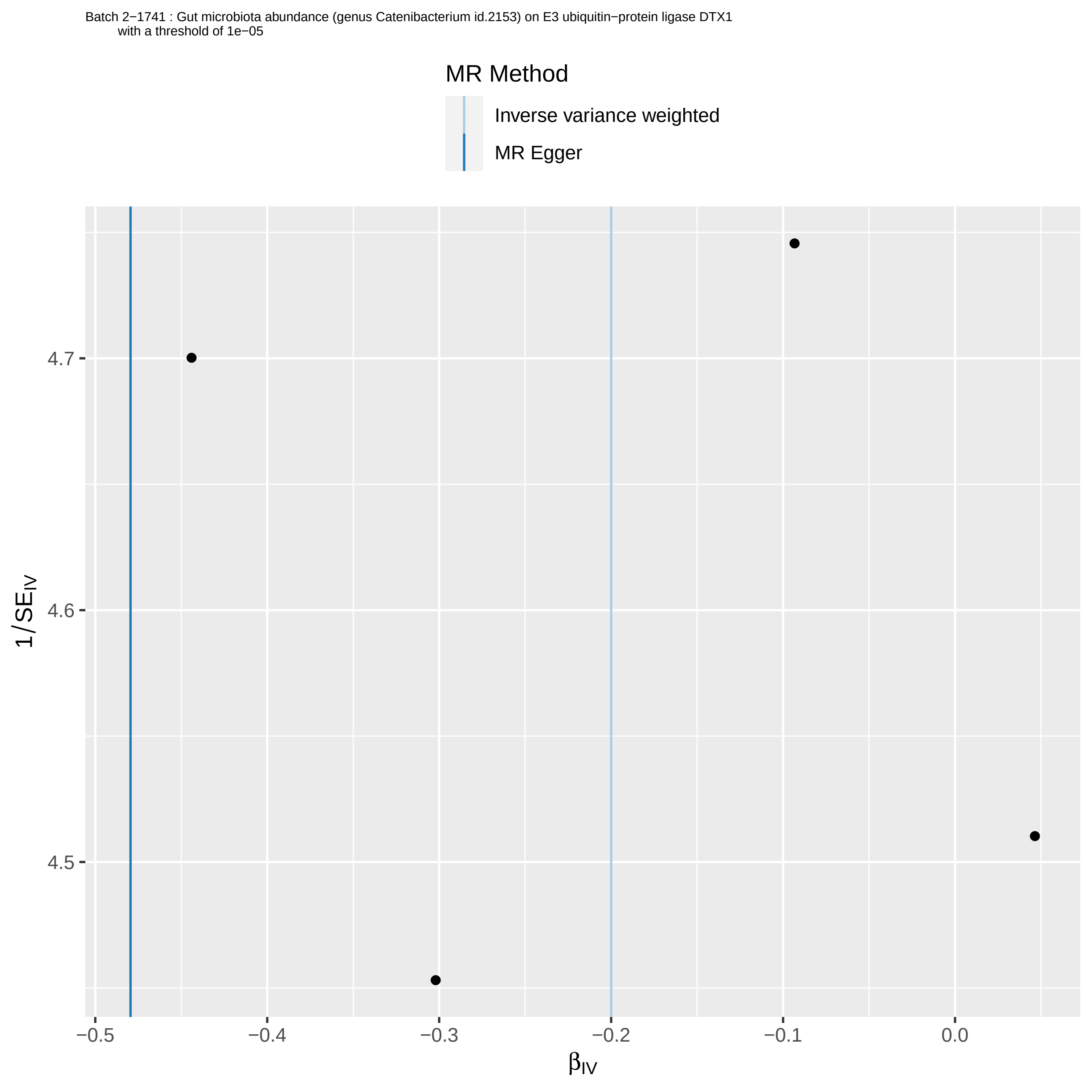
Batch 2−1741 : Gut microbiota abundance (genus Catenibacterium id.2153) on E3 ubiquitin−protein ligase DTX1
with a threshold of 1e−05
MR Method
Inverse variance weighted
MR Egger
4.7
4.6
4.5
1/SE$_{IV}$
−0.5
−0.4
−0.3
−0.2
−0.1
0.0
β$_{IV}$

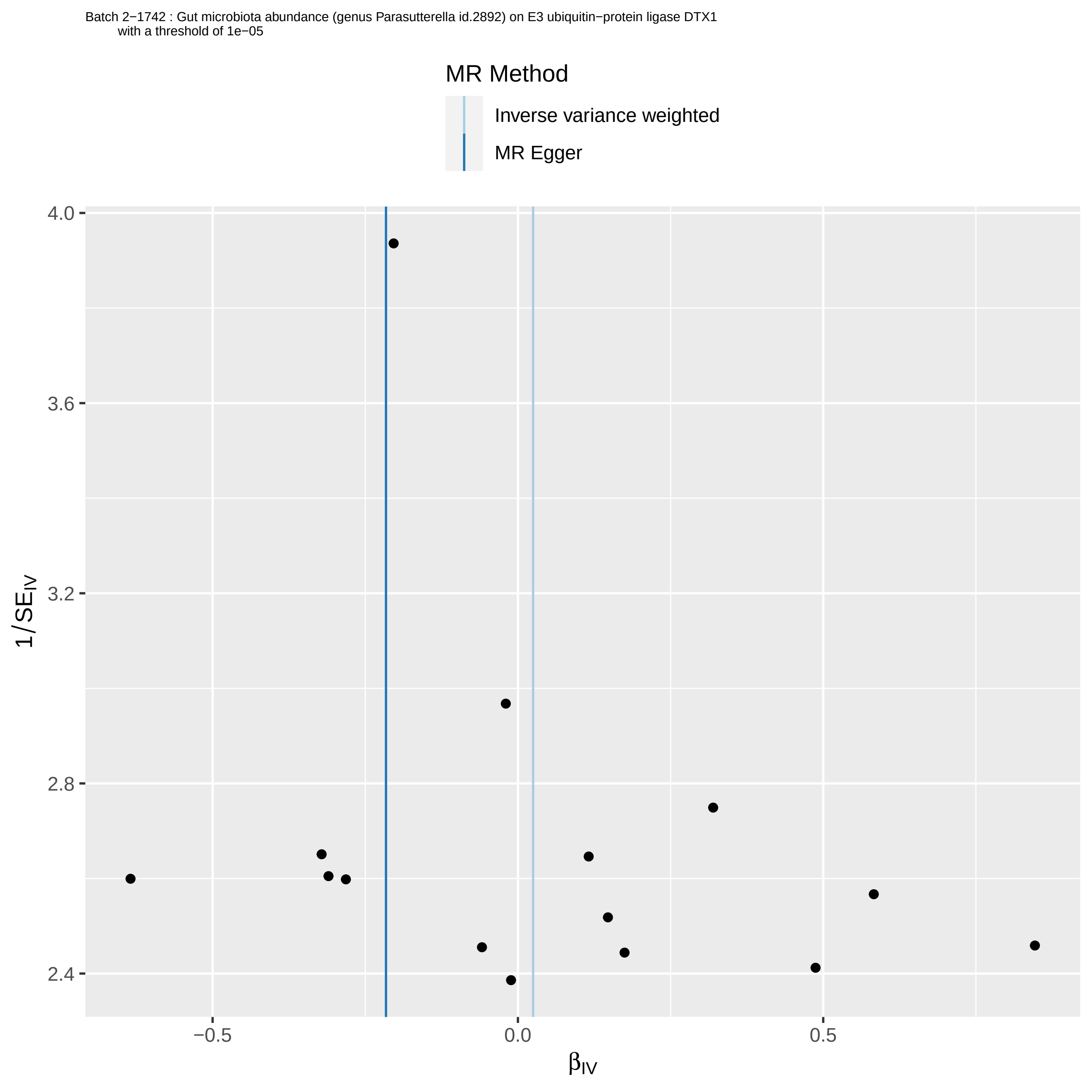
Batch 2−1742 : Gut microbiota abundance (genus Parasutterella id.2892) on E3 ubiquitin−protein ligase DTX1
with a threshold of 1e−05
MR Method
Inverse variance weighted
MR Egger
4.0
3.6
3.2
2.8
2.4
1/SE_IV
−0.5
0.0
0.5
β_IV

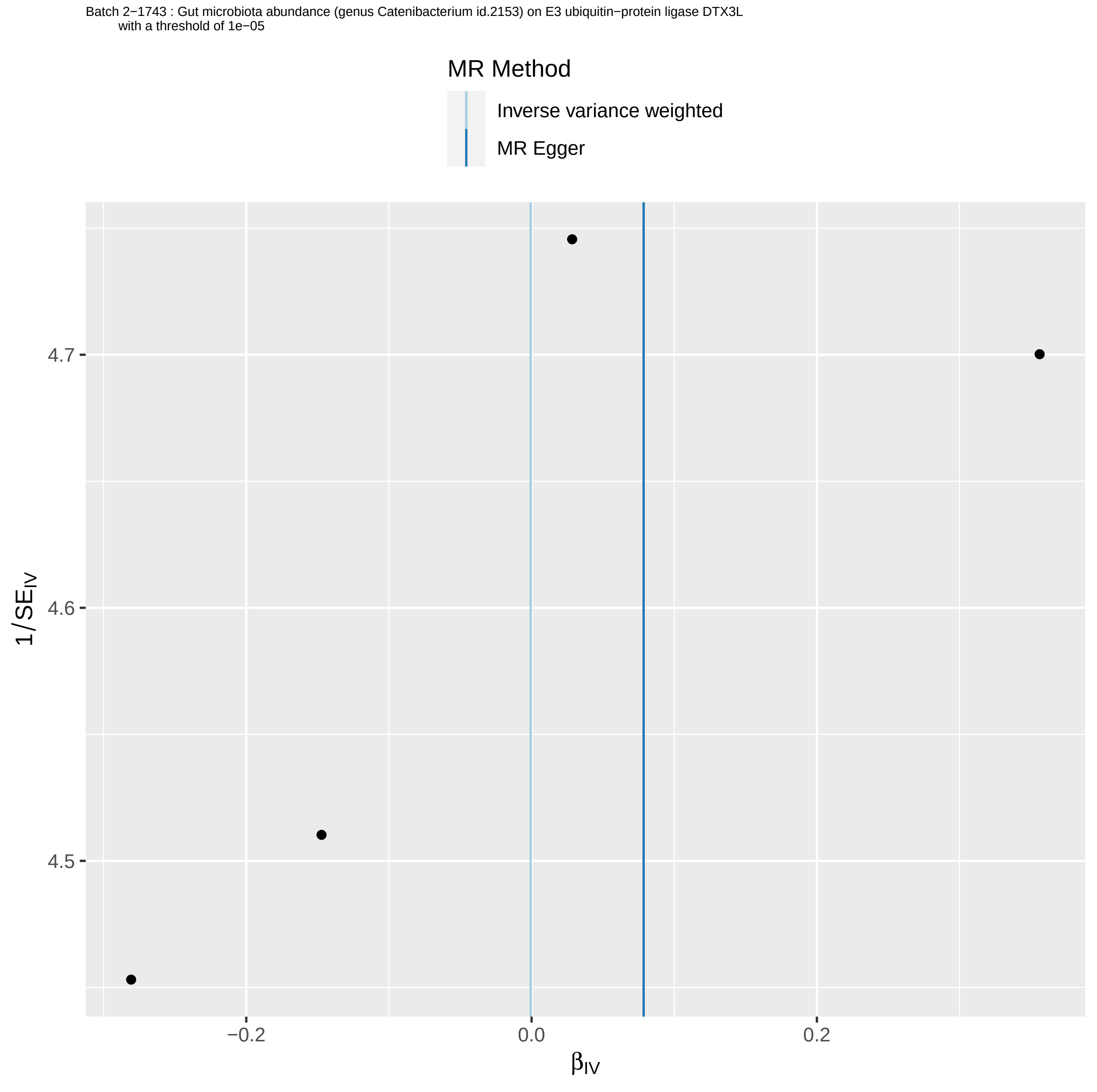

Batch 2−1743 : Gut microbiota abundance (genus Catenibacterium id.2153) on E3 ubiquitin−protein ligase DTX3L
with a threshold of 1e−05
MR Method
Inverse variance weighted
MR Egger
4.7
4.6
4.5
$1/SE_{IV}$
−0.2
0.0
0.2
β_{IV}

Batch 2−1744 : Gut microbiota abundance (genus Parasutterella id.2892) on E3 ubiquitin−protein ligase DTX3L
with a threshold of 1e−05

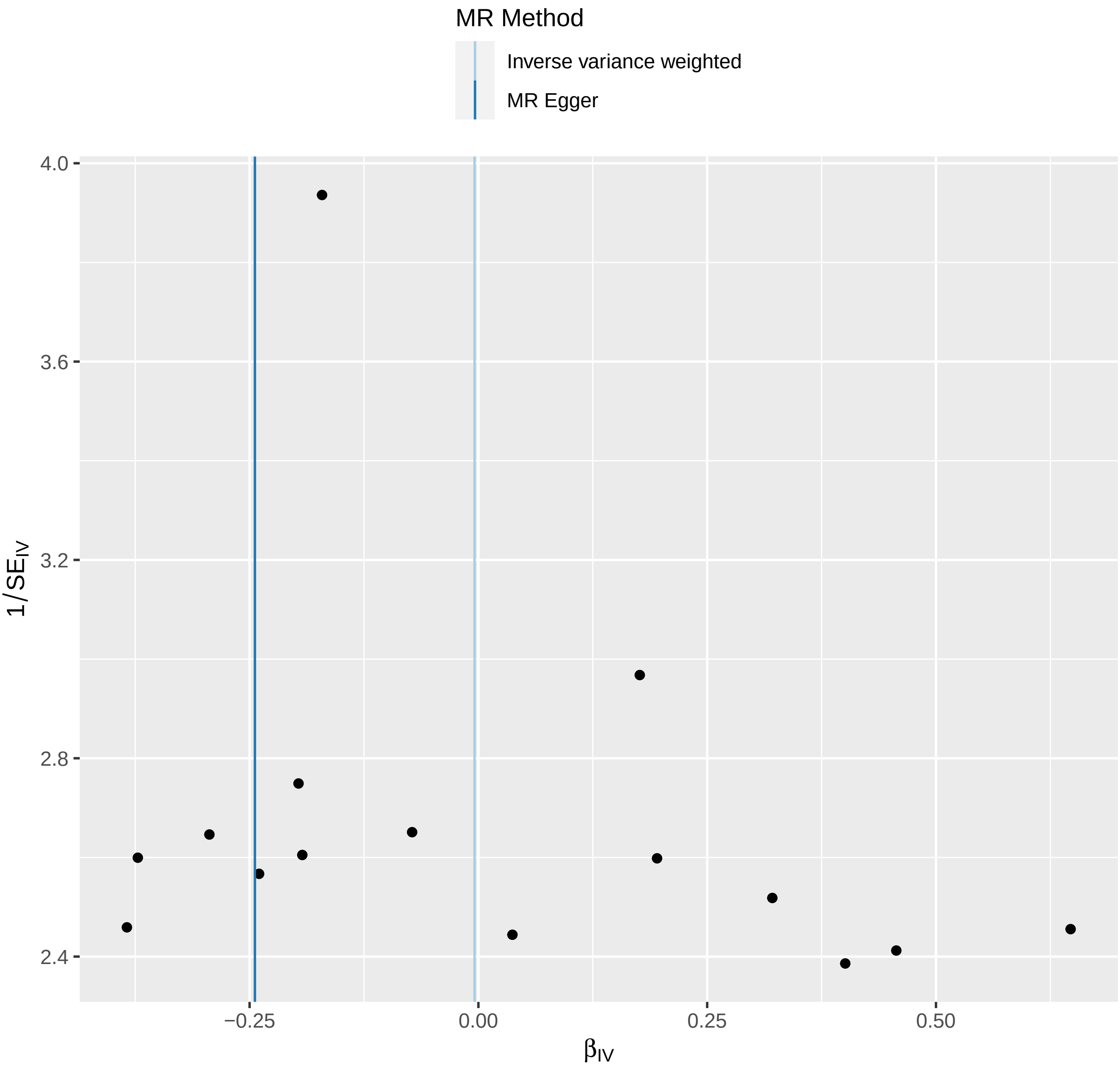

Batch 2−1745 : Gut microbiota abundance (genus Catenibacterium id.2153) on Dual specificity protein phosphatase 13 isoform A
with a threshold of 1e−05

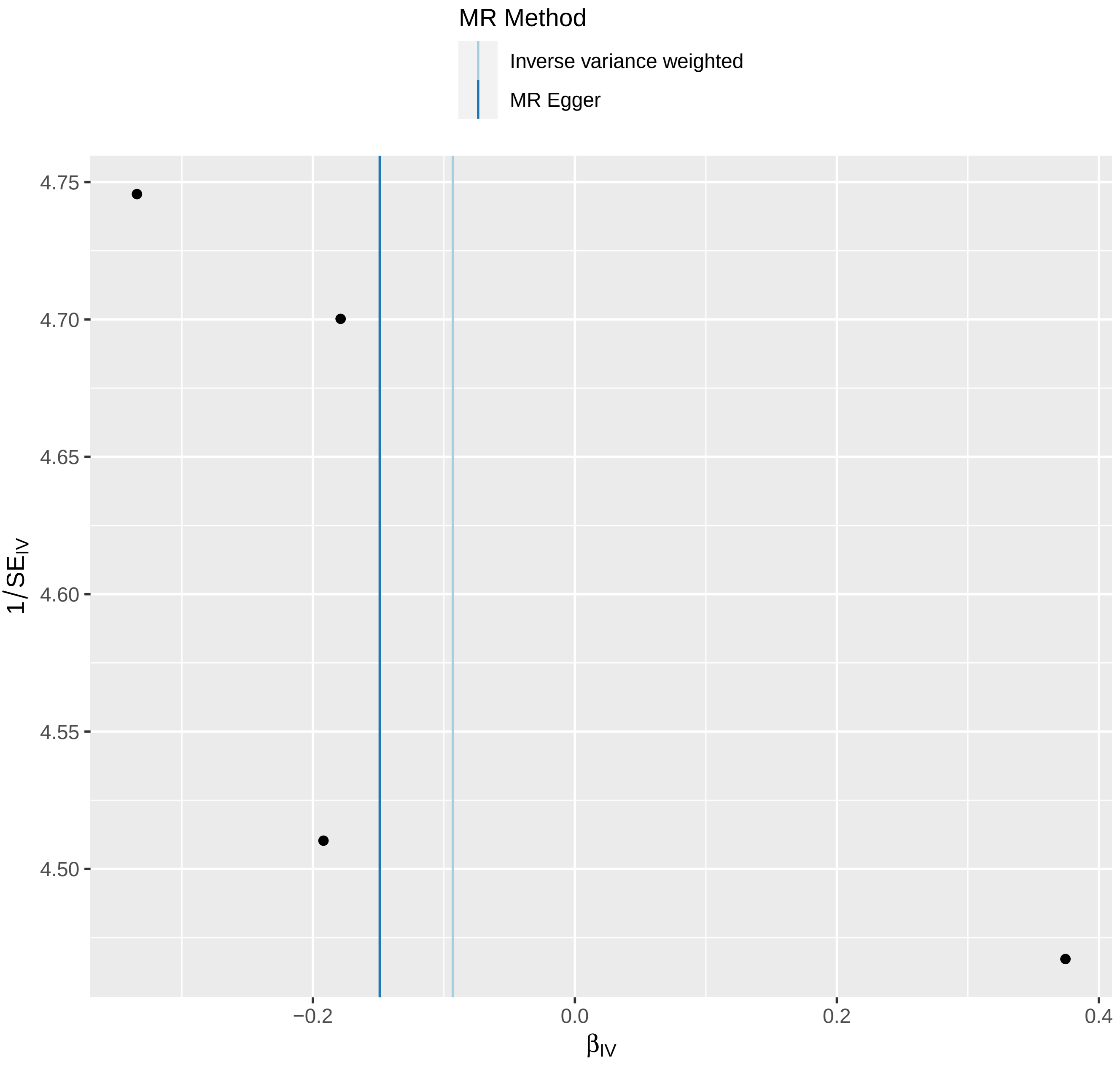

Batch 2−1746 : Gut microbiota abundance (genus Parasutterella id.2892) on Dual specificity protein phosphatase 13 isoform A
with a threshold of 1e−05

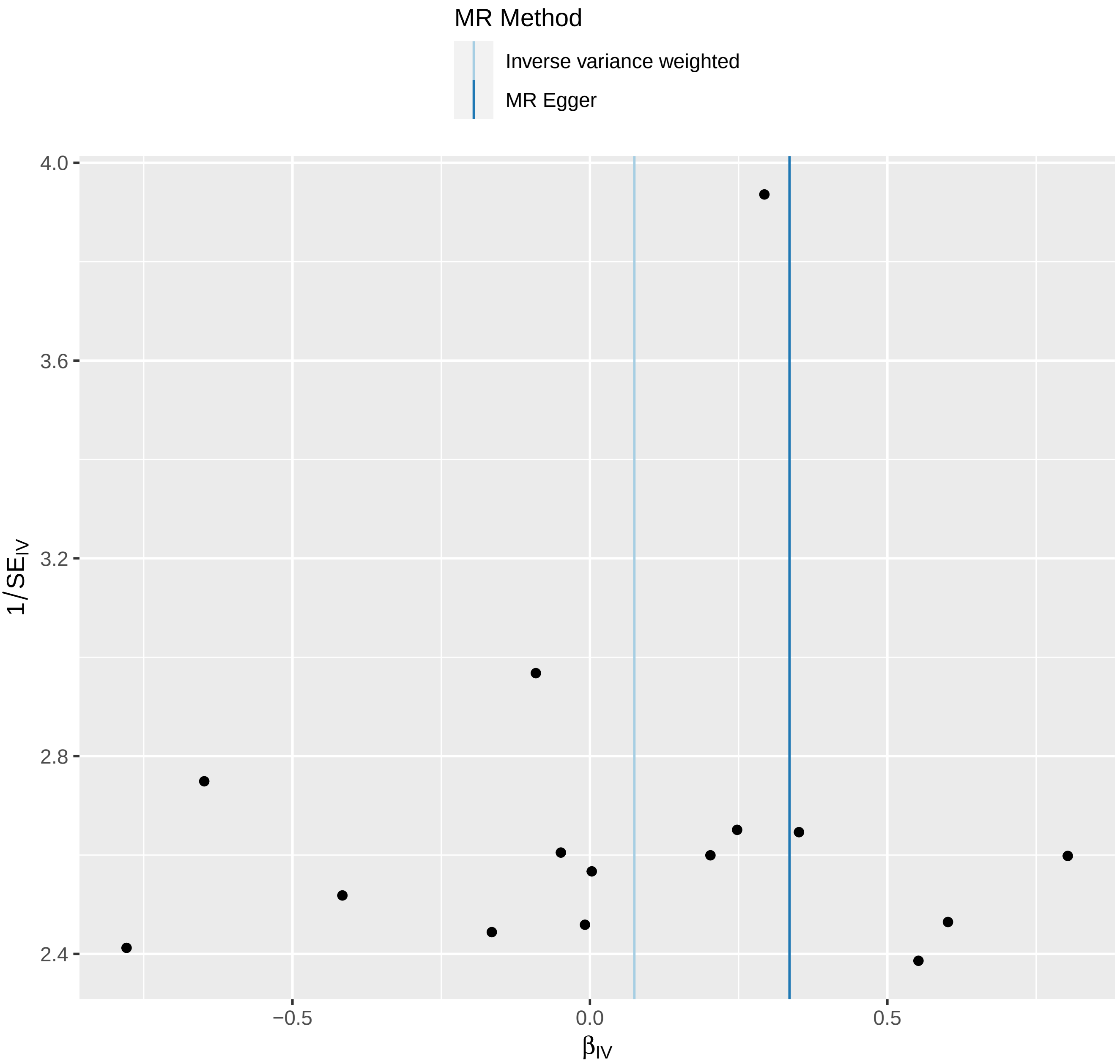

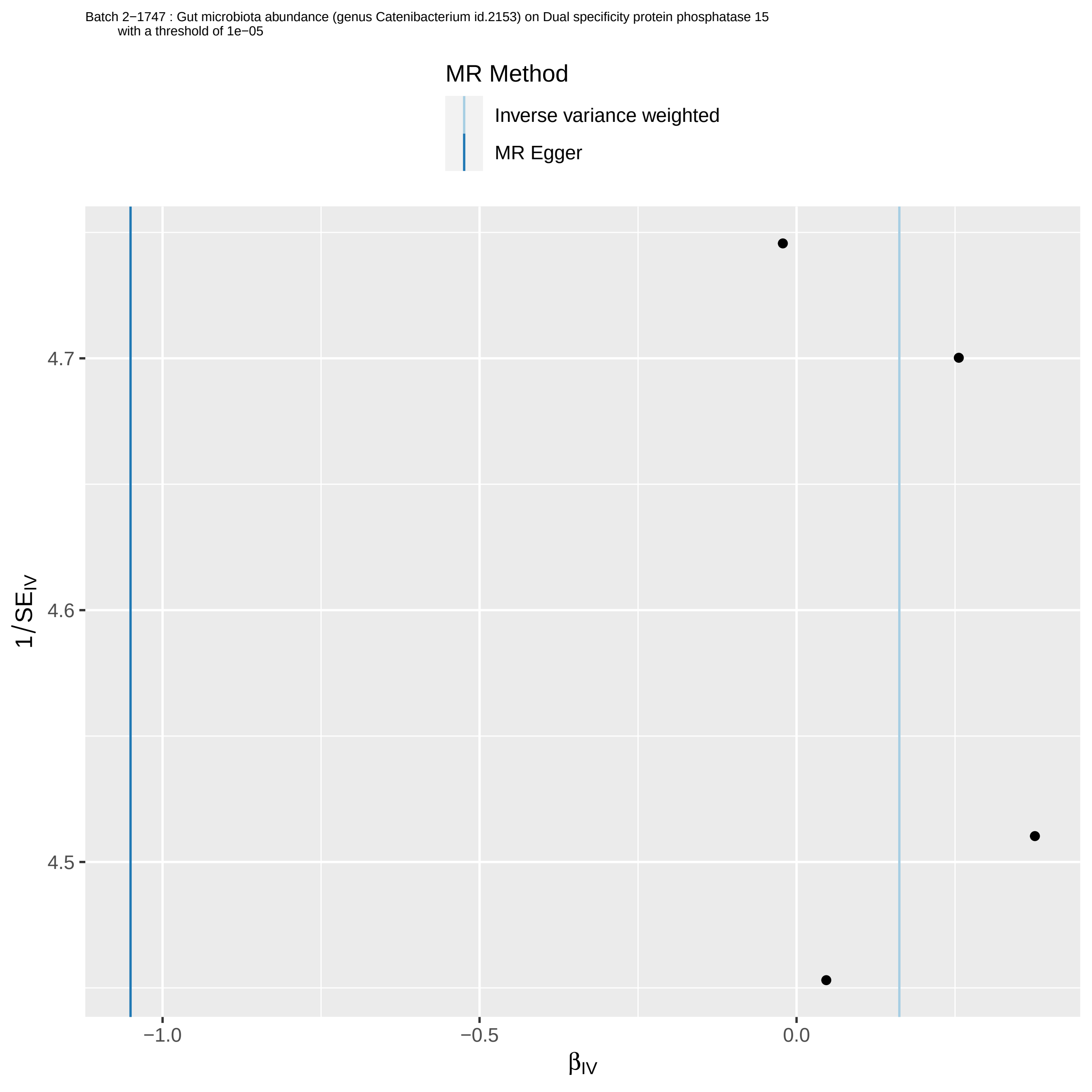

Batch 2−1747 : Gut microbiota abundance (genus Catenibacterium id.2153) on Dual specificity protein phosphatase 15
with a threshold of 1e−05
MR Method
Inverse variance weighted
MR Egger
4.7
4.6
4.5
1/SE_IV
−1.0
−0.5
0.0
β_IV

Batch 2−1748 : Gut microbiota abundance (genus Parasutterella id.2892) on Dual specificity protein phosphatase 15
with a threshold of 1e−05

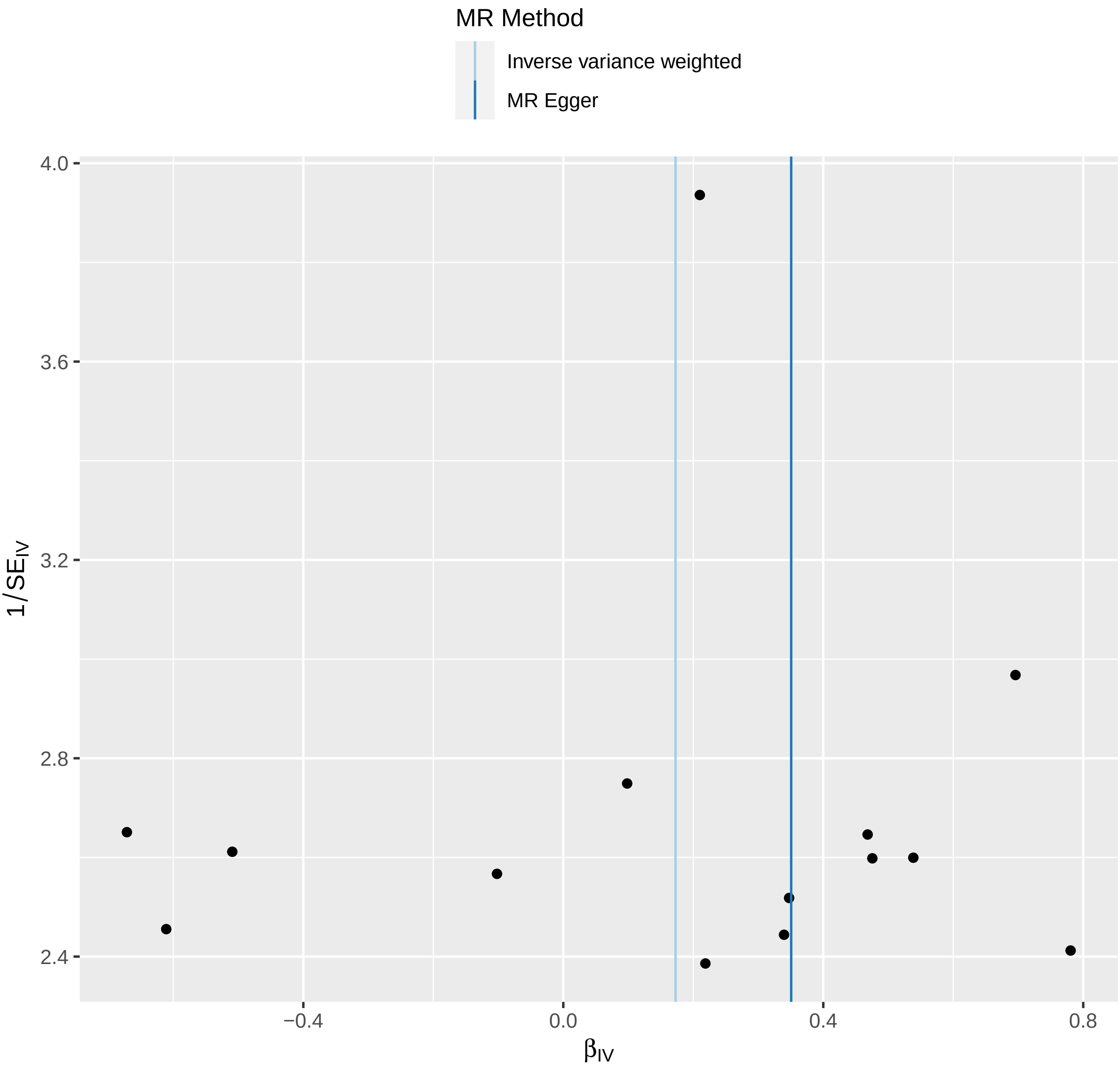

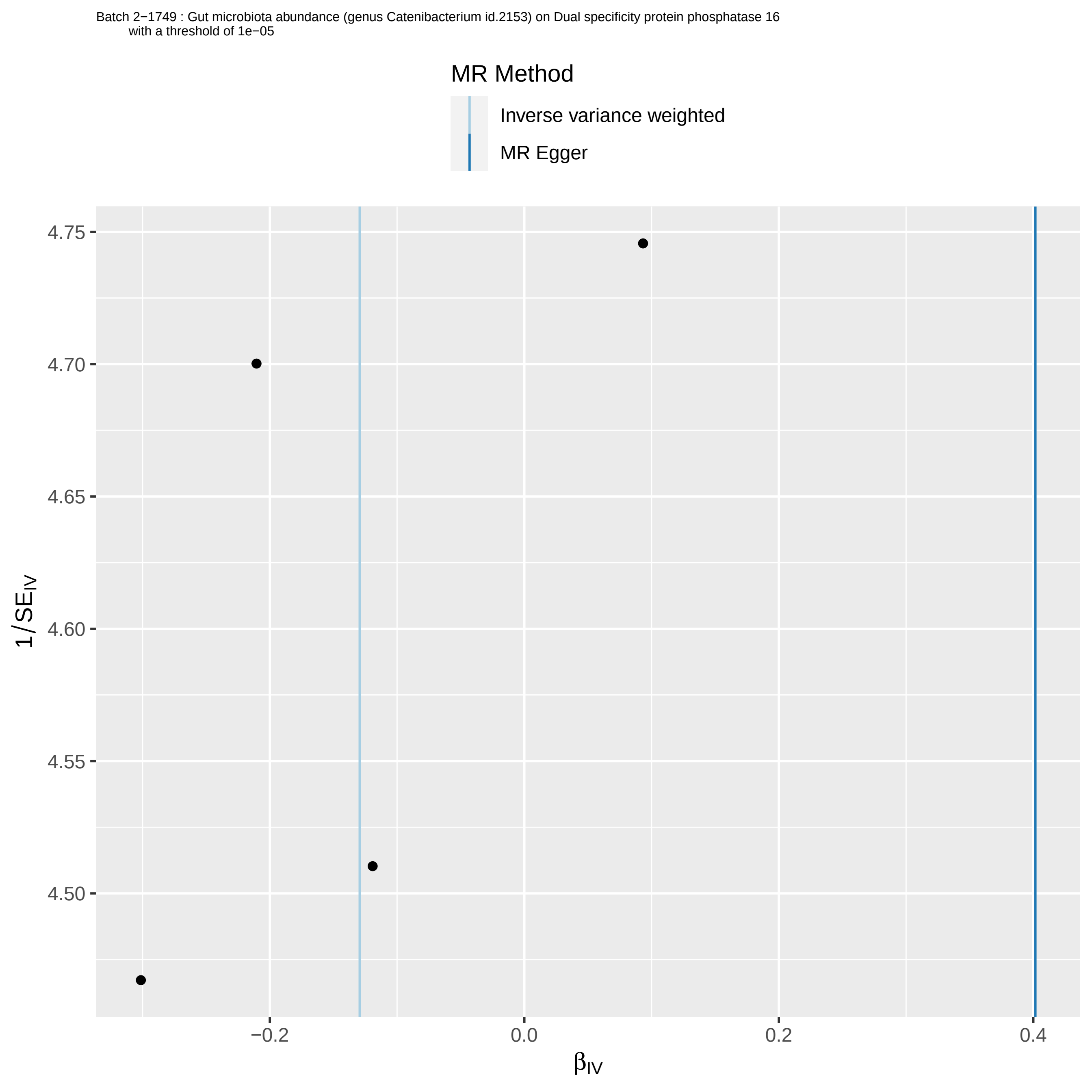

Batch 2−1749 : Gut microbiota abundance (genus Catenibacterium id.2153) on Dual specificity protein phosphatase 16
with a threshold of 1e−05
MR Method
Inverse variance weighted
MR Egger
4.75
4.70
4.65
4.60
4.55
4.50
1/SE$_{IV}$
−0.2
0.0
0.2
0.4
β$_{IV}$

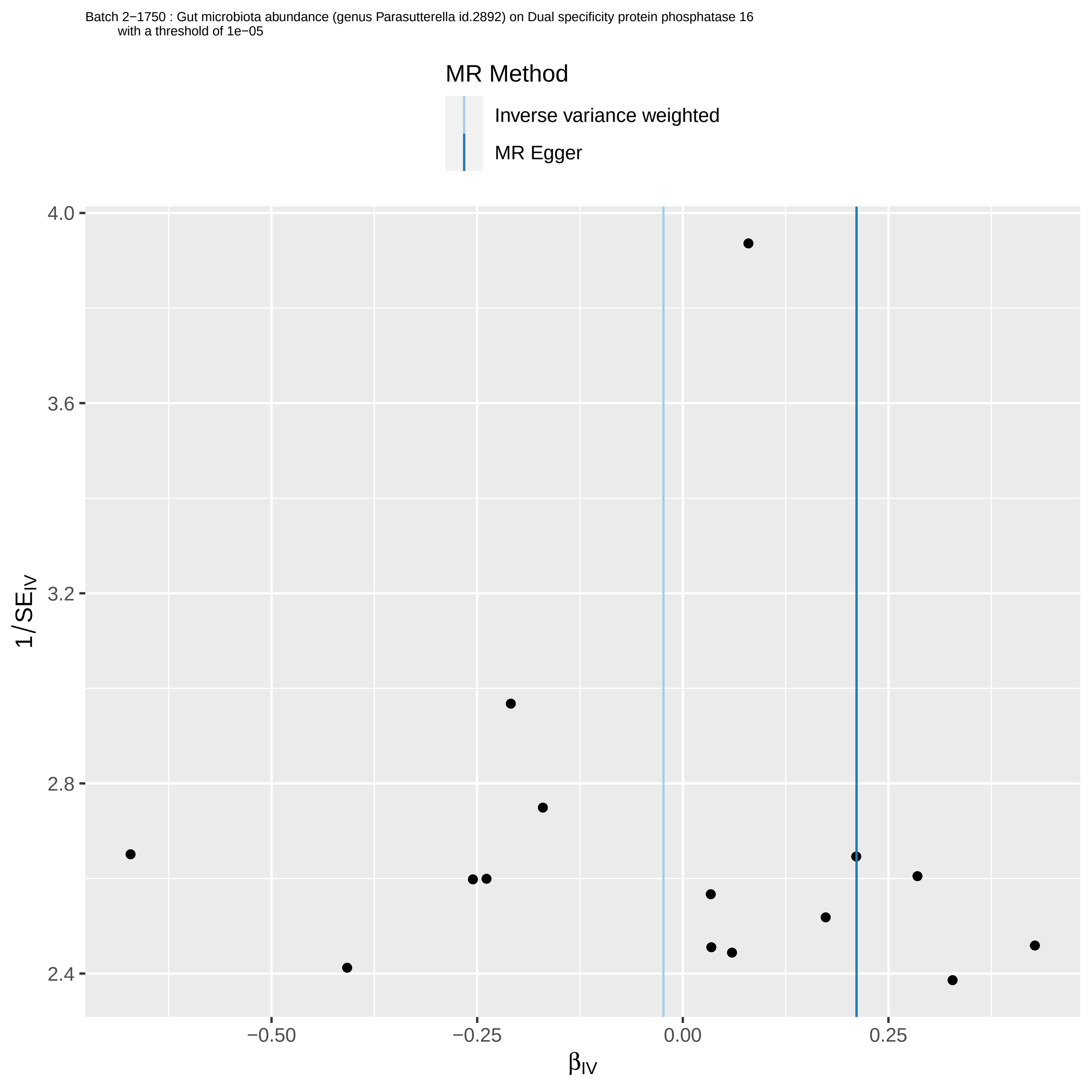
Batch 2−1750 : Gut microbiota abundance (genus Parasutterella id.2892) on Dual specificity protein phosphatase 16
with a threshold of 1e−05
MR Method
Inverse variance weighted
MR Egger
4.0
3.6
3.2
2.8
2.4
1/SE_IV
−0.50
−0.25
0.00
0.25
β_IV

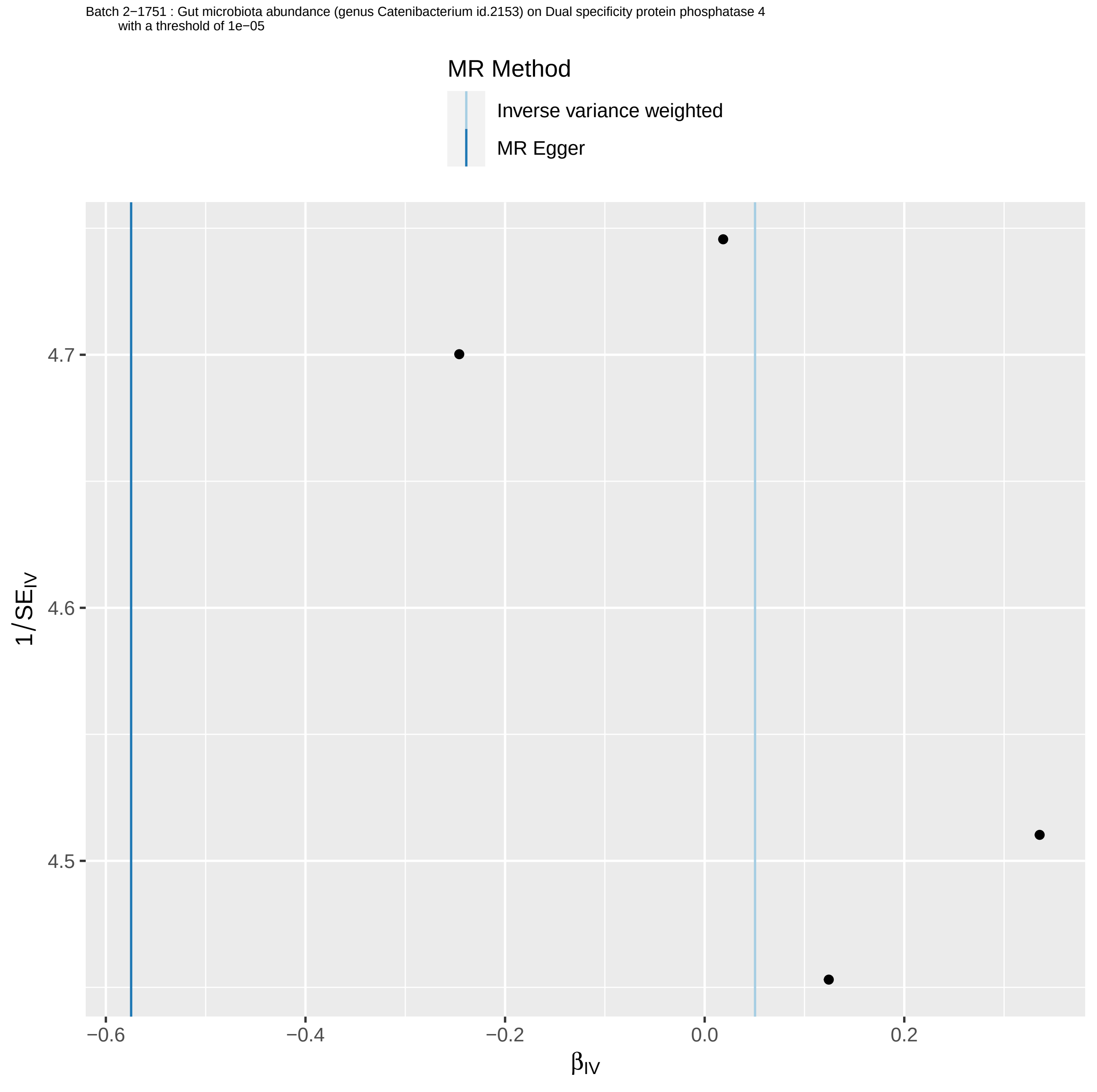
Batch 2−1751 : Gut microbiota abundance (genus Catenibacterium id.2153) on Dual specificity protein phosphatase 4
with a threshold of 1e−05
MR Method
Inverse variance weighted
MR Egger
4.7
4.6
4.5
1/SE_IV
−0.6
−0.4
−0.2
0.0
0.2
β_IV

Batch 2−1752 : Gut microbiota abundance (genus Parasutterella id.2892) on Dual specificity protein phosphatase 4
with a threshold of 1e−05

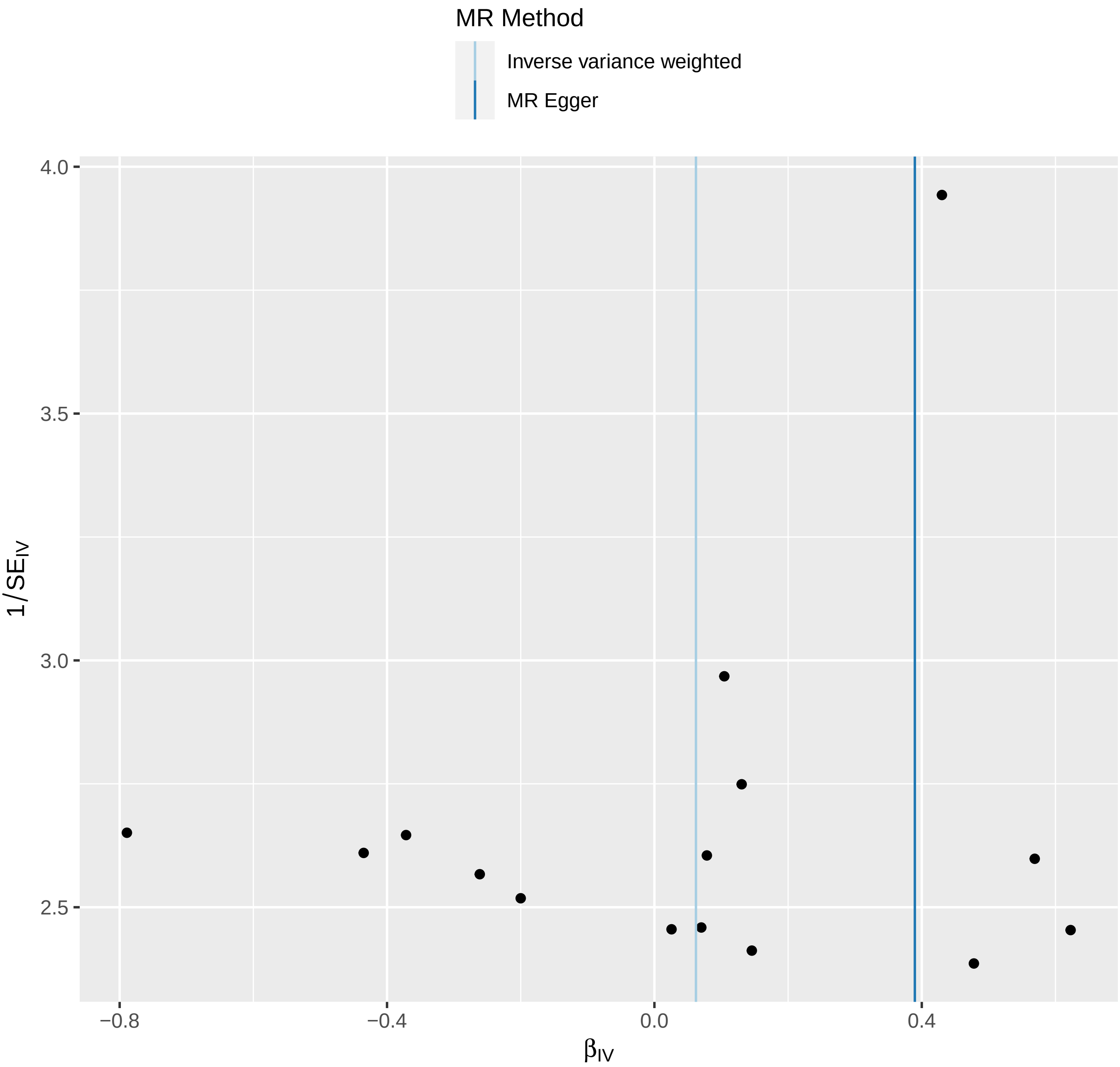

Batch 2−1753 : Gut microbiota abundance (genus Catenibacterium id.2153) on Dual specificity protein phosphatase 6 with a threshold of 1e−05

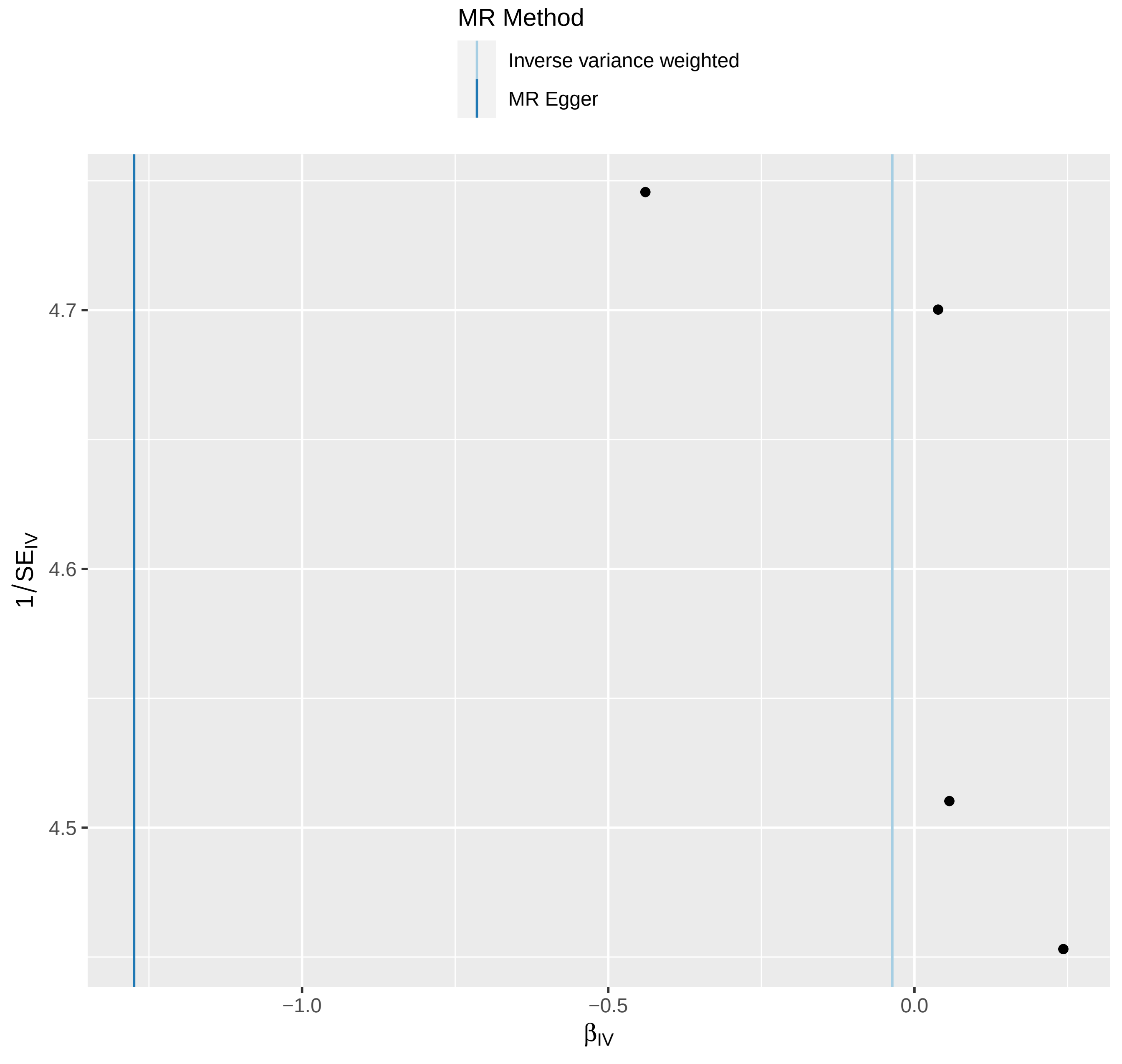

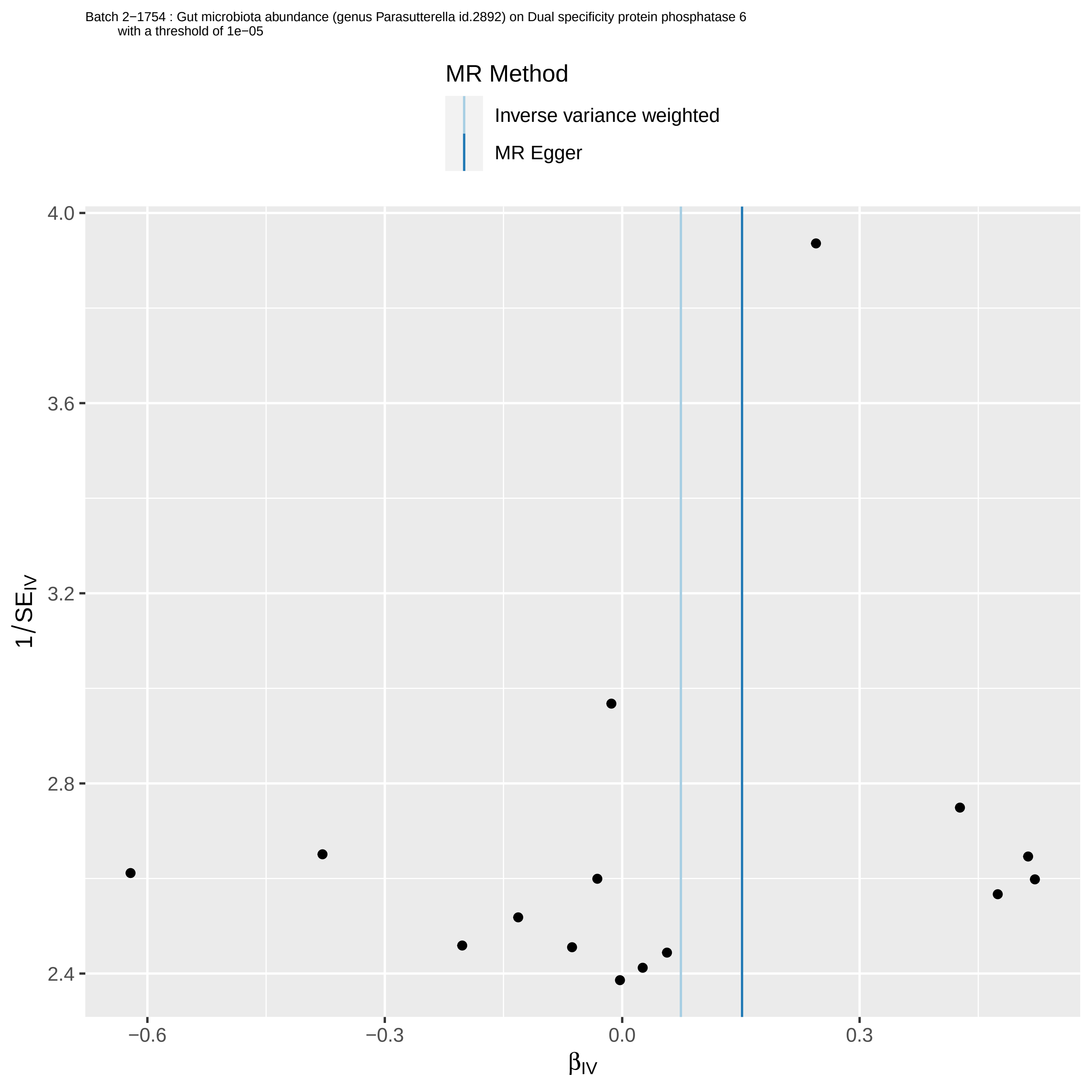

Batch 2−1754 : Gut microbiota abundance (genus Parasutterella id.2892) on Dual specificity protein phosphatase 6
with a threshold of 1e−05
MR Method
Inverse variance weighted
MR Egger
4.0
3.6
3.2
2.8
2.4
1/SE_IV
−0.6
−0.3
0.0
0.3
β_IV

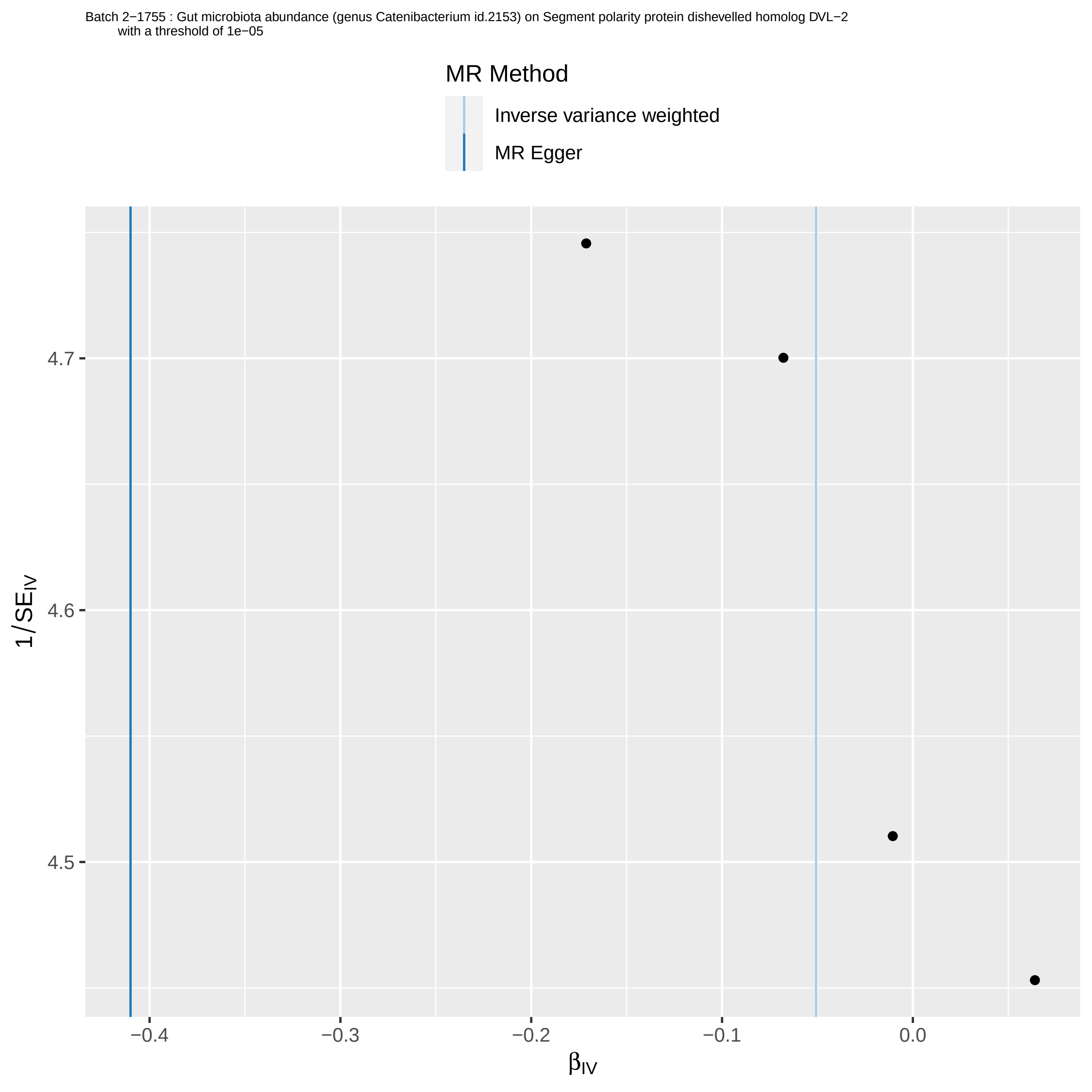
Batch 2−1755 : Gut microbiota abundance (genus Catenibacterium id.2153) on Segment polarity protein dishevelled homolog DVL−2
with a threshold of 1e−05
MR Method
Inverse variance weighted
MR Egger
4.7
4.6
4.5
1/SE_IV
−0.4
−0.3
−0.2
−0.1
0.0
β_IV

Batch 2−1756 : Gut microbiota abundance (genus Parasutterella id.2892) on Segment polarity protein dishevelled homolog DVL−2
with a threshold of 1e−05

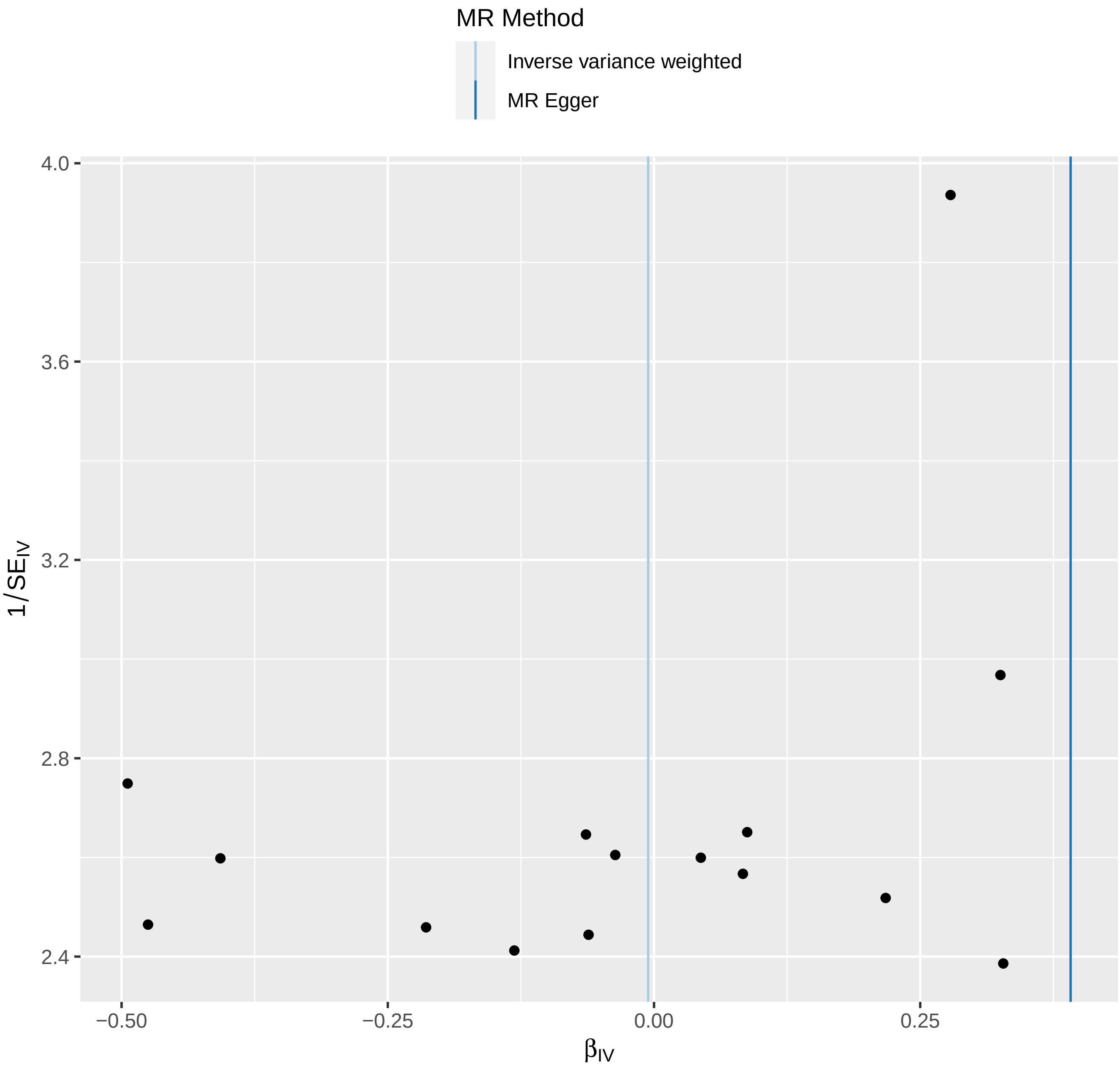

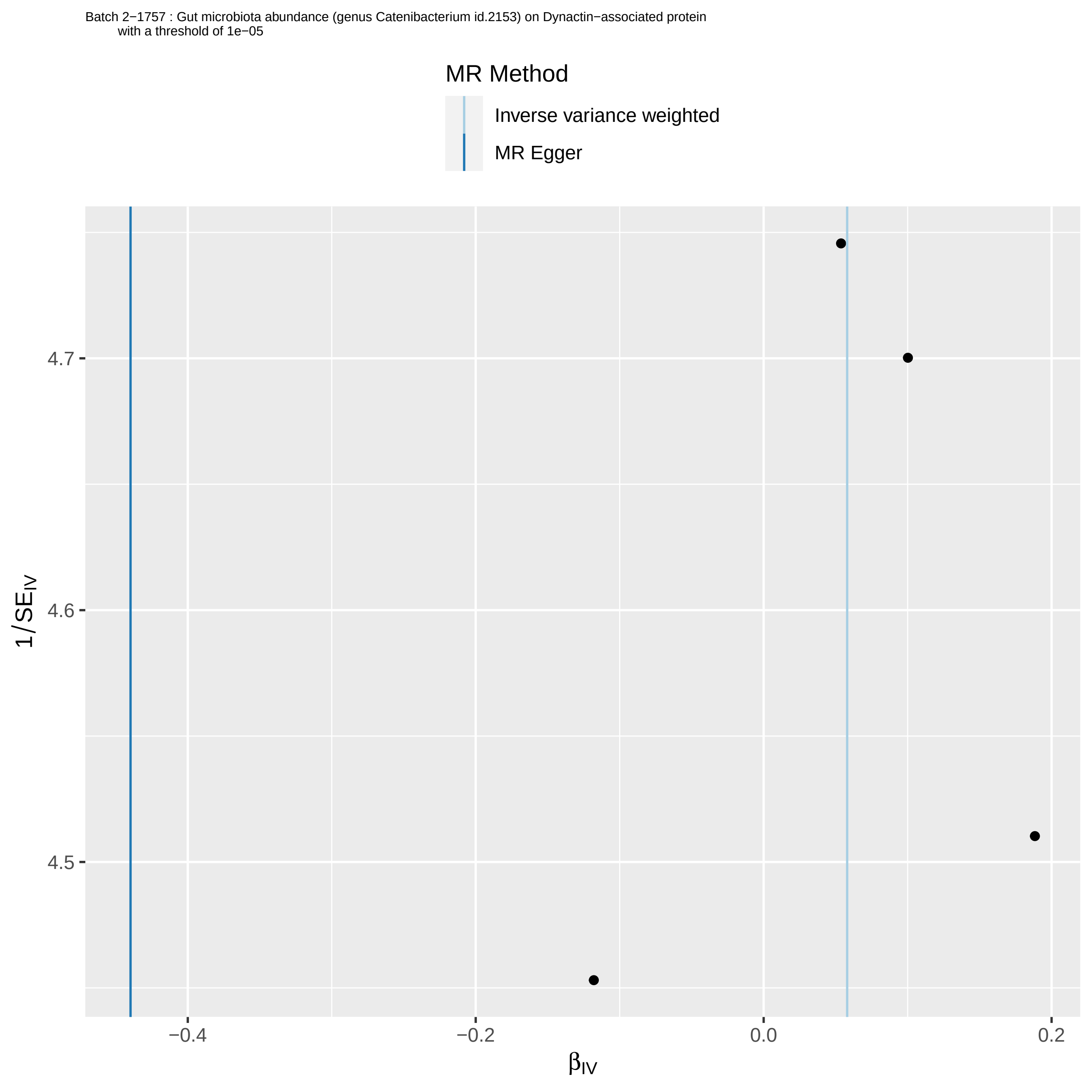
Batch 2−1757 : Gut microbiota abundance (genus Catenibacterium id.2153) on Dynactin−associated protein
with a threshold of 1e−05
MR Method
Inverse variance weighted
MR Egger
4.7
4.6
4.5
1/SE_IV
−0.4
−0.2
0.0
0.2
β_IV

Batch 2−1758 : Gut microbiota abundance (genus Parasutterella id.2892) on Dynactin−associated protein
with a threshold of 1e−05

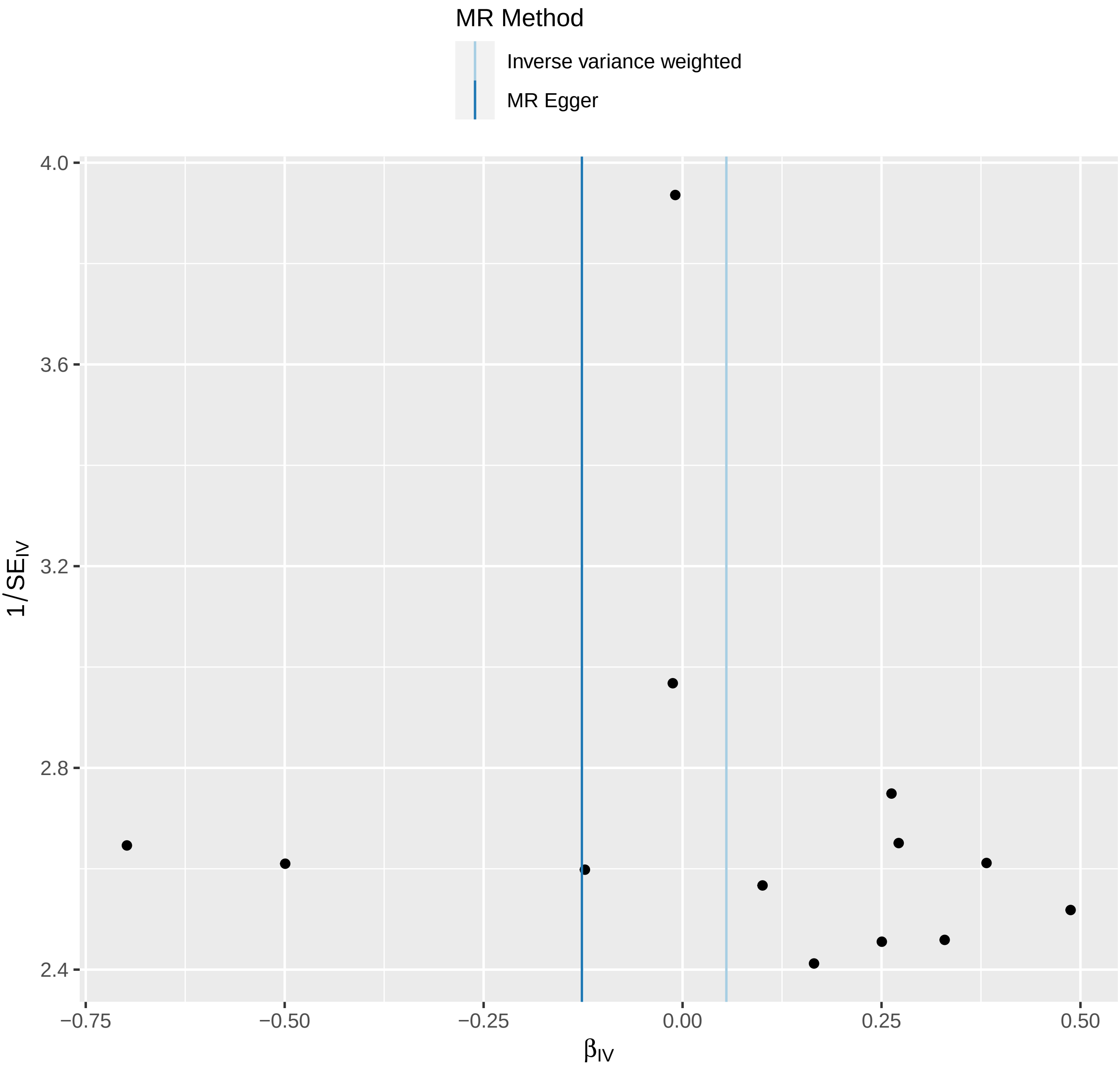

Batch 2−1759 : Gut microbiota abundance (genus Catenibacterium id.2153) on Dynein light chain 1, cytoplasmic with a threshold of 1e−05

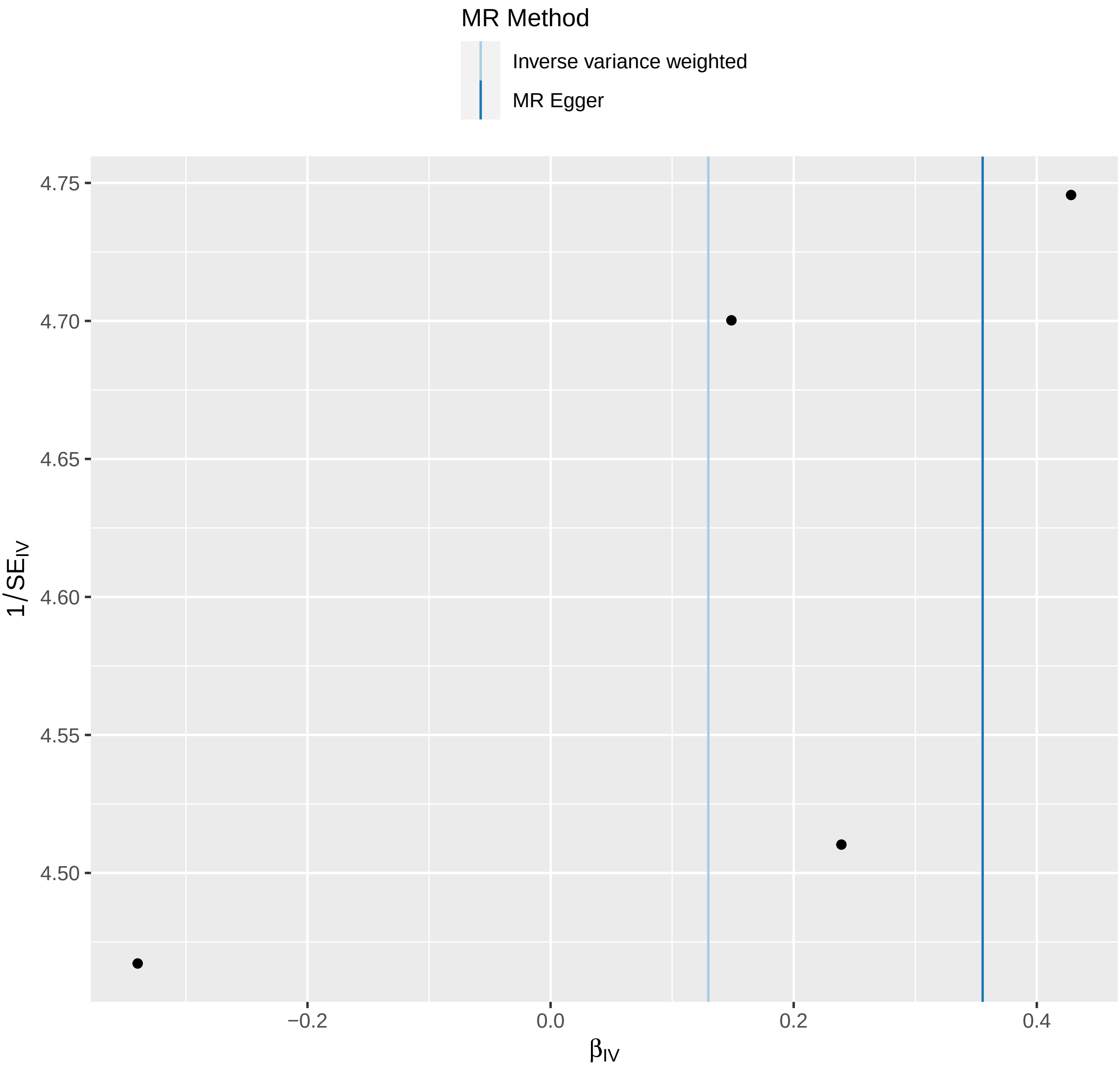

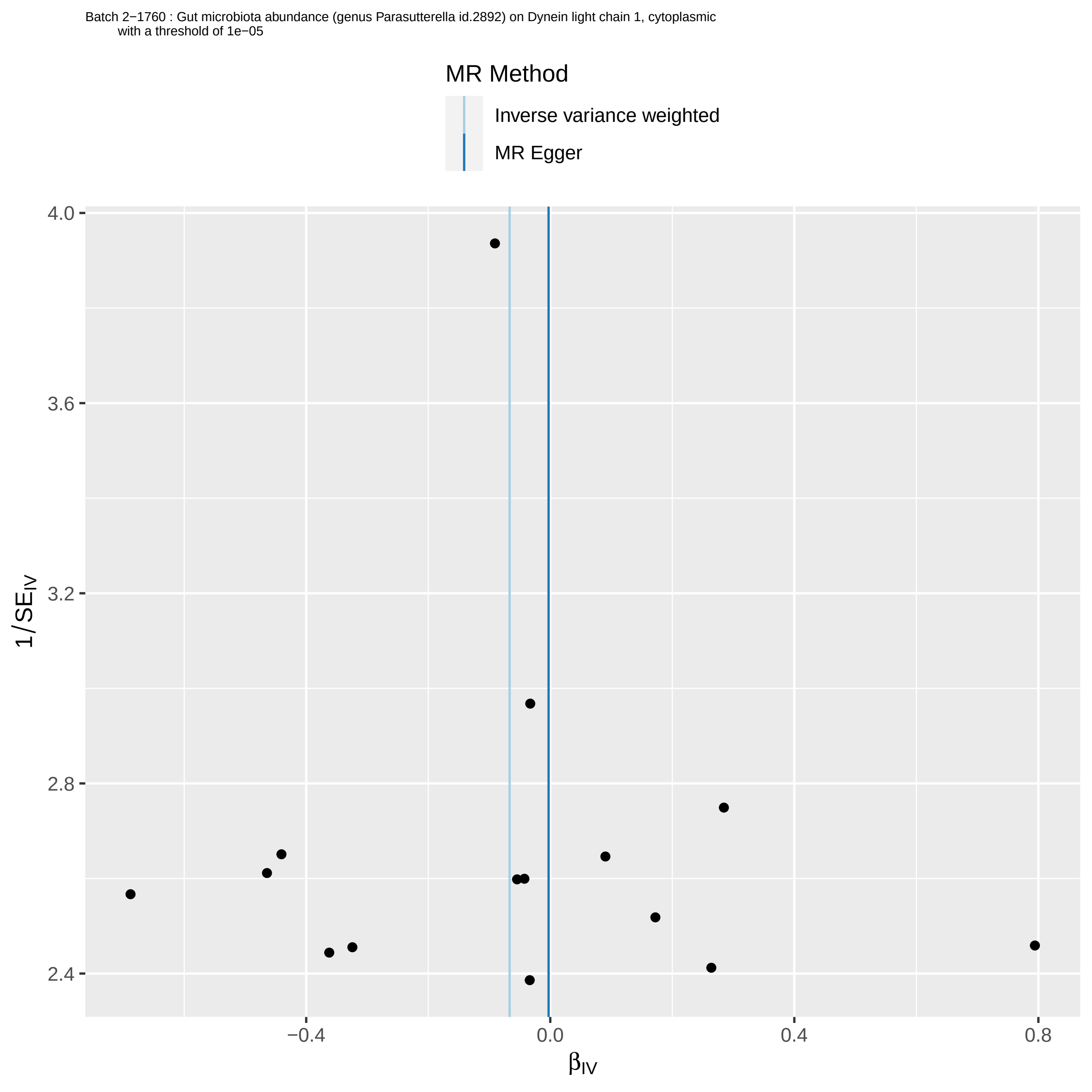
Batch 2−1760 : Gut microbiota abundance (genus Parasutterella id.2892) on Dynein light chain 1, cytoplasmic
with a threshold of 1e−05
MR Method
Inverse variance weighted
MR Egger
4.0
3.6
3.2
2.8
2.4
1/SE_IV
−0.4
0.0
0.4
0.8
β_IV

Batch 2−1761 : Gut microbiota abundance (genus Catenibacterium id.2153) on Dynein light chain Tctex−type 3
with a threshold of 1e−05

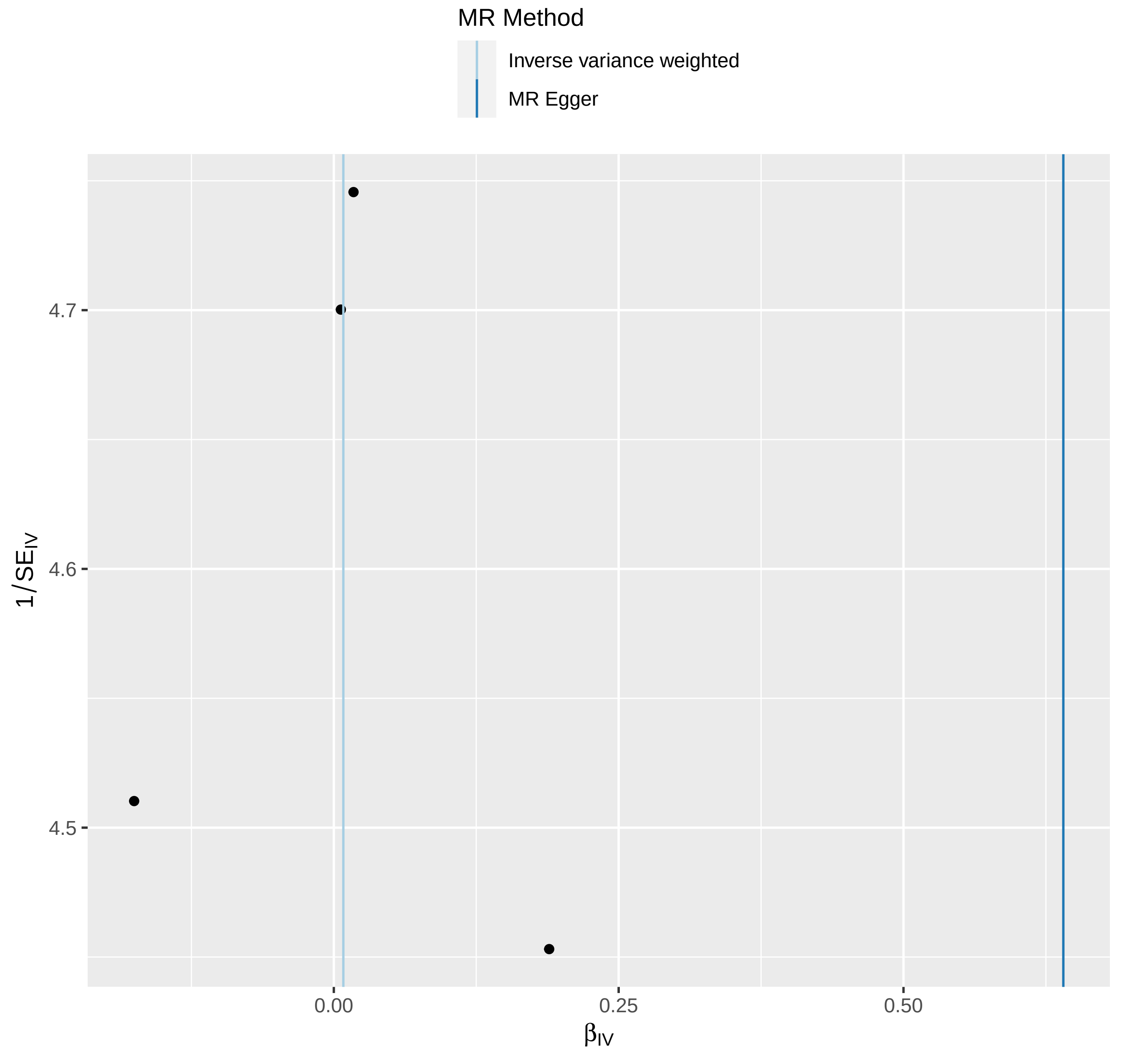

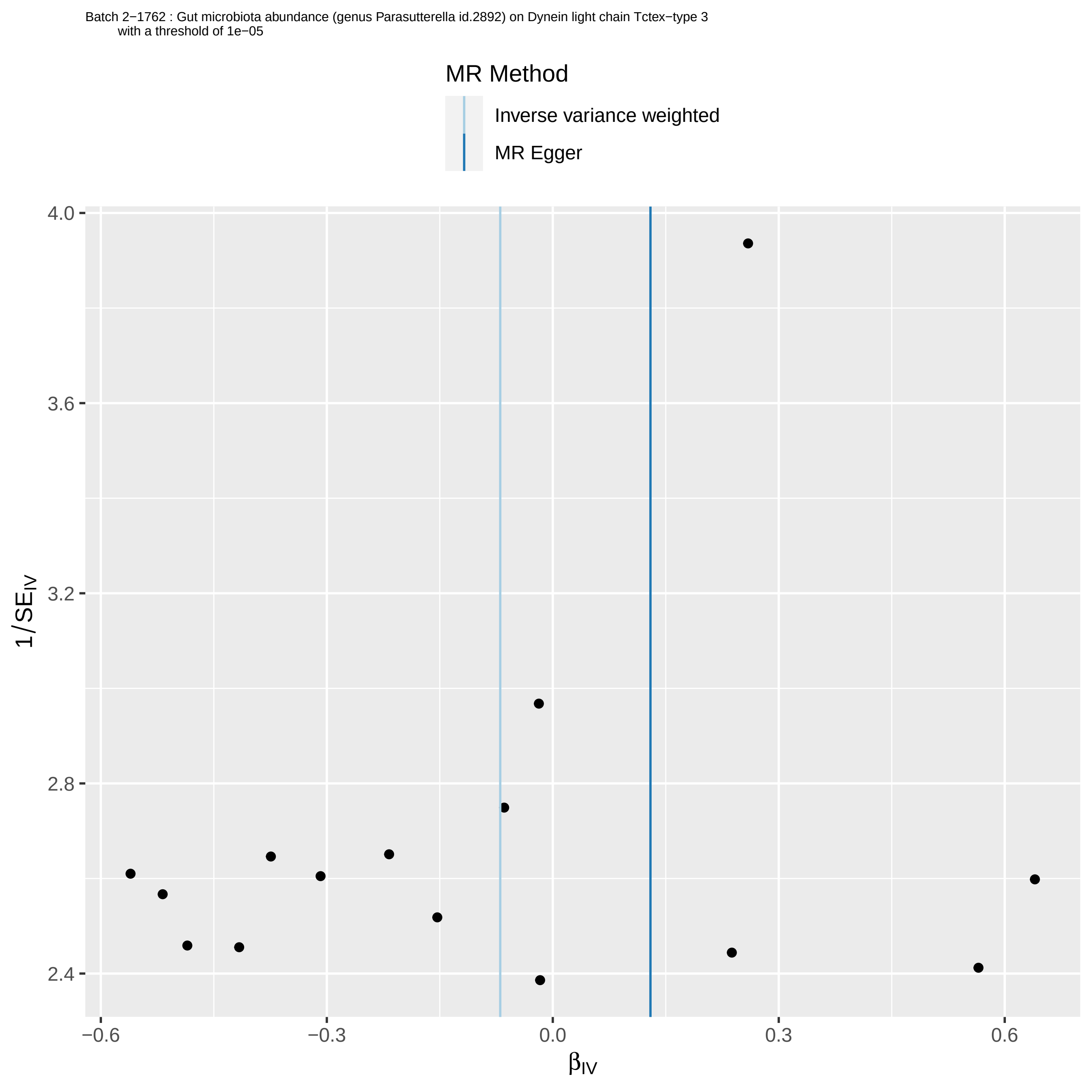
Batch 2−1762 : Gut microbiota abundance (genus Parasutterella id.2892) on Dynein light chain Tctex−type 3
with a threshold of 1e−05
MR Method
Inverse variance weighted
MR Egger
4.0
3.6
3.2
2.8
2.4
1/SE_IV
−0.6
−0.3
0.0
0.3
0.6
β_IV

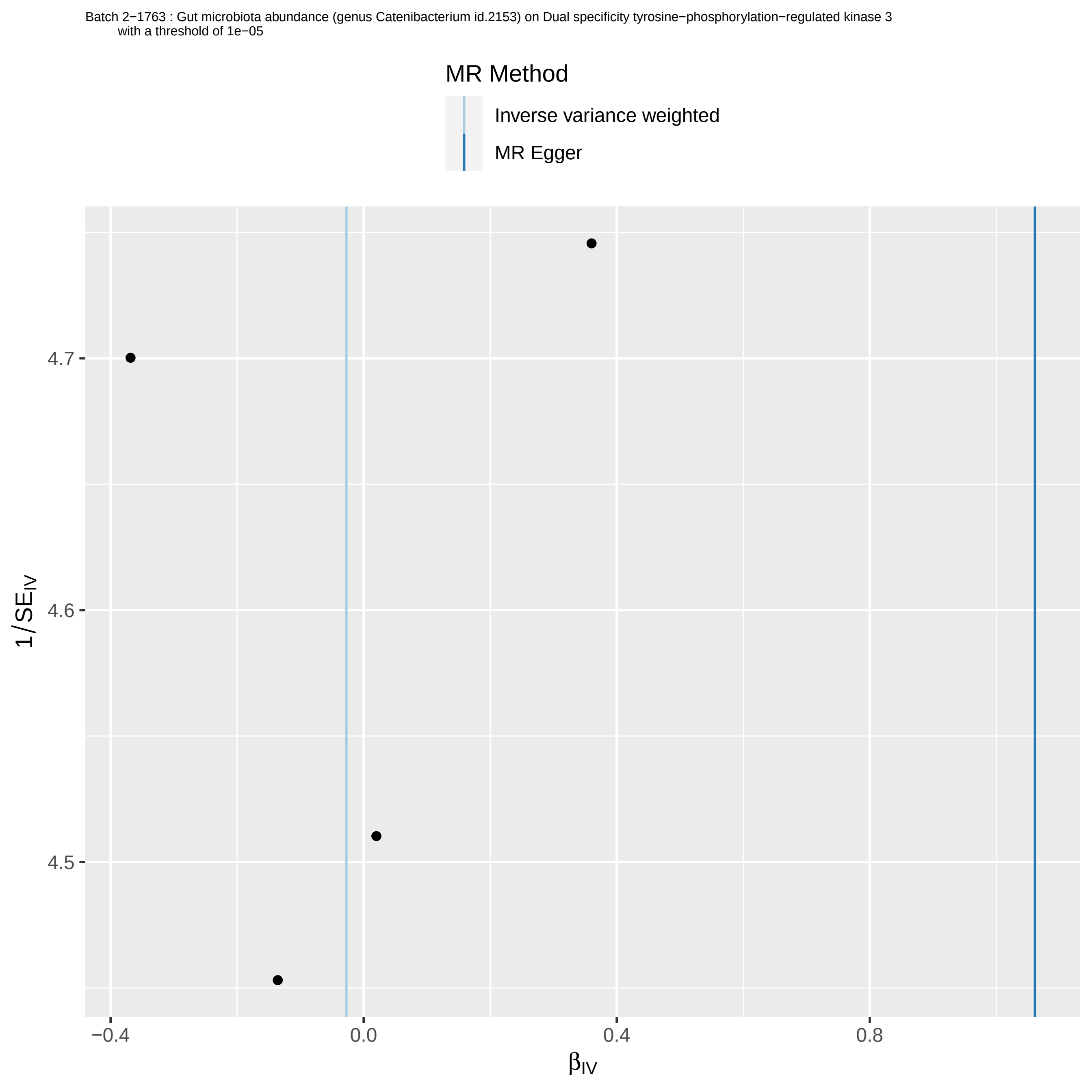
Batch 2−1763 : Gut microbiota abundance (genus Catenibacterium id.2153) on Dual specificity tyrosine−phosphorylation−regulated kinase 3
with a threshold of 1e−05
MR Method
Inverse variance weighted
MR Egger
4.7
4.6
4.5
1/SE_IV
−0.4
0.0
0.4
0.8
β_IV

Batch 2−1764 : Gut microbiota abundance (genus Parasutterella id.2892) on Dual specificity tyrosine−phosphorylation−regulated kinase 3 with a threshold of 1e−05

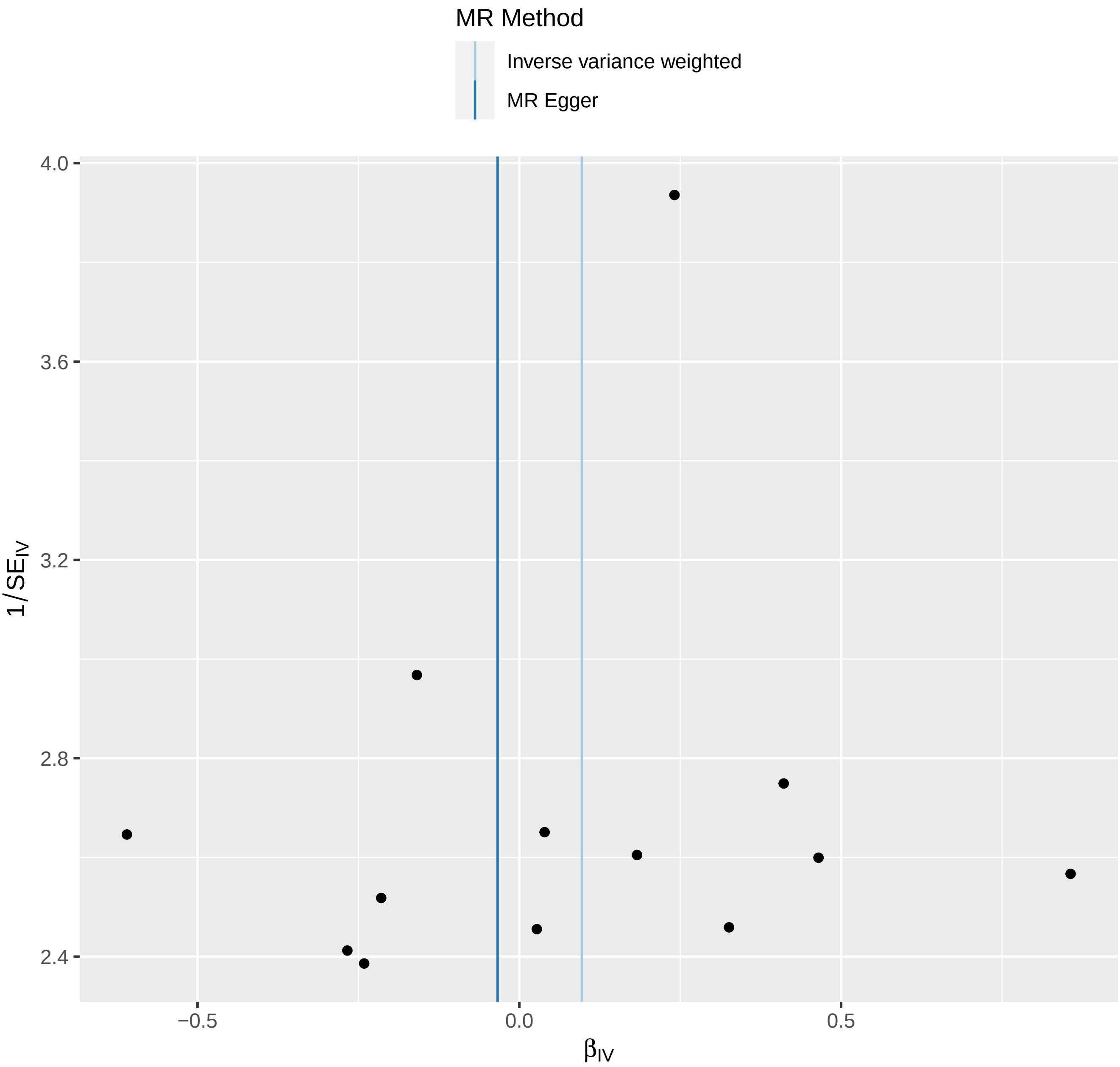

Batch 2−1765 : Gut microbiota abundance (genus Catenibacterium id.2153) on Endothelin−converting enzyme 1
with a threshold of 1e−05

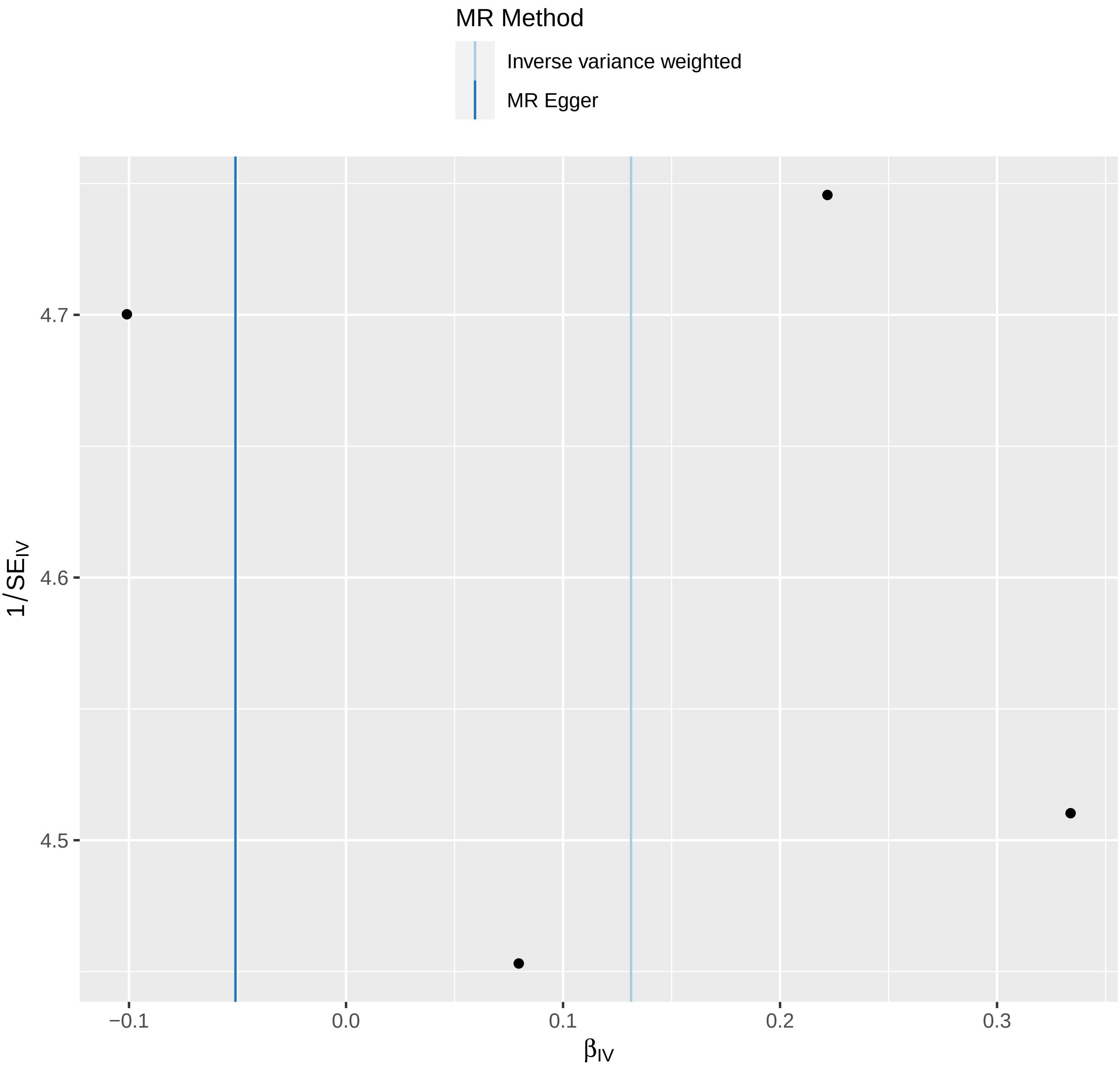

Batch 2−1766 : Gut microbiota abundance (genus Parasutterella id.2892) on Endothelin−converting enzyme 1
with a threshold of 1e−05

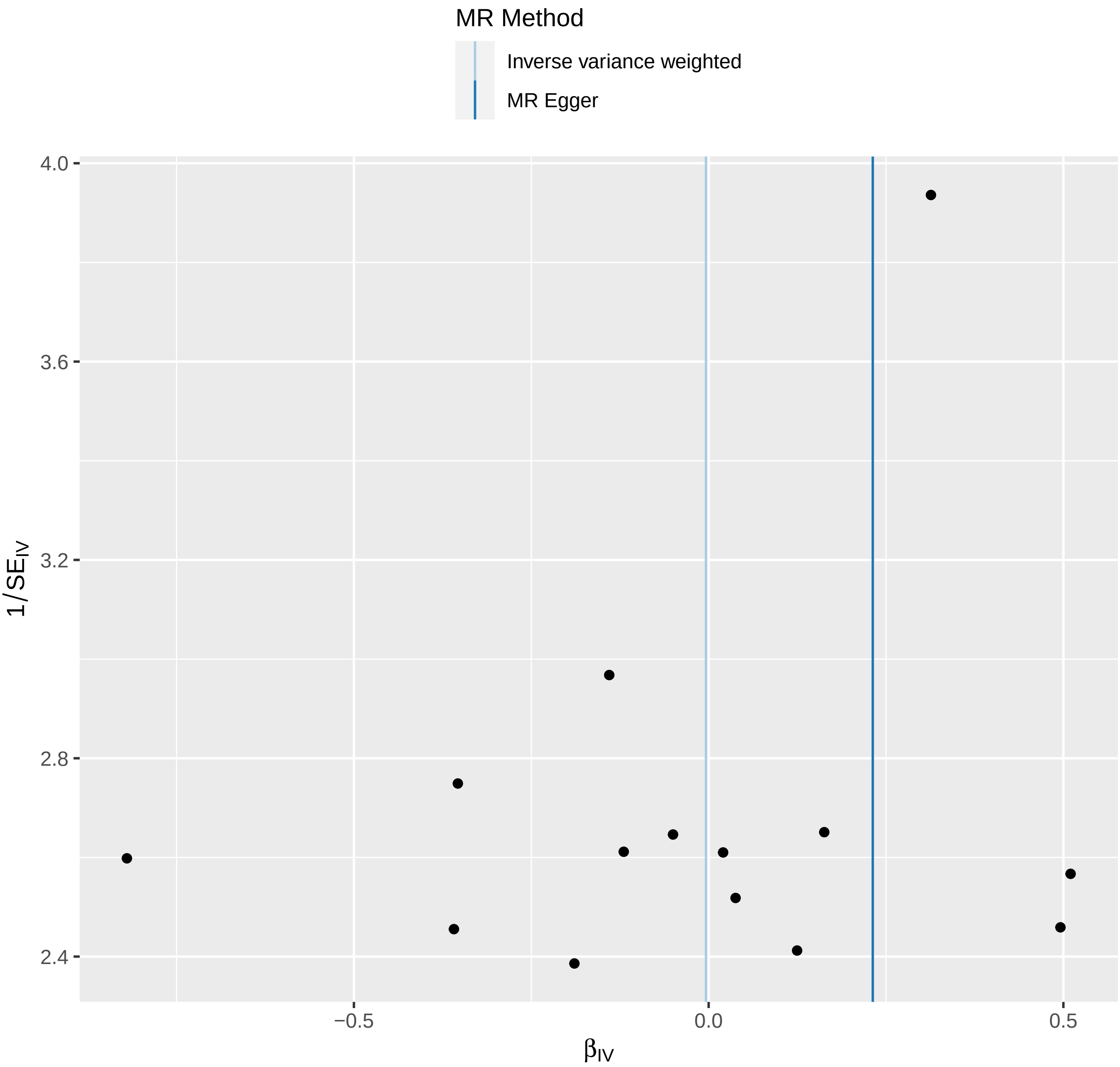

Batch 2−1767 : Gut microbiota abundance (genus Catenibacterium id.2153) on Endothelin−converting enzyme 1
with a threshold of 1e−05

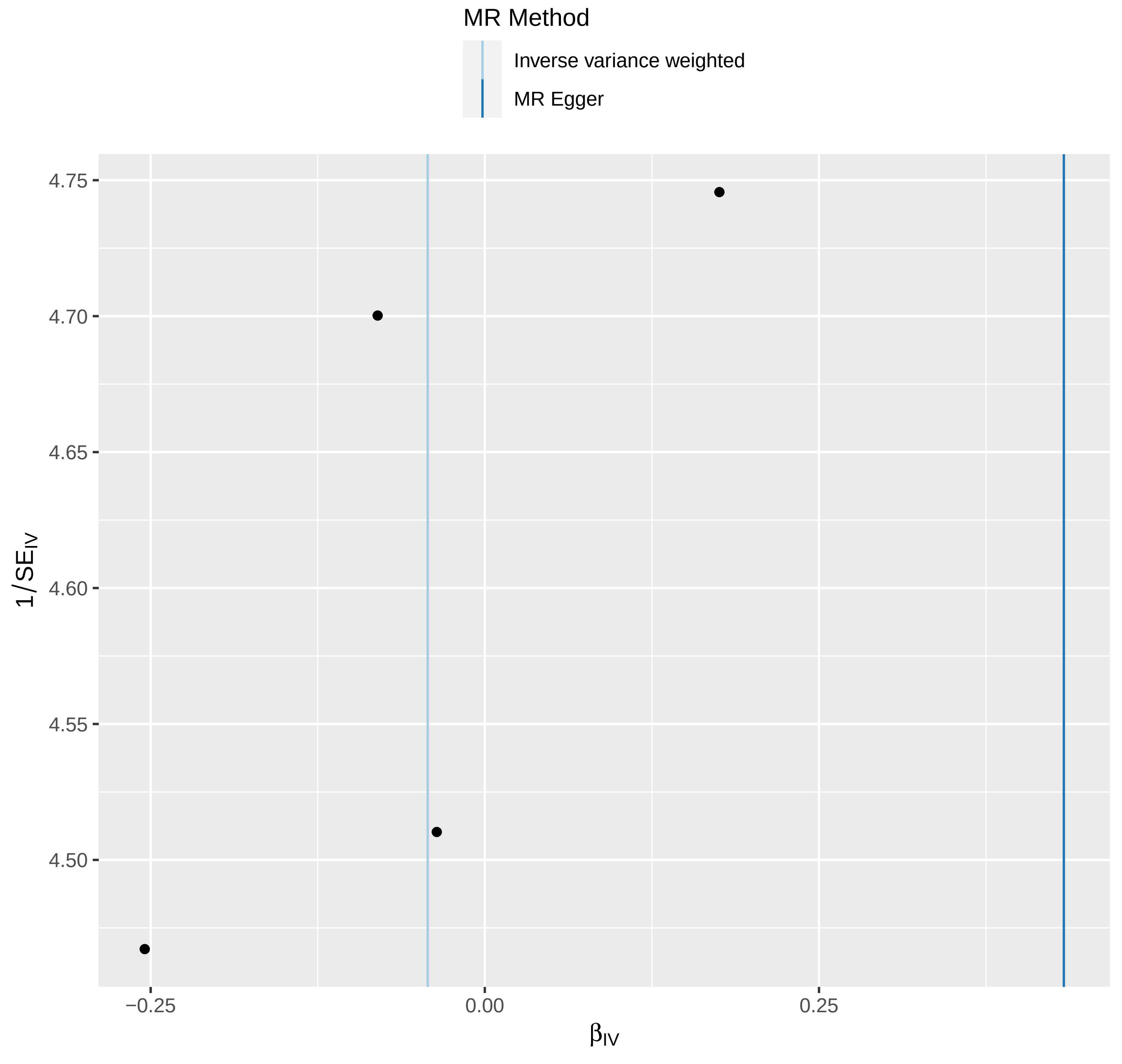

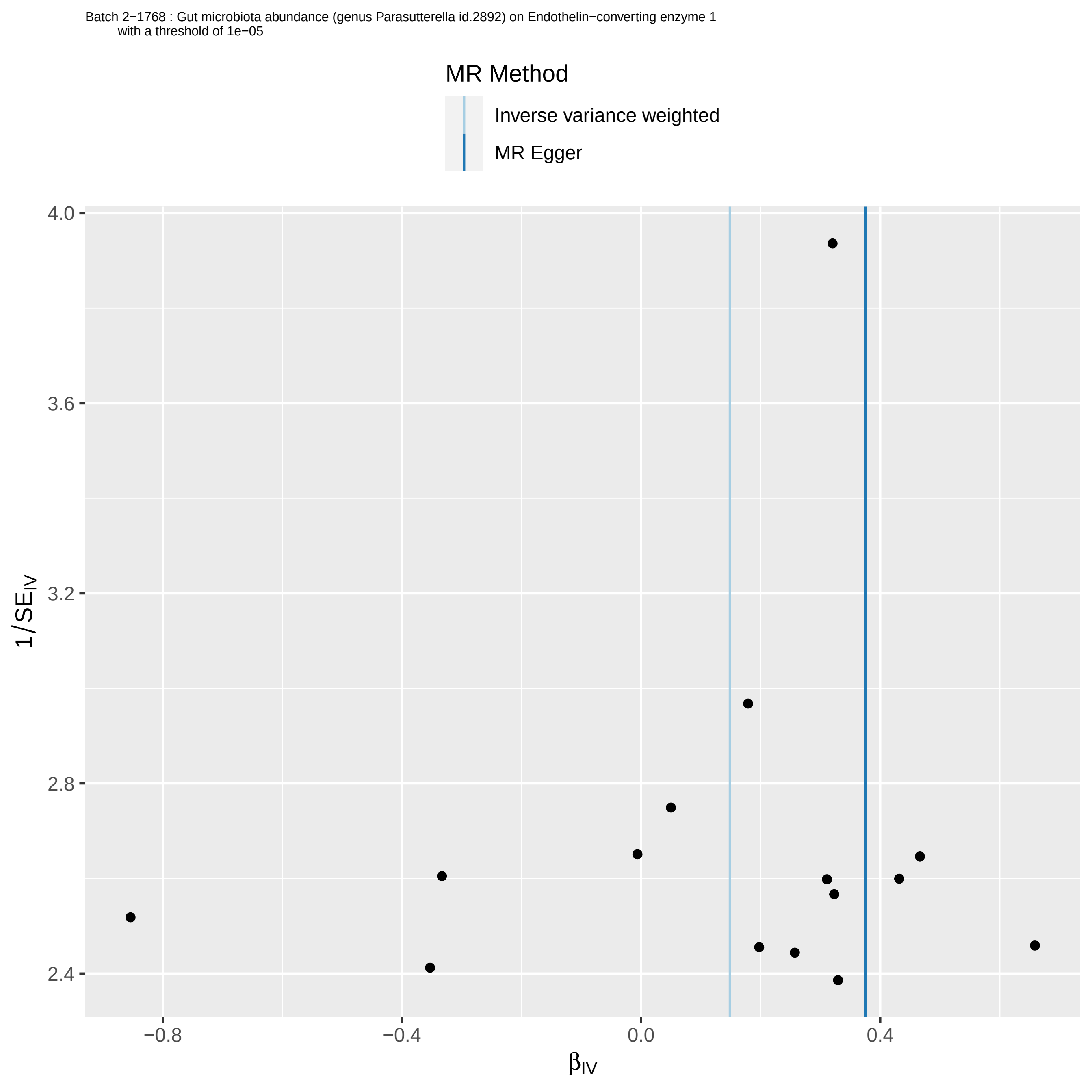

Batch 2−1768 : Gut microbiota abundance (genus Parasutterella id.2892) on Endothelin−converting enzyme 1
with a threshold of 1e−05
MR Method
Inverse variance weighted
MR Egger
4.0
3.6
3.2
2.8
2.4
1/SE_IV
−0.8
−0.4
0.0
0.4
β_IV

Batch 2−1769 : Gut microbiota abundance (genus Catenibacterium id.2153) on Ectodysplasin−A, secreted form
with a threshold of 1e−05

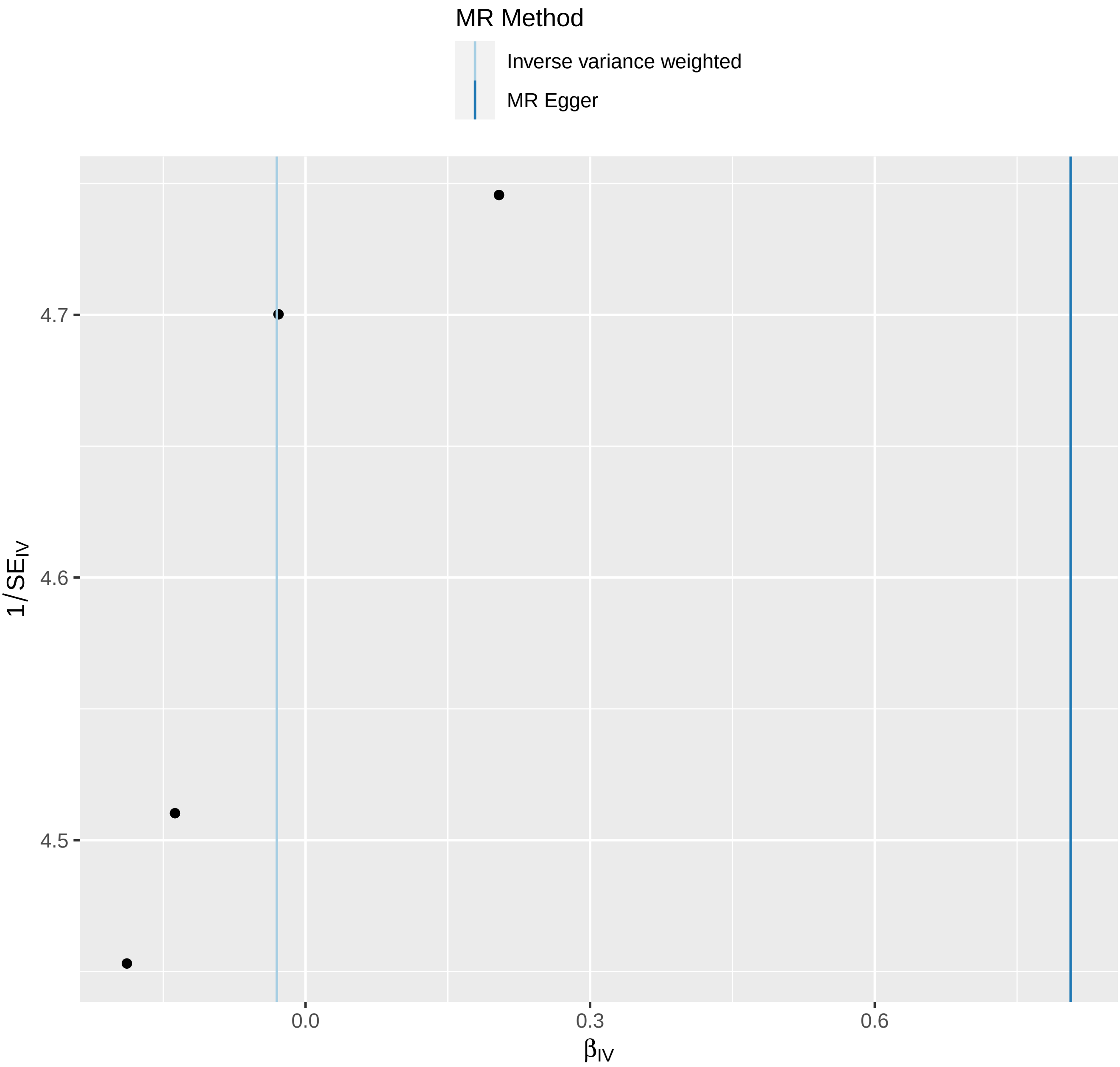

Batch 2−1770 : Gut microbiota abundance (genus Parasutterella id.2892) on Ectodysplasin−A, secreted form
with a threshold of 1e−05

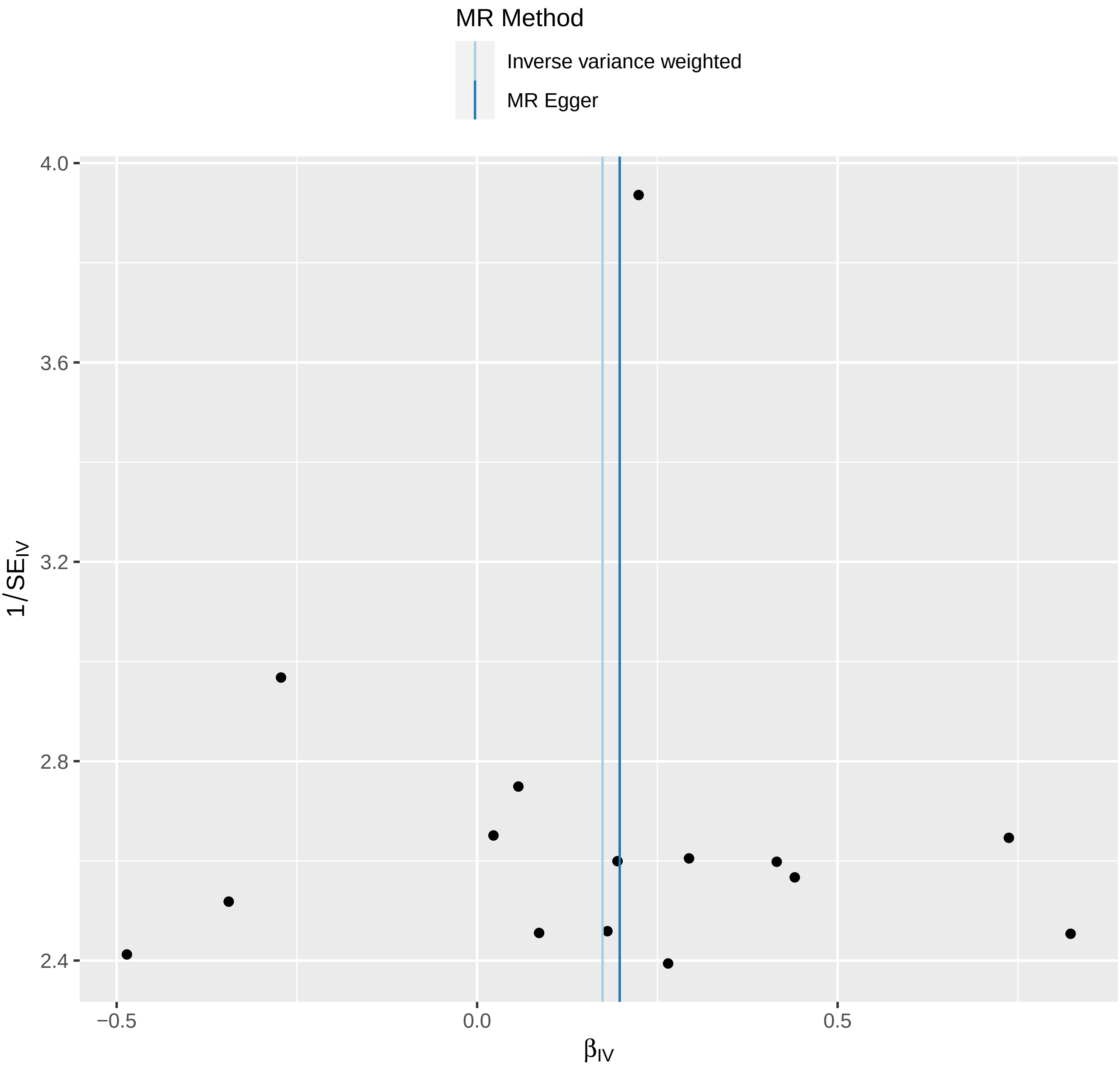

Batch 2−1771 : Gut microbiota abundance (genus Catenibacterium id.2153) on Ectodysplasin−A, secreted form
with a threshold of 1e−05

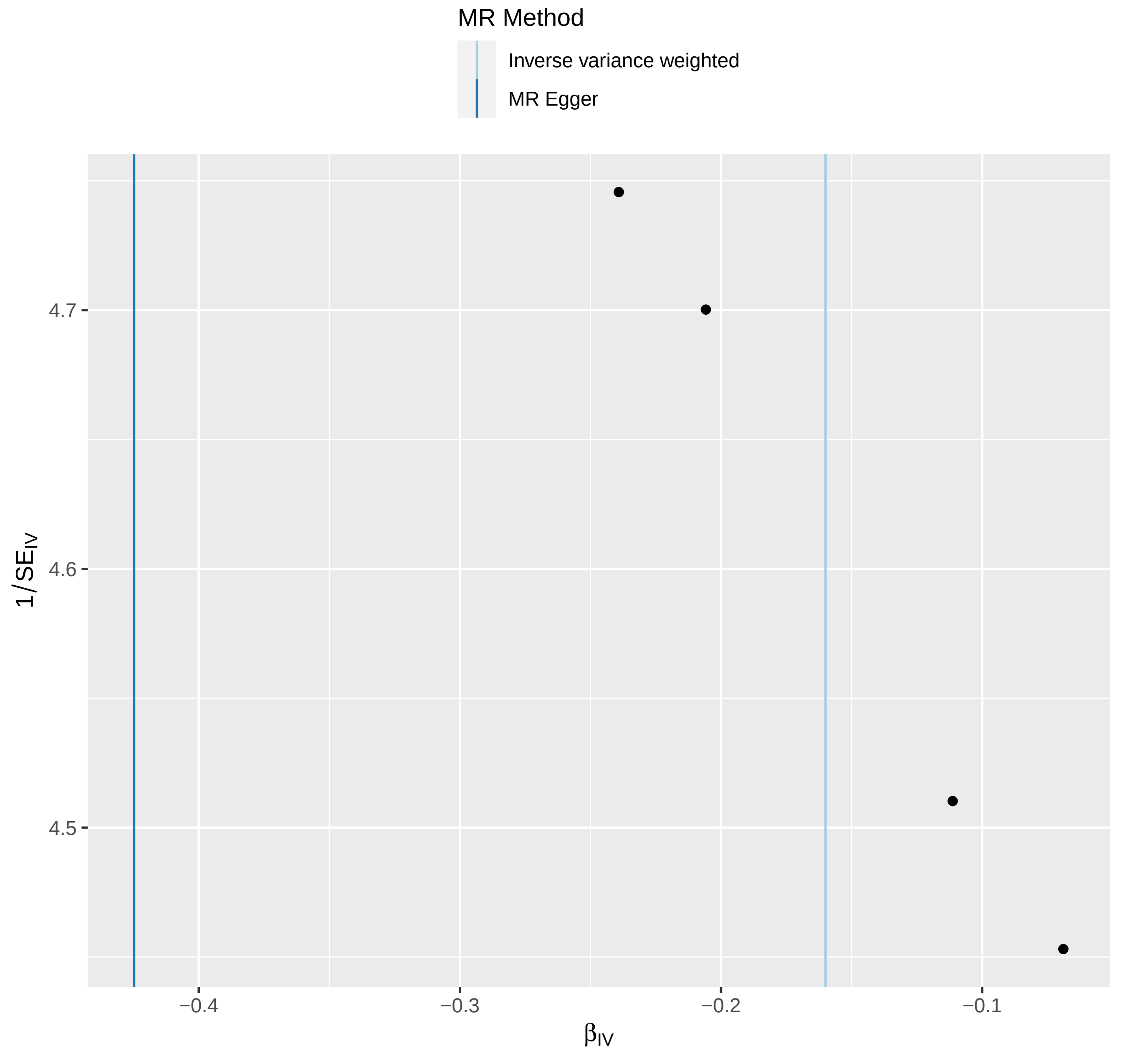

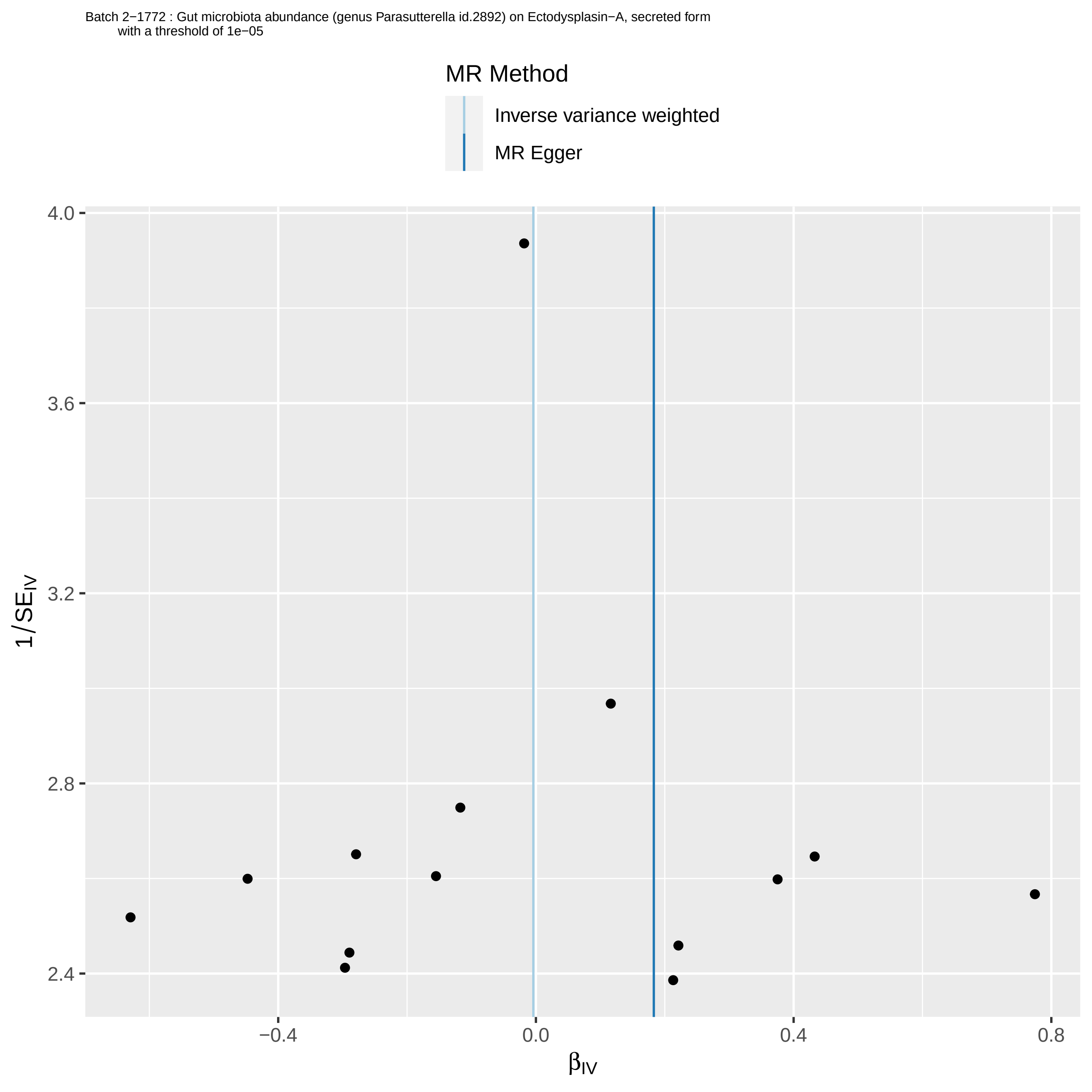
Batch 2−1772 : Gut microbiota abundance (genus Parasutterella id.2892) on Ectodysplasin−A, secreted form
with a threshold of 1e−05
MR Method
Inverse variance weighted
MR Egger
4.0
3.6
3.2
2.8
2.4
1/SE_IV
−0.4
0.0
0.4
0.8
β_IV

Batch 2−1773 : Gut microbiota abundance (genus Catenibacterium id.2153) on Tumor necrosis factor receptor superfamily member 27 with a threshold of 1e−05

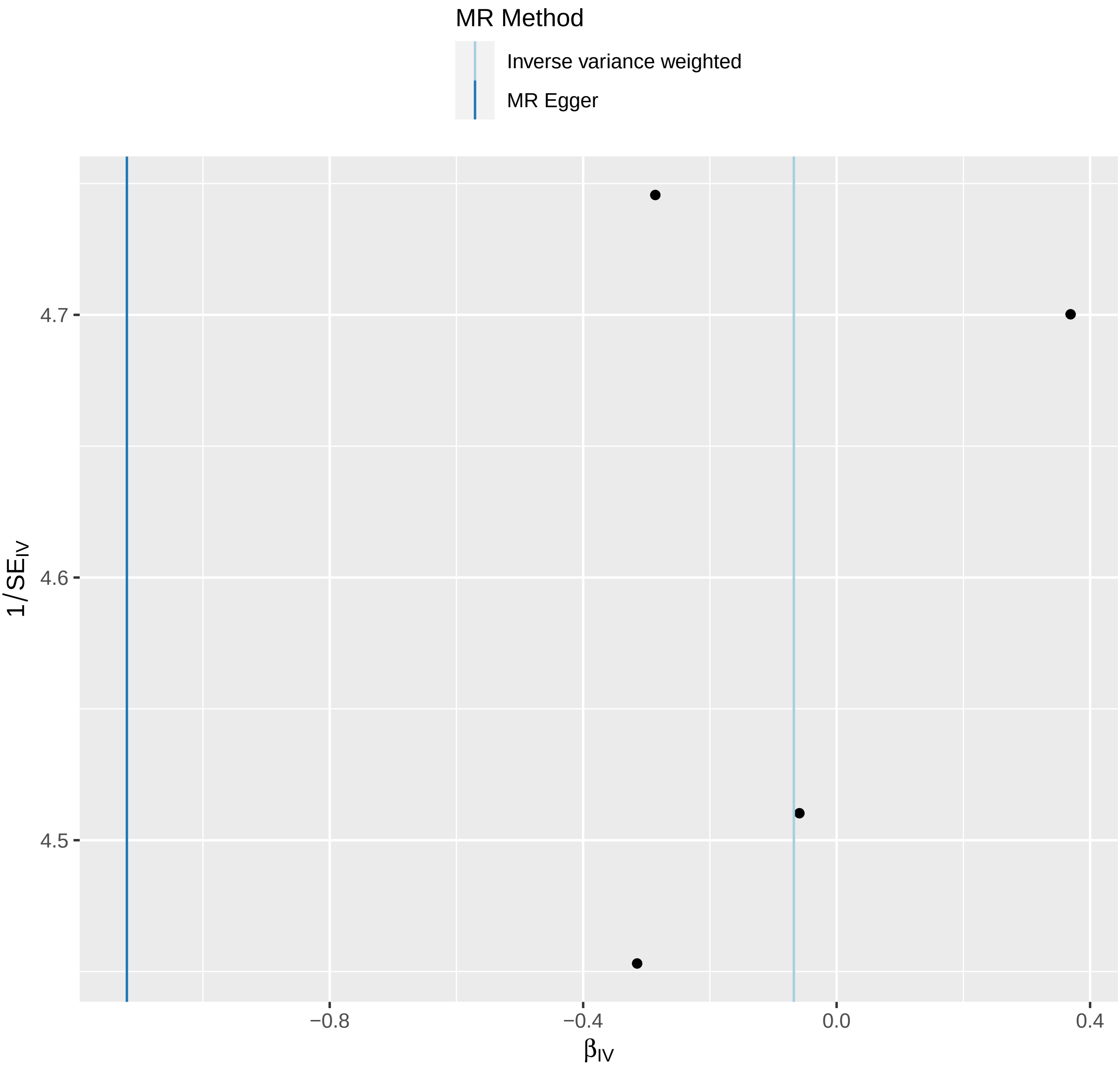

Batch 2−1774 : Gut microbiota abundance (genus Parasutterella id.2892) on Tumor necrosis factor receptor superfamily member 27 with a threshold of 1e−05

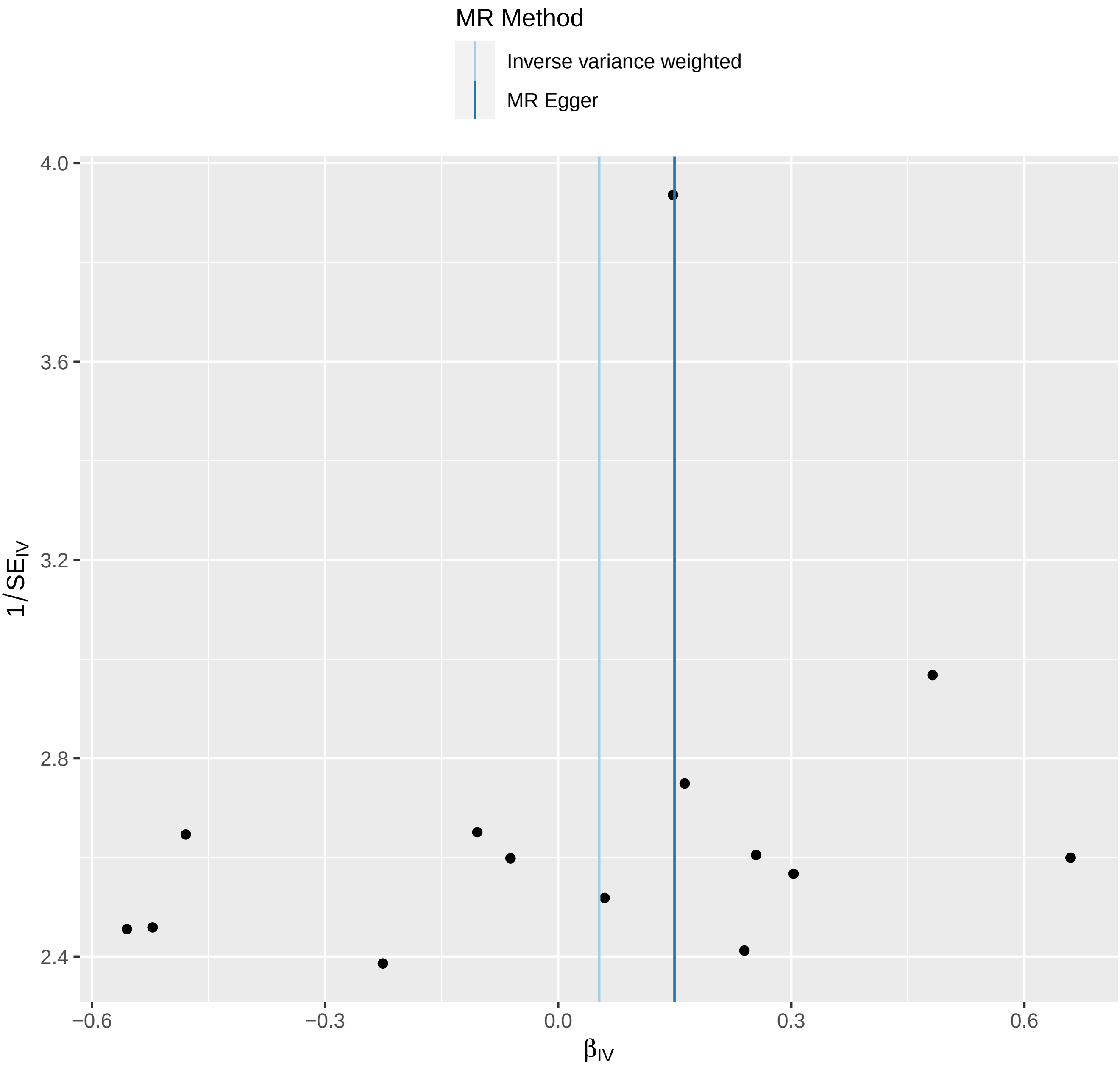

Batch 2−1775 : Gut microbiota abundance (genus Catenibacterium id.2153) on Tumor necrosis factor receptor superfamily member EDAR
with a threshold of 1e−05

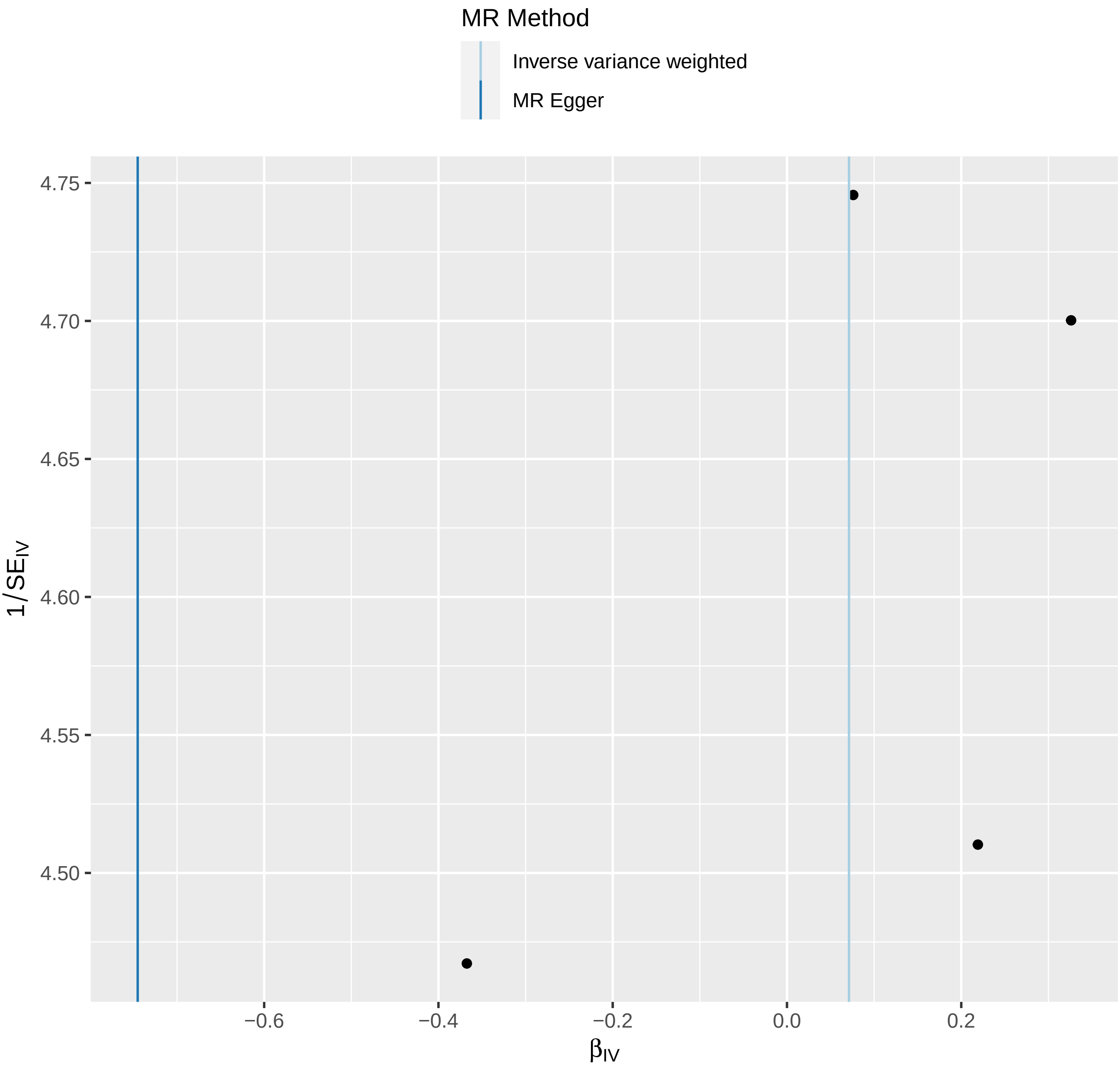

Batch 2−1776 : Gut microbiota abundance (genus Parasutterella id.2892) on Tumor necrosis factor receptor superfamily member EDAR with a threshold of 1e−05

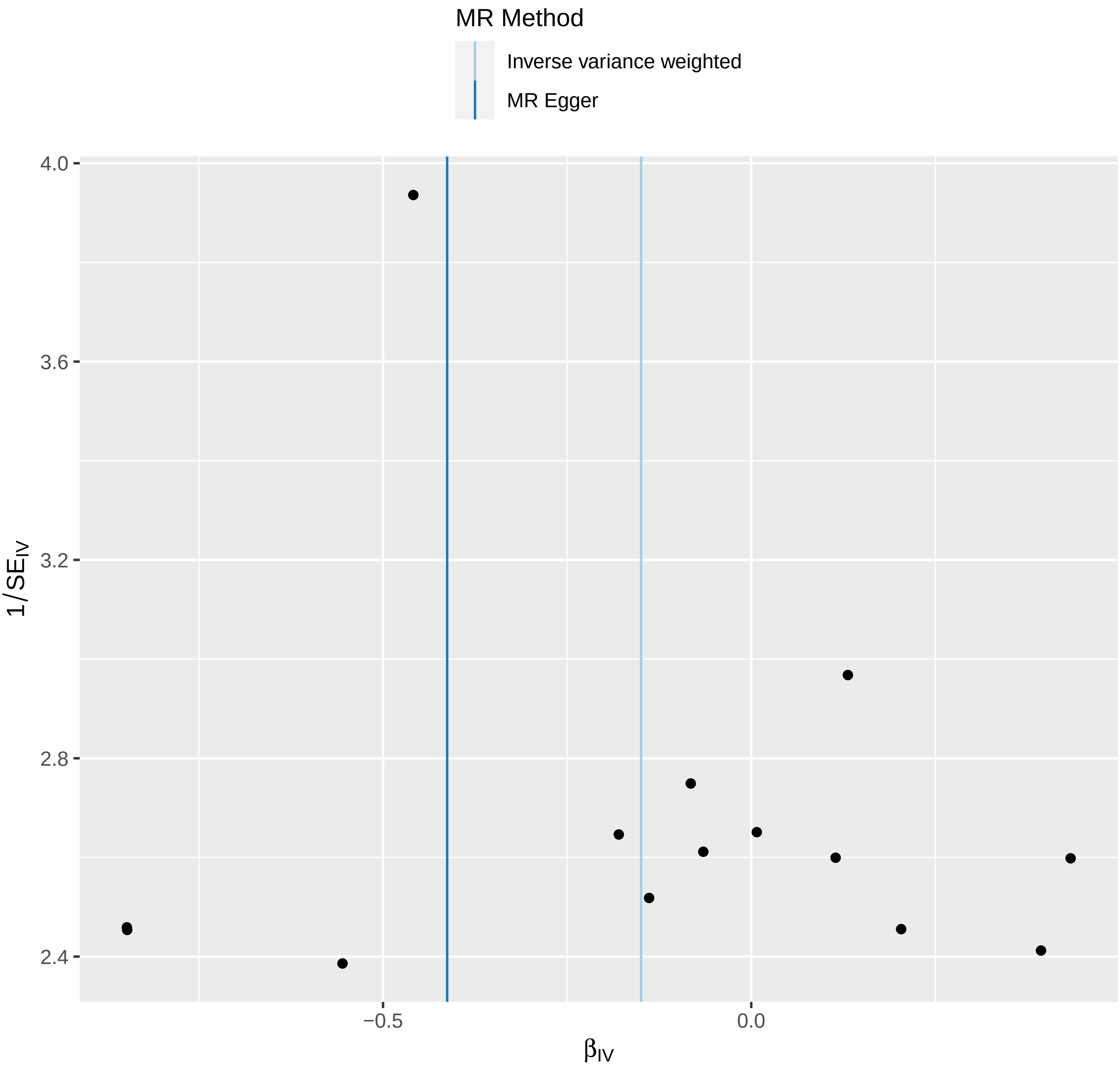

Batch 2−1777 : Gut microbiota abundance (genus Catenibacterium id.2153) on Enhancer of mRNA−decapping protein 4 with a threshold of 1e−05

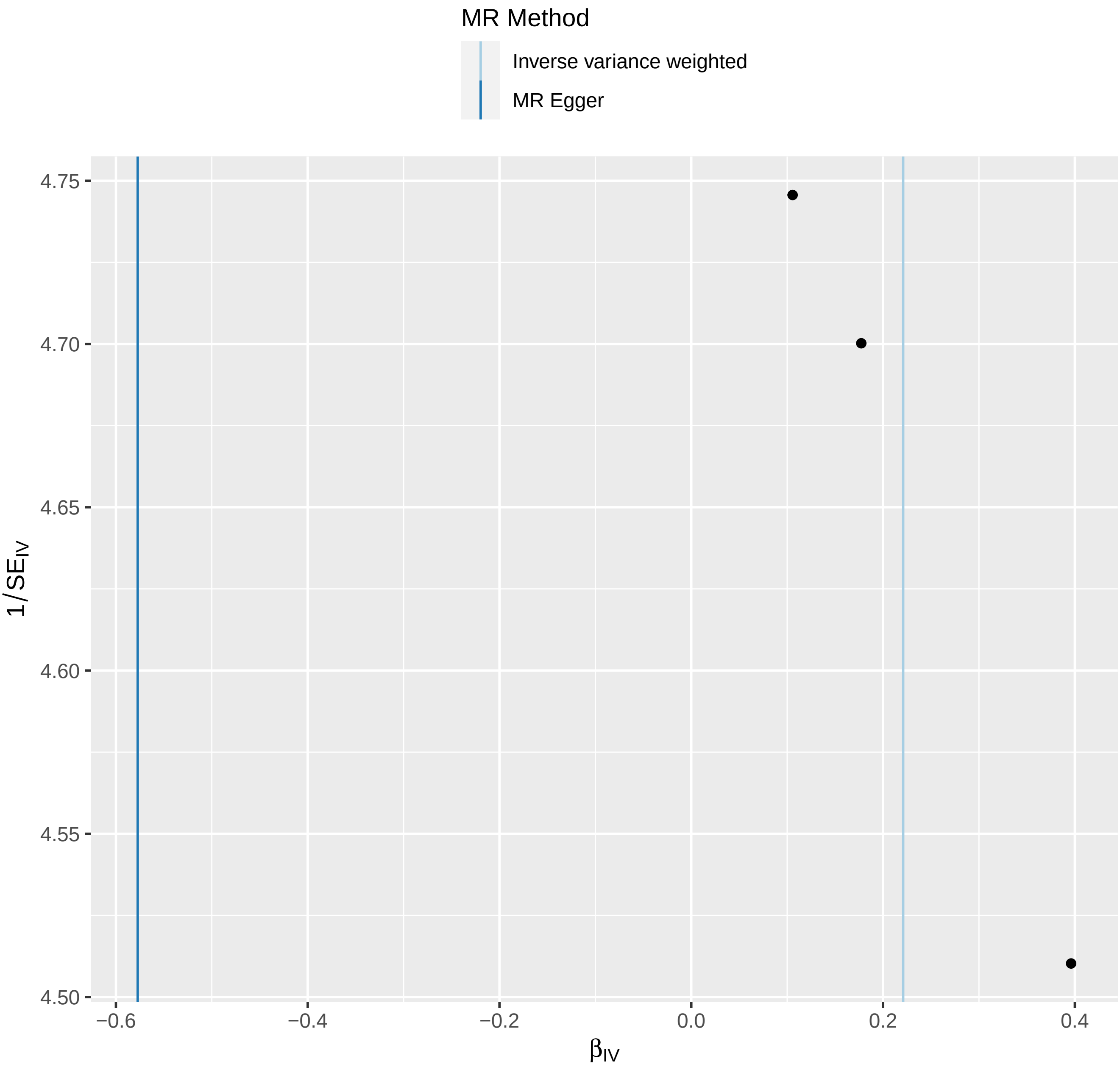

Batch 2−1778 : Gut microbiota abundance (genus Parasutterella id.2892) on Enhancer of mRNA−decapping protein 4 with a threshold of 1e−05

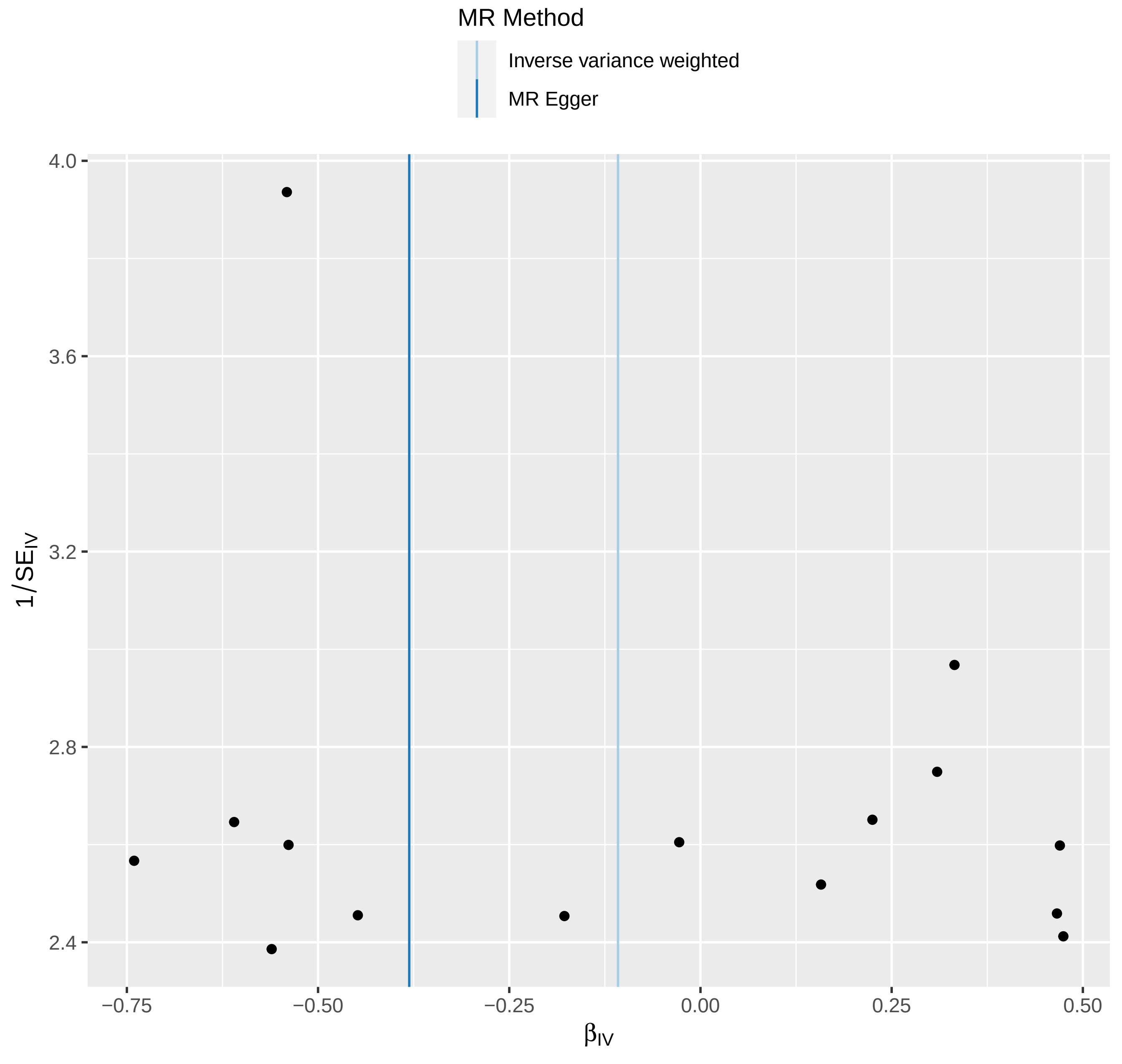

Batch 2−1779 : Gut microbiota abundance (genus Catenibacterium id.2153) on Epididymal secretory protein E3−beta
with a threshold of 1e−05

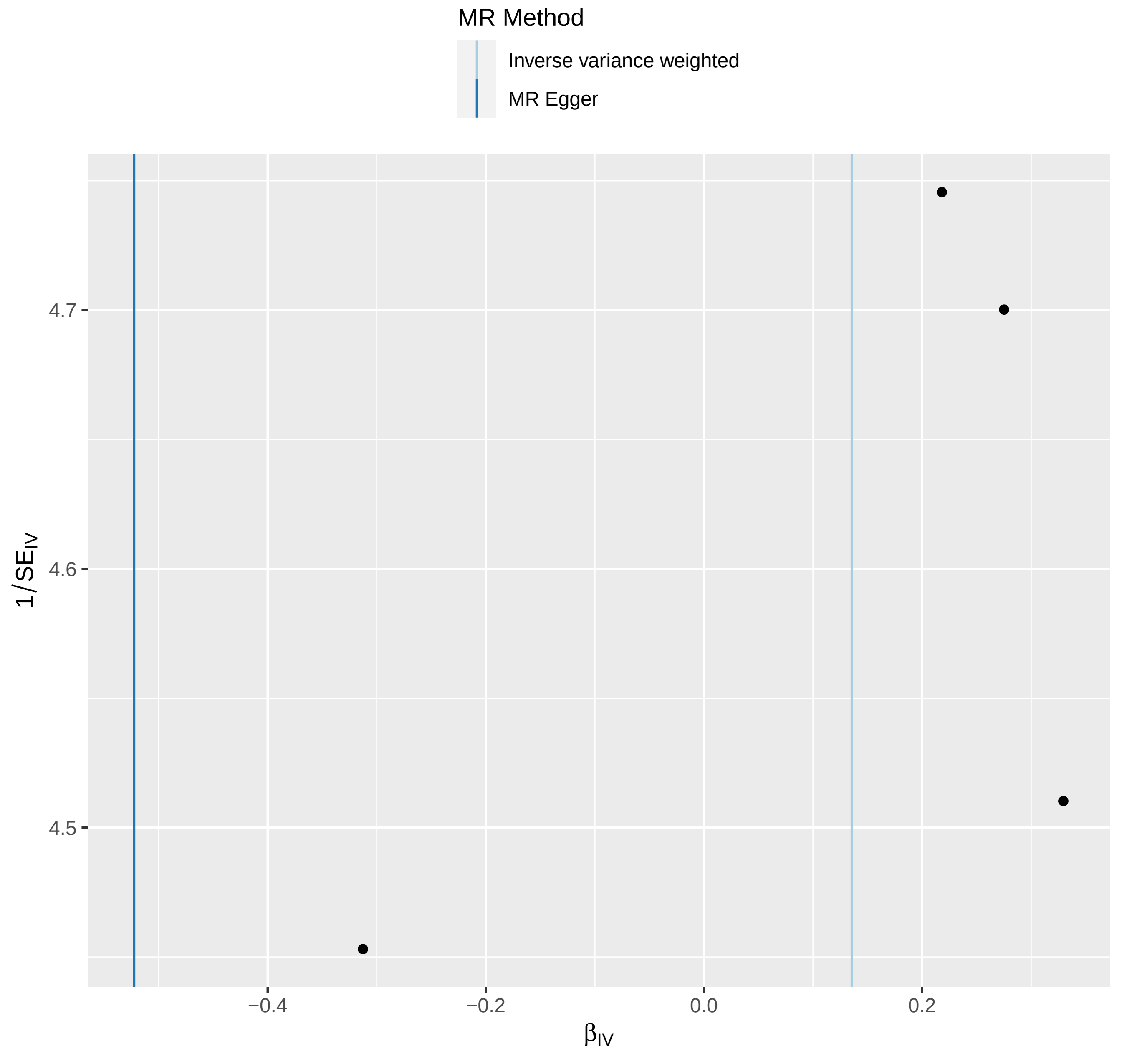

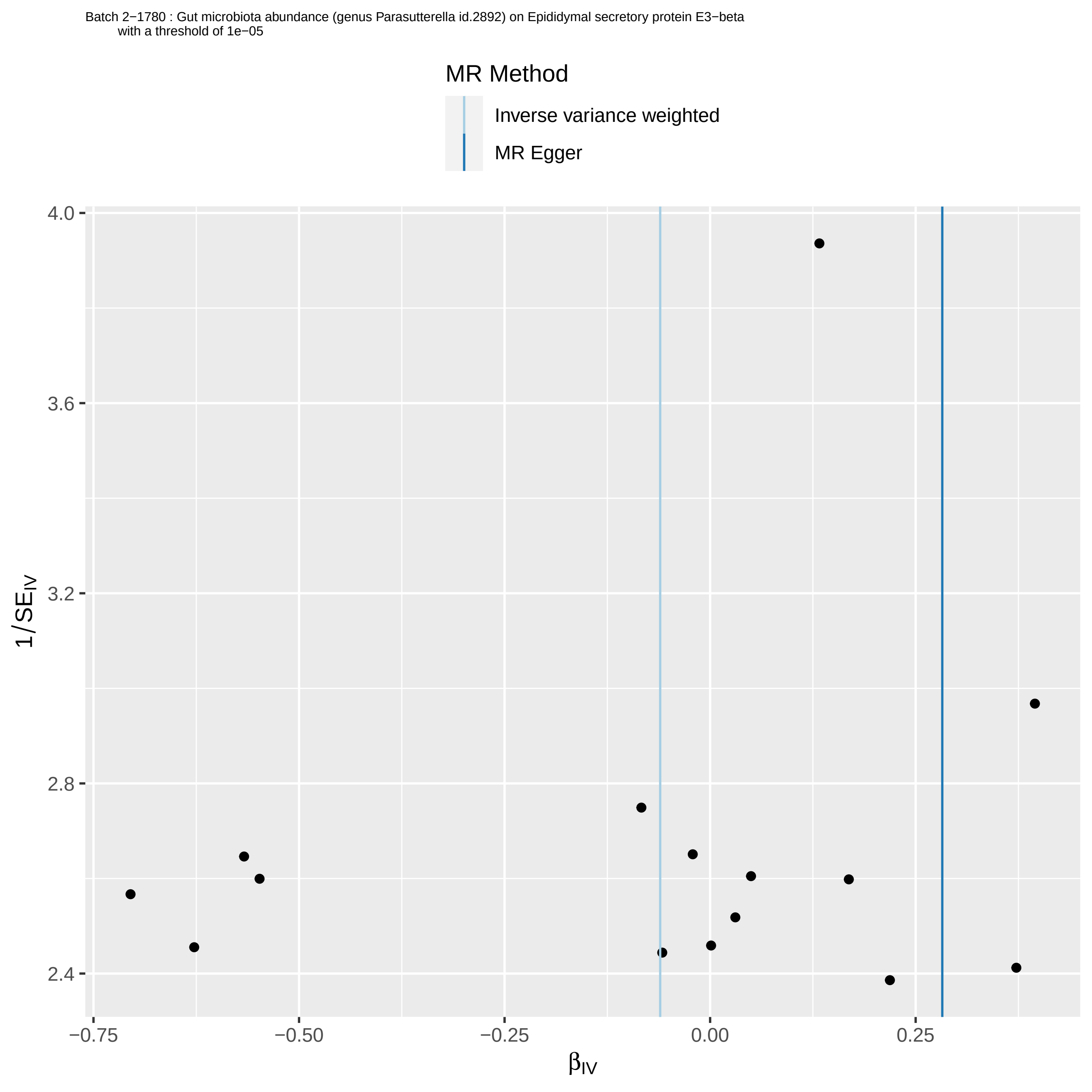
Batch 2−1780 : Gut microbiota abundance (genus Parasutterella id.2892) on Epididymal secretory protein E3−beta
with a threshold of 1e−05
MR Method
Inverse variance weighted
MR Egger
4.0
3.6
3.2
2.8
2.4
1/SE_IV
−0.75
−0.50
−0.25
0.00
0.25
β_IV

Batch 2−1781 : Gut microbiota abundance (genus Catenibacterium id.2153) on Endothelial differentiation−related factor 1 with a threshold of 1e−05

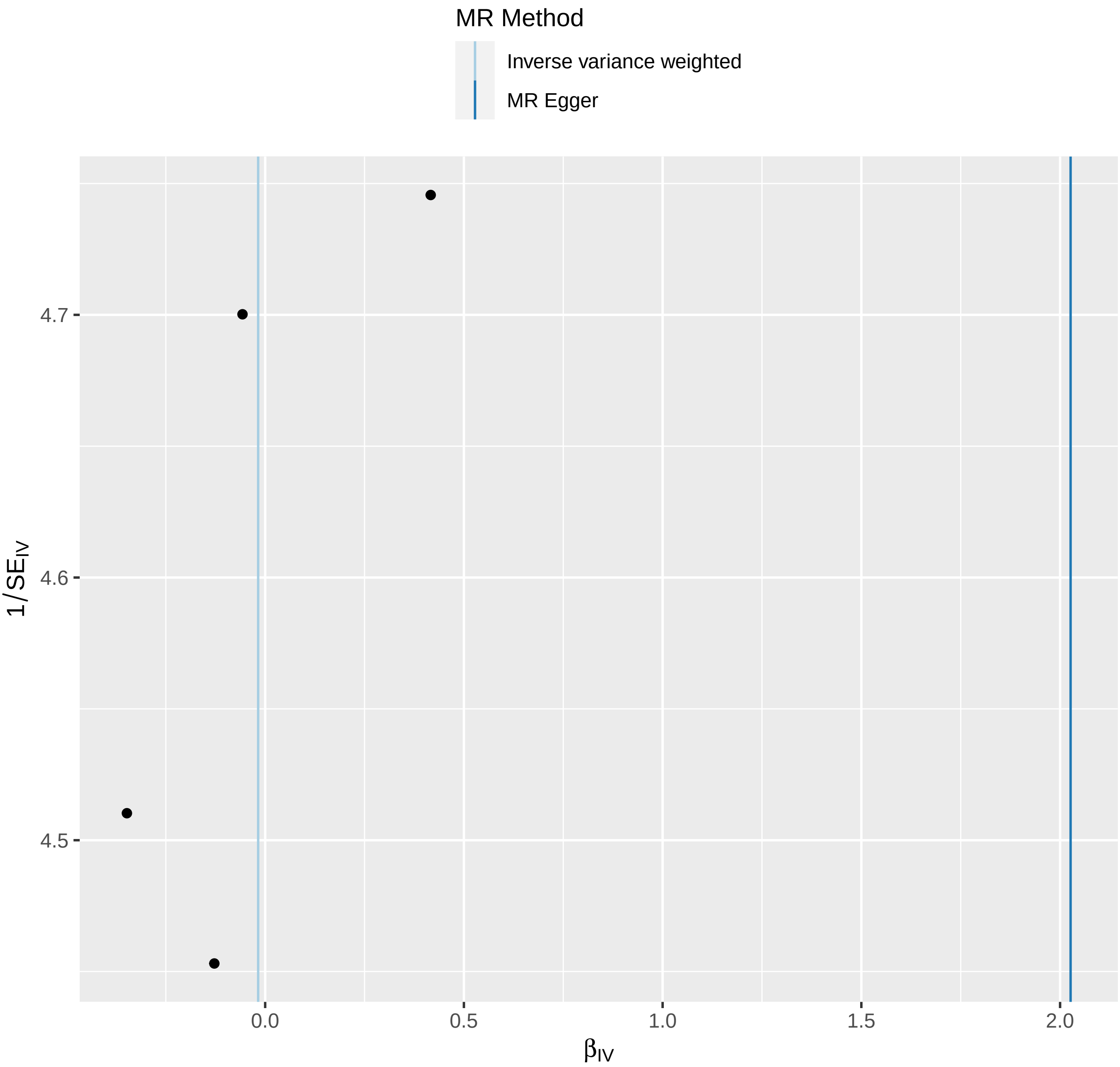

Batch 2−1782 : Gut microbiota abundance (genus Parasutterella id.2892) on Endothelial differentiation−related factor 1
with a threshold of 1e−05

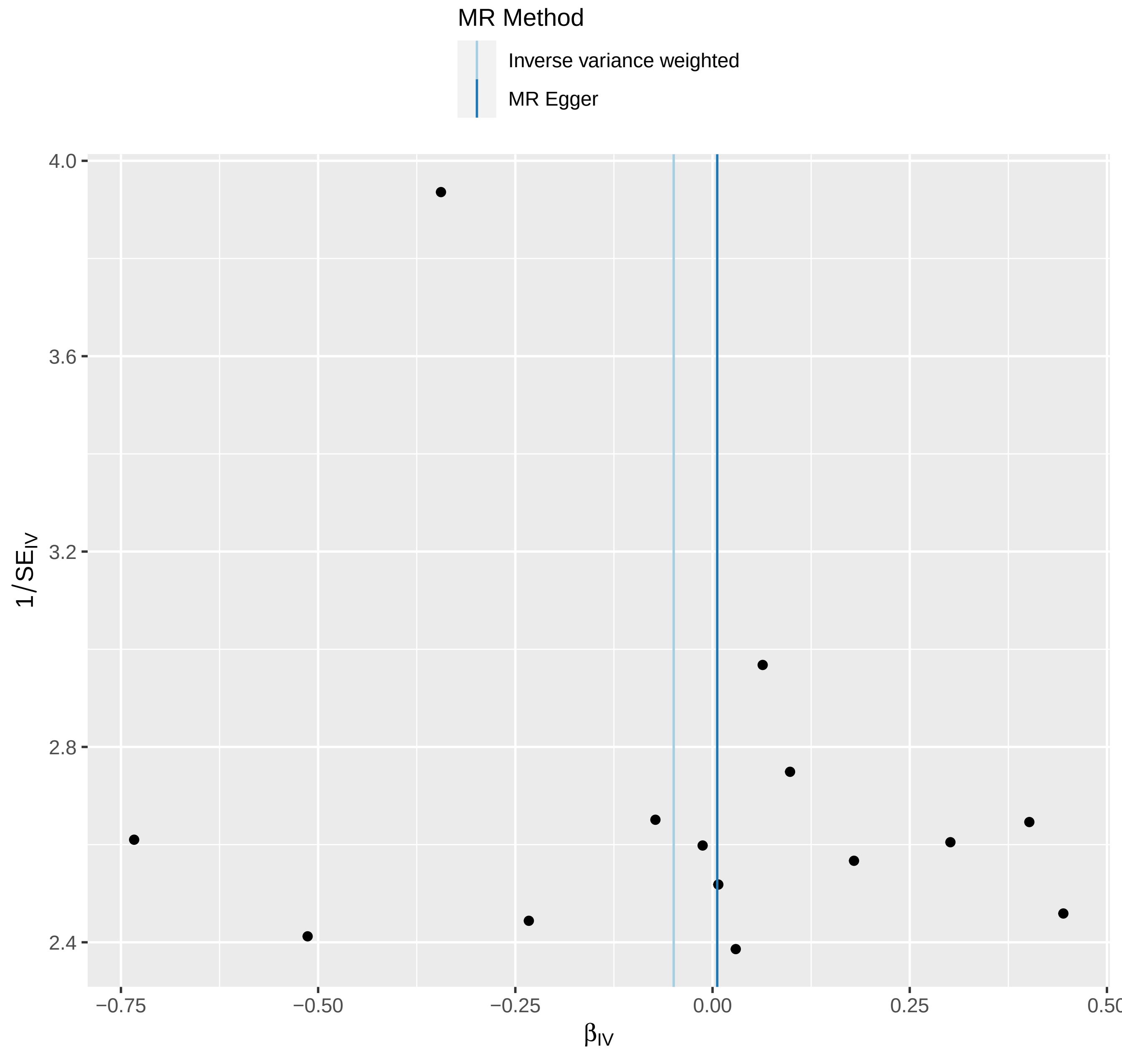

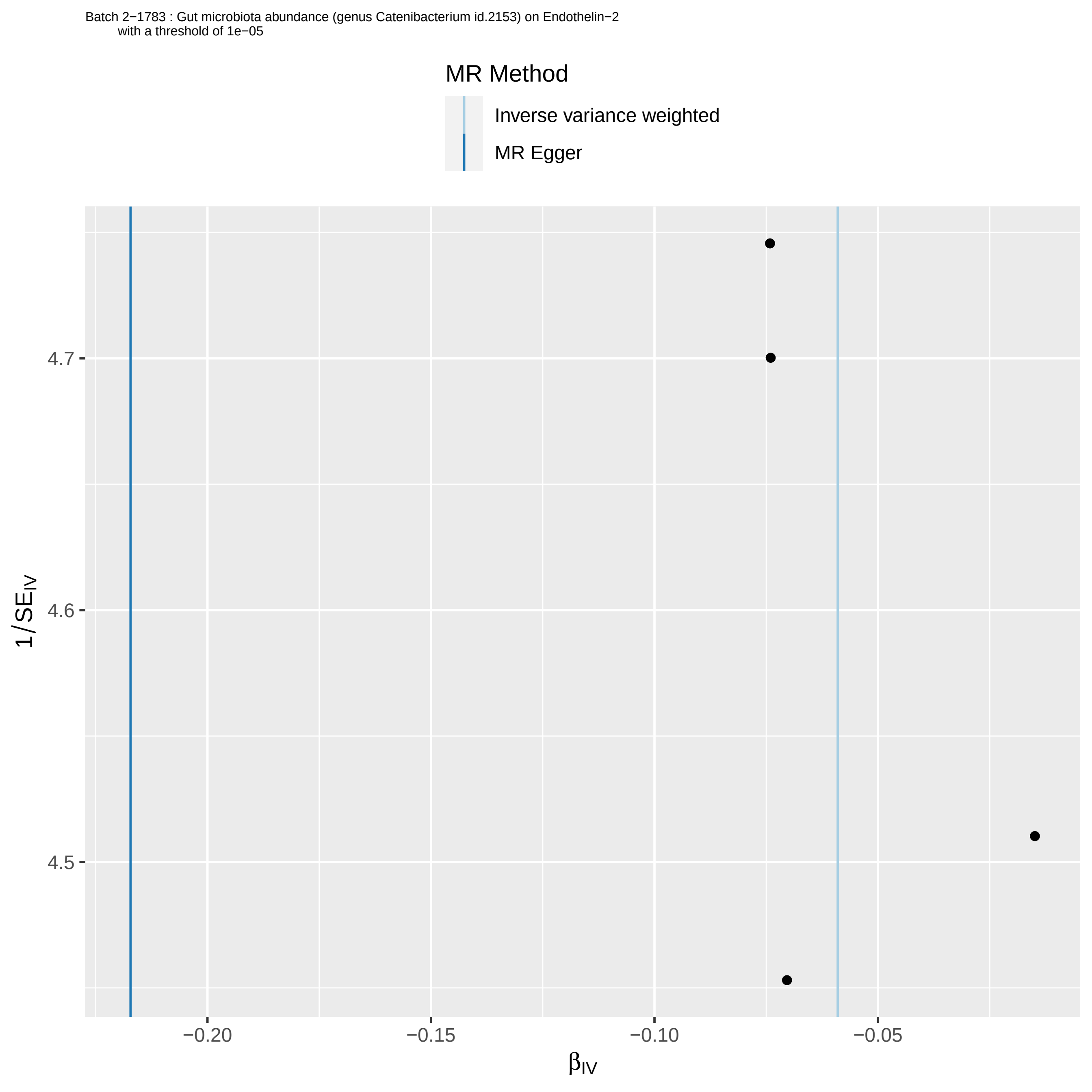
Batch 2−1783 : Gut microbiota abundance (genus Catenibacterium id.2153) on Endothelin−2
with a threshold of 1e−05
MR Method
Inverse variance weighted
MR Egger
4.7
4.6
4.5
1/SE_IV
−0.20
−0.15
−0.10
−0.05
β_IV

Batch 2−1784 : Gut microbiota abundance (genus Parasutterella id.2892) on Endothelin−2 with a threshold of 1e−05

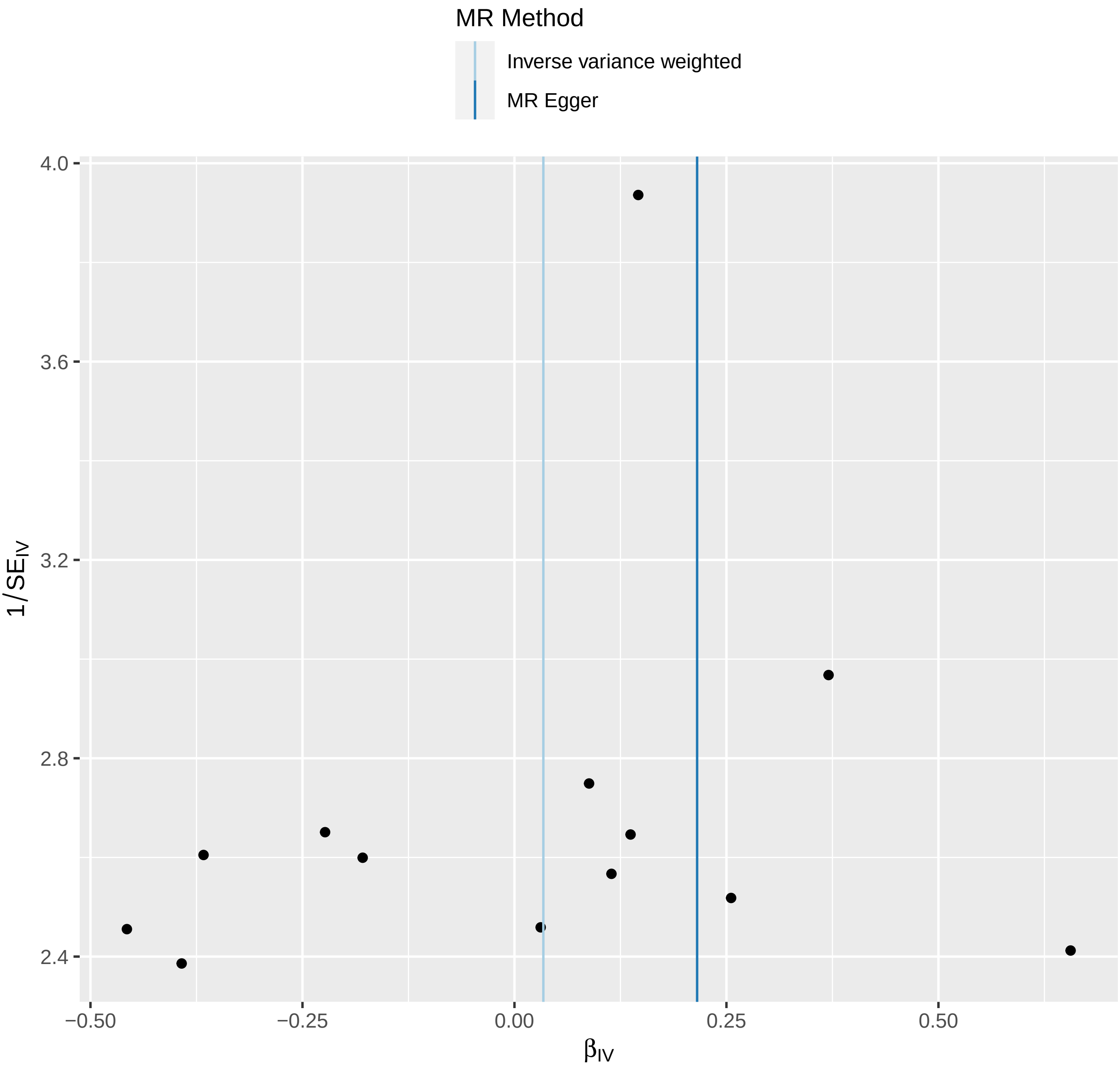

Batch 2−1785 : Gut microbiota abundance (genus Catenibacterium id.2153) on Early endosome antigen 1
with a threshold of 1e−05

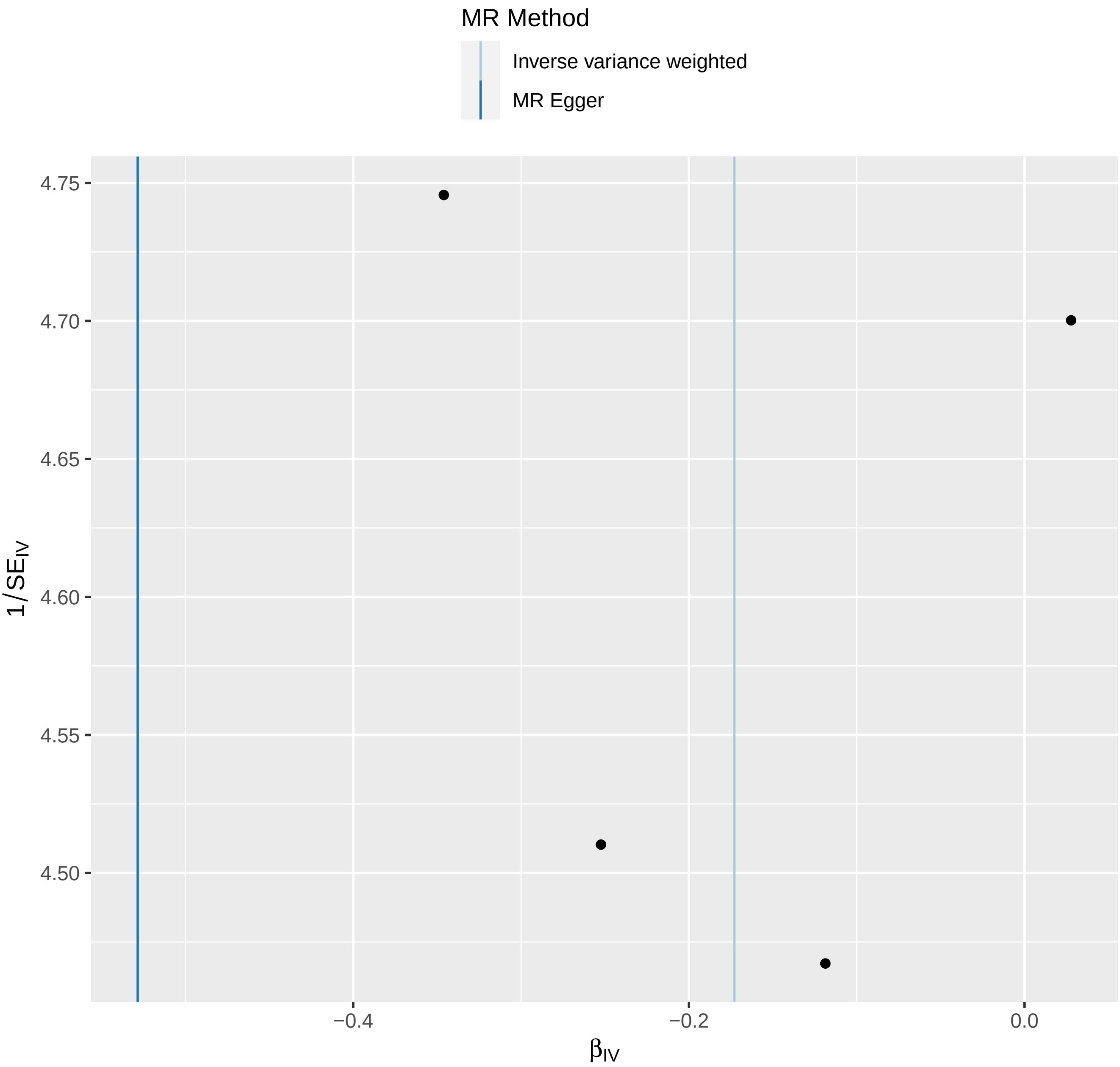

Batch 2−1786 : Gut microbiota abundance (genus Parasutterella id.2892) on Early endosome antigen 1
with a threshold of 1e−05

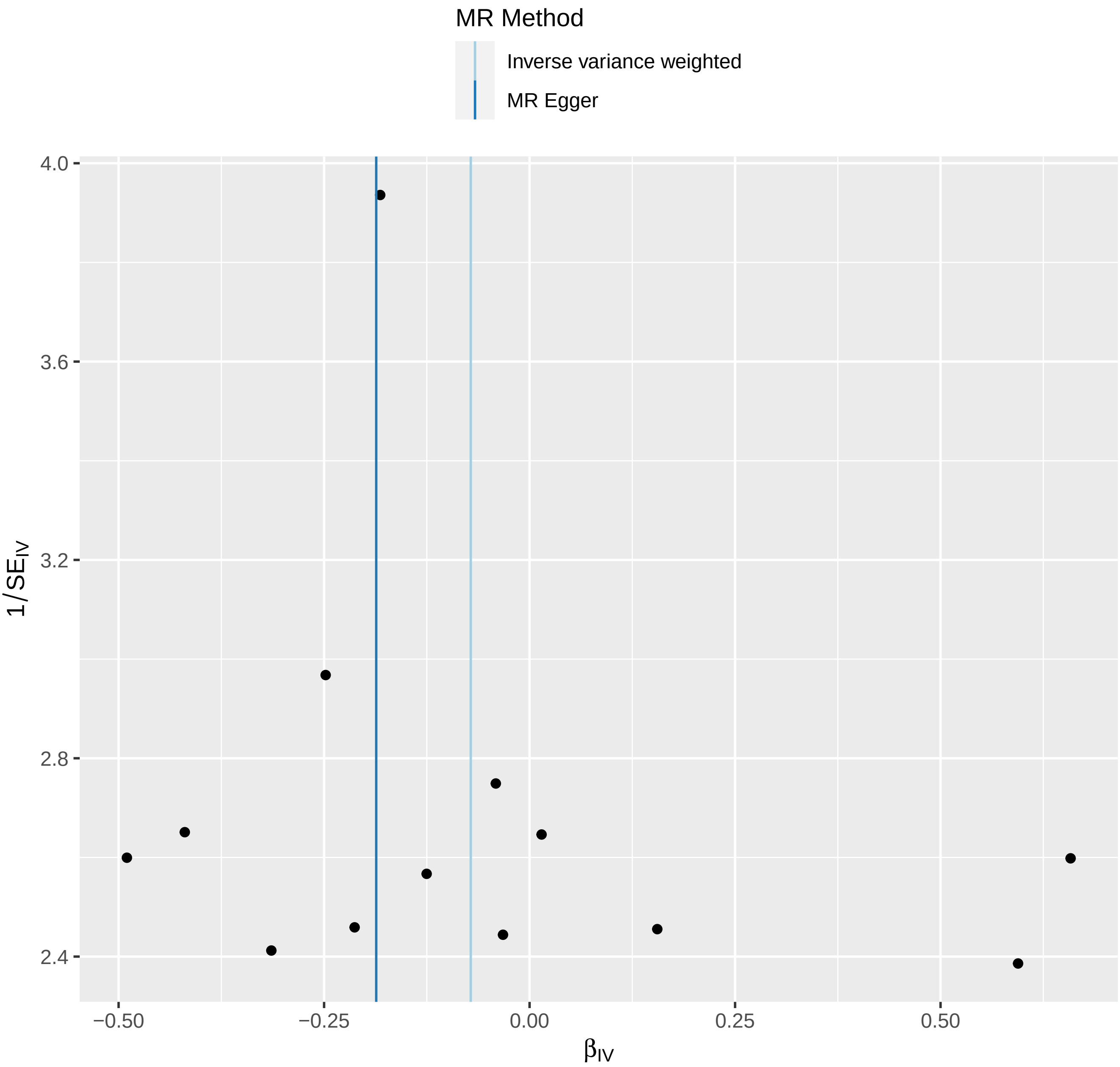

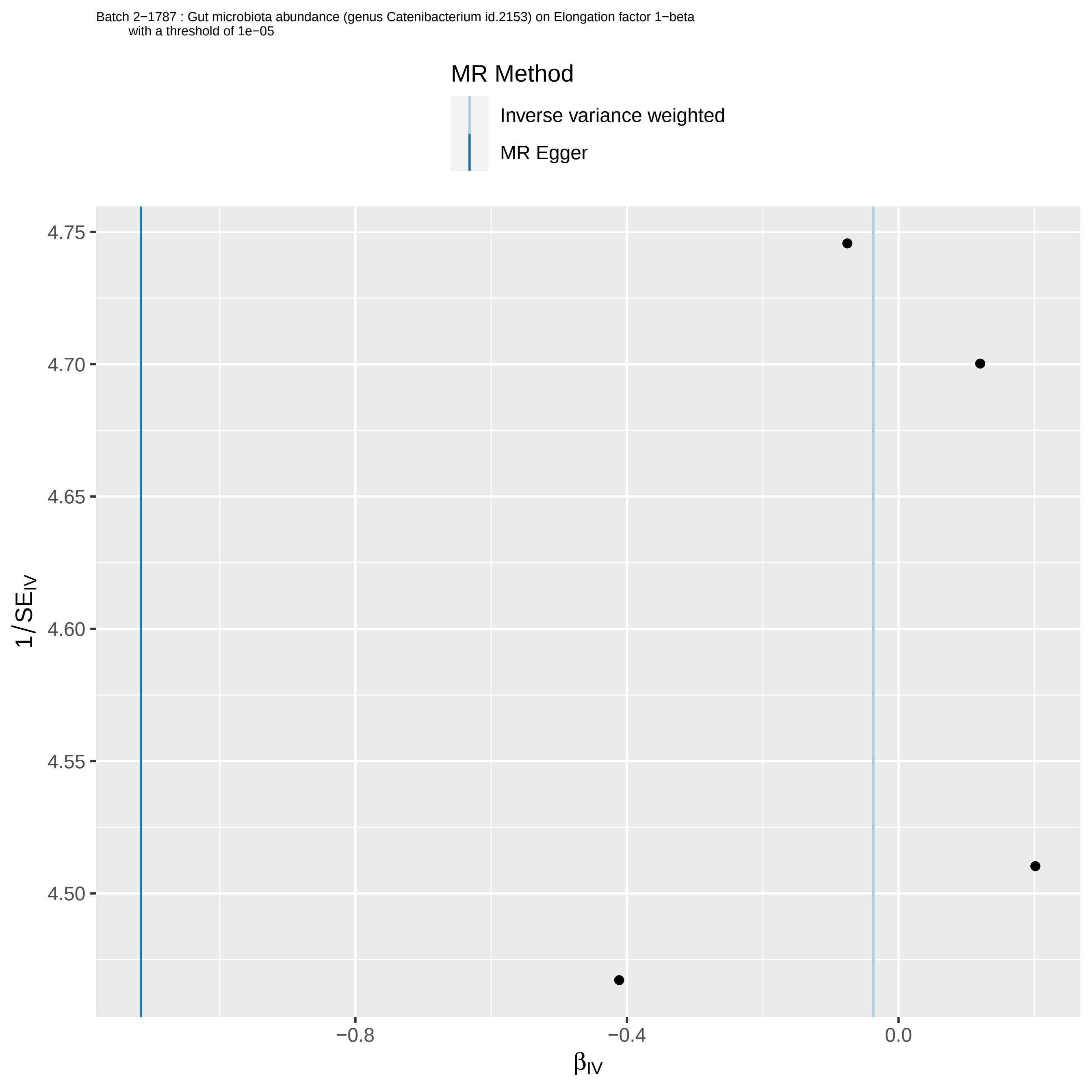

Batch 2−1787 : Gut microbiota abundance (genus Catenibacterium id.2153) on Elongation factor 1−beta
with a threshold of 1e−05
MR Method
Inverse variance weighted
MR Egger
4.75
4.70
4.65
4.60
4.55
4.50
1/SE_IV
−0.8
−0.4
0.0
β_IV

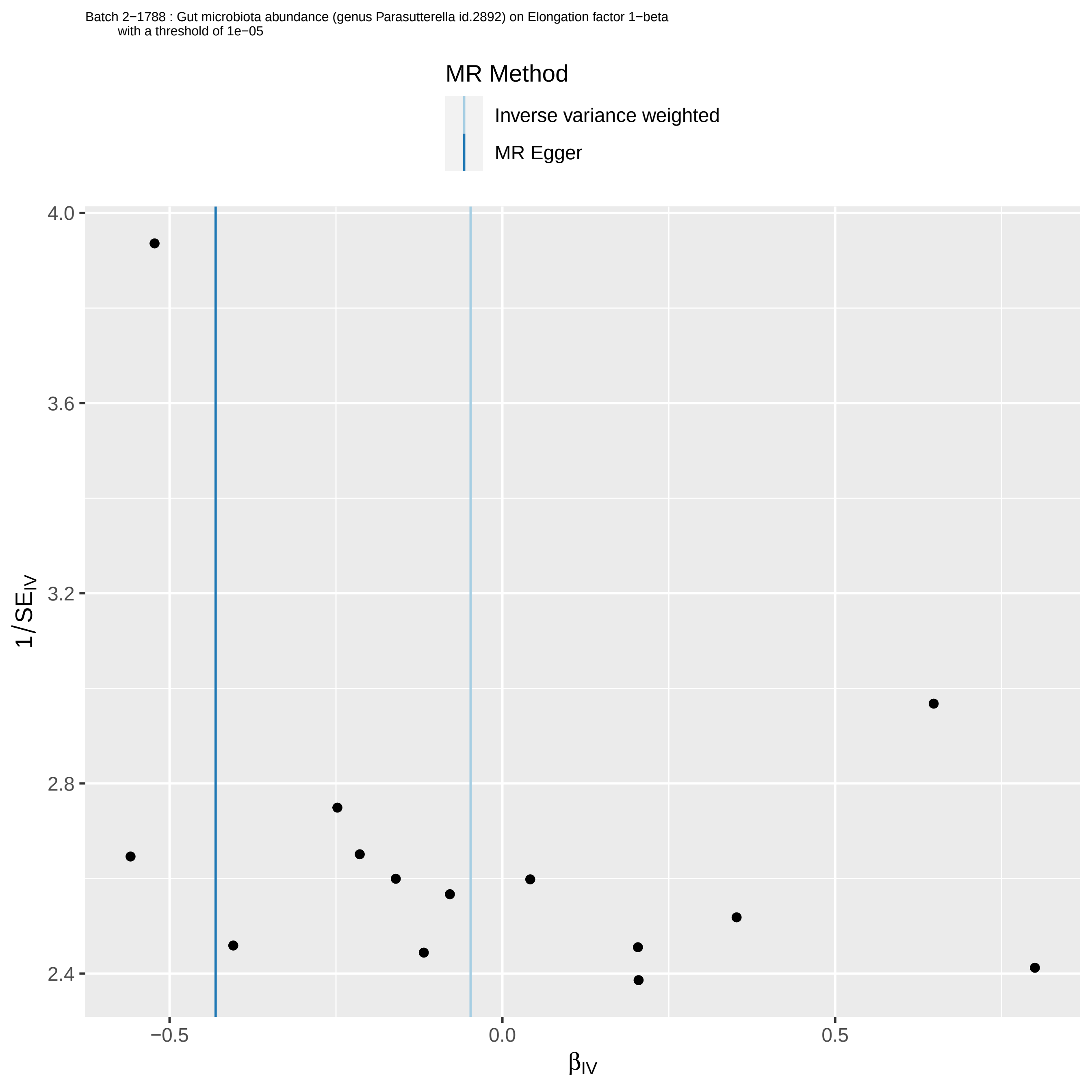

Batch 2−1788 : Gut microbiota abundance (genus Parasutterella id.2892) on Elongation factor 1−beta
with a threshold of 1e−05
MR Method
Inverse variance weighted
MR Egger
4.0
3.6
3.2
2.8
2.4
1/SE_IV
−0.5
0.0
0.5
β_IV

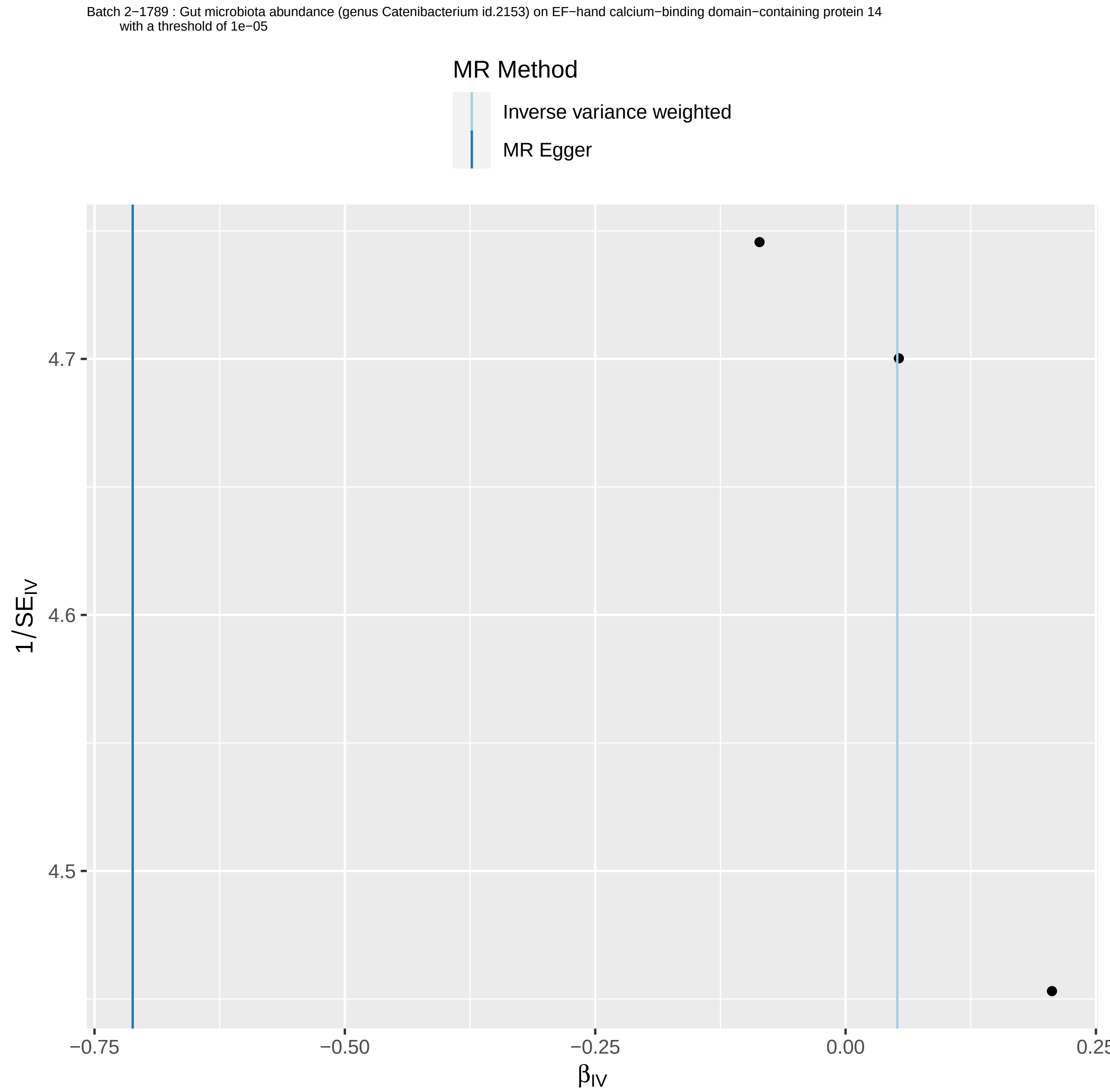
Batch 2−1789 : Gut microbiota abundance (genus Catenibacterium id.2153) on EF−hand calcium−binding domain−containing protein 14
with a threshold of 1e−05
MR Method
Inverse variance weighted
MR Egger
4.7
4.6
4.5
1/SE_IV
−0.75
−0.50
−0.25
0.00
0.25
β_IV

Batch 2−1790 : Gut microbiota abundance (genus Parasutterella id.2892) on EF−hand calcium−binding domain−containing protein 14
with a threshold of 1e−05

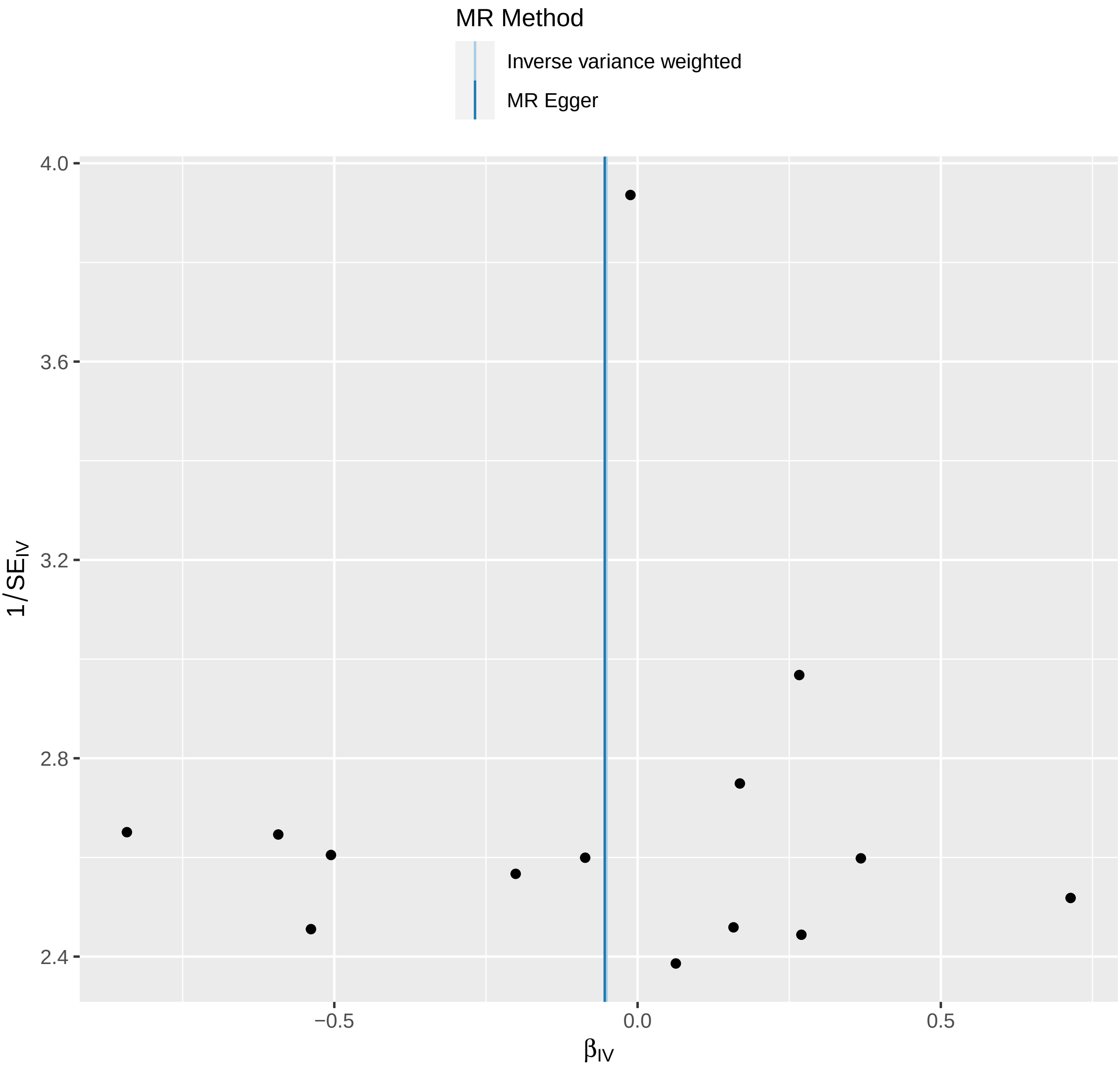

Batch 2−1791 : Gut microbiota abundance (genus Catenibacterium id.2153) on Calcium uptake protein 3, mitochondrial
with a threshold of 1e−05

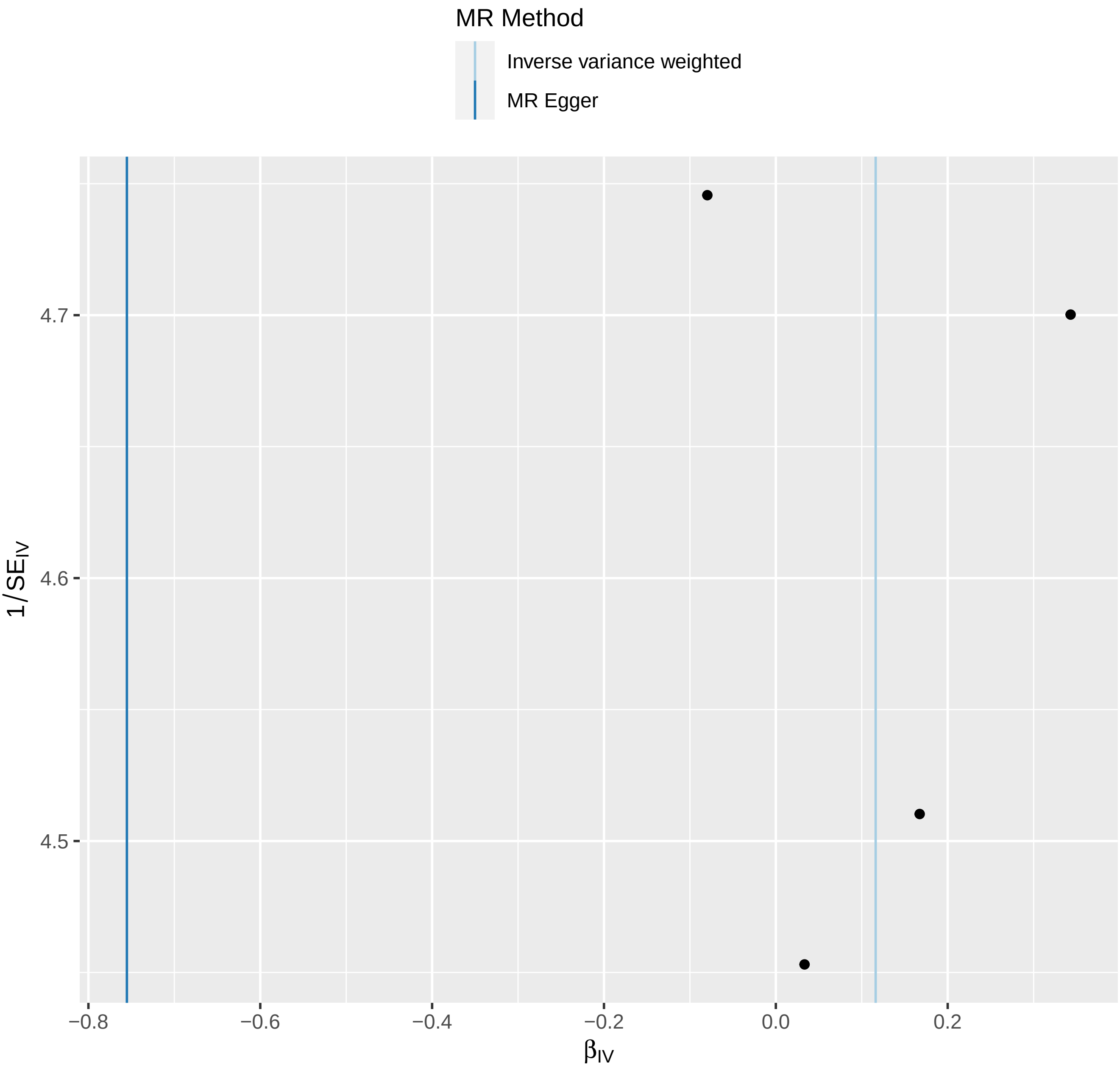

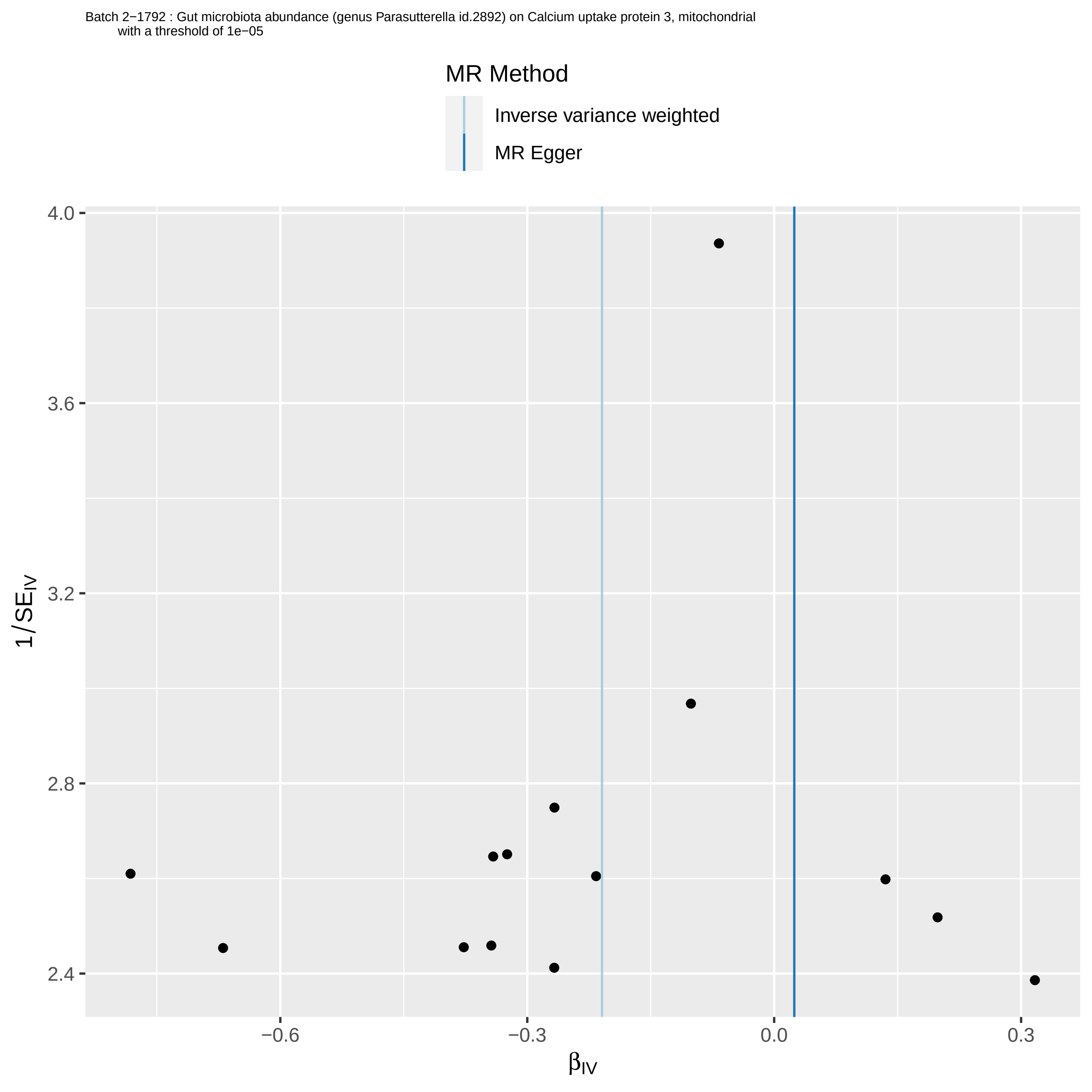
Batch 2−1792 : Gut microbiota abundance (genus Parasutterella id.2892) on Calcium uptake protein 3, mitochondrial
with a threshold of 1e−05
MR Method
Inverse variance weighted
MR Egger
4.0
3.6
3.2
2.8
2.4
1/SE_IV
−0.6
−0.3
0.0
0.3
β_IV

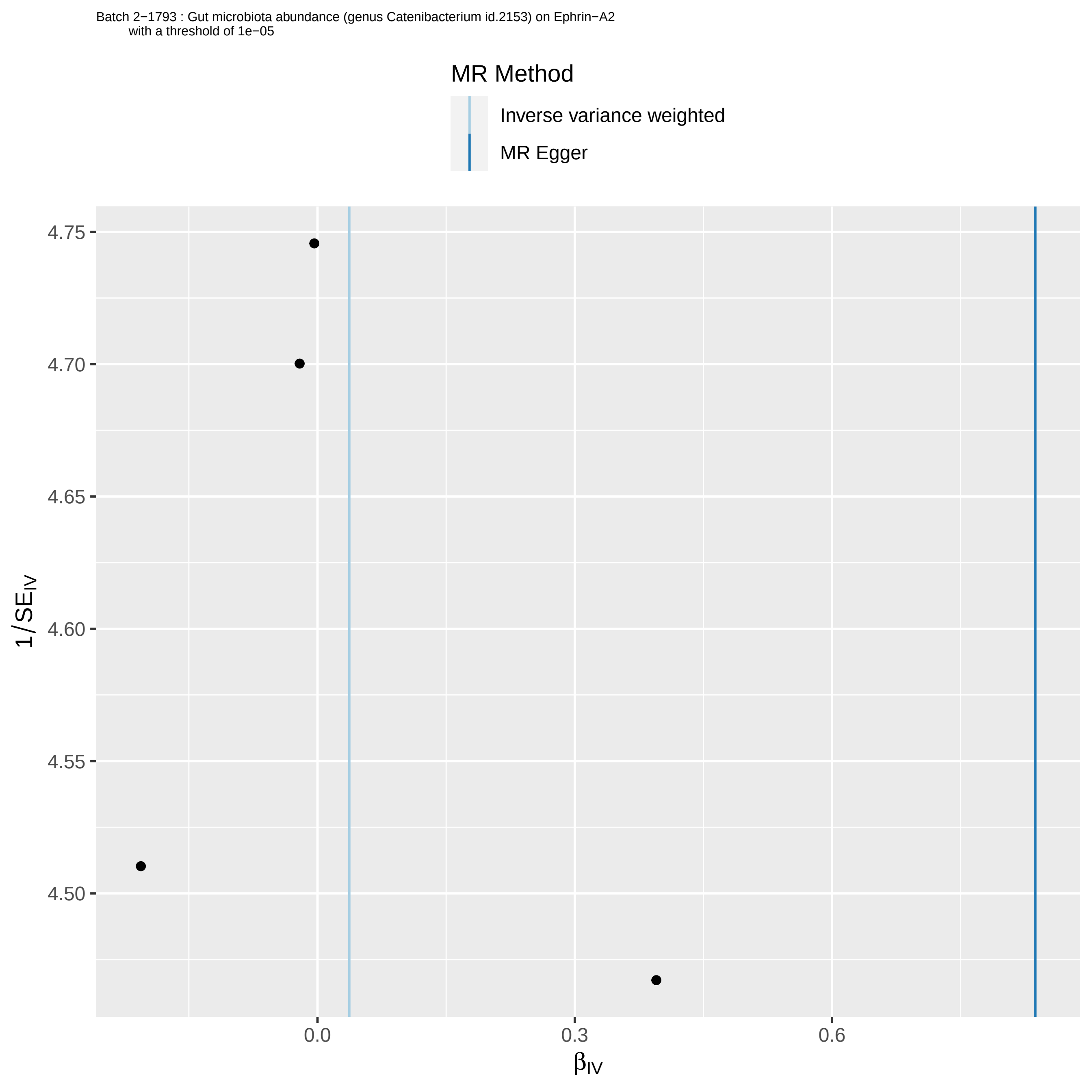
Batch 2−1793 : Gut microbiota abundance (genus Catenibacterium id.2153) on Ephrin−A2
with a threshold of 1e−05
MR Method
Inverse variance weighted
MR Egger
4.75
4.70
4.65
4.60
4.55
4.50
1/SE_IV
0.0
0.3
0.6
β_IV

Batch 2−1794 : Gut microbiota abundance (genus Parasutterella id.2892) on Ephrin−A2
with a threshold of 1e−05

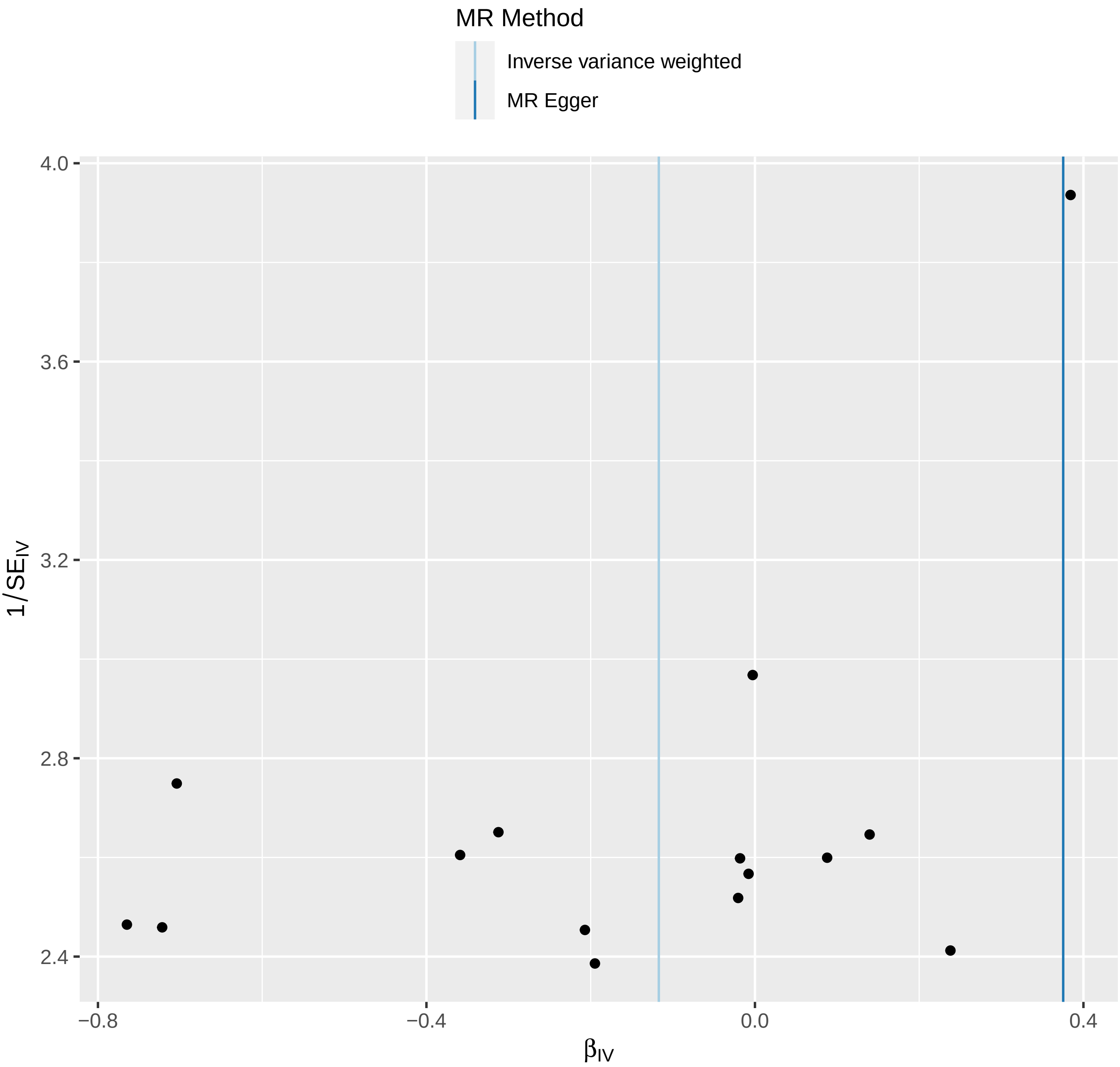

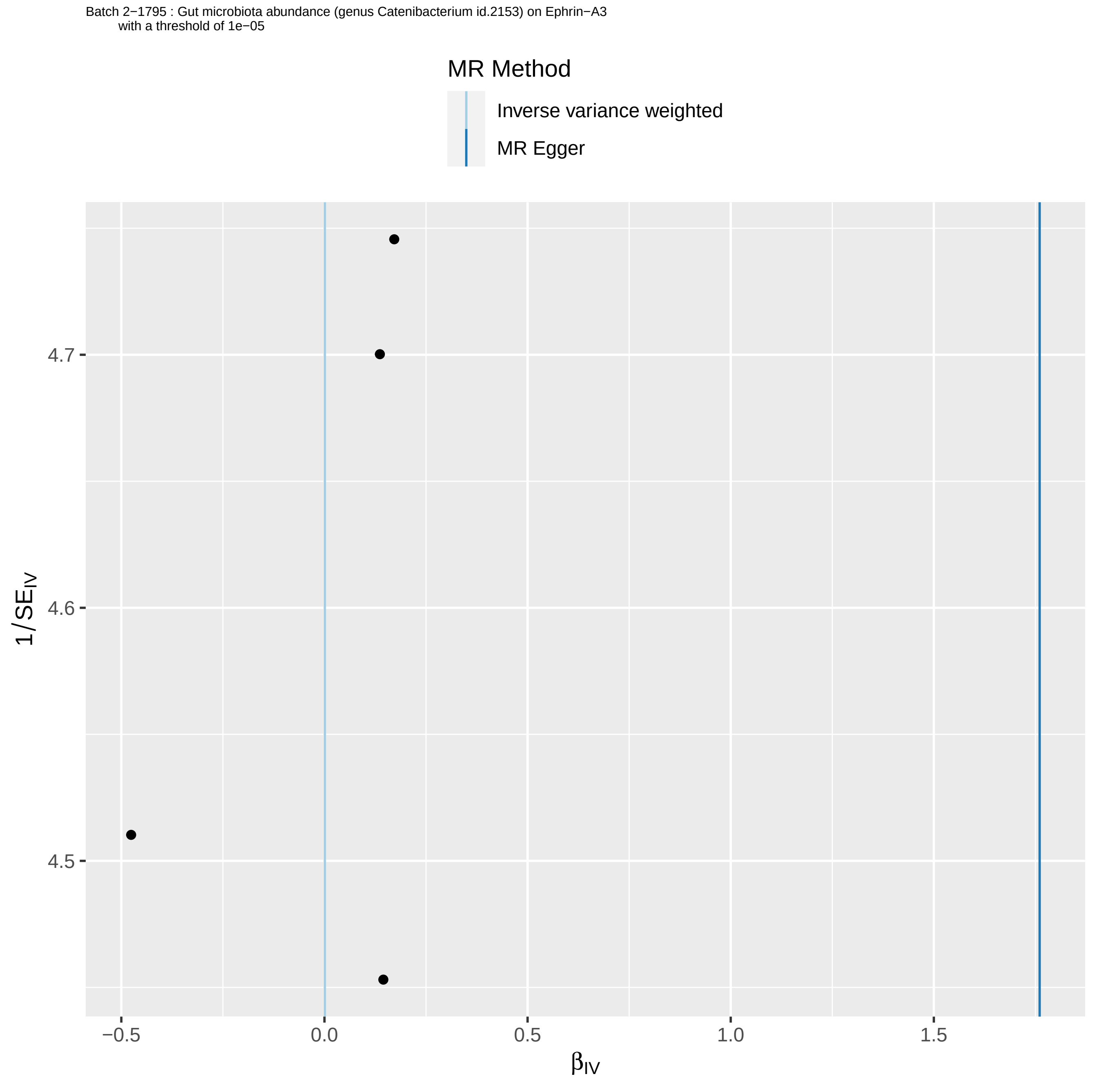
Batch 2−1795 : Gut microbiota abundance (genus Catenibacterium id.2153) on Ephrin−A3
with a threshold of 1e−05
MR Method
Inverse variance weighted
MR Egger
4.7
4.6
4.5
1/SE$_{IV}$
−0.5
0.0
0.5
1.0
1.5
β$_{IV}$

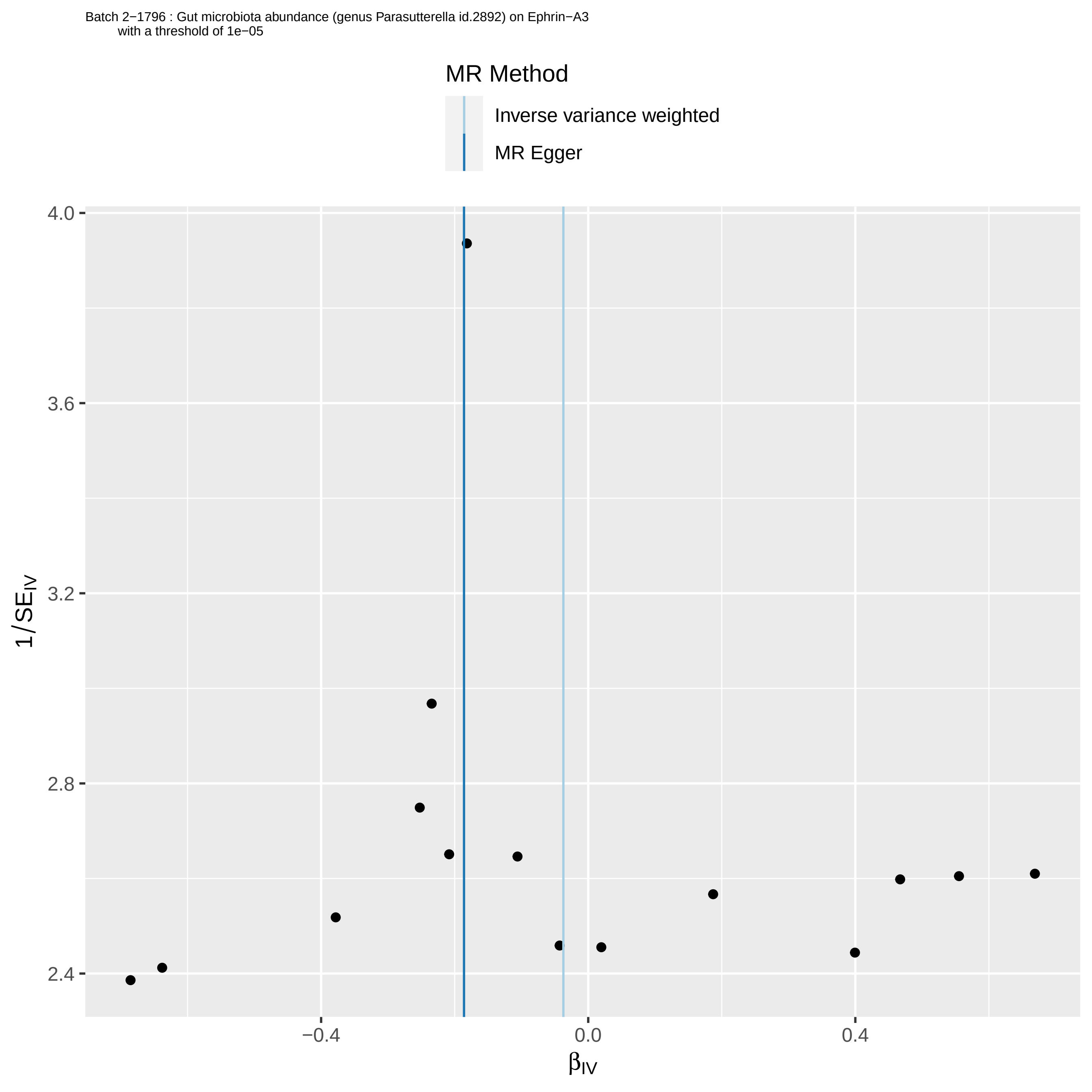

Batch 2−1796 : Gut microbiota abundance (genus Parasutterella id.2892) on Ephrin−A3
with a threshold of 1e−05
MR Method
Inverse variance weighted
MR Egger
4.0
3.6
3.2
2.8
2.4
1/SE_IV
−0.4
0.0
0.4
β_IV

Batch 2−1797 : Gut microbiota abundance (genus Catenibacterium id.2153) on Ephrin−A3
with a threshold of 1e−05

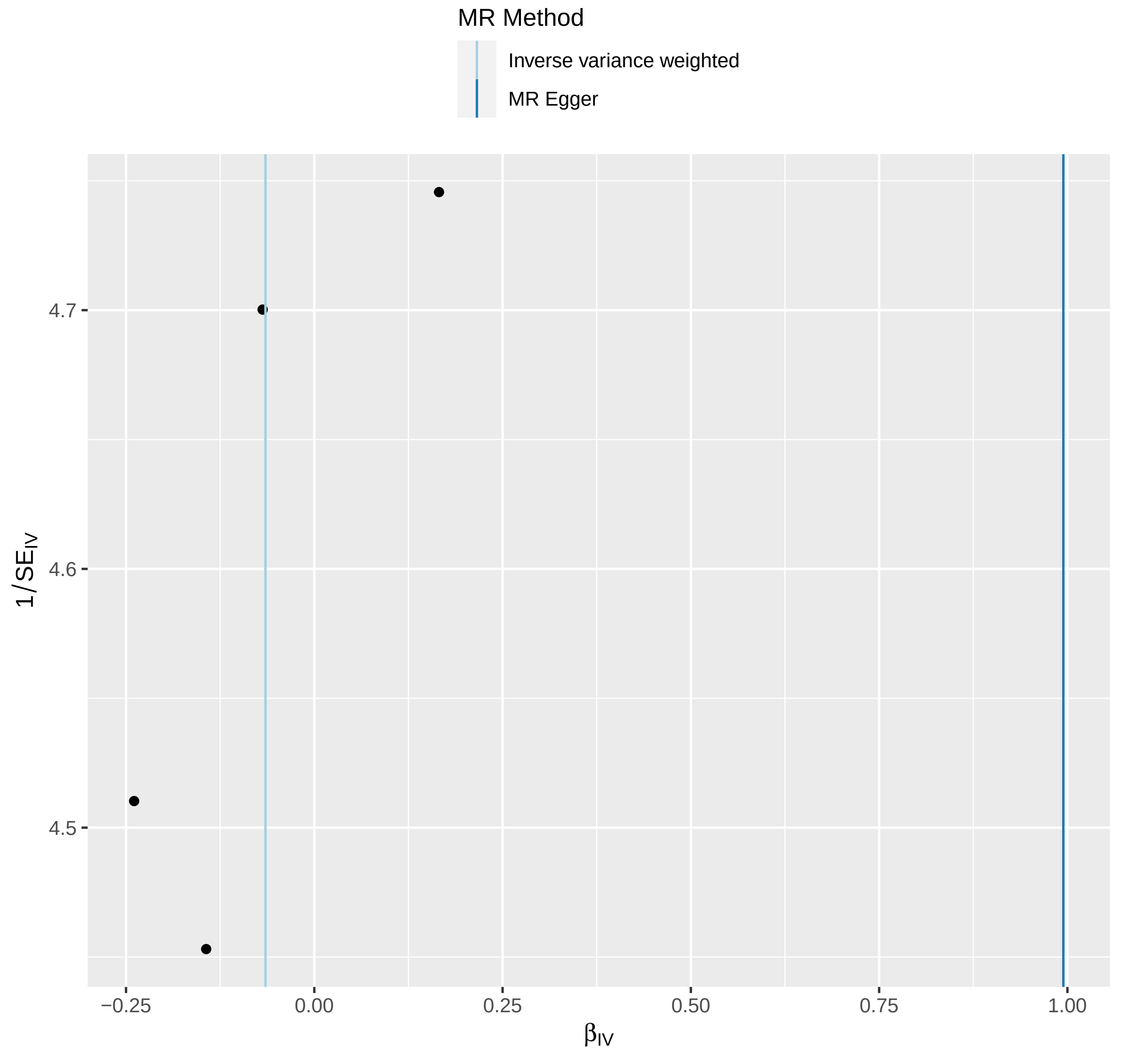

Batch 2−1798 : Gut microbiota abundance (genus Parasutterella id.2892) on Ephrin−A3
with a threshold of 1e−05

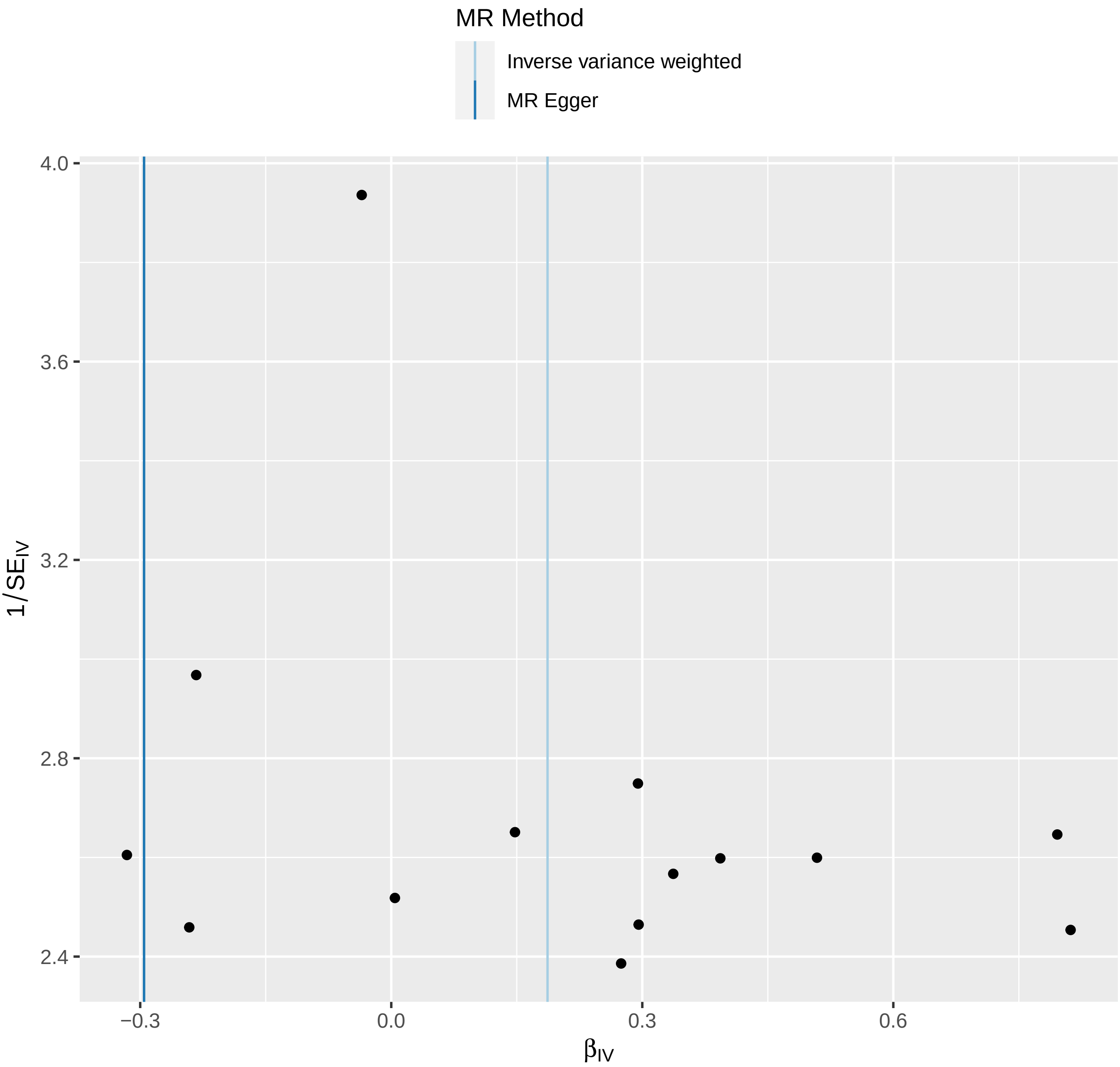

Batch 2−1799 : Gut microbiota abundance (genus Catenibacterium id.2153) on Ephrin−A4
with a threshold of 1e−05

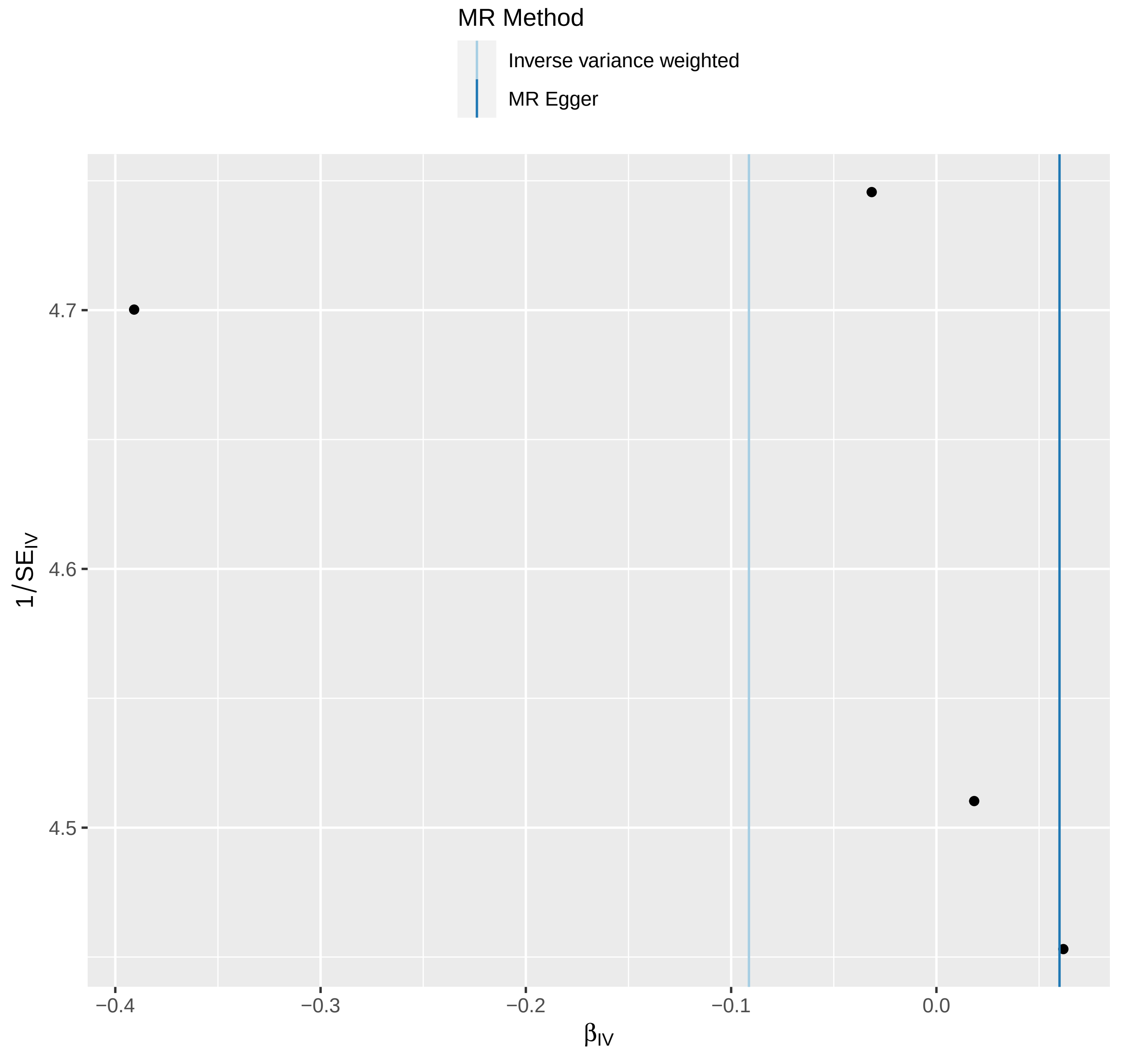

Batch 2−1800 : Gut microbiota abundance (genus Parasutterella id.2892) on Ephrin−A4
with a threshold of 1e−05

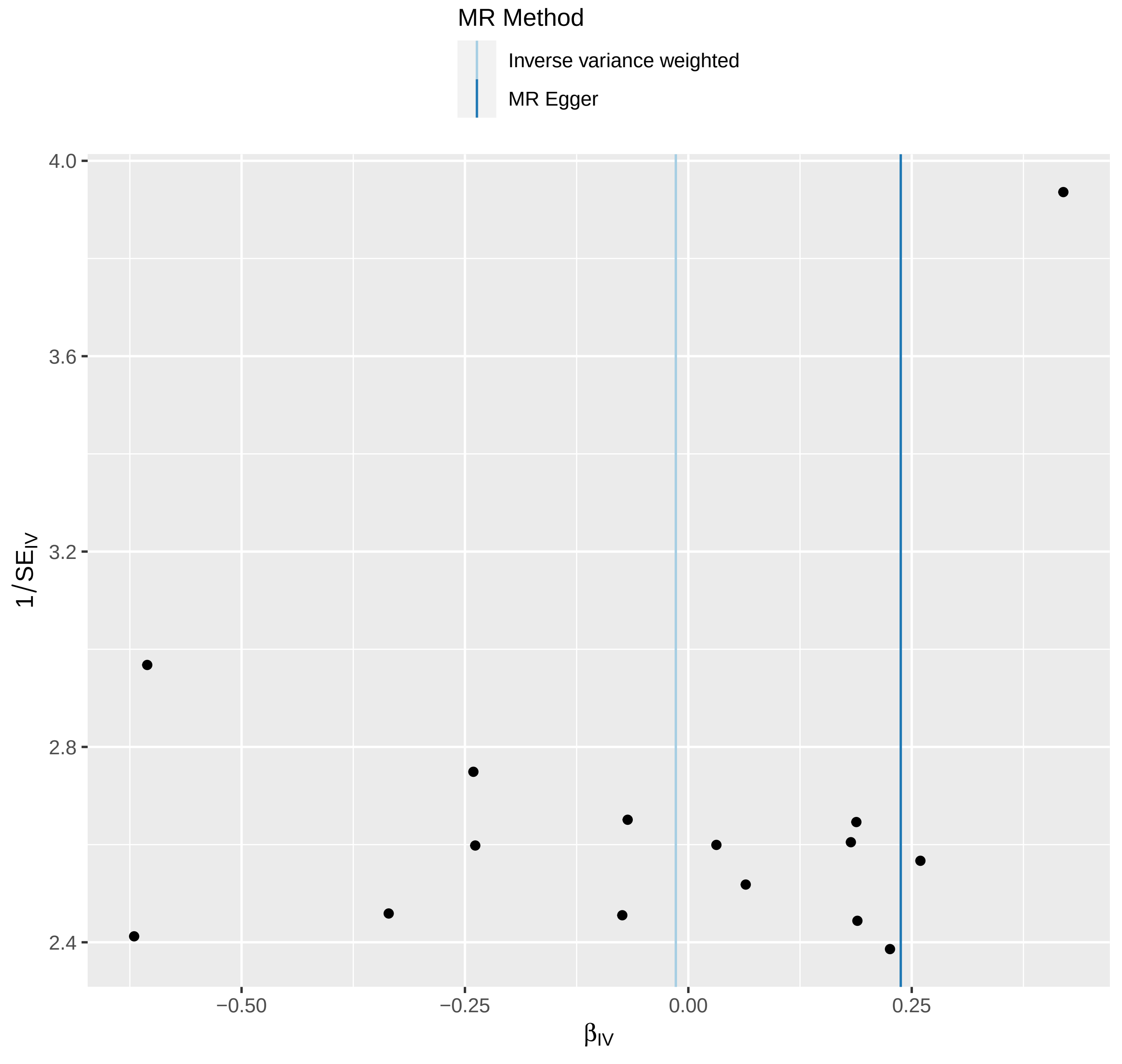

Batch 2−1801 : Gut microbiota abundance (genus Catenibacterium id.2153) on Ephrin−A5
with a threshold of 1e−05

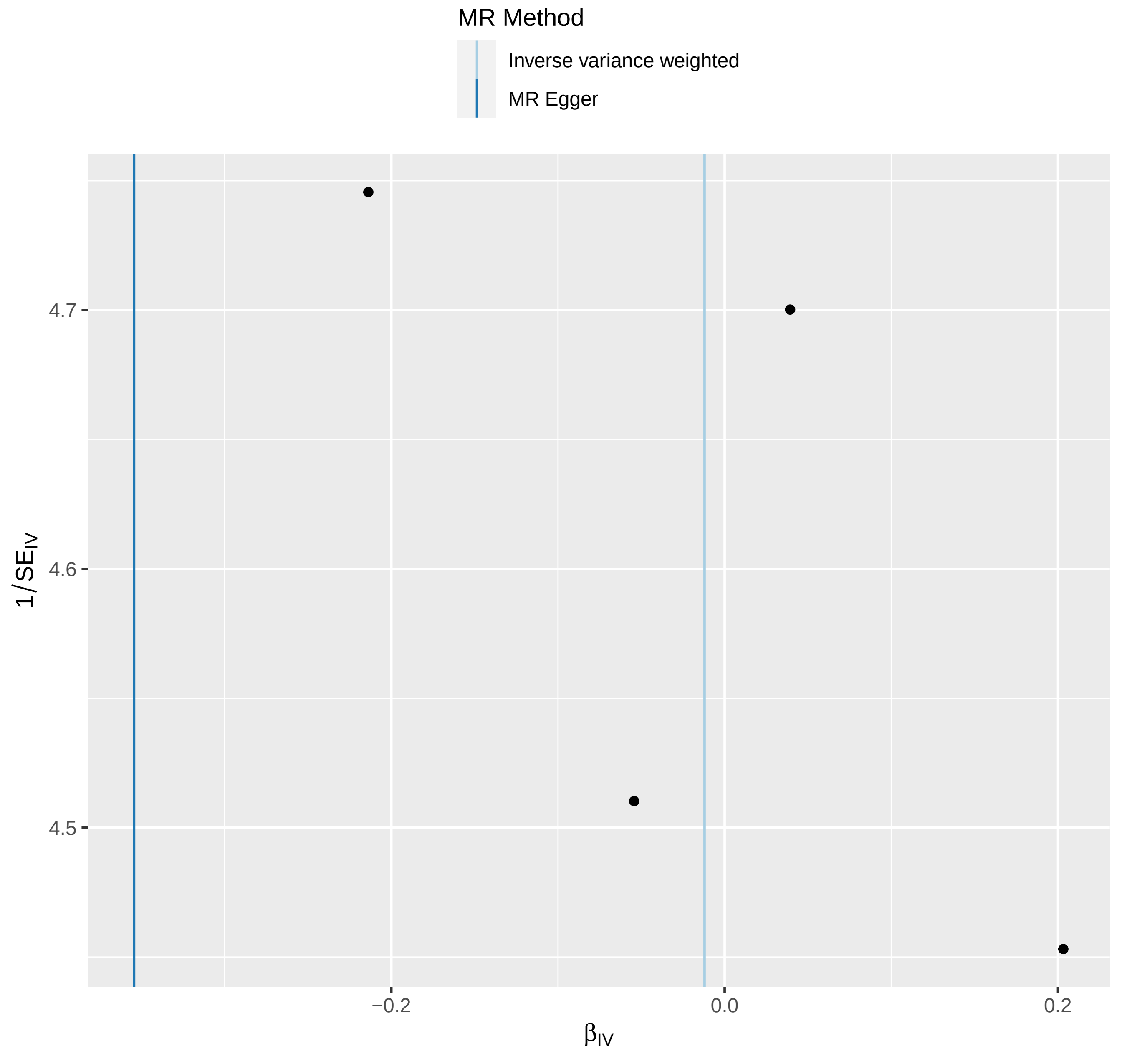

Batch 2−1802 : Gut microbiota abundance (genus Parasutterella id.2892) on Ephrin−A5
with a threshold of 1e−05

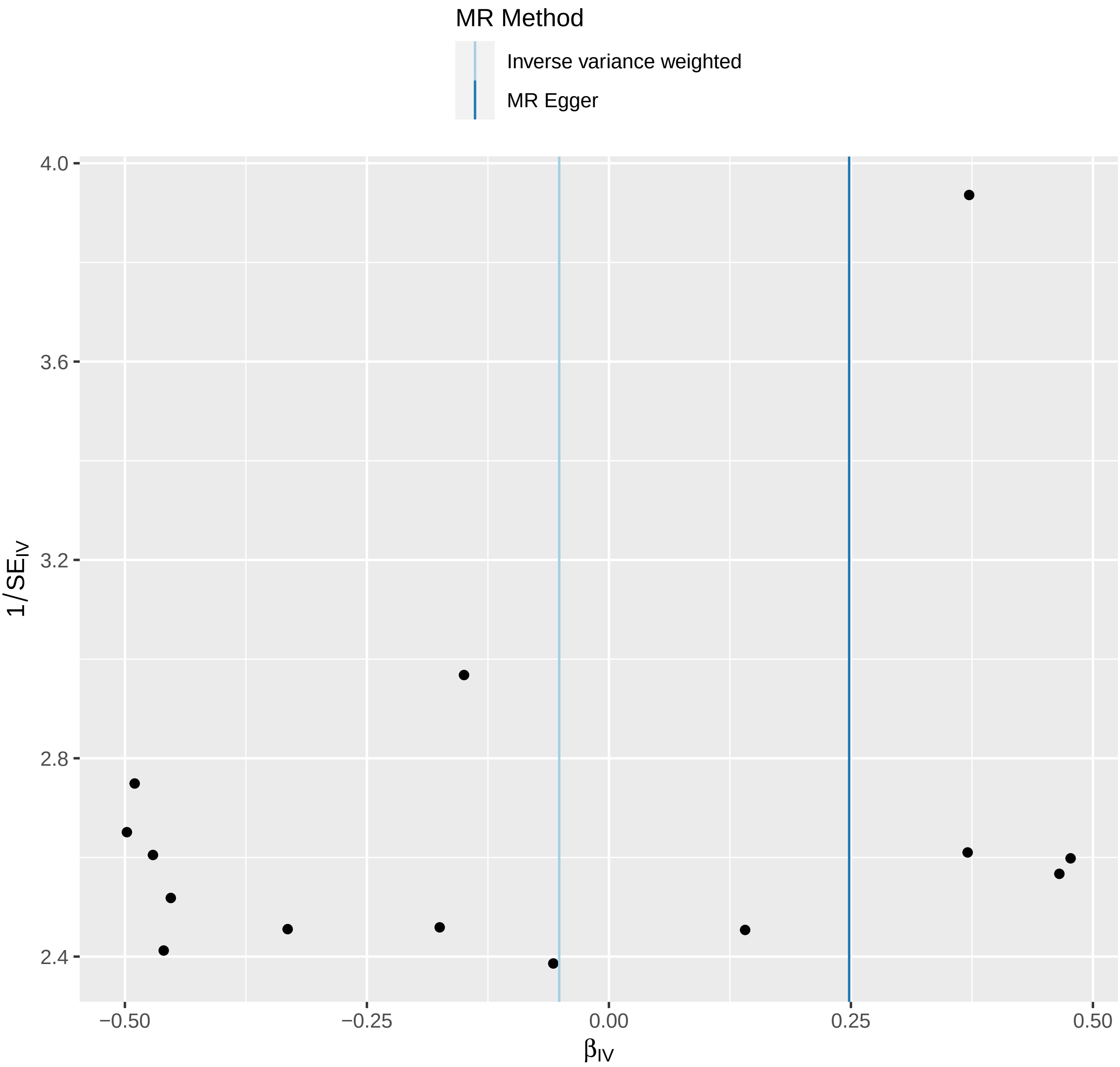

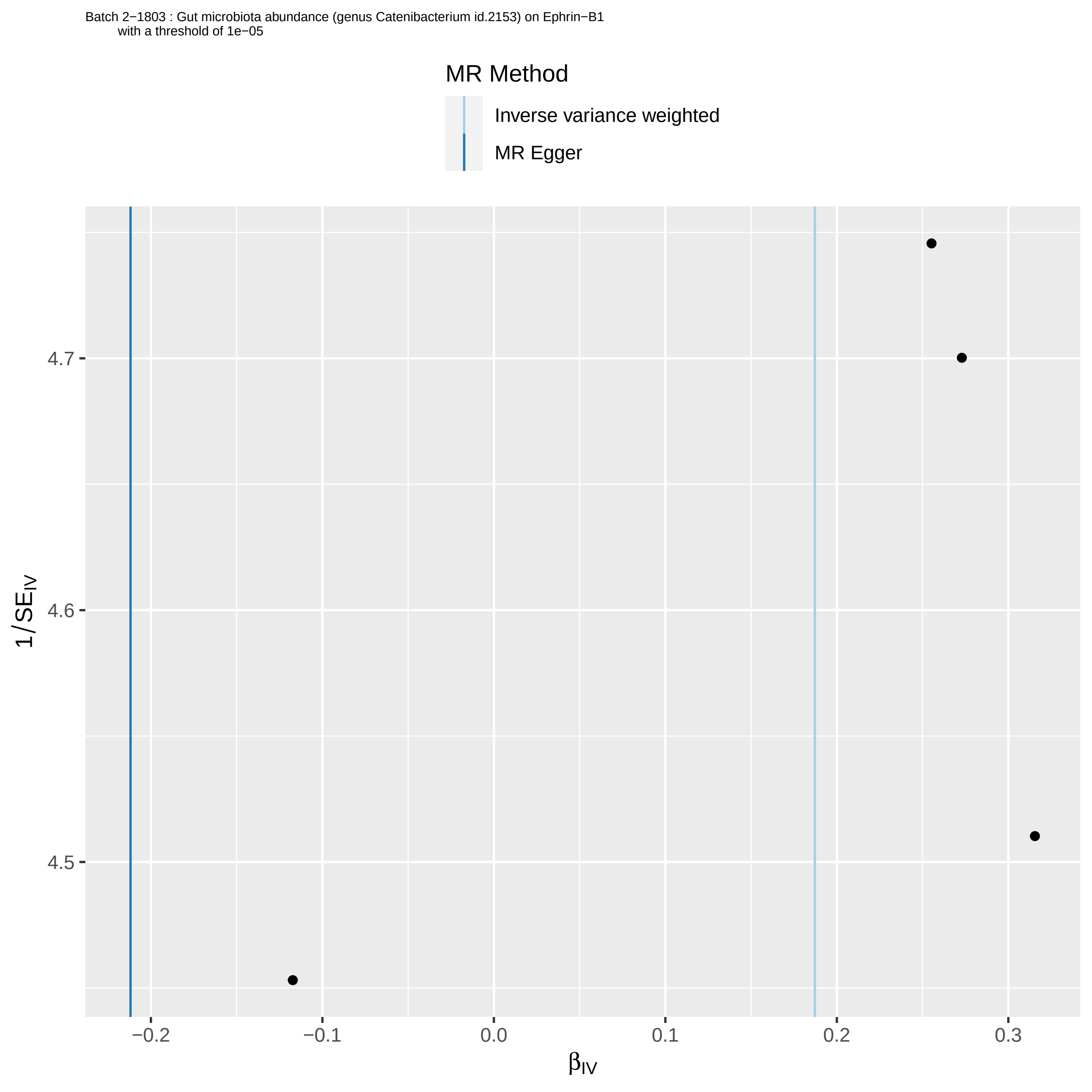
Batch 2−1803 : Gut microbiota abundance (genus Catenibacterium id.2153) on Ephrin−B1
with a threshold of 1e−05
MR Method
Inverse variance weighted
MR Egger
4.7
4.6
4.5
1/SE_IV
−0.2
−0.1
0.0
0.1
0.2
0.3
β_IV

Batch 2−1804 : Gut microbiota abundance (genus Parasutterella id.2892) on Ephrin−B1
with a threshold of 1e−05

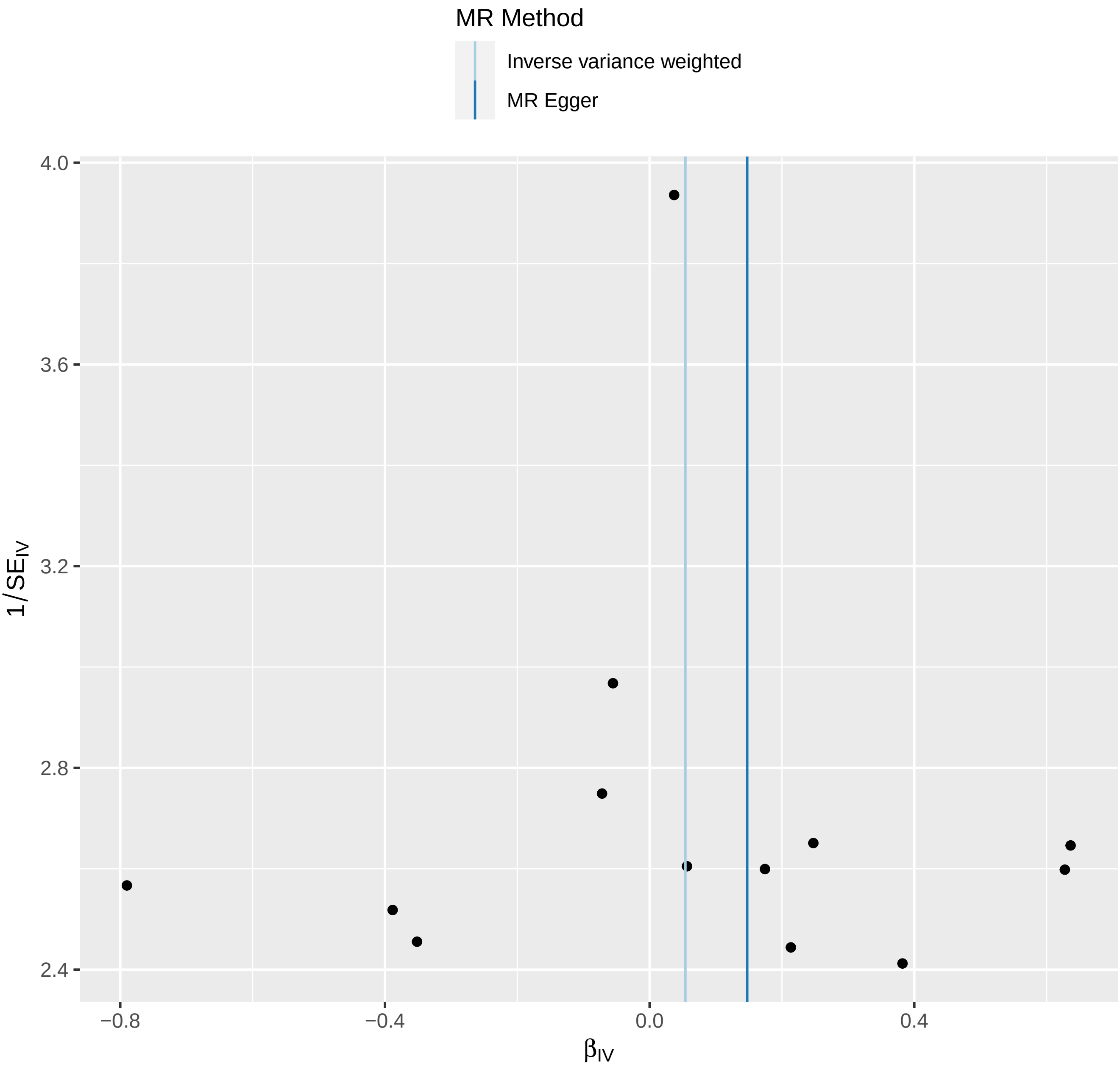

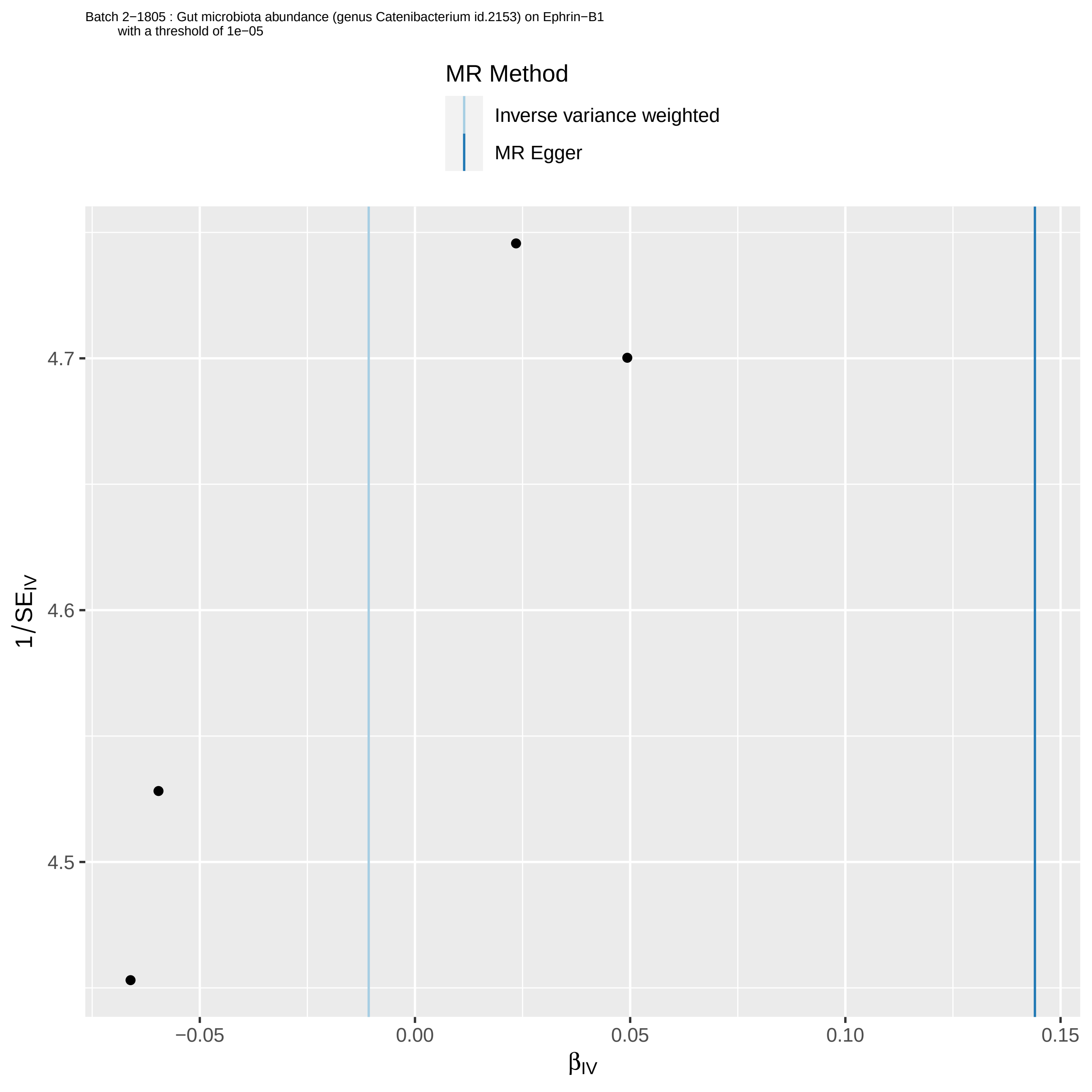

Batch 2−1805 : Gut microbiota abundance (genus Catenibacterium id.2153) on Ephrin−B1
with a threshold of 1e−05
MR Method
Inverse variance weighted
MR Egger
4.7
4.6
4.5
1/SE_IV
−0.05
0.00
0.05
0.10
0.15
β_IV

Batch 2−1806 : Gut microbiota abundance (genus Parasutterella id.2892) on Ephrin−B1
with a threshold of 1e−05

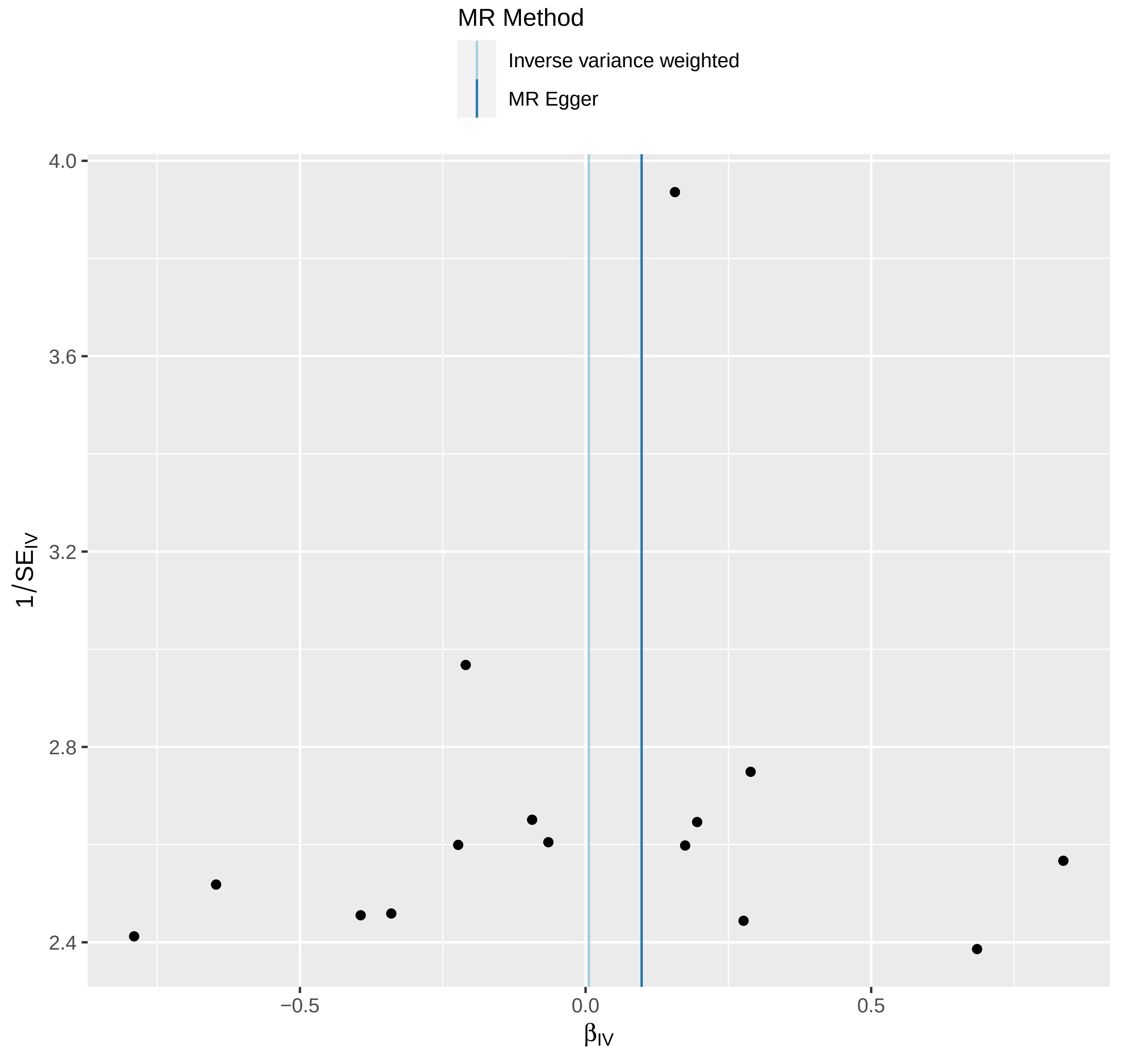

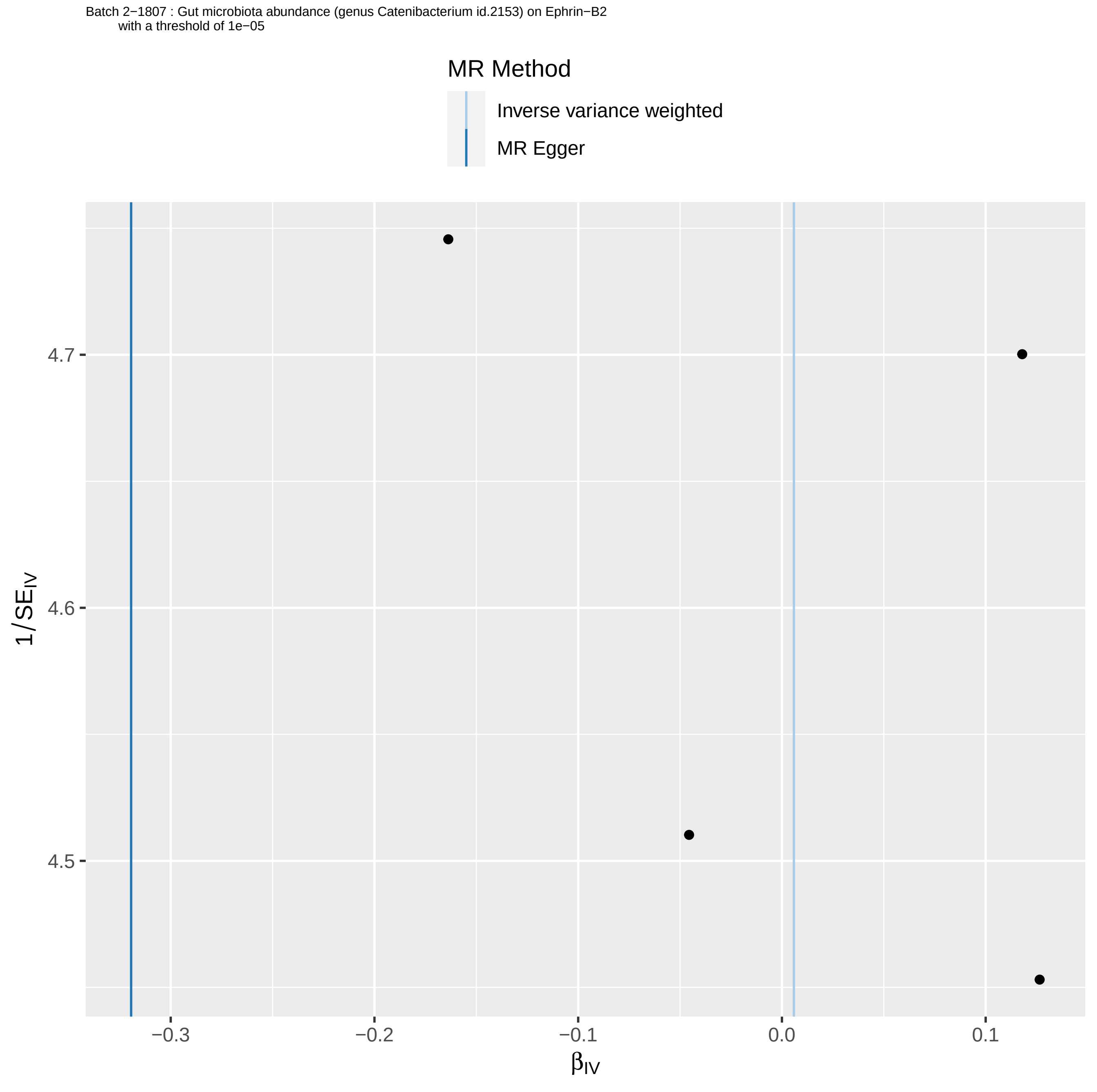

Batch 2−1807 : Gut microbiota abundance (genus Catenibacterium id.2153) on Ephrin−B2
with a threshold of 1e−05
MR Method
Inverse variance weighted
MR Egger
4.7
4.6
4.5
1/SE_IV
−0.3
−0.2
−0.1
0.0
0.1
β_IV

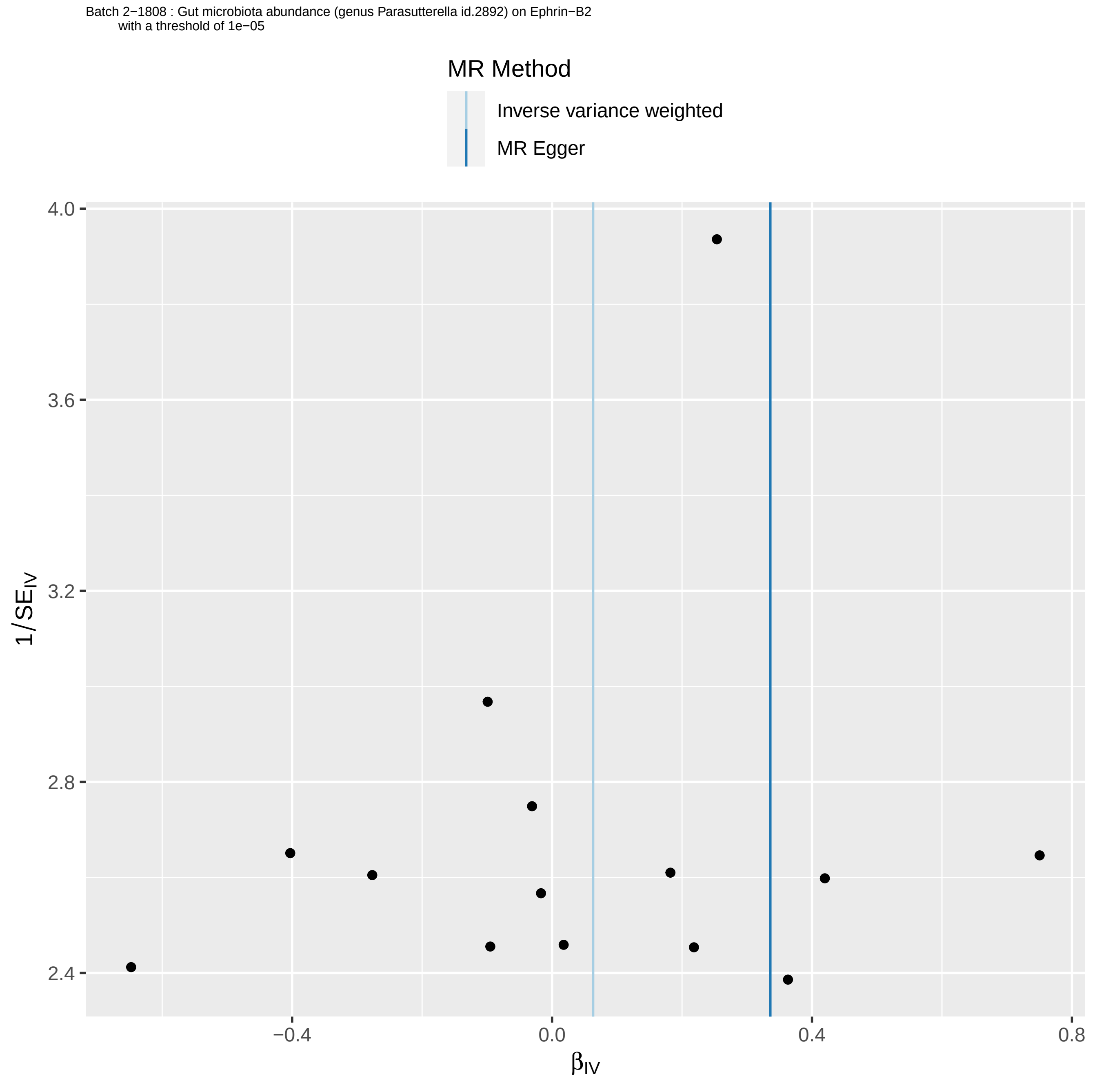

Batch 2−1808 : Gut microbiota abundance (genus Parasutterella id.2892) on Ephrin−B2
with a threshold of 1e−05
MR Method
Inverse variance weighted
MR Egger
4.0
3.6
3.2
2.8
2.4
1/SE_IV
−0.4
0.0
0.4
0.8
β_IV

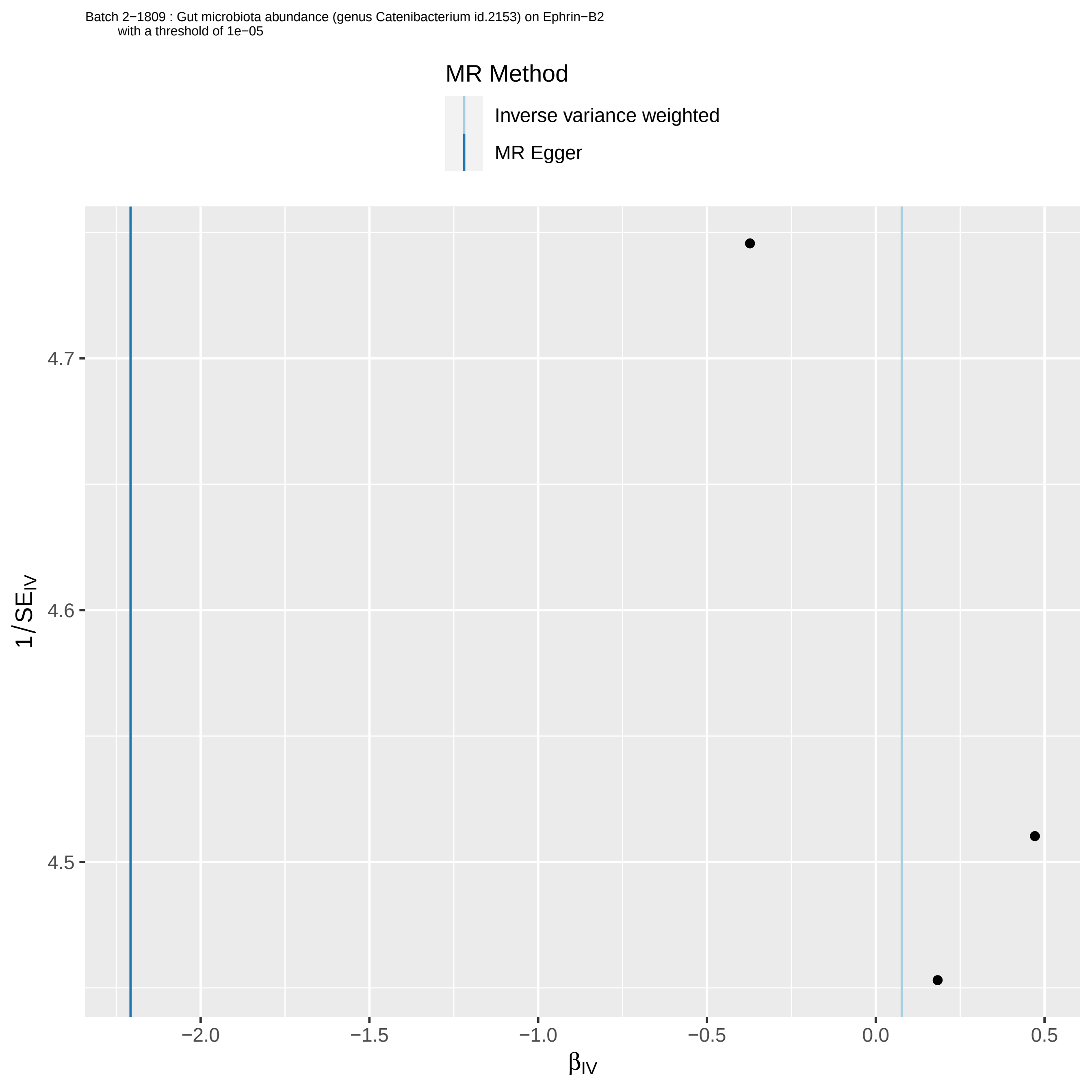

Batch 2−1809 : Gut microbiota abundance (genus Catenibacterium id.2153) on Ephrin−B2
with a threshold of 1e−05
MR Method
Inverse variance weighted
MR Egger
4.7
4.6
4.5
1/SE_IV
−2.0
−1.5
−1.0
−0.5
0.0
0.5
β_IV

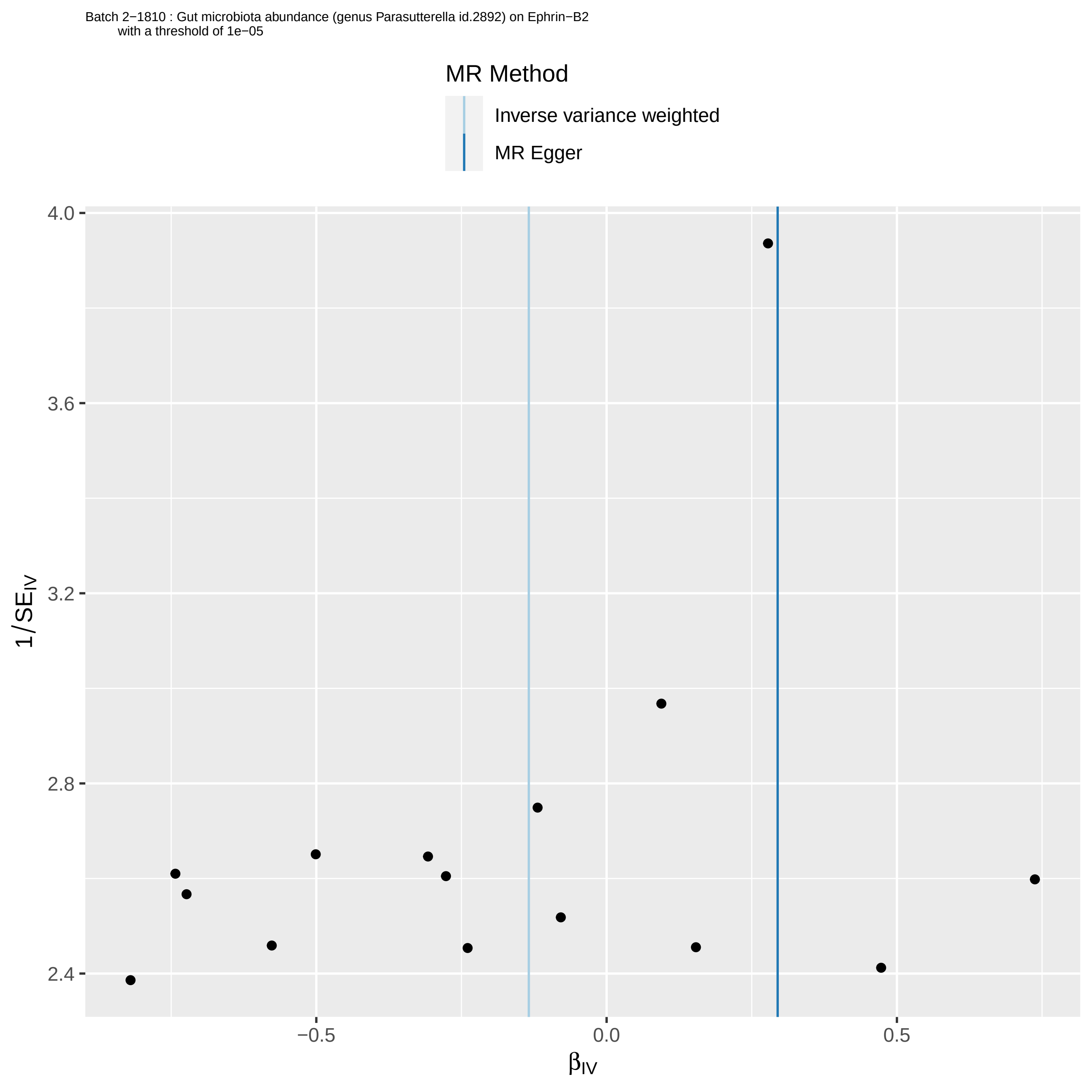

Batch 2−1810 : Gut microbiota abundance (genus Parasutterella id.2892) on Ephrin−B2
with a threshold of 1e−05
MR Method
Inverse variance weighted
MR Egger
4.0
3.6
3.2
2.8
2.4
1/SE$_{IV}$
−0.5
0.0
0.5
β_{IV}

Batch 2−1811 : Gut microbiota abundance (genus Catenibacterium id.2153) on Ephrin−B3
with a threshold of 1e−05

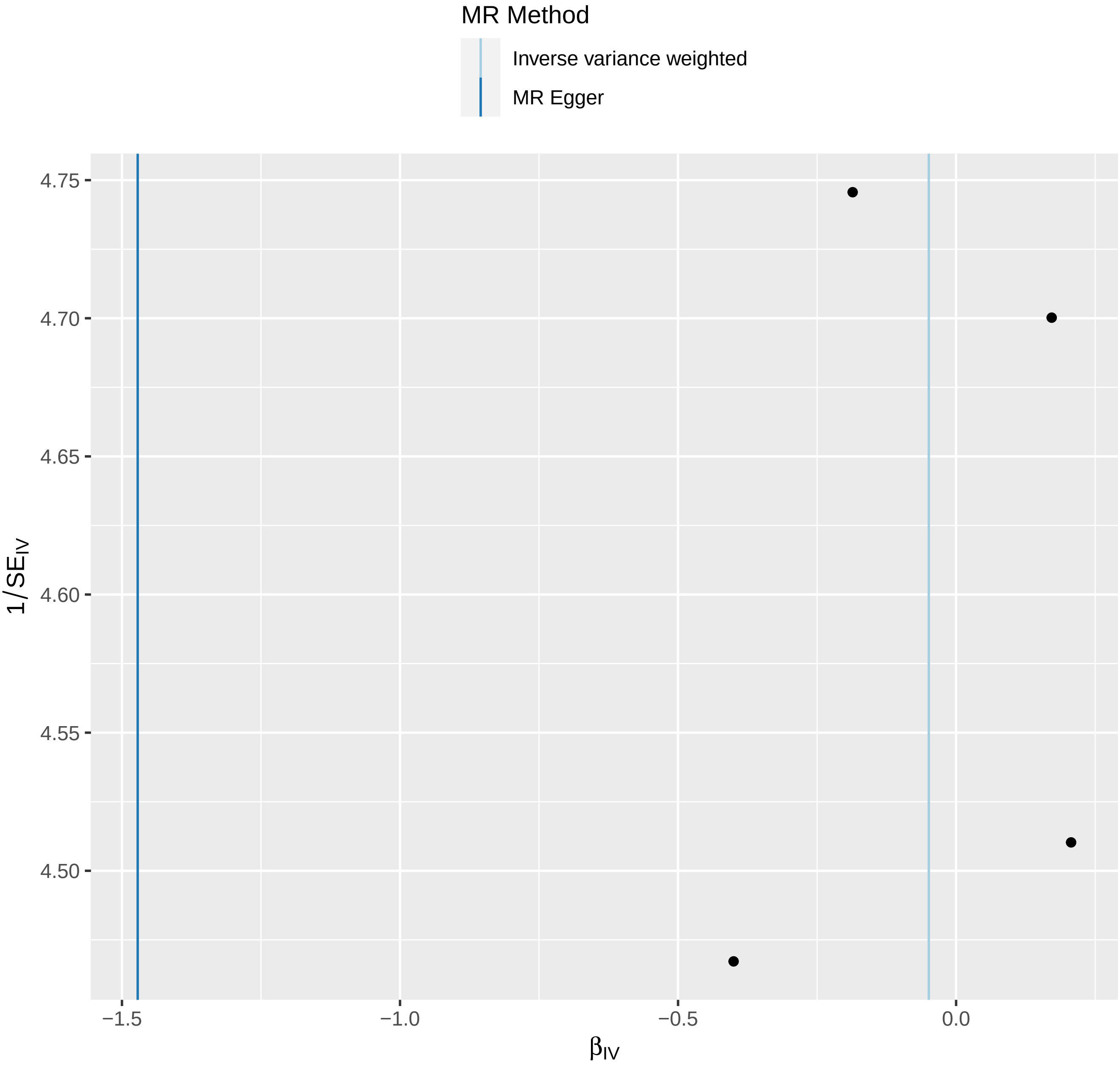

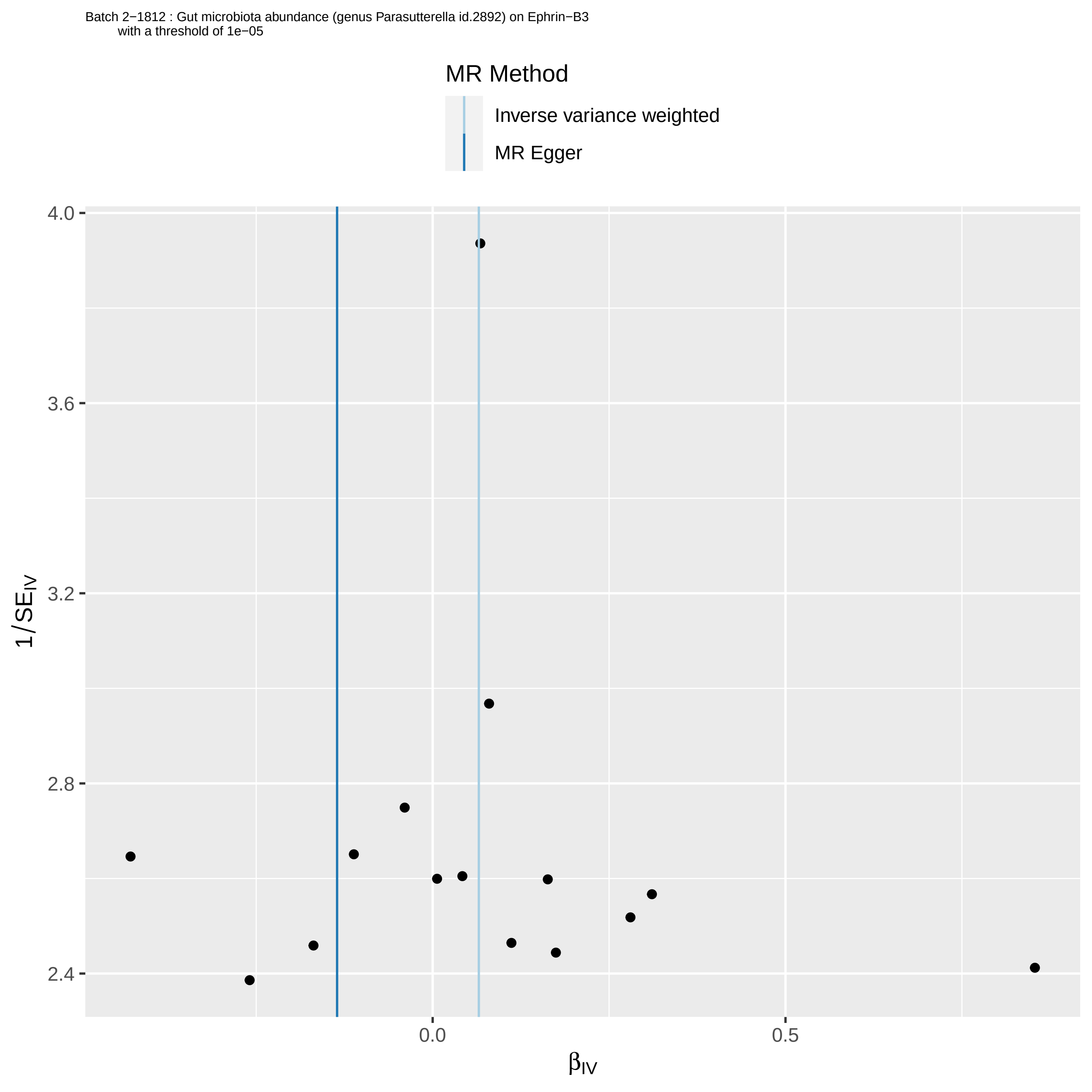
Batch 2−1812 : Gut microbiota abundance (genus Parasutterella id.2892) on Ephrin−B3
with a threshold of 1e−05
MR Method
Inverse variance weighted
MR Egger
4.0
3.6
3.2
2.8
2.4
$1/SE_{IV}$
0.0
0.5
β_{IV}

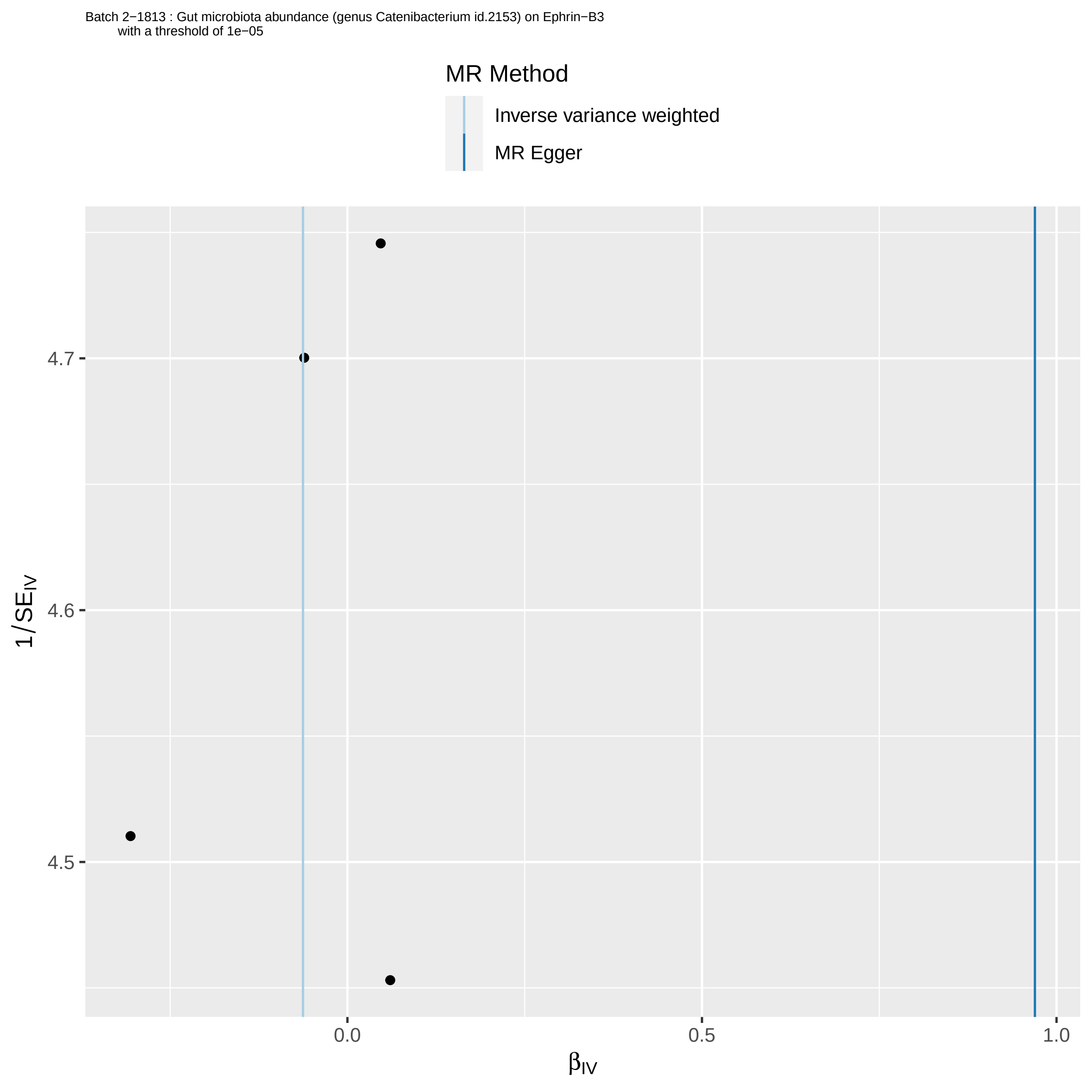
Batch 2−1813 : Gut microbiota abundance (genus Catenibacterium id.2153) on Ephrin−B3
with a threshold of 1e−05
MR Method
Inverse variance weighted
MR Egger
4.7
4.6
4.5
1/SE_IV
0.0
0.5
1.0
β_IV

Batch 2−1814 : Gut microbiota abundance (genus Parasutterella id.2892) on Ephrin−B3
with a threshold of 1e−05

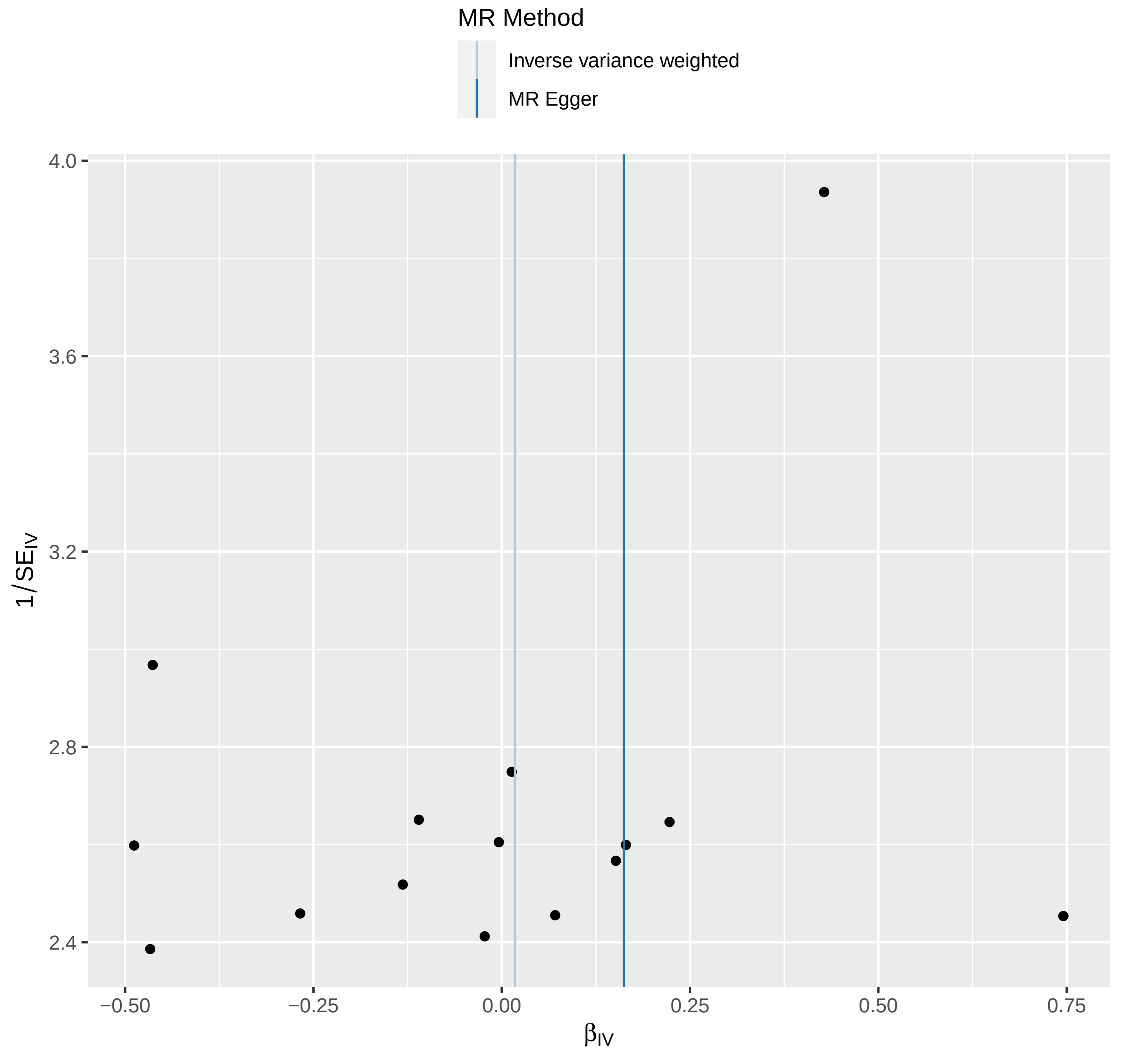

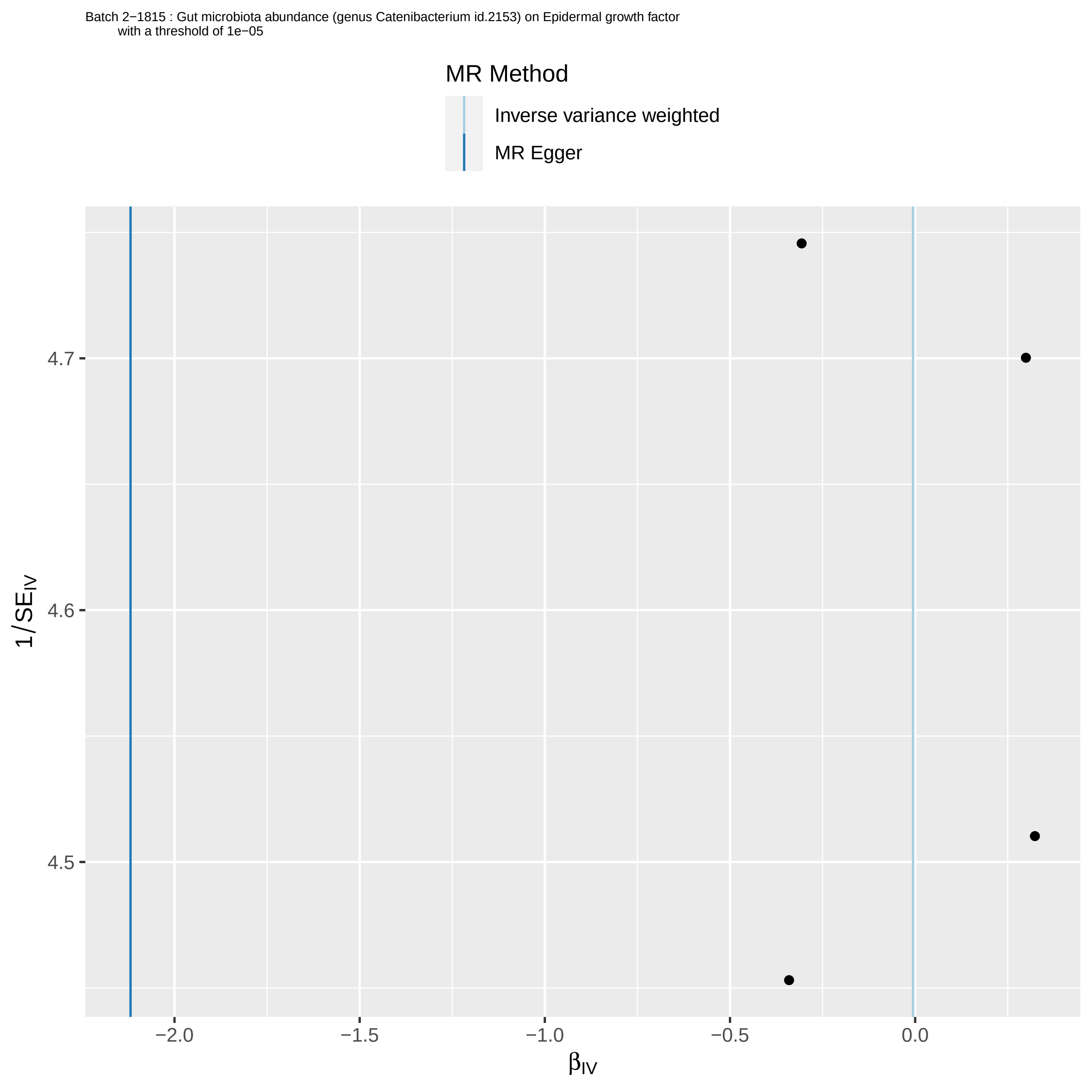

Batch 2−1815 : Gut microbiota abundance (genus Catenibacterium id.2153) on Epidermal growth factor
with a threshold of 1e−05
MR Method
Inverse variance weighted
MR Egger
1/SE_IV
4.7
4.6
4.5
−2.0
−1.5
−1.0
−0.5
0.0
β_IV

Batch 2−1816 : Gut microbiota abundance (genus Parasutterella id.2892) on Epidermal growth factor with a threshold of 1e−05

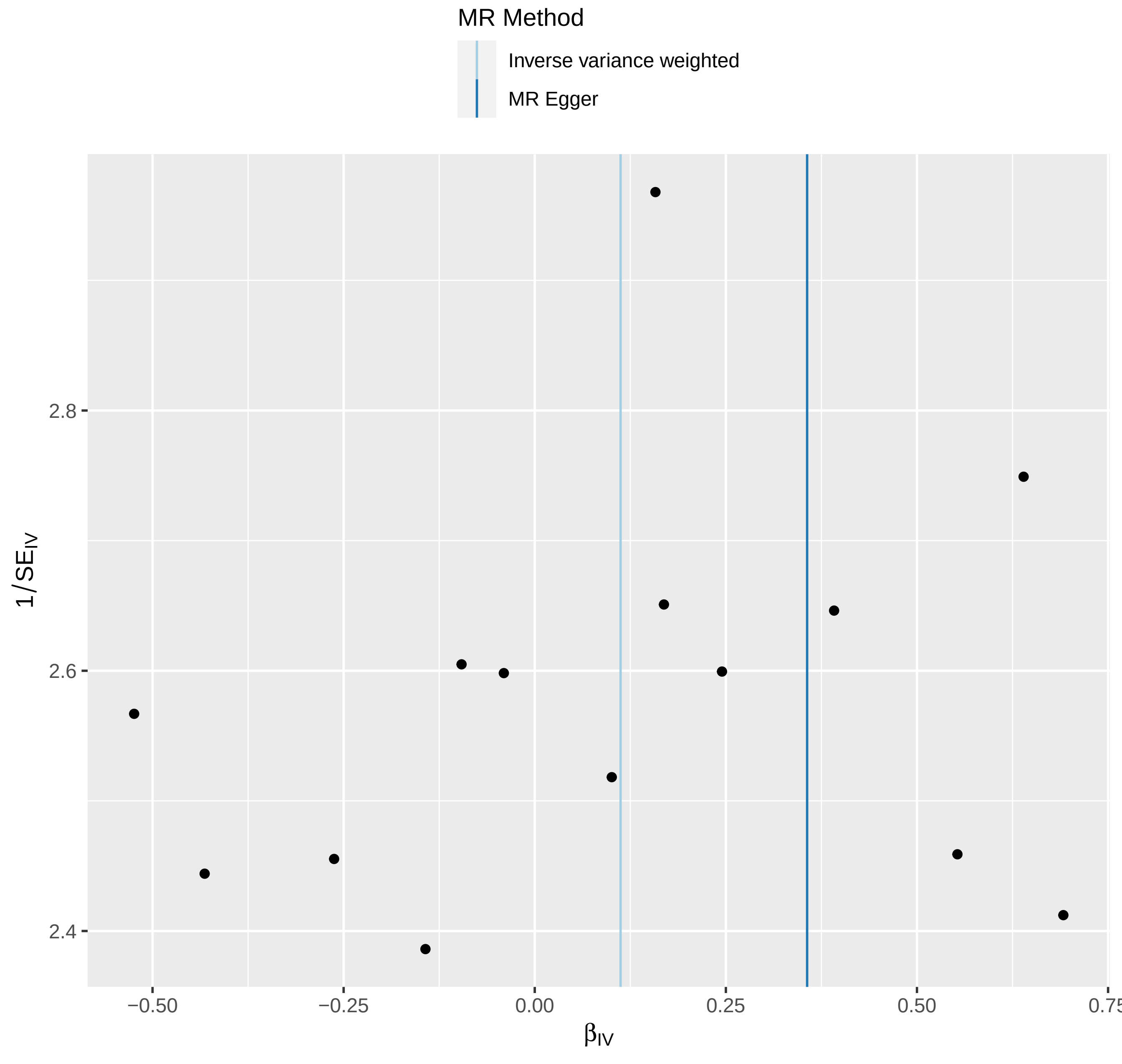

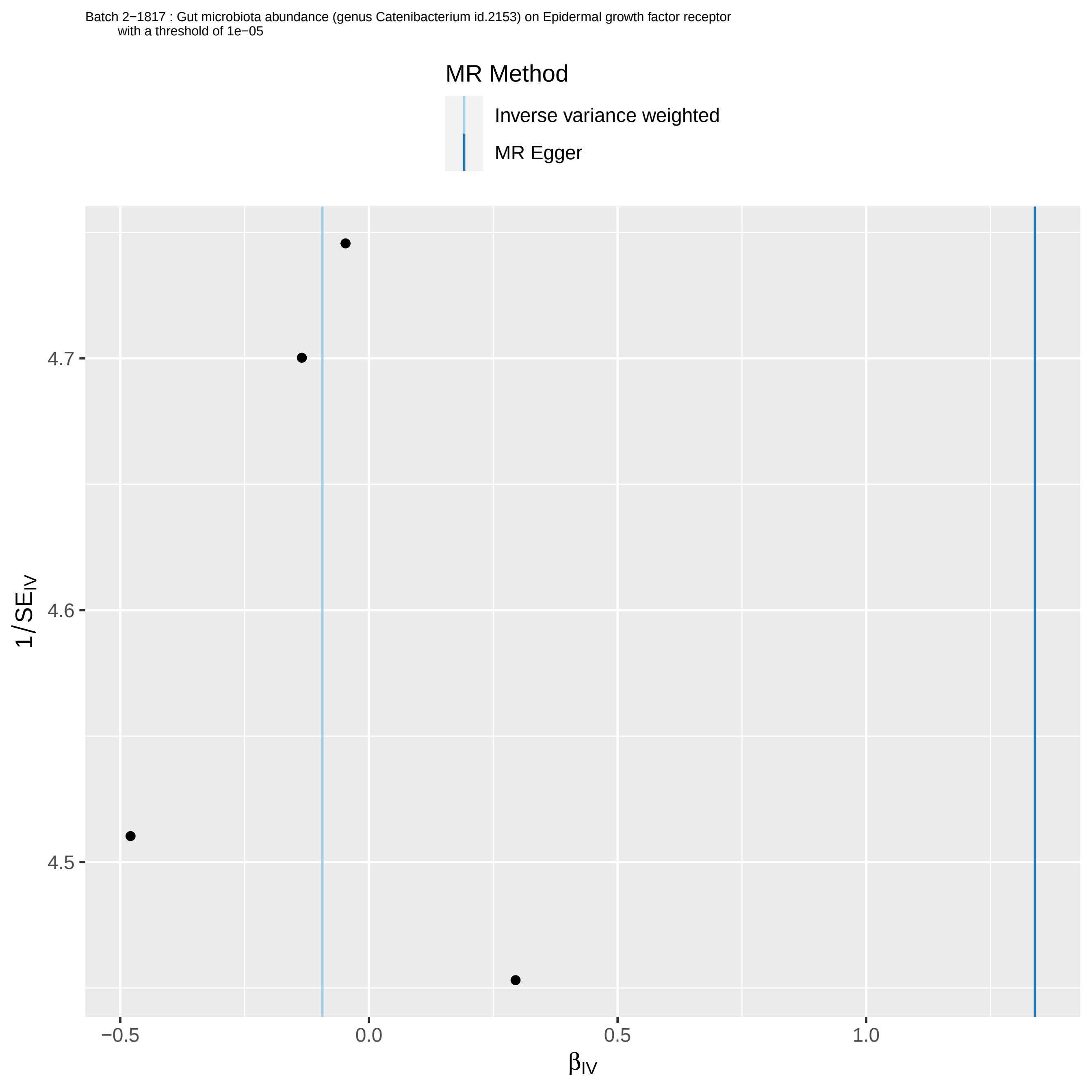
Batch 2−1817 : Gut microbiota abundance (genus Catenibacterium id.2153) on Epidermal growth factor receptor
with a threshold of 1e−05
MR Method
Inverse variance weighted
MR Egger
4.7
4.6
4.5
1/SE_IV
−0.5
0.0
0.5
1.0
β_IV

Batch 2−1818 : Gut microbiota abundance (genus Parasutterella id.2892) on Epidermal growth factor receptor
with a threshold of 1e−05

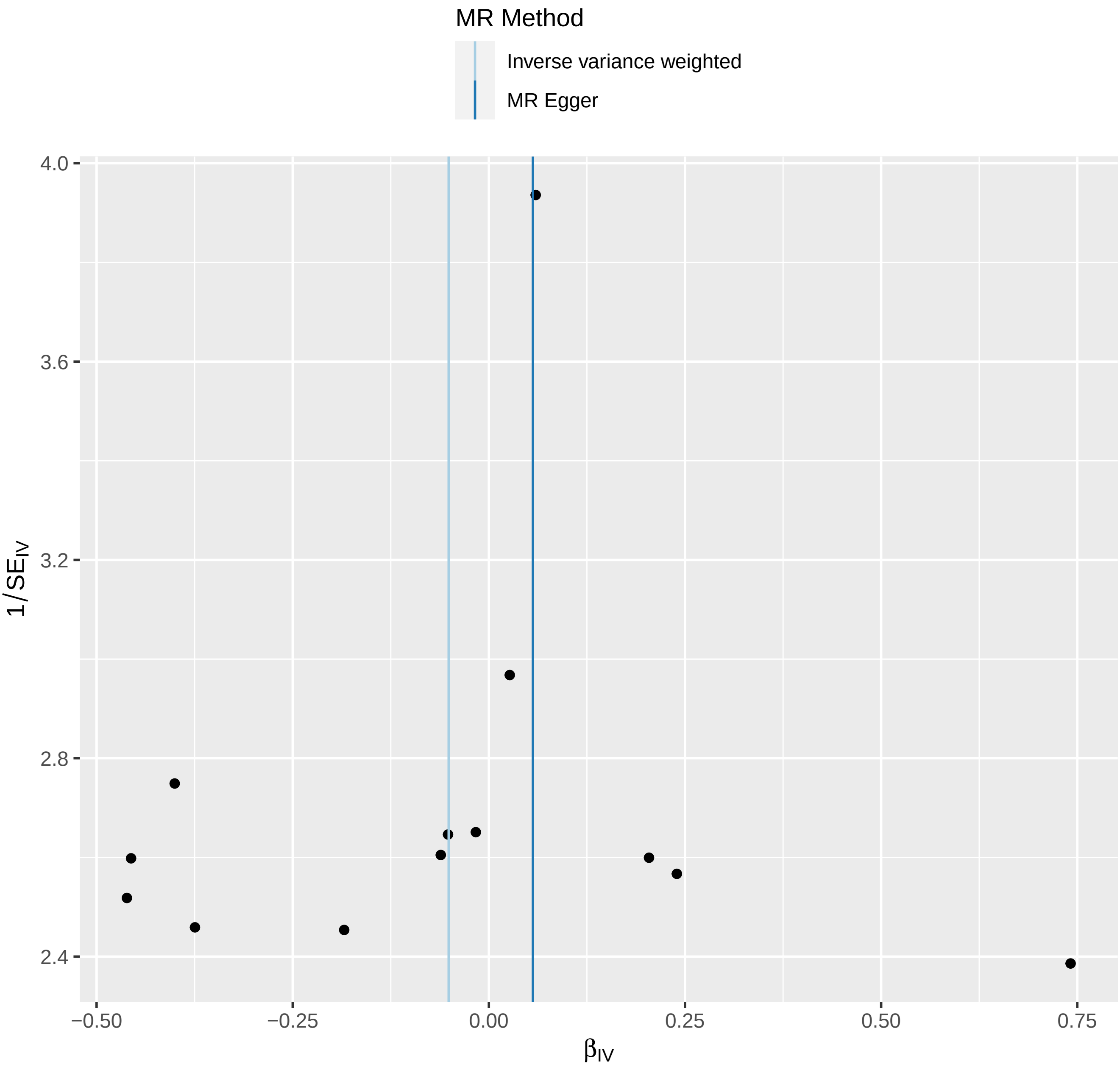

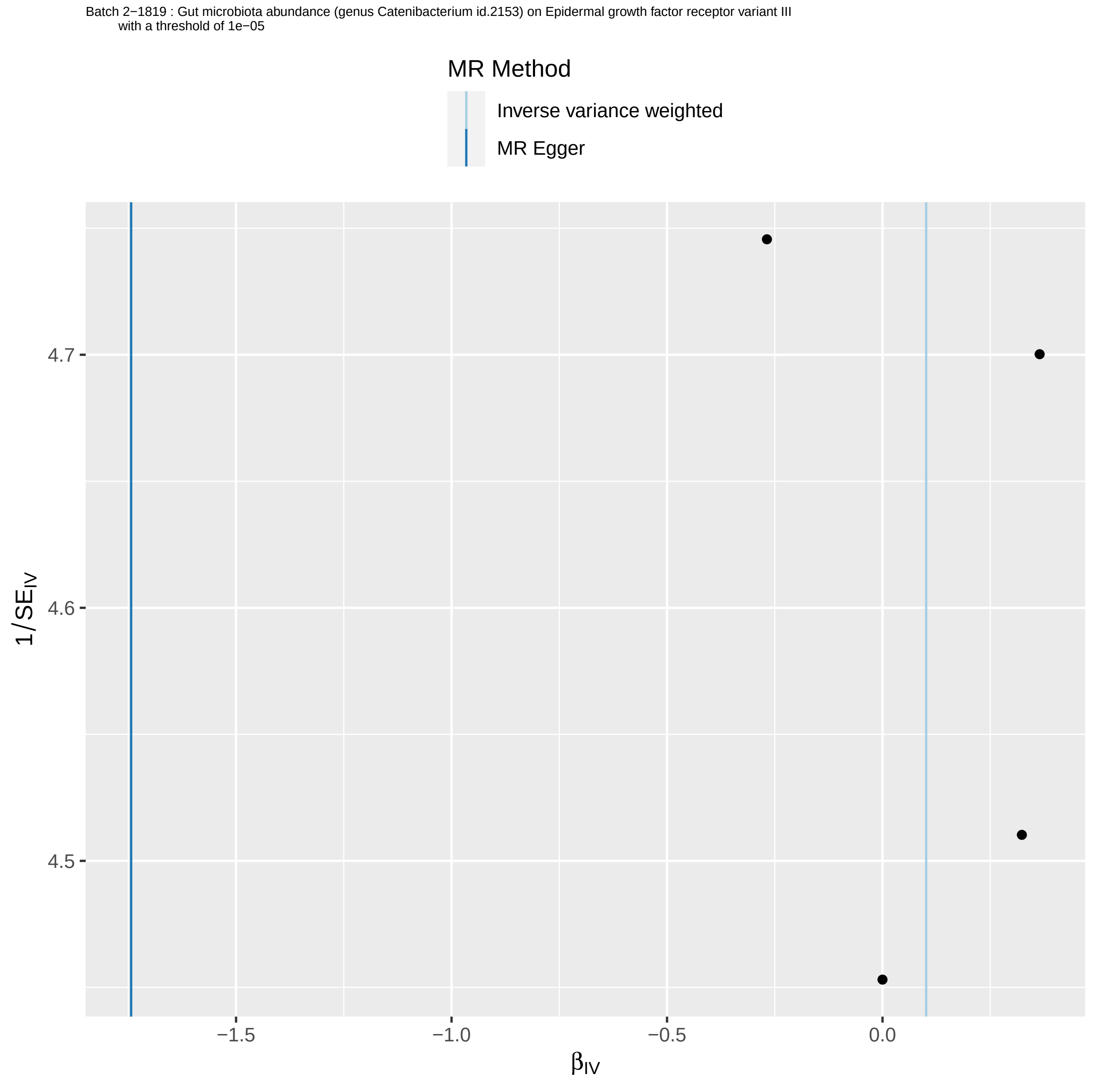
Batch 2−1819 : Gut microbiota abundance (genus Catenibacterium id.2153) on Epidermal growth factor receptor variant III
with a threshold of 1e−05
MR Method
Inverse variance weighted
MR Egger
4.7
4.6
4.5
1/SE_IV
−1.5
−1.0
−0.5
0.0
β_IV

Batch 2−1820 : Gut microbiota abundance (genus Parasutterella id.2892) on Epidermal growth factor receptor variant III
with a threshold of 1e−05

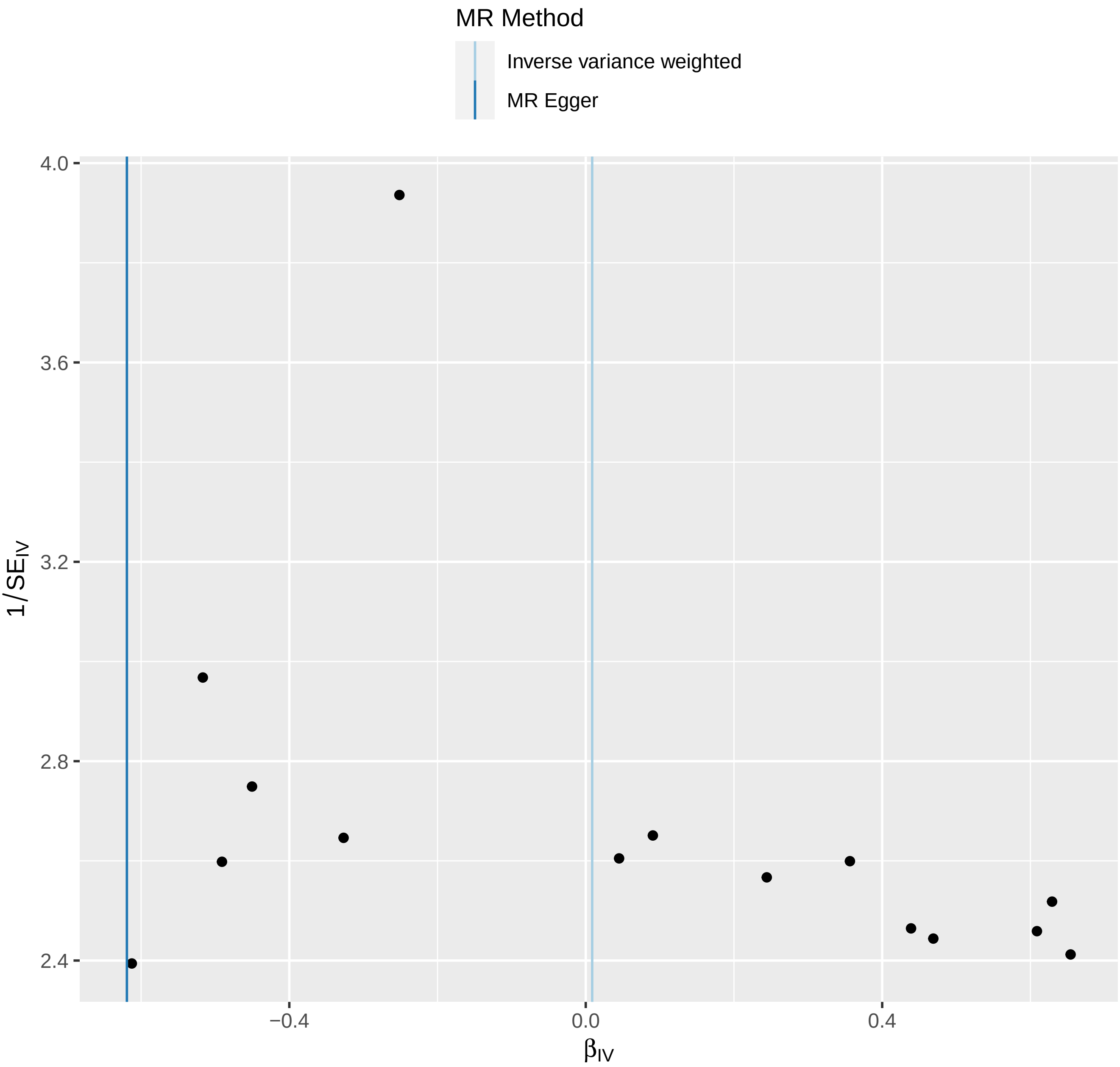

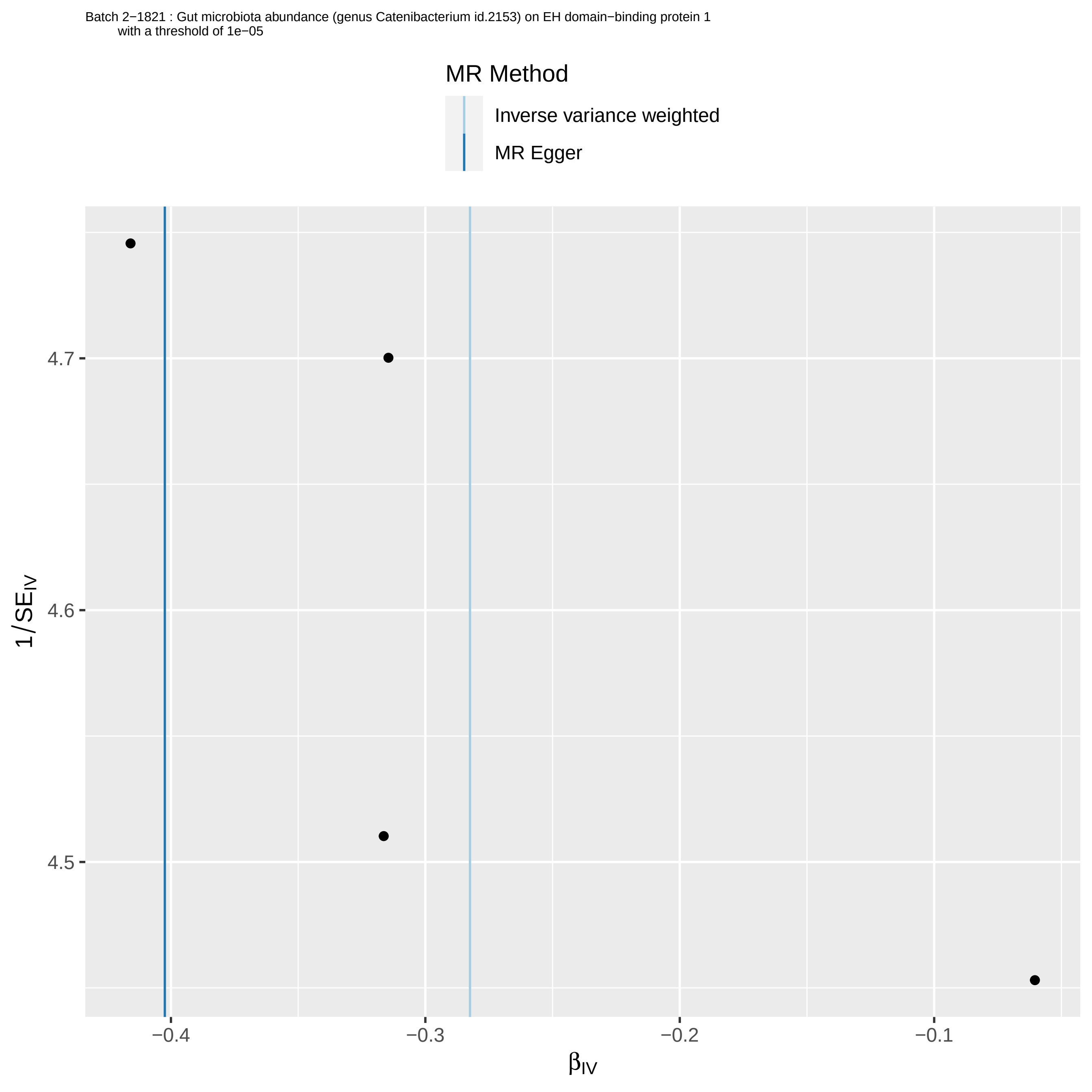
Batch 2−1821 : Gut microbiota abundance (genus Catenibacterium id.2153) on EH domain−binding protein 1
with a threshold of 1e−05
MR Method
Inverse variance weighted
MR Egger
4.7
4.6
4.5
1/SE_IV
−0.4
−0.3
−0.2
−0.1
β_IV

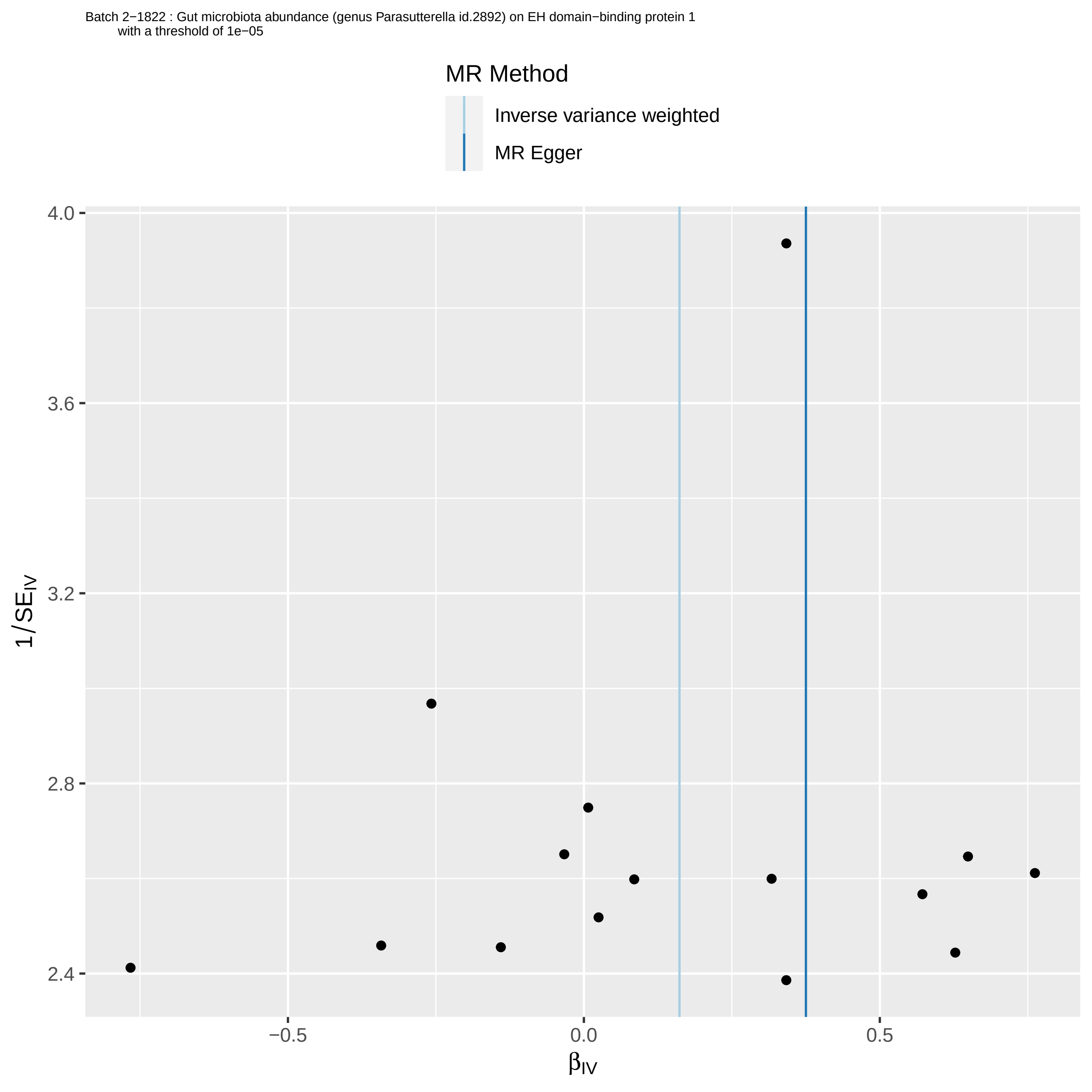

Batch 2−1822 : Gut microbiota abundance (genus Parasutterella id.2892) on EH domain−binding protein 1
with a threshold of 1e−05
MR Method
Inverse variance weighted
MR Egger
4.0
3.6
3.2
2.8
2.4
1/SE$_{IV}$
−0.5
0.0
0.5
β$_{IV}$

Batch 2−1823 : Gut microbiota abundance (genus Catenibacterium id.2153) on EH domain−containing protein 4
with a threshold of 1e−05

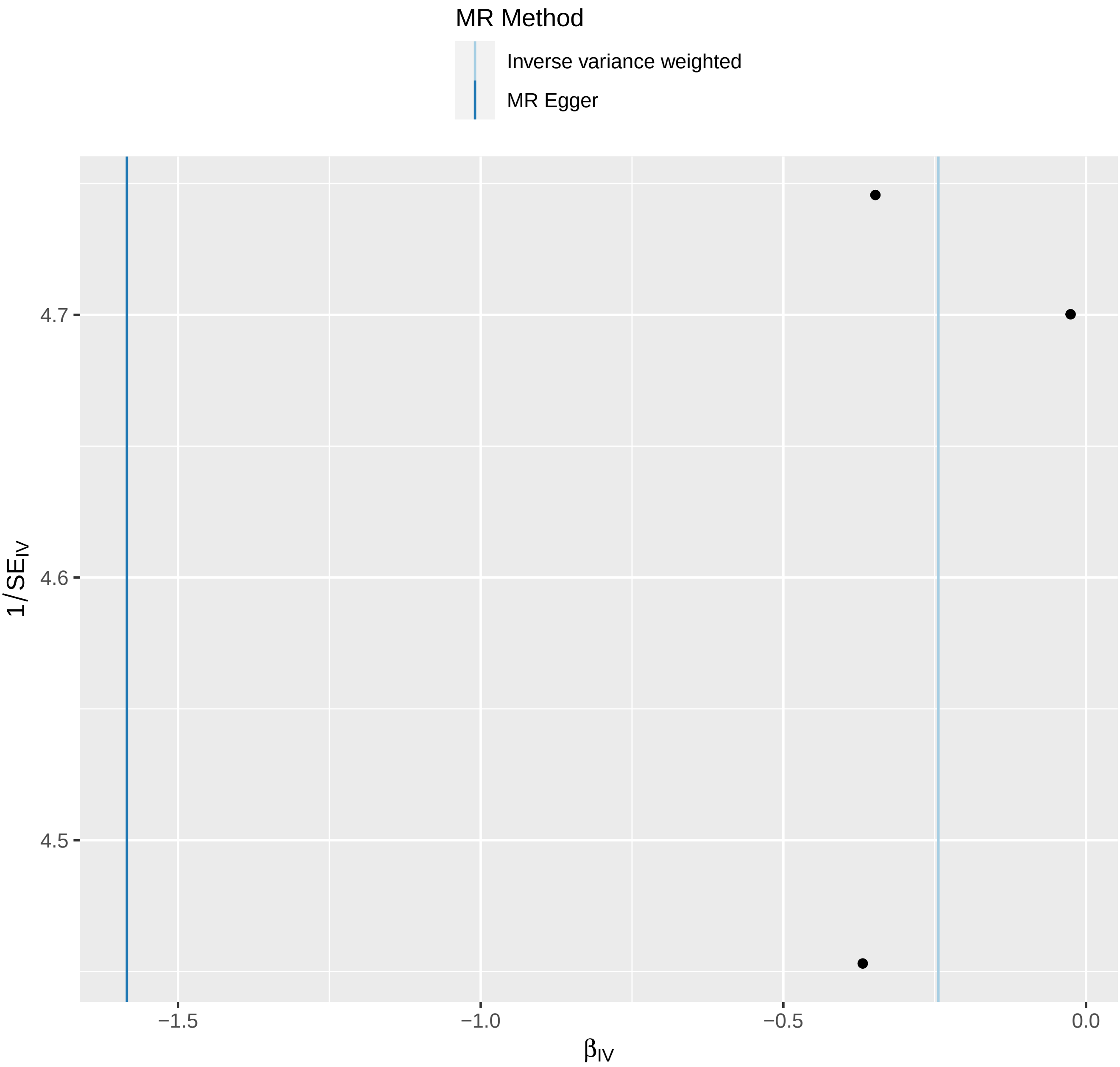

Batch 2−1824 : Gut microbiota abundance (genus Parasutterella id.2892) on EH domain−containing protein 4
with a threshold of 1e−05

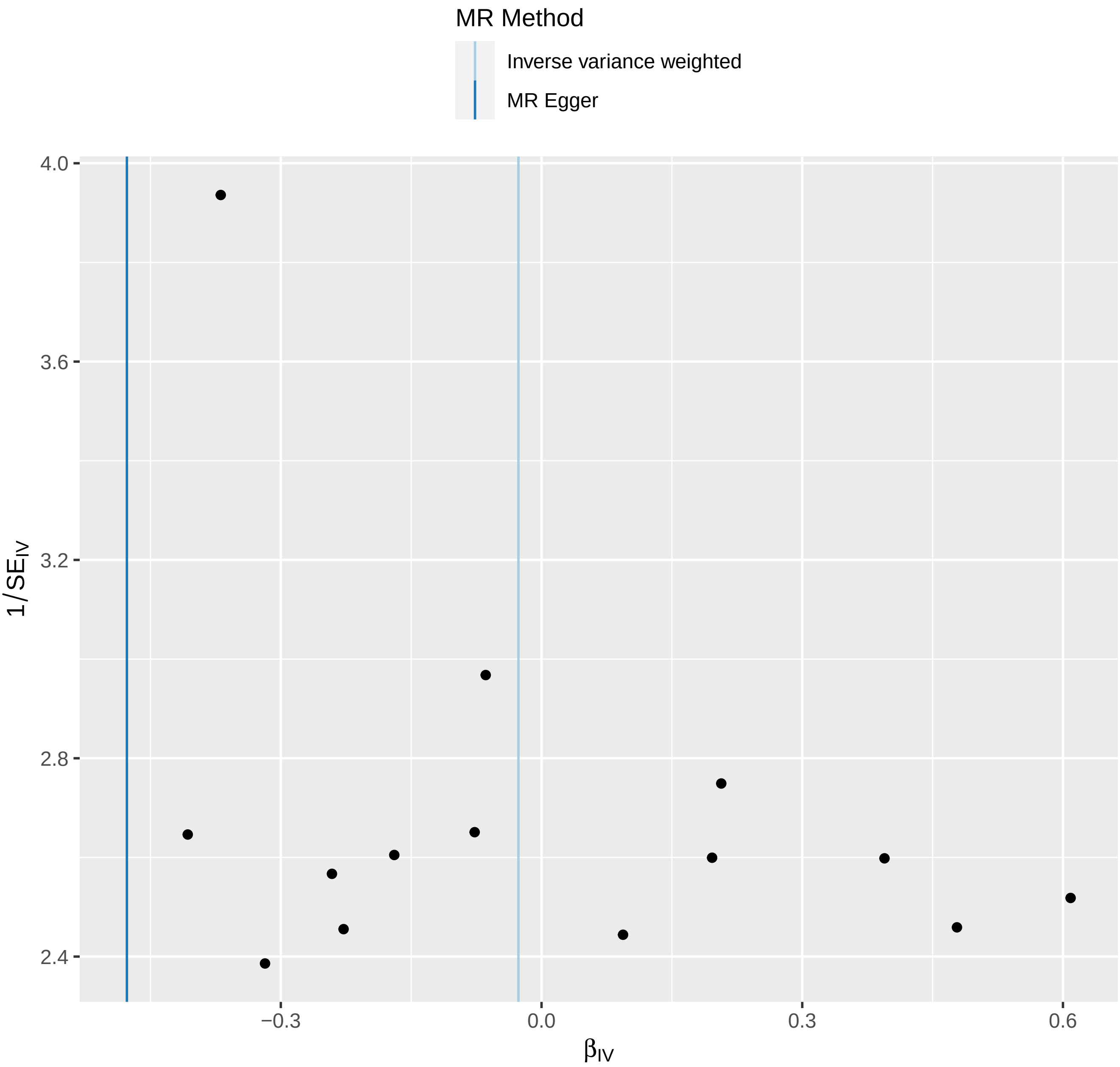

Batch 2−1825 : Gut microbiota abundance (genus Catenibacterium id.2153) on ETS homologous factor
with a threshold of 1e−05

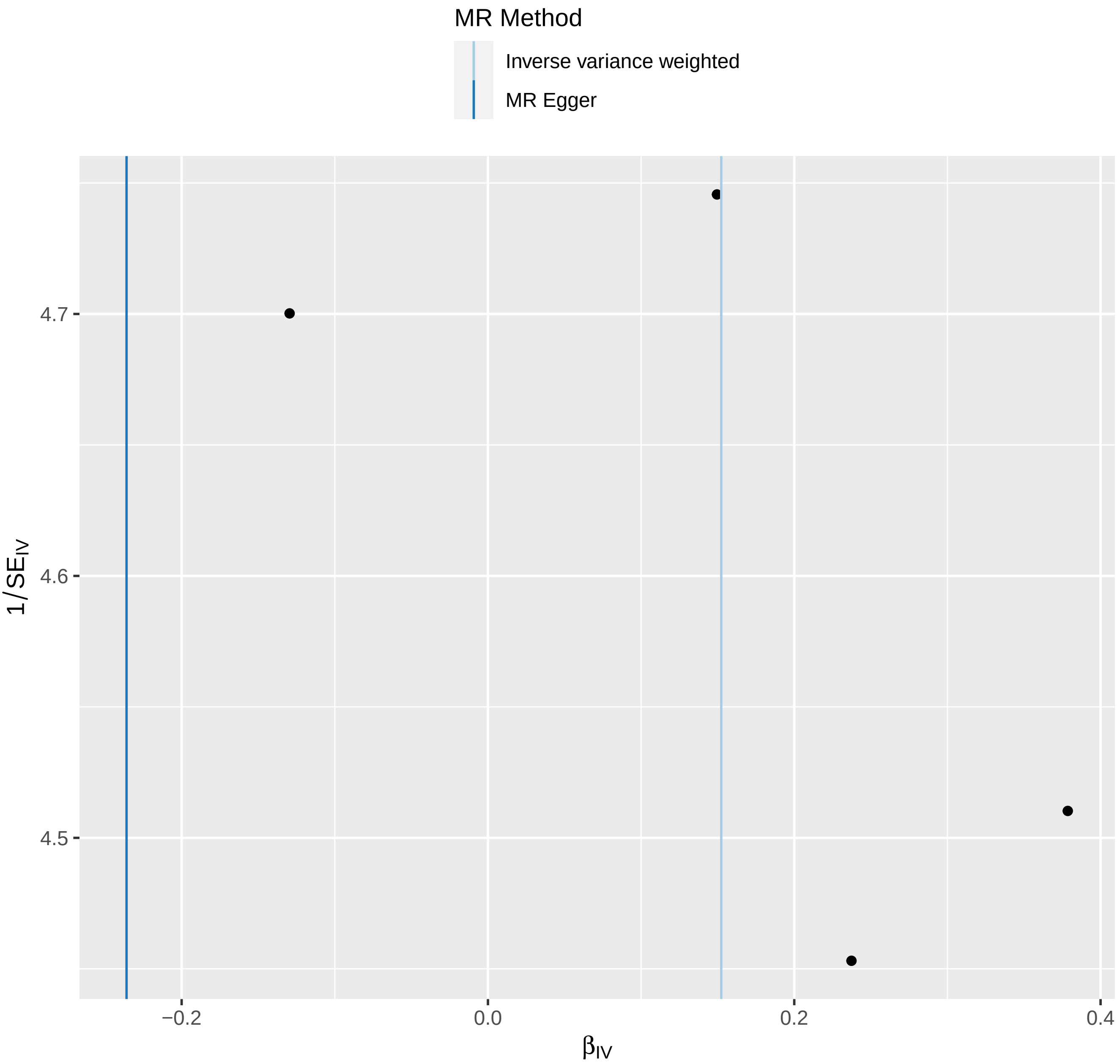

Batch 2−1826 : Gut microbiota abundance (genus Parasutterella id.2892) on ETS homologous factor
with a threshold of 1e−05

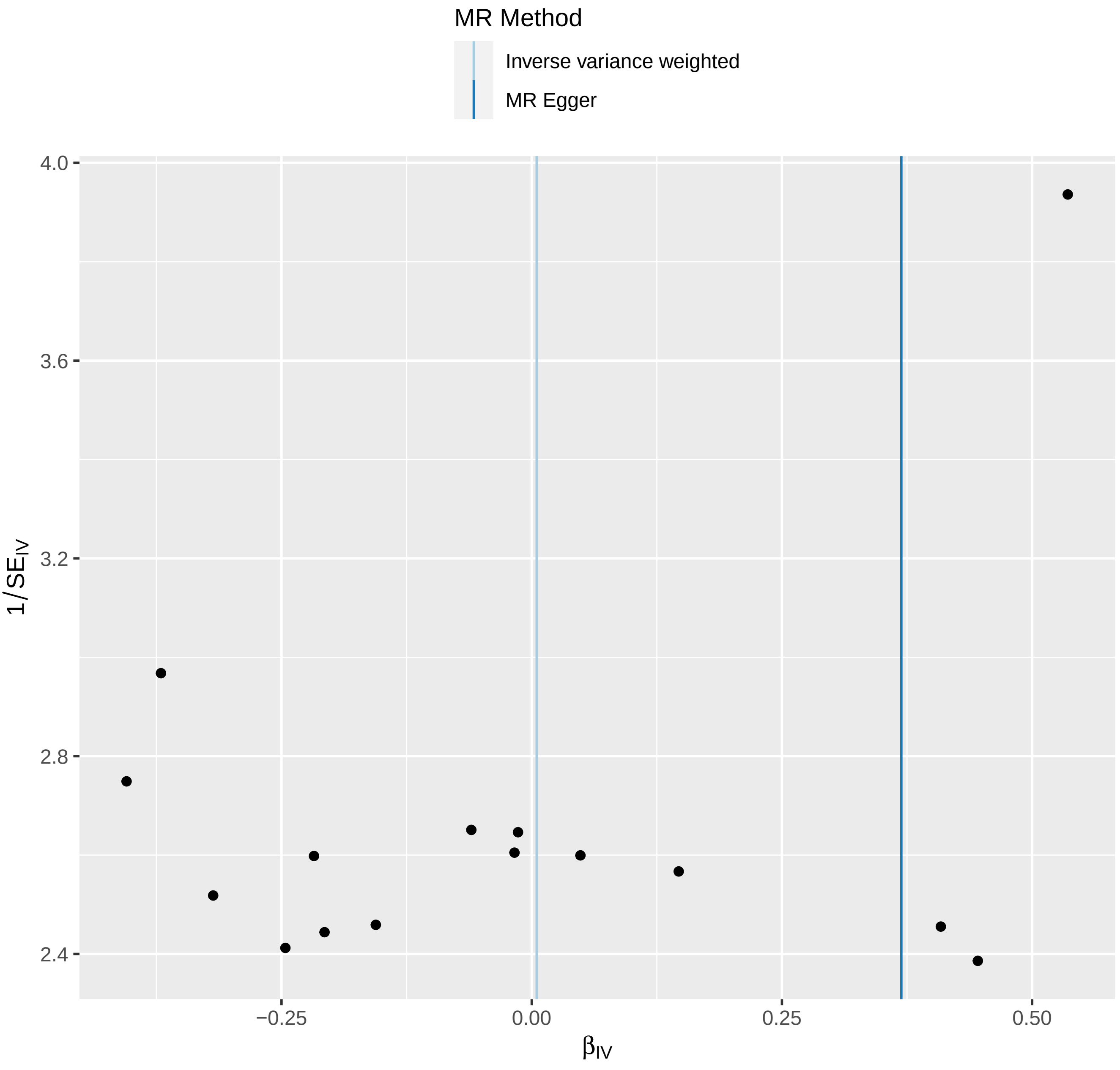

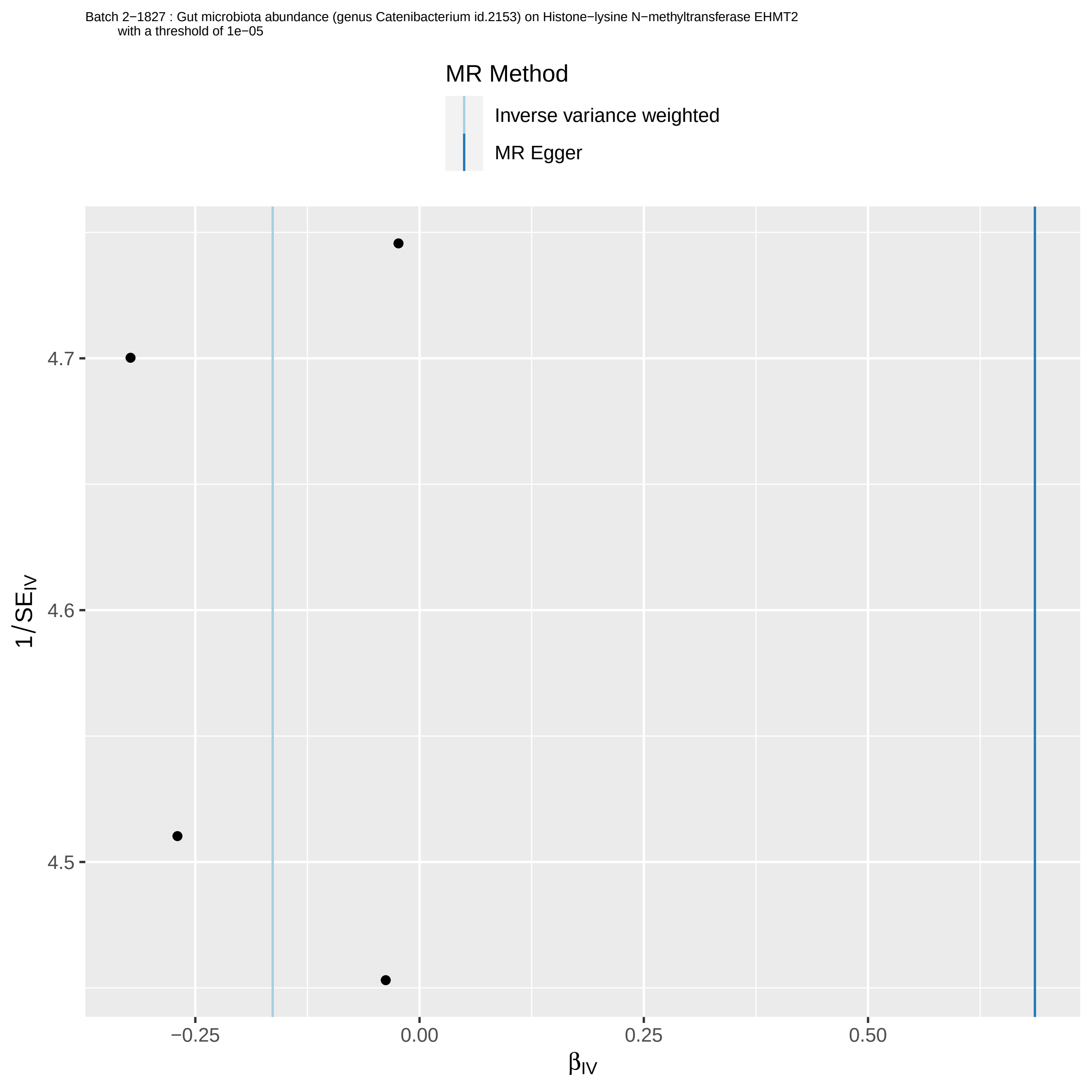
Batch 2−1827 : Gut microbiota abundance (genus Catenibacterium id.2153) on Histone−lysine N−methyltransferase EHMT2
with a threshold of 1e−05
MR Method
Inverse variance weighted
MR Egger
4.7
4.6
4.5
$1/SE_{IV}$
−0.25
0.00
0.25
0.50
β_{IV}

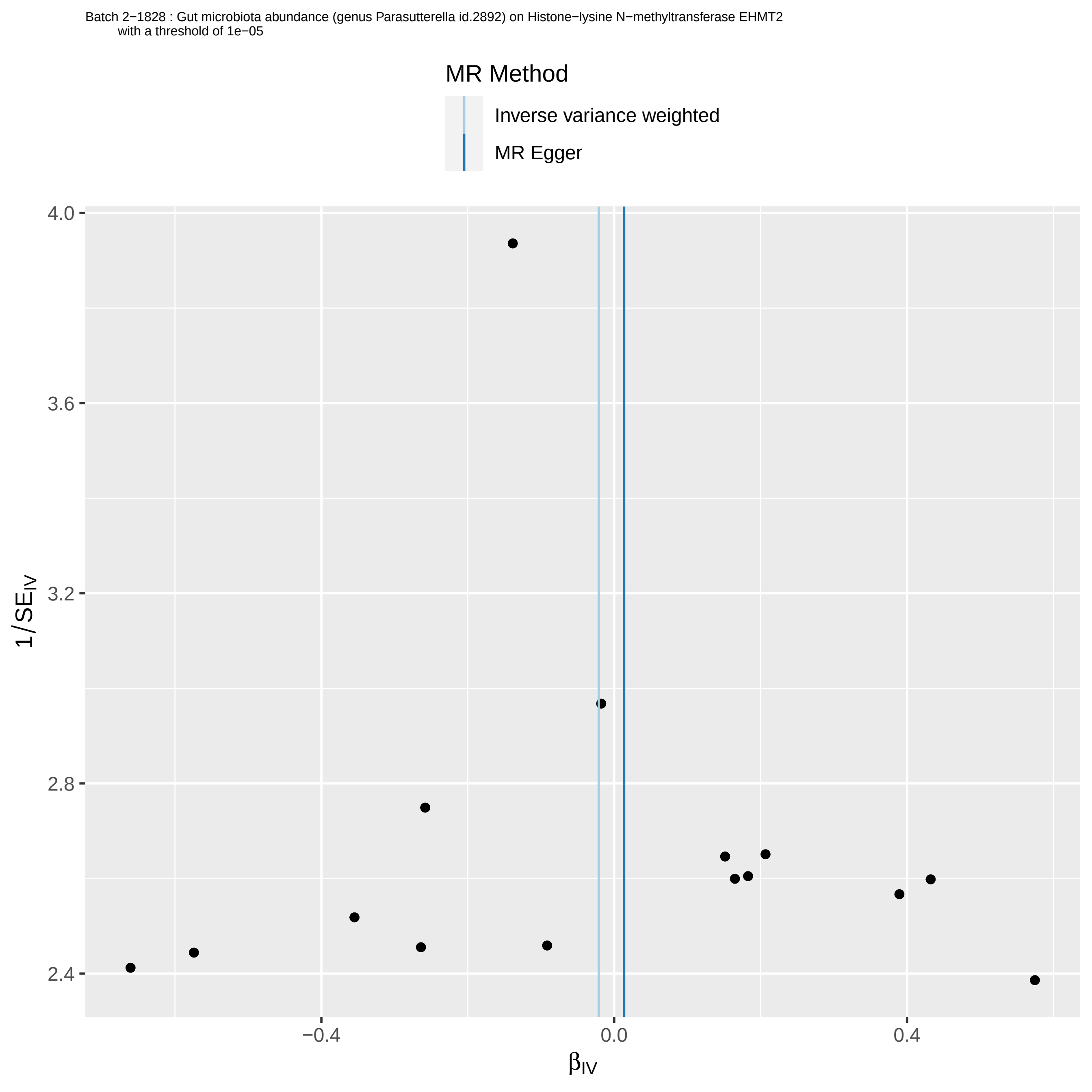

Batch 2−1828 : Gut microbiota abundance (genus Parasutterella id.2892) on Histone−lysine N−methyltransferase EHMT2
with a threshold of 1e−05
MR Method
Inverse variance weighted
MR Egger
4.0
3.6
3.2
2.8
2.4
1/SE_IV
−0.4
0.0
0.4
β_IV

Batch 2−1829 : Gut microbiota abundance (genus Catenibacterium id.2153) on EP300−interacting inhibitor of differentiation 3
with a threshold of 1e−05

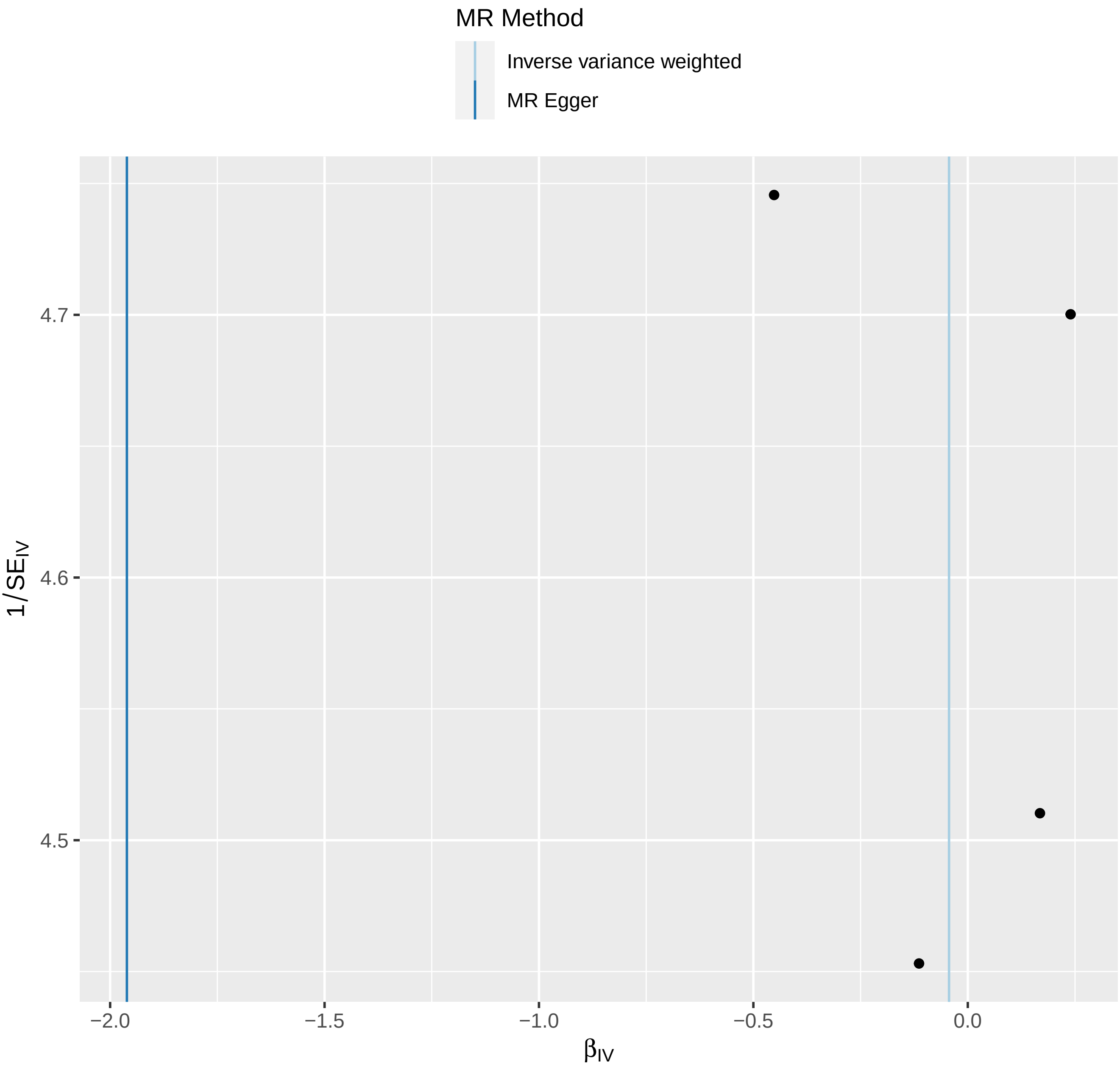

Batch 2−1830 : Gut microbiota abundance (genus Parasutterella id.2892) on EP300−interacting inhibitor of differentiation 3
with a threshold of 1e−05

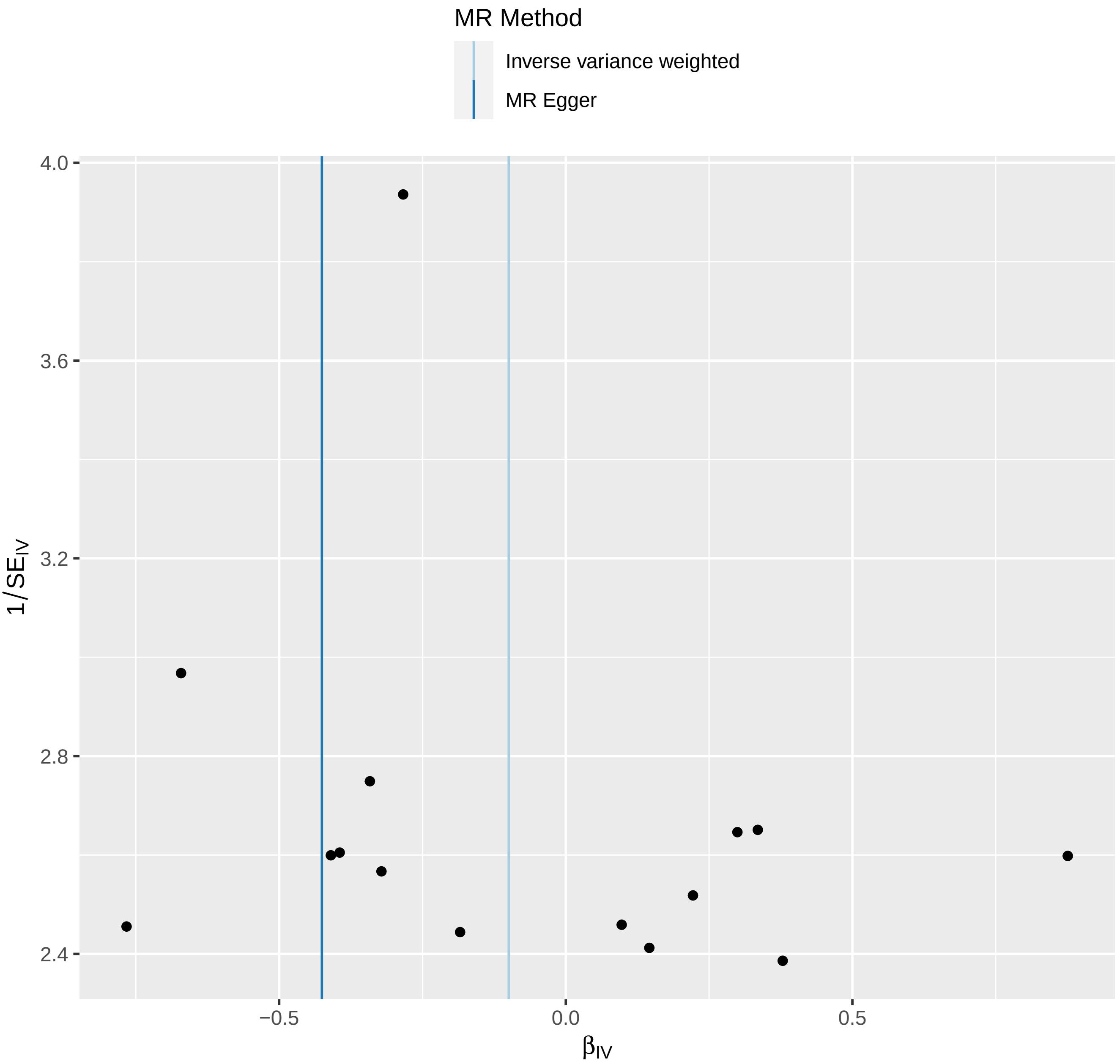

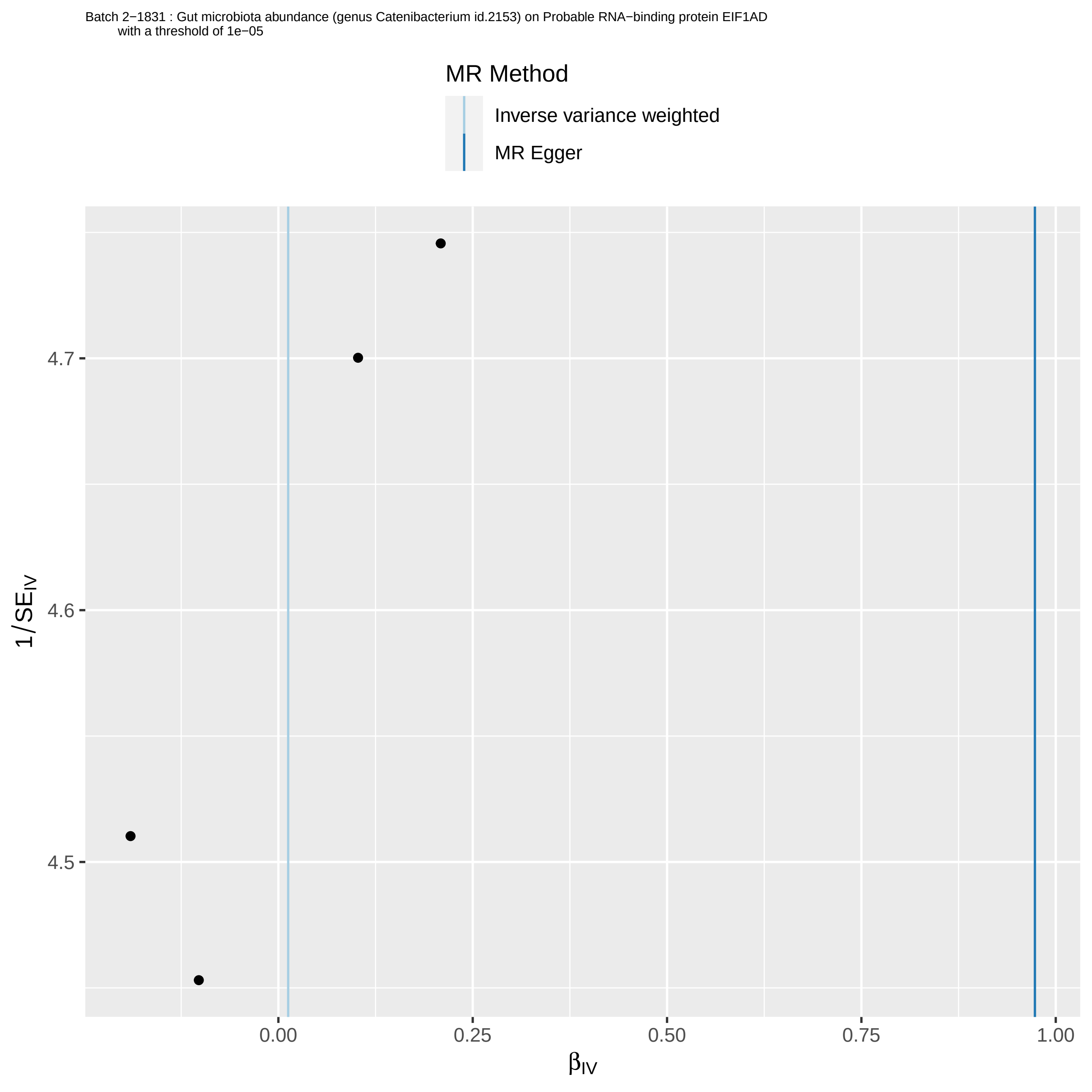
Batch 2−1831 : Gut microbiota abundance (genus Catenibacterium id.2153) on Probable RNA−binding protein EIF1AD
with a threshold of 1e−05
MR Method
Inverse variance weighted
MR Egger
4.7
4.6
4.5
1/SE_IV
0.00
0.25
0.50
0.75
1.00
β_IV

Batch 2−1832 : Gut microbiota abundance (genus Parasutterella id.2892) on Probable RNA−binding protein EIF1AD
with a threshold of 1e−05

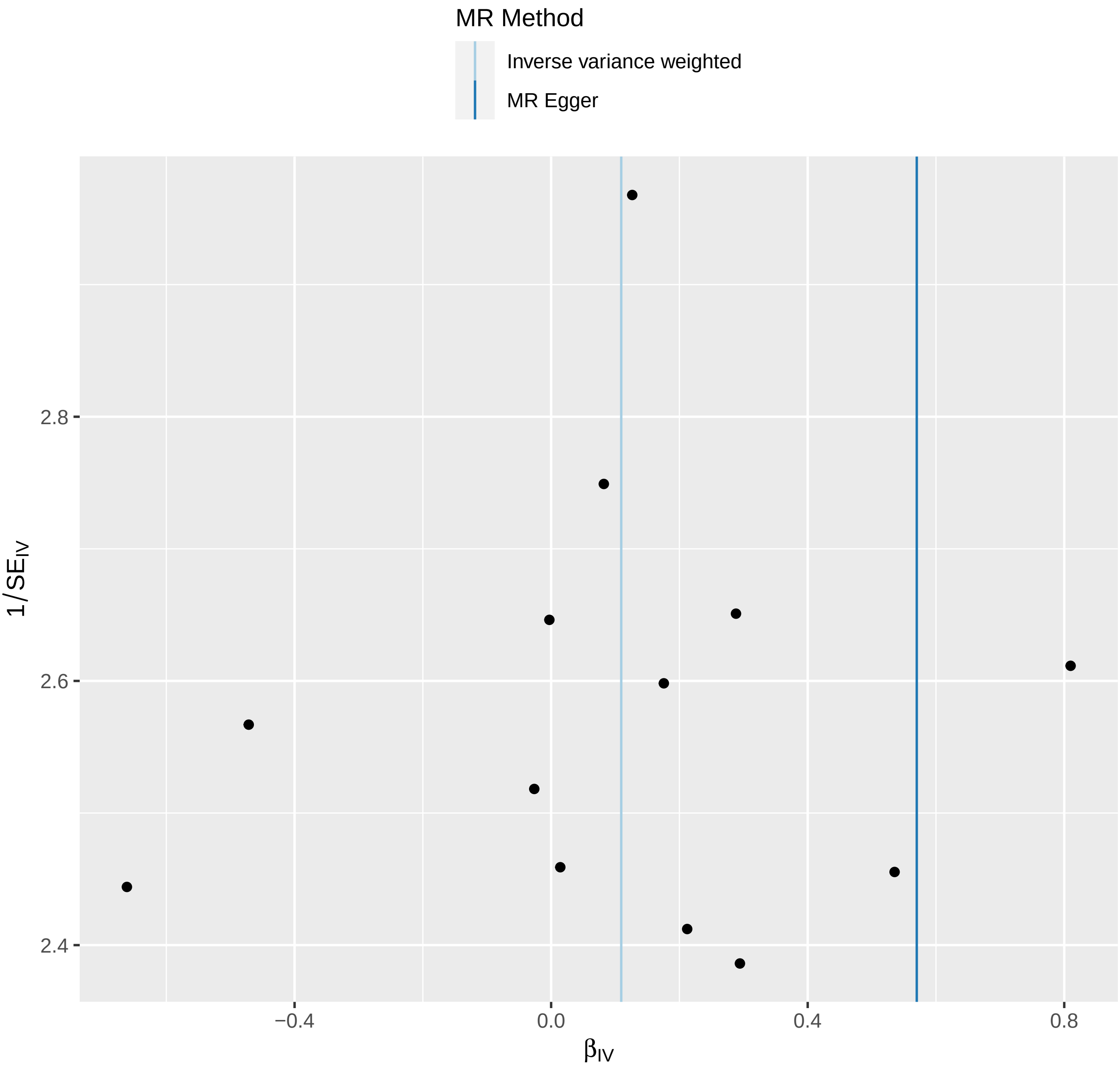

Batch 2−1833 : Gut microbiota abundance (genus Catenibacterium id.2153) on Eukaryotic translation initiation factor 1A, X−chromosomal with a threshold of 1e−05

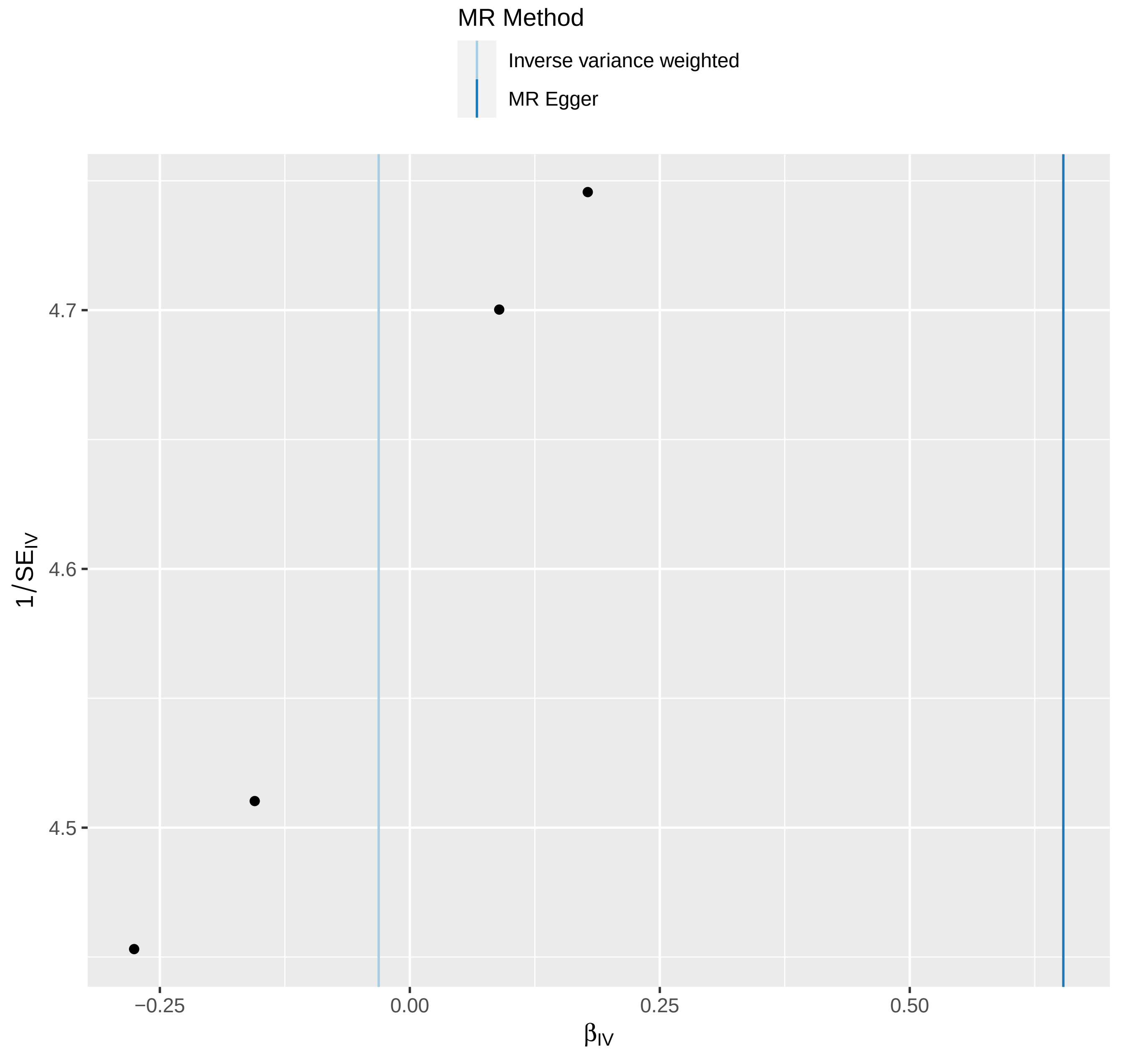

Batch 2−1834 : Gut microbiota abundance (genus Parasutterella id.2892) on Eukaryotic translation initiation factor 1A, X−chromosomal
with a threshold of 1e−05

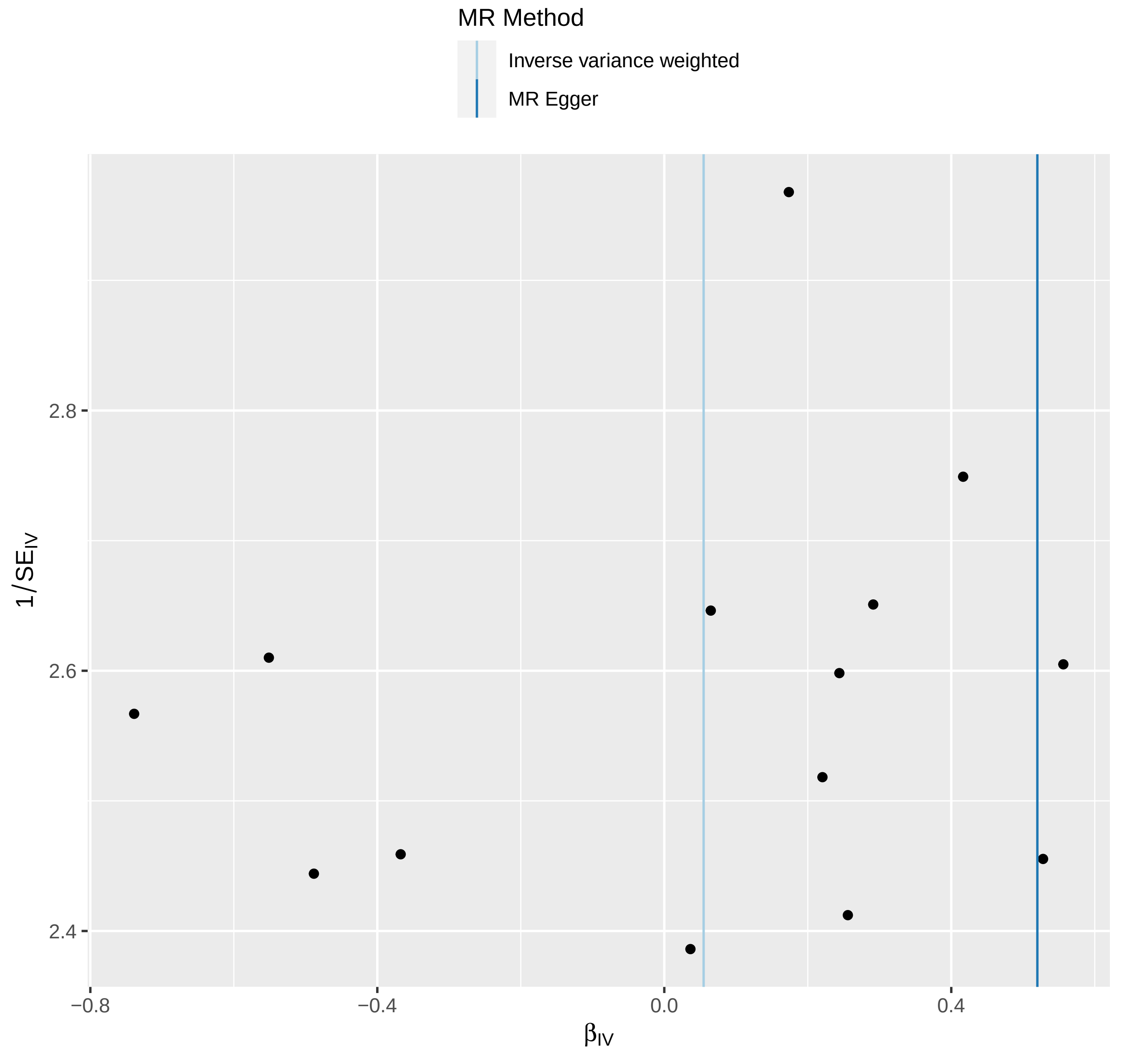

Batch 2−1835 : Gut microbiota abundance (genus Catenibacterium id.2153) on Translation initiation factor eIF−2B subunit alpha
with a threshold of 1e−05

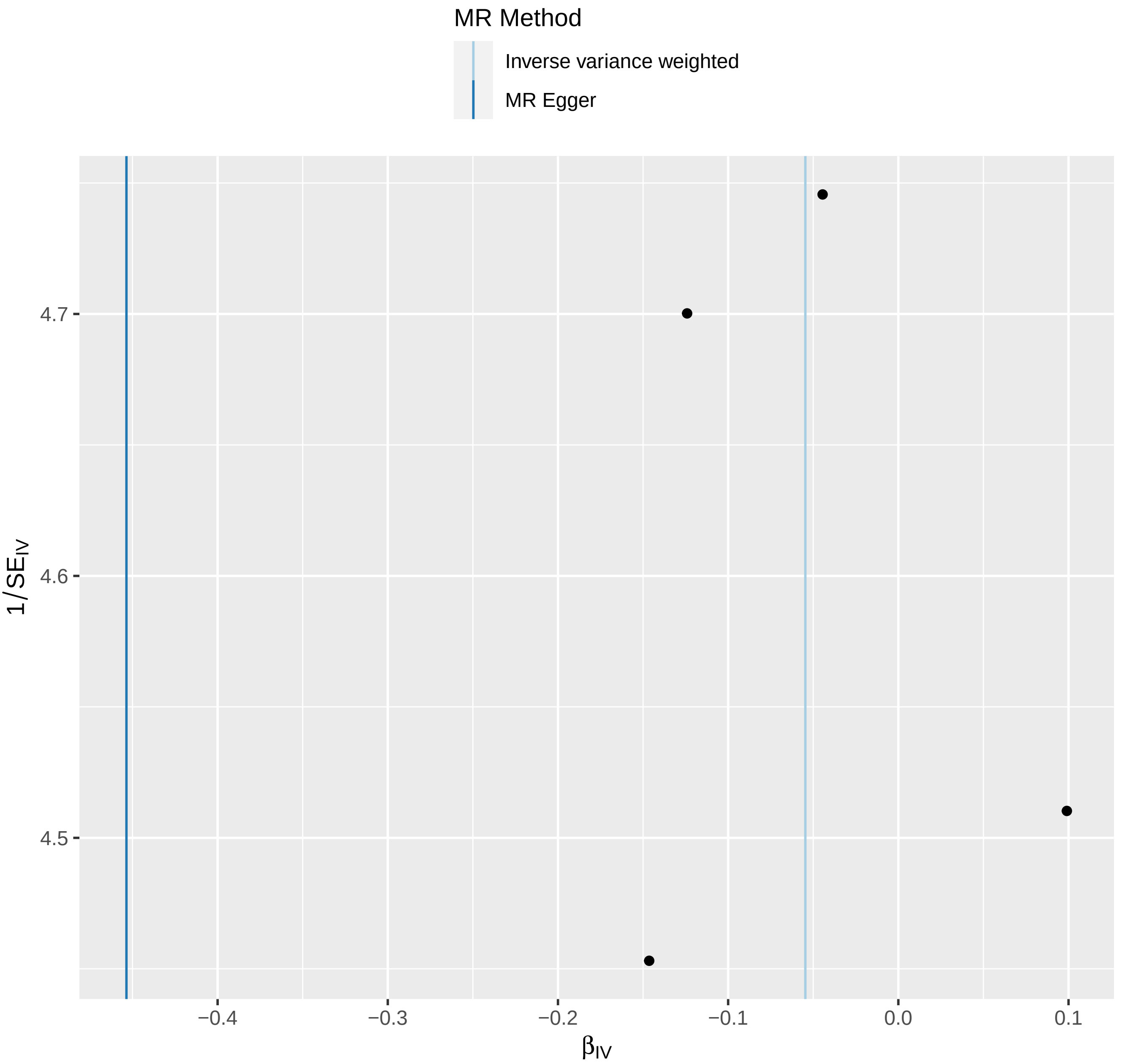

Batch 2−1836 : Gut microbiota abundance (genus Parasutterella id.2892) on Translation initiation factor eIF−2B subunit alpha
with a threshold of 1e−05

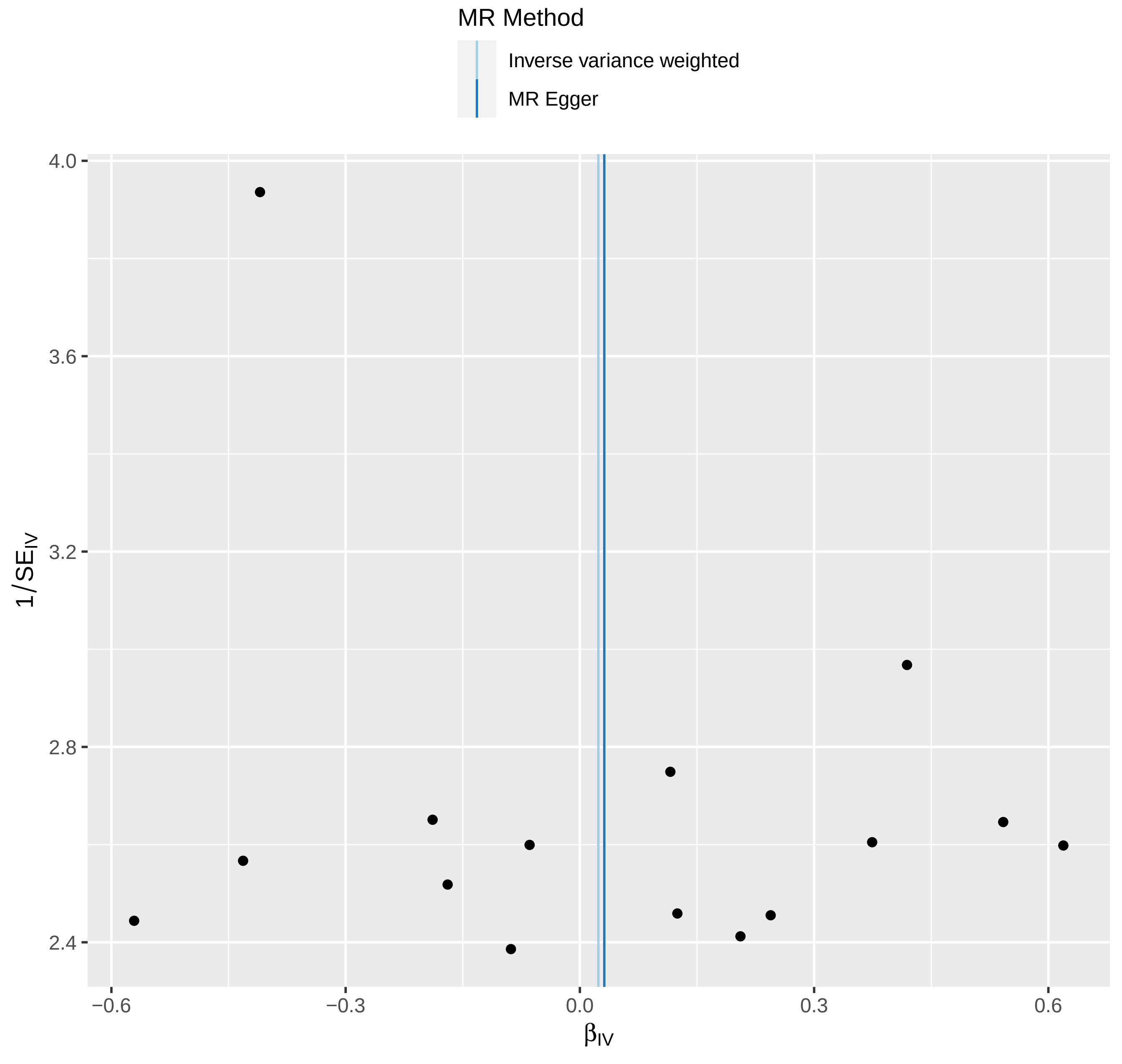

Batch 2−1837 : Gut microbiota abundance (genus Catenibacterium id.2153) on Eukaryotic translation initiation factor 3 subunit G
with a threshold of 1e−05

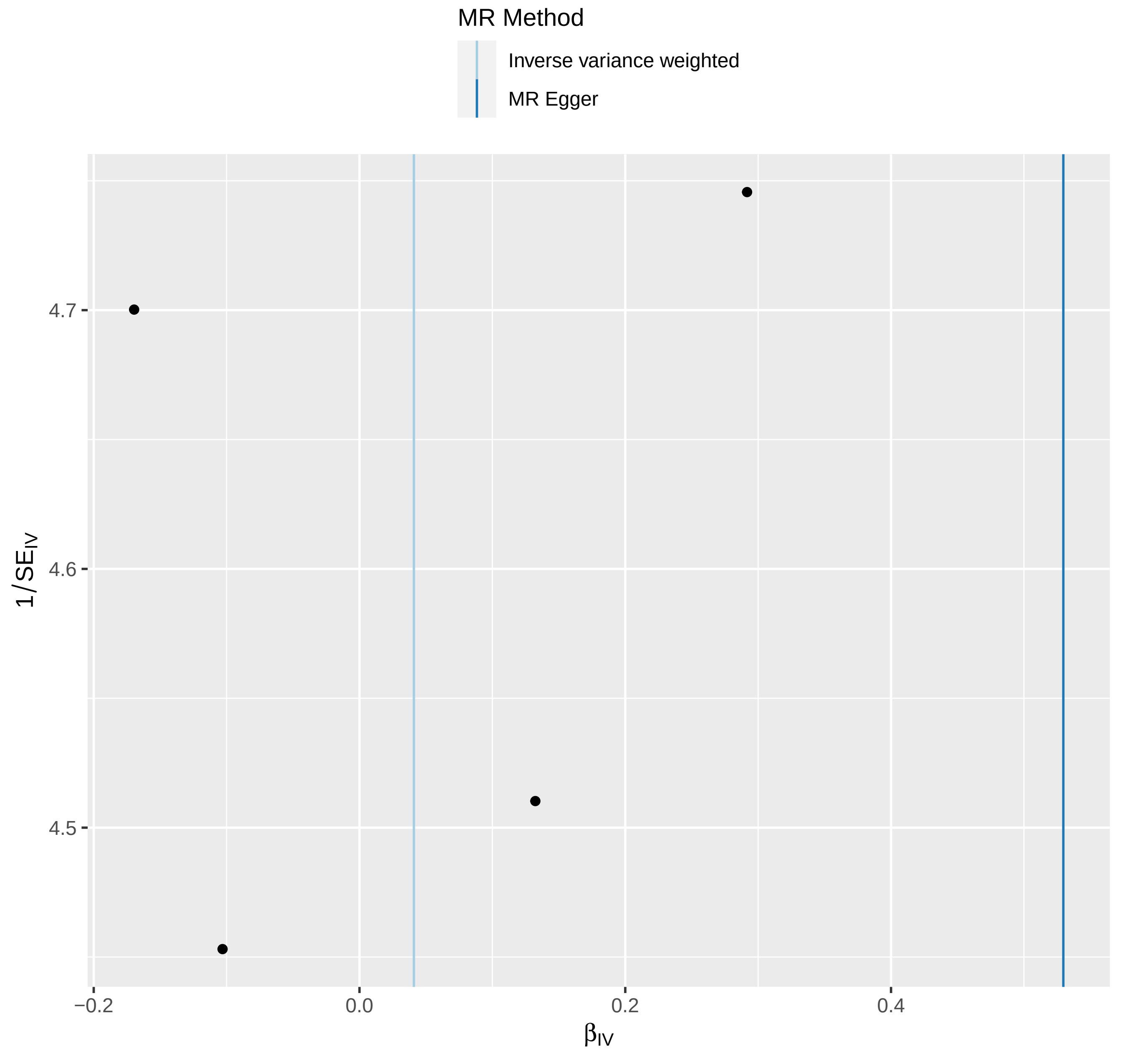

Batch 2−1838 : Gut microbiota abundance (genus Parasutterella id.2892) on Eukaryotic translation initiation factor 3 subunit G with a threshold of 1e−05

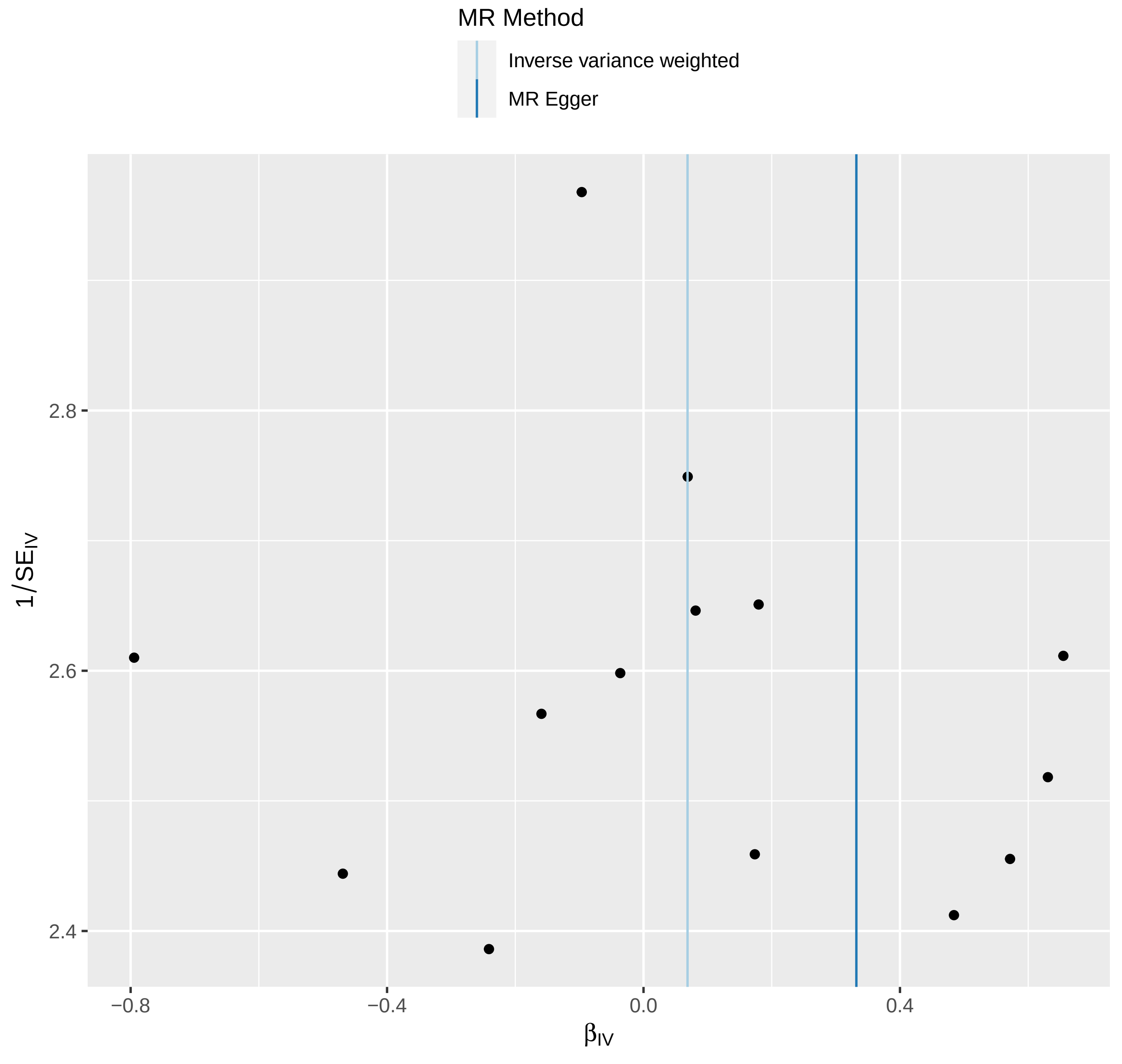

Batch 2−1839 : Gut microbiota abundance (genus Catenibacterium id.2153) on Eukaryotic translation initiation factor 3 subunit J with a threshold of 1e−05

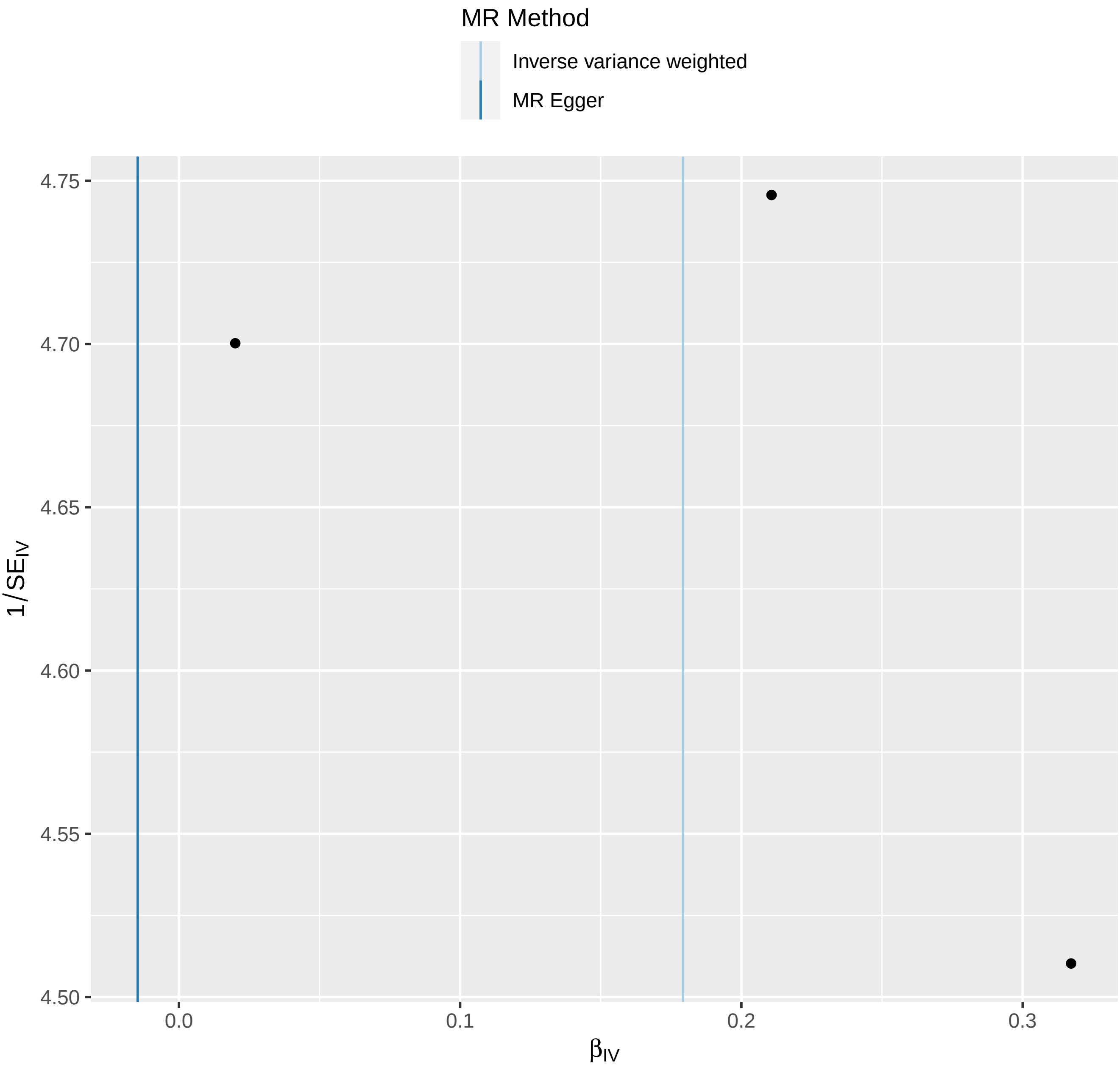

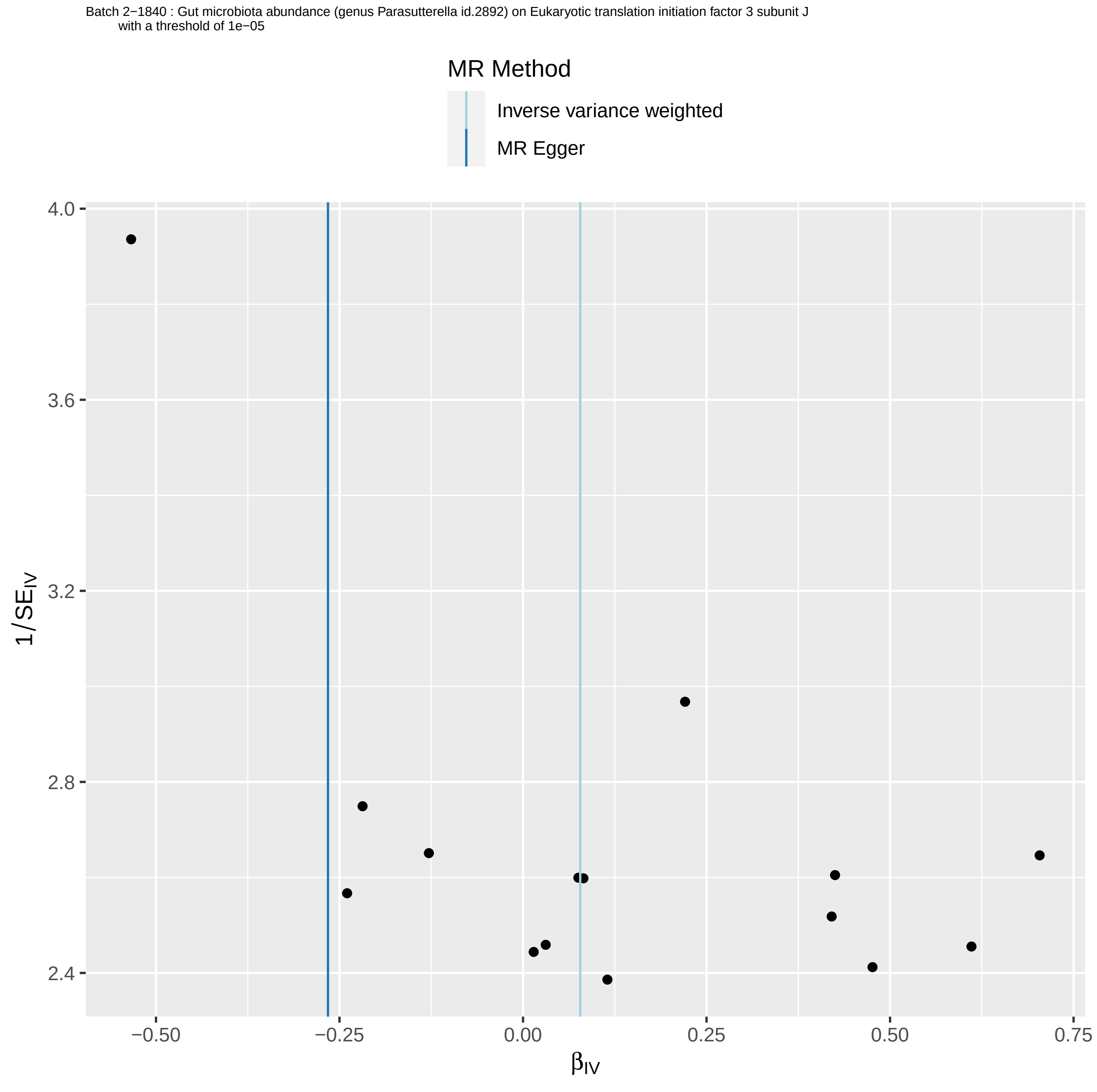

Batch 2−1840 : Gut microbiota abundance (genus Parasutterella id.2892) on Eukaryotic translation initiation factor 3 subunit J
with a threshold of 1e−05
MR Method
Inverse variance weighted
MR Egger
4.0
3.6
3.2
2.8
2.4
1/SE$_{IV}$
−0.50
−0.25
0.00
0.25
0.50
0.75
β_{IV}

Batch 2−1841 : Gut microbiota abundance (genus Catenibacterium id.2153) on Eukaryotic initiation factor 4A−III
with a threshold of 1e−05

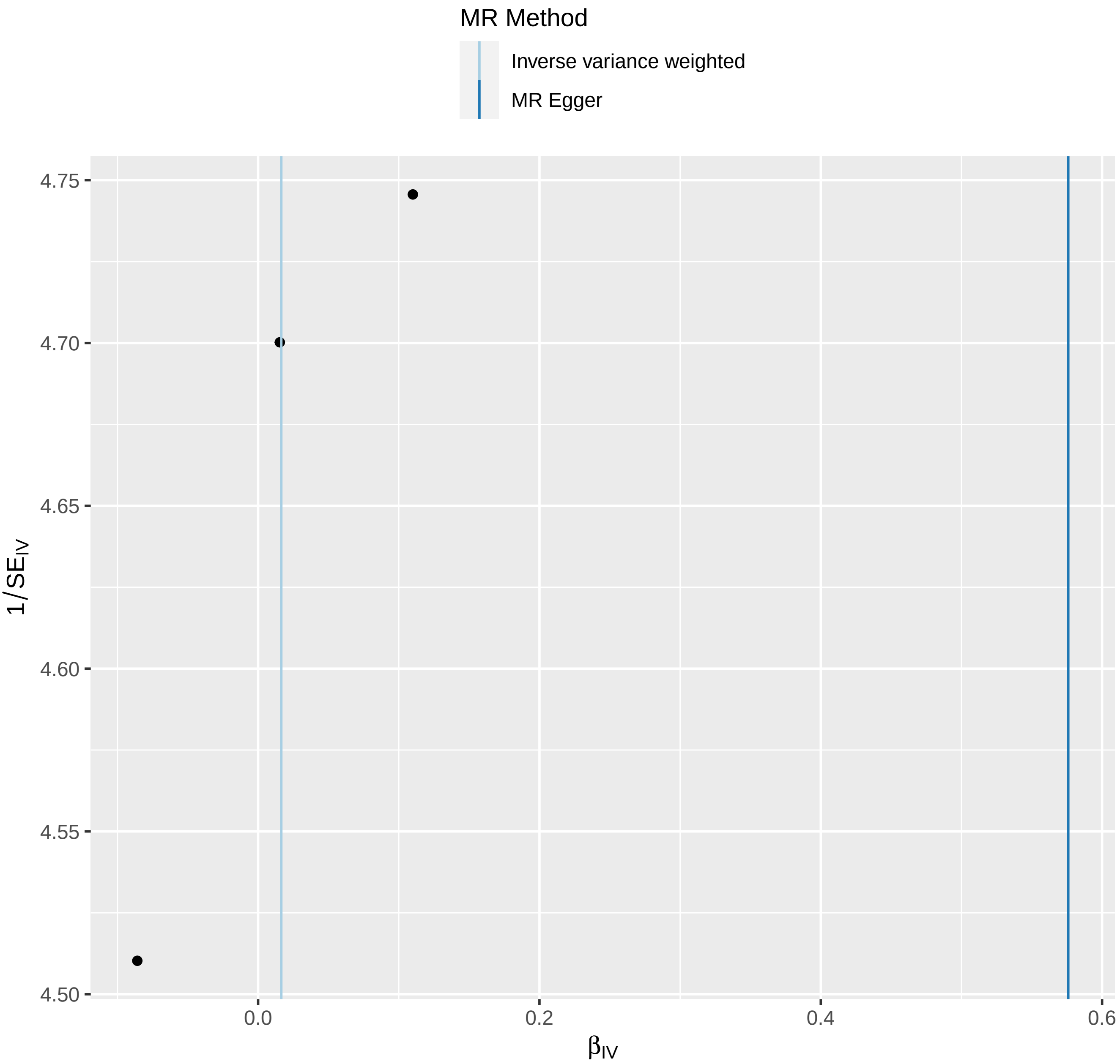

Batch 2−1842 : Gut microbiota abundance (genus Parasutterella id.2892) on Eukaryotic initiation factor 4A−III
with a threshold of 1e−05

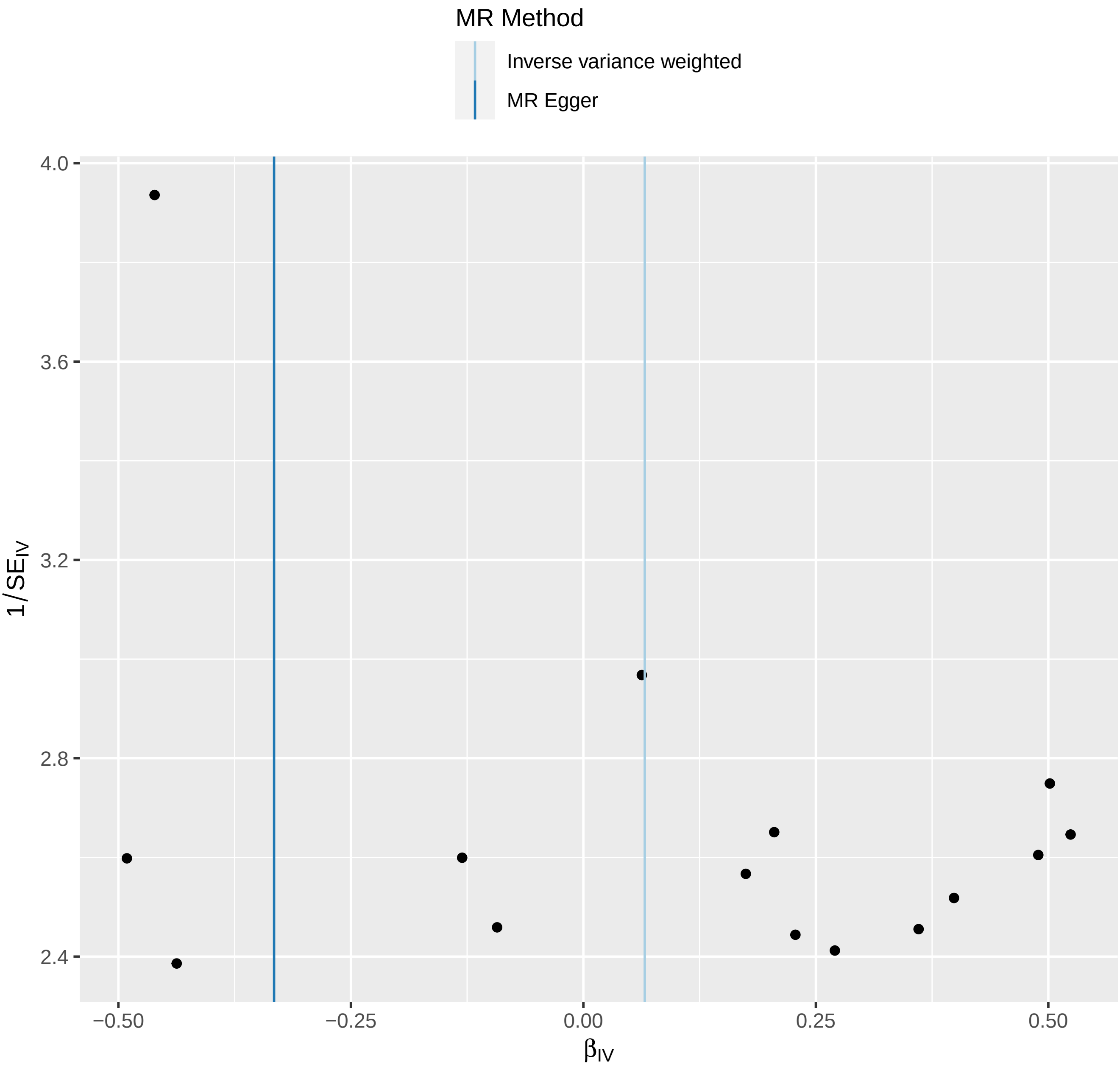

Batch 2−1843 : Gut microbiota abundance (genus Catenibacterium id.2153) on Eukaryotic translation initiation factor 4B
with a threshold of 1e−05

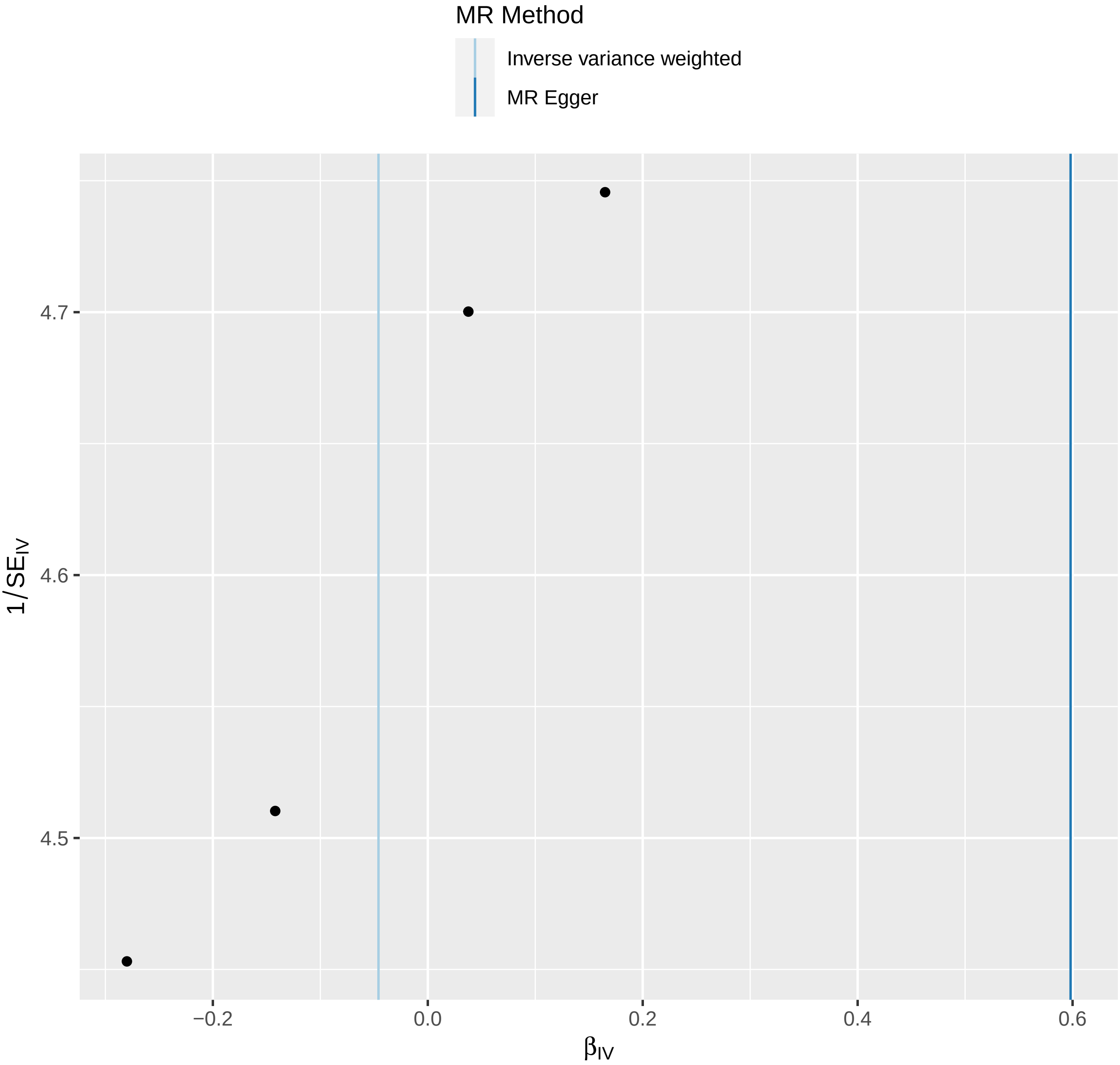

Batch 2−1844 : Gut microbiota abundance (genus Parasutterella id.2892) on Eukaryotic translation initiation factor 4B with a threshold of 1e−05

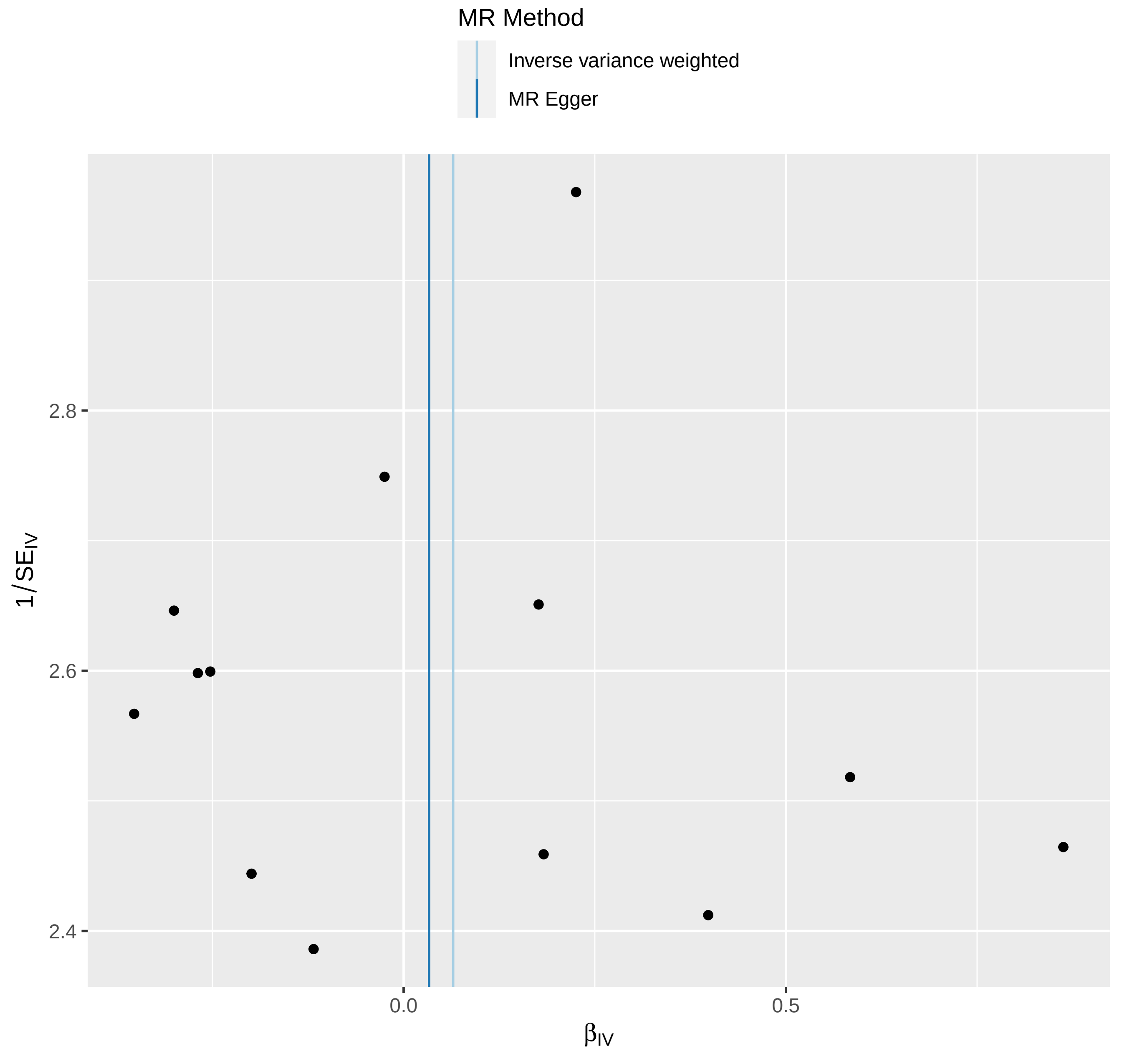

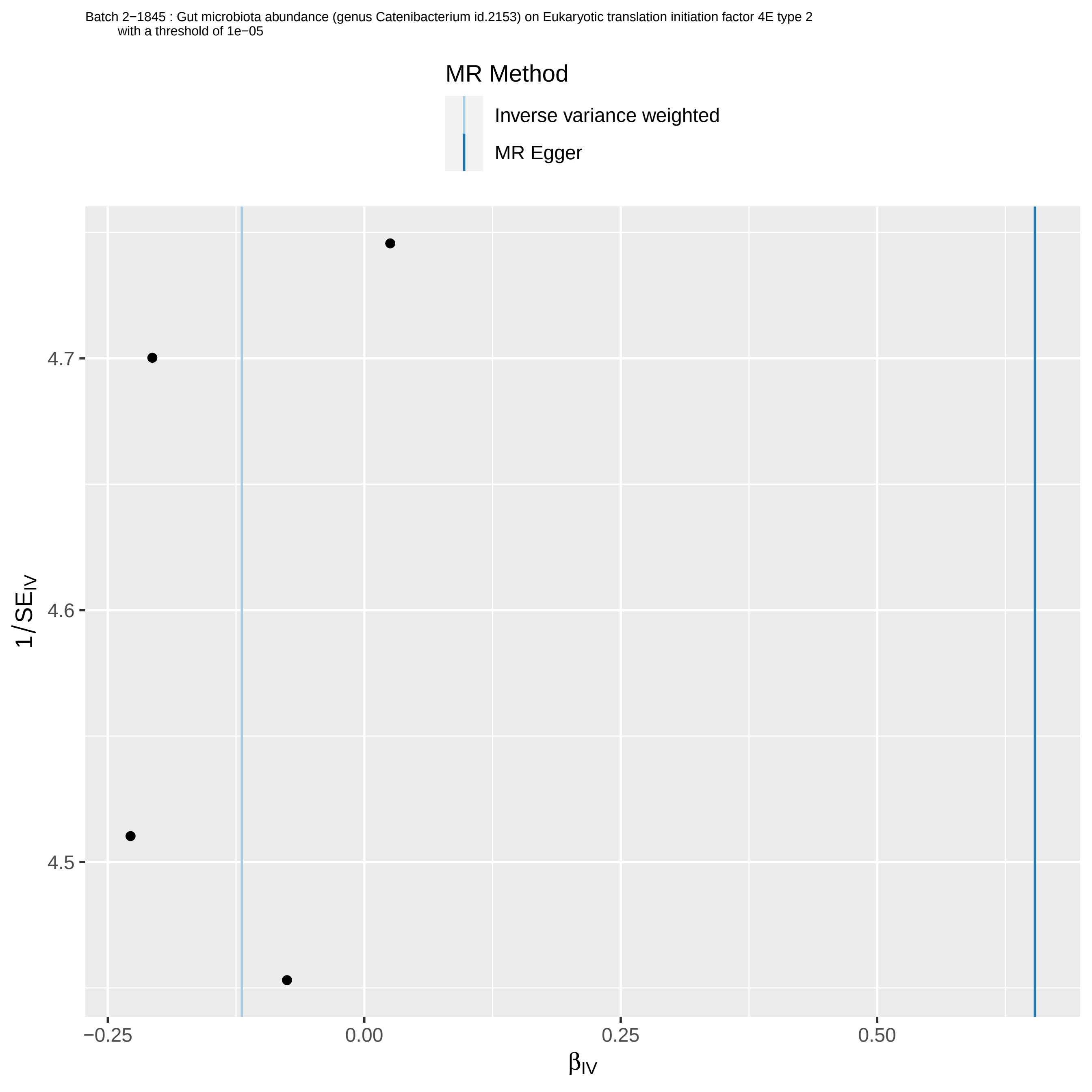

Batch 2−1845 : Gut microbiota abundance (genus Catenibacterium id.2153) on Eukaryotic translation initiation factor 4E type 2
with a threshold of 1e−05
MR Method
Inverse variance weighted
MR Egger
4.7
4.6
4.5
1/SE_IV
−0.25
0.00
0.25
0.50
β_IV

Batch 2−1846 : Gut microbiota abundance (genus Parasutterella id.2892) on Eukaryotic translation initiation factor 4E type 2
with a threshold of 1e−05

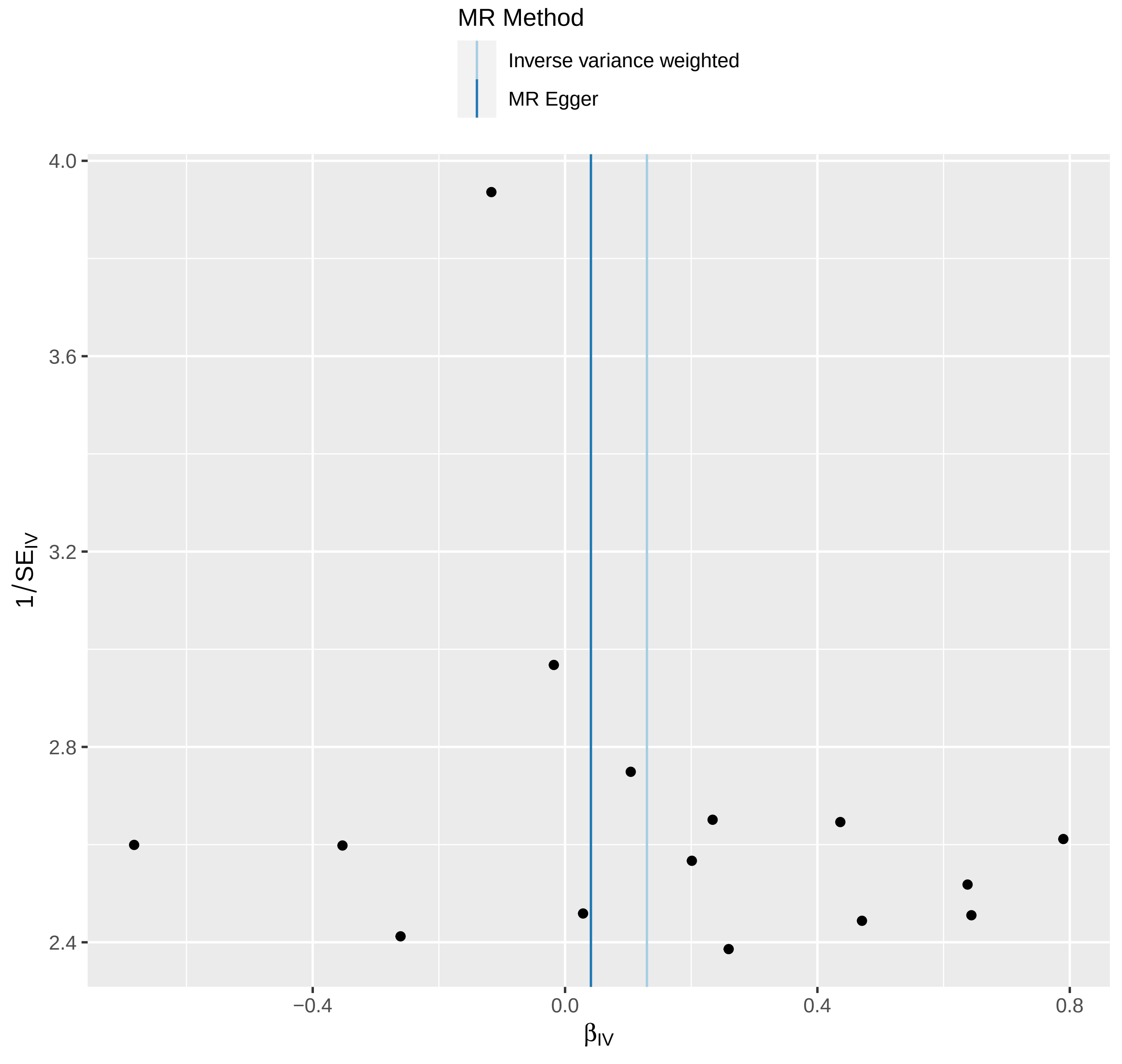

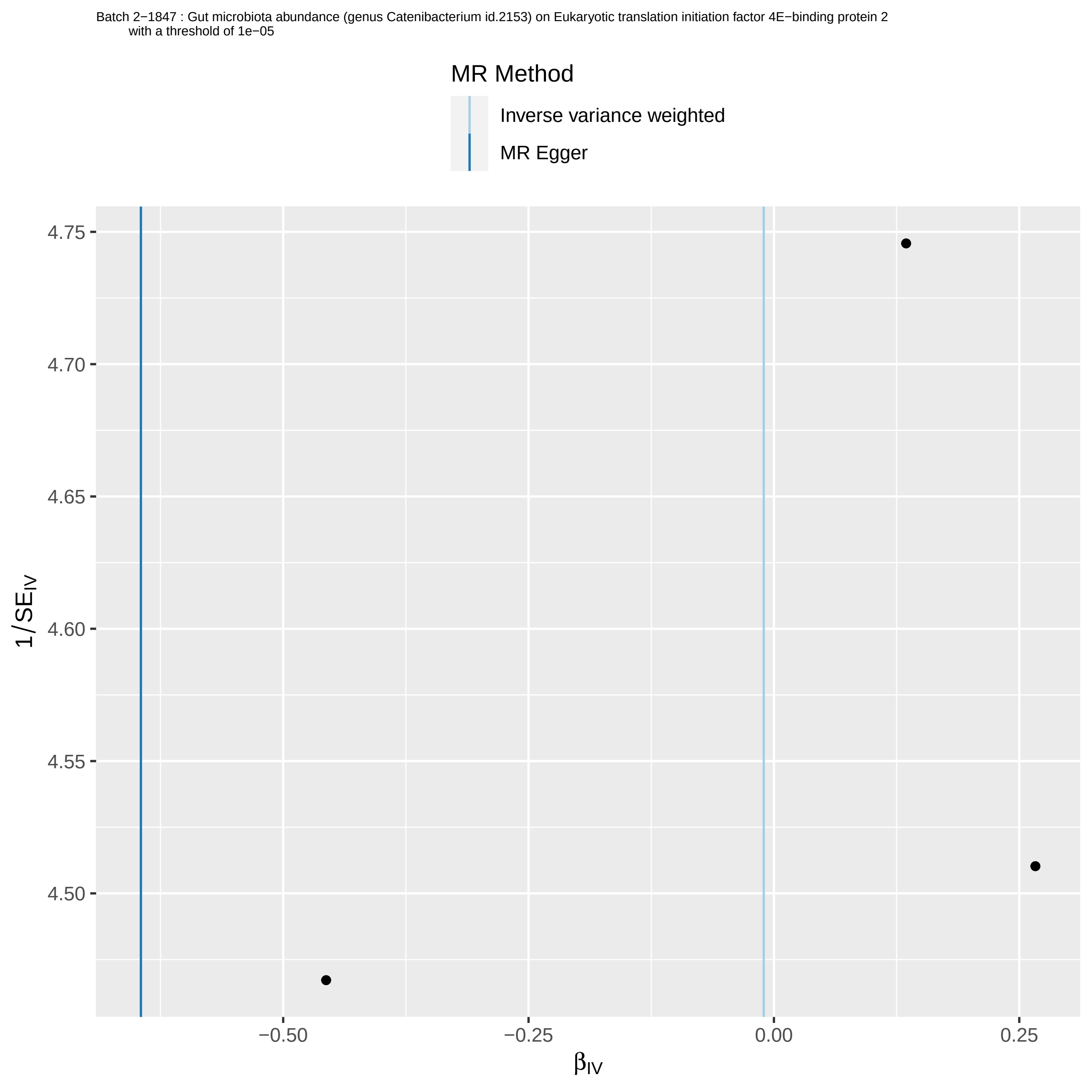

Batch 2−1847 : Gut microbiota abundance (genus Catenibacterium id.2153) on Eukaryotic translation initiation factor 4E−binding protein 2
with a threshold of 1e−05
MR Method
Inverse variance weighted
MR Egger
4.75
4.70
4.65
4.60
4.55
4.50
1/SE_IV
−0.50
−0.25
0.00
0.25
β_IV

Batch 2−1848 : Gut microbiota abundance (genus Parasutterella id.2892) on Eukaryotic translation initiation factor 4E−binding protein 2 with a threshold of 1e−05

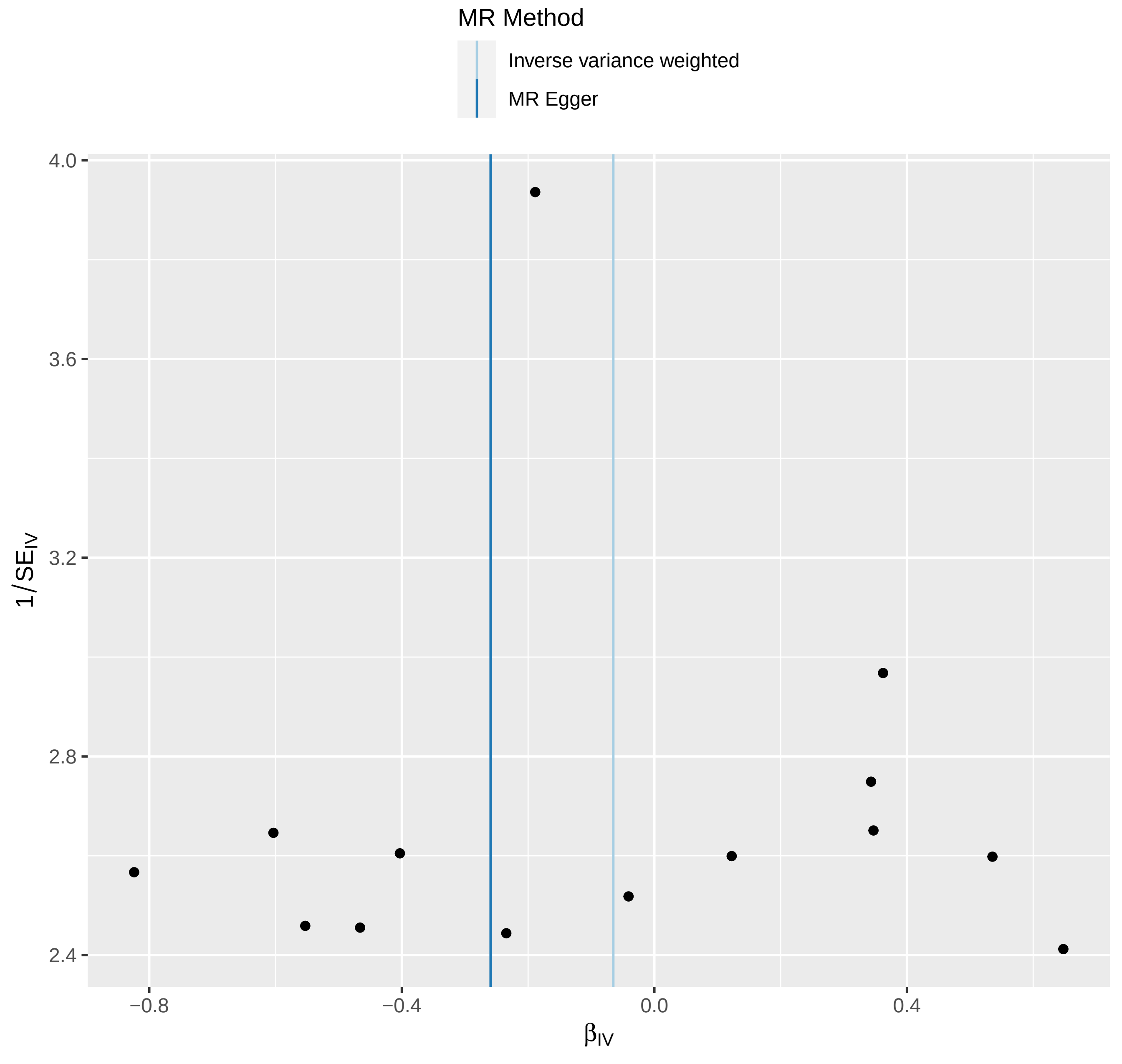

Batch 2−1849 : Gut microbiota abundance (genus Catenibacterium id.2153) on Eukaryotic translation initiation factor 4 gamma 3
with a threshold of 1e−05

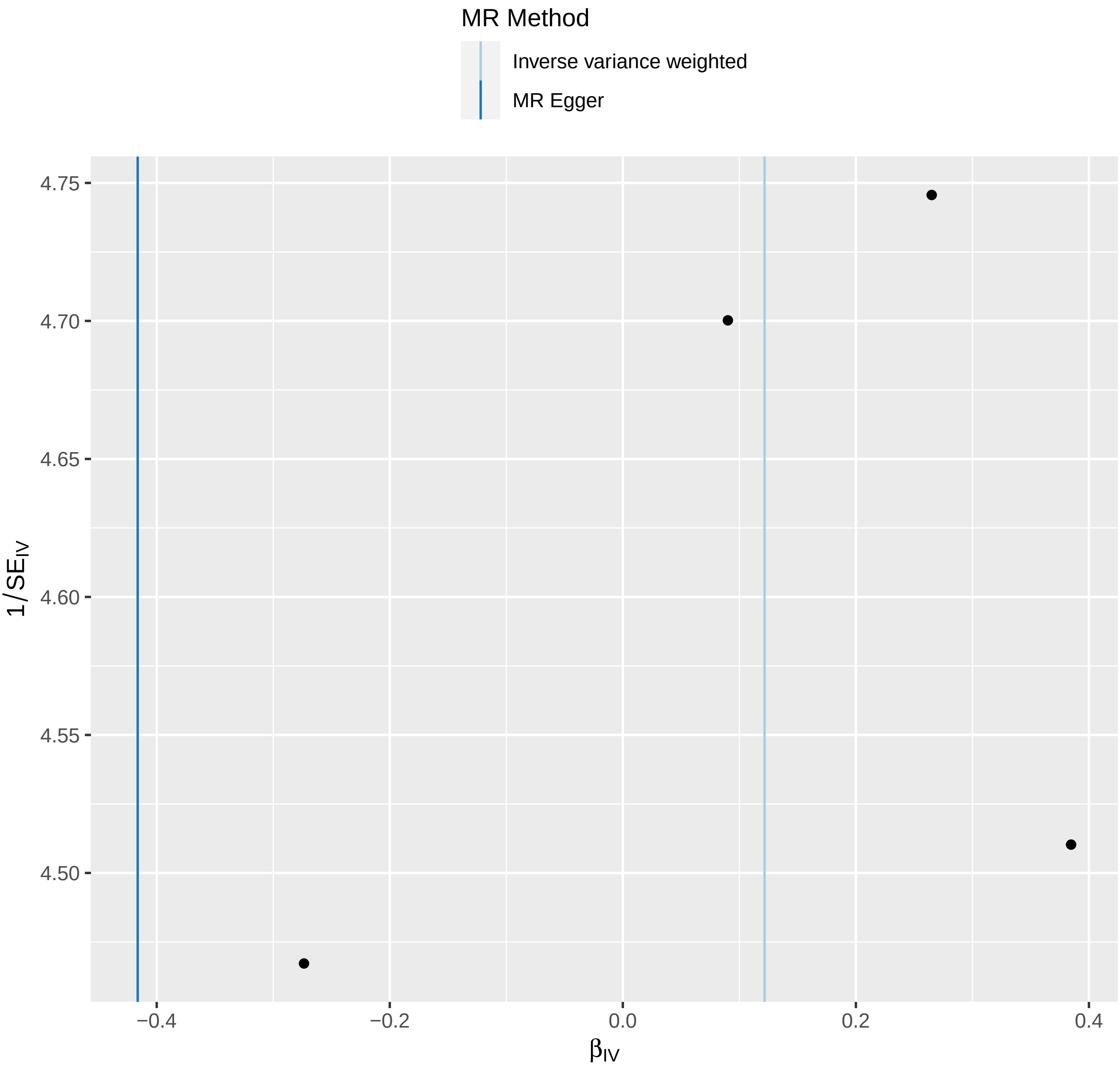

Batch 2−1850 : Gut microbiota abundance (genus Parasutterella id.2892) on Eukaryotic translation initiation factor 4 gamma 3
with a threshold of 1e−05

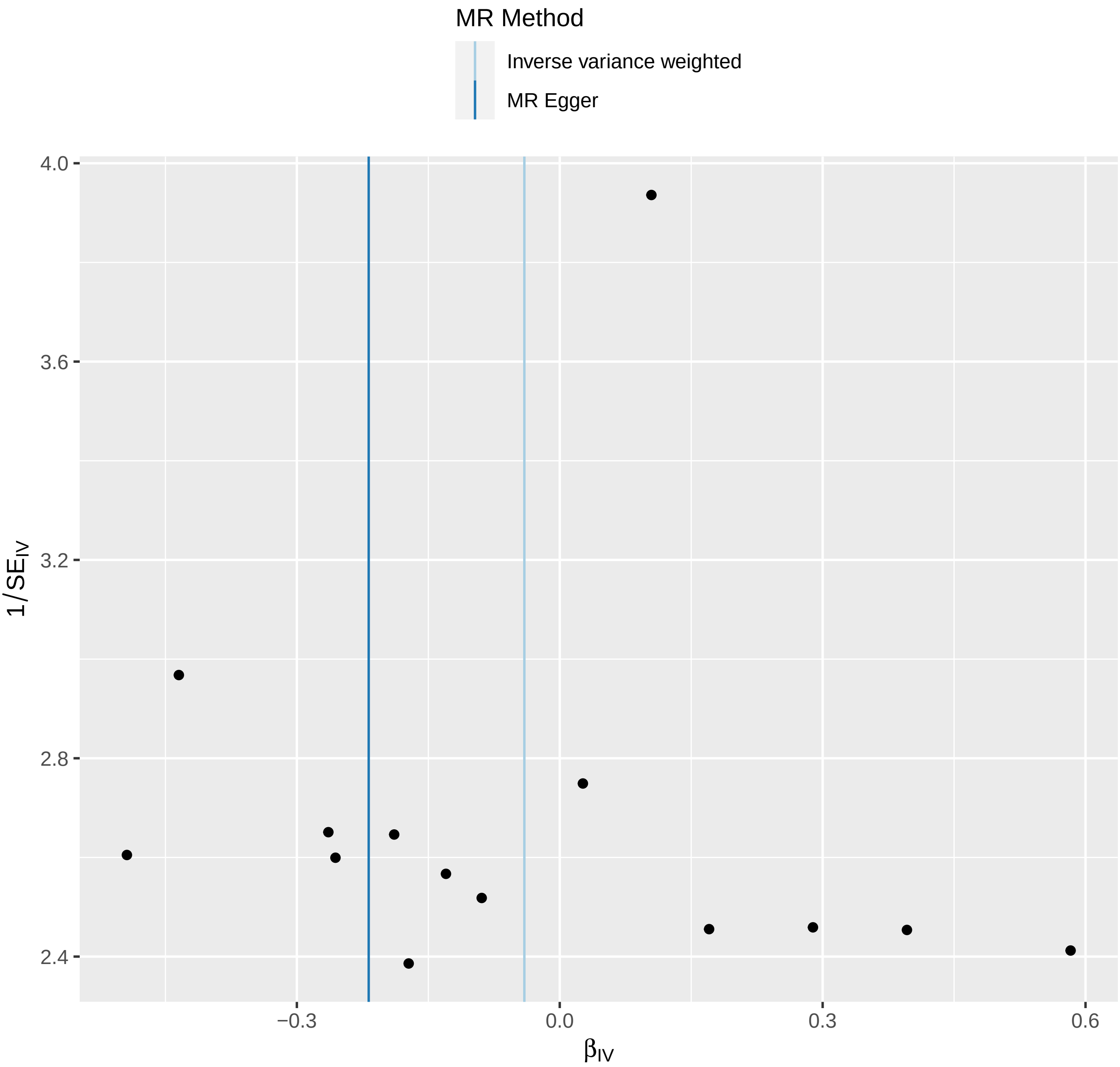

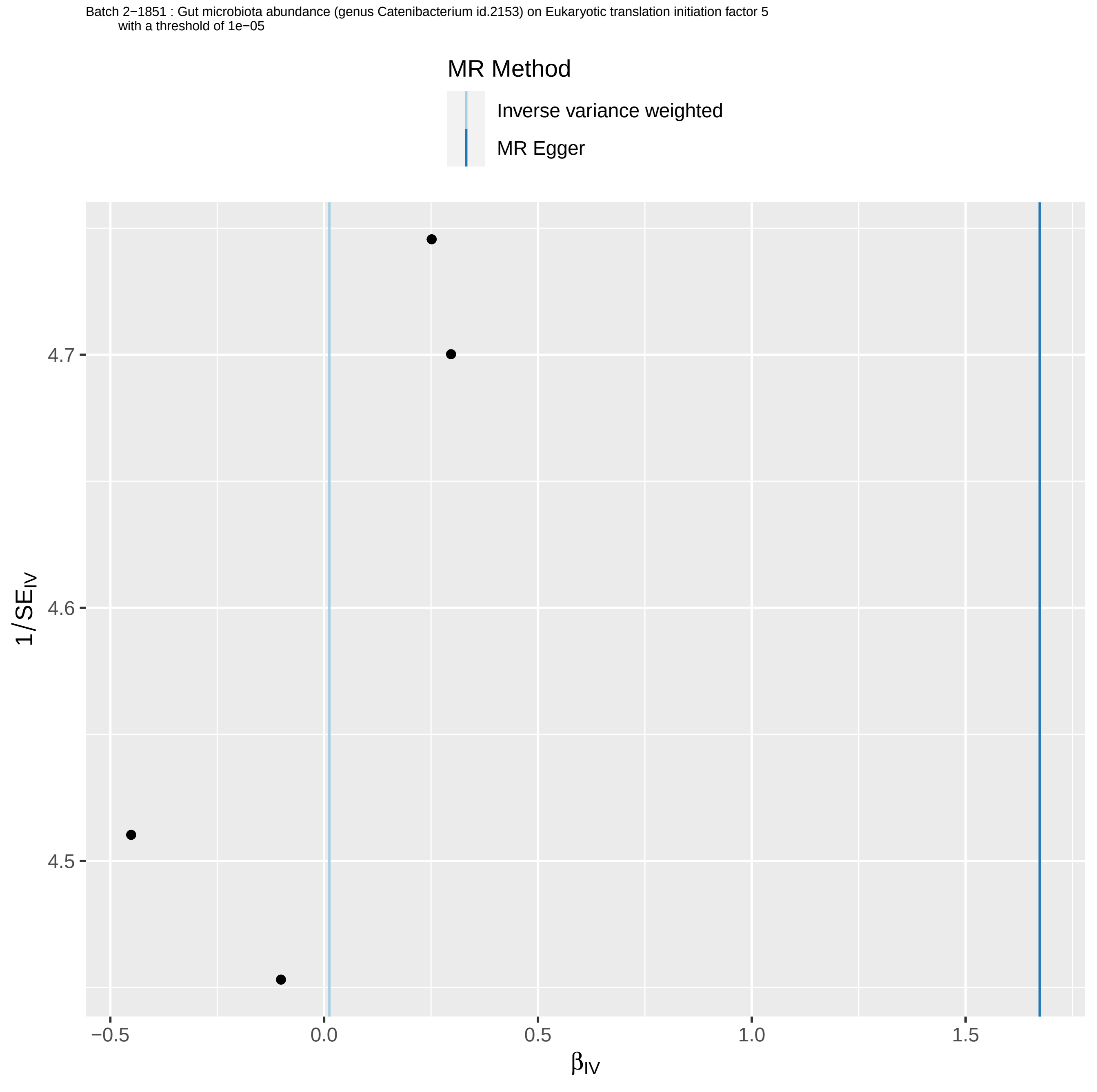
Batch 2−1851 : Gut microbiota abundance (genus Catenibacterium id.2153) on Eukaryotic translation initiation factor 5
with a threshold of 1e−05
MR Method
Inverse variance weighted
MR Egger
4.7
4.6
4.5
1/SE_IV
−0.5
0.0
0.5
1.0
1.5
β_IV

Batch 2−1852 : Gut microbiota abundance (genus Parasutterella id.2892) on Eukaryotic translation initiation factor 5
with a threshold of 1e−05

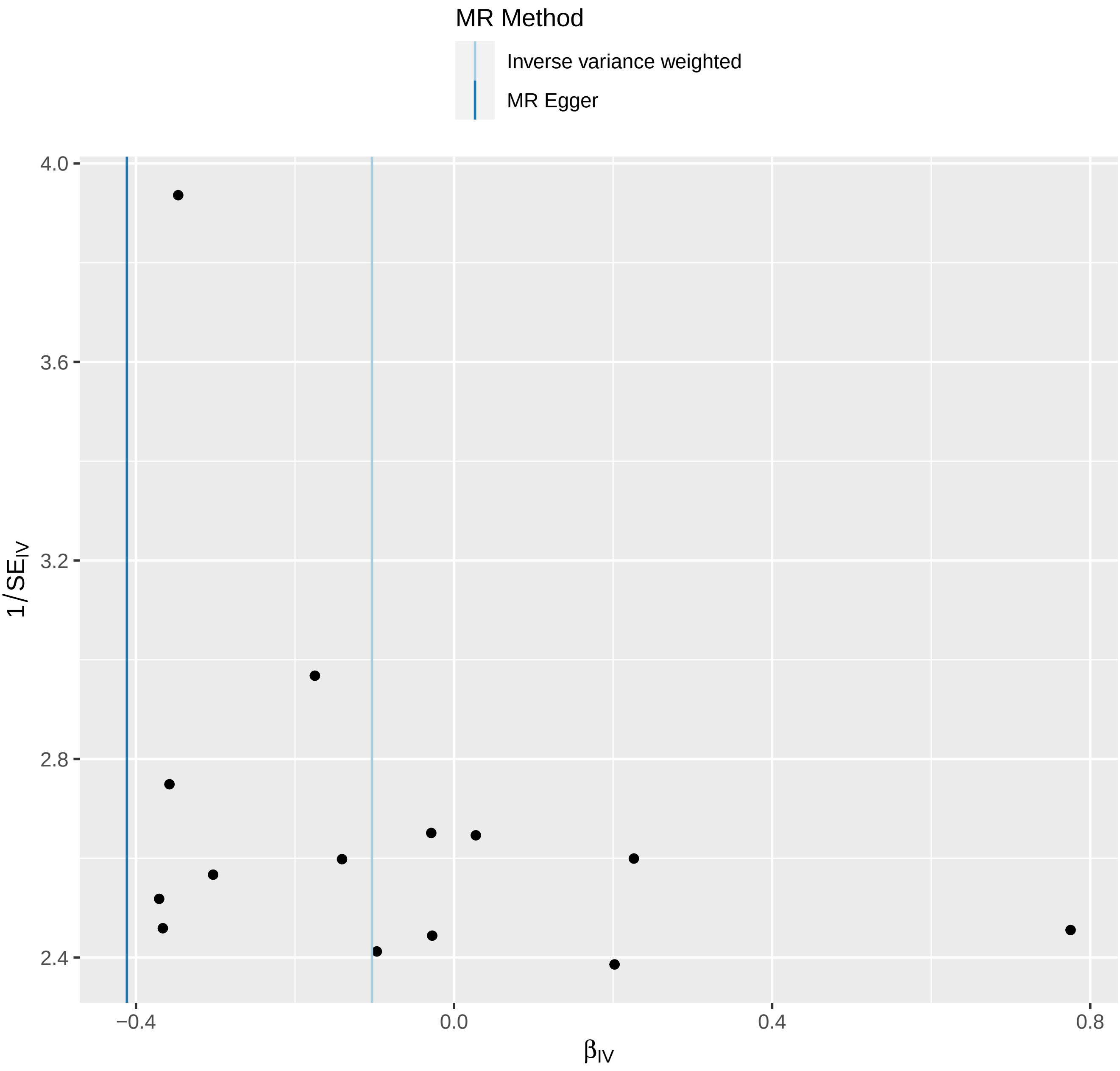

Batch 2−1853 : Gut microbiota abundance (genus Catenibacterium id.2153) on Eukaryotic translation initiation factor 5A−1
with a threshold of 1e−05

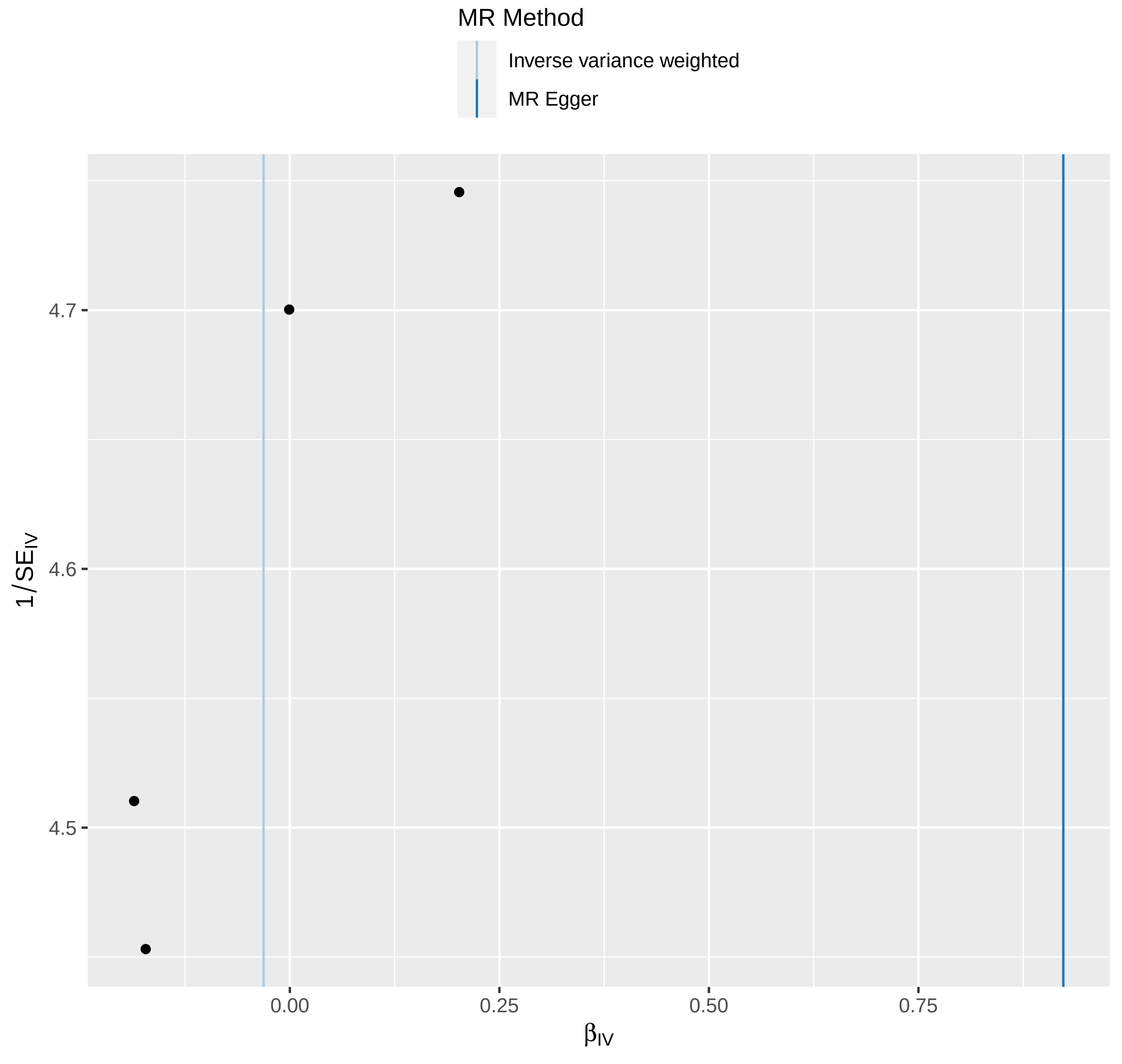

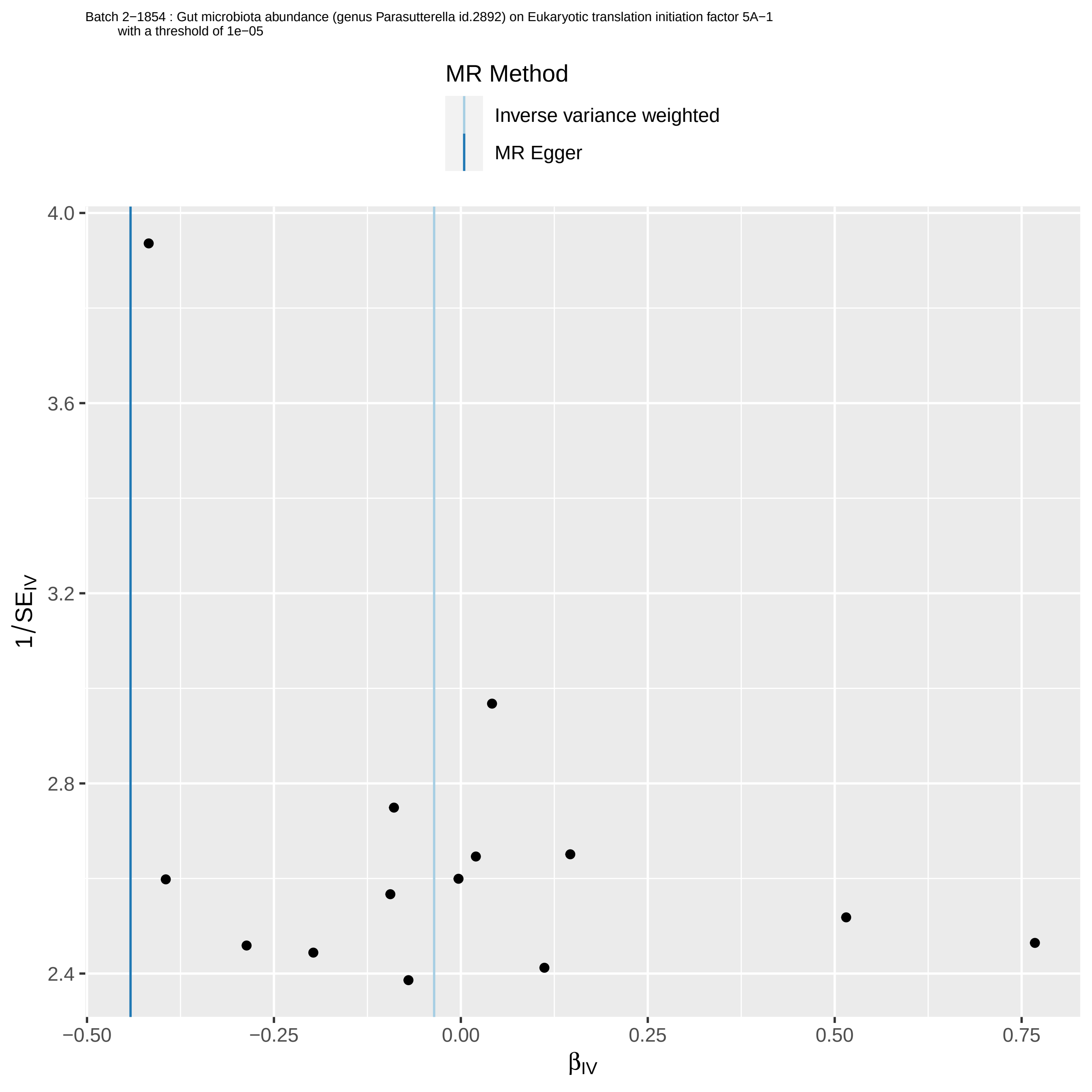

Batch 2−1854 : Gut microbiota abundance (genus Parasutterella id.2892) on Eukaryotic translation initiation factor 5A−1
with a threshold of 1e−05
MR Method
Inverse variance weighted
MR Egger
4.0
3.6
3.2
2.8
2.4
1/SE_IV
−0.50
−0.25
0.00
0.25
0.50
0.75
β_IV

Batch 2−1855 : Gut microbiota abundance (genus Catenibacterium id.2153) on ELAV−like protein 1
with a threshold of 1e−05

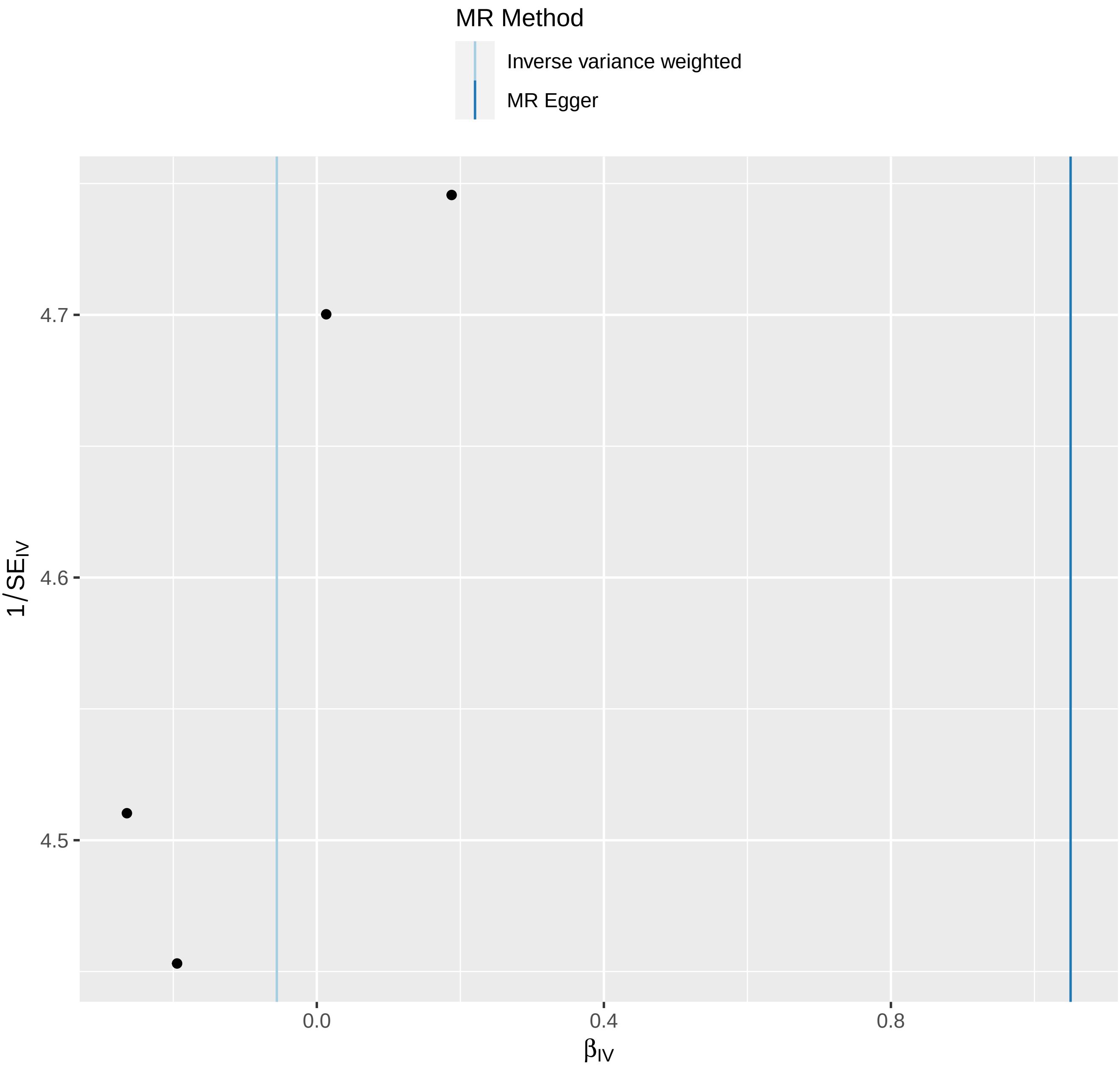

Batch 2−1856 : Gut microbiota abundance (genus Parasutterella id.2892) on ELAV−like protein 1
with a threshold of 1e−05

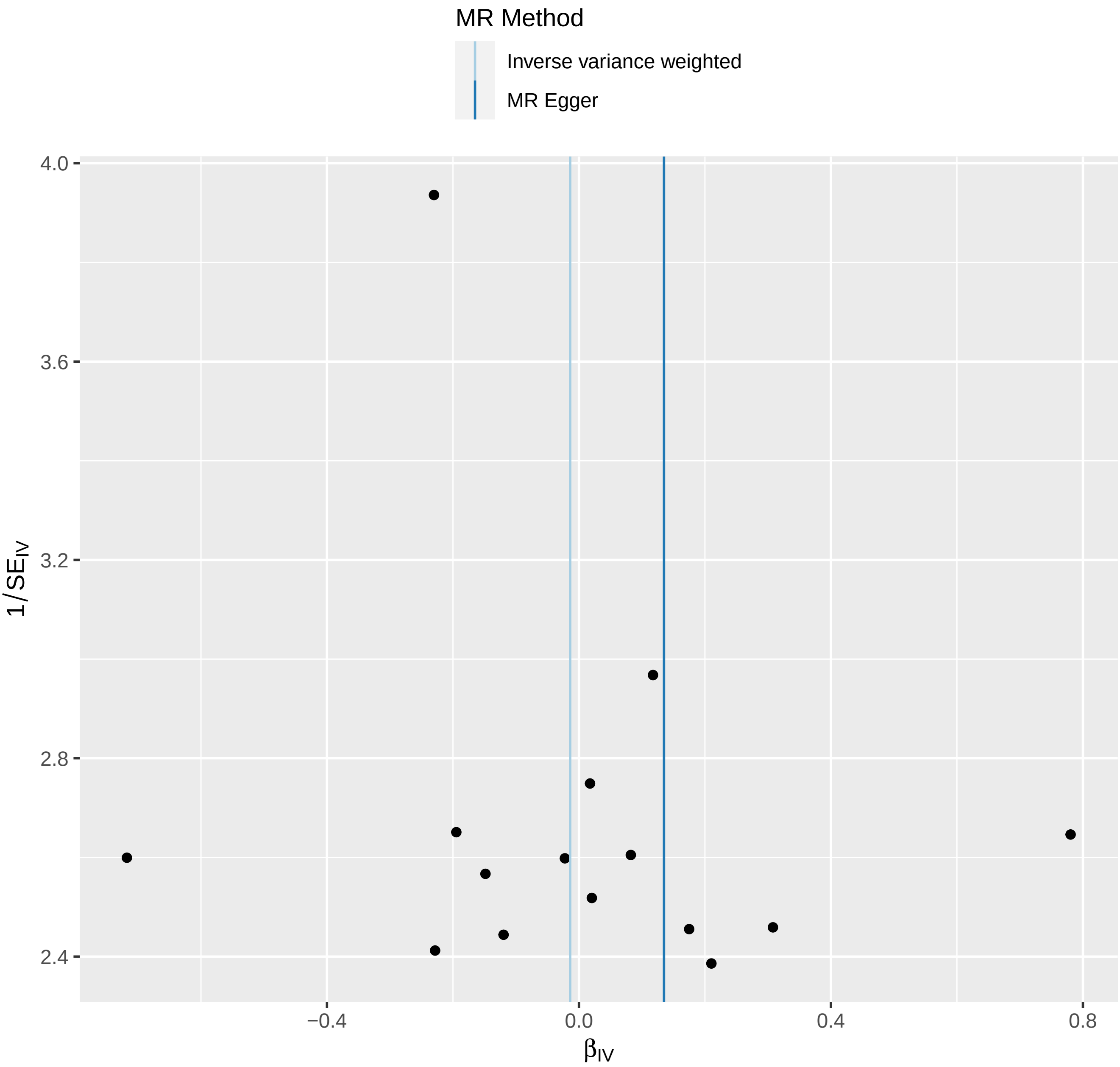

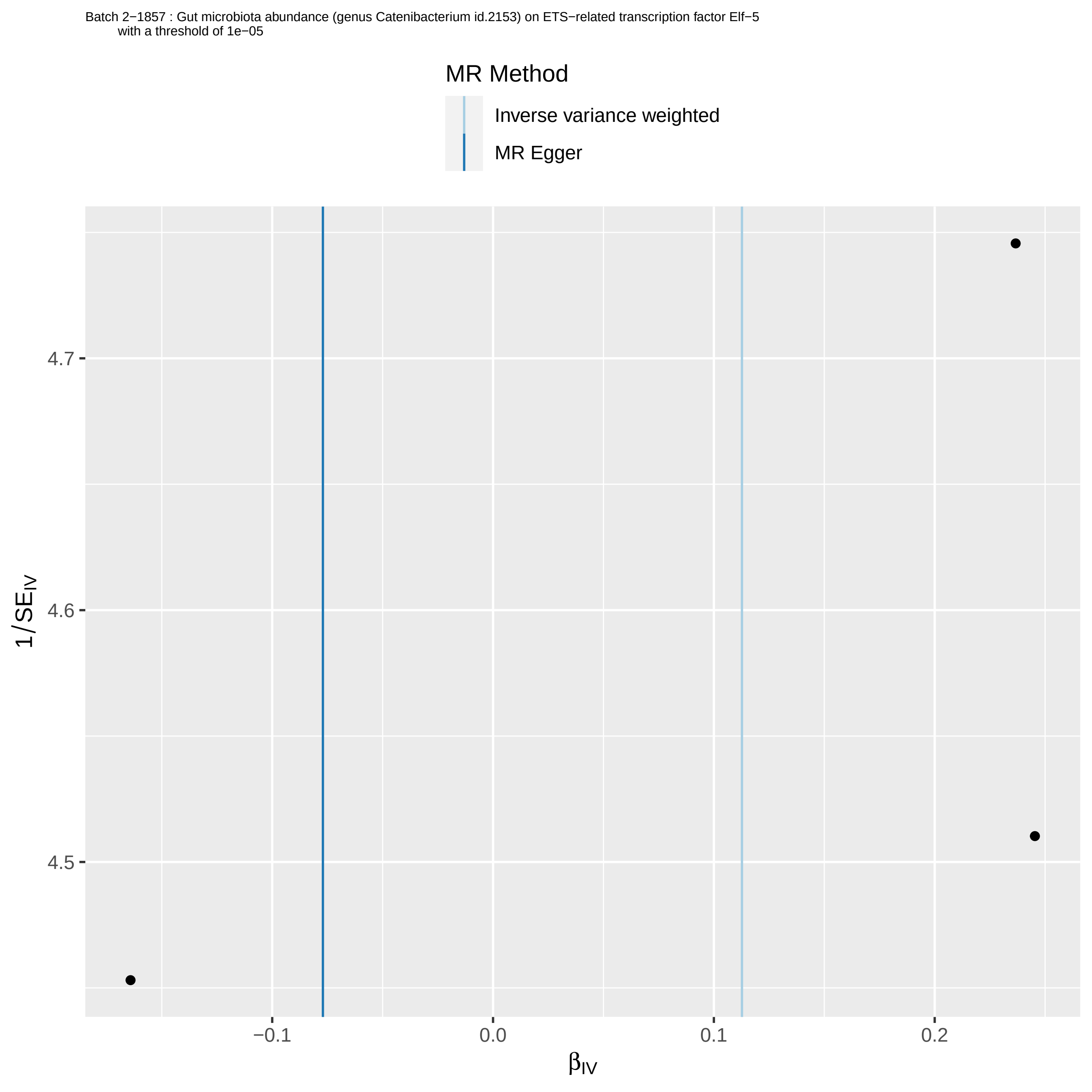

Batch 2−1857 : Gut microbiota abundance (genus Catenibacterium id.2153) on ETS−related transcription factor Elf−5
with a threshold of 1e−05
MR Method
Inverse variance weighted
MR Egger
4.7
4.6
4.5
1/SE_IV
−0.1
0.0
0.1
0.2
β_IV

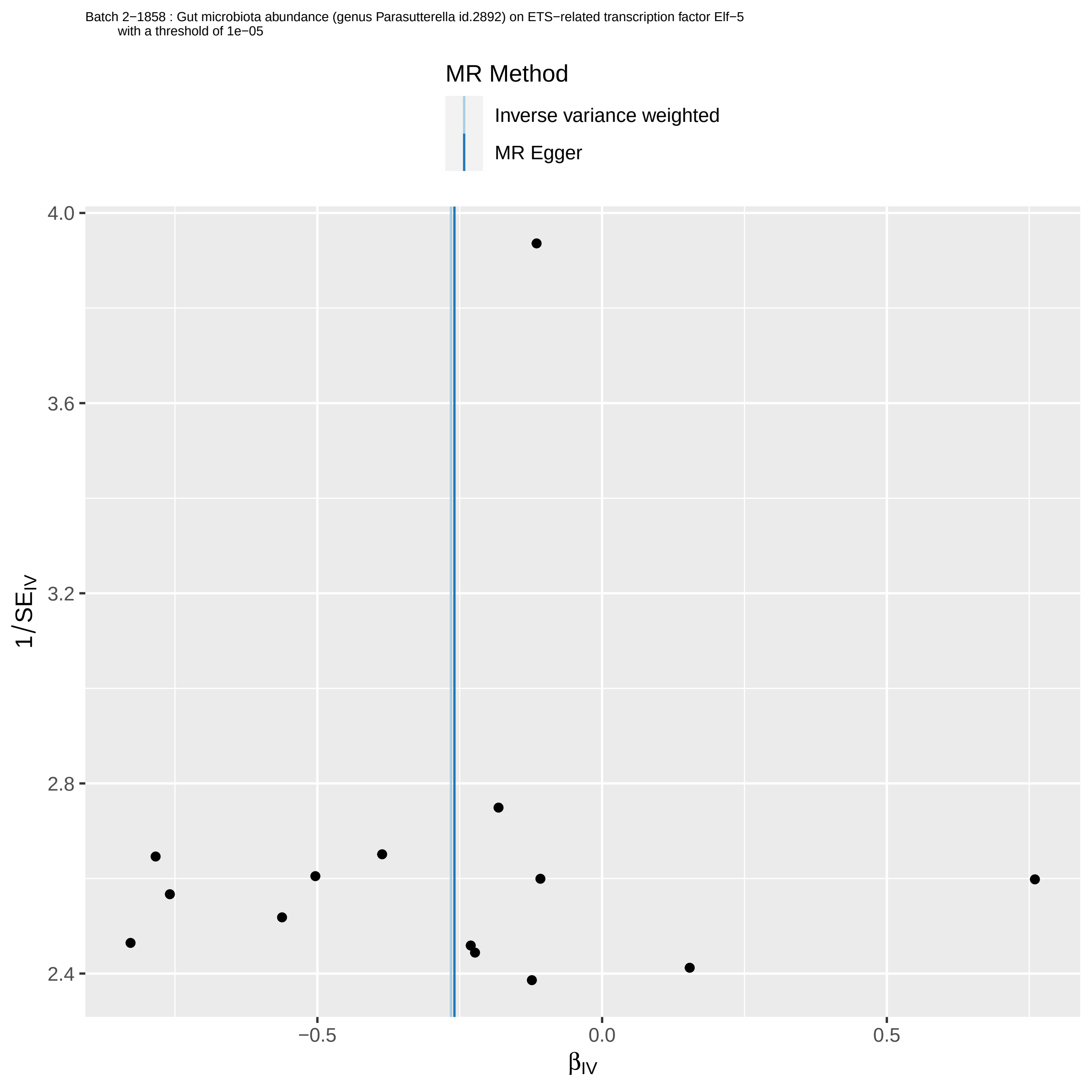
Batch 2−1858 : Gut microbiota abundance (genus Parasutterella id.2892) on ETS−related transcription factor Elf−5
with a threshold of 1e−05
MR Method
Inverse variance weighted
MR Egger
4.0
3.6
3.2
2.8
2.4
1/SE$_{IV}$
−0.5
0.0
0.5
β$_{IV}$

Batch 2−1859 : Gut microbiota abundance (genus Catenibacterium id.2153) on ETS domain−containing protein Elk−1
with a threshold of 1e−05

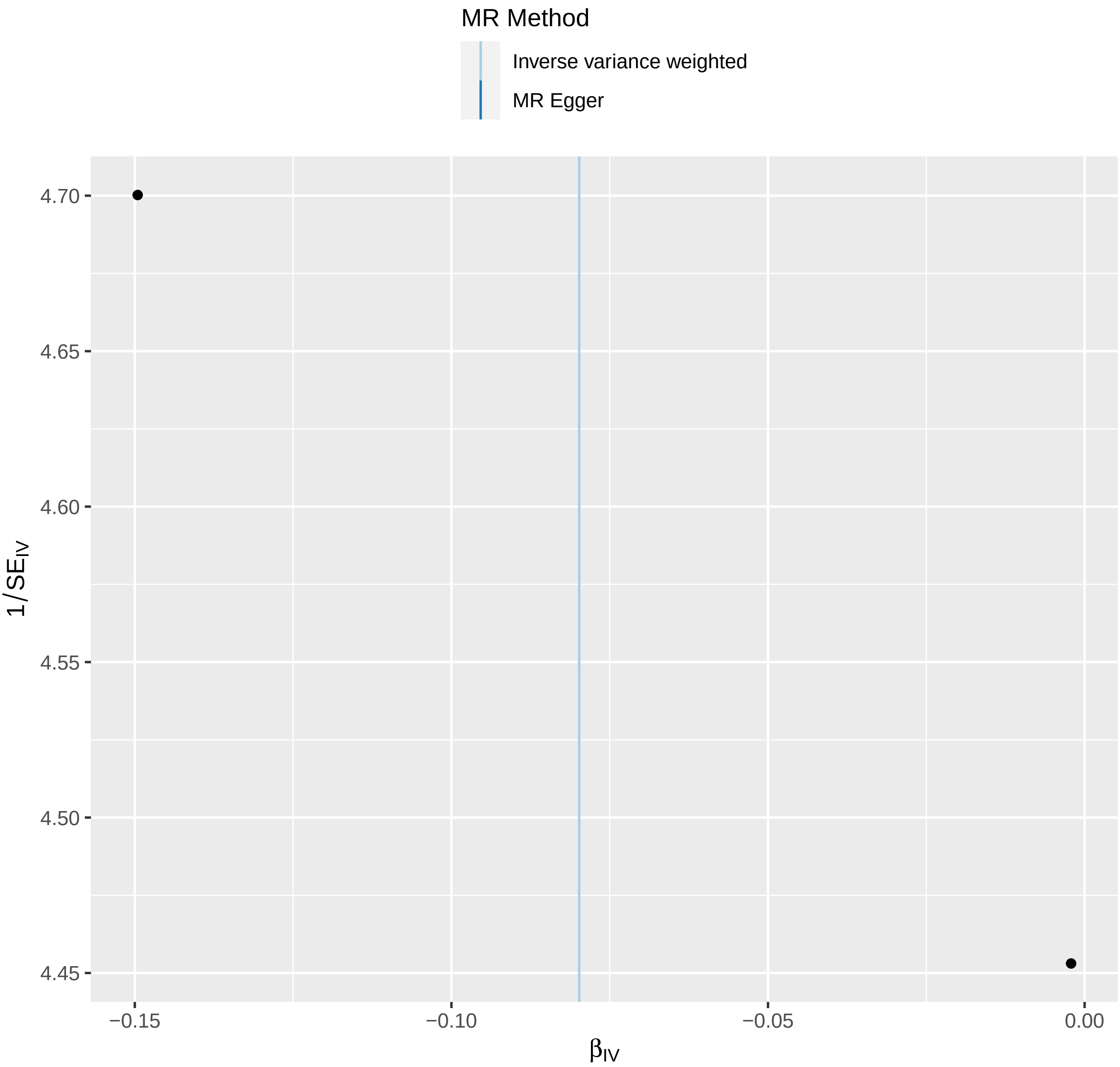

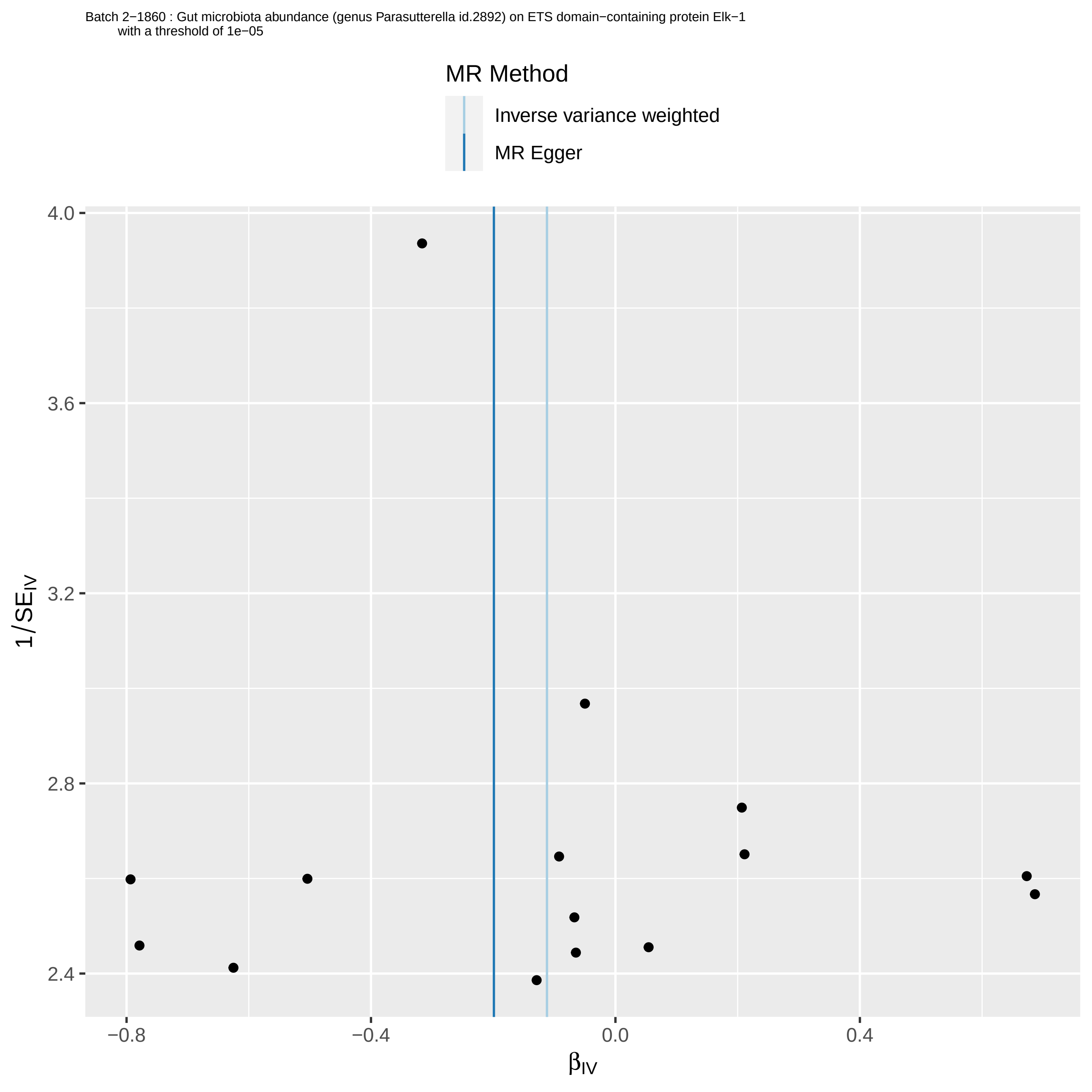
Batch 2−1860 : Gut microbiota abundance (genus Parasutterella id.2892) on ETS domain−containing protein Elk−1
with a threshold of 1e−05
MR Method
Inverse variance weighted
MR Egger
4.0
3.6
3.2
2.8
2.4
1/SE_IV
−0.8
−0.4
0.0
0.4
β_IV

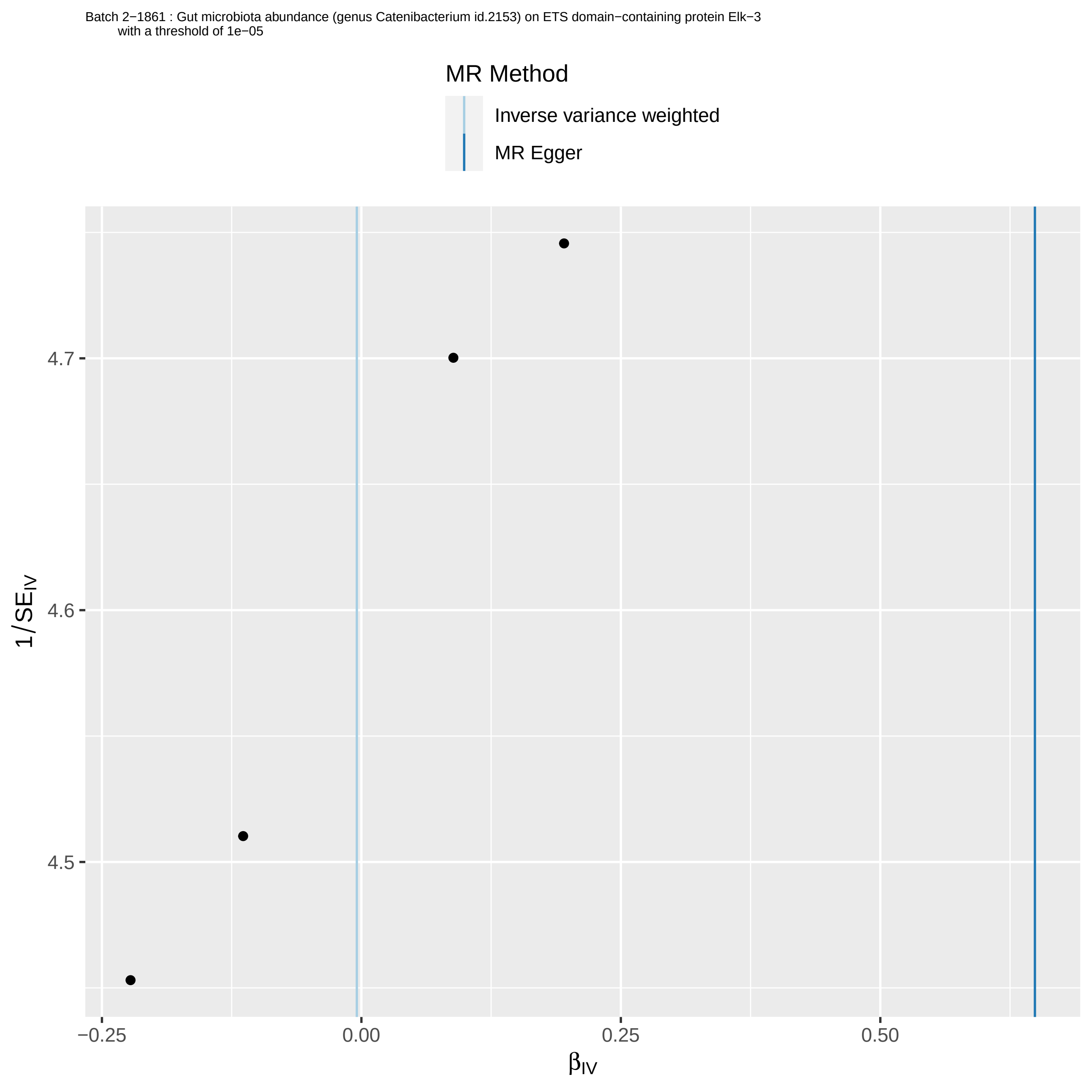

Batch 2−1861 : Gut microbiota abundance (genus Catenibacterium id.2153) on ETS domain−containing protein Elk−3
with a threshold of 1e−05
MR Method
Inverse variance weighted
MR Egger
4.7
4.6
4.5
1/SE_IV
−0.25
0.00
0.25
0.50
β_IV

Batch 2−1862 : Gut microbiota abundance (genus Parasutterella id.2892) on ETS domain−containing protein Elk−3
with a threshold of 1e−05

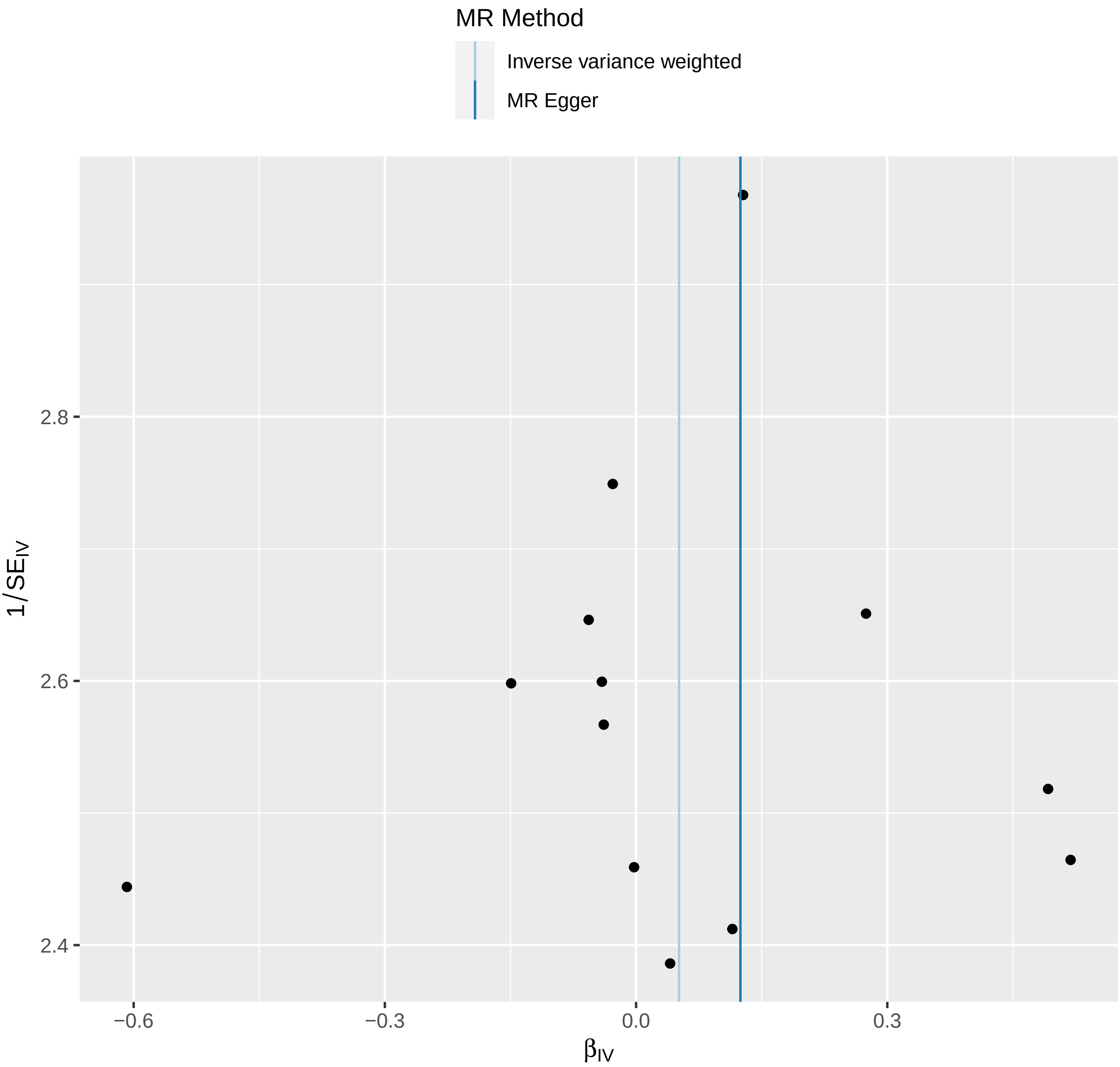

Batch 2−1863 : Gut microbiota abundance (genus Catenibacterium id.2153) on RNA polymerase II elongation factor ELL
with a threshold of 1e−05

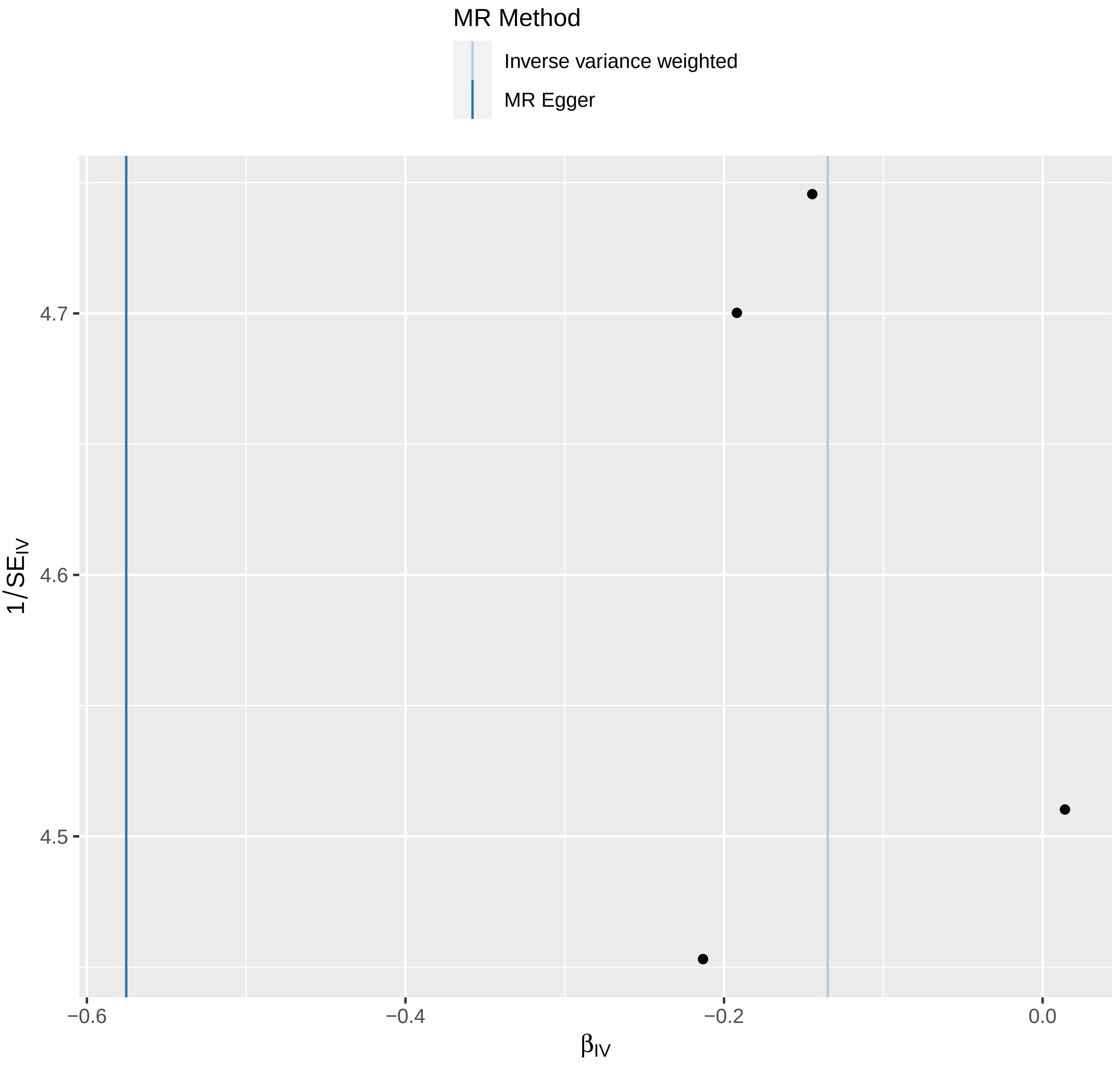

Batch 2−1864 : Gut microbiota abundance (genus Parasutterella id.2892) on RNA polymerase II elongation factor ELL
with a threshold of 1e−05

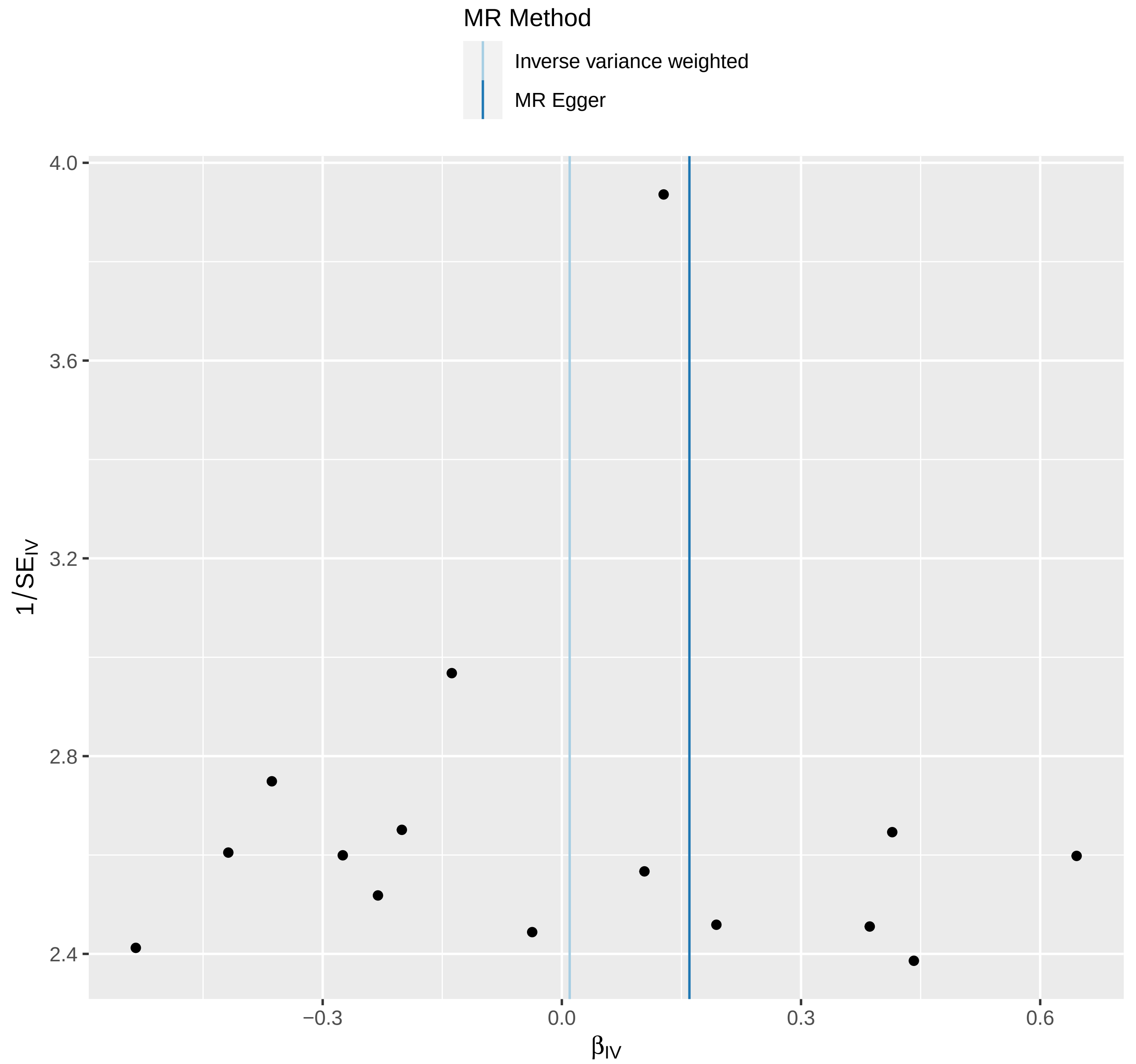

Batch 2−1865 : Gut microbiota abundance (genus Catenibacterium id.2153) on RNA polymerase II elongation factor ELL2
with a threshold of 1e−05

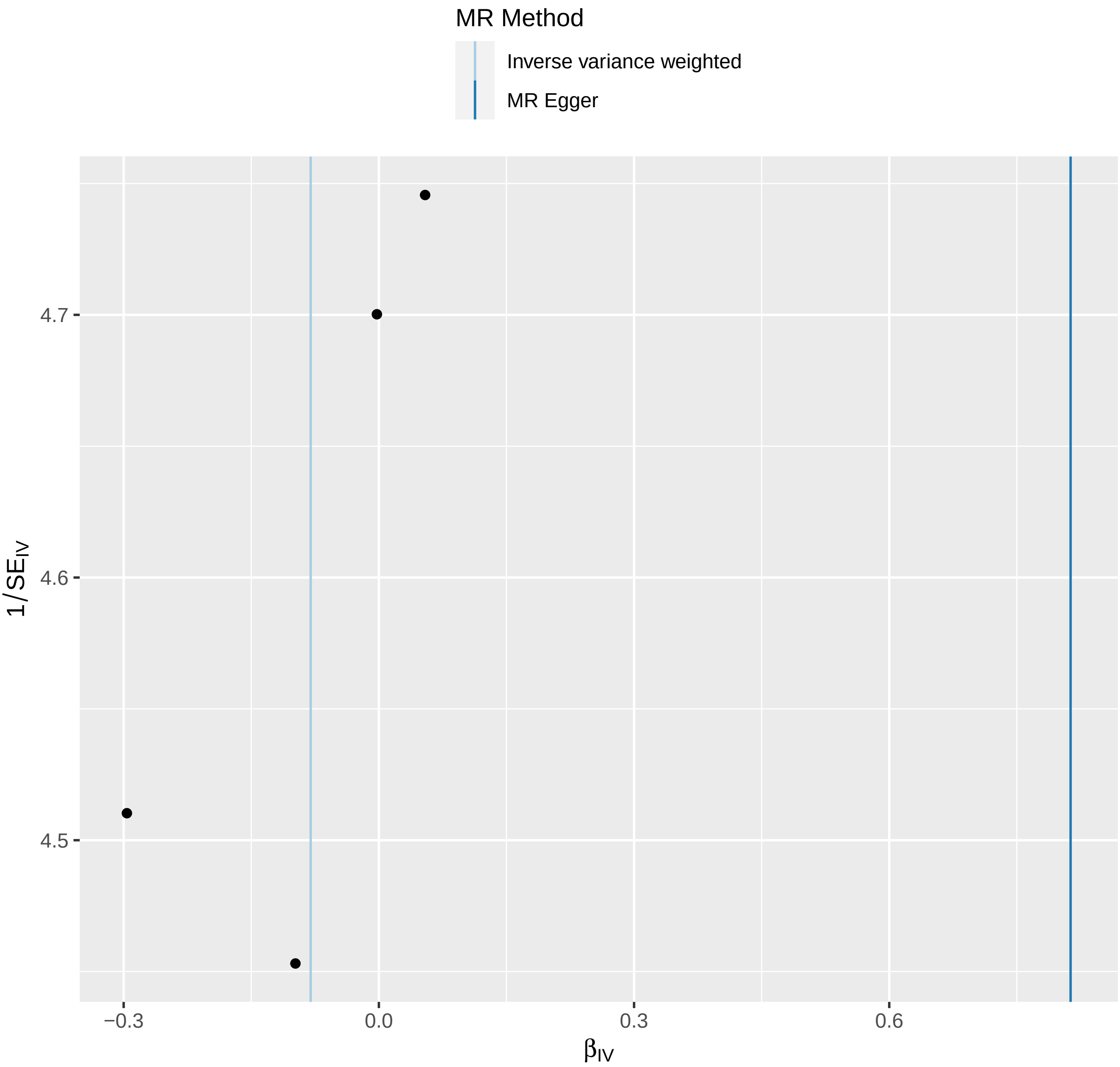

Batch 2−1866 : Gut microbiota abundance (genus Parasutterella id.2892) on RNA polymerase II elongation factor ELL2
with a threshold of 1e−05

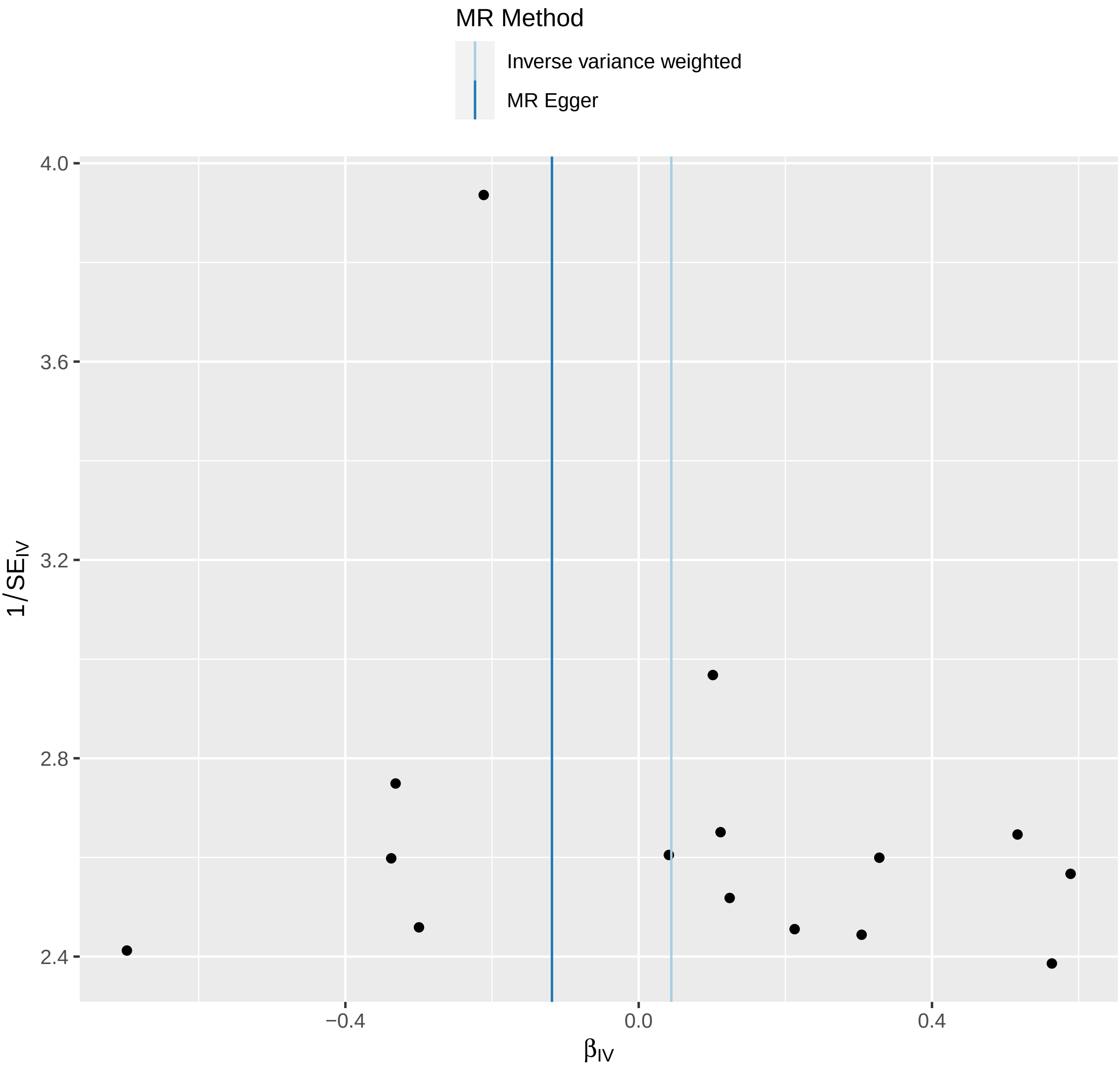

Batch 2−1867 : Gut microbiota abundance (genus Catenibacterium id.2153) on Engulfment and cell motility protein 1 with a threshold of 1e−05

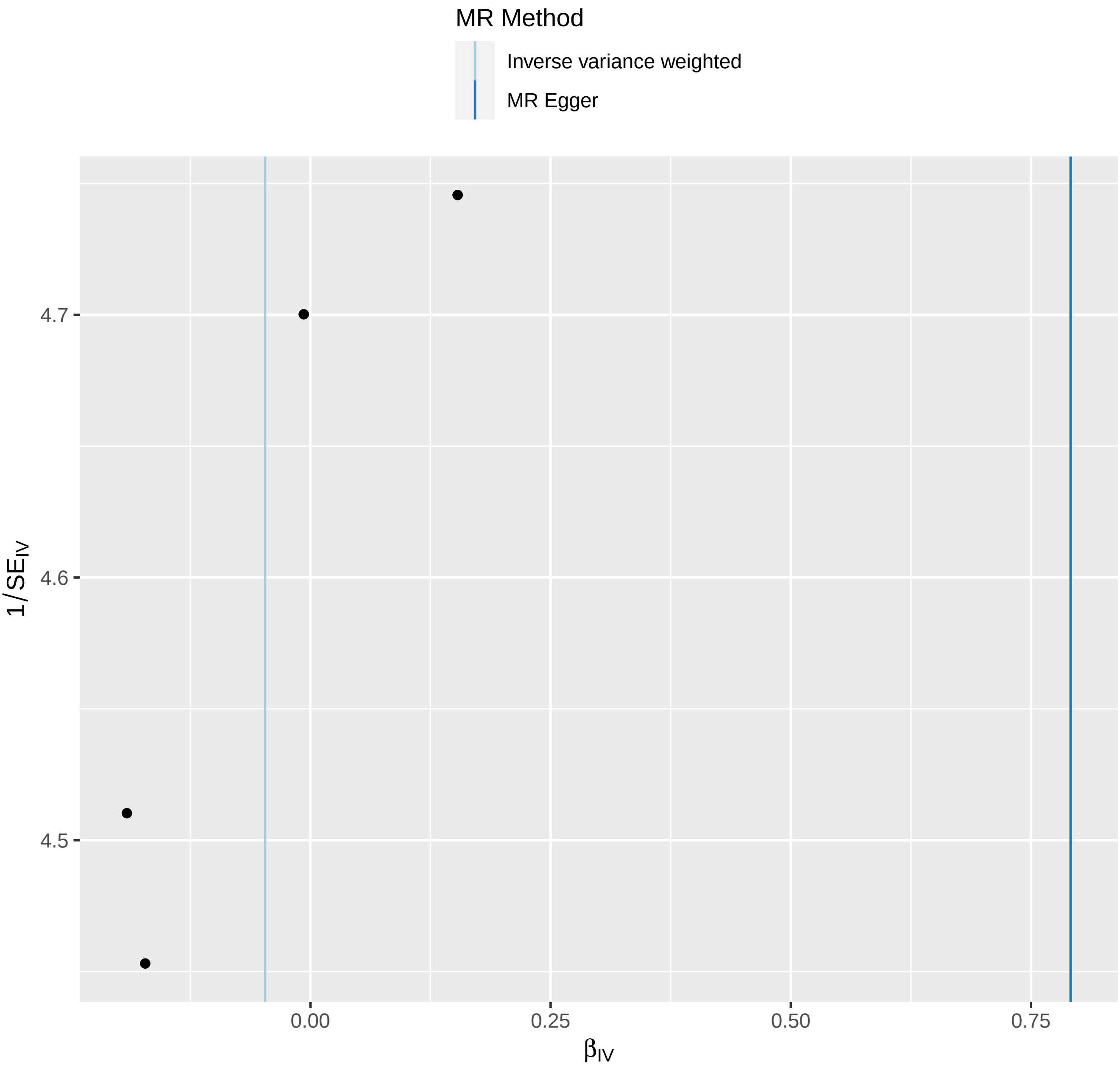

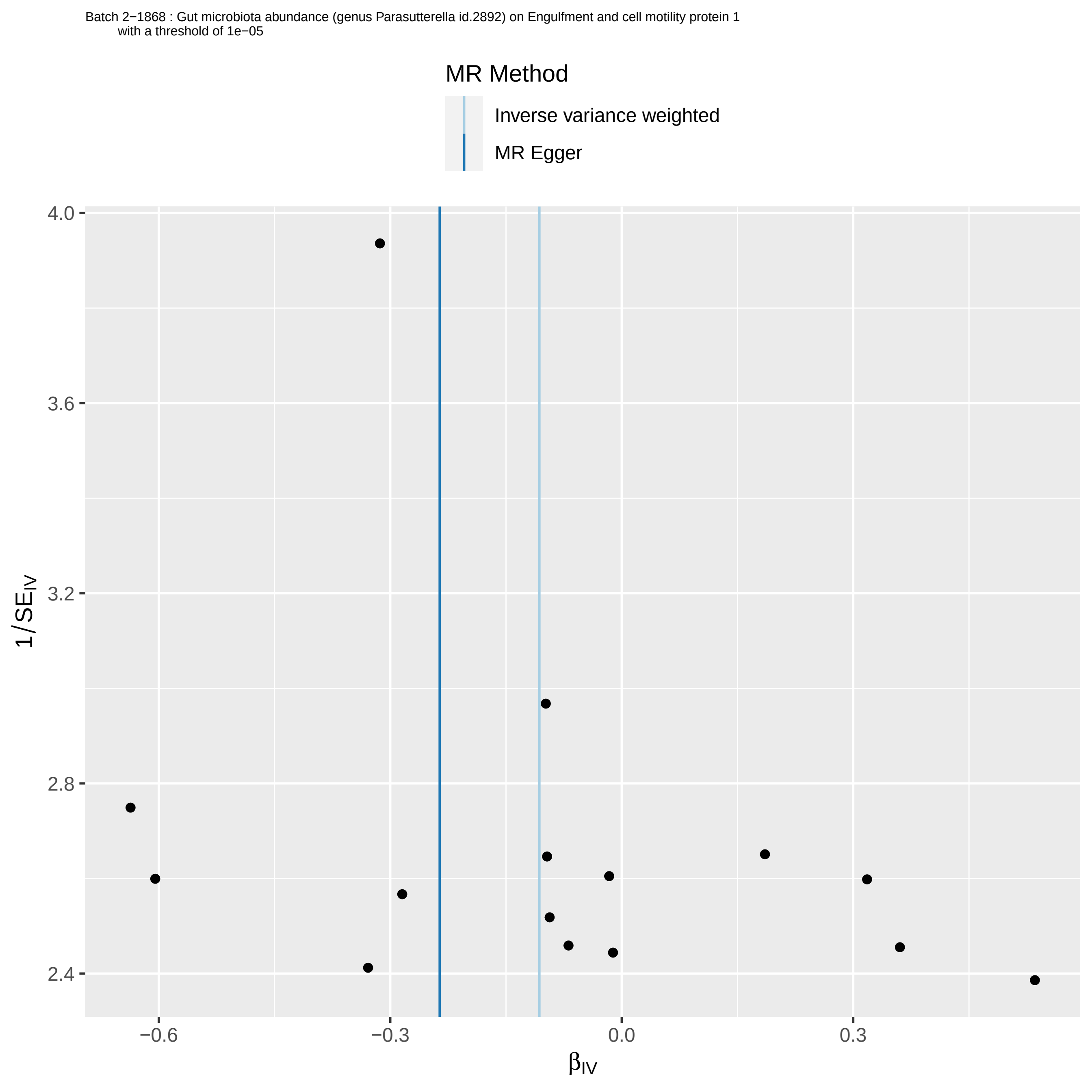
Batch 2−1868 : Gut microbiota abundance (genus Parasutterella id.2892) on Engulfment and cell motility protein 1
with a threshold of 1e−05
MR Method
Inverse variance weighted
MR Egger
4.0
3.6
3.2
2.8
2.4
1/SE_IV
−0.6
−0.3
0.0
0.3
β_IV

Batch 2−1869 : Gut microbiota abundance (genus Catenibacterium id.2153) on ER membrane protein complex subunit 1
with a threshold of 1e−05

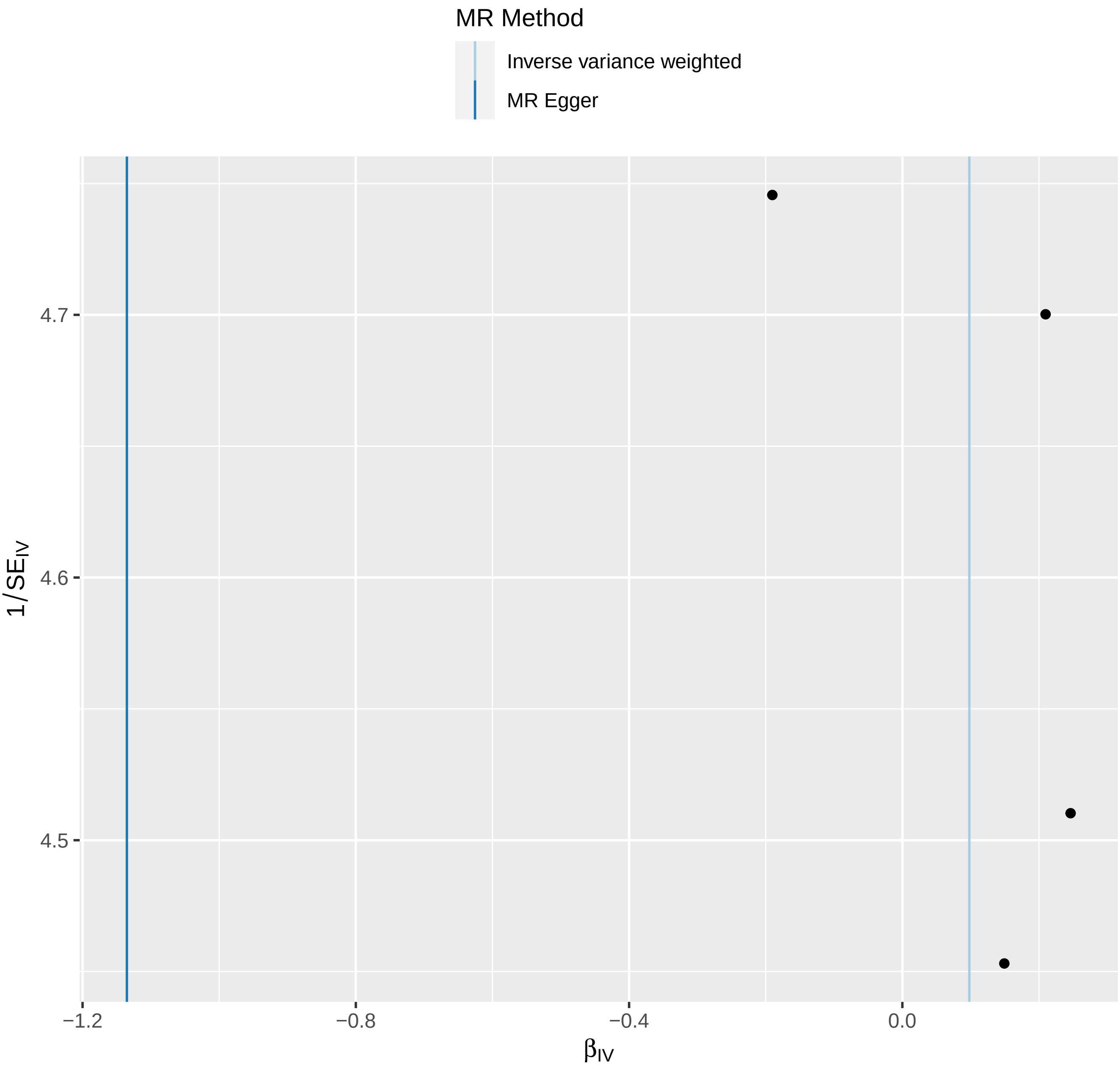

Batch 2−1870 : Gut microbiota abundance (genus Parasutterella id.2892) on ER membrane protein complex subunit 1
with a threshold of 1e−05

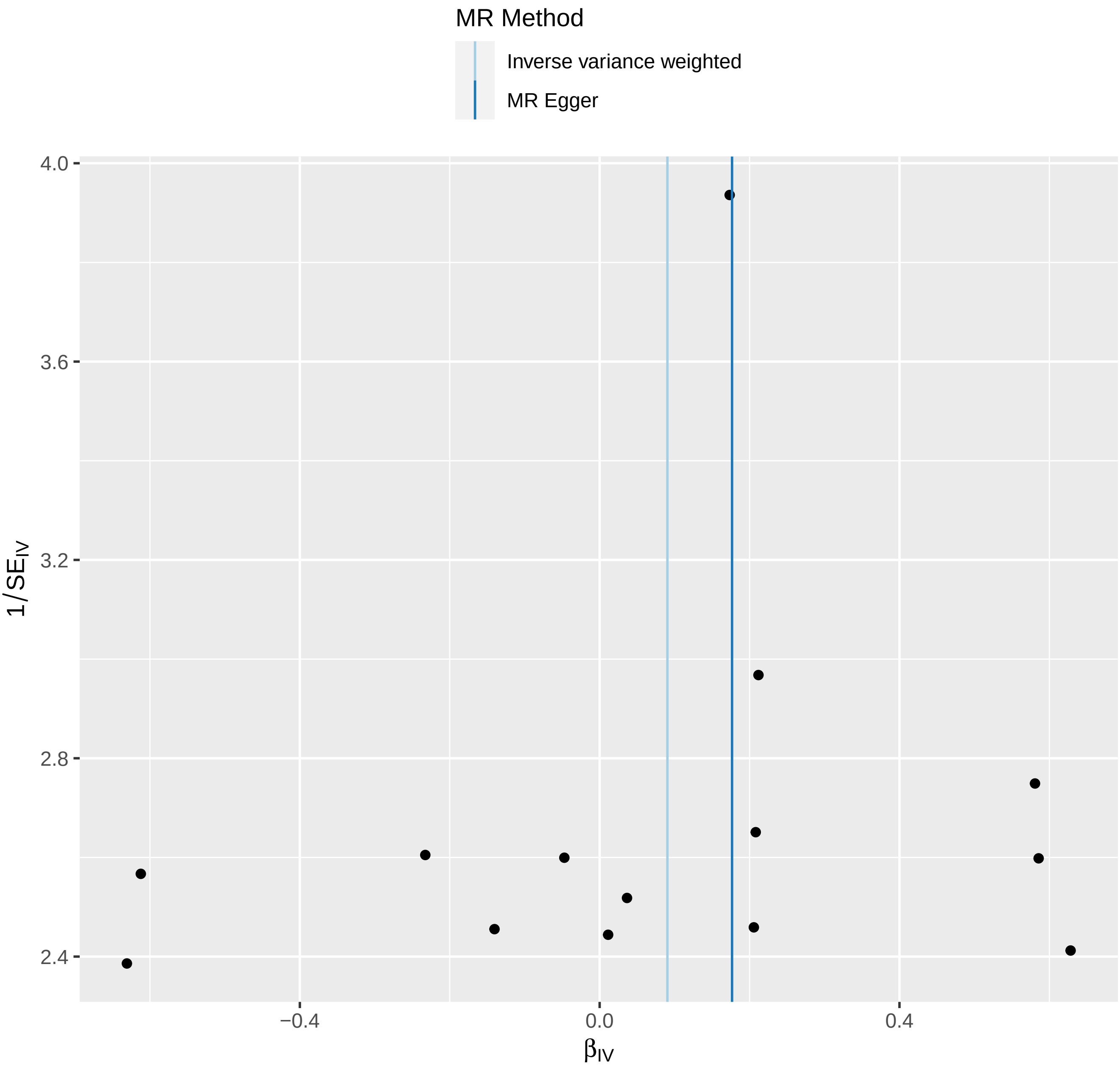

Batch 2−1871 : Gut microbiota abundance (genus Catenibacterium id.2153) on ER membrane protein complex subunit 4
with a threshold of 1e−05

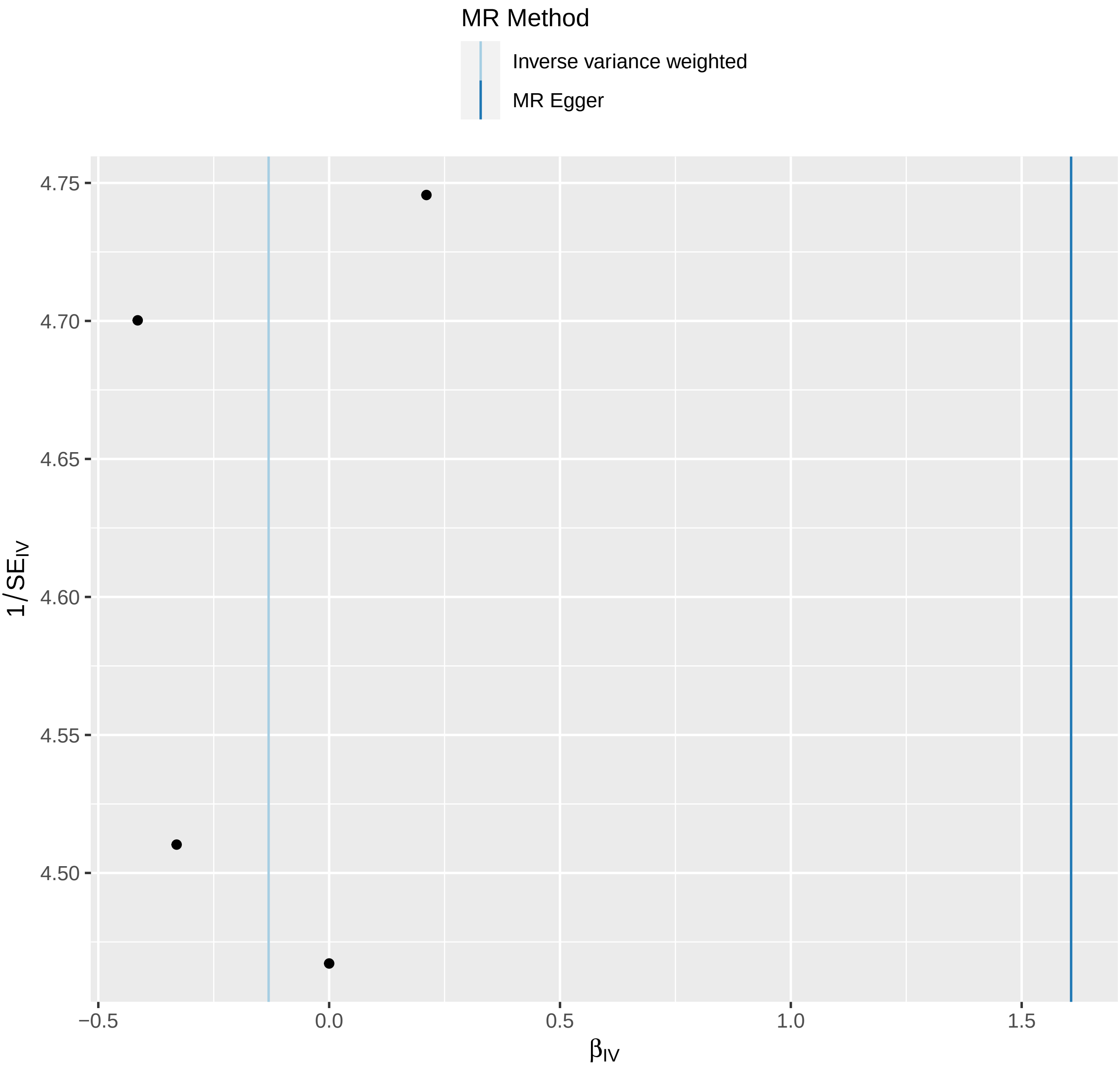

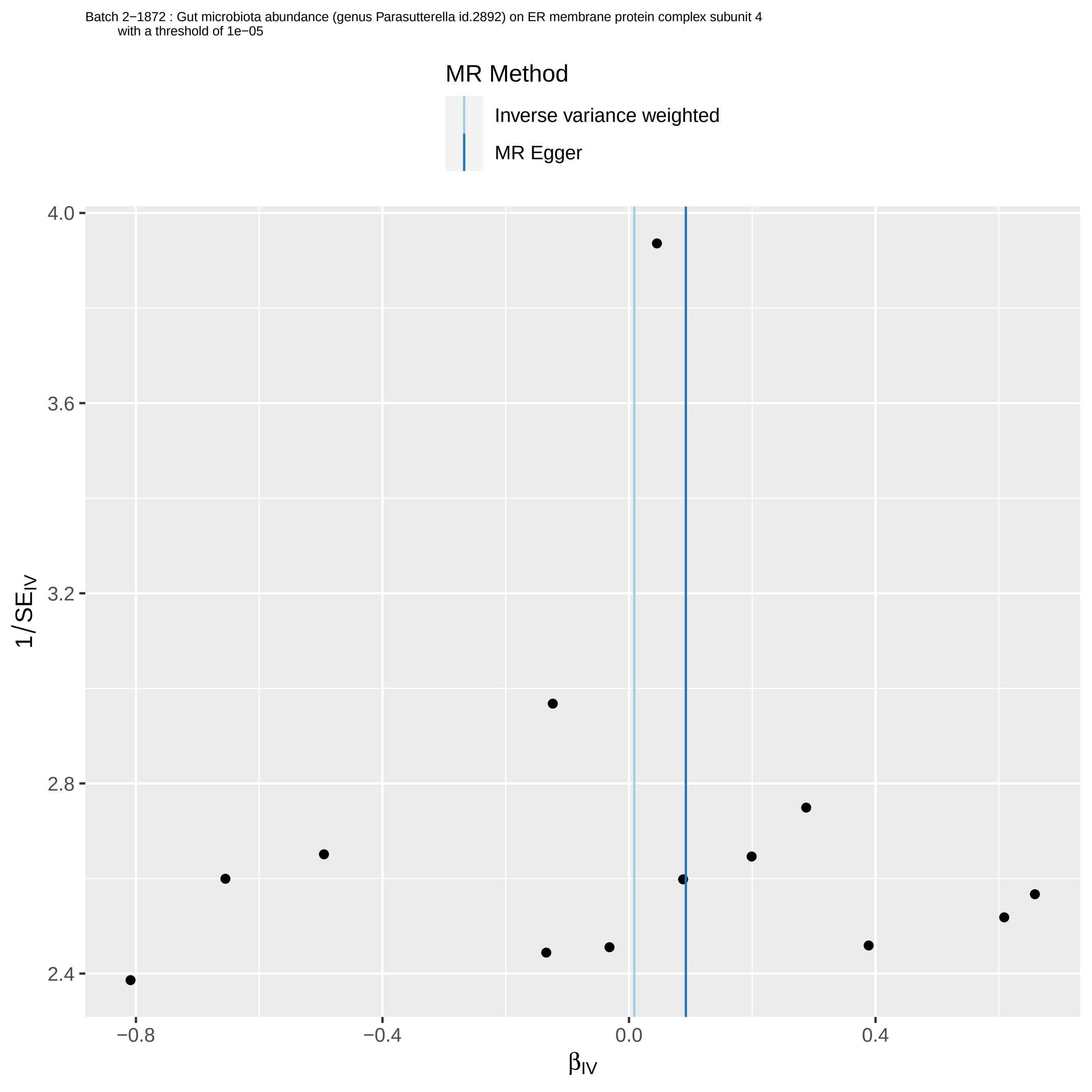
Batch 2−1872 : Gut microbiota abundance (genus Parasutterella id.2892) on ER membrane protein complex subunit 4
with a threshold of 1e−05
MR Method
Inverse variance weighted
MR Egger
4.0
3.6
3.2
2.8
2.4
1/SE_IV
−0.8
−0.4
0.0
0.4
β_IV

Batch 2−1873 : Gut microbiota abundance (genus Catenibacterium id.2153) on EMI domain−containing protein 1
with a threshold of 1e−05

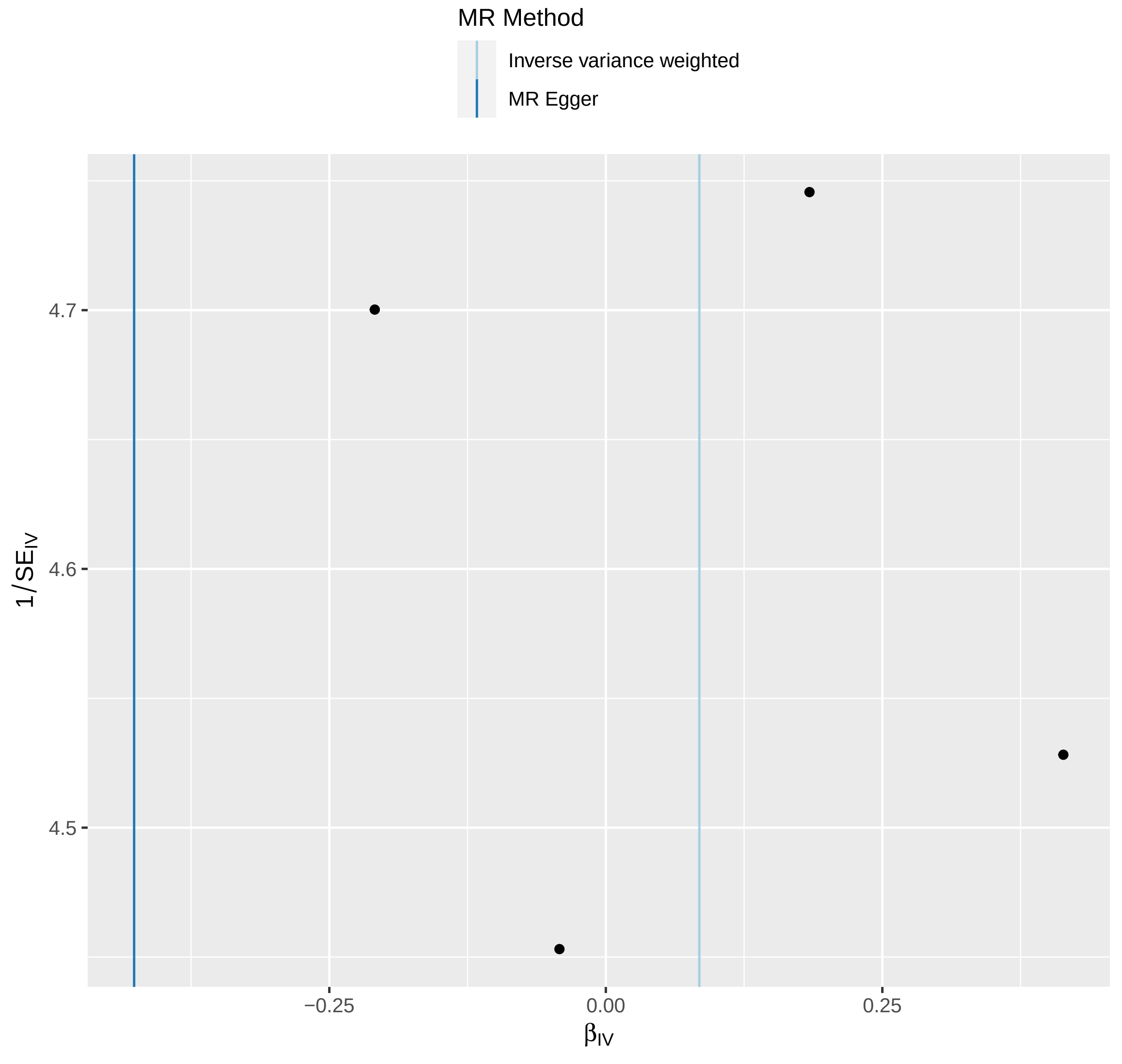

Batch 2−1874 : Gut microbiota abundance (genus Parasutterella id.2892) on EMI domain−containing protein 1
with a threshold of 1e−05

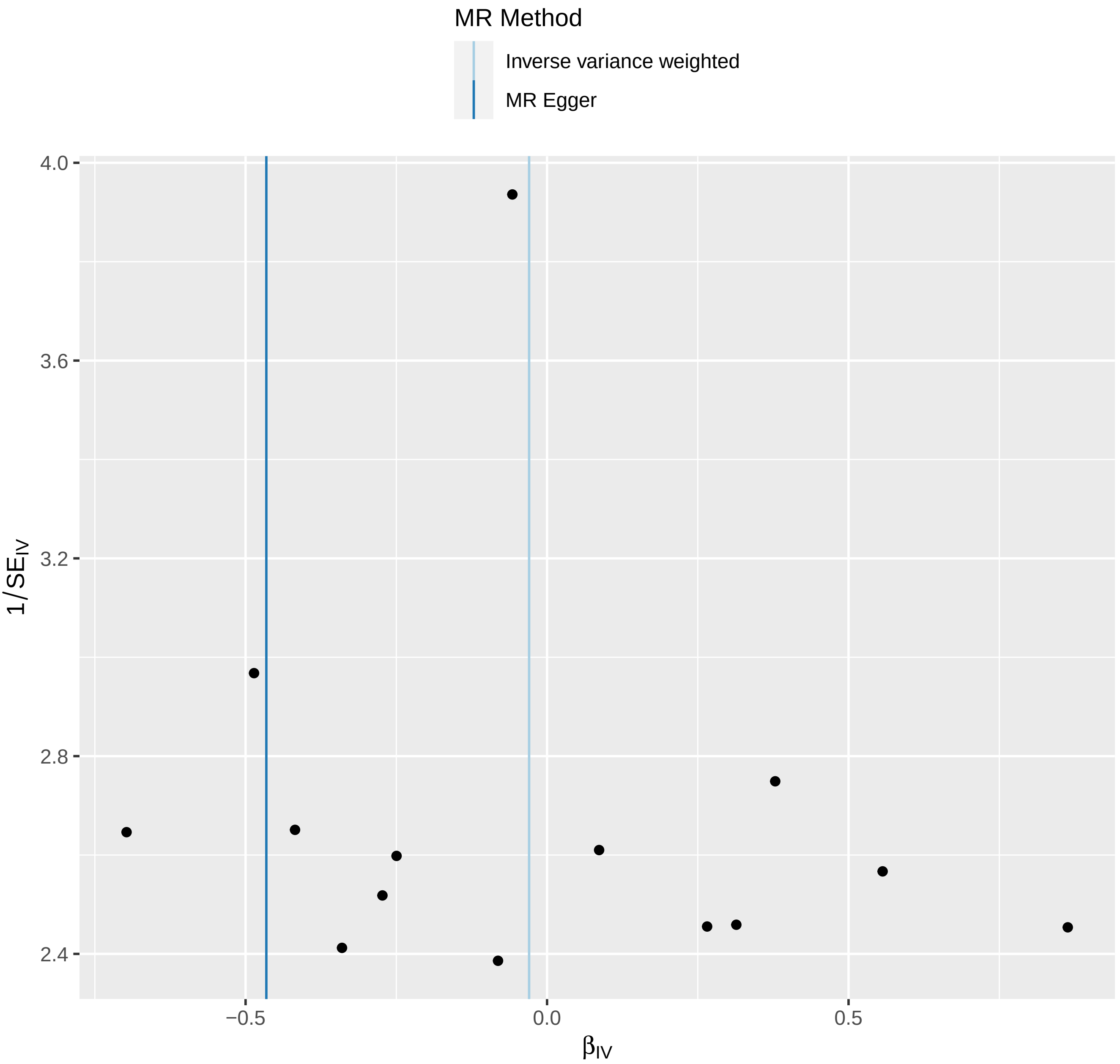

Batch 2−1875 : Gut microbiota abundance (genus Catenibacterium id.2153) on EMILIN−3
with a threshold of 1e−05

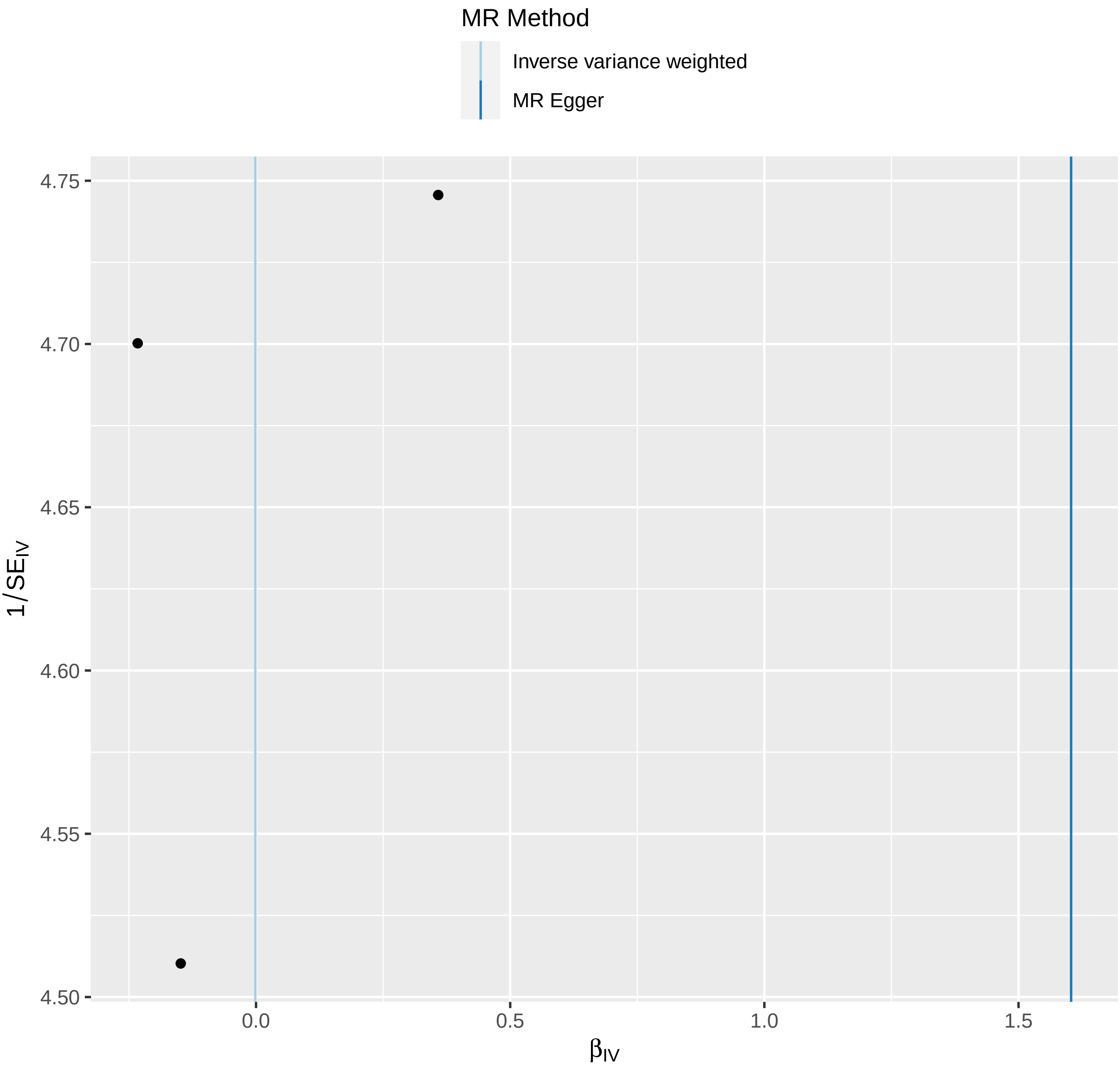

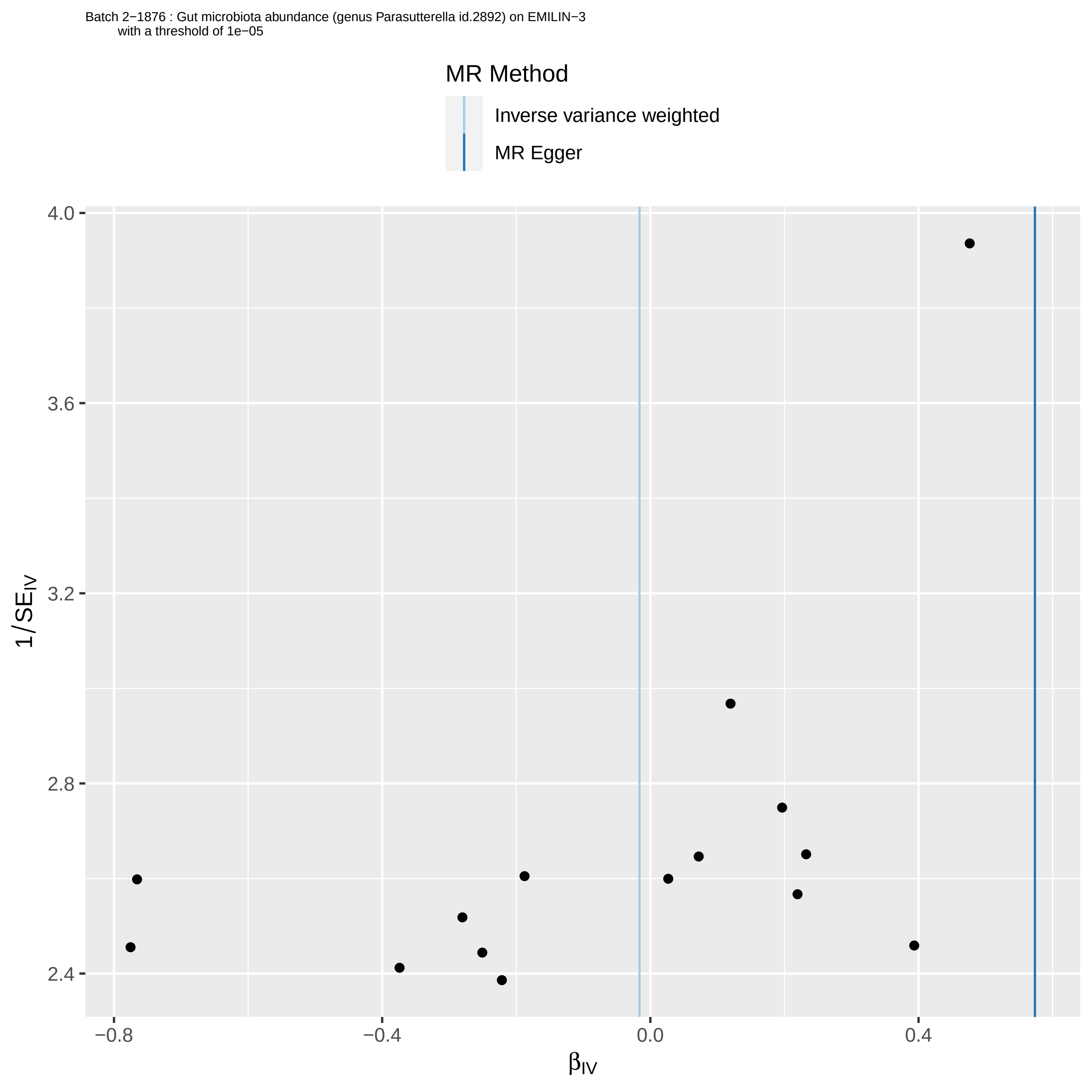

Batch 2−1876 : Gut microbiota abundance (genus Parasutterella id.2892) on EMILIN−3
with a threshold of 1e−05
MR Method
Inverse variance weighted
MR Egger
4.0
3.6
3.2
2.8
2.4
1/SE$_{IV}$
−0.8
−0.4
0.0
0.4
β$_{IV}$

Batch 2−1877 : Gut microbiota abundance (genus Catenibacterium id.2153) on EMILIN−3
with a threshold of 1e−05

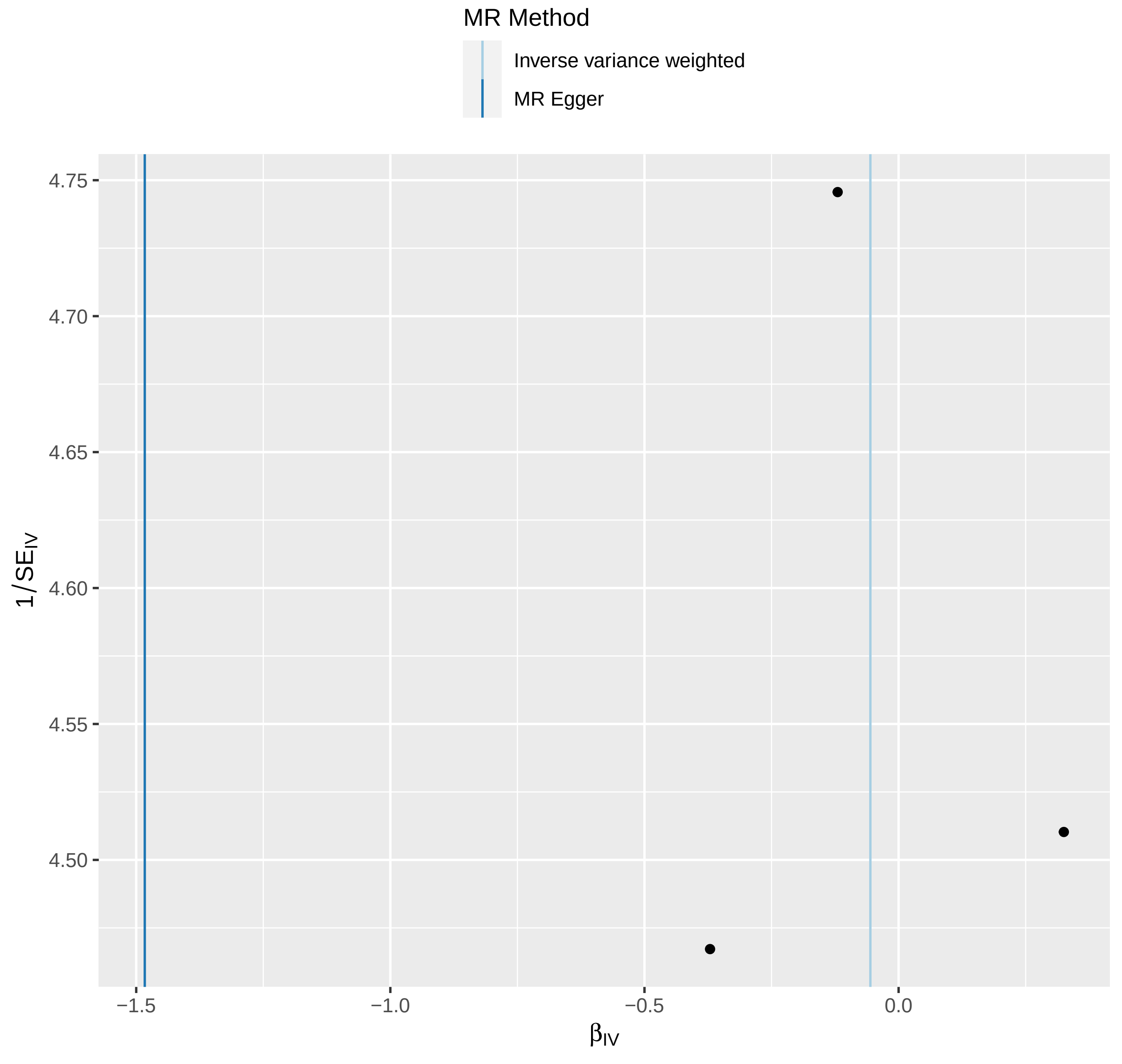

Batch 2−1878 : Gut microbiota abundance (genus Parasutterella id.2892) on EMILIN−3
with a threshold of 1e−05

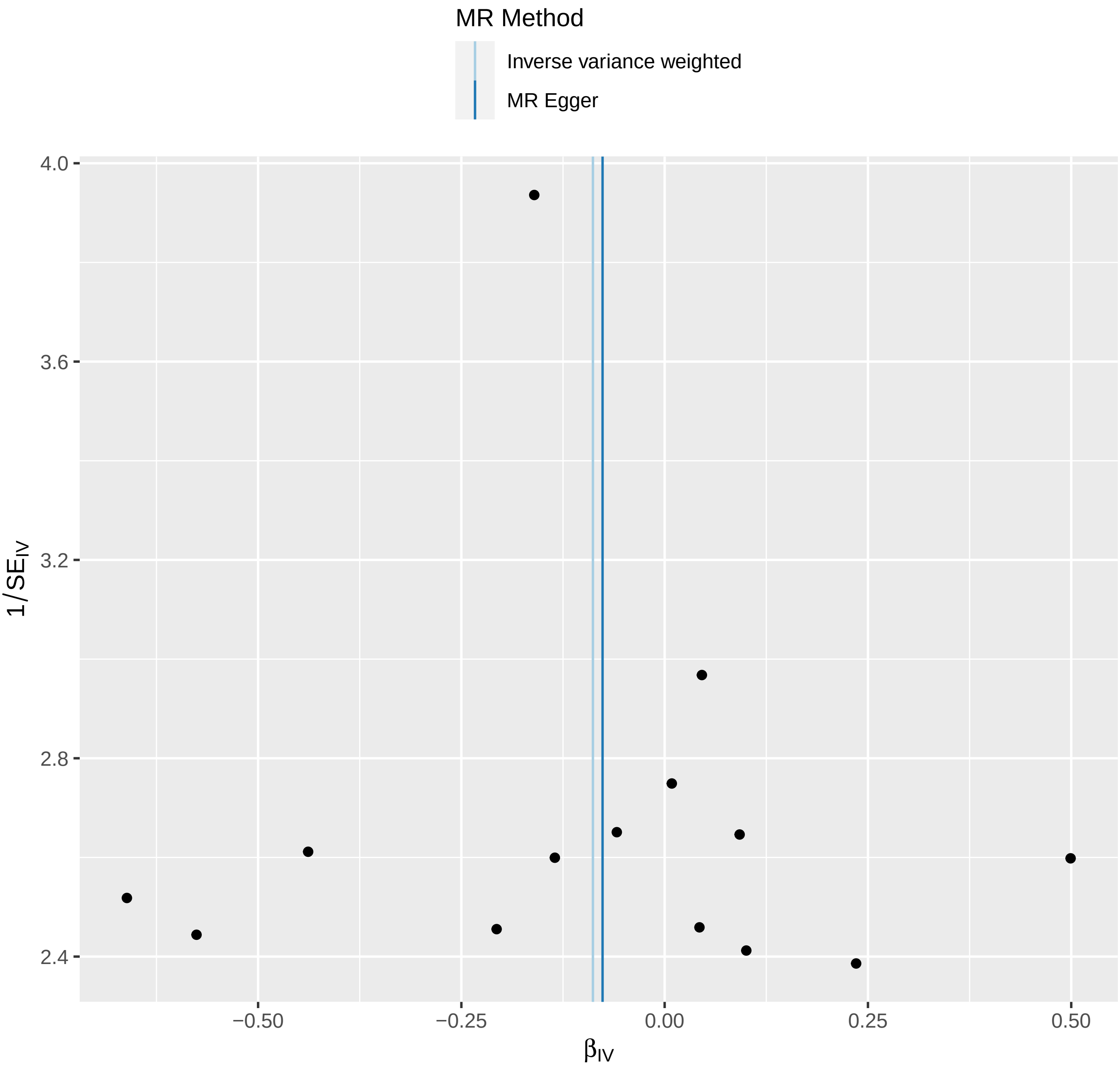

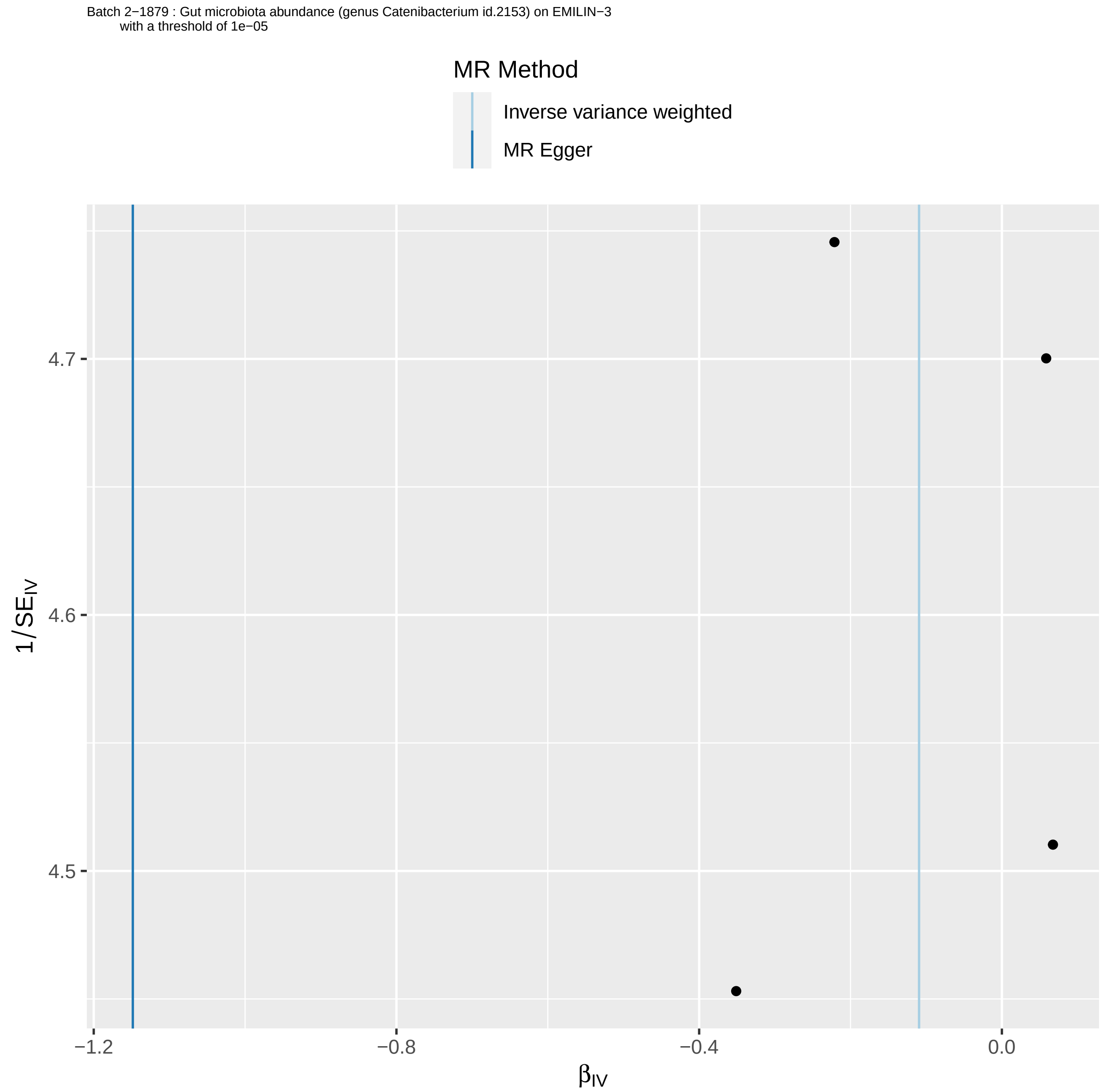
Batch 2−1879 : Gut microbiota abundance (genus Catenibacterium id.2153) on EMILIN−3
with a threshold of 1e−05
MR Method
Inverse variance weighted
MR Egger
4.7
4.6
4.5
1/SE$_{IV}$
−1.2
−0.8
−0.4
0.0
β_{IV}

Batch 2−1880 : Gut microbiota abundance (genus Parasutterella id.2892) on EMILIN−3
with a threshold of 1e−05

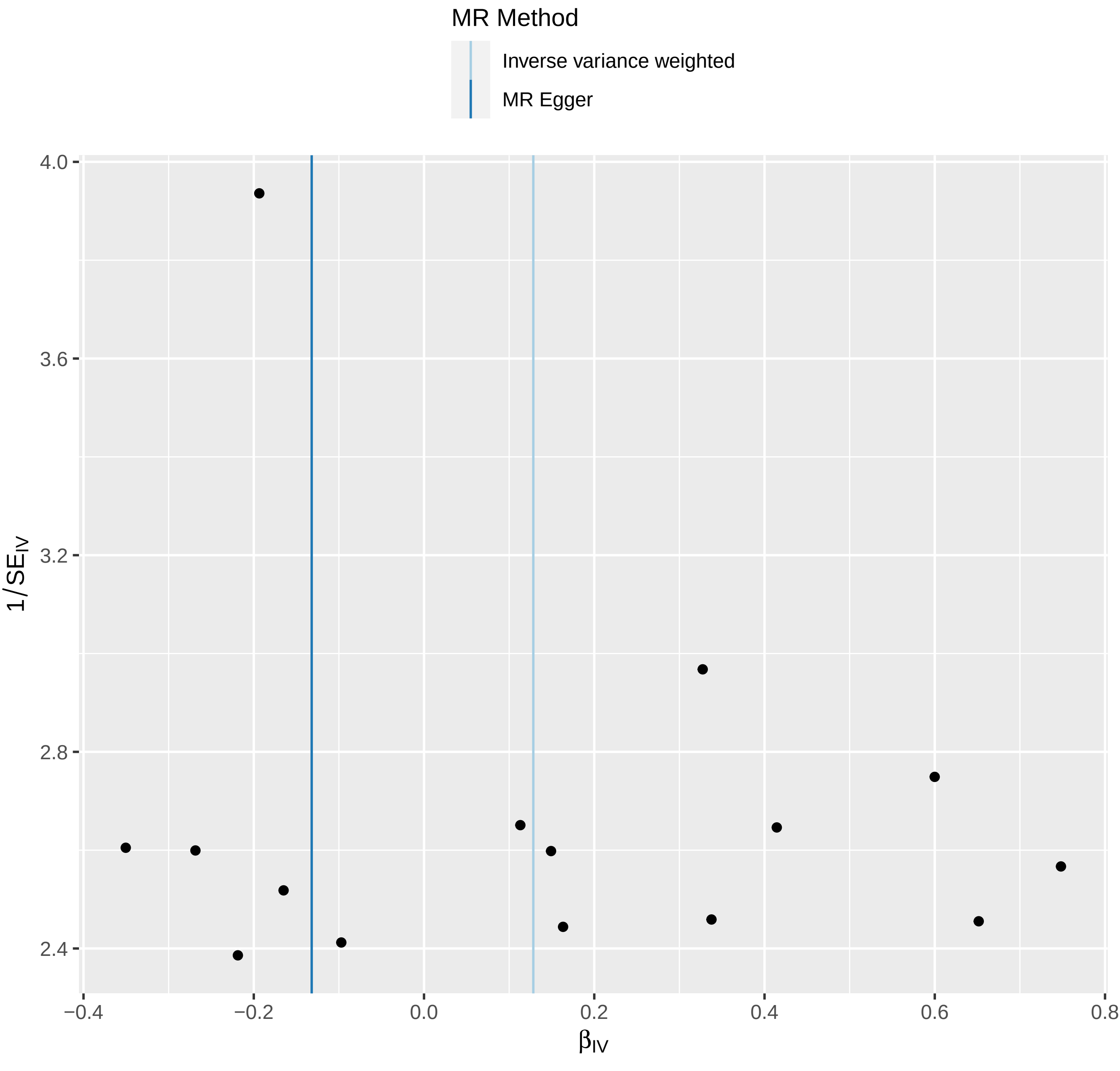

Batch 2−1881 : Gut microbiota abundance (genus Catenibacterium id.2153) on Protein enabled homolog with a threshold of 1e−05

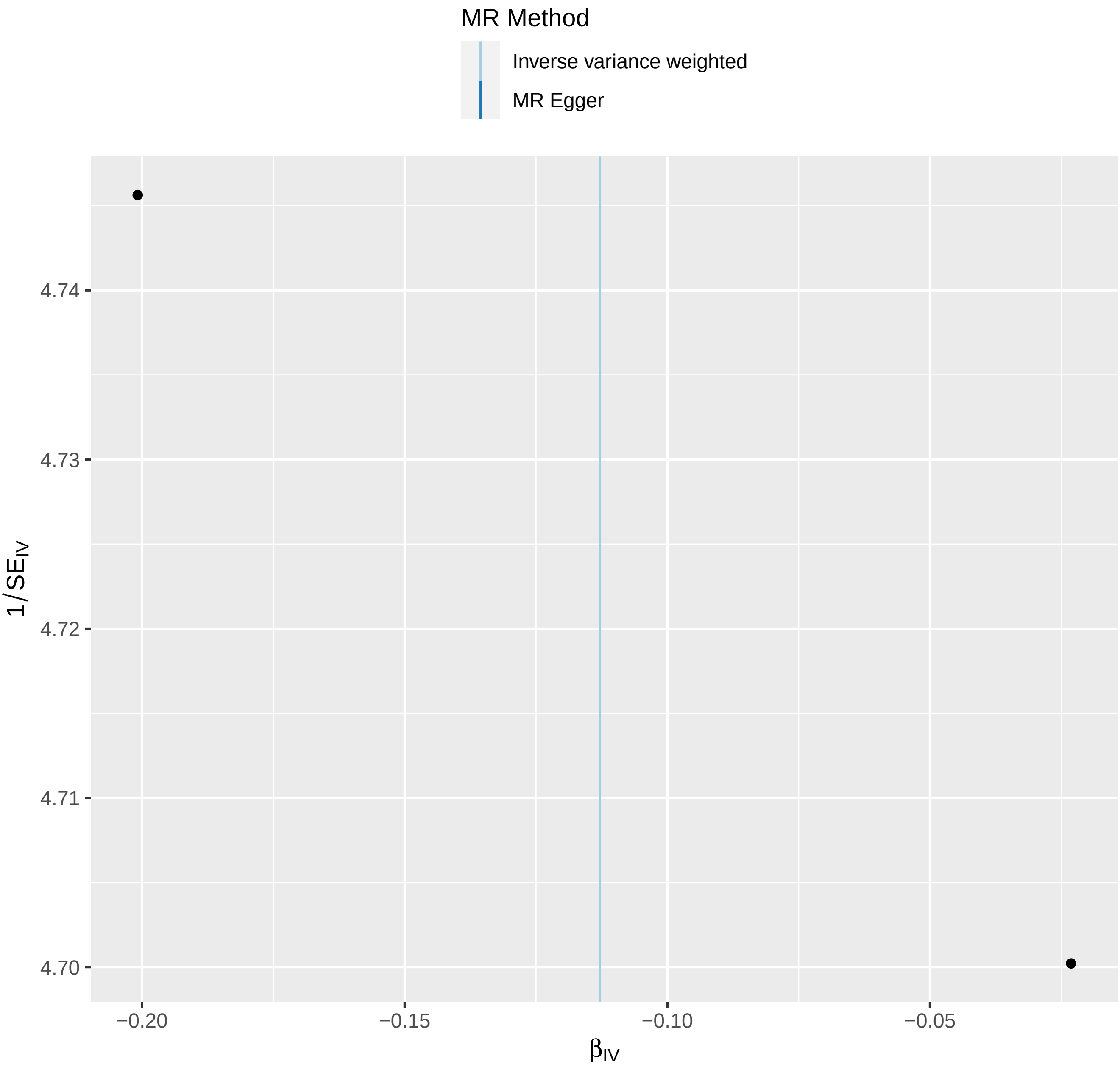

Batch 2−1882 : Gut microbiota abundance (genus Parasutterella id.2892) on Protein enabled homolog
with a threshold of 1e−05

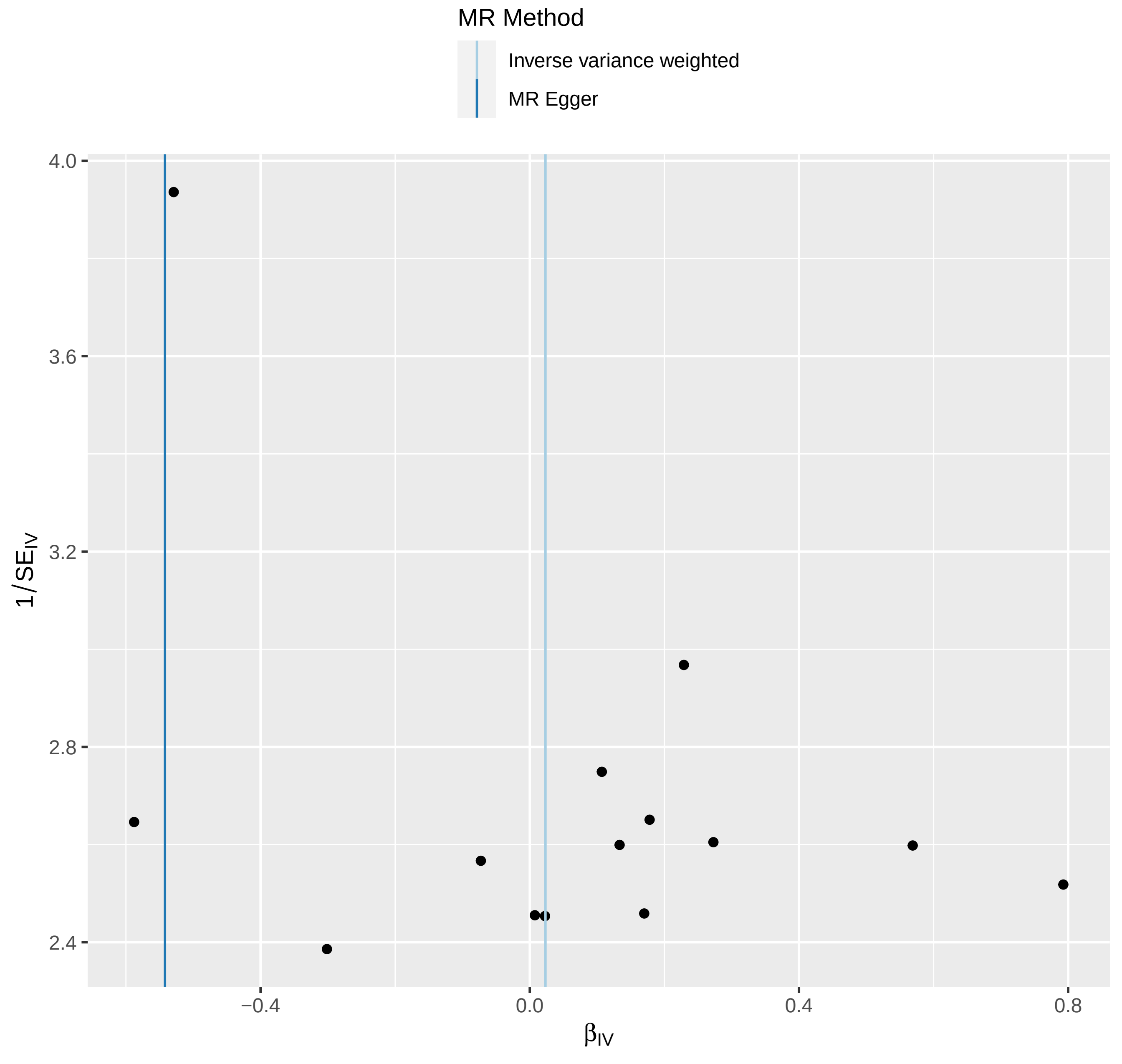

Batch 2−1883 : Gut microbiota abundance (genus Catenibacterium id.2153) on Poly(U)−specific endoribonuclease
with a threshold of 1e−05

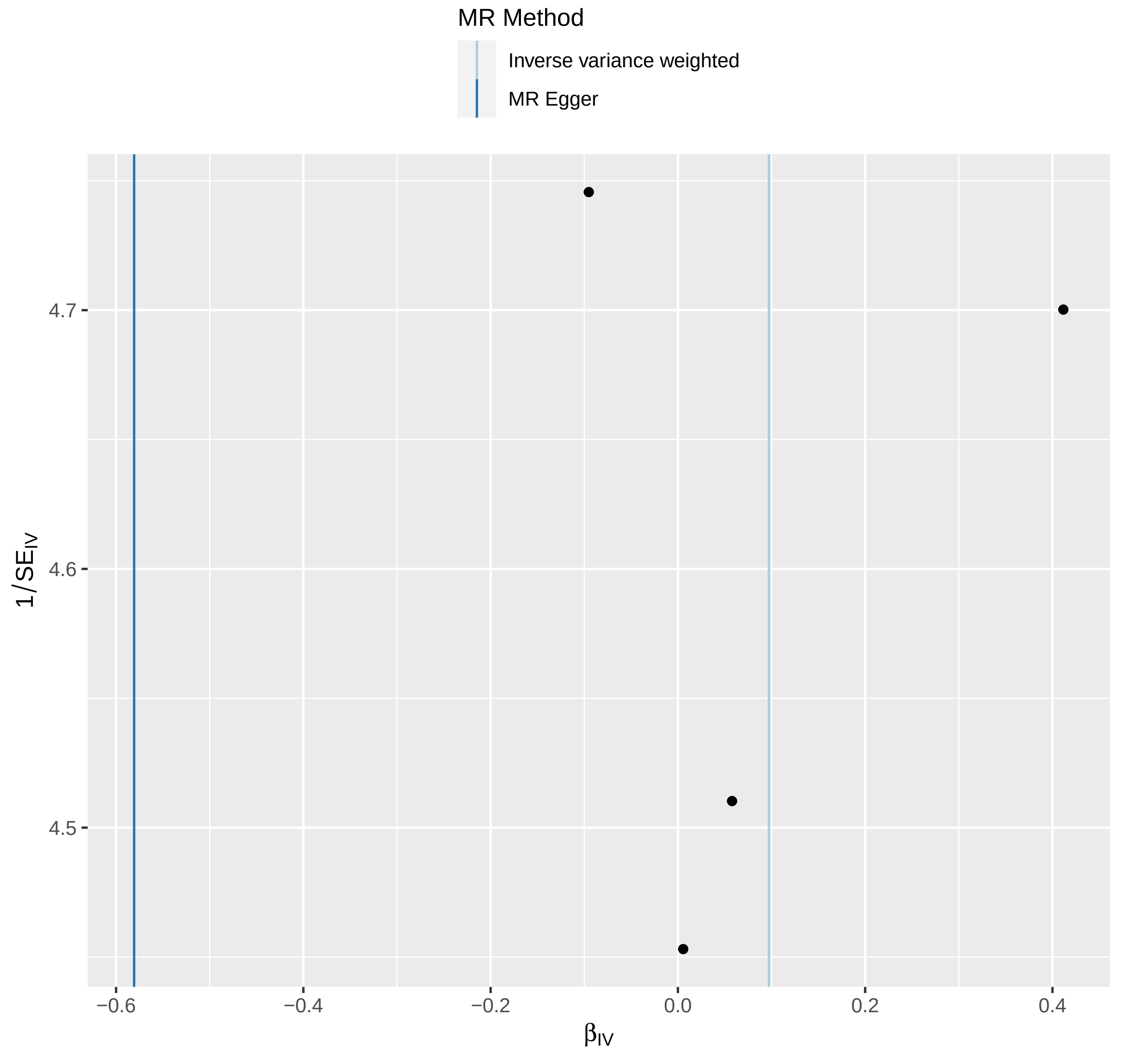

Batch 2−1884 : Gut microbiota abundance (genus Parasutterella id.2892) on Poly(U)−specific endoribonuclease
with a threshold of 1e−05

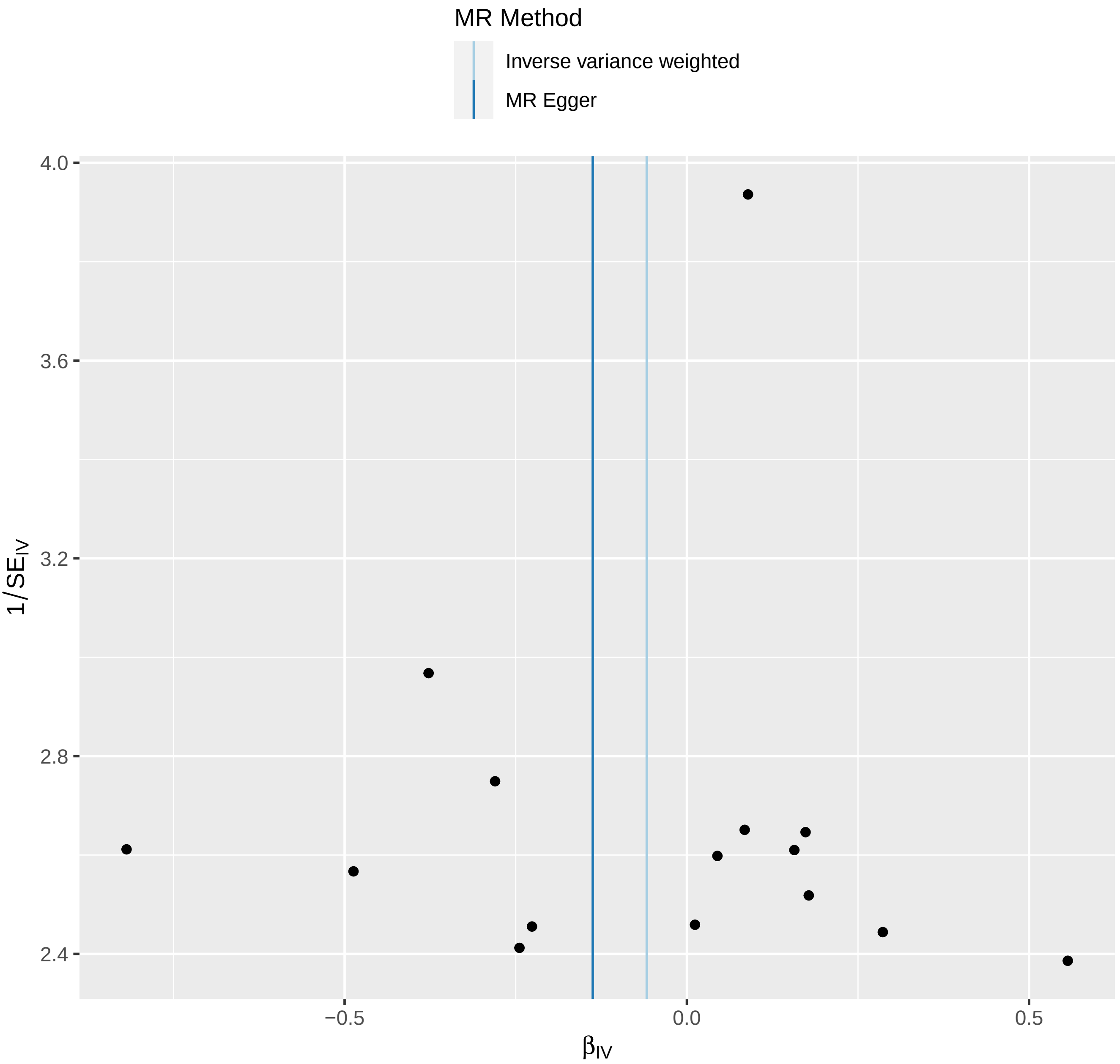

Batch 2−1885 : Gut microbiota abundance (genus Catenibacterium id.2153) on Endoglin
with a threshold of 1e−05

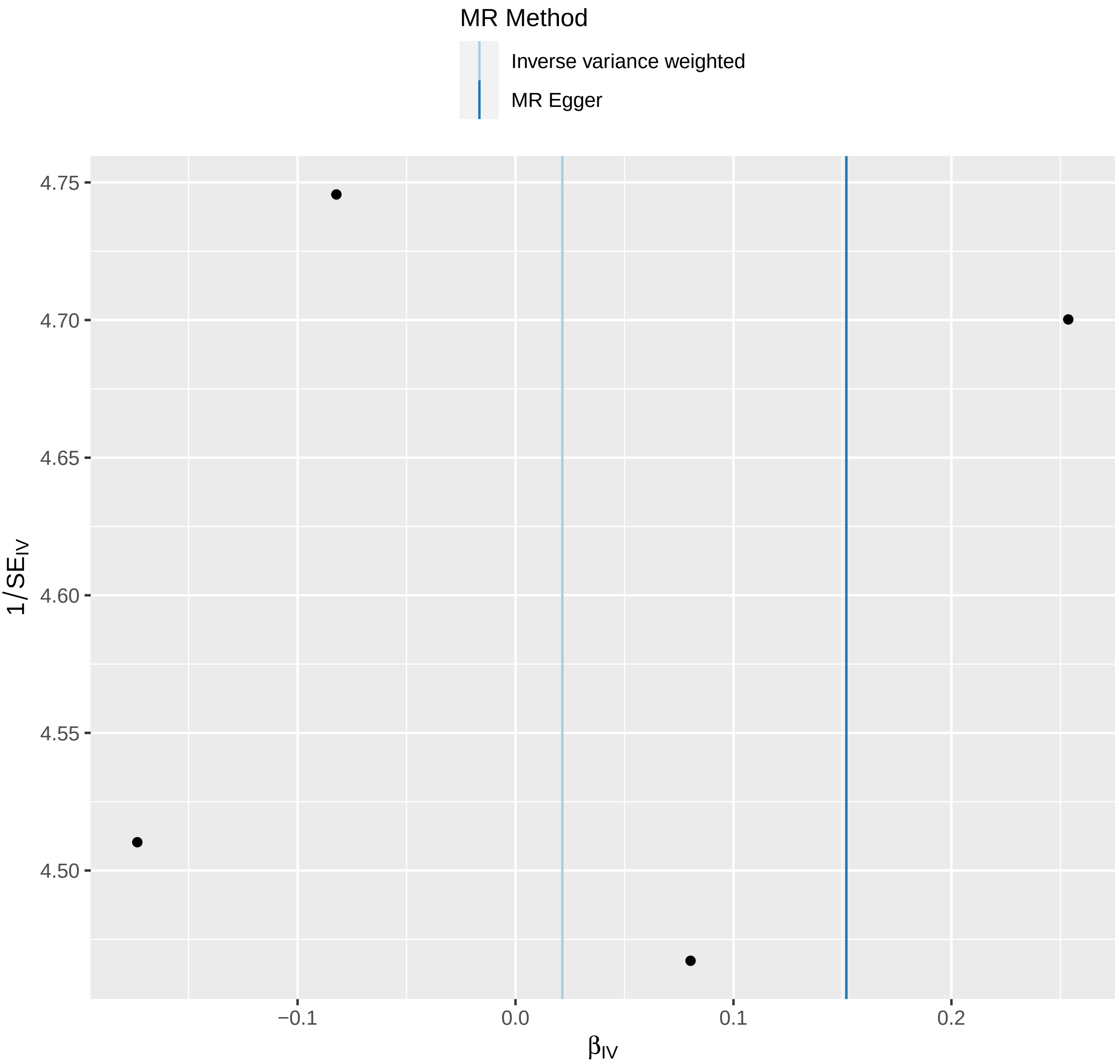

Batch 2−1886 : Gut microbiota abundance (genus Parasutterella id.2892) on Endoglin
with a threshold of 1e−05

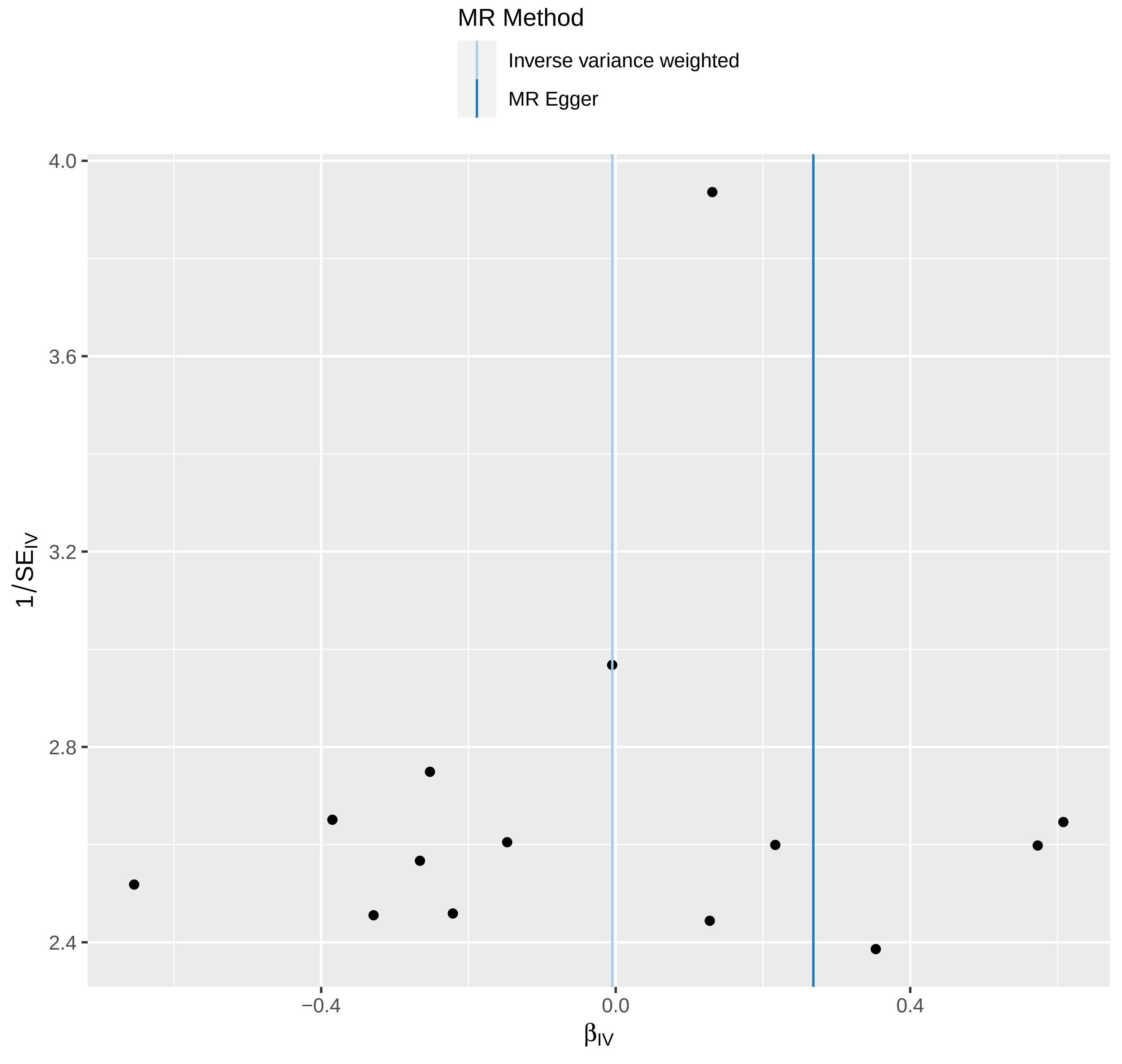

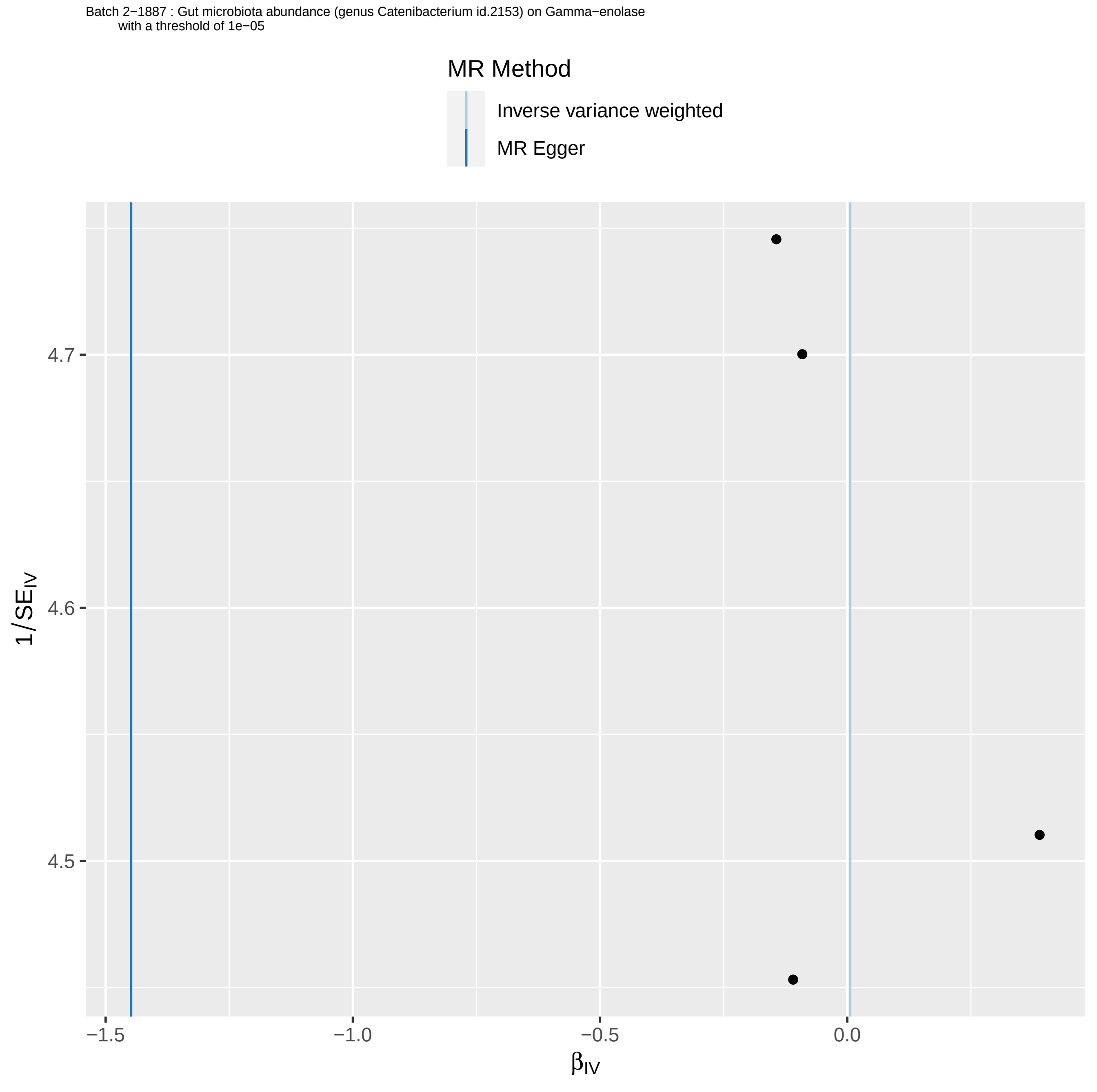

Batch 2−1887 : Gut microbiota abundance (genus Catenibacterium id.2153) on Gamma−enolase
with a threshold of 1e−05
MR Method
Inverse variance weighted
MR Egger
4.7
4.6
4.5
1/SE$_{IV}$
−1.5
−1.0
−0.5
0.0
β$_{IV}$

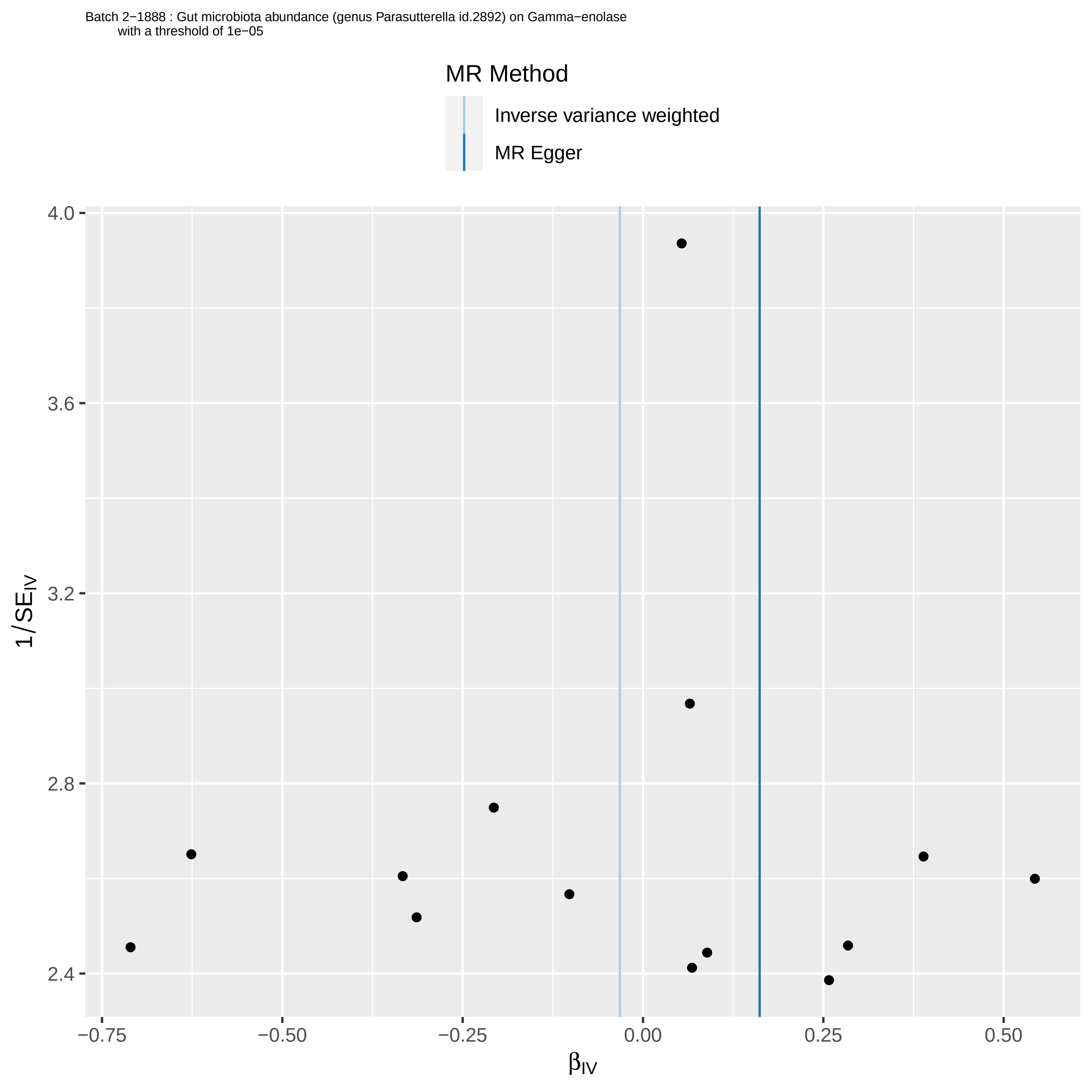

Batch 2−1888 : Gut microbiota abundance (genus Parasutterella id.2892) on Gamma−enolase
with a threshold of 1e−05
MR Method
Inverse variance weighted
MR Egger
4.0
3.6
3.2
2.8
2.4
1/SE_IV
−0.75
−0.50
−0.25
0.00
0.25
0.50
β_IV

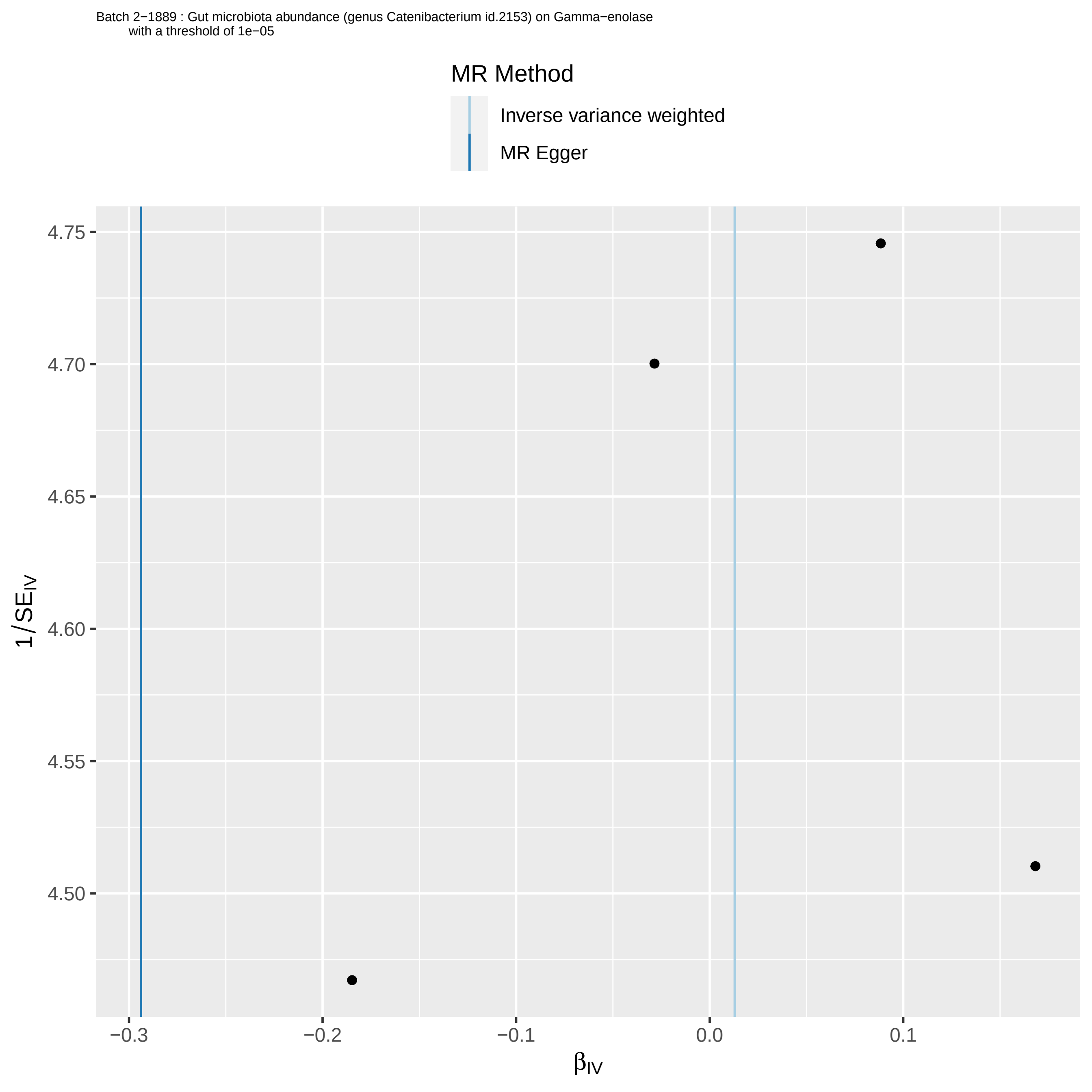
Batch 2−1889 : Gut microbiota abundance (genus Catenibacterium id.2153) on Gamma−enolase
with a threshold of 1e−05
MR Method
Inverse variance weighted
MR Egger
1/SE_IV
4.75
4.70
4.65
4.60
4.55
4.50
−0.3
−0.2
−0.1
0.0
0.1
β_IV

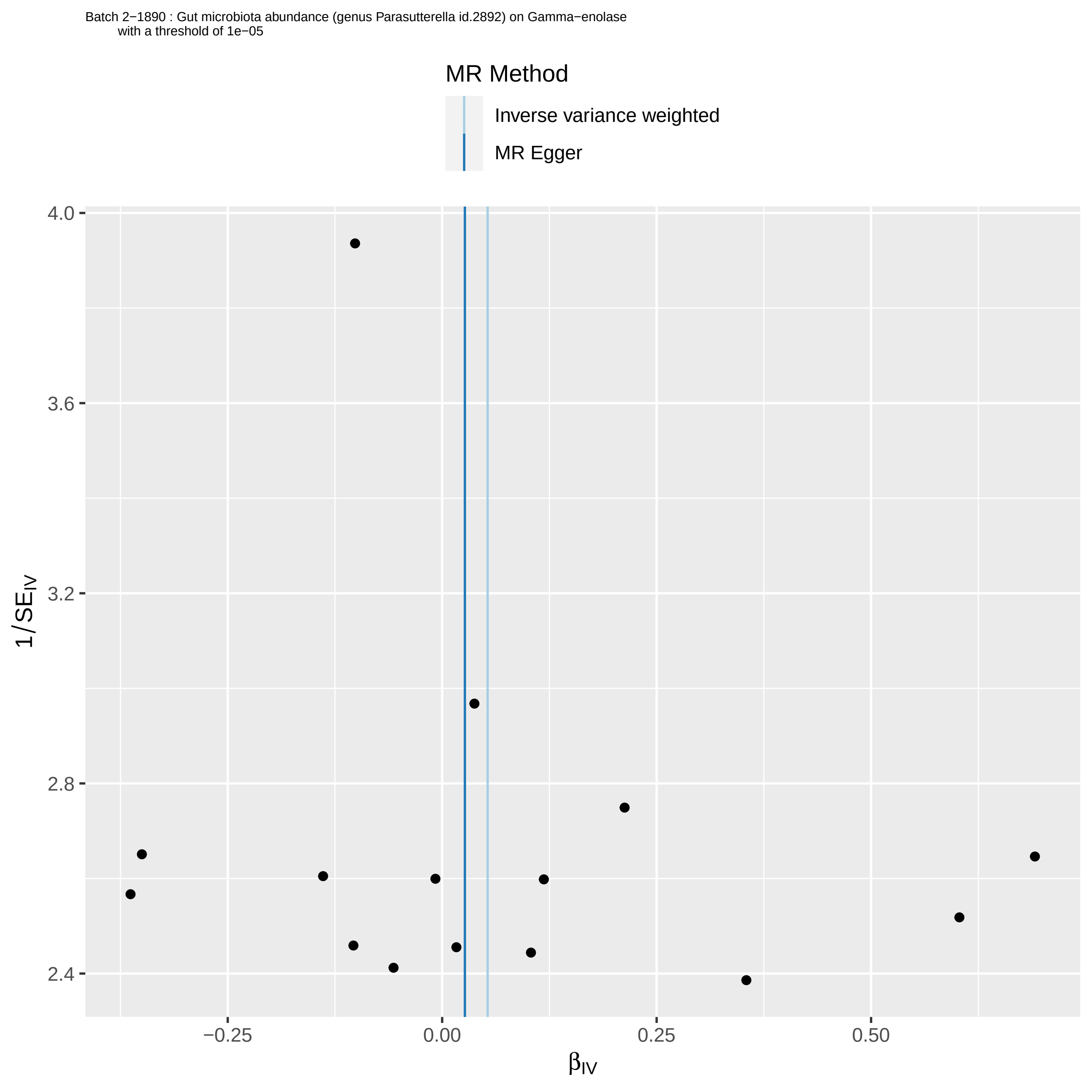

Batch 2−1890 : Gut microbiota abundance (genus Parasutterella id.2892) on Gamma−enolase
with a threshold of 1e−05
MR Method
Inverse variance weighted
MR Egger
4.0
3.6
3.2
2.8
2.4
1/SE_IV
−0.25
0.00
0.25
0.50
β_IV

Batch 2−1891 : Gut microbiota abundance (genus Catenibacterium id.2153) on Ecto−NOX disulfide−thiol exchanger 2
with a threshold of 1e−05

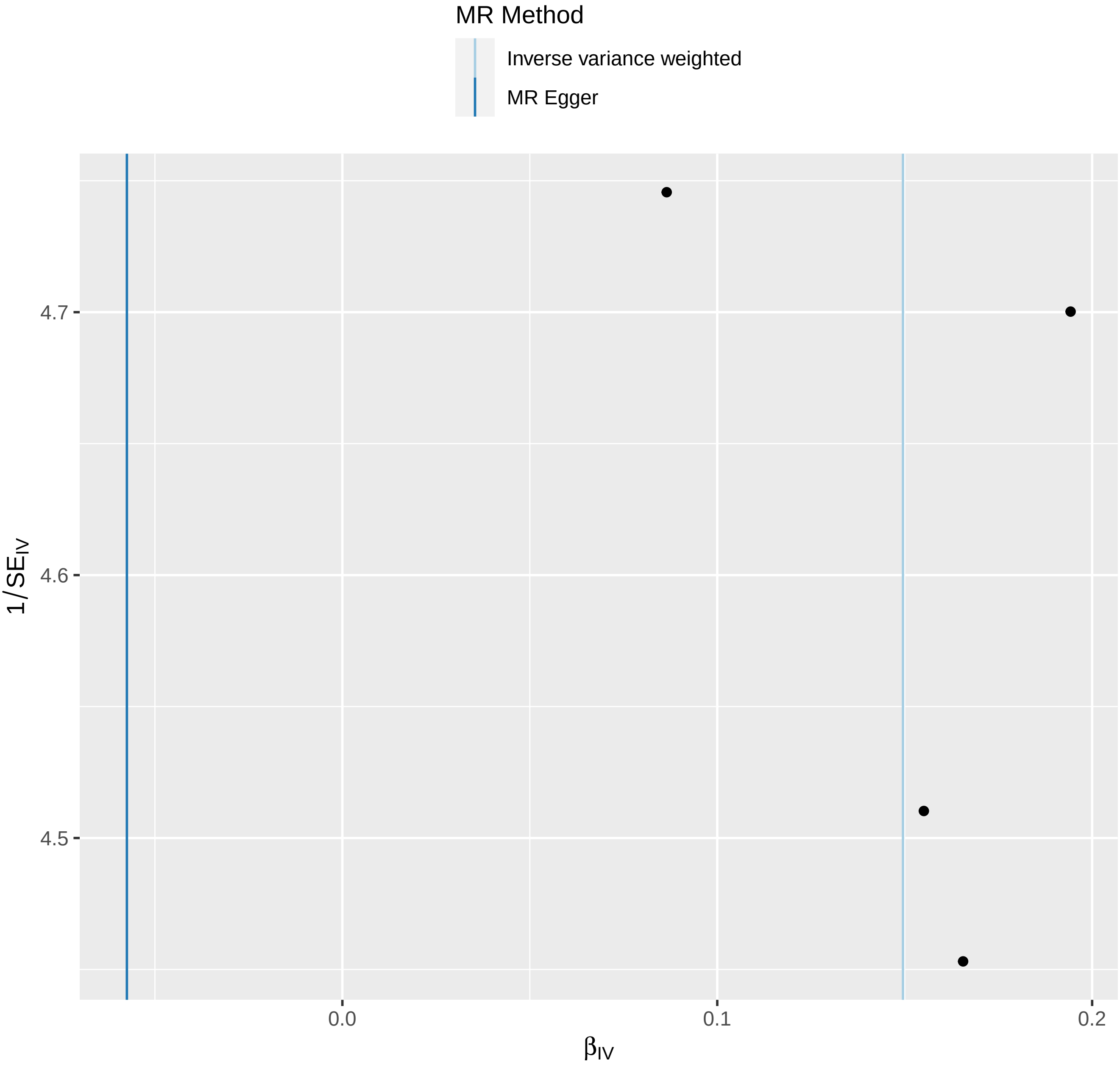

Batch 2−1892 : Gut microbiota abundance (genus Parasutterella id.2892) on Ecto−NOX disulfide−thiol exchanger 2
with a threshold of 1e−05

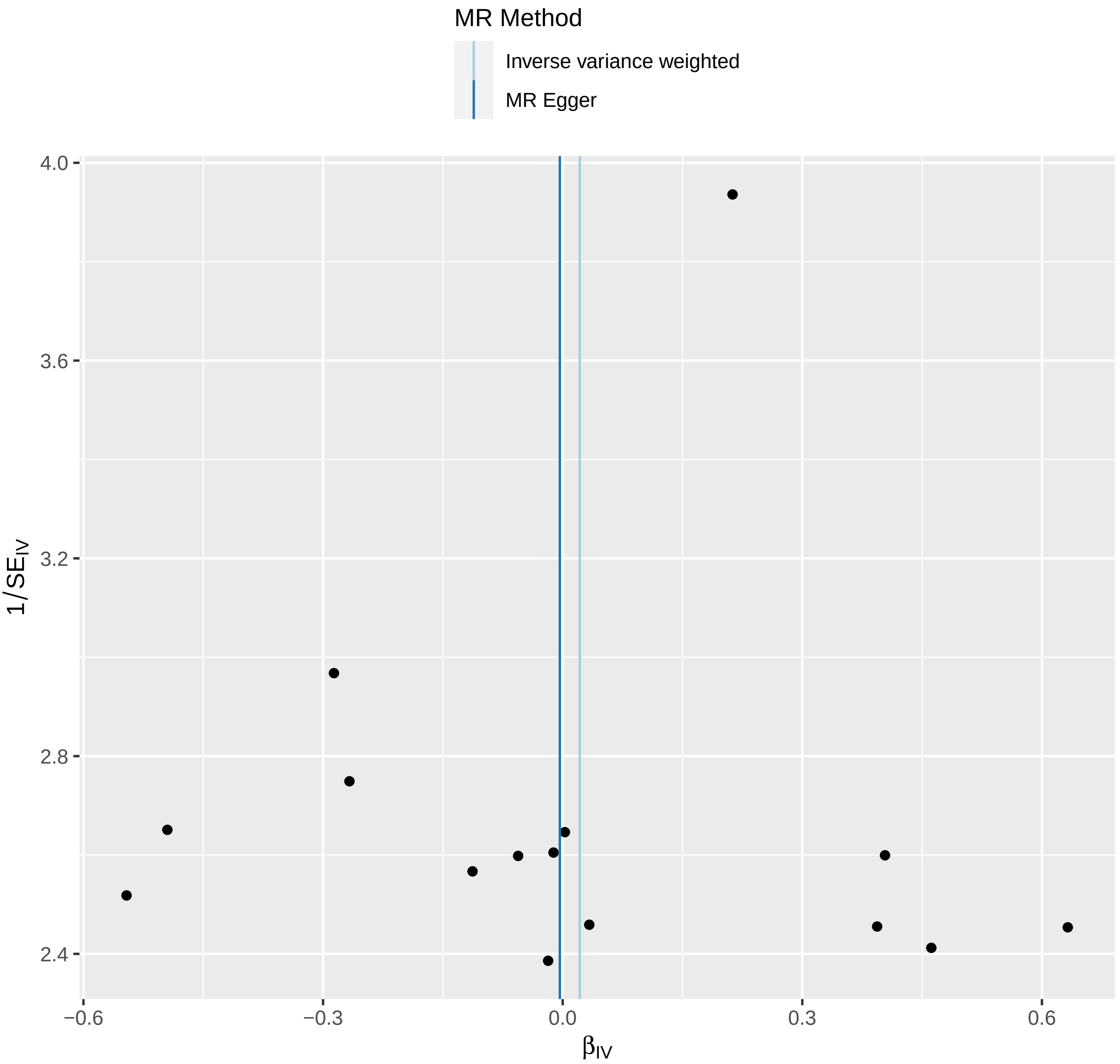

Batch 2−1893 : Gut microbiota abundance (genus Catenibacterium id.2153) on Ectonucleotide pyrophosphatase/phosphodiesterase family member 5 with a threshold of 1e−05

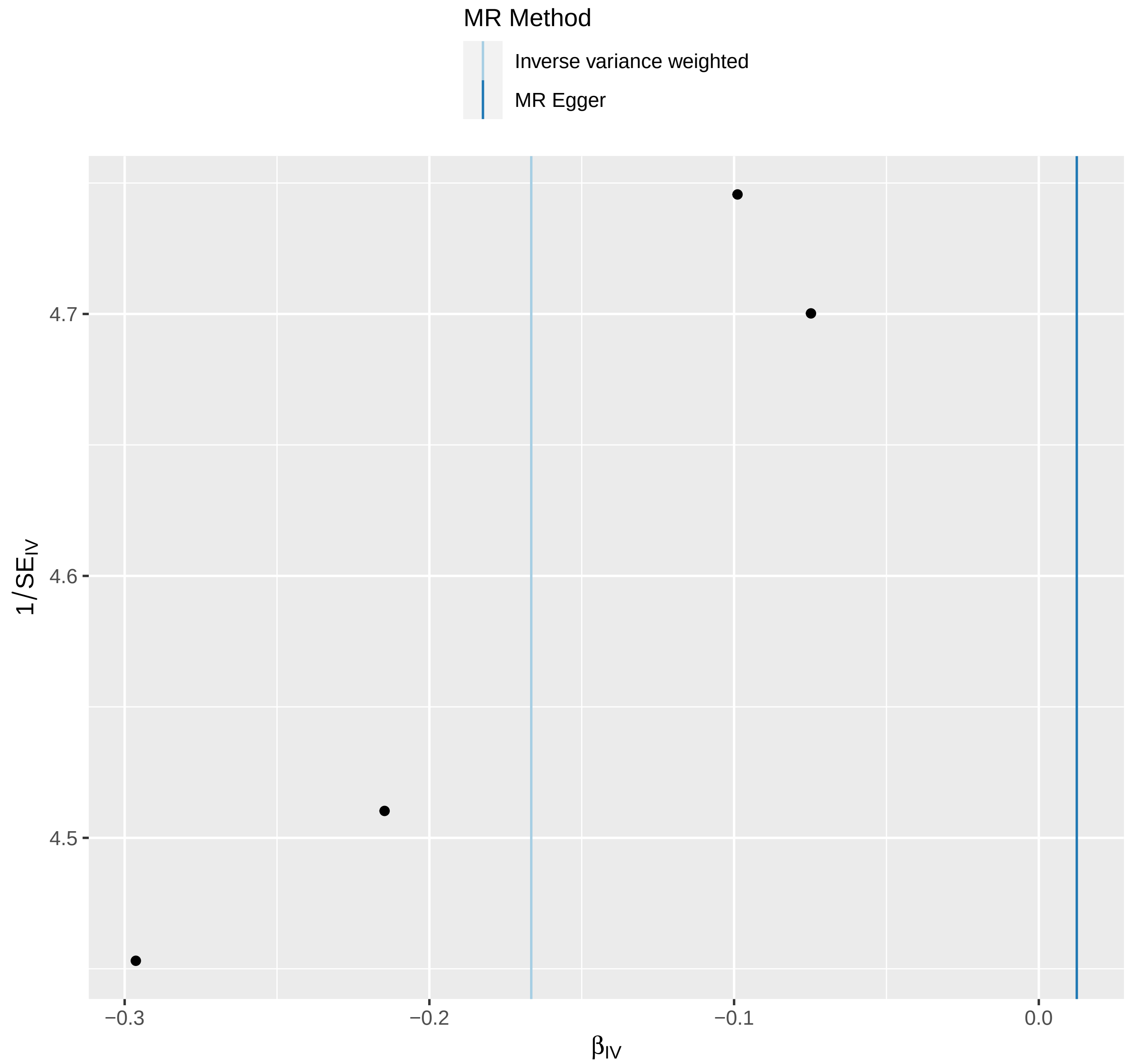

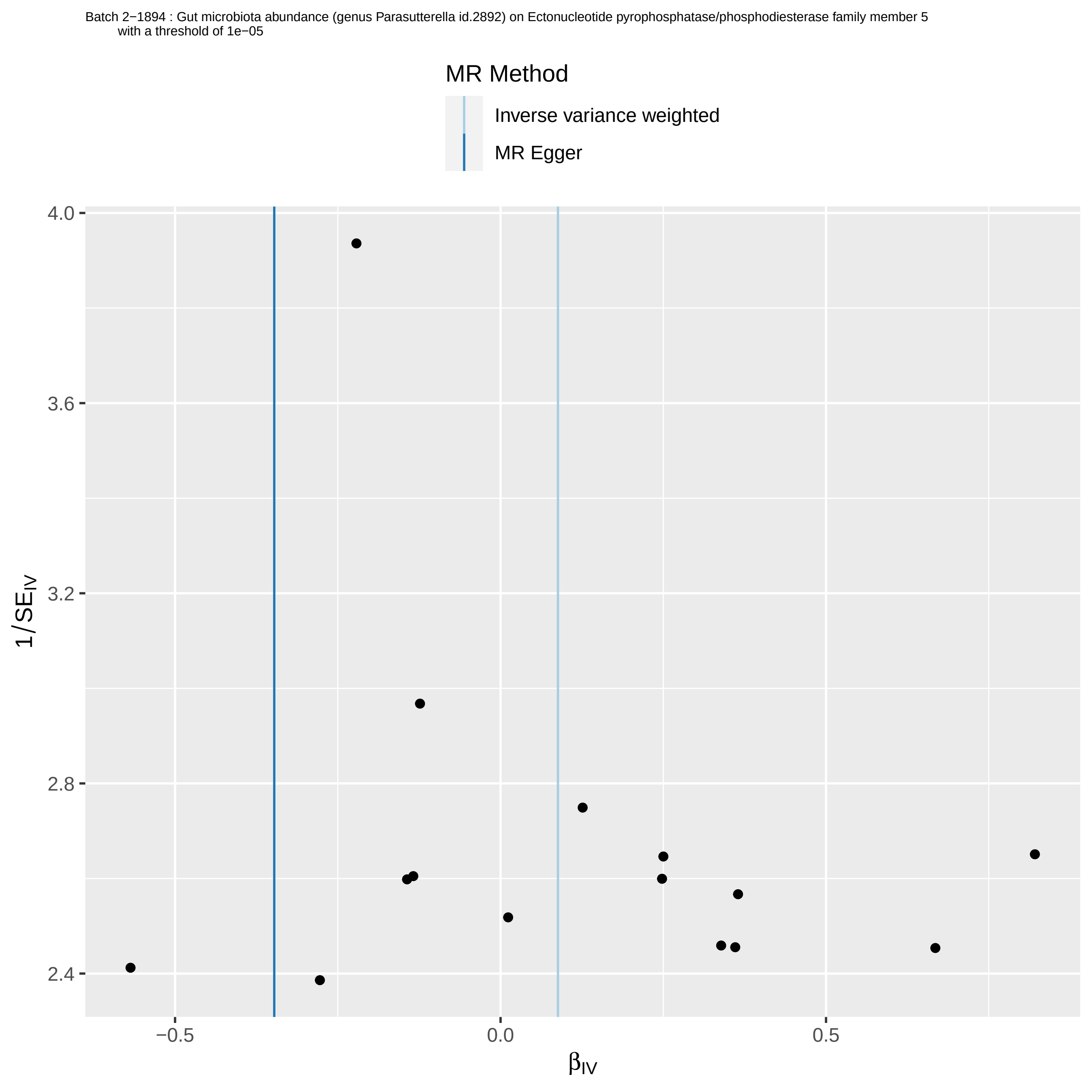

Batch 2−1894 : Gut microbiota abundance (genus Parasutterella id.2892) on Ectonucleotide pyrophosphatase/phosphodiesterase family member 5
with a threshold of 1e−05
MR Method
Inverse variance weighted
MR Egger
4.0
3.6
3.2
2.8
2.4
1/SE_IV
−0.5
0.0
0.5
β_IV

Batch 2−1895 : Gut microbiota abundance (genus Catenibacterium id.2153) on Ectonucleotide pyrophosphatase/phosphodiesterase family member 7 with a threshold of 1e−05

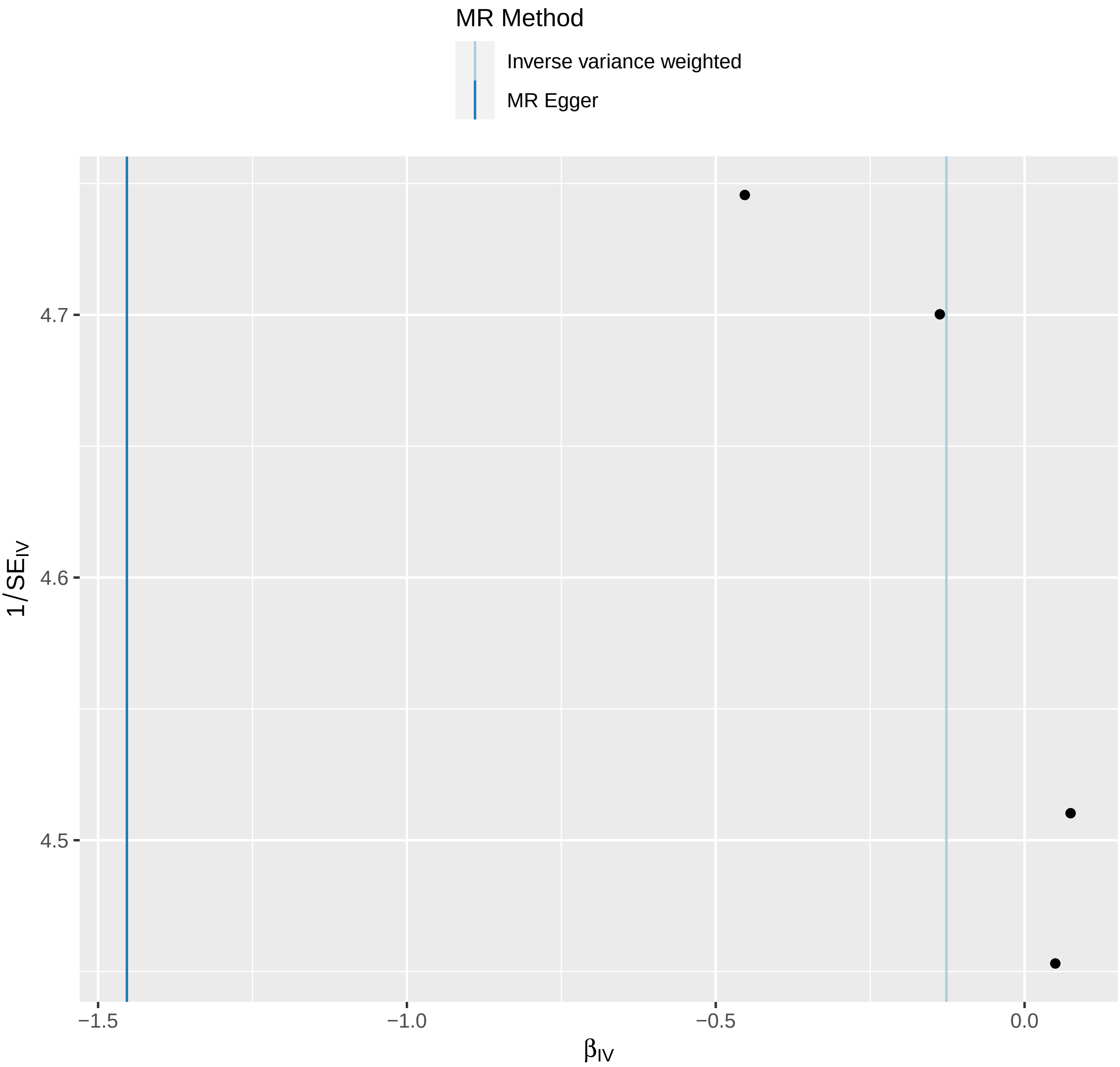

Batch 2−1896 : Gut microbiota abundance (genus Parasutterella id.2892) on Ectonucleotide pyrophosphatase/phosphodiesterase family member 7 with a threshold of 1e−05

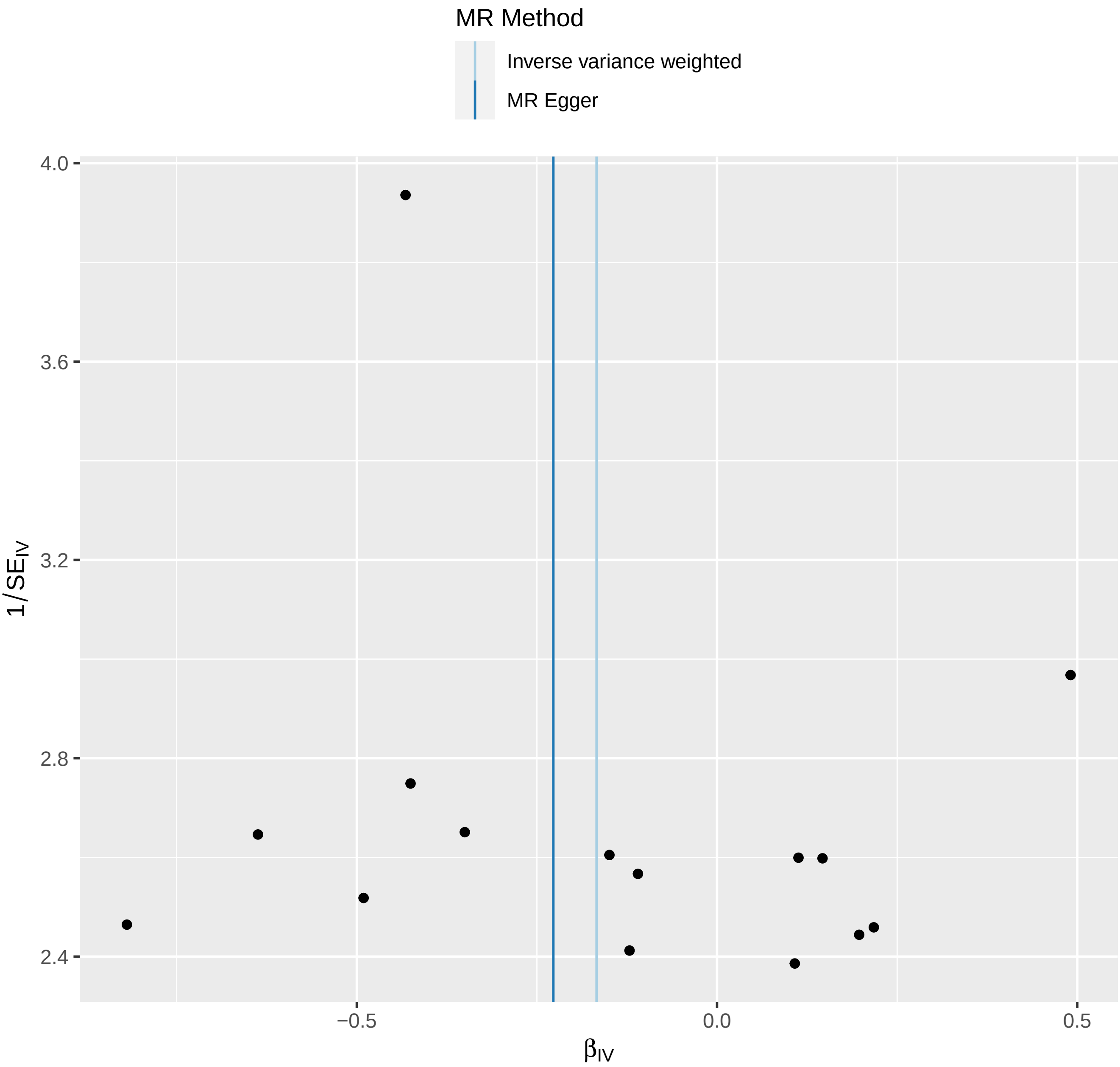

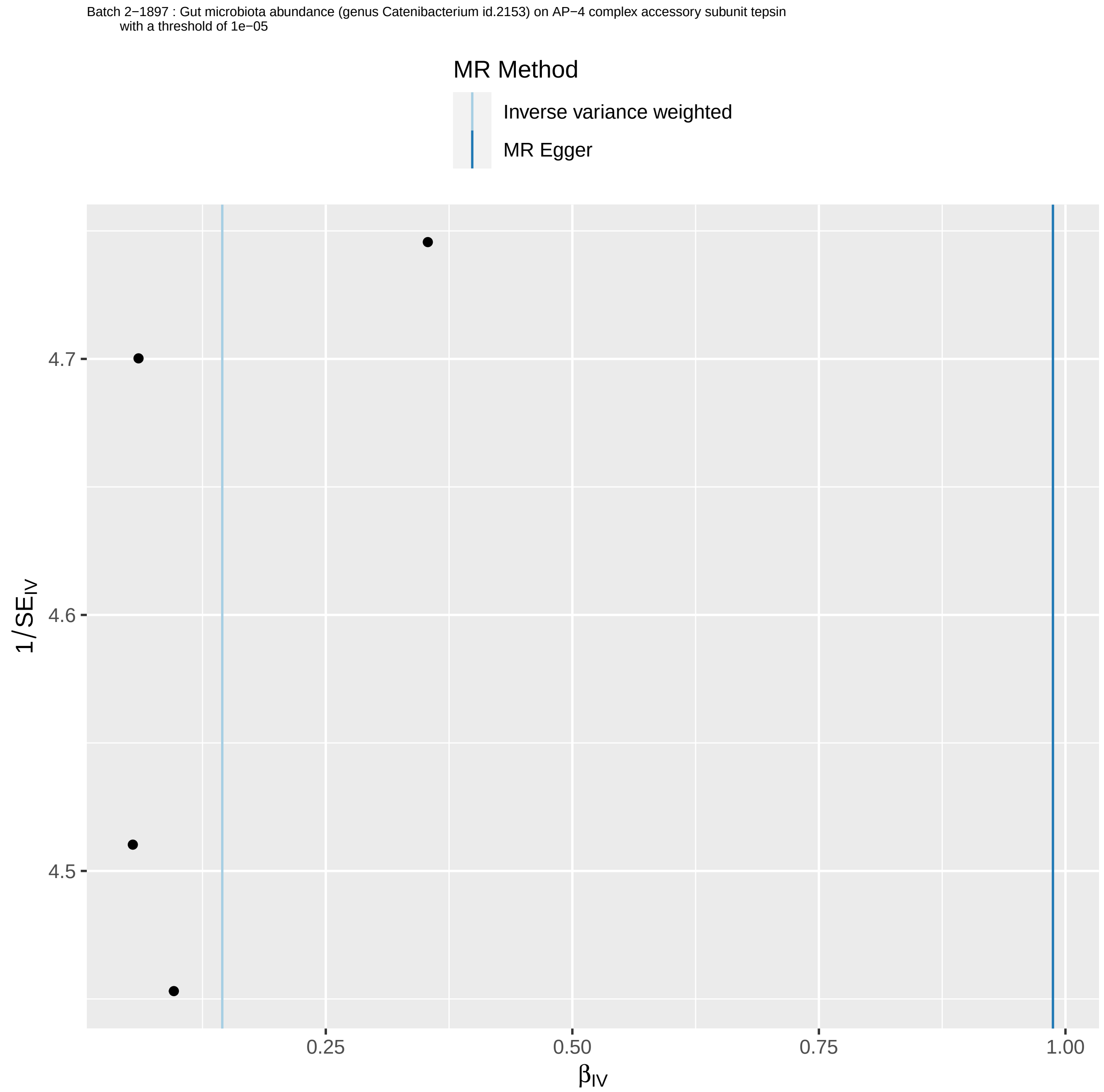

Batch 2−1897 : Gut microbiota abundance (genus Catenibacterium id.2153) on AP−4 complex accessory subunit tepsin
with a threshold of 1e−05
MR Method
Inverse variance weighted
MR Egger
4.7
4.6
4.5
1/SE$_{IV}$
0.25
0.50
0.75
1.00
β$_{IV}$

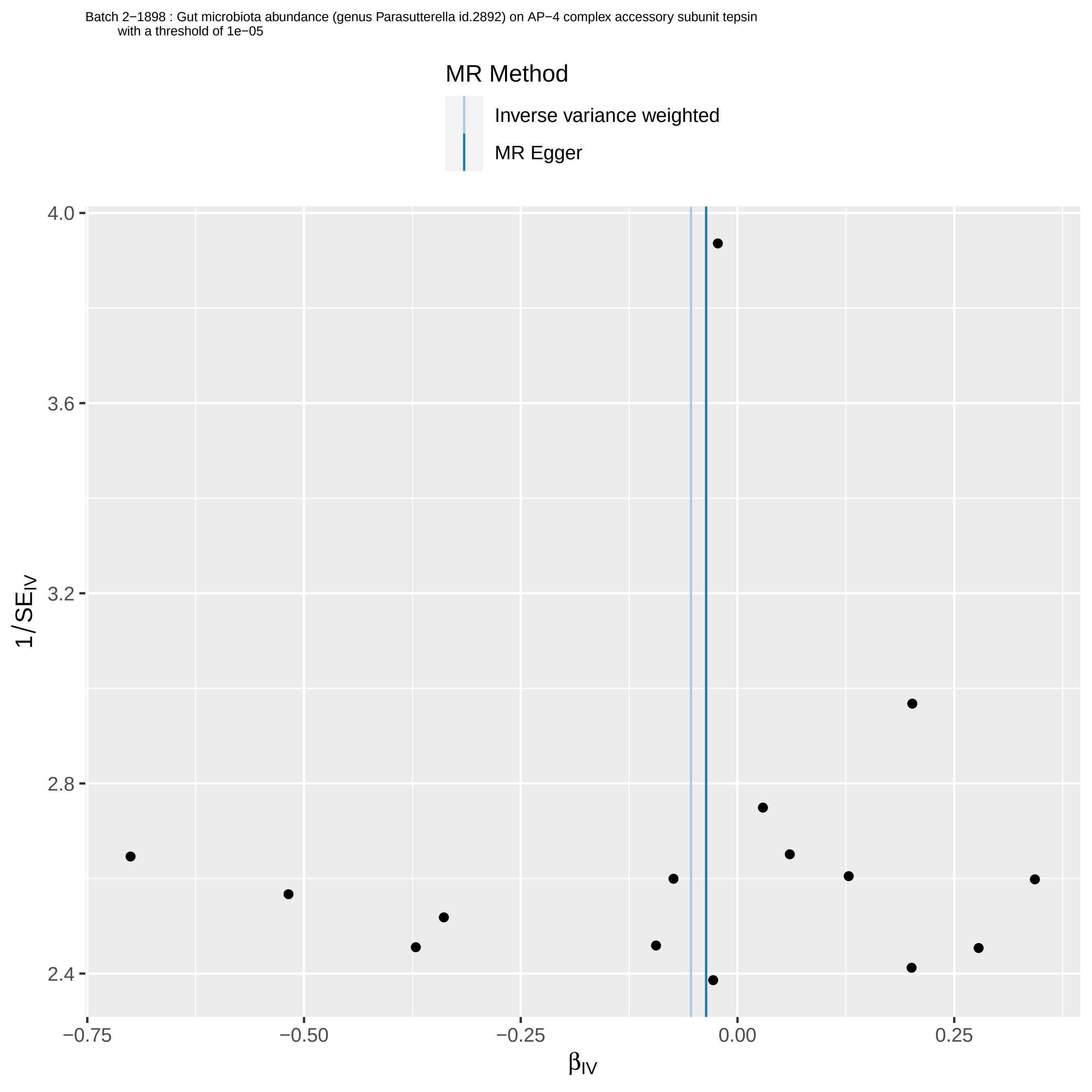

Batch 2−1898 : Gut microbiota abundance (genus Parasutterella id.2892) on AP−4 complex accessory subunit tepsin
with a threshold of 1e−05
MR Method
Inverse variance weighted
MR Egger
4.0
3.6
3.2
2.8
2.4
1/SE_IV
−0.75
−0.50
−0.25
0.00
0.25
β_IV

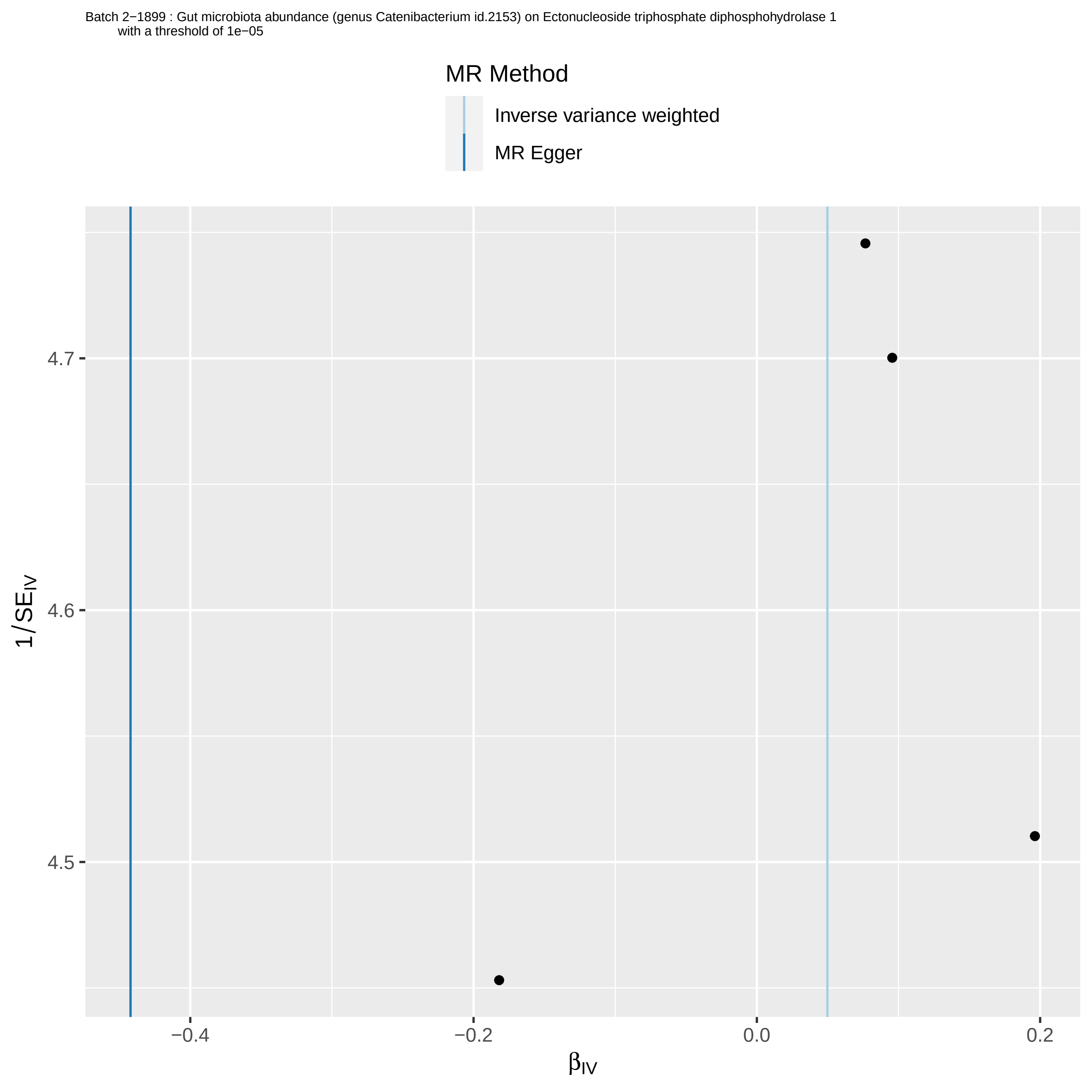

Batch 2−1899 : Gut microbiota abundance (genus Catenibacterium id.2153) on Ectonucleoside triphosphate diphosphohydrolase 1
with a threshold of 1e−05
MR Method
Inverse variance weighted
MR Egger
4.7
4.6
4.5
1/SE_IV
−0.4
−0.2
0.0
0.2
β_IV

Batch 2−1900 : Gut microbiota abundance (genus Parasutterella id.2892) on Ectonucleoside triphosphate diphosphohydrolase 1
with a threshold of 1e−05

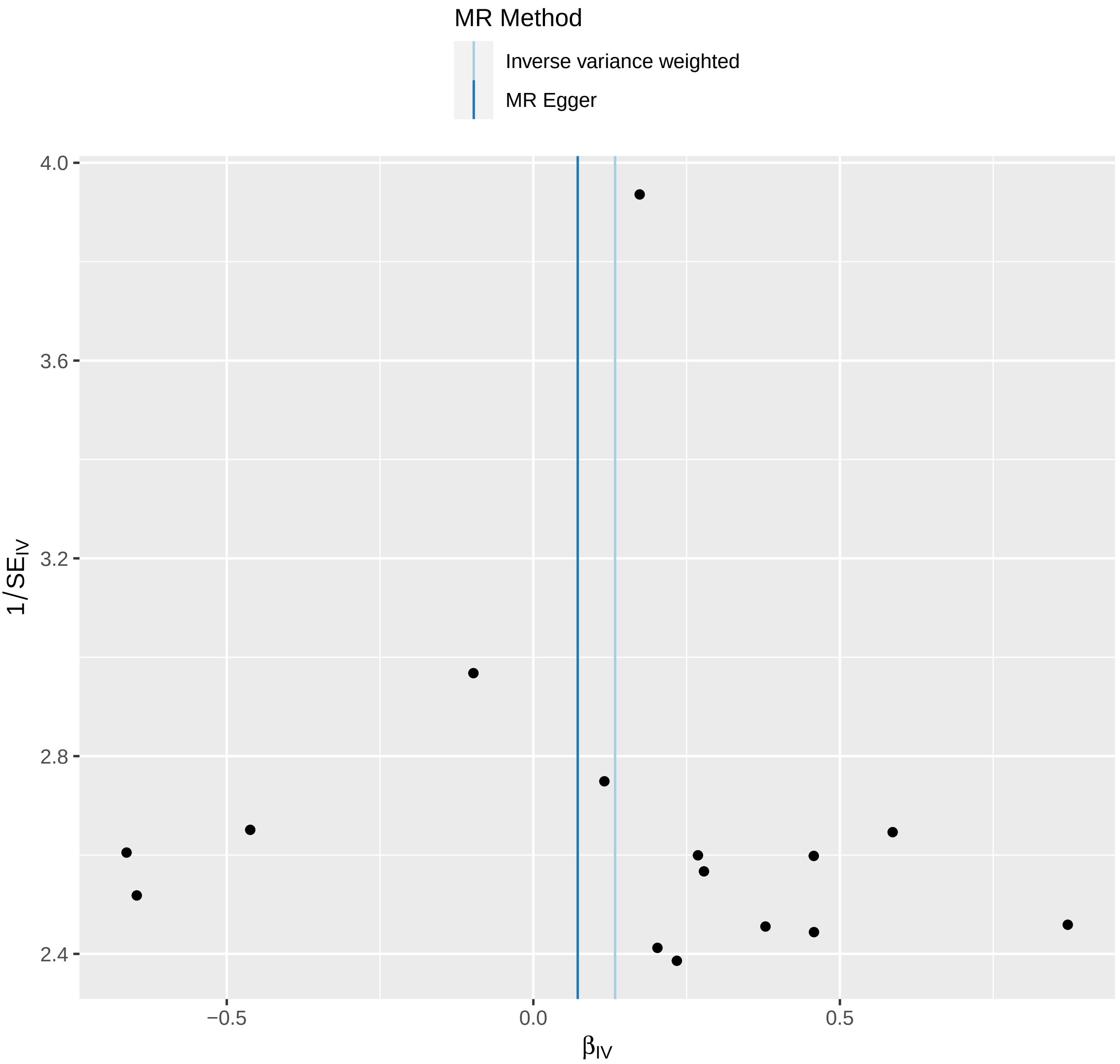

Batch 2−1901 : Gut microbiota abundance (genus Catenibacterium id.2153) on Ectonucleoside triphosphate diphosphohydrolase 1
with a threshold of 1e−05

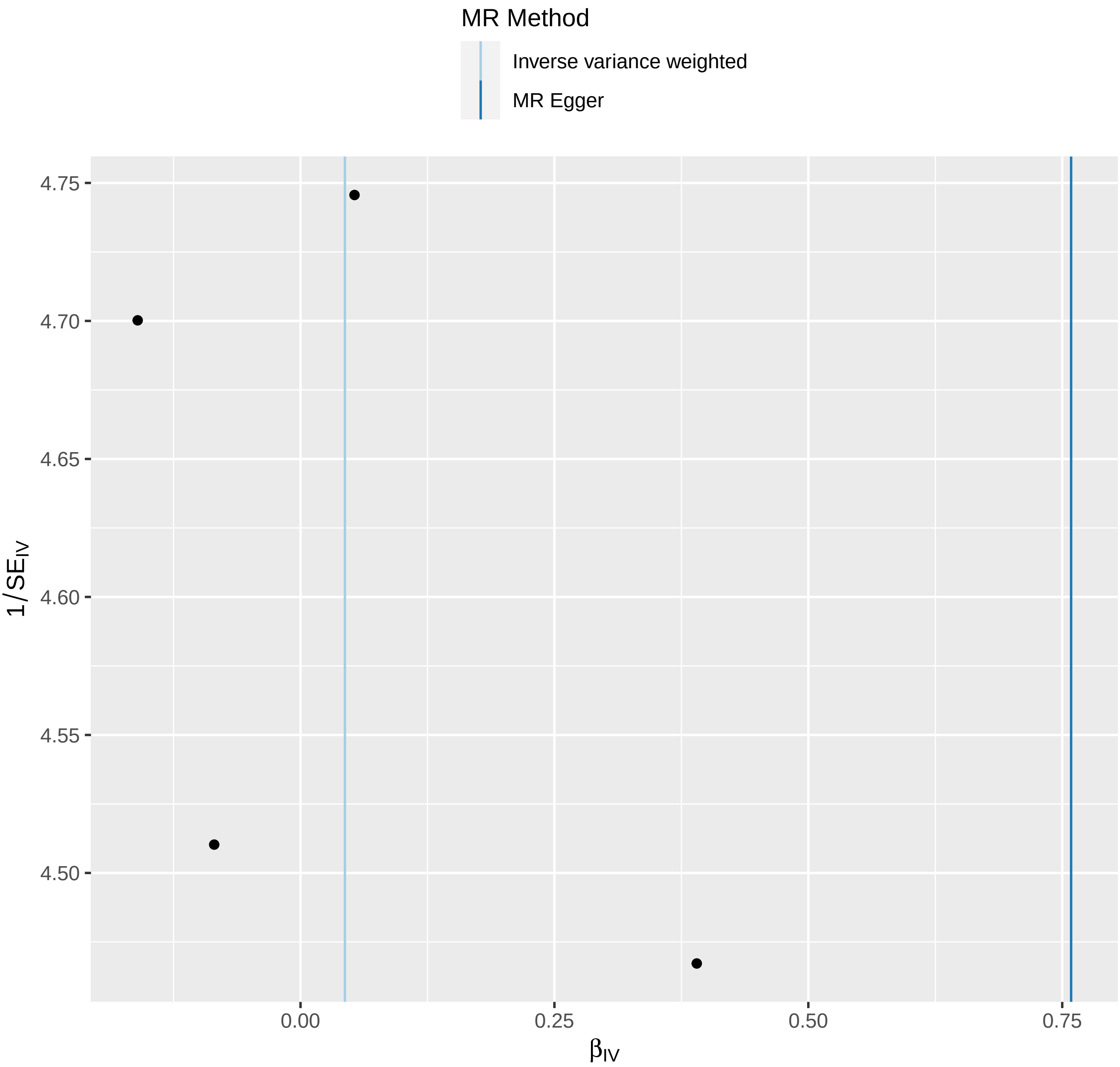

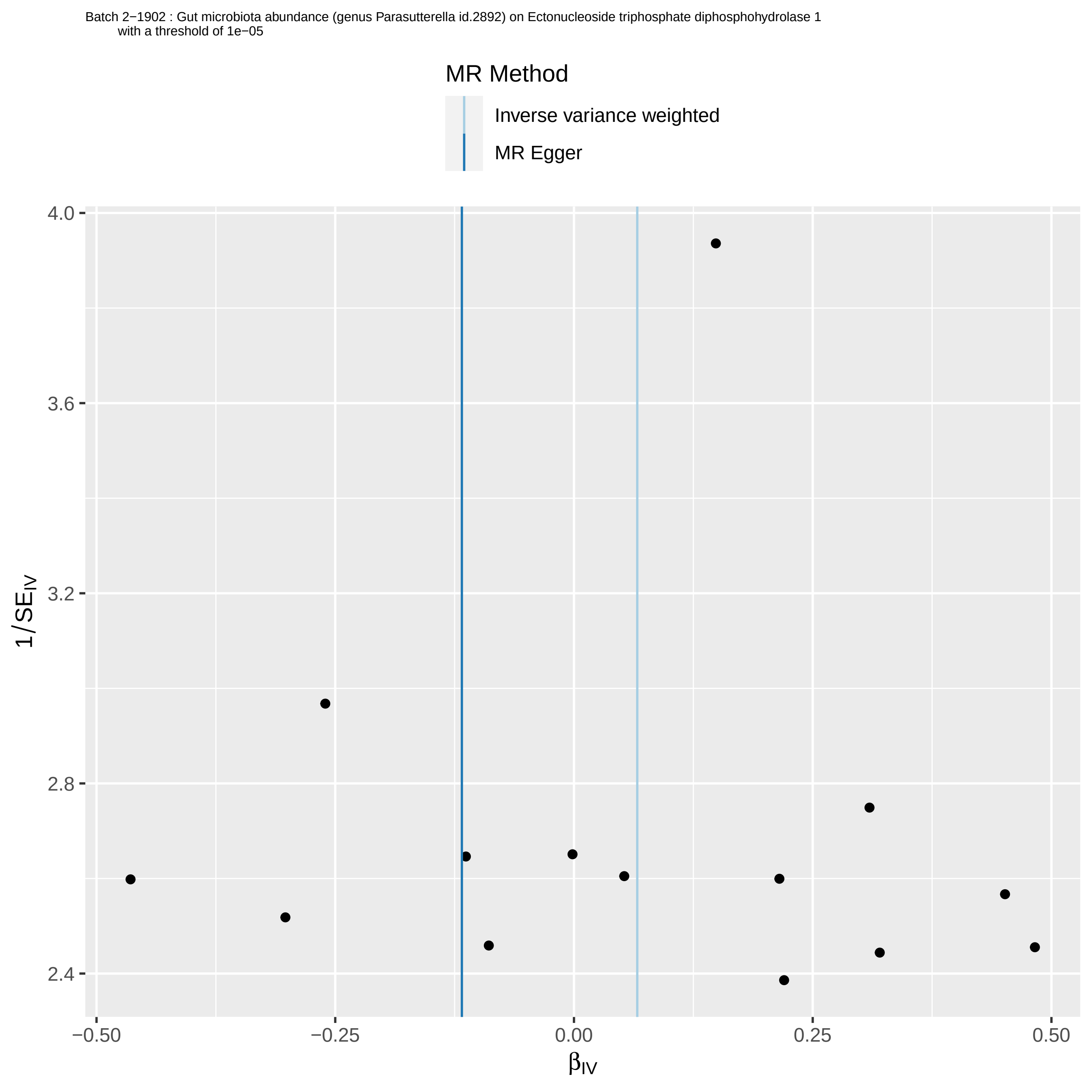

Batch 2−1902 : Gut microbiota abundance (genus Parasutterella id.2892) on Ectonucleoside triphosphate diphosphohydrolase 1
with a threshold of 1e−05
MR Method
Inverse variance weighted
MR Egger
4.0
3.6
3.2
2.8
2.4
1/SE$_{IV}$
−0.50
−0.25
0.00
0.25
0.50
β$_{IV}$

Batch 2−1903 : Gut microbiota abundance (genus Catenibacterium id.2153) on Ectonucleoside triphosphate diphosphohydrolase 3
with a threshold of 1e−05

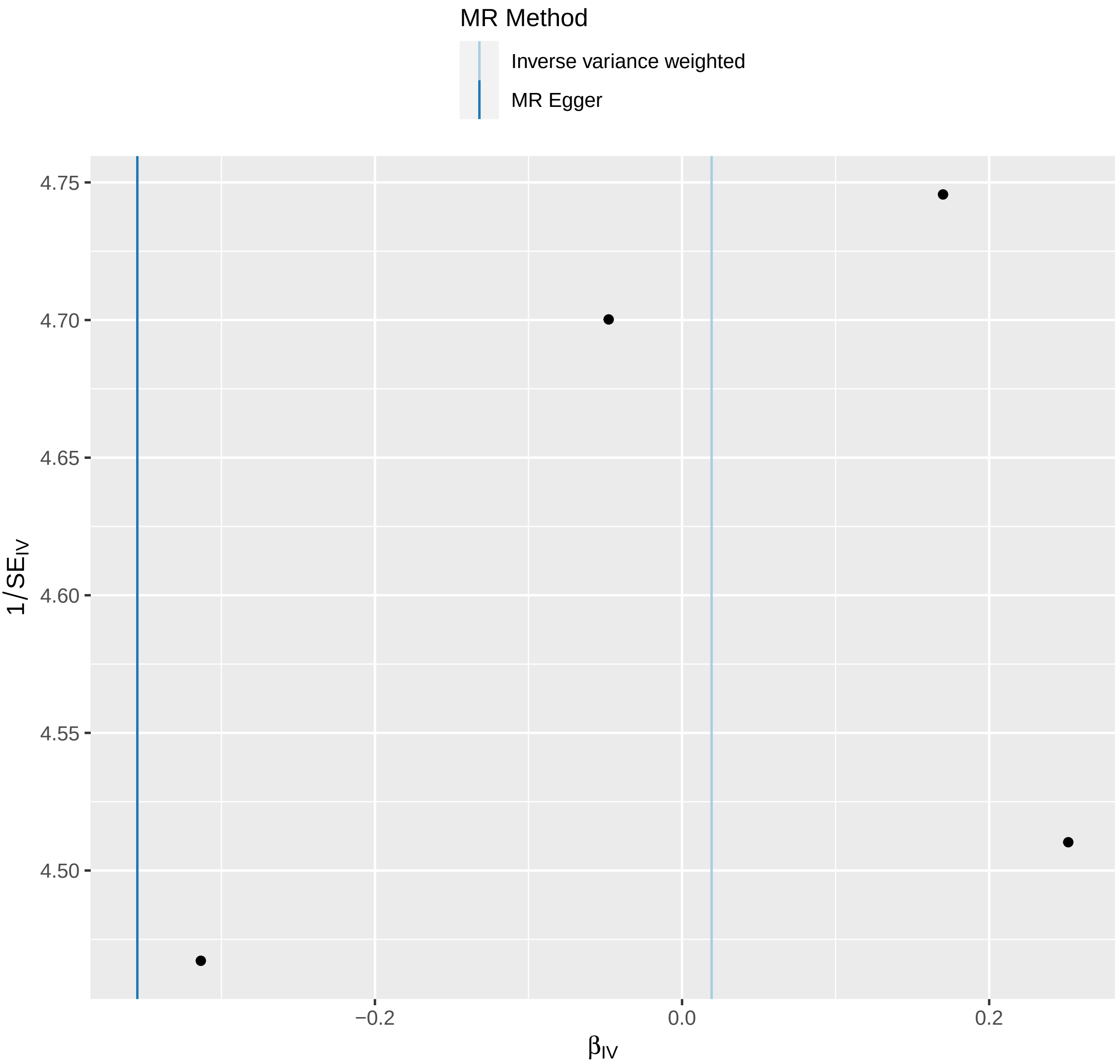

Batch 2−1904 : Gut microbiota abundance (genus Parasutterella id.2892) on Ectonucleoside triphosphate diphosphohydrolase 3 with a threshold of 1e−05

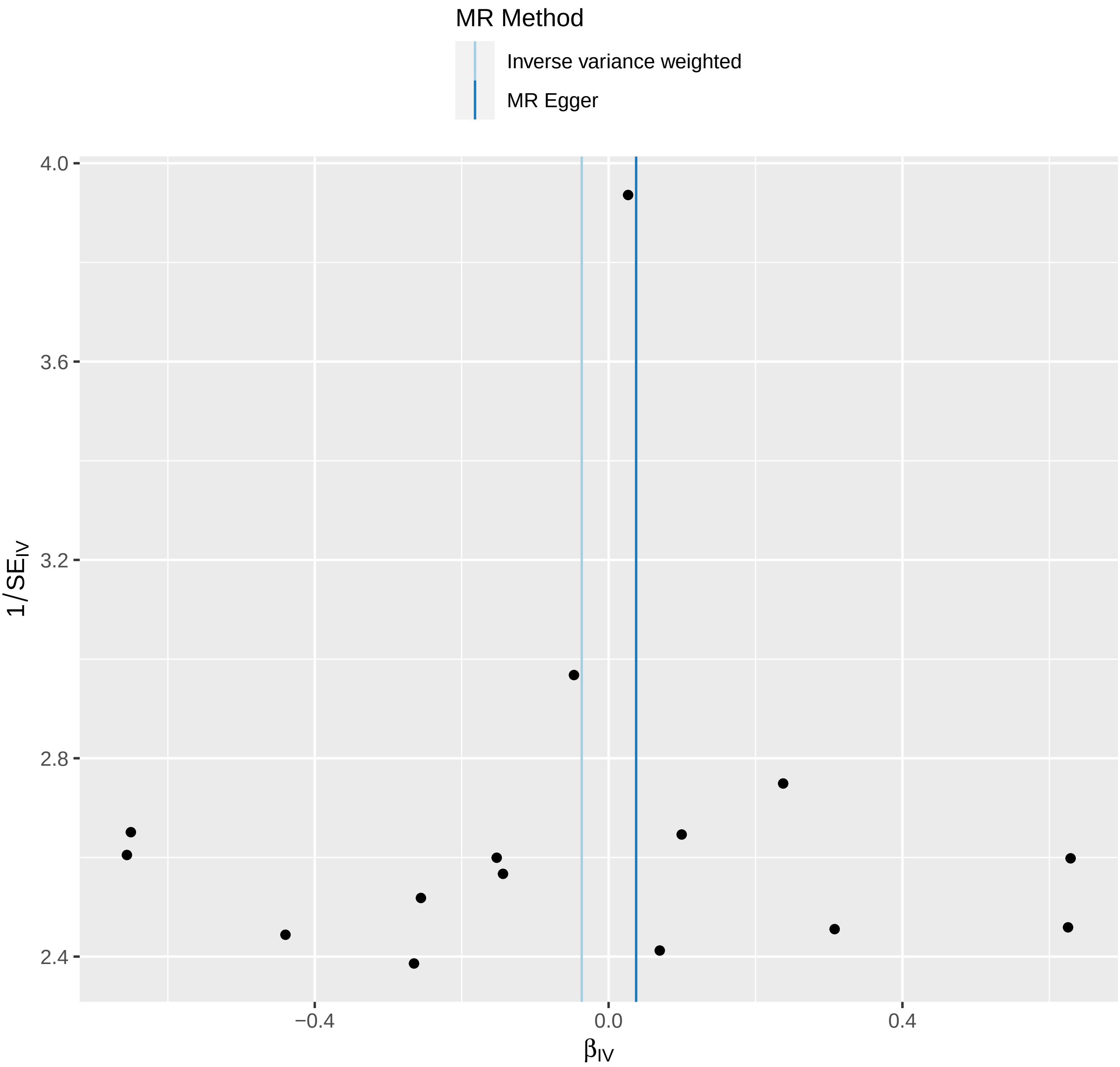

Batch 2−1905 : Gut microbiota abundance (genus Catenibacterium id.2153) on Ectonucleoside triphosphate diphosphohydrolase 5 with a threshold of 1e−05

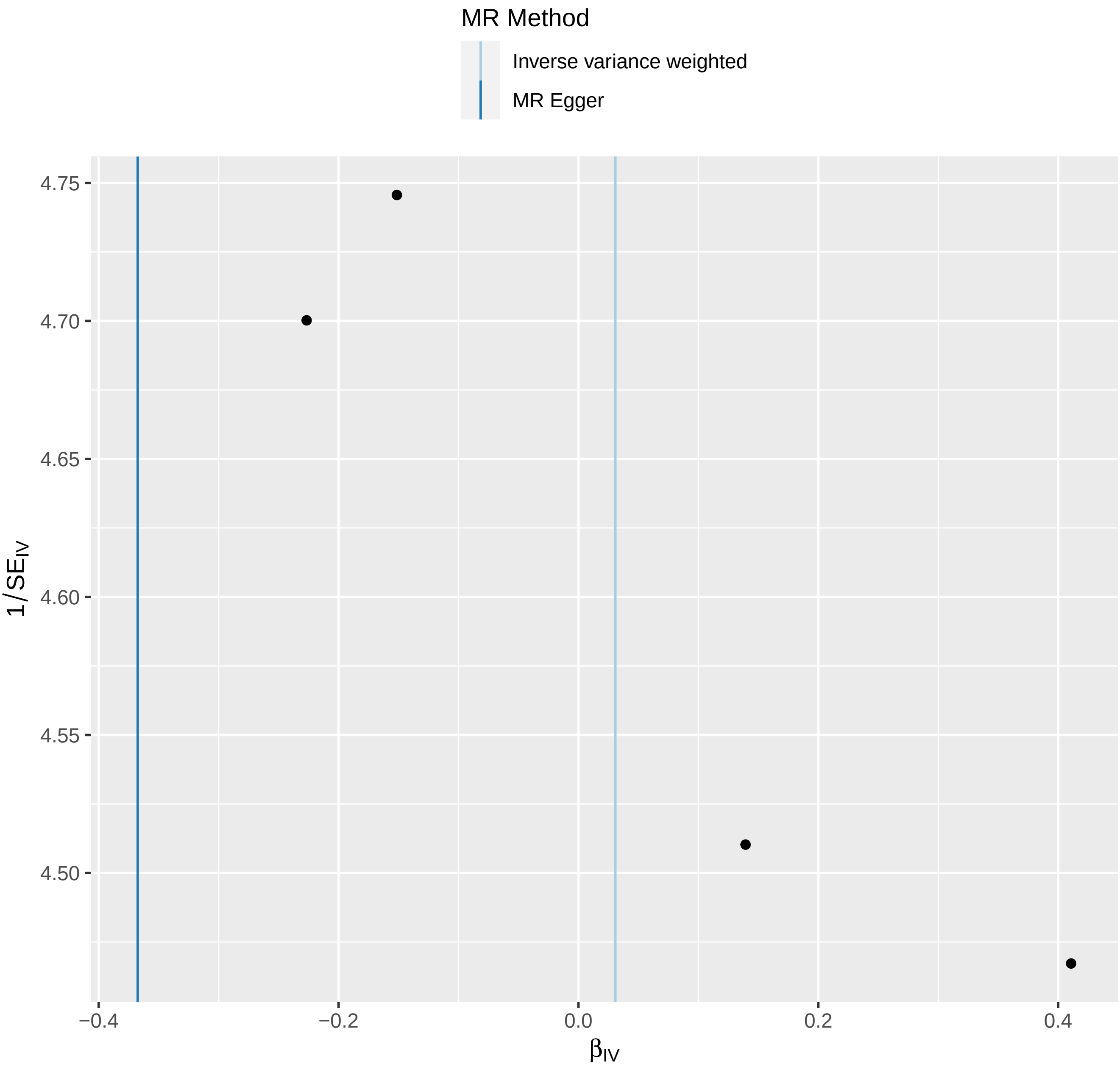

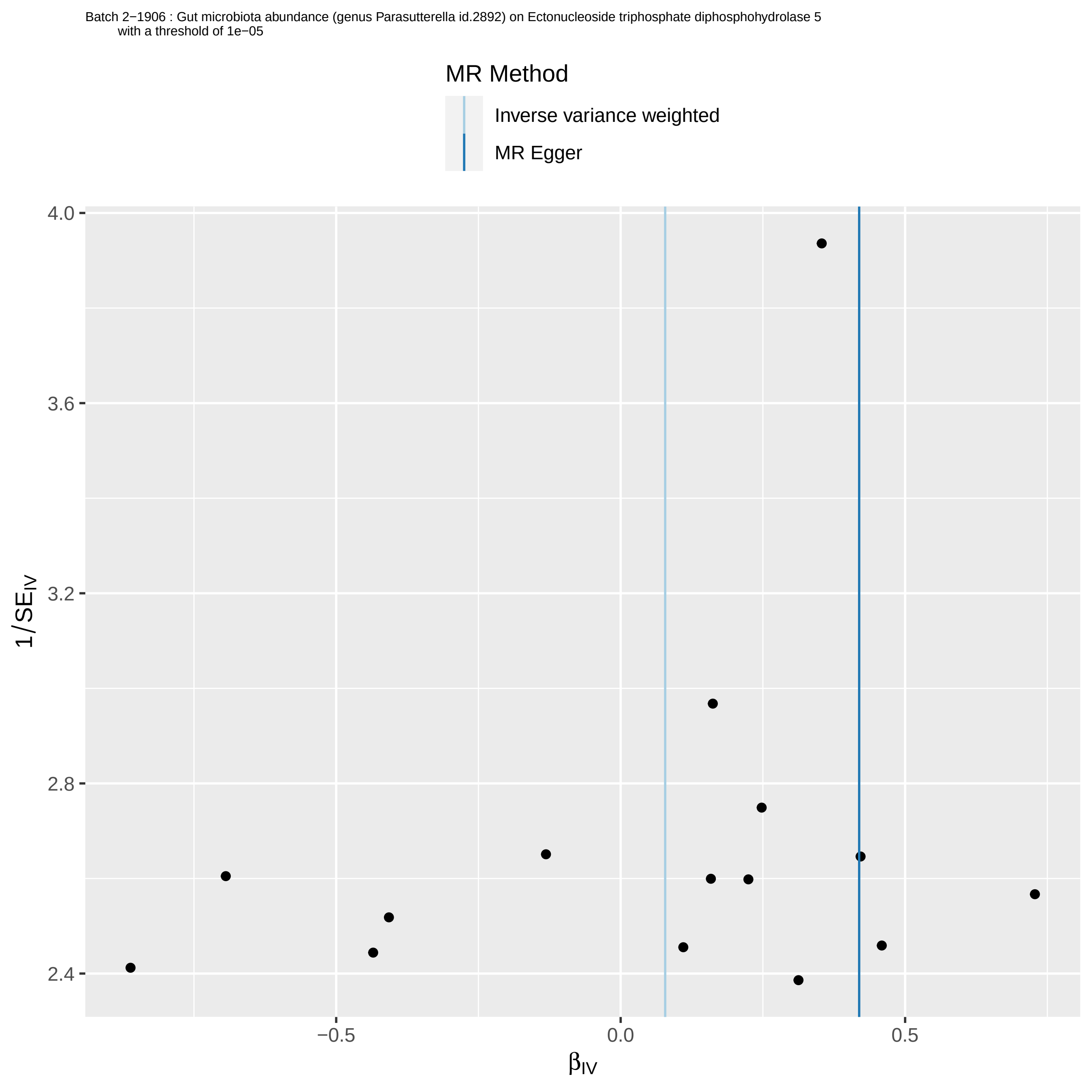

Batch 2−1906 : Gut microbiota abundance (genus Parasutterella id.2892) on Ectonucleoside triphosphate diphosphohydrolase 5
with a threshold of 1e−05
MR Method
Inverse variance weighted
MR Egger
4.0
3.6
3.2
2.8
2.4
1/SE_IV
−0.5
0.0
0.5
β_IV

Batch 2−1907 : Gut microbiota abundance (genus Catenibacterium id.2153) on Ectonucleoside triphosphate diphosphohydrolase 6
with a threshold of 1e−05

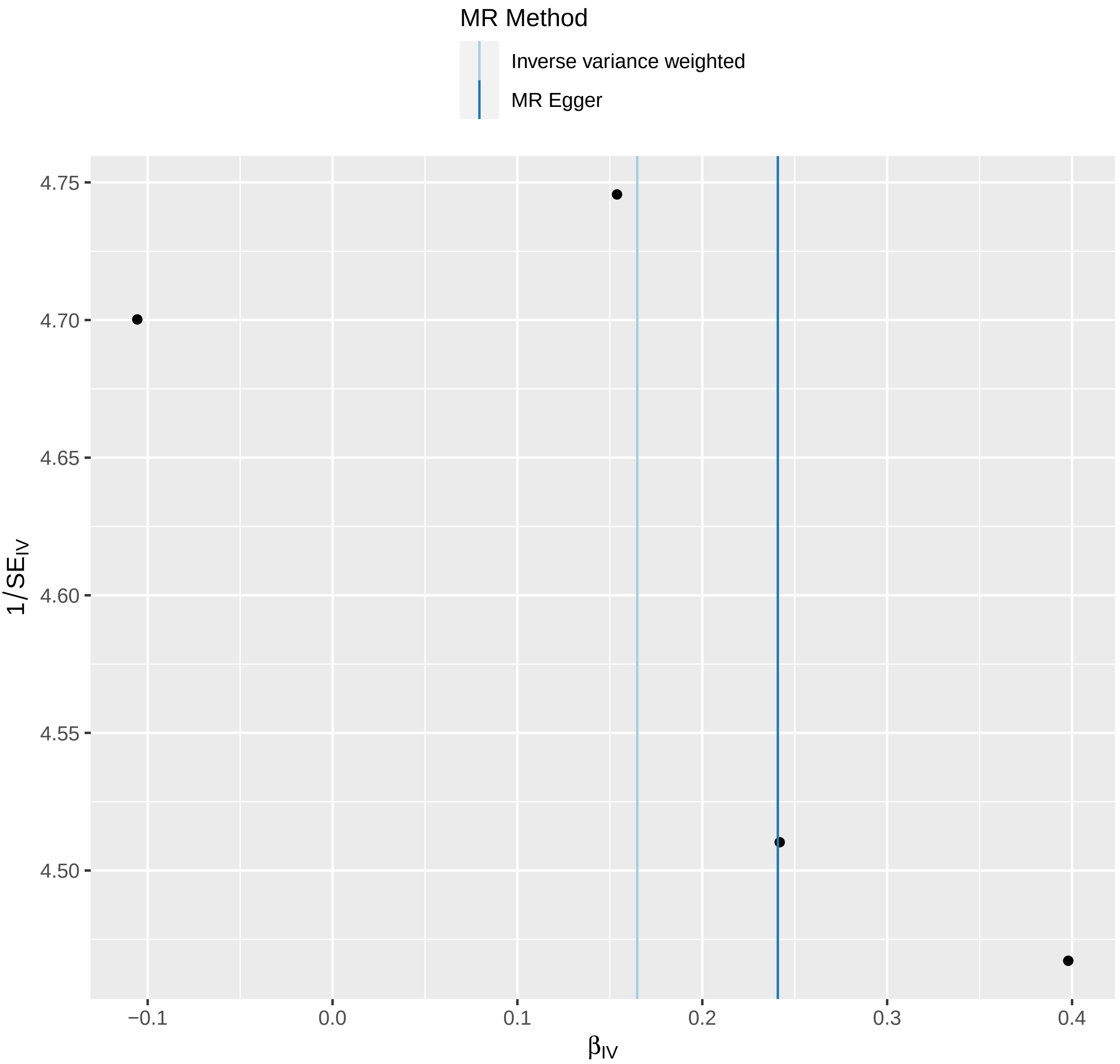

Batch 2−1908 : Gut microbiota abundance (genus Parasutterella id.2892) on Ectonucleoside triphosphate diphosphohydrolase 6
with a threshold of 1e−05

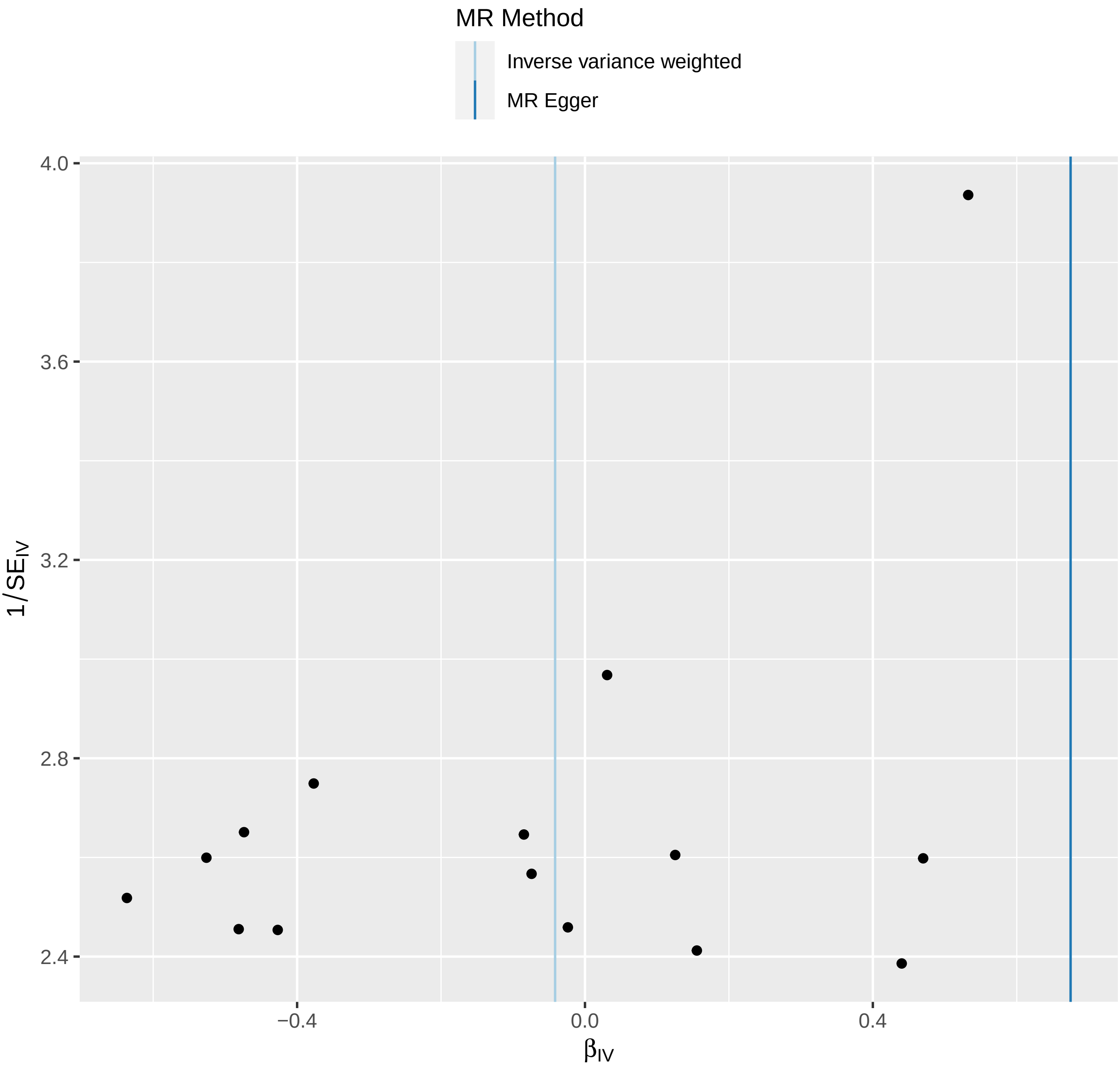

Batch 2−1909 : Gut microbiota abundance (genus Catenibacterium id.2153) on Ephrin type−A receptor 10
with a threshold of 1e−05

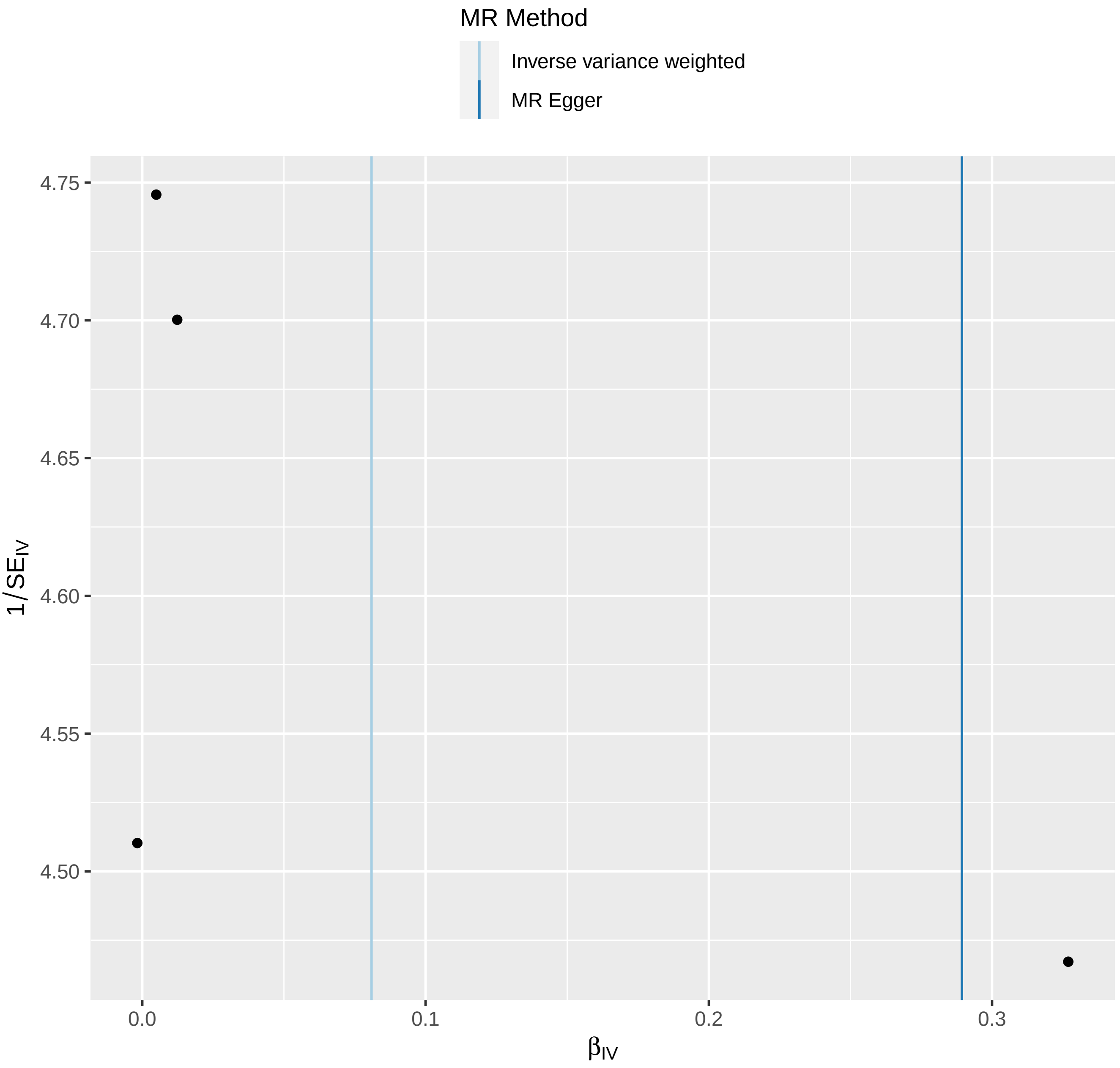

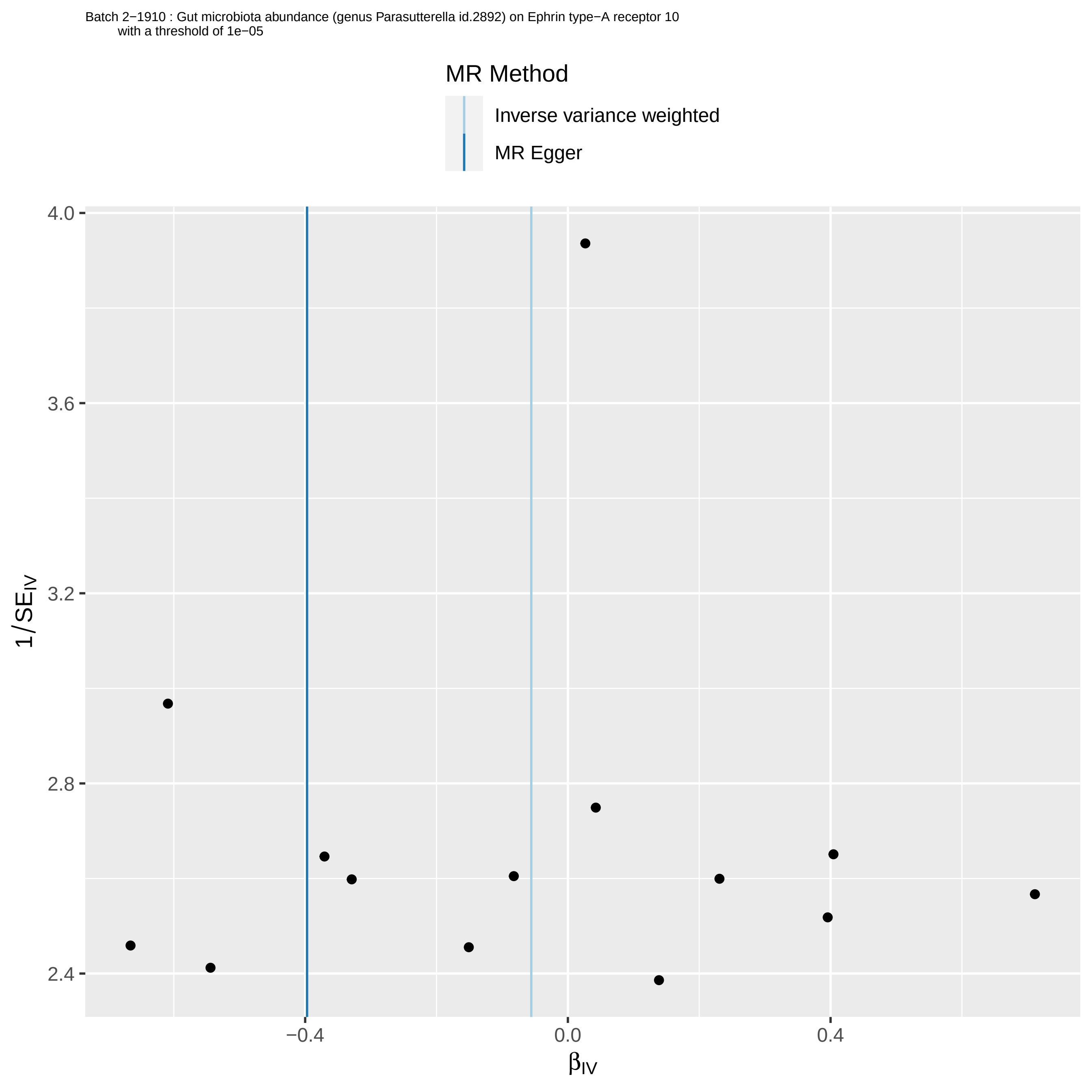
Batch 2−1910 : Gut microbiota abundance (genus Parasutterella id.2892) on Ephrin type−A receptor 10
with a threshold of 1e−05
MR Method
Inverse variance weighted
MR Egger
4.0
3.6
3.2
2.8
2.4
1/SE_IV
−0.4
0.0
0.4
β_IV

Batch 2−1911 : Gut microbiota abundance (genus Catenibacterium id.2153) on Ephrin type−A receptor 1
with a threshold of 1e−05

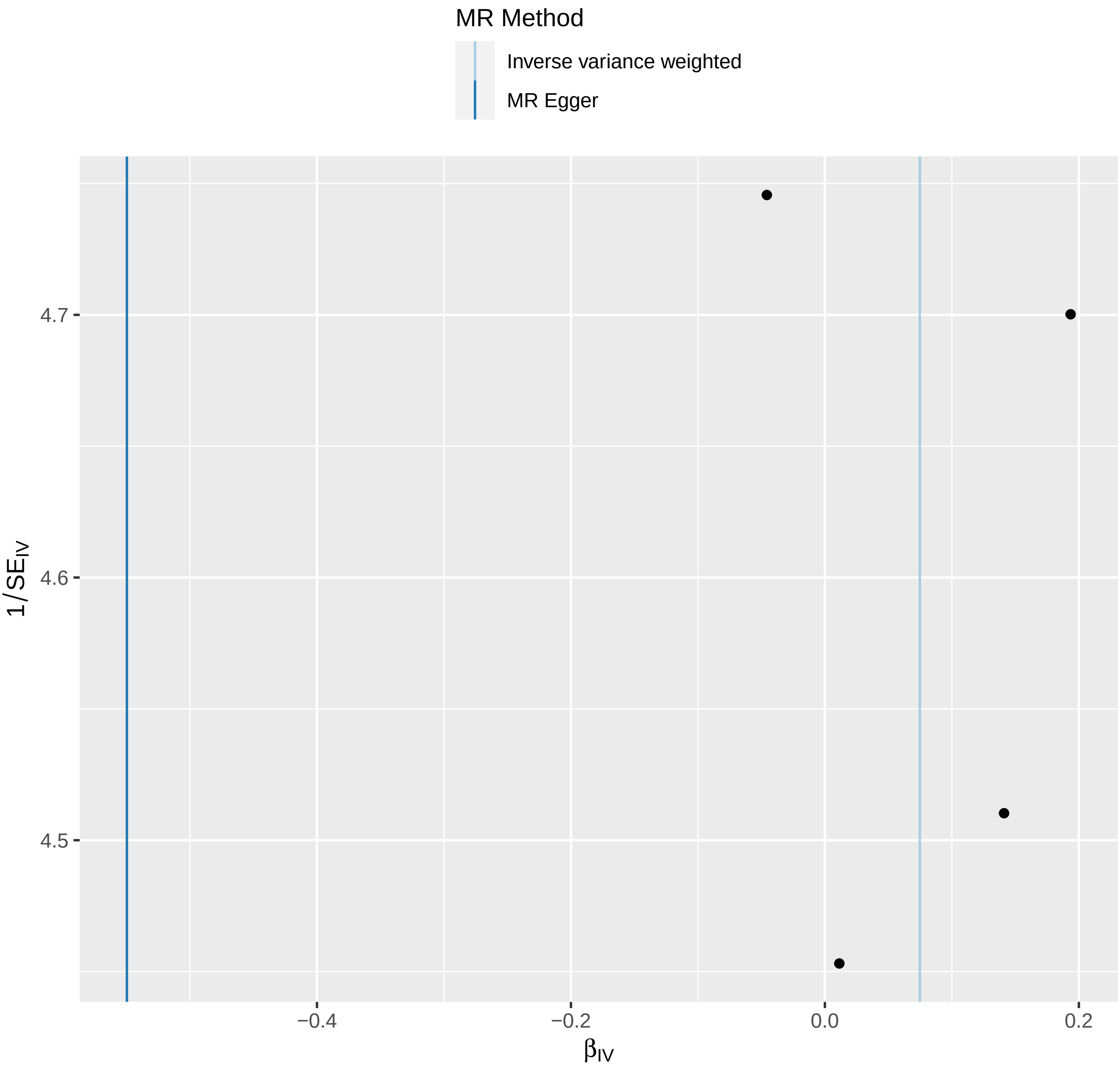

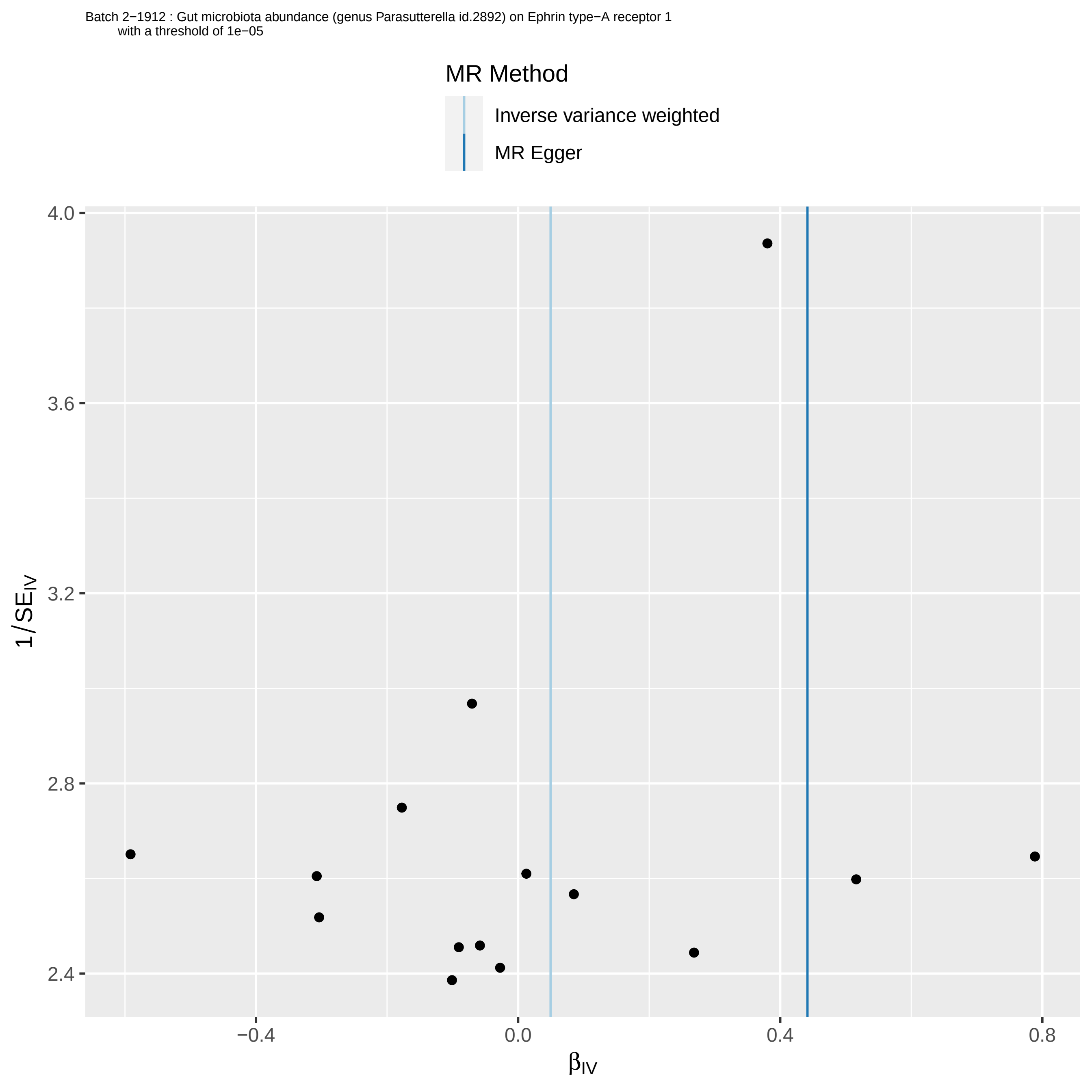

Batch 2−1912 : Gut microbiota abundance (genus Parasutterella id.2892) on Ephrin type−A receptor 1
with a threshold of 1e−05
MR Method
Inverse variance weighted
MR Egger
4.0
3.6
3.2
2.8
2.4
1/SE_IV
−0.4
0.0
0.4
0.8
β_IV

Batch 2−1913 : Gut microbiota abundance (genus Catenibacterium id.2153) on Ephrin type−A receptor 2
with a threshold of 1e−05

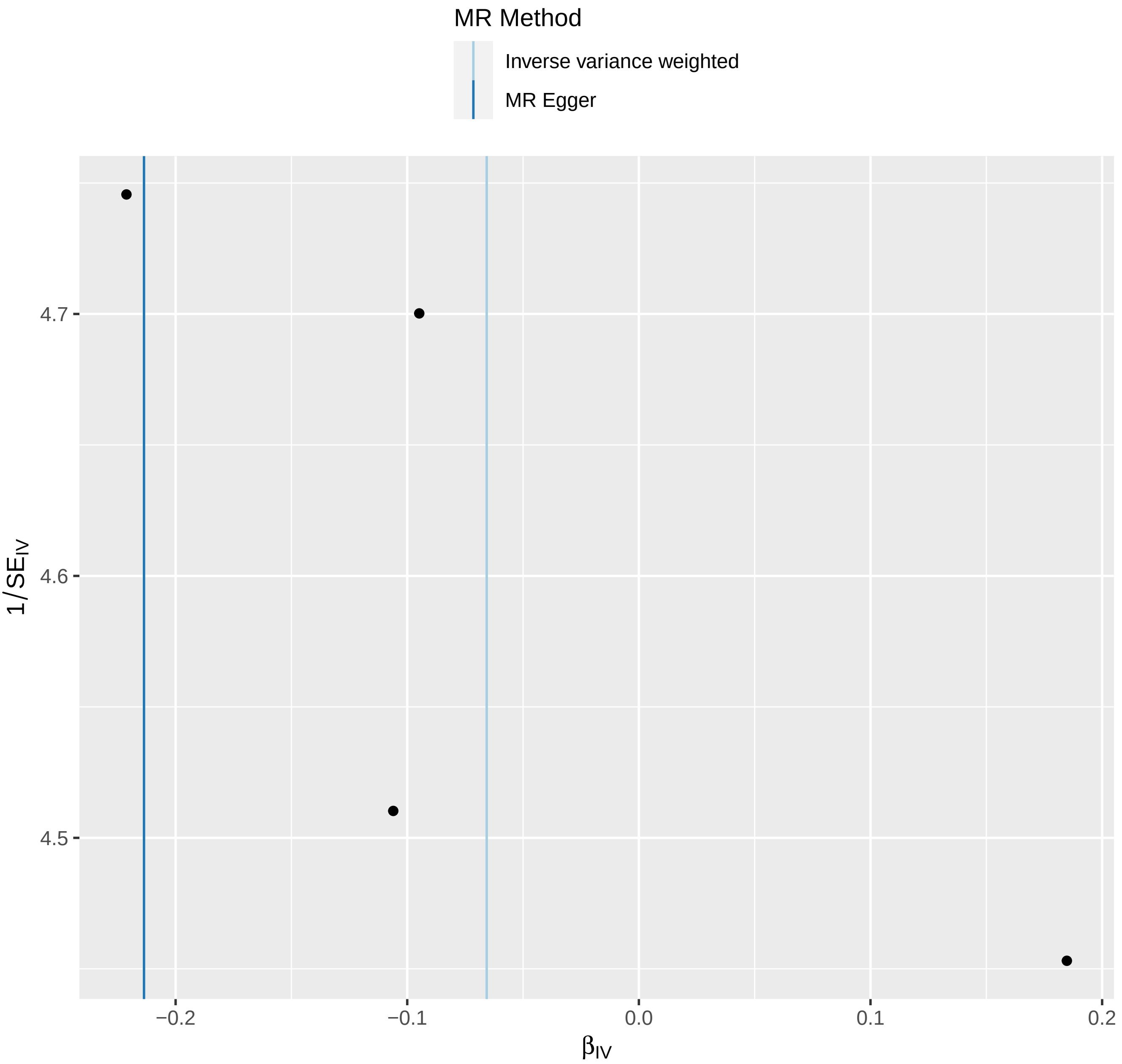

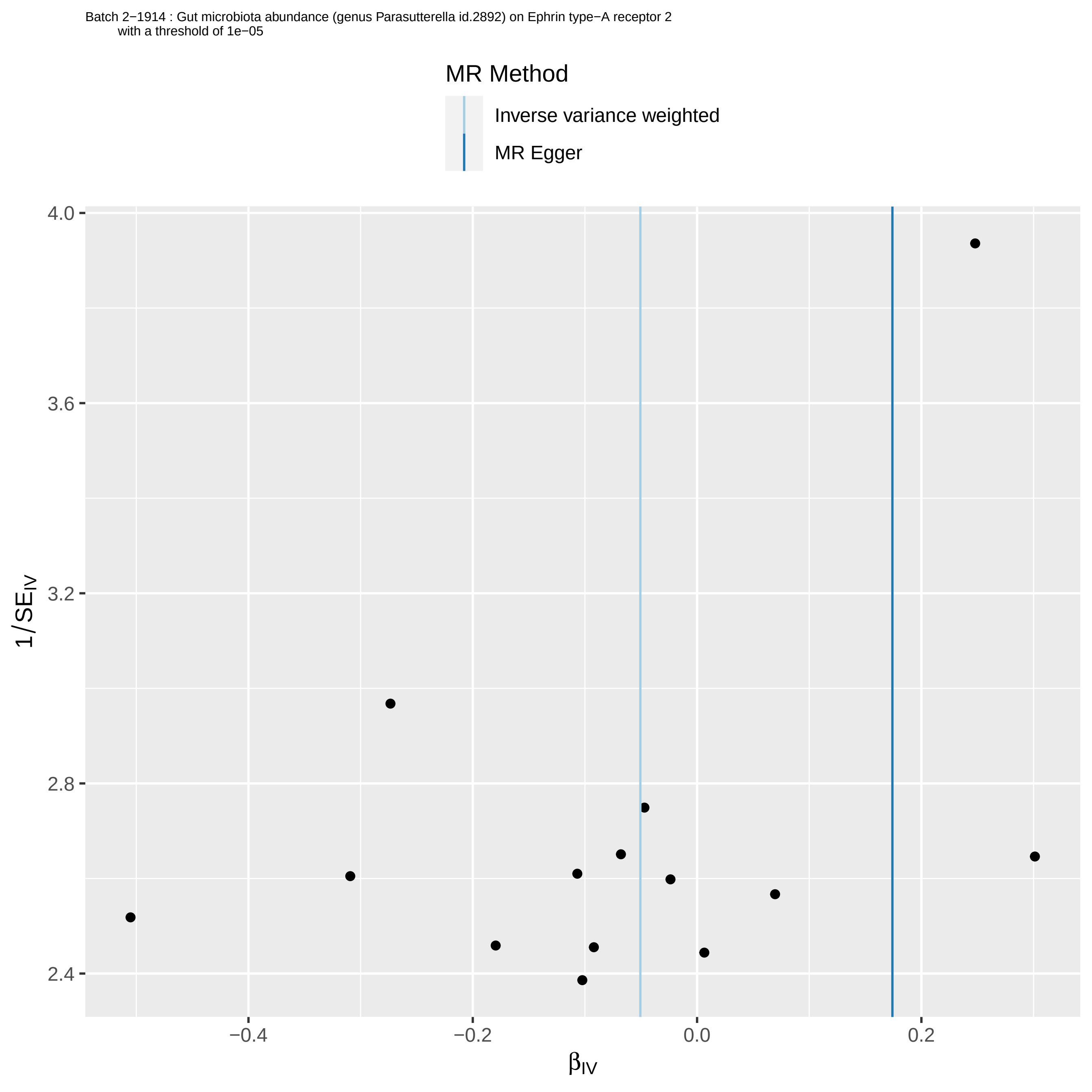

Batch 2−1914 : Gut microbiota abundance (genus Parasutterella id.2892) on Ephrin type−A receptor 2
with a threshold of 1e−05
MR Method
Inverse variance weighted
MR Egger
4.0
3.6
3.2
2.8
2.4
1/SE_IV
−0.4
−0.2
0.0
0.2
β_IV

Batch 2−1915 : Gut microbiota abundance (genus Catenibacterium id.2153) on Ephrin type−A receptor 5 with a threshold of 1e−05

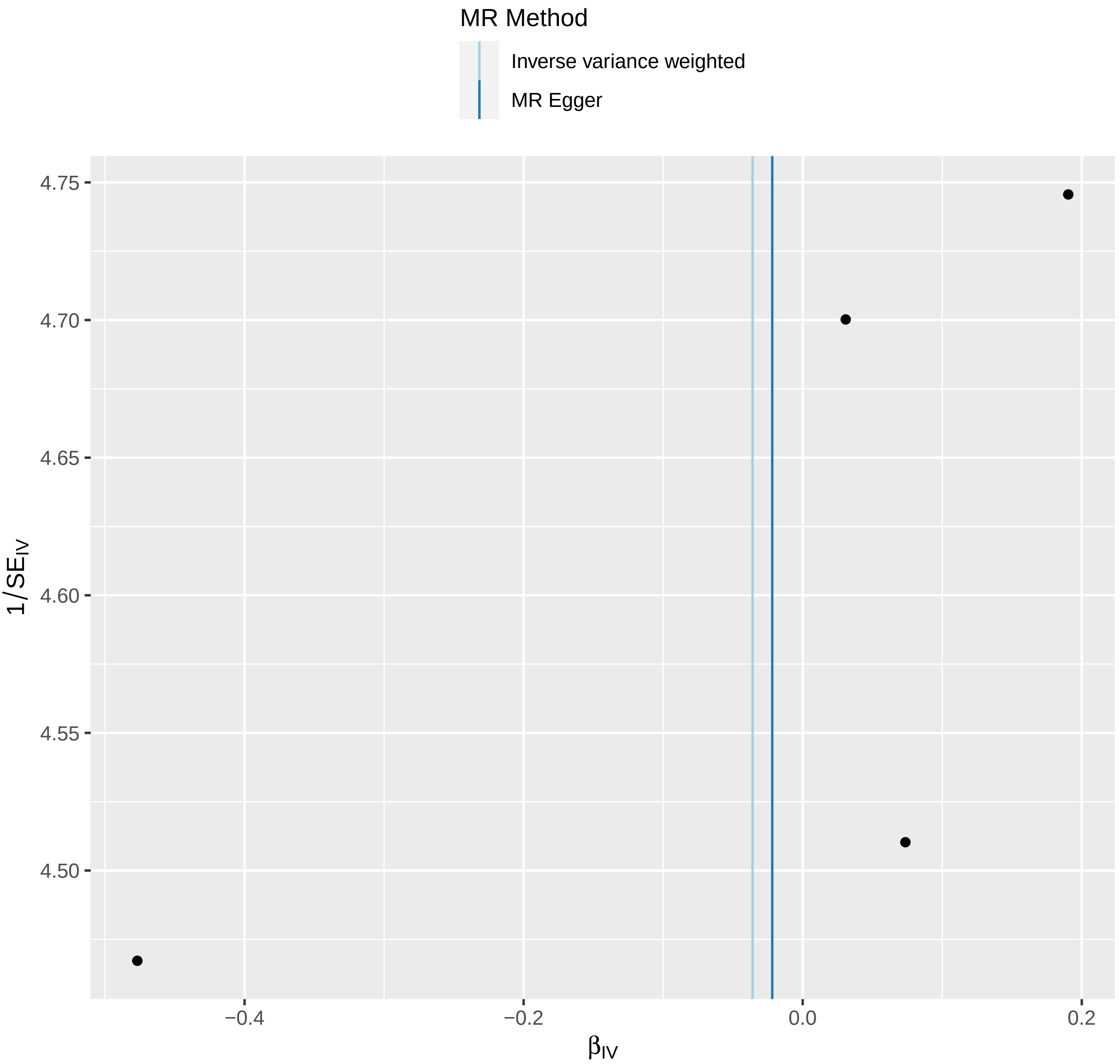

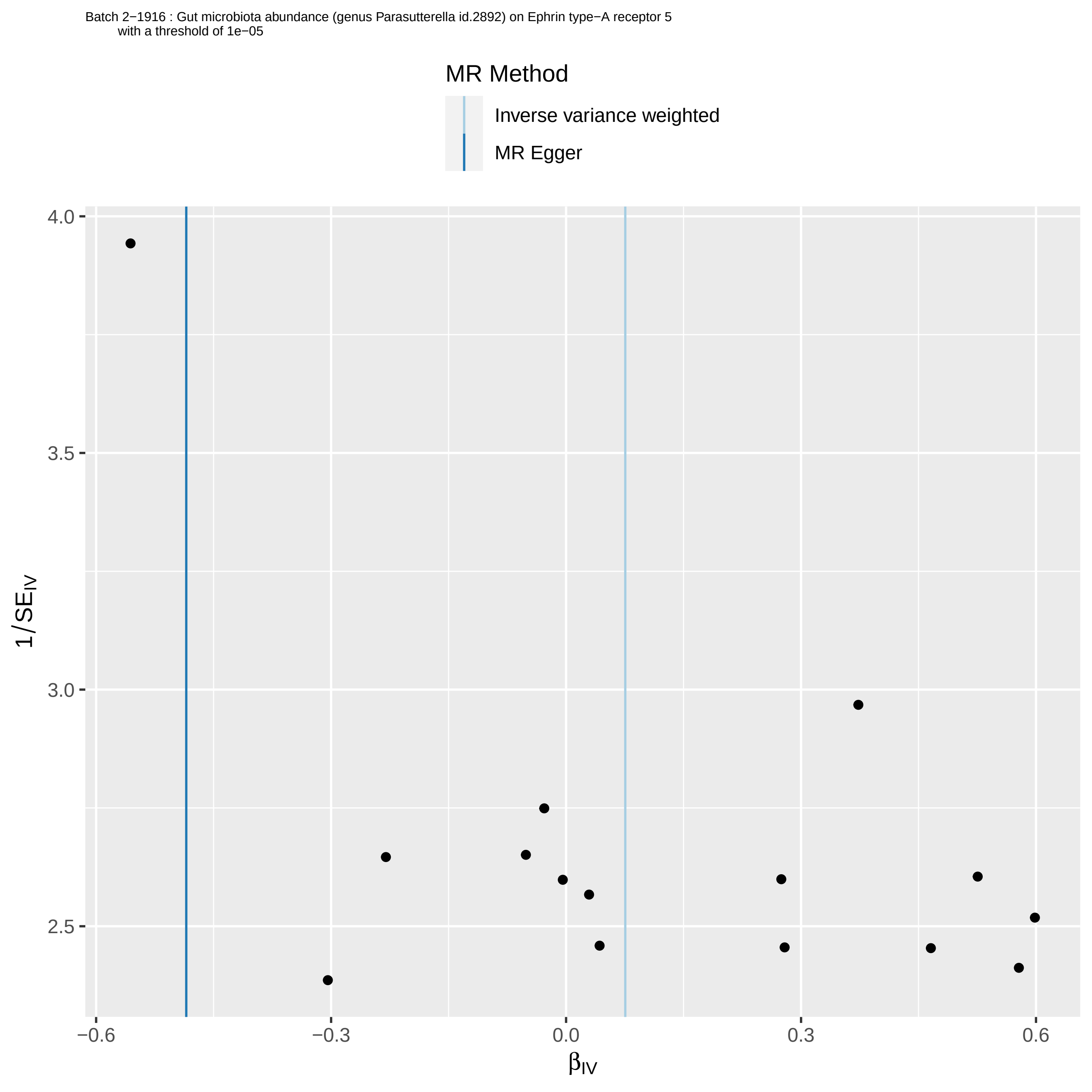

Batch 2−1916 : Gut microbiota abundance (genus Parasutterella id.2892) on Ephrin type−A receptor 5
with a threshold of 1e−05
MR Method
Inverse variance weighted
MR Egger
4.0
3.5
3.0
2.5
1/SE_IV
−0.6
−0.3
0.0
0.3
0.6
β_IV

Batch 2−1917 : Gut microbiota abundance (genus Catenibacterium id.2153) on Ephrin type−A receptor 5
with a threshold of 1e−05

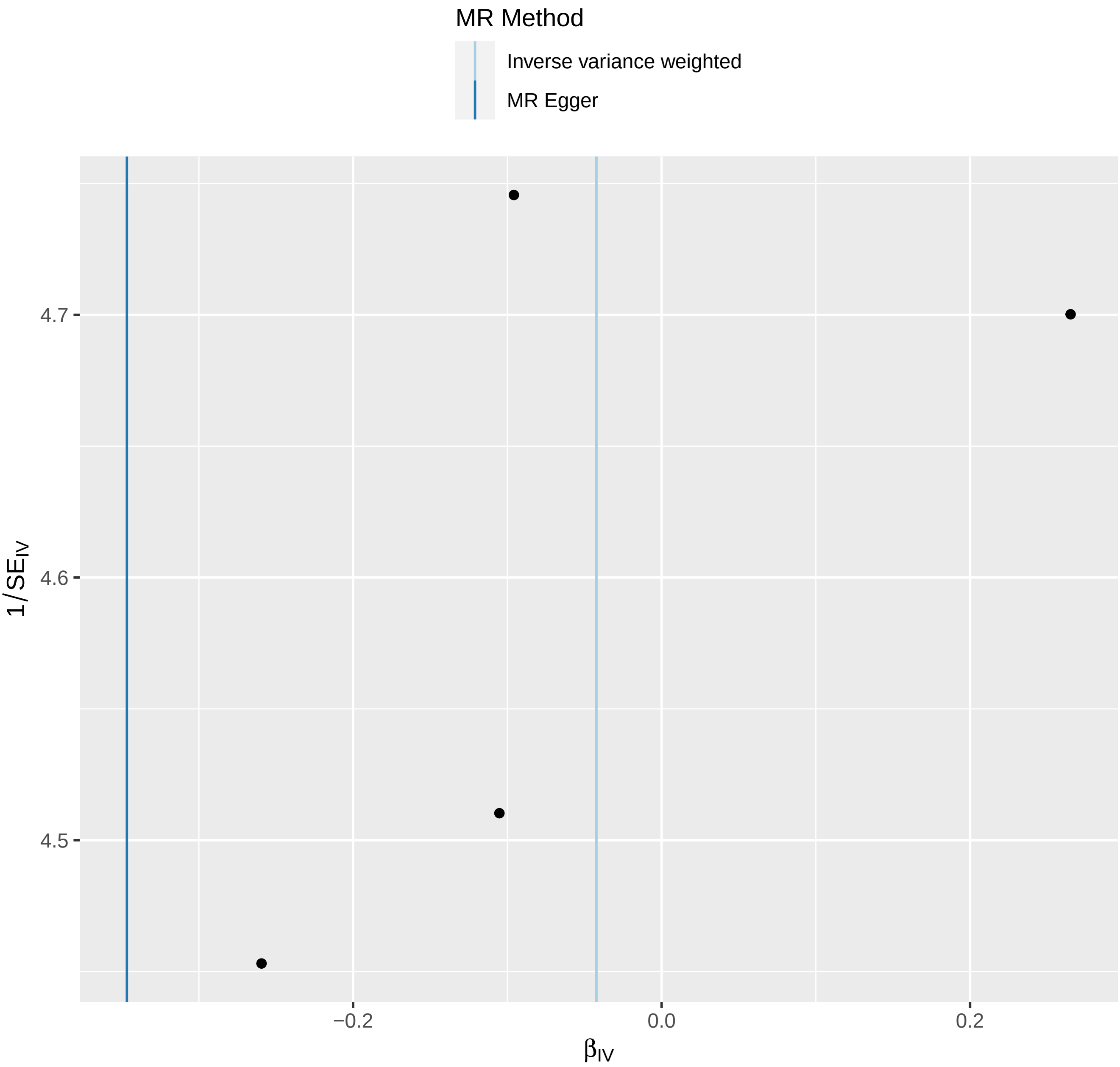

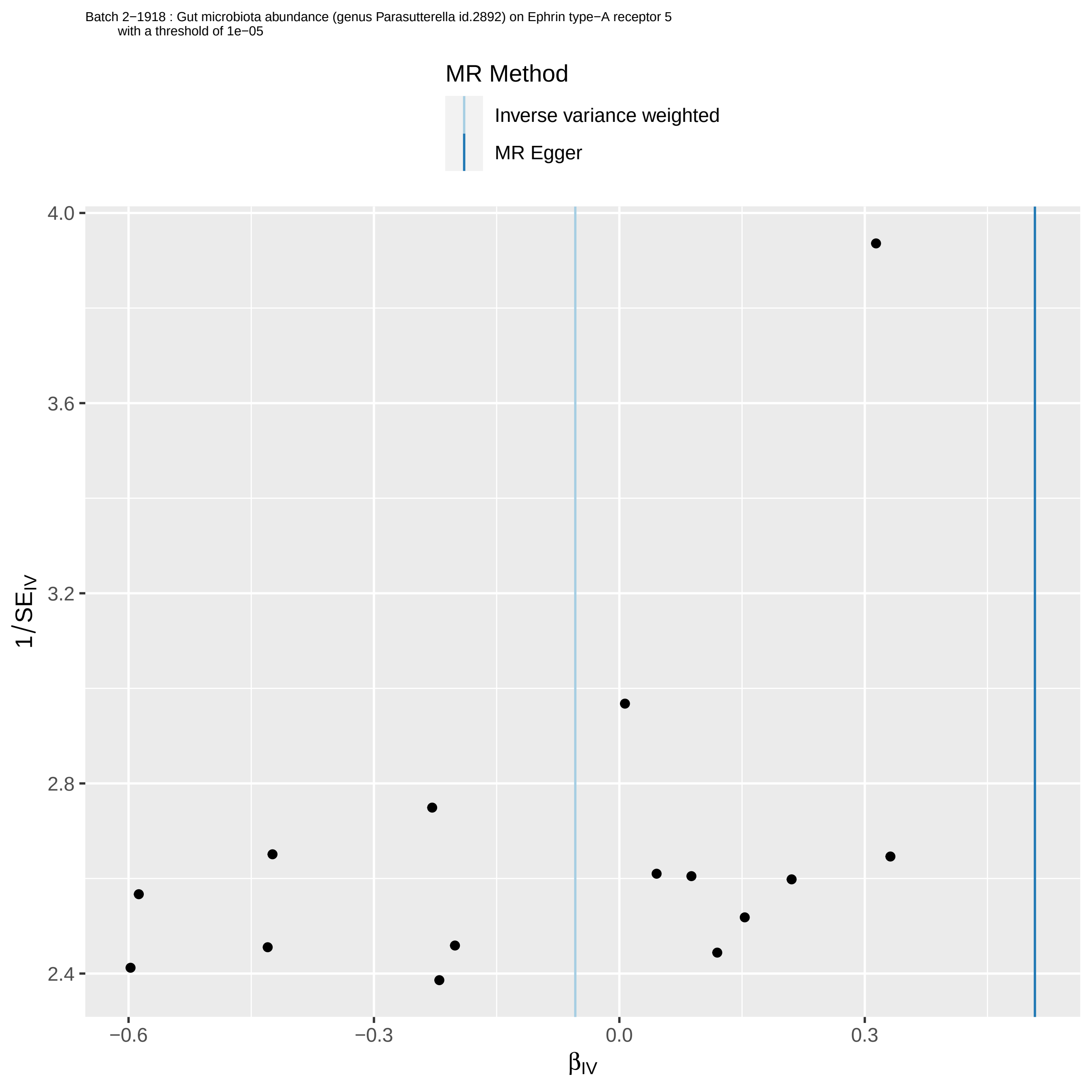

Batch 2−1918 : Gut microbiota abundance (genus Parasutterella id.2892) on Ephrin type−A receptor 5
with a threshold of 1e−05
MR Method
Inverse variance weighted
MR Egger
4.0
3.6
3.2
2.8
2.4
1/SE_IV
−0.6
−0.3
0.0
0.3
β_IV

Batch 2−1919 : Gut microbiota abundance (genus Catenibacterium id.2153) on Ephrin type−B receptor 2
with a threshold of 1e−05

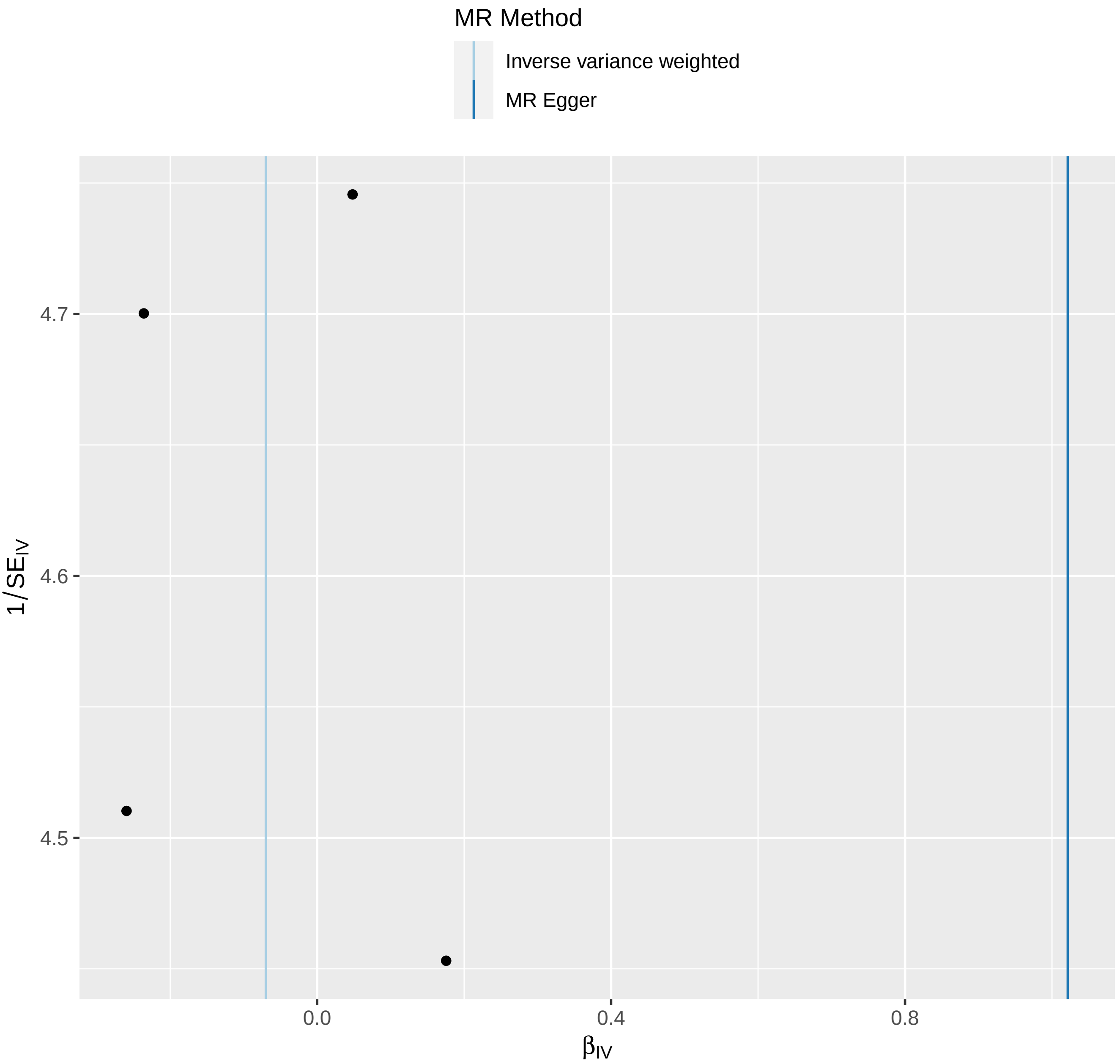

Batch 2−1920 : Gut microbiota abundance (genus Parasutterella id.2892) on Ephrin type−B receptor 2 with a threshold of 1e−05

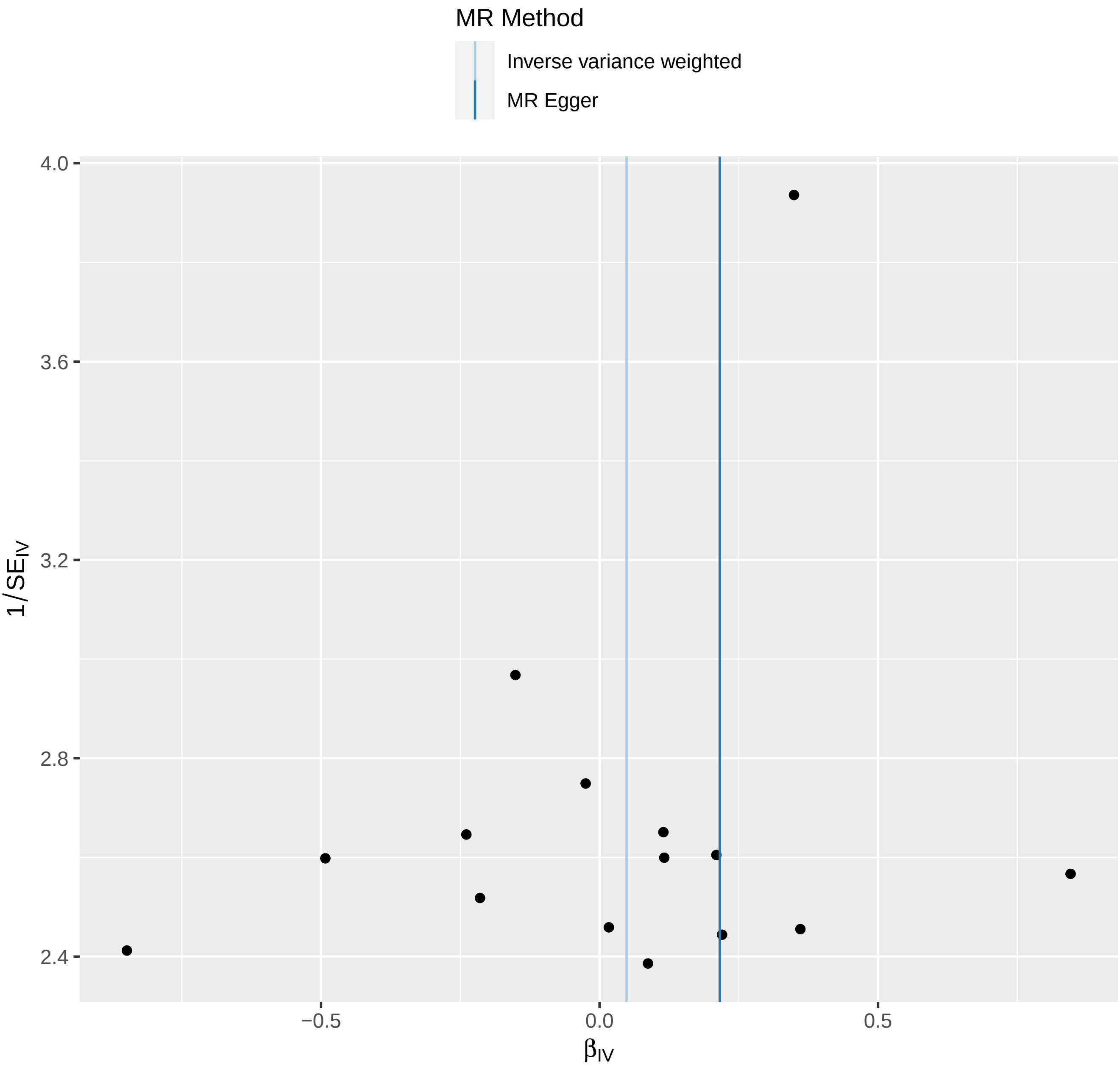

Batch 2−1921 : Gut microbiota abundance (genus Catenibacterium id.2153) on Ephrin type−B receptor 2
with a threshold of 1e−05

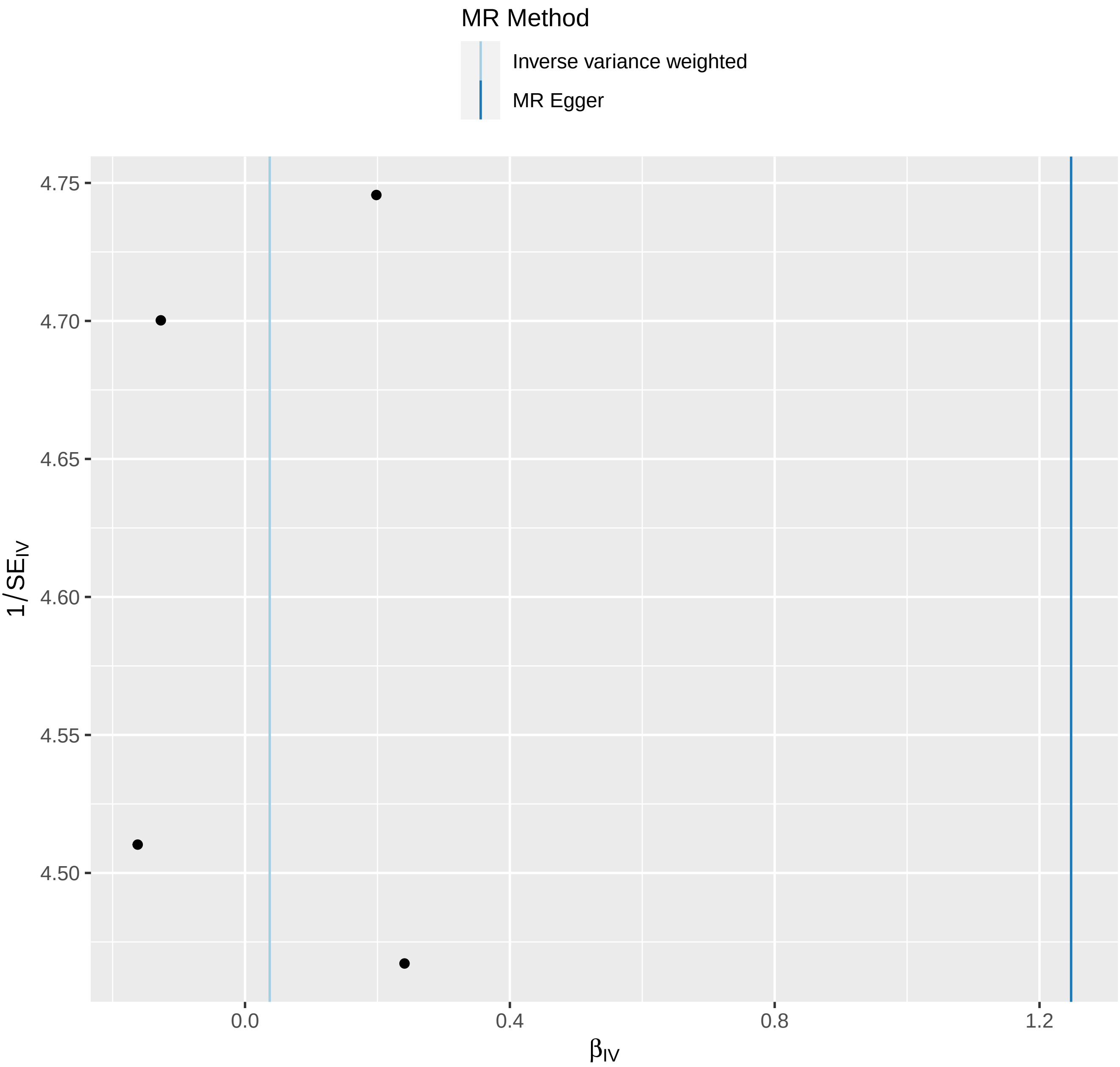

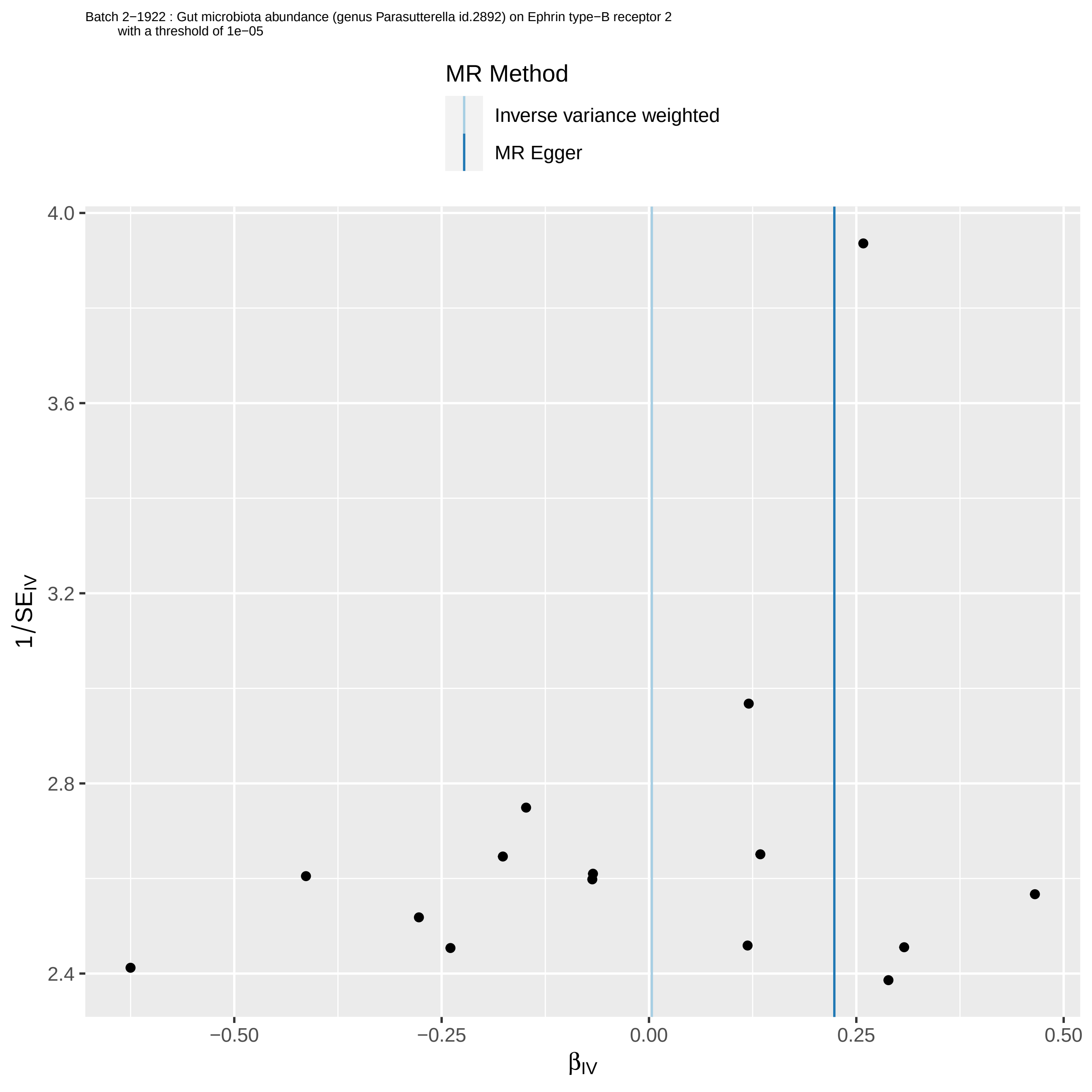
Batch 2−1922 : Gut microbiota abundance (genus Parasutterella id.2892) on Ephrin type−B receptor 2
with a threshold of 1e−05
MR Method
Inverse variance weighted
MR Egger
4.0
3.6
3.2
2.8
2.4
1/SE_IV
−0.50
−0.25
0.00
0.25
0.50
β_IV

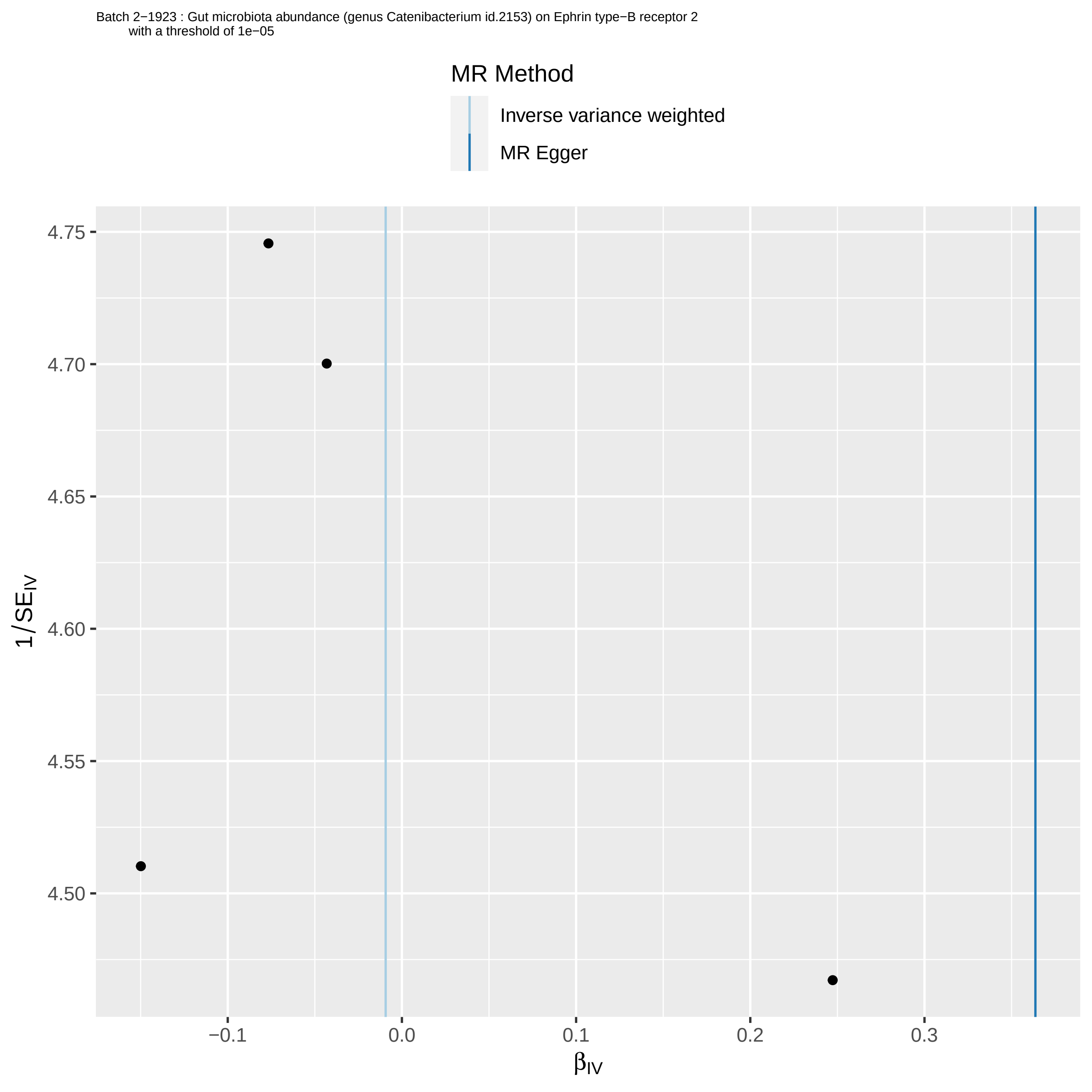
Batch 2−1923 : Gut microbiota abundance (genus Catenibacterium id.2153) on Ephrin type−B receptor 2
with a threshold of 1e−05
MR Method
Inverse variance weighted
MR Egger
4.75
4.70
4.65
4.60
4.55
4.50
1/SE_IV
−0.1
0.0
0.1
0.2
0.3
β_IV

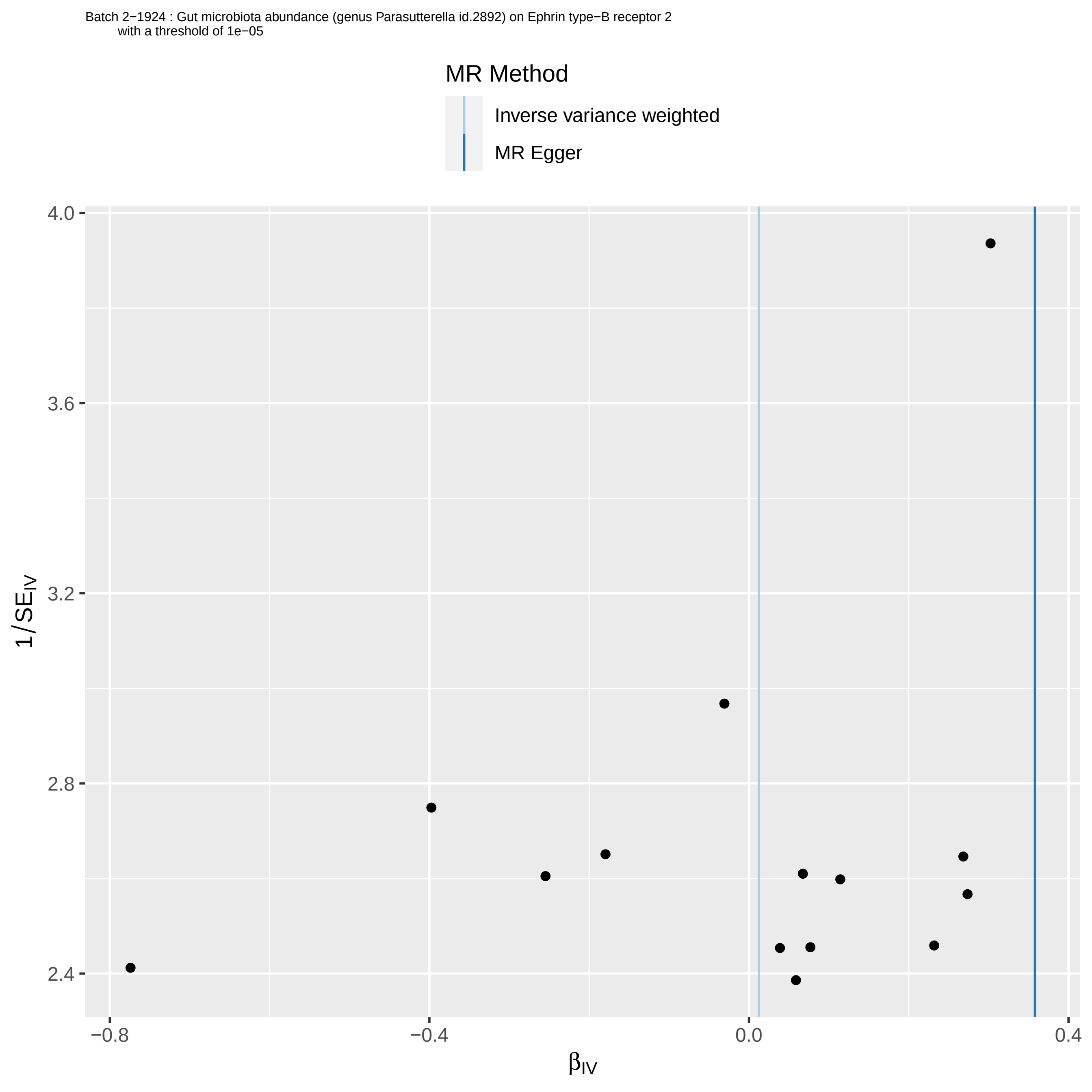

Batch 2−1924 : Gut microbiota abundance (genus Parasutterella id.2892) on Ephrin type−B receptor 2
with a threshold of 1e−05
MR Method
Inverse variance weighted
MR Egger
4.0
3.6
3.2
2.8
2.4
1/SE$_{IV}$
−0.8
−0.4
0.0
0.4
β$_{IV}$

Batch 2−1925 : Gut microbiota abundance (genus Catenibacterium id.2153) on Ephrin type−B receptor 3
with a threshold of 1e−05

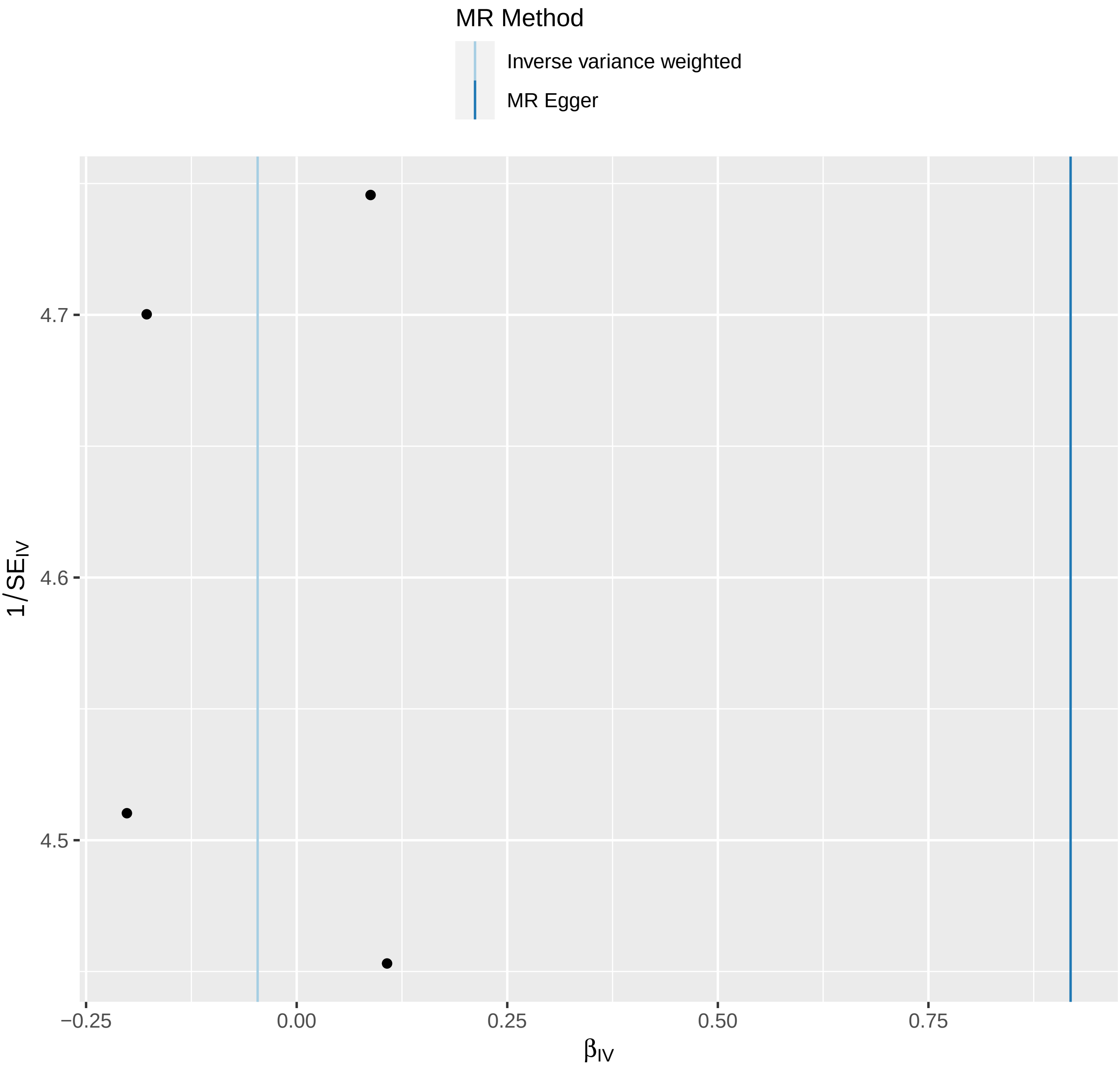

Batch 2−1926 : Gut microbiota abundance (genus Parasutterella id.2892) on Ephrin type−B receptor 3
with a threshold of 1e−05

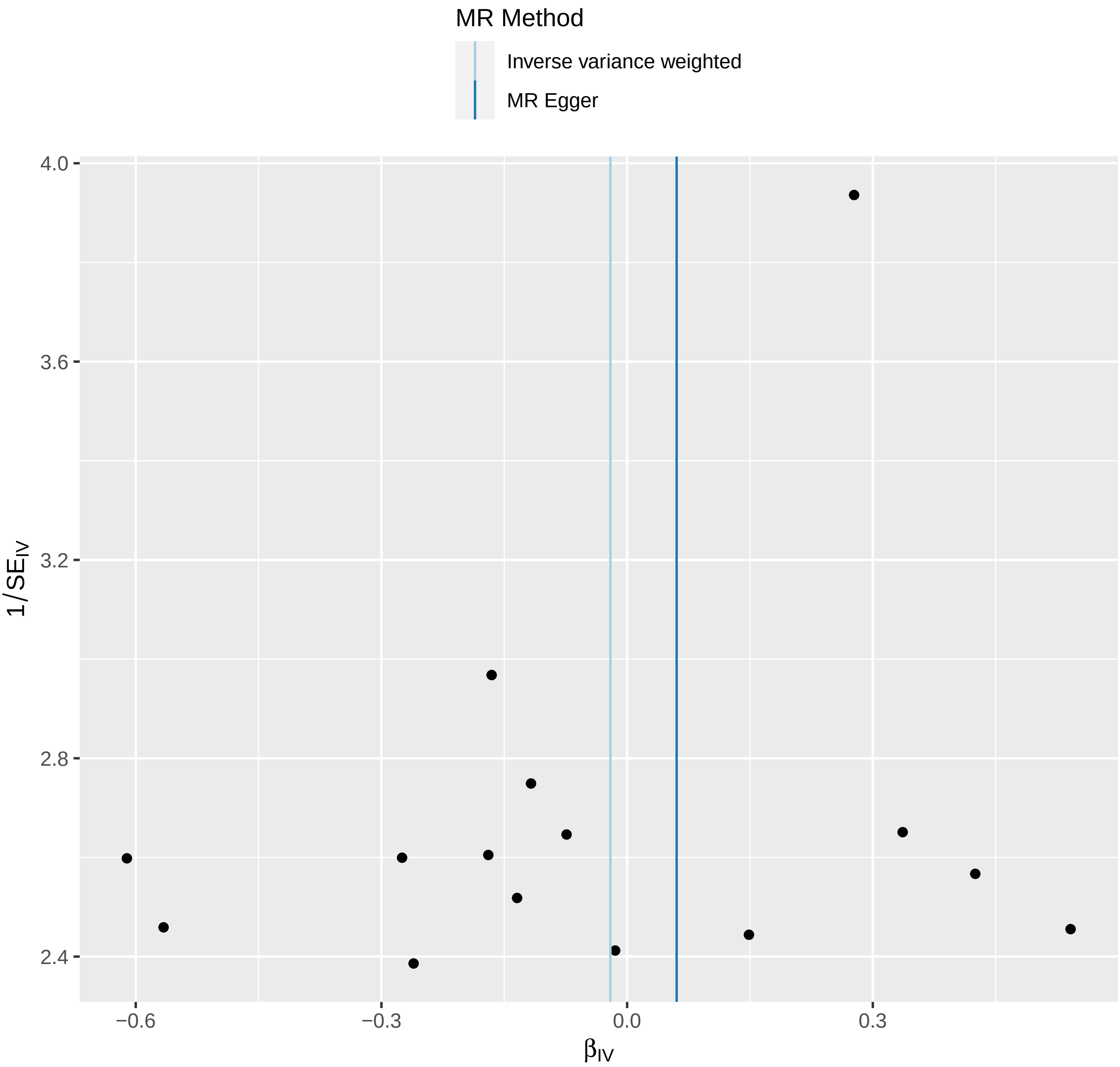

Batch 2−1927 : Gut microbiota abundance (genus Catenibacterium id.2153) on Ephrin type−B receptor 4
with a threshold of 1e−05

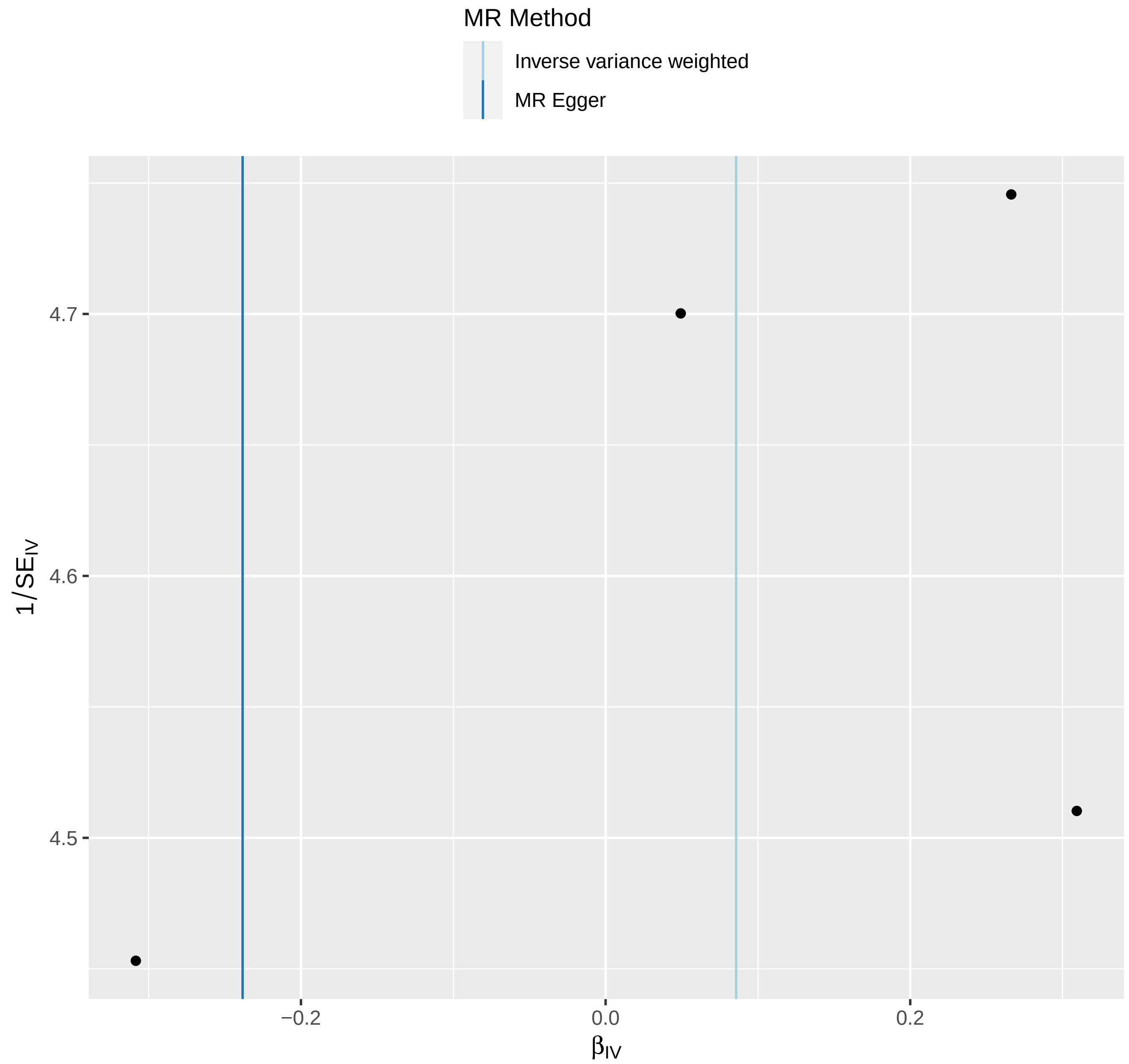

Batch 2−1928 : Gut microbiota abundance (genus Parasutterella id.2892) on Ephrin type−B receptor 4
with a threshold of 1e−05

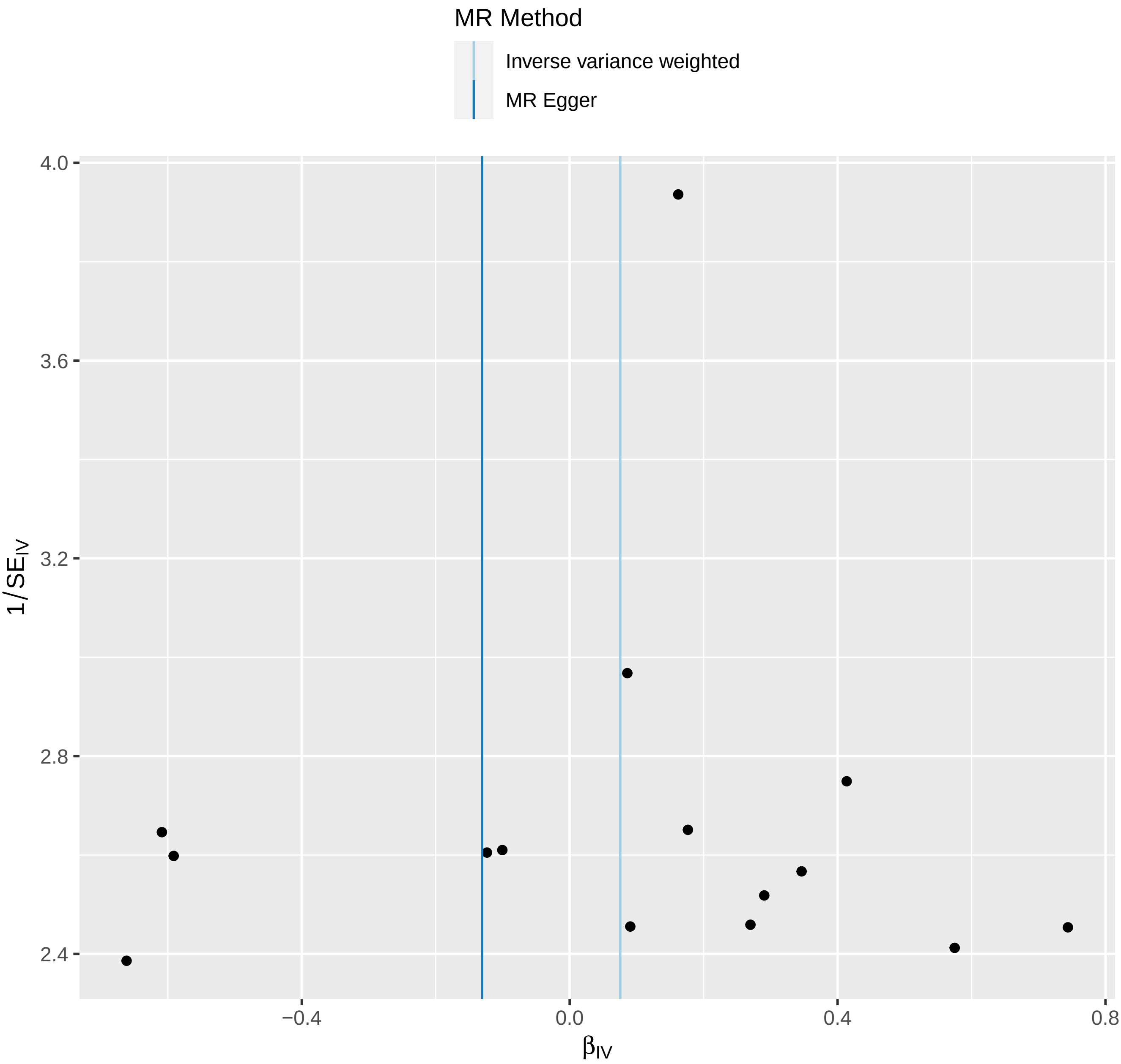

Batch 2−1929 : Gut microbiota abundance (genus Catenibacterium id.2153) on Ephrin type−B receptor 6
with a threshold of 1e−05

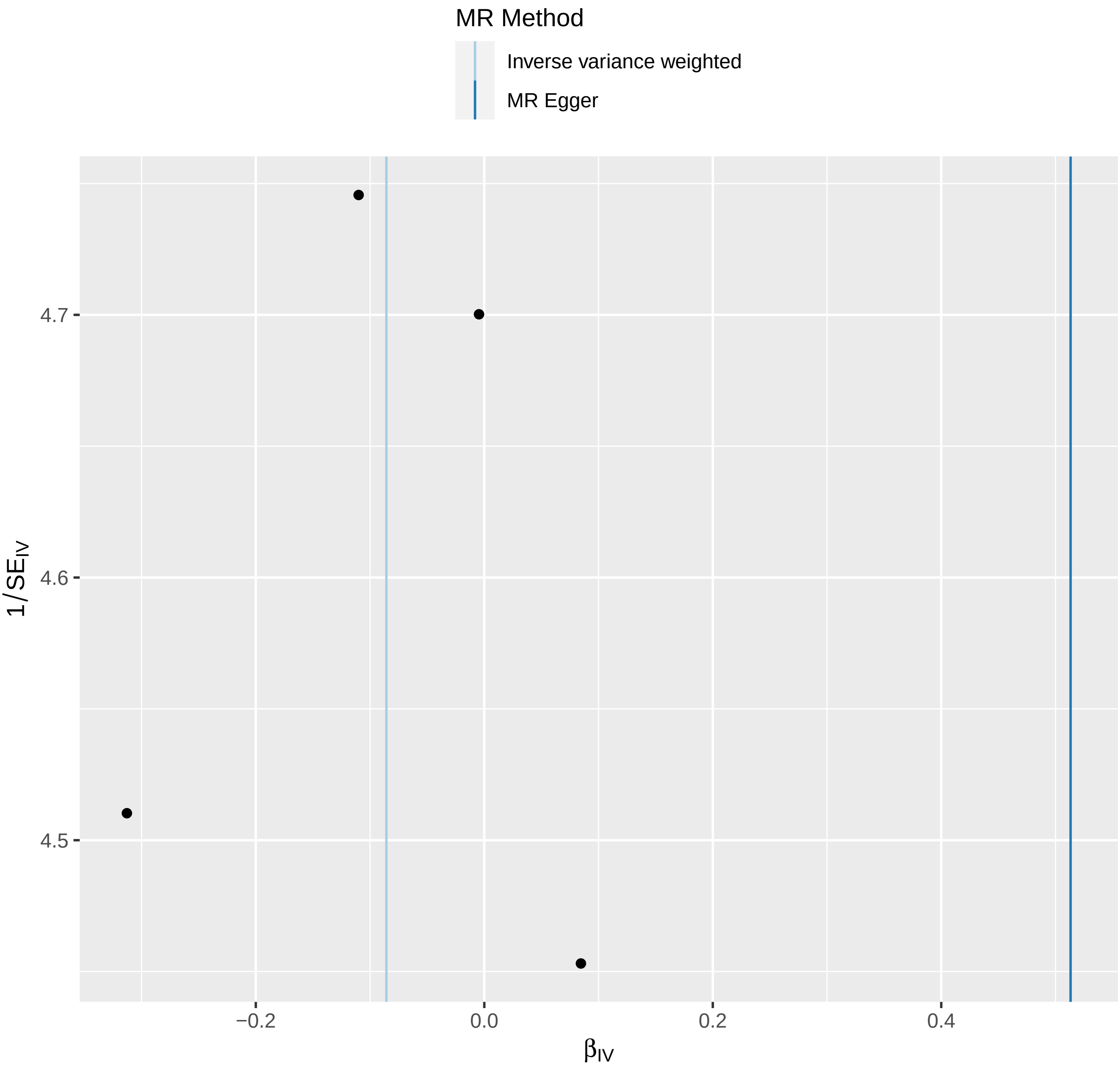

Batch 2−1930 : Gut microbiota abundance (genus Parasutterella id.2892) on Ephrin type−B receptor 6 with a threshold of 1e−05

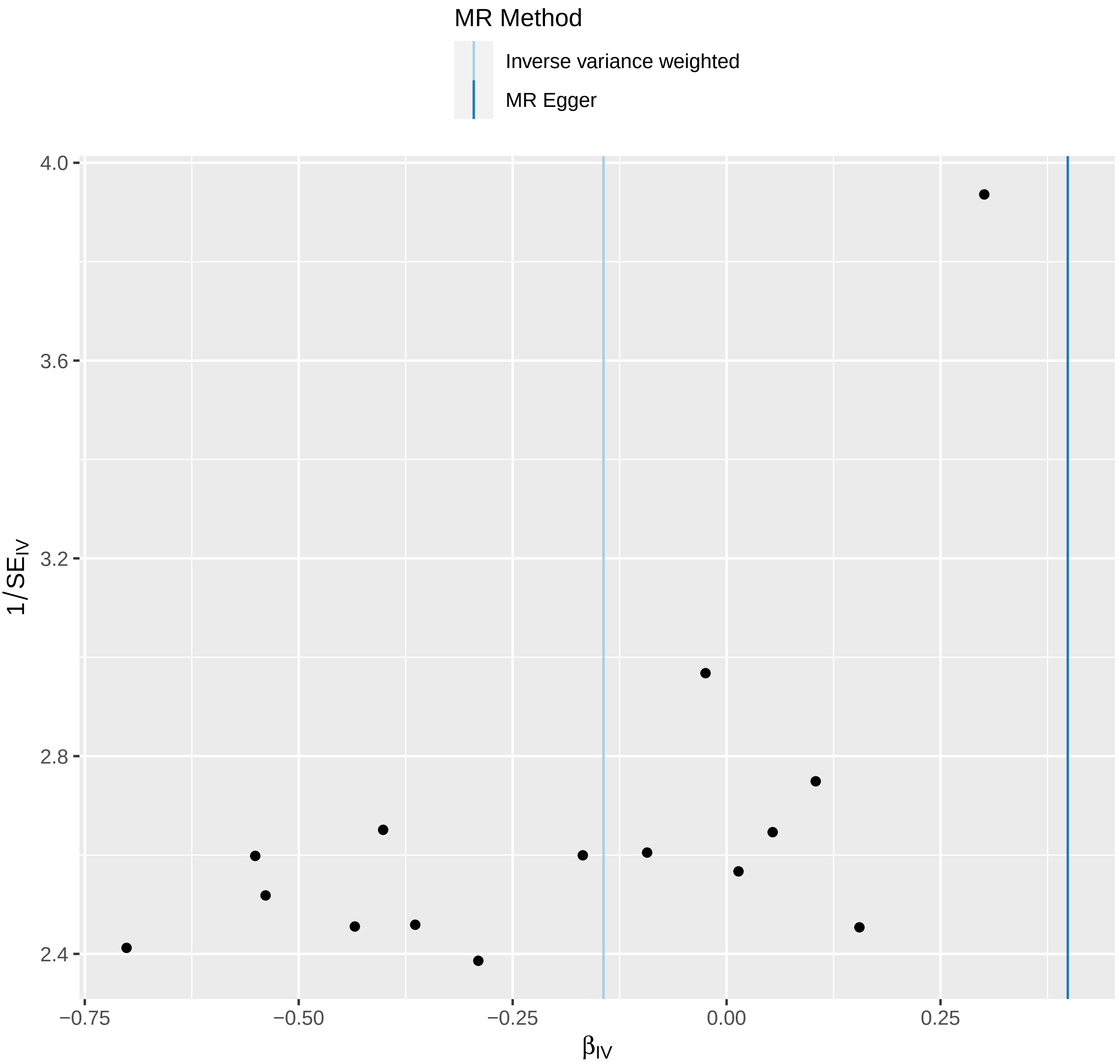

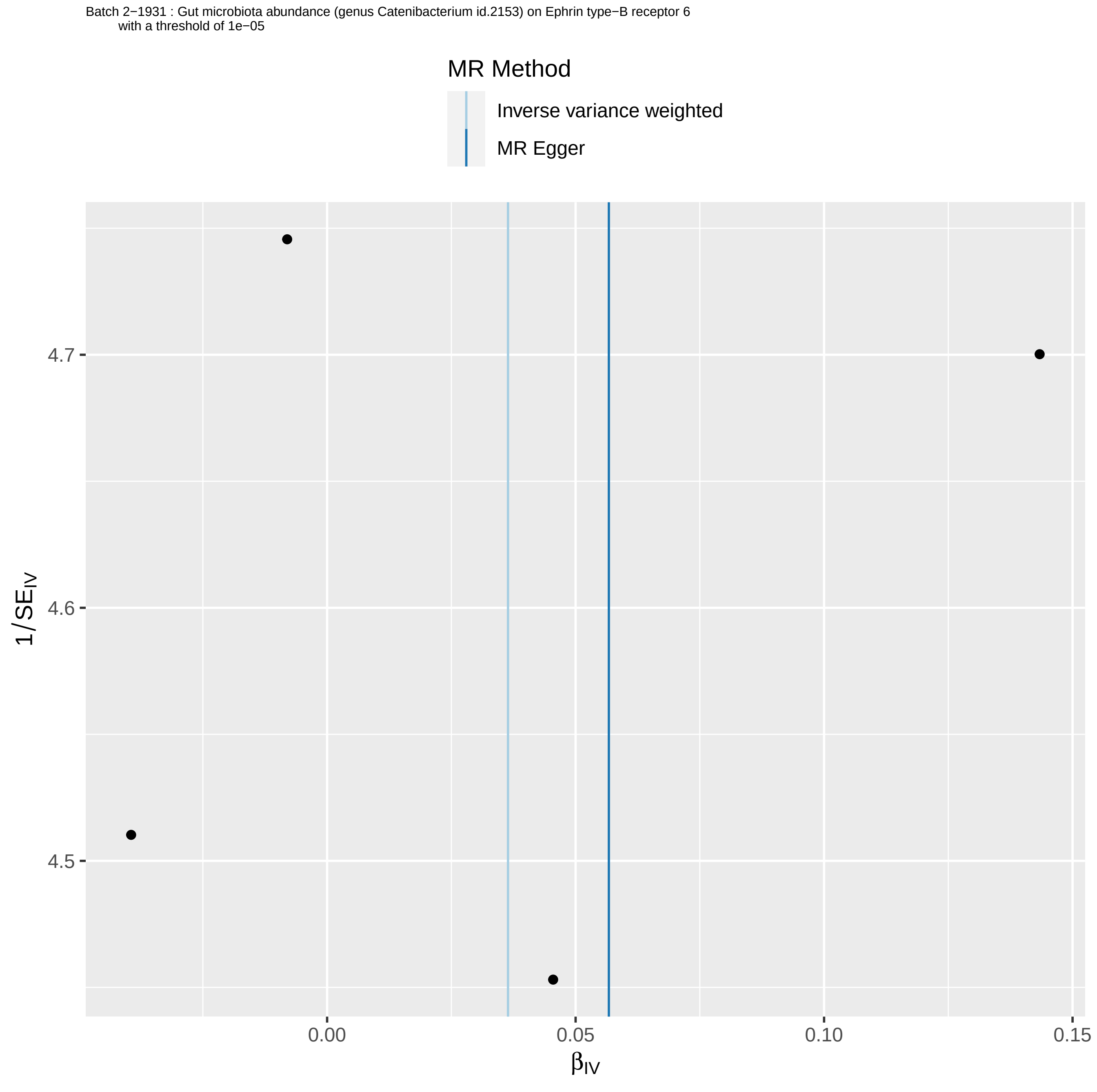
Batch 2−1931 : Gut microbiota abundance (genus Catenibacterium id.2153) on Ephrin type−B receptor 6
with a threshold of 1e−05
MR Method
Inverse variance weighted
MR Egger
4.7
4.6
4.5
1/SE_IV
0.00
0.05
0.10
0.15
β_IV

Batch 2−1932 : Gut microbiota abundance (genus Parasutterella id.2892) on Ephrin type−B receptor 6
with a threshold of 1e−05

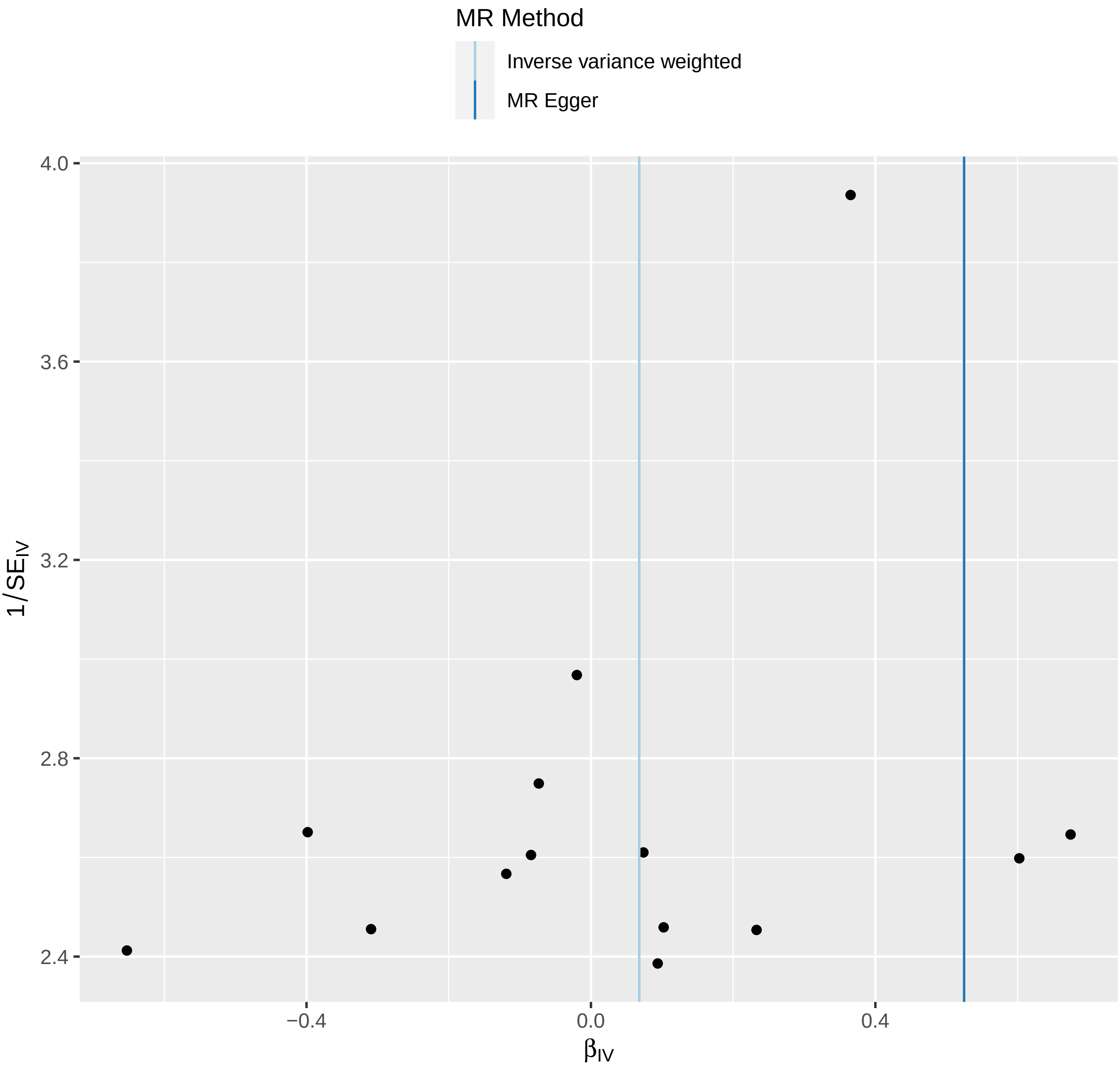

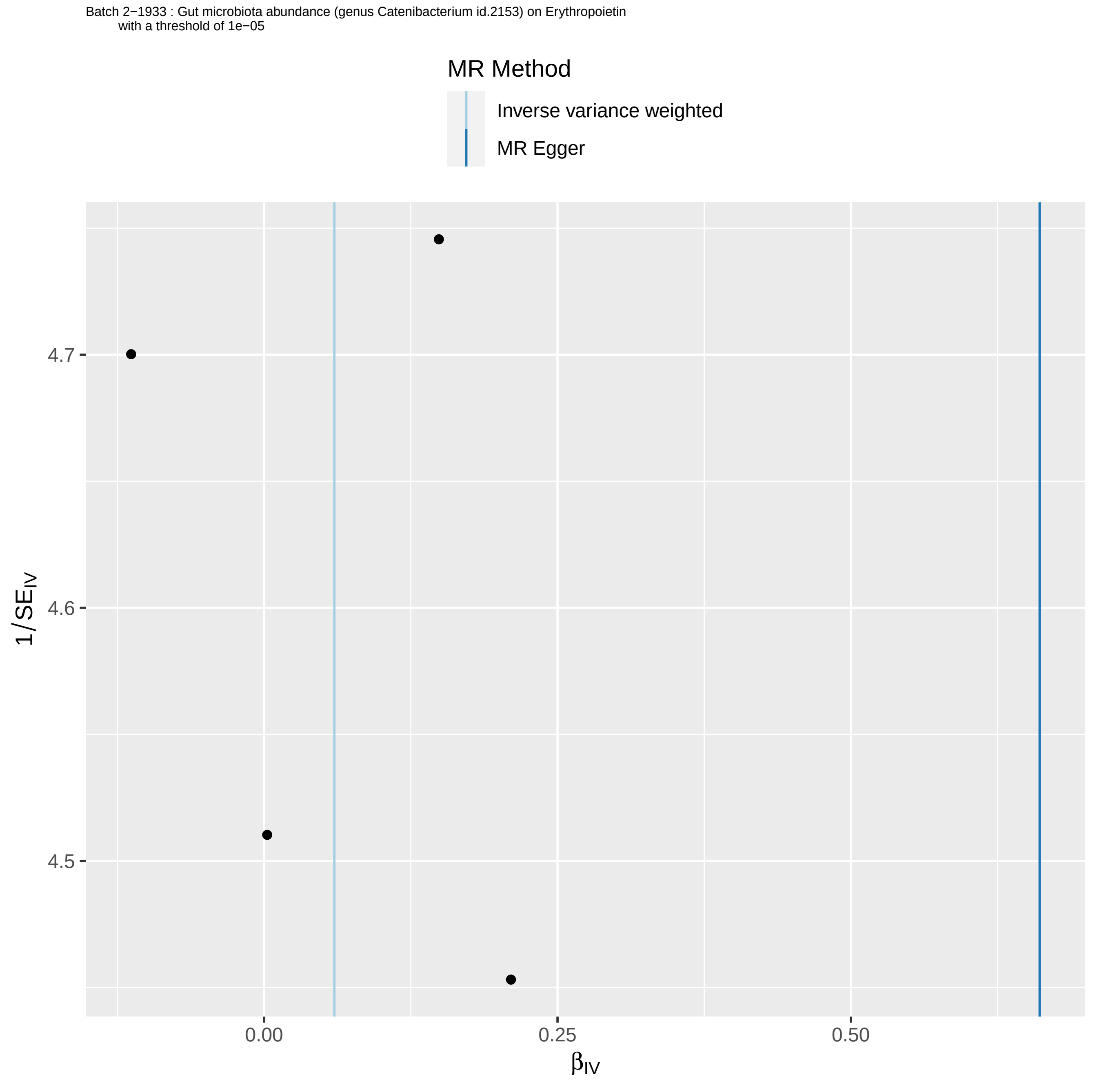

Batch 2−1933 : Gut microbiota abundance (genus Catenibacterium id.2153) on Erythropoietin
with a threshold of 1e−05
MR Method
Inverse variance weighted
MR Egger
4.7
4.6
4.5
1/SE_IV
0.00
0.25
0.50
β_IV

Batch 2−1934 : Gut microbiota abundance (genus Parasutterella id.2892) on Erythropoietin
with a threshold of 1e−05

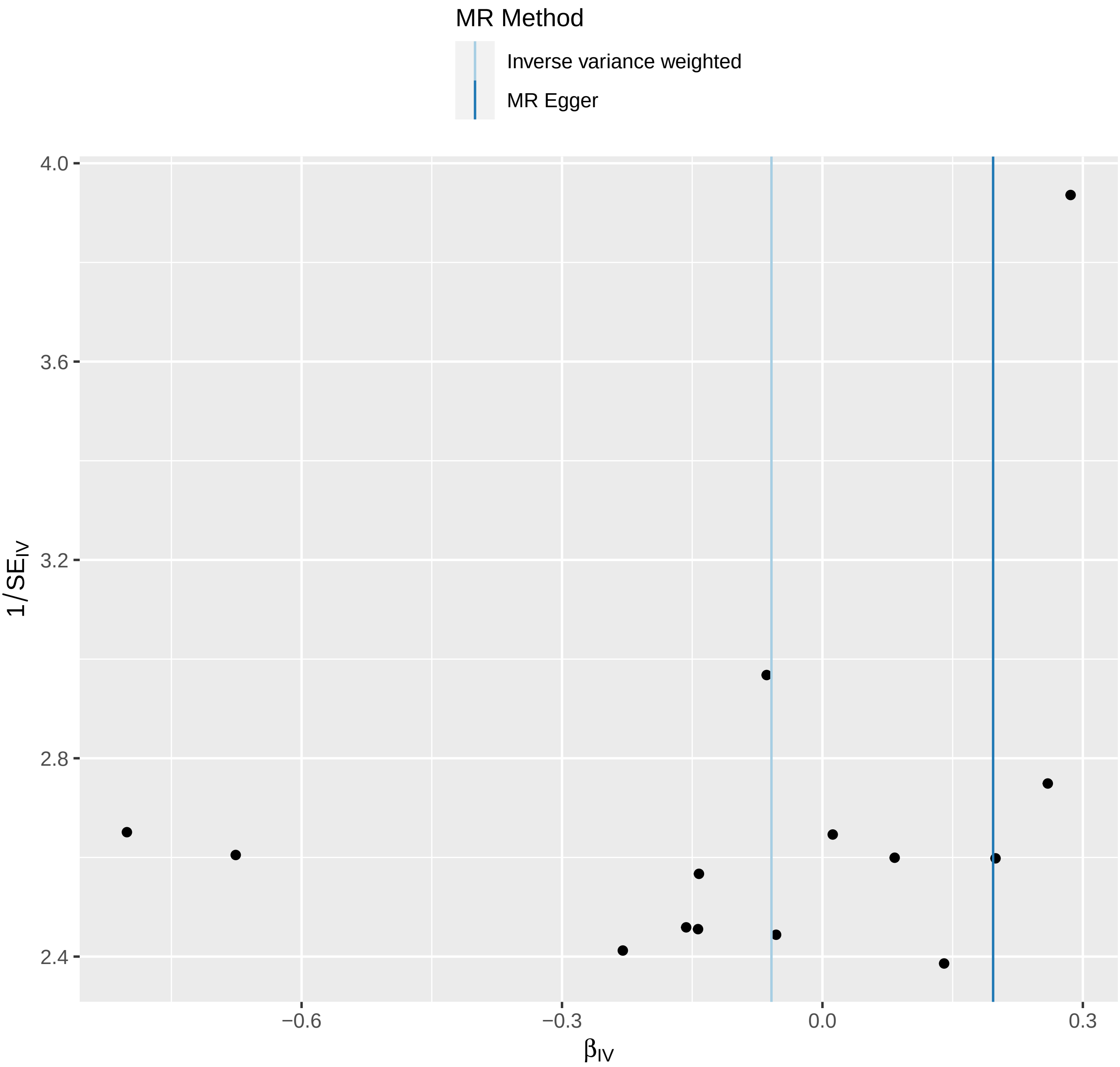

Batch 2−1935 : Gut microbiota abundance (genus Catenibacterium id.2153) on Erythropoietin receptor
with a threshold of 1e−05

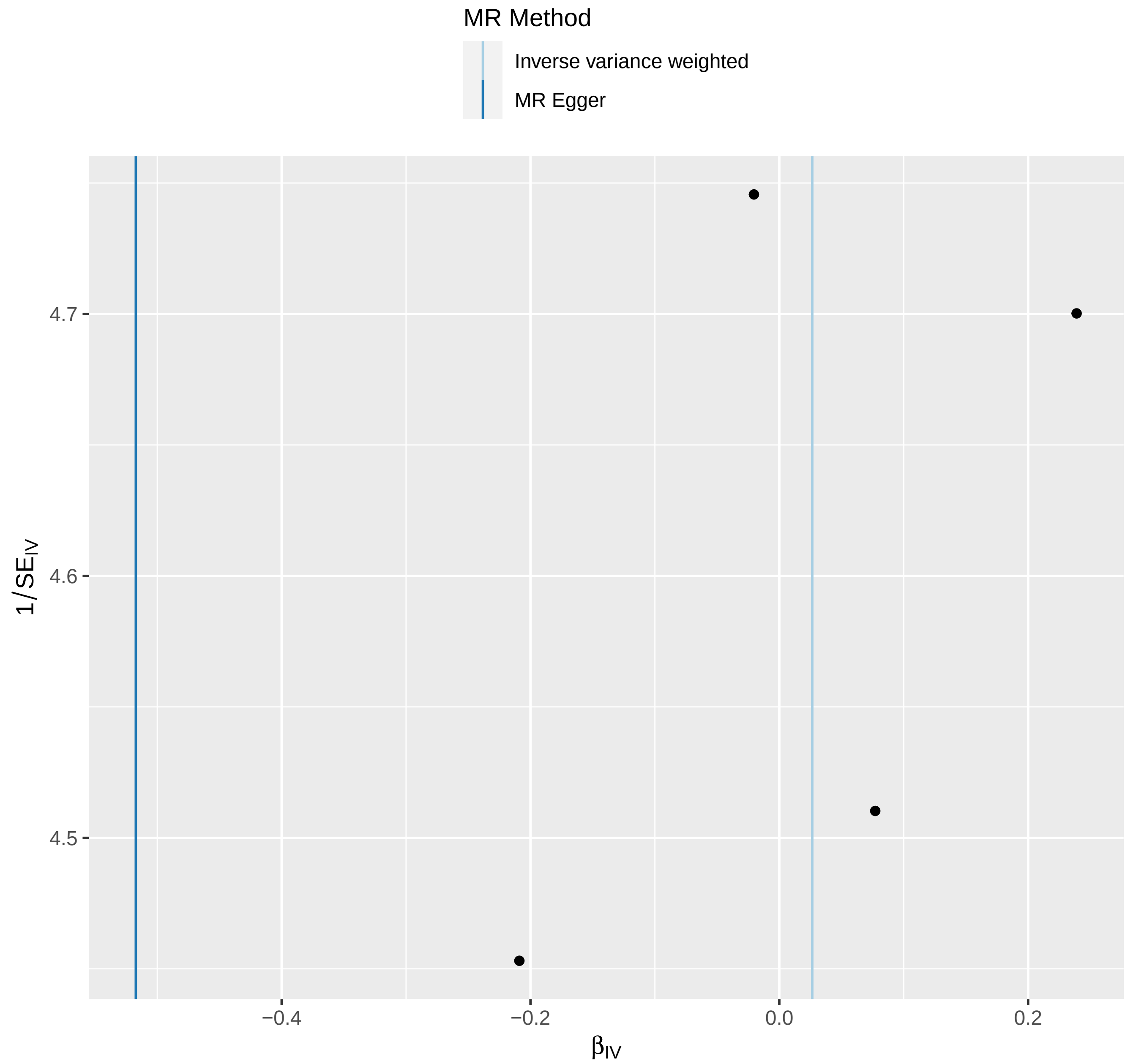

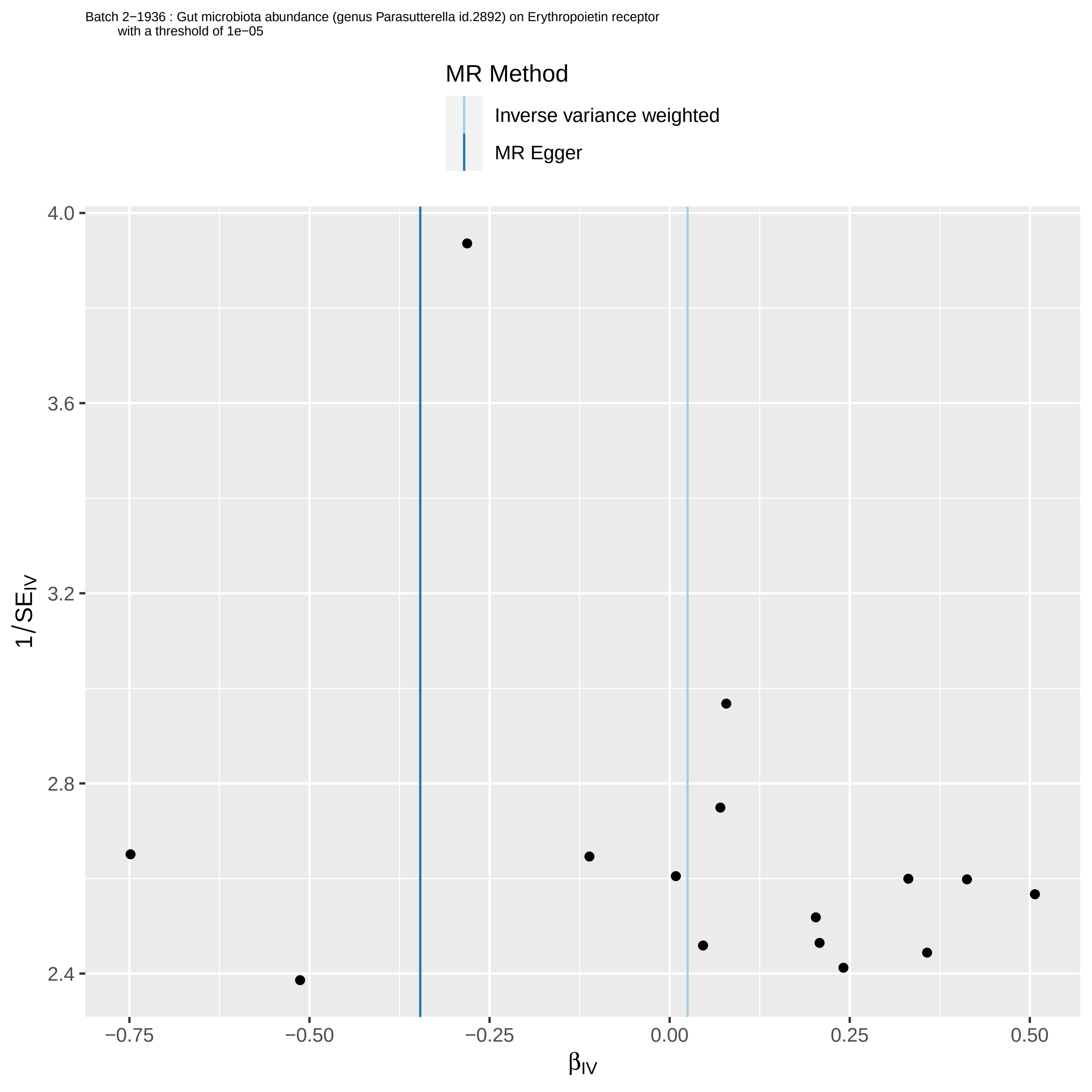
Batch 2−1936 : Gut microbiota abundance (genus Parasutterella id.2892) on Erythropoietin receptor
with a threshold of 1e−05
MR Method
Inverse variance weighted
MR Egger
4.0
3.6
3.2
2.8
2.4
1/SE_IV
−0.75
−0.50
−0.25
0.00
0.25
0.50
β_IV

Batch 2−1937 : Gut microbiota abundance (genus Catenibacterium id.2153) on Epidermal growth factor receptor substrate 15−like 1
with a threshold of 1e−05

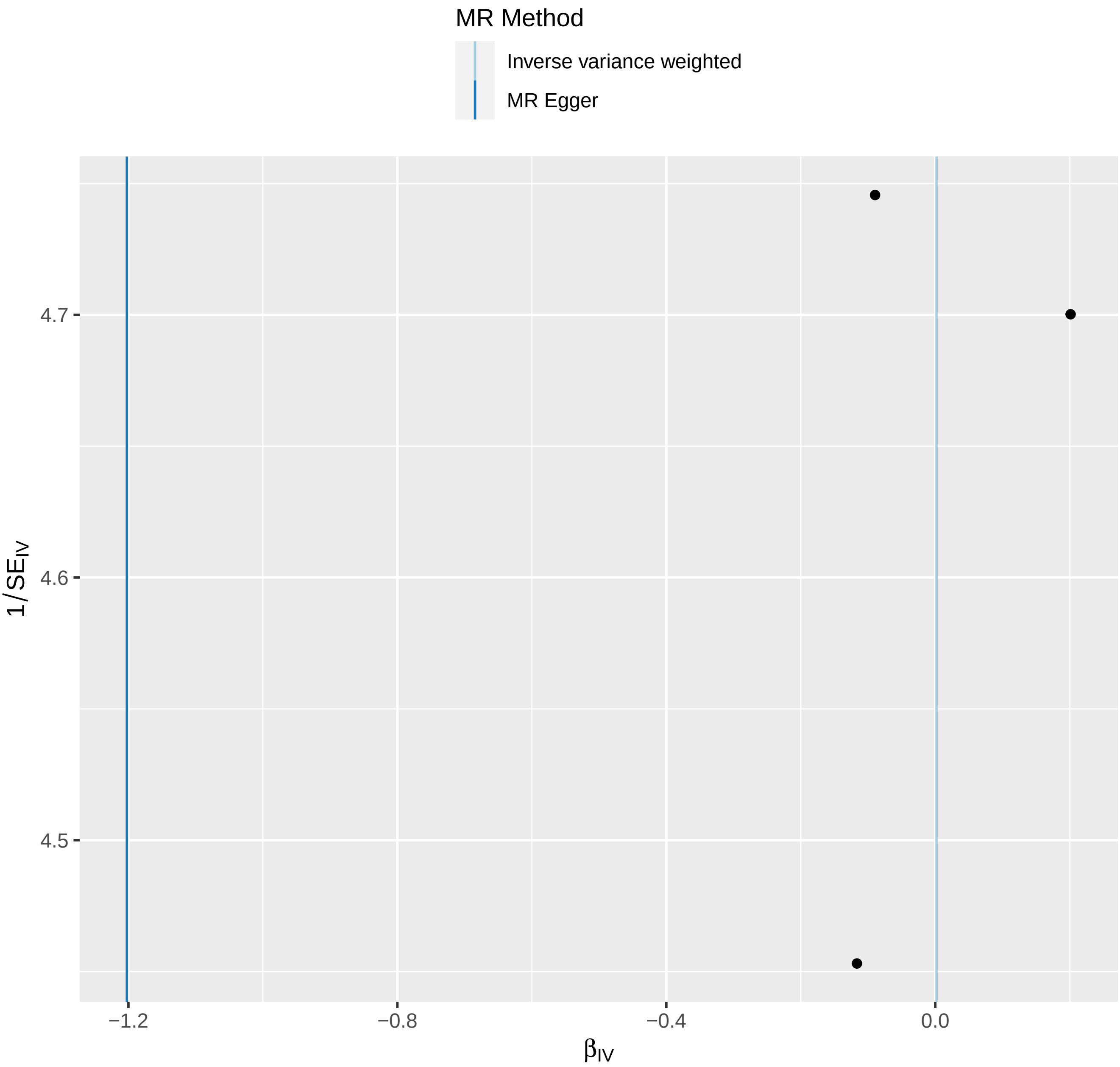

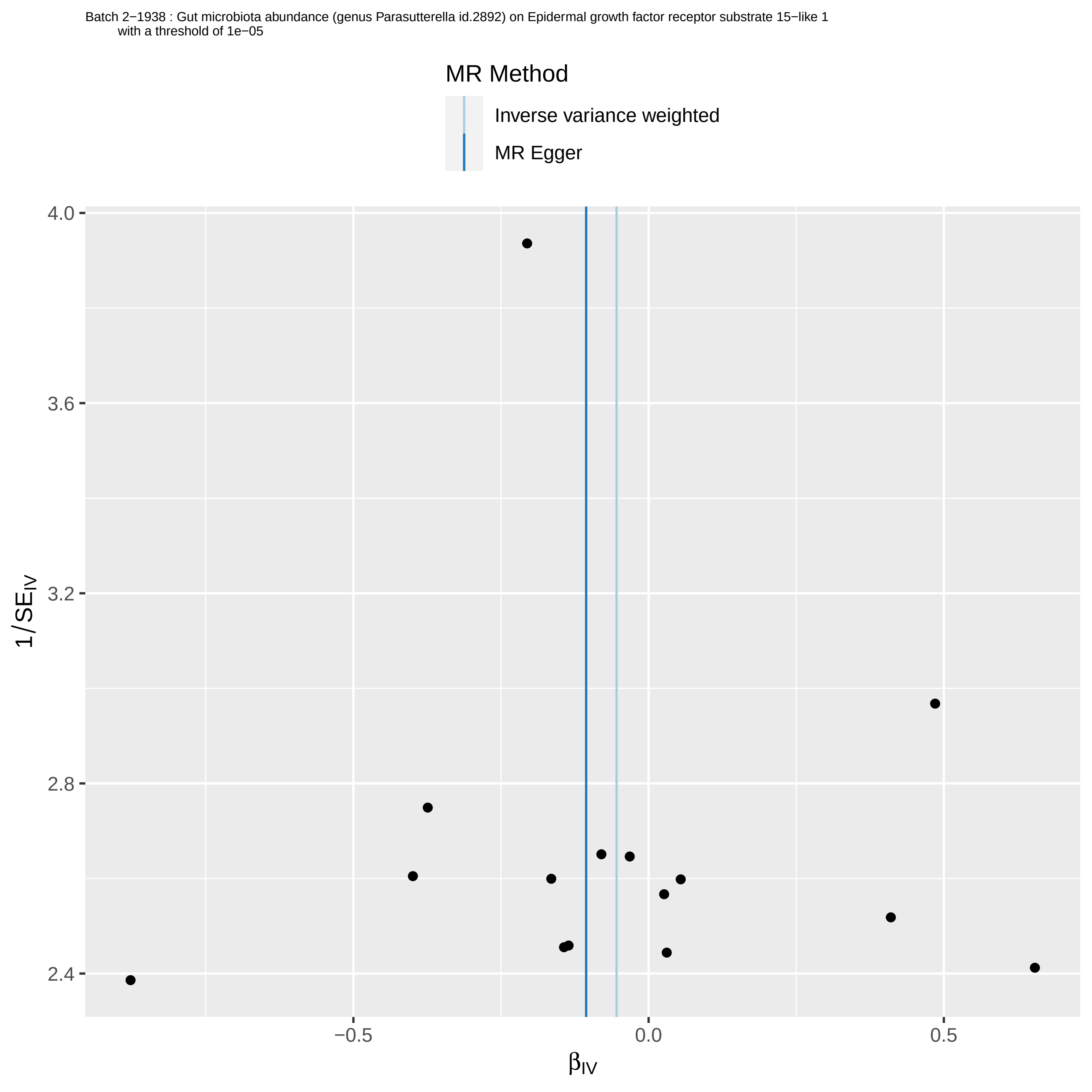

Batch 2−1938 : Gut microbiota abundance (genus Parasutterella id.2892) on Epidermal growth factor receptor substrate 15−like 1
with a threshold of 1e−05
MR Method
Inverse variance weighted
MR Egger
4.0
3.6
3.2
2.8
2.4
1/SE$_{IV}$
−0.5
0.0
0.5
β$_{IV}$

Batch 2−1939 : Gut microbiota abundance (genus Catenibacterium id.2153) on Epiphycan
with a threshold of 1e−05

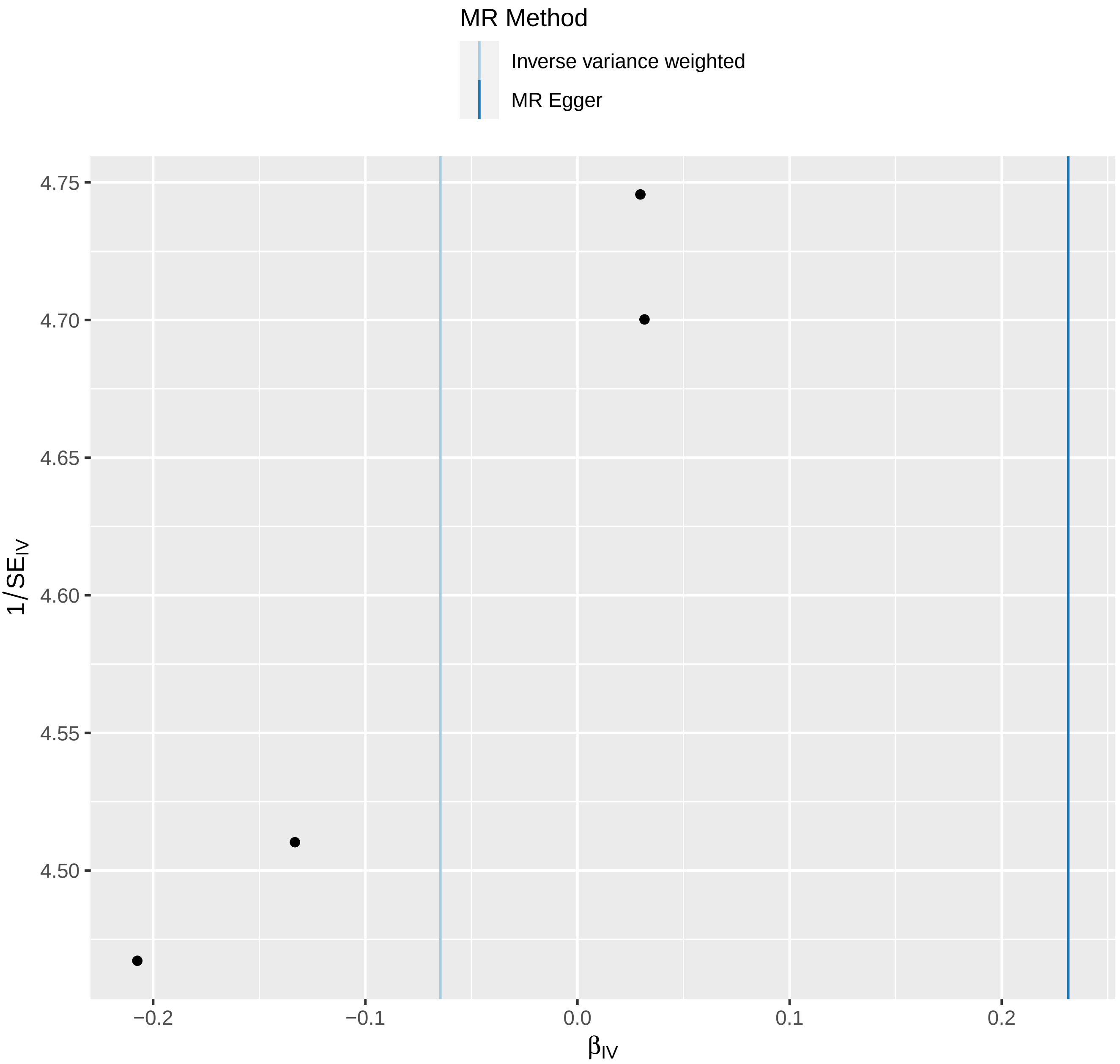

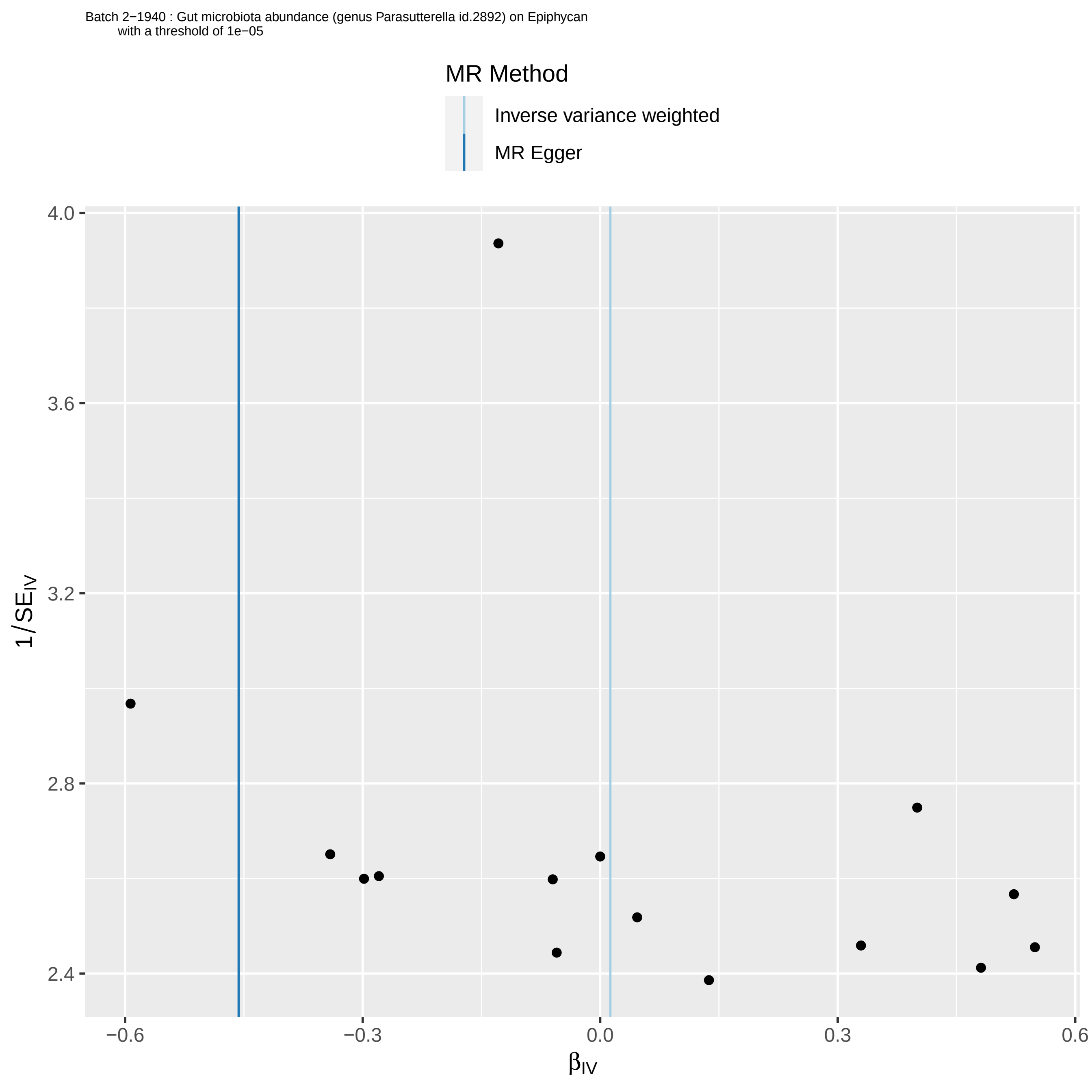
Batch 2−1940 : Gut microbiota abundance (genus Parasutterella id.2892) on Epiphycan
with a threshold of 1e−05
MR Method
Inverse variance weighted
MR Egger
4.0
3.6
3.2
2.8
2.4
1/SE$_{IV}$
−0.6
−0.3
0.0
0.3
0.6
β$_{IV}$

Batch 2−1941 : Gut microbiota abundance (genus Catenibacterium id.2153) on Endoplasmic reticulum aminopeptidase 1
with a threshold of 1e−05

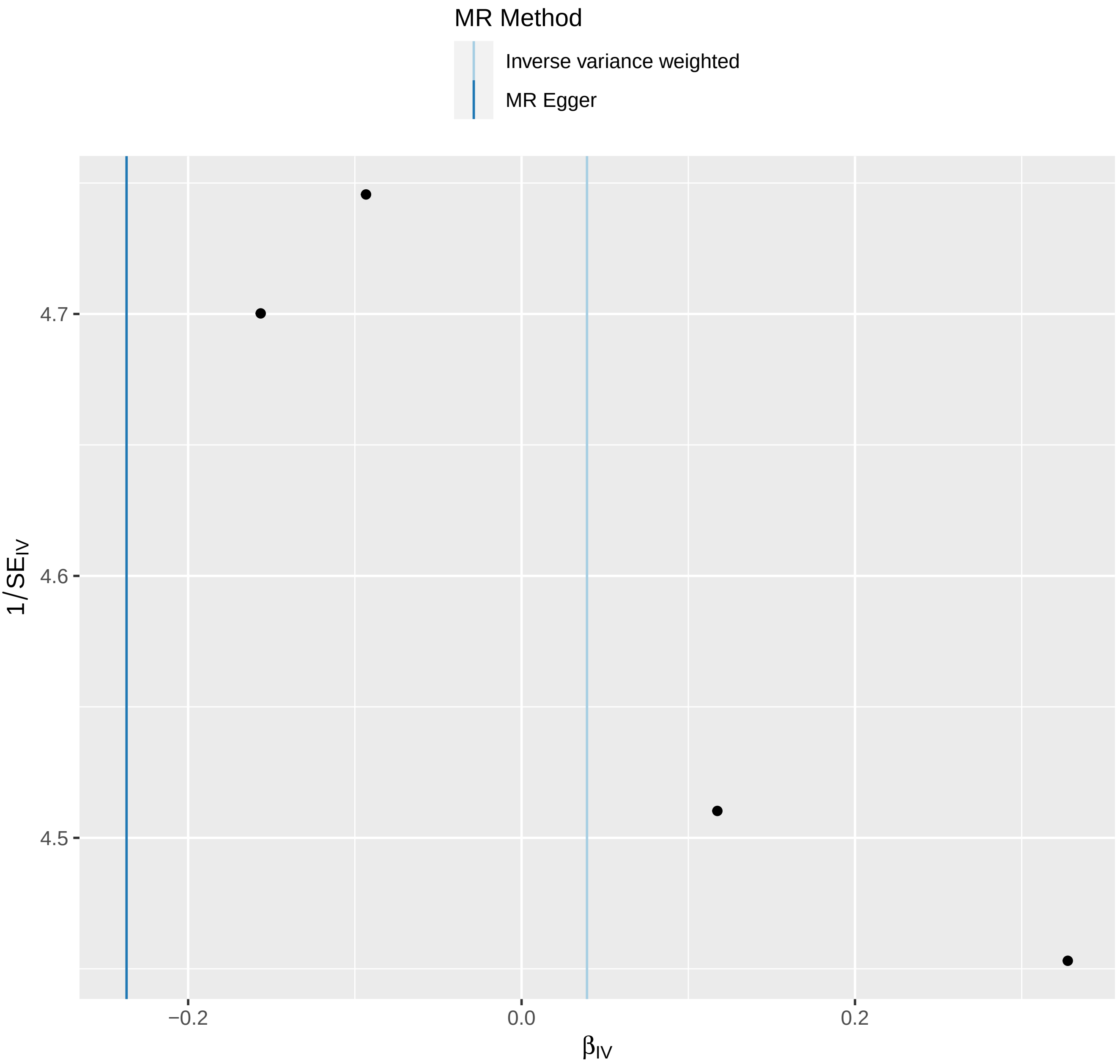

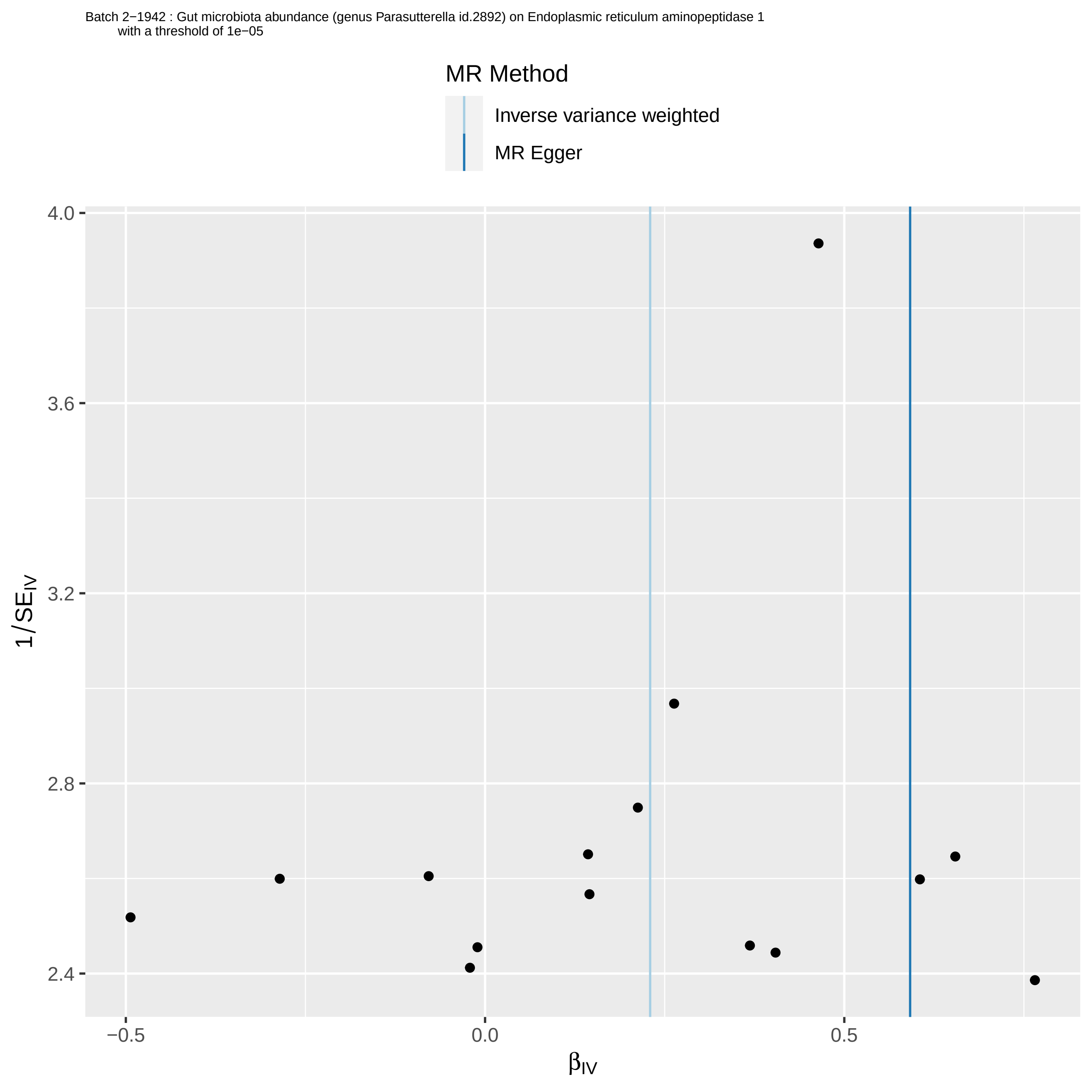
Batch 2−1942 : Gut microbiota abundance (genus Parasutterella id.2892) on Endoplasmic reticulum aminopeptidase 1
with a threshold of 1e−05
MR Method
Inverse variance weighted
MR Egger
4.0
3.6
3.2
2.8
2.4
1/SE_IV
−0.5
0.0
0.5
β_IV

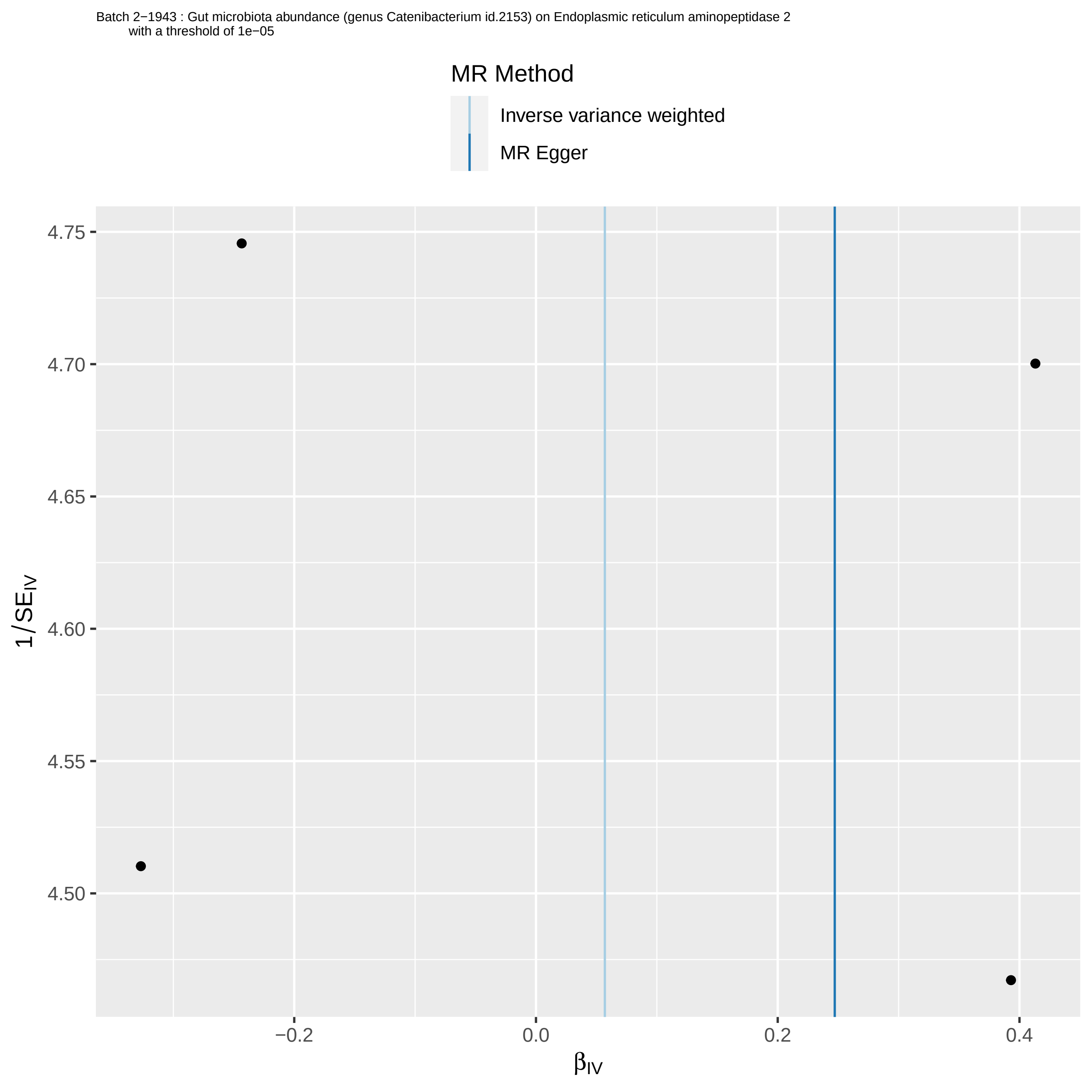
Batch 2−1943 : Gut microbiota abundance (genus Catenibacterium id.2153) on Endoplasmic reticulum aminopeptidase 2
with a threshold of 1e−05
MR Method
Inverse variance weighted
MR Egger
4.75
4.70
4.65
4.60
4.55
4.50
1/SE_IV
−0.2
0.0
0.2
0.4
β_IV

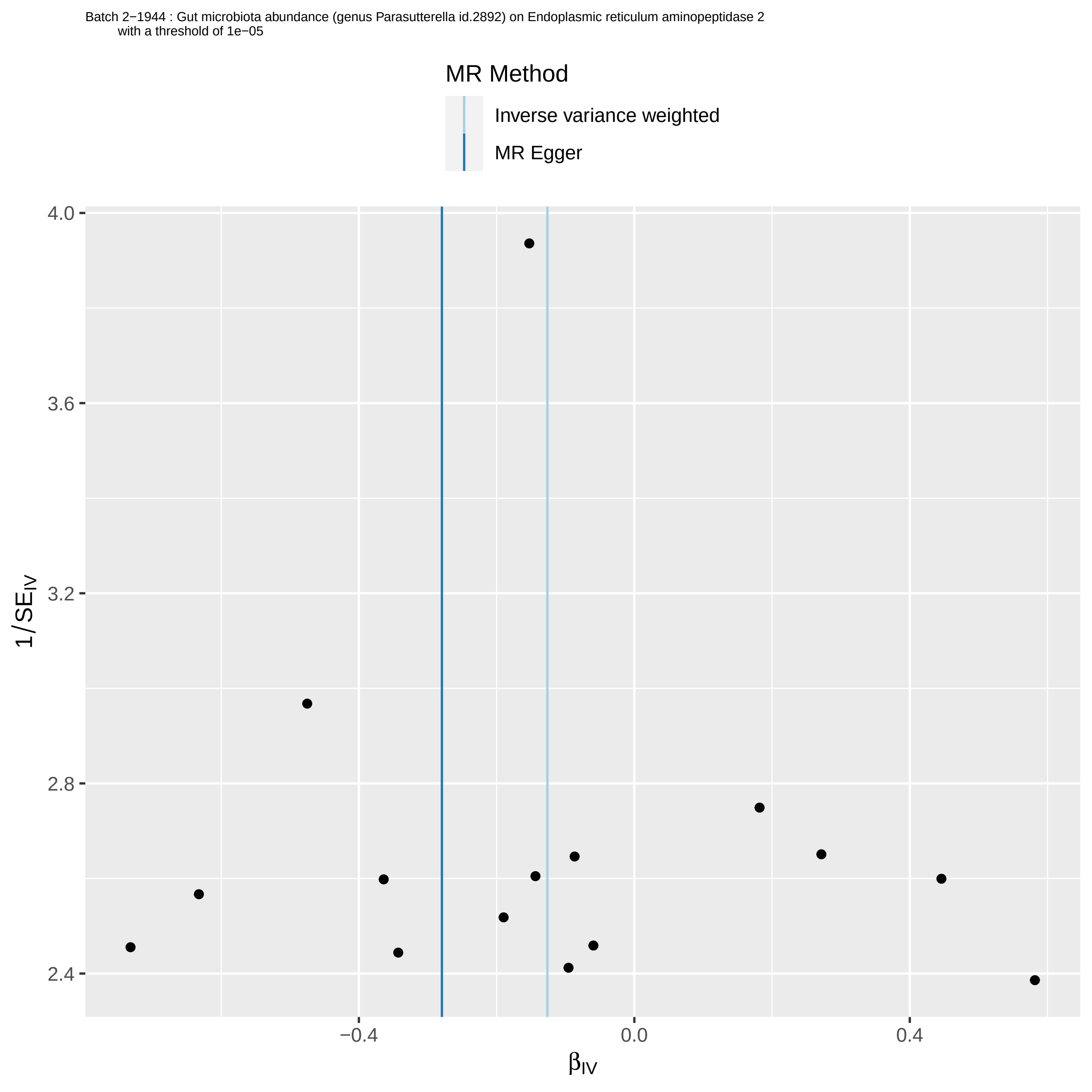

Batch 2−1944 : Gut microbiota abundance (genus Parasutterella id.2892) on Endoplasmic reticulum aminopeptidase 2
with a threshold of 1e−05
MR Method
Inverse variance weighted
MR Egger
4.0
3.6
3.2
2.8
2.4
1/SE_IV
−0.4
0.0
0.4
β_IV

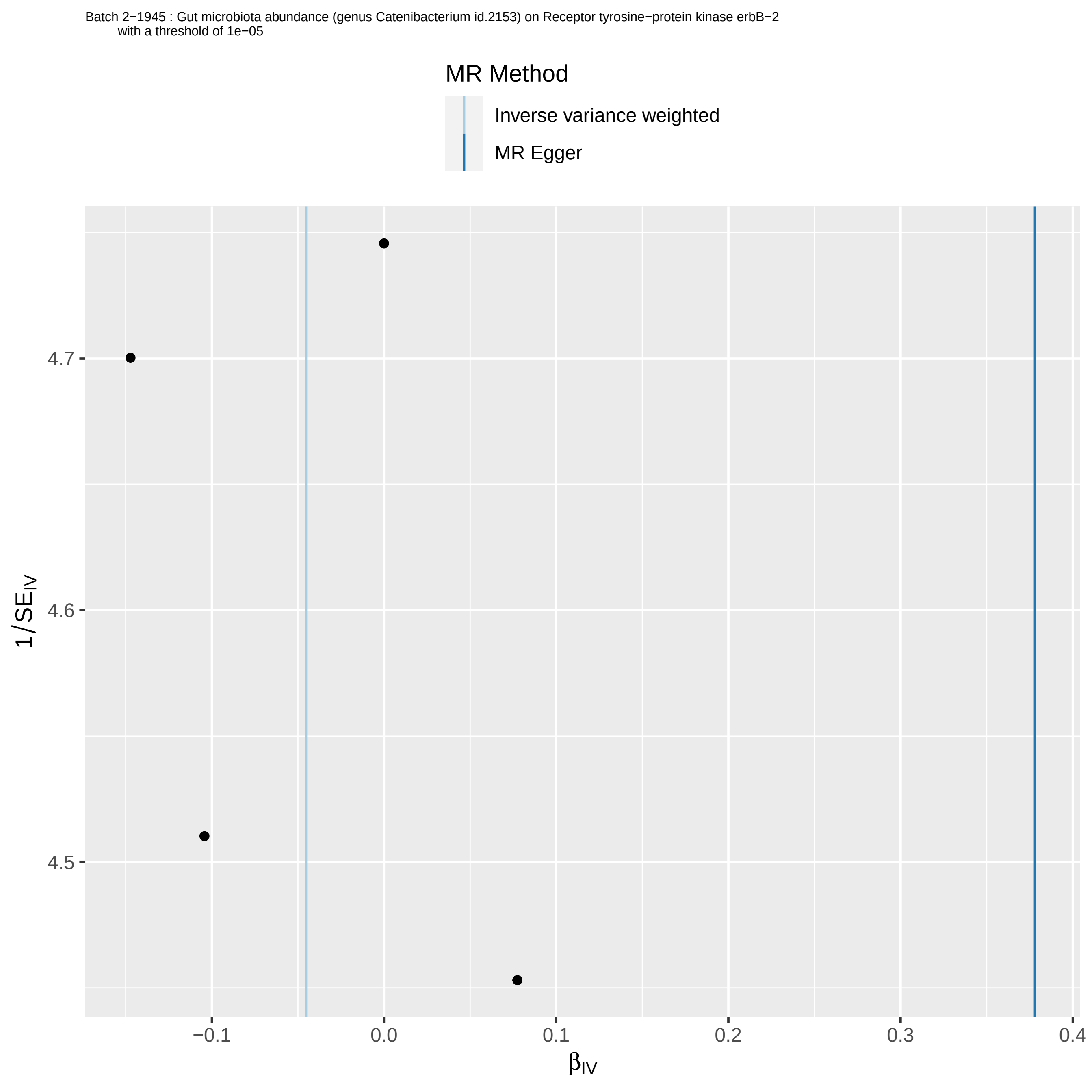

Batch 2−1945 : Gut microbiota abundance (genus Catenibacterium id.2153) on Receptor tyrosine−protein kinase erbB−2
with a threshold of 1e−05
MR Method
Inverse variance weighted
MR Egger
4.7
4.6
4.5
1/SE_IV
−0.1
0.0
0.1
0.2
0.3
0.4
β_IV

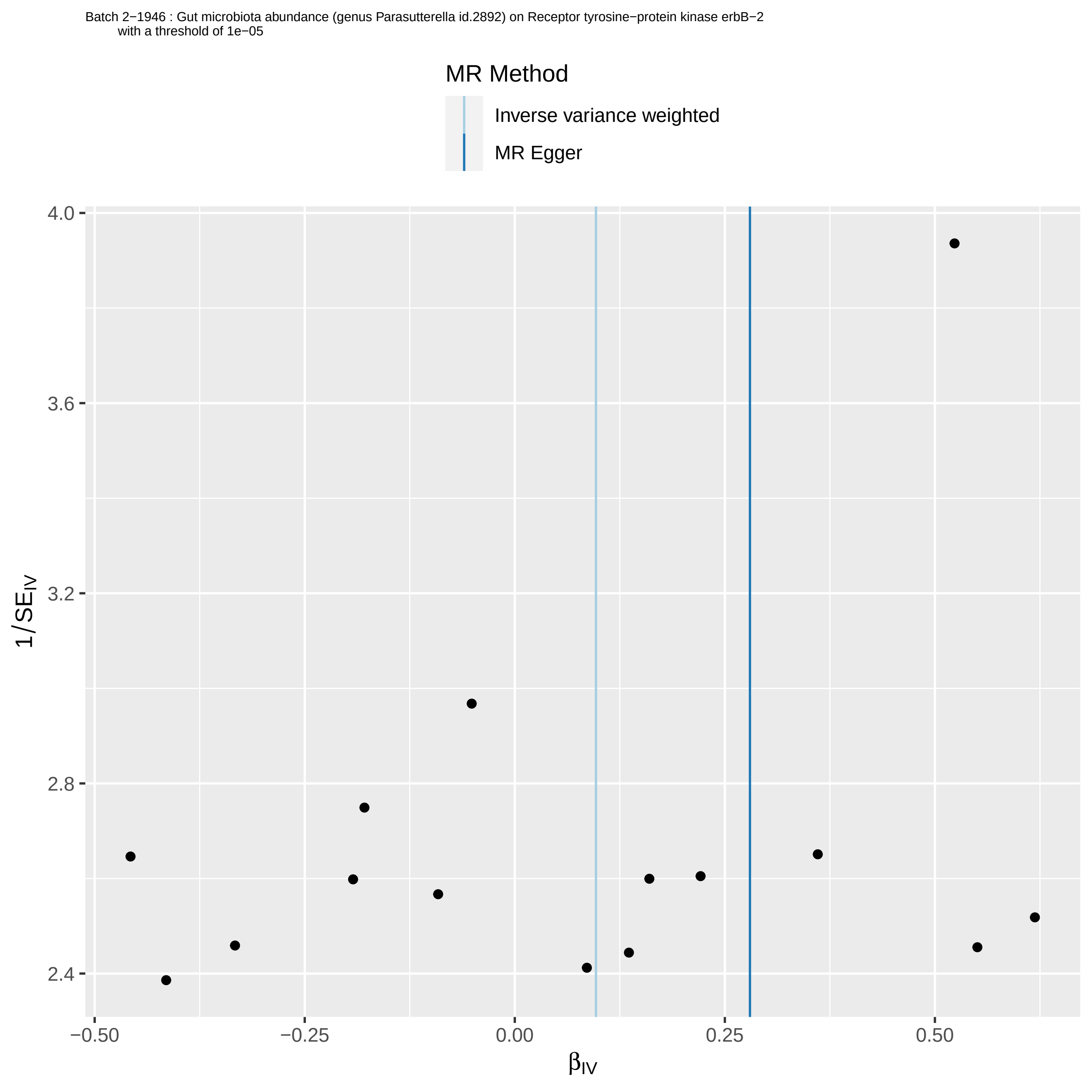

Batch 2−1946 : Gut microbiota abundance (genus Parasutterella id.2892) on Receptor tyrosine−protein kinase erbB−2
with a threshold of 1e−05
MR Method
Inverse variance weighted
MR Egger
4.0
3.6
3.2
2.8
2.4
1/SE_IV
−0.50
−0.25
0.00
0.25
0.50
β_IV

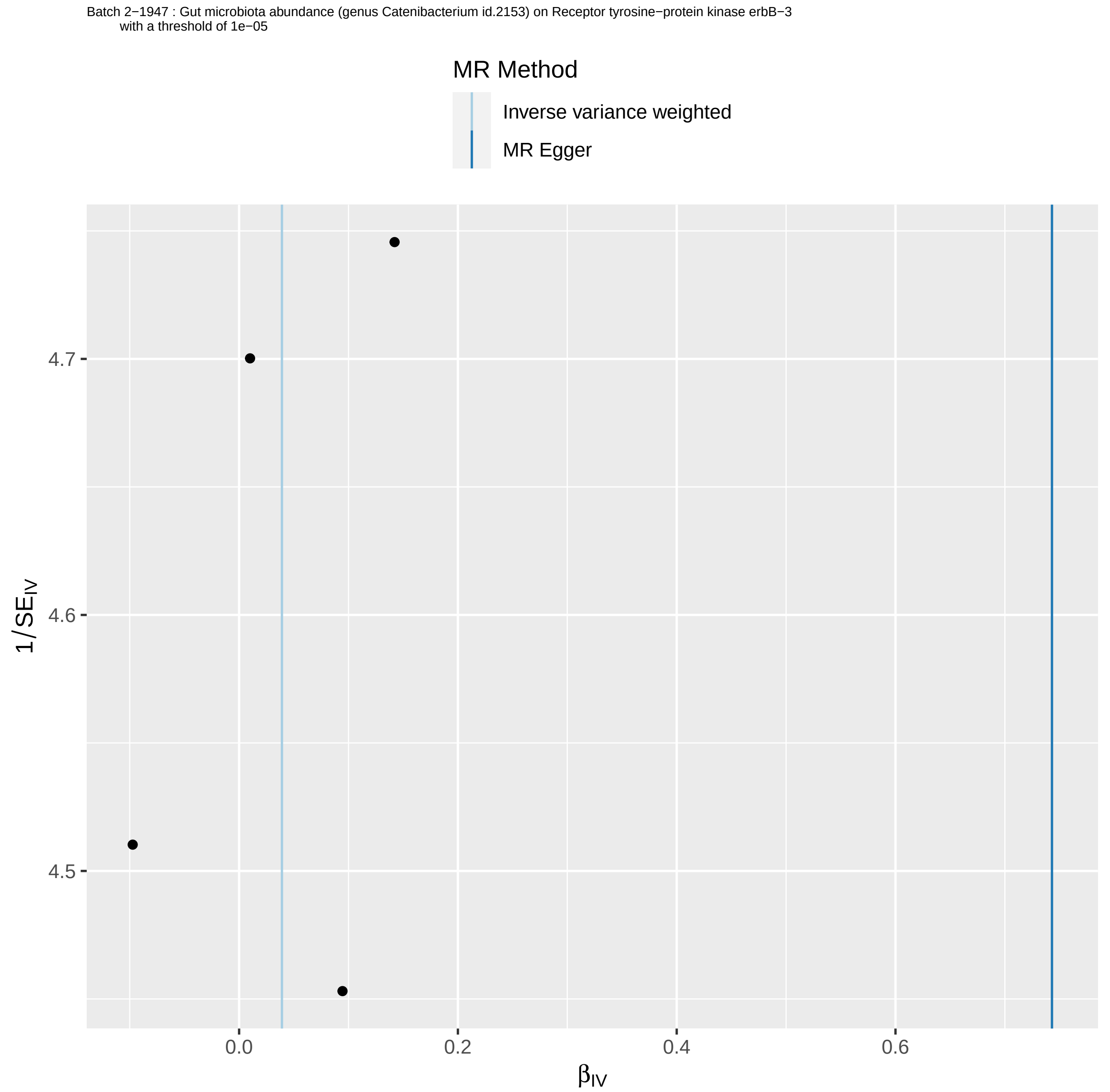
Batch 2−1947 : Gut microbiota abundance (genus Catenibacterium id.2153) on Receptor tyrosine−protein kinase erbB−3
with a threshold of 1e−05
MR Method
Inverse variance weighted
MR Egger
4.7
4.6
4.5
1/SE_IV
0.0
0.2
0.4
0.6
β_IV

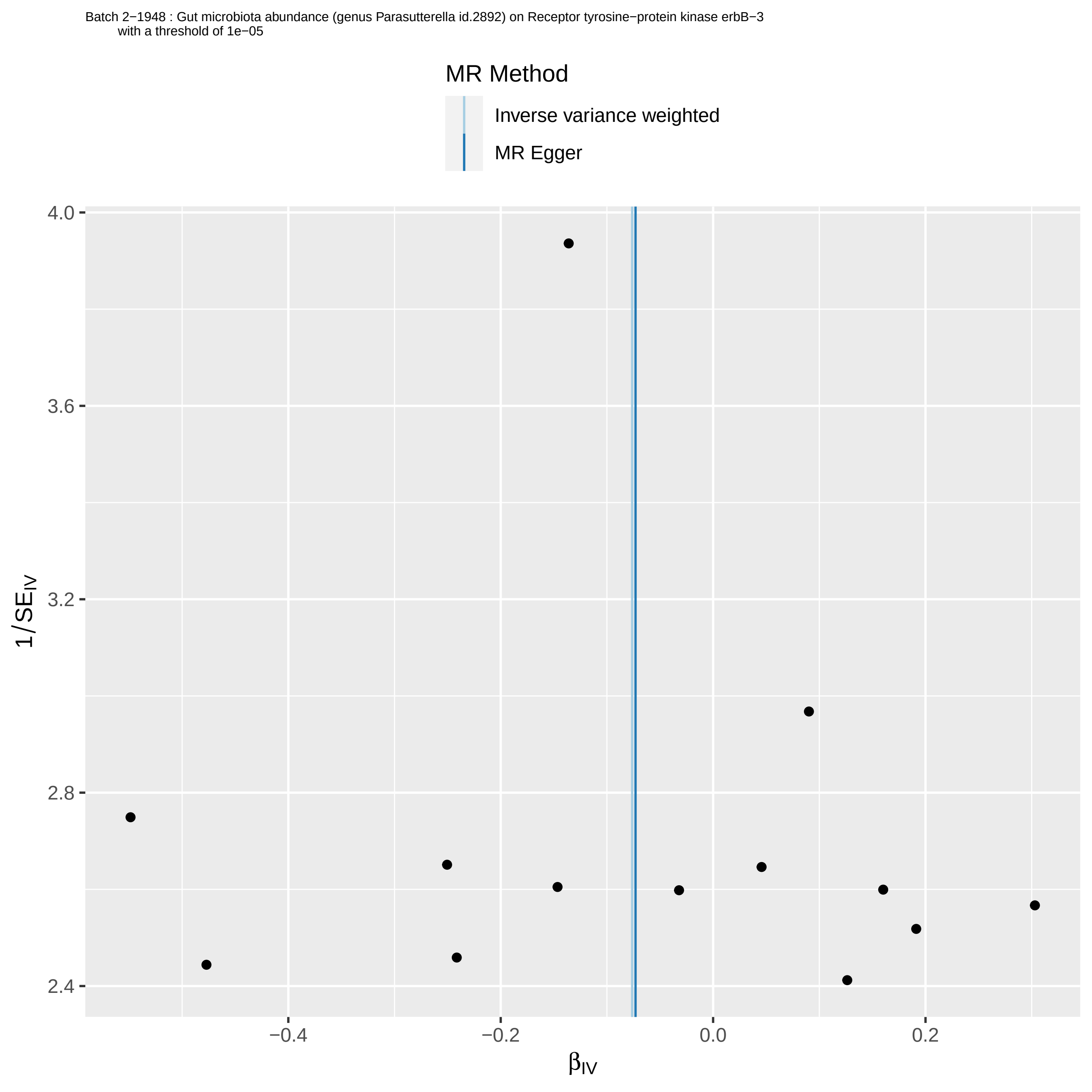
Batch 2−1948 : Gut microbiota abundance (genus Parasutterella id.2892) on Receptor tyrosine−protein kinase erbB−3
with a threshold of 1e−05
MR Method
Inverse variance weighted
MR Egger
4.0
3.6
3.2
2.8
2.4
1/SE_IV
−0.4
−0.2
0.0
0.2
β_IV

Batch 2−1949 : Gut microbiota abundance (genus Catenibacterium id.2153) on DNA excision repair protein ERCC−1
with a threshold of 1e−05

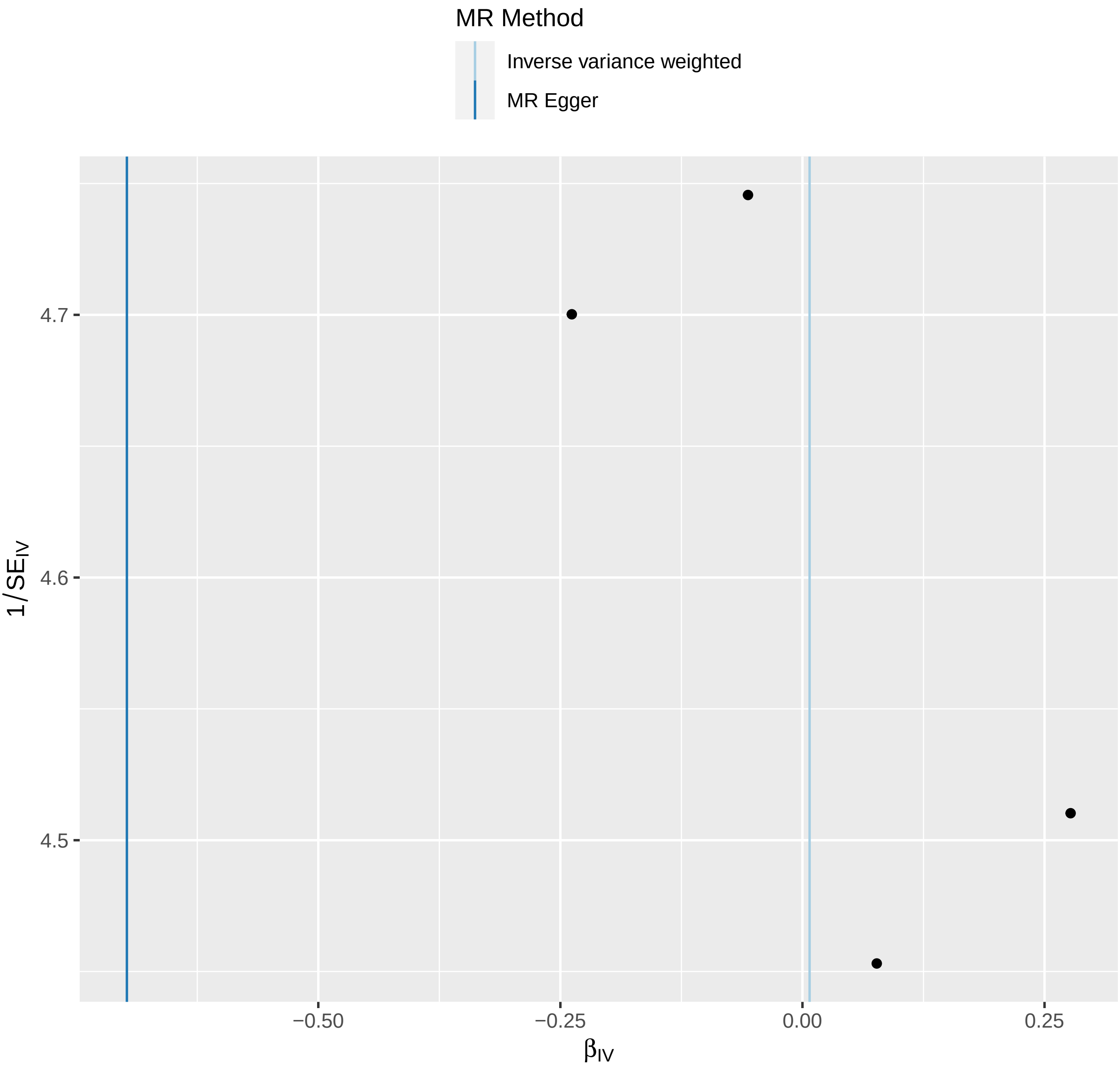

Batch 2−1950 : Gut microbiota abundance (genus Parasutterella id.2892) on DNA excision repair protein ERCC−1
with a threshold of 1e−05

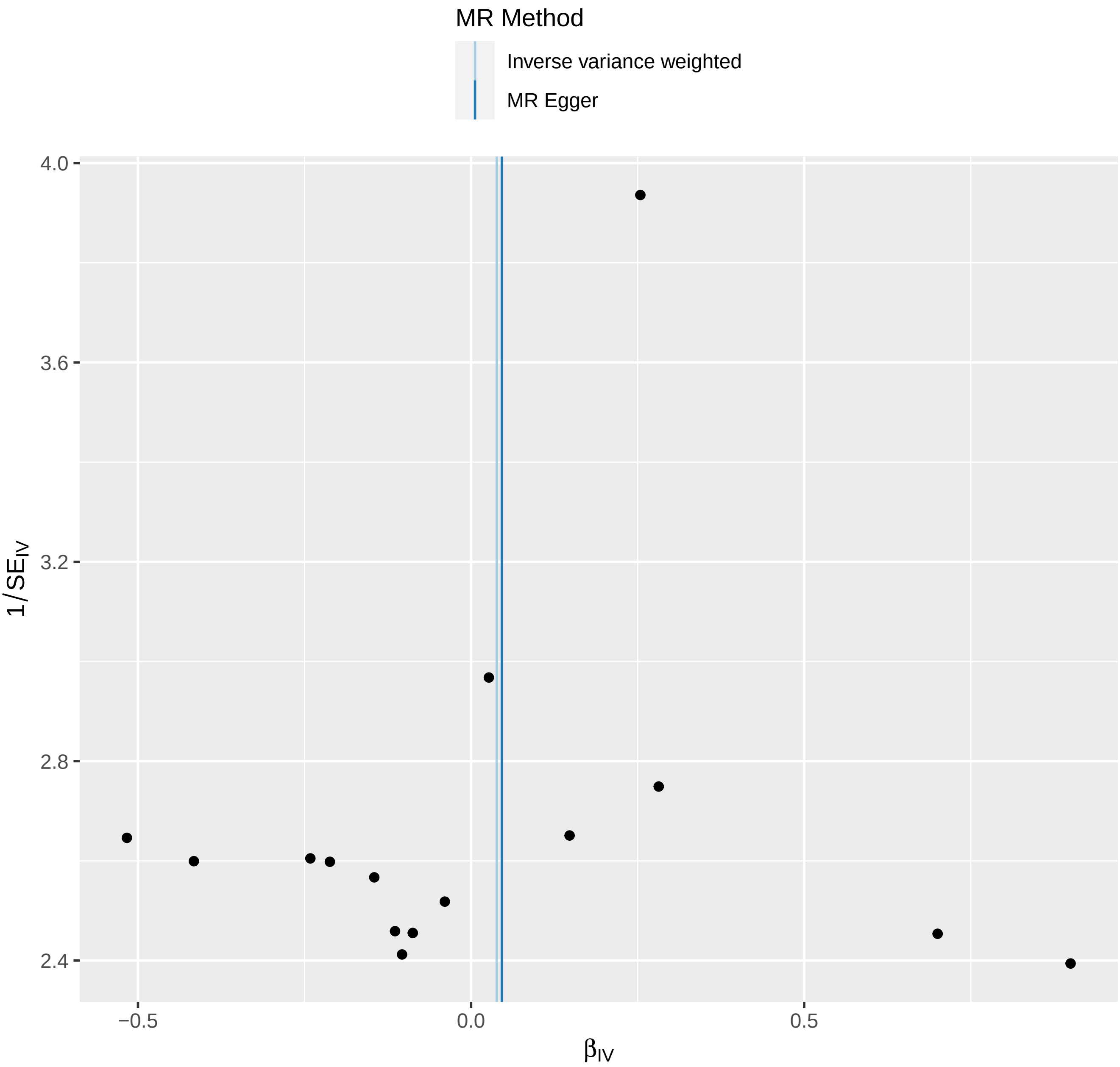

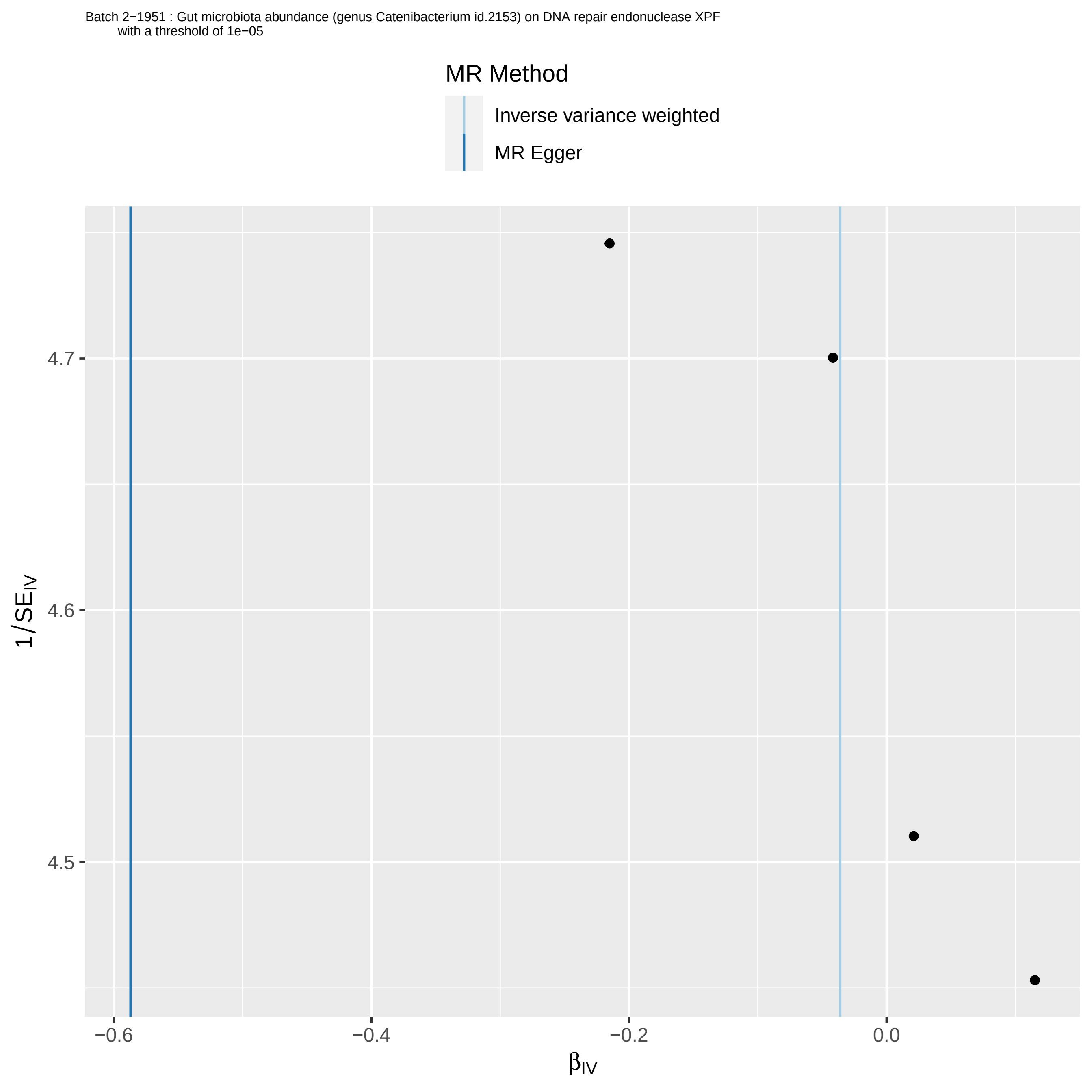
Batch 2−1951 : Gut microbiota abundance (genus Catenibacterium id.2153) on DNA repair endonuclease XPF
with a threshold of 1e−05
MR Method
Inverse variance weighted
MR Egger
4.7
4.6
4.5
1/SE_IV
−0.6
−0.4
−0.2
0.0
β_IV

Batch 2−1952 : Gut microbiota abundance (genus Parasutterella id.2892) on DNA repair endonuclease XPF
with a threshold of 1e−05

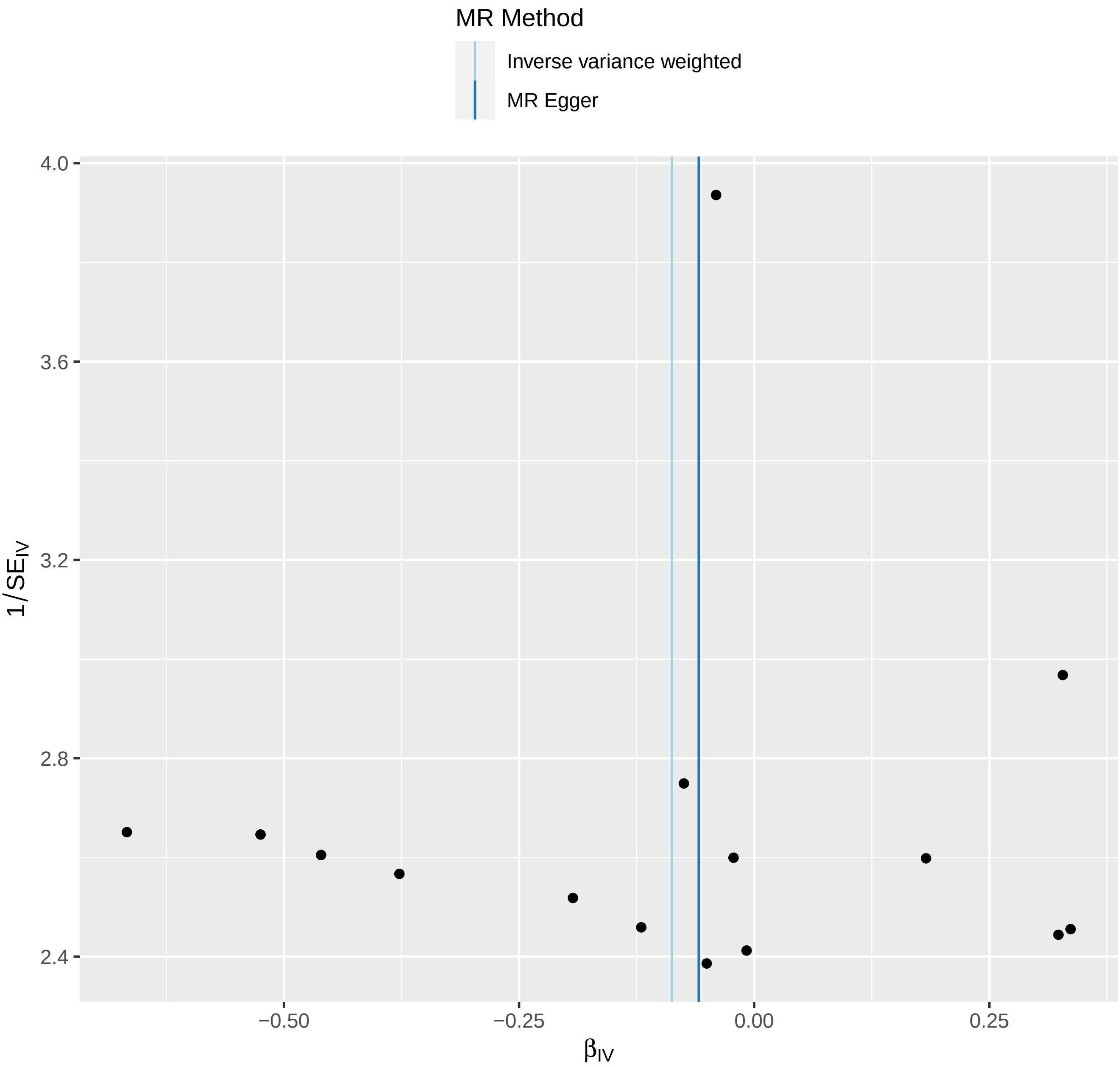

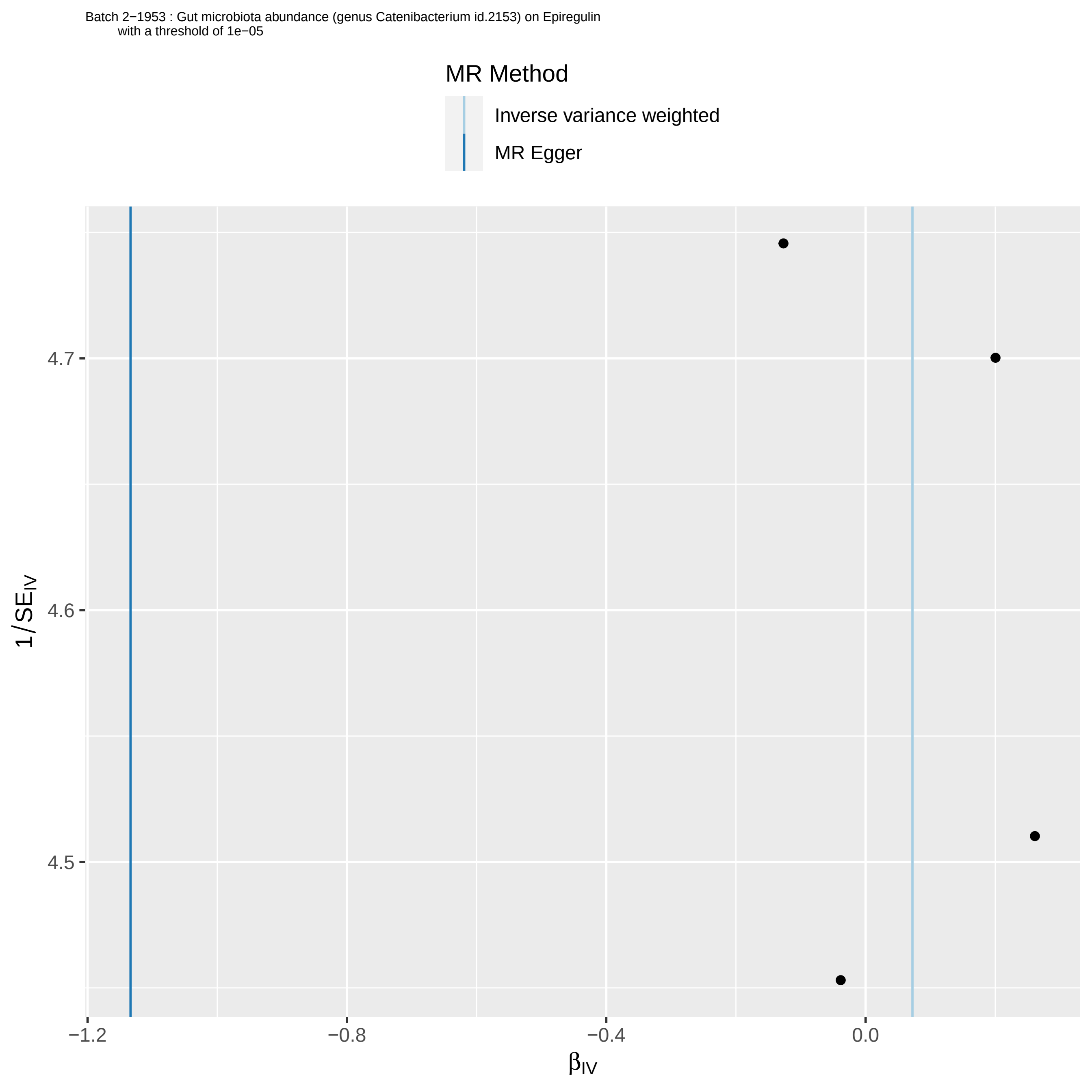
Batch 2−1953 : Gut microbiota abundance (genus Catenibacterium id.2153) on Epiregulin
with a threshold of 1e−05
MR Method
Inverse variance weighted
MR Egger
4.7
4.6
4.5
1/SE_IV
−1.2
−0.8
−0.4
0.0
β_IV

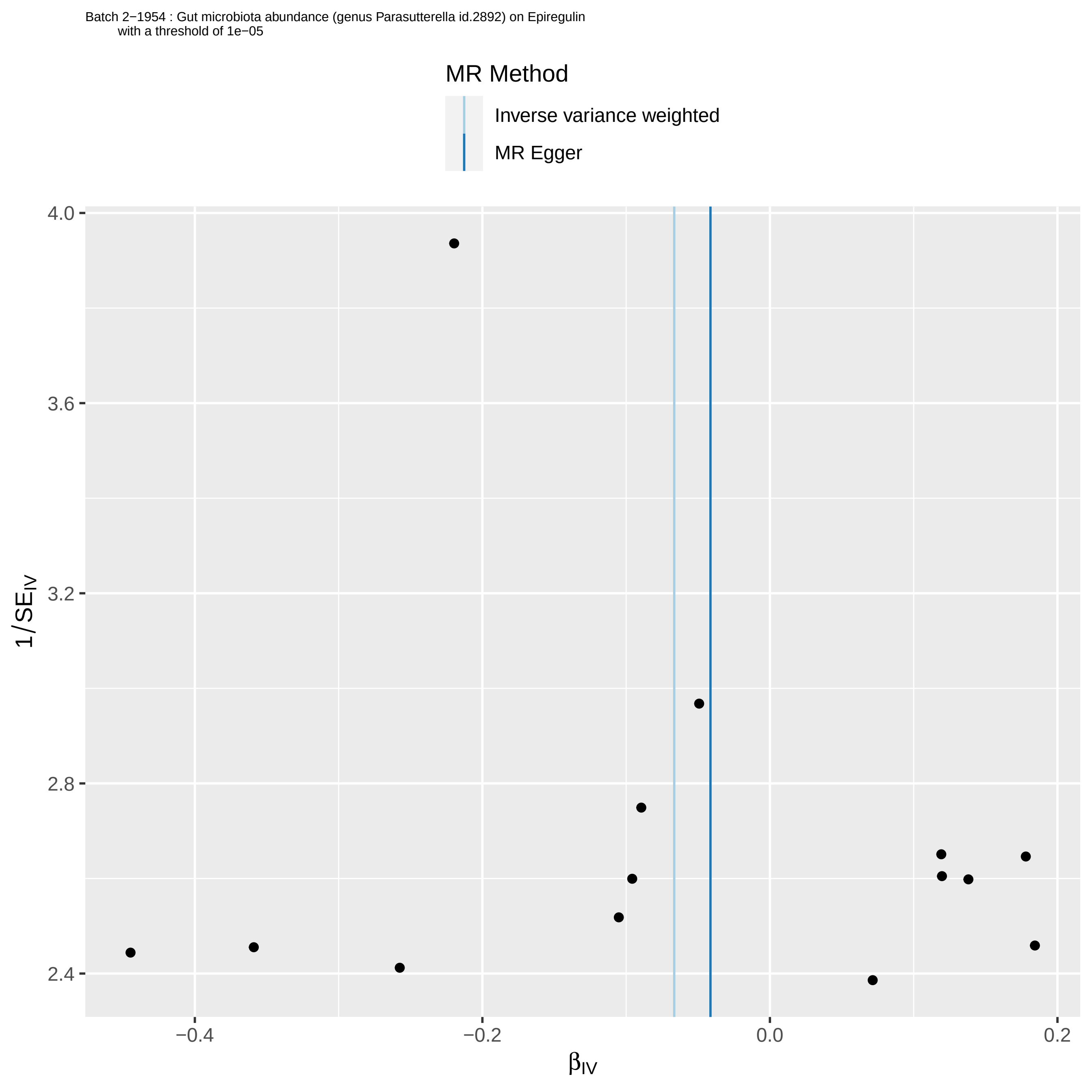
Batch 2−1954 : Gut microbiota abundance (genus Parasutterella id.2892) on Epiregulin
with a threshold of 1e−05
MR Method
Inverse variance weighted
MR Egger
4.0
3.6
3.2
2.8
2.4
1/SE_IV
−0.4
−0.2
0.0
0.2
β_IV

Batch 2−1955 : Gut microbiota abundance (genus Catenibacterium id.2153) on Enhancer of rudimentary homolog with a threshold of 1e−05

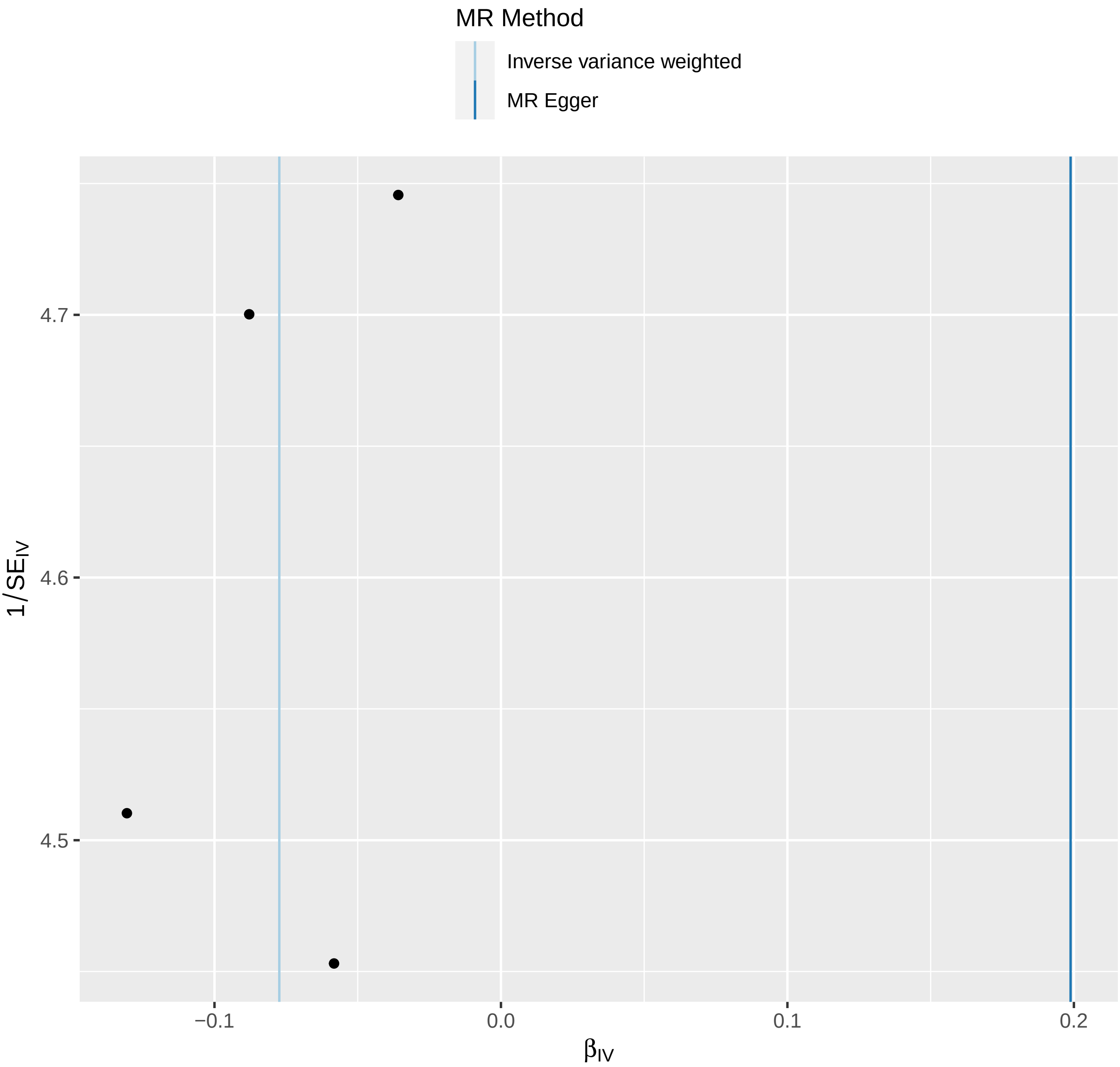

Batch 2−1956 : Gut microbiota abundance (genus Parasutterella id.2892) on Enhancer of rudimentary homolog with a threshold of 1e−05

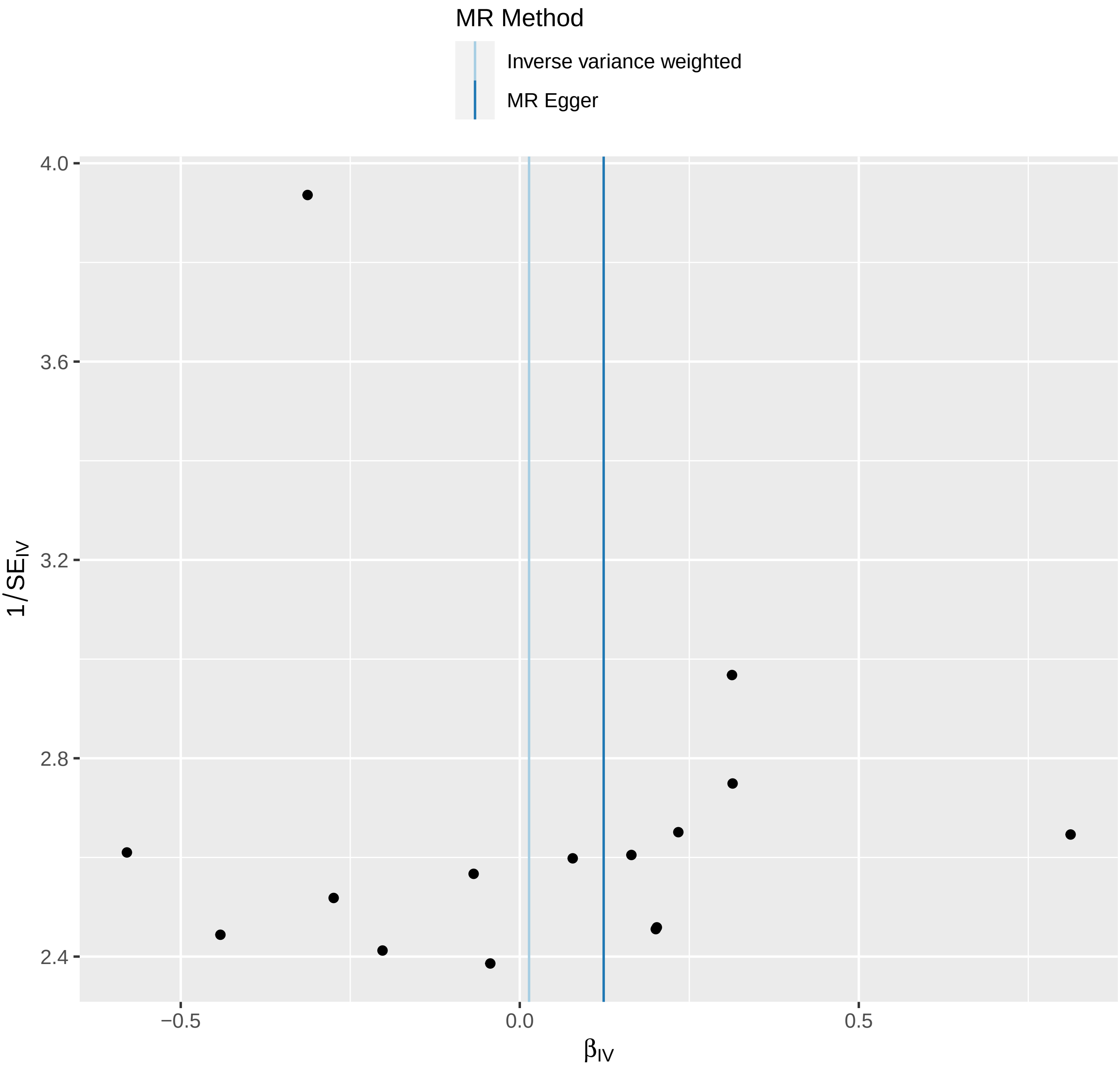

Batch 2−1957 : Gut microbiota abundance (genus Catenibacterium id.2153) on Endoplasmic reticulum lectin 1
with a threshold of 1e−05

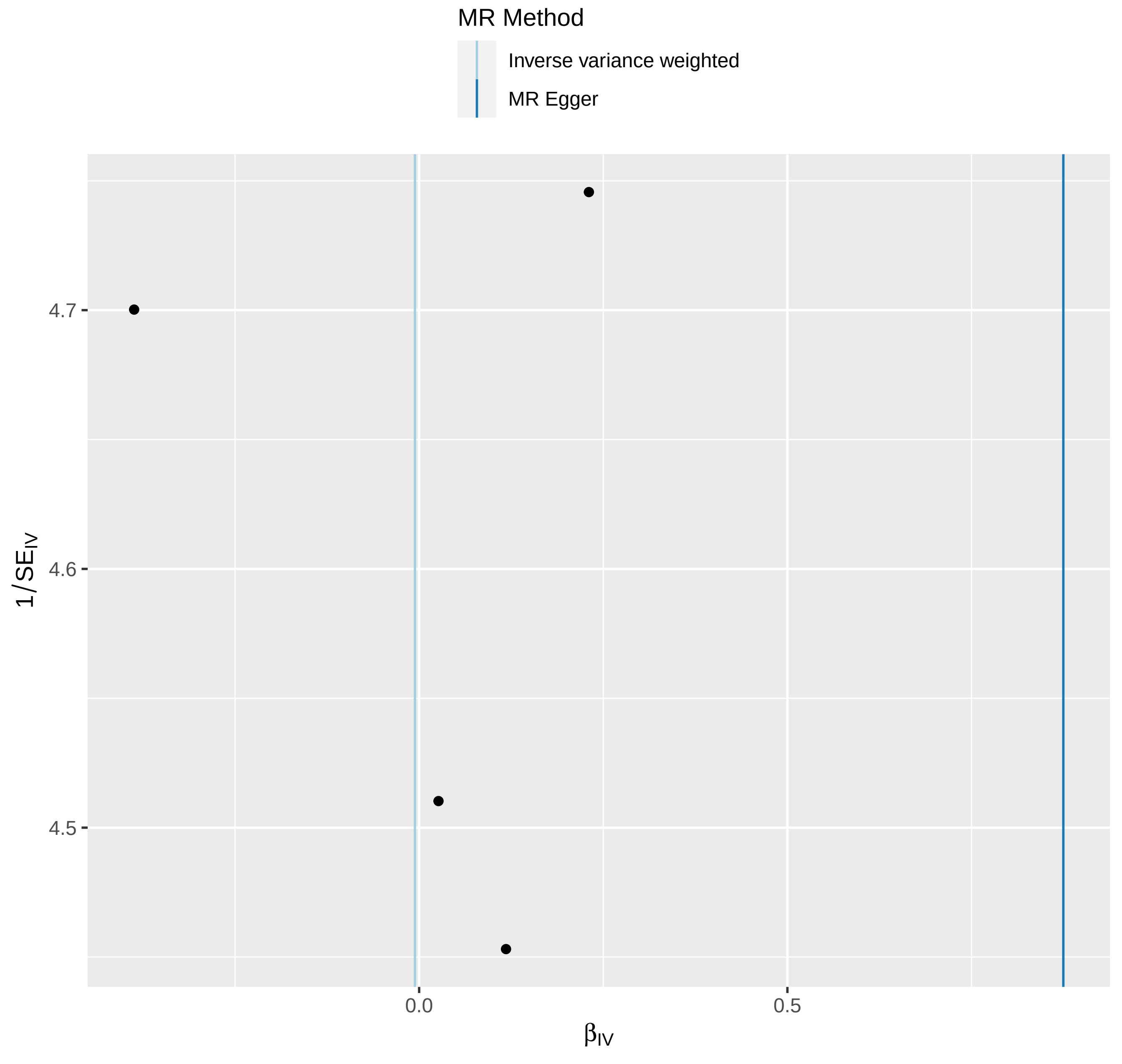

Batch 2−1958 : Gut microbiota abundance (genus Parasutterella id.2892) on Endoplasmic reticulum lectin 1
with a threshold of 1e−05

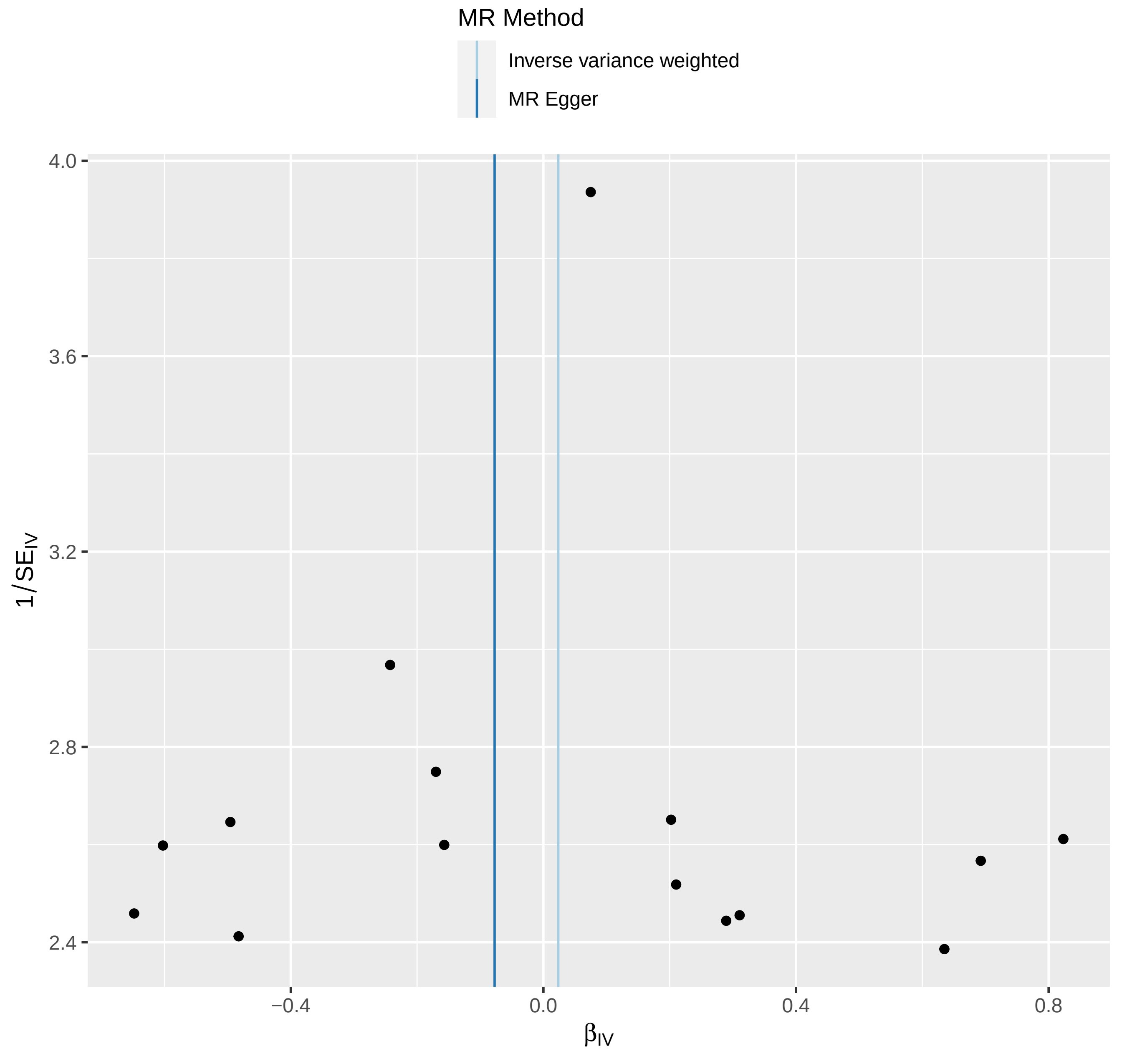

Batch 2−1959 : Gut microbiota abundance (genus Catenibacterium id.2153) on Erlin−1
with a threshold of 1e−05

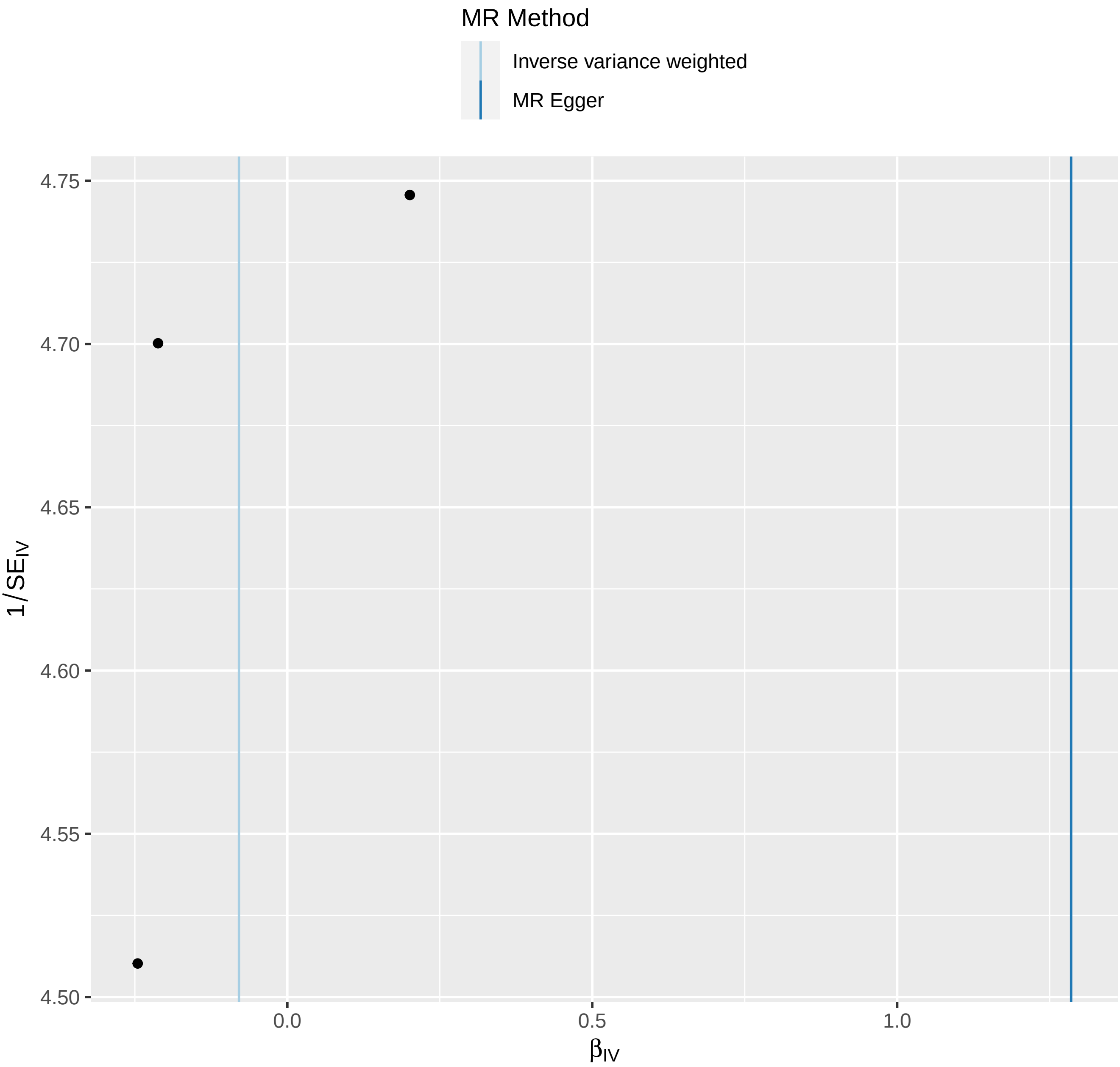

Batch 2−1960 : Gut microbiota abundance (genus Parasutterella id.2892) on Erlin−1
with a threshold of 1e−05

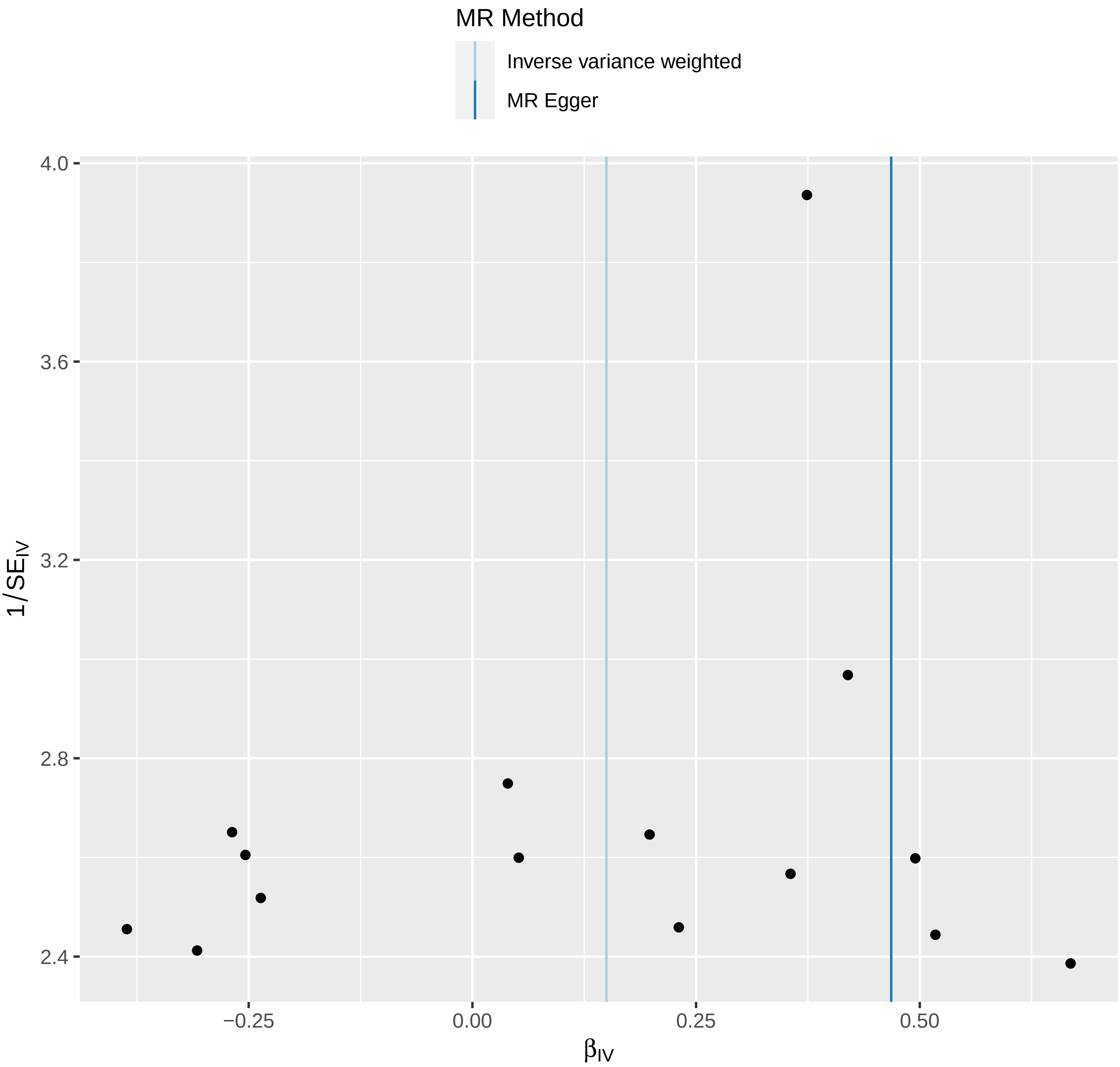

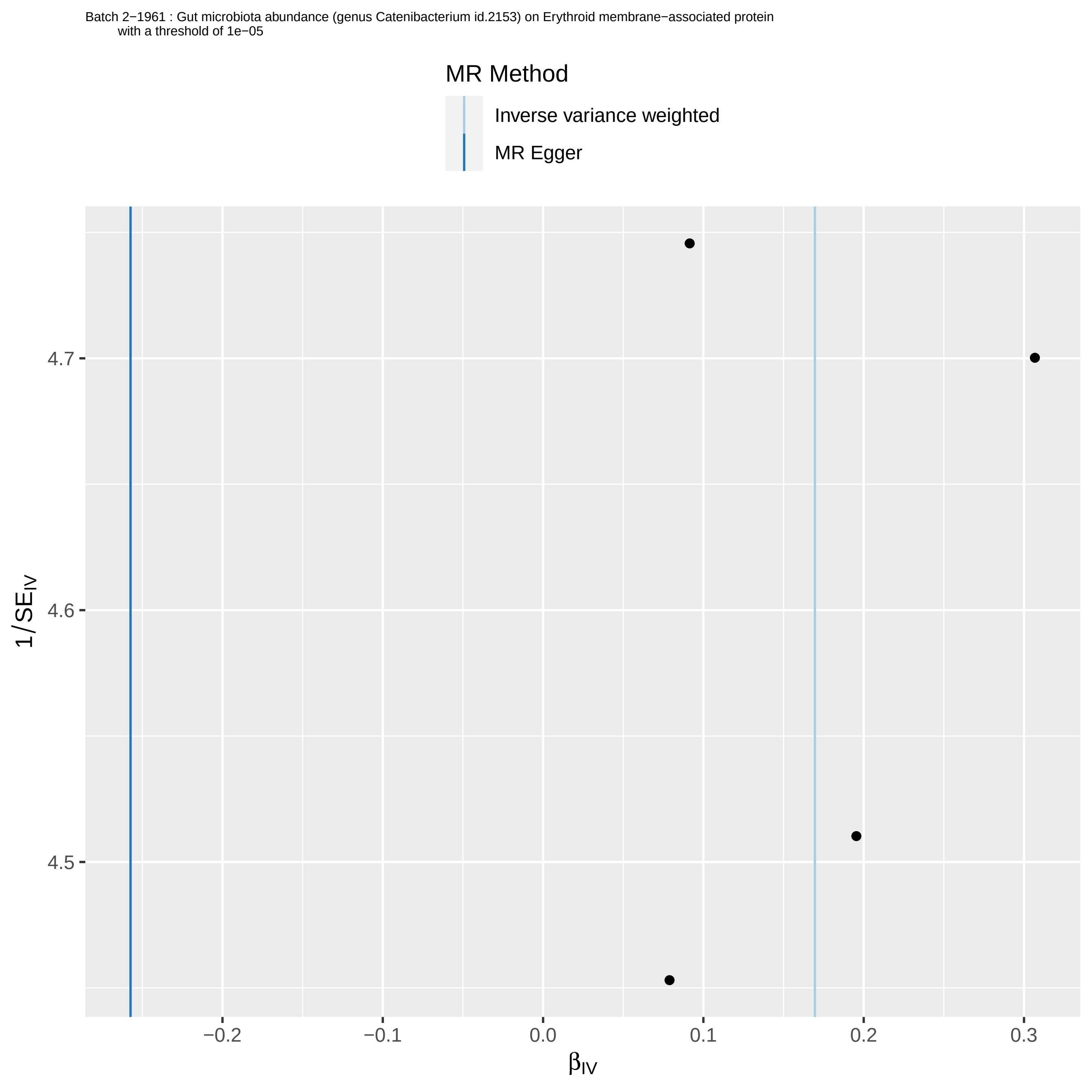
Batch 2−1961 : Gut microbiota abundance (genus Catenibacterium id.2153) on Erythroid membrane−associated protein
with a threshold of 1e−05
MR Method
Inverse variance weighted
MR Egger
4.7
4.6
4.5
1/SE_IV
−0.2
−0.1
0.0
0.1
0.2
0.3
β_IV

Batch 2−1962 : Gut microbiota abundance (genus Parasutterella id.2892) on Erythroid membrane−associated protein with a threshold of 1e−05

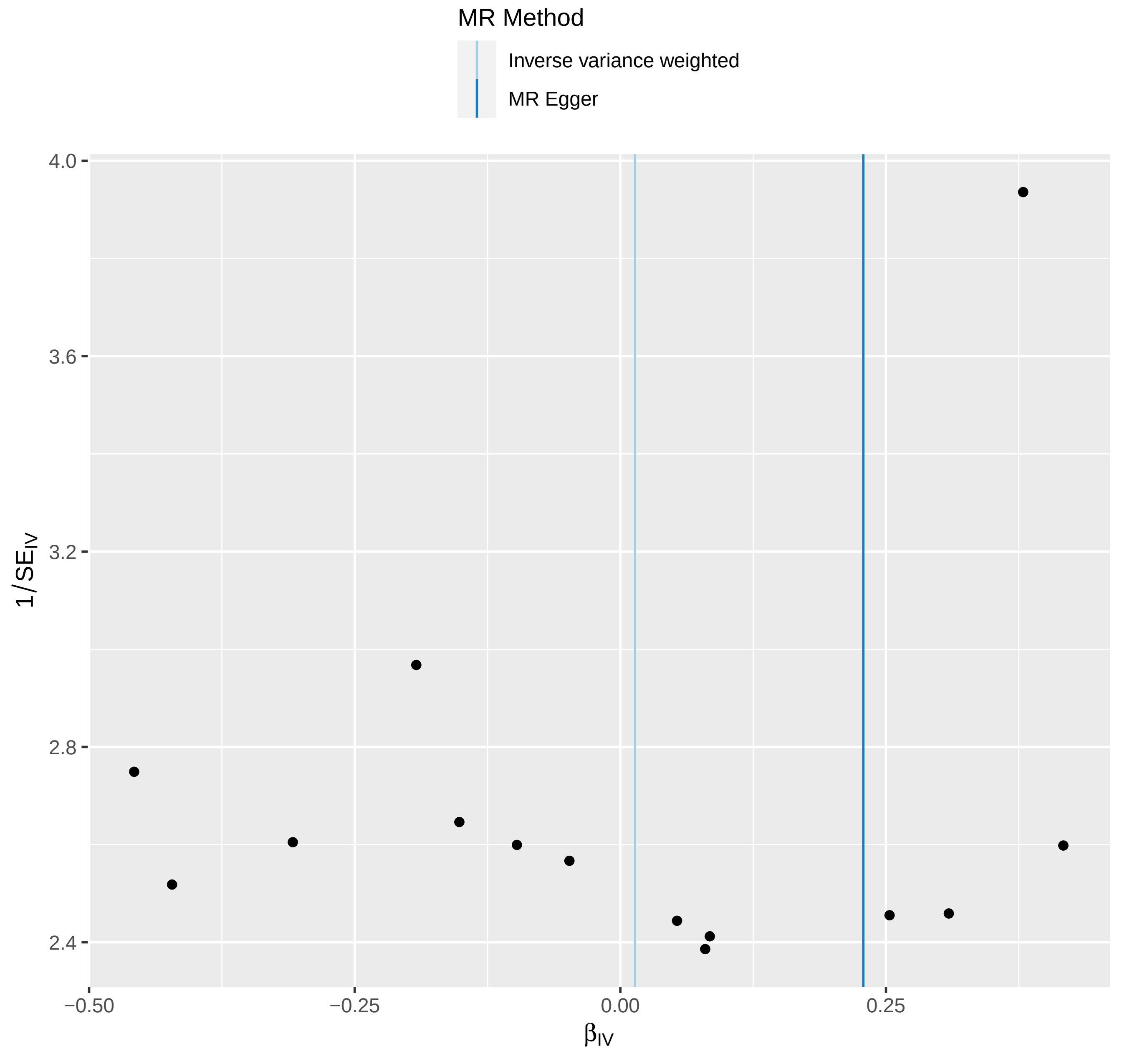

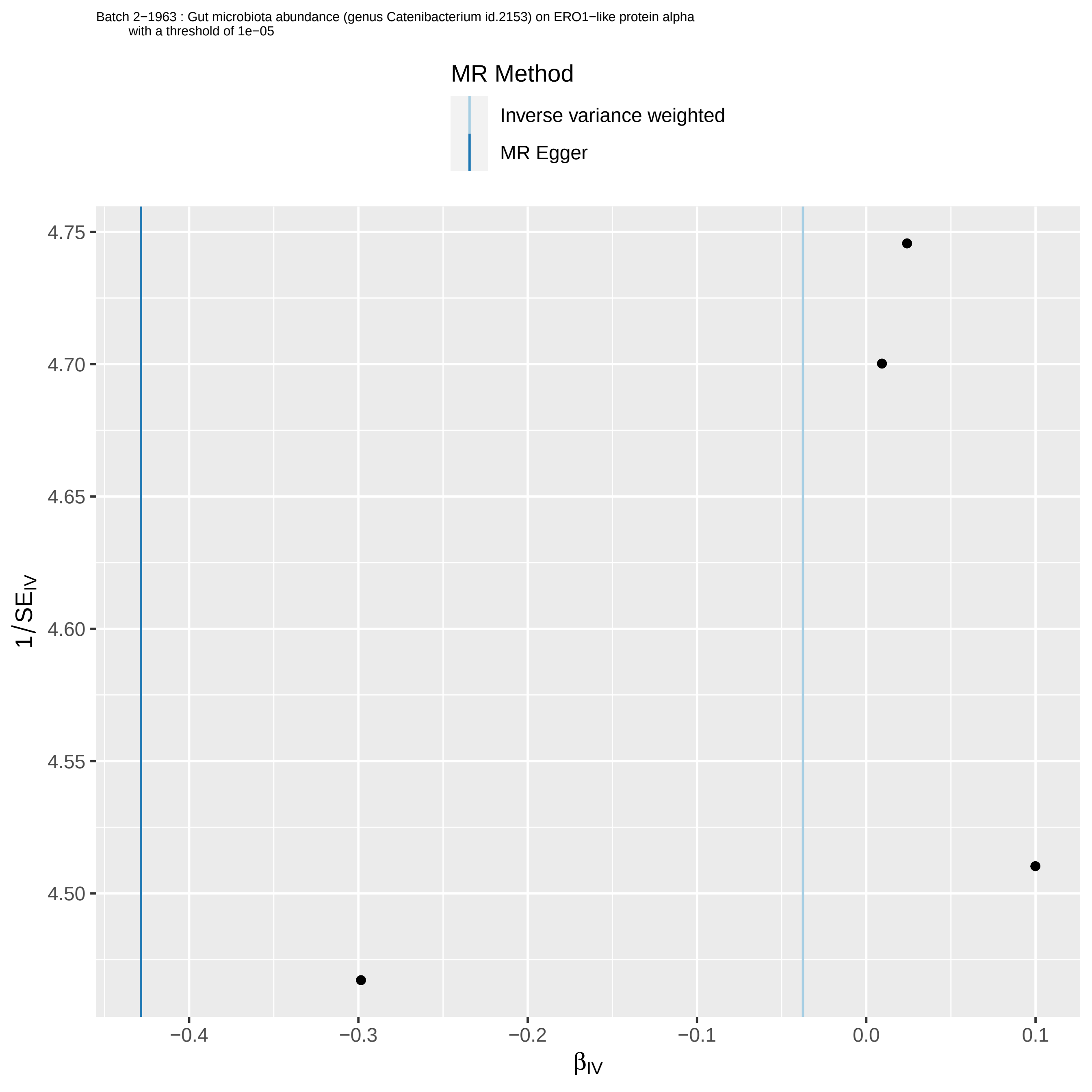
Batch 2−1963 : Gut microbiota abundance (genus Catenibacterium id.2153) on ERO1−like protein alpha
with a threshold of 1e−05
MR Method
Inverse variance weighted
MR Egger
4.75
4.70
4.65
4.60
4.55
4.50
1/SE_IV
−0.4
−0.3
−0.2
−0.1
0.0
0.1
β_IV

Batch 2−1964 : Gut microbiota abundance (genus Parasutterella id.2892) on ERO1−like protein alpha
with a threshold of 1e−05

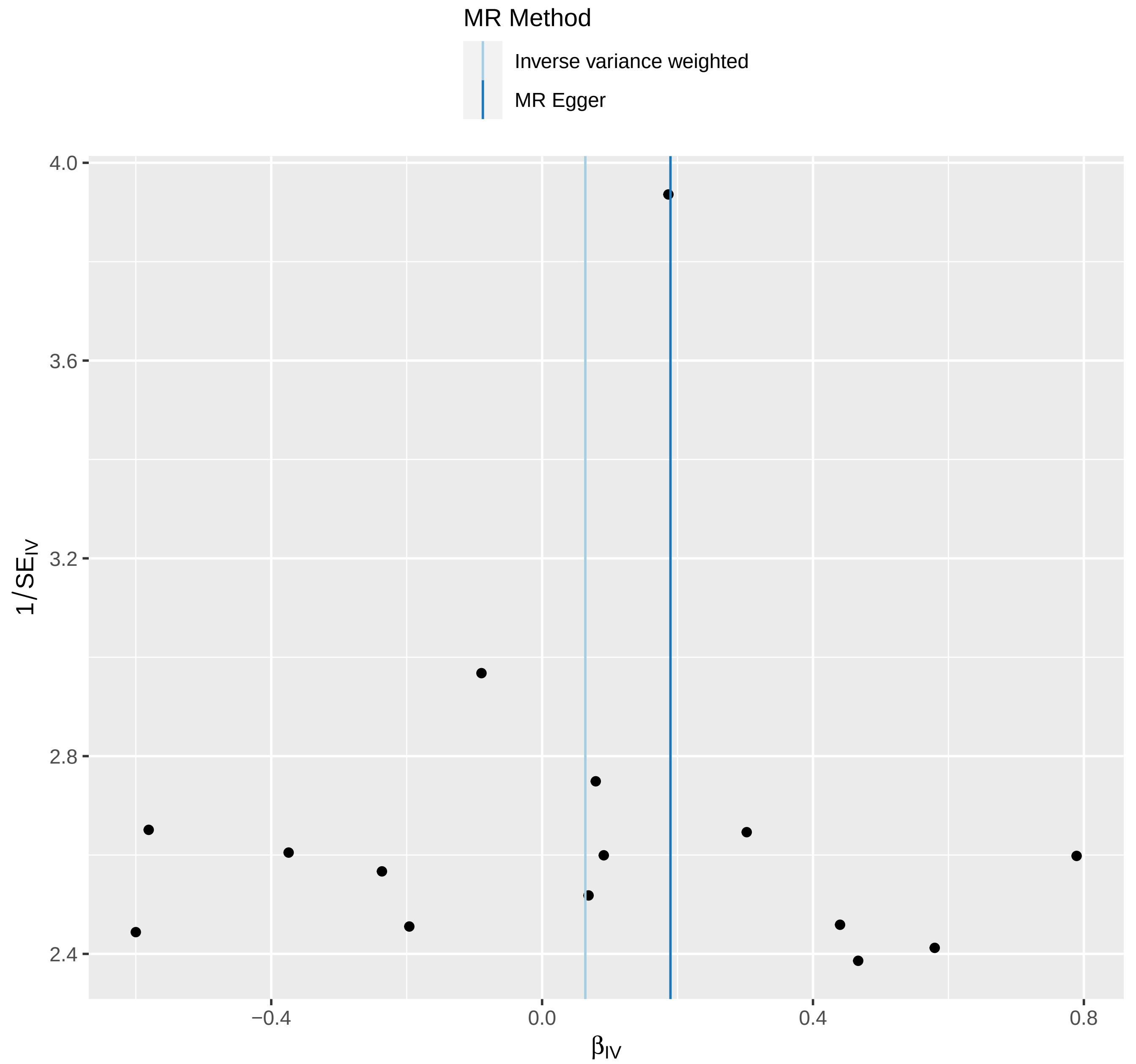

Batch 2−1965 : Gut microbiota abundance (genus Catenibacterium id.2153) on ERO1−like protein beta
with a threshold of 1e−05

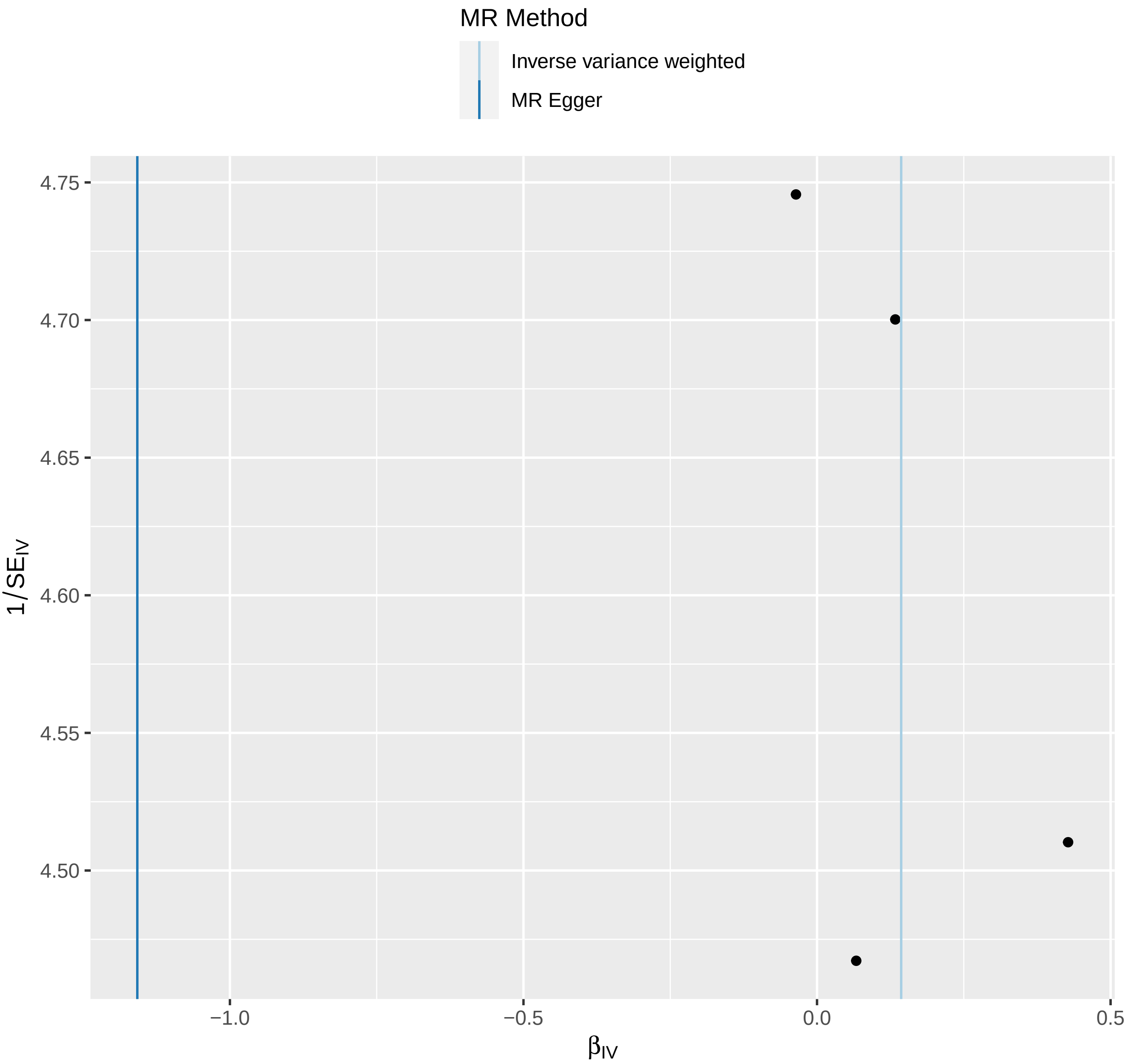

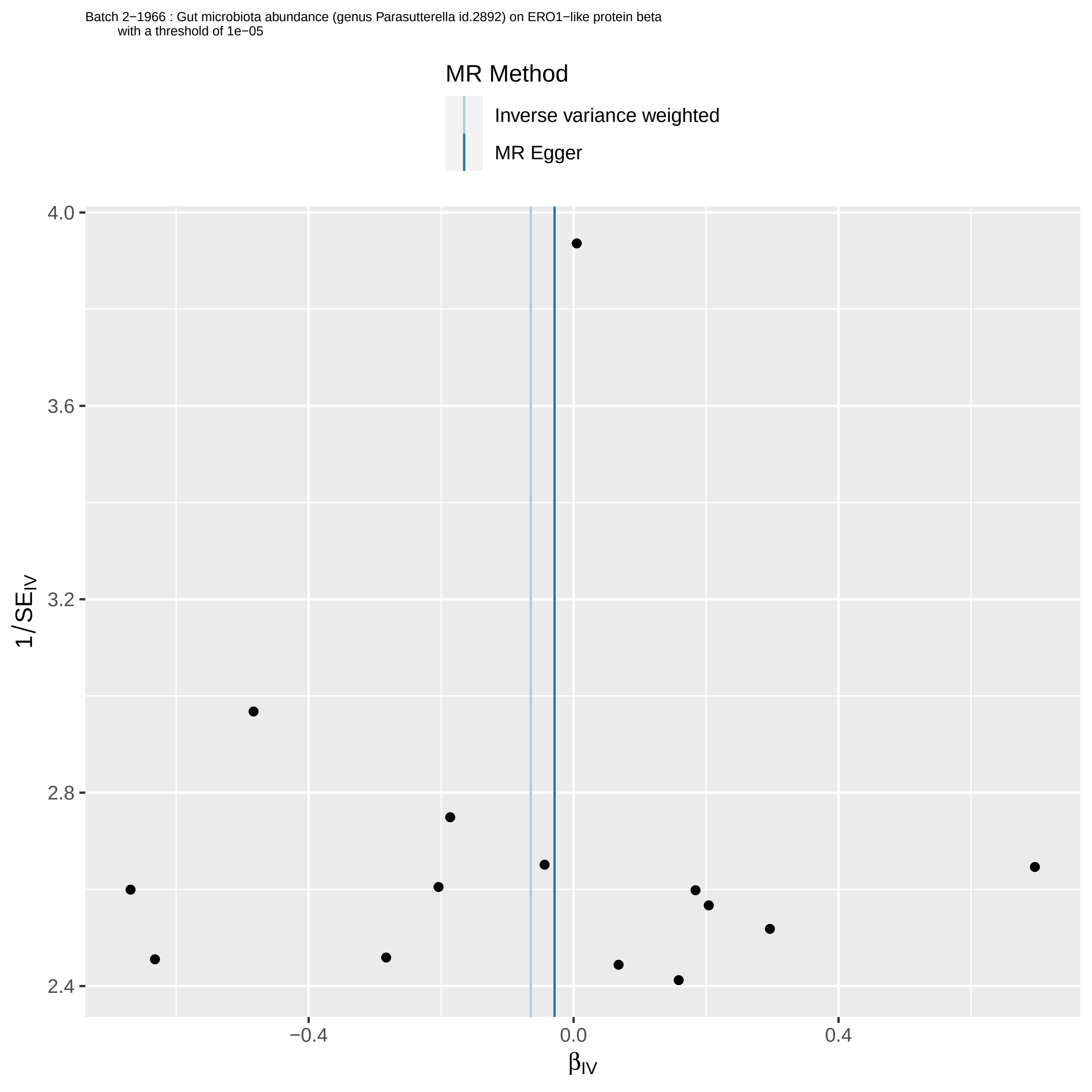
Batch 2−1966 : Gut microbiota abundance (genus Parasutterella id.2892) on ERO1−like protein beta
with a threshold of 1e−05
MR Method
Inverse variance weighted
MR Egger
4.0
3.6
3.2
2.8
2.4
1/SE_IV
−0.4
0.0
0.4
β_IV

Batch 2−1967 : Gut microbiota abundance (genus Catenibacterium id.2153) on Endoplasmic reticulum resident protein 29
with a threshold of 1e−05

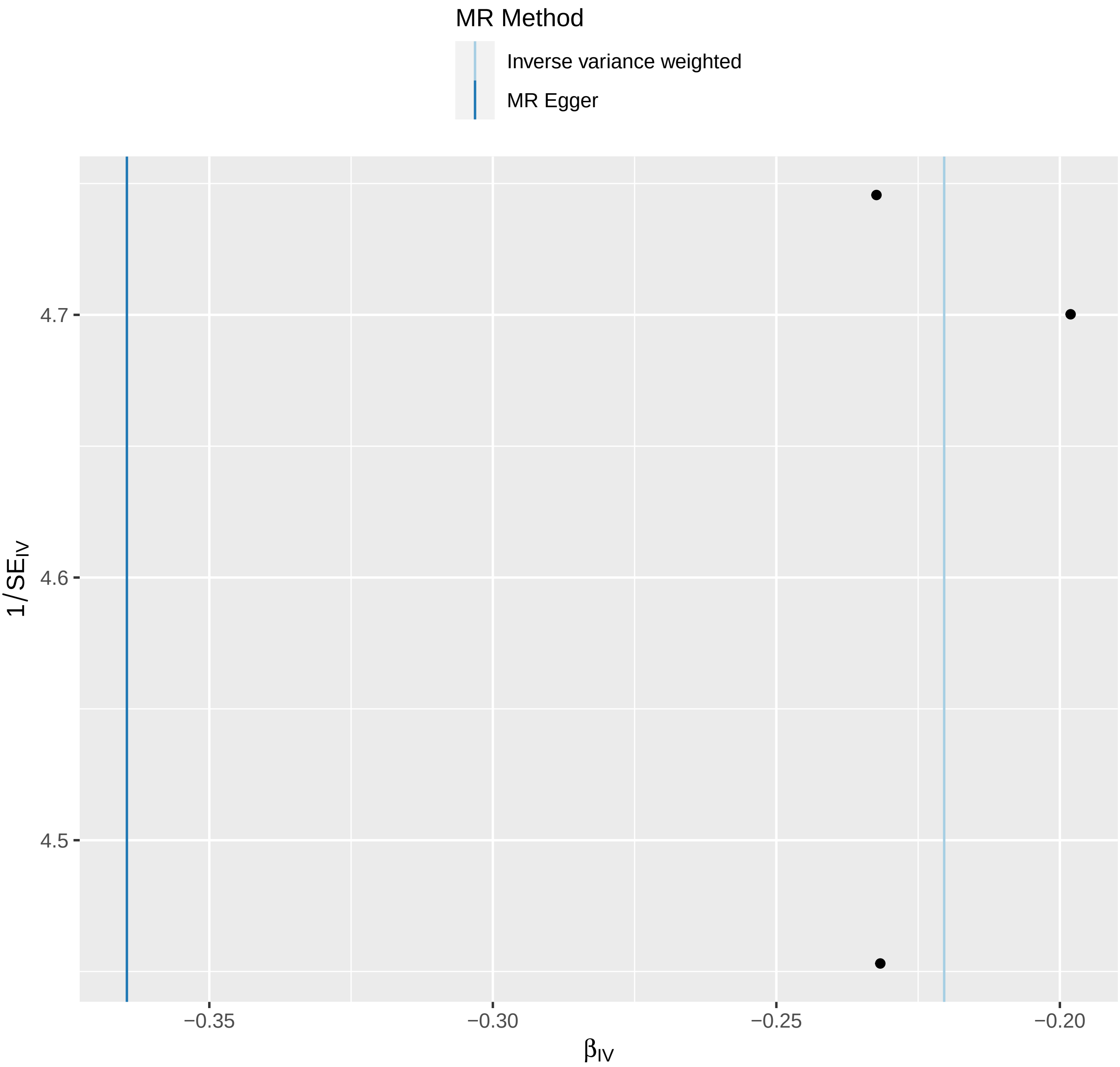

Batch 2−1968 : Gut microbiota abundance (genus Parasutterella id.2892) on Endoplasmic reticulum resident protein 29 with a threshold of 1e−05

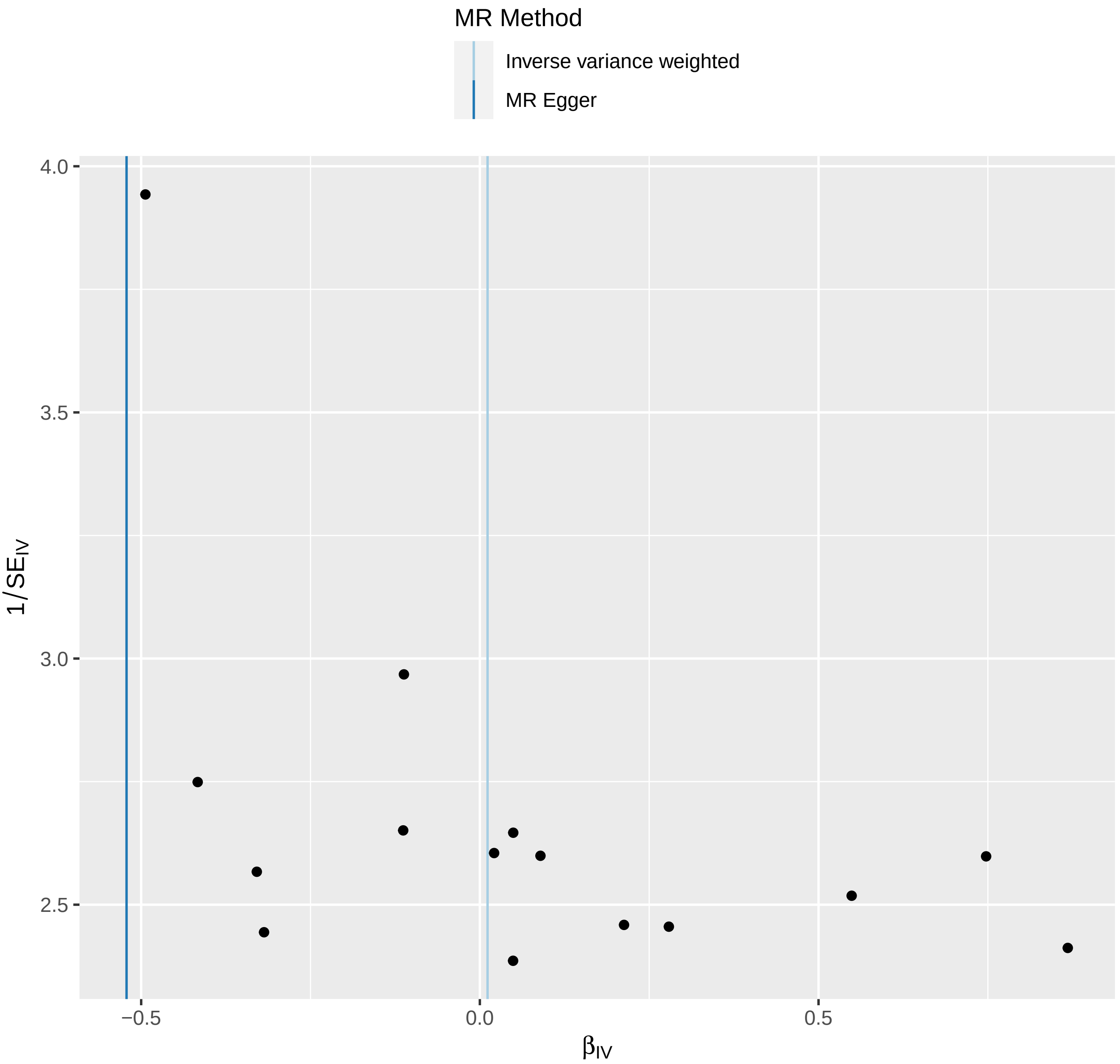

Batch 2−1969 : Gut microbiota abundance (genus Catenibacterium id.2153) on Endoplasmic reticulum resident protein 44
with a threshold of 1e−05

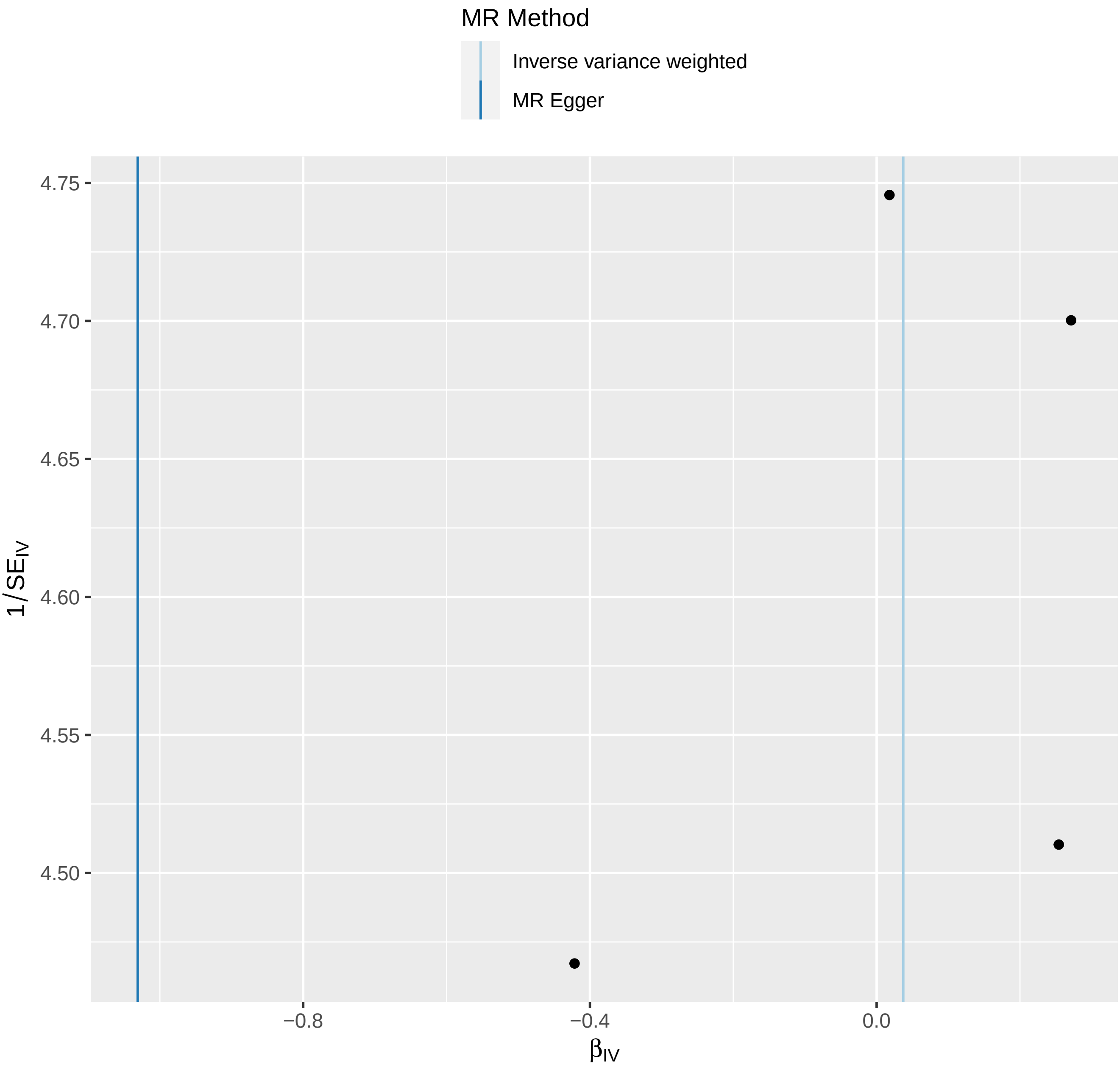

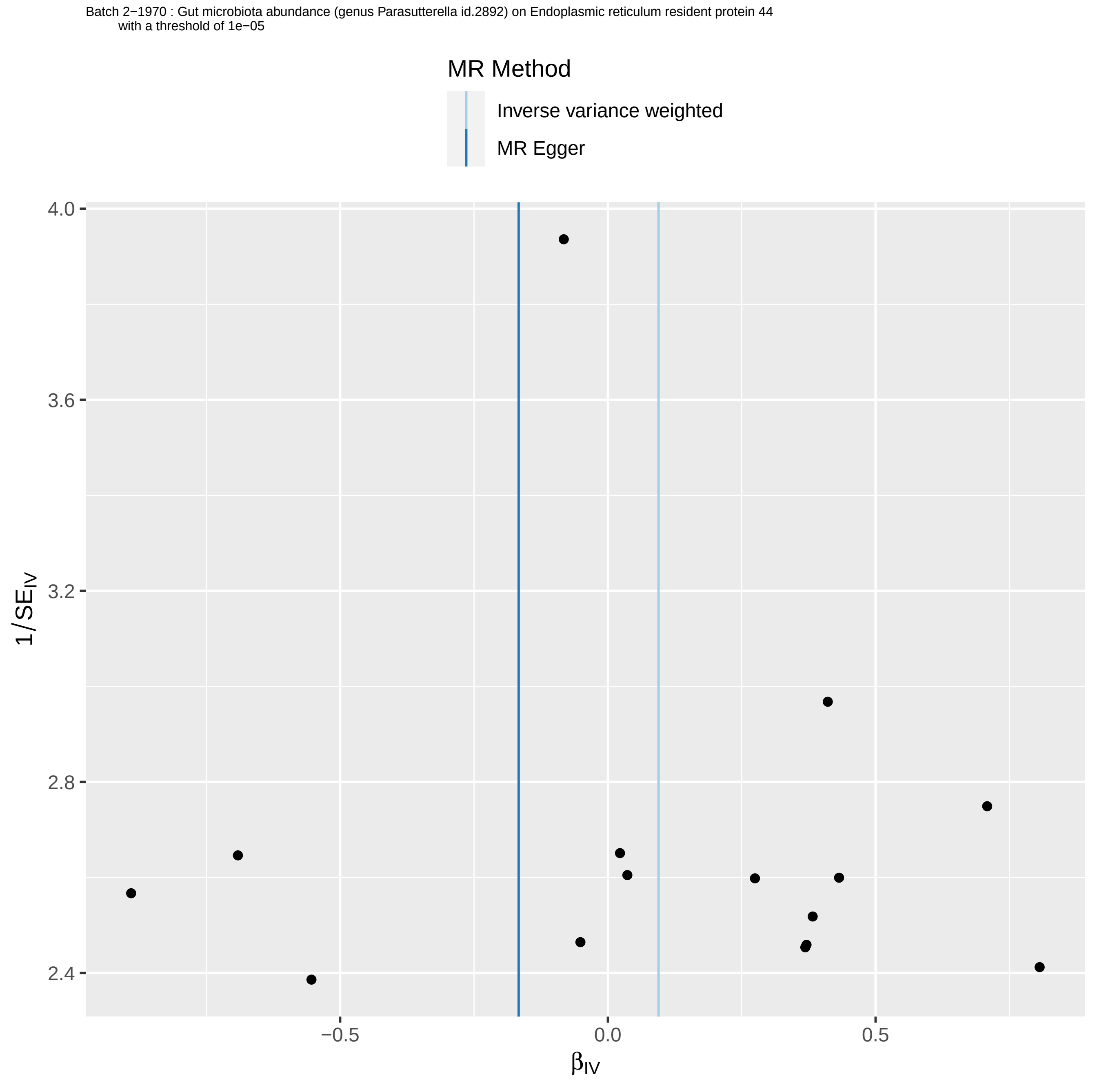
Batch 2−1970 : Gut microbiota abundance (genus Parasutterella id.2892) on Endoplasmic reticulum resident protein 44
with a threshold of 1e−05
MR Method
Inverse variance weighted
MR Egger
4.0
3.6
3.2
2.8
2.4
1/SE_IV
−0.5
0.0
0.5
β_IV

Batch 2−1971 : Gut microbiota abundance (genus Catenibacterium id.2153) on Endogenous retrovirus group V member 1 Env polyprotein with a threshold of 1e−05

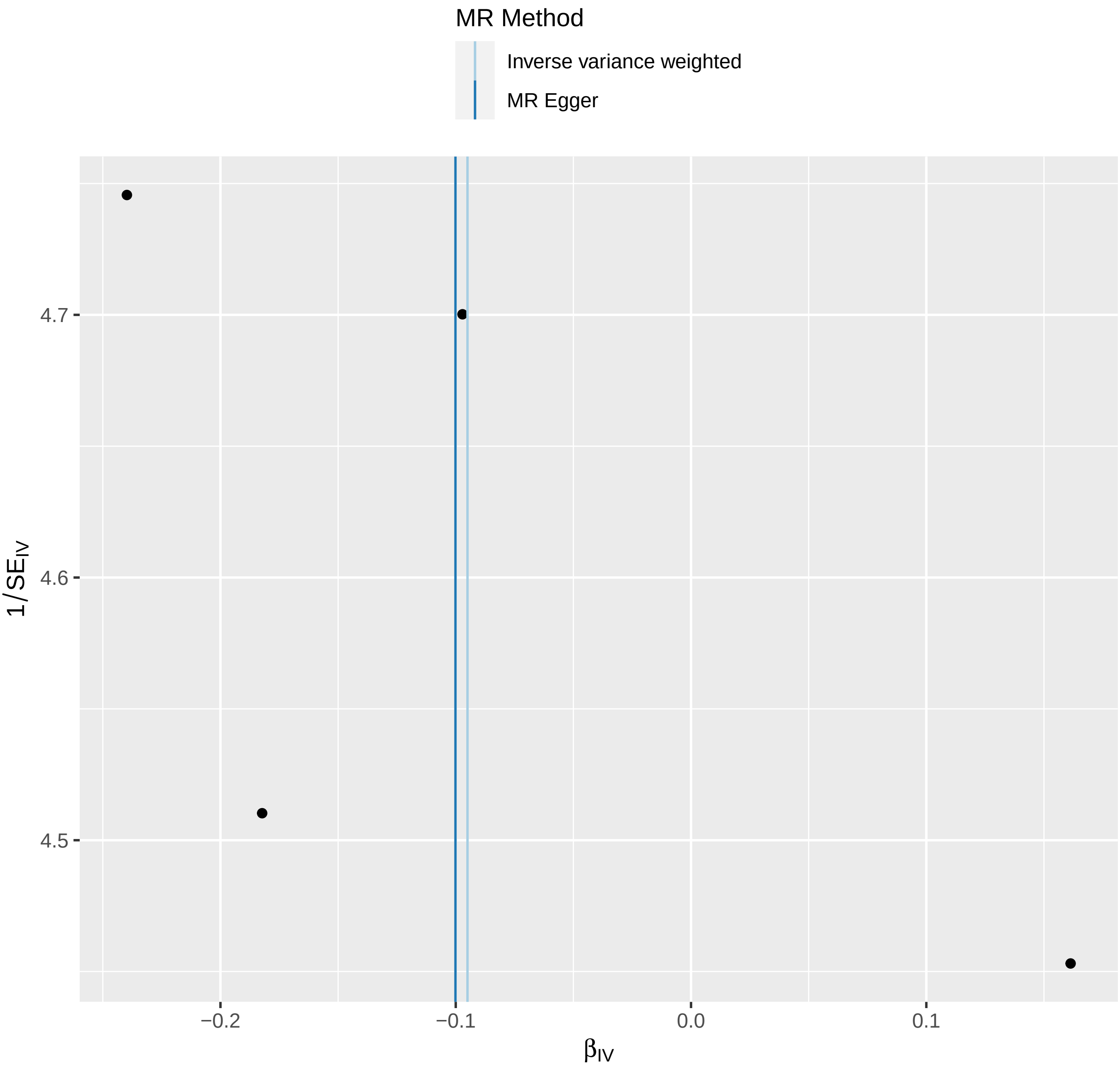

Batch 2−1972 : Gut microbiota abundance (genus Parasutterella id.2892) on Endogenous retrovirus group V member 1 Env polyprotein with a threshold of 1e−05

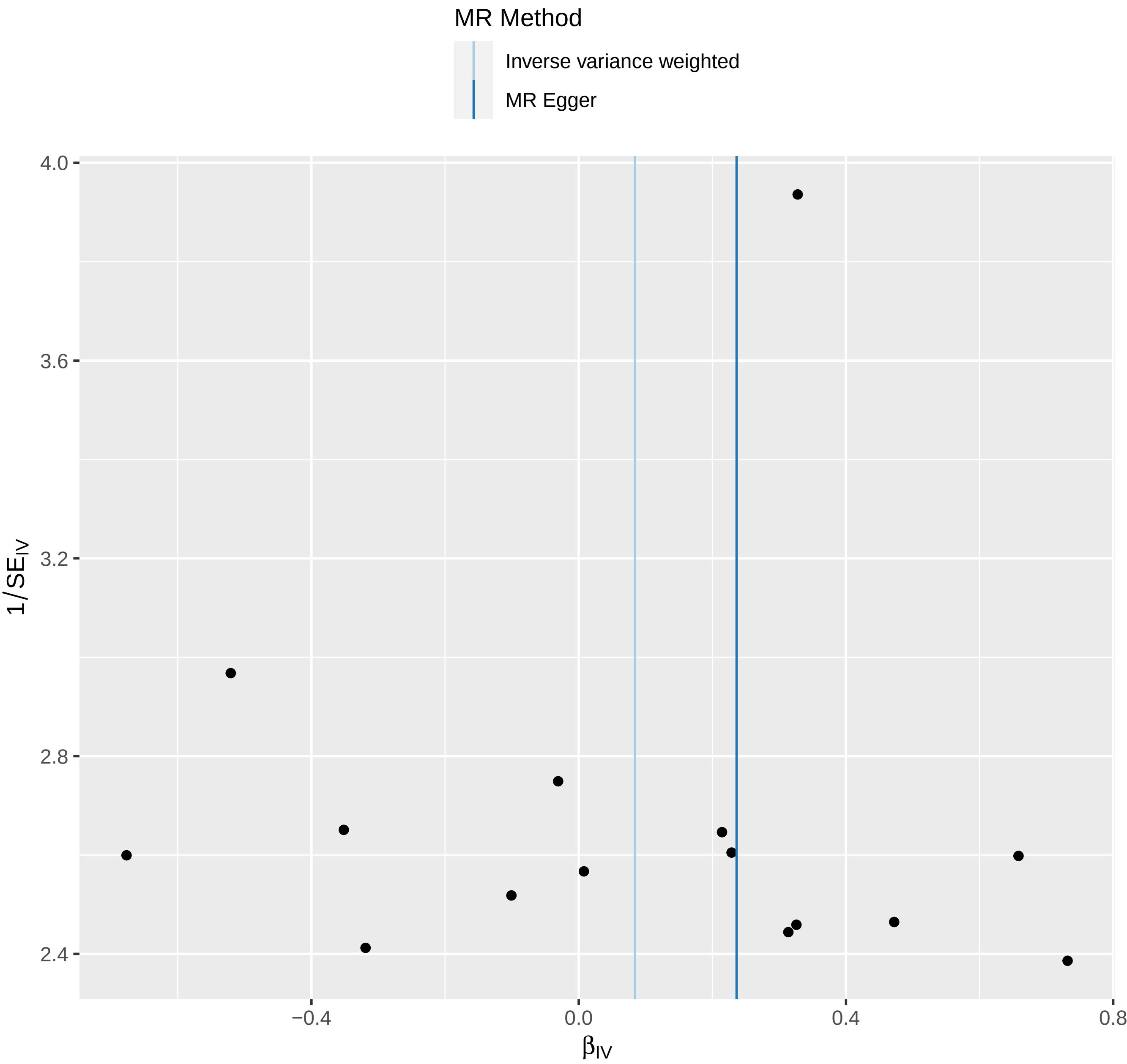

Batch 2−1973 : Gut microbiota abundance (genus Catenibacterium id.2153) on Endothelial cell−selective adhesion molecule
with a threshold of 1e−05

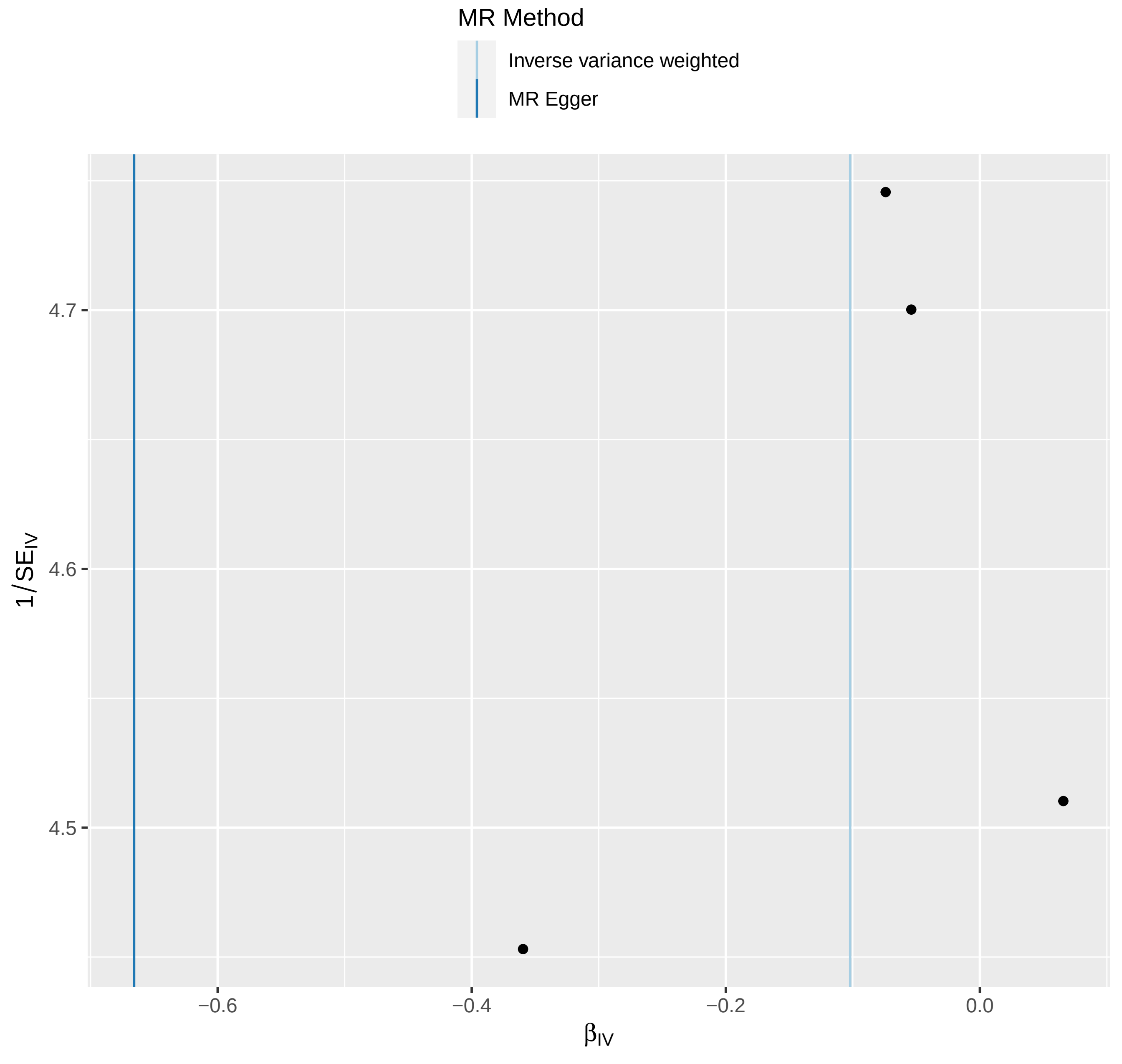

Batch 2−1974 : Gut microbiota abundance (genus Parasutterella id.2892) on Endothelial cell−selective adhesion molecule
with a threshold of 1e−05

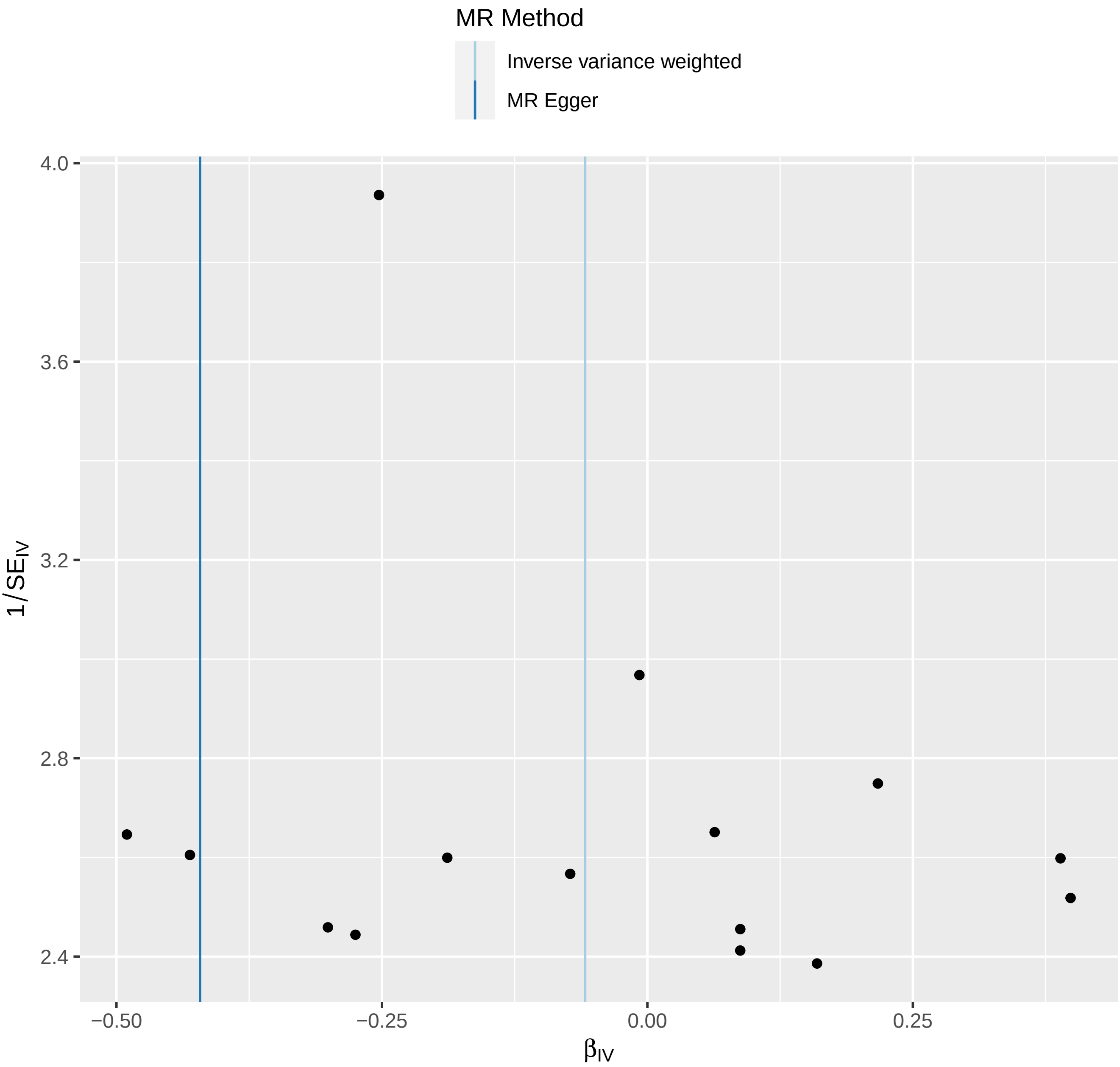

Batch 2−1975 : Gut microbiota abundance (genus Catenibacterium id.2153) on Endothelial cell−selective adhesion molecule
with a threshold of 1e−05

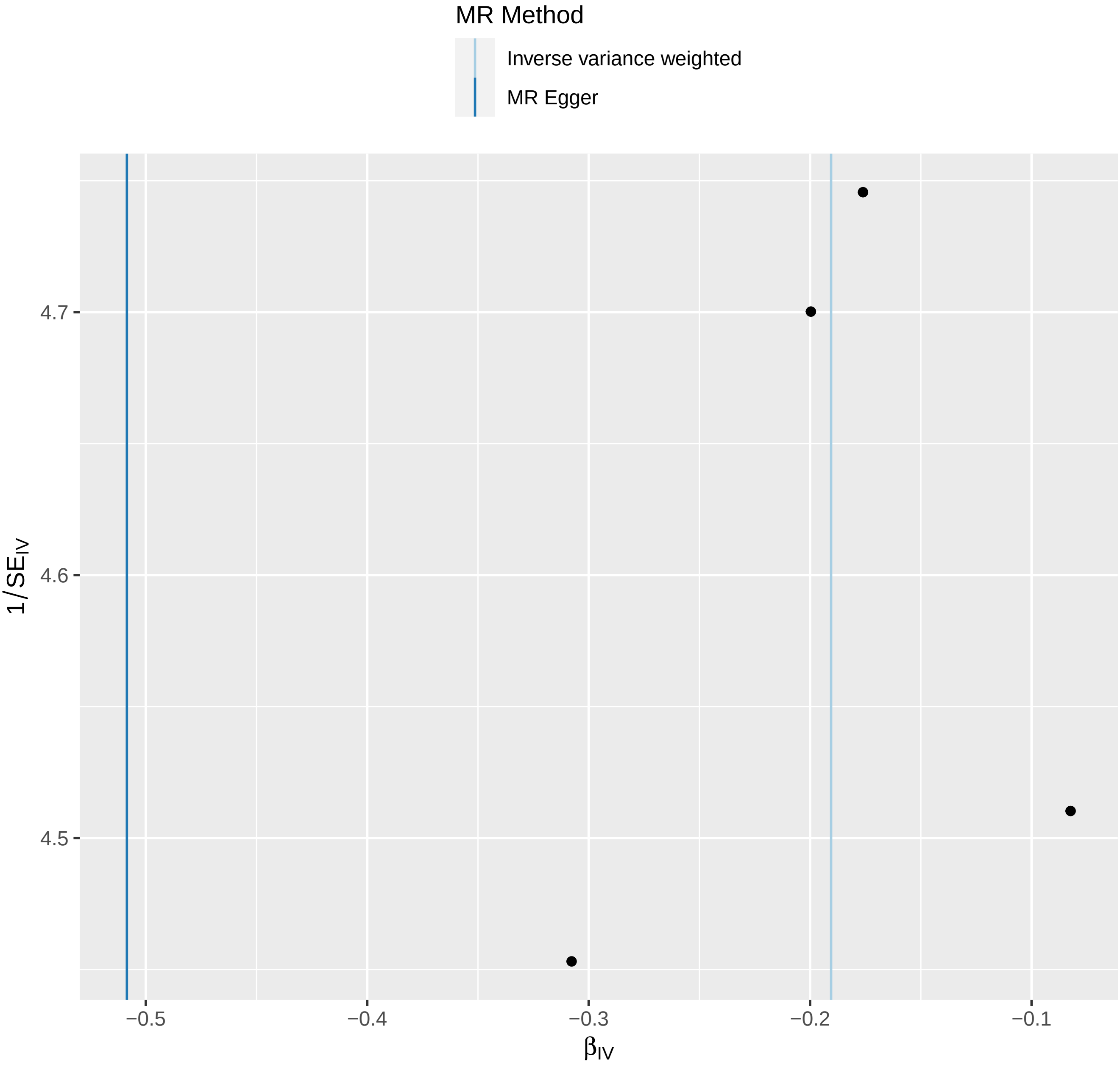

Batch 2−1976 : Gut microbiota abundance (genus Parasutterella id.2892) on Endothelial cell−selective adhesion molecule
with a threshold of 1e−05

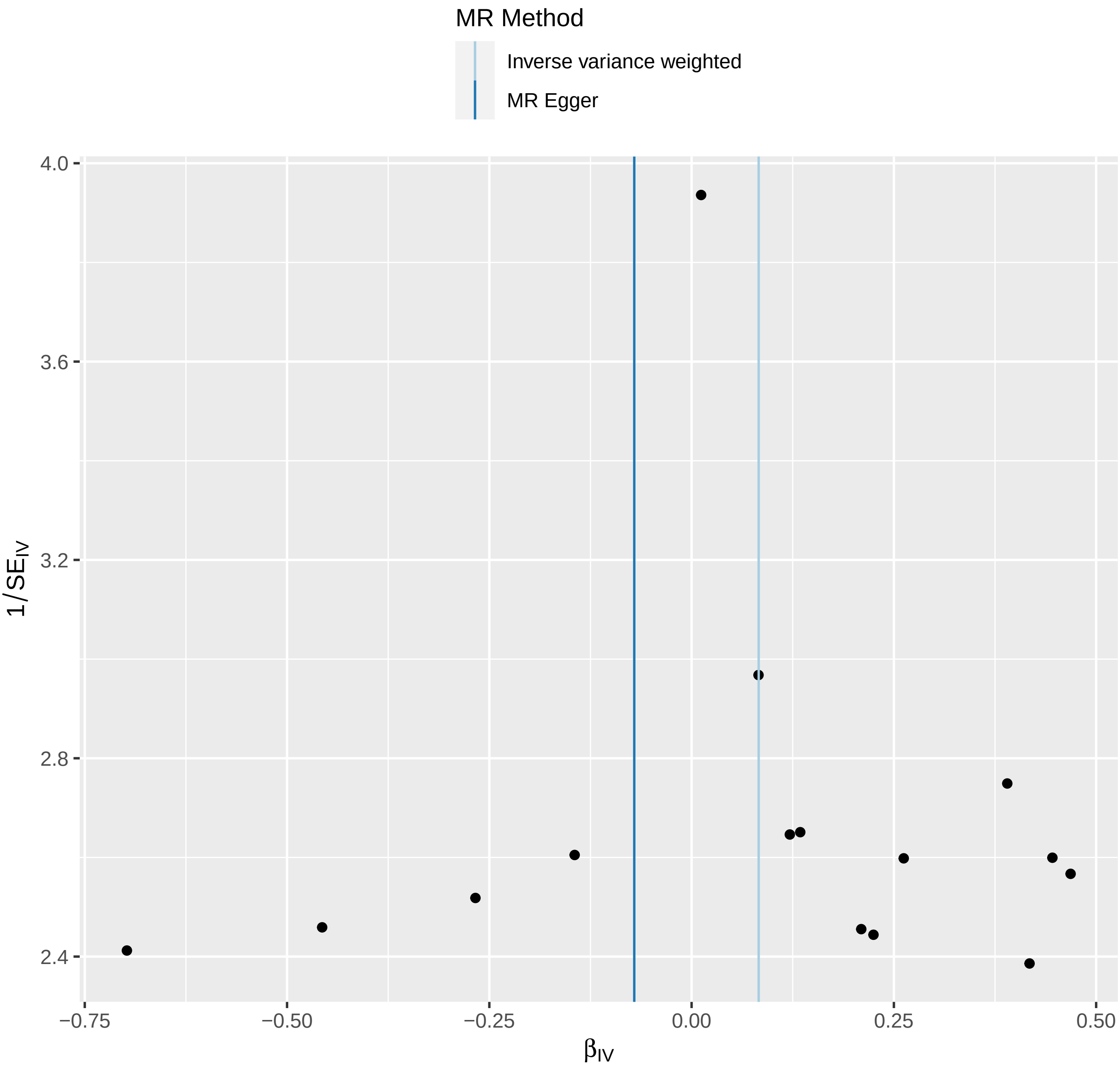

Batch 2−1977 : Gut microbiota abundance (genus Catenibacterium id.2153) on S−formylglutathione hydrolase
with a threshold of 1e−05

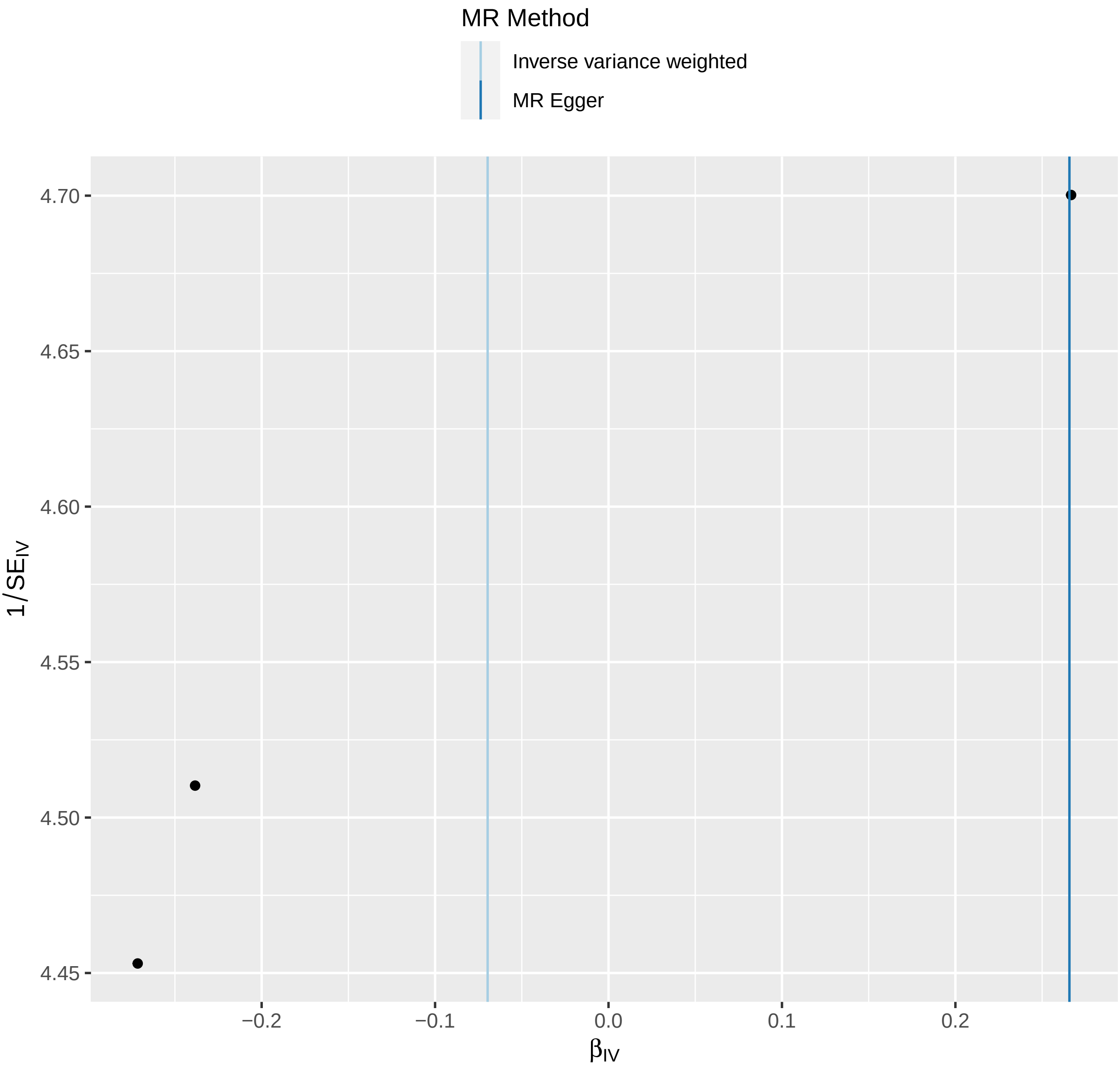

Batch 2−1978 : Gut microbiota abundance (genus Parasutterella id.2892) on S−formylglutathione hydrolase
with a threshold of 1e−05

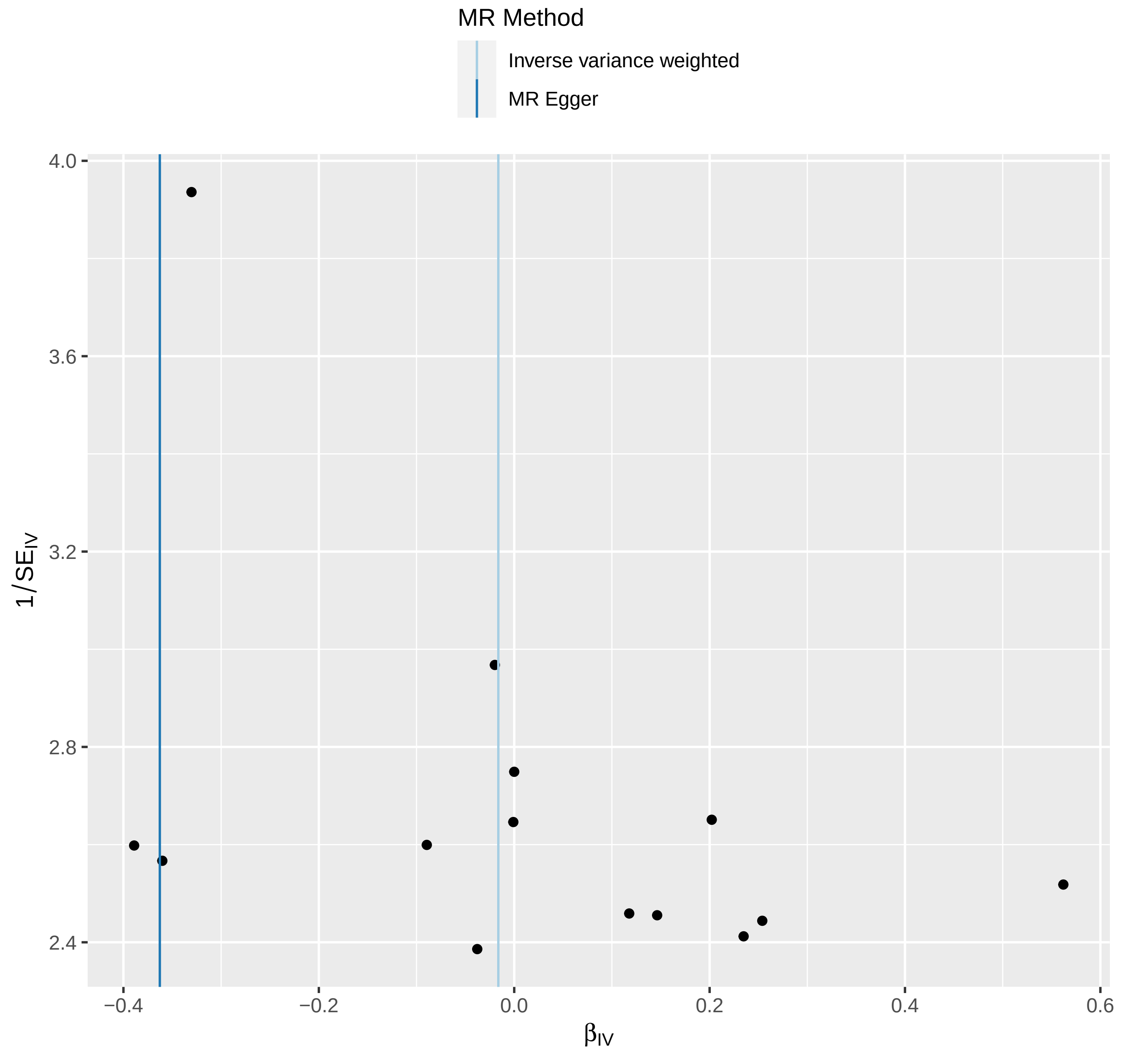

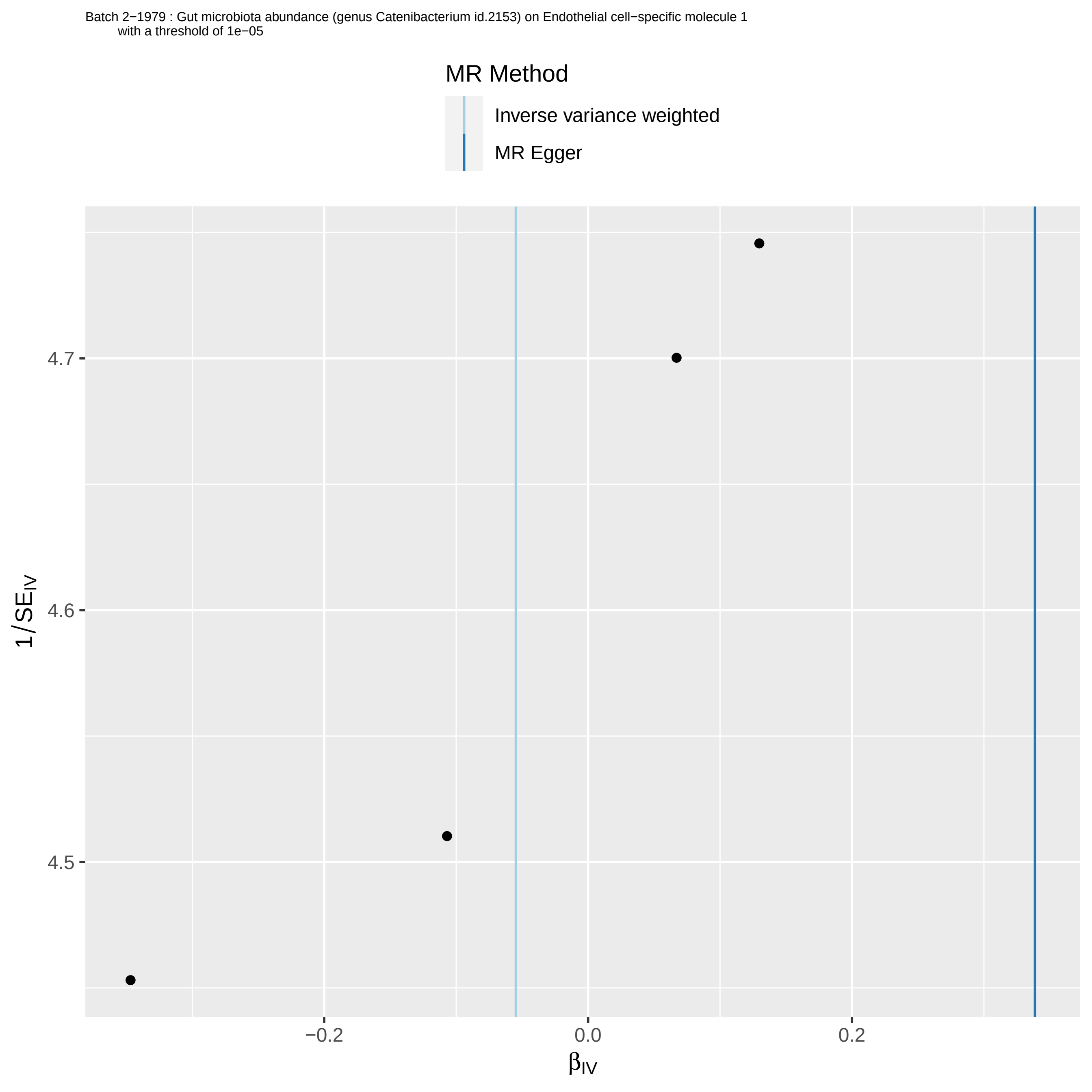
Batch 2−1979 : Gut microbiota abundance (genus Catenibacterium id.2153) on Endothelial cell−specific molecule 1
with a threshold of 1e−05
MR Method
Inverse variance weighted
MR Egger
4.7
4.6
4.5
1/SE_IV
−0.2
0.0
0.2
β_IV

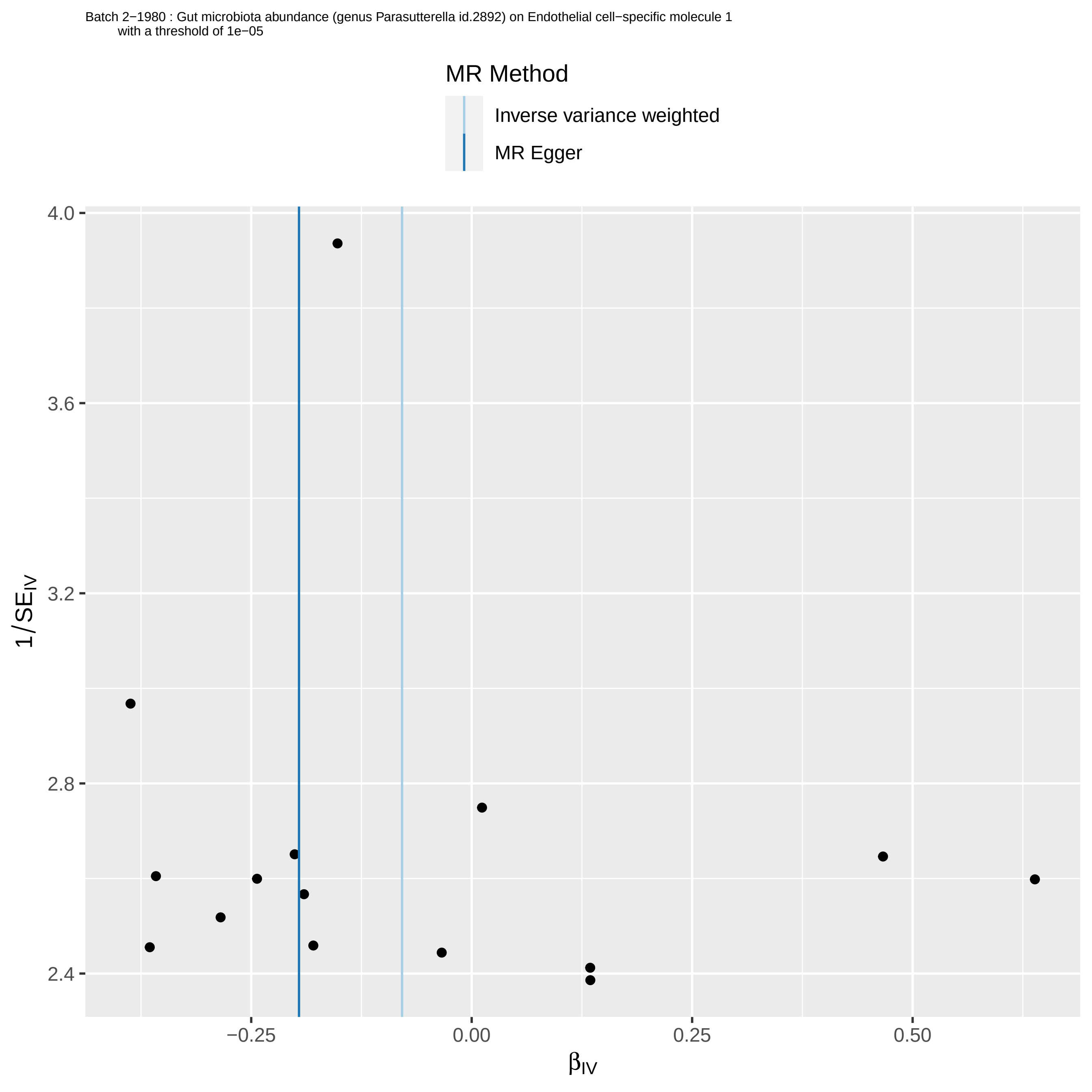
Batch 2−1980 : Gut microbiota abundance (genus Parasutterella id.2892) on Endothelial cell−specific molecule 1
with a threshold of 1e−05
MR Method
Inverse variance weighted
MR Egger
4.0
3.6
3.2
2.8
2.4
1/SE_IV
−0.25
0.00
0.25
0.50
β_IV

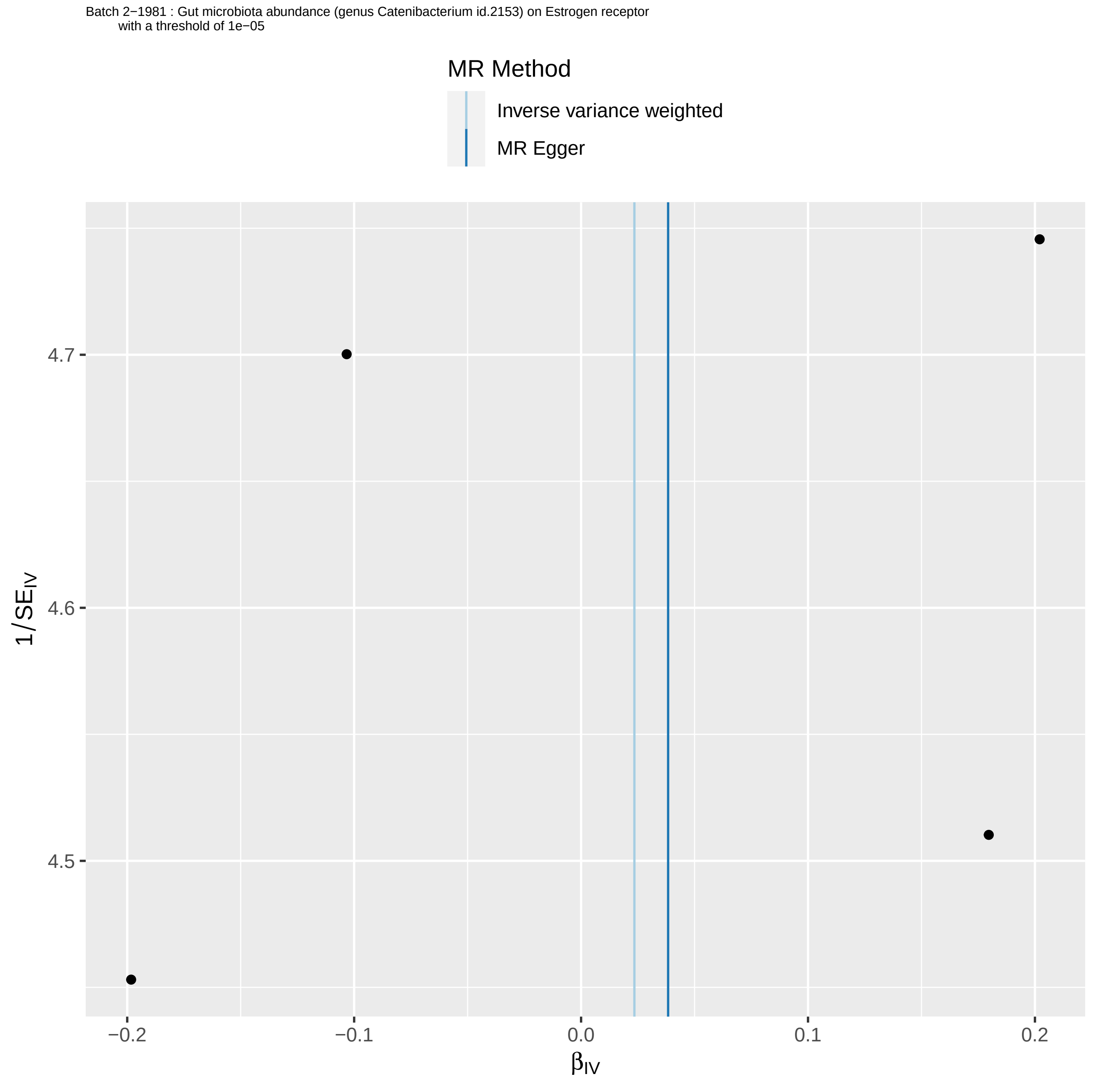
Batch 2−1981 : Gut microbiota abundance (genus Catenibacterium id.2153) on Estrogen receptor
with a threshold of 1e−05
MR Method
Inverse variance weighted
MR Egger
4.7
4.6
4.5
1/SE_IV
−0.2
−0.1
0.0
0.1
0.2
β_IV

Batch 2−1982 : Gut microbiota abundance (genus Parasutterella id.2892) on Estrogen receptor
with a threshold of 1e−05

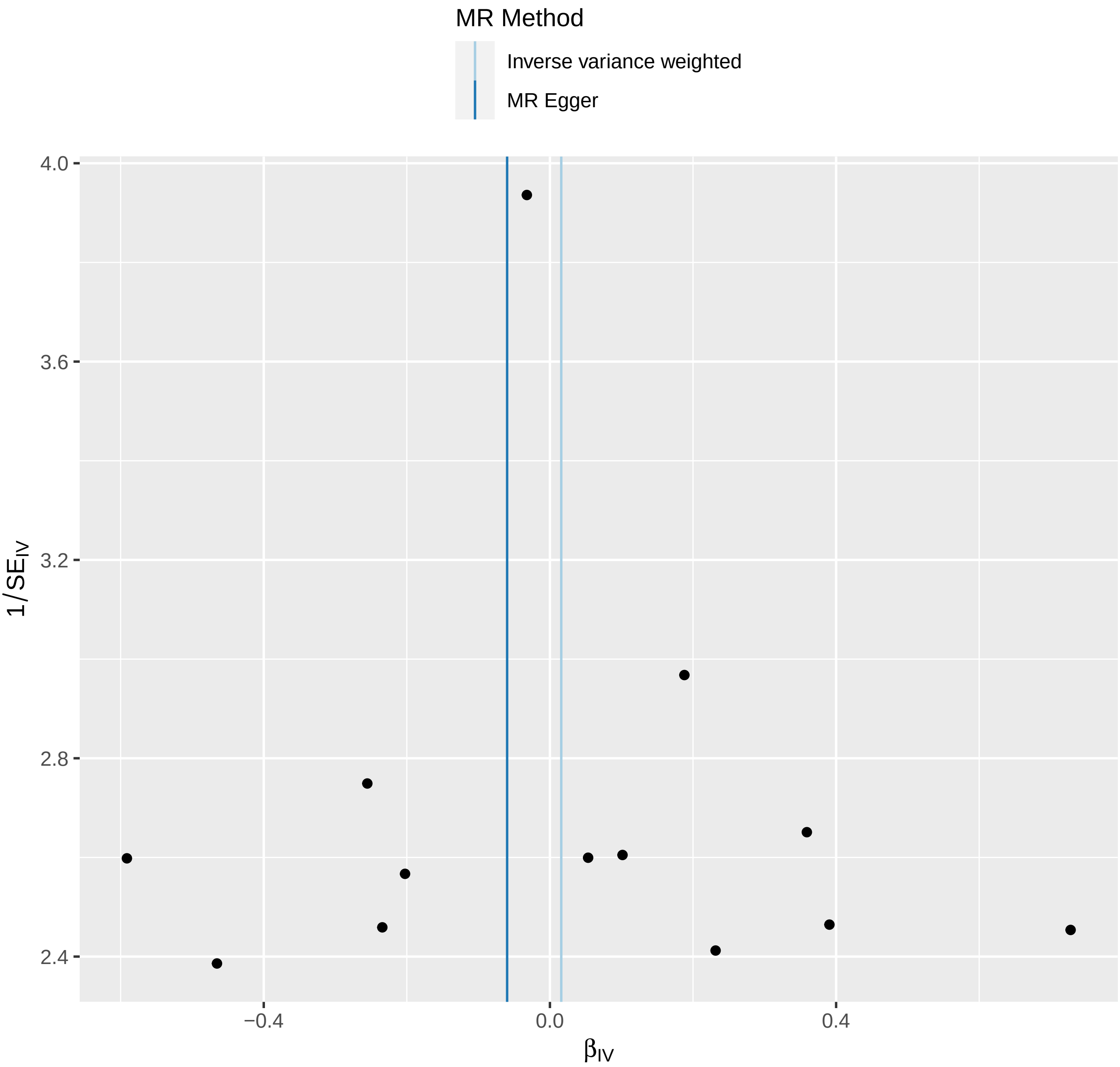

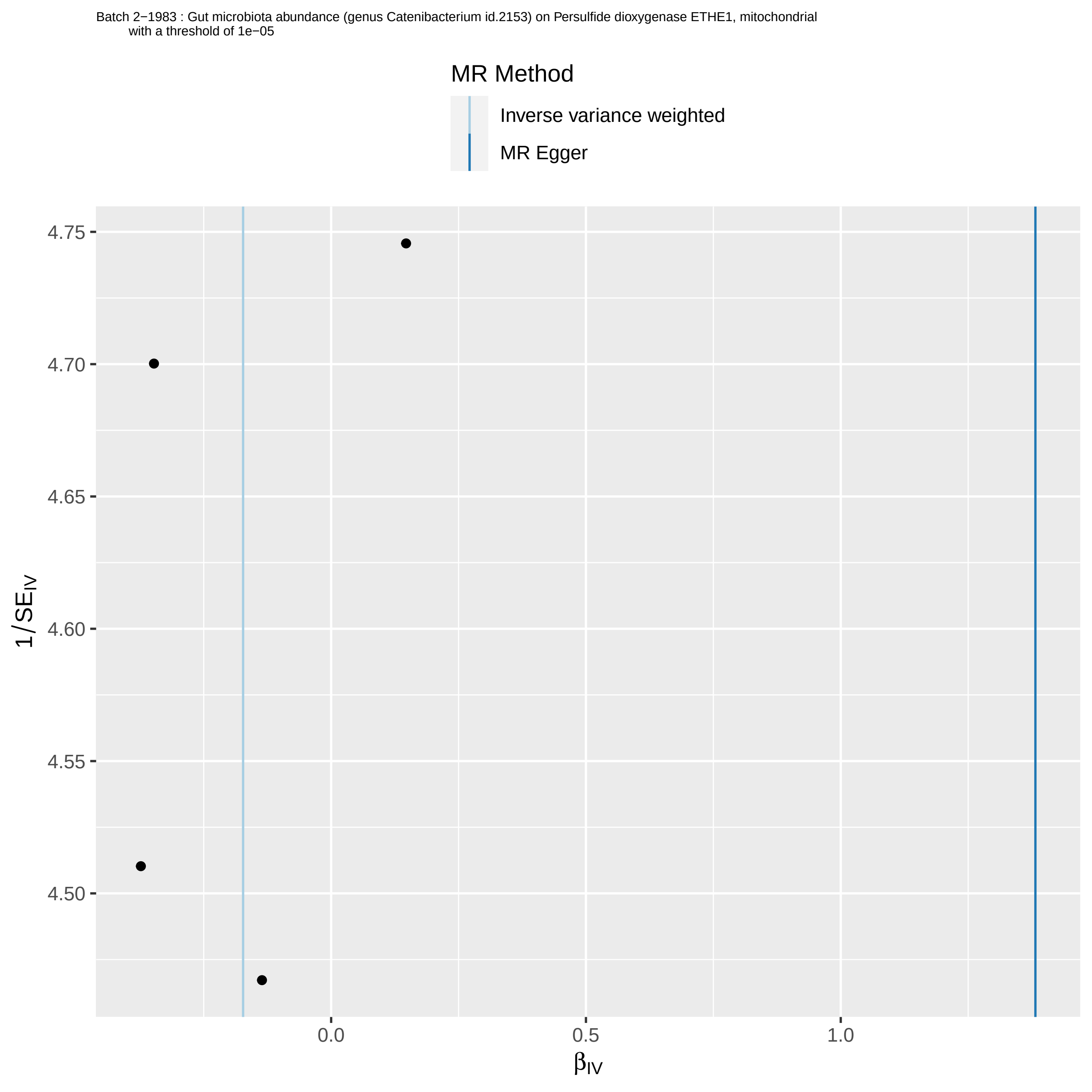

Batch 2−1983 : Gut microbiota abundance (genus Catenibacterium id.2153) on Persulfide dioxygenase ETHE1, mitochondrial with a threshold of 1e−05
MR Method
Inverse variance weighted
MR Egger
4.75
4.70
4.65
4.60
4.55
4.50
1/SE_IV
0.0
0.5
1.0
β_IV

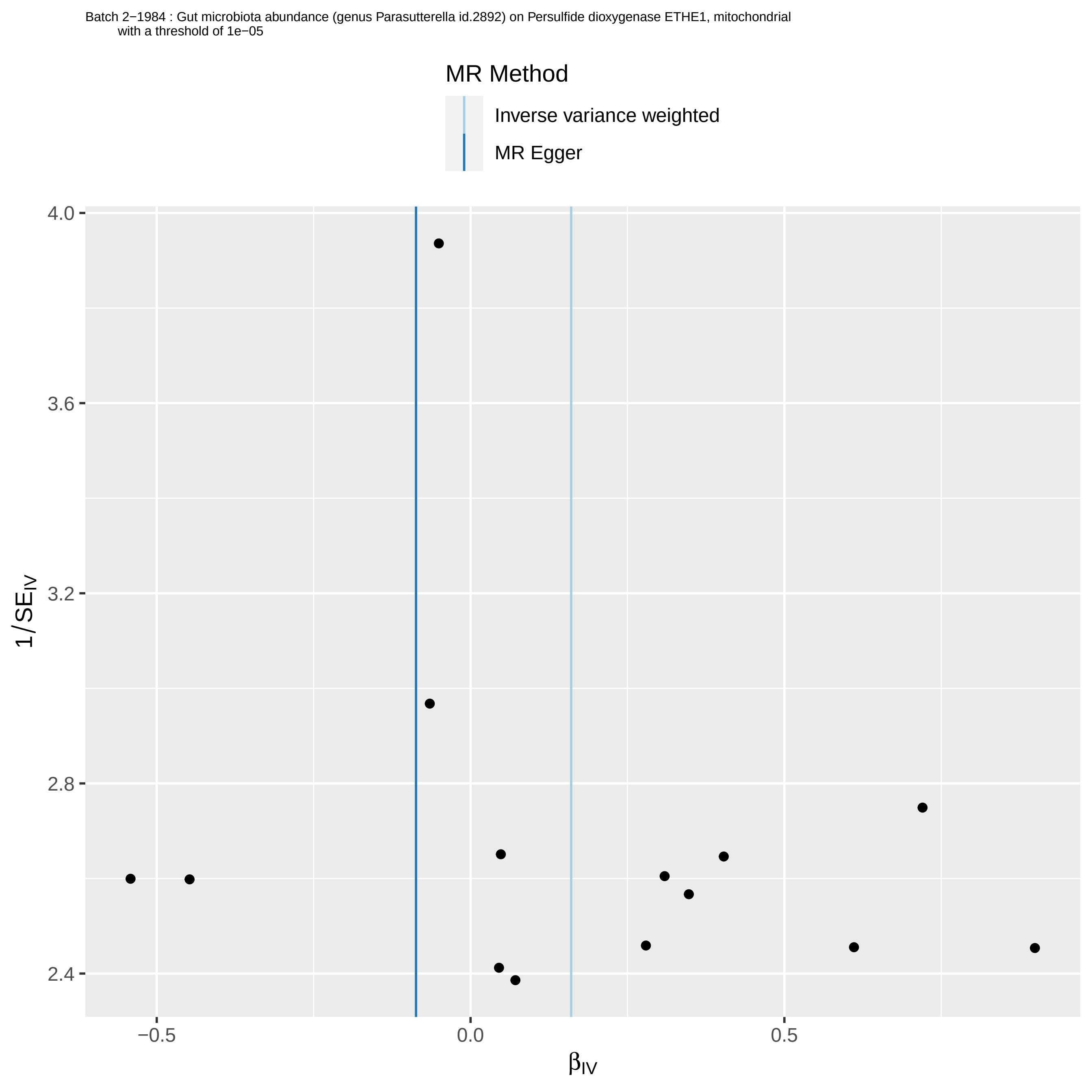
Batch 2−1984 : Gut microbiota abundance (genus Parasutterella id.2892) on Persulfide dioxygenase ETHE1, mitochondrial
with a threshold of 1e−05
MR Method
Inverse variance weighted
MR Egger
4.0
3.6
3.2
2.8
2.4
1/SE_IV
−0.5
0.0
0.5
β_IV

Batch 2−1985 : Gut microbiota abundance (genus Catenibacterium id.2153) on Ethanolamine kinase 1
with a threshold of 1e−05

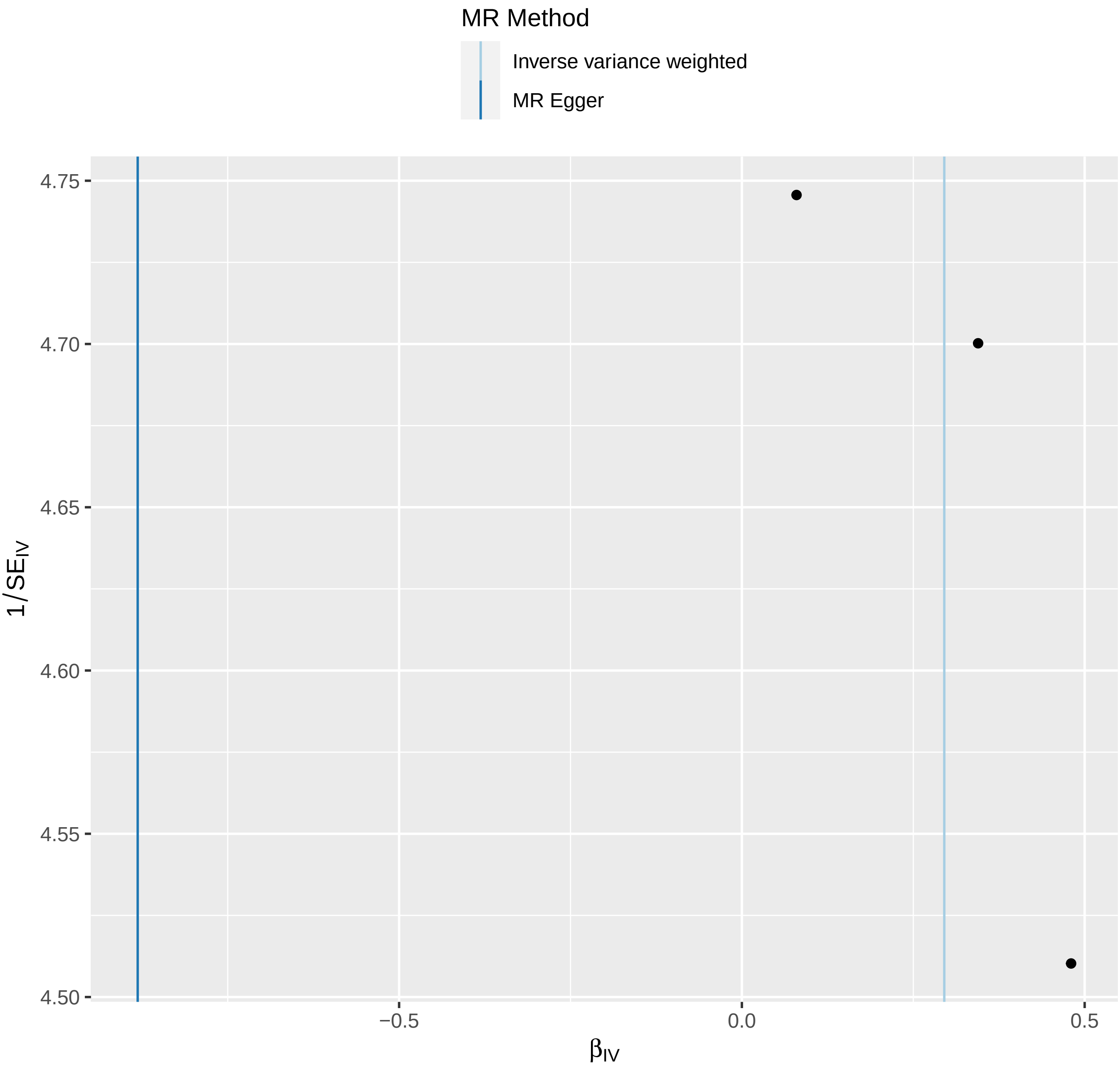

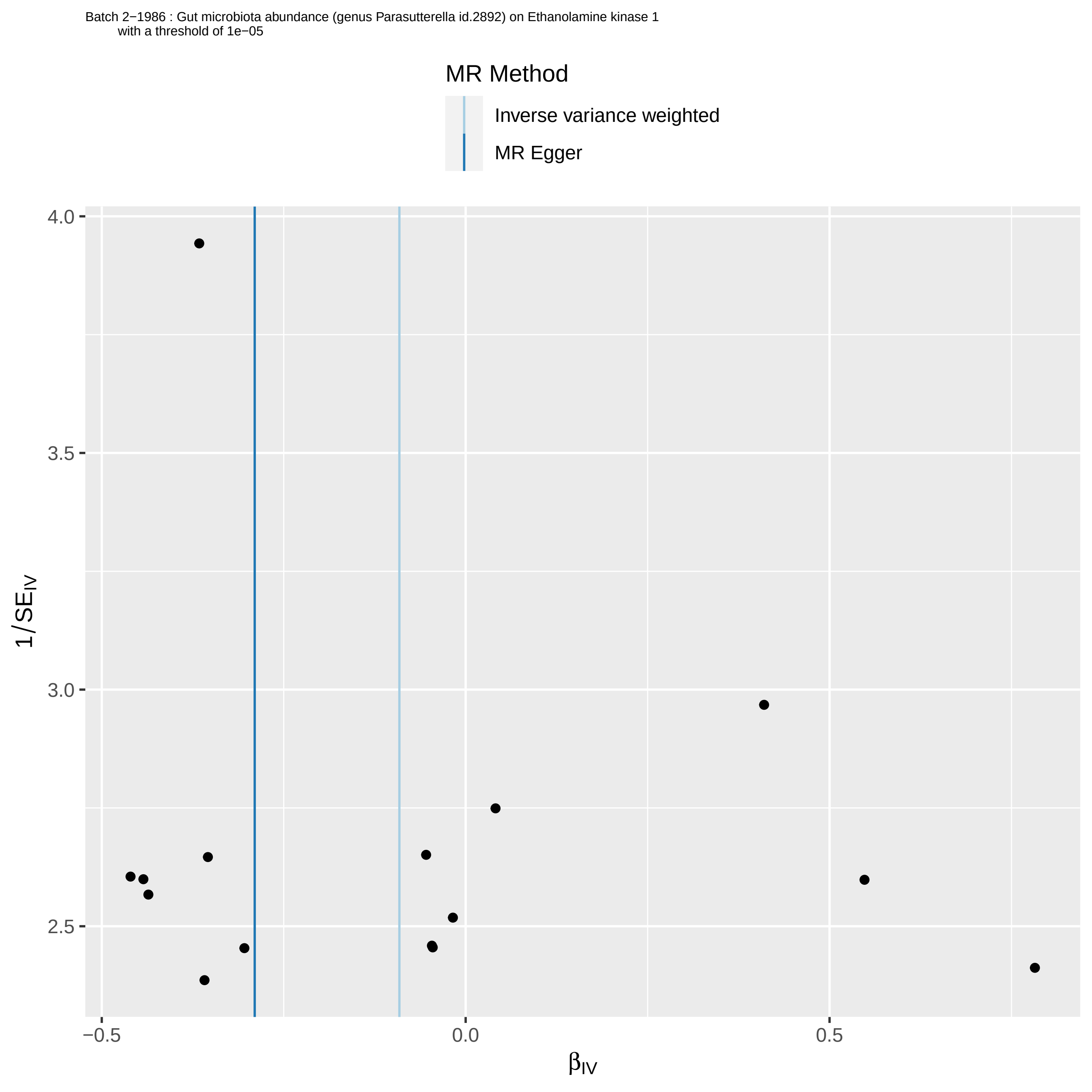
Batch 2−1986 : Gut microbiota abundance (genus Parasutterella id.2892) on Ethanolamine kinase 1
with a threshold of 1e−05
MR Method
Inverse variance weighted
MR Egger
4.0
3.5
3.0
2.5
1/SE_IV
−0.5
0.0
0.5
β_IV

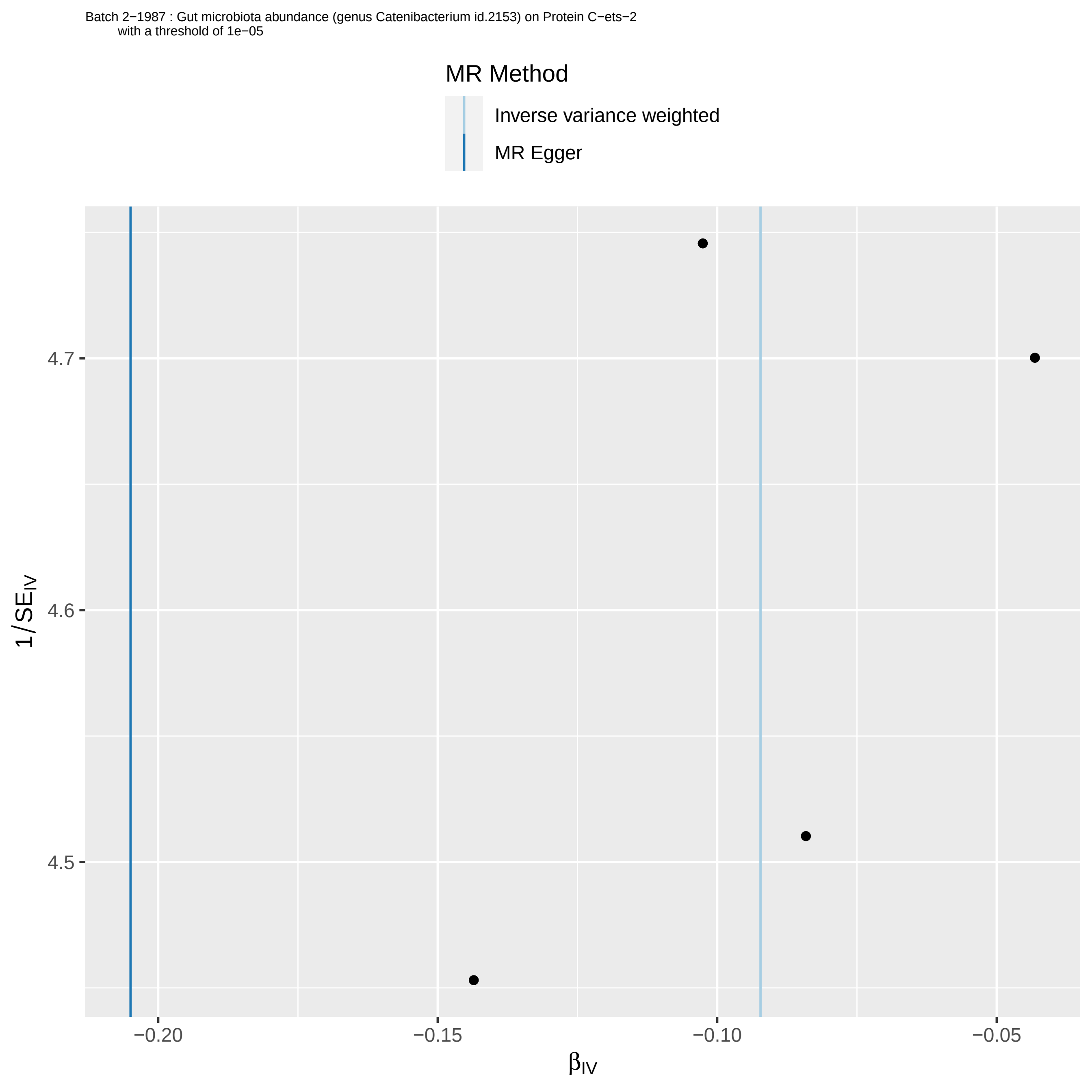
Batch 2−1987 : Gut microbiota abundance (genus Catenibacterium id.2153) on Protein C−ets−2
with a threshold of 1e−05
MR Method
Inverse variance weighted
MR Egger
4.7
4.6
4.5
1/SE_IV
−0.20
−0.15
−0.10
−0.05
β_IV

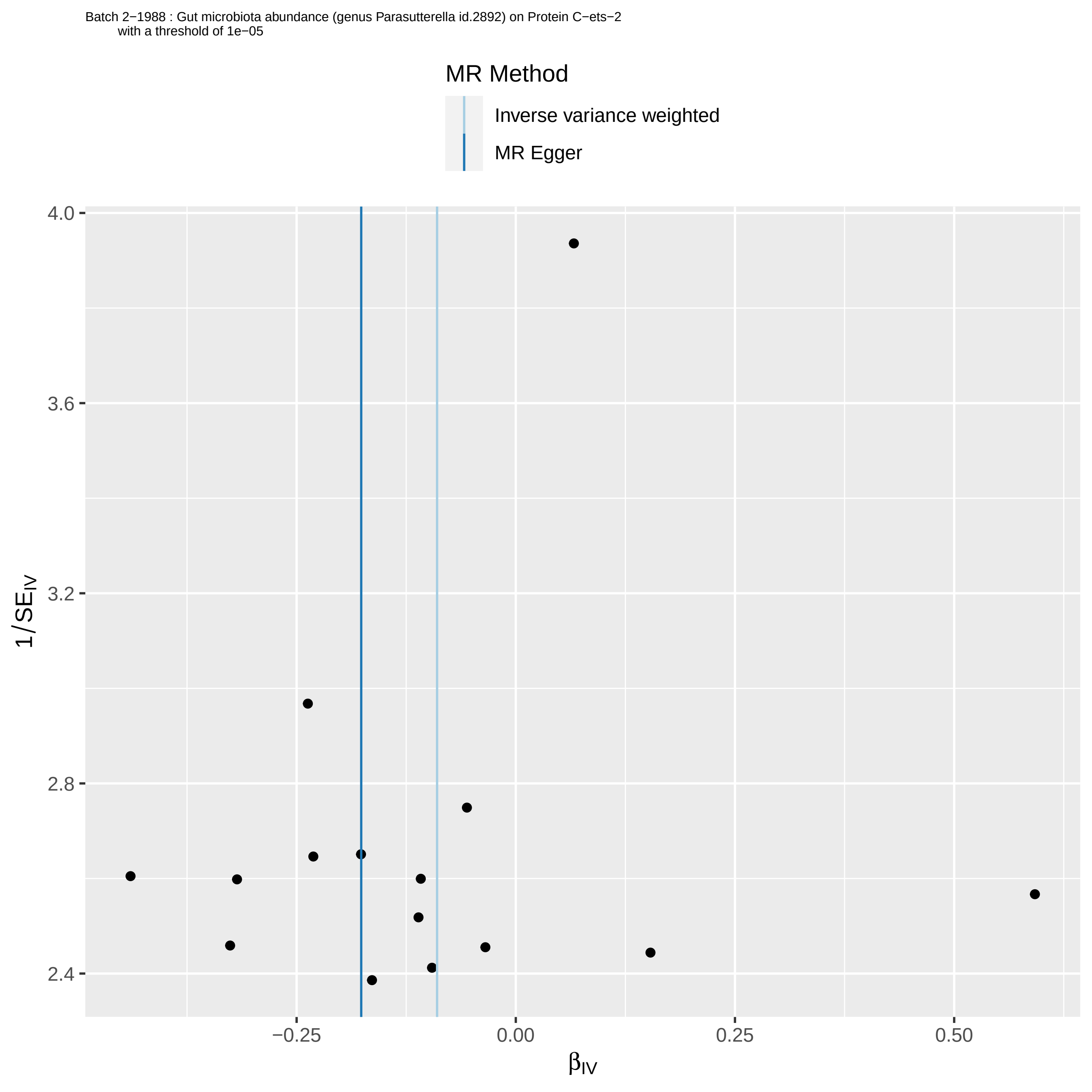
Batch 2−1988 : Gut microbiota abundance (genus Parasutterella id.2892) on Protein C−ets−2
with a threshold of 1e−05
MR Method
Inverse variance weighted
MR Egger
4.0
3.6
3.2
2.8
2.4
1/SE$_{IV}$
−0.25
0.00
0.25
0.50
β$_{IV}$

Batch 2−1989 : Gut microbiota abundance (genus Catenibacterium id.2153) on Protein eva−1 homolog C
with a threshold of 1e−05

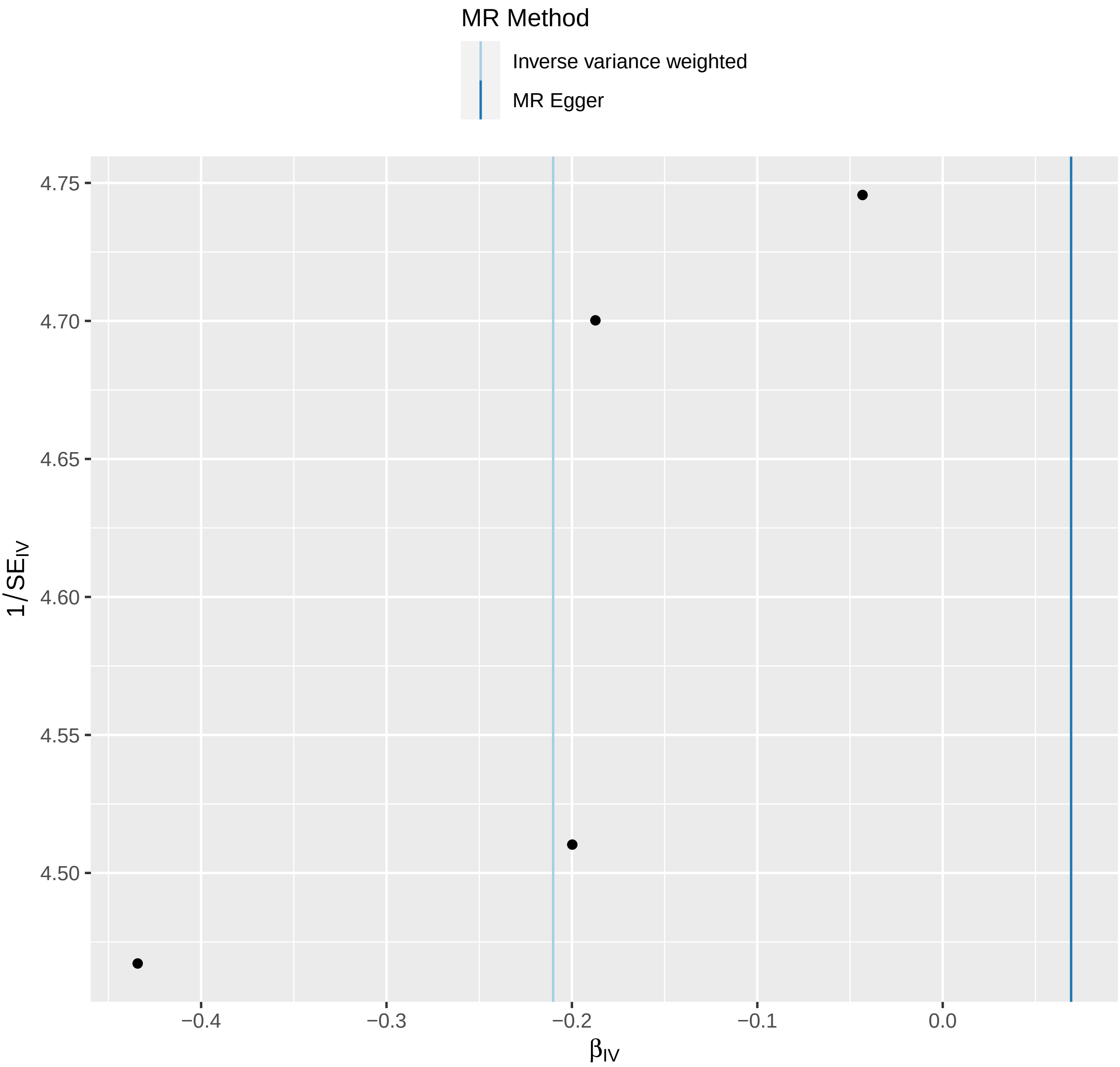

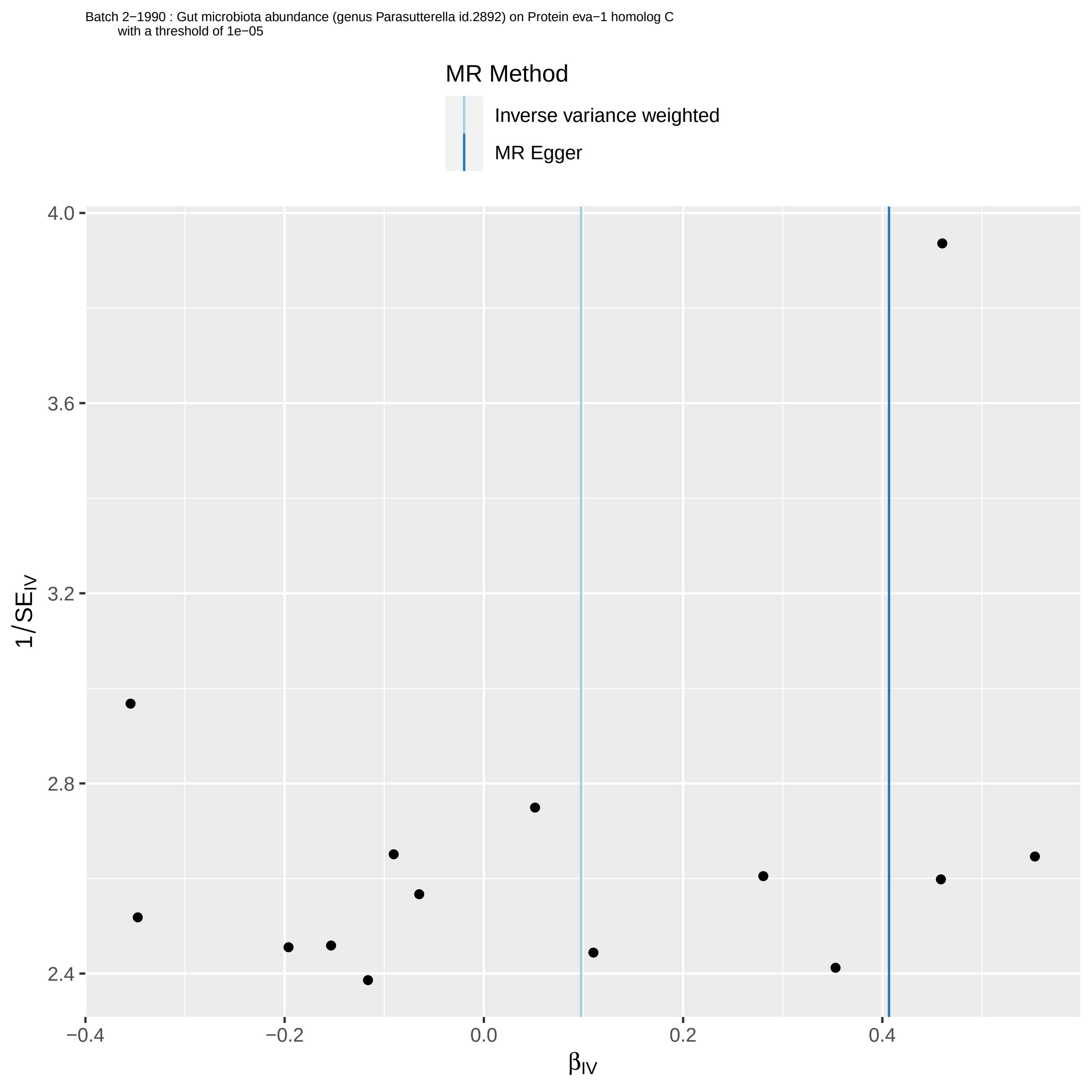

Batch 2−1990 : Gut microbiota abundance (genus Parasutterella id.2892) on Protein eva−1 homolog C
with a threshold of 1e−05
MR Method
Inverse variance weighted
MR Egger
4.0
3.6
3.2
2.8
2.4
1/SE_IV
−0.4
−0.2
0.0
0.2
0.4
β_IV

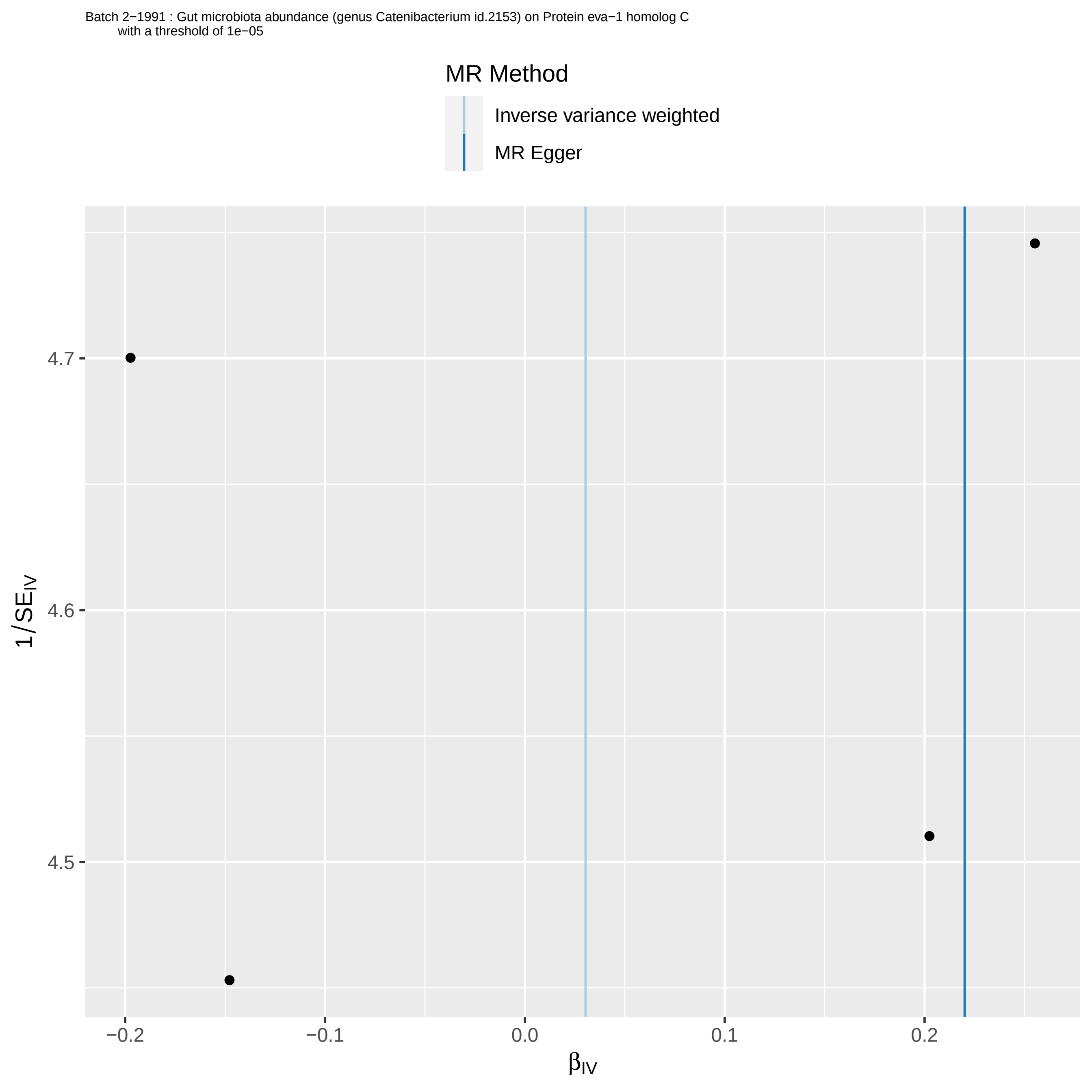

Batch 2−1991 : Gut microbiota abundance (genus Catenibacterium id.2153) on Protein eva−1 homolog C
with a threshold of 1e−05
MR Method
Inverse variance weighted
MR Egger
4.7
4.6
4.5
1/SE_IV
−0.2
−0.1
0.0
0.1
0.2
β_IV

Batch 2−1992 : Gut microbiota abundance (genus Parasutterella id.2892) on Protein eva−1 homolog C
with a threshold of 1e−05

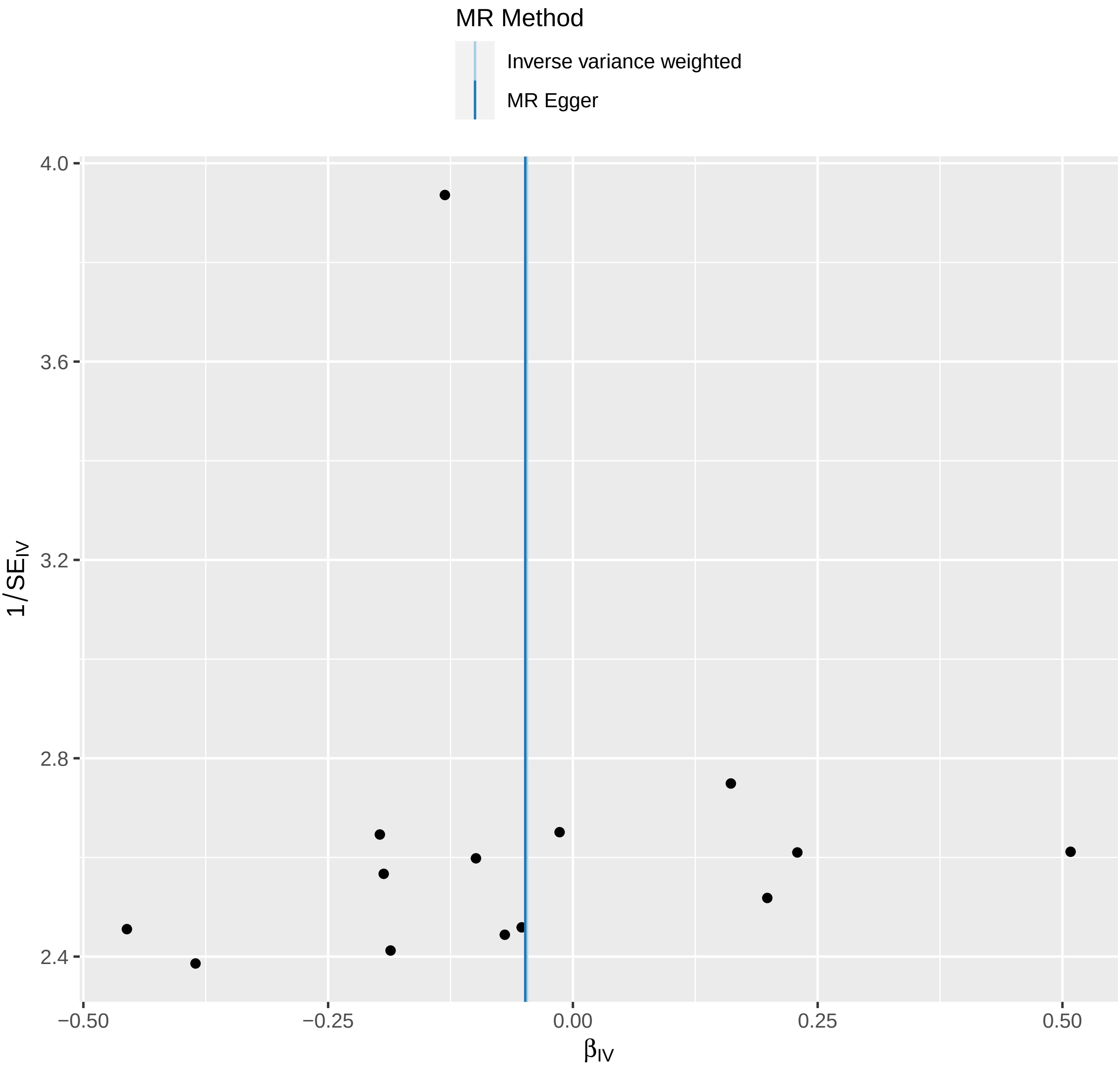

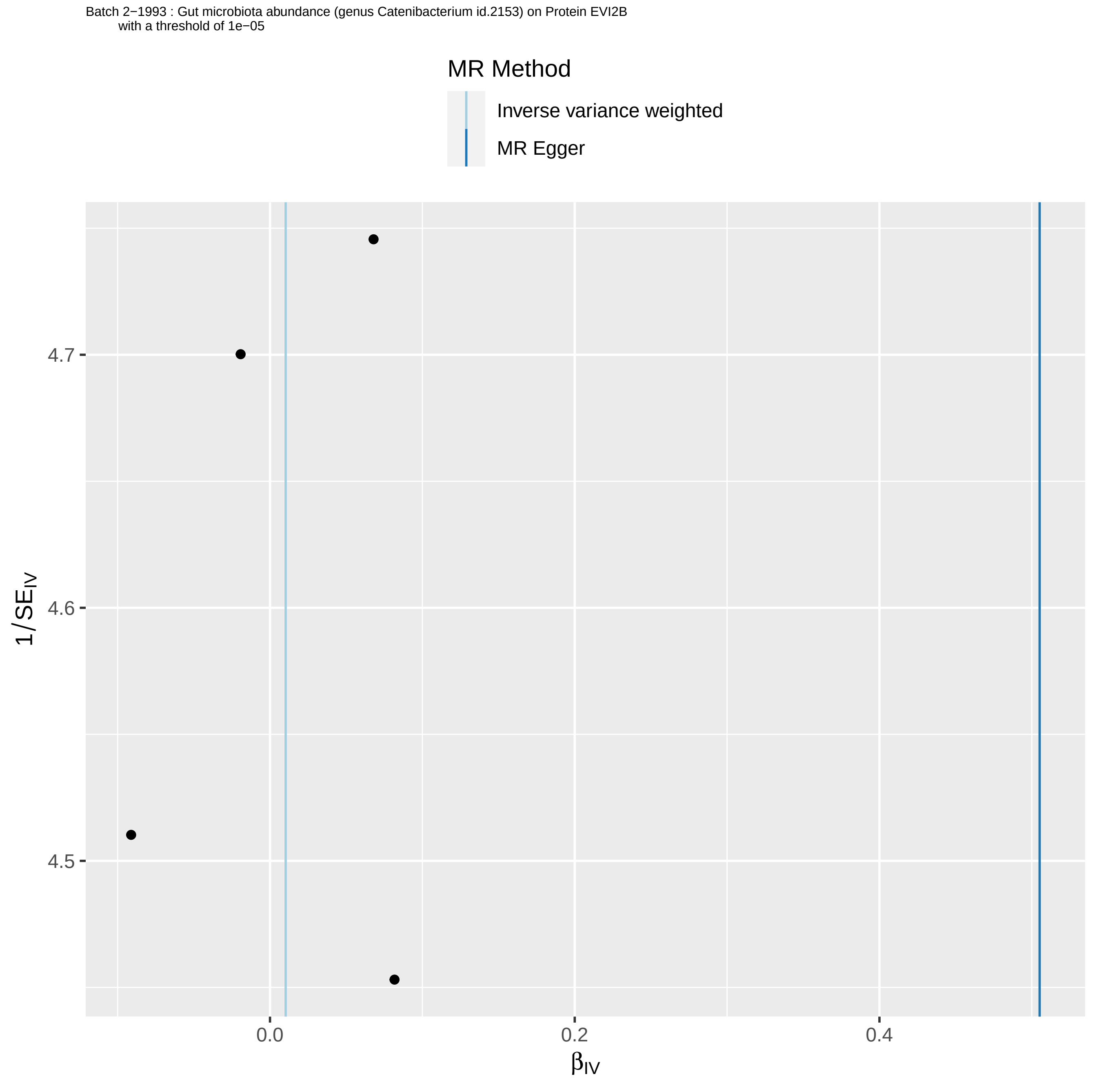

Batch 2−1993 : Gut microbiota abundance (genus Catenibacterium id.2153) on Protein EVI2B
with a threshold of 1e−05
MR Method
Inverse variance weighted
MR Egger
1/SE$_{IV}$
4.7
4.6
4.5
0.0
0.2
0.4
β$_{IV}$

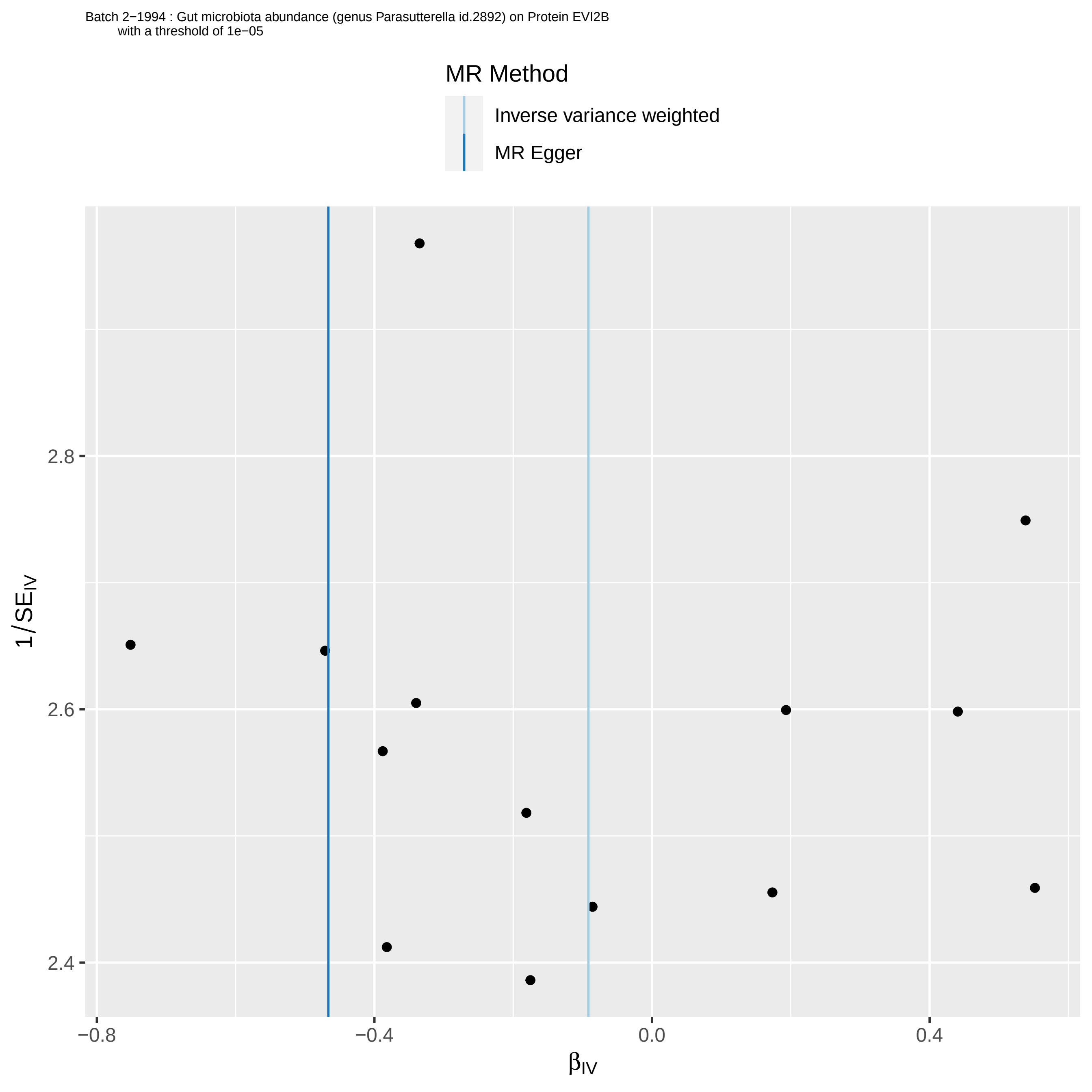

Batch 2−1994 : Gut microbiota abundance (genus Parasutterella id.2892) on Protein EVI2B
with a threshold of 1e−05
MR Method
Inverse variance weighted
MR Egger
2.8
2.6
2.4
1/SE_IV
−0.8
−0.4
0.0
0.4
β_IV

Batch 2−1995 : Gut microbiota abundance (genus Catenibacterium id.2153) on Ena/VASP−like protein
with a threshold of 1e−05

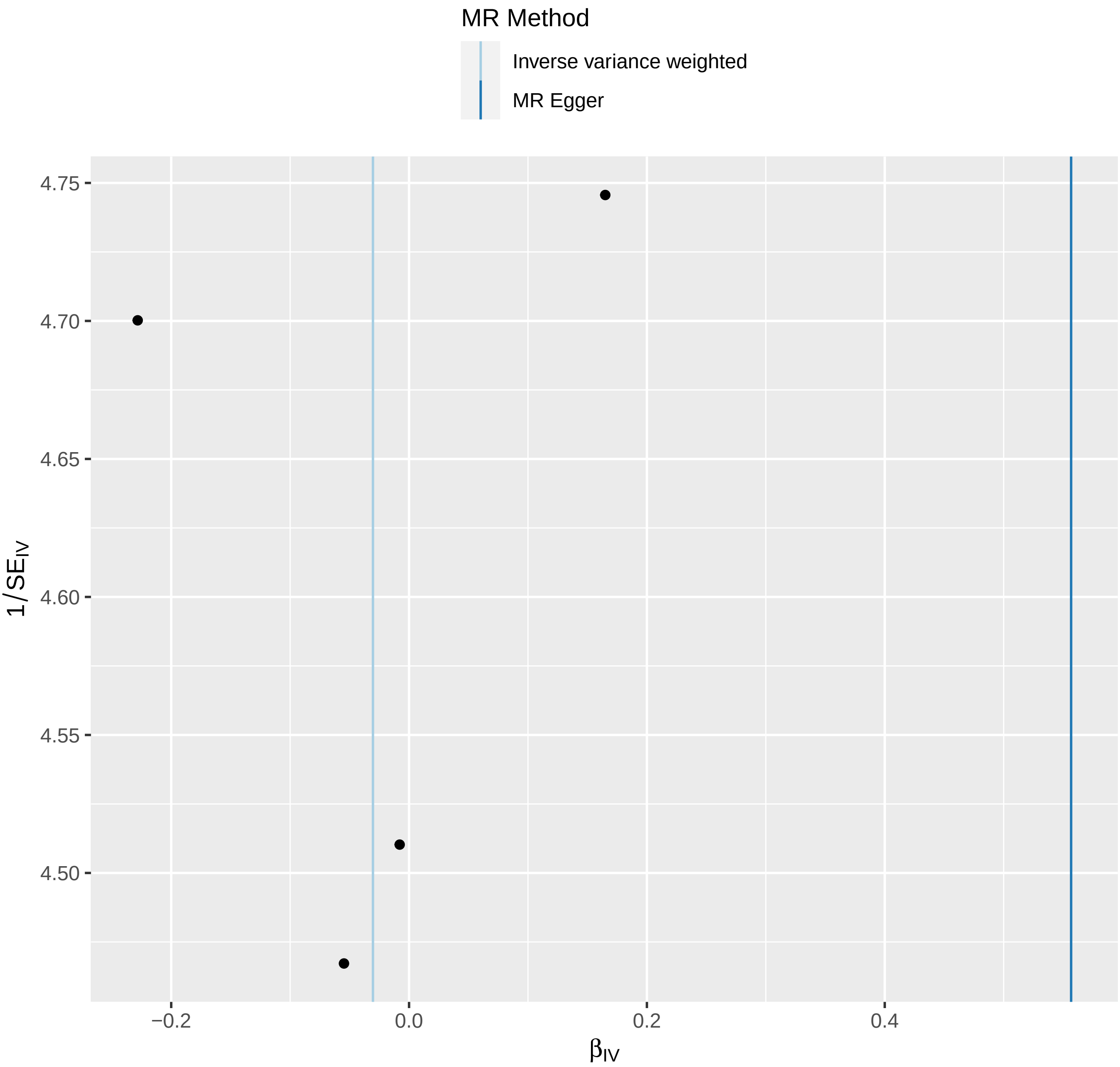

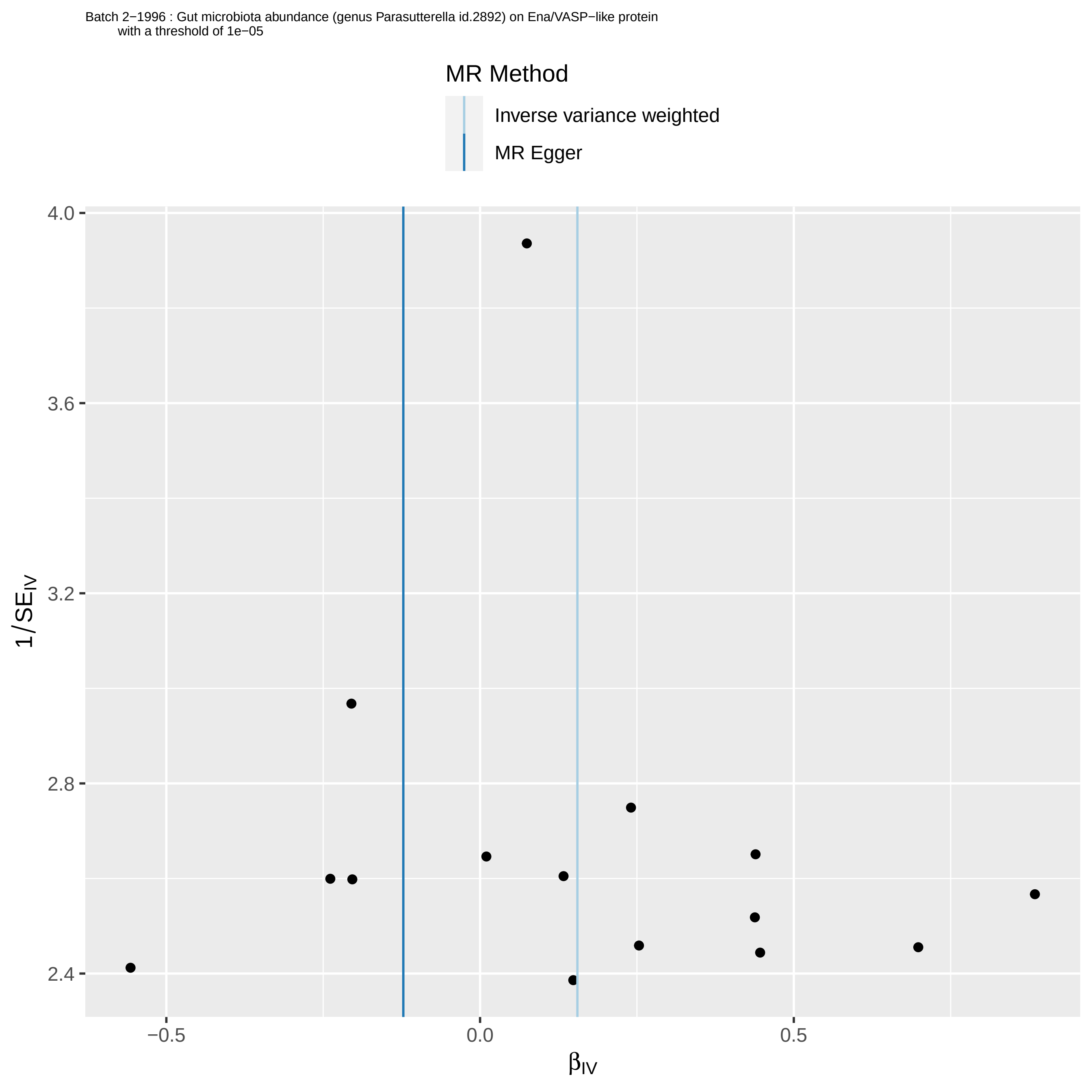
Batch 2−1996 : Gut microbiota abundance (genus Parasutterella id.2892) on Ena/VASP−like protein
with a threshold of 1e−05
MR Method
Inverse variance weighted
MR Egger
4.0
3.6
3.2
2.8
2.4
1/SE_IV
−0.5
0.0
0.5
β_IV

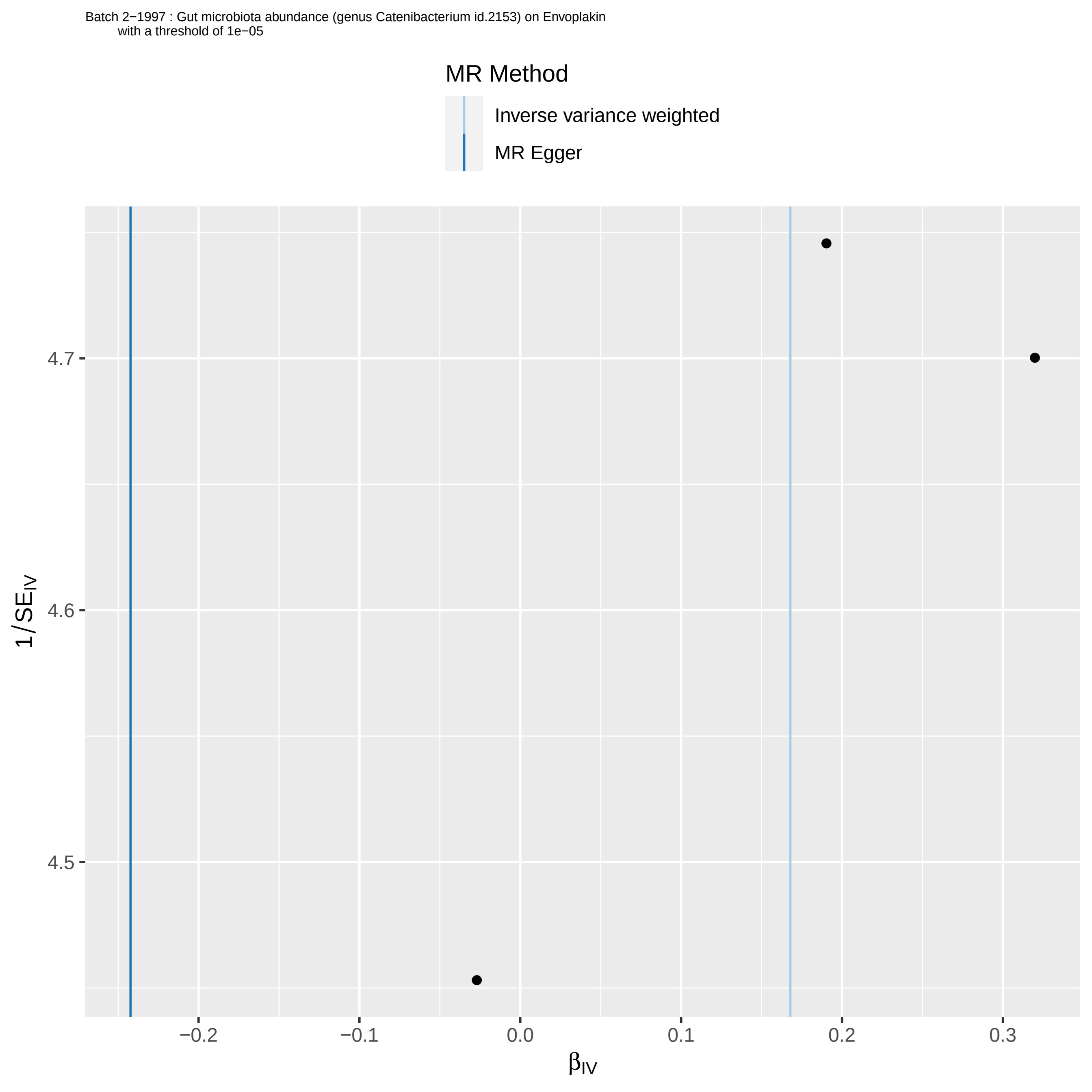

Batch 2−1997 : Gut microbiota abundance (genus Catenibacterium id.2153) on Envoplakin
with a threshold of 1e−05
MR Method
Inverse variance weighted
MR Egger
4.7
4.6
4.5
1/SE_IV
−0.2
−0.1
0.0
0.1
0.2
0.3
β_IV

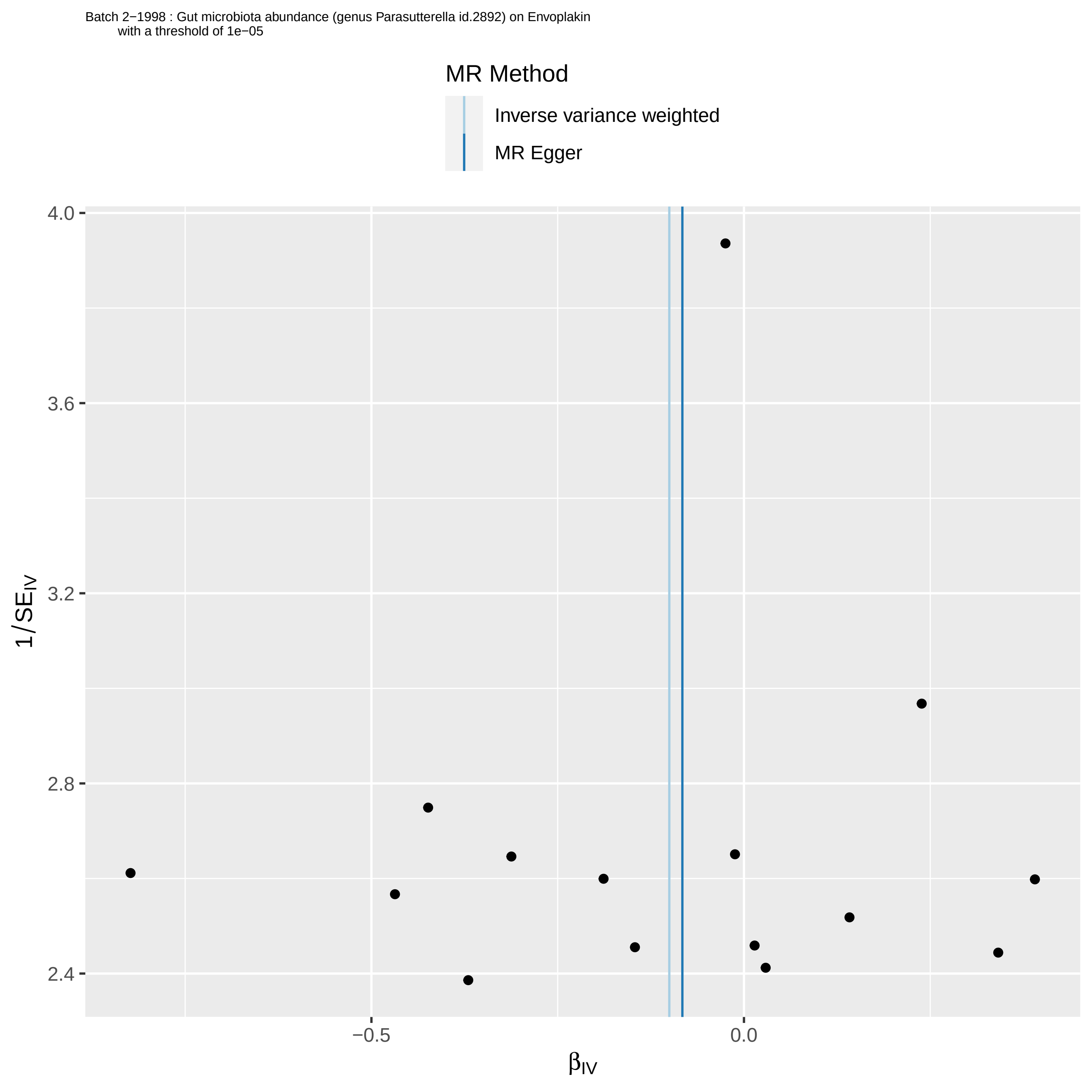

Batch 2−1998 : Gut microbiota abundance (genus Parasutterella id.2892) on Envoplakin
with a threshold of 1e−05
MR Method
Inverse variance weighted
MR Egger
4.0
3.6
3.2
2.8
2.4
1/SE_IV
−0.5
0.0
β_IV

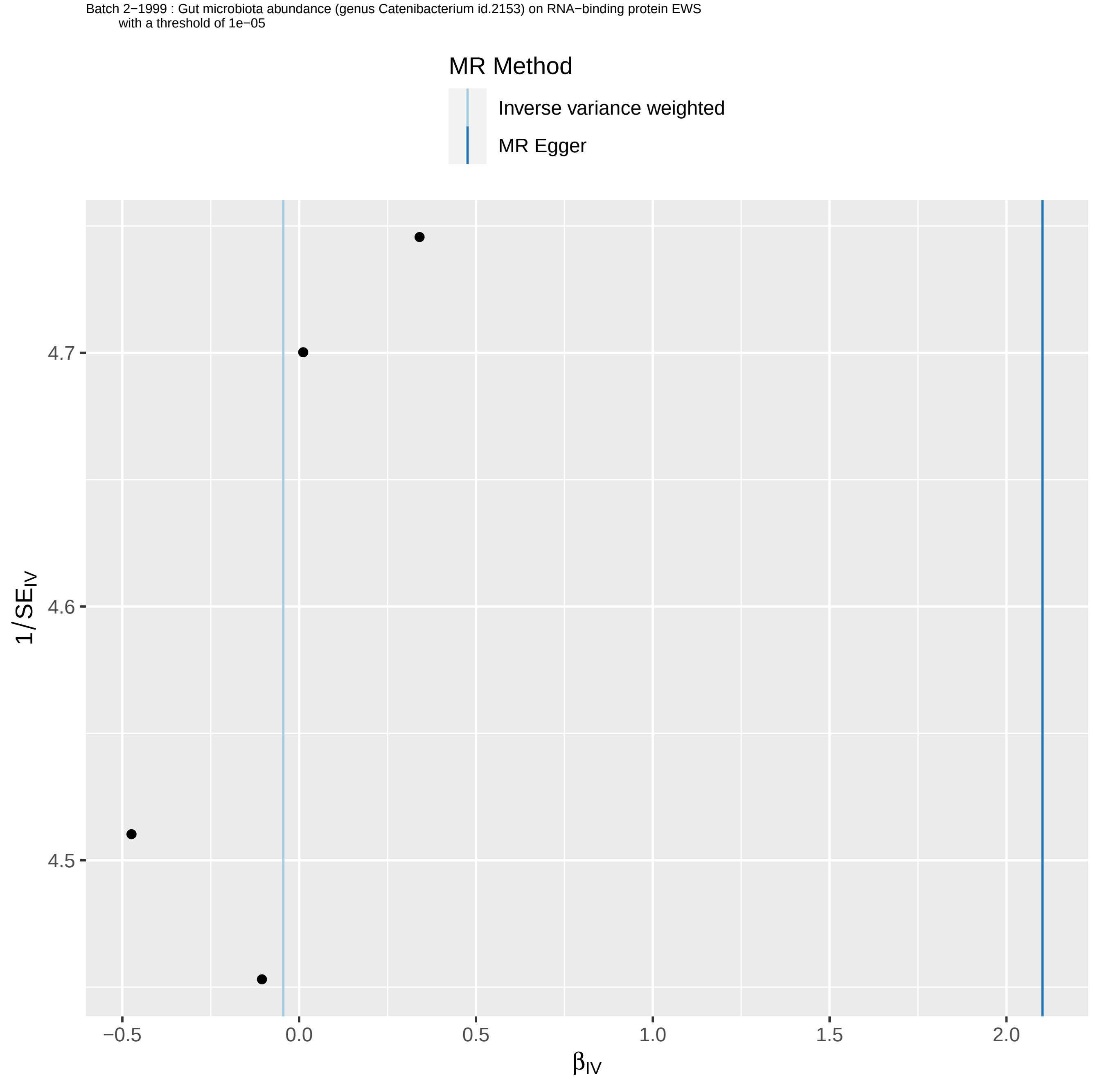

Batch 2−1999 : Gut microbiota abundance (genus Catenibacterium id.2153) on RNA−binding protein EWS
with a threshold of 1e−05
MR Method
Inverse variance weighted
MR Egger
4.7
4.6
4.5
1/SE_IV
−0.5
0.0
0.5
1.0
1.5
2.0
β_IV

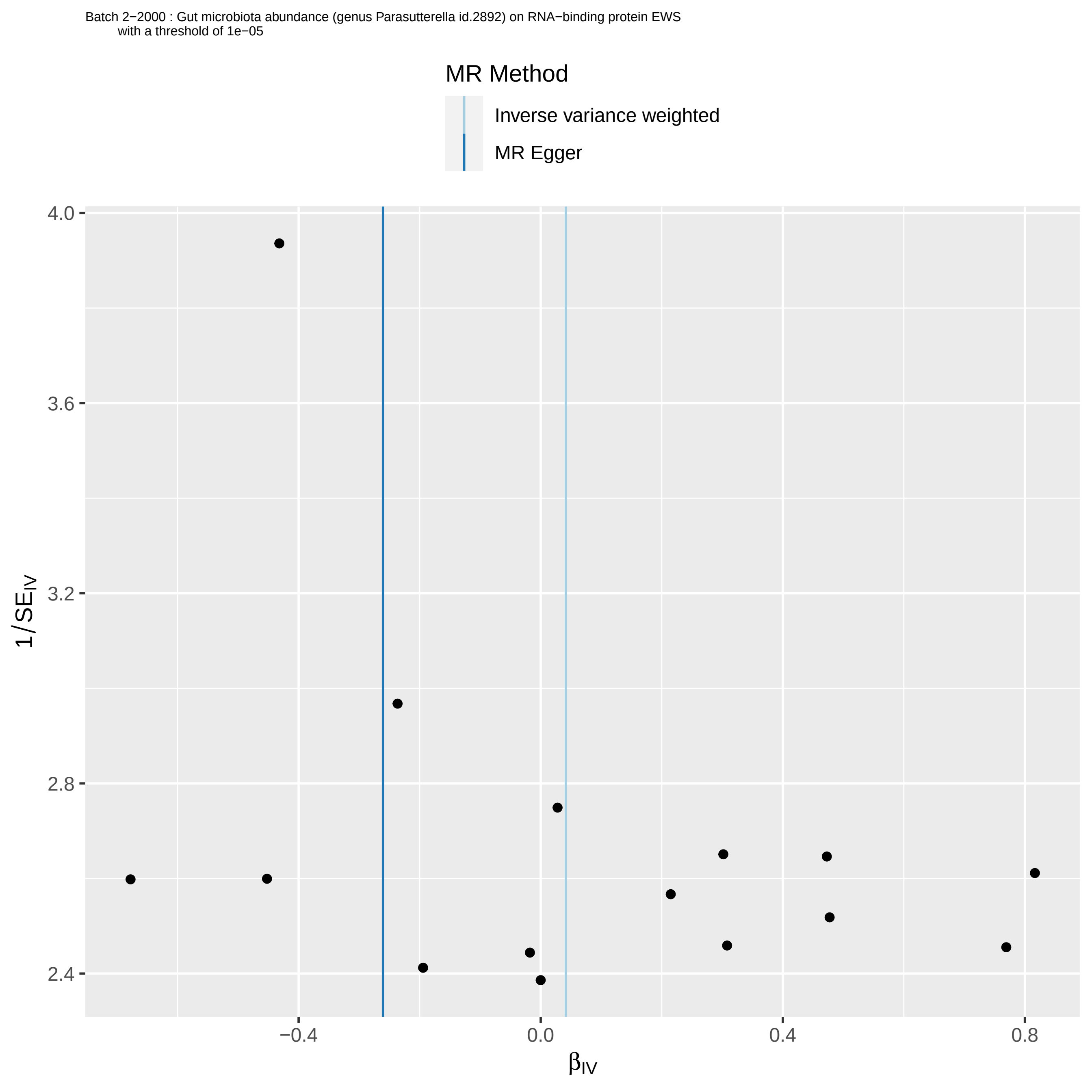

Batch 2−2000 : Gut microbiota abundance (genus Parasutterella id.2892) on RNA−binding protein EWS
with a threshold of 1e−05
MR Method
Inverse variance weighted
MR Egger
4.0
3.6
3.2
2.8
2.4
1/SE_IV
−0.4
0.0
0.4
0.8
β_IV

Batch 2−2001 : Gut microbiota abundance (genus Catenibacterium id.2153) on Exosome complex component CSL4
with a threshold of 1e−05

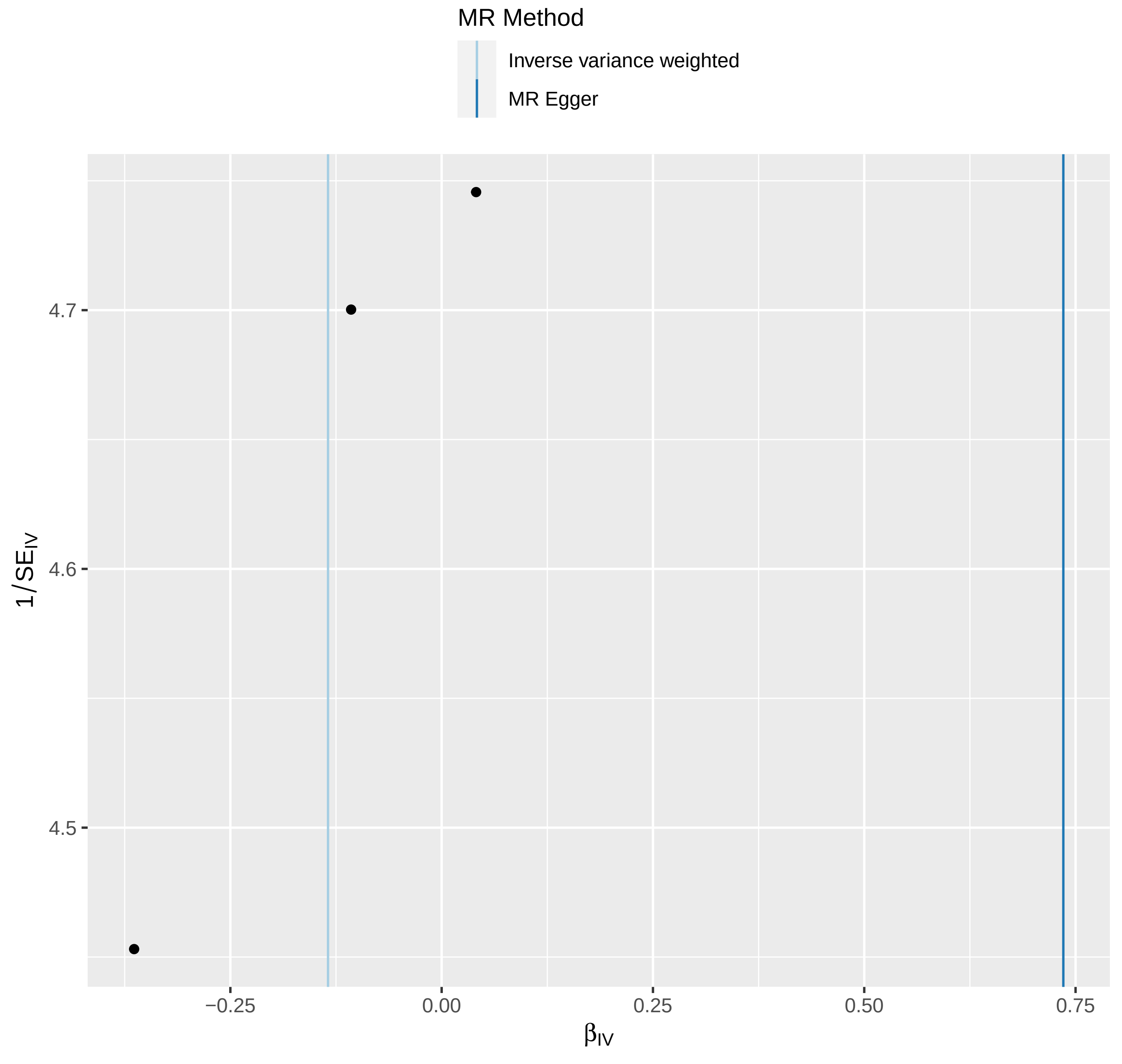

Batch 2−2002 : Gut microbiota abundance (genus Parasutterella id.2892) on Exosome complex component CSL4
with a threshold of 1e−05

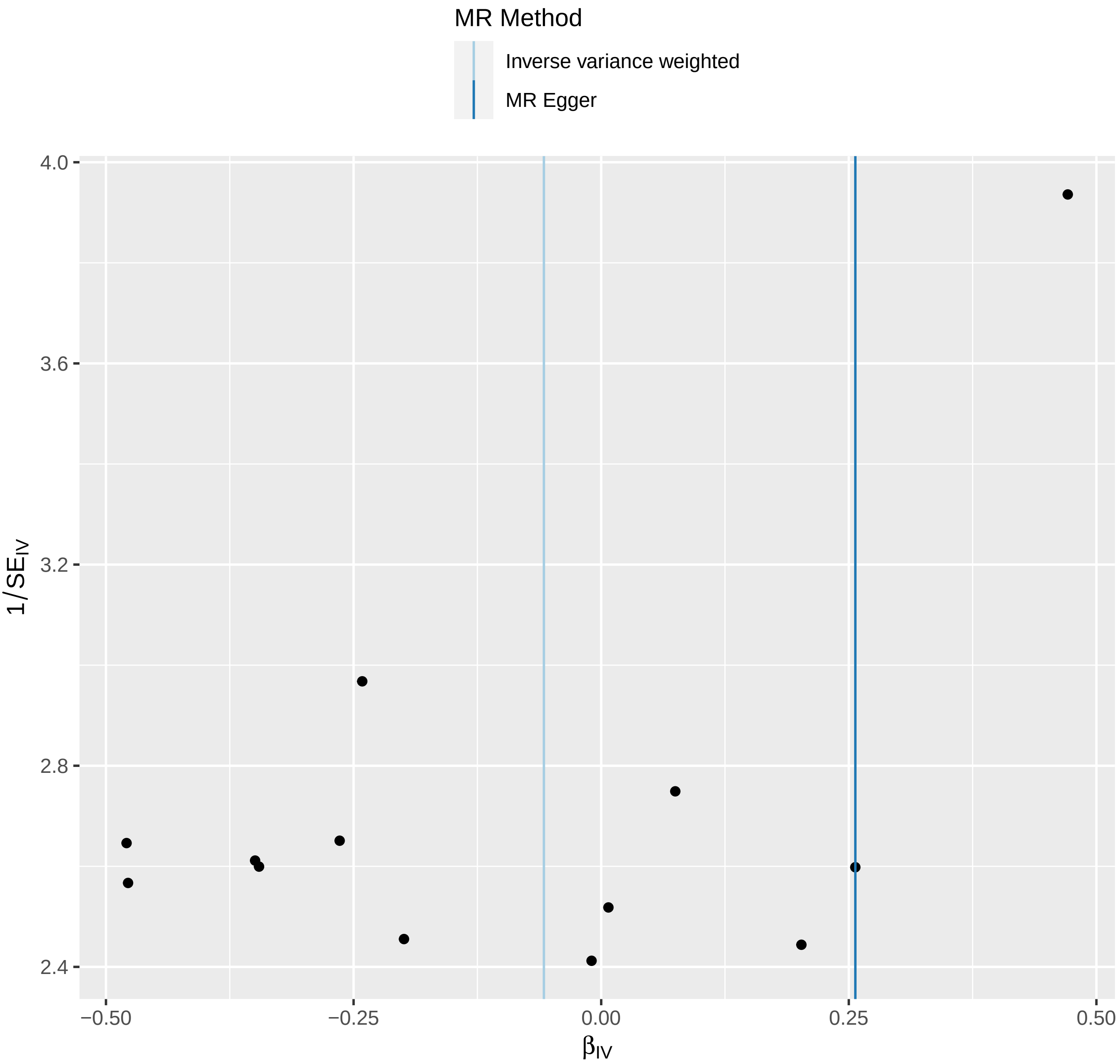

Batch 2−2003 : Gut microbiota abundance (genus Catenibacterium id.2153) on Exosome complex component RRP40
with a threshold of 1e−05

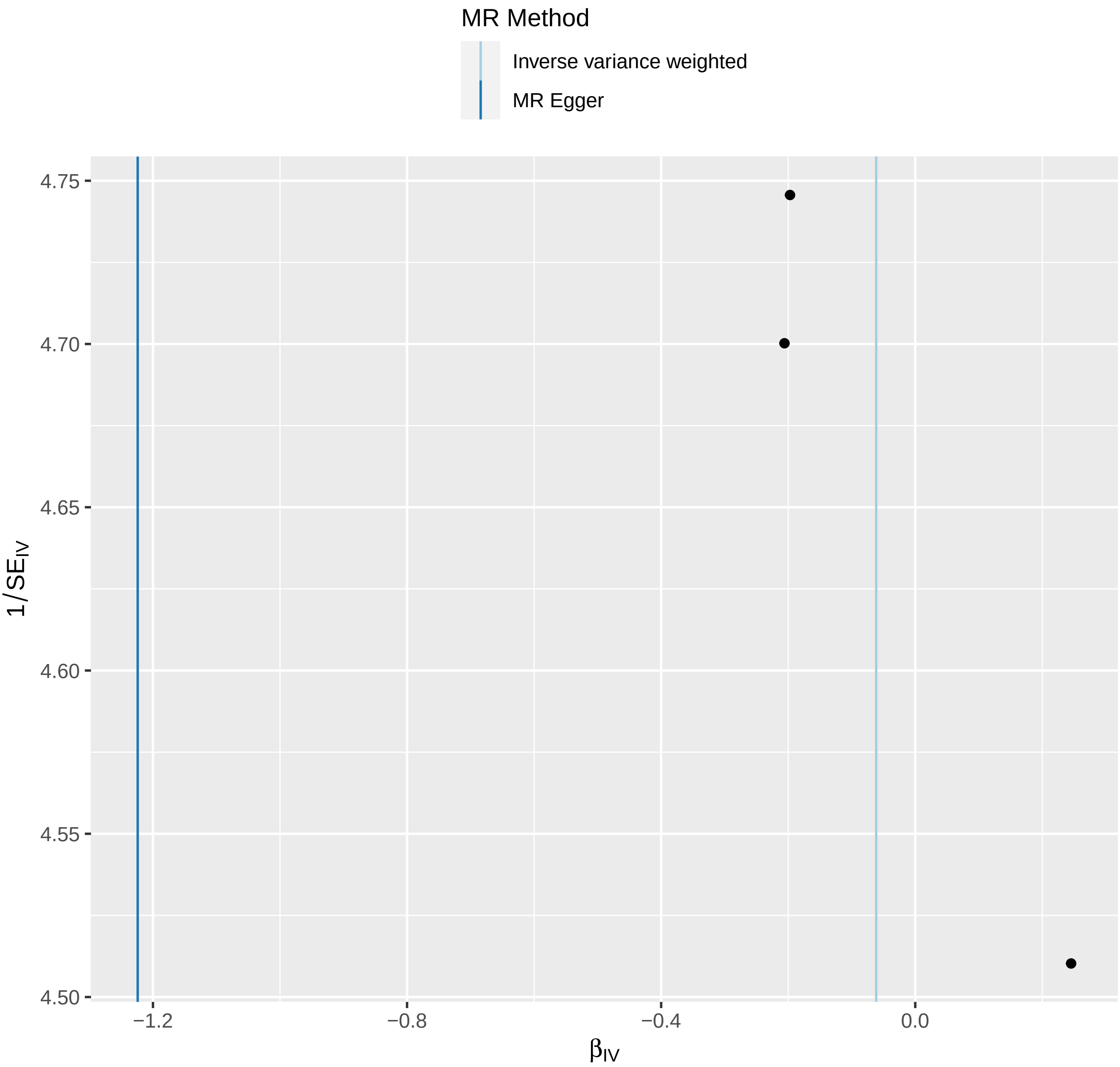

Batch 2−2004 : Gut microbiota abundance (genus Parasutterella id.2892) on Exosome complex component RRP40
with a threshold of 1e−05

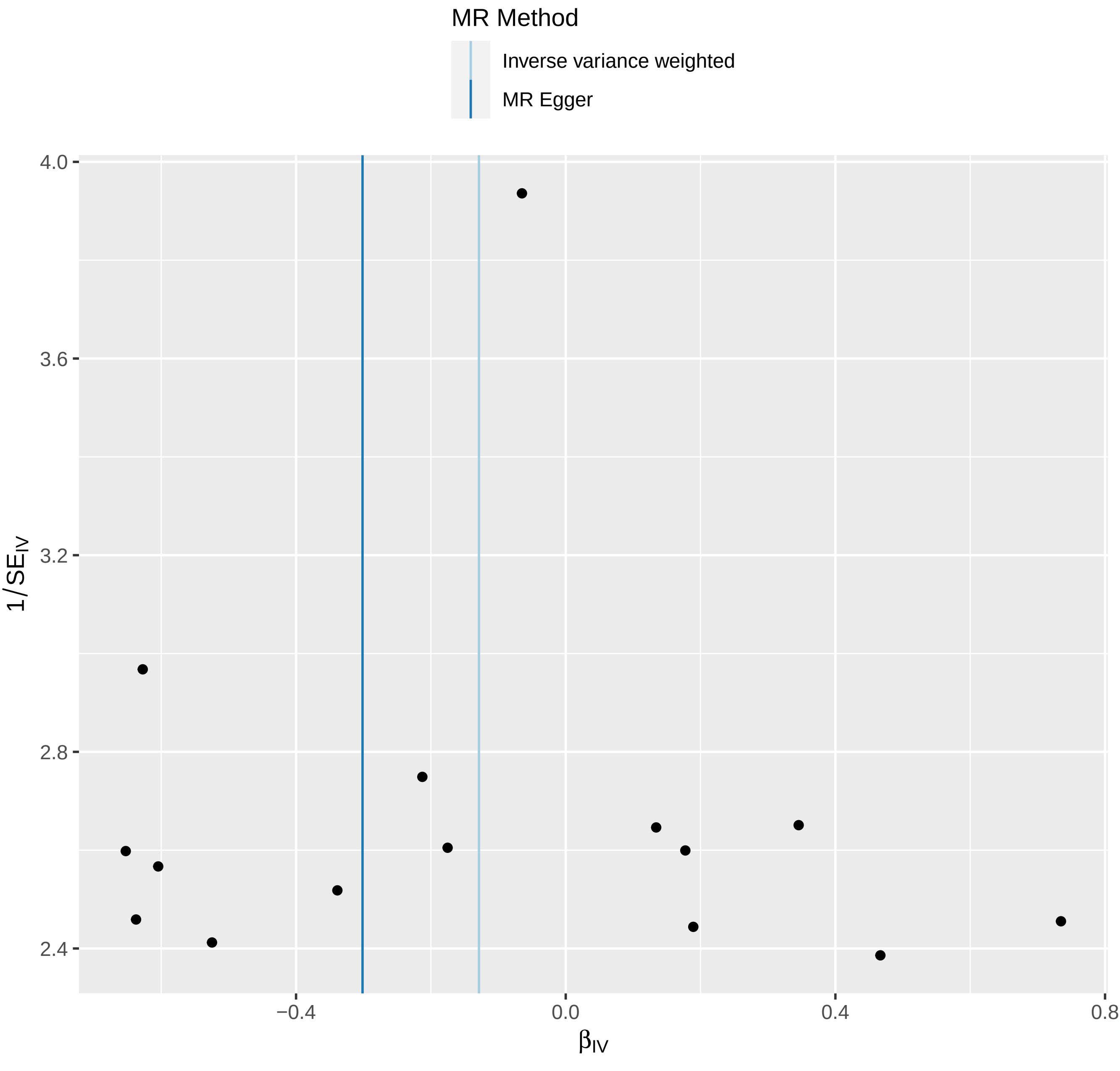

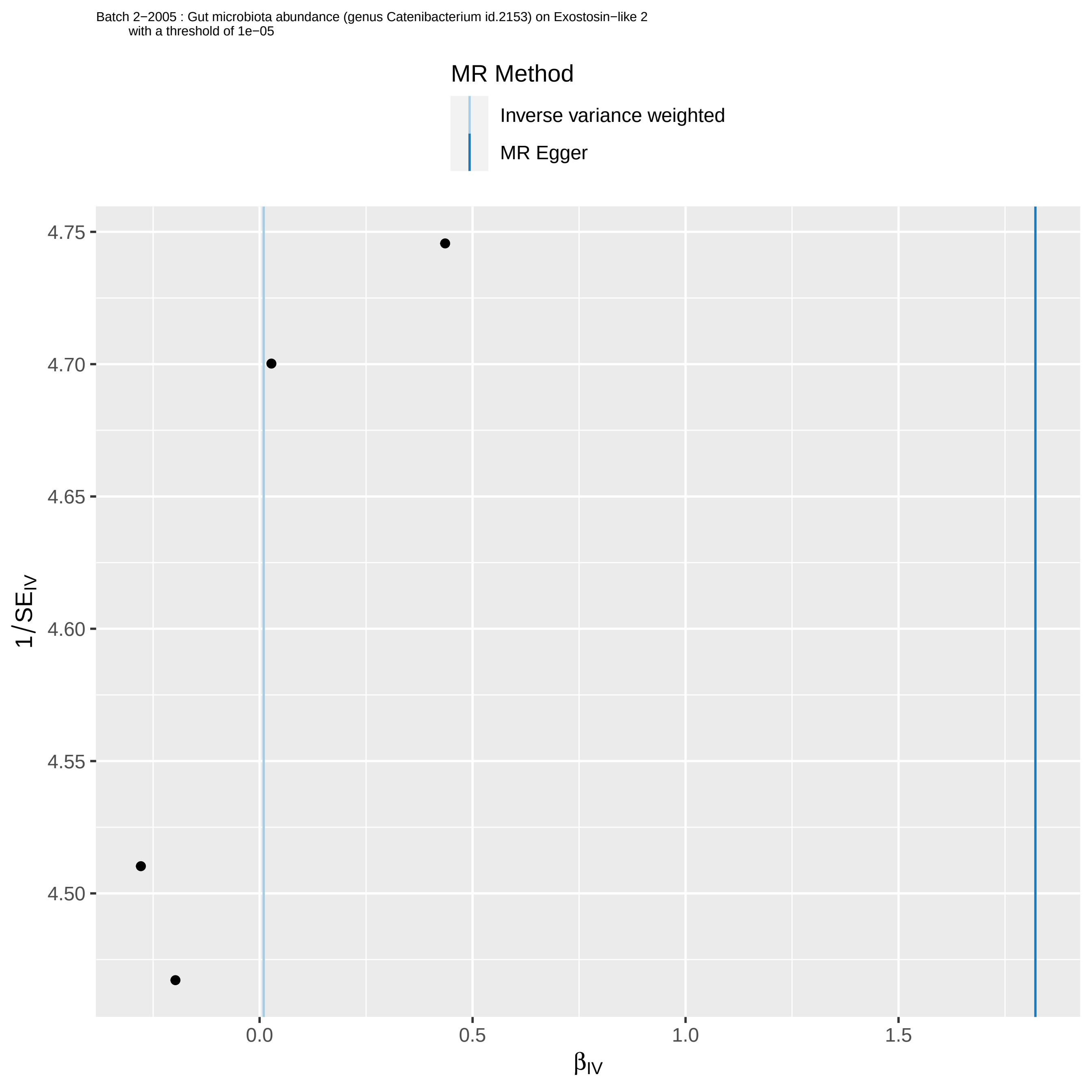
Batch 2−2005 : Gut microbiota abundance (genus Catenibacterium id.2153) on Exostosin−like 2
with a threshold of 1e−05
MR Method
Inverse variance weighted
MR Egger
4.75
4.70
4.65
4.60
4.55
4.50
$1/SE_{IV}$
0.0
0.5
1.0
1.5
β_{IV}

Batch 2−2006 : Gut microbiota abundance (genus Parasutterella id.2892) on Exostosin−like 2
with a threshold of 1e−05

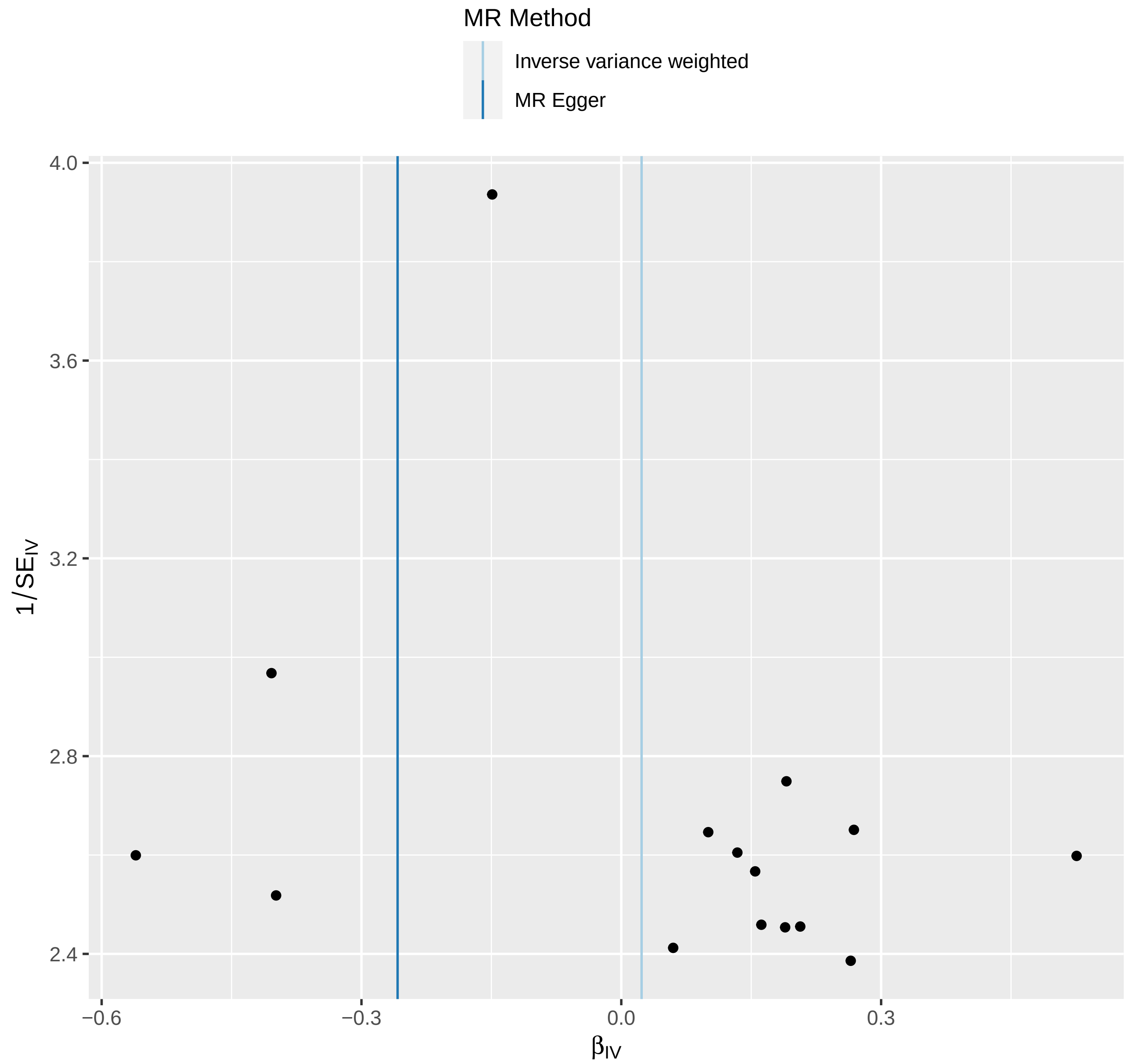

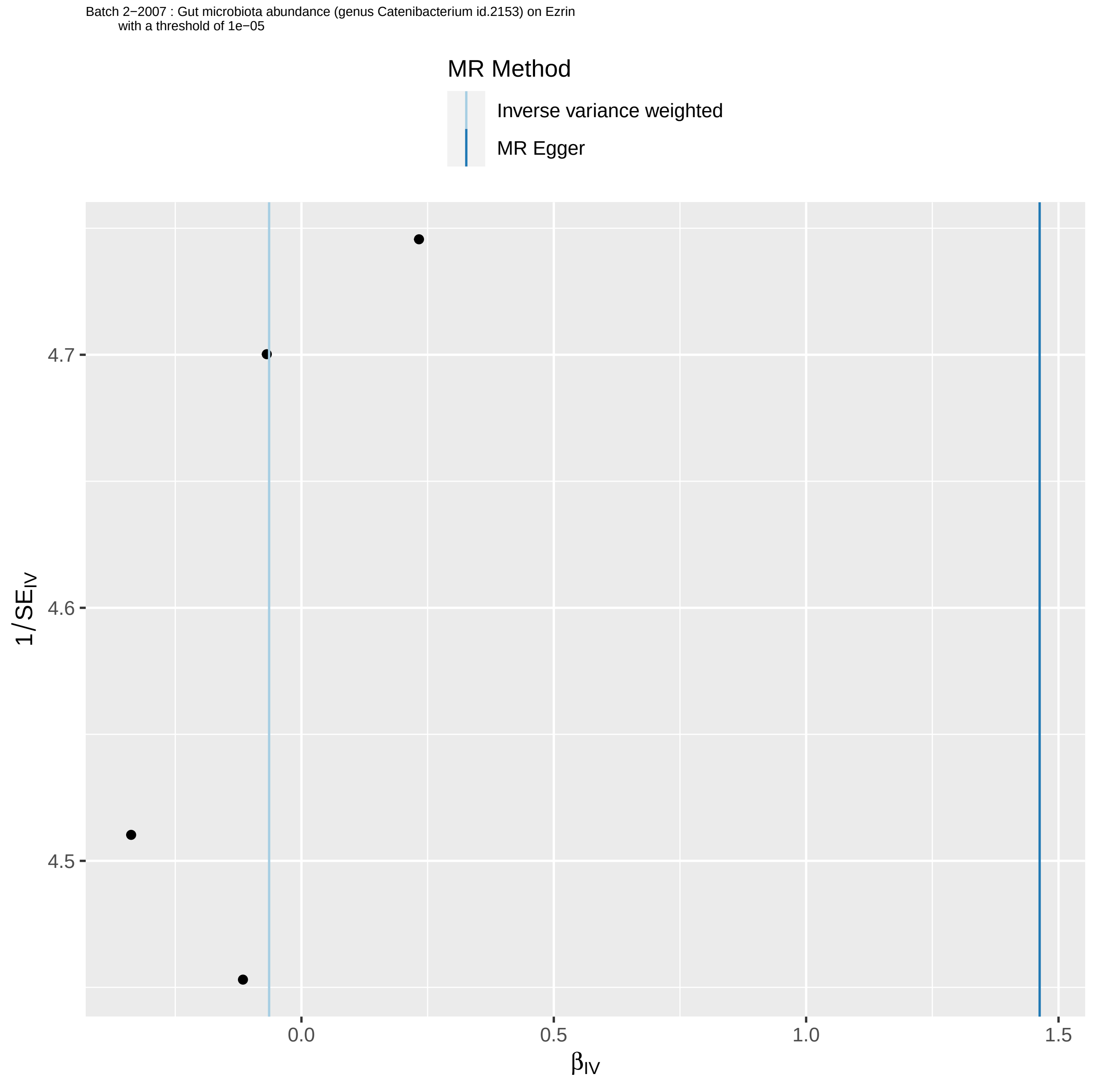
Batch 2−2007 : Gut microbiota abundance (genus Catenibacterium id.2153) on Ezrin
with a threshold of 1e−05
MR Method
Inverse variance weighted
MR Egger
4.7
4.6
4.5
1/SE_IV
0.0
0.5
1.0
1.5
β_IV

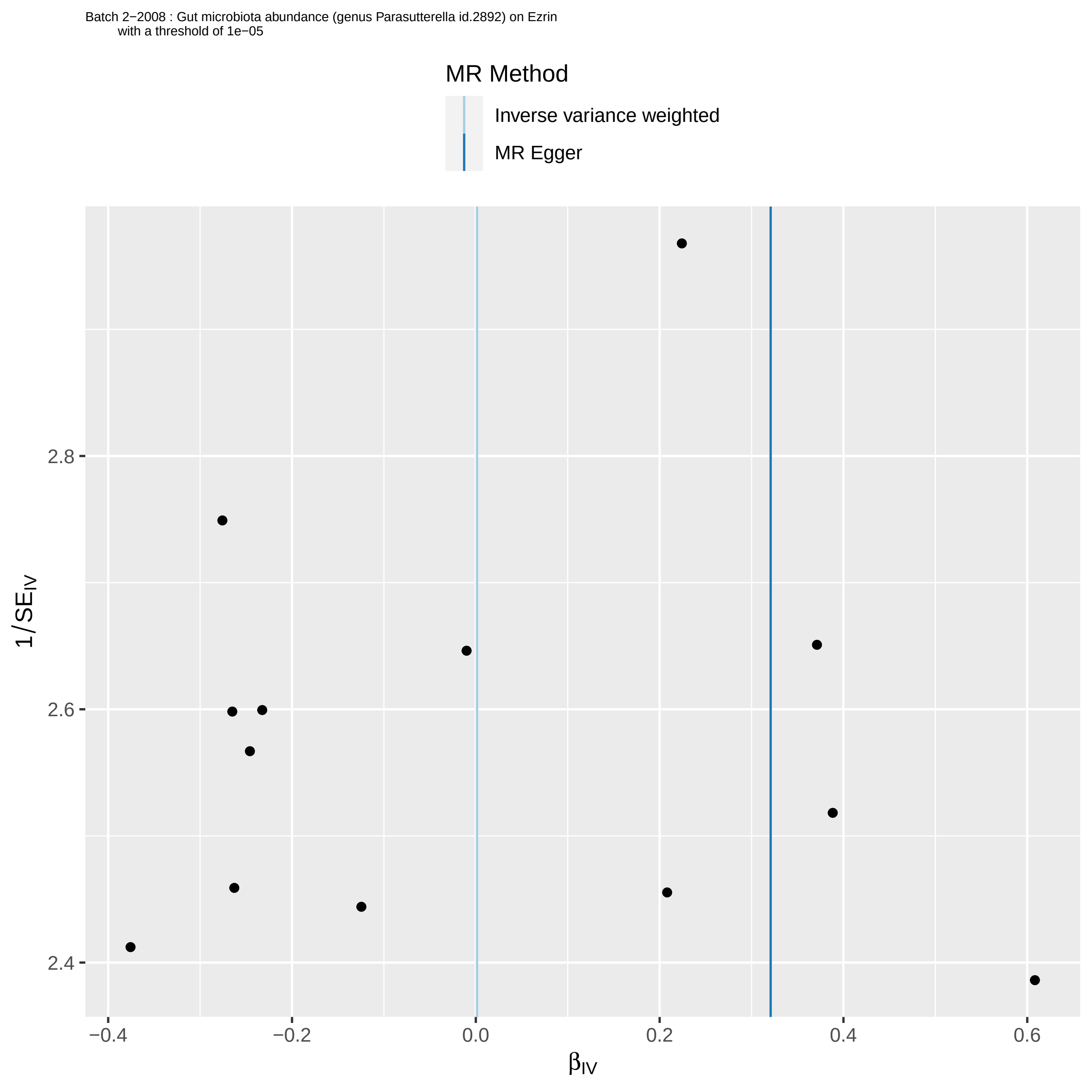

Batch 2−2008 : Gut microbiota abundance (genus Parasutterella id.2892) on Ezrin
with a threshold of 1e−05
MR Method
Inverse variance weighted
MR Egger
2.8
2.6
2.4
1/SE_IV
−0.4
−0.2
0.0
0.2
0.4
0.6
β_IV

Batch 2−2009 : Gut microbiota abundance (genus Catenibacterium id.2153) on Coagulation factor Xa
with a threshold of 1e−05

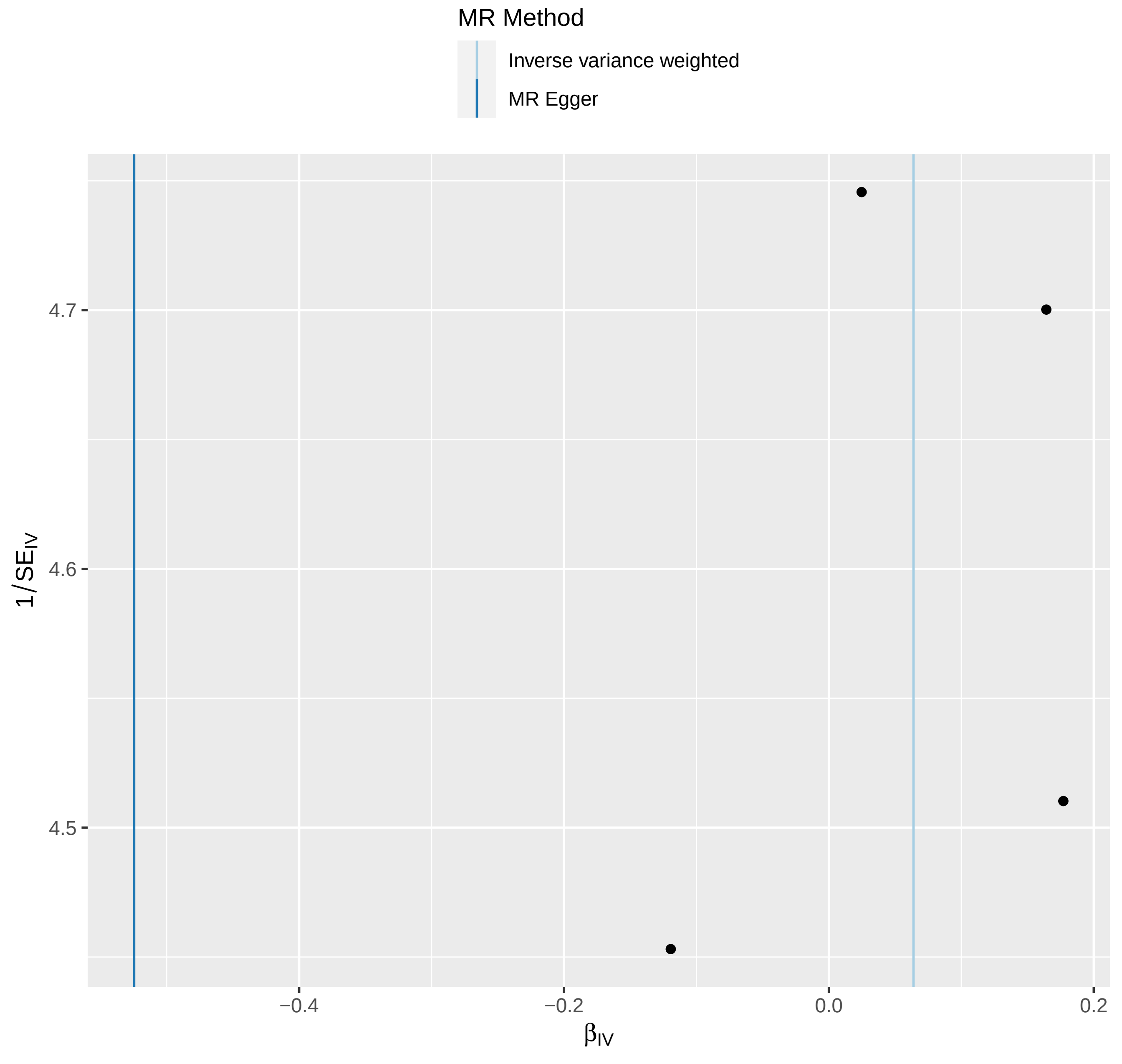

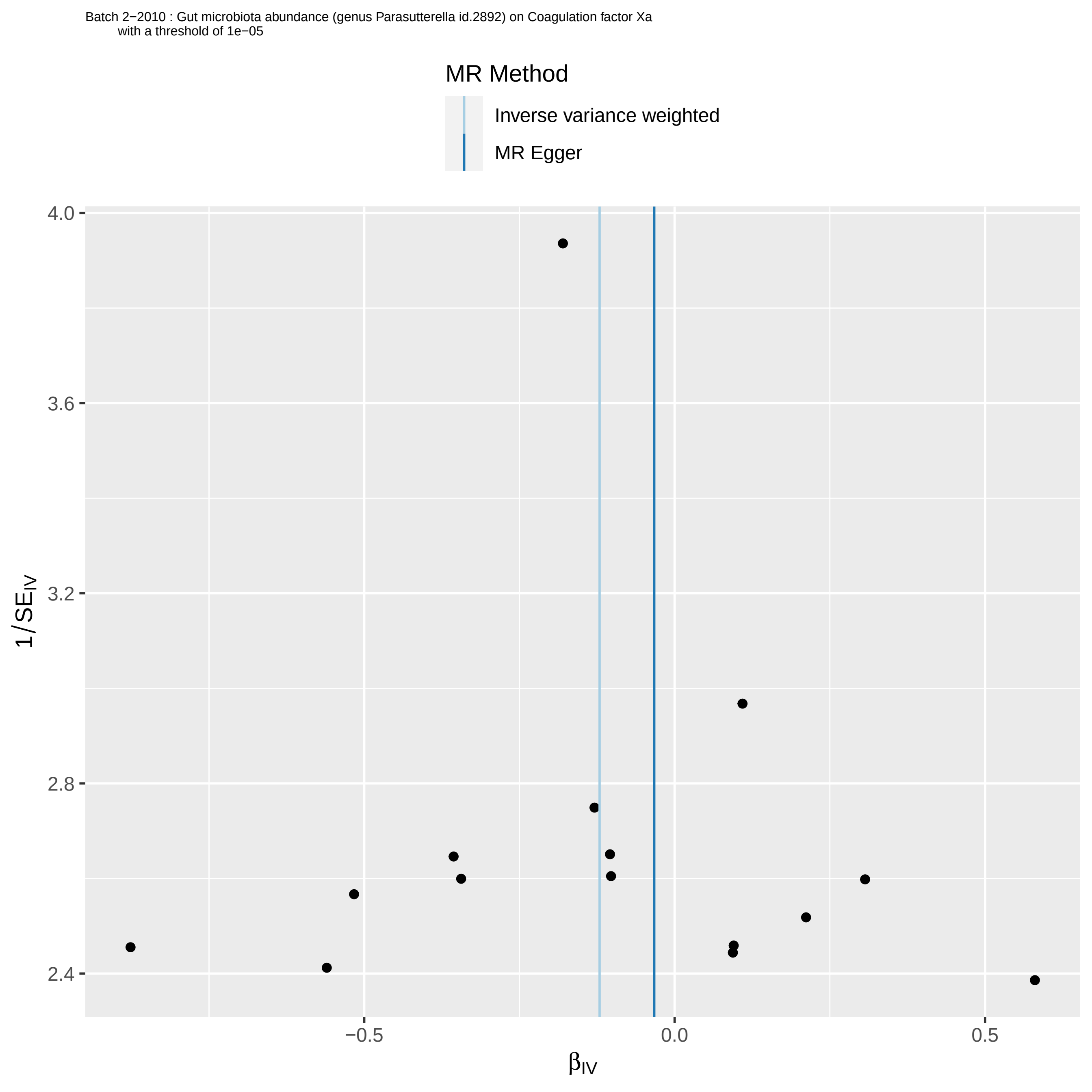
Batch 2−2010 : Gut microbiota abundance (genus Parasutterella id.2892) on Coagulation factor Xa
with a threshold of 1e−05
MR Method
Inverse variance weighted
MR Egger
4.0
3.6
3.2
2.8
2.4
1/SE_IV
−0.5
0.0
0.5
β_IV

Batch 2−2011 : Gut microbiota abundance (genus Catenibacterium id.2153) on Coagulation Factor X
with a threshold of 1e−05

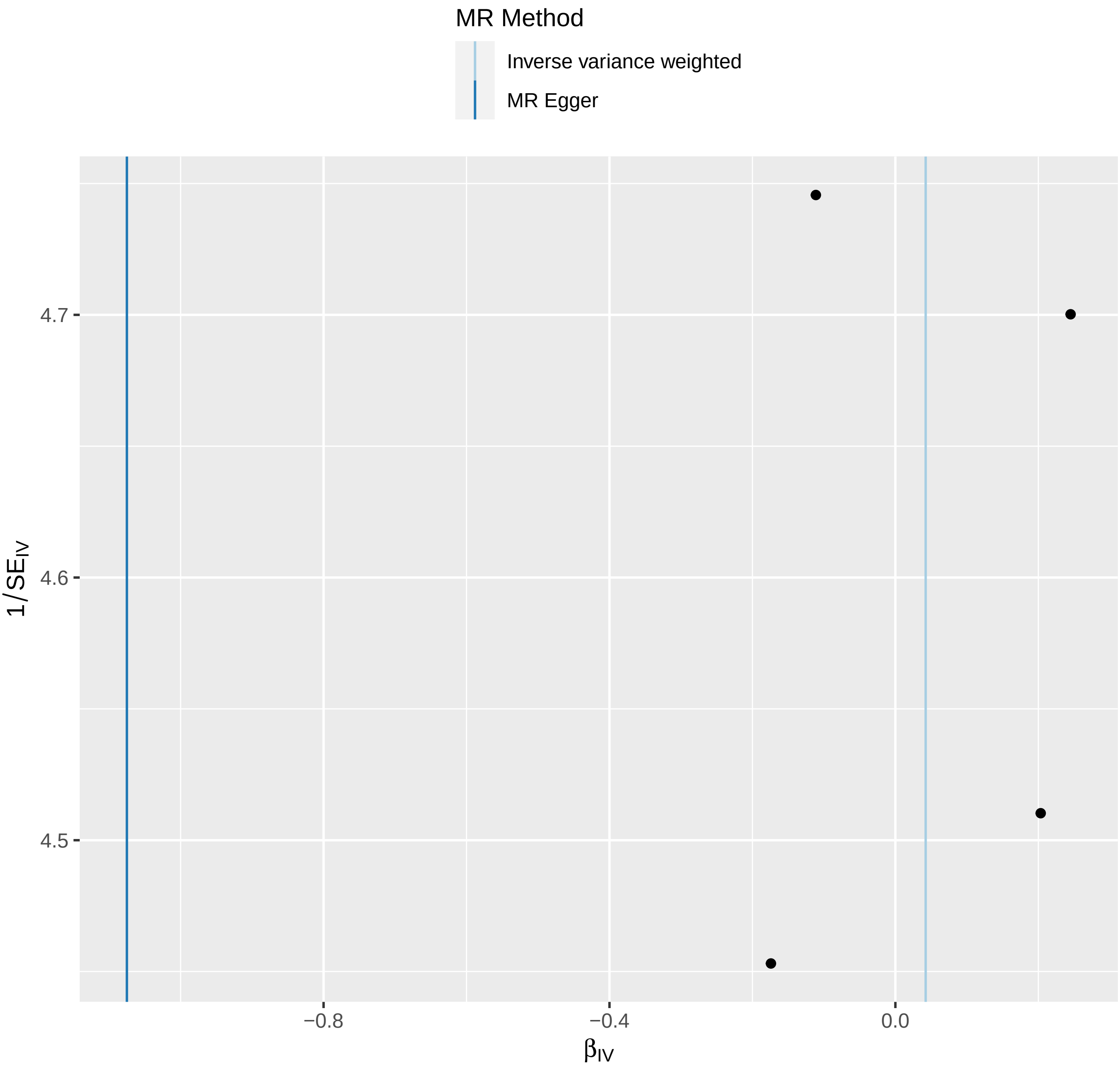

Batch 2−2012 : Gut microbiota abundance (genus Parasutterella id.2892) on Coagulation Factor X
with a threshold of 1e−05

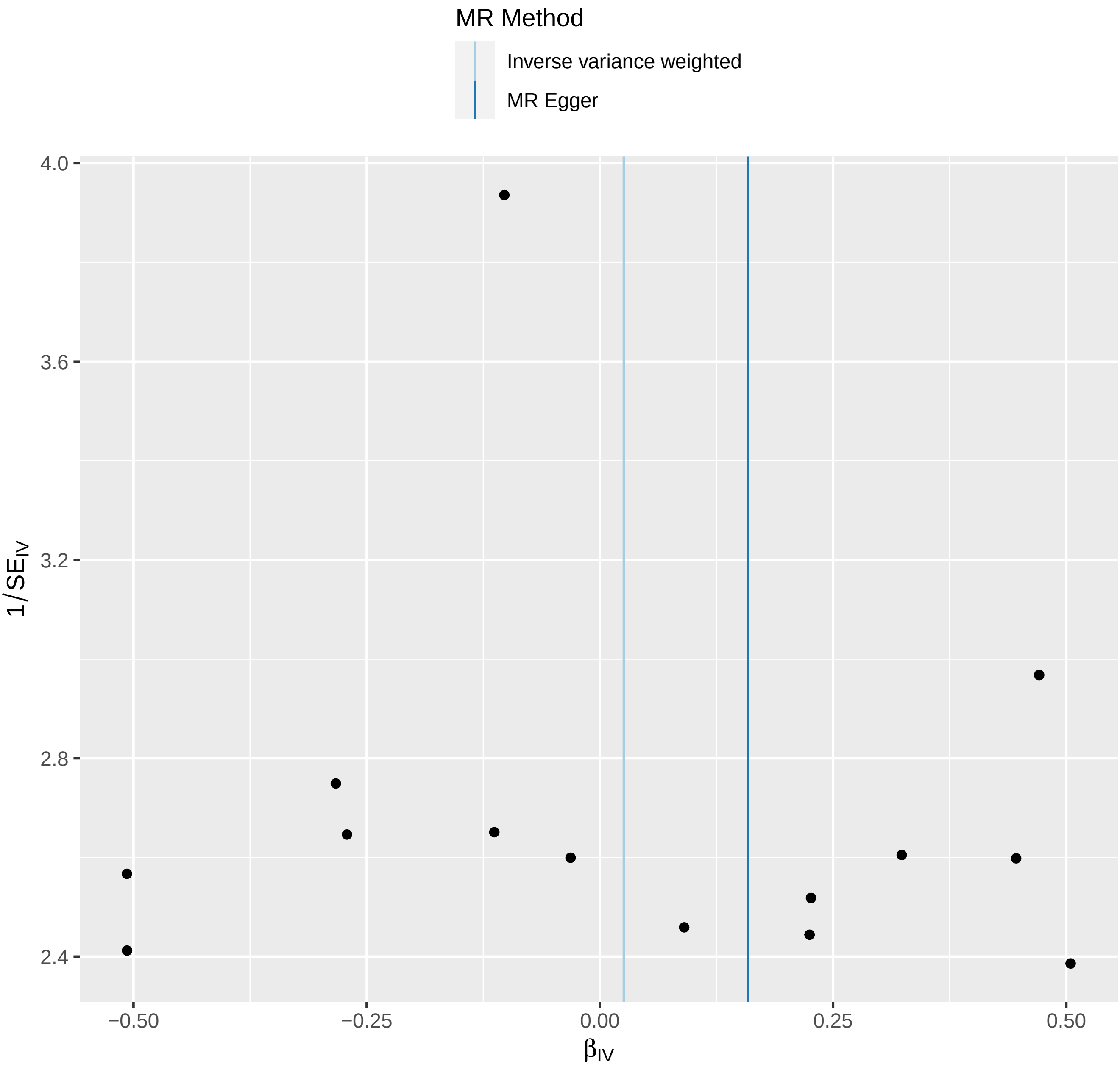

Batch 2−2013 : Gut microbiota abundance (genus Catenibacterium id.2153) on Prothrombin
with a threshold of 1e−05

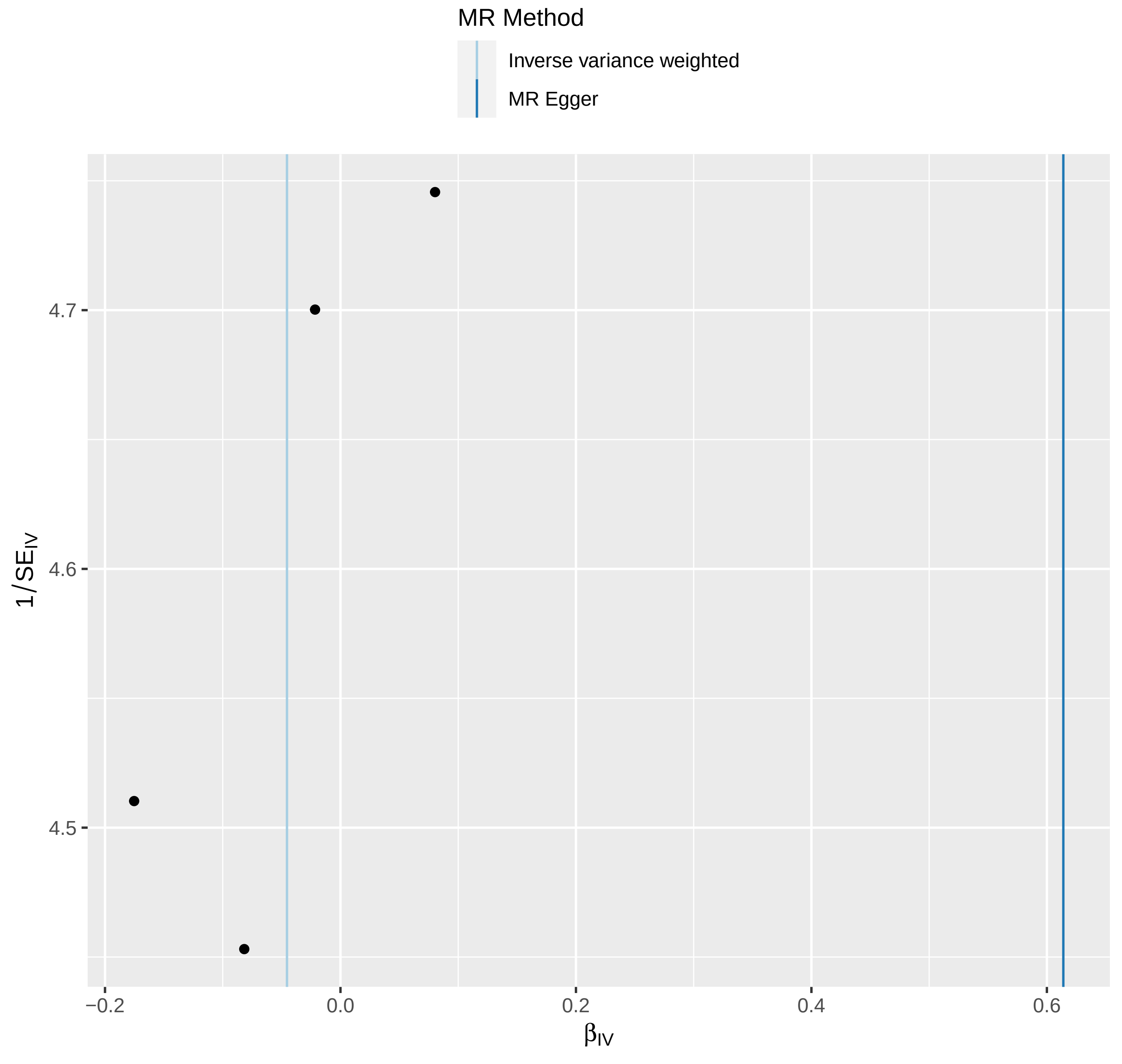

Batch 2−2014 : Gut microbiota abundance (genus Parasutterella id.2892) on Prothrombin
with a threshold of 1e−05

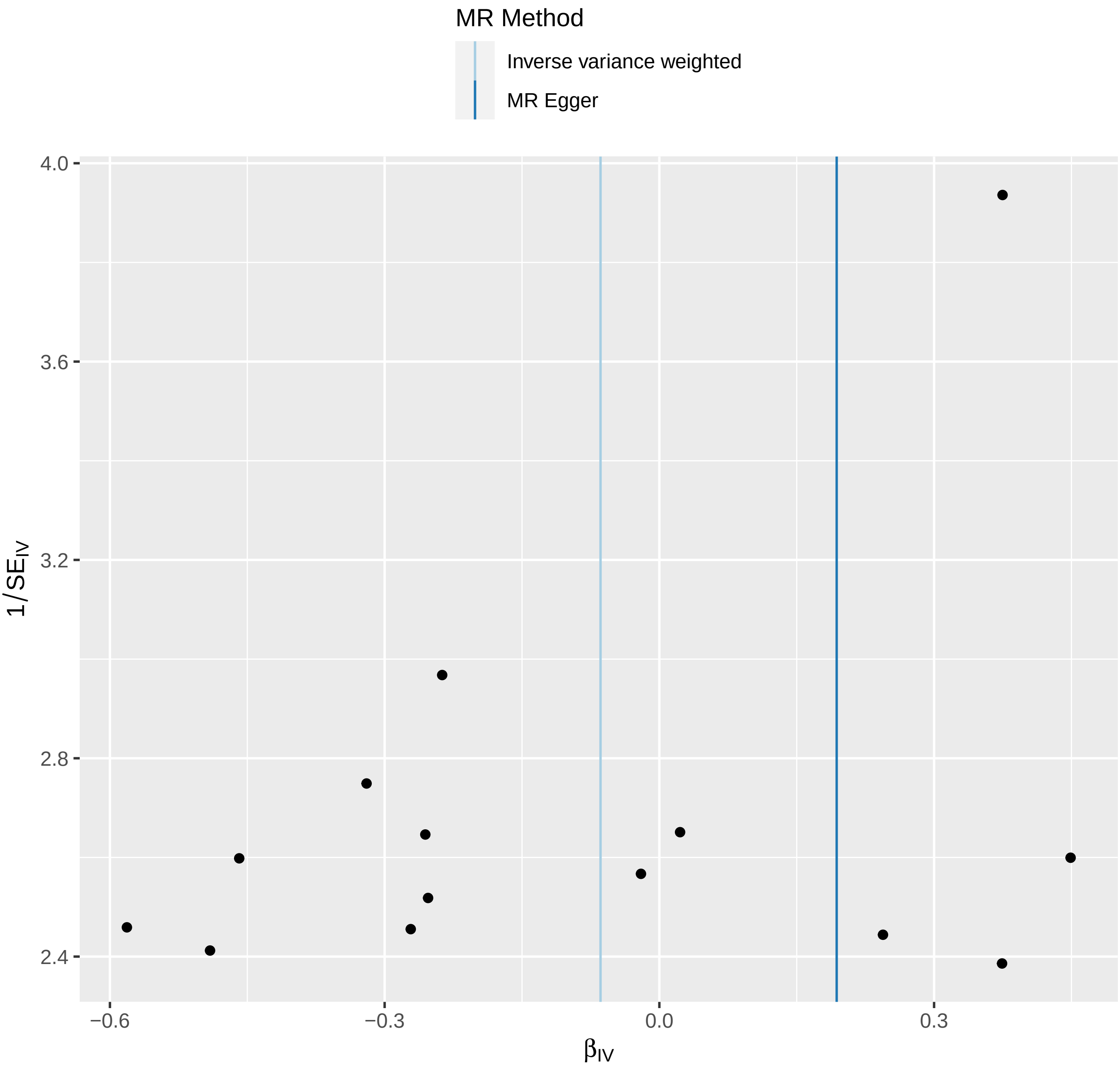

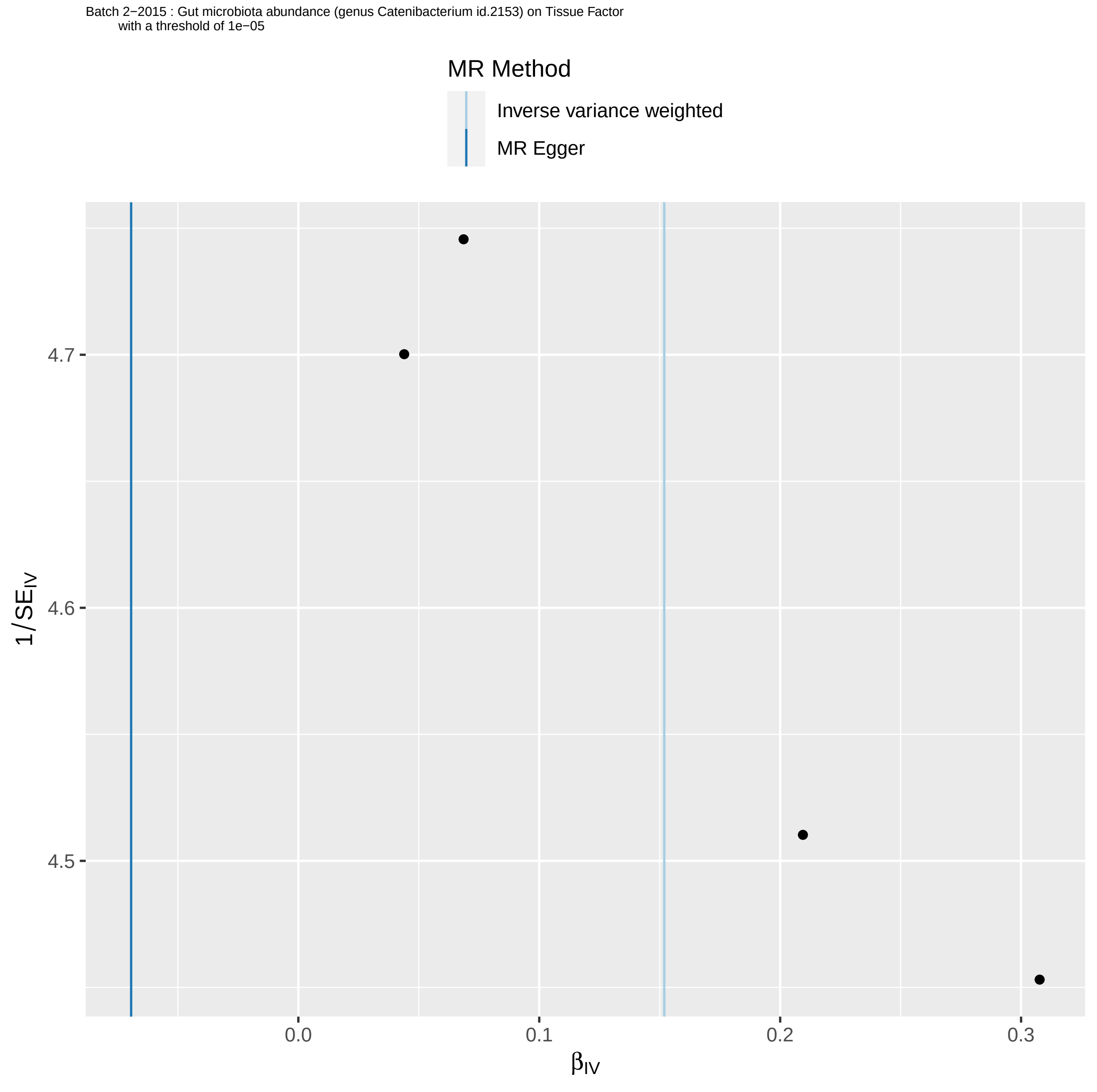
Batch 2−2015 : Gut microbiota abundance (genus Catenibacterium id.2153) on Tissue Factor
with a threshold of 1e−05
MR Method
Inverse variance weighted
MR Egger
4.7
4.6
4.5
1/SE_IV
0.0
0.1
0.2
0.3
β_IV

Batch 2−2016 : Gut microbiota abundance (genus Parasutterella id.2892) on Tissue Factor
with a threshold of 1e−05

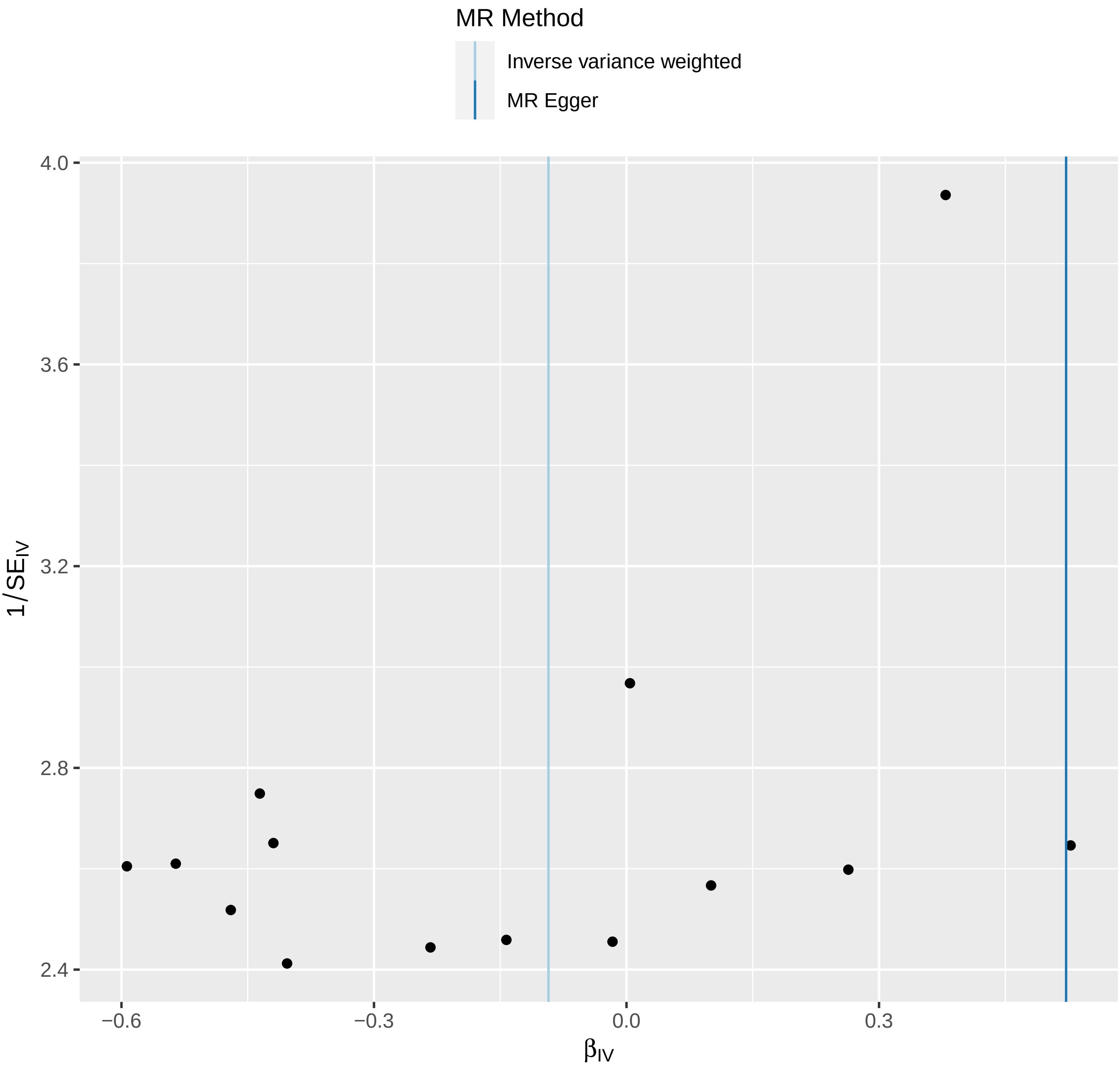

Batch 2−2017 : Gut microbiota abundance (genus Catenibacterium id.2153) on Coagulation Factor VIII
with a threshold of 1e−05

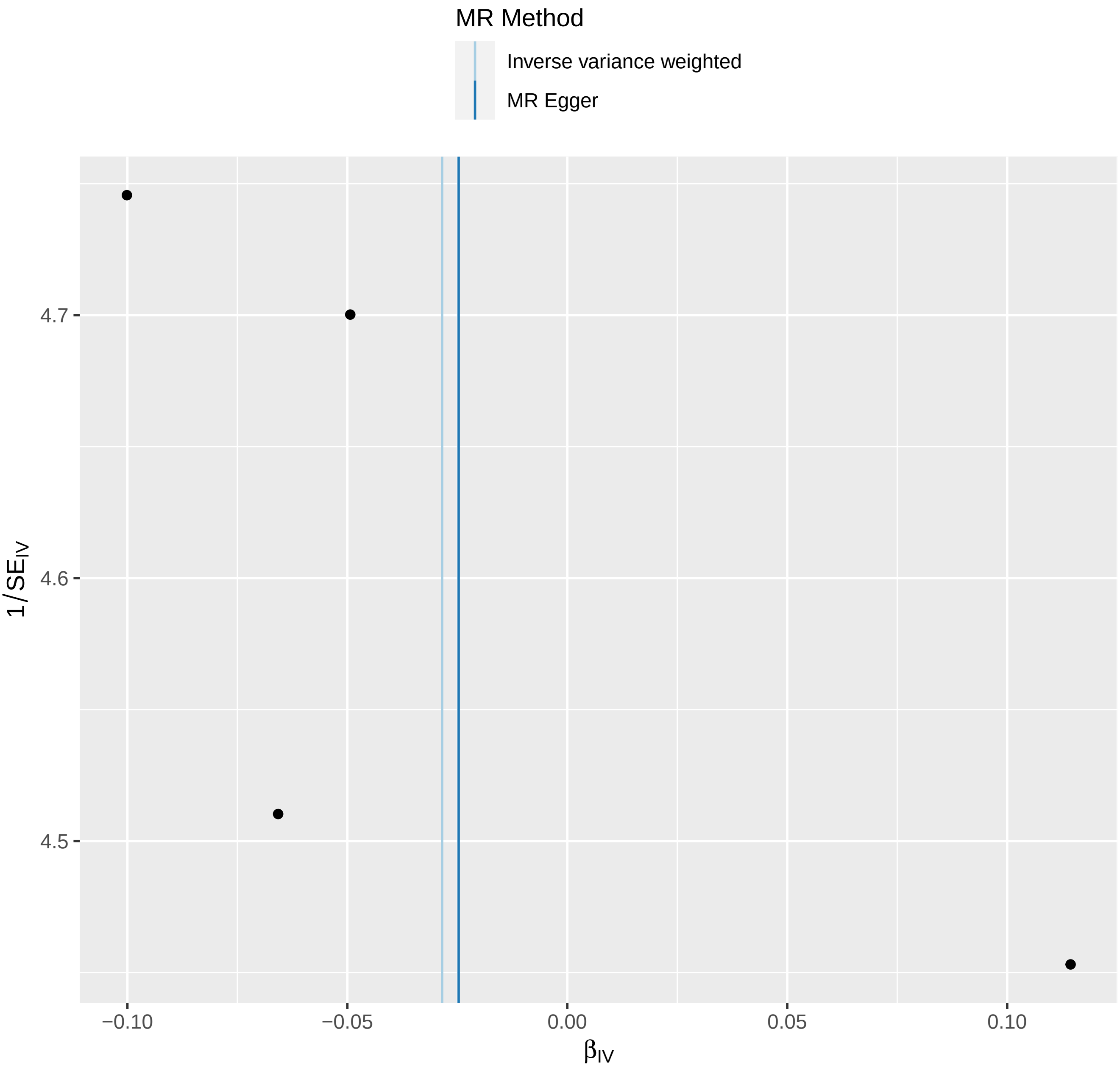

Batch 2−2018 : Gut microbiota abundance (genus Parasutterella id.2892) on Coagulation Factor VIII
with a threshold of 1e−05

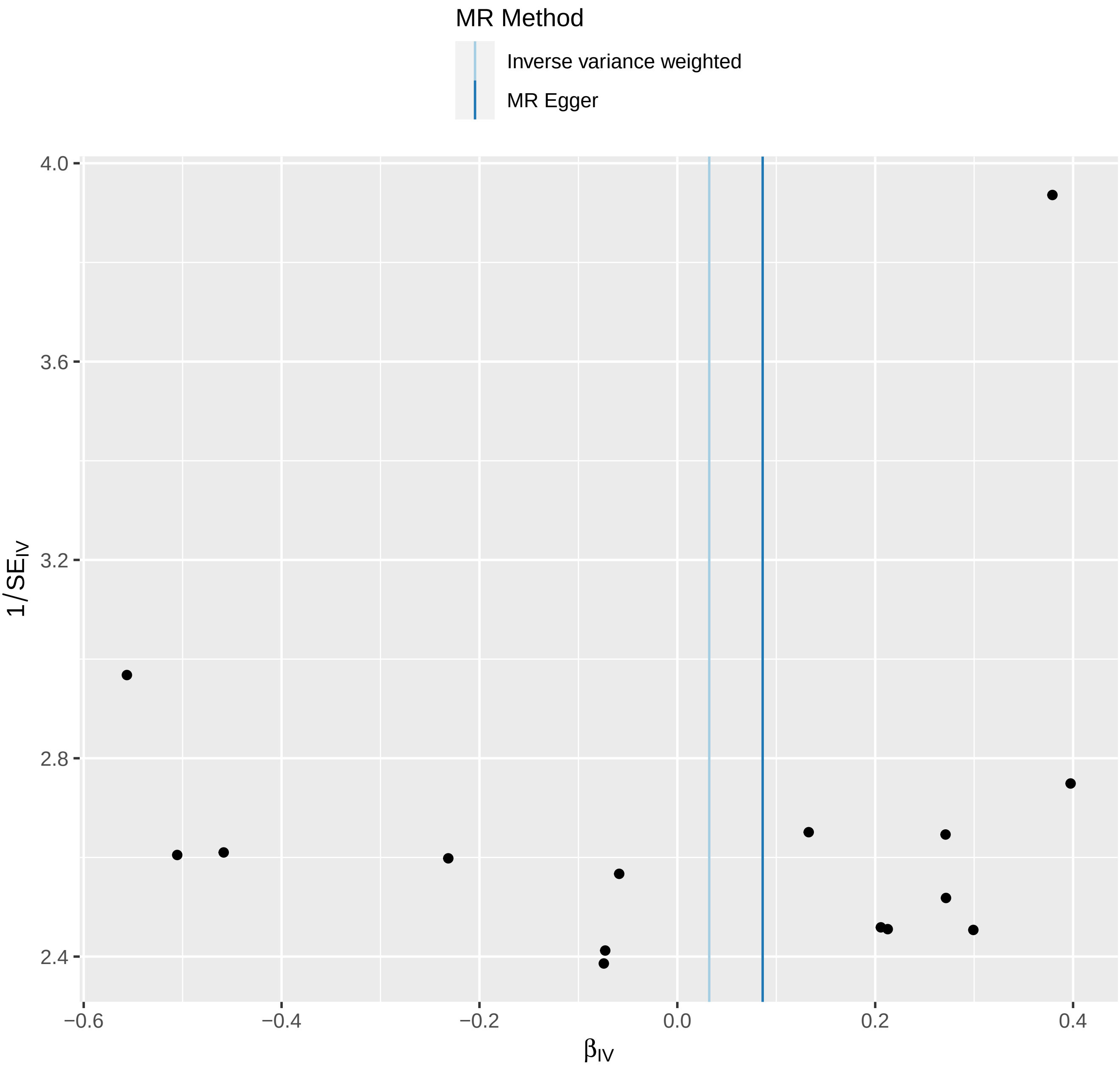

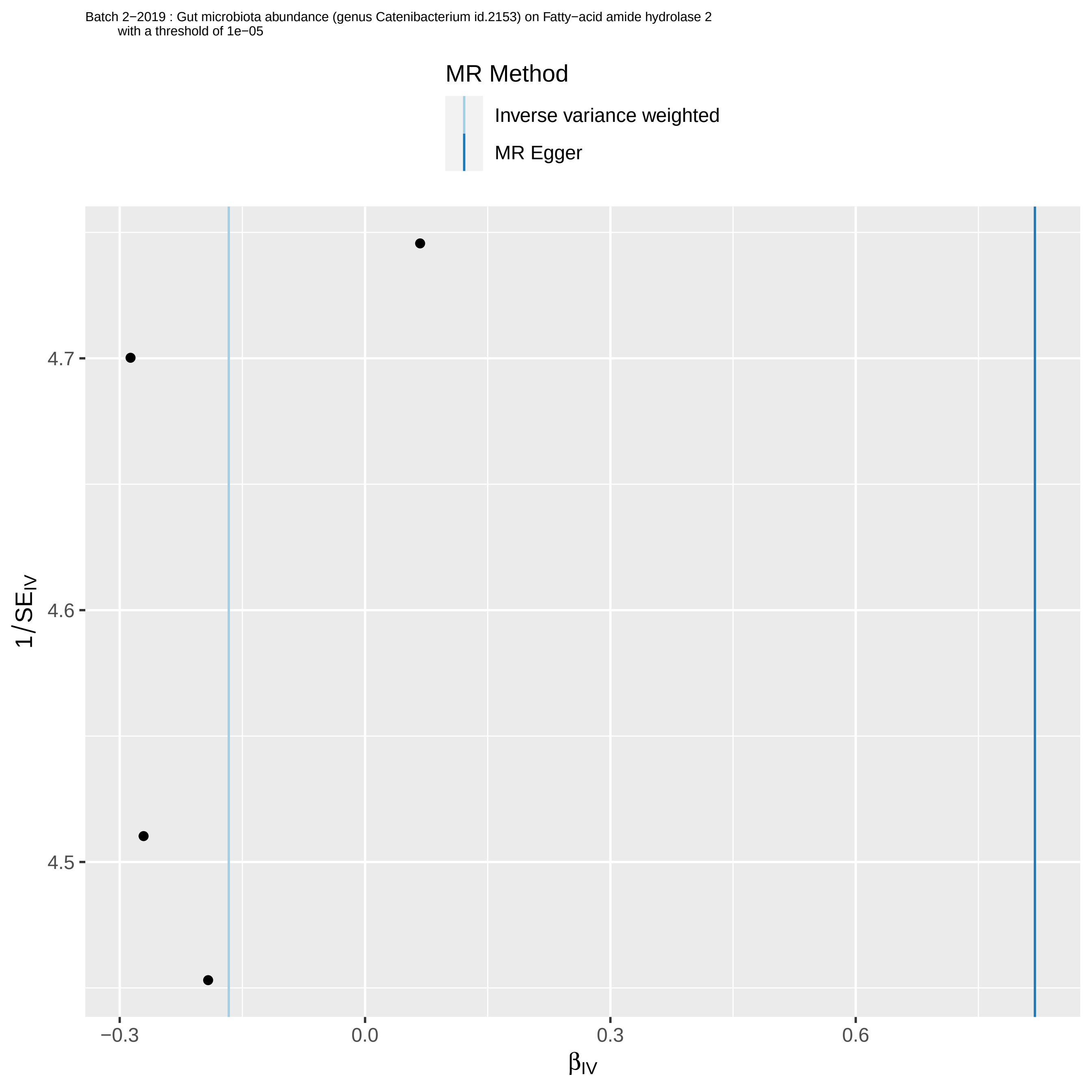
Batch 2−2019 : Gut microbiota abundance (genus Catenibacterium id.2153) on Fatty−acid amide hydrolase 2
with a threshold of 1e−05
MR Method
Inverse variance weighted
MR Egger
4.7
4.6
4.5
1/SE_IV
−0.3
0.0
0.3
0.6
β_IV

Batch 2−2020 : Gut microbiota abundance (genus Parasutterella id.2892) on Fatty−acid amide hydrolase 2
with a threshold of 1e−05

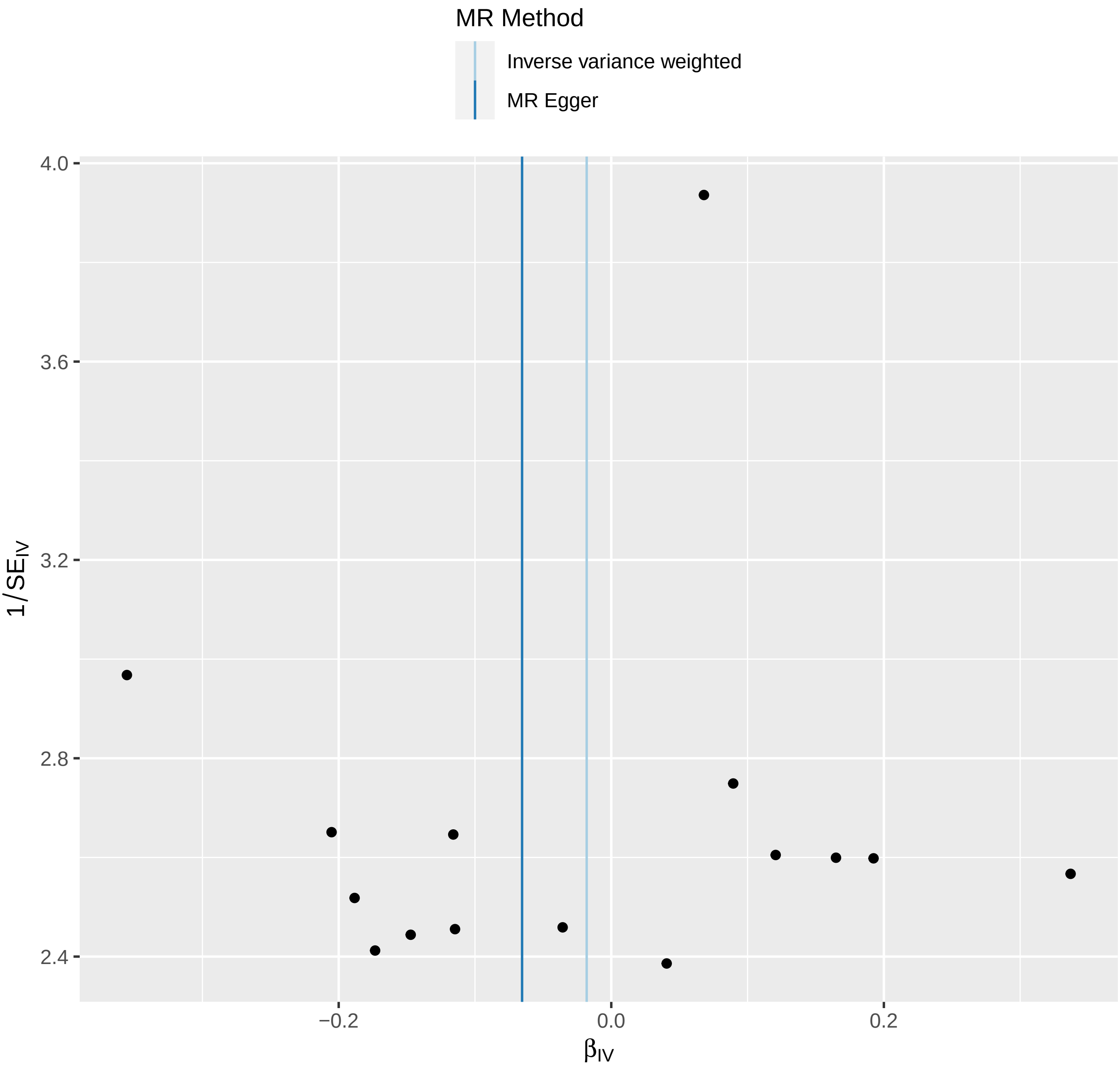

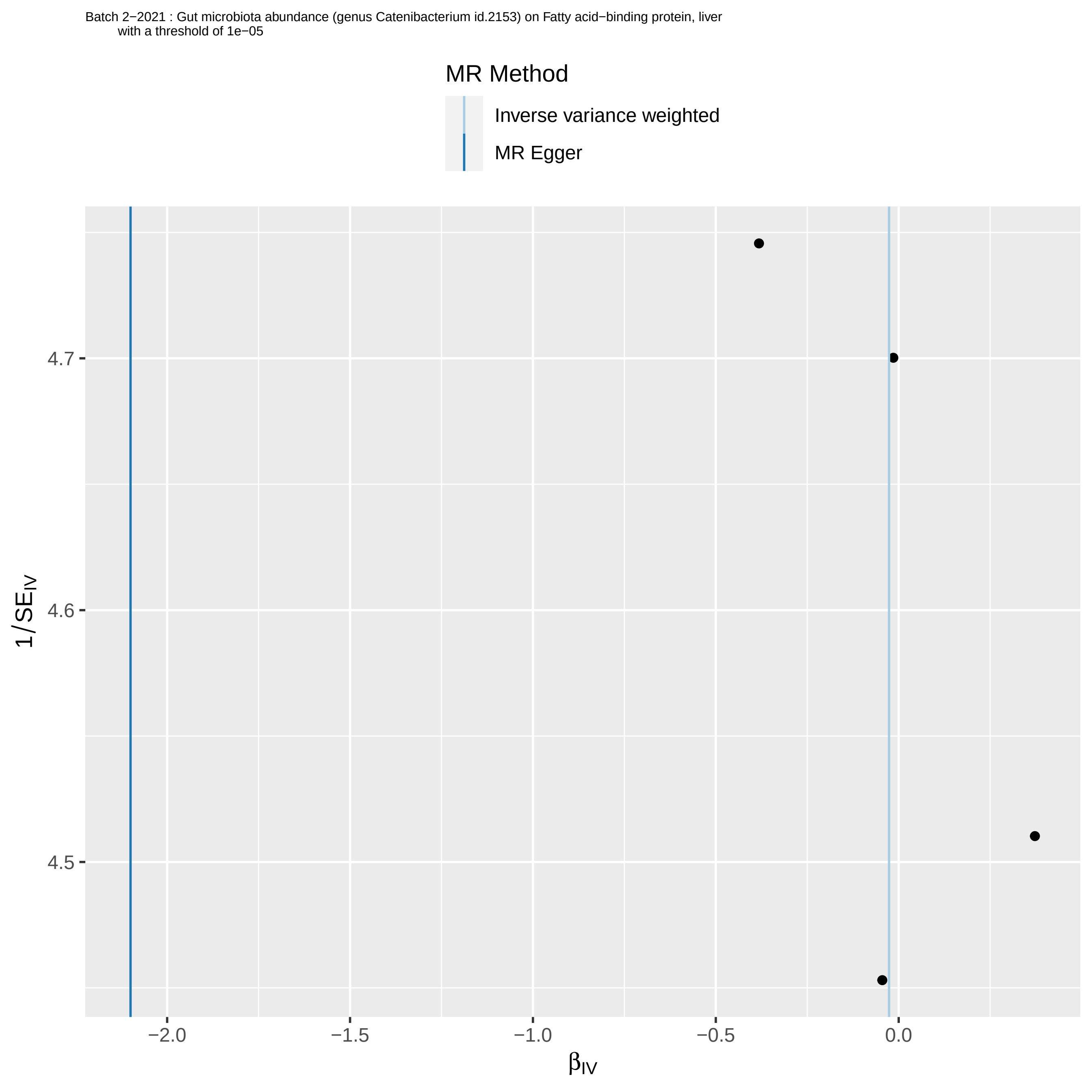
Batch 2−2021 : Gut microbiota abundance (genus Catenibacterium id.2153) on Fatty acid−binding protein, liver
with a threshold of 1e−05
MR Method
Inverse variance weighted
MR Egger
4.7
4.6
4.5
1/SE_IV
−2.0
−1.5
−1.0
−0.5
0.0
β_IV

Batch 2−2022 : Gut microbiota abundance (genus Parasutterella id.2892) on Fatty acid−binding protein, liver
with a threshold of 1e−05

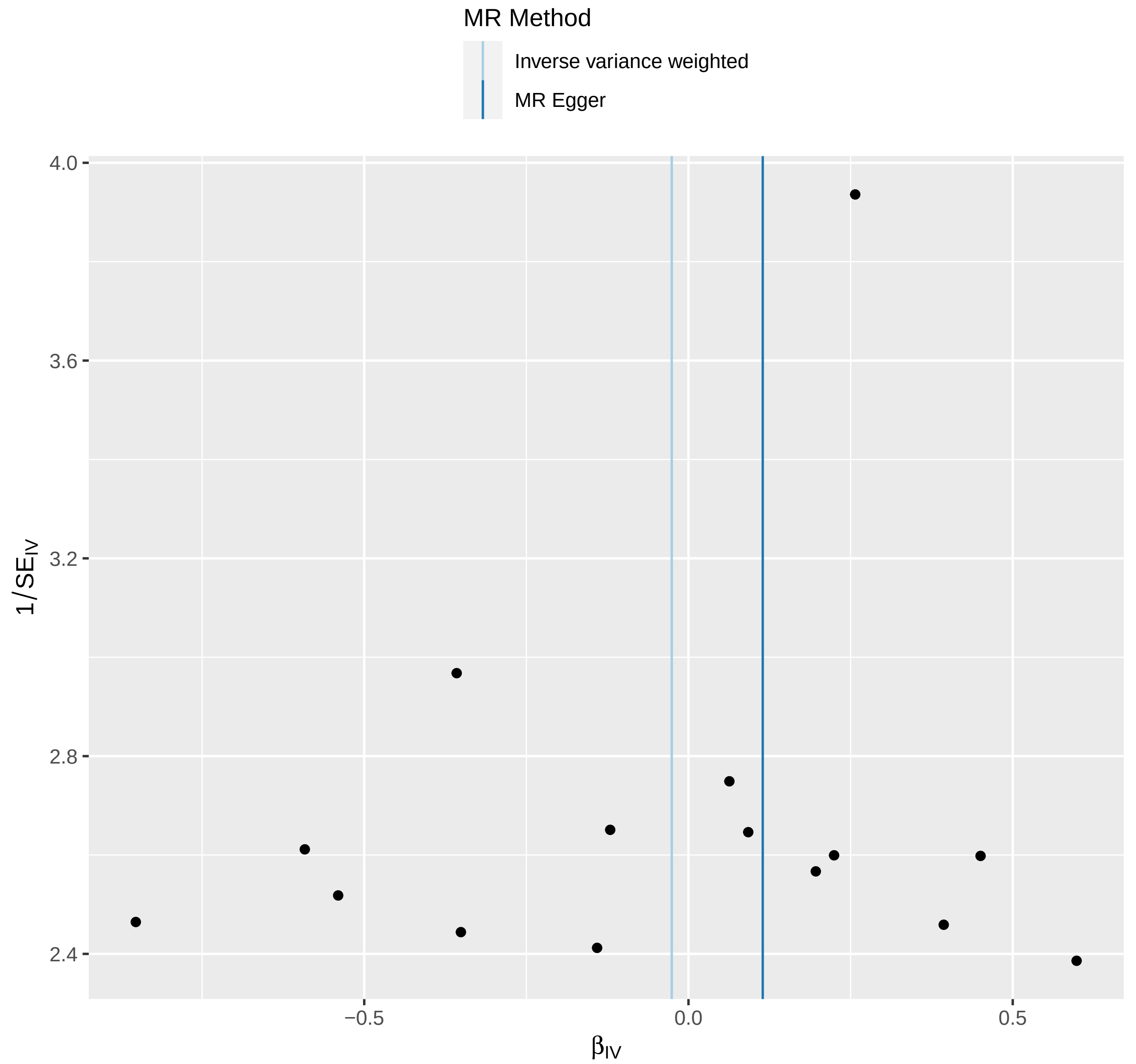

Batch 2−2023 : Gut microbiota abundance (genus Catenibacterium id.2153) on Fatty acid−binding protein, heart
with a threshold of 1e−05

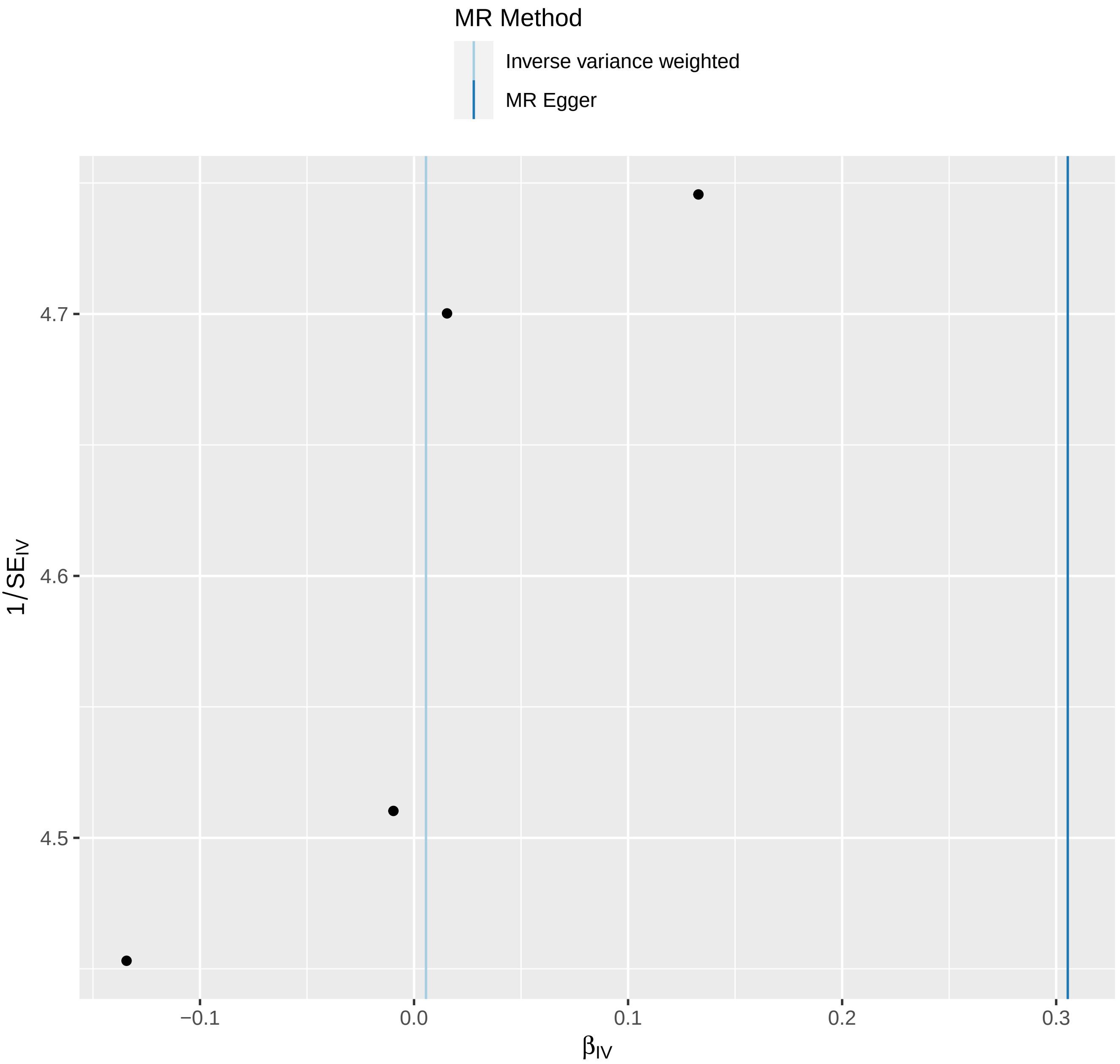

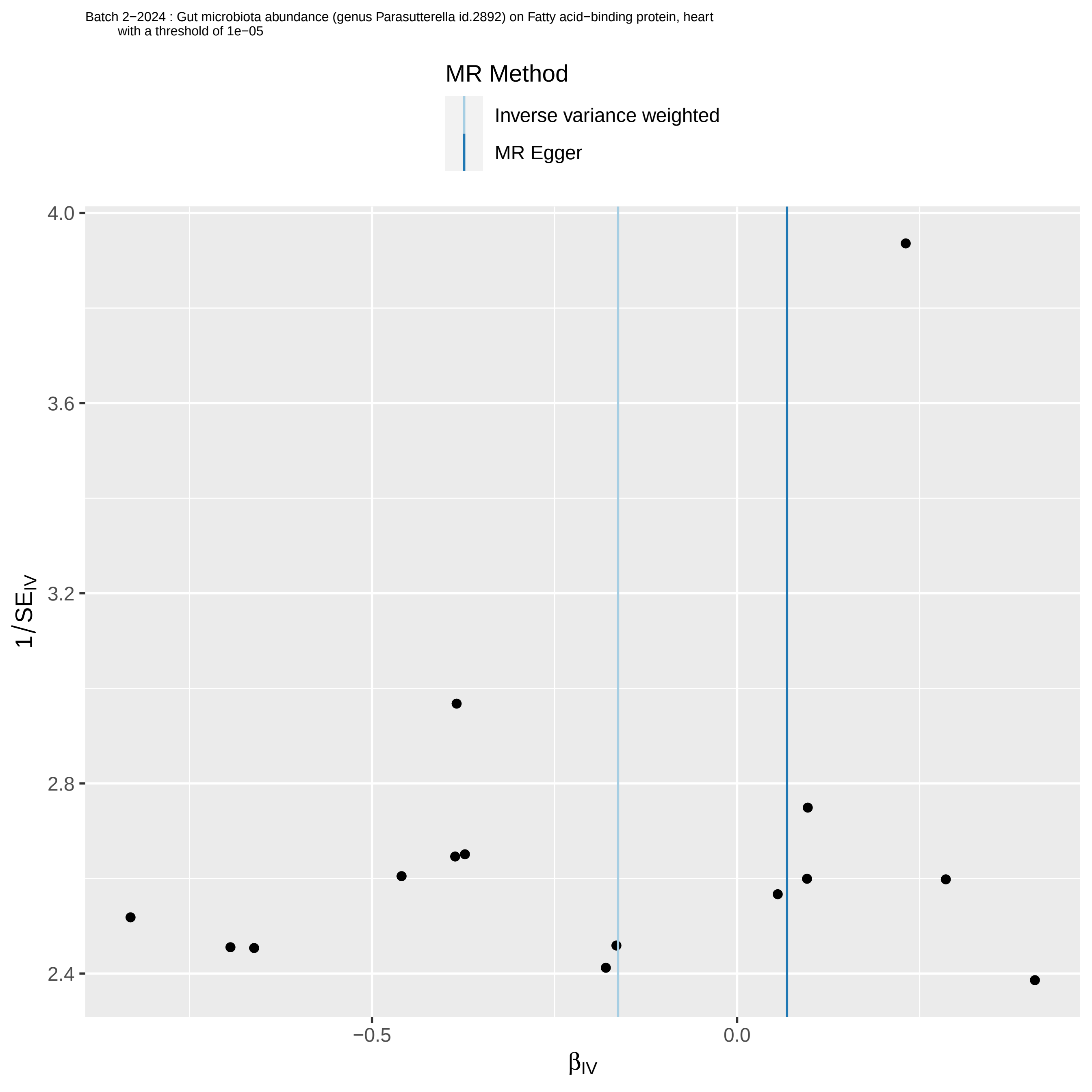
Batch 2−2024 : Gut microbiota abundance (genus Parasutterella id.2892) on Fatty acid−binding protein, heart
with a threshold of 1e−05
MR Method
Inverse variance weighted
MR Egger
4.0
3.6
3.2
2.8
2.4
1/SE_IV
−0.5
0.0
β_IV

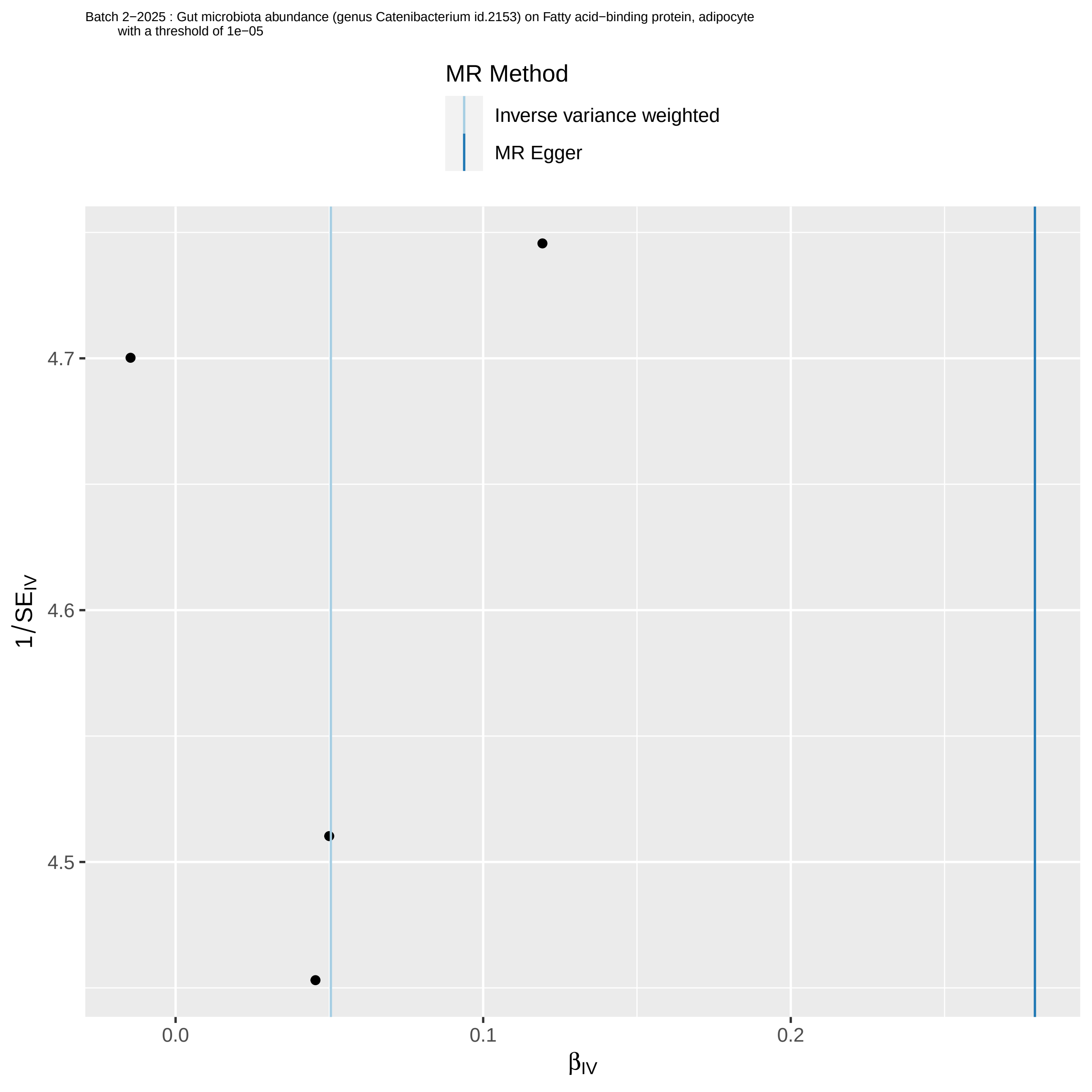
Batch 2−2025 : Gut microbiota abundance (genus Catenibacterium id.2153) on Fatty acid−binding protein, adipocyte
with a threshold of 1e−05
MR Method
Inverse variance weighted
MR Egger
4.7
4.6
4.5
1/SE_IV
0.0
0.1
0.2
β_IV

Batch 2−2026 : Gut microbiota abundance (genus Parasutterella id.2892) on Fatty acid−binding protein, adipocyte
with a threshold of 1e−05

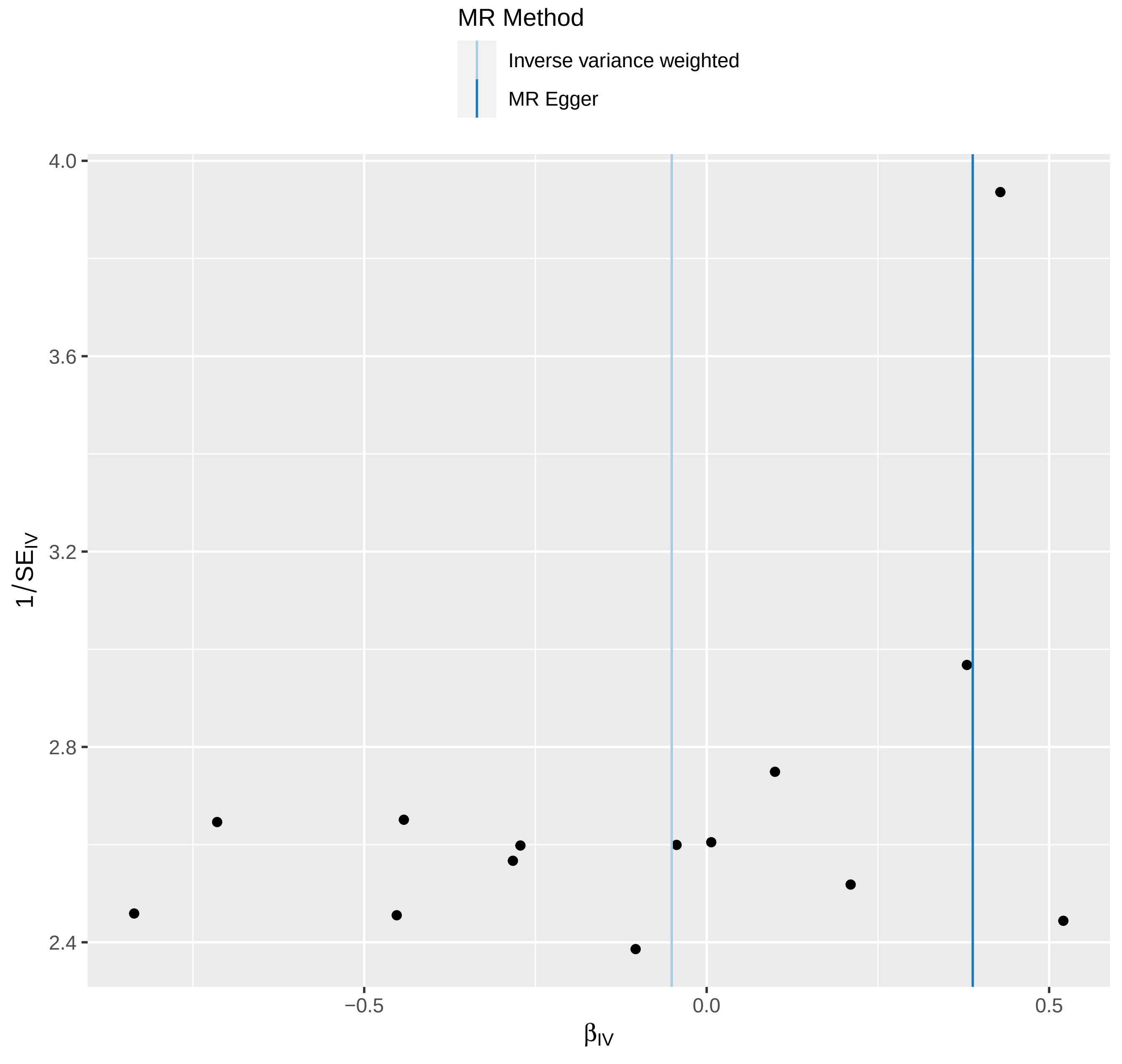

Batch 2−2027 : Gut microbiota abundance (genus Catenibacterium id.2153) on Fatty acid−binding protein, epidermal
with a threshold of 1e−05

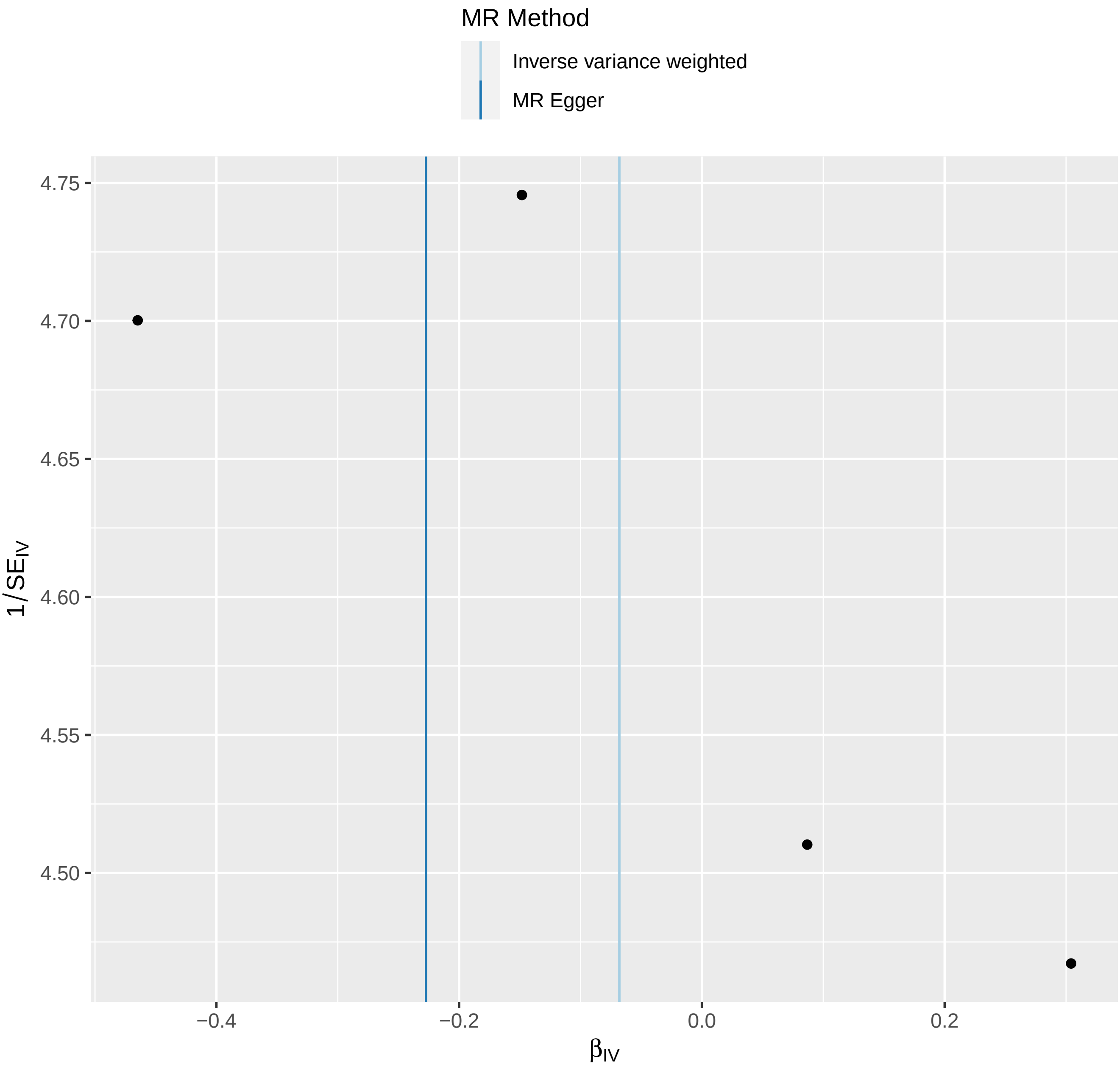

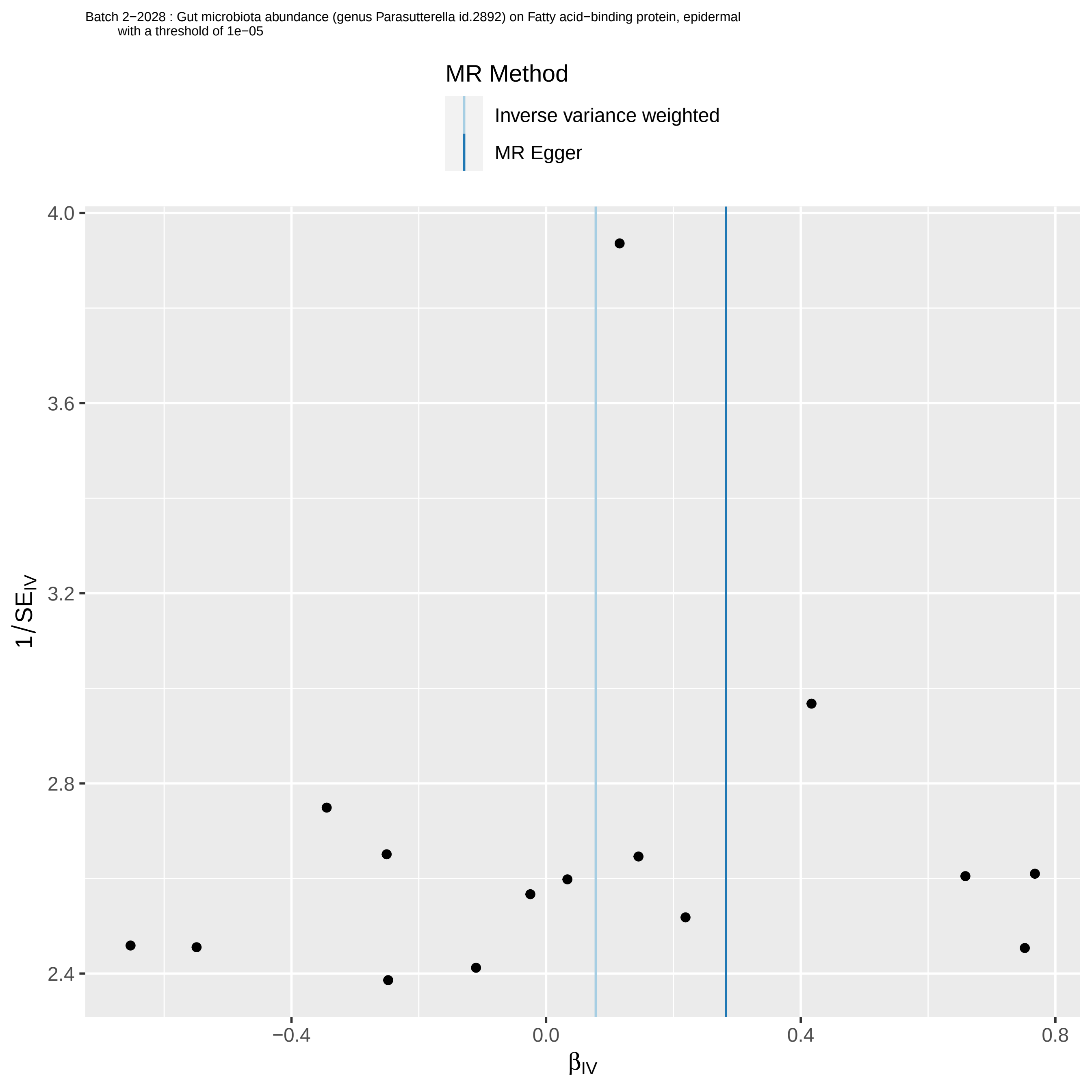

Batch 2−2028 : Gut microbiota abundance (genus Parasutterella id.2892) on Fatty acid−binding protein, epidermal
with a threshold of 1e−05
MR Method
Inverse variance weighted
MR Egger
4.0
3.6
3.2
2.8
2.4
1/SE_IV
−0.4
0.0
0.4
0.8
β_IV

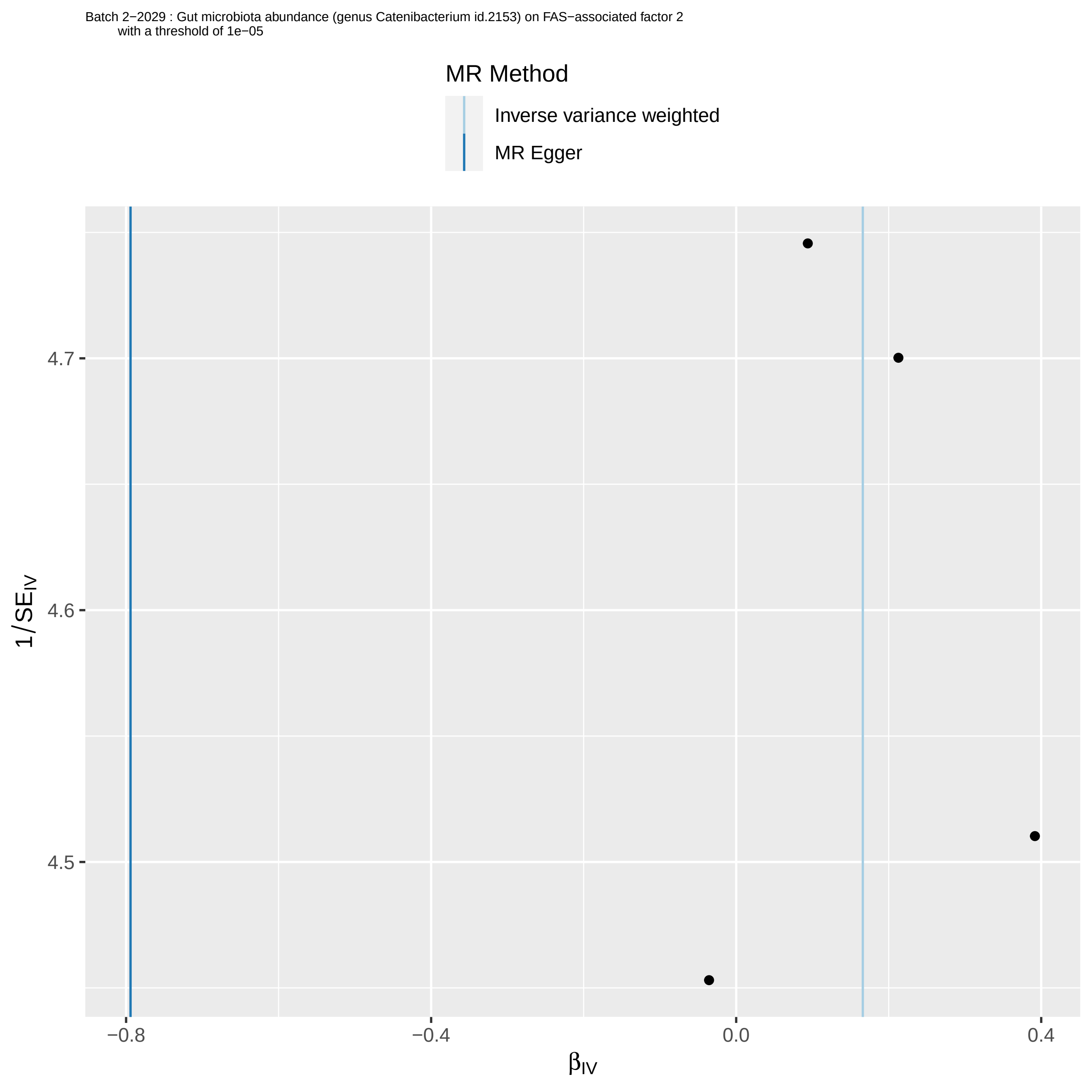

Batch 2−2029 : Gut microbiota abundance (genus Catenibacterium id.2153) on FAS−associated factor 2
with a threshold of 1e−05
MR Method
Inverse variance weighted
MR Egger
4.7
4.6
4.5
1/SE_IV
−0.8
−0.4
0.0
0.4
β_IV

Batch 2−2030 : Gut microbiota abundance (genus Parasutterella id.2892) on FAS−associated factor 2
with a threshold of 1e−05

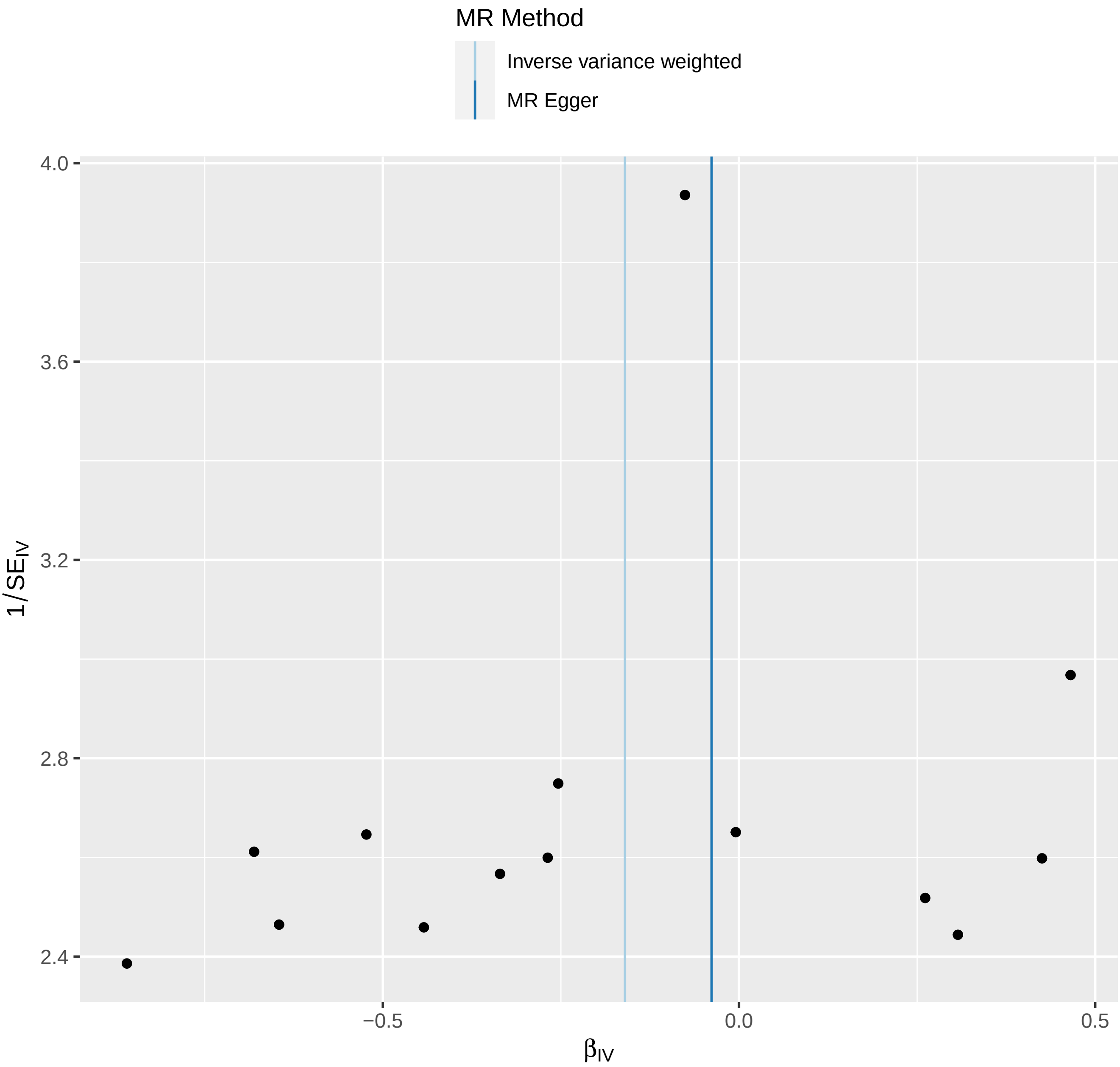

Batch 2−2031 : Gut microbiota abundance (genus Catenibacterium id.2153) on FAS−associated factor 2
with a threshold of 1e−05

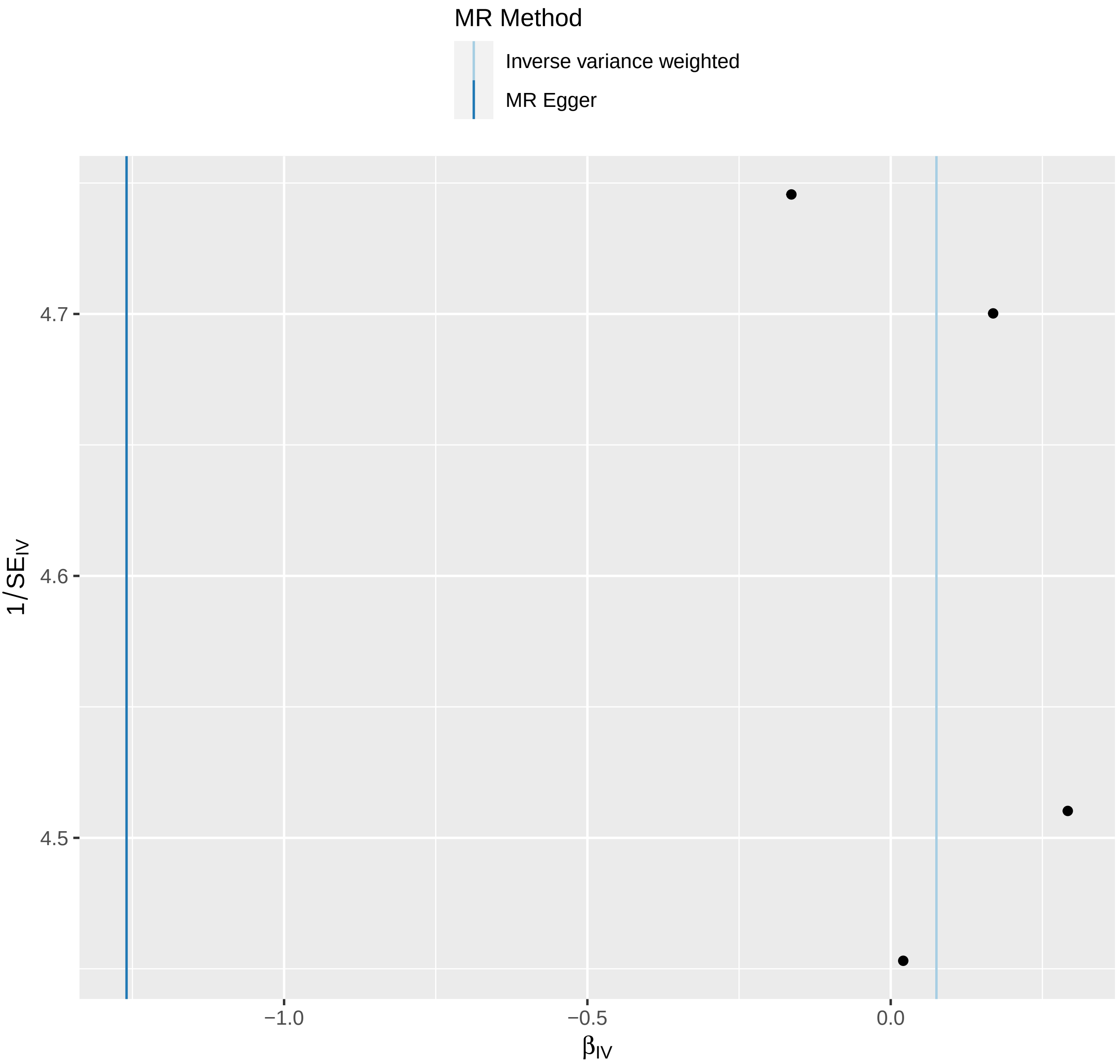

Batch 2−2032 : Gut microbiota abundance (genus Parasutterella id.2892) on FAS−associated factor 2
with a threshold of 1e−05

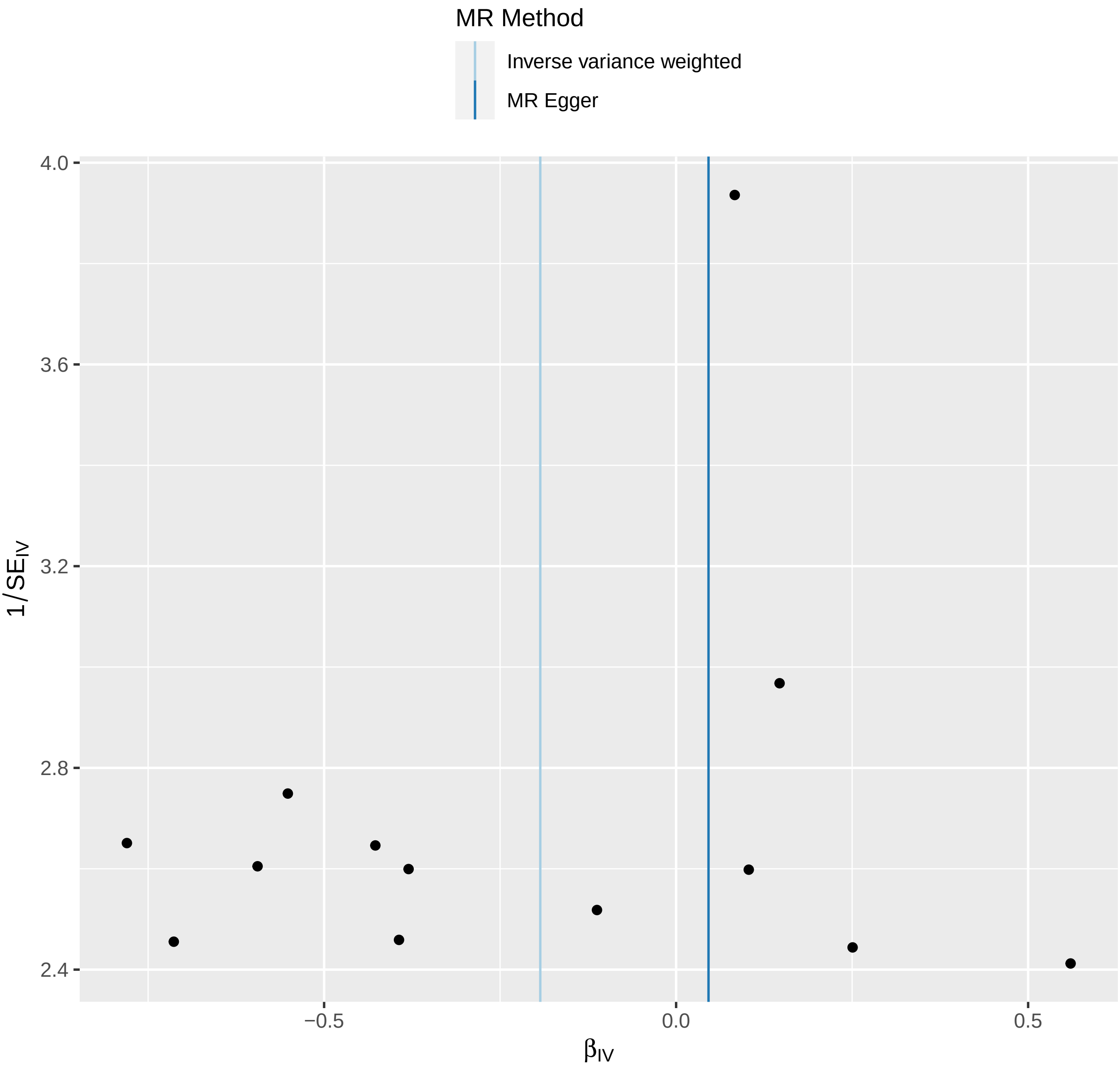

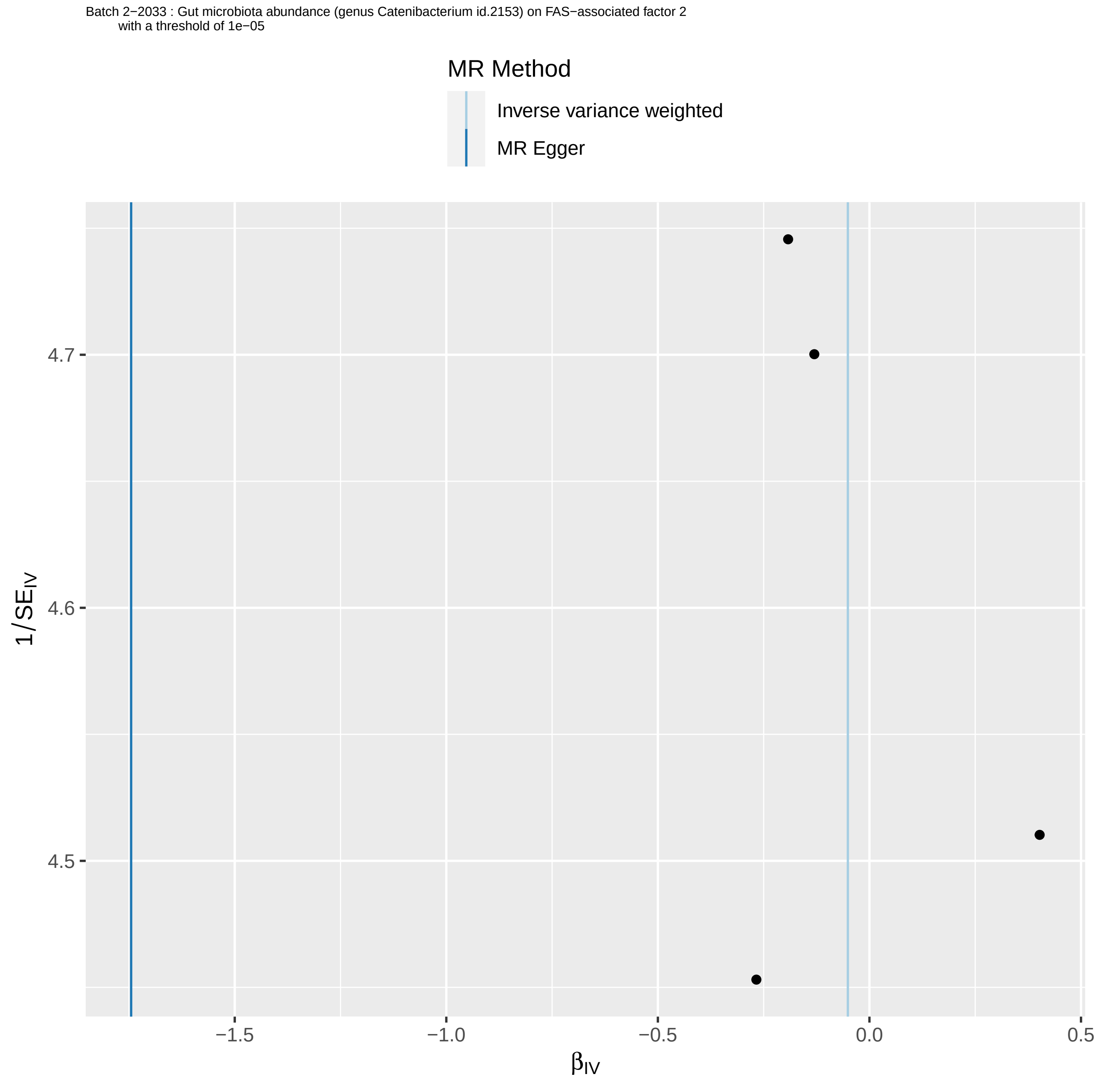
Batch 2−2033 : Gut microbiota abundance (genus Catenibacterium id.2153) on FAS−associated factor 2
with a threshold of 1e−05
MR Method
Inverse variance weighted
MR Egger
4.7
4.6
4.5
1/SE_IV
−1.5
−1.0
−0.5
0.0
0.5
β_IV

Batch 2−2034 : Gut microbiota abundance (genus Parasutterella id.2892) on FAS−associated factor 2
with a threshold of 1e−05

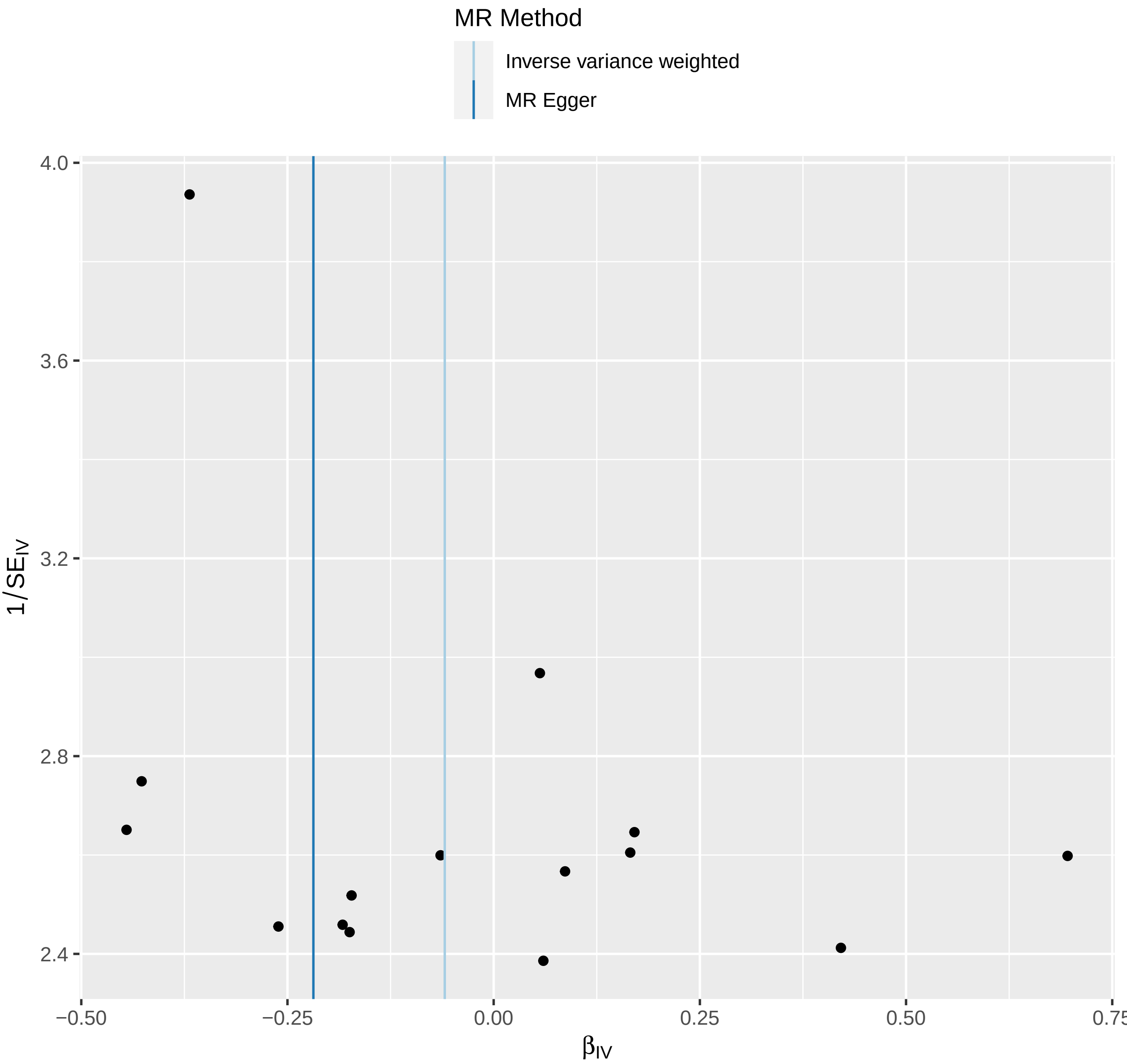

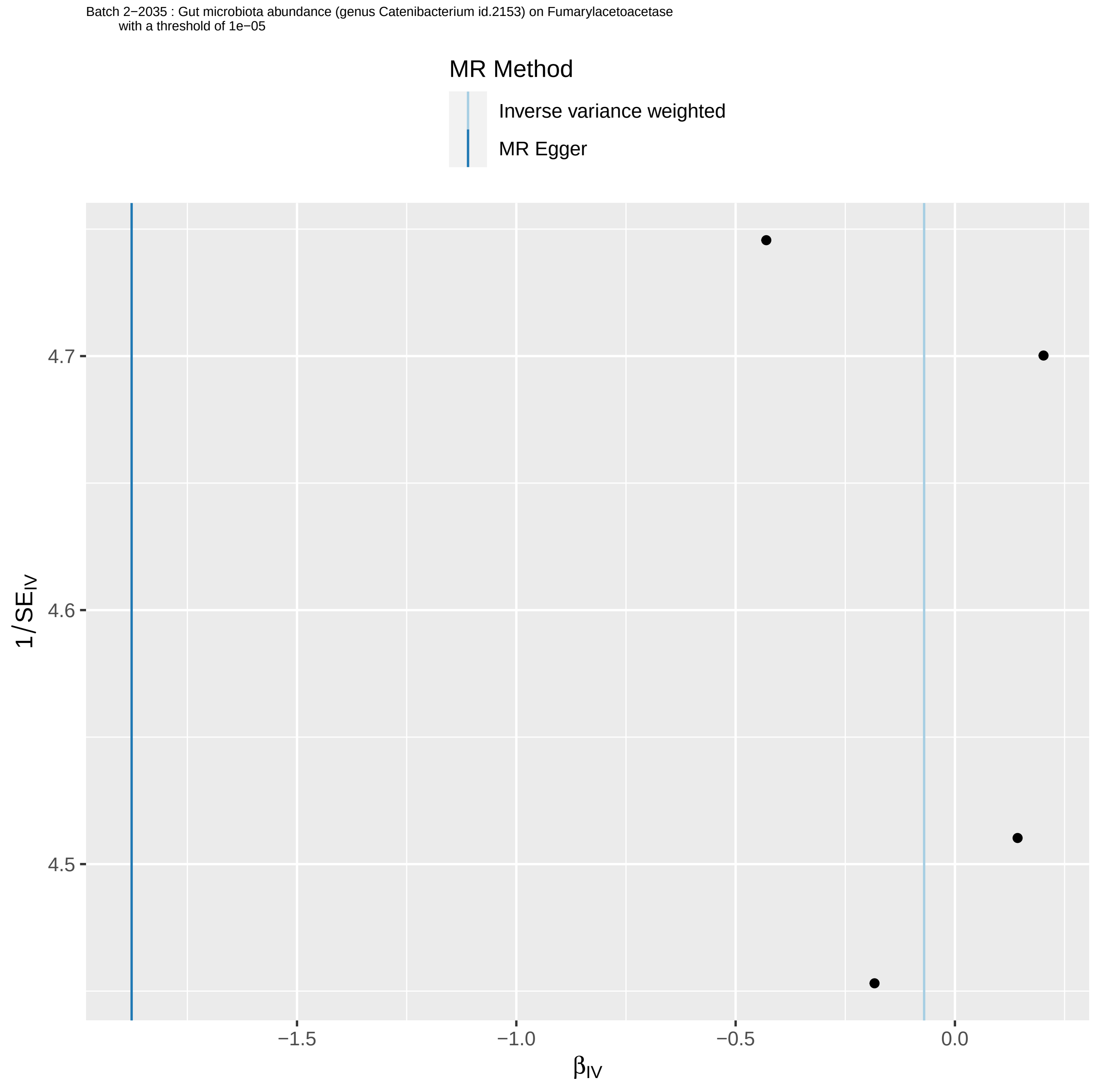

Batch 2−2035 : Gut microbiota abundance (genus Catenibacterium id.2153) on Fumarylacetoacetase
with a threshold of 1e−05
MR Method
Inverse variance weighted
MR Egger
4.7
4.6
4.5
1/SE_IV
−1.5
−1.0
−0.5
0.0
β_IV

Batch 2−2036 : Gut microbiota abundance (genus Parasutterella id.2892) on Fumarylacetoacetase
with a threshold of 1e−05

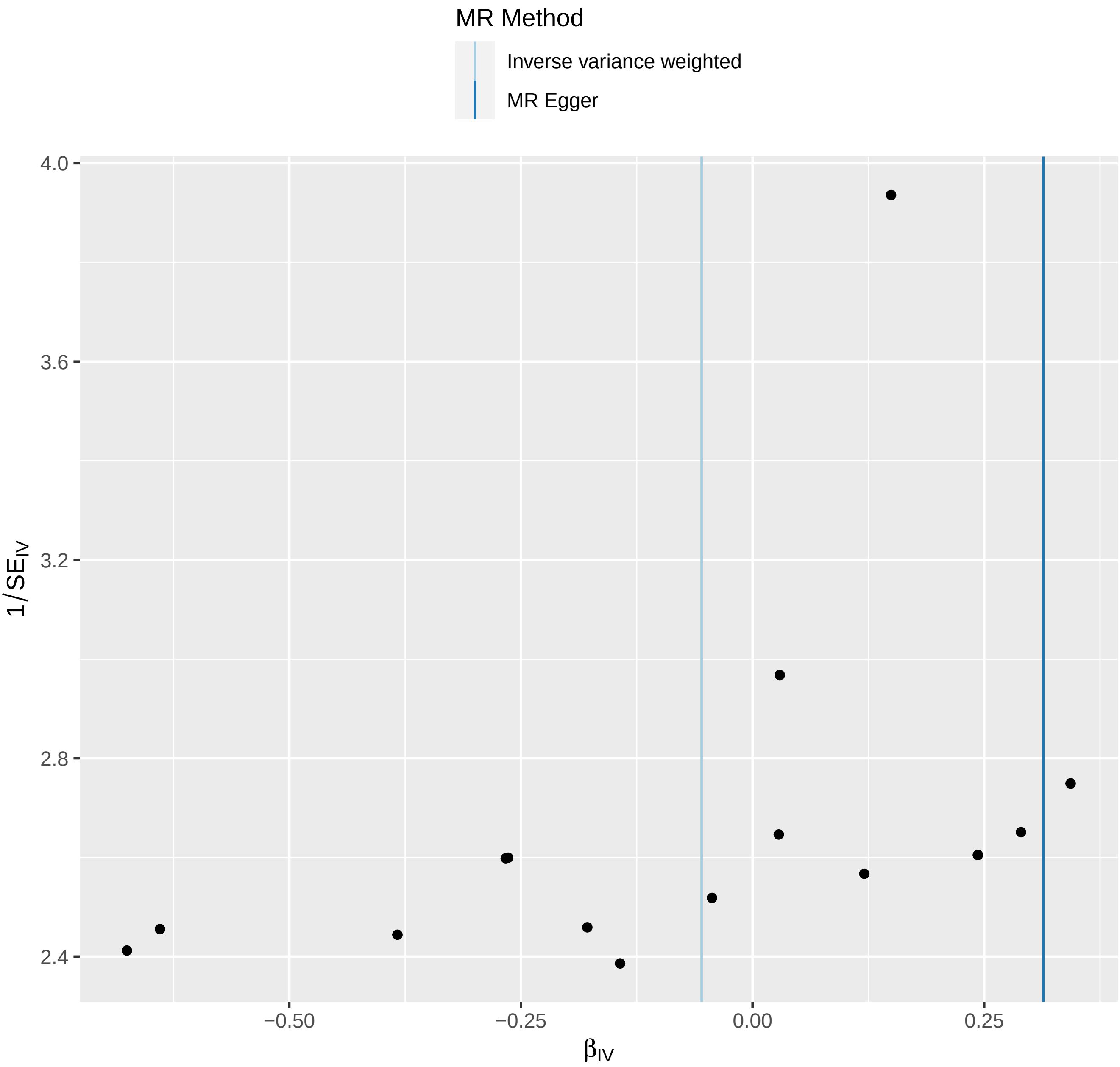

Batch 2−2037 : Gut microbiota abundance (genus Catenibacterium id.2153) on Fas apoptotic inhibitory molecule 3
with a threshold of 1e−05

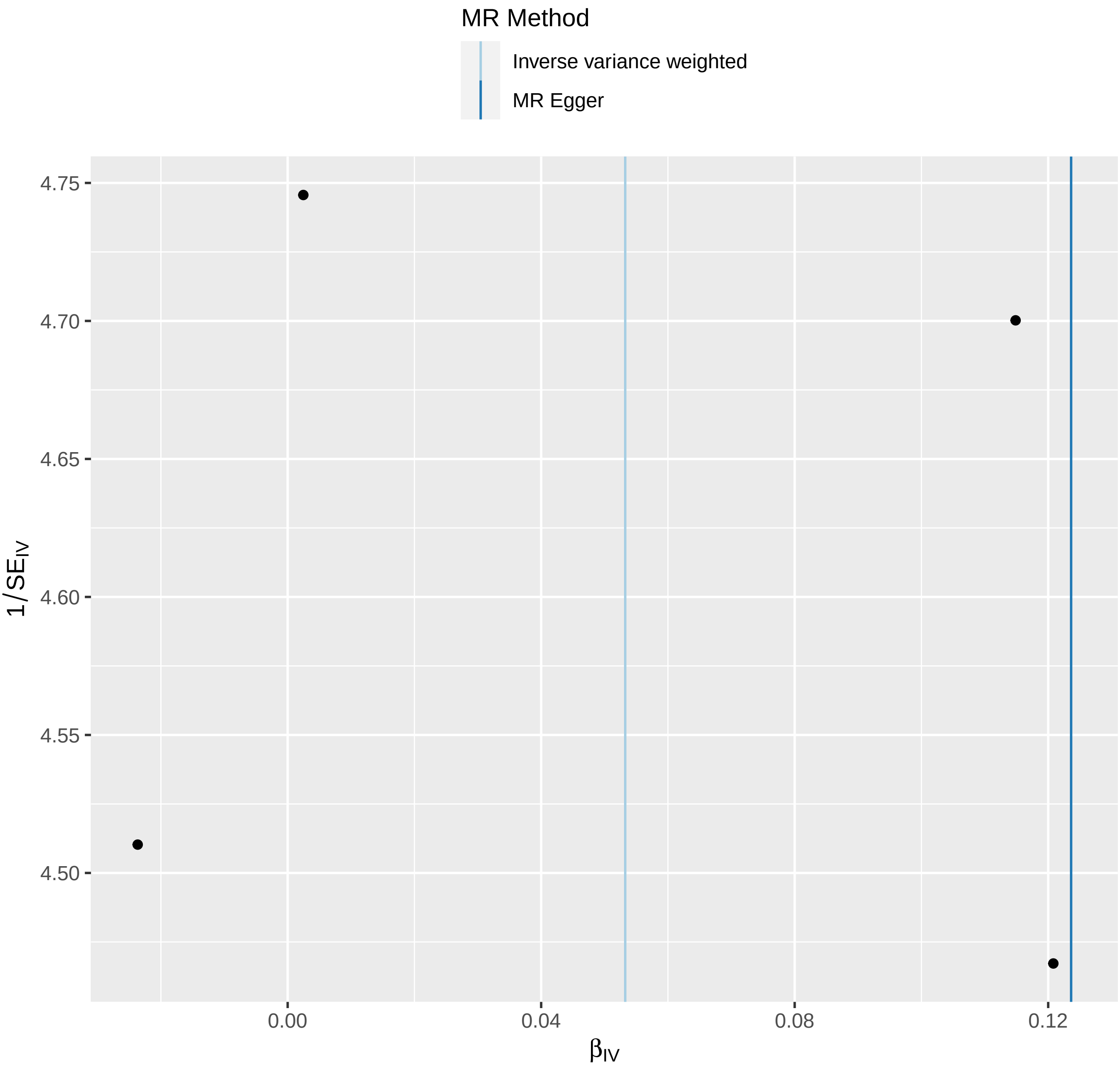

Batch 2−2038 : Gut microbiota abundance (genus Parasutterella id.2892) on Fas apoptotic inhibitory molecule 3
with a threshold of 1e−05

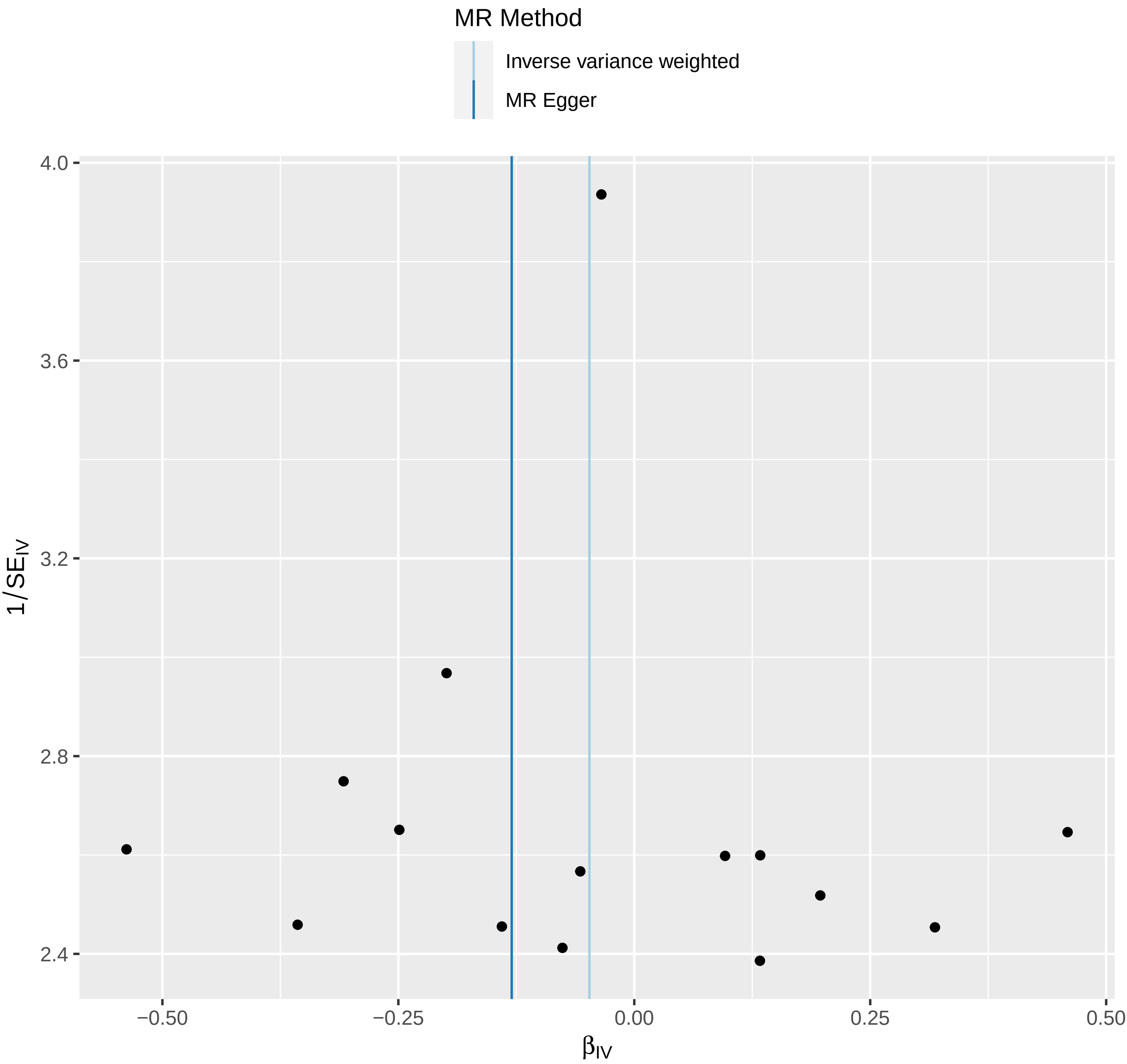

Batch 2−2039 : Gut microbiota abundance (genus Catenibacterium id.2153) on Protein FAM107A
with a threshold of 1e−05

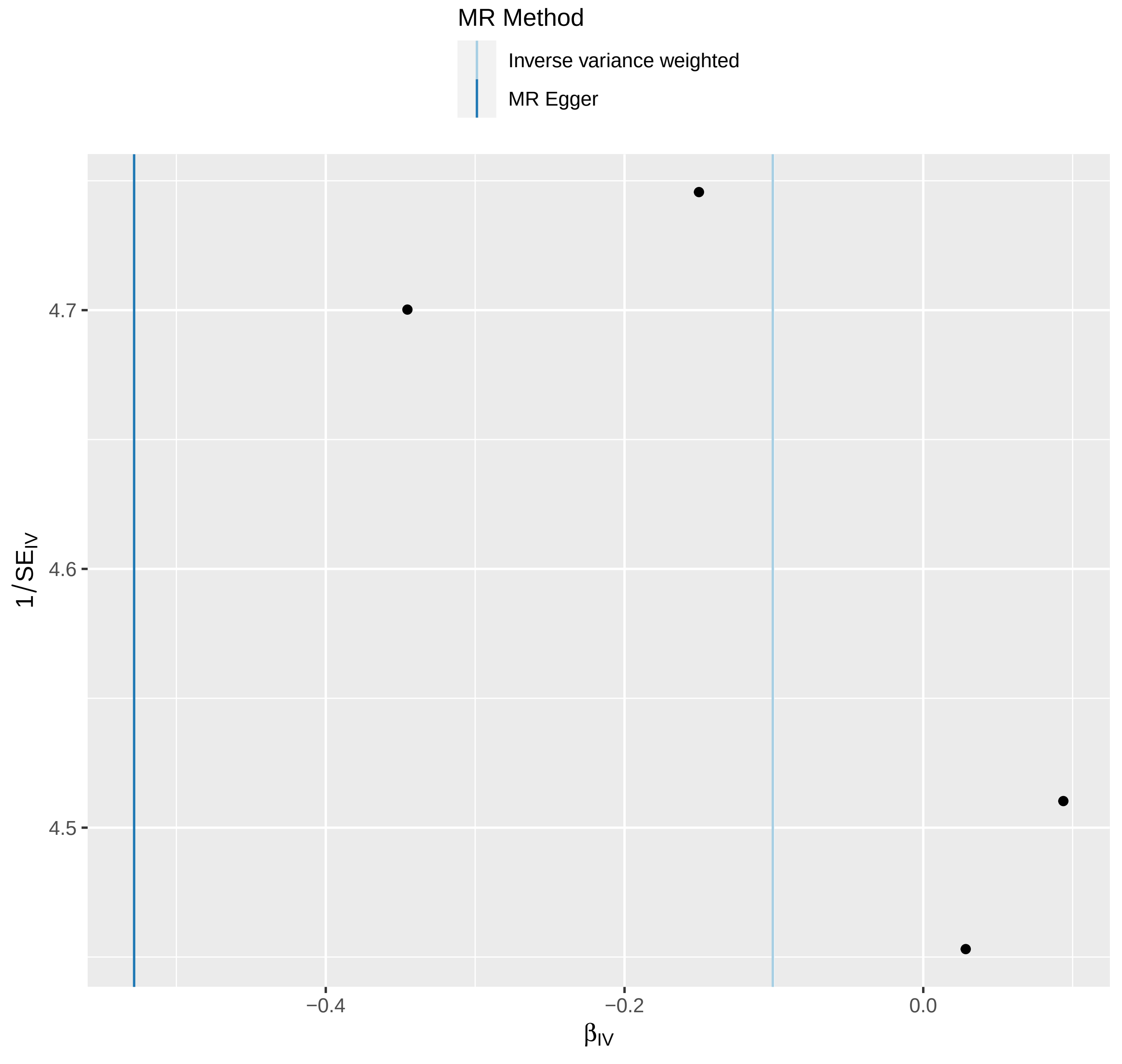

Batch 2−2040 : Gut microbiota abundance (genus Parasutterella id.2892) on Protein FAM107A
with a threshold of 1e−05

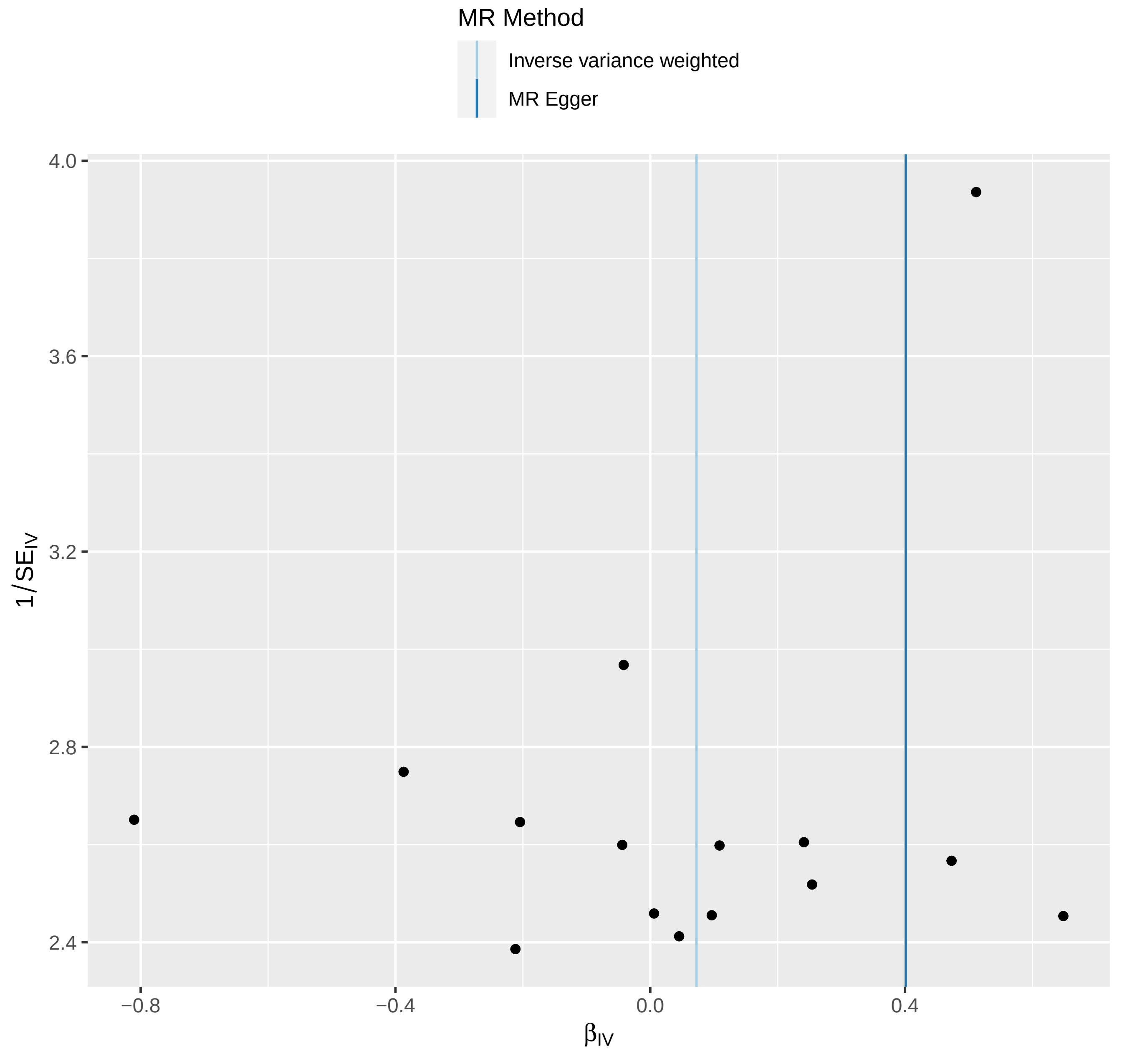

Batch 2−2041 : Gut microbiota abundance (genus Catenibacterium id.2153) on Protein FAM107B
with a threshold of 1e−05

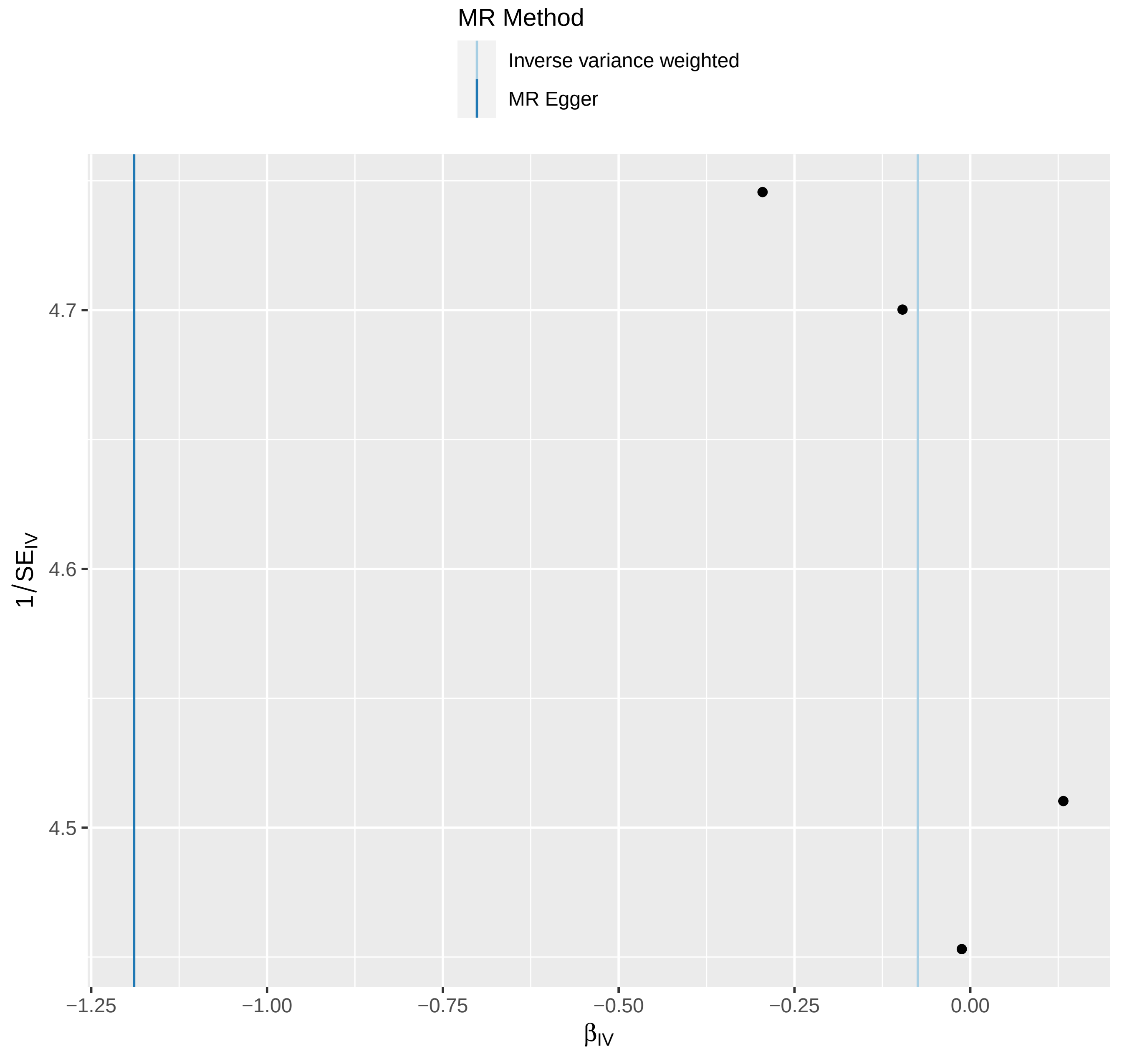

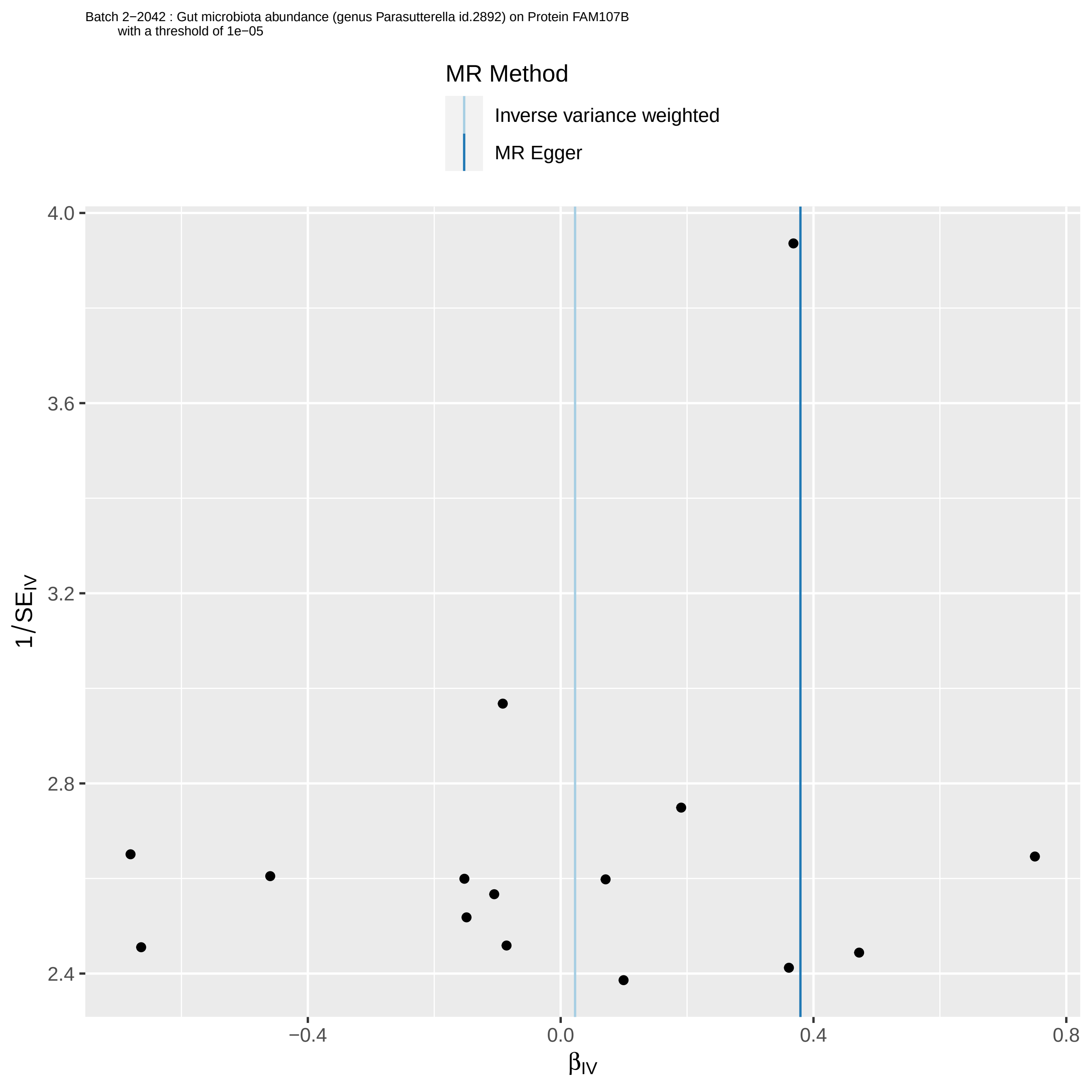
Batch 2−2042 : Gut microbiota abundance (genus Parasutterella id.2892) on Protein FAM107B
with a threshold of 1e−05
MR Method
Inverse variance weighted
MR Egger
4.0
3.6
3.2
2.8
2.4
1/SE$_{IV}$
−0.4
0.0
0.4
0.8
β_{IV}

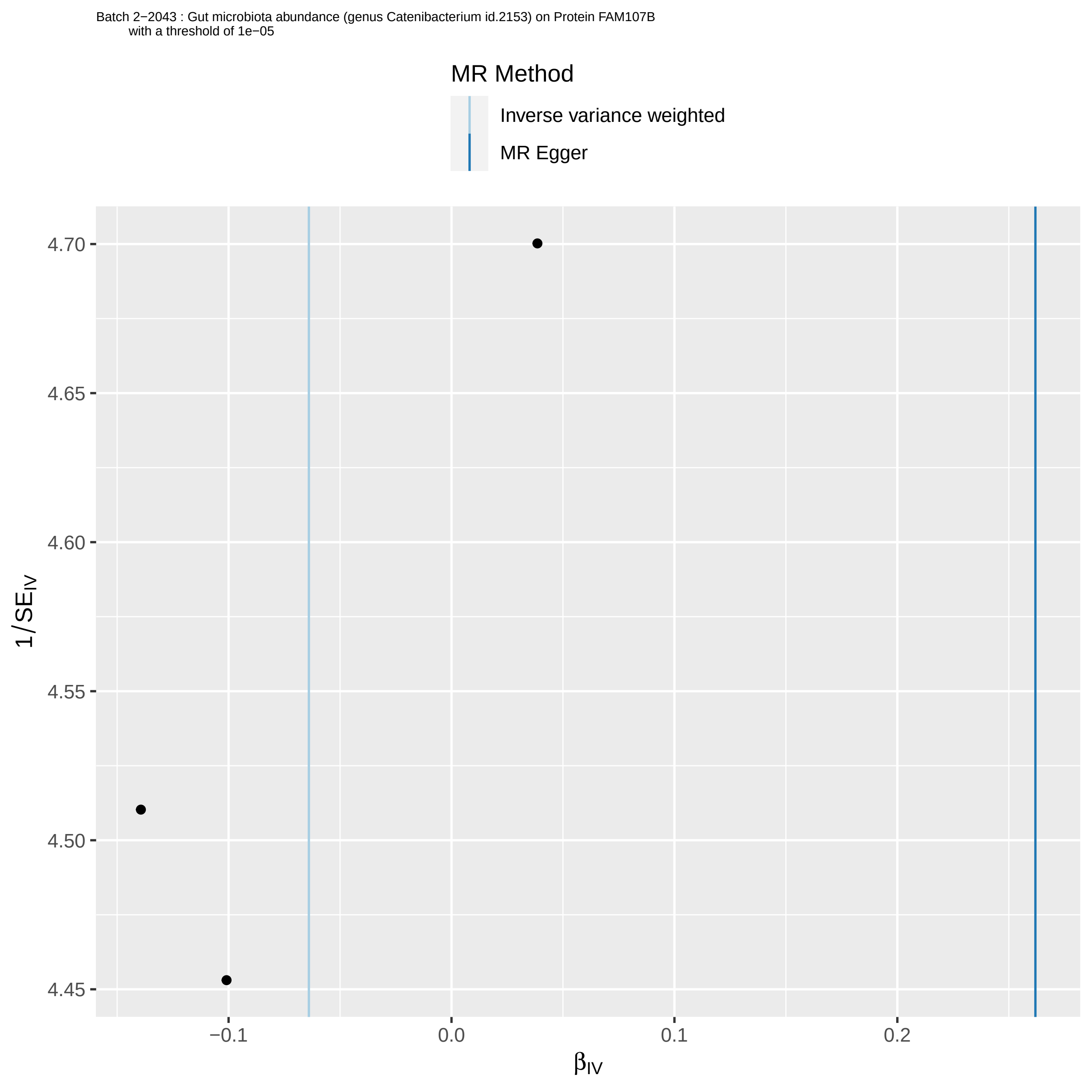

Batch 2−2043 : Gut microbiota abundance (genus Catenibacterium id.2153) on Protein FAM107B
with a threshold of 1e−05
MR Method
Inverse variance weighted
MR Egger
4.70
4.65
4.60
4.55
4.50
4.45
1/SE$_{IV}$
−0.1
0.0
0.1
0.2
β$_{IV}$

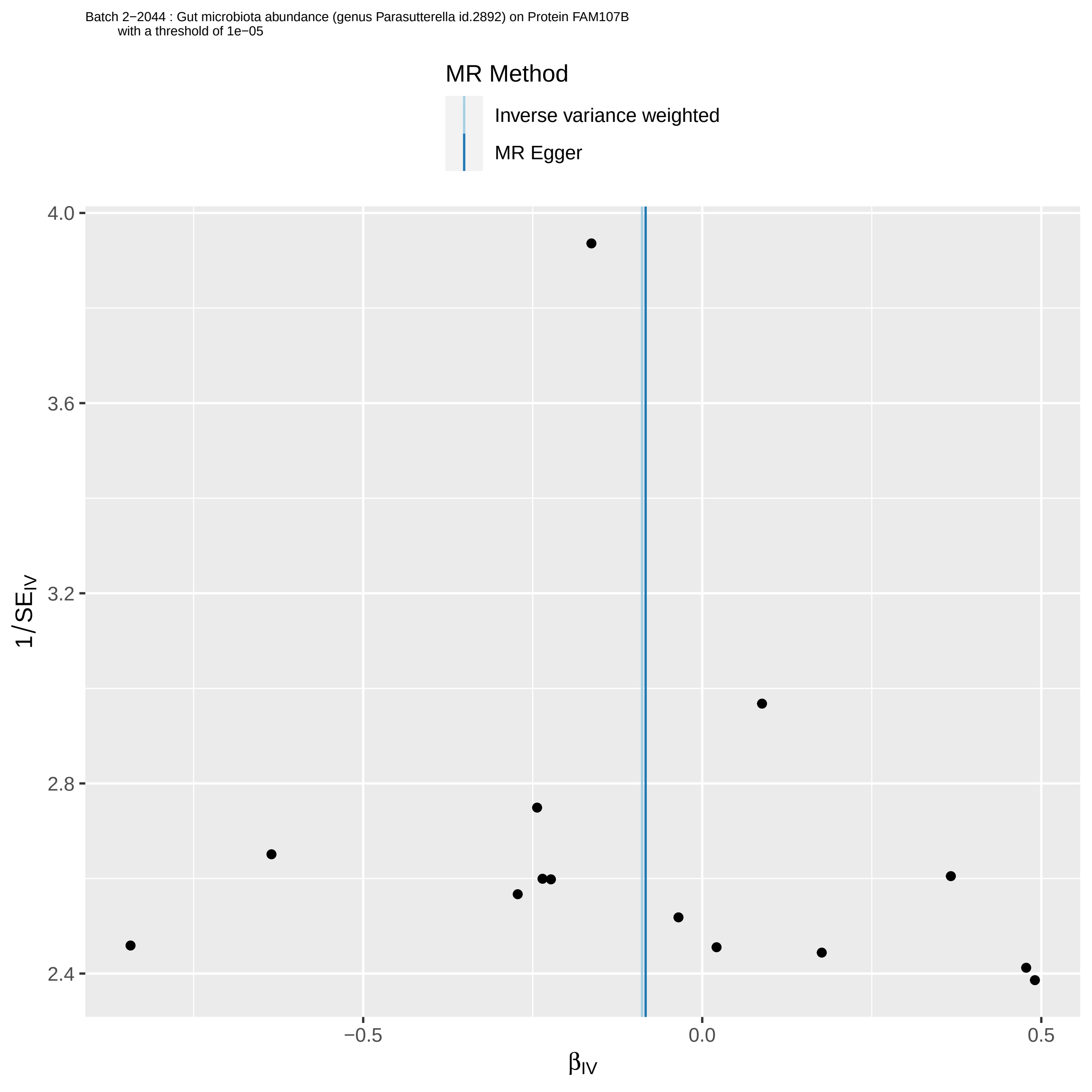

Batch 2−2044 : Gut microbiota abundance (genus Parasutterella id.2892) on Protein FAM107B
with a threshold of 1e−05
MR Method
Inverse variance weighted
MR Egger
4.0
3.6
3.2
2.8
2.4
1/SE_IV
−0.5
0.0
0.5
β_IV

Batch 2−2045 : Gut microbiota abundance (genus Catenibacterium id.2153) on Protein FAM134B
with a threshold of 1e−05

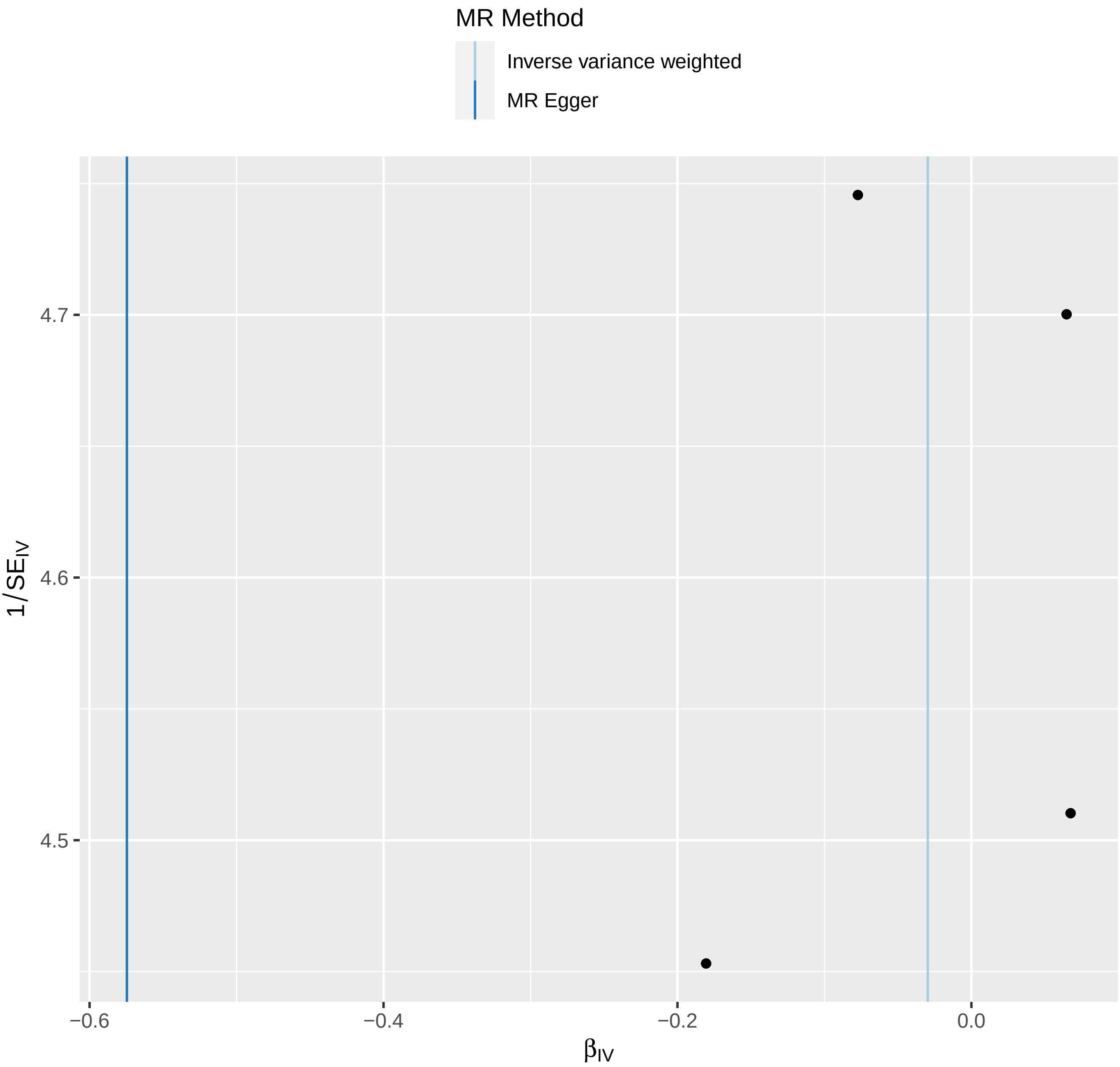

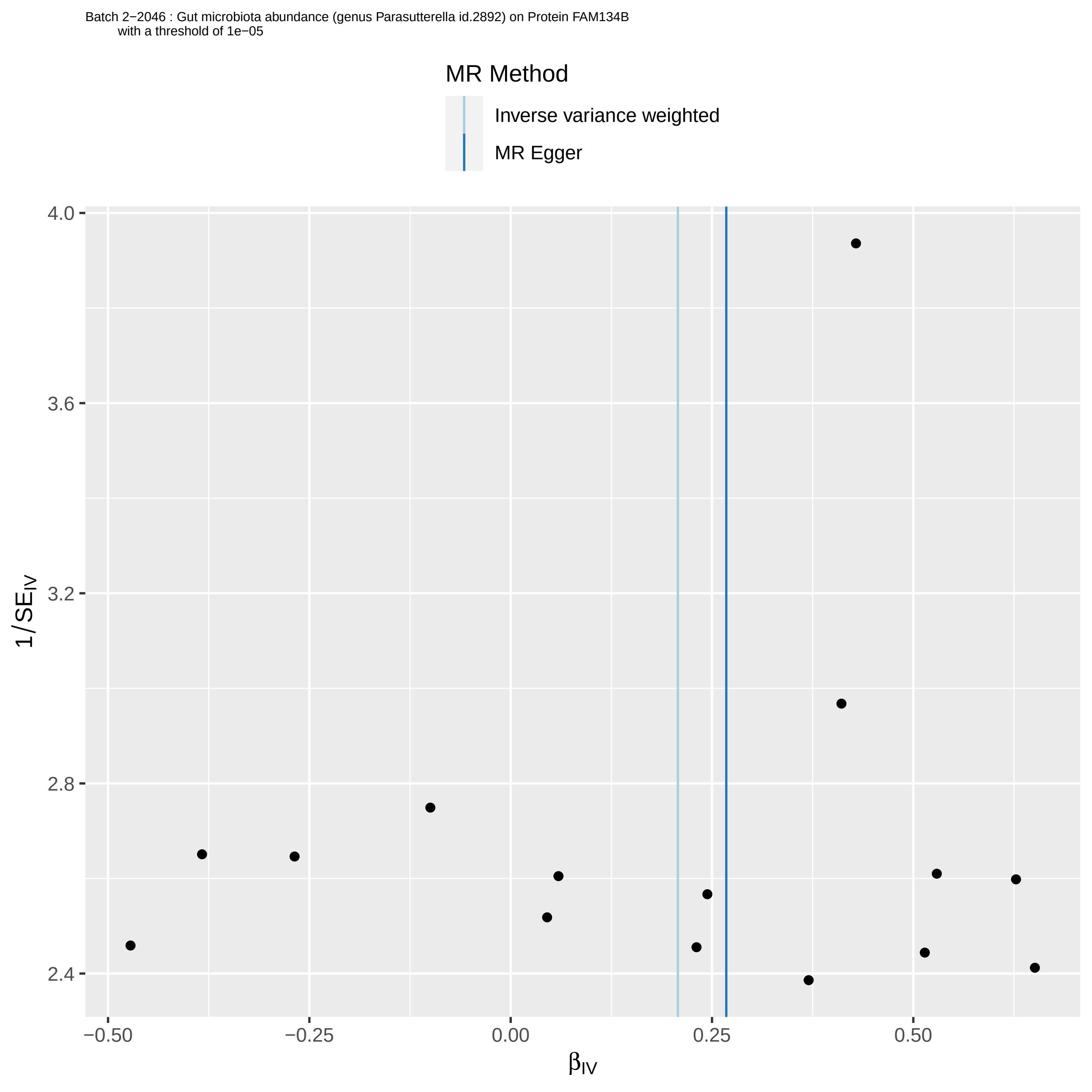

Batch 2−2046 : Gut microbiota abundance (genus Parasutterella id.2892) on Protein FAM134B
with a threshold of 1e−05
MR Method
Inverse variance weighted
MR Egger
4.0
3.6
3.2
2.8
2.4
1/SE$_{IV}$
−0.50
−0.25
0.00
0.25
0.50
β$_{IV}$

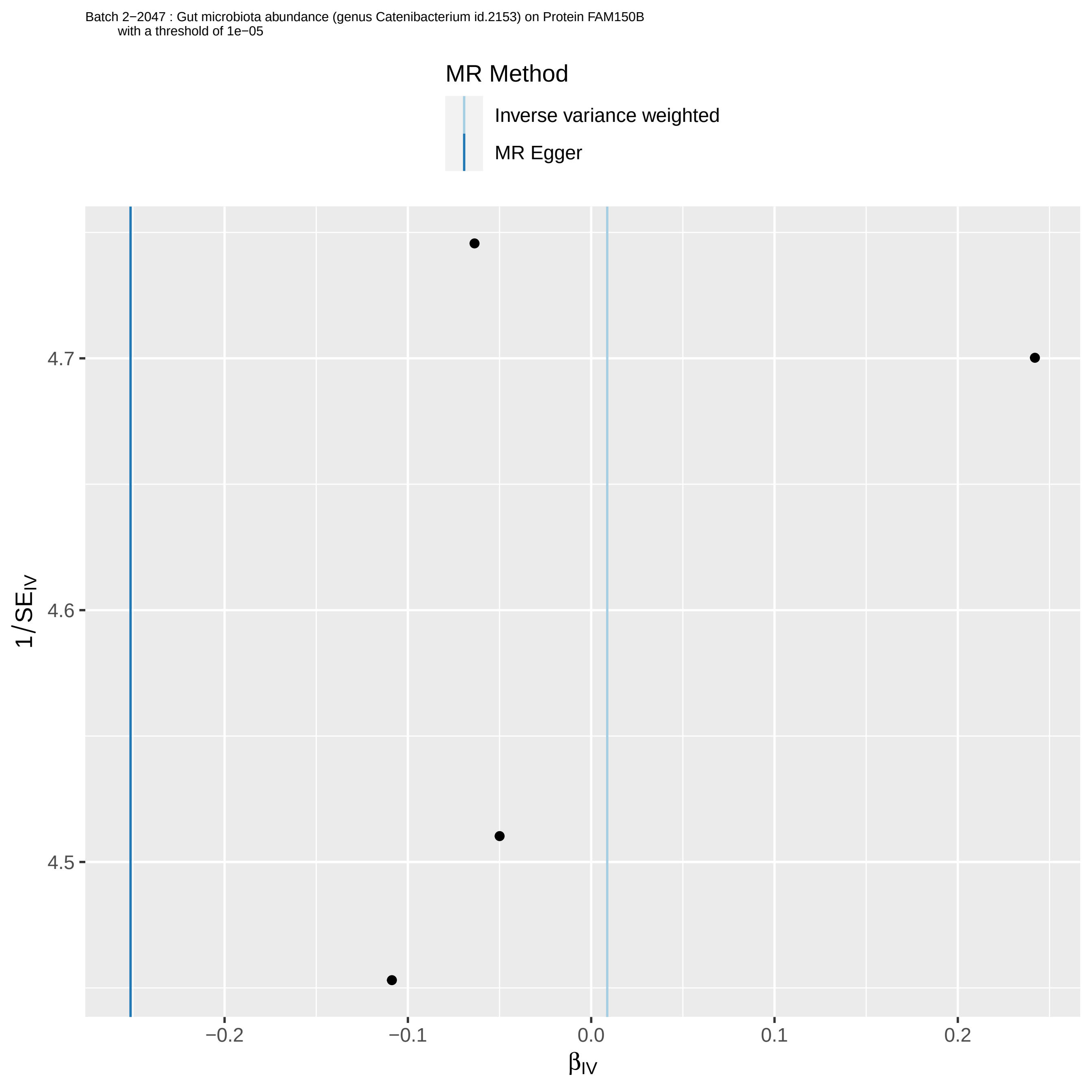

Batch 2−2047 : Gut microbiota abundance (genus Catenibacterium id.2153) on Protein FAM150B
with a threshold of 1e−05
MR Method
Inverse variance weighted
MR Egger
4.7
4.6
4.5
1/SE_IV
−0.2
−0.1
0.0
0.1
0.2
β_IV

Batch 2−2048 : Gut microbiota abundance (genus Parasutterella id.2892) on Protein FAM150B
with a threshold of 1e−05

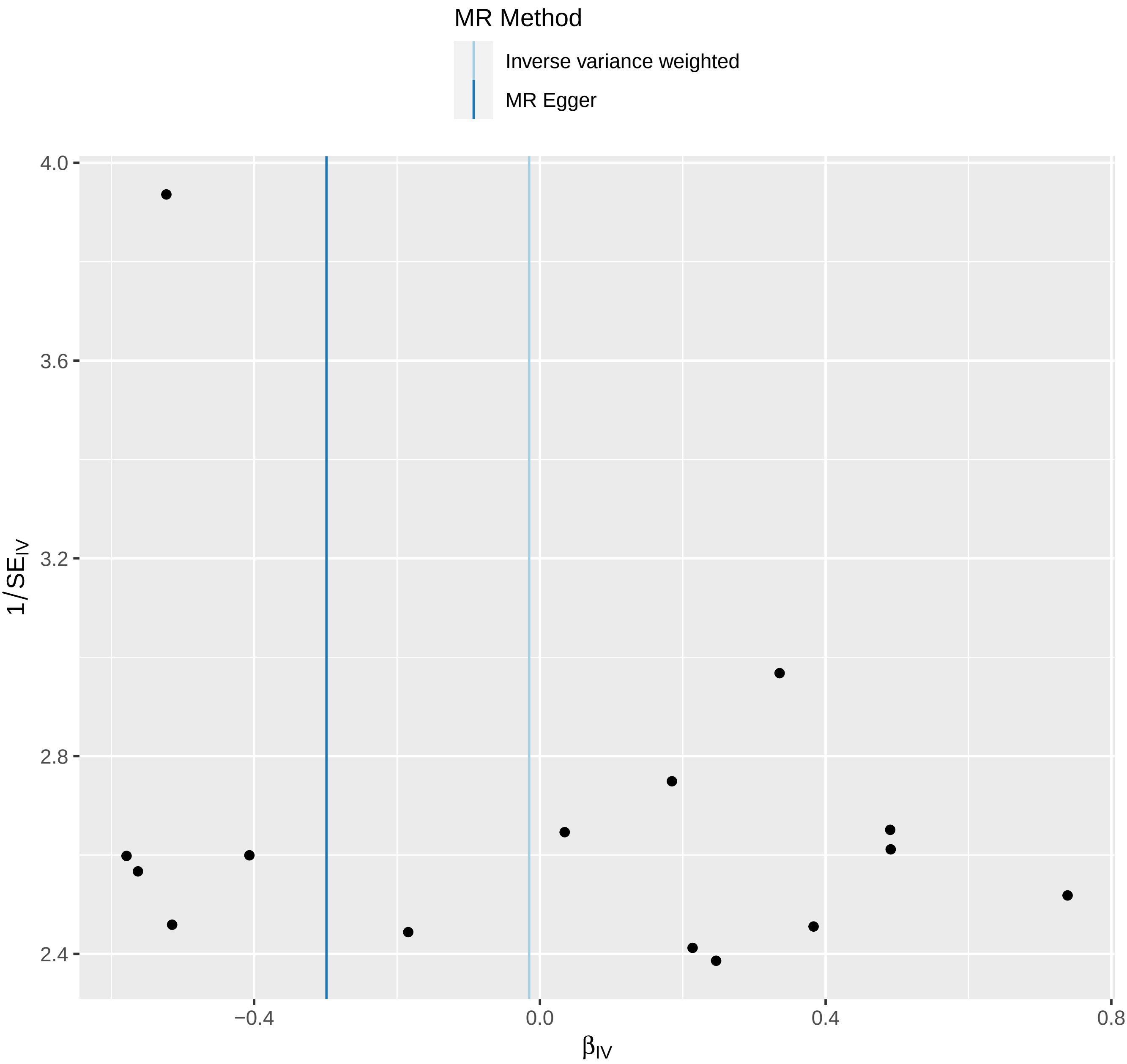

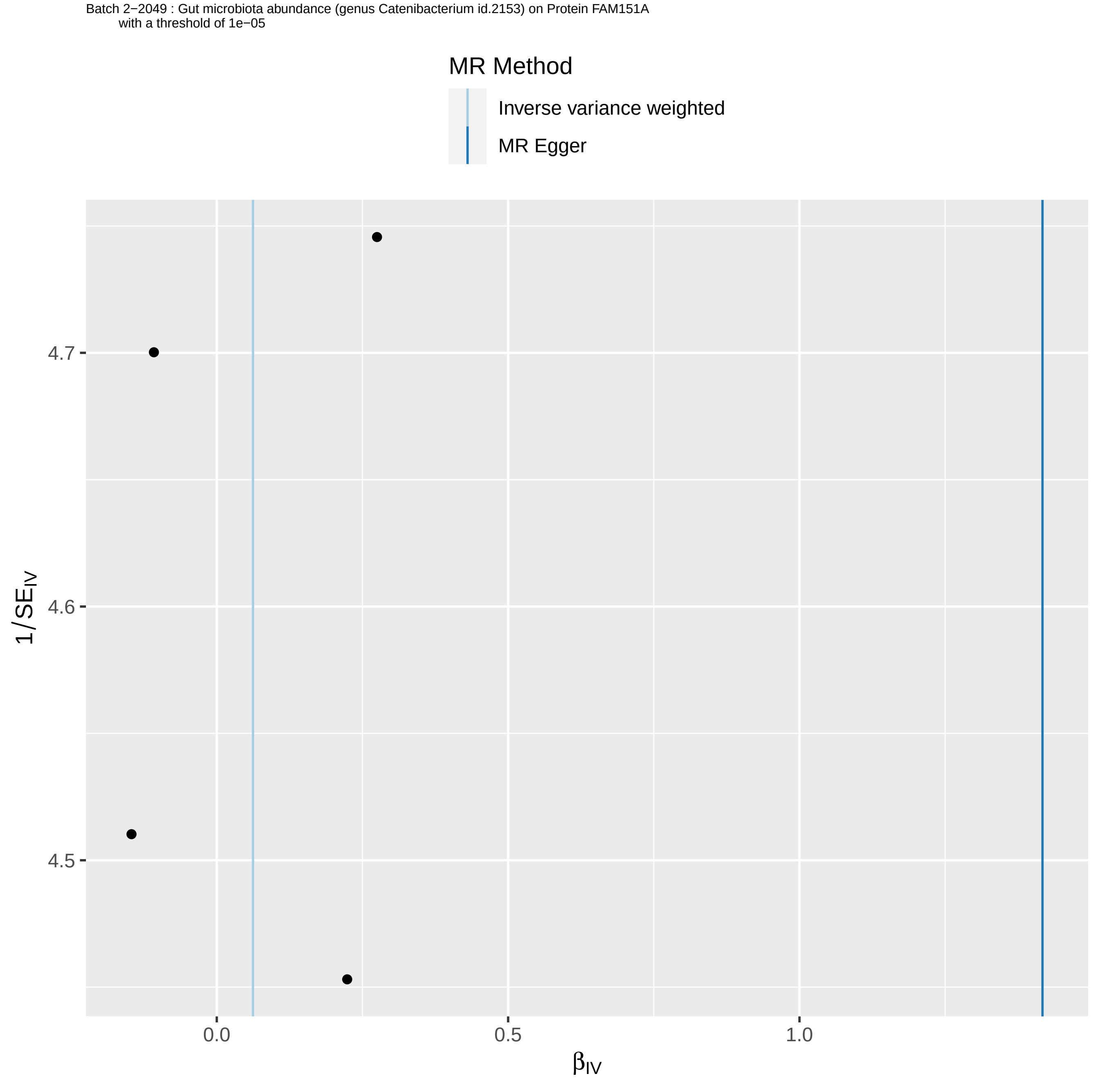
Batch 2−2049 : Gut microbiota abundance (genus Catenibacterium id.2153) on Protein FAM151A
with a threshold of 1e−05
MR Method
Inverse variance weighted
MR Egger
4.7
4.6
4.5
0.0
0.5
1.0
β_{IV}
$1/SE_{IV}$

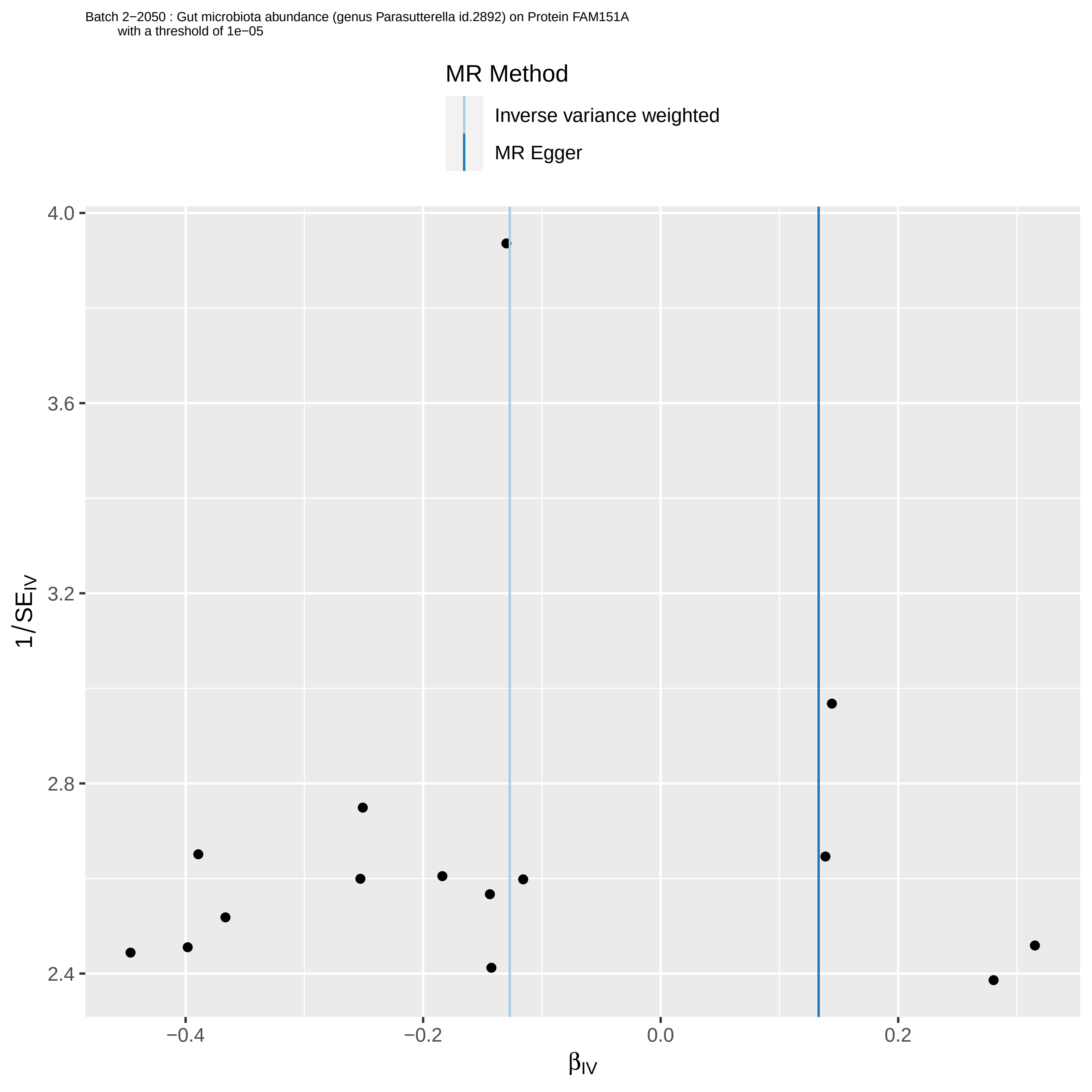
Batch 2−2050 : Gut microbiota abundance (genus Parasutterella id.2892) on Protein FAM151A
with a threshold of 1e−05
MR Method
Inverse variance weighted
MR Egger
4.0
3.6
3.2
2.8
2.4
1/SE_IV
−0.4
−0.2
0.0
0.2
β_IV

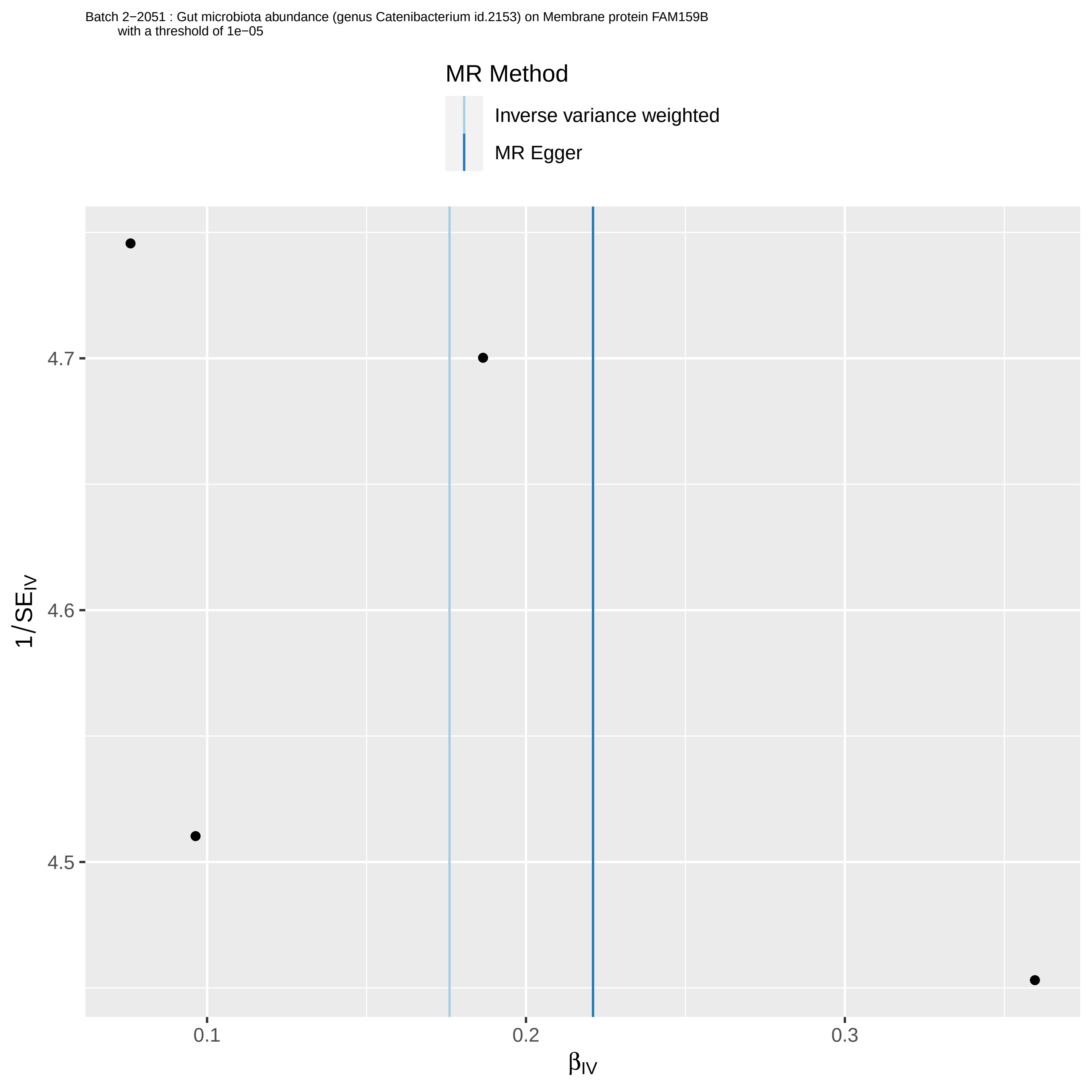
Batch 2−2051 : Gut microbiota abundance (genus Catenibacterium id.2153) on Membrane protein FAM159B
with a threshold of 1e−05
MR Method
Inverse variance weighted
MR Egger
4.7
4.6
4.5
0.1
0.2
0.3
1/SE_IV
β_IV

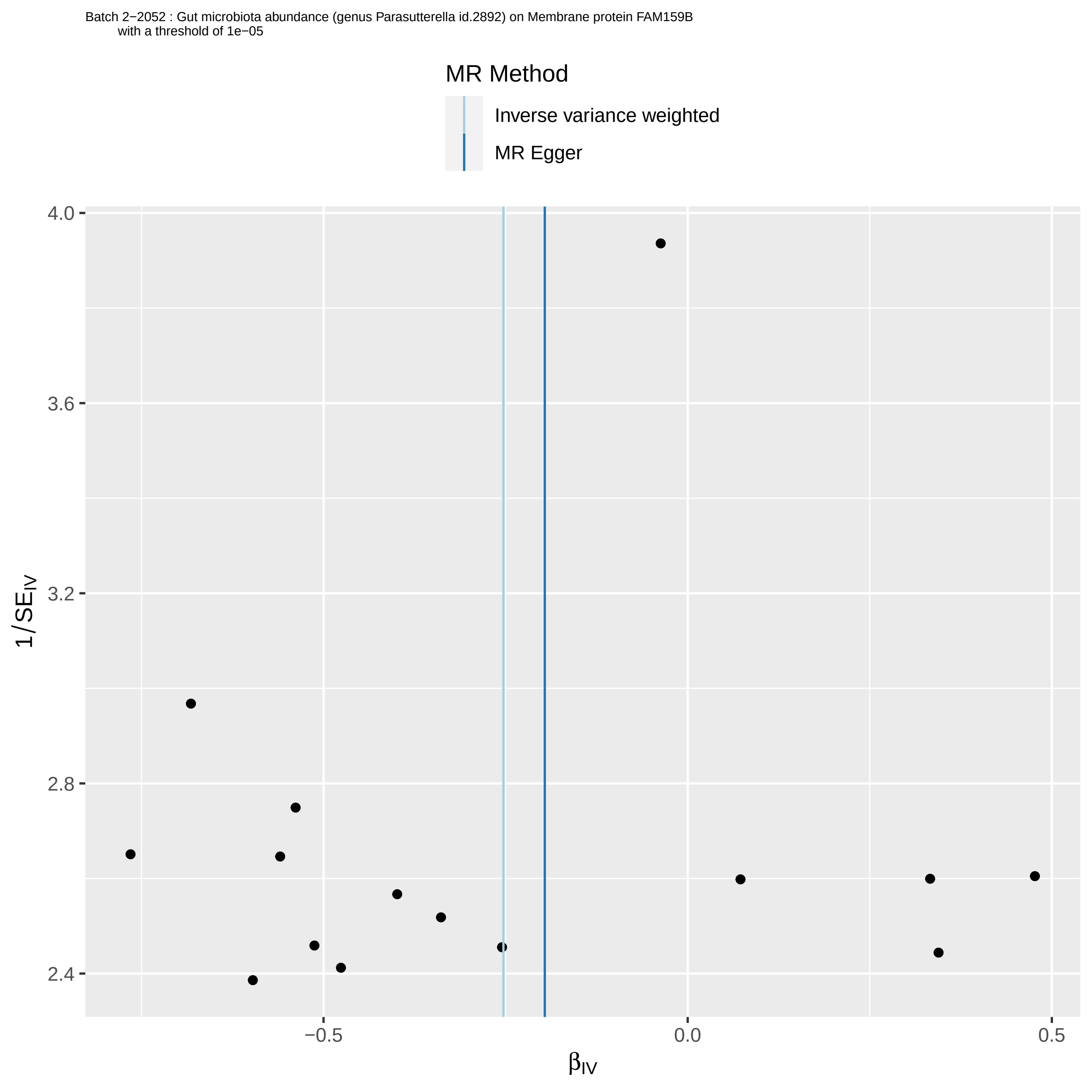
Batch 2−2052 : Gut microbiota abundance (genus Parasutterella id.2892) on Membrane protein FAM159B
with a threshold of 1e−05
MR Method
Inverse variance weighted
MR Egger
4.0
3.6
3.2
2.8
2.4
1/SE_IV
−0.5
0.0
0.5
β_IV

Batch 2−2053 : Gut microbiota abundance (genus Catenibacterium id.2153) on Protein FAM163A
with a threshold of 1e−05

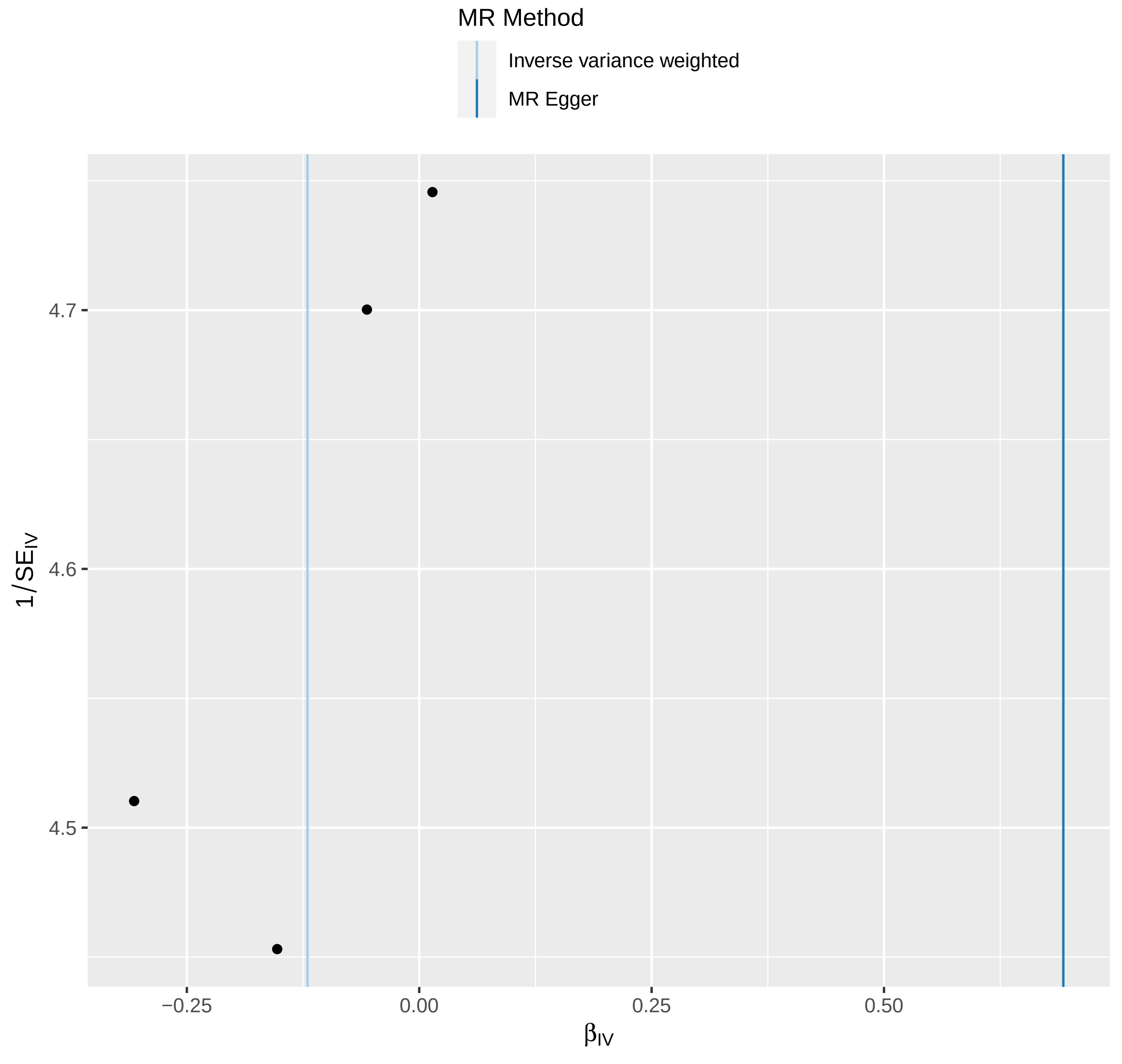

Batch 2−2054 : Gut microbiota abundance (genus Parasutterella id.2892) on Protein FAM163A
with a threshold of 1e−05

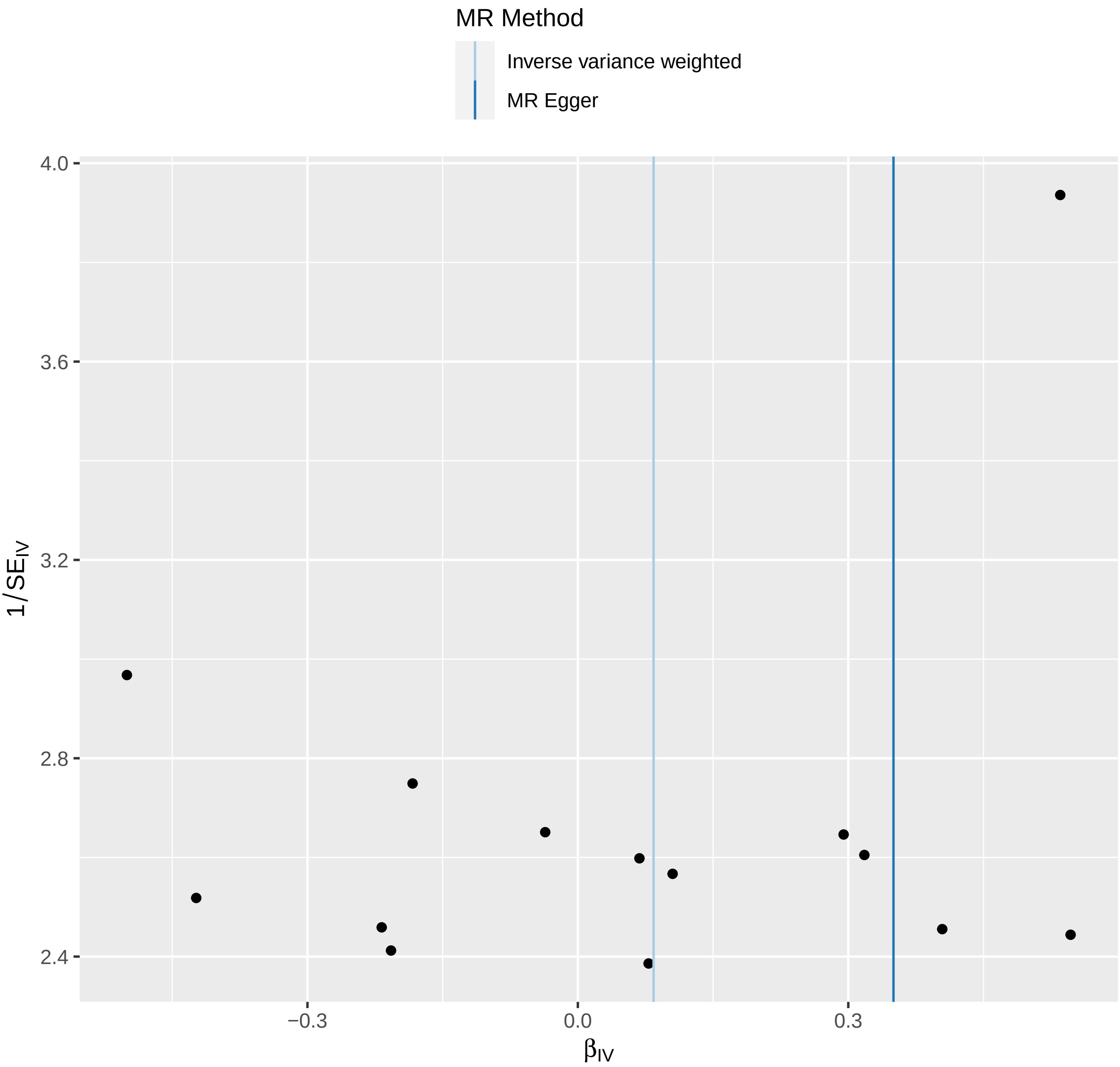

Batch 2−2055 : Gut microbiota abundance (genus Catenibacterium id.2153) on Protein FAM163B
with a threshold of 1e−05

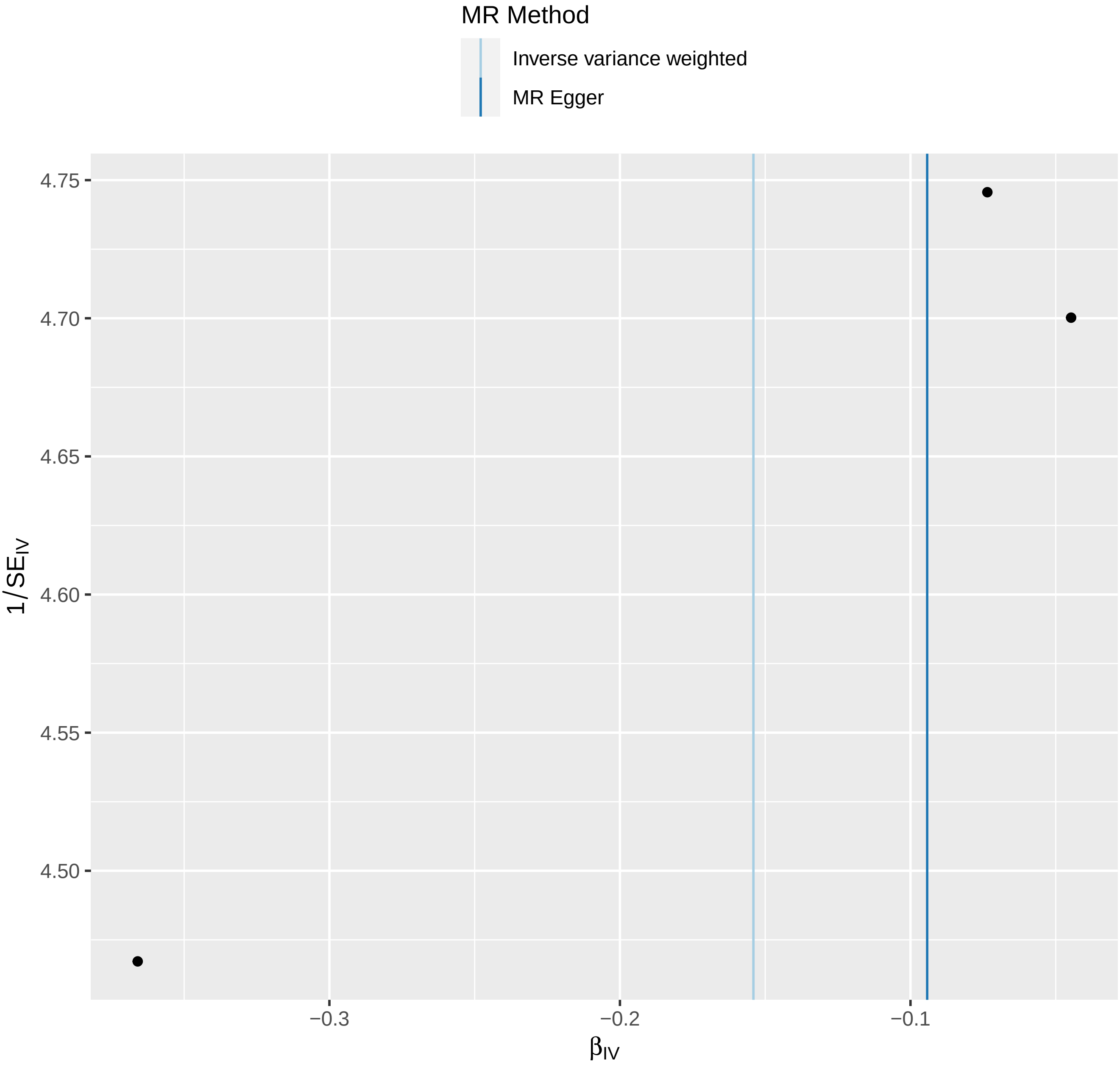

Batch 2−2056 : Gut microbiota abundance (genus Parasutterella id.2892) on Protein FAM163B
with a threshold of 1e−05

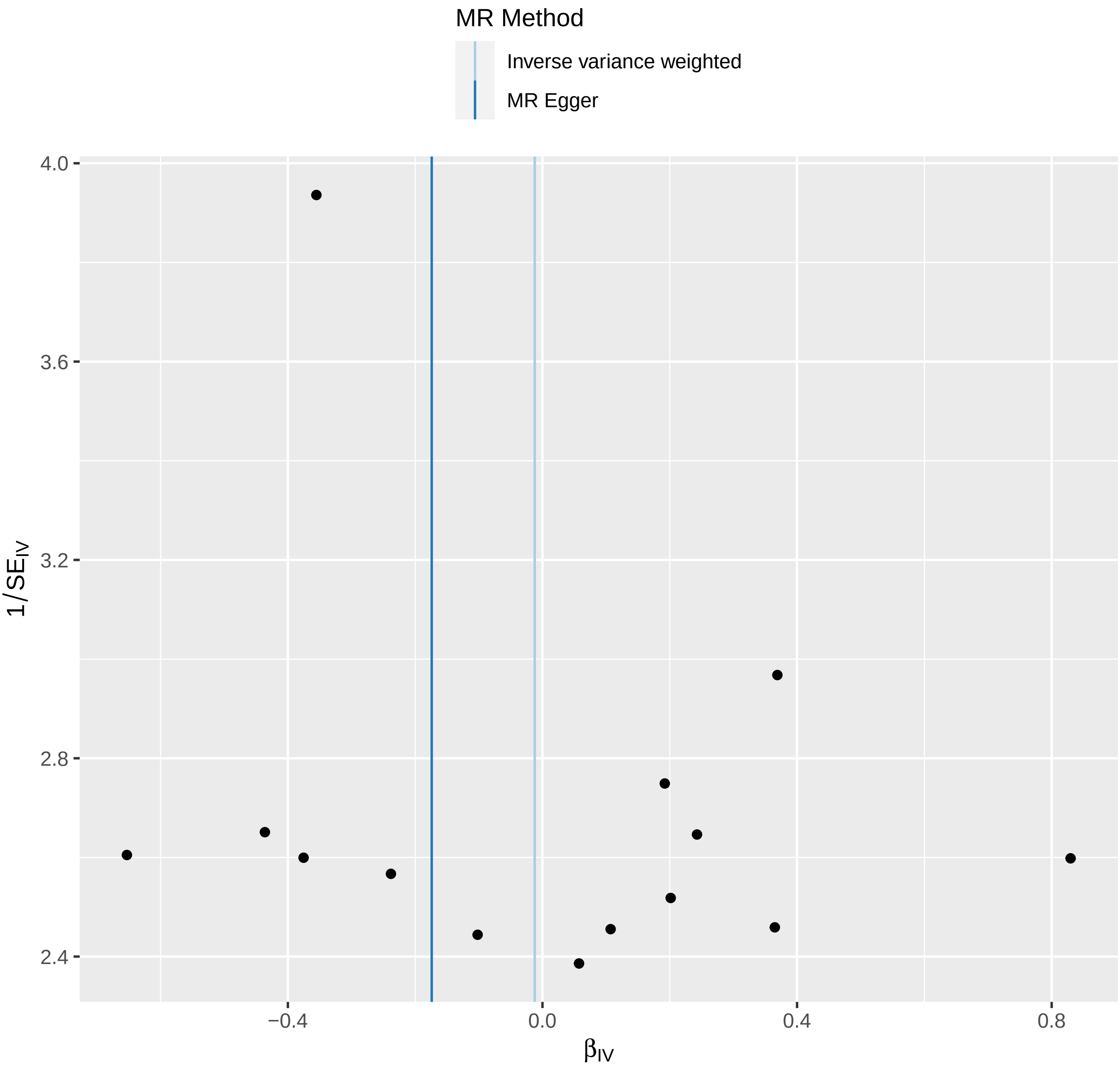

Batch 2−2057 : Gut microbiota abundance (genus Catenibacterium id.2153) on Protein FAM171B
with a threshold of 1e−05

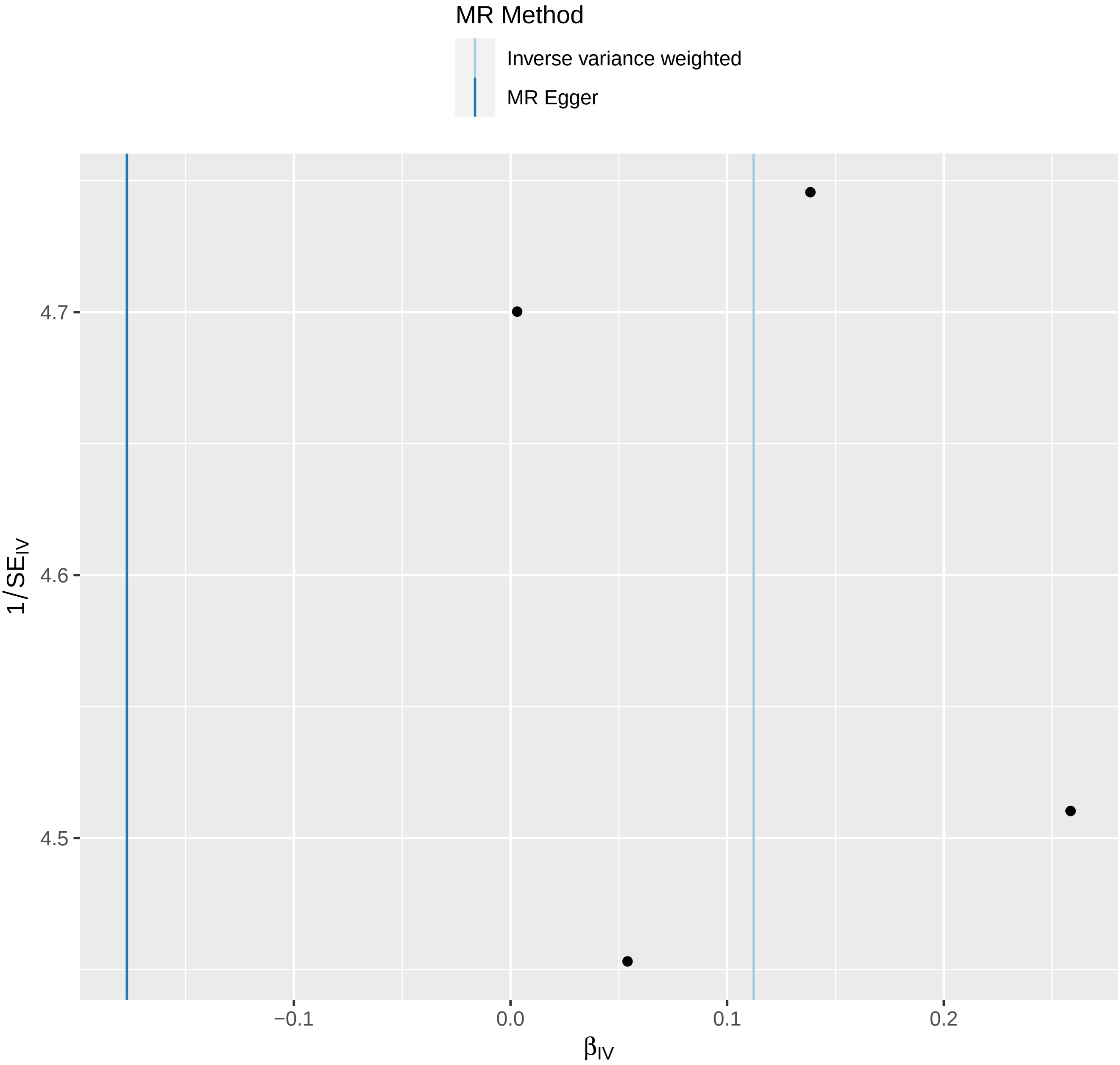

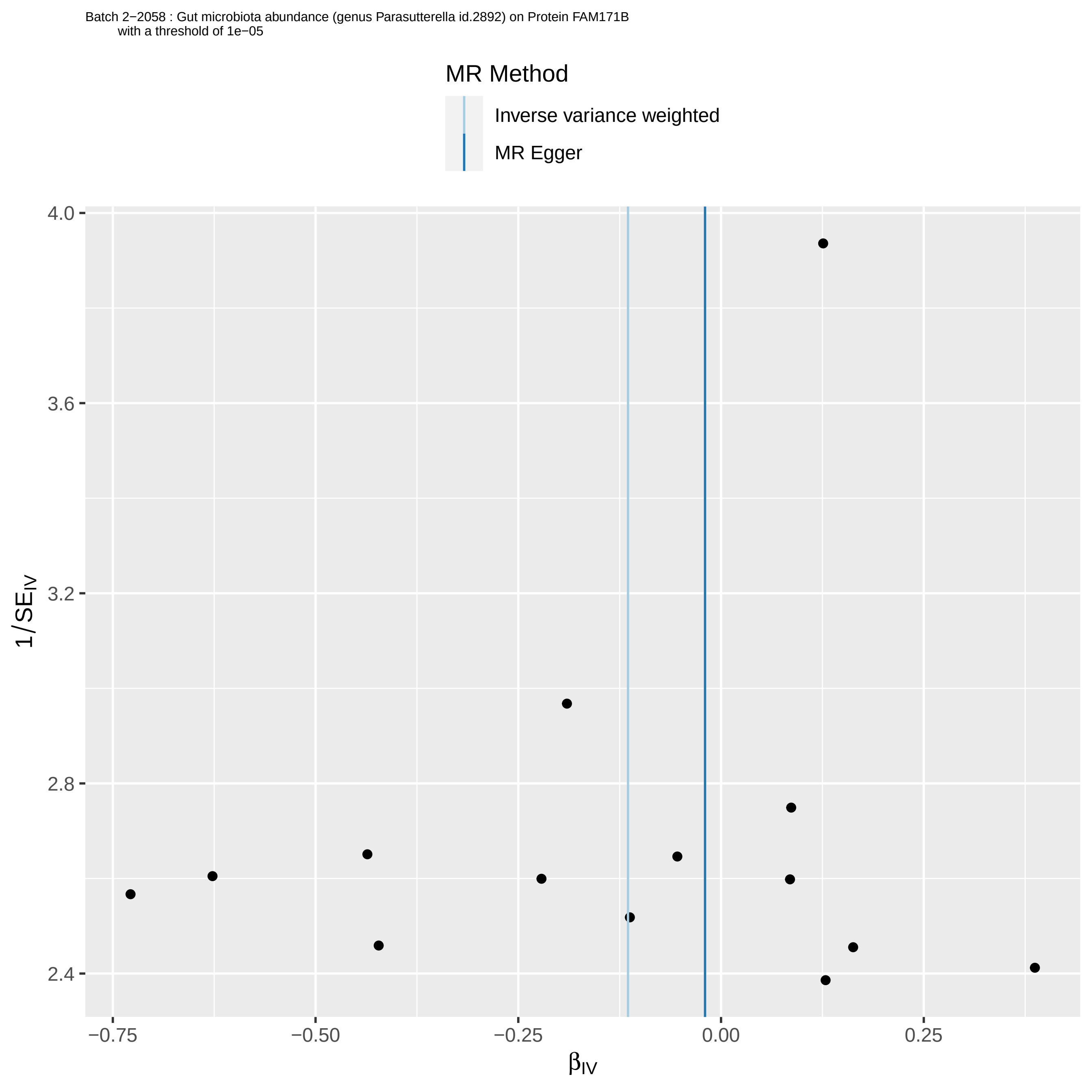

Batch 2−2058 : Gut microbiota abundance (genus Parasutterella id.2892) on Protein FAM171B
with a threshold of 1e−05
MR Method
Inverse variance weighted
MR Egger
4.0
3.6
3.2
2.8
2.4
1/SE_IV
−0.75
−0.50
−0.25
0.00
0.25
β_IV

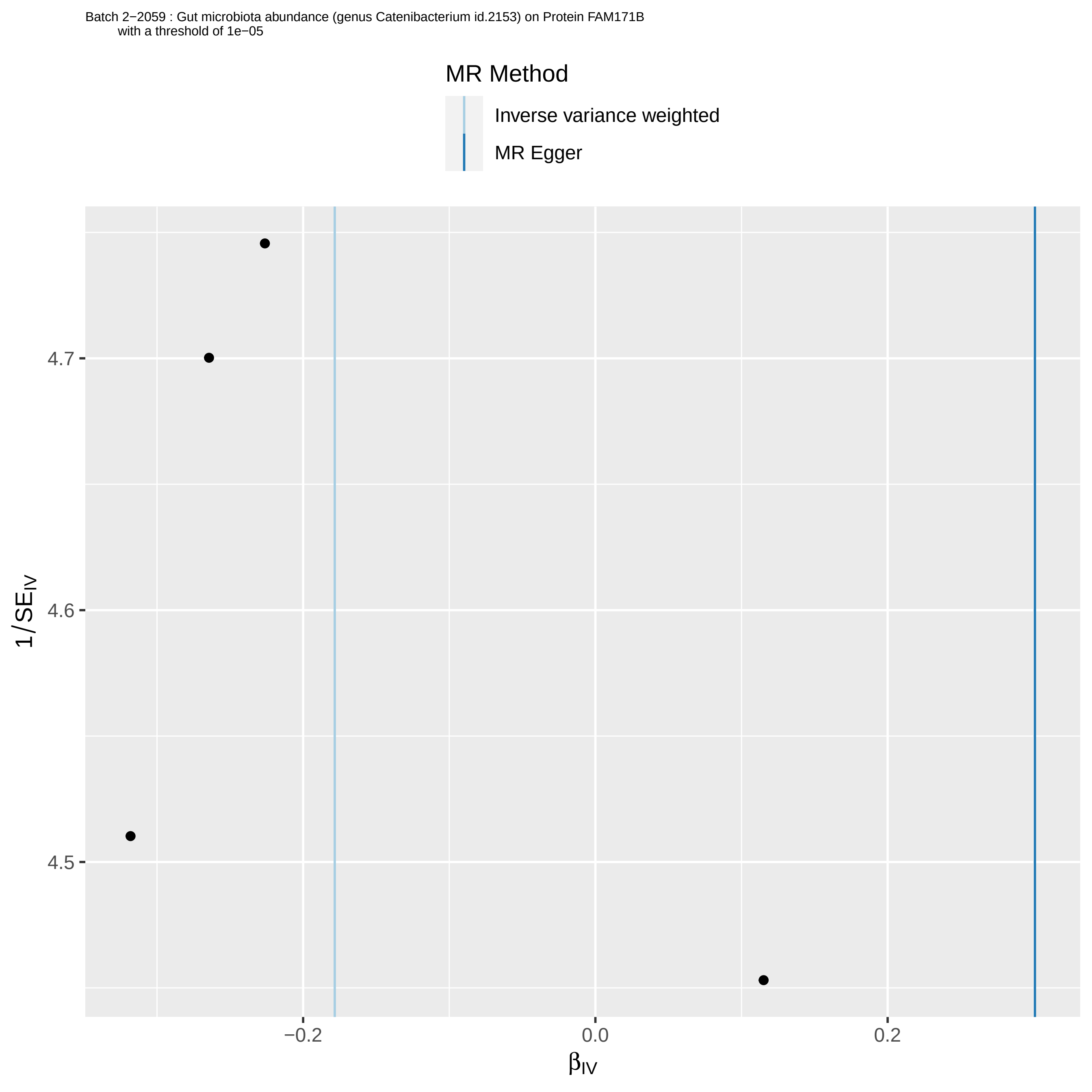

Batch 2−2059 : Gut microbiota abundance (genus Catenibacterium id.2153) on Protein FAM171B
with a threshold of 1e−05
MR Method
Inverse variance weighted
MR Egger
4.7
4.6
4.5
$1/SE_{IV}$
−0.2
0.0
0.2
β_{IV}

Batch 2−2060 : Gut microbiota abundance (genus Parasutterella id.2892) on Protein FAM171B
with a threshold of 1e−05

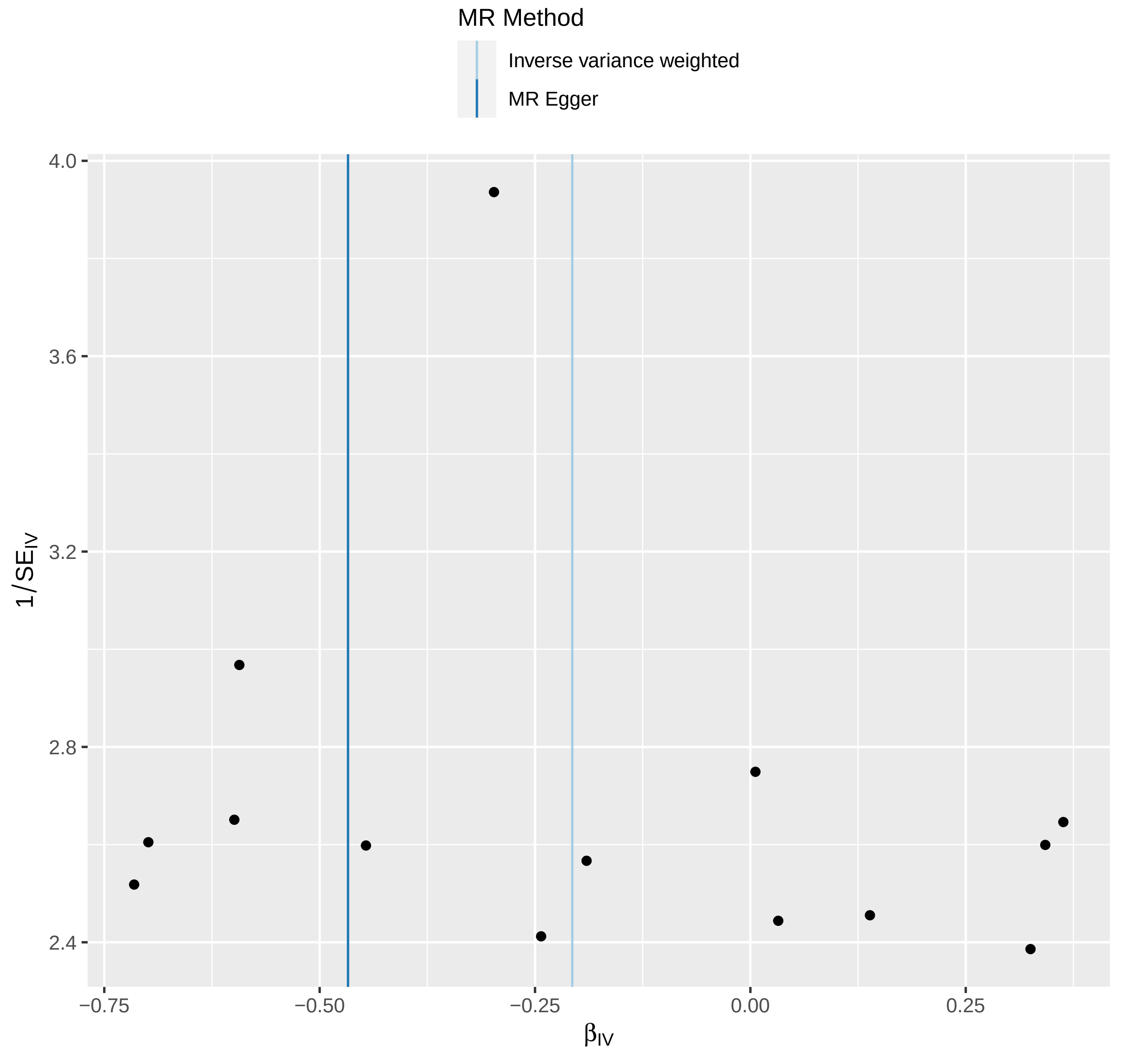

Batch 2−2061 : Gut microbiota abundance (genus Catenibacterium id.2153) on Protein FAM171B
with a threshold of 1e−05

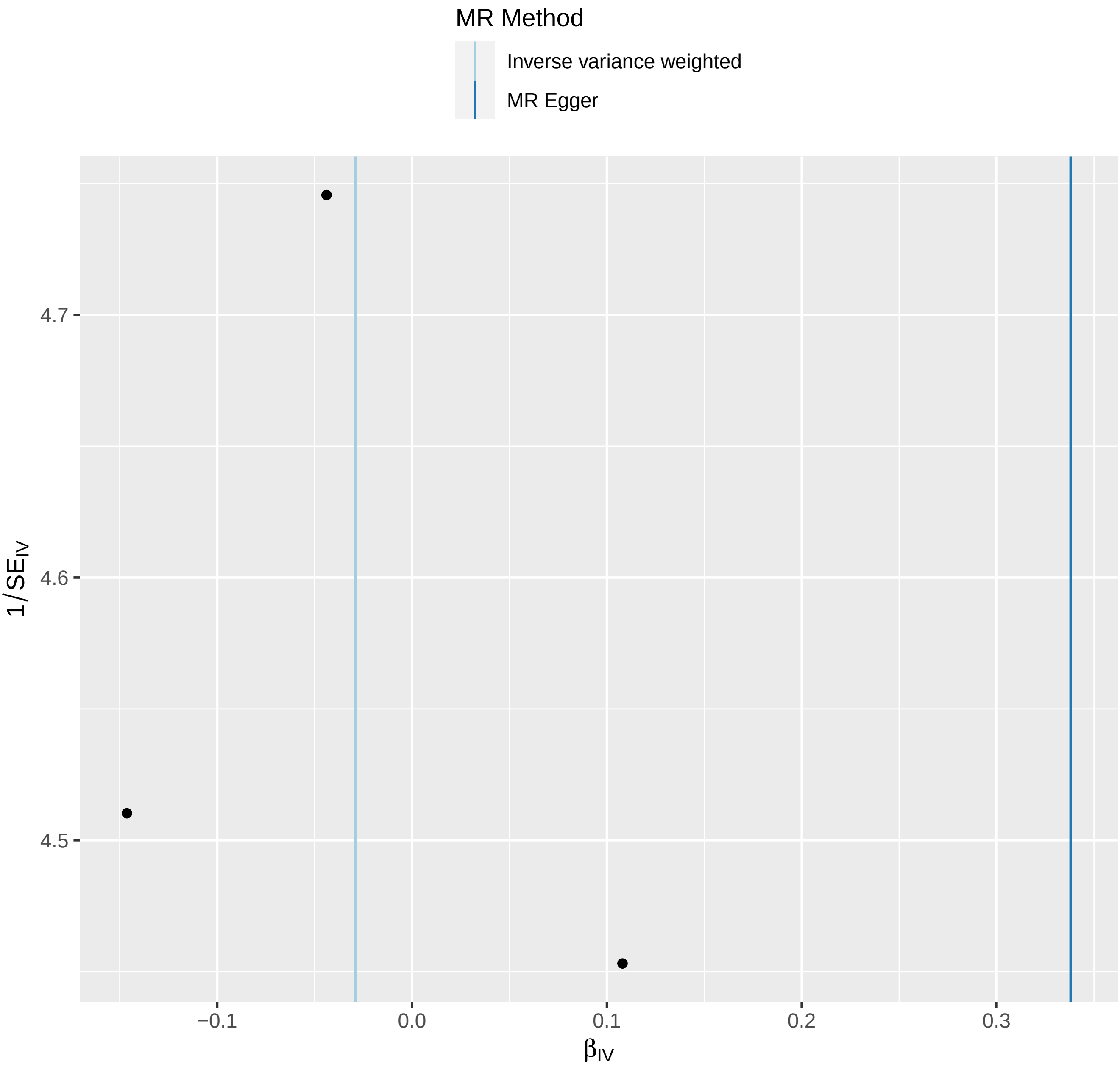

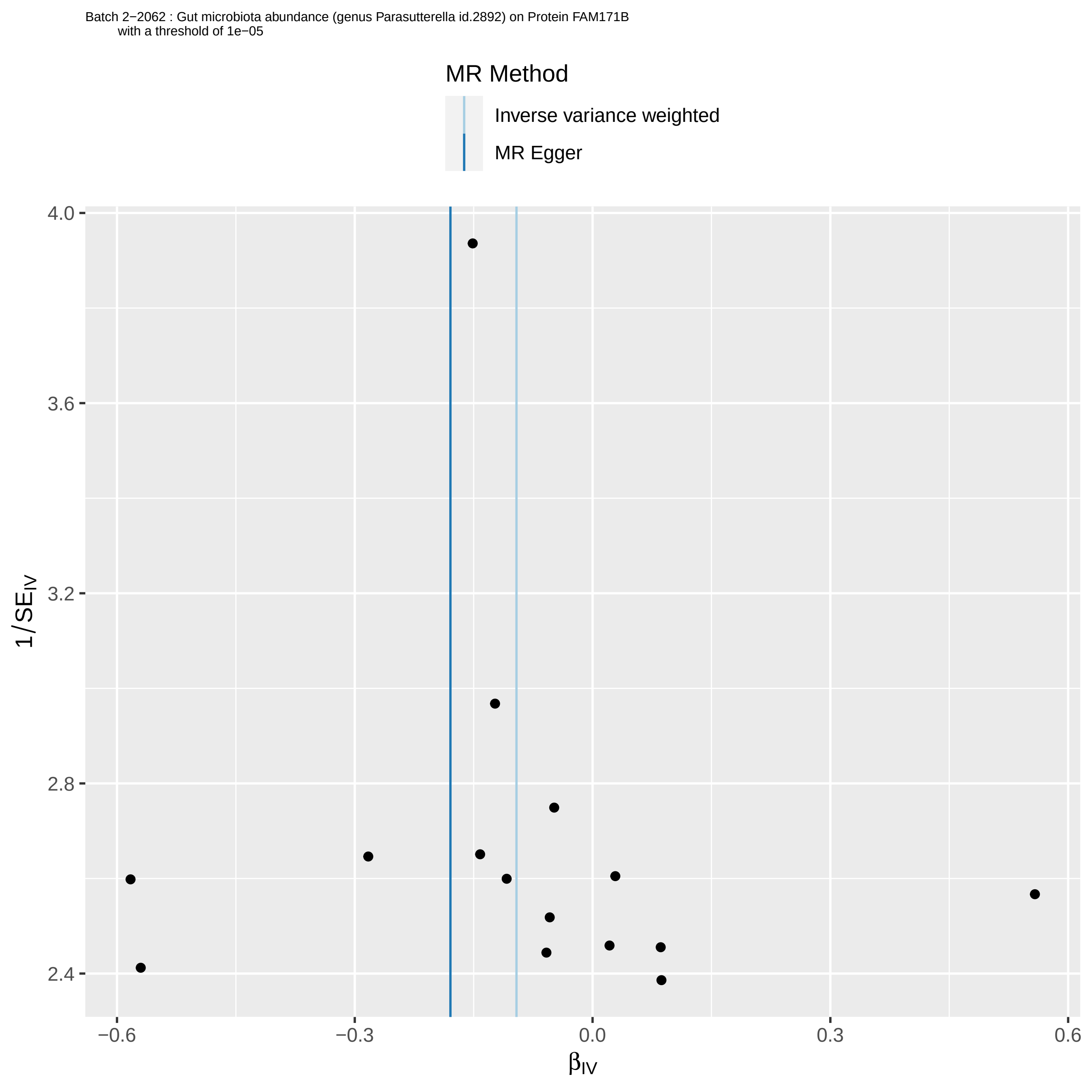

Batch 2−2062 : Gut microbiota abundance (genus Parasutterella id.2892) on Protein FAM171B
with a threshold of 1e−05
MR Method
Inverse variance weighted
MR Egger
1/SE_IV
4.0
3.6
3.2
2.8
2.4
−0.6
−0.3
0.0
0.3
0.6
β_IV

Batch 2−2063 : Gut microbiota abundance (genus Catenibacterium id.2153) on Protein FAM172A
with a threshold of 1e−05

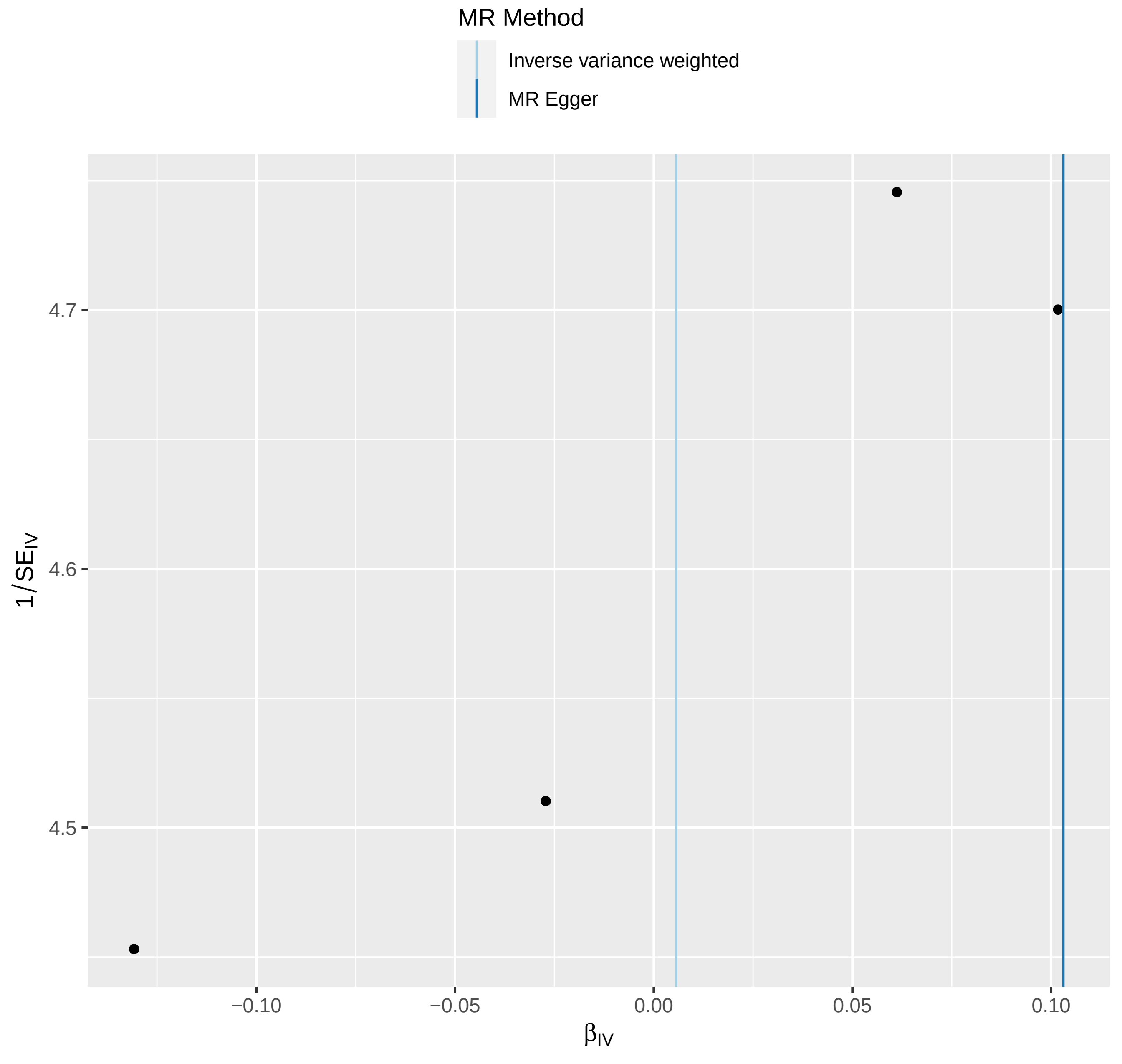

Batch 2−2064 : Gut microbiota abundance (genus Parasutterella id.2892) on Protein FAM172A
with a threshold of 1e−05

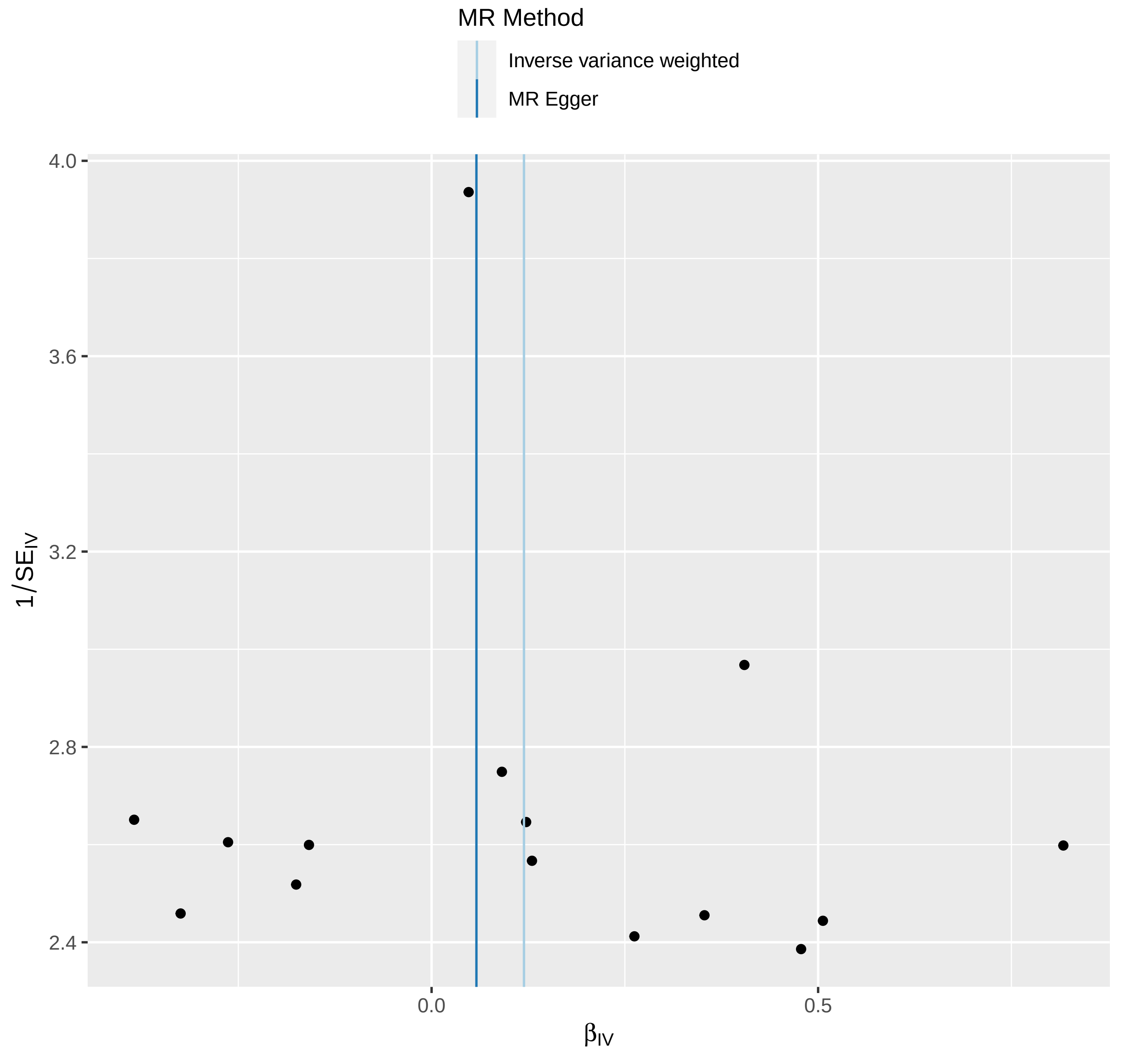

Batch 2−2065 : Gut microbiota abundance (genus Catenibacterium id.2153) on Protein FAM173A
with a threshold of 1e−05

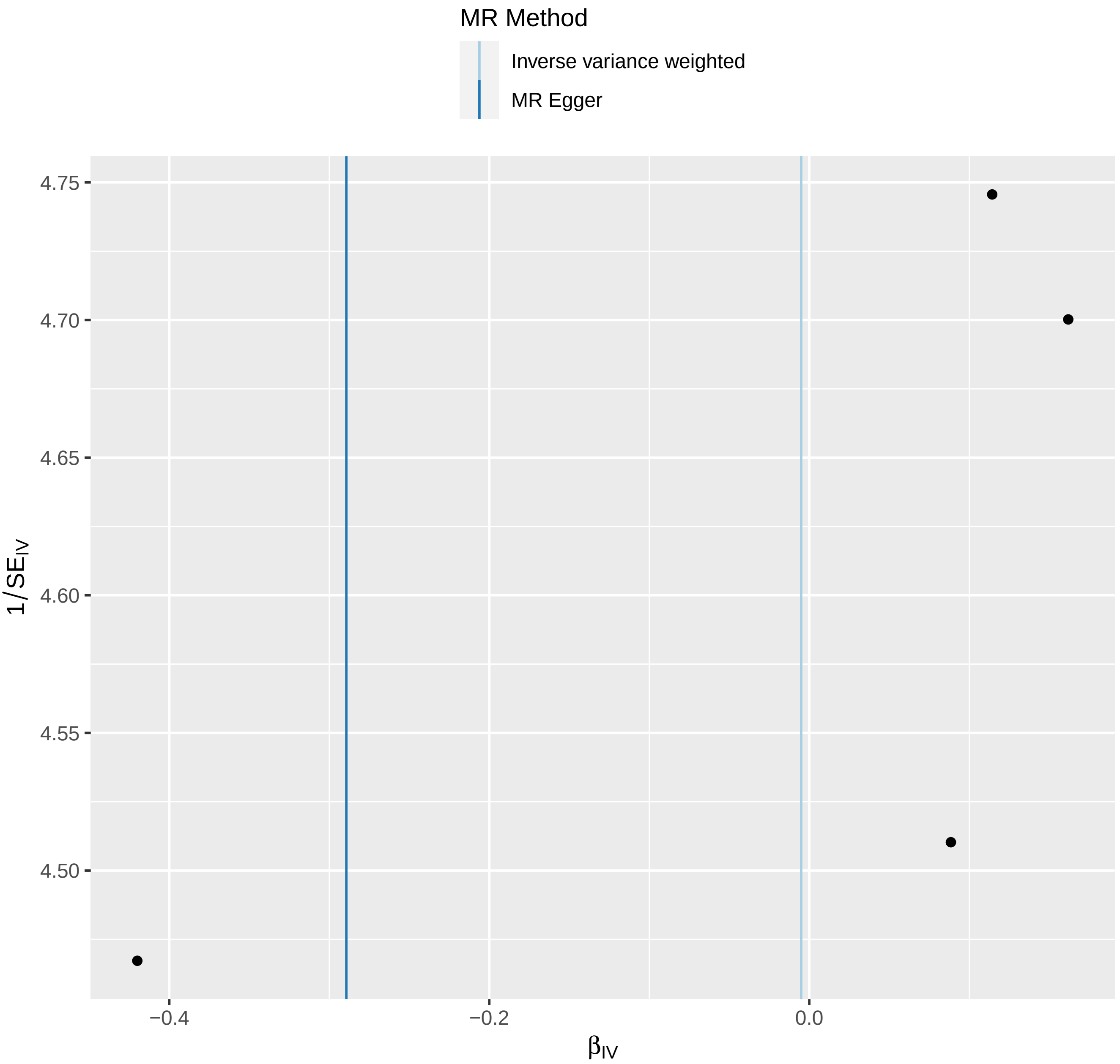

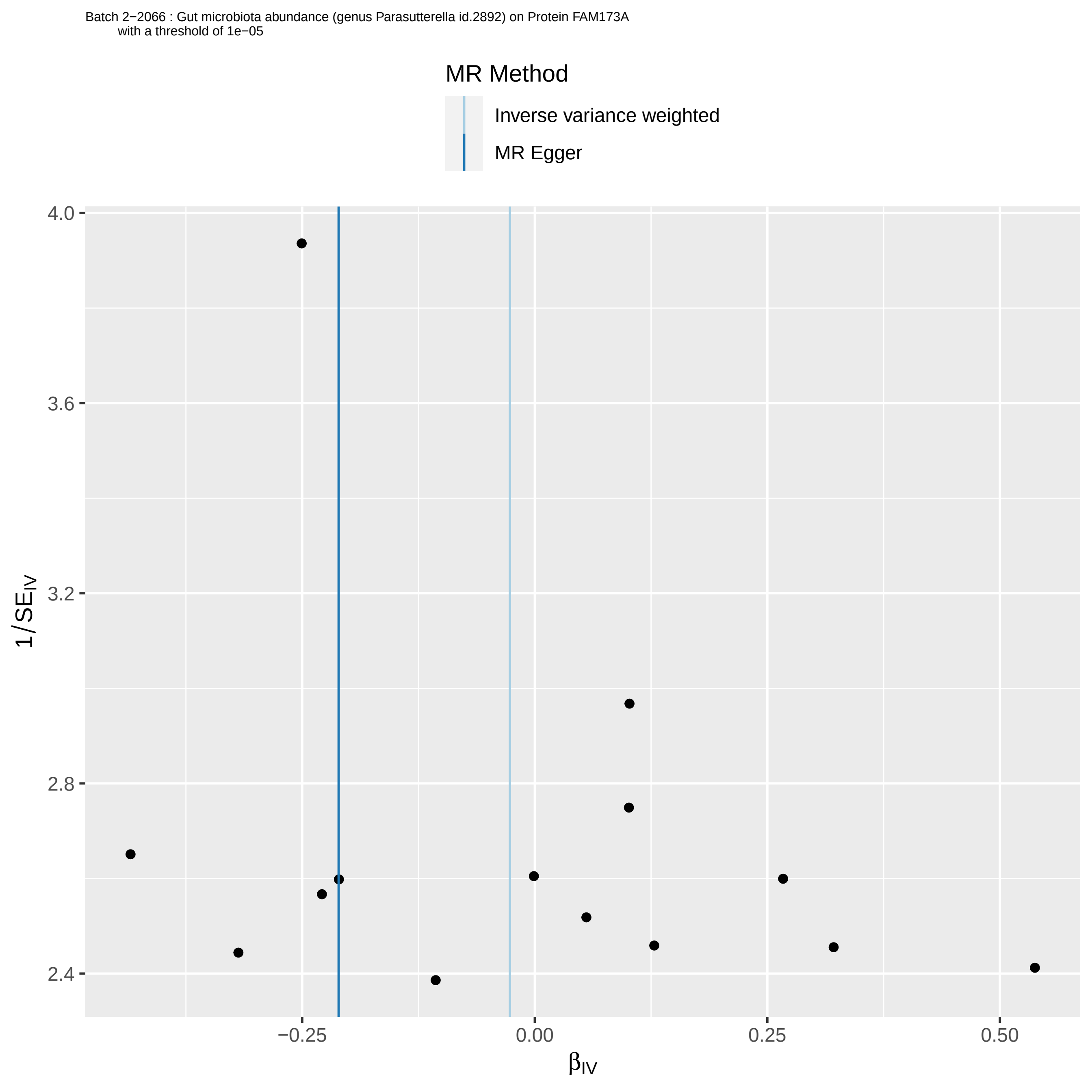
Batch 2−2066 : Gut microbiota abundance (genus Parasutterella id.2892) on Protein FAM173A
with a threshold of 1e−05
MR Method
Inverse variance weighted
MR Egger
4.0
3.6
3.2
2.8
2.4
1/SE$_{IV}$
−0.25
0.00
0.25
0.50
β_{IV}

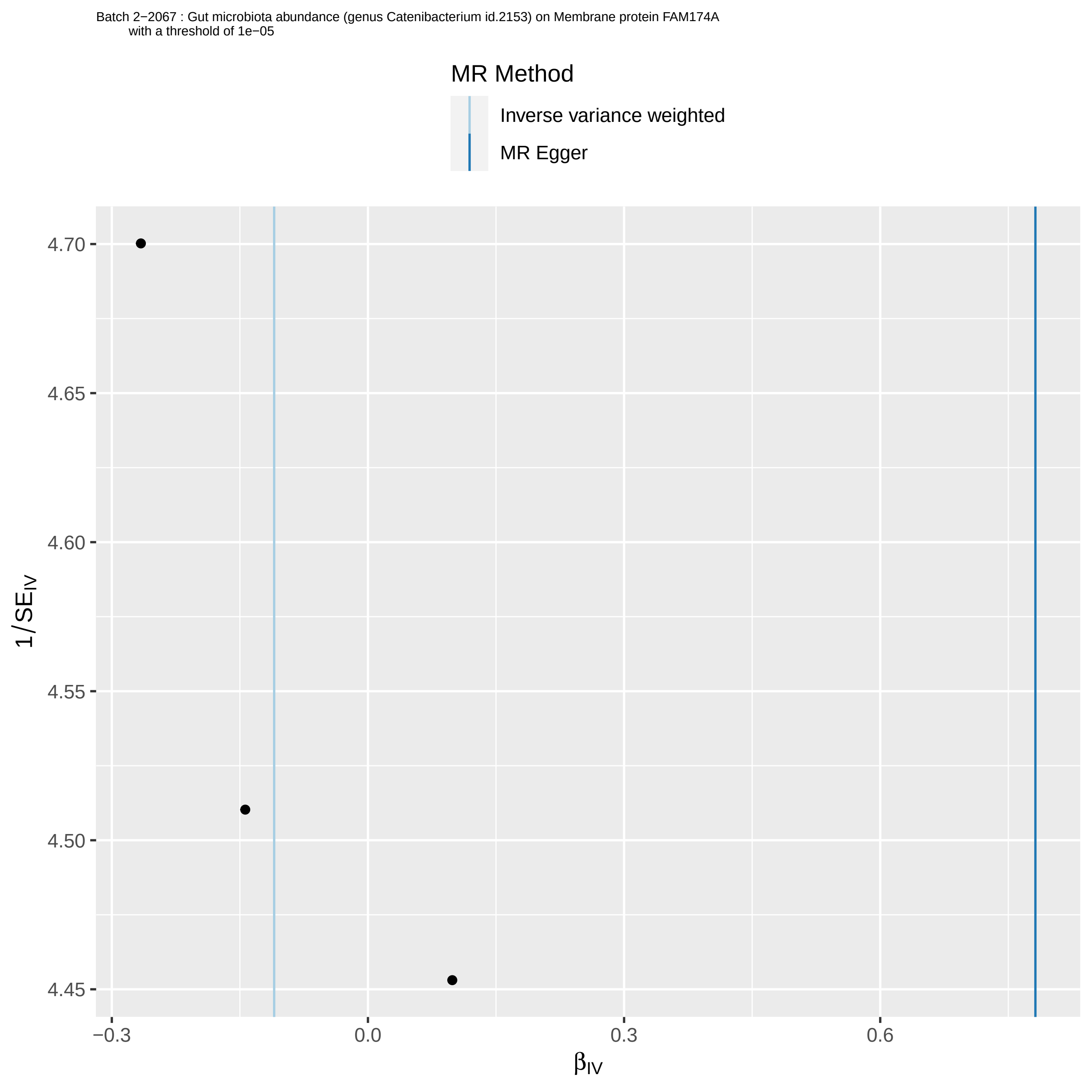

Batch 2−2067 : Gut microbiota abundance (genus Catenibacterium id.2153) on Membrane protein FAM174A
with a threshold of 1e−05
MR Method
Inverse variance weighted
MR Egger
4.70
4.65
4.60
4.55
4.50
4.45
1/SE_IV
−0.3
0.0
0.3
0.6
β_IV

Batch 2−2068 : Gut microbiota abundance (genus Parasutterella id.2892) on Membrane protein FAM174A
with a threshold of 1e−05

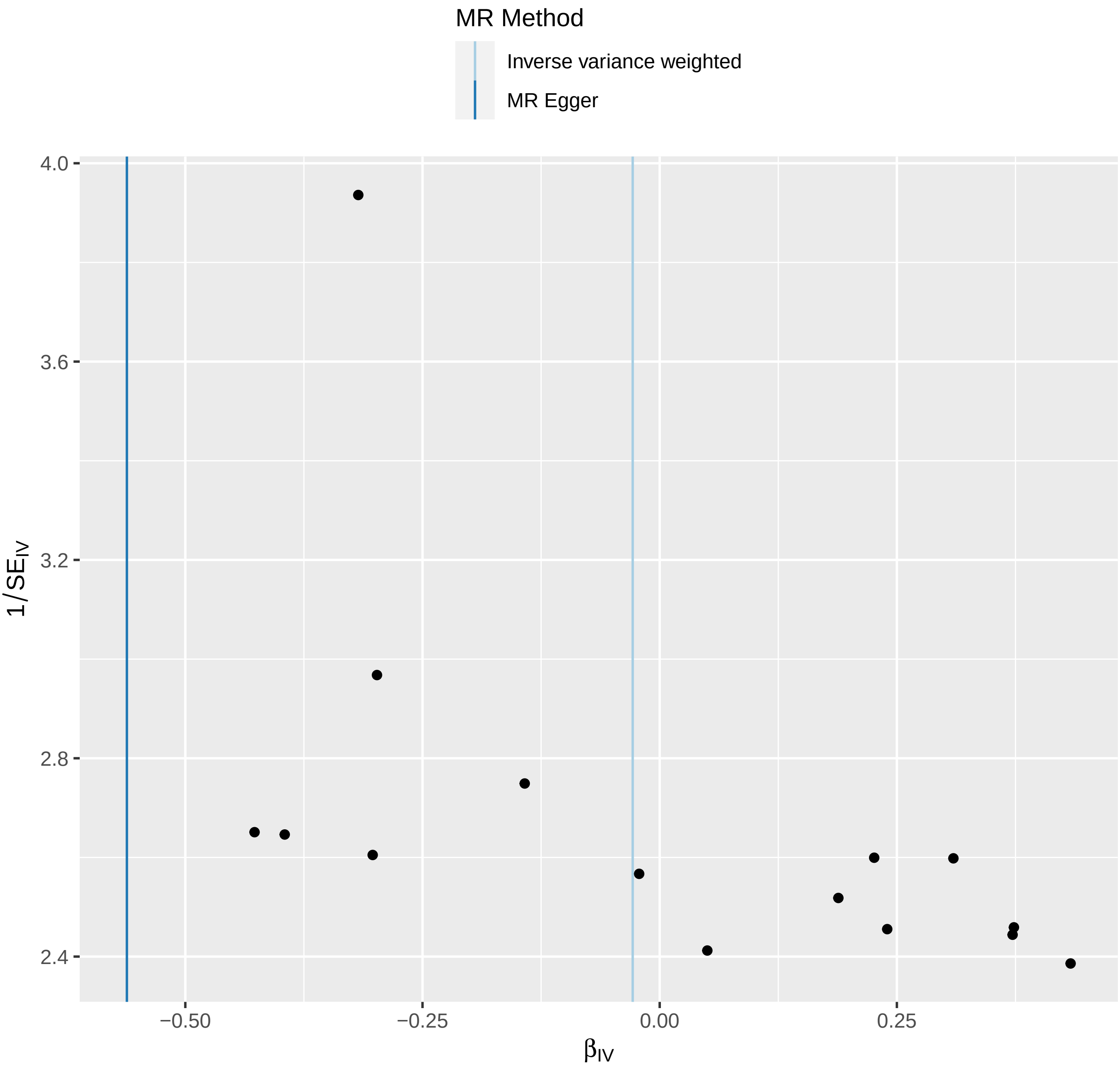

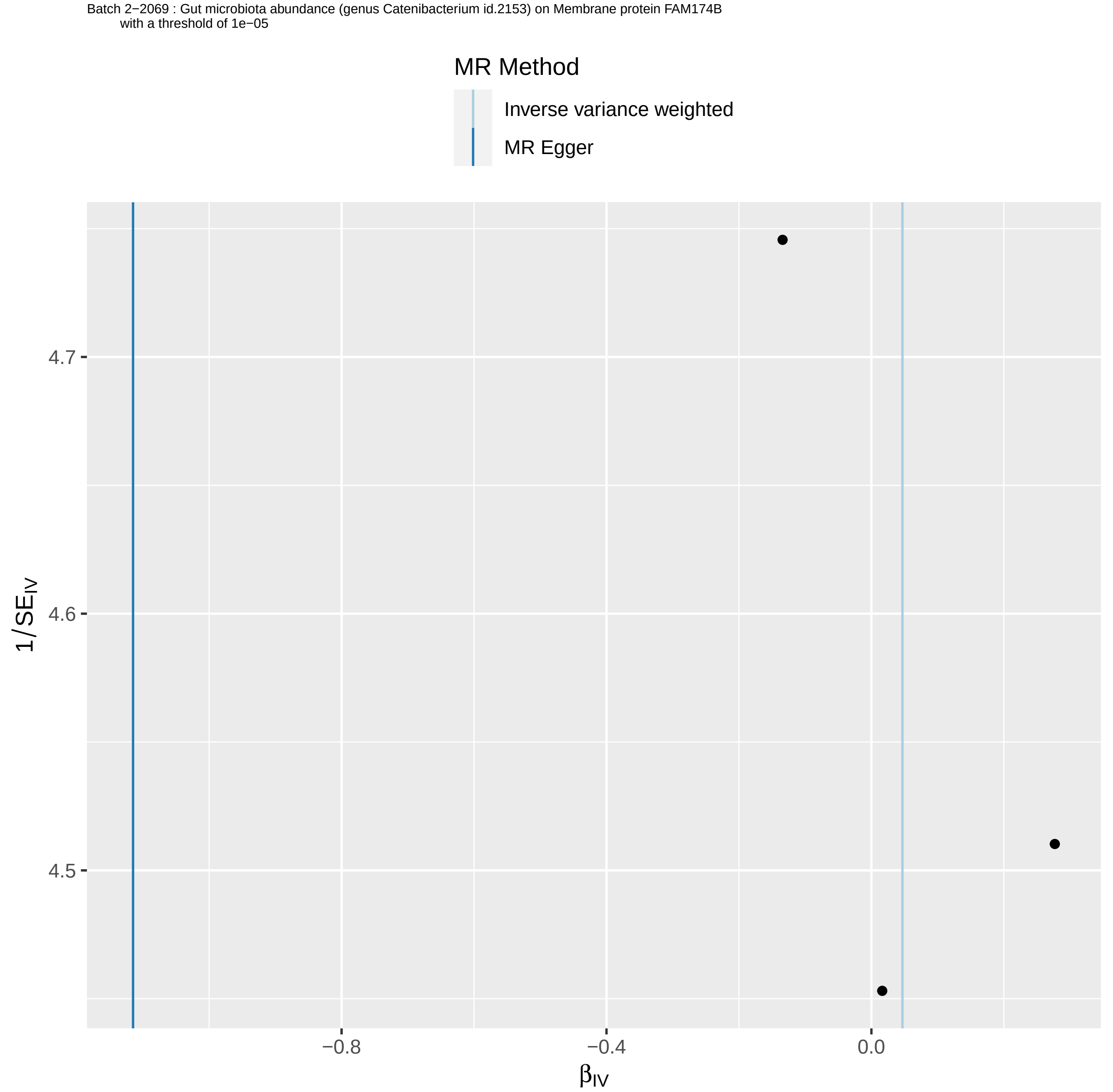
Batch 2−2069 : Gut microbiota abundance (genus Catenibacterium id.2153) on Membrane protein FAM174B
with a threshold of 1e−05
MR Method
Inverse variance weighted
MR Egger
4.7
4.6
4.5
1/SE_IV
−0.8
−0.4
0.0
β_IV

Batch 2−2070 : Gut microbiota abundance (genus Parasutterella id.2892) on Membrane protein FAM174B
with a threshold of 1e−05

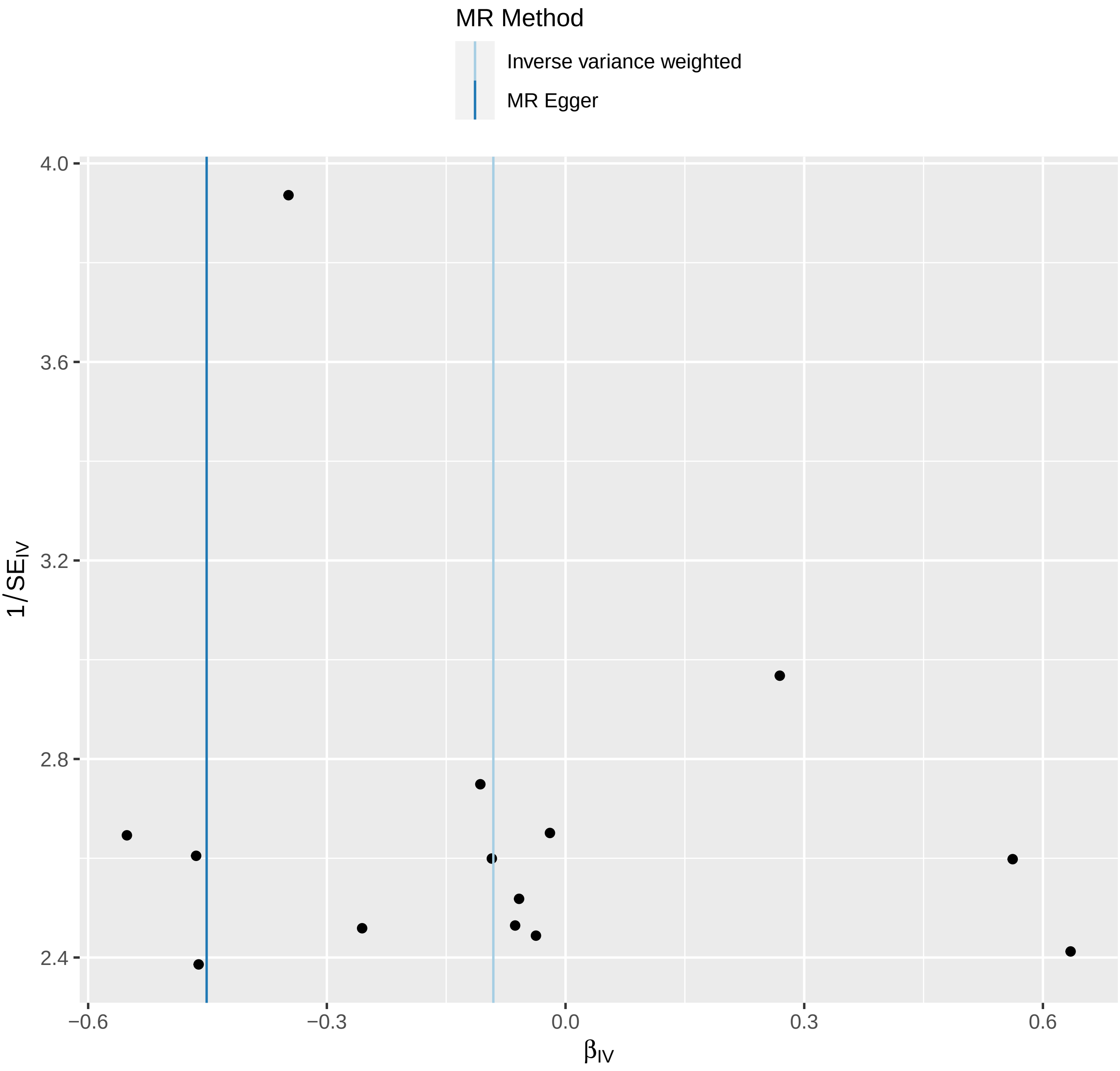

Batch 2−2071 : Gut microbiota abundance (genus Catenibacterium id.2153) on BRISC complex subunit Abro1
with a threshold of 1e−05

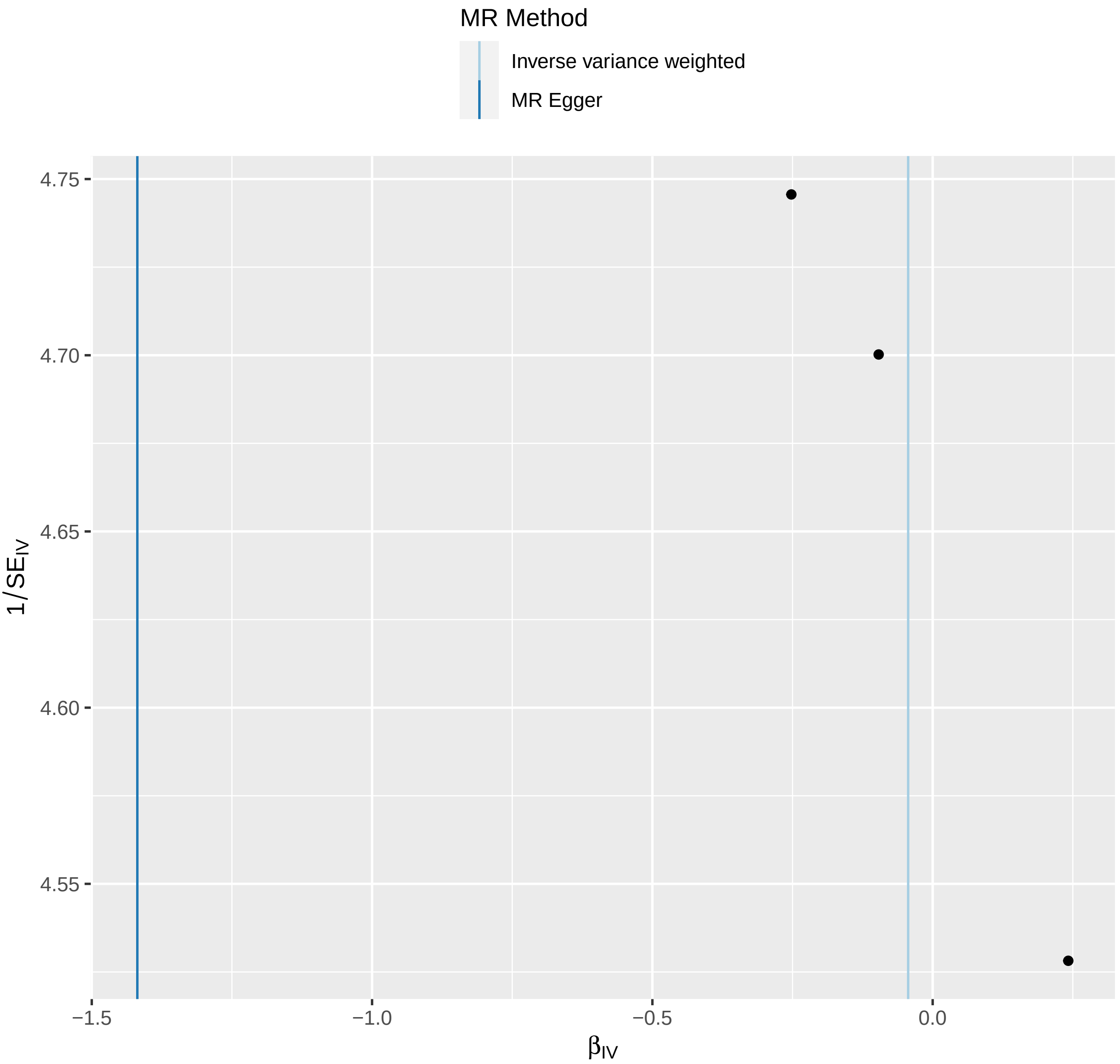

Batch 2−2072 : Gut microbiota abundance (genus Parasutterella id.2892) on BRISC complex subunit Abro1
with a threshold of 1e−05

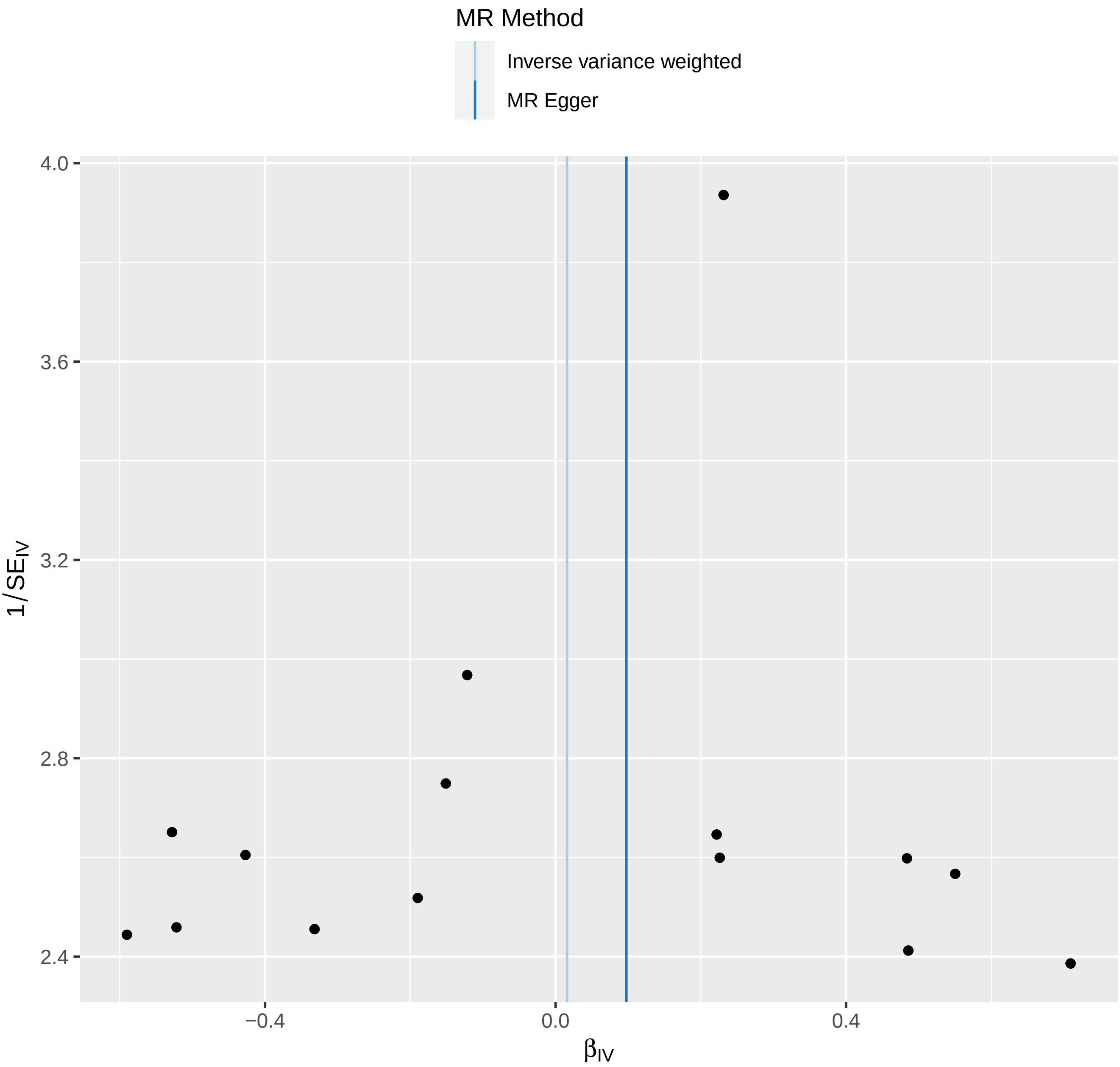

Batch 2−2073 : Gut microbiota abundance (genus Catenibacterium id.2153) on Protein FAM177A1
with a threshold of 1e−05

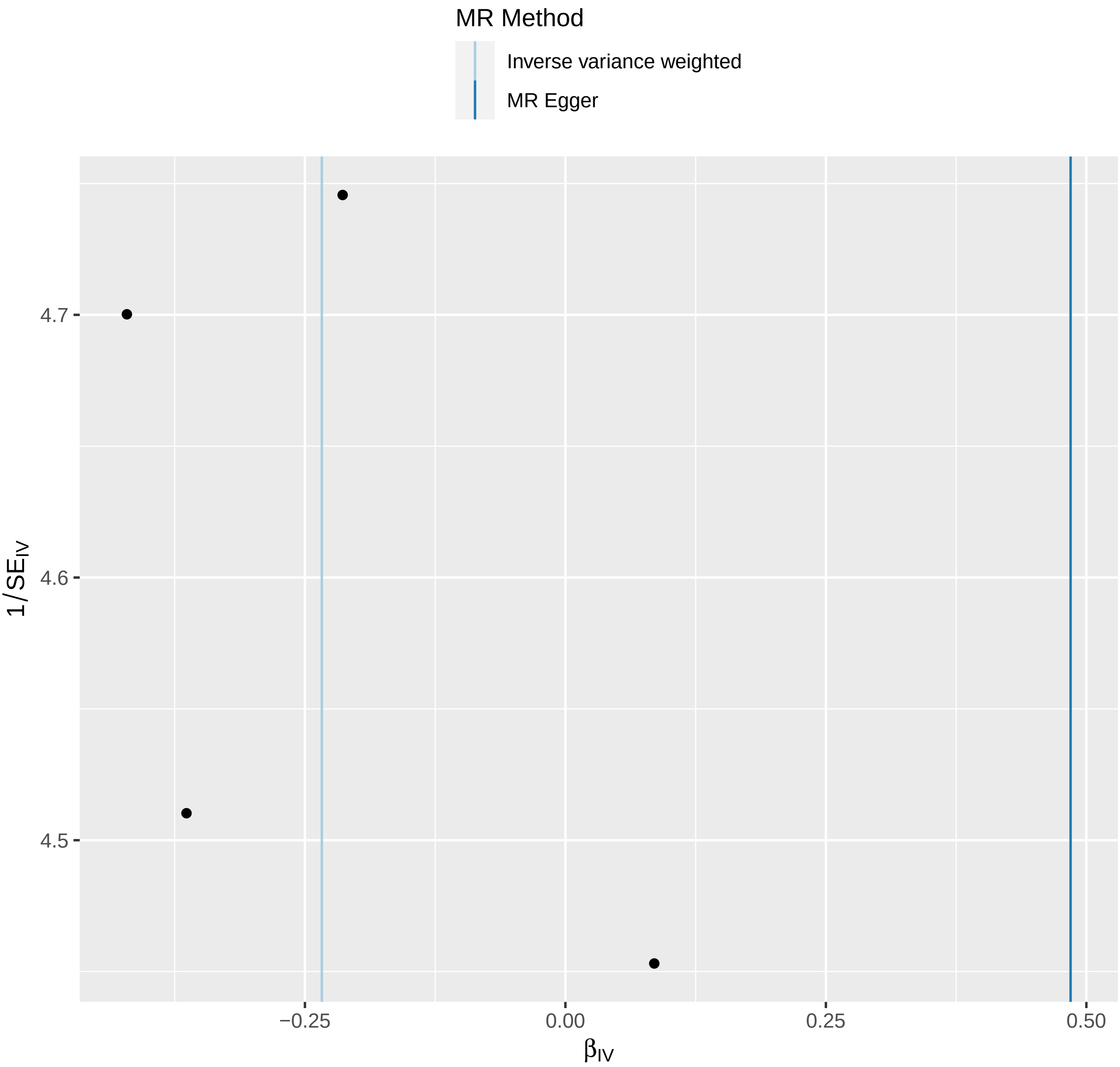

Batch 2−2074 : Gut microbiota abundance (genus Parasutterella id.2892) on Protein FAM177A1
with a threshold of 1e−05

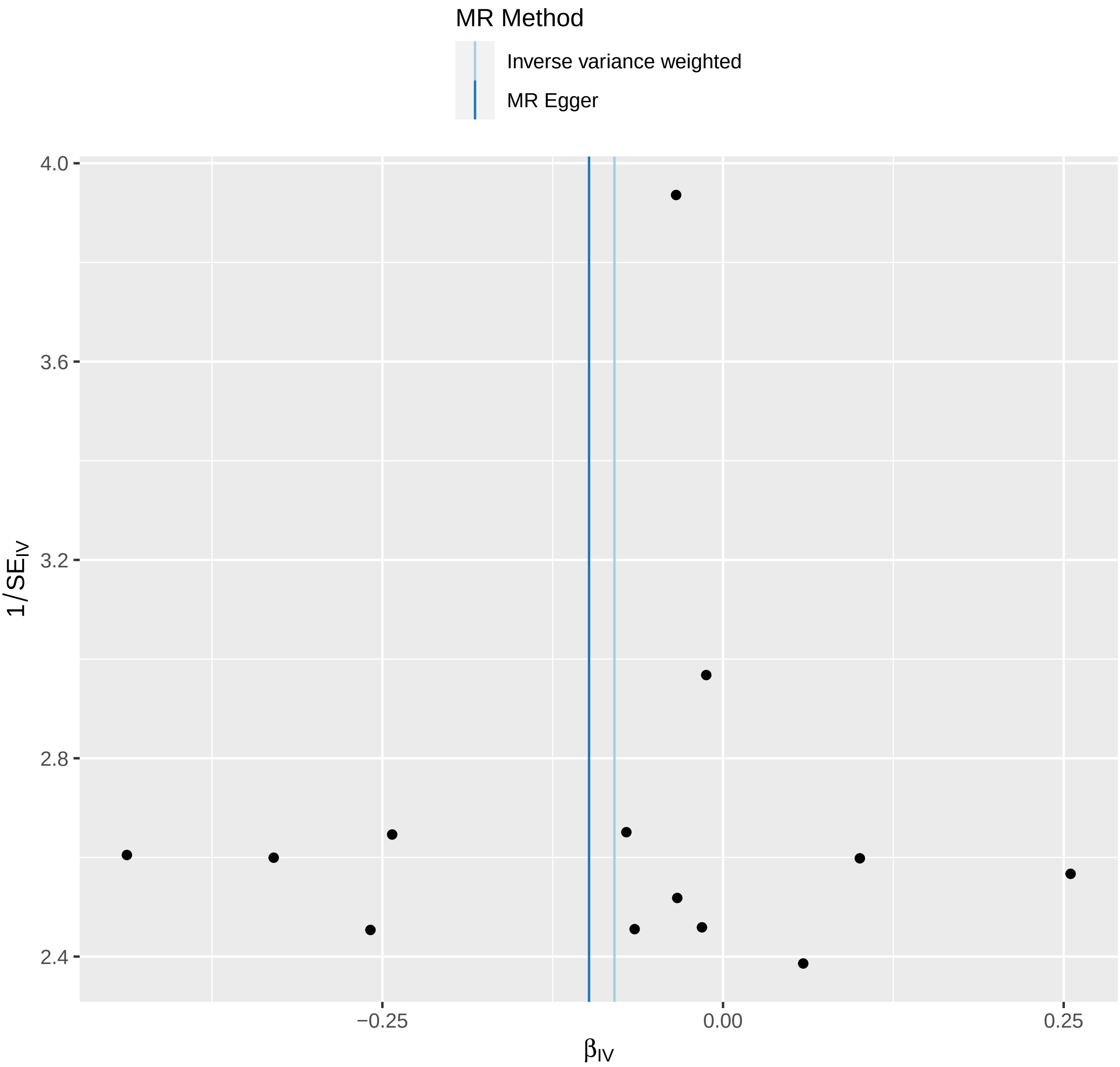

Batch 2−2075 : Gut microbiota abundance (genus Catenibacterium id.2153) on Protein FAM189A2
with a threshold of 1e−05

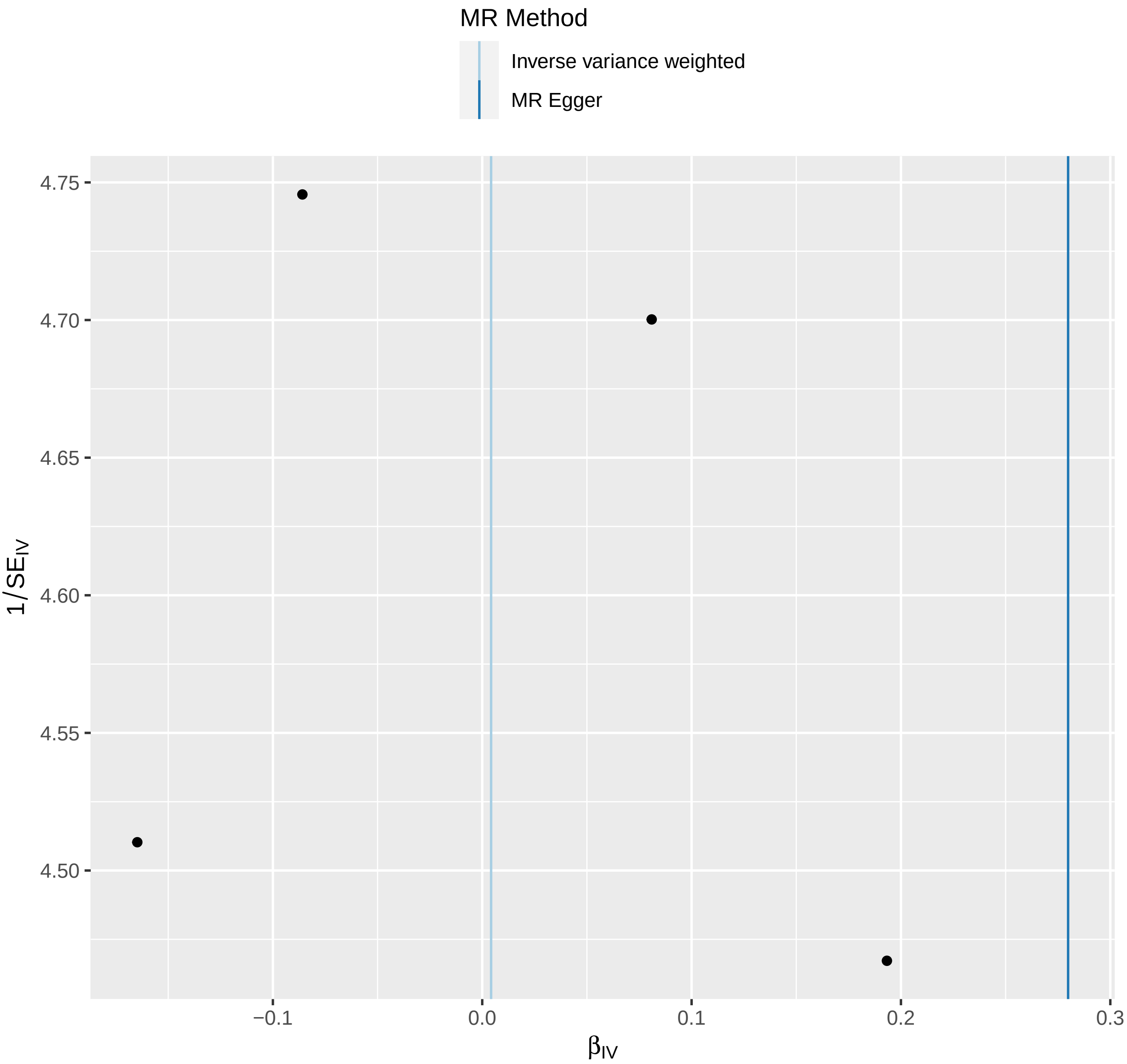

Batch 2−2076 : Gut microbiota abundance (genus Parasutterella id.2892) on Protein FAM189A2
with a threshold of 1e−05

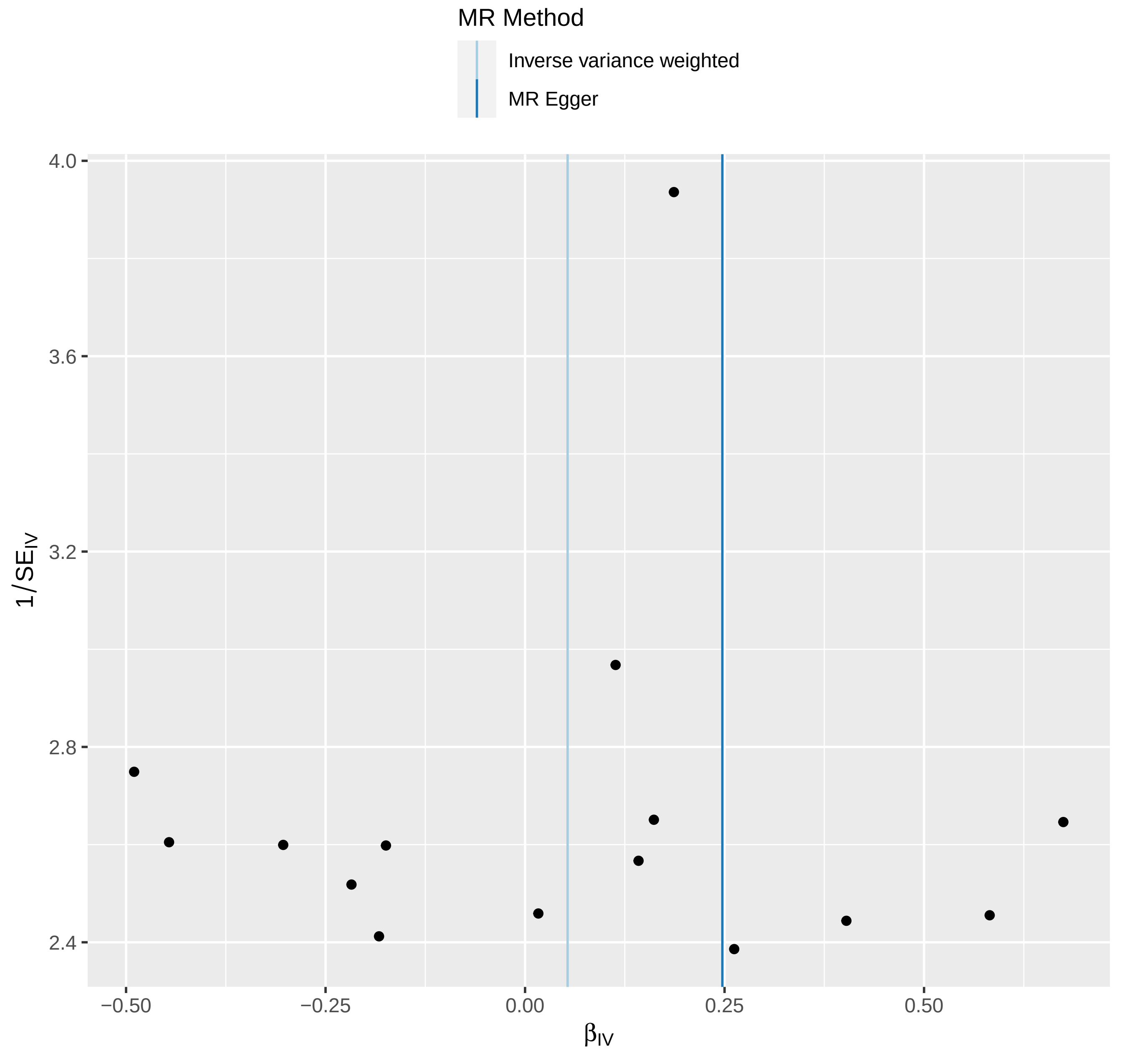

Batch 2−2077 : Gut microbiota abundance (genus Catenibacterium id.2153) on Protein FAM189A2
with a threshold of 1e−05

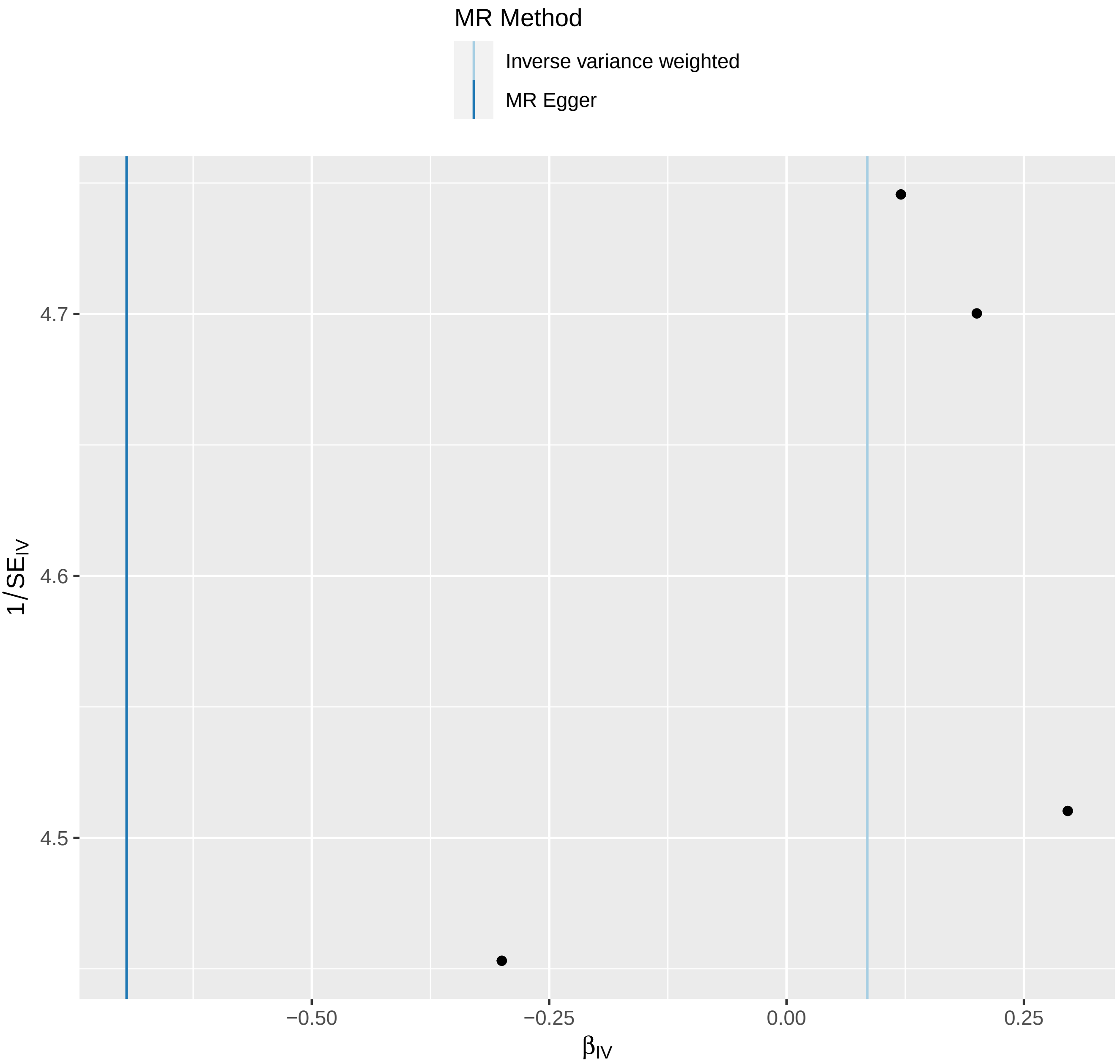

Batch 2−2078 : Gut microbiota abundance (genus Parasutterella id.2892) on Protein FAM189A2
with a threshold of 1e−05

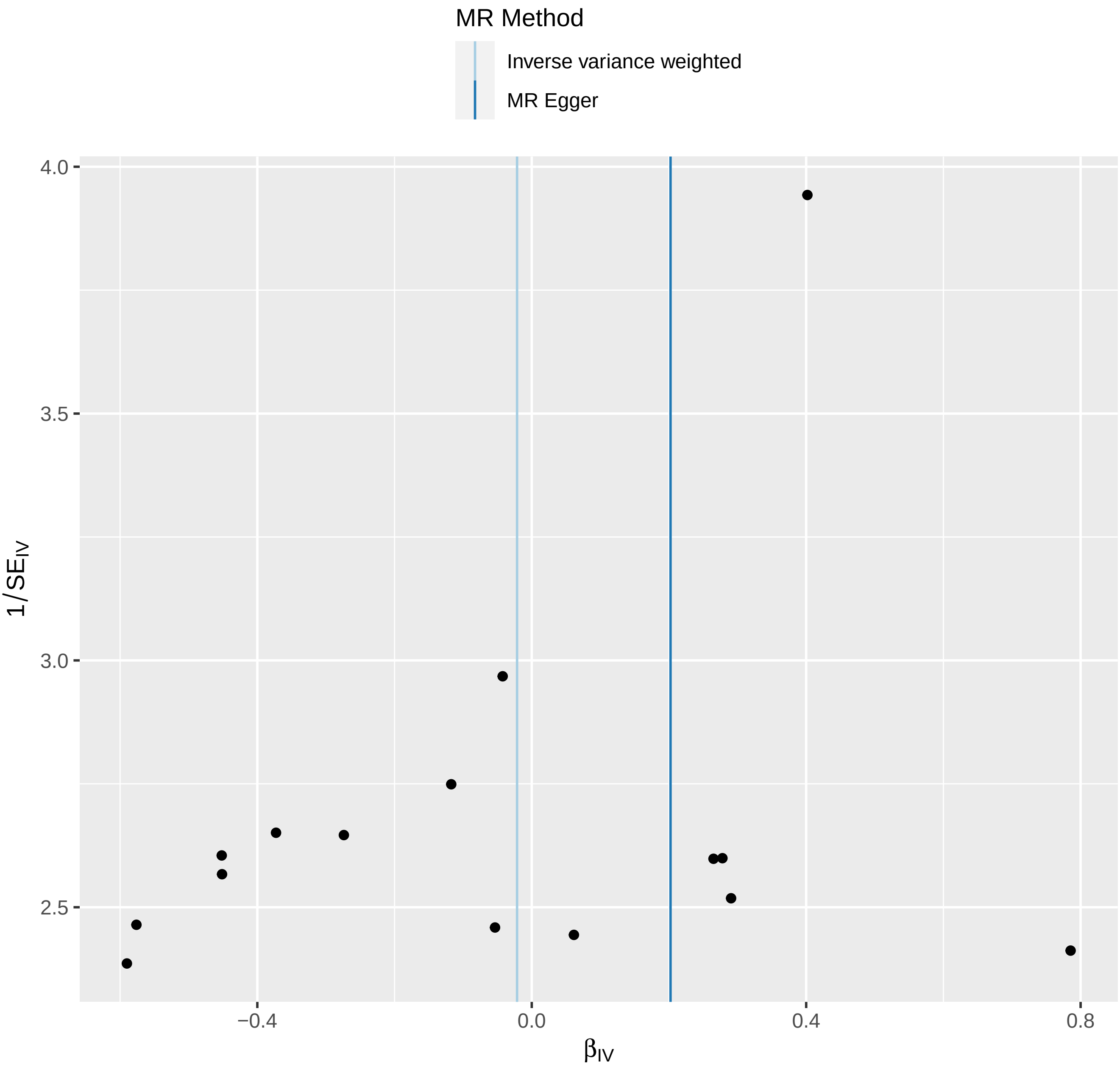

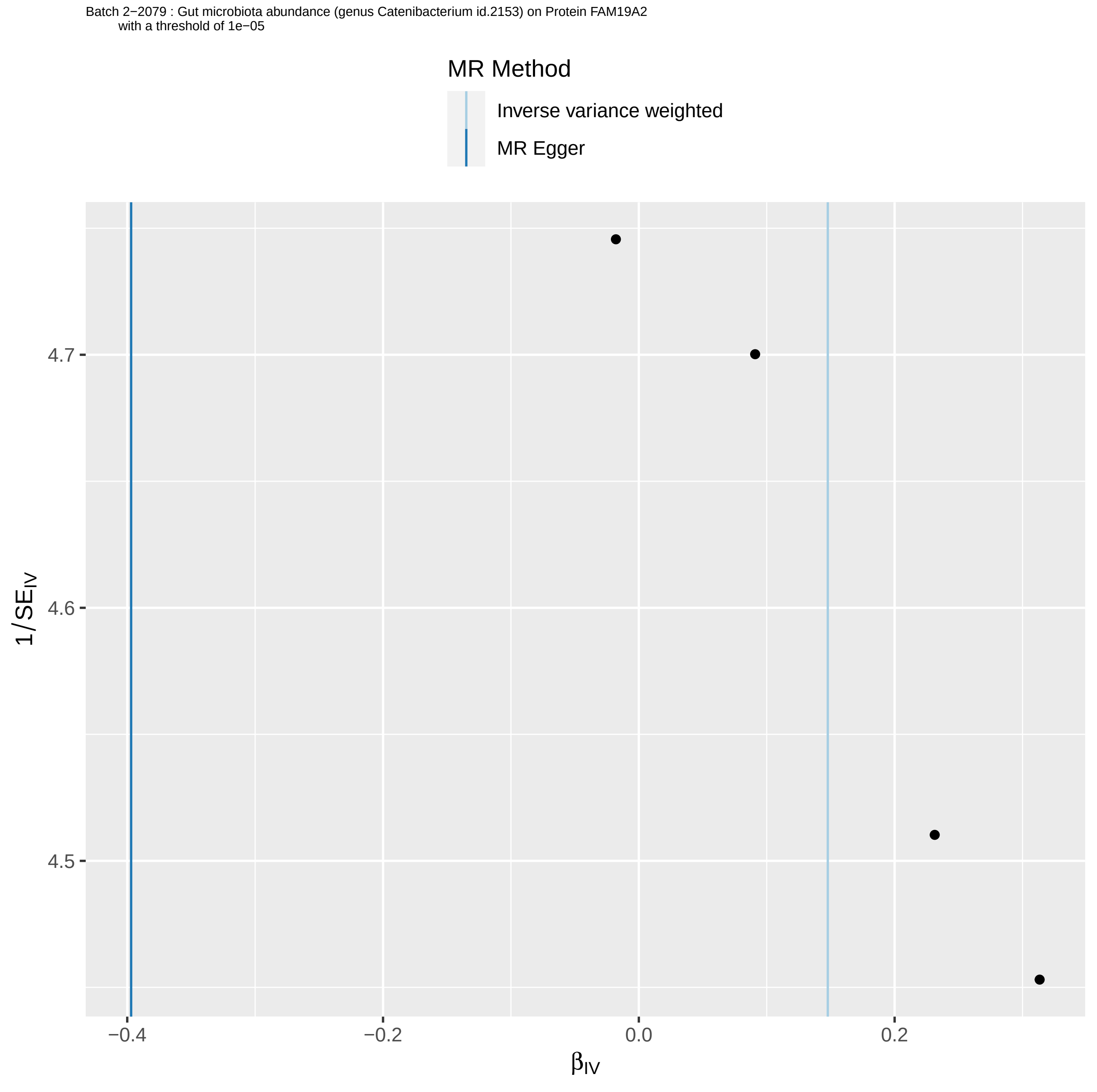

Batch 2−2079 : Gut microbiota abundance (genus Catenibacterium id.2153) on Protein FAM19A2
with a threshold of 1e−05
MR Method
Inverse variance weighted
MR Egger
4.7
4.6
4.5
1/SE_IV
−0.4
−0.2
0.0
0.2
β_IV

Batch 2−2080 : Gut microbiota abundance (genus Parasutterella id.2892) on Protein FAM19A2
with a threshold of 1e−05

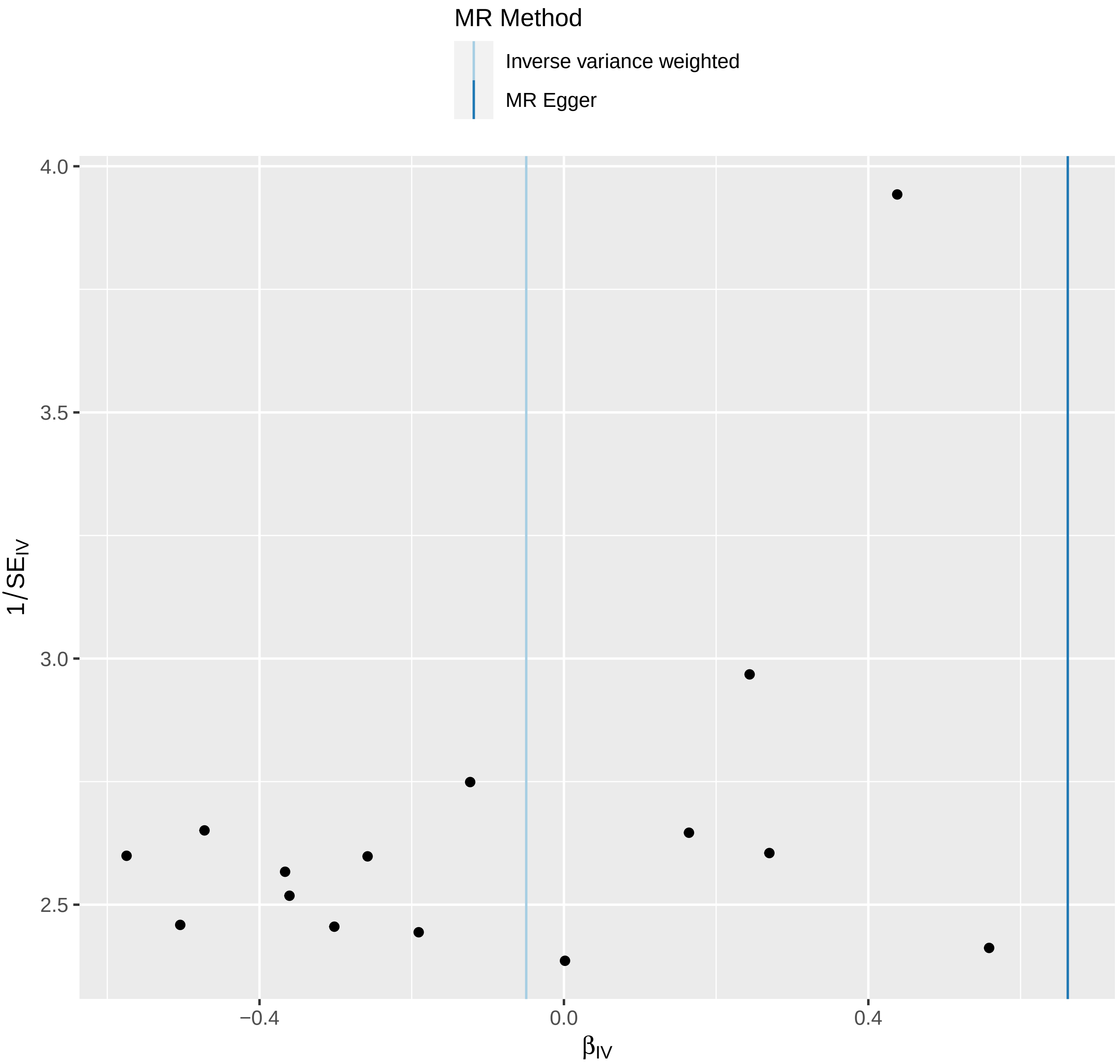

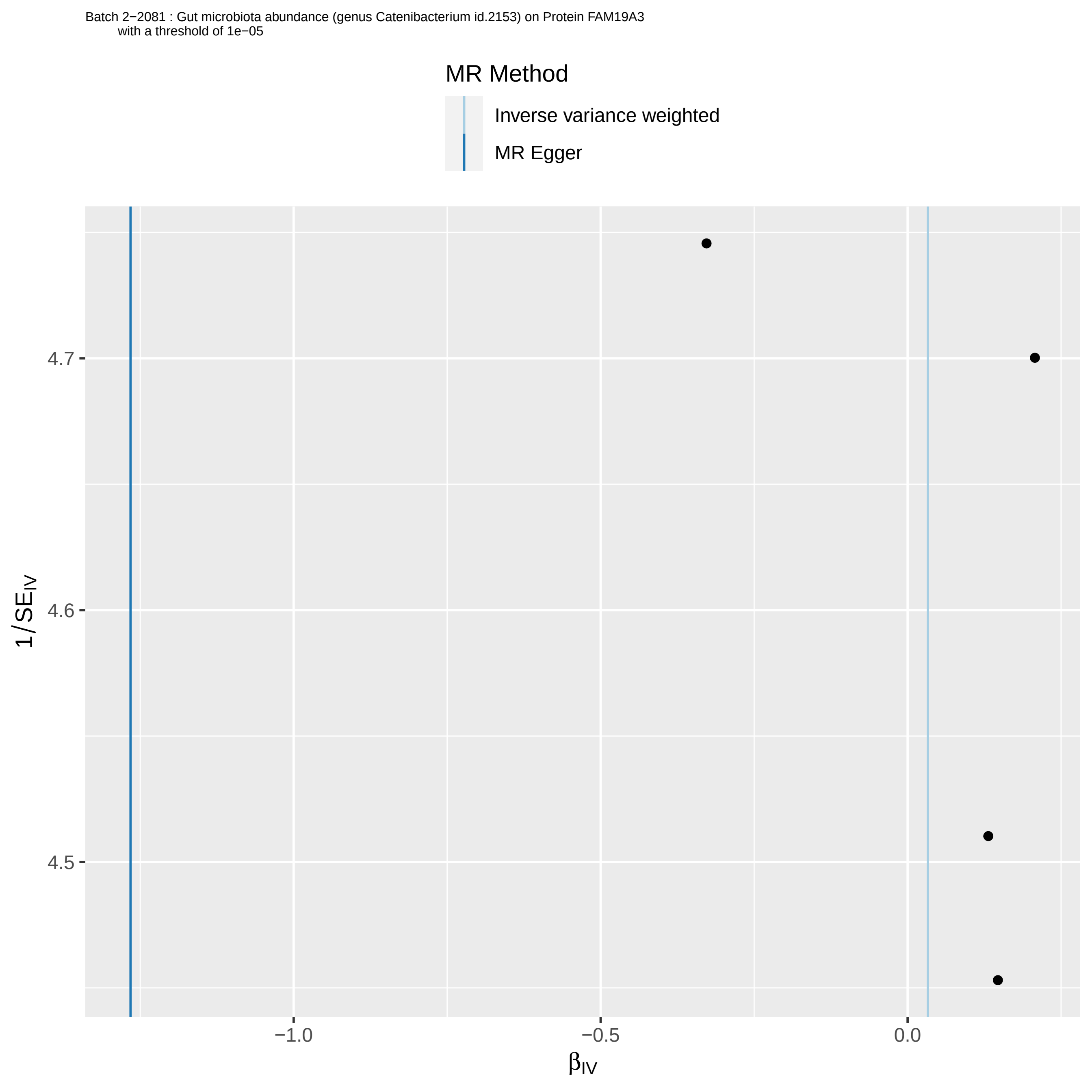

Batch 2−2081 : Gut microbiota abundance (genus Catenibacterium id.2153) on Protein FAM19A3
with a threshold of 1e−05
MR Method
Inverse variance weighted
MR Egger
4.7
4.6
4.5
1/SE_IV
−1.0
−0.5
0.0
β_IV

Batch 2−2082 : Gut microbiota abundance (genus Parasutterella id.2892) on Protein FAM19A3
with a threshold of 1e−05

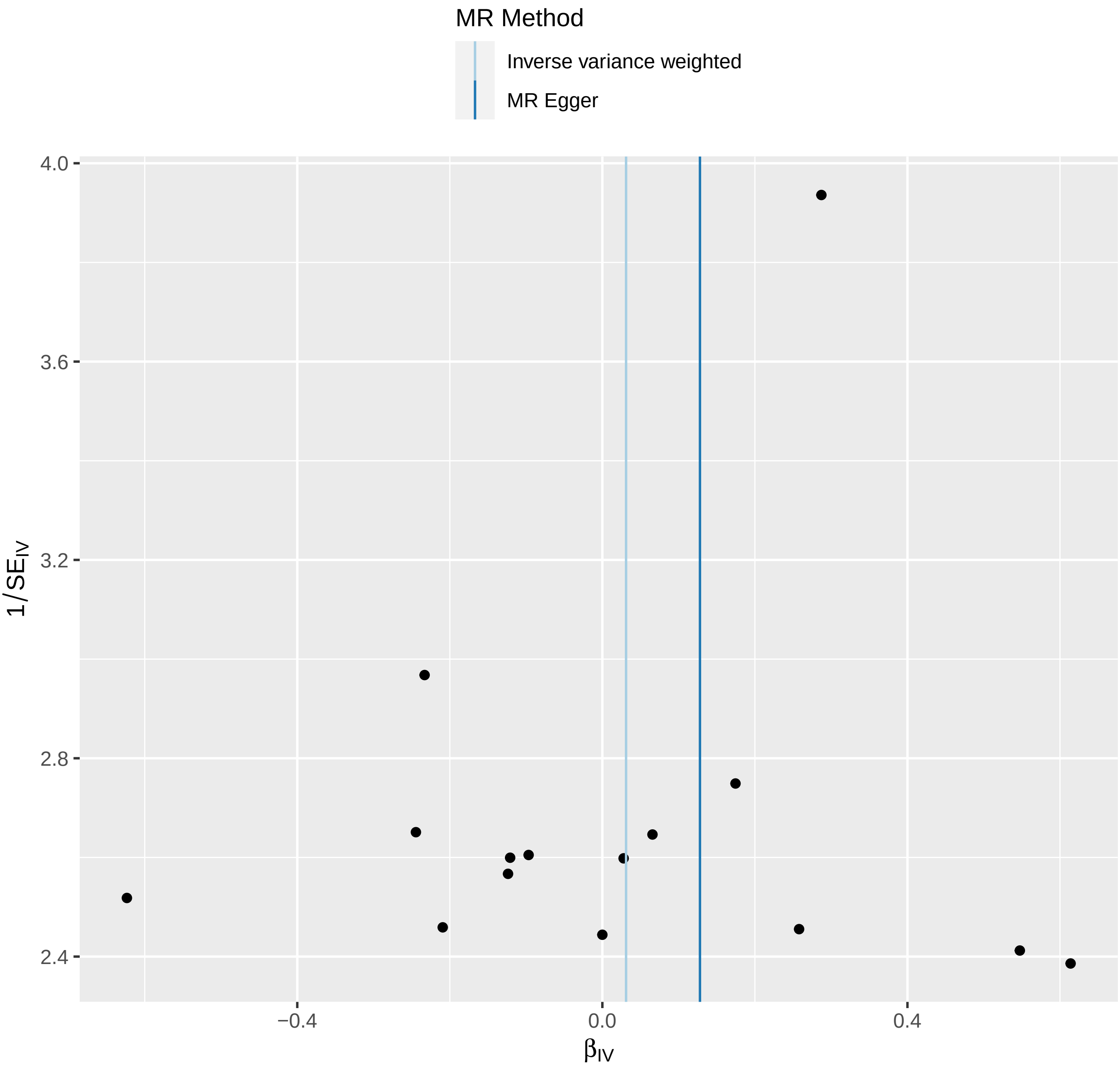

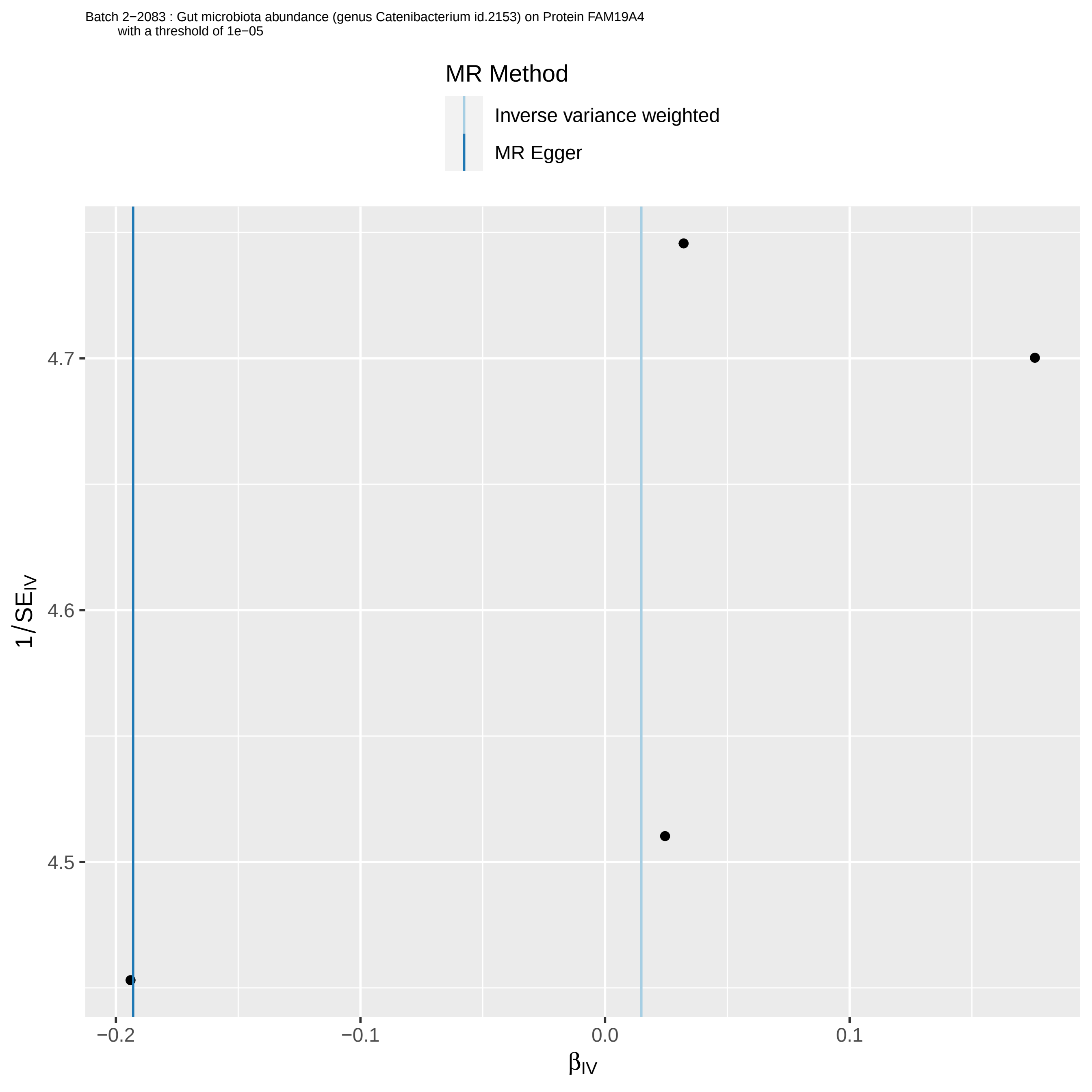
Batch 2−2083 : Gut microbiota abundance (genus Catenibacterium id.2153) on Protein FAM19A4
with a threshold of 1e−05
MR Method
Inverse variance weighted
MR Egger
4.7
4.6
4.5
1/SE_IV
−0.2
−0.1
0.0
0.1
β_IV

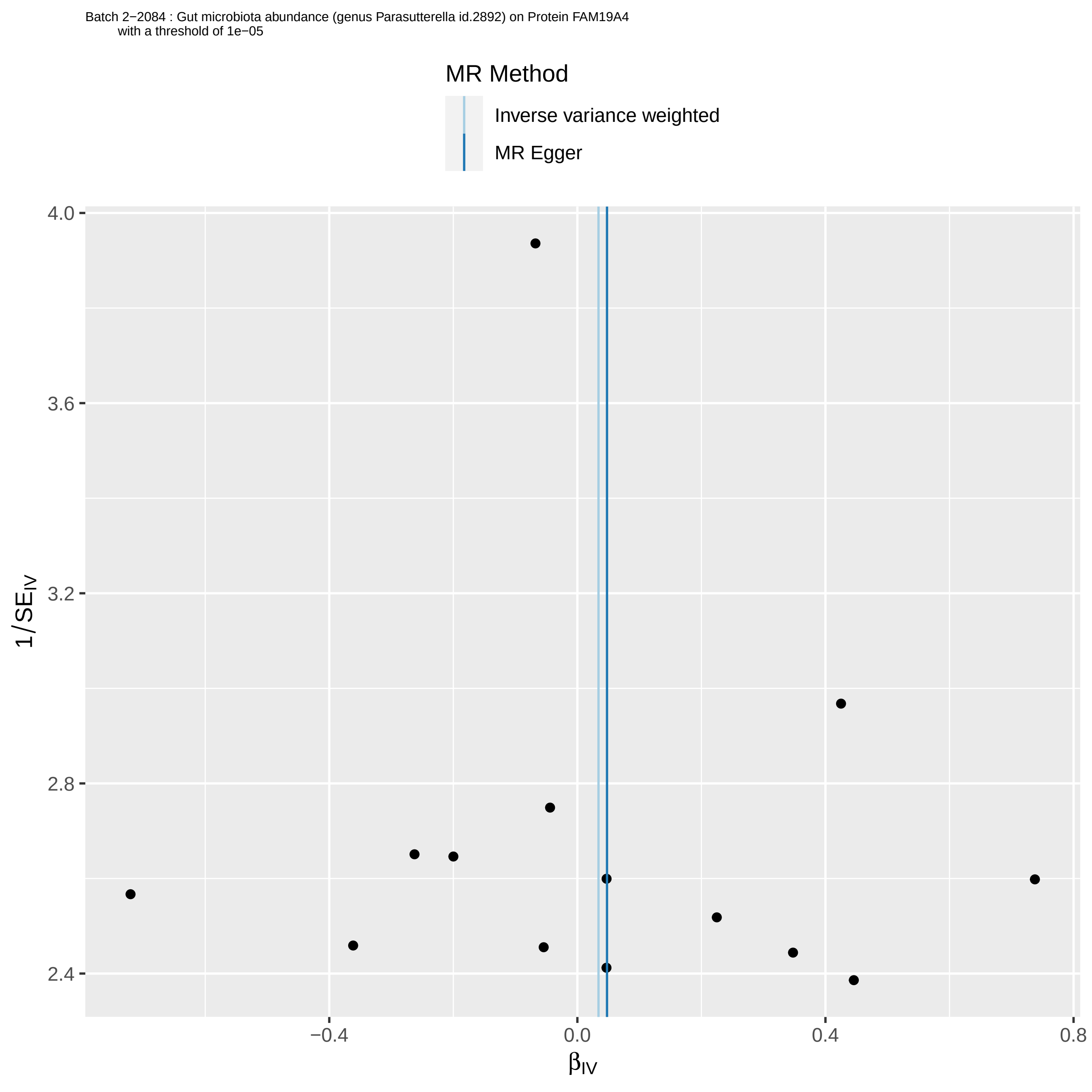
Batch 2−2084 : Gut microbiota abundance (genus Parasutterella id.2892) on Protein FAM19A4
with a threshold of 1e−05
MR Method
Inverse variance weighted
MR Egger
4.0
3.6
3.2
2.8
2.4
1/SE_IV
−0.4
0.0
0.4
0.8
β_IV

Batch 2−2085 : Gut microbiota abundance (genus Catenibacterium id.2153) on Protein FAM19A5
with a threshold of 1e−05

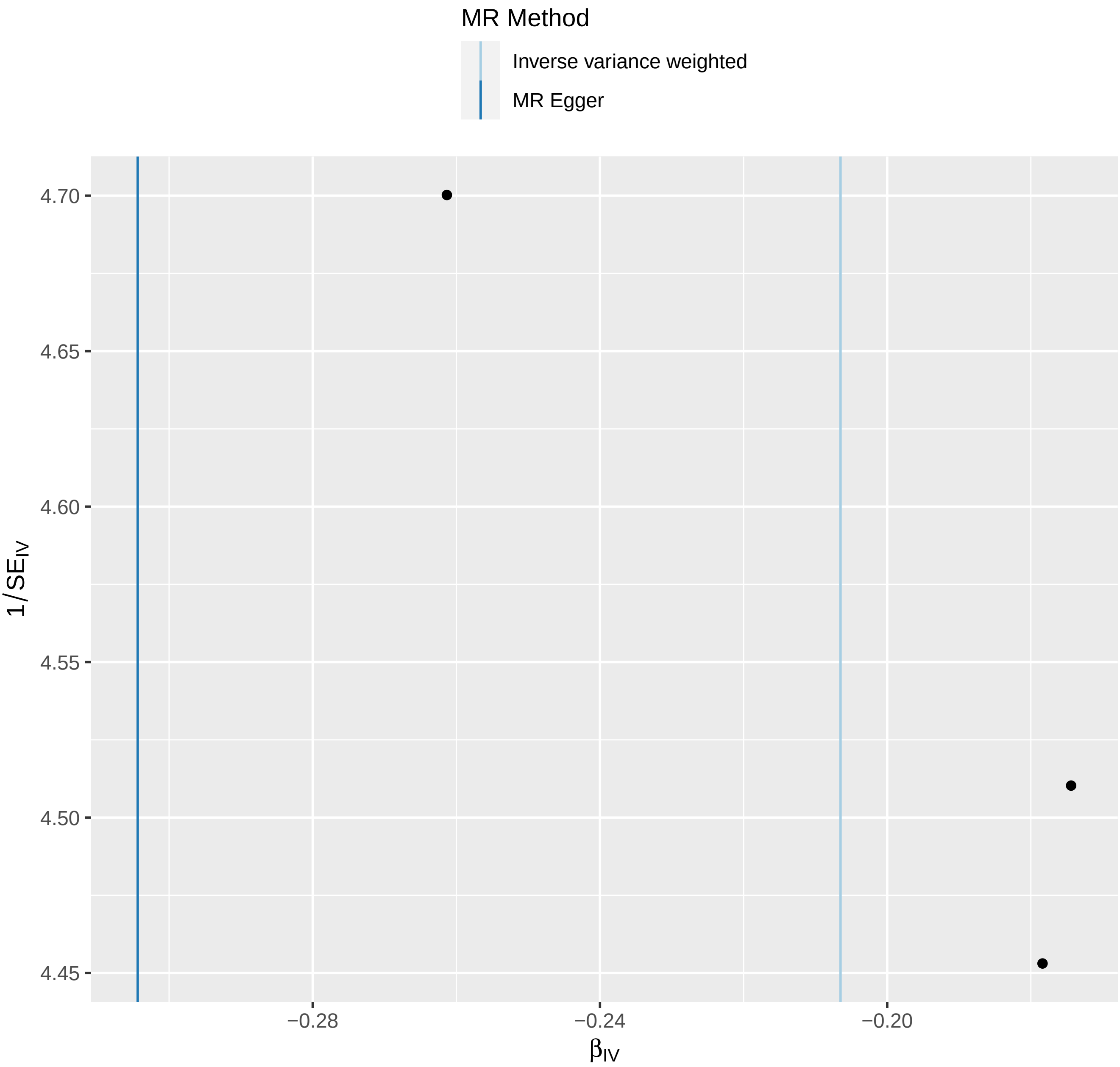

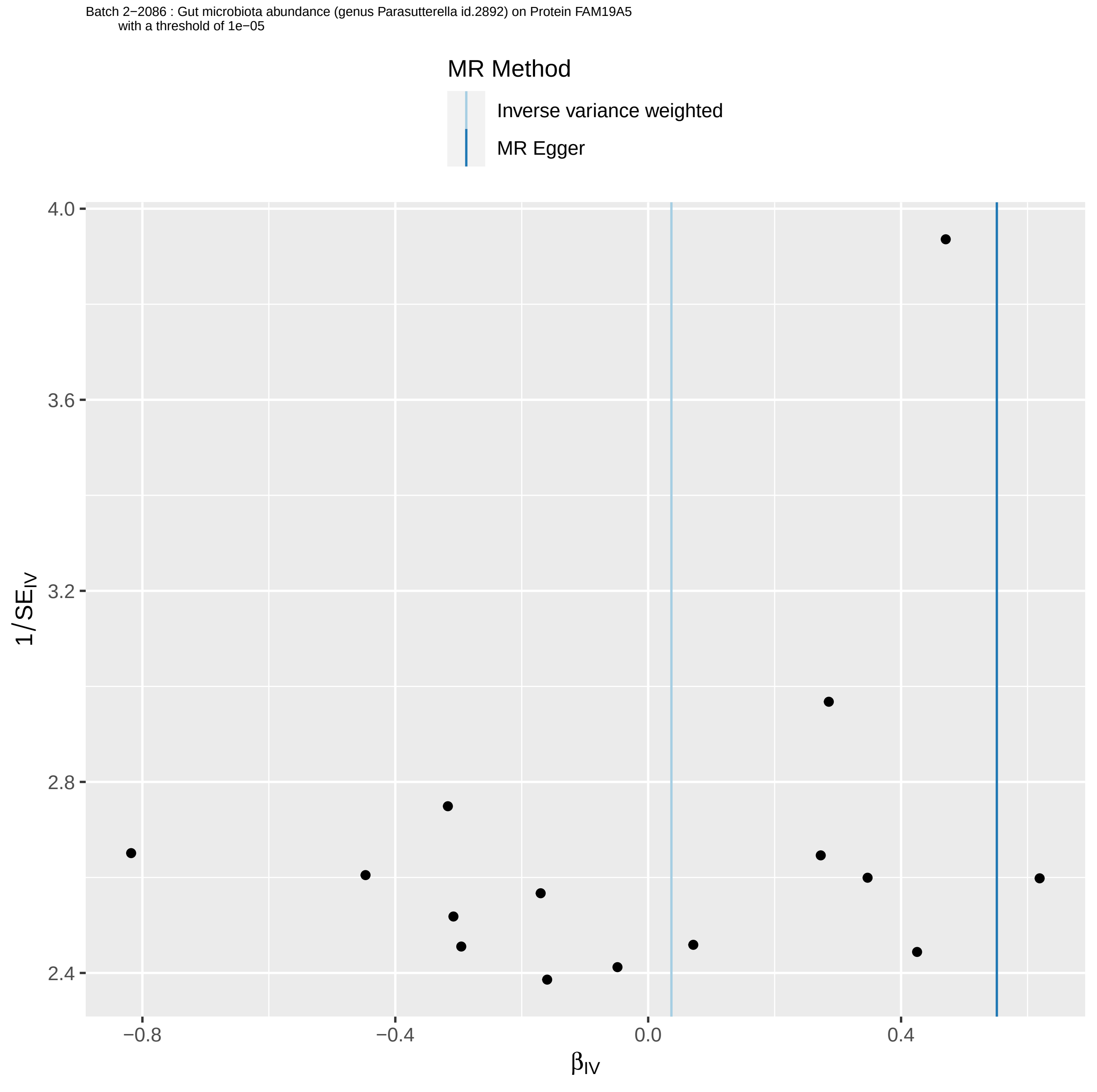

Batch 2−2086 : Gut microbiota abundance (genus Parasutterella id.2892) on Protein FAM19A5
with a threshold of 1e−05
MR Method
Inverse variance weighted
MR Egger
4.0
3.6
3.2
2.8
2.4
1/SE_IV
−0.8
−0.4
0.0
0.4
β_IV

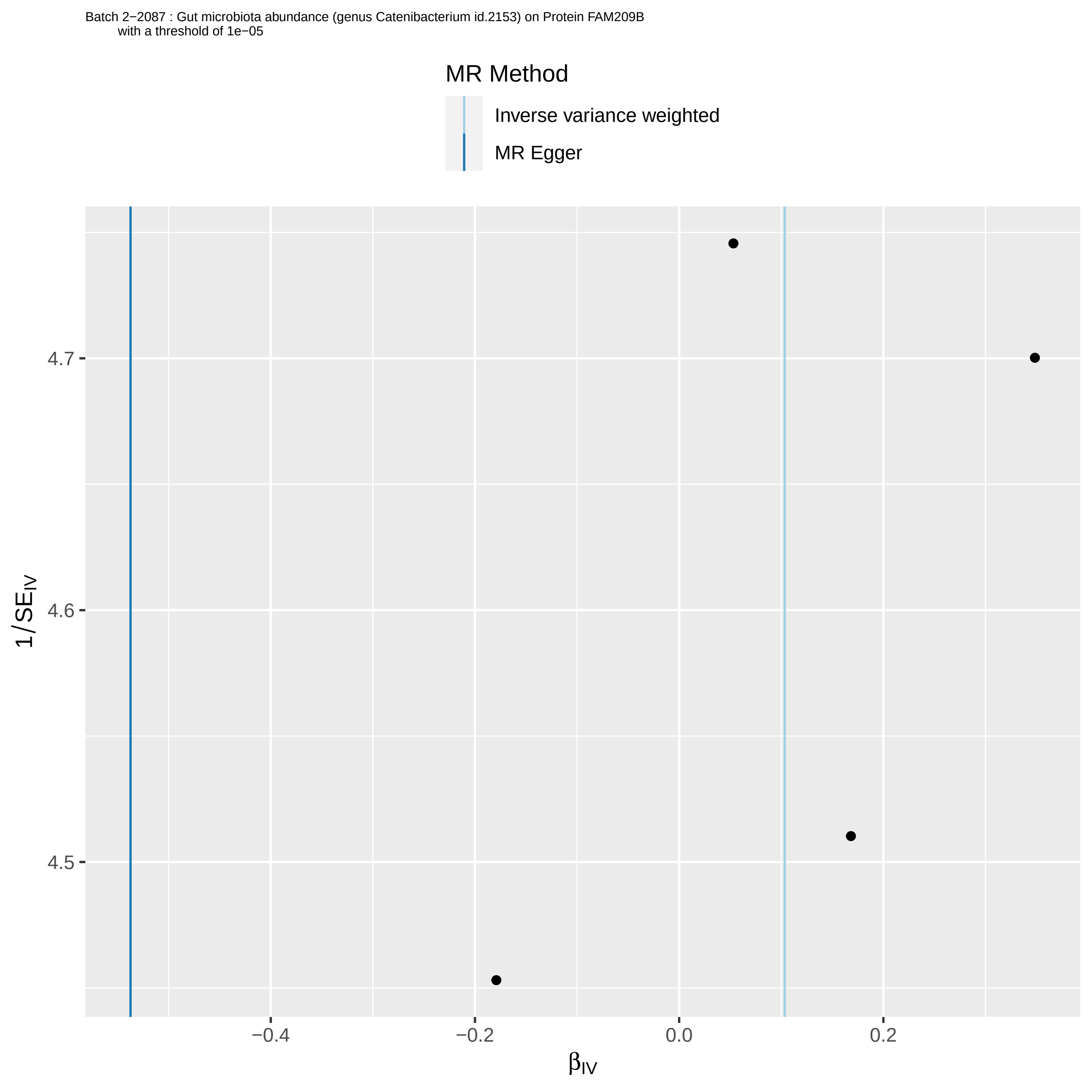

Batch 2−2087 : Gut microbiota abundance (genus Catenibacterium id.2153) on Protein FAM209B
with a threshold of 1e−05
MR Method
Inverse variance weighted
MR Egger
4.7
4.6
4.5
−0.4
−0.2
0.0
0.2
$1/SE_{IV}$
β_{IV}

Batch 2−2088 : Gut microbiota abundance (genus Parasutterella id.2892) on Protein FAM209B
with a threshold of 1e−05

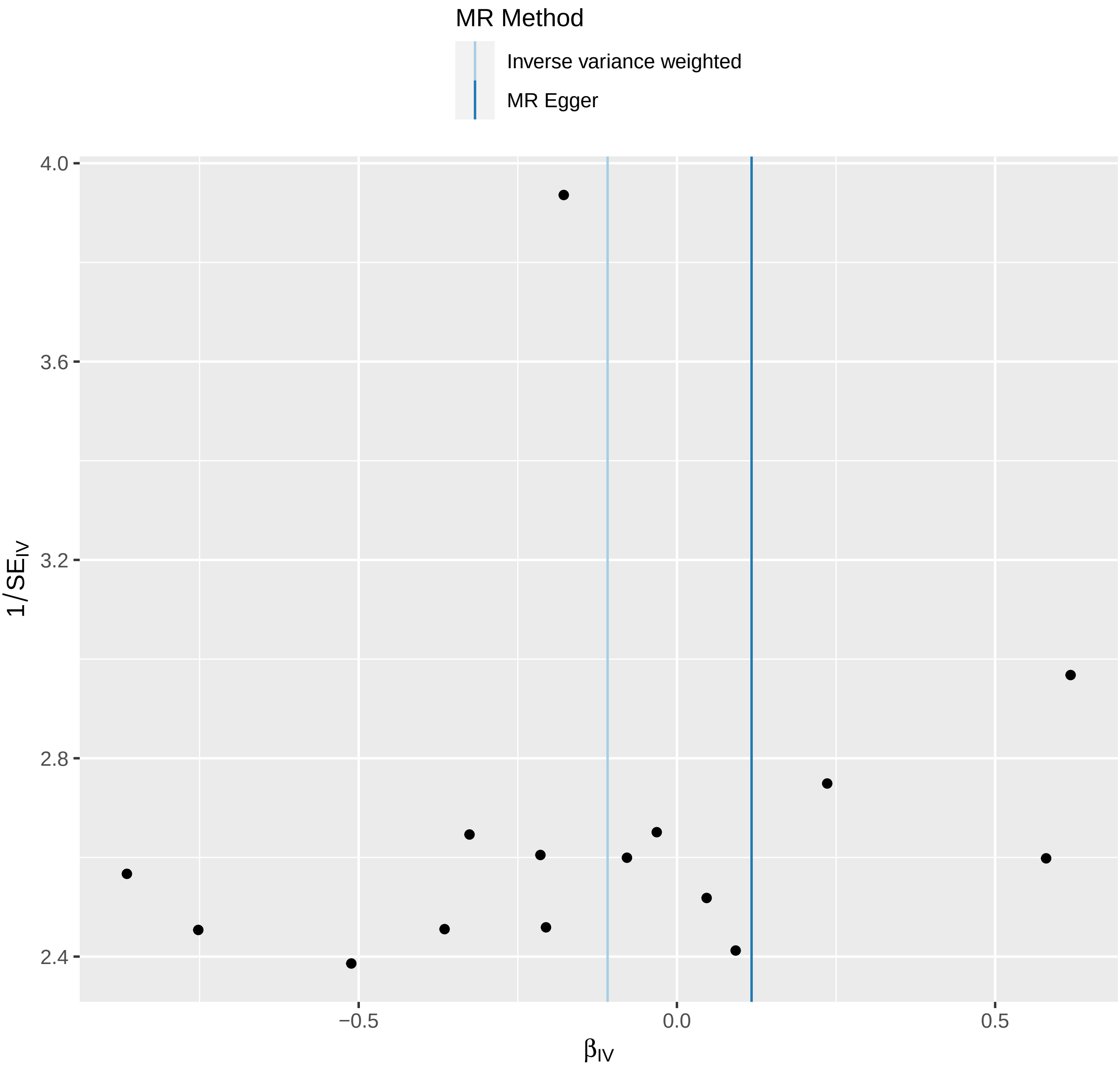

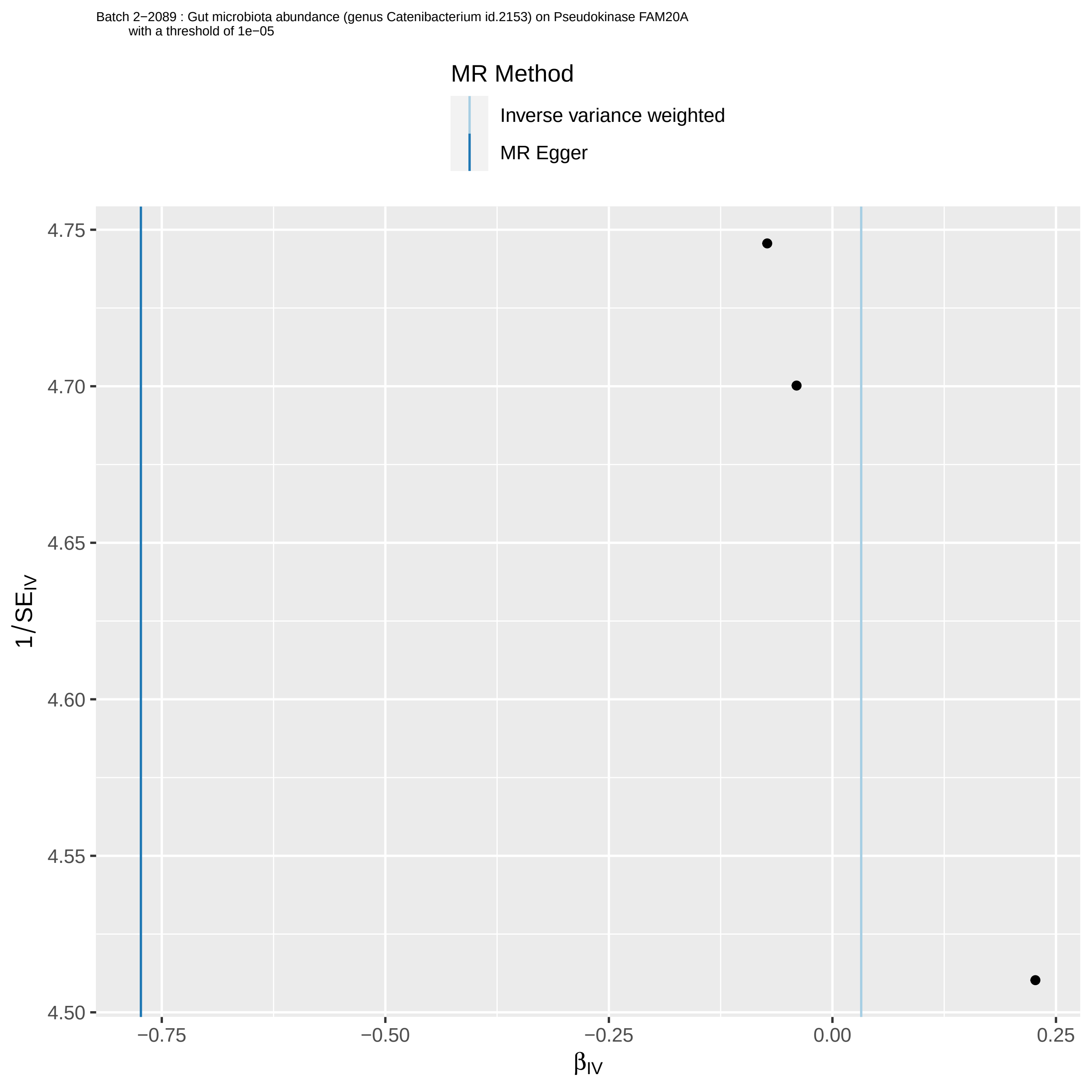

Batch 2−2089 : Gut microbiota abundance (genus Catenibacterium id.2153) on Pseudokinase FAM20A
with a threshold of 1e−05
MR Method
Inverse variance weighted
MR Egger
4.75
4.70
4.65
4.60
4.55
4.50
1/SE$_{IV}$
−0.75
−0.50
−0.25
0.00
0.25
β$_{IV}$

Batch 2−2090 : Gut microbiota abundance (genus Parasutterella id.2892) on Pseudokinase FAM20A
with a threshold of 1e−05

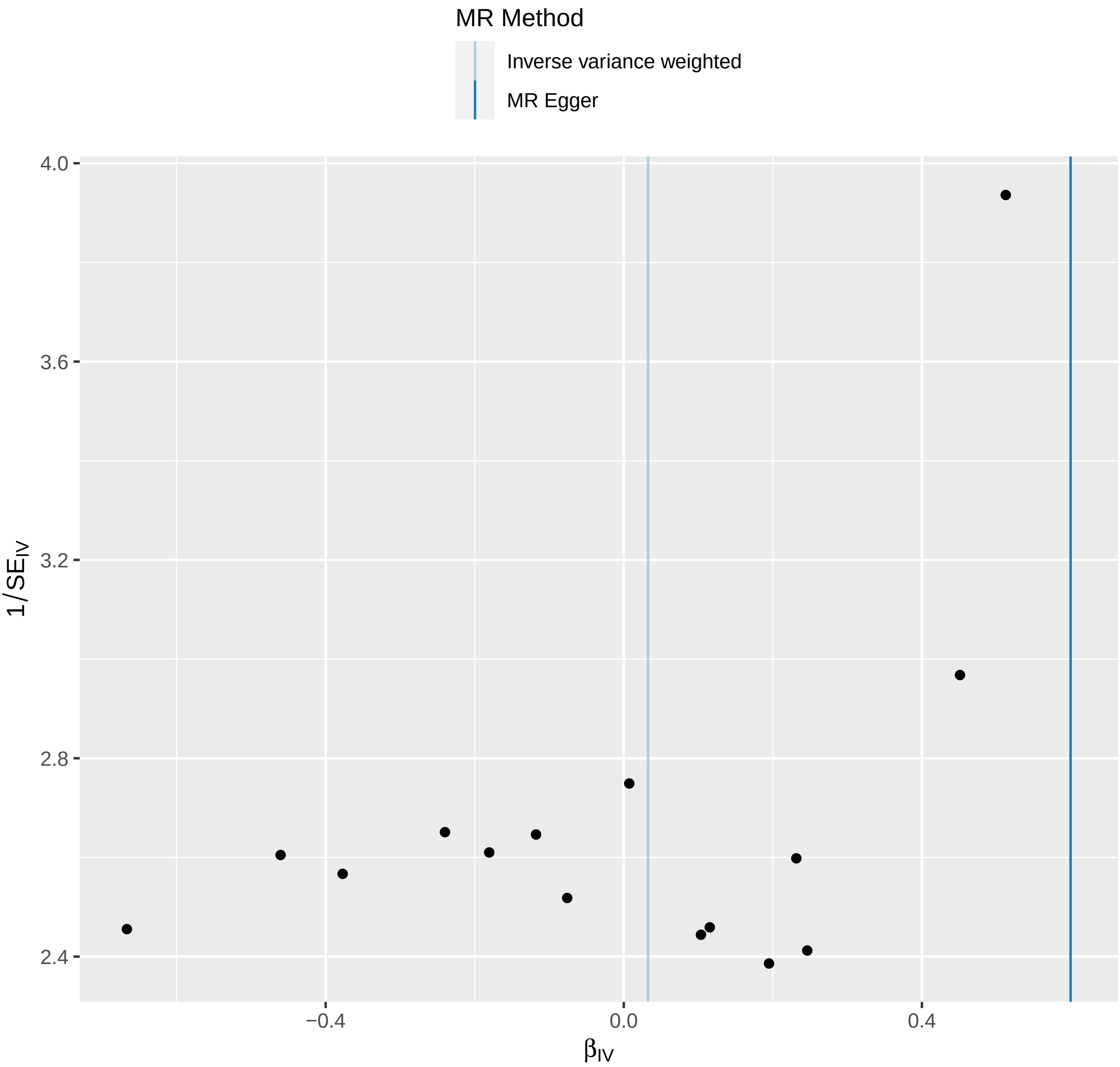

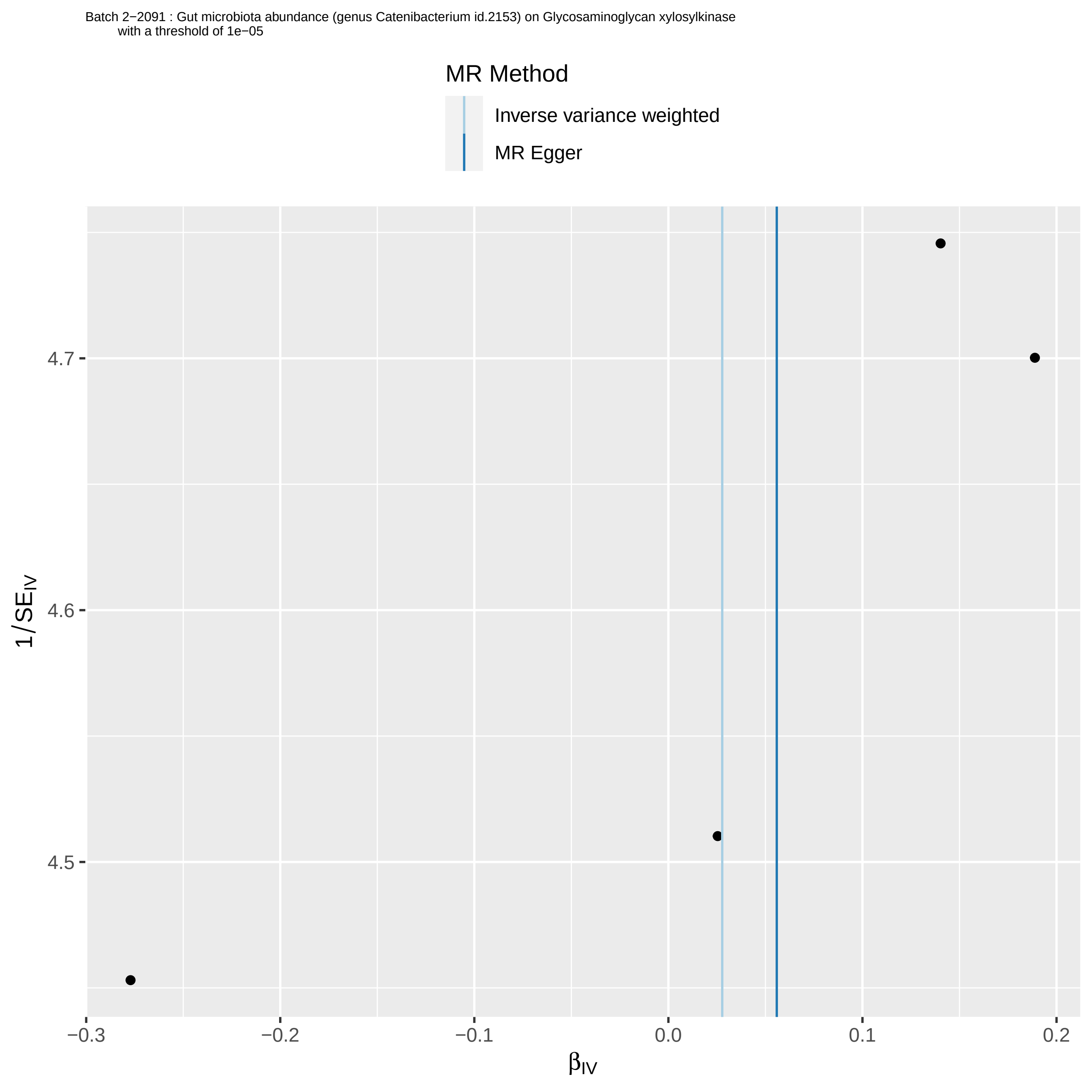
Batch 2−2091 : Gut microbiota abundance (genus Catenibacterium id.2153) on Glycosaminoglycan xylosylkinase
with a threshold of 1e−05
MR Method
Inverse variance weighted
MR Egger
4.7
4.6
4.5
1/SE_IV
−0.3
−0.2
−0.1
0.0
0.1
0.2
β_IV

Batch 2−2092 : Gut microbiota abundance (genus Parasutterella id.2892) on Glycosaminoglycan xylosylkinase
with a threshold of 1e−05

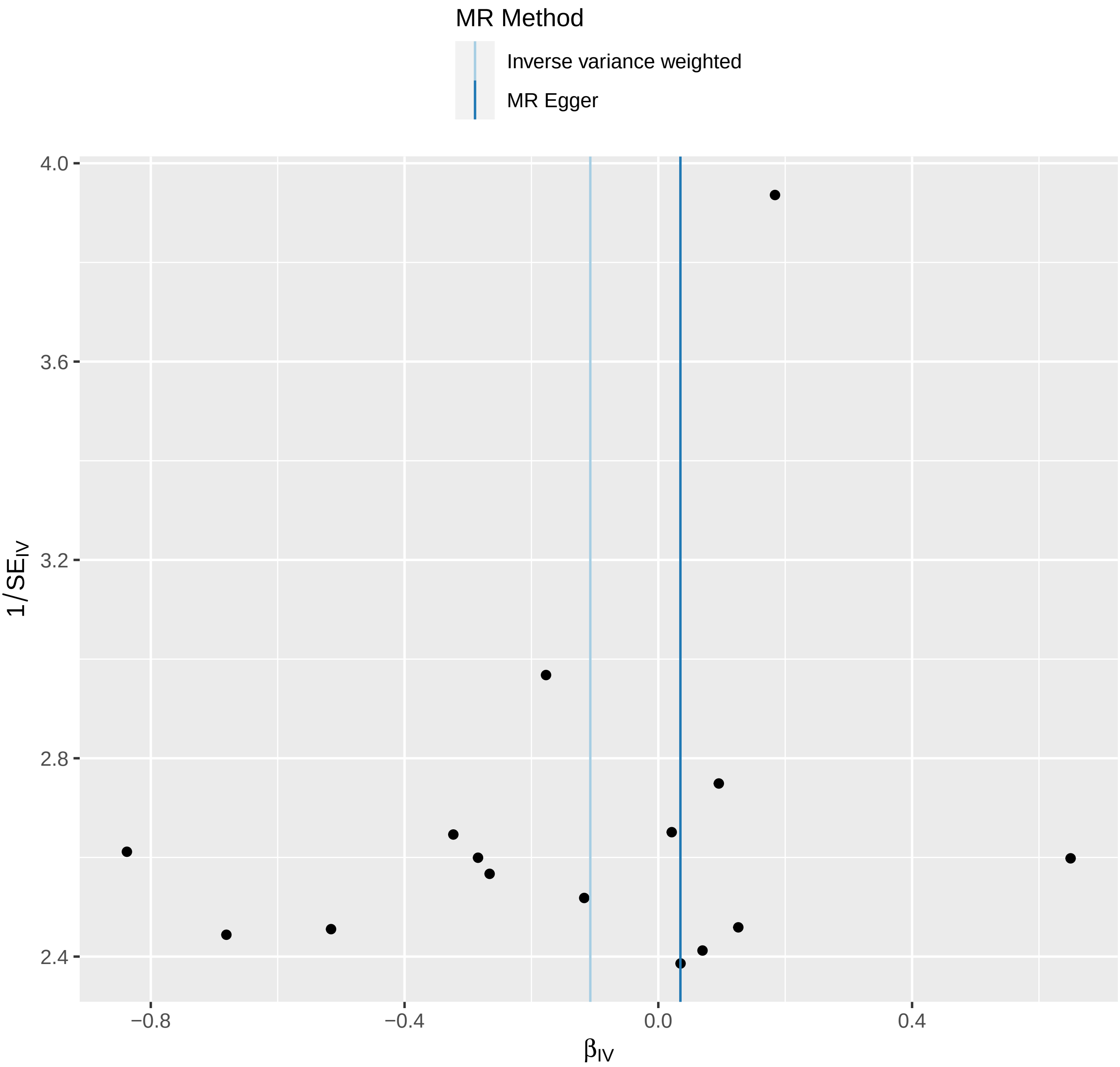

Batch 2−2093 : Gut microbiota abundance (genus Catenibacterium id.2153) on Redox−regulatory protein FAM213A
with a threshold of 1e−05

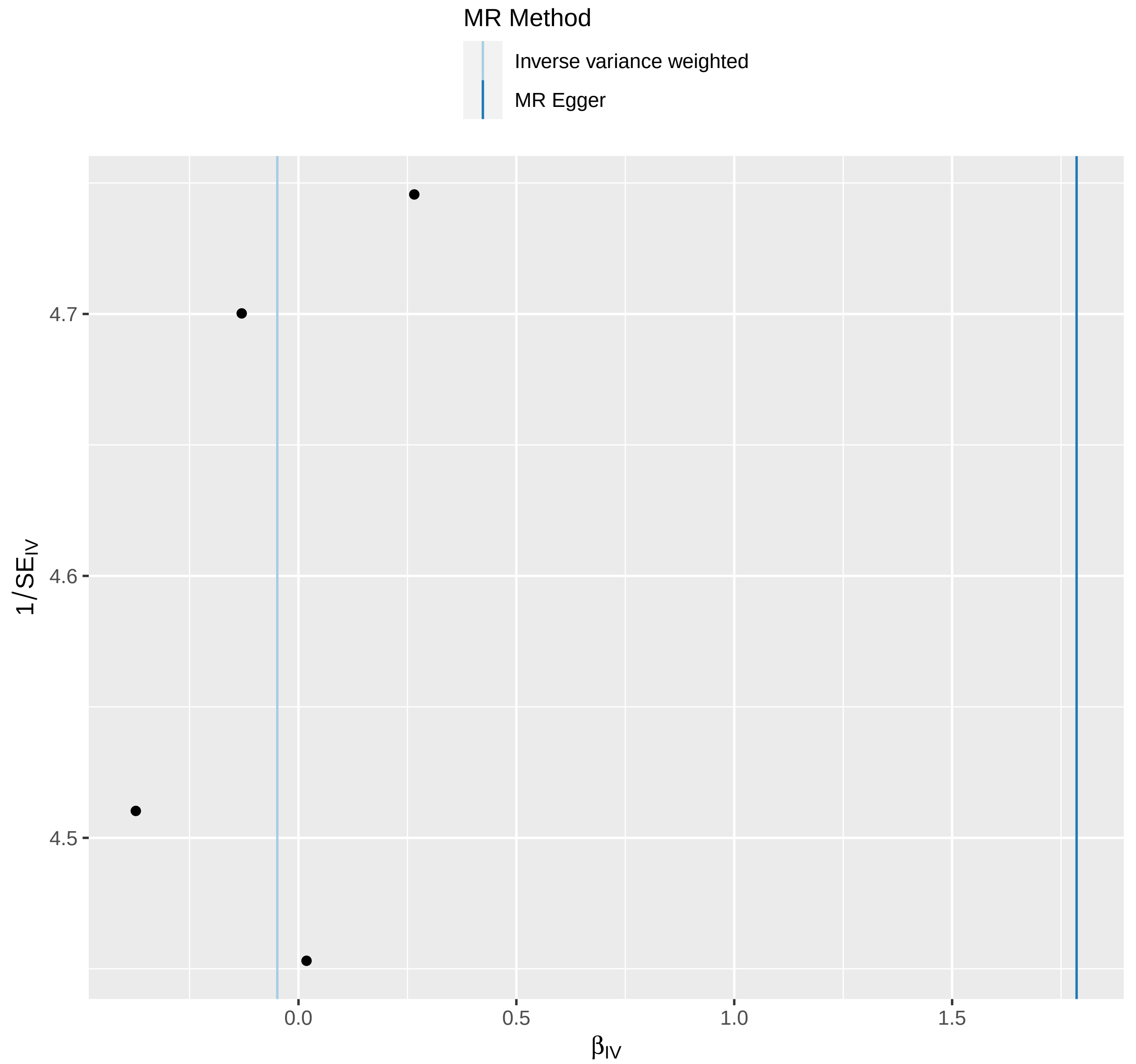

Batch 2−2094 : Gut microbiota abundance (genus Parasutterella id.2892) on Redox−regulatory protein FAM213A
with a threshold of 1e−05

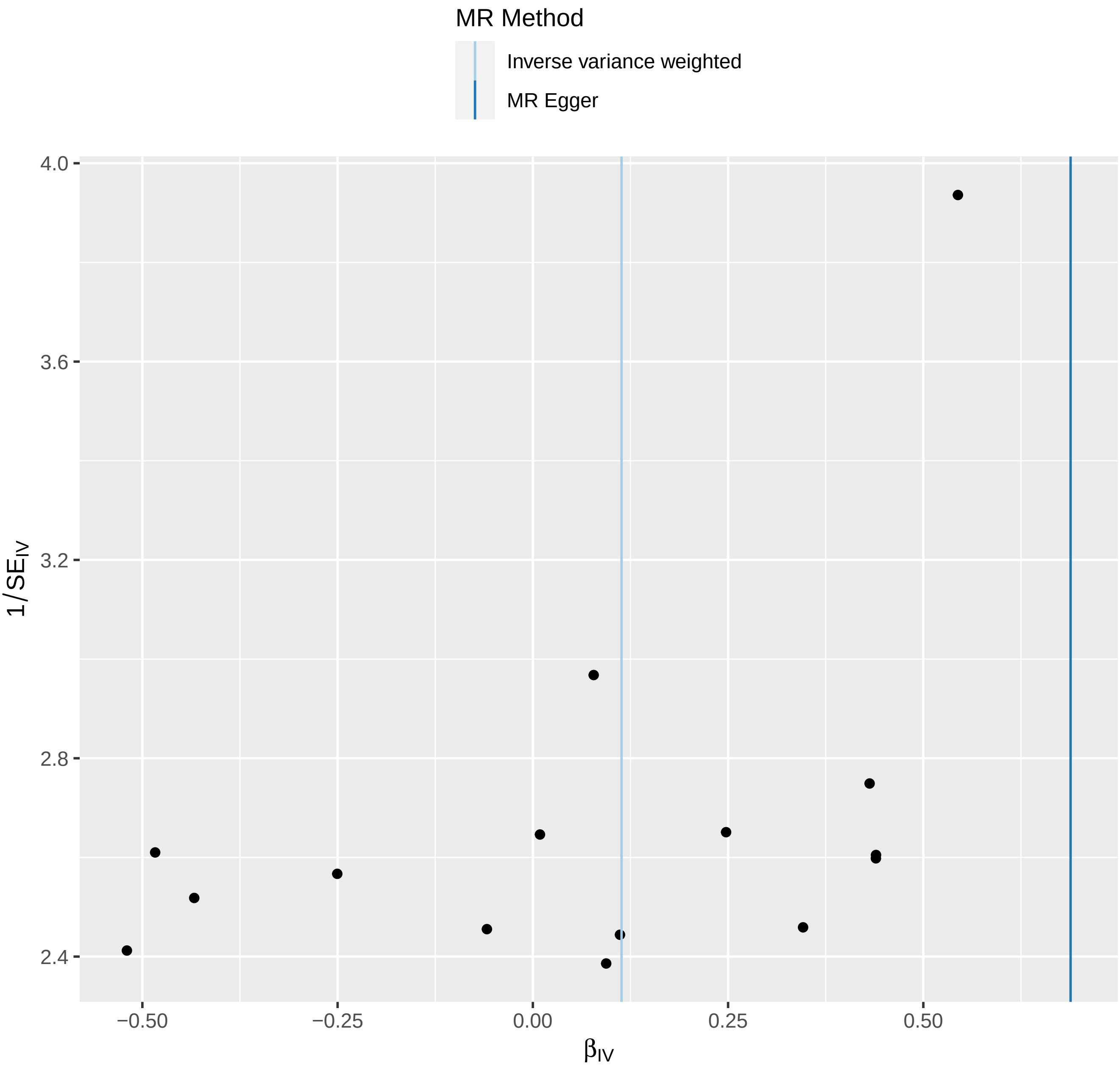

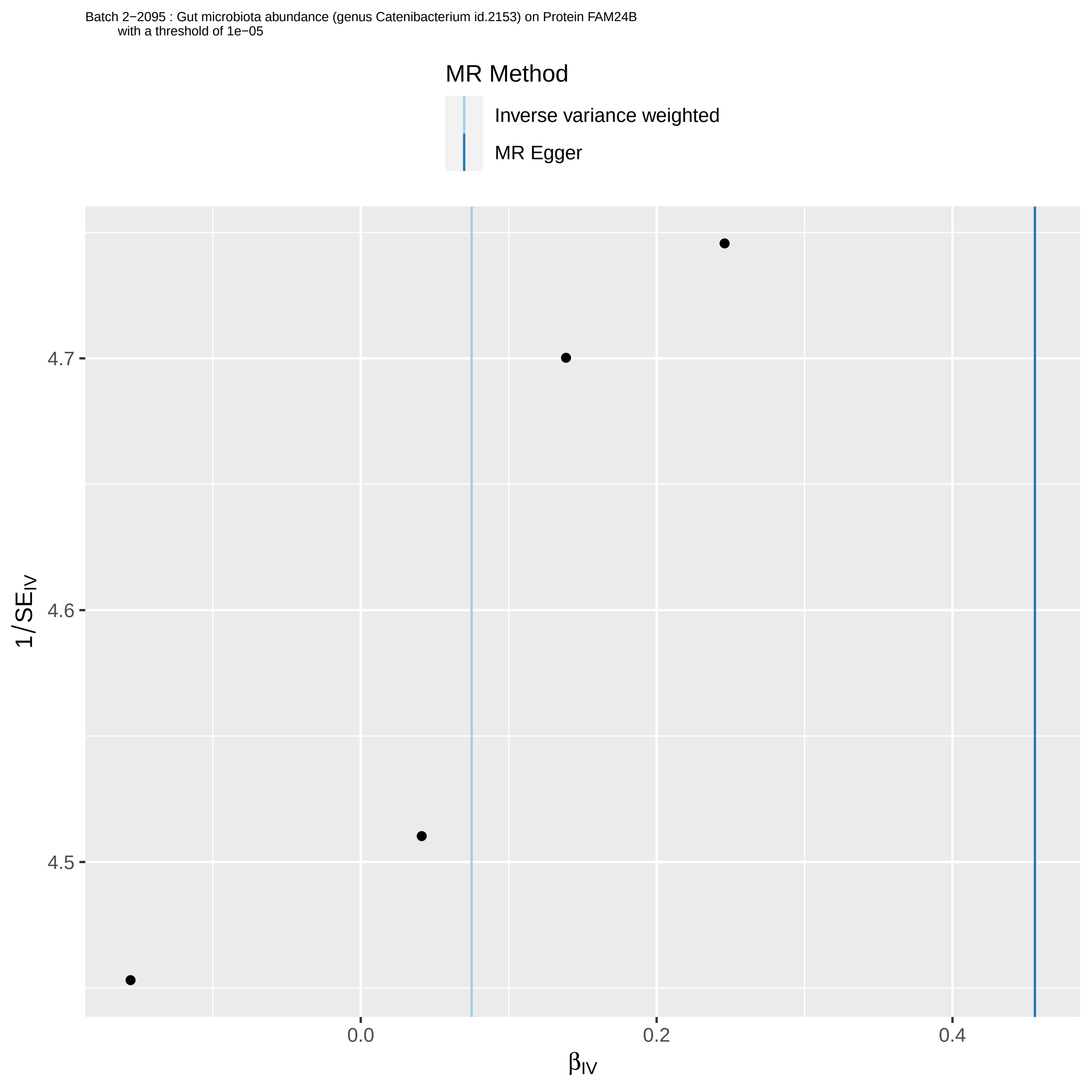
Batch 2−2095 : Gut microbiota abundance (genus Catenibacterium id.2153) on Protein FAM24B
with a threshold of 1e−05
MR Method
Inverse variance weighted
MR Egger
4.7
4.6
4.5
1/SE_IV
0.0
0.2
0.4
β_IV

Batch 2−2096 : Gut microbiota abundance (genus Parasutterella id.2892) on Protein FAM24B
with a threshold of 1e−05

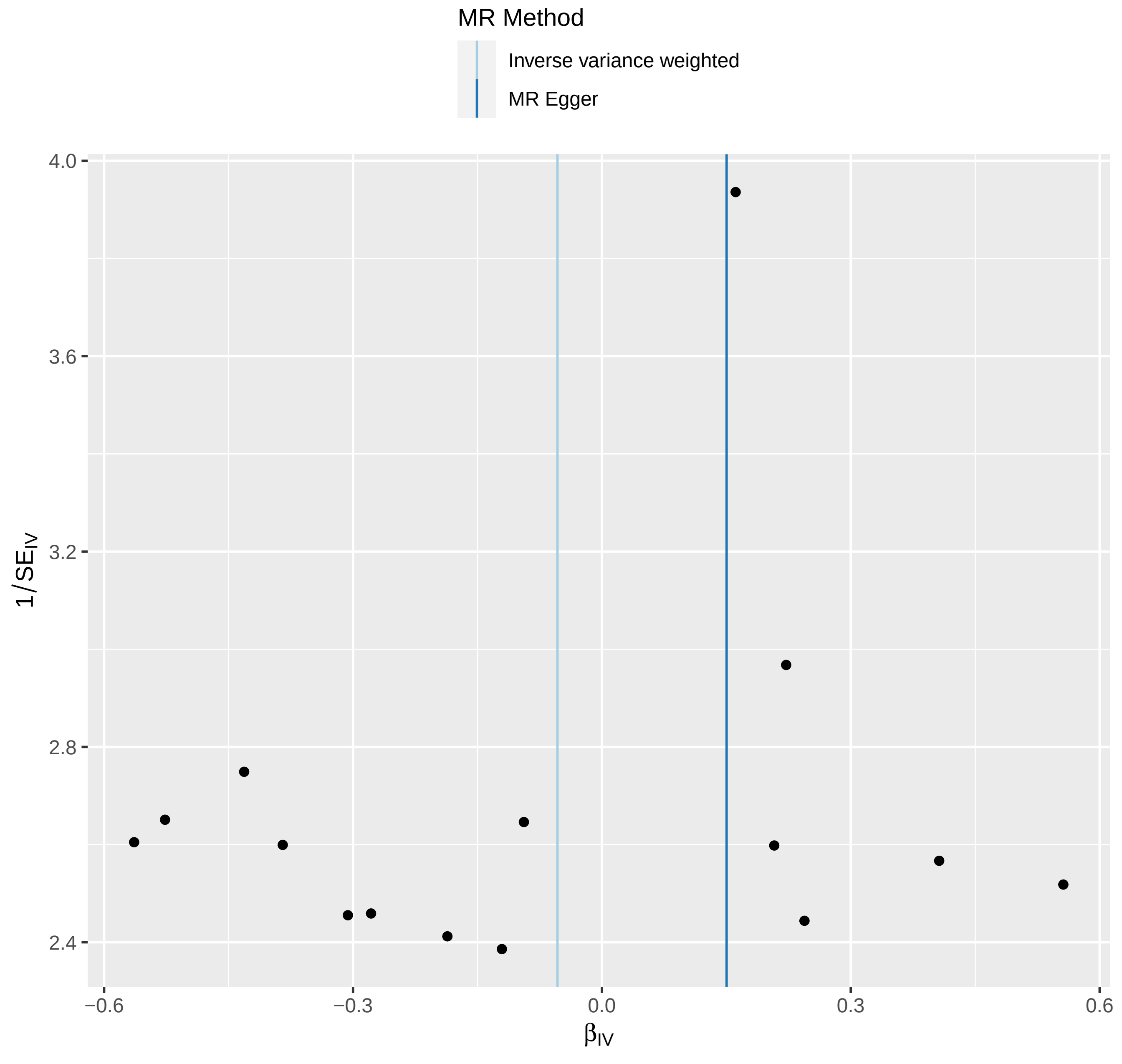

Batch 2−2097 : Gut microbiota abundance (genus Catenibacterium id.2153) on Protein FAM3B
with a threshold of 1e−05

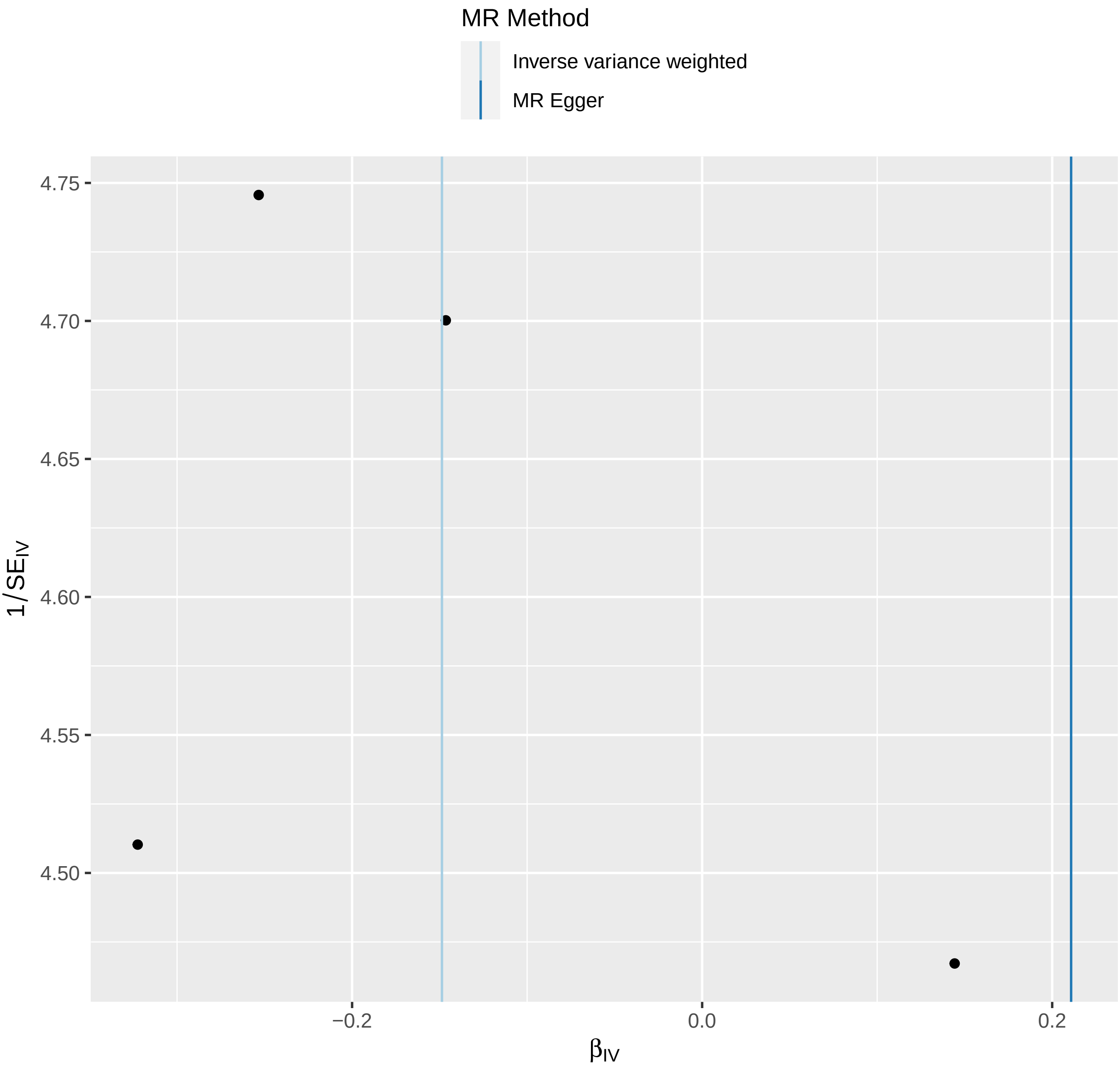

Batch 2−2098 : Gut microbiota abundance (genus Parasutterella id.2892) on Protein FAM3B
with a threshold of 1e−05

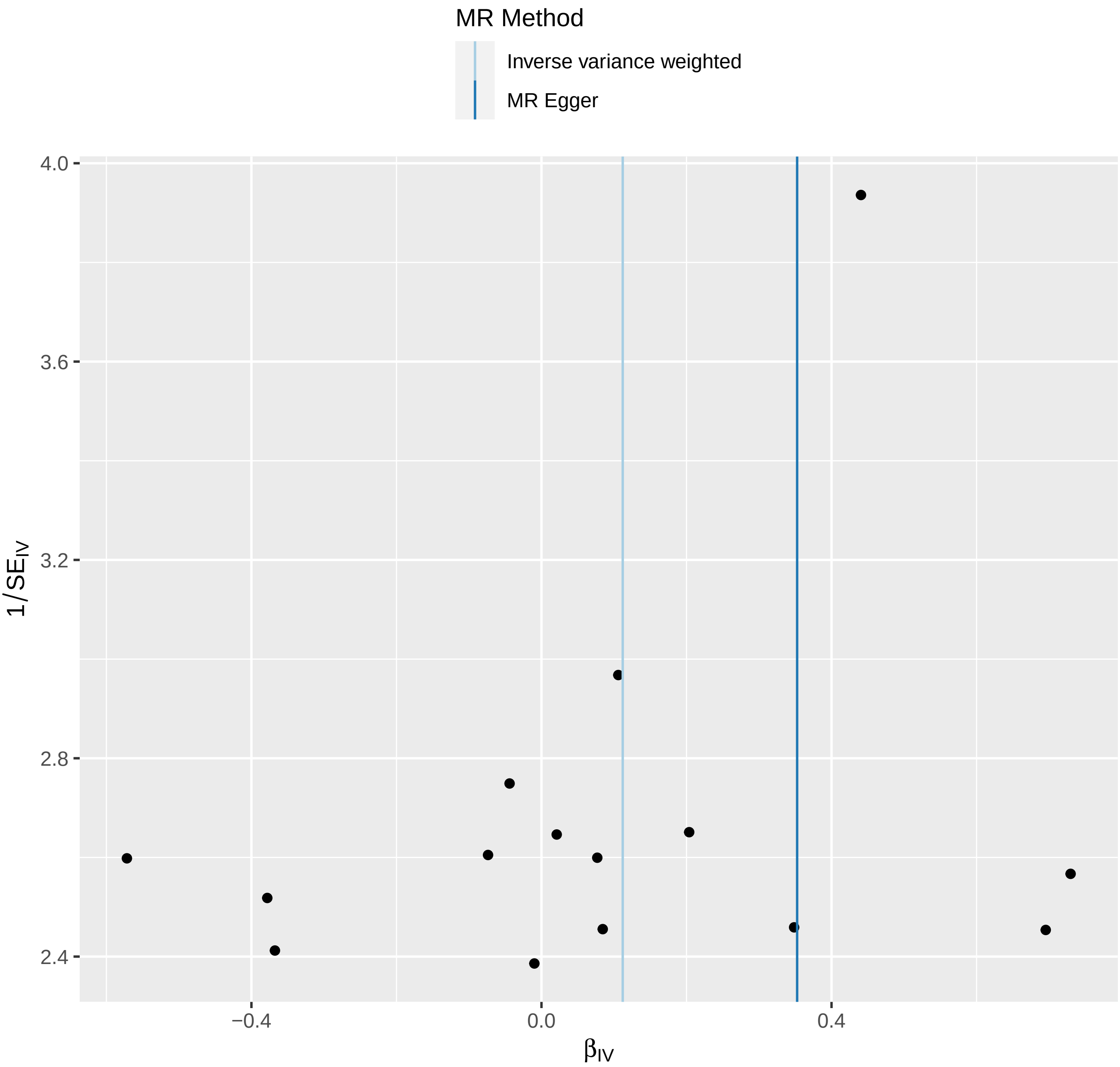

Batch 2−2099 : Gut microbiota abundance (genus Catenibacterium id.2153) on Protein FAM3B
with a threshold of 1e−05

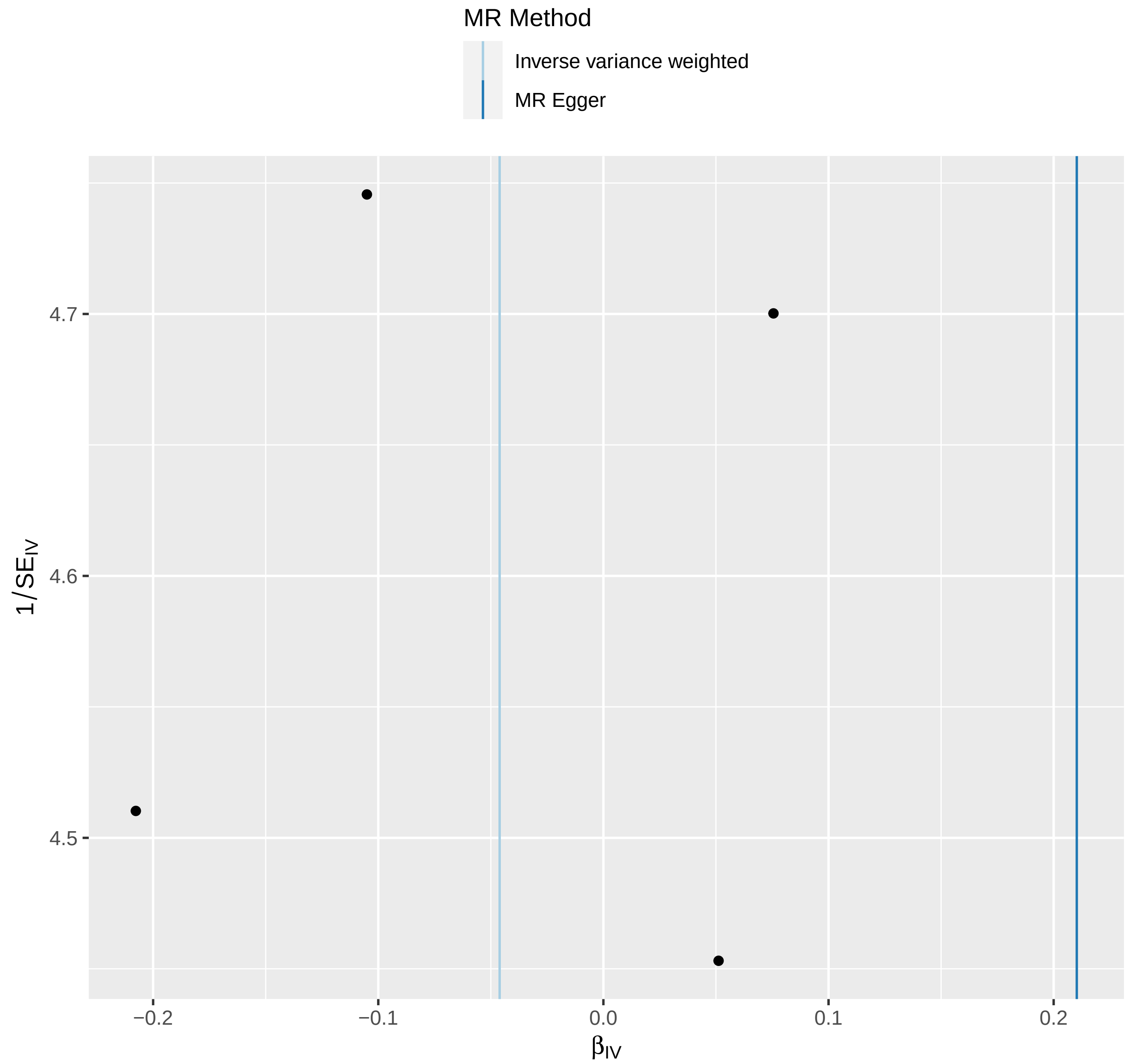

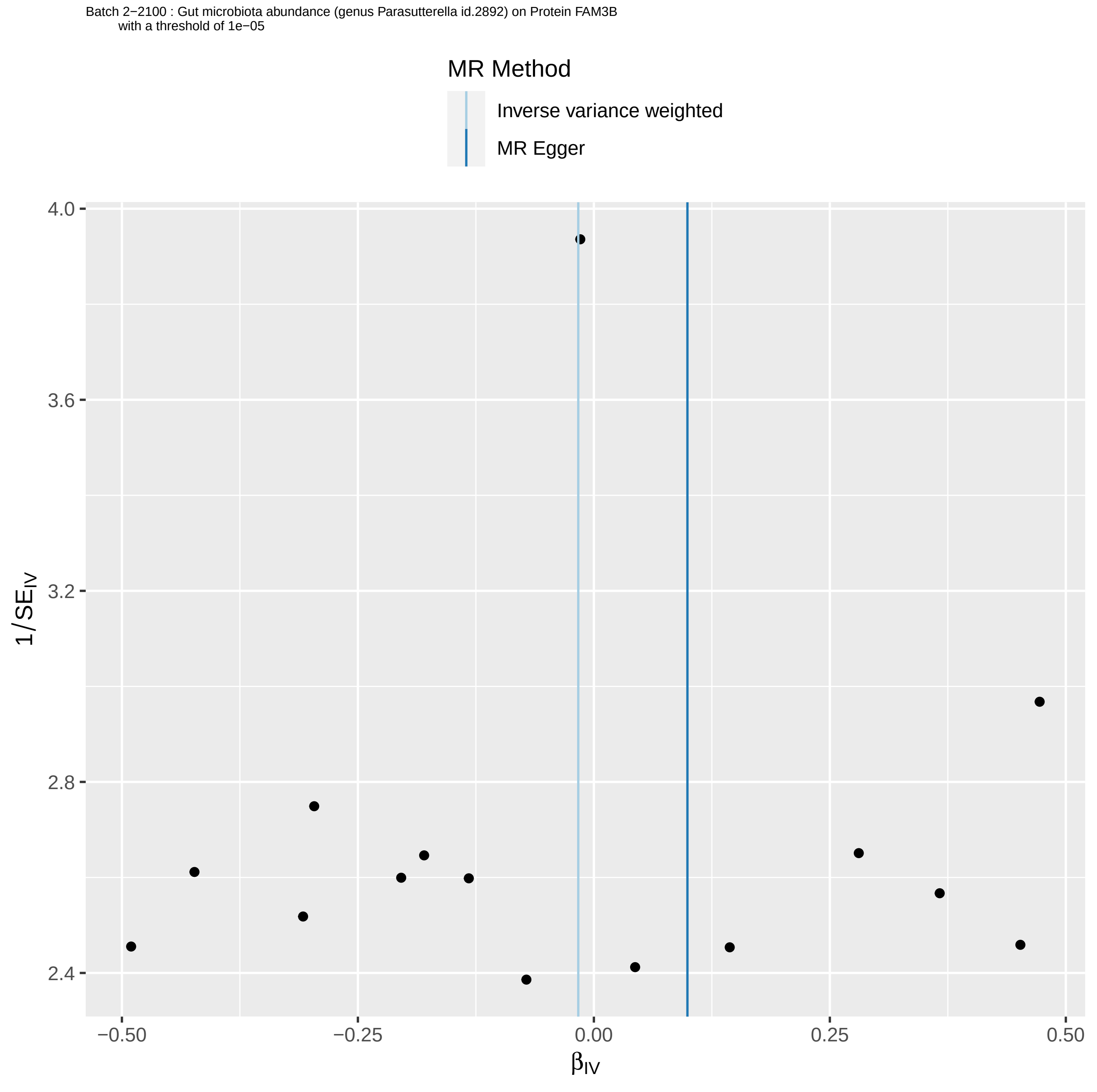

Batch 2−2100 : Gut microbiota abundance (genus Parasutterella id.2892) on Protein FAM3B
with a threshold of 1e−05
MR Method
Inverse variance weighted
MR Egger
4.0
3.6
3.2
2.8
2.4
1/SE_IV
−0.50
−0.25
0.00
0.25
0.50
β_IV

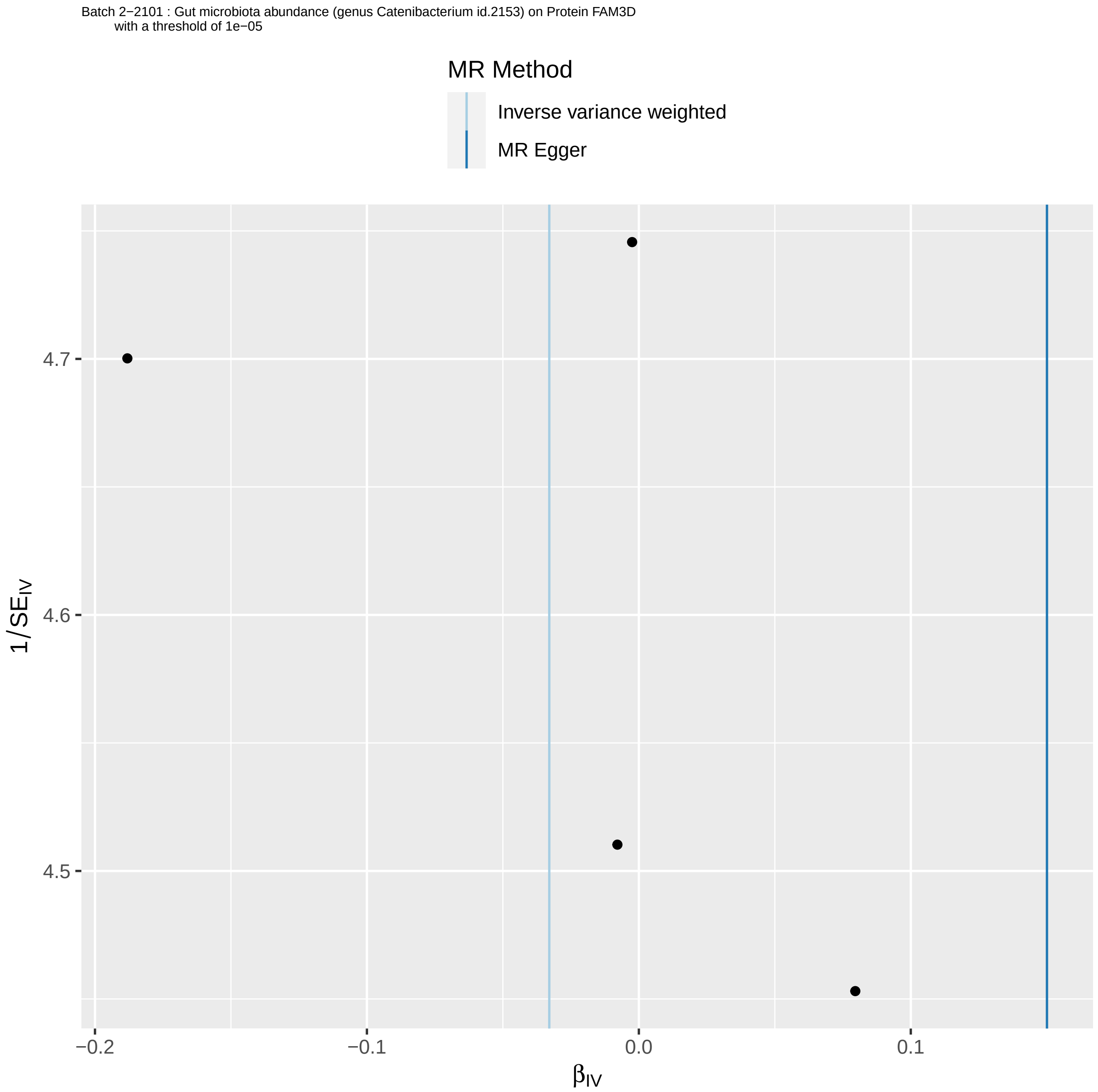
Batch 2−2101 : Gut microbiota abundance (genus Catenibacterium id.2153) on Protein FAM3D
with a threshold of 1e−05
MR Method
Inverse variance weighted
MR Egger
4.7
4.6
4.5
1/SE_IV
−0.2
−0.1
0.0
0.1
β_IV

Batch 2−2102 : Gut microbiota abundance (genus Parasutterella id.2892) on Protein FAM3D
with a threshold of 1e−05

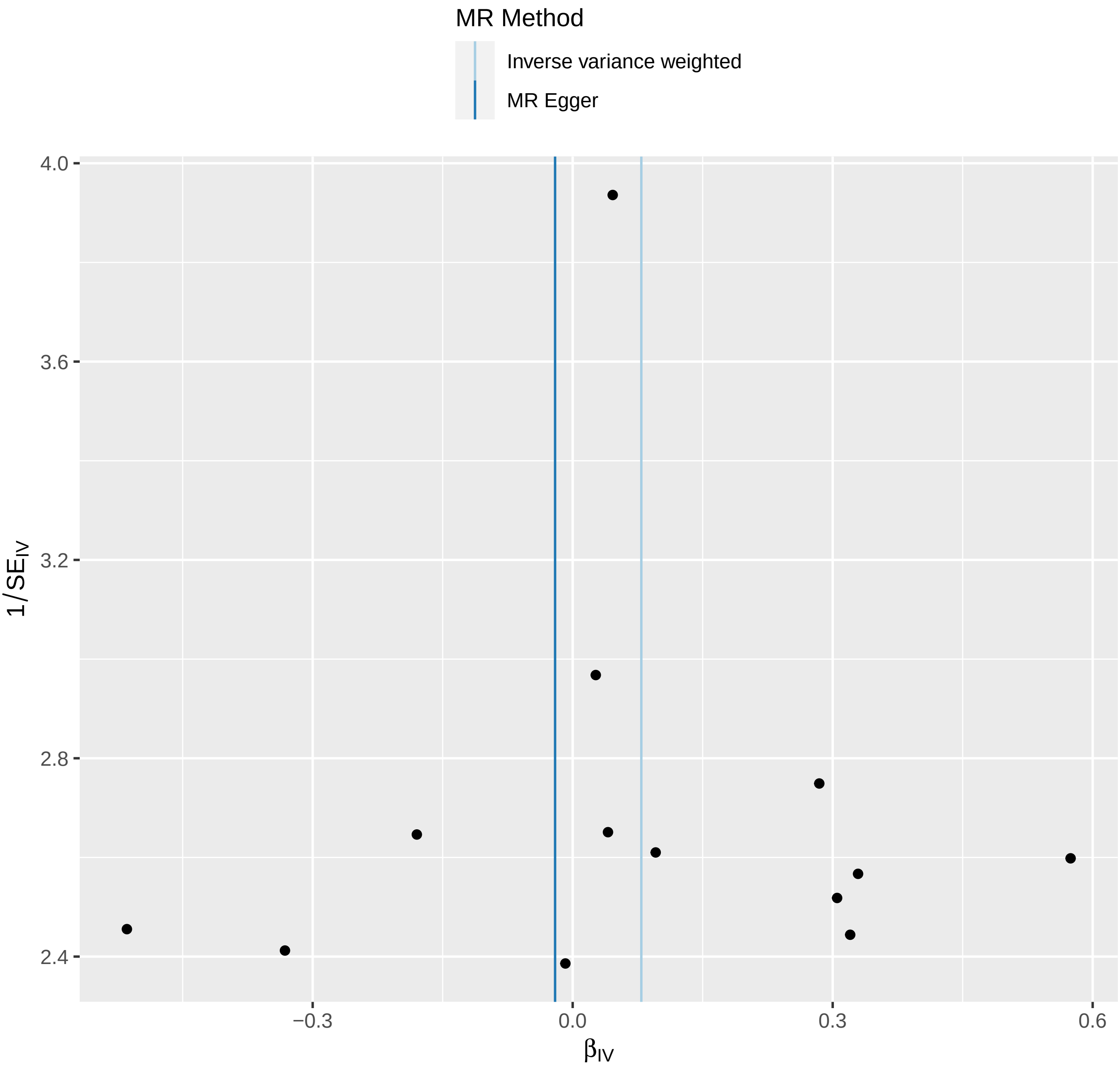

Batch 2−2103 : Gut microbiota abundance (genus Catenibacterium id.2153) on MIP18 family protein FAM96A
with a threshold of 1e−05

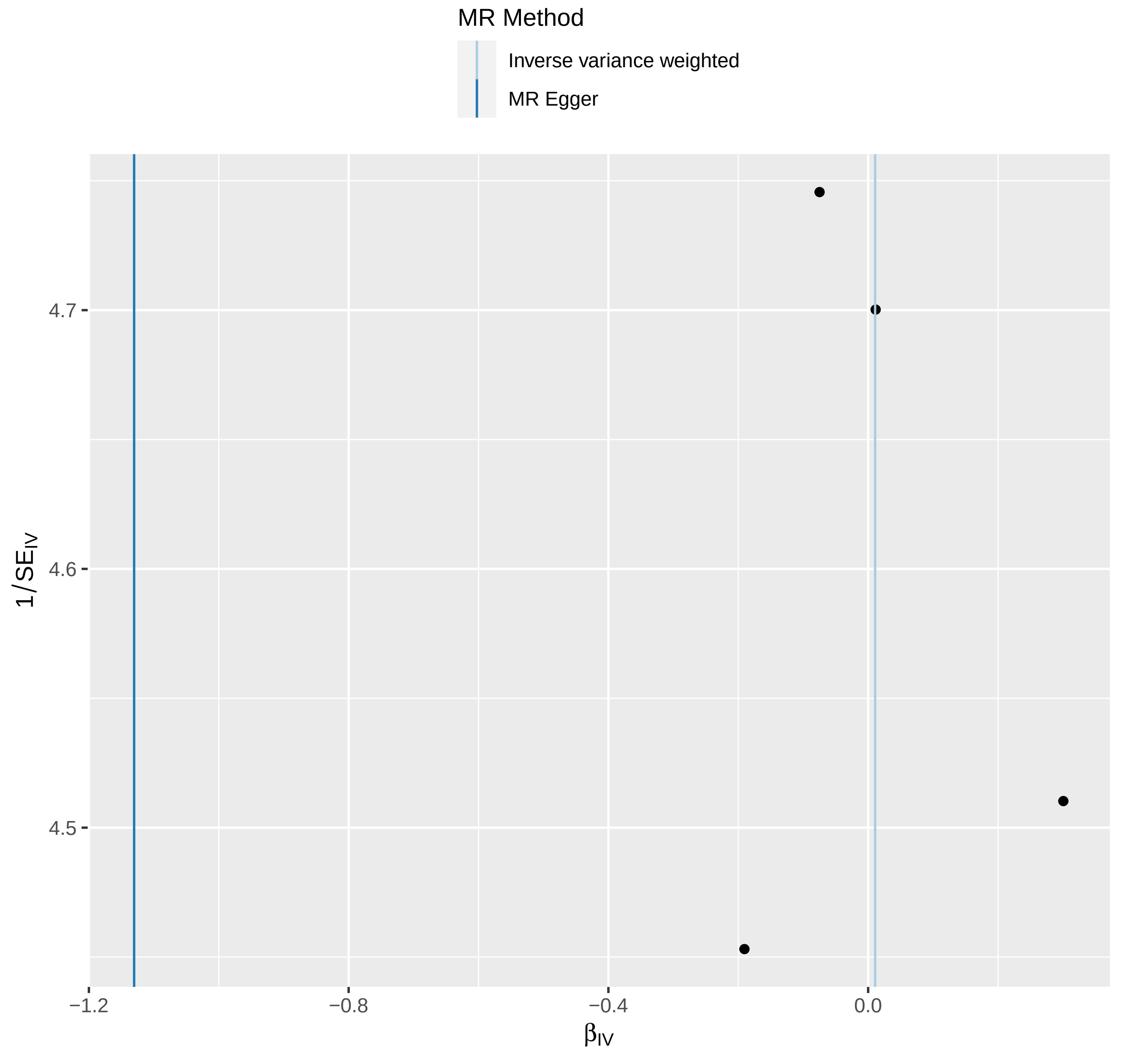

Batch 2−2104 : Gut microbiota abundance (genus Parasutterella id.2892) on MIP18 family protein FAM96A
with a threshold of 1e−05

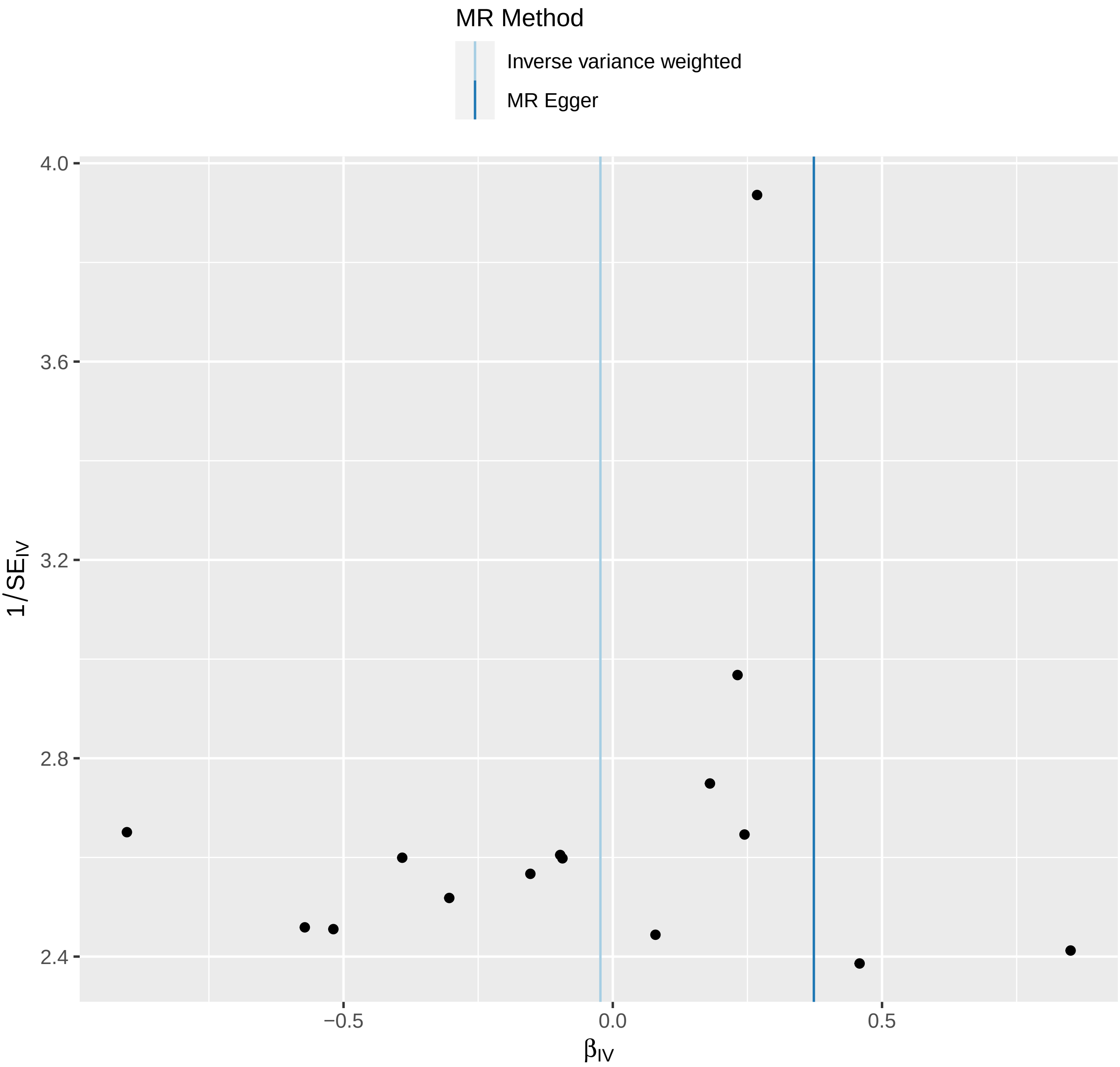

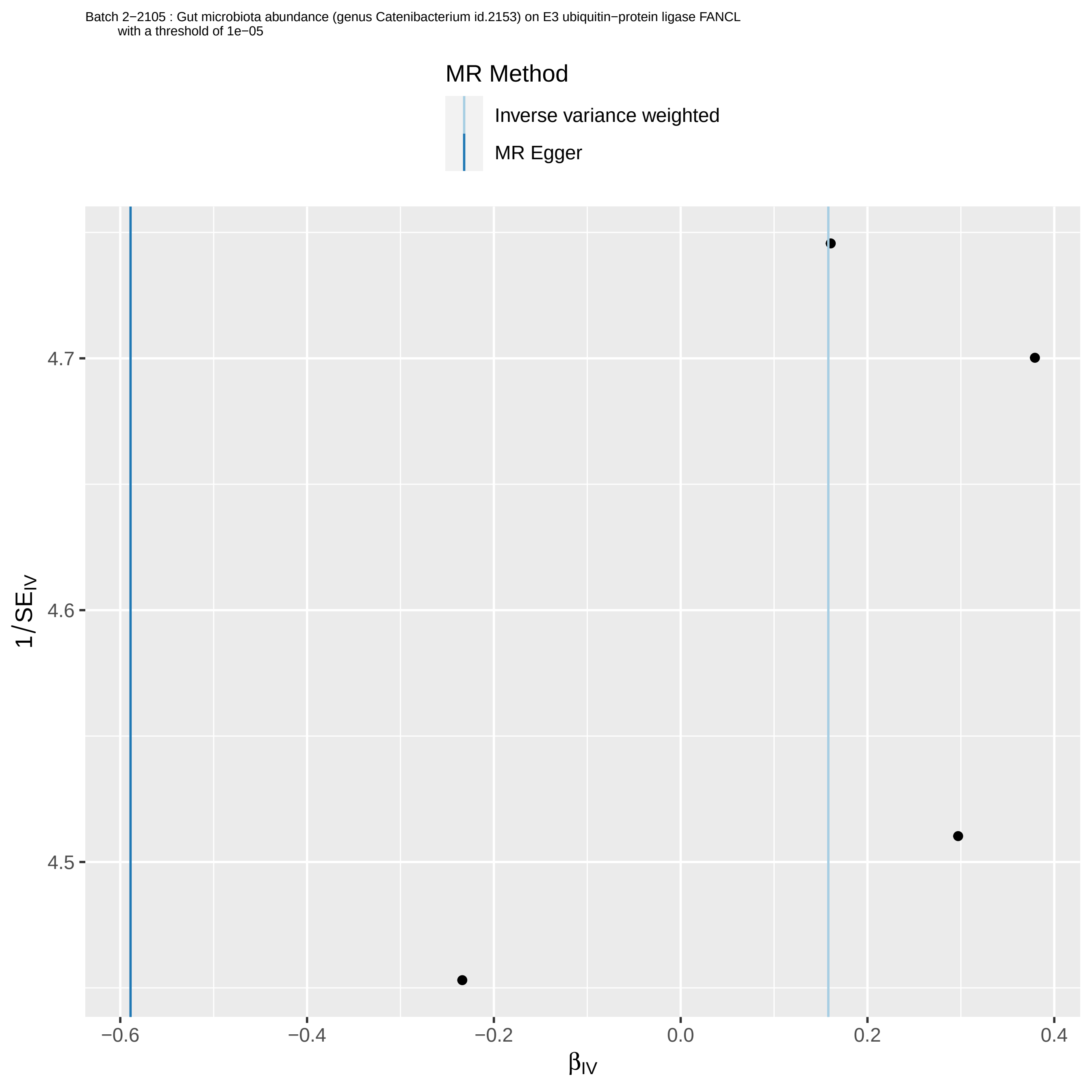

Batch 2−2105 : Gut microbiota abundance (genus Catenibacterium id.2153) on E3 ubiquitin−protein ligase FANCL
with a threshold of 1e−05
MR Method
Inverse variance weighted
MR Egger
1/SE$_{IV}$
4.7
4.6
4.5
−0.6
−0.4
−0.2
0.0
0.2
0.4
β$_{IV}$

Batch 2−2106 : Gut microbiota abundance (genus Parasutterella id.2892) on E3 ubiquitin−protein ligase FANCL
with a threshold of 1e−05

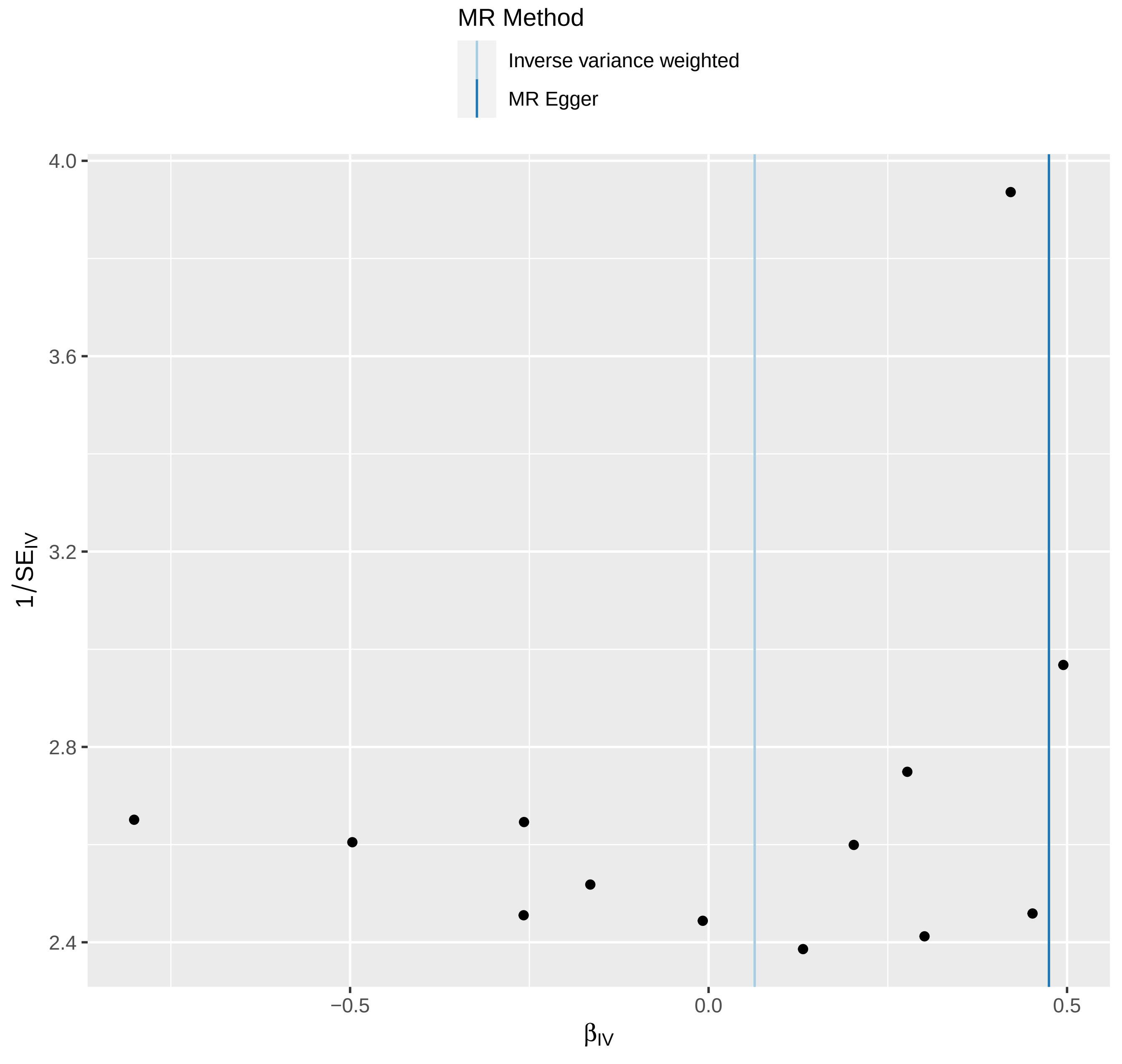

Batch 2−2107 : Gut microbiota abundance (genus Catenibacterium id.2153) on Prolyl endopeptidase FAP
with a threshold of 1e−05

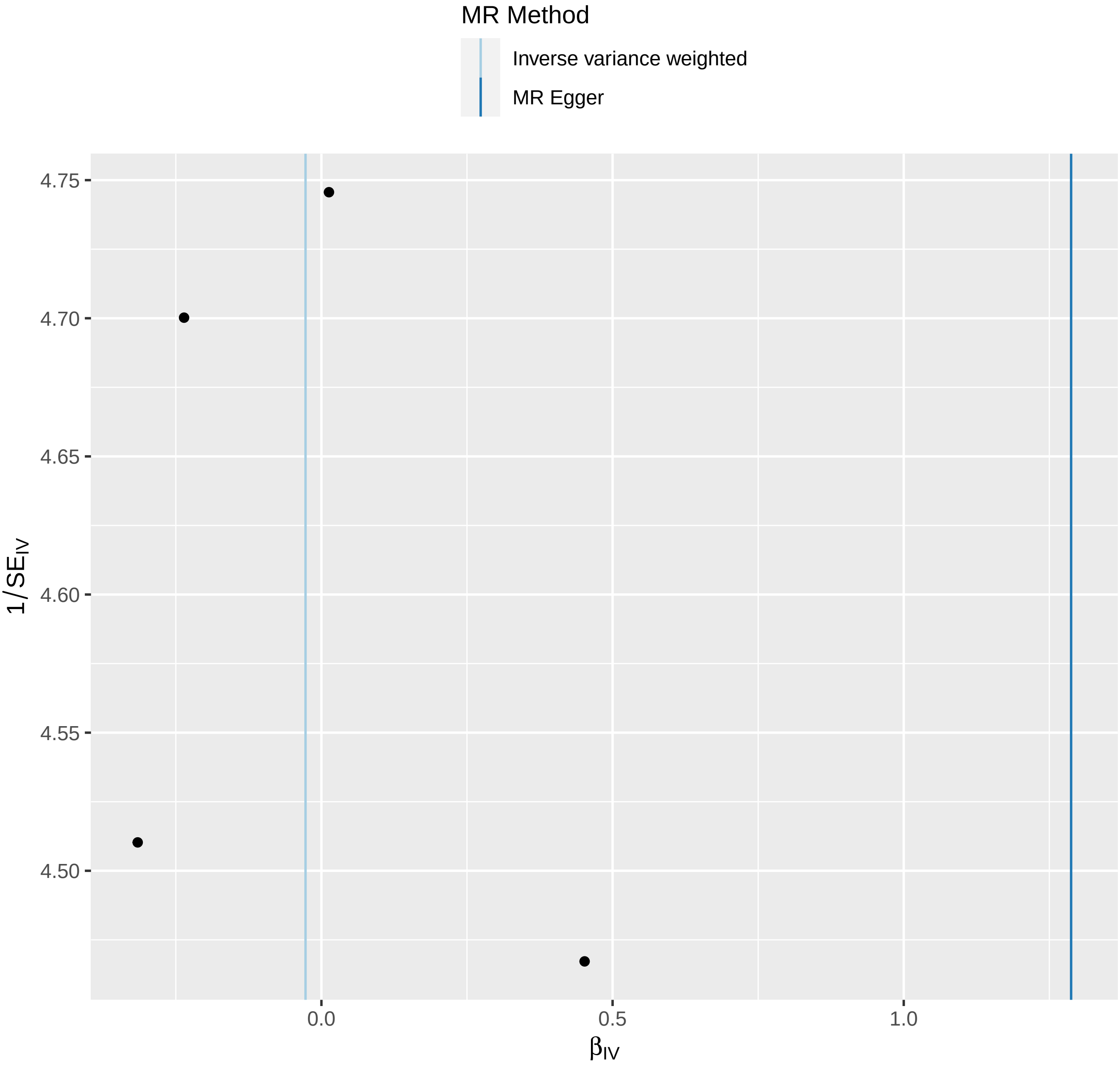

Batch 2−2108 : Gut microbiota abundance (genus Parasutterella id.2892) on Prolyl endopeptidase FAP
with a threshold of 1e−05

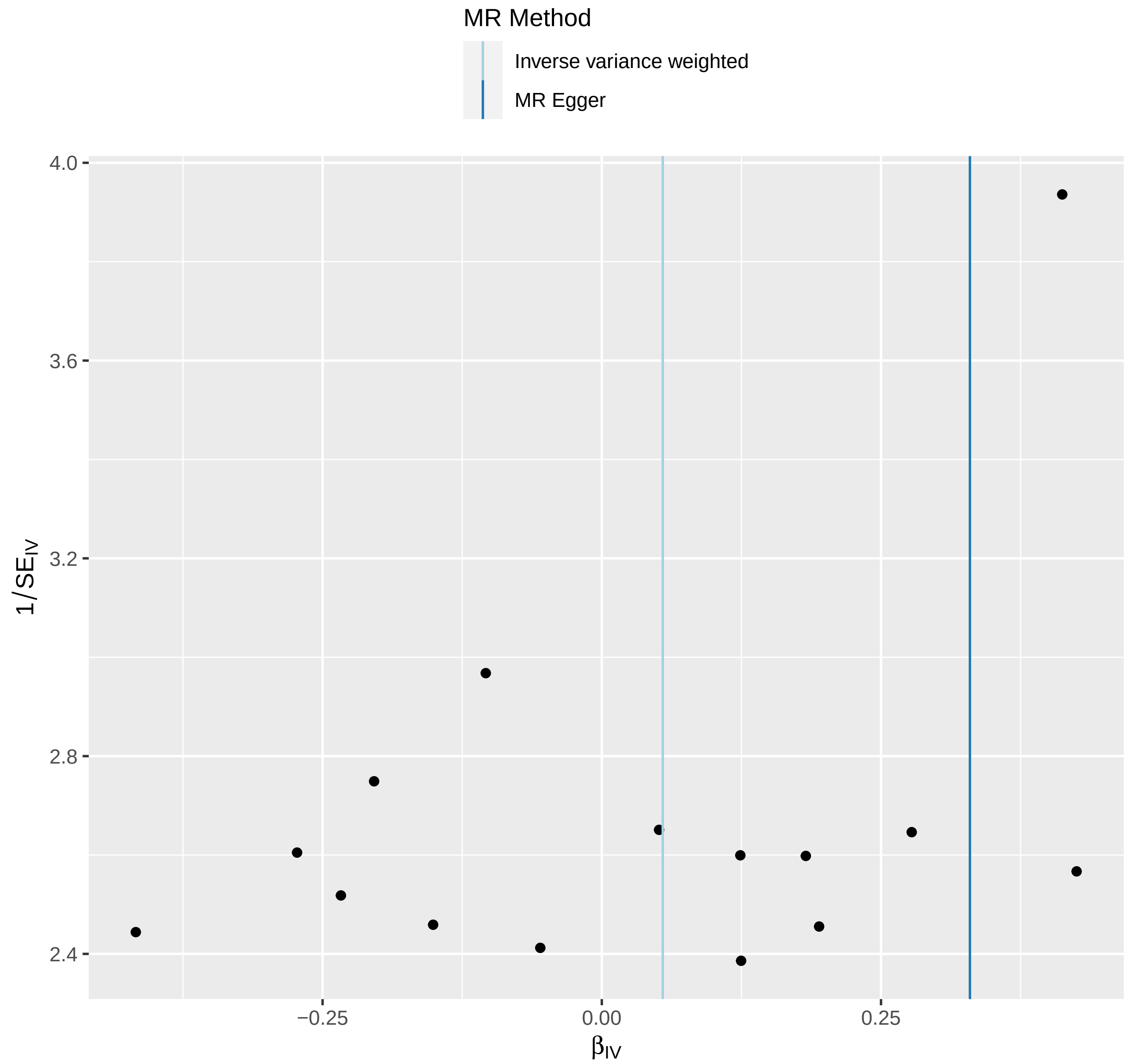

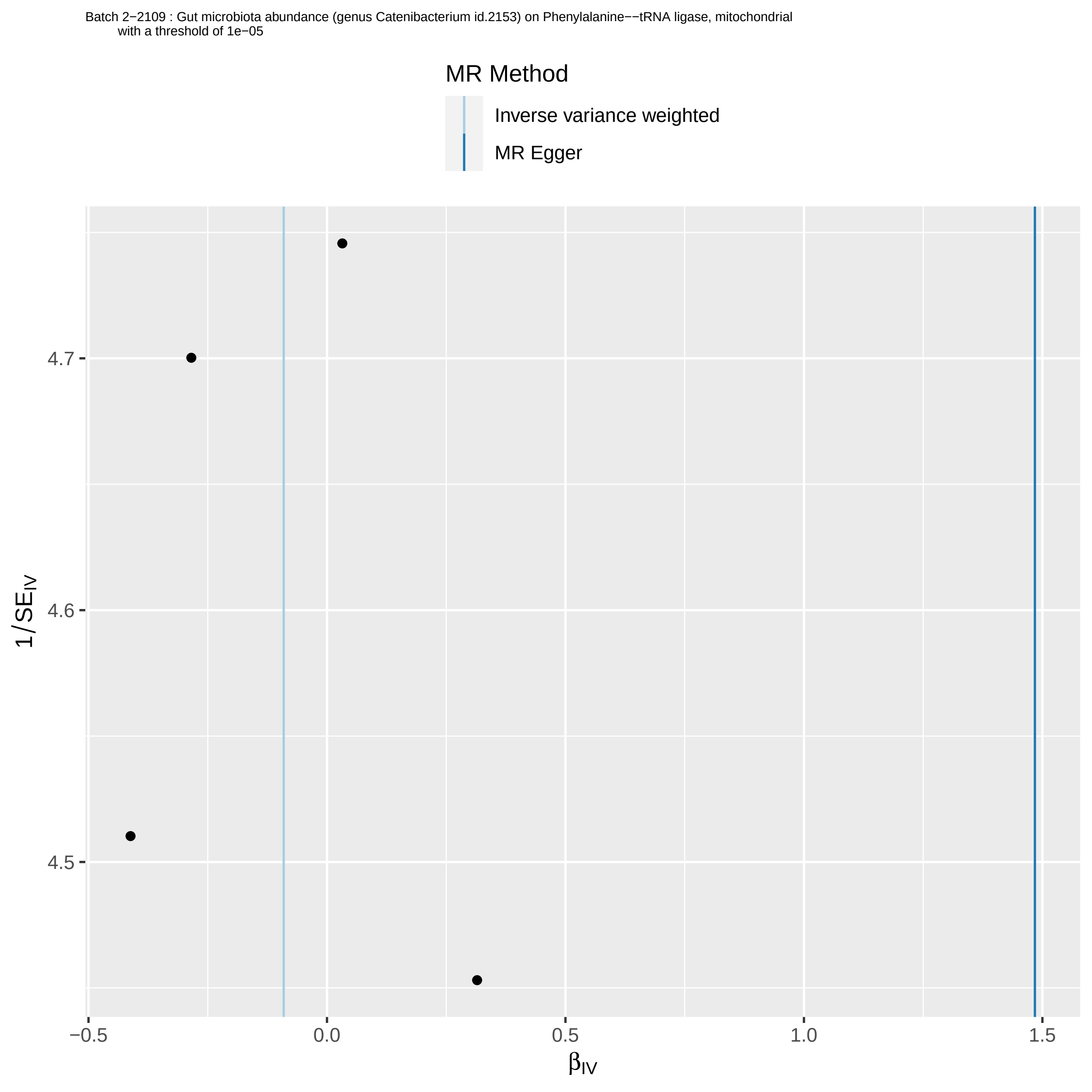

Batch 2−2109 : Gut microbiota abundance (genus Catenibacterium id.2153) on Phenylalanine−−tRNA ligase, mitochondrial
with a threshold of 1e−05
MR Method
Inverse variance weighted
MR Egger
4.7
4.6
4.5
1/SE_IV
−0.5
0.0
0.5
1.0
1.5
β_IV

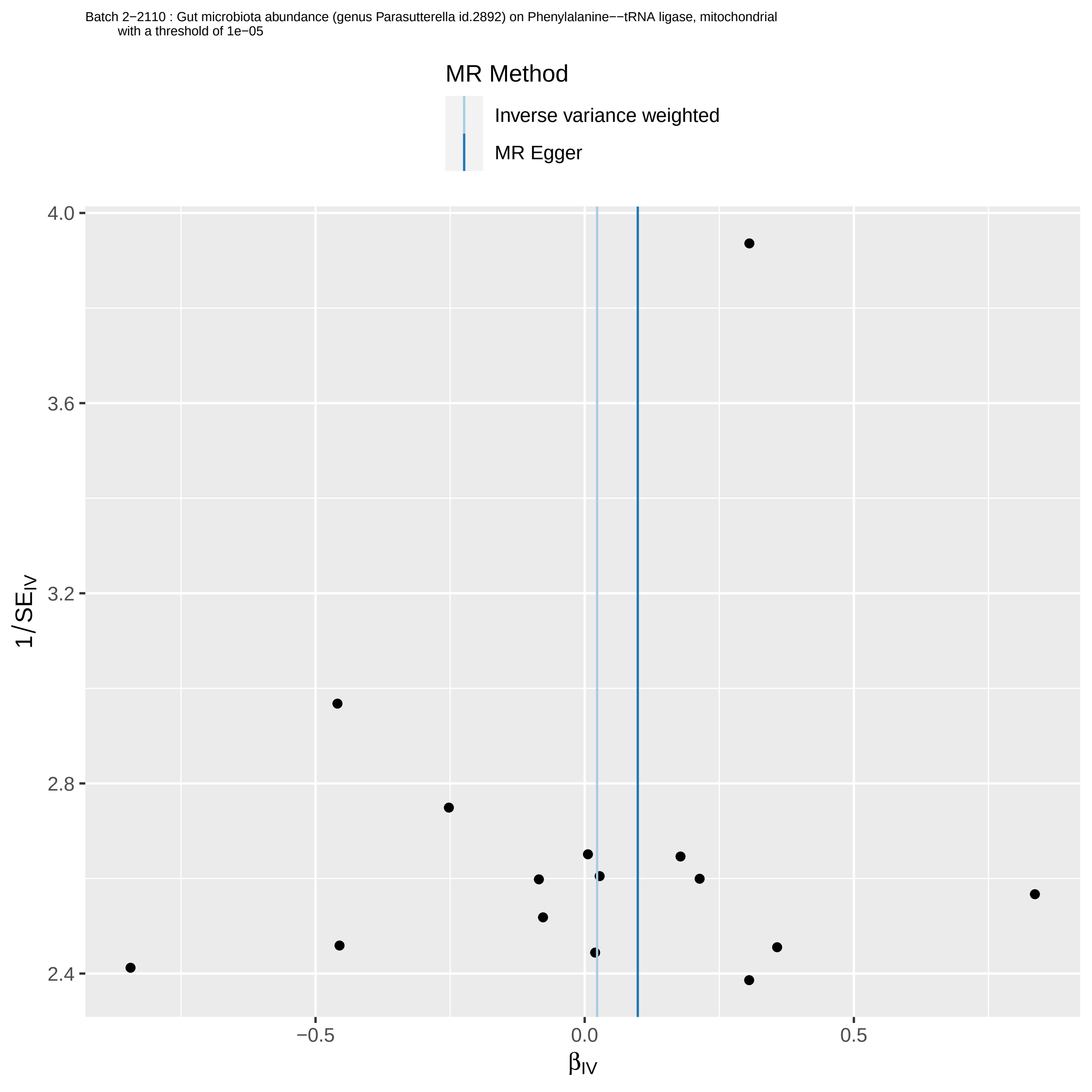

Batch 2−2110 : Gut microbiota abundance (genus Parasutterella id.2892) on Phenylalanine−−tRNA ligase, mitochondrial
with a threshold of 1e−05
MR Method
Inverse variance weighted
MR Egger
4.0
3.6
3.2
2.8
2.4
1/SE_IV
−0.5
0.0
0.5
β_IV

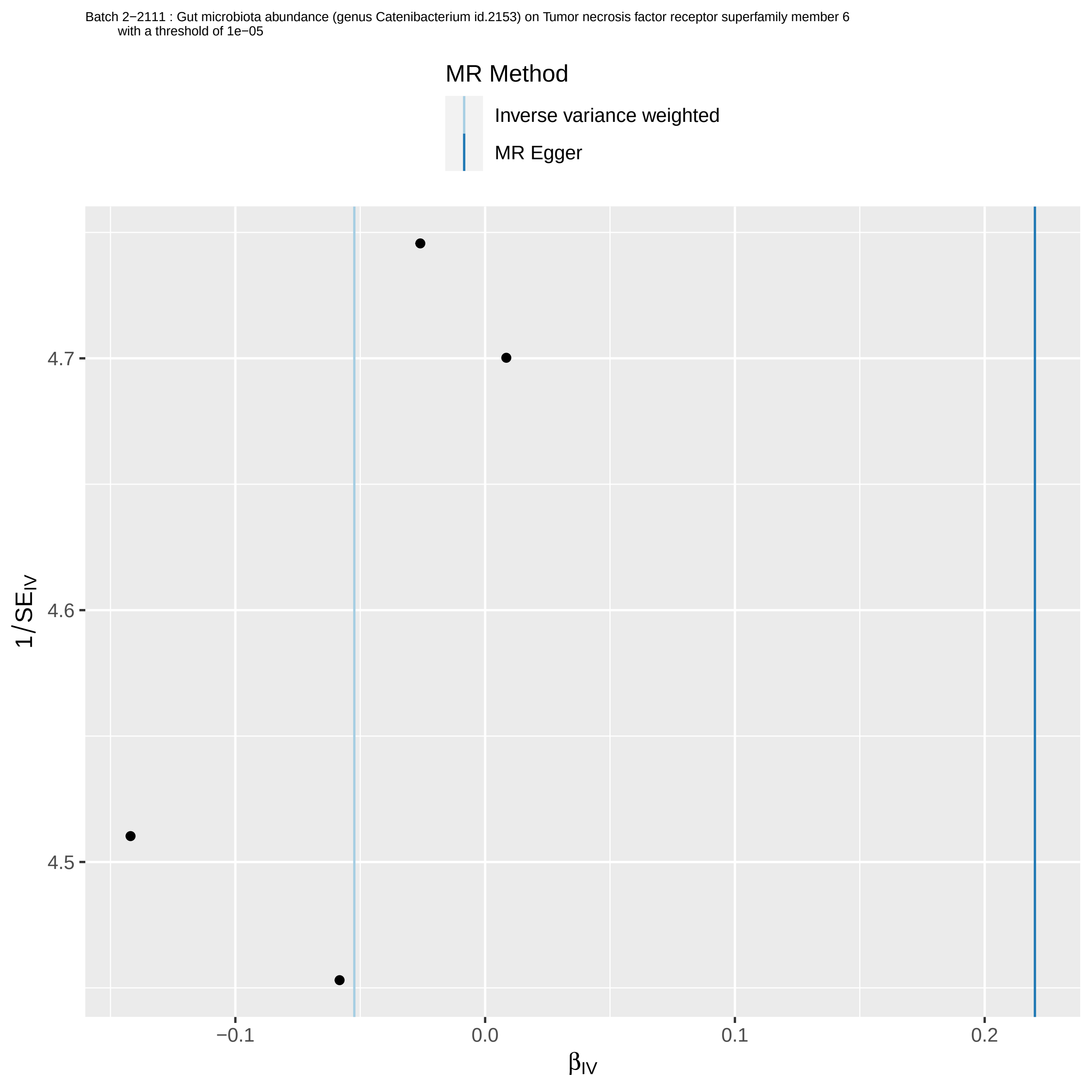
Batch 2−2111 : Gut microbiota abundance (genus Catenibacterium id.2153) on Tumor necrosis factor receptor superfamily member 6
with a threshold of 1e−05
MR Method
Inverse variance weighted
MR Egger
4.7
4.6
4.5
1/SE_IV
−0.1
0.0
0.1
0.2
β_IV

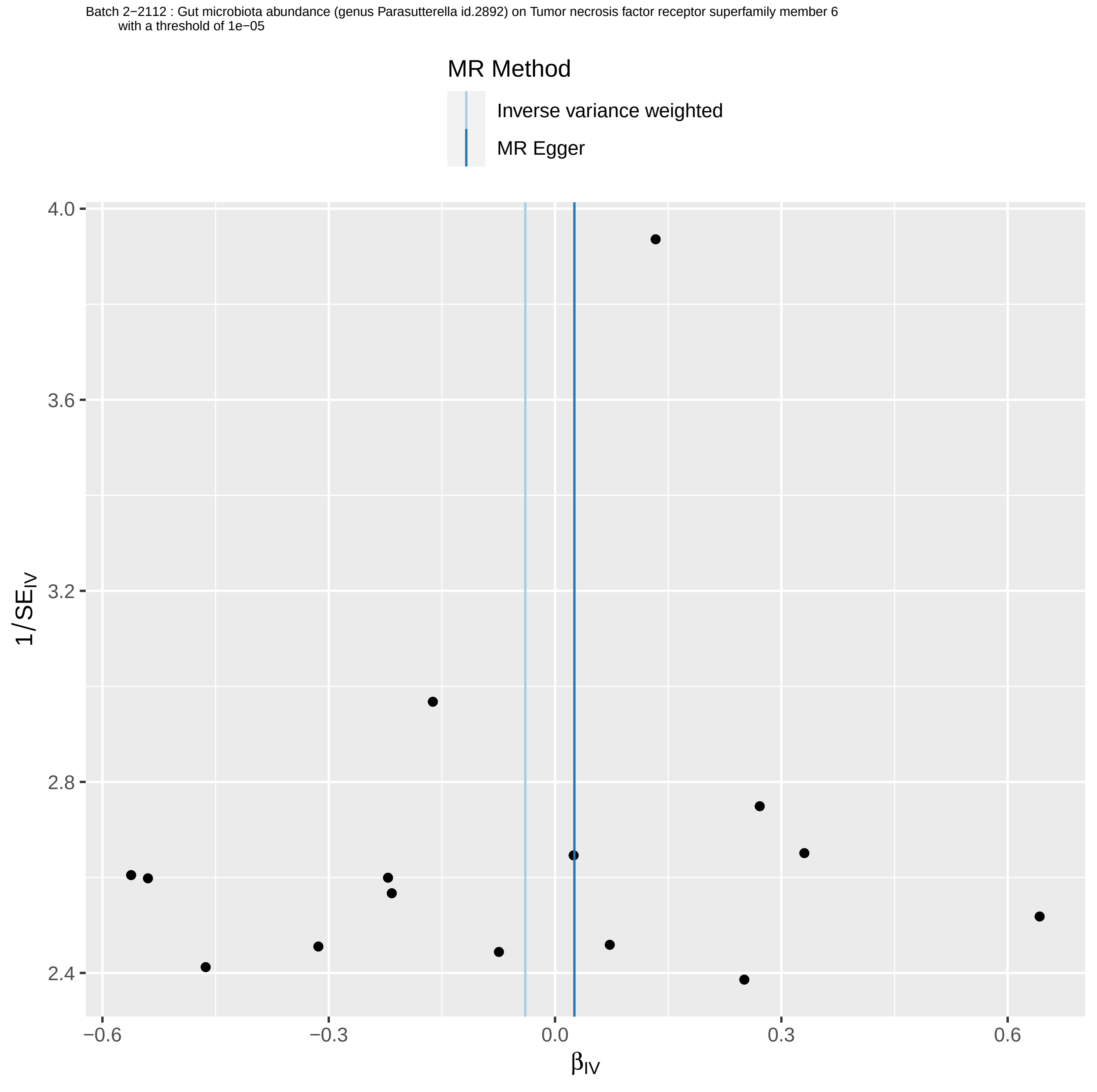

Batch 2−2112 : Gut microbiota abundance (genus Parasutterella id.2892) on Tumor necrosis factor receptor superfamily member 6
with a threshold of 1e−05
MR Method
Inverse variance weighted
MR Egger
4.0
3.6
3.2
2.8
2.4
1/SE_IV
−0.6
−0.3
0.0
0.3
0.6
β_IV

Batch 2−2113 : Gut microbiota abundance (genus Catenibacterium id.2153) on Tumor necrosis factor receptor superfamily member 6 with a threshold of 1e−05

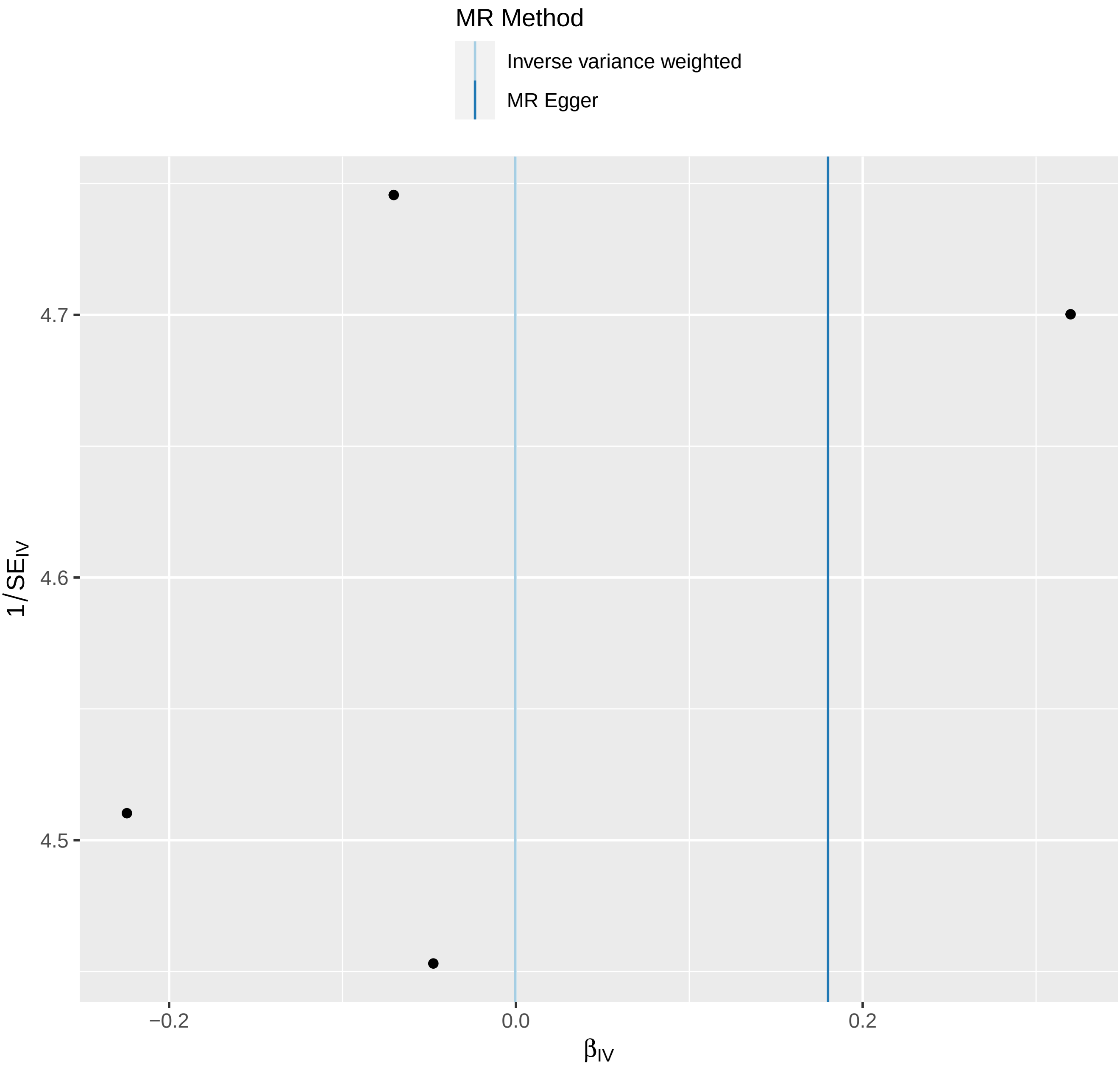

Batch 2−2114 : Gut microbiota abundance (genus Parasutterella id.2892) on Tumor necrosis factor receptor superfamily member 6 with a threshold of 1e−05

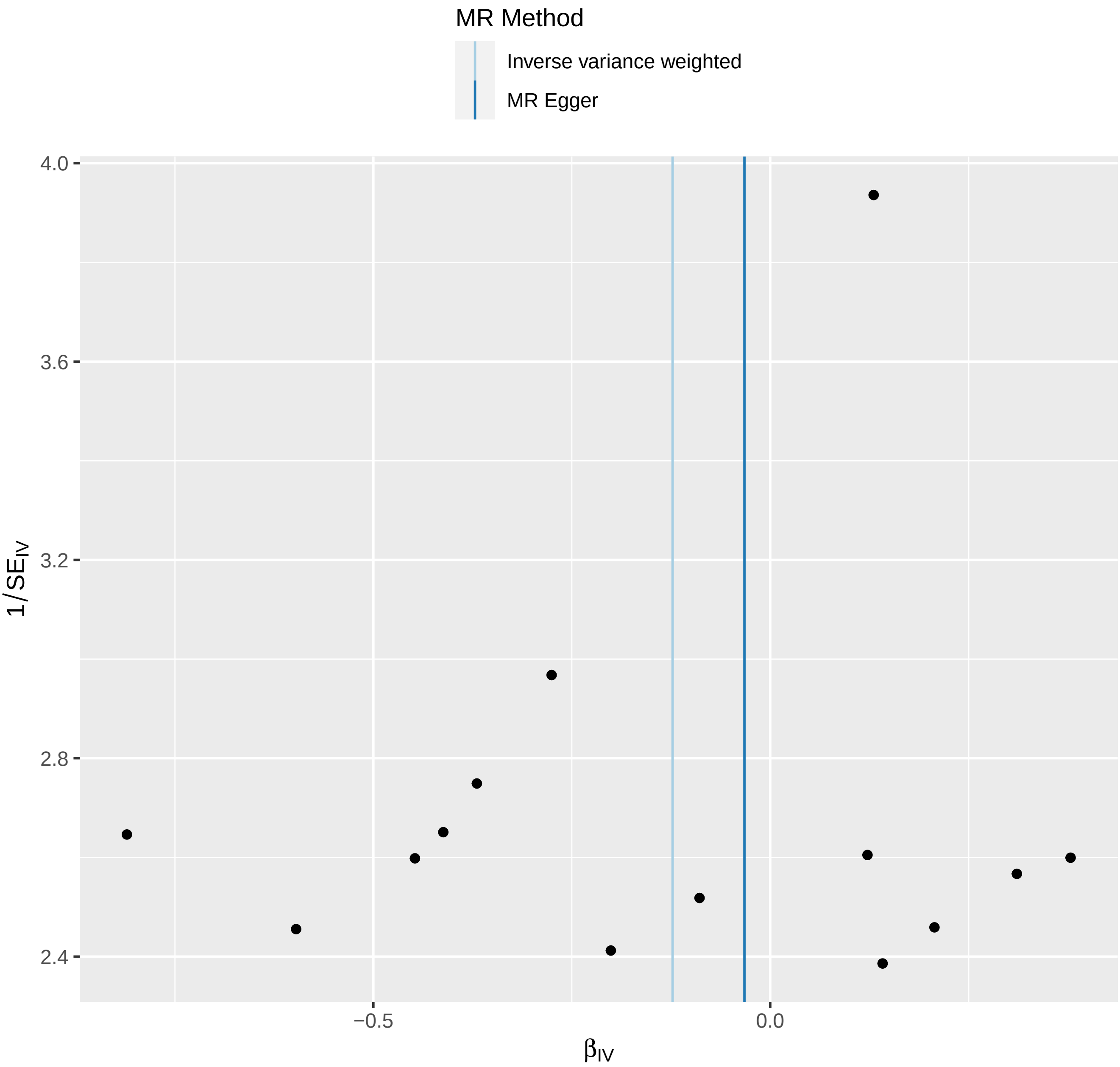

Batch 2−2115 : Gut microbiota abundance (genus Catenibacterium id.2153) on Tumor necrosis factor receptor superfamily member 6
with a threshold of 1e−05

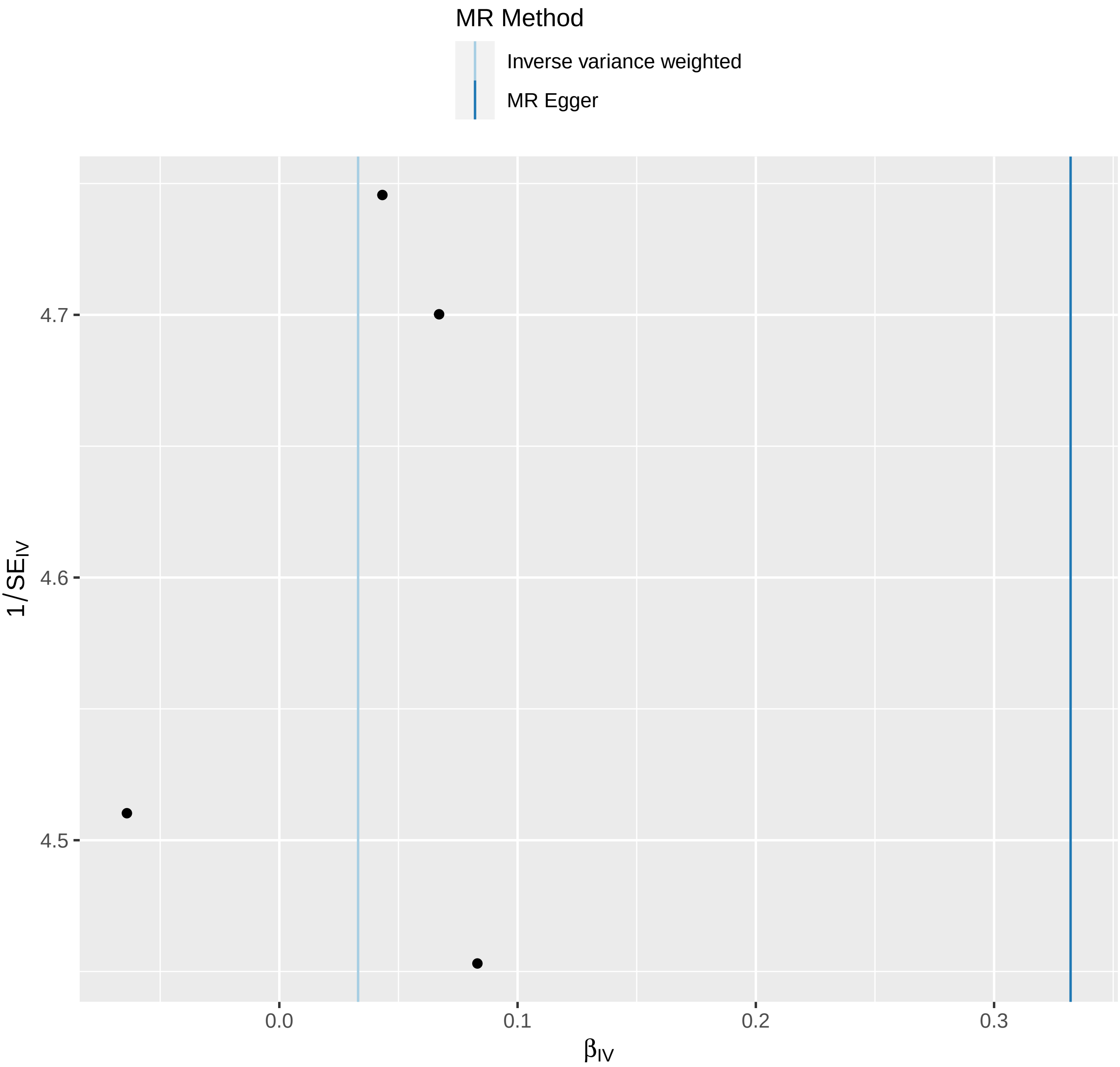

Batch 2−2116 : Gut microbiota abundance (genus Parasutterella id.2892) on Tumor necrosis factor receptor superfamily member 6 with a threshold of 1e−05

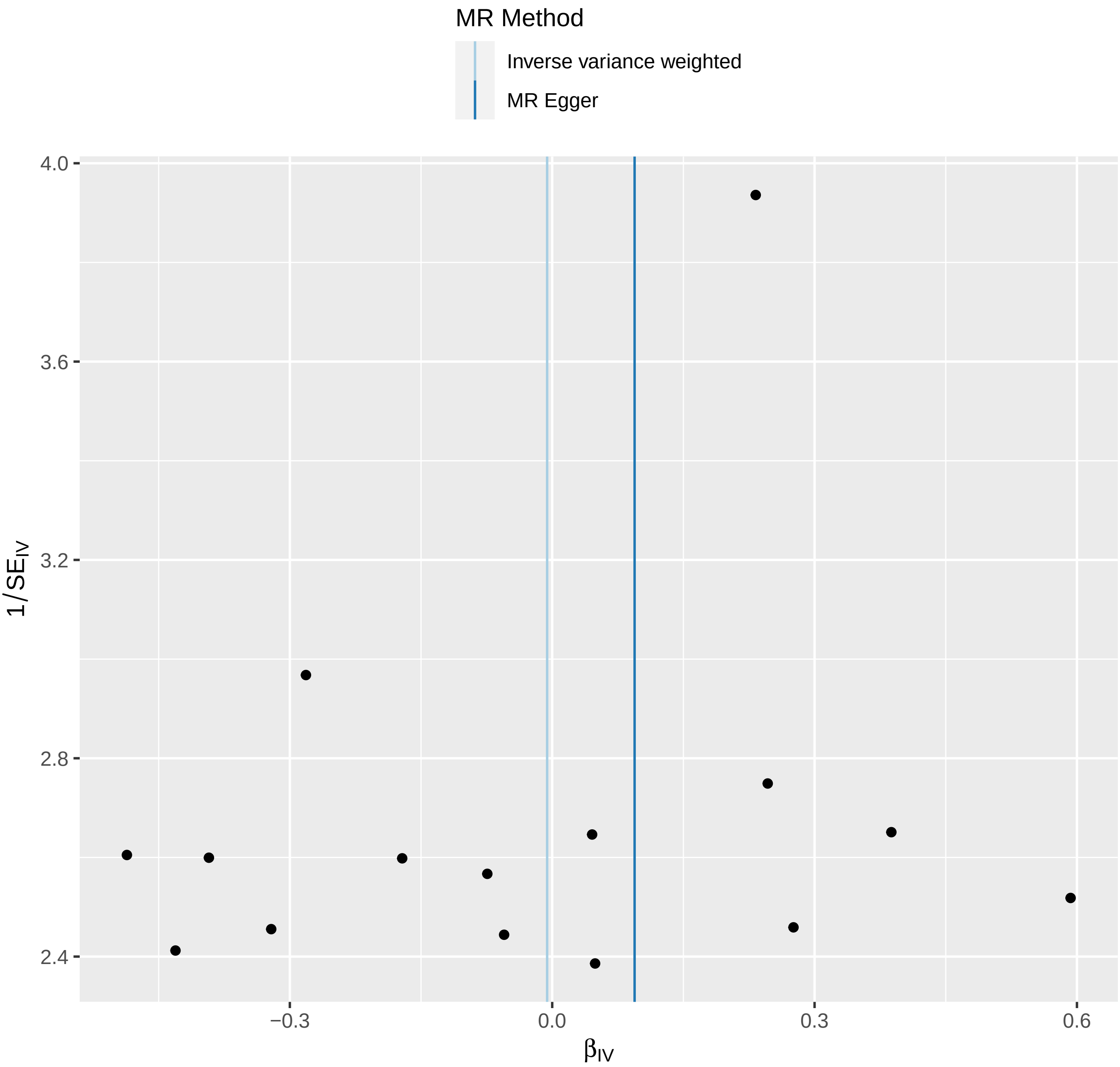

Batch 2−2117 : Gut microbiota abundance (genus Catenibacterium id.2153) on Tumor necrosis factor ligand superfamily member 6, soluble form with a threshold of 1e−05

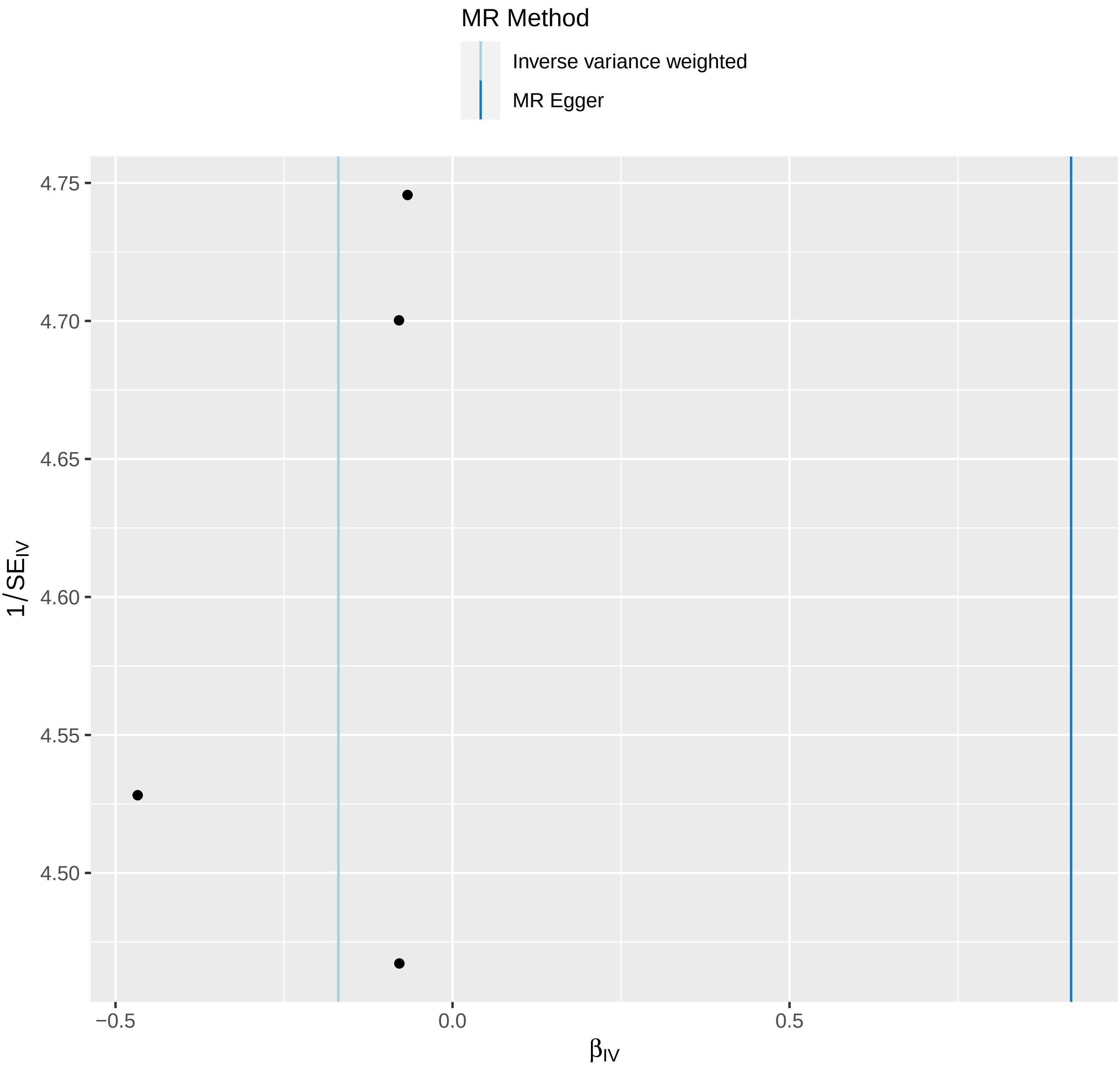

Batch 2−2118 : Gut microbiota abundance (genus Parasutterella id.2892) on Tumor necrosis factor ligand superfamily member 6, soluble form
with a threshold of 1e−05

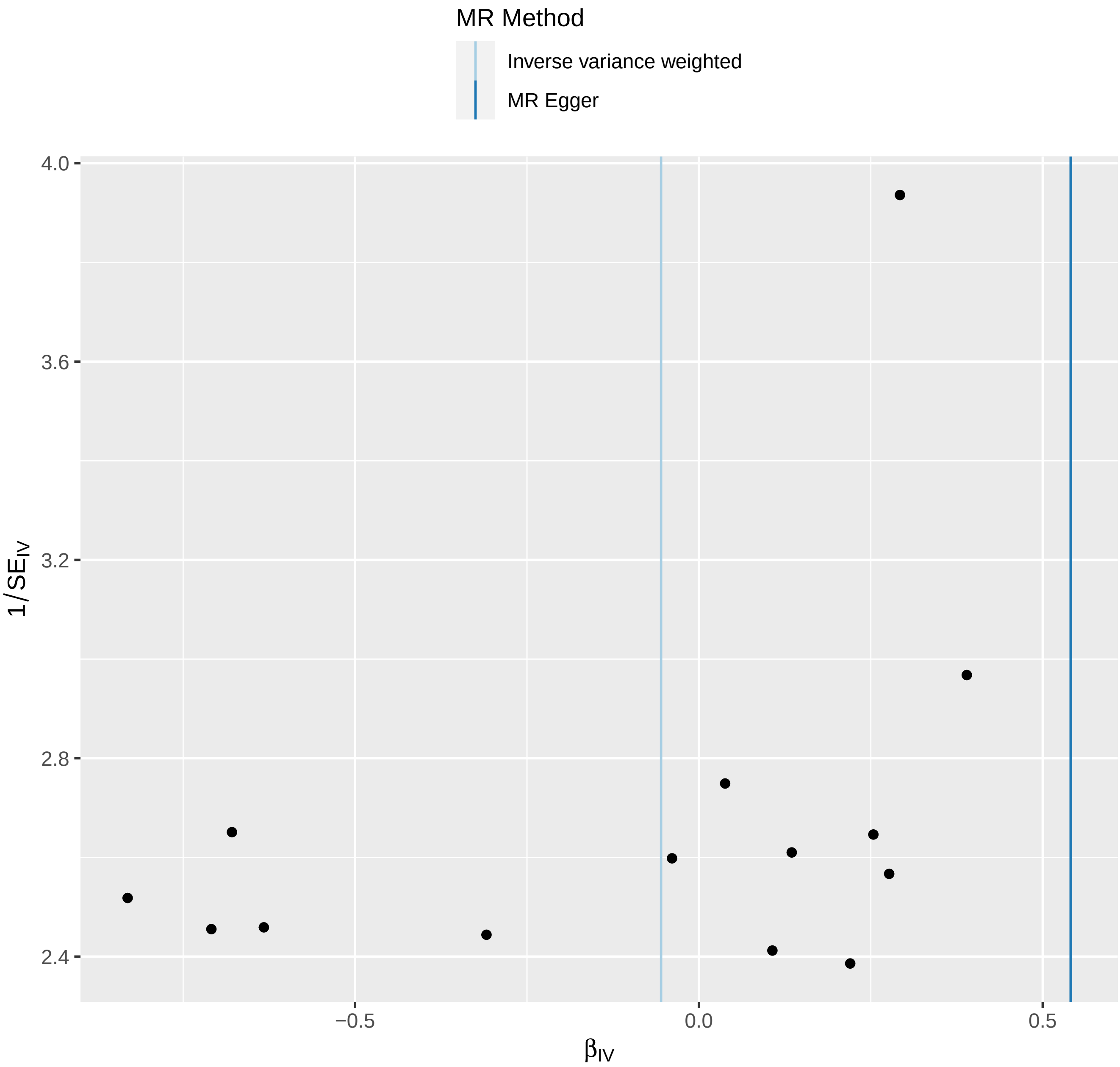

Batch 2−2119 : Gut microbiota abundance (genus Catenibacterium id.2153) on Fetal and adult testis−expressed transcript protein
with a threshold of 1e−05

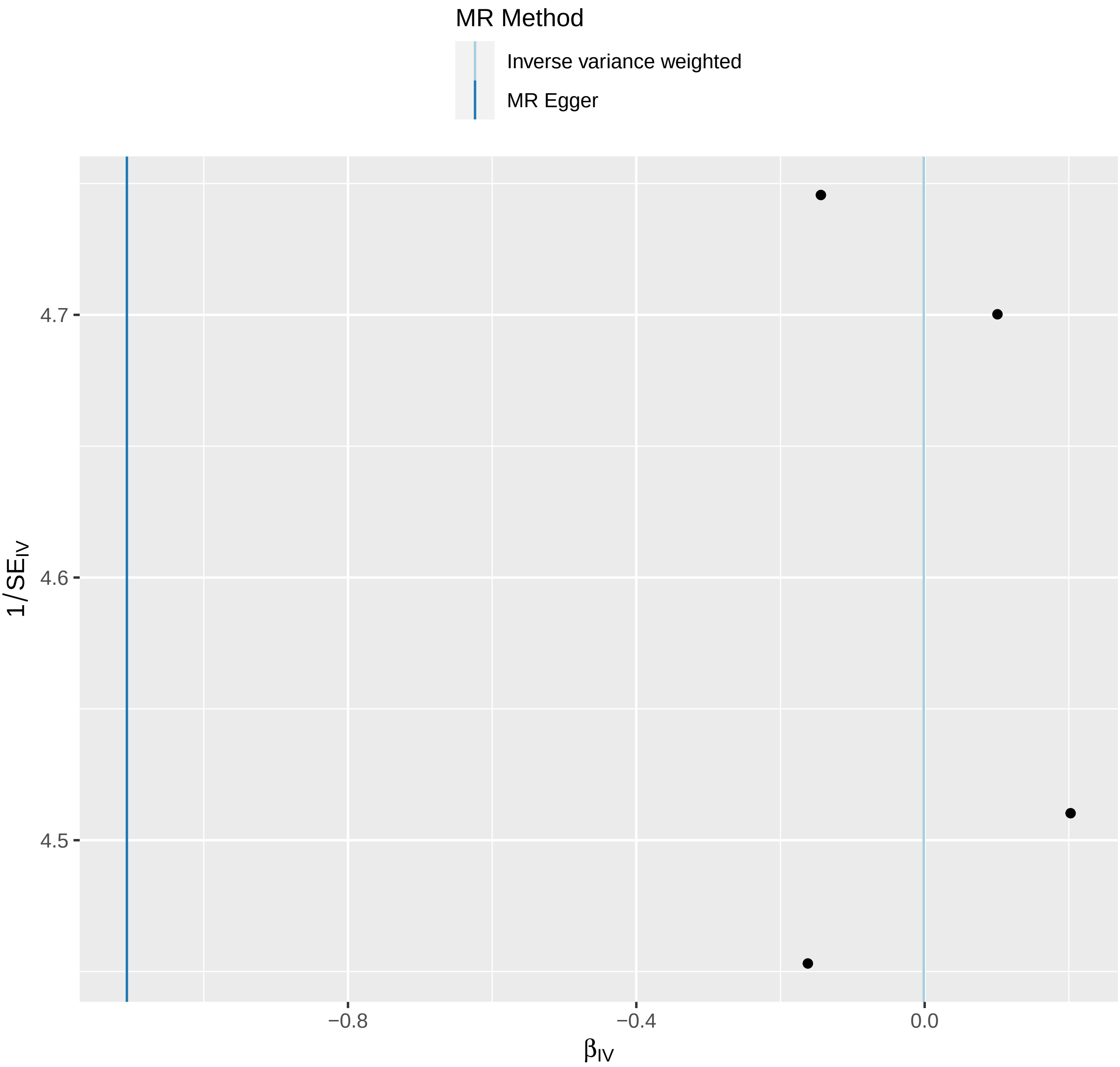

Batch 2−2120 : Gut microbiota abundance (genus Parasutterella id.2892) on Fetal and adult testis−expressed transcript protein
with a threshold of 1e−05

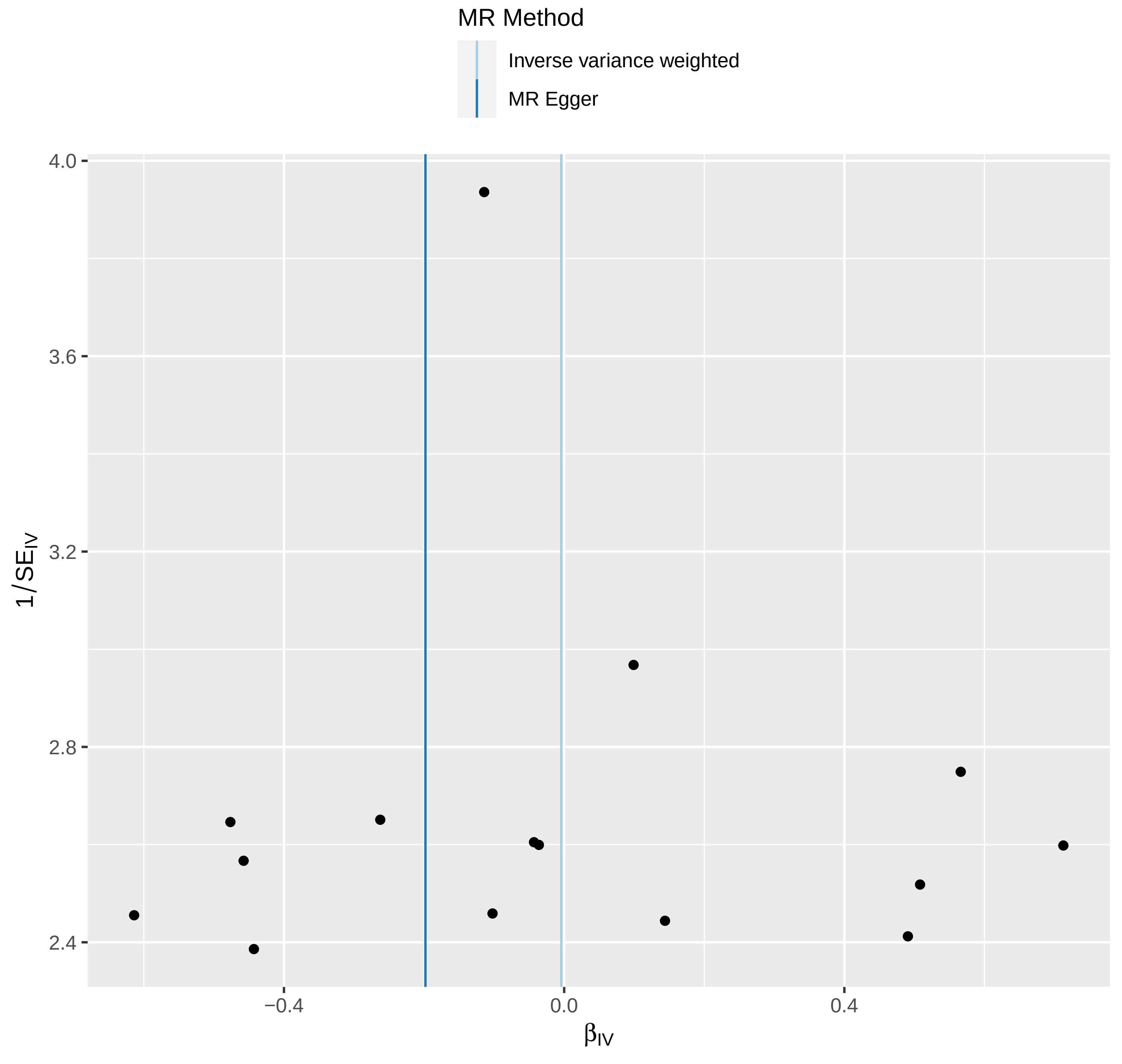

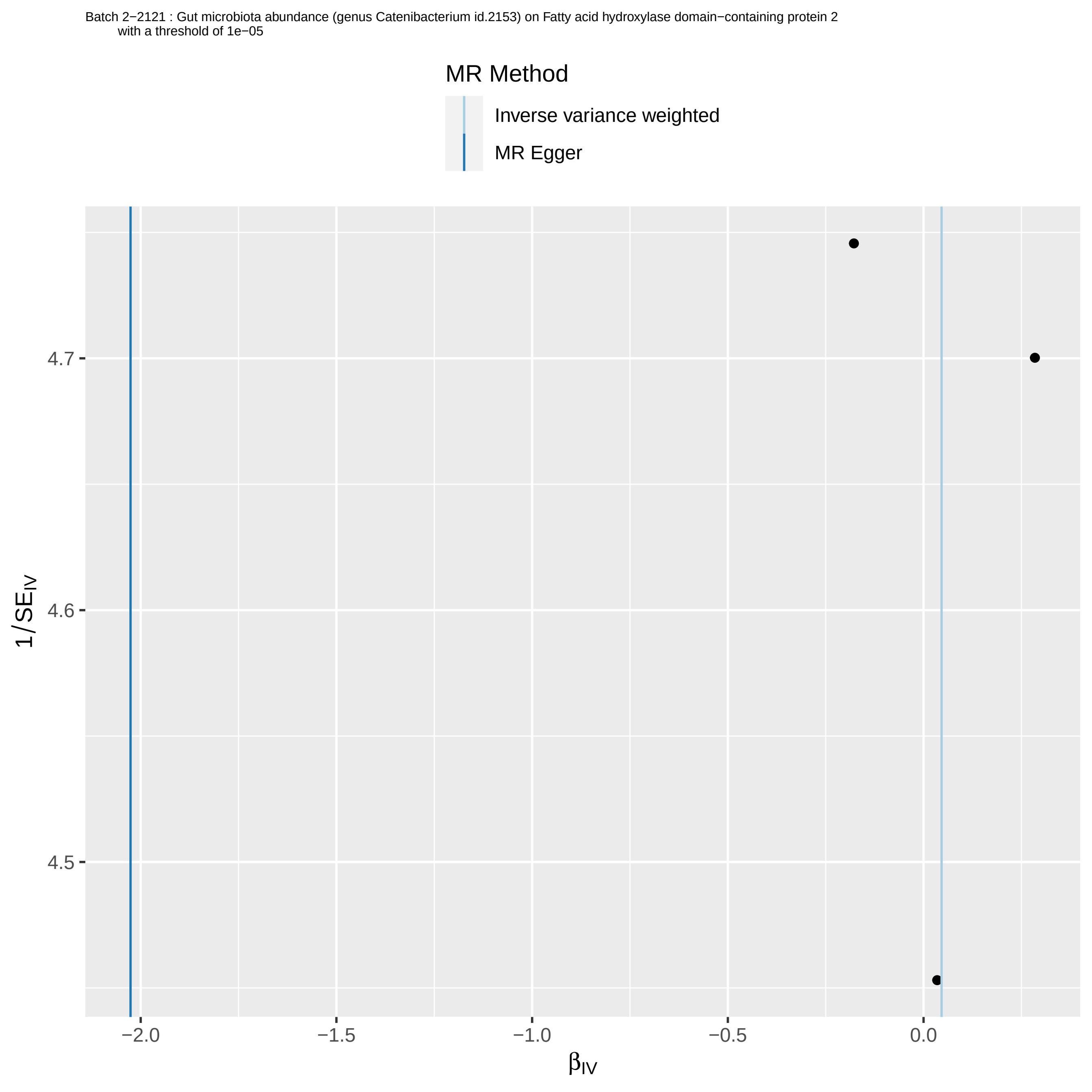

Batch 2−2121 : Gut microbiota abundance (genus Catenibacterium id.2153) on Fatty acid hydroxylase domain−containing protein 2
with a threshold of 1e−05
MR Method
Inverse variance weighted
MR Egger
4.7
4.6
4.5
1/SE_IV
−2.0
−1.5
−1.0
−0.5
0.0
β_IV

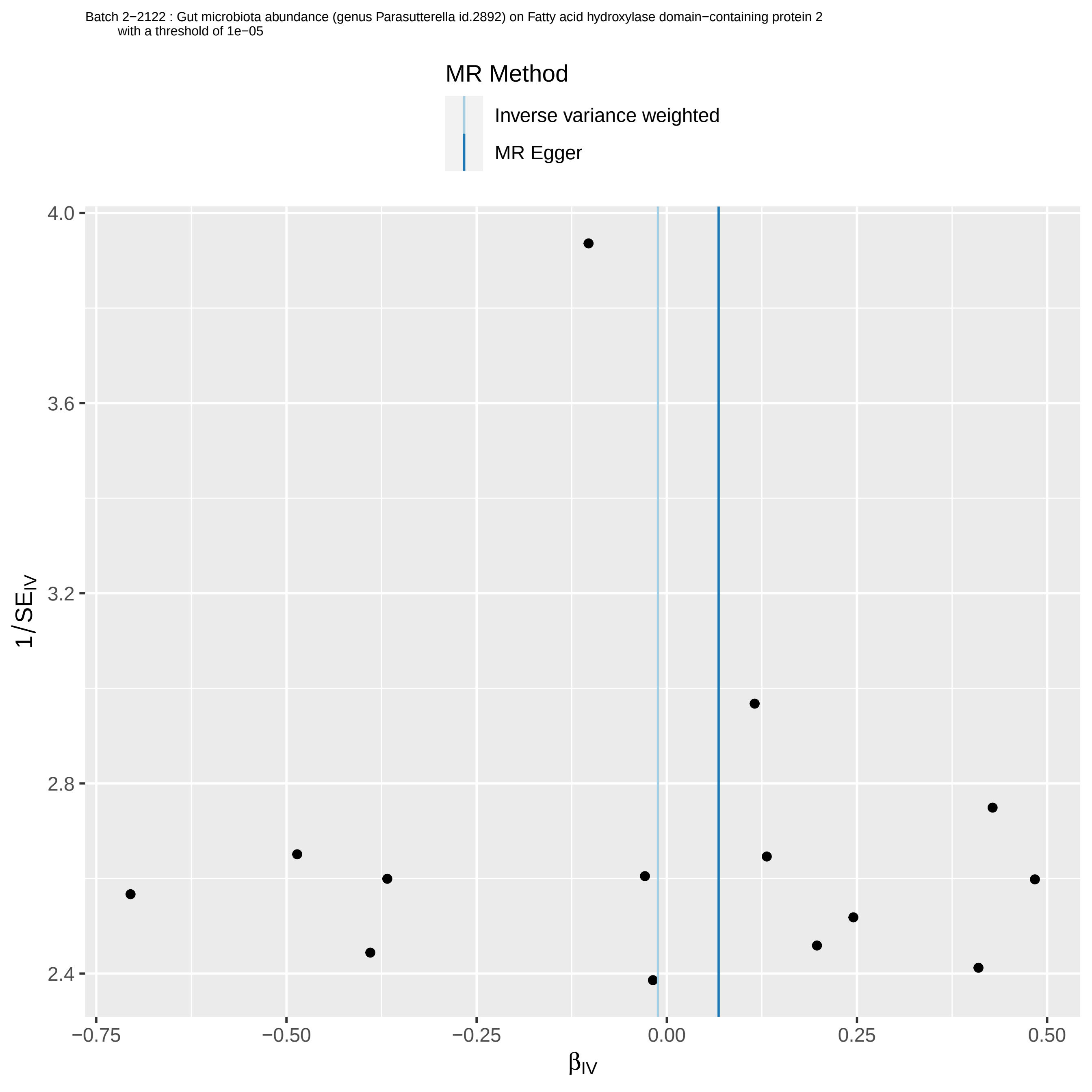

Batch 2−2122 : Gut microbiota abundance (genus Parasutterella id.2892) on Fatty acid hydroxylase domain−containing protein 2
with a threshold of 1e−05
MR Method
Inverse variance weighted
MR Egger
4.0
3.6
3.2
2.8
2.4
1/SE_IV
−0.75
−0.50
−0.25
0.00
0.25
0.50
β_IV

Batch 2−2123 : Gut microbiota abundance (genus Catenibacterium id.2153) on Fibulin−1
with a threshold of 1e−05

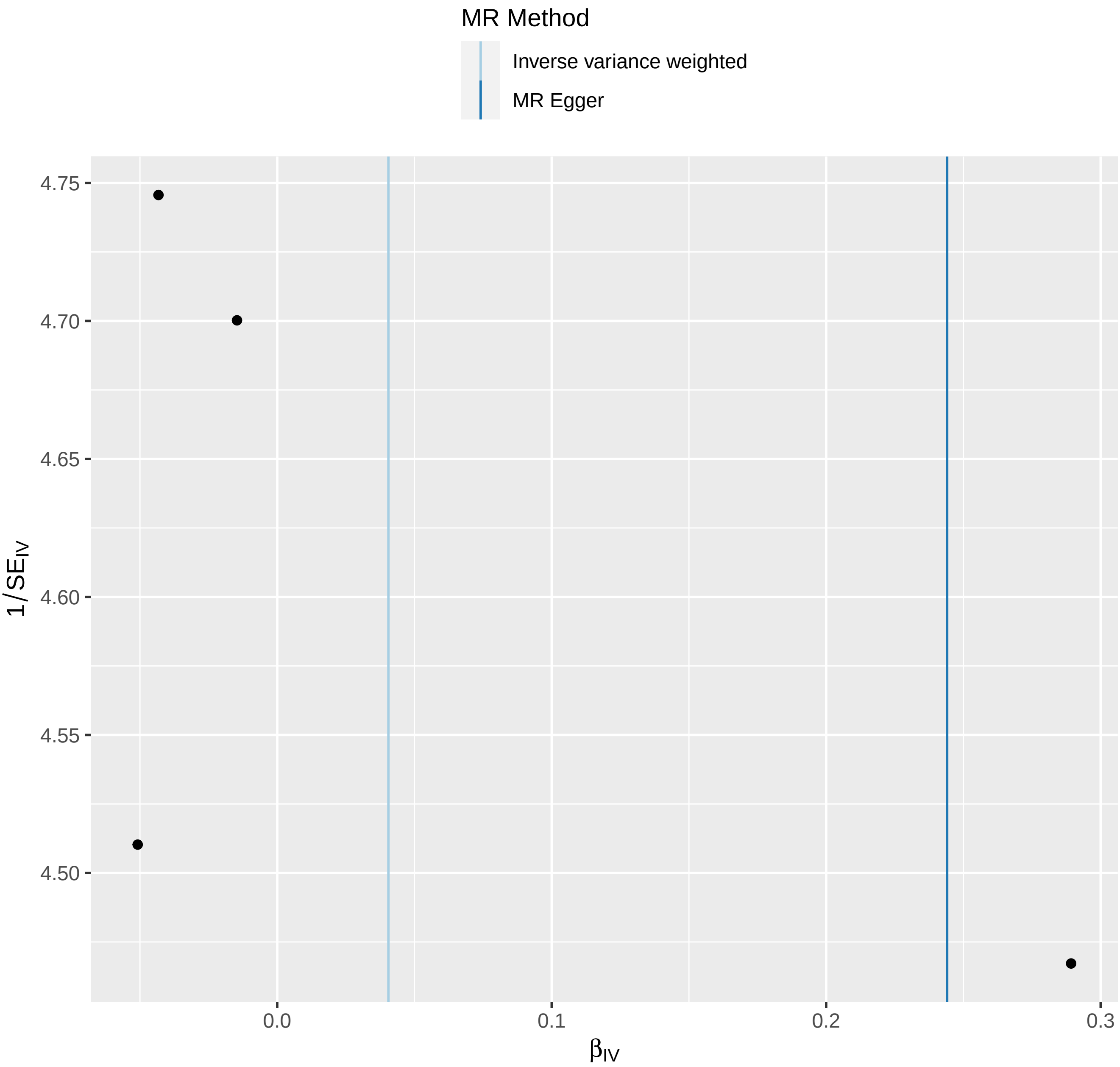

Batch 2−2124 : Gut microbiota abundance (genus Parasutterella id.2892) on Fibulin−1
with a threshold of 1e−05

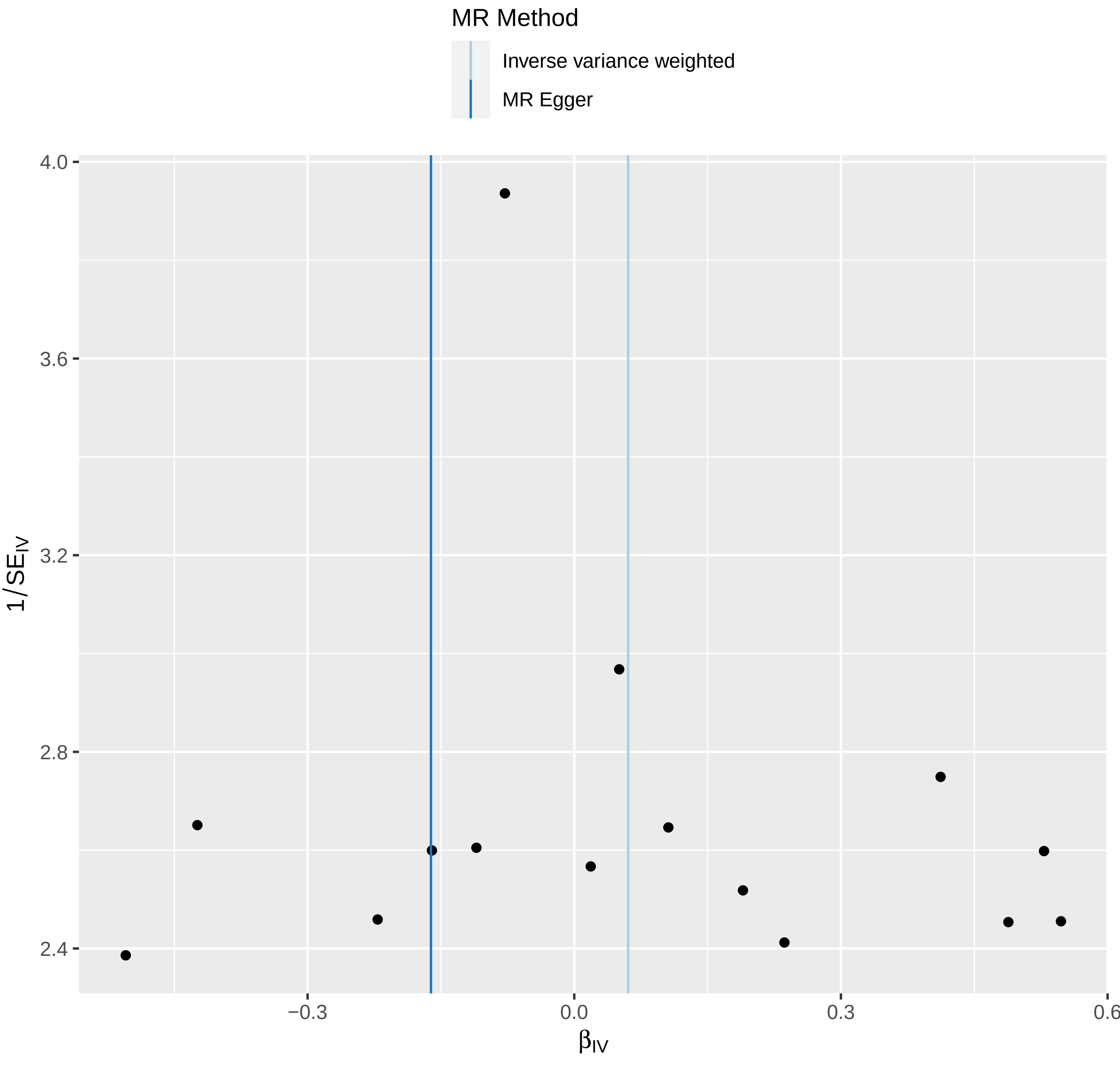

Batch 2−2125 : Gut microbiota abundance (genus Catenibacterium id.2153) on Fructose−1,6−bisphosphatase 1
with a threshold of 1e−05

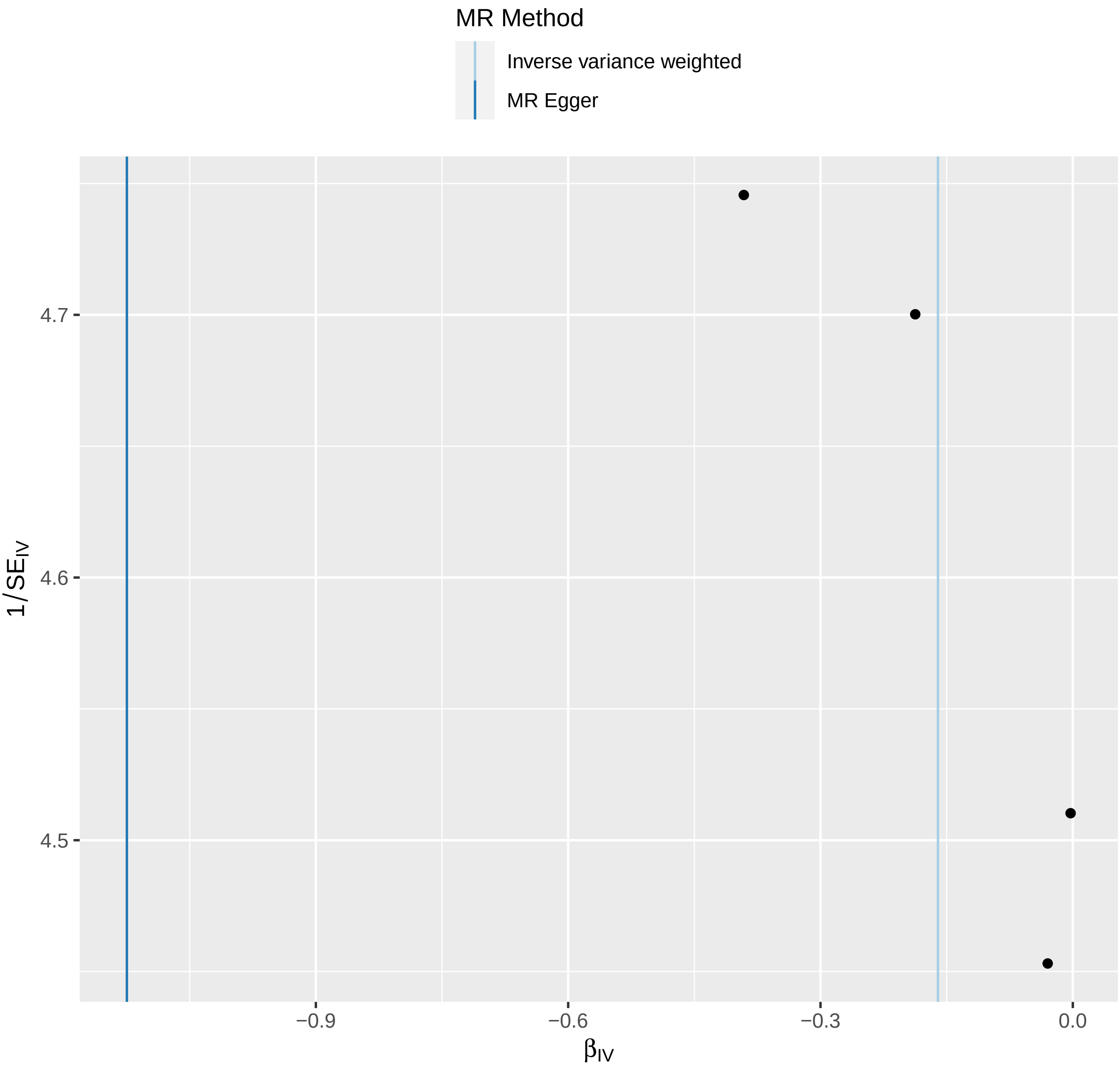

Batch 2−2126 : Gut microbiota abundance (genus Parasutterella id.2892) on Fructose−1,6−bisphosphatase 1 with a threshold of 1e−05

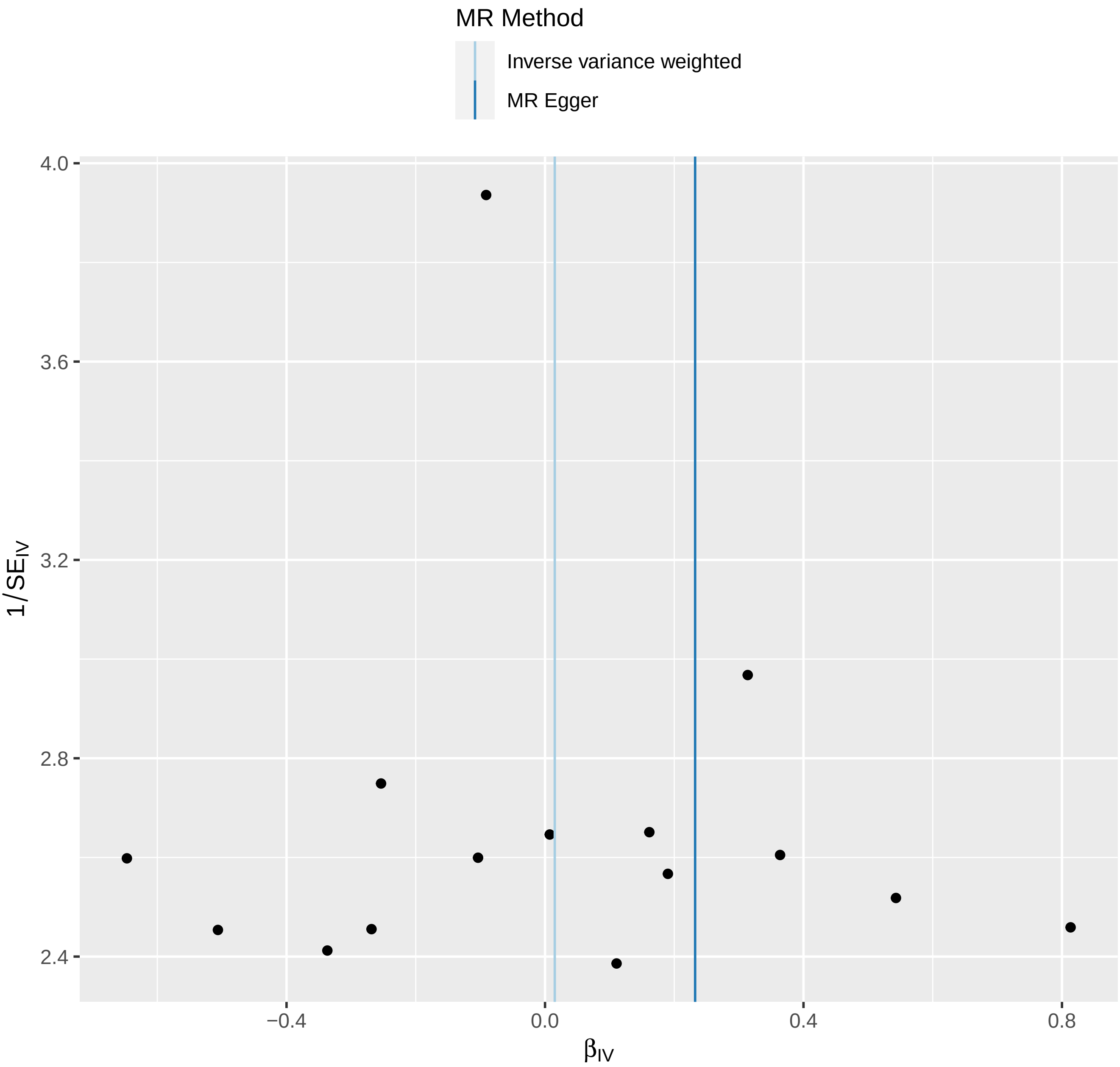

Batch 2−2127 : Gut microbiota abundance (genus Catenibacterium id.2153) on Fructose−1,6−bisphosphatase isozyme 2 with a threshold of 1e−05

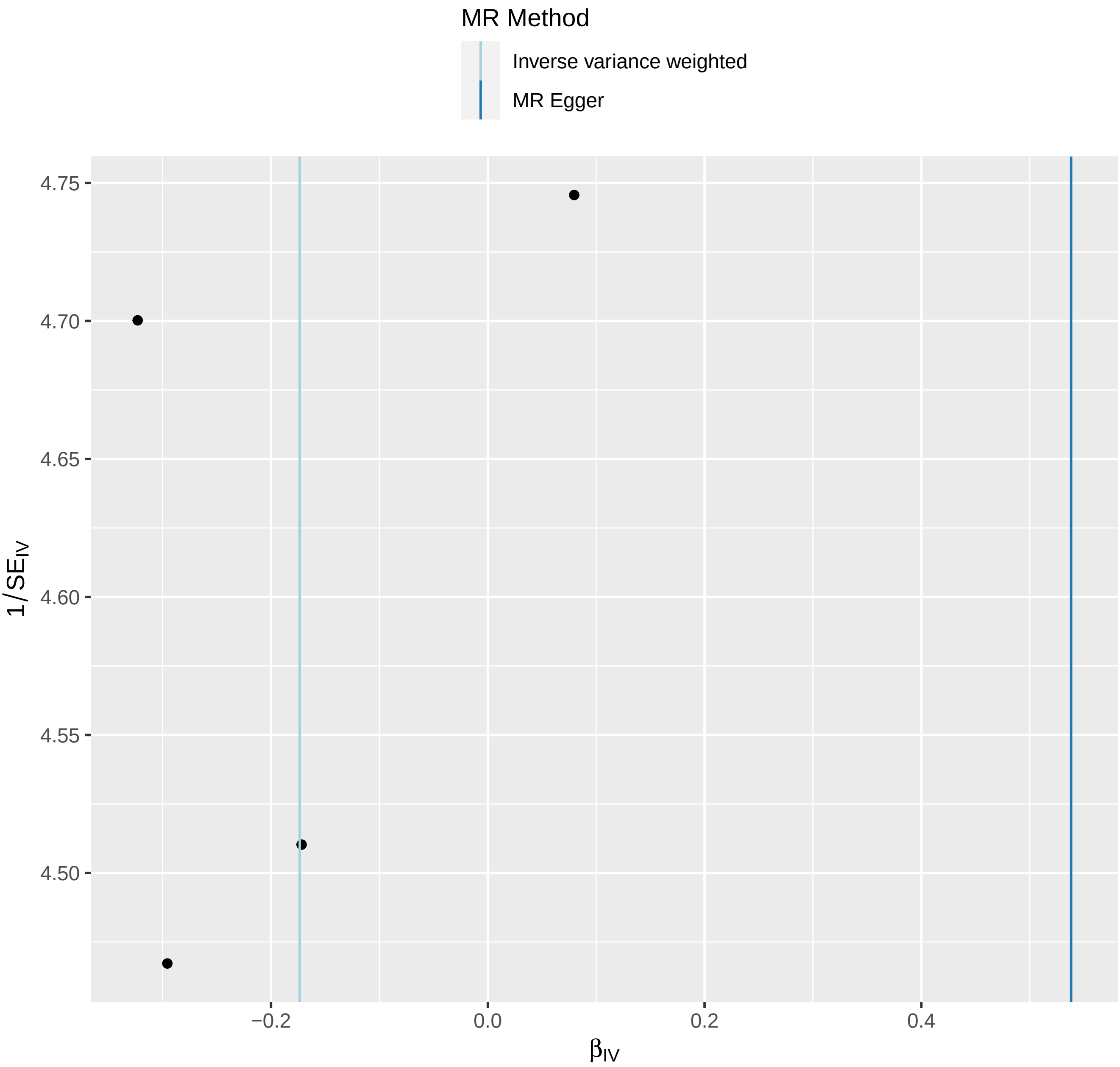

Batch 2−2128 : Gut microbiota abundance (genus Parasutterella id.2892) on Fructose−1,6−bisphosphatase isozyme 2 with a threshold of 1e−05

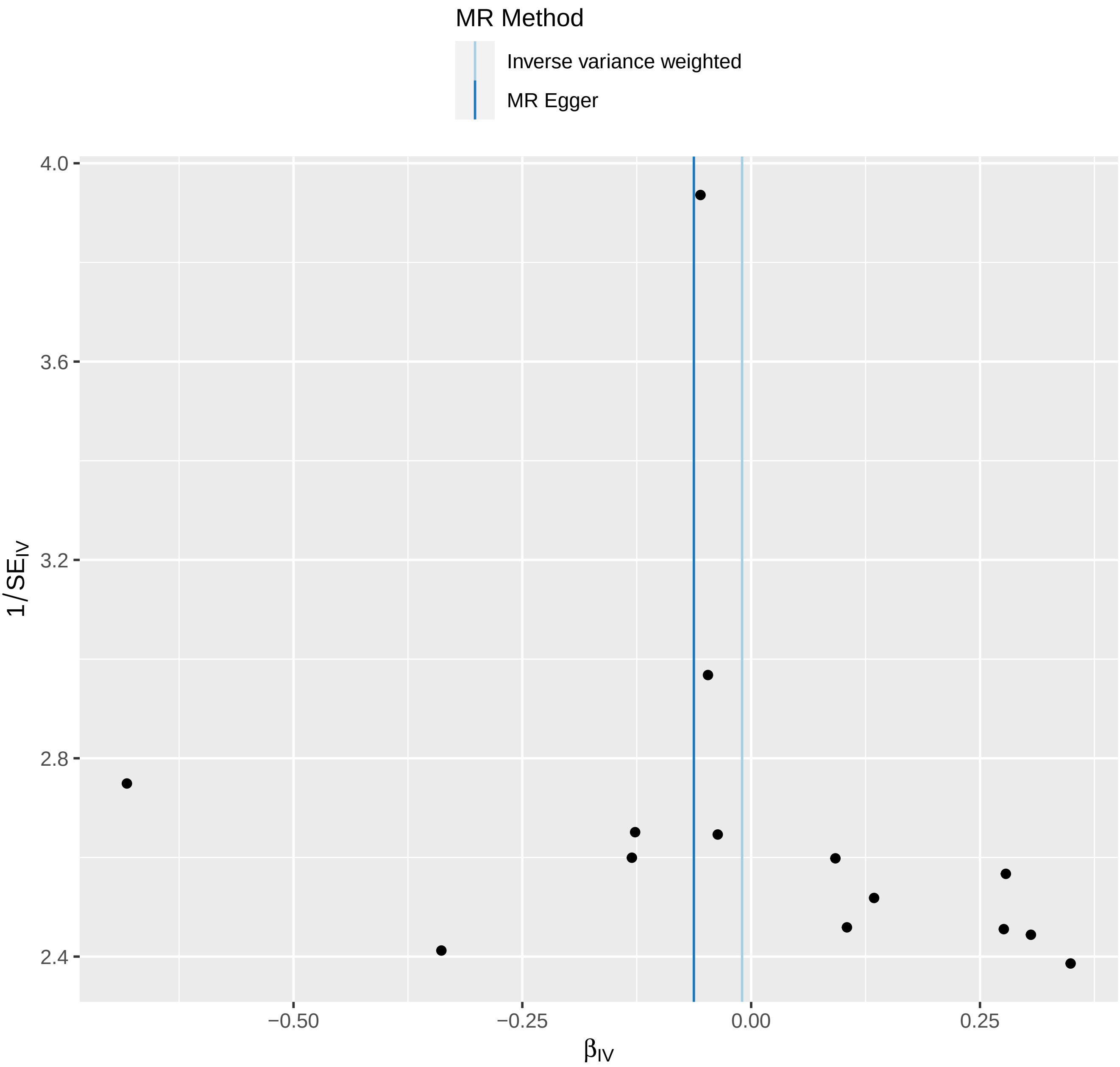

Batch 2−2129 : Gut microbiota abundance (genus Catenibacterium id.2153) on F−box/LRR−repeat protein 4
with a threshold of 1e−05

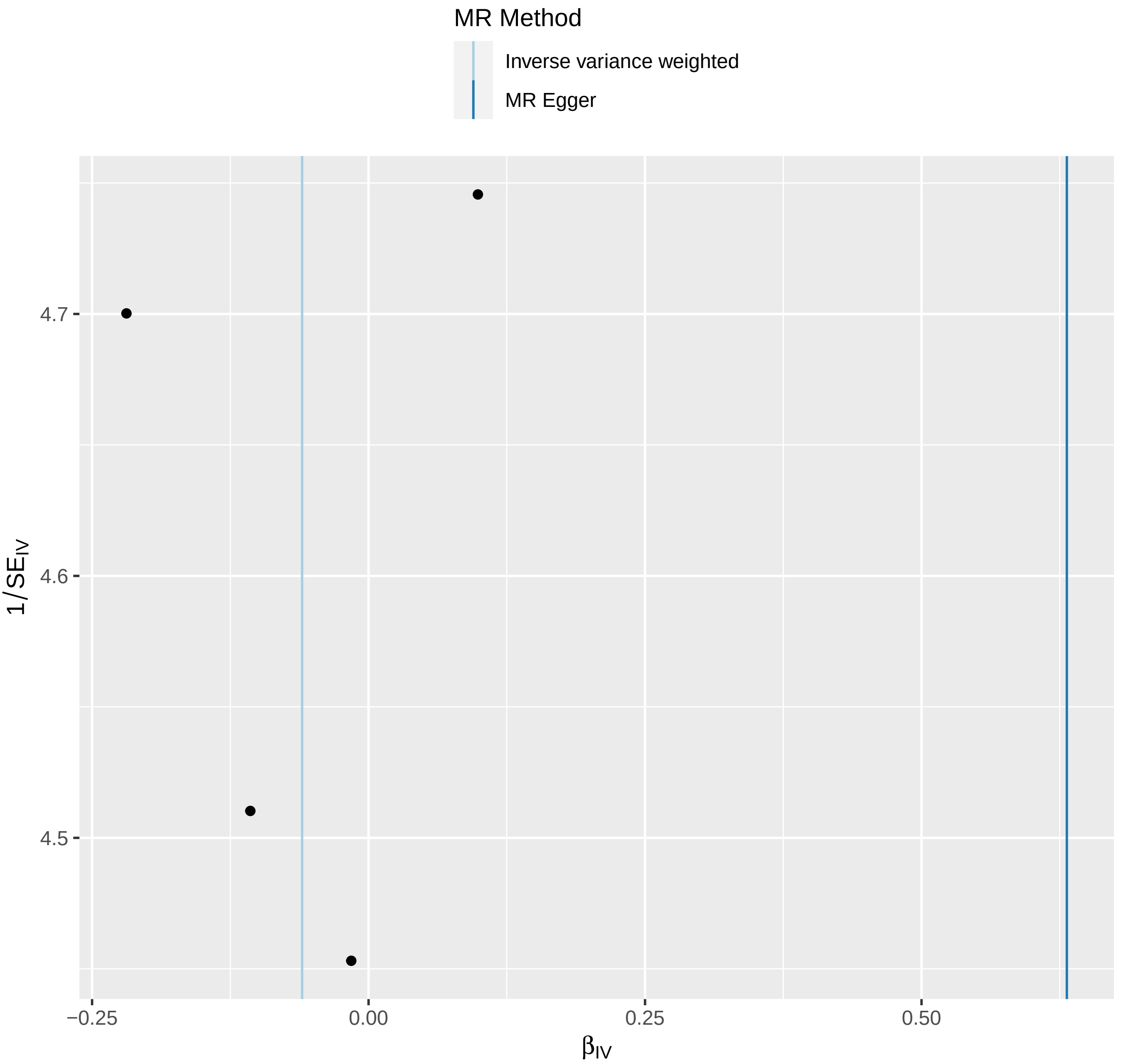

Batch 2−2130 : Gut microbiota abundance (genus Parasutterella id.2892) on F−box/LRR−repeat protein 4
with a threshold of 1e−05

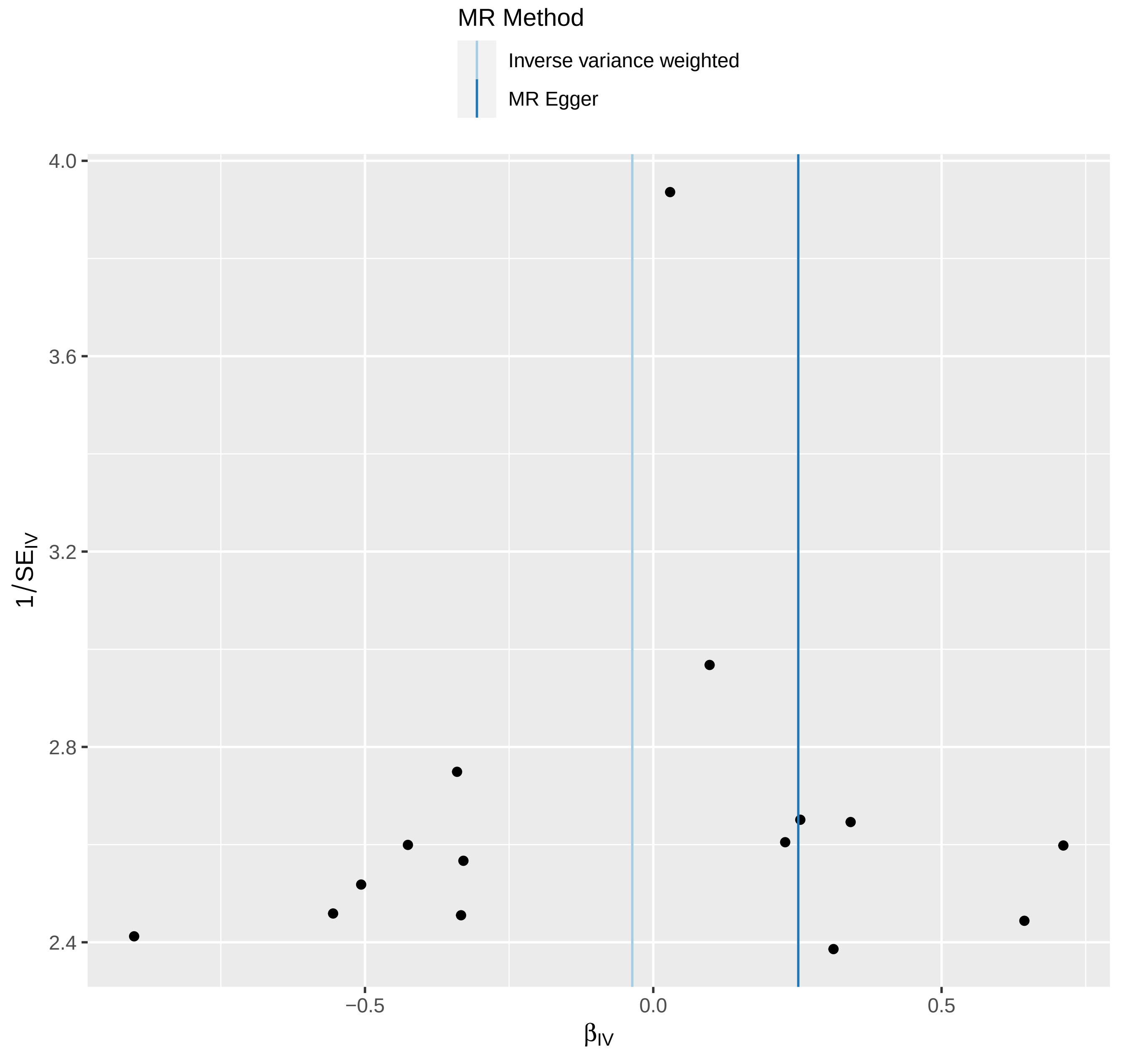

Batch 2−2131 : Gut microbiota abundance (genus Catenibacterium id.2153) on F−box/LRR−repeat protein 4
with a threshold of 1e−05

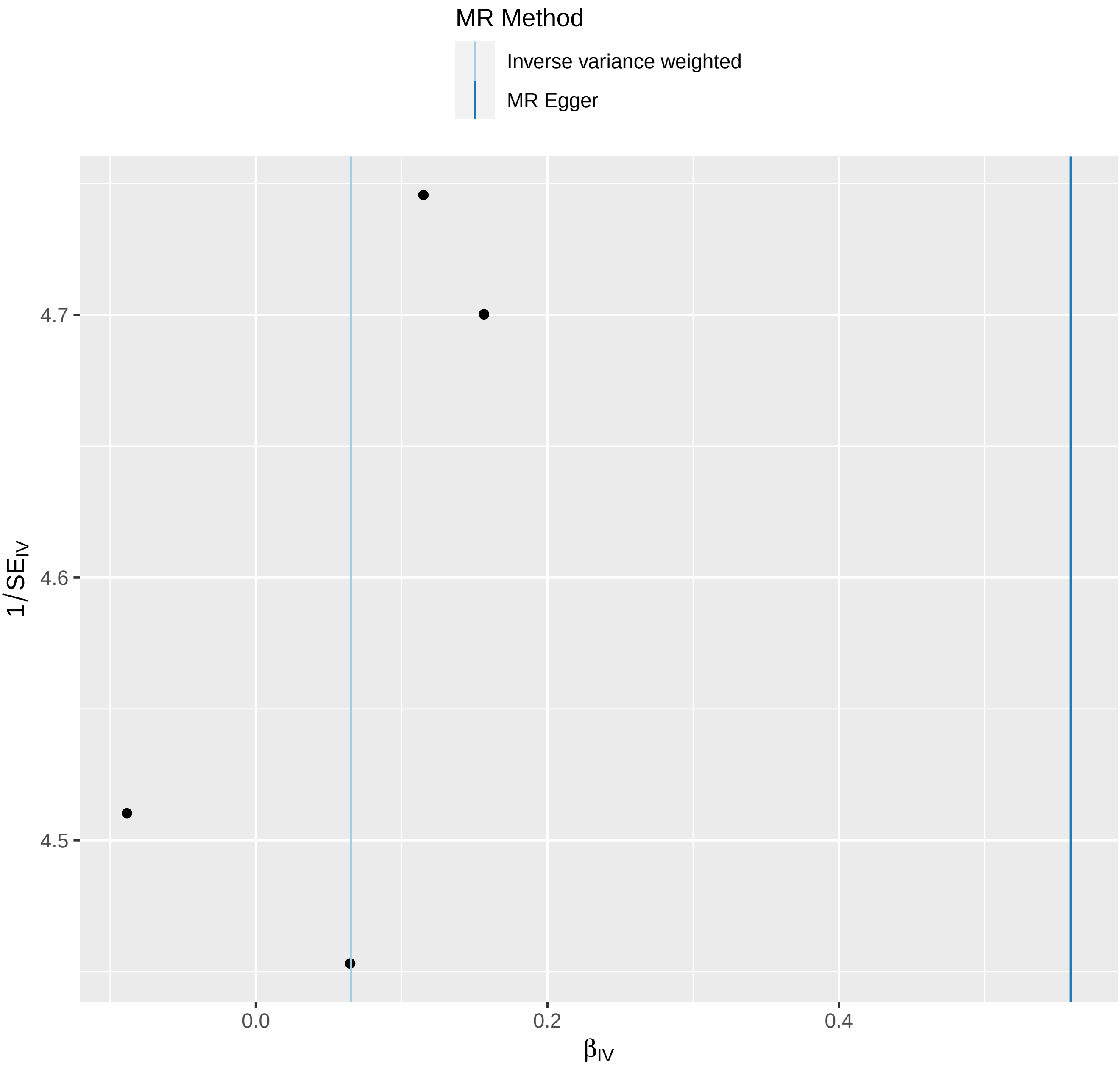

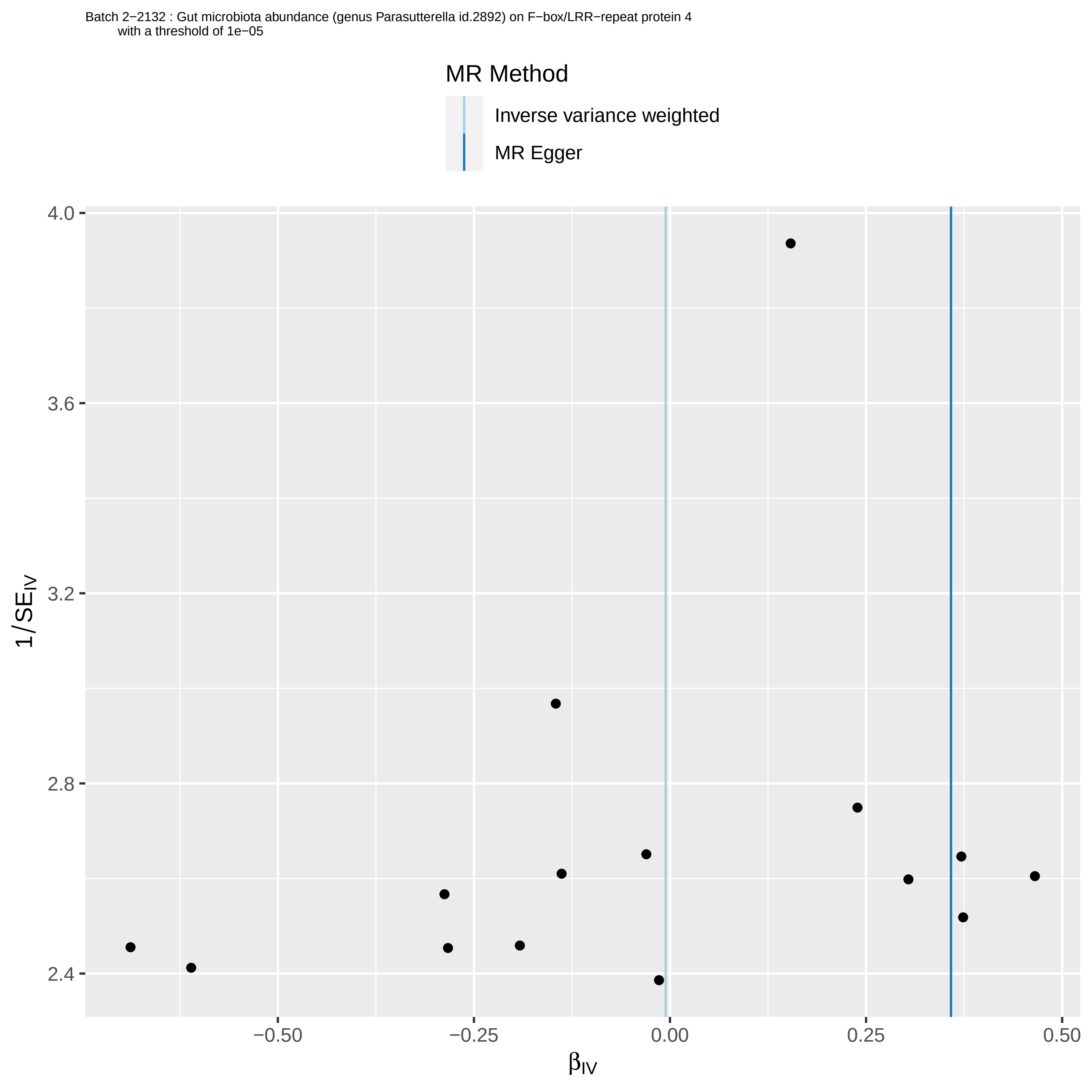

Batch 2−2132 : Gut microbiota abundance (genus Parasutterella id.2892) on F−box/LRR−repeat protein 4
with a threshold of 1e−05
MR Method
Inverse variance weighted
MR Egger
4.0
3.6
3.2
2.8
2.4
1/SE$_{IV}$
−0.50
−0.25
0.00
0.25
0.50
β$_{IV}$

Batch 2−2133 : Gut microbiota abundance (genus Catenibacterium id.2153) on F−box/LRR−repeat protein 4
with a threshold of 1e−05

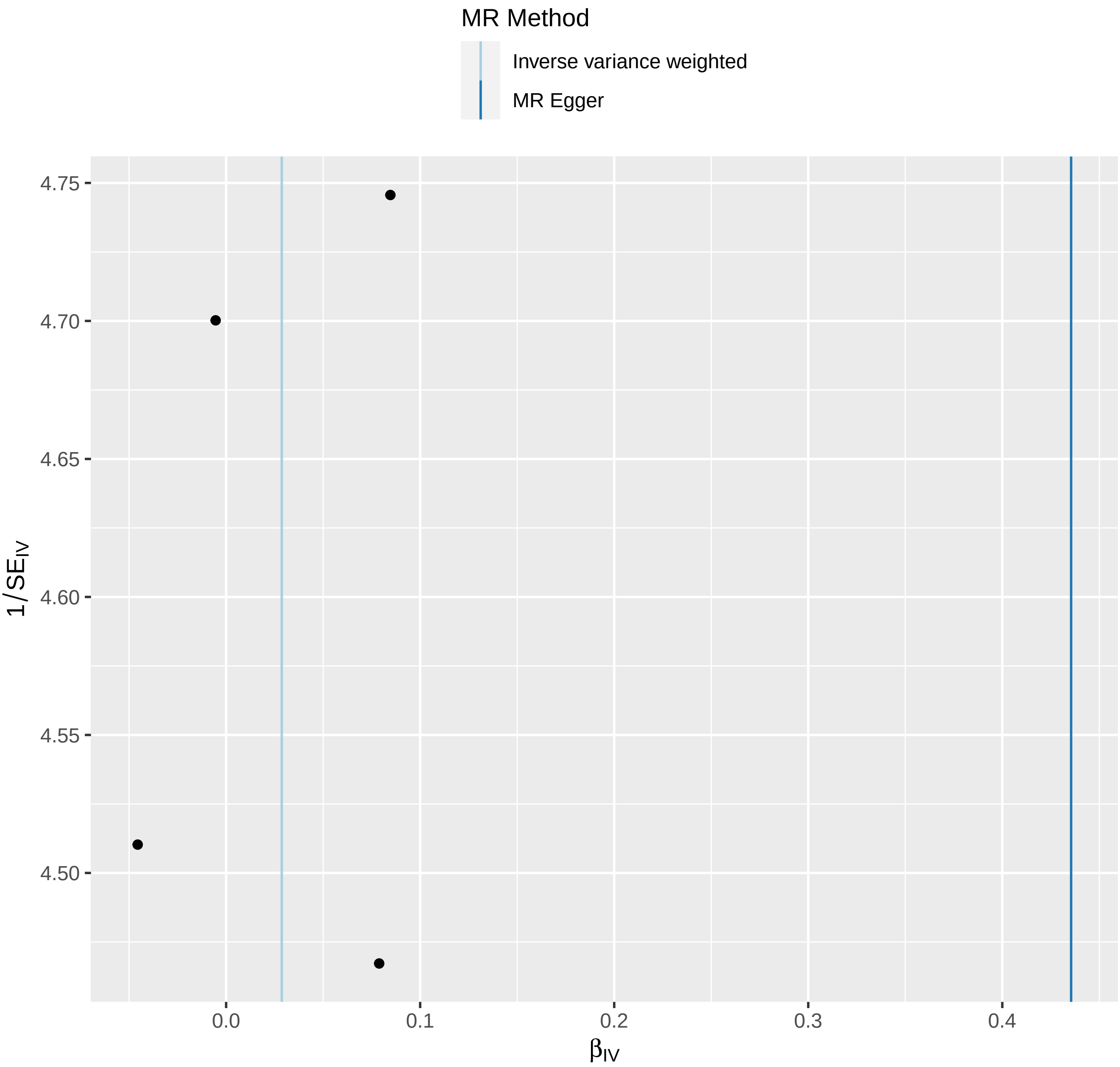

Batch 2−2134 : Gut microbiota abundance (genus Parasutterella id.2892) on F−box/LRR−repeat protein 4
with a threshold of 1e−05

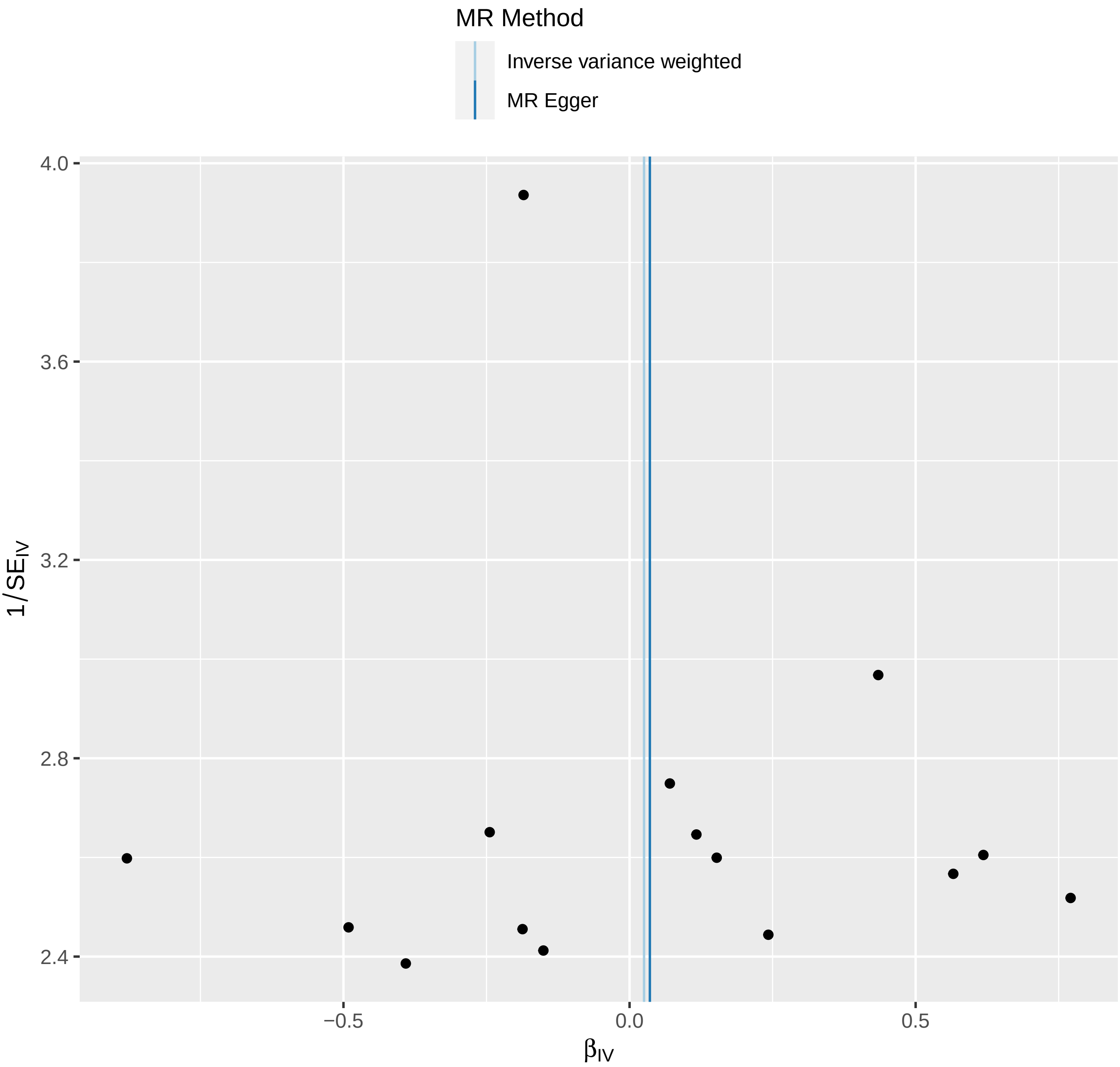

Batch 2−2135 : Gut microbiota abundance (genus Catenibacterium id.2153) on F−box/LRR−repeat protein 5 with a threshold of 1e−05

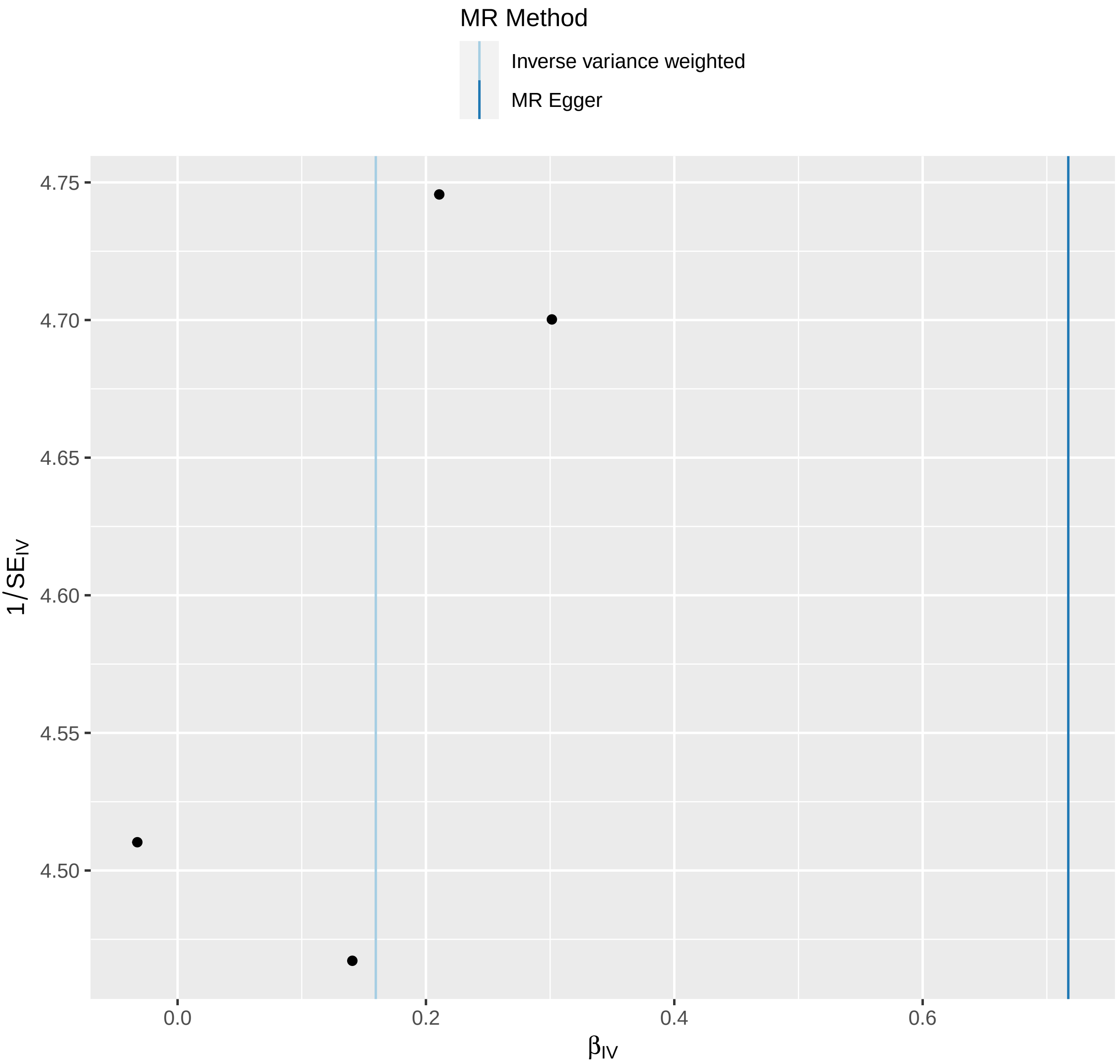

Batch 2−2136 : Gut microbiota abundance (genus Parasutterella id.2892) on F−box/LRR−repeat protein 5
with a threshold of 1e−05

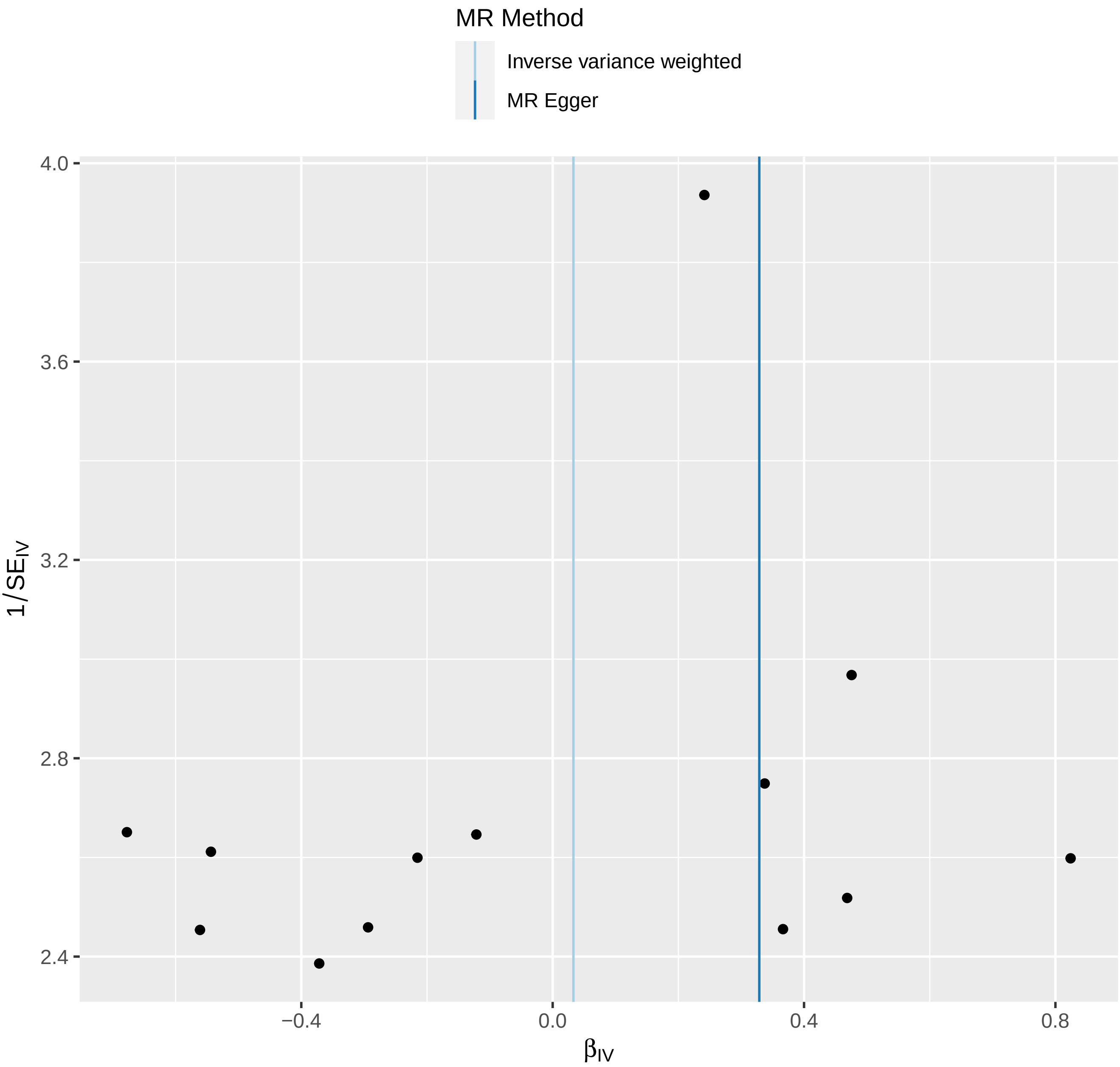

Batch 2−2137 : Gut microbiota abundance (genus Catenibacterium id.2153) on F−box only protein 3
with a threshold of 1e−05

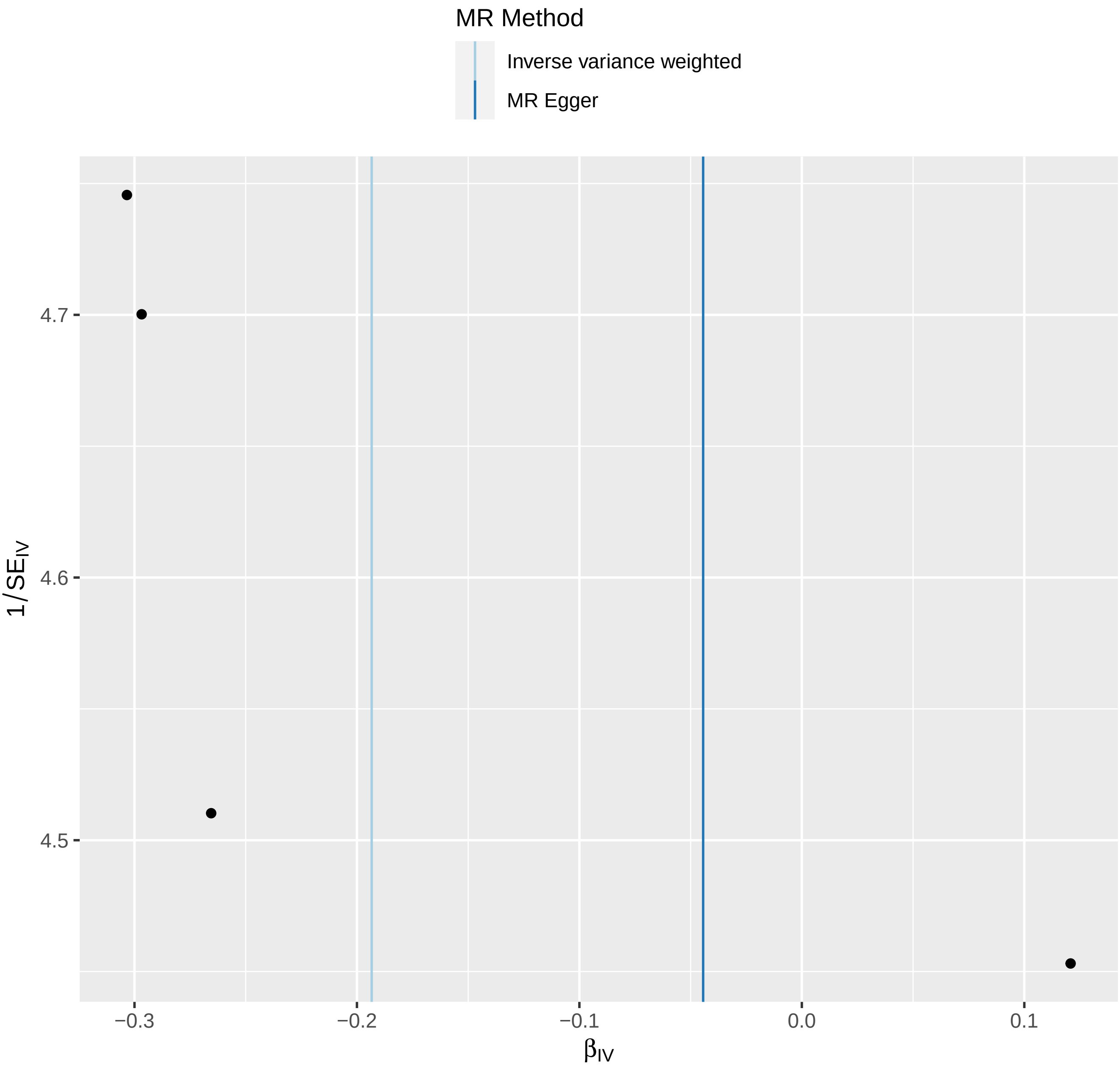

Batch 2−2138 : Gut microbiota abundance (genus Parasutterella id.2892) on F−box only protein 3
with a threshold of 1e−05

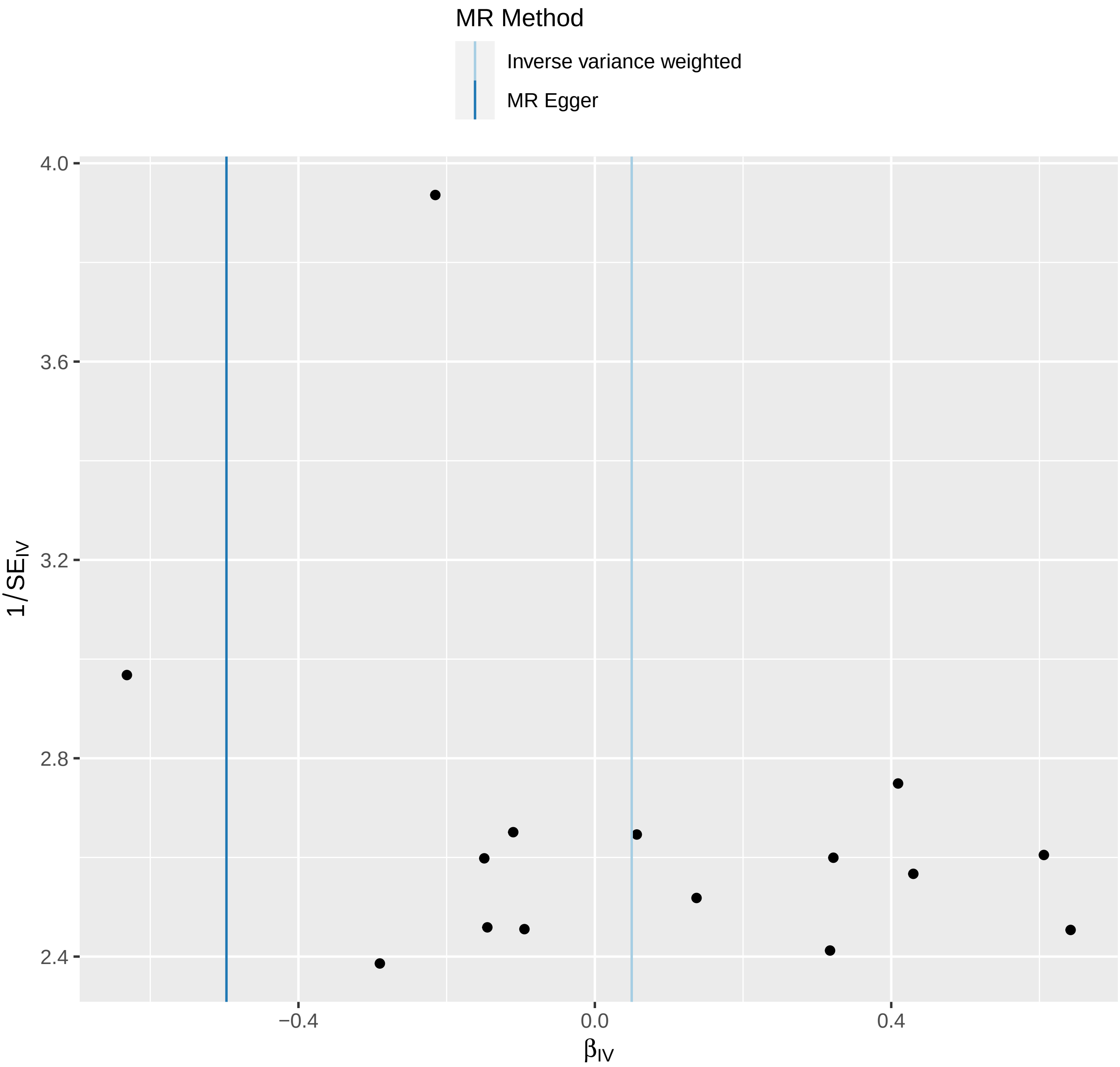

Batch 2−2139 : Gut microbiota abundance (genus Catenibacterium id.2153) on High affinity immunoglobulin alpha and immunoglobulin mu Fc receptor with a threshold of 1e−05

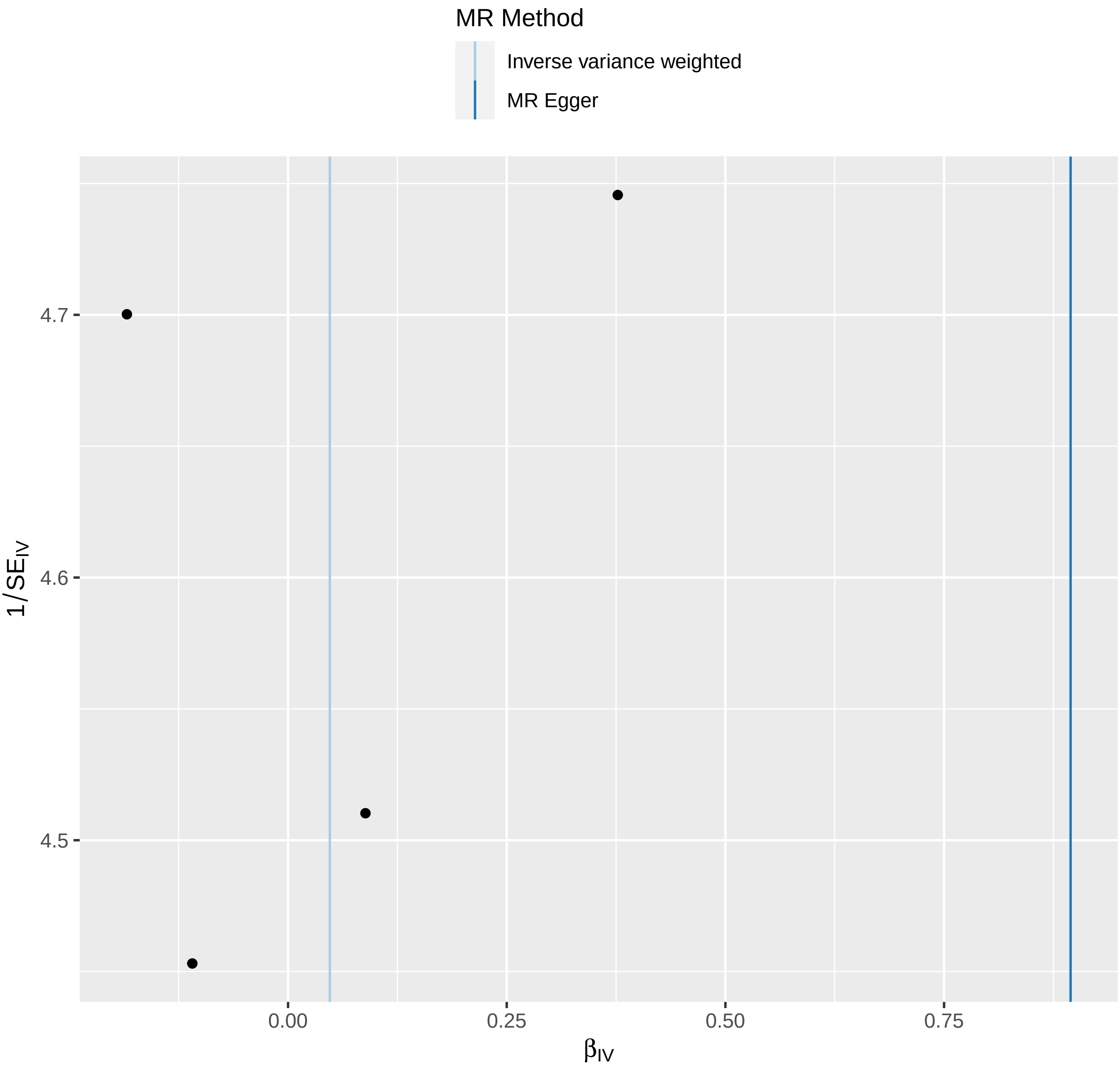

Batch 2−2140 : Gut microbiota abundance (genus Parasutterella id.2892) on High affinity immunoglobulin alpha and immunoglobulin mu Fc receptor with a threshold of 1e−05

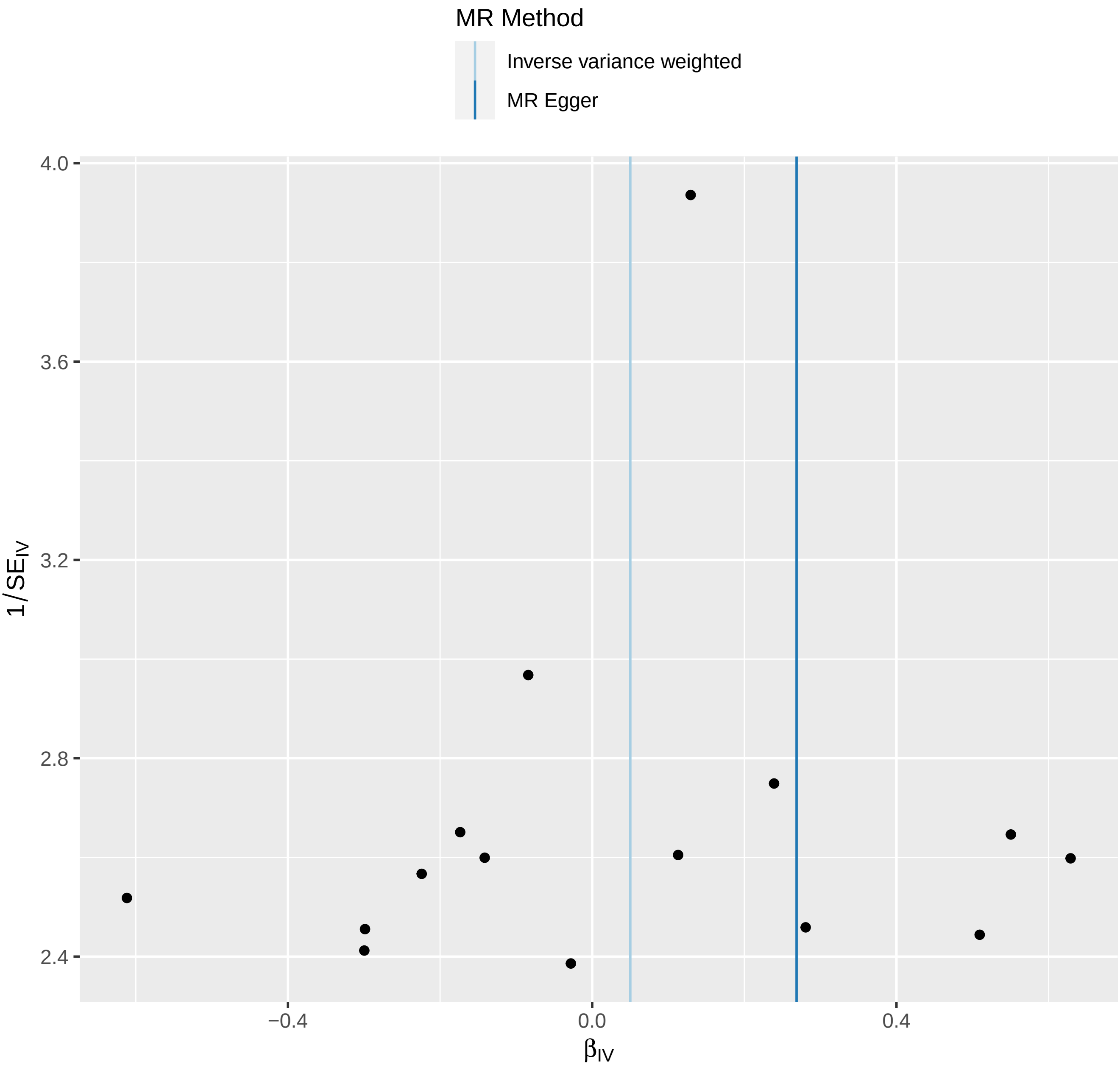

Batch 2−2141 : Gut microbiota abundance (genus Catenibacterium id.2153) on Immunoglobulin alpha Fc receptor
with a threshold of 1e−05

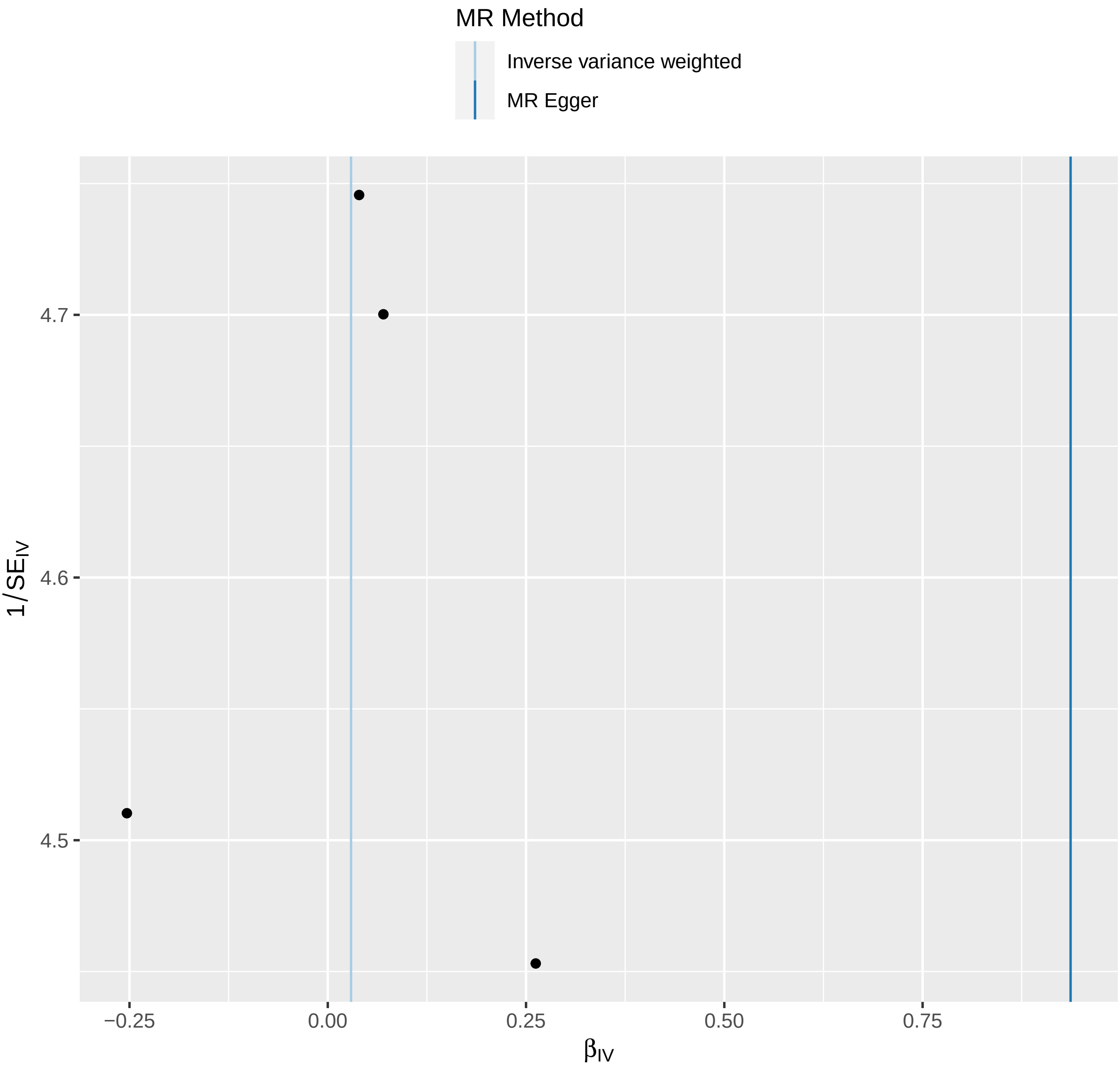

Batch 2−2142 : Gut microbiota abundance (genus Parasutterella id.2892) on Immunoglobulin alpha Fc receptor
with a threshold of 1e−05

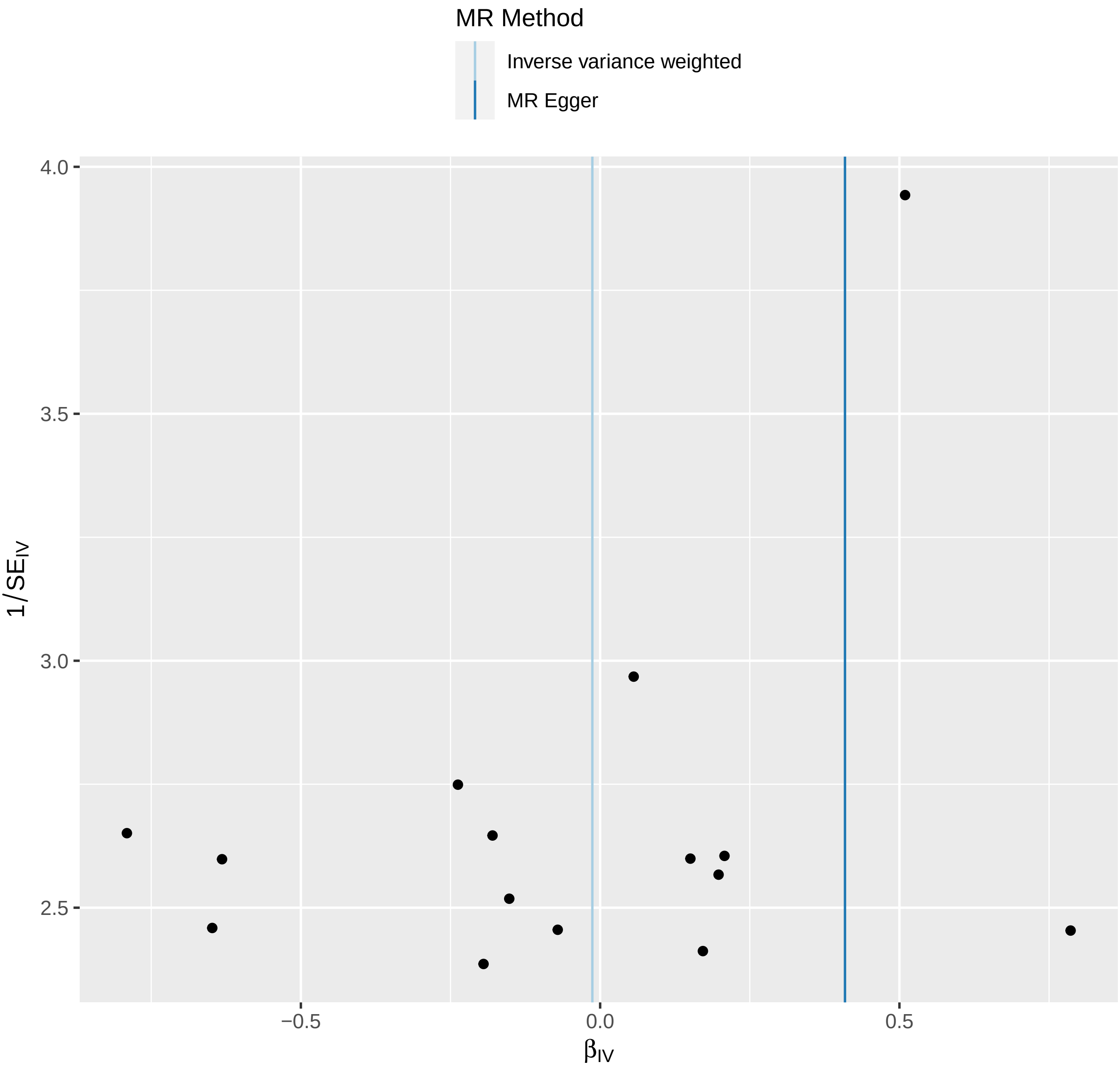

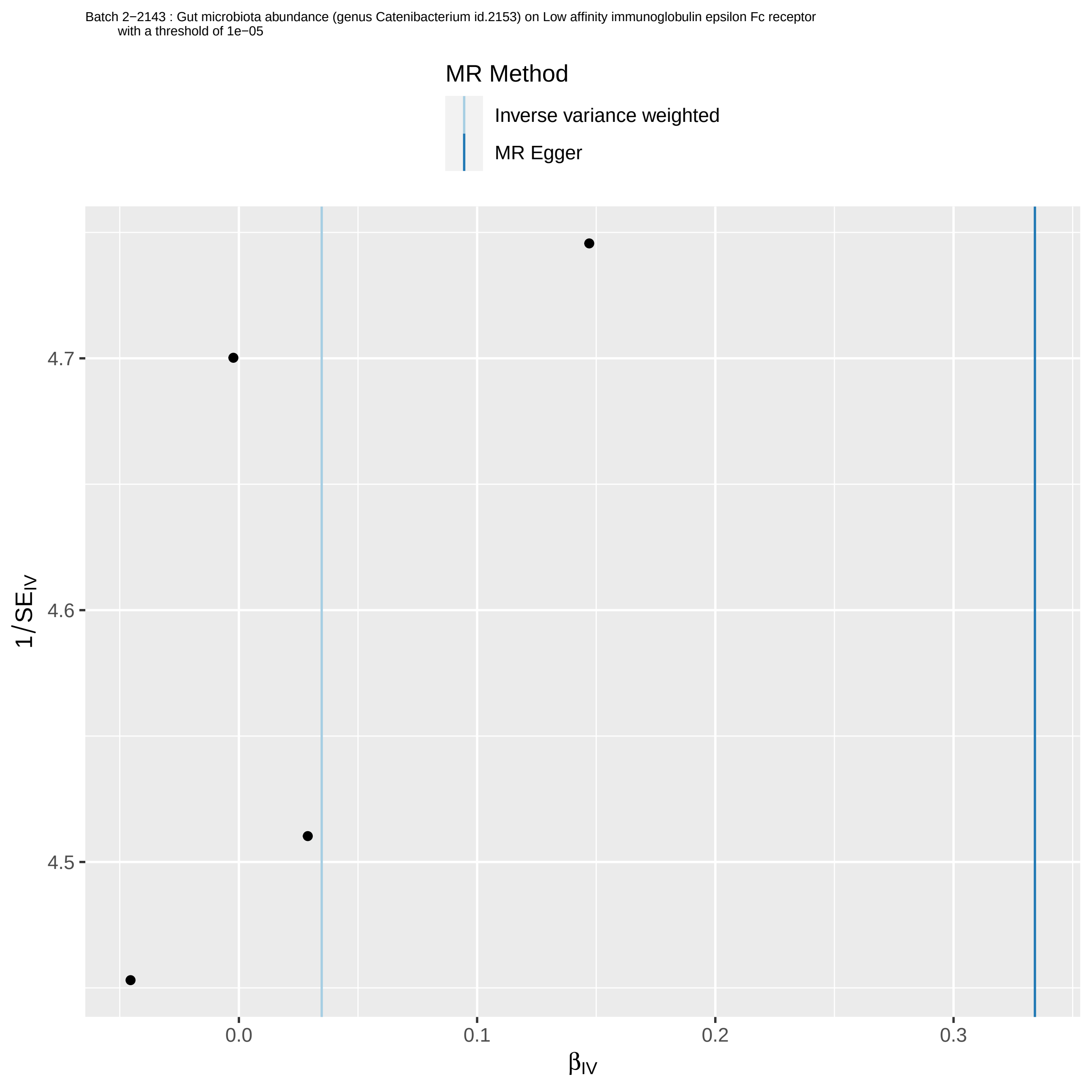

Batch 2−2143 : Gut microbiota abundance (genus Catenibacterium id.2153) on Low affinity immunoglobulin epsilon Fc receptor with a threshold of 1e−05
MR Method
Inverse variance weighted
MR Egger
4.7
4.6
4.5
1/SE$_{IV}$
0.0
0.1
0.2
0.3
β_{IV}

Batch 2−2144 : Gut microbiota abundance (genus Parasutterella id.2892) on Low affinity immunoglobulin epsilon Fc receptor
with a threshold of 1e−05

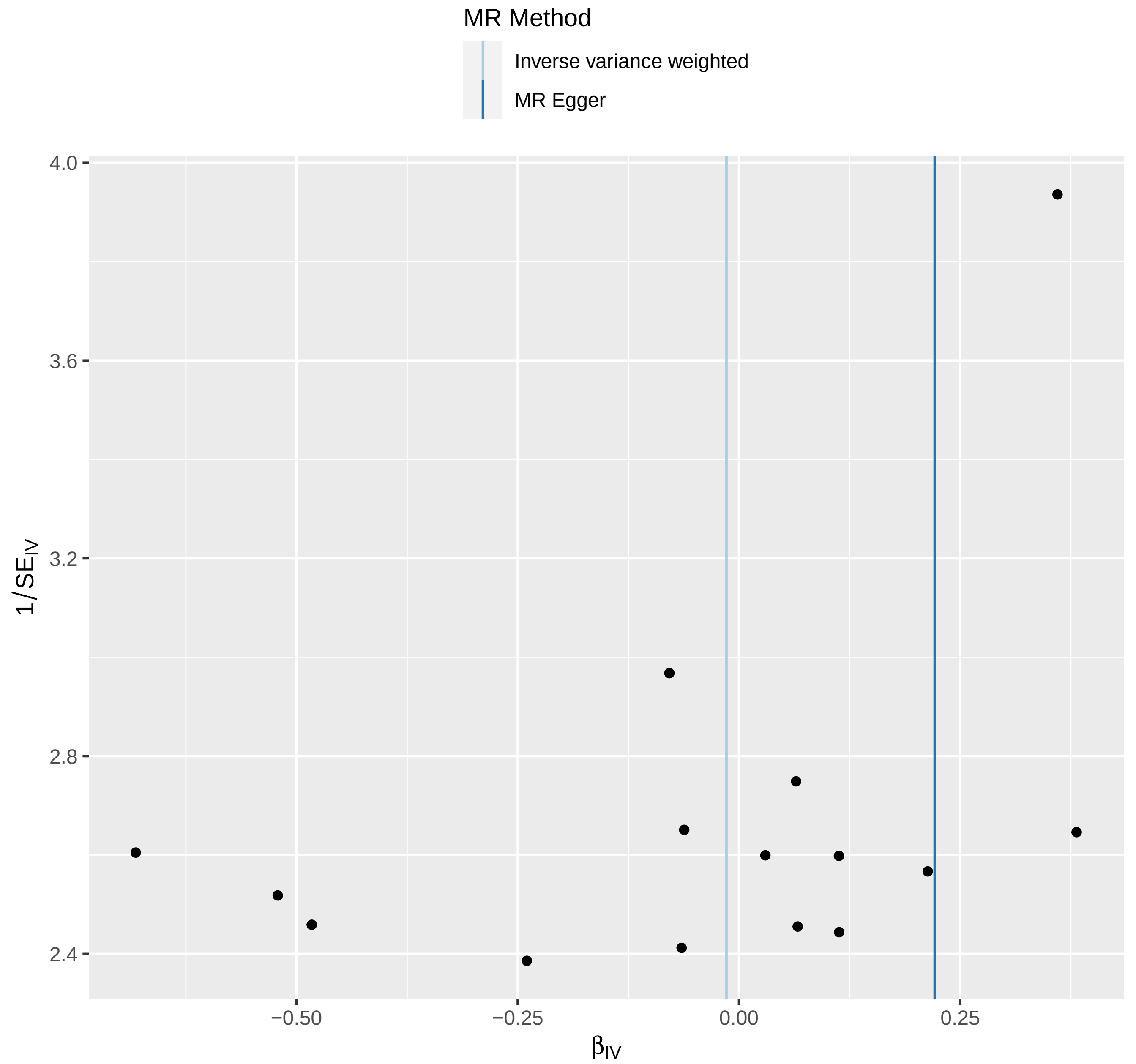

Batch 2−2145 : Gut microbiota abundance (genus Catenibacterium id.2153) on High affinity immunoglobulin gamma Fc receptor I
with a threshold of 1e−05

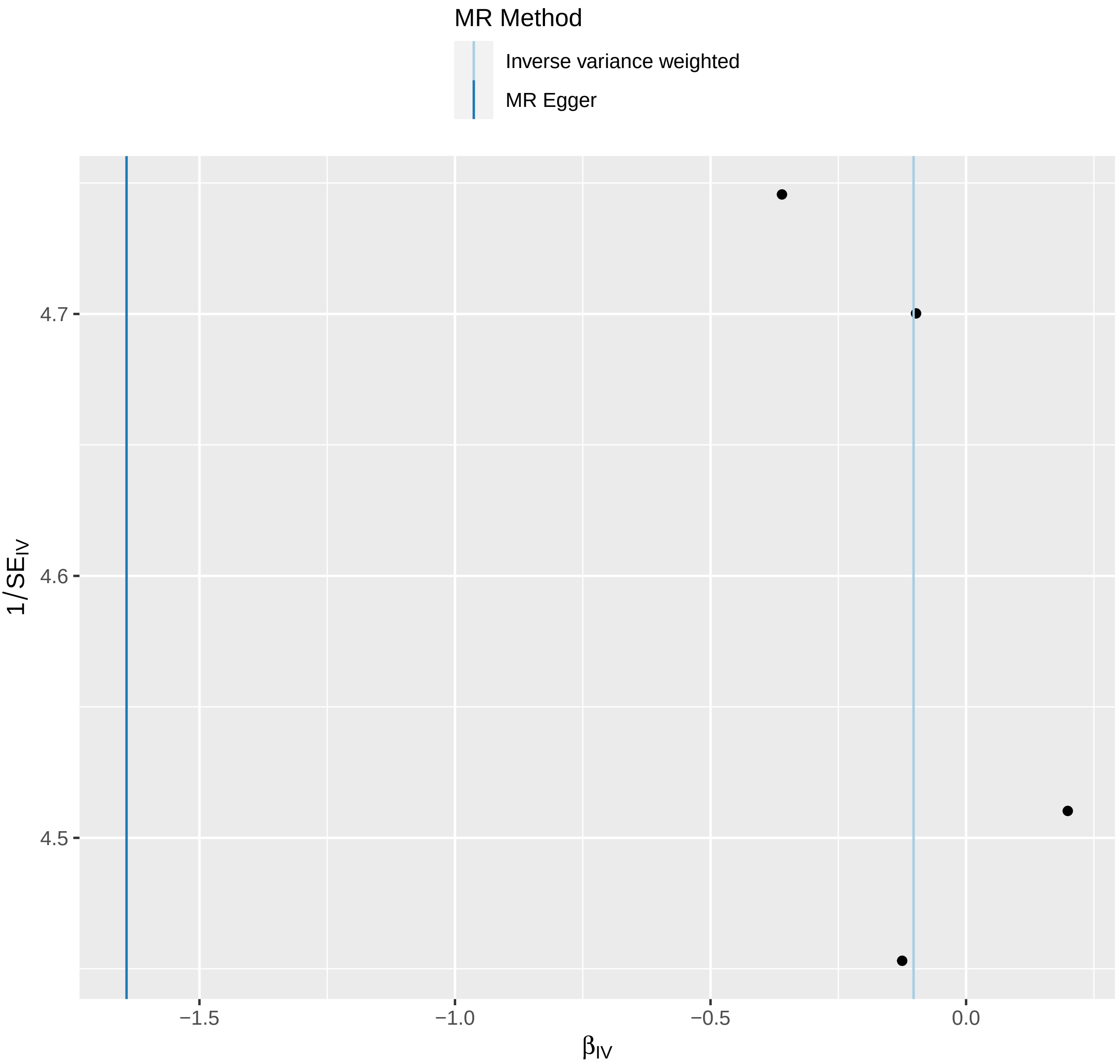

Batch 2−2146 : Gut microbiota abundance (genus Parasutterella id.2892) on High affinity immunoglobulin gamma Fc receptor I
with a threshold of 1e−05

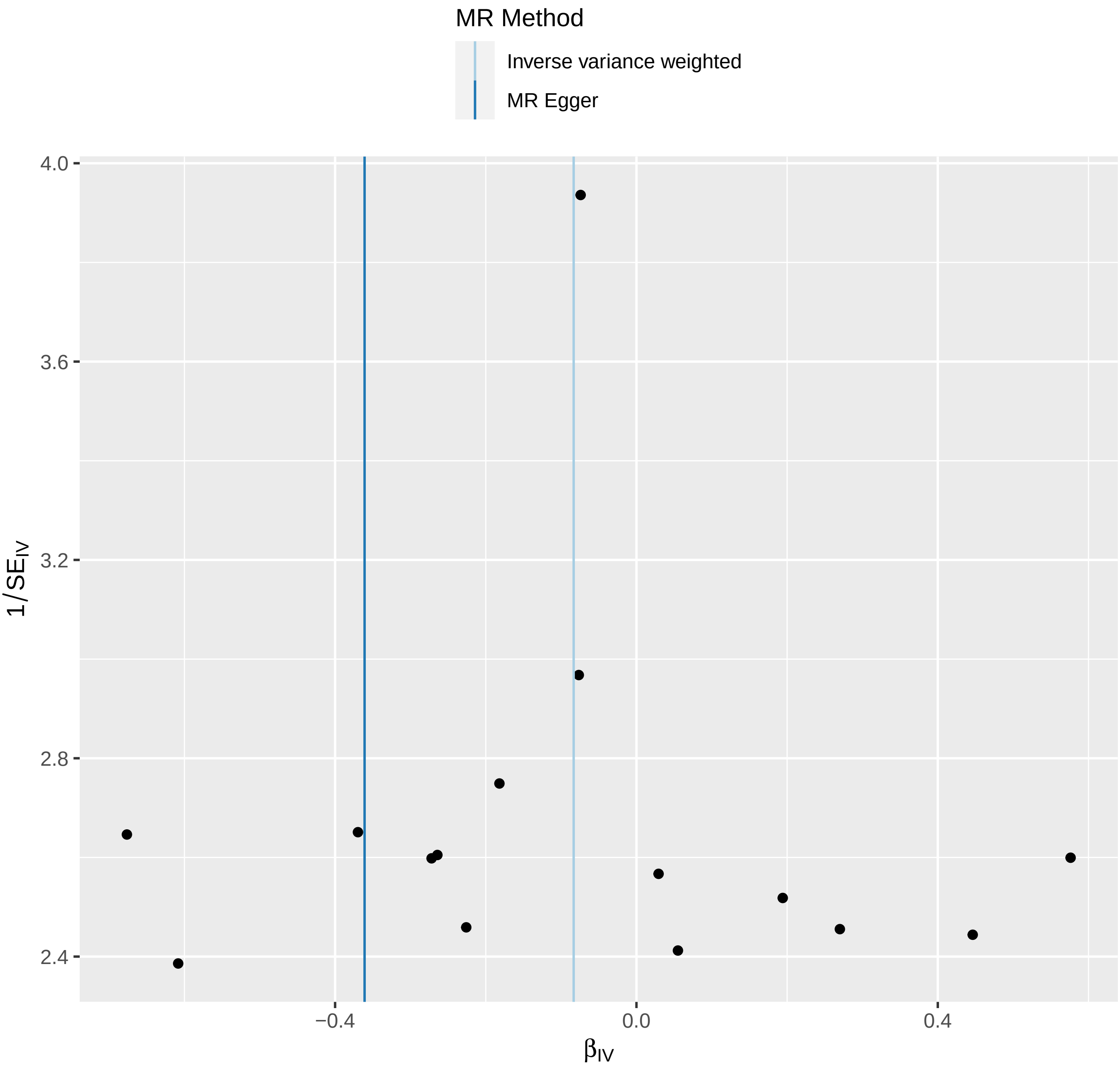

Batch 2−2147 : Gut microbiota abundance (genus Catenibacterium id.2153) on Low affinity immunoglobulin gamma Fc region receptor II−a
with a threshold of 1e−05

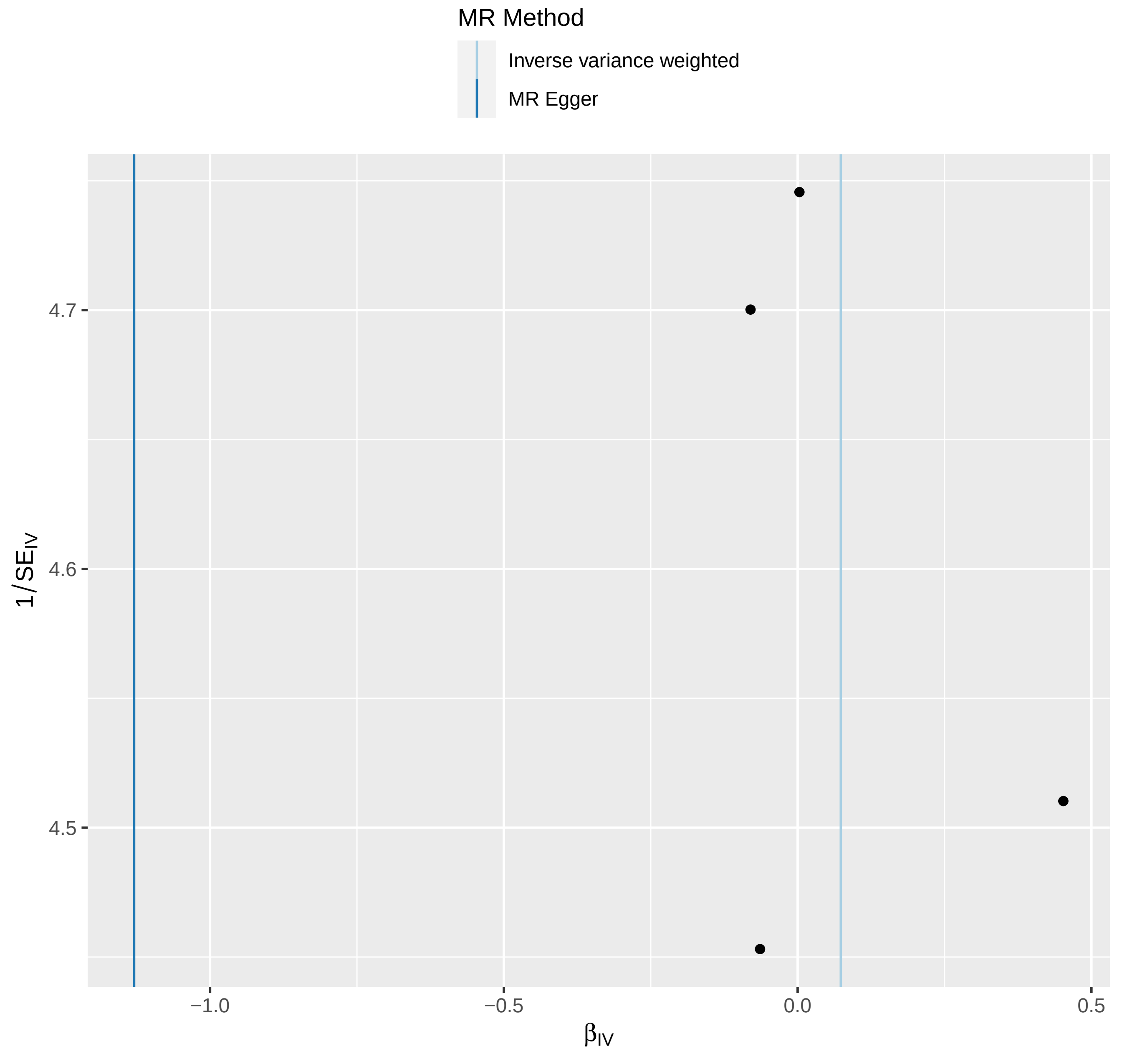

Batch 2−2148 : Gut microbiota abundance (genus Parasutterella id.2892) on Low affinity immunoglobulin gamma Fc region receptor II−a
with a threshold of 1e−05

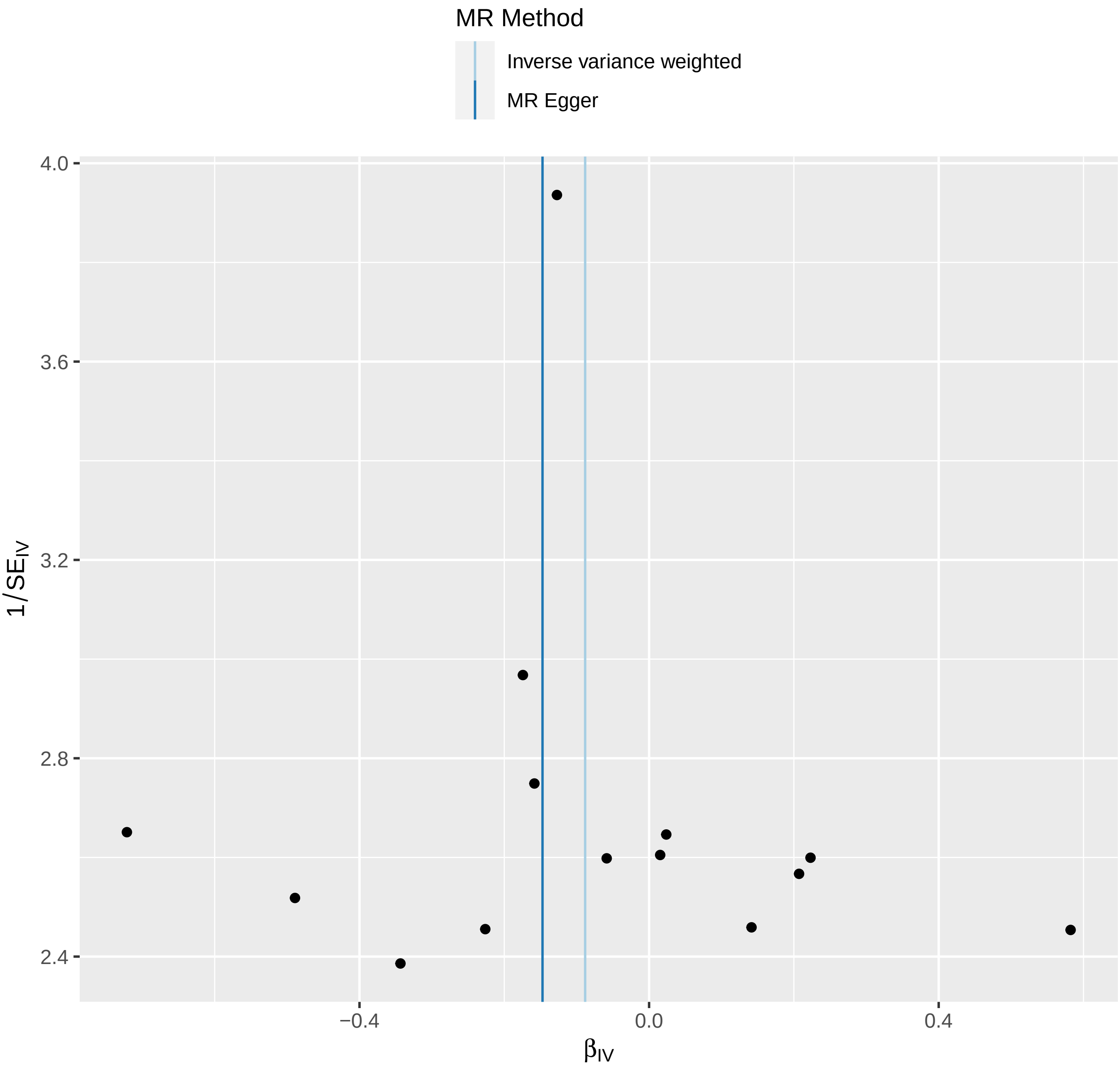

Batch 2−2149 : Gut microbiota abundance (genus Catenibacterium id.2153) on Low affinity immunoglobulin gamma Fc region receptor II−b with a threshold of 1e−05

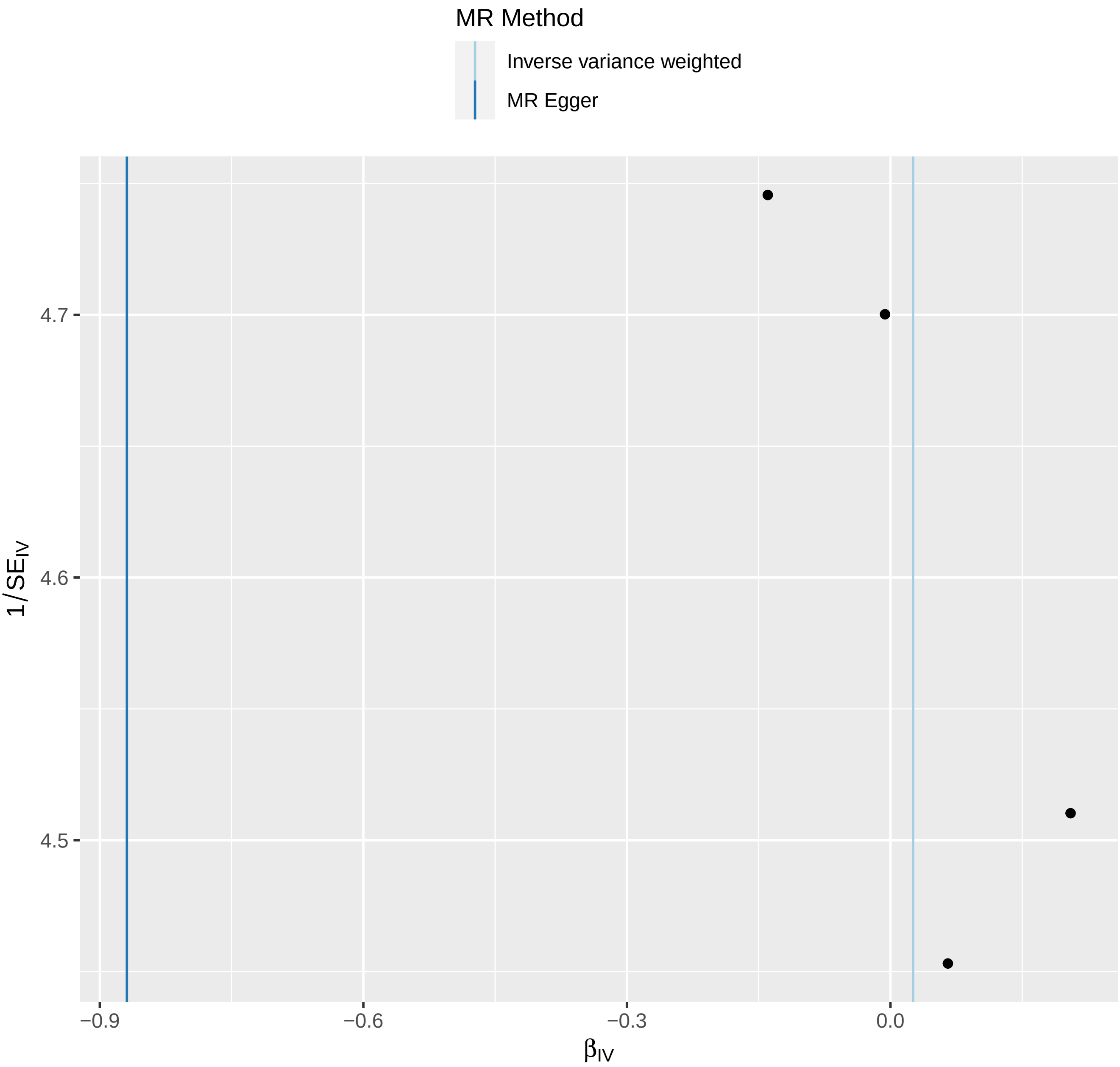

Batch 2−2150 : Gut microbiota abundance (genus Parasutterella id.2892) on Low affinity immunoglobulin gamma Fc region receptor II−b with a threshold of 1e−05

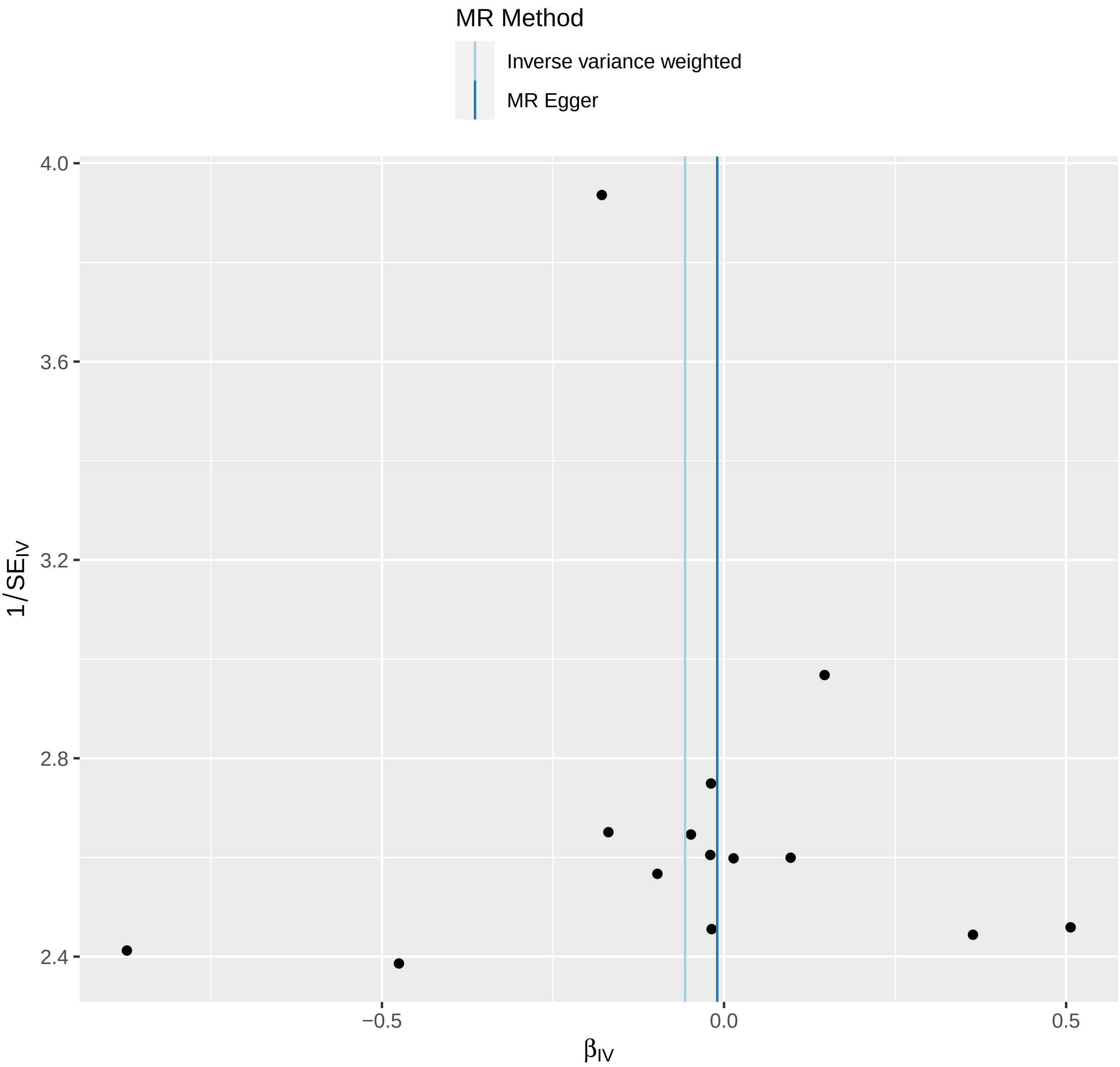

Batch 2−2151 : Gut microbiota abundance (genus Catenibacterium id.2153) on Low affinity immunoglobulin gamma Fc region receptor III−B
with a threshold of 1e−05

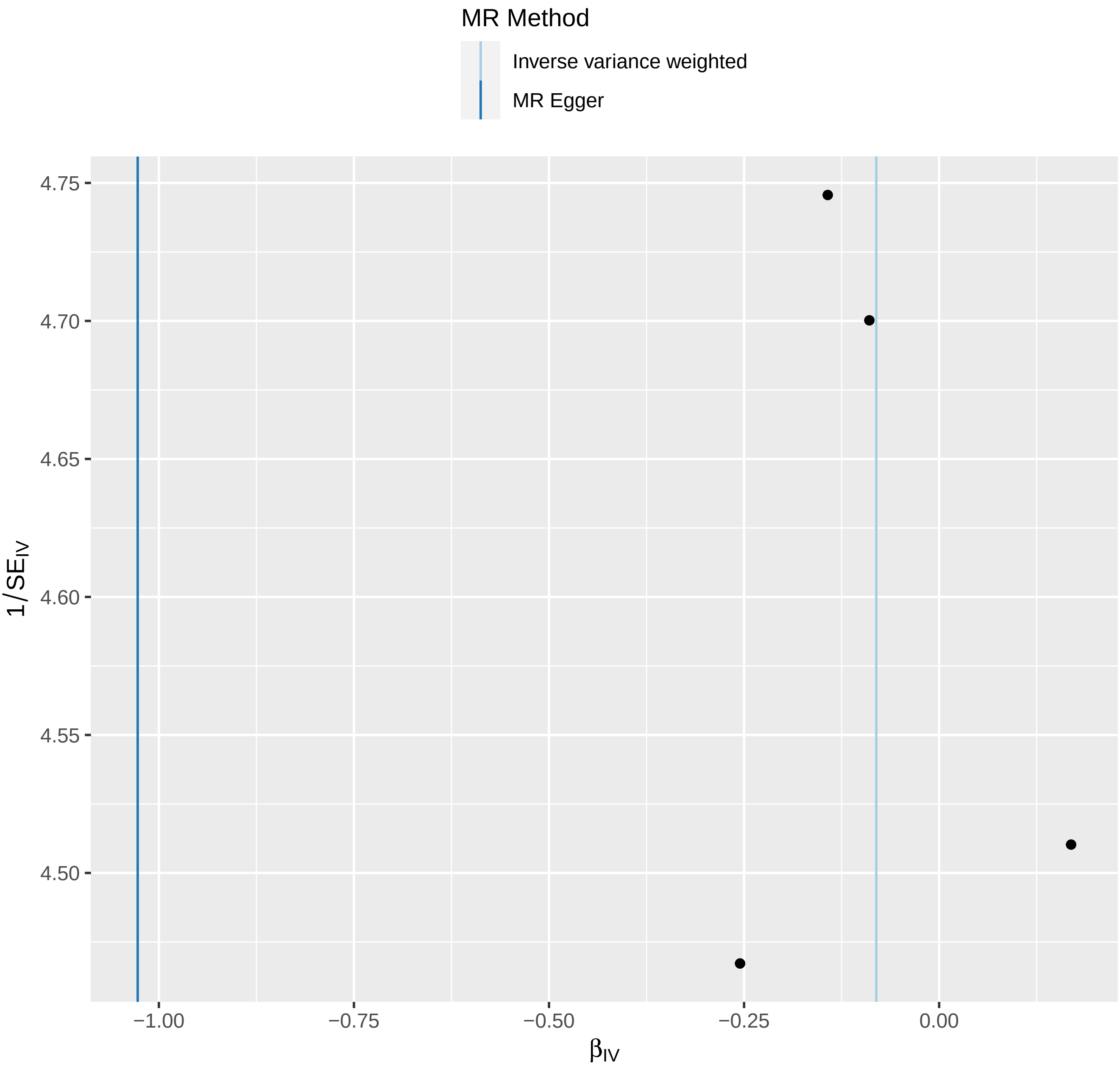

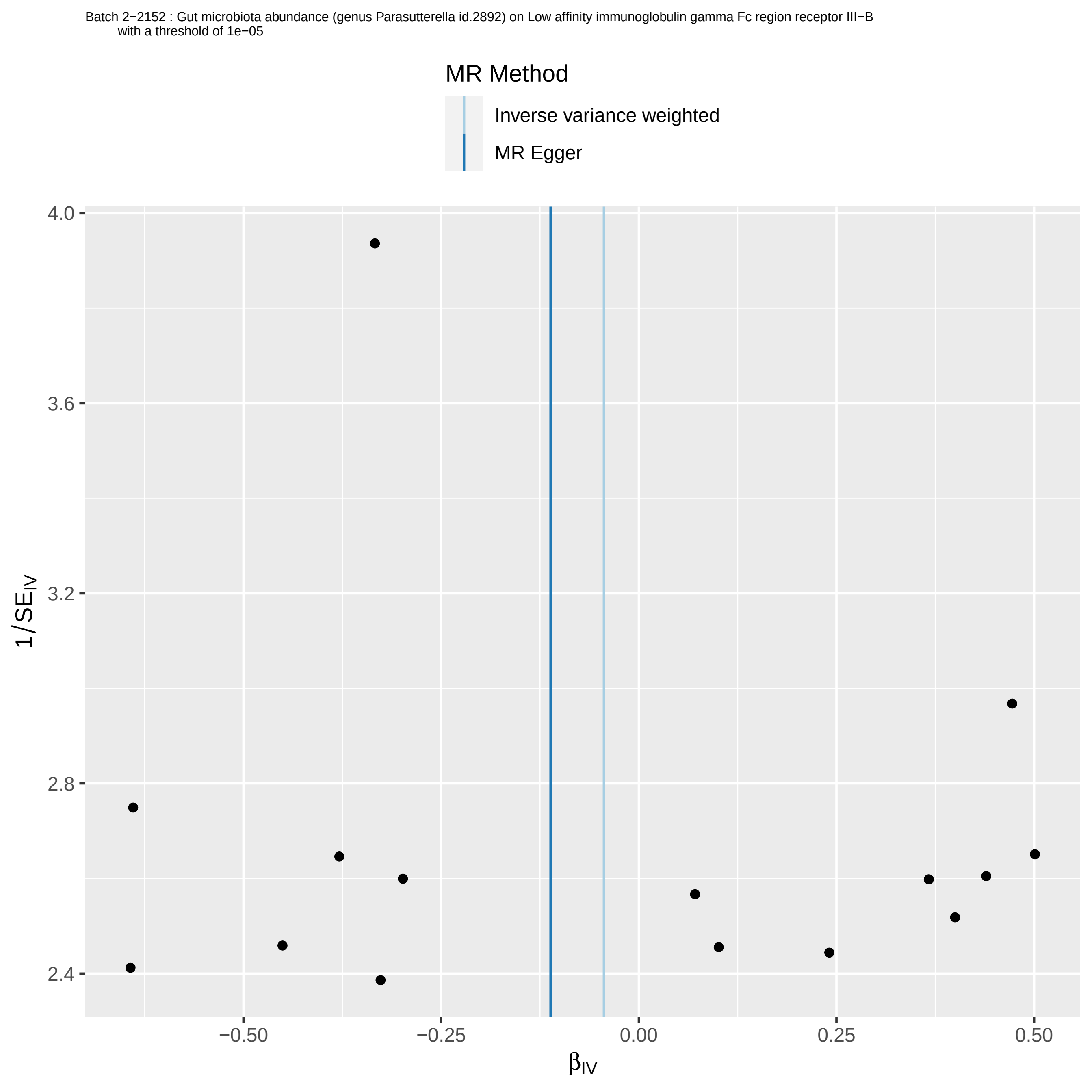

Batch 2−2152 : Gut microbiota abundance (genus Parasutterella id.2892) on Low affinity immunoglobulin gamma Fc region receptor III−B
with a threshold of 1e−05
MR Method
Inverse variance weighted
MR Egger
4.0
3.6
3.2
2.8
2.4
1/SE_IV
−0.50
−0.25
0.00
0.25
0.50
β_IV

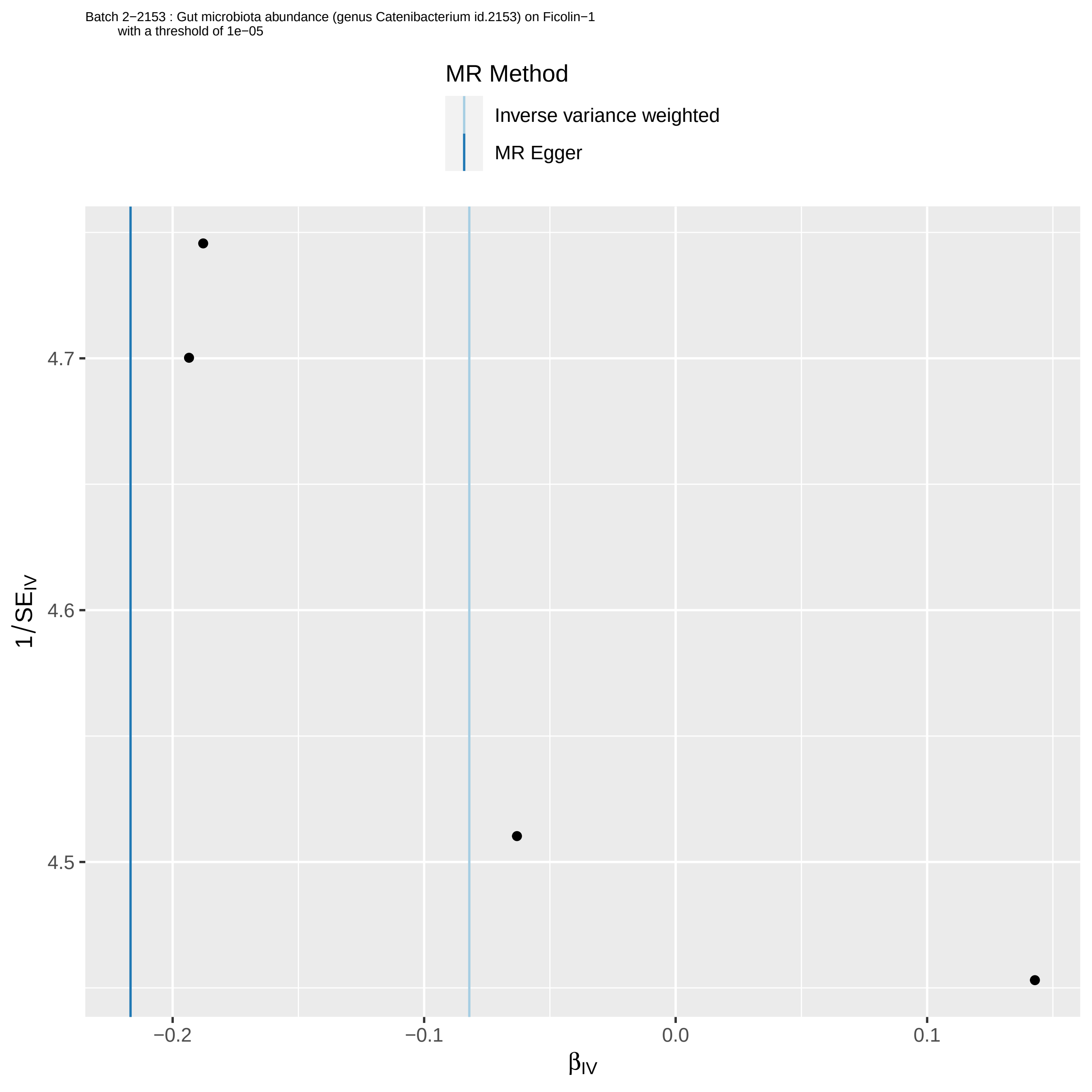
Batch 2−2153 : Gut microbiota abundance (genus Catenibacterium id.2153) on Ficolin−1
with a threshold of 1e−05
MR Method
Inverse variance weighted
MR Egger
4.7
4.6
4.5
1/SE$_{IV}$
−0.2
−0.1
0.0
0.1
β$_{IV}$

Batch 2−2154 : Gut microbiota abundance (genus Parasutterella id.2892) on Ficolin−1
with a threshold of 1e−05

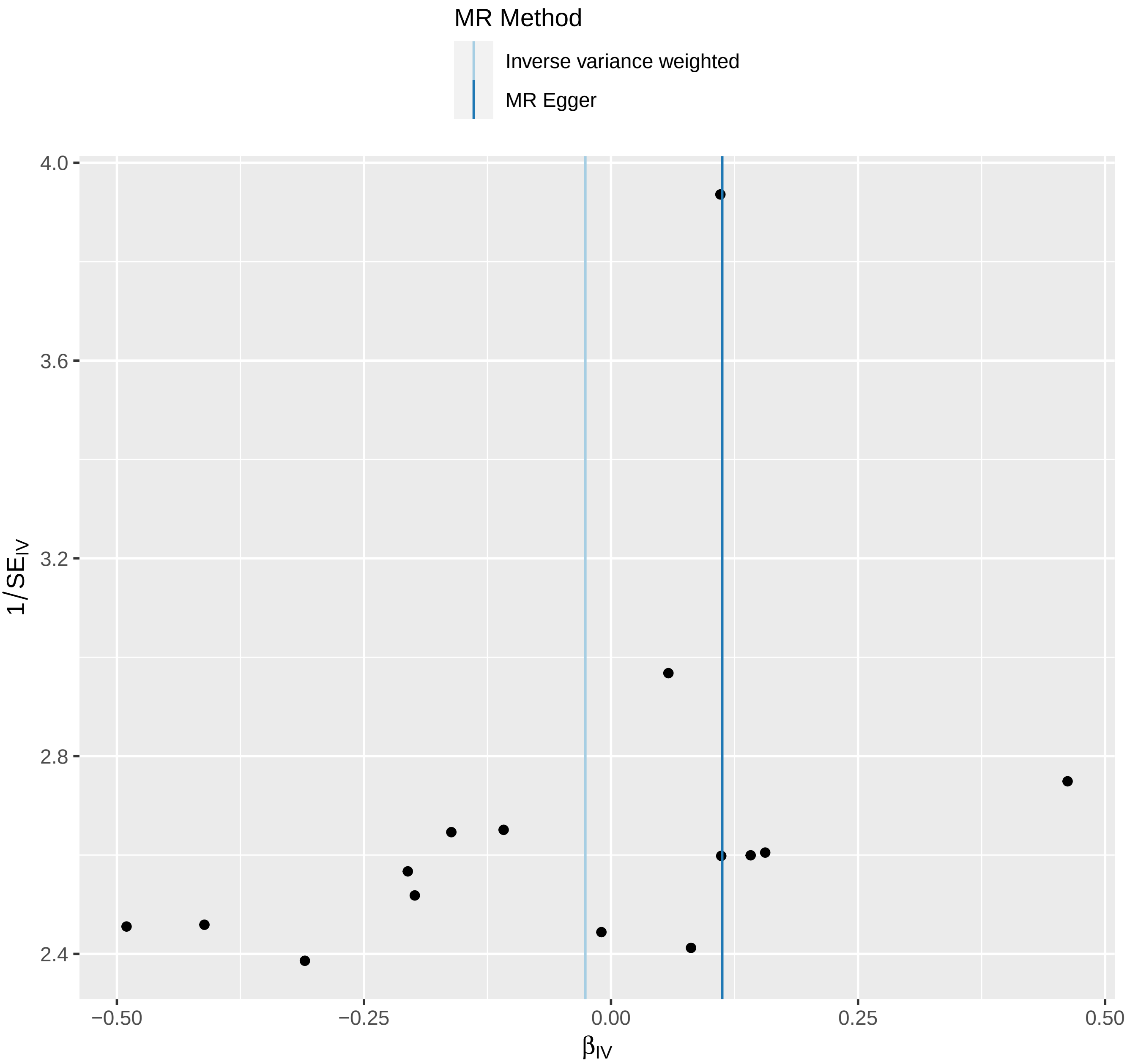

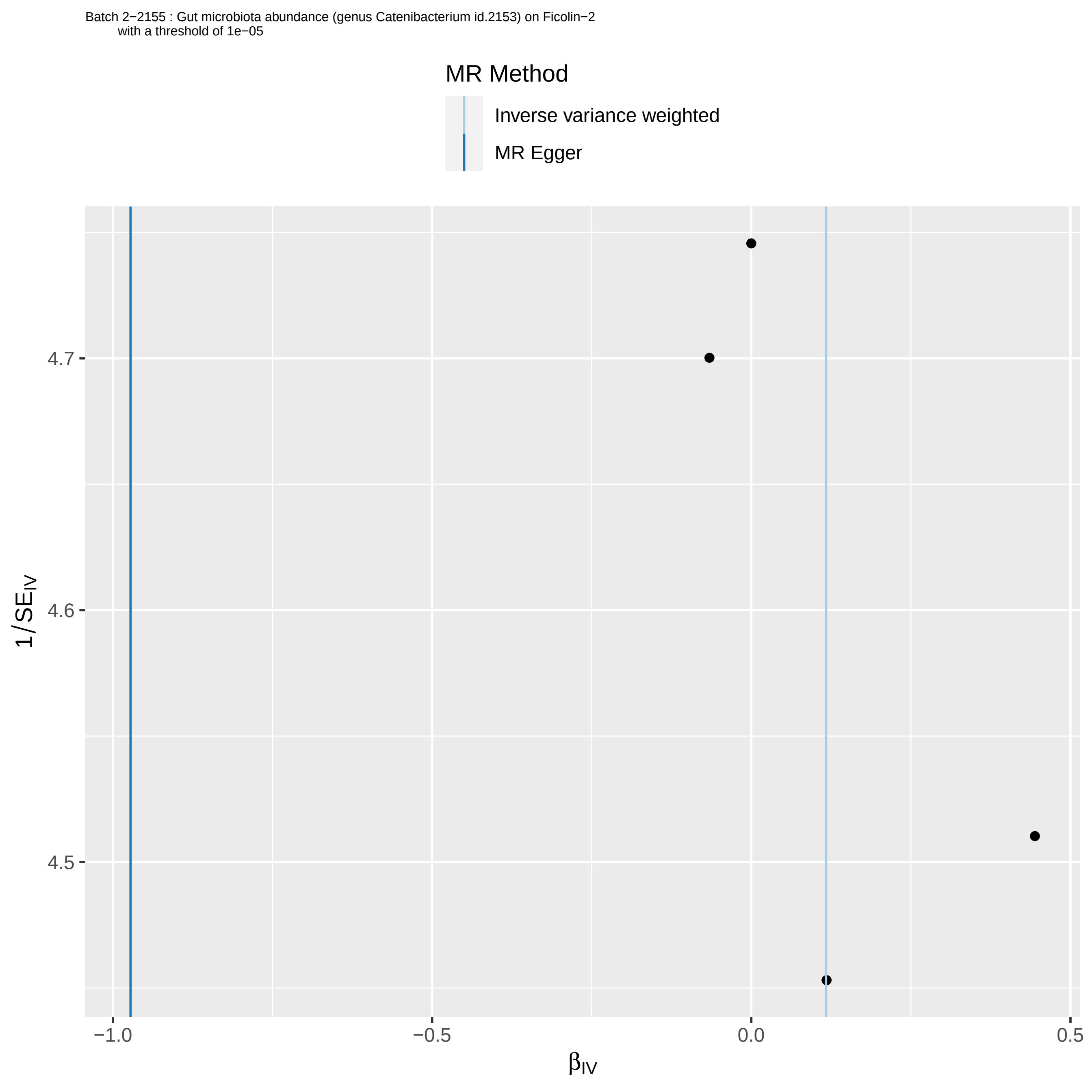
Batch 2−2155 : Gut microbiota abundance (genus Catenibacterium id.2153) on Ficolin−2
with a threshold of 1e−05
MR Method
Inverse variance weighted
MR Egger
4.7
4.6
4.5
1/SE_IV
−1.0
−0.5
0.0
0.5
β_IV

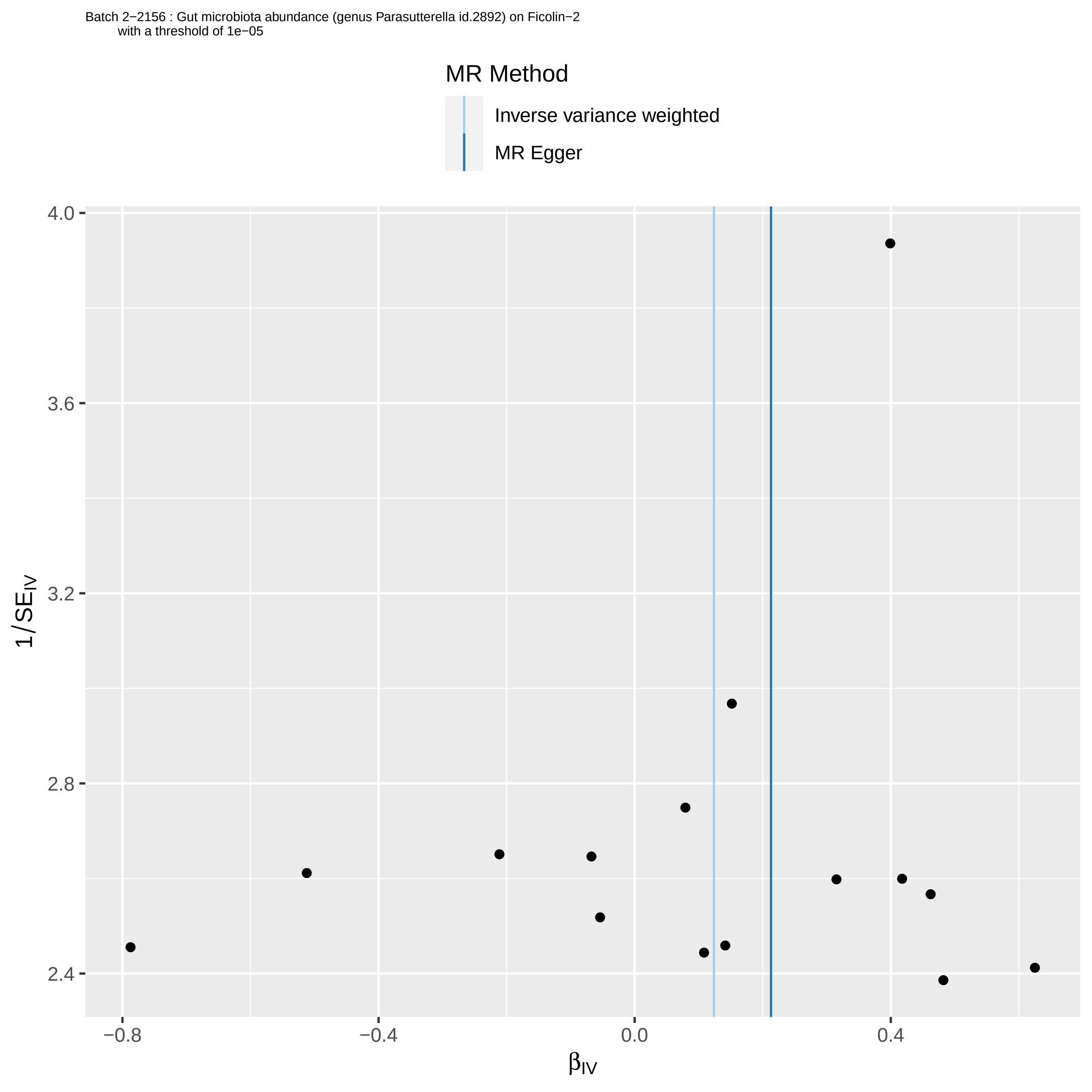

Batch 2−2156 : Gut microbiota abundance (genus Parasutterella id.2892) on Ficolin−2
with a threshold of 1e−05
MR Method
Inverse variance weighted
MR Egger
4.0
3.6
3.2
2.8
2.4
1/SE$_{IV}$
−0.8
−0.4
0.0
0.4
β$_{IV}$

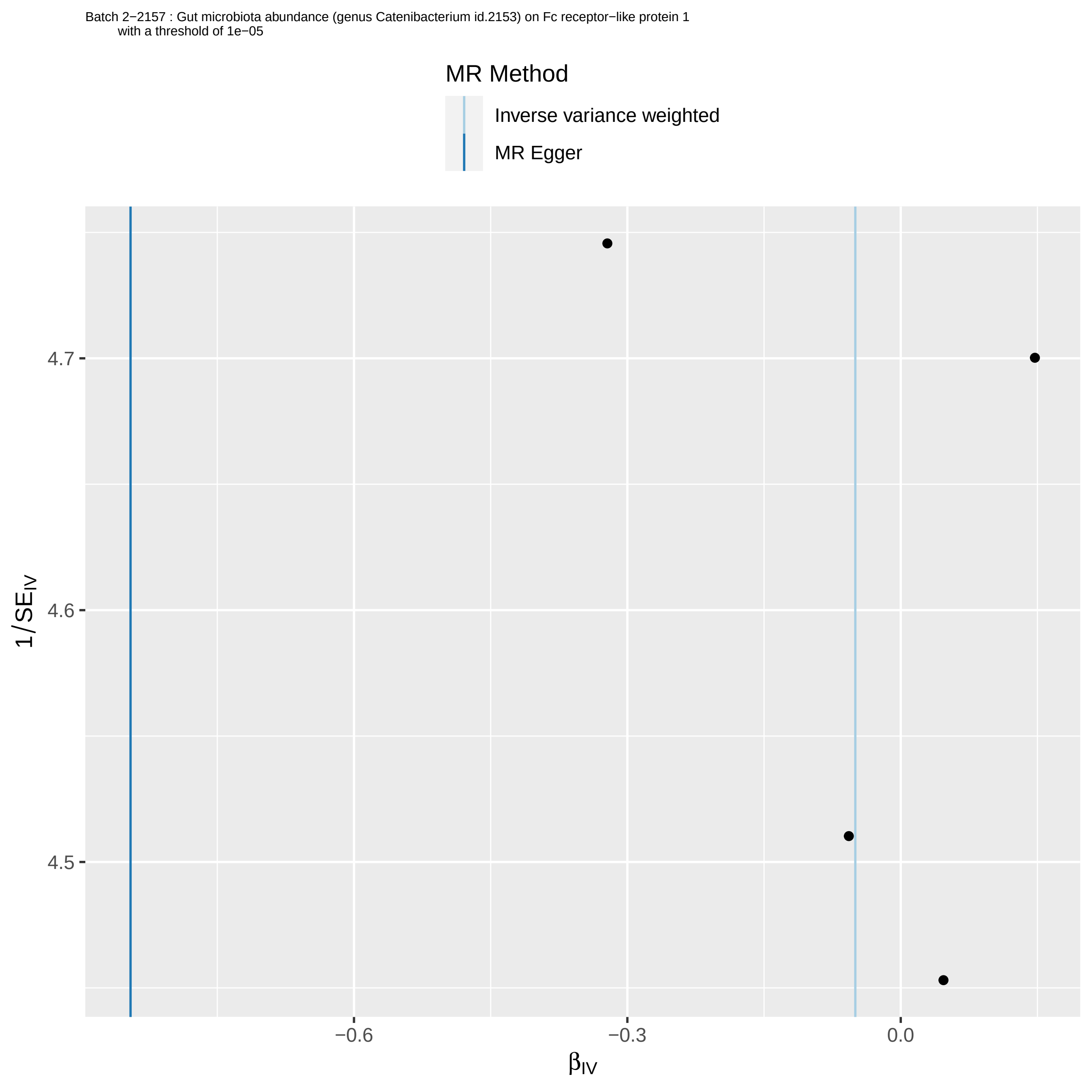

Batch 2−2157 : Gut microbiota abundance (genus Catenibacterium id.2153) on Fc receptor−like protein 1
with a threshold of 1e−05
MR Method
Inverse variance weighted
MR Egger
4.7
4.6
4.5
1/SE_IV
−0.6
−0.3
0.0
β_IV

Batch 2−2158 : Gut microbiota abundance (genus Parasutterella id.2892) on Fc receptor−like protein 1
with a threshold of 1e−05

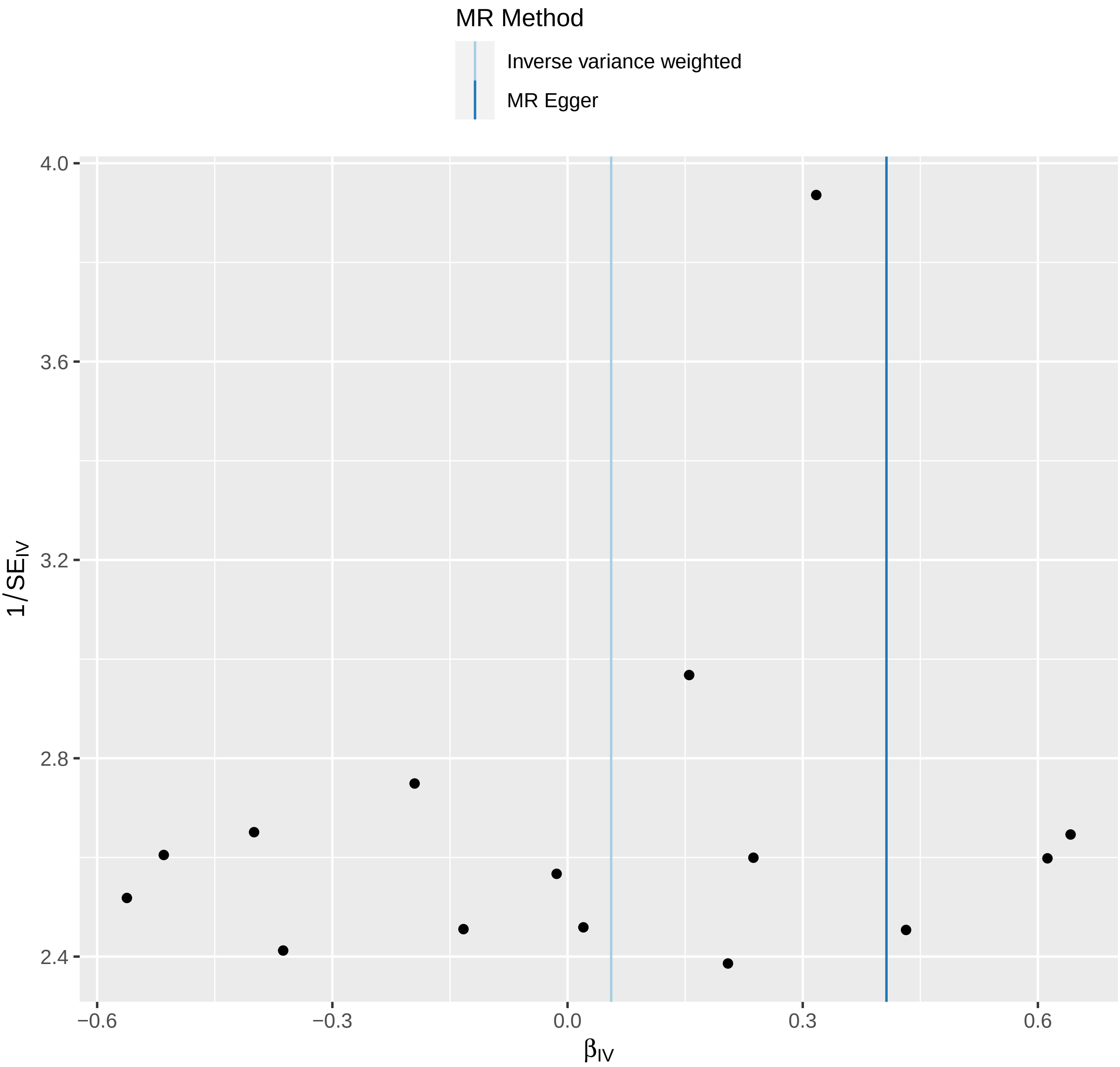

Batch 2−2159 : Gut microbiota abundance (genus Catenibacterium id.2153) on Fc receptor−like protein 2
with a threshold of 1e−05

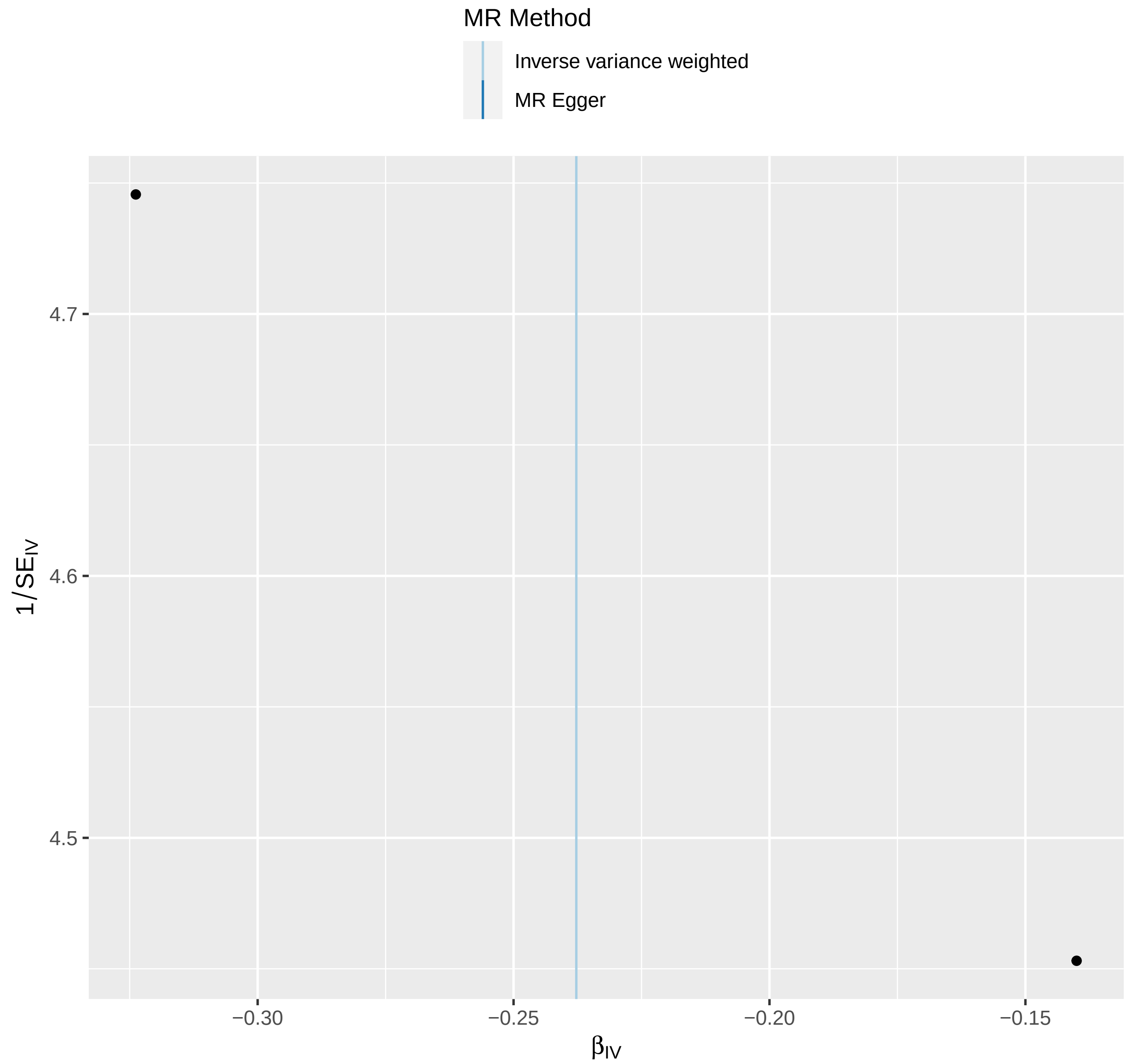

Batch 2−2160 : Gut microbiota abundance (genus Parasutterella id.2892) on Fc receptor−like protein 2
with a threshold of 1e−05

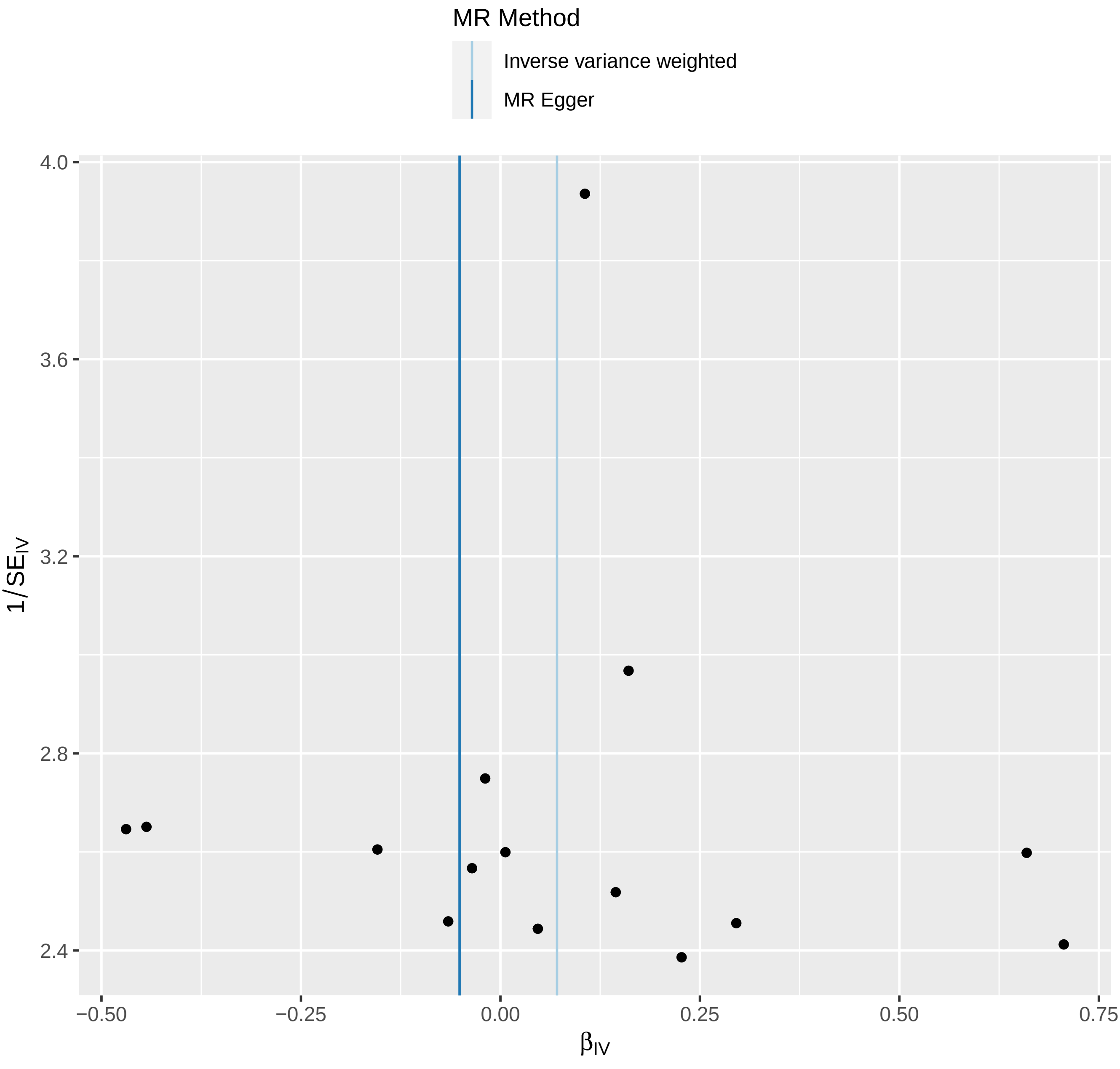

Batch 2−2161 : Gut microbiota abundance (genus Catenibacterium id.2153) on Fc receptor−like protein 3
with a threshold of 1e−05

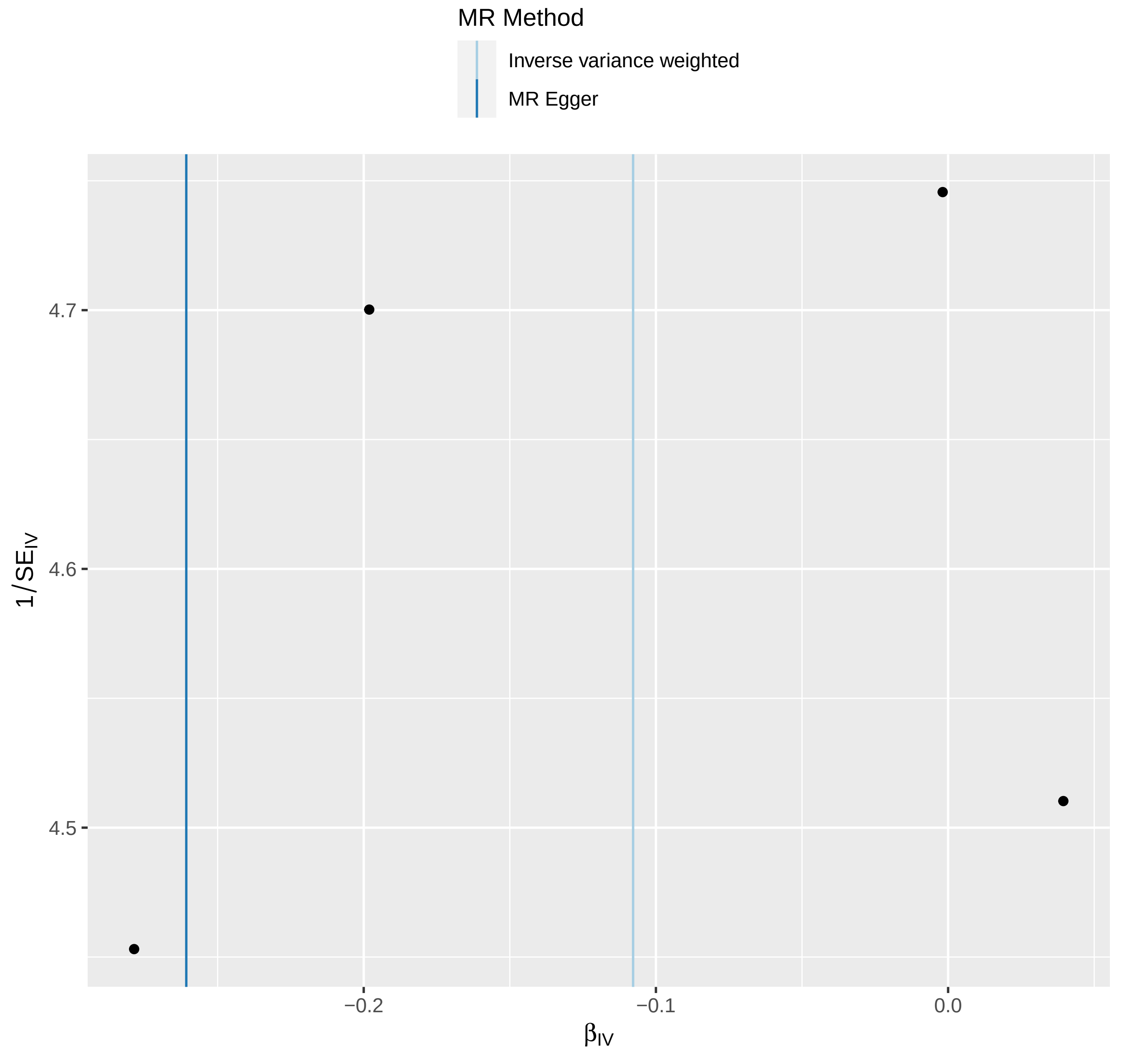

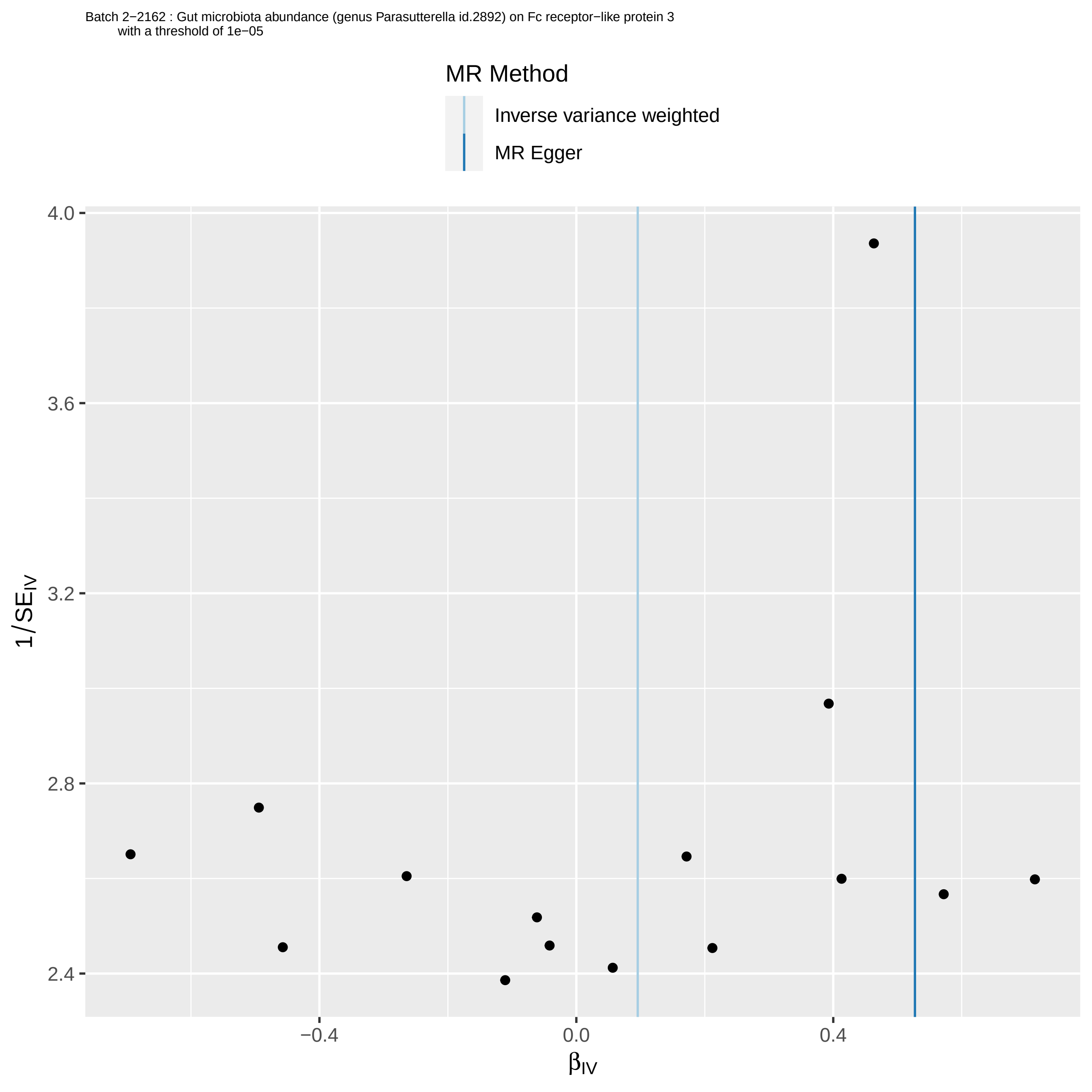
Batch 2−2162 : Gut microbiota abundance (genus Parasutterella id.2892) on Fc receptor−like protein 3
with a threshold of 1e−05
MR Method
Inverse variance weighted
MR Egger
4.0
3.6
3.2
2.8
2.4
$1/SE_{IV}$
−0.4
0.0
0.4
β_{IV}

Batch 2−2163 : Gut microbiota abundance (genus Catenibacterium id.2153) on Fc receptor−like protein 4
with a threshold of 1e−05

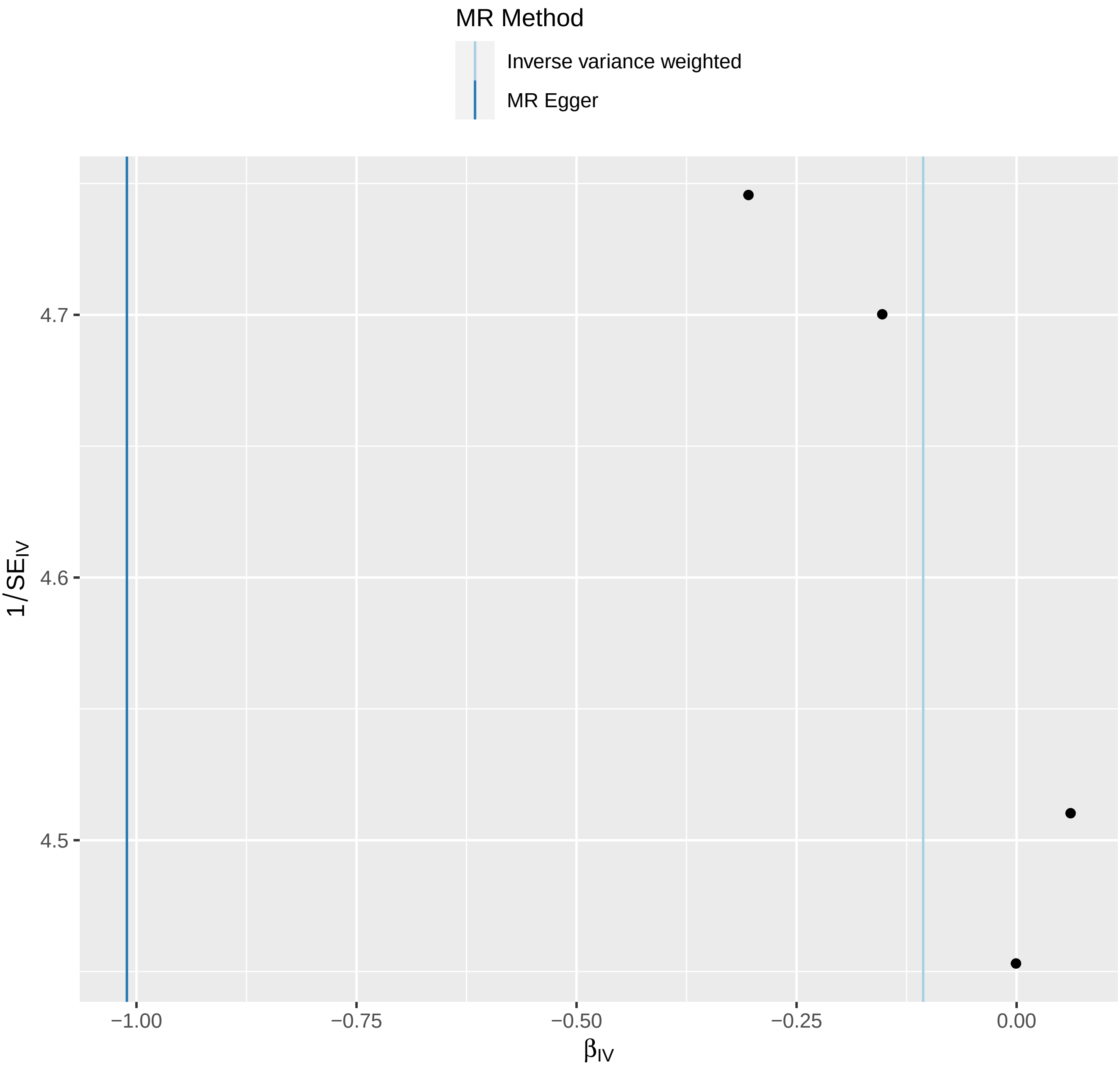

Batch 2−2164 : Gut microbiota abundance (genus Parasutterella id.2892) on Fc receptor−like protein 4
with a threshold of 1e−05

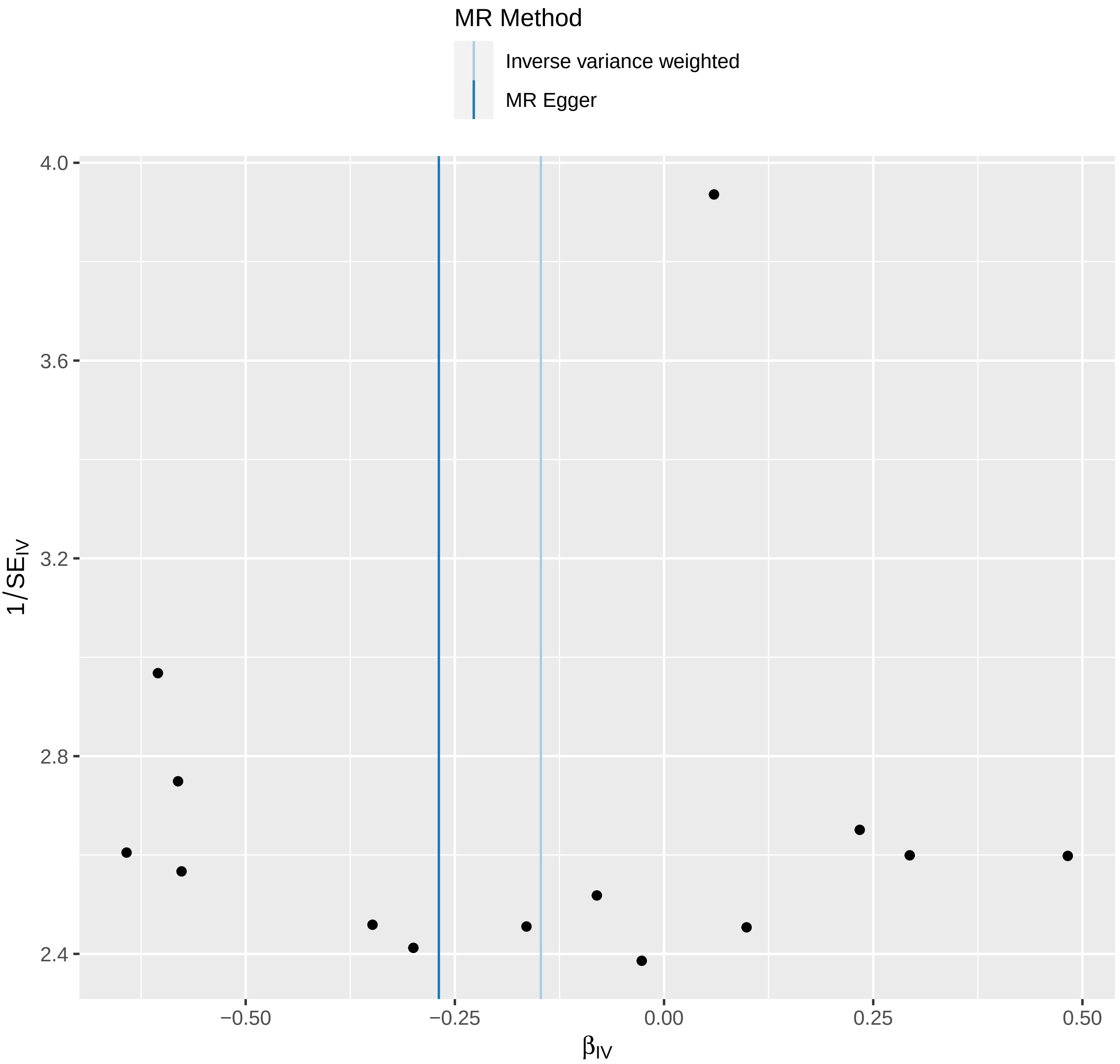

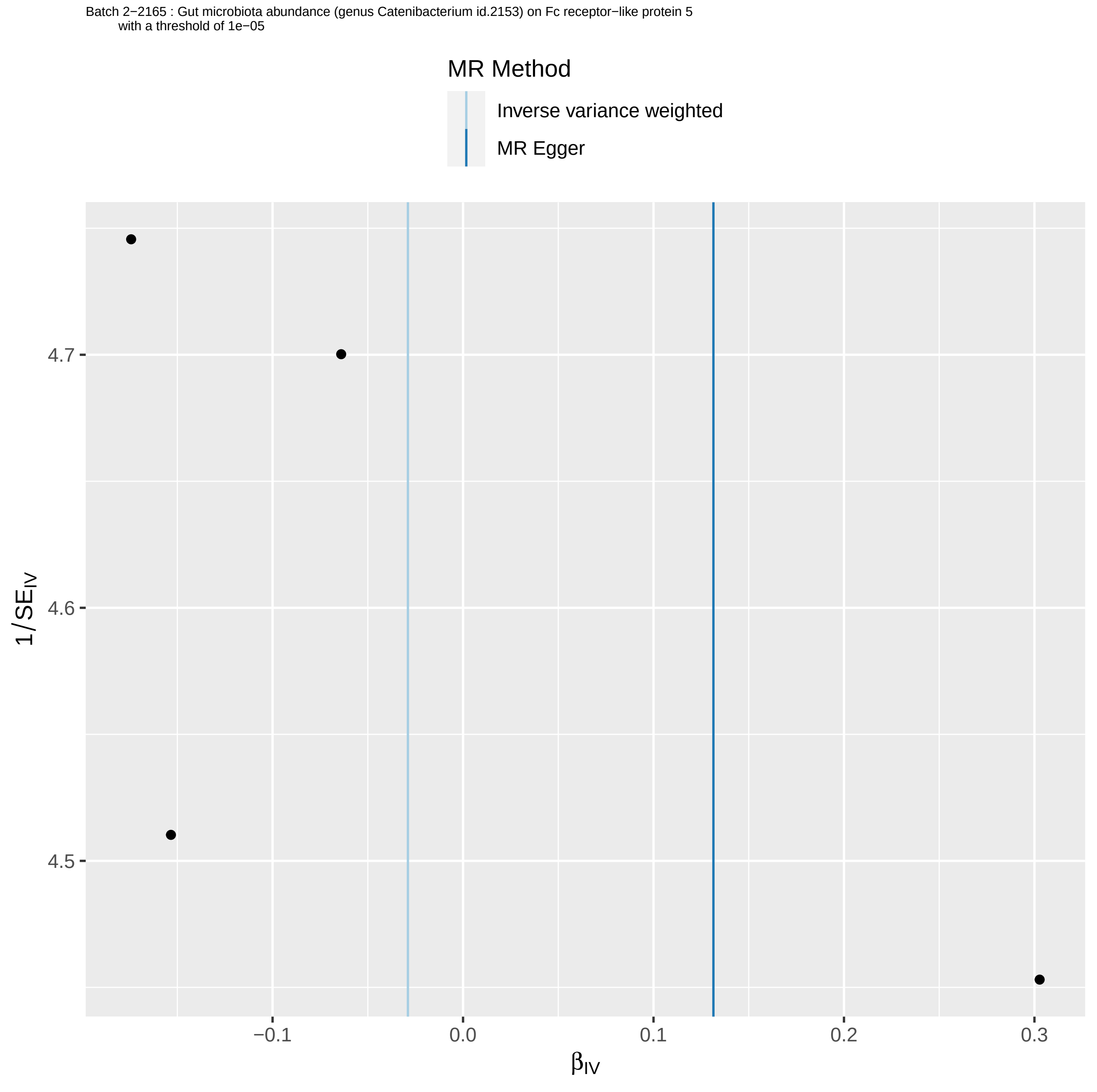

Batch 2−2165 : Gut microbiota abundance (genus Catenibacterium id.2153) on Fc receptor−like protein 5
with a threshold of 1e−05
MR Method
Inverse variance weighted
MR Egger
4.7
4.6
4.5
1/SE_IV
−0.1
0.0
0.1
0.2
0.3
β_IV

Batch 2−2166 : Gut microbiota abundance (genus Parasutterella id.2892) on Fc receptor−like protein 5
with a threshold of 1e−05

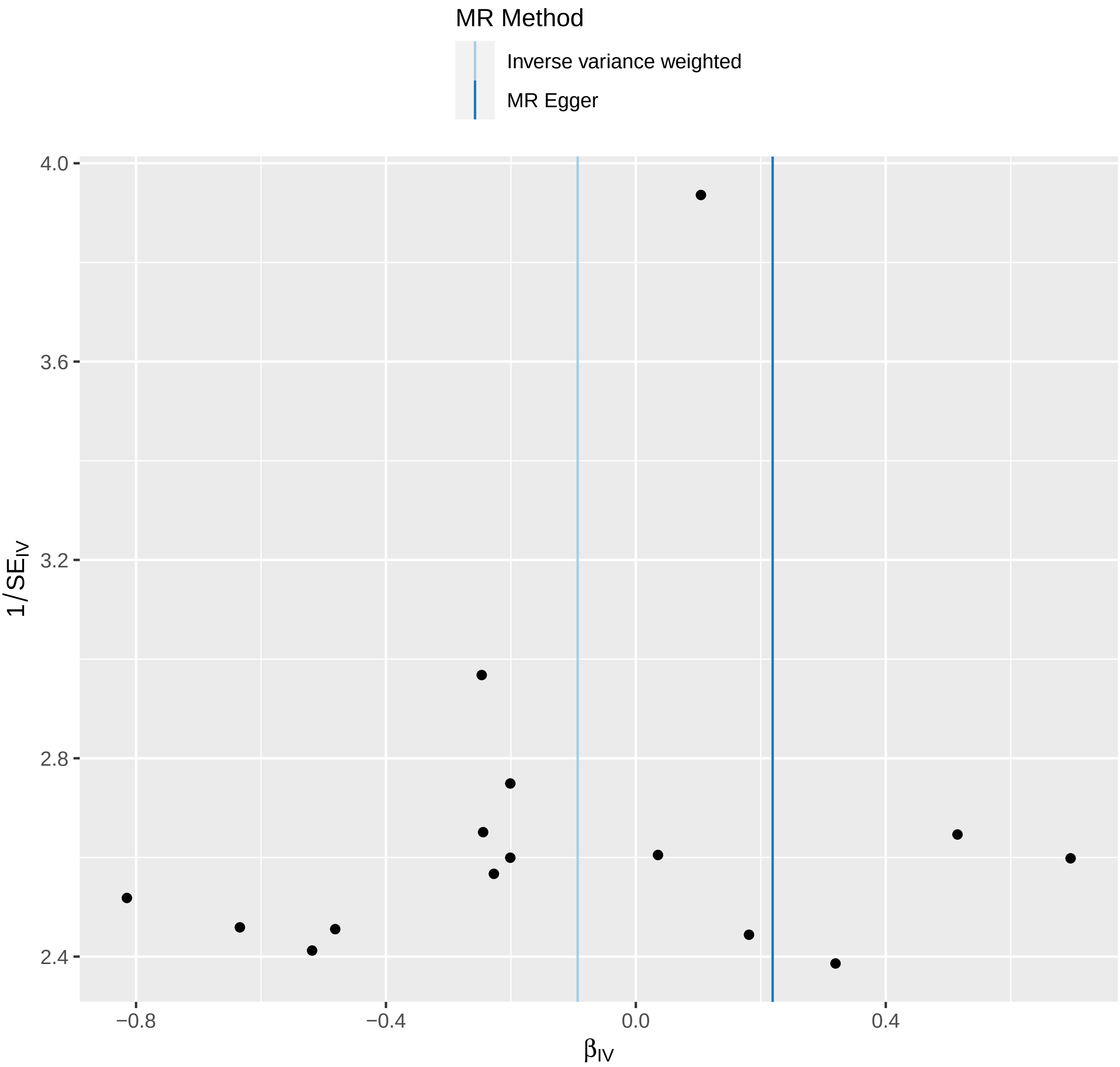

Batch 2−2167 : Gut microbiota abundance (genus Catenibacterium id.2153) on Fc receptor−like protein 6
with a threshold of 1e−05

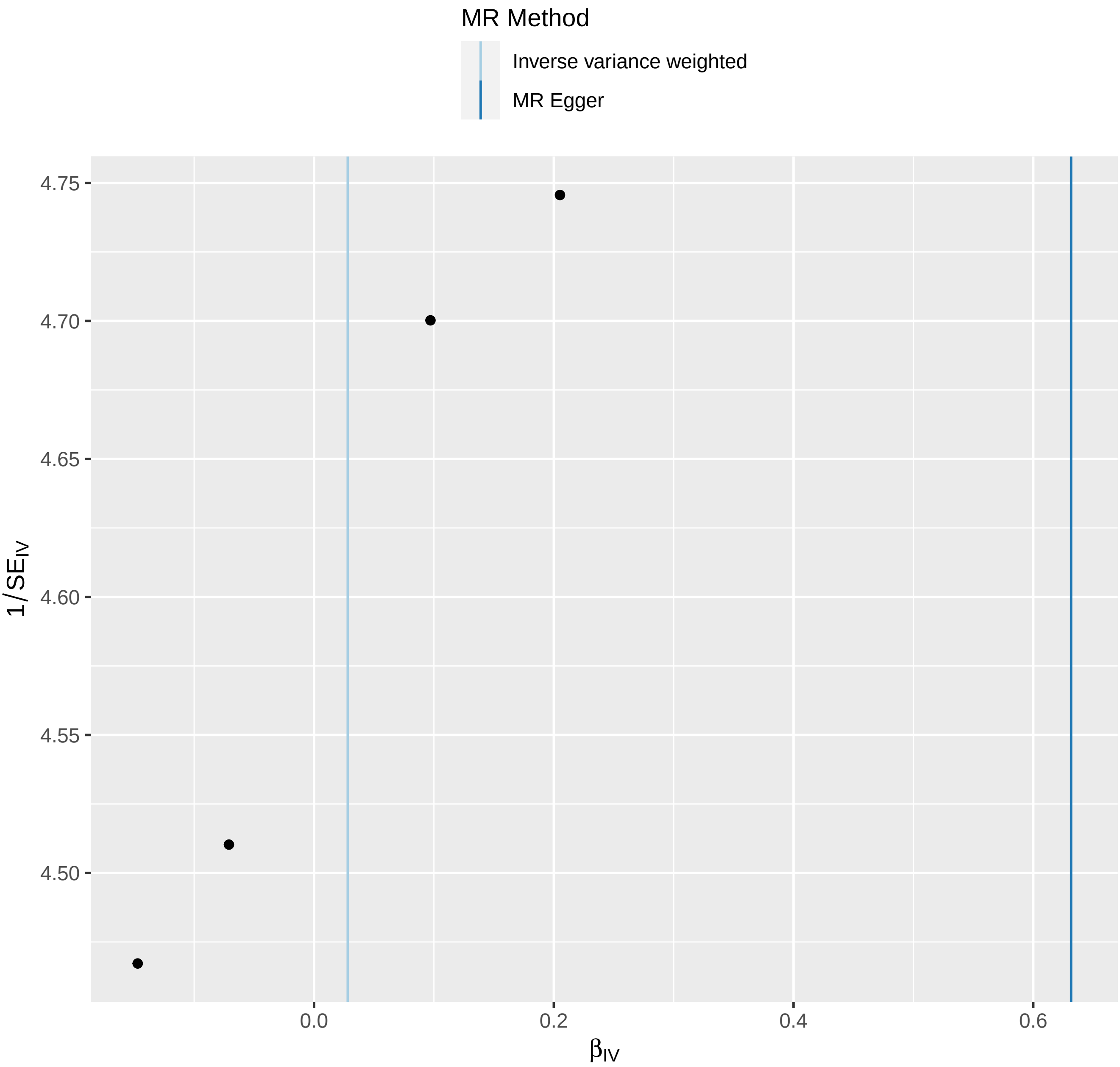

Batch 2−2168 : Gut microbiota abundance (genus Parasutterella id.2892) on Fc receptor−like protein 6
with a threshold of 1e−05

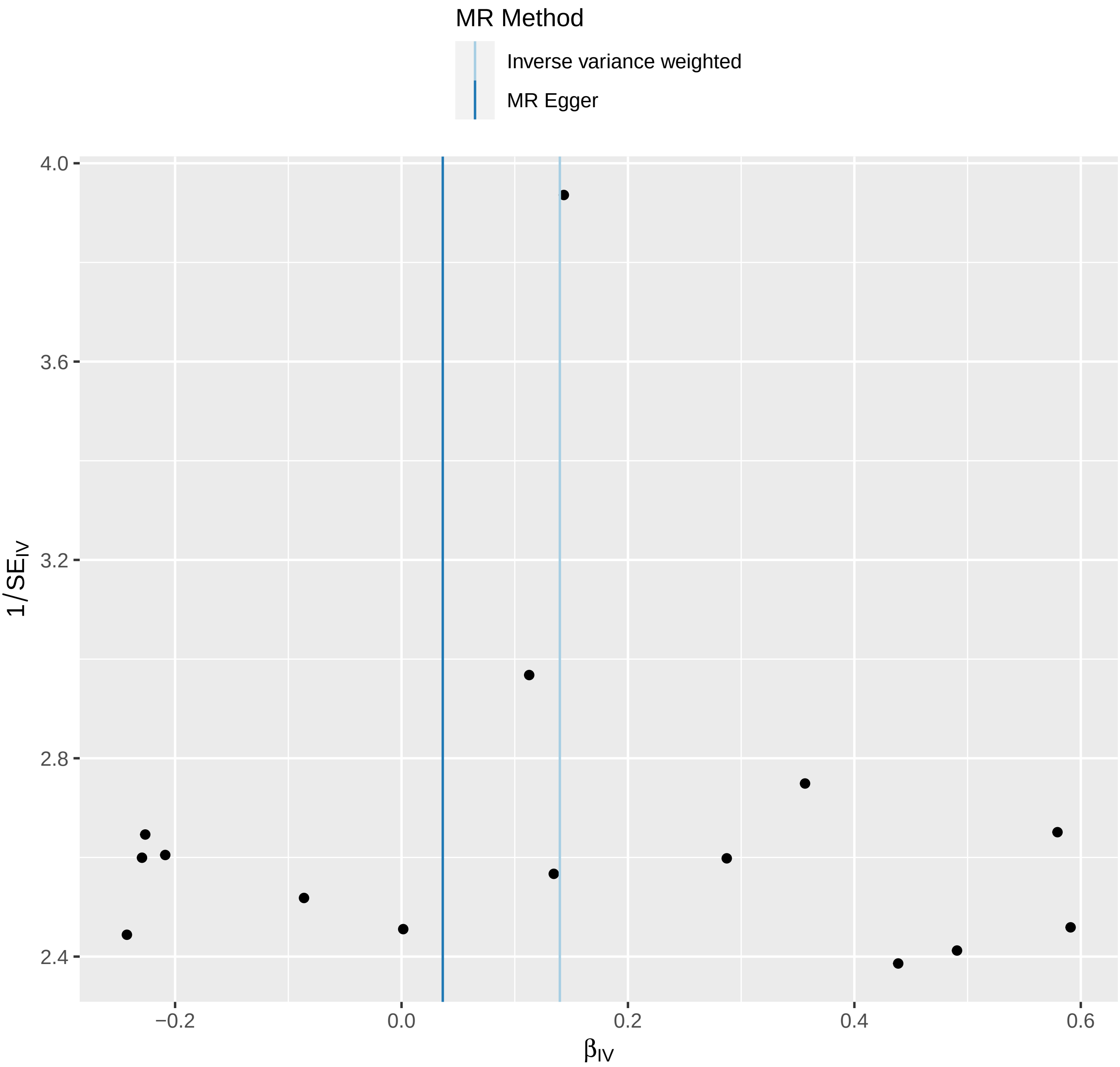

Batch 2−2169 : Gut microbiota abundance (genus Catenibacterium id.2153) on Flap endonuclease 1
with a threshold of 1e−05

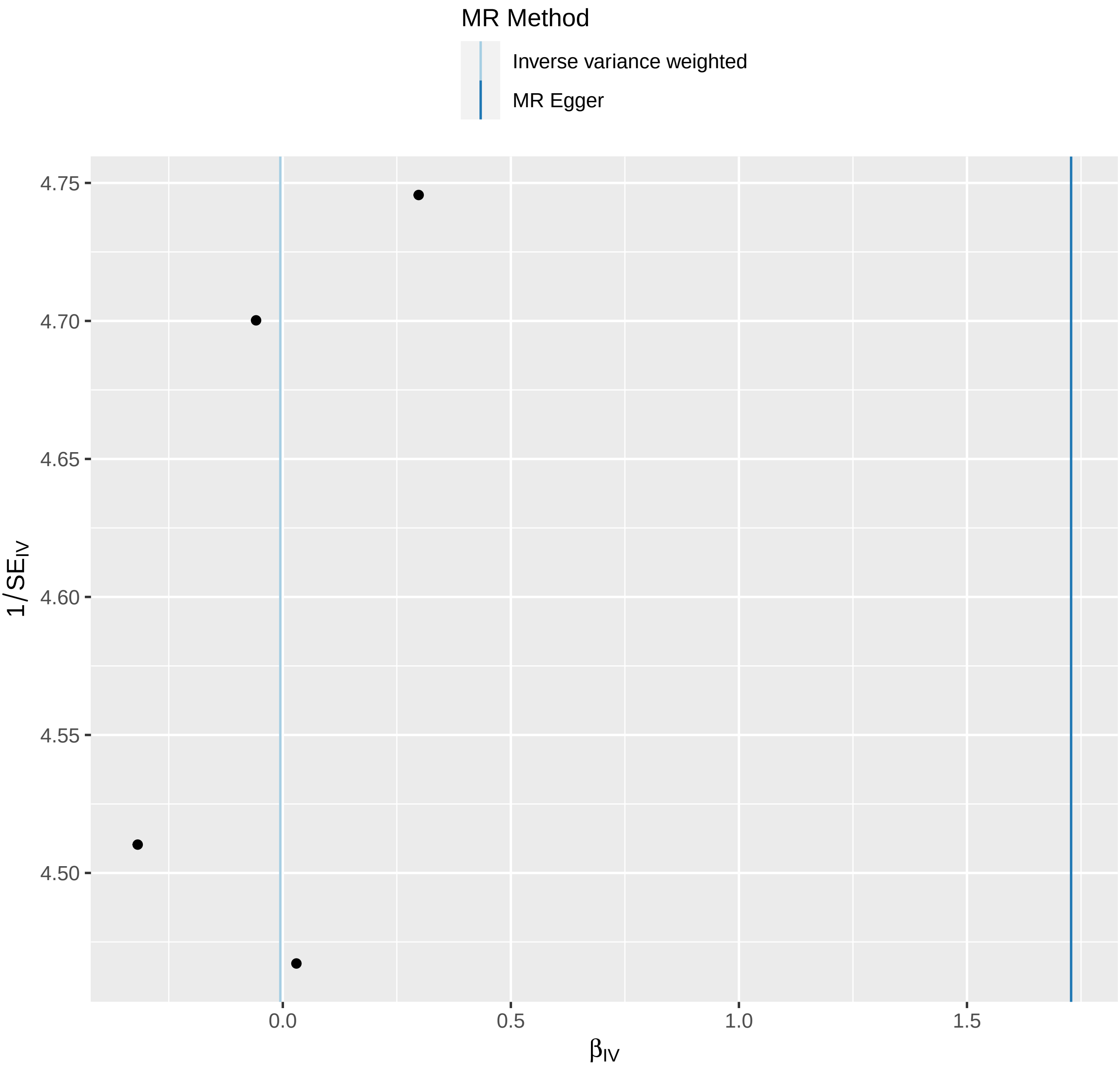

Batch 2−2170 : Gut microbiota abundance (genus Parasutterella id.2892) on Flap endonuclease 1
with a threshold of 1e−05

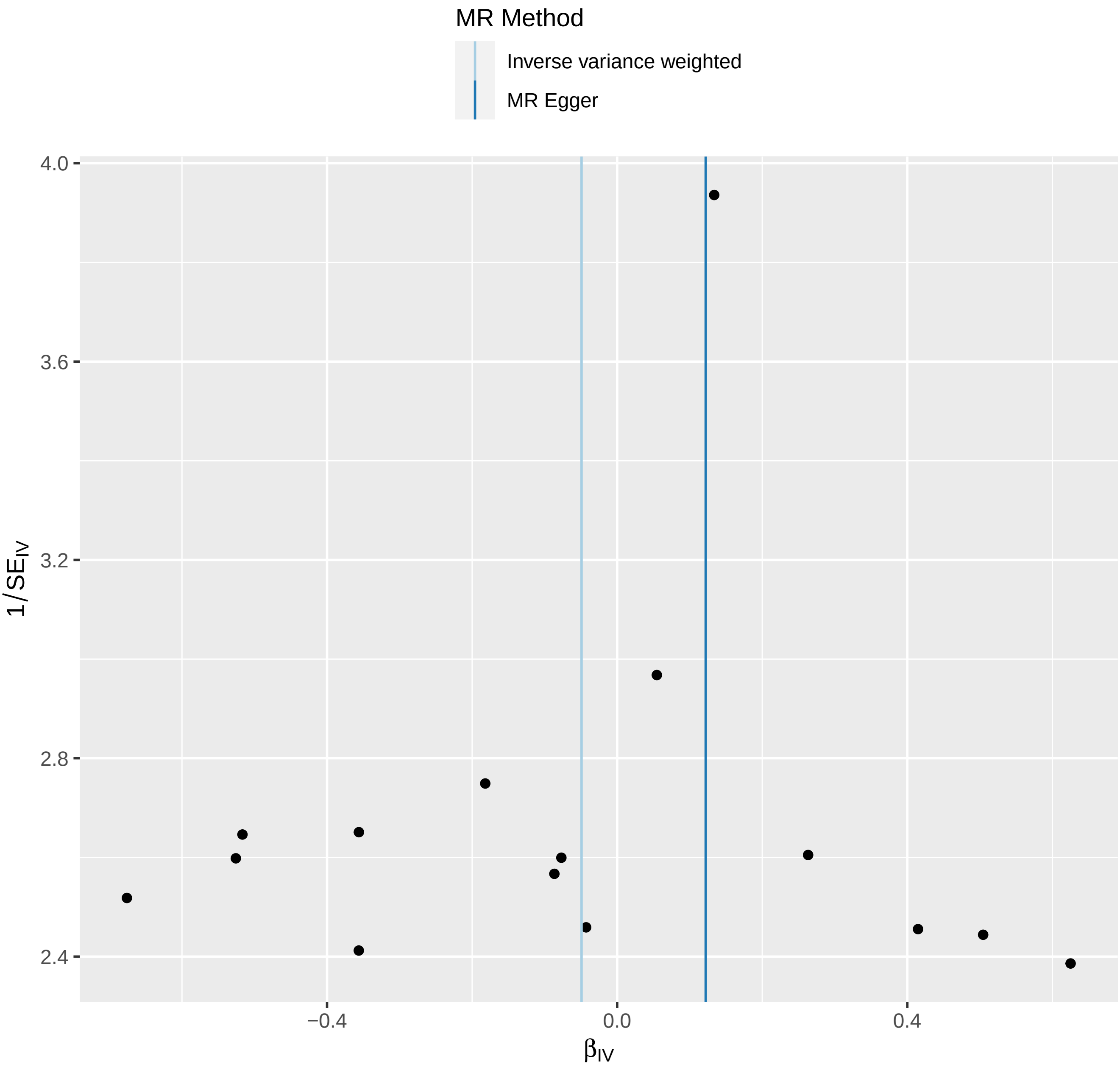

Batch 2−2171 : Gut microbiota abundance (genus Catenibacterium id.2153) on D−dimer
with a threshold of 1e−05

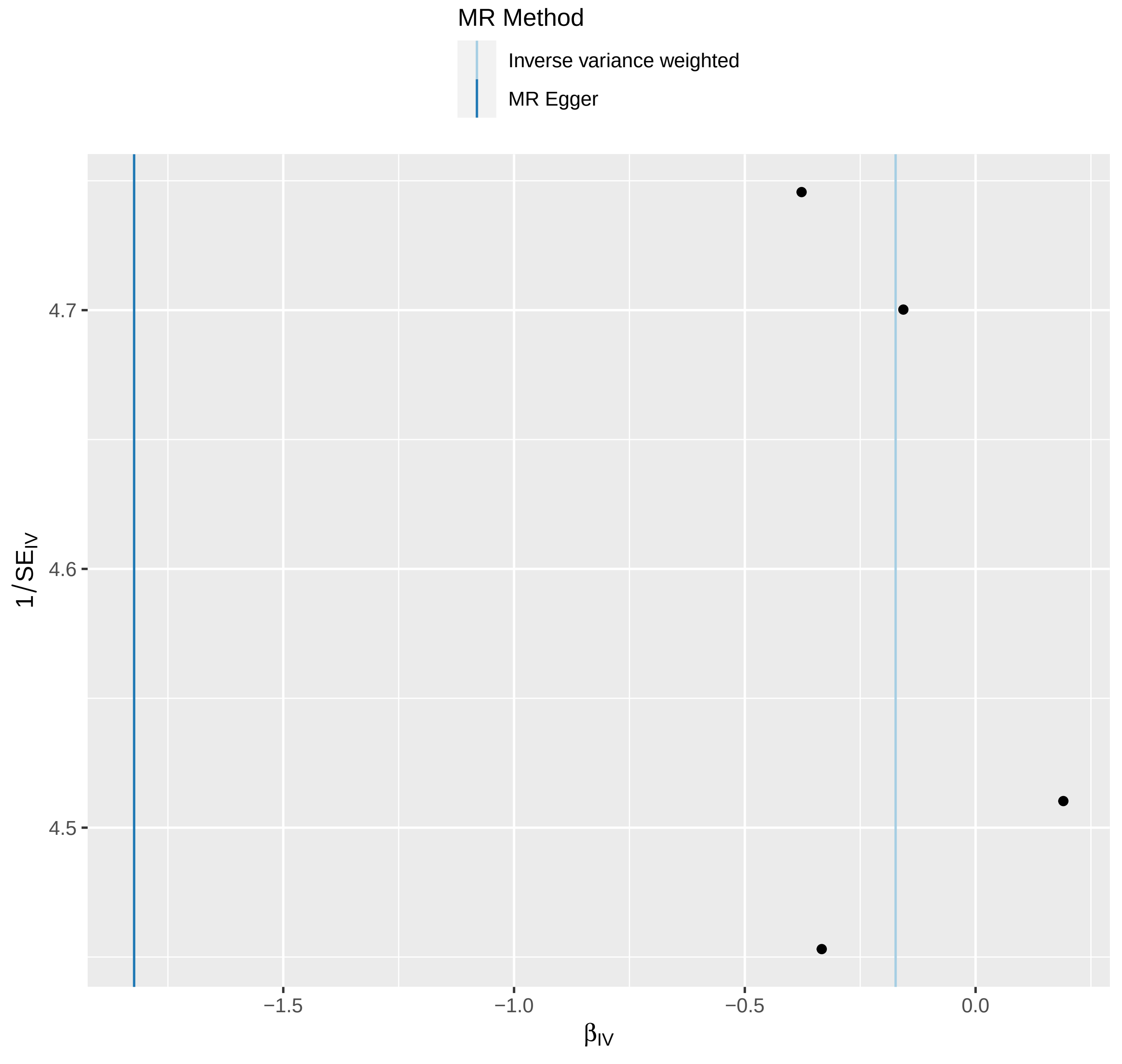

Batch 2−2172 : Gut microbiota abundance (genus Parasutterella id.2892) on D−dimer
with a threshold of 1e−05

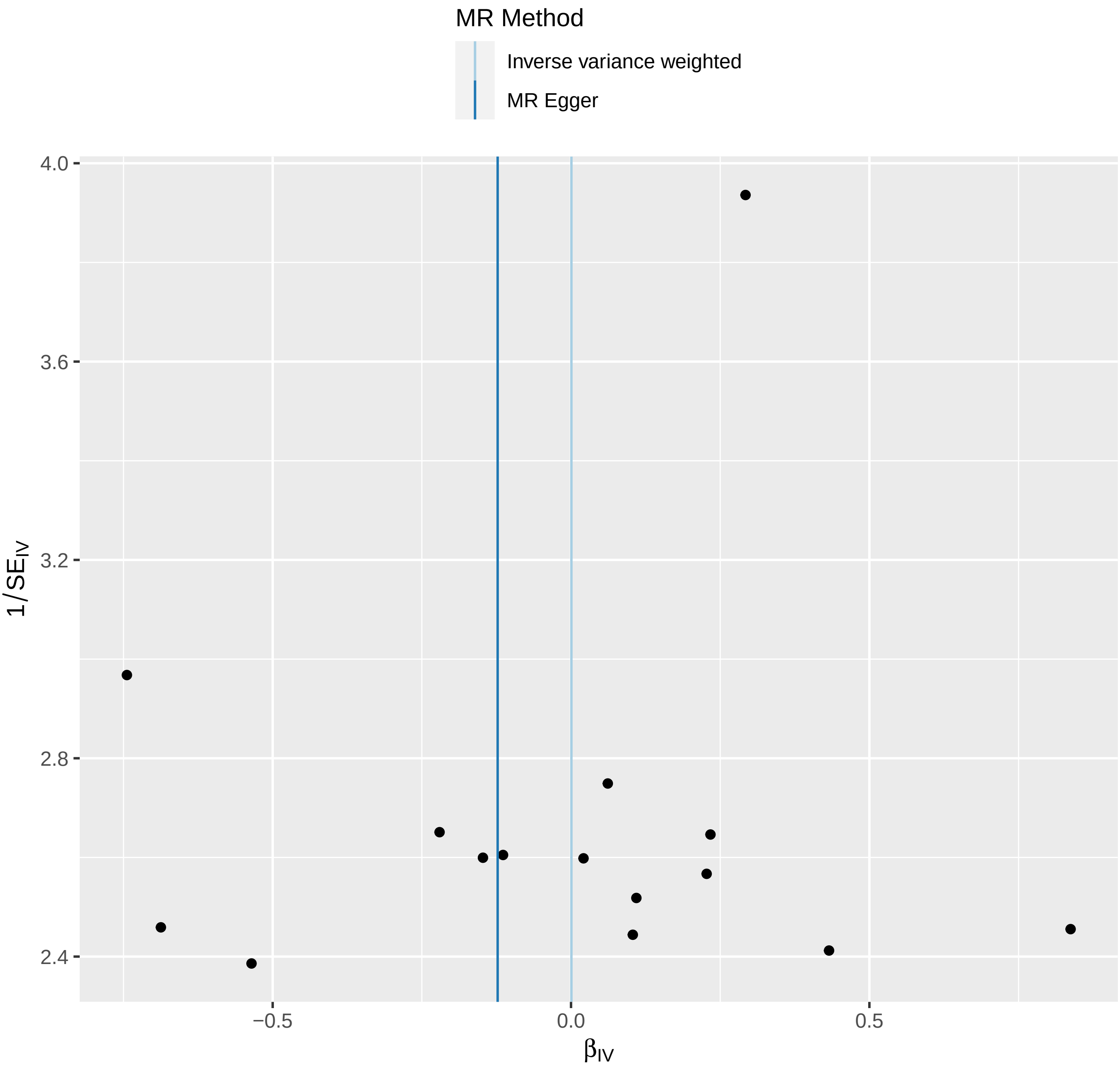

Batch 2−2173 : Gut microbiota abundance (genus Catenibacterium id.2153) on Fibroblast growth factor 10
with a threshold of 1e−05

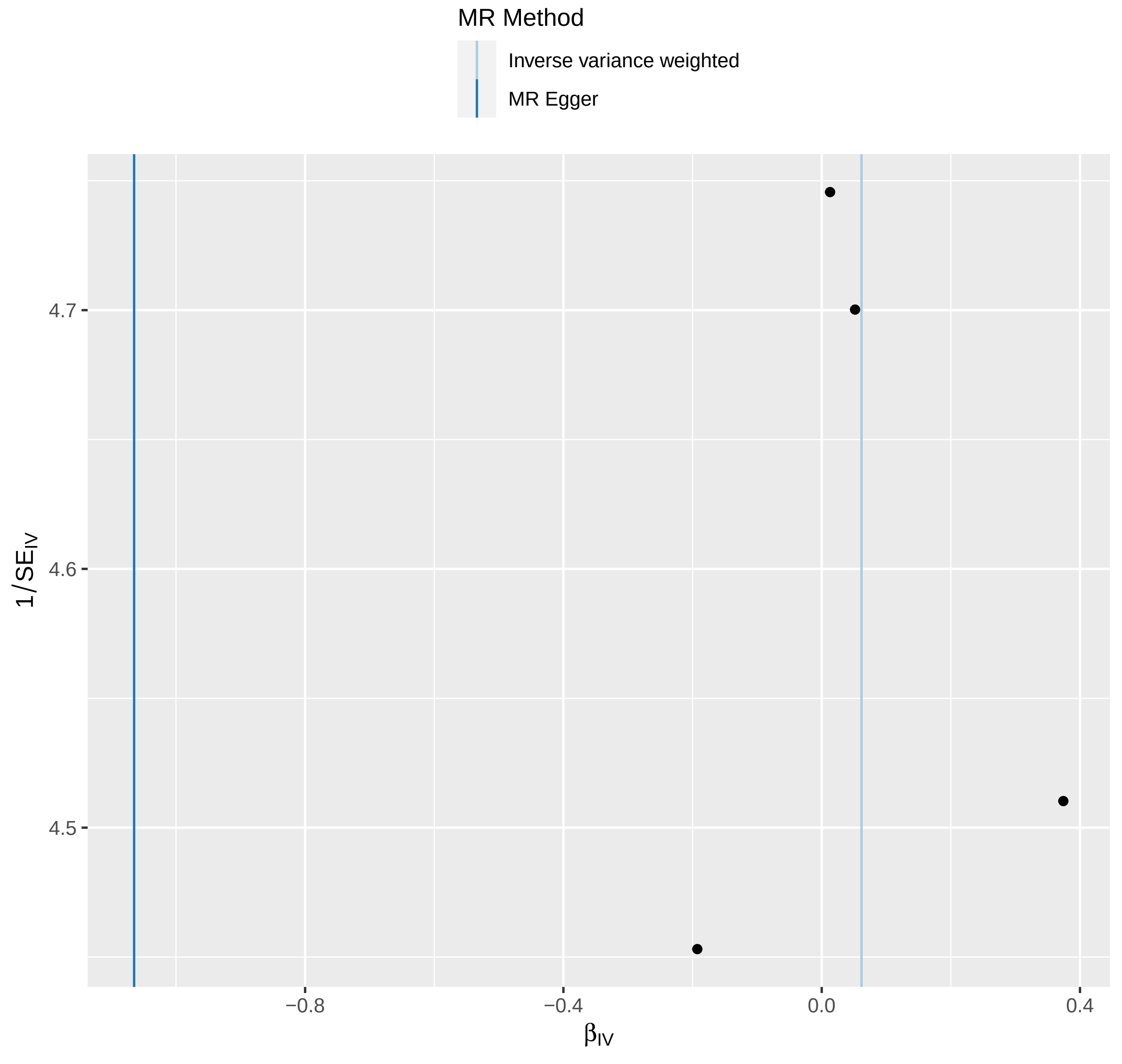

Batch 2−2174 : Gut microbiota abundance (genus Parasutterella id.2892) on Fibroblast growth factor 10
with a threshold of 1e−05

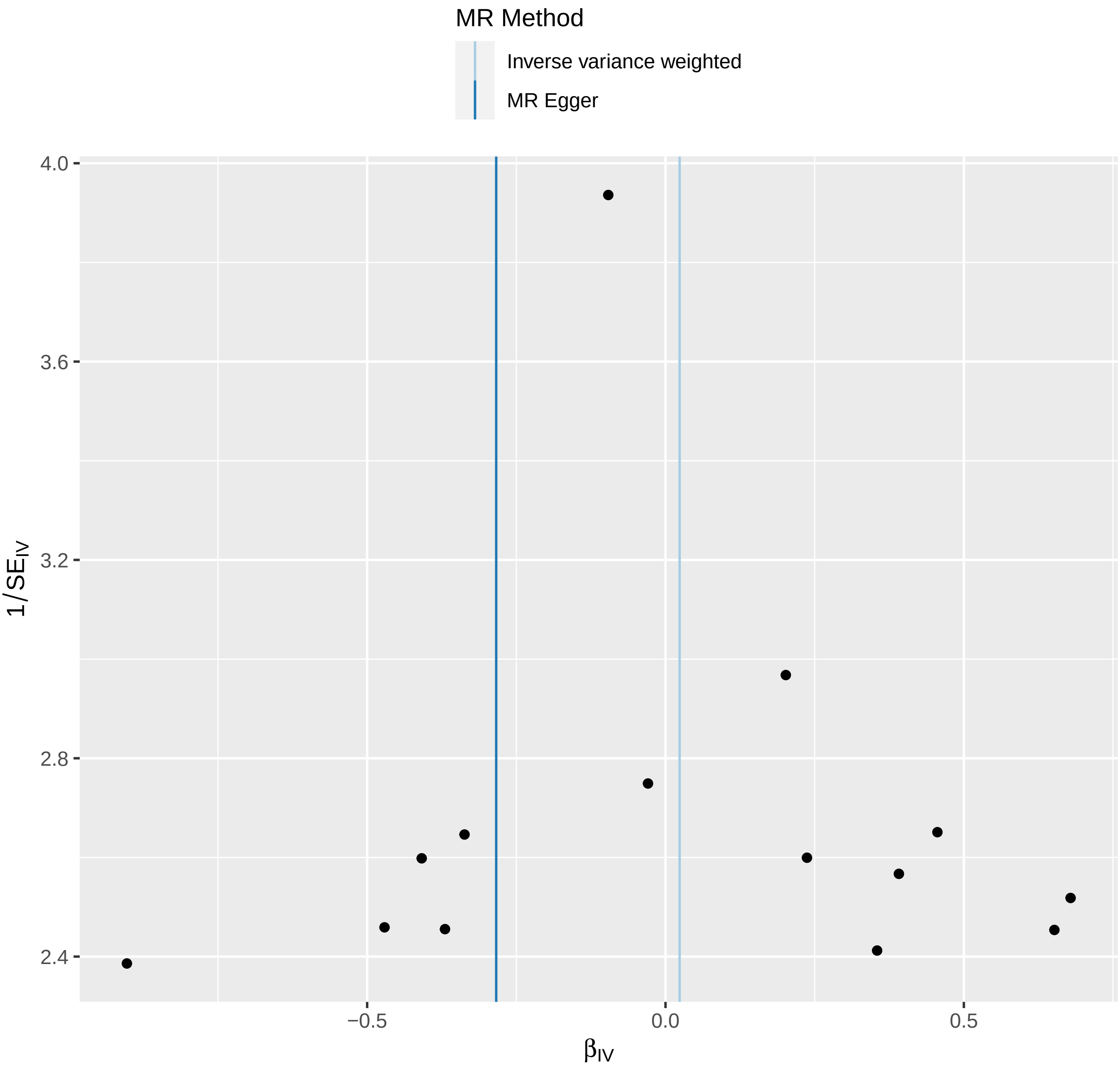

Batch 2−2175 : Gut microbiota abundance (genus Catenibacterium id.2153) on Fibroblast growth factor 1
with a threshold of 1e−05

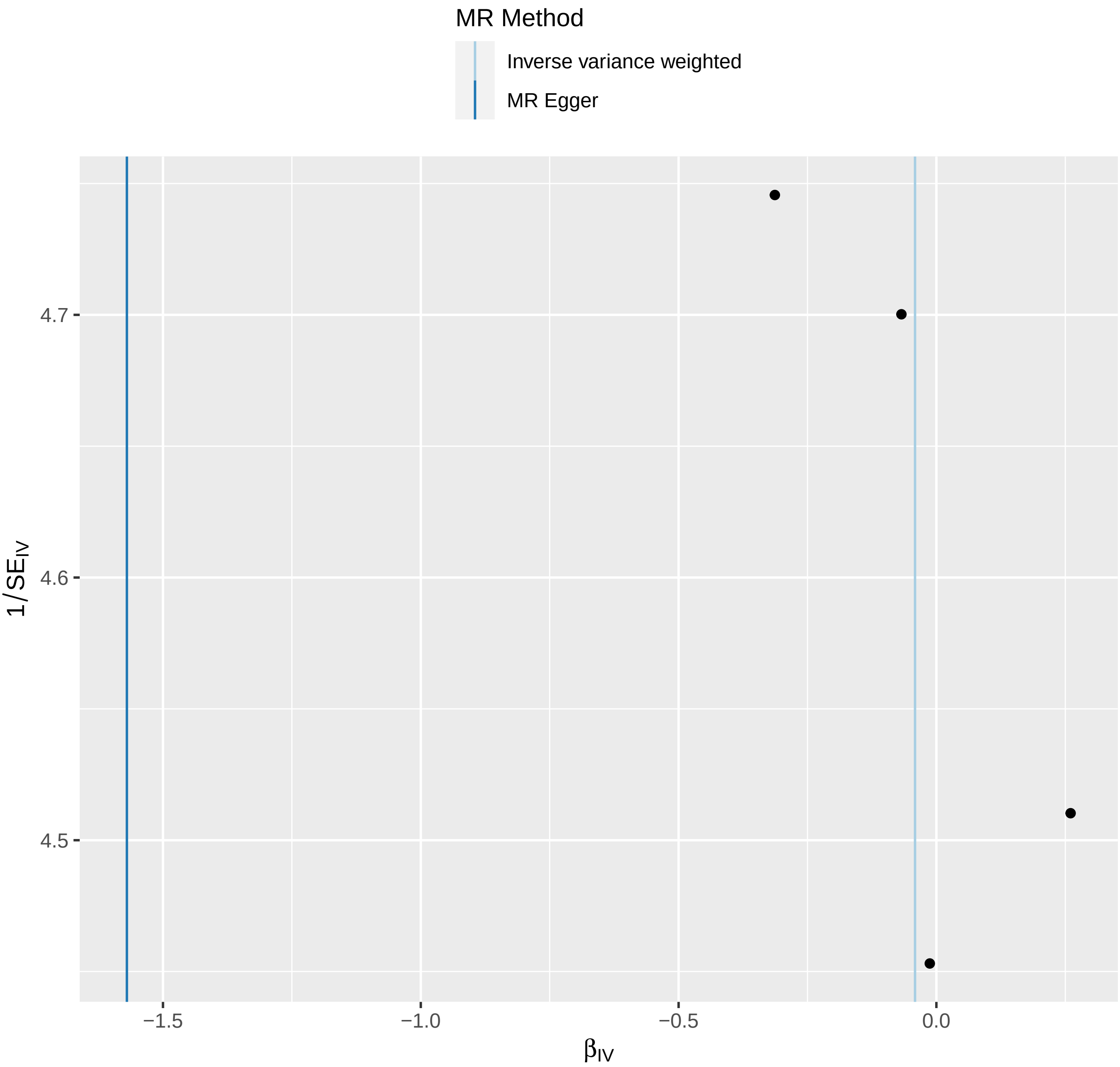

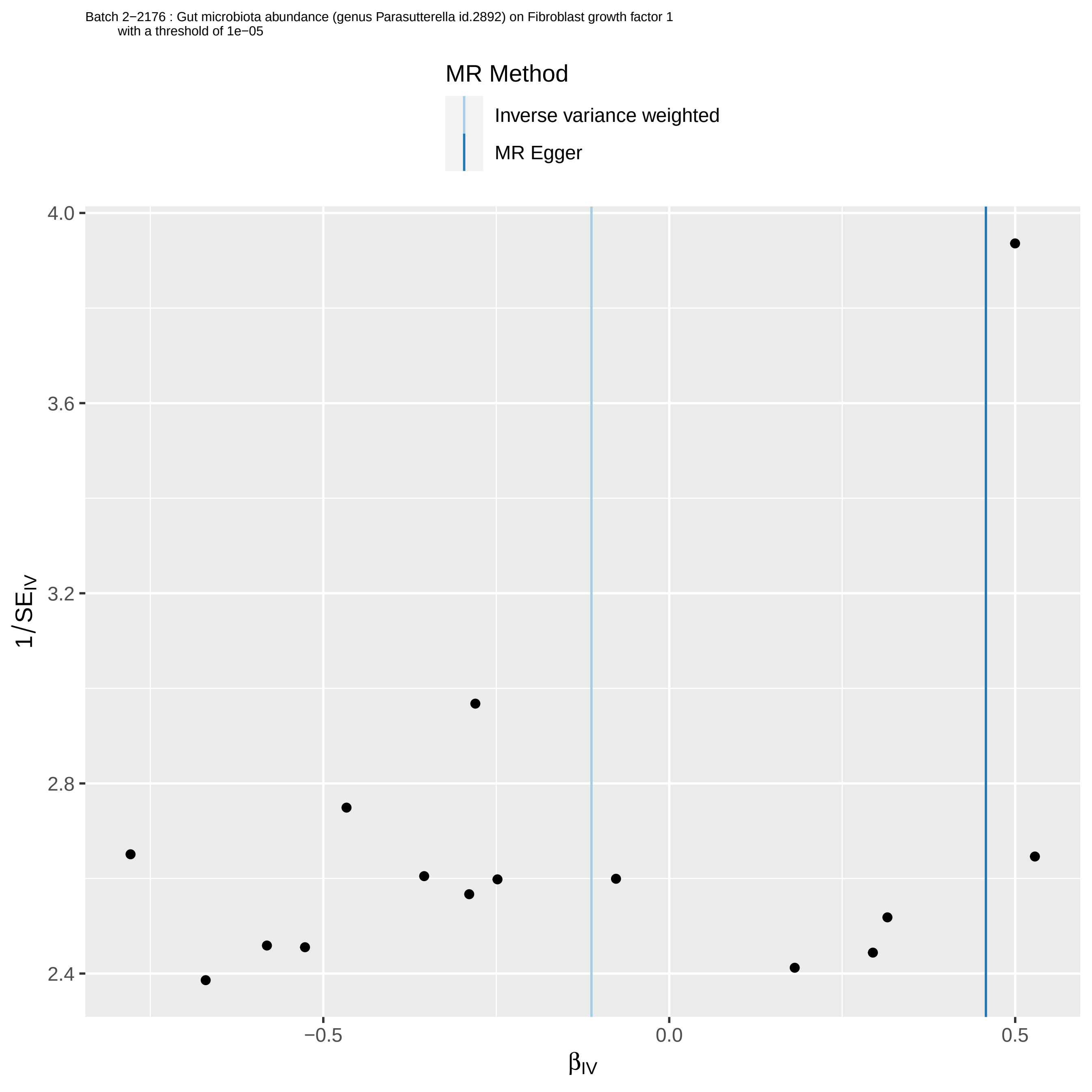
Batch 2−2176 : Gut microbiota abundance (genus Parasutterella id.2892) on Fibroblast growth factor 1
with a threshold of 1e−05
MR Method
Inverse variance weighted
MR Egger
4.0
3.6
3.2
2.8
2.4
1/SE_IV
−0.5
0.0
0.5
β_IV

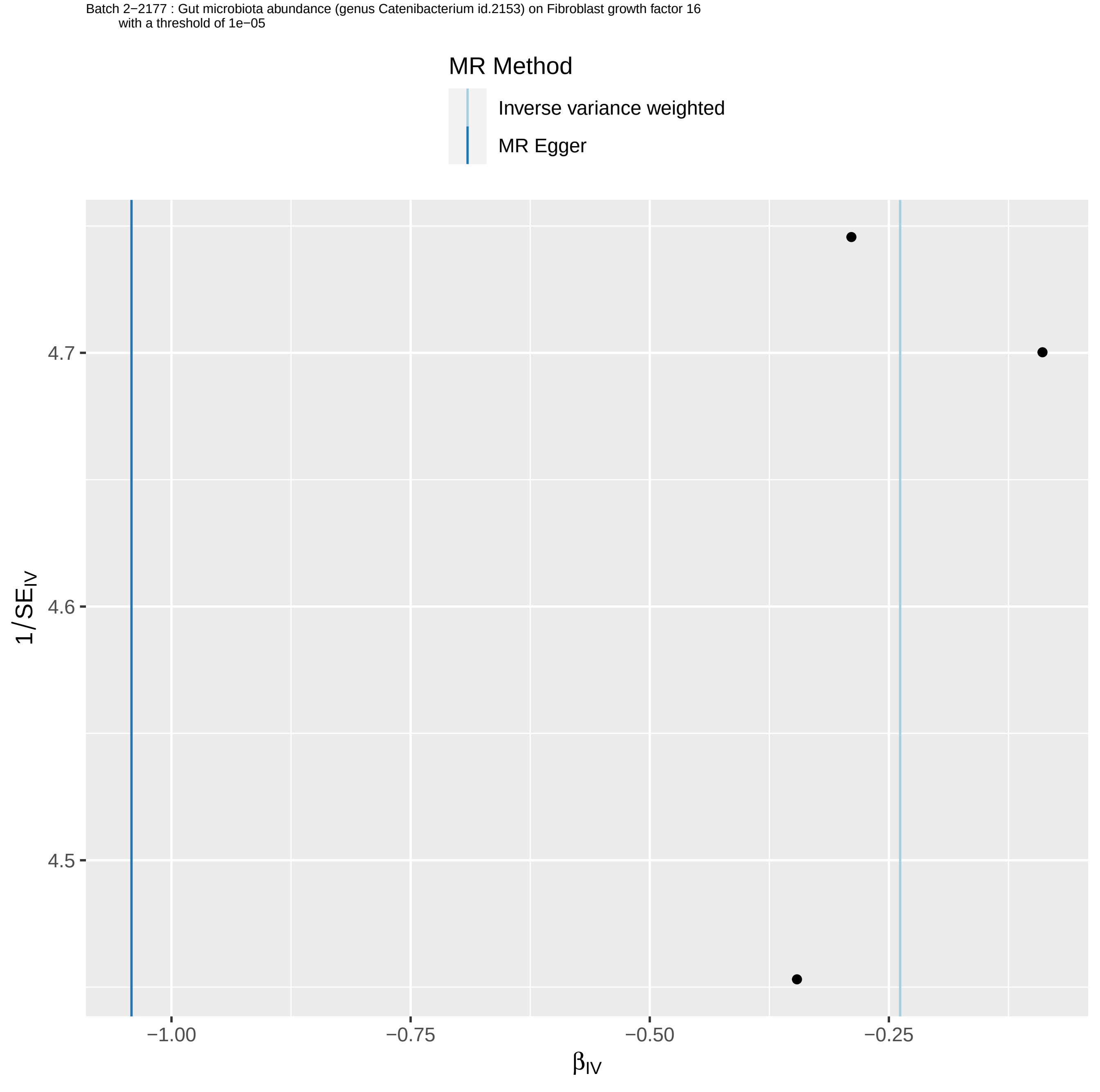

Batch 2−2177 : Gut microbiota abundance (genus Catenibacterium id.2153) on Fibroblast growth factor 16
with a threshold of 1e−05
MR Method
Inverse variance weighted
MR Egger
4.7
4.6
4.5
1/SE_IV
−1.00
−0.75
−0.50
−0.25
β_IV

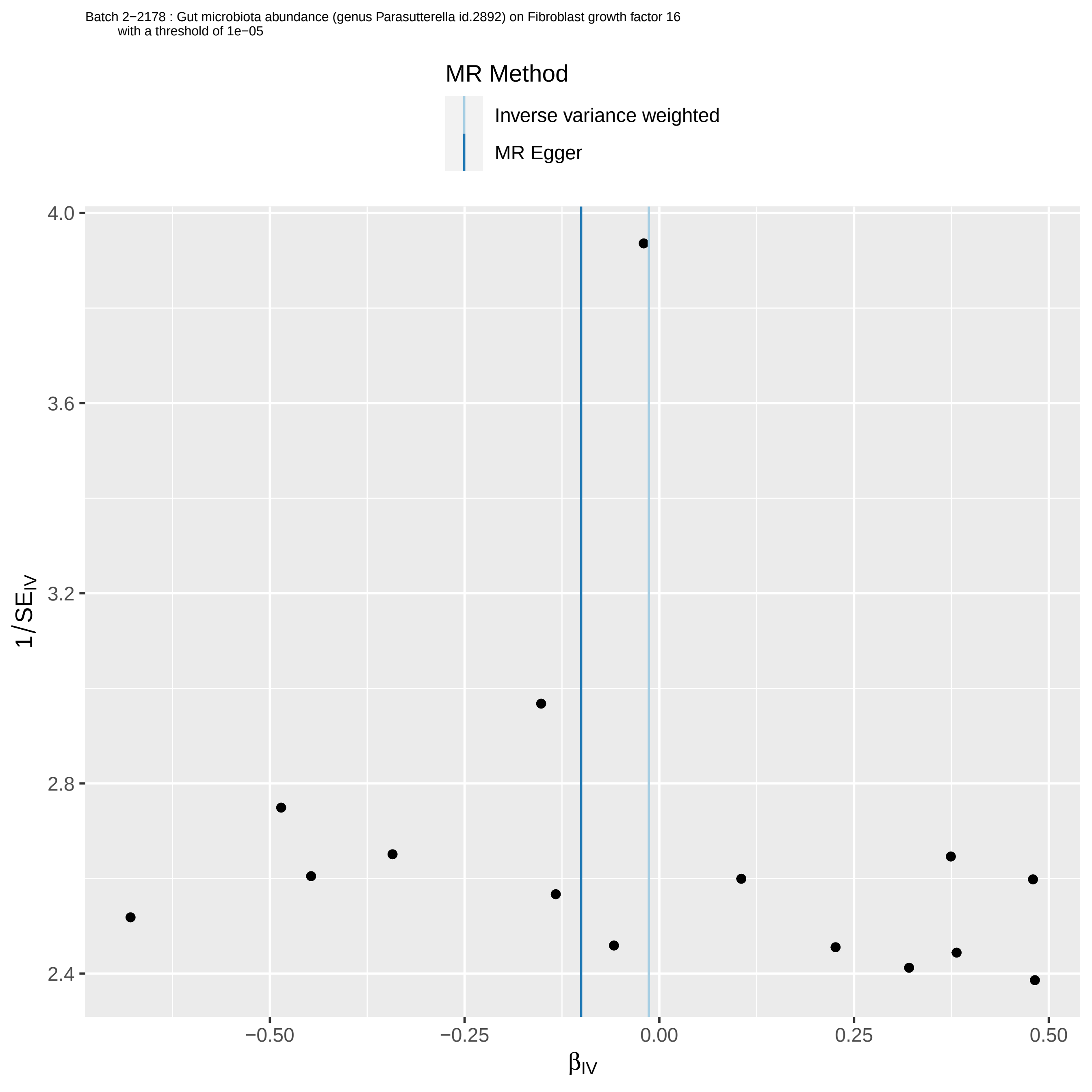
Batch 2−2178 : Gut microbiota abundance (genus Parasutterella id.2892) on Fibroblast growth factor 16
with a threshold of 1e−05
MR Method
Inverse variance weighted
MR Egger
4.0
3.6
3.2
2.8
2.4
1/SE$_{IV}$
−0.50
−0.25
0.00
0.25
0.50
β_{IV}

Batch 2−2179 : Gut microbiota abundance (genus Catenibacterium id.2153) on Fibroblast growth factor 16
with a threshold of 1e−05

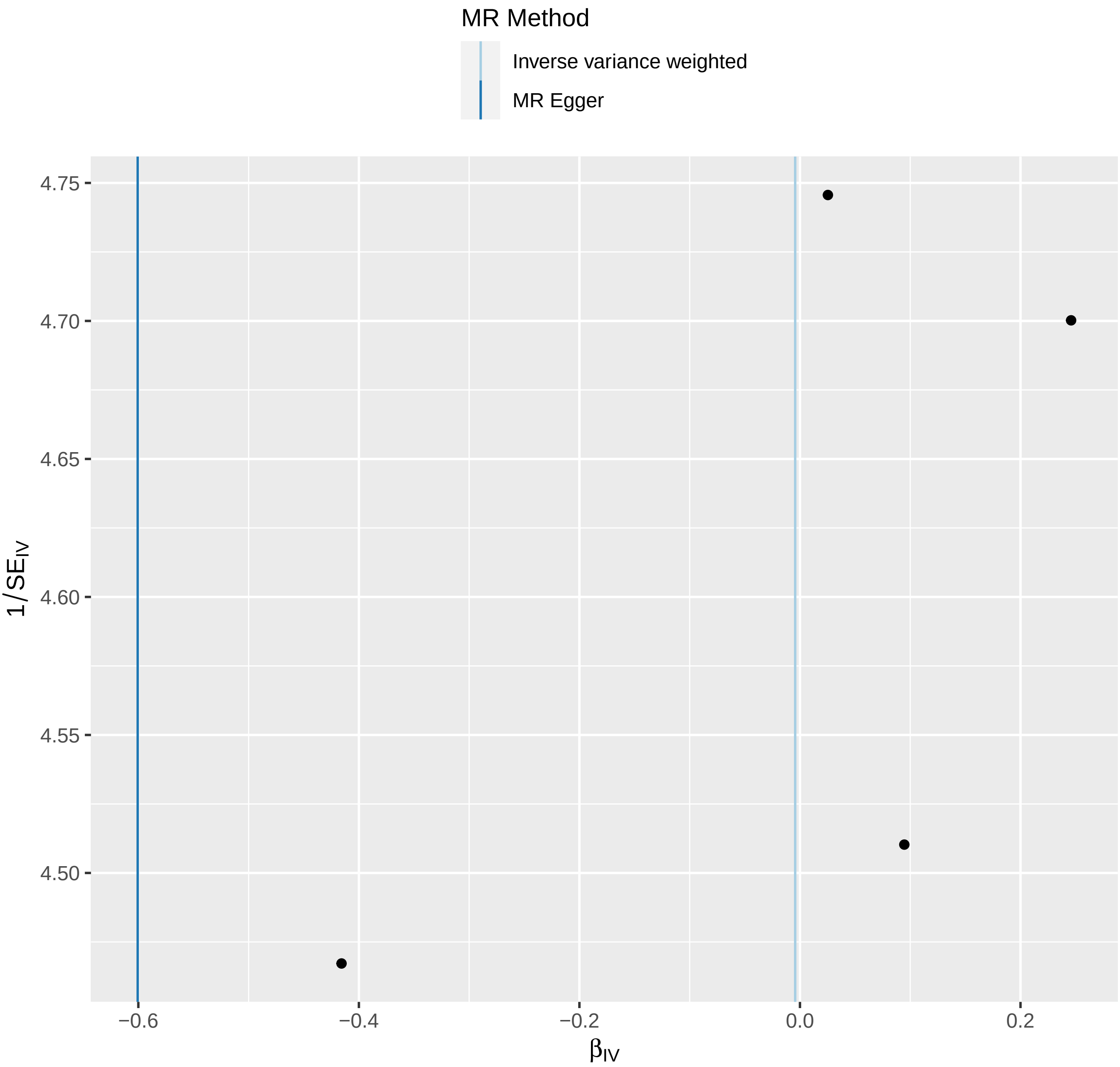

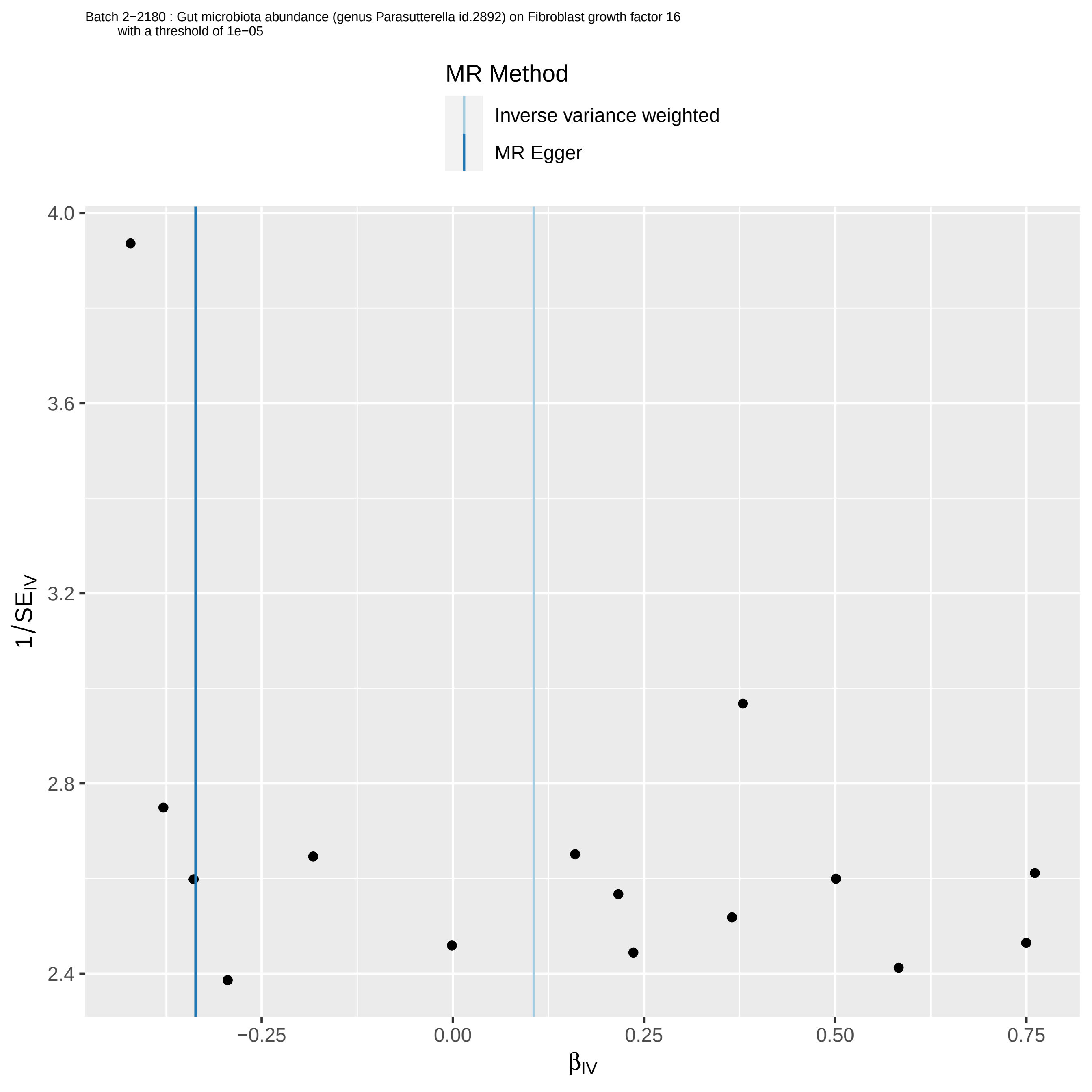
Batch 2−2180 : Gut microbiota abundance (genus Parasutterella id.2892) on Fibroblast growth factor 16
with a threshold of 1e−05
MR Method
Inverse variance weighted
MR Egger
4.0
3.6
3.2
2.8
2.4
1/SE_IV
−0.25
0.00
0.25
0.50
0.75
β_IV

Batch 2−2181 : Gut microbiota abundance (genus Catenibacterium id.2153) on Fibroblast growth factor 19
with a threshold of 1e−05

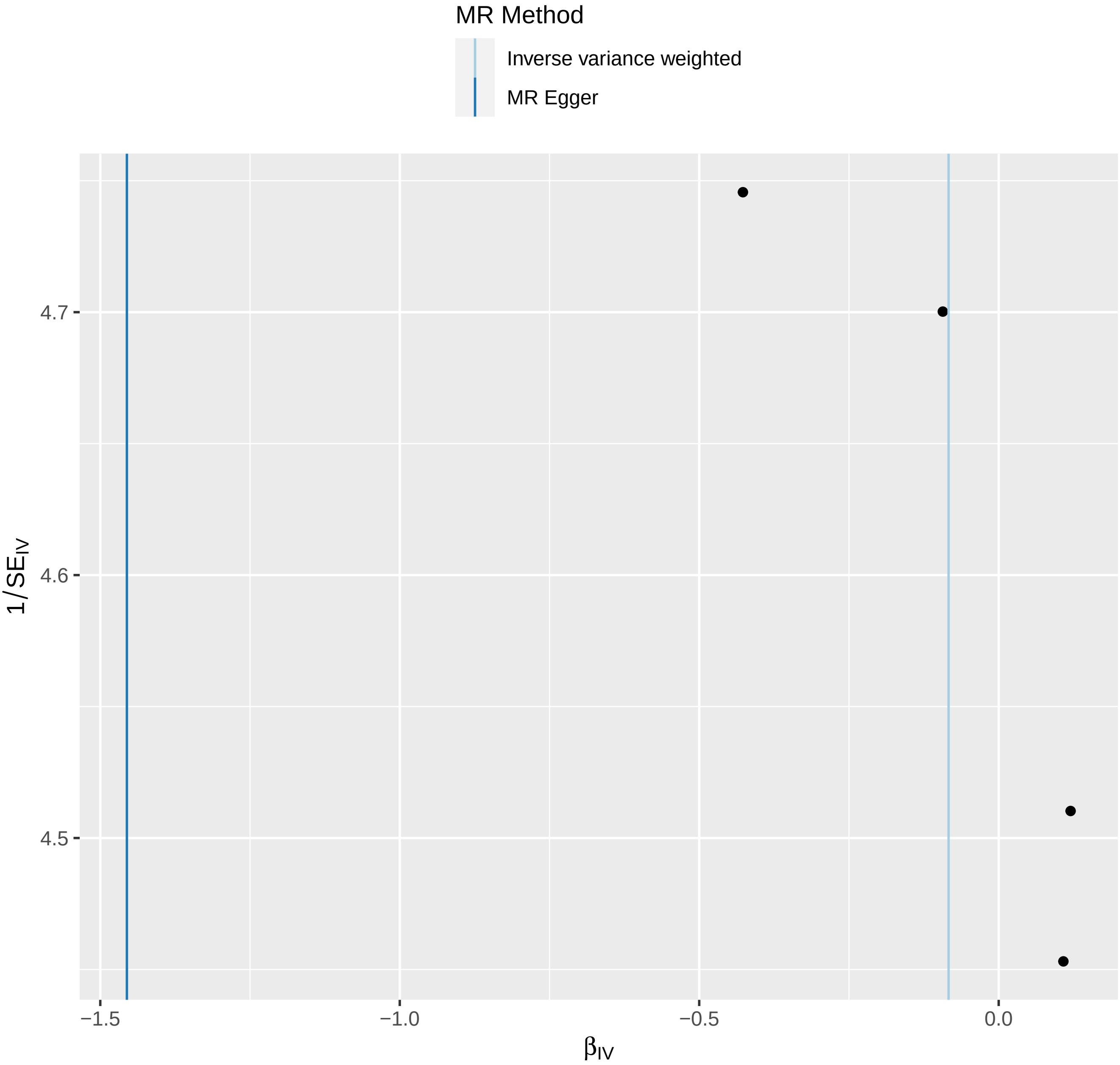

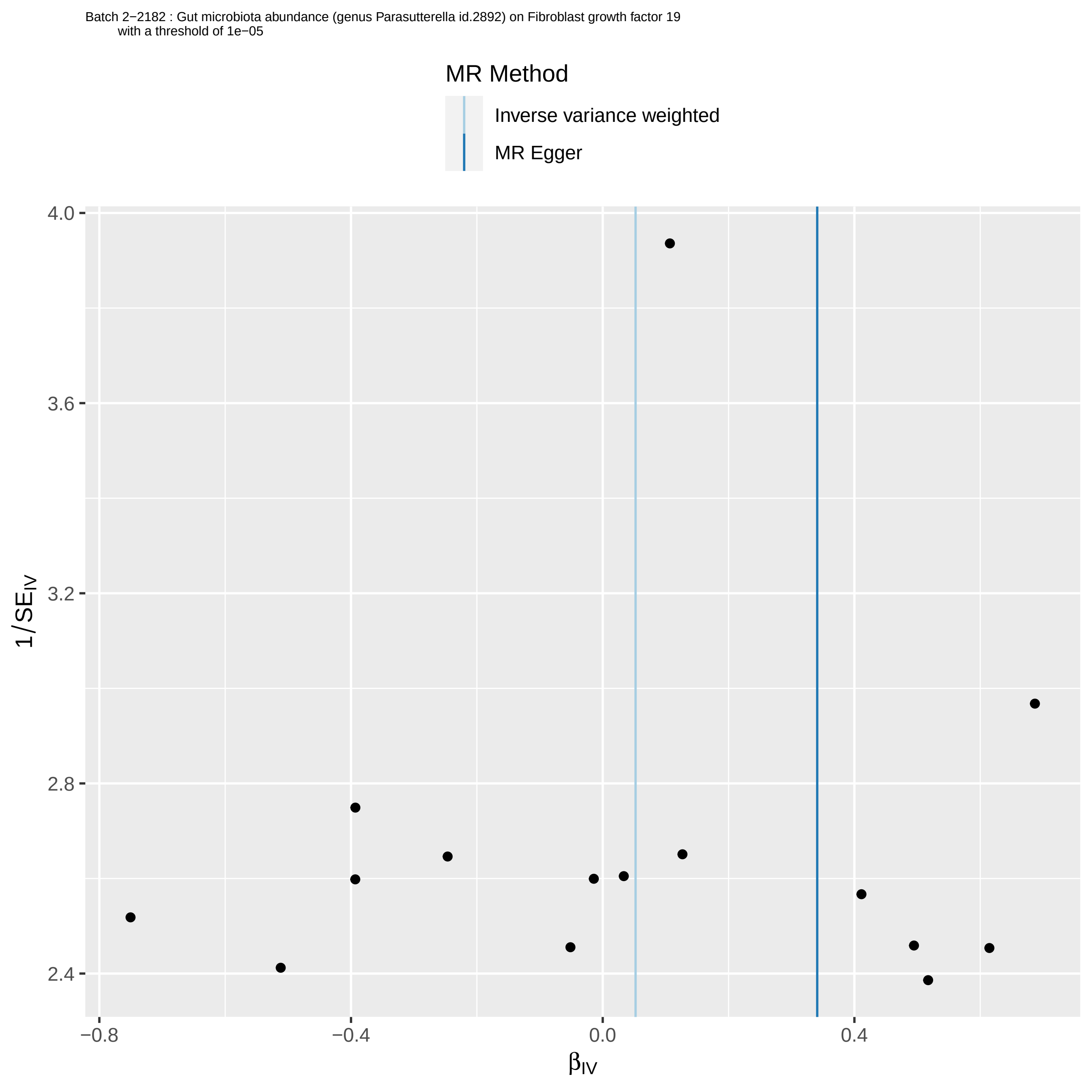

Batch 2−2182 : Gut microbiota abundance (genus Parasutterella id.2892) on Fibroblast growth factor 19
with a threshold of 1e−05
MR Method
Inverse variance weighted
MR Egger
4.0
3.6
3.2
2.8
2.4
1/SE_IV
−0.8
−0.4
0.0
0.4
β_IV

Batch 2−2183 : Gut microbiota abundance (genus Catenibacterium id.2153) on Fibroblast growth factor 20
with a threshold of 1e−05

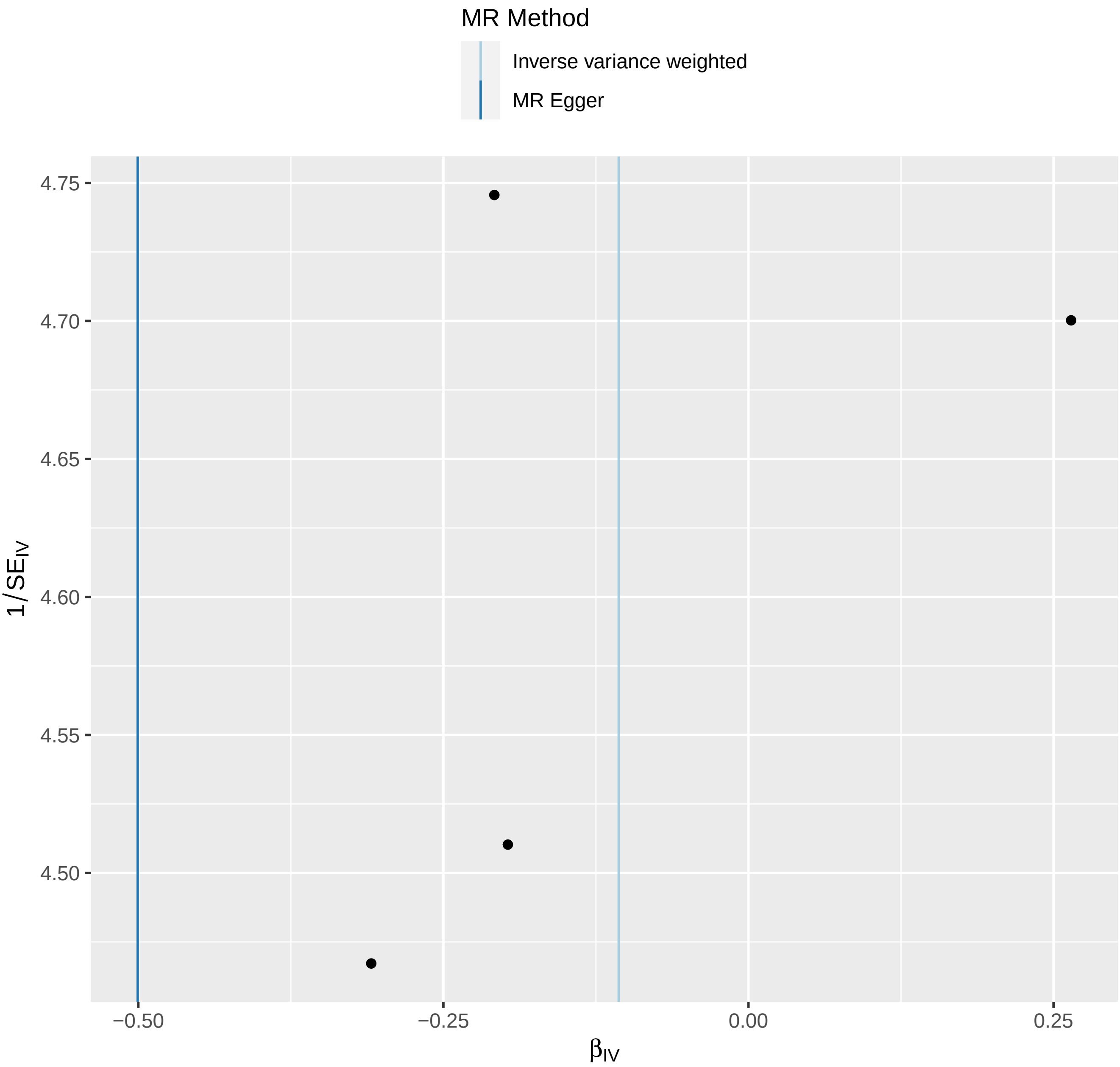

Batch 2−2184 : Gut microbiota abundance (genus Parasutterella id.2892) on Fibroblast growth factor 20
with a threshold of 1e−05

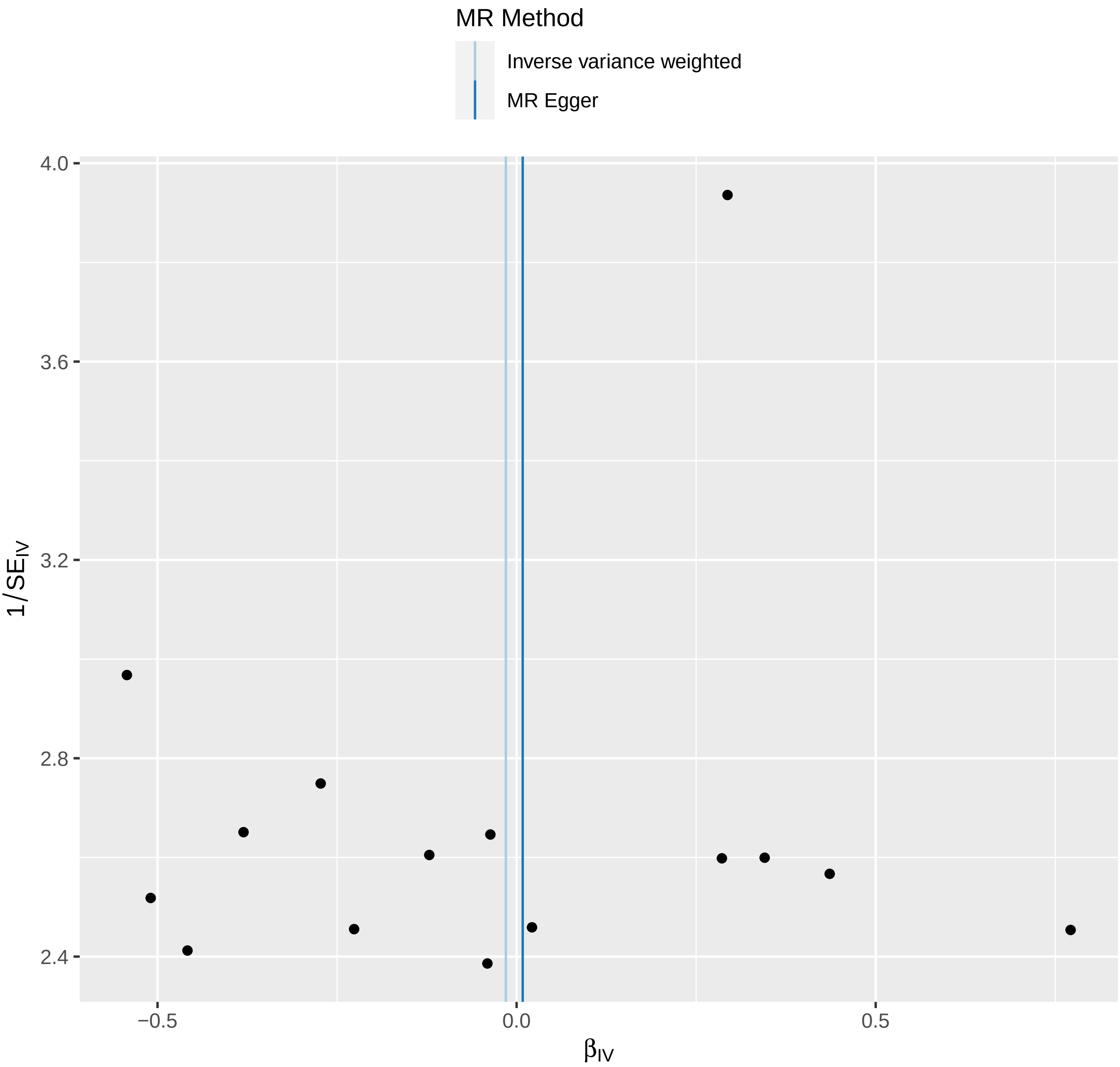

Batch 2−2185 : Gut microbiota abundance (genus Catenibacterium id.2153) on Fibroblast growth factor 23
with a threshold of 1e−05

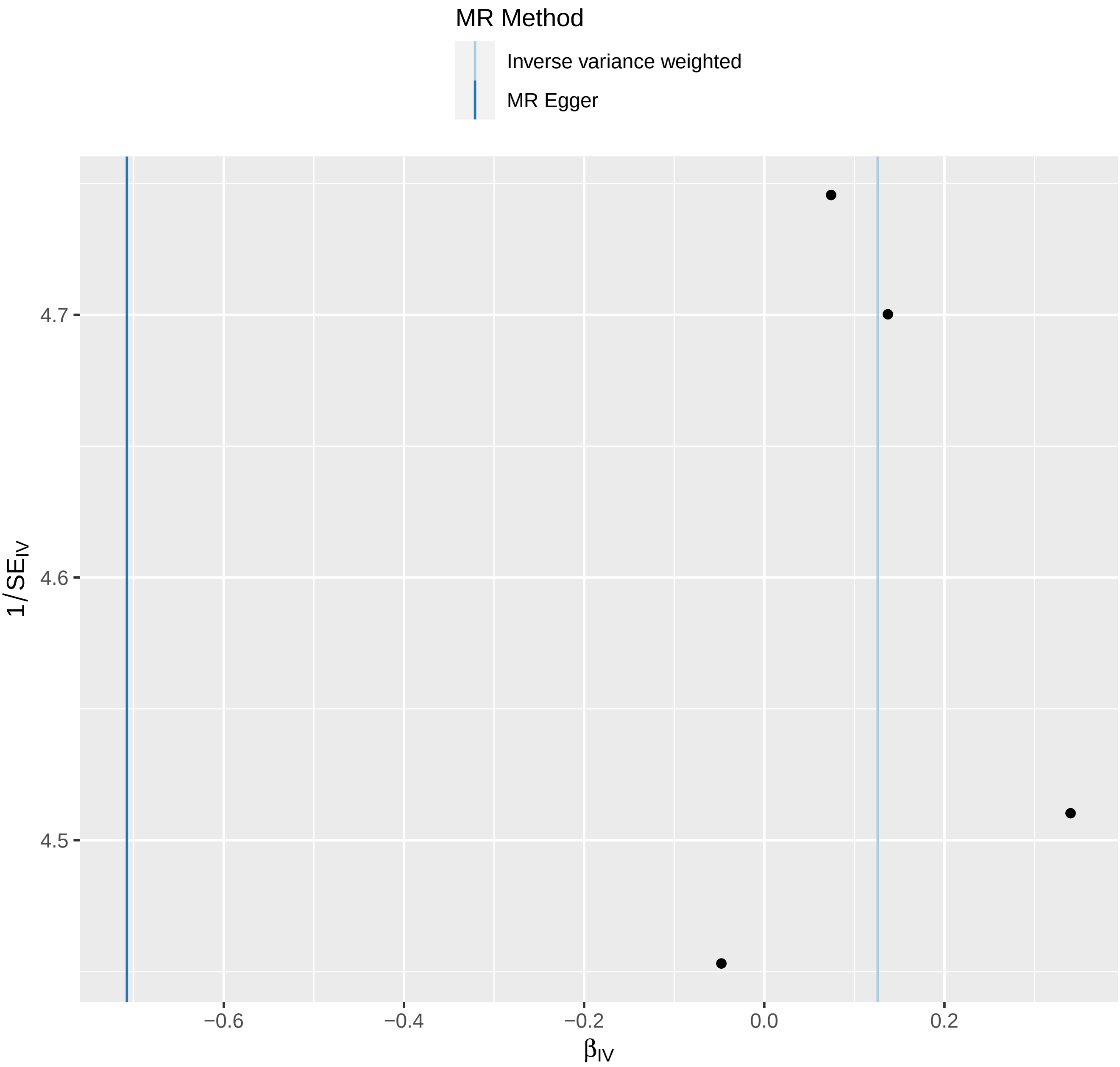

Batch 2−2186 : Gut microbiota abundance (genus Parasutterella id.2892) on Fibroblast growth factor 23
with a threshold of 1e−05

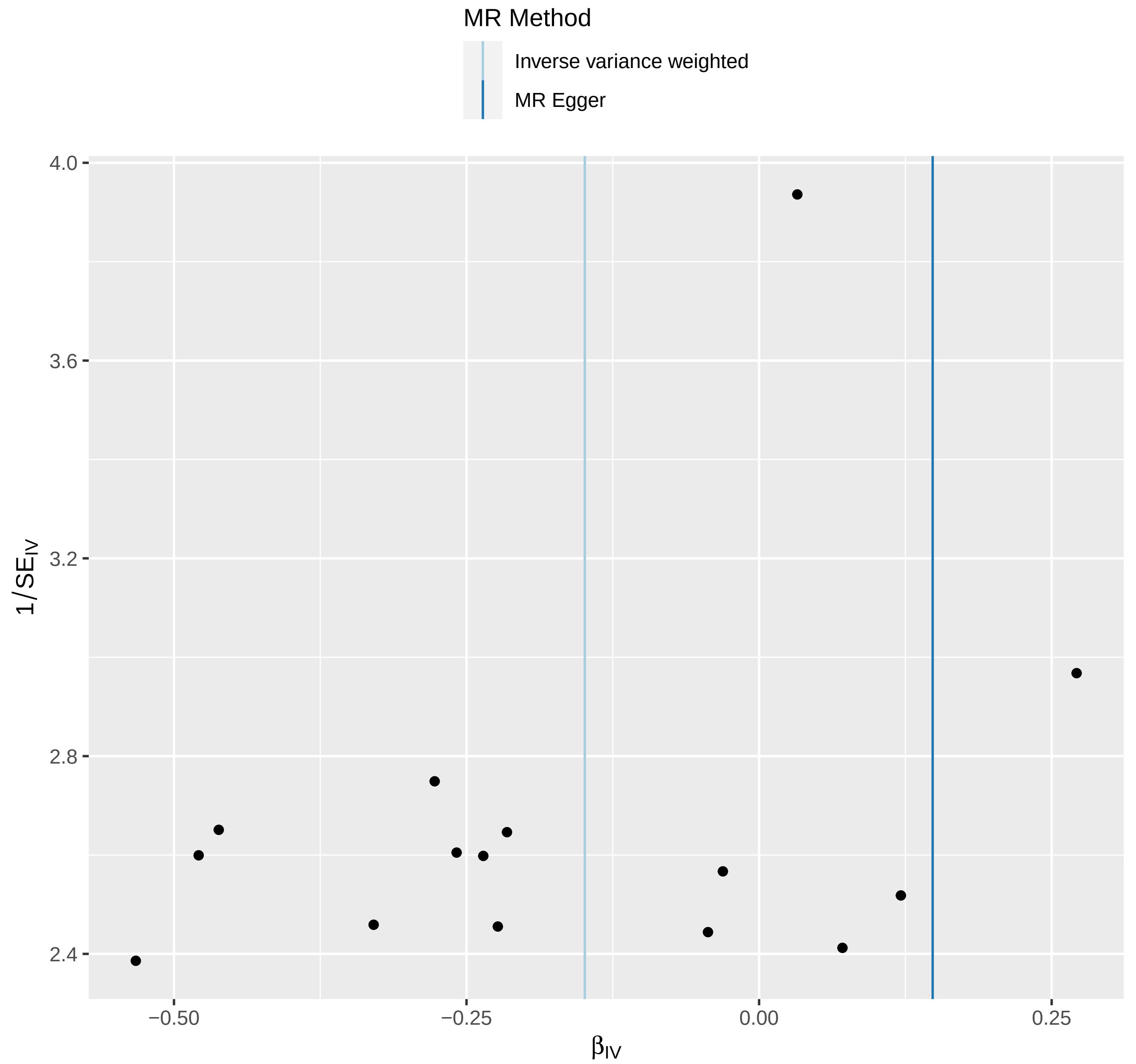

Batch 2−2187 : Gut microbiota abundance (genus Catenibacterium id.2153) on Fibroblast growth factor 4
with a threshold of 1e−05

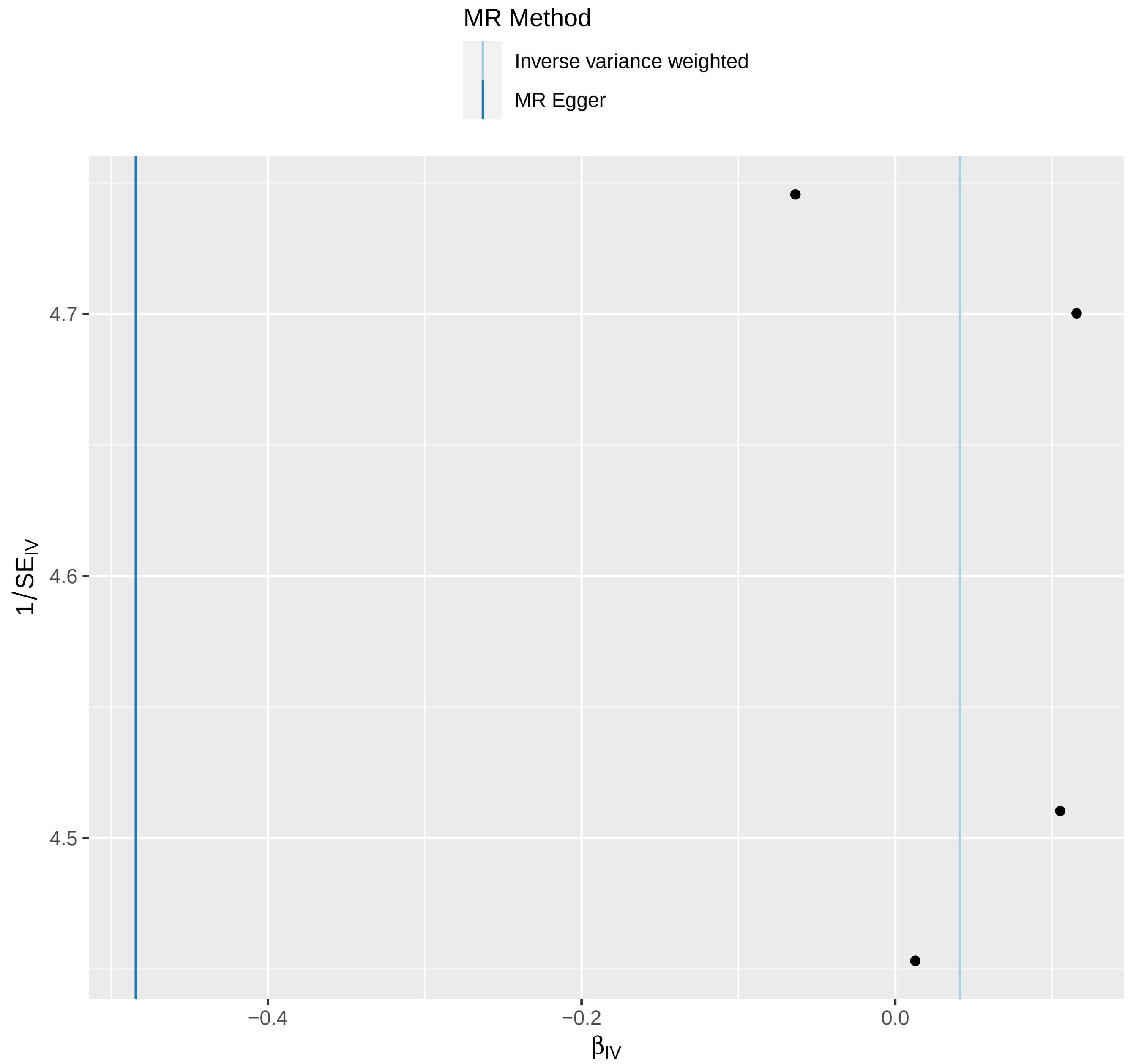

Batch 2−2188 : Gut microbiota abundance (genus Parasutterella id.2892) on Fibroblast growth factor 4
with a threshold of 1e−05

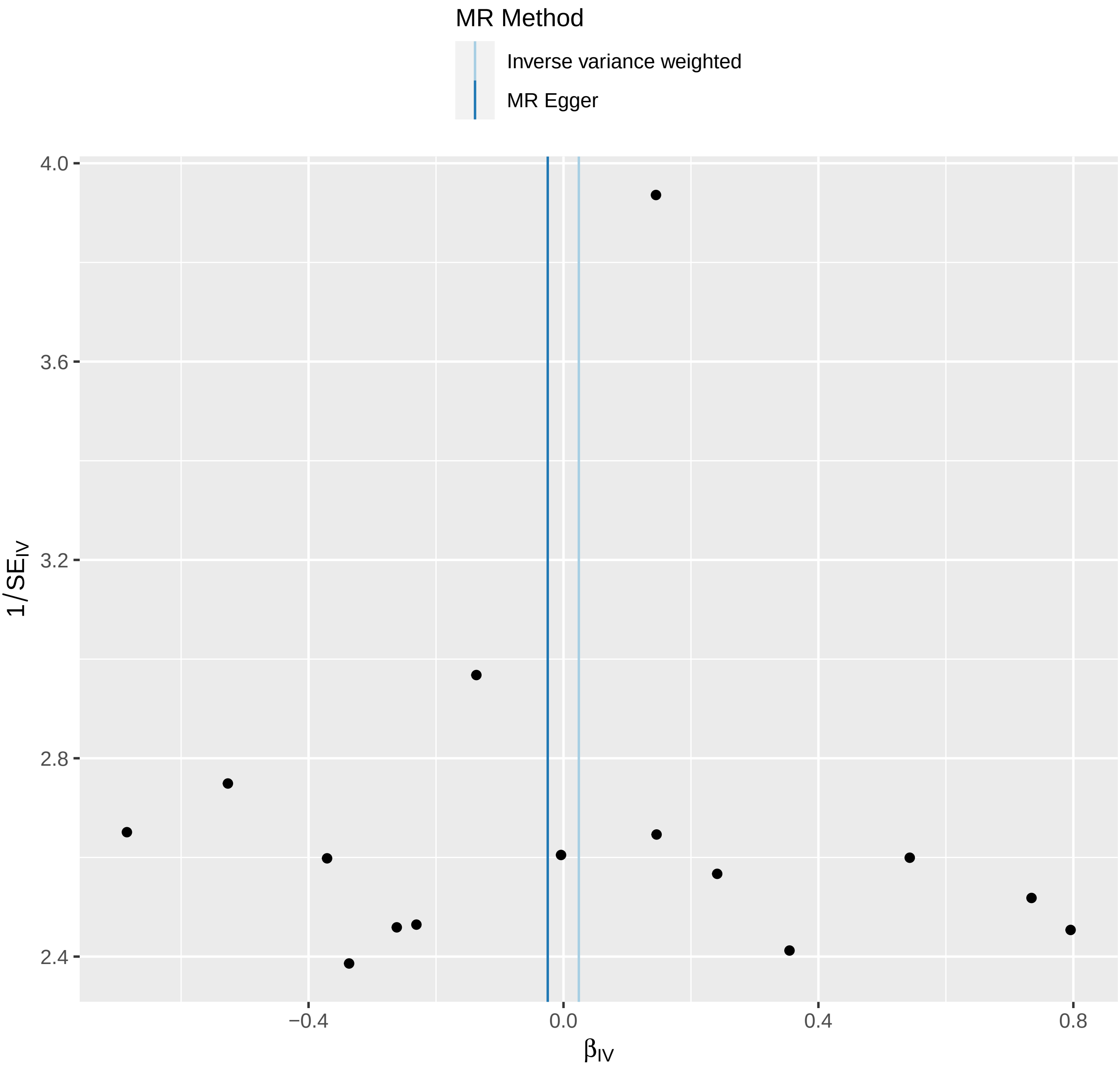

Batch 2−2189 : Gut microbiota abundance (genus Catenibacterium id.2153) on Fibroblast growth factor 5
with a threshold of 1e−05

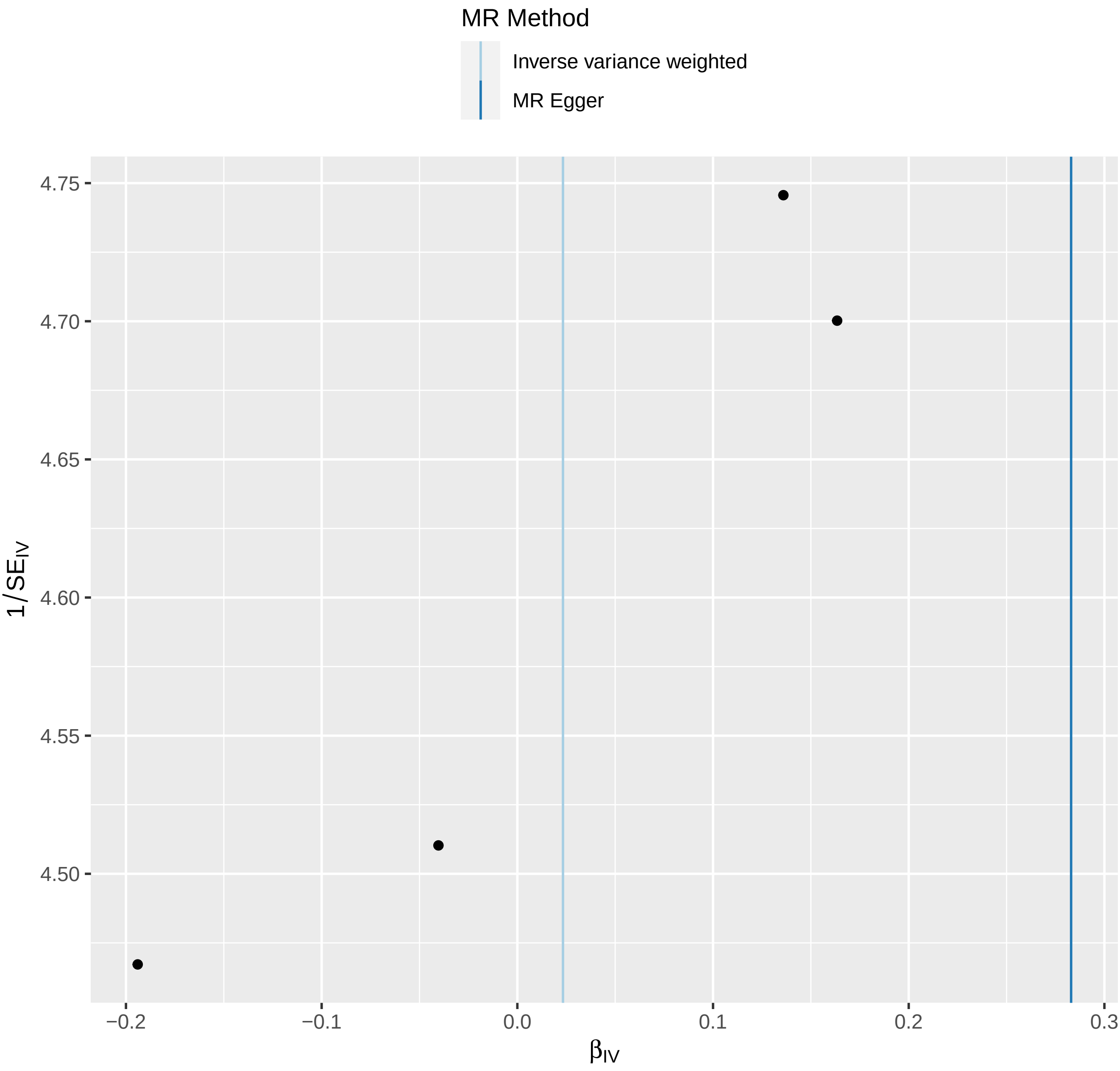

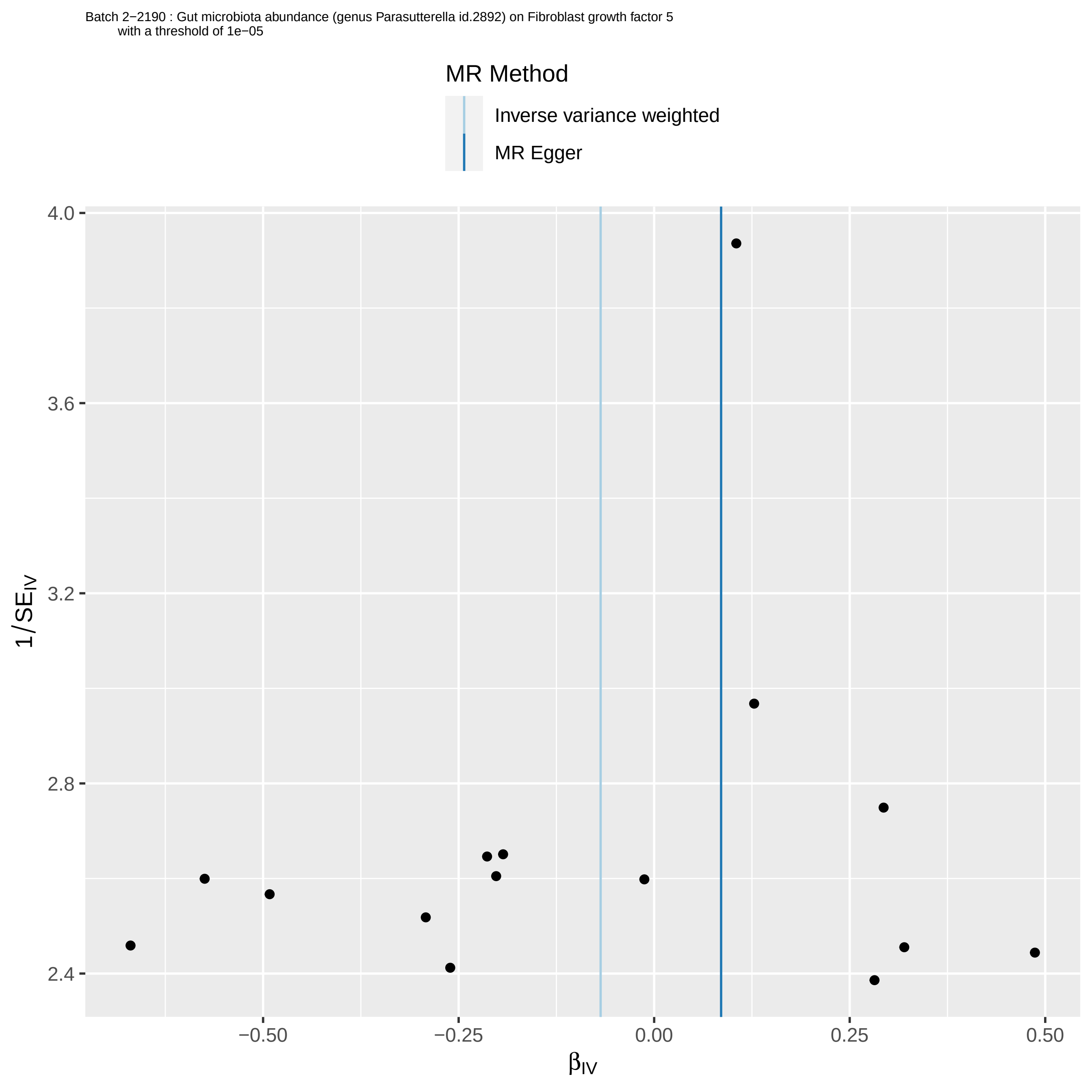

Batch 2−2190 : Gut microbiota abundance (genus Parasutterella id.2892) on Fibroblast growth factor 5
with a threshold of 1e−05
MR Method
Inverse variance weighted
MR Egger
4.0
3.6
3.2
2.8
2.4
1/SE_IV
−0.50
−0.25
0.00
0.25
0.50
β_IV

Batch 2−2191 : Gut microbiota abundance (genus Catenibacterium id.2153) on Fibroblast growth factor 6
with a threshold of 1e−05

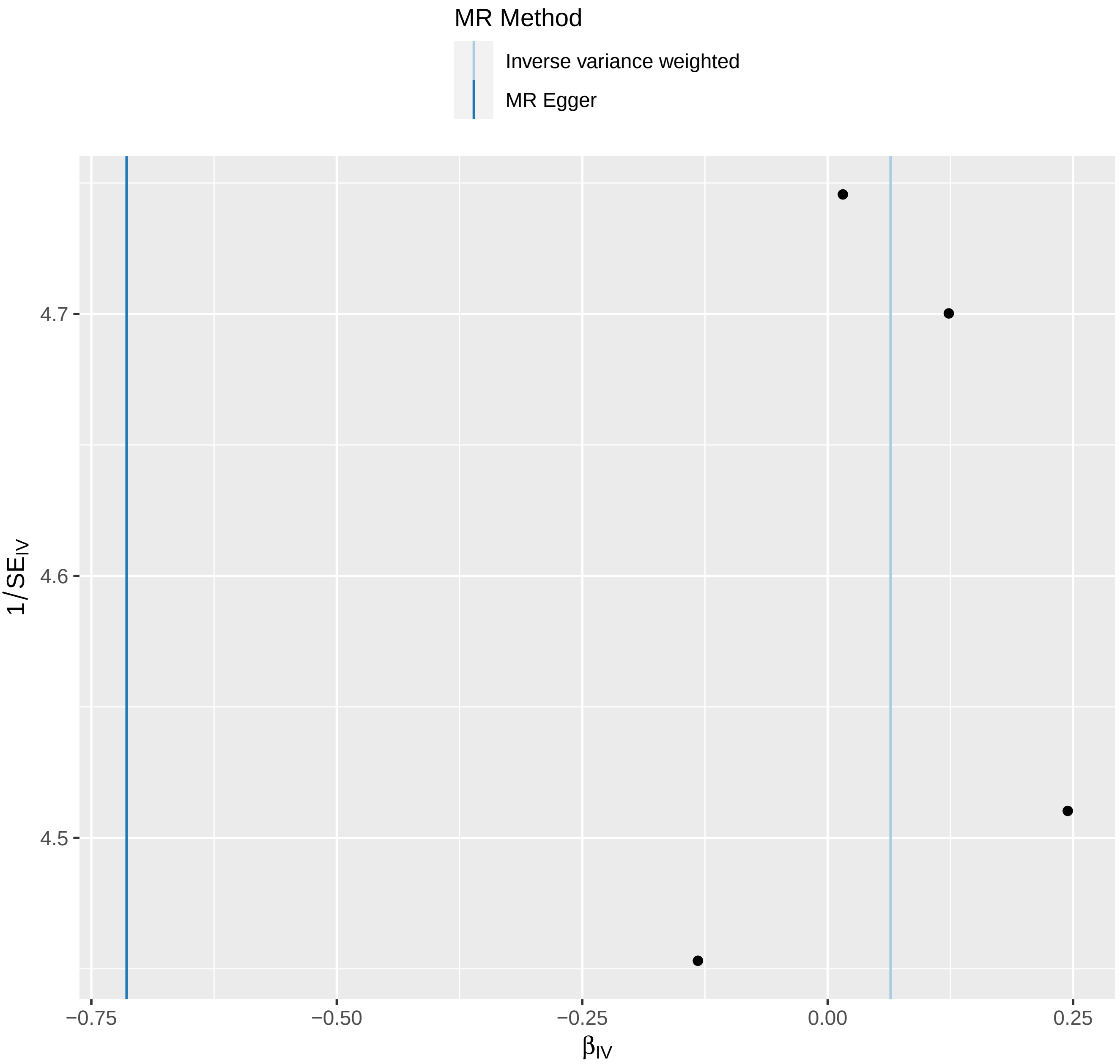

Batch 2−2192 : Gut microbiota abundance (genus Parasutterella id.2892) on Fibroblast growth factor 6
with a threshold of 1e−05

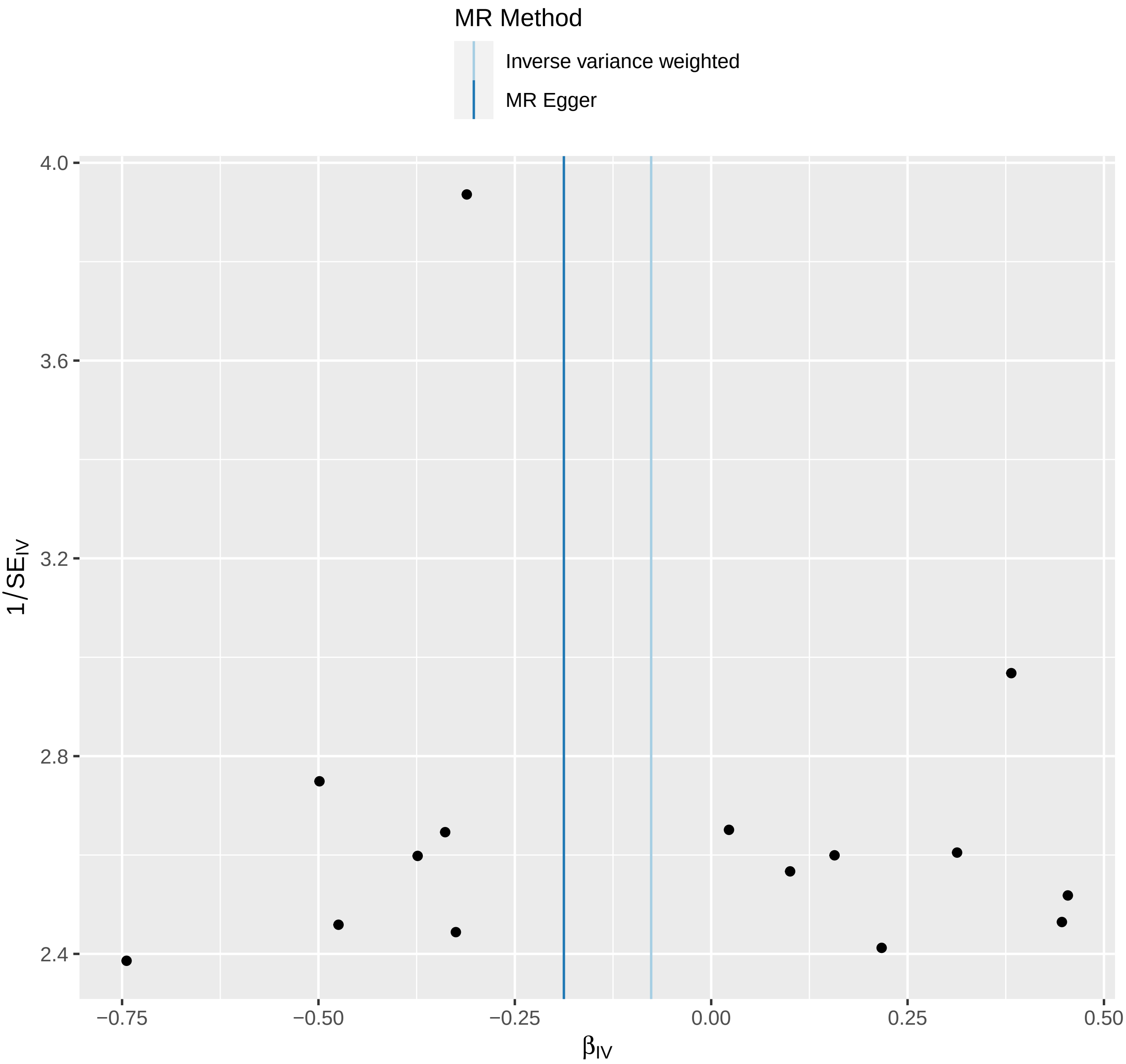

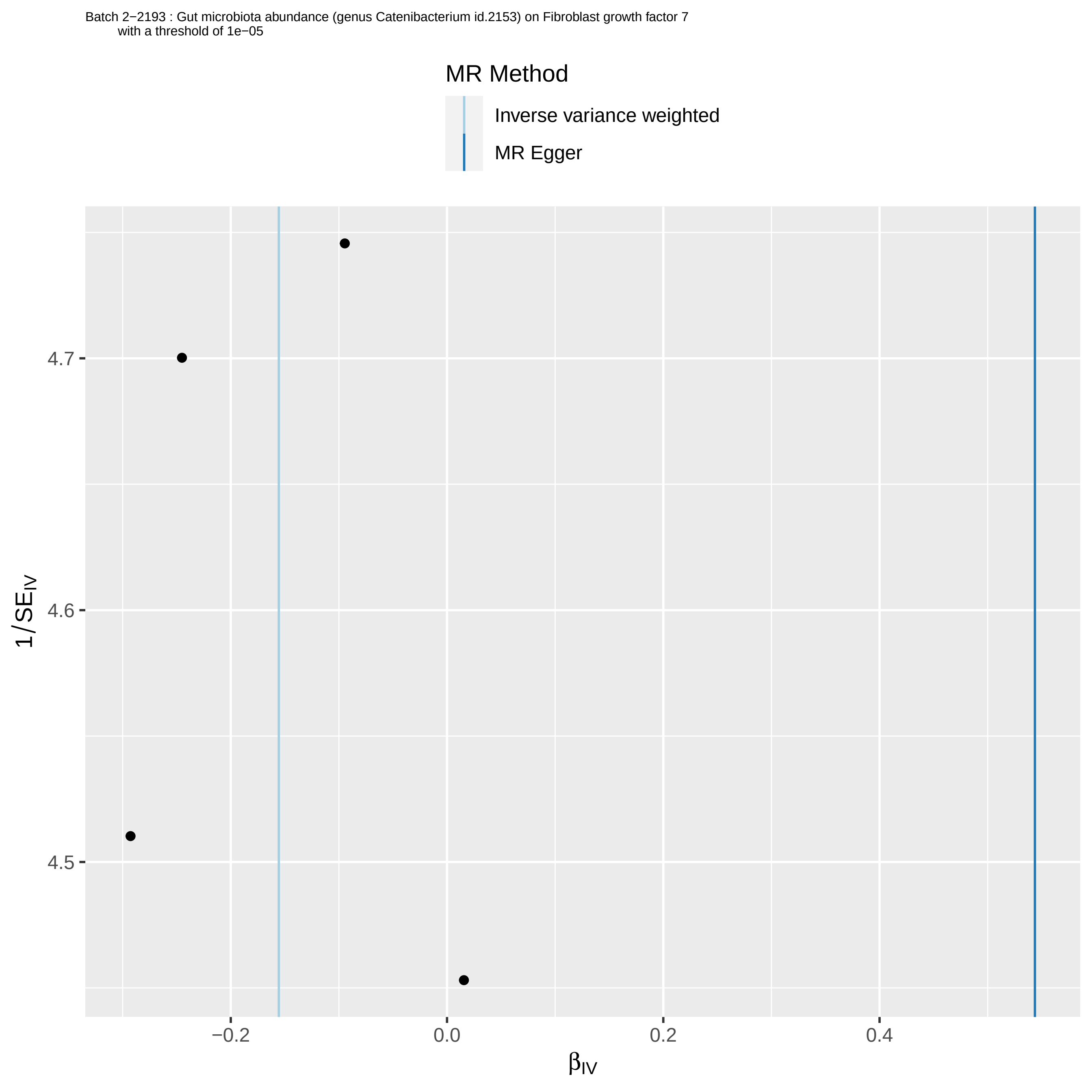
Batch 2−2193 : Gut microbiota abundance (genus Catenibacterium id.2153) on Fibroblast growth factor 7
with a threshold of 1e−05
MR Method
Inverse variance weighted
MR Egger
4.7
4.6
4.5
1/SE_IV
−0.2
0.0
0.2
0.4
β_IV

Batch 2−2194 : Gut microbiota abundance (genus Parasutterella id.2892) on Fibroblast growth factor 7
with a threshold of 1e−05

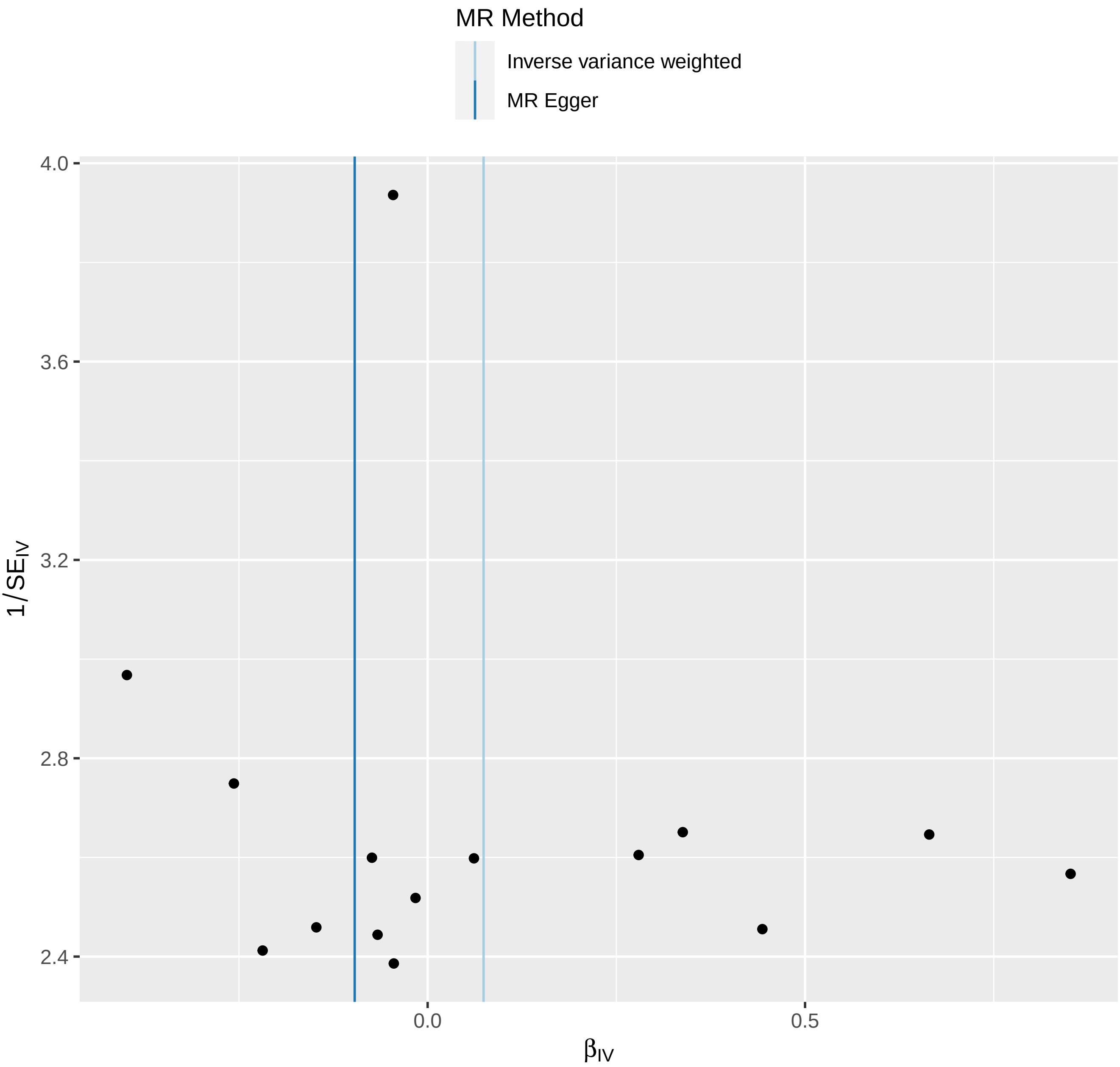

Batch 2−2195 : Gut microbiota abundance (genus Catenibacterium id.2153) on Fibroblast growth factor 7
with a threshold of 1e−05

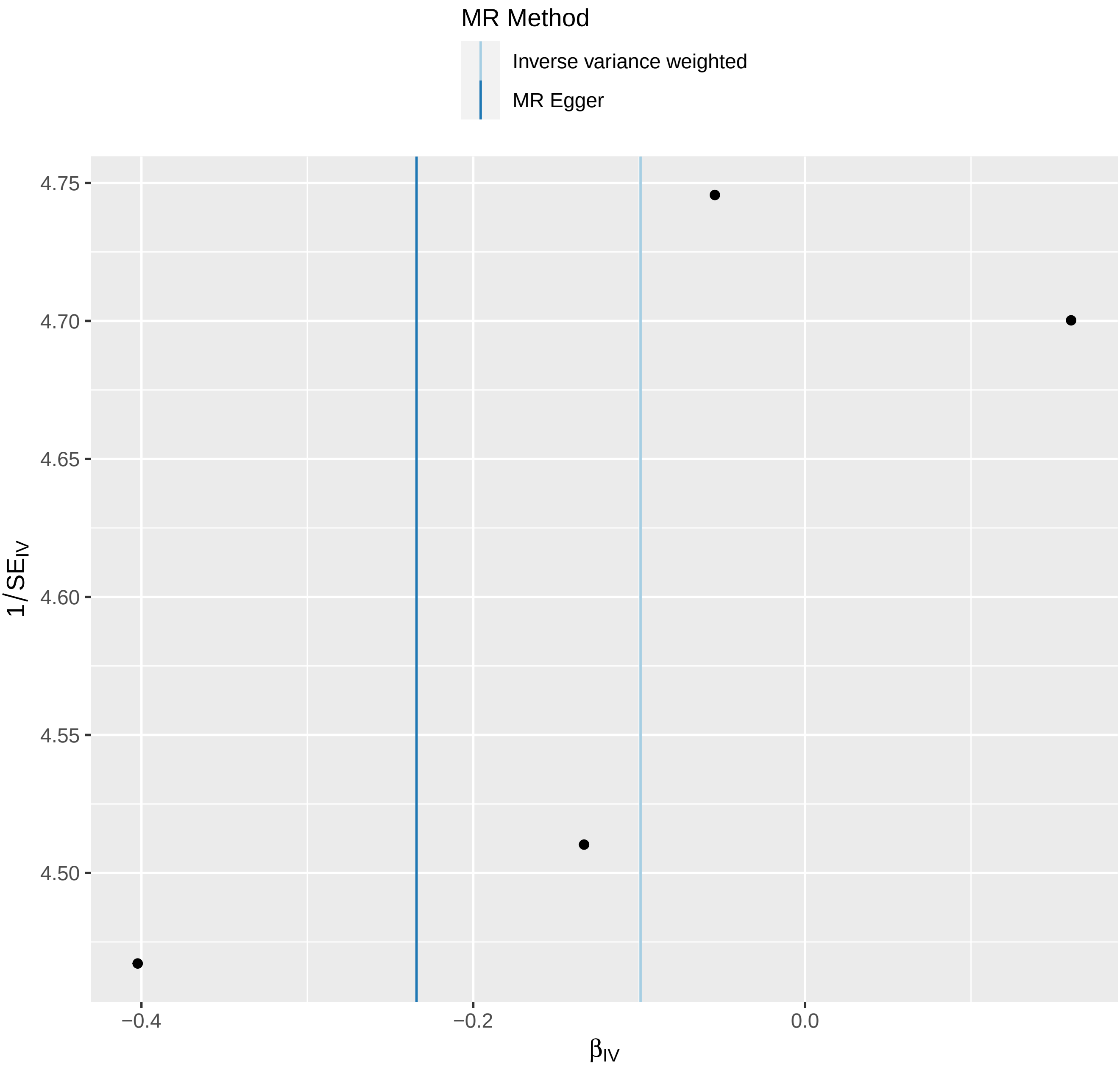

Batch 2−2196 : Gut microbiota abundance (genus Parasutterella id.2892) on Fibroblast growth factor 7
with a threshold of 1e−05

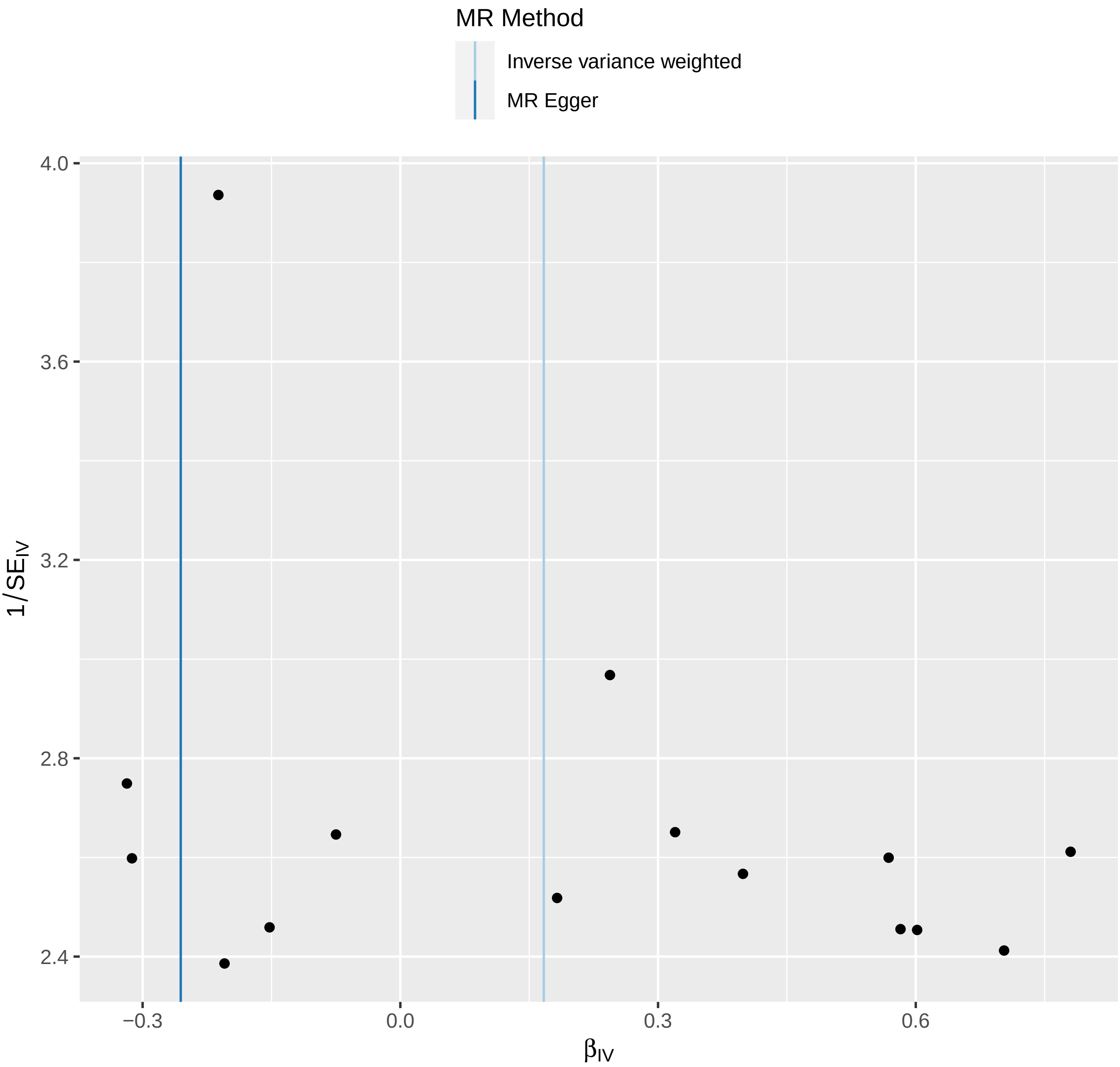

Batch 2−2197 : Gut microbiota abundance (genus Catenibacterium id.2153) on Fibroblast growth factor 8 isoform B
with a threshold of 1e−05

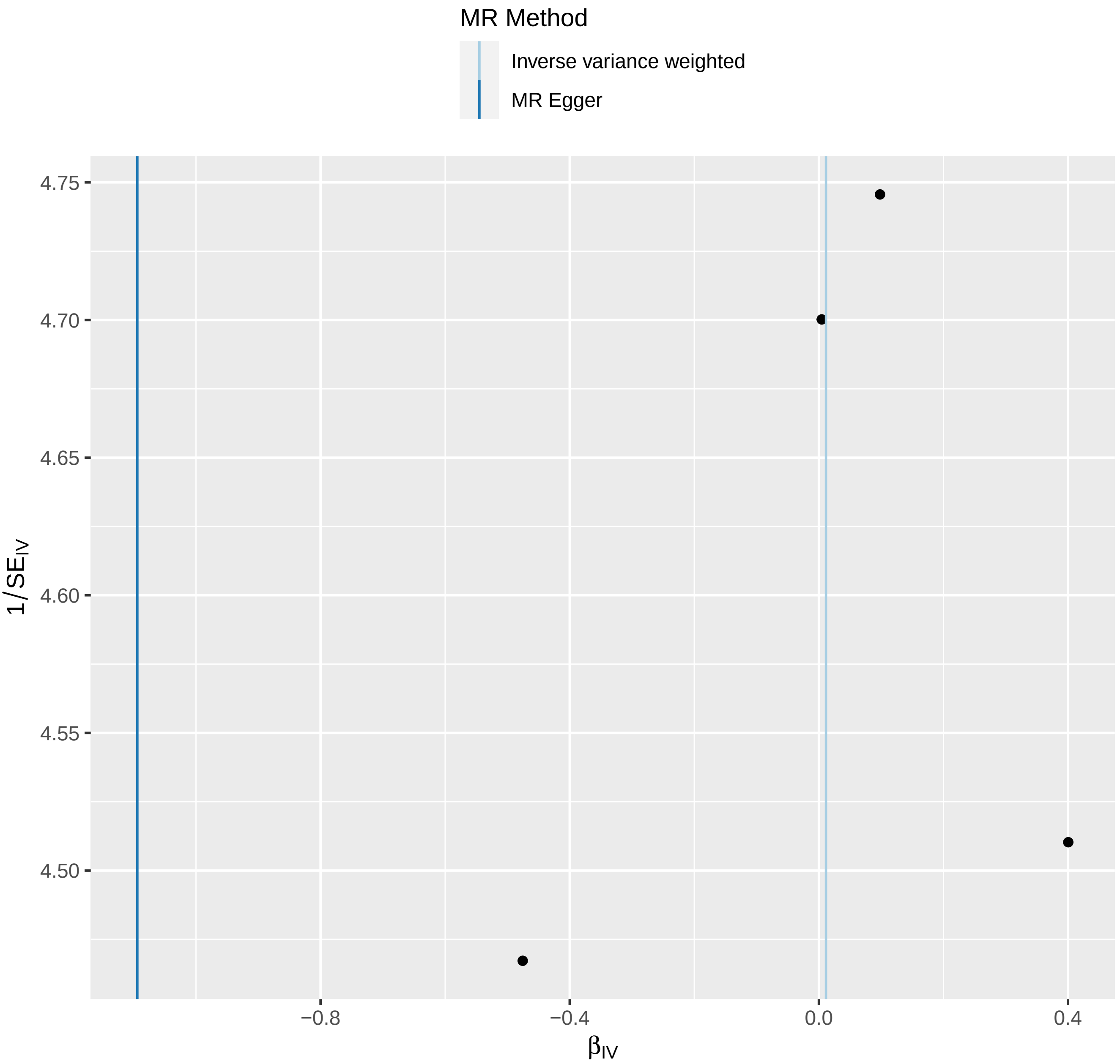

Batch 2−2198 : Gut microbiota abundance (genus Parasutterella id.2892) on Fibroblast growth factor 8 isoform B
with a threshold of 1e−05

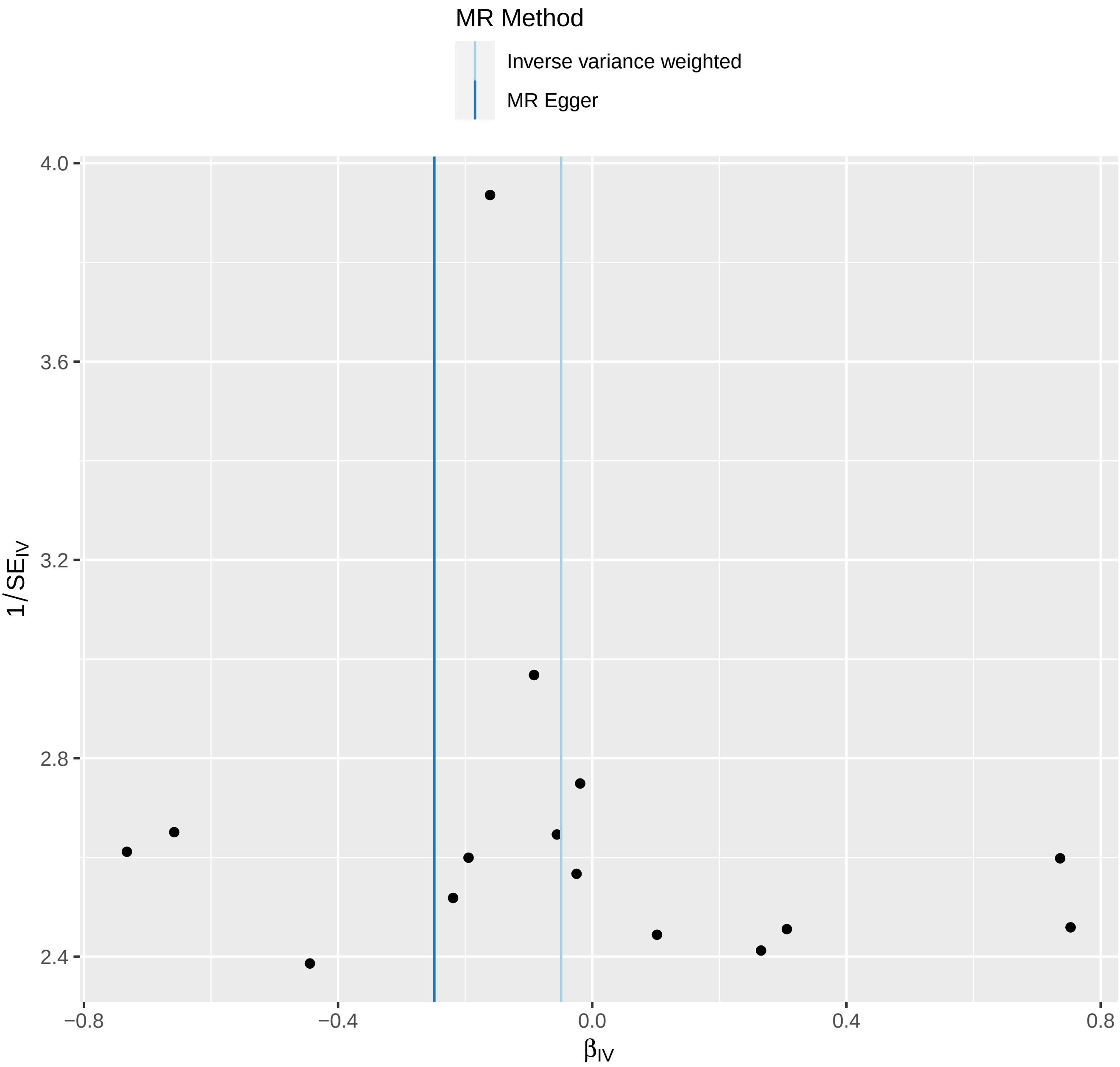

Batch 2−2199 : Gut microbiota abundance (genus Catenibacterium id.2153) on Fibroblast growth factor 8 isoform A
with a threshold of 1e−05

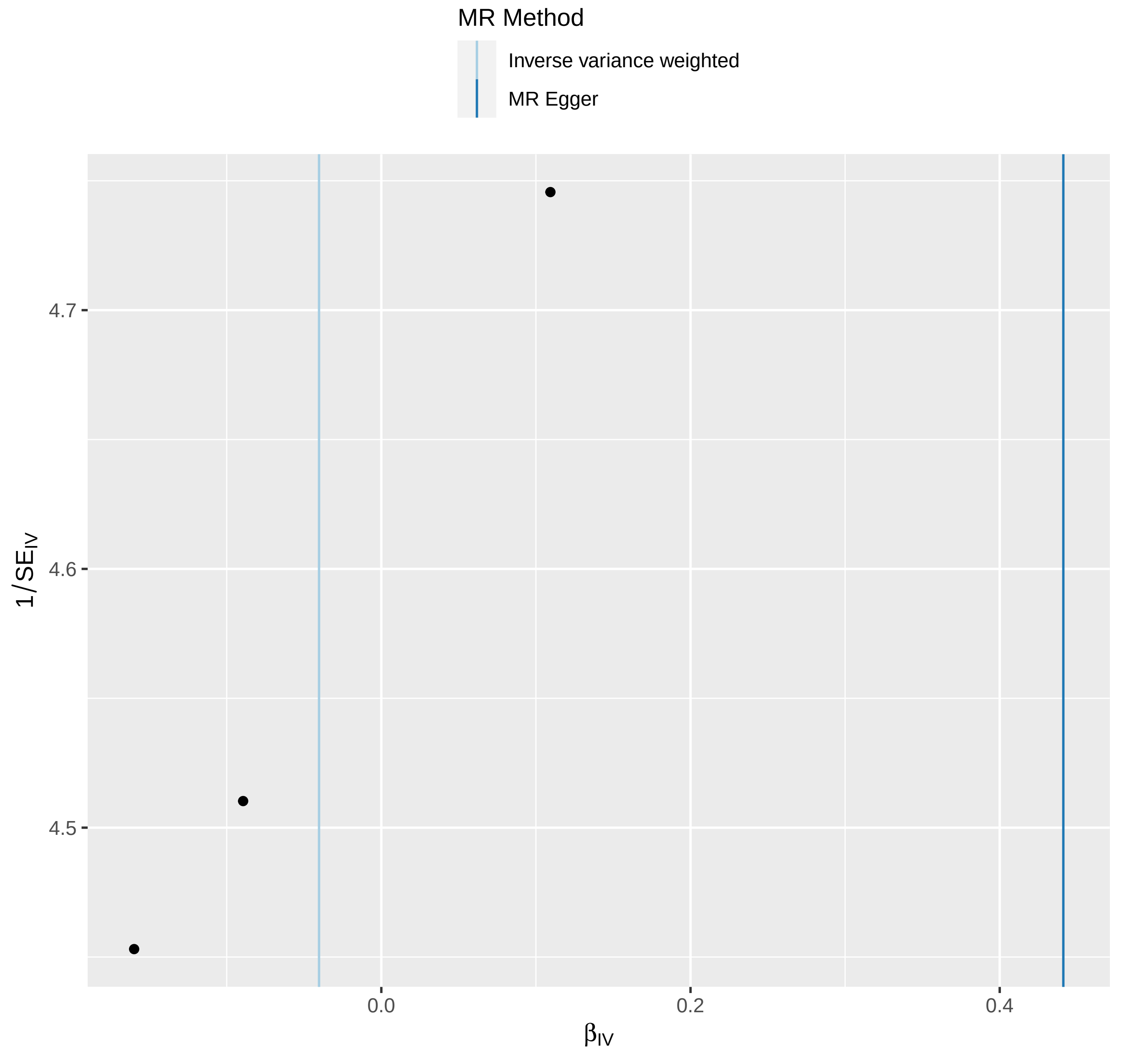

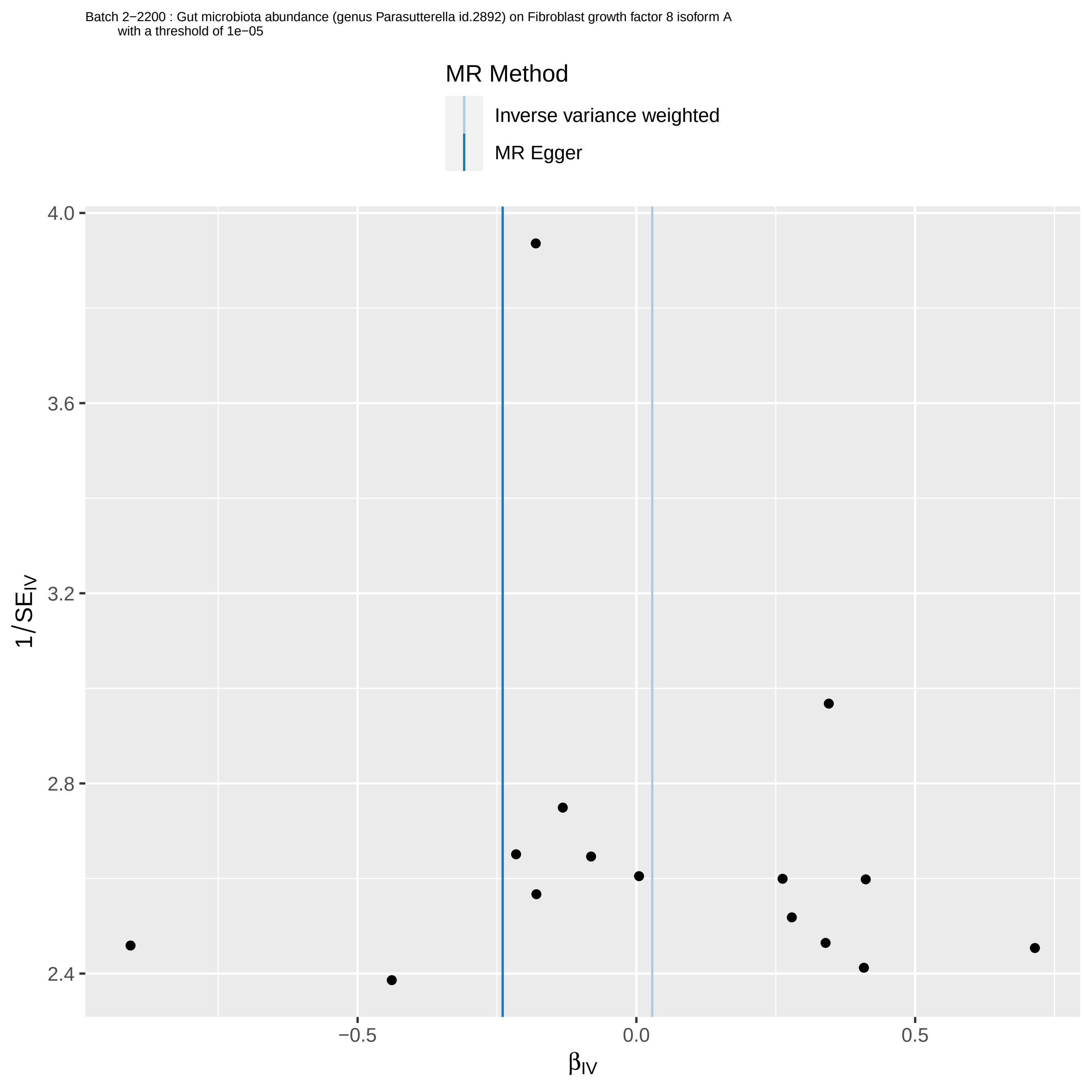
Batch 2−2200 : Gut microbiota abundance (genus Parasutterella id.2892) on Fibroblast growth factor 8 isoform A
with a threshold of 1e−05
MR Method
Inverse variance weighted
MR Egger
4.0
3.6
3.2
2.8
2.4
1/SE$_{IV}$
−0.5
0.0
0.5
β$_{IV}$

Batch 2−2201 : Gut microbiota abundance (genus Catenibacterium id.2153) on Fibroblast growth factor 9
with a threshold of 1e−05

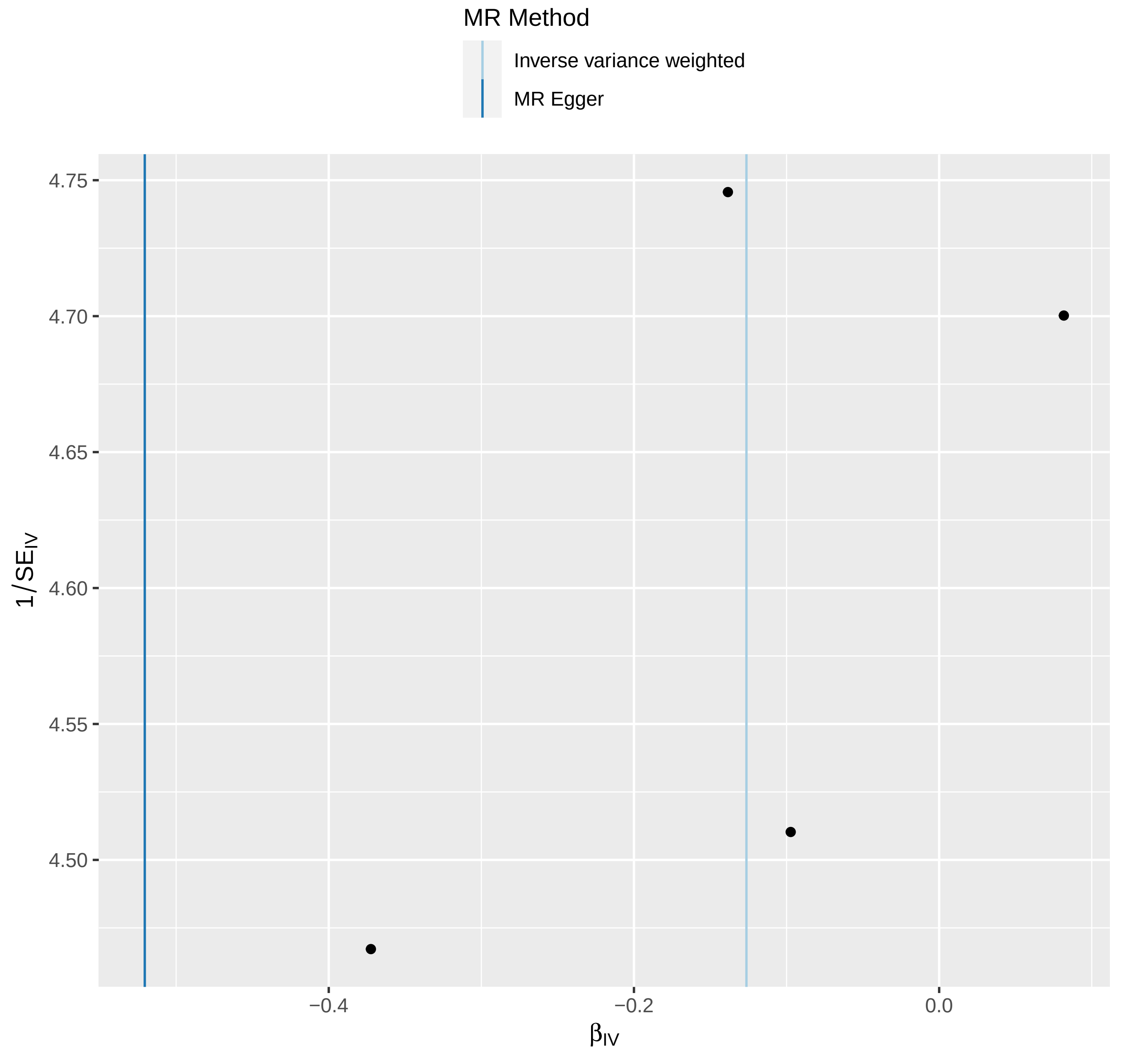

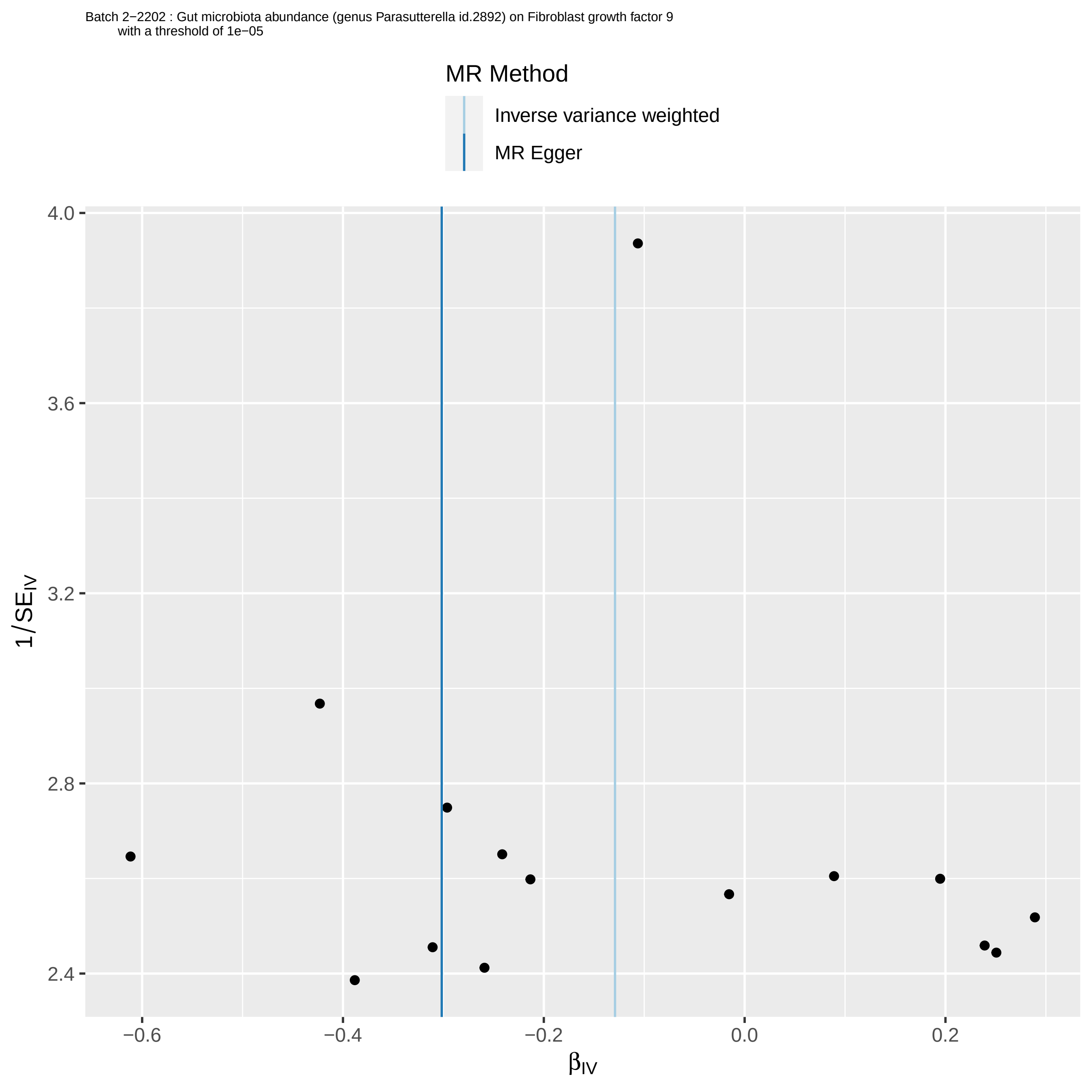

Batch 2−2202 : Gut microbiota abundance (genus Parasutterella id.2892) on Fibroblast growth factor 9
with a threshold of 1e−05
MR Method
Inverse variance weighted
MR Egger
4.0
3.6
3.2
2.8
2.4
1/SE_IV
−0.6
−0.4
−0.2
0.0
0.2
β_IV

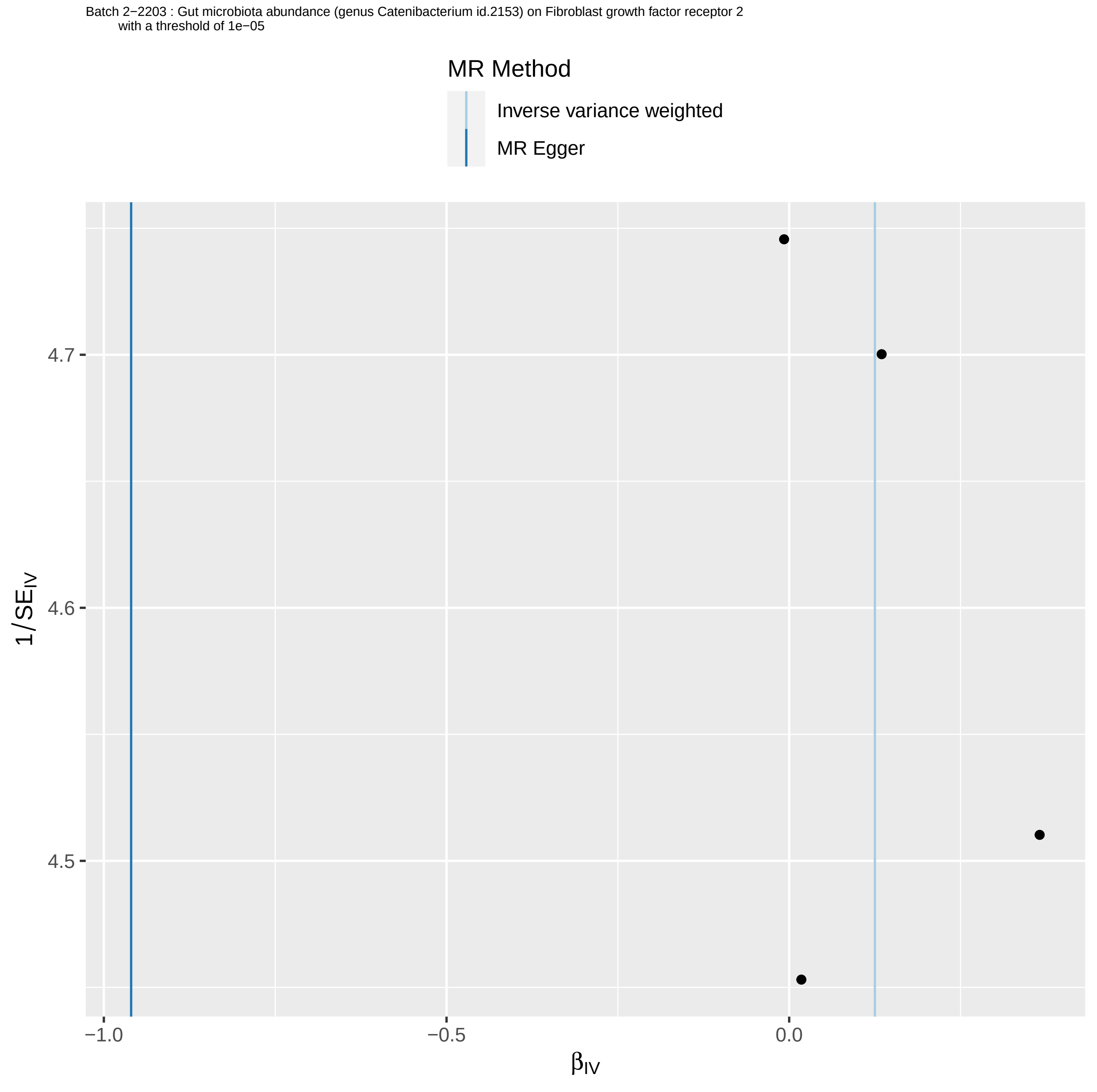
Batch 2−2203 : Gut microbiota abundance (genus Catenibacterium id.2153) on Fibroblast growth factor receptor 2
with a threshold of 1e−05
MR Method
Inverse variance weighted
MR Egger
4.7
4.6
4.5
1/SE_IV
−1.0
−0.5
0.0
β_IV

Batch 2−2204 : Gut microbiota abundance (genus Parasutterella id.2892) on Fibroblast growth factor receptor 2 with a threshold of 1e−05

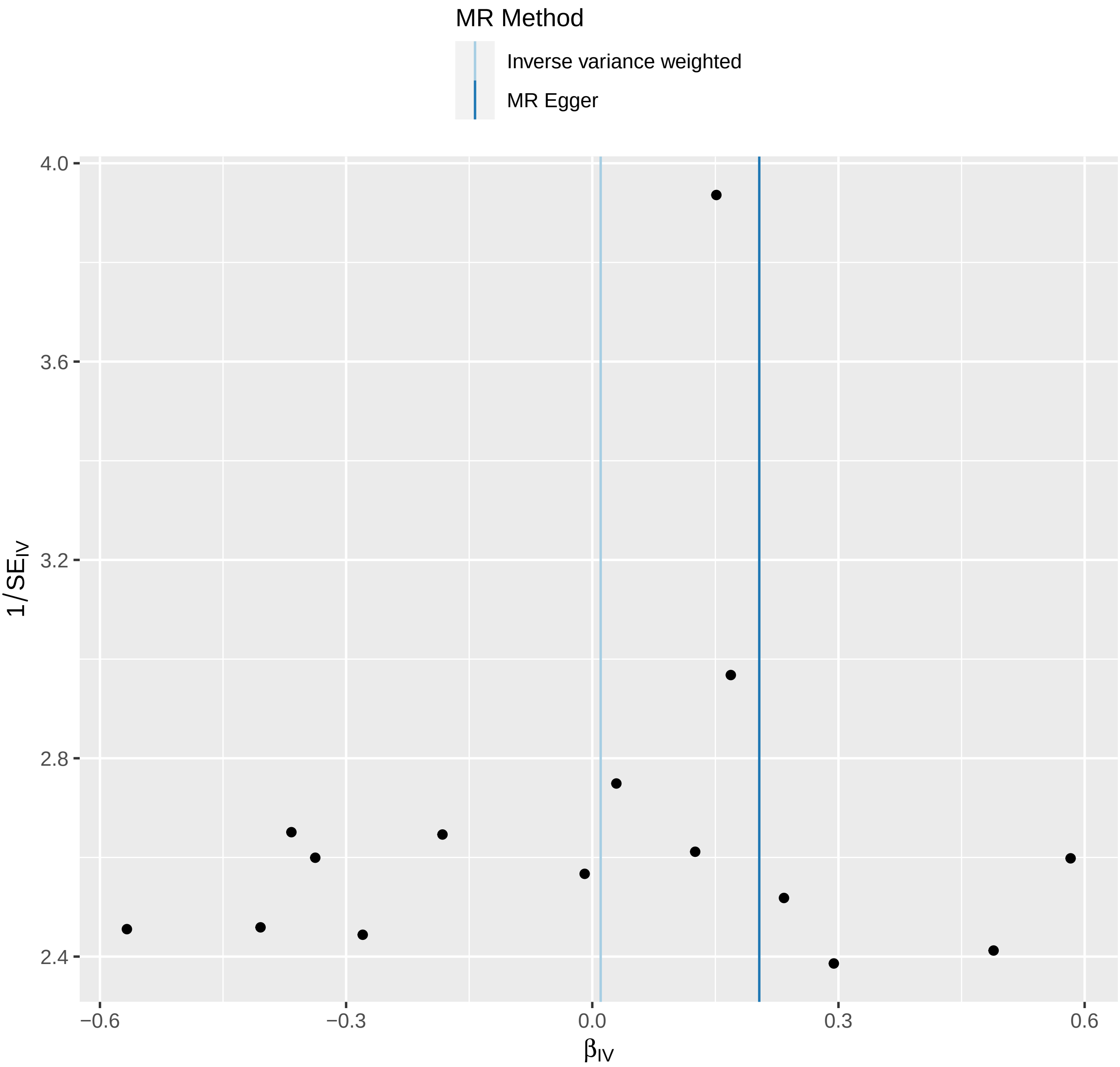

Batch 2−2205 : Gut microbiota abundance (genus Catenibacterium id.2153) on Fibroblast growth factor receptor 3
with a threshold of 1e−05

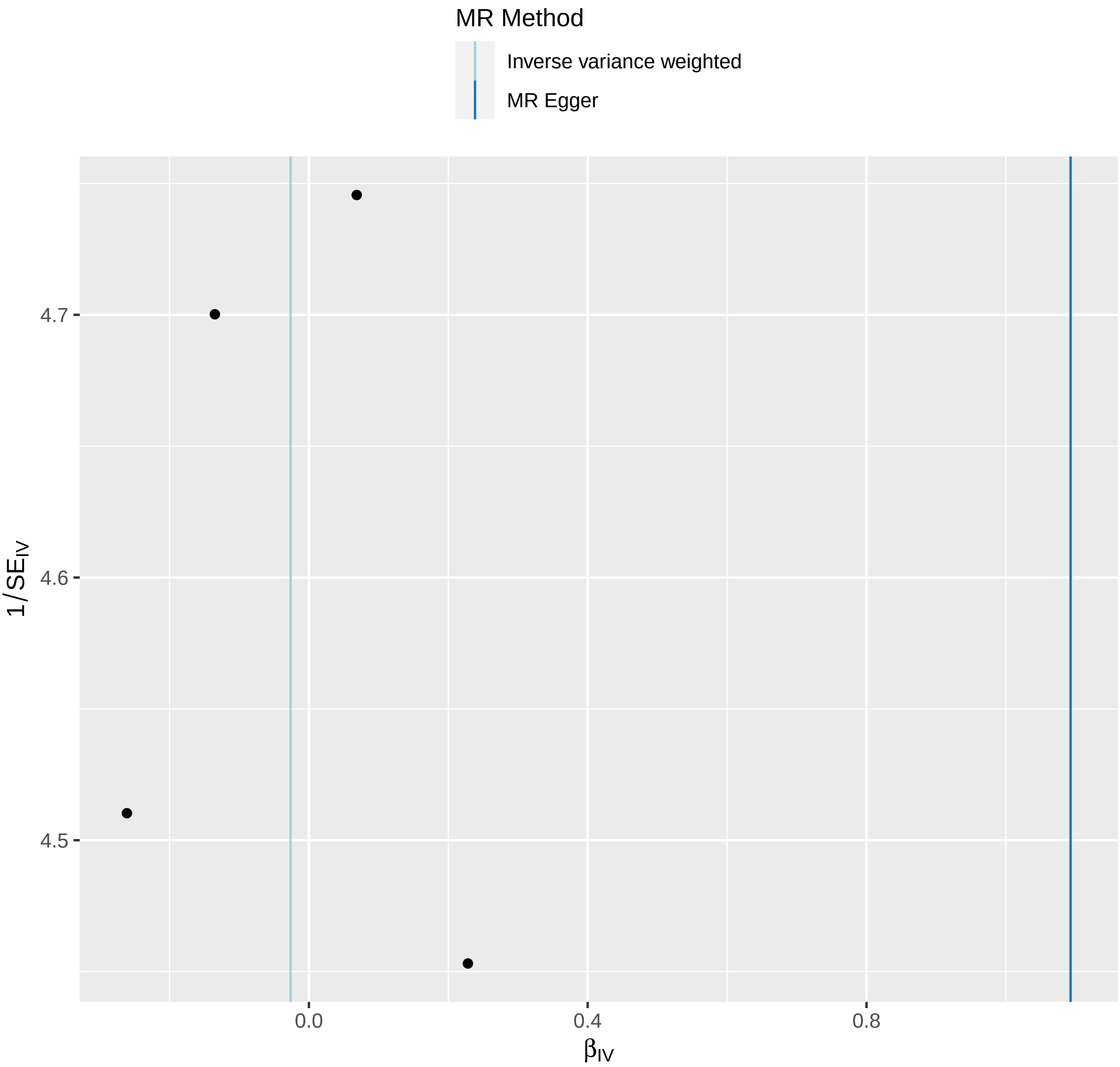

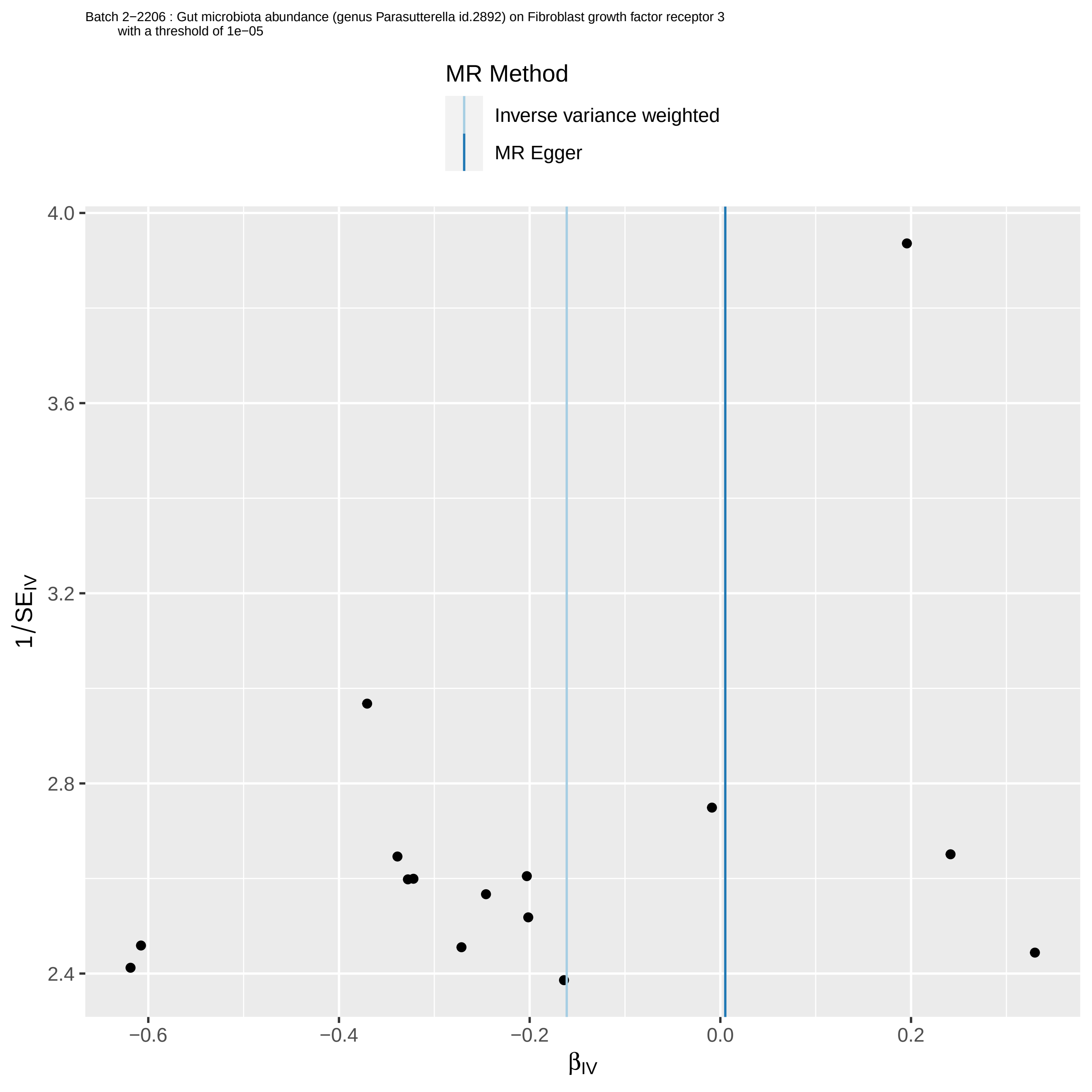
Batch 2−2206 : Gut microbiota abundance (genus Parasutterella id.2892) on Fibroblast growth factor receptor 3
with a threshold of 1e−05
MR Method
Inverse variance weighted
MR Egger
4.0
3.6
3.2
2.8
2.4
$1/SE_{IV}$
−0.6
−0.4
−0.2
0.0
0.2
β_{IV}

Batch 2−2207 : Gut microbiota abundance (genus Catenibacterium id.2153) on Fibroblast growth factor receptor 3 with a threshold of 1e−05

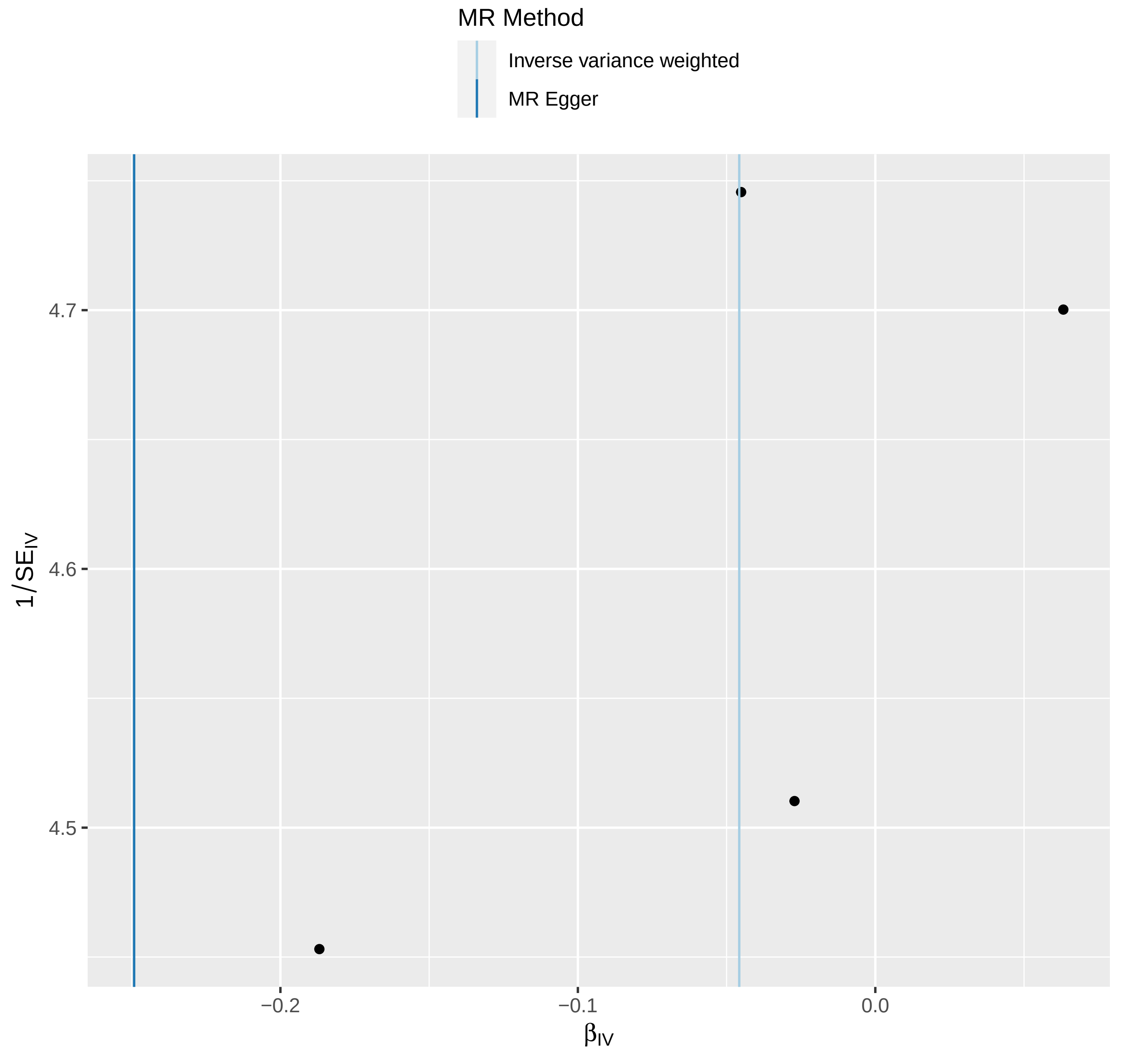

Batch 2−2208 : Gut microbiota abundance (genus Parasutterella id.2892) on Fibroblast growth factor receptor 3
with a threshold of 1e−05

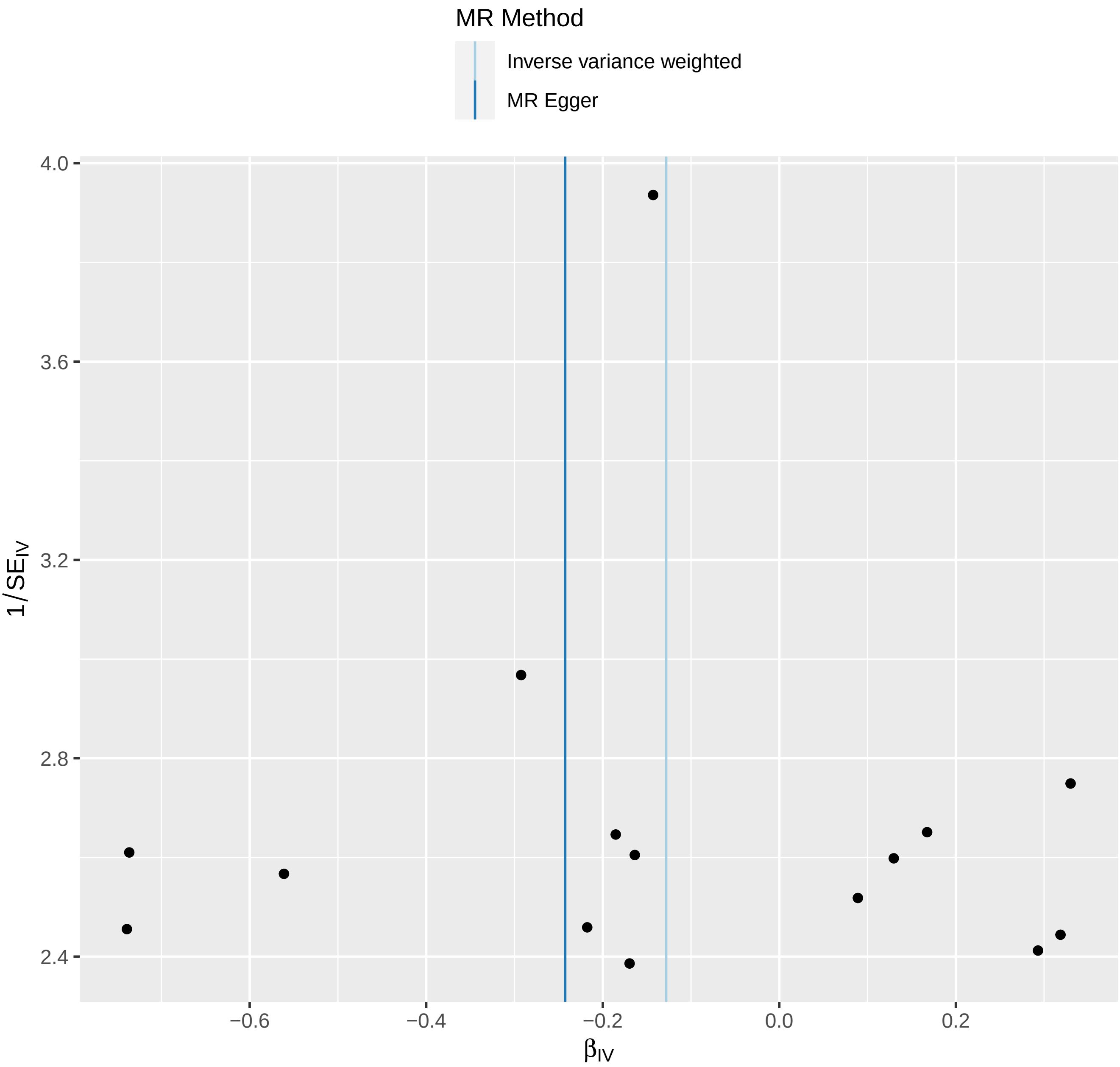

Batch 2−2209 : Gut microbiota abundance (genus Catenibacterium id.2153) on Fibroblast growth factor receptor 4
with a threshold of 1e−05

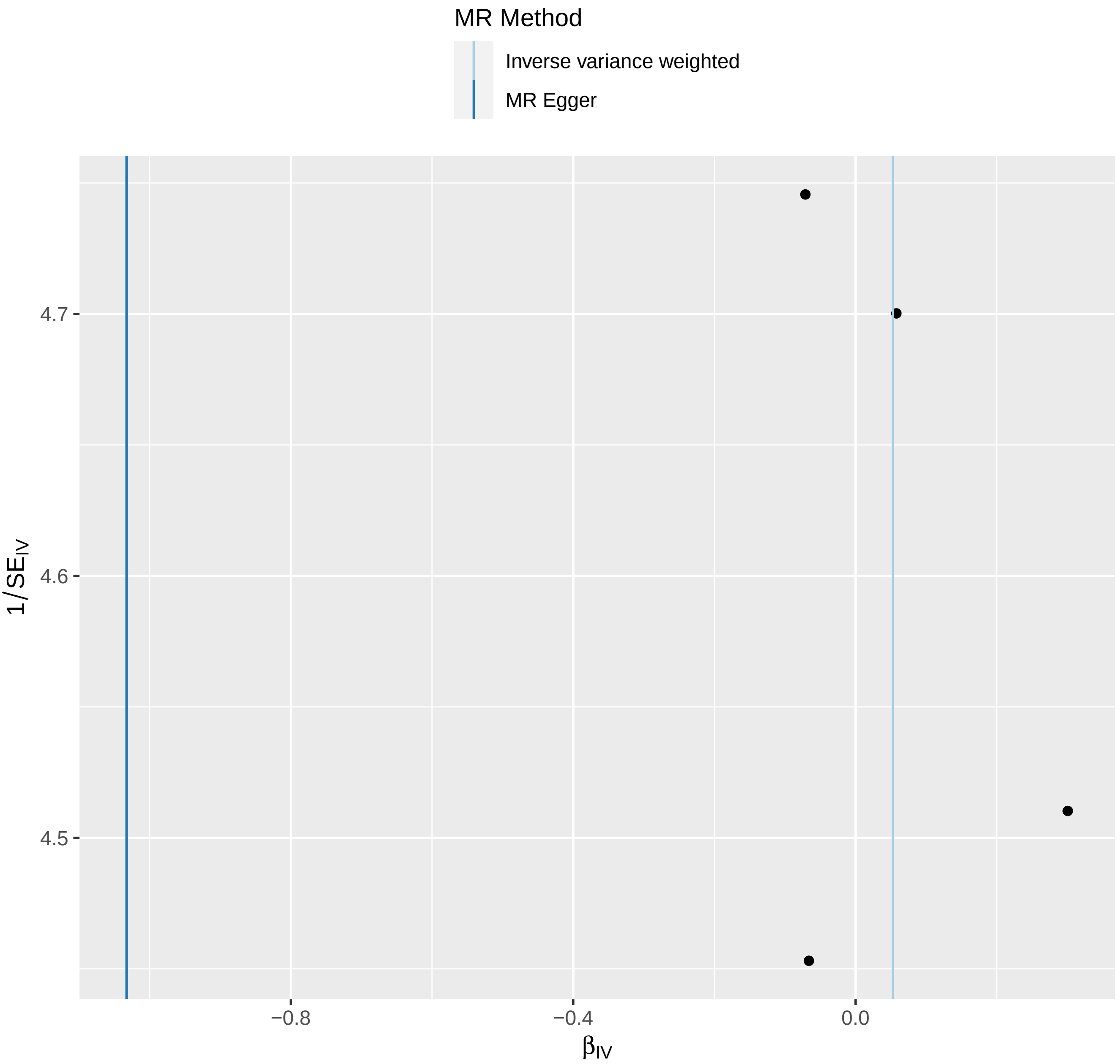

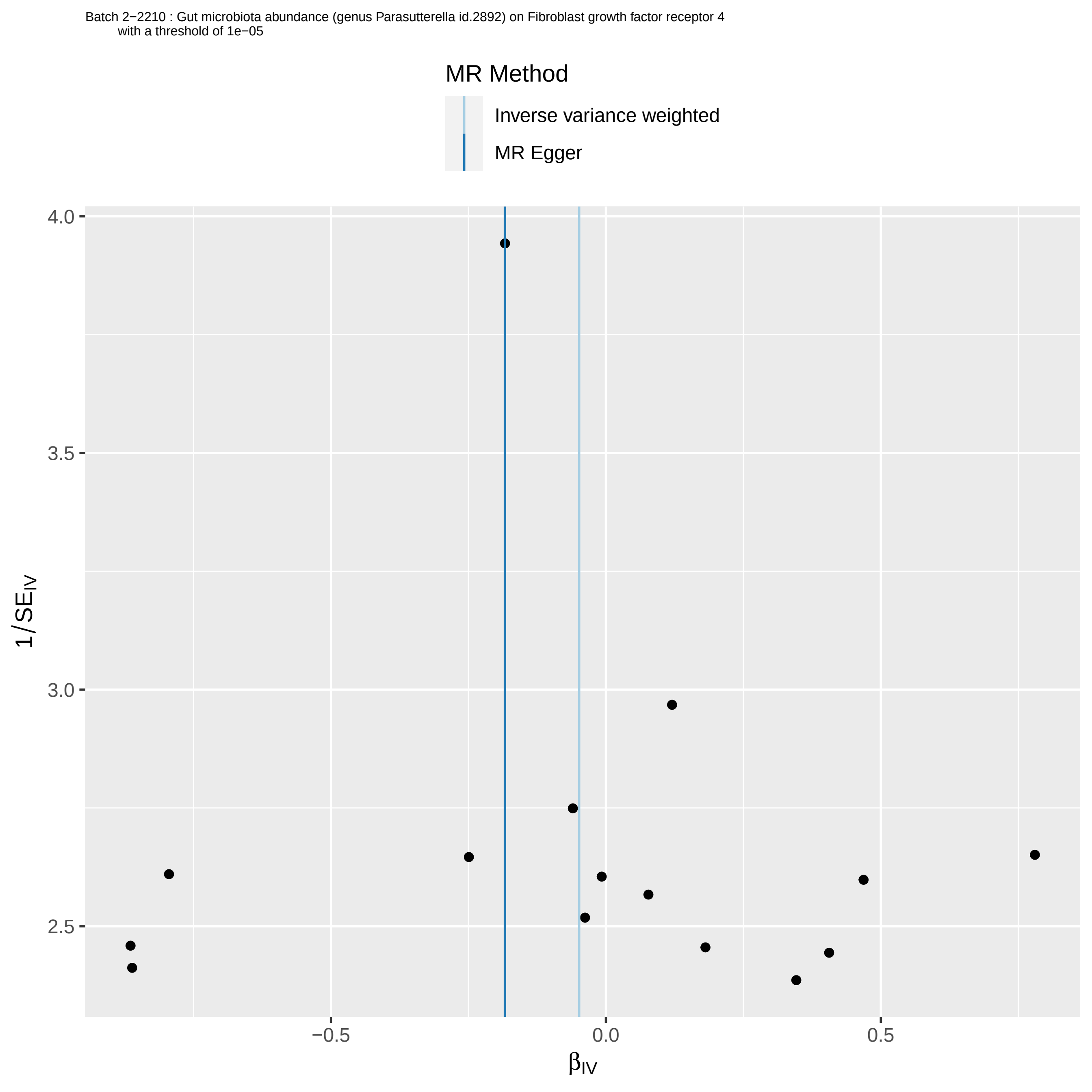
Batch 2−2210 : Gut microbiota abundance (genus Parasutterella id.2892) on Fibroblast growth factor receptor 4
with a threshold of 1e−05
MR Method
Inverse variance weighted
MR Egger
4.0
3.5
3.0
2.5
1/SE_IV
−0.5
0.0
0.5
β_IV

Batch 2−2211 : Gut microbiota abundance (genus Catenibacterium id.2153) on Tyrosine−protein kinase Fgr
with a threshold of 1e−05

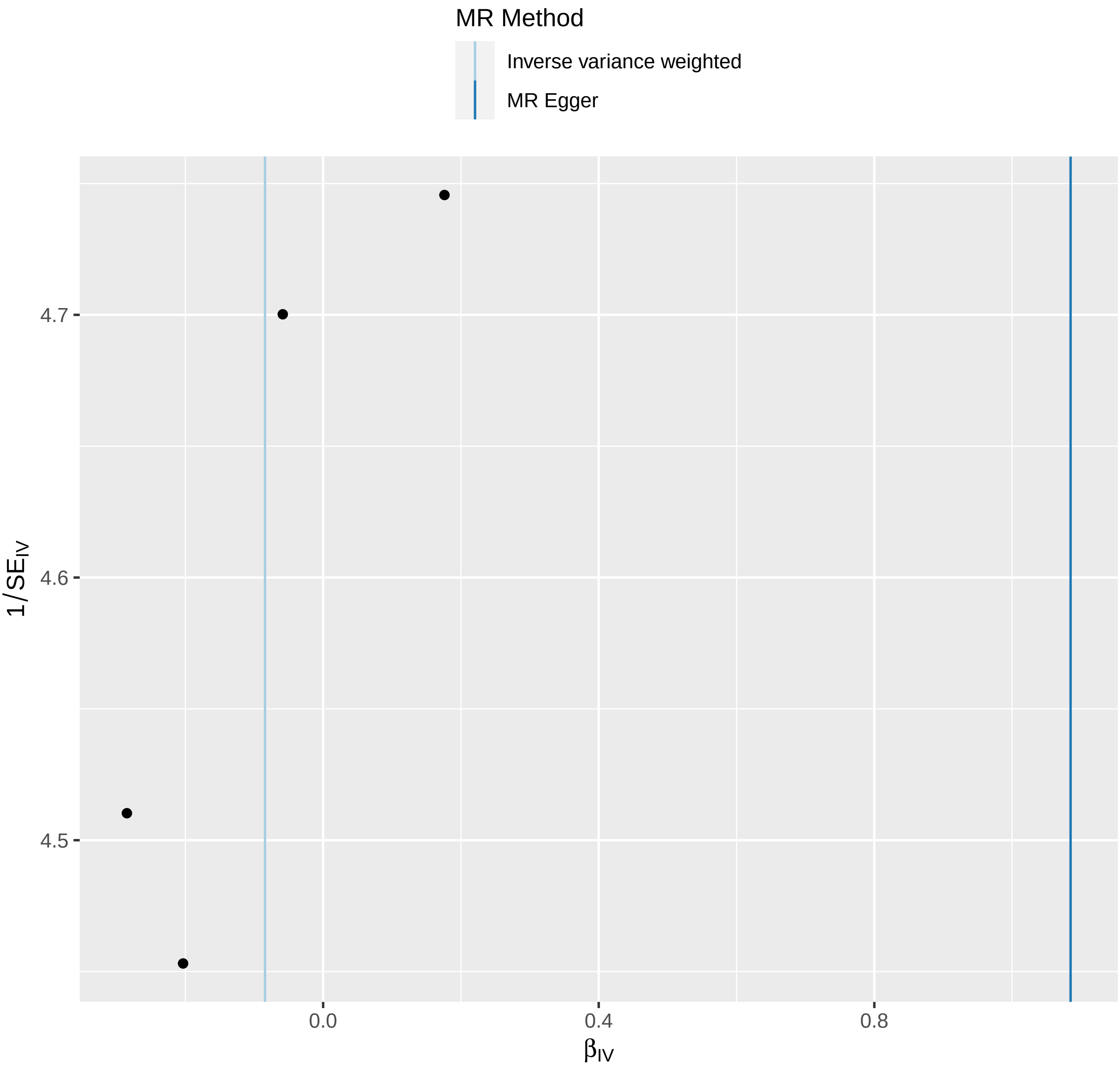

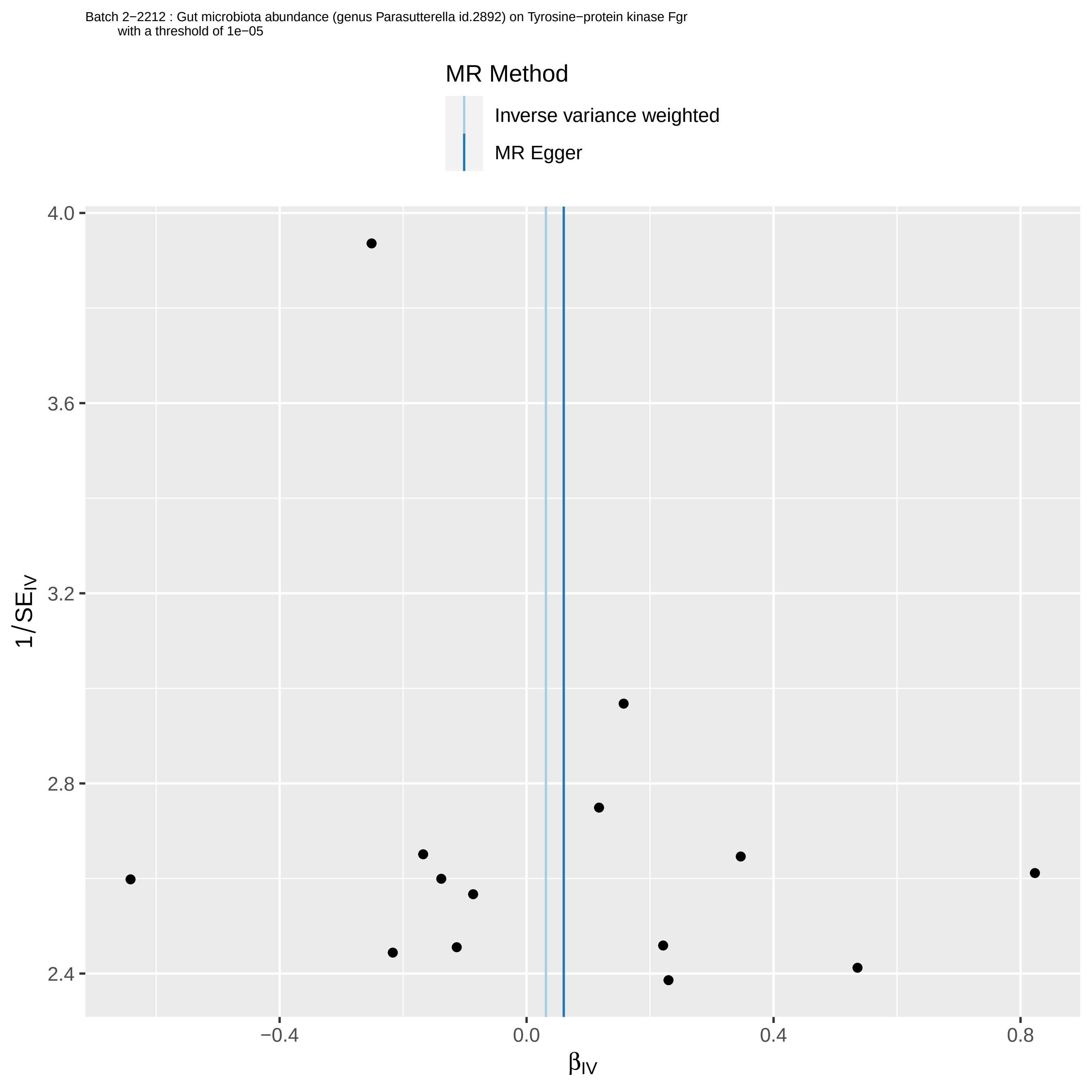

Batch 2−2212 : Gut microbiota abundance (genus Parasutterella id.2892) on Tyrosine−protein kinase Fgr
with a threshold of 1e−05
MR Method
Inverse variance weighted
MR Egger
4.0
3.6
3.2
2.8
2.4
1/SE_IV
−0.4
0.0
0.4
0.8
β_IV

Batch 2−2213 : Gut microbiota abundance (genus Catenibacterium id.2153) on Bis(5'−adenosyl)−triphosphatase
with a threshold of 1e−05

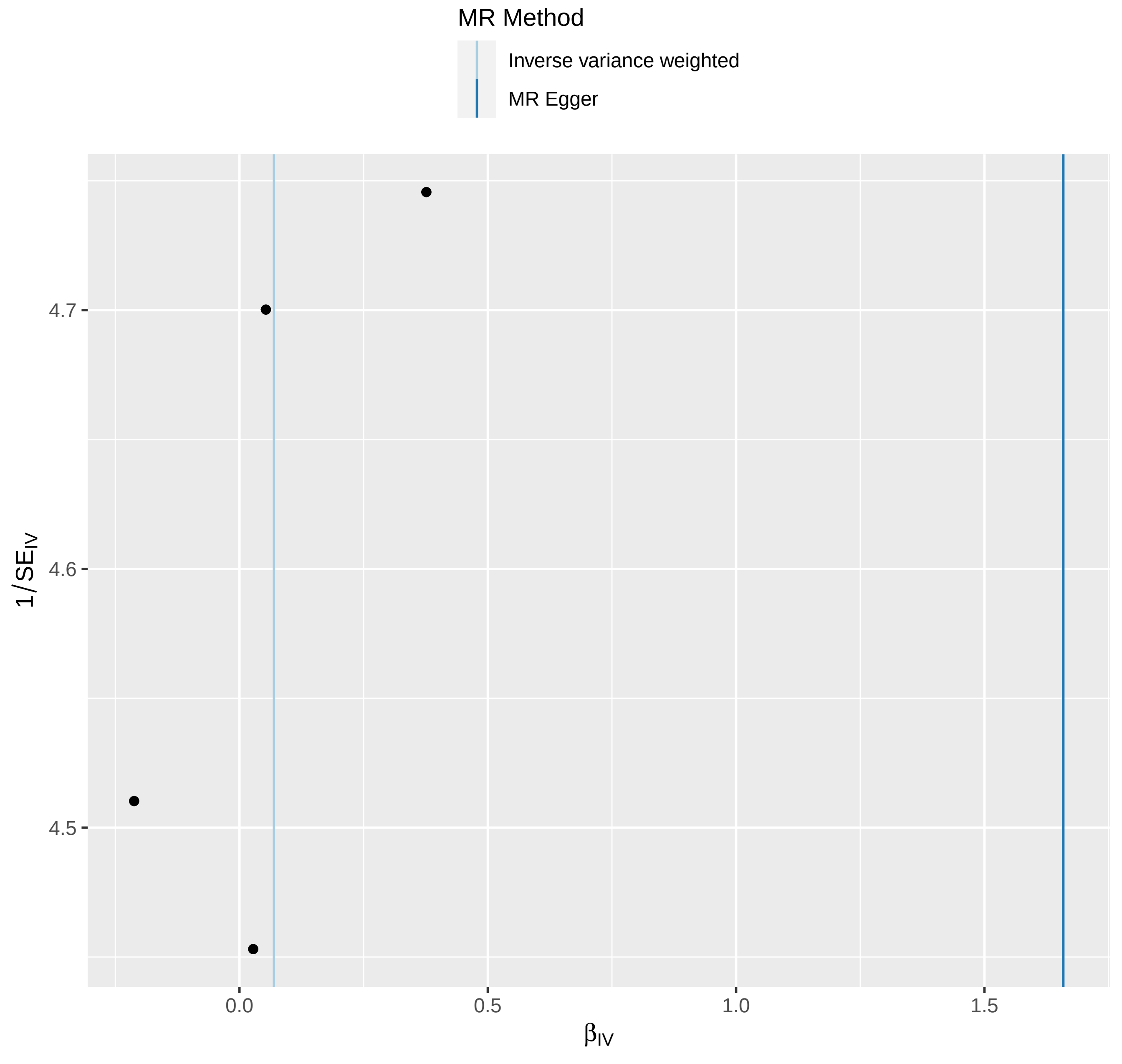

Batch 2−2214 : Gut microbiota abundance (genus Parasutterella id.2892) on Bis(5'−adenosyl)−triphosphatase with a threshold of 1e−05

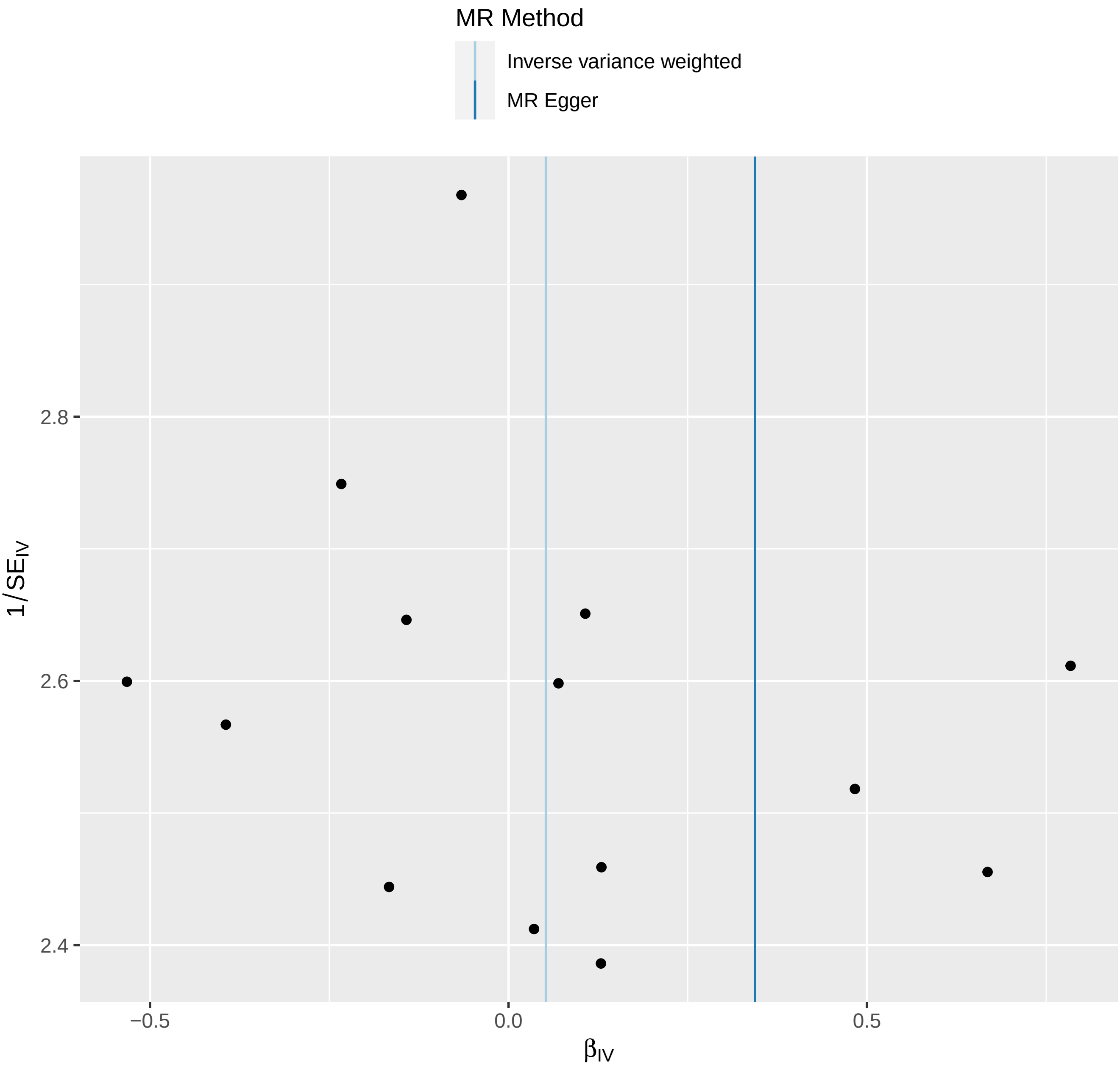

Batch 2−2215 : Gut microbiota abundance (genus Catenibacterium id.2153) on Fibrinogen C domain−containing protein 1
with a threshold of 1e−05

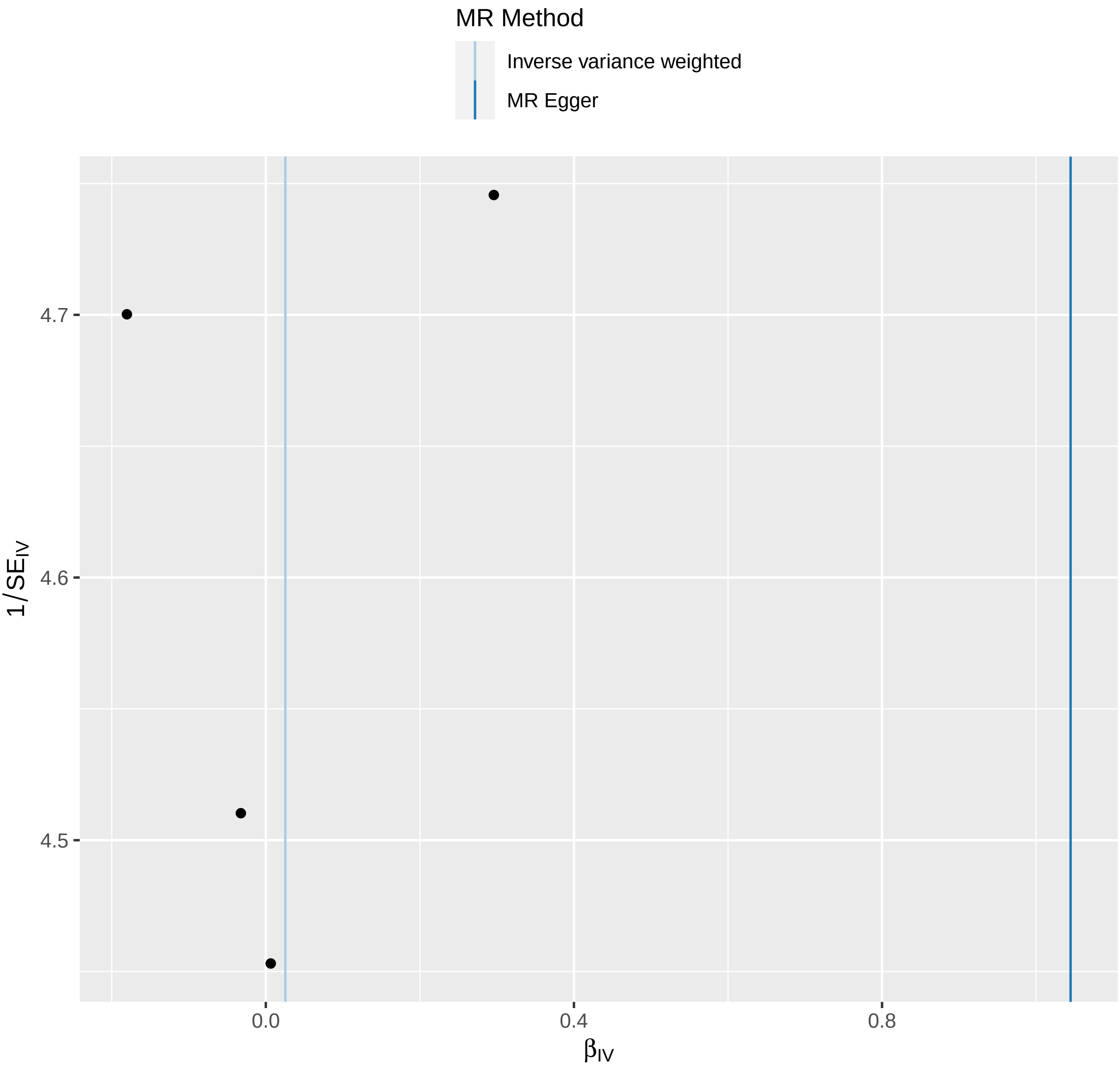

Batch 2−2216 : Gut microbiota abundance (genus Parasutterella id.2892) on Fibrinogen C domain−containing protein 1
with a threshold of 1e−05

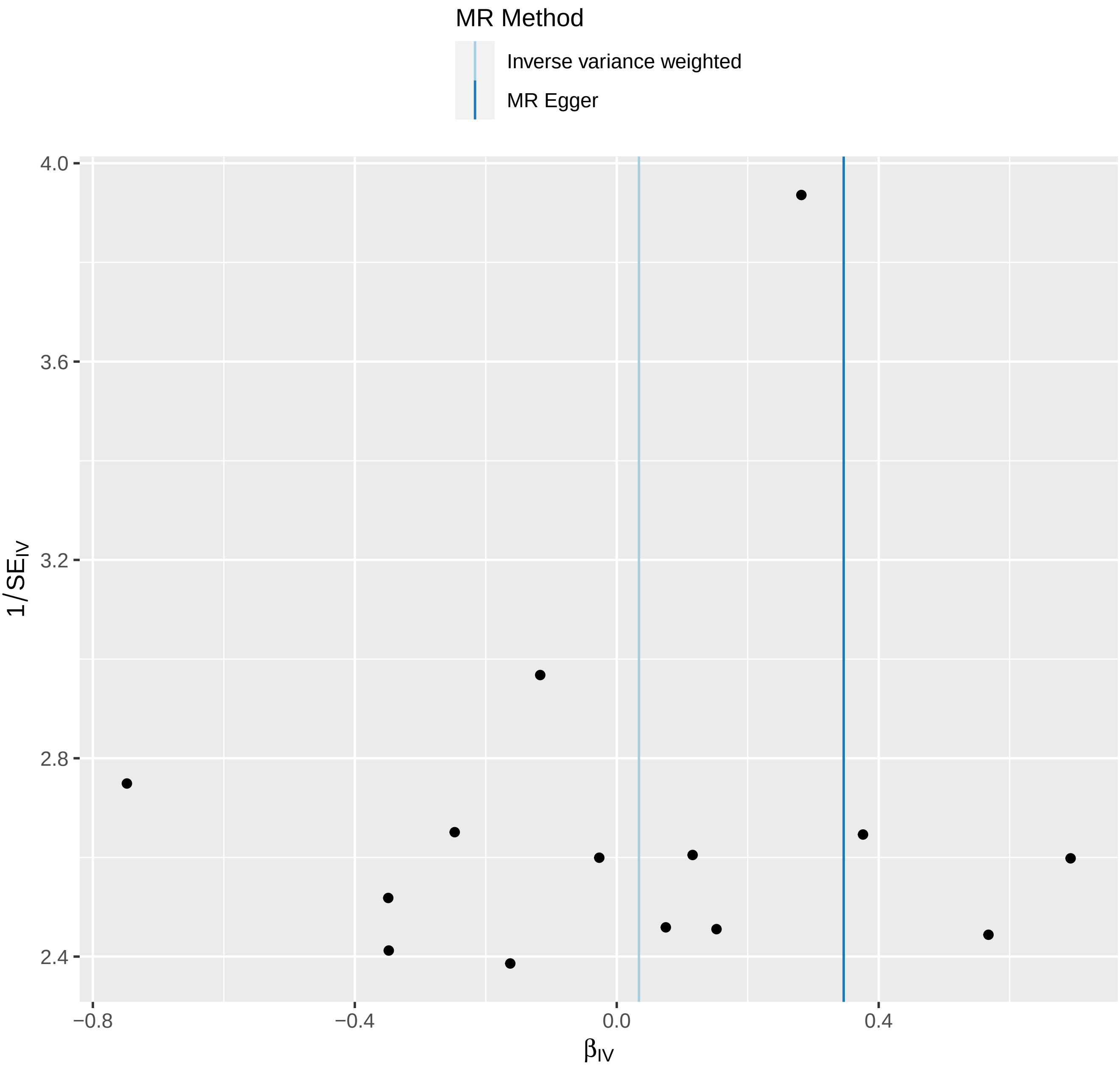

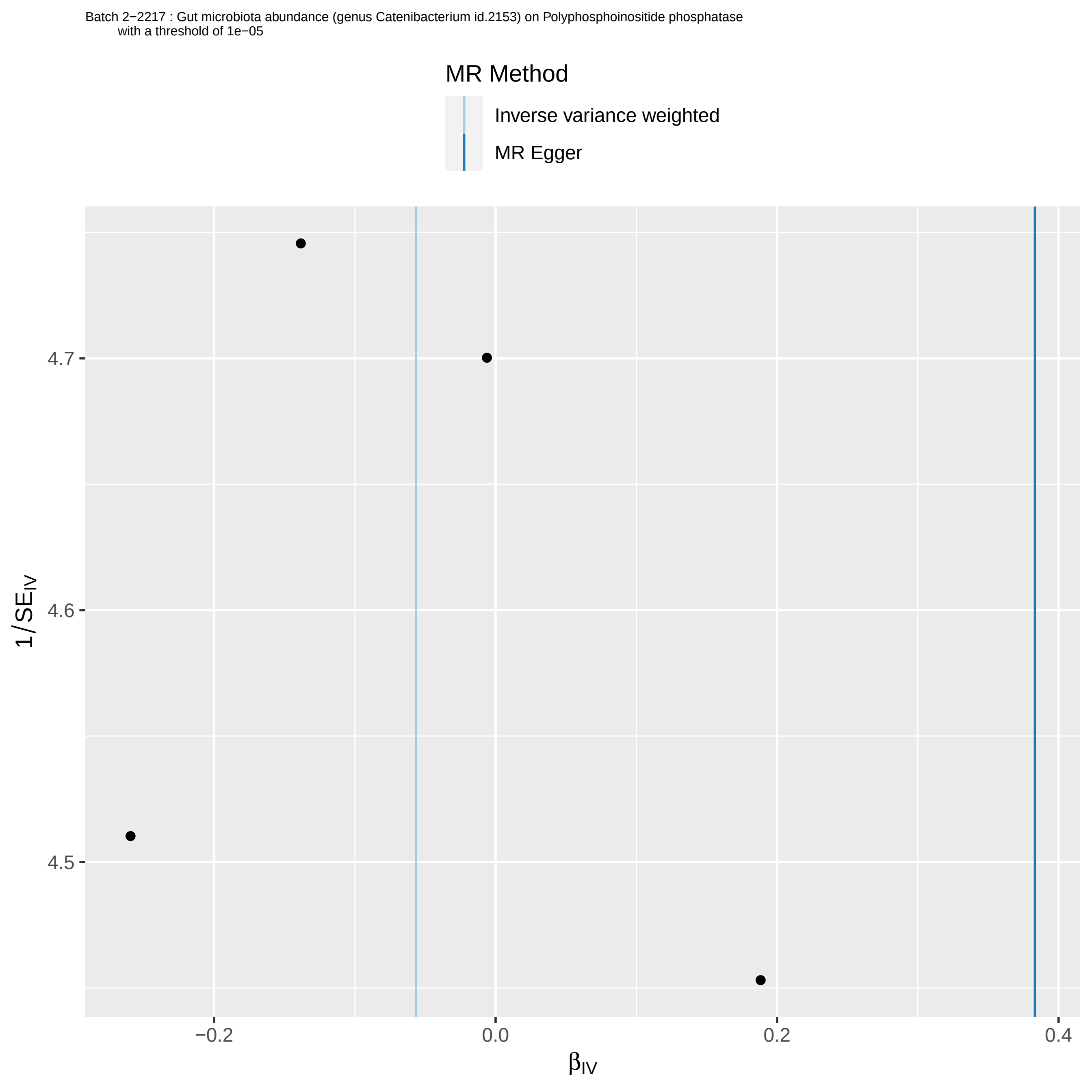

Batch 2−2217 : Gut microbiota abundance (genus Catenibacterium id.2153) on Polyphosphoinositide phosphatase
with a threshold of 1e−05
MR Method
Inverse variance weighted
MR Egger
4.7
4.6
4.5
1/SE_IV
−0.2
0.0
0.2
0.4
β_IV

Batch 2−2218 : Gut microbiota abundance (genus Parasutterella id.2892) on Polyphosphoinositide phosphatase
with a threshold of 1e−05

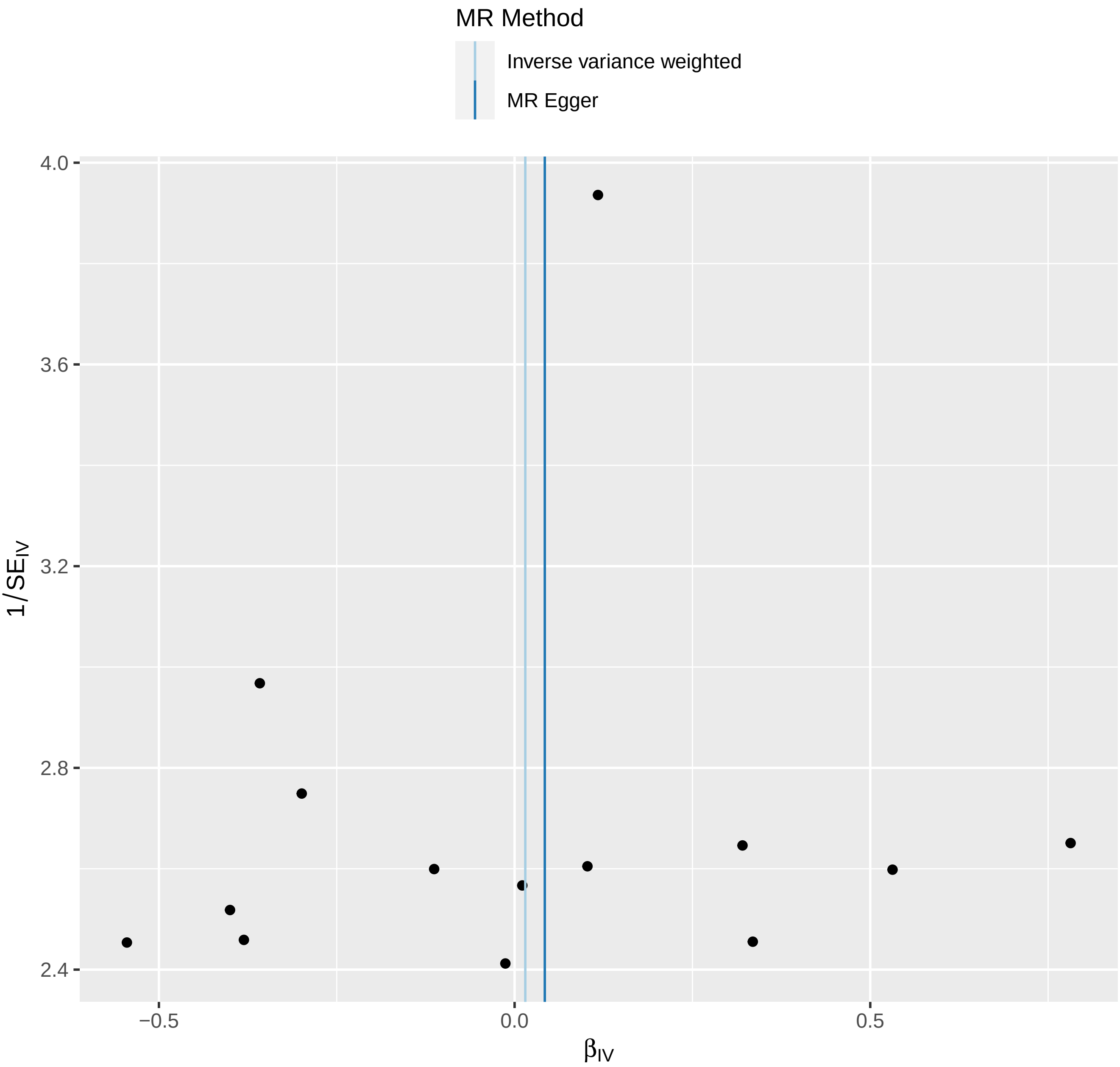

Batch 2−2219 : Gut microbiota abundance (genus Catenibacterium id.2153) on Vascular endothelial growth factor D
with a threshold of 1e−05

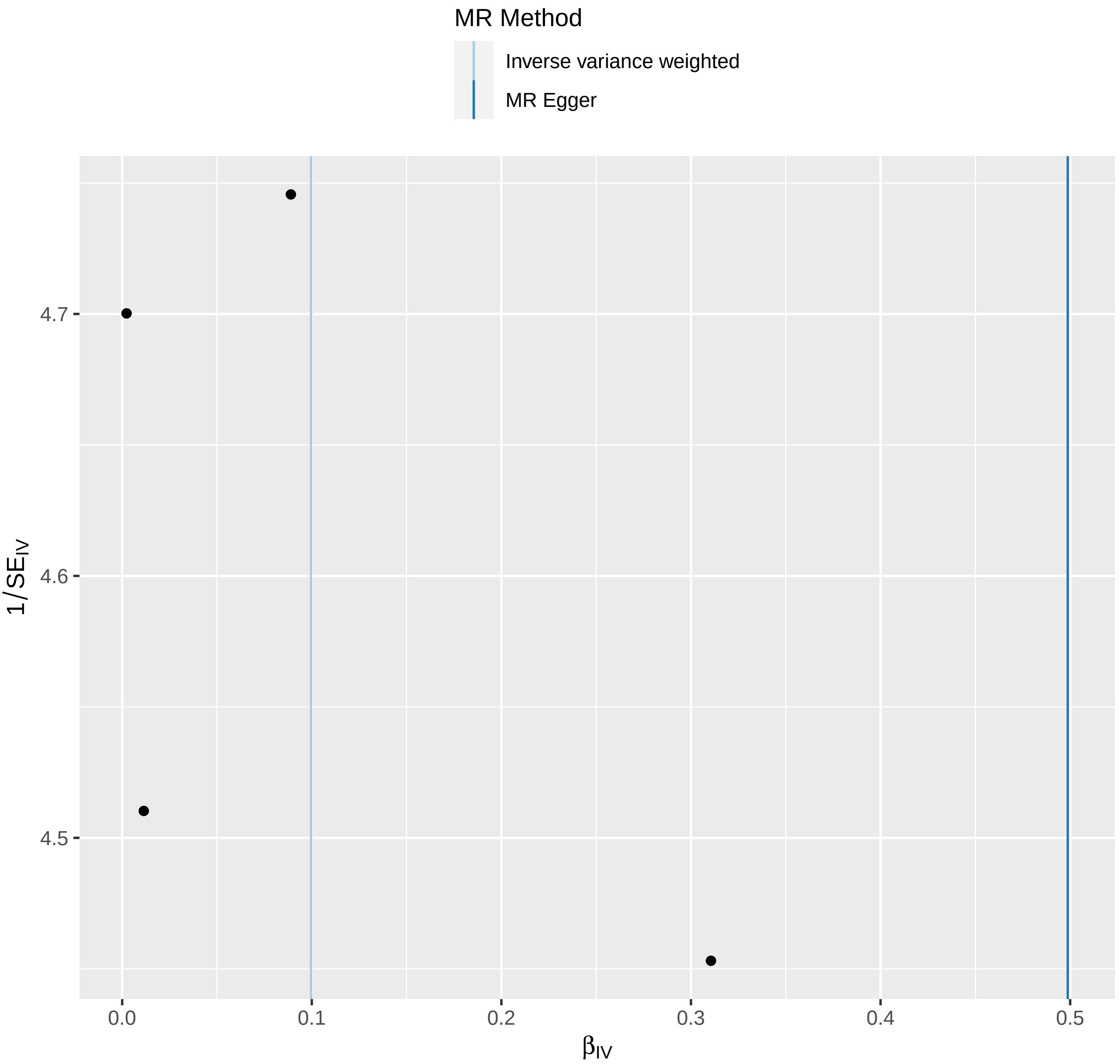

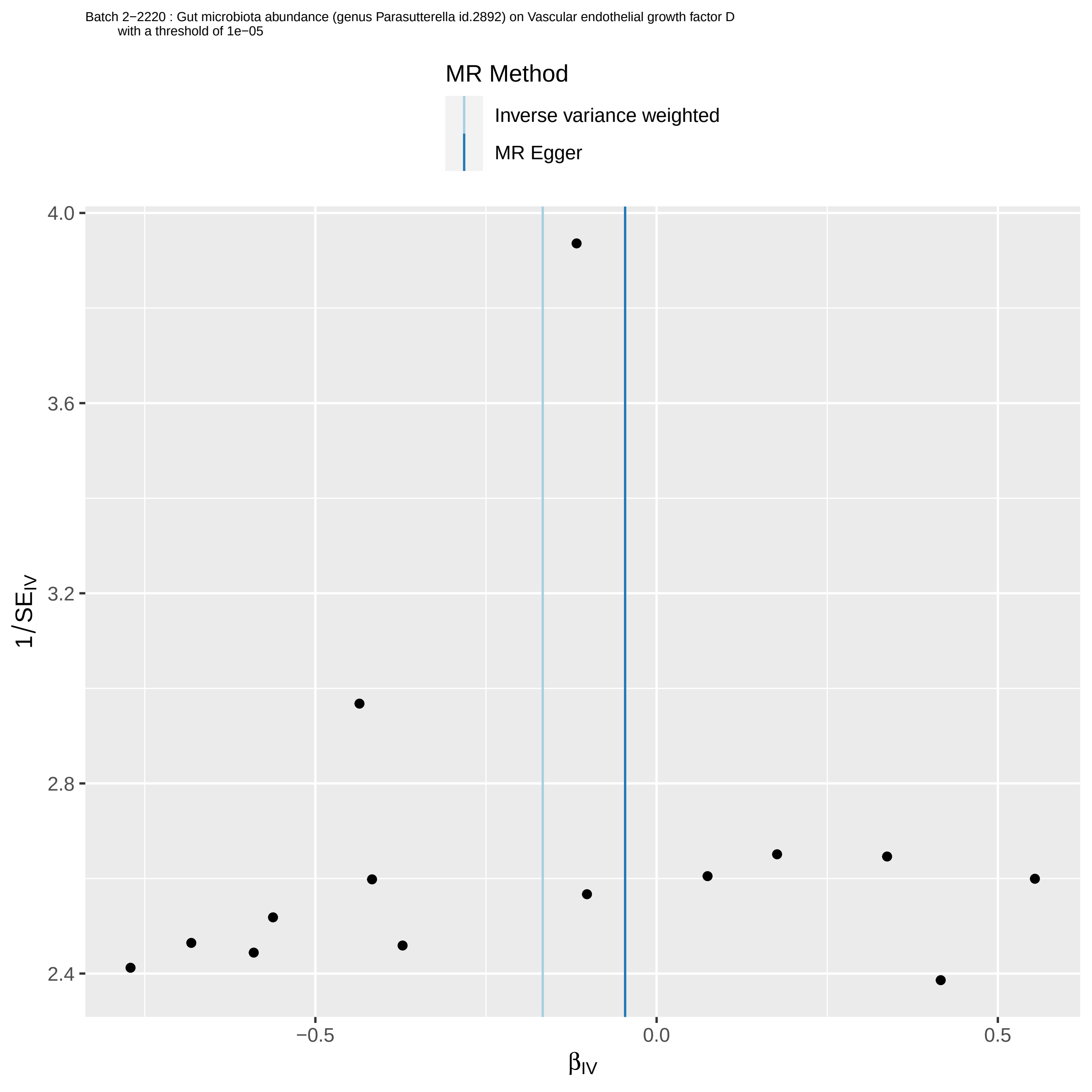

Batch 2−2220 : Gut microbiota abundance (genus Parasutterella id.2892) on Vascular endothelial growth factor D
with a threshold of 1e−05
MR Method
Inverse variance weighted
MR Egger
4.0
3.6
3.2
2.8
2.4
1/SE_IV
−0.5
0.0
0.5
β_IV

Batch 2−2221 : Gut microbiota abundance (genus Catenibacterium id.2153) on Vascular endothelial growth factor D
with a threshold of 1e−05

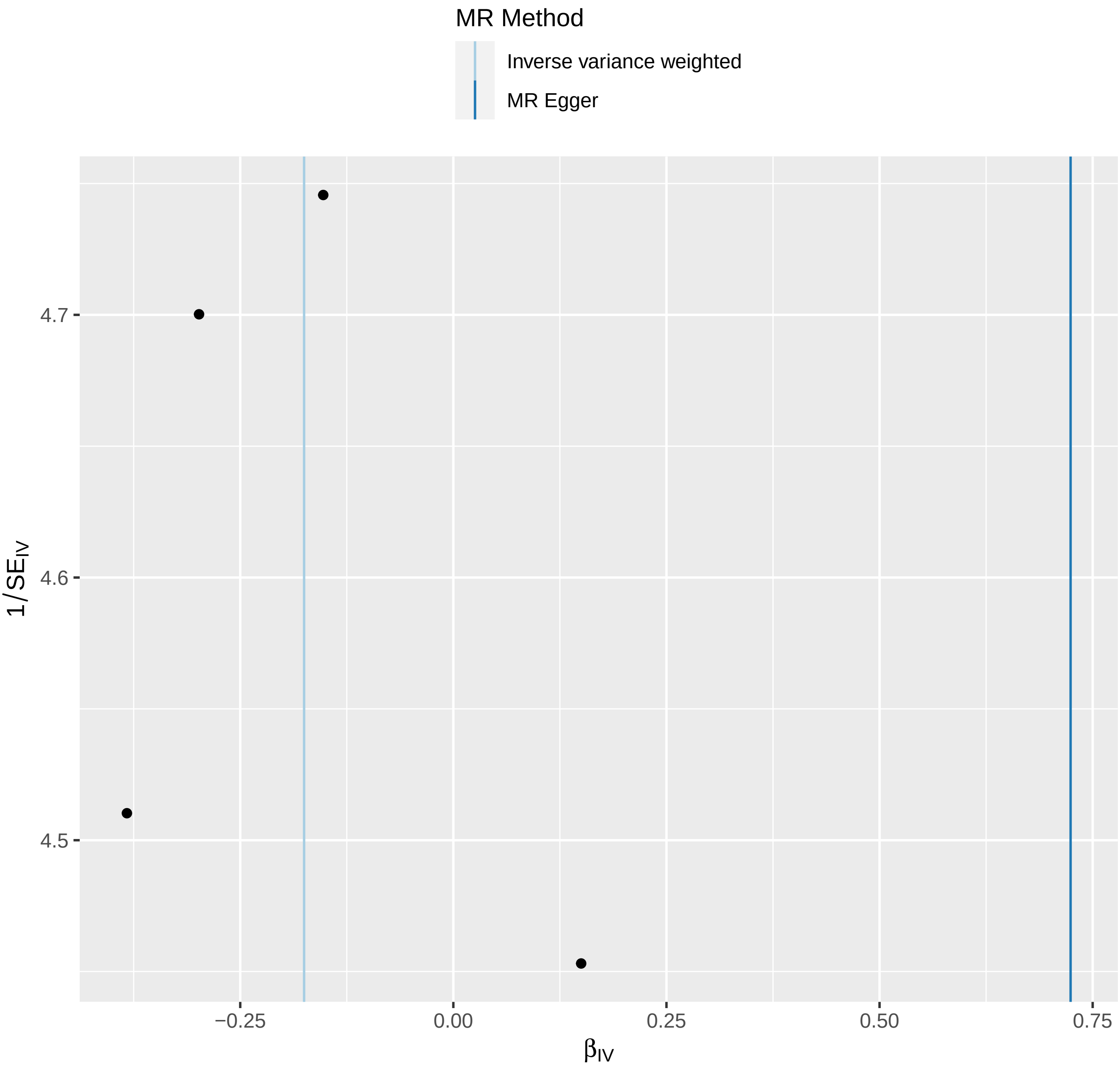

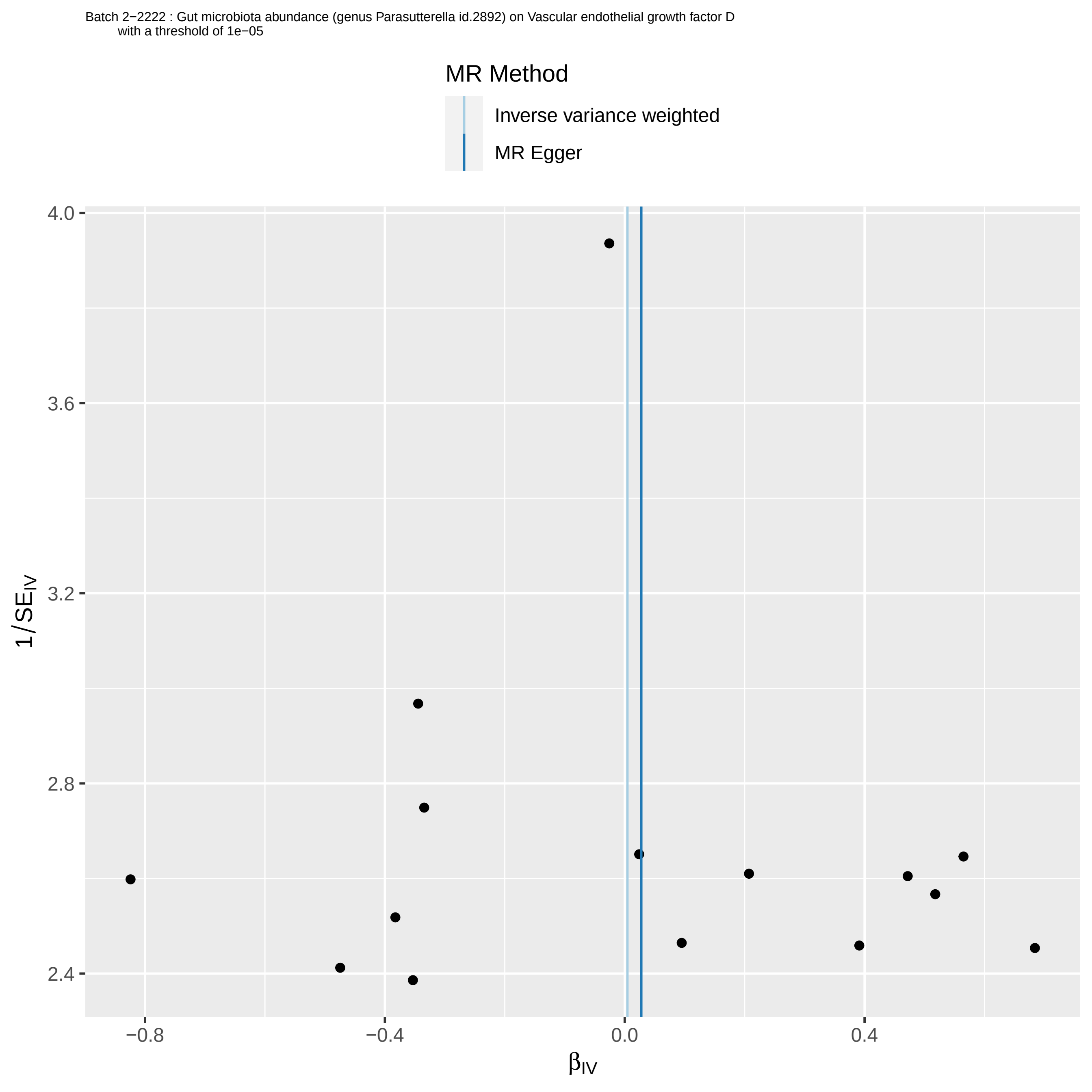

Batch 2−2222 : Gut microbiota abundance (genus Parasutterella id.2892) on Vascular endothelial growth factor D
with a threshold of 1e−05
MR Method
Inverse variance weighted
MR Egger
4.0
3.6
3.2
2.8
2.4
1/SE_IV
−0.8
−0.4
0.0
0.4
β_IV

Batch 2−2223 : Gut microbiota abundance (genus Catenibacterium id.2153) on Four−jointed box protein 1
with a threshold of 1e−05

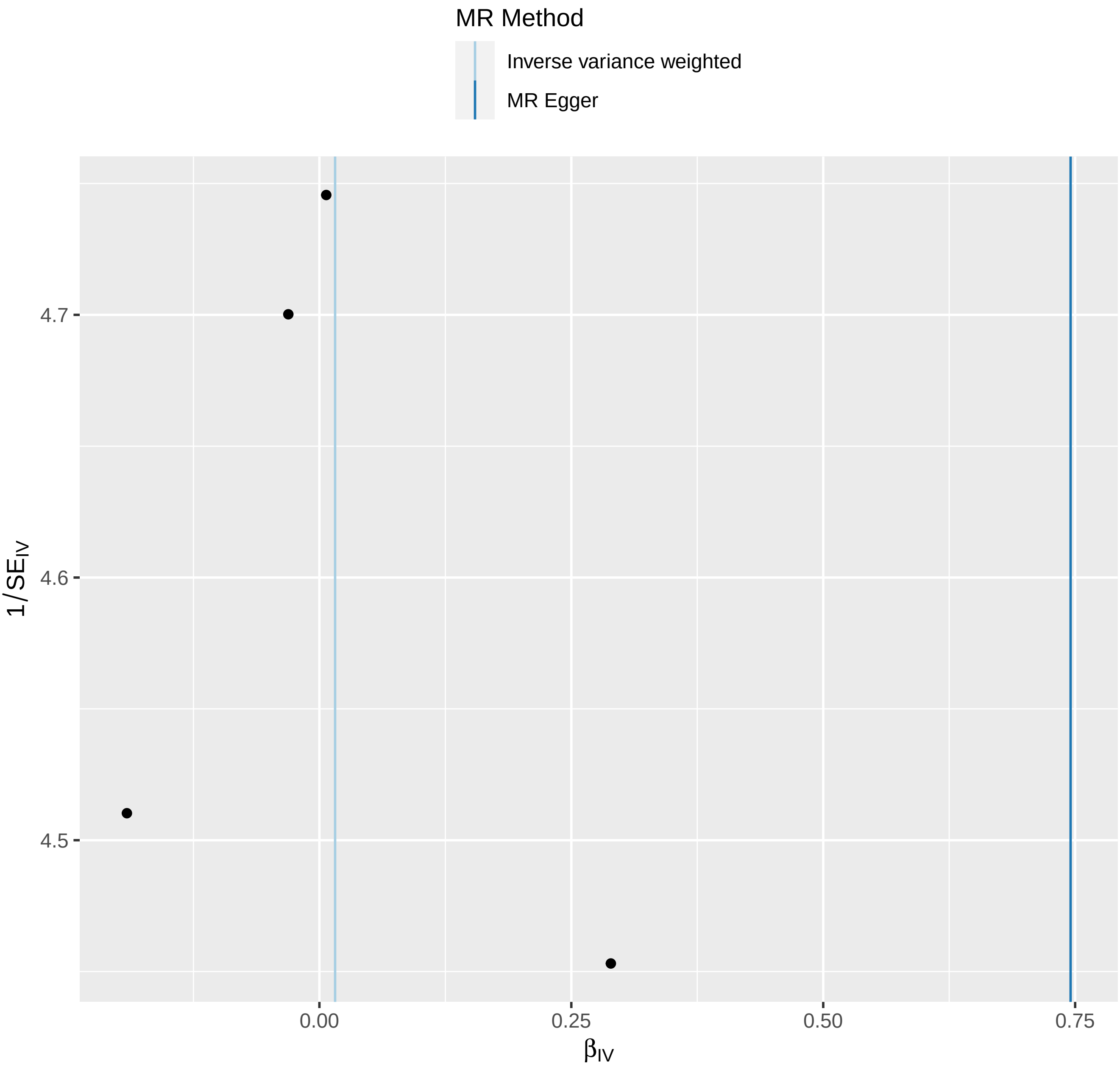

Batch 2−2224 : Gut microbiota abundance (genus Parasutterella id.2892) on Four−jointed box protein 1
with a threshold of 1e−05

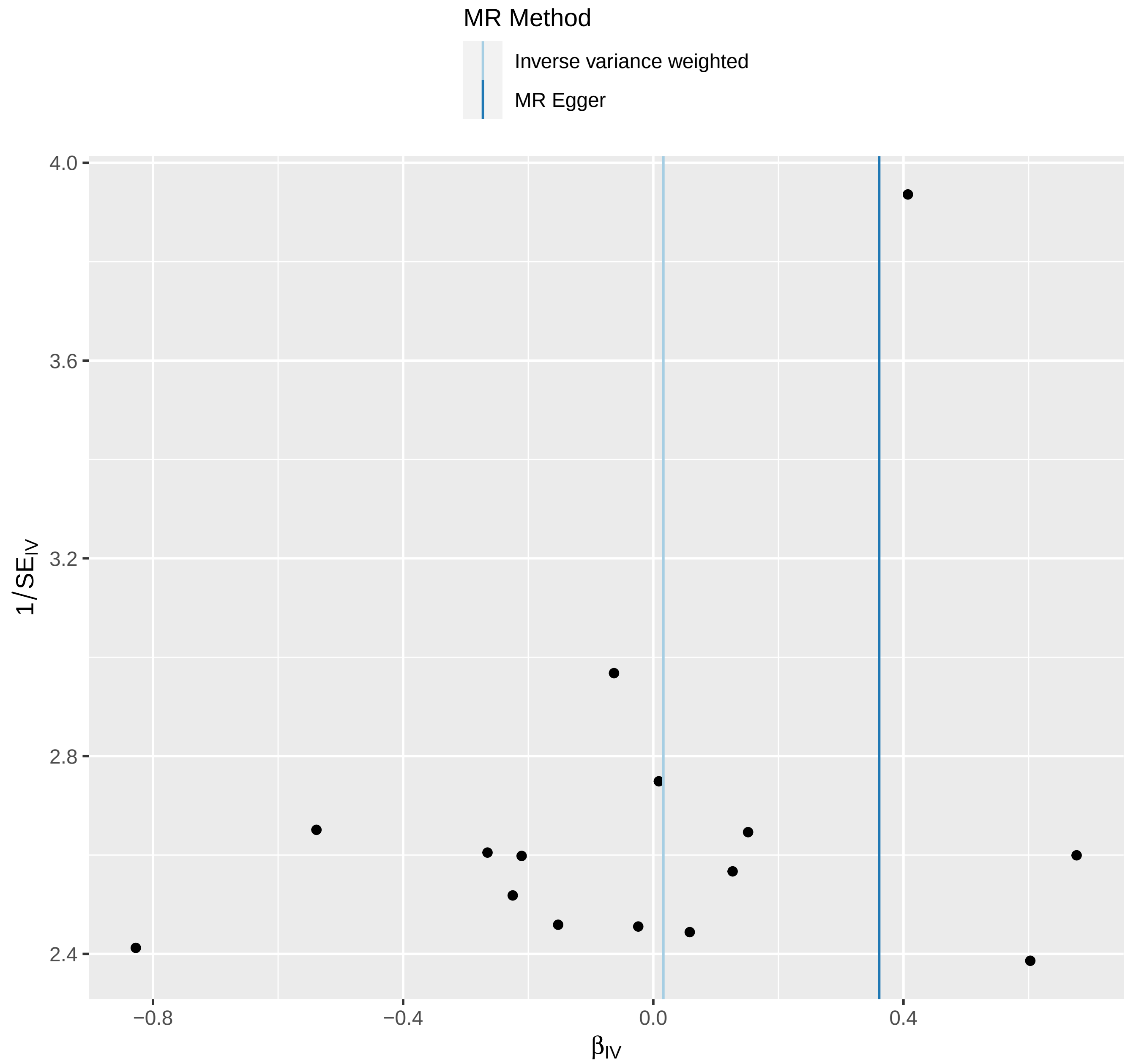

Batch 2−2225 : Gut microbiota abundance (genus Catenibacterium id.2153) on Peptidyl−prolyl cis−trans isomerase FKBP14
with a threshold of 1e−05

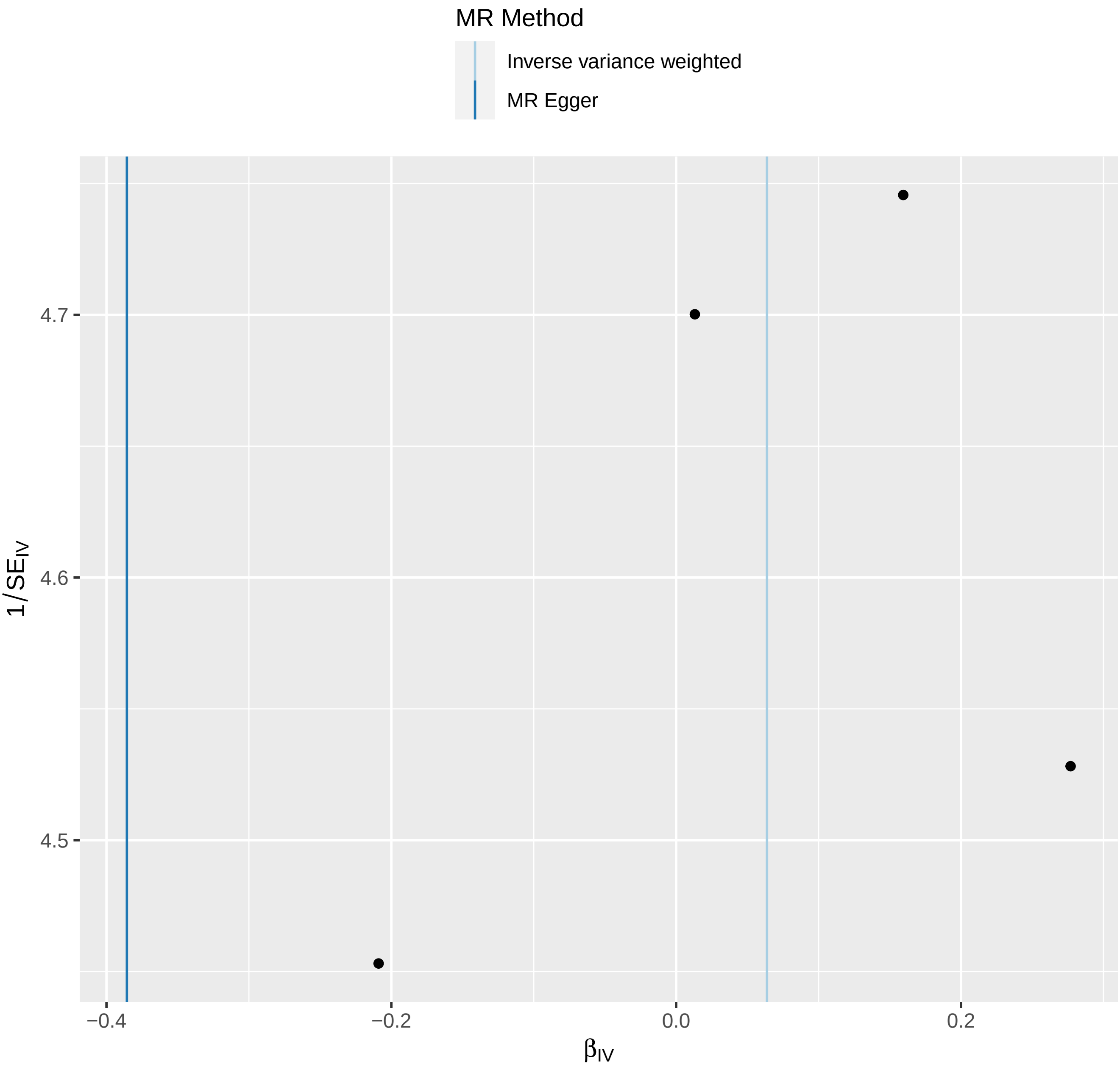

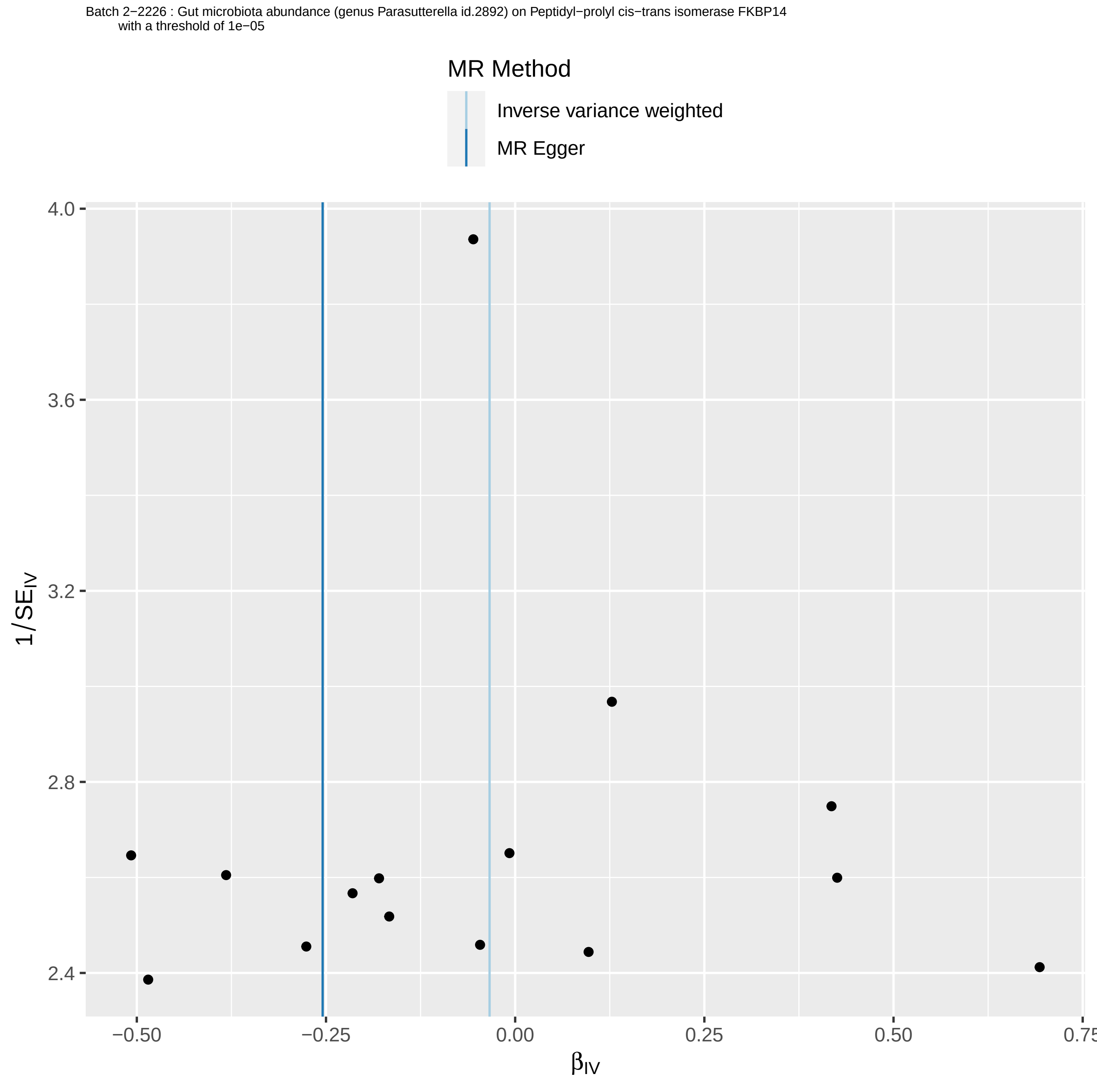
Batch 2−2226 : Gut microbiota abundance (genus Parasutterella id.2892) on Peptidyl−prolyl cis−trans isomerase FKBP14
with a threshold of 1e−05
MR Method
Inverse variance weighted
MR Egger
4.0
3.6
3.2
2.8
2.4
1/SE_IV
−0.50
−0.25
0.00
0.25
0.50
0.75
β_IV

Batch 2−2227 : Gut microbiota abundance (genus Catenibacterium id.2153) on Peptidyl−prolyl cis−trans isomerase FKBP2
with a threshold of 1e−05

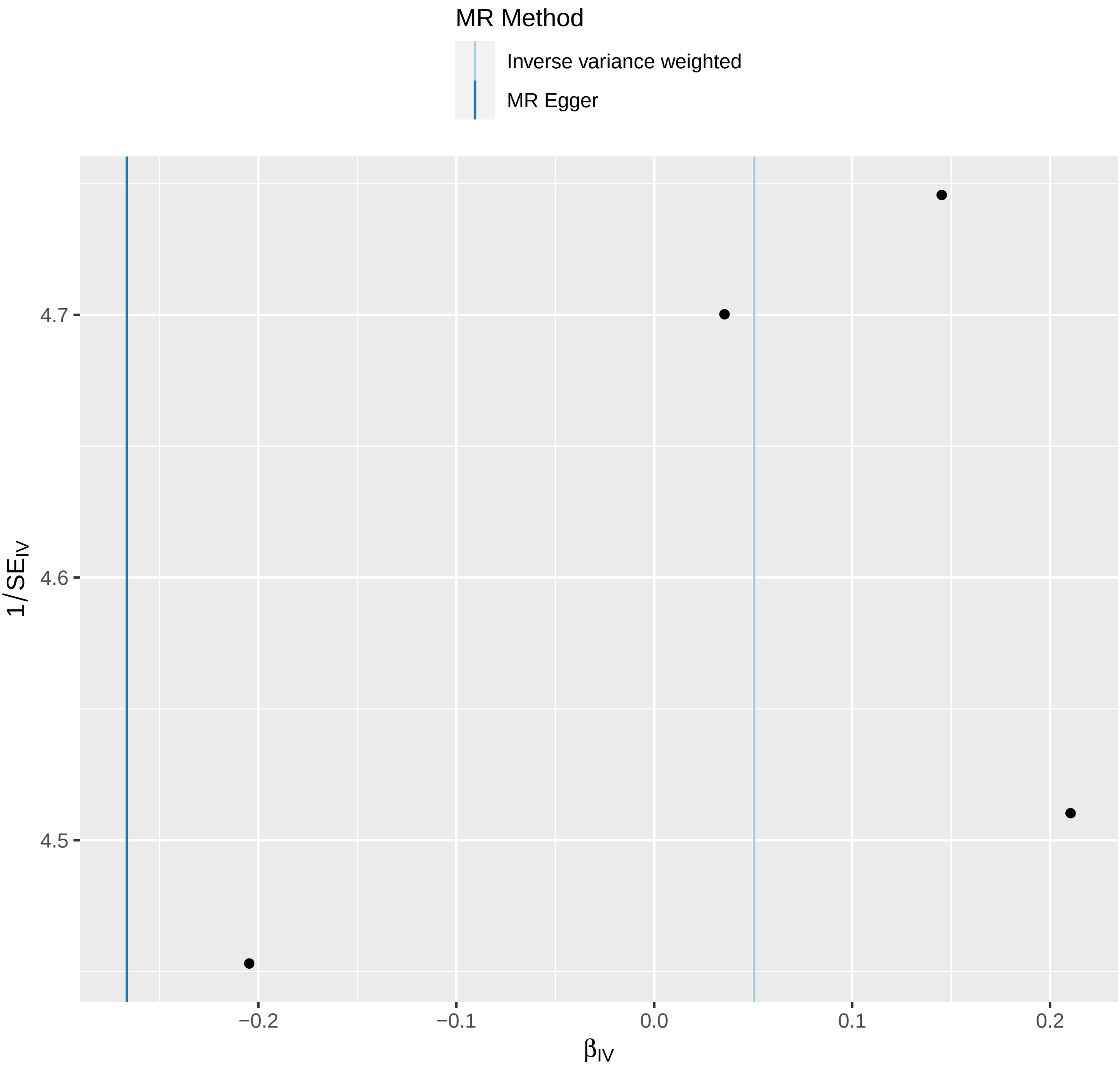

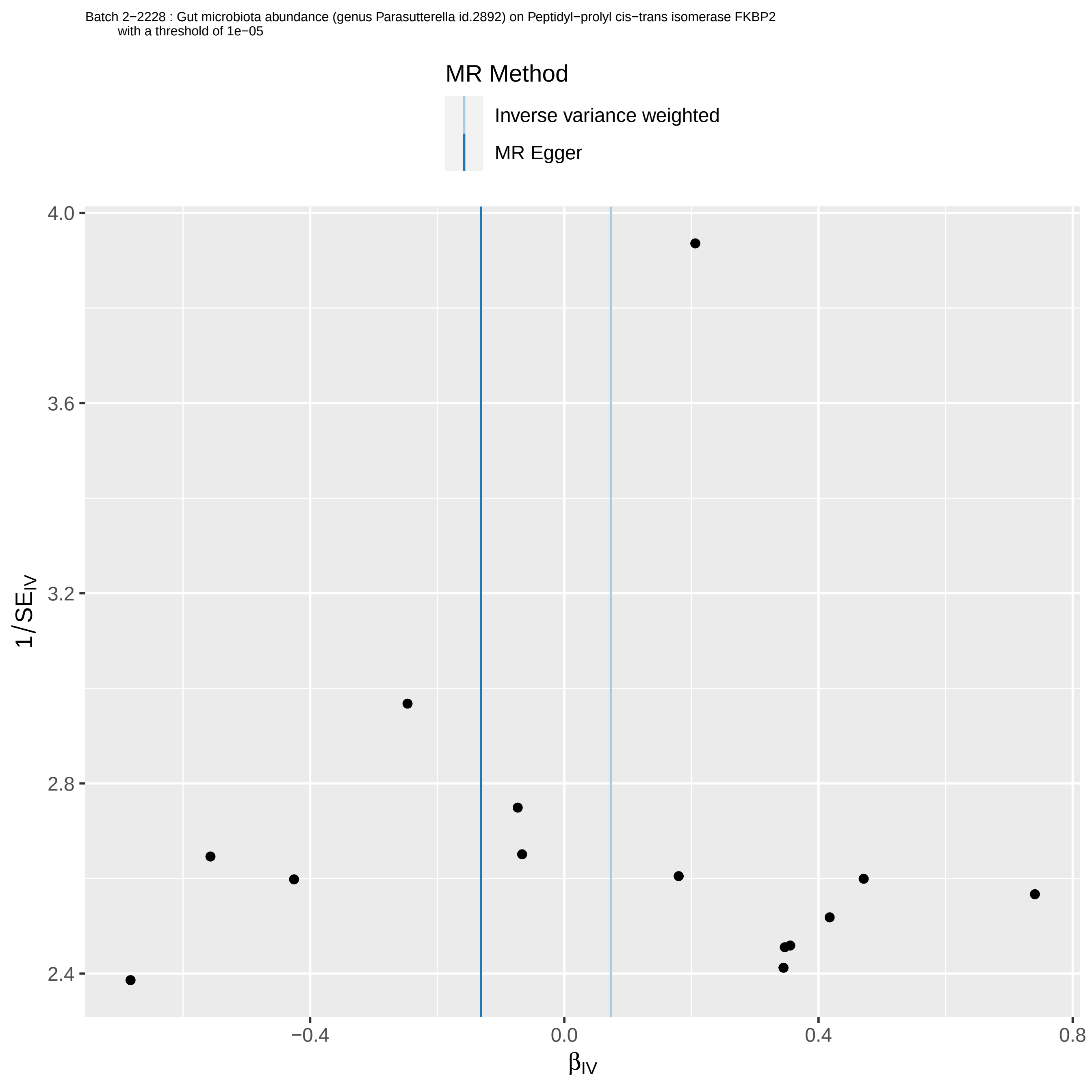

Batch 2−2228 : Gut microbiota abundance (genus Parasutterella id.2892) on Peptidyl−prolyl cis−trans isomerase FKBP2
with a threshold of 1e−05
MR Method
Inverse variance weighted
MR Egger
4.0
3.6
3.2
2.8
2.4
1/SE_IV
−0.4
0.0
0.4
0.8
β_IV

Batch 2−2229 : Gut microbiota abundance (genus Catenibacterium id.2153) on Inactive peptidyl−prolyl cis−trans isomerase FKBP6
with a threshold of 1e−05

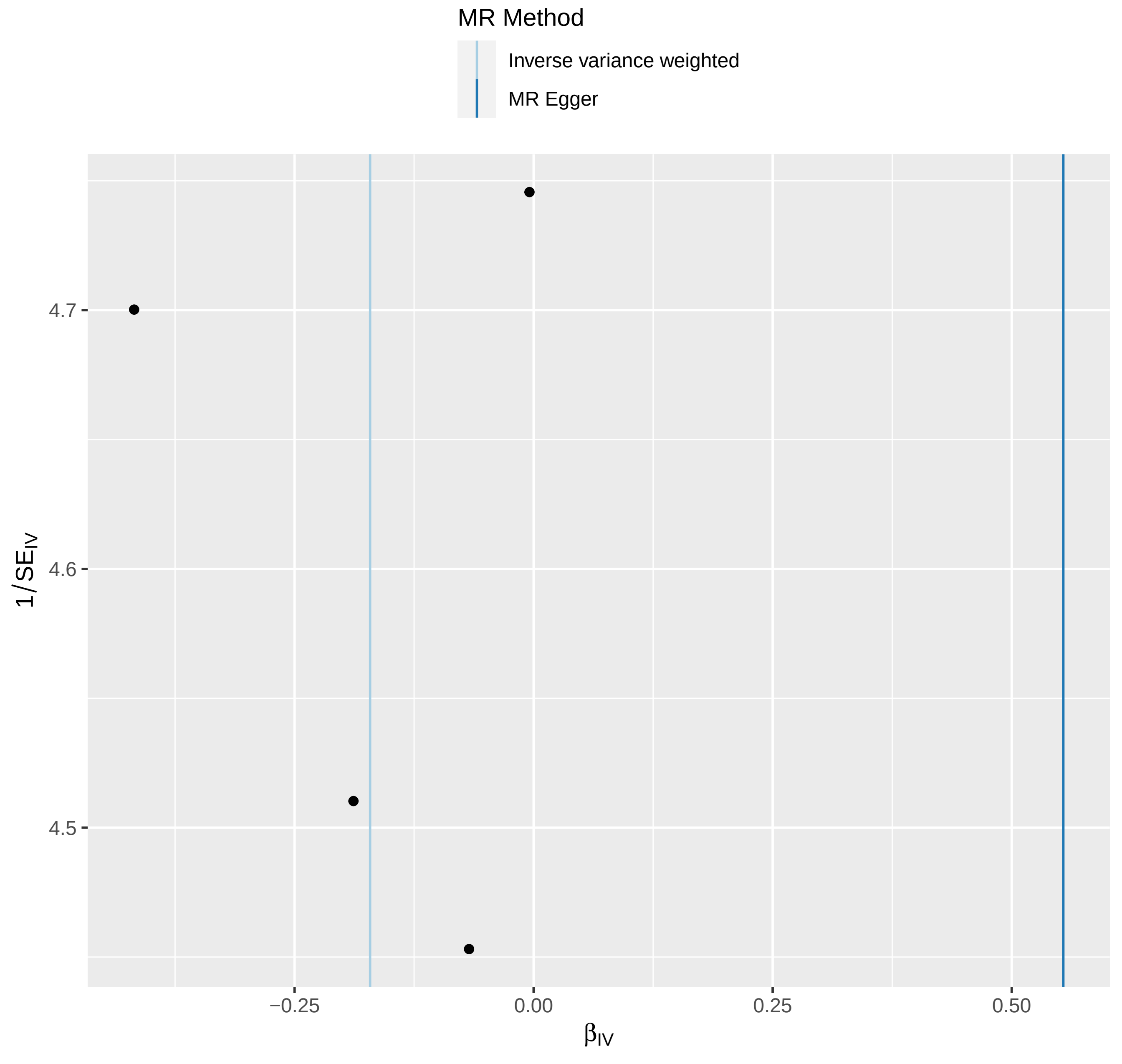

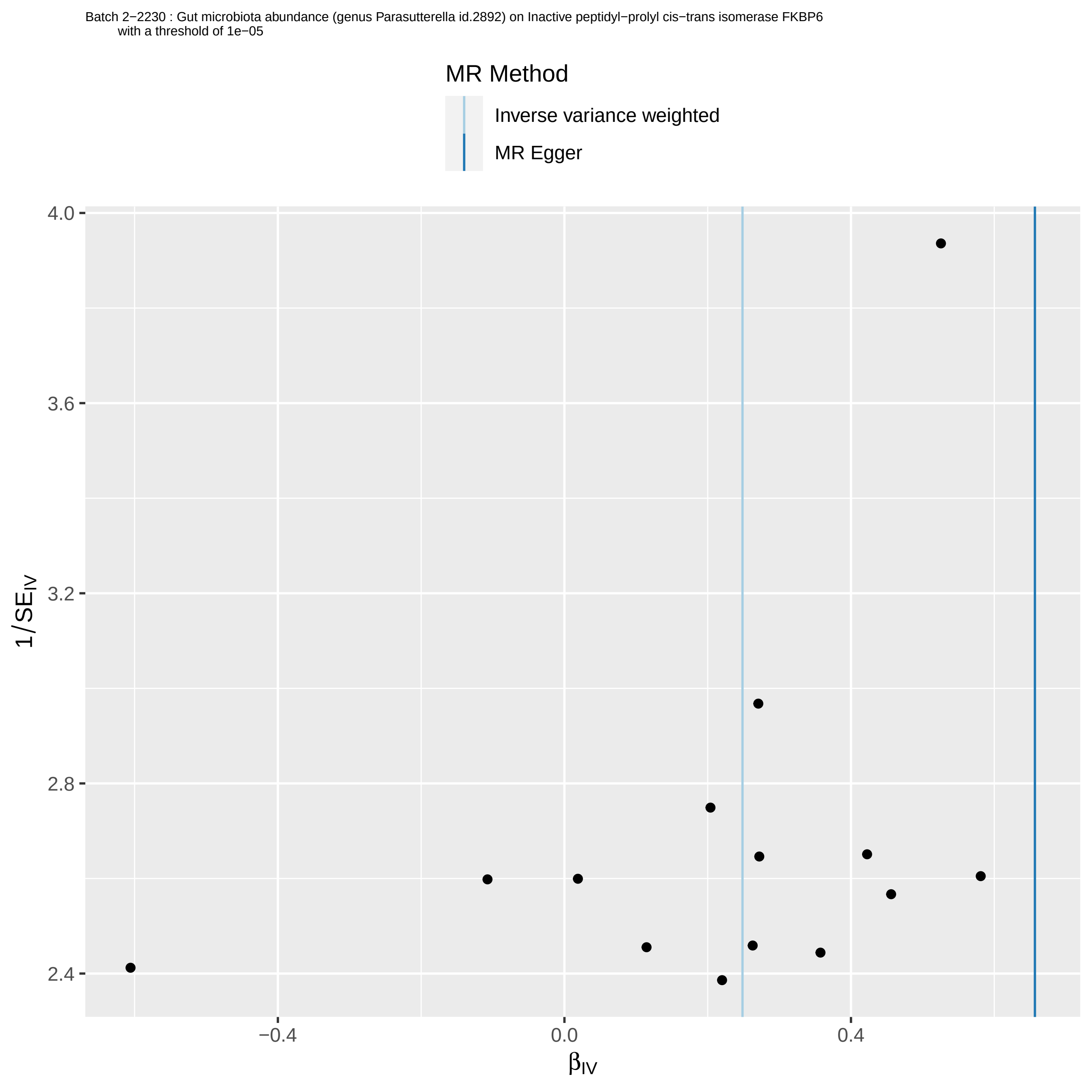
Batch 2−2230 : Gut microbiota abundance (genus Parasutterella id.2892) on Inactive peptidyl−prolyl cis−trans isomerase FKBP6
with a threshold of 1e−05
MR Method
Inverse variance weighted
MR Egger
4.0
3.6
3.2
2.8
2.4
1/SE_IV
−0.4
0.0
0.4
β_IV

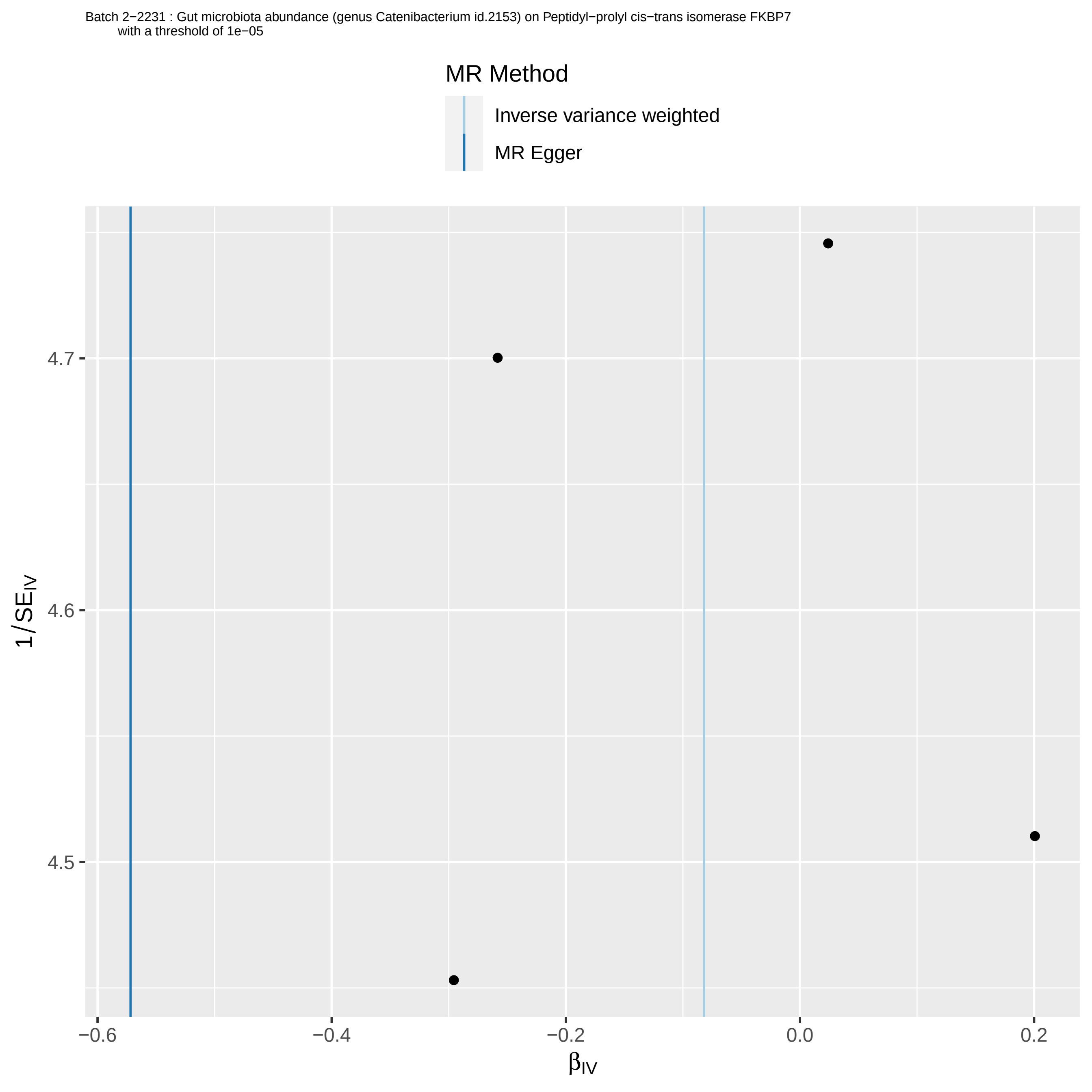

Batch 2−2231 : Gut microbiota abundance (genus Catenibacterium id.2153) on Peptidyl−prolyl cis−trans isomerase FKBP7
with a threshold of 1e−05
MR Method
Inverse variance weighted
MR Egger
4.7
4.6
4.5
1/SE$_{IV}$
−0.6
−0.4
−0.2
0.0
0.2
β$_{IV}$

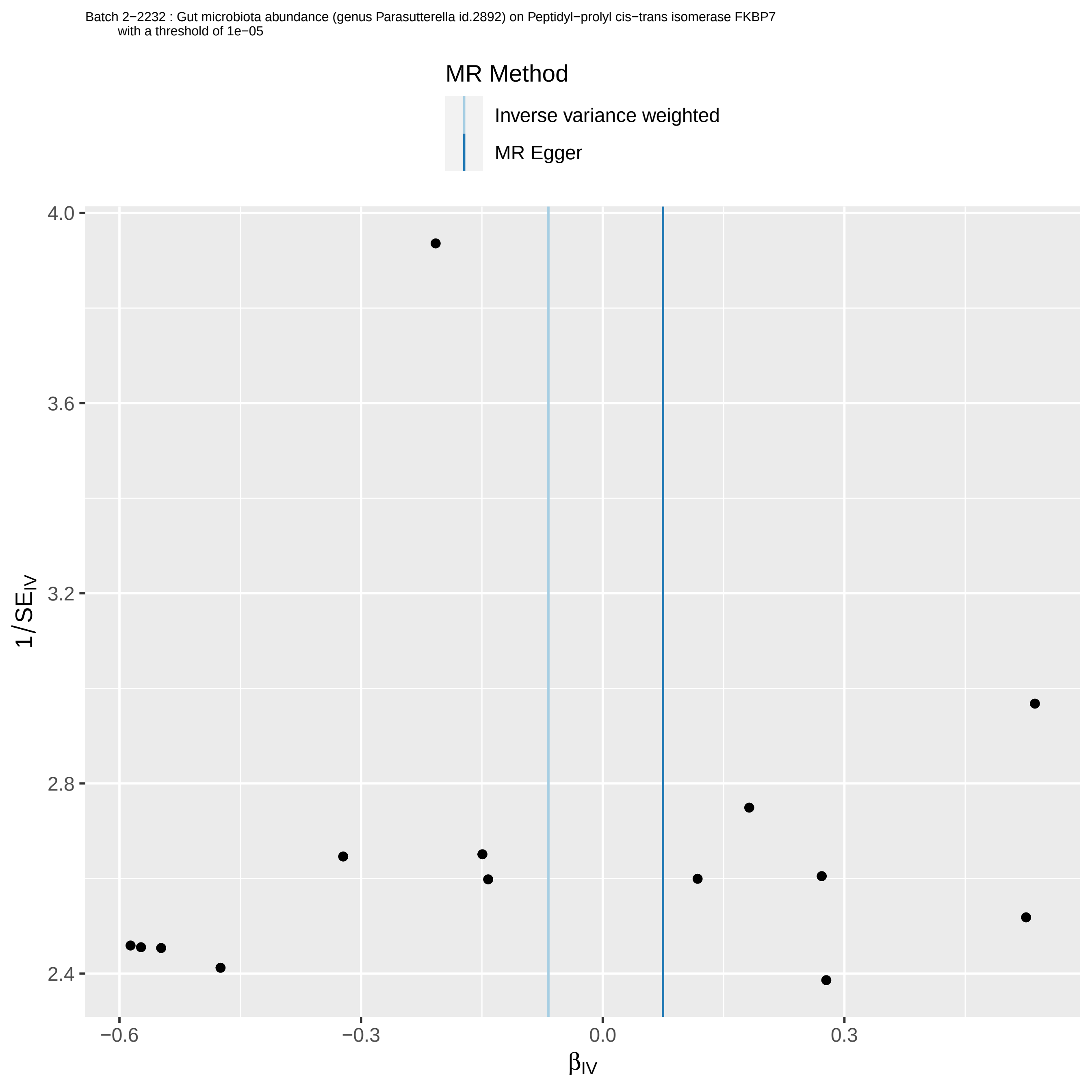
Batch 2−2232 : Gut microbiota abundance (genus Parasutterella id.2892) on Peptidyl−prolyl cis−trans isomerase FKBP7
with a threshold of 1e−05
MR Method
Inverse variance weighted
MR Egger
4.0
3.6
3.2
2.8
2.4
1/SE_IV
−0.6
−0.3
0.0
0.3
β_IV

Batch 2−2233 : Gut microbiota abundance (genus Catenibacterium id.2153) on Filamin−A
with a threshold of 1e−05

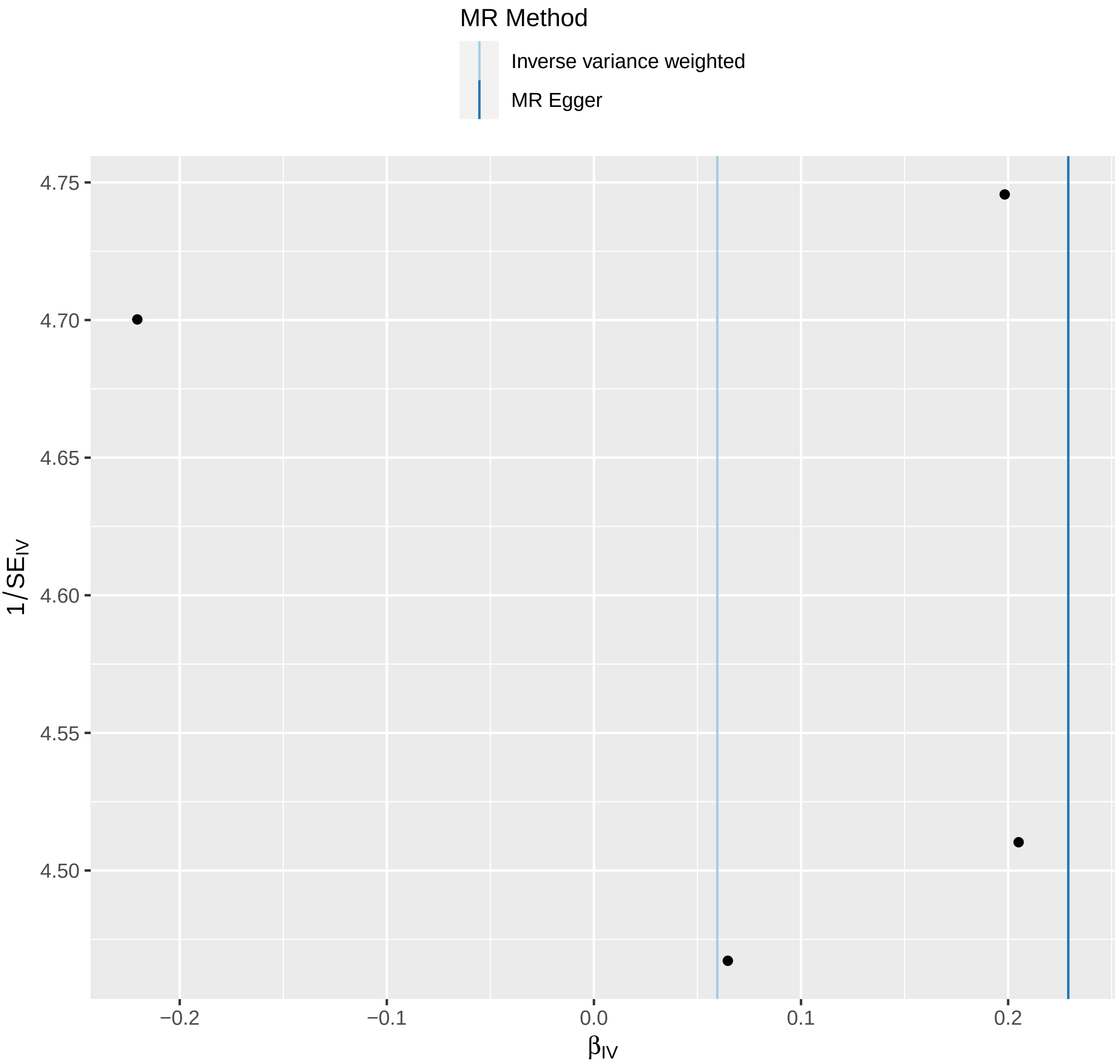

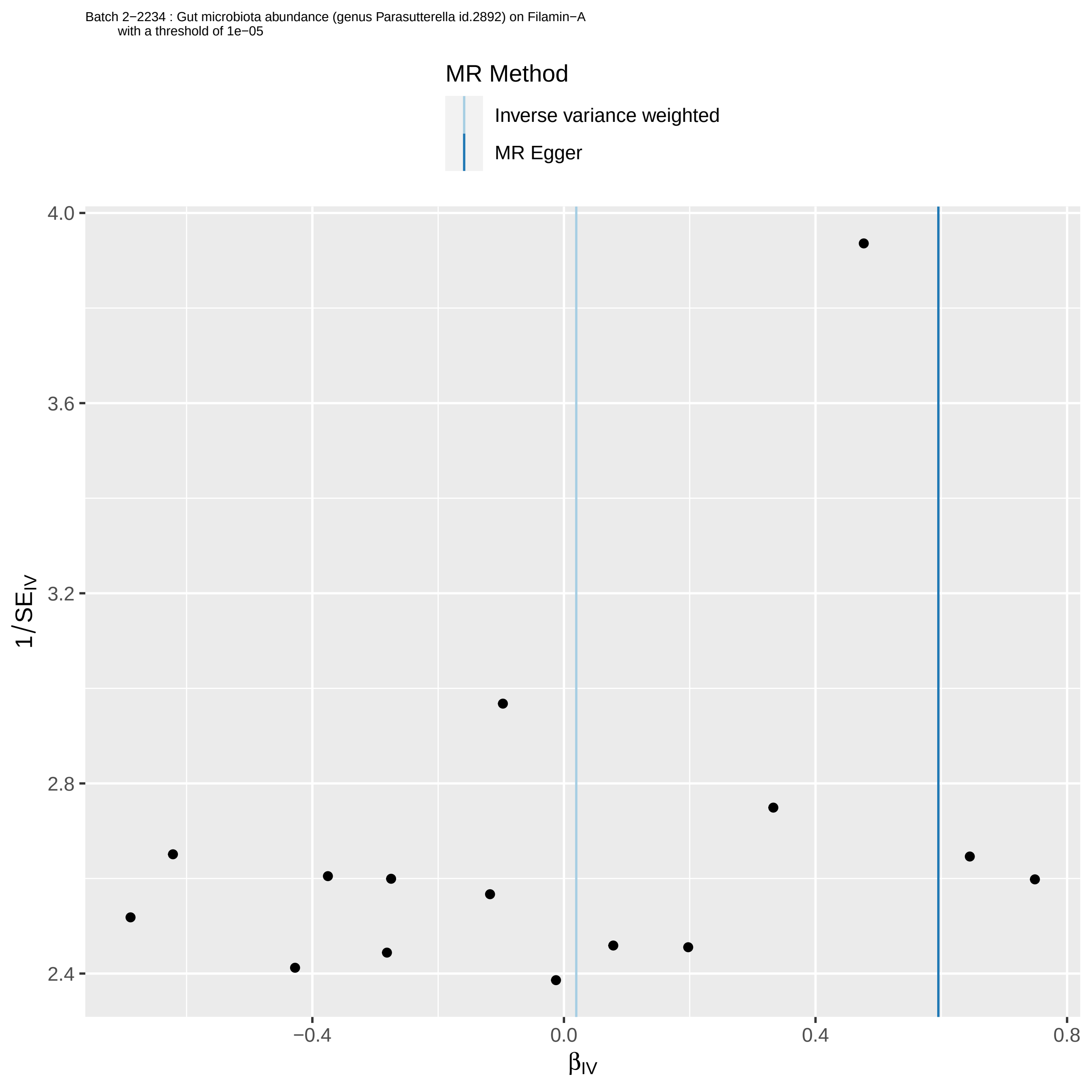
Batch 2−2234 : Gut microbiota abundance (genus Parasutterella id.2892) on Filamin−A
with a threshold of 1e−05
MR Method
Inverse variance weighted
MR Egger
4.0
3.6
3.2
2.8
2.4
1/SE_IV
−0.4
0.0
0.4
0.8
β_IV

Batch 2−2235 : Gut microbiota abundance (genus Catenibacterium id.2153) on Filamin−A
with a threshold of 1e−05

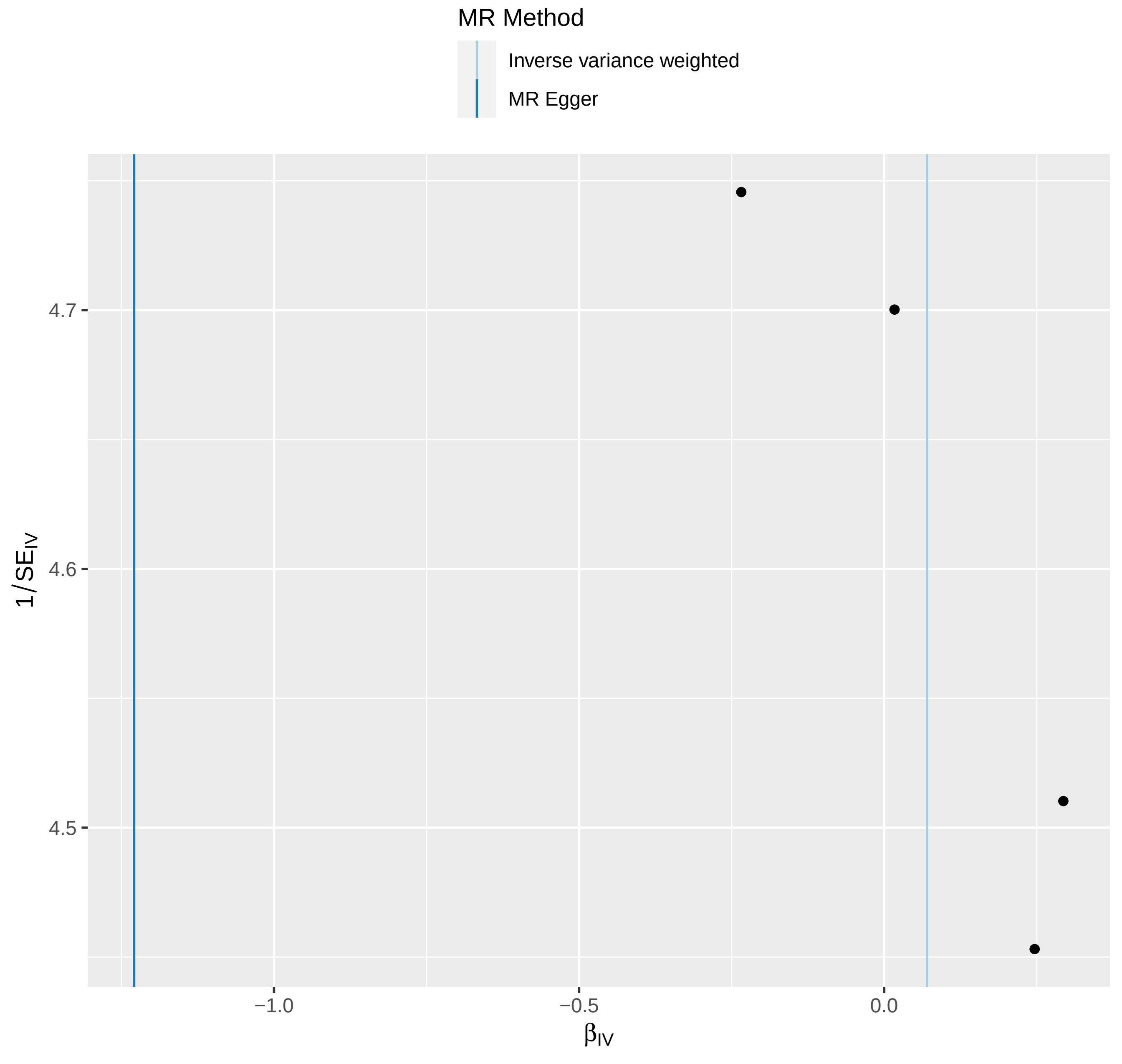

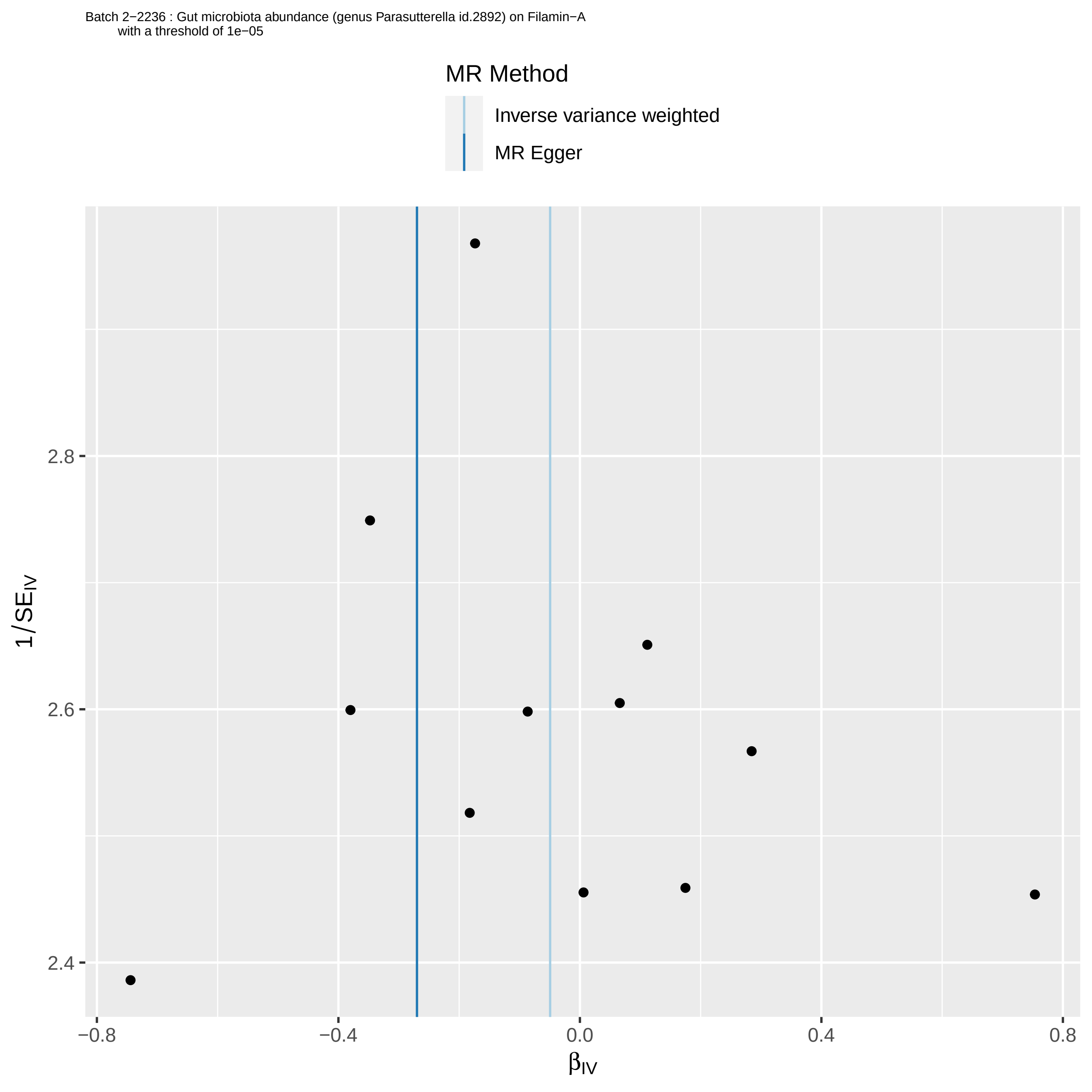
Batch 2−2236 : Gut microbiota abundance (genus Parasutterella id.2892) on Filamin−A
with a threshold of 1e−05
MR Method
Inverse variance weighted
MR Egger
2.8
2.6
2.4
1/SE_IV
−0.8
−0.4
0.0
0.4
0.8
β_IV

Batch 2−2237 : Gut microbiota abundance (genus Catenibacterium id.2153) on Filamin−A
with a threshold of 1e−05

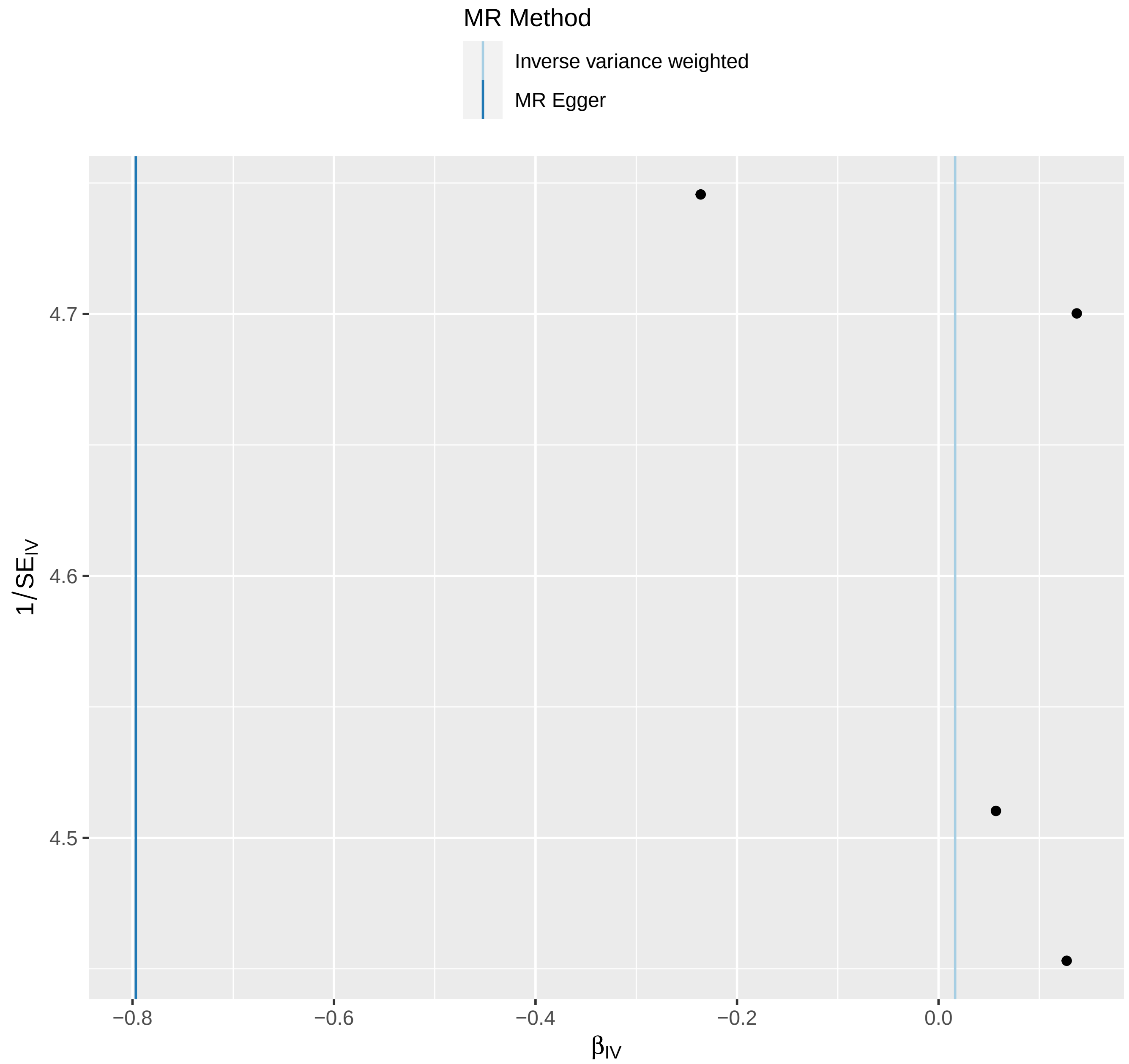

Batch 2−2238 : Gut microbiota abundance (genus Parasutterella id.2892) on Filamin−A
with a threshold of 1e−05

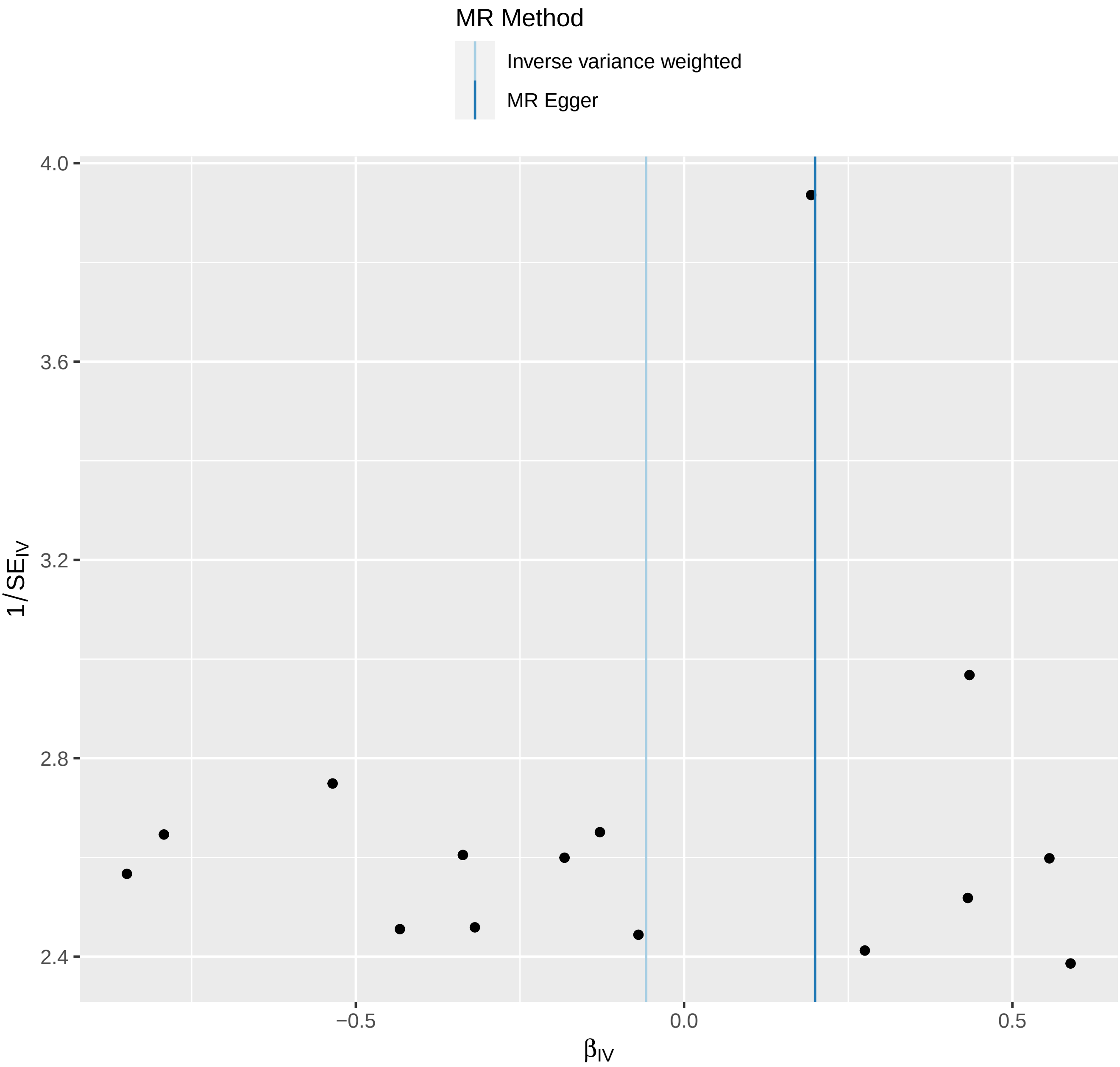

Batch 2−2239 : Gut microbiota abundance (genus Catenibacterium id.2153) on Filamin−A
with a threshold of 1e−05

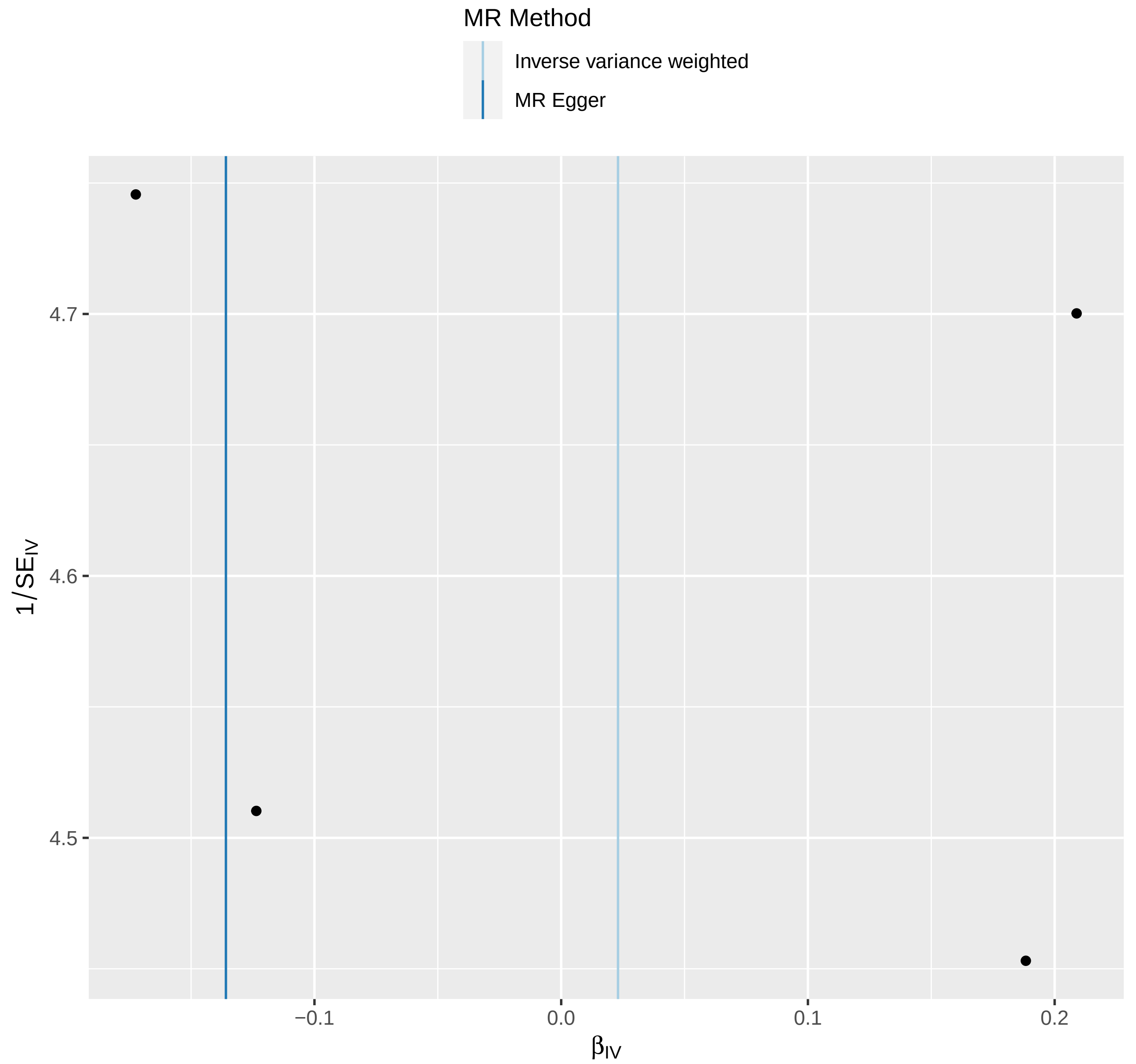

Batch 2−2240 : Gut microbiota abundance (genus Parasutterella id.2892) on Filamin−A
with a threshold of 1e−05

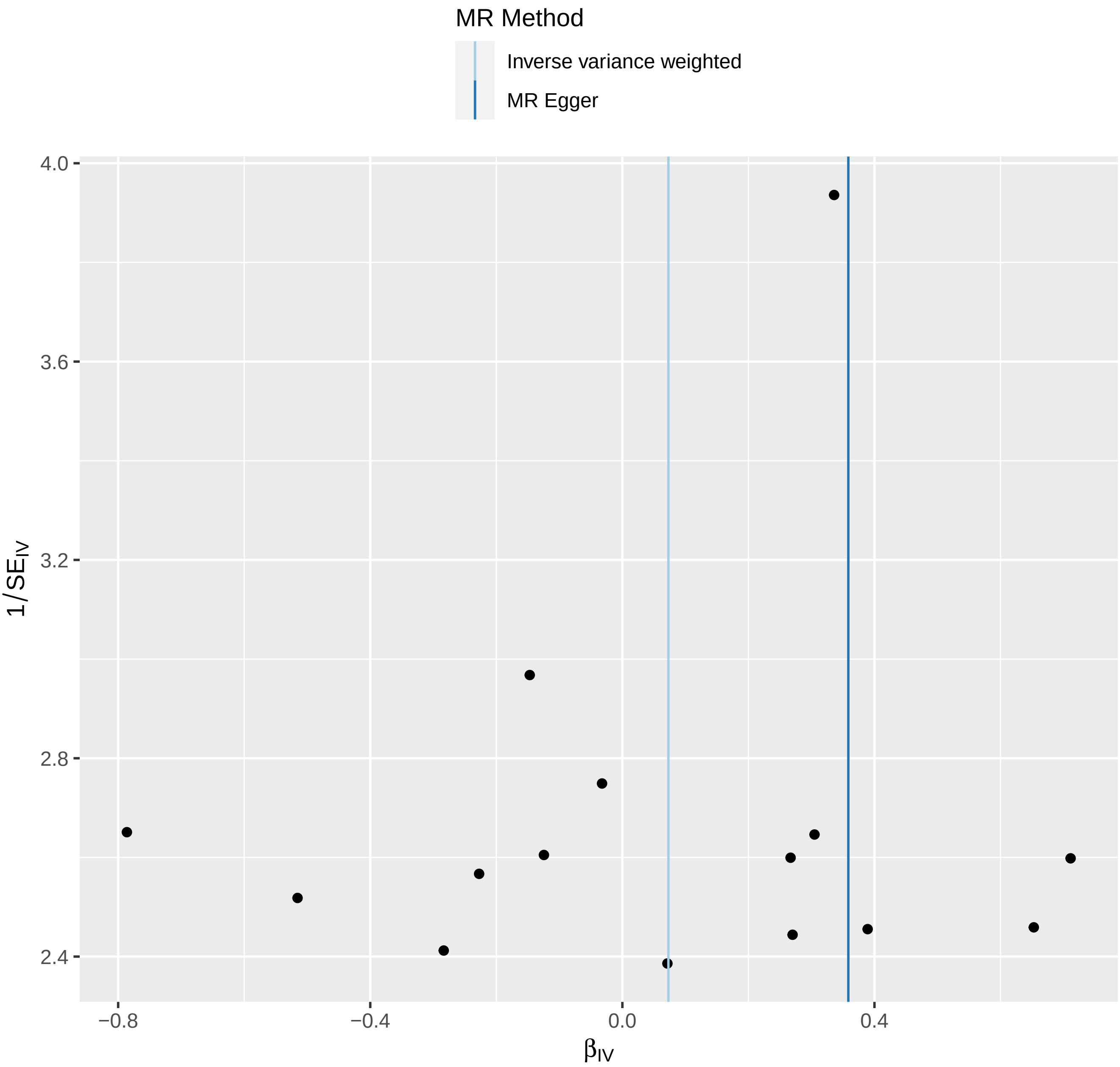

Batch 2−2241 : Gut microbiota abundance (genus Catenibacterium id.2153) on Leucine−rich repeat transmembrane protein FLRT1 with a threshold of 1e−05

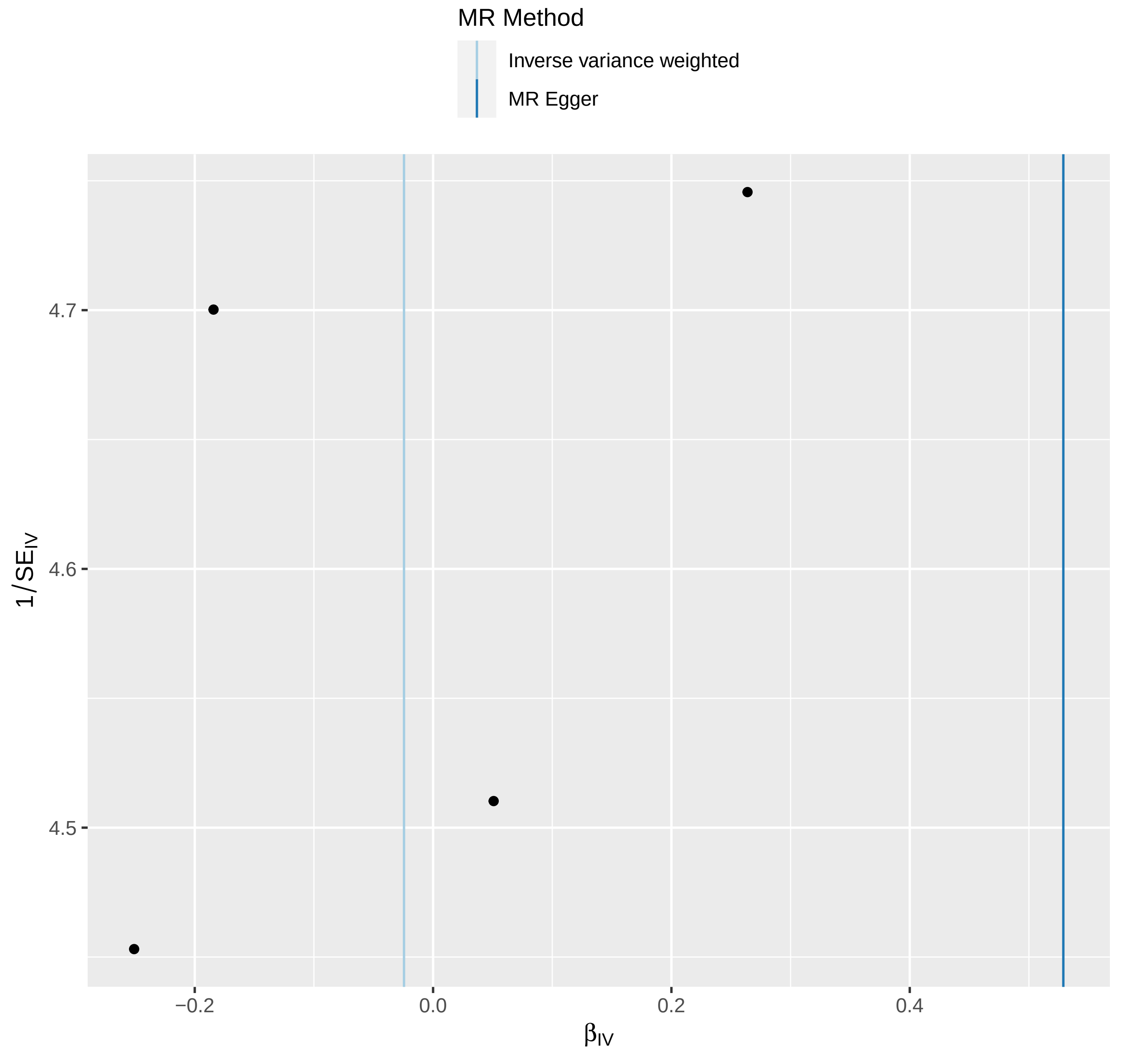

Batch 2−2242 : Gut microbiota abundance (genus Parasutterella id.2892) on Leucine−rich repeat transmembrane protein FLRT1
with a threshold of 1e−05

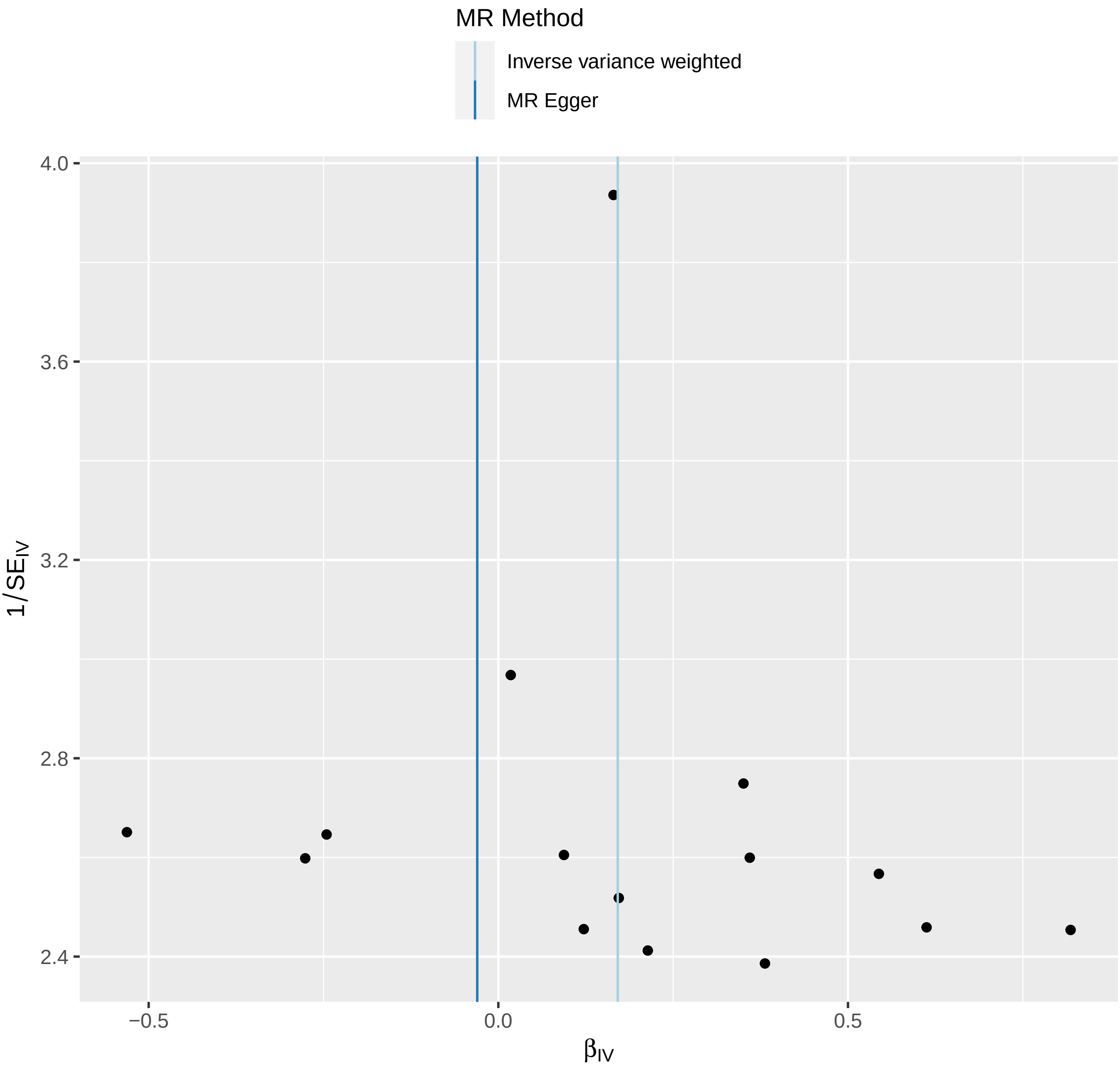

Batch 2−2243 : Gut microbiota abundance (genus Catenibacterium id.2153) on Leucine−rich repeat transmembrane protein FLRT1
with a threshold of 1e−05

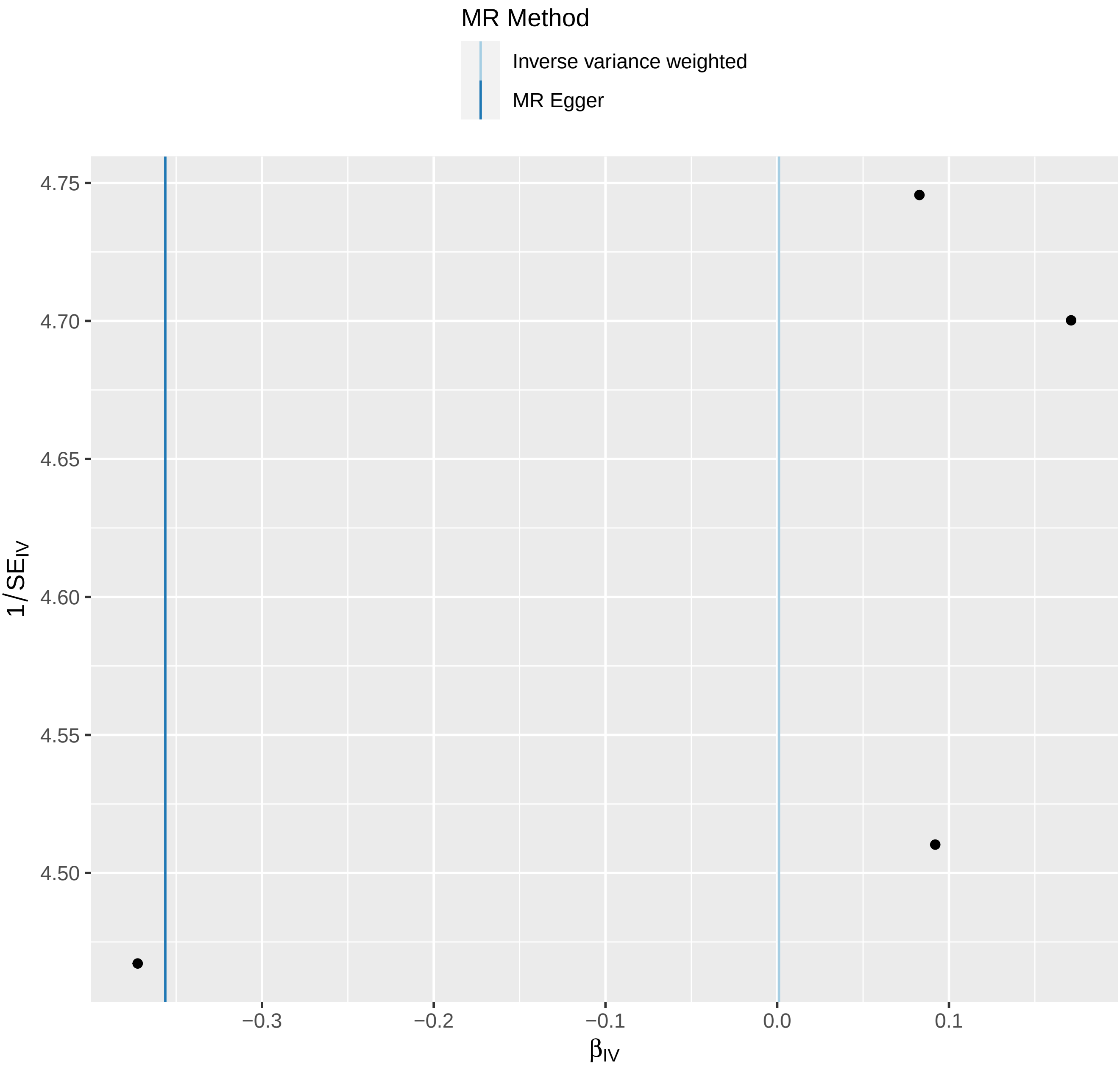

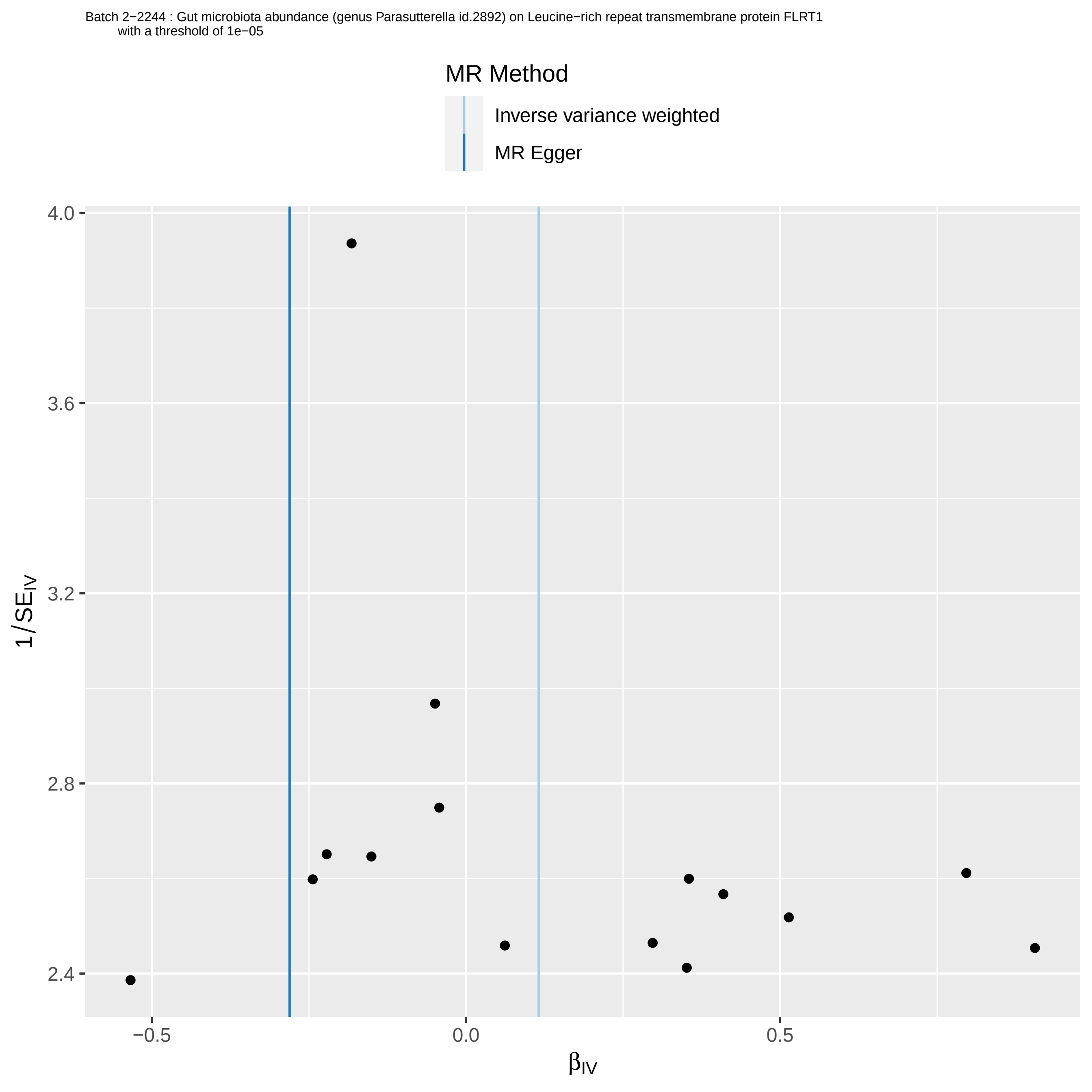
Batch 2−2244 : Gut microbiota abundance (genus Parasutterella id.2892) on Leucine−rich repeat transmembrane protein FLRT1
with a threshold of 1e−05
MR Method
Inverse variance weighted
MR Egger
4.0
3.6
3.2
2.8
2.4
1/SE_IV
−0.5
0.0
0.5
β_IV

Batch 2−2245 : Gut microbiota abundance (genus Catenibacterium id.2153) on Leucine−rich repeat transmembrane protein FLRT2
with a threshold of 1e−05

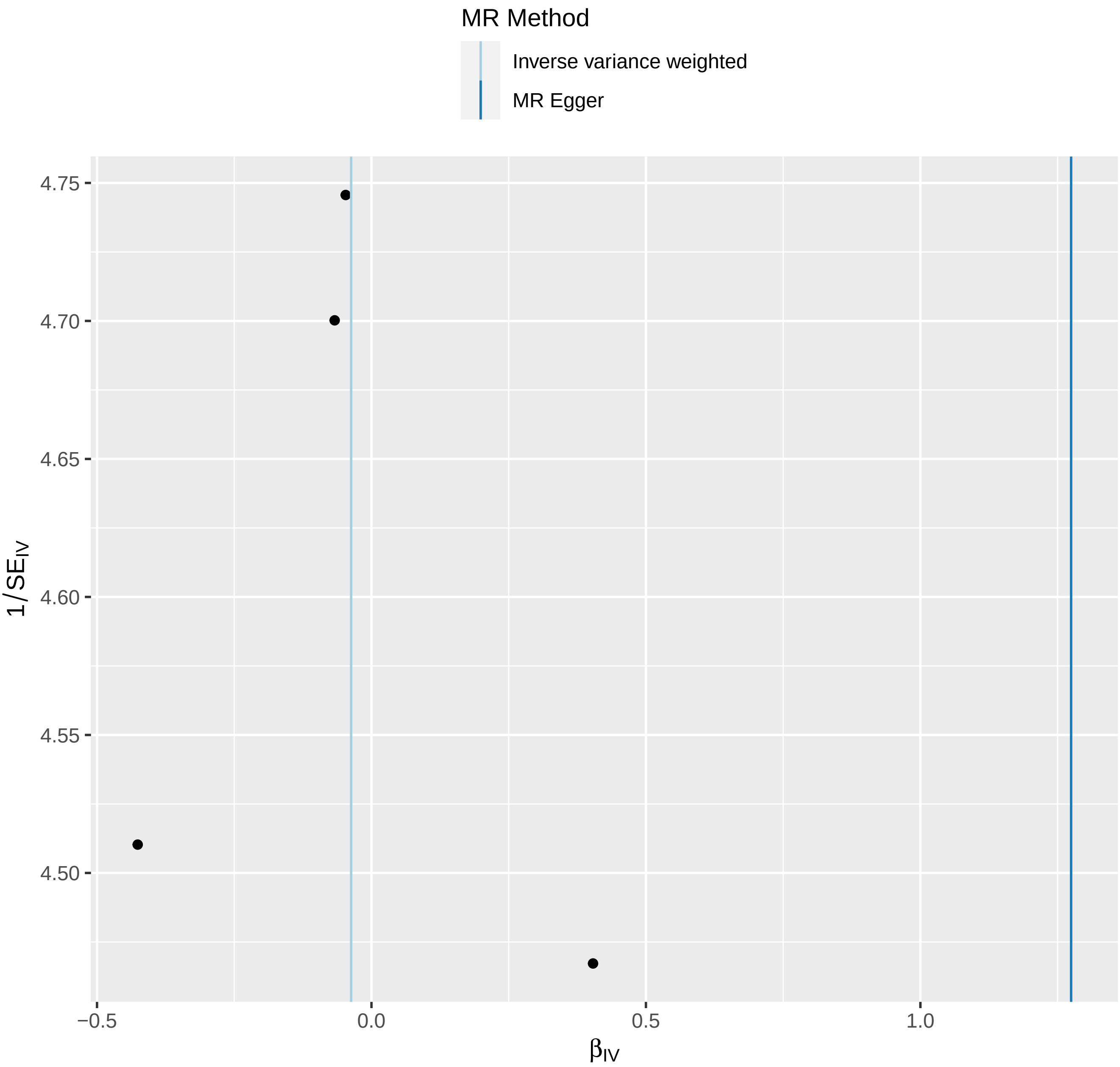

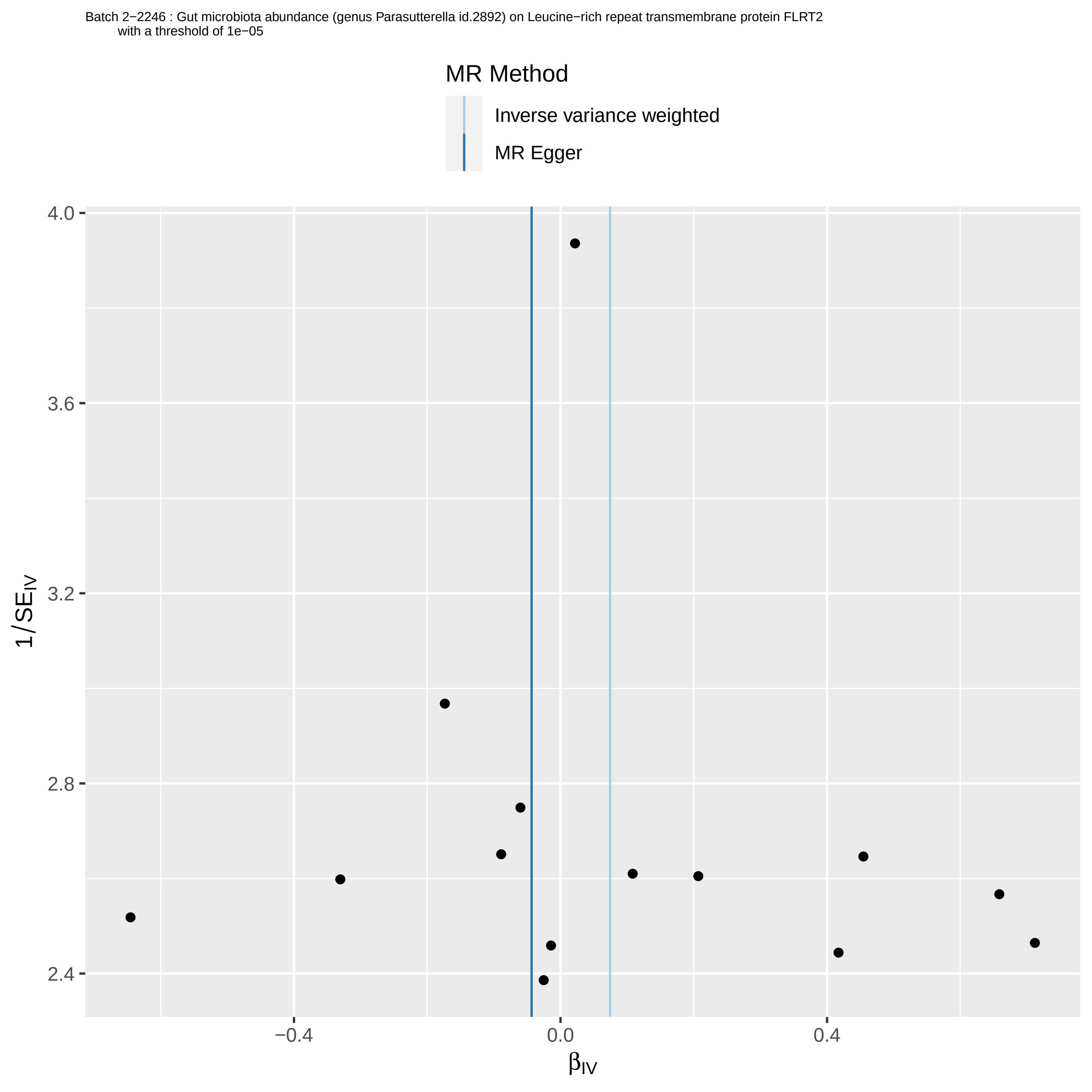
Batch 2−2246 : Gut microbiota abundance (genus Parasutterella id.2892) on Leucine−rich repeat transmembrane protein FLRT2
with a threshold of 1e−05
MR Method
Inverse variance weighted
MR Egger
4.0
3.6
3.2
2.8
2.4
1/SE_IV
−0.4
0.0
0.4
β_IV

Batch 2−2247 : Gut microbiota abundance (genus Catenibacterium id.2153) on Leucine−rich repeat transmembrane protein FLRT3
with a threshold of 1e−05

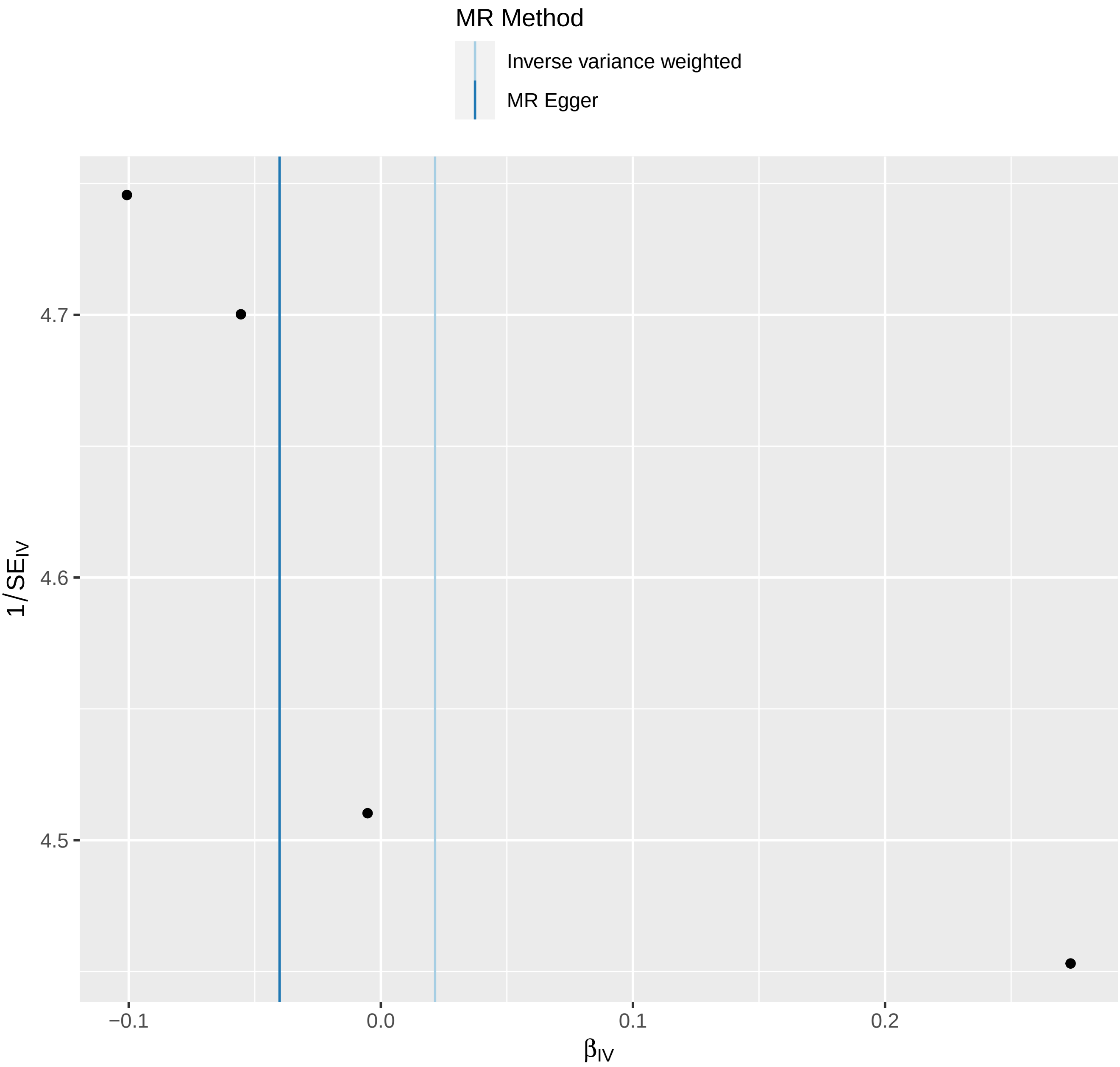

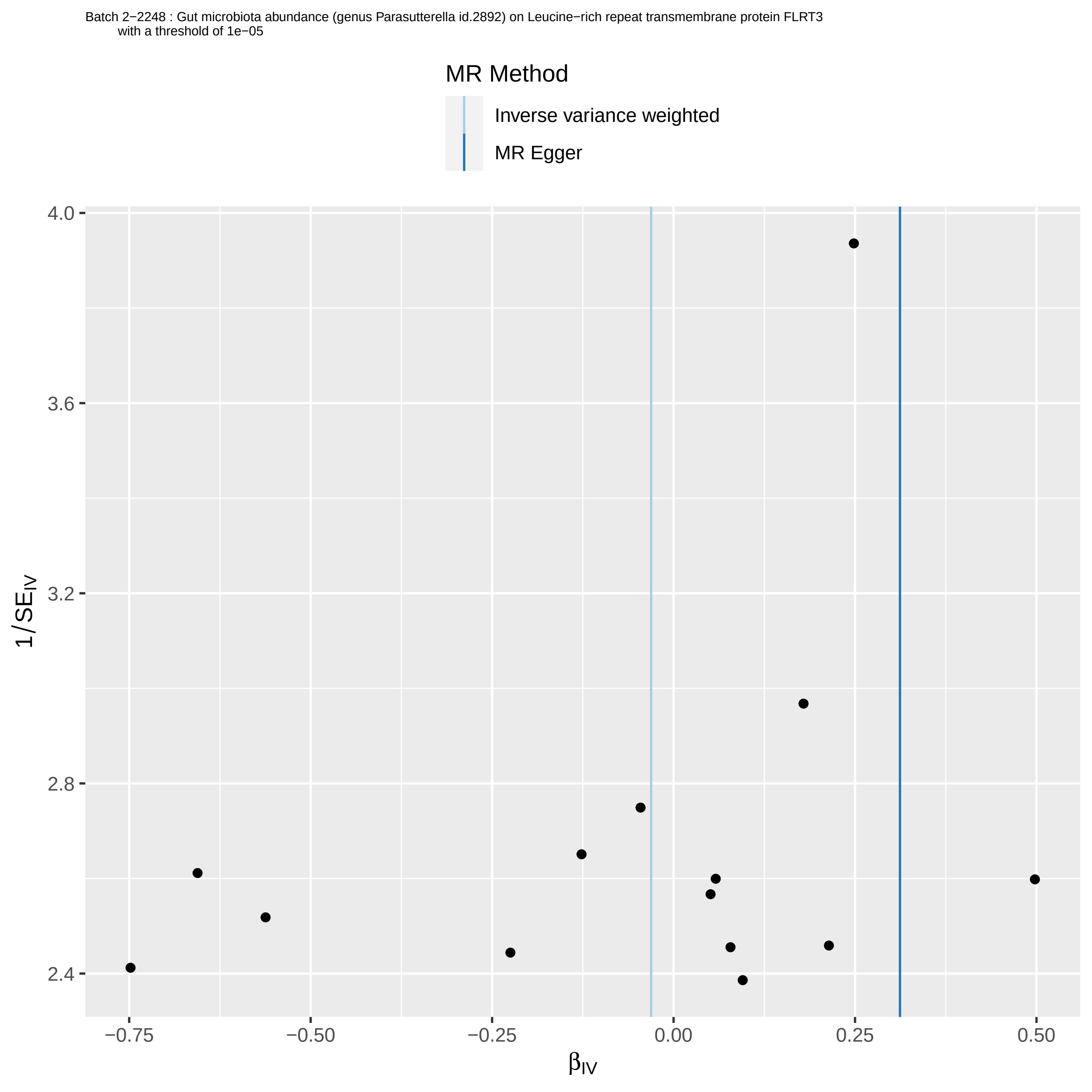

Batch 2−2248 : Gut microbiota abundance (genus Parasutterella id.2892) on Leucine−rich repeat transmembrane protein FLRT3
with a threshold of 1e−05
MR Method
Inverse variance weighted
MR Egger
4.0
3.6
3.2
2.8
2.4
1/SE_IV
−0.75
−0.50
−0.25
0.00
0.25
0.50
β_IV

Batch 2−2249 : Gut microbiota abundance (genus Catenibacterium id.2153) on Leucine−rich repeat transmembrane protein FLRT3
with a threshold of 1e−05

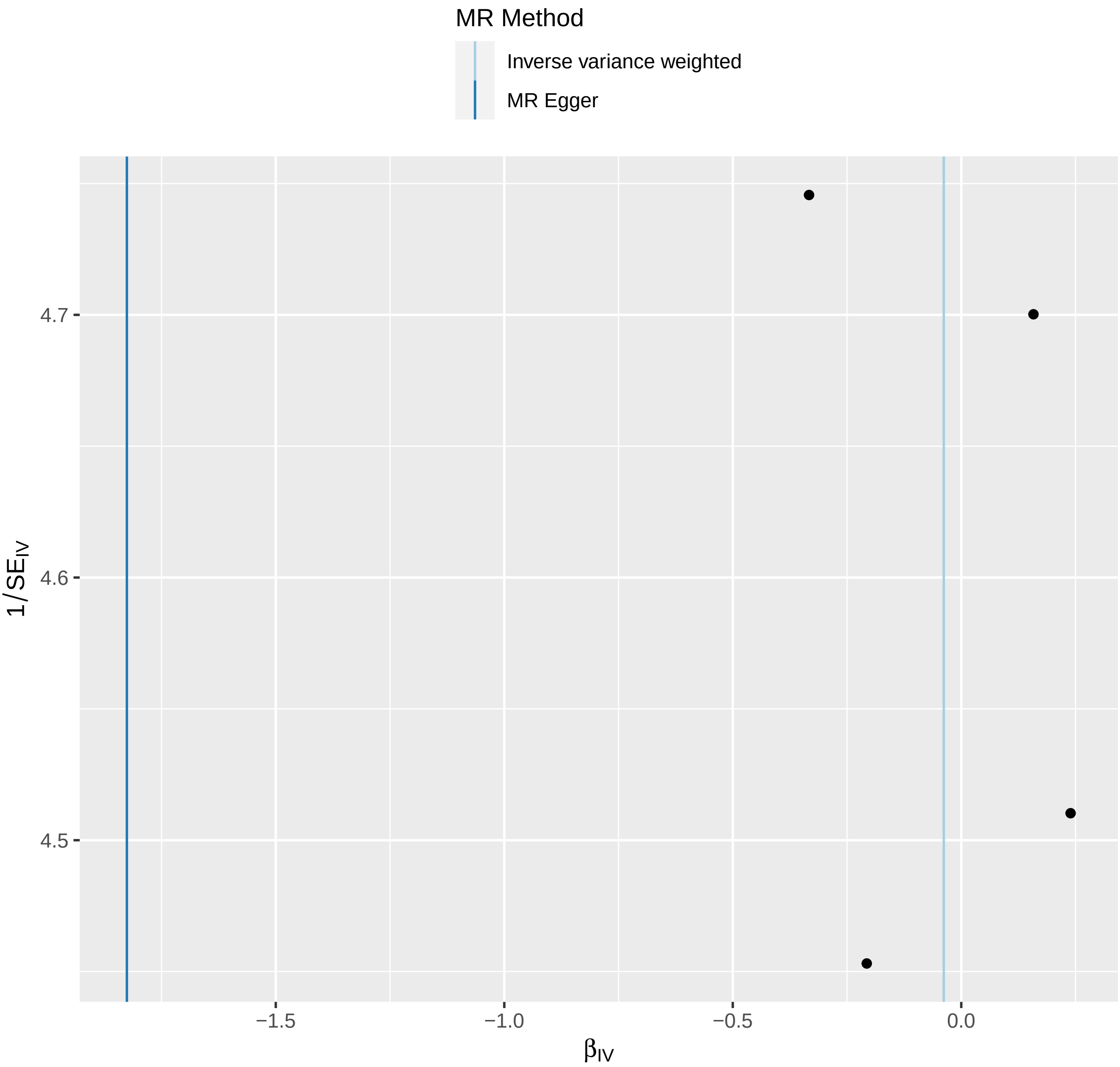

Batch 2−2250 : Gut microbiota abundance (genus Parasutterella id.2892) on Leucine−rich repeat transmembrane protein FLRT3
with a threshold of 1e−05

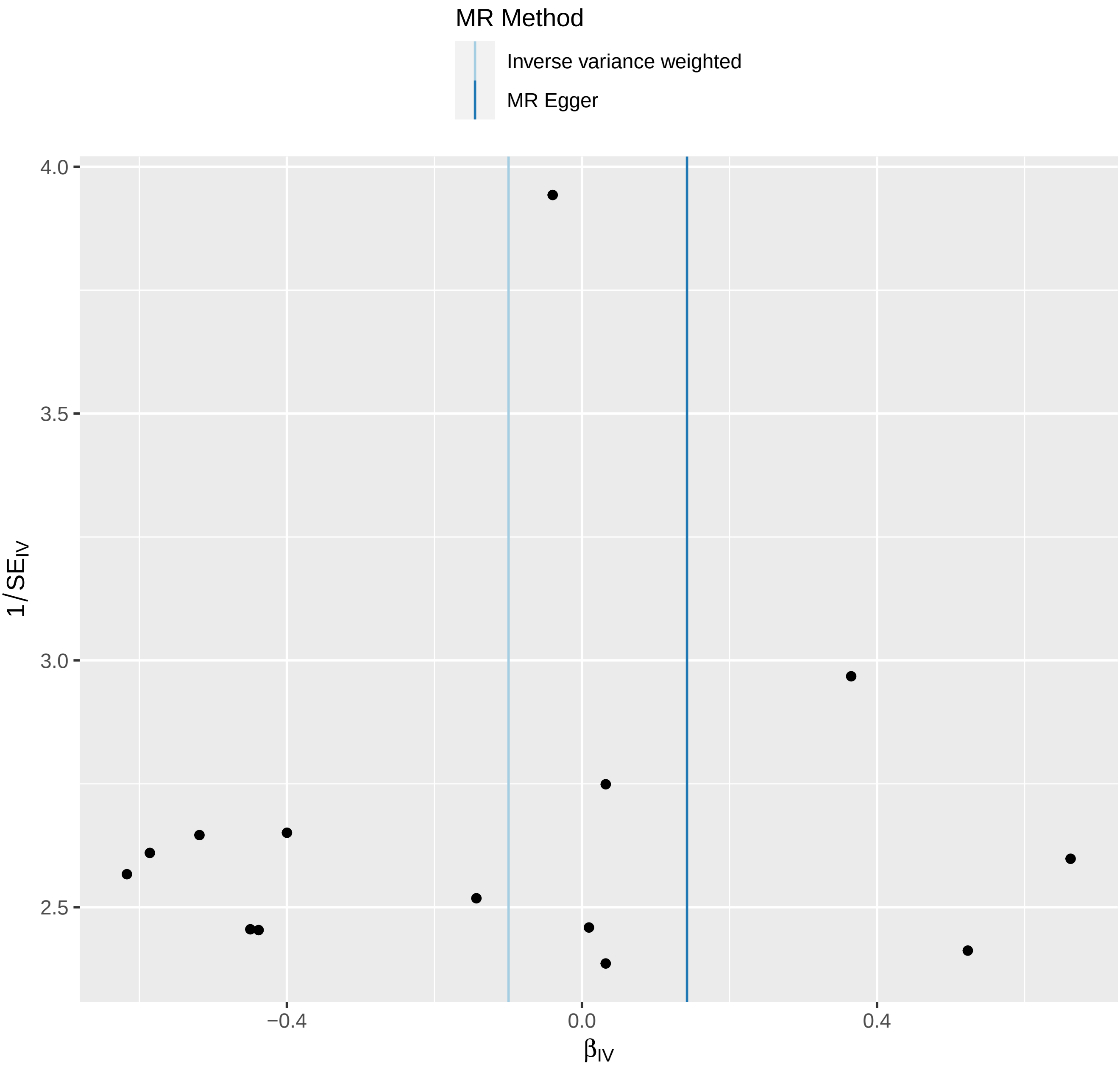

Batch 2−2251 : Gut microbiota abundance (genus Catenibacterium id.2153) on Receptor−type tyrosine−protein kinase FLT3
with a threshold of 1e−05

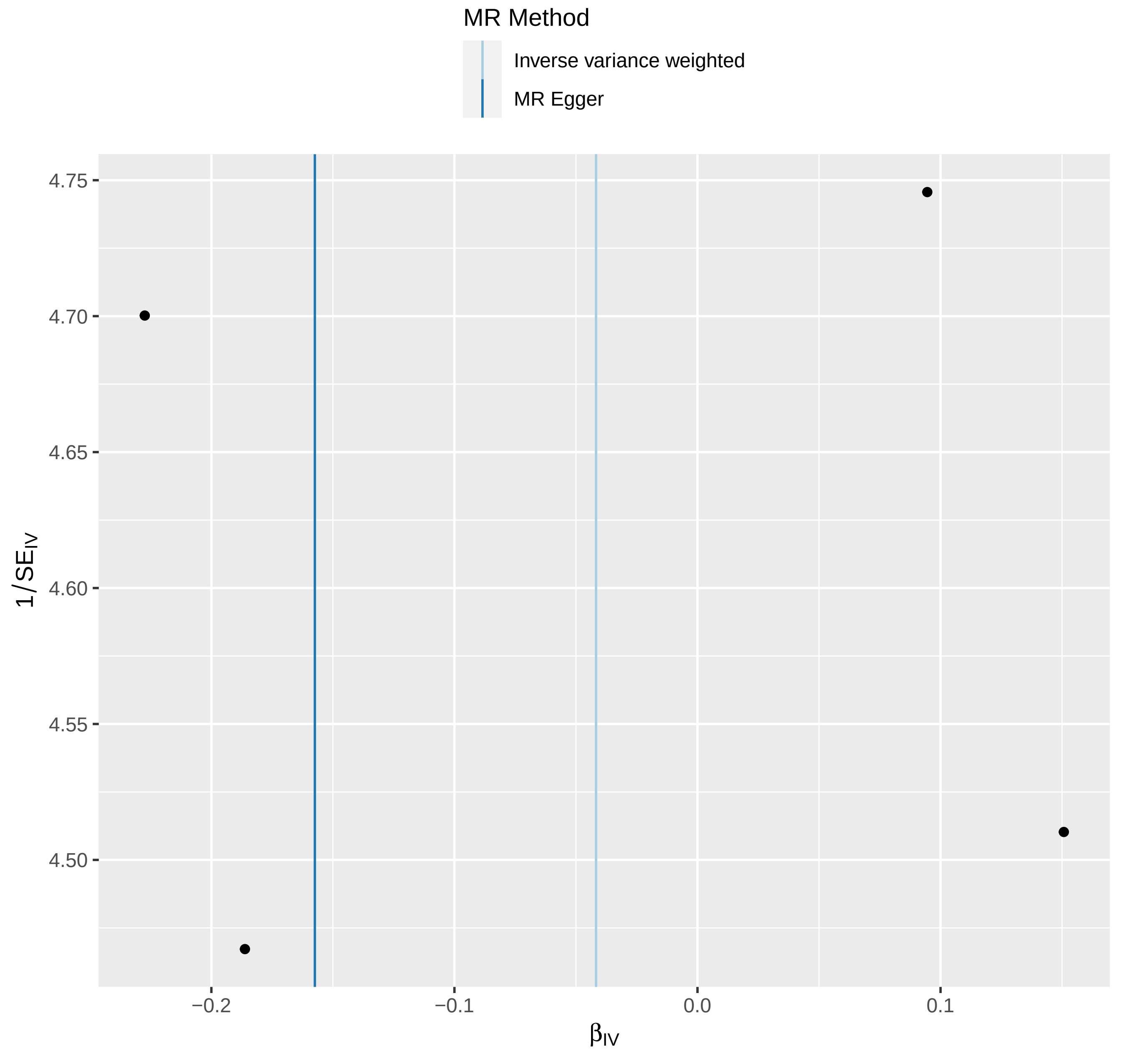

Batch 2−2252 : Gut microbiota abundance (genus Parasutterella id.2892) on Receptor−type tyrosine−protein kinase FLT3
with a threshold of 1e−05

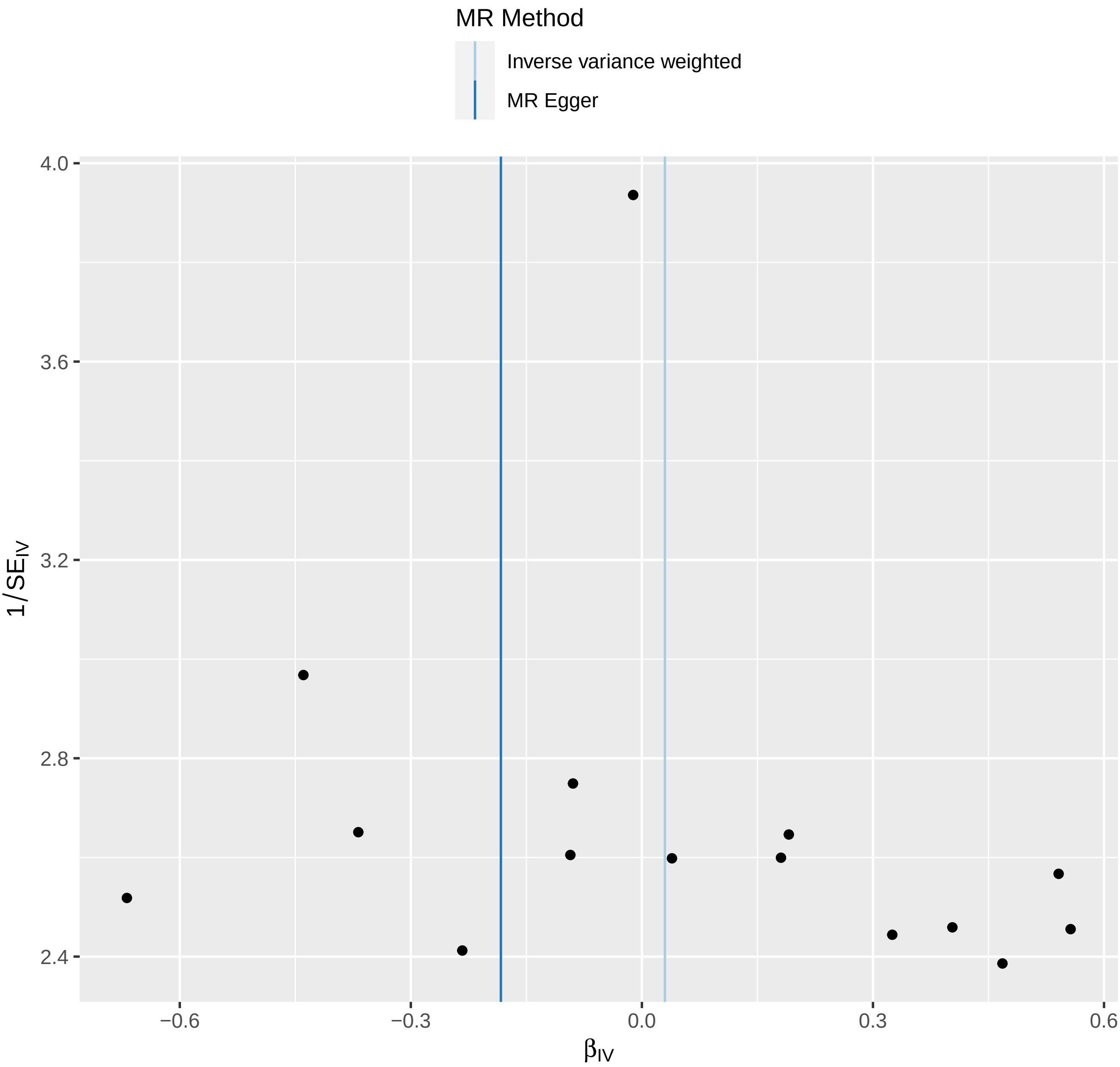

Batch 2−2253 : Gut microbiota abundance (genus Catenibacterium id.2153) on Fms−related tyrosine kinase 3 ligand
with a threshold of 1e−05

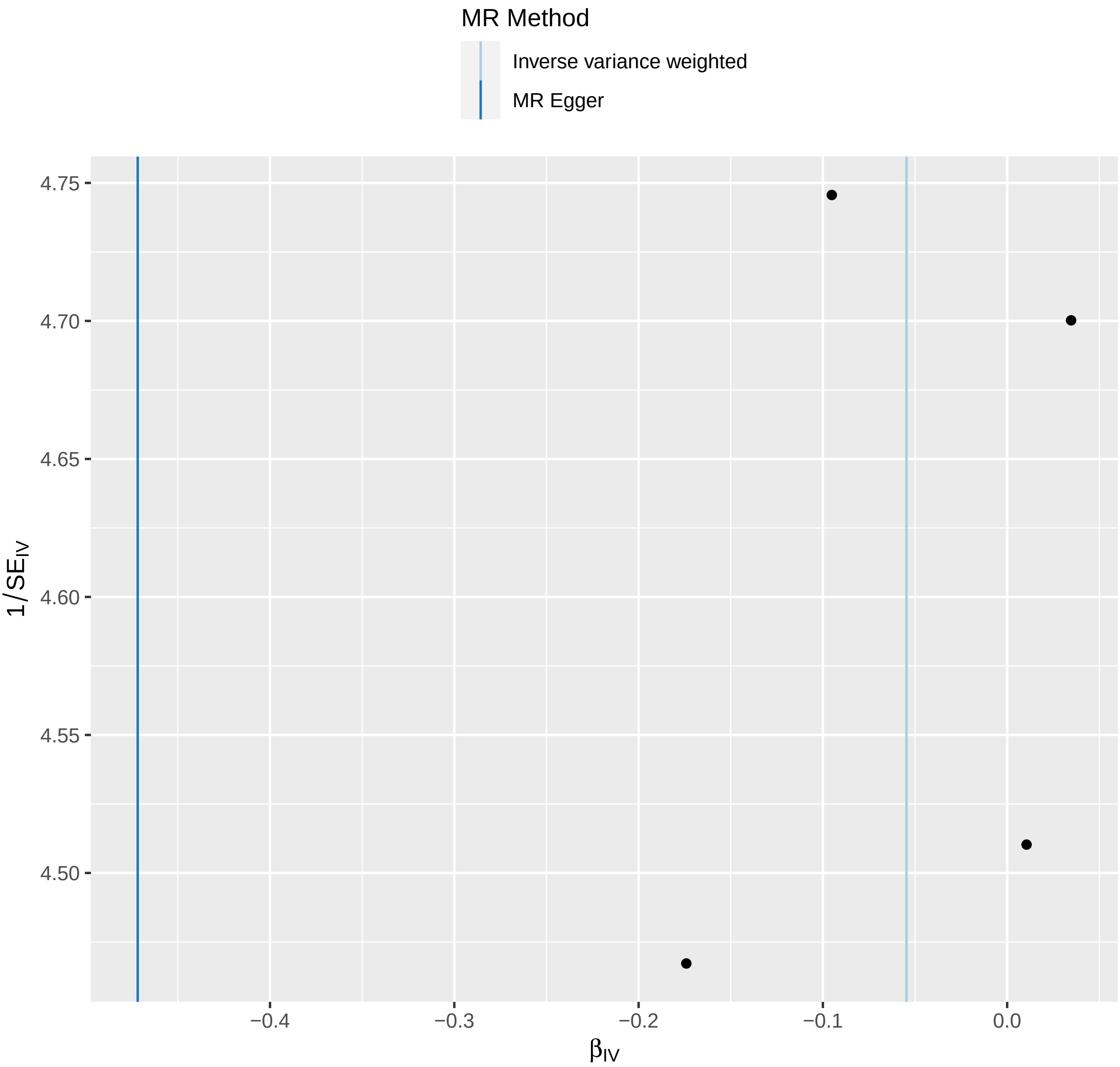

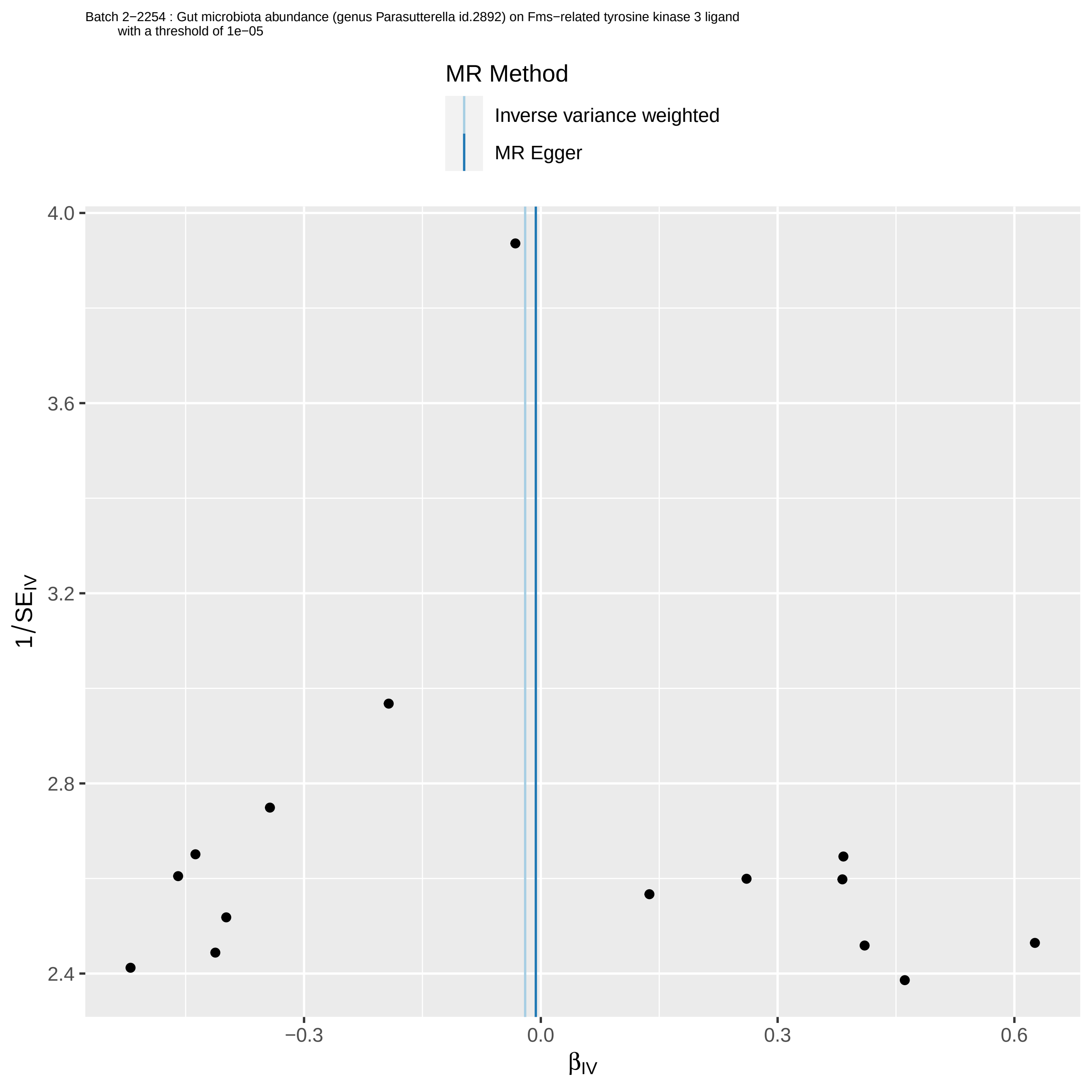
Batch 2−2254 : Gut microbiota abundance (genus Parasutterella id.2892) on Fms−related tyrosine kinase 3 ligand
with a threshold of 1e−05
MR Method
Inverse variance weighted
MR Egger
4.0
3.6
3.2
2.8
2.4
1/SE_IV
−0.3
0.0
0.3
0.6
β_IV

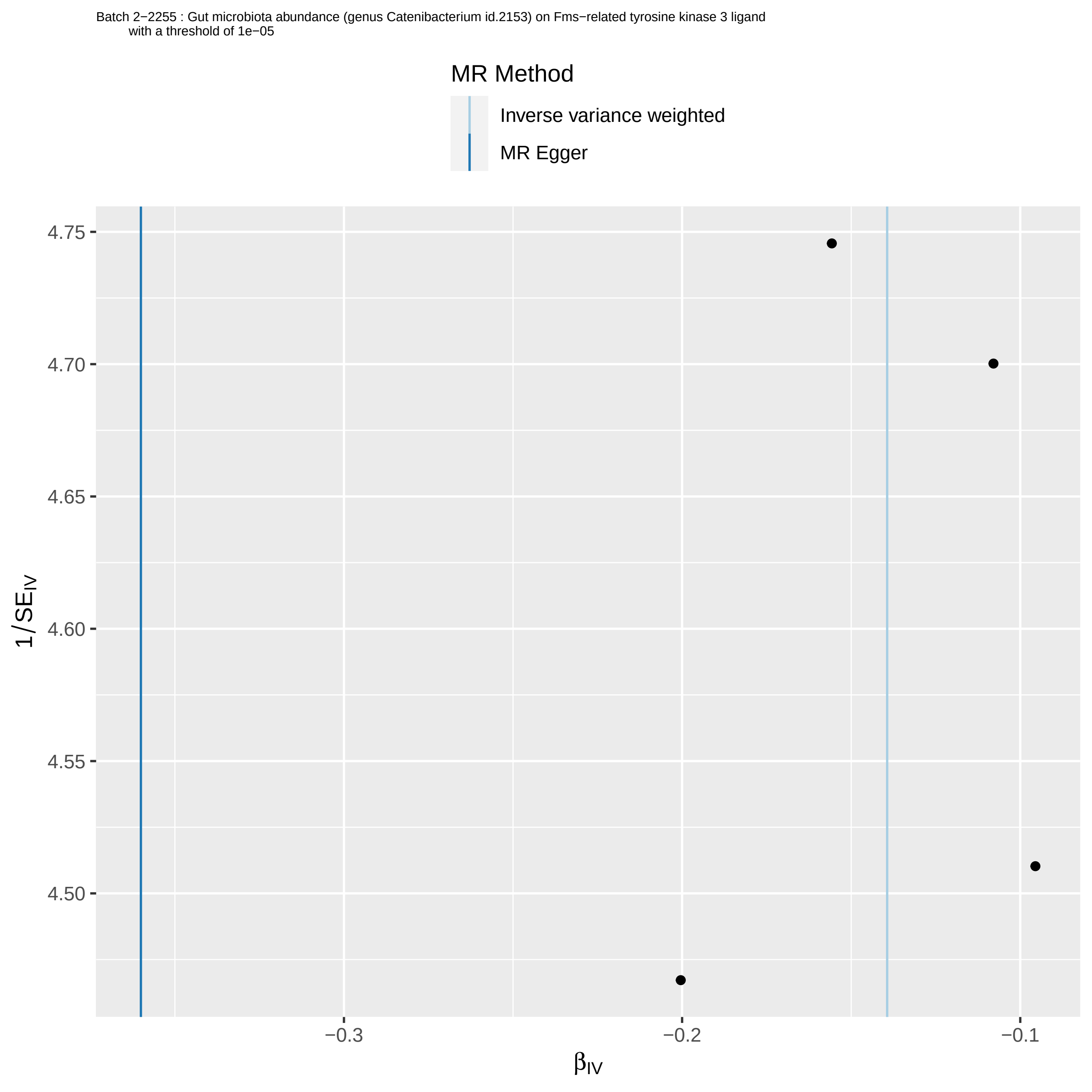
Batch 2−2255 : Gut microbiota abundance (genus Catenibacterium id.2153) on Fms−related tyrosine kinase 3 ligand with a threshold of 1e−05
MR Method
Inverse variance weighted
MR Egger
4.75
4.70
4.65
4.60
4.55
4.50
1/SE_IV
−0.3
−0.2
−0.1
β_IV

Batch 2−2256 : Gut microbiota abundance (genus Parasutterella id.2892) on Fms−related tyrosine kinase 3 ligand
with a threshold of 1e−05

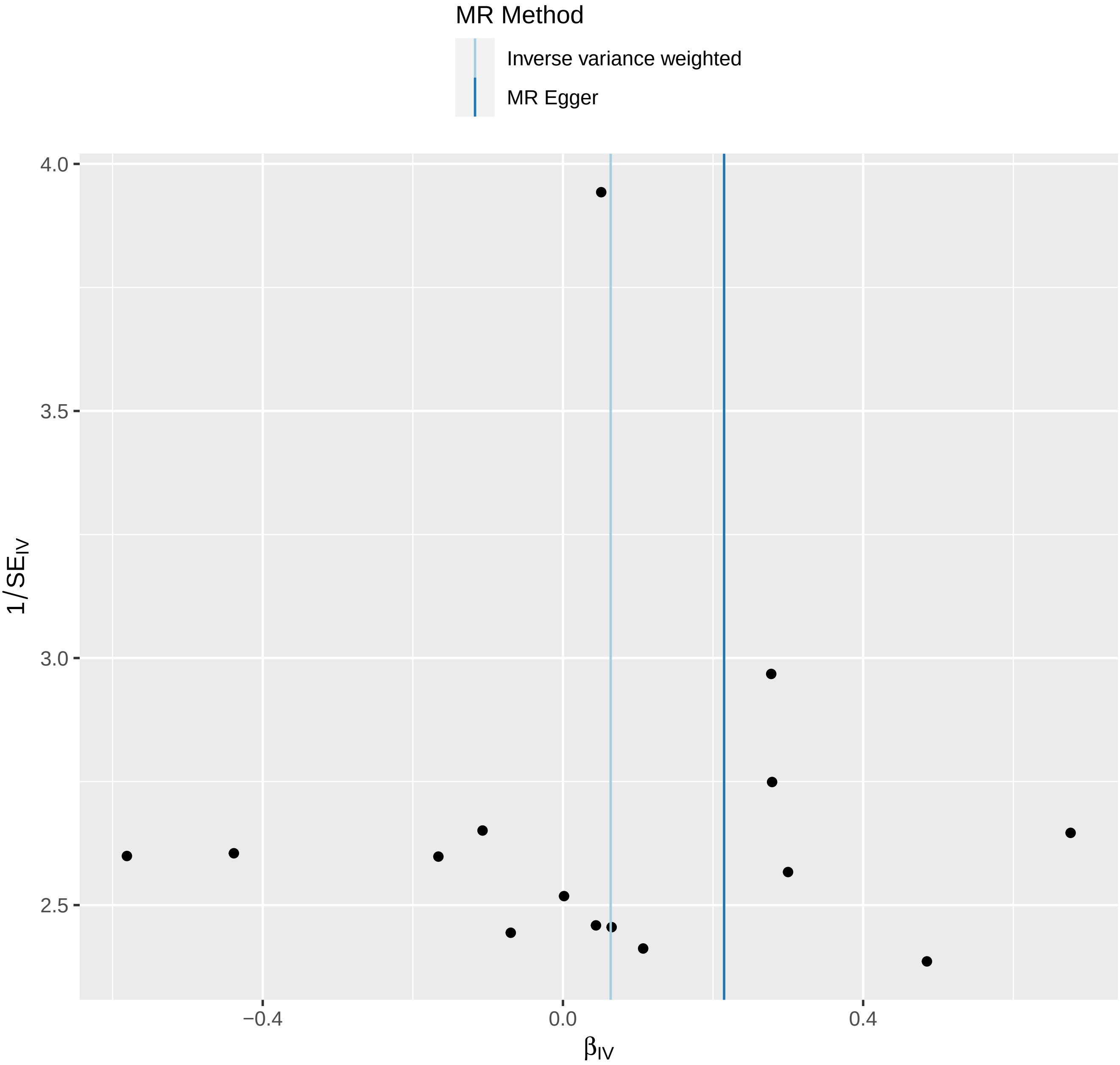

Batch 2−2257 : Gut microbiota abundance (genus Catenibacterium id.2153) on Vascular endothelial growth factor receptor 3
with a threshold of 1e−05

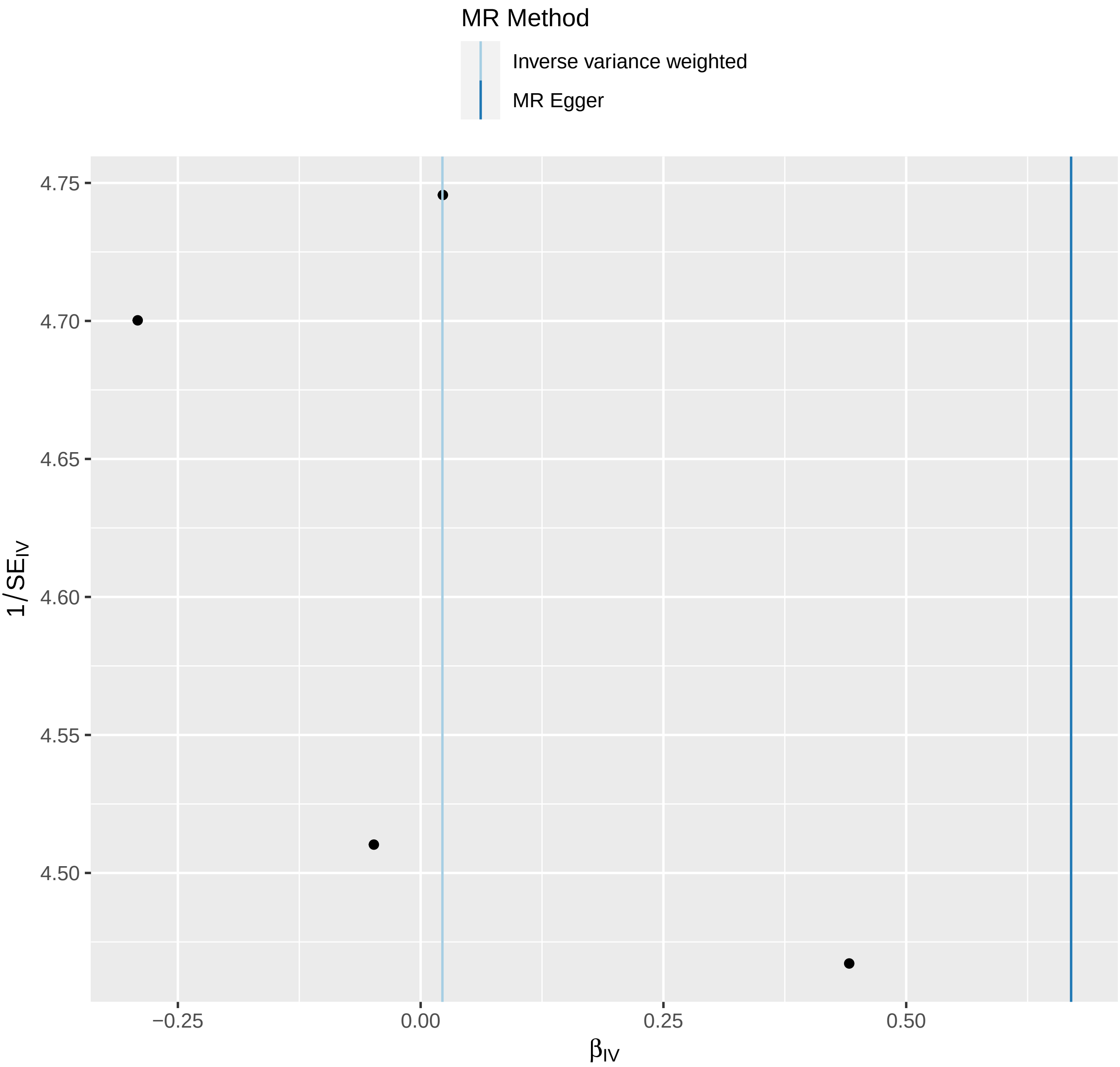

Batch 2−2258 : Gut microbiota abundance (genus Parasutterella id.2892) on Vascular endothelial growth factor receptor 3 with a threshold of 1e−05

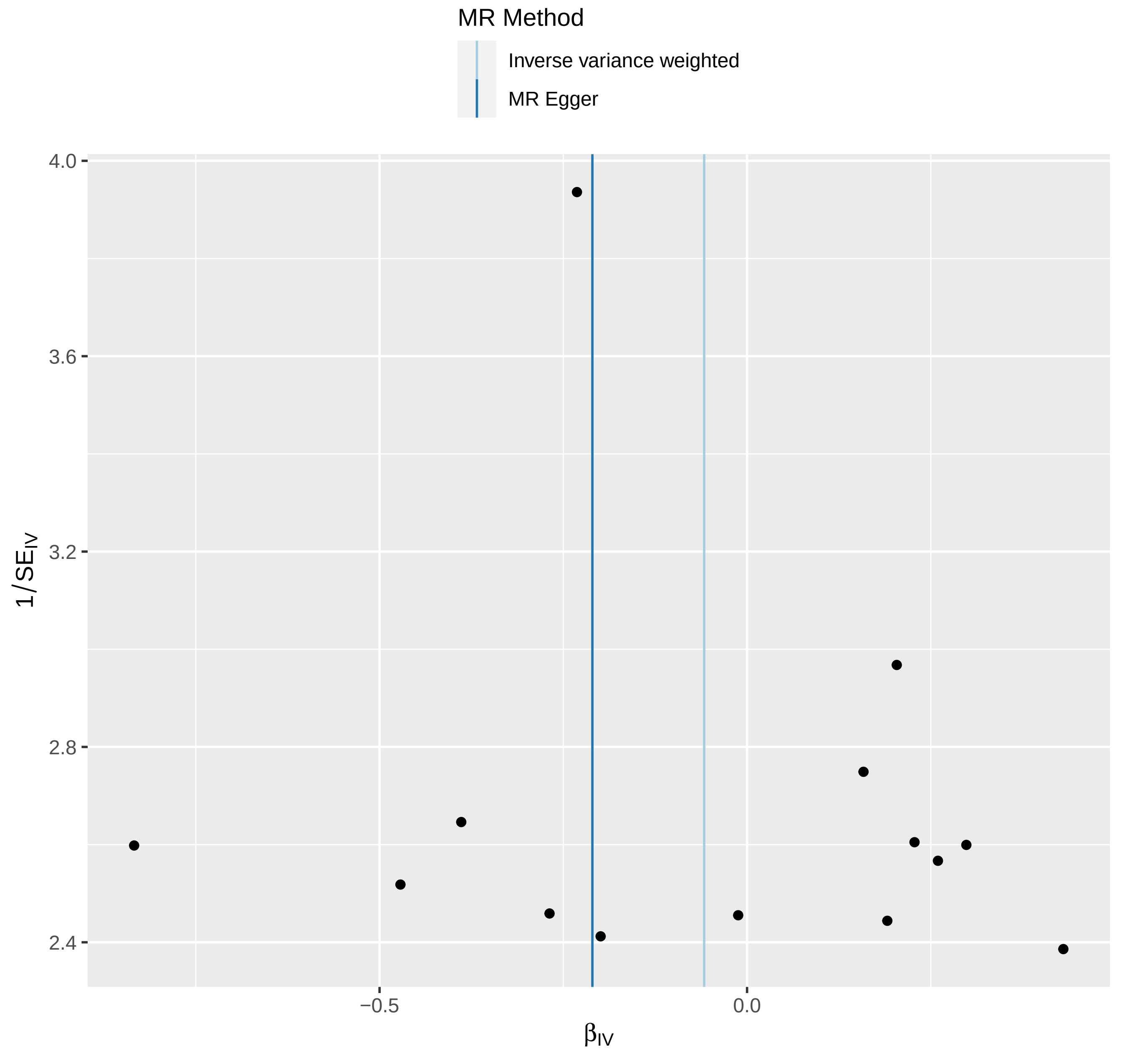

Batch 2−2259 : Gut microbiota abundance (genus Catenibacterium id.2153) on Feline leukemia virus subgroup C receptor−related protein 1 with a threshold of 1e−05

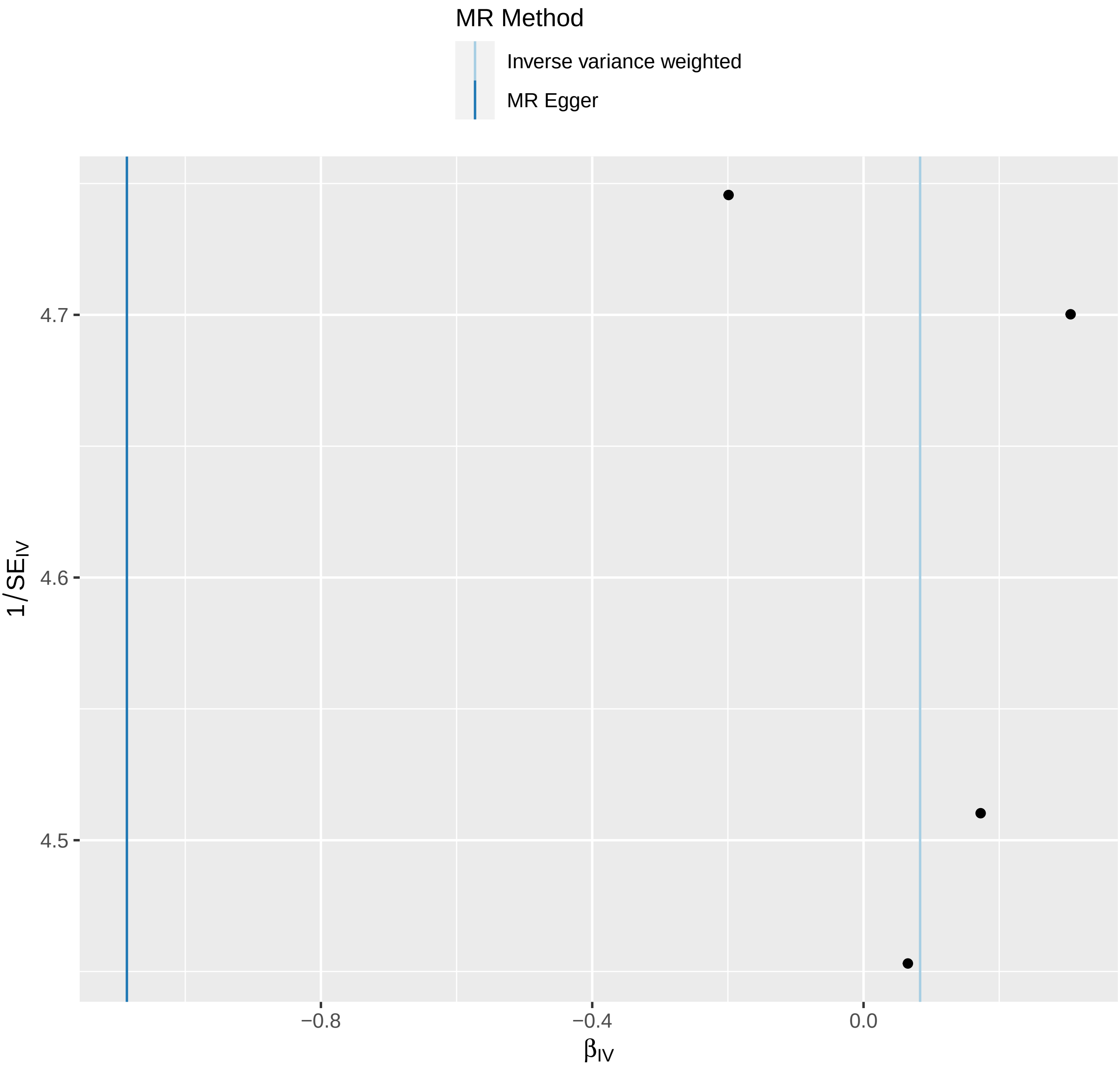

Batch 2−2260 : Gut microbiota abundance (genus Parasutterella id.2892) on Feline leukemia virus subgroup C receptor−related protein 1
with a threshold of 1e−05

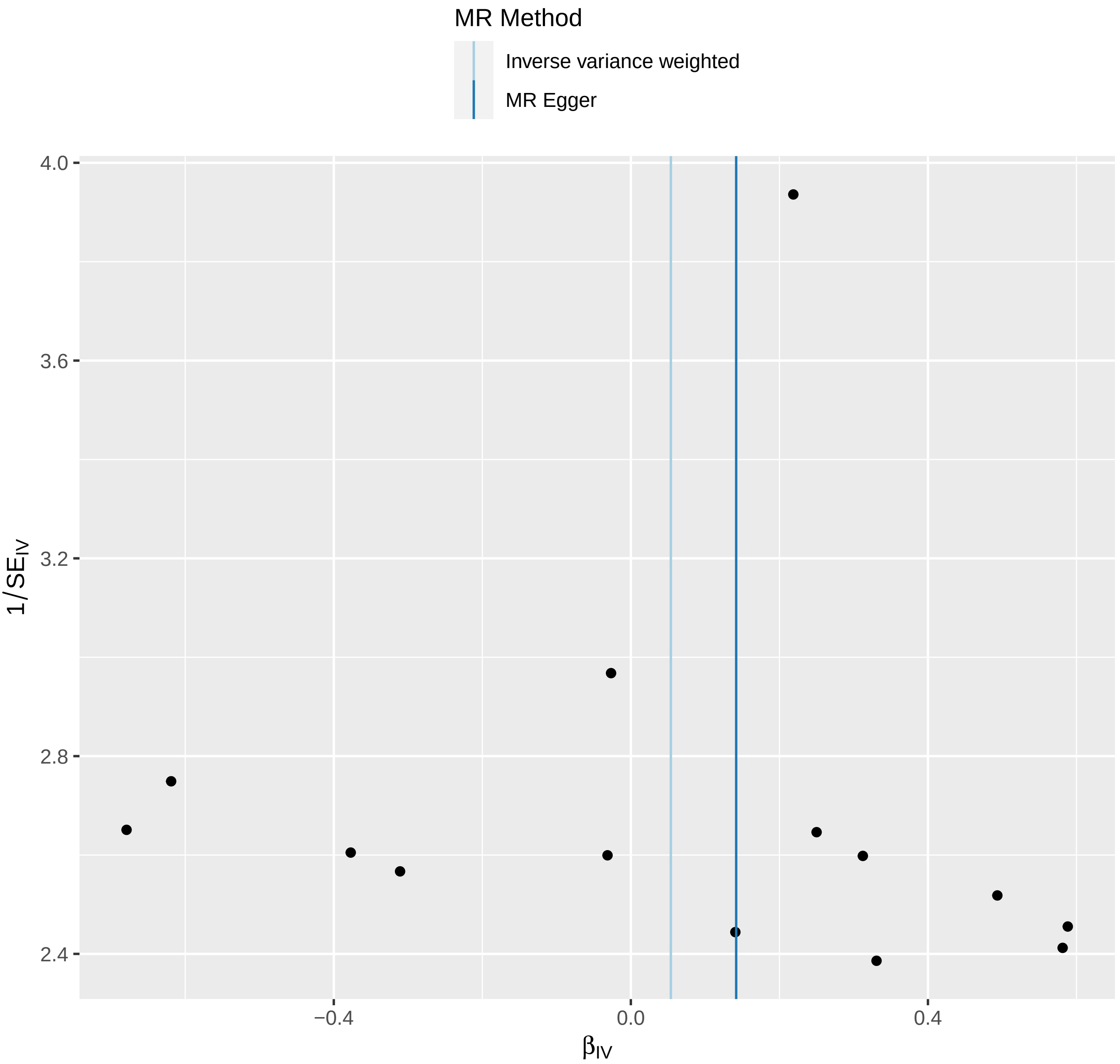

Batch 2−2261 : Gut microbiota abundance (genus Catenibacterium id.2153) on Fibronectin Fragment 4
with a threshold of 1e−05

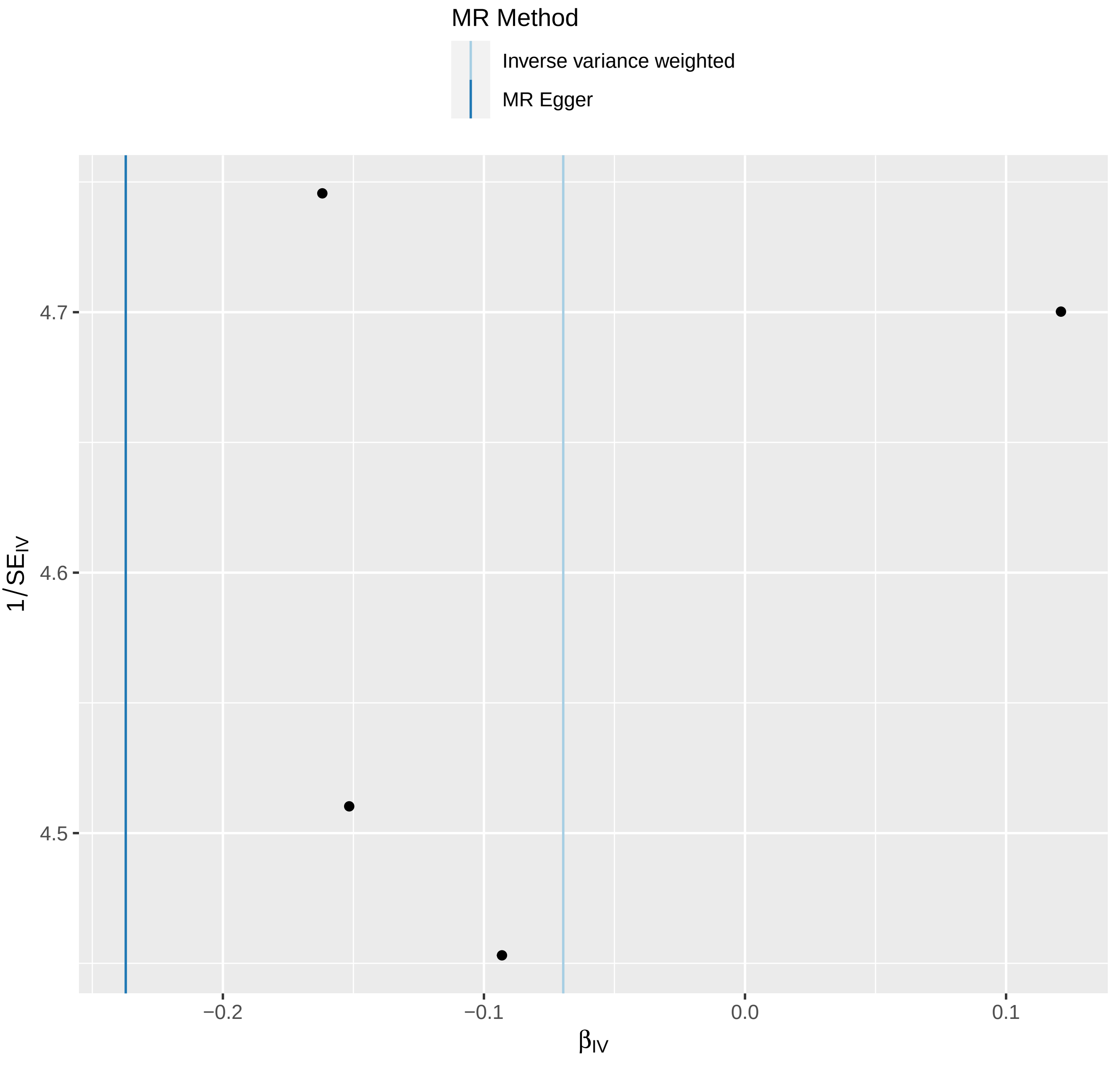

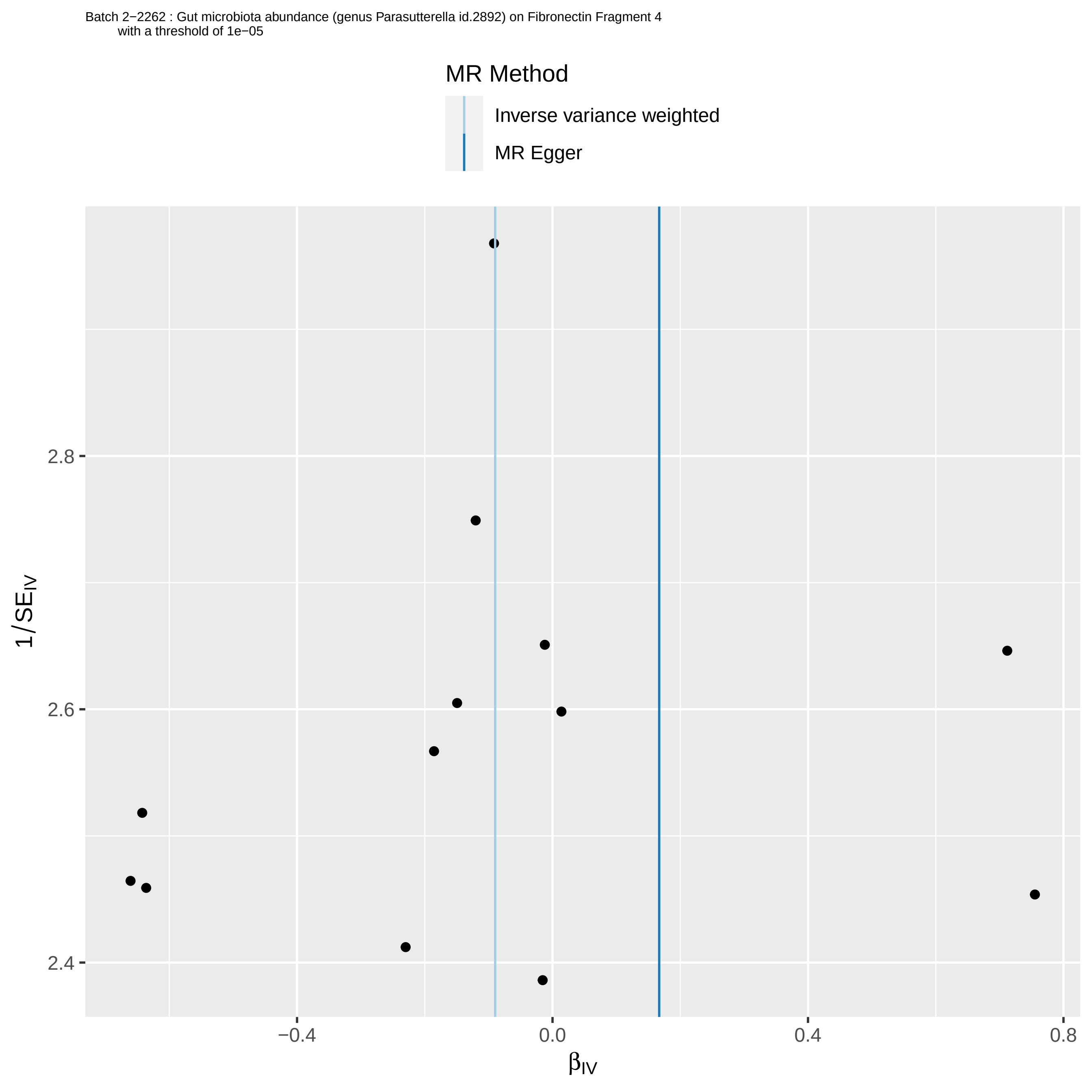
Batch 2−2262 : Gut microbiota abundance (genus Parasutterella id.2892) on Fibronectin Fragment 4
with a threshold of 1e−05
MR Method
Inverse variance weighted
MR Egger
2.8
2.6
2.4
1/SE_IV
−0.4
0.0
0.4
0.8
β_IV

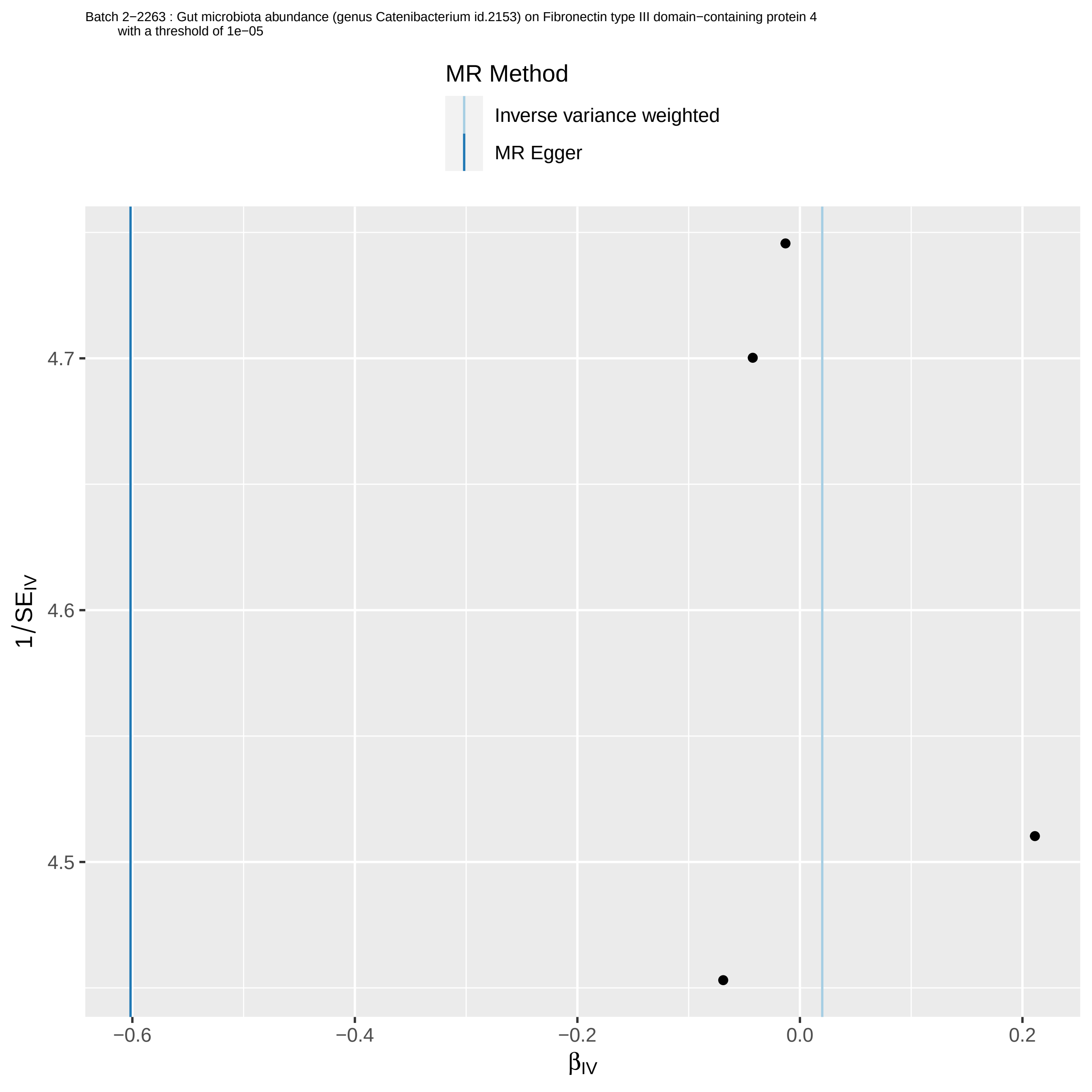

Batch 2−2263 : Gut microbiota abundance (genus Catenibacterium id.2153) on Fibronectin type III domain−containing protein 4
with a threshold of 1e−05
MR Method
Inverse variance weighted
MR Egger
4.7
4.6
4.5
1/SE_IV
−0.6
−0.4
−0.2
0.0
0.2
β_IV

Batch 2−2264 : Gut microbiota abundance (genus Parasutterella id.2892) on Fibronectin type III domain−containing protein 4
with a threshold of 1e−05

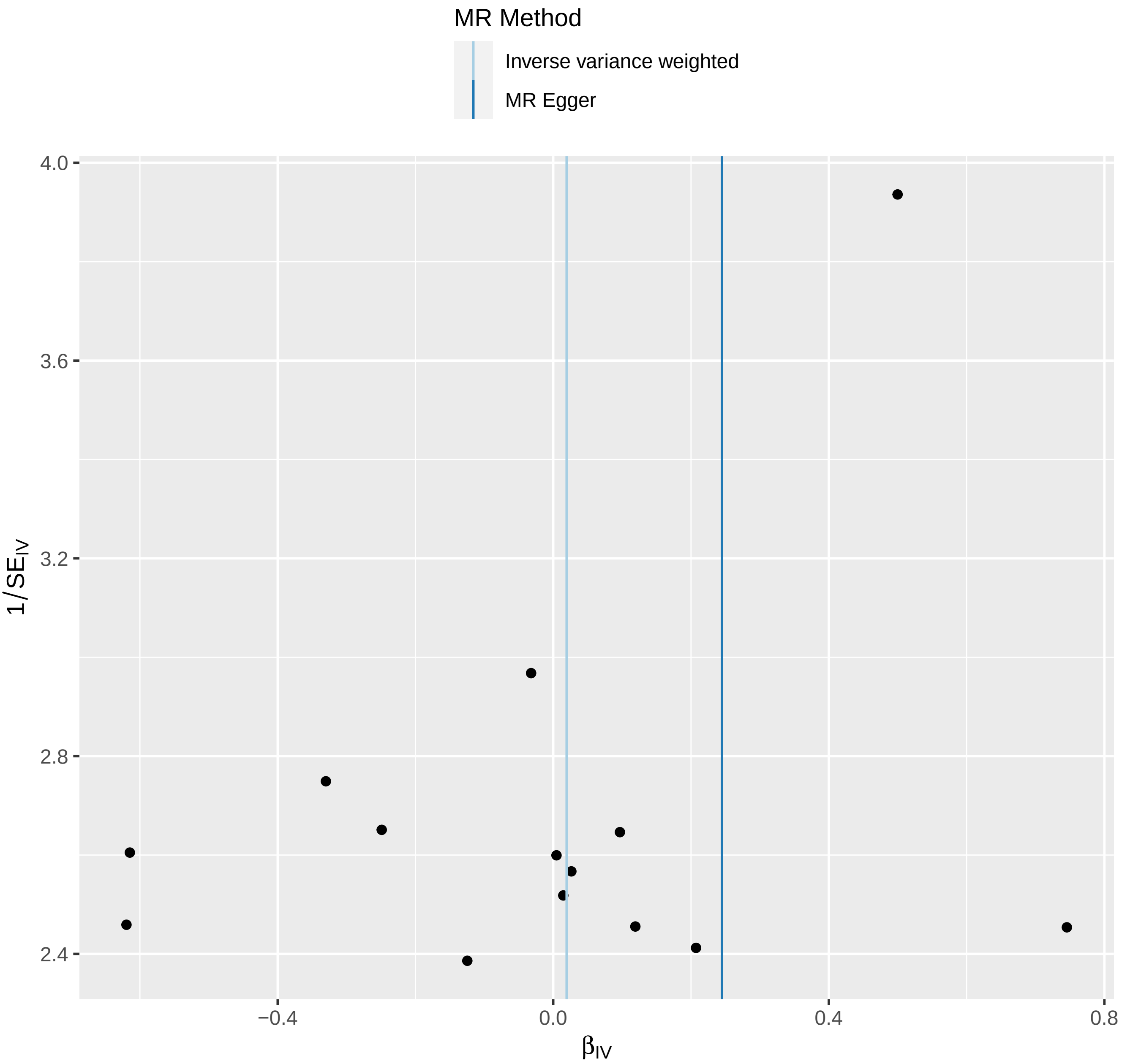

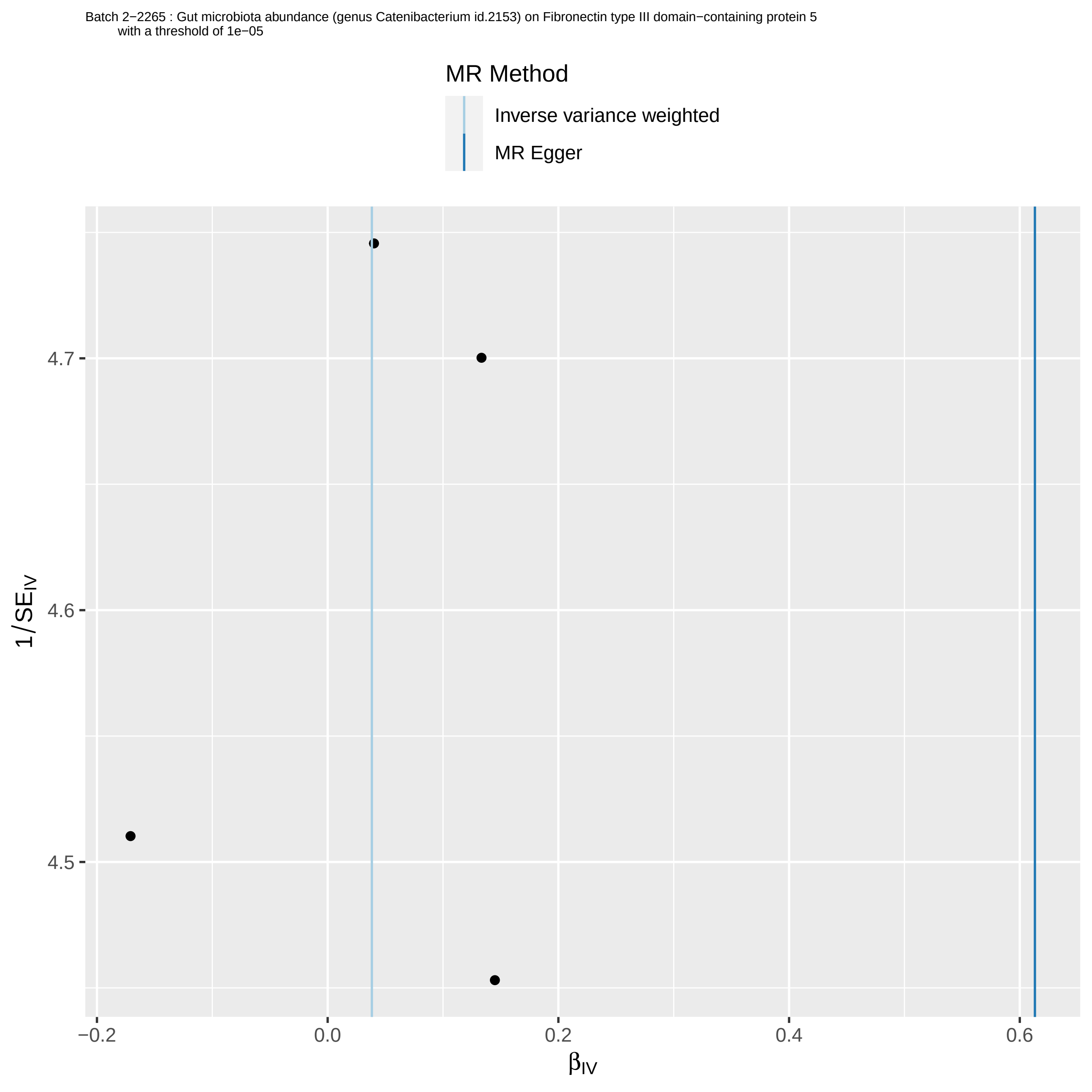

Batch 2−2265 : Gut microbiota abundance (genus Catenibacterium id.2153) on Fibronectin type III domain−containing protein 5
with a threshold of 1e−05
MR Method
Inverse variance weighted
MR Egger
4.7
4.6
4.5
$1/SE_{IV}$
−0.2
0.0
0.2
0.4
0.6
β_{IV}

Batch 2−2266 : Gut microbiota abundance (genus Parasutterella id.2892) on Fibronectin type III domain−containing protein 5
with a threshold of 1e−05

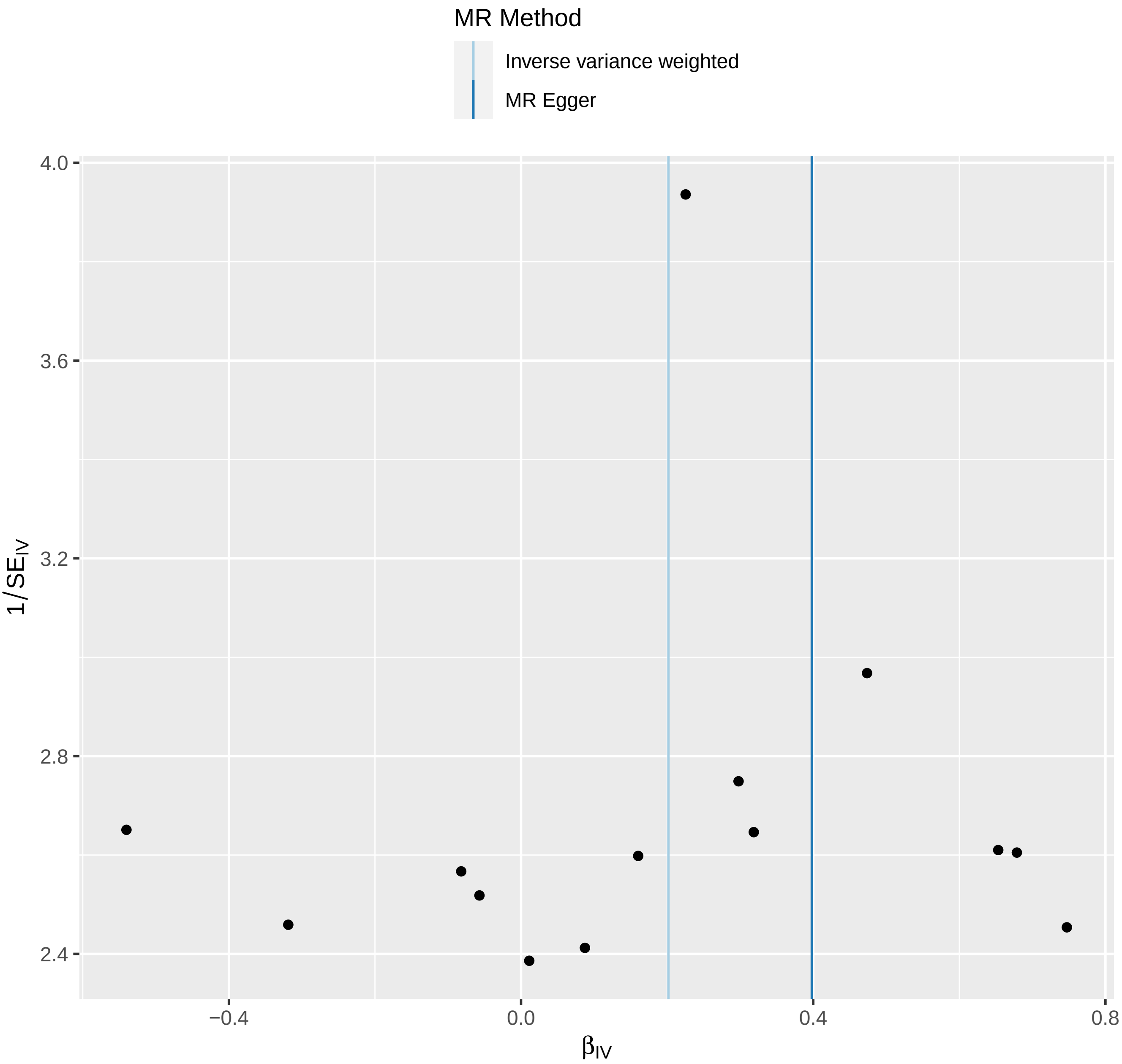

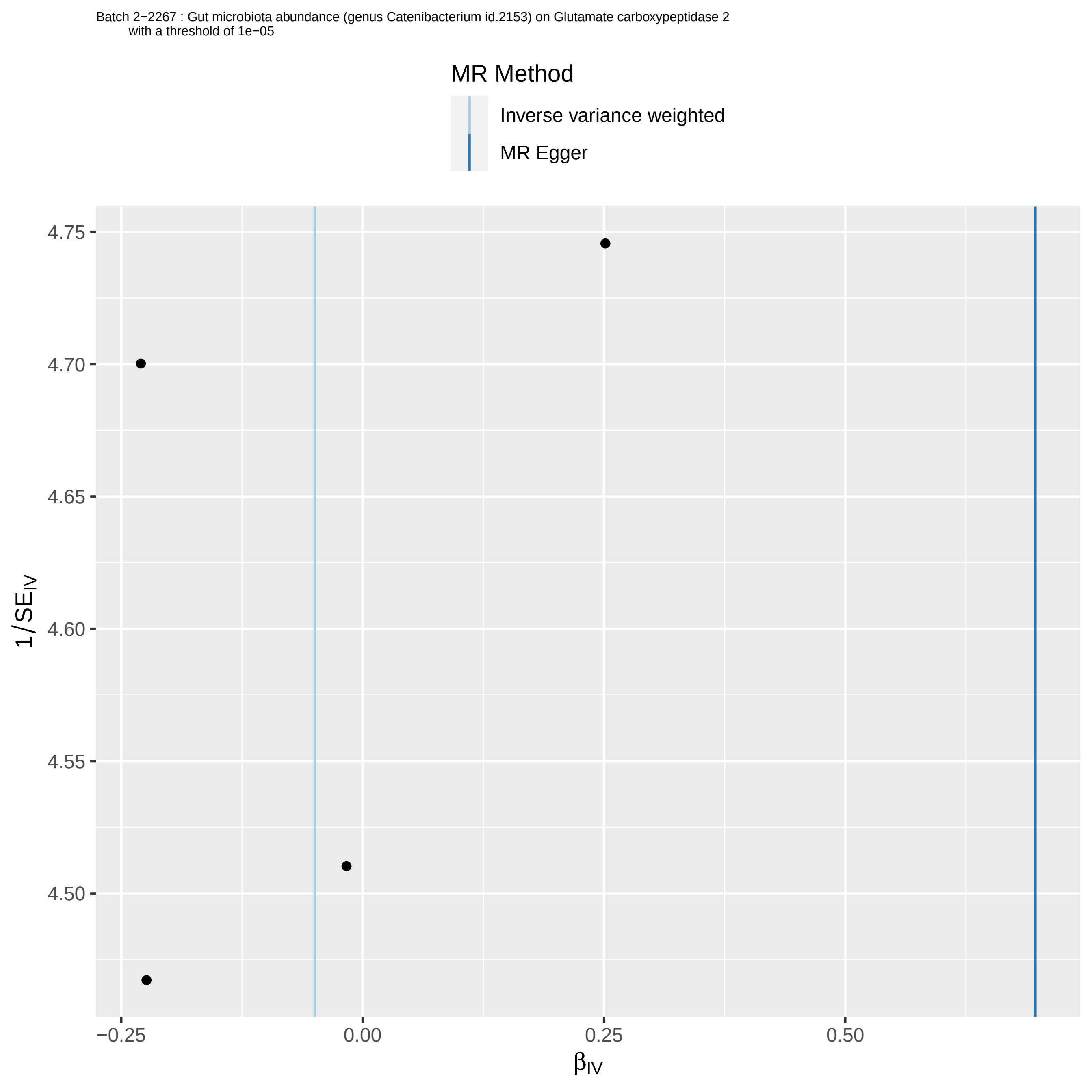
Batch 2−2267 : Gut microbiota abundance (genus Catenibacterium id.2153) on Glutamate carboxypeptidase 2
with a threshold of 1e−05
MR Method
Inverse variance weighted
MR Egger
4.75
4.70
4.65
4.60
4.55
4.50
1/SE_IV
−0.25
0.00
0.25
0.50
β_IV

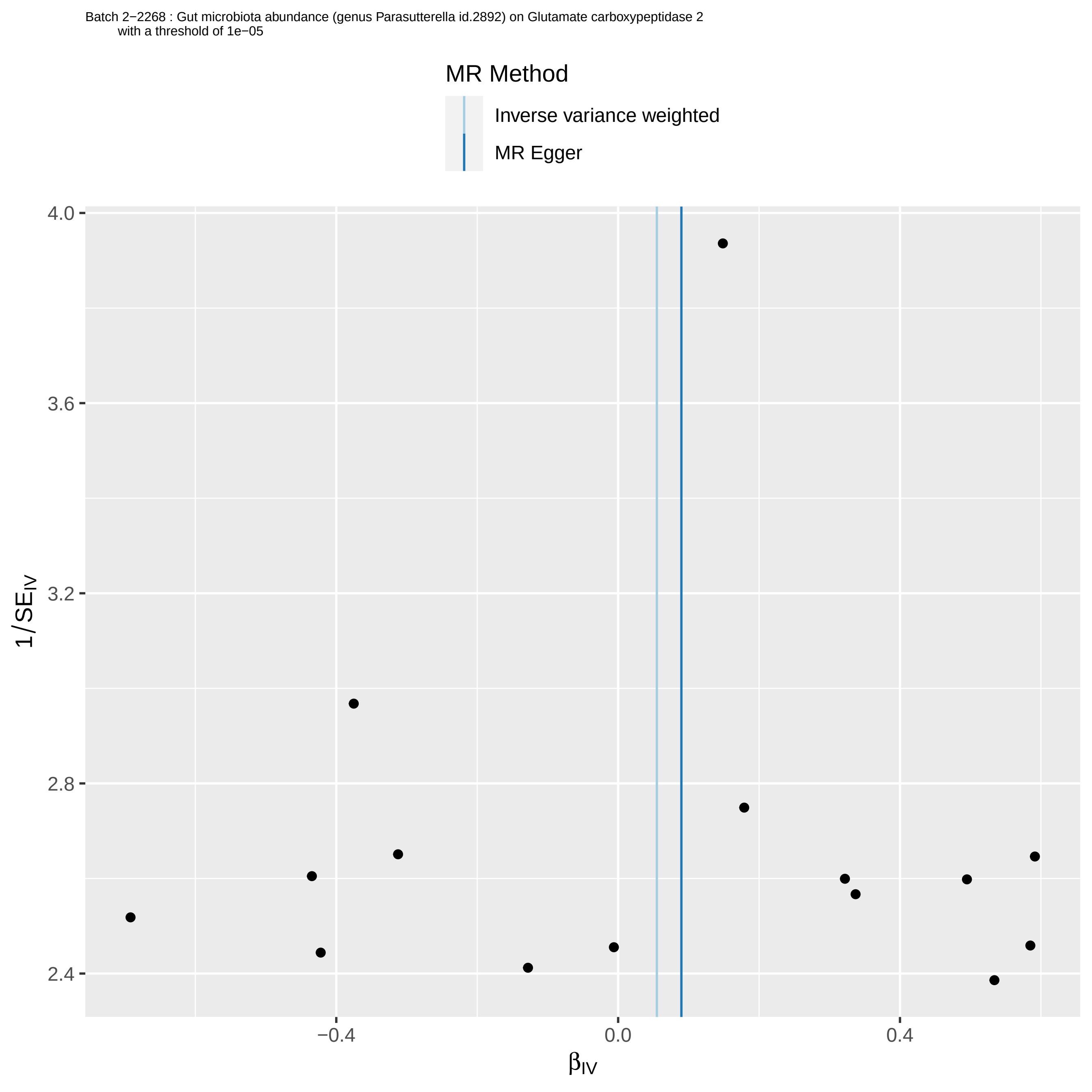

Batch 2−2268 : Gut microbiota abundance (genus Parasutterella id.2892) on Glutamate carboxypeptidase 2
with a threshold of 1e−05
MR Method
Inverse variance weighted
MR Egger
4.0
3.6
3.2
2.8
2.4
1/SE_IV
−0.4
0.0
0.4
β_IV

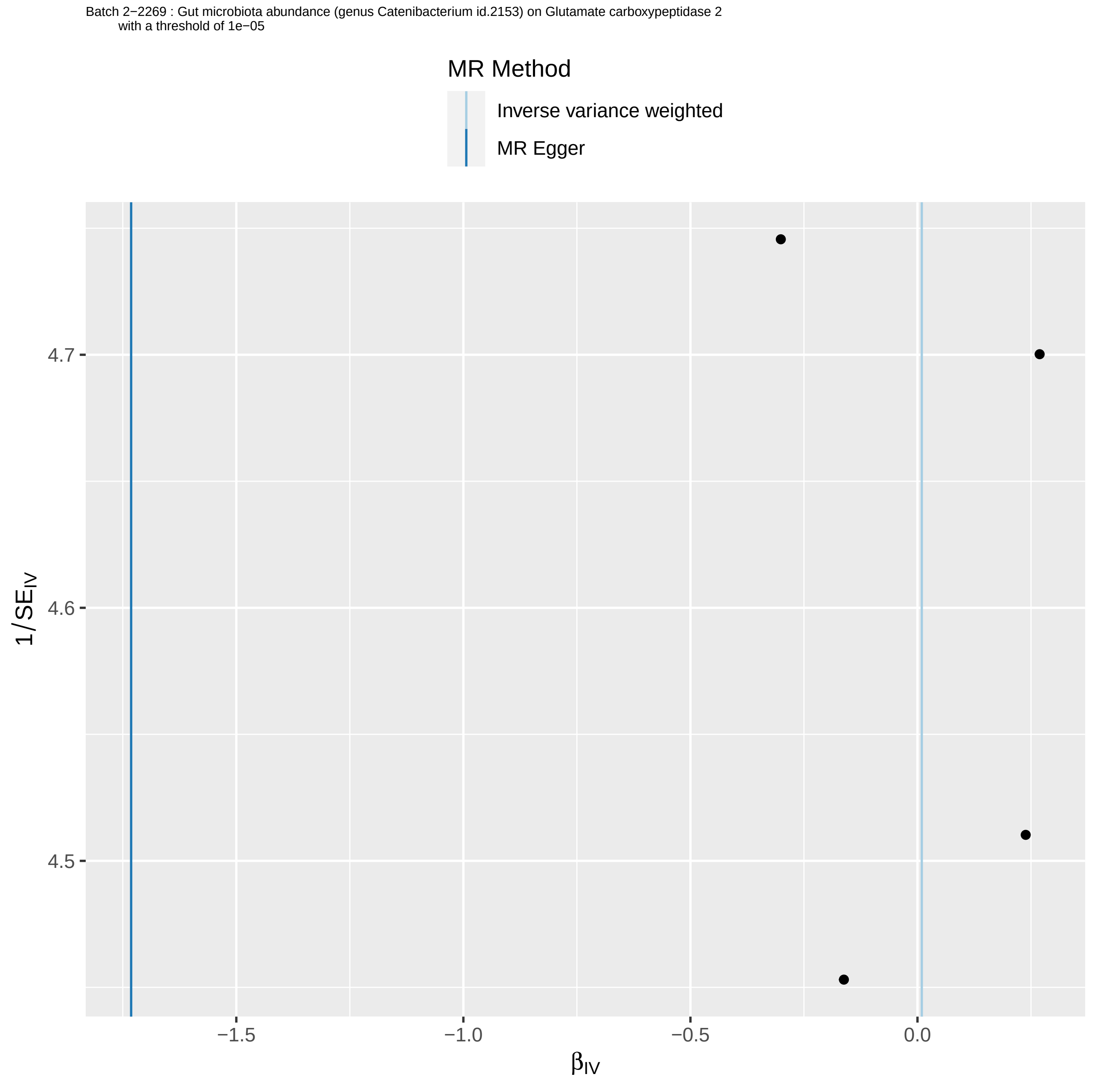
Batch 2−2269 : Gut microbiota abundance (genus Catenibacterium id.2153) on Glutamate carboxypeptidase 2
with a threshold of 1e−05
MR Method
Inverse variance weighted
MR Egger
4.7
4.6
4.5
1/SE_IV
−1.5
−1.0
−0.5
0.0
β_IV

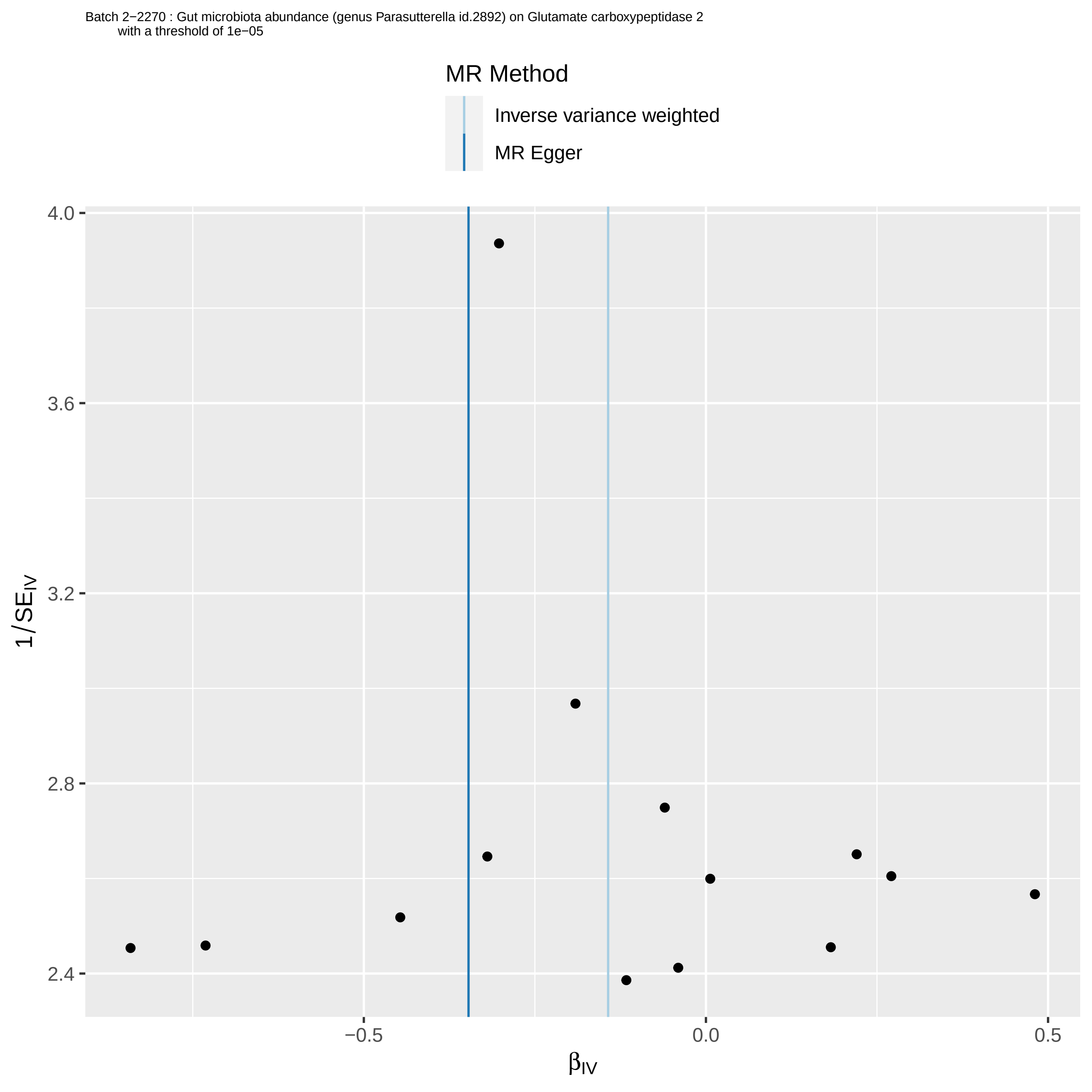

Batch 2−2270 : Gut microbiota abundance (genus Parasutterella id.2892) on Glutamate carboxypeptidase 2
with a threshold of 1e−05
MR Method
Inverse variance weighted
MR Egger
4.0
3.6
3.2
2.8
2.4
1/SE$_{IV}$
−0.5
0.0
0.5
β$_{IV}$

Batch 2−2271 : Gut microbiota abundance (genus Catenibacterium id.2153) on Forkhead box protein C2
with a threshold of 1e−05

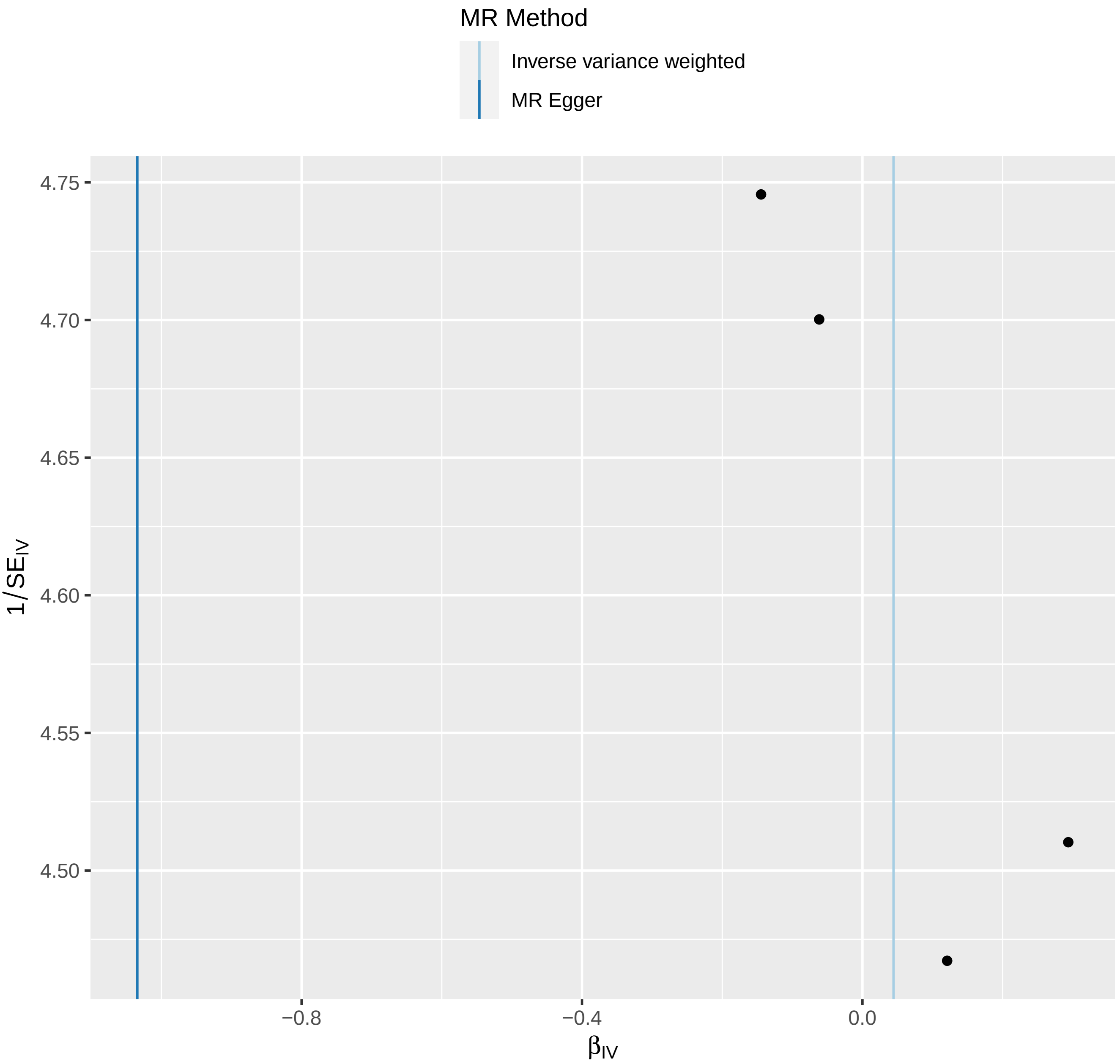

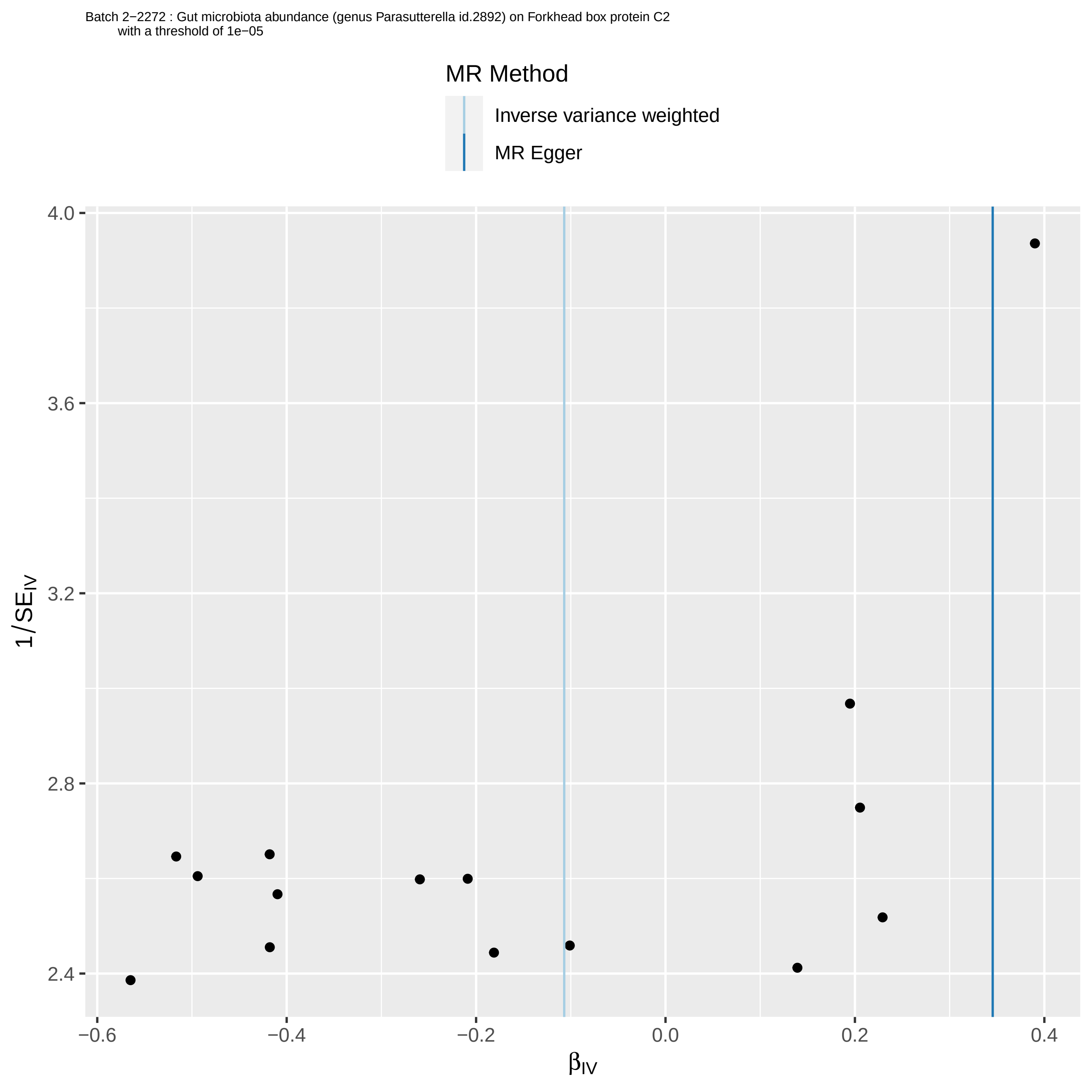

Batch 2−2272 : Gut microbiota abundance (genus Parasutterella id.2892) on Forkhead box protein C2
with a threshold of 1e−05
MR Method
Inverse variance weighted
MR Egger
4.0
3.6
3.2
2.8
2.4
1/SE$_{IV}$
−0.6
−0.4
−0.2
0.0
0.2
0.4
β$_{IV}$

Batch 2−2273 : Gut microbiota abundance (genus Catenibacterium id.2153) on Forkhead box protein J2
with a threshold of 1e−05

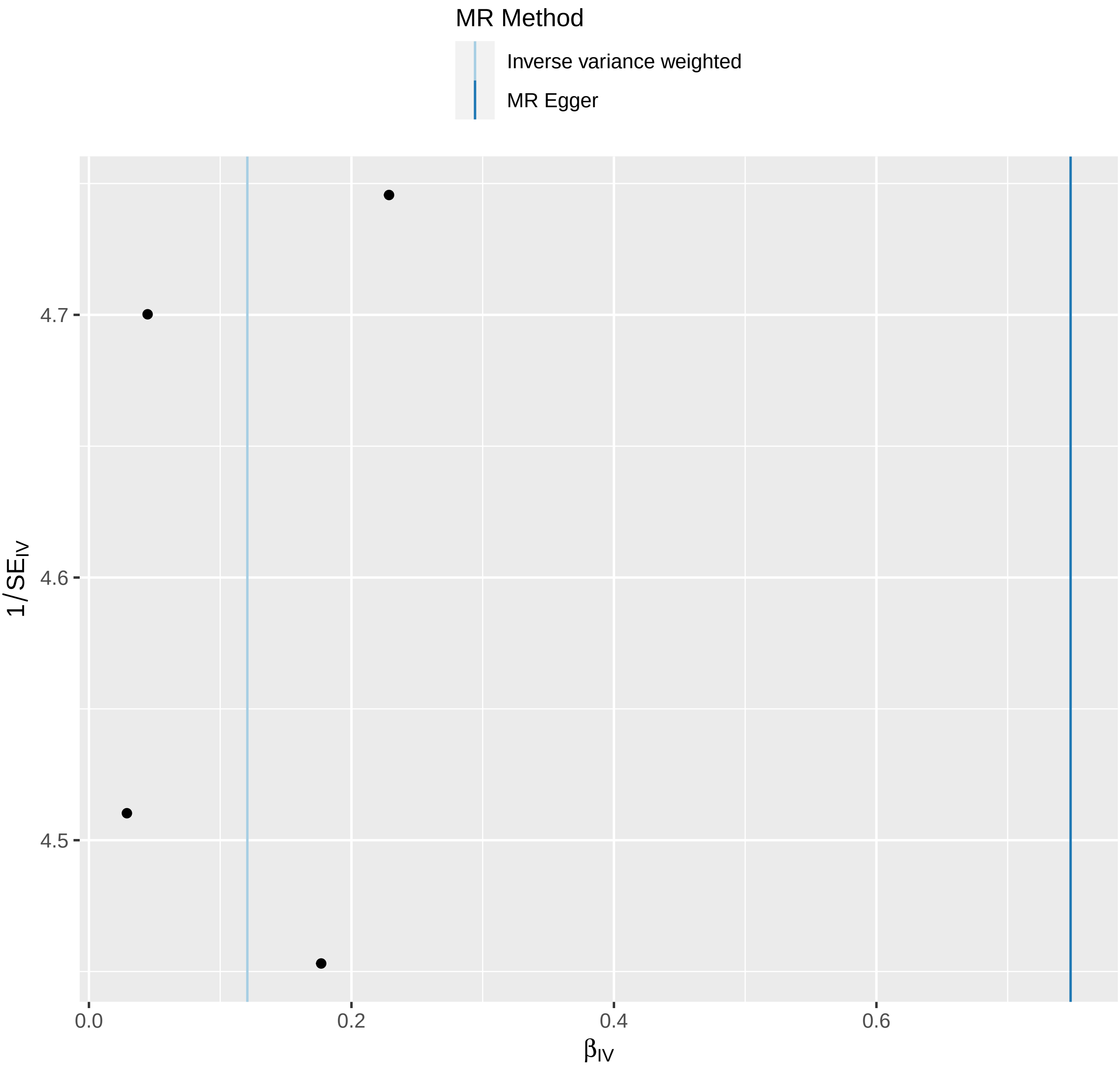

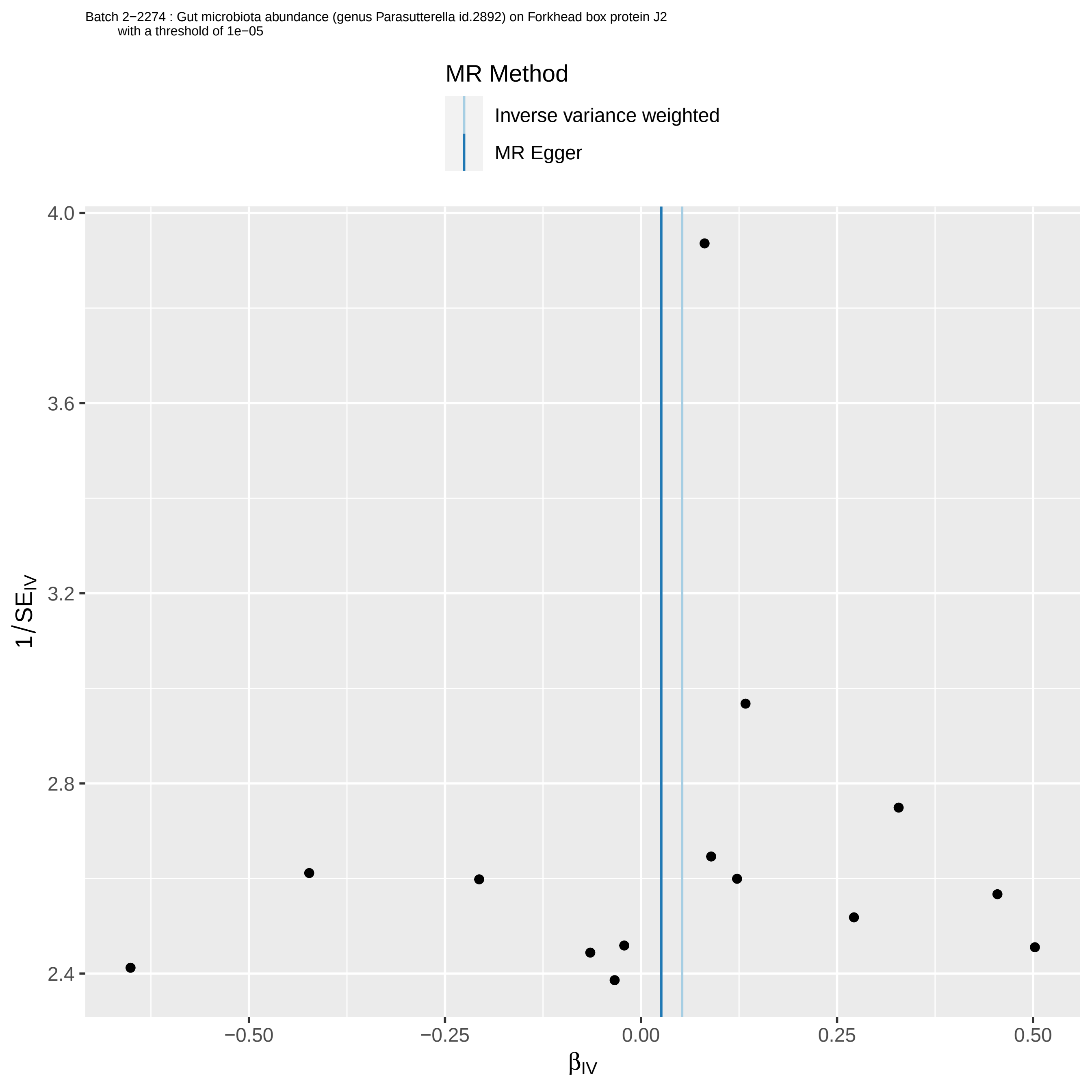

Batch 2−2274 : Gut microbiota abundance (genus Parasutterella id.2892) on Forkhead box protein J2
with a threshold of 1e−05
MR Method
Inverse variance weighted
MR Egger
4.0
3.6
3.2
2.8
2.4
1/SE_IV
−0.50
−0.25
0.00
0.25
0.50
β_IV

Batch 2−2275 : Gut microbiota abundance (genus Catenibacterium id.2153) on Forkhead box protein L2
with a threshold of 1e−05

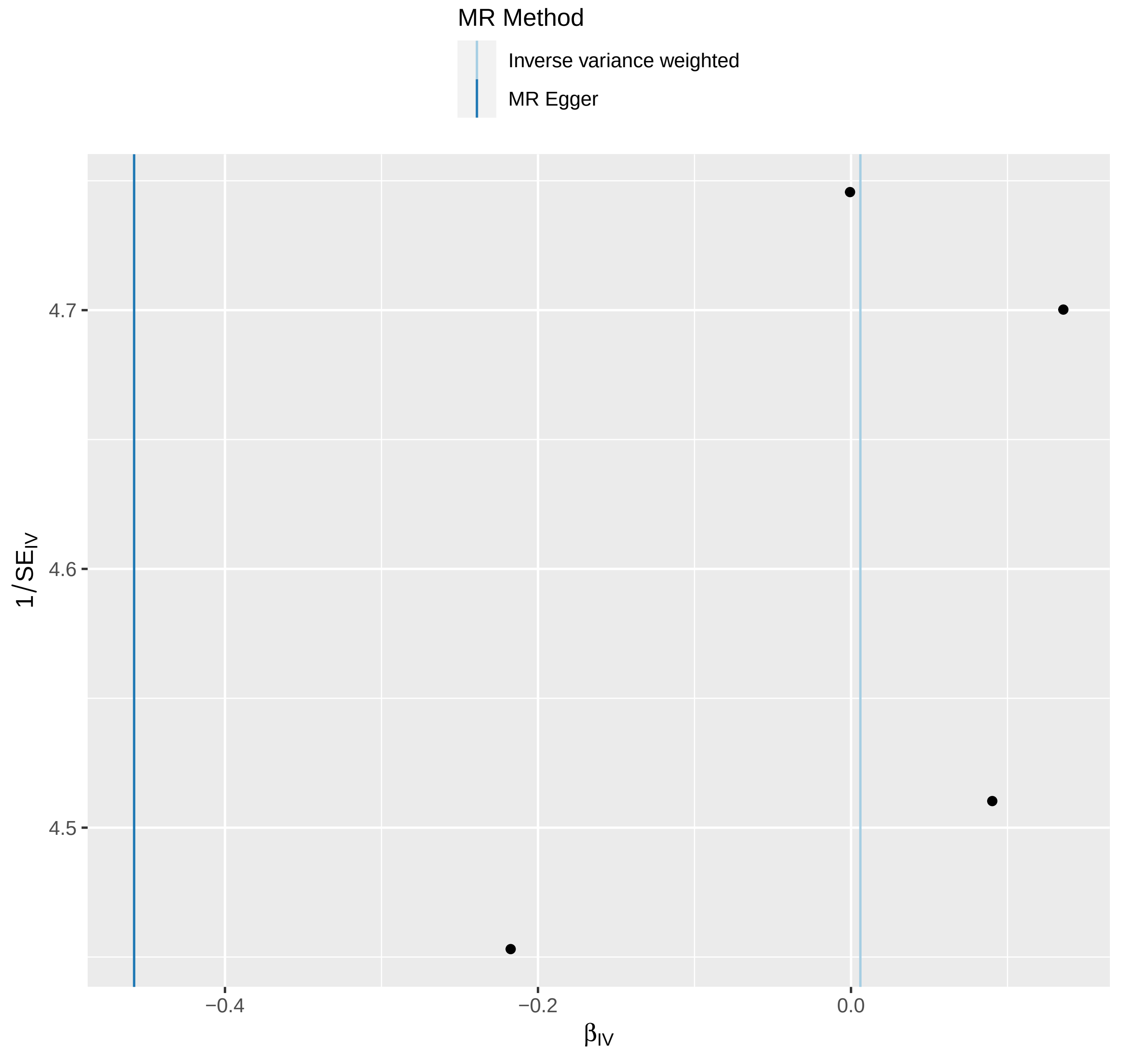

Batch 2−2276 : Gut microbiota abundance (genus Parasutterella id.2892) on Forkhead box protein L2
with a threshold of 1e−05

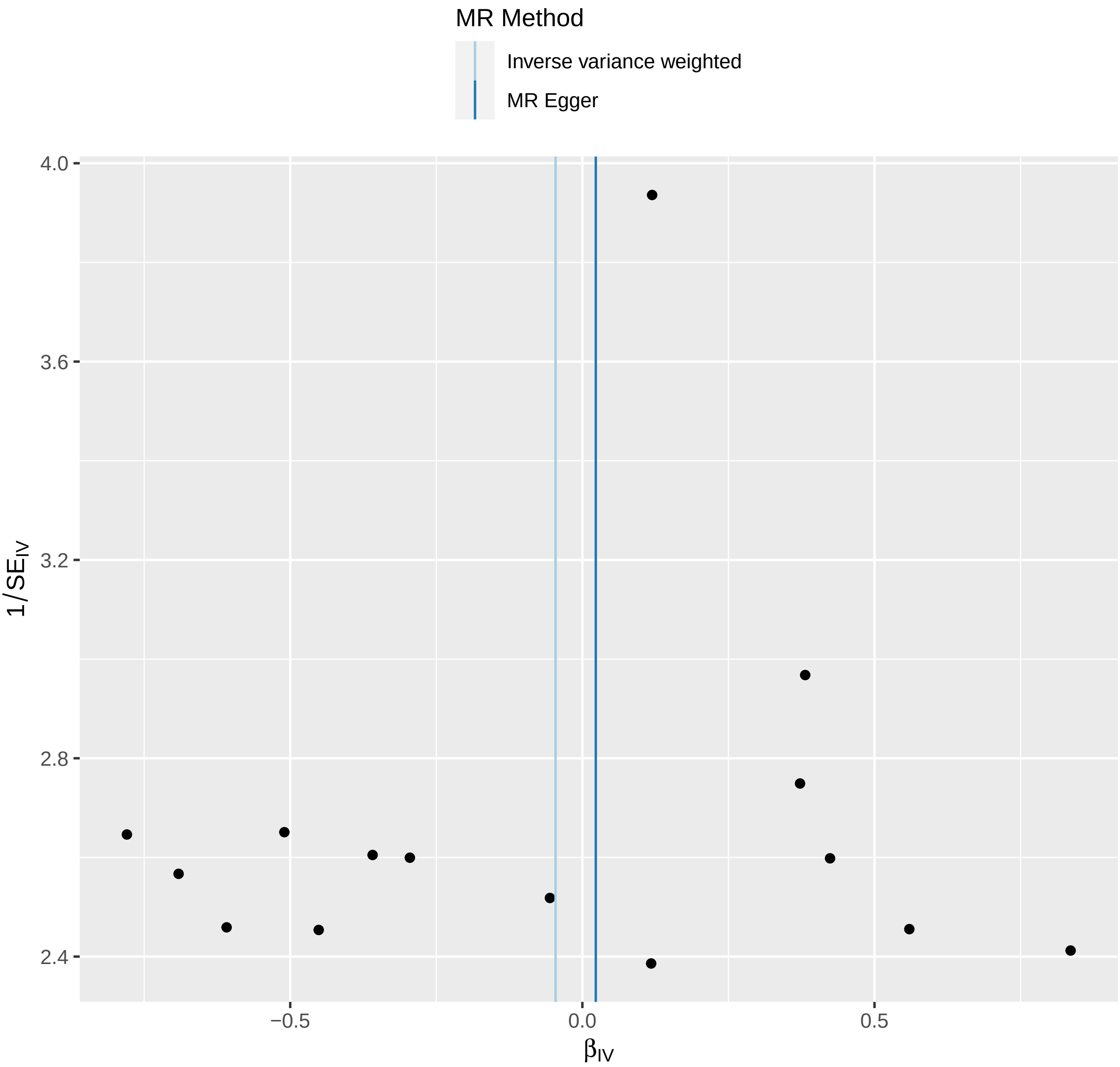

Batch 2−2277 : Gut microbiota abundance (genus Catenibacterium id.2153) on Tyrosine−protein kinase FRK
with a threshold of 1e−05

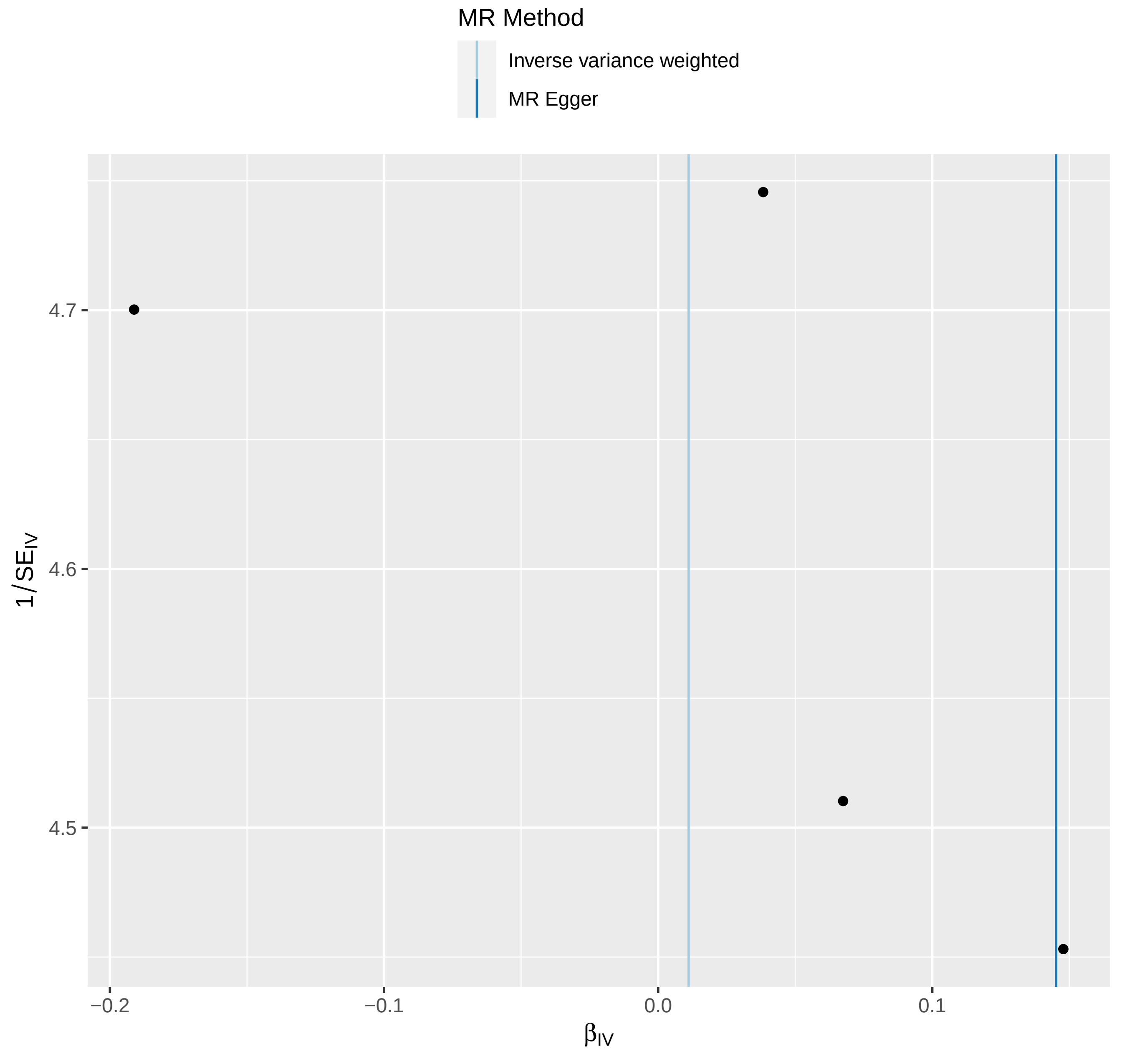

Batch 2−2278 : Gut microbiota abundance (genus Parasutterella id.2892) on Tyrosine−protein kinase FRK
with a threshold of 1e−05

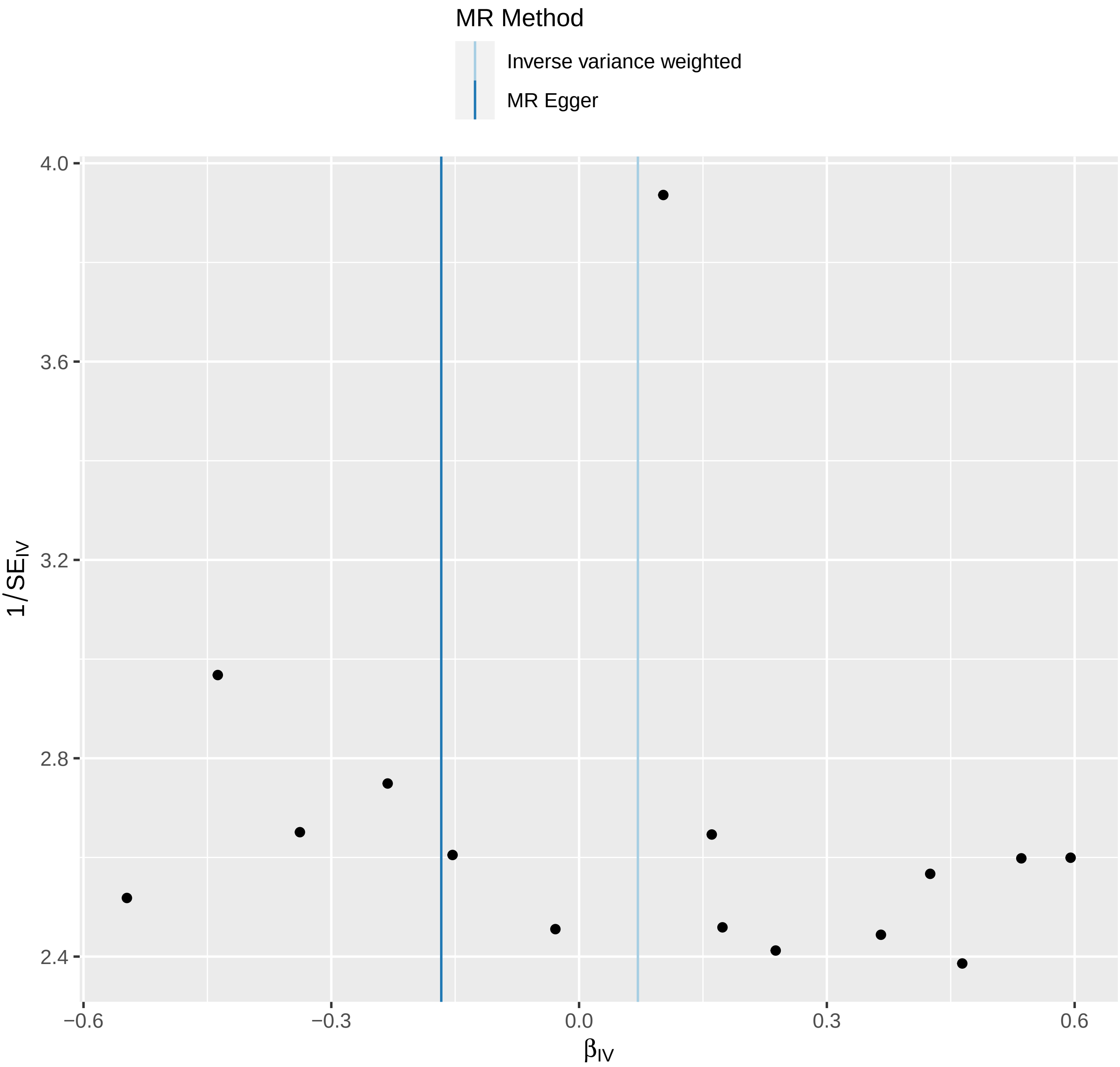

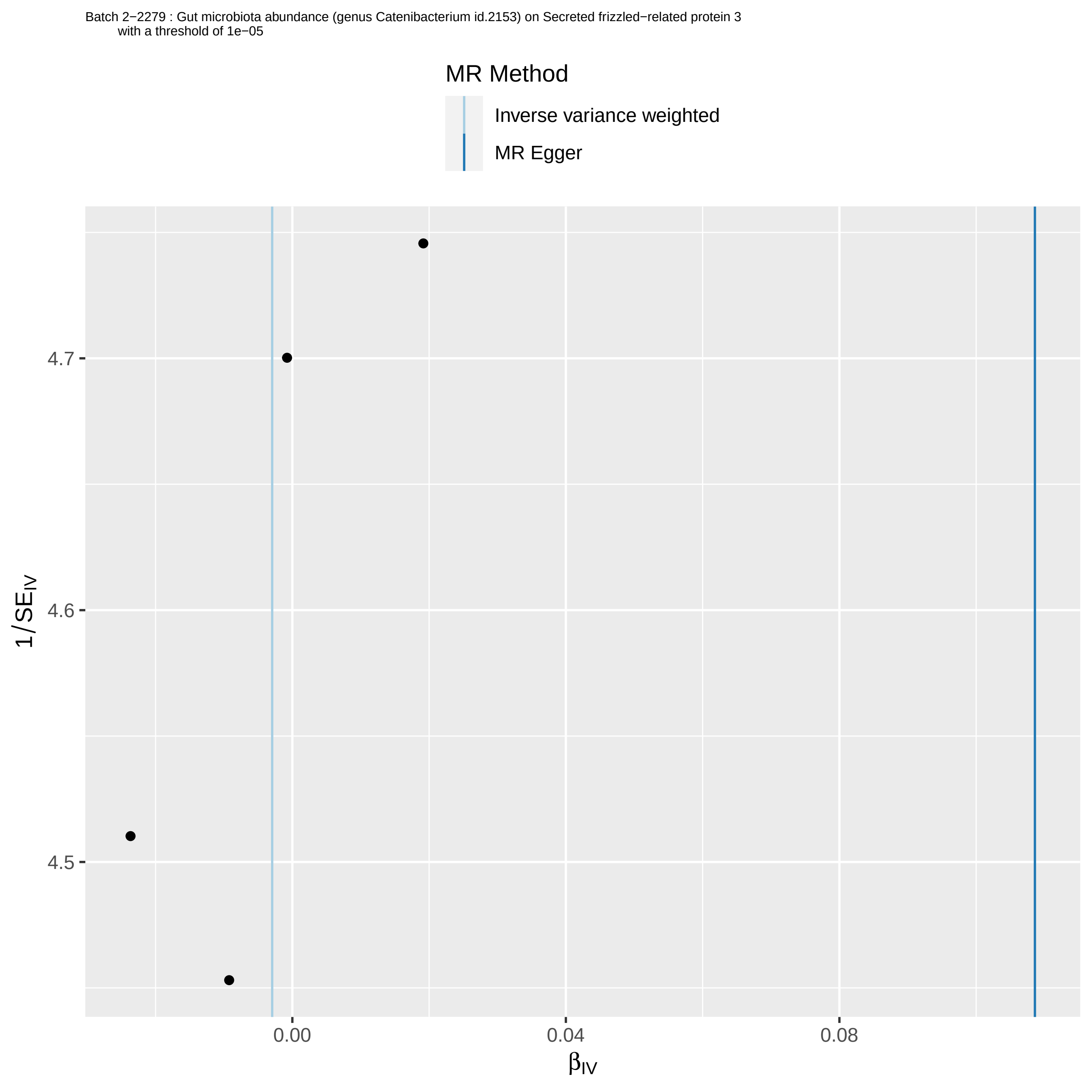
Batch 2−2279 : Gut microbiota abundance (genus Catenibacterium id.2153) on Secreted frizzled−related protein 3
with a threshold of 1e−05
MR Method
Inverse variance weighted
MR Egger
4.7
4.6
4.5
1/SE$_{IV}$
0.00
0.04
0.08
β_{IV}

Batch 2−2280 : Gut microbiota abundance (genus Parasutterella id.2892) on Secreted frizzled−related protein 3
with a threshold of 1e−05

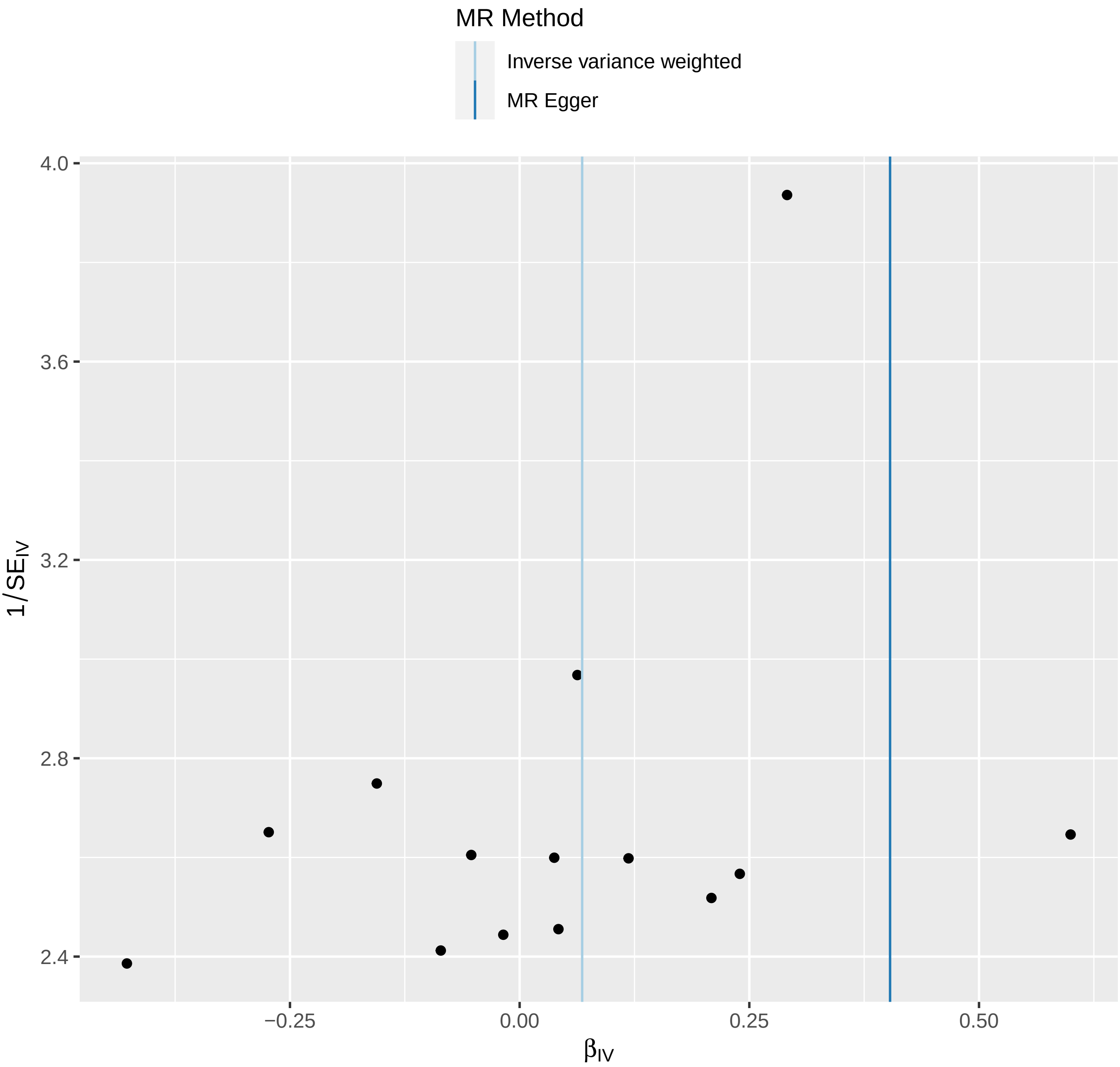

Batch 2−2281 : Gut microbiota abundance (genus Catenibacterium id.2153) on Secreted frizzled−related protein 3 with a threshold of 1e−05

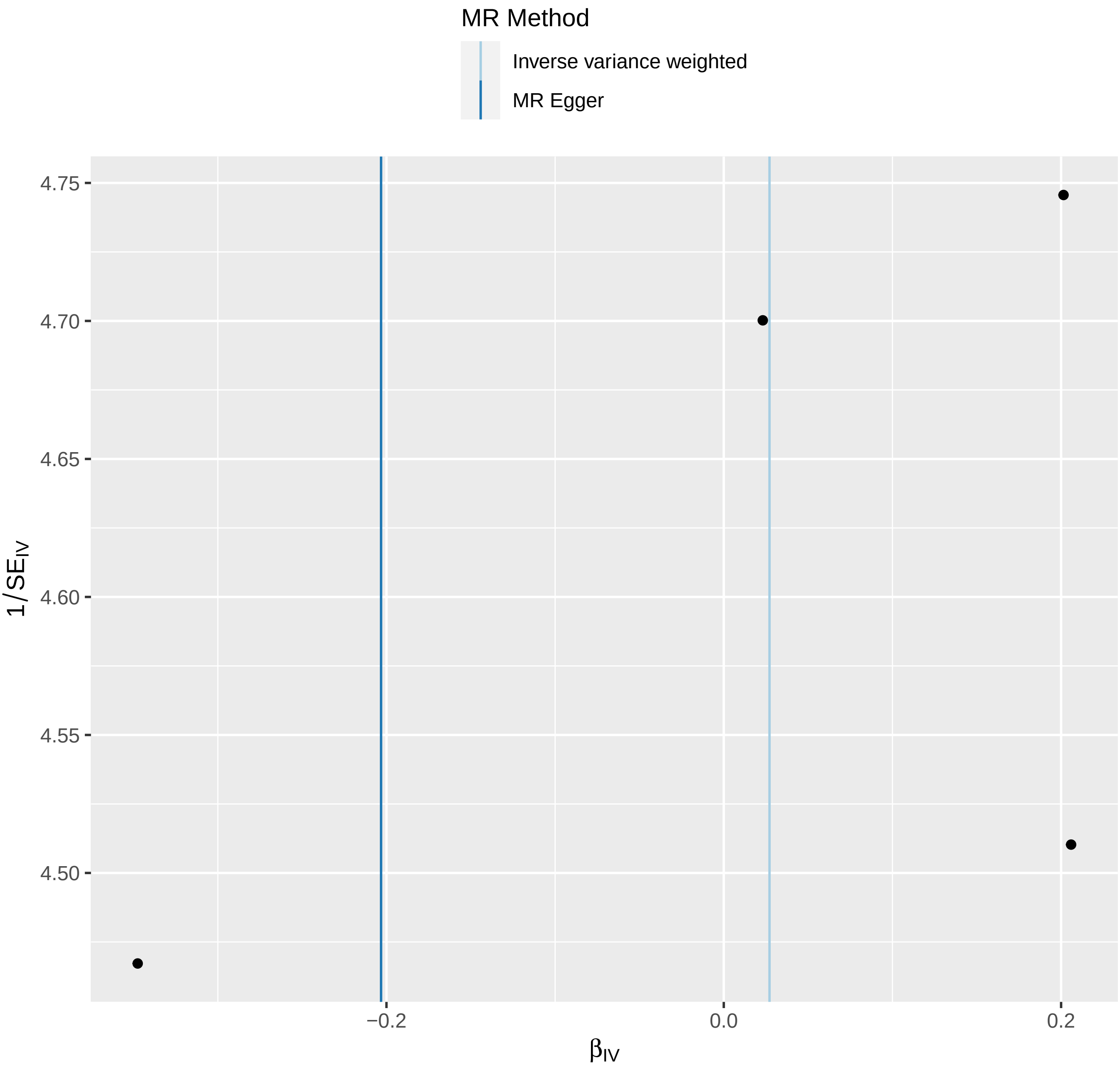

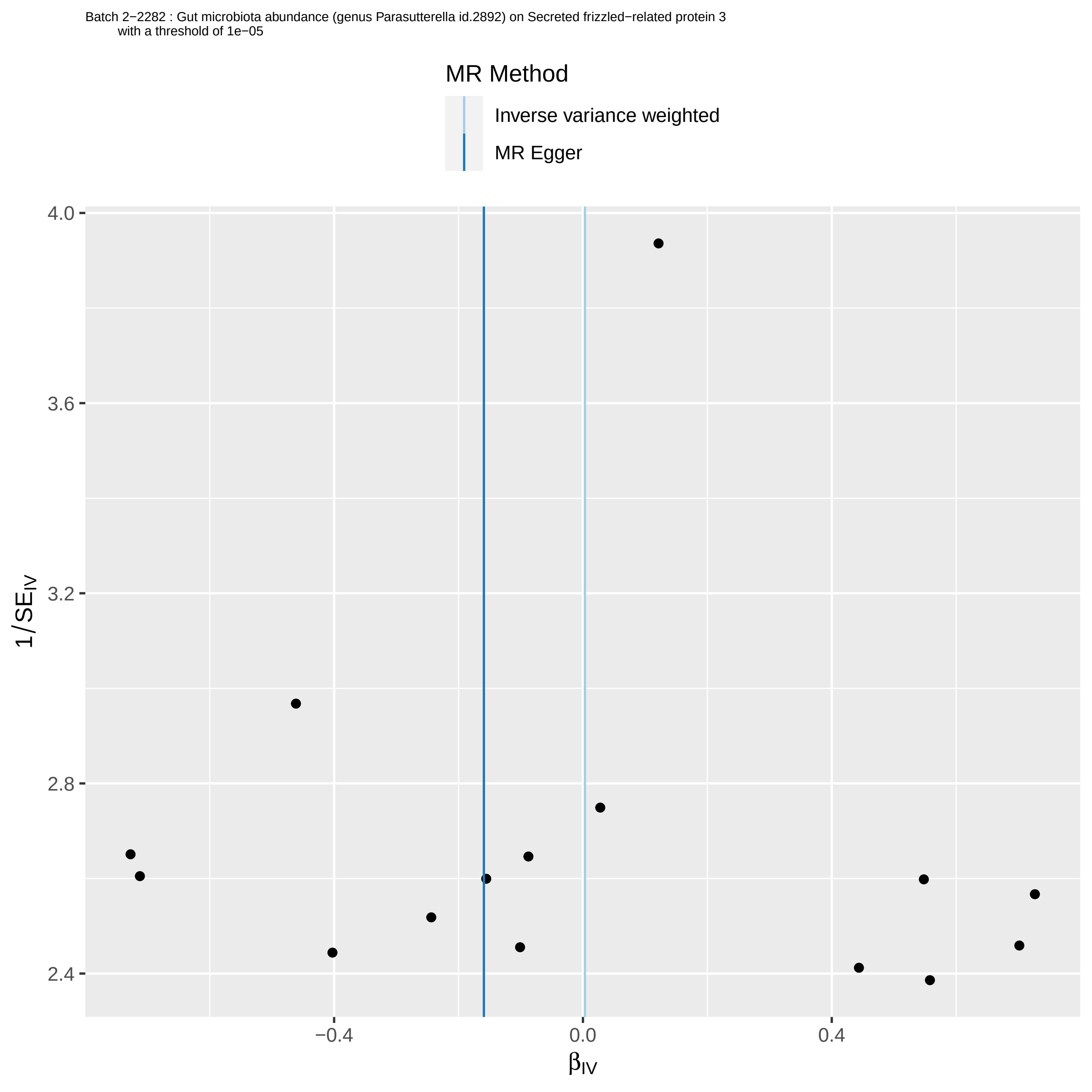
Batch 2−2282 : Gut microbiota abundance (genus Parasutterella id.2892) on Secreted frizzled−related protein 3
with a threshold of 1e−05
MR Method
Inverse variance weighted
MR Egger
4.0
3.6
3.2
2.8
2.4
1/SE$_{IV}$
−0.4
0.0
0.4
β_{IV}

Batch 2−2283 : Gut microbiota abundance (genus Catenibacterium id.2153) on Follistatin
with a threshold of 1e−05

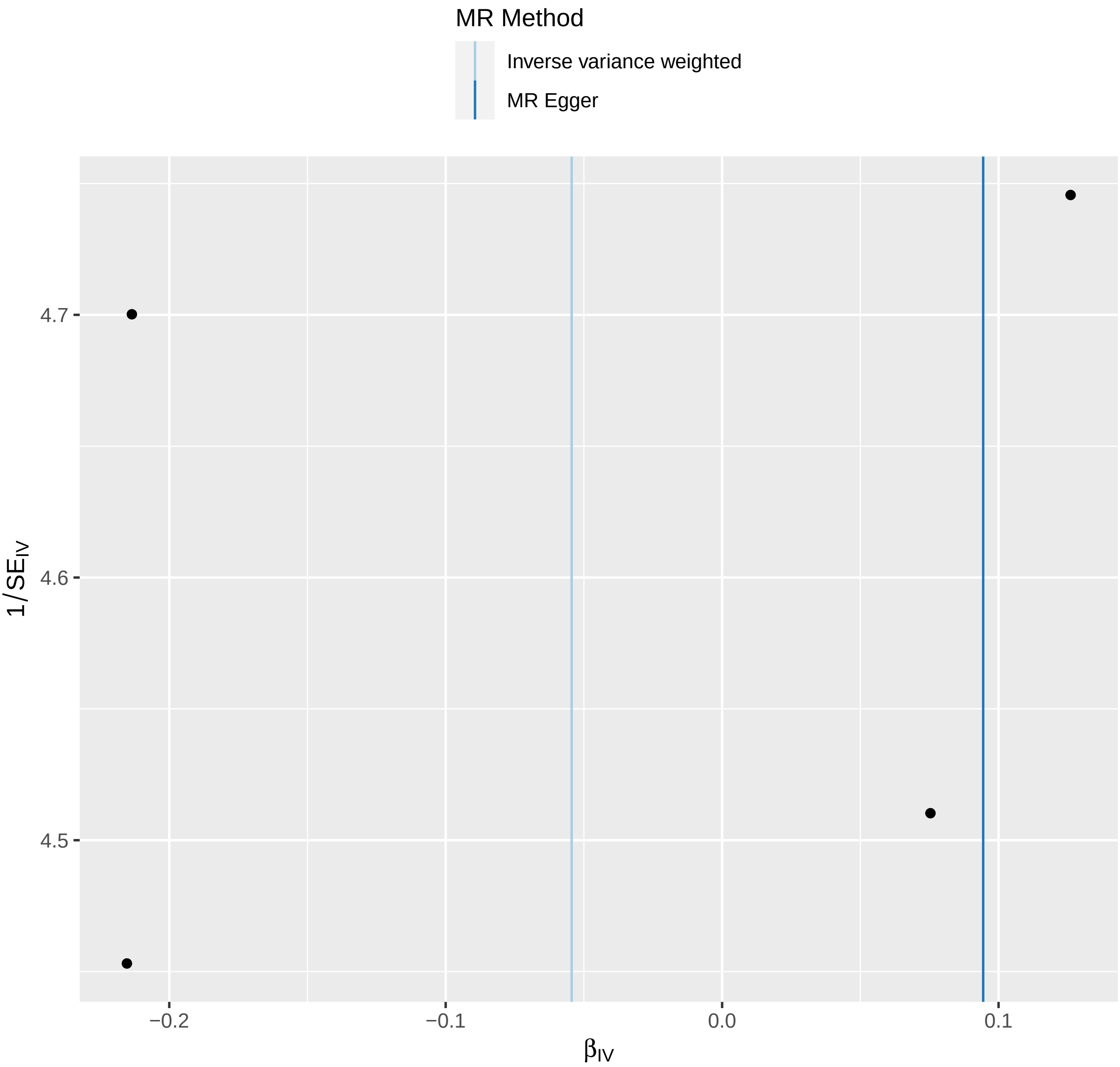

Batch 2−2284 : Gut microbiota abundance (genus Parasutterella id.2892) on Follistatin
with a threshold of 1e−05

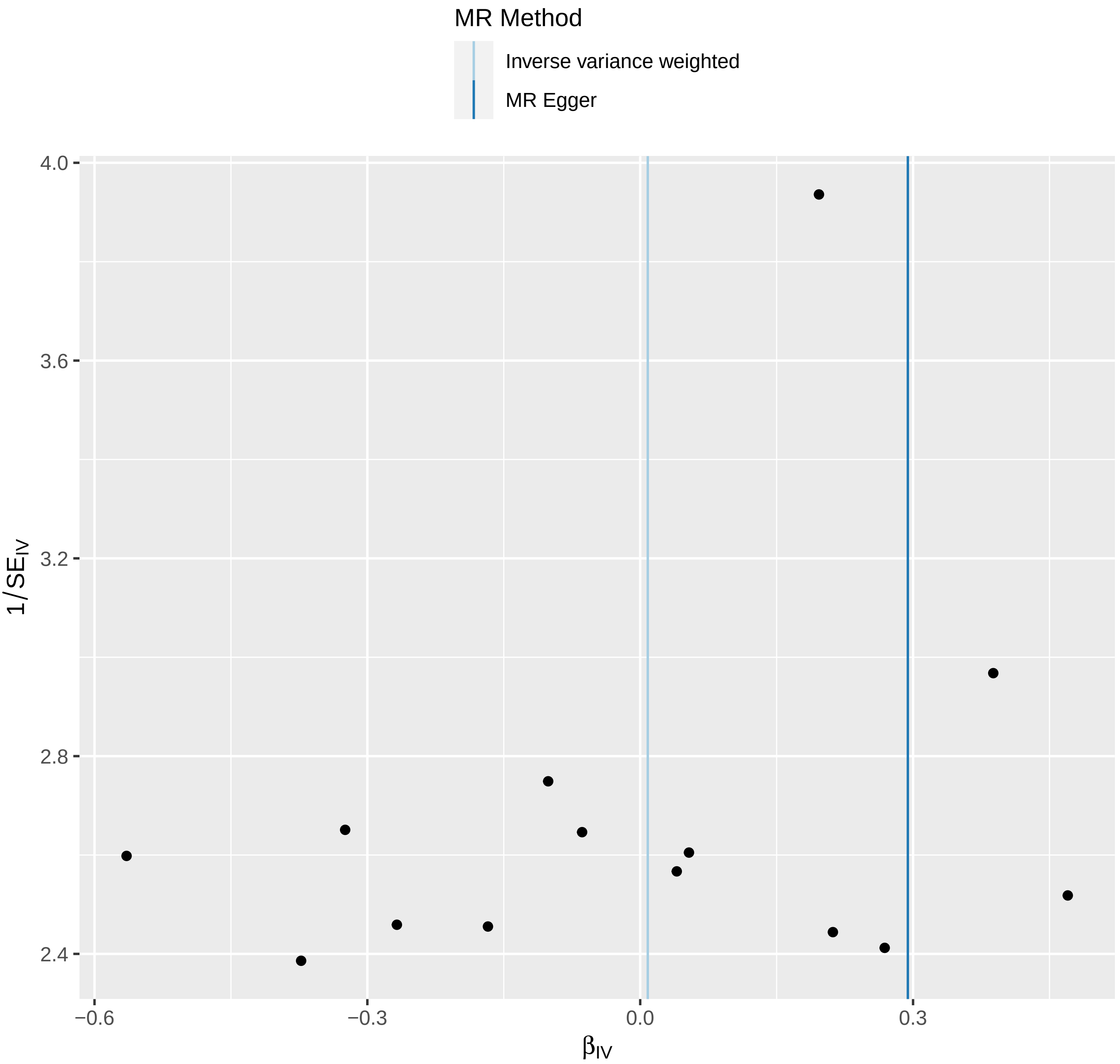

Batch 2−2285 : Gut microbiota abundance (genus Catenibacterium id.2153) on Follistatin−related protein 1
with a threshold of 1e−05

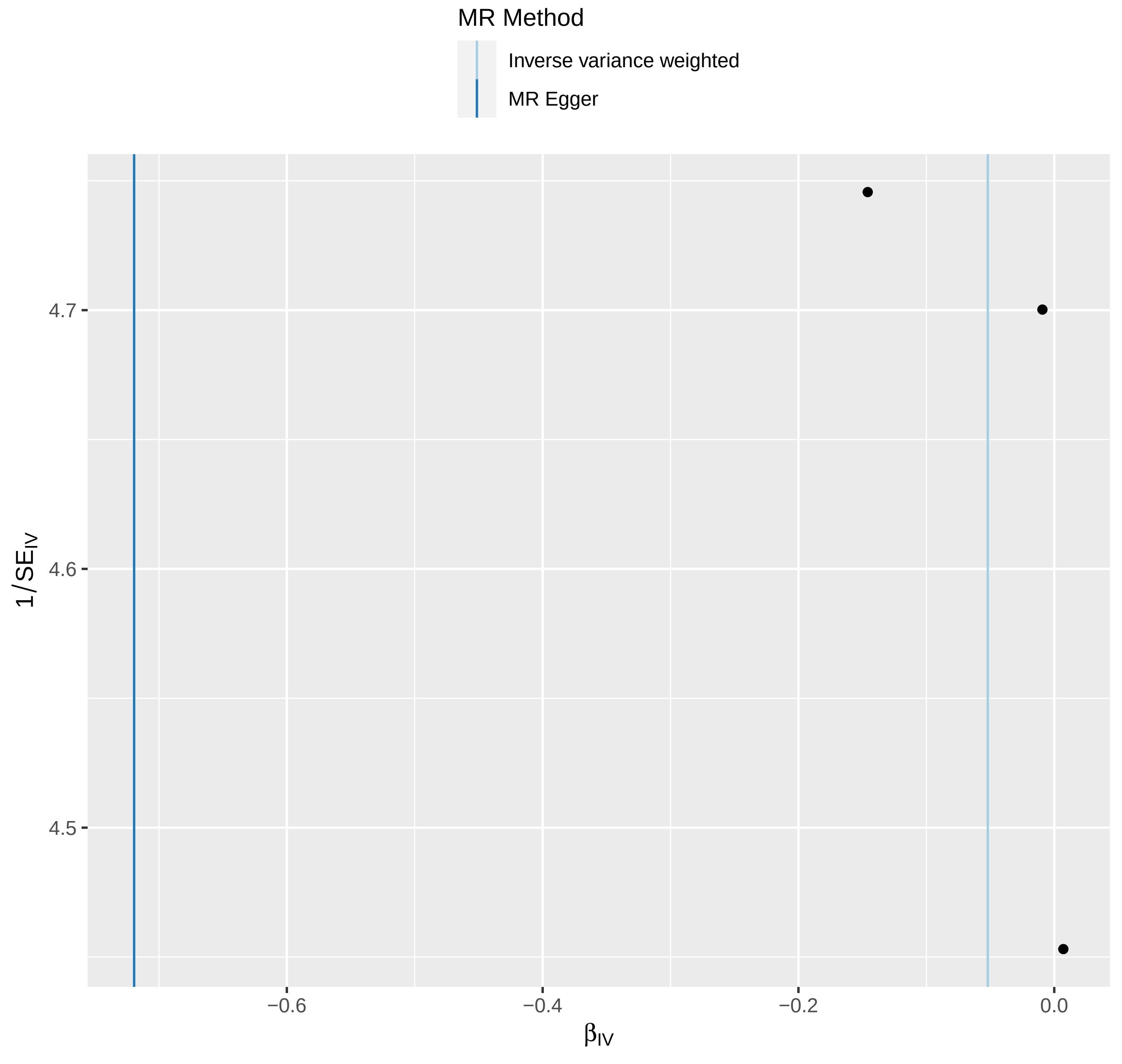

Batch 2−2286 : Gut microbiota abundance (genus Parasutterella id.2892) on Follistatin−related protein 1
with a threshold of 1e−05

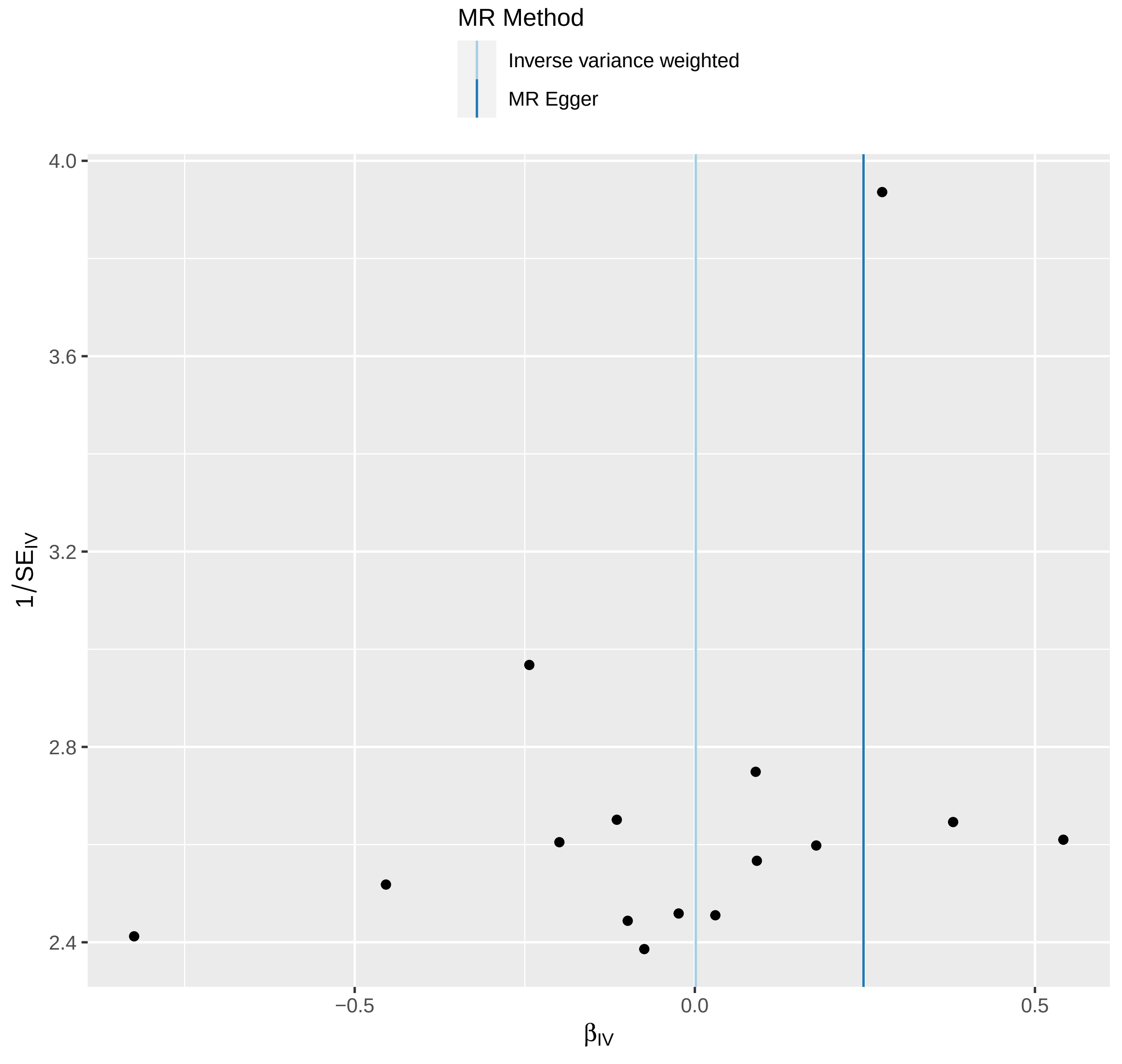

Batch 2−2287 : Gut microbiota abundance (genus Catenibacterium id.2153) on Follistatin−related protein 1
with a threshold of 1e−05

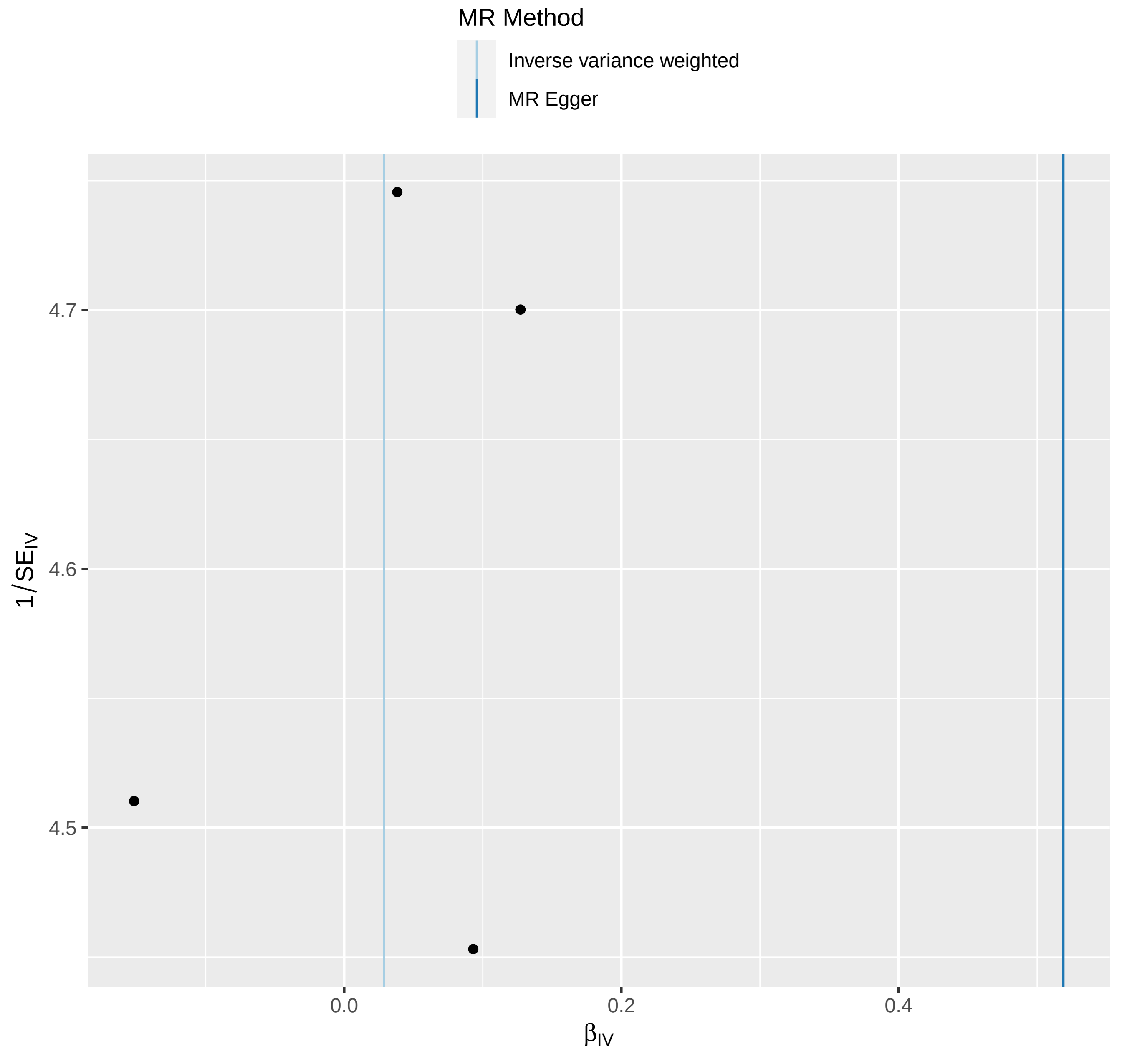

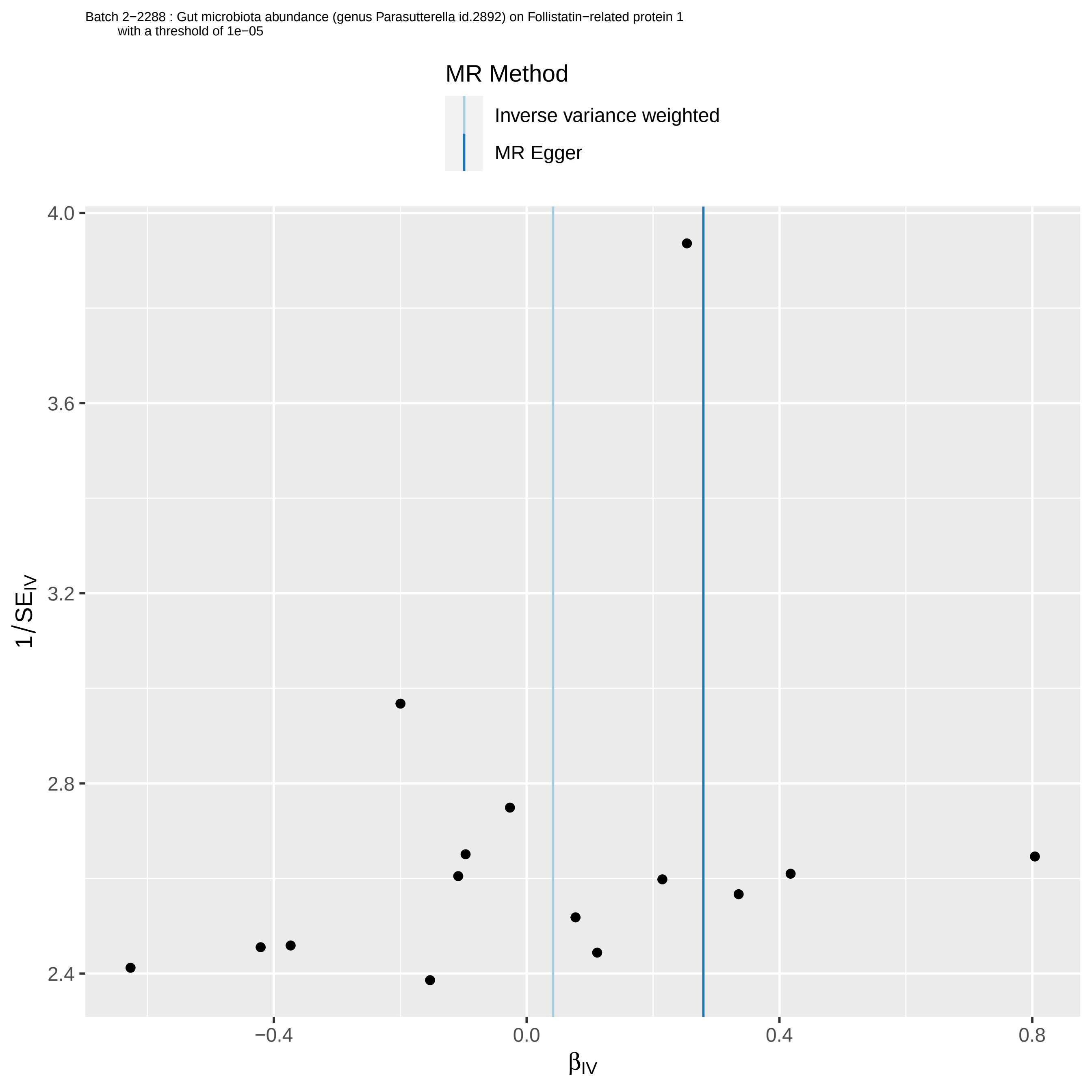

Batch 2−2288 : Gut microbiota abundance (genus Parasutterella id.2892) on Follistatin−related protein 1
with a threshold of 1e−05
MR Method
Inverse variance weighted
MR Egger
4.0
3.6
3.2
2.8
2.4
1/SE$_{IV}$
−0.4
0.0
0.4
0.8
β$_{IV}$

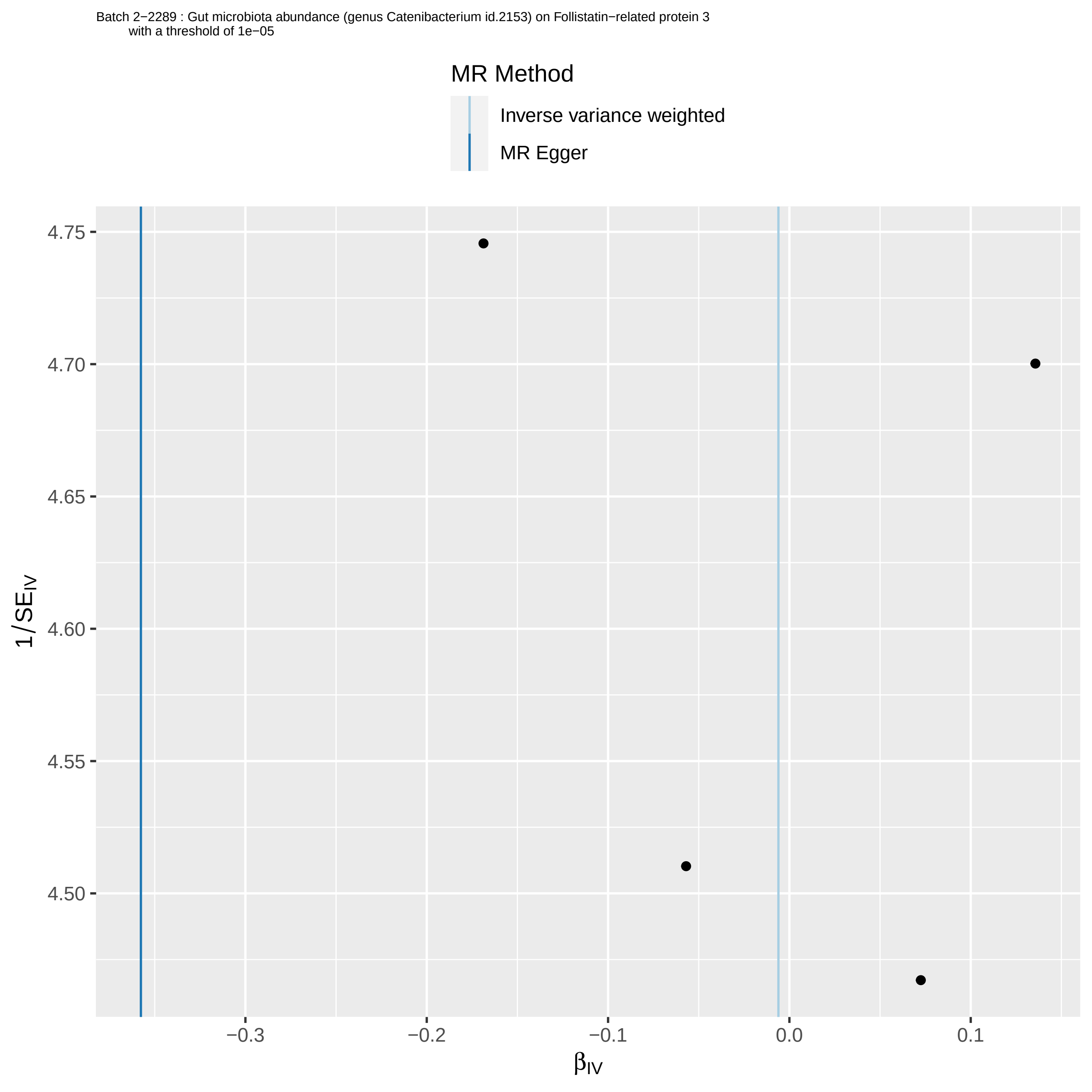

Batch 2−2289 : Gut microbiota abundance (genus Catenibacterium id.2153) on Follistatin−related protein 3
with a threshold of 1e−05
MR Method
Inverse variance weighted
MR Egger
4.75
4.70
4.65
4.60
4.55
4.50
1/SE_IV
−0.3
−0.2
−0.1
0.0
0.1
β_IV

Batch 2−2290 : Gut microbiota abundance (genus Parasutterella id.2892) on Follistatin−related protein 3
with a threshold of 1e−05

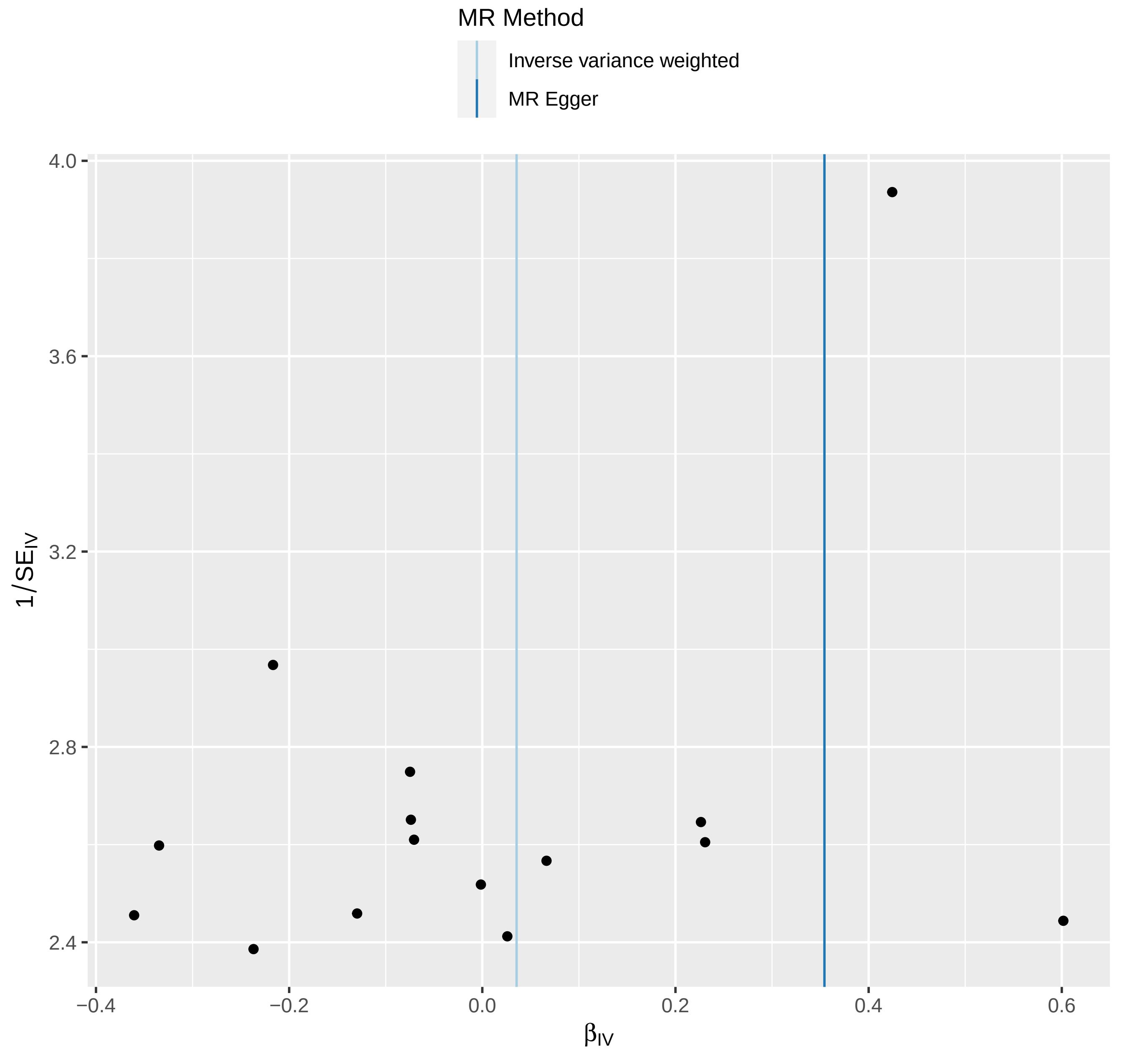

Batch 2−2291 : Gut microbiota abundance (genus Catenibacterium id.2153) on Follistatin−related protein 4
with a threshold of 1e−05

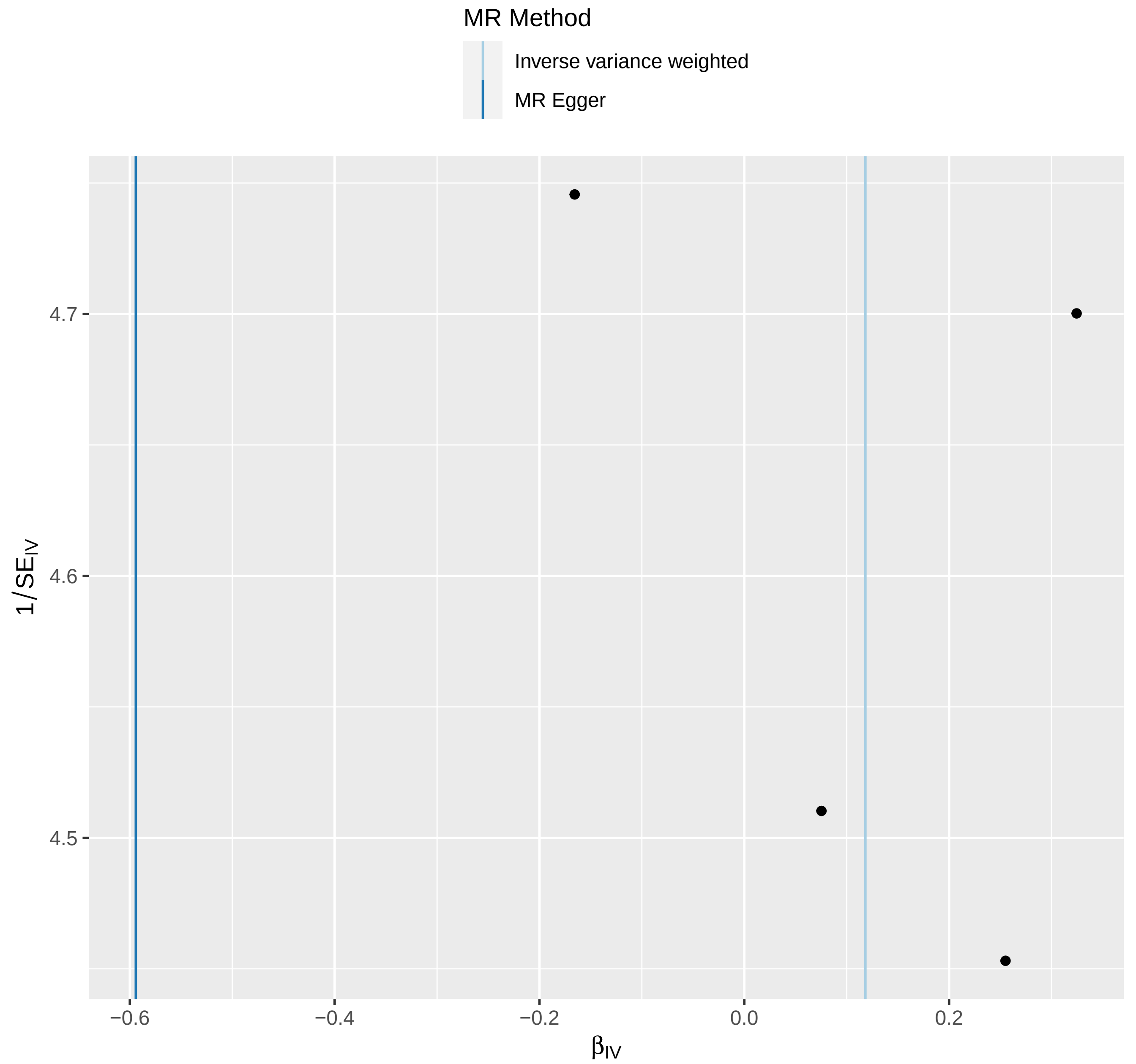

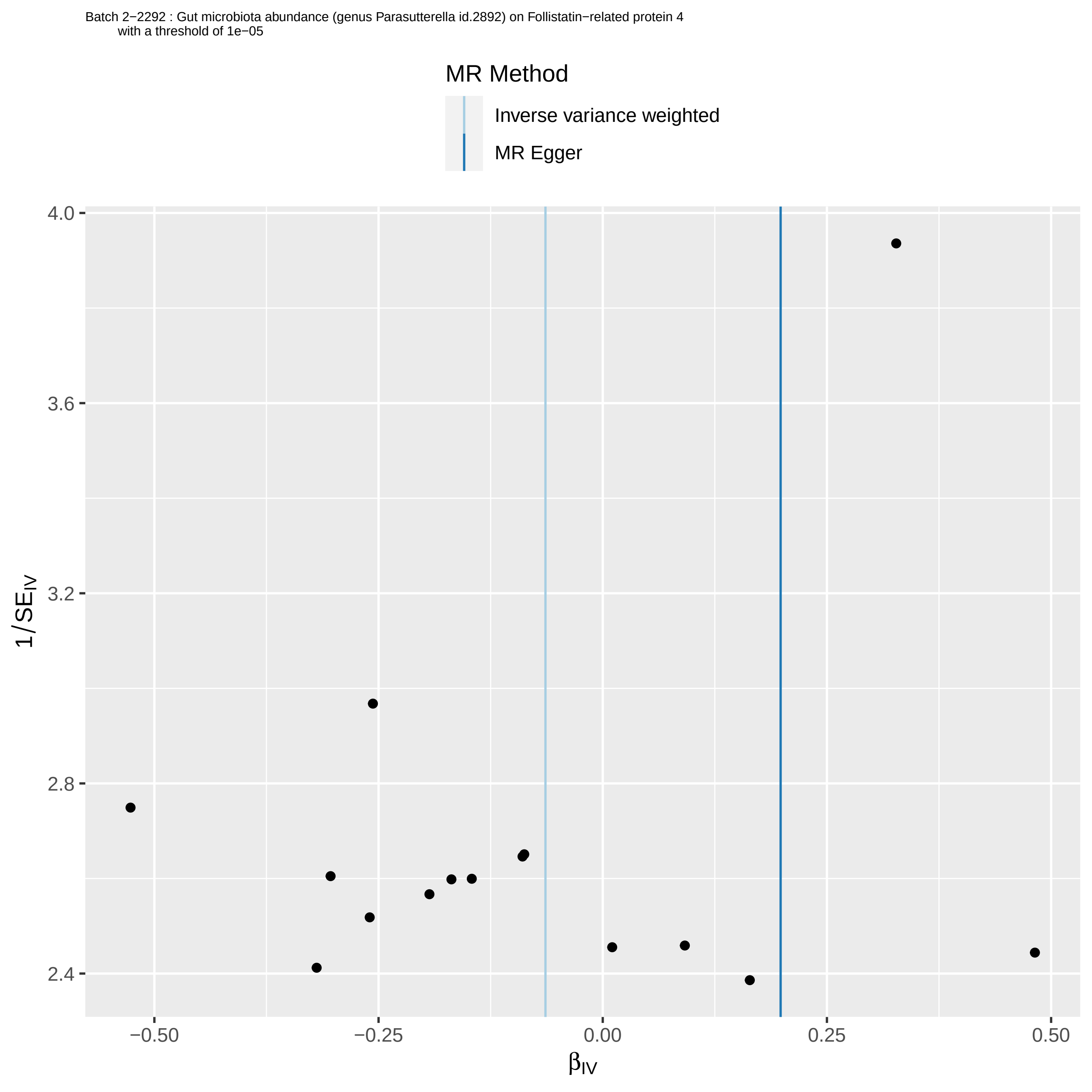
Batch 2−2292 : Gut microbiota abundance (genus Parasutterella id.2892) on Follistatin−related protein 4
with a threshold of 1e−05
MR Method
Inverse variance weighted
MR Egger
4.0
3.6
3.2
2.8
2.4
1/SE_IV
−0.50
−0.25
0.00
0.25
0.50
β_IV

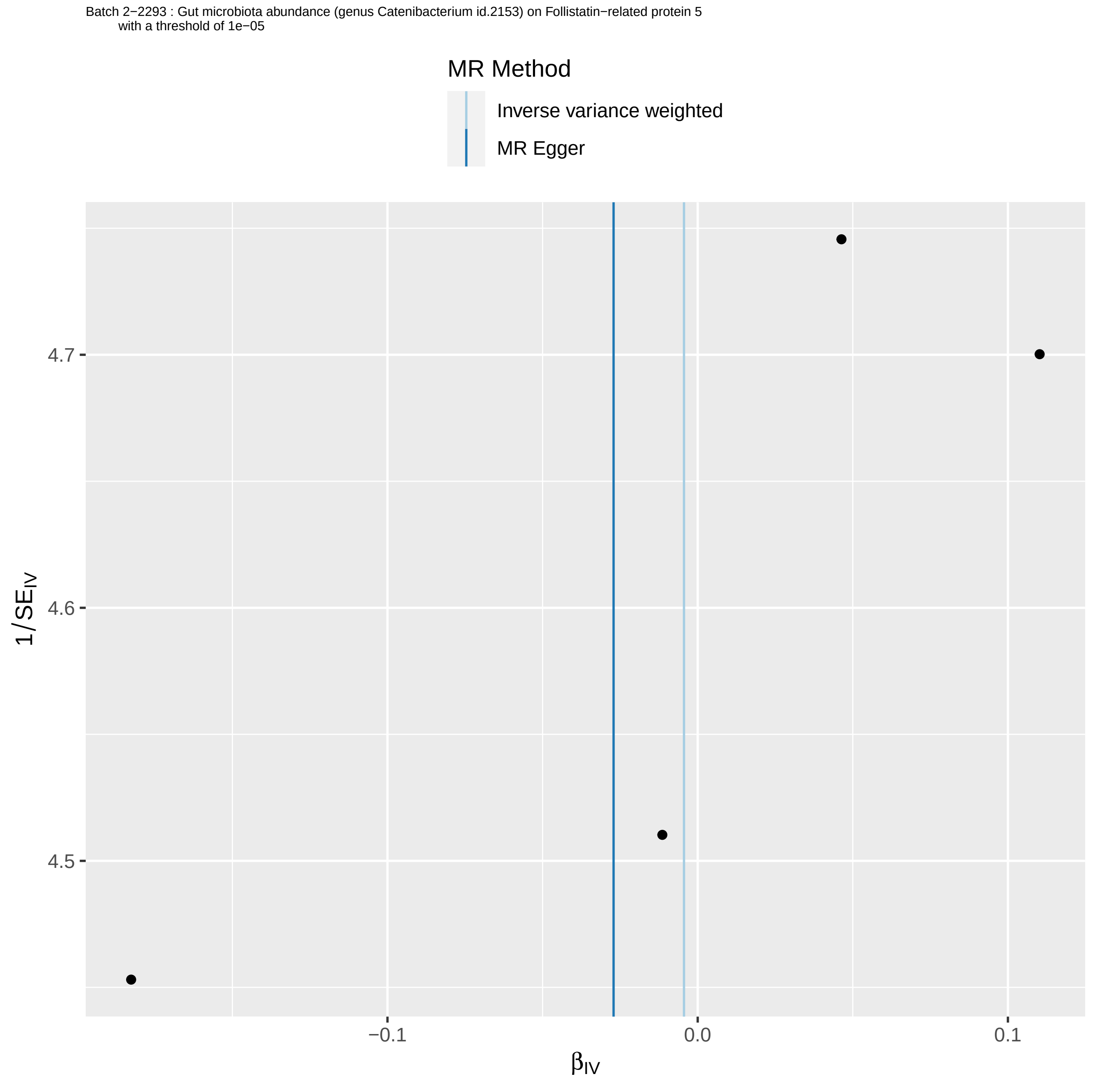
Batch 2−2293 : Gut microbiota abundance (genus Catenibacterium id.2153) on Follistatin−related protein 5
with a threshold of 1e−05
MR Method
Inverse variance weighted
MR Egger
4.7
4.6
4.5
1/SE$_{IV}$
−0.1
0.0
0.1
β_{IV}

Batch 2−2294 : Gut microbiota abundance (genus Parasutterella id.2892) on Follistatin−related protein 5
with a threshold of 1e−05

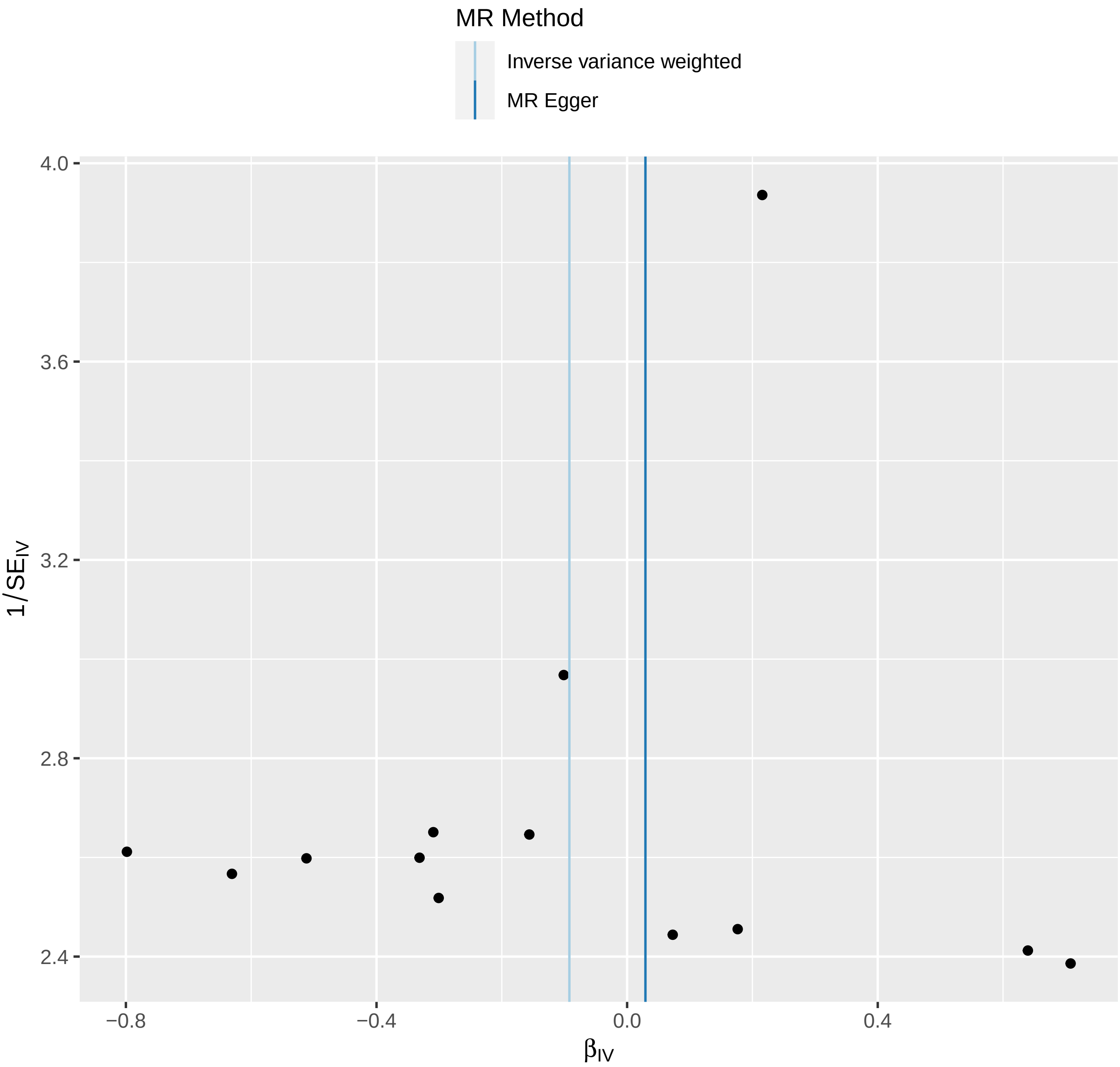

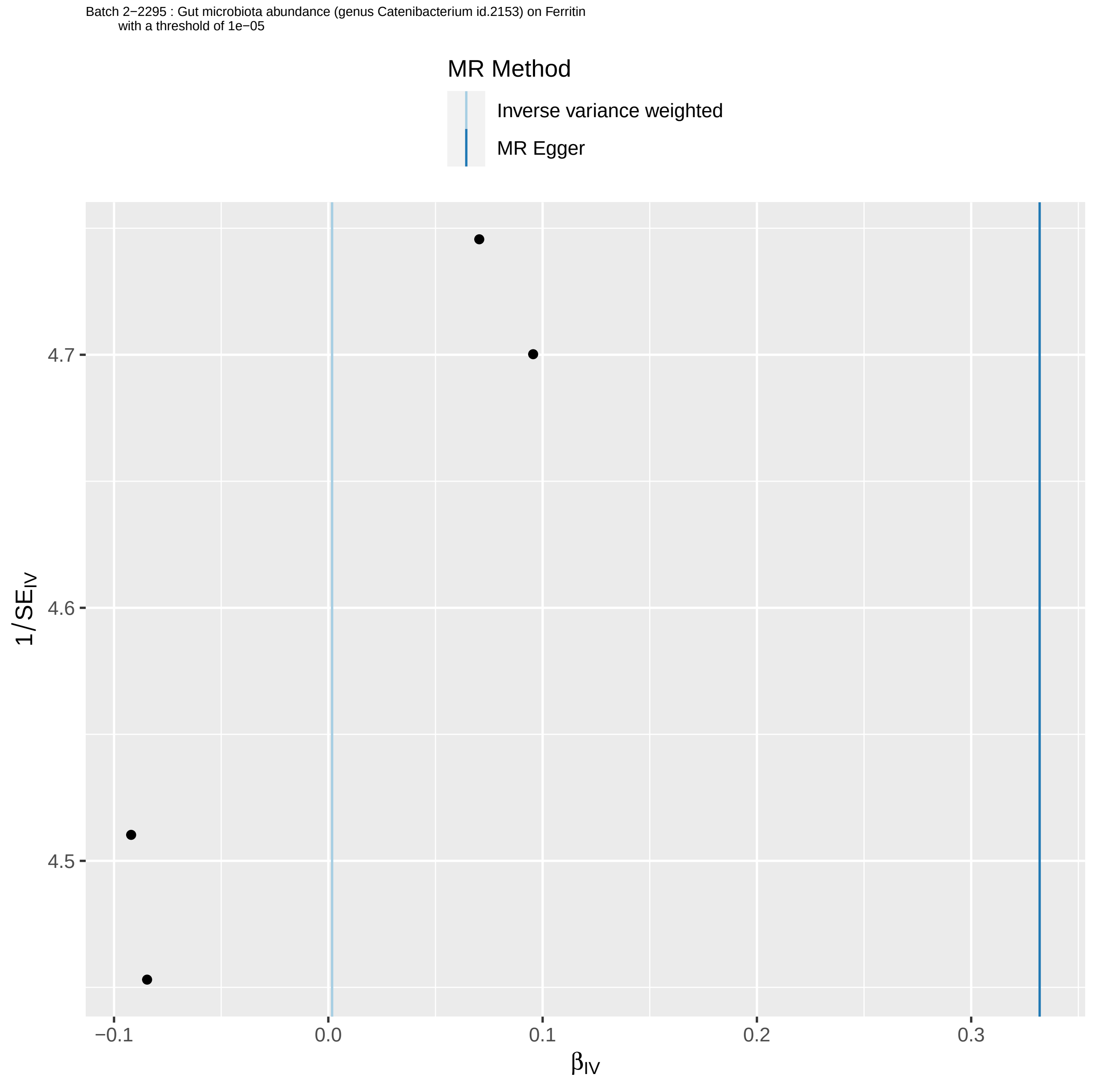
Batch 2−2295 : Gut microbiota abundance (genus Catenibacterium id.2153) on Ferritin
with a threshold of 1e−05
MR Method
Inverse variance weighted
MR Egger
4.7
4.6
4.5
1/SE_IV
−0.1
0.0
0.1
0.2
0.3
β_IV

Batch 2−2296 : Gut microbiota abundance (genus Parasutterella id.2892) on Ferritin
with a threshold of 1e−05

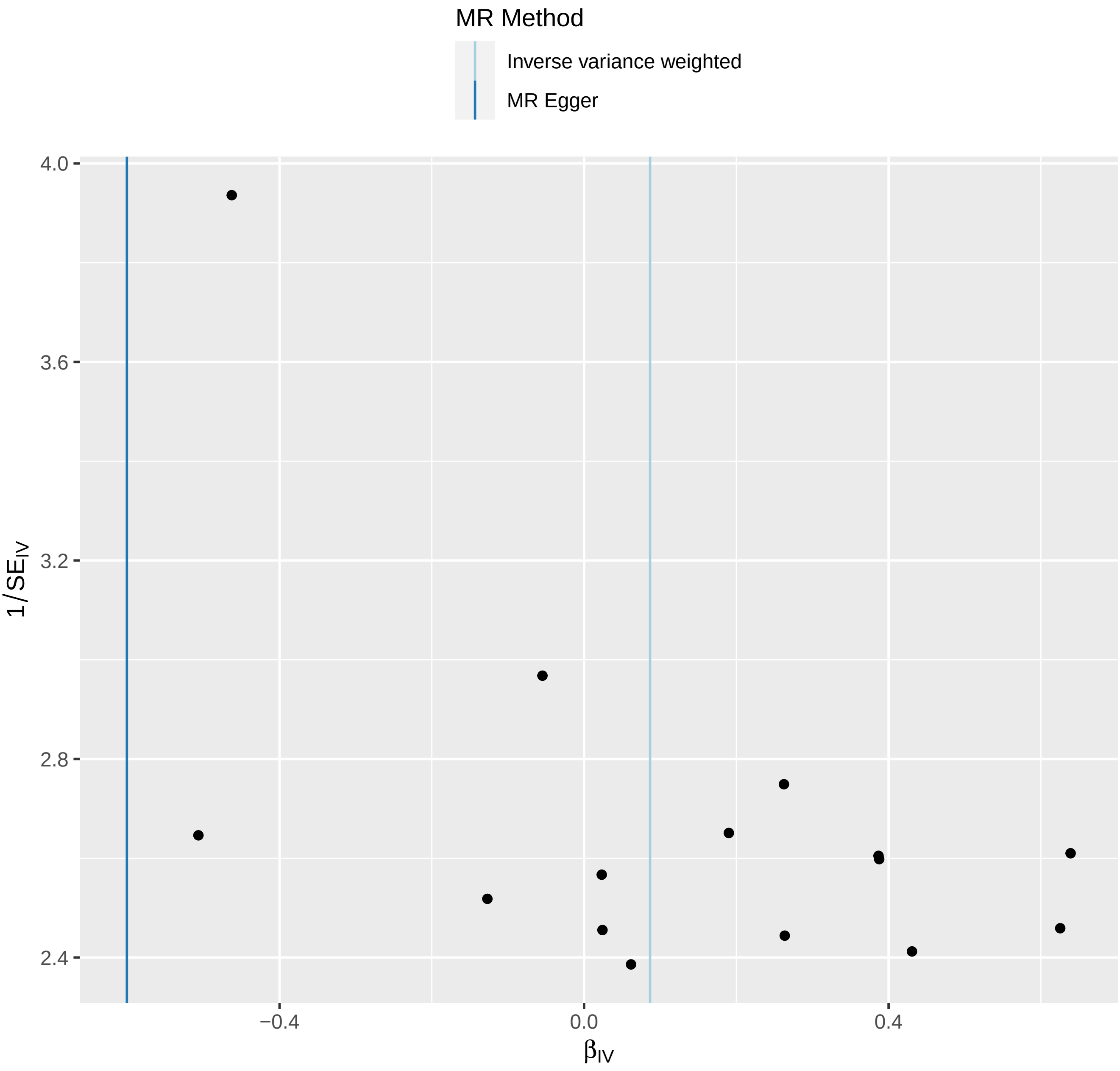

Batch 2−2297 : Gut microbiota abundance (genus Catenibacterium id.2153) on Ferritin
with a threshold of 1e−05

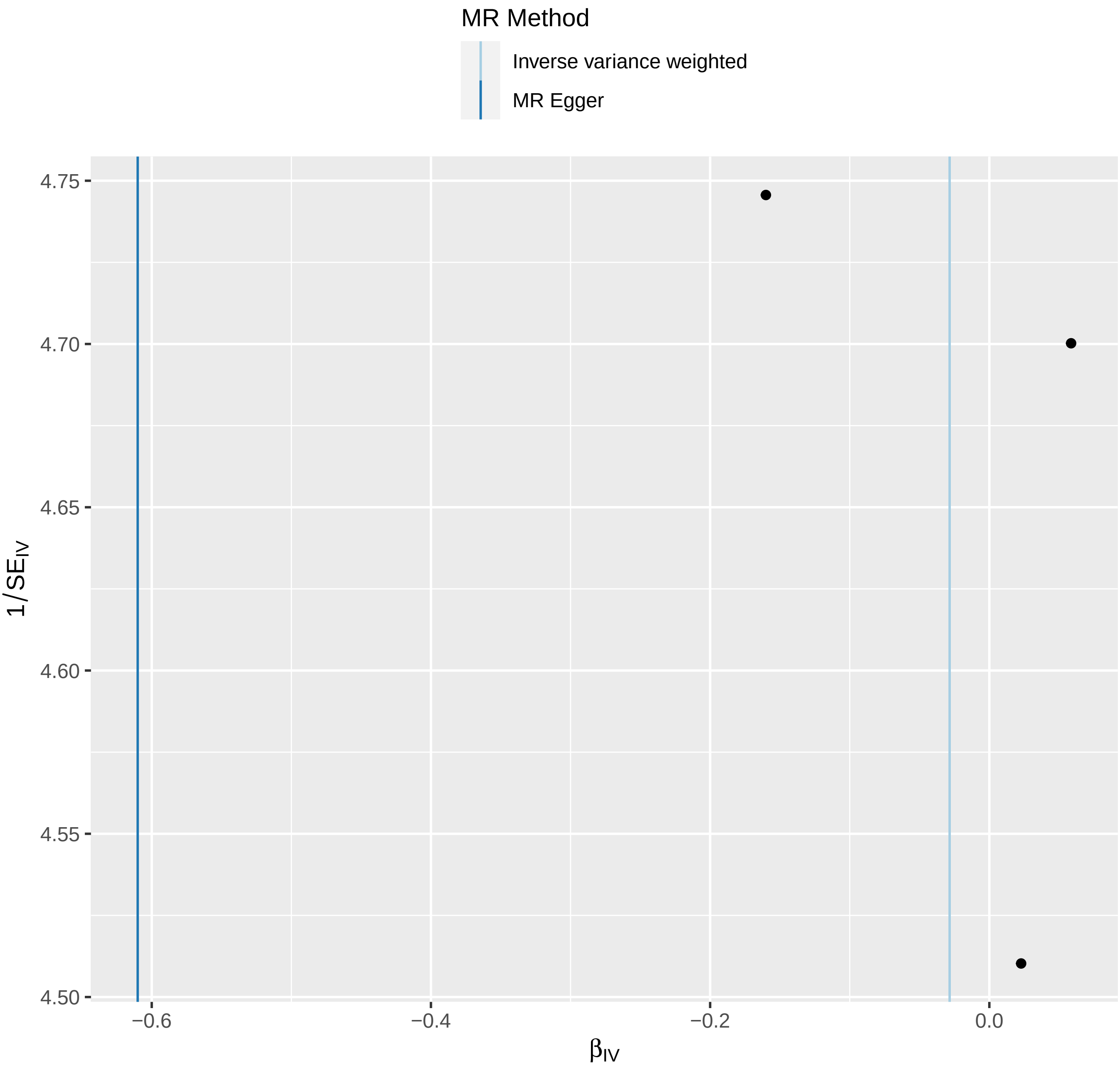

Batch 2−2298 : Gut microbiota abundance (genus Parasutterella id.2892) on Ferritin
with a threshold of 1e−05

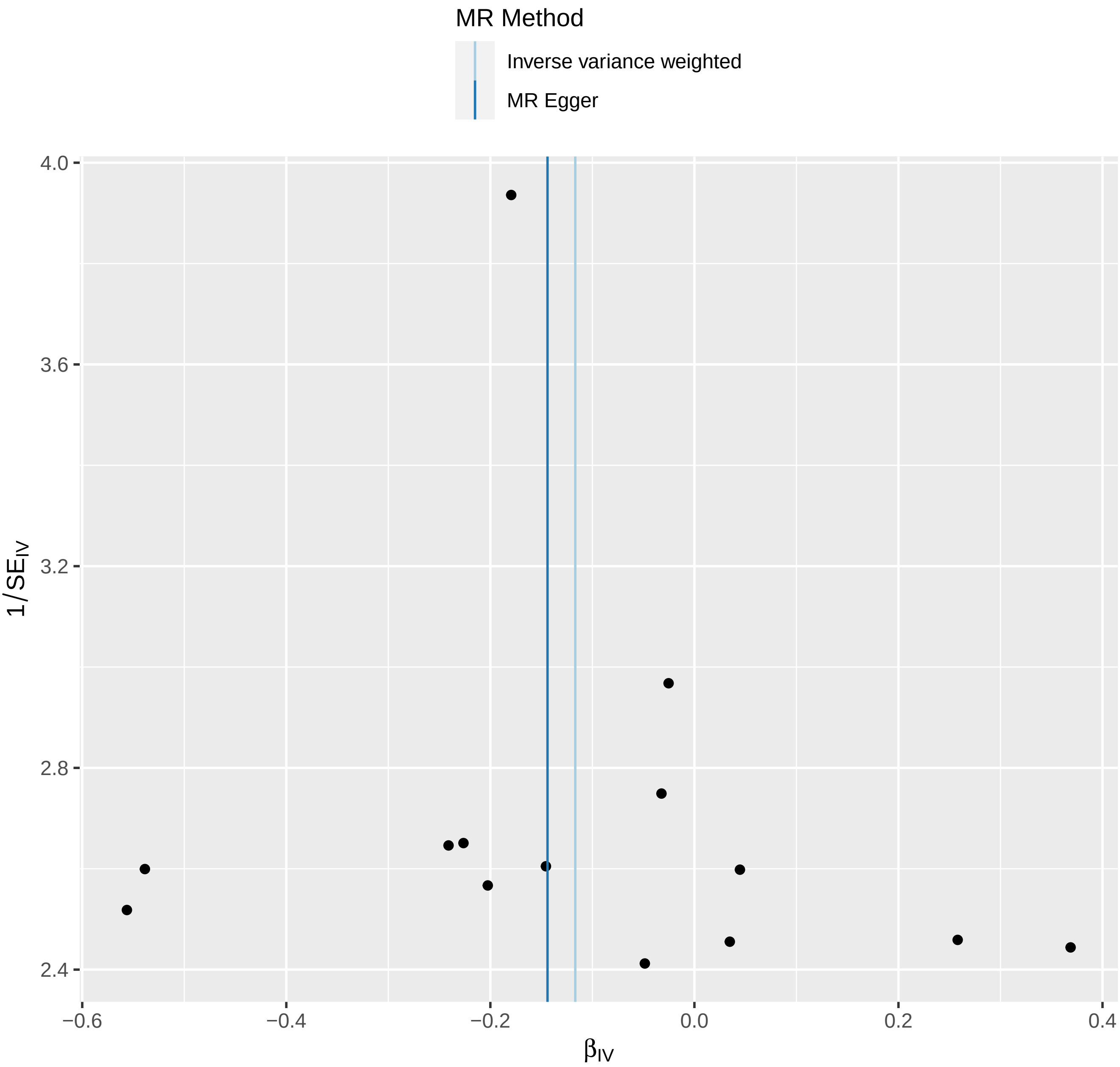

Batch 2−2299 : Gut microbiota abundance (genus Catenibacterium id.2153) on Furin
with a threshold of 1e−05

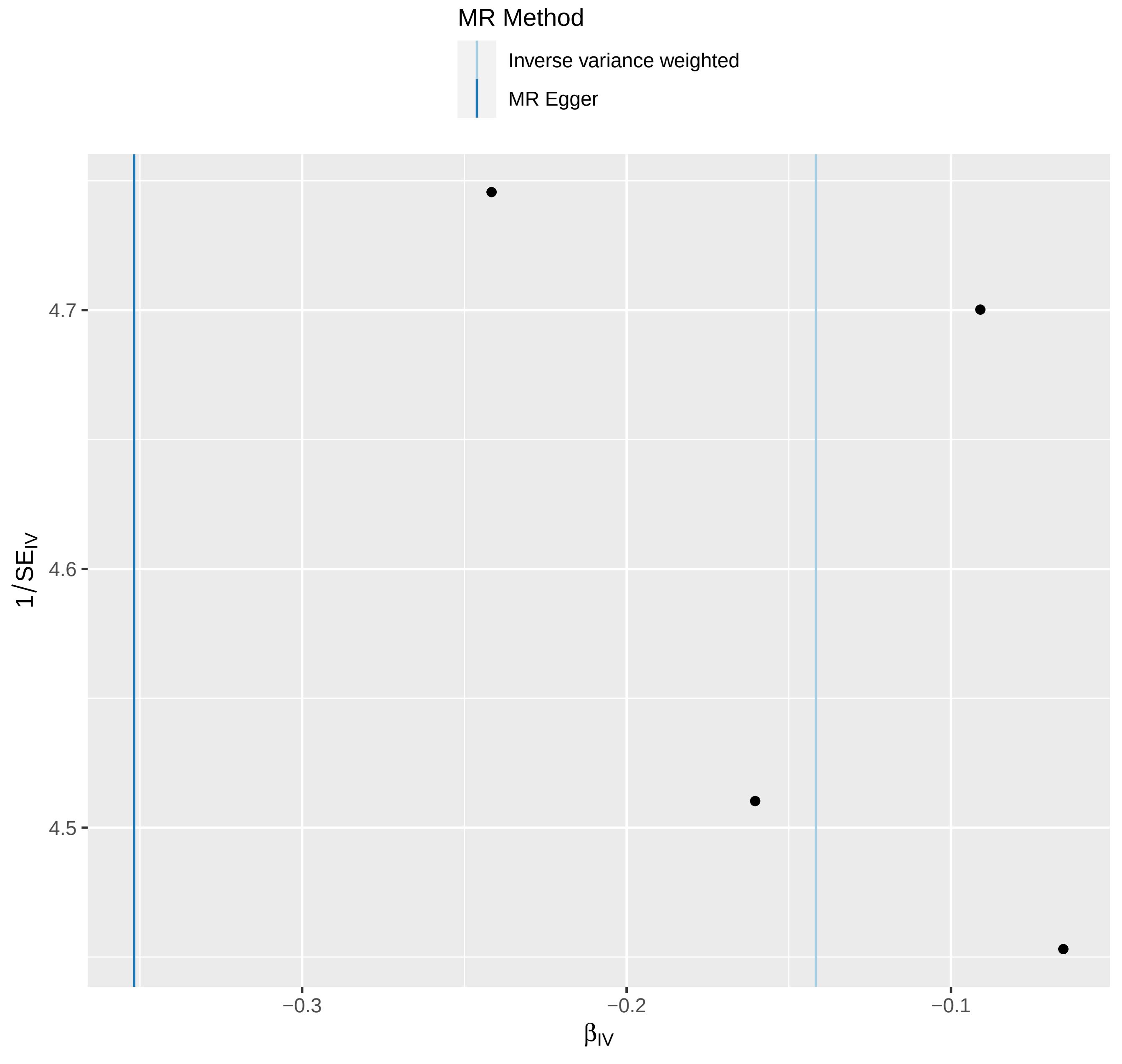

Batch 2−2300 : Gut microbiota abundance (genus Parasutterella id.2892) on Furin
with a threshold of 1e−05

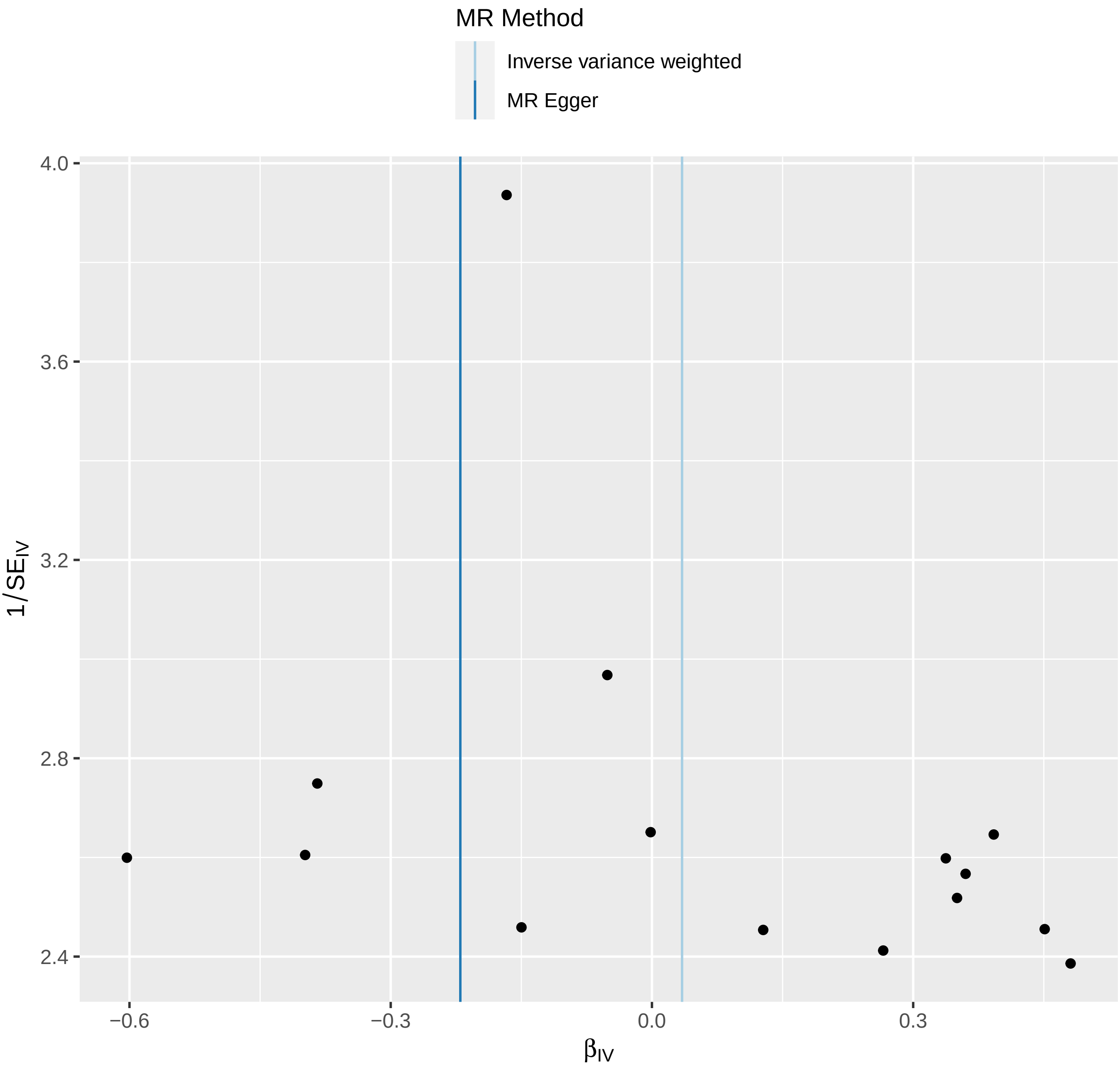

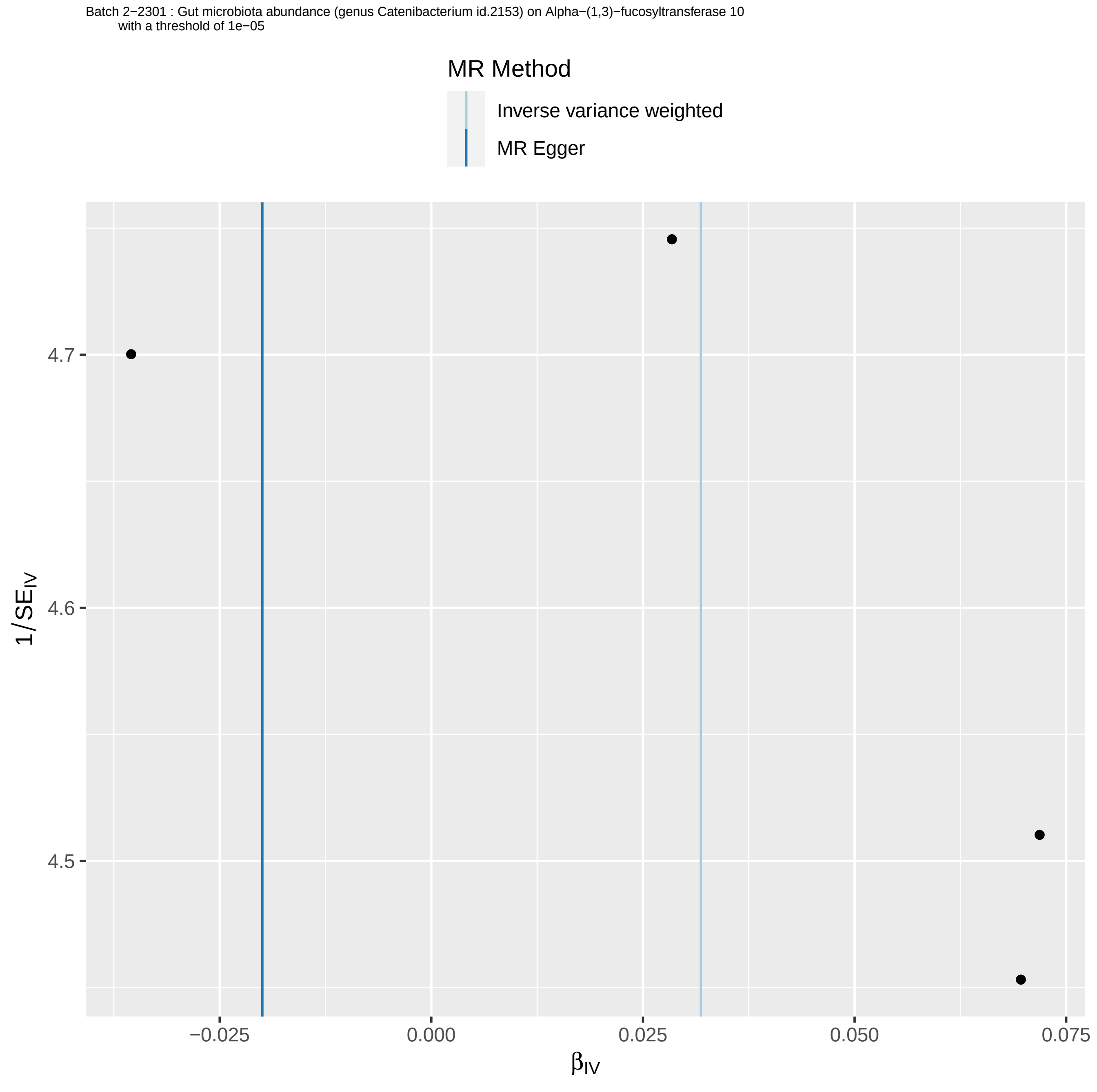

Batch 2−2301 : Gut microbiota abundance (genus Catenibacterium id.2153) on Alpha−(1,3)−fucosyltransferase 10
with a threshold of 1e−05
MR Method
Inverse variance weighted
MR Egger
4.7
4.6
4.5
1/SE_IV
−0.025
0.000
0.025
0.050
0.075
β_IV

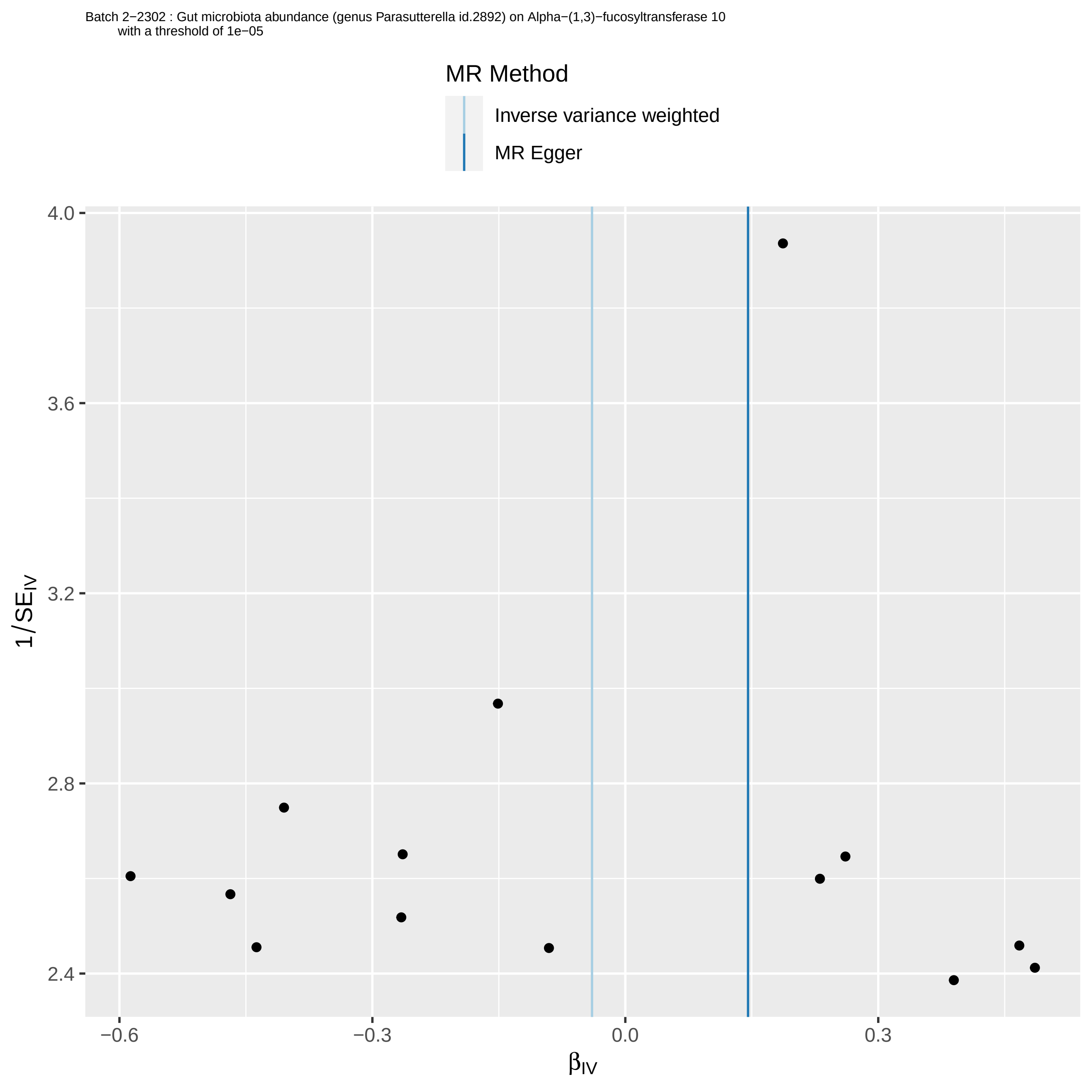
Batch 2−2302 : Gut microbiota abundance (genus Parasutterella id.2892) on Alpha−(1,3)−fucosyltransferase 10
with a threshold of 1e−05
MR Method
Inverse variance weighted
MR Egger
4.0
3.6
3.2
2.8
2.4
$1/SE_{IV}$
−0.6
−0.3
0.0
0.3
β_{IV}

Batch 2−2303 : Gut microbiota abundance (genus Catenibacterium id.2153) on Galactoside 3(4)−L−fucosyltransferase
with a threshold of 1e−05

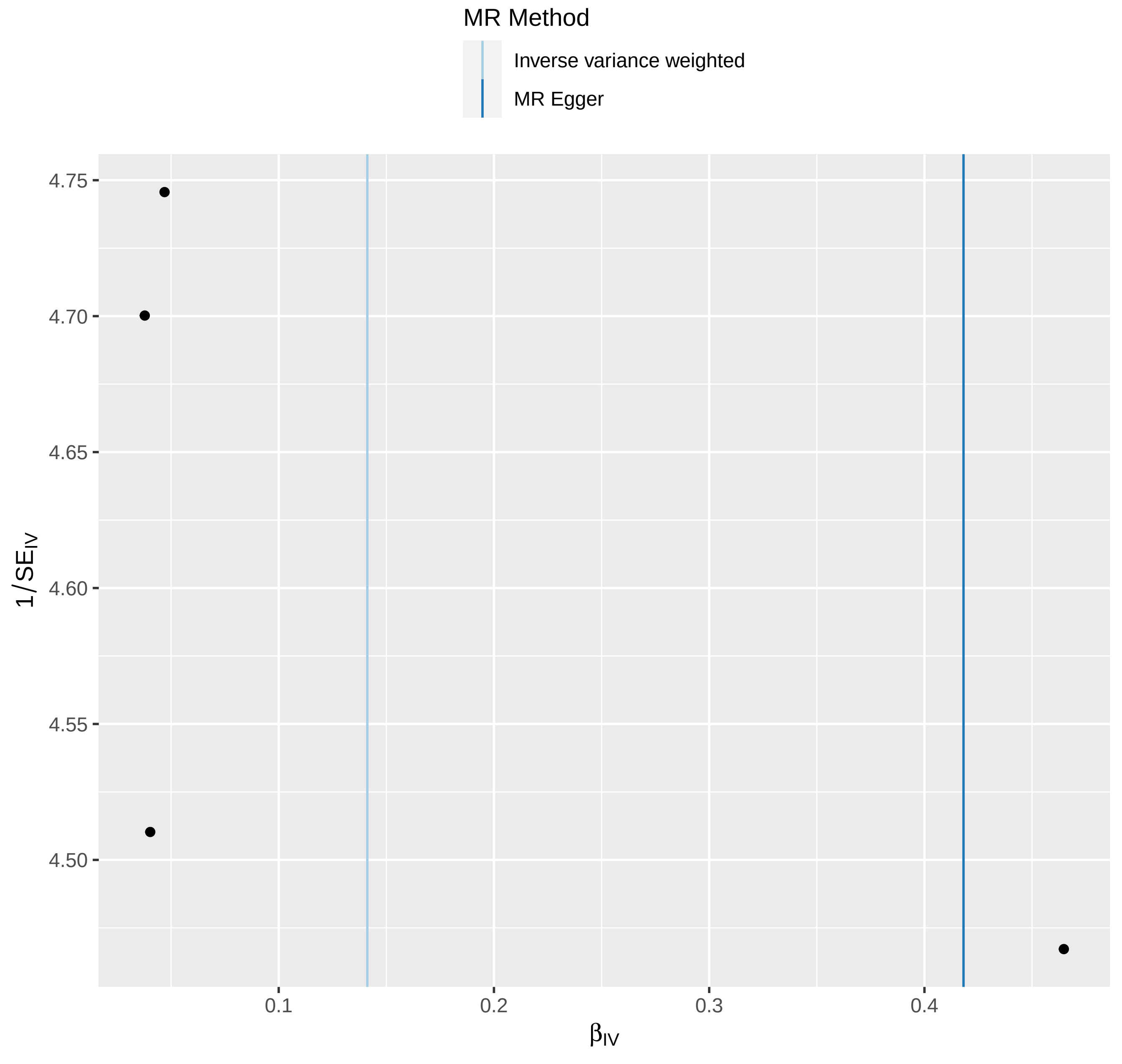

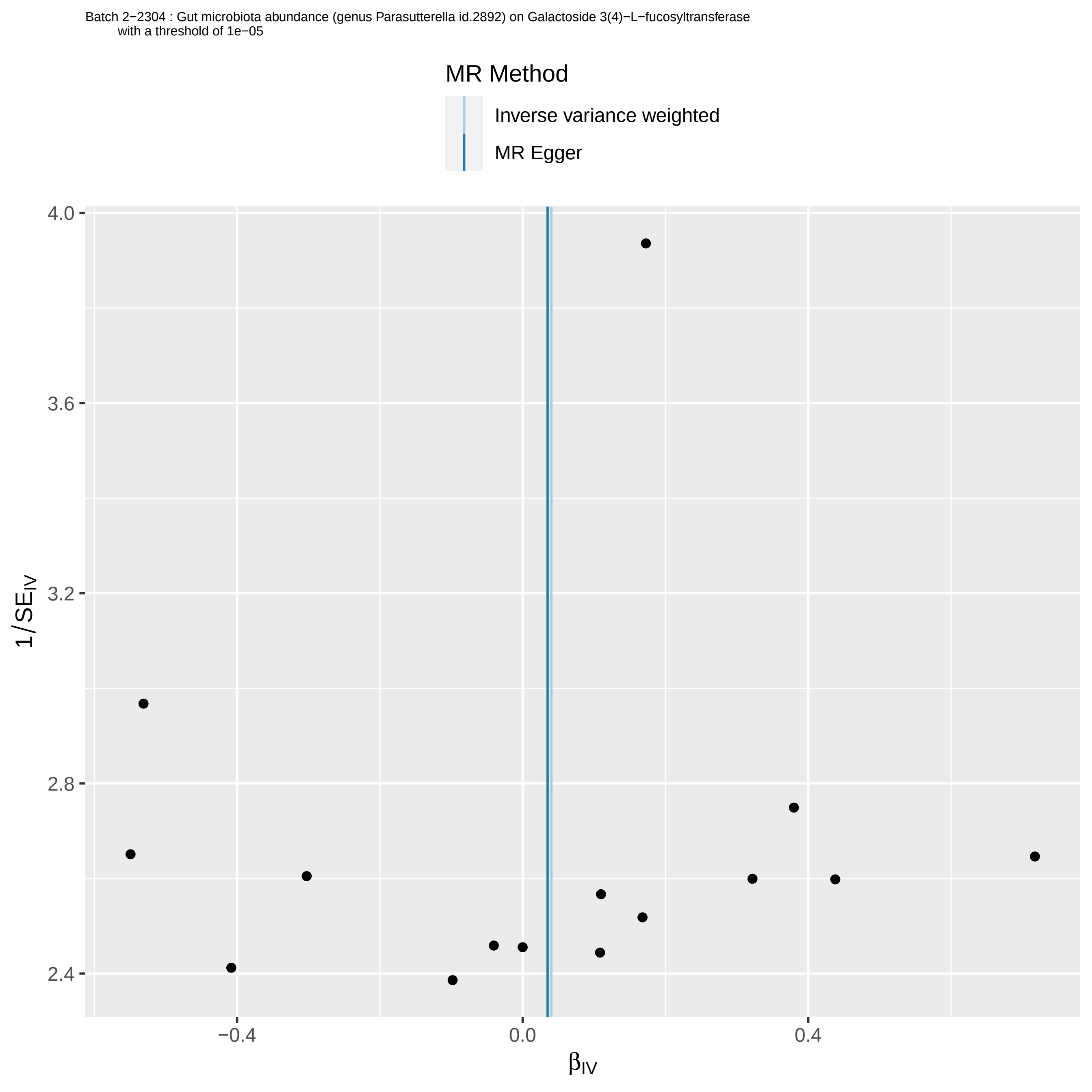
Batch 2−2304 : Gut microbiota abundance (genus Parasutterella id.2892) on Galactoside 3(4)−L−fucosyltransferase
with a threshold of 1e−05
MR Method
Inverse variance weighted
MR Egger
4.0
3.6
3.2
2.8
2.4
1/SE_IV
−0.4
0.0
0.4
β_IV

Batch 2−2305 : Gut microbiota abundance (genus Catenibacterium id.2153) on Alpha−(1,3)−fucosyltransferase 5
with a threshold of 1e−05

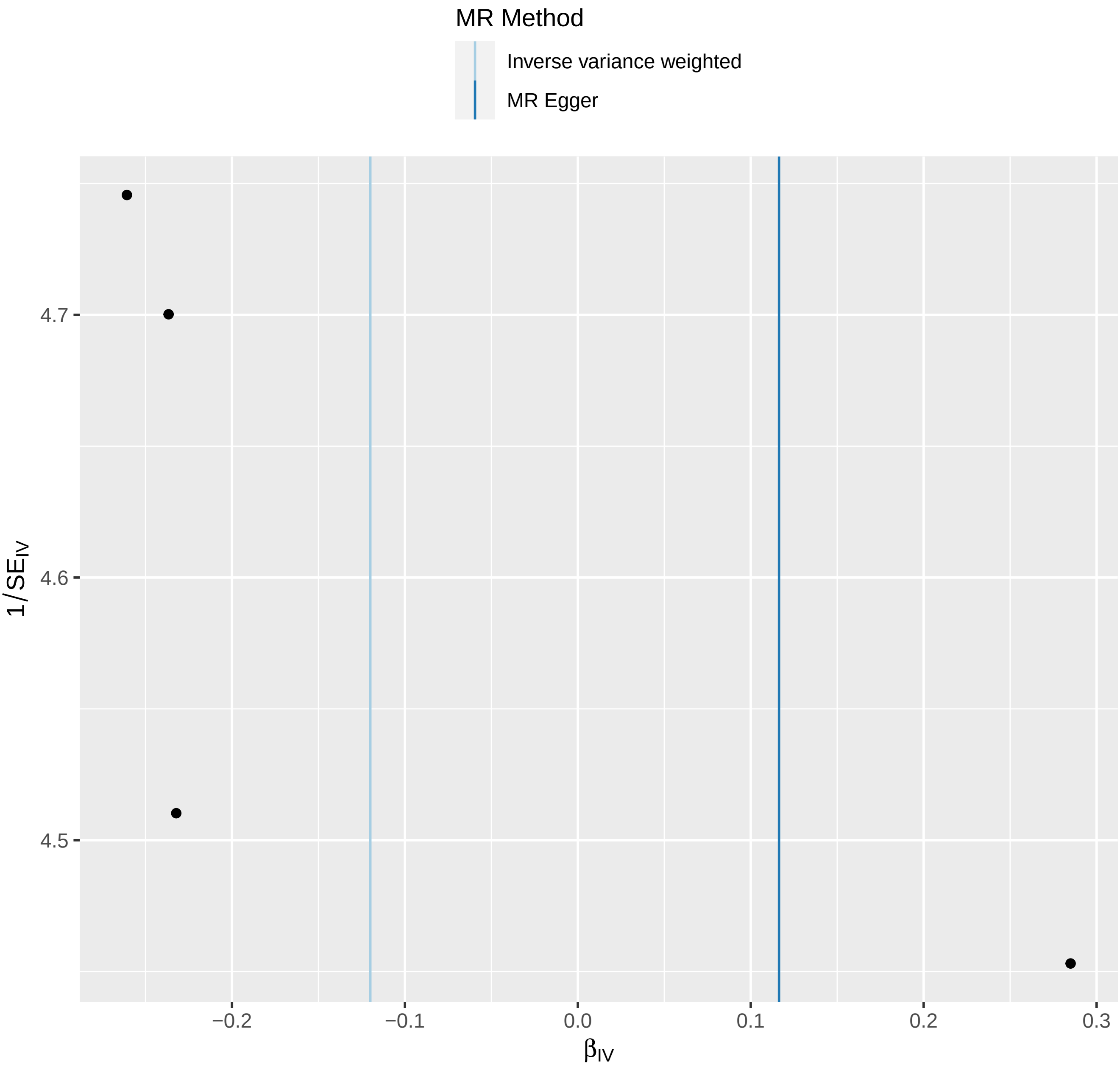

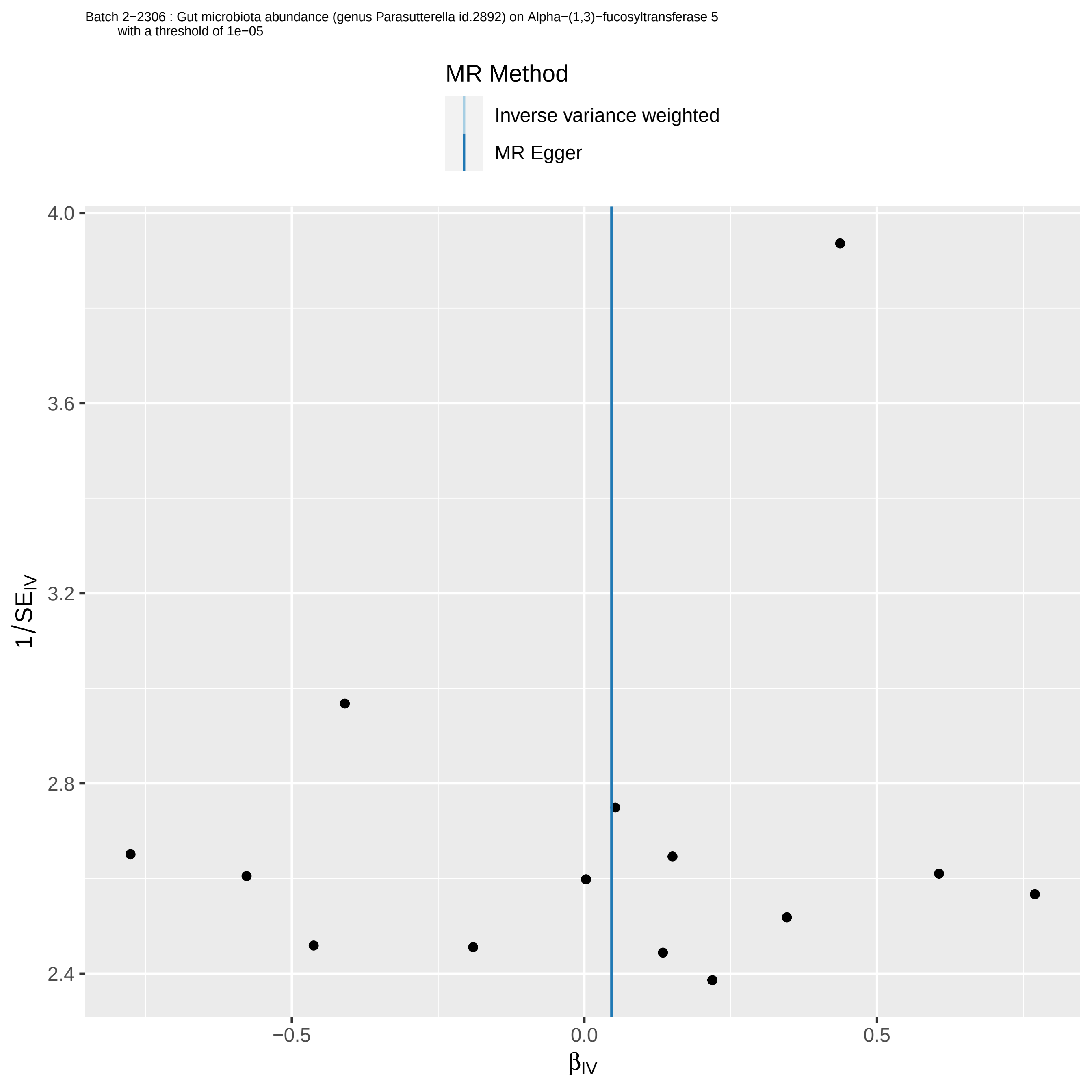

Batch 2−2306 : Gut microbiota abundance (genus Parasutterella id.2892) on Alpha−(1,3)−fucosyltransferase 5
with a threshold of 1e−05
MR Method
Inverse variance weighted
MR Egger
4.0
3.6
3.2
2.8
2.4
1/SE_IV
−0.5
0.0
0.5
β_IV

Batch 2−2307 : Gut microbiota abundance (genus Catenibacterium id.2153) on Alpha−(1,6)−fucosyltransferase
with a threshold of 1e−05

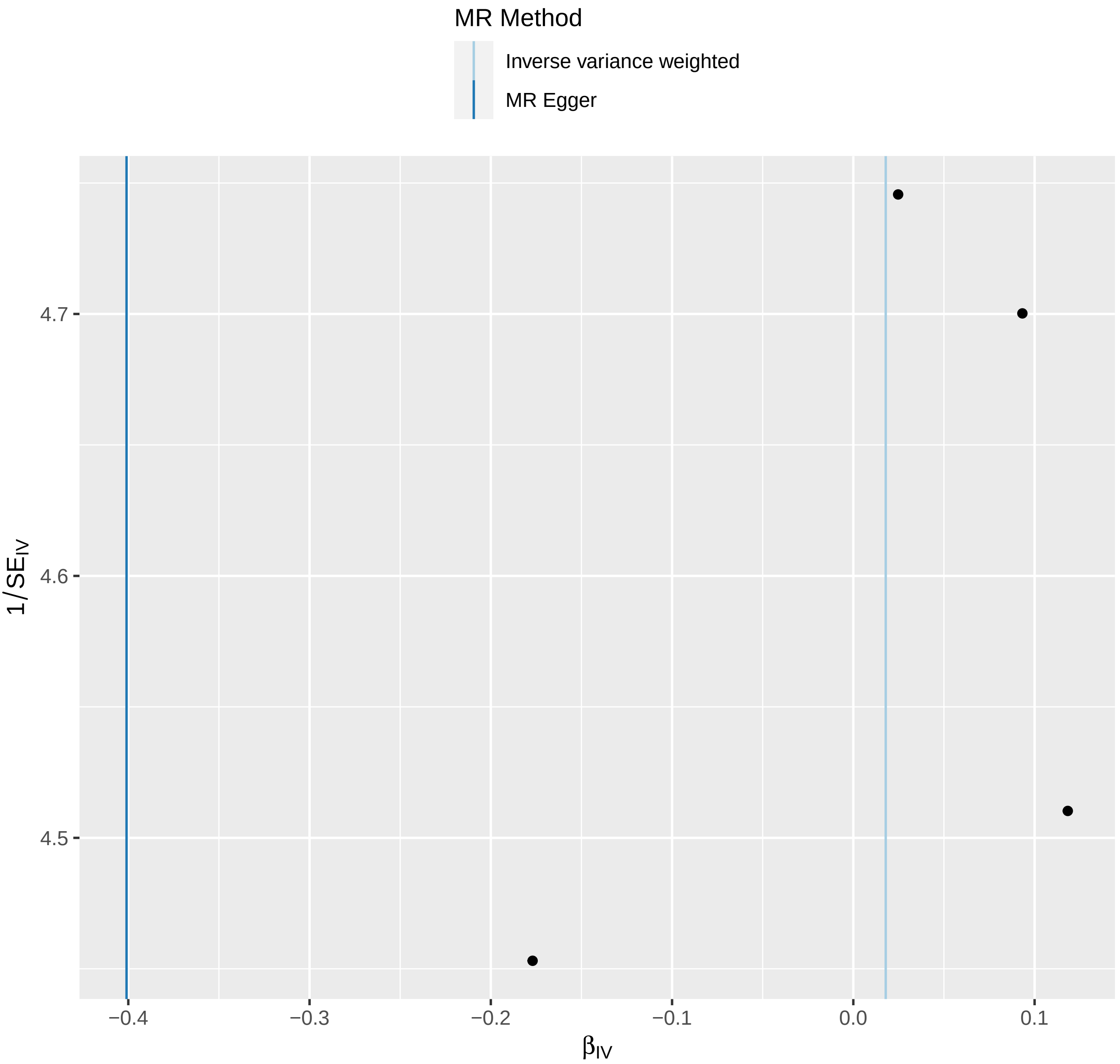

Batch 2−2308 : Gut microbiota abundance (genus Parasutterella id.2892) on Alpha−(1,6)−fucosyltransferase
with a threshold of 1e−05

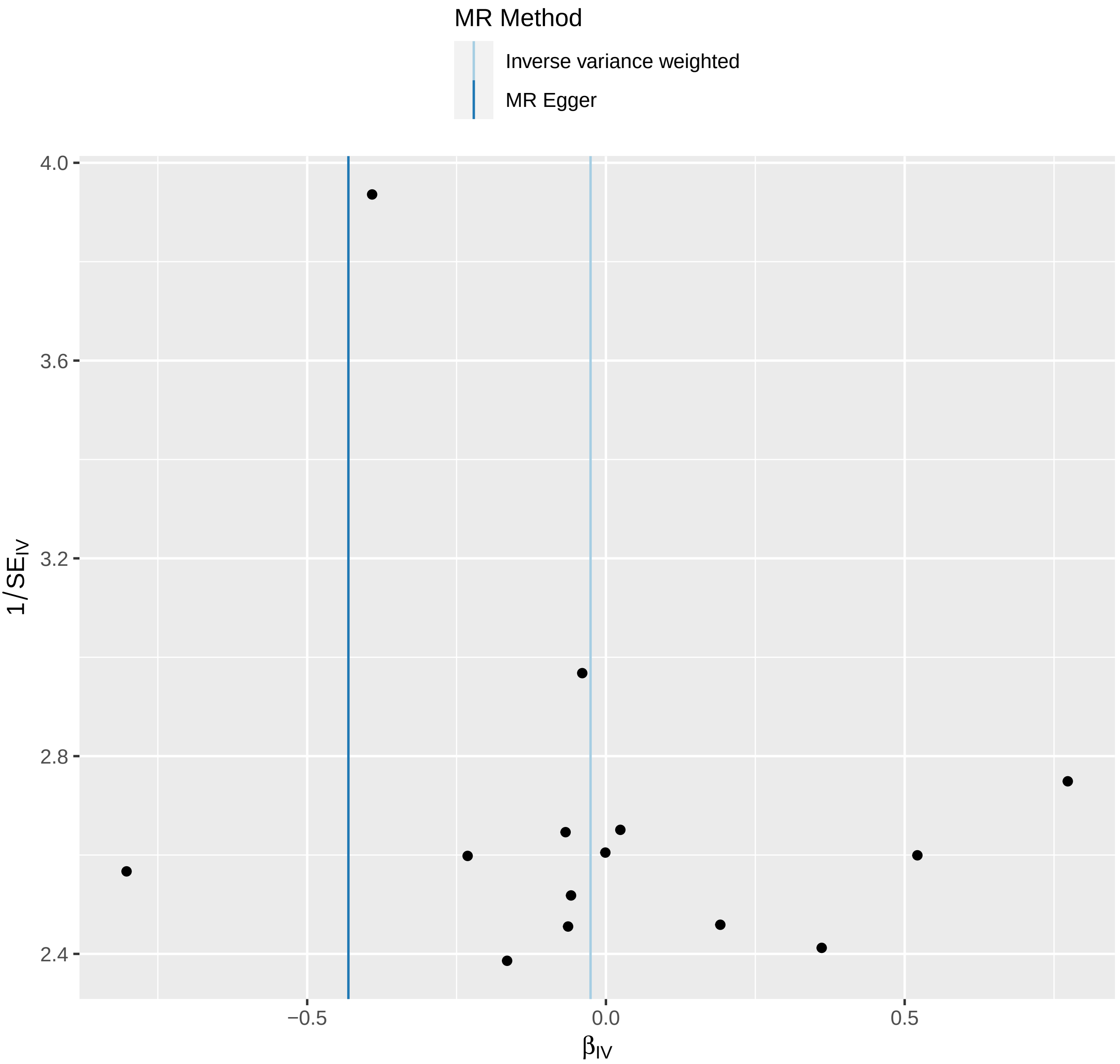

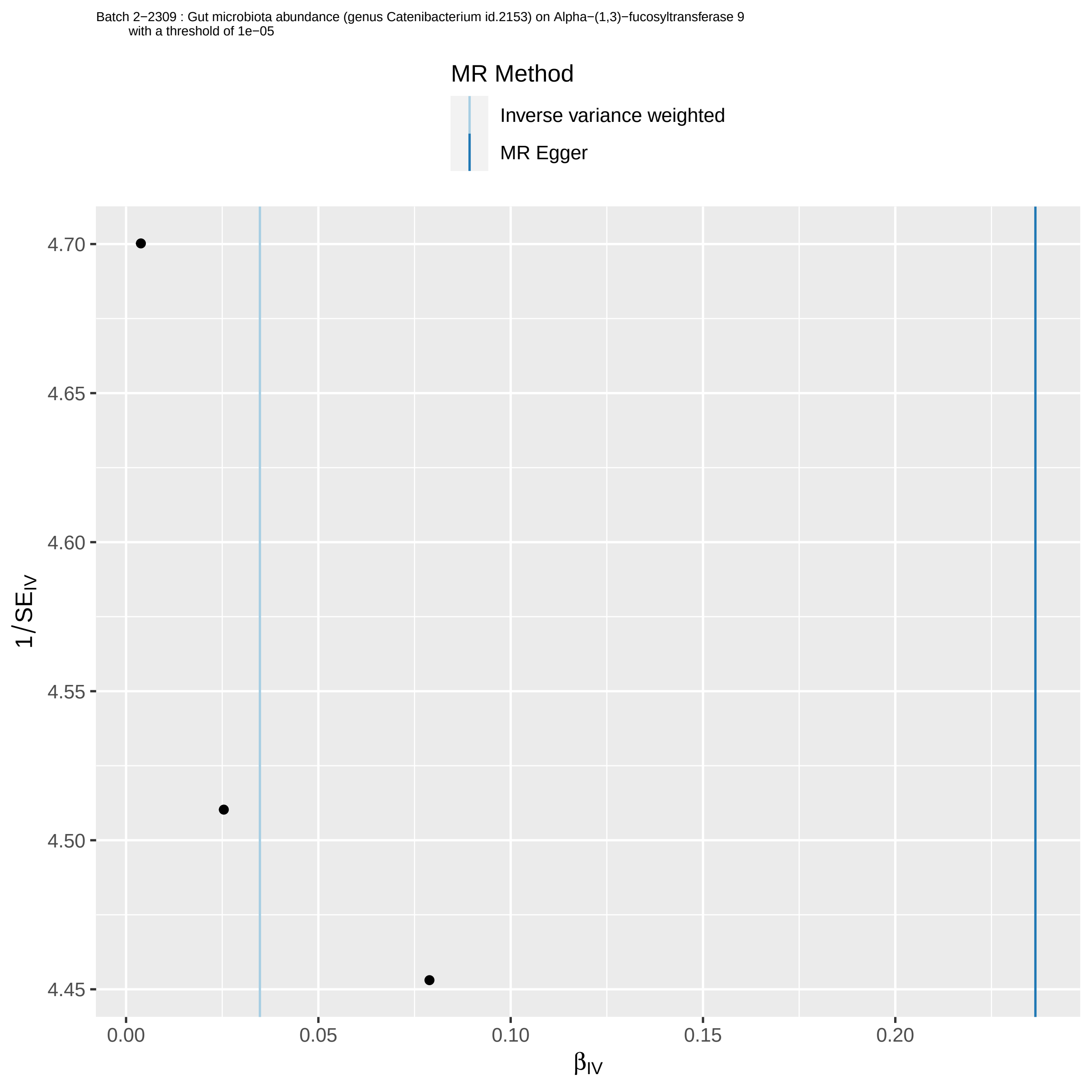

Batch 2−2309 : Gut microbiota abundance (genus Catenibacterium id.2153) on Alpha−(1,3)−fucosyltransferase 9
with a threshold of 1e−05
MR Method
Inverse variance weighted
MR Egger
4.70
4.65
4.60
4.55
4.50
4.45
1/SE_IV
0.00
0.05
0.10
0.15
0.20
β_IV

Batch 2−2310 : Gut microbiota abundance (genus Parasutterella id.2892) on Alpha−(1,3)−fucosyltransferase 9
with a threshold of 1e−05

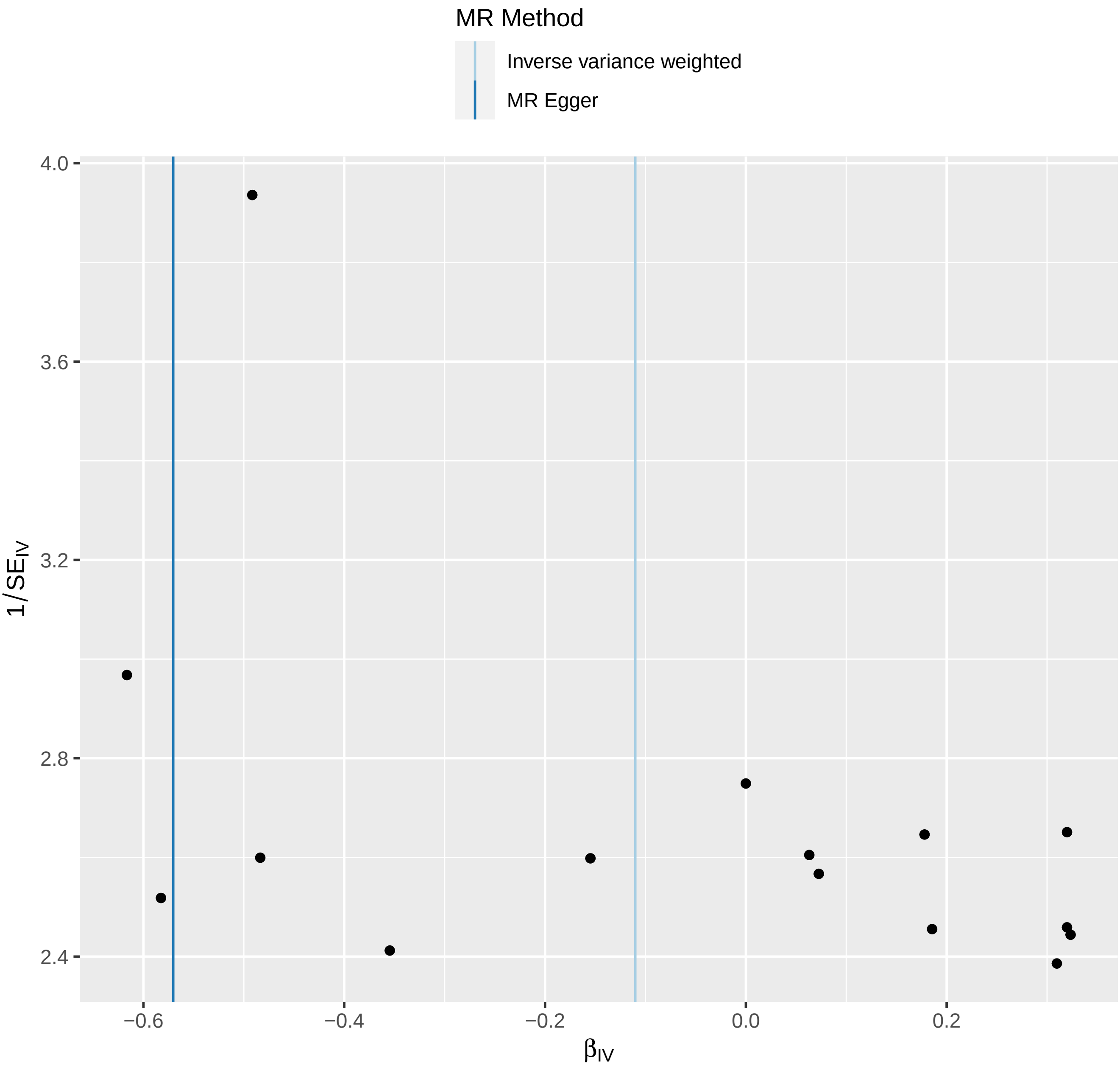

Batch 2−2311 : Gut microbiota abundance (genus Catenibacterium id.2153) on Fragile X mental retardation syndrome−related protein 1 with a threshold of 1e−05

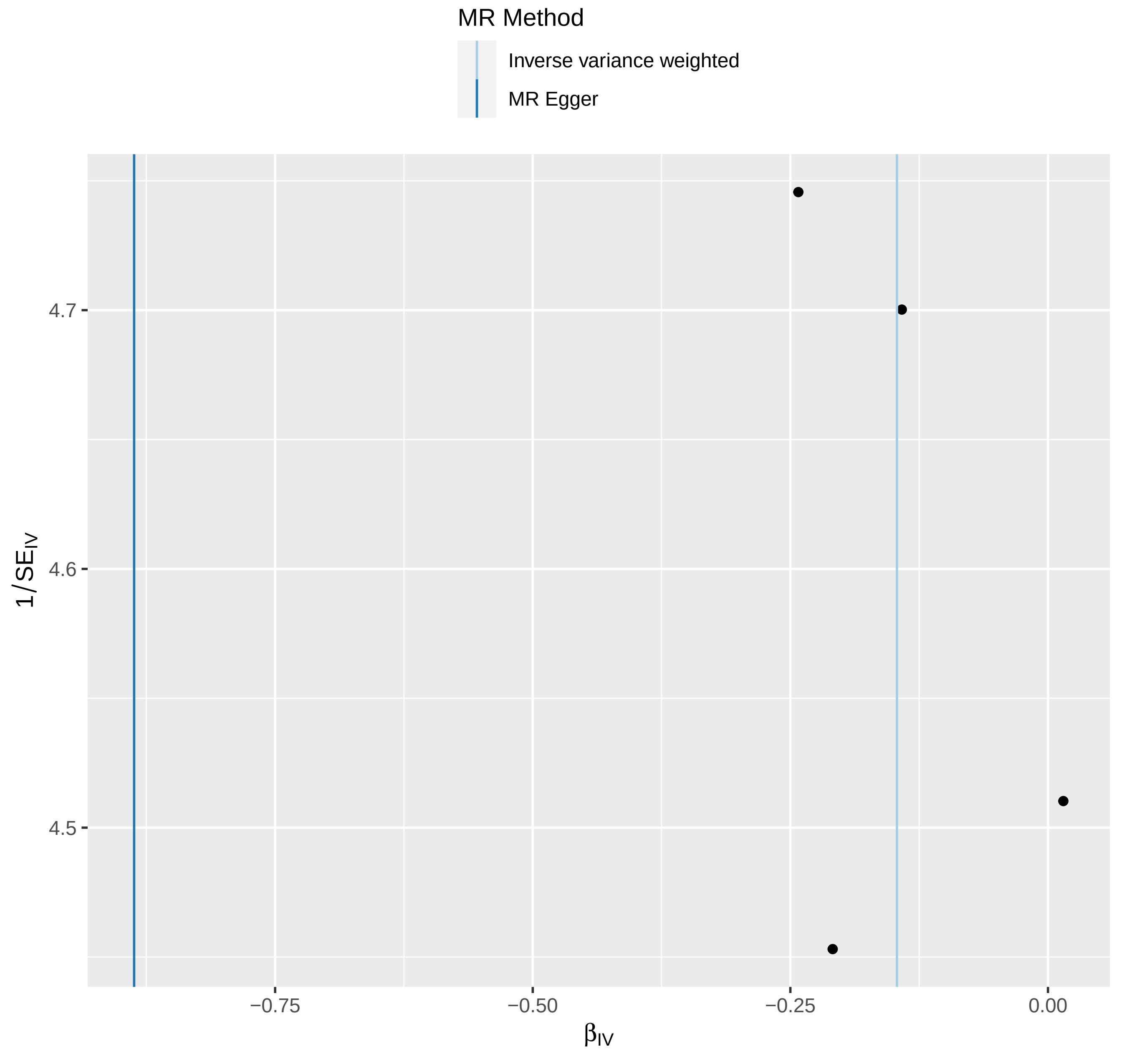

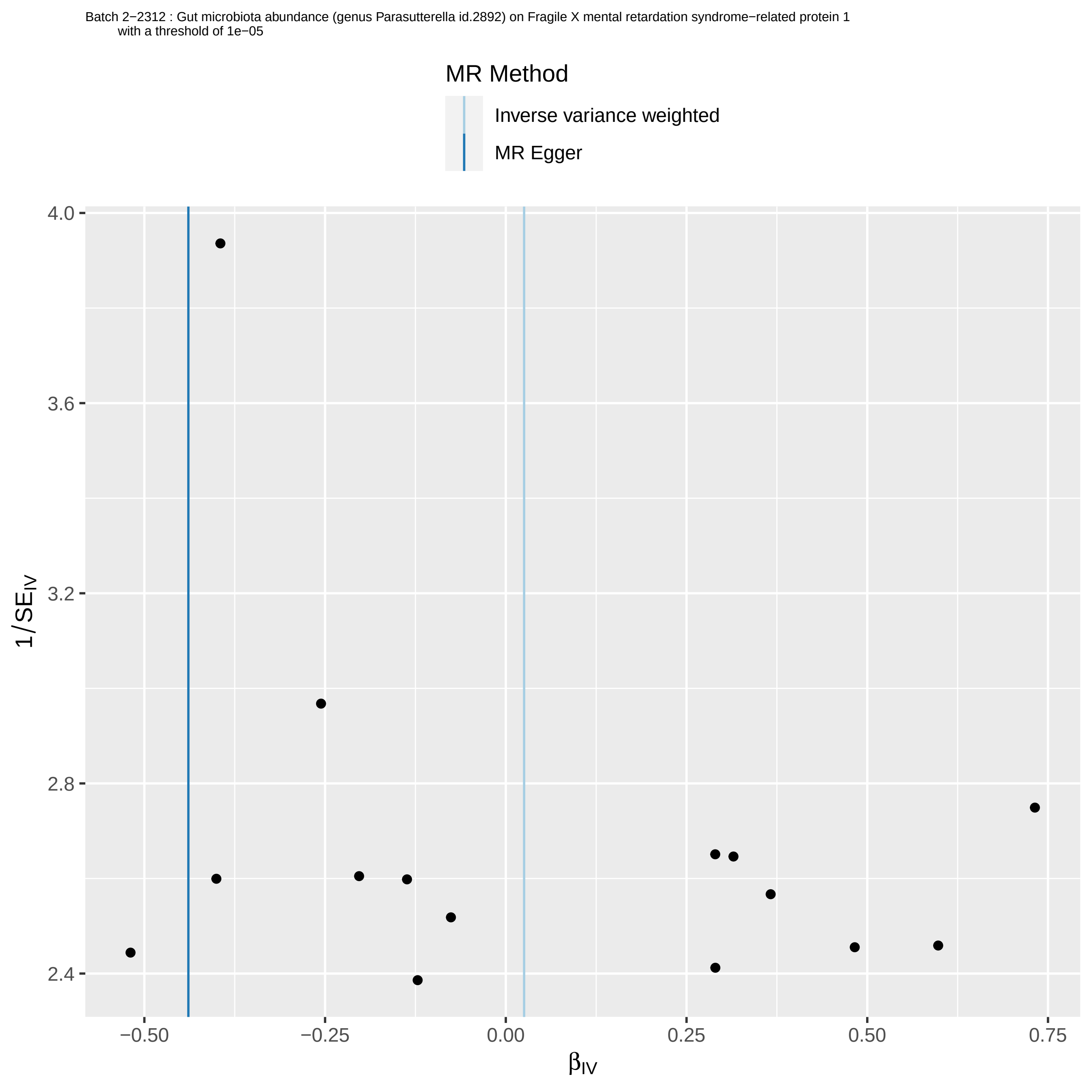

Batch 2−2312 : Gut microbiota abundance (genus Parasutterella id.2892) on Fragile X mental retardation syndrome−related protein 1 with a threshold of 1e−05
MR Method
Inverse variance weighted
MR Egger
4.0
3.6
3.2
2.8
2.4
1/SE$_{IV}$
−0.50
−0.25
0.00
0.25
0.50
0.75
β$_{IV}$

Batch 2−2313 : Gut microbiota abundance (genus Catenibacterium id.2153) on Tyrosine−protein kinase Fyn with a threshold of 1e−05

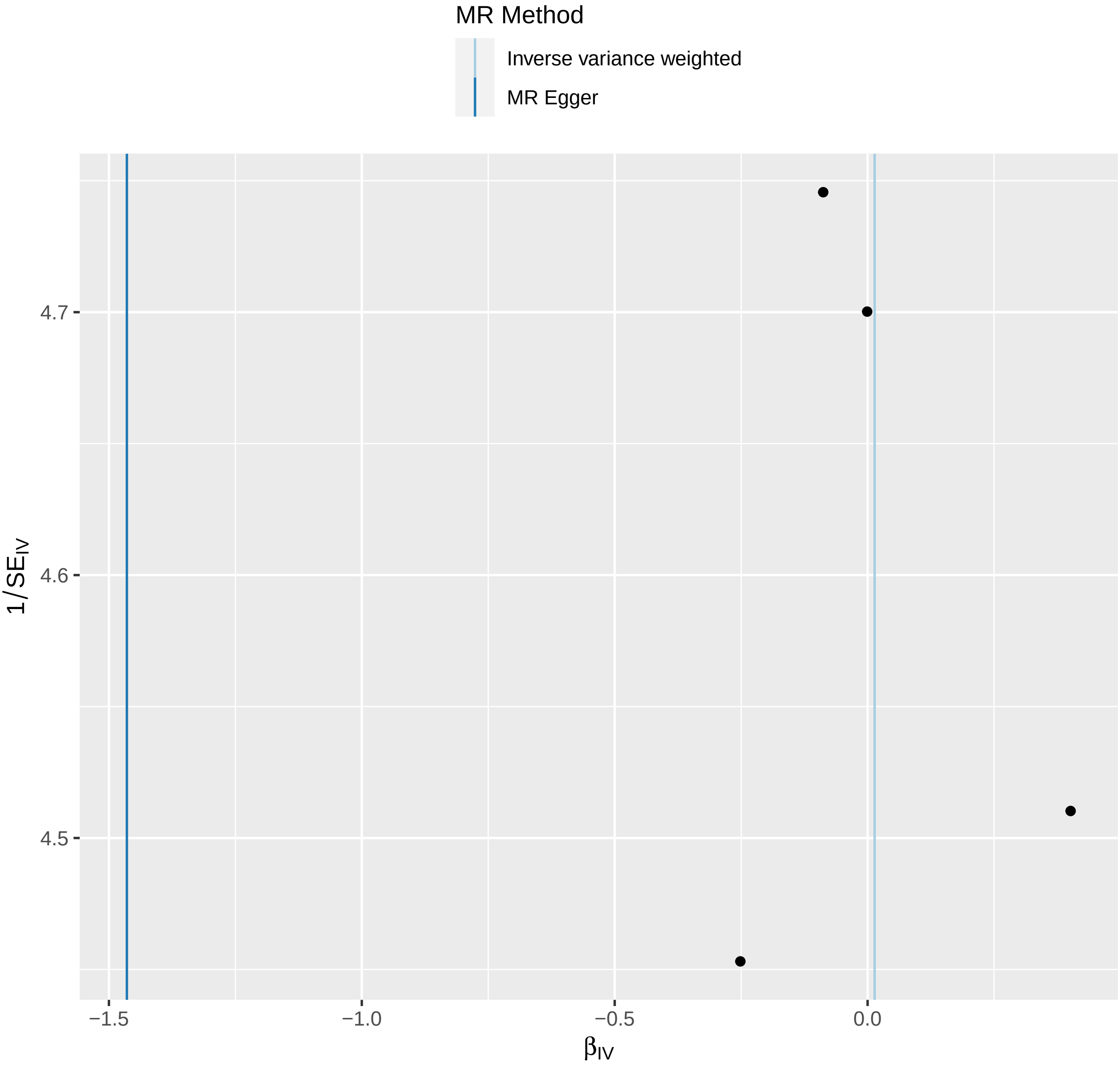

Batch 2−2314 : Gut microbiota abundance (genus Parasutterella id.2892) on Tyrosine−protein kinase Fyn
with a threshold of 1e−05

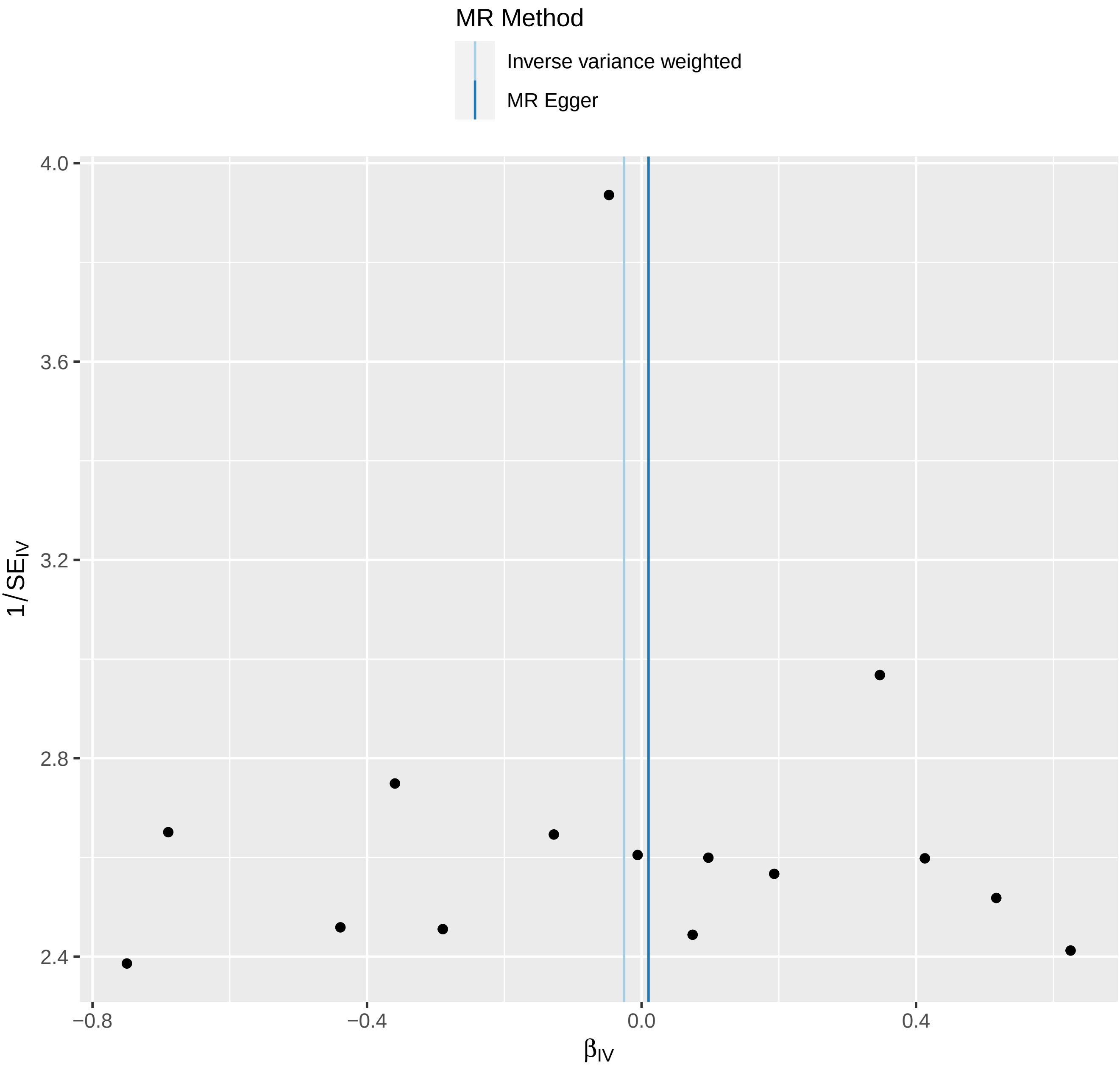

Batch 2−2315 : Gut microbiota abundance (genus Catenibacterium id.2153) on G0/G1 switch protein 2
with a threshold of 1e−05

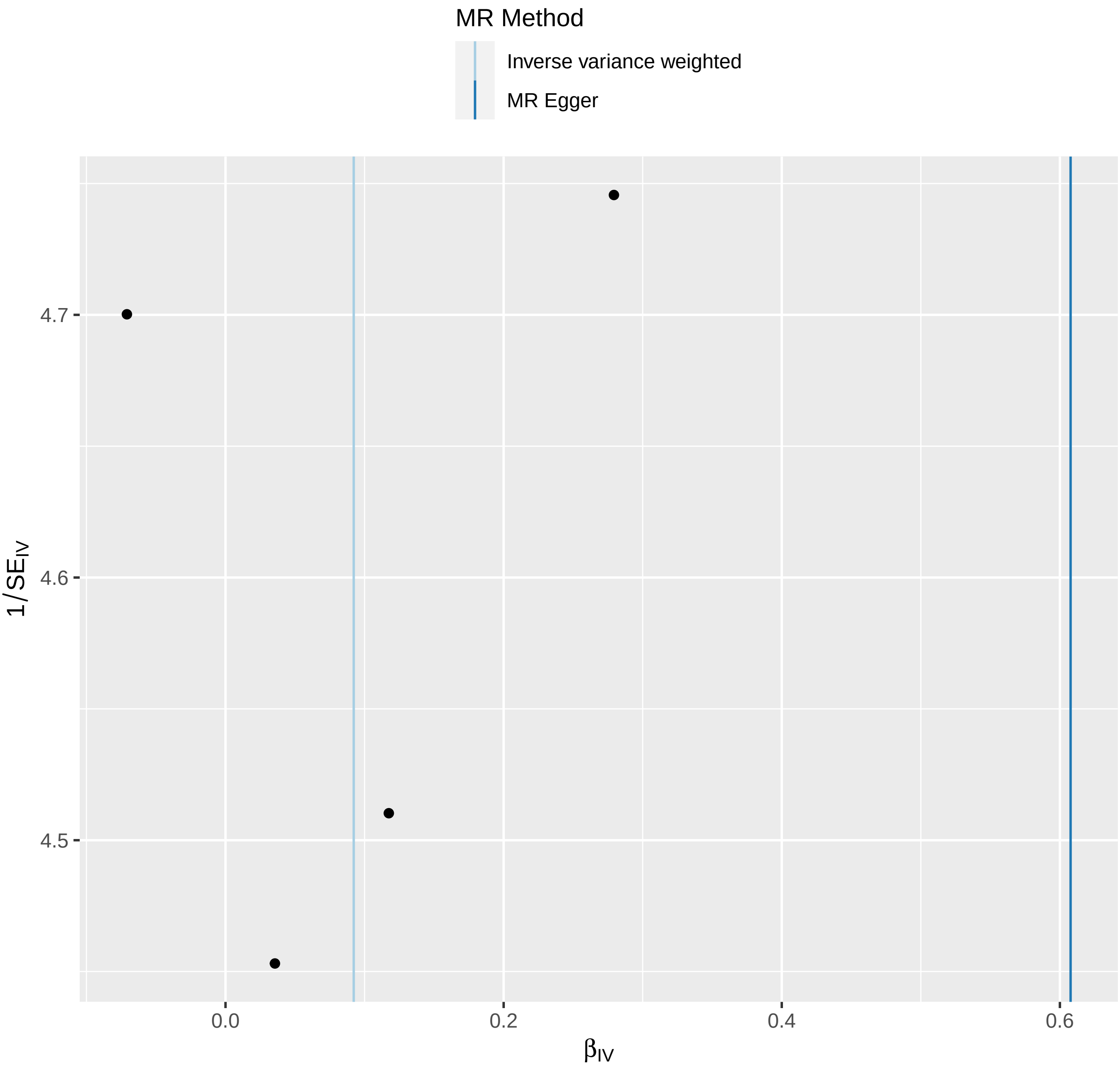

Batch 2−2316 : Gut microbiota abundance (genus Parasutterella id.2892) on G0/G1 switch protein 2
with a threshold of 1e−05

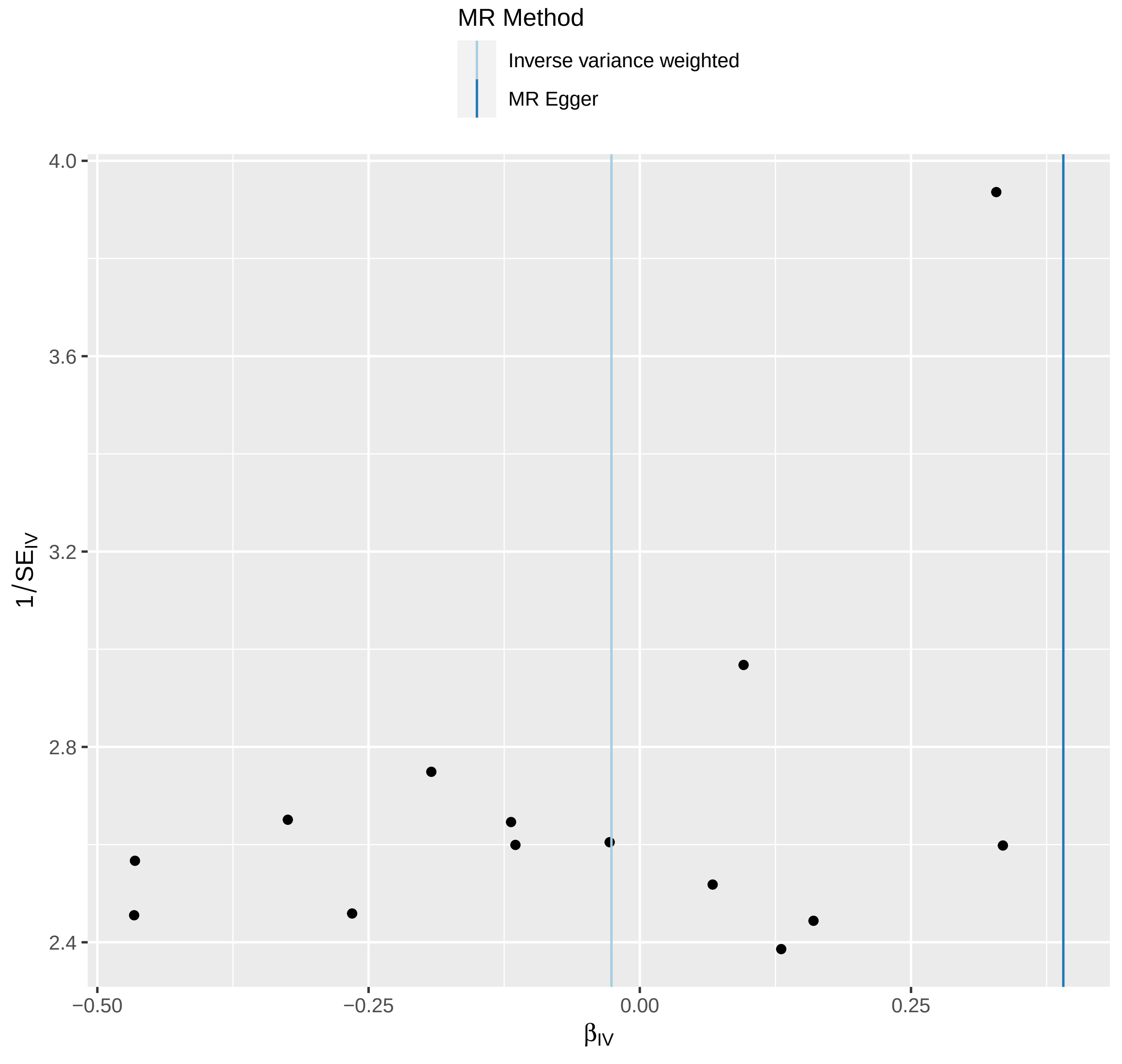

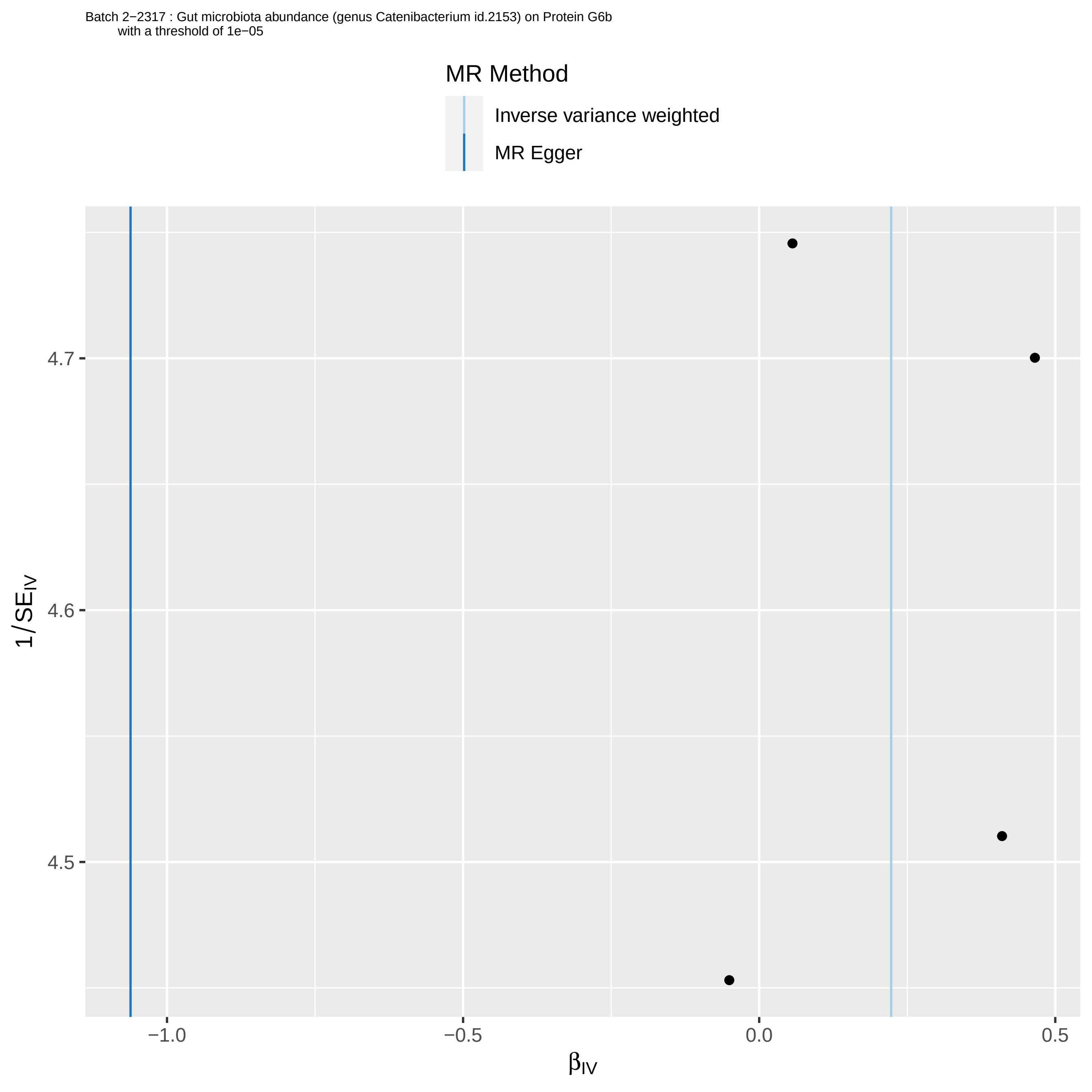

Batch 2−2317 : Gut microbiota abundance (genus Catenibacterium id.2153) on Protein G6b
with a threshold of 1e−05
MR Method
Inverse variance weighted
MR Egger
4.7
4.6
4.5
1/SE_IV
−1.0
−0.5
0.0
0.5
β_IV

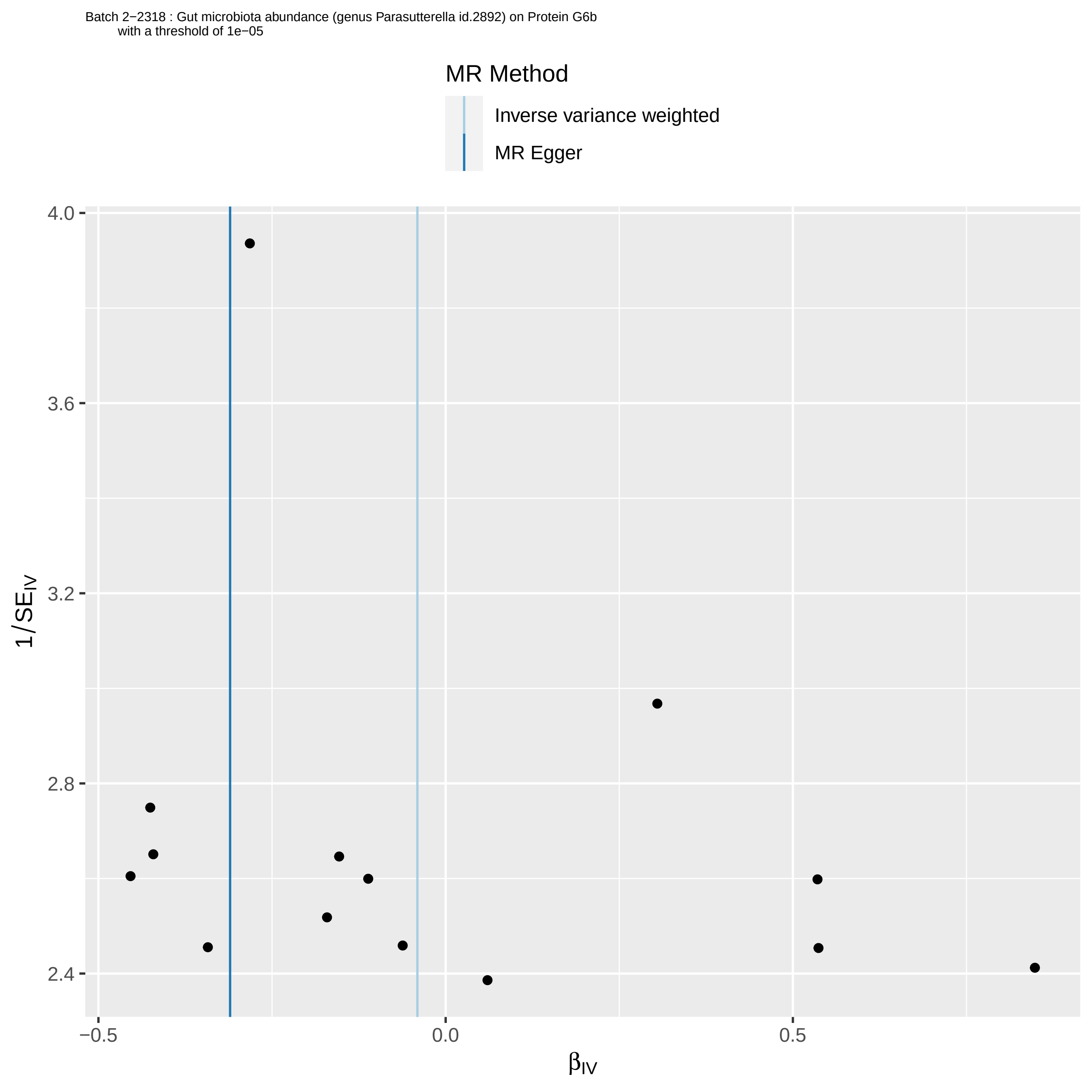

Batch 2−2318 : Gut microbiota abundance (genus Parasutterella id.2892) on Protein G6b
with a threshold of 1e−05
MR Method
Inverse variance weighted
MR Egger
4.0
3.6
3.2
2.8
2.4
1/SE_IV
−0.5
0.0
0.5
β_IV

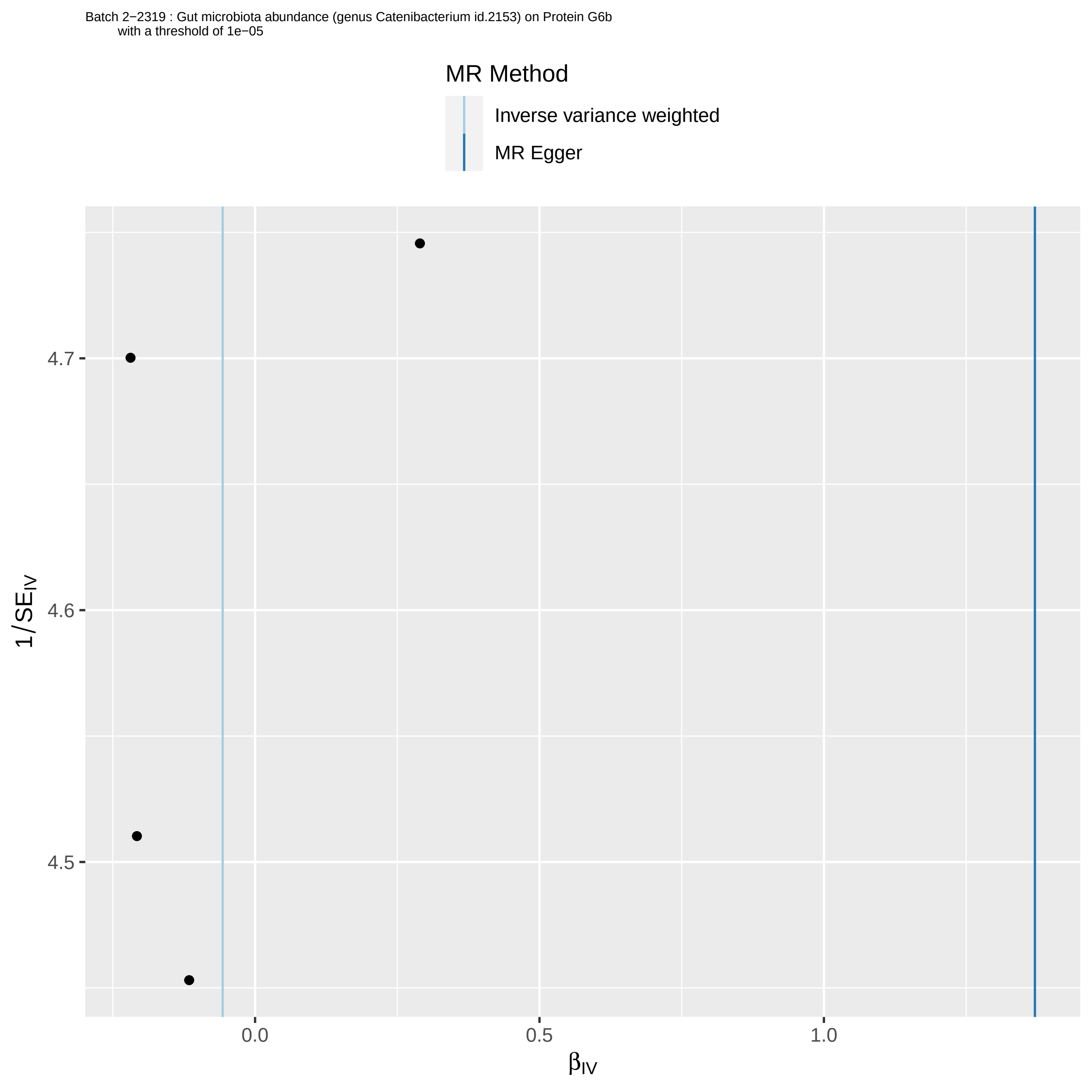

Batch 2−2319 : Gut microbiota abundance (genus Catenibacterium id.2153) on Protein G6b
with a threshold of 1e−05
MR Method
Inverse variance weighted
MR Egger
4.7
4.6
4.5
1/SE$_{IV}$
0.0
0.5
1.0
β_{IV}

Batch 2−2320 : Gut microbiota abundance (genus Parasutterella id.2892) on Protein G6b
with a threshold of 1e−05

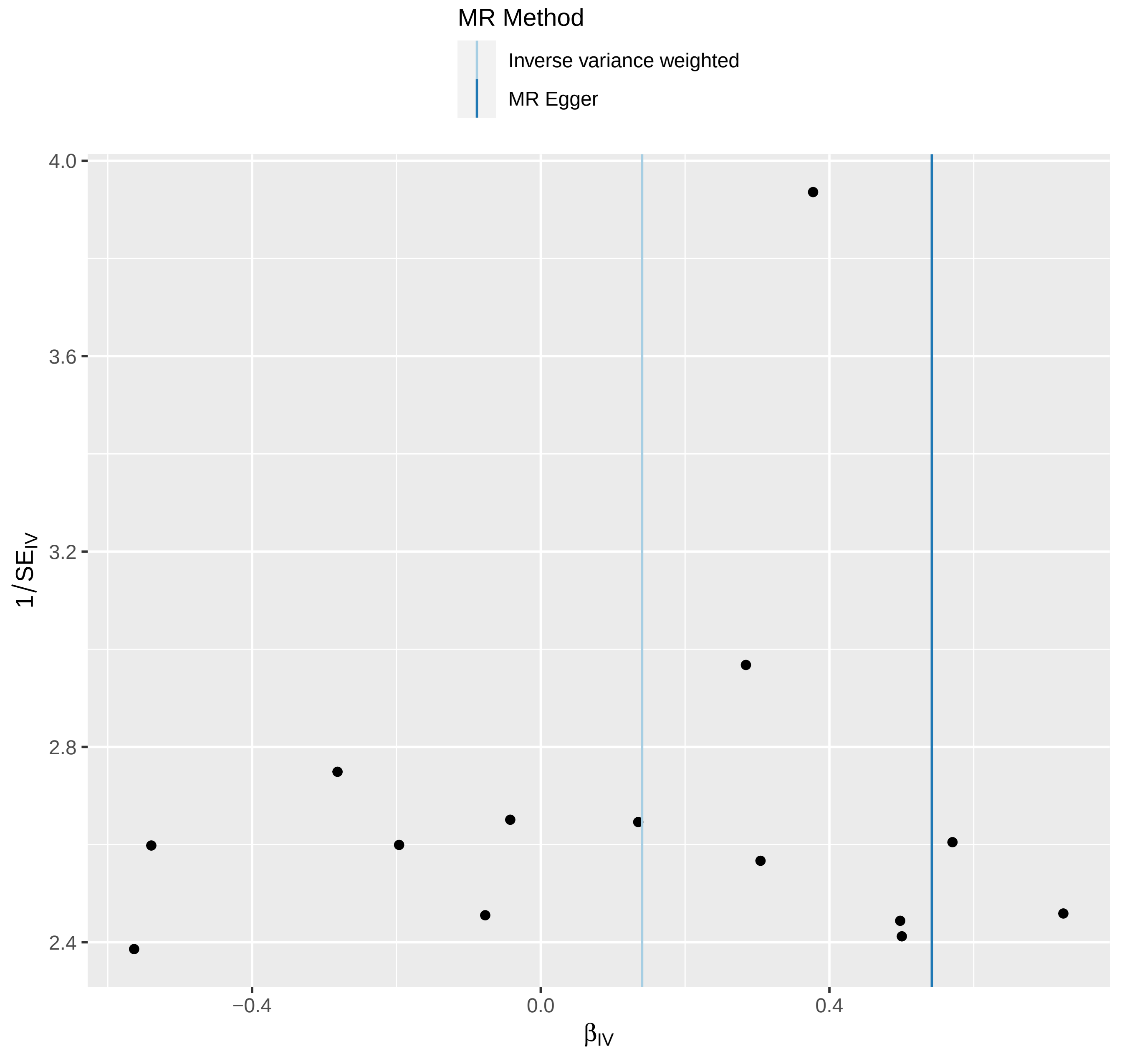

Batch 2−2321 : Gut microbiota abundance (genus Catenibacterium id.2153) on Gamma−aminobutyric acid receptor−associated protein−like 1 with a threshold of 1e−05

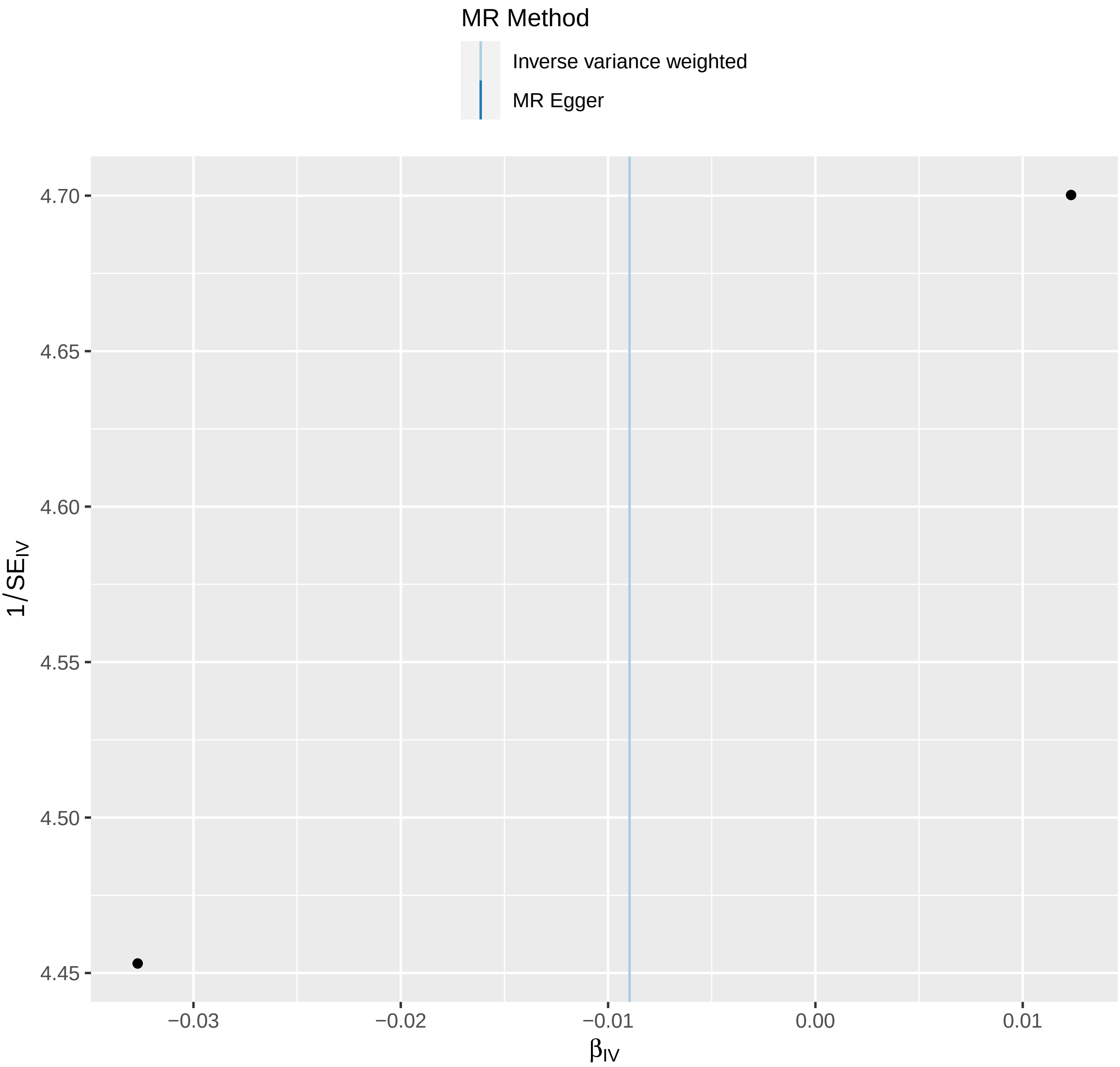

Batch 2−2322 : Gut microbiota abundance (genus Parasutterella id.2892) on Gamma−aminobutyric acid receptor−associated protein−like 1
with a threshold of 1e−05

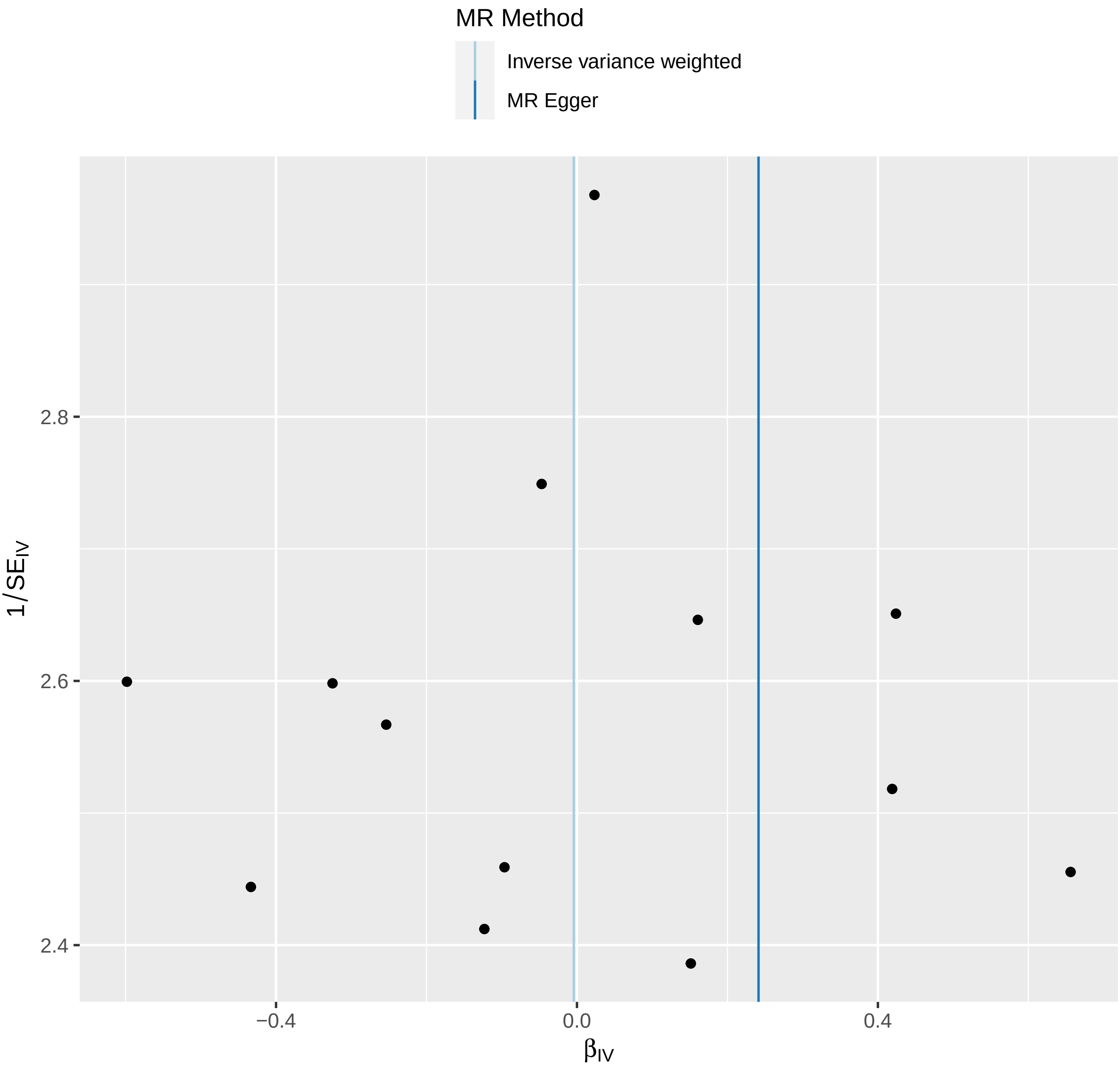

Batch 2−2323 : Gut microbiota abundance (genus Catenibacterium id.2153) on Gamma−aminobutyric acid receptor−associated protein−like 2 with a threshold of 1e−05

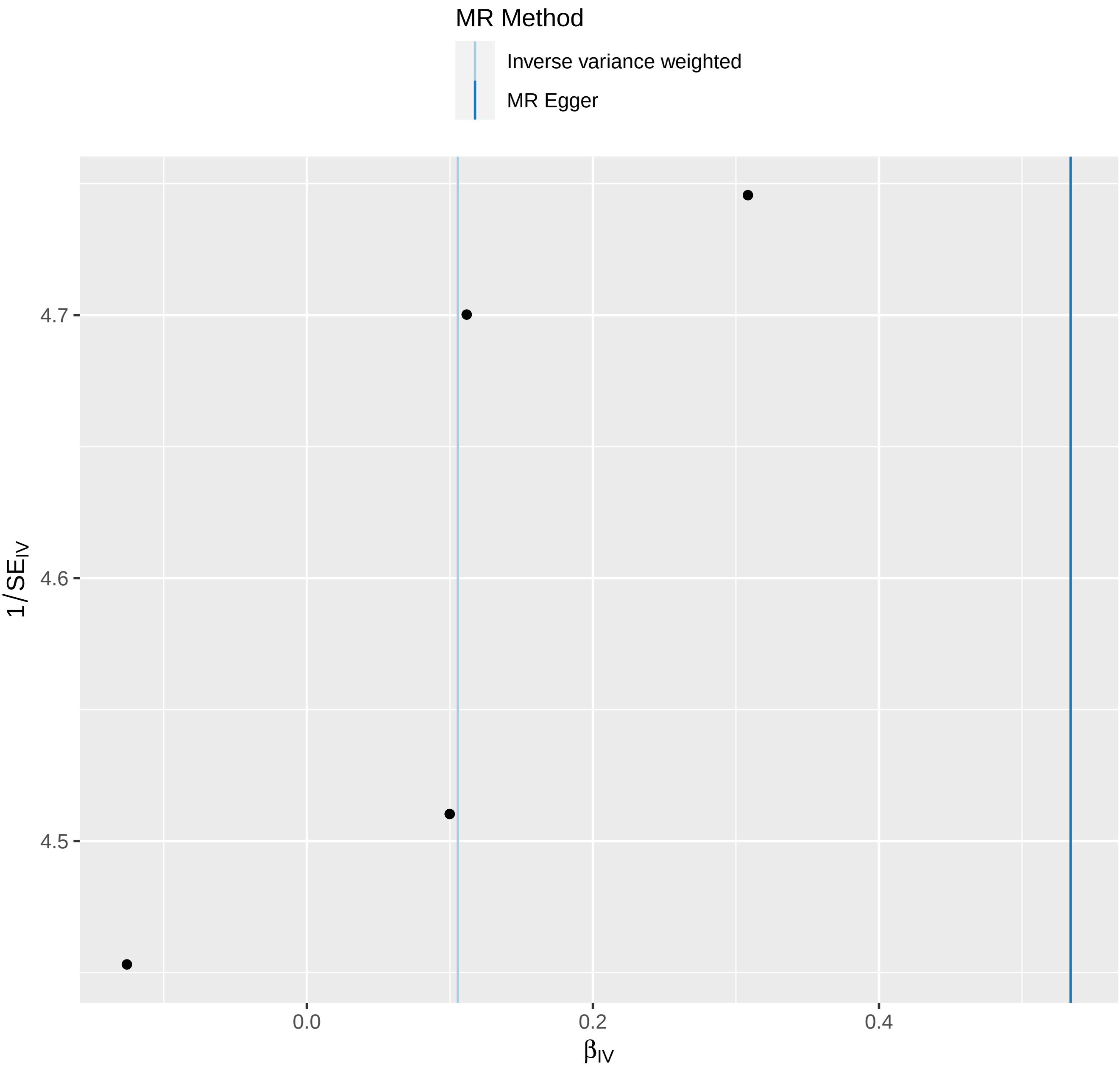

Batch 2−2324 : Gut microbiota abundance (genus Parasutterella id.2892) on Gamma−aminobutyric acid receptor−associated protein−like 2
with a threshold of 1e−05

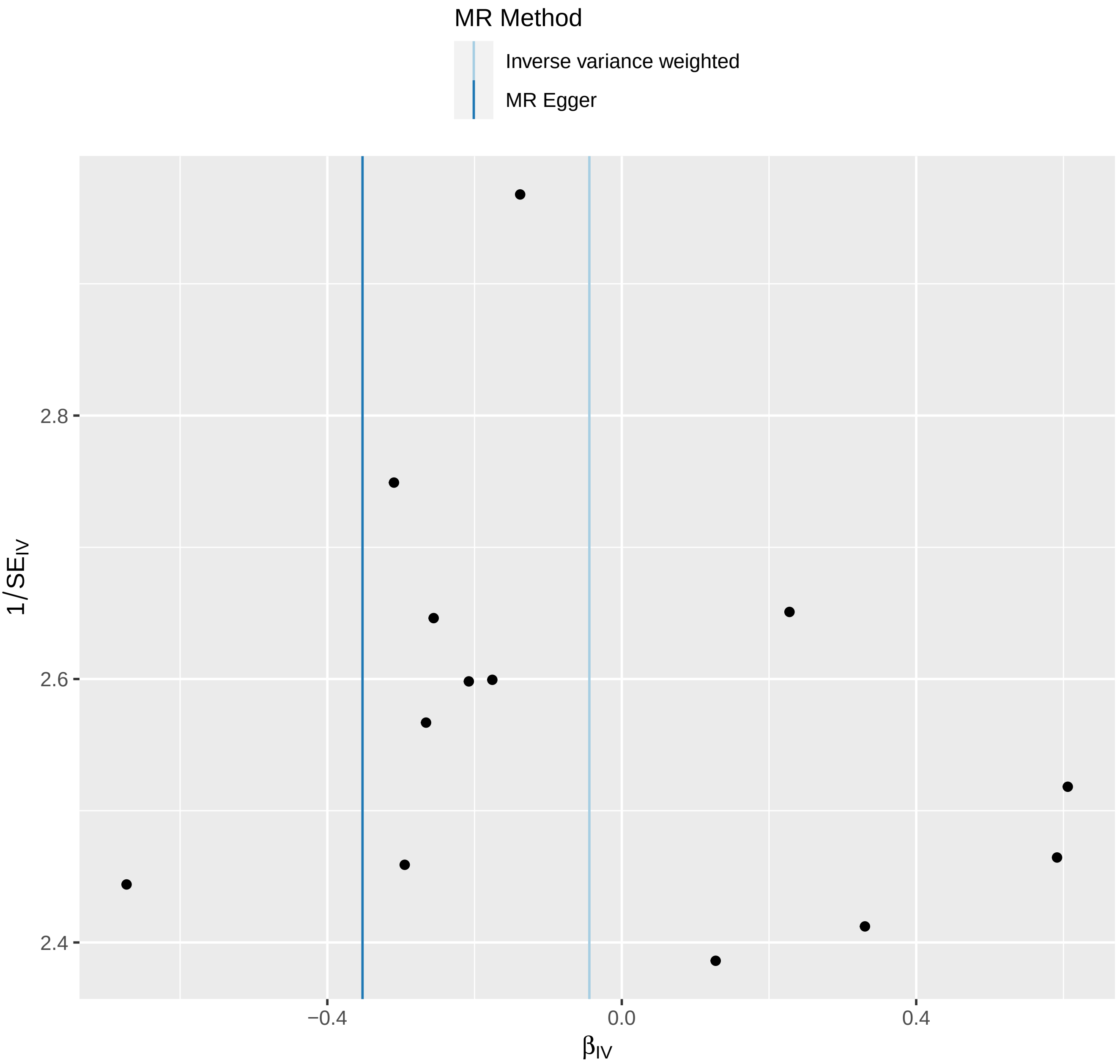

Batch 2−2325 : Gut microbiota abundance (genus Catenibacterium id.2153) on Gamma−aminobutyric acid type B receptor subunit 2
with a threshold of 1e−05

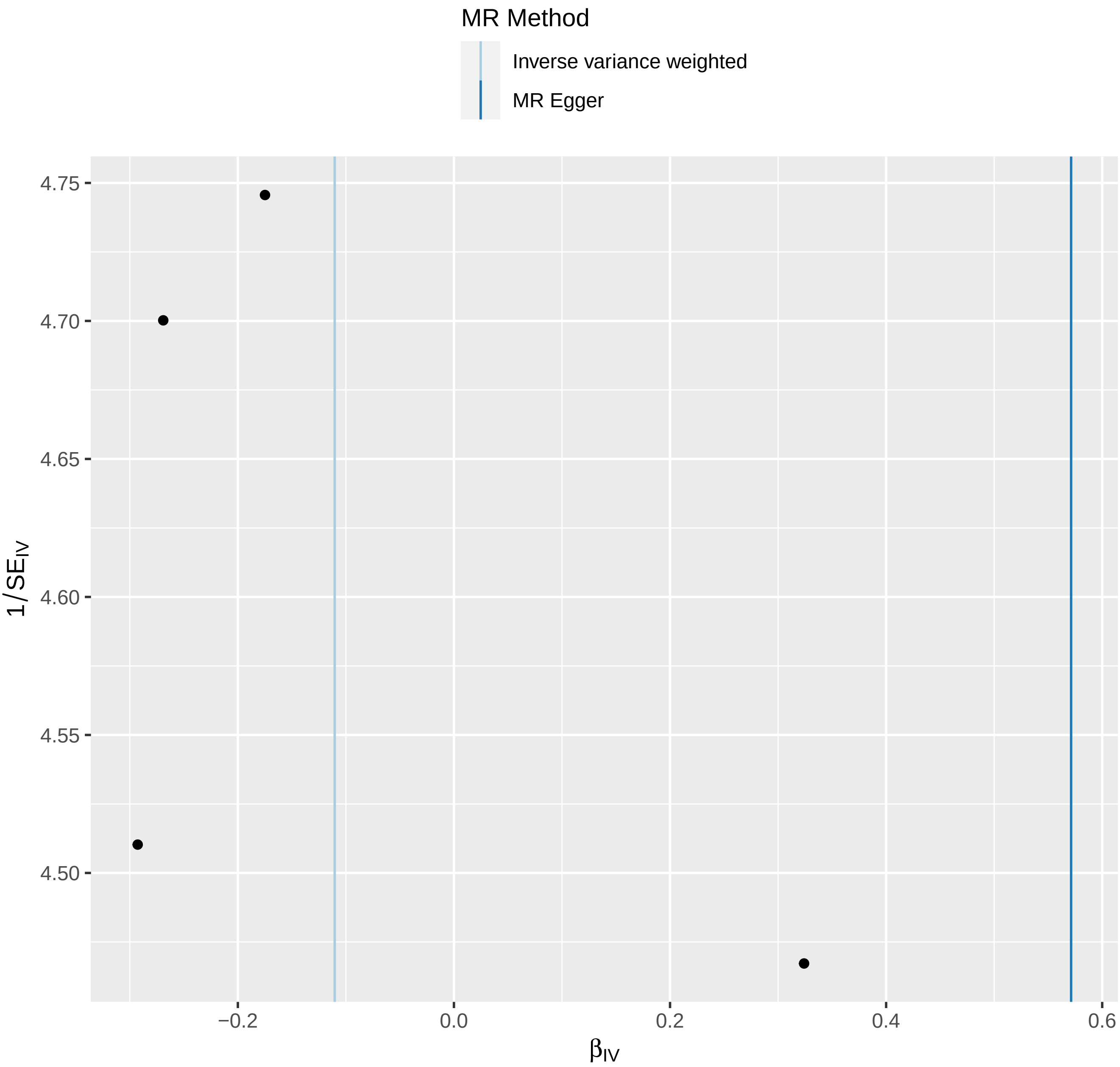

Batch 2−2326 : Gut microbiota abundance (genus Parasutterella id.2892) on Gamma−aminobutyric acid type B receptor subunit 2
with a threshold of 1e−05

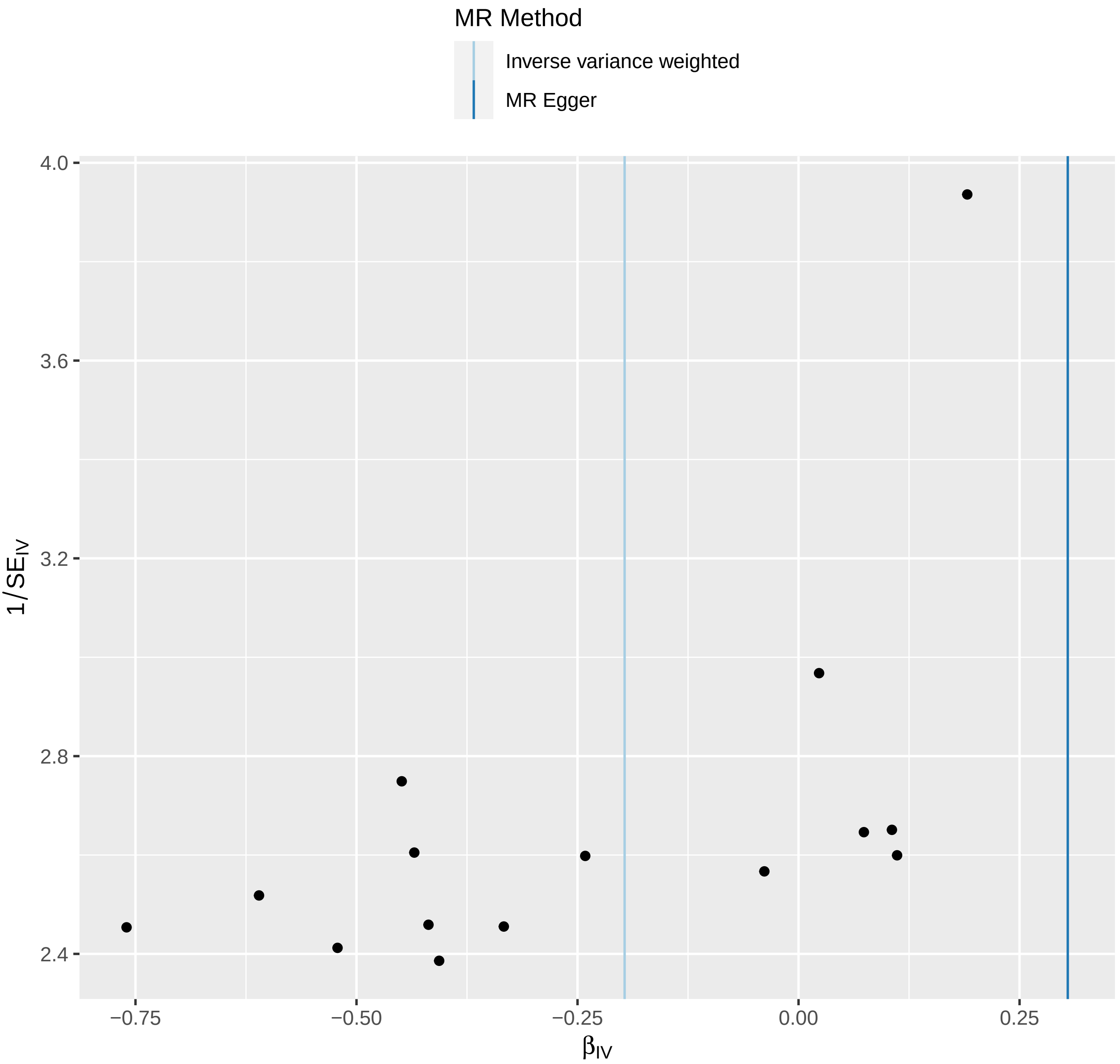

Batch 2−2327 : Gut microbiota abundance (genus Catenibacterium id.2153) on Gamma−aminobutyric acid type B receptor subunit 2
with a threshold of 1e−05

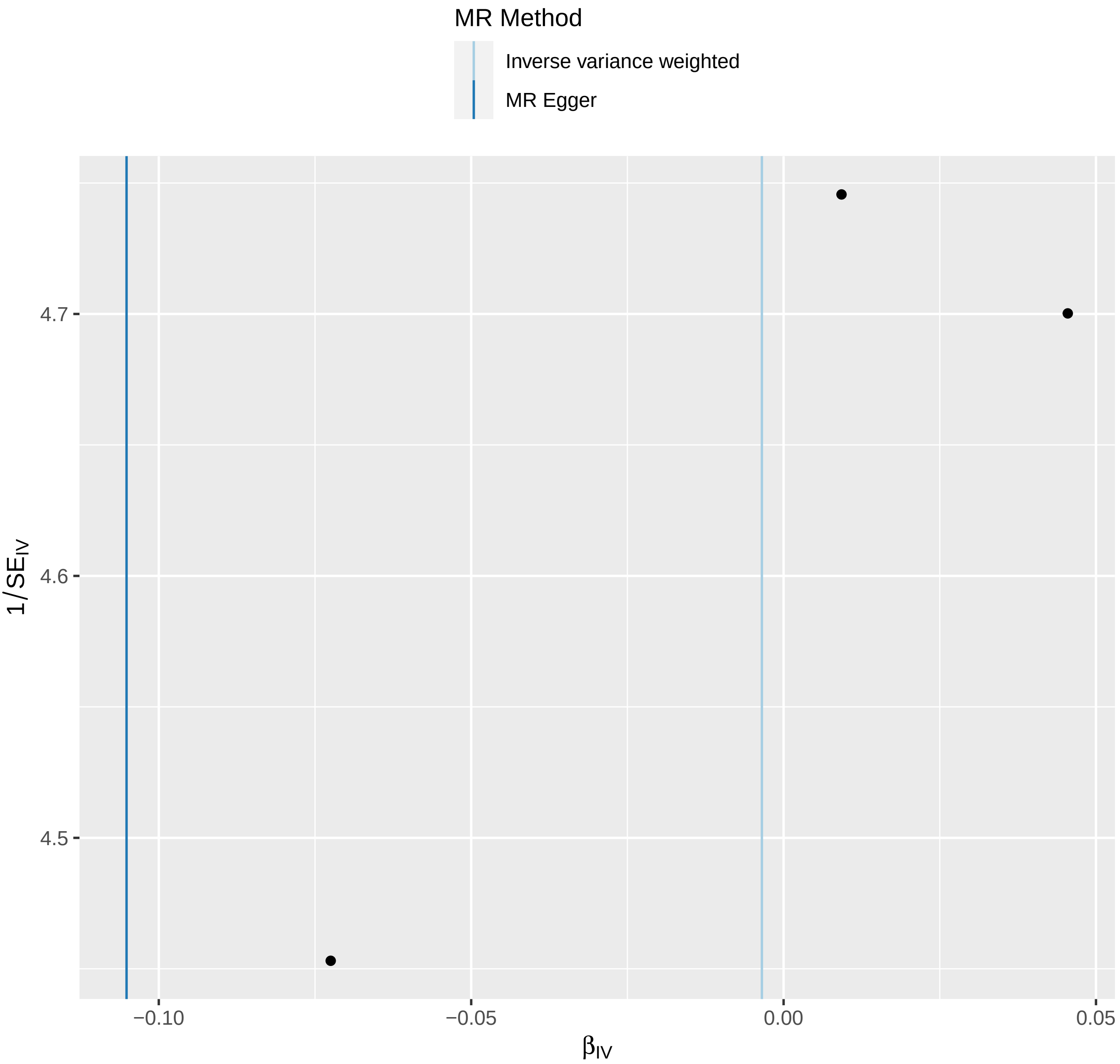

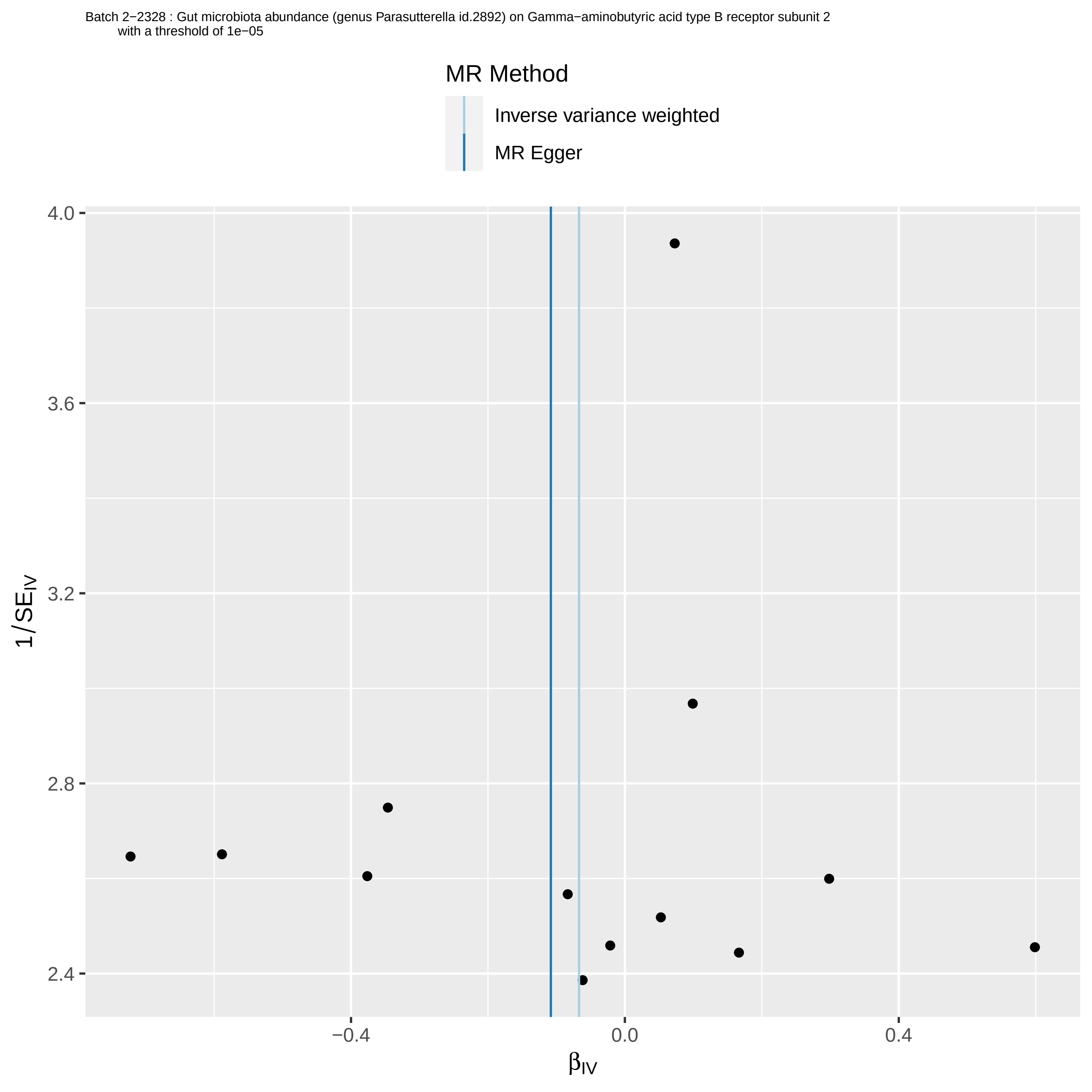
Batch 2−2328 : Gut microbiota abundance (genus Parasutterella id.2892) on Gamma−aminobutyric acid type B receptor subunit 2
with a threshold of 1e−05
MR Method
Inverse variance weighted
MR Egger
4.0
3.6
3.2
2.8
2.4
1/SE_IV
−0.4
0.0
0.4
β_IV

Batch 2−2329 : Gut microbiota abundance (genus Catenibacterium id.2153) on Growth arrest and DNA damage−inducible proteins−interacting protein 1 with a threshold of 1e−05

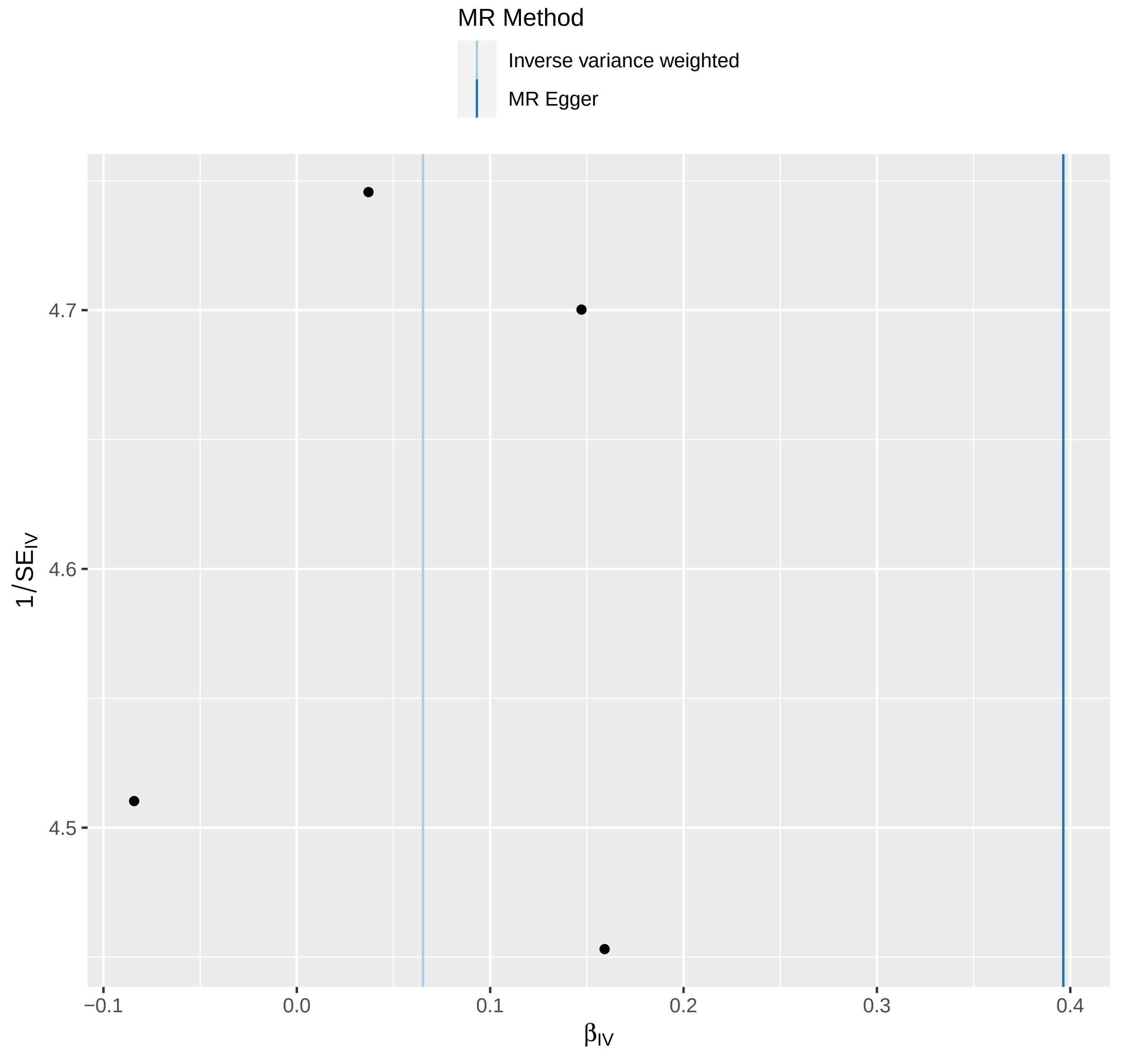

Batch 2−2330 : Gut microbiota abundance (genus Parasutterella id.2892) on Growth arrest and DNA damage−inducible proteins−interacting protein 1 with a threshold of 1e−05

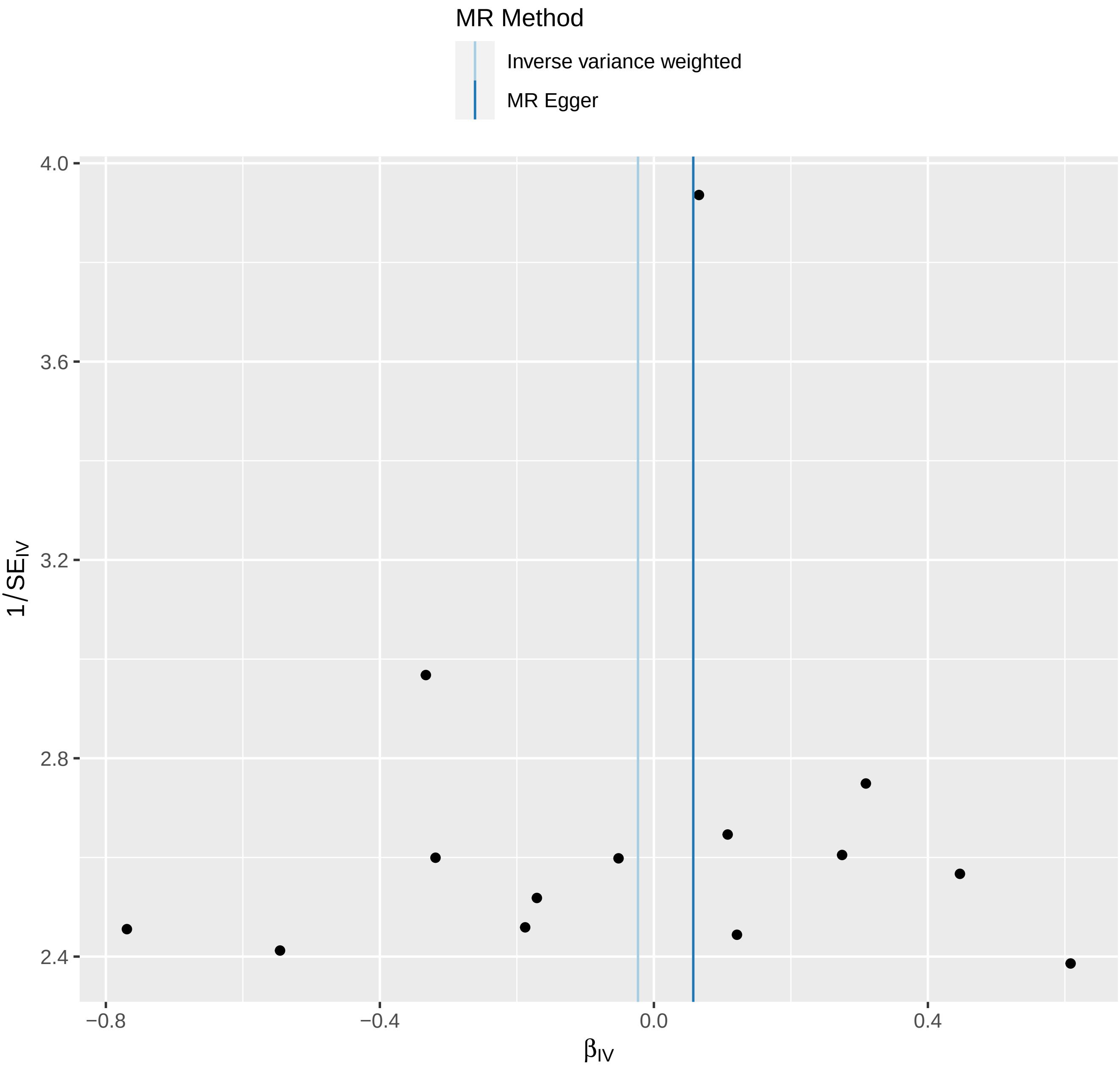

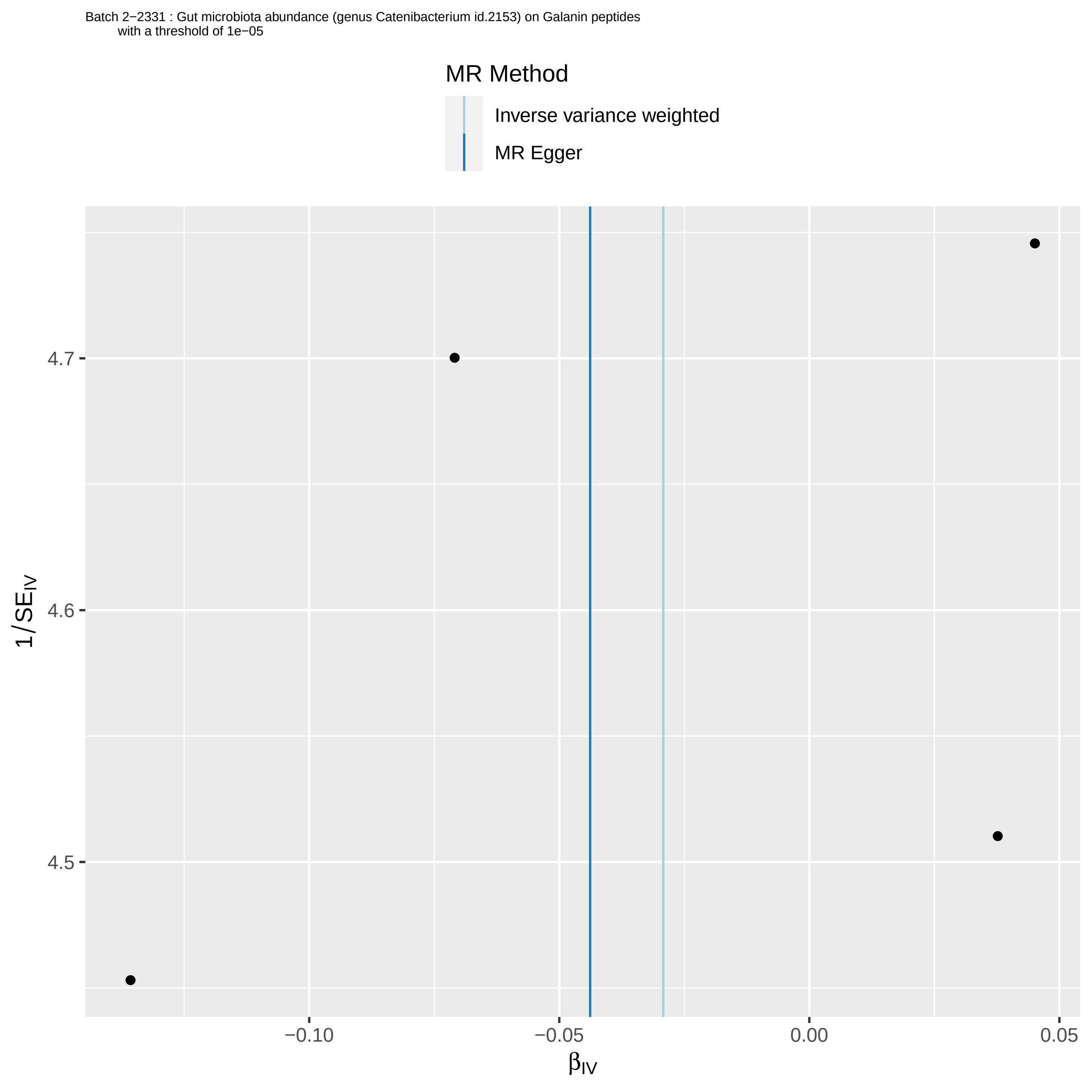
Batch 2−2331 : Gut microbiota abundance (genus Catenibacterium id.2153) on Galanin peptides
with a threshold of 1e−05
MR Method
Inverse variance weighted
MR Egger
4.7
4.6
4.5
$1/SE_{IV}$
−0.10
−0.05
0.00
0.05
β_{IV}

Batch 2−2332 : Gut microbiota abundance (genus Parasutterella id.2892) on Galanin peptides
with a threshold of 1e−05

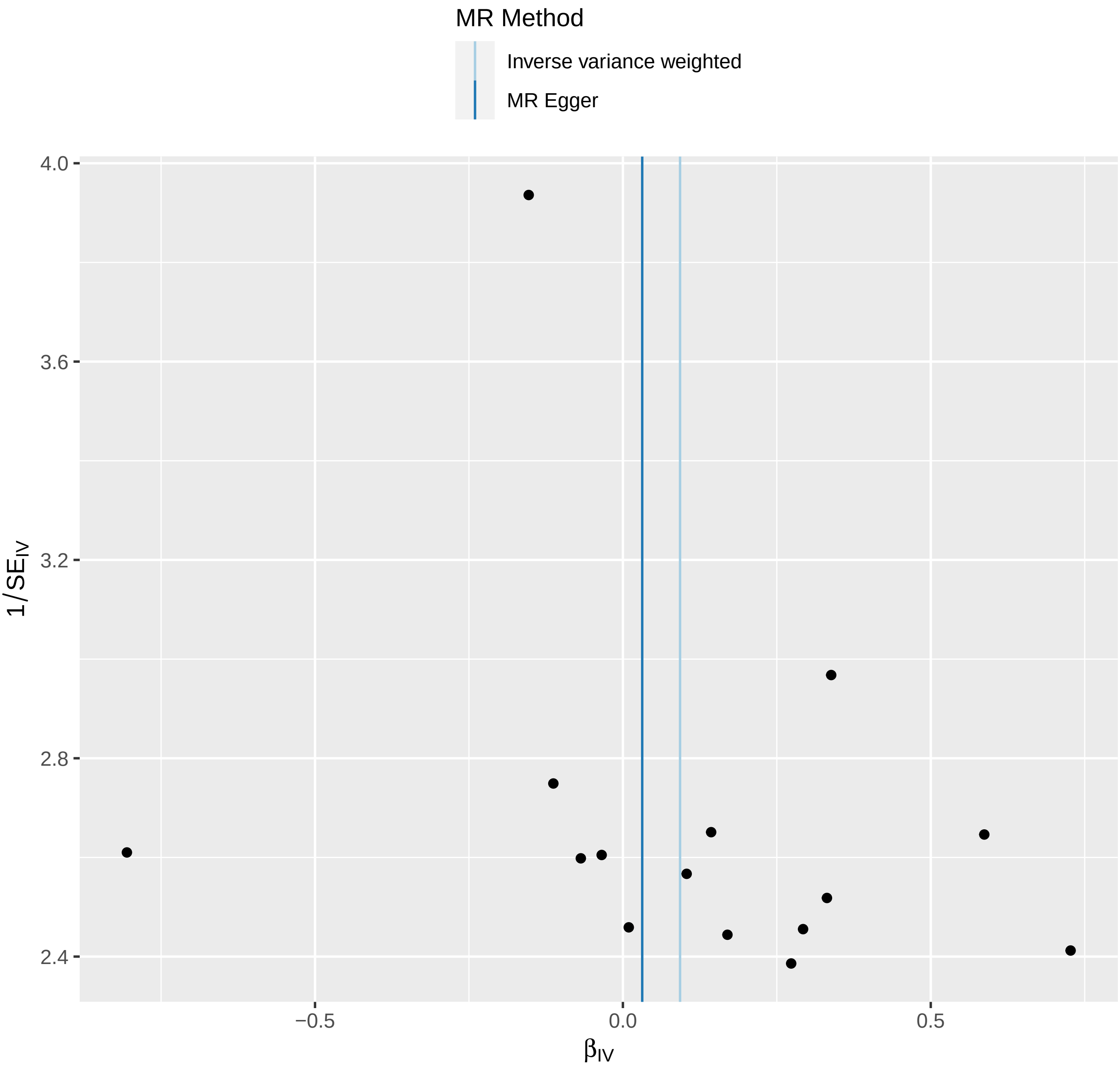

Batch 2−2333 : Gut microbiota abundance (genus Catenibacterium id.2153) on Polypeptide N−acetylgalactosaminyltransferase 10 with a threshold of 1e−05

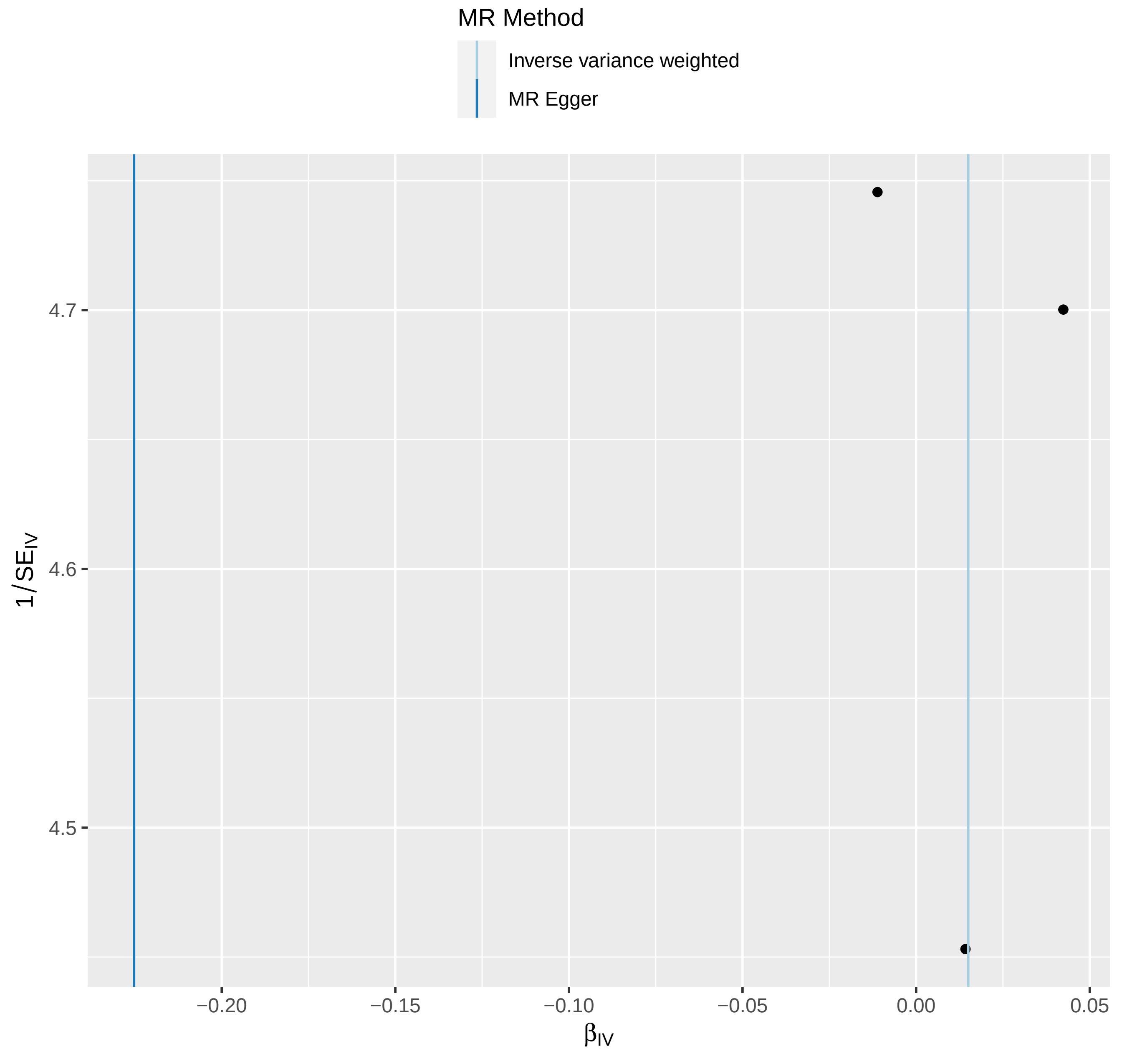

Batch 2−2334 : Gut microbiota abundance (genus Parasutterella id.2892) on Polypeptide N−acetylgalactosaminyltransferase 10 with a threshold of 1e−05

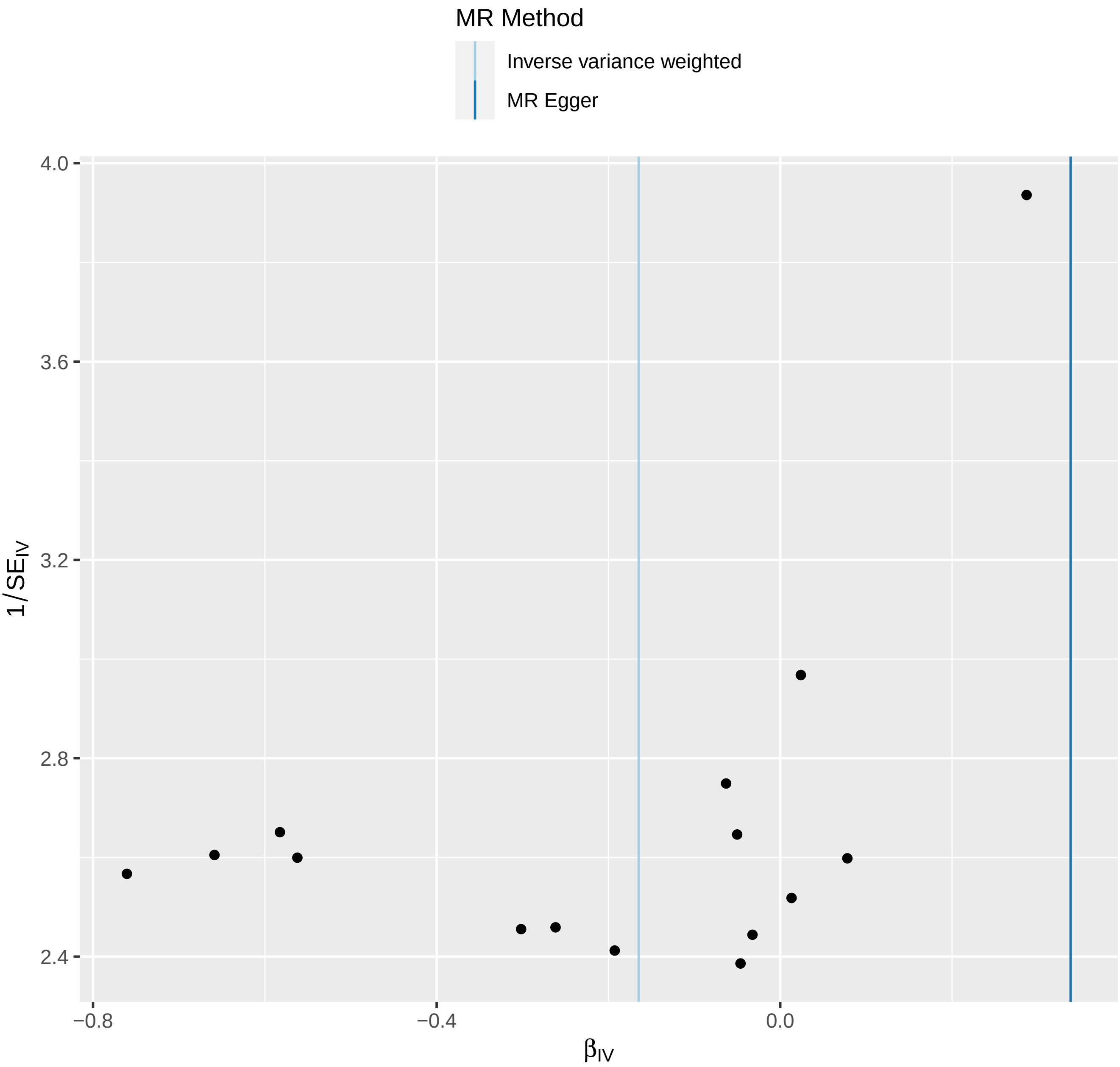

Batch 2−2335 : Gut microbiota abundance (genus Catenibacterium id.2153) on Polypeptide N−acetylgalactosaminyltransferase 11 with a threshold of 1e−05

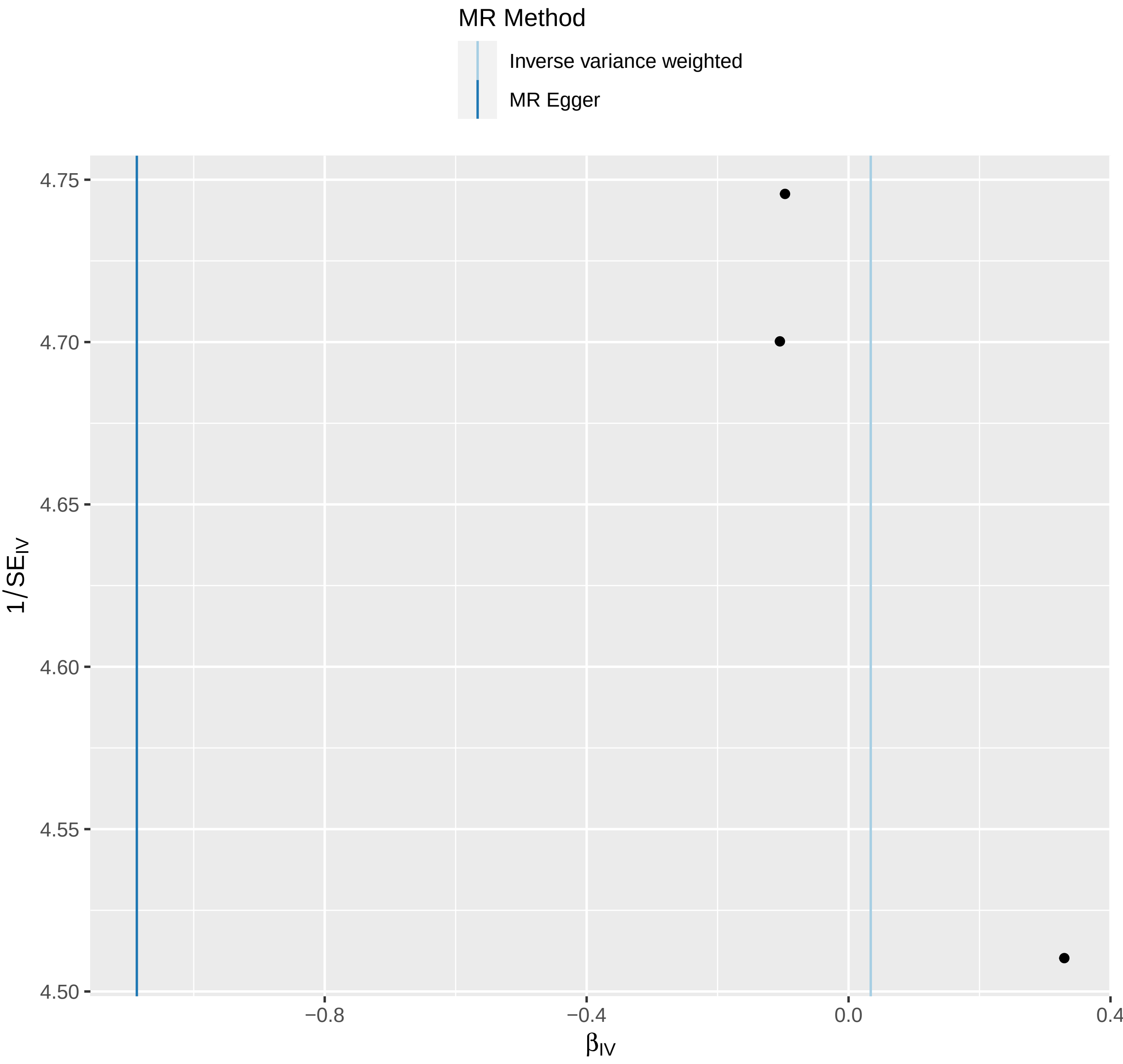

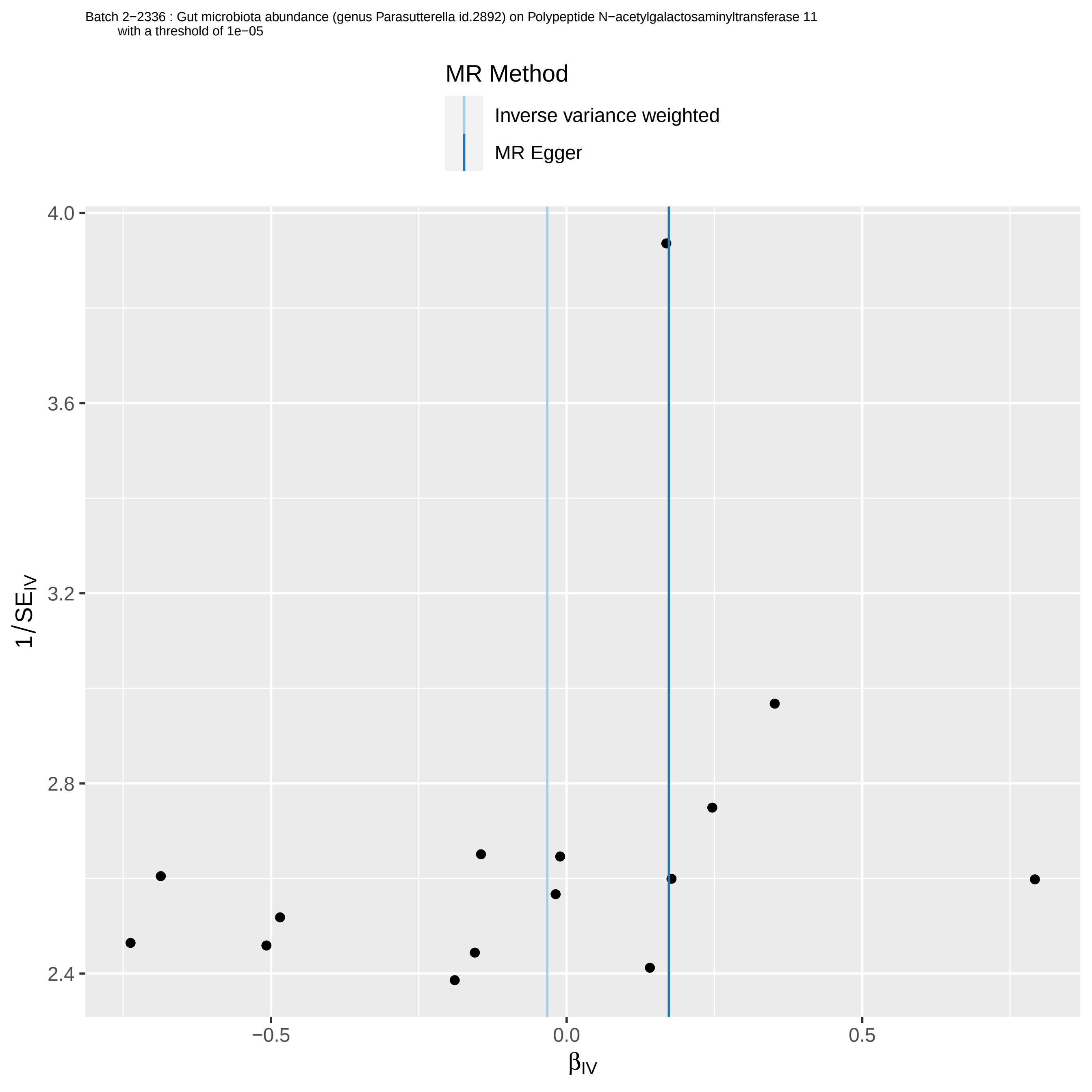
Batch 2−2336 : Gut microbiota abundance (genus Parasutterella id.2892) on Polypeptide N−acetylgalactosaminyltransferase 11
with a threshold of 1e−05
MR Method
Inverse variance weighted
MR Egger
4.0
3.6
3.2
2.8
2.4
1/SE_IV
−0.5
0.0
0.5
β_IV

Batch 2−2337 : Gut microbiota abundance (genus Catenibacterium id.2153) on Polypeptide N−acetylgalactosaminyltransferase 16
with a threshold of 1e−05

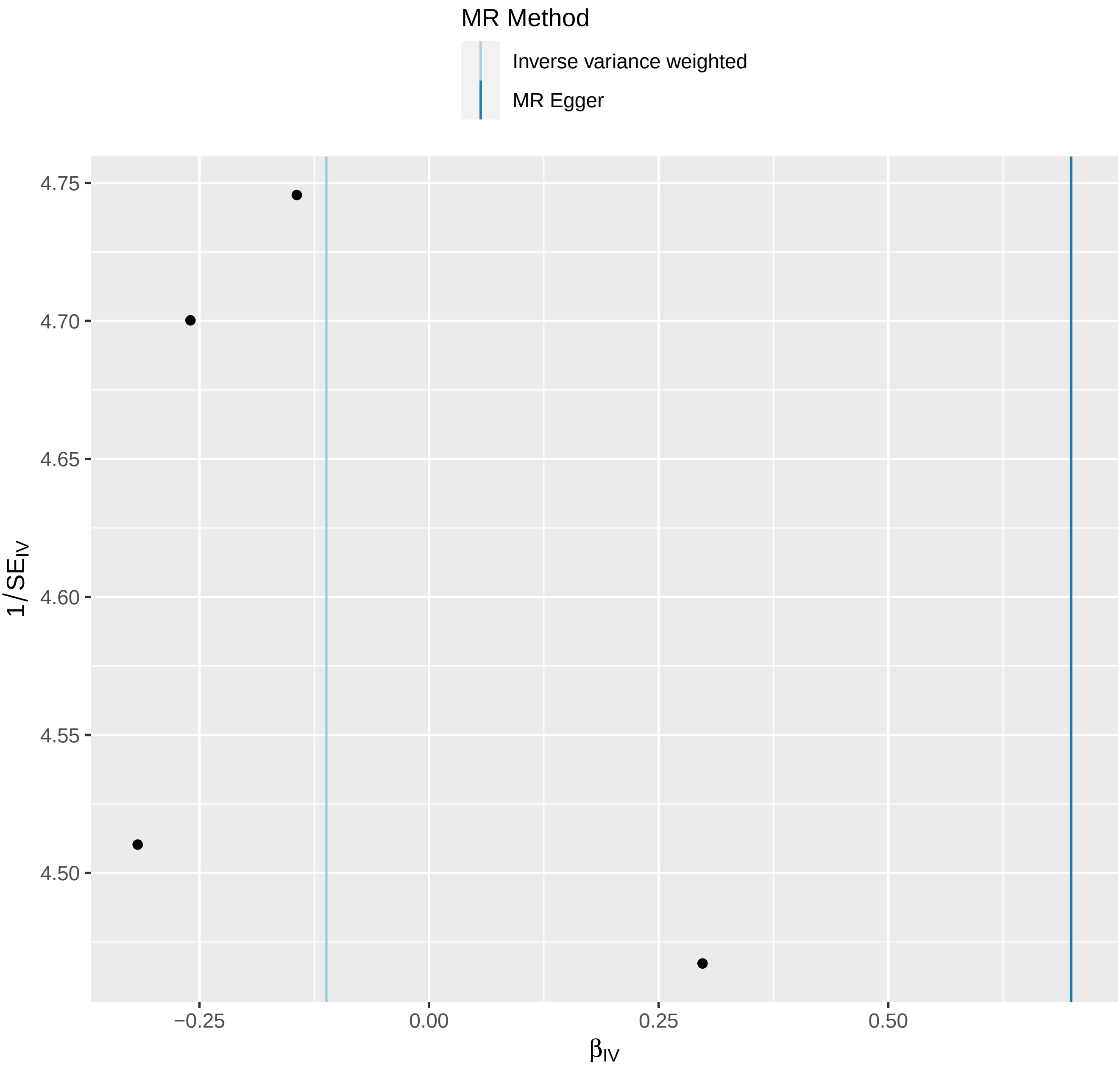

Batch 2−2338 : Gut microbiota abundance (genus Parasutterella id.2892) on Polypeptide N−acetylgalactosaminyltransferase 16
with a threshold of 1e−05

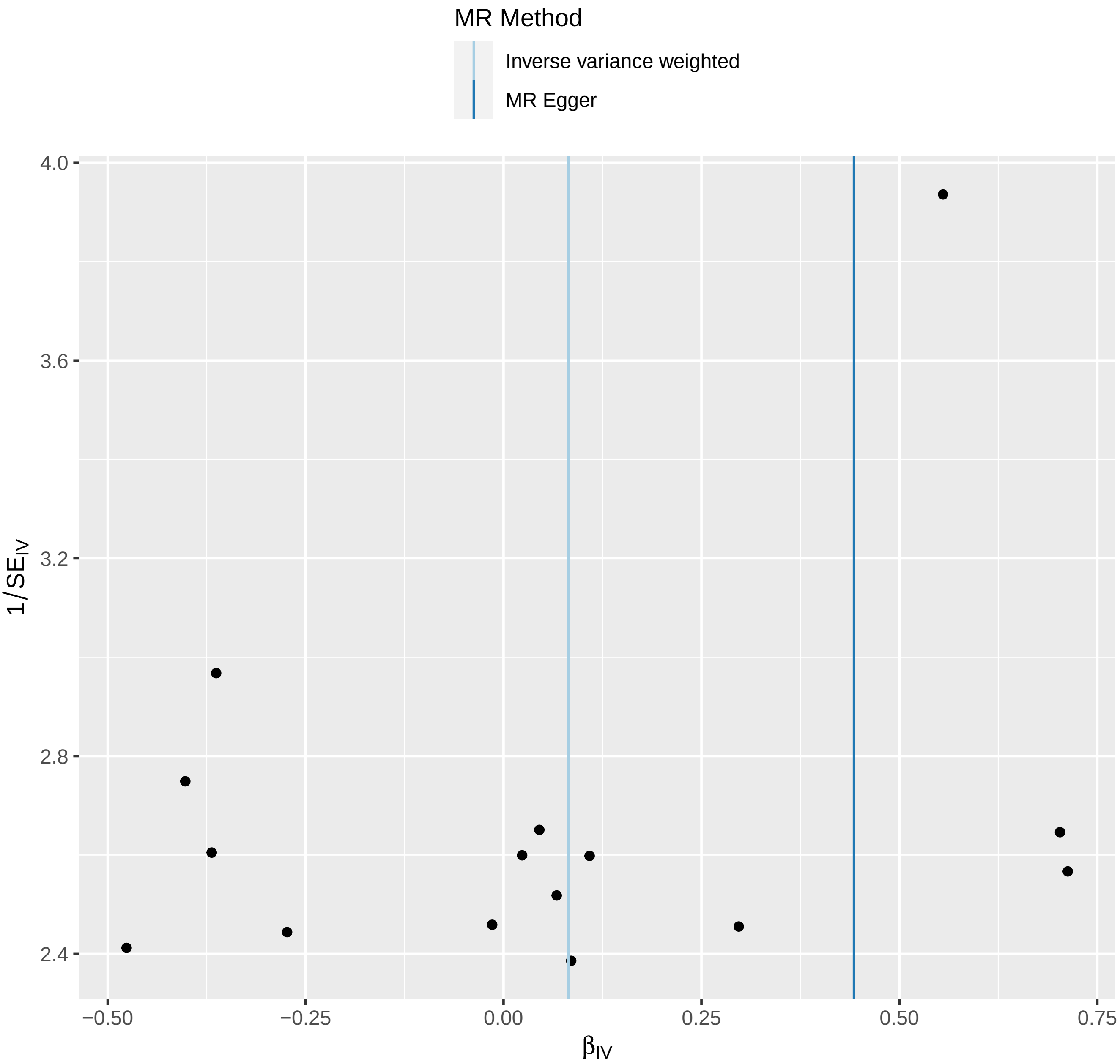

Batch 2−2339 : Gut microbiota abundance (genus Catenibacterium id.2153) on Polypeptide N−acetylgalactosaminyltransferase 1
with a threshold of 1e−05

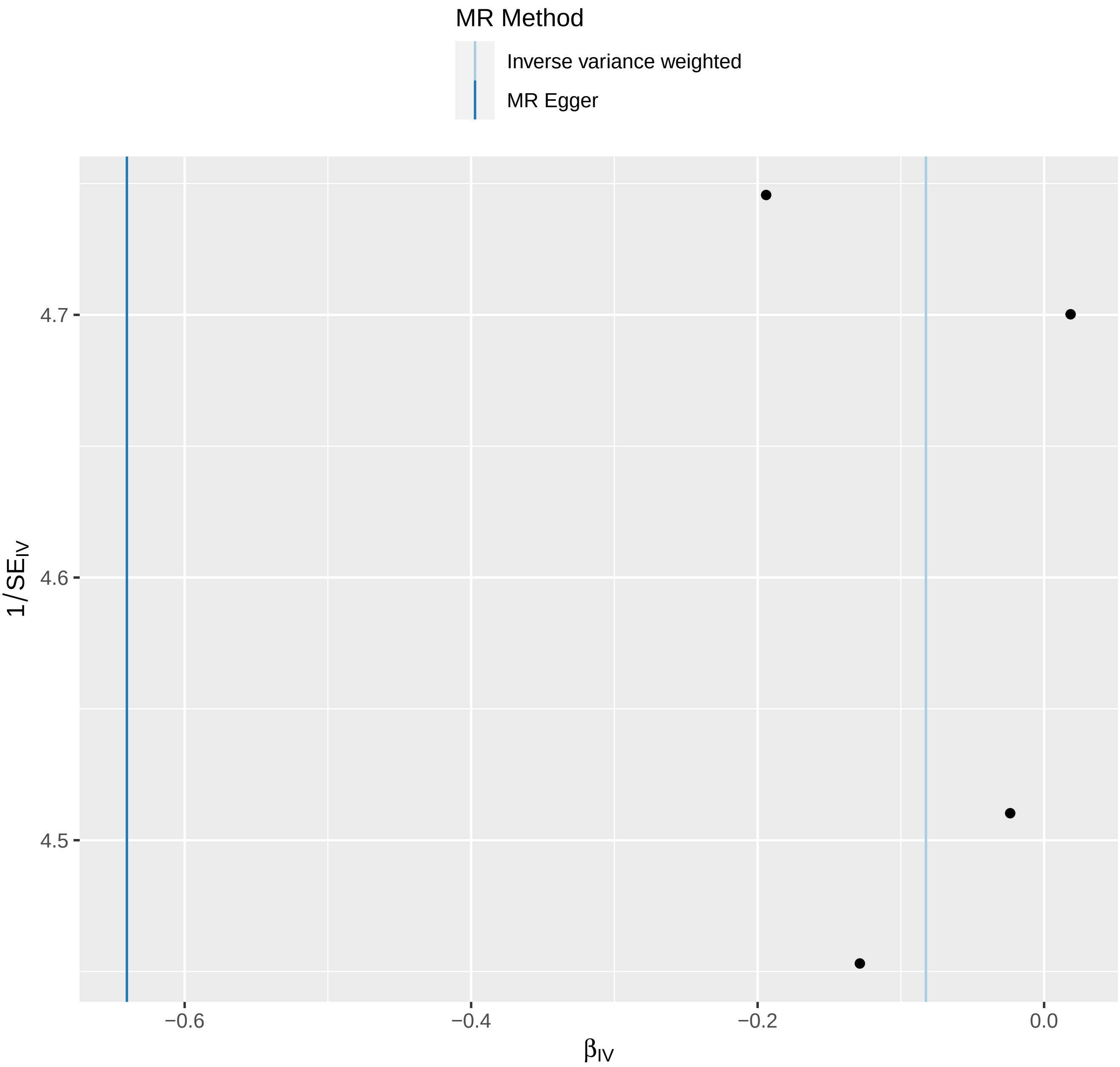

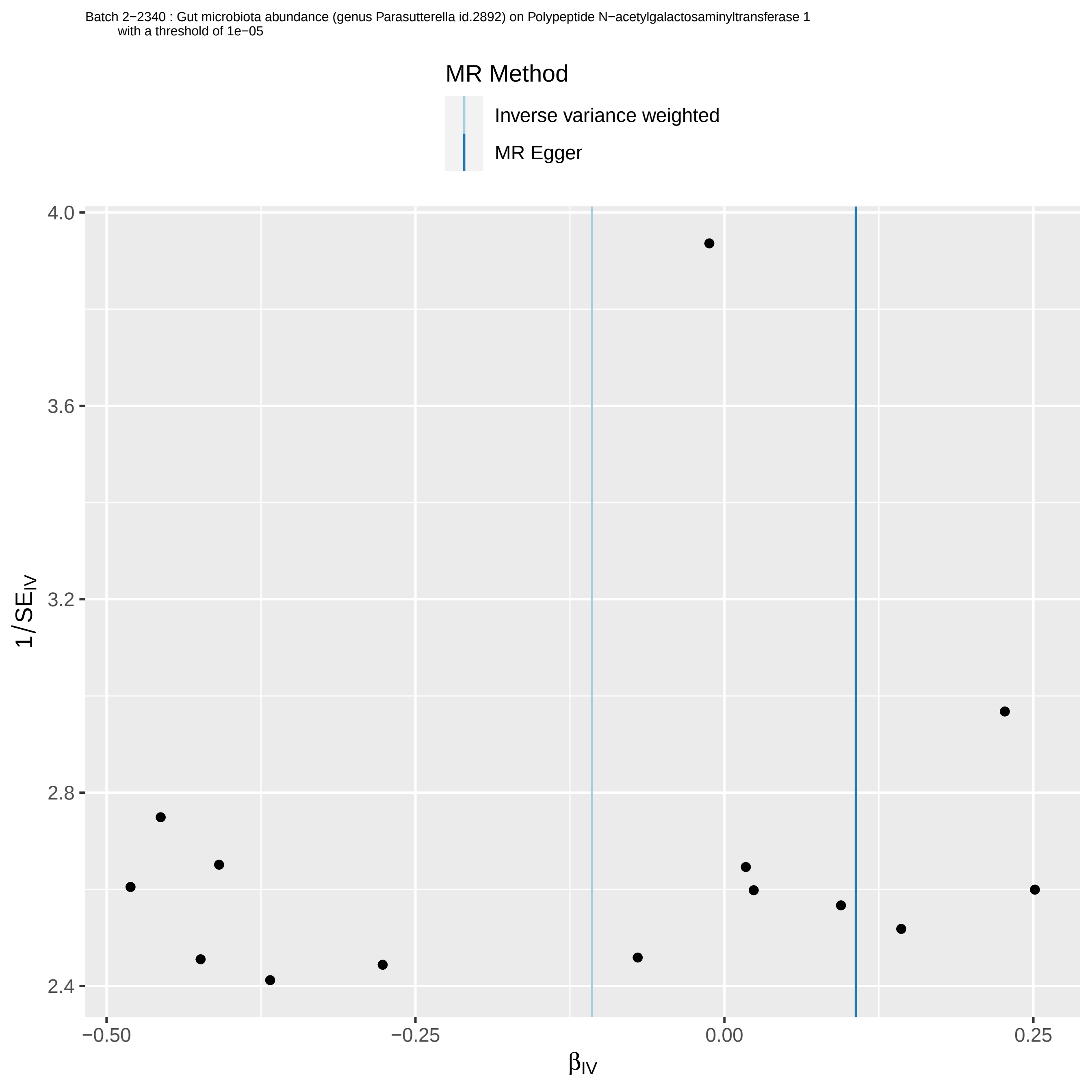

Batch 2−2340 : Gut microbiota abundance (genus Parasutterella id.2892) on Polypeptide N−acetylgalactosaminyltransferase 1
with a threshold of 1e−05
MR Method
Inverse variance weighted
MR Egger
4.0
3.6
3.2
2.8
2.4
1/SE_IV
−0.50
−0.25
0.00
0.25
β_IV

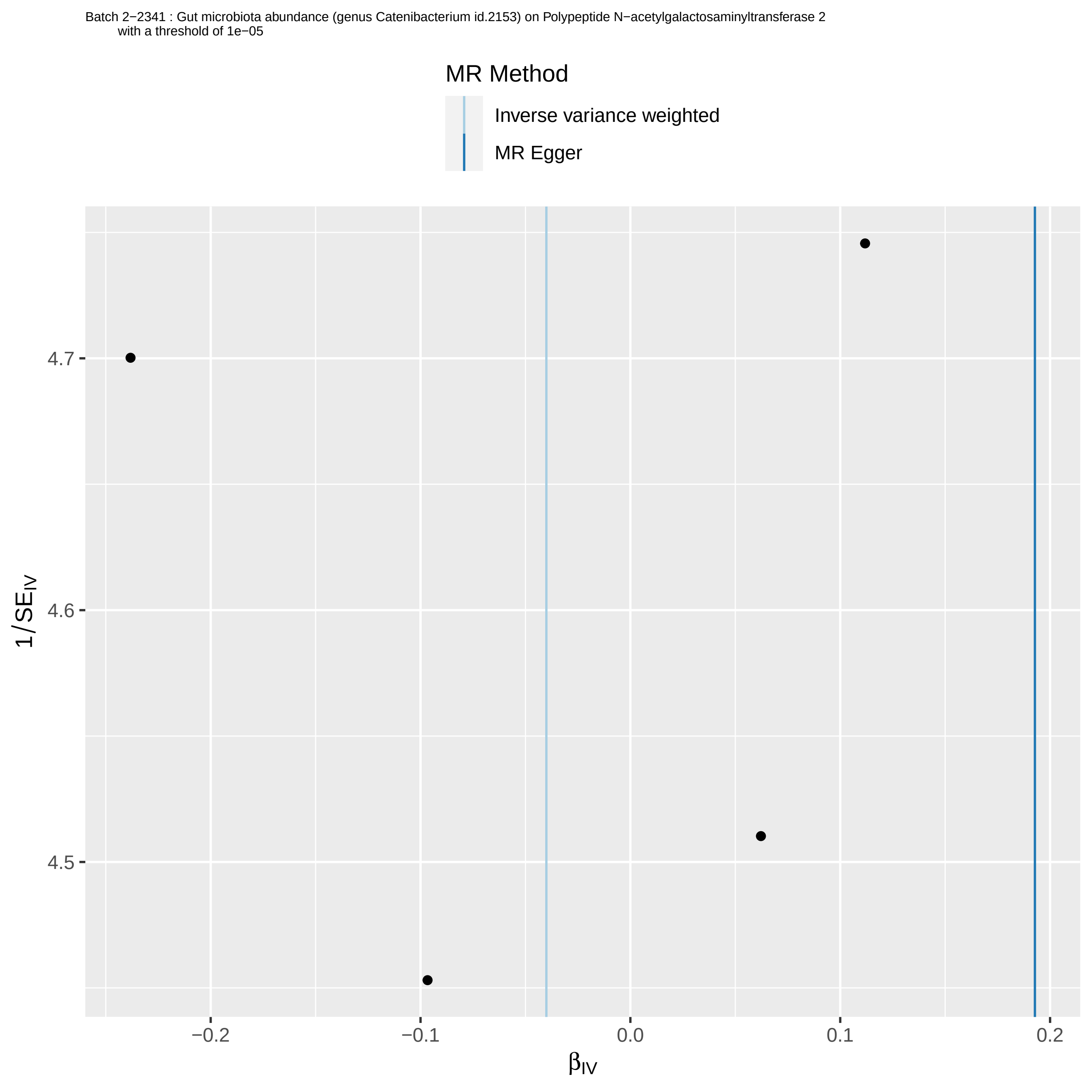

Batch 2−2341 : Gut microbiota abundance (genus Catenibacterium id.2153) on Polypeptide N−acetylgalactosaminyltransferase 2
with a threshold of 1e−05
MR Method
Inverse variance weighted
MR Egger
4.7
4.6
4.5
$1/SE_{IV}$
−0.2
−0.1
0.0
0.1
0.2
β_{IV}

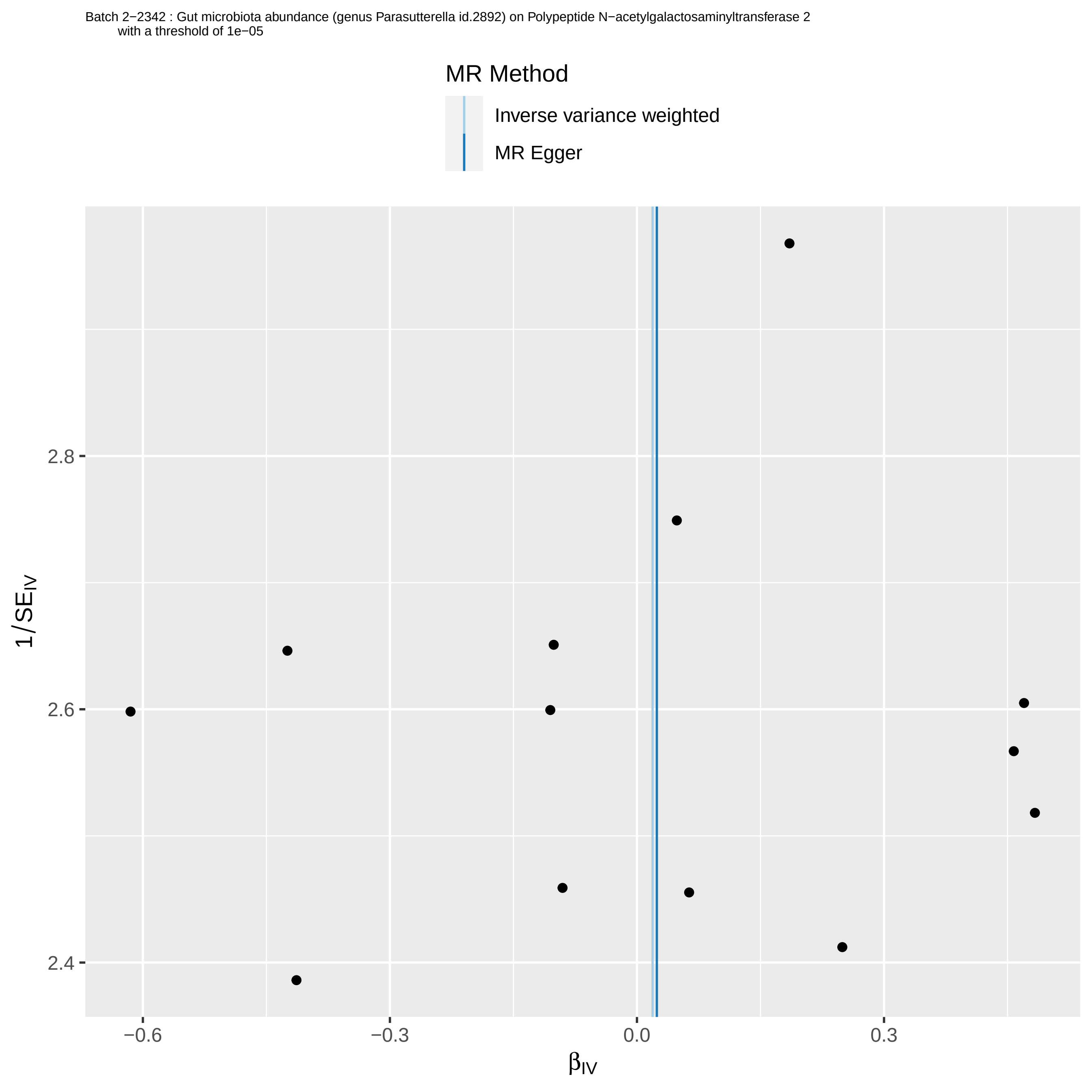
Batch 2−2342 : Gut microbiota abundance (genus Parasutterella id.2892) on Polypeptide N−acetylgalactosaminyltransferase 2
with a threshold of 1e−05
MR Method
Inverse variance weighted
MR Egger
2.8
2.6
2.4
1/SE$_{IV}$
−0.6
−0.3
0.0
0.3
β$_{IV}$

Batch 2−2343 : Gut microbiota abundance (genus Catenibacterium id.2153) on Polypeptide N−acetylgalactosaminyltransferase 3
with a threshold of 1e−05

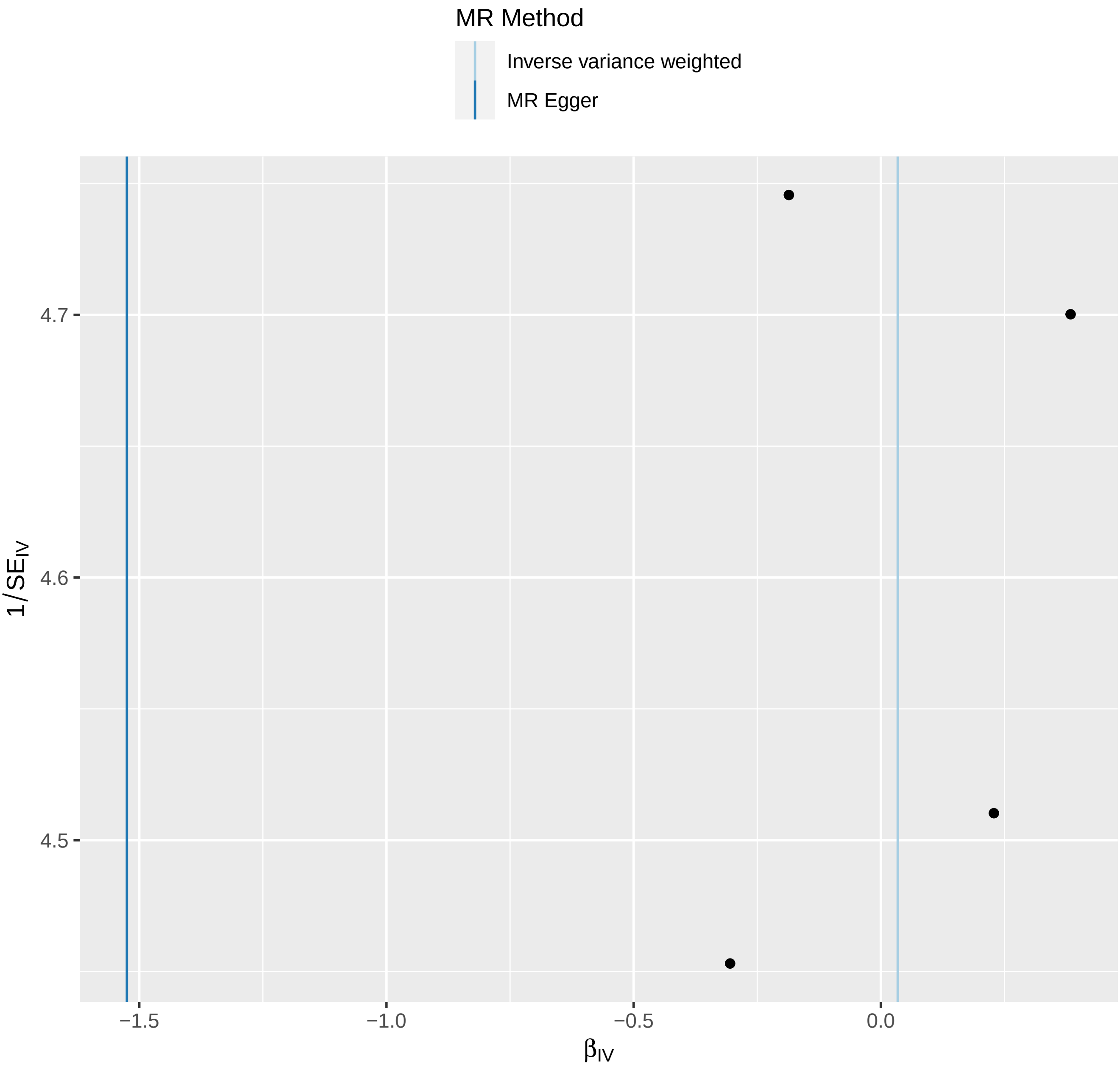

Batch 2−2344 : Gut microbiota abundance (genus Parasutterella id.2892) on Polypeptide N−acetylgalactosaminyltransferase 3
with a threshold of 1e−05

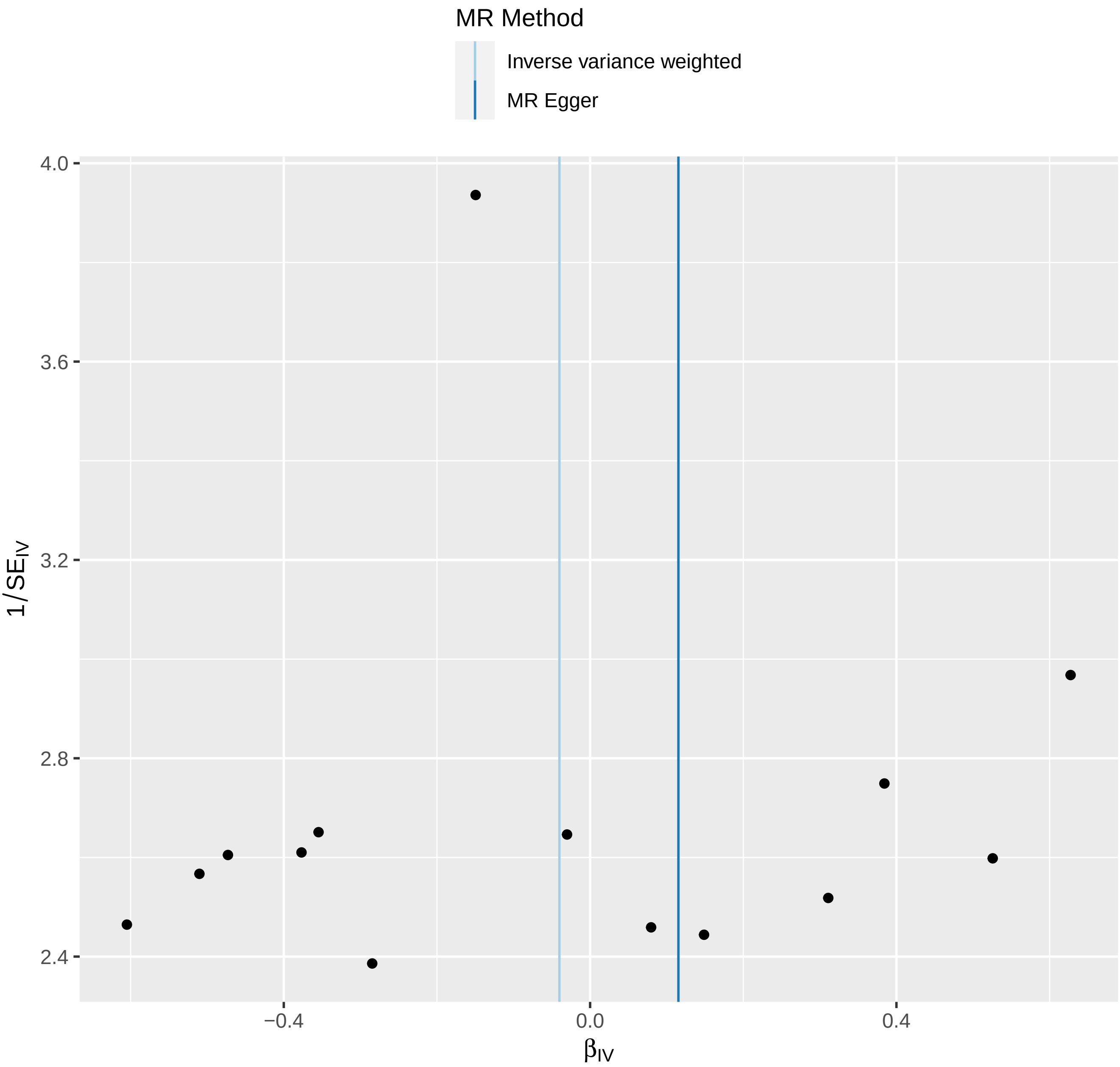

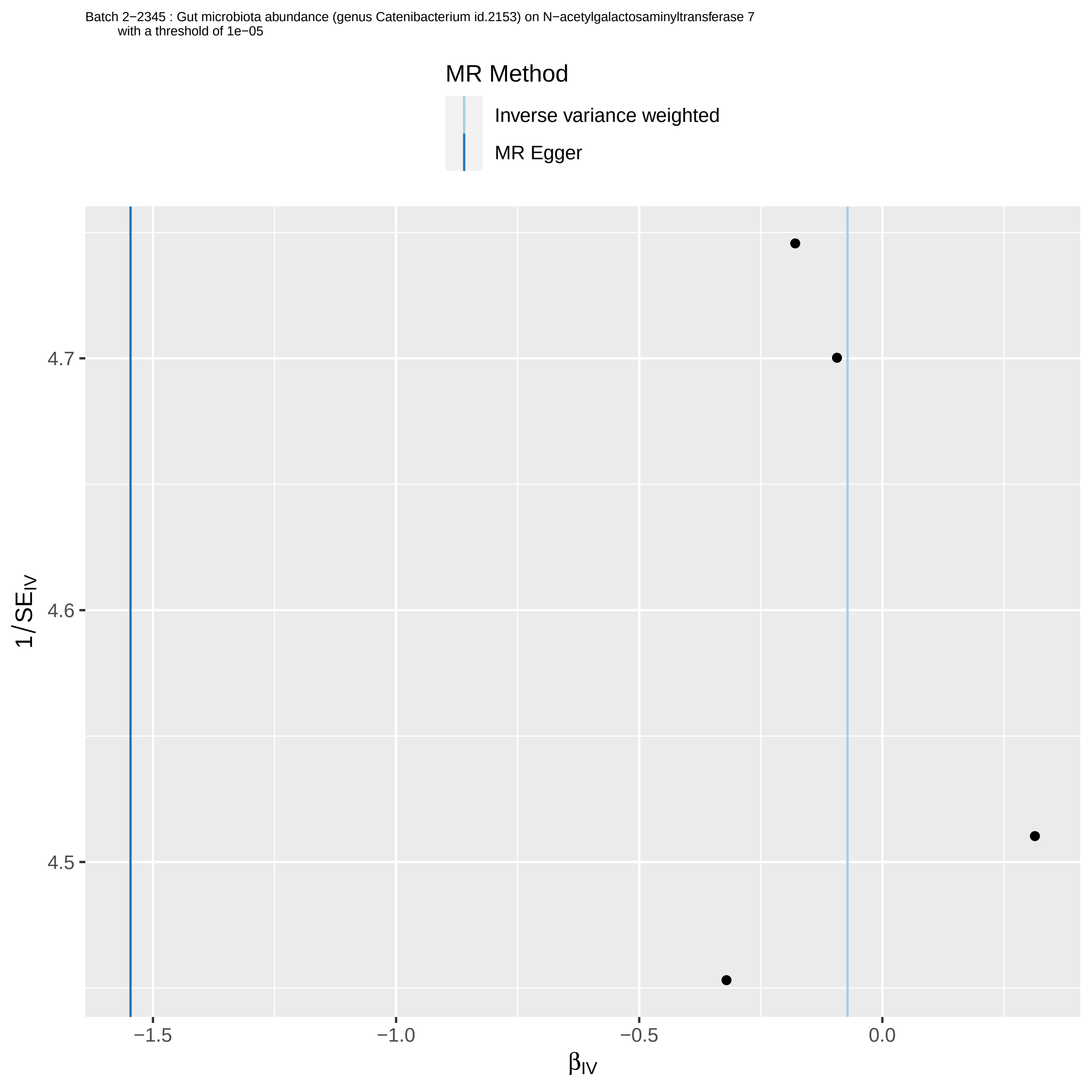

Batch 2−2345 : Gut microbiota abundance (genus Catenibacterium id.2153) on N−acetylgalactosaminyltransferase 7
with a threshold of 1e−05
MR Method
Inverse variance weighted
MR Egger
4.7
4.6
4.5
−1.5
−1.0
−0.5
0.0
$1/SE_{IV}$
β_{IV}

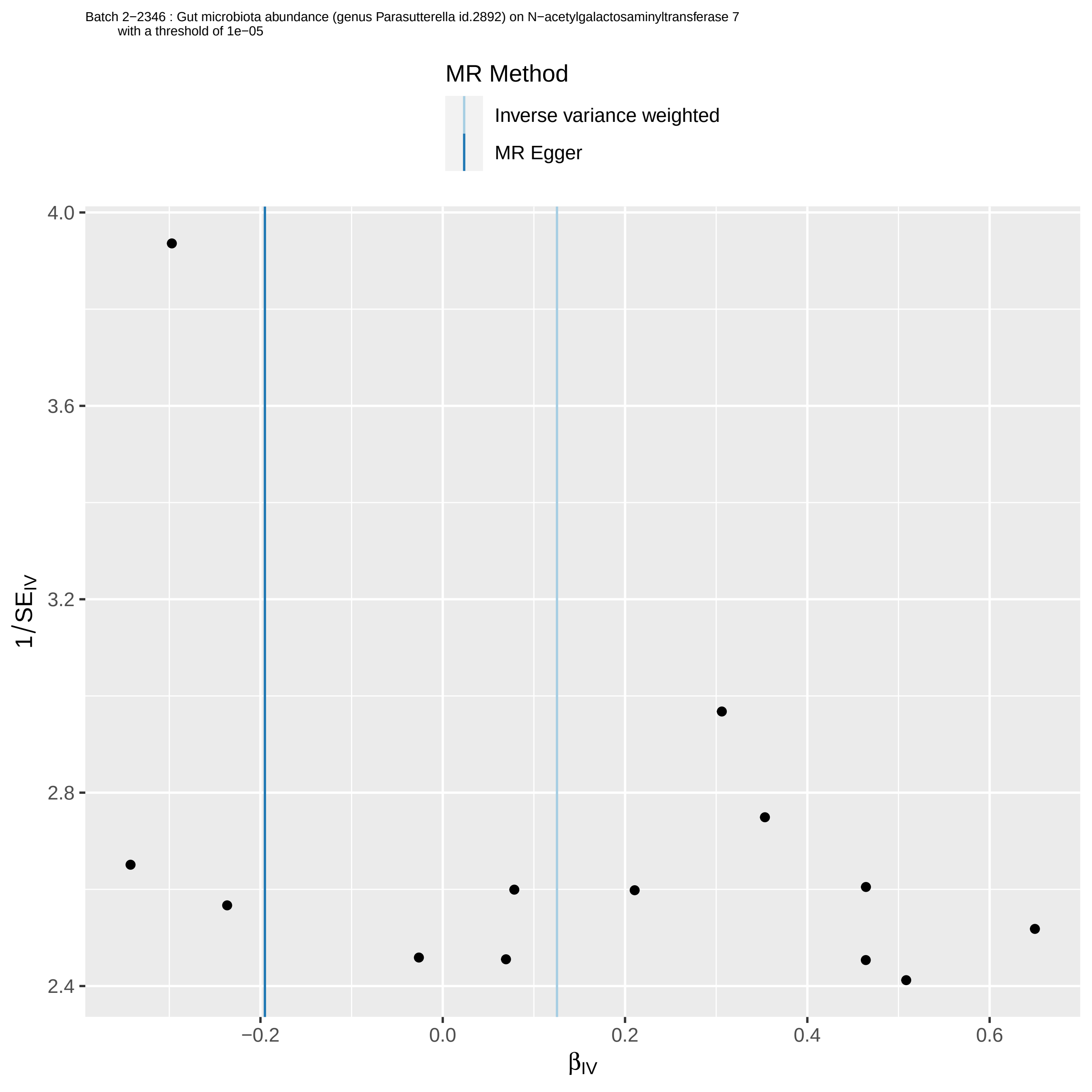
Batch 2−2346 : Gut microbiota abundance (genus Parasutterella id.2892) on N−acetylgalactosaminyltransferase 7
with a threshold of 1e−05
MR Method
Inverse variance weighted
MR Egger
4.0
3.6
3.2
2.8
2.4
1/SE_IV
−0.2
0.0
0.2
0.4
0.6
β_IV

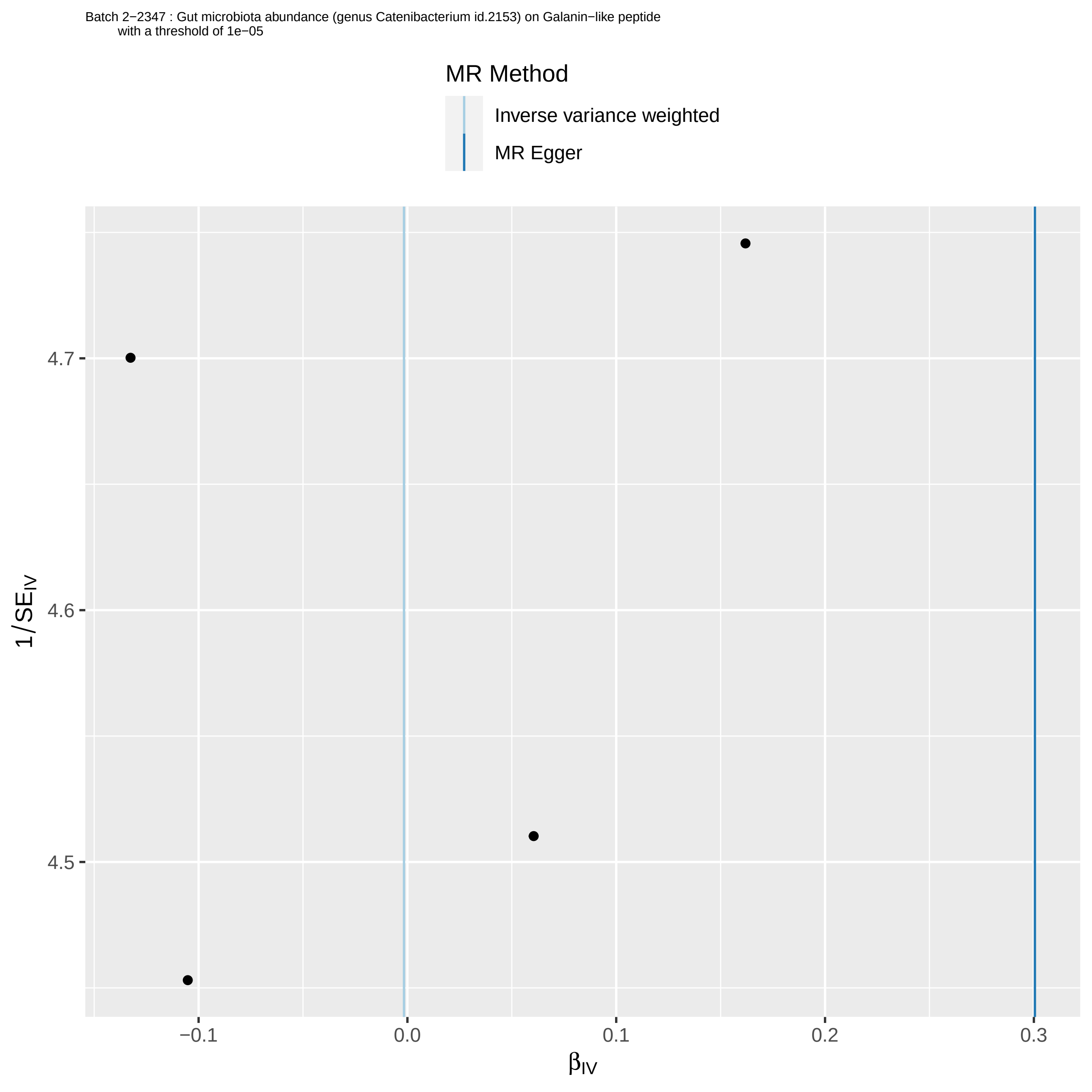
Batch 2−2347 : Gut microbiota abundance (genus Catenibacterium id.2153) on Galanin−like peptide
with a threshold of 1e−05
MR Method
Inverse variance weighted
MR Egger
4.7
4.6
4.5
−0.1
0.0
0.1
0.2
0.3
$1/SE_{IV}$
β_{IV}

Batch 2−2348 : Gut microbiota abundance (genus Parasutterella id.2892) on Galanin−like peptide
with a threshold of 1e−05

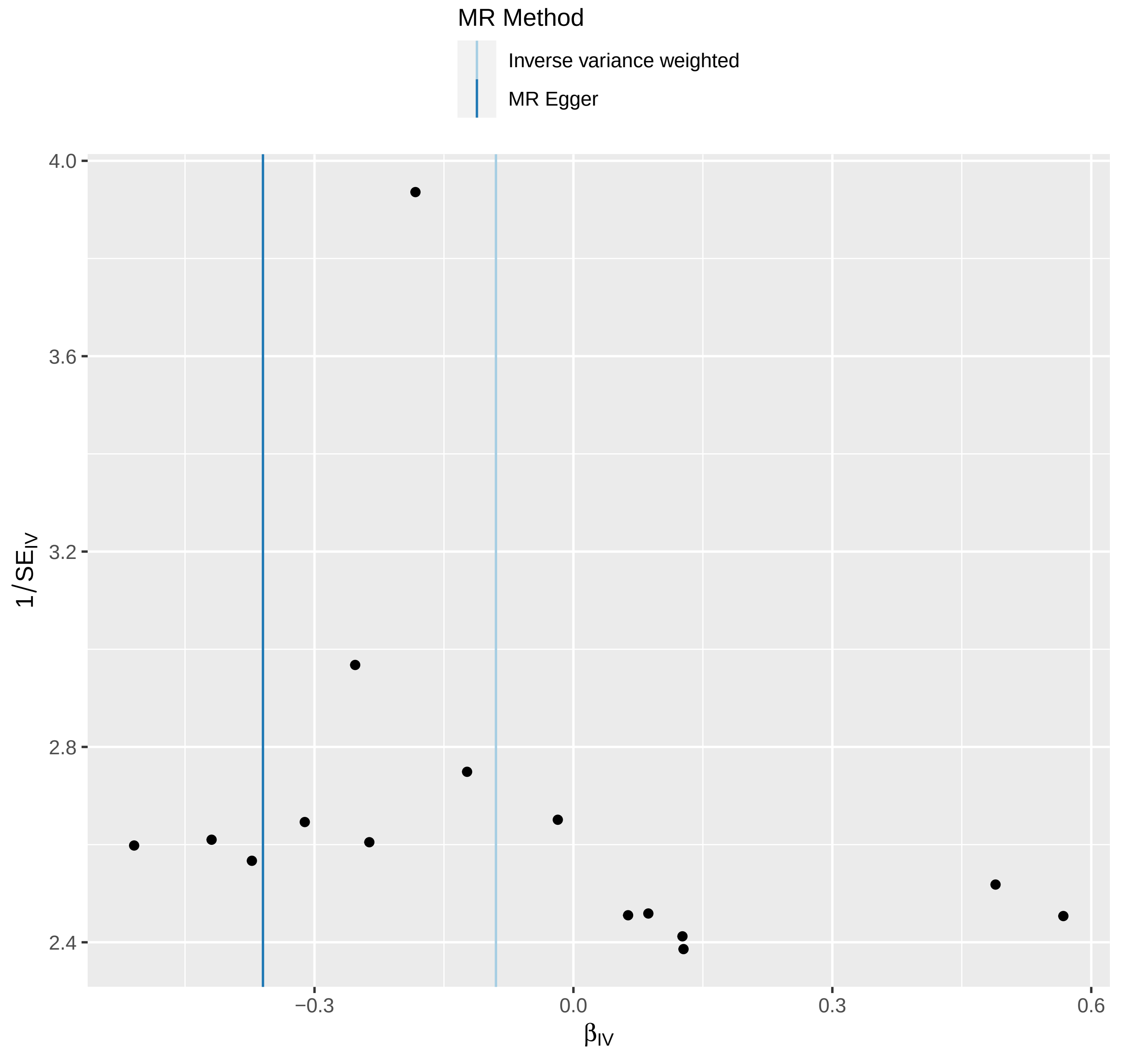

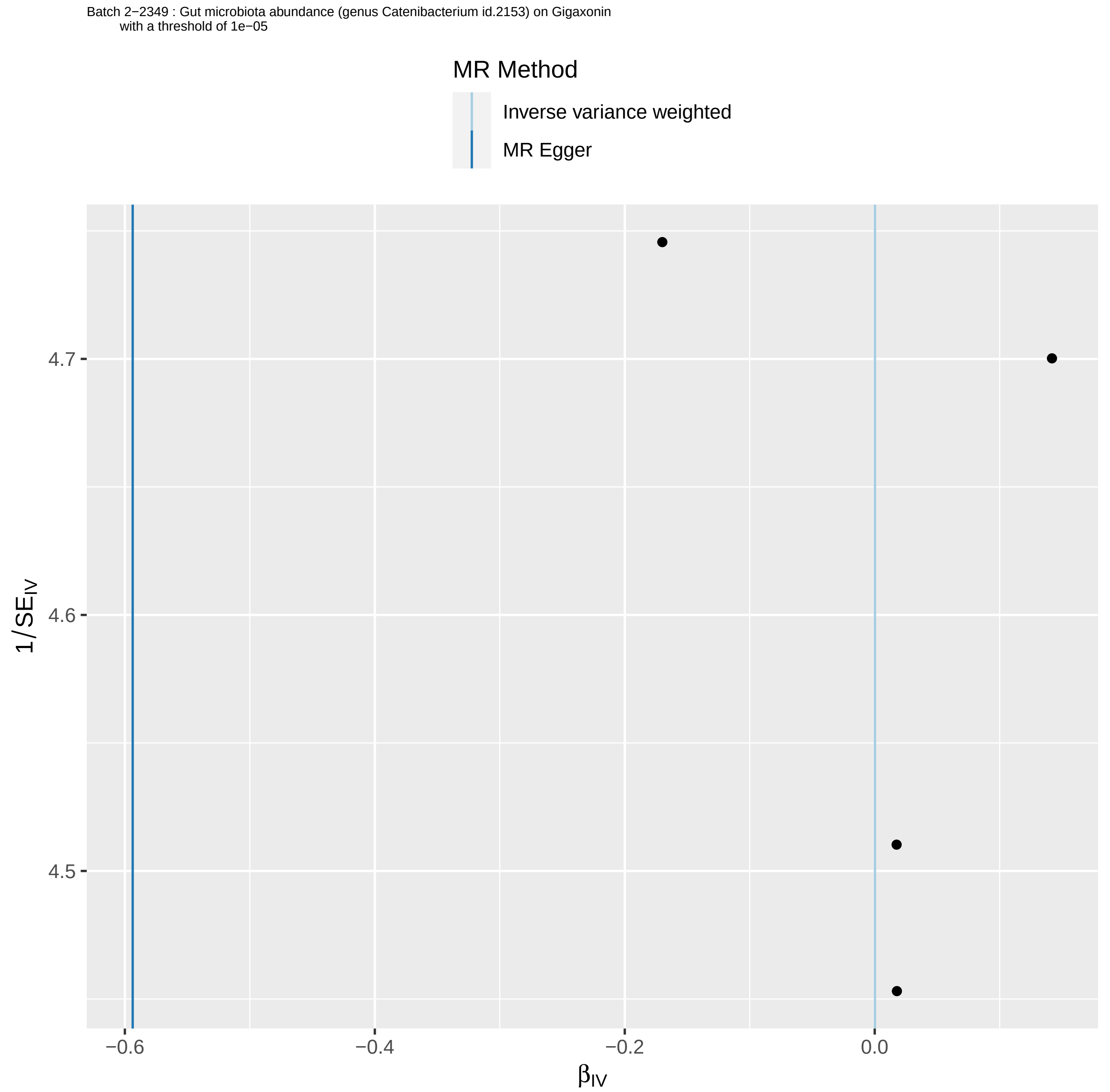
Batch 2−2349 : Gut microbiota abundance (genus Catenibacterium id.2153) on Gigaxonin
with a threshold of 1e−05
MR Method
Inverse variance weighted
MR Egger
4.7
4.6
4.5
1/SE_IV
−0.6
−0.4
−0.2
0.0
β_IV

Batch 2−2350 : Gut microbiota abundance (genus Parasutterella id.2892) on Gigaxonin
with a threshold of 1e−05

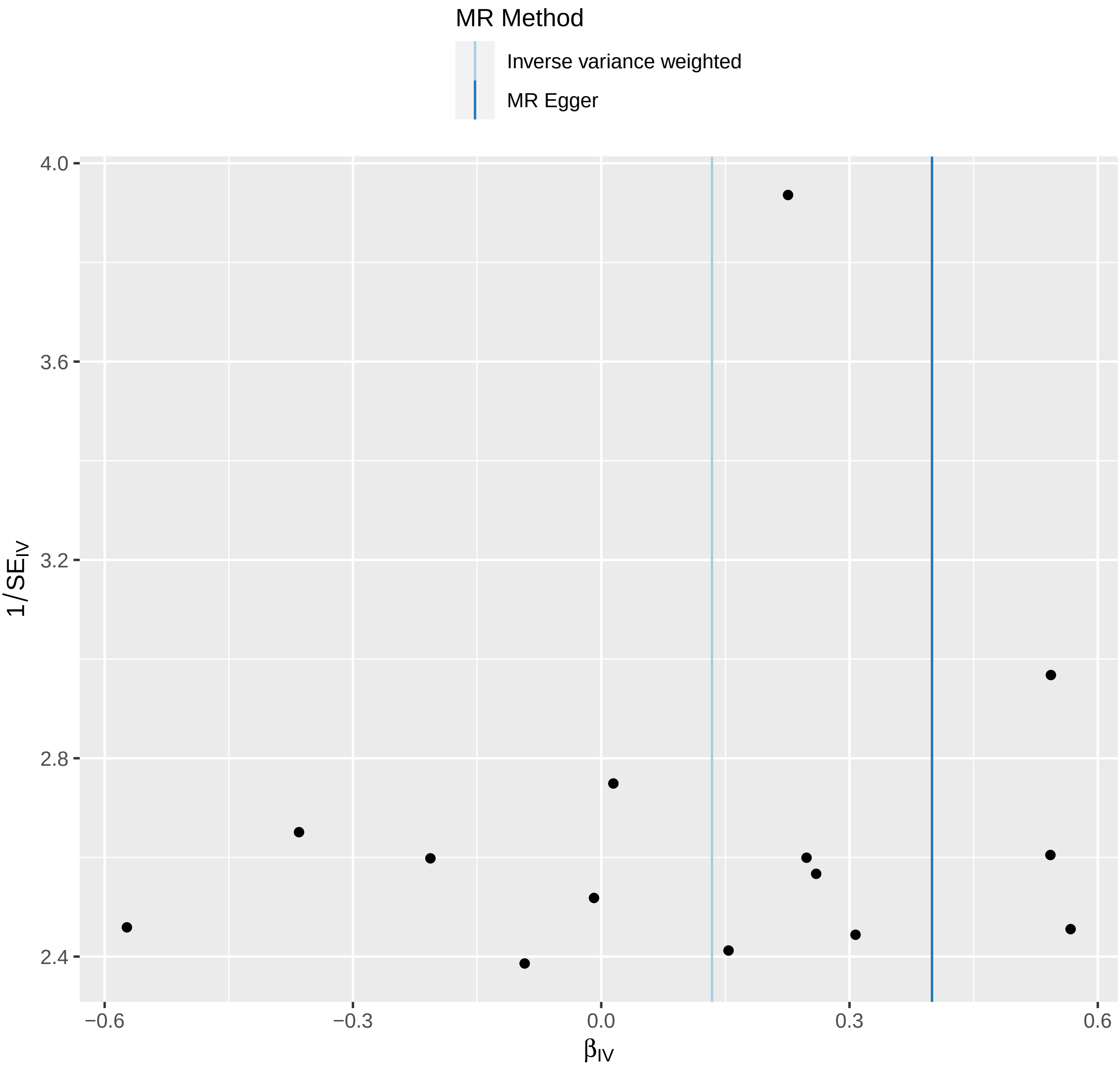

Batch 2−2351 : Gut microbiota abundance (genus Catenibacterium id.2153) on Glyceraldehyde−3−phosphate dehydrogenase, testis−specific
with a threshold of 1e−05

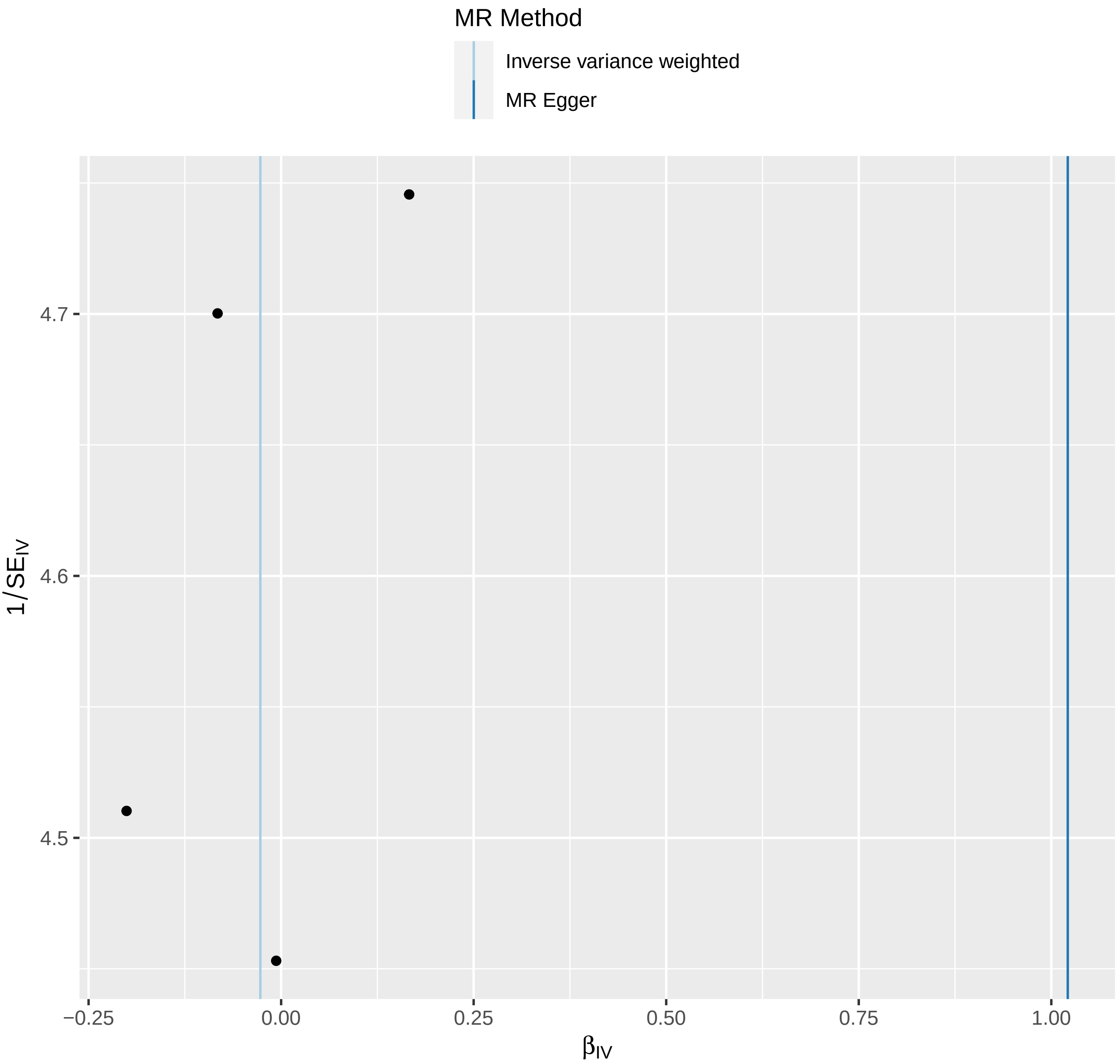

Batch 2−2352 : Gut microbiota abundance (genus Parasutterella id.2892) on Glyceraldehyde−3−phosphate dehydrogenase, testis−specific
with a threshold of 1e−05

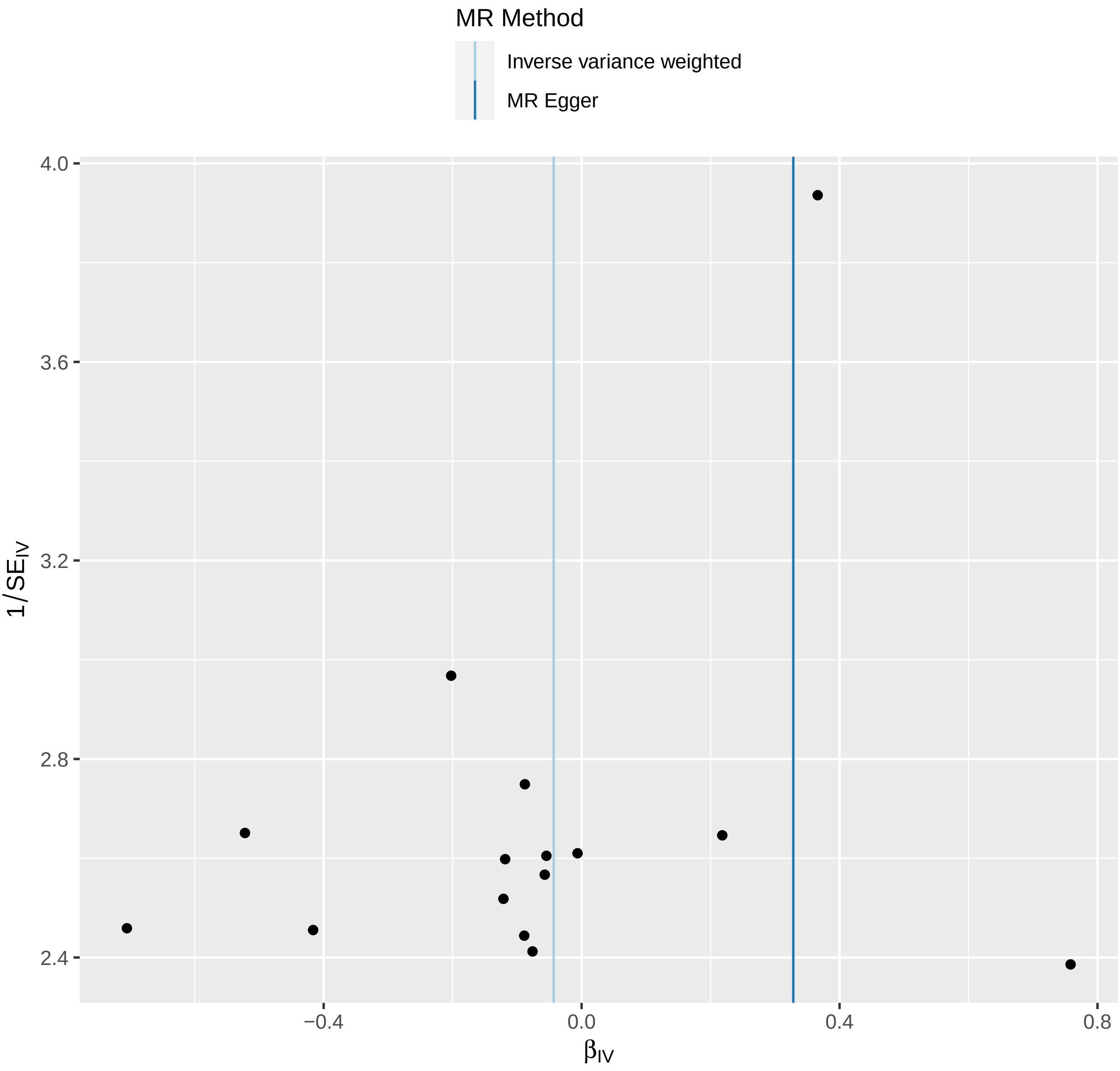

Batch 2−2353 : Gut microbiota abundance (genus Catenibacterium id.2153) on Growth arrest−specific protein 1
with a threshold of 1e−05

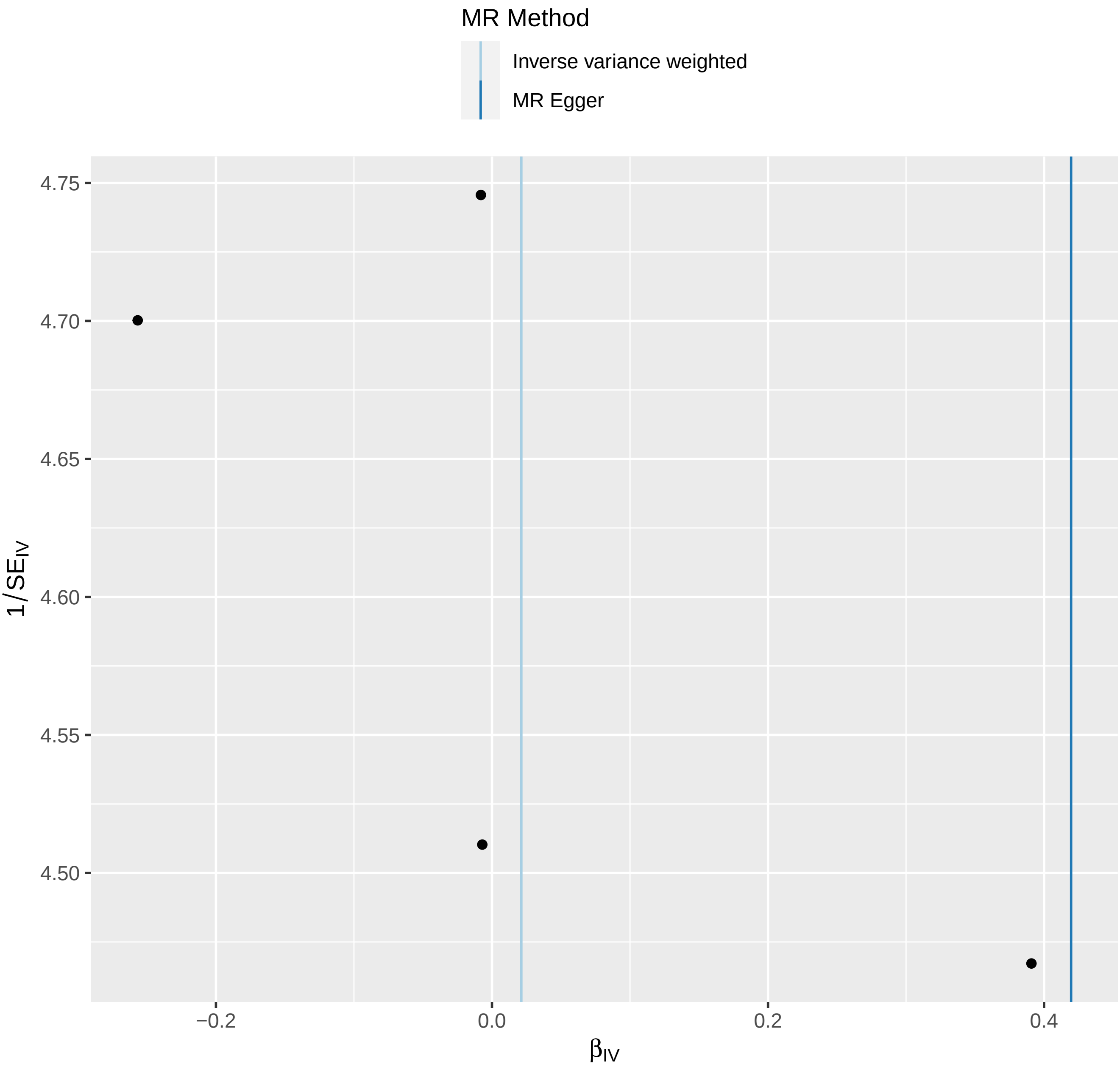

Batch 2−2354 : Gut microbiota abundance (genus Parasutterella id.2892) on Growth arrest−specific protein 1
with a threshold of 1e−05

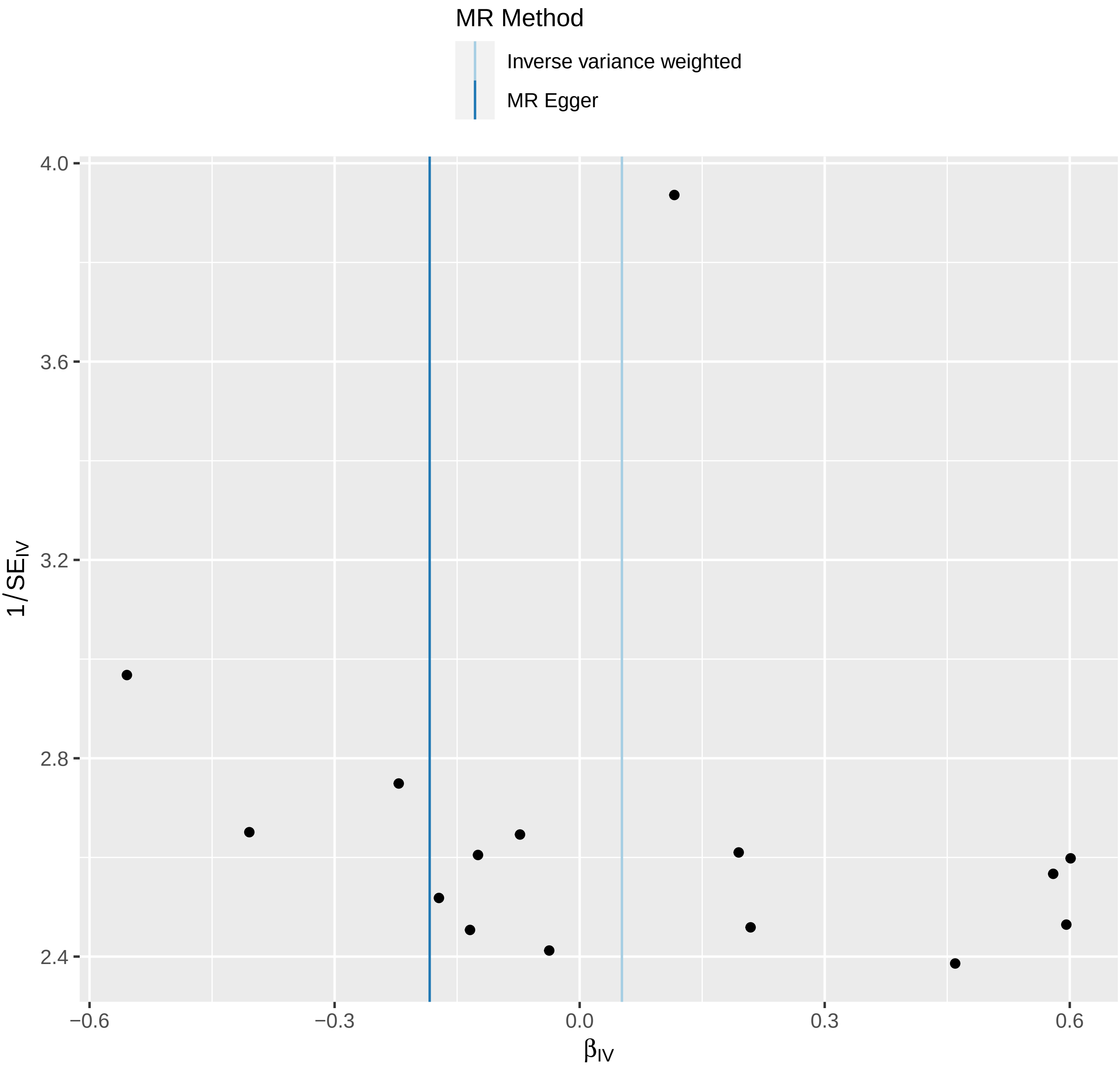

Batch 2−2355 : Gut microbiota abundance (genus Catenibacterium id.2153) on Guanylate−binding protein 6
with a threshold of 1e−05

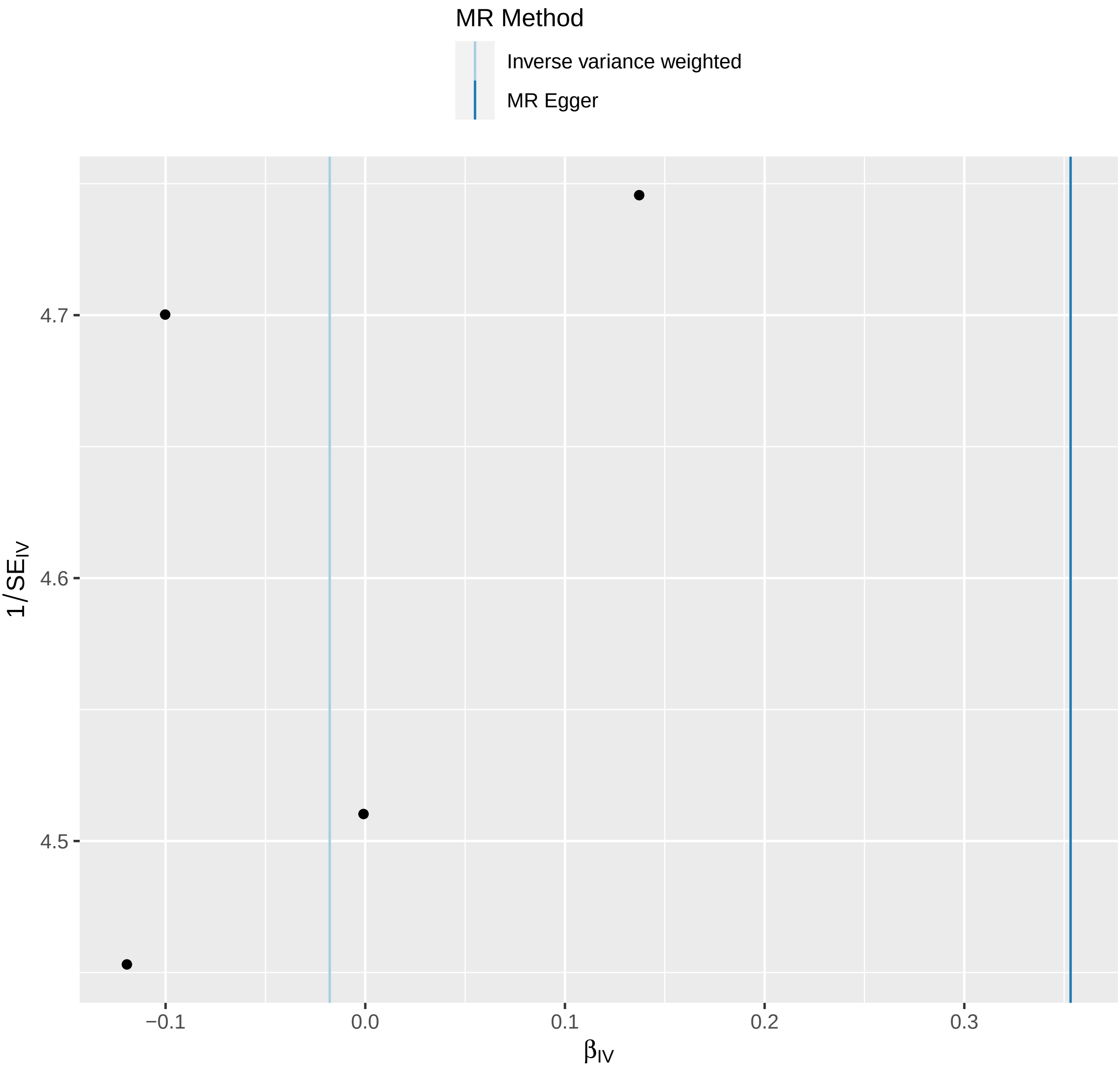

Batch 2−2356 : Gut microbiota abundance (genus Parasutterella id.2892) on Guanylate−binding protein 6
with a threshold of 1e−05

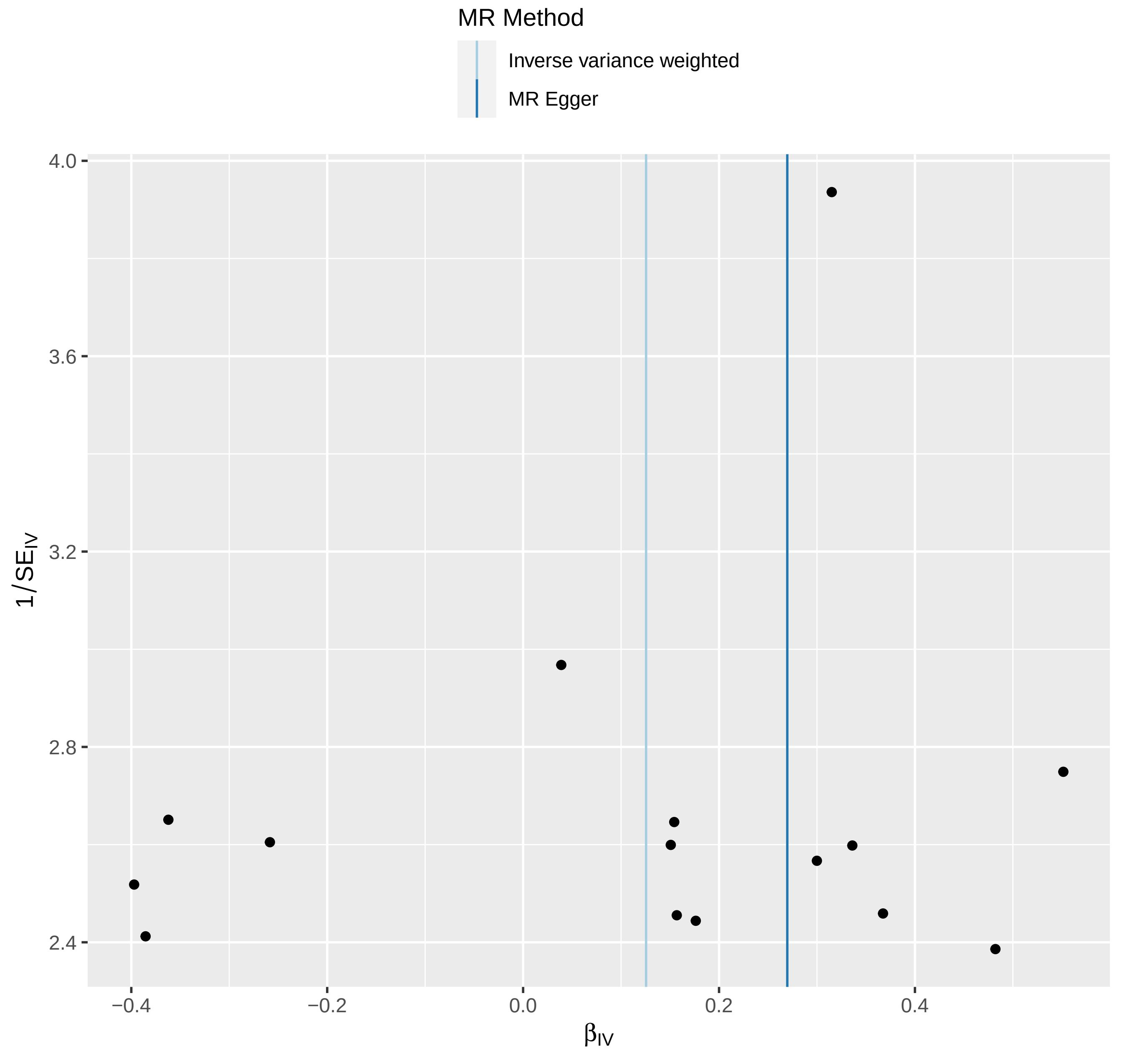

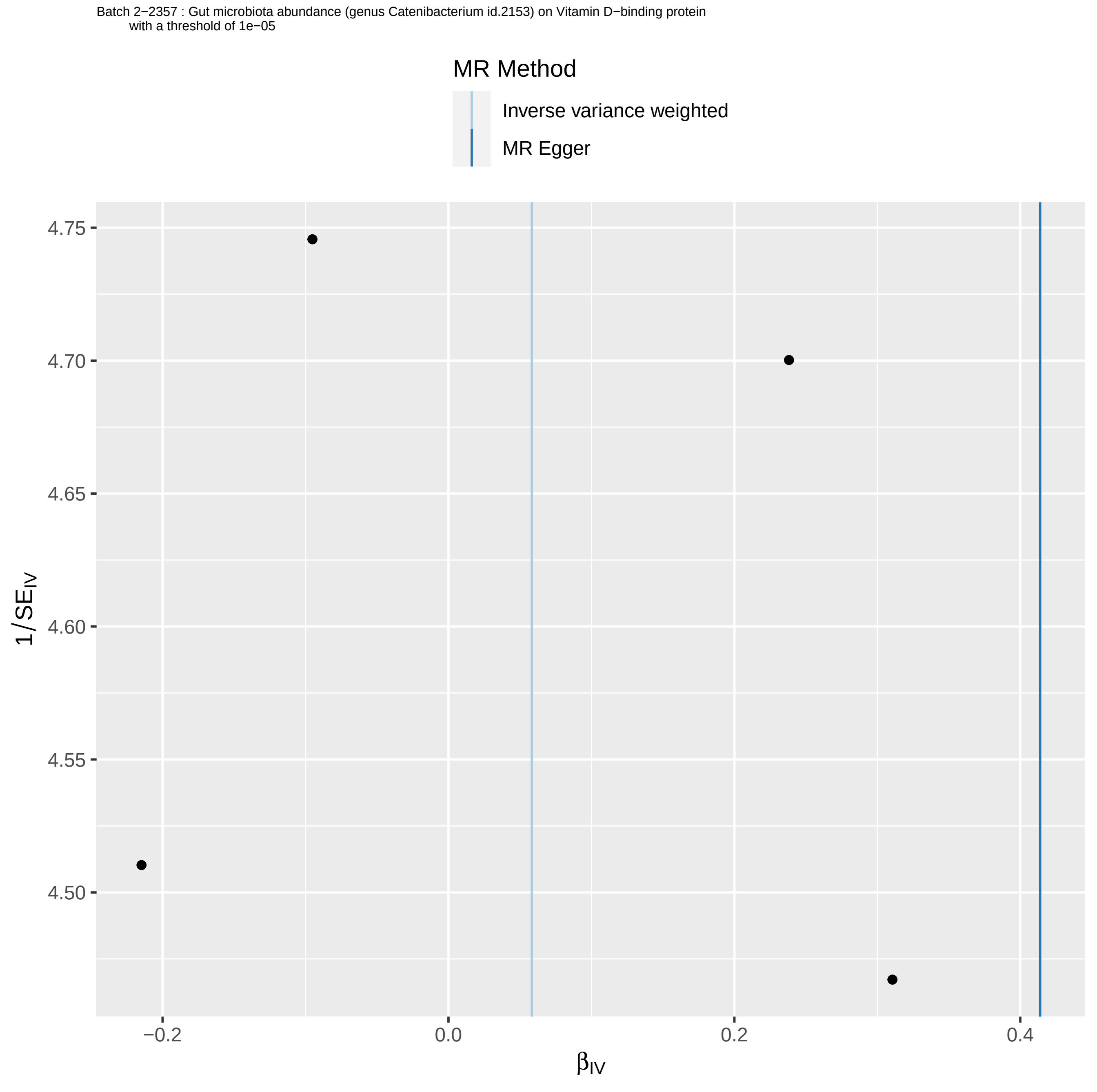

Batch 2−2357 : Gut microbiota abundance (genus Catenibacterium id.2153) on Vitamin D−binding protein
with a threshold of 1e−05
MR Method
Inverse variance weighted
MR Egger
4.75
4.70
4.65
4.60
4.55
4.50
1/SE_IV
−0.2
0.0
0.2
0.4
β_IV

Batch 2−2358 : Gut microbiota abundance (genus Parasutterella id.2892) on Vitamin D−binding protein
with a threshold of 1e−05

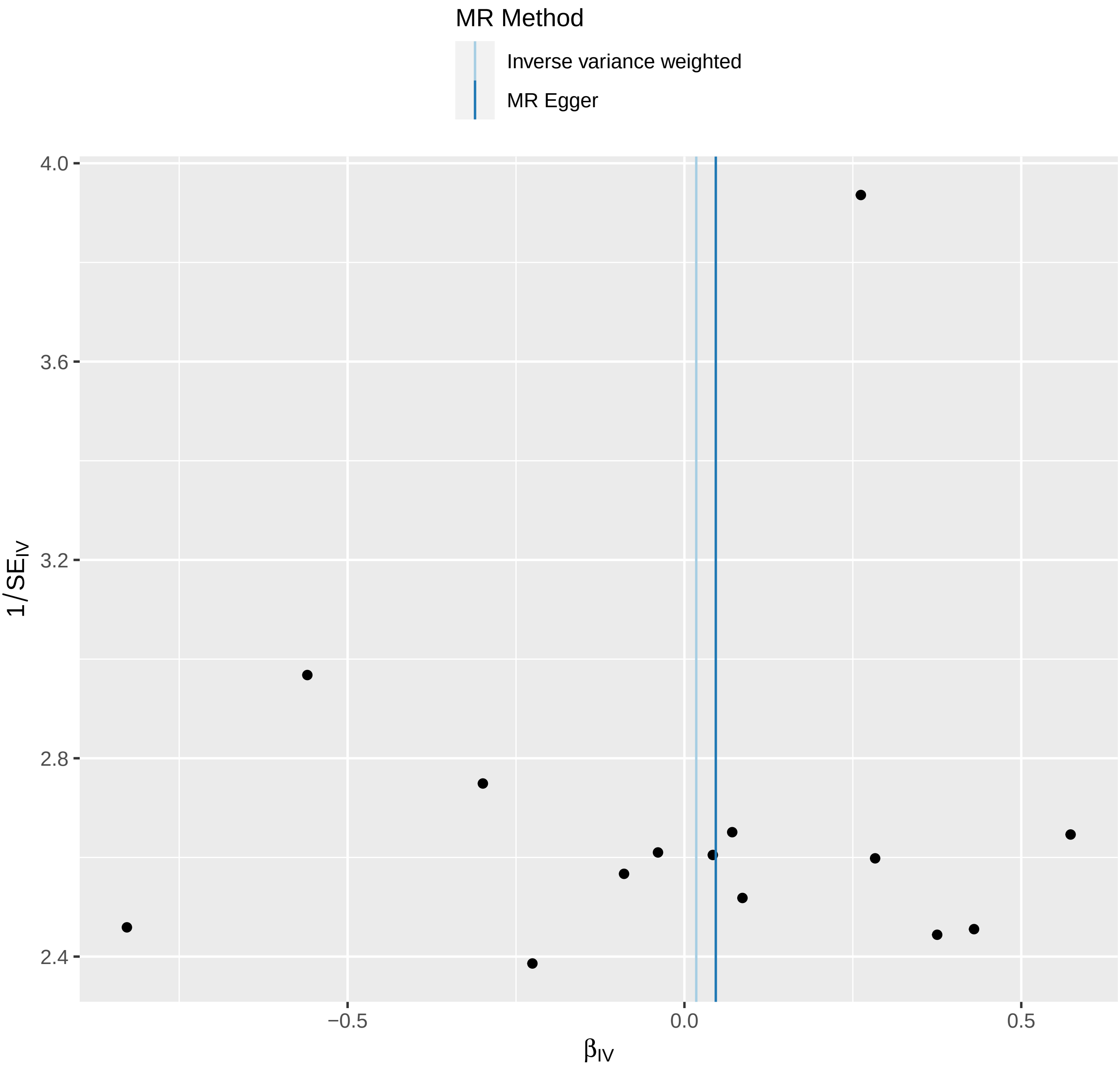

Batch 2−2359 : Gut microbiota abundance (genus Catenibacterium id.2153) on Grancalcin
with a threshold of 1e−05

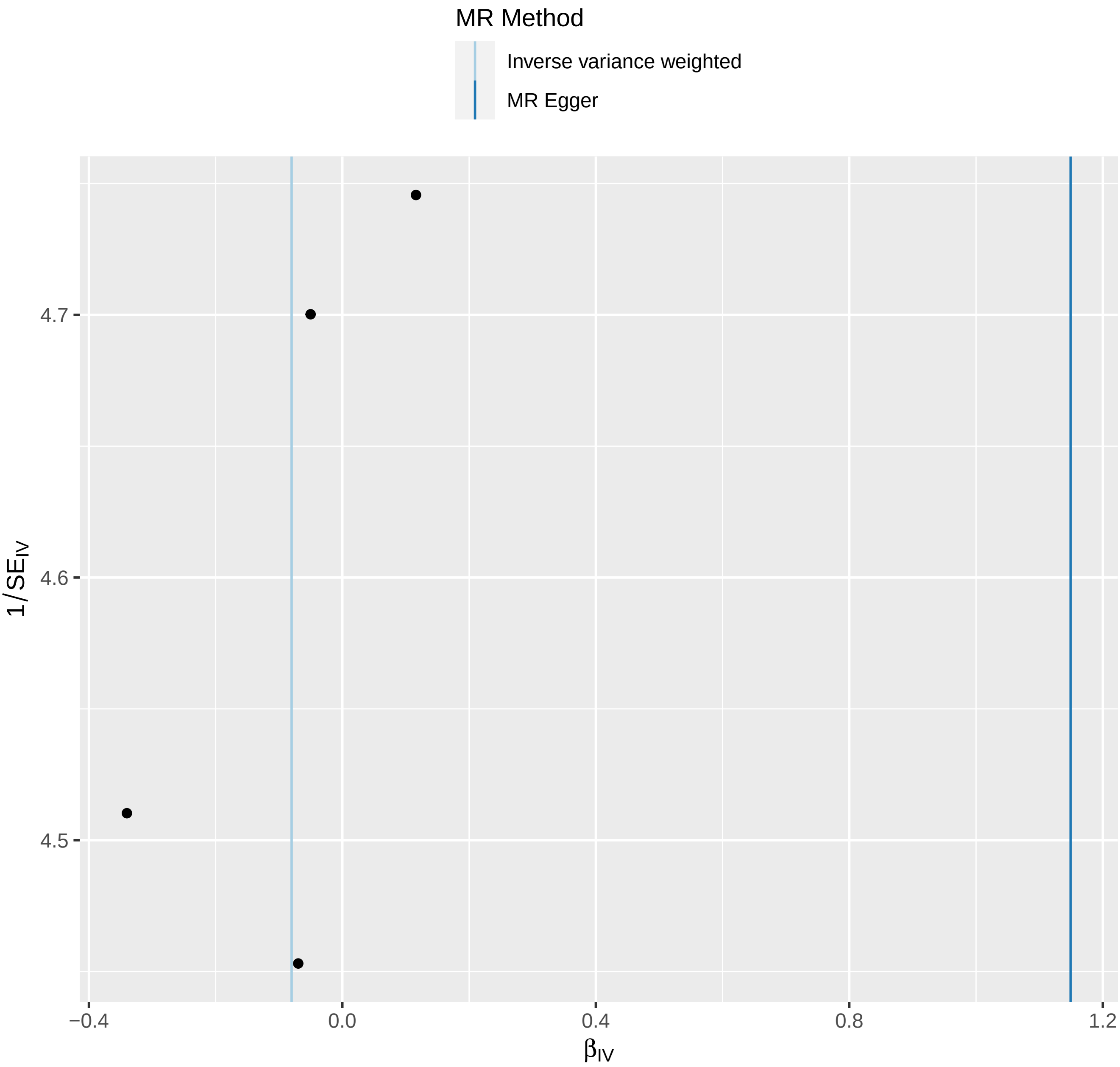

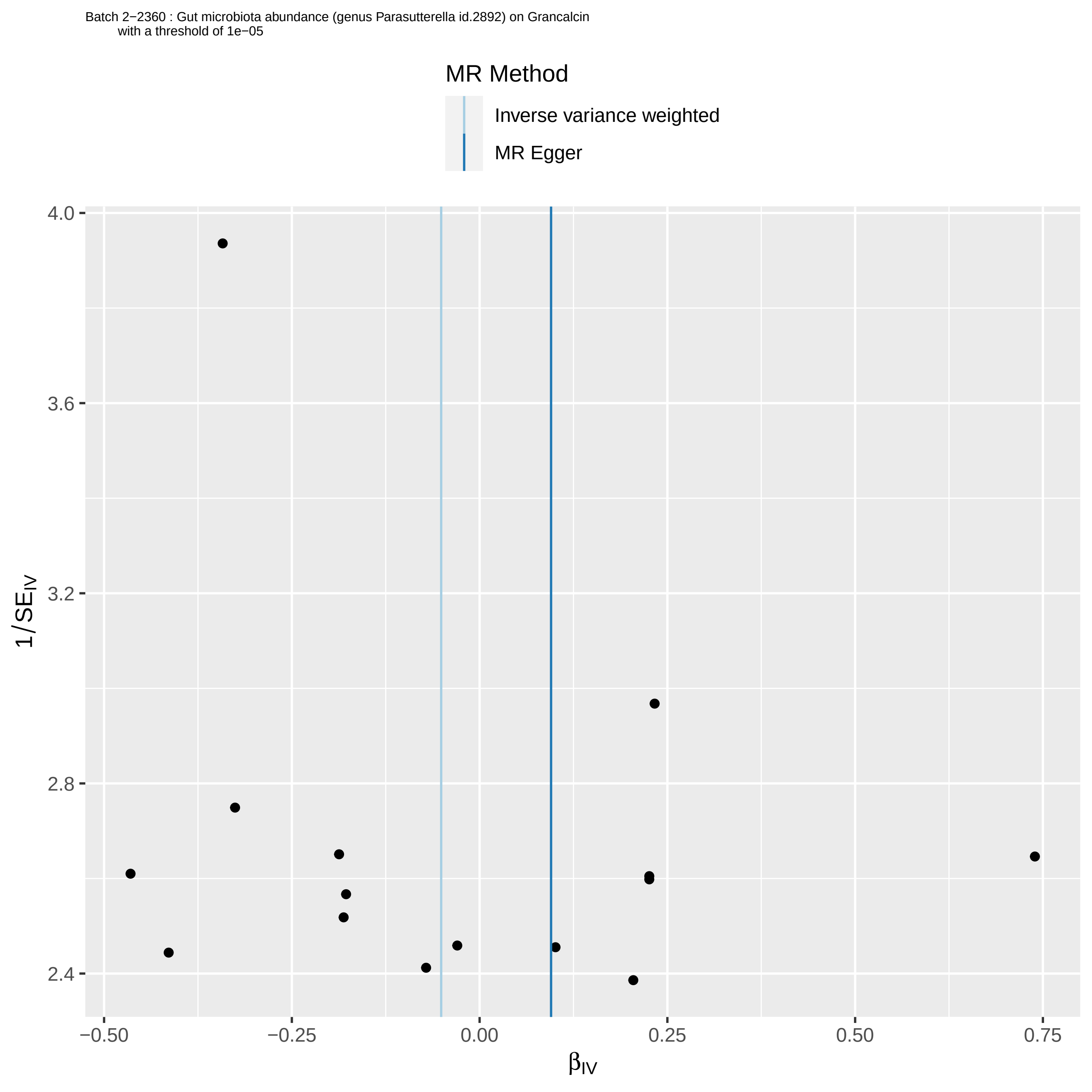
Batch 2−2360 : Gut microbiota abundance (genus Parasutterella id.2892) on Grancalcin
with a threshold of 1e−05
MR Method
Inverse variance weighted
MR Egger
4.0
3.6
3.2
2.8
2.4
1/SE_IV
−0.50
−0.25
0.00
0.25
0.50
0.75
β_IV

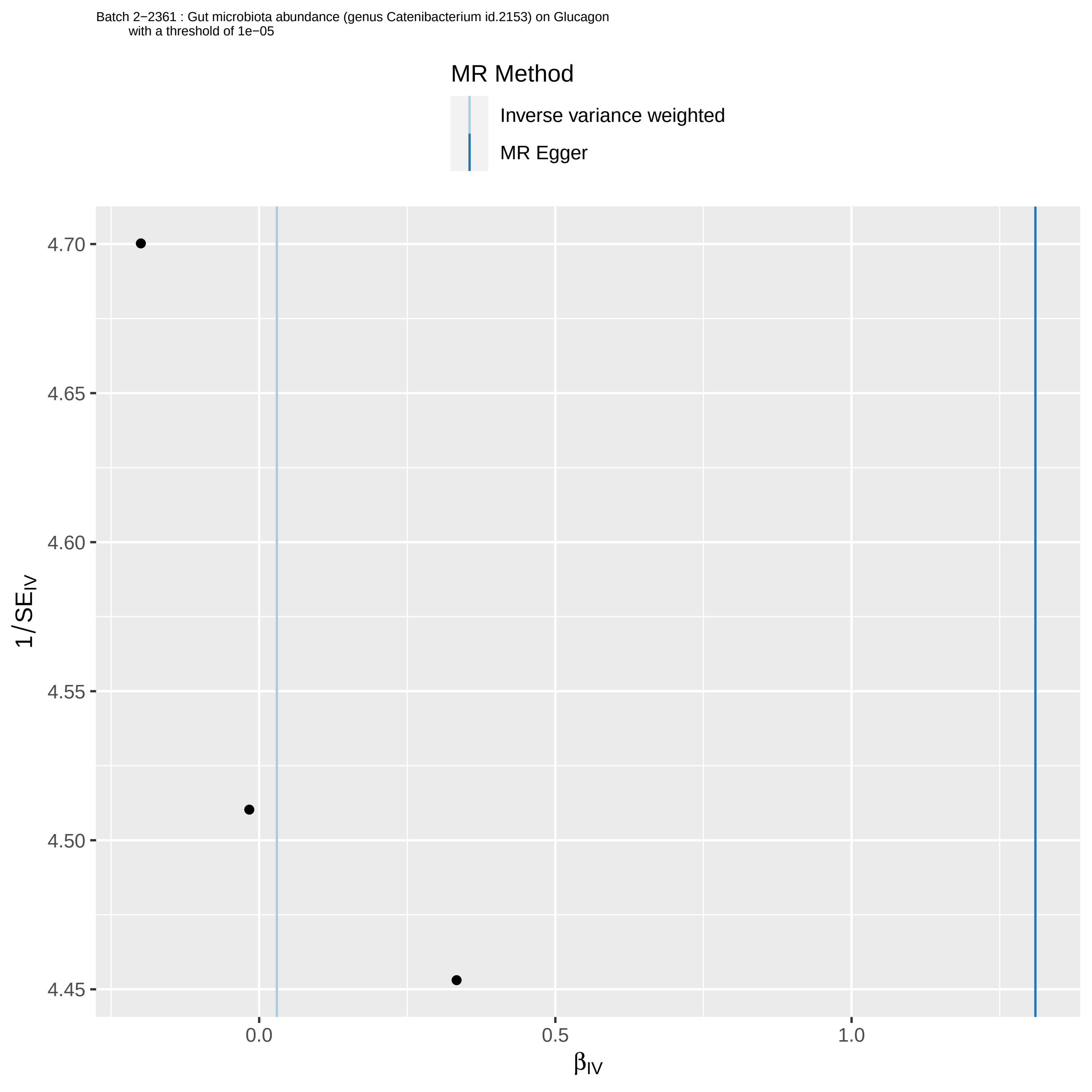
Batch 2−2361 : Gut microbiota abundance (genus Catenibacterium id.2153) on Glucagon
with a threshold of 1e−05
MR Method
Inverse variance weighted
MR Egger
4.70
4.65
4.60
4.55
4.50
4.45
1/SE_IV
0.0
0.5
1.0
β_IV

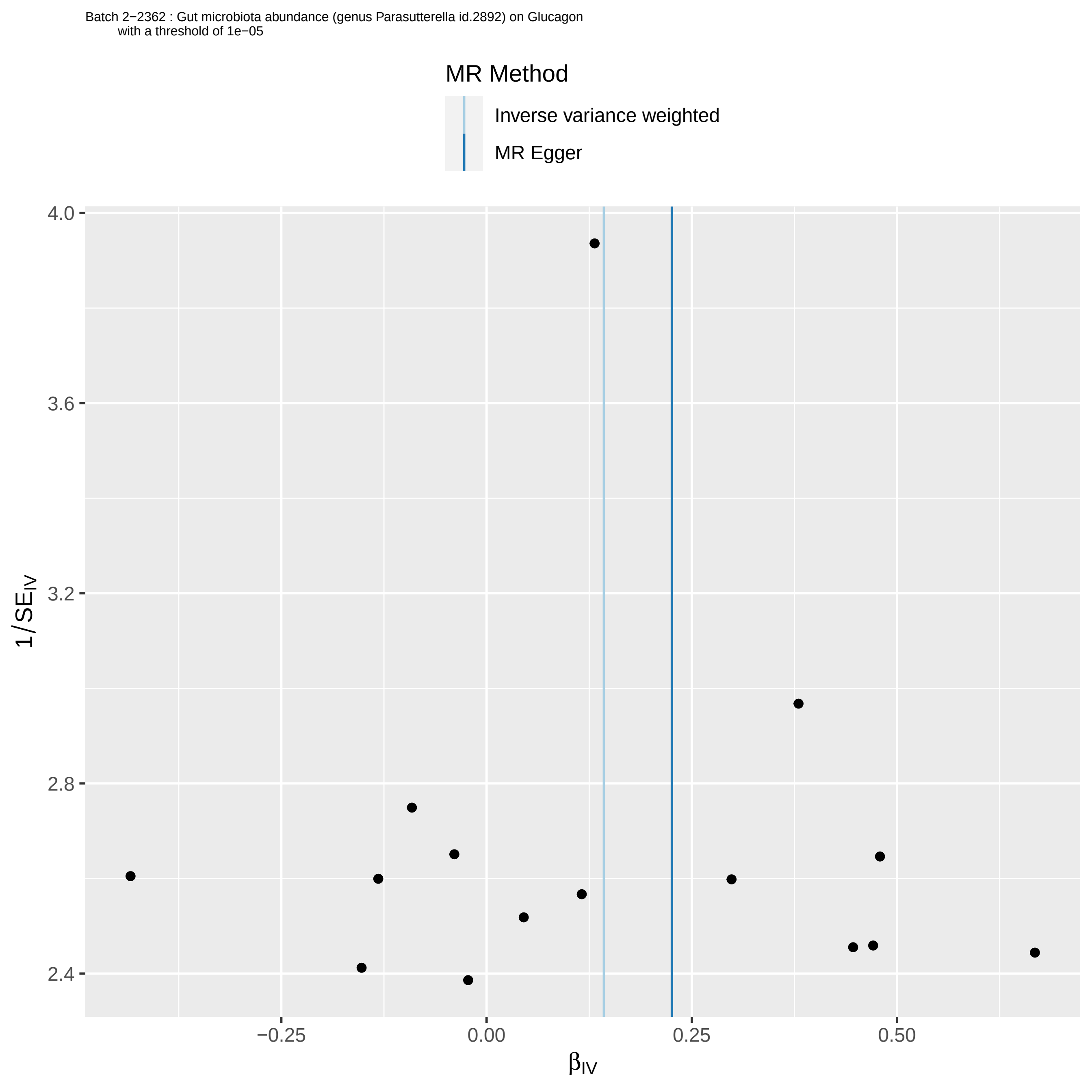

Batch 2−2362 : Gut microbiota abundance (genus Parasutterella id.2892) on Glucagon
with a threshold of 1e−05
MR Method
Inverse variance weighted
MR Egger
4.0
3.6
3.2
2.8
2.4
1/SE$_{IV}$
−0.25
0.00
0.25
0.50
β$_{IV}$

Batch 2−2363 : Gut microbiota abundance (genus Catenibacterium id.2153) on GTP cyclohydrolase 1
with a threshold of 1e−05

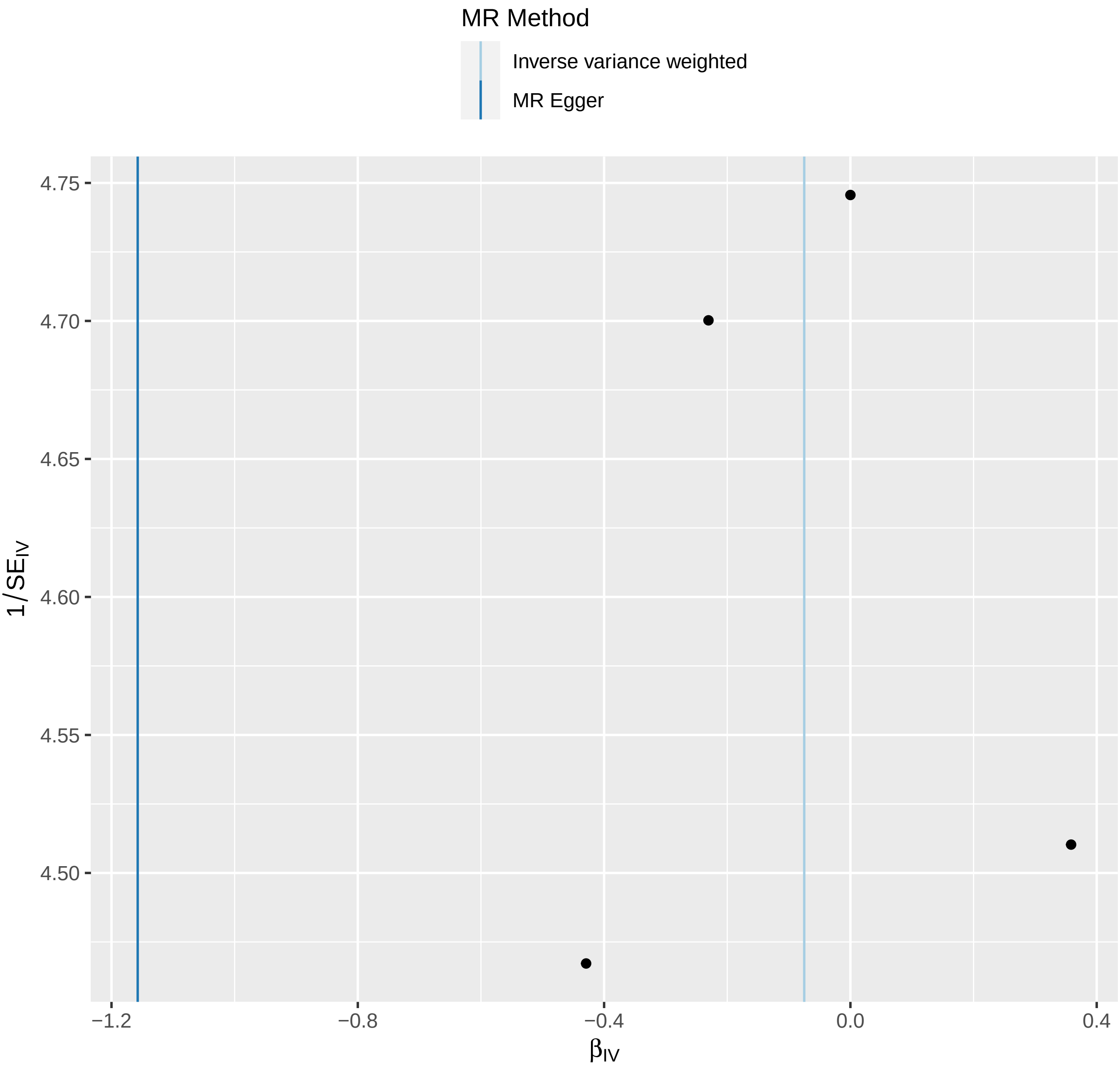

Batch 2−2364 : Gut microbiota abundance (genus Parasutterella id.2892) on GTP cyclohydrolase 1
with a threshold of 1e−05

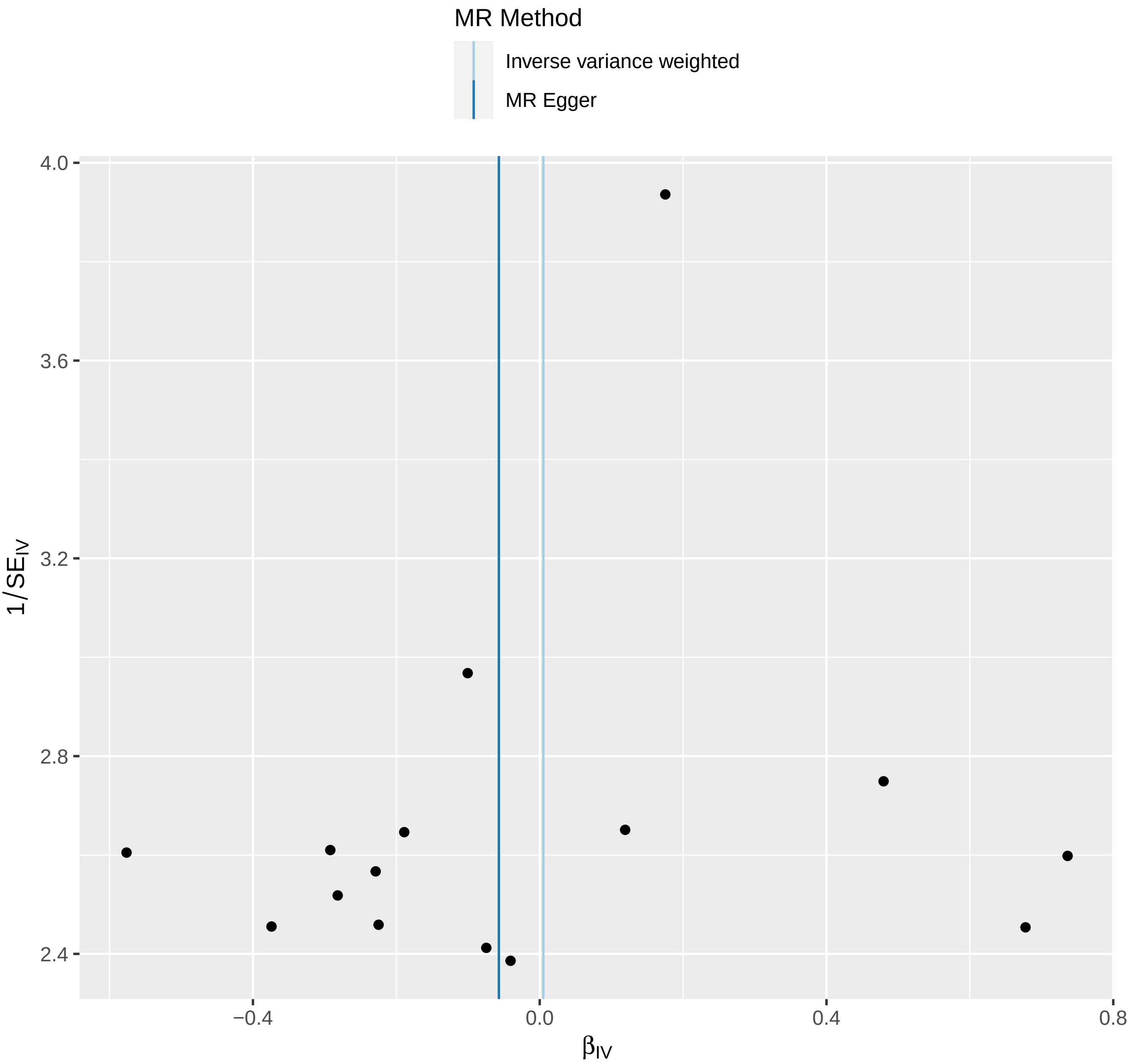

Batch 2−2365 : Gut microbiota abundance (genus Catenibacterium id.2153) on Glucokinase
with a threshold of 1e−05

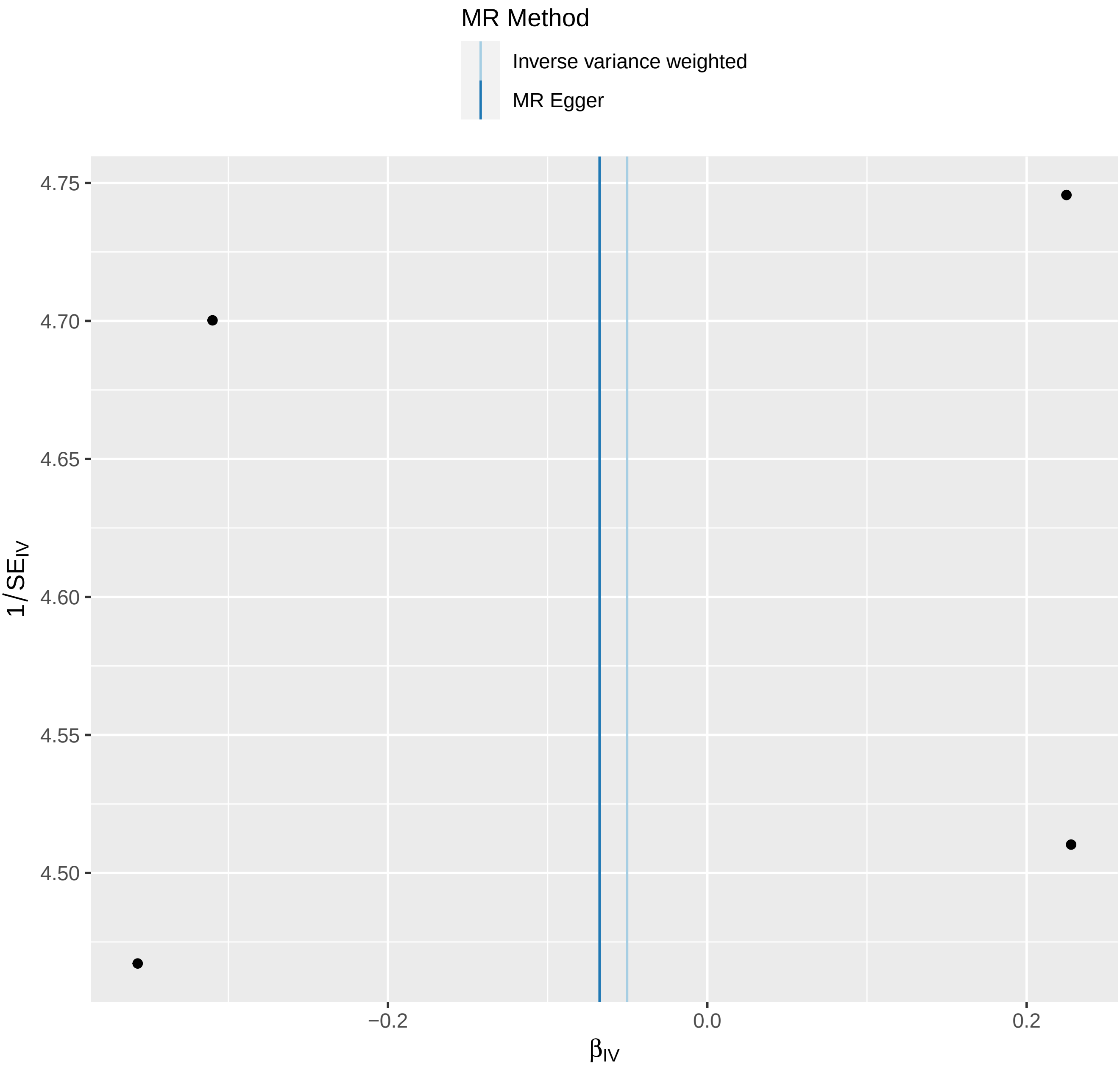

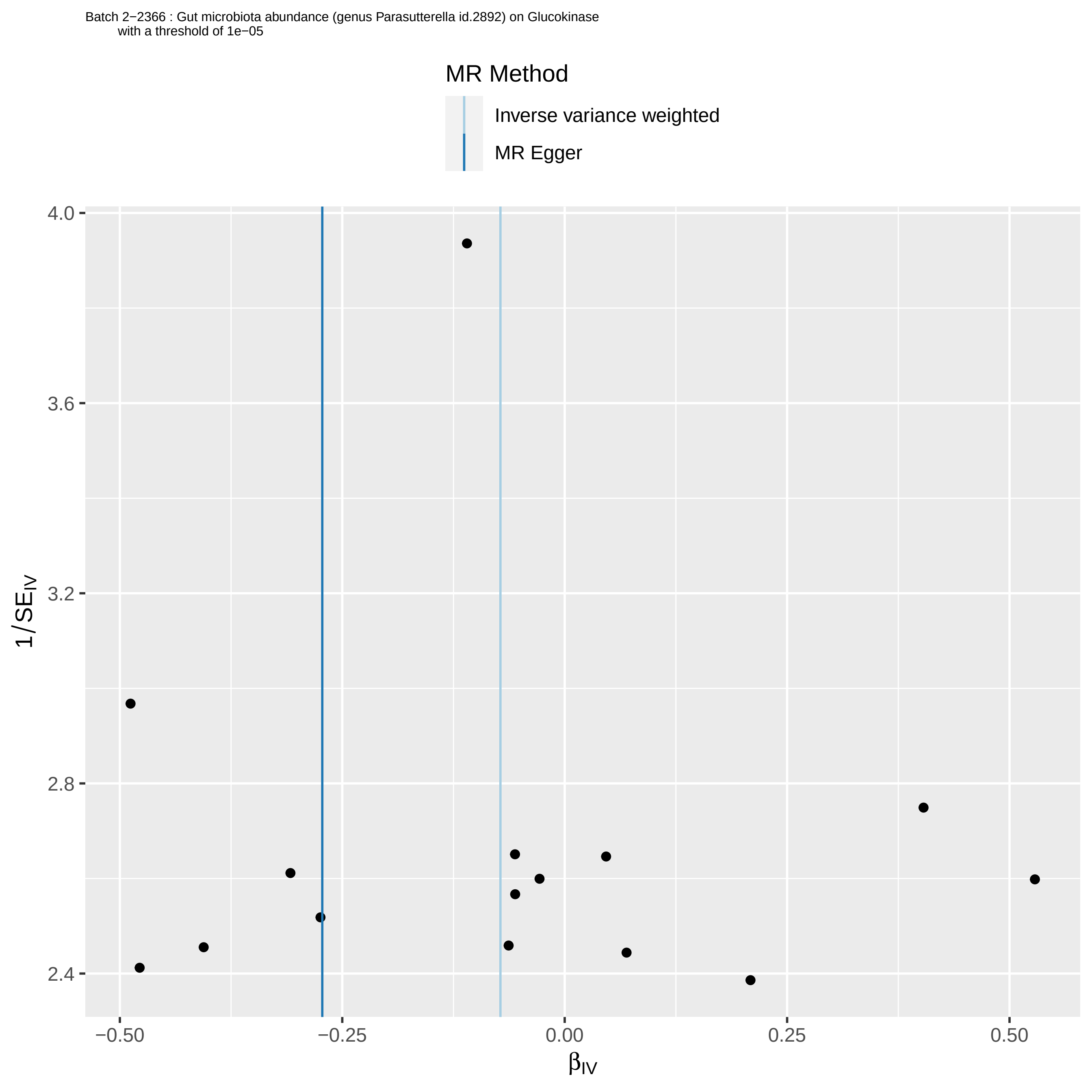
Batch 2−2366 : Gut microbiota abundance (genus Parasutterella id.2892) on Glucokinase
with a threshold of 1e−05
MR Method
Inverse variance weighted
MR Egger
4.0
3.6
3.2
2.8
2.4
1/SE$_{IV}$
−0.50
−0.25
0.00
0.25
0.50
β$_{IV}$

Batch 2−2367 : Gut microbiota abundance (genus Catenibacterium id.2153) on Glucokinase regulatory protein
with a threshold of 1e−05

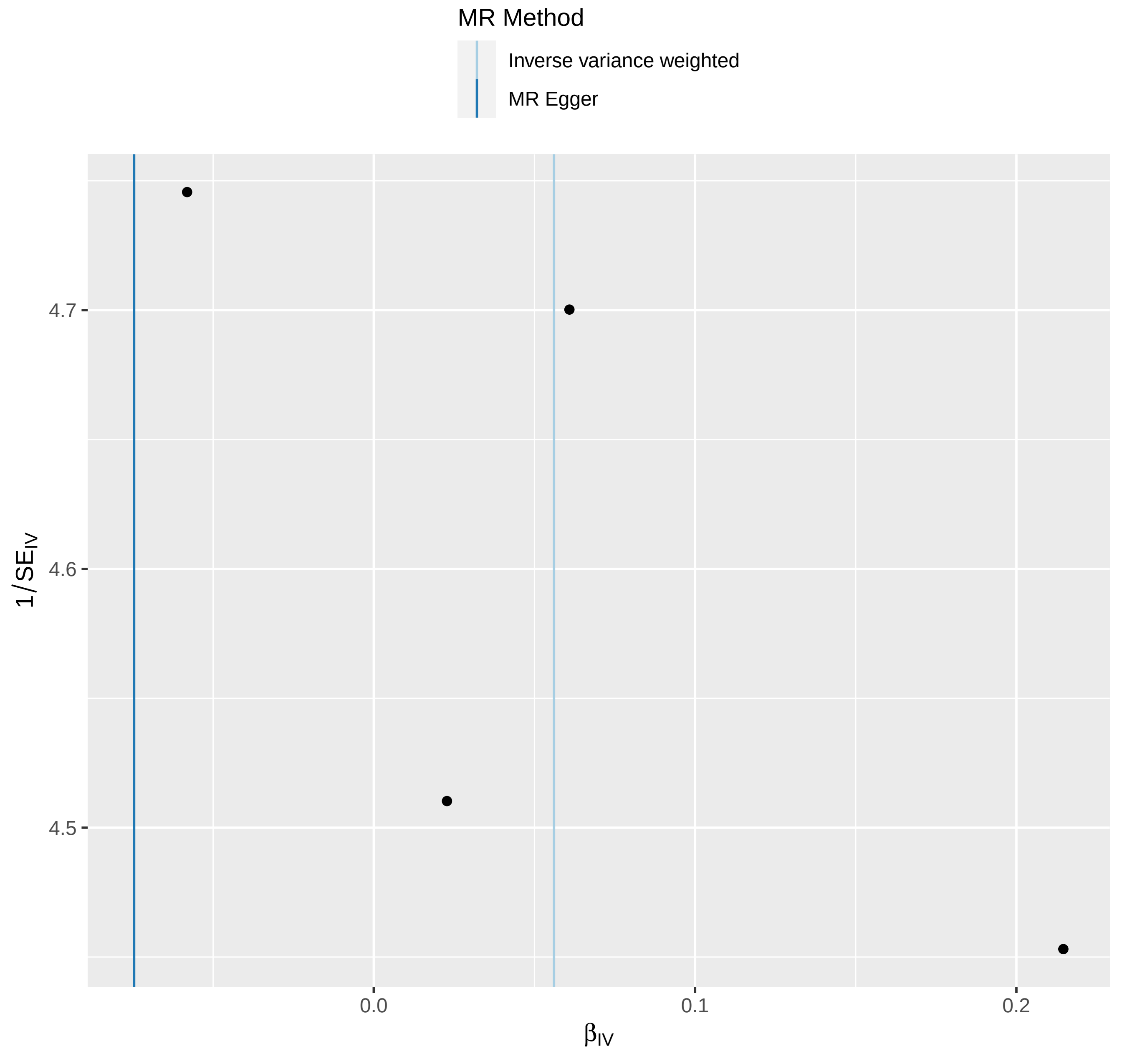

Batch 2−2368 : Gut microbiota abundance (genus Parasutterella id.2892) on Glucokinase regulatory protein with a threshold of 1e−05

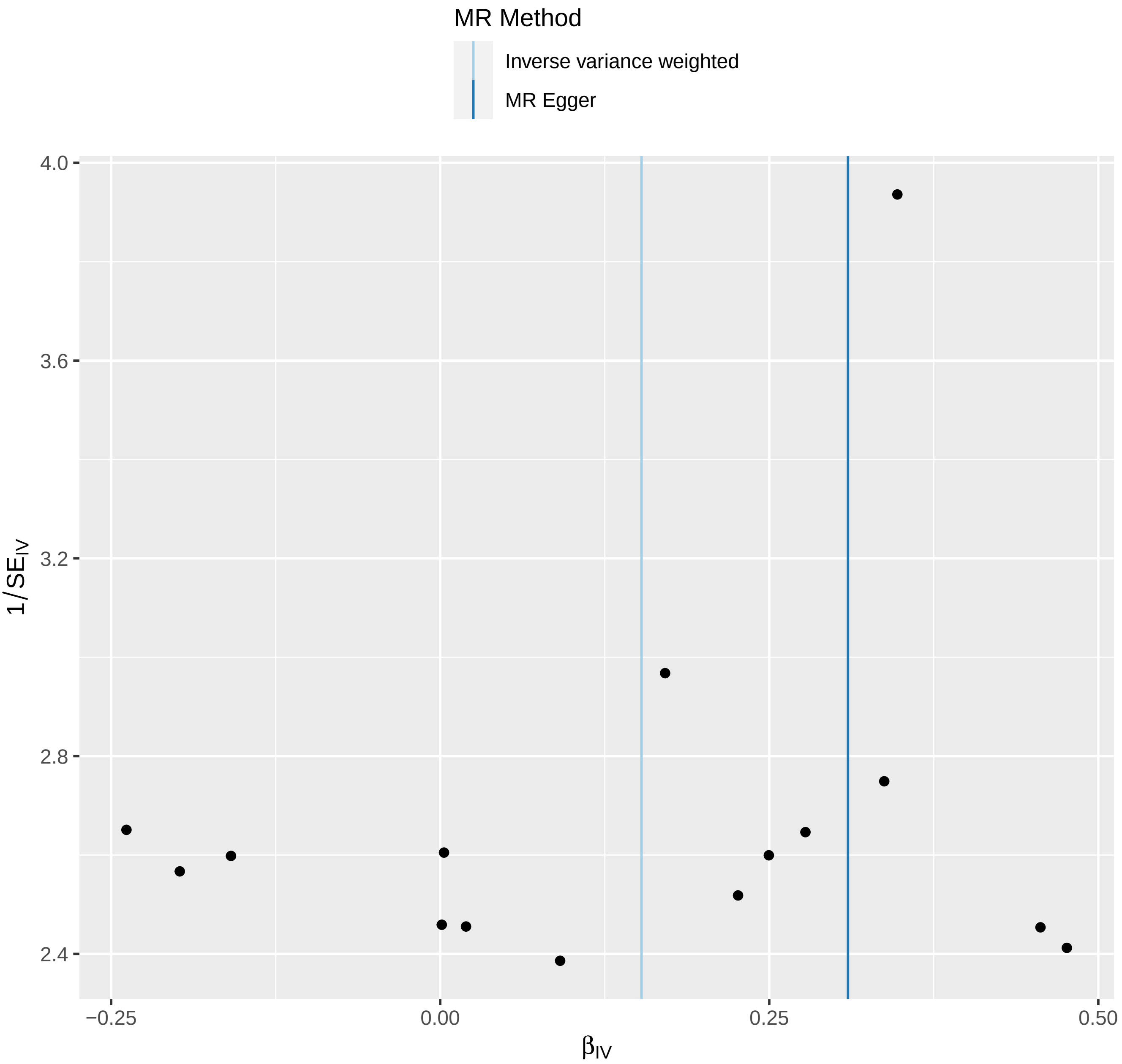

Batch 2−2369 : Gut microbiota abundance (genus Catenibacterium id.2153) on Beta−1,3−galactosyl−O−glycosyl−glycoprotein beta−1,6−N−acetylglucosaminyltransferase with a threshold of 1e−05

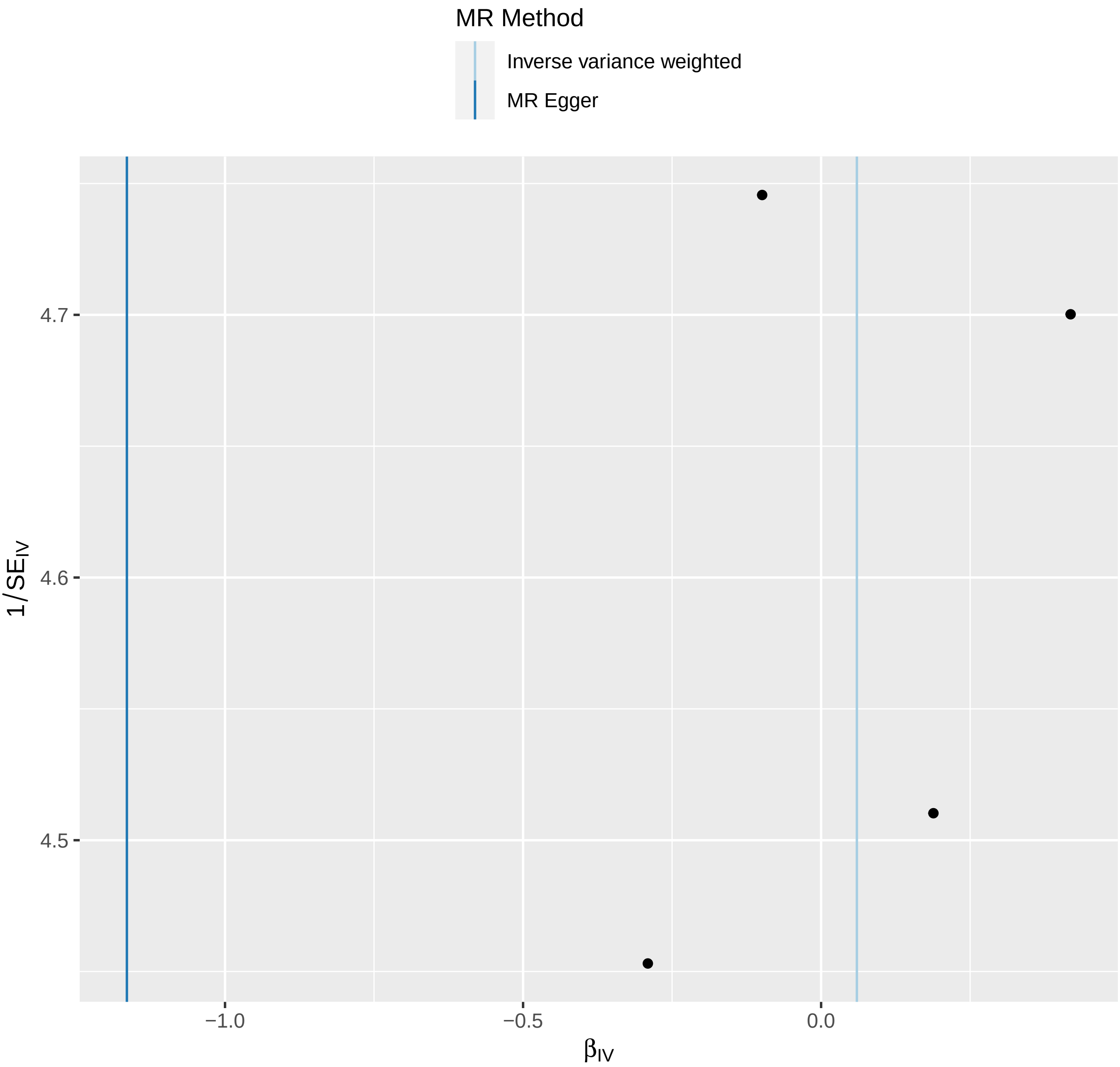

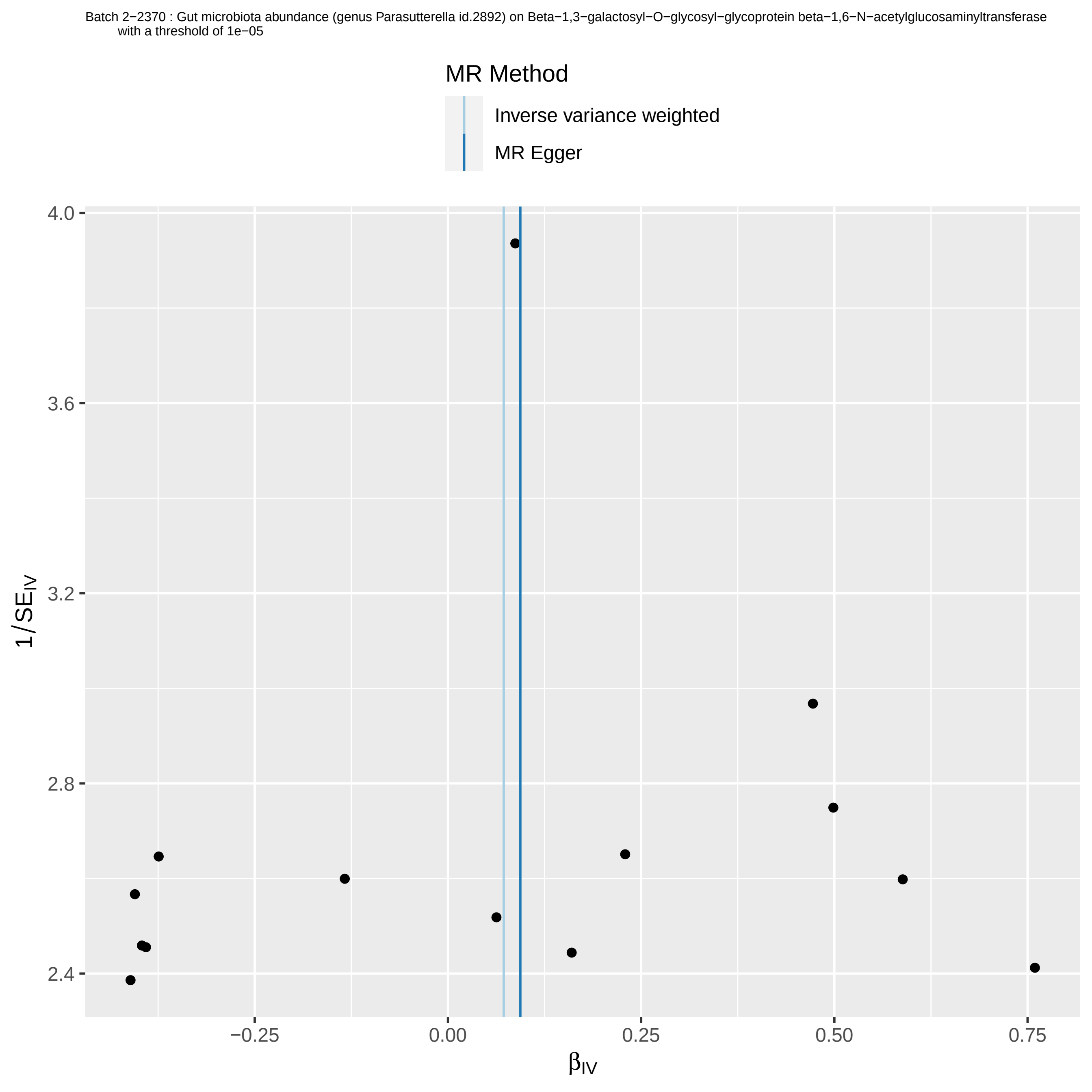

Batch 2−2370 : Gut microbiota abundance (genus Parasutterella id.2892) on Beta−1,3−galactosyl−O−glycosyl−glycoprotein beta−1,6−N−acetylglucosaminyltransferase with a threshold of 1e−05
MR Method
Inverse variance weighted
MR Egger
4.0
3.6
3.2
2.8
2.4
1/SE_IV
−0.25
0.00
0.25
0.50
0.75
β_IV

Batch 2−2371 : Gut microbiota abundance (genus Catenibacterium id.2153) on N−acetyllactosaminide beta−1,6−N−acetylglucosaminyl−transferase, isoform C with a threshold of 1e−05

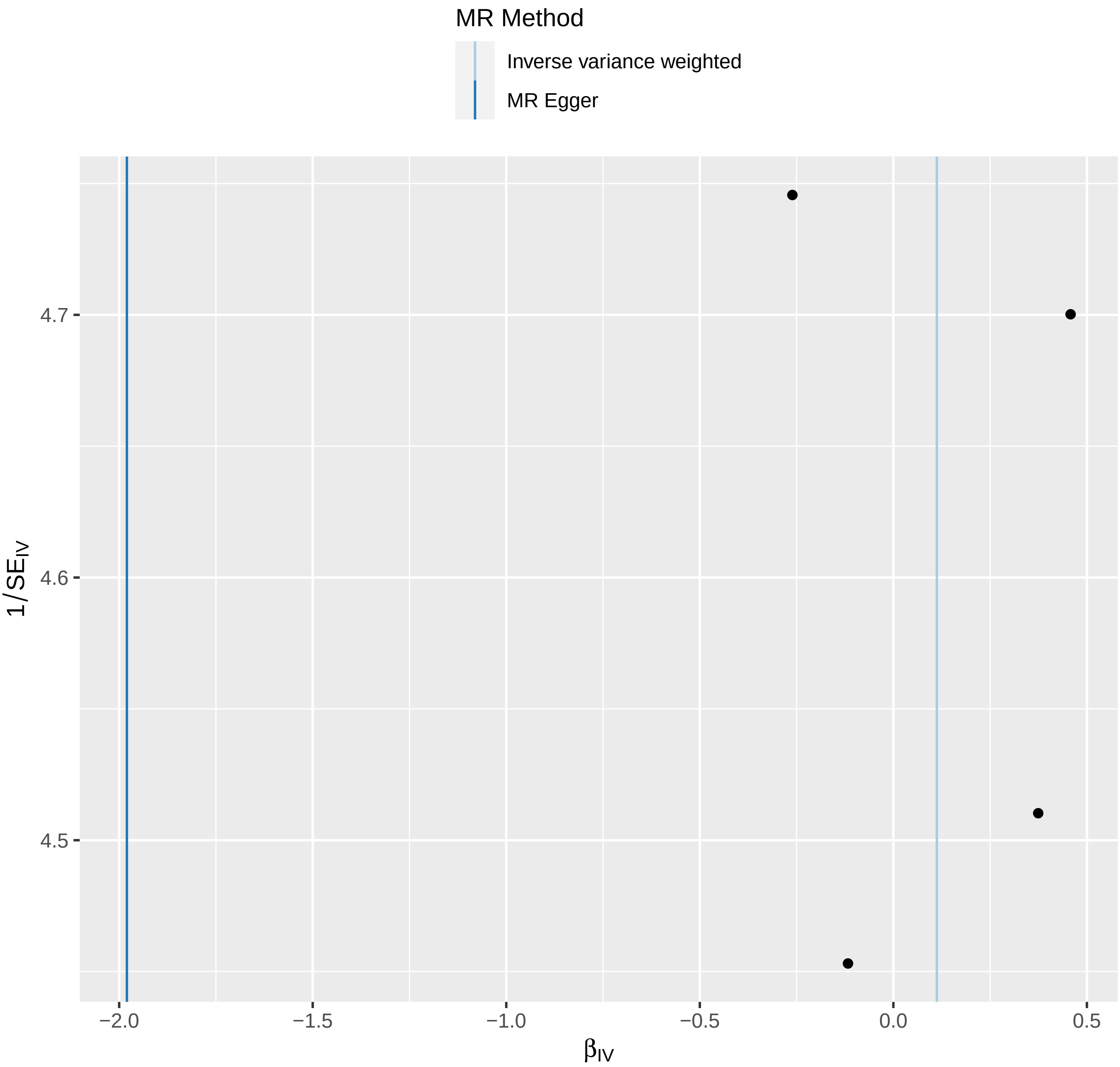

Batch 2−2372 : Gut microbiota abundance (genus Parasutterella id.2892) on N−acetyllactosaminide beta−1,6−N−acetylglucosaminyl−transferase, isoform C with a threshold of 1e−05

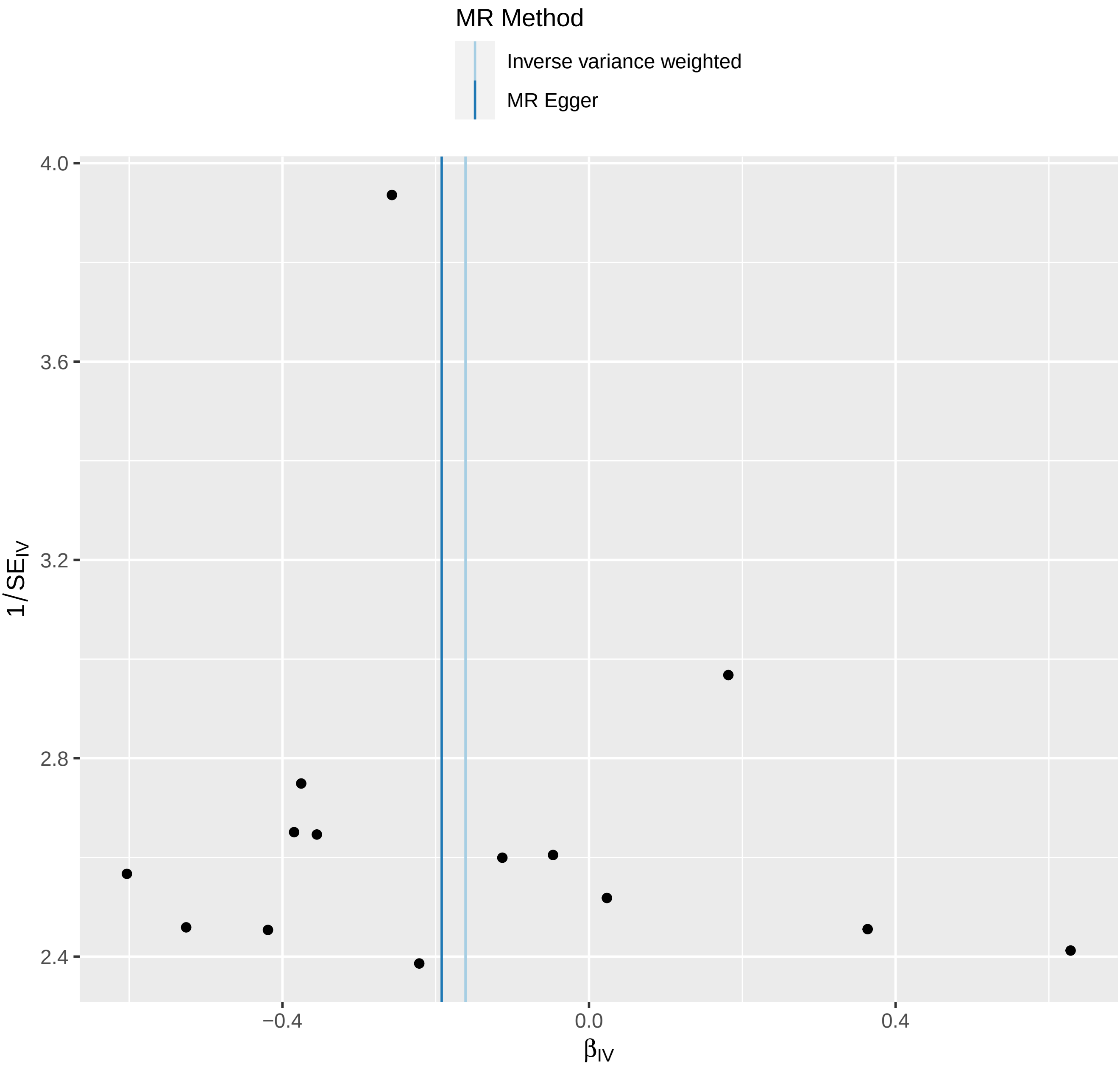

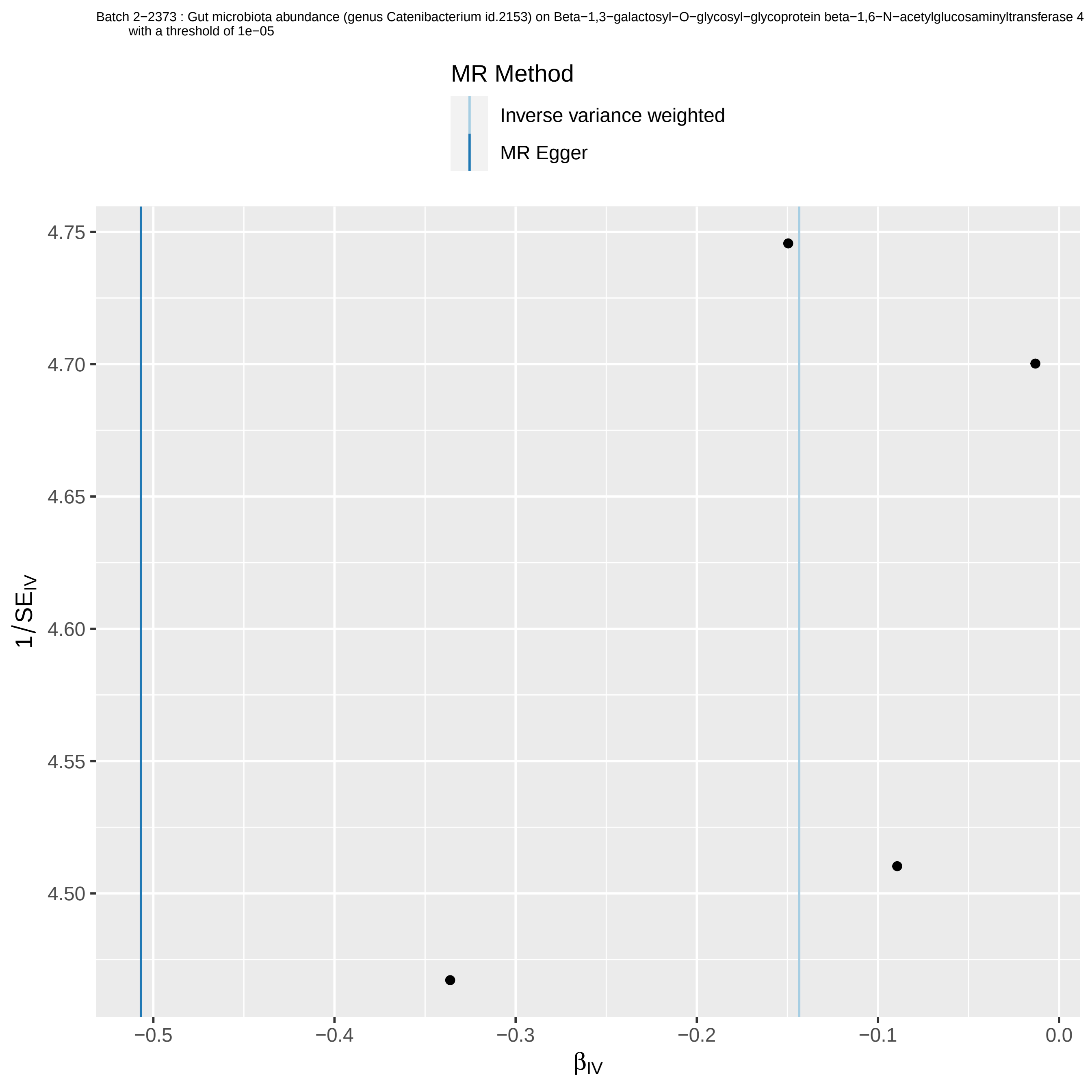
Batch 2−2373 : Gut microbiota abundance (genus Catenibacterium id.2153) on Beta−1,3−galactosyl−O−glycosyl−glycoprotein beta−1,6−N−acetylglucosaminyltransferase 4 with a threshold of 1e−05
MR Method
Inverse variance weighted
MR Egger
4.75
4.70
4.65
4.60
4.55
4.50
1/SE_IV
−0.5
−0.4
−0.3
−0.2
−0.1
0.0
β_IV

Batch 2−2374 : Gut microbiota abundance (genus Parasutterella id.2892) on Beta−1,3−galactosyl−O−glycosyl−glycoprotein beta−1,6−N−acetylglucosaminyltransferase 4 with a threshold of 1e−05

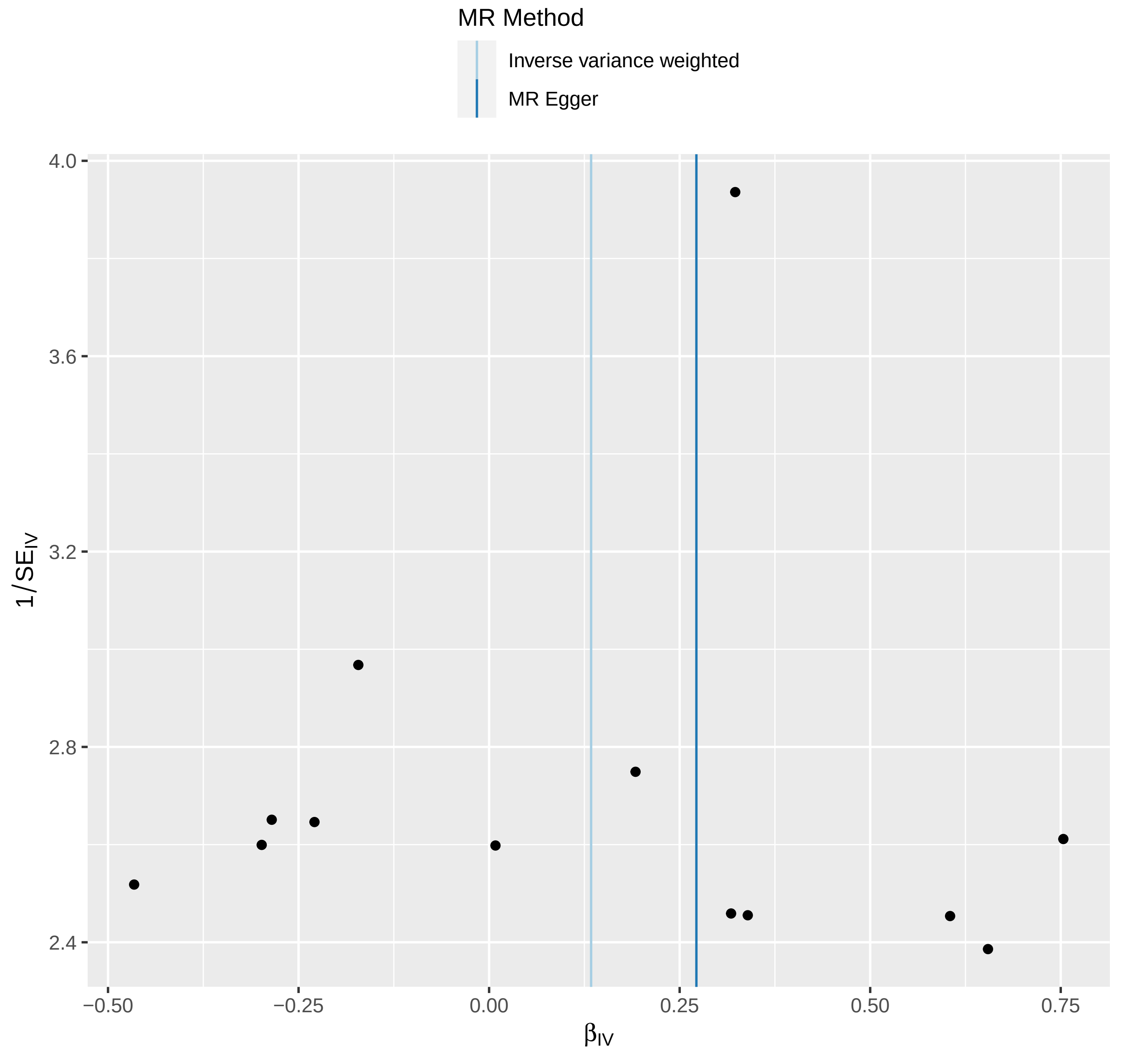

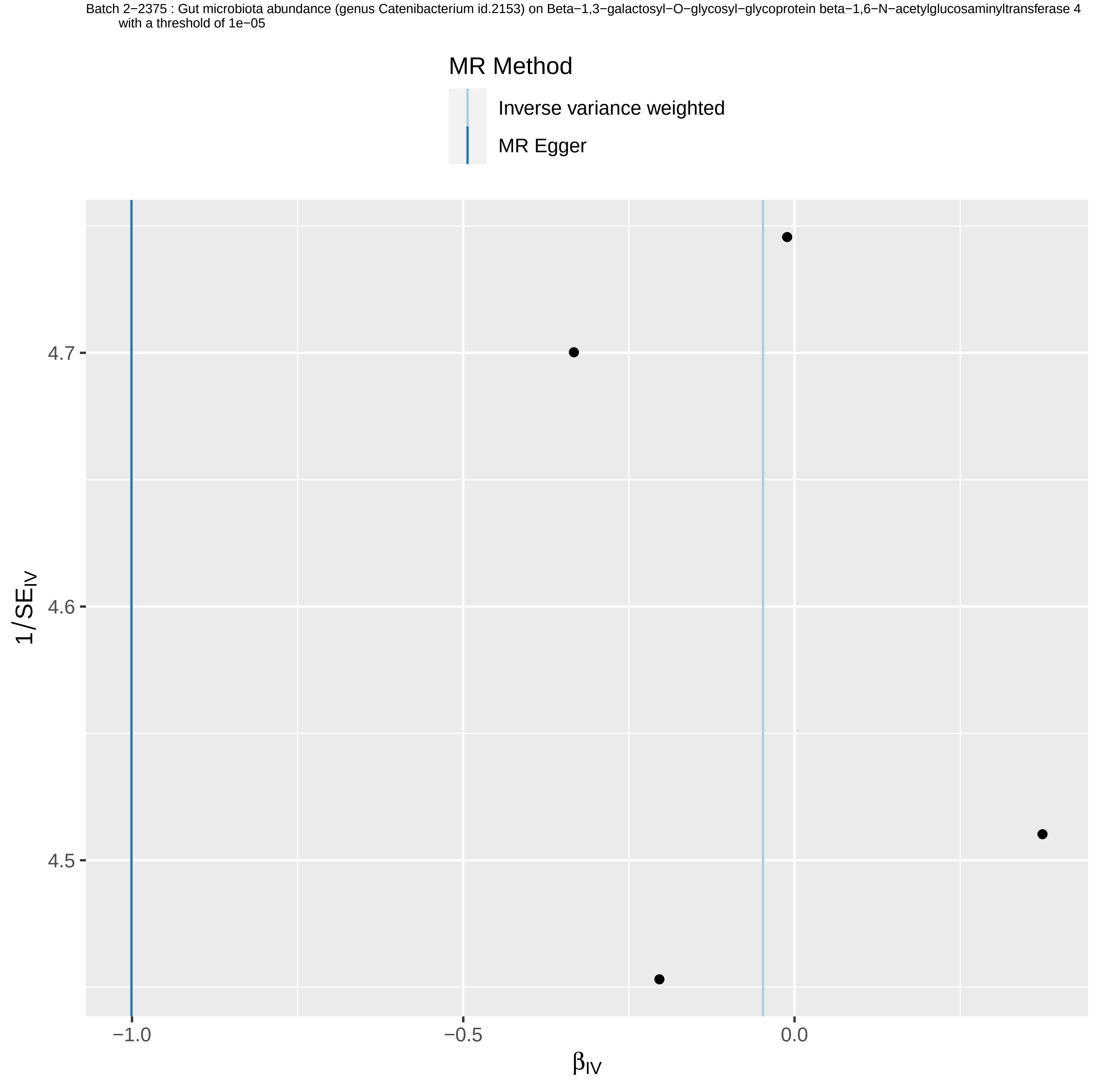
Batch 2−2375 : Gut microbiota abundance (genus Catenibacterium id.2153) on Beta−1,3−galactosyl−O−glycosyl−glycoprotein beta−1,6−N−acetylglucosaminyltransferase 4
with a threshold of 1e−05
MR Method
Inverse variance weighted
MR Egger
4.7
4.6
4.5
1/SE_IV
−1.0
−0.5
0.0
β_IV

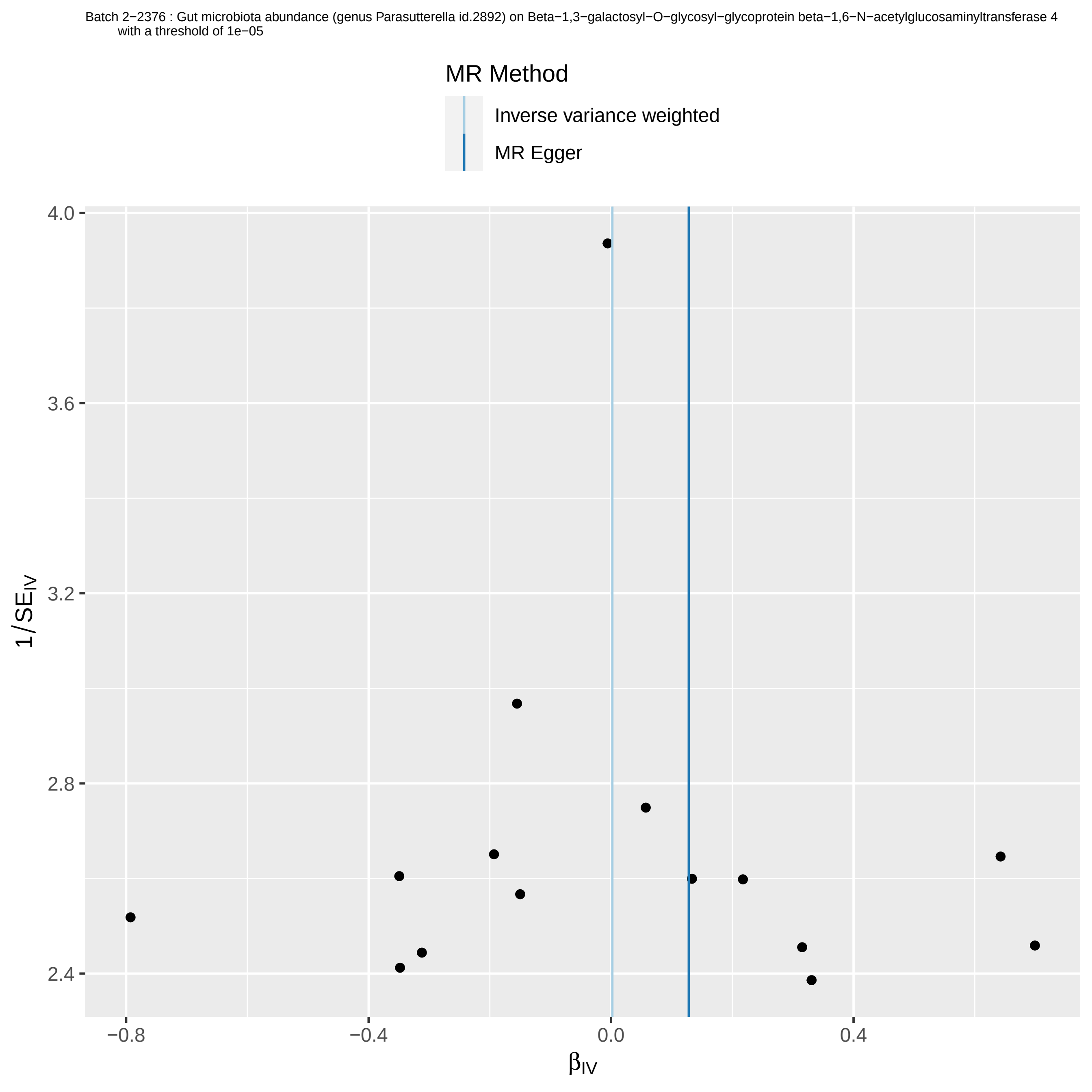
Batch 2−2376 : Gut microbiota abundance (genus Parasutterella id.2892) on Beta−1,3−galactosyl−O−glycosyl−glycoprotein beta−1,6−N−acetylglucosaminyltransferase 4
with a threshold of 1e−05
MR Method
Inverse variance weighted
MR Egger
4.0
3.6
3.2
2.8
2.4
1/SE_IV
−0.8
−0.4
0.0
0.4
β_IV

Batch 2−2377 : Gut microbiota abundance (genus Catenibacterium id.2153) on Guanine deaminase
with a threshold of 1e−05

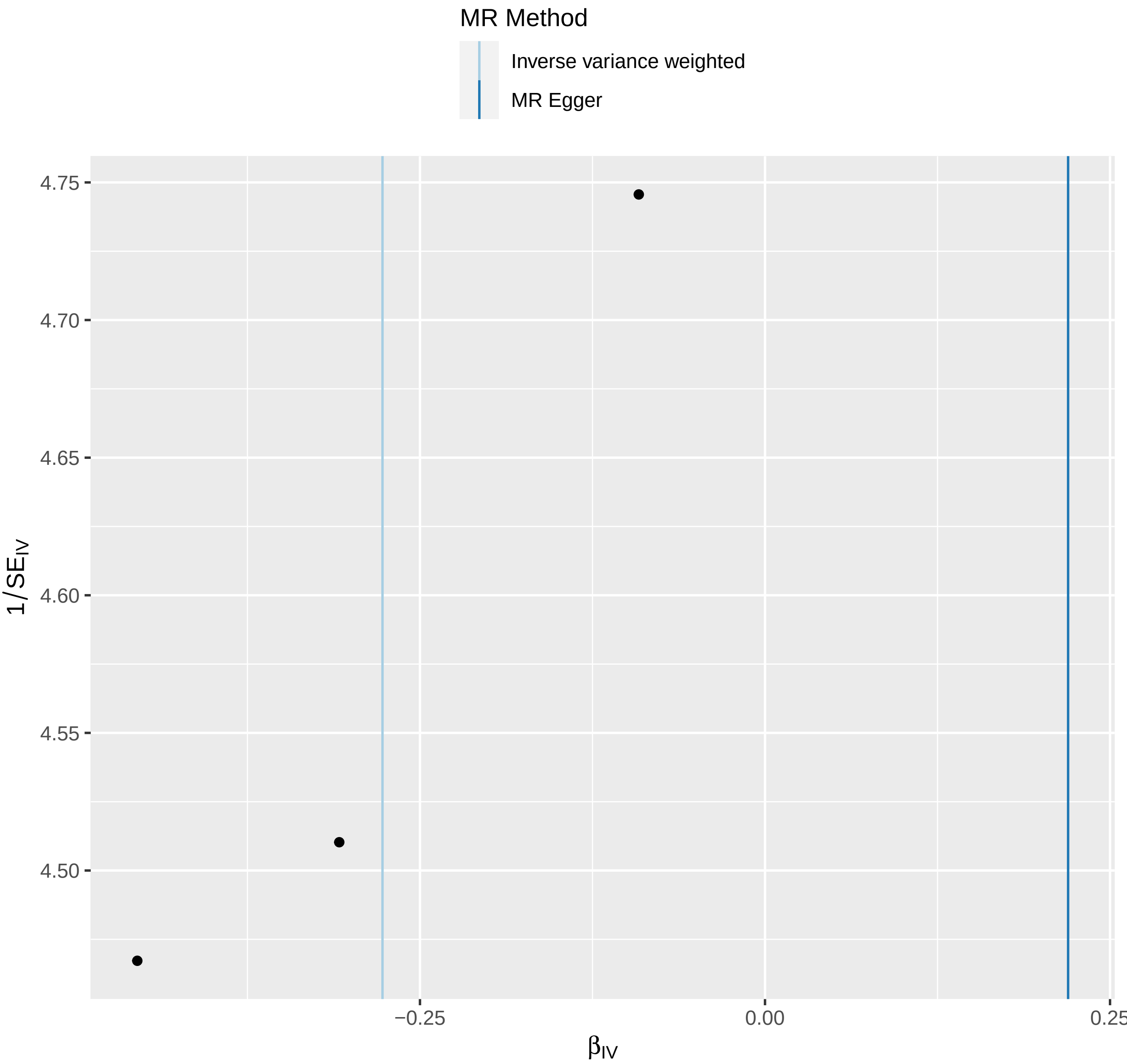

Batch 2−2378 : Gut microbiota abundance (genus Parasutterella id.2892) on Guanine deaminase
with a threshold of 1e−05

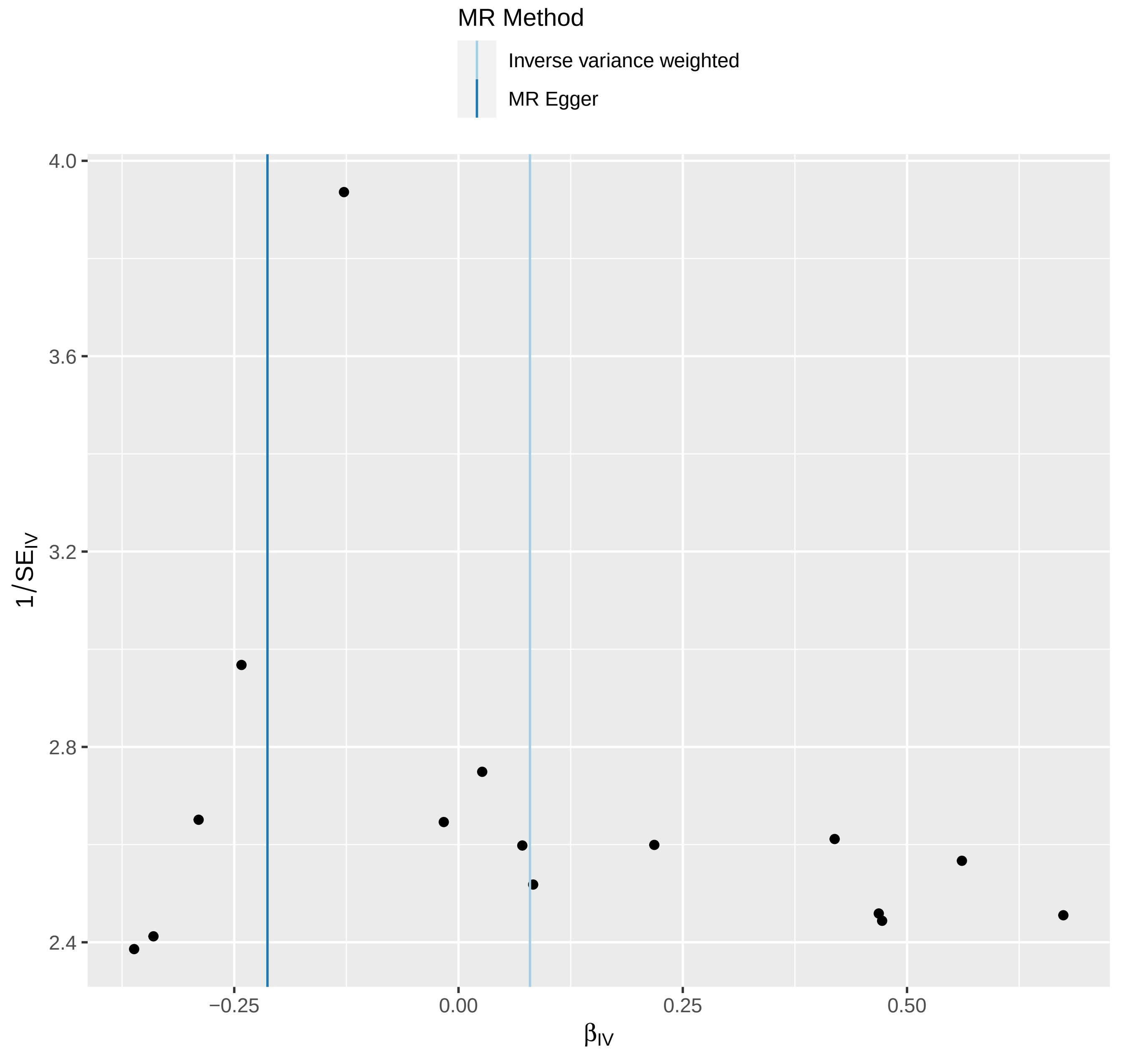

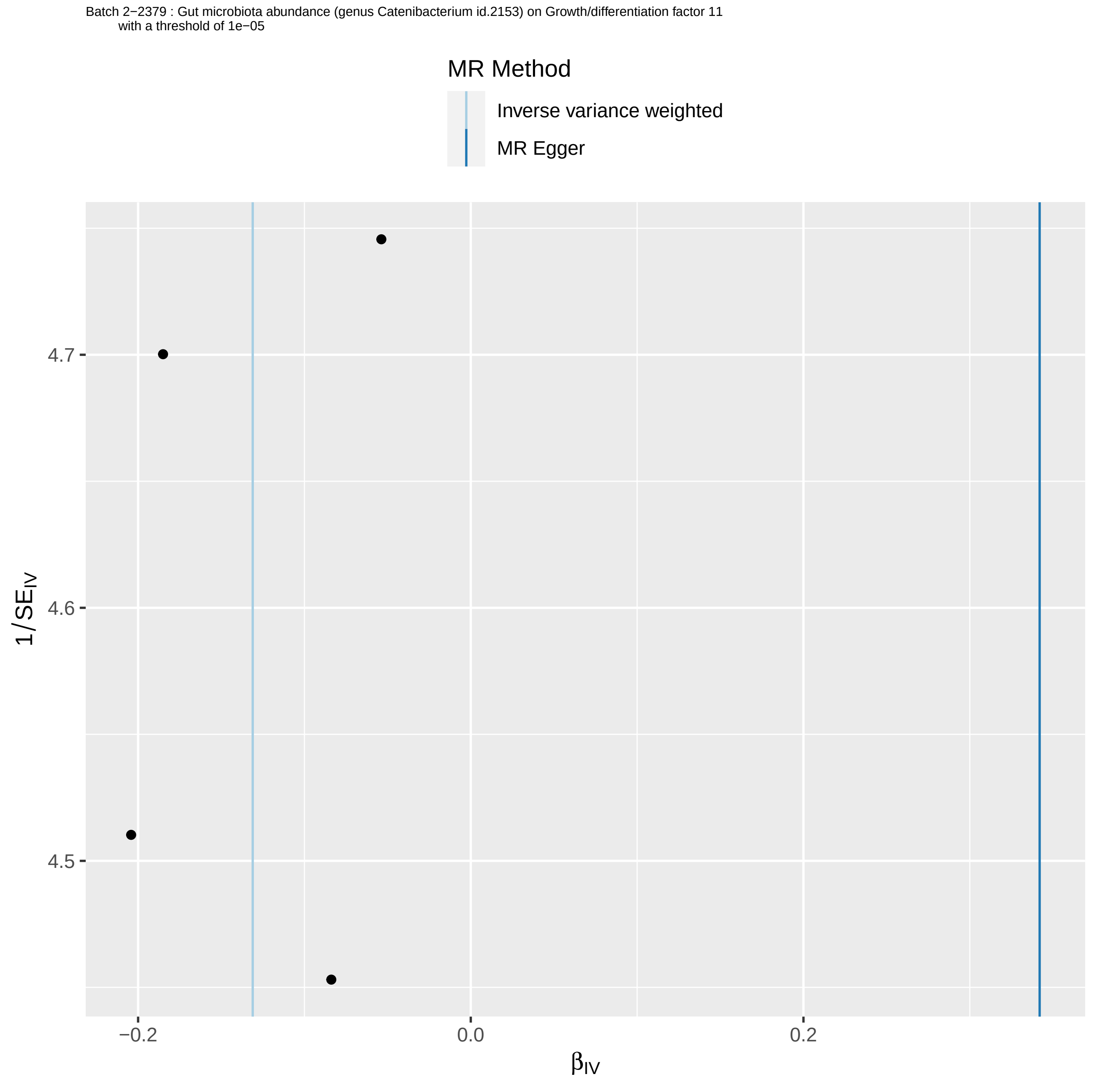

Batch 2−2379 : Gut microbiota abundance (genus Catenibacterium id.2153) on Growth/differentiation factor 11
with a threshold of 1e−05
MR Method
Inverse variance weighted
MR Egger
4.7
4.6
4.5
1/SE_IV
−0.2
0.0
0.2
β_IV

Batch 2−2380 : Gut microbiota abundance (genus Parasutterella id.2892) on Growth/differentiation factor 11
with a threshold of 1e−05

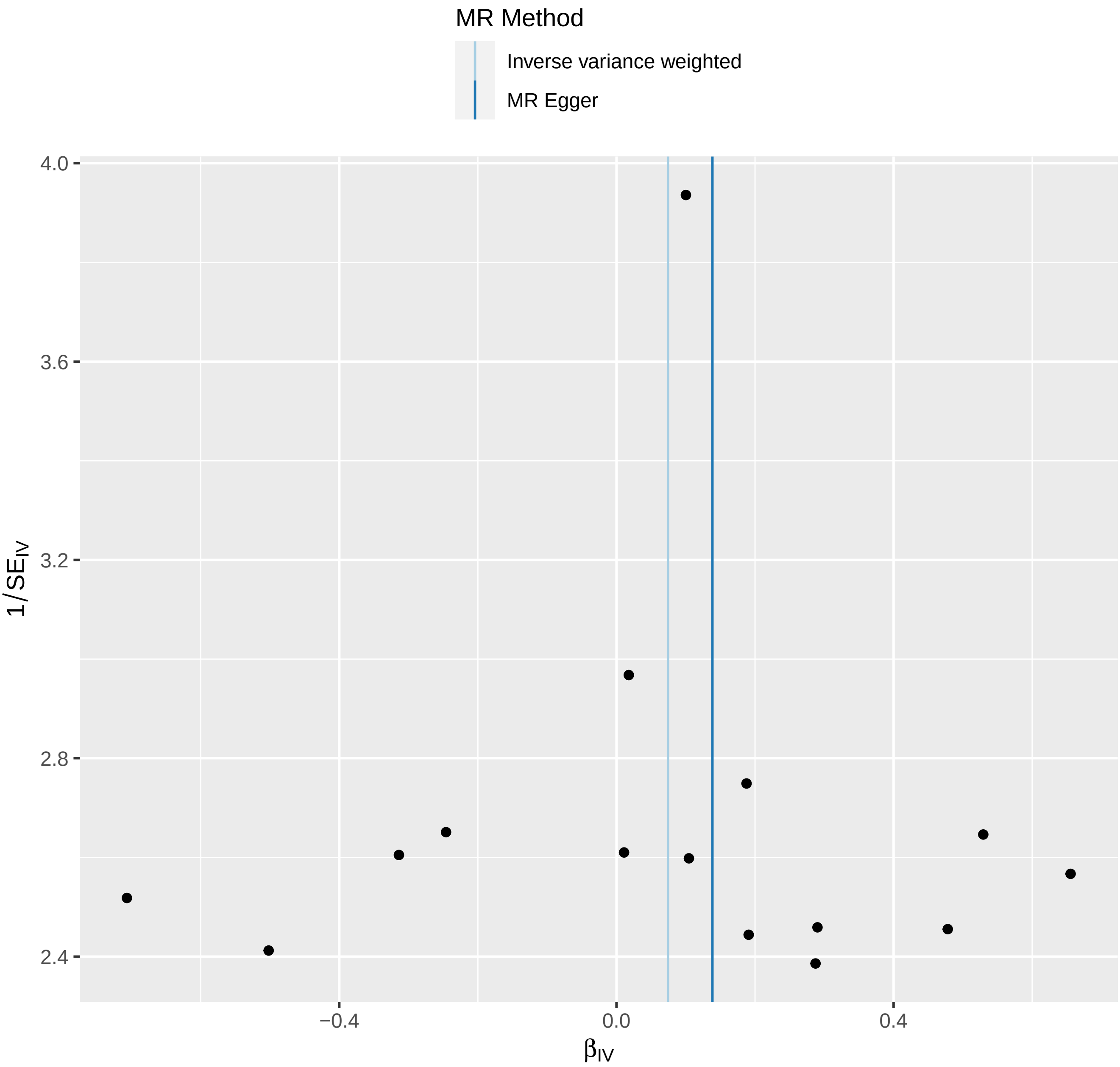

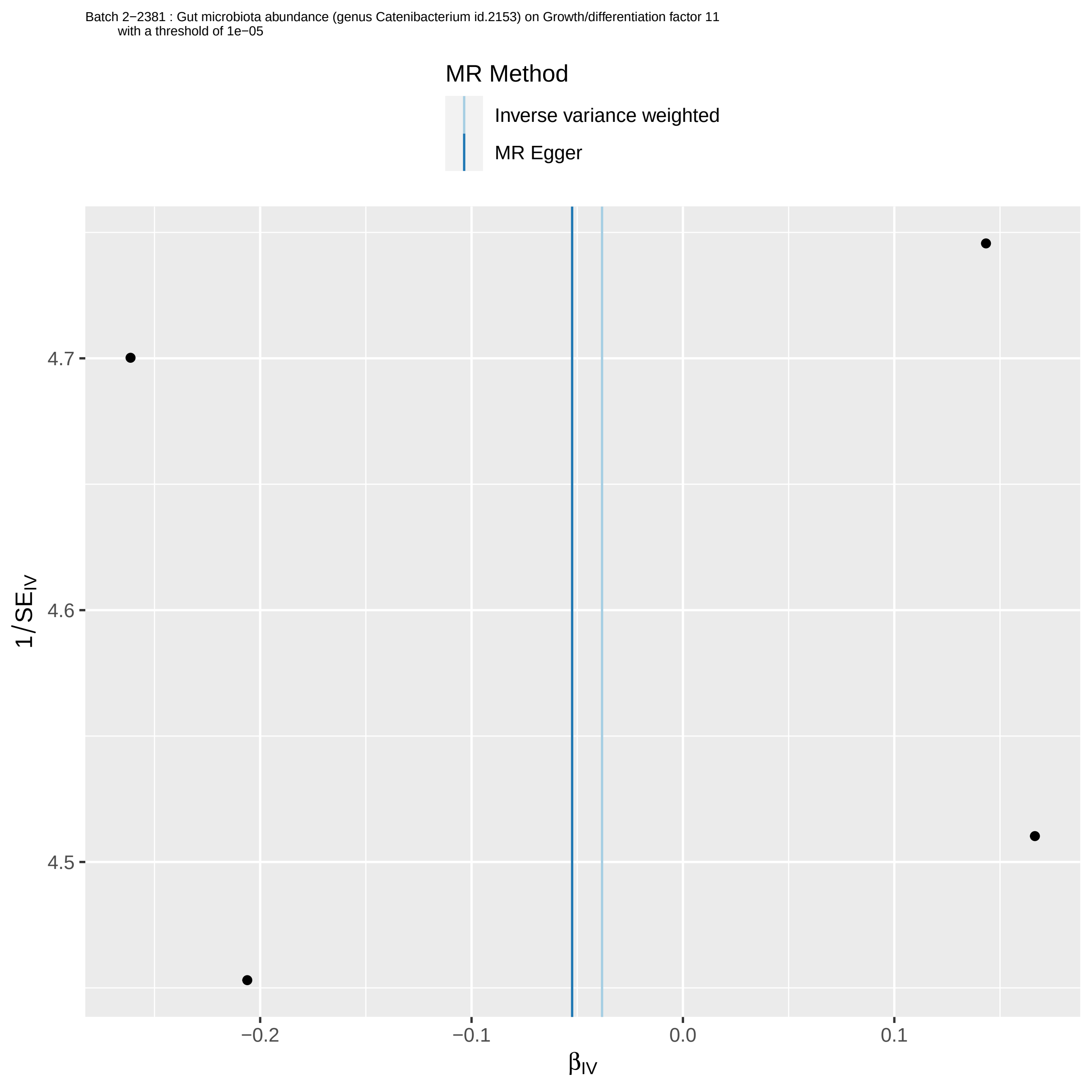
Batch 2−2381 : Gut microbiota abundance (genus Catenibacterium id.2153) on Growth/differentiation factor 11
with a threshold of 1e−05
MR Method
Inverse variance weighted
MR Egger
4.7
4.6
4.5
1/SE$_{IV}$
−0.2
−0.1
0.0
0.1
β$_{IV}$

Batch 2−2382 : Gut microbiota abundance (genus Parasutterella id.2892) on Growth/differentiation factor 11
with a threshold of 1e−05

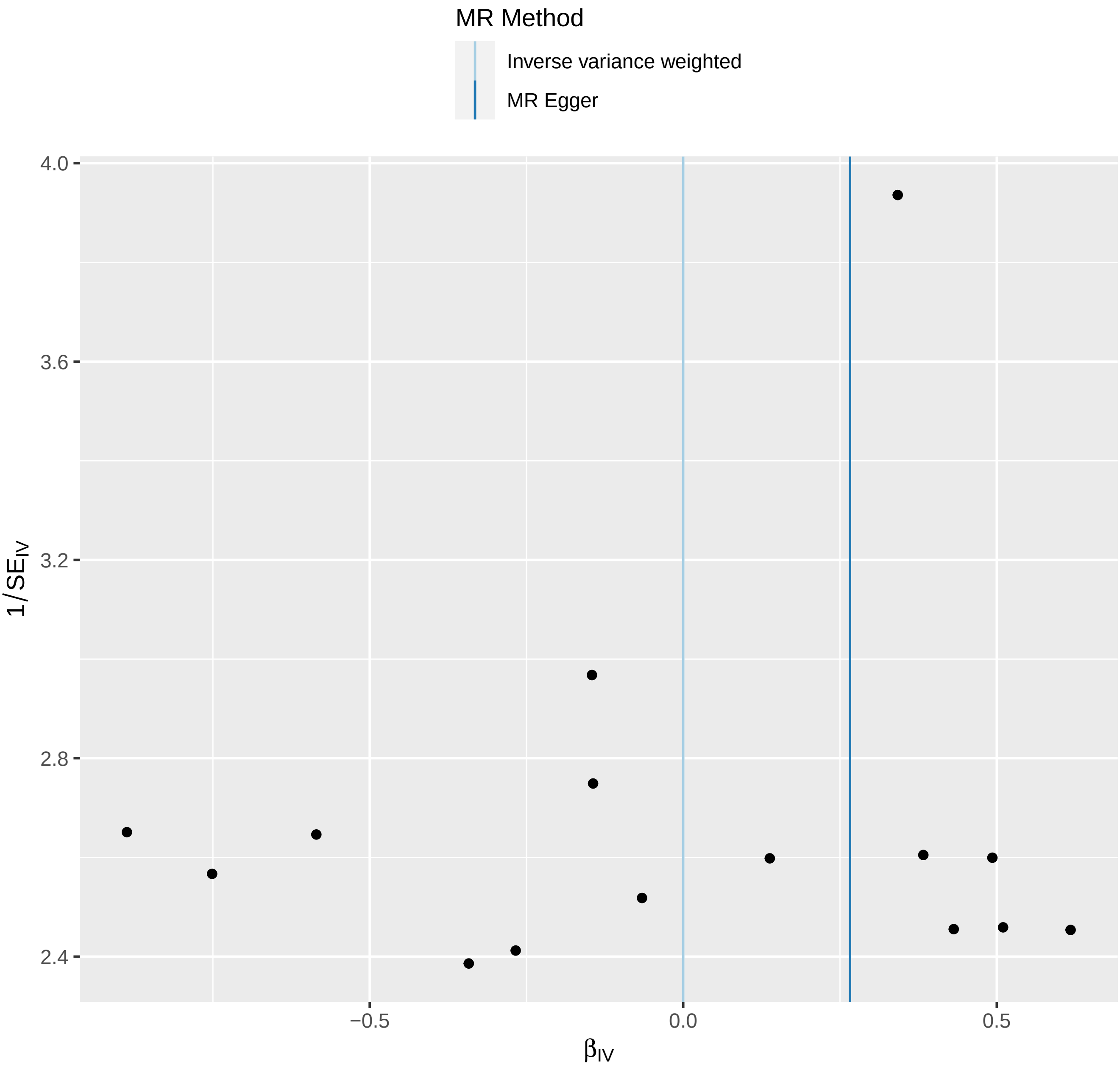

Batch 2−2383 : Gut microbiota abundance (genus Catenibacterium id.2153) on Growth/differentiation factor 11
with a threshold of 1e−05

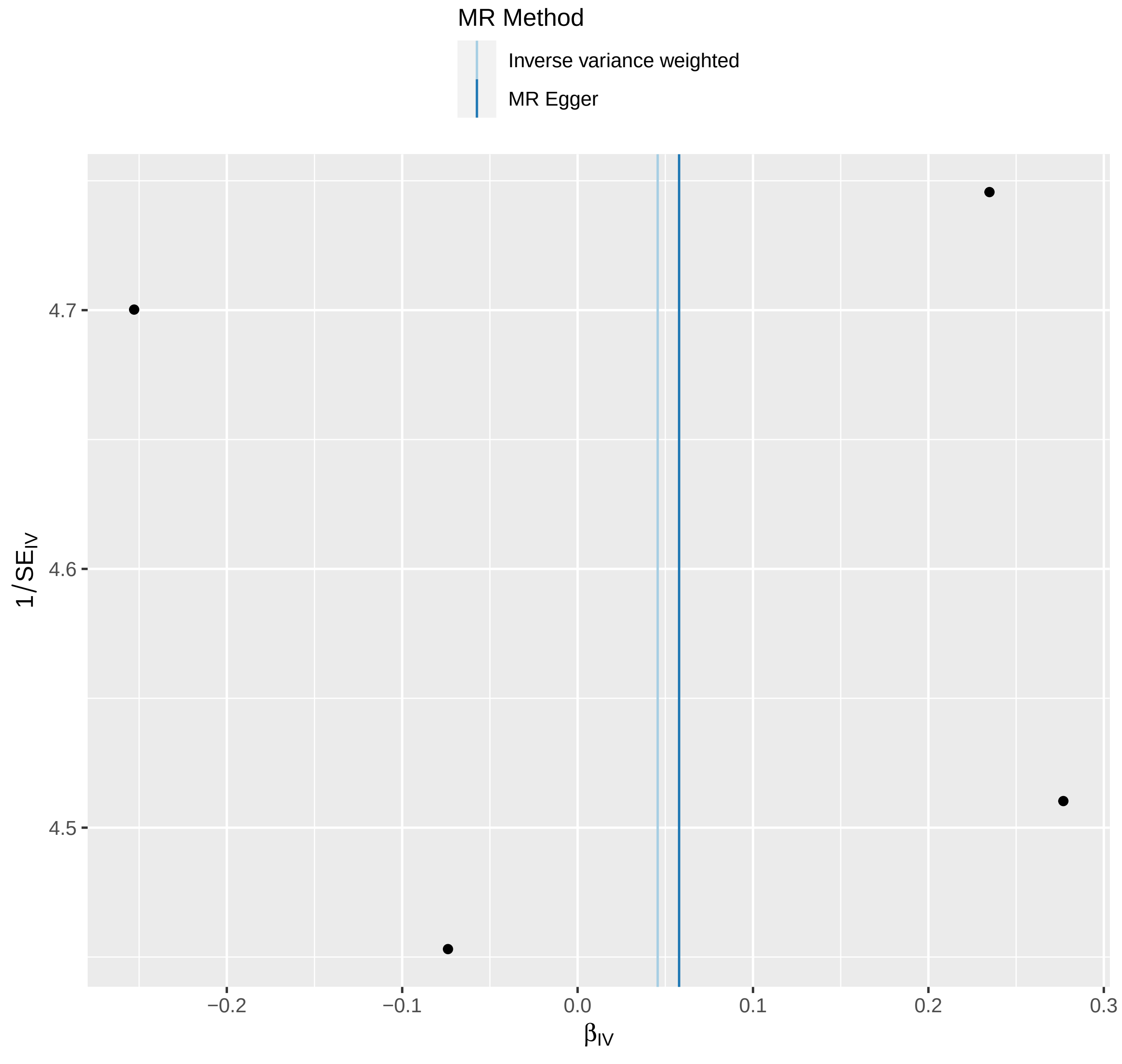

Batch 2−2384 : Gut microbiota abundance (genus Parasutterella id.2892) on Growth/differentiation factor 11
with a threshold of 1e−05

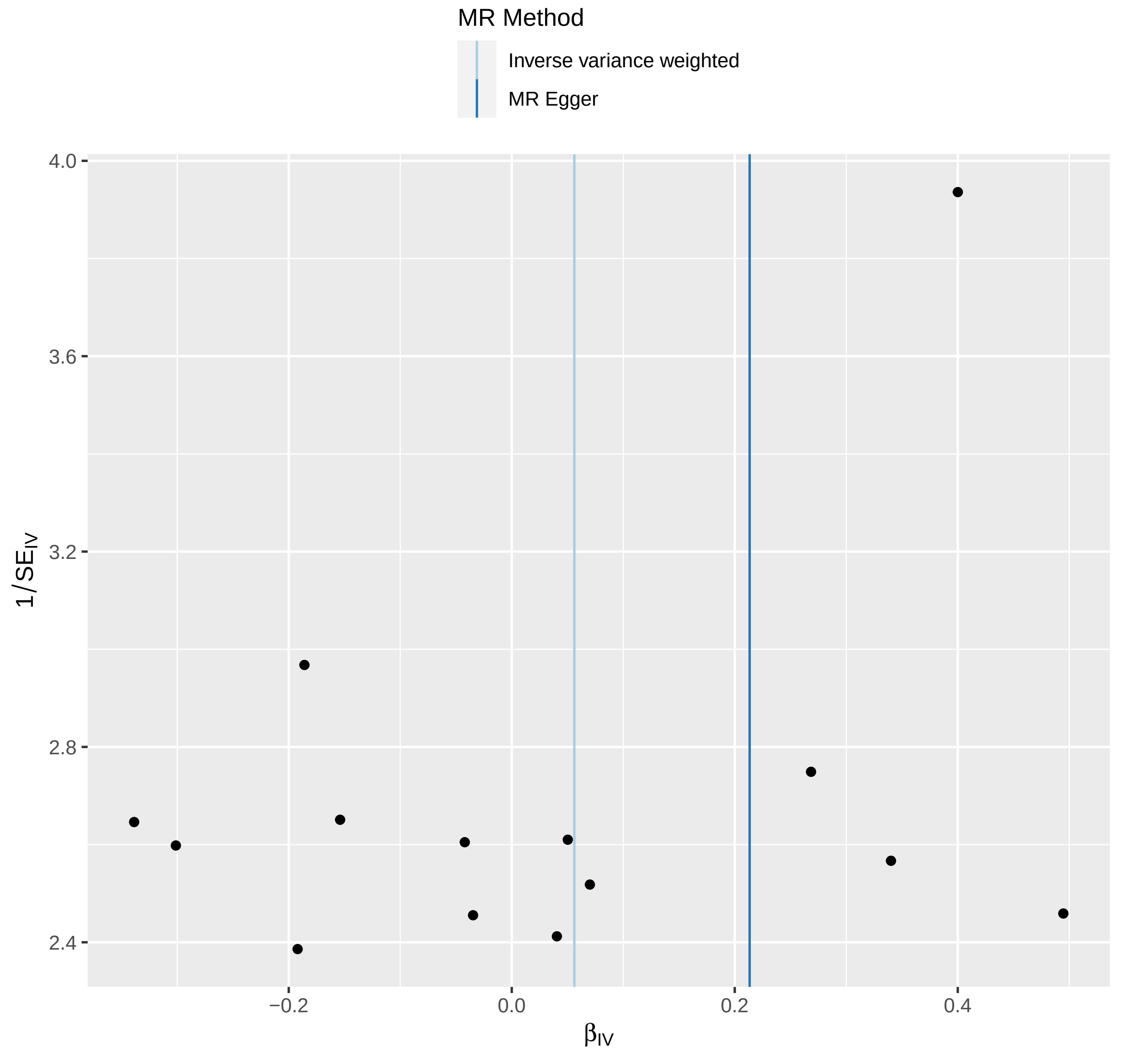

Batch 2−2385 : Gut microbiota abundance (genus Catenibacterium id.2153) on Growth/differentiation factor 11
with a threshold of 1e−05

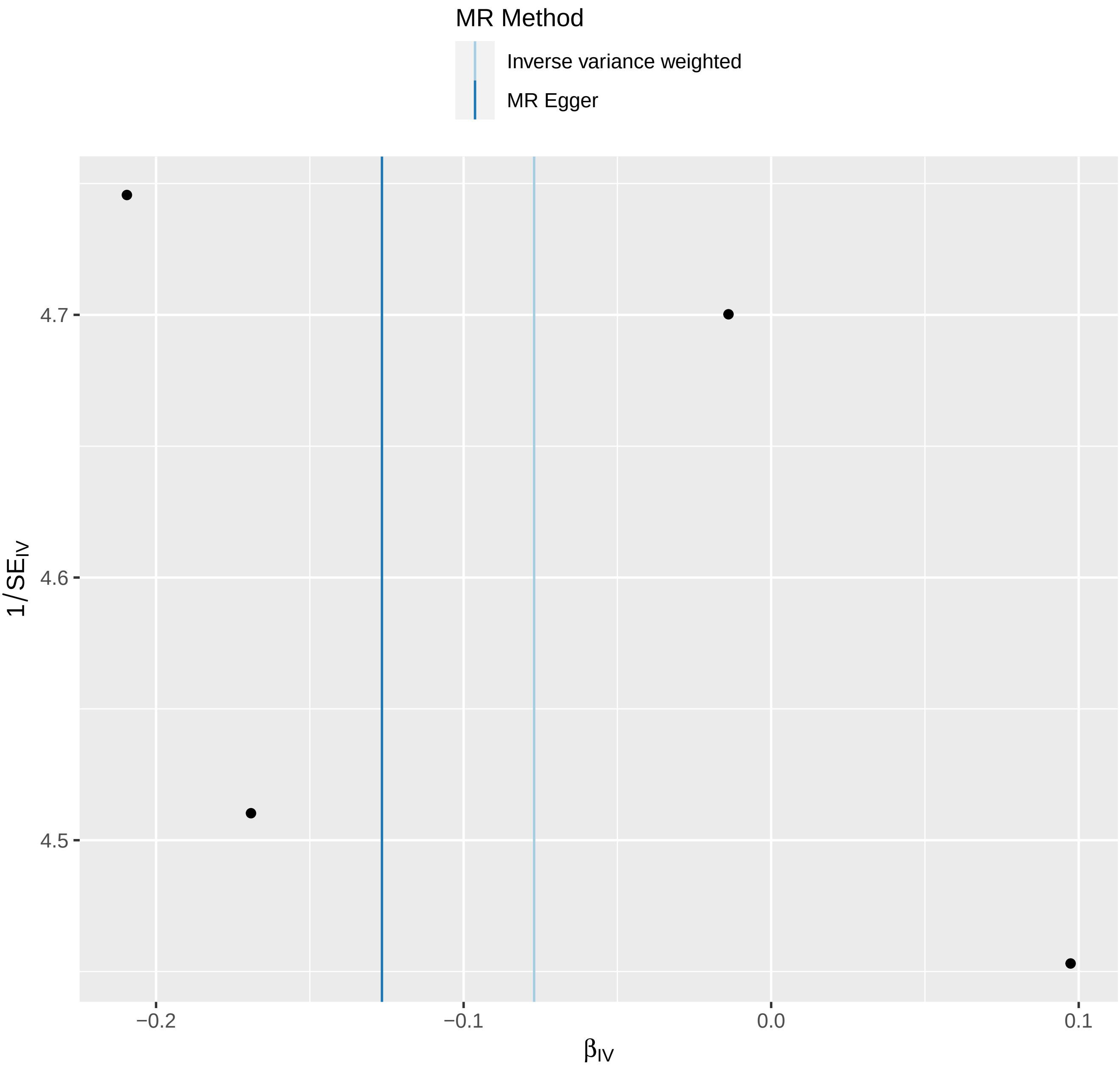

Batch 2−2386 : Gut microbiota abundance (genus Parasutterella id.2892) on Growth/differentiation factor 11
with a threshold of 1e−05

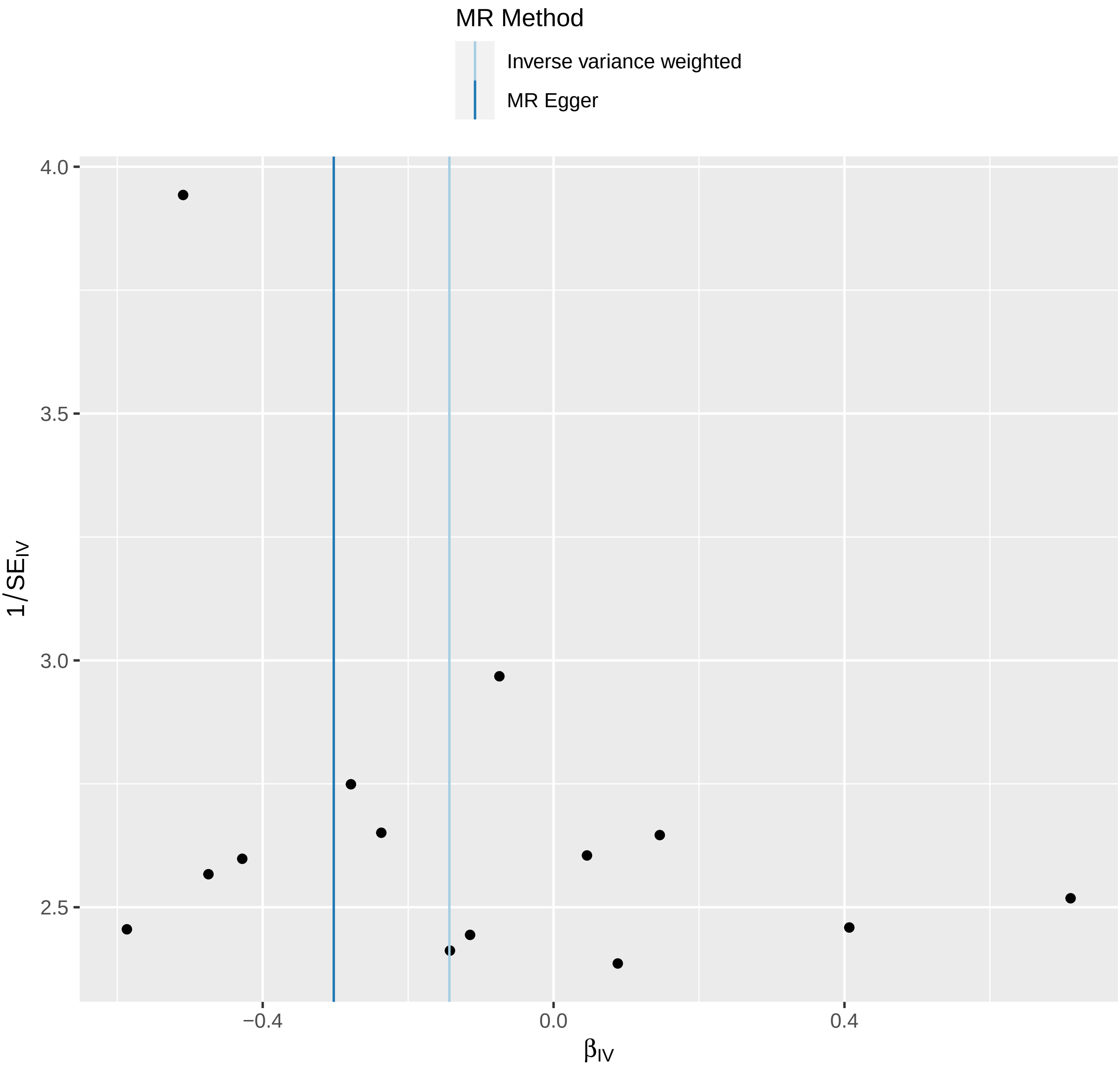

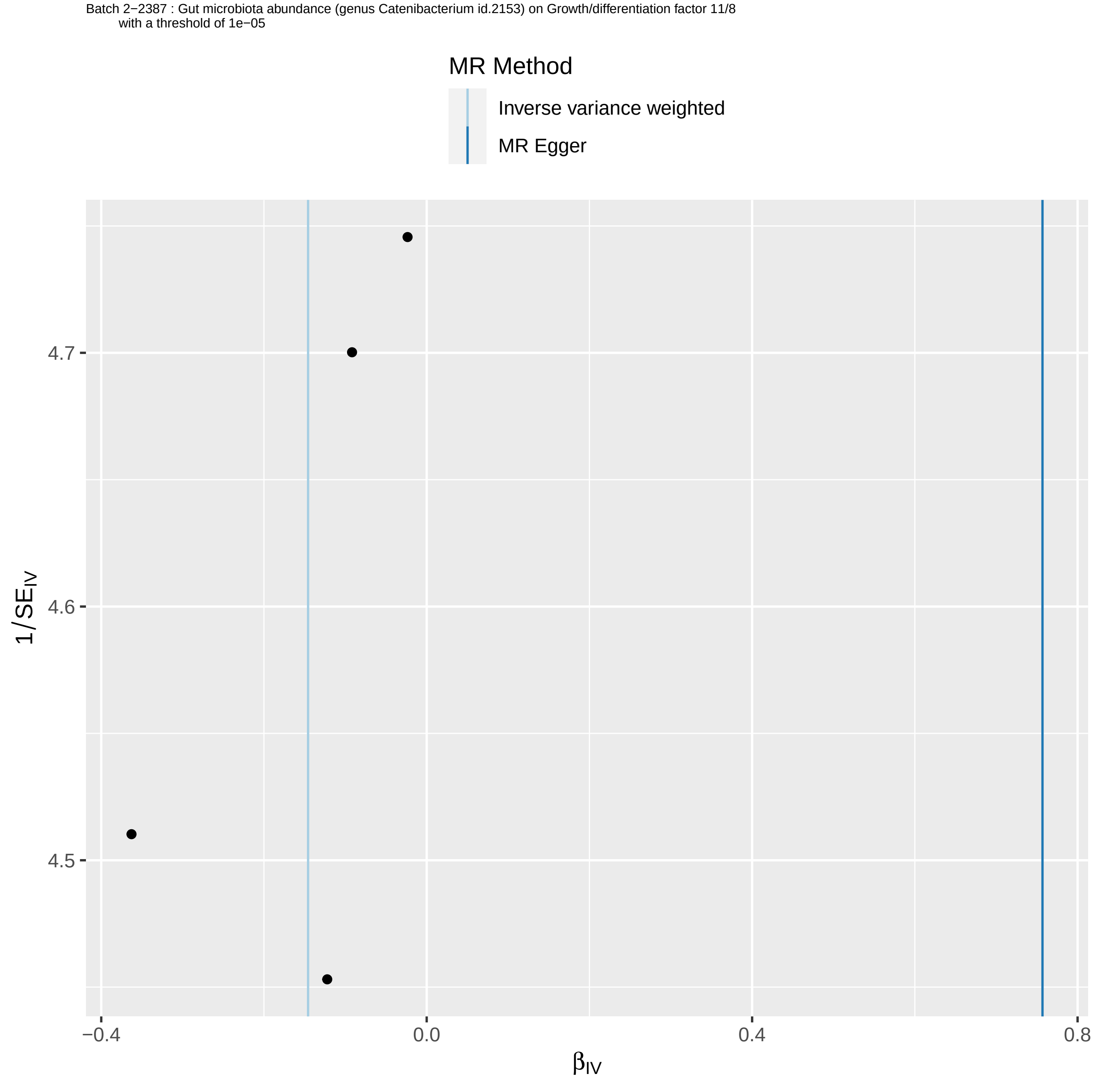

Batch 2−2387 : Gut microbiota abundance (genus Catenibacterium id.2153) on Growth/differentiation factor 11/8
with a threshold of 1e−05
MR Method
Inverse variance weighted
MR Egger
4.7
4.6
4.5
1/SE_IV
−0.4
0.0
0.4
0.8
β_IV

Batch 2−2388 : Gut microbiota abundance (genus Parasutterella id.2892) on Growth/differentiation factor 11/8
with a threshold of 1e−05

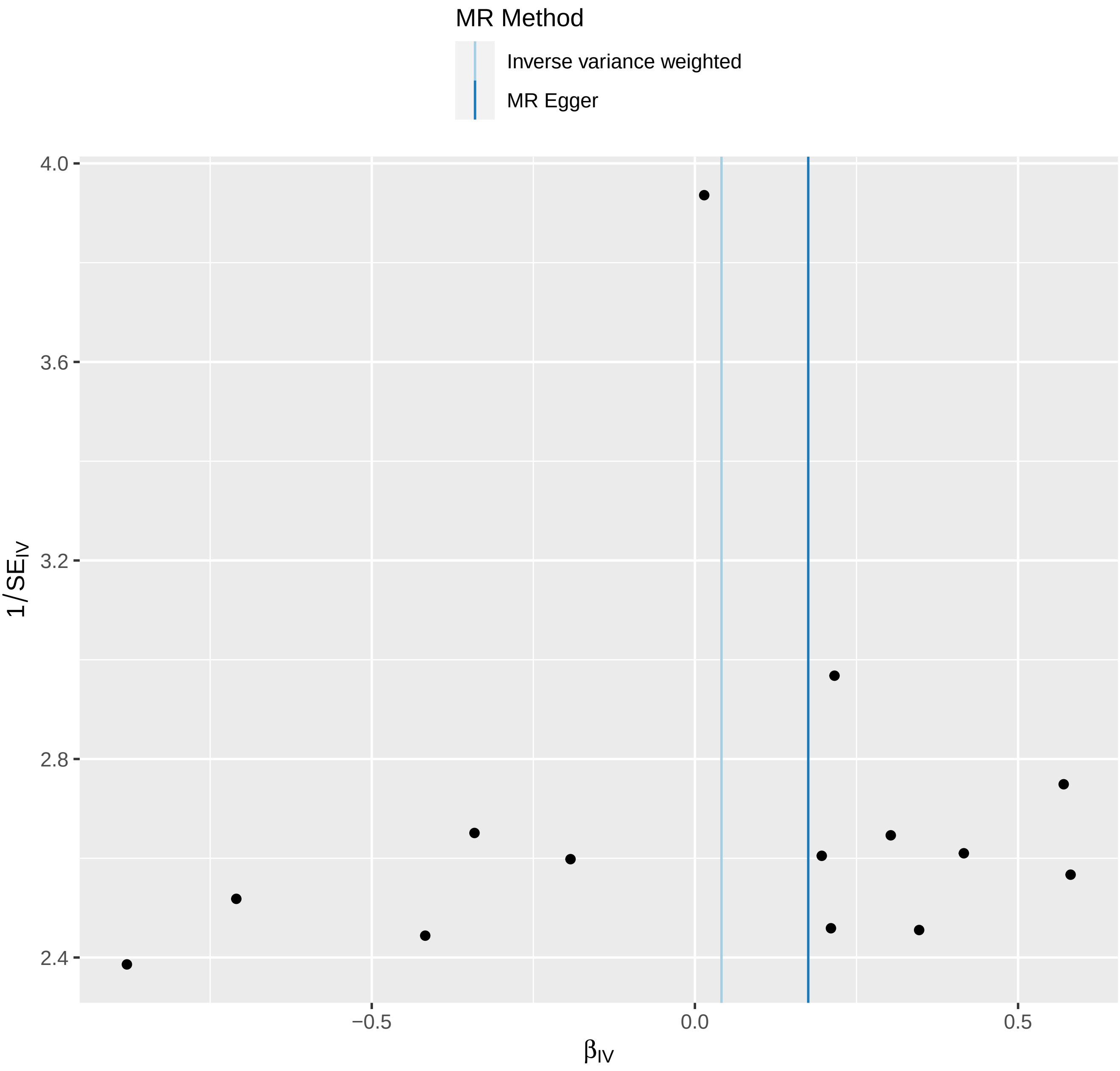

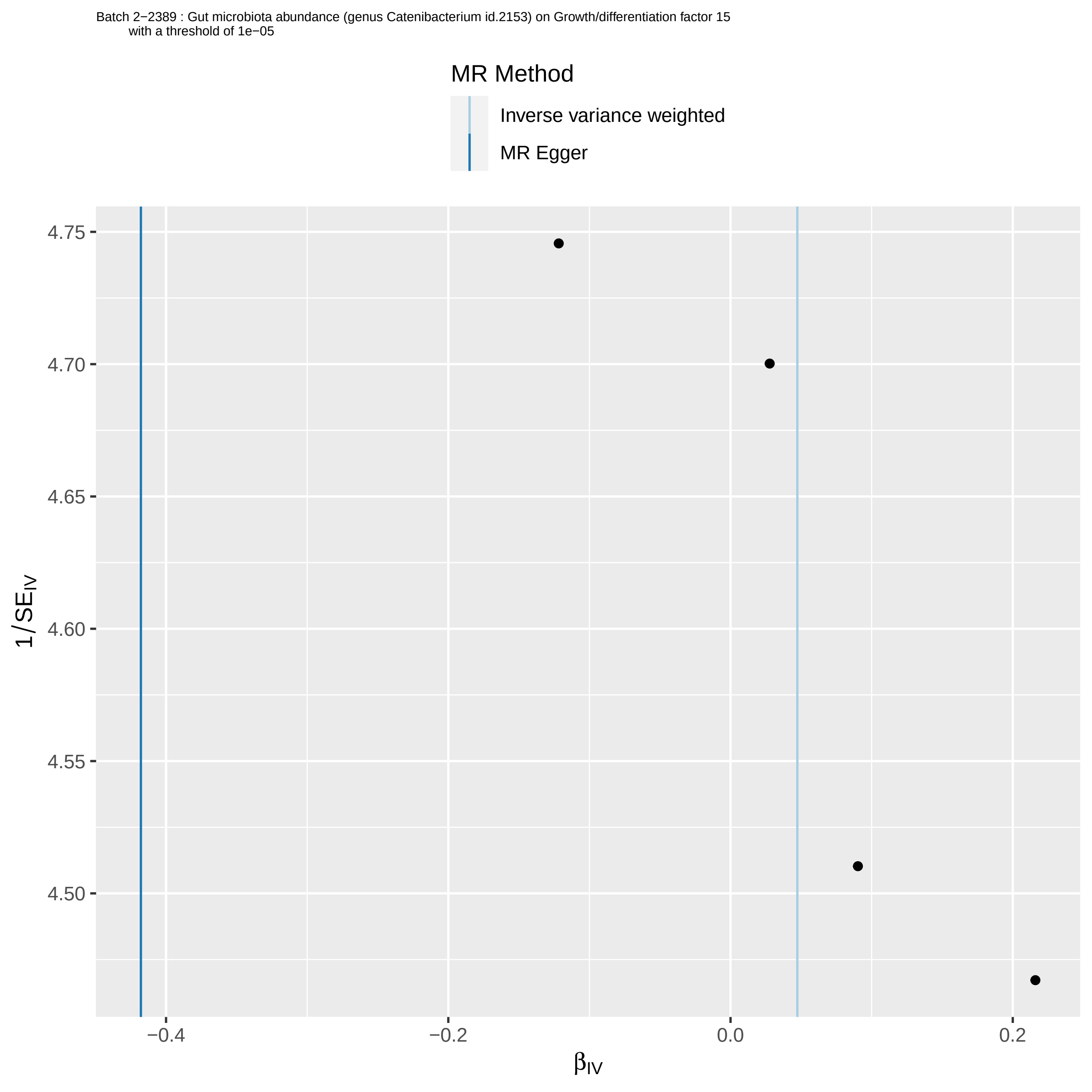
Batch 2−2389 : Gut microbiota abundance (genus Catenibacterium id.2153) on Growth/differentiation factor 15
with a threshold of 1e−05
MR Method
Inverse variance weighted
MR Egger
4.75
4.70
4.65
4.60
4.55
4.50
1/SE_IV
−0.4
−0.2
0.0
0.2
β_IV

Batch 2−2390 : Gut microbiota abundance (genus Parasutterella id.2892) on Growth/differentiation factor 15
with a threshold of 1e−05

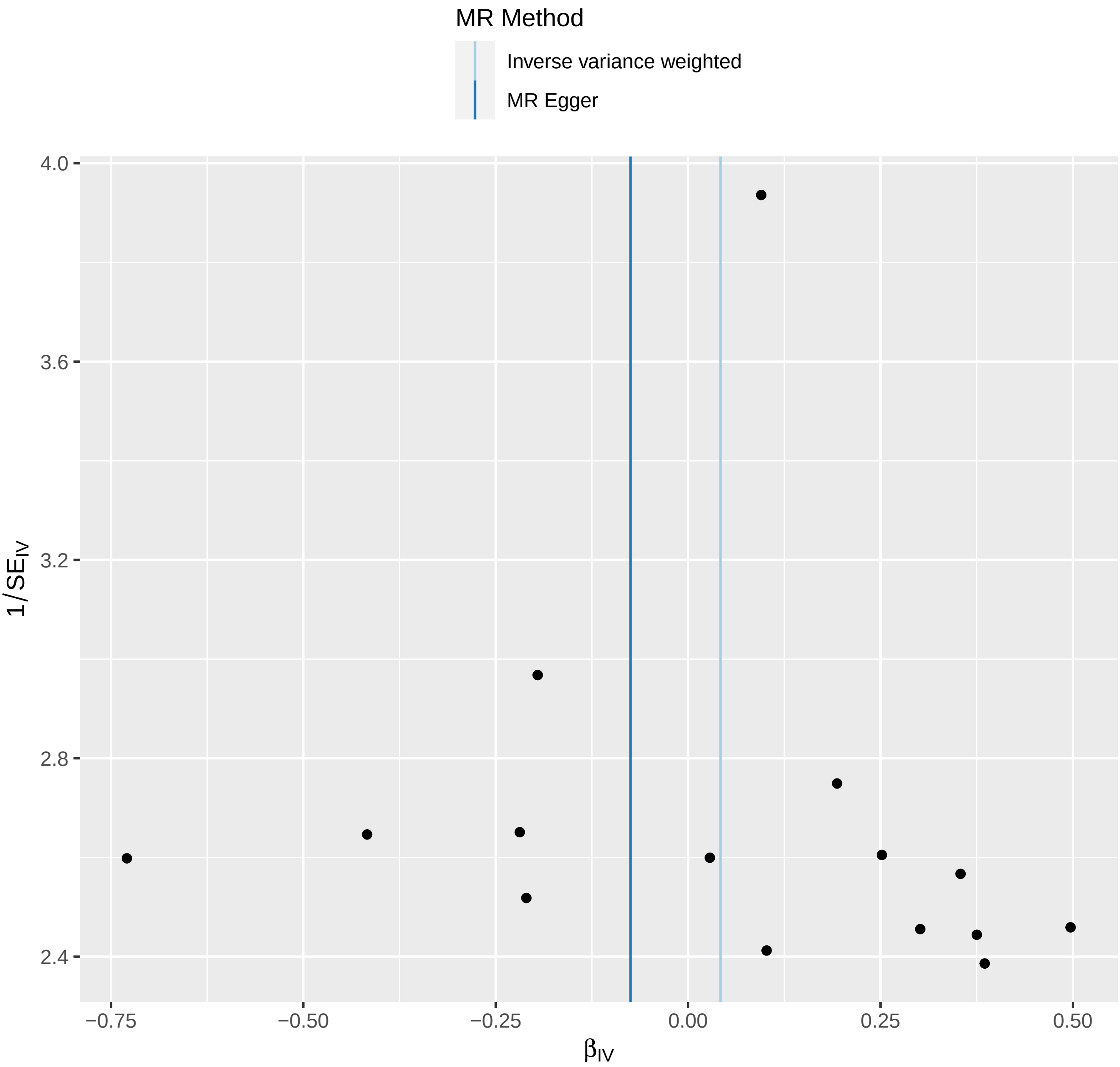

Batch 2−2391 : Gut microbiota abundance (genus Catenibacterium id.2153) on Growth/differentiation factor 5
with a threshold of 1e−05

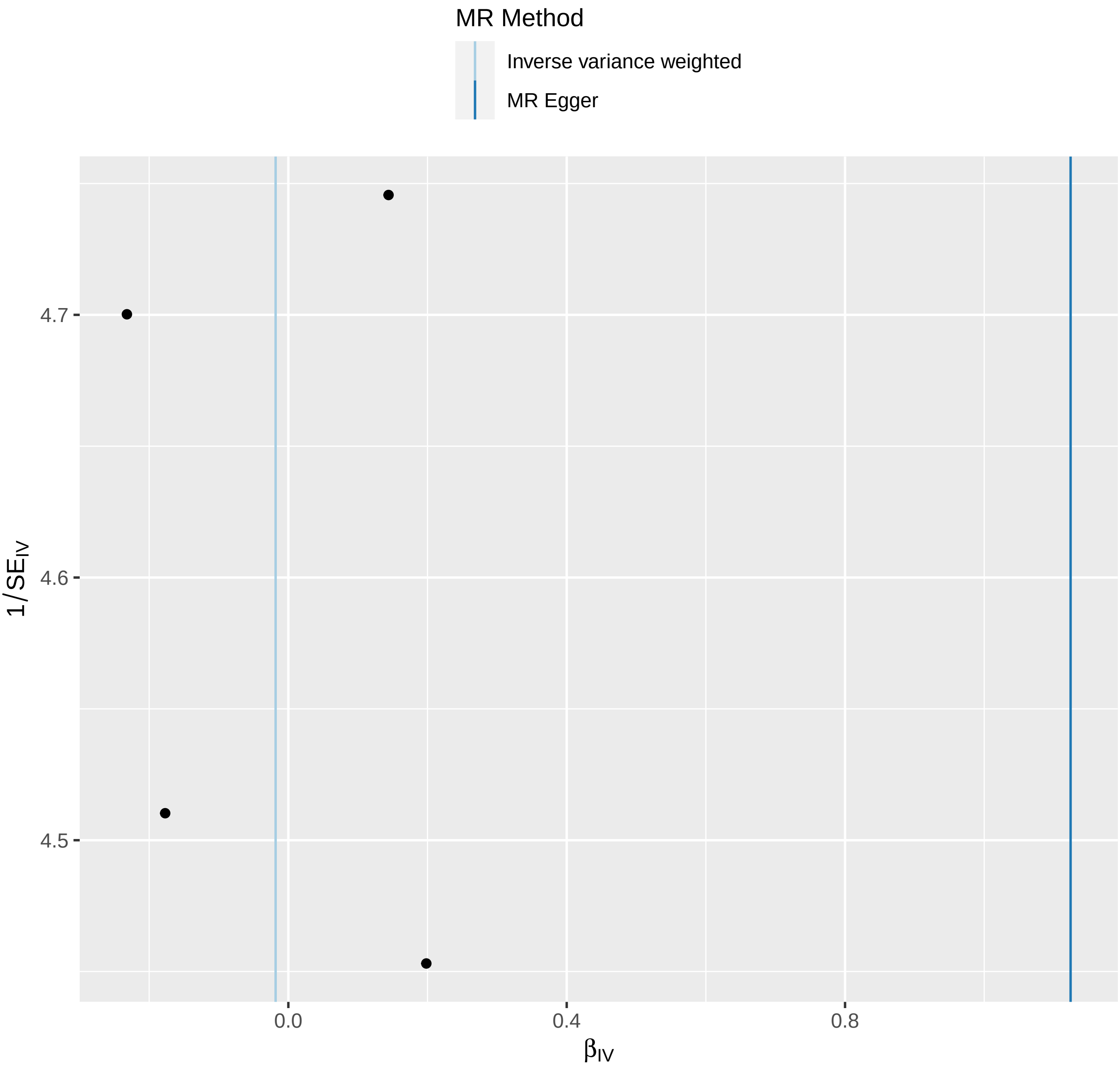

Batch 2−2392 : Gut microbiota abundance (genus Parasutterella id.2892) on Growth/differentiation factor 5
with a threshold of 1e−05

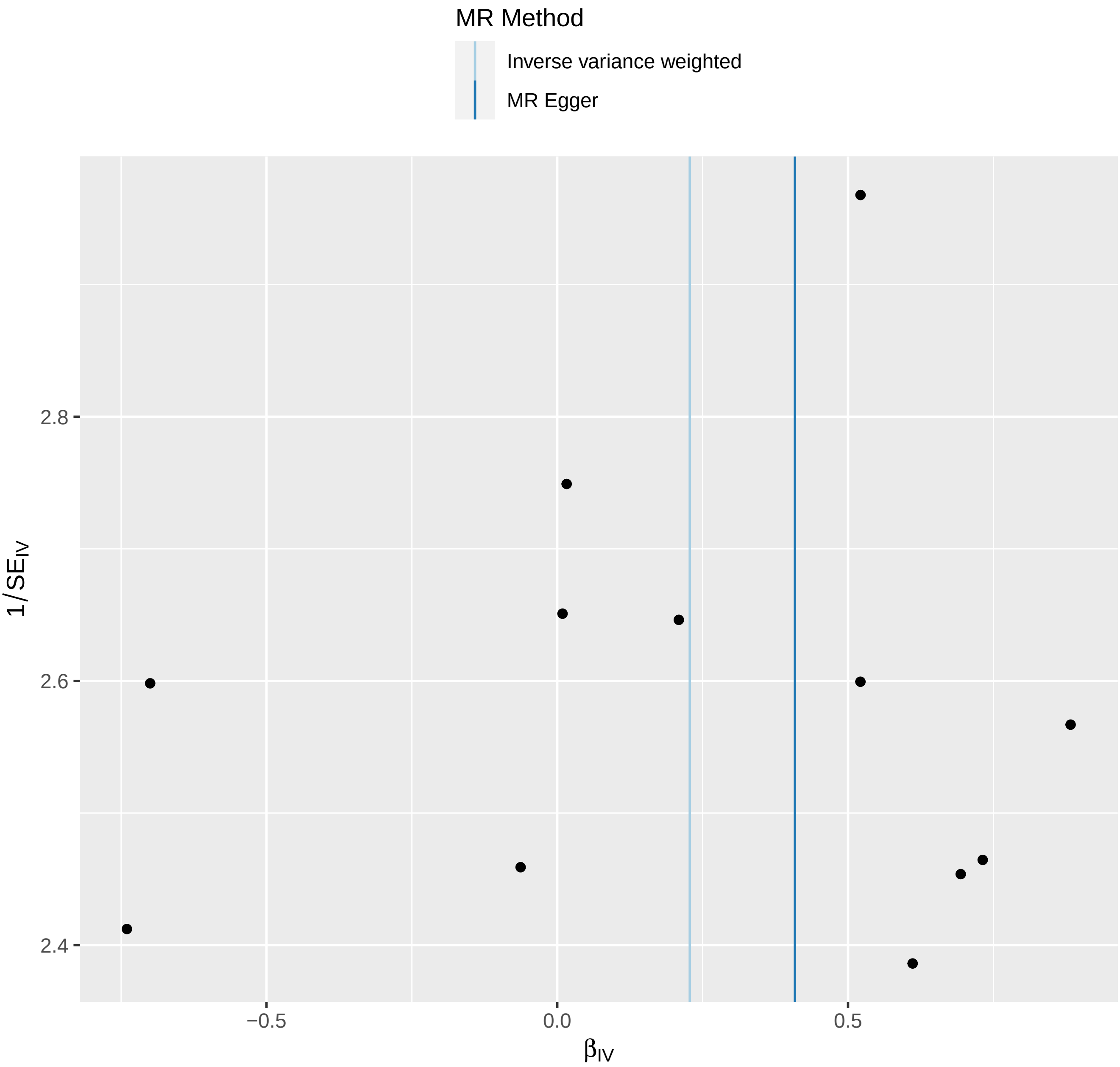

Batch 2−2393 : Gut microbiota abundance (genus Catenibacterium id.2153) on Growth/differentiation factor 9
with a threshold of 1e−05

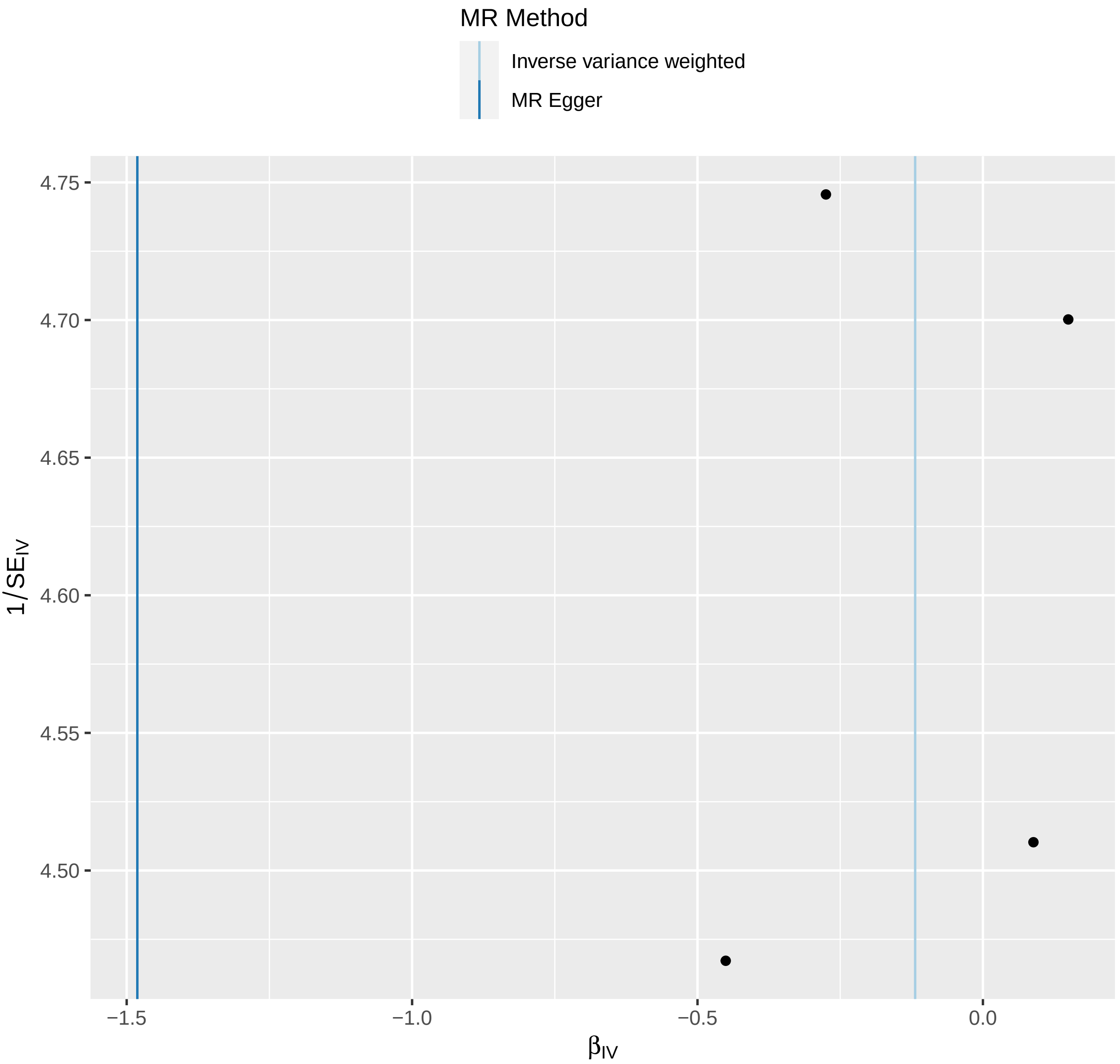

Batch 2−2394 : Gut microbiota abundance (genus Parasutterella id.2892) on Growth/differentiation factor 9
with a threshold of 1e−05

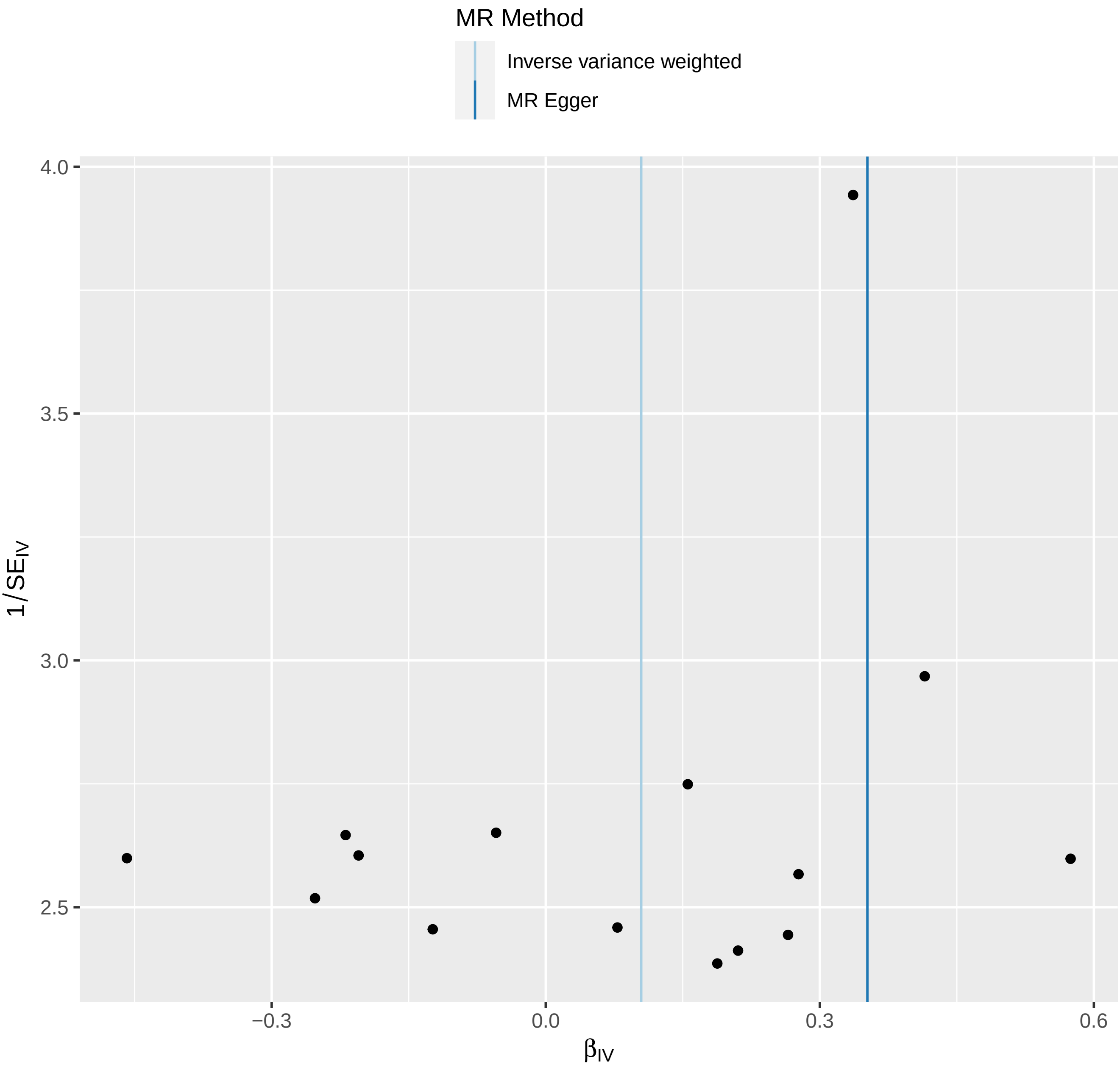

Batch 2−2395 : Gut microbiota abundance (genus Catenibacterium id.2153) on Glial cell line−derived neurotrophic factor with a threshold of 1e−05

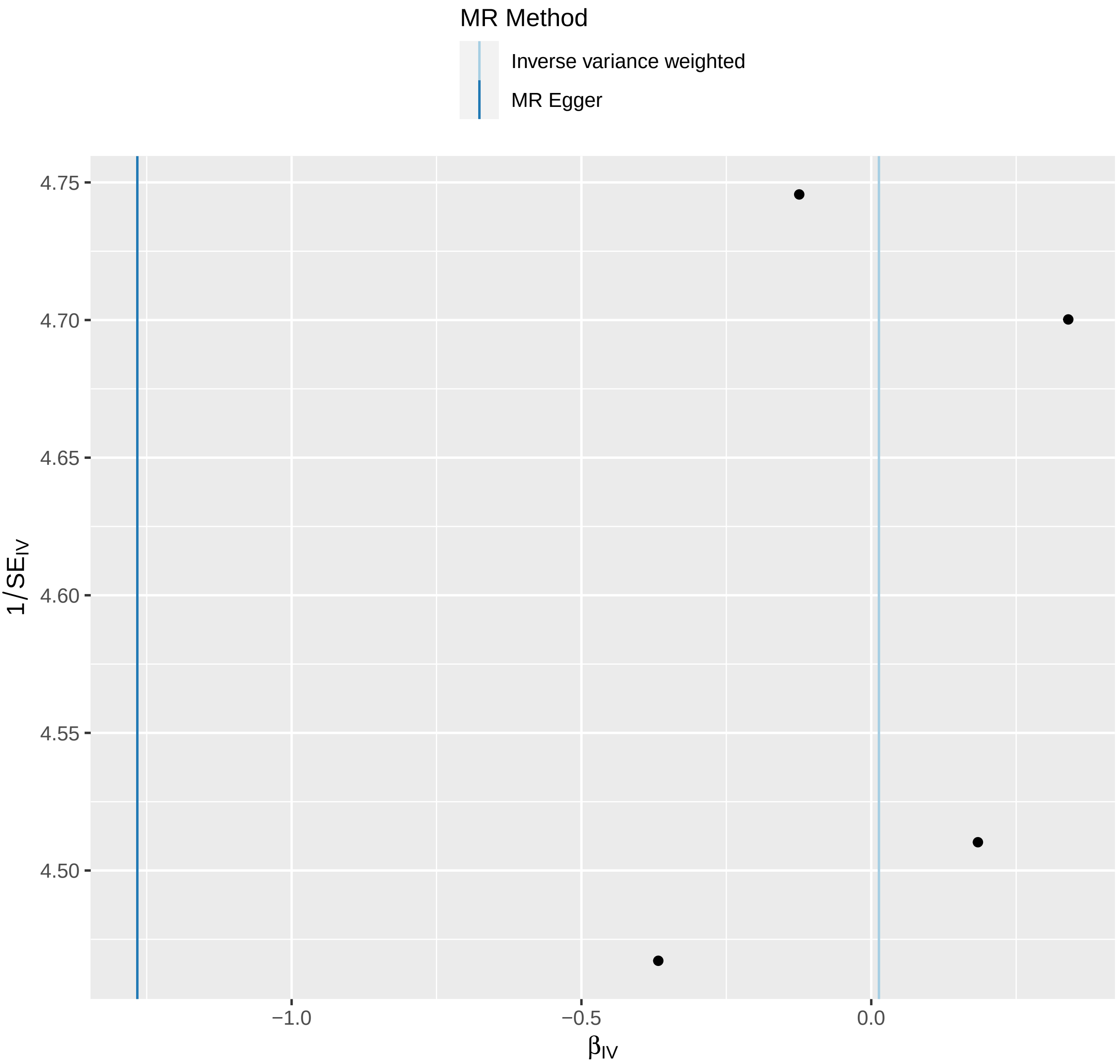

Batch 2−2396 : Gut microbiota abundance (genus Parasutterella id.2892) on Glial cell line−derived neurotrophic factor
with a threshold of 1e−05

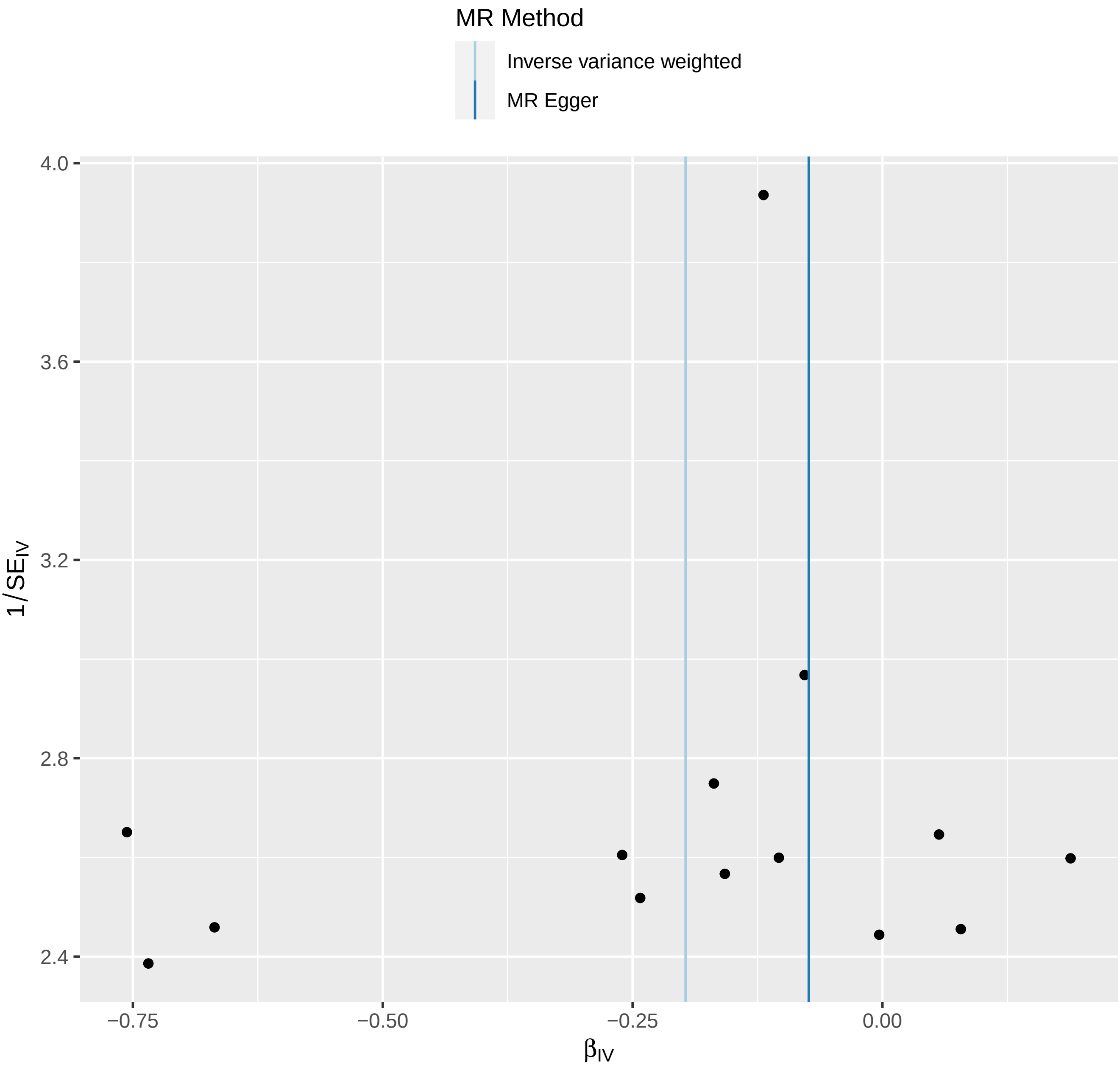

Batch 2−2397 : Gut microbiota abundance (genus Catenibacterium id.2153) on GTP−binding protein GEM
with a threshold of 1e−05

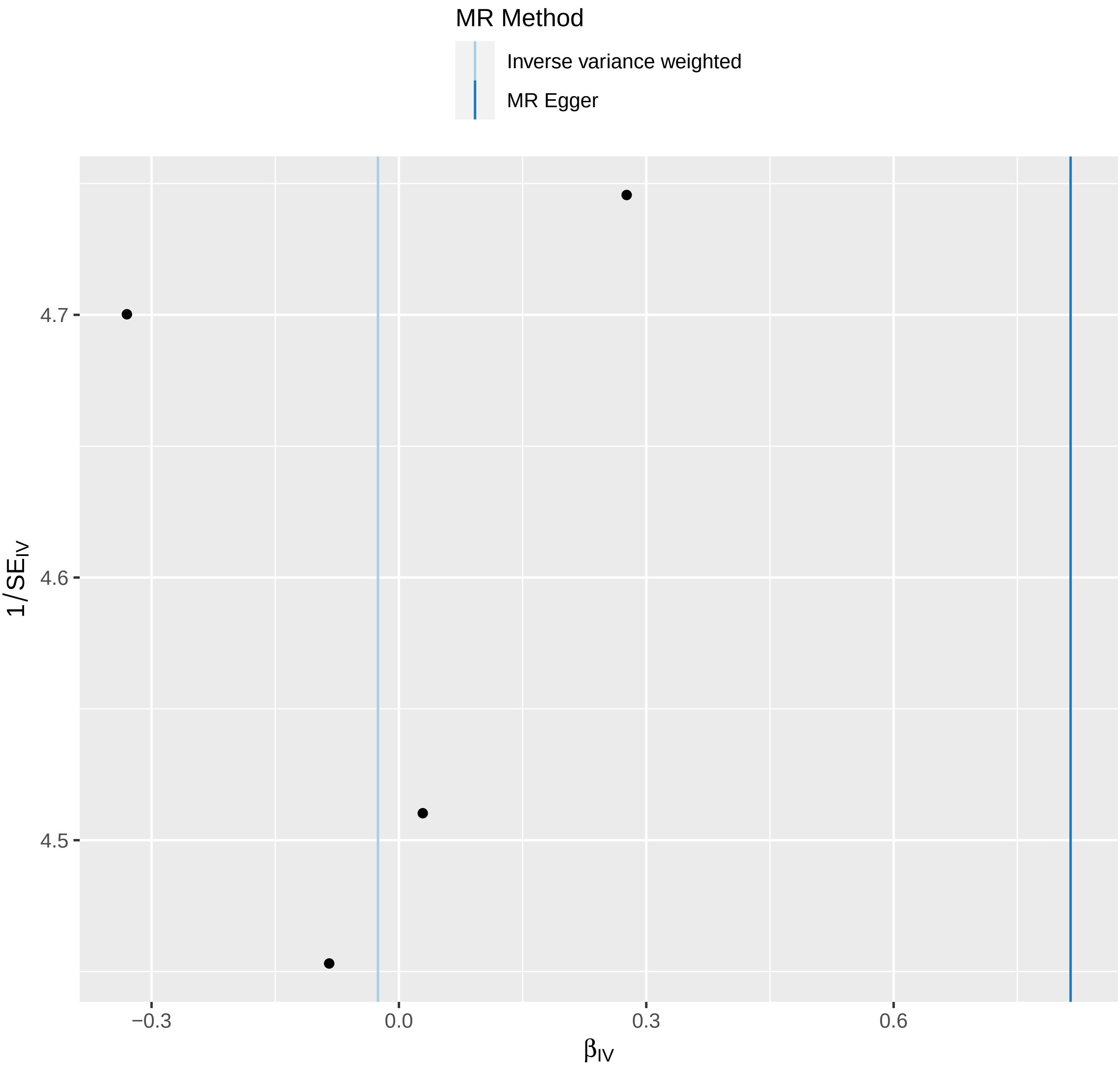

Batch 2−2398 : Gut microbiota abundance (genus Parasutterella id.2892) on GTP−binding protein GEM
with a threshold of 1e−05

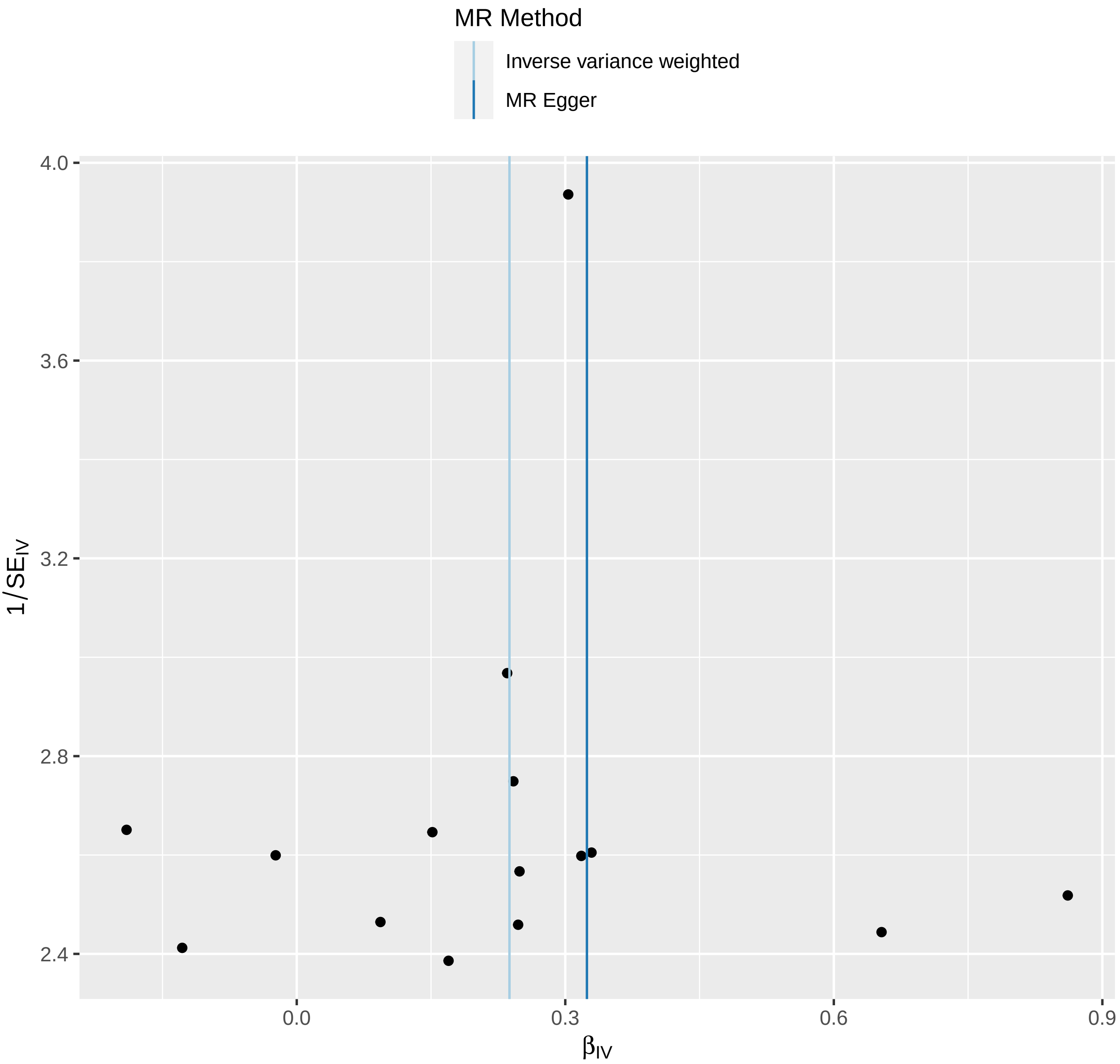

Batch 2−2399 : Gut microbiota abundance (genus Catenibacterium id.2153) on Glial fibrillary acidic protein
with a threshold of 1e−05

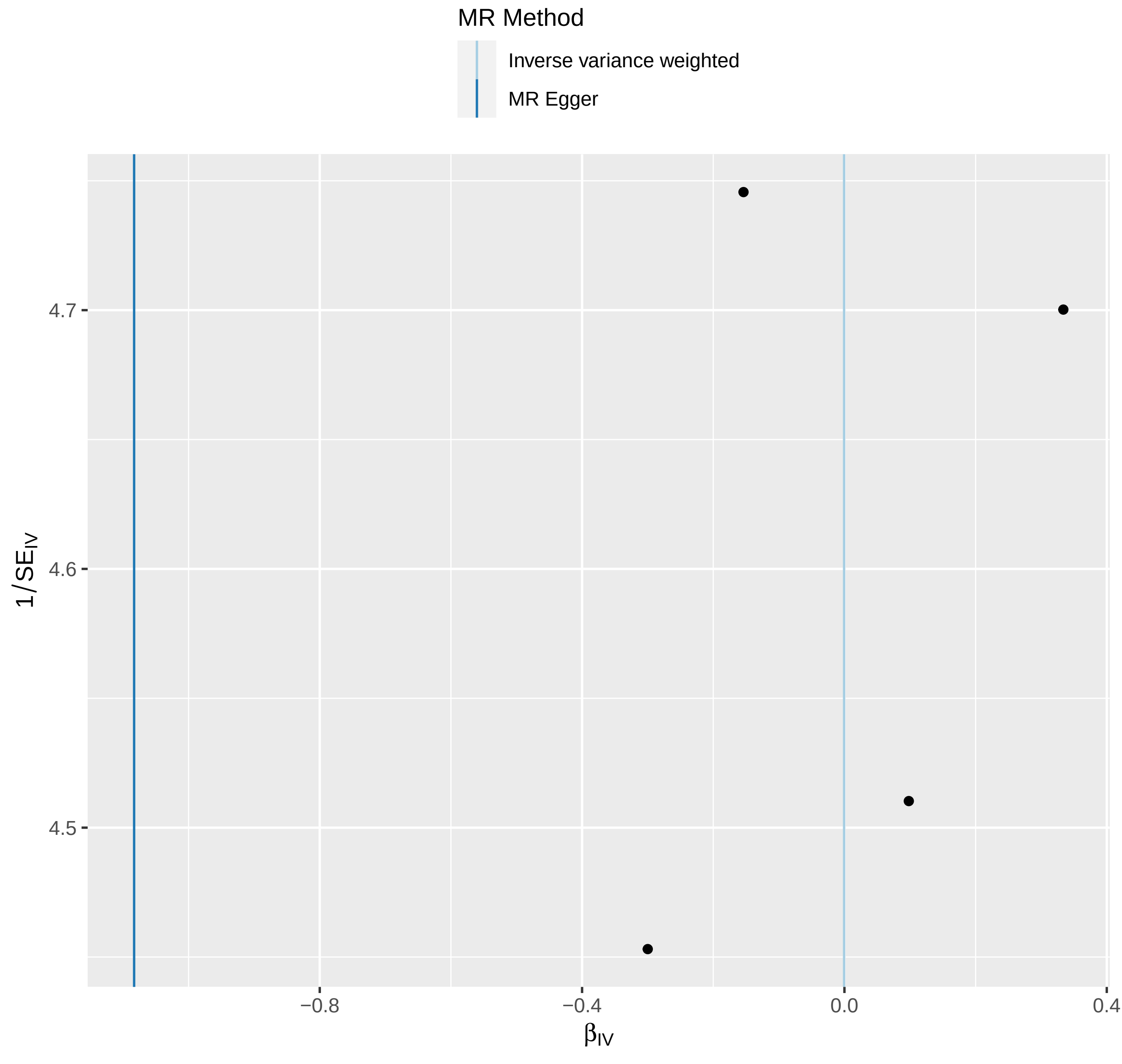

Batch 2−2400 : Gut microbiota abundance (genus Parasutterella id.2892) on Glial fibrillary acidic protein
with a threshold of 1e−05

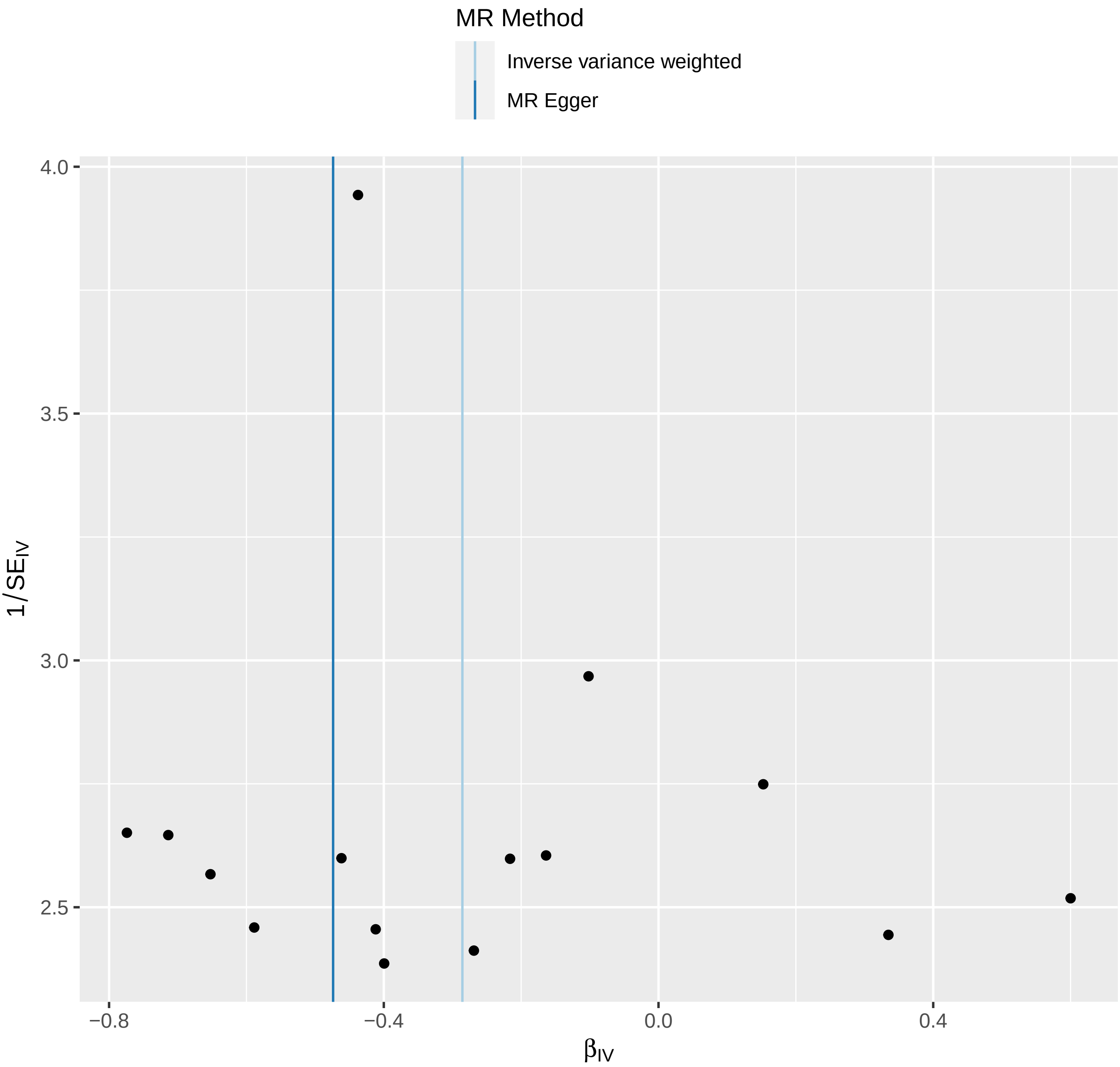

Batch 2−2401 : Gut microbiota abundance (genus Catenibacterium id.2153) on GDNF family receptor alpha−1
with a threshold of 1e−05

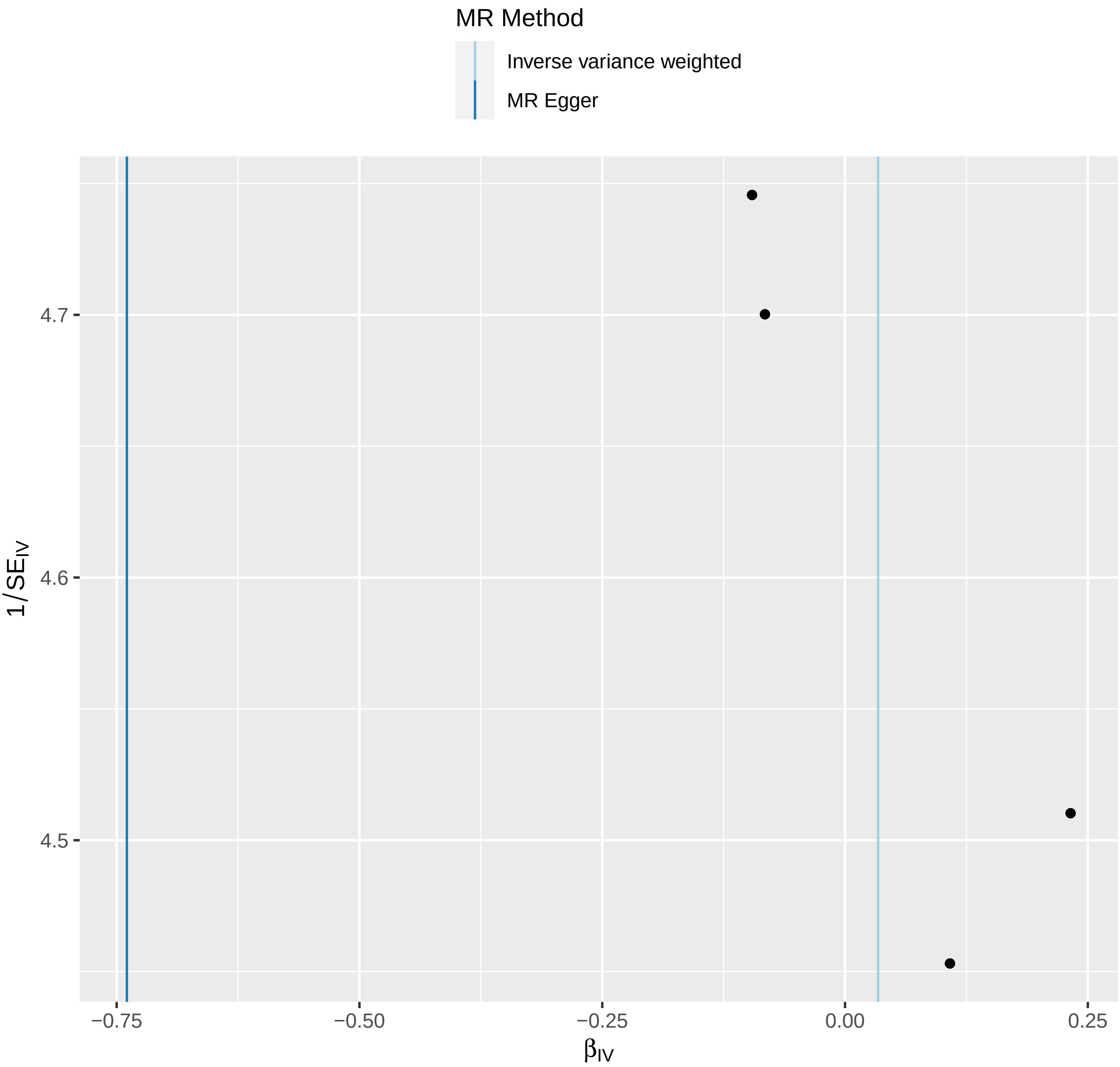

Batch 2−2402 : Gut microbiota abundance (genus Parasutterella id.2892) on GDNF family receptor alpha−1
with a threshold of 1e−05

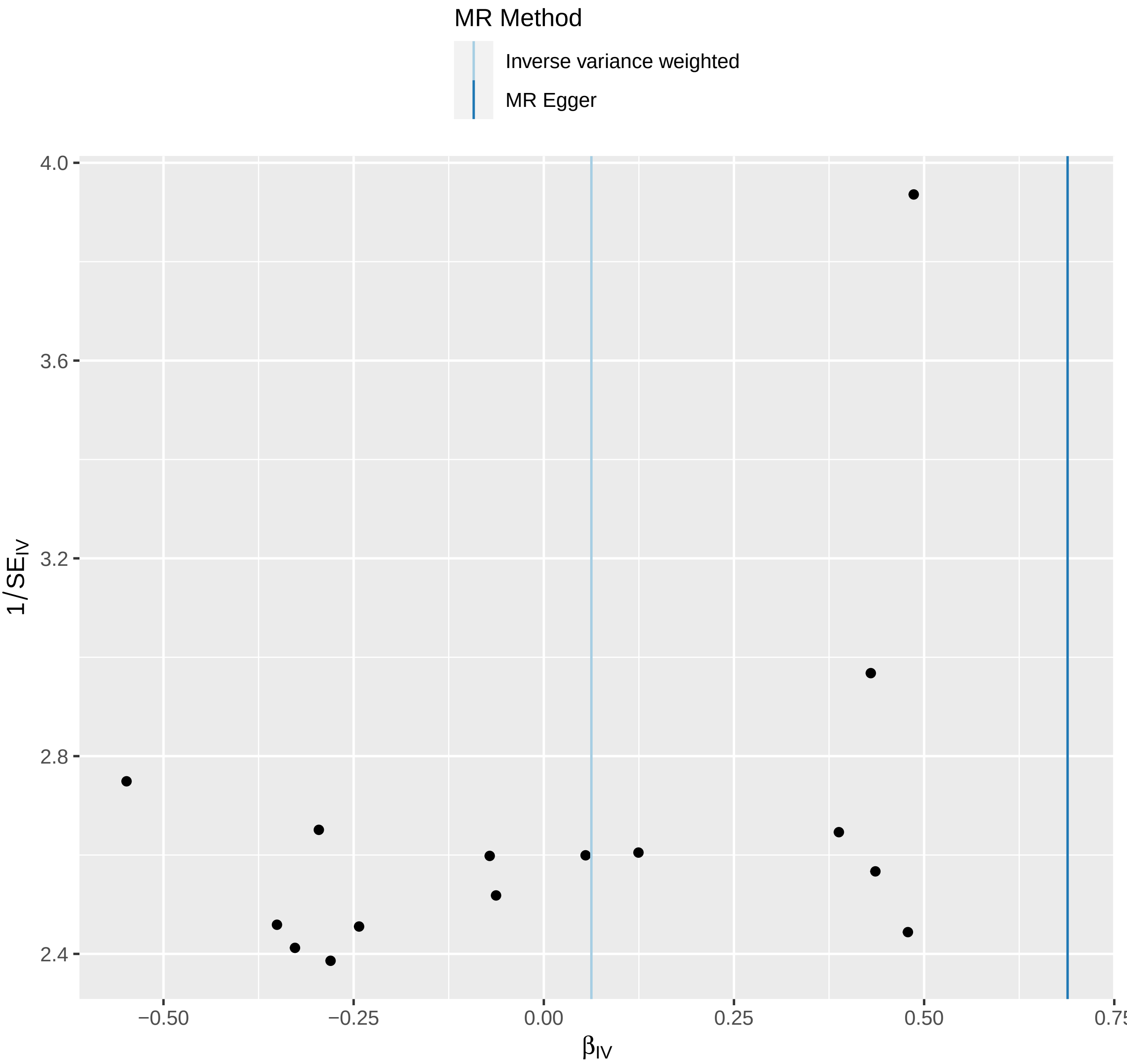

Batch 2−2403 : Gut microbiota abundance (genus Catenibacterium id.2153) on GDNF family receptor alpha−2
with a threshold of 1e−05

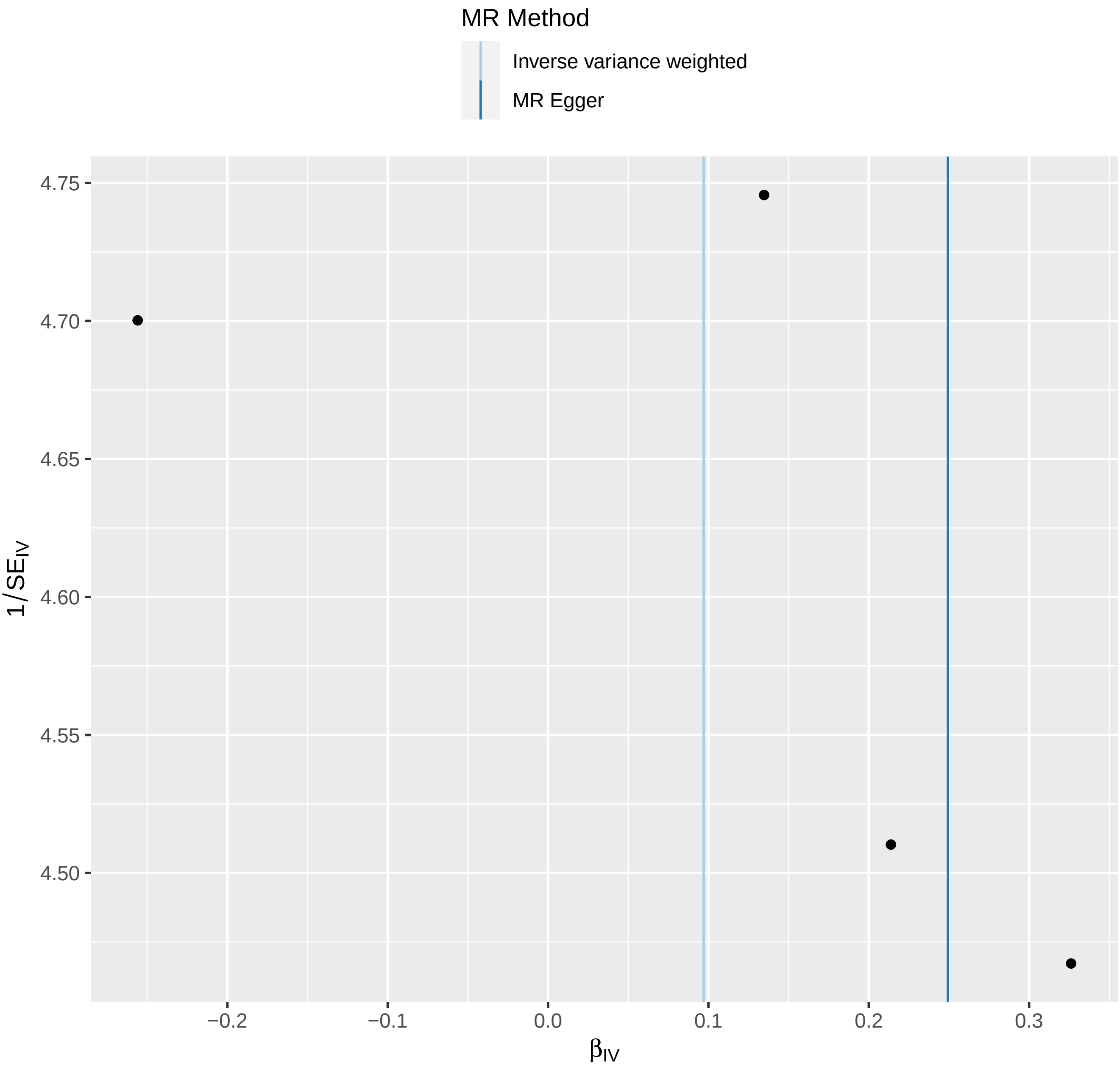

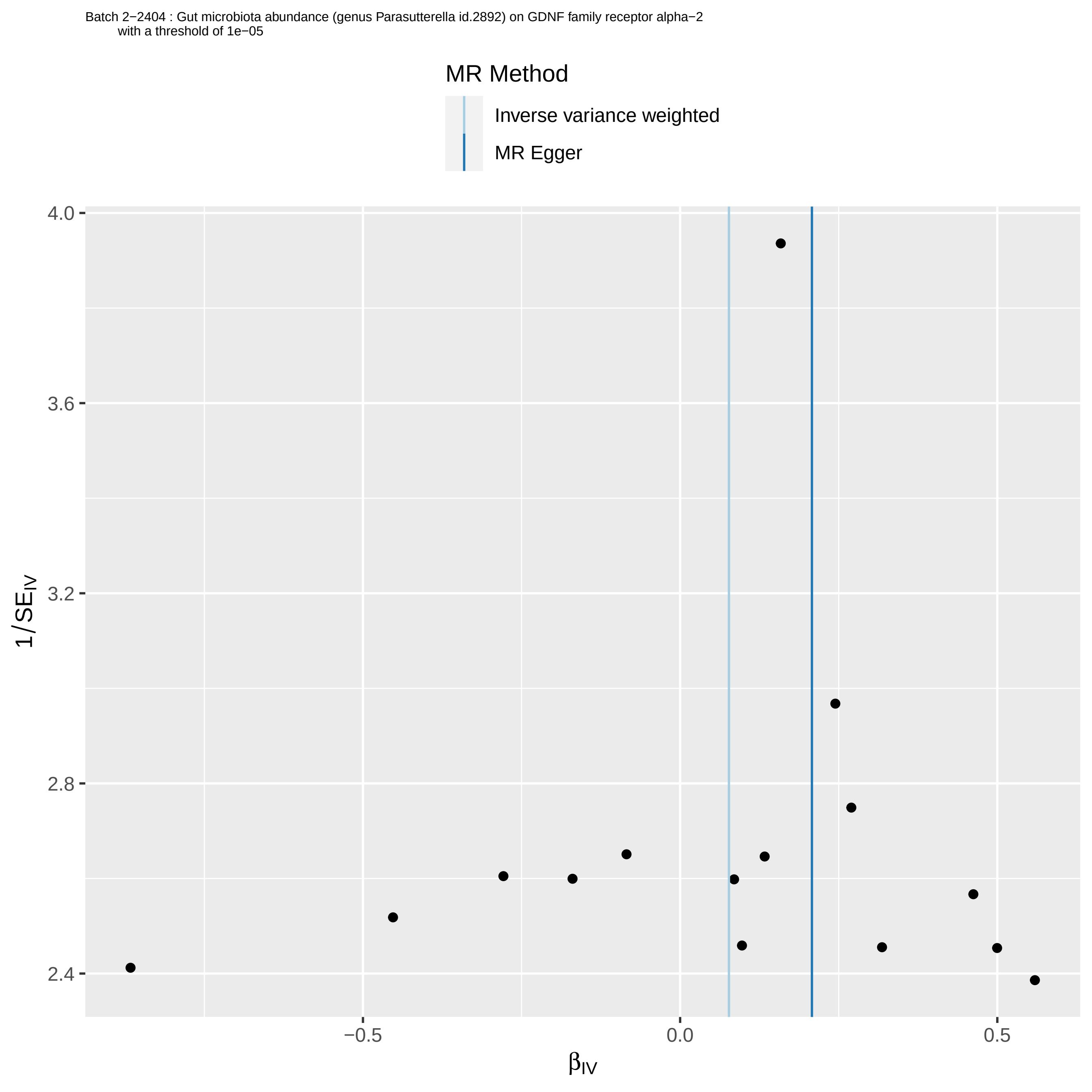

Batch 2−2404 : Gut microbiota abundance (genus Parasutterella id.2892) on GDNF family receptor alpha−2
with a threshold of 1e−05
MR Method
Inverse variance weighted
MR Egger
4.0
3.6
3.2
2.8
2.4
$1/SE_{IV}$
−0.5
0.0
0.5
β_{IV}

Batch 2−2405 : Gut microbiota abundance (genus Catenibacterium id.2153) on GDNF family receptor alpha−3
with a threshold of 1e−05

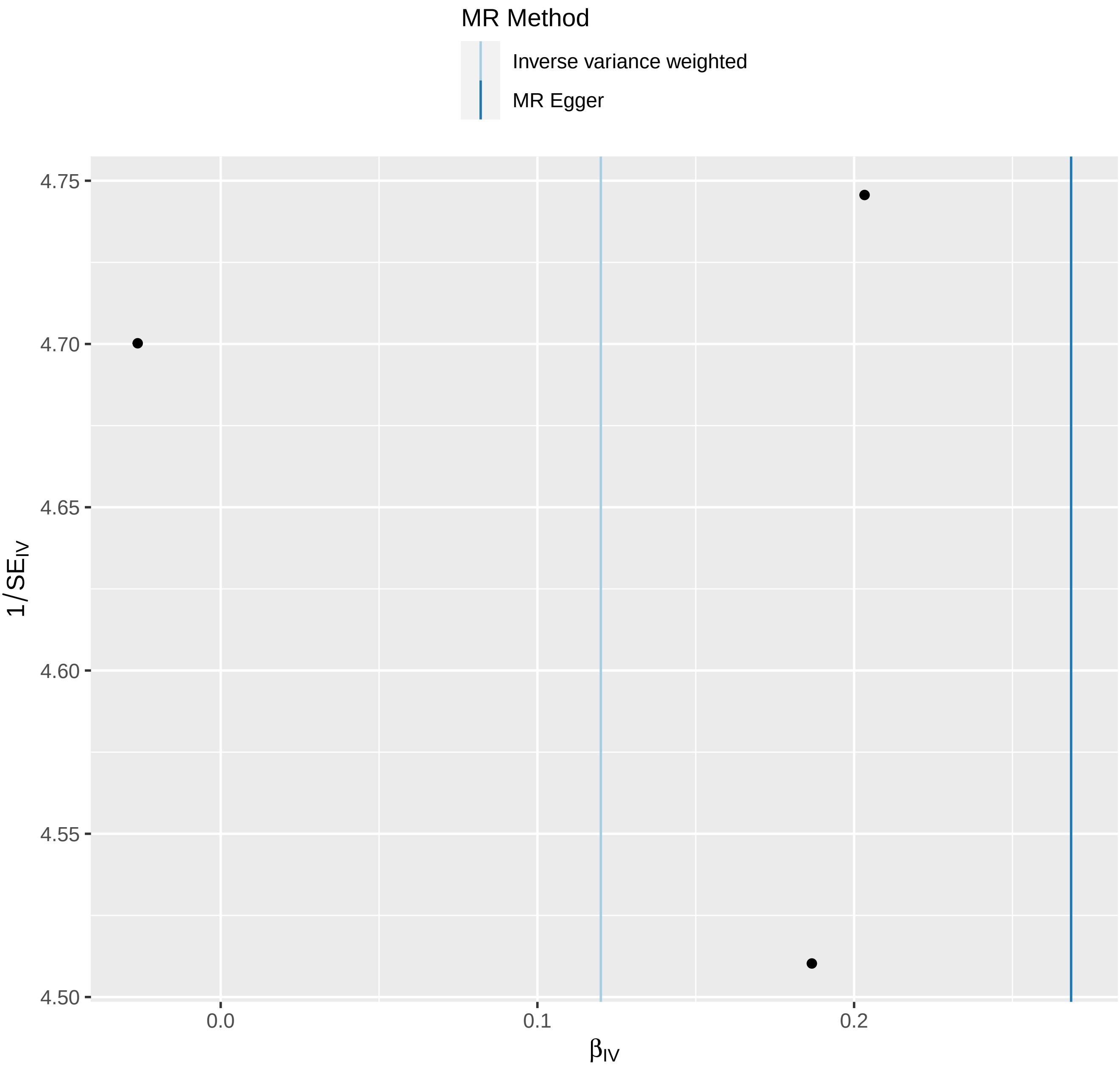

Batch 2−2406 : Gut microbiota abundance (genus Parasutterella id.2892) on GDNF family receptor alpha−3
with a threshold of 1e−05

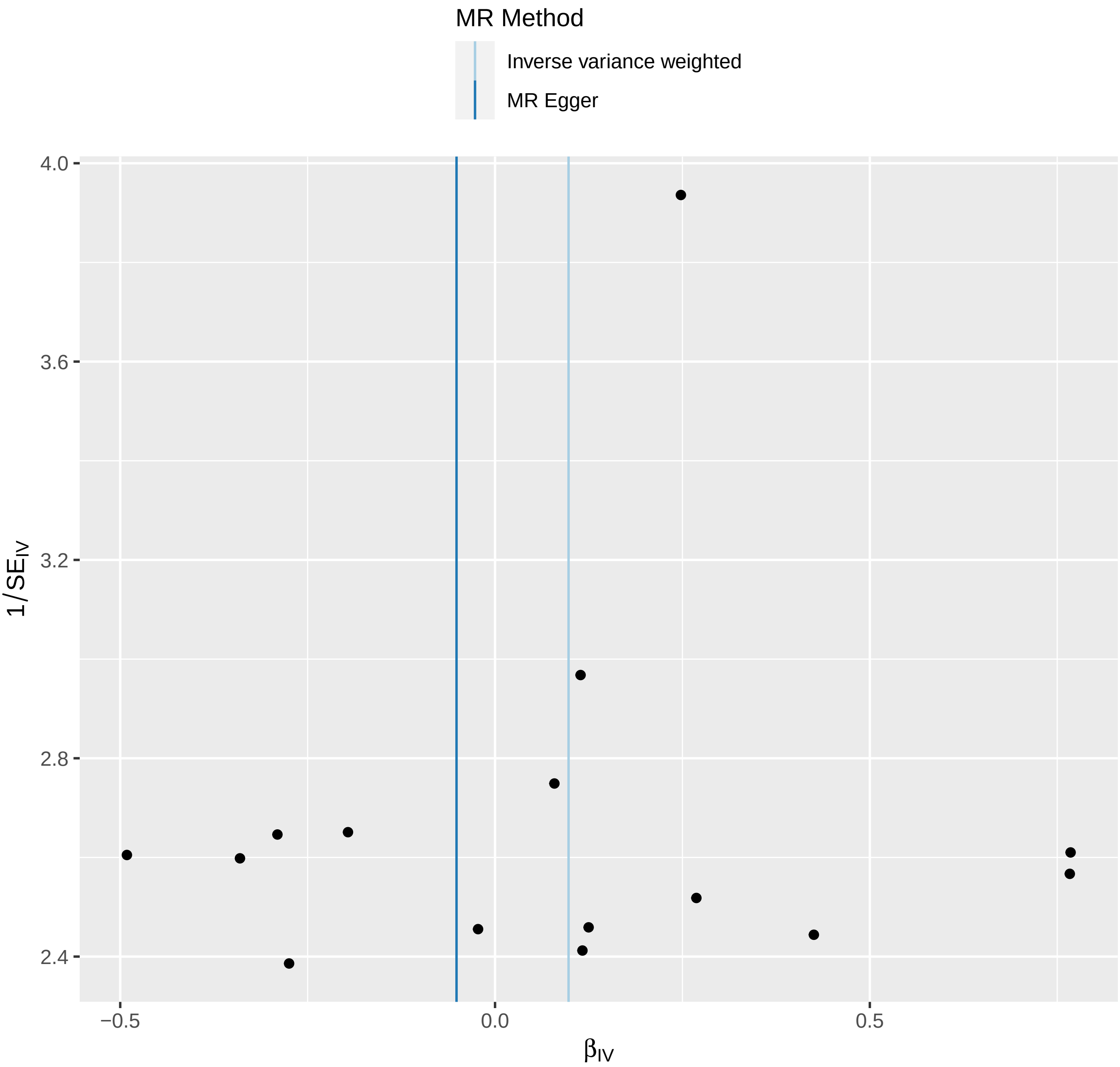

Batch 2−2407 : Gut microbiota abundance (genus Catenibacterium id.2153) on GDNF family receptor alpha−like
with a threshold of 1e−05

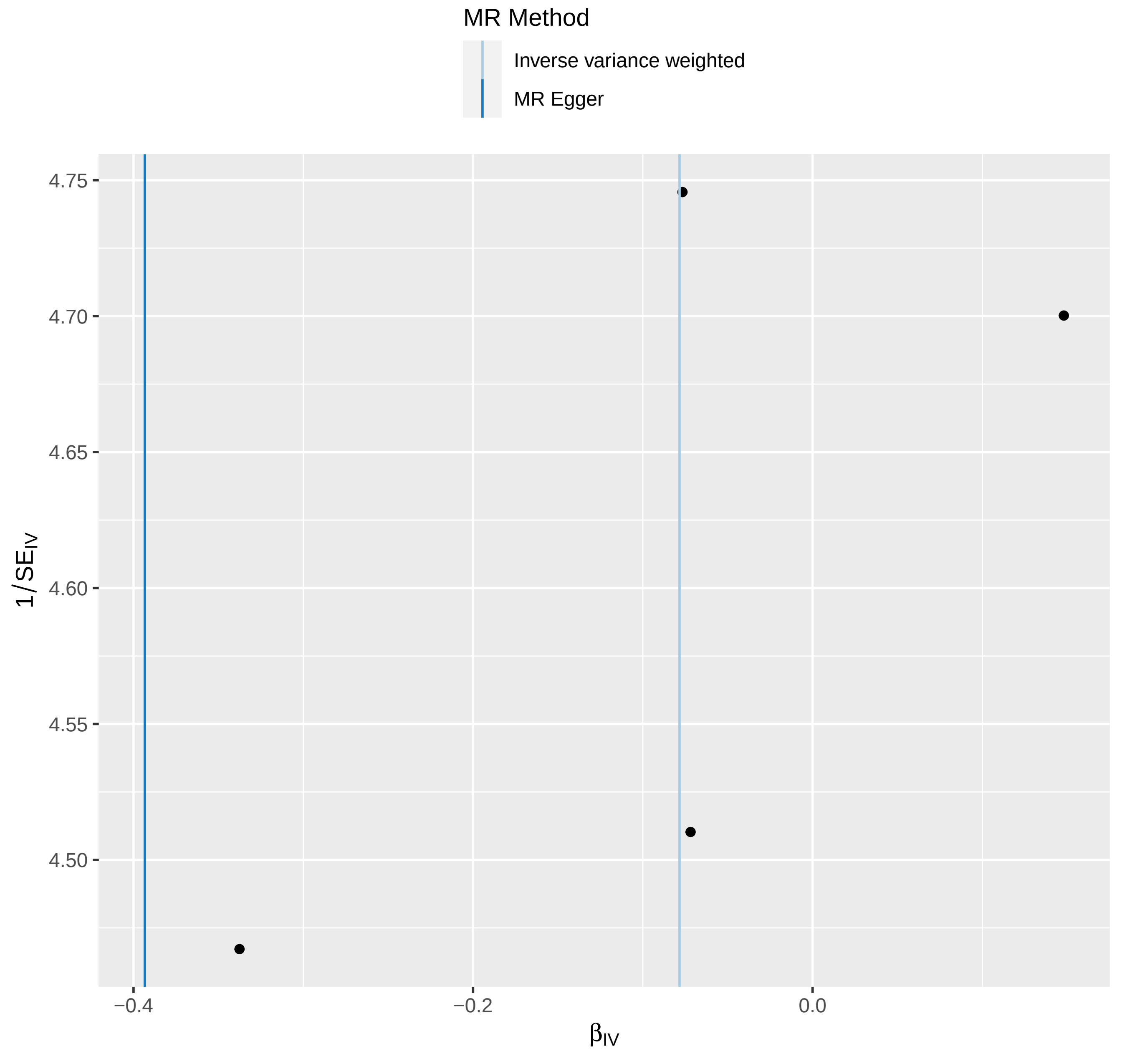

Batch 2−2408 : Gut microbiota abundance (genus Parasutterella id.2892) on GDNF family receptor alpha−like
with a threshold of 1e−05

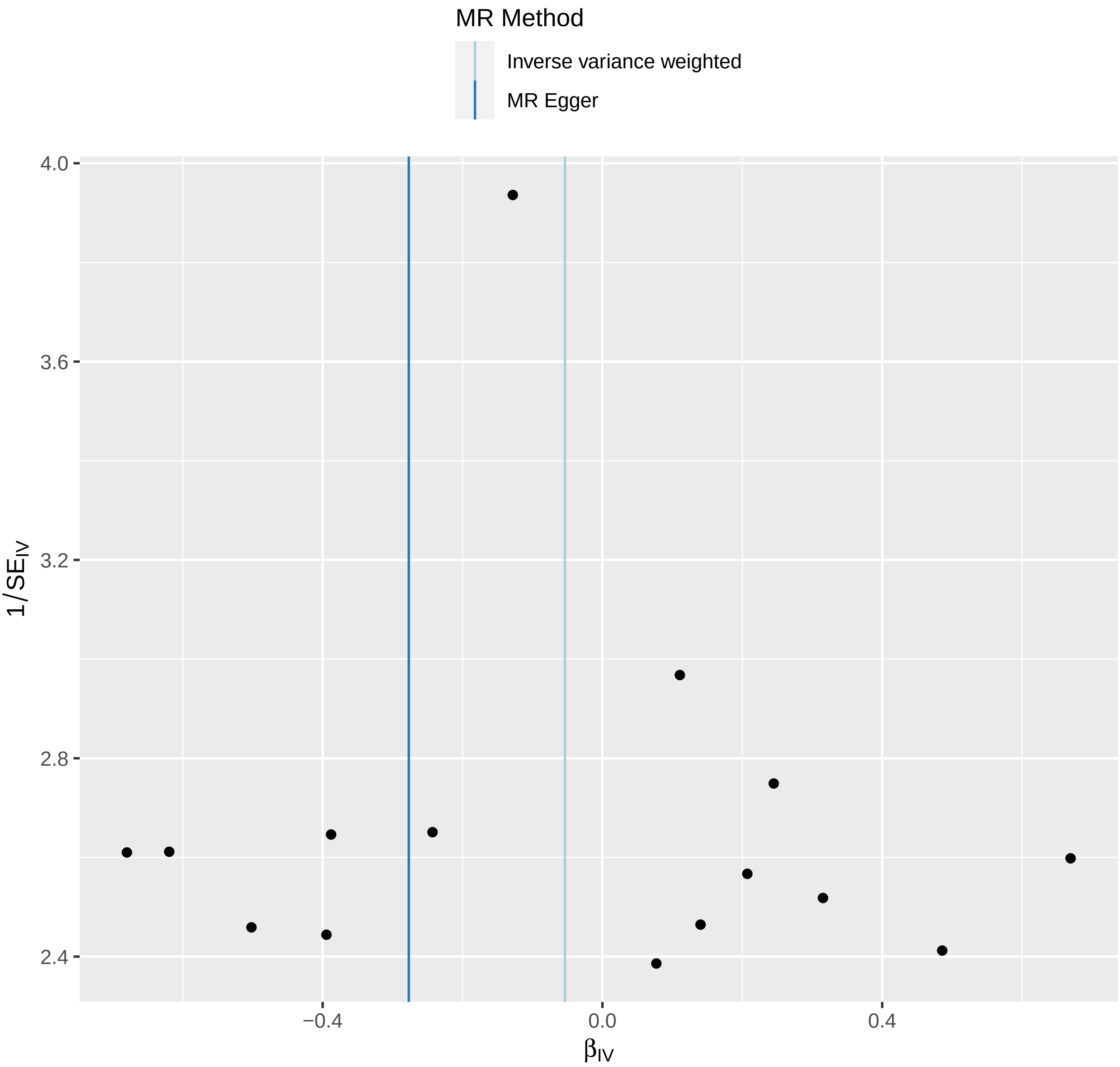

Batch 2−2409 : Gut microbiota abundance (genus Catenibacterium id.2153) on ADP−ribosylation factor−binding protein GGA1 with a threshold of 1e−05

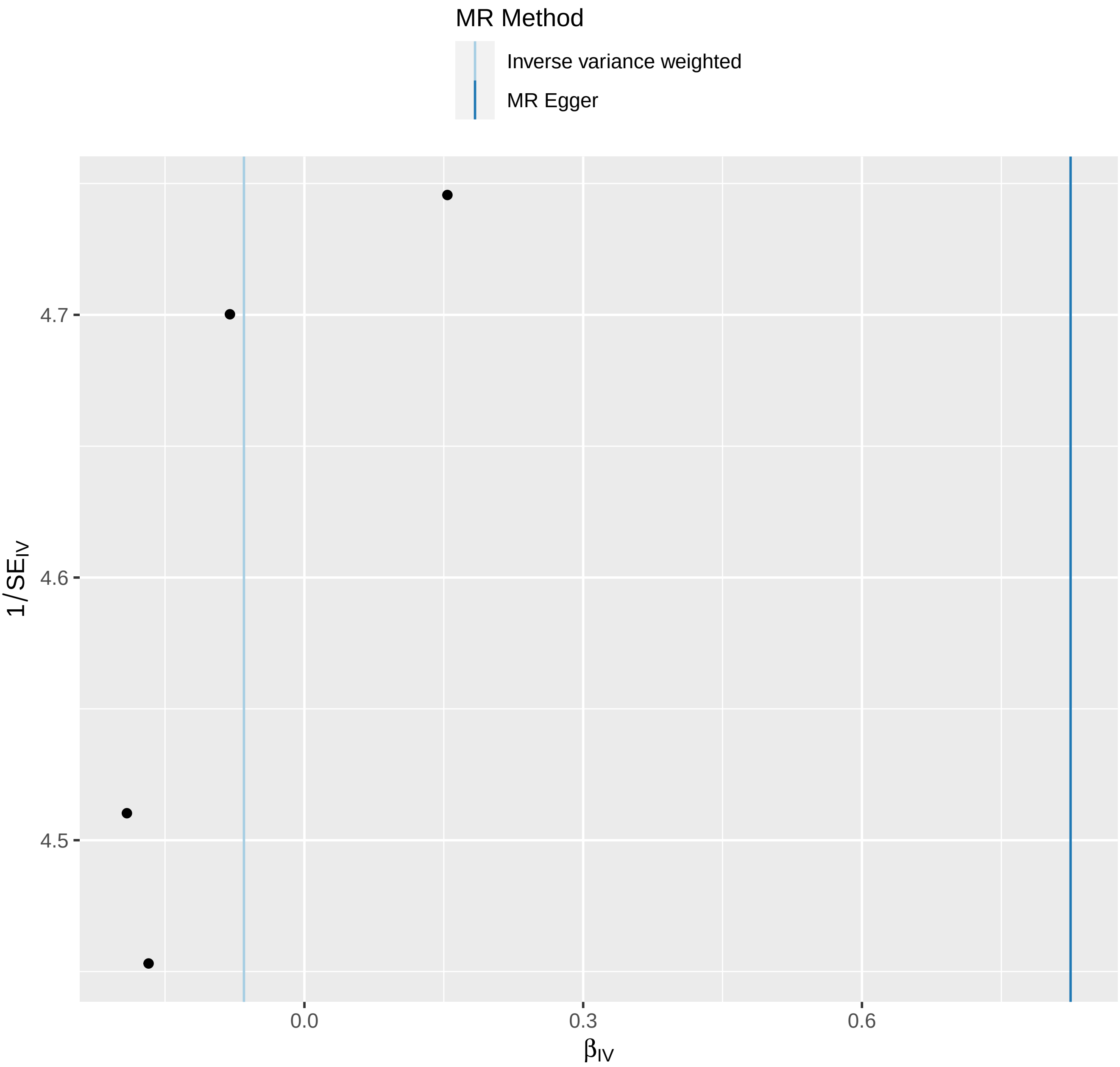

Batch 2−2410 : Gut microbiota abundance (genus Parasutterella id.2892) on ADP−ribosylation factor−binding protein GGA1
with a threshold of 1e−05

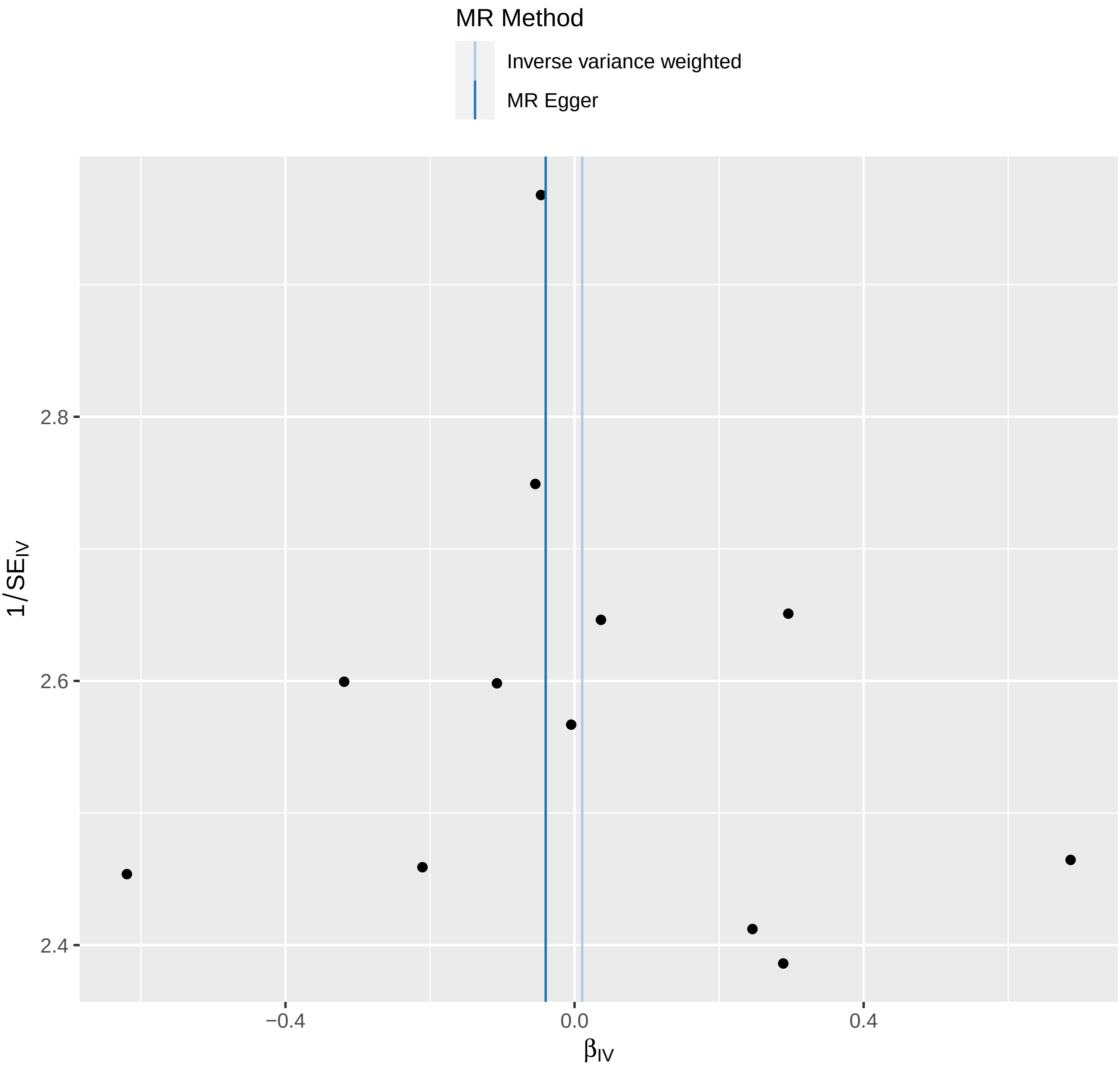

Batch 2−2411 : Gut microbiota abundance (genus Catenibacterium id.2153) on ADP−ribosylation factor−binding protein GGA3
with a threshold of 1e−05

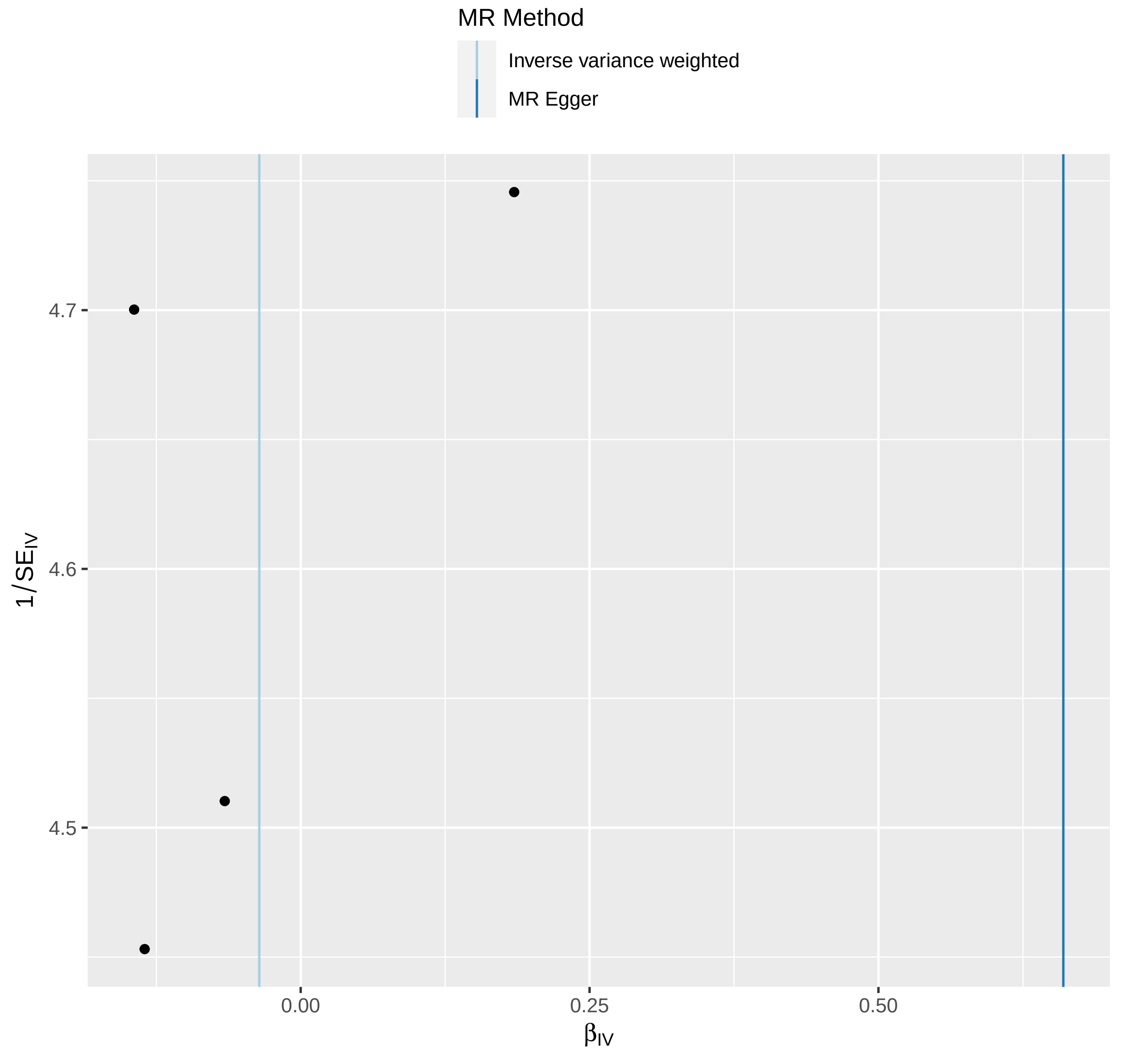

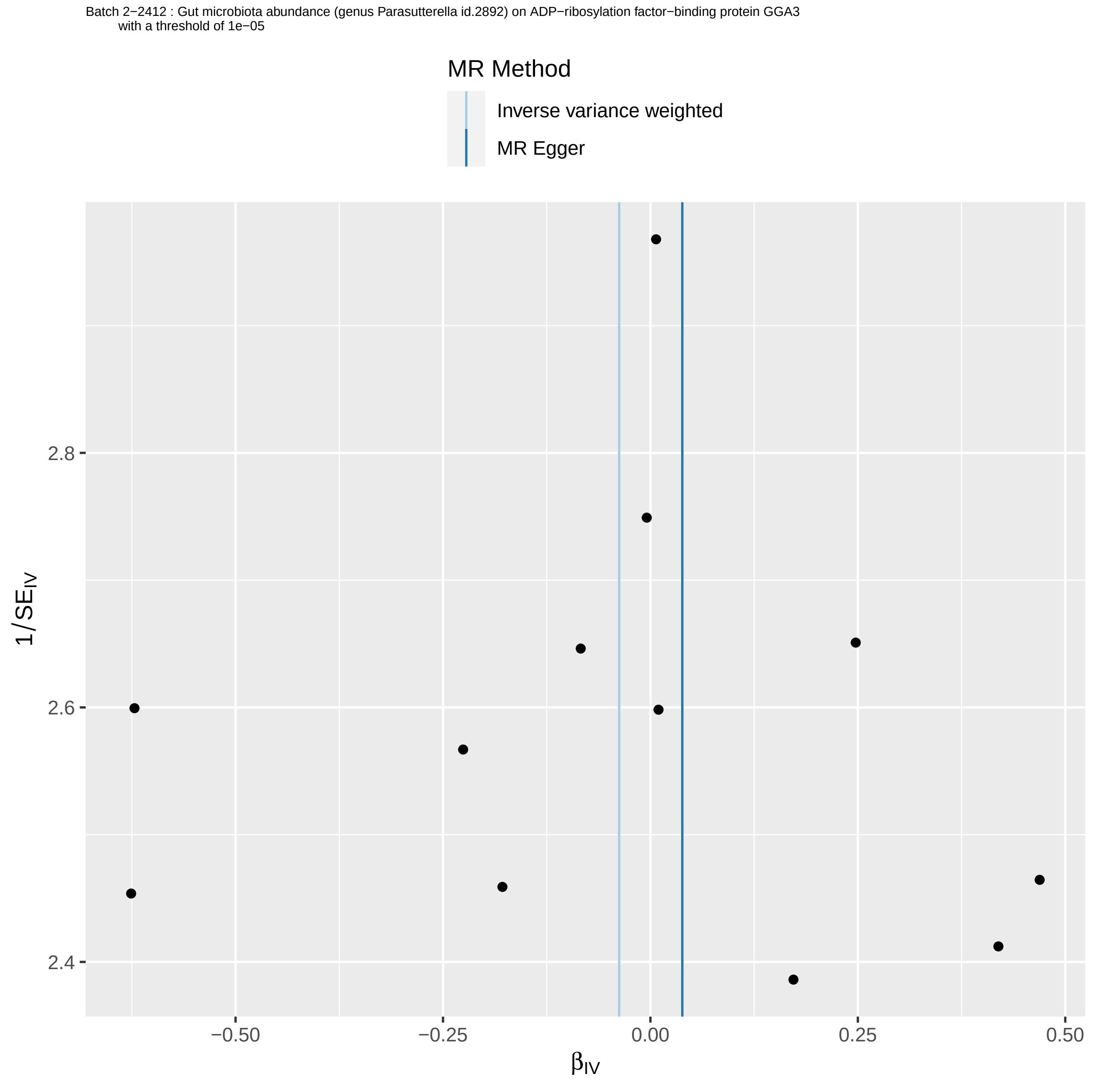
Batch 2−2412 : Gut microbiota abundance (genus Parasutterella id.2892) on ADP−ribosylation factor−binding protein GGA3
with a threshold of 1e−05
MR Method
Inverse variance weighted
MR Egger
2.8
2.6
2.4
1/SE_IV
−0.50
−0.25
0.00
0.25
0.50
β_IV

Batch 2−2413 : Gut microbiota abundance (genus Catenibacterium id.2153) on Gamma−glutamyl hydrolase
with a threshold of 1e−05

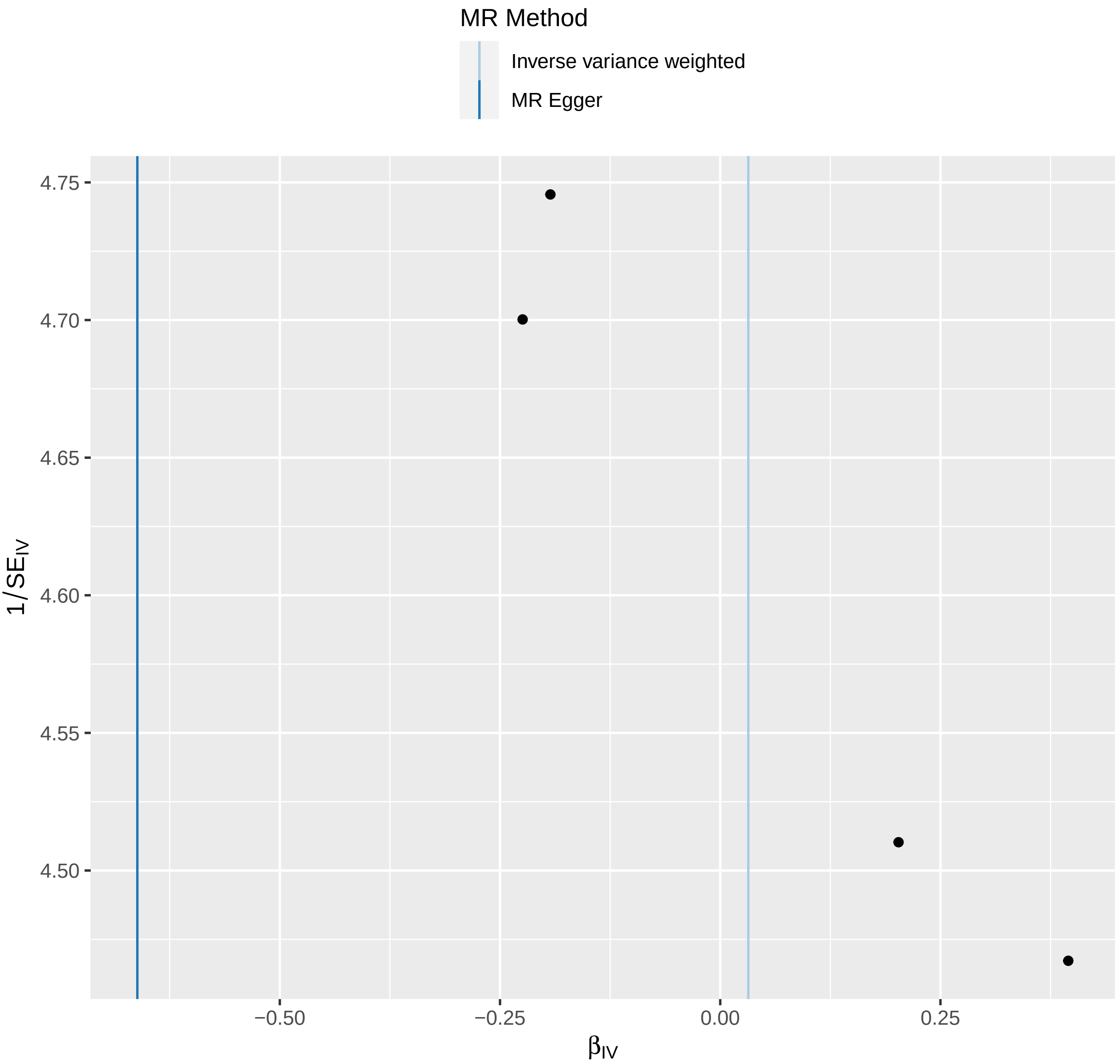

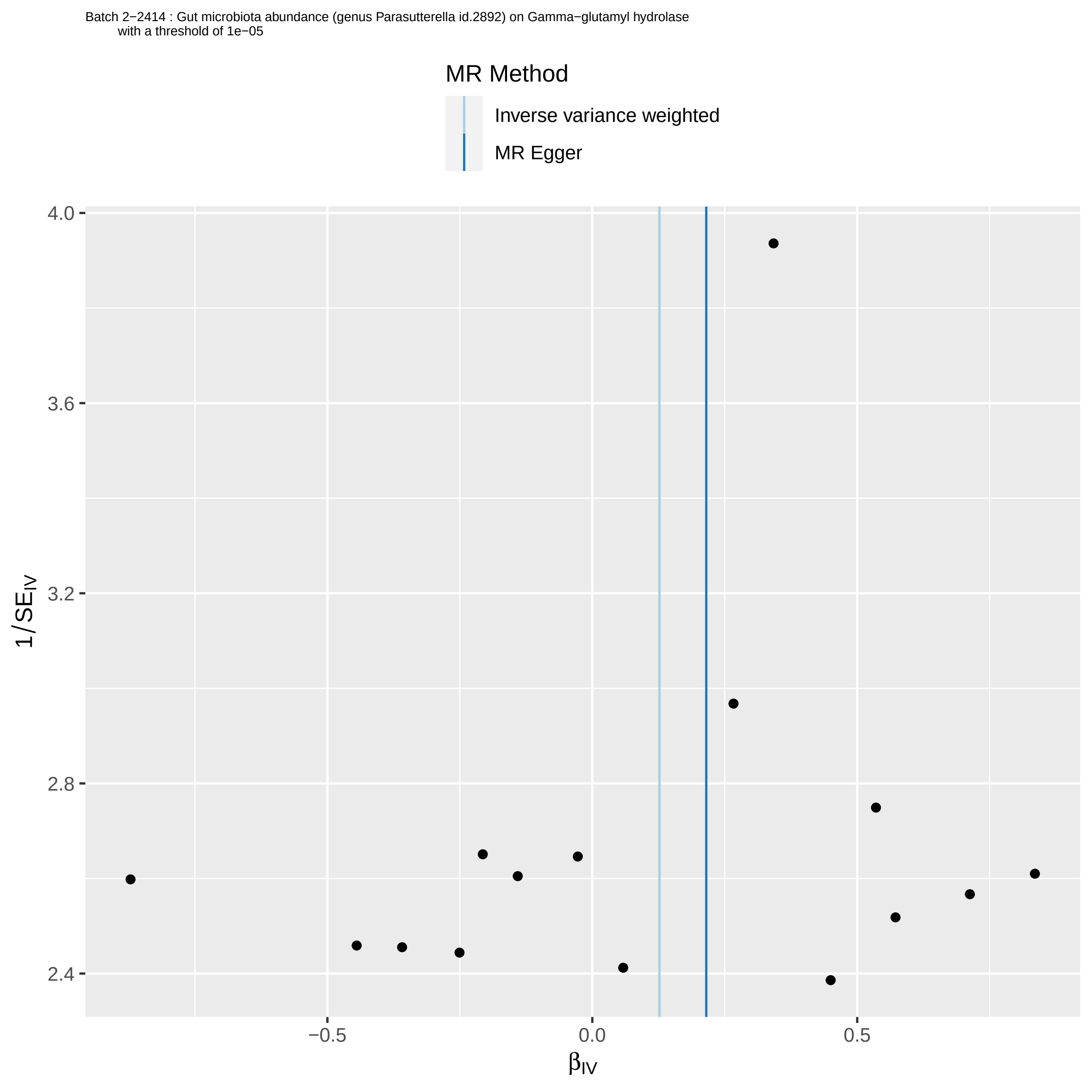
Batch 2−2414 : Gut microbiota abundance (genus Parasutterella id.2892) on Gamma−glutamyl hydrolase
with a threshold of 1e−05
MR Method
Inverse variance weighted
MR Egger
4.0
3.6
3.2
2.8
2.4
1/SE$_{IV}$
−0.5
0.0
0.5
β_{IV}

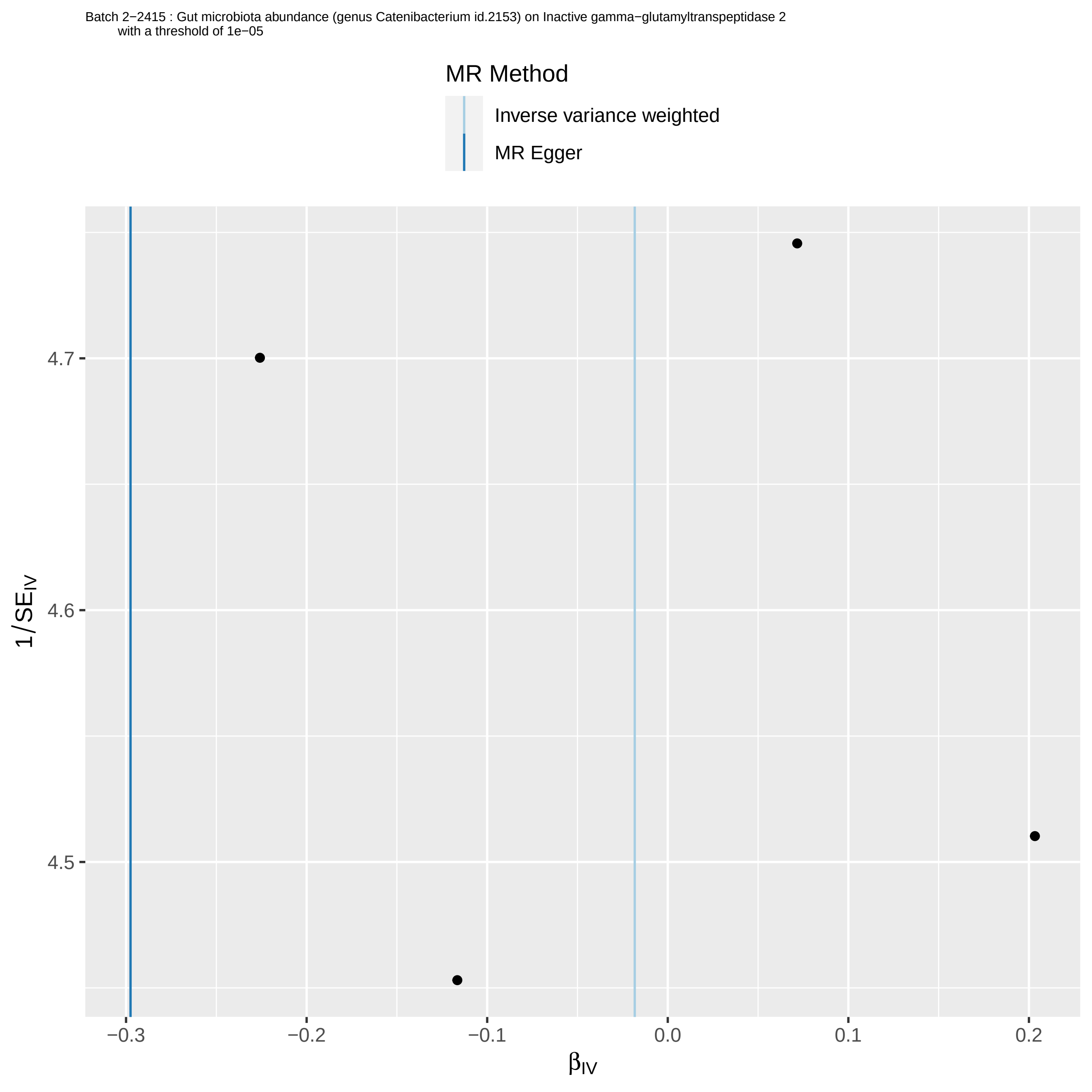
Batch 2−2415 : Gut microbiota abundance (genus Catenibacterium id.2153) on Inactive gamma−glutamyltranspeptidase 2
with a threshold of 1e−05
MR Method
Inverse variance weighted
MR Egger
4.7
4.6
4.5
1/SE_IV
−0.3
−0.2
−0.1
0.0
0.1
0.2
β_IV

Batch 2−2416 : Gut microbiota abundance (genus Parasutterella id.2892) on Inactive gamma−glutamyltranspeptidase 2
with a threshold of 1e−05

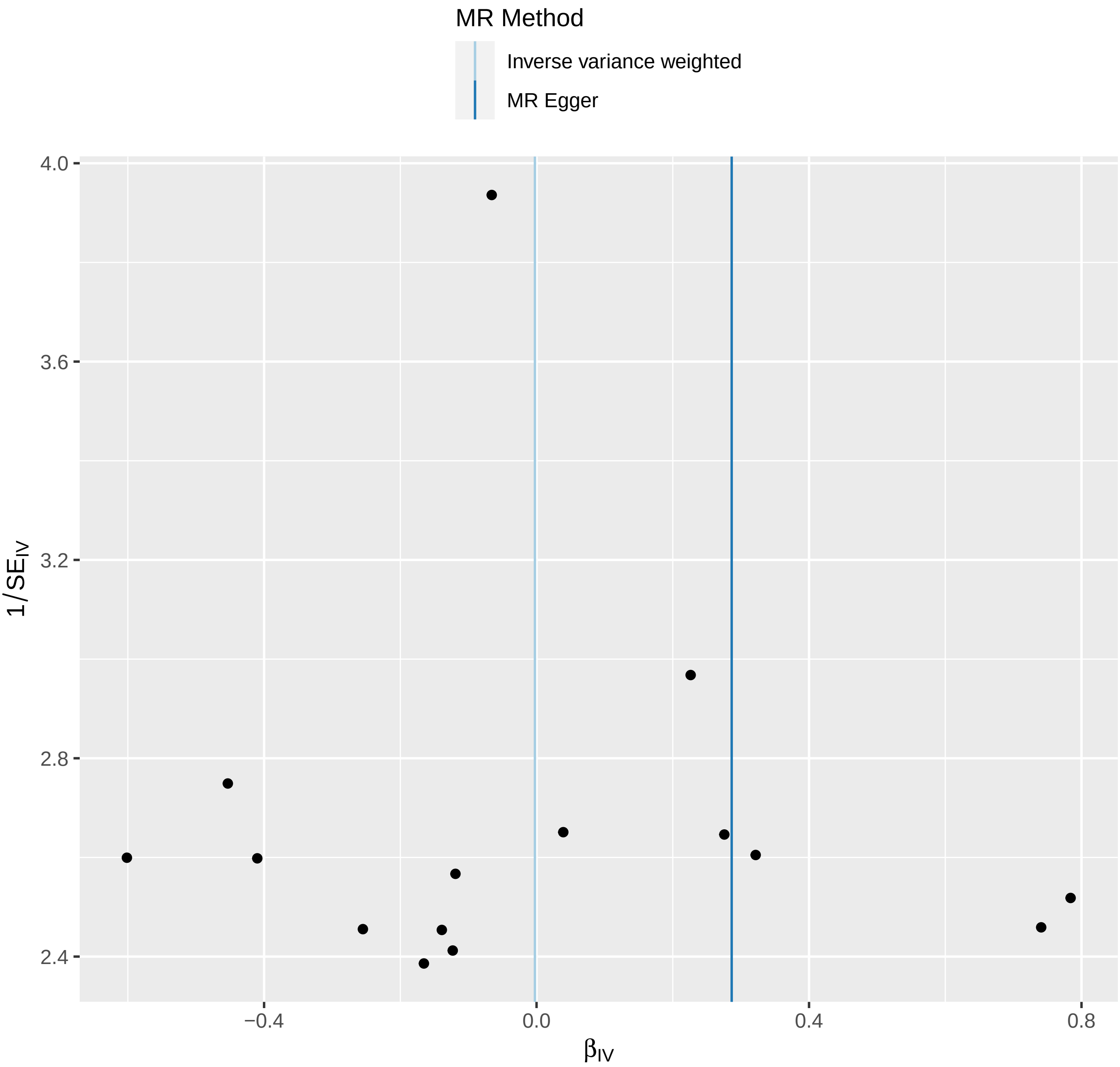

Batch 2−2417 : Gut microbiota abundance (genus Catenibacterium id.2153) on Somatotropin
with a threshold of 1e−05

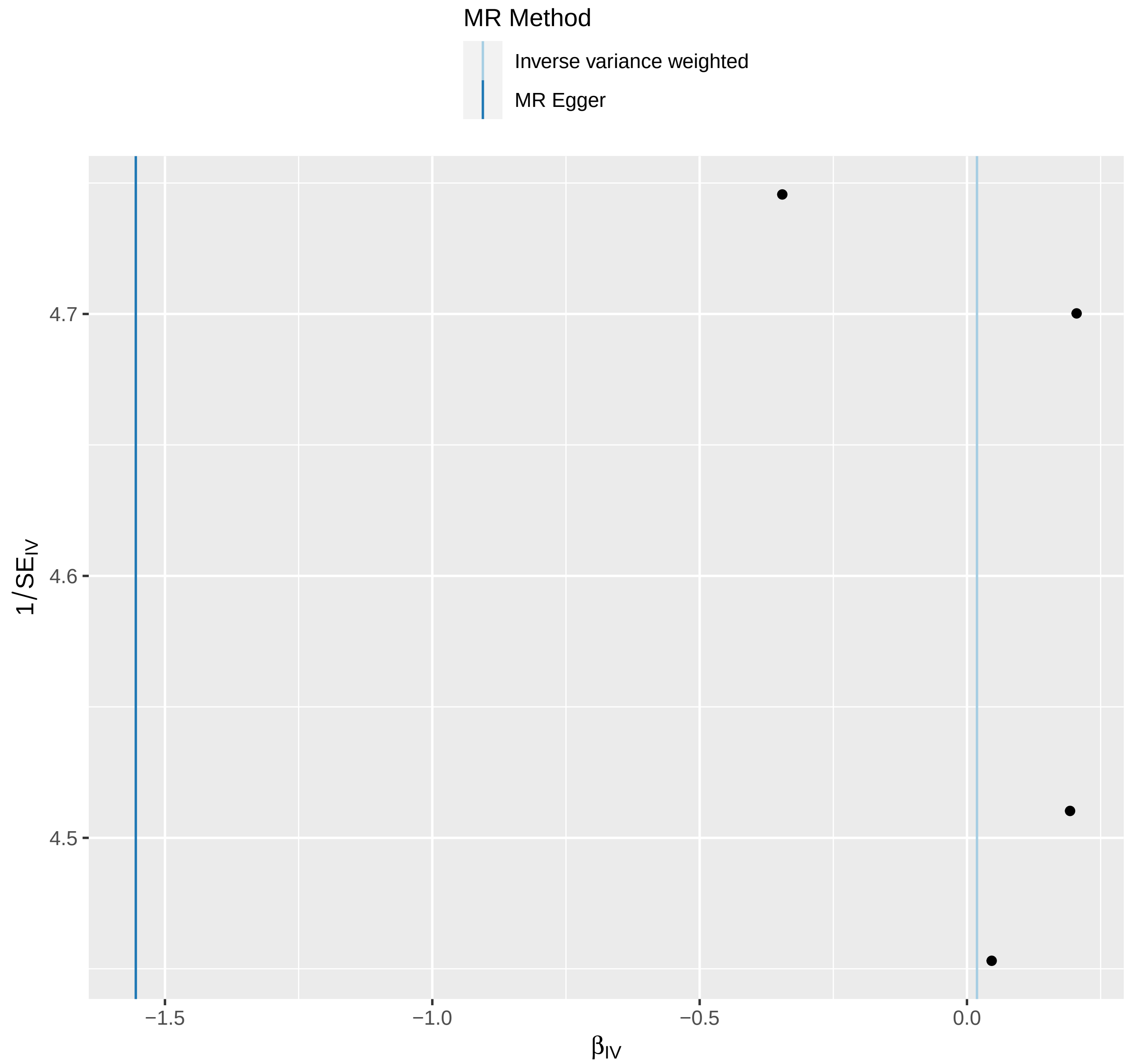

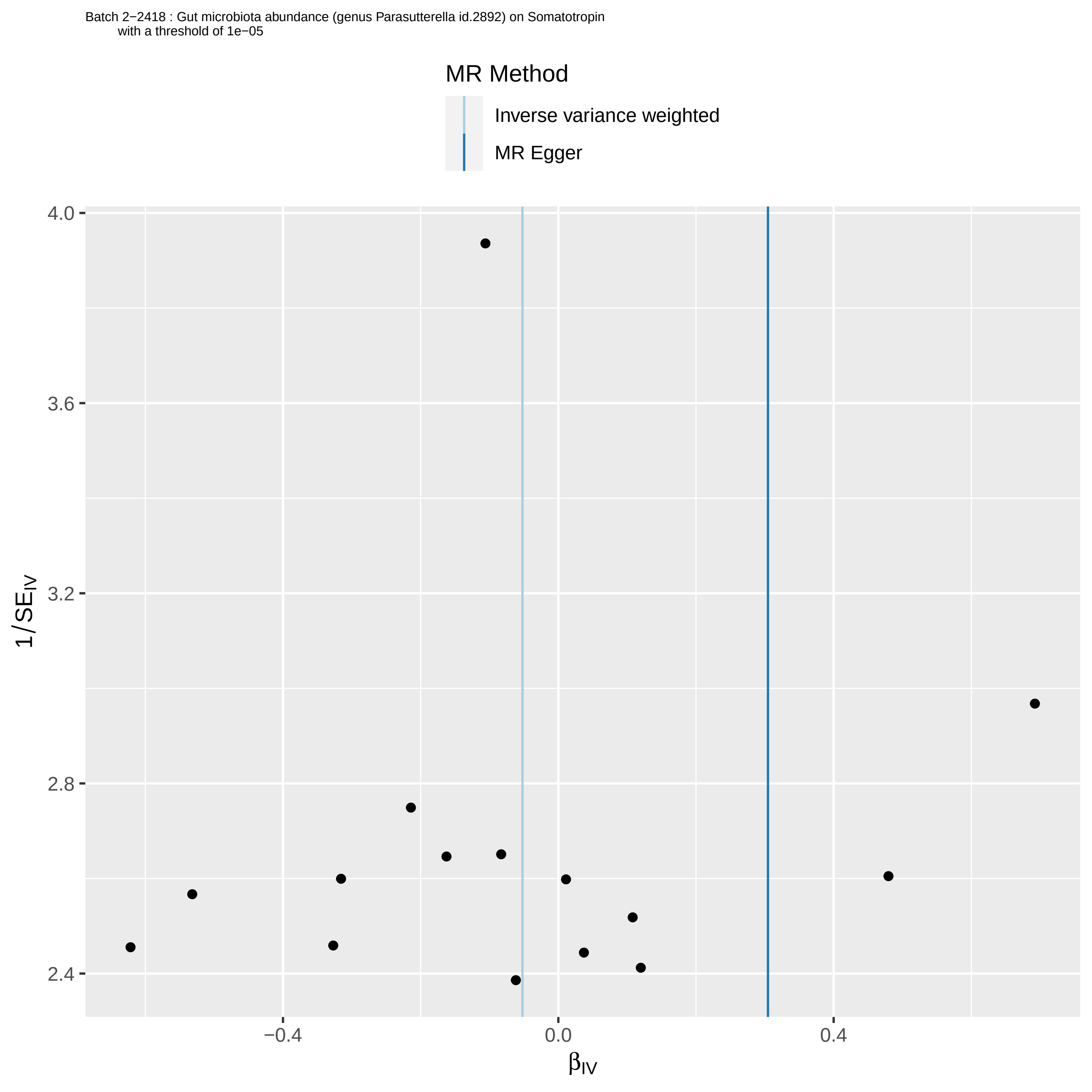

Batch 2−2418 : Gut microbiota abundance (genus Parasutterella id.2892) on Somatotropin
with a threshold of 1e−05
MR Method
Inverse variance weighted
MR Egger
4.0
3.6
3.2
2.8
2.4
1/SE_IV
−0.4
0.0
0.4
β_IV

Batch 2−2419 : Gut microbiota abundance (genus Catenibacterium id.2153) on GH3 domain−containing protein
with a threshold of 1e−05

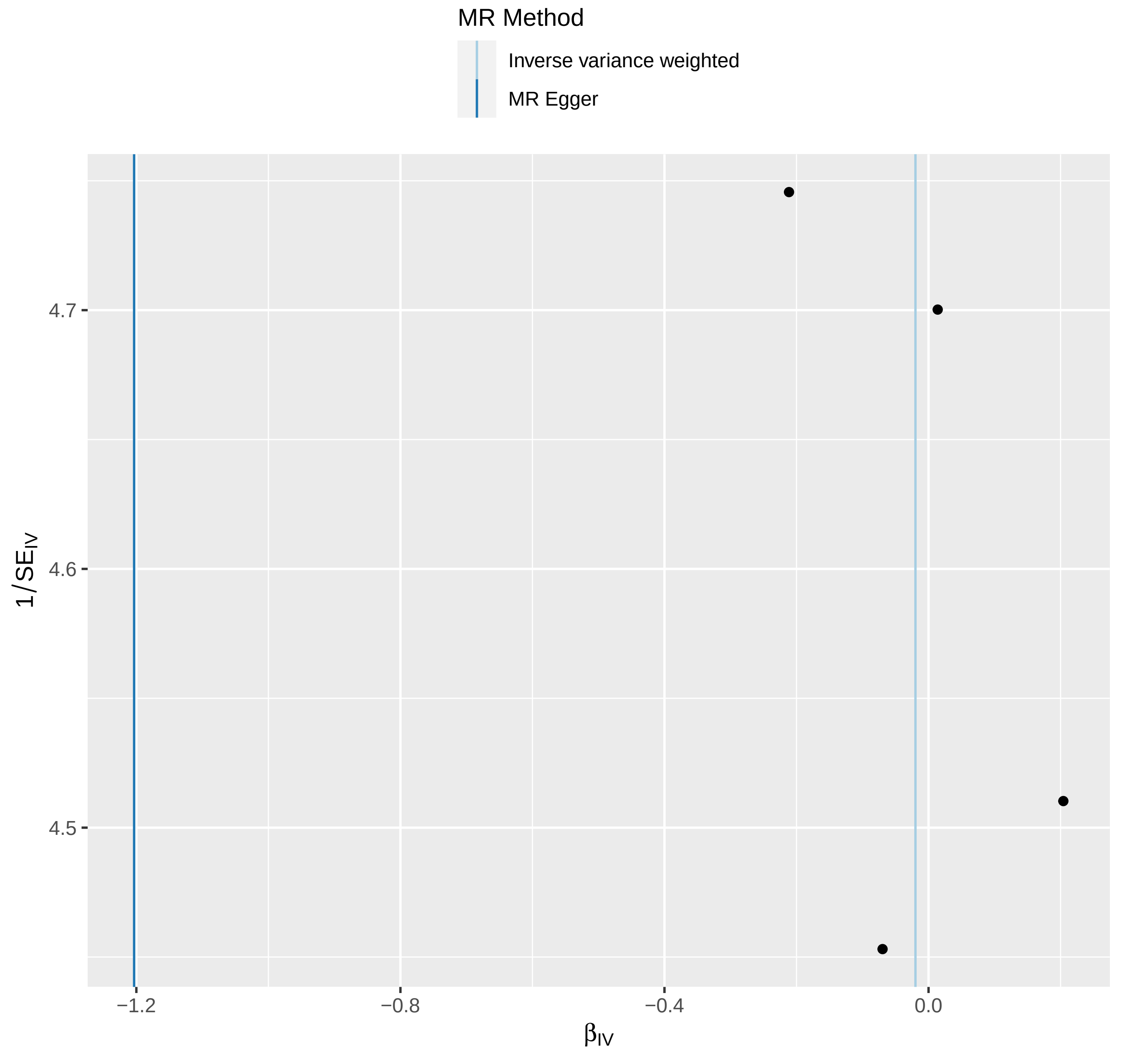

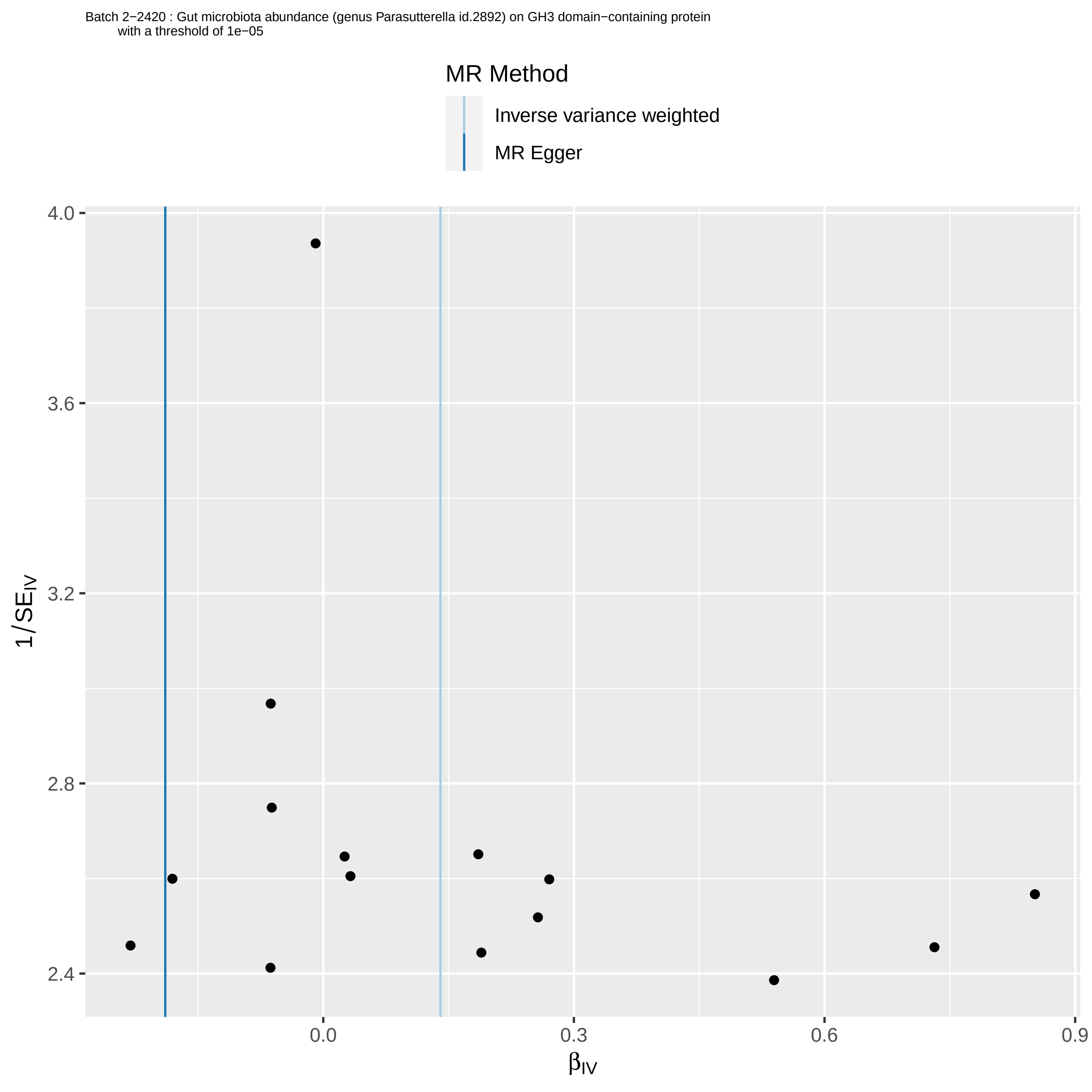

Batch 2−2420 : Gut microbiota abundance (genus Parasutterella id.2892) on GH3 domain−containing protein
with a threshold of 1e−05
MR Method
Inverse variance weighted
MR Egger
4.0
3.6
3.2
2.8
2.4
1/SE_IV
0.0
0.3
0.6
0.9
β_IV

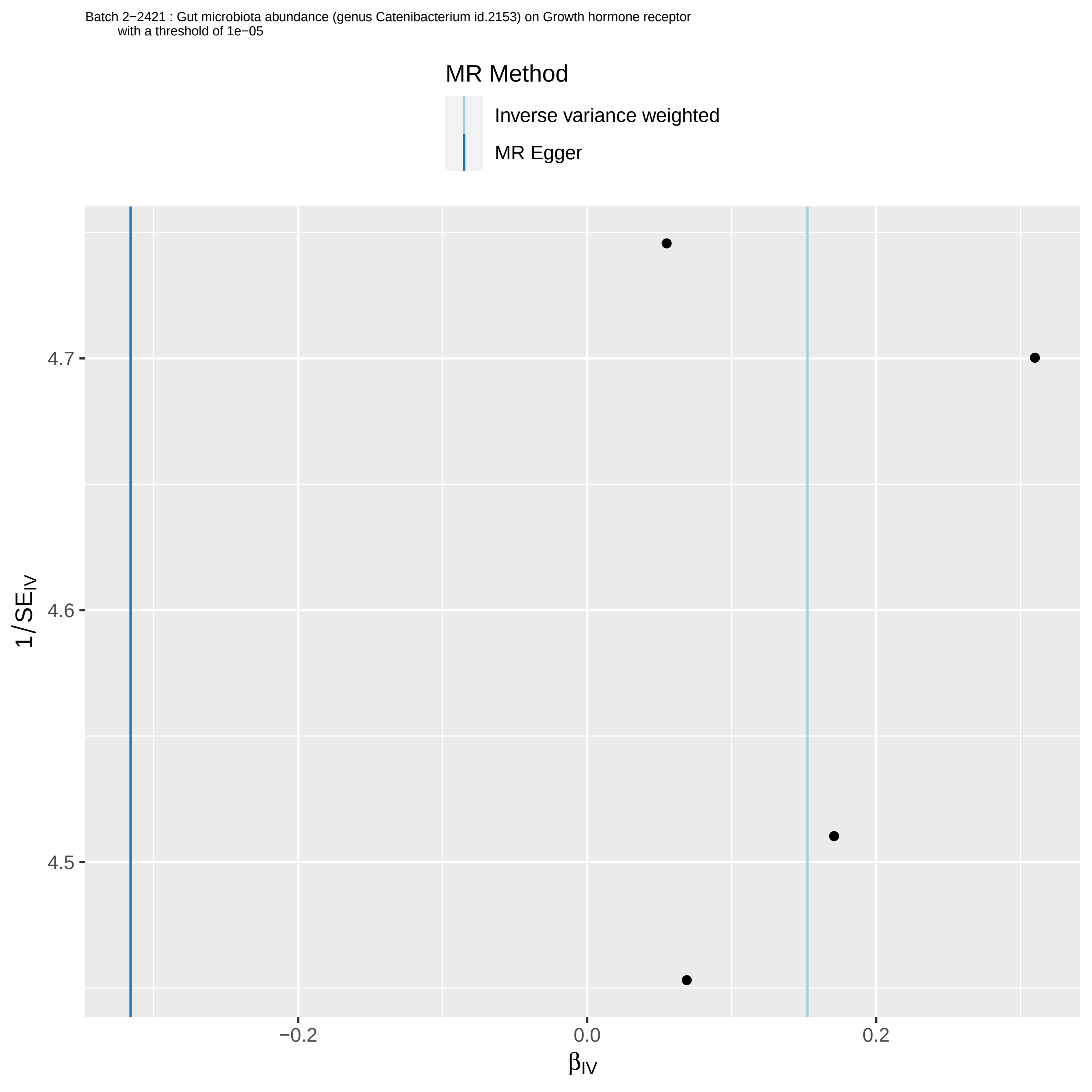
Batch 2−2421 : Gut microbiota abundance (genus Catenibacterium id.2153) on Growth hormone receptor
with a threshold of 1e−05
MR Method
Inverse variance weighted
MR Egger
4.7
4.6
4.5
1/SE_IV
−0.2
0.0
0.2
β_IV

Batch 2−2422 : Gut microbiota abundance (genus Parasutterella id.2892) on Growth hormone receptor
with a threshold of 1e−05

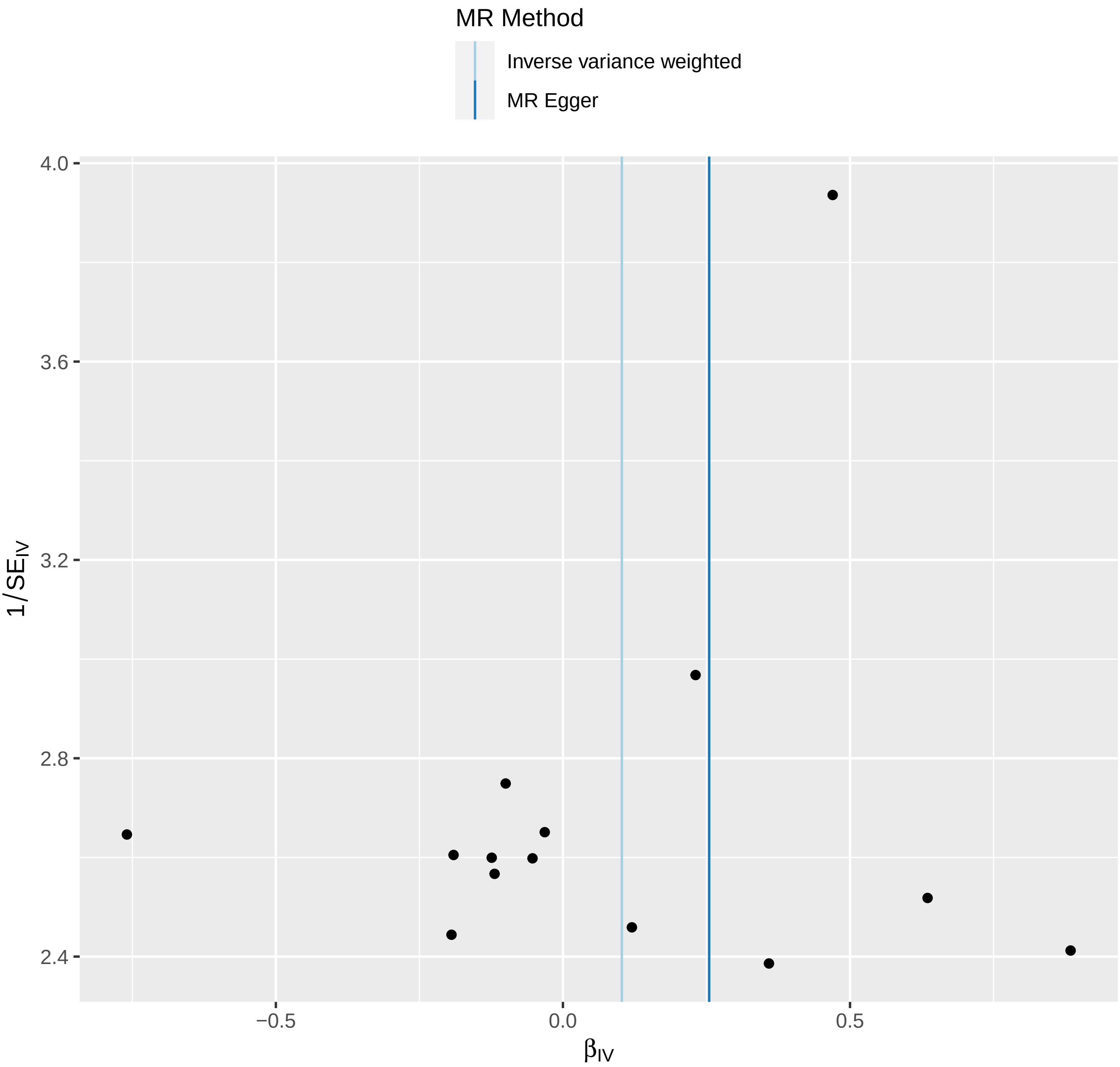

Batch 2−2423 : Gut microbiota abundance (genus Catenibacterium id.2153) on Appetite−regulating hormone
with a threshold of 1e−05

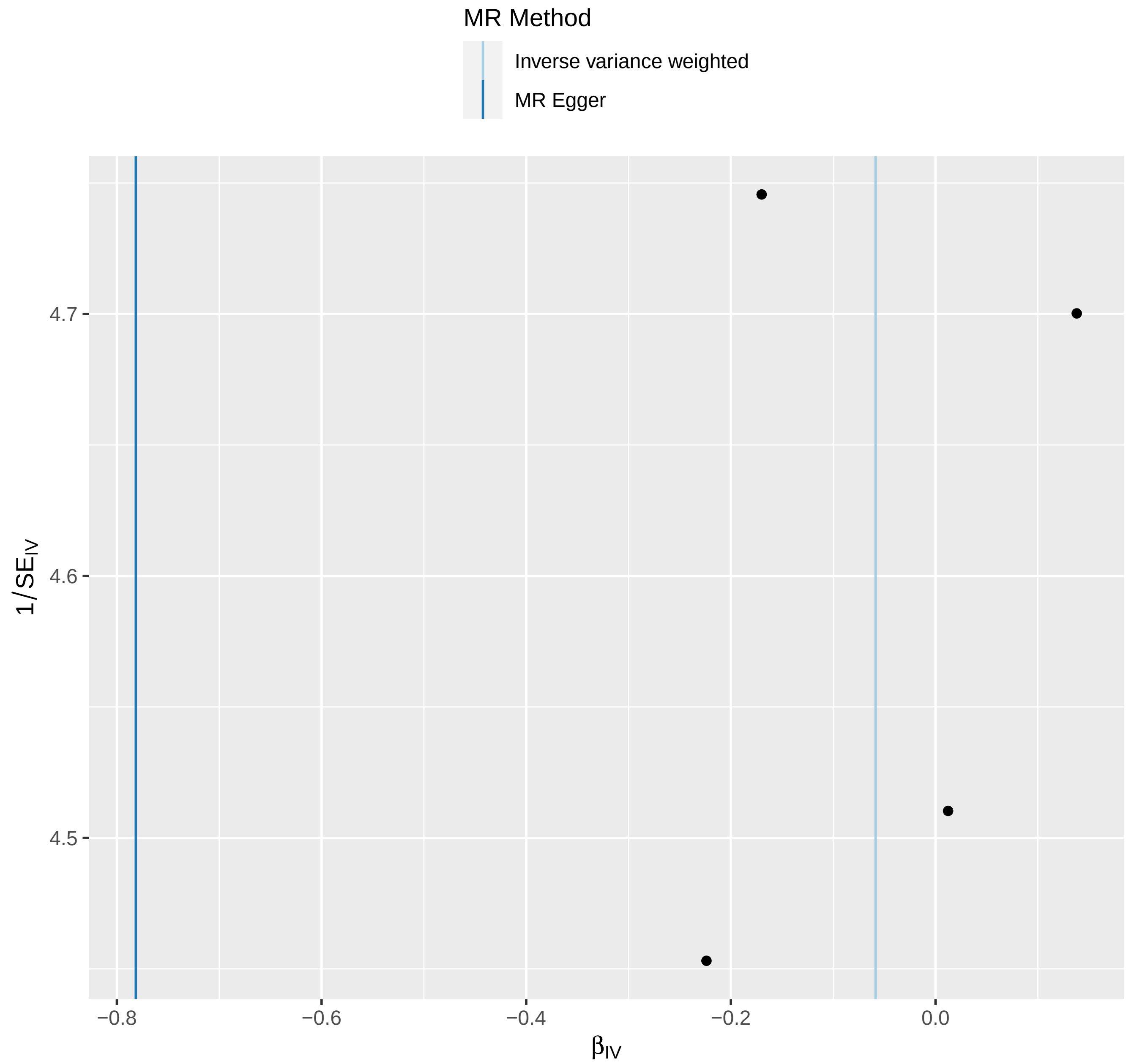

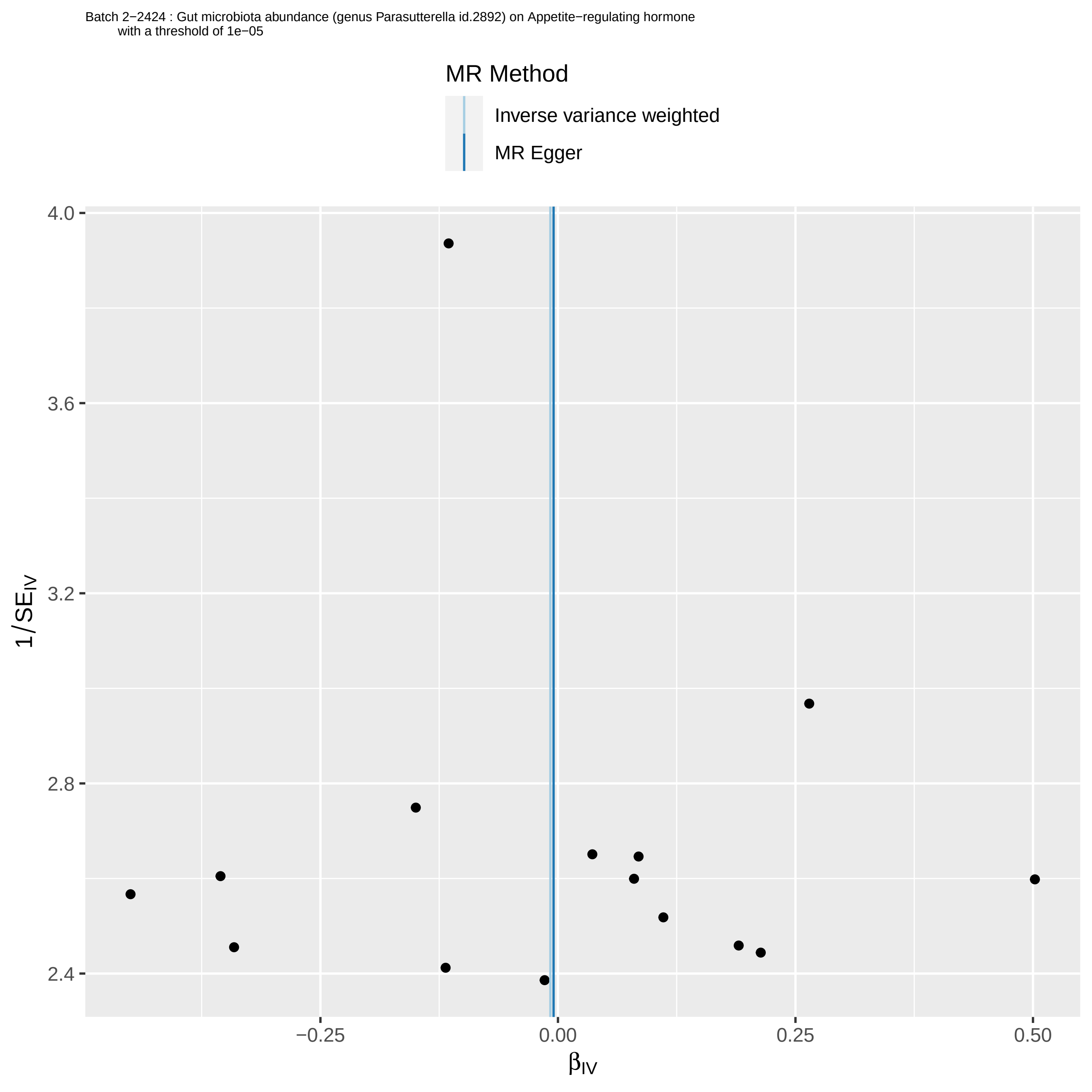
Batch 2−2424 : Gut microbiota abundance (genus Parasutterella id.2892) on Appetite−regulating hormone
with a threshold of 1e−05
MR Method
Inverse variance weighted
MR Egger
4.0
3.6
3.2
2.8
2.4
1/SE$_{IV}$
−0.25
0.00
0.25
0.50
β_{IV}

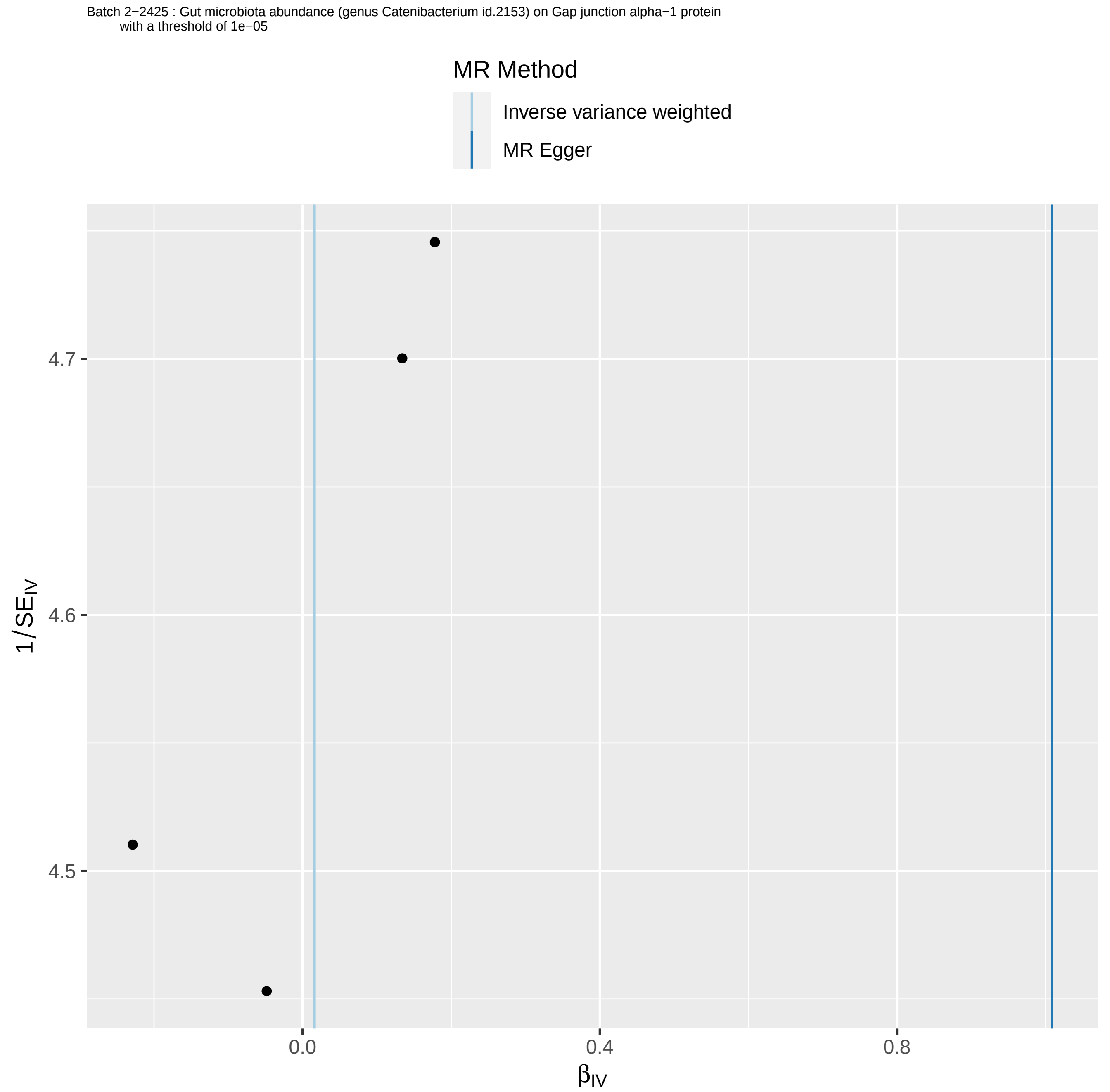
Batch 2−2425 : Gut microbiota abundance (genus Catenibacterium id.2153) on Gap junction alpha−1 protein
with a threshold of 1e−05
MR Method
Inverse variance weighted
MR Egger
4.7
4.6
4.5
1/SE$_{IV}$
0.0
0.4
0.8
β_{IV}

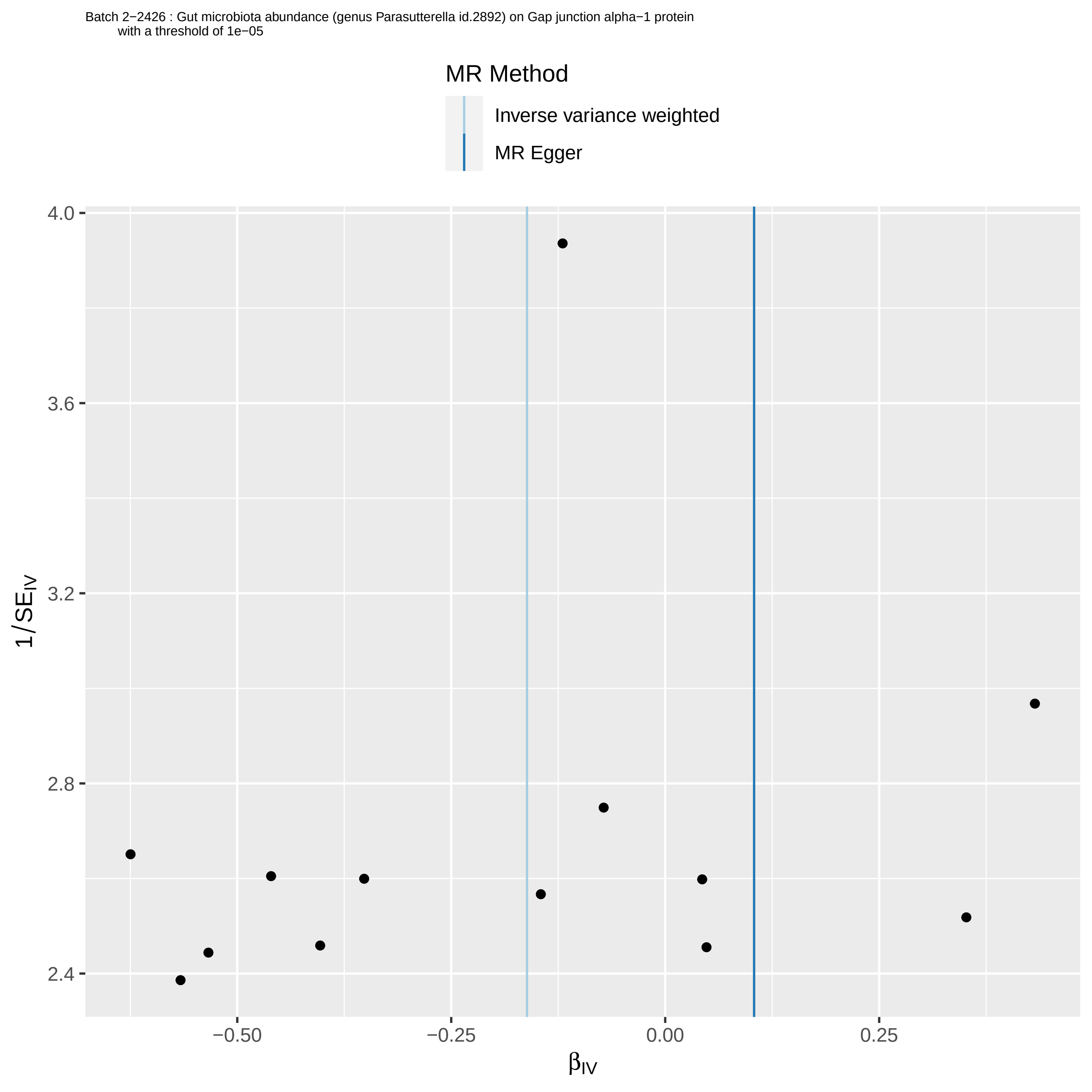
Batch 2−2426 : Gut microbiota abundance (genus Parasutterella id.2892) on Gap junction alpha−1 protein
with a threshold of 1e−05
MR Method
Inverse variance weighted
MR Egger
4.0
3.6
3.2
2.8
2.4
1/SE_IV
−0.50
−0.25
0.00
0.25
β_IV

Batch 2−2427 : Gut microbiota abundance (genus Catenibacterium id.2153) on Gap junction alpha−8 protein
with a threshold of 1e−05

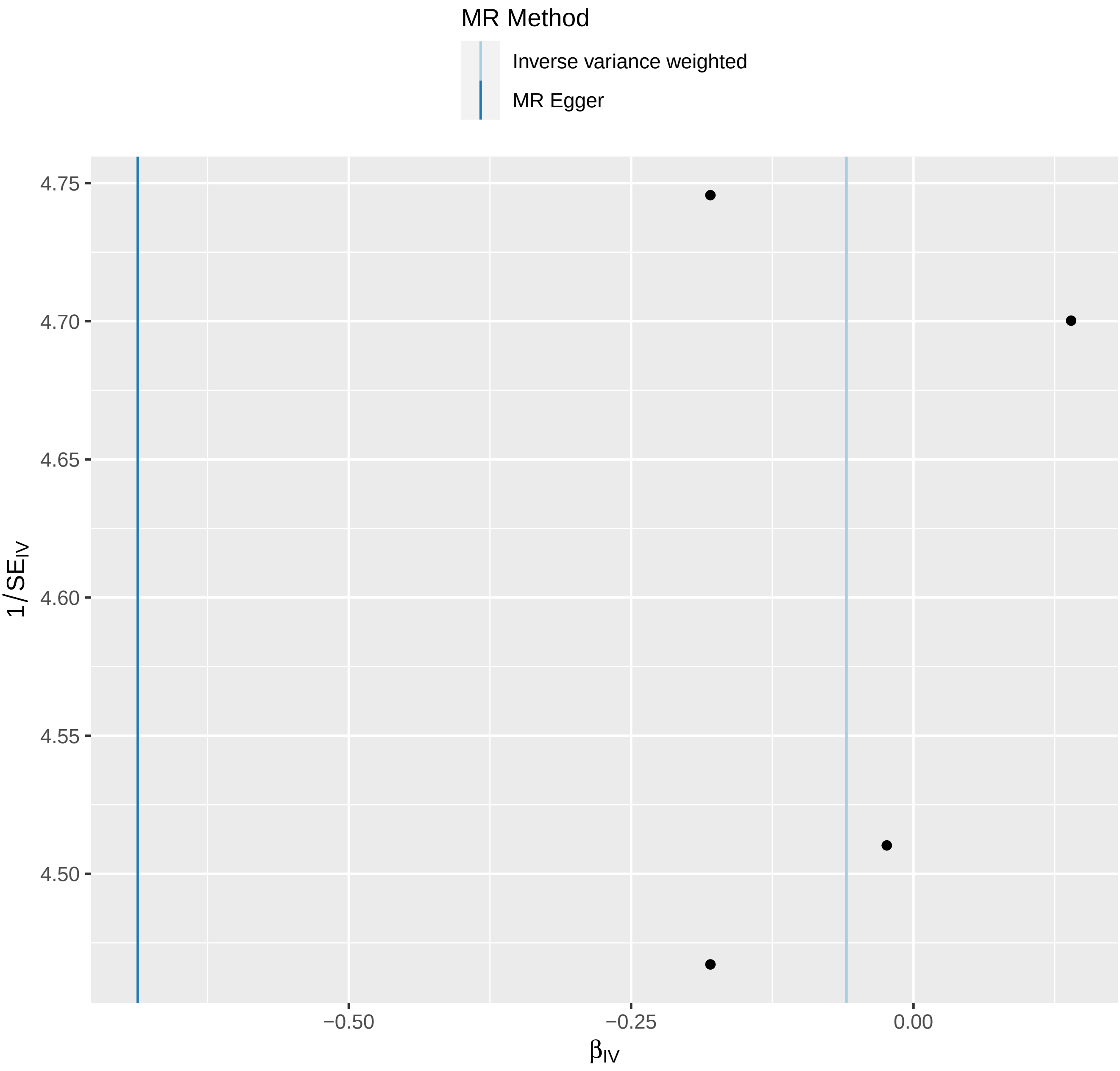

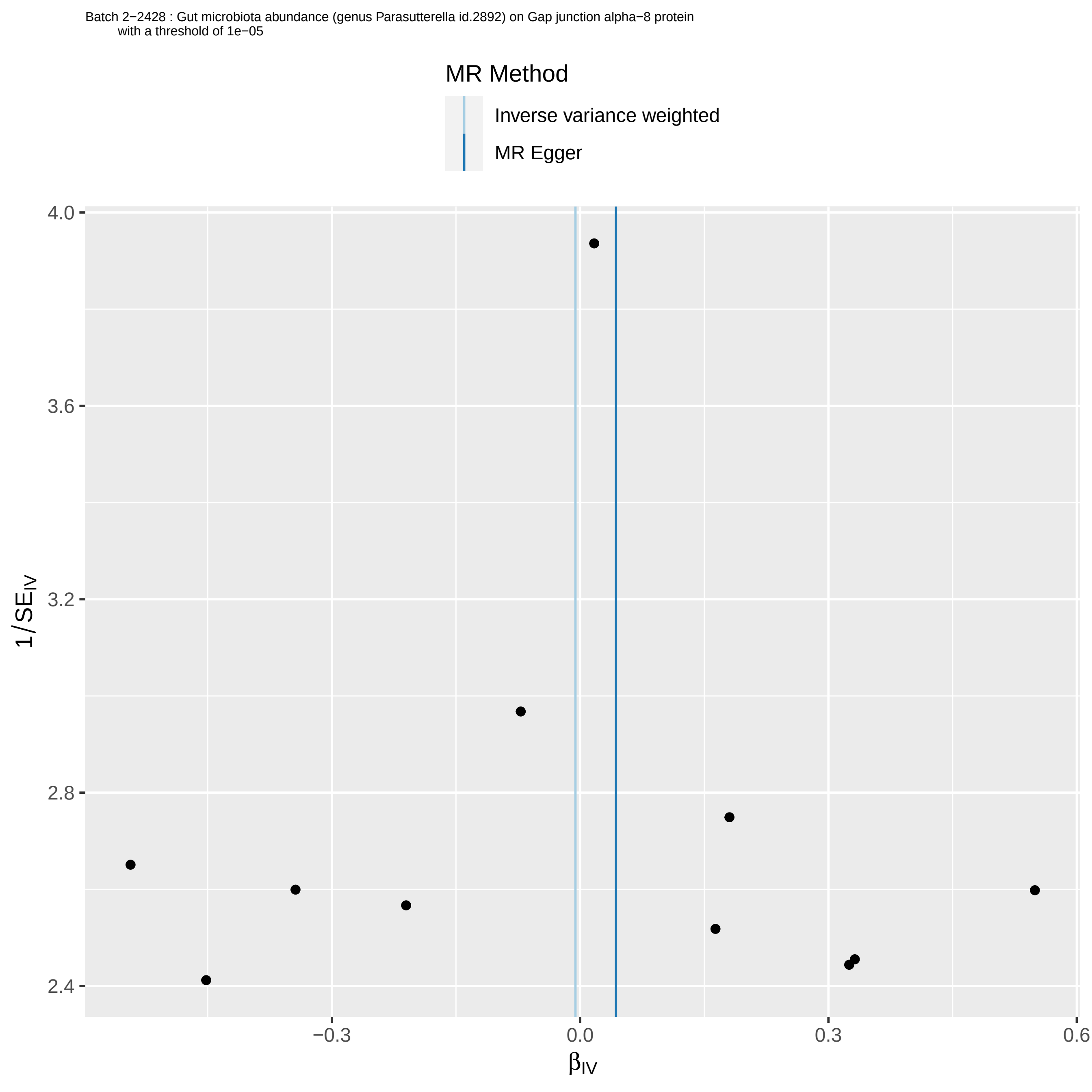
Batch 2−2428 : Gut microbiota abundance (genus Parasutterella id.2892) on Gap junction alpha−8 protein
with a threshold of 1e−05
MR Method
Inverse variance weighted
MR Egger
4.0
3.6
3.2
2.8
2.4
1/SEIV
−0.3
0.0
0.3
0.6
βIV

Batch 2−2429 : Gut microbiota abundance (genus Catenibacterium id.2153) on Gap junction delta−2 protein
with a threshold of 1e−05

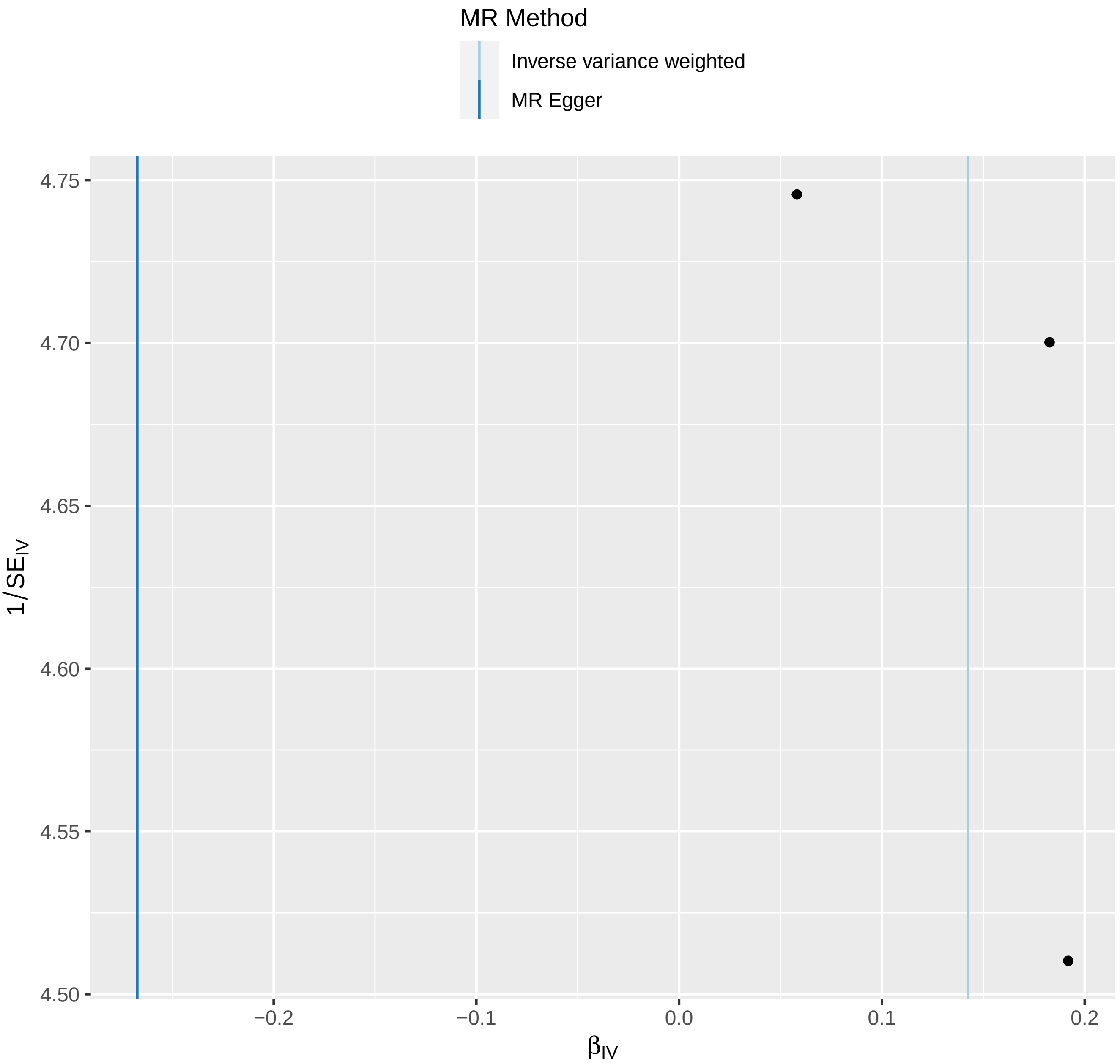

Batch 2−2430 : Gut microbiota abundance (genus Parasutterella id.2892) on Gap junction delta−2 protein
with a threshold of 1e−05

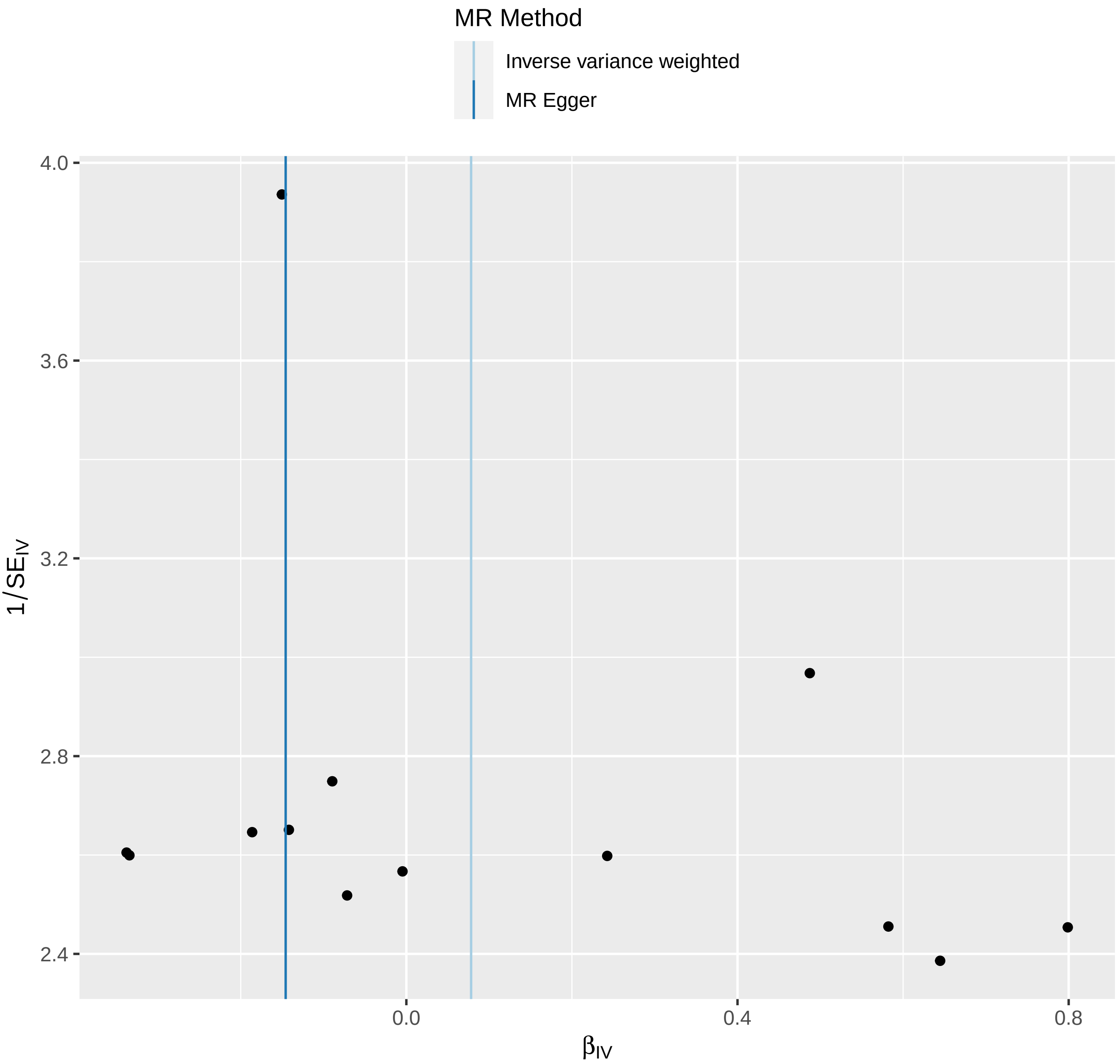

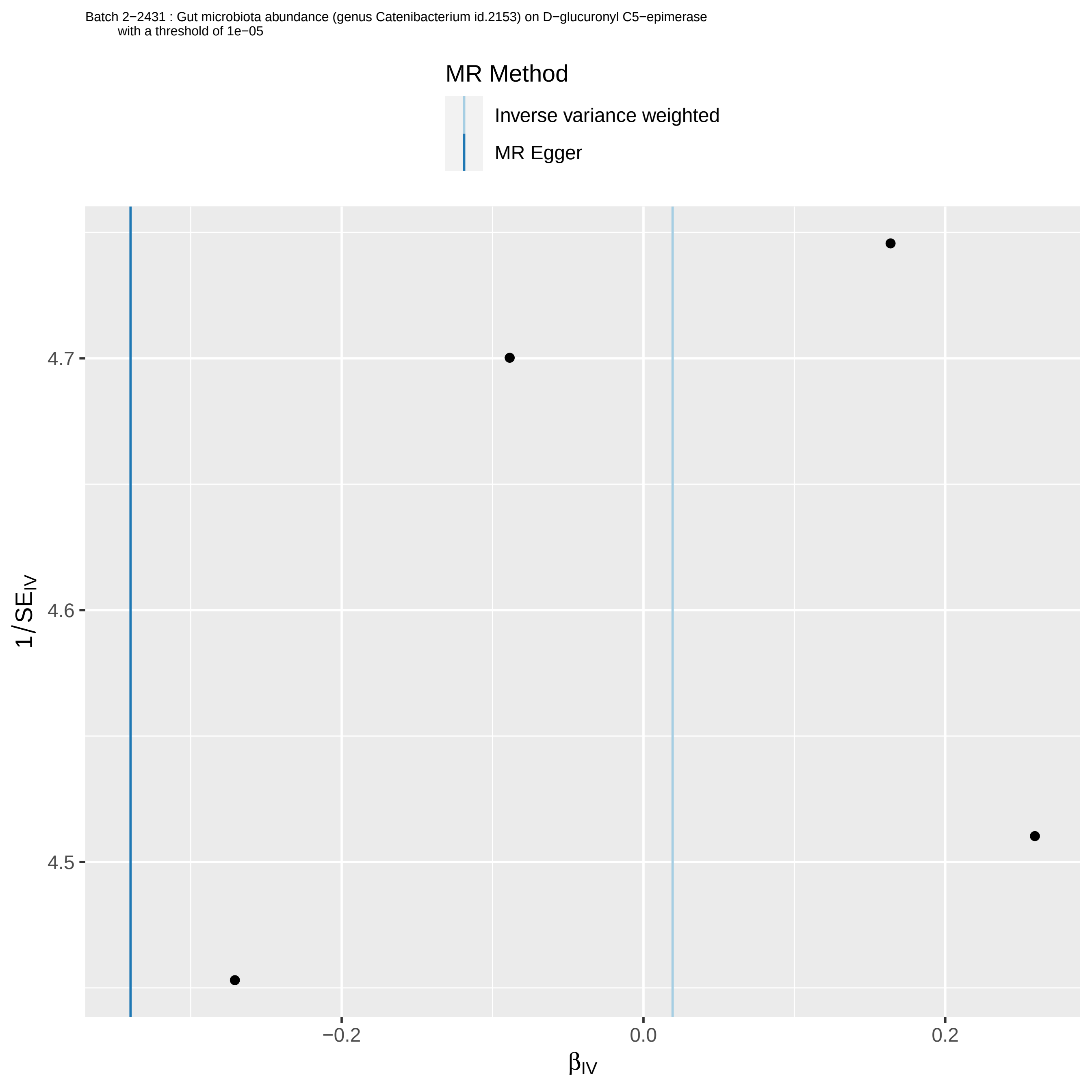
Batch 2−2431 : Gut microbiota abundance (genus Catenibacterium id.2153) on D−glucuronyl C5−epimerase
with a threshold of 1e−05
MR Method
Inverse variance weighted
MR Egger
4.7
4.6
4.5
1/SE_IV
−0.2
0.0
0.2
β_IV

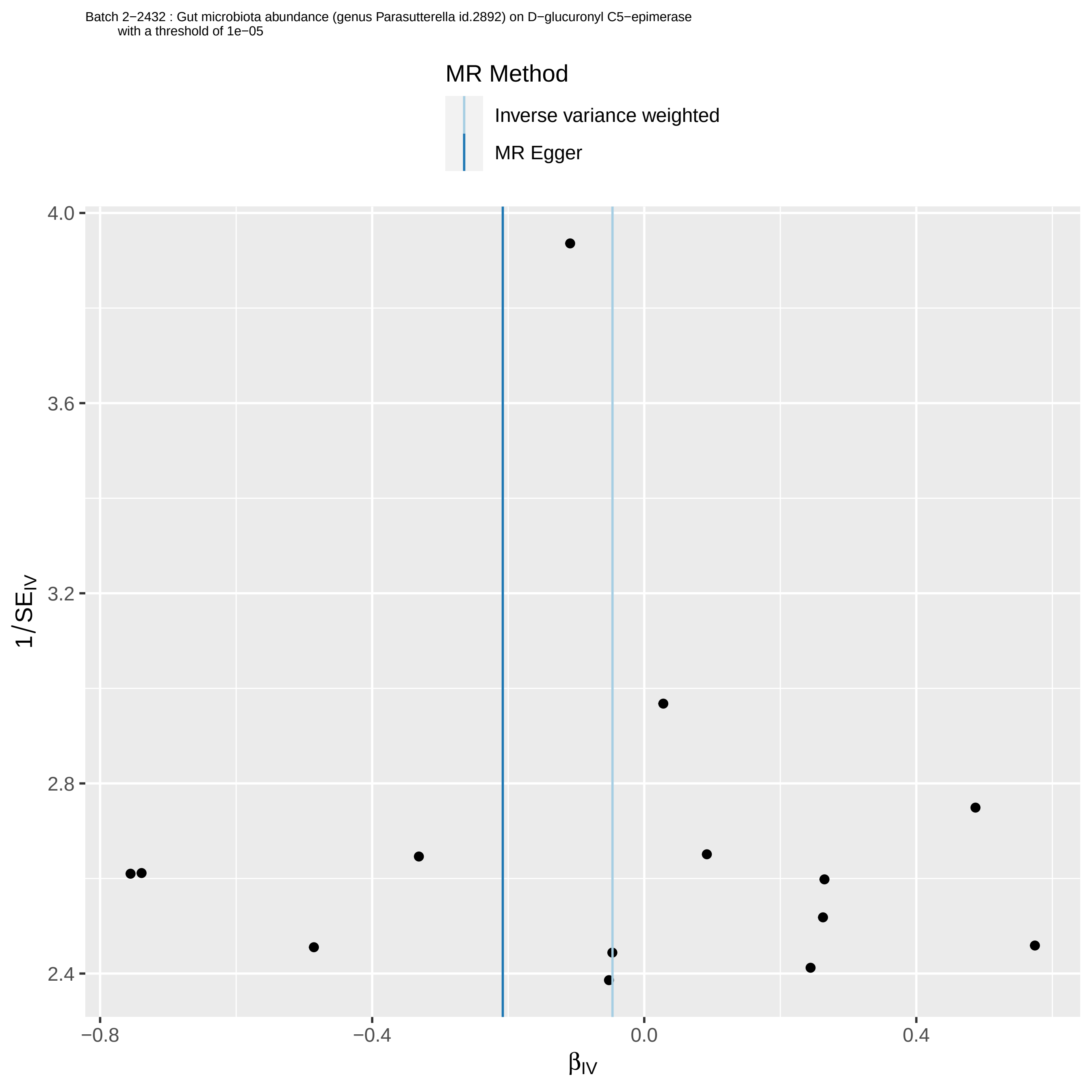
Batch 2−2432 : Gut microbiota abundance (genus Parasutterella id.2892) on D−glucuronyl C5−epimerase
with a threshold of 1e−05
MR Method
Inverse variance weighted
MR Egger
4.0
3.6
3.2
2.8
2.4
1/SE_IV
−0.8
−0.4
0.0
0.4
β_IV

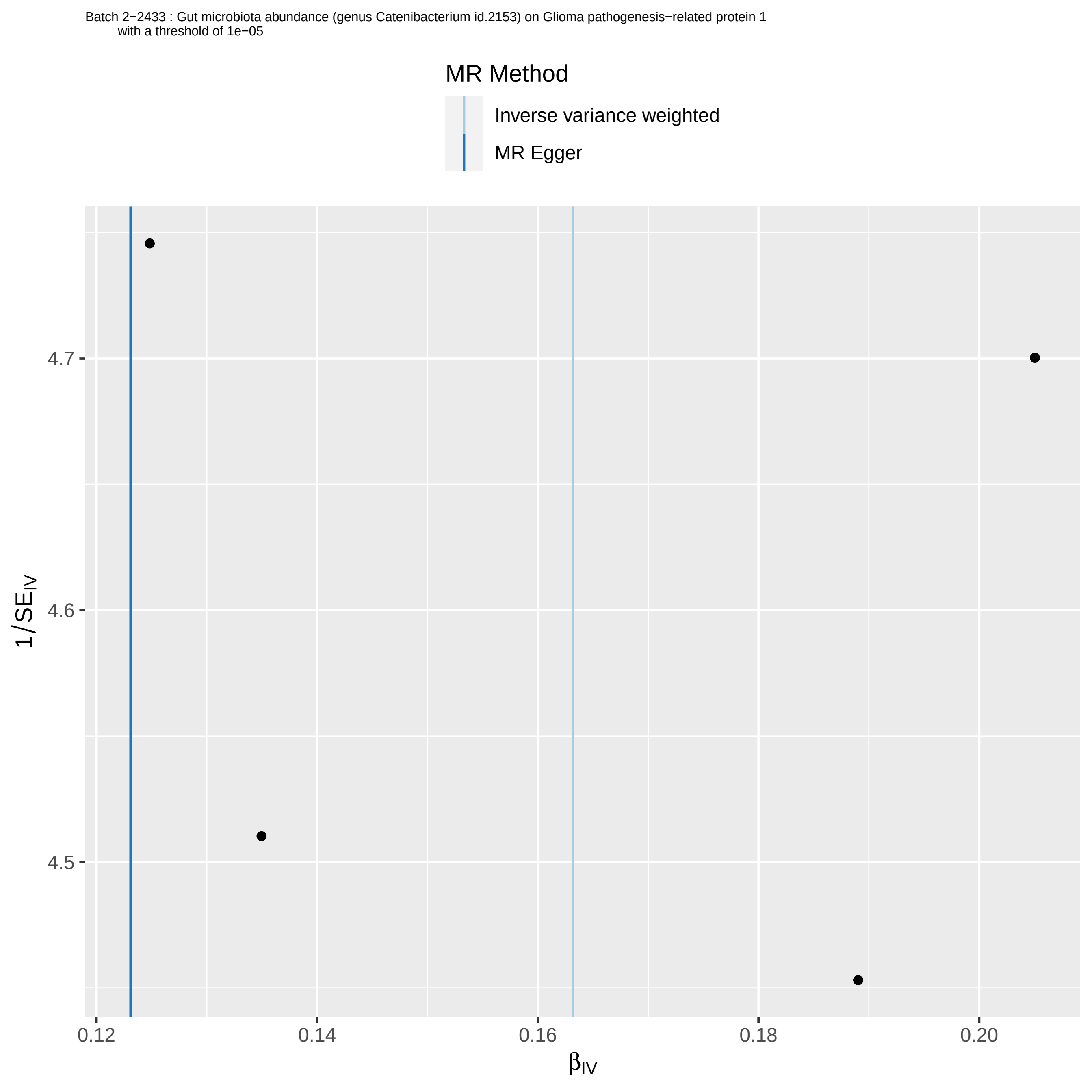
Batch 2−2433 : Gut microbiota abundance (genus Catenibacterium id.2153) on Glioma pathogenesis−related protein 1
with a threshold of 1e−05
MR Method
Inverse variance weighted
MR Egger
4.7
4.6
4.5
1/SE$_{IV}$
0.12
0.14
0.16
0.18
0.20
β$_{IV}$

Batch 2−2434 : Gut microbiota abundance (genus Parasutterella id.2892) on Glioma pathogenesis−related protein 1
with a threshold of 1e−05

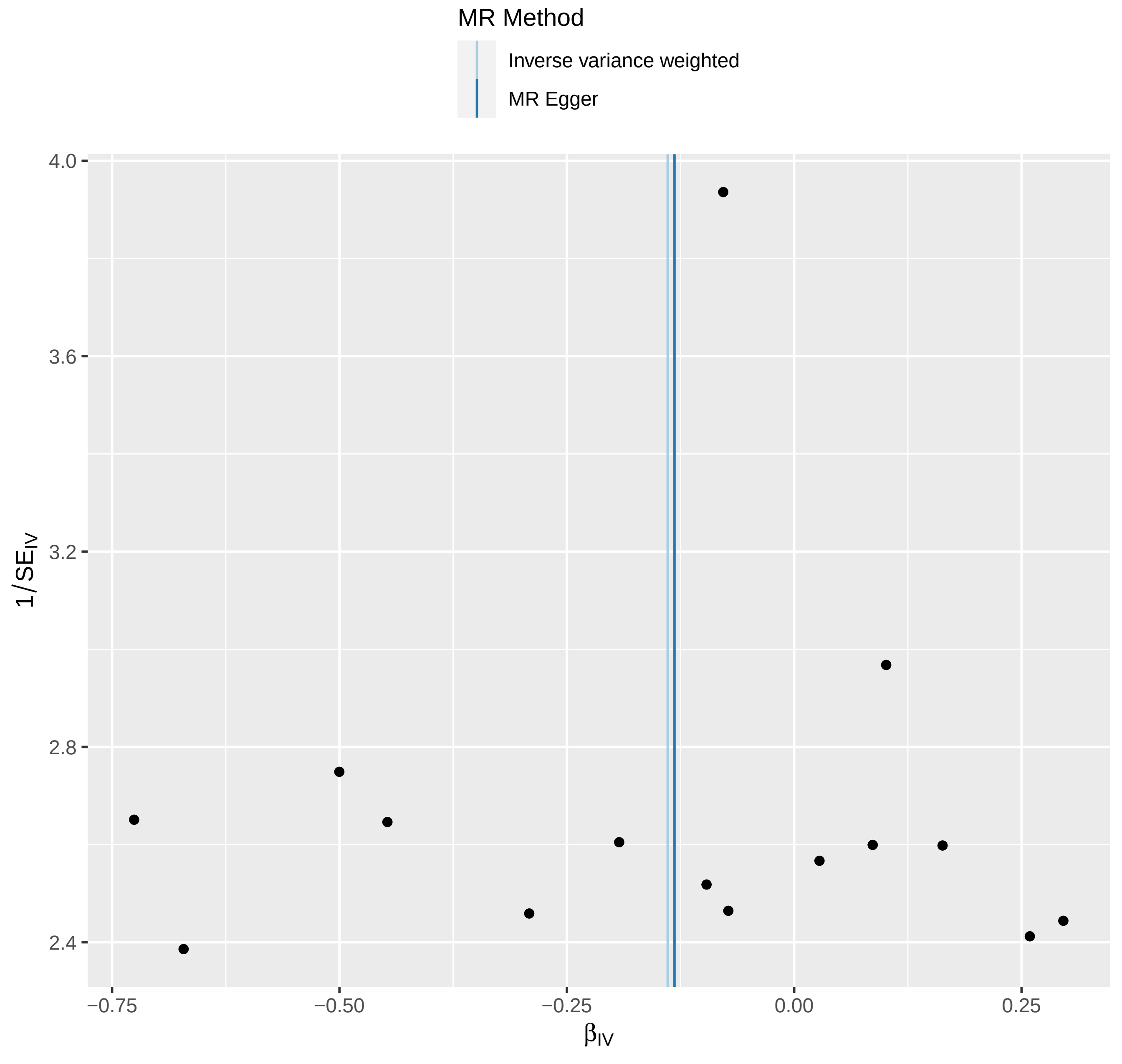

Batch 2−2435 : Gut microbiota abundance (genus Catenibacterium id.2153) on Lactoylglutathione lyase
with a threshold of 1e−05

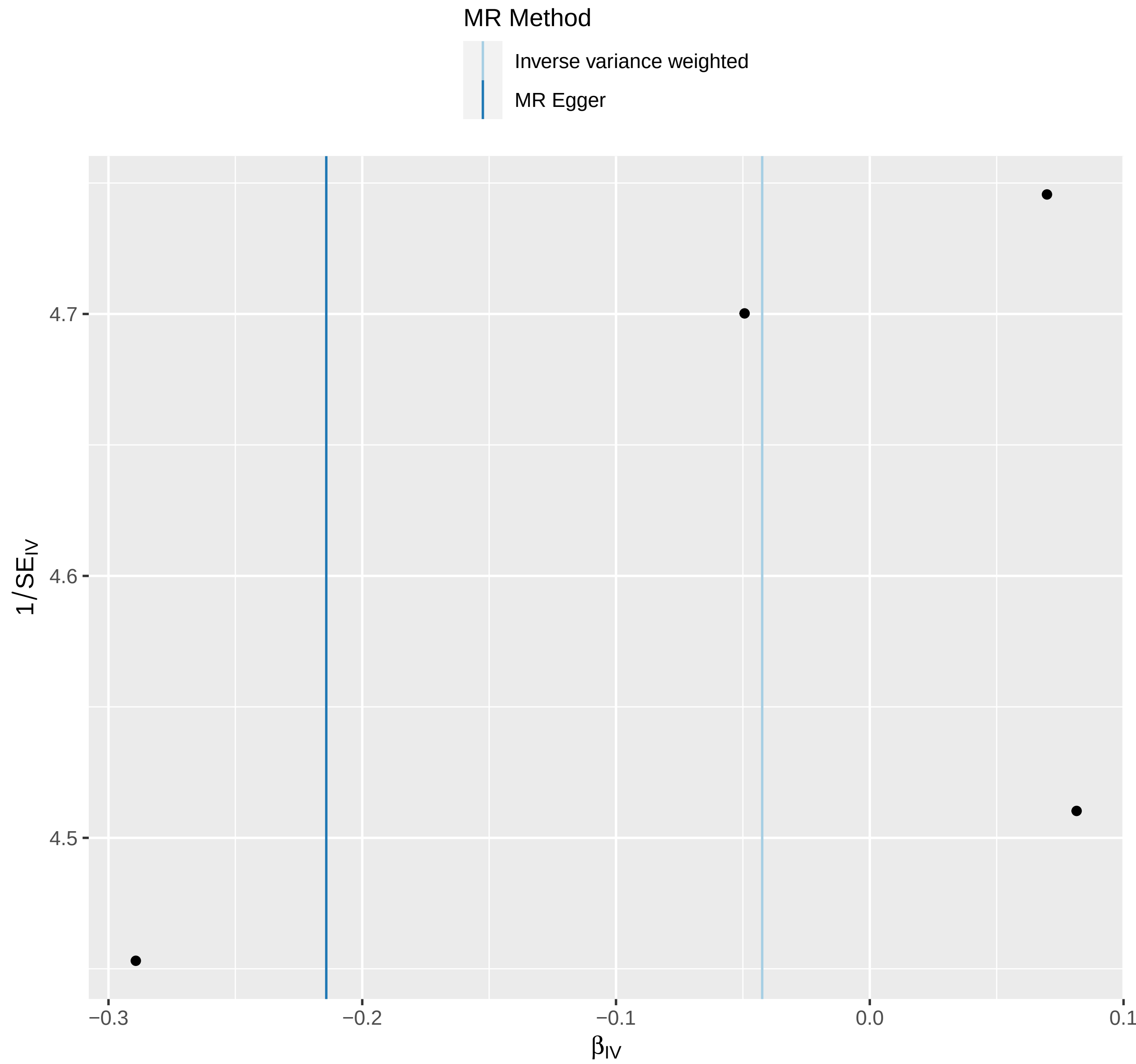

Batch 2−2436 : Gut microbiota abundance (genus Parasutterella id.2892) on Lactoylglutathione lyase
with a threshold of 1e−05

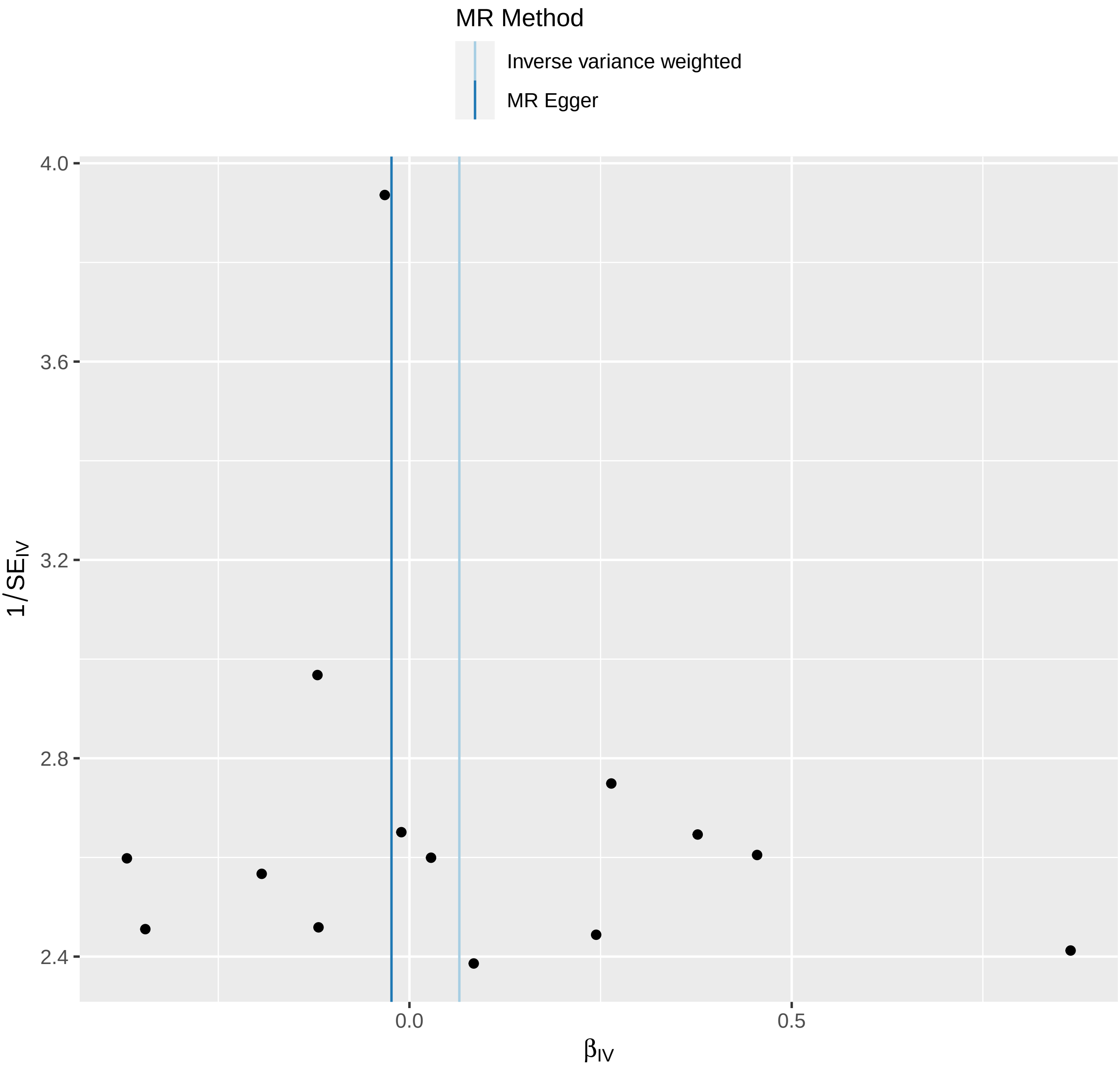

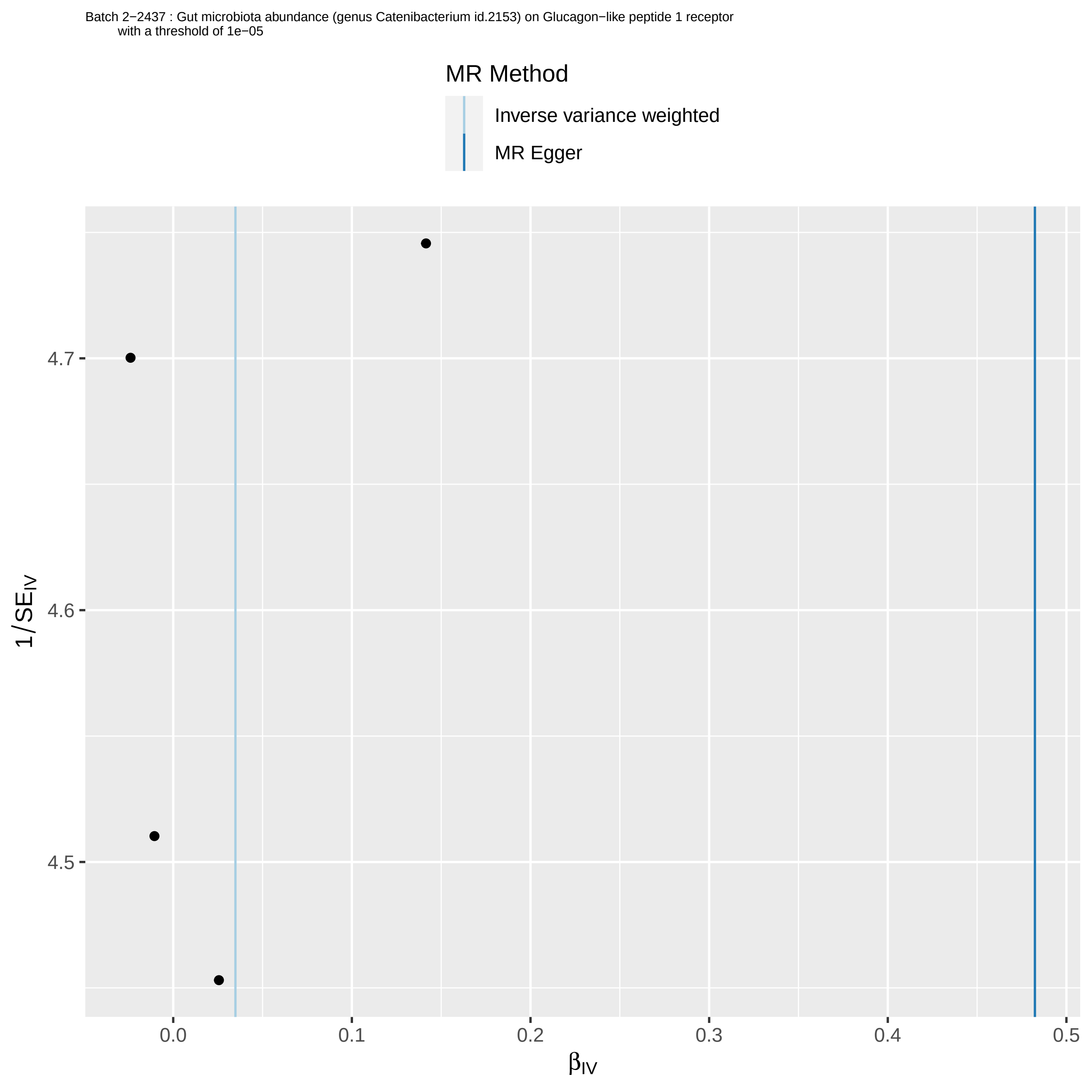

Batch 2−2437 : Gut microbiota abundance (genus Catenibacterium id.2153) on Glucagon−like peptide 1 receptor
with a threshold of 1e−05
MR Method
Inverse variance weighted
MR Egger
4.7
4.6
4.5
1/SE_IV
0.0
0.1
0.2
0.3
0.4
0.5
β_IV

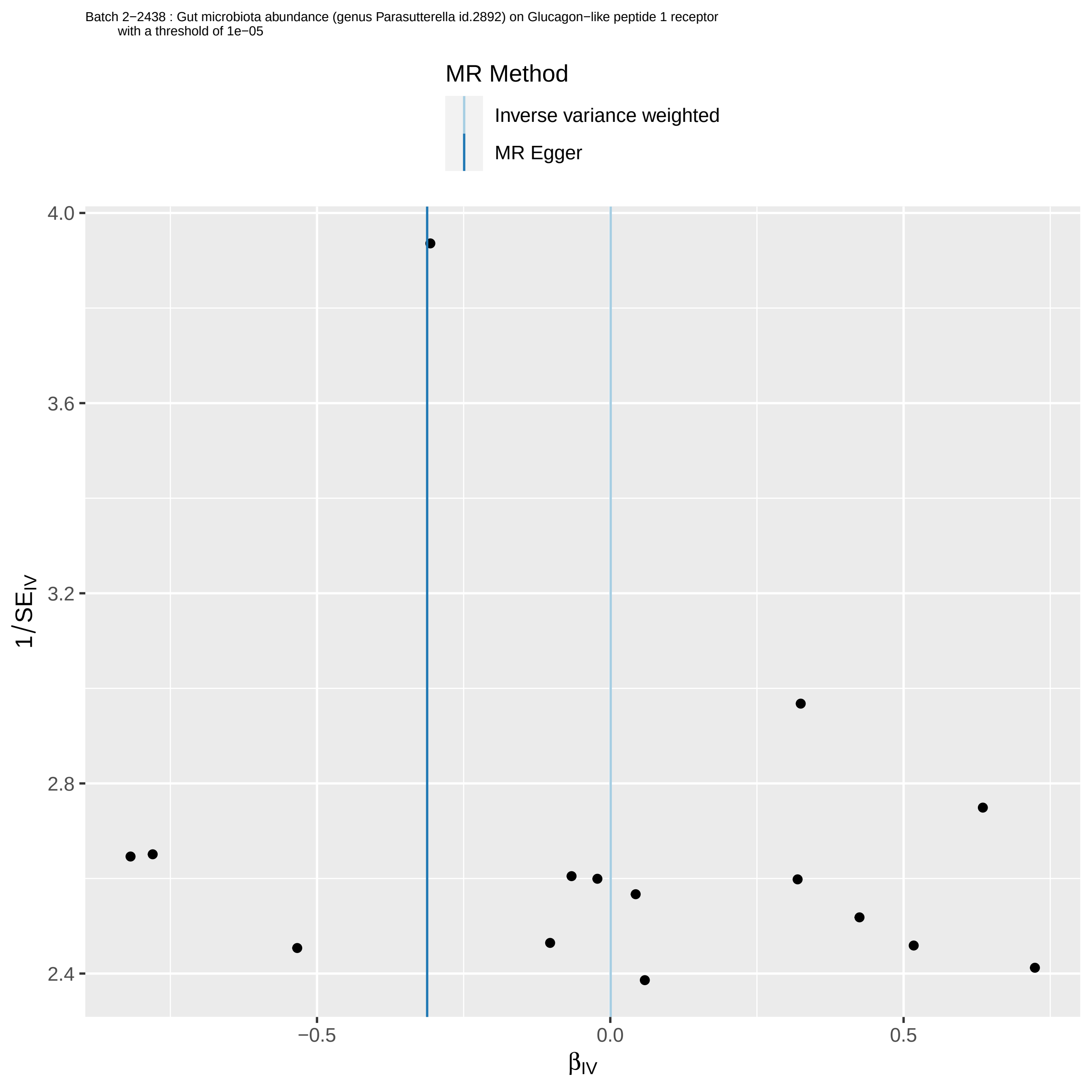

Batch 2−2438 : Gut microbiota abundance (genus Parasutterella id.2892) on Glucagon−like peptide 1 receptor with a threshold of 1e−05
MR Method
Inverse variance weighted
MR Egger
4.0
3.6
3.2
2.8
2.4
1/SE_IV
−0.5
0.0
0.5
β_IV

Batch 2−2439 : Gut microbiota abundance (genus Catenibacterium id.2153) on Glutaredoxin−2, mitochondrial
with a threshold of 1e−05

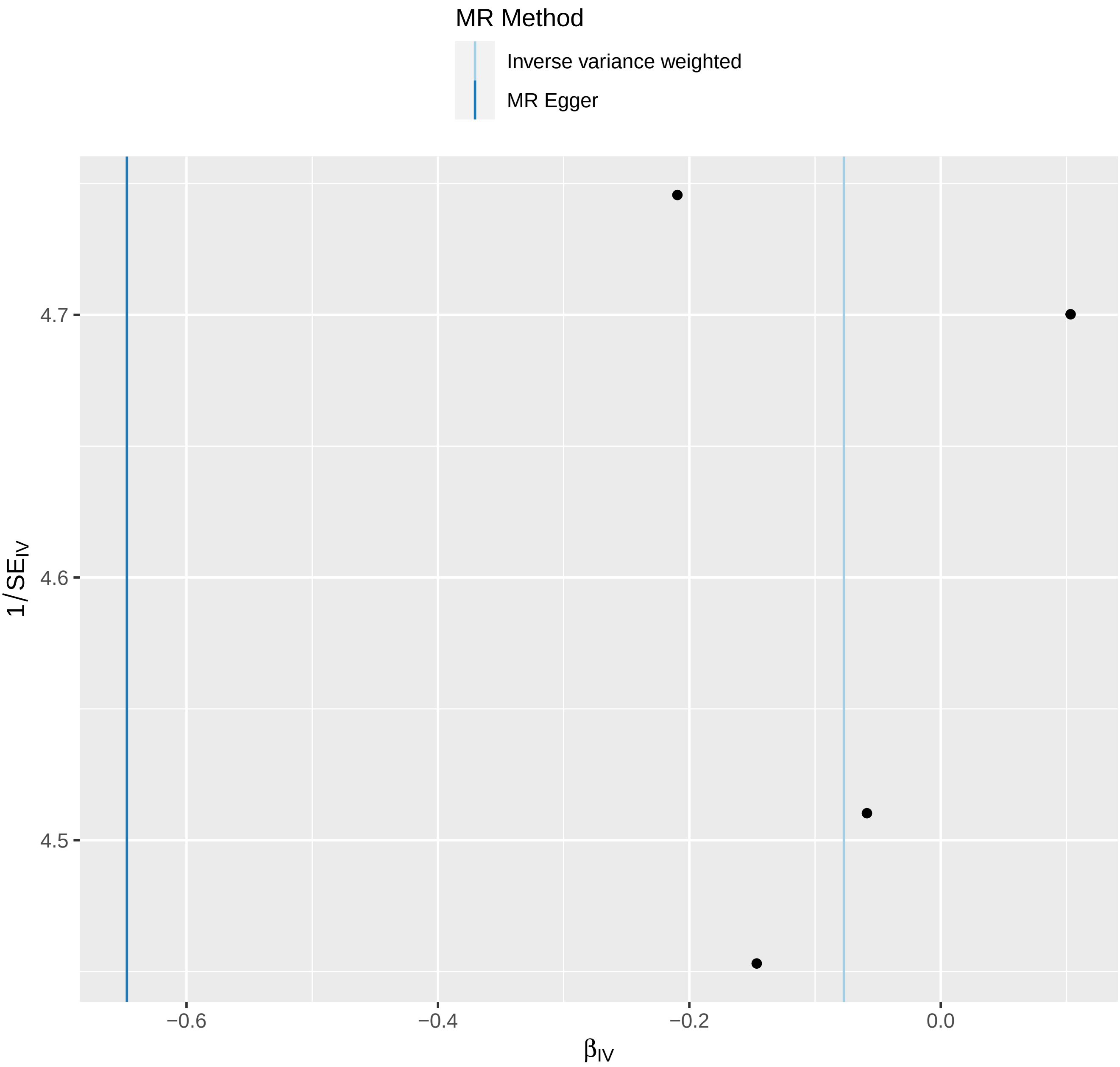

Batch 2−2440 : Gut microbiota abundance (genus Parasutterella id.2892) on Glutaredoxin−2, mitochondrial
with a threshold of 1e−05

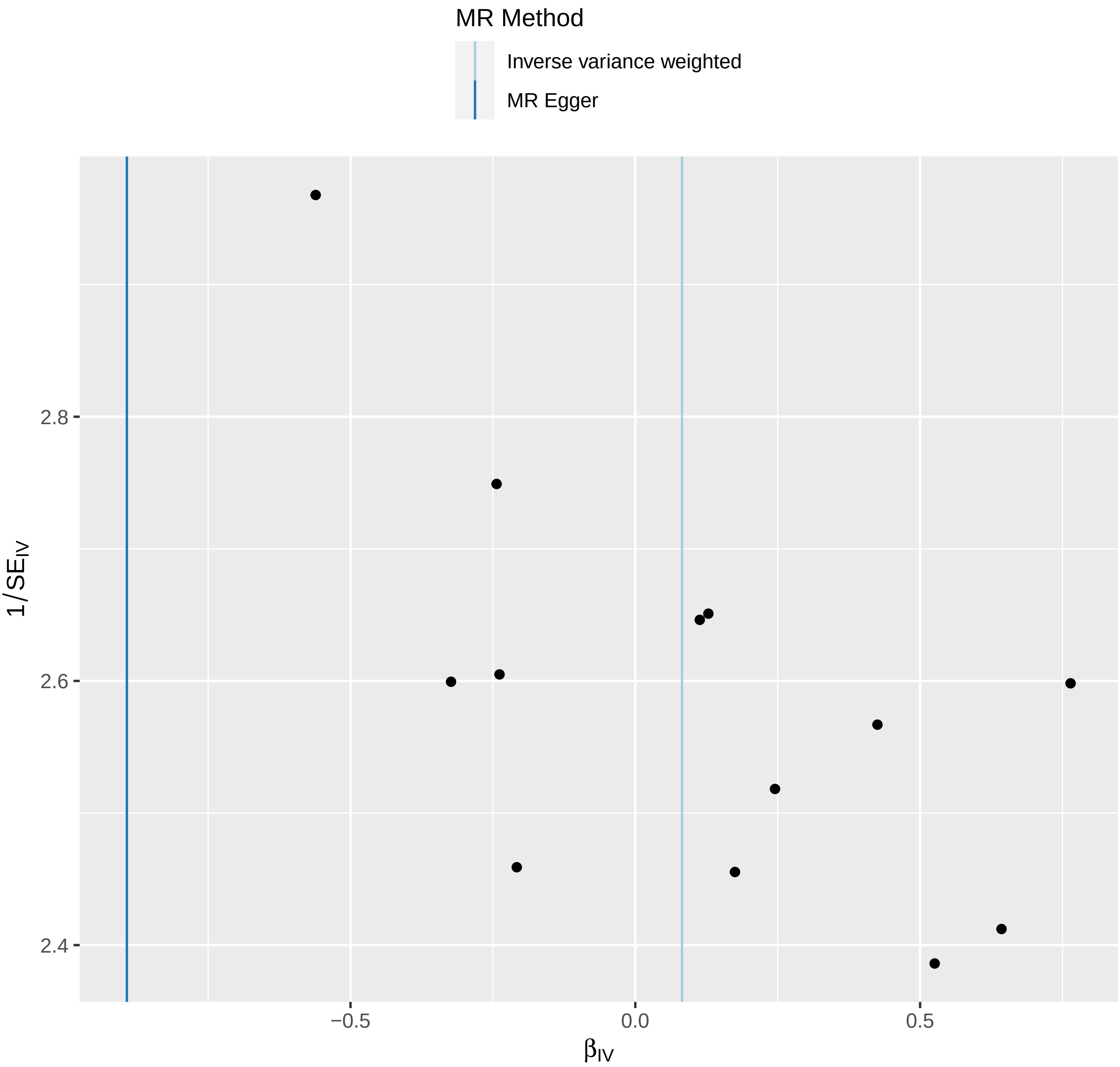

Batch 2−2441 : Gut microbiota abundance (genus Catenibacterium id.2153) on Glycosyltransferase 8 domain−containing protein 1 with a threshold of 1e−05

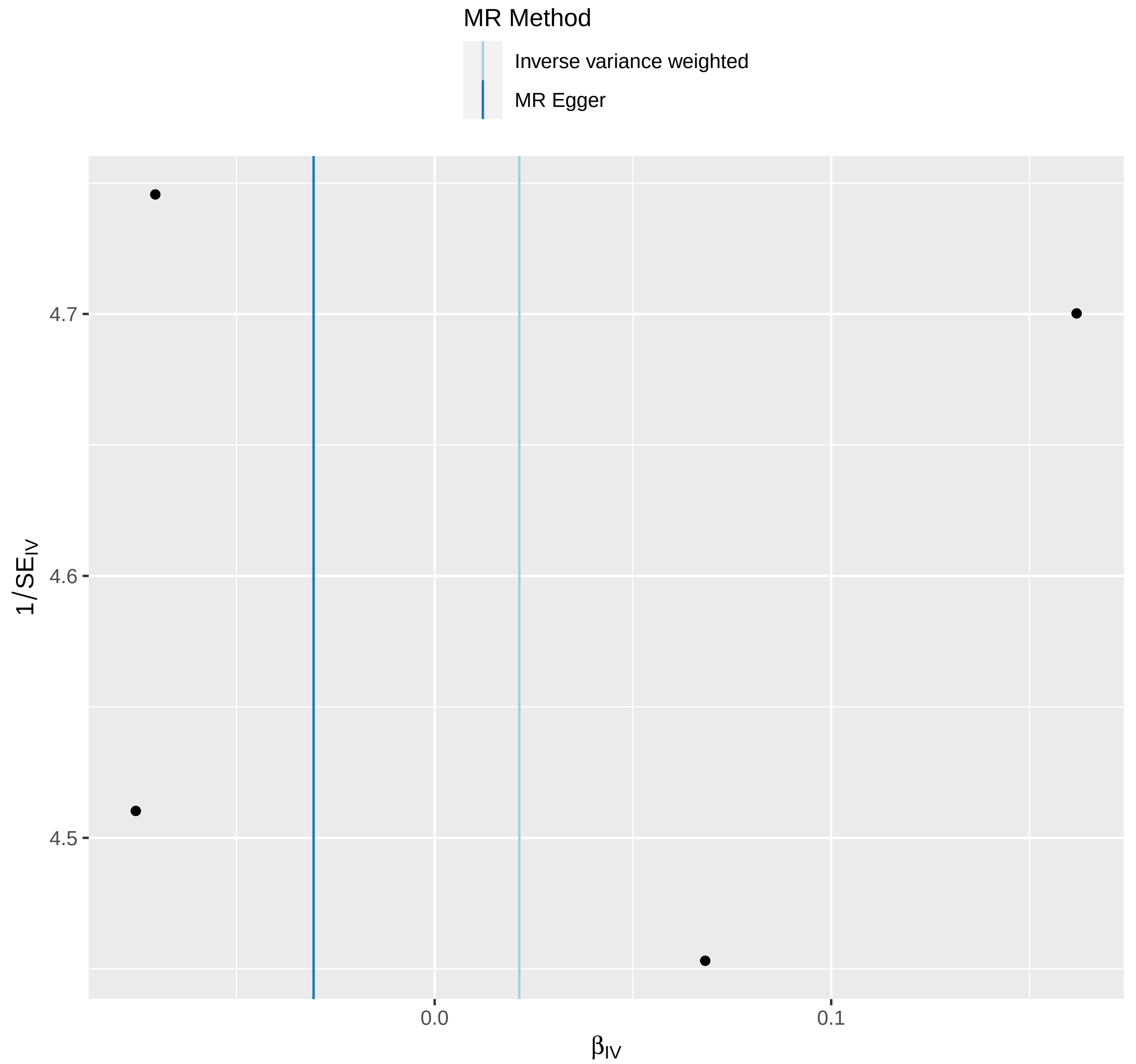

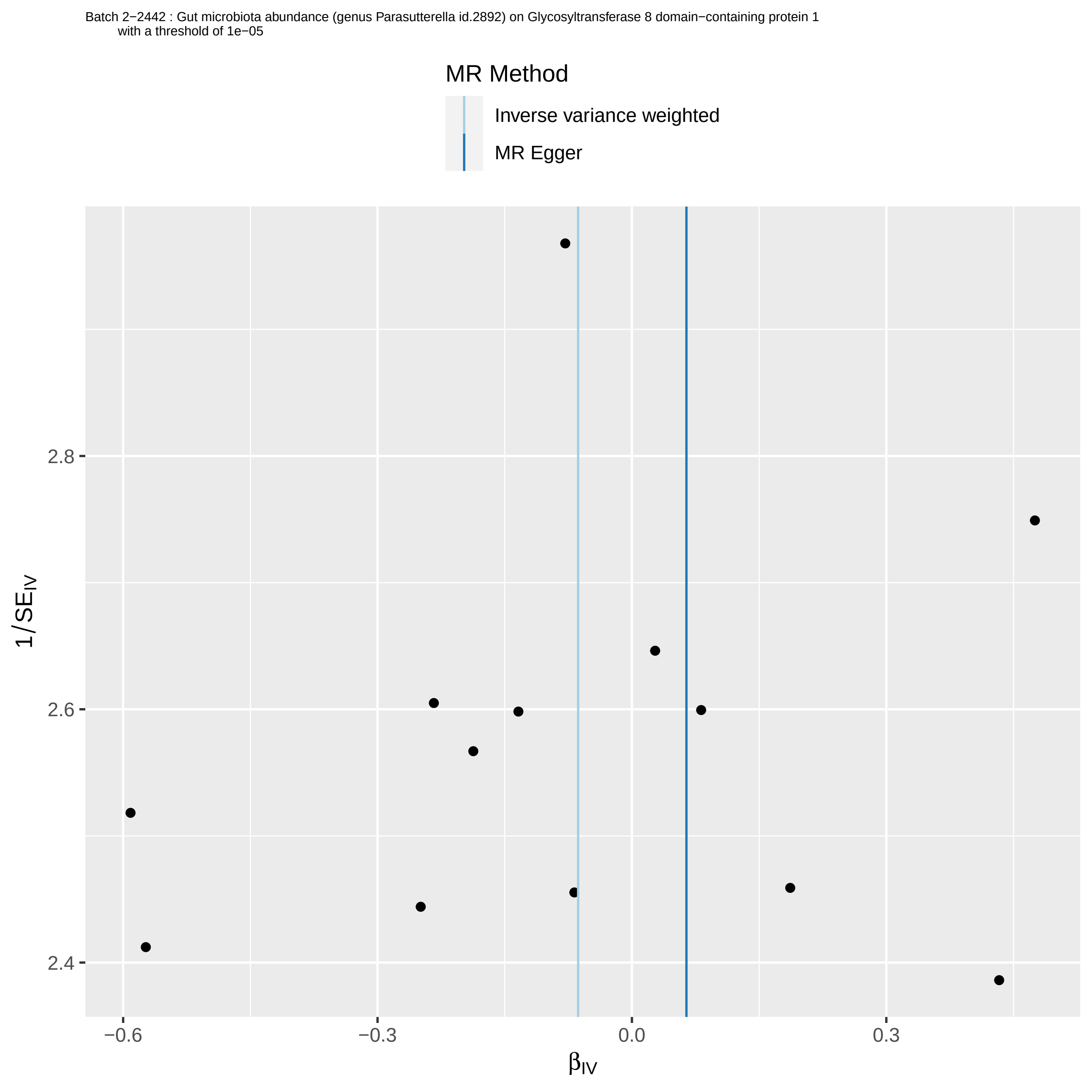
Batch 2−2442 : Gut microbiota abundance (genus Parasutterella id.2892) on Glycosyltransferase 8 domain−containing protein 1
with a threshold of 1e−05
MR Method
Inverse variance weighted
MR Egger
2.8
2.6
2.4
$1/SE_{IV}$
−0.6
−0.3
0.0
0.3
β_{IV}

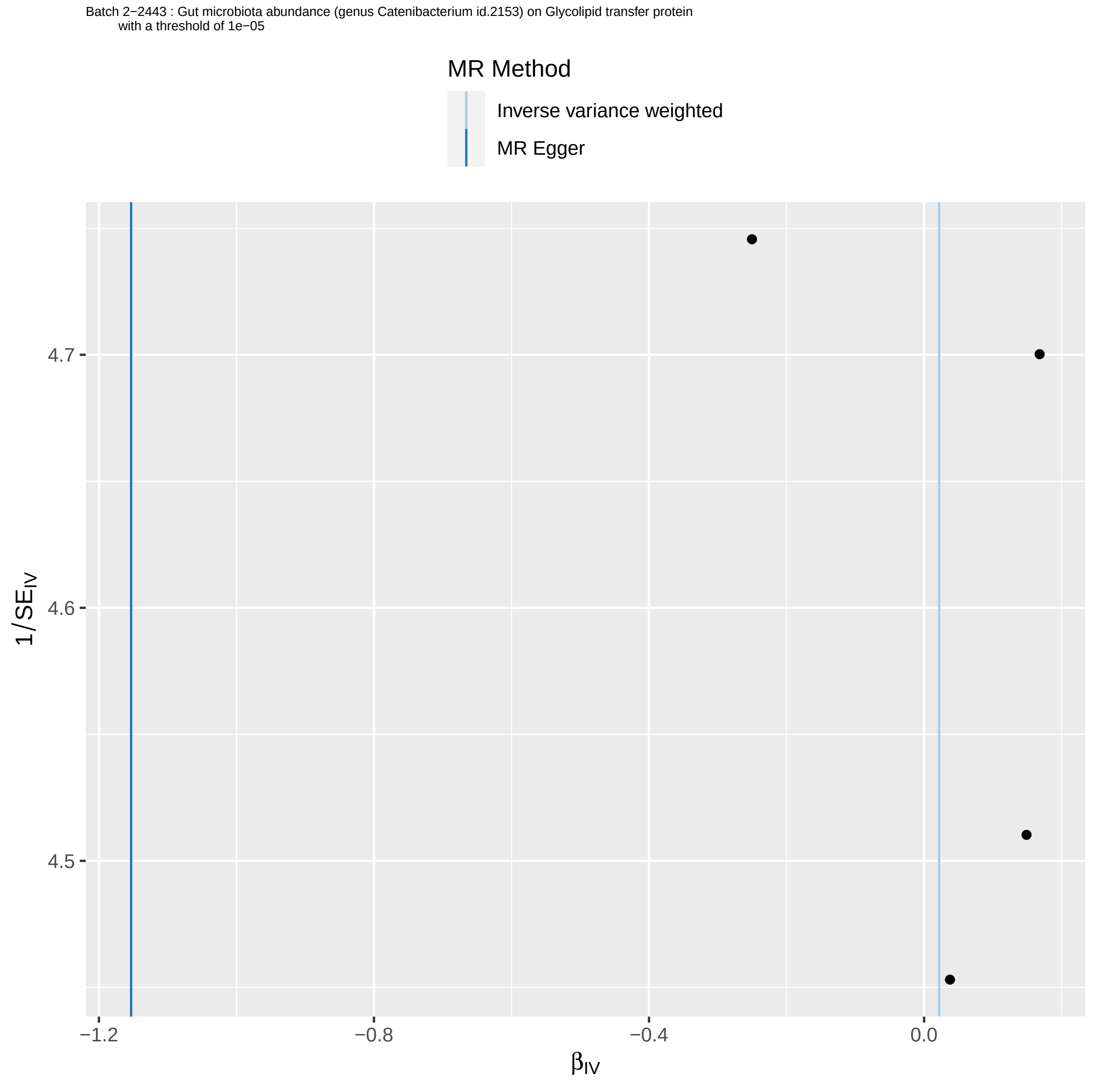

Batch 2−2443 : Gut microbiota abundance (genus Catenibacterium id.2153) on Glycolipid transfer protein
with a threshold of 1e−05
MR Method
Inverse variance weighted
MR Egger
4.7
4.6
4.5
1/SE_IV
−1.2
−0.8
−0.4
0.0
β_IV

Batch 2−2444 : Gut microbiota abundance (genus Parasutterella id.2892) on Glycolipid transfer protein
with a threshold of 1e−05

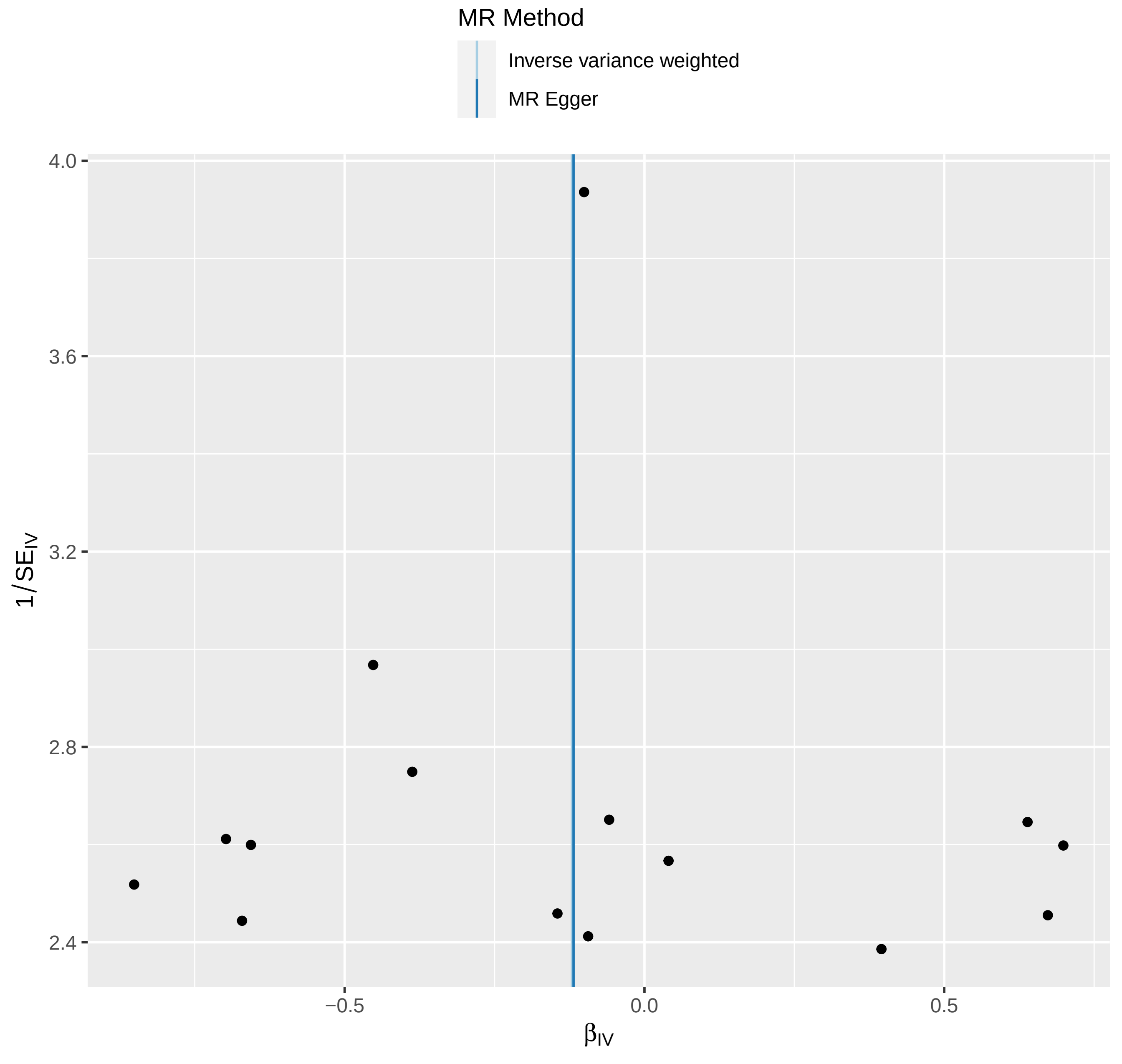

Batch 2−2445 : Gut microbiota abundance (genus Catenibacterium id.2153) on Glycolipid transfer protein domain−containing protein 2 with a threshold of 1e−05

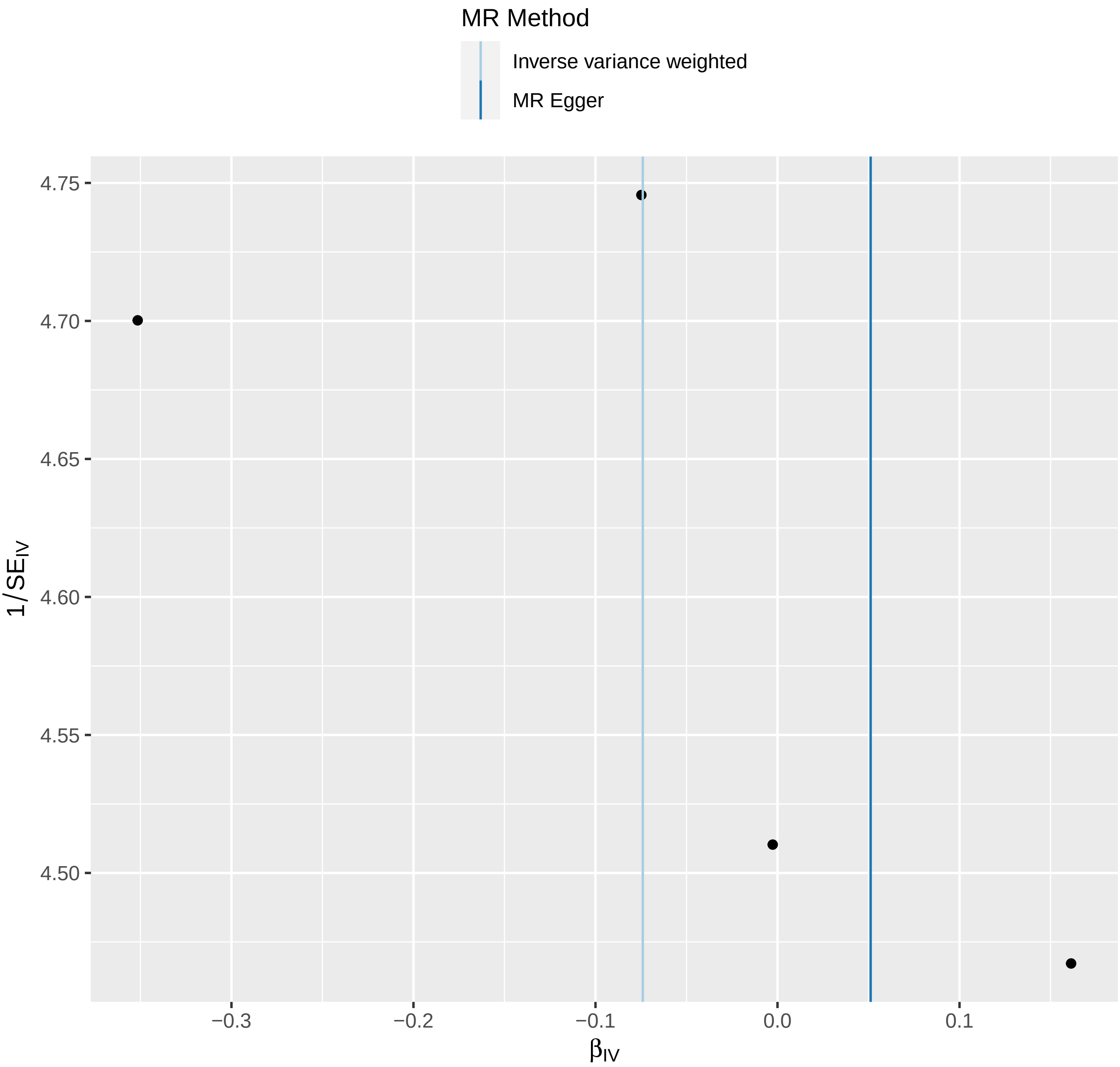

Batch 2−2446 : Gut microbiota abundance (genus Parasutterella id.2892) on Glycolipid transfer protein domain−containing protein 2
with a threshold of 1e−05

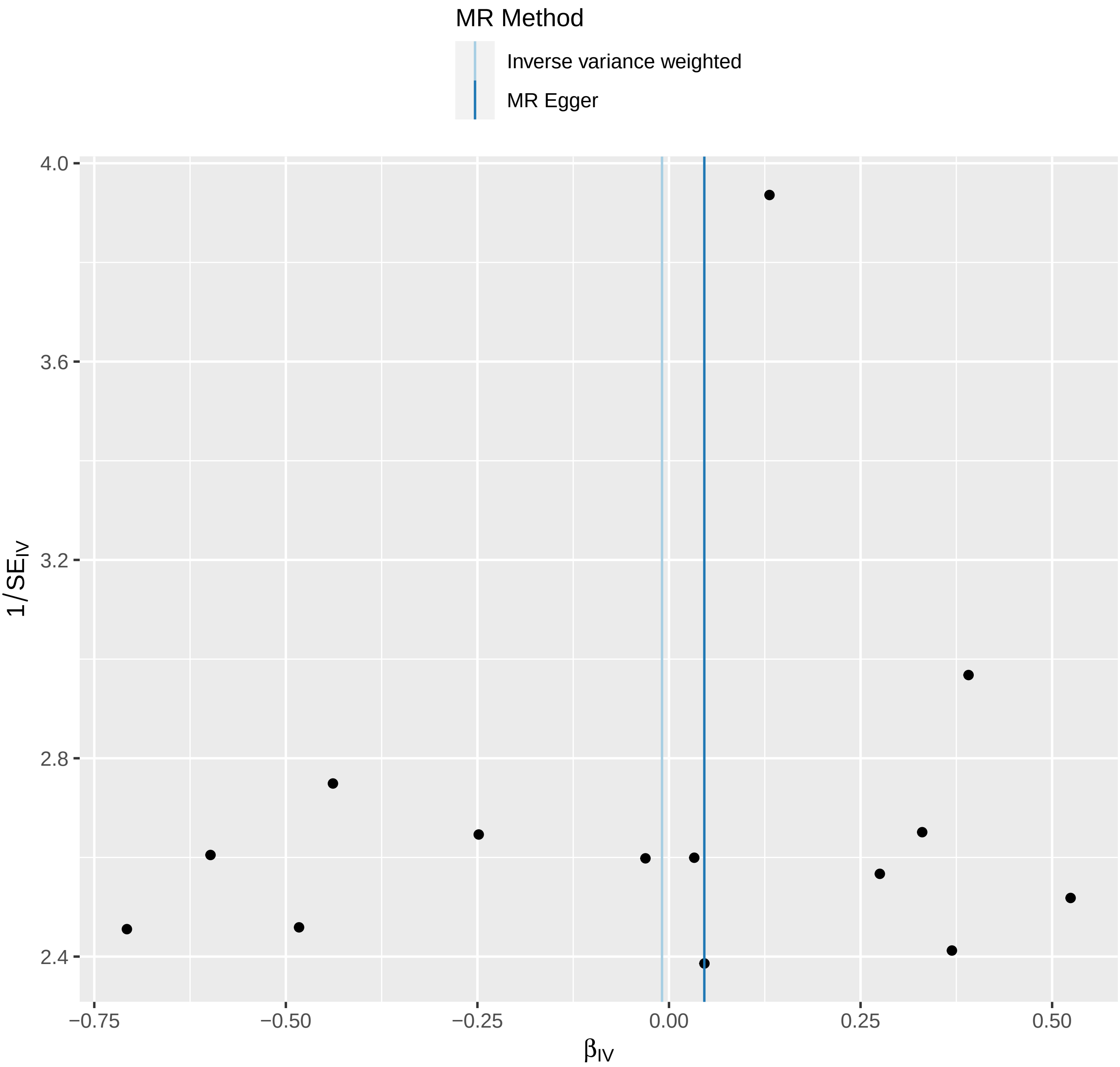

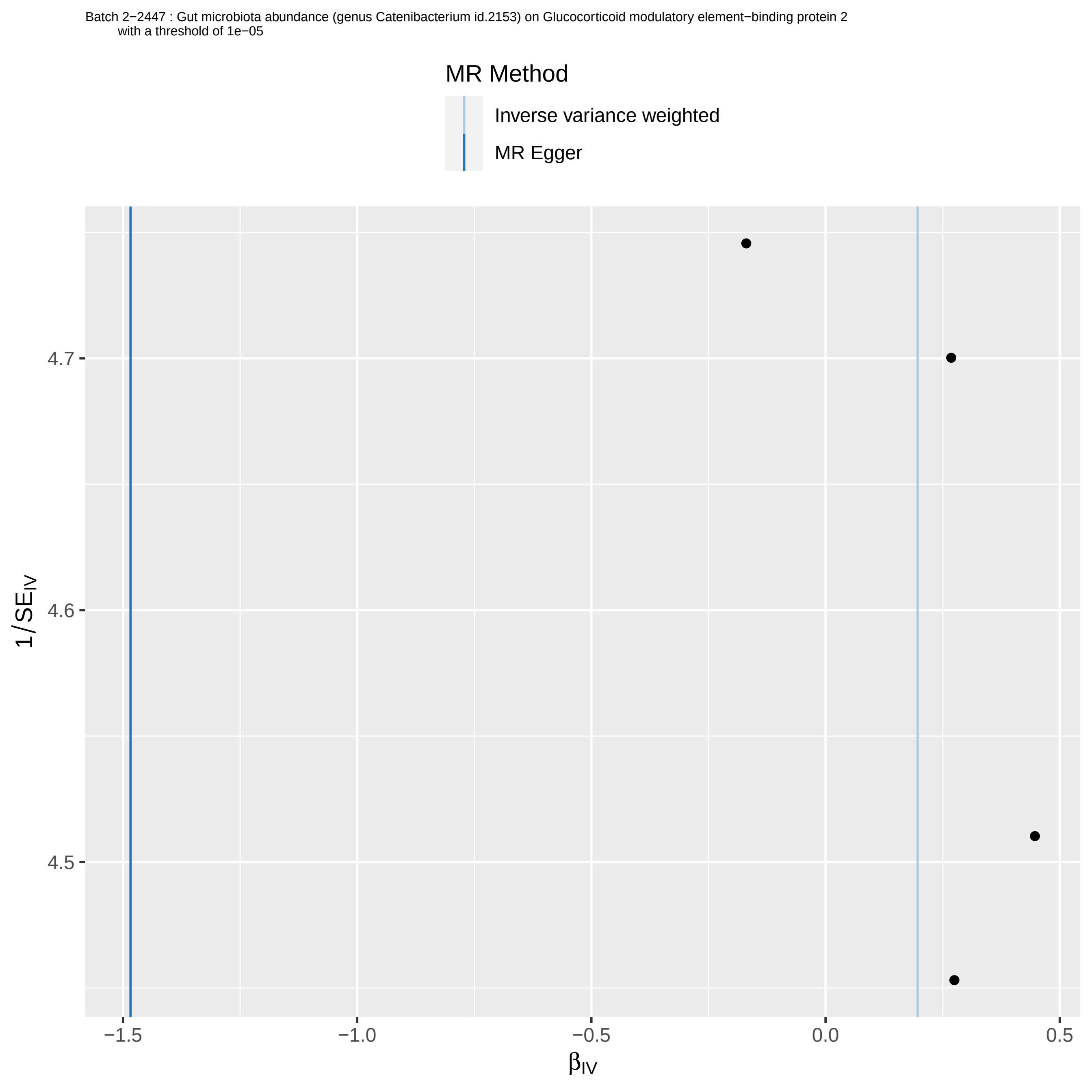
Batch 2−2447 : Gut microbiota abundance (genus Catenibacterium id.2153) on Glucocorticoid modulatory element−binding protein 2
with a threshold of 1e−05
MR Method
Inverse variance weighted
MR Egger
4.7
4.6
4.5
1/SE_IV
−1.5
−1.0
−0.5
0.0
0.5
β_IV

Batch 2−2448 : Gut microbiota abundance (genus Parasutterella id.2892) on Glucocorticoid modulatory element−binding protein 2
with a threshold of 1e−05

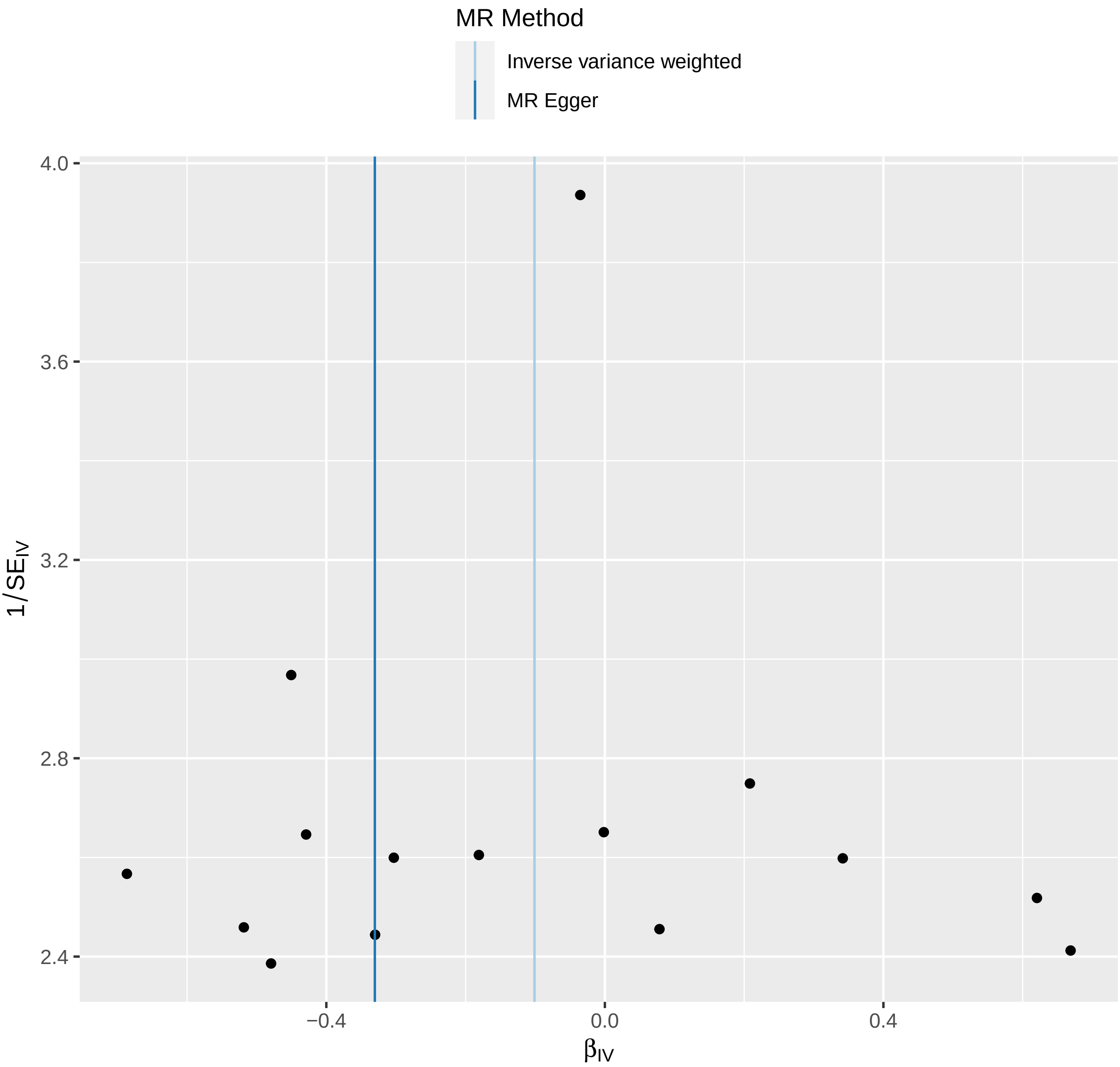

Batch 2−2449 : Gut microbiota abundance (genus Catenibacterium id.2153) on Glia maturation factor gamma
with a threshold of 1e−05

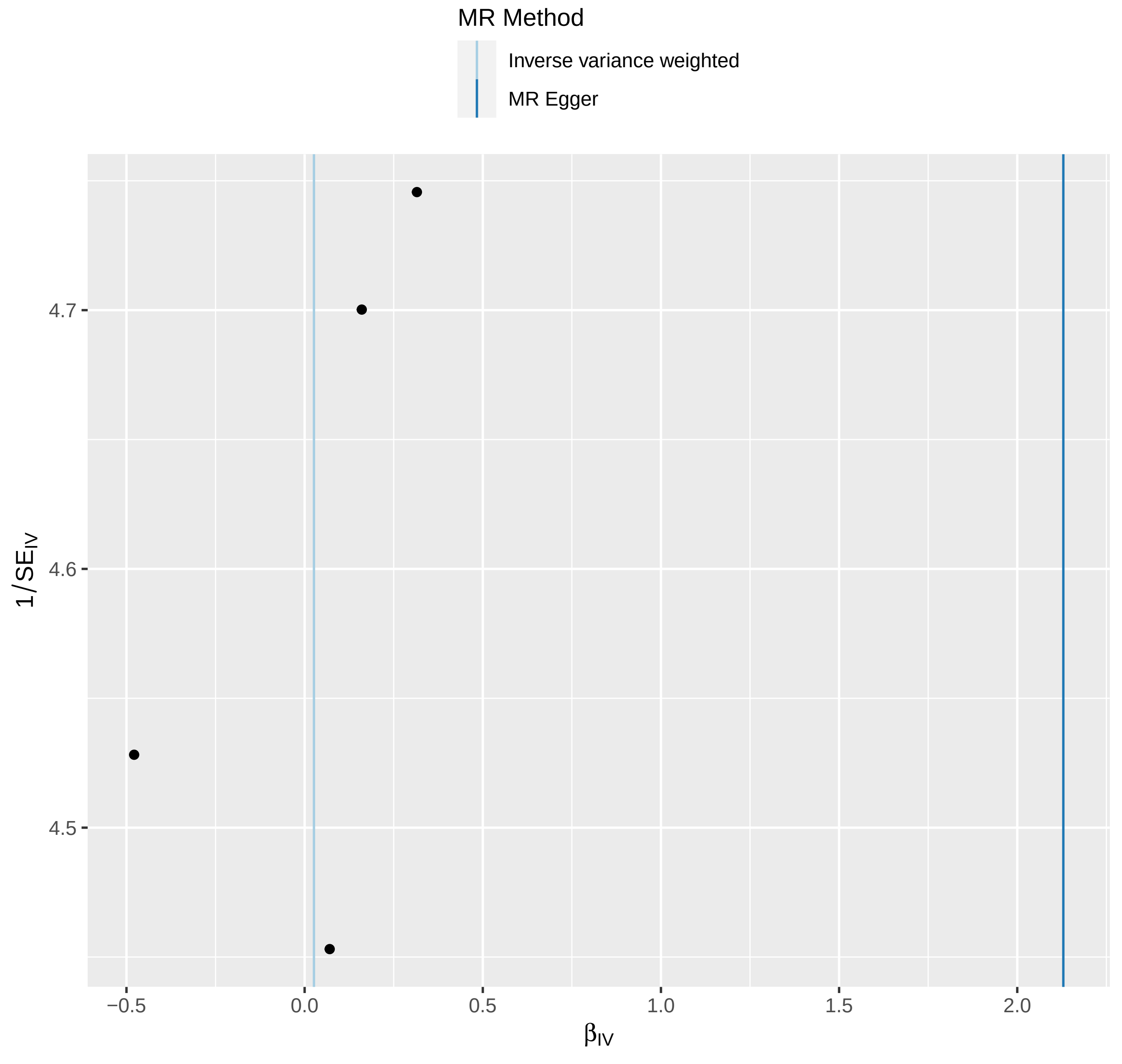

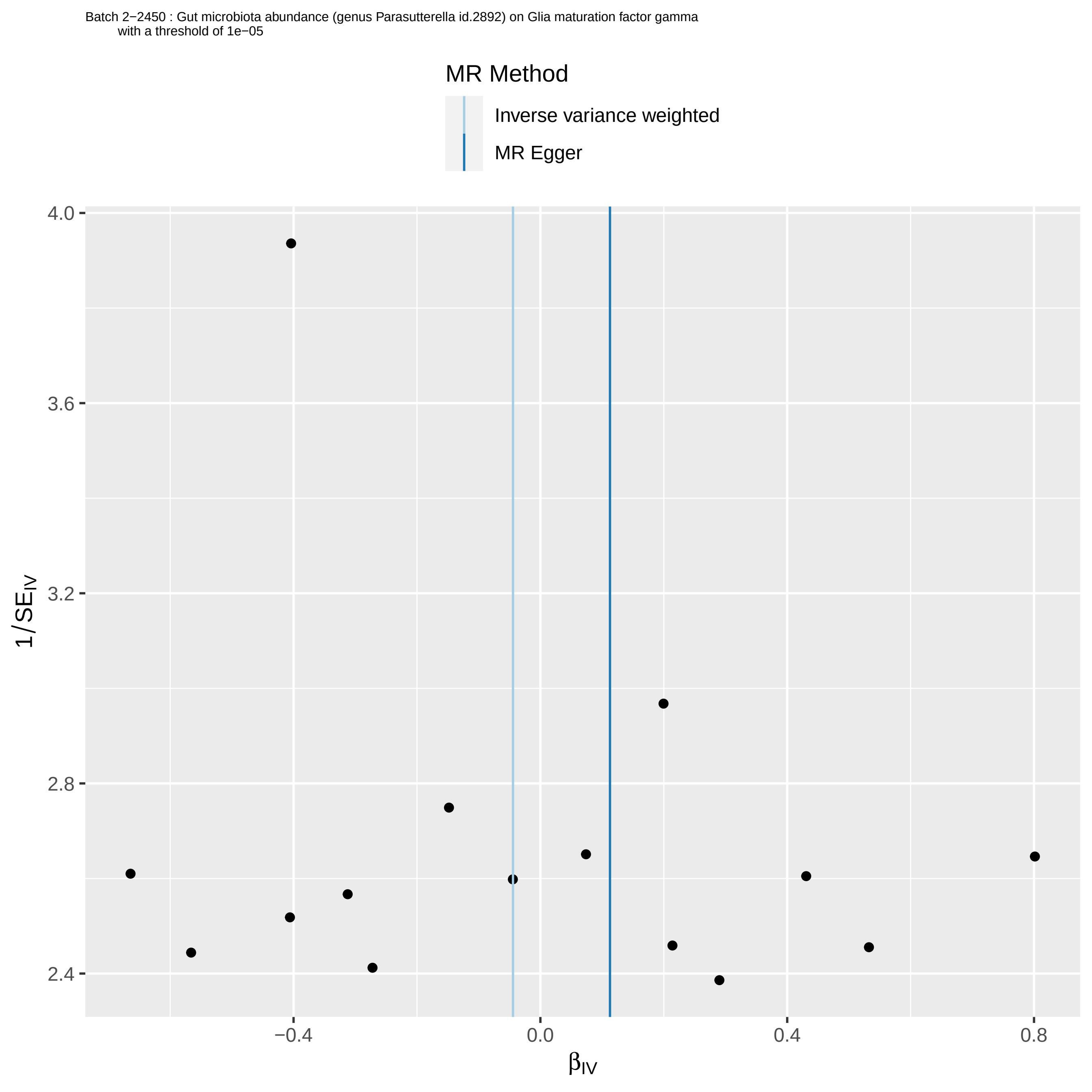

Batch 2−2450 : Gut microbiota abundance (genus Parasutterella id.2892) on Glia maturation factor gamma
with a threshold of 1e−05
MR Method
Inverse variance weighted
MR Egger
4.0
3.6
3.2
2.8
2.4
1/SE_IV
−0.4
0.0
0.4
0.8
β_IV

Batch 2−2451 : Gut microbiota abundance (genus Catenibacterium id.2153) on Guanine nucleotide−binding protein G(k) subunit alpha
with a threshold of 1e−05

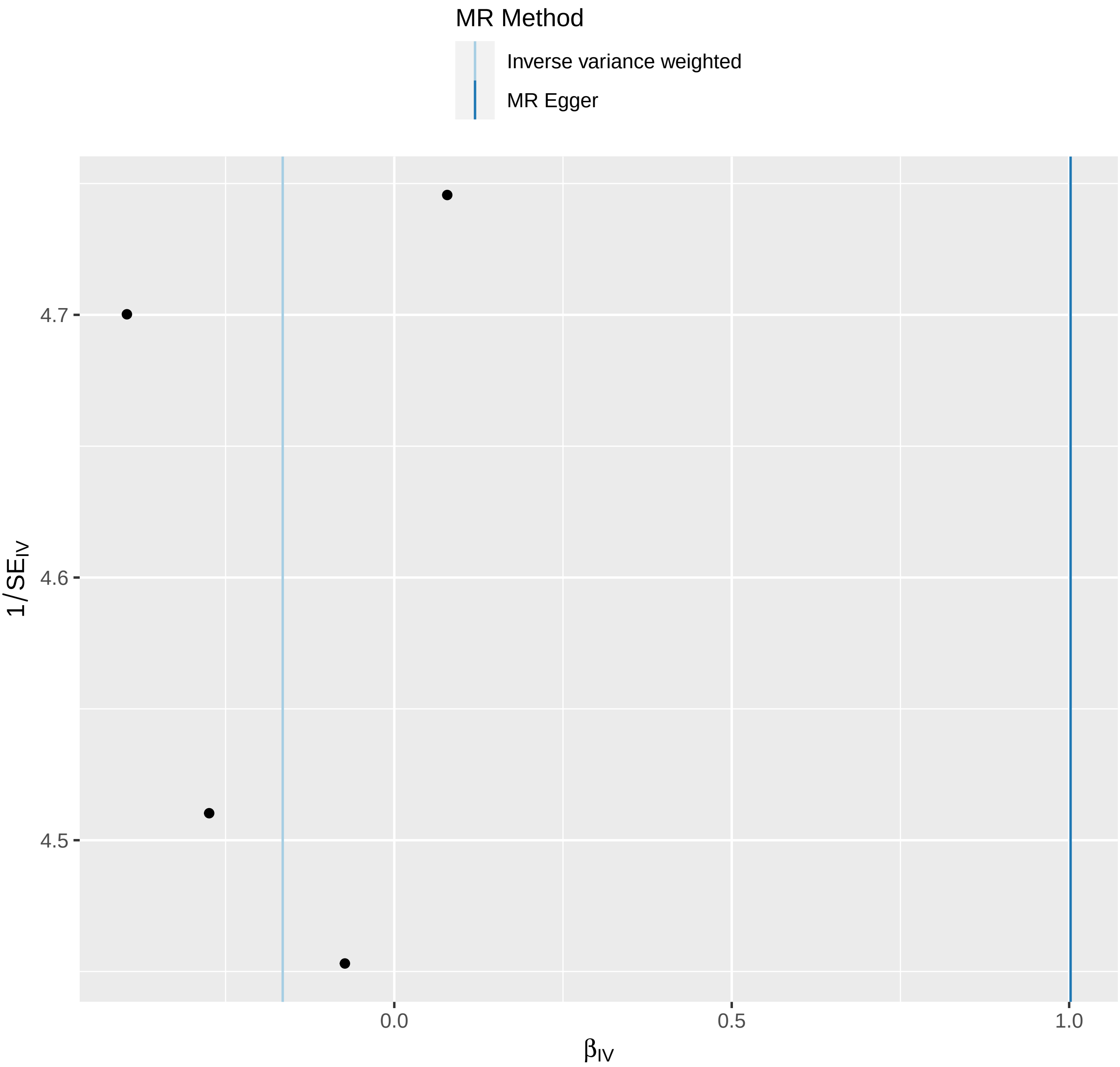

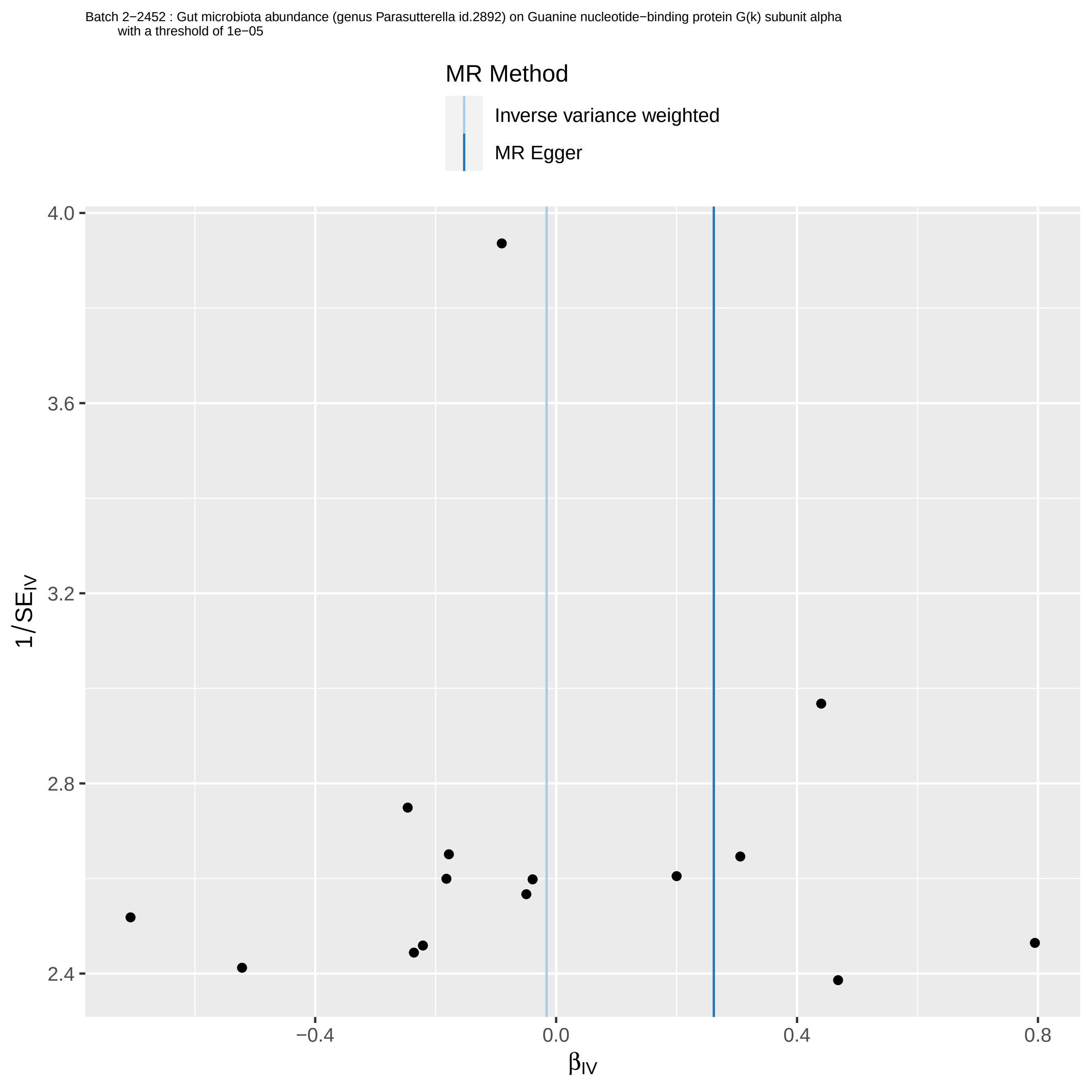
Batch 2−2452 : Gut microbiota abundance (genus Parasutterella id.2892) on Guanine nucleotide−binding protein G(k) subunit alpha
with a threshold of 1e−05
MR Method
Inverse variance weighted
MR Egger
4.0
3.6
3.2
2.8
2.4
1/SE_IV
−0.4
0.0
0.4
0.8
β_IV

Batch 2−2453 : Gut microbiota abundance (genus Catenibacterium id.2153) on Guanine nucleotide−binding protein G(I)/G(S)/G(O) subunit gamma−T2
with a threshold of 1e−05

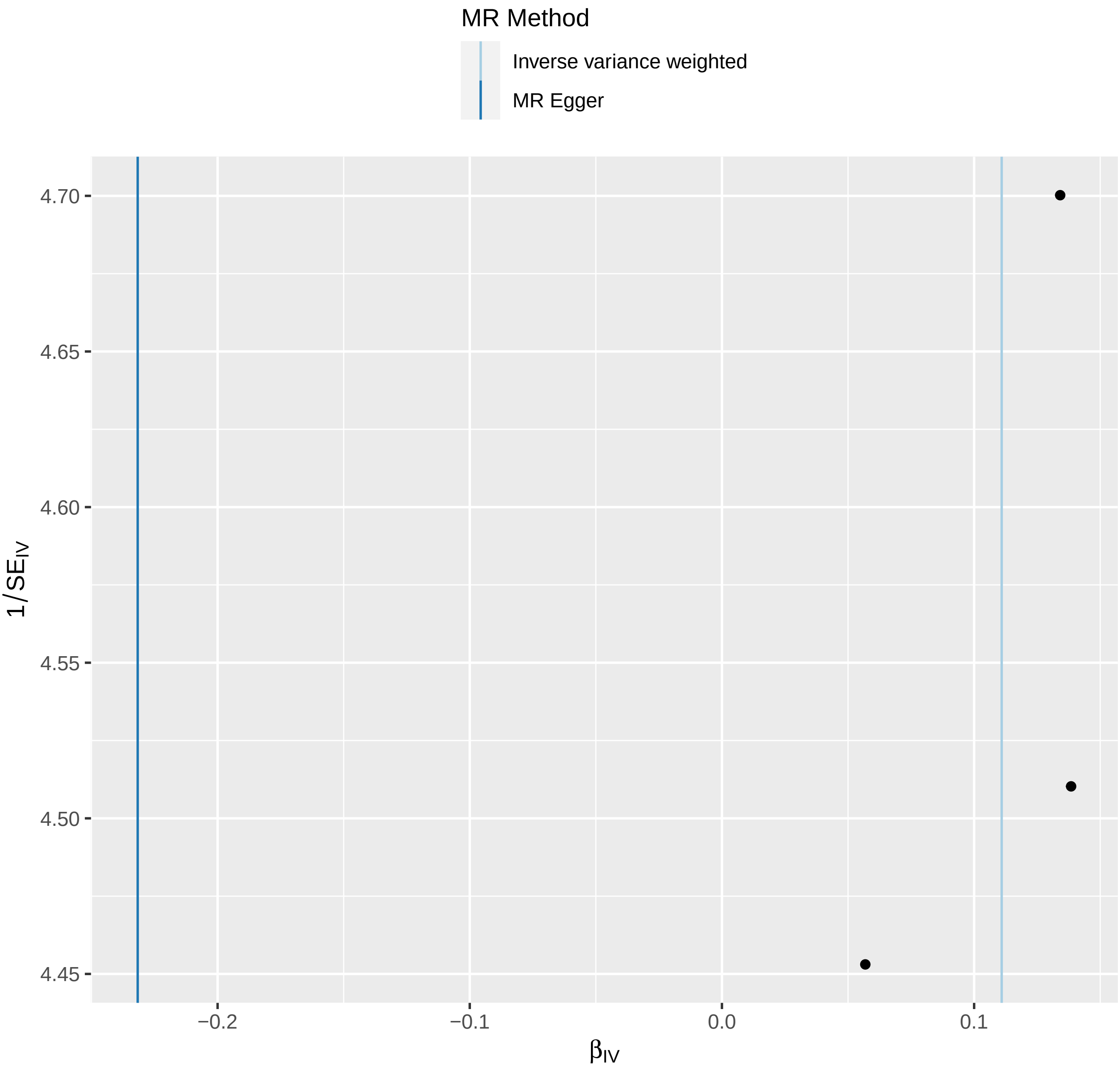

Batch 2−2454 : Gut microbiota abundance (genus Parasutterella id.2892) on Guanine nucleotide−binding protein G(I)/G(S)/G(O) subunit gamma−T2 with a threshold of 1e−05

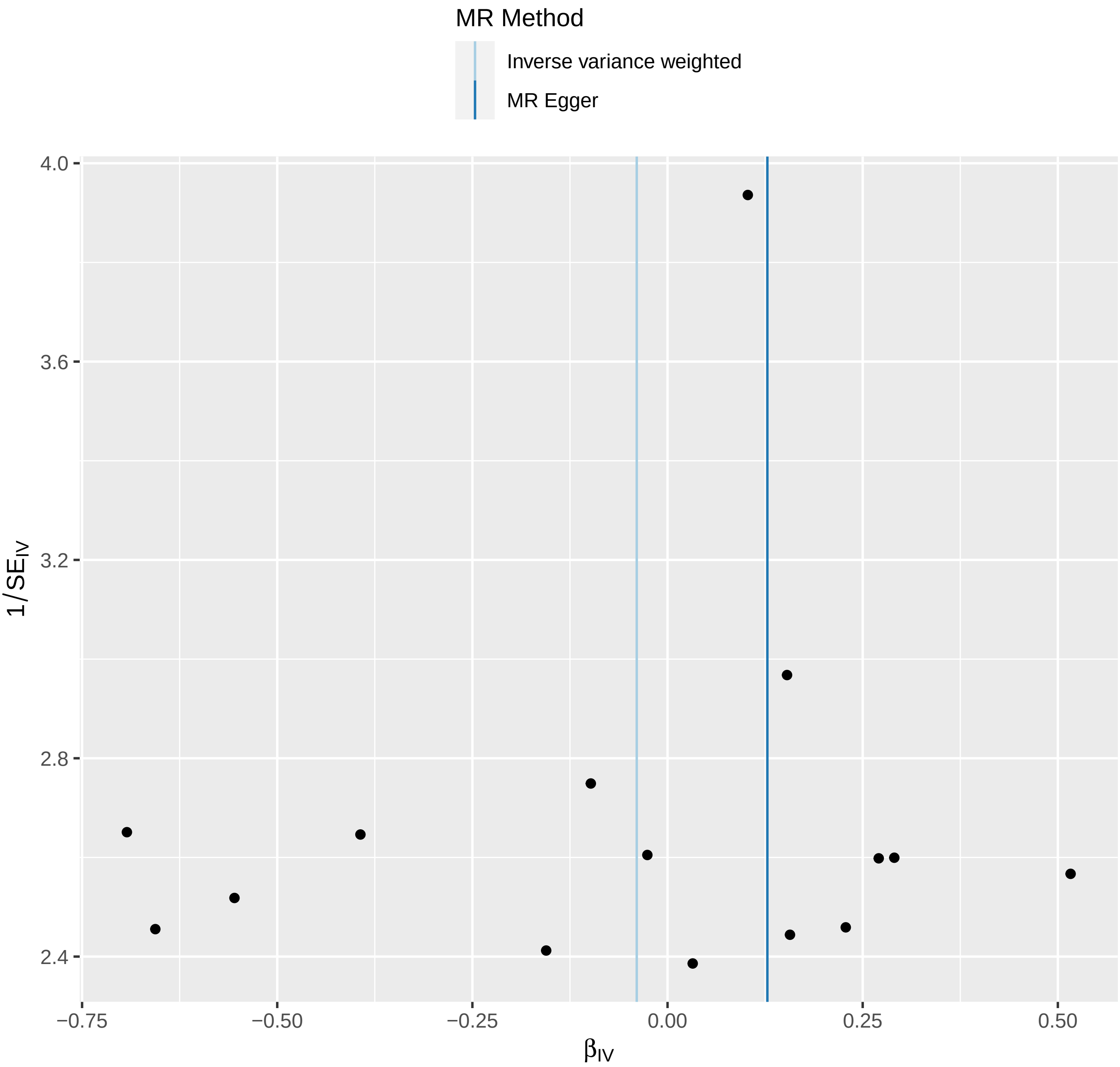

Batch 2−2455 : Gut microbiota abundance (genus Catenibacterium id.2153) on Granulysin
with a threshold of 1e−05

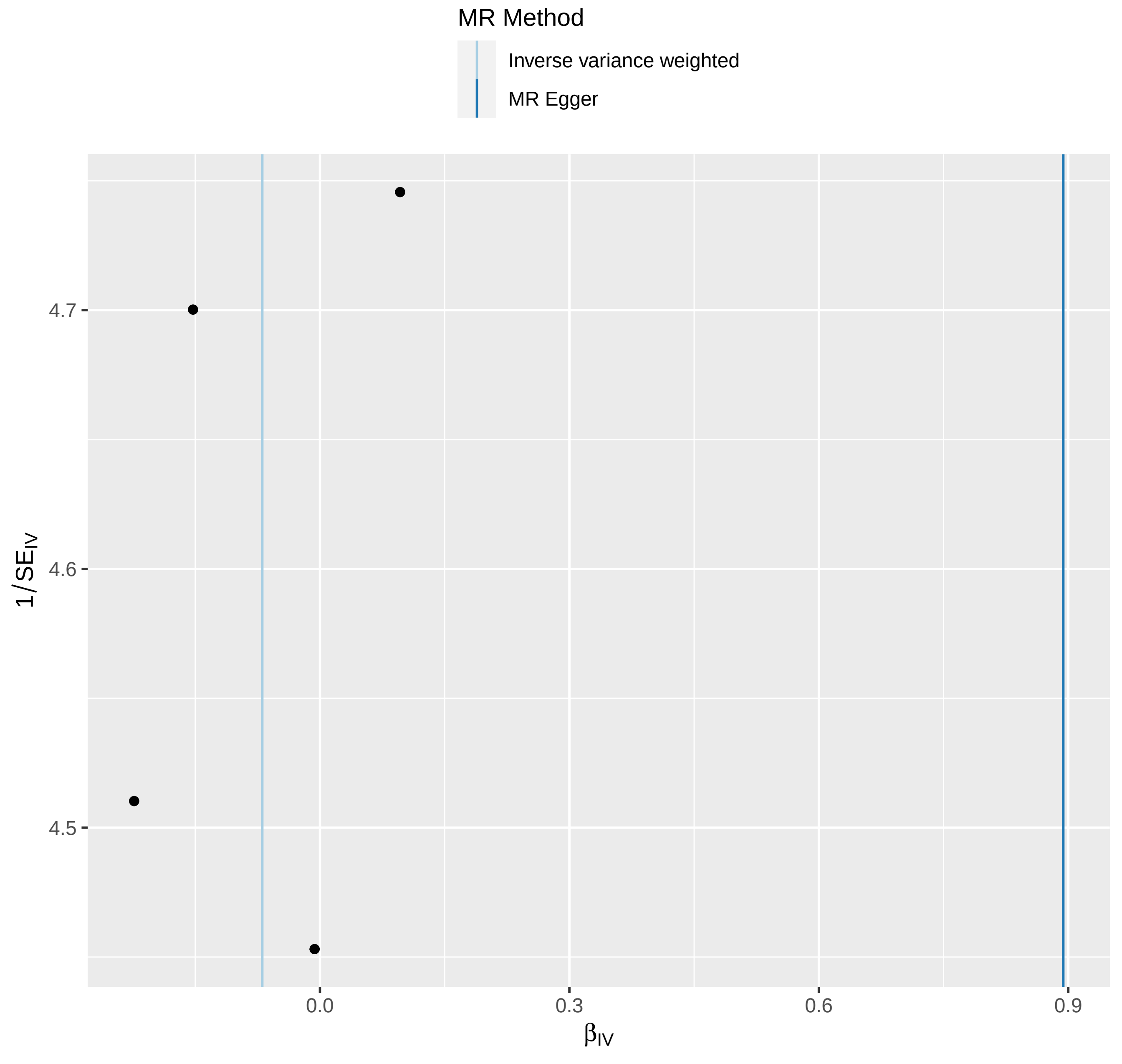

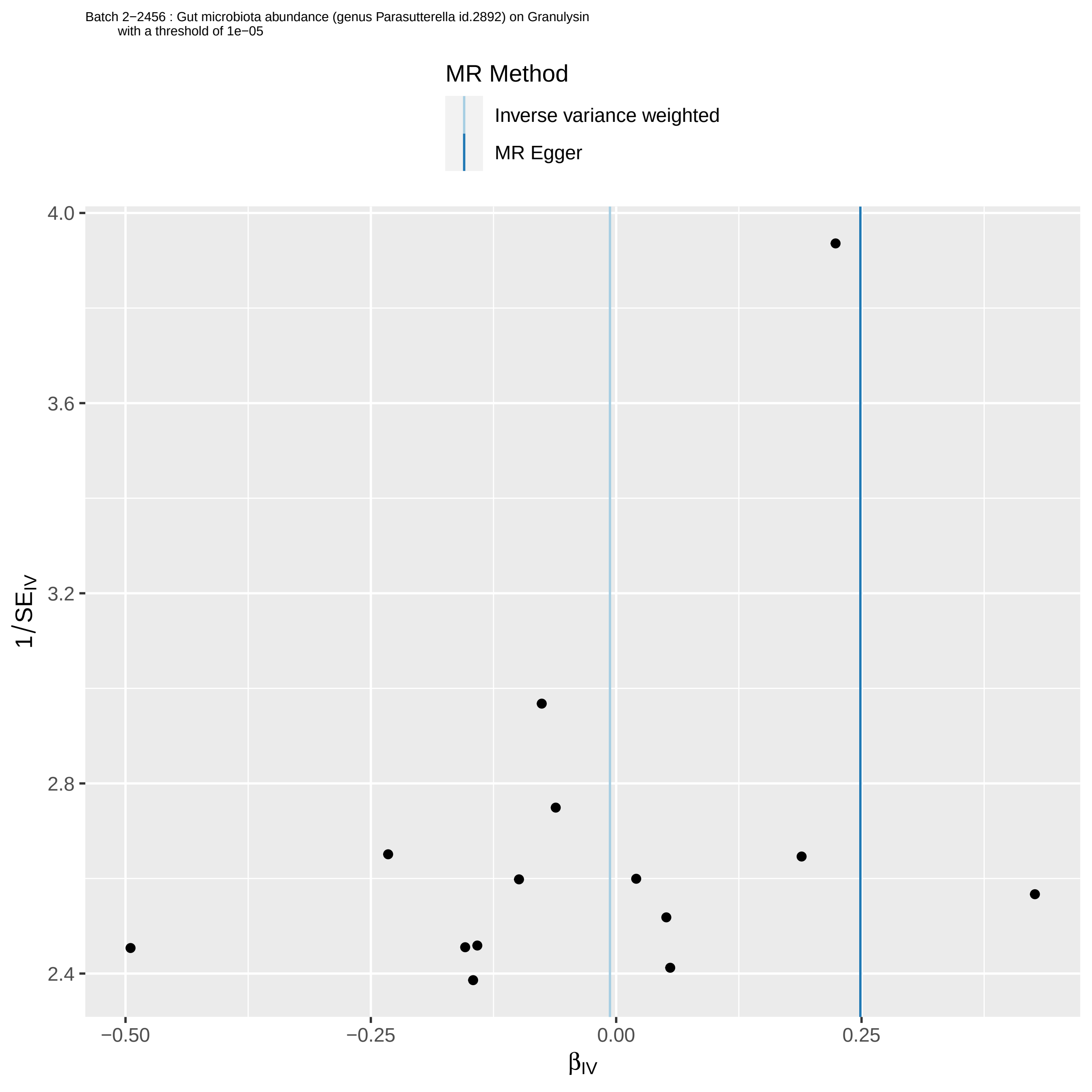
Batch 2−2456 : Gut microbiota abundance (genus Parasutterella id.2892) on Granulysin
with a threshold of 1e−05
MR Method
Inverse variance weighted
MR Egger
4.0
3.6
3.2
2.8
2.4
1/SE$_{IV}$
−0.50
−0.25
0.00
0.25
β$_{IV}$

Batch 2−2457 : Gut microbiota abundance (genus Catenibacterium id.2153) on Glycine N−methyltransferase with a threshold of 1e−05

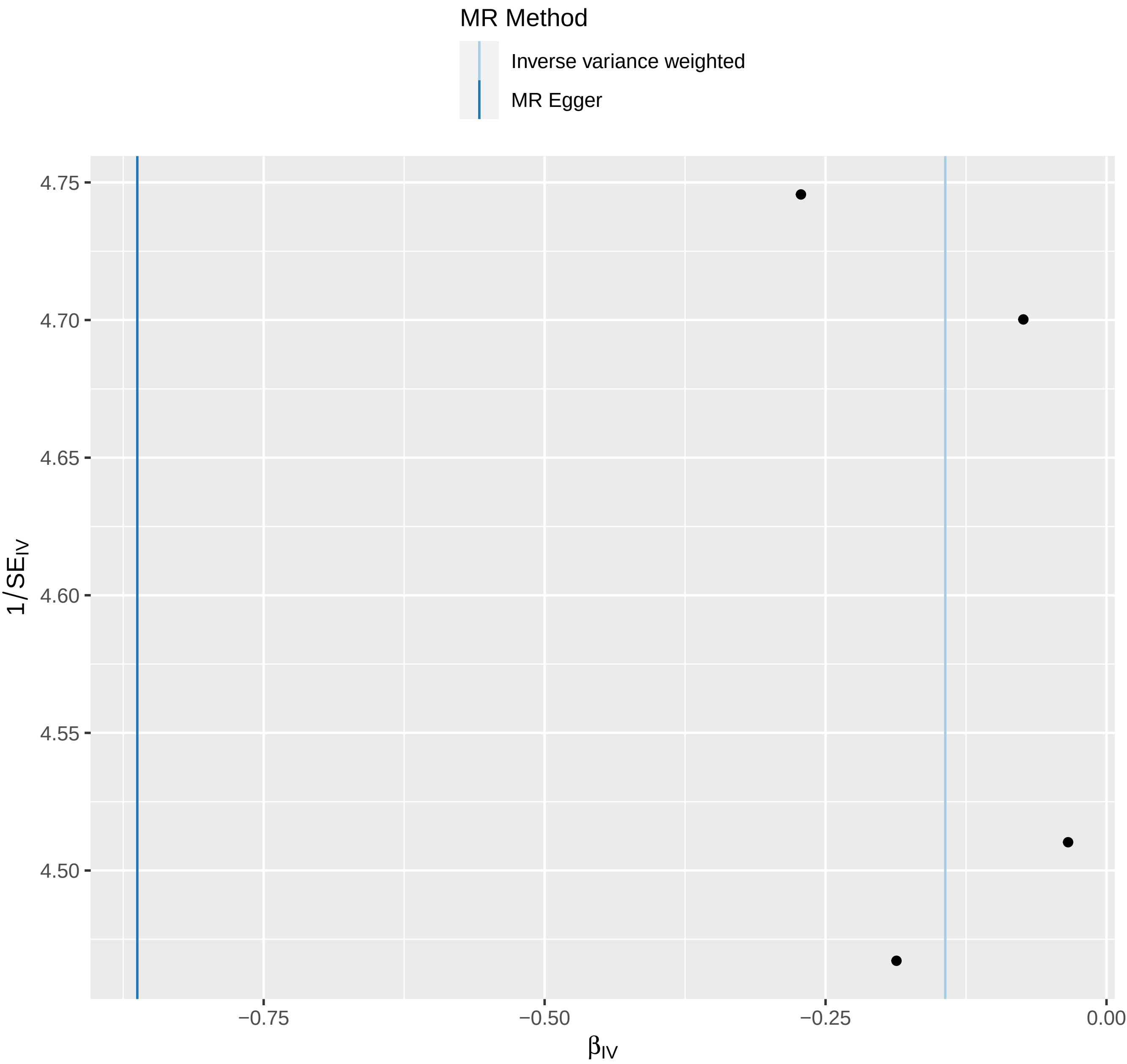

Batch 2−2458 : Gut microbiota abundance (genus Parasutterella id.2892) on Glycine N−methyltransferase
with a threshold of 1e−05

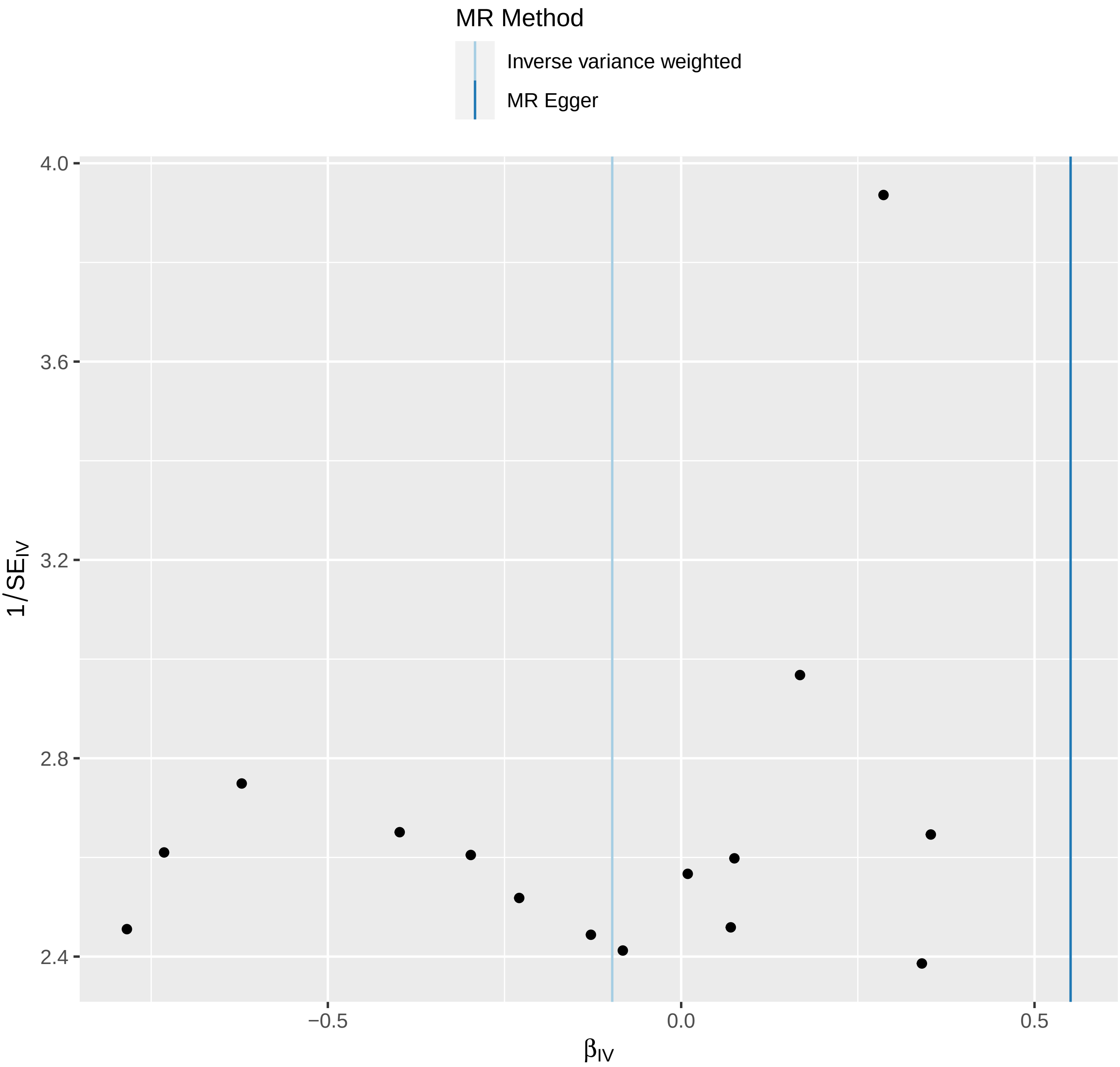

Batch 2−2459 : Gut microbiota abundance (genus Catenibacterium id.2153) on Glucosamine−6−phosphate isomerase 1
with a threshold of 1e−05

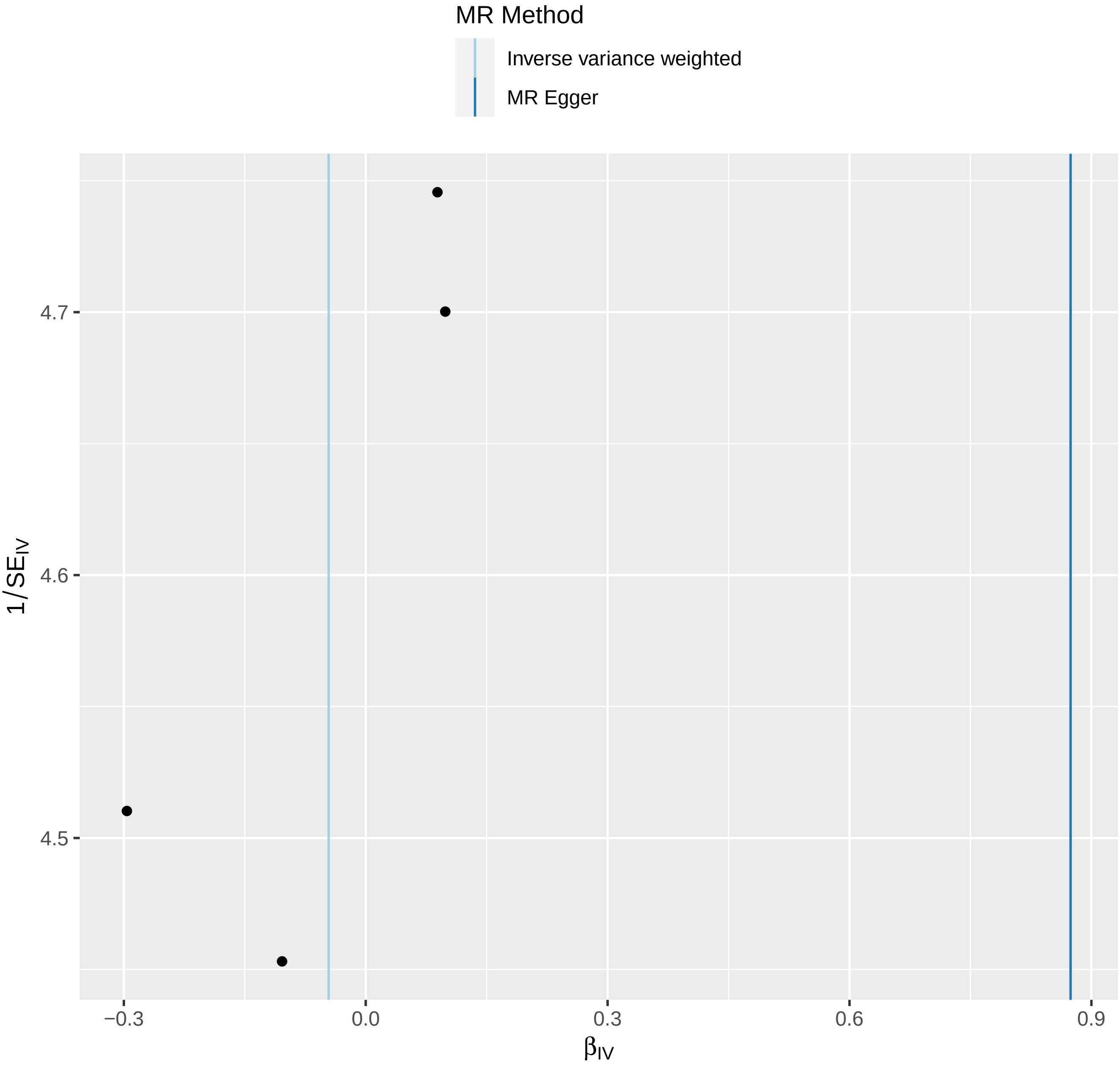

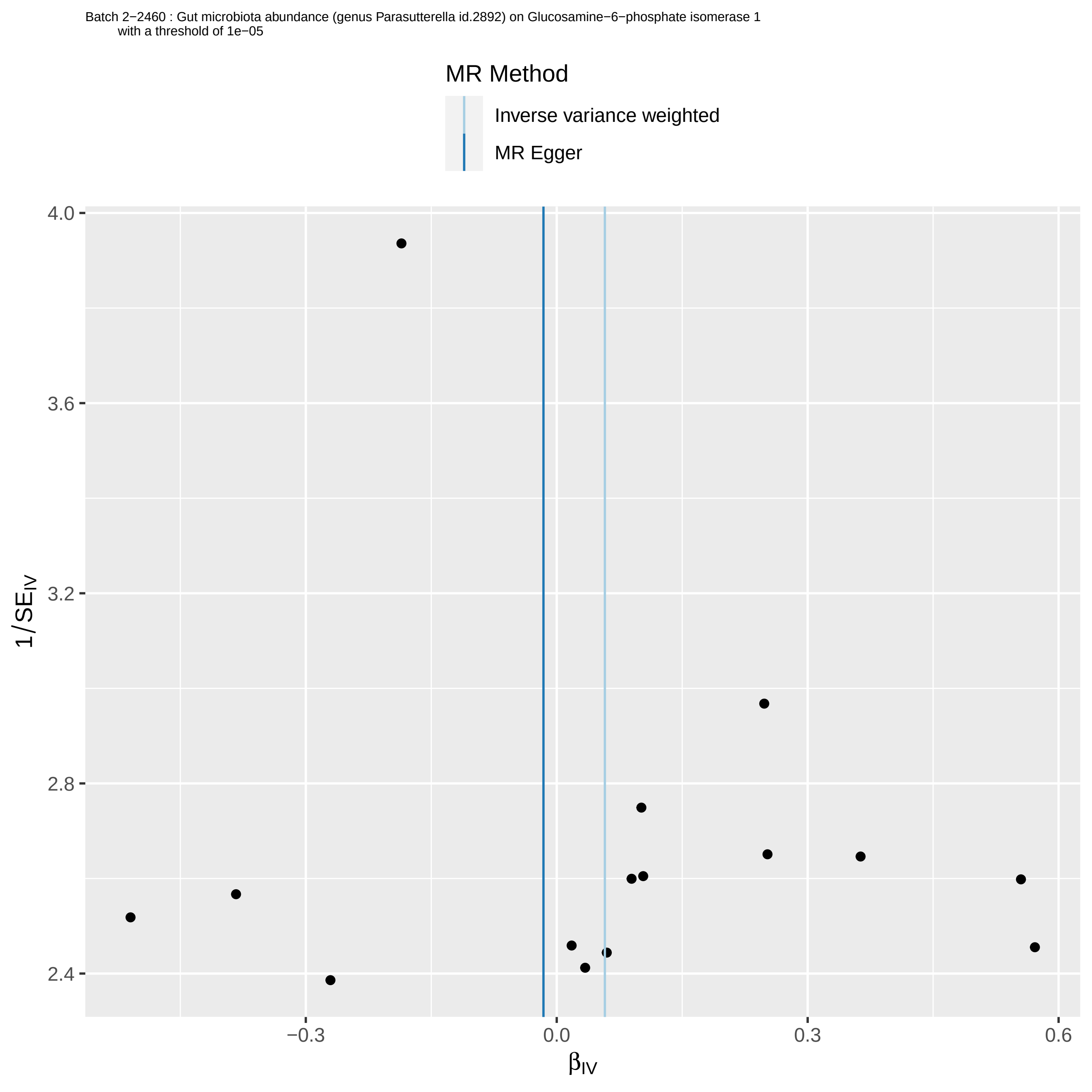
Batch 2−2460 : Gut microbiota abundance (genus Parasutterella id.2892) on Glucosamine−6−phosphate isomerase 1
with a threshold of 1e−05
MR Method
Inverse variance weighted
MR Egger
4.0
3.6
3.2
2.8
2.4
1/SE$_{IV}$
−0.3
0.0
0.3
0.6
β$_{IV}$

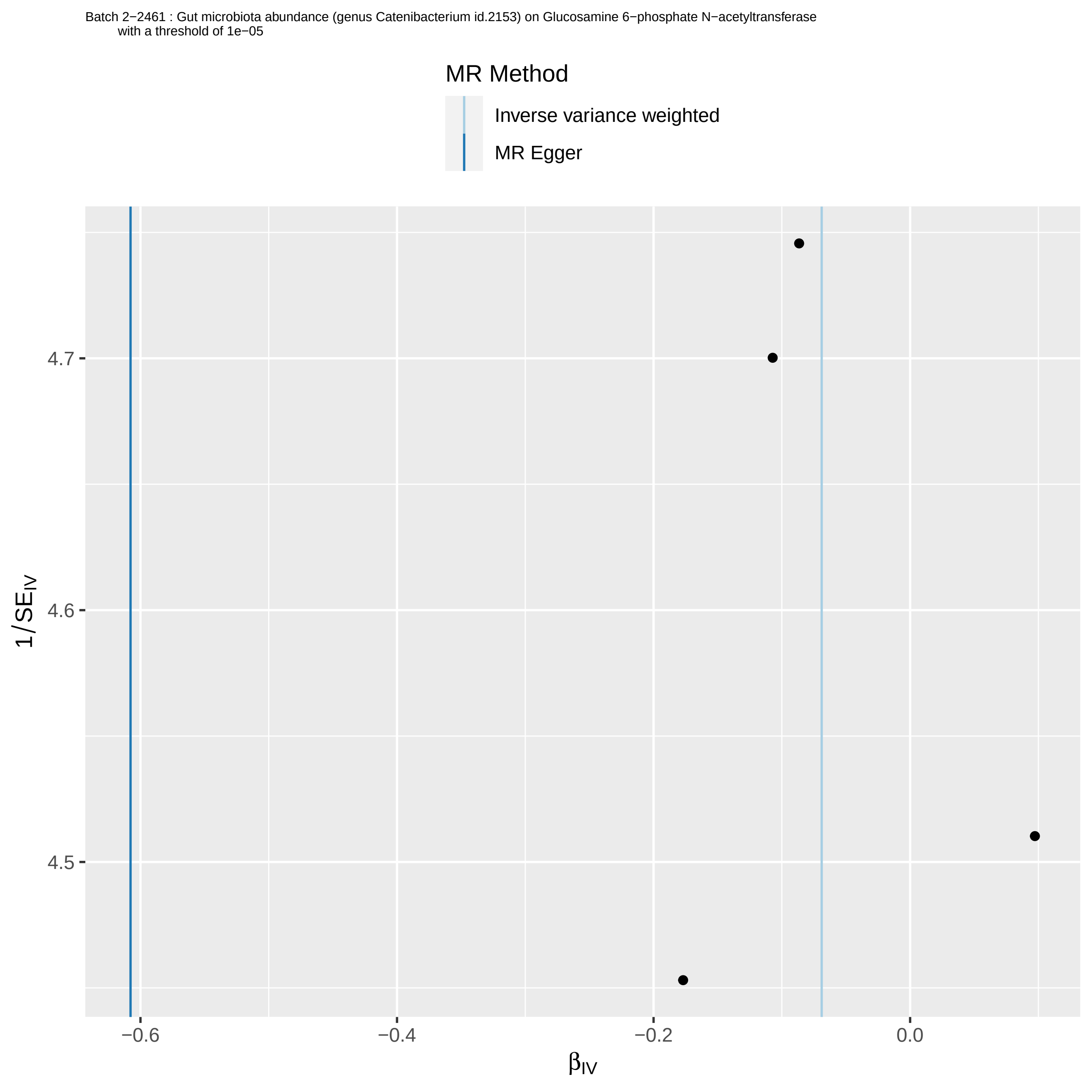
Batch 2−2461 : Gut microbiota abundance (genus Catenibacterium id.2153) on Glucosamine 6−phosphate N−acetyltransferase
with a threshold of 1e−05
MR Method
Inverse variance weighted
MR Egger
4.7
4.6
4.5
1/SE_IV
−0.6
−0.4
−0.2
0.0
β_IV

Batch 2−2462 : Gut microbiota abundance (genus Parasutterella id.2892) on Glucosamine 6−phosphate N−acetyltransferase
with a threshold of 1e−05

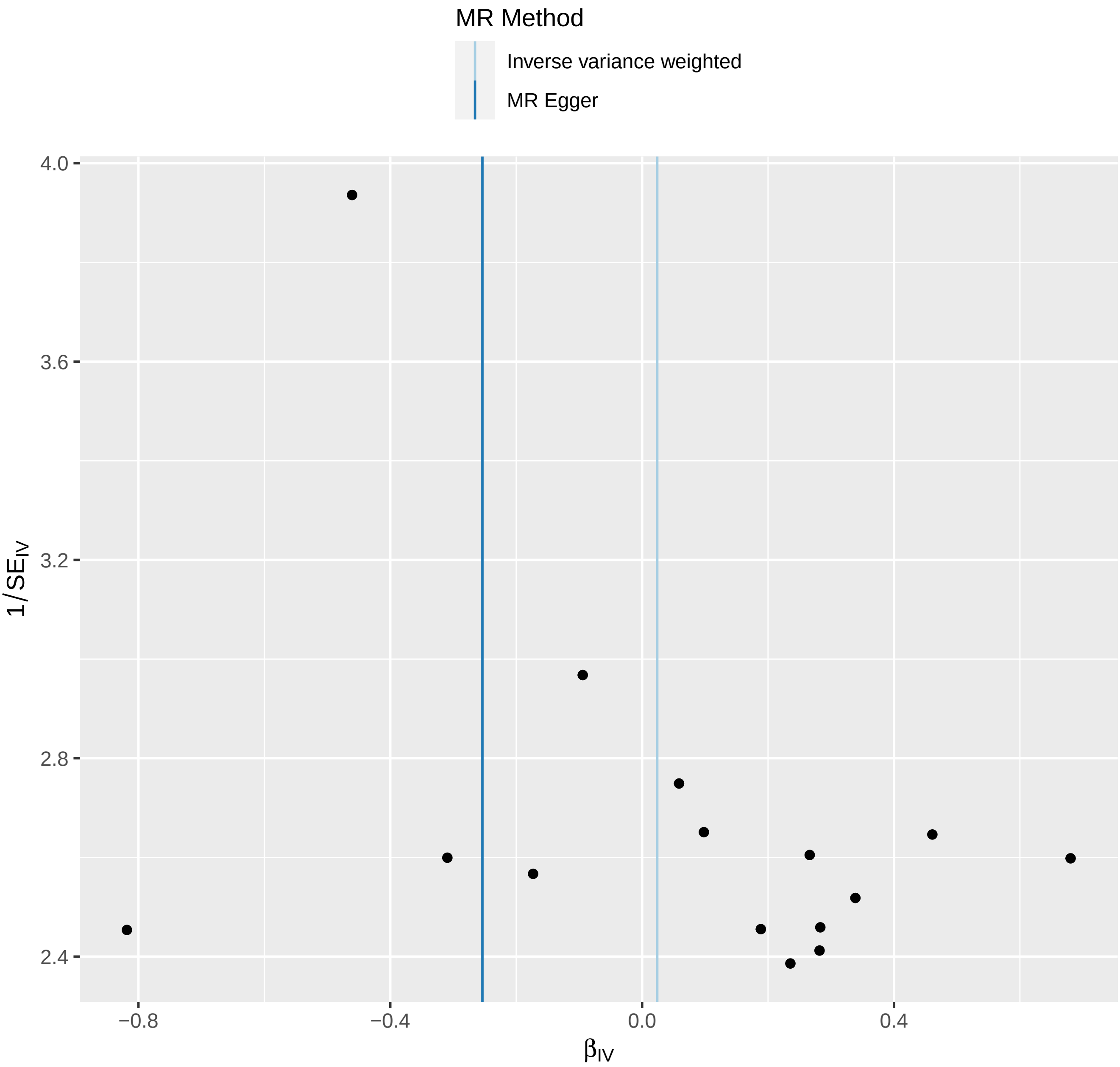

Batch 2−2463 : Gut microbiota abundance (genus Catenibacterium id.2153) on N−acetylglucosamine−1−phosphotransferase subunit gamma with a threshold of 1e−05

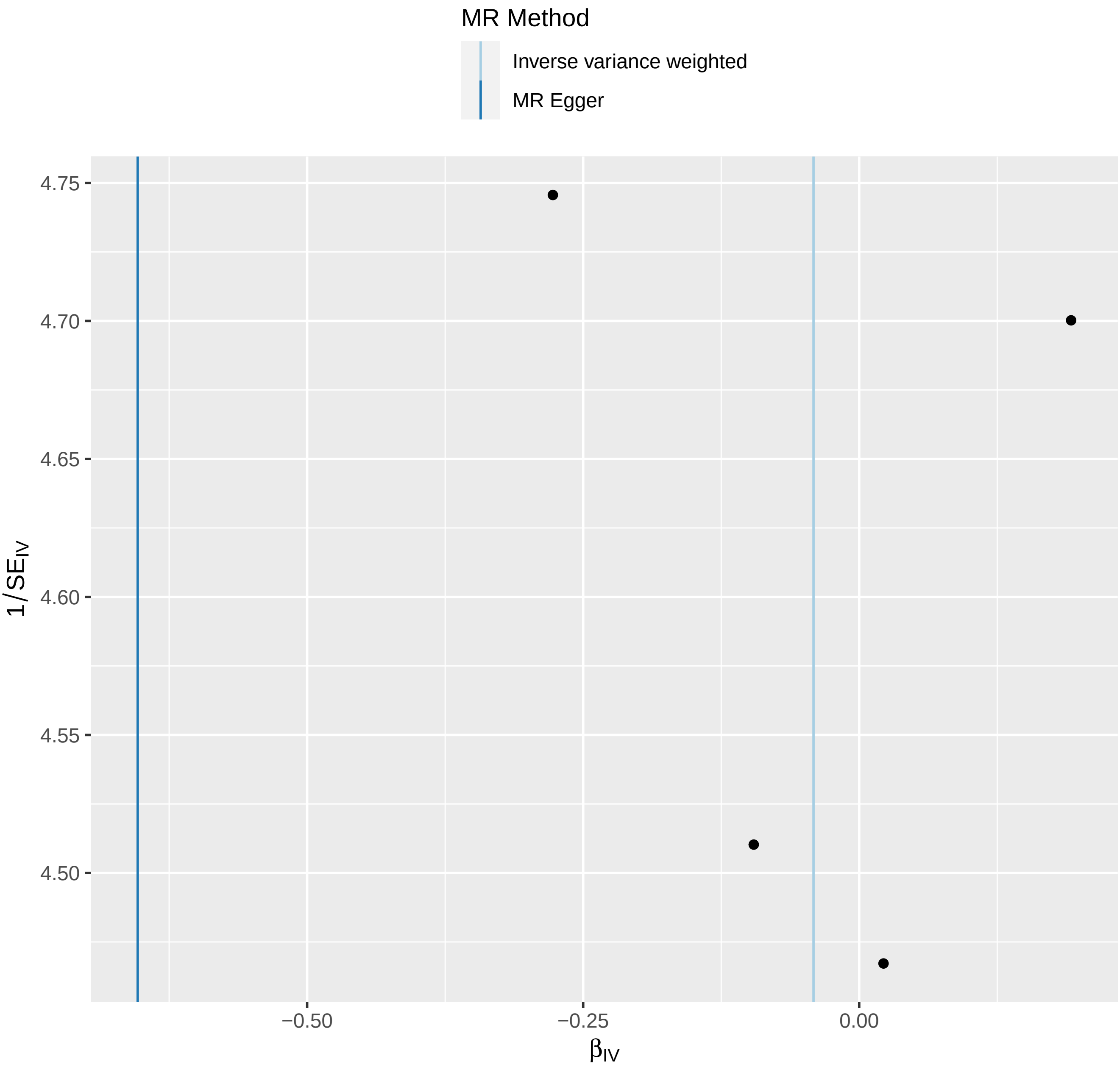

Batch 2−2464 : Gut microbiota abundance (genus Parasutterella id.2892) on N−acetylglucosamine−1−phosphotransferase subunit gamma with a threshold of 1e−05

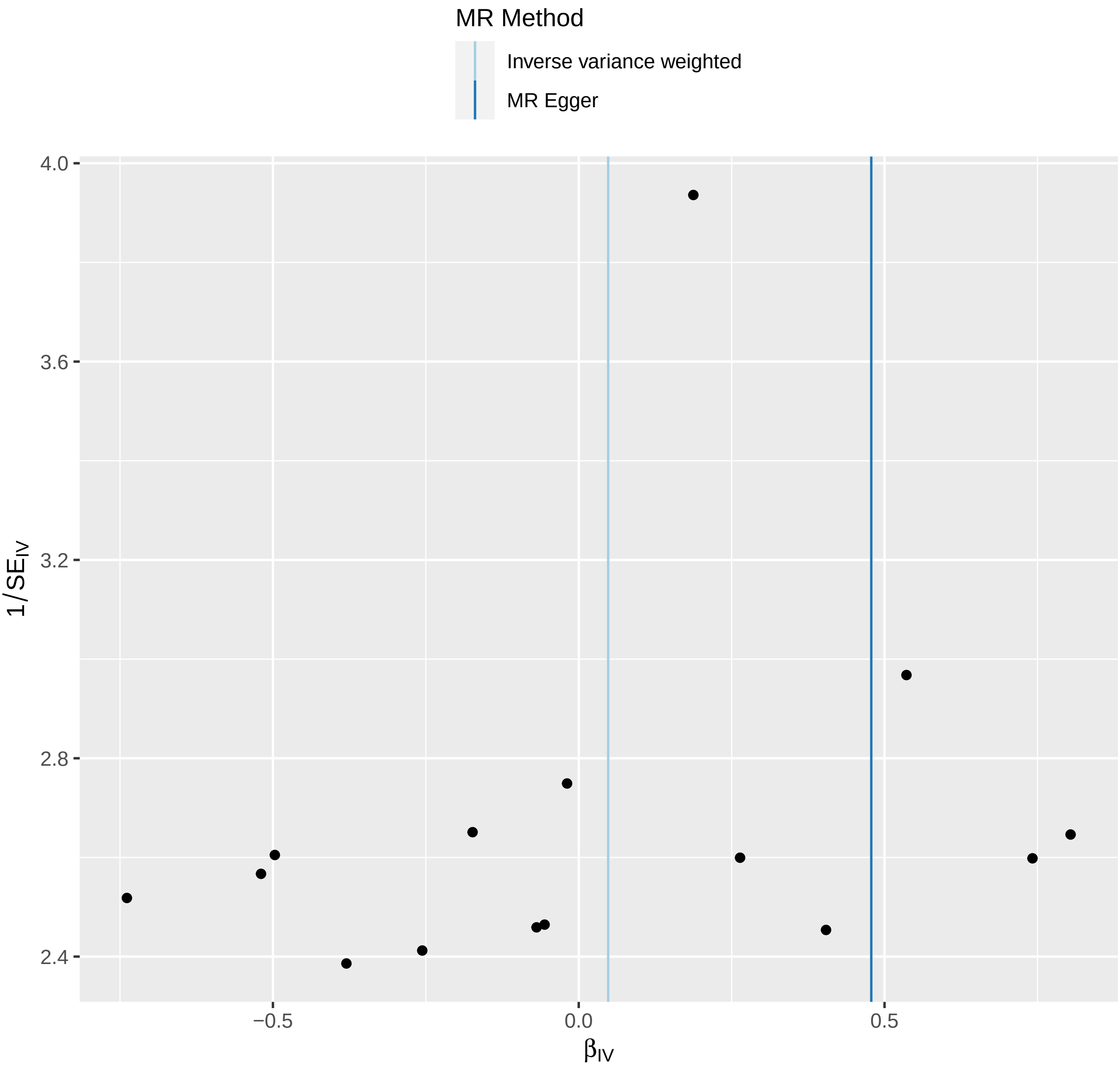

Batch 2−2465 : Gut microbiota abundance (genus Catenibacterium id.2153) on Progonadoliberin−1
with a threshold of 1e−05

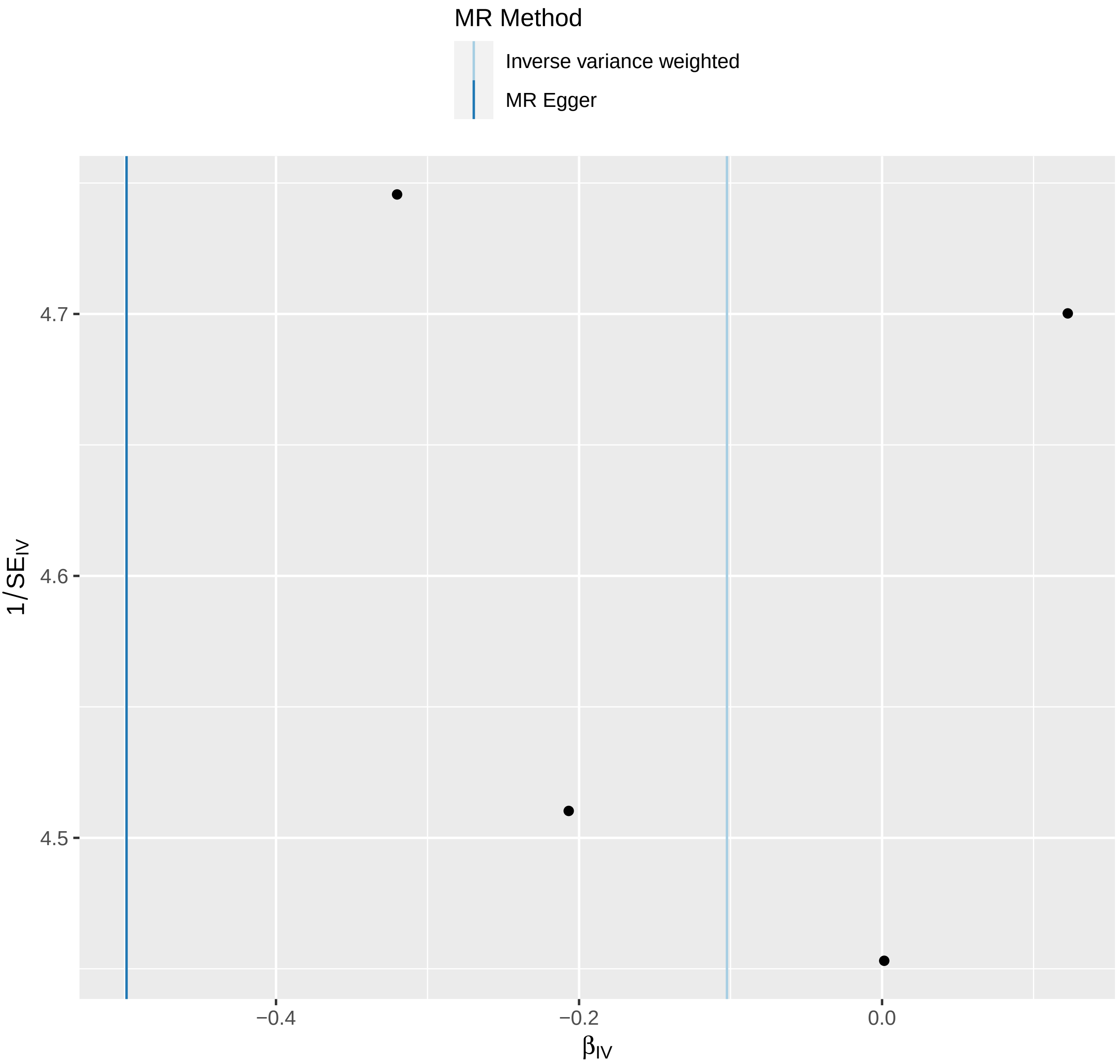

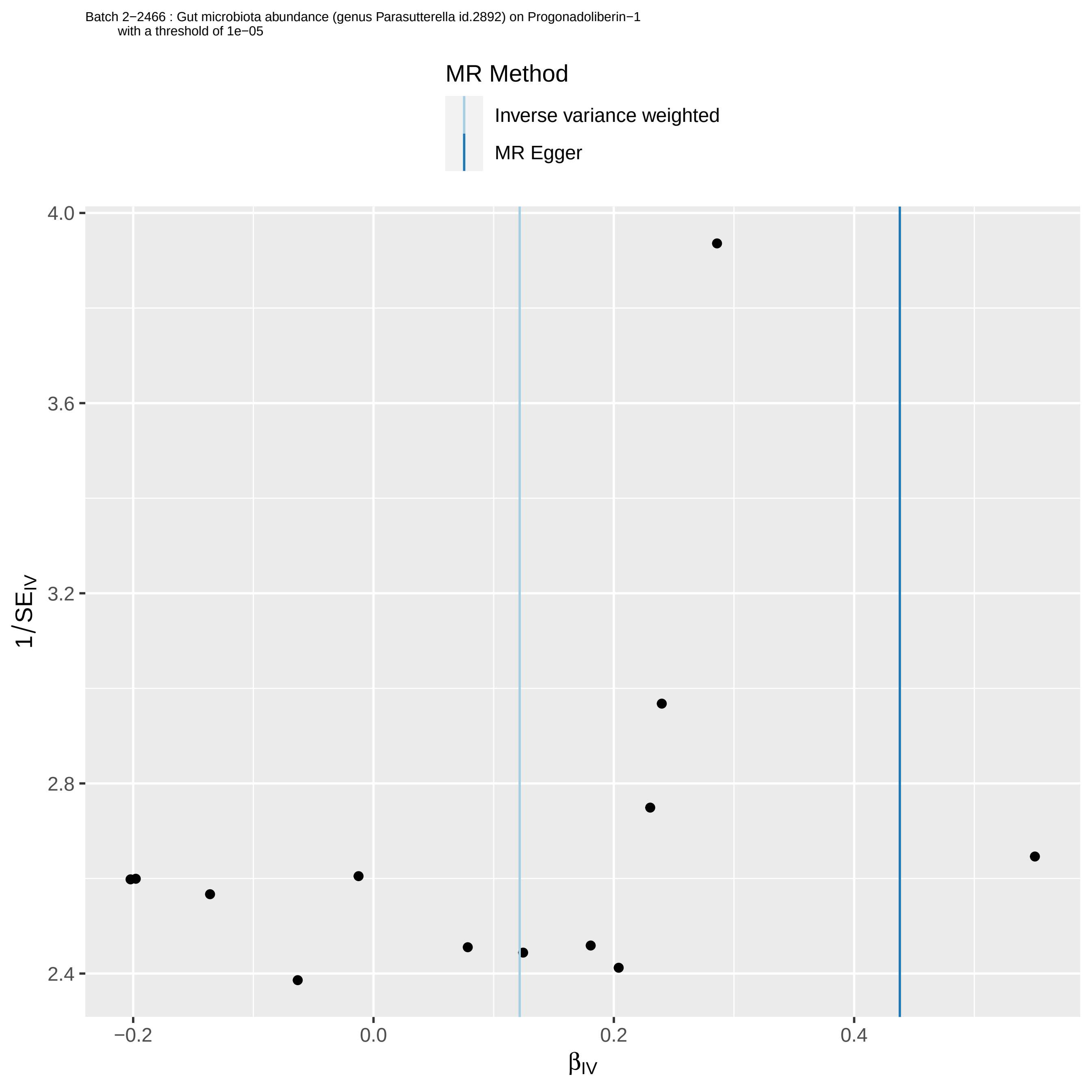

Batch 2−2466 : Gut microbiota abundance (genus Parasutterella id.2892) on Progonadoliberin−1
with a threshold of 1e−05
MR Method
Inverse variance weighted
MR Egger
4.0
3.6
3.2
2.8
2.4
1/SE$_{IV}$
−0.2
0.0
0.2
0.4
β$_{IV}$

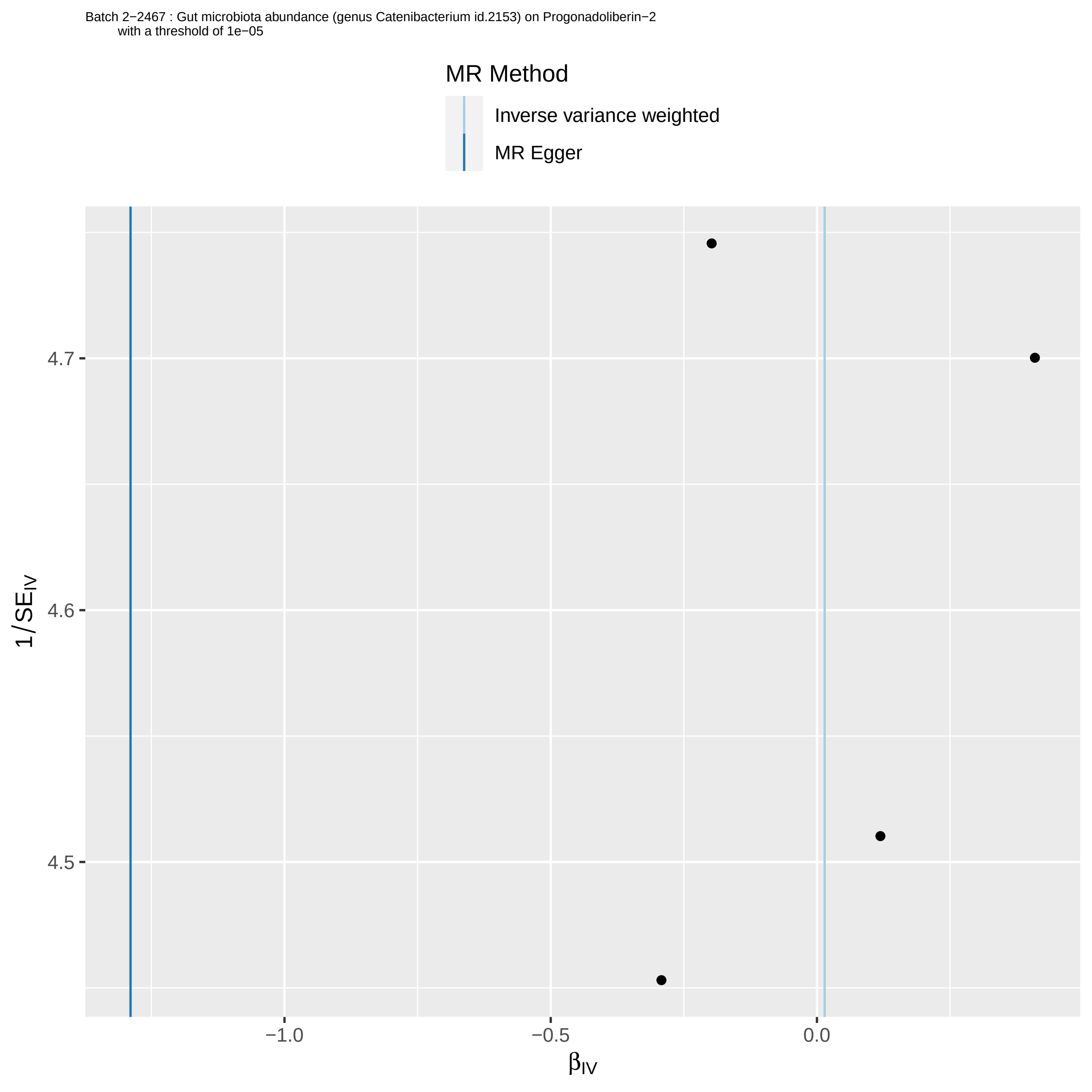

Batch 2−2467 : Gut microbiota abundance (genus Catenibacterium id.2153) on Progonadoliberin−2
with a threshold of 1e−05
MR Method
Inverse variance weighted
MR Egger
4.7
4.6
4.5
1/SE_IV
−1.0
−0.5
0.0
β_IV

Batch 2−2468 : Gut microbiota abundance (genus Parasutterella id.2892) on Progonadoliberin−2
with a threshold of 1e−05

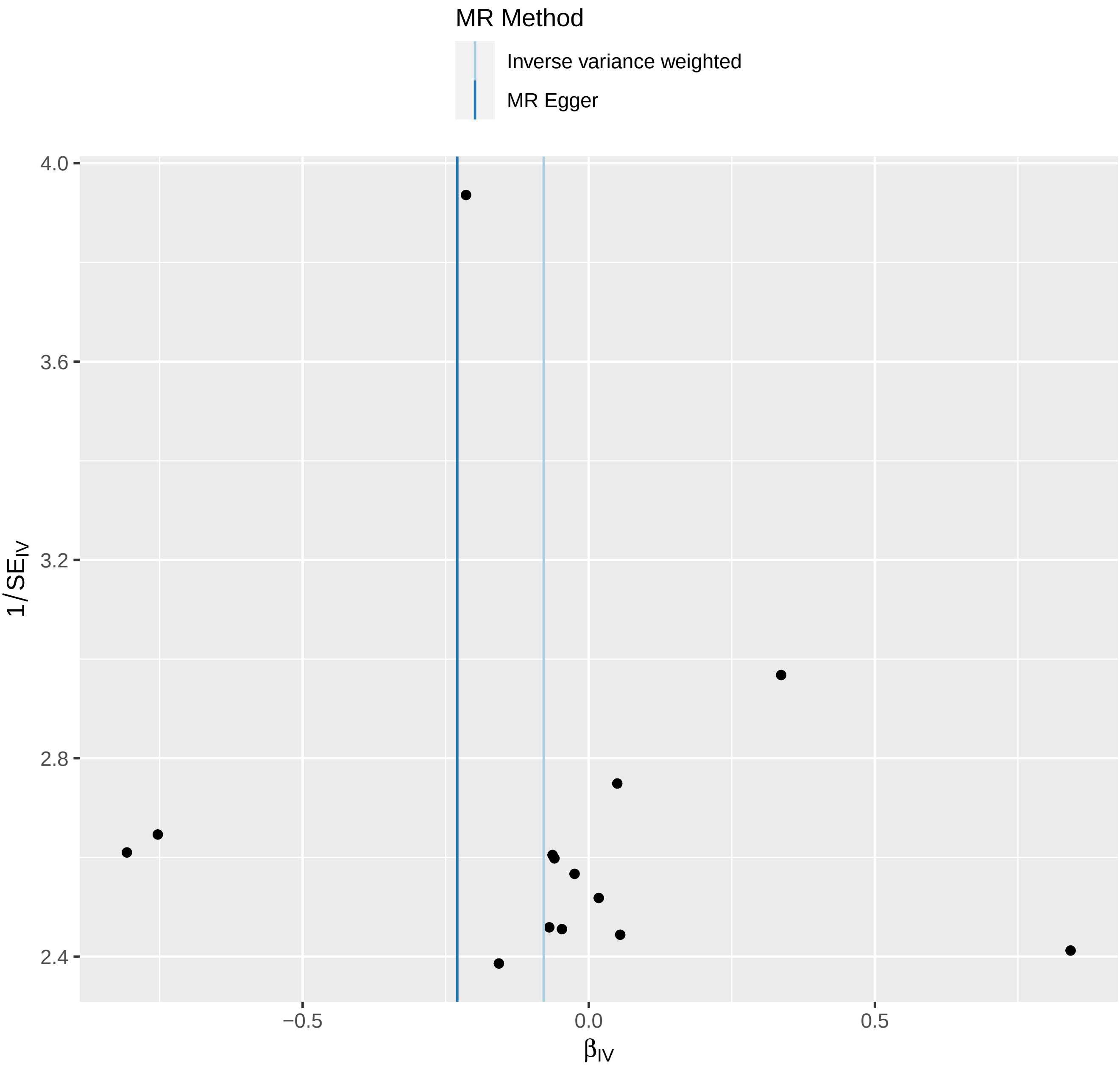

Batch 2−2469 : Gut microbiota abundance (genus Catenibacterium id.2153) on N−acetylglucosamine−6−sulfatase
with a threshold of 1e−05

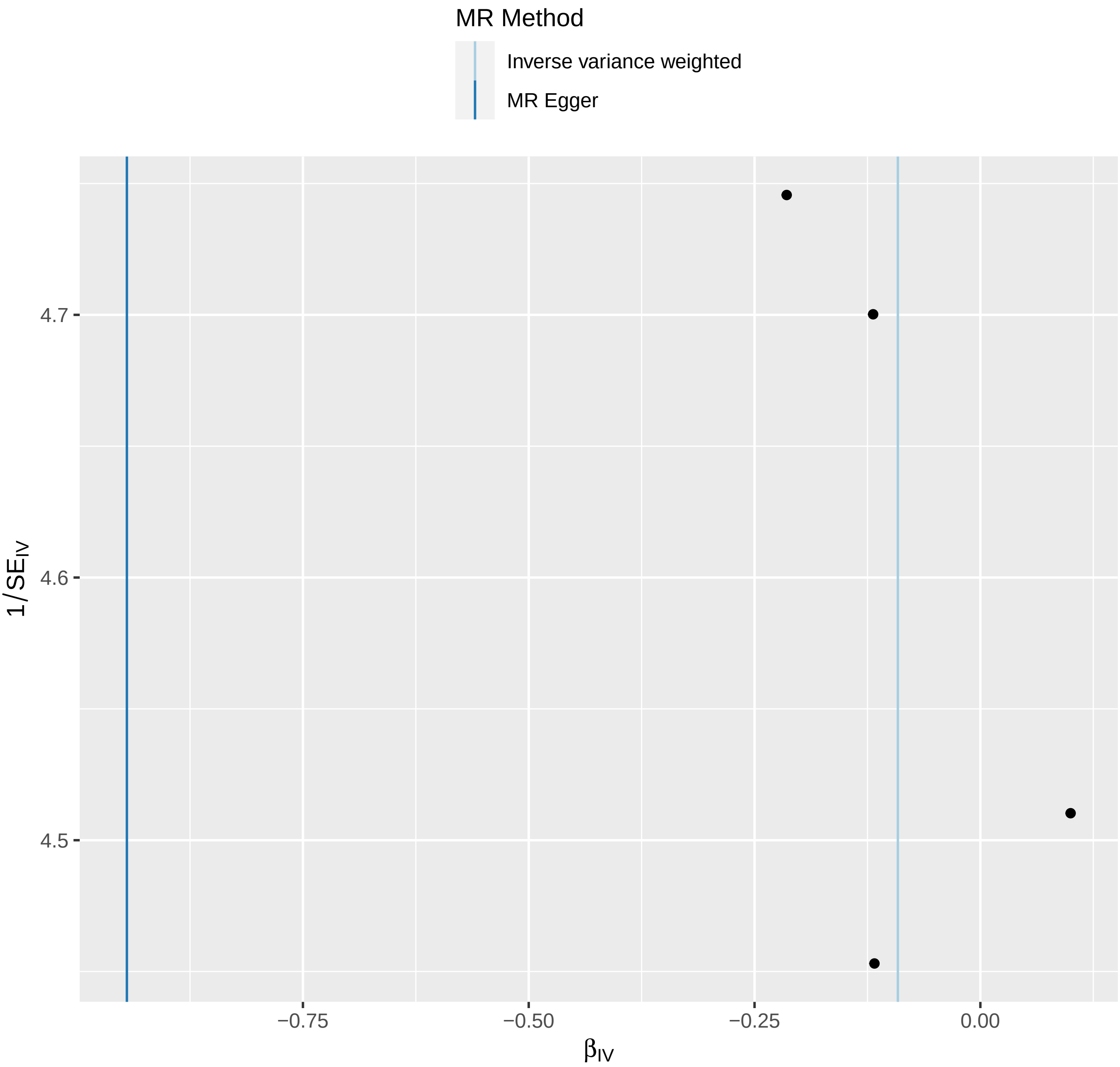

Batch 2−2470 : Gut microbiota abundance (genus Parasutterella id.2892) on N−acetylglucosamine−6−sulfatase
with a threshold of 1e−05

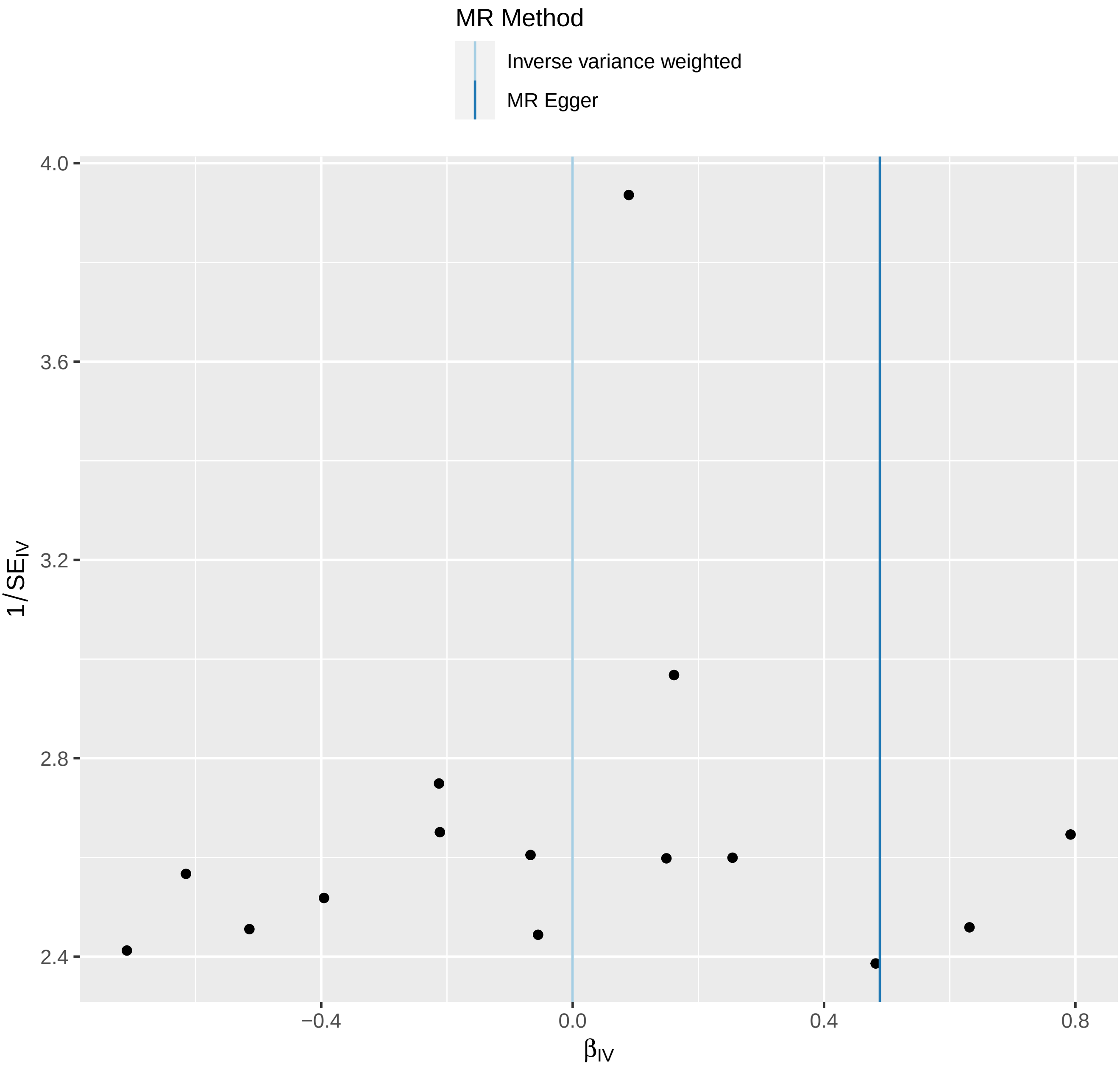

Batch 2−2471 : Gut microbiota abundance (genus Catenibacterium id.2153) on N−acetylglucosamine−6−sulfatase
with a threshold of 1e−05

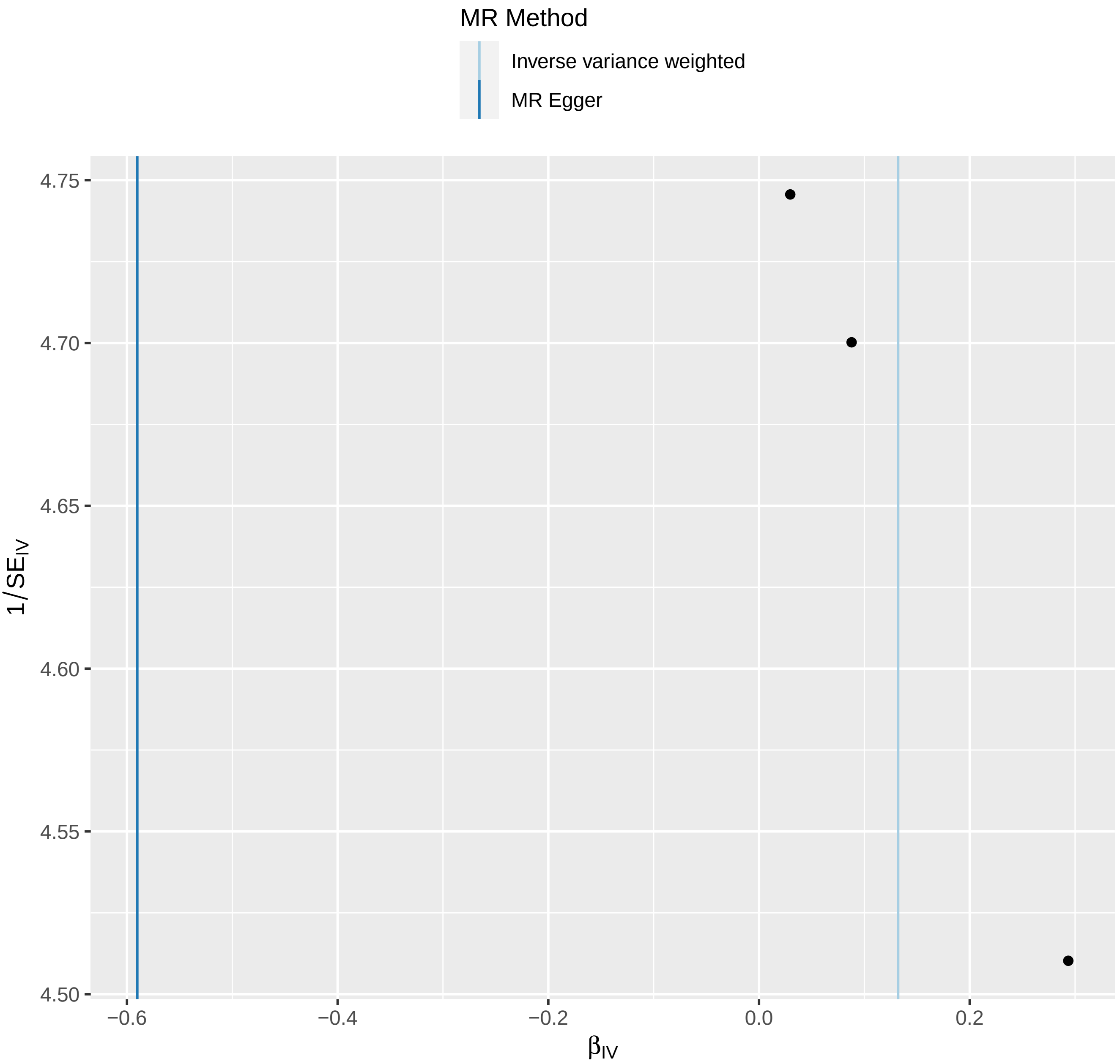

Batch 2−2472 : Gut microbiota abundance (genus Parasutterella id.2892) on N−acetylglucosamine−6−sulfatase
with a threshold of 1e−05

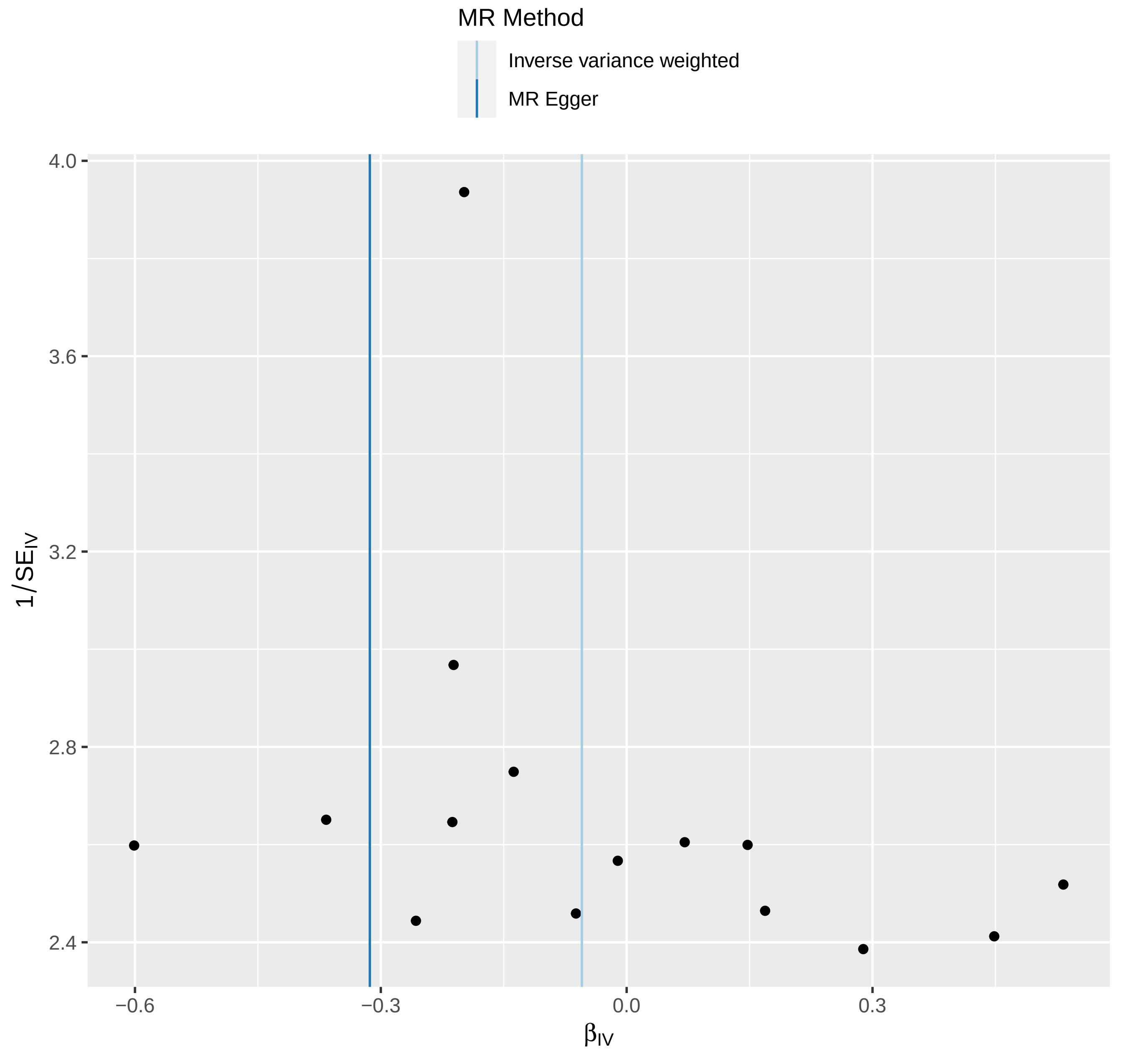

Batch 2−2473 : Gut microbiota abundance (genus Catenibacterium id.2153) on Golgi membrane protein 1
with a threshold of 1e−05

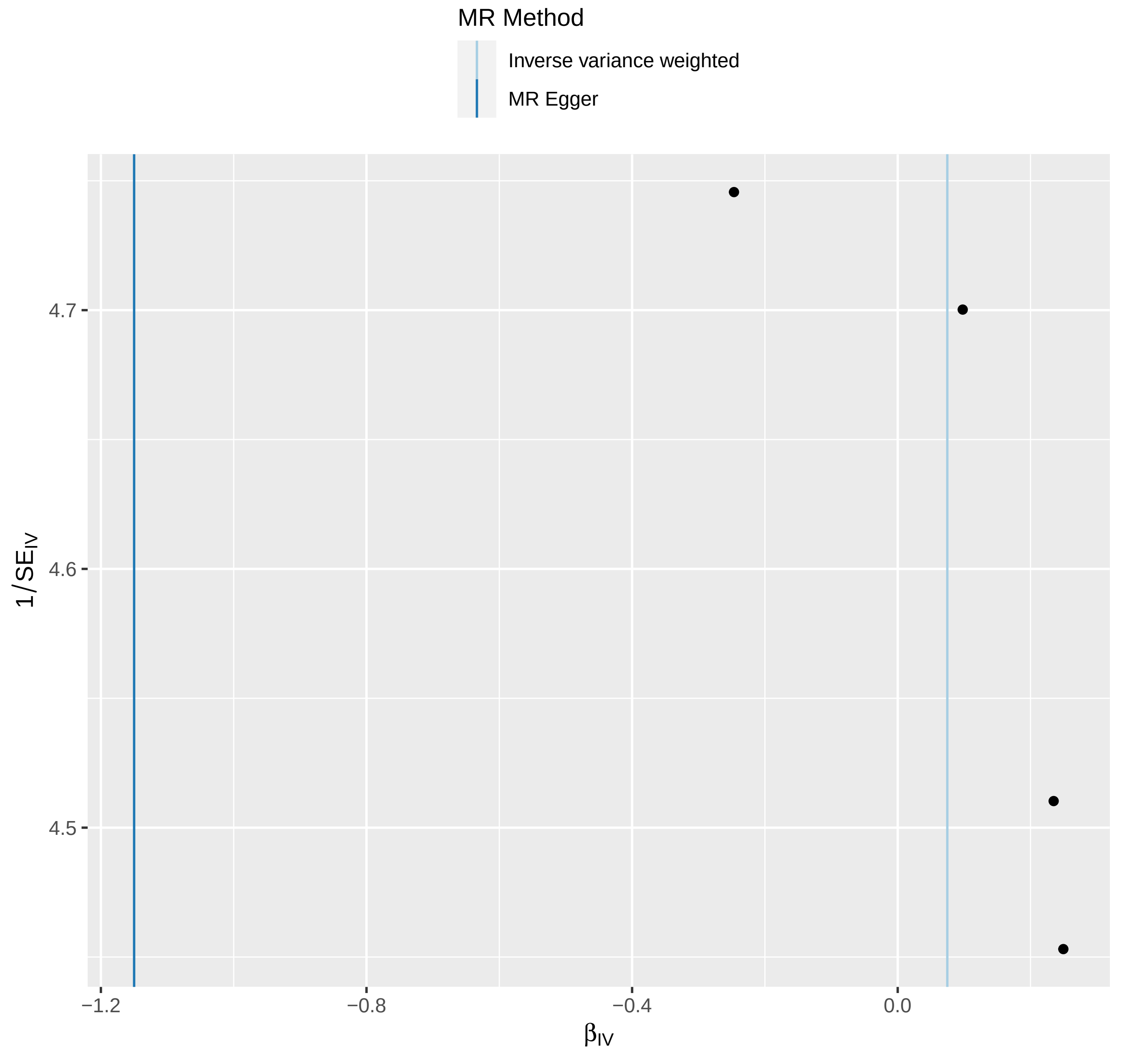

Batch 2−2474 : Gut microbiota abundance (genus Parasutterella id.2892) on Golgi membrane protein 1
with a threshold of 1e−05

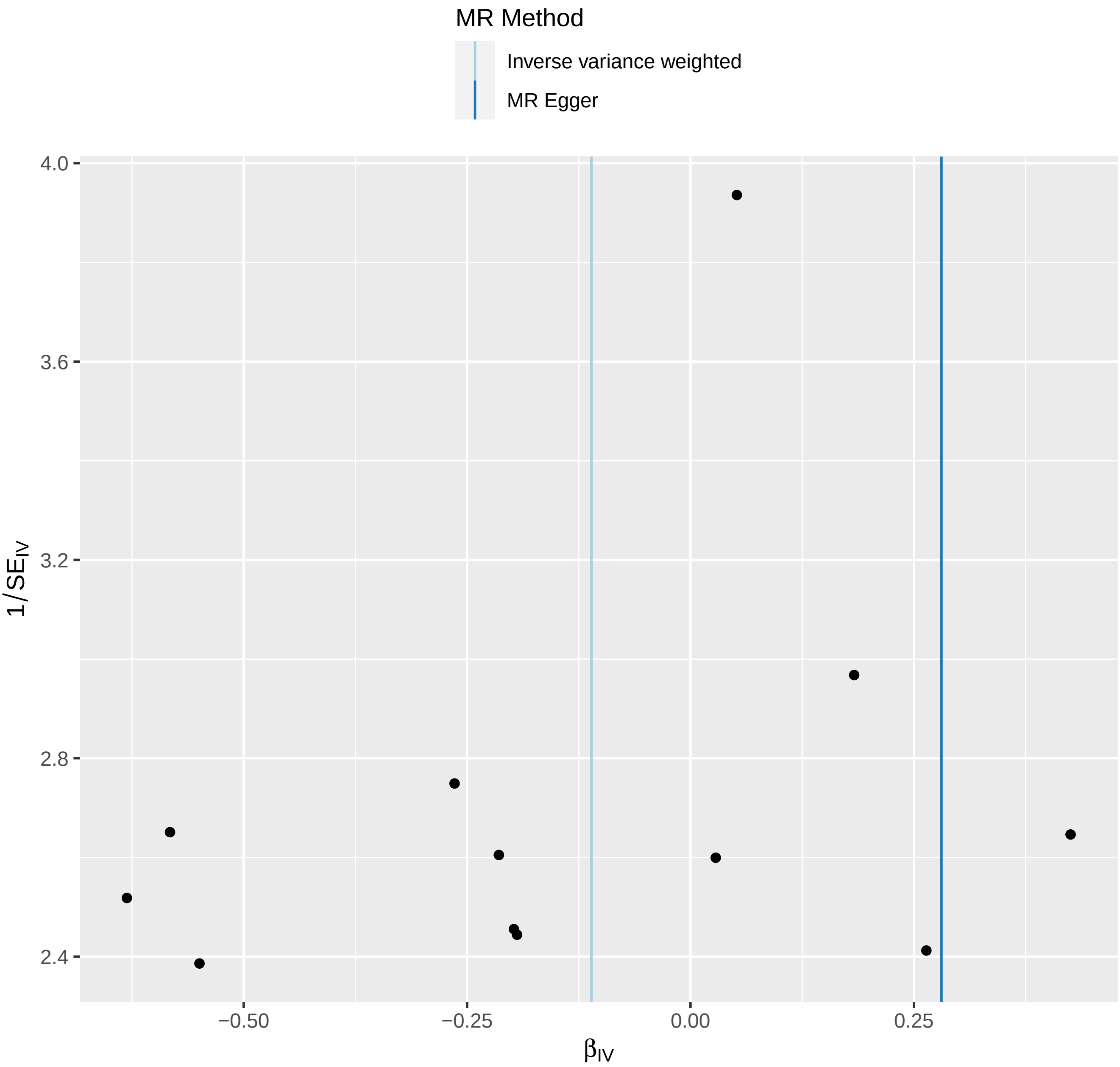

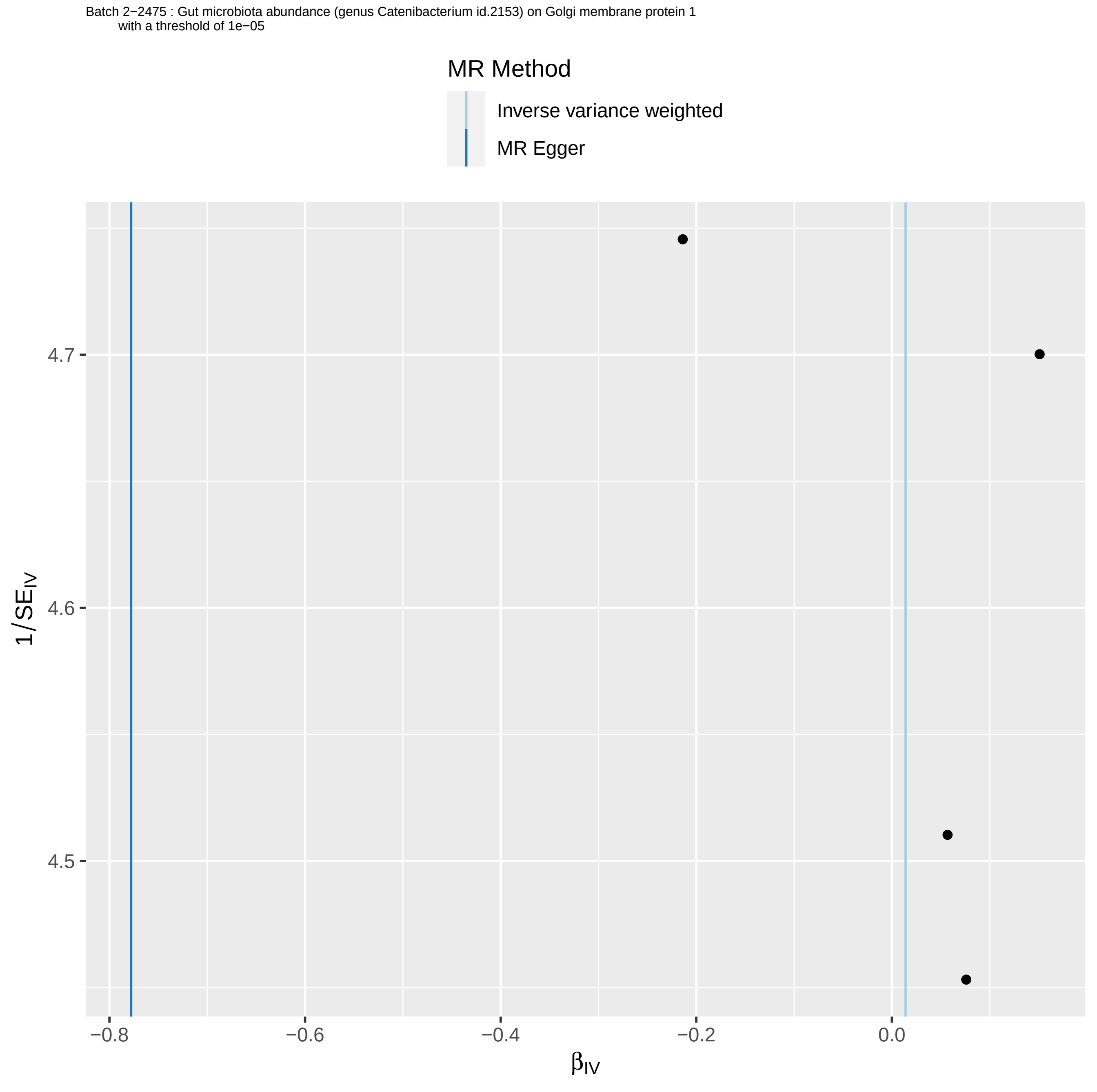
Batch 2−2475 : Gut microbiota abundance (genus Catenibacterium id.2153) on Golgi membrane protein 1
with a threshold of 1e−05
MR Method
Inverse variance weighted
MR Egger
4.7
4.6
4.5
$1/SE_{IV}$
−0.8
−0.6
−0.4
−0.2
0.0
β_{IV}

Batch 2−2476 : Gut microbiota abundance (genus Parasutterella id.2892) on Golgi membrane protein 1
with a threshold of 1e−05

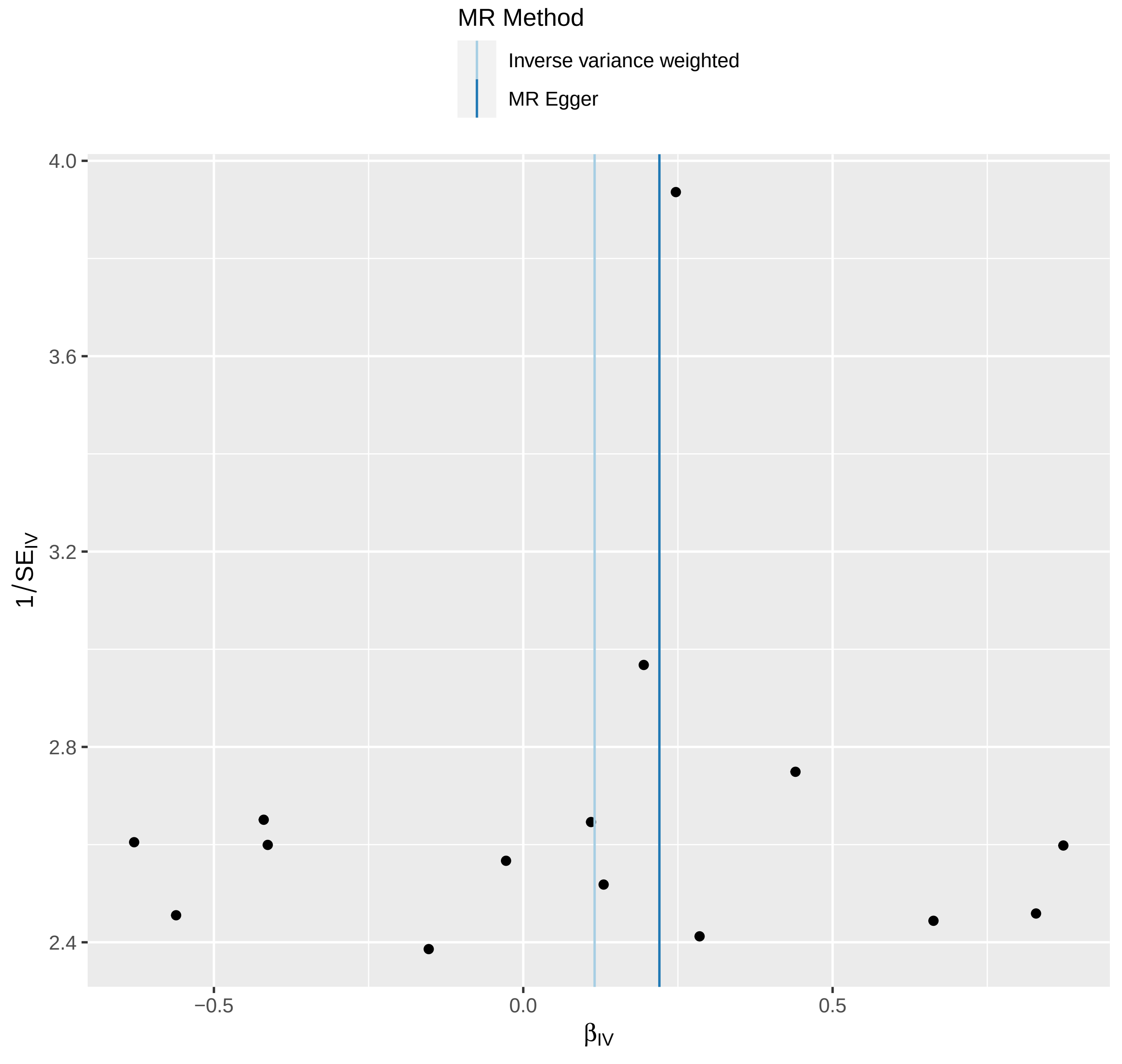

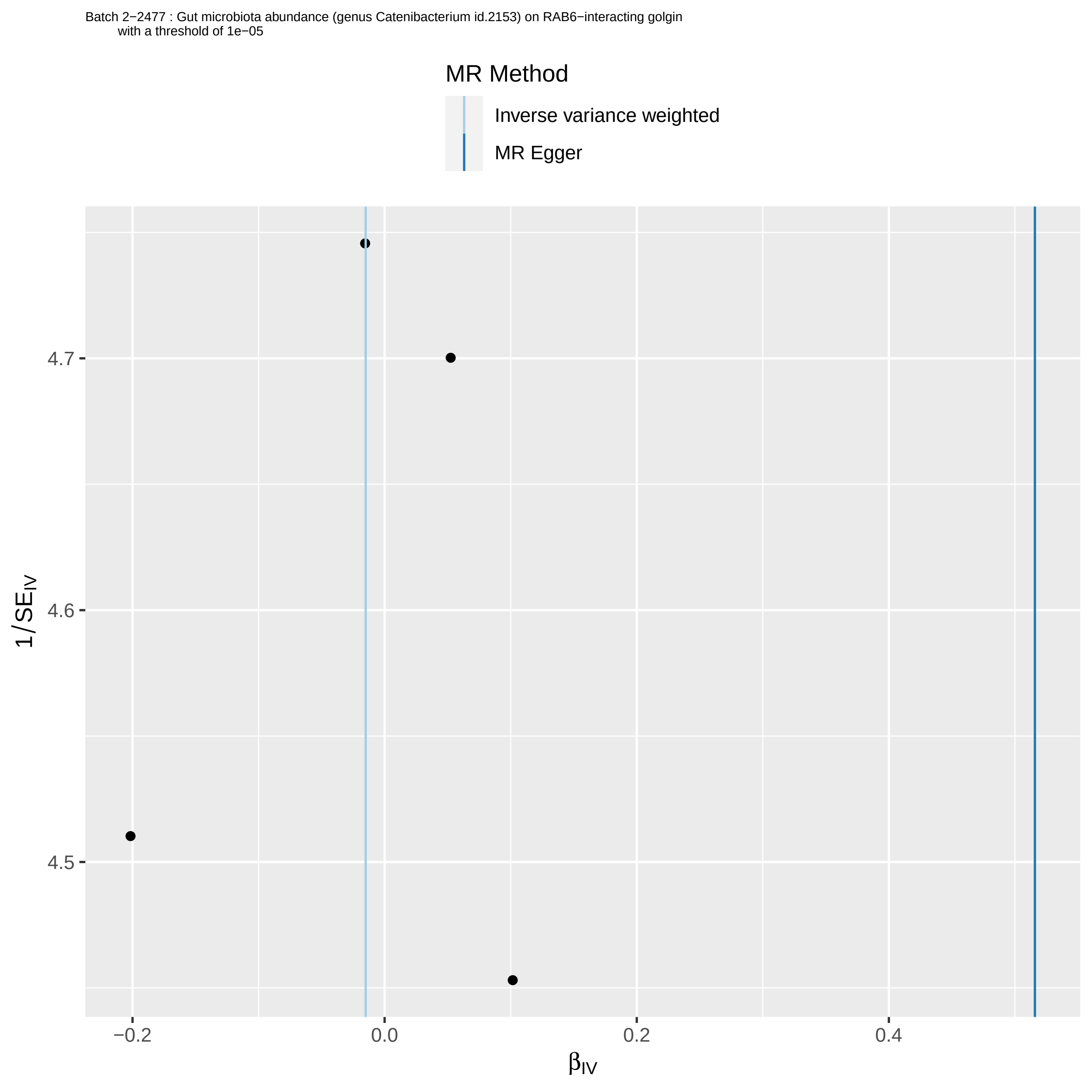
Batch 2−2477 : Gut microbiota abundance (genus Catenibacterium id.2153) on RAB6−interacting golgin
with a threshold of 1e−05
MR Method
Inverse variance weighted
MR Egger
4.7
4.6
4.5
$1/SE_{IV}$
−0.2
0.0
0.2
0.4
β_{IV}

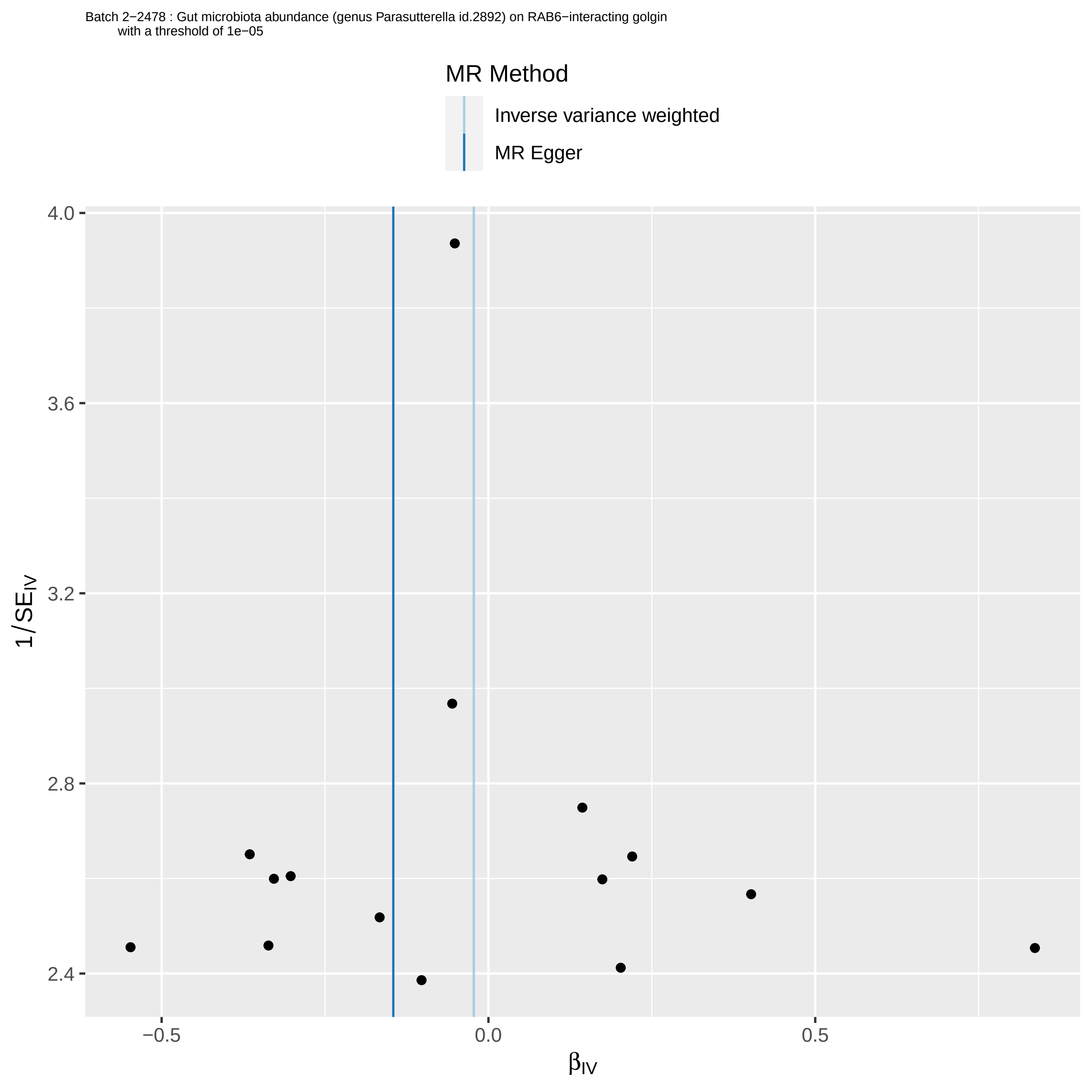

Batch 2−2478 : Gut microbiota abundance (genus Parasutterella id.2892) on RAB6−interacting golgin with a threshold of 1e−05
MR Method
Inverse variance weighted
MR Egger
4.0
3.6
3.2
2.8
2.4
1/SE_IV
−0.5
0.0
0.5
β_IV

Batch 2−2479 : Gut microbiota abundance (genus Catenibacterium id.2153) on Golgi SNAP receptor complex member 1
with a threshold of 1e−05

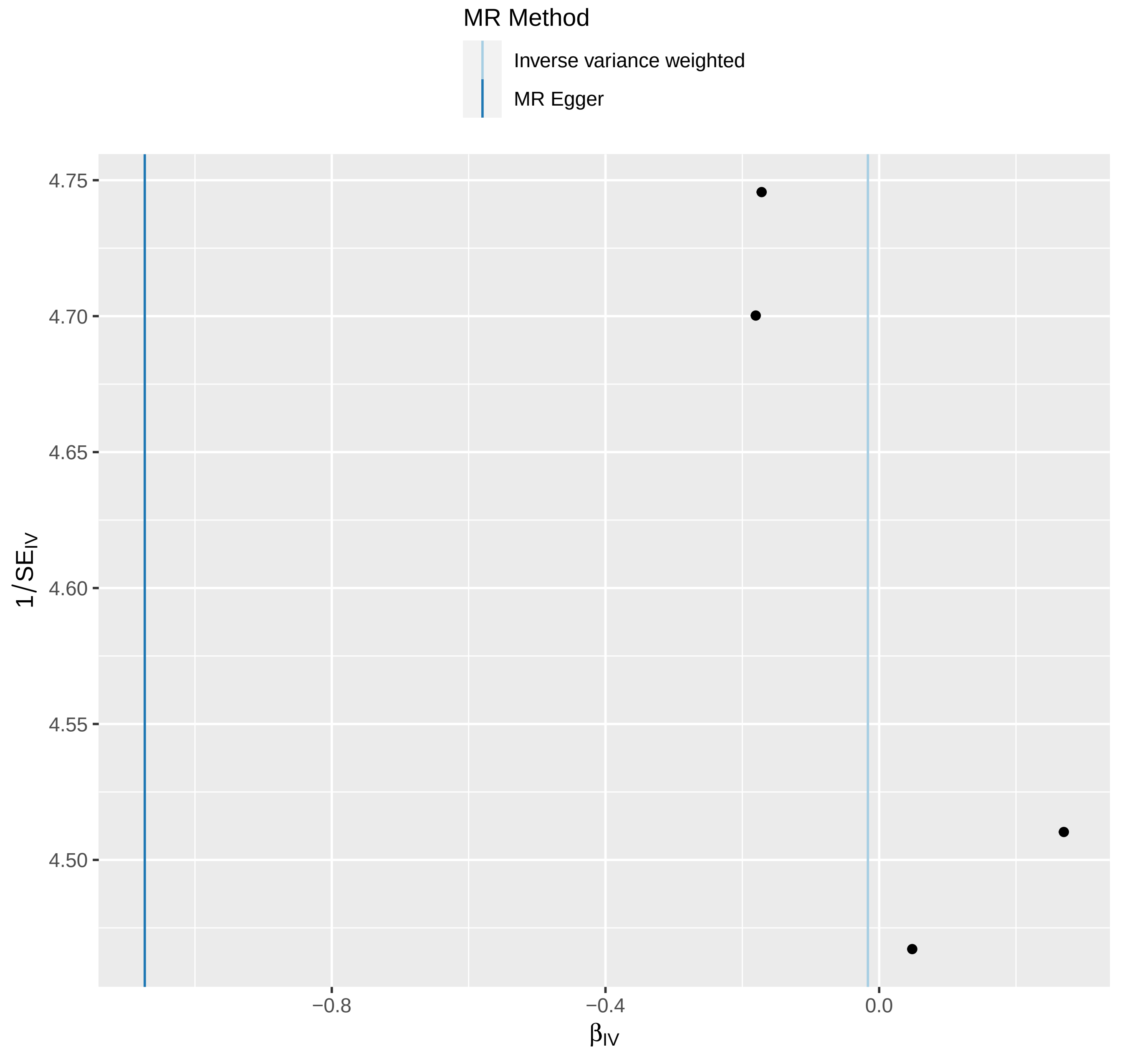

Batch 2−2480 : Gut microbiota abundance (genus Parasutterella id.2892) on Golgi SNAP receptor complex member 1
with a threshold of 1e−05

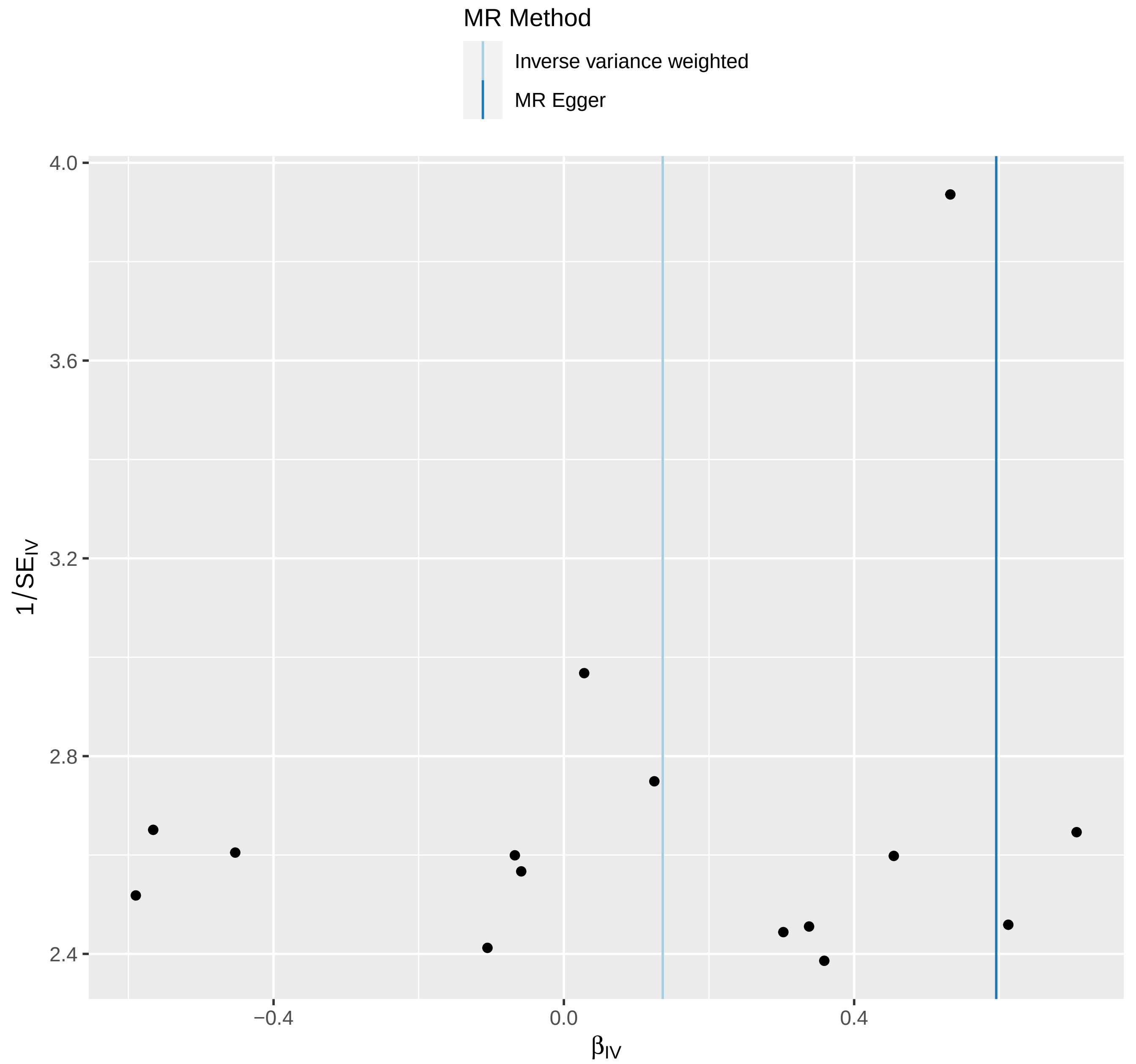

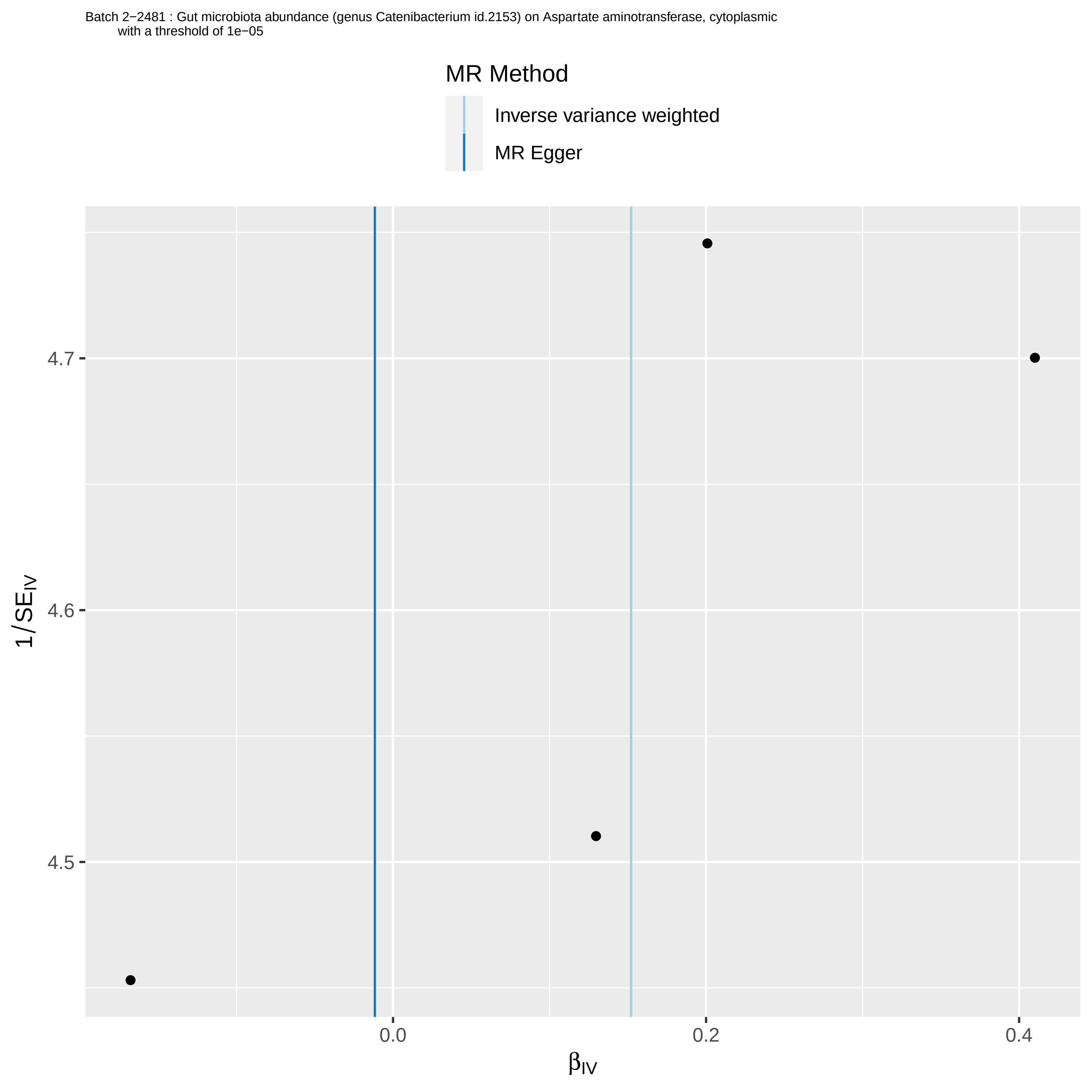

Batch 2−2481 : Gut microbiota abundance (genus Catenibacterium id.2153) on Aspartate aminotransferase, cytoplasmic
with a threshold of 1e−05
MR Method
Inverse variance weighted
MR Egger
4.7
4.6
4.5
1/SE_IV
0.0
0.2
0.4
β_IV

Batch 2−2482 : Gut microbiota abundance (genus Parasutterella id.2892) on Aspartate aminotransferase, cytoplasmic
with a threshold of 1e−05

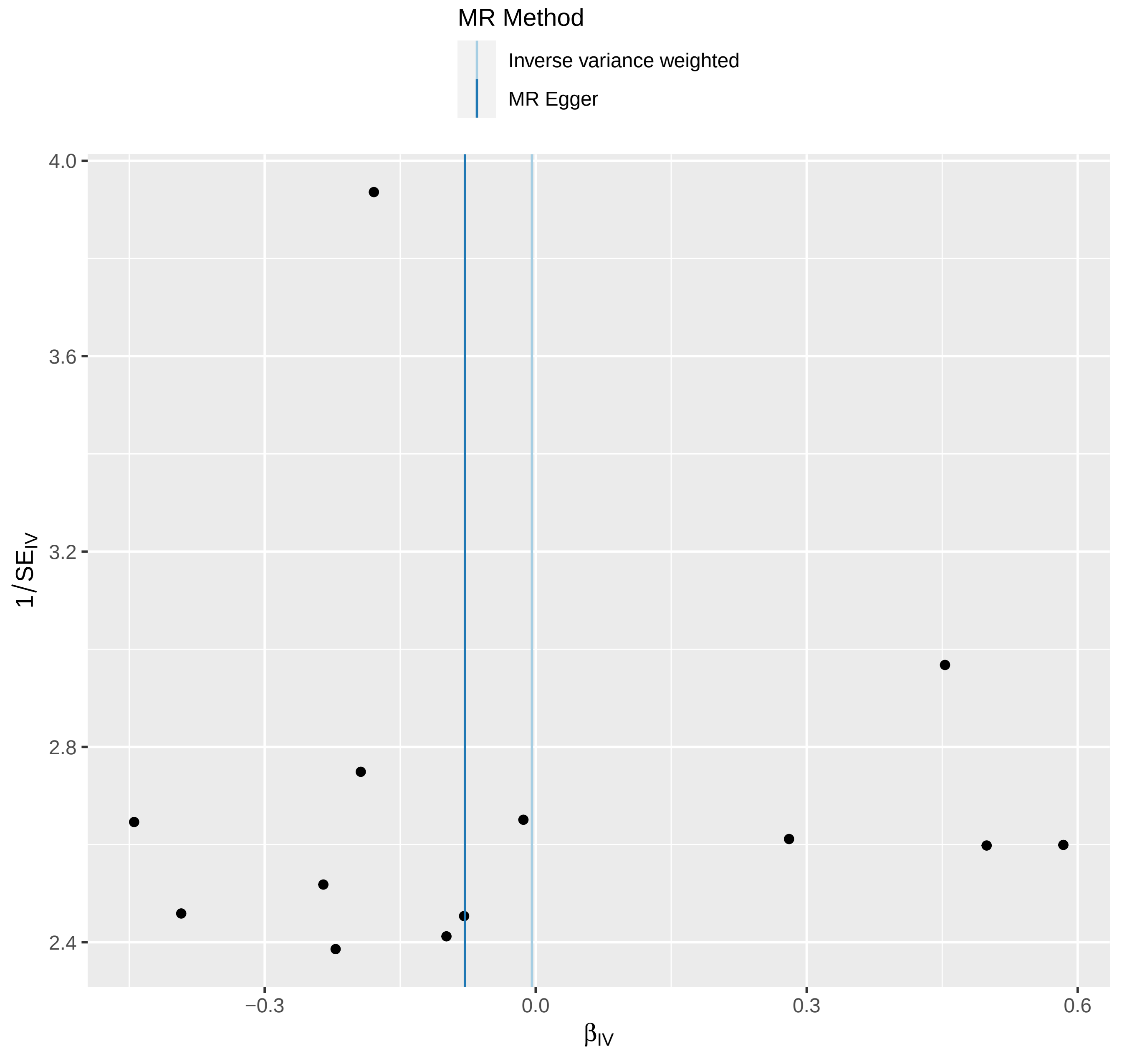

Batch 2−2483 : Gut microbiota abundance (genus Catenibacterium id.2153) on Platelet glycoprotein Ib alpha chain with a threshold of 1e−05

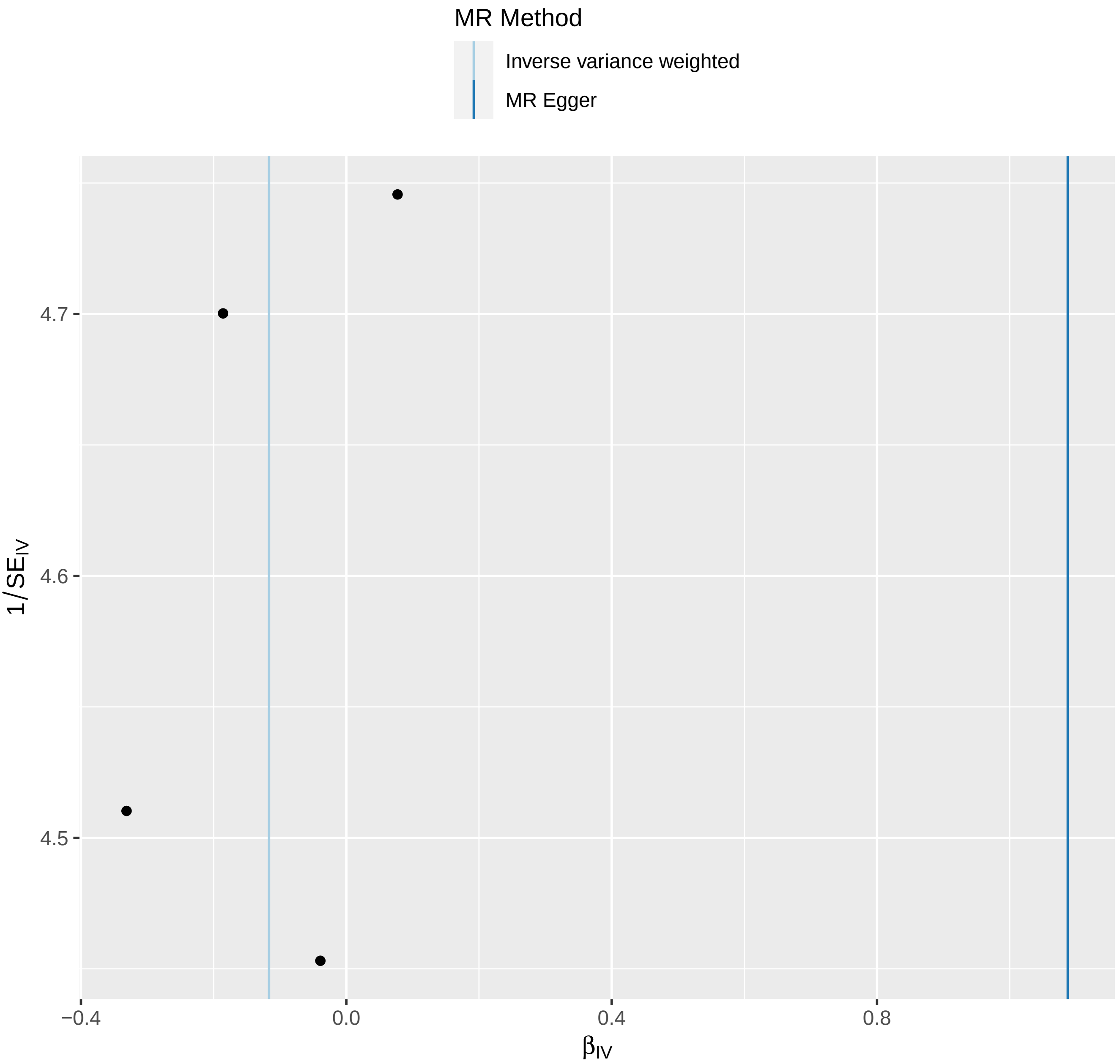

Batch 2−2484 : Gut microbiota abundance (genus Parasutterella id.2892) on Platelet glycoprotein Ib alpha chain
with a threshold of 1e−05

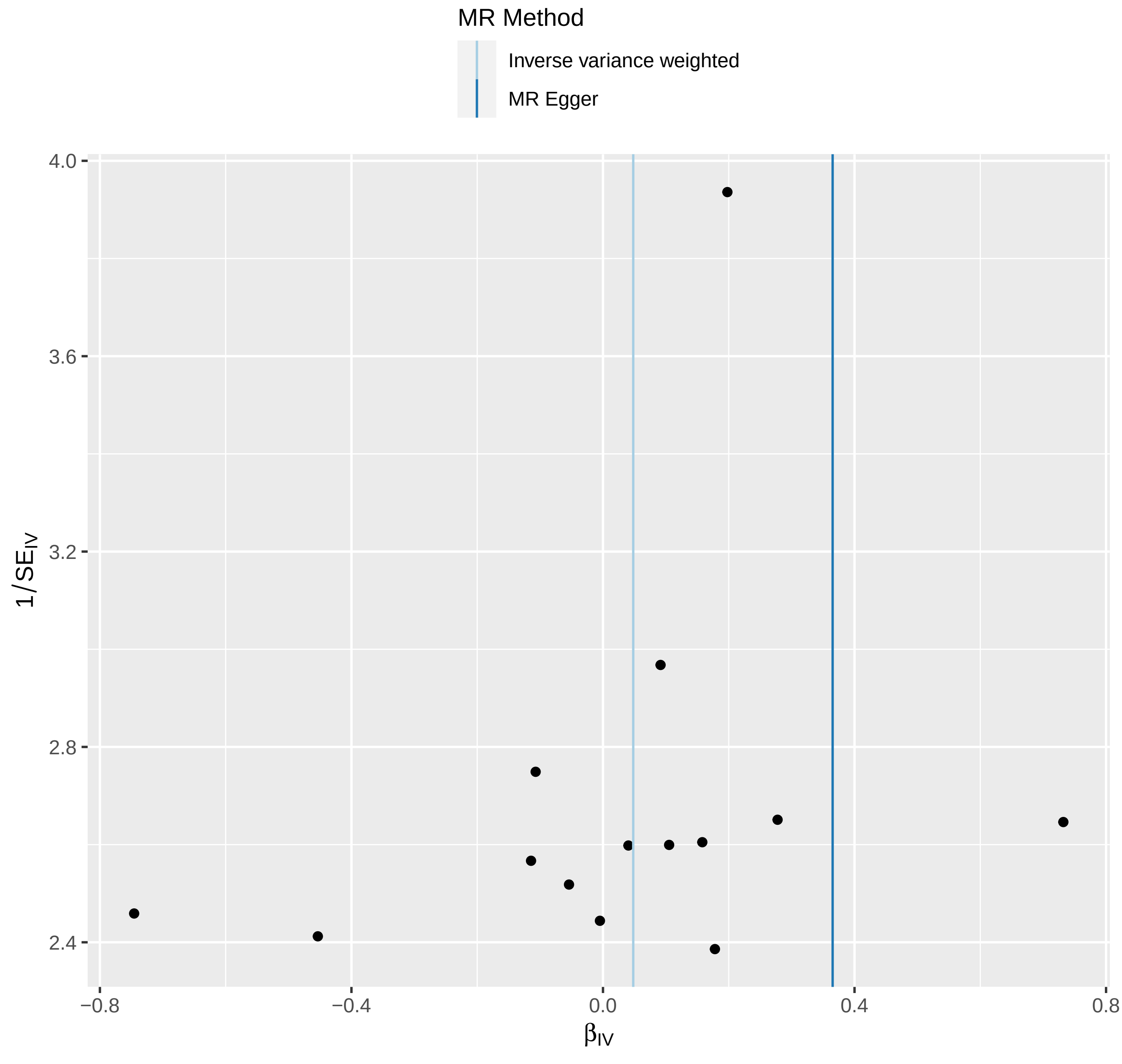

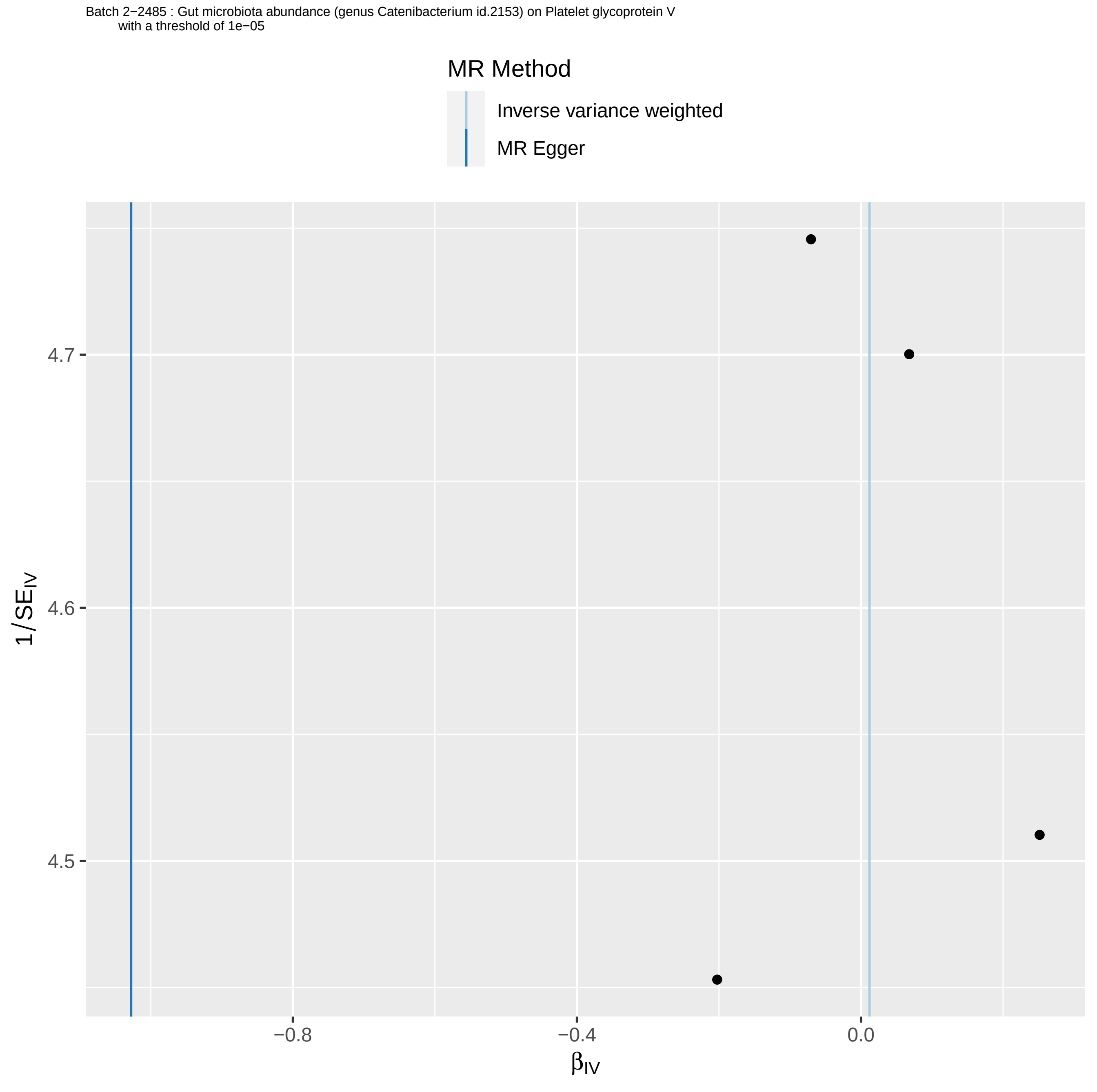
Batch 2−2485 : Gut microbiota abundance (genus Catenibacterium id.2153) on Platelet glycoprotein V
with a threshold of 1e−05
MR Method
Inverse variance weighted
MR Egger
4.7
4.6
4.5
1/SE_IV
−0.8
−0.4
0.0
β_IV

Batch 2−2486 : Gut microbiota abundance (genus Parasutterella id.2892) on Platelet glycoprotein V
with a threshold of 1e−05

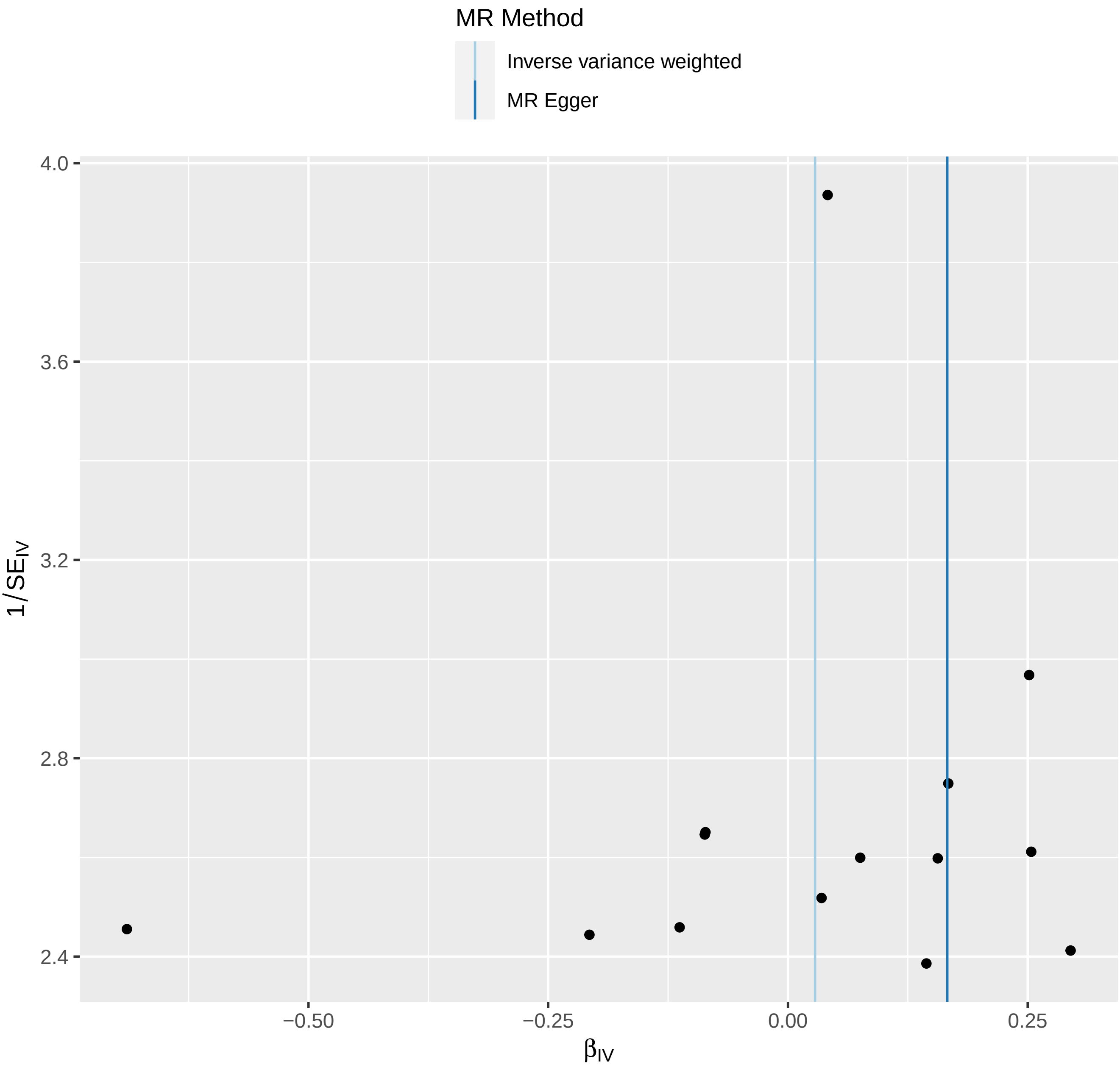

Batch 2−2487 : Gut microbiota abundance (genus Catenibacterium id.2153) on Cell surface A33 antigen
with a threshold of 1e−05

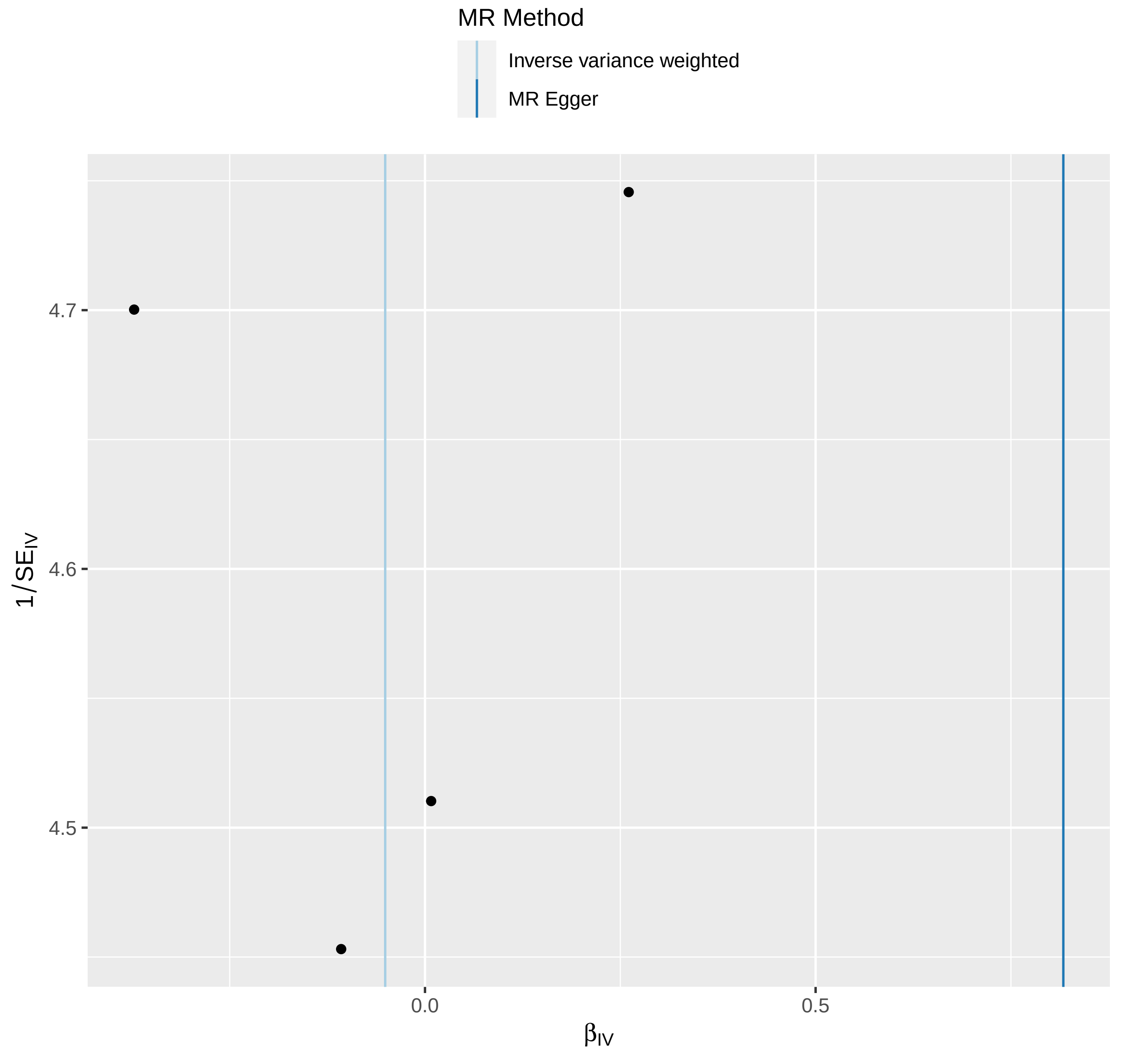

Batch 2−2488 : Gut microbiota abundance (genus Parasutterella id.2892) on Cell surface A33 antigen
with a threshold of 1e−05

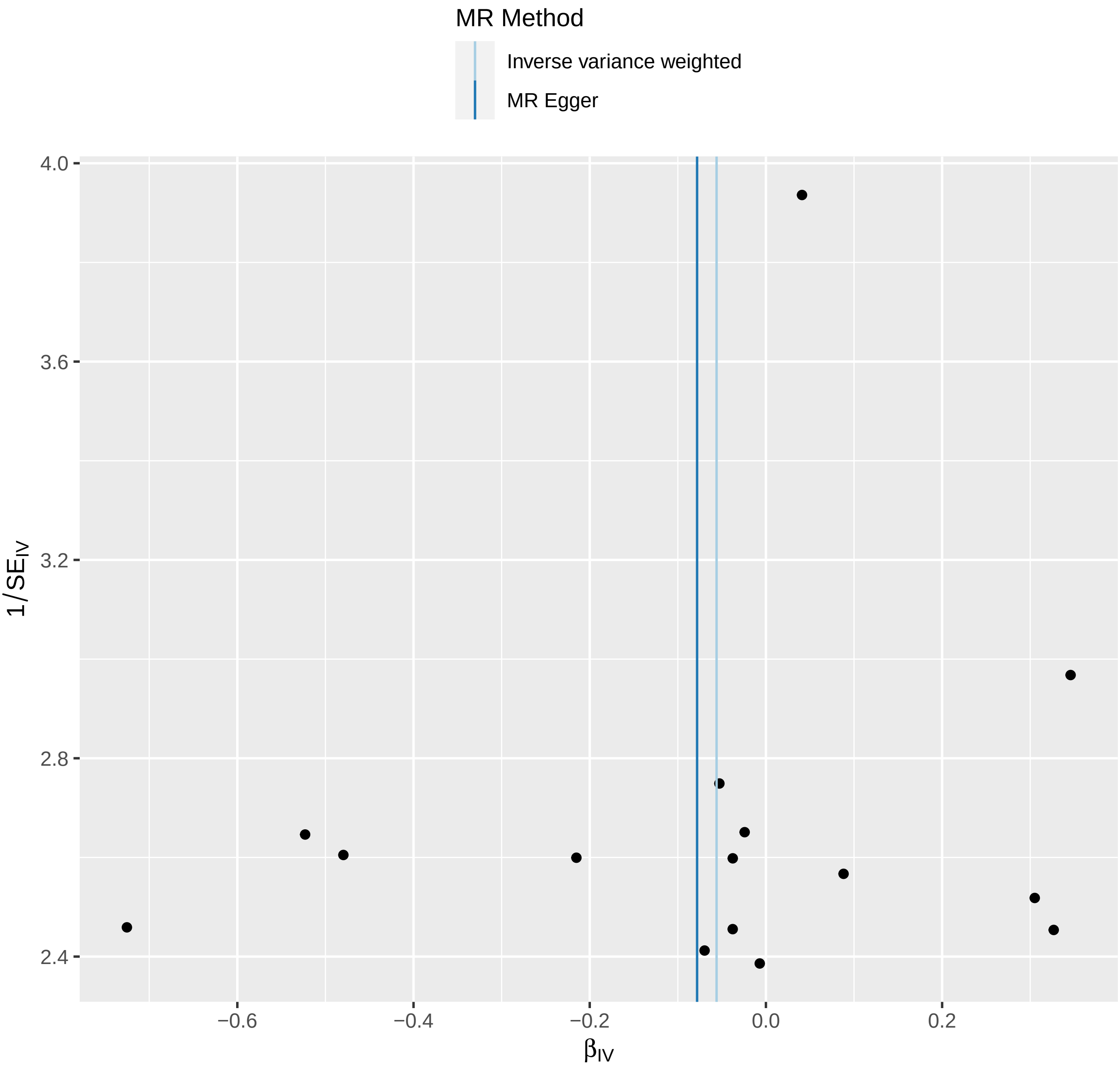

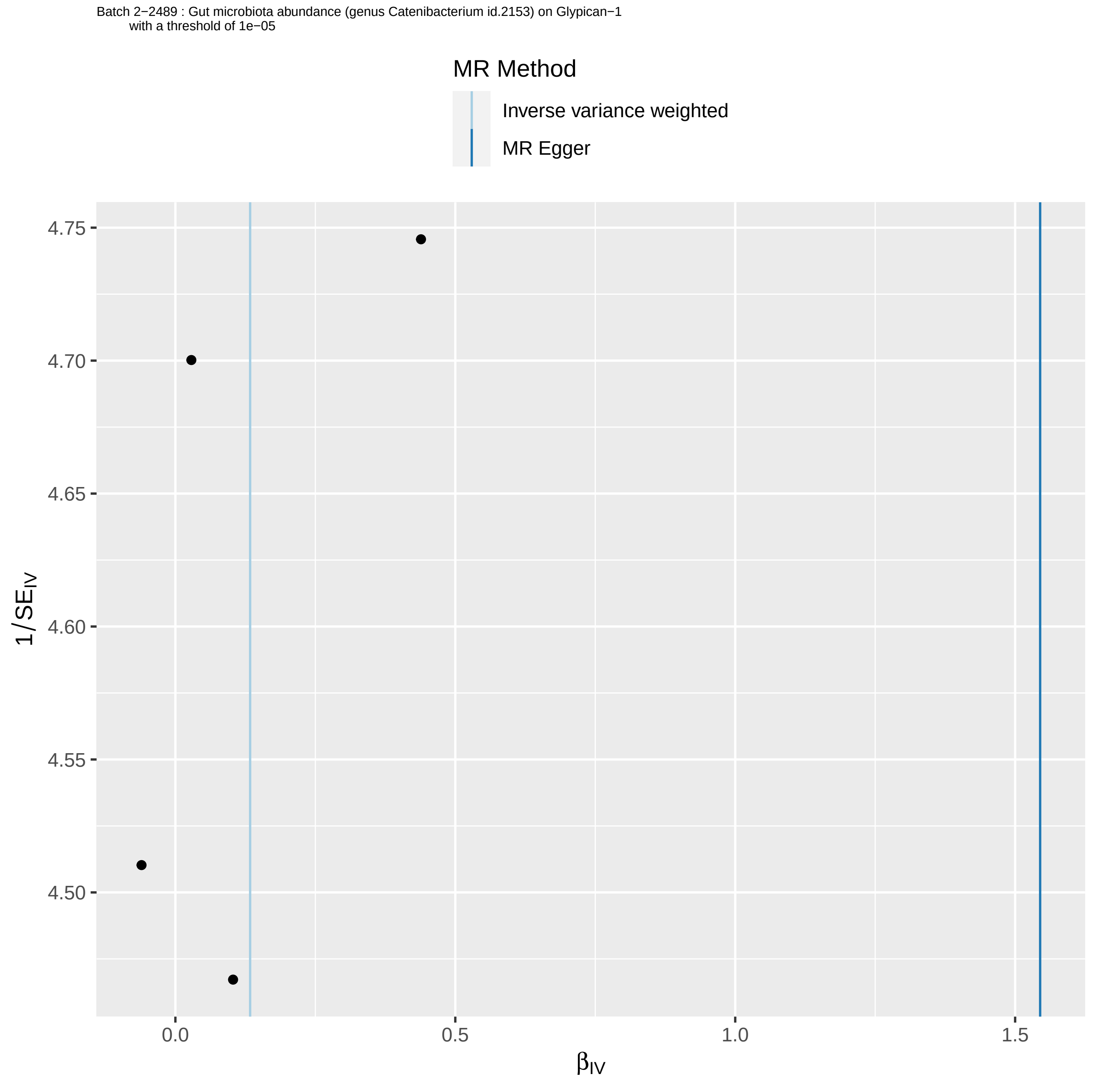
Batch 2−2489 : Gut microbiota abundance (genus Catenibacterium id.2153) on Glypican−1
with a threshold of 1e−05
MR Method
Inverse variance weighted
MR Egger
4.75
4.70
4.65
4.60
4.55
4.50
1/SE_IV
0.0
0.5
1.0
1.5
β_IV

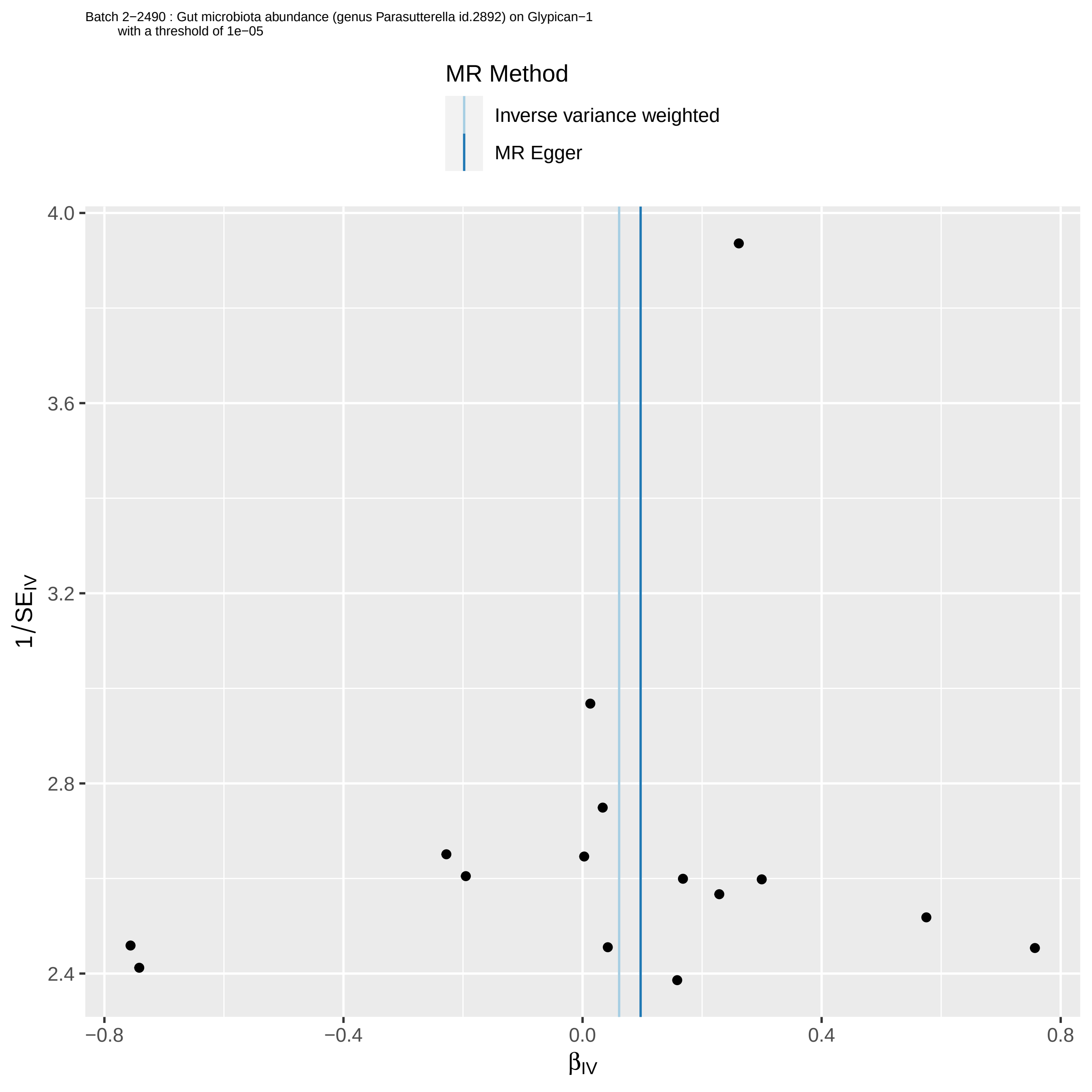

Batch 2−2490 : Gut microbiota abundance (genus Parasutterella id.2892) on Glypican−1
with a threshold of 1e−05
MR Method
Inverse variance weighted
MR Egger
4.0
3.6
3.2
2.8
2.4
1/SE_IV
−0.8
−0.4
0.0
0.4
0.8
β_IV

Batch 2−2491 : Gut microbiota abundance (genus Catenibacterium id.2153) on Glypican−3
with a threshold of 1e−05

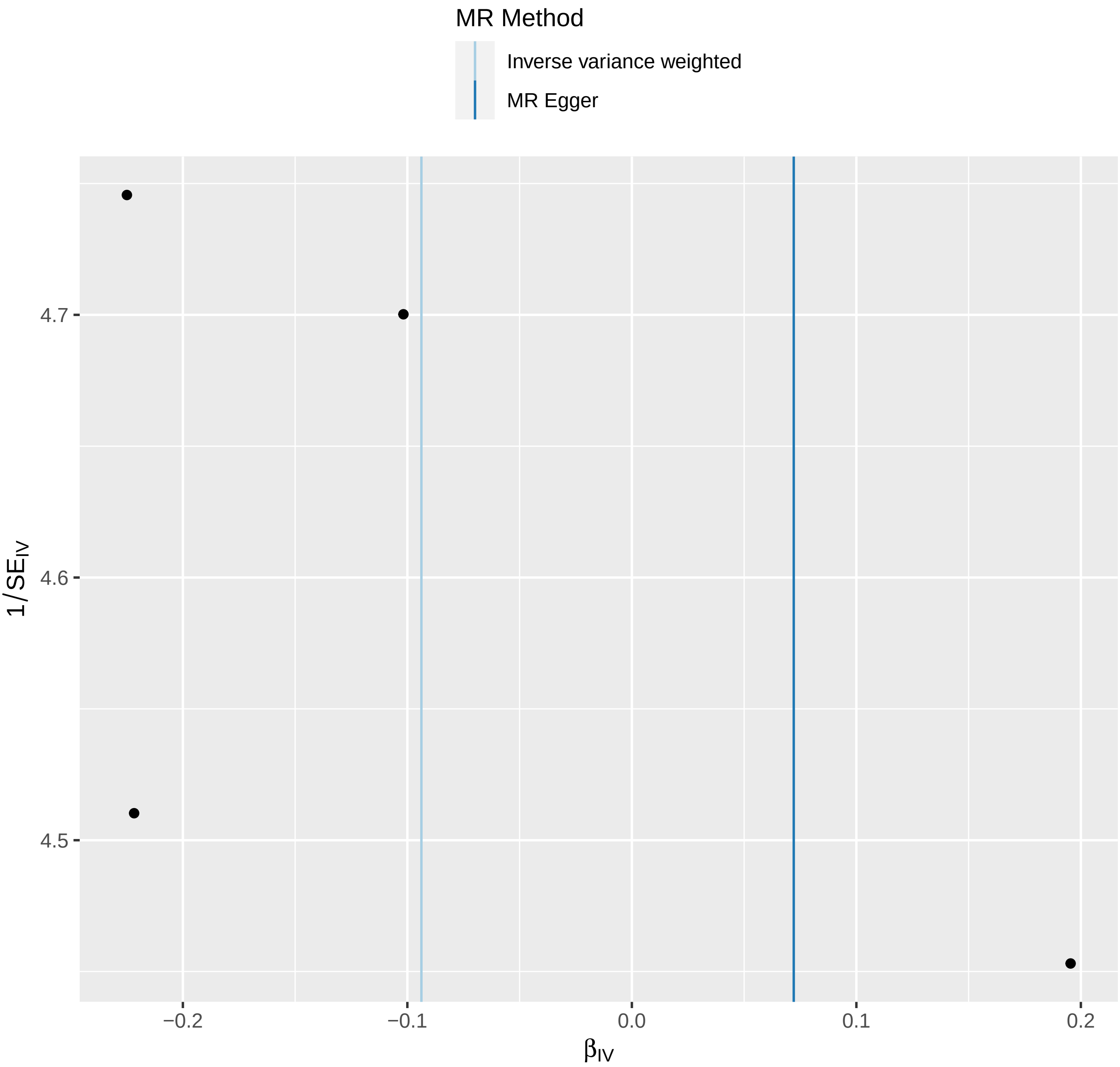

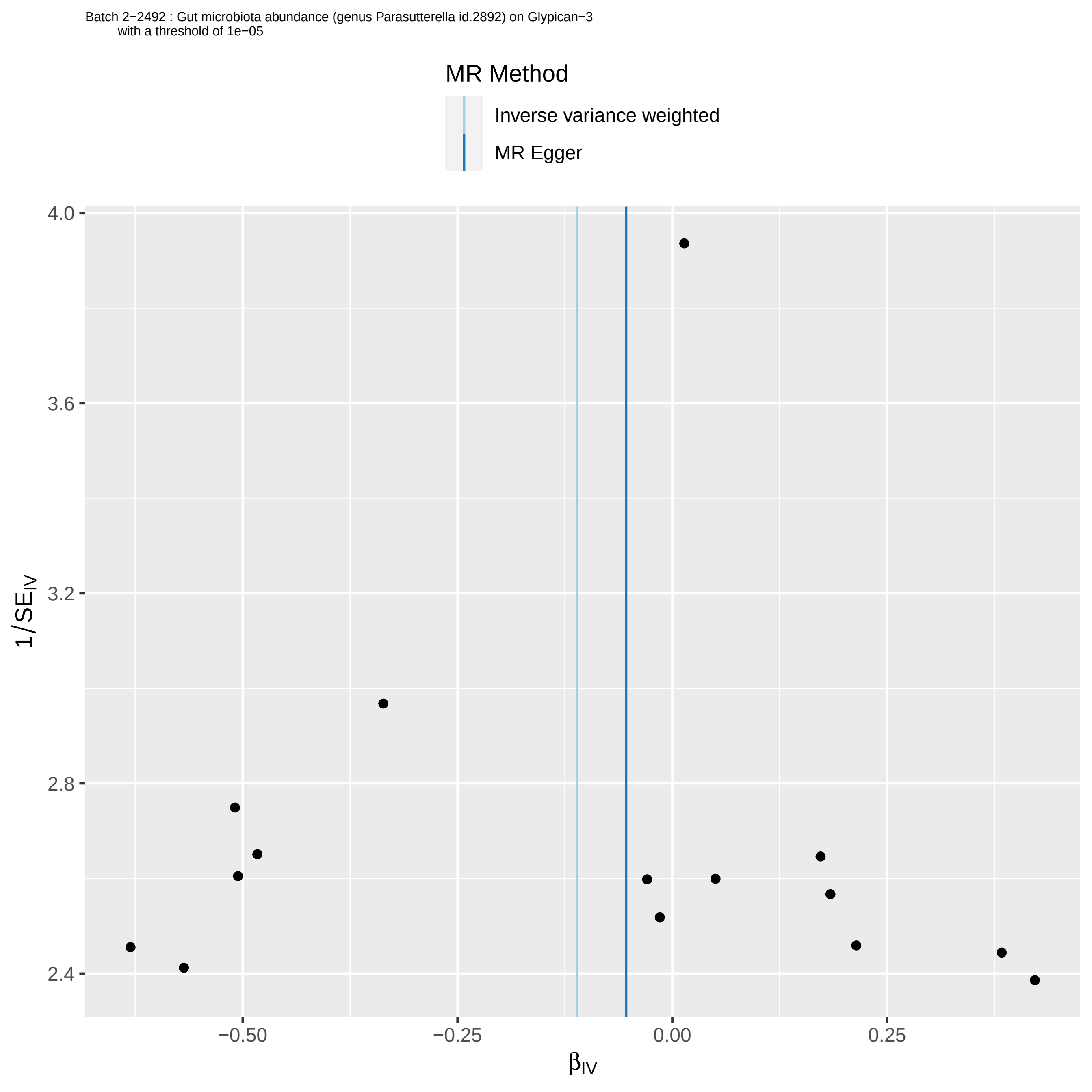

Batch 2−2492 : Gut microbiota abundance (genus Parasutterella id.2892) on Glypican−3
with a threshold of 1e−05
MR Method
Inverse variance weighted
MR Egger
4.0
3.6
3.2
2.8
2.4
1/SE_IV
−0.50
−0.25
0.00
0.25
β_IV

Batch 2−2493 : Gut microbiota abundance (genus Catenibacterium id.2153) on Glypican−5
with a threshold of 1e−05

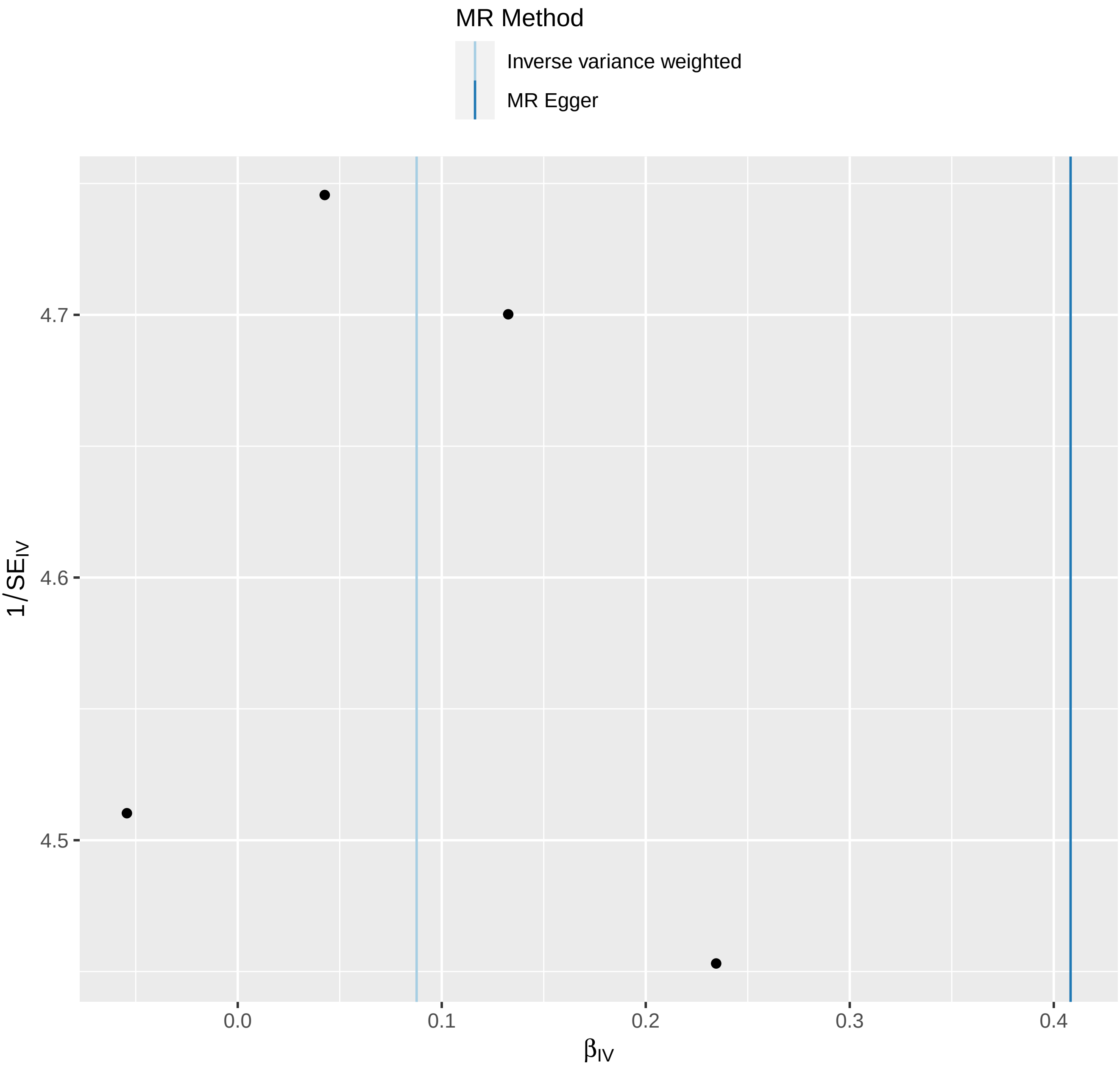

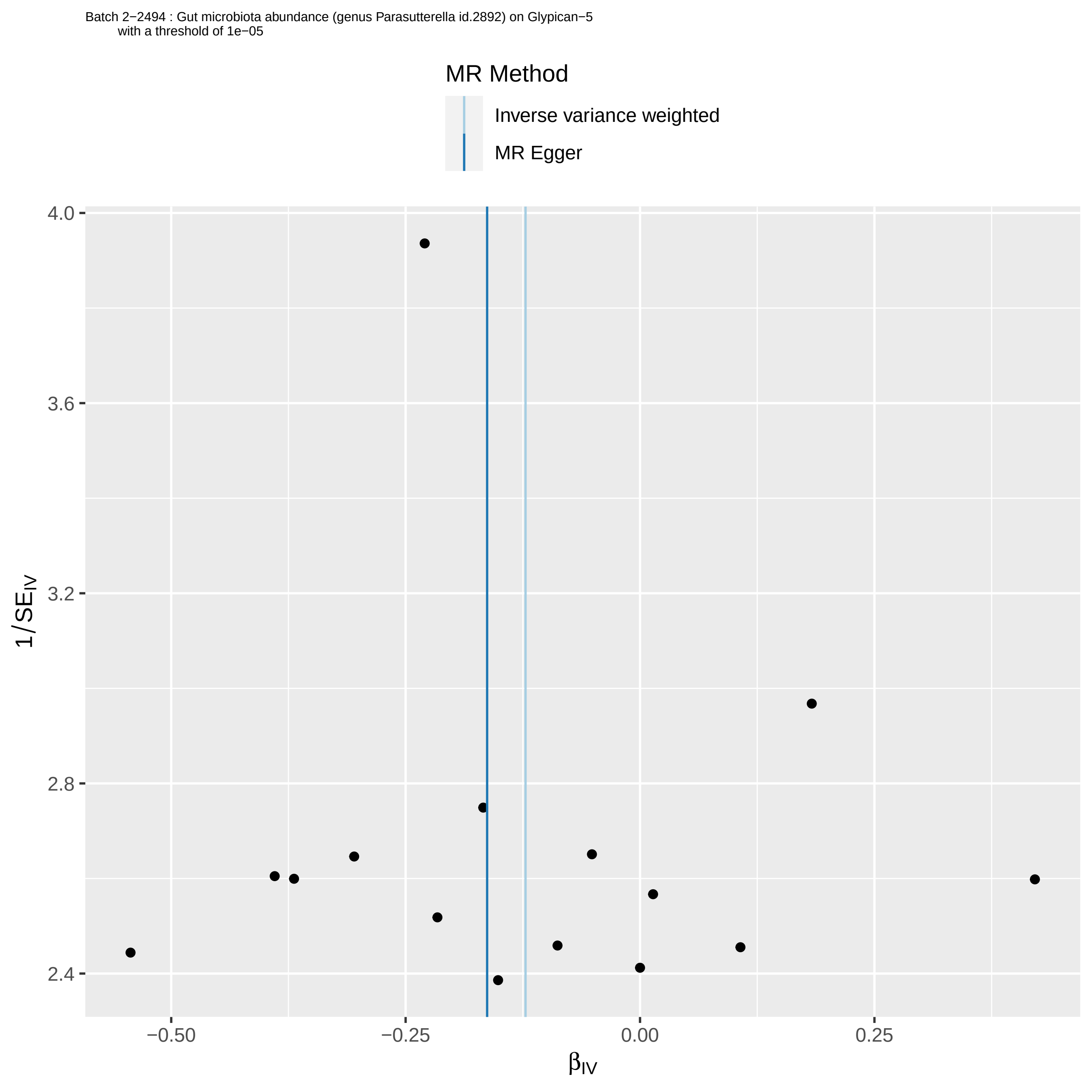

Batch 2−2494 : Gut microbiota abundance (genus Parasutterella id.2892) on Glypican−5
with a threshold of 1e−05
MR Method
Inverse variance weighted
MR Egger
4.0
3.6
3.2
2.8
2.4
1/SE_IV
−0.50
−0.25
0.00
0.25
β_IV

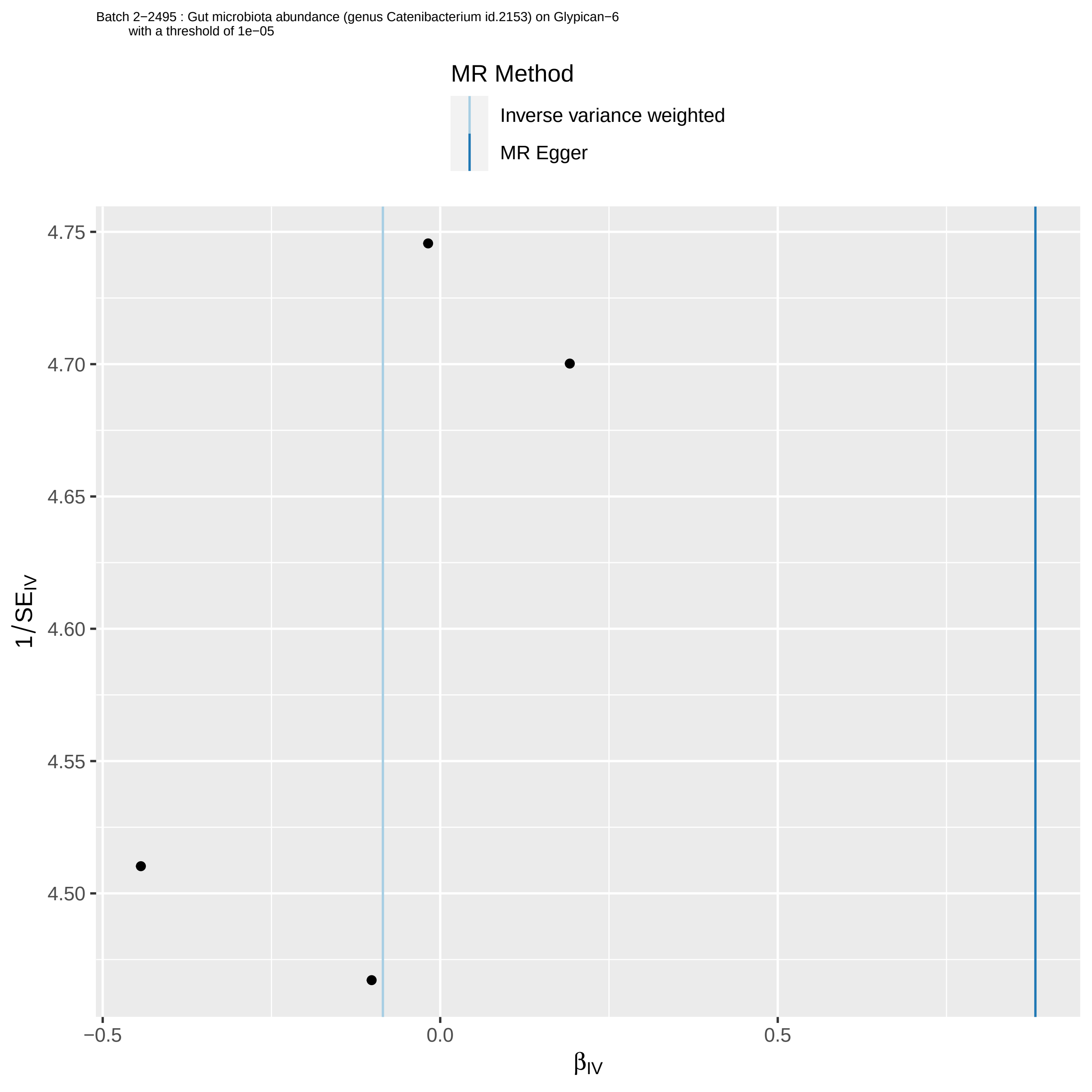

Batch 2−2495 : Gut microbiota abundance (genus Catenibacterium id.2153) on Glypican−6
with a threshold of 1e−05
MR Method
Inverse variance weighted
MR Egger
4.75
4.70
4.65
4.60
4.55
4.50
1/SE_IV
−0.5
0.0
0.5
β_IV

Batch 2−2496 : Gut microbiota abundance (genus Parasutterella id.2892) on Glypican−6
with a threshold of 1e−05

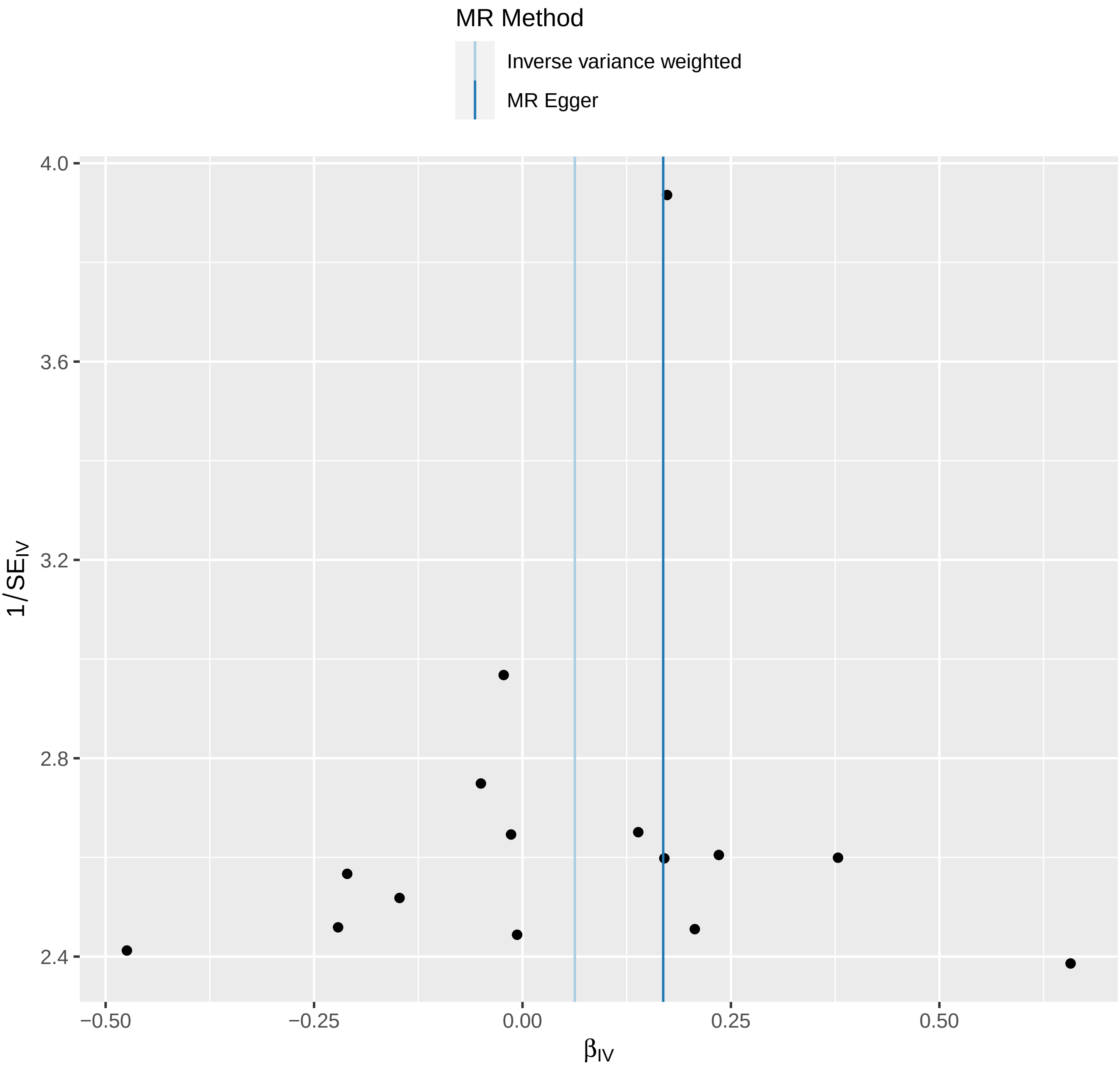

Batch 2−2497 : Gut microbiota abundance (genus Catenibacterium id.2153) on Glycerophosphocholine phosphodiesterase GPCPD1
with a threshold of 1e−05

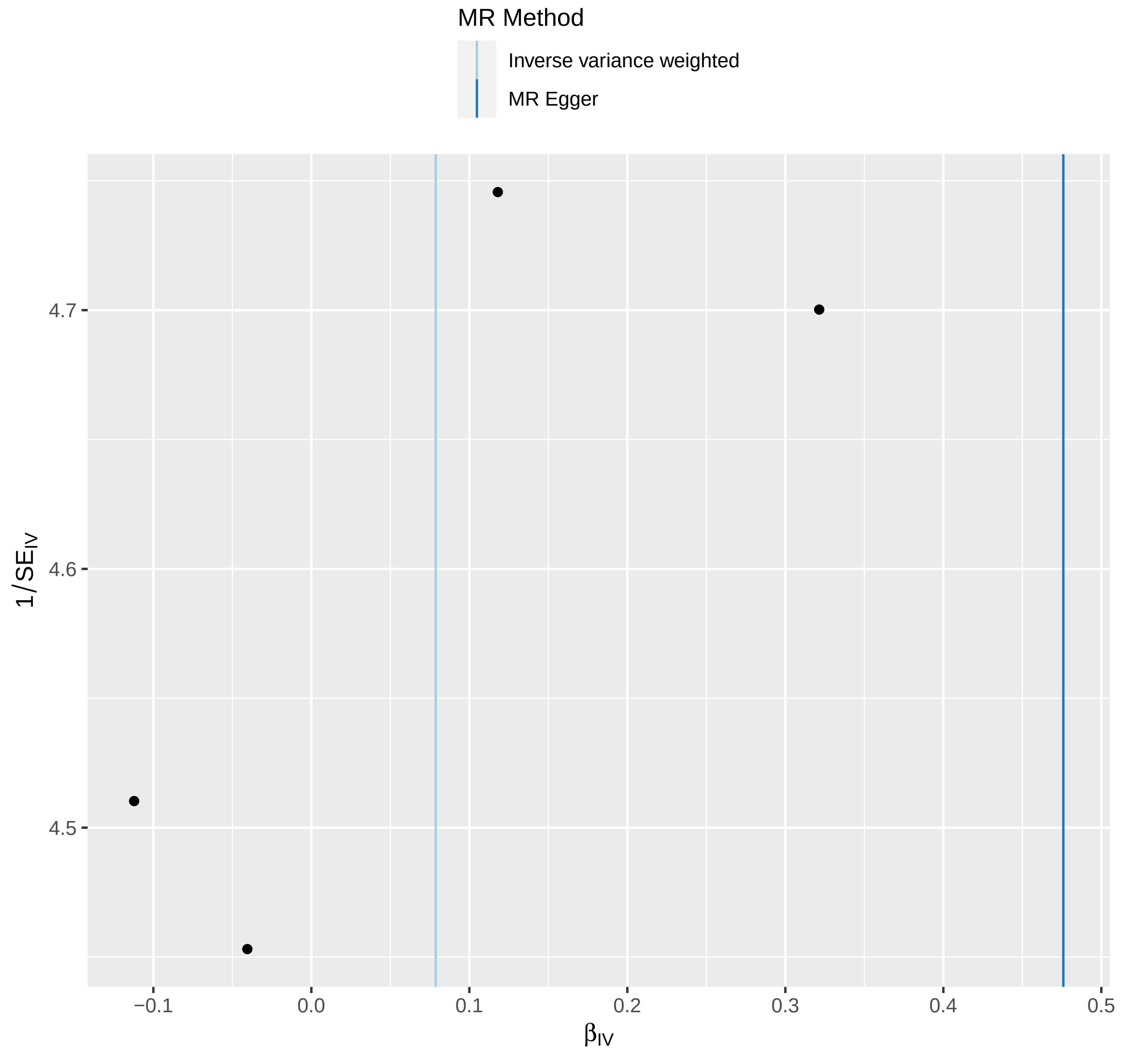

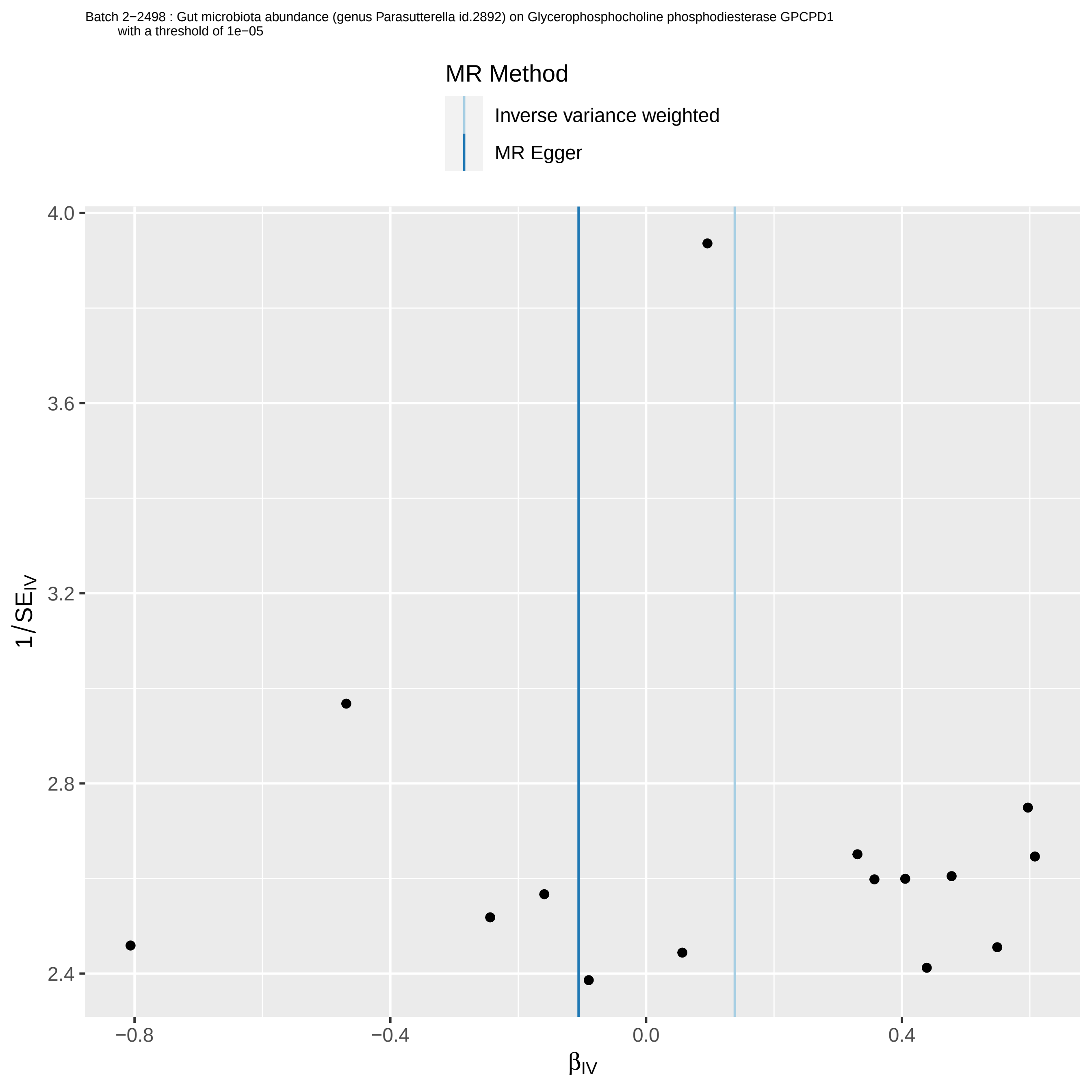
Batch 2−2498 : Gut microbiota abundance (genus Parasutterella id.2892) on Glycerophosphocholine phosphodiesterase GPCPD1
with a threshold of 1e−05
MR Method
Inverse variance weighted
MR Egger
4.0
3.6
3.2
2.8
2.4
1/SE_IV
−0.8
−0.4
0.0
0.4
β_IV

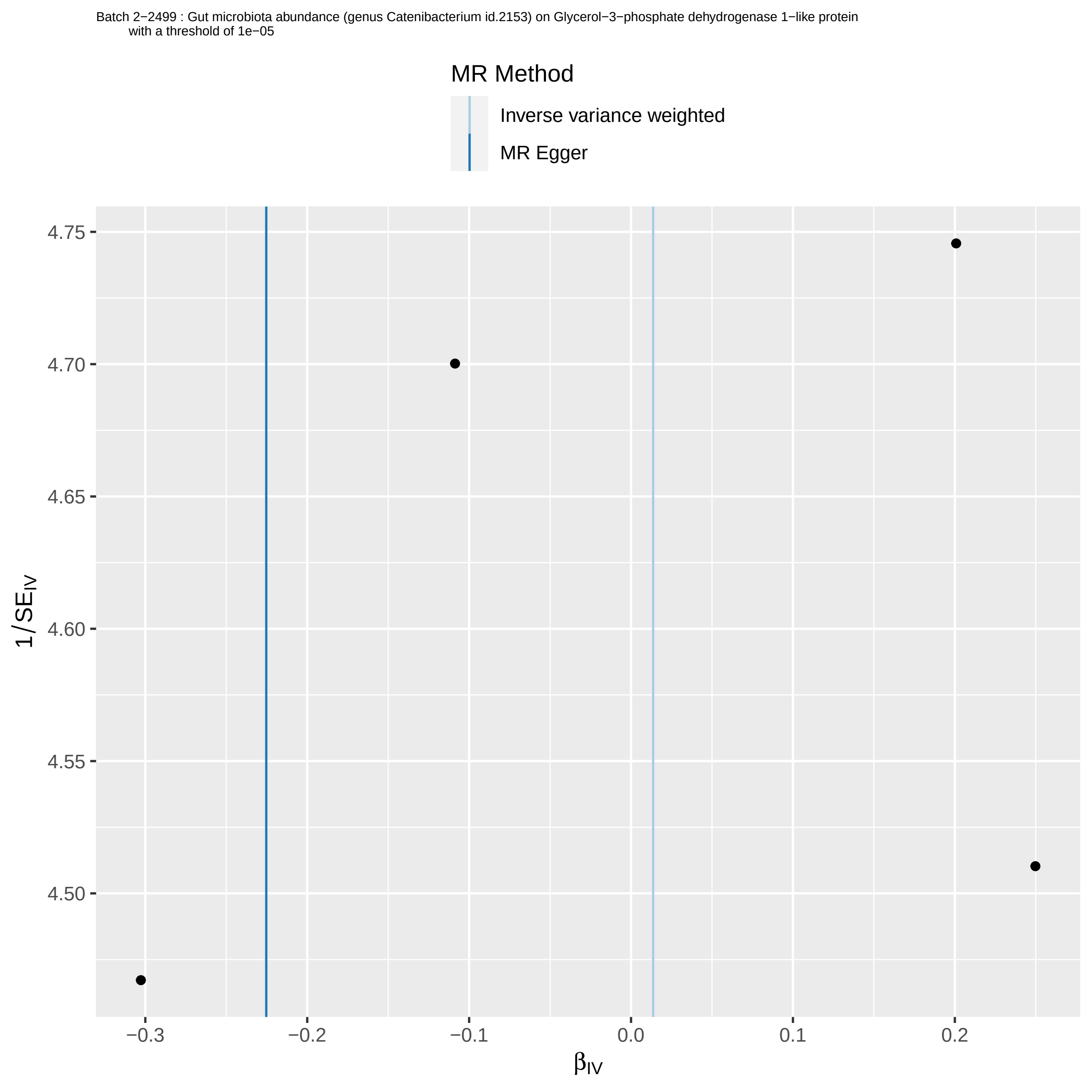

Batch 2−2499 : Gut microbiota abundance (genus Catenibacterium id.2153) on Glycerol−3−phosphate dehydrogenase 1−like protein
with a threshold of 1e−05
MR Method
Inverse variance weighted
MR Egger
4.75
4.70
4.65
4.60
4.55
4.50
1/SE_IV
−0.3
−0.2
−0.1
0.0
0.1
0.2
β_IV

Batch 2−2500 : Gut microbiota abundance (genus Parasutterella id.2892) on Glycerol−3−phosphate dehydrogenase 1−like protein
with a threshold of 1e−05

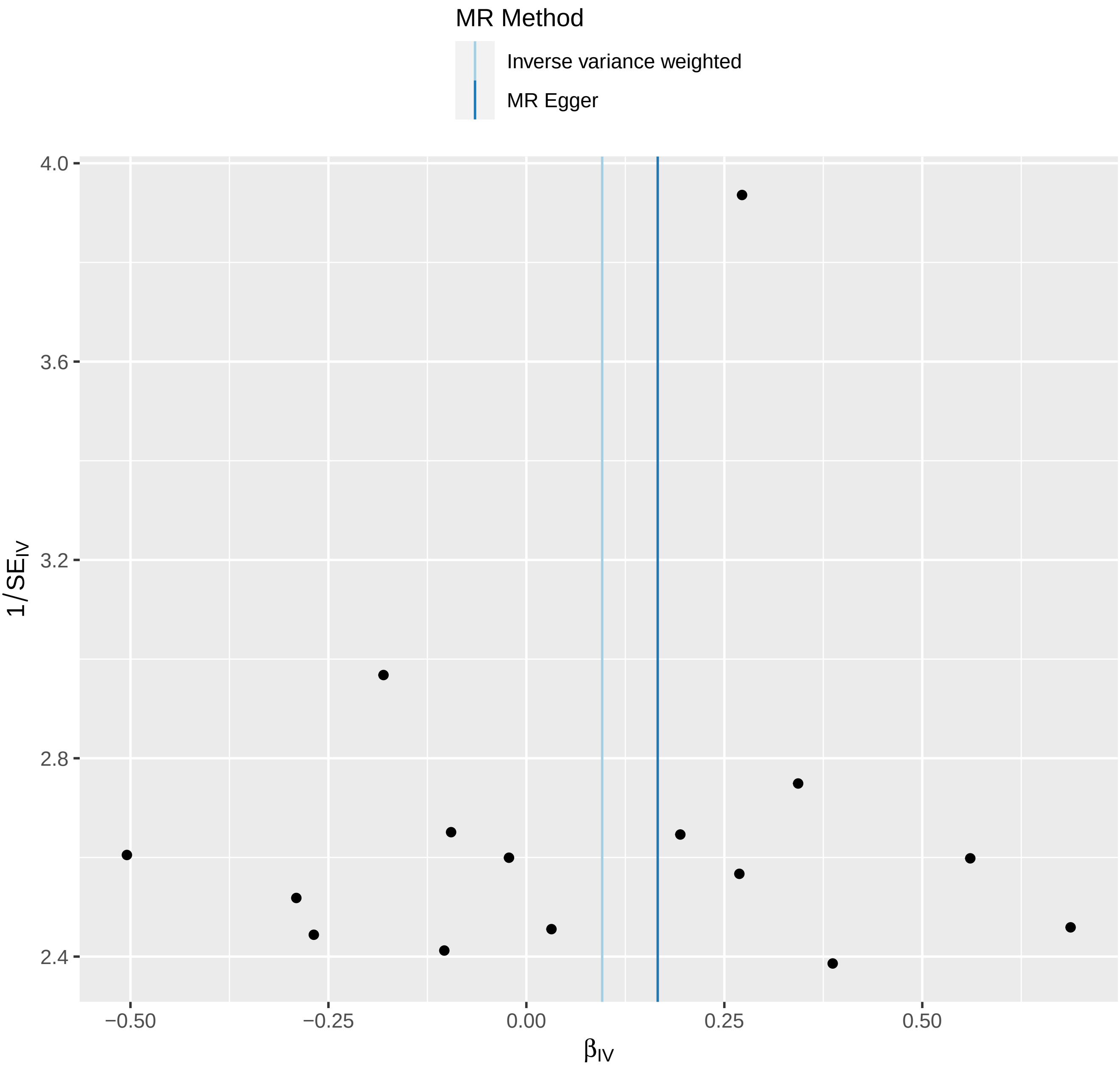

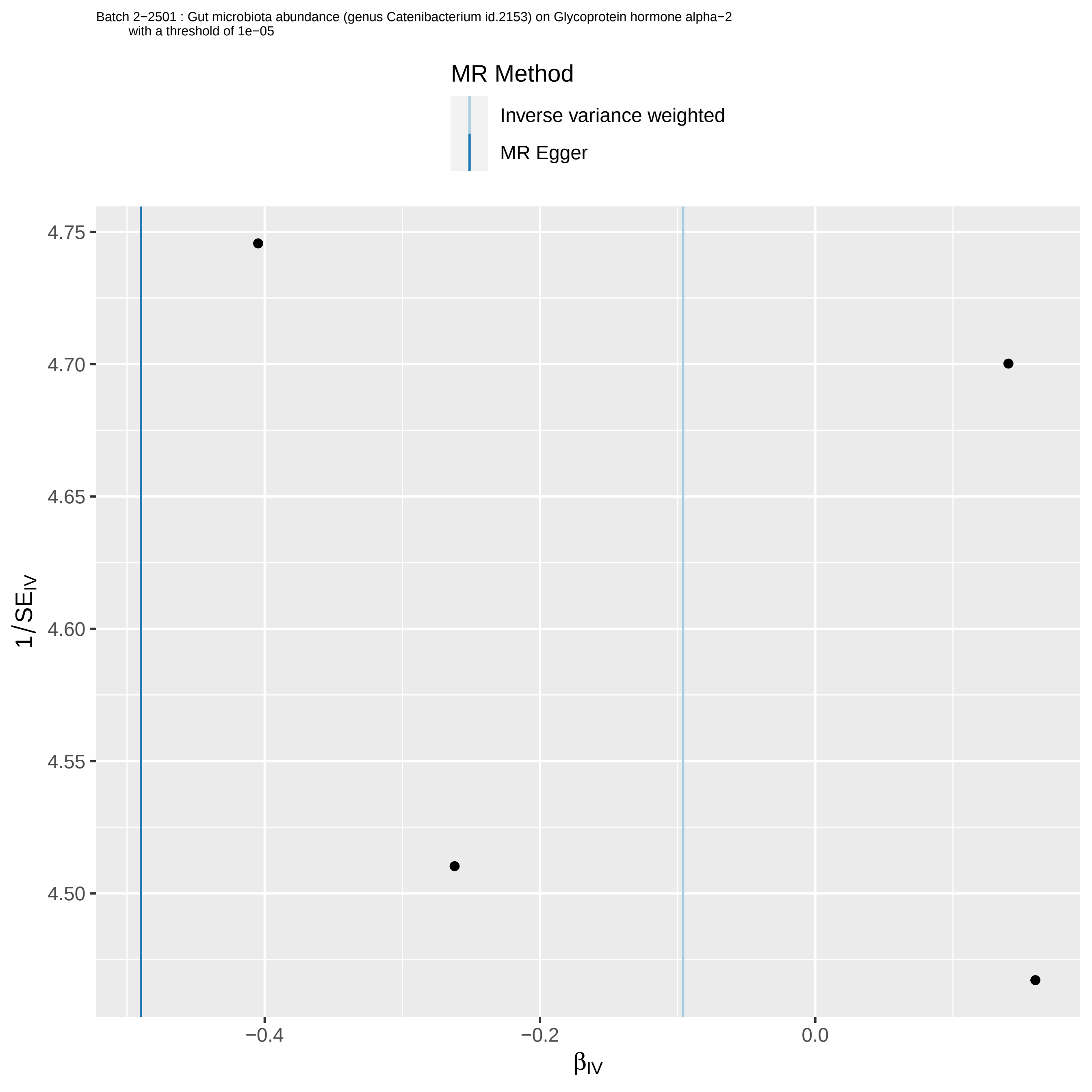
Batch 2−2501 : Gut microbiota abundance (genus Catenibacterium id.2153) on Glycoprotein hormone alpha−2
with a threshold of 1e−05
MR Method
Inverse variance weighted
MR Egger
4.75
4.70
4.65
4.60
4.55
4.50
1/SE$_{IV}$
−0.4
−0.2
0.0
β$_{IV}$

Batch 2−2502 : Gut microbiota abundance (genus Parasutterella id.2892) on Glycoprotein hormone alpha−2
with a threshold of 1e−05

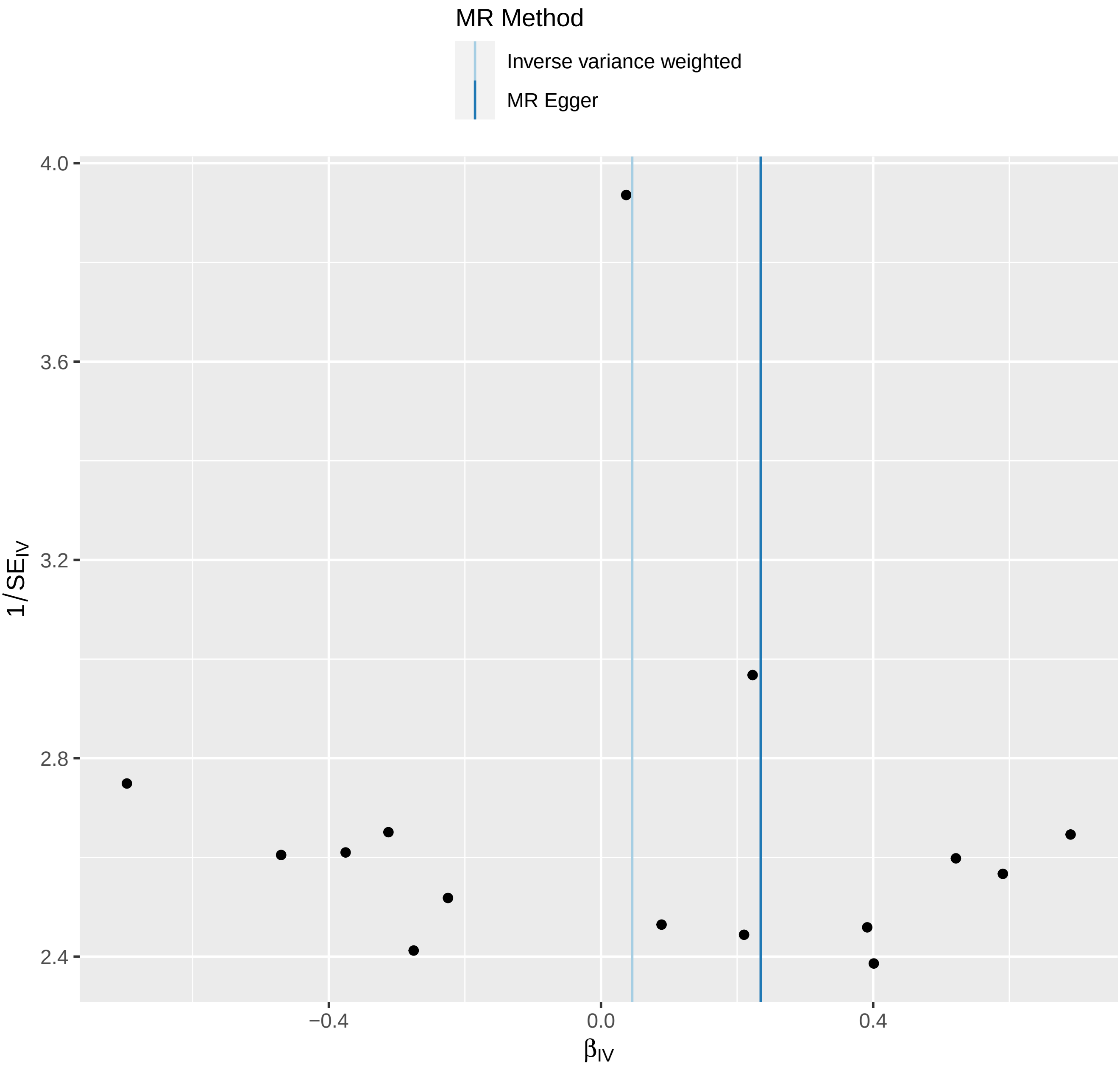

Batch 2−2503 : Gut microbiota abundance (genus Catenibacterium id.2153) on Glucose−6−phosphate isomerase
with a threshold of 1e−05

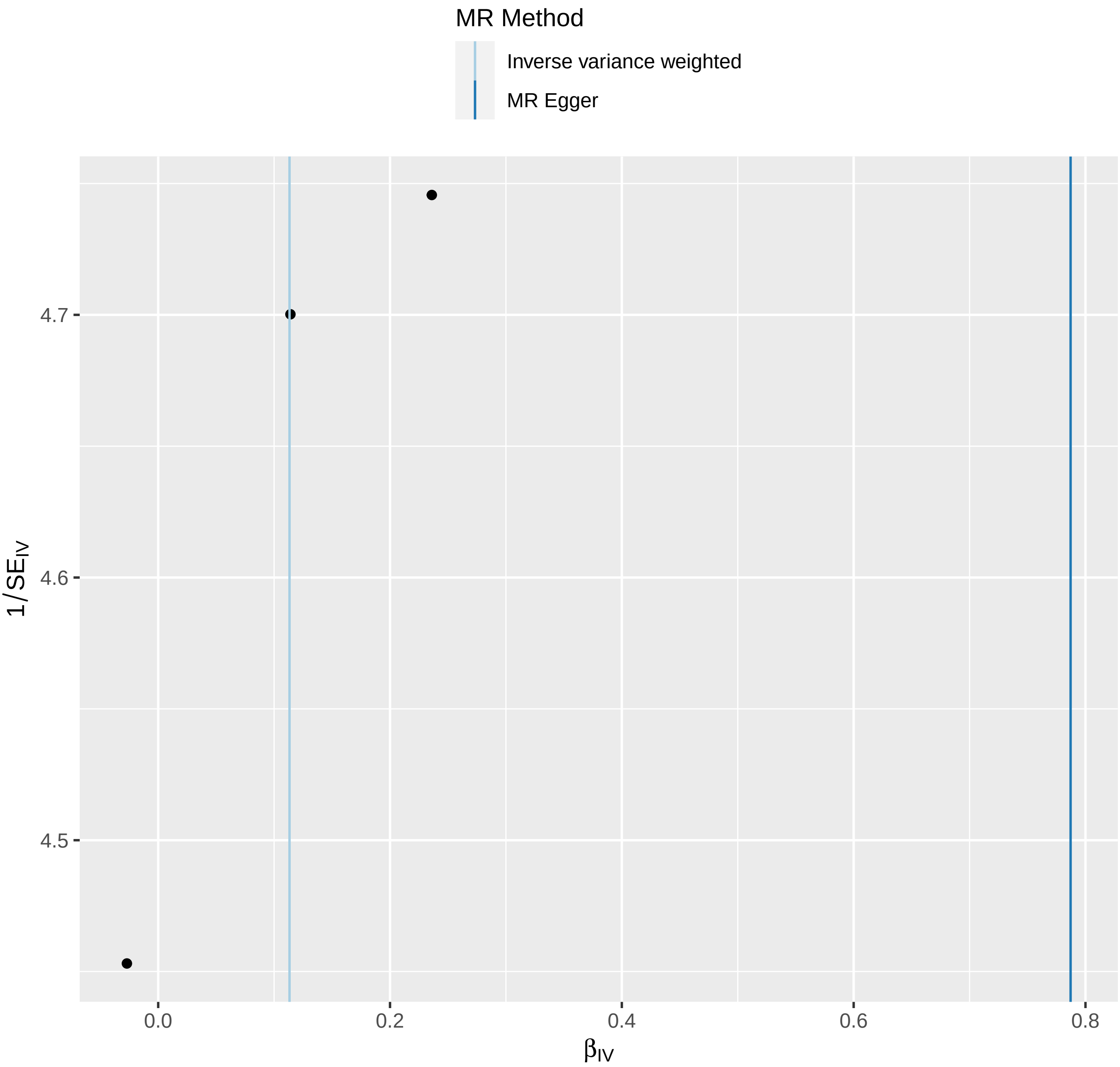

Batch 2−2504 : Gut microbiota abundance (genus Parasutterella id.2892) on Glucose−6−phosphate isomerase
with a threshold of 1e−05

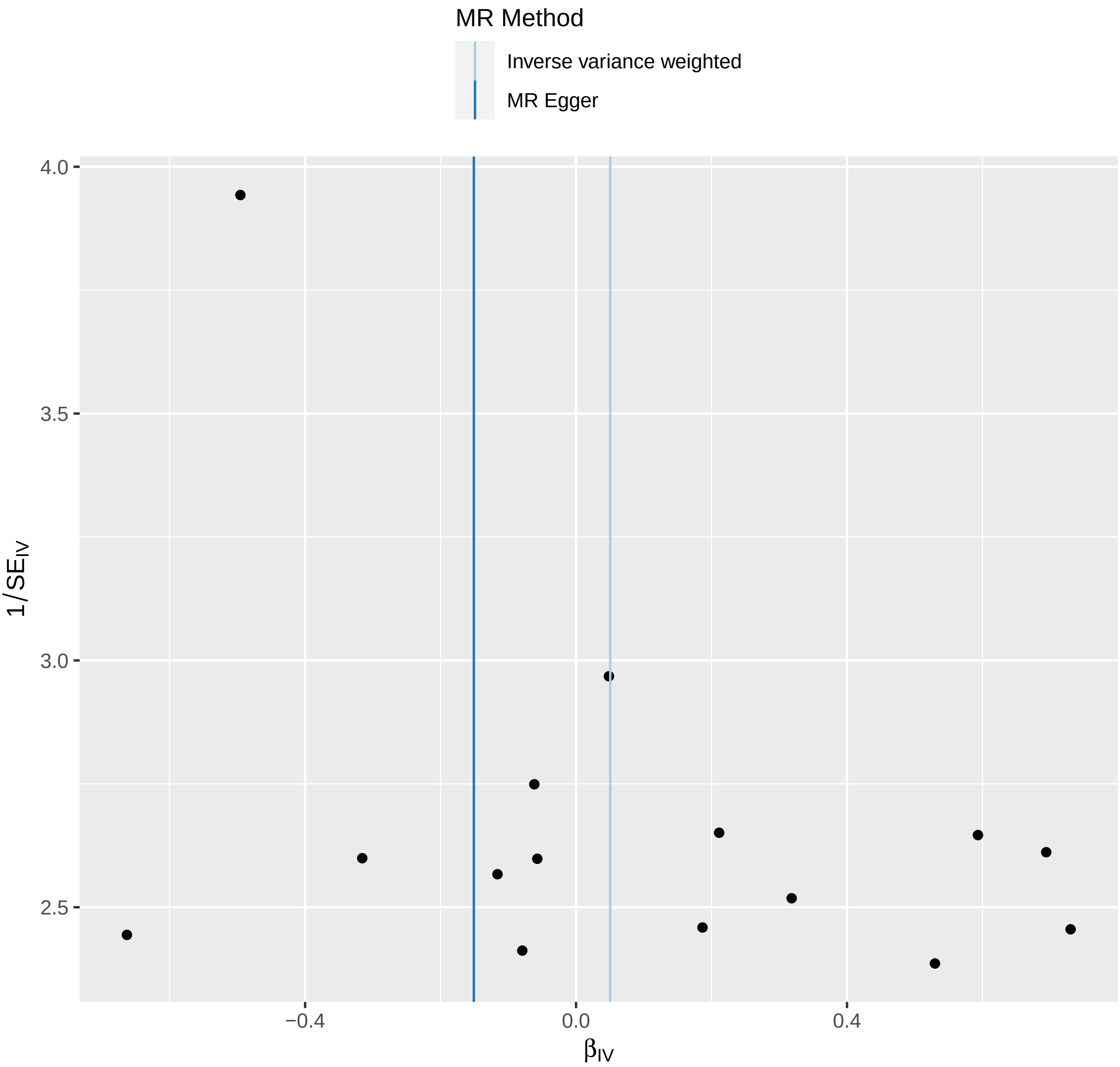

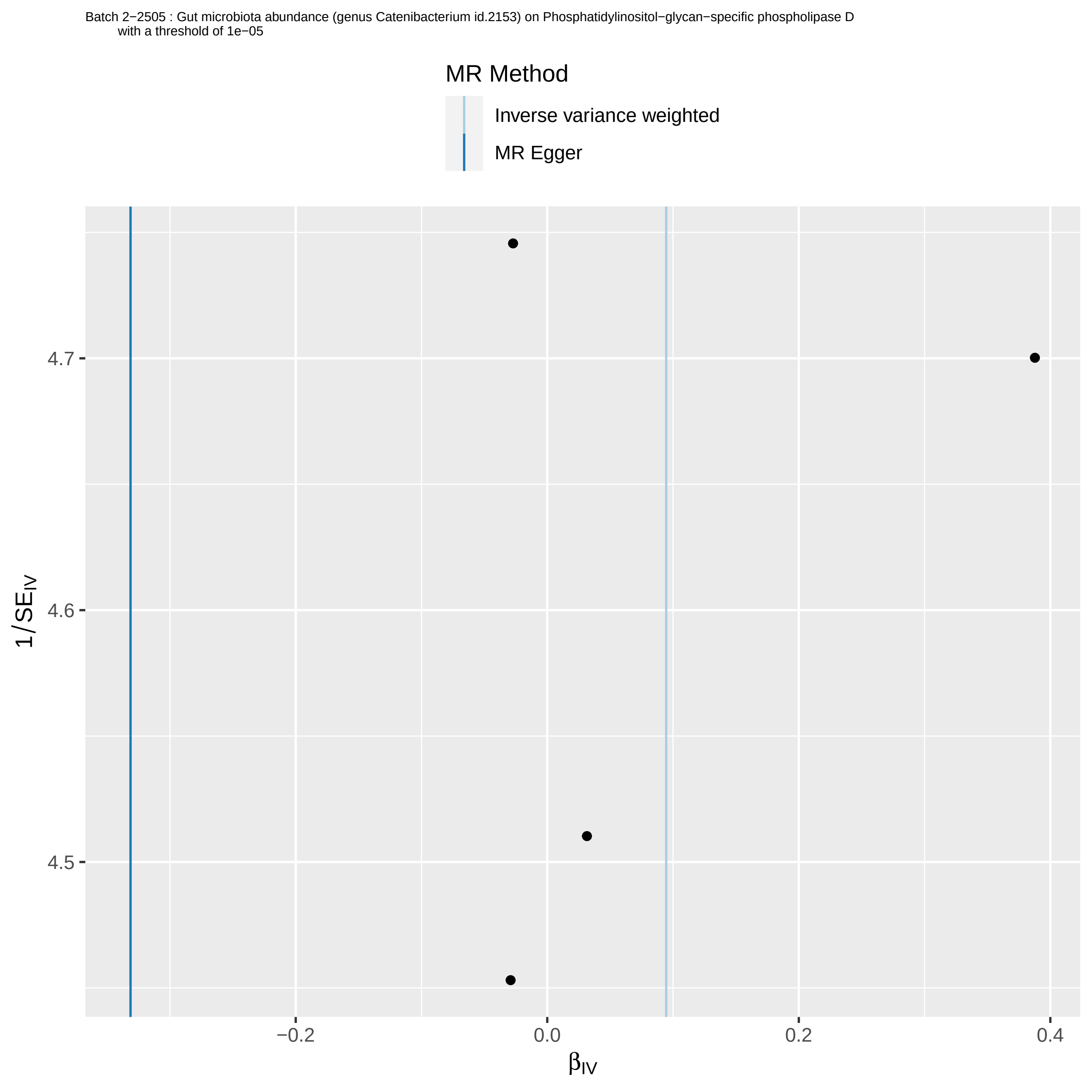

Batch 2−2505 : Gut microbiota abundance (genus Catenibacterium id.2153) on Phosphatidylinositol−glycan−specific phospholipase D
with a threshold of 1e−05
MR Method
Inverse variance weighted
MR Egger
4.7
4.6
4.5
1/SE$_{IV}$
−0.2
0.0
0.2
0.4
β$_{IV}$

Batch 2−2506 : Gut microbiota abundance (genus Parasutterella id.2892) on Phosphatidylinositol−glycan−specific phospholipase D
with a threshold of 1e−05

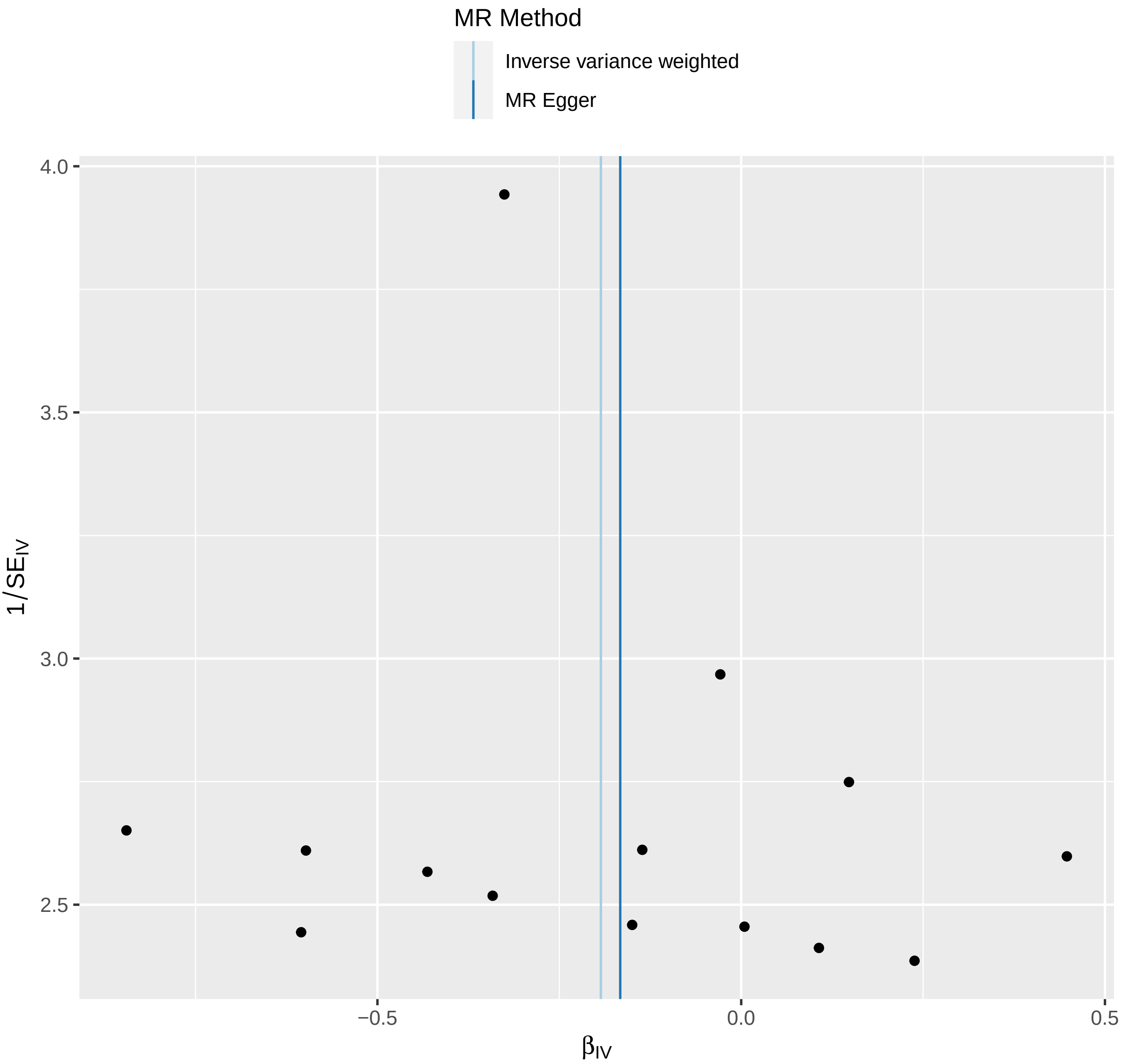

Batch 2−2507 : Gut microbiota abundance (genus Catenibacterium id.2153) on Phosphatidylinositol−glycan−specific phospholipase D
with a threshold of 1e−05

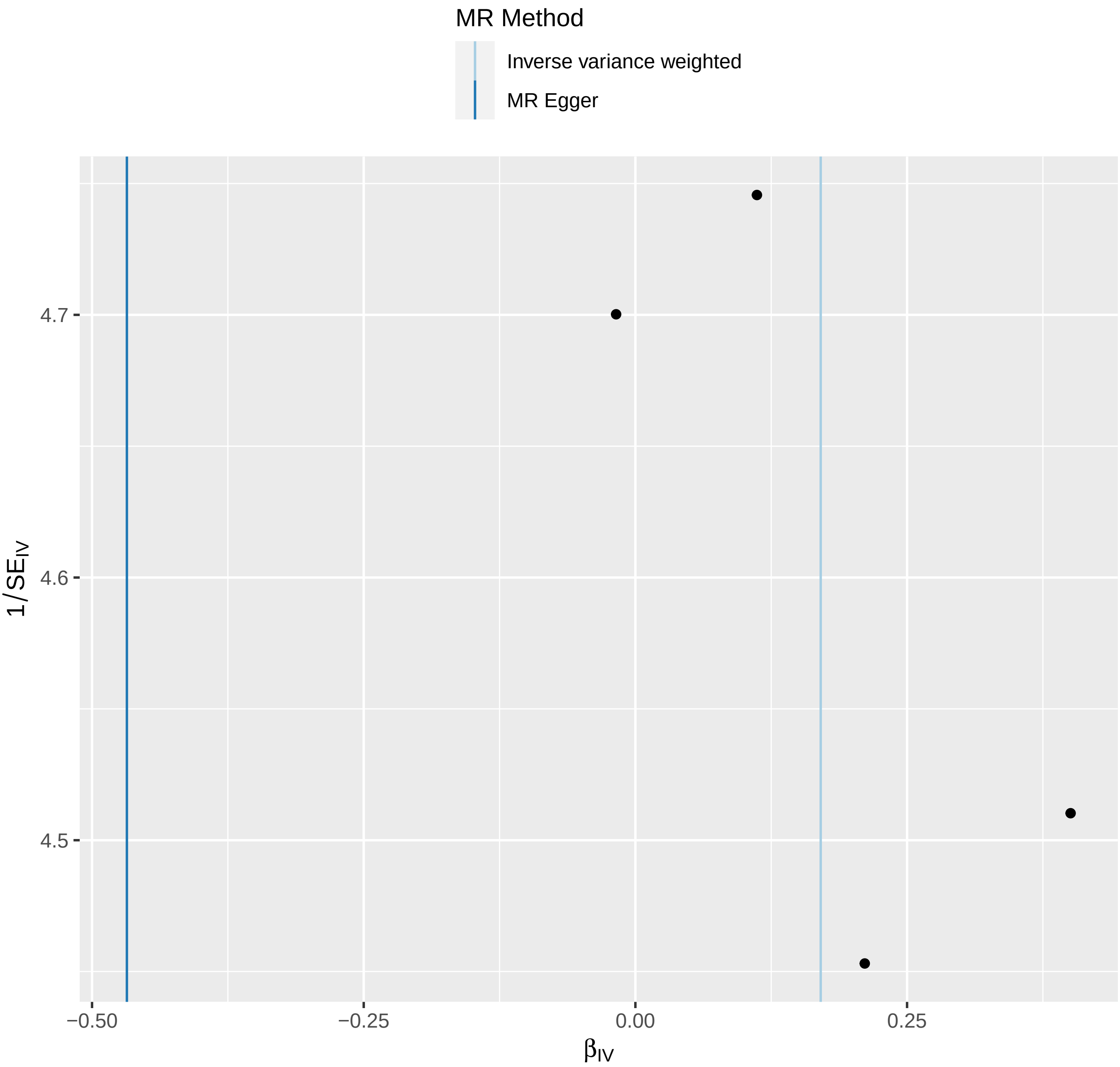

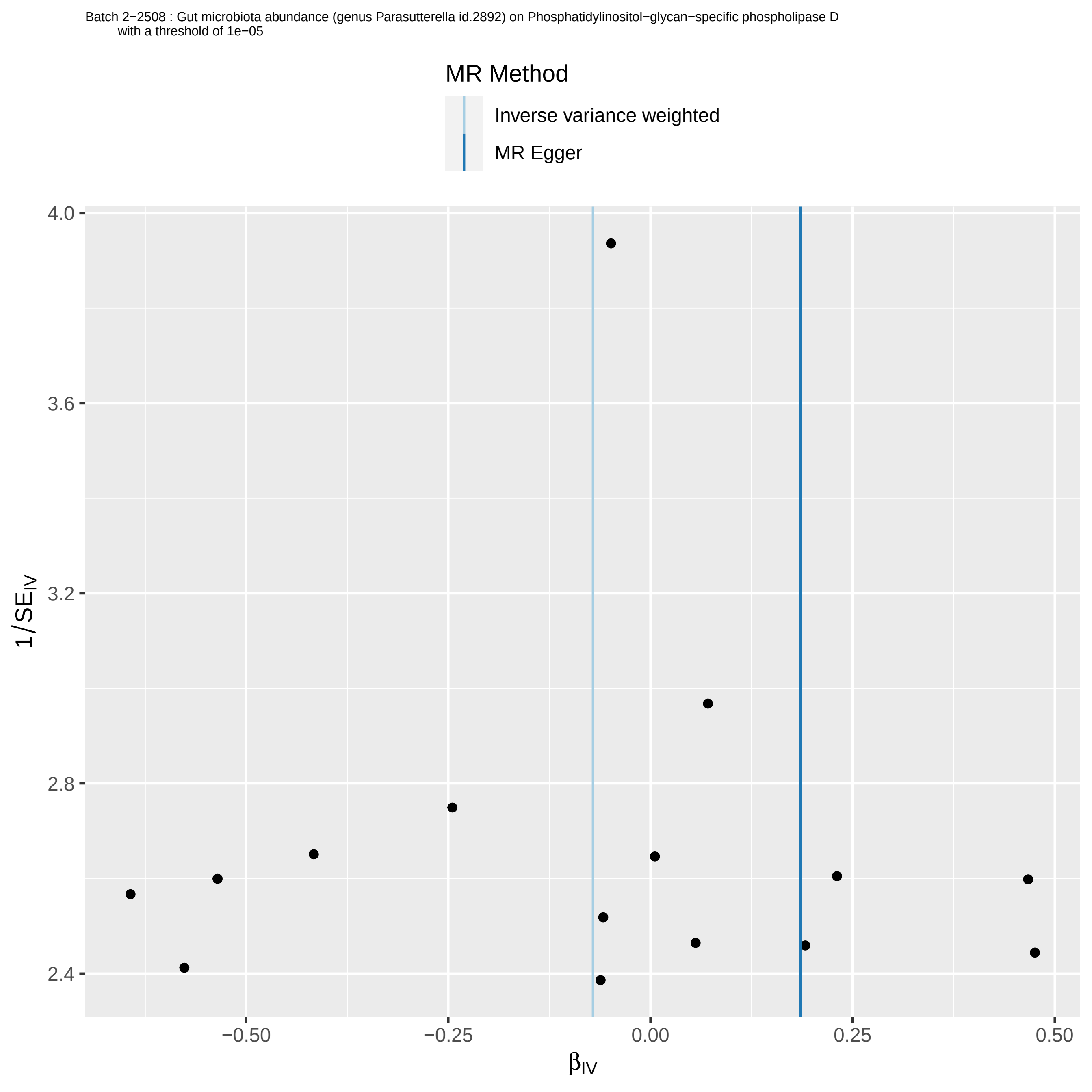

Batch 2−2508 : Gut microbiota abundance (genus Parasutterella id.2892) on Phosphatidylinositol−glycan−specific phospholipase D
with a threshold of 1e−05
MR Method
Inverse variance weighted
MR Egger
4.0
3.6
3.2
2.8
2.4
1/SE_IV
−0.50
−0.25
0.00
0.25
0.50
β_IV

Batch 2−2509 : Gut microbiota abundance (genus Catenibacterium id.2153) on Transmembrane glycoprotein NMB
with a threshold of 1e−05

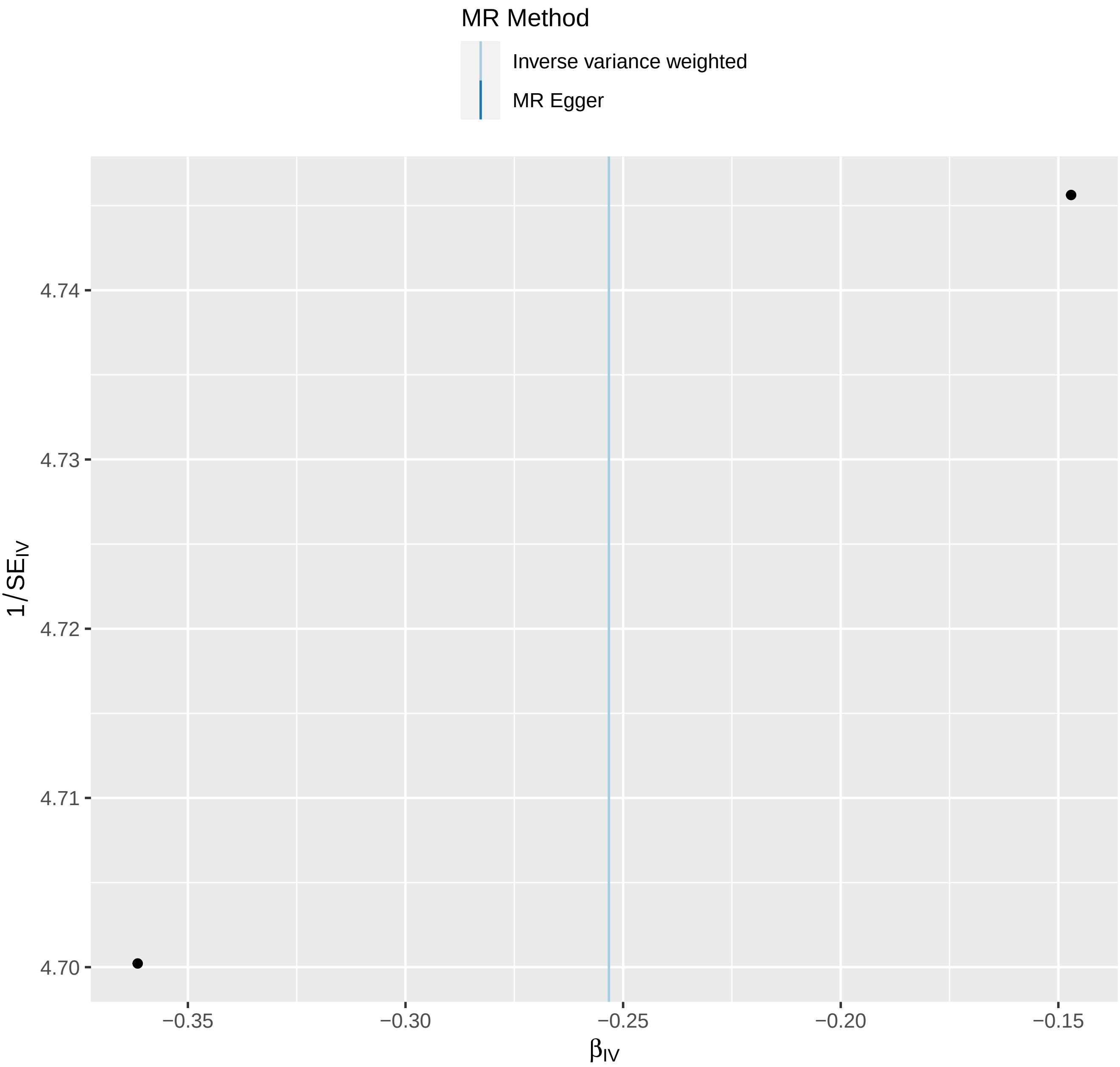

Batch 2−2510 : Gut microbiota abundance (genus Parasutterella id.2892) on Transmembrane glycoprotein NMB
with a threshold of 1e−05

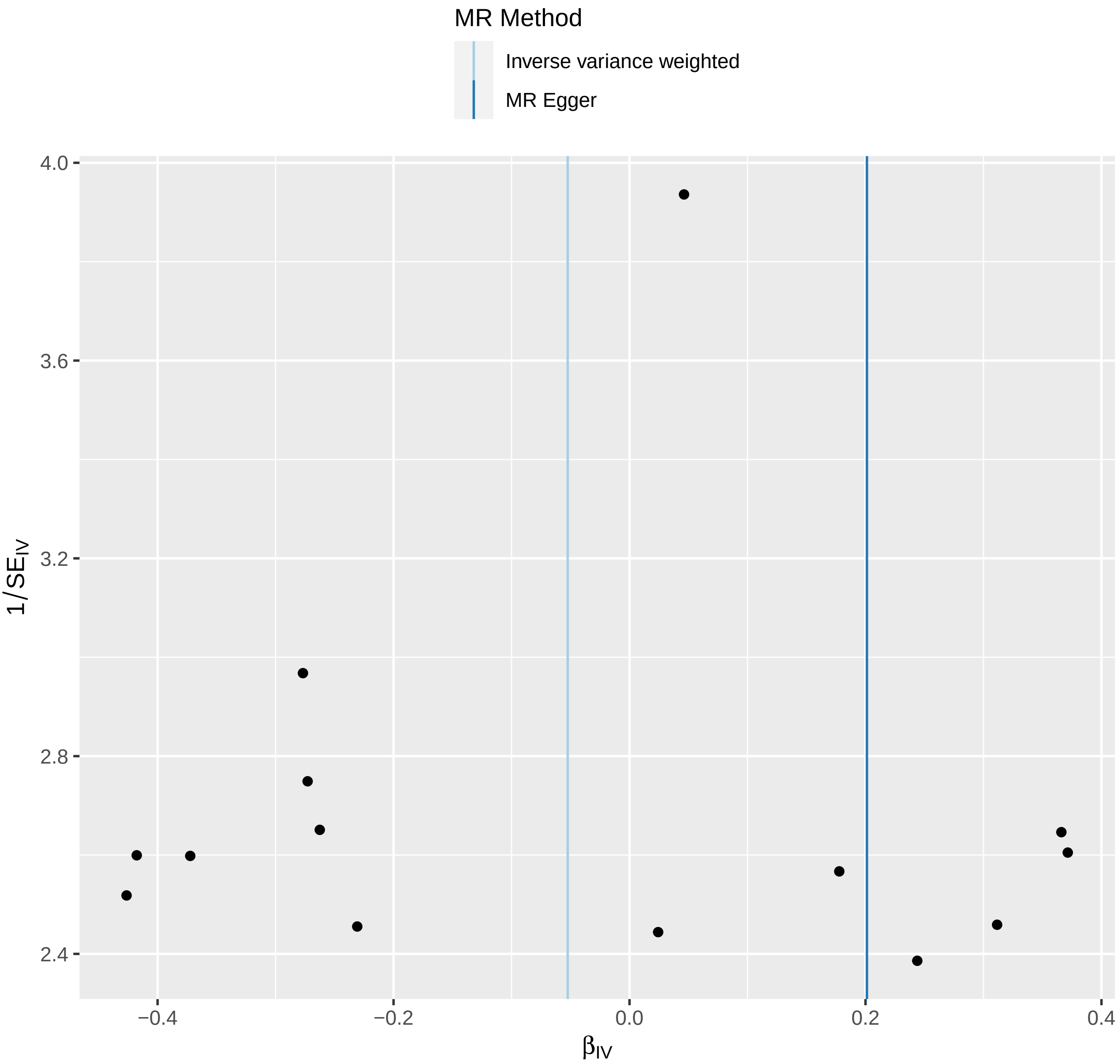

Batch 2−2511 : Gut microbiota abundance (genus Catenibacterium id.2153) on Transmembrane glycoprotein NMB
with a threshold of 1e−05

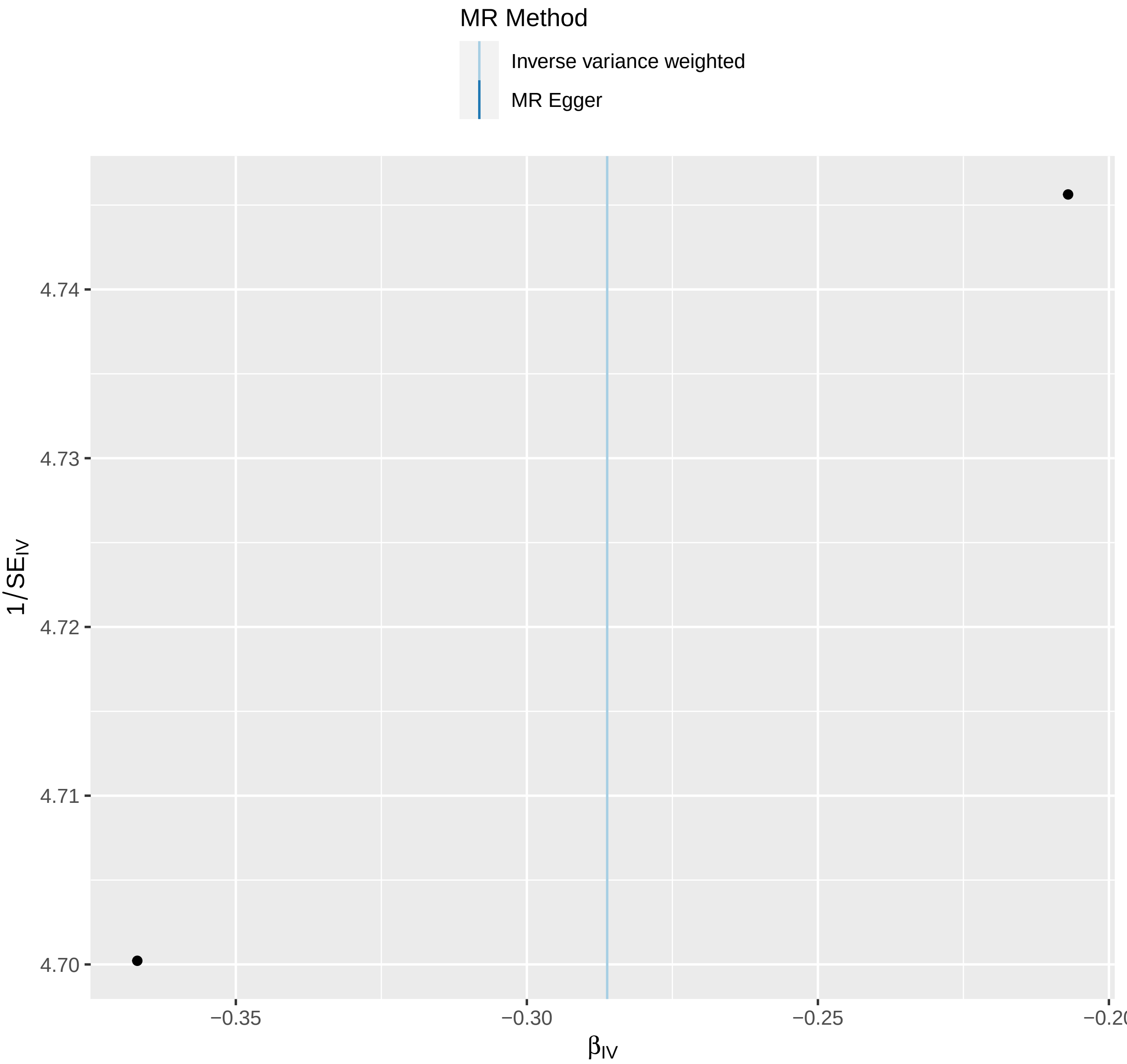

Batch 2−2512 : Gut microbiota abundance (genus Parasutterella id.2892) on Transmembrane glycoprotein NMB
with a threshold of 1e−05

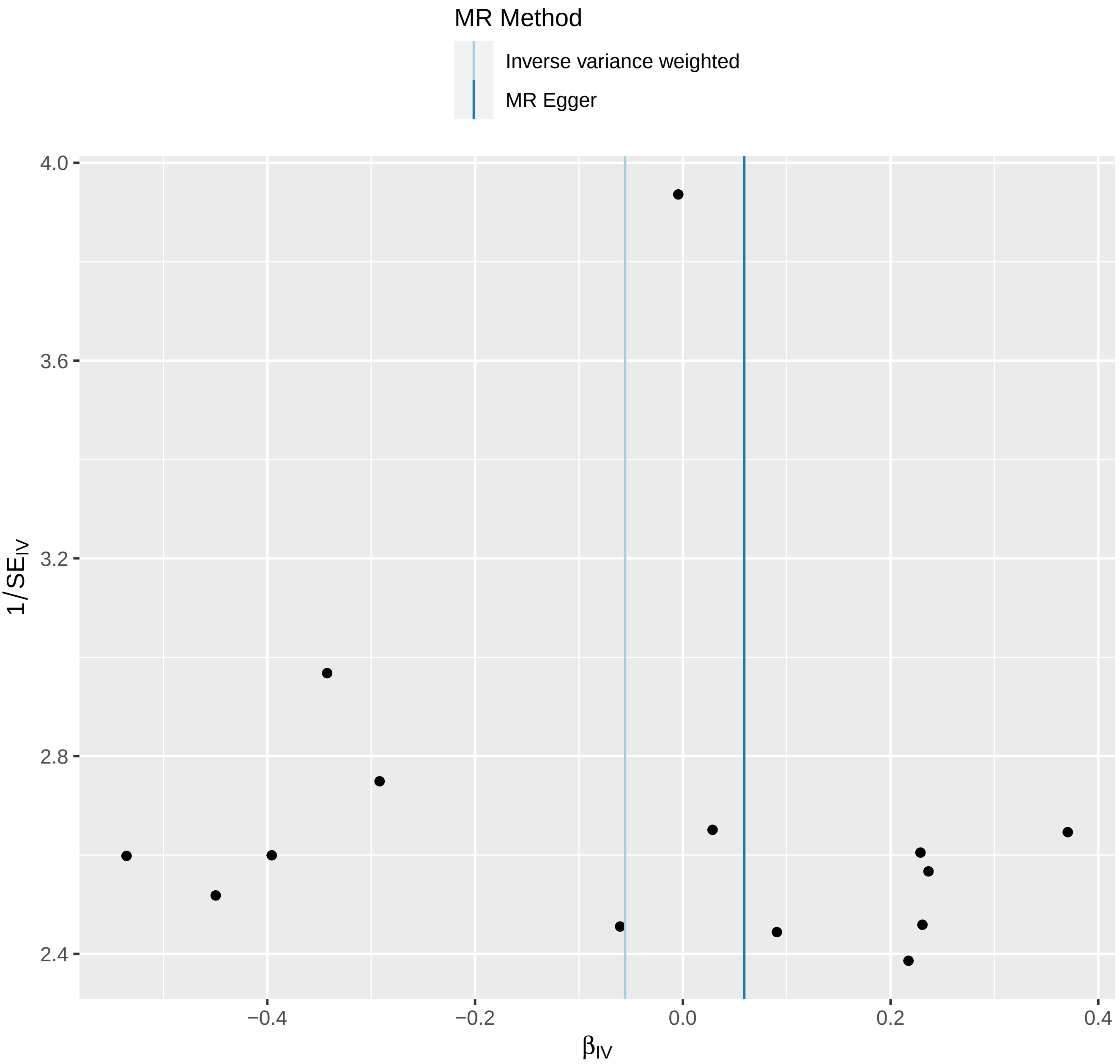

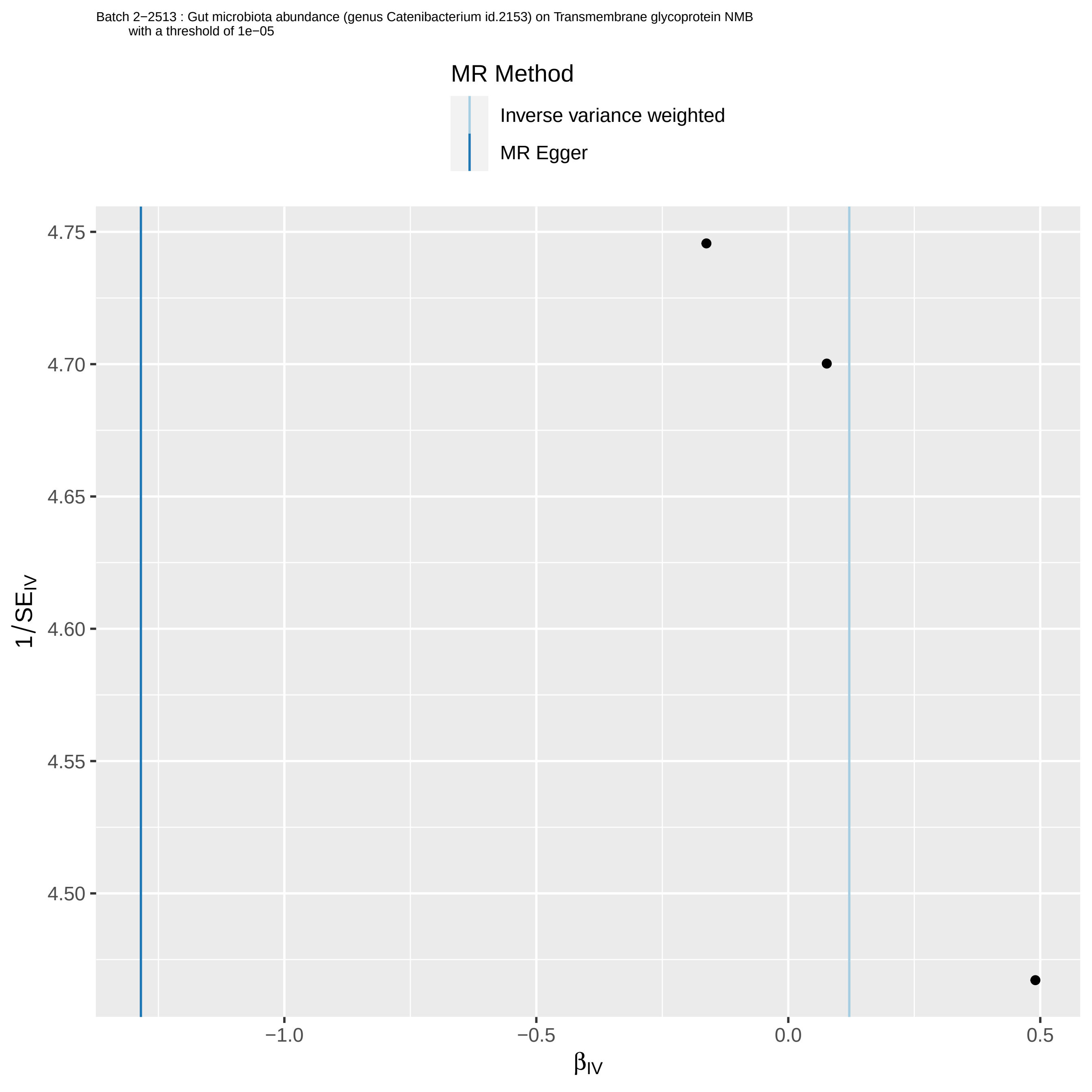
Batch 2−2513 : Gut microbiota abundance (genus Catenibacterium id.2153) on Transmembrane glycoprotein NMB
with a threshold of 1e−05
MR Method
Inverse variance weighted
MR Egger
4.75
4.70
4.65
4.60
4.55
4.50
1/SE_IV
−1.0
−0.5
0.0
0.5
β_IV

Batch 2−2514 : Gut microbiota abundance (genus Parasutterella id.2892) on Transmembrane glycoprotein NMB
with a threshold of 1e−05

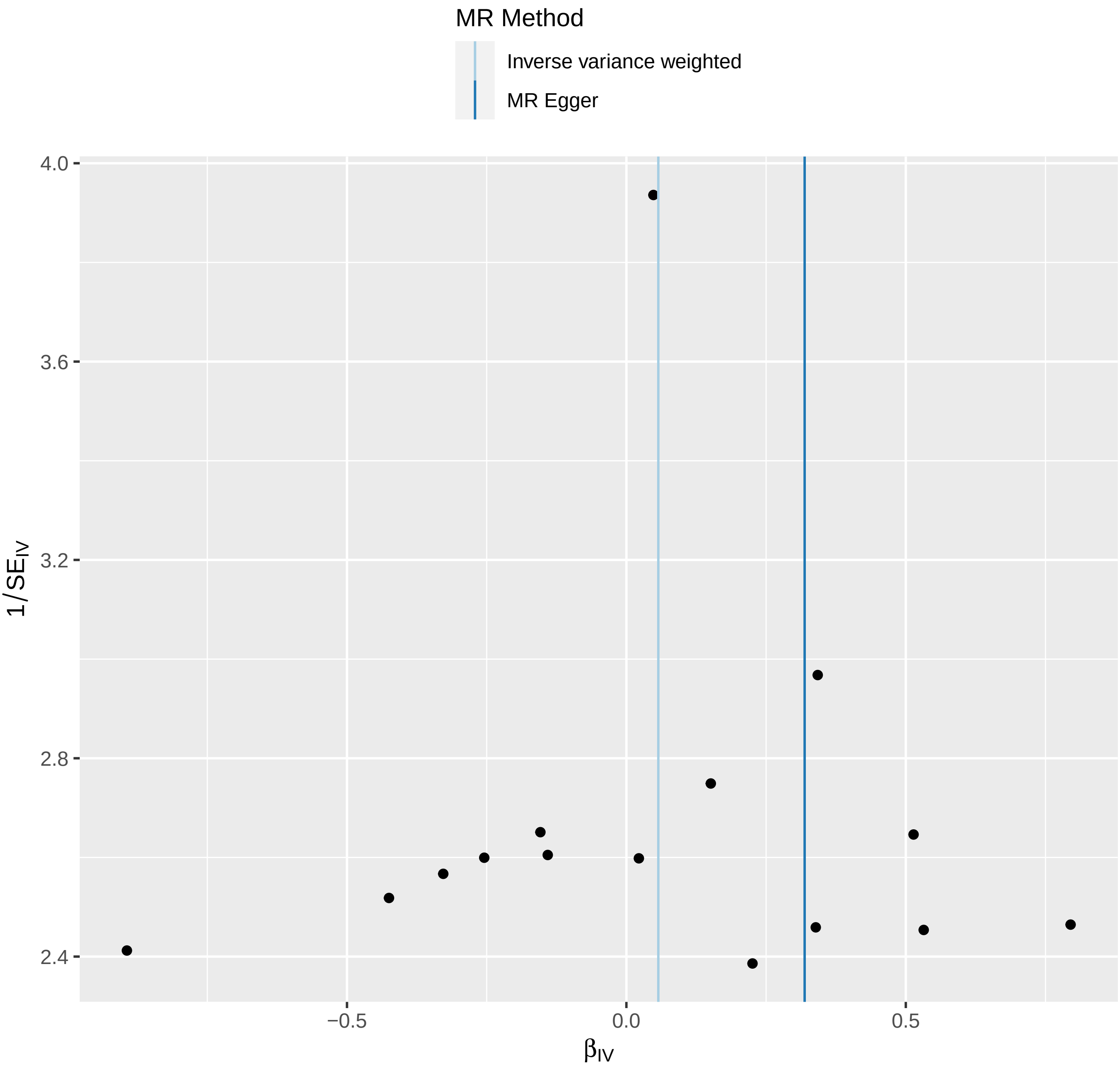

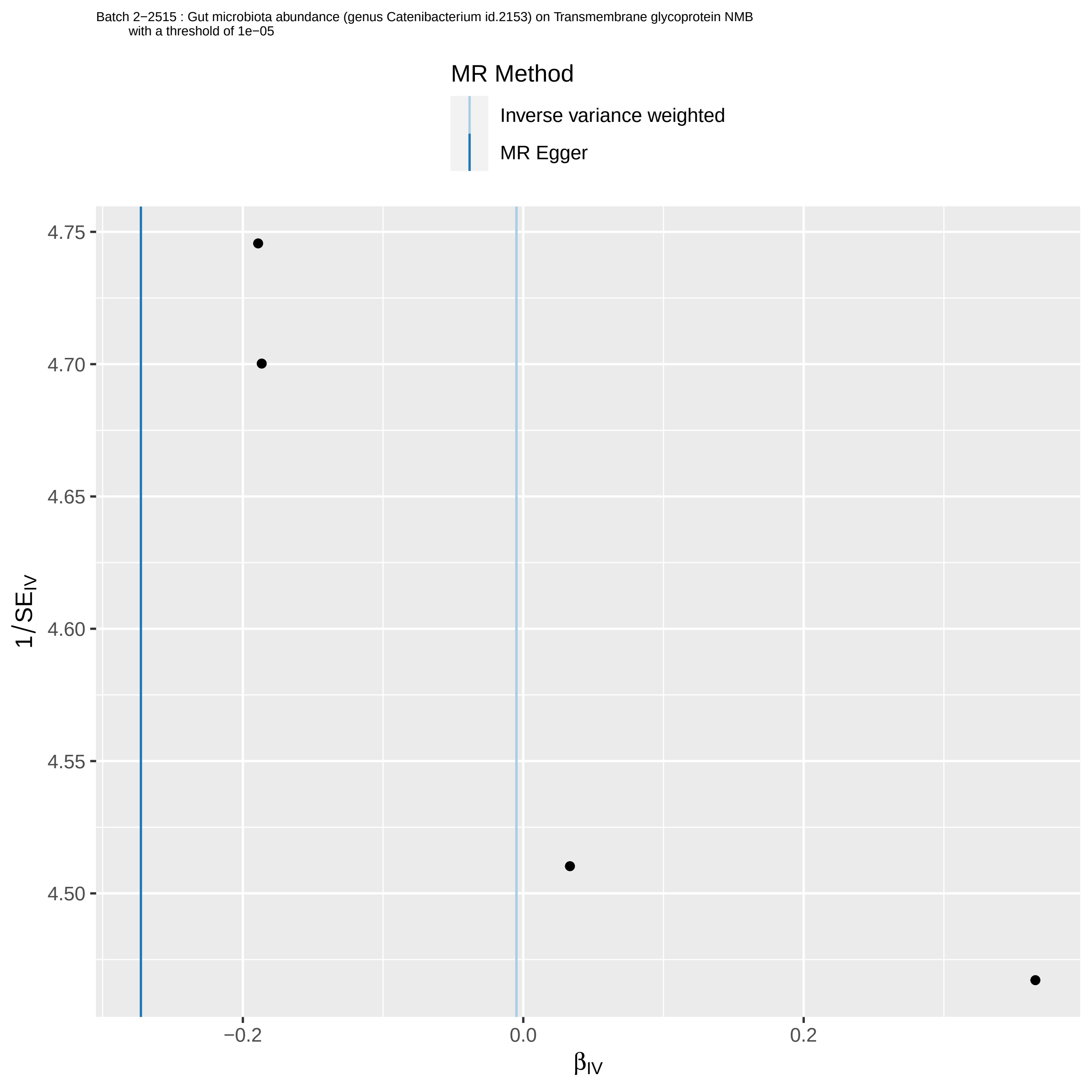

Batch 2−2515 : Gut microbiota abundance (genus Catenibacterium id.2153) on Transmembrane glycoprotein NMB
with a threshold of 1e−05
MR Method
Inverse variance weighted
MR Egger
4.75
4.70
4.65
4.60
4.55
4.50
1/SE_IV
−0.2
0.0
0.2
β_IV

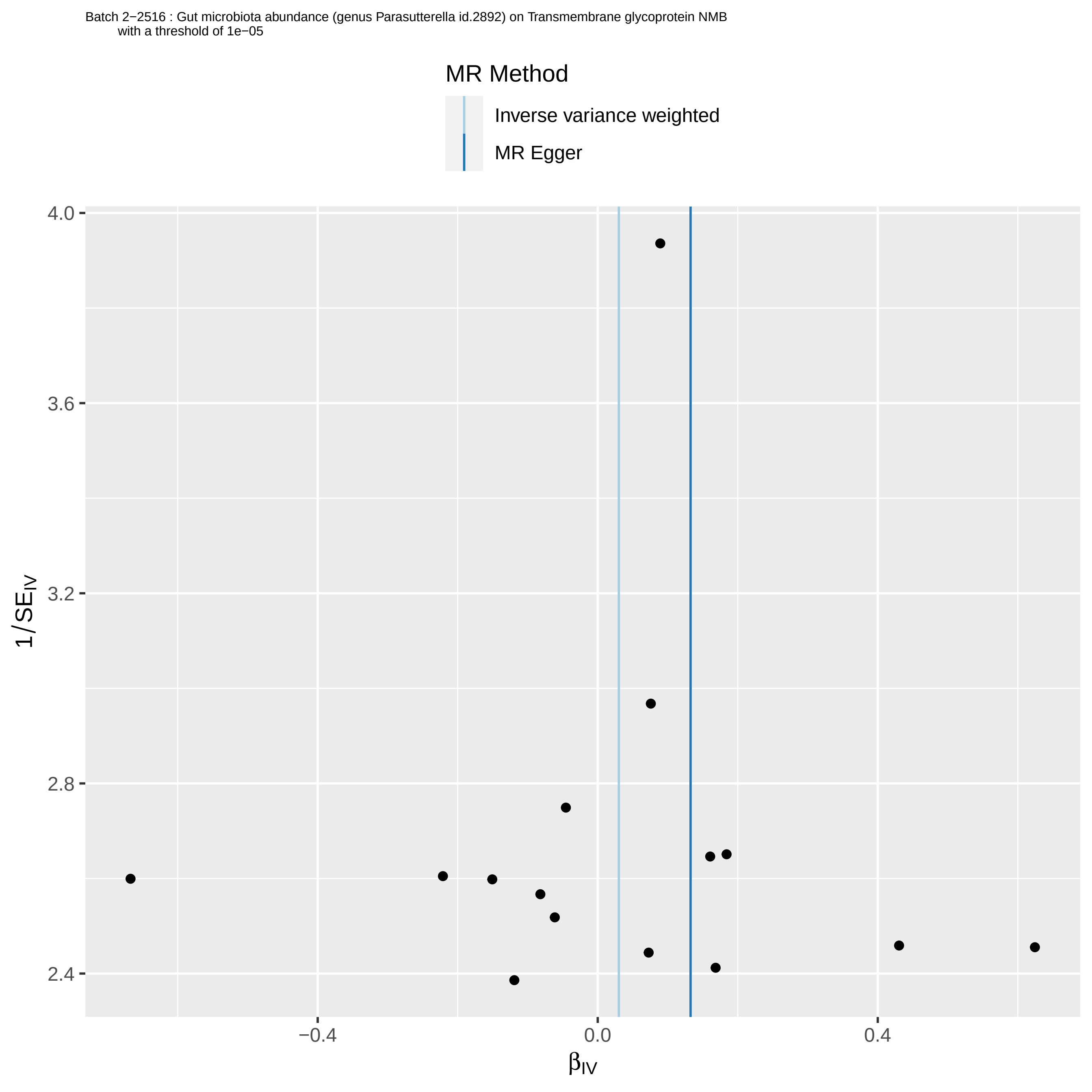

Batch 2−2516 : Gut microbiota abundance (genus Parasutterella id.2892) on Transmembrane glycoprotein NMB
with a threshold of 1e−05
MR Method
Inverse variance weighted
MR Egger
4.0
3.6
3.2
2.8
2.4
1/SE_IV
−0.4
0.0
0.4
β_IV

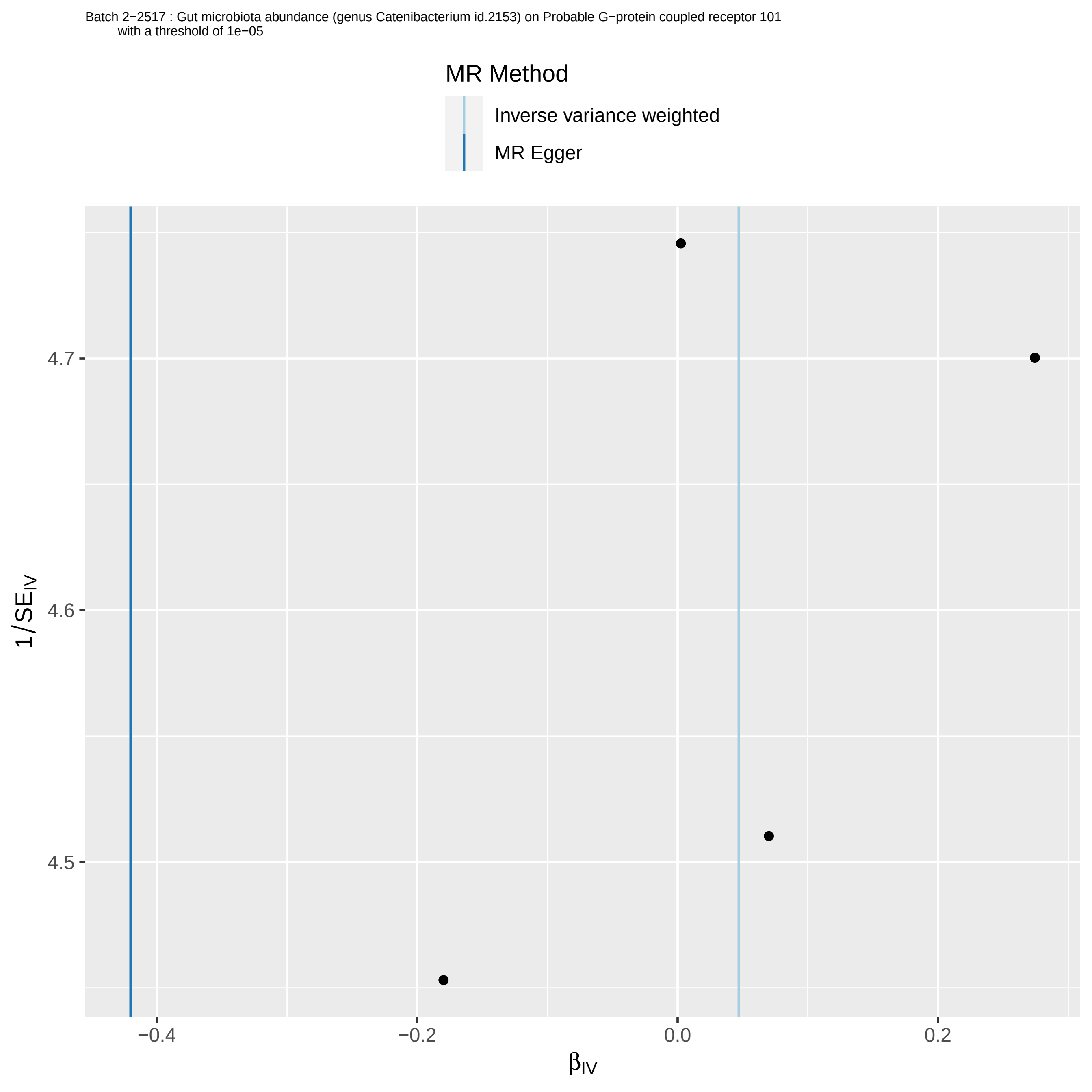

Batch 2−2517 : Gut microbiota abundance (genus Catenibacterium id.2153) on Probable G−protein coupled receptor 101
with a threshold of 1e−05
MR Method
Inverse variance weighted
MR Egger
4.7
4.6
4.5
1/SE_IV
−0.4
−0.2
0.0
0.2
β_IV

Batch 2−2518 : Gut microbiota abundance (genus Parasutterella id.2892) on Probable G−protein coupled receptor 101
with a threshold of 1e−05

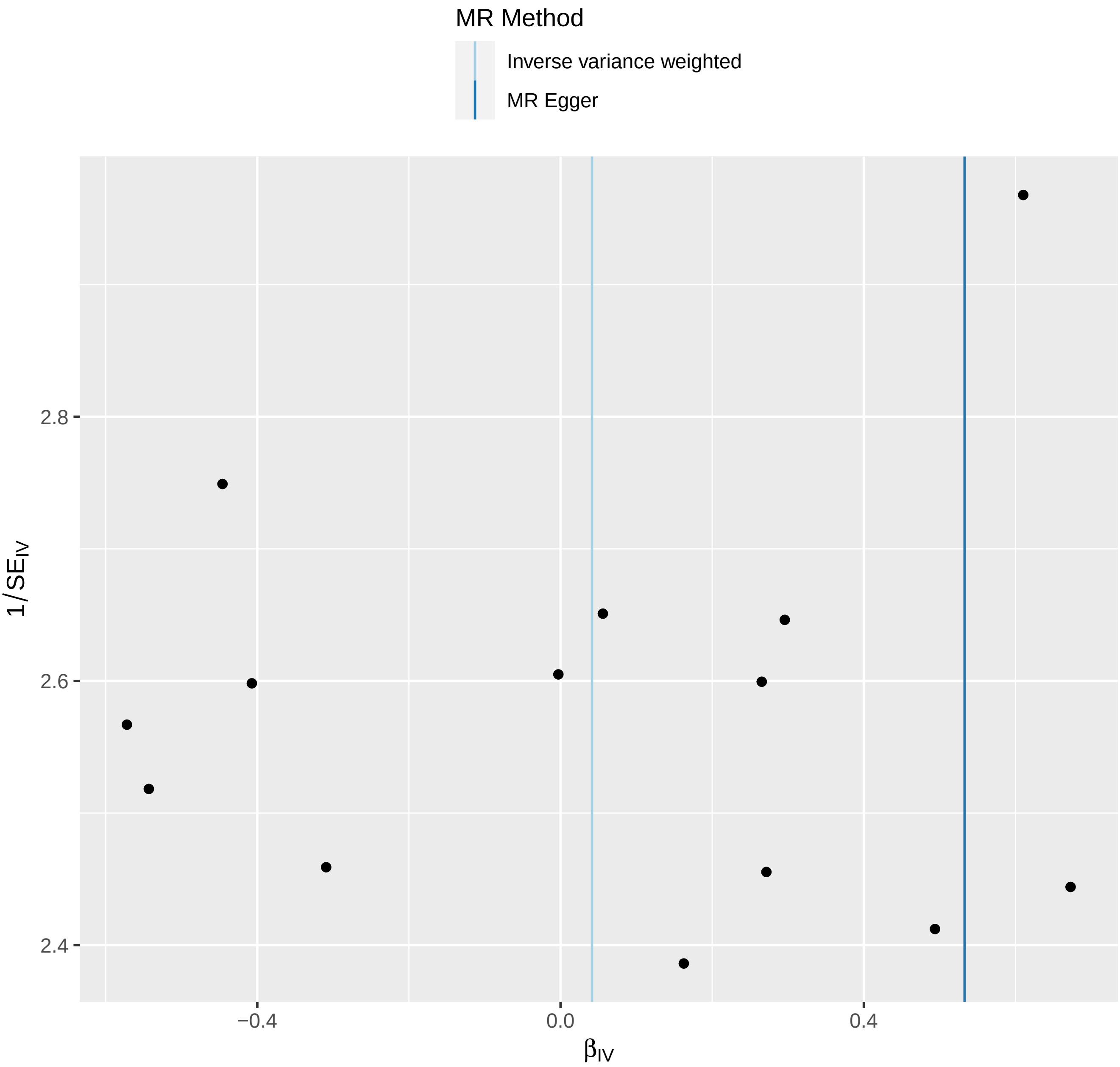

Batch 2−2519 : Gut microbiota abundance (genus Catenibacterium id.2153) on Probable G−protein coupled receptor 101
with a threshold of 1e−05

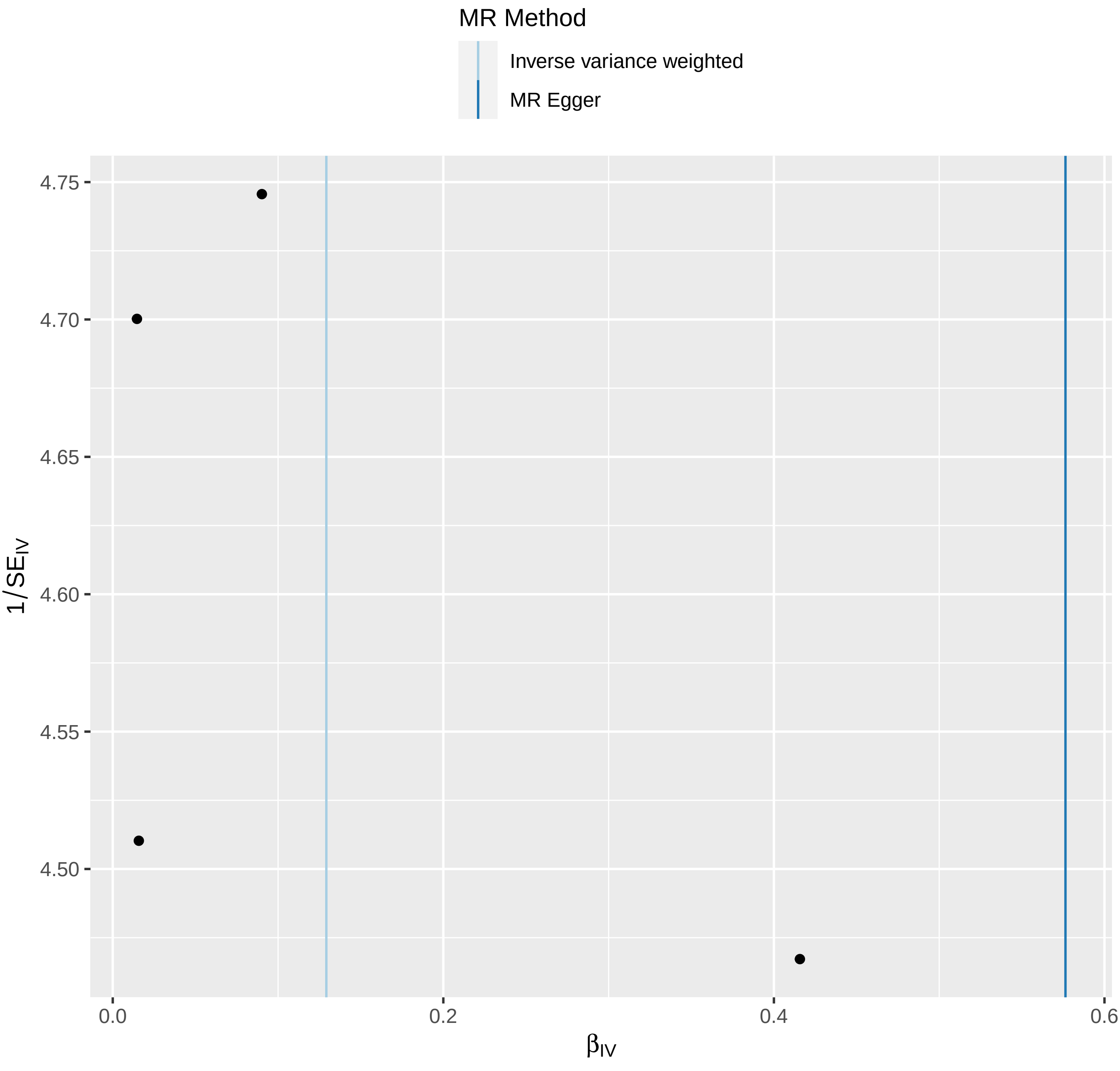

Batch 2−2520 : Gut microbiota abundance (genus Parasutterella id.2892) on Probable G−protein coupled receptor 101
with a threshold of 1e−05

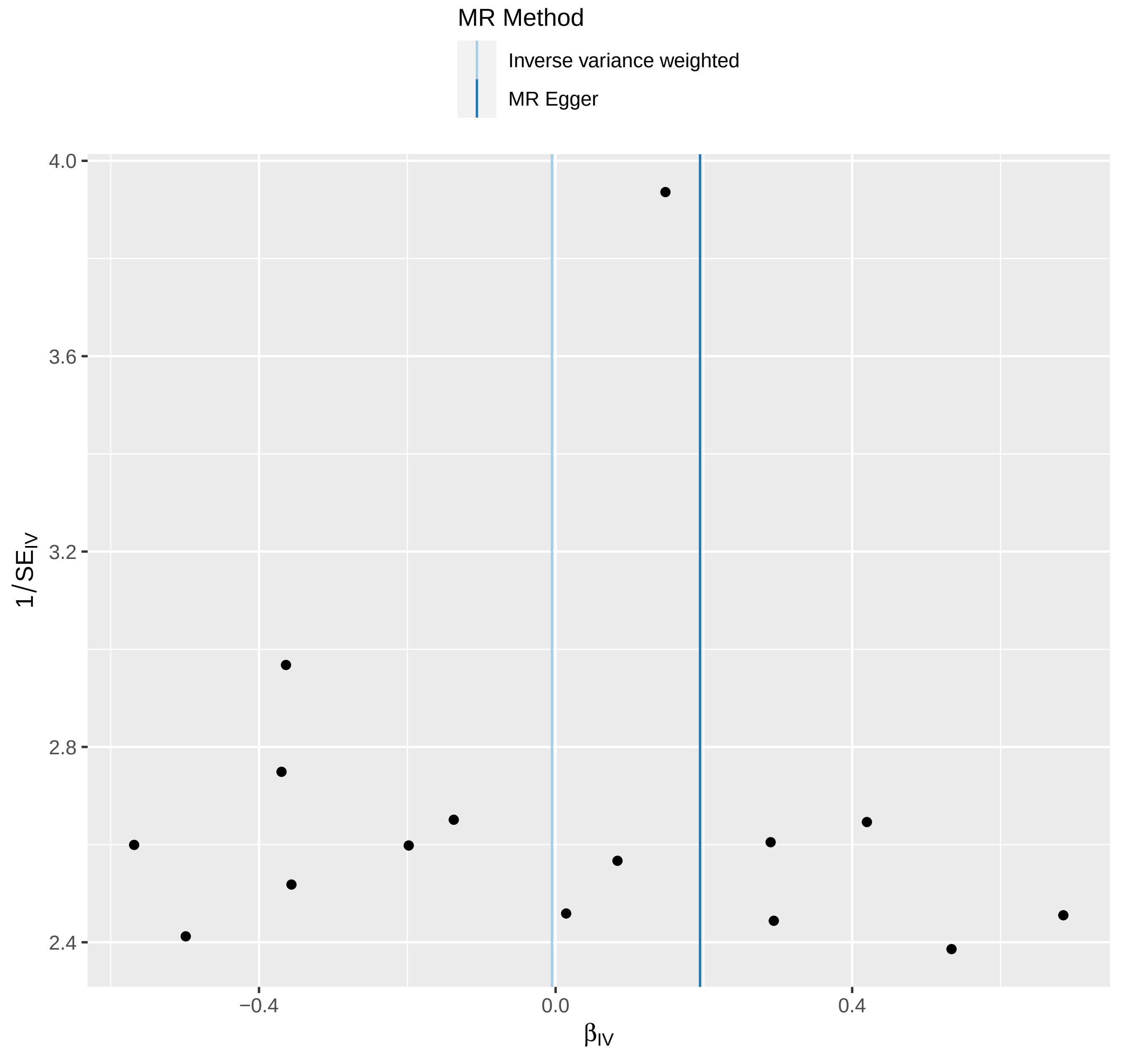

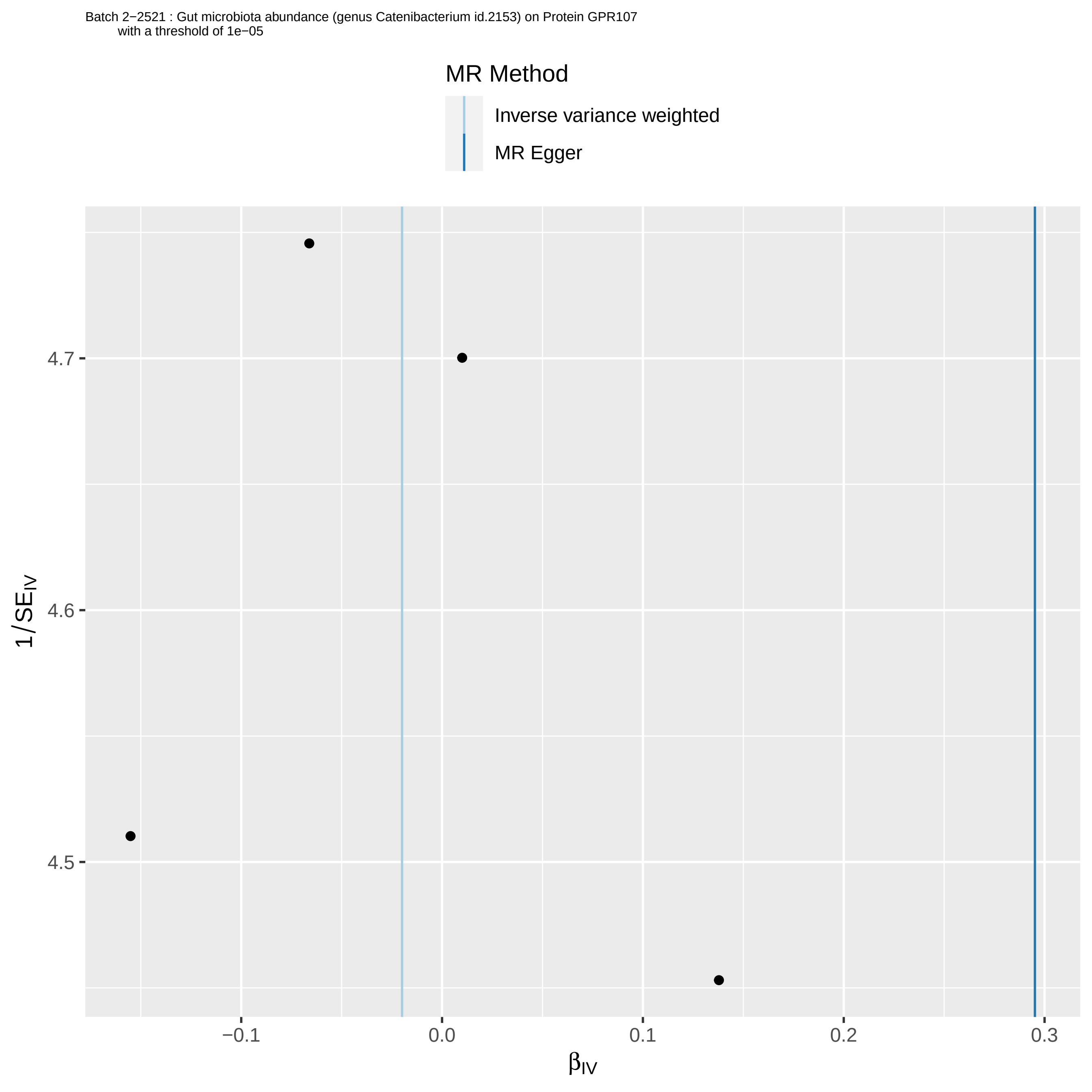

Batch 2−2521 : Gut microbiota abundance (genus Catenibacterium id.2153) on Protein GPR107
with a threshold of 1e−05
MR Method
Inverse variance weighted
MR Egger
4.7
4.6
4.5
1/SE_IV
−0.1
0.0
0.1
0.2
0.3
β_IV

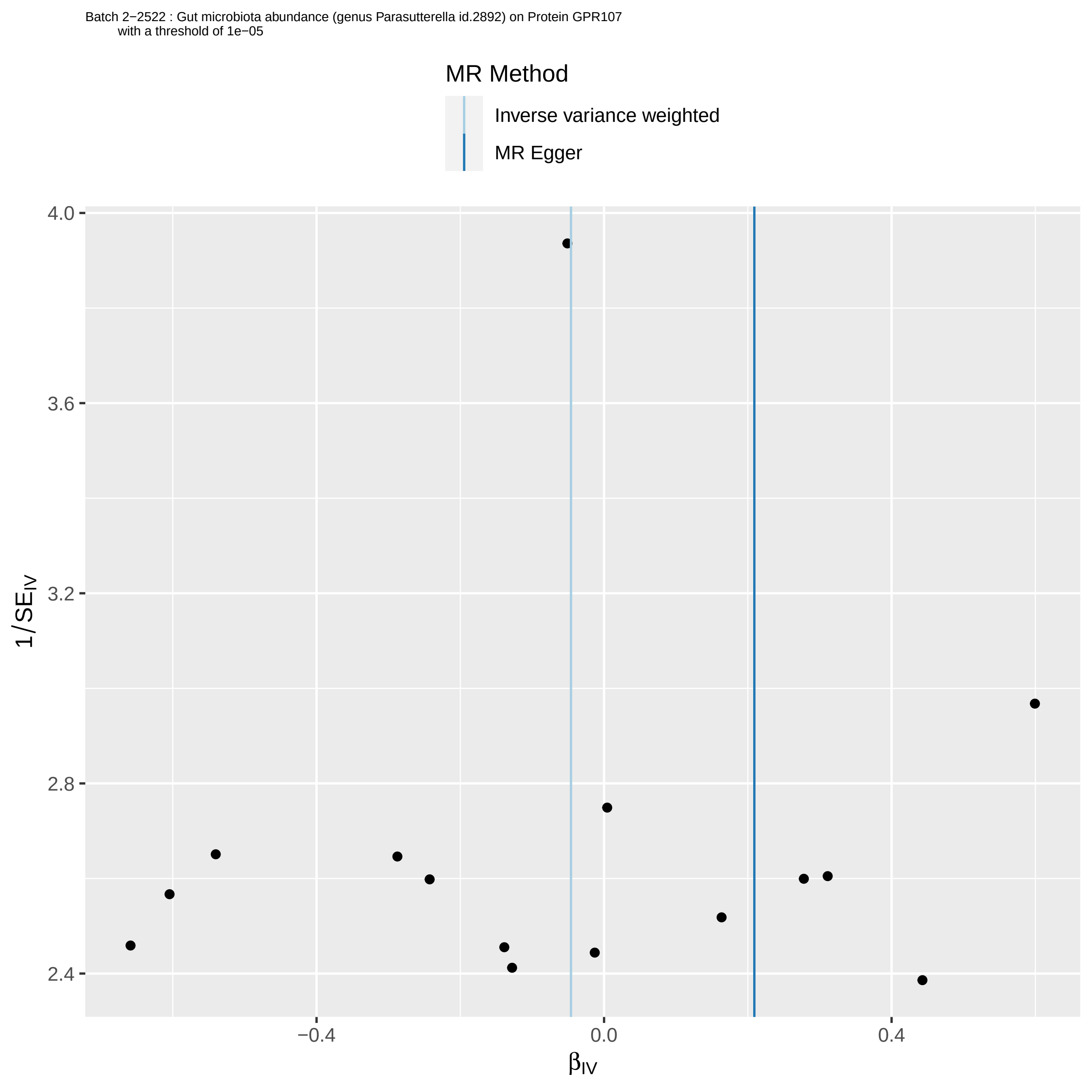
Batch 2−2522 : Gut microbiota abundance (genus Parasutterella id.2892) on Protein GPR107
with a threshold of 1e−05
MR Method
Inverse variance weighted
MR Egger
4.0
3.6
3.2
2.8
2.4
1/SE_IV
−0.4
0.0
0.4
β_IV

Batch 2−2523 : Gut microbiota abundance (genus Catenibacterium id.2153) on Probable G−protein coupled receptor 135
with a threshold of 1e−05

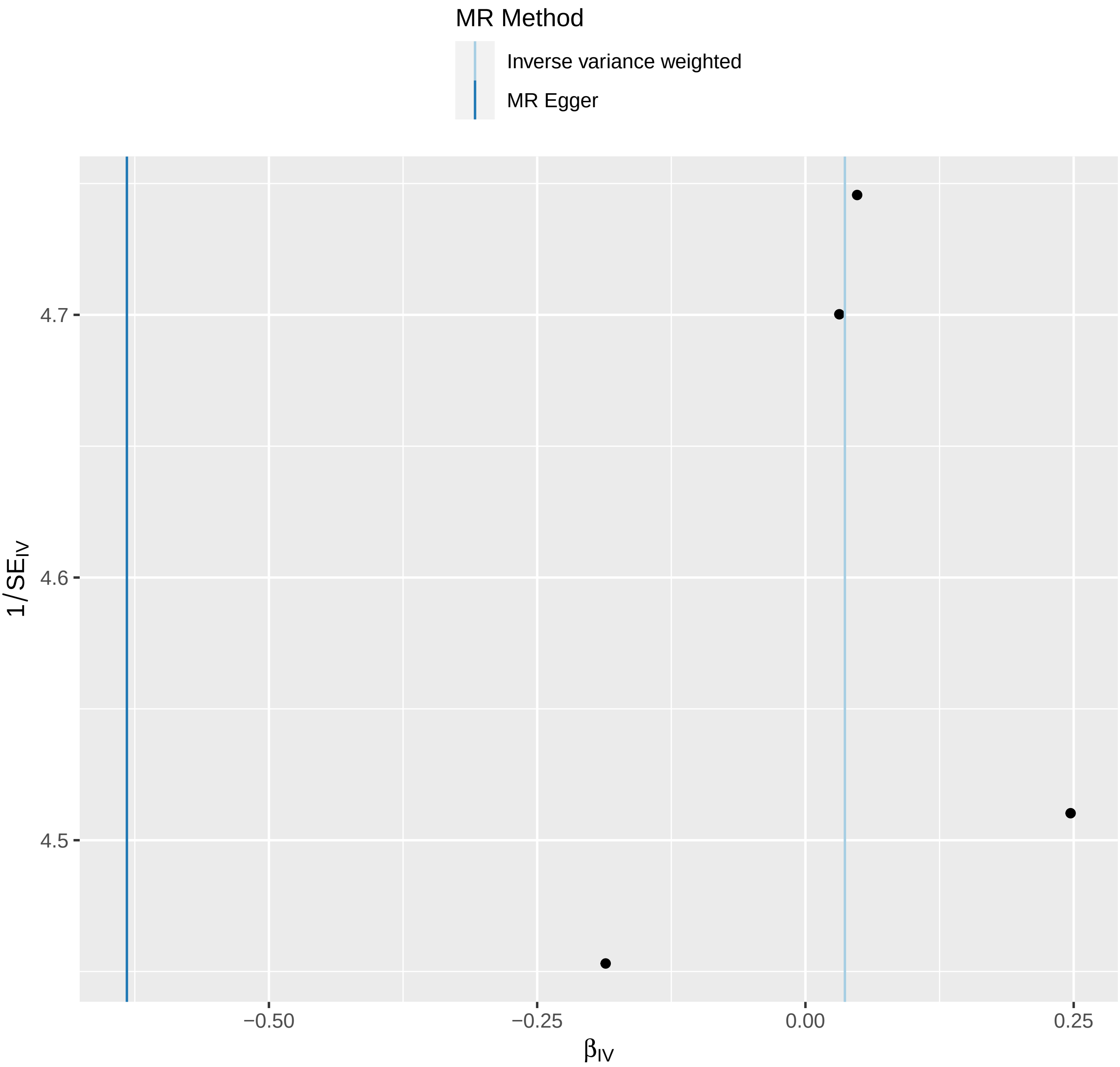

Batch 2−2524 : Gut microbiota abundance (genus Parasutterella id.2892) on Probable G−protein coupled receptor 135
with a threshold of 1e−05

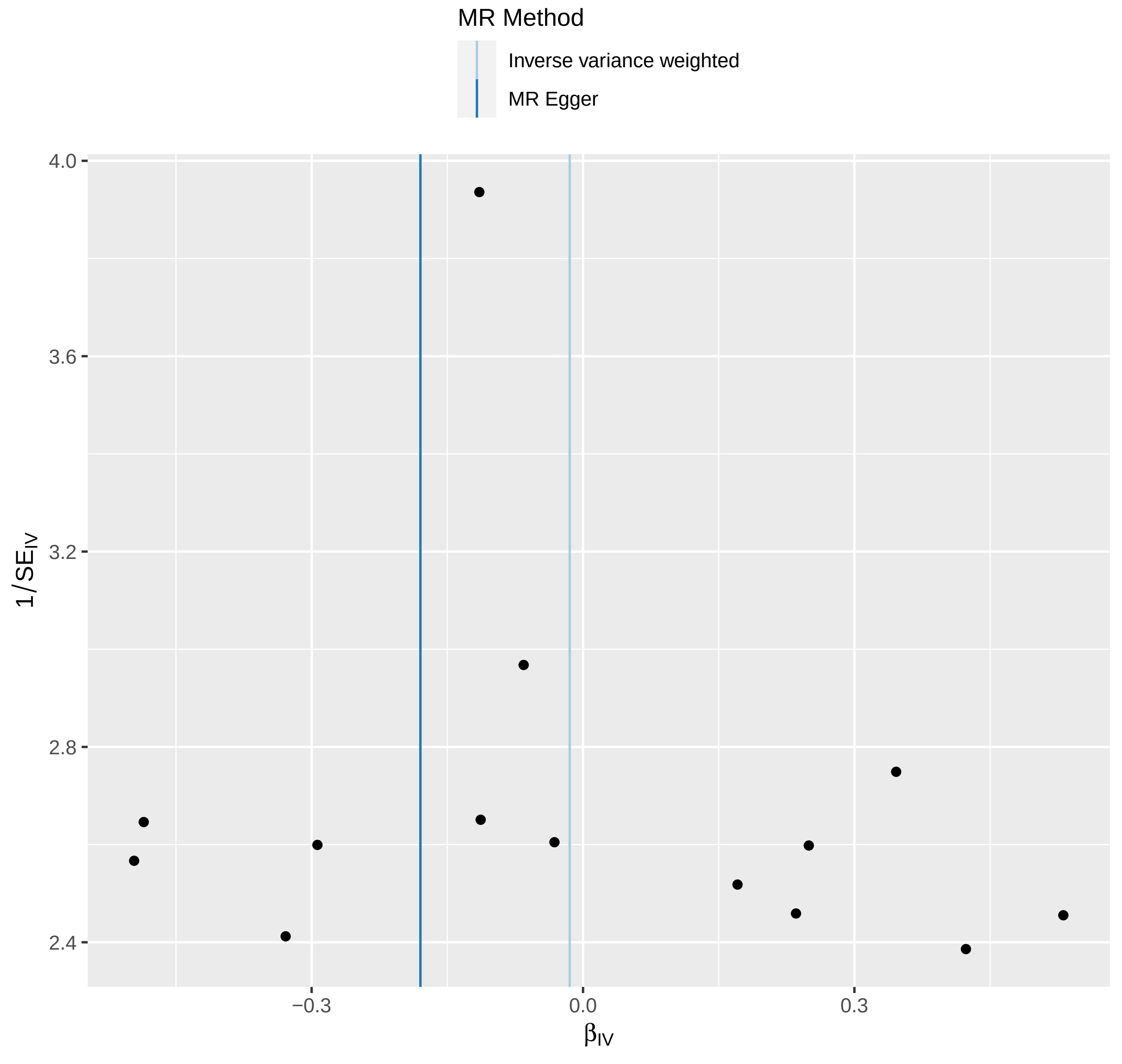

Batch 2−2525 : Gut microbiota abundance (genus Catenibacterium id.2153) on G−protein coupled receptor 26
with a threshold of 1e−05

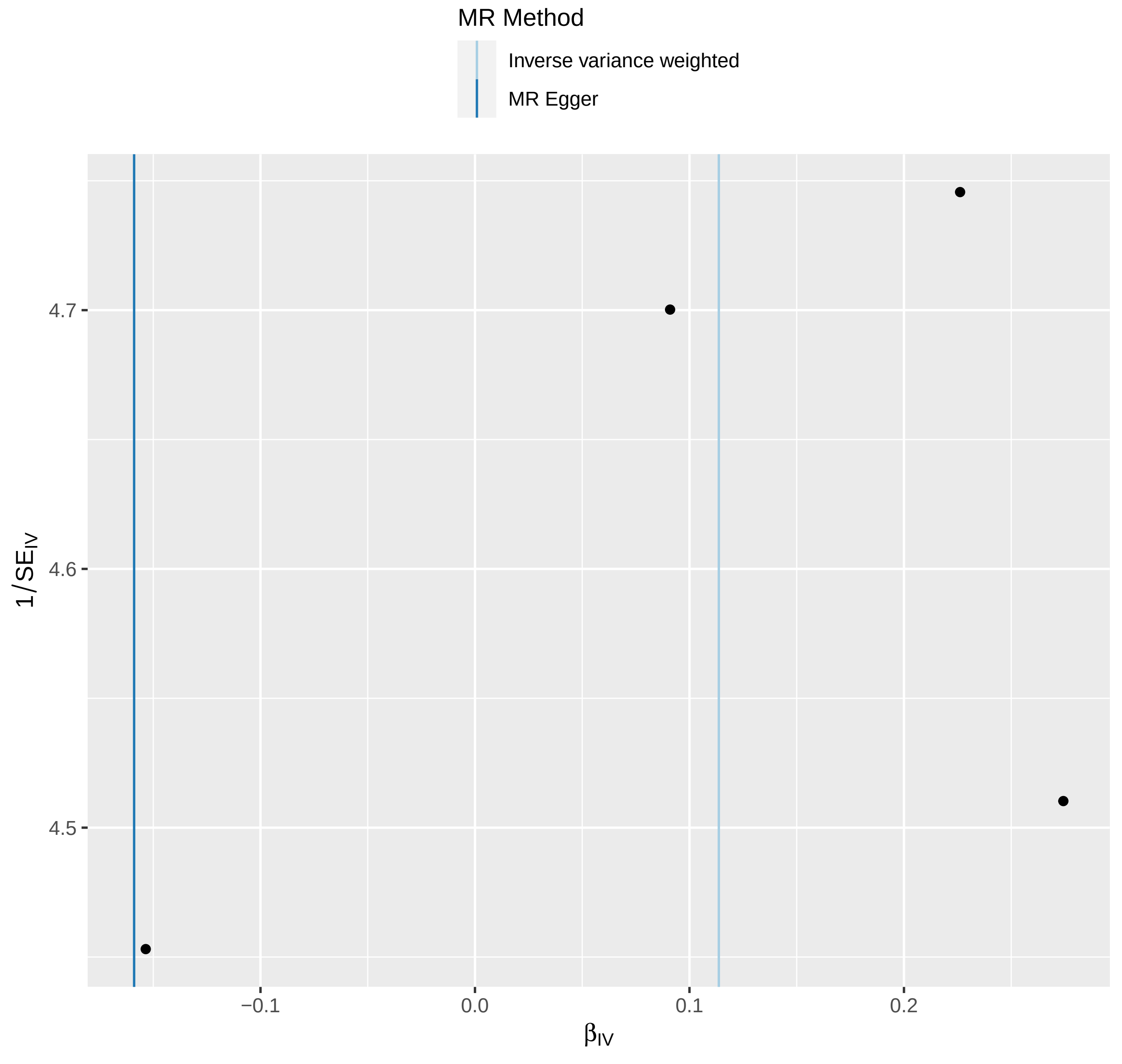

Batch 2−2526 : Gut microbiota abundance (genus Parasutterella id.2892) on G−protein coupled receptor 26
with a threshold of 1e−05

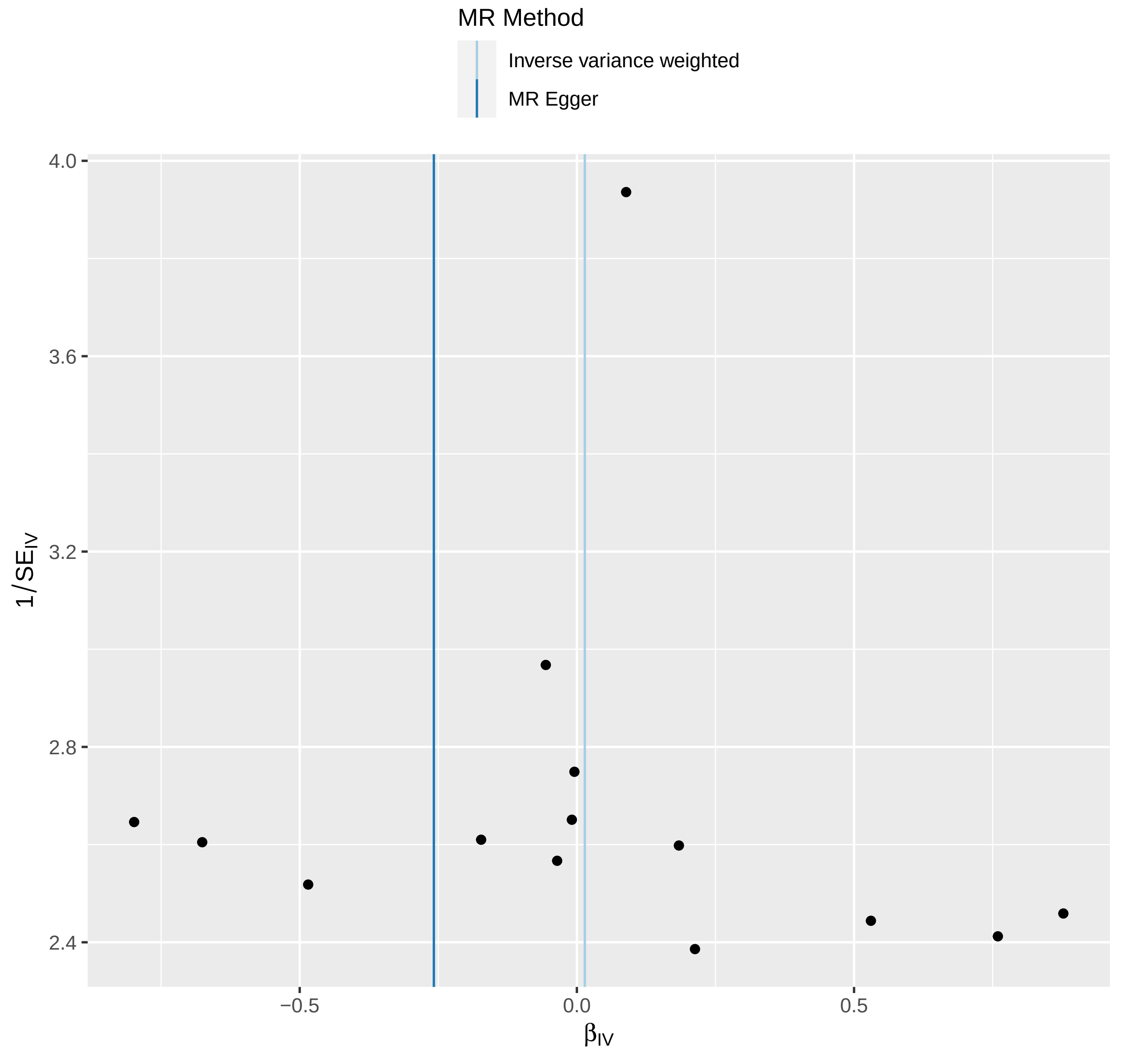

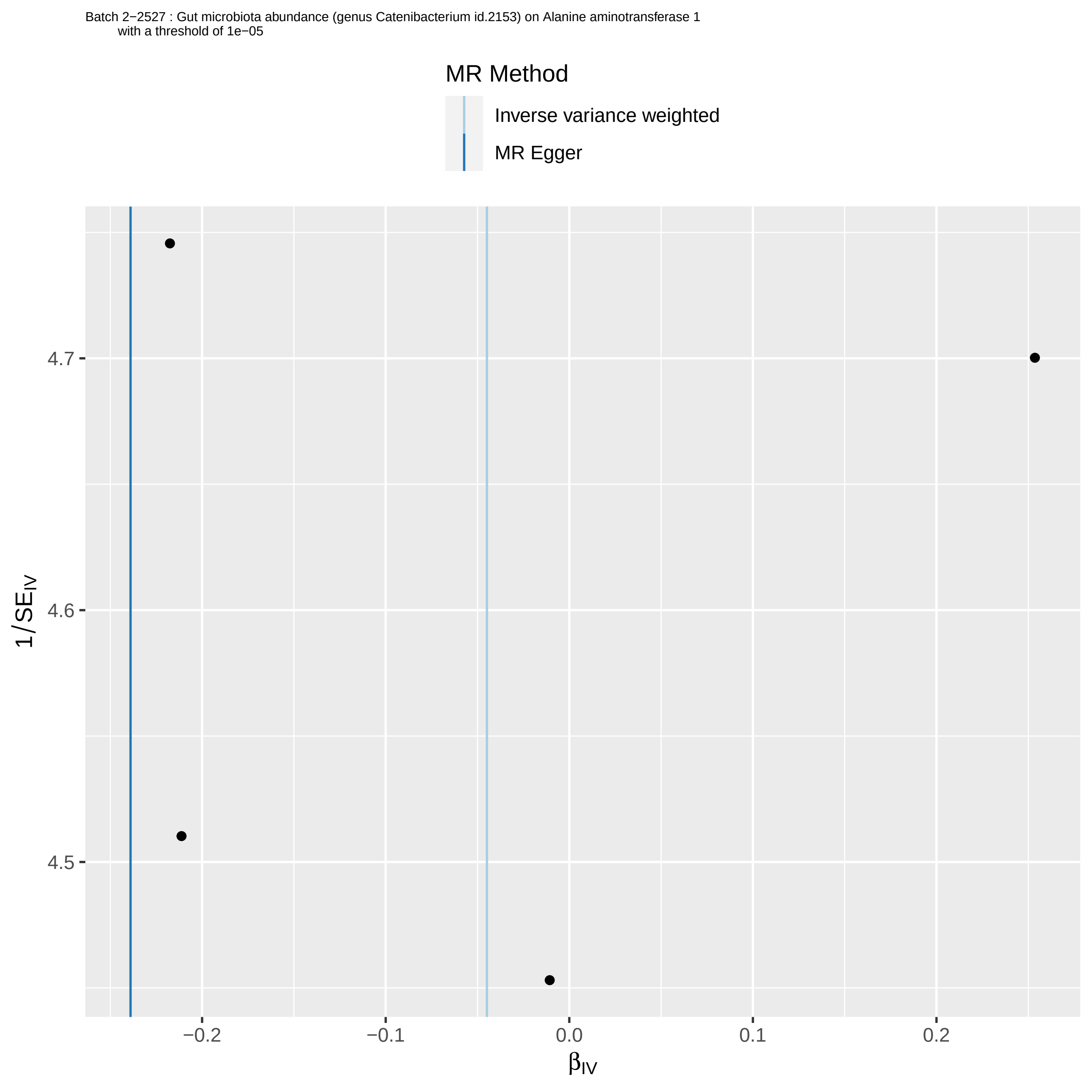

Batch 2−2527 : Gut microbiota abundance (genus Catenibacterium id.2153) on Alanine aminotransferase 1
with a threshold of 1e−05
MR Method
Inverse variance weighted
MR Egger
4.7
4.6
4.5
1/SE_IV
−0.2
−0.1
0.0
0.1
0.2
β_IV

Batch 2−2528 : Gut microbiota abundance (genus Parasutterella id.2892) on Alanine aminotransferase 1
with a threshold of 1e−05

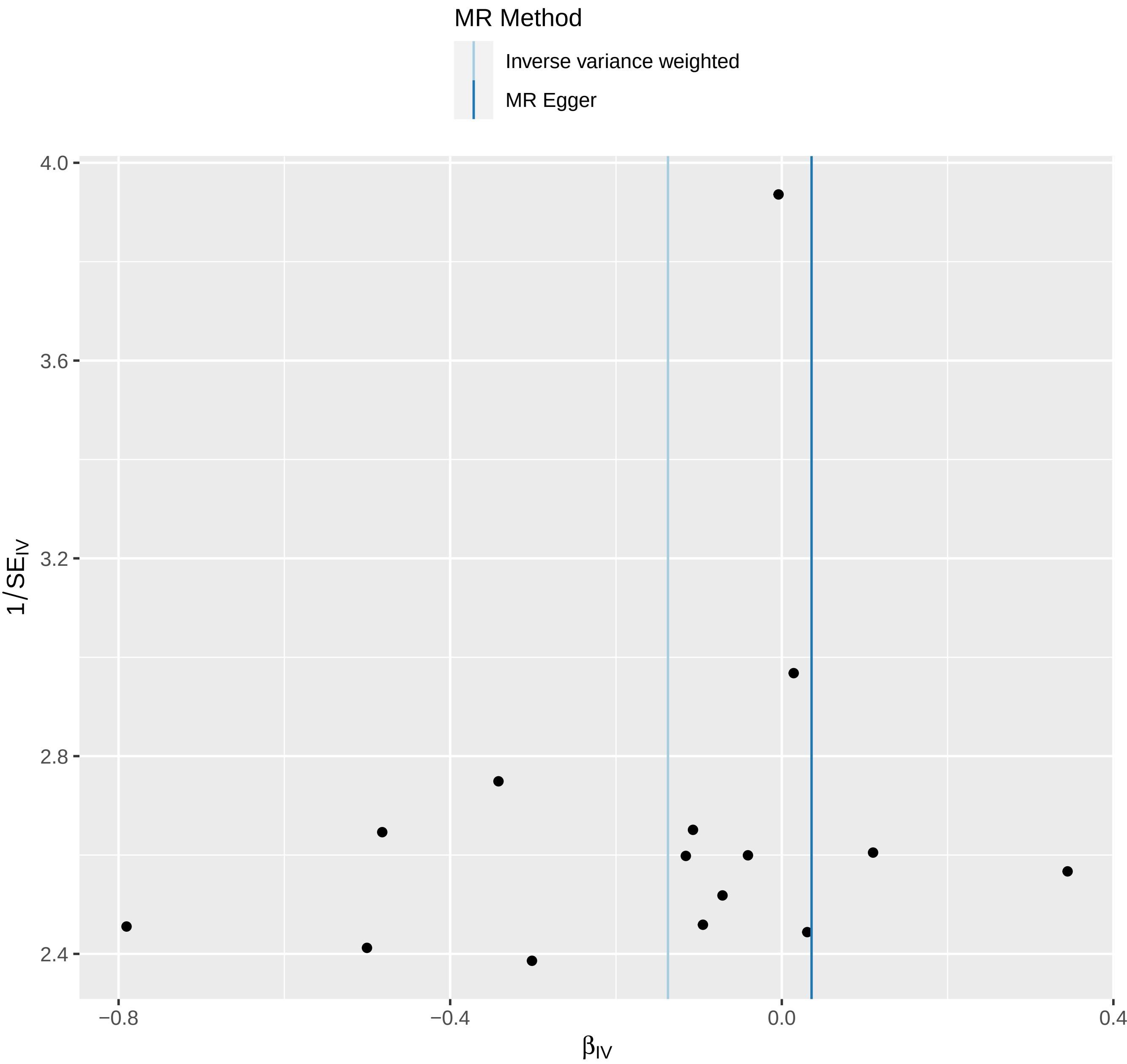

Batch 2−2529 : Gut microbiota abundance (genus Catenibacterium id.2153) on Glutathione peroxidase 7
with a threshold of 1e−05

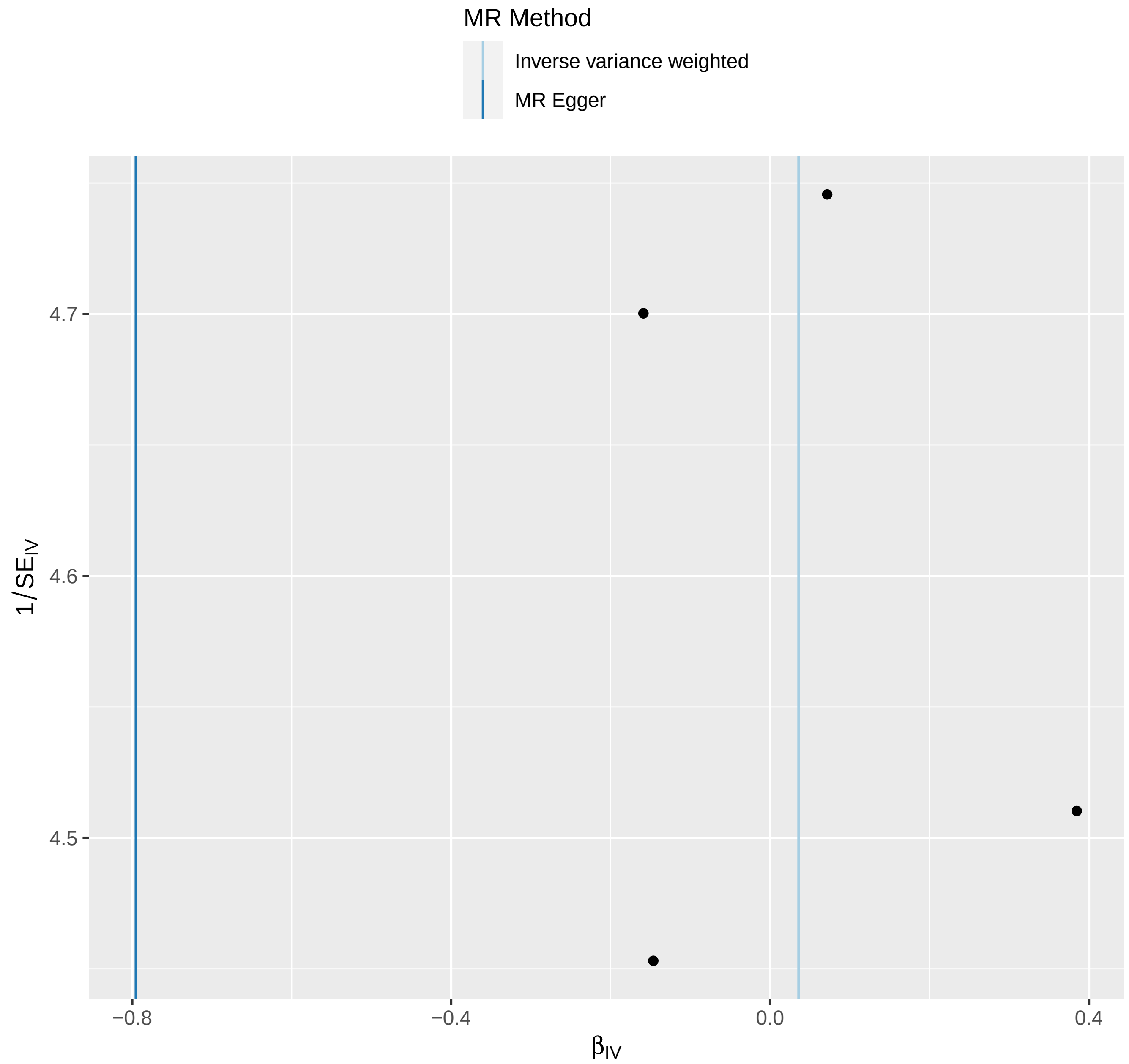

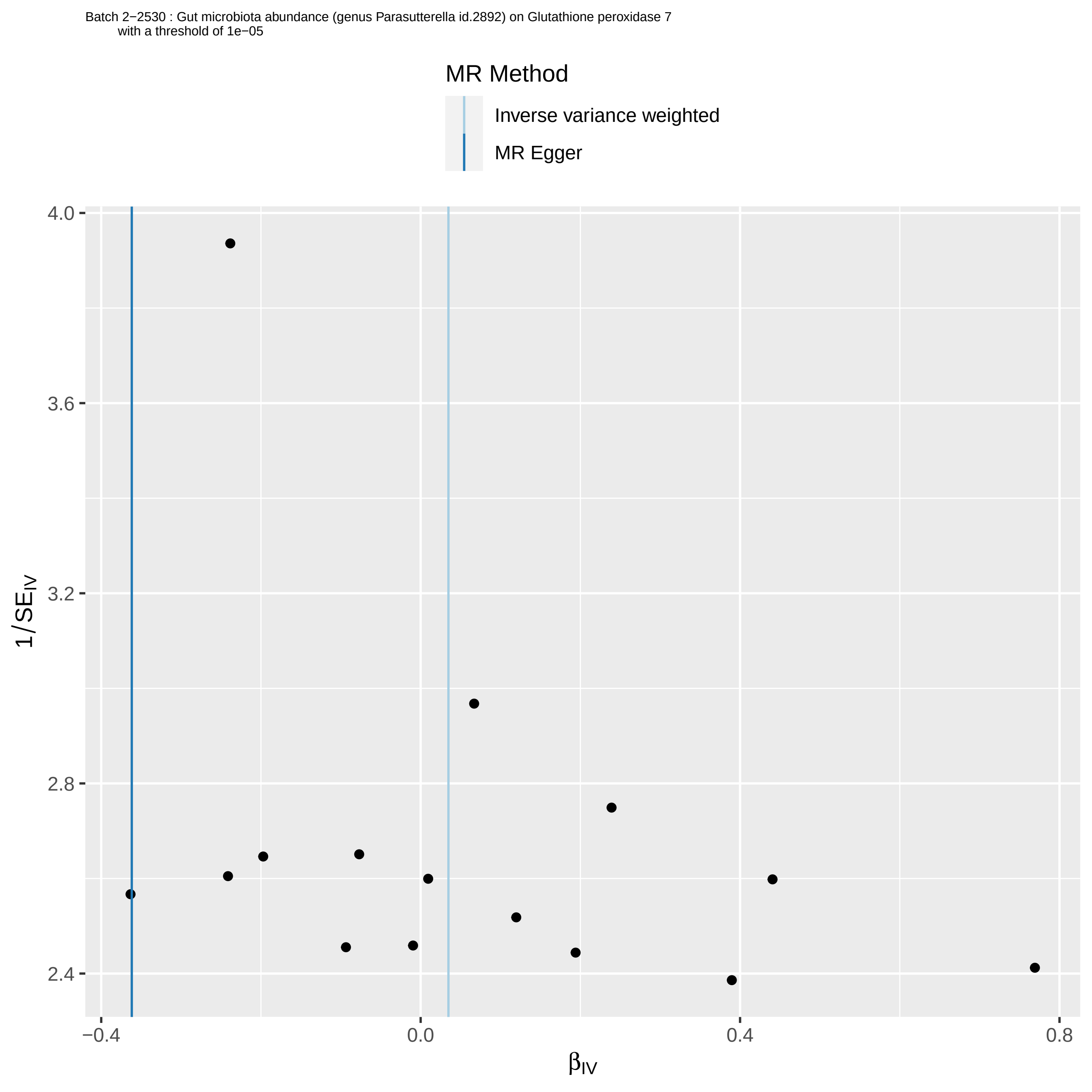

Batch 2−2530 : Gut microbiota abundance (genus Parasutterella id.2892) on Glutathione peroxidase 7
with a threshold of 1e−05
MR Method
Inverse variance weighted
MR Egger
4.0
3.6
3.2
2.8
2.4
1/SE_IV
−0.4
0.0
0.4
0.8
β_IV

Batch 2−2531 : Gut microbiota abundance (genus Catenibacterium id.2153) on GRAM domain−containing protein 1C
with a threshold of 1e−05

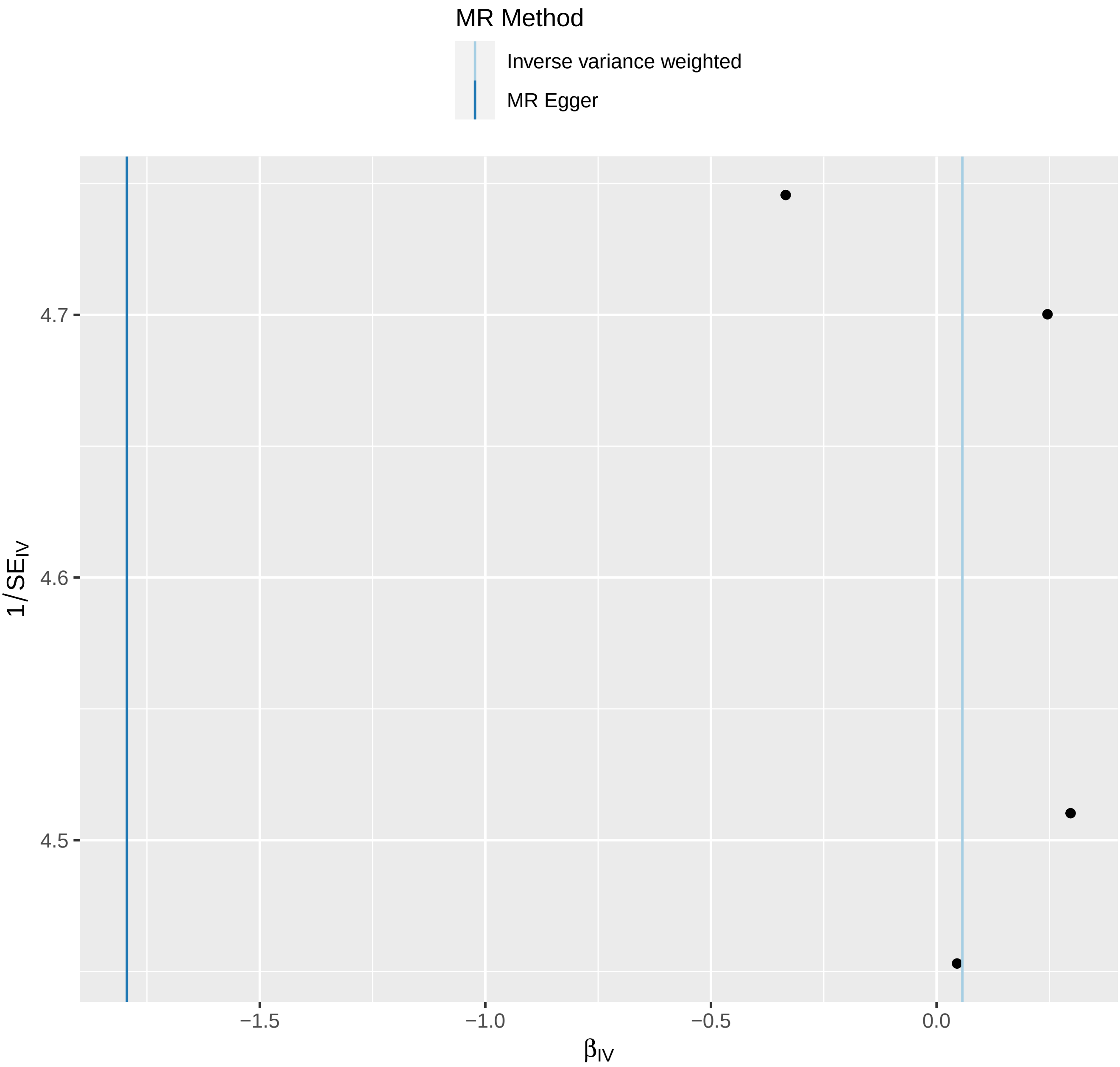

Batch 2−2532 : Gut microbiota abundance (genus Parasutterella id.2892) on GRAM domain−containing protein 1C
with a threshold of 1e−05

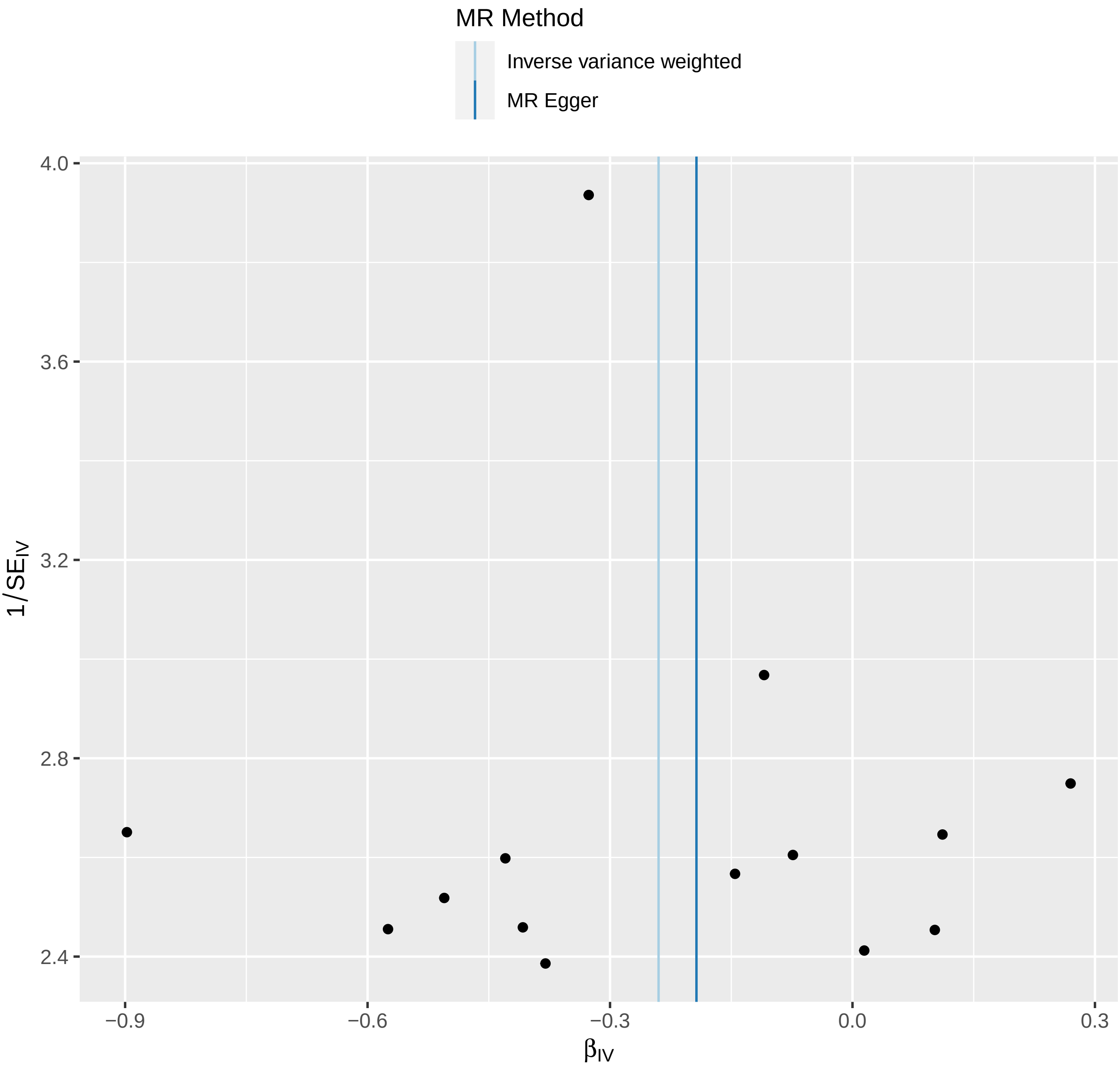

Batch 2−2533 : Gut microbiota abundance (genus Catenibacterium id.2153) on GRAM domain−containing protein 1C
with a threshold of 1e−05

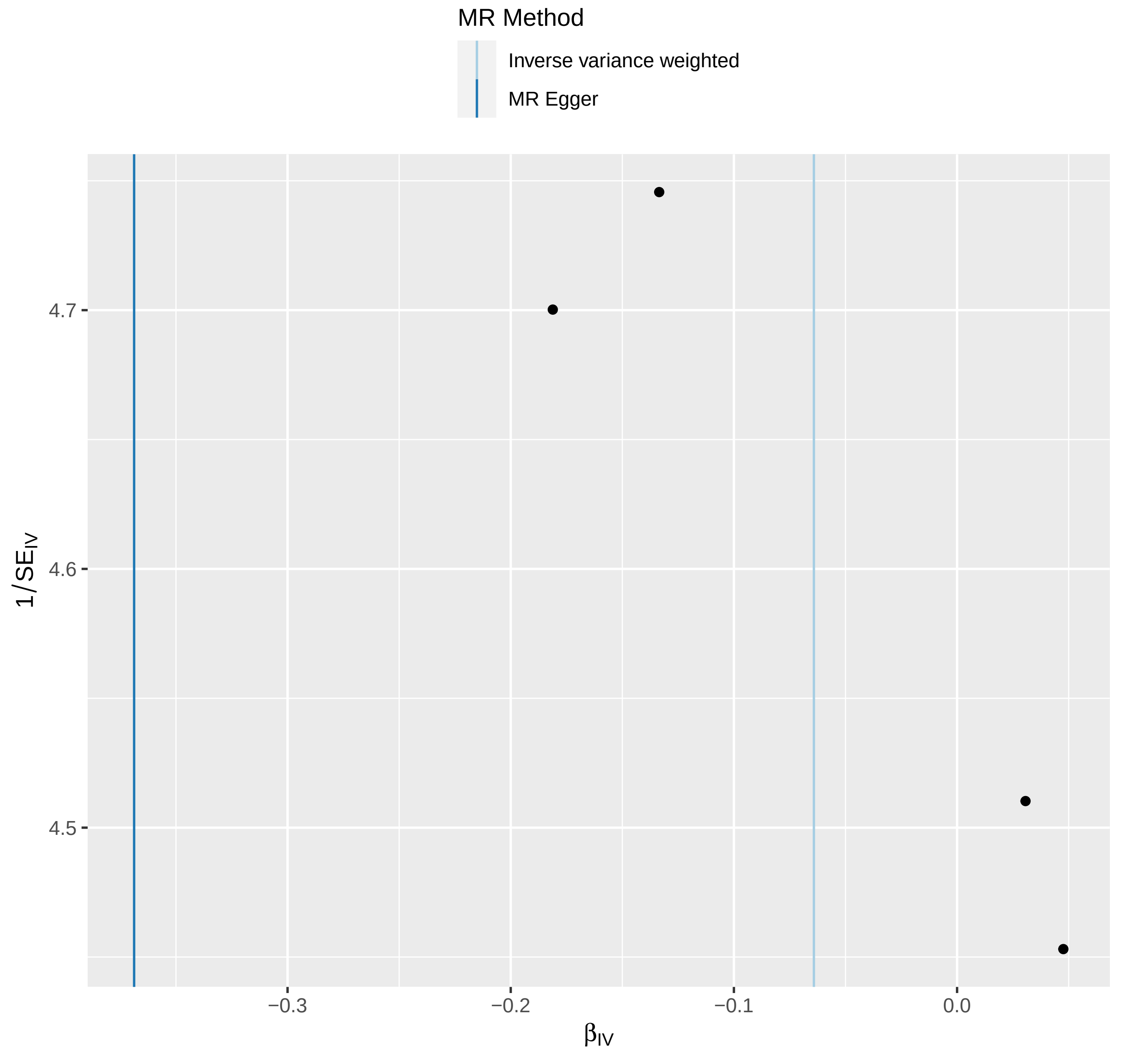

Batch 2−2534 : Gut microbiota abundance (genus Parasutterella id.2892) on GRAM domain−containing protein 1C
with a threshold of 1e−05

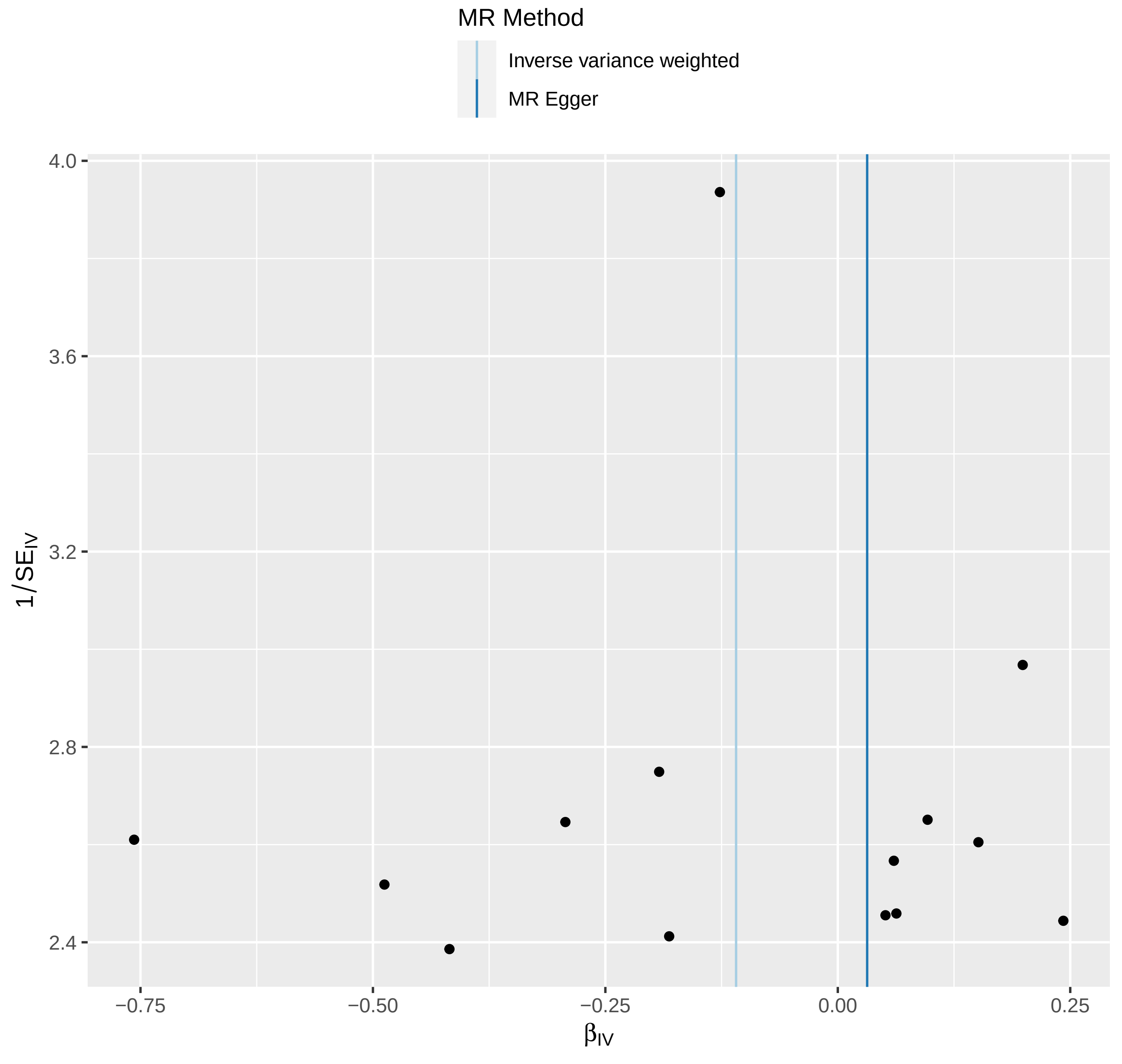

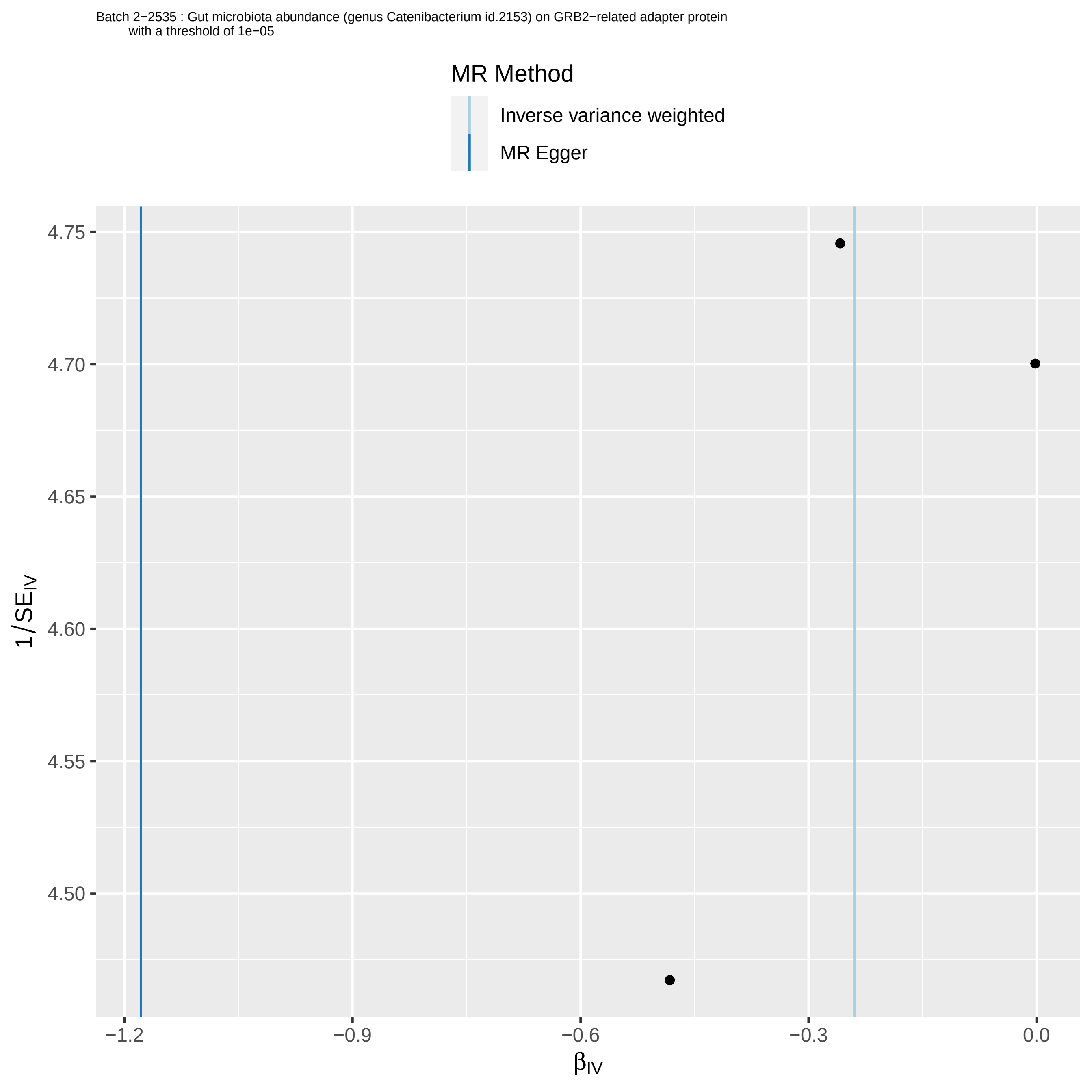
Batch 2−2535 : Gut microbiota abundance (genus Catenibacterium id.2153) on GRB2−related adapter protein
with a threshold of 1e−05
MR Method
Inverse variance weighted
MR Egger
4.75
4.70
4.65
4.60
4.55
4.50
1/SE_IV
−1.2
−0.9
−0.6
−0.3
0.0
β_IV

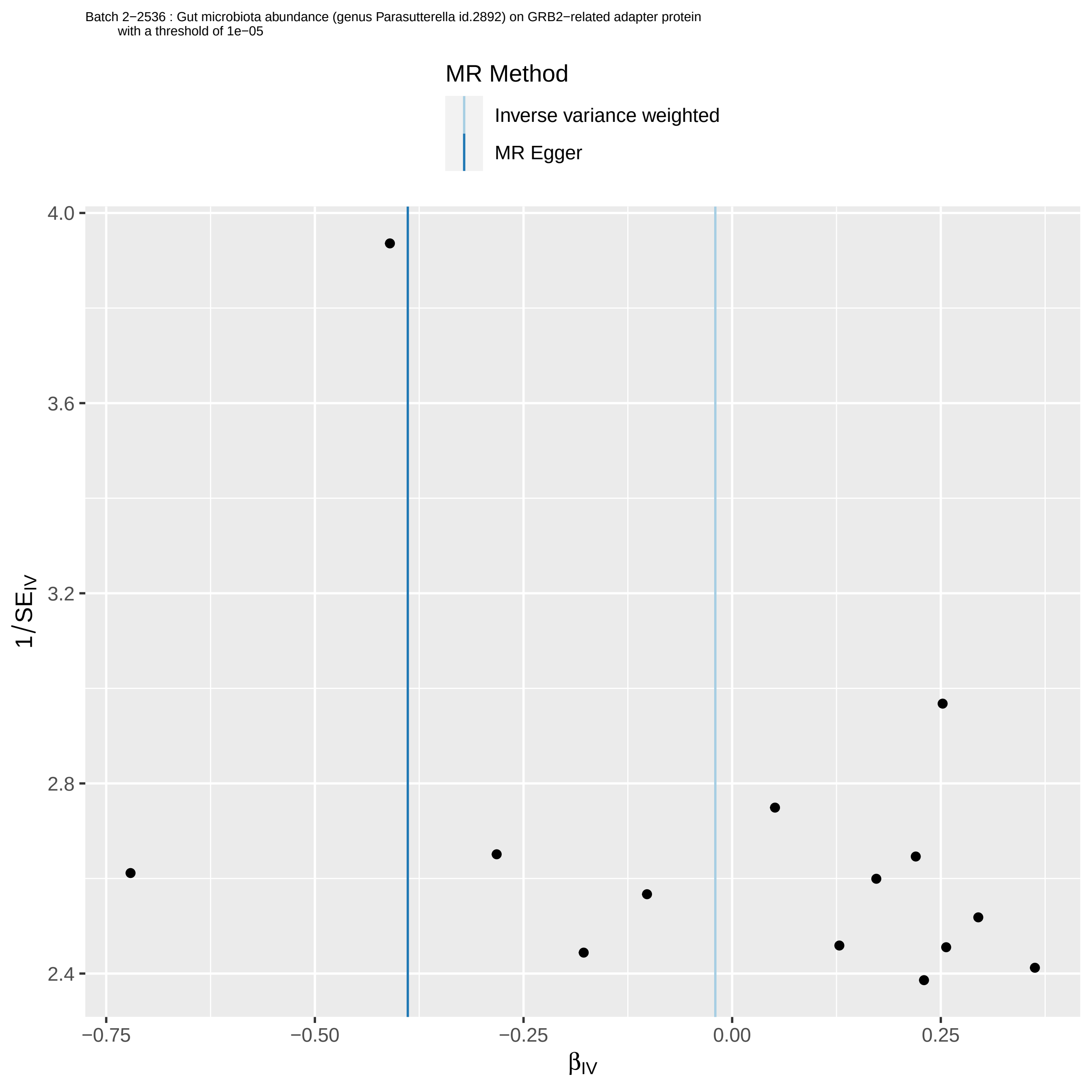

Batch 2−2536 : Gut microbiota abundance (genus Parasutterella id.2892) on GRB2−related adapter protein
with a threshold of 1e−05
MR Method
Inverse variance weighted
MR Egger
4.0
3.6
3.2
2.8
2.4
1/SE_IV
−0.75
−0.50
−0.25
0.00
0.25
β_IV

Batch 2−2537 : Gut microbiota abundance (genus Catenibacterium id.2153) on GRB2−related adapter protein 2
with a threshold of 1e−05

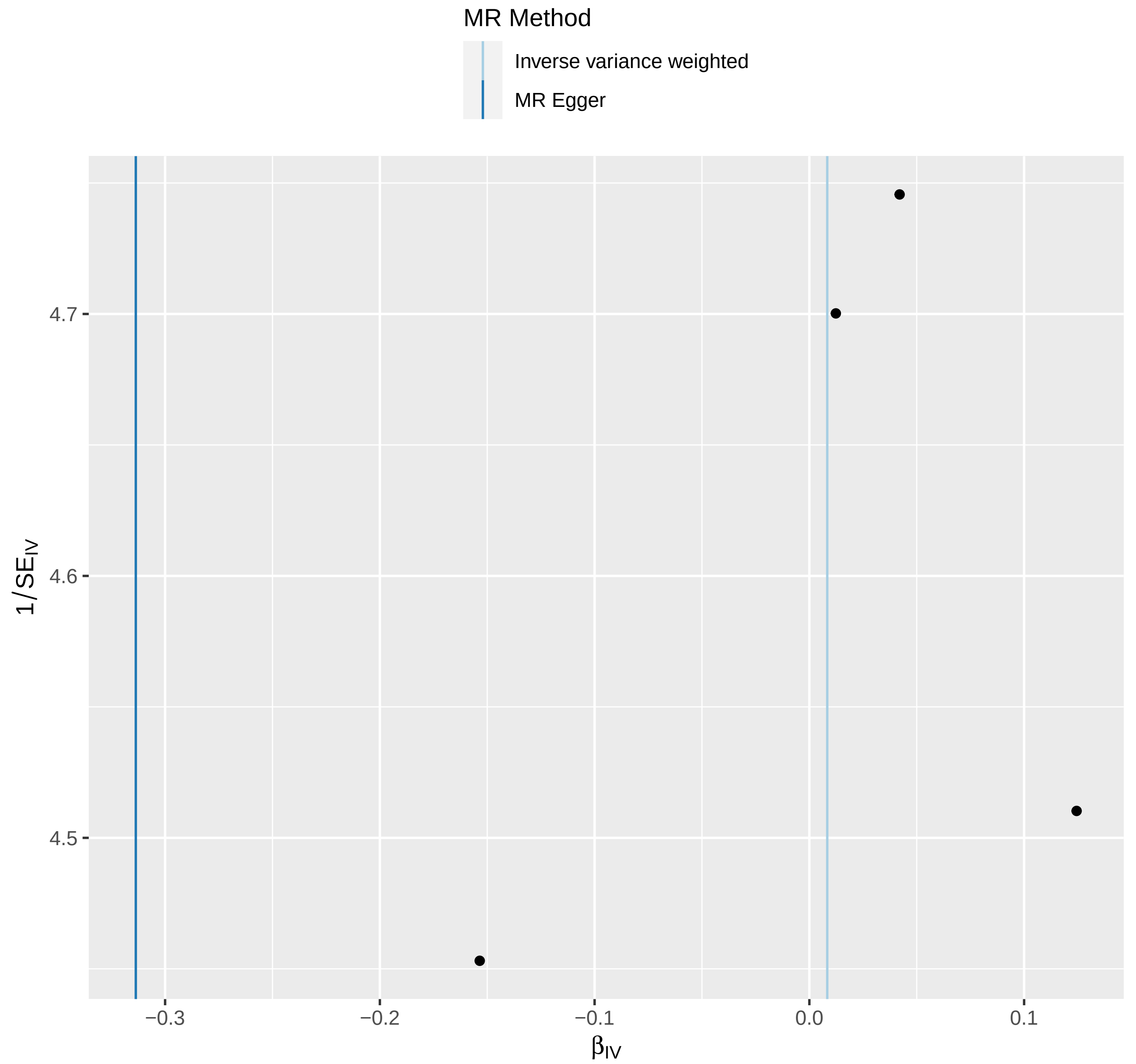

Batch 2−2538 : Gut microbiota abundance (genus Parasutterella id.2892) on GRB2−related adapter protein 2
with a threshold of 1e−05

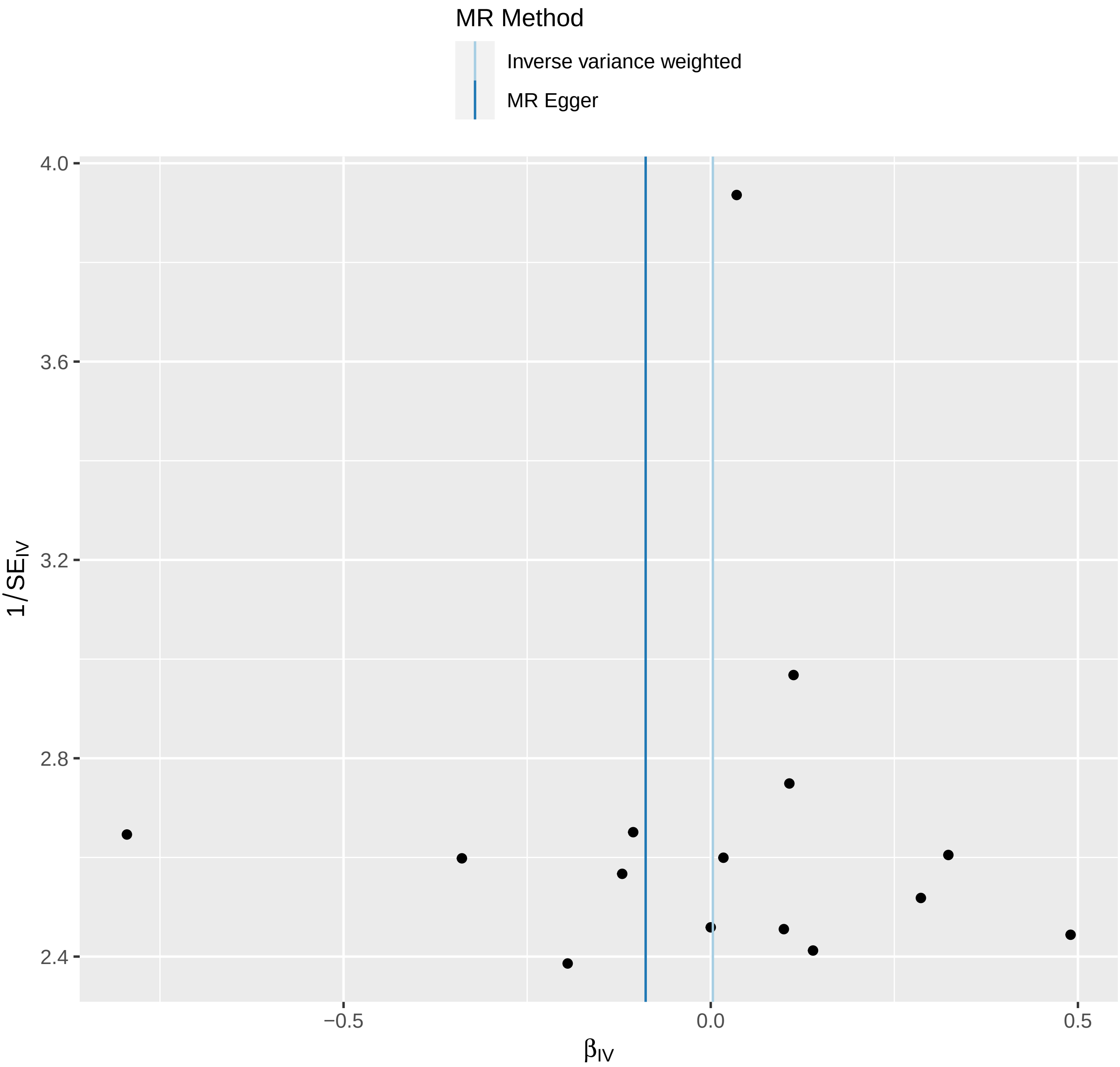

Batch 2−2539 : Gut microbiota abundance (genus Catenibacterium id.2153) on Growth factor receptor−bound protein 14
with a threshold of 1e−05

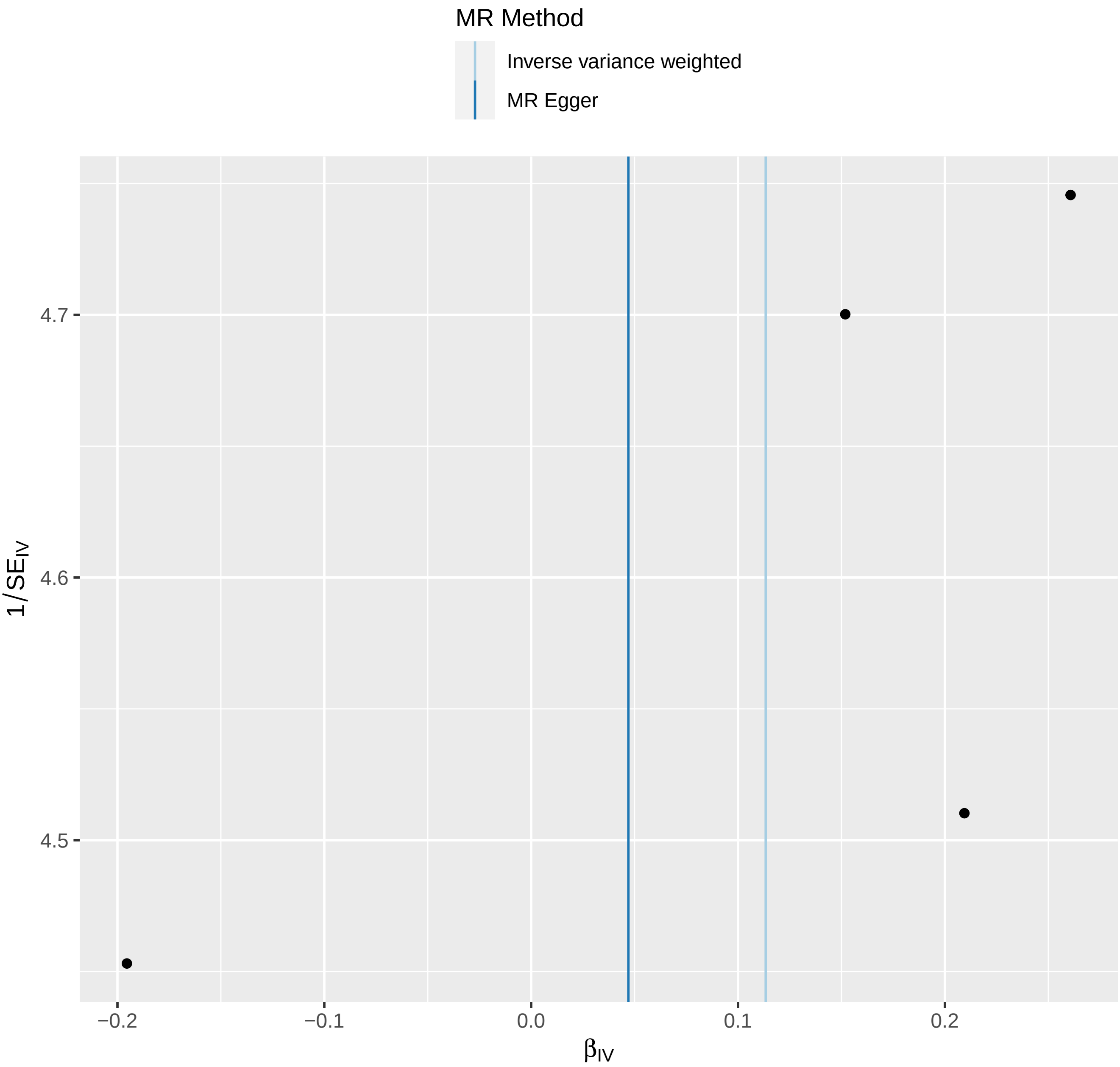

Batch 2−2540 : Gut microbiota abundance (genus Parasutterella id.2892) on Growth factor receptor−bound protein 14
with a threshold of 1e−05

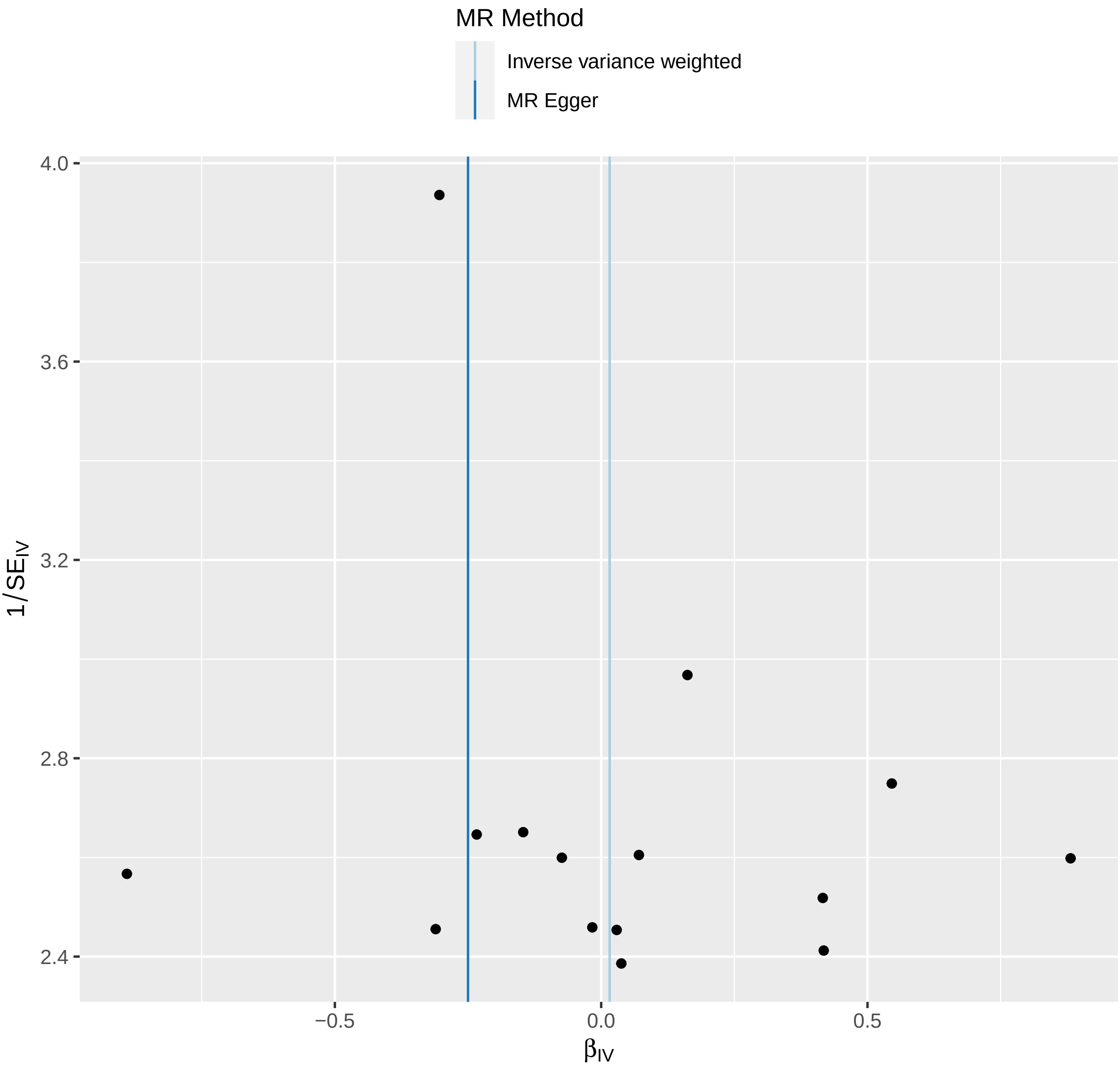

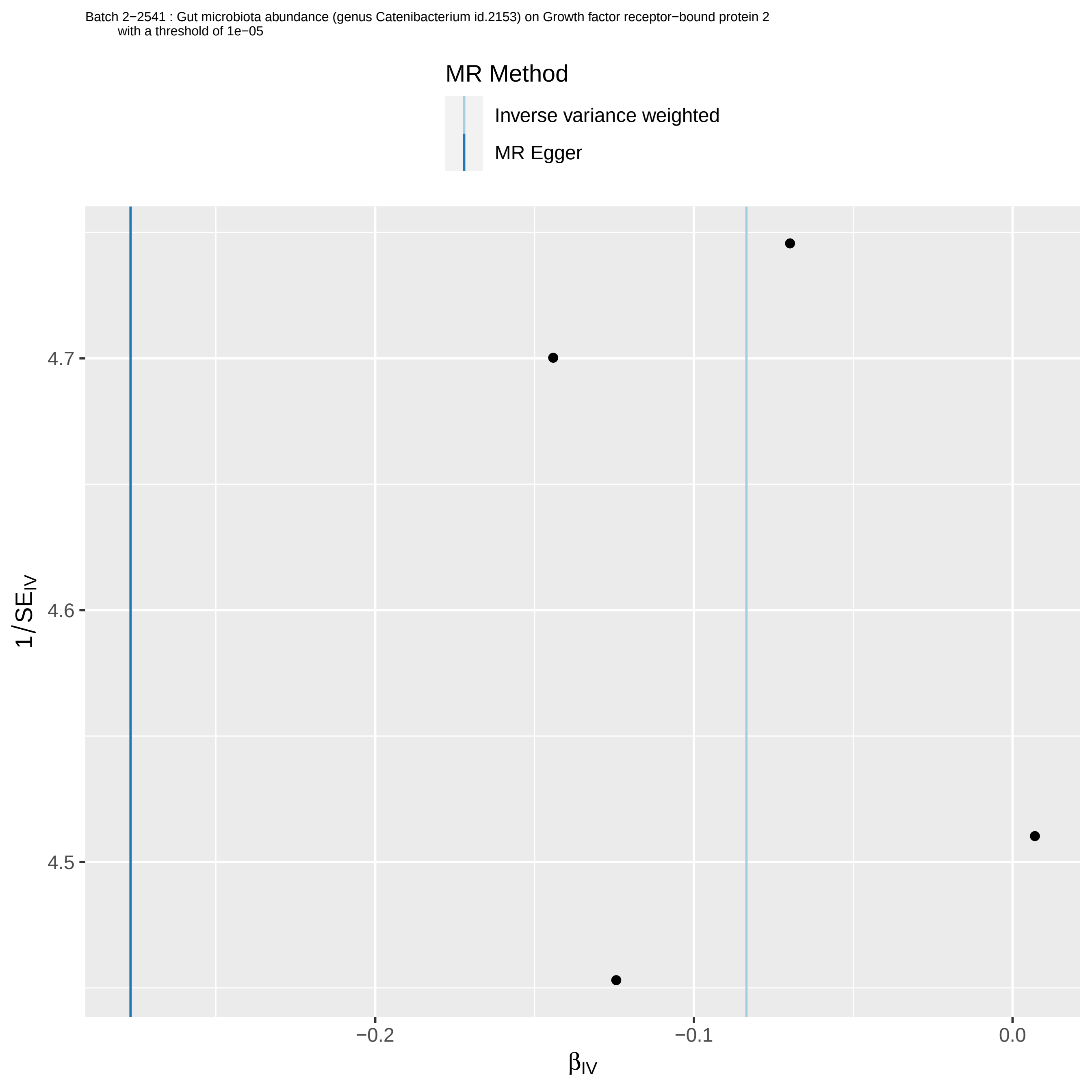

Batch 2−2541 : Gut microbiota abundance (genus Catenibacterium id.2153) on Growth factor receptor−bound protein 2
with a threshold of 1e−05
MR Method
Inverse variance weighted
MR Egger
4.7
4.6
4.5
1/SE_IV
−0.2
−0.1
0.0
β_IV

Batch 2−2542 : Gut microbiota abundance (genus Parasutterella id.2892) on Growth factor receptor−bound protein 2 with a threshold of 1e−05

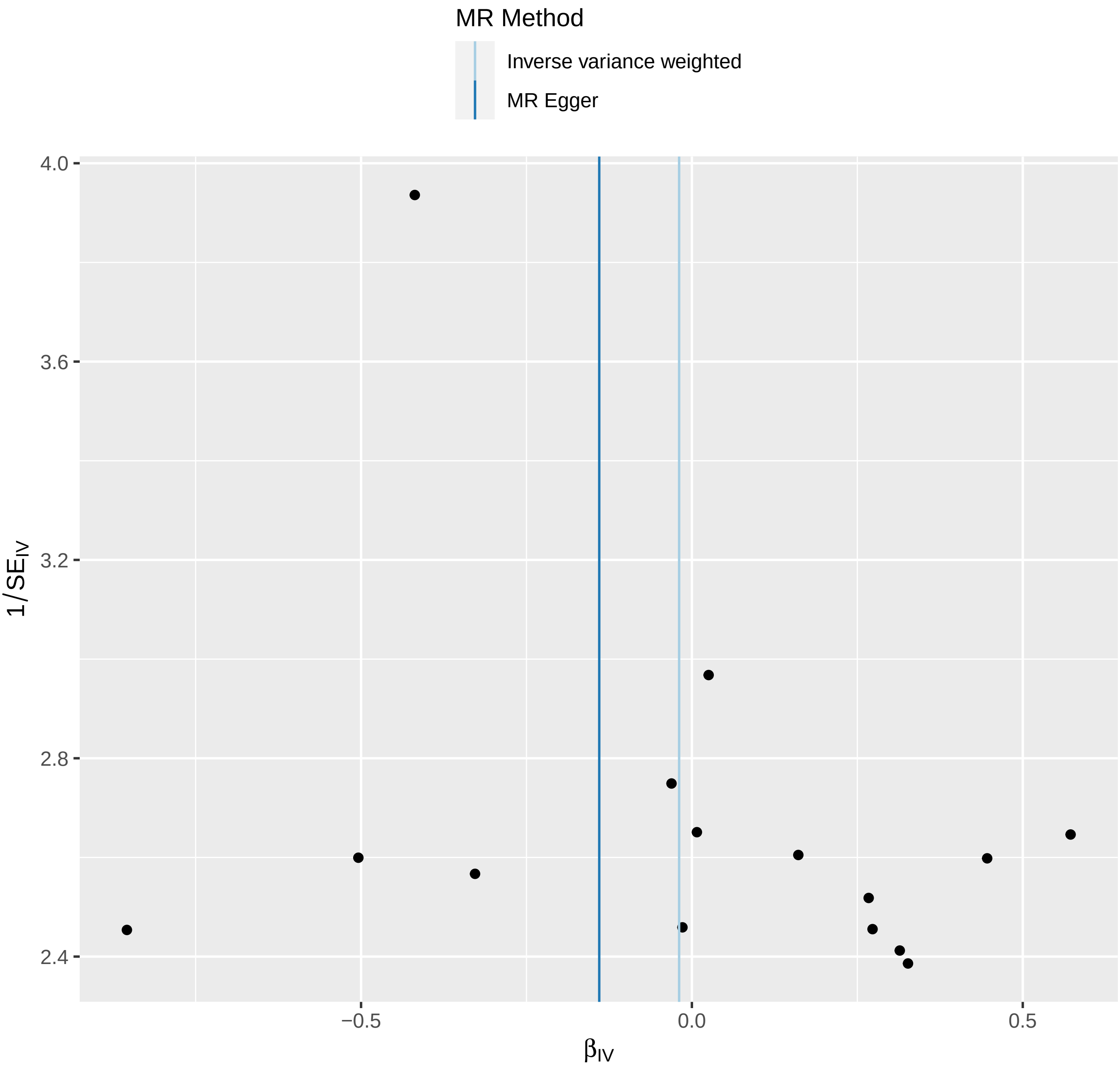

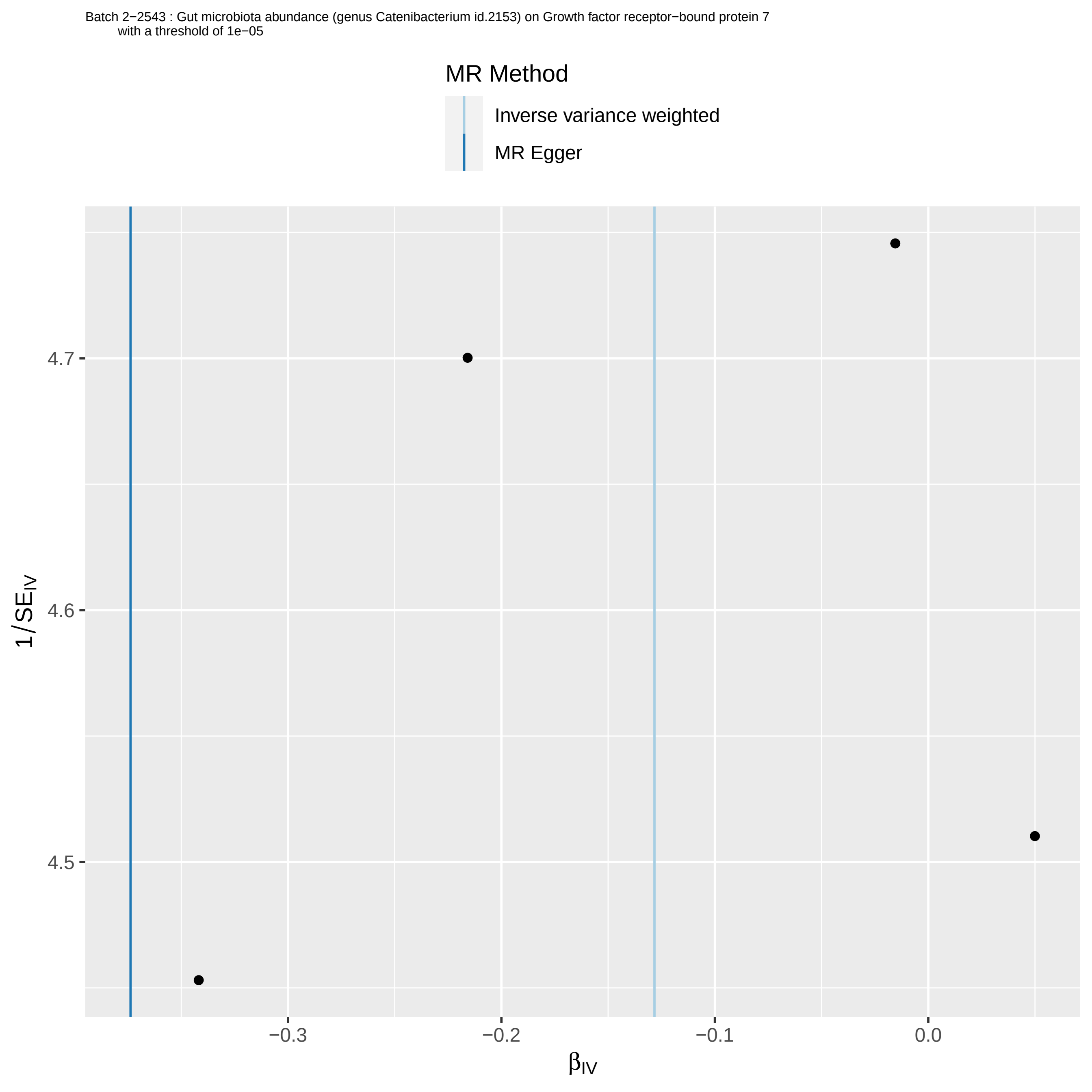

Batch 2−2543 : Gut microbiota abundance (genus Catenibacterium id.2153) on Growth factor receptor−bound protein 7
with a threshold of 1e−05
MR Method
Inverse variance weighted
MR Egger
4.7
4.6
4.5
−0.3
−0.2
−0.1
0.0
$1/SE_{IV}$
β_{IV}

Batch 2−2544 : Gut microbiota abundance (genus Parasutterella id.2892) on Growth factor receptor−bound protein 7 with a threshold of 1e−05

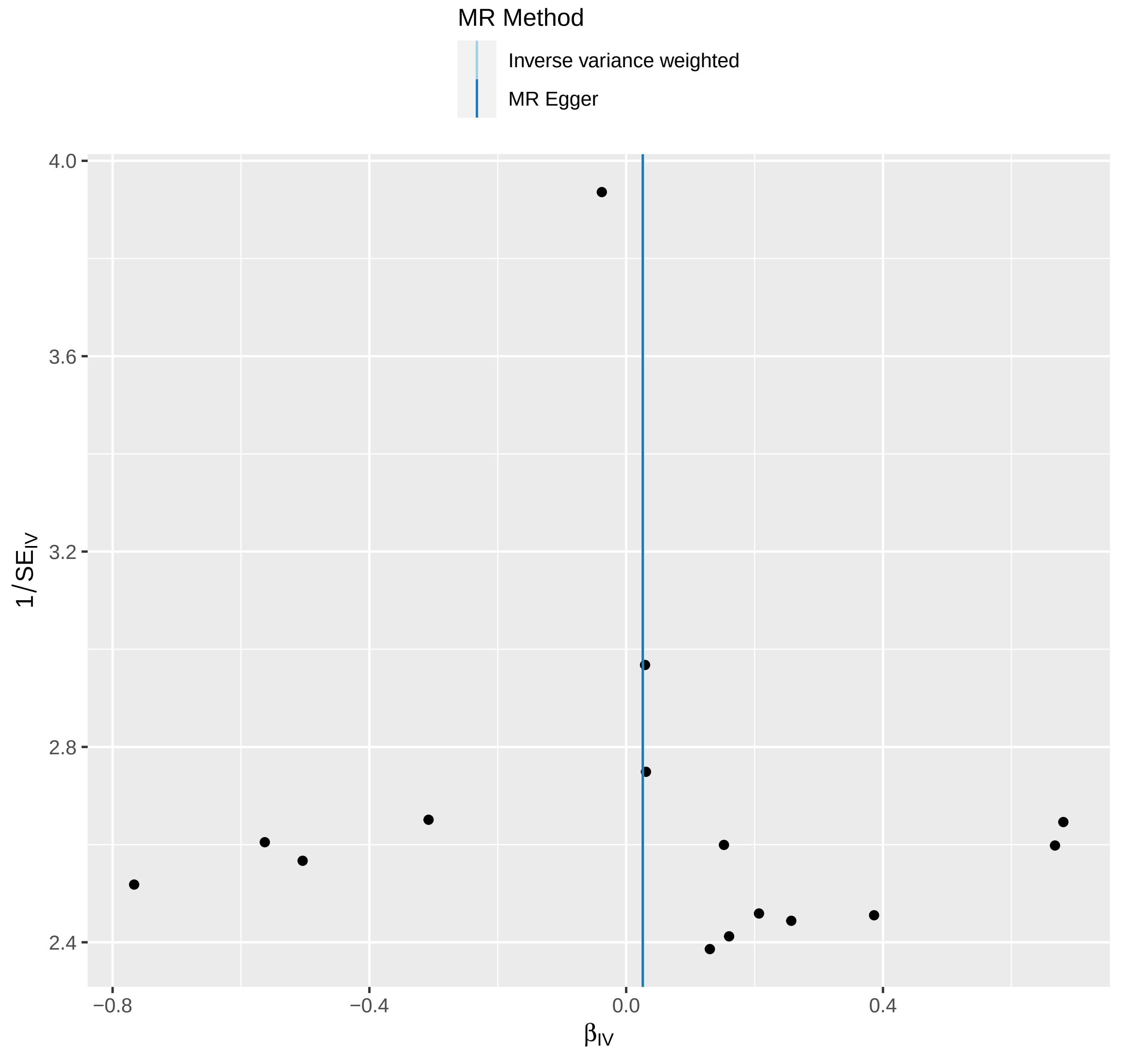

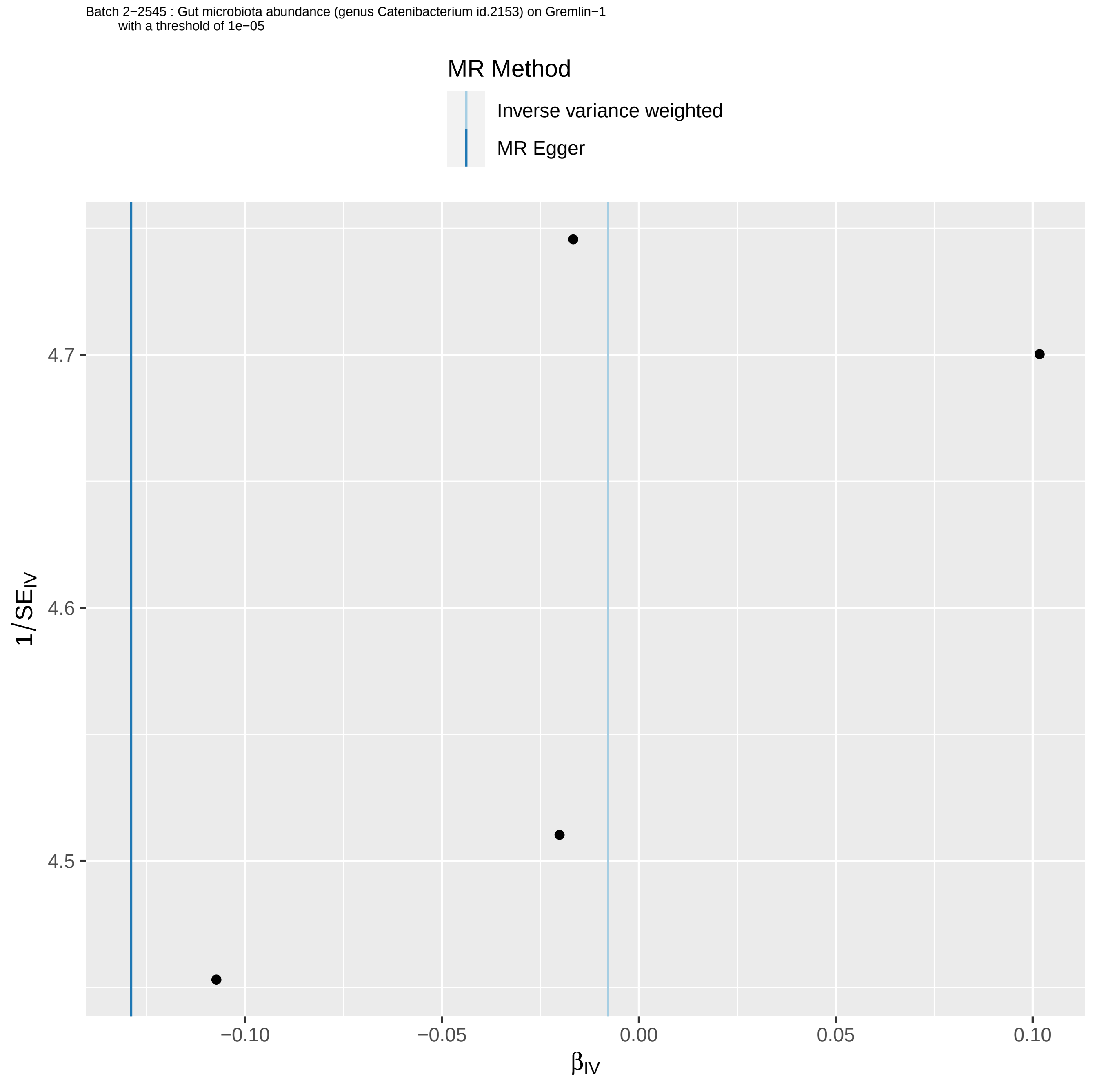

Batch 2−2545 : Gut microbiota abundance (genus Catenibacterium id.2153) on Gremlin−1
with a threshold of 1e−05
MR Method
Inverse variance weighted
MR Egger
4.7
4.6
4.5
1/SE_IV
−0.10
−0.05
0.00
0.05
0.10
β_IV

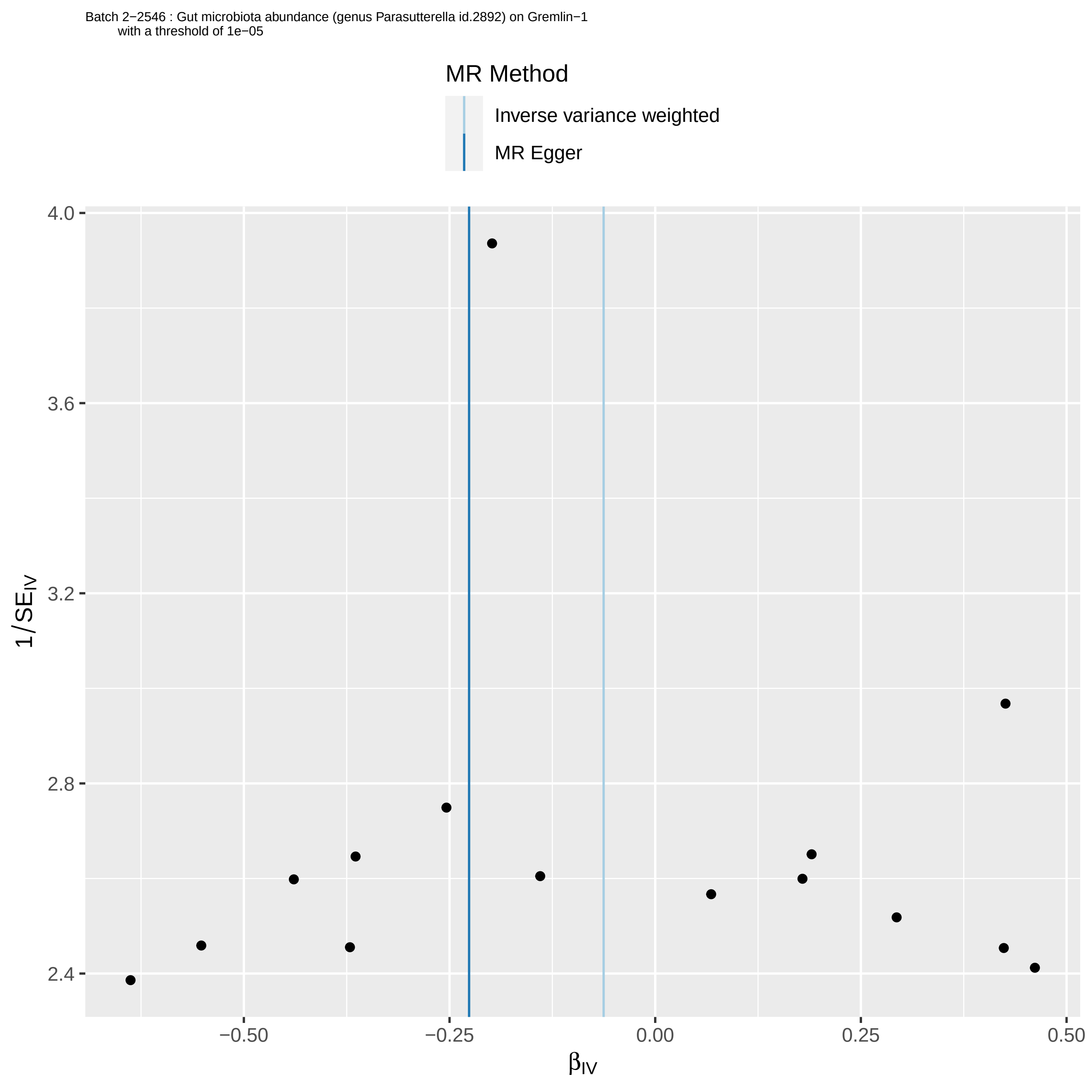

Batch 2−2546 : Gut microbiota abundance (genus Parasutterella id.2892) on Gremlin−1
with a threshold of 1e−05
MR Method
Inverse variance weighted
MR Egger
4.0
3.6
3.2
2.8
2.4
1/SE_IV
−0.50
−0.25
0.00
0.25
0.50
β_IV

Batch 2−2547 : Gut microbiota abundance (genus Catenibacterium id.2153) on Gremlin−2
with a threshold of 1e−05

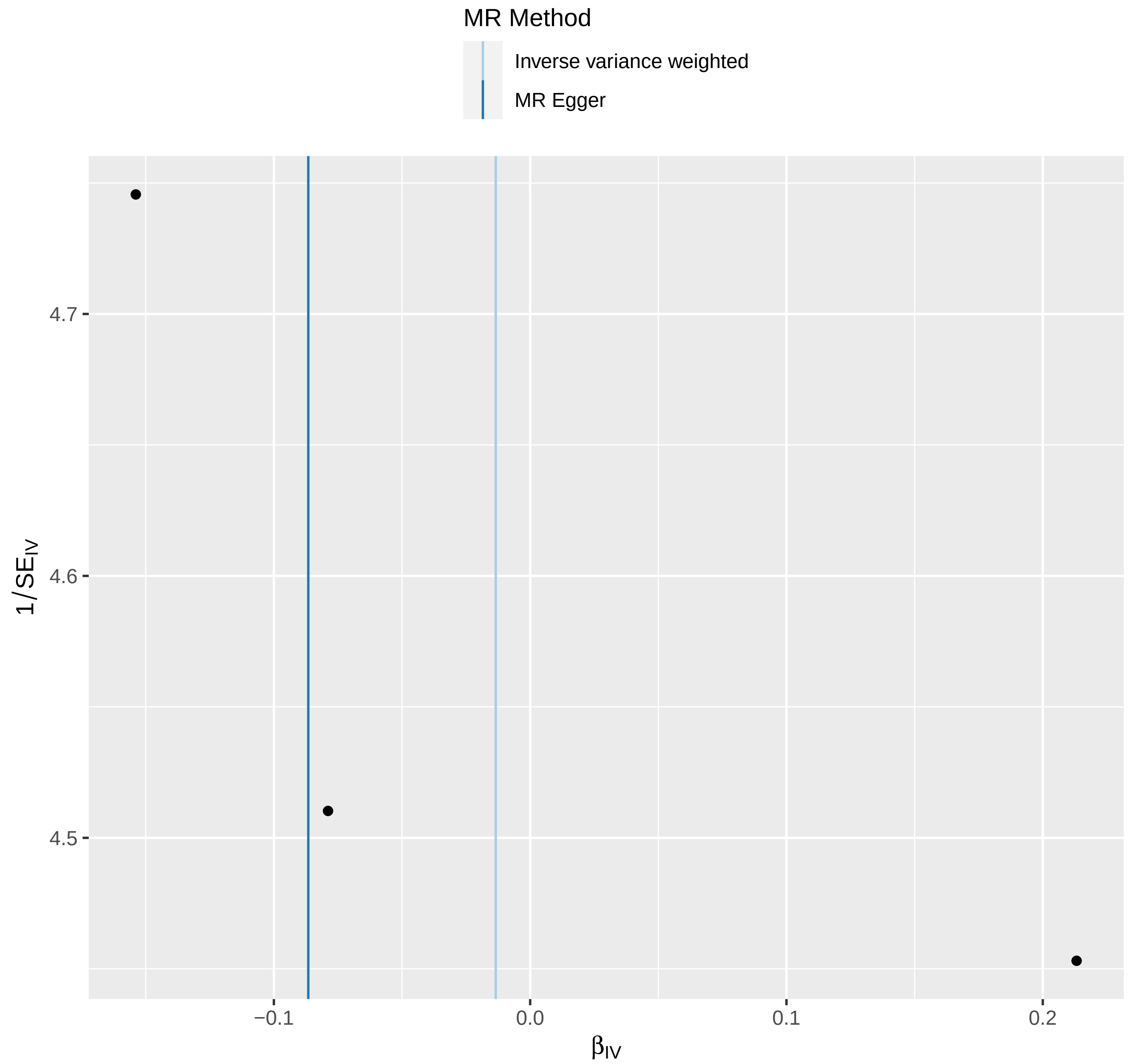

Batch 2−2548 : Gut microbiota abundance (genus Parasutterella id.2892) on Gremlin−2
with a threshold of 1e−05

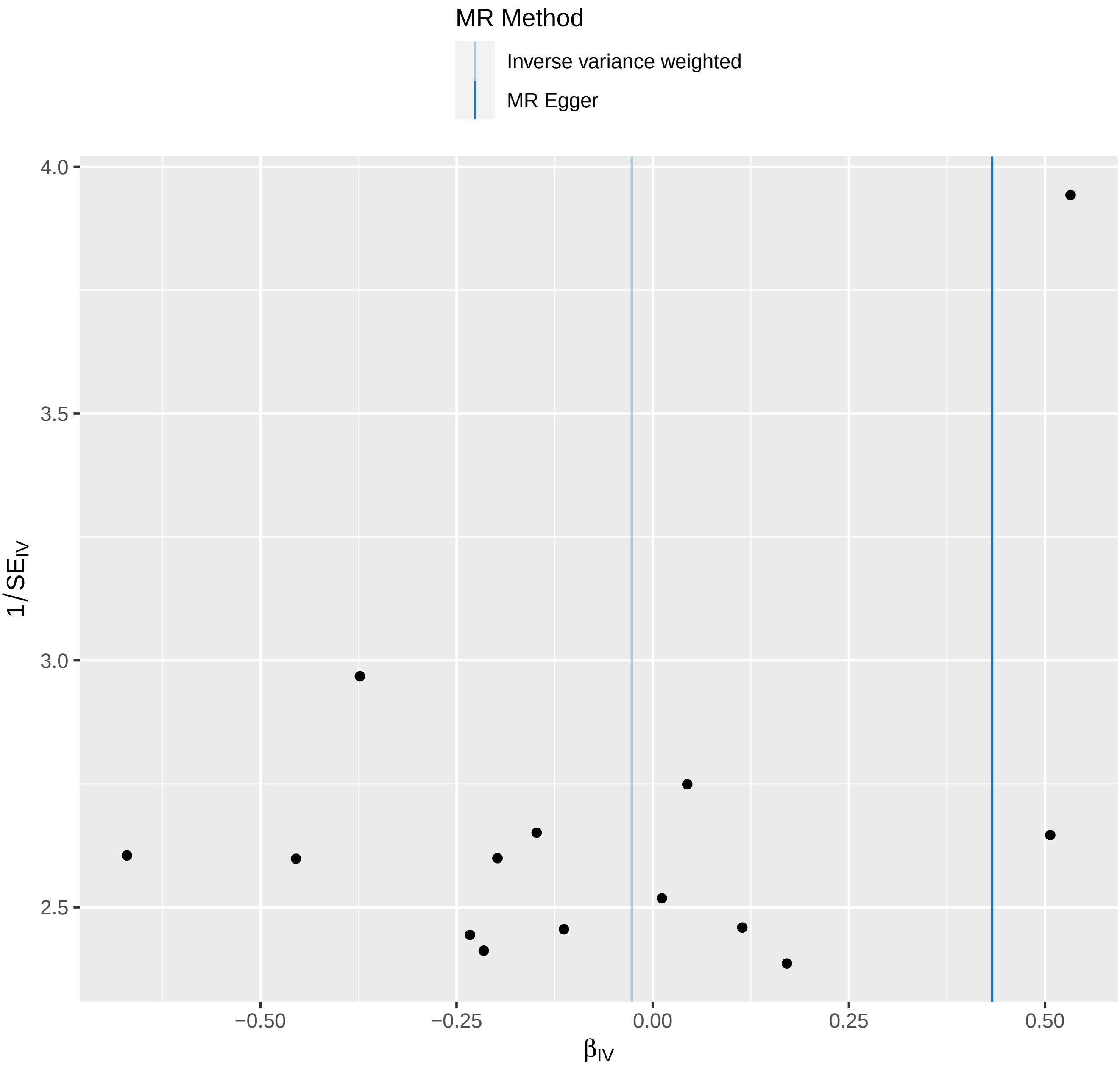

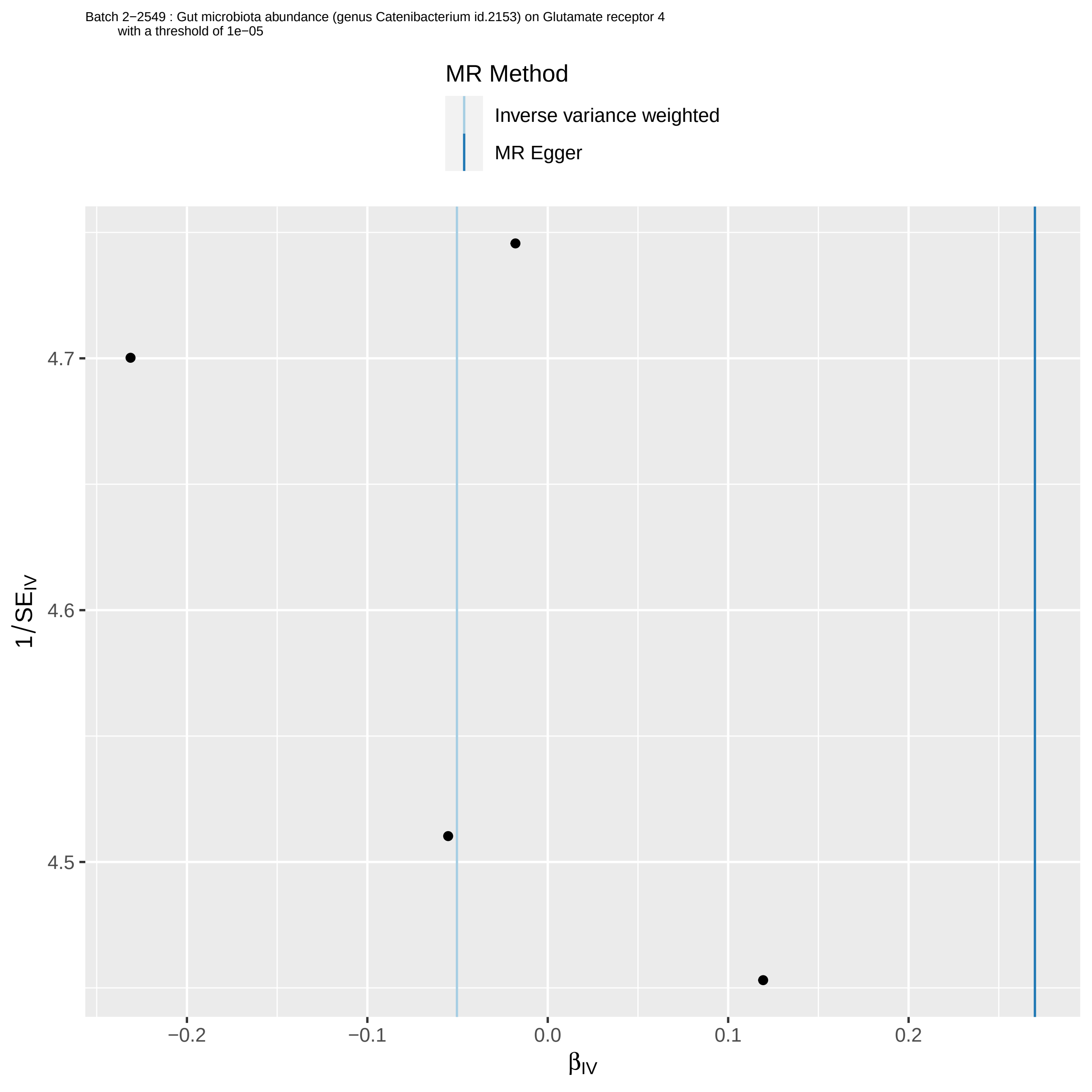

Batch 2−2549 : Gut microbiota abundance (genus Catenibacterium id.2153) on Glutamate receptor 4
with a threshold of 1e−05
MR Method
Inverse variance weighted
MR Egger
4.7
4.6
4.5
$1/SE_{IV}$
−0.2
−0.1
0.0
0.1
0.2
β_{IV}

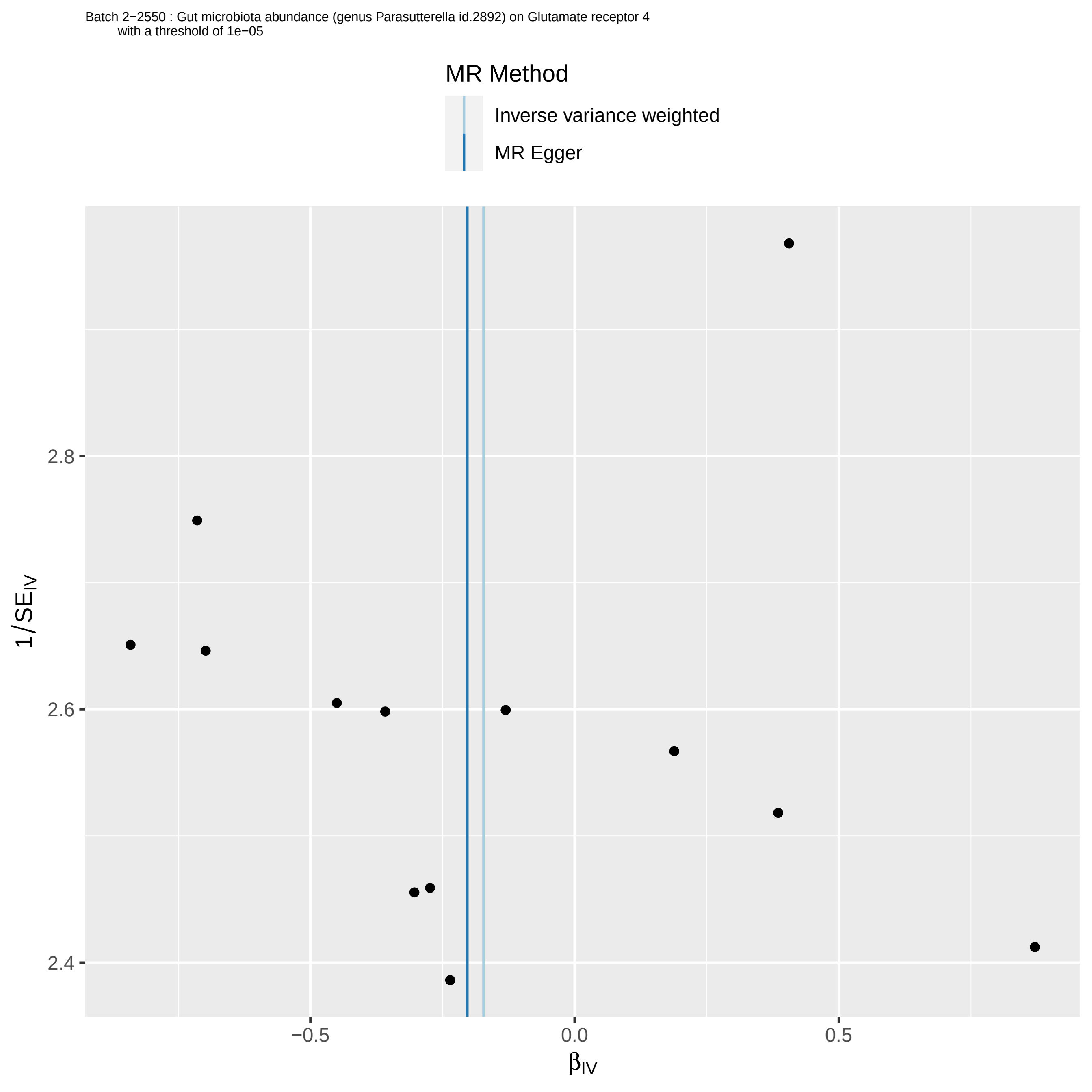
Batch 2−2550 : Gut microbiota abundance (genus Parasutterella id.2892) on Glutamate receptor 4
with a threshold of 1e−05
MR Method
Inverse variance weighted
MR Egger
2.8
2.6
2.4
1/SE_IV
−0.5
0.0
0.5
β_IV

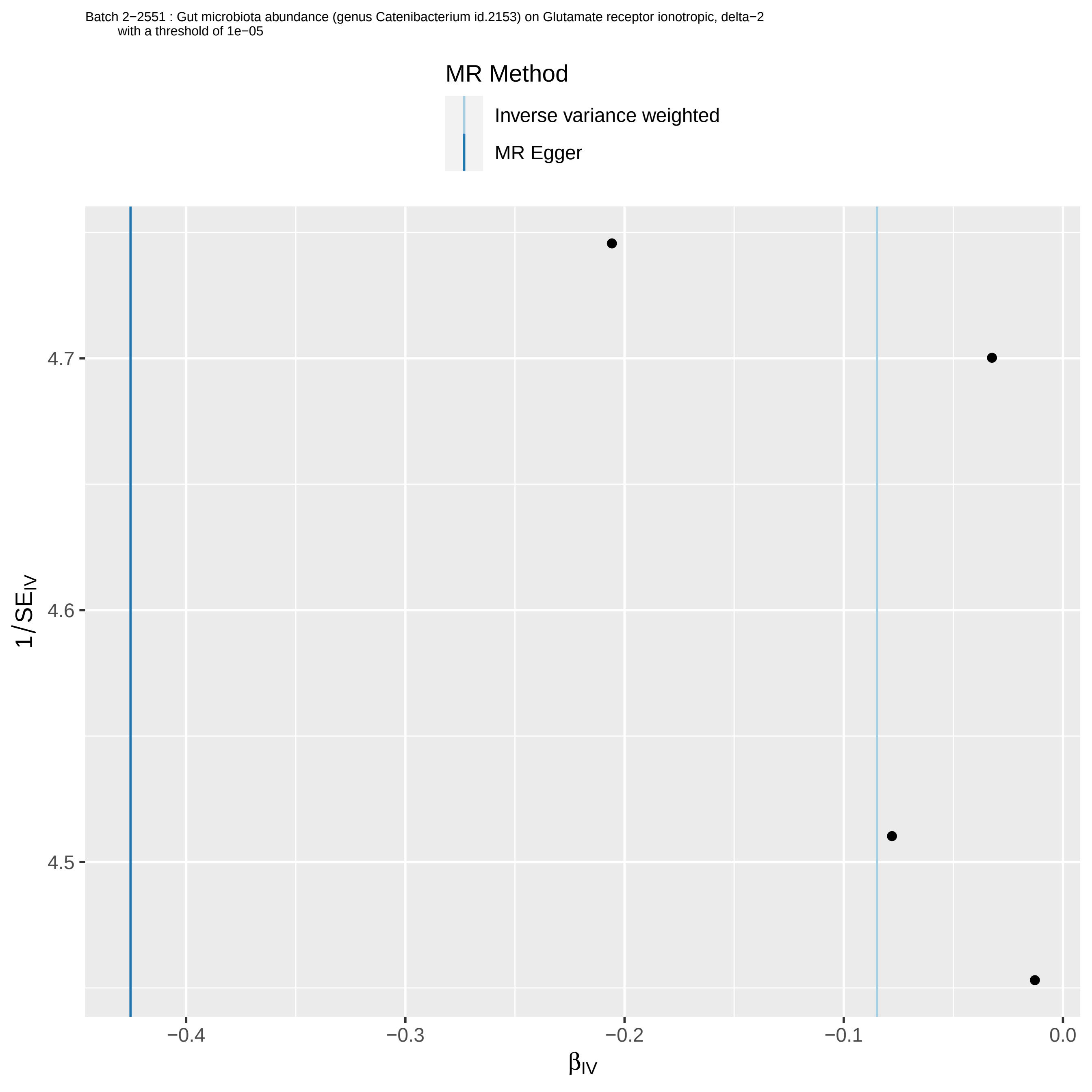

Batch 2−2551 : Gut microbiota abundance (genus Catenibacterium id.2153) on Glutamate receptor ionotropic, delta−2
with a threshold of 1e−05
MR Method
Inverse variance weighted
MR Egger
4.7
4.6
4.5
1/SE_IV
−0.4
−0.3
−0.2
−0.1
0.0
β_IV

Batch 2−2552 : Gut microbiota abundance (genus Parasutterella id.2892) on Glutamate receptor ionotropic, delta−2
with a threshold of 1e−05

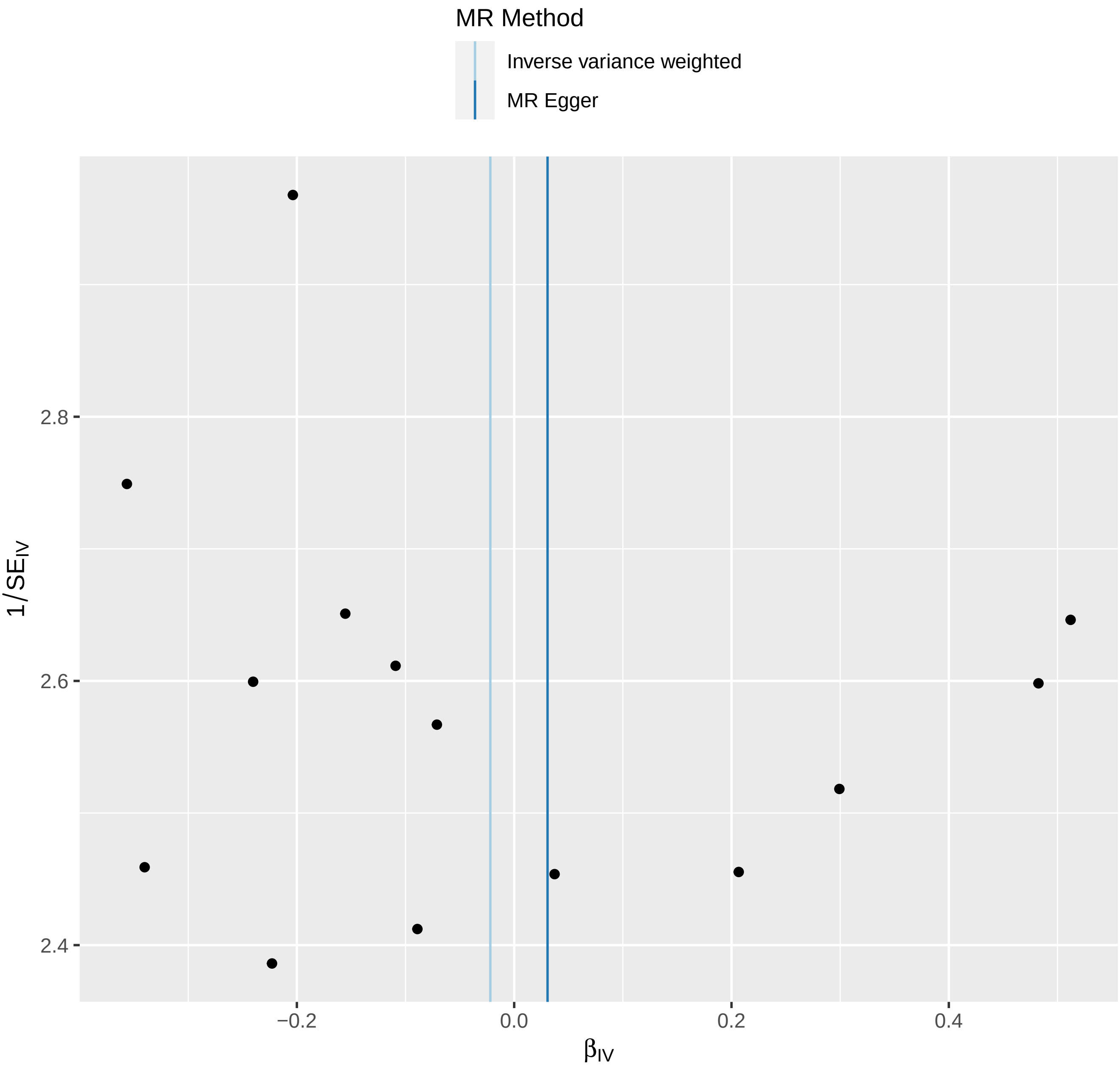

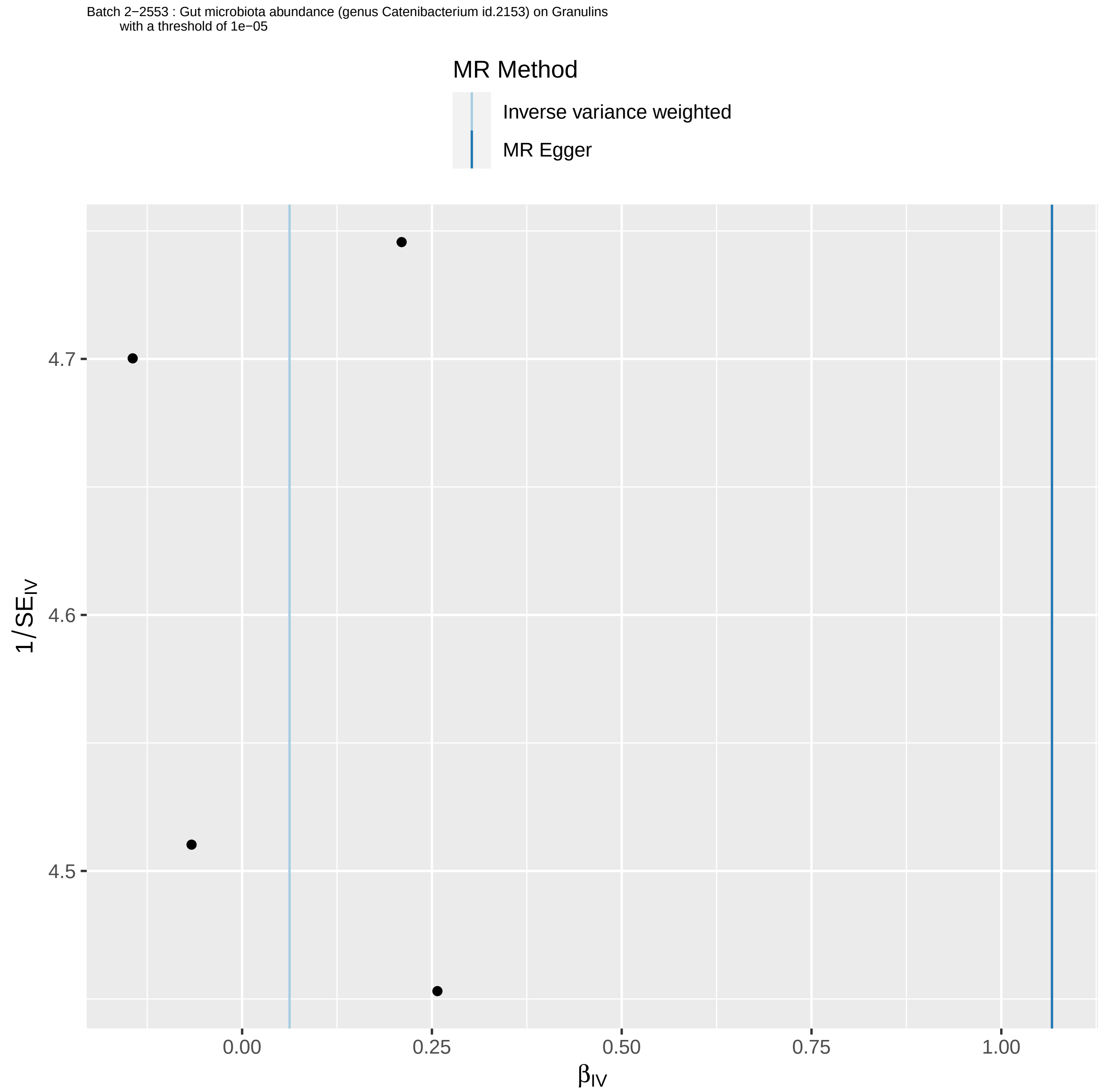
Batch 2−2553 : Gut microbiota abundance (genus Catenibacterium id.2153) on Granulins
with a threshold of 1e−05
MR Method
Inverse variance weighted
MR Egger
4.7
4.6
4.5
$1/SE_{IV}$
0.00
0.25
0.50
0.75
1.00
β_{IV}

Batch 2−2554 : Gut microbiota abundance (genus Parasutterella id.2892) on Granulins
with a threshold of 1e−05

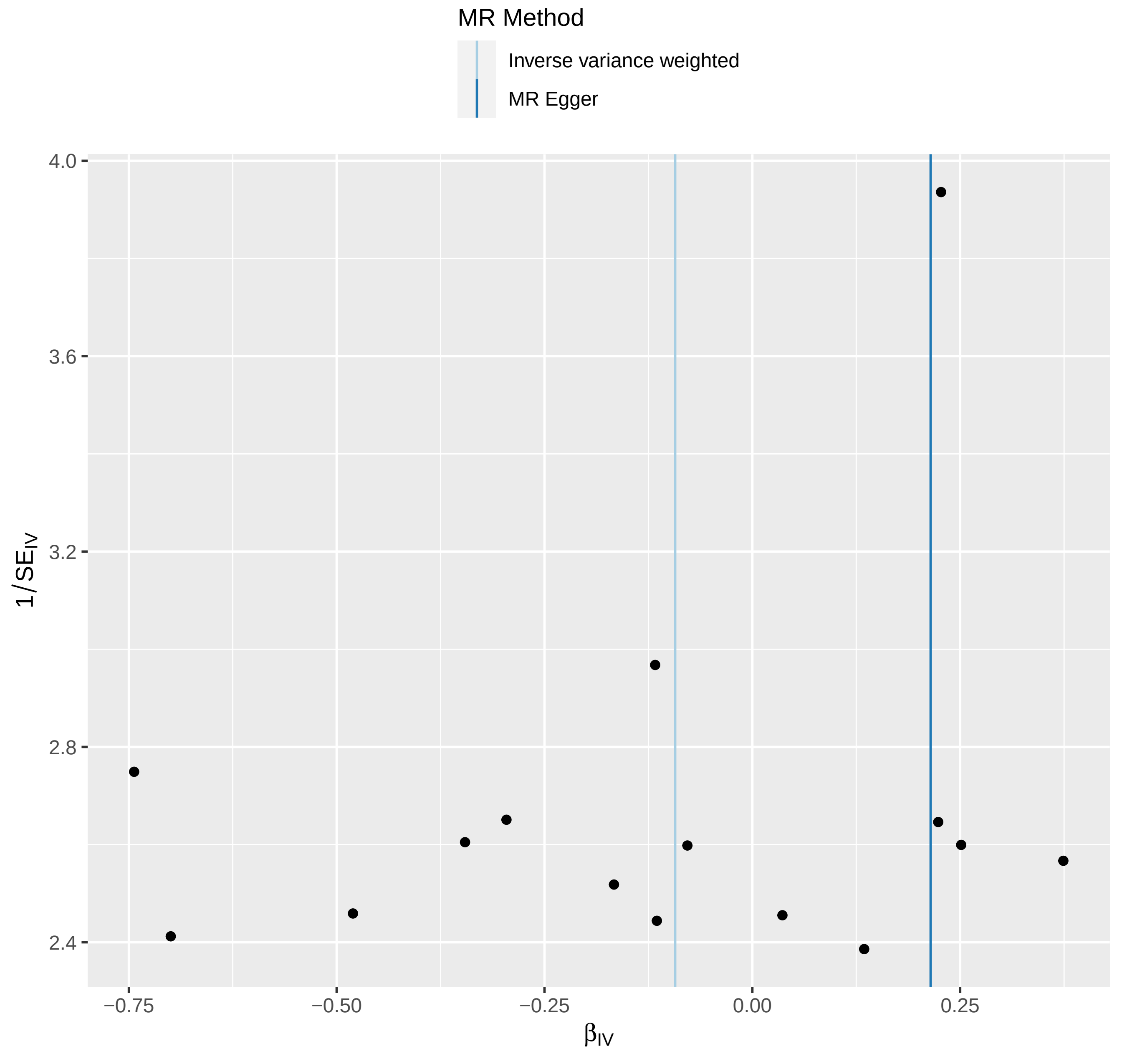

Batch 2−2555 : Gut microbiota abundance (genus Catenibacterium id.2153) on Gastrin−releasing peptide
with a threshold of 1e−05

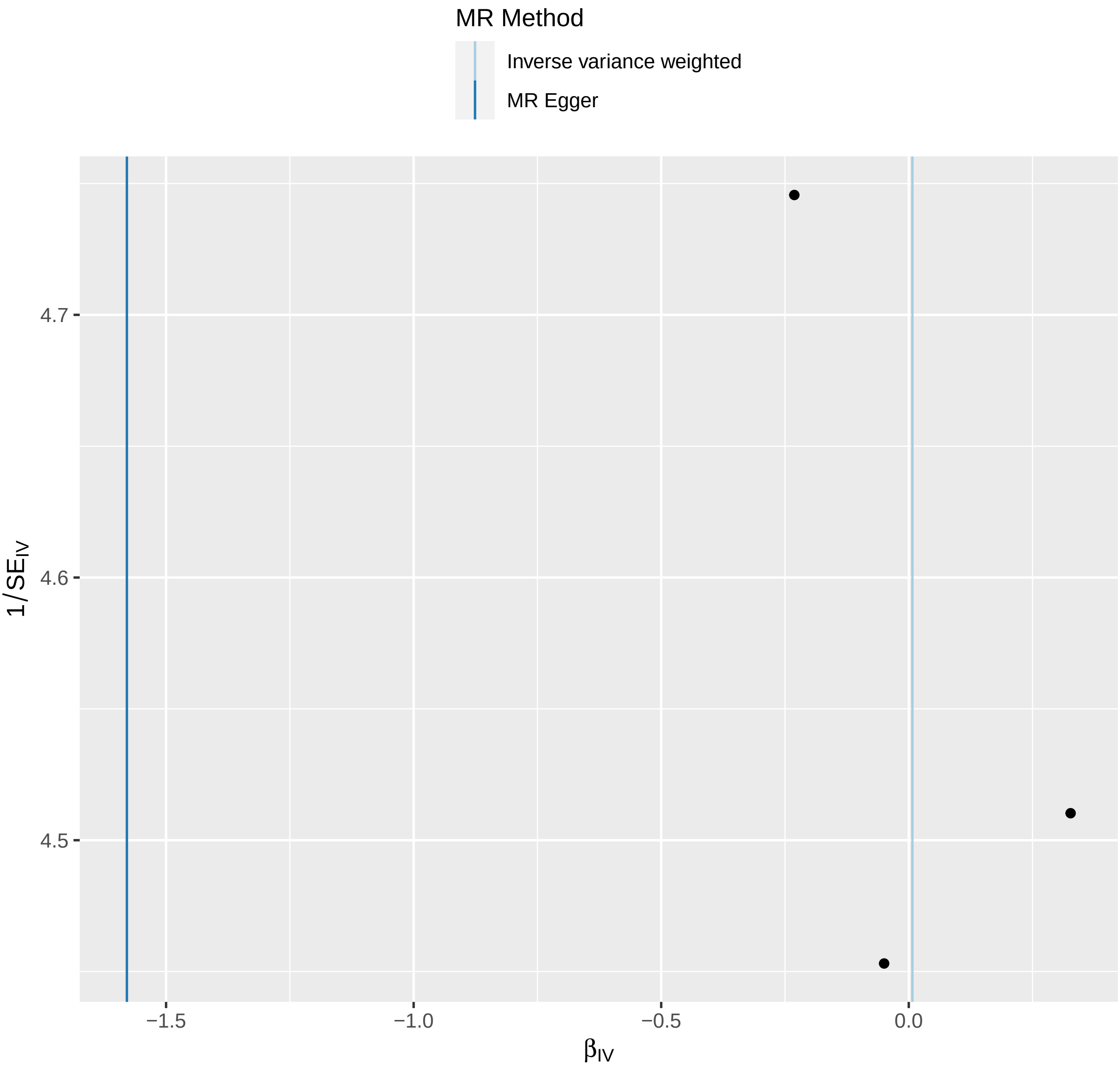

Batch 2−2556 : Gut microbiota abundance (genus Parasutterella id.2892) on Gastrin−releasing peptide
with a threshold of 1e−05

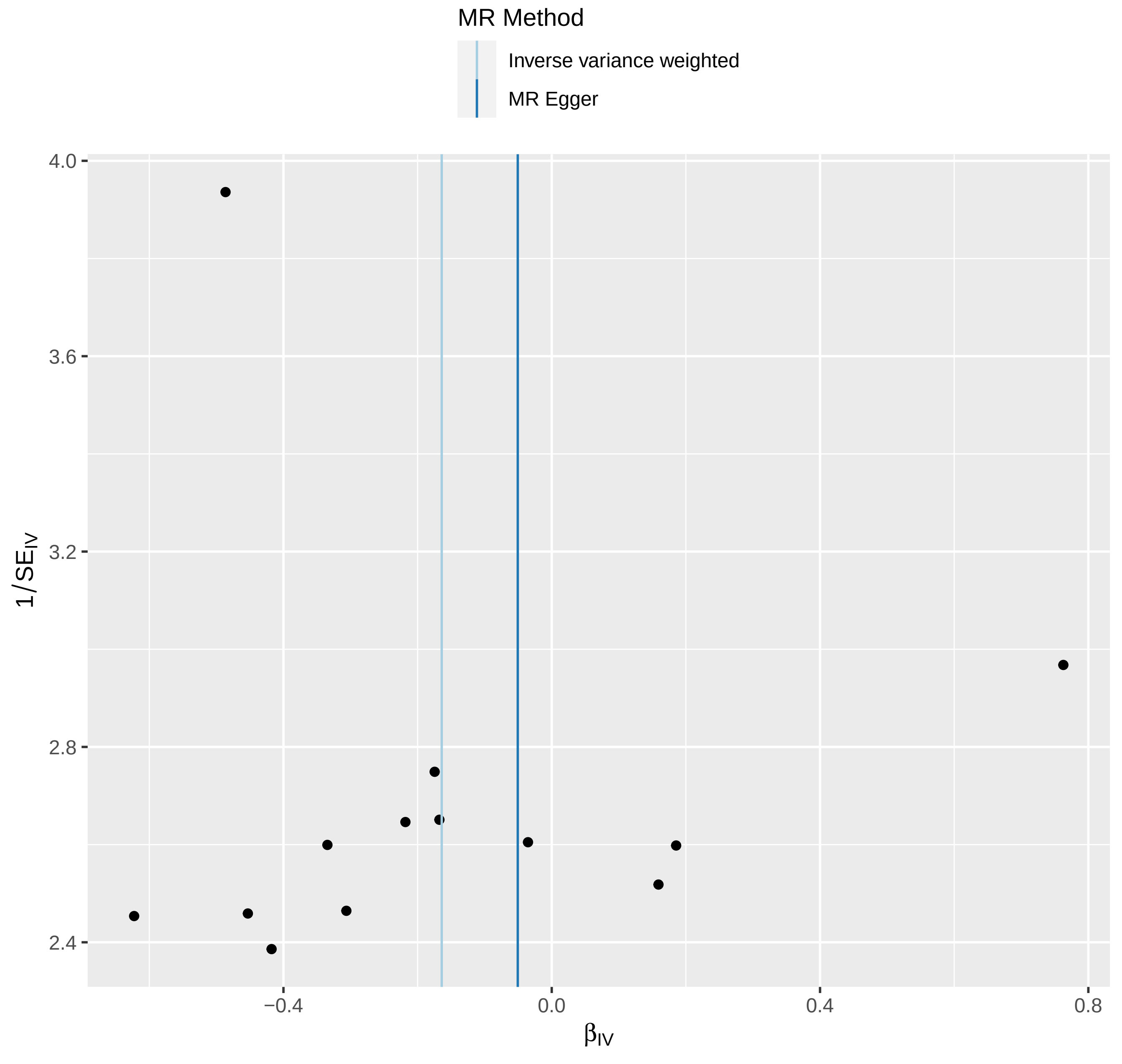

Batch 2−2557 : Gut microbiota abundance (genus Catenibacterium id.2153) on Gastrin−releasing peptide
with a threshold of 1e−05

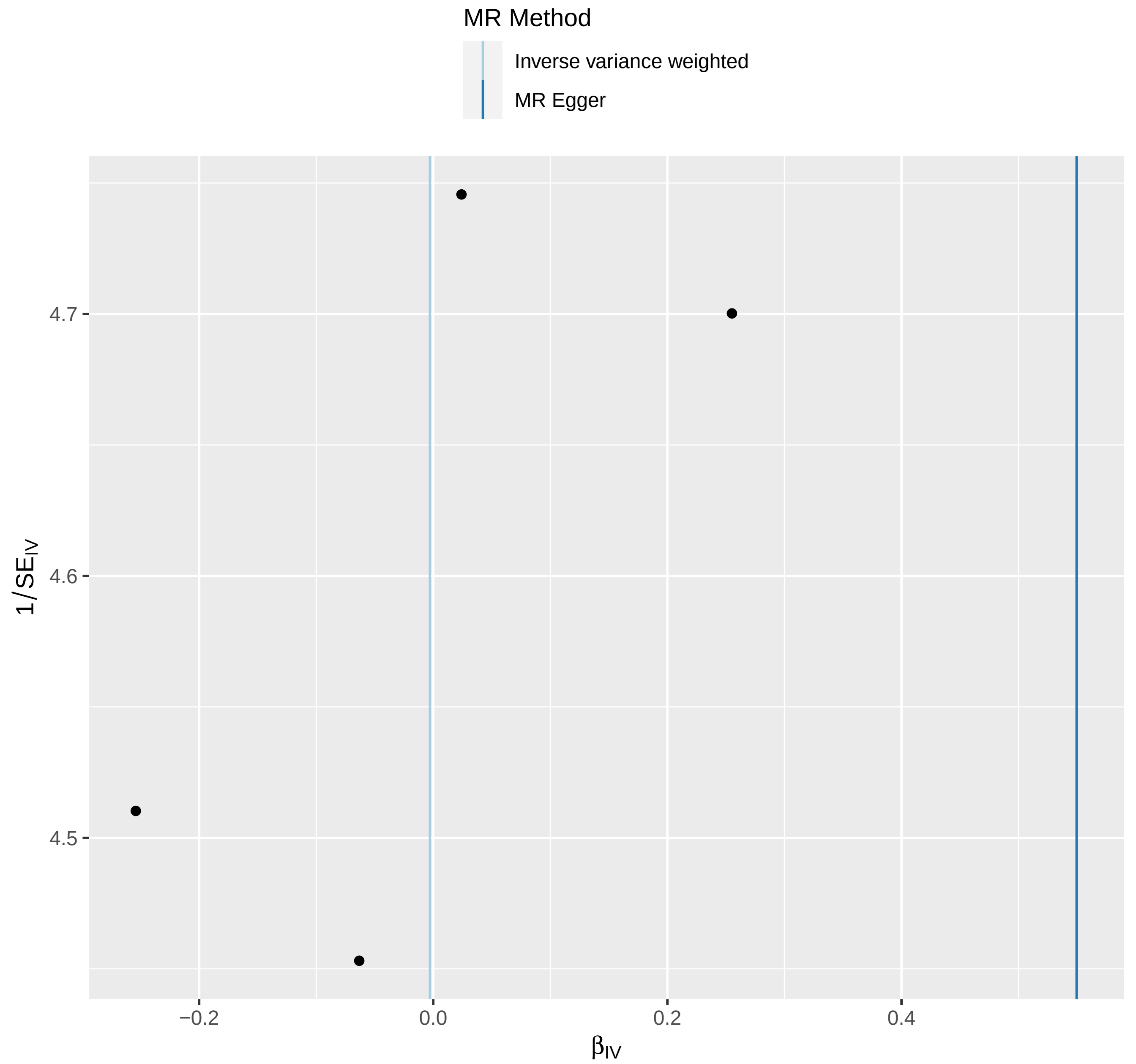

Batch 2−2558 : Gut microbiota abundance (genus Parasutterella id.2892) on Gastrin−releasing peptide
with a threshold of 1e−05

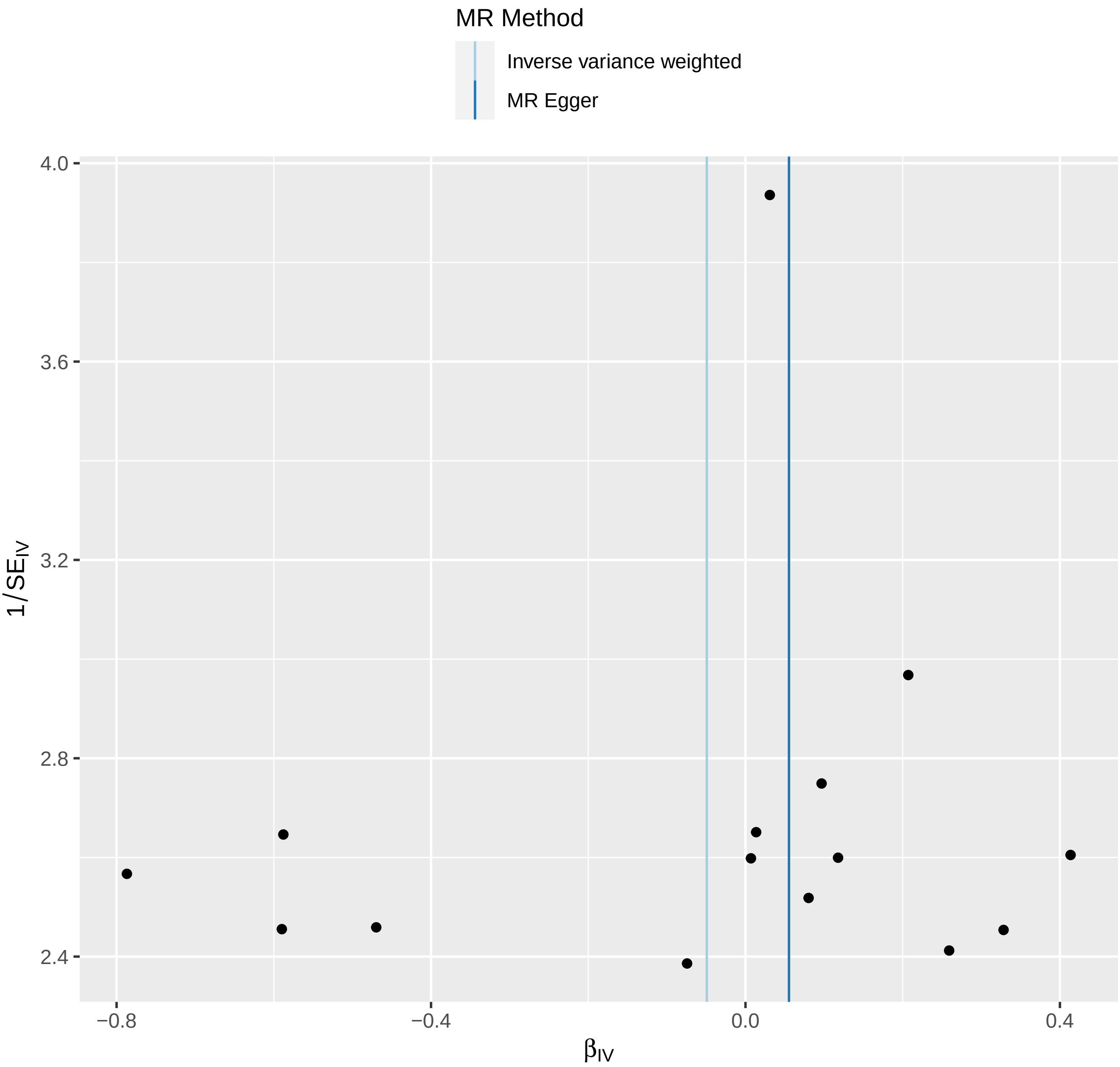

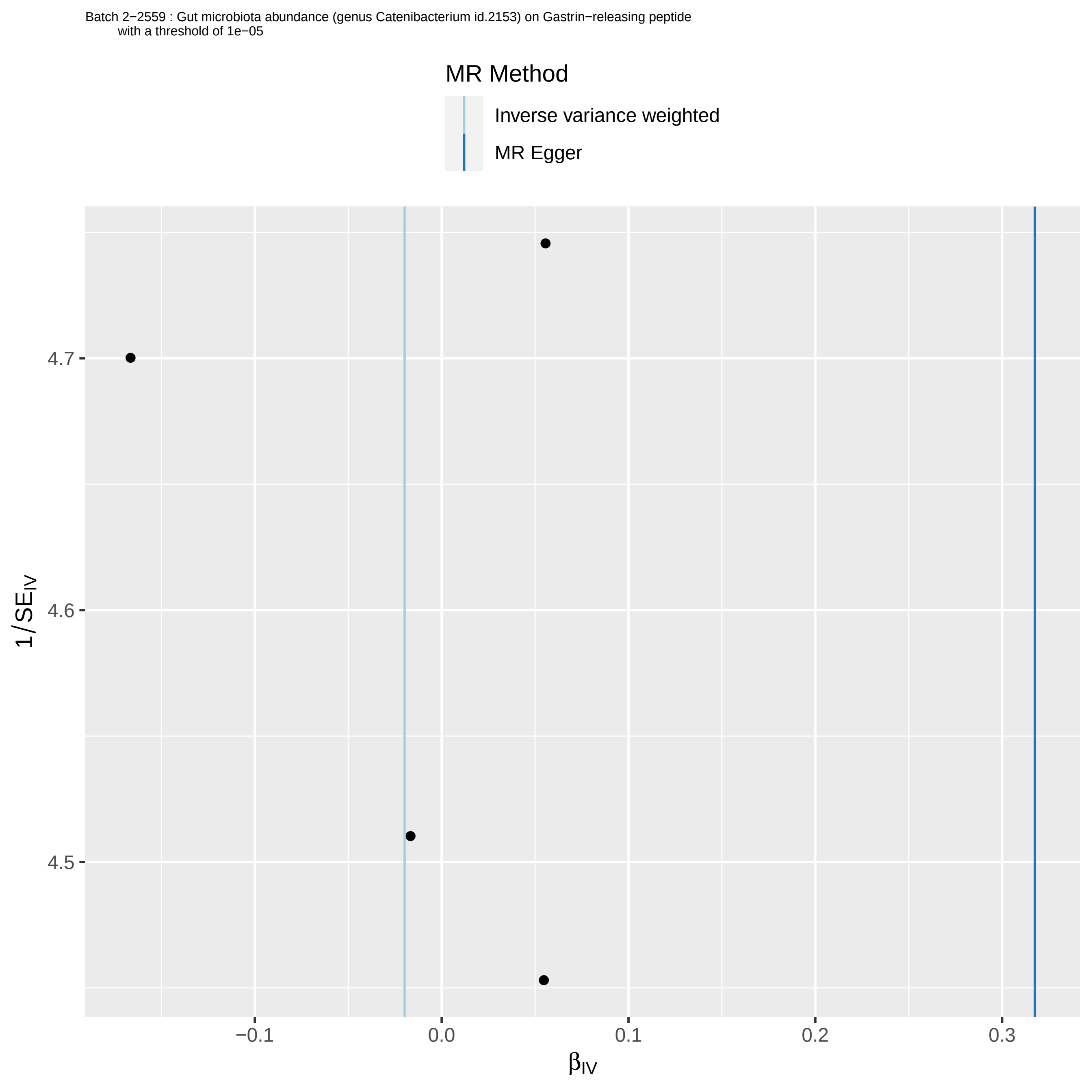

Batch 2−2559 : Gut microbiota abundance (genus Catenibacterium id.2153) on Gastrin−releasing peptide
with a threshold of 1e−05
MR Method
Inverse variance weighted
MR Egger
4.7
4.6
4.5
1/SE_IV
−0.1
0.0
0.1
0.2
0.3
β_IV

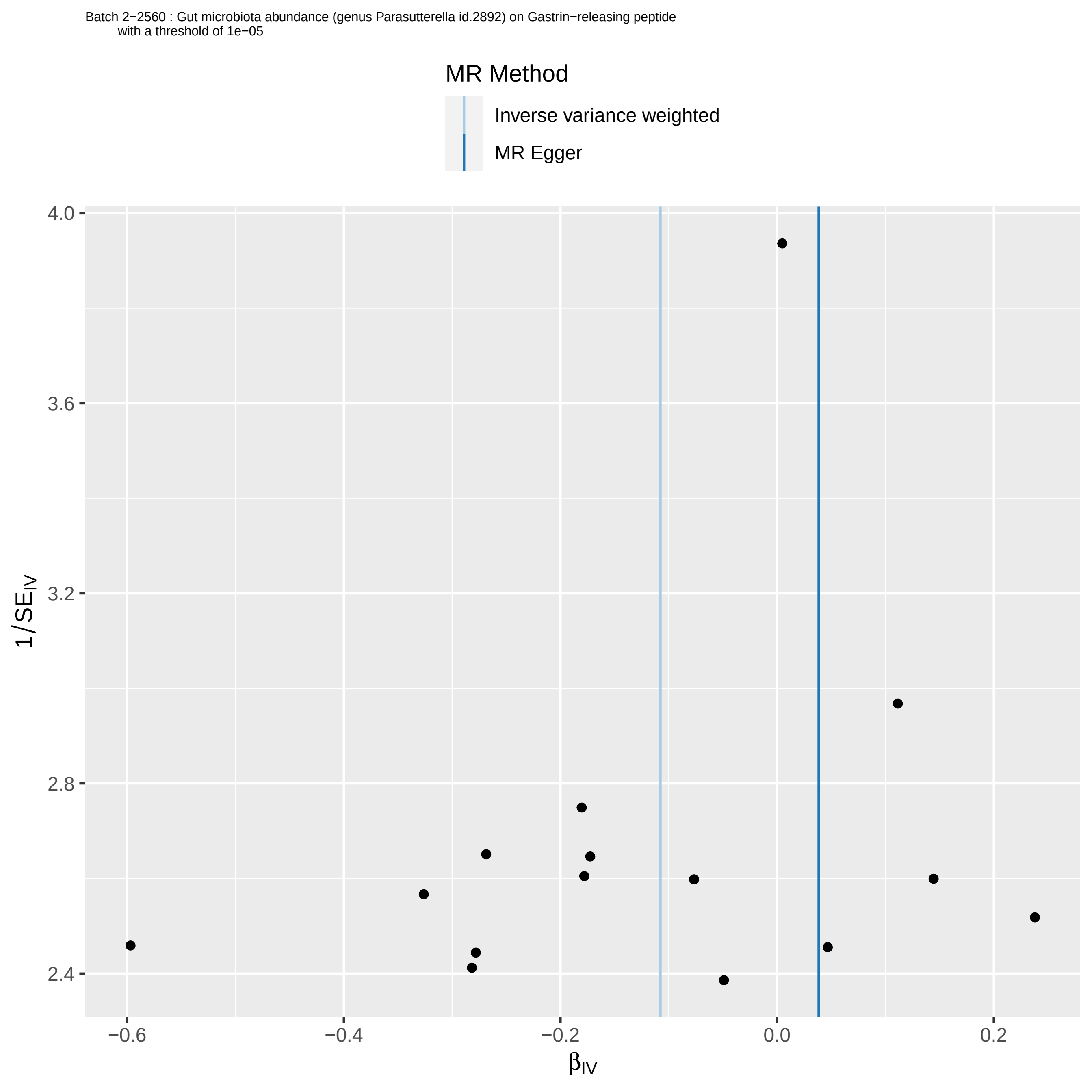
Batch 2−2560 : Gut microbiota abundance (genus Parasutterella id.2892) on Gastrin−releasing peptide
with a threshold of 1e−05
MR Method
Inverse variance weighted
MR Egger
1/SE_IV
4.0
3.6
3.2
2.8
2.4
−0.6
−0.4
−0.2
0.0
0.2
β_IV

Batch 2−2561 : Gut microbiota abundance (genus Catenibacterium id.2153) on GrpE protein homolog 1, mitochondrial with a threshold of 1e−05

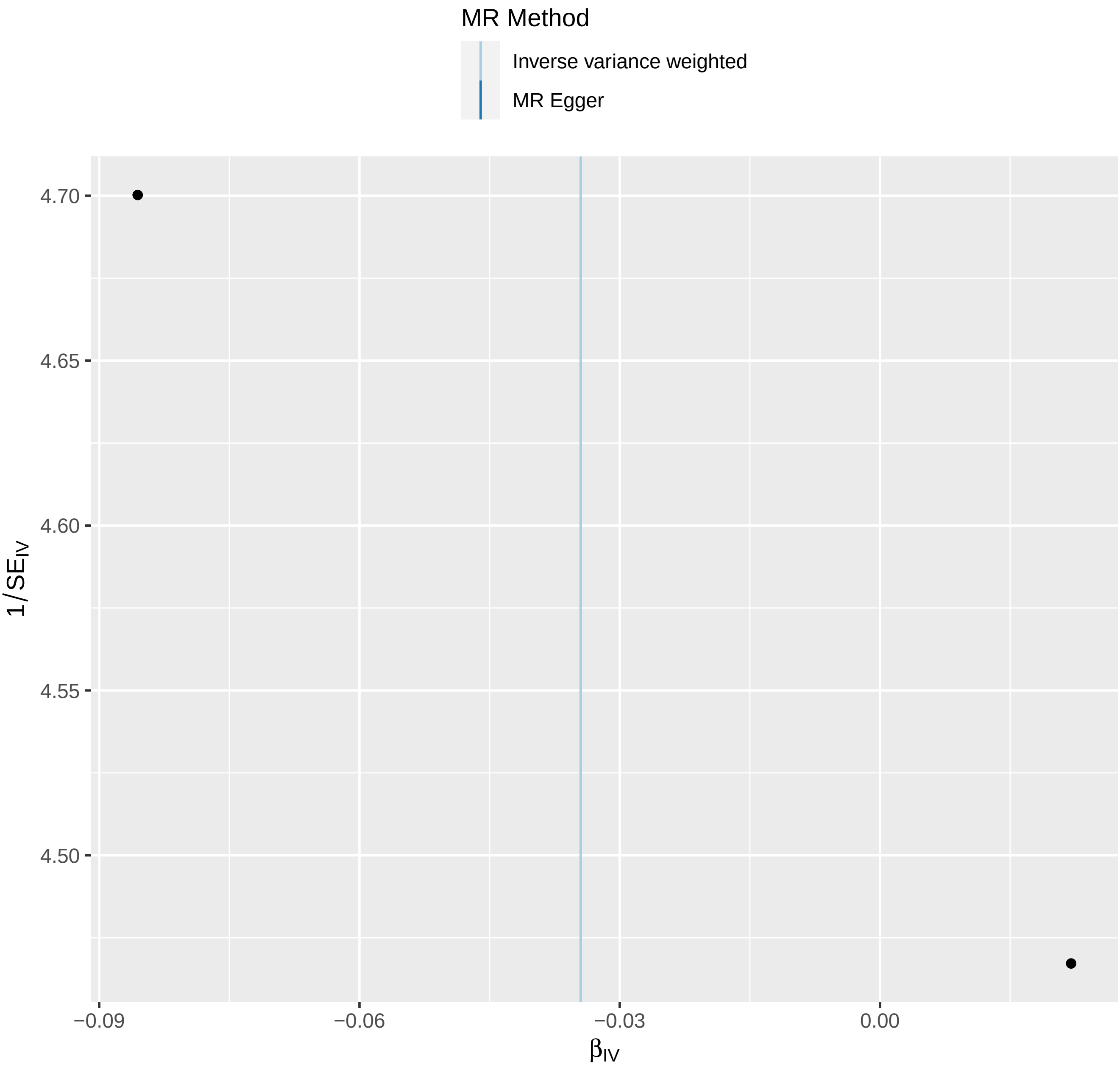

Batch 2−2562 : Gut microbiota abundance (genus Parasutterella id.2892) on GrpE protein homolog 1, mitochondrial
with a threshold of 1e−05

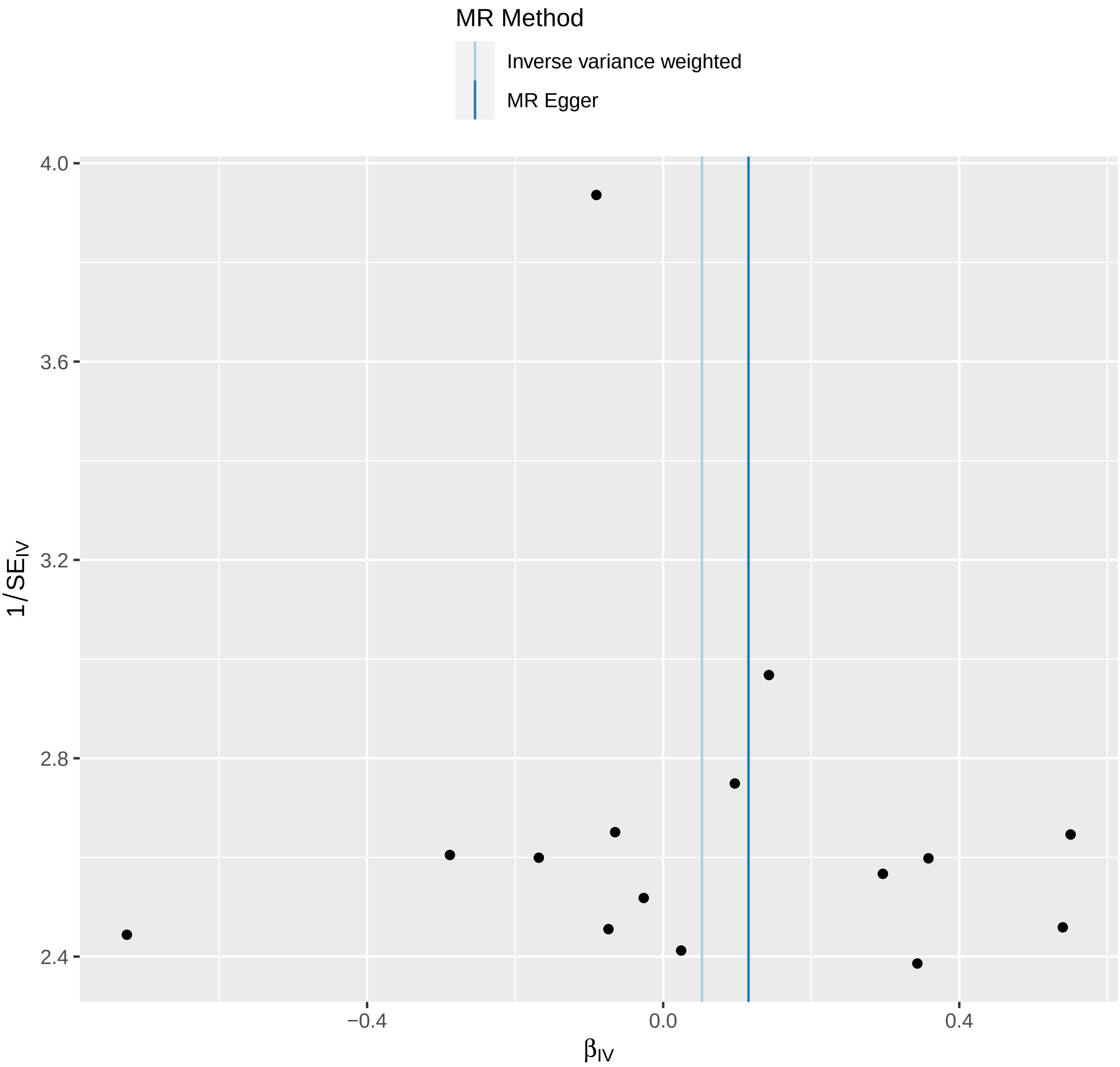

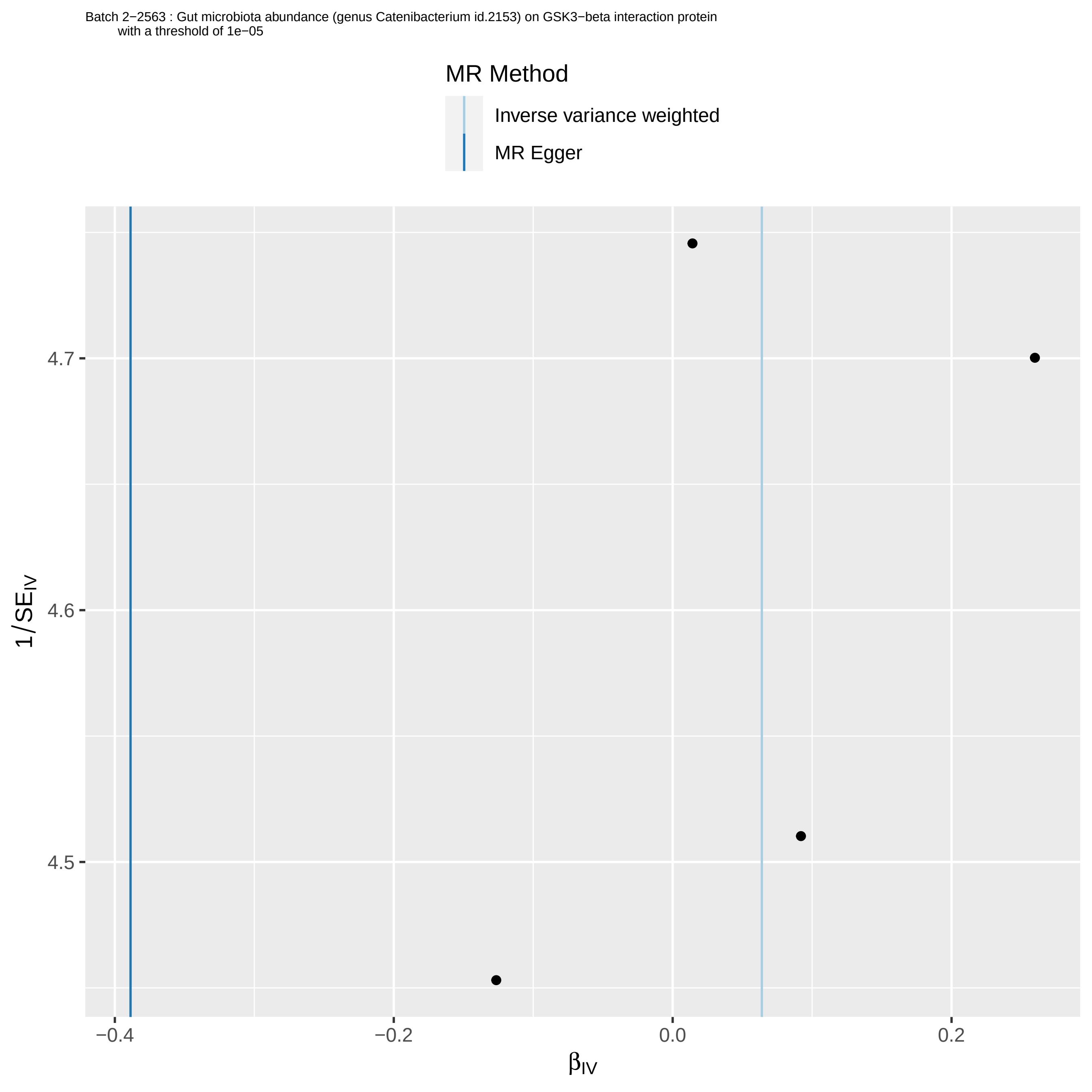

Batch 2−2563 : Gut microbiota abundance (genus Catenibacterium id.2153) on GSK3−beta interaction protein
with a threshold of 1e−05
MR Method
Inverse variance weighted
MR Egger
4.7
4.6
4.5
1/SE_IV
−0.4
−0.2
0.0
0.2
β_IV

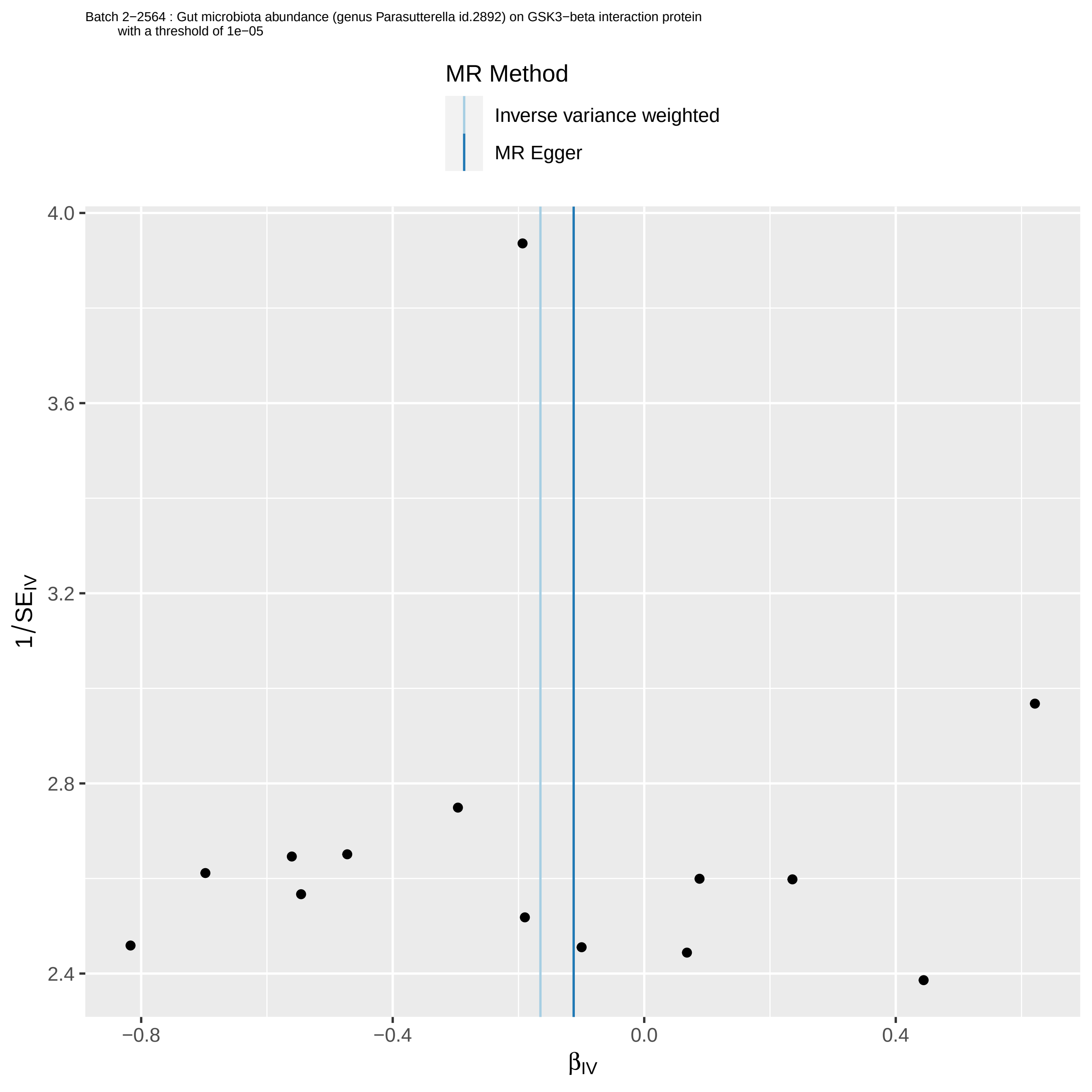
Batch 2−2564 : Gut microbiota abundance (genus Parasutterella id.2892) on GSK3−beta interaction protein
with a threshold of 1e−05
MR Method
Inverse variance weighted
MR Egger
4.0
3.6
3.2
2.8
2.4
1/SE_IV
−0.8
−0.4
0.0
0.4
β_IV

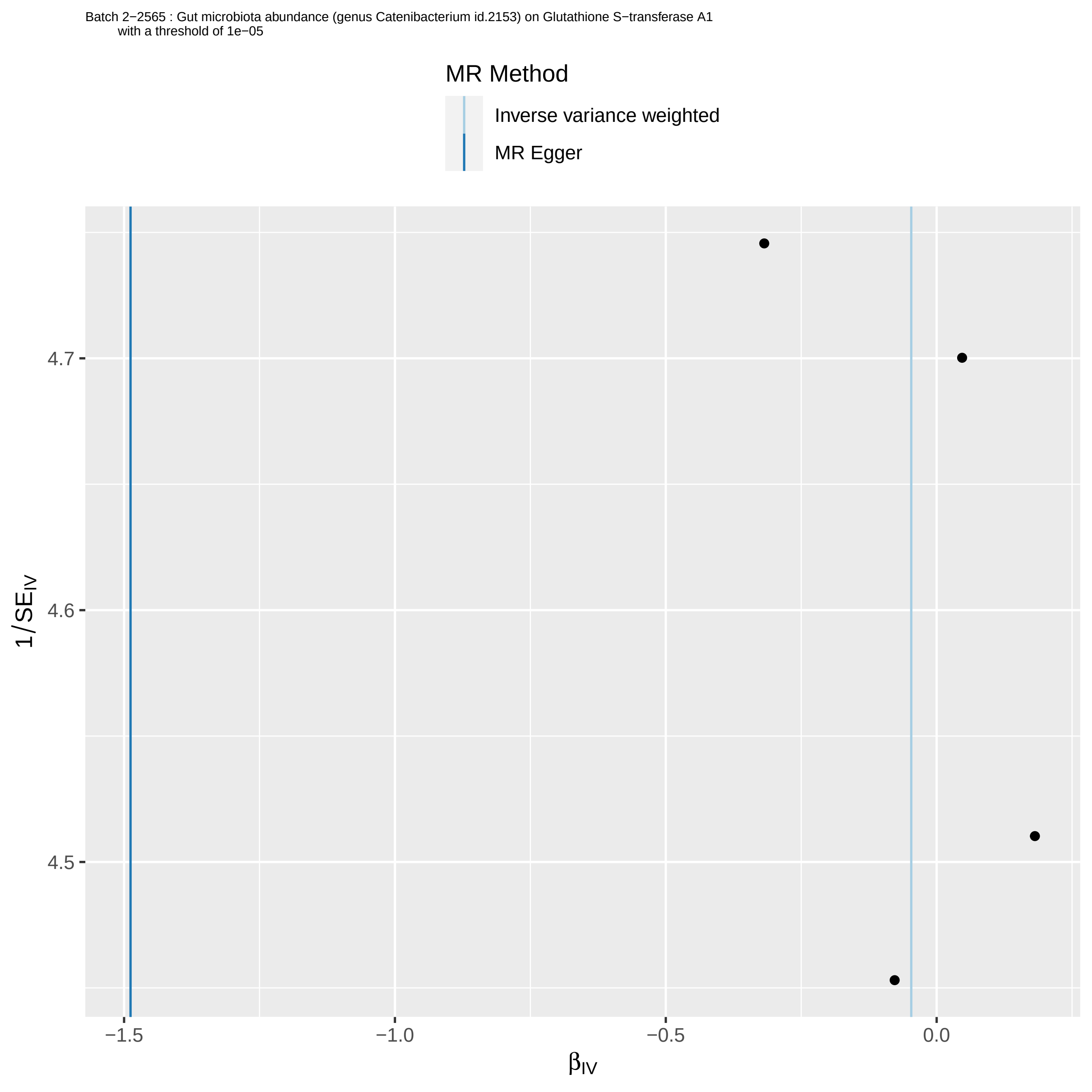
Batch 2−2565 : Gut microbiota abundance (genus Catenibacterium id.2153) on Glutathione S−transferase A1
with a threshold of 1e−05
MR Method
Inverse variance weighted
MR Egger
4.7
4.6
4.5
1/SE_IV
−1.5
−1.0
−0.5
0.0
β_IV

Batch 2−2566 : Gut microbiota abundance (genus Parasutterella id.2892) on Glutathione S−transferase A1
with a threshold of 1e−05

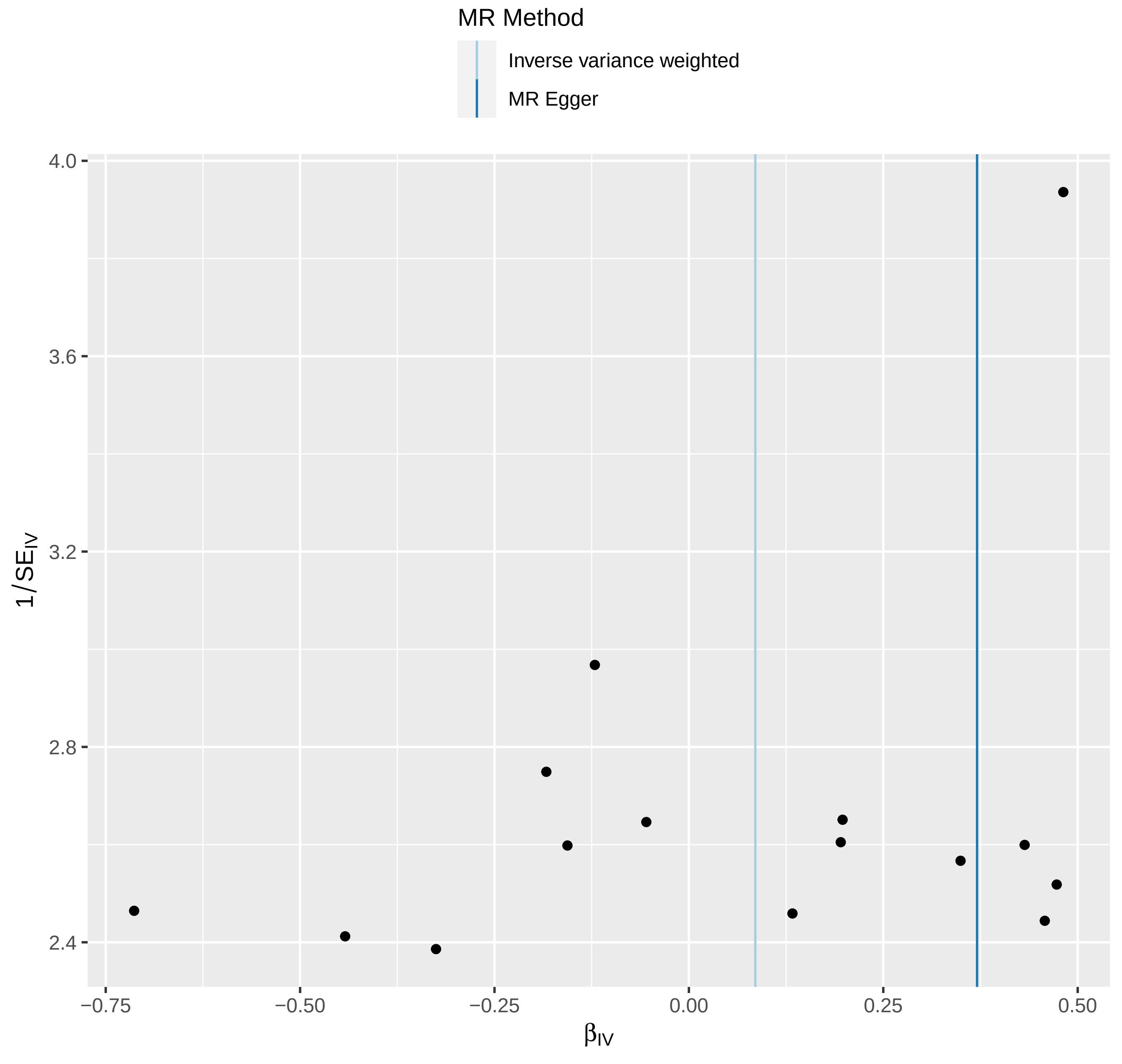

Batch 2−2567 : Gut microbiota abundance (genus Catenibacterium id.2153) on Glutathione S−transferase A3
with a threshold of 1e−05

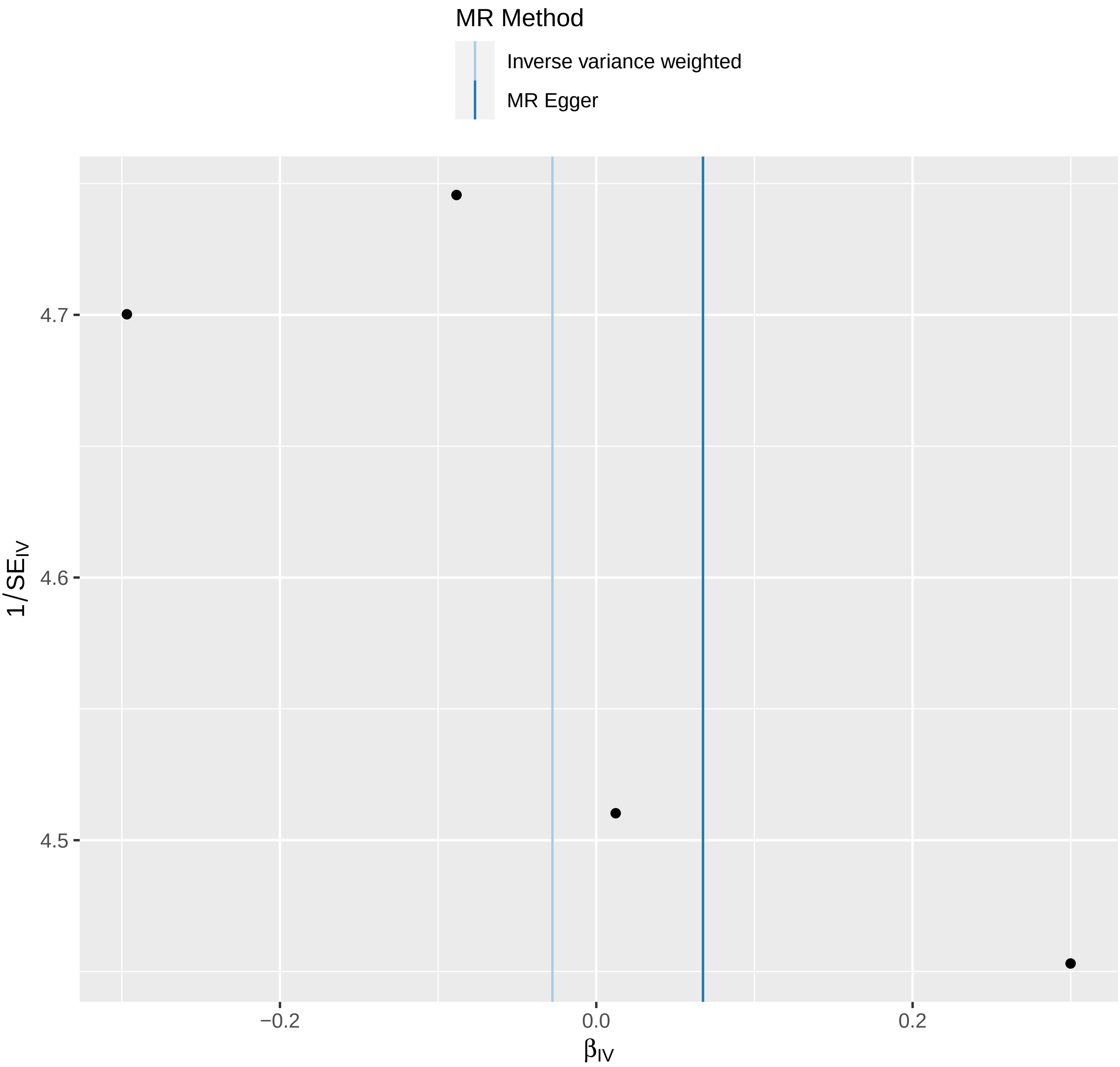

Batch 2−2568 : Gut microbiota abundance (genus Parasutterella id.2892) on Glutathione S−transferase A3
with a threshold of 1e−05

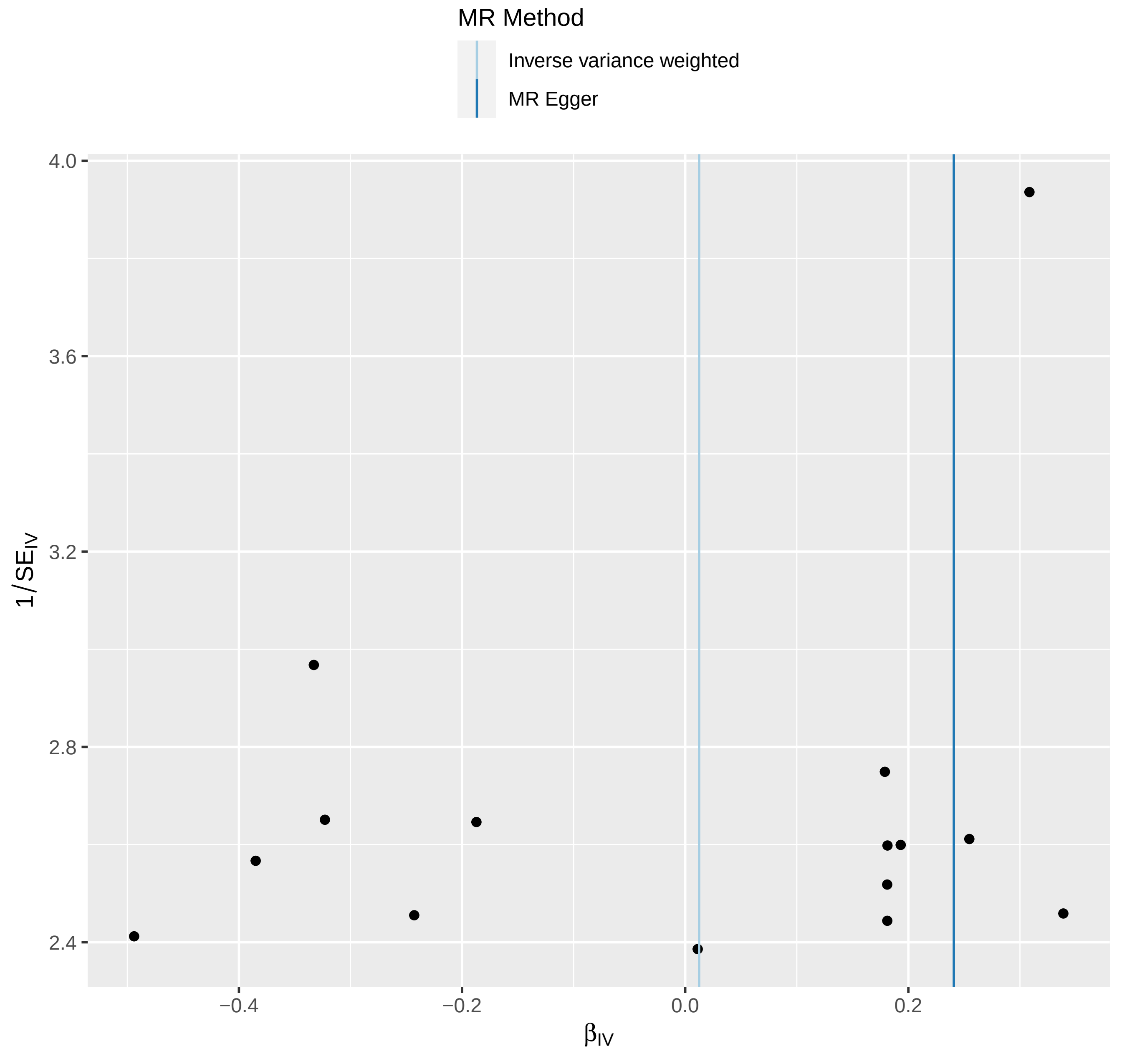

Batch 2−2569 : Gut microbiota abundance (genus Catenibacterium id.2153) on Glutathione S−transferase A4
with a threshold of 1e−05

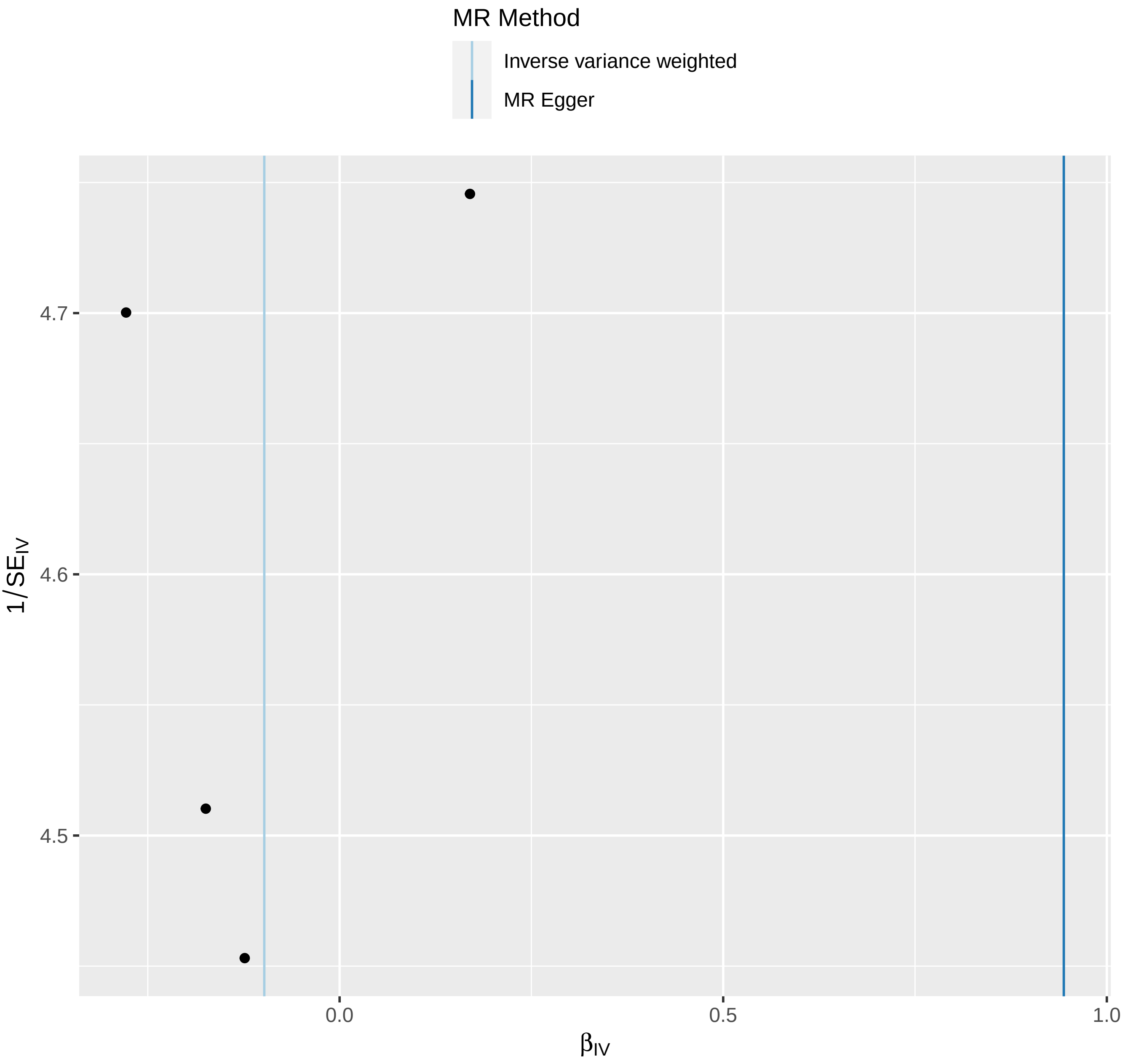

Batch 2−2570 : Gut microbiota abundance (genus Parasutterella id.2892) on Glutathione S−transferase A4
with a threshold of 1e−05

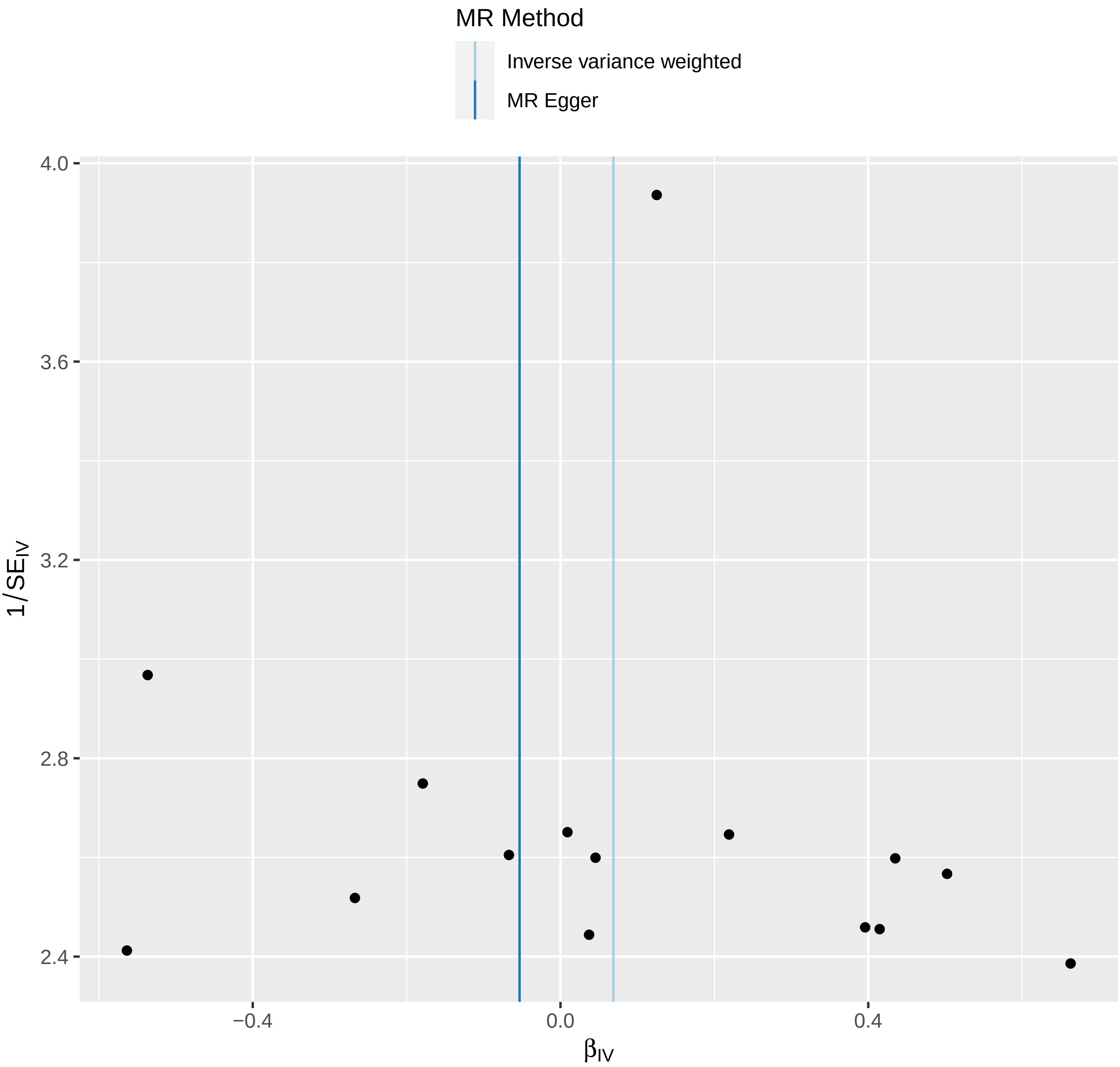

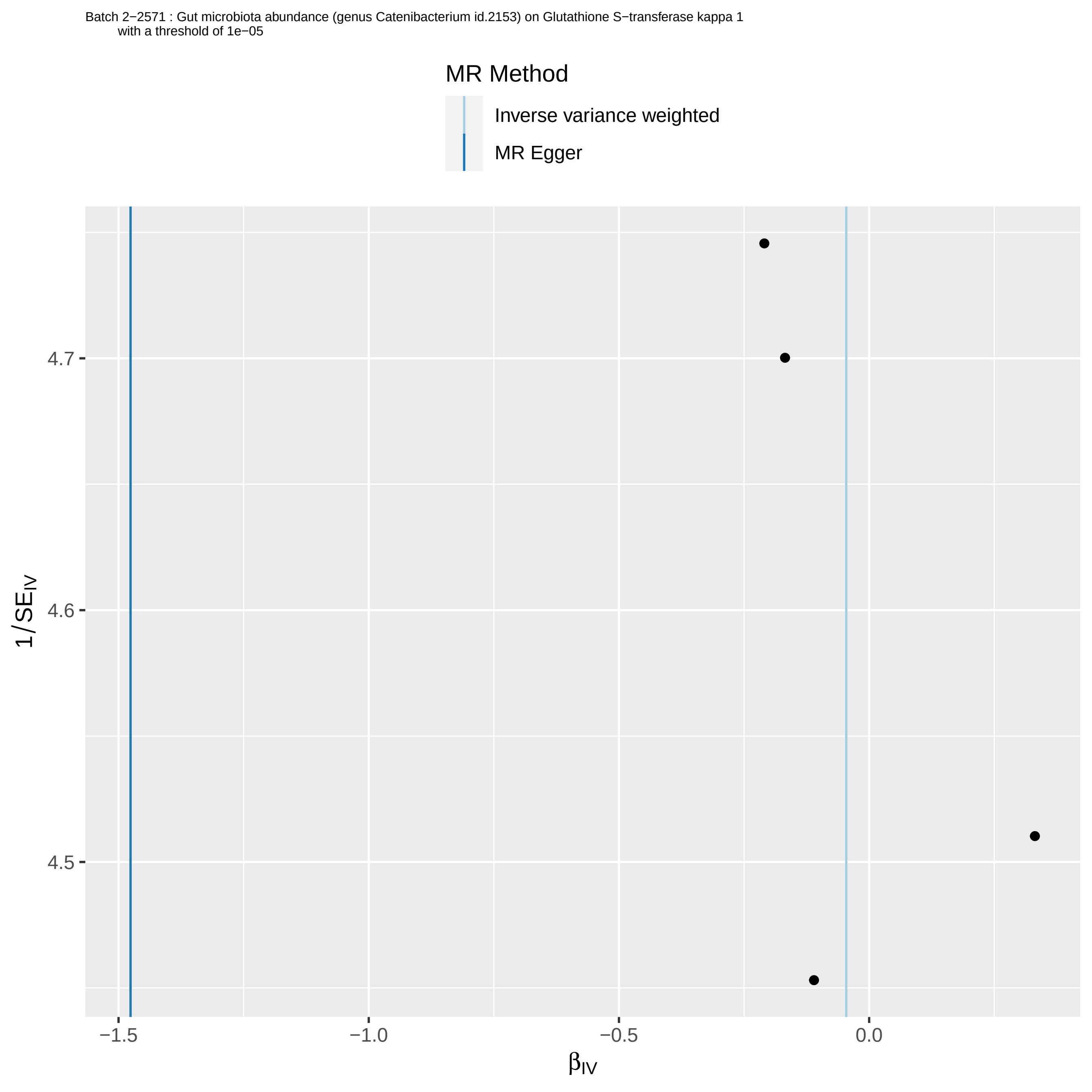

Batch 2−2571 : Gut microbiota abundance (genus Catenibacterium id.2153) on Glutathione S−transferase kappa 1
with a threshold of 1e−05
MR Method
Inverse variance weighted
MR Egger
4.7
4.6
4.5
$1/SE_{IV}$
−1.5
−1.0
−0.5
0.0
β_{IV}

Batch 2−2572 : Gut microbiota abundance (genus Parasutterella id.2892) on Glutathione S−transferase kappa 1
with a threshold of 1e−05

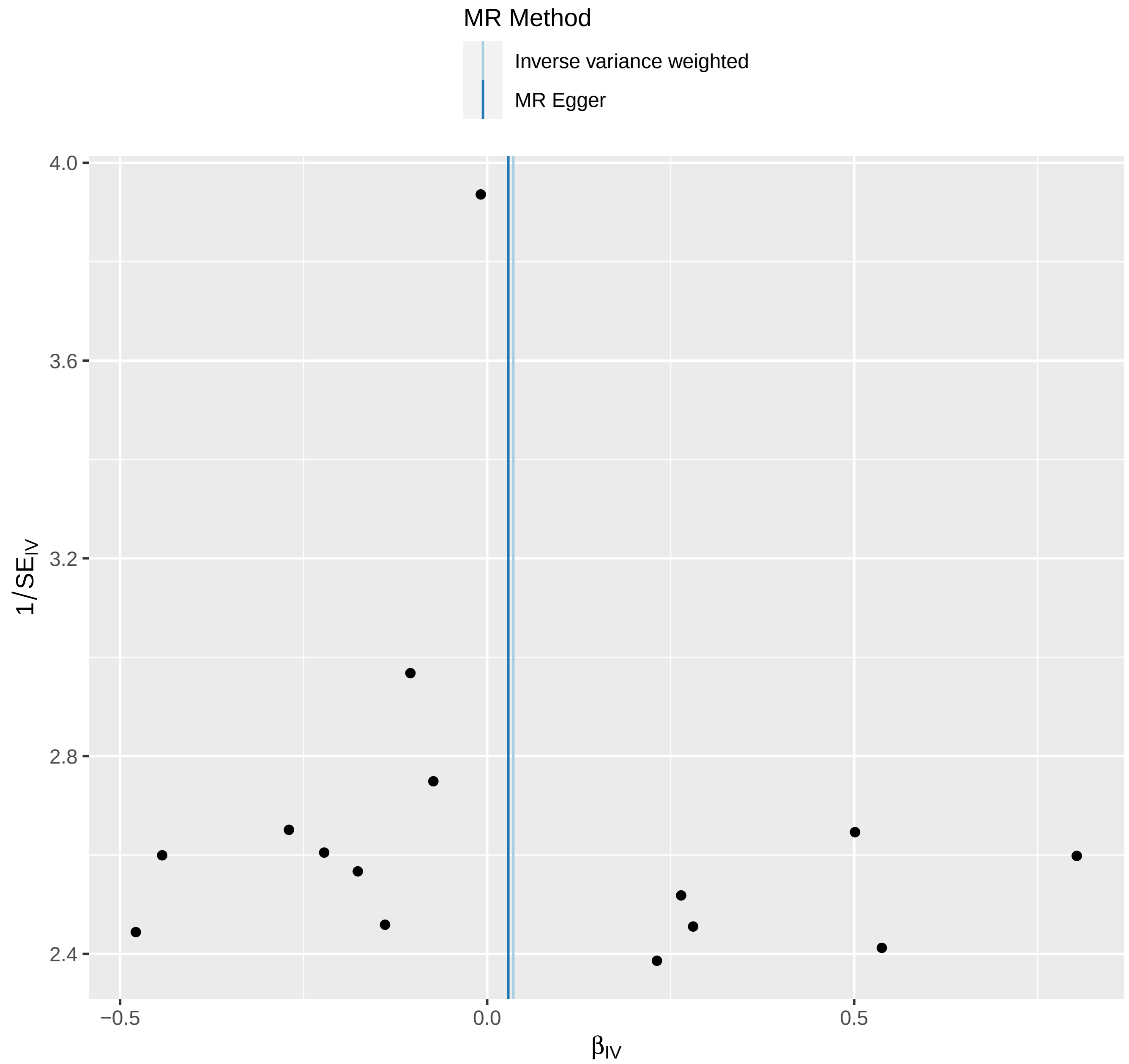

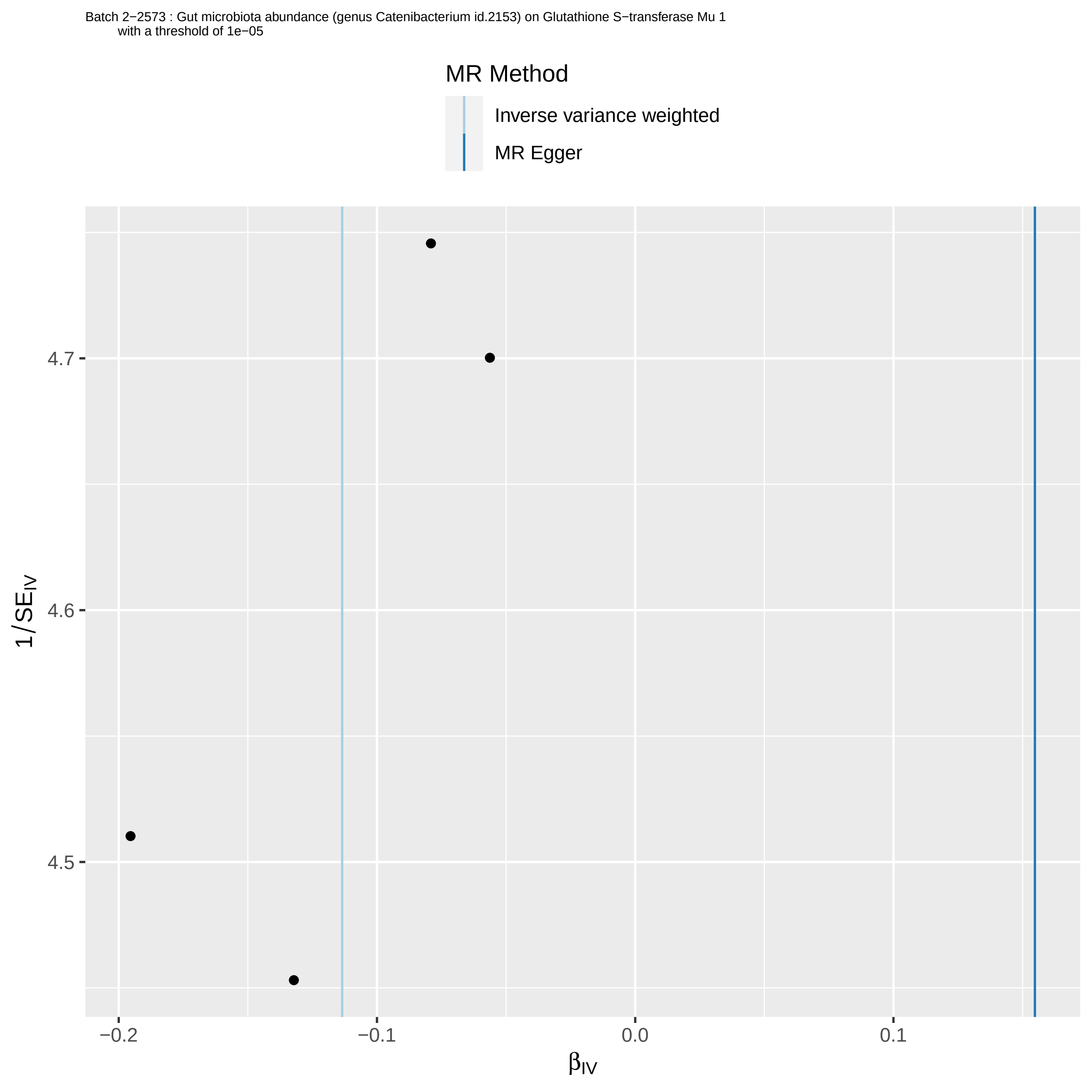
Batch 2−2573 : Gut microbiota abundance (genus Catenibacterium id.2153) on Glutathione S−transferase Mu 1
with a threshold of 1e−05
MR Method
Inverse variance weighted
MR Egger
4.7
4.6
4.5
1/SE_IV
−0.2
−0.1
0.0
0.1
β_IV

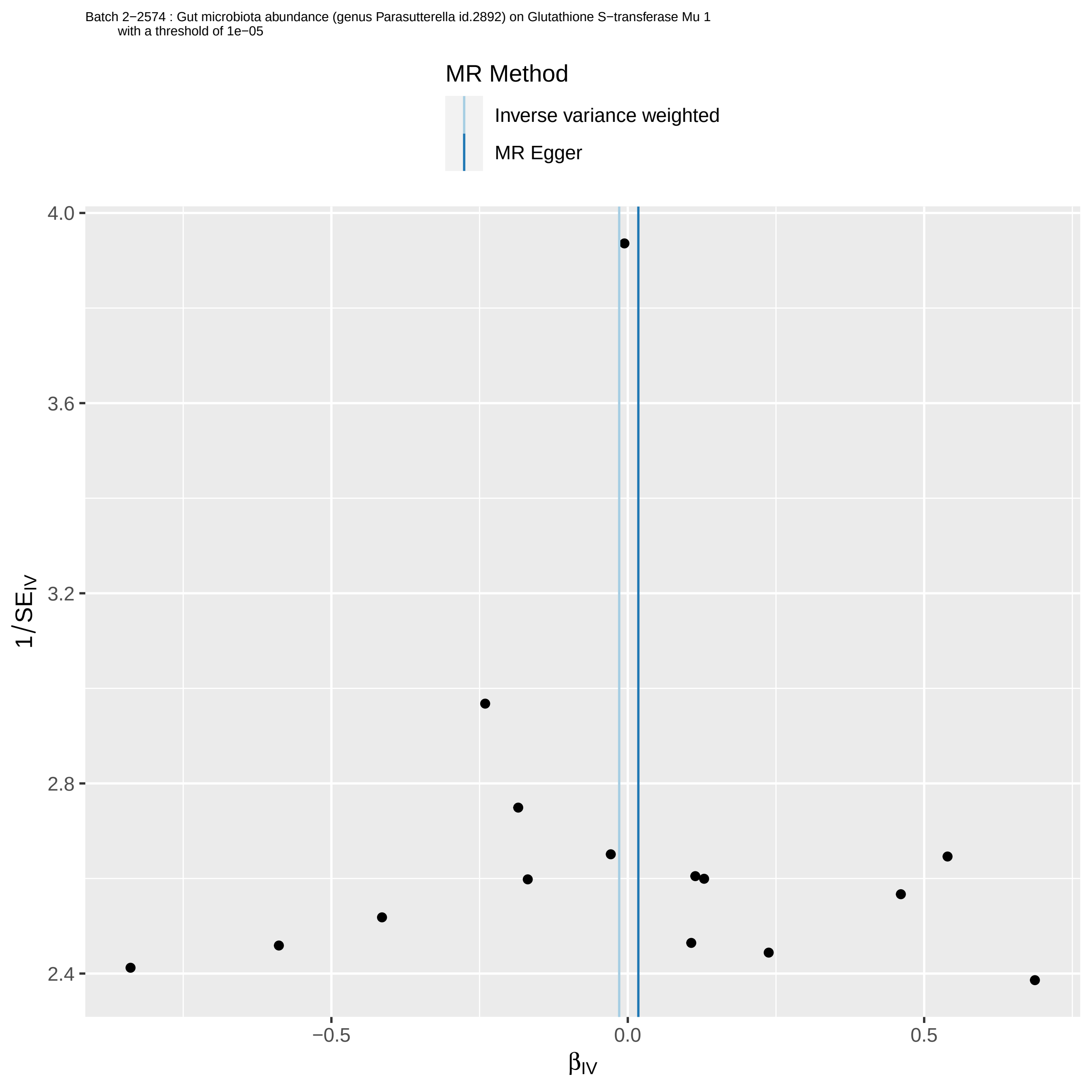
Batch 2−2574 : Gut microbiota abundance (genus Parasutterella id.2892) on Glutathione S−transferase Mu 1
with a threshold of 1e−05
MR Method
Inverse variance weighted
MR Egger
4.0
3.6
3.2
2.8
2.4
1/SE_IV
−0.5
0.0
0.5
β_IV

Batch 2−2575 : Gut microbiota abundance (genus Catenibacterium id.2153) on Glutathione S−transferase omega−1 with a threshold of 1e−05

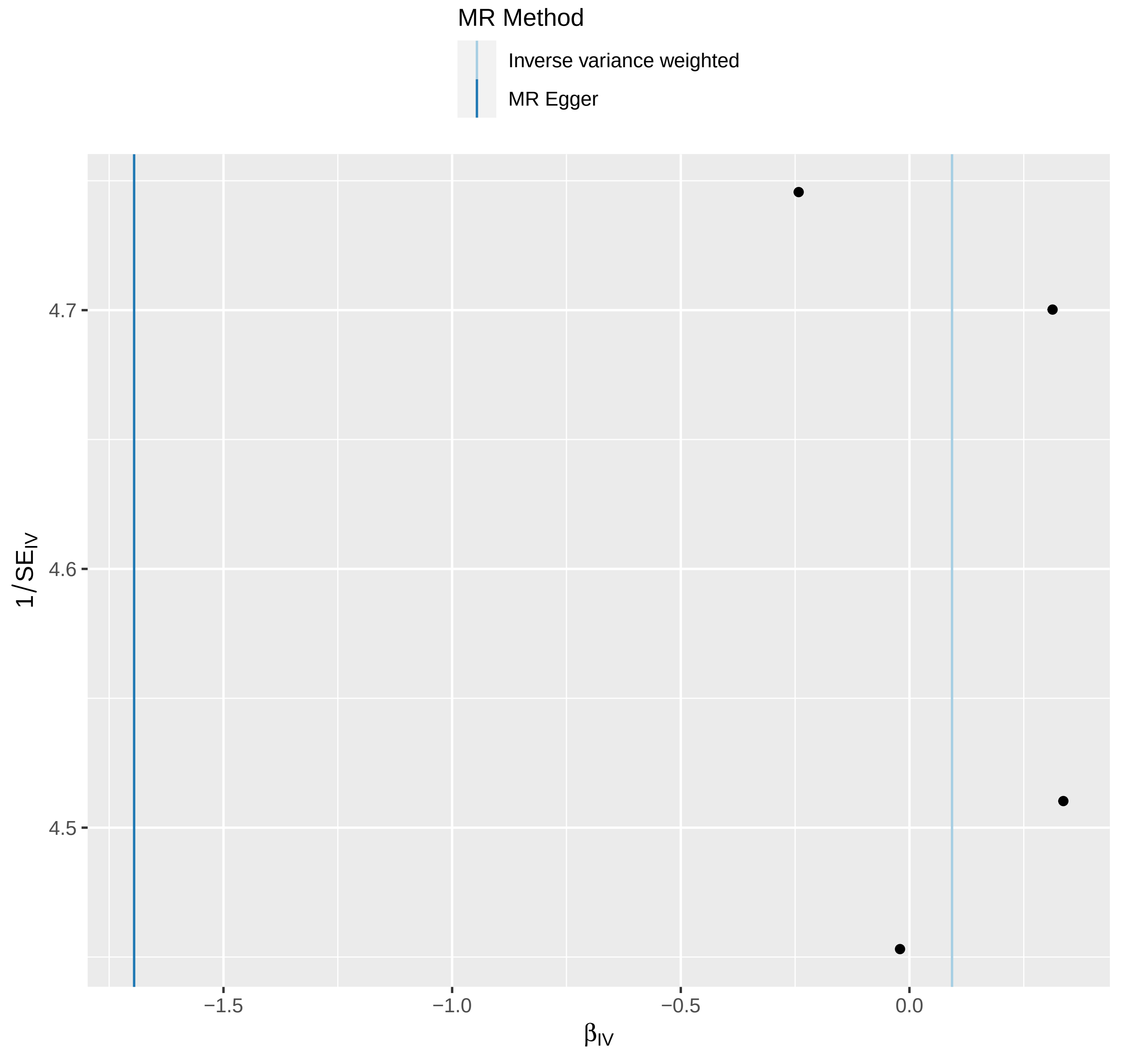

Batch 2−2576 : Gut microbiota abundance (genus Parasutterella id.2892) on Glutathione S−transferase omega−1 with a threshold of 1e−05

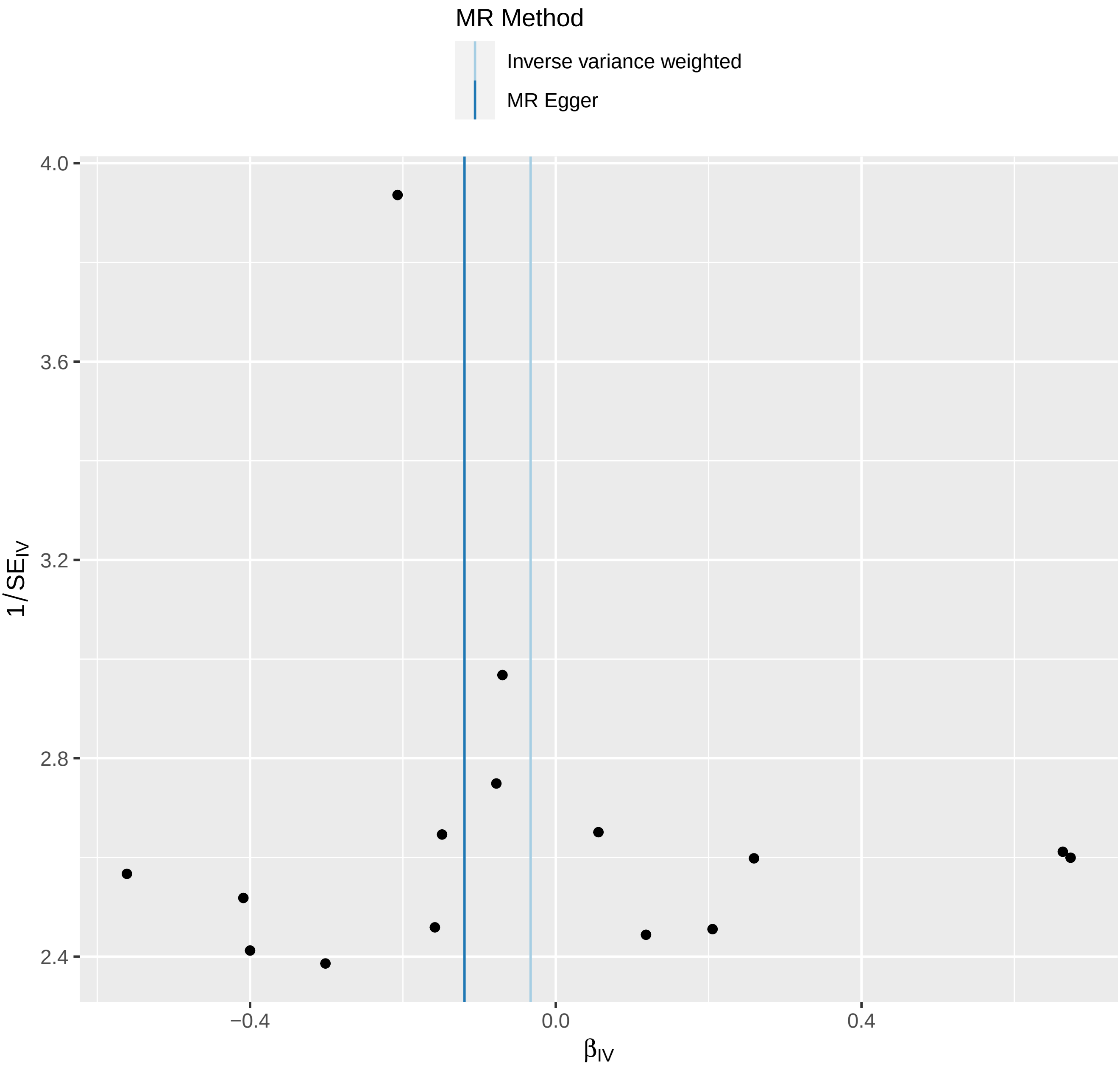

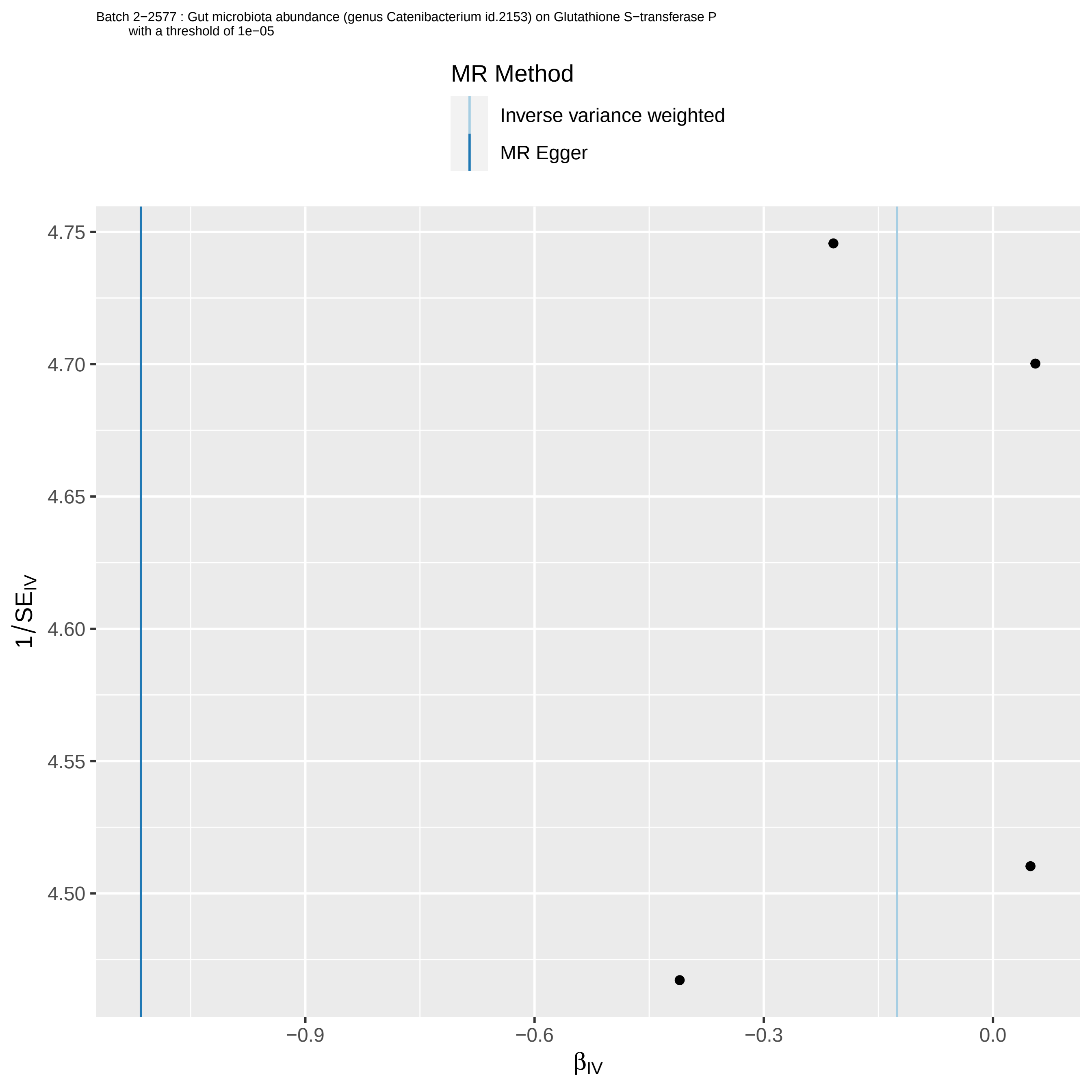
Batch 2−2577 : Gut microbiota abundance (genus Catenibacterium id.2153) on Glutathione S−transferase P
with a threshold of 1e−05
MR Method
Inverse variance weighted
MR Egger
4.75
4.70
4.65
4.60
4.55
4.50
1/SE$_{IV}$
−0.9
−0.6
−0.3
0.0
β$_{IV}$

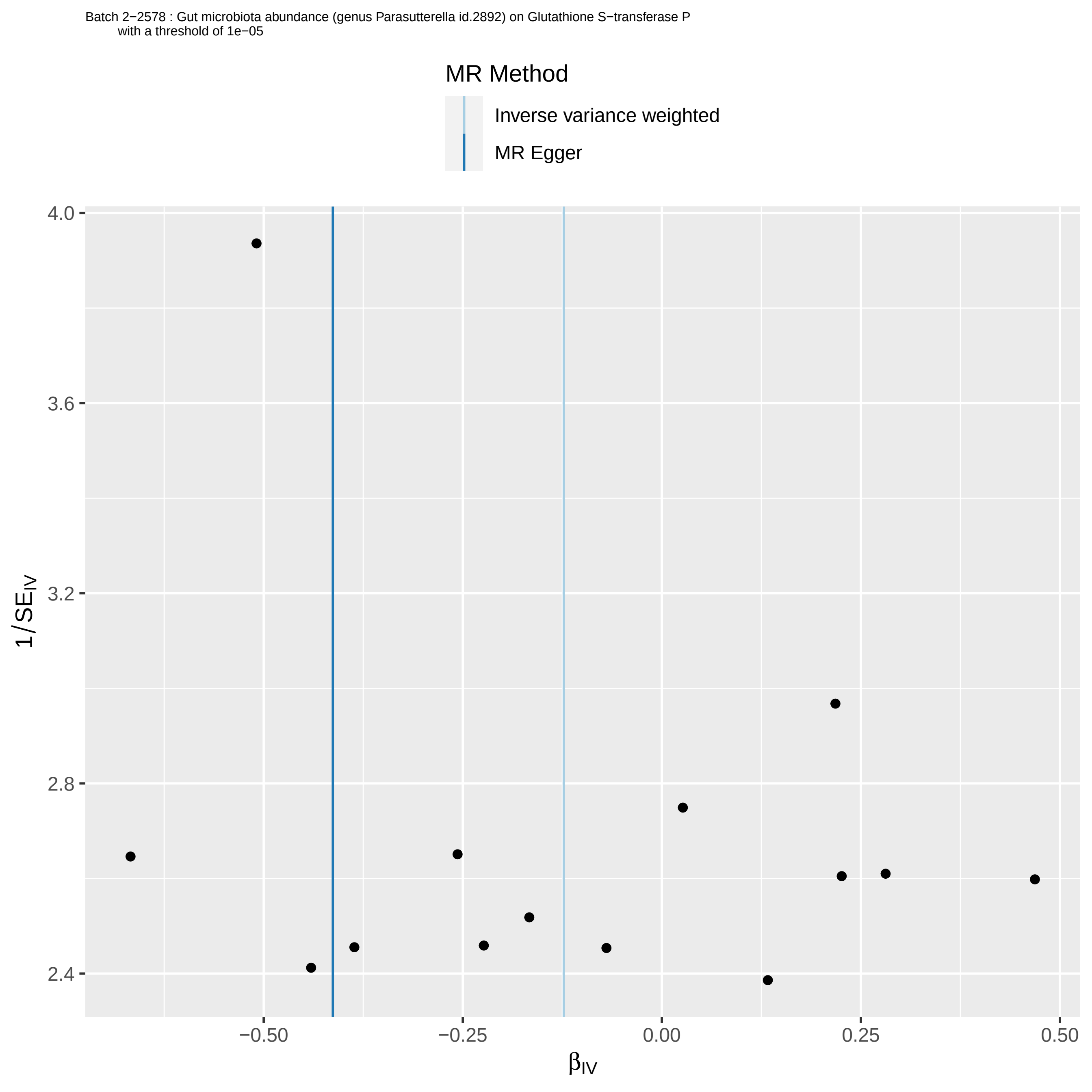
Batch 2−2578 : Gut microbiota abundance (genus Parasutterella id.2892) on Glutathione S−transferase P
with a threshold of 1e−05
MR Method
Inverse variance weighted
MR Egger
4.0
3.6
3.2
2.8
2.4
1/SE_IV
−0.50
−0.25
0.00
0.25
0.50
β_IV

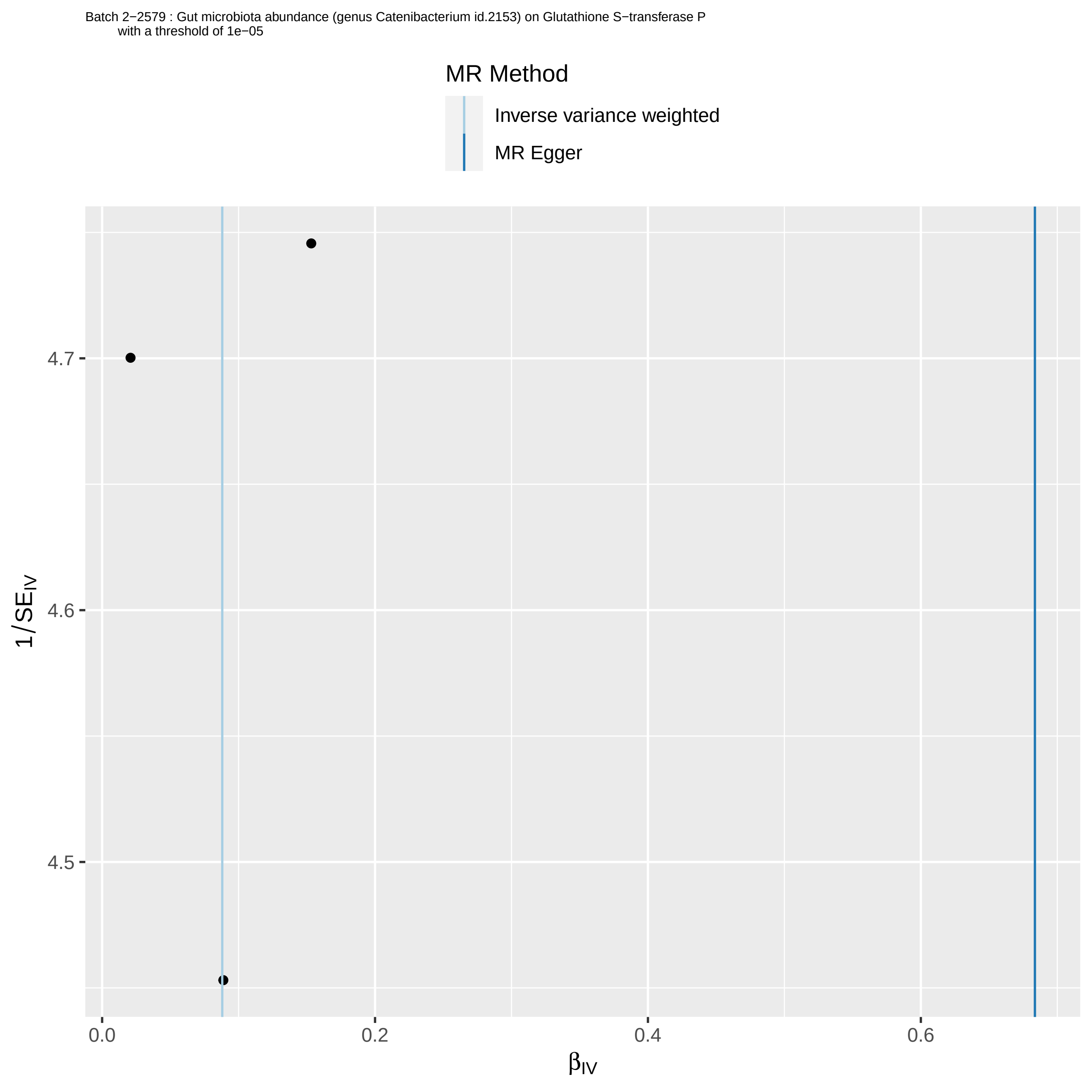

Batch 2−2579 : Gut microbiota abundance (genus Catenibacterium id.2153) on Glutathione S−transferase P
with a threshold of 1e−05
MR Method
Inverse variance weighted
MR Egger
4.7
4.6
4.5
1/SE_IV
0.0
0.2
0.4
0.6
β_IV

Batch 2−2580 : Gut microbiota abundance (genus Parasutterella id.2892) on Glutathione S−transferase P
with a threshold of 1e−05

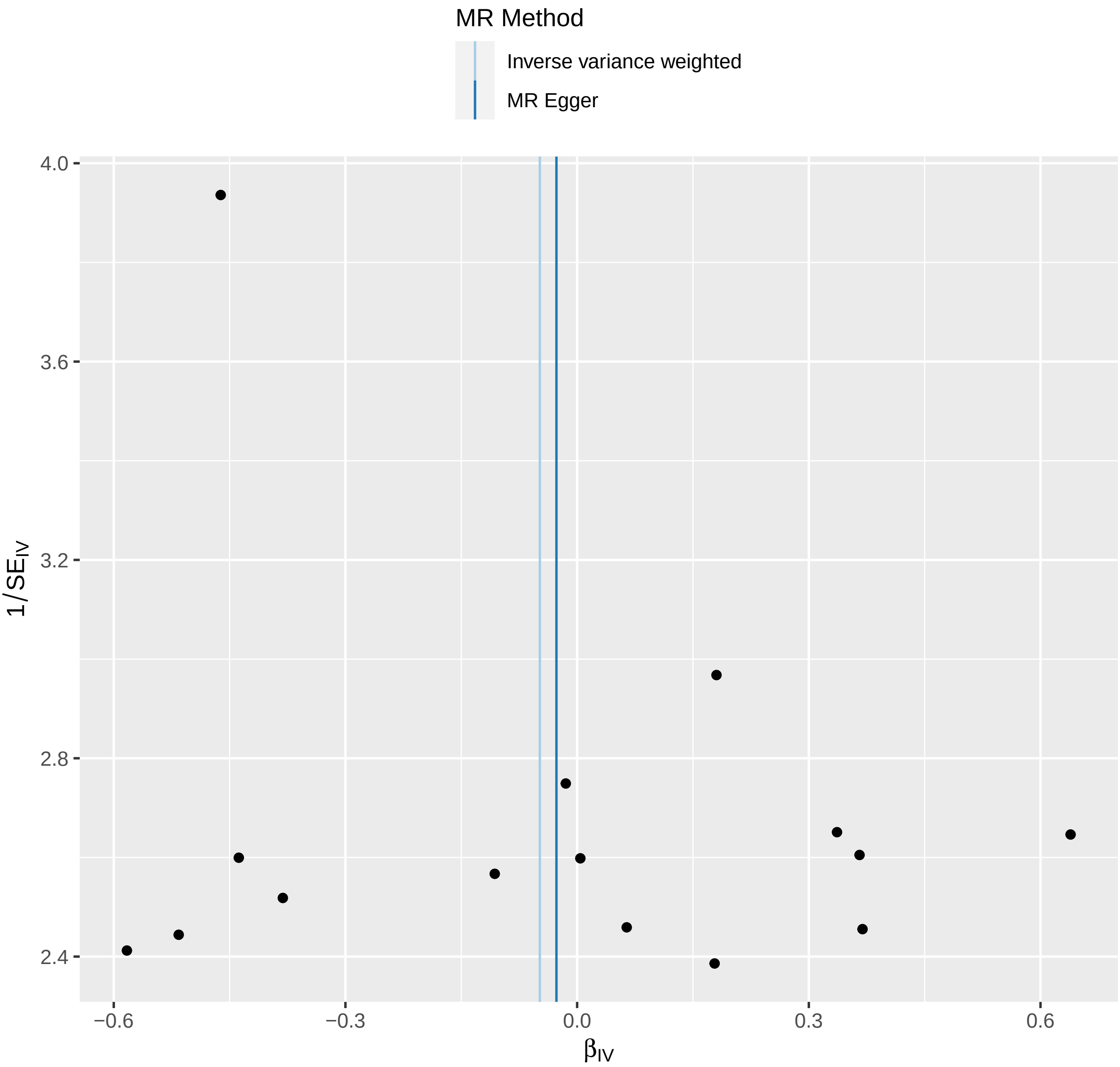

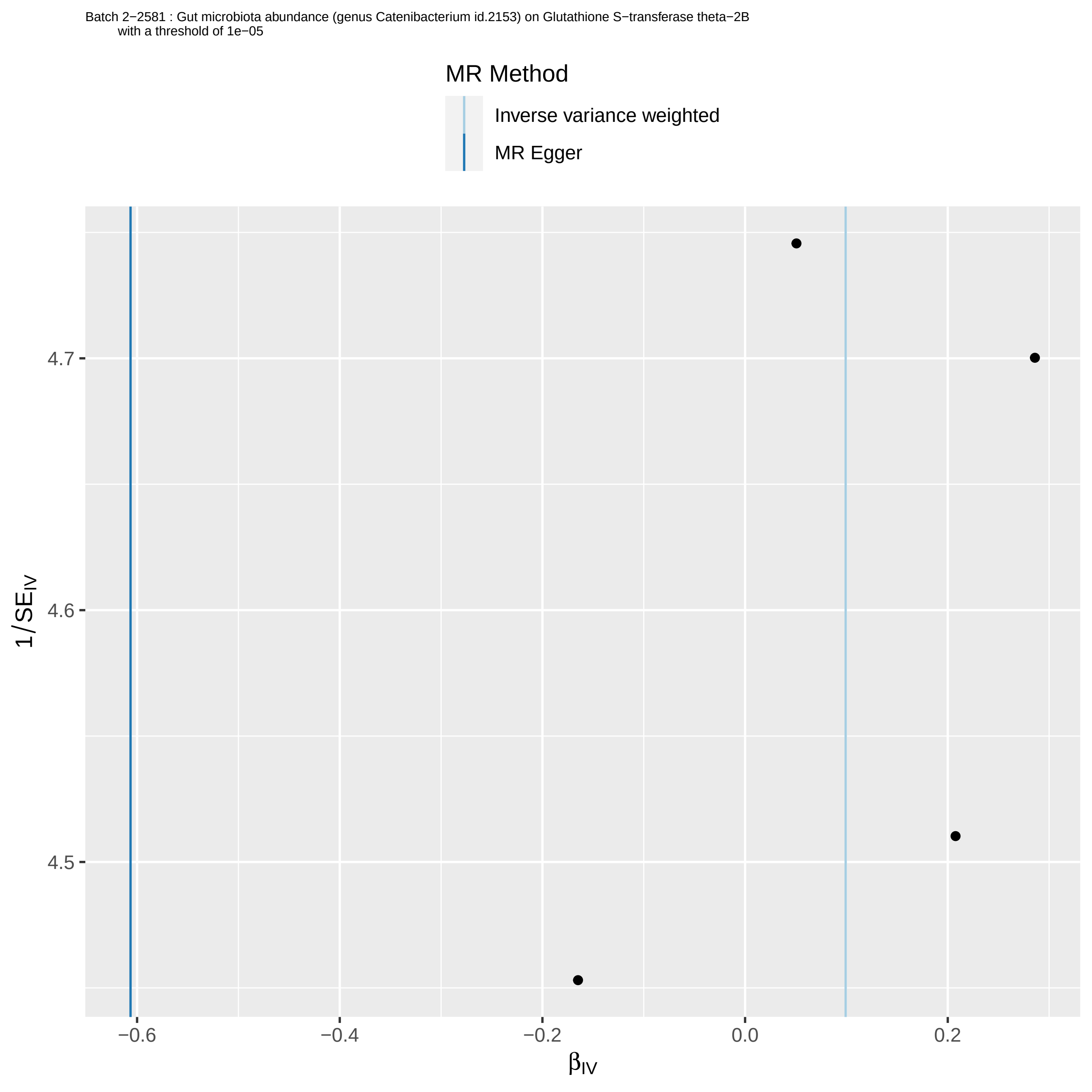

Batch 2−2581 : Gut microbiota abundance (genus Catenibacterium id.2153) on Glutathione S−transferase theta−2B
with a threshold of 1e−05
MR Method
Inverse variance weighted
MR Egger
4.7
4.6
4.5
1/SE$_{IV}$
−0.6
−0.4
−0.2
0.0
0.2
β_{IV}

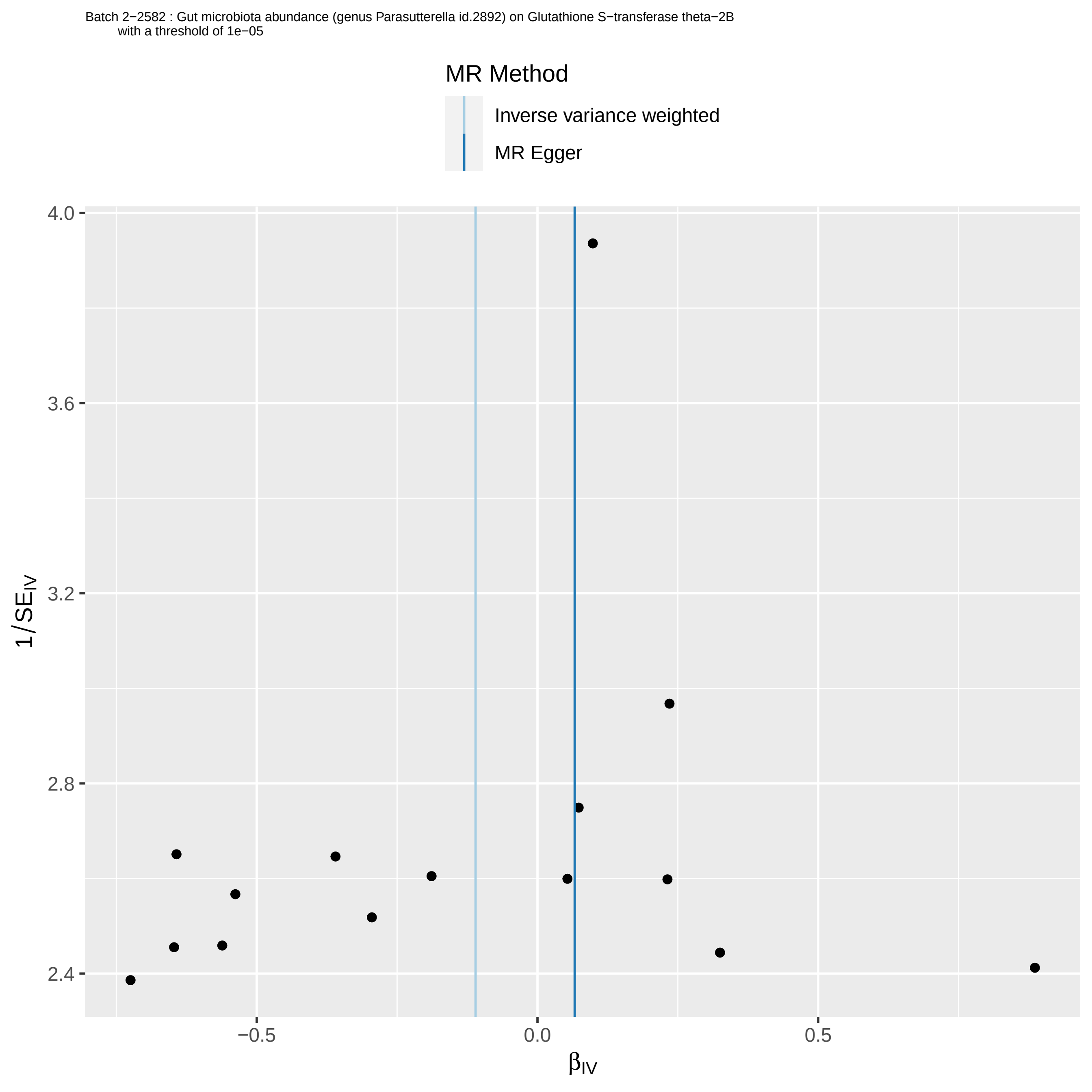

Batch 2−2582 : Gut microbiota abundance (genus Parasutterella id.2892) on Glutathione S−transferase theta−2B
with a threshold of 1e−05
MR Method
Inverse variance weighted
MR Egger
4.0
3.6
3.2
2.8
2.4
1/SE_IV
−0.5
0.0
0.5
β_IV

Batch 2−2583 : Gut microbiota abundance (genus Catenibacterium id.2153) on General transcription factor II−I
with a threshold of 1e−05

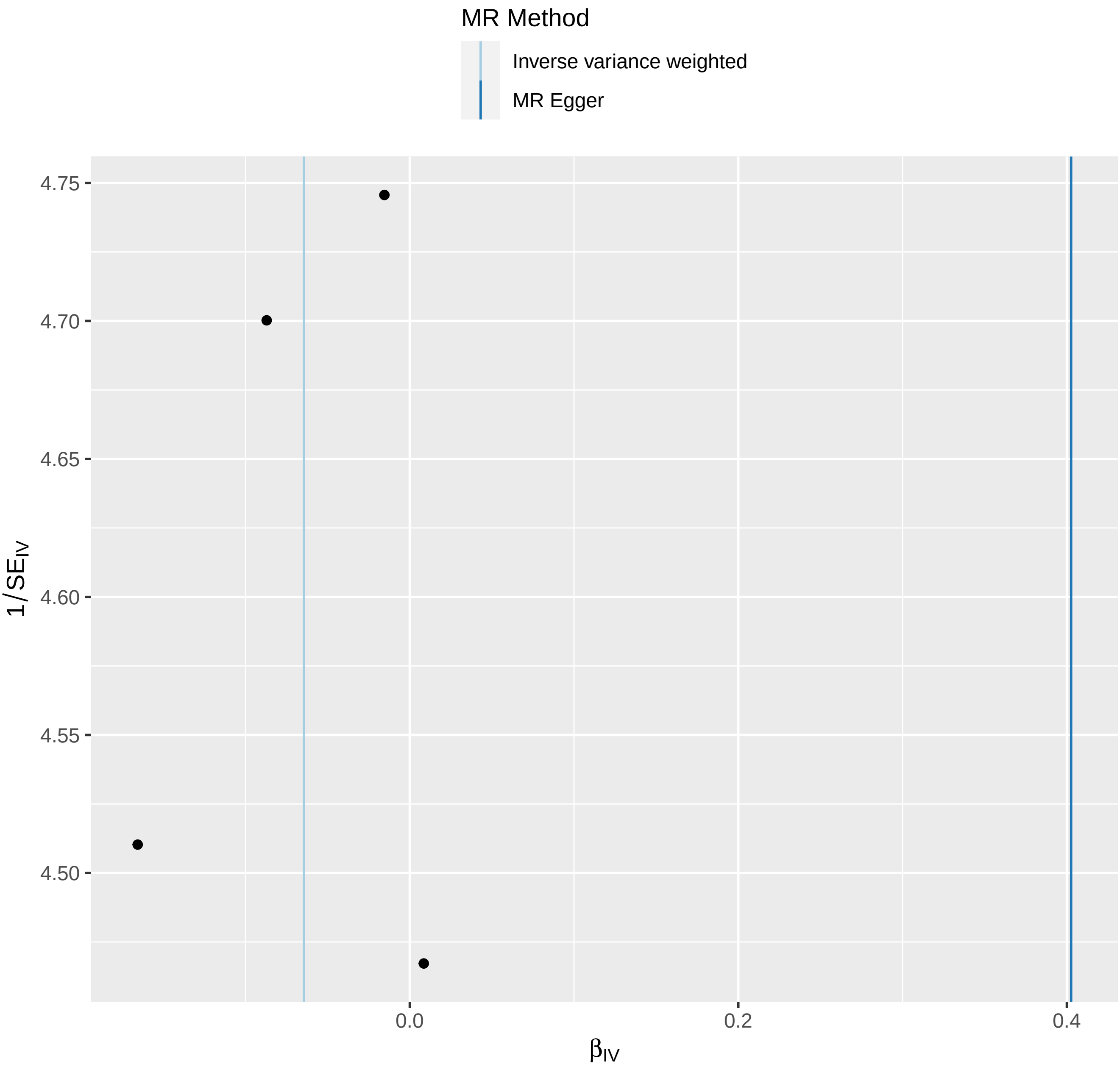

Batch 2−2584 : Gut microbiota abundance (genus Parasutterella id.2892) on General transcription factor II−I
with a threshold of 1e−05

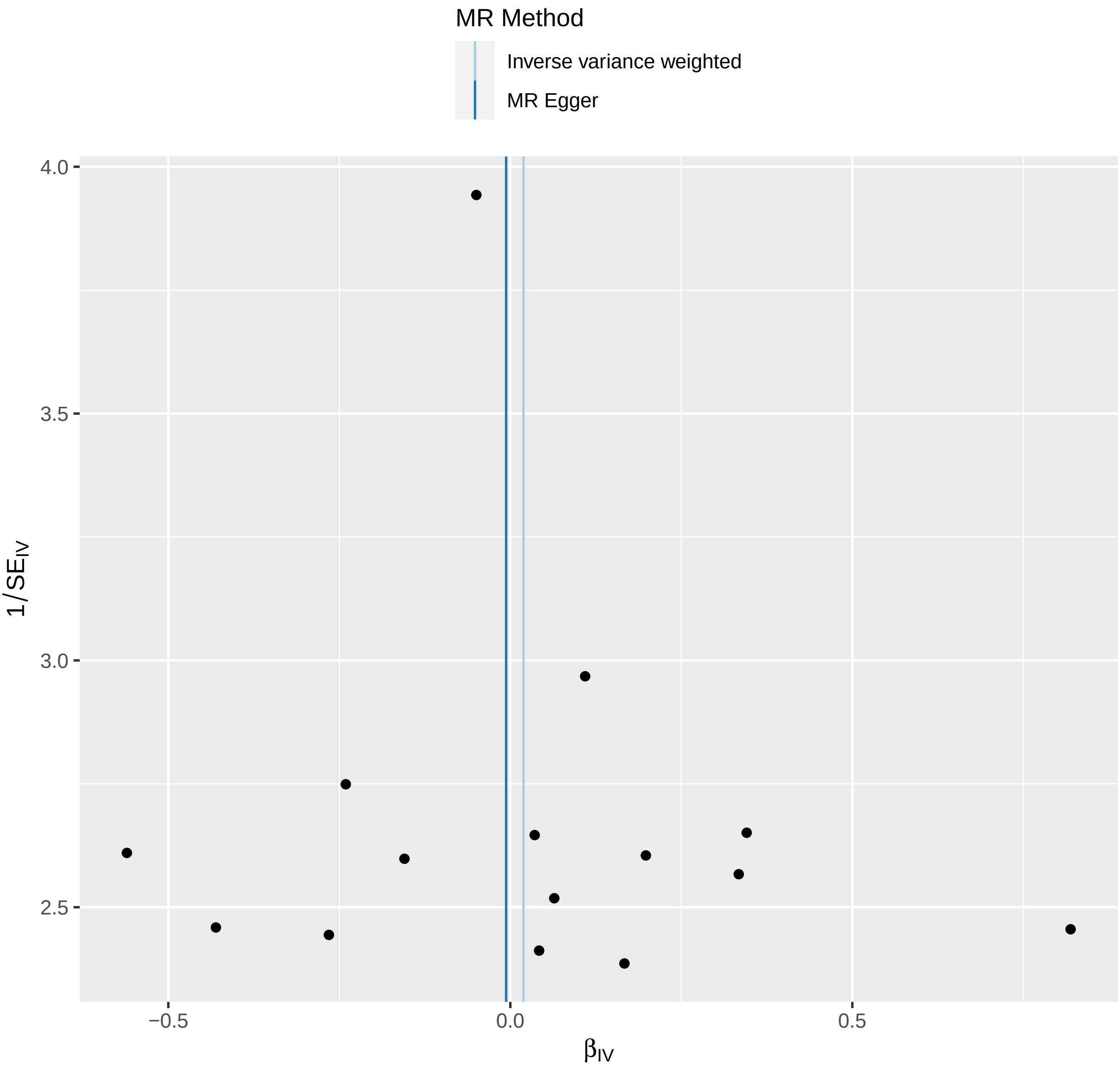

Batch 2−2585 : Gut microbiota abundance (genus Catenibacterium id.2153) on Guanylyl cyclase−activating protein 1
with a threshold of 1e−05

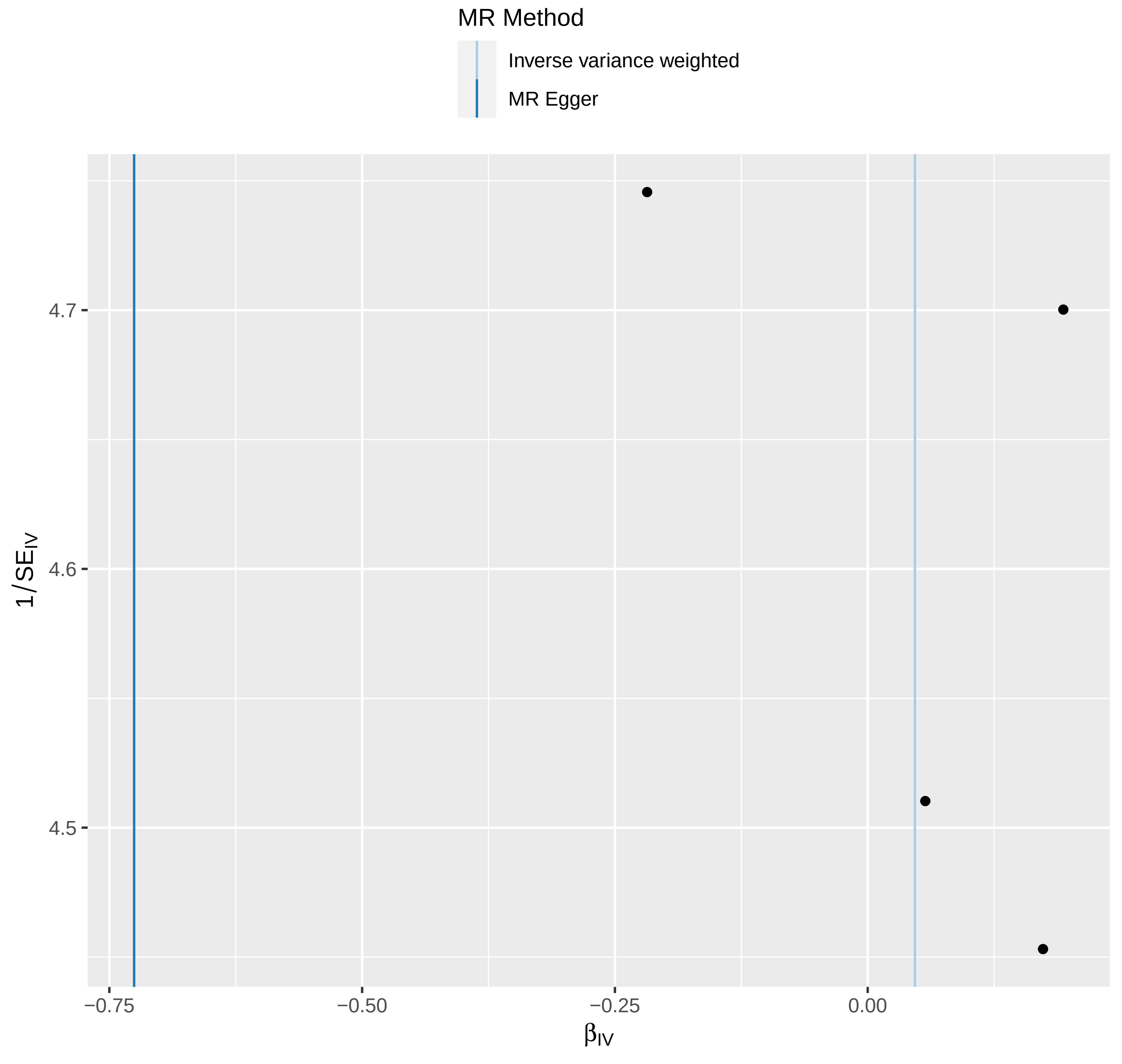

Batch 2−2586 : Gut microbiota abundance (genus Parasutterella id.2892) on Guanylyl cyclase−activating protein 1
with a threshold of 1e−05

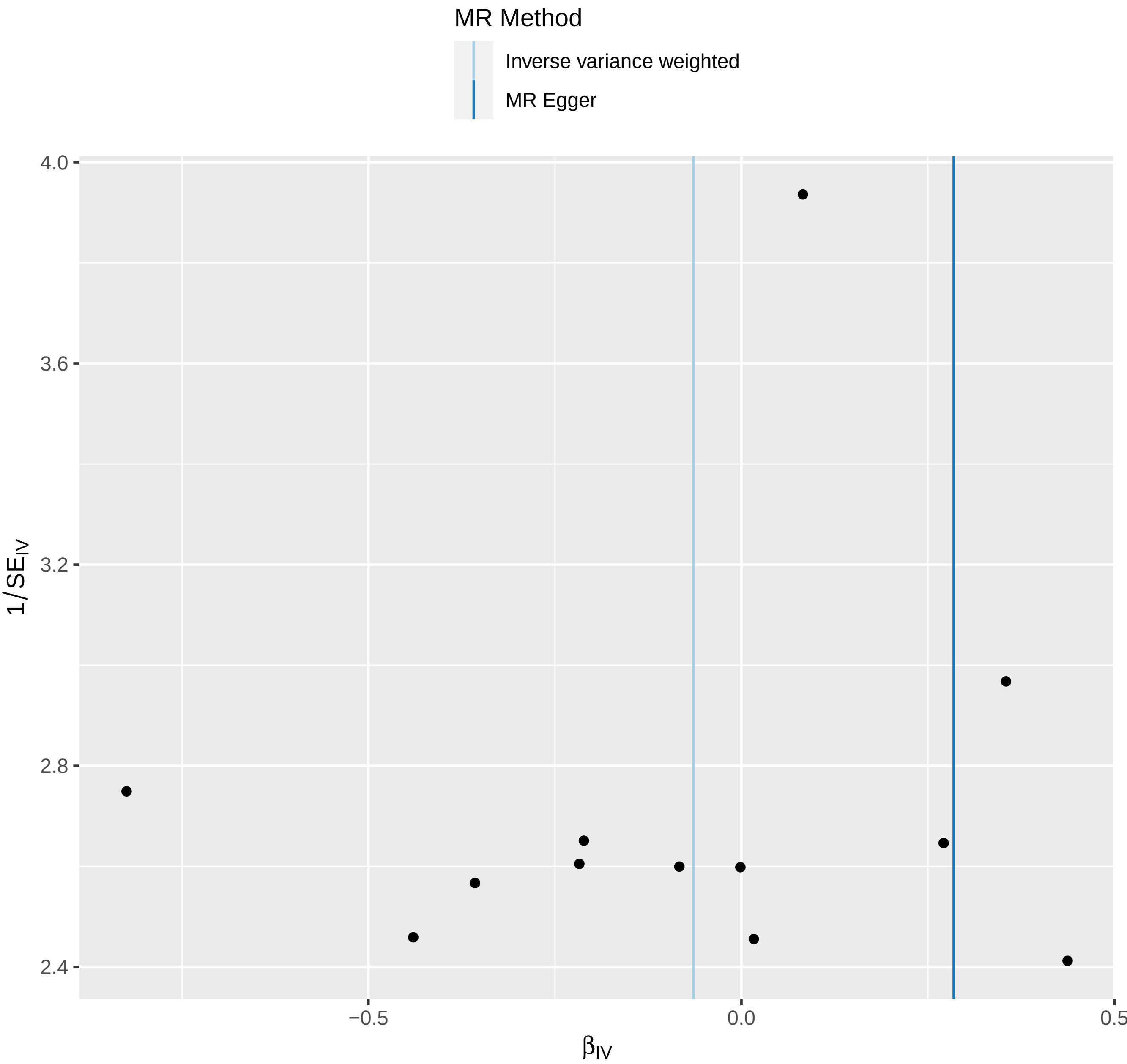

Batch 2−2587 : Gut microbiota abundance (genus Catenibacterium id.2153) on Guanylate cyclase activator 2B
with a threshold of 1e−05

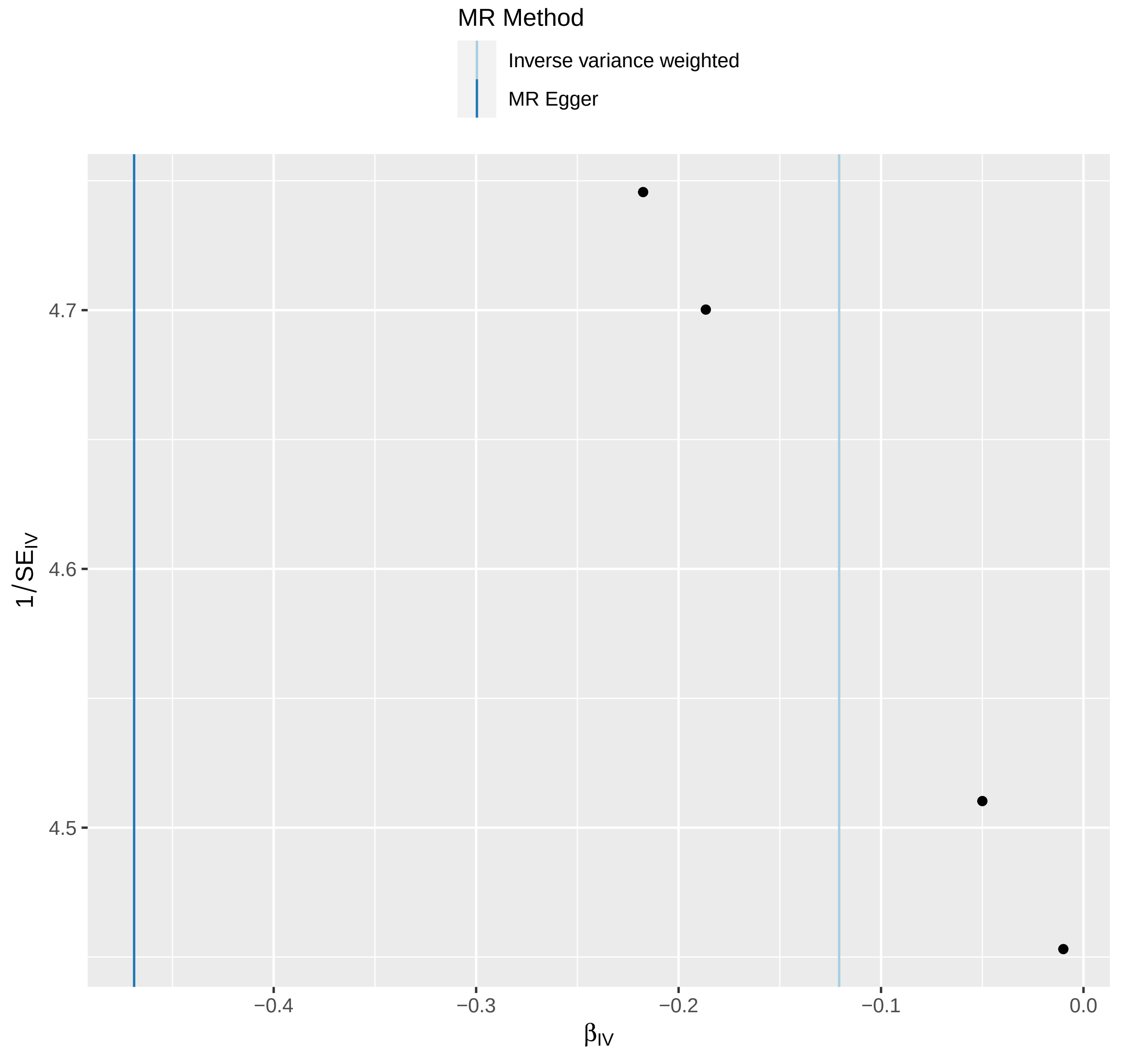

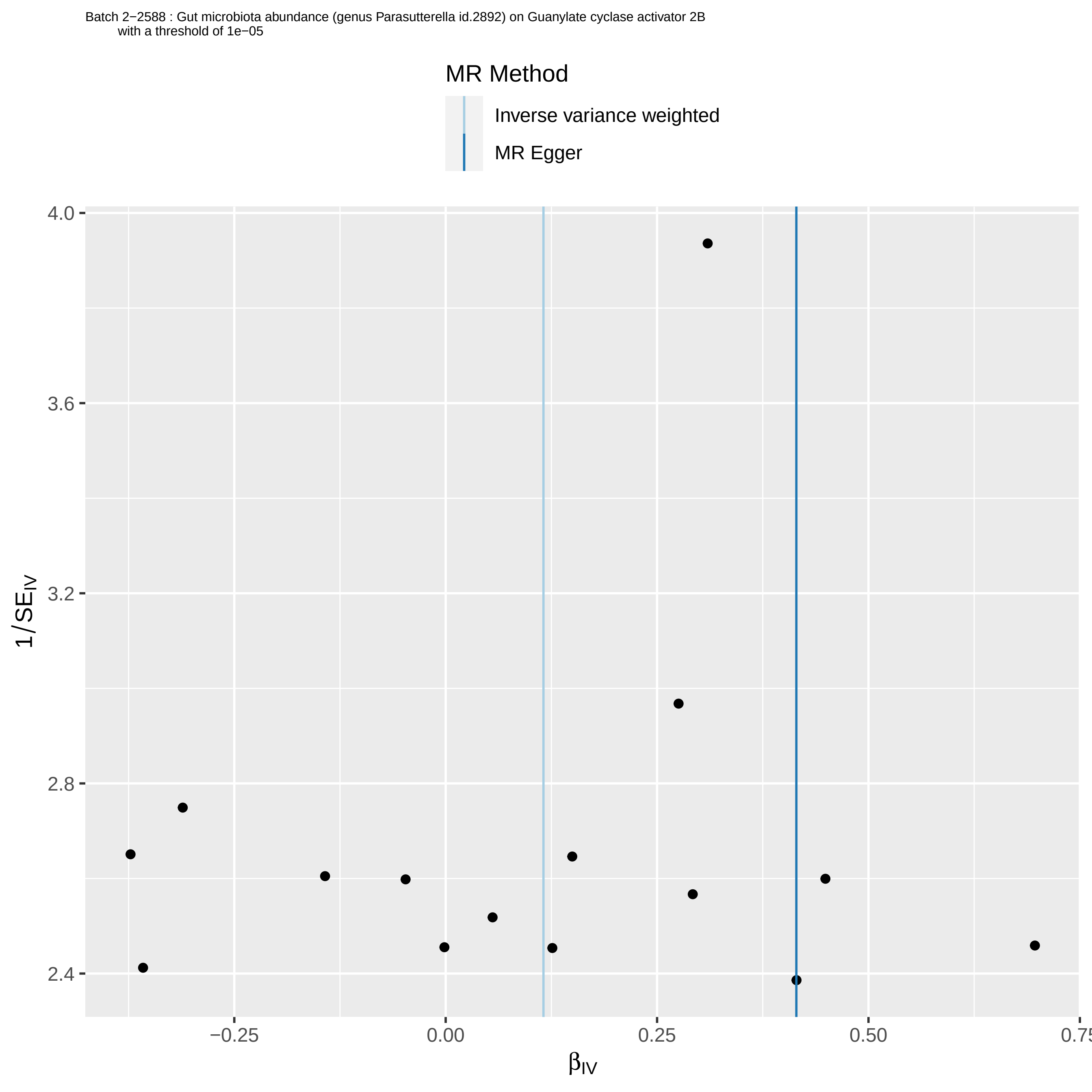
Batch 2−2588 : Gut microbiota abundance (genus Parasutterella id.2892) on Guanylate cyclase activator 2B
with a threshold of 1e−05
MR Method
Inverse variance weighted
MR Egger
4.0
3.6
3.2
2.8
2.4
1/SE_IV
−0.25
0.00
0.25
0.50
0.75
β_IV

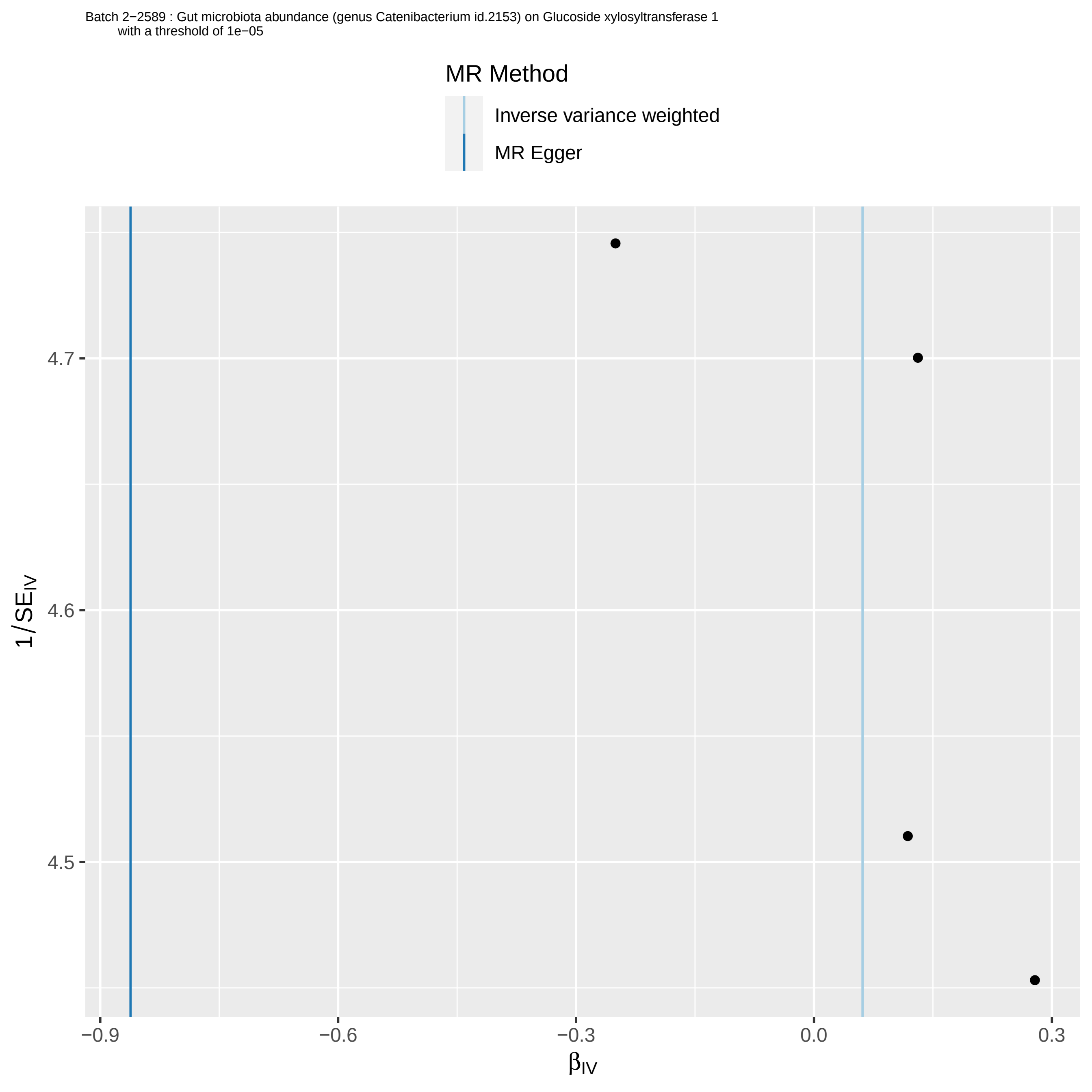
Batch 2−2589 : Gut microbiota abundance (genus Catenibacterium id.2153) on Glucoside xylosyltransferase 1
with a threshold of 1e−05
MR Method
Inverse variance weighted
MR Egger
4.7
4.6
4.5
1/SE_IV
−0.9
−0.6
−0.3
0.0
0.3
β_IV

Batch 2−2590 : Gut microbiota abundance (genus Parasutterella id.2892) on Glucoside xylosyltransferase 1
with a threshold of 1e−05

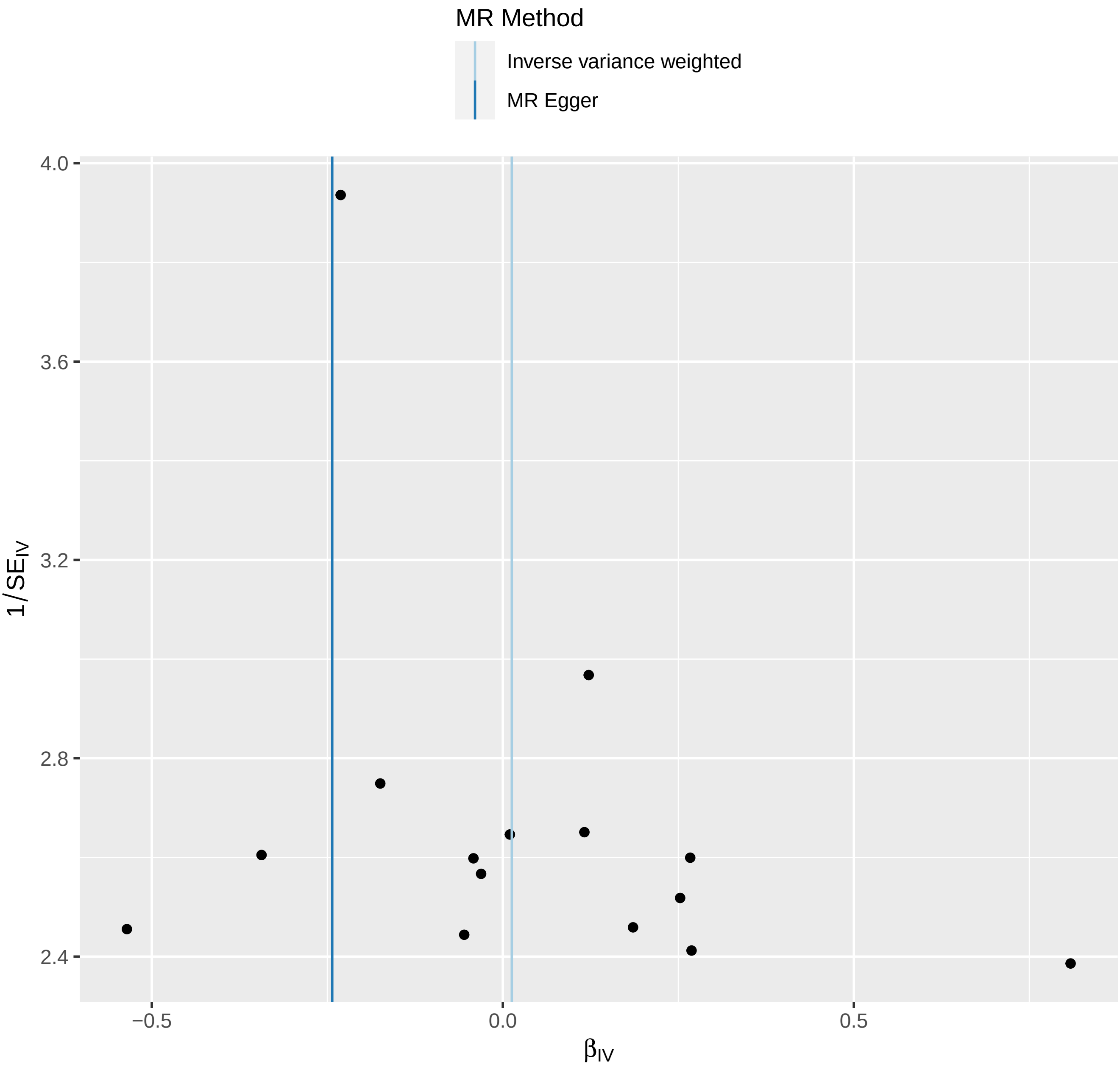

Batch 2−2591 : Gut microbiota abundance (genus Catenibacterium id.2153) on Glycophorin−A
with a threshold of 1e−05

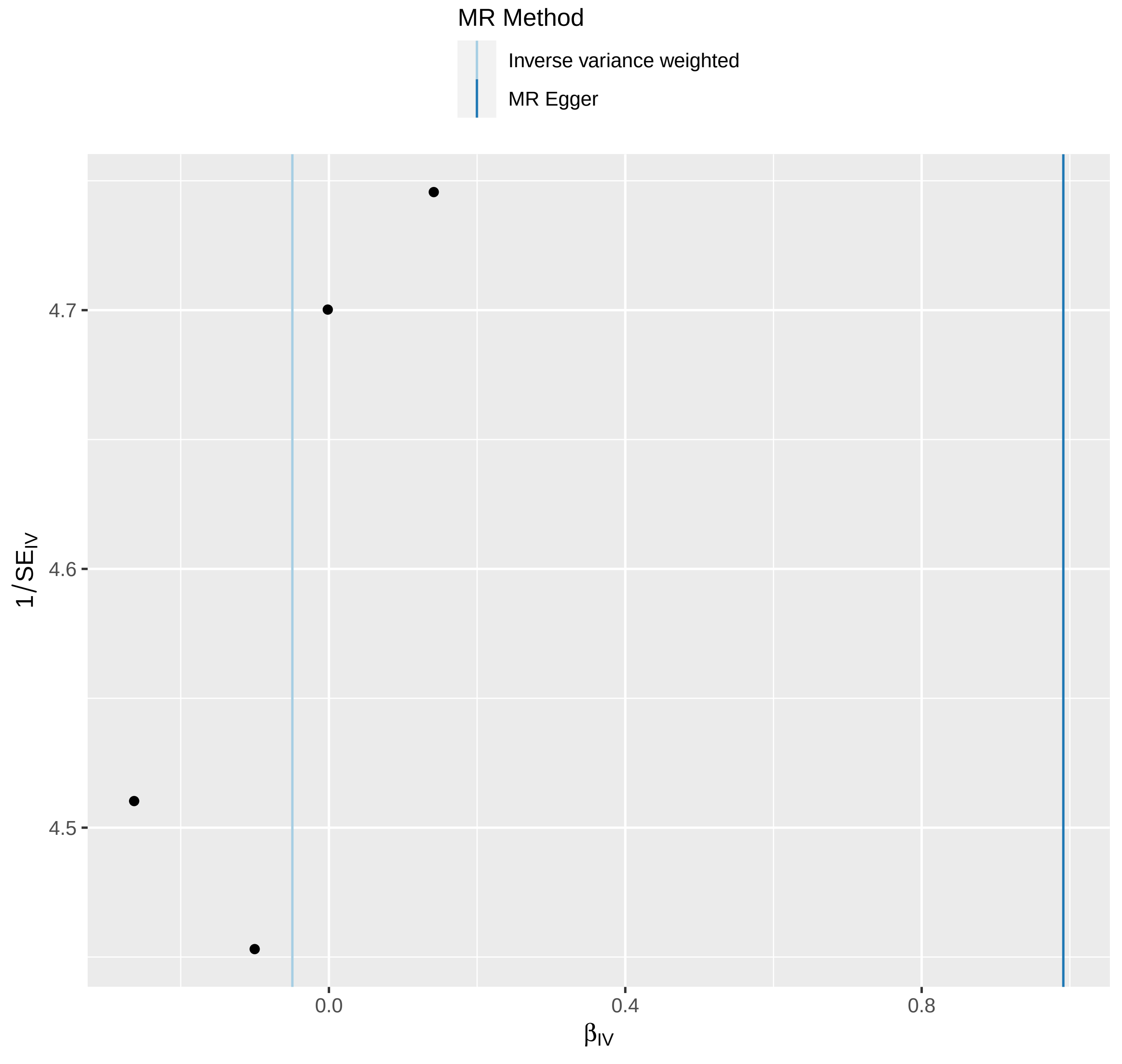

Batch 2−2592 : Gut microbiota abundance (genus Parasutterella id.2892) on Glycophorin−A
with a threshold of 1e−05

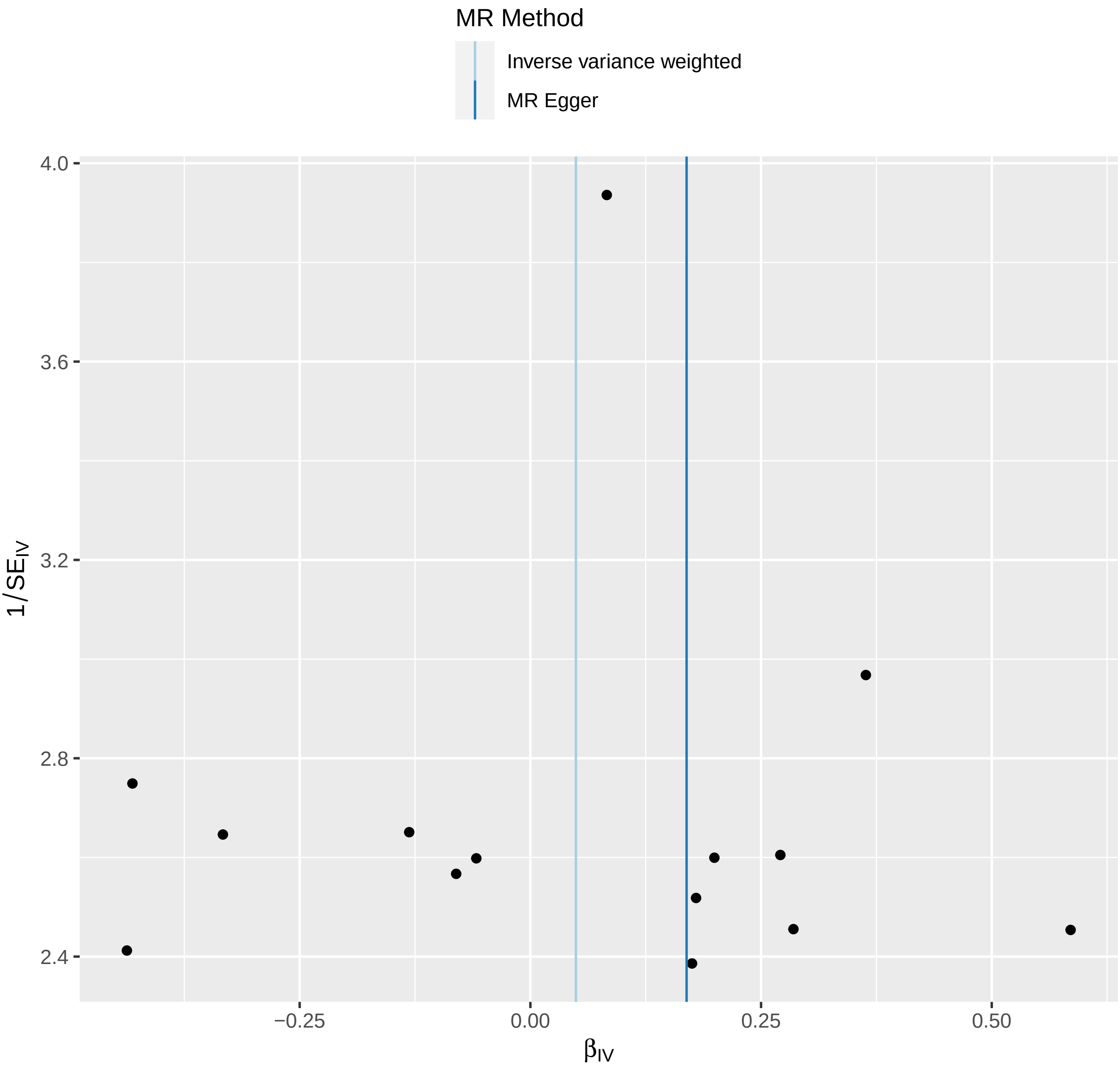

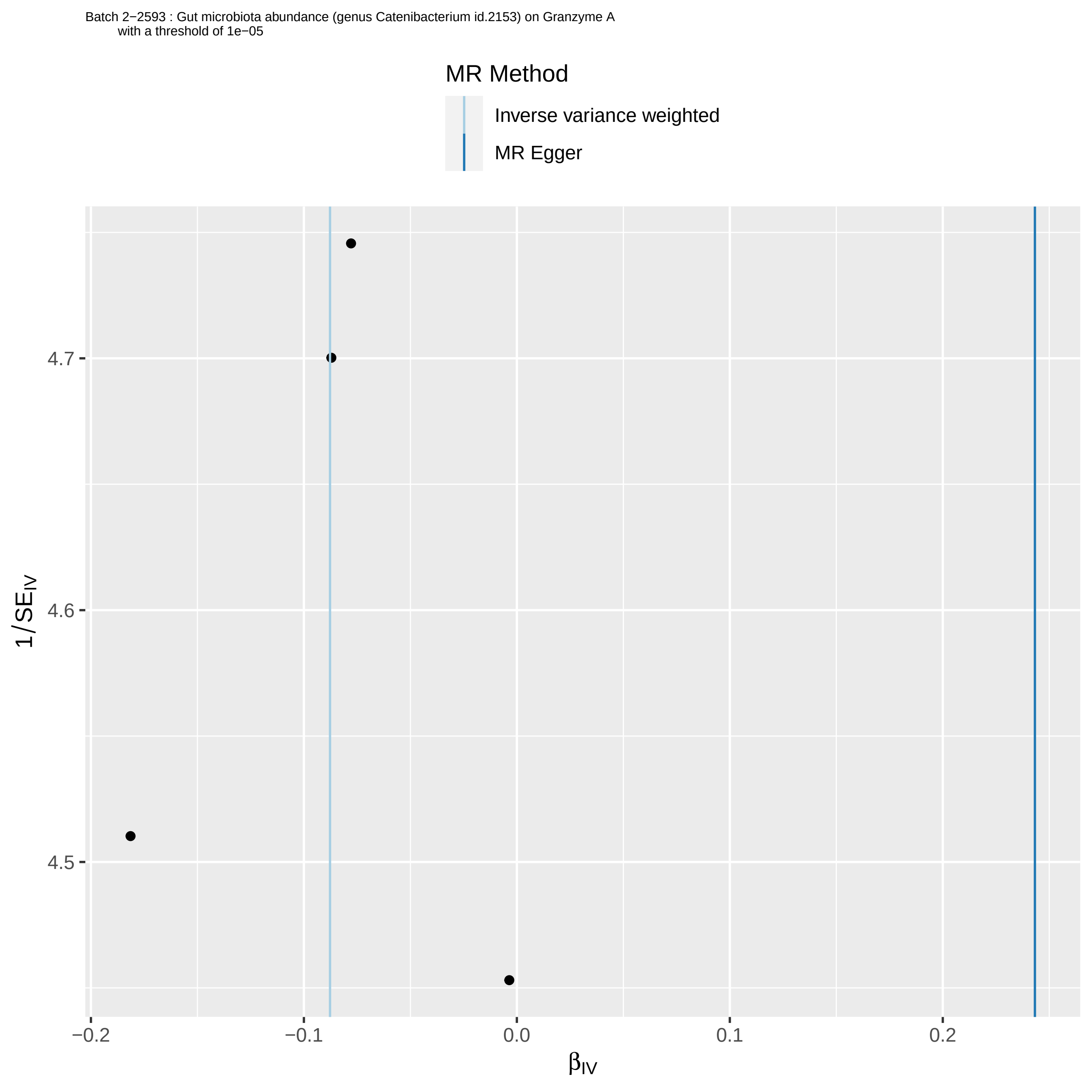

Batch 2−2593 : Gut microbiota abundance (genus Catenibacterium id.2153) on Granzyme A
with a threshold of 1e−05
MR Method
Inverse variance weighted
MR Egger
4.7
4.6
4.5
1/SE$_{IV}$
−0.2
−0.1
0.0
0.1
0.2
β$_{IV}$

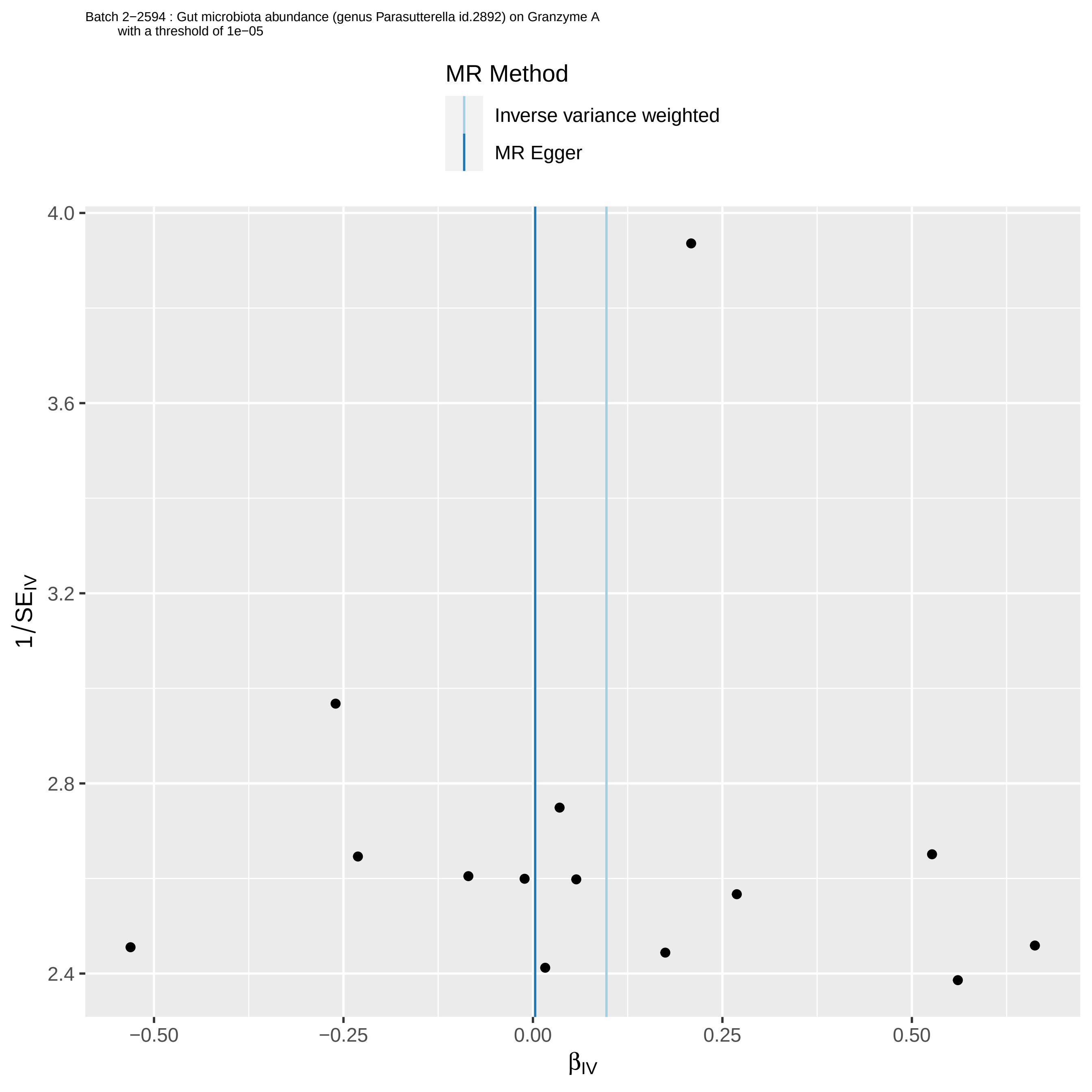

Batch 2−2594 : Gut microbiota abundance (genus Parasutterella id.2892) on Granzyme A
with a threshold of 1e−05
MR Method
Inverse variance weighted
MR Egger
4.0
3.6
3.2
2.8
2.4
1/SE_IV
−0.50
−0.25
0.00
0.25
0.50
β_IV

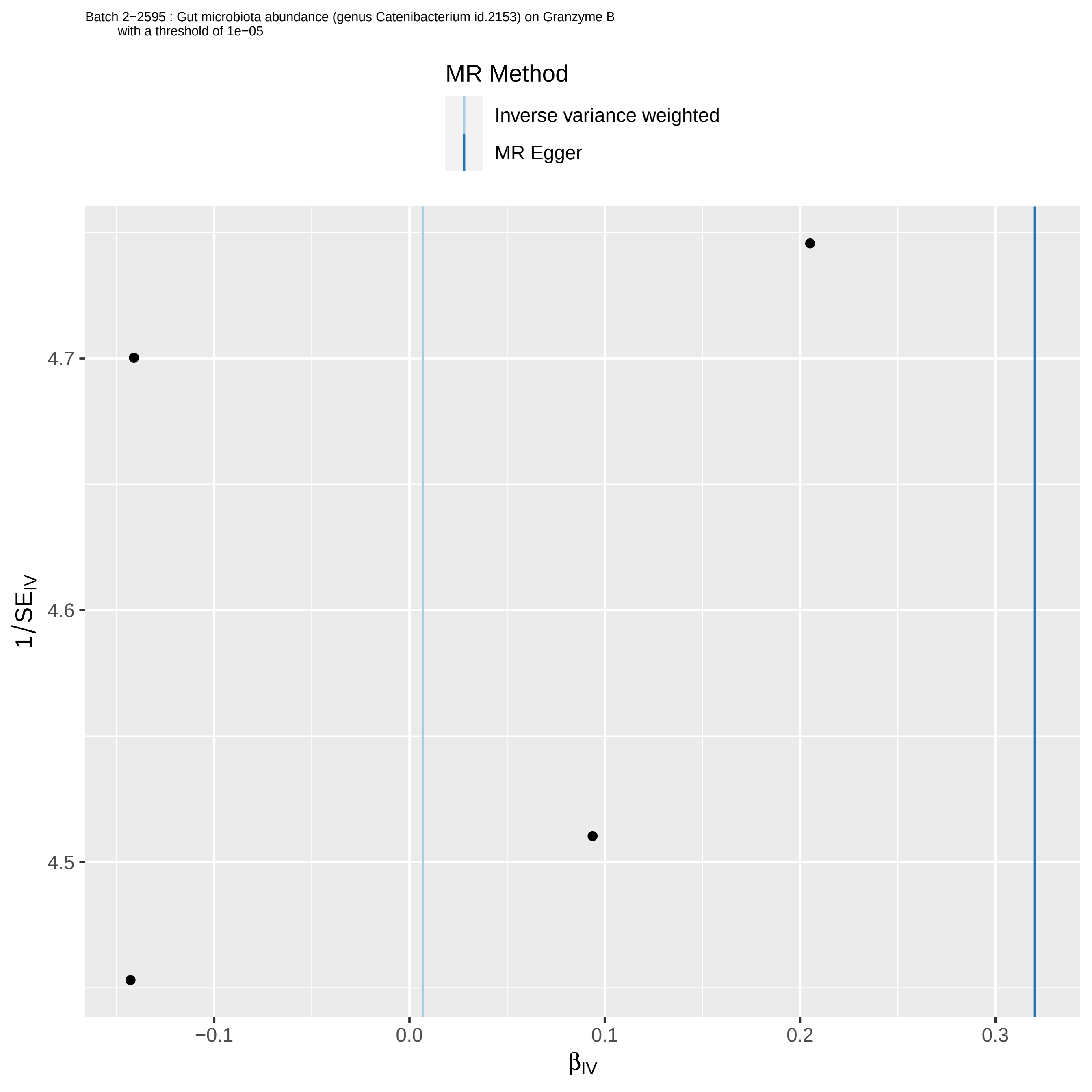

Batch 2−2595 : Gut microbiota abundance (genus Catenibacterium id.2153) on Granzyme B
with a threshold of 1e−05
MR Method
Inverse variance weighted
MR Egger
4.7
4.6
4.5
1/SE_IV
−0.1
0.0
0.1
0.2
0.3
β_IV

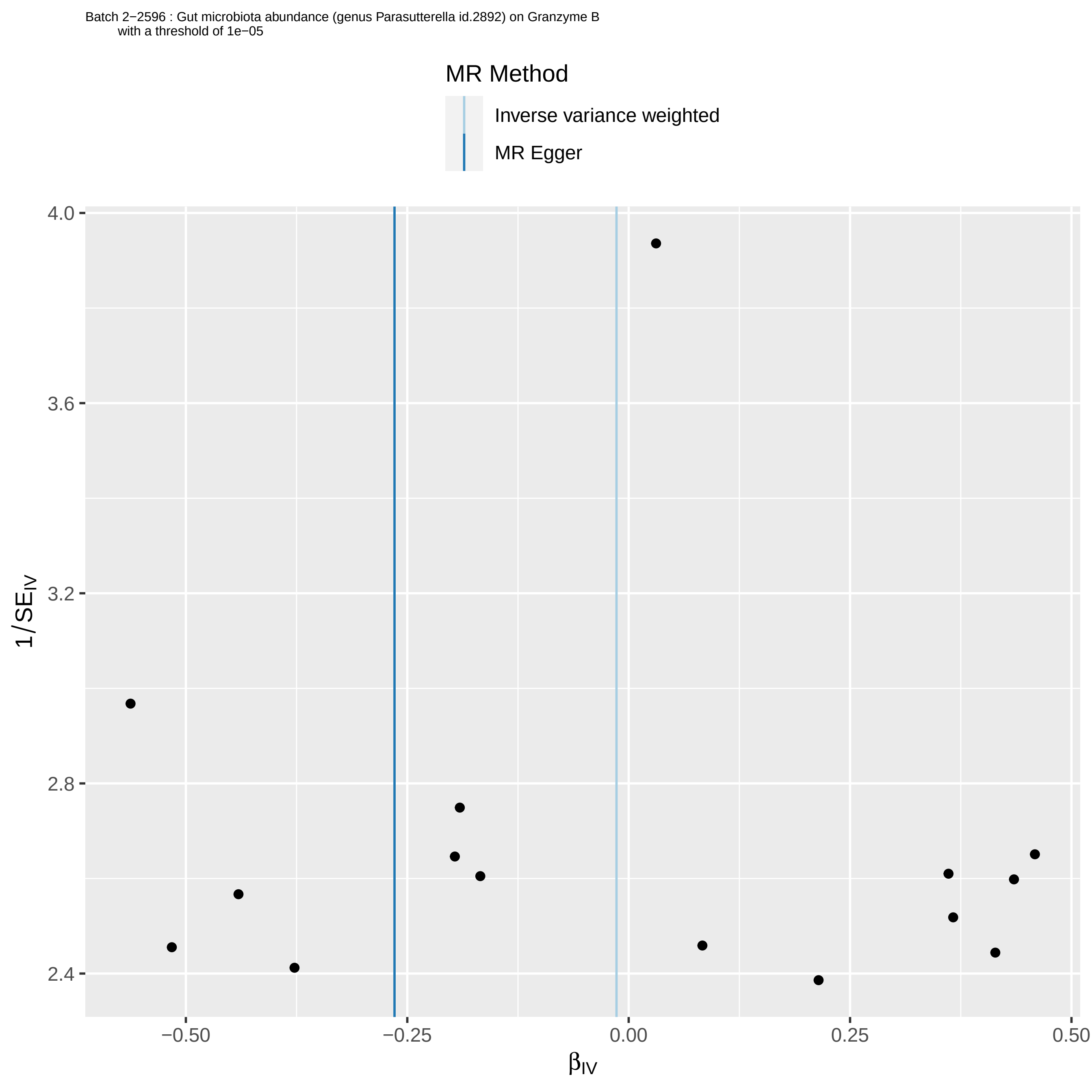

Batch 2−2596 : Gut microbiota abundance (genus Parasutterella id.2892) on Granzyme B
with a threshold of 1e−05
MR Method
Inverse variance weighted
MR Egger
4.0
3.6
3.2
2.8
2.4
1/SE_IV
−0.50
−0.25
0.00
0.25
0.50
β_IV

Batch 2−2597 : Gut microbiota abundance (genus Catenibacterium id.2153) on Granzyme B
with a threshold of 1e−05

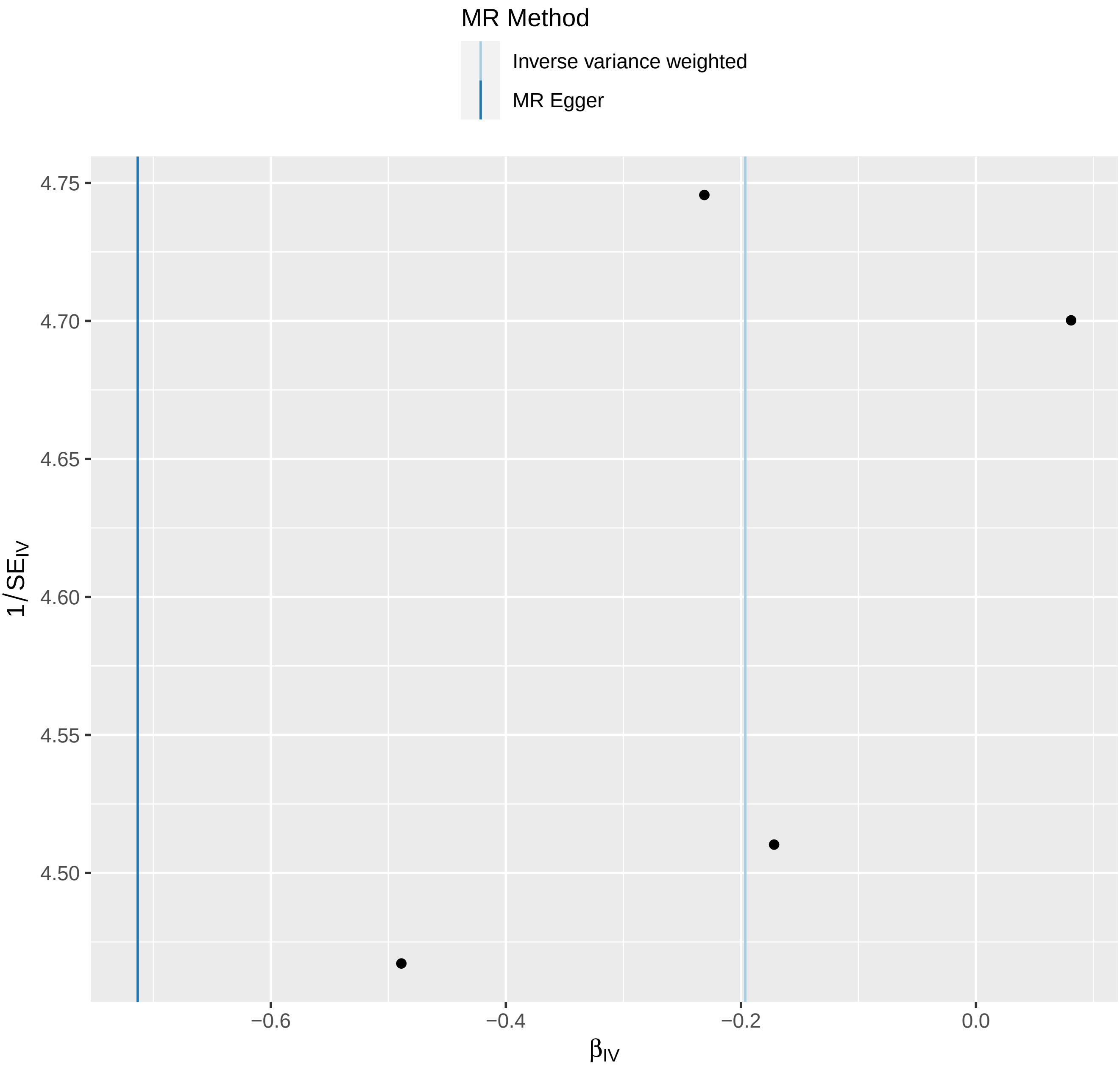

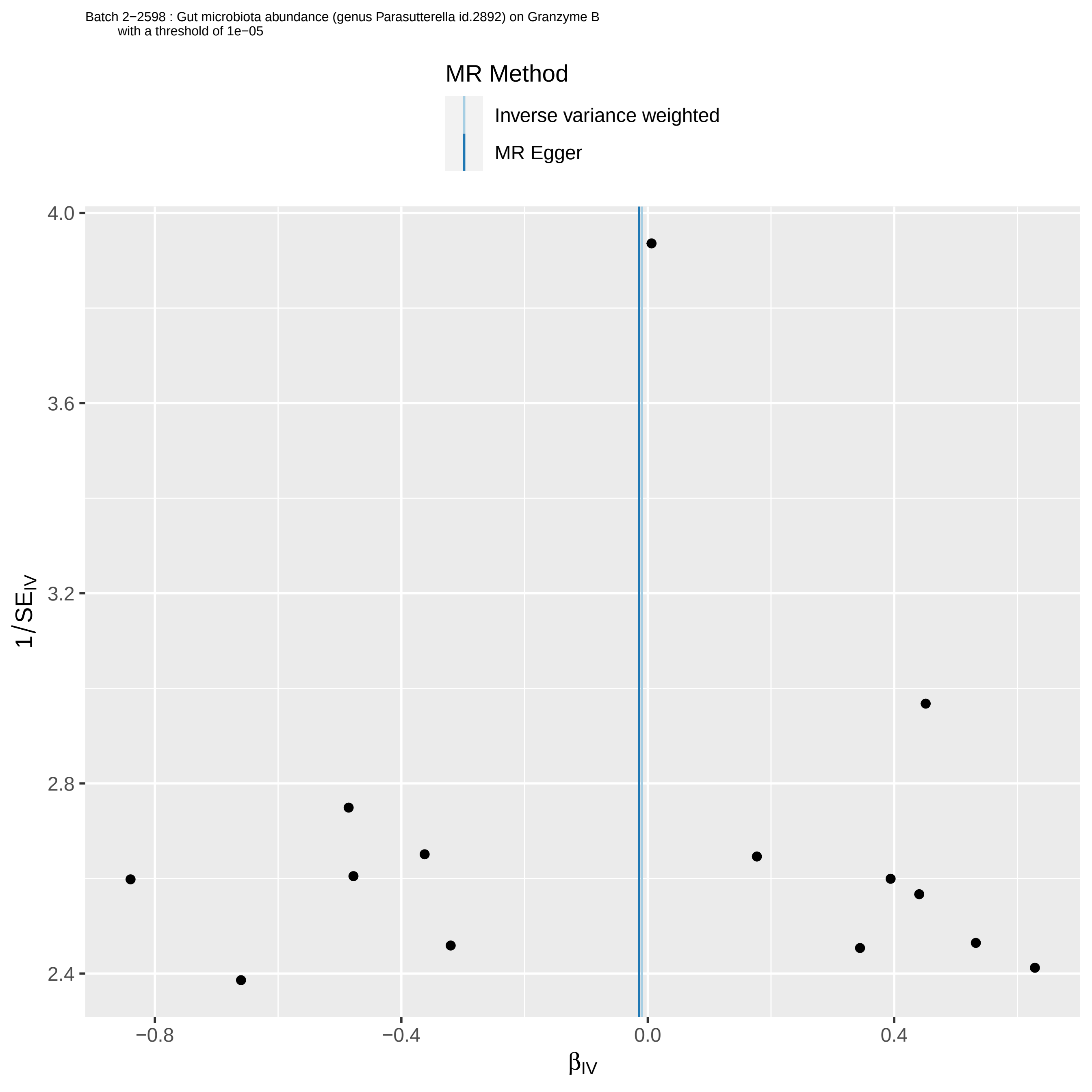
Batch 2−2598 : Gut microbiota abundance (genus Parasutterella id.2892) on Granzyme B
with a threshold of 1e−05
MR Method
Inverse variance weighted
MR Egger
4.0
3.6
3.2
2.8
2.4
1/SE$_{IV}$
−0.8
−0.4
0.0
0.4
β$_{IV}$

Batch 2−2599 : Gut microbiota abundance (genus Catenibacterium id.2153) on Granzyme H
with a threshold of 1e−05

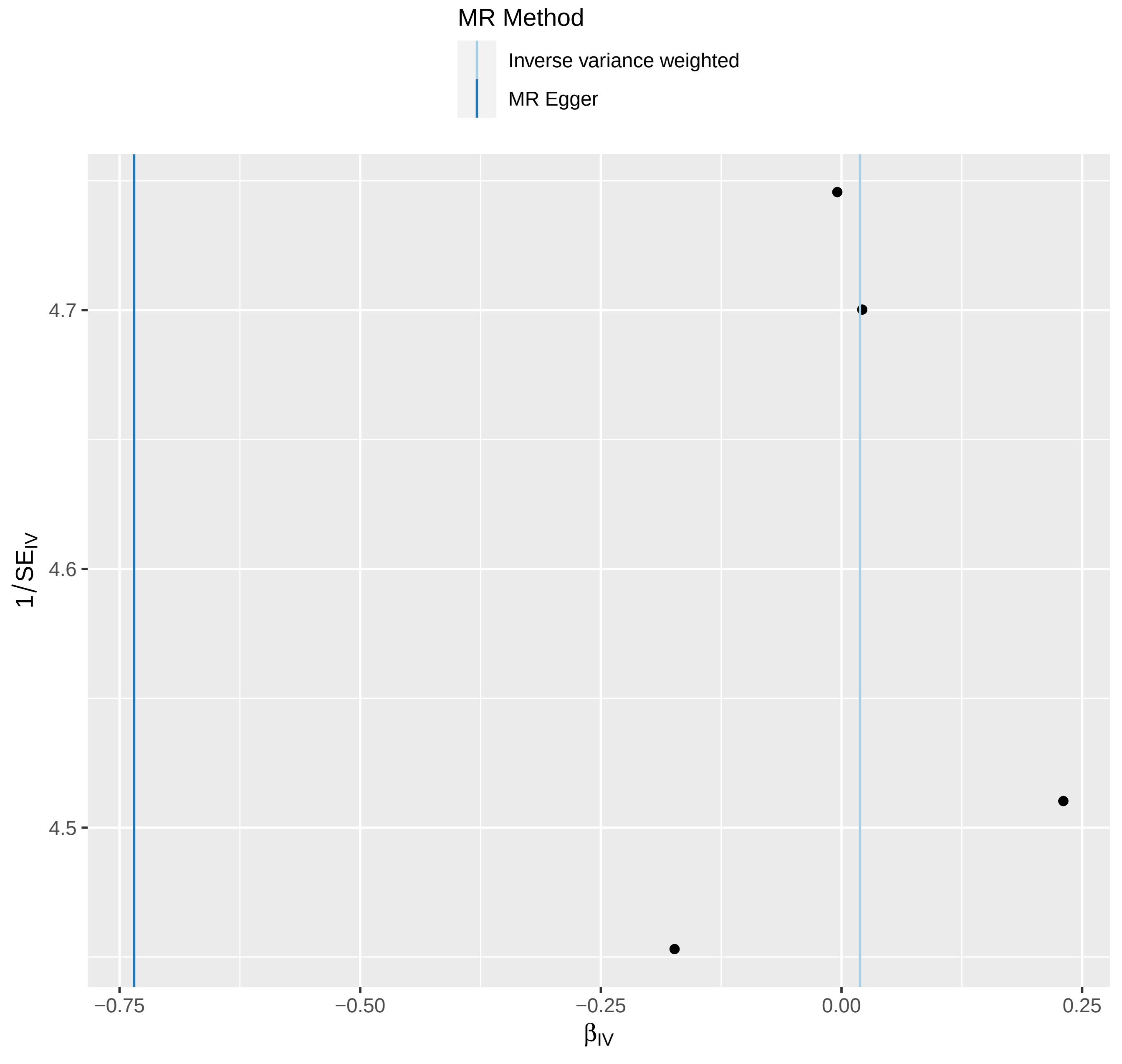

Batch 2−2600 : Gut microbiota abundance (genus Parasutterella id.2892) on Granzyme H
with a threshold of 1e−05

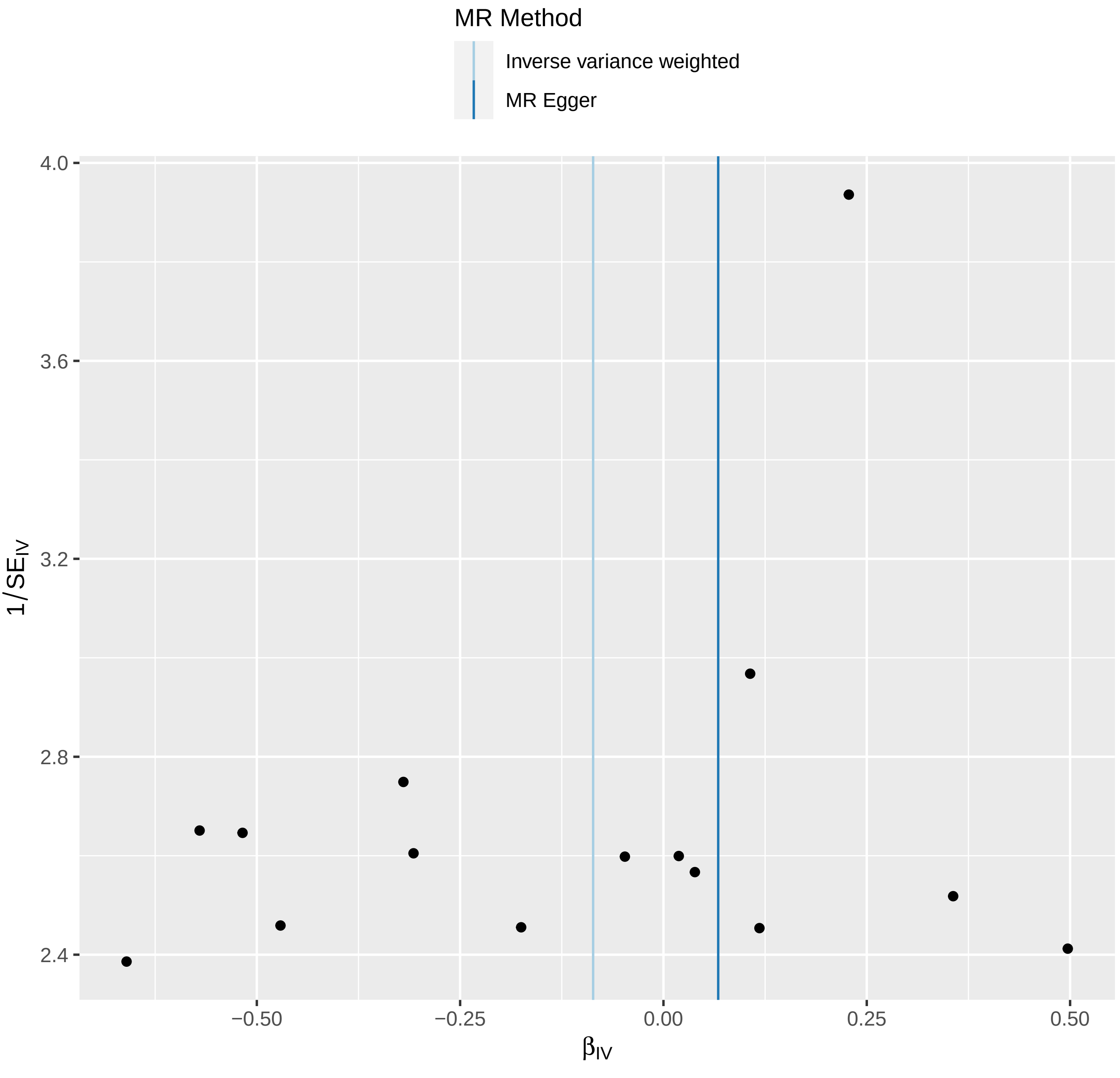

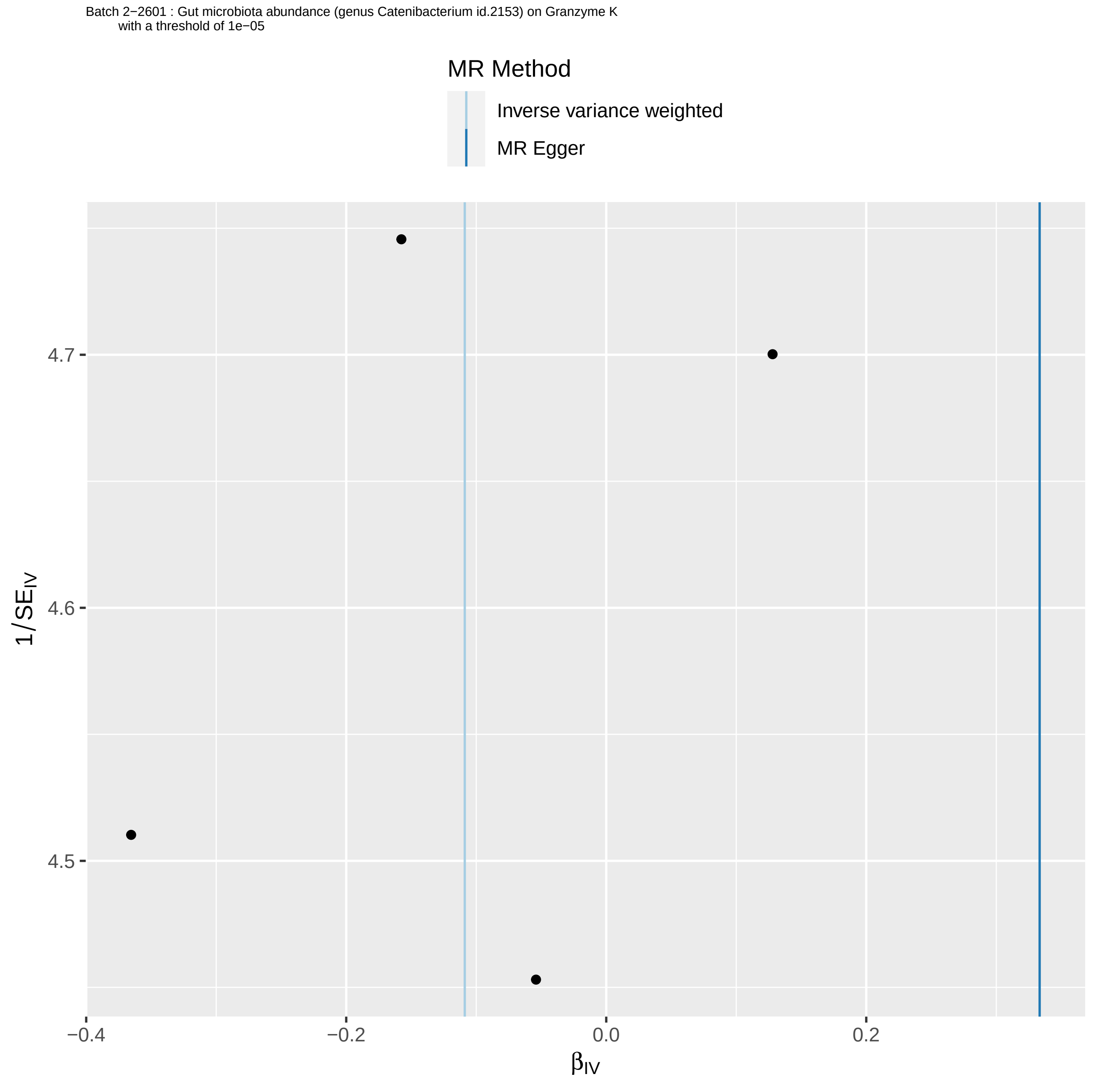
Batch 2−2601 : Gut microbiota abundance (genus Catenibacterium id.2153) on Granzyme K
with a threshold of 1e−05
MR Method
Inverse variance weighted
MR Egger
4.7
4.6
4.5
1/SE_IV
−0.4
−0.2
0.0
0.2
β_IV

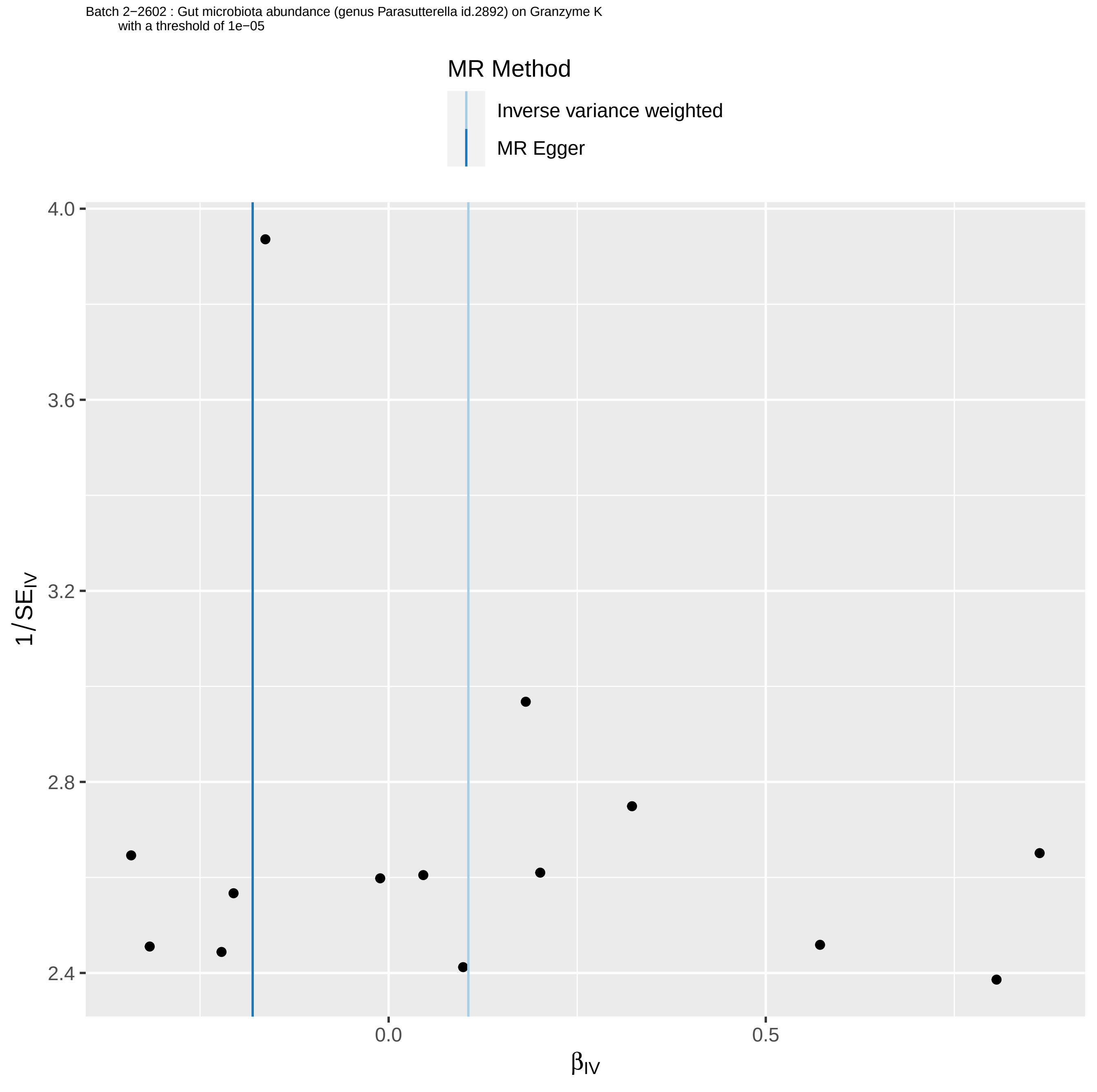

Batch 2−2602 : Gut microbiota abundance (genus Parasutterella id.2892) on Granzyme K
with a threshold of 1e−05
MR Method
Inverse variance weighted
MR Egger
4.0
3.6
3.2
2.8
2.4
1/SE_IV
0.0
0.5
β_IV

Batch 2−2603 : Gut microbiota abundance (genus Catenibacterium id.2153) on Granzyme M
with a threshold of 1e−05

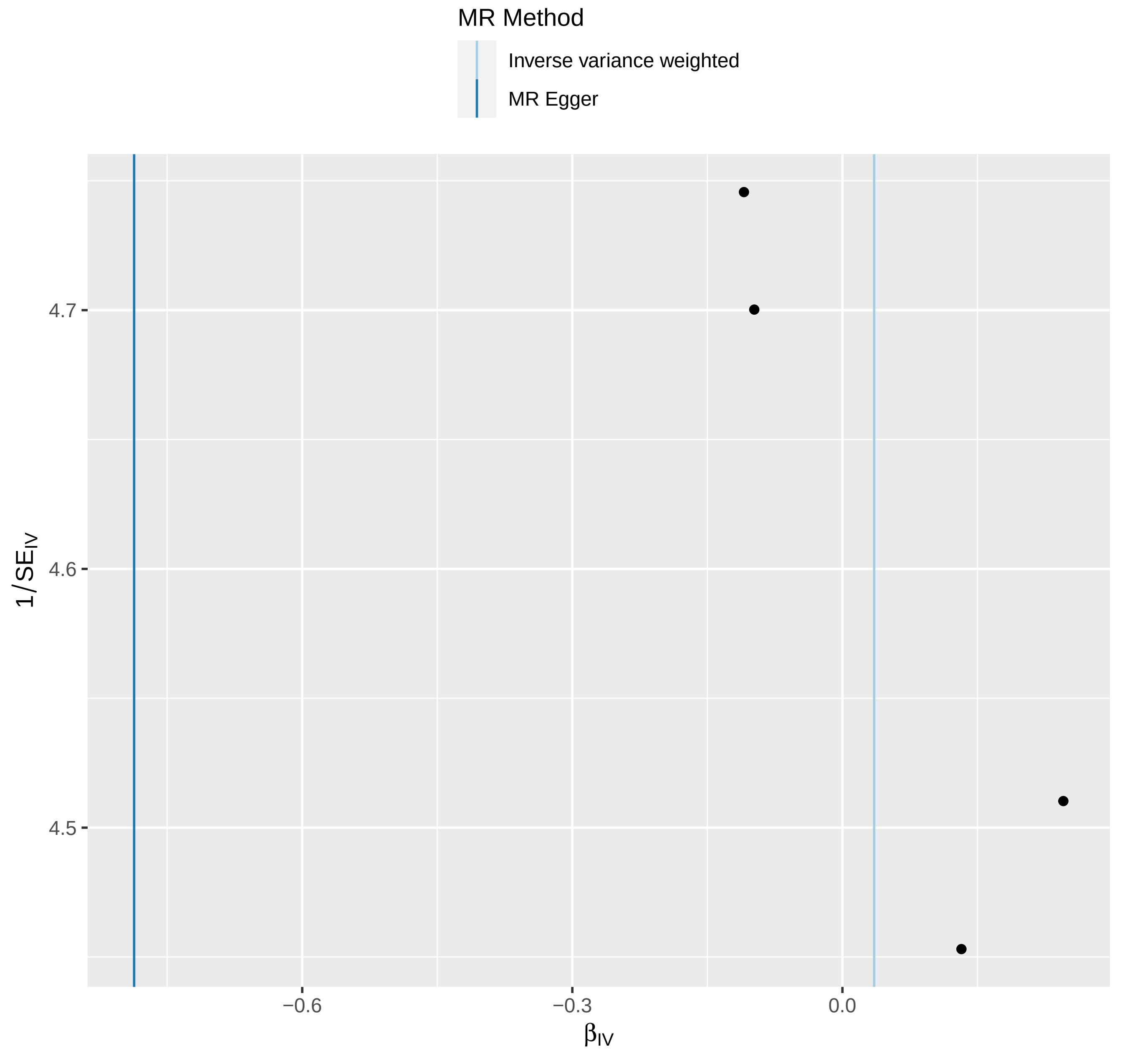

Batch 2−2604 : Gut microbiota abundance (genus Parasutterella id.2892) on Granzyme M
with a threshold of 1e−05

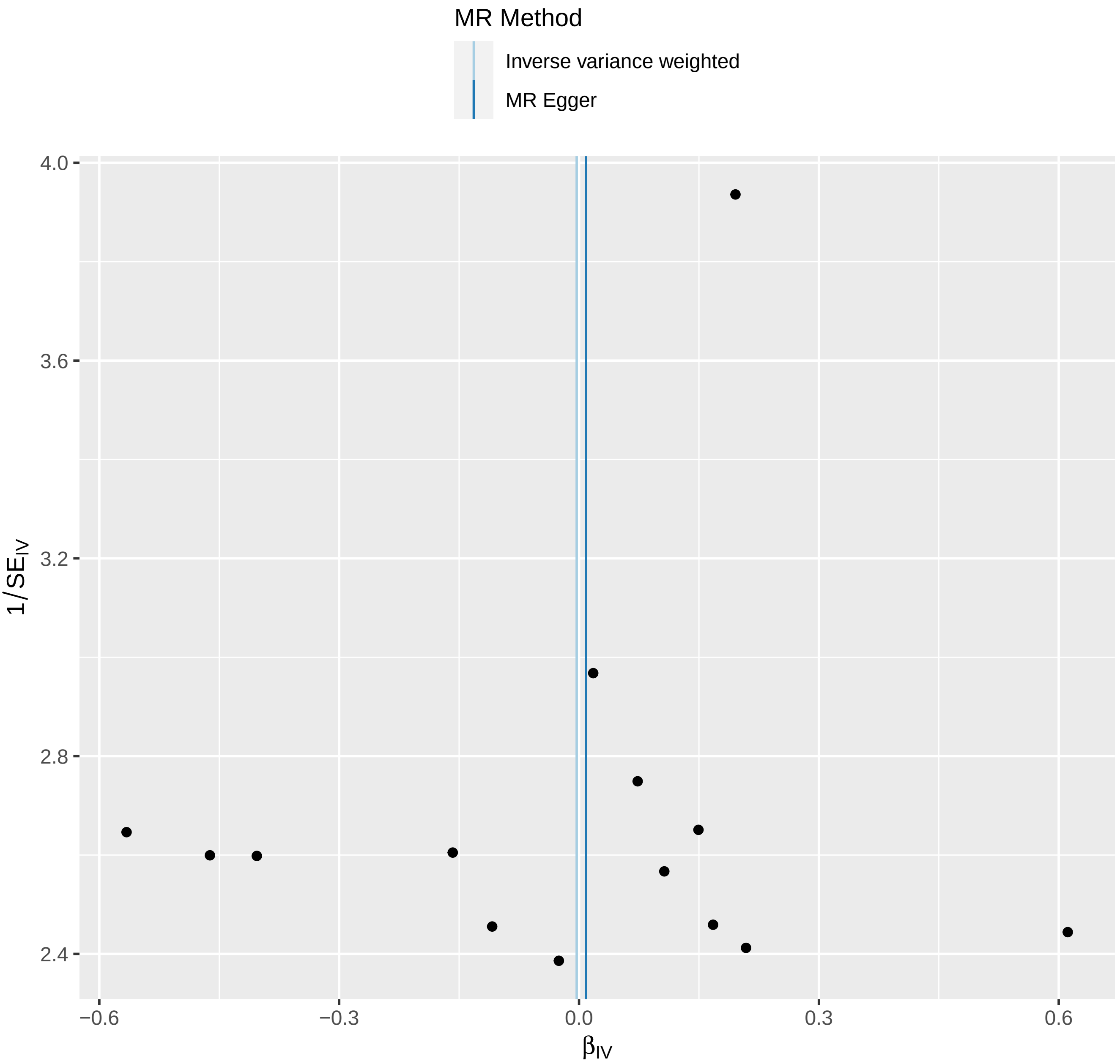

Batch 2−2605 : Gut microbiota abundance (genus Catenibacterium id.2153) on Histone H1x
with a threshold of 1e−05

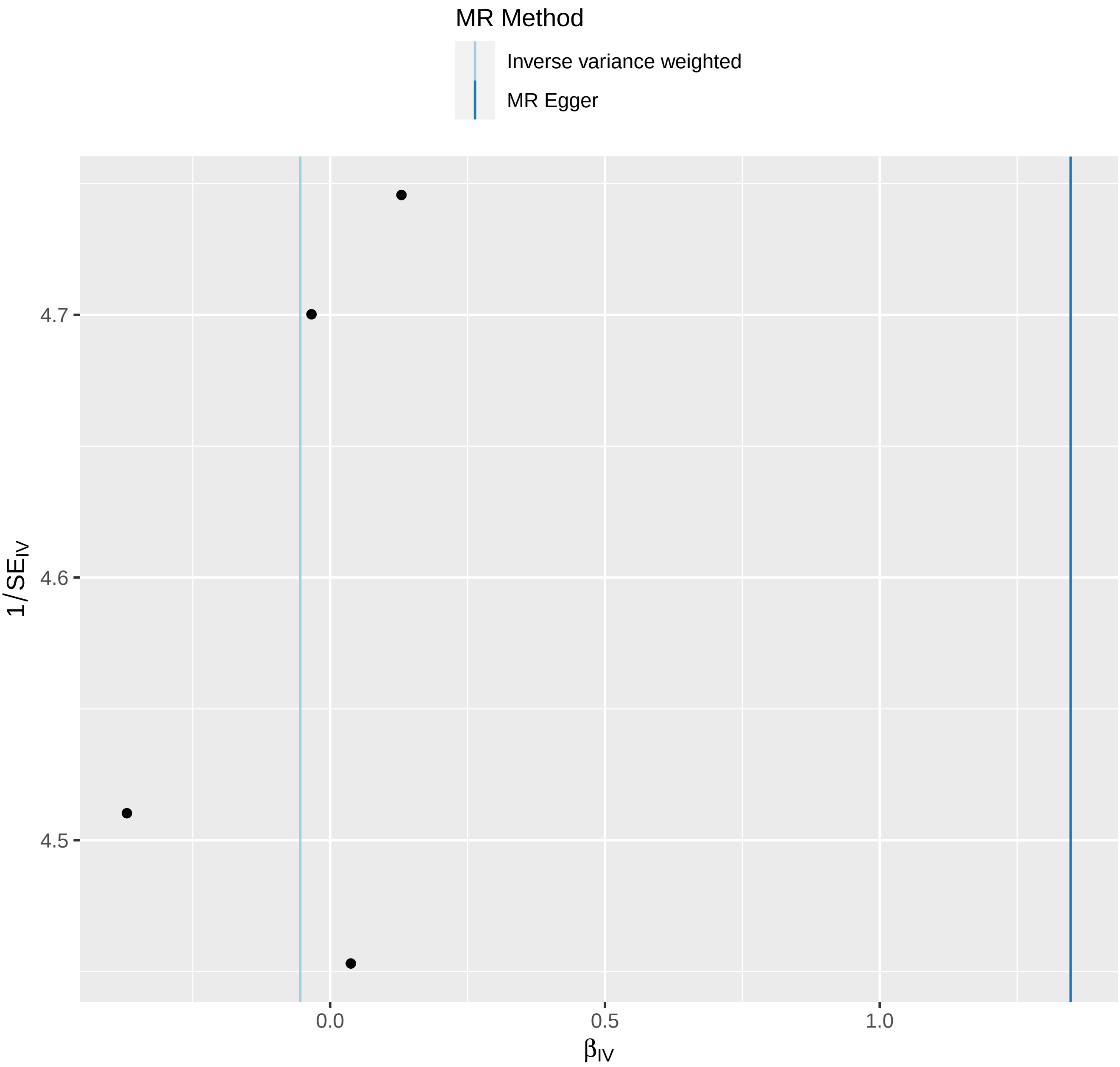

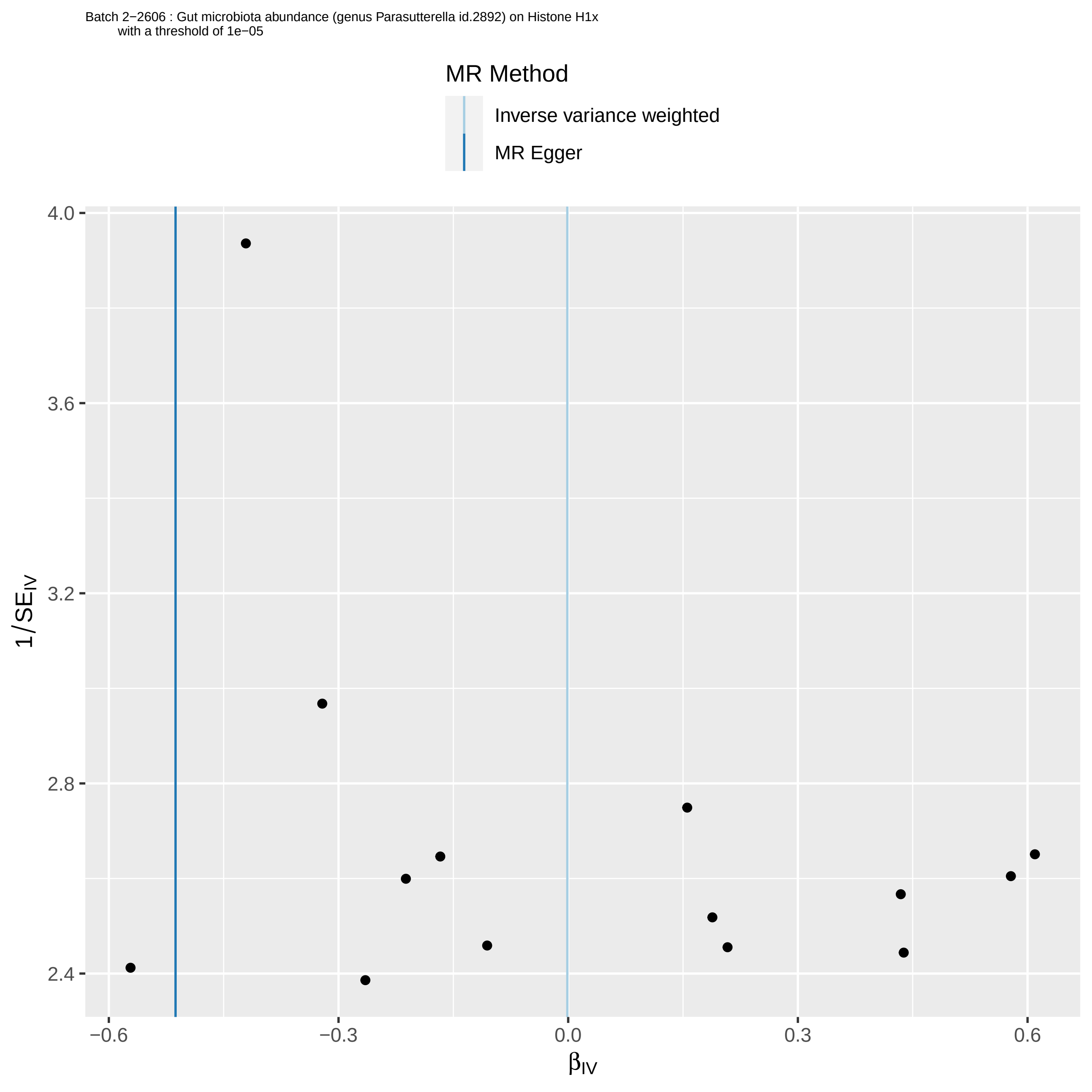

Batch 2−2606 : Gut microbiota abundance (genus Parasutterella id.2892) on Histone H1x
with a threshold of 1e−05
MR Method
Inverse variance weighted
MR Egger
4.0
3.6
3.2
2.8
2.4
1/SE_IV
−0.6
−0.3
0.0
0.3
0.6
β_IV

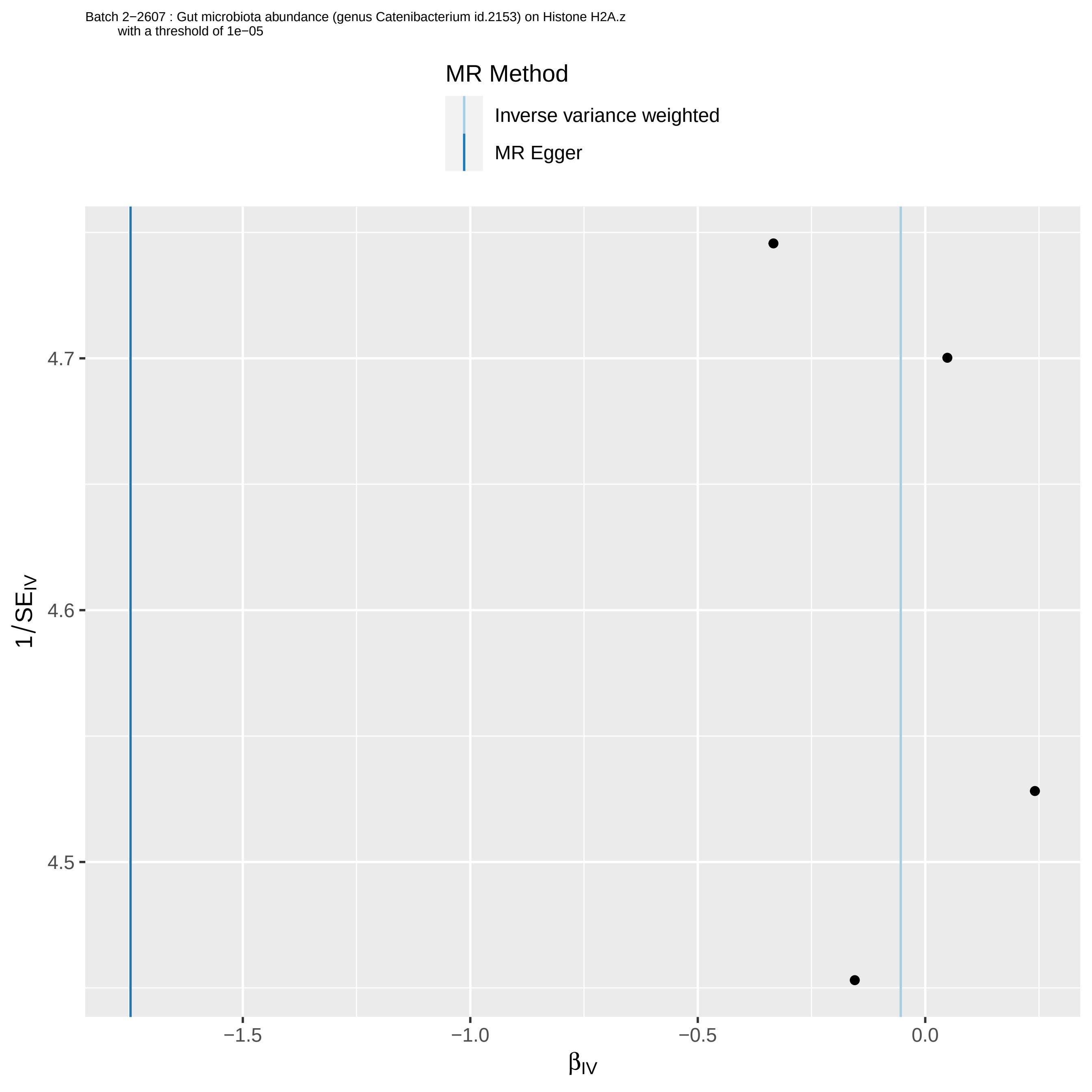
Batch 2−2607 : Gut microbiota abundance (genus Catenibacterium id.2153) on Histone H2A.z
with a threshold of 1e−05
MR Method
Inverse variance weighted
MR Egger
4.7
4.6
4.5
1/SE_IV
−1.5
−1.0
−0.5
0.0
β_IV

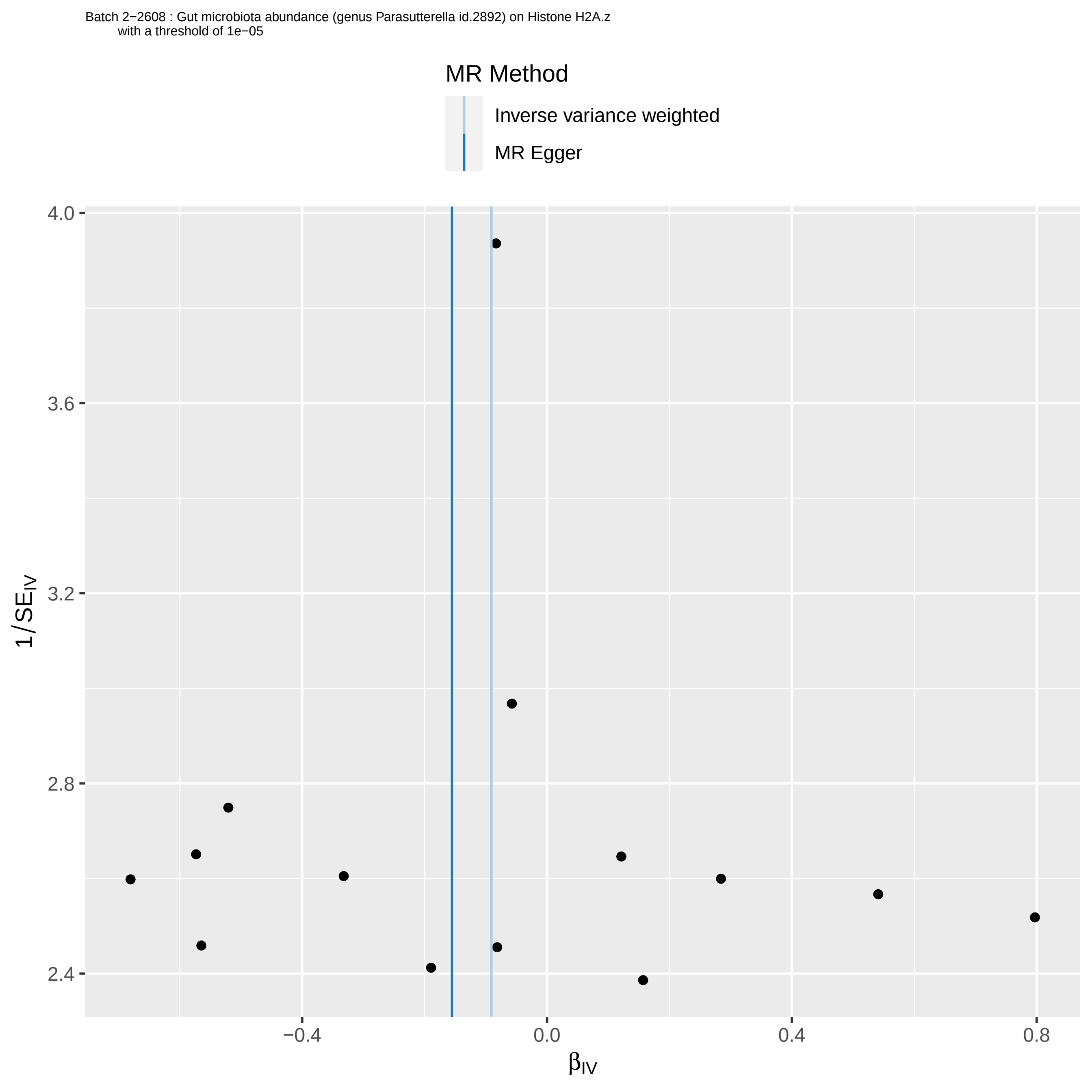

Batch 2−2608 : Gut microbiota abundance (genus Parasutterella id.2892) on Histone H2A.z
with a threshold of 1e−05
MR Method
Inverse variance weighted
MR Egger
4.0
3.6
3.2
2.8
2.4
1/SE_IV
−0.4
0.0
0.4
0.8
β_IV

Batch 2−2609 : Gut microbiota abundance (genus Catenibacterium id.2153) on GDH/6PGL endoplasmic bifunctional protein with a threshold of 1e−05

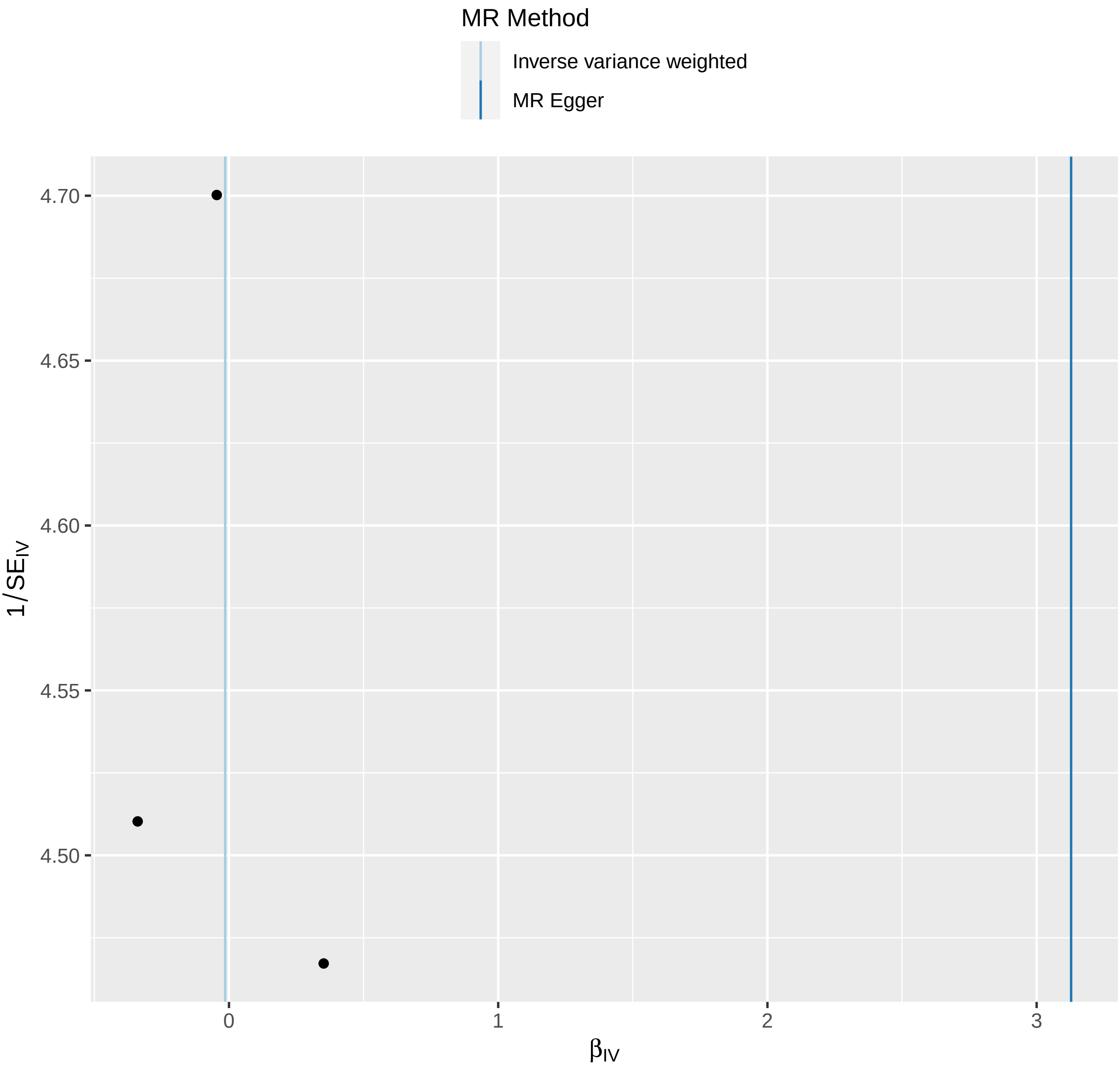

Batch 2−2610 : Gut microbiota abundance (genus Parasutterella id.2892) on GDH/6PGL endoplasmic bifunctional protein
with a threshold of 1e−05

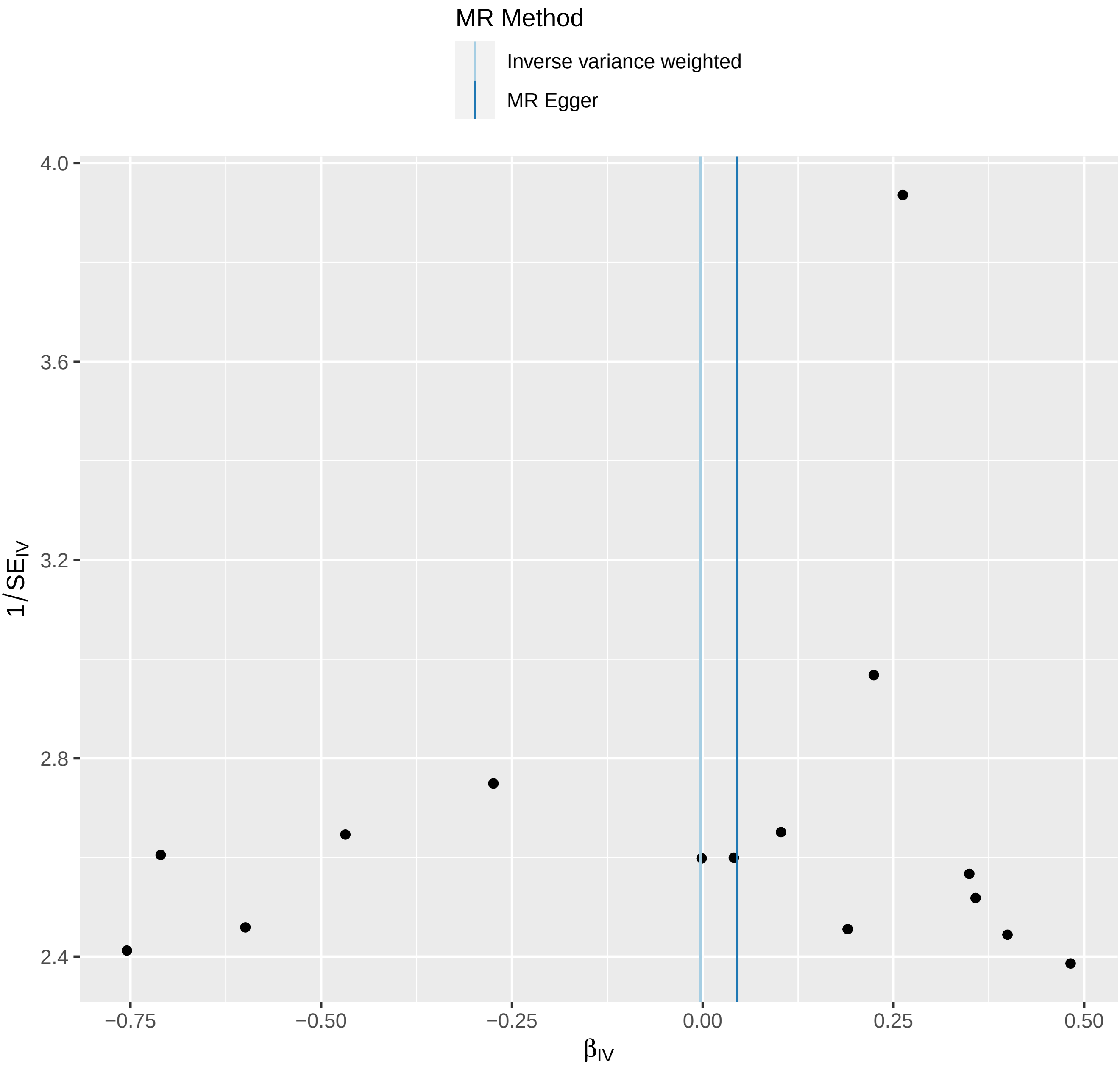

Batch 2−2611 : Gut microbiota abundance (genus Catenibacterium id.2153) on 3−hydroxyanthranilate 3,4−dioxygenase with a threshold of 1e−05

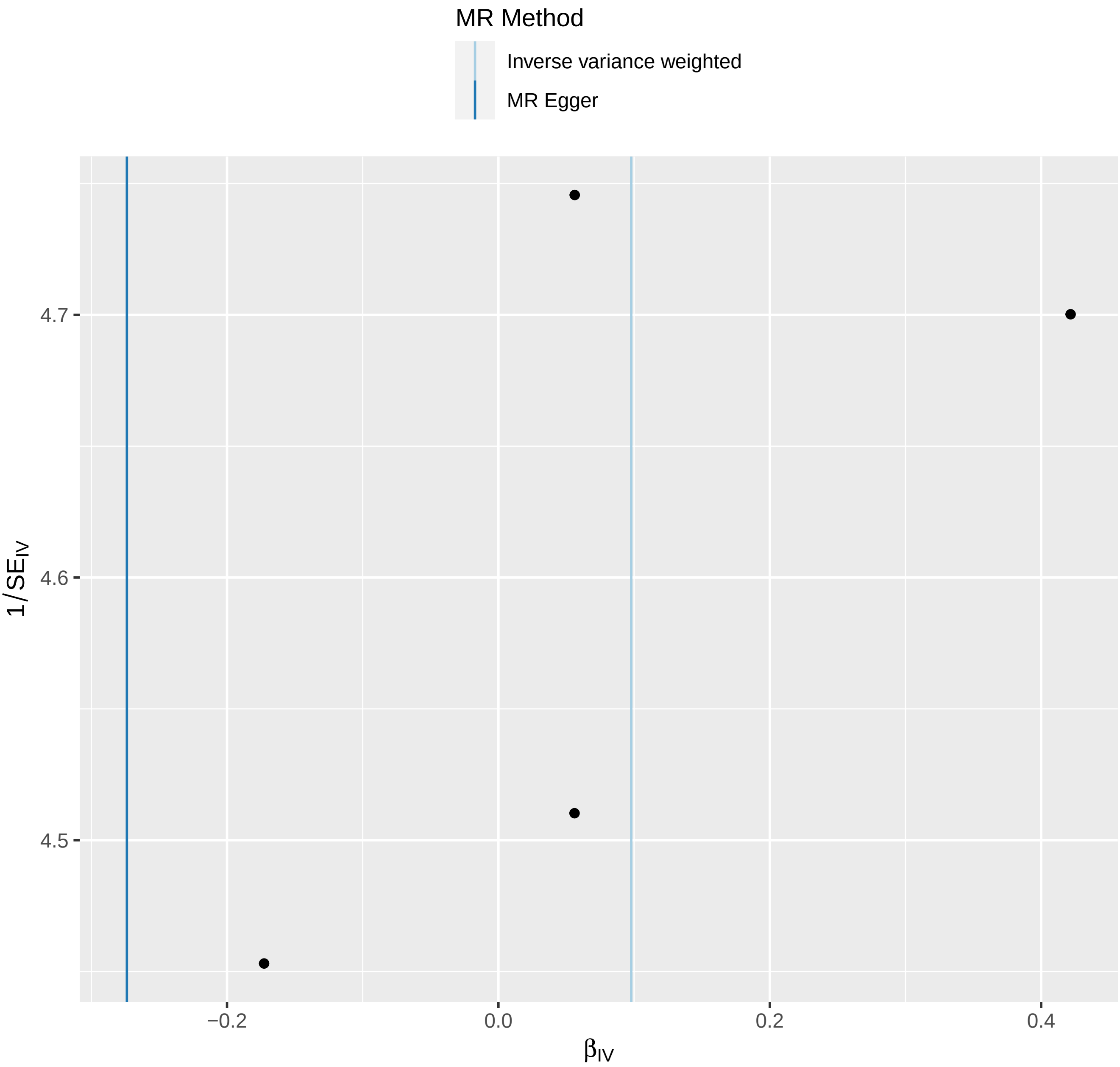

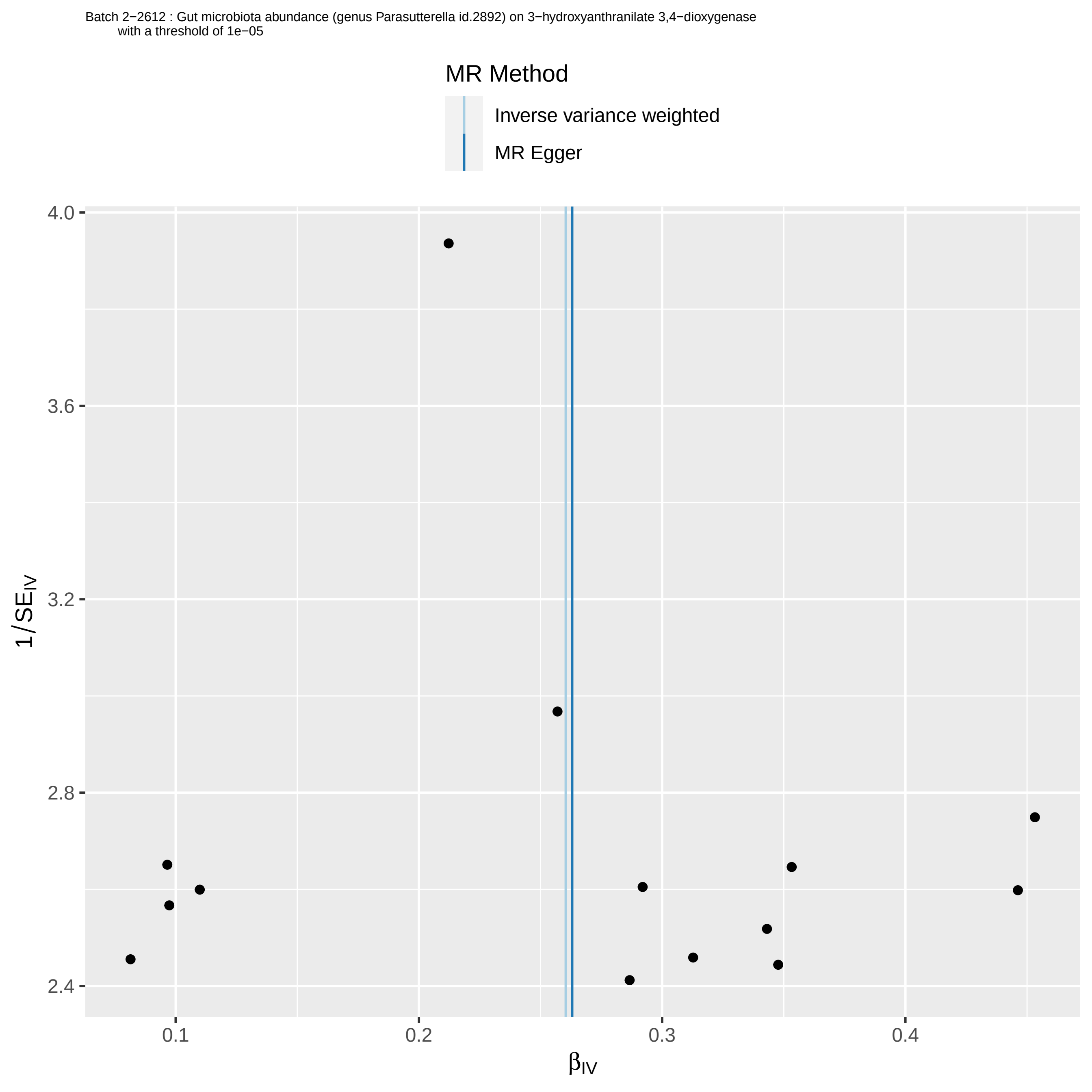
Batch 2−2612 : Gut microbiota abundance (genus Parasutterella id.2892) on 3−hydroxyanthranilate 3,4−dioxygenase
with a threshold of 1e−05
MR Method
Inverse variance weighted
MR Egger
4.0
3.6
3.2
2.8
2.4
1/SE_IV
0.1
0.2
0.3
0.4
β_IV

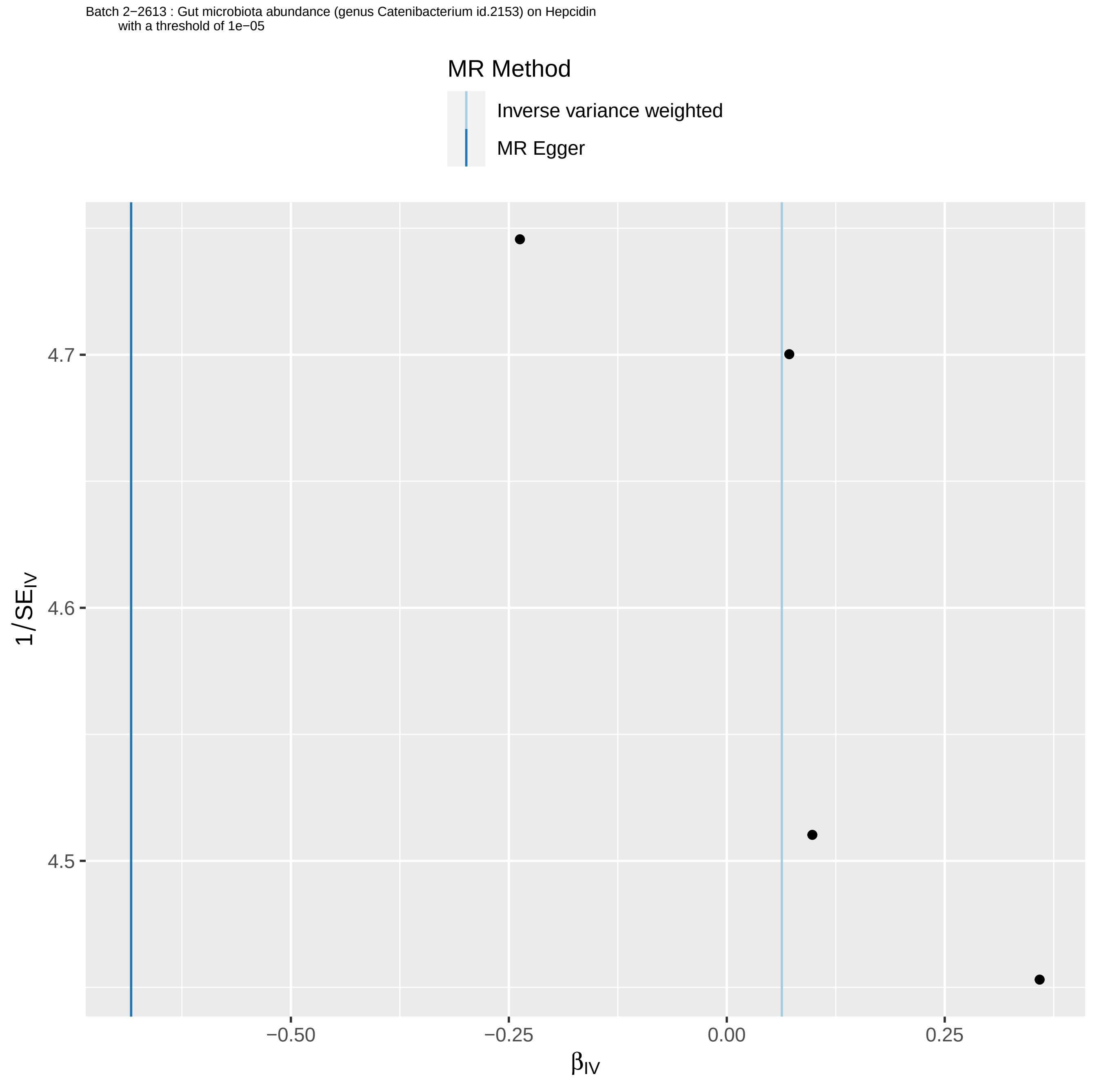
Batch 2−2613 : Gut microbiota abundance (genus Catenibacterium id.2153) on Hepcidin
with a threshold of 1e−05
MR Method
Inverse variance weighted
MR Egger
4.7
4.6
4.5
1/SE_IV
−0.50
−0.25
0.00
0.25
β_IV

Batch 2−2614 : Gut microbiota abundance (genus Parasutterella id.2892) on Hepcidin
with a threshold of 1e−05

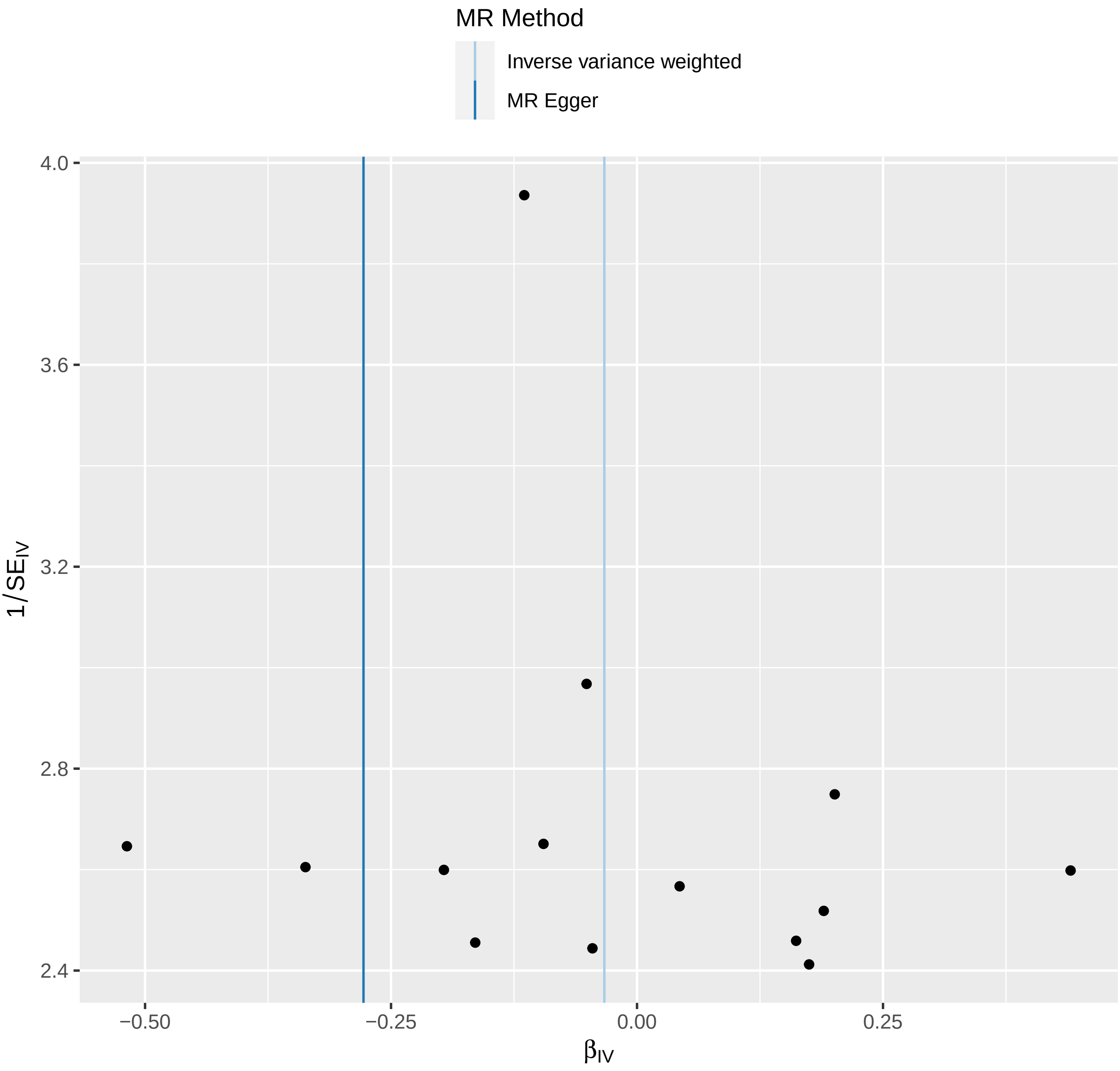

Batch 2−2615 : Gut microbiota abundance (genus Catenibacterium id.2153) on Hyaluronan and proteoglycan link protein 1
with a threshold of 1e−05

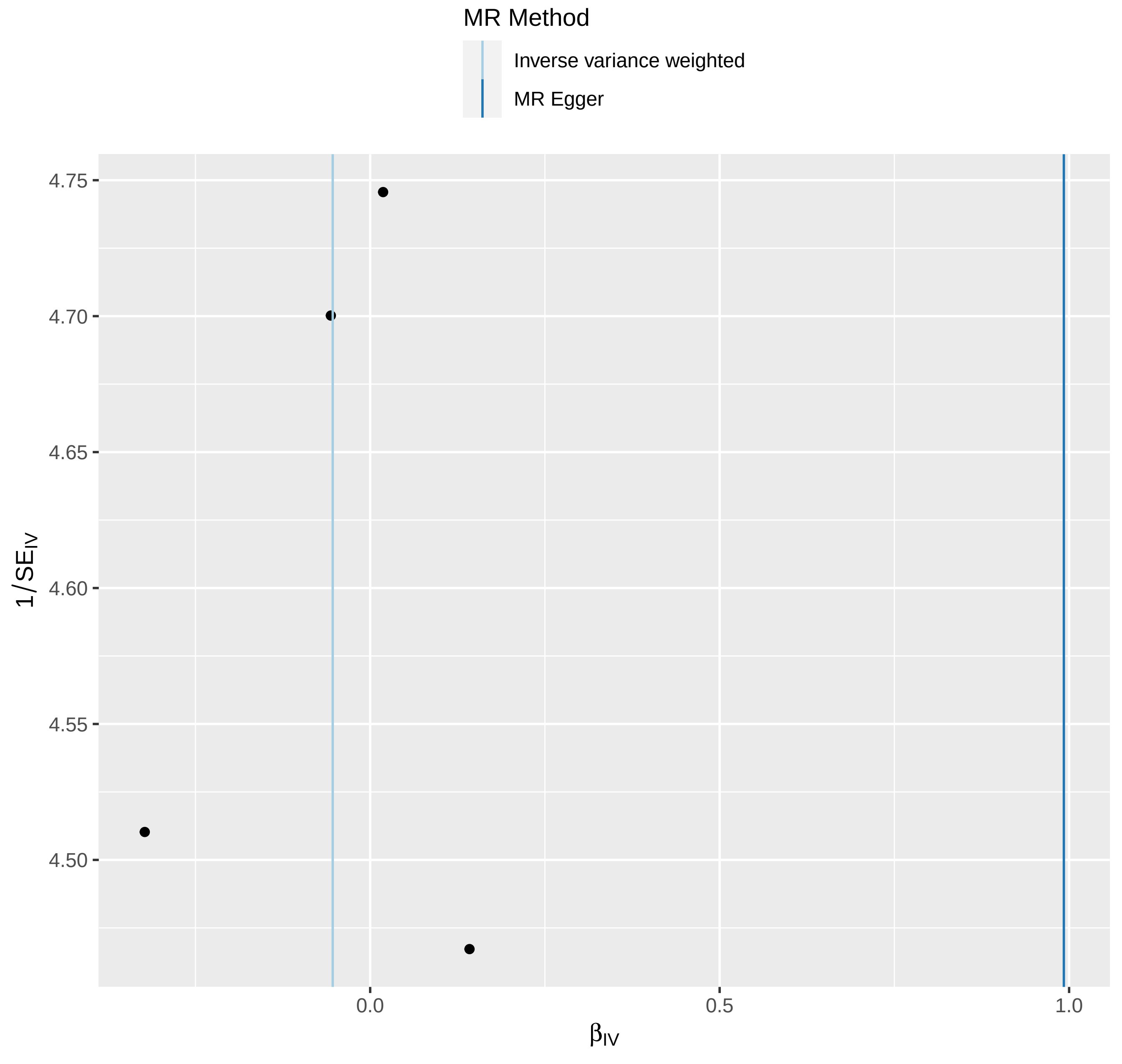

Batch 2−2616 : Gut microbiota abundance (genus Parasutterella id.2892) on Hyaluronan and proteoglycan link protein 1
with a threshold of 1e−05

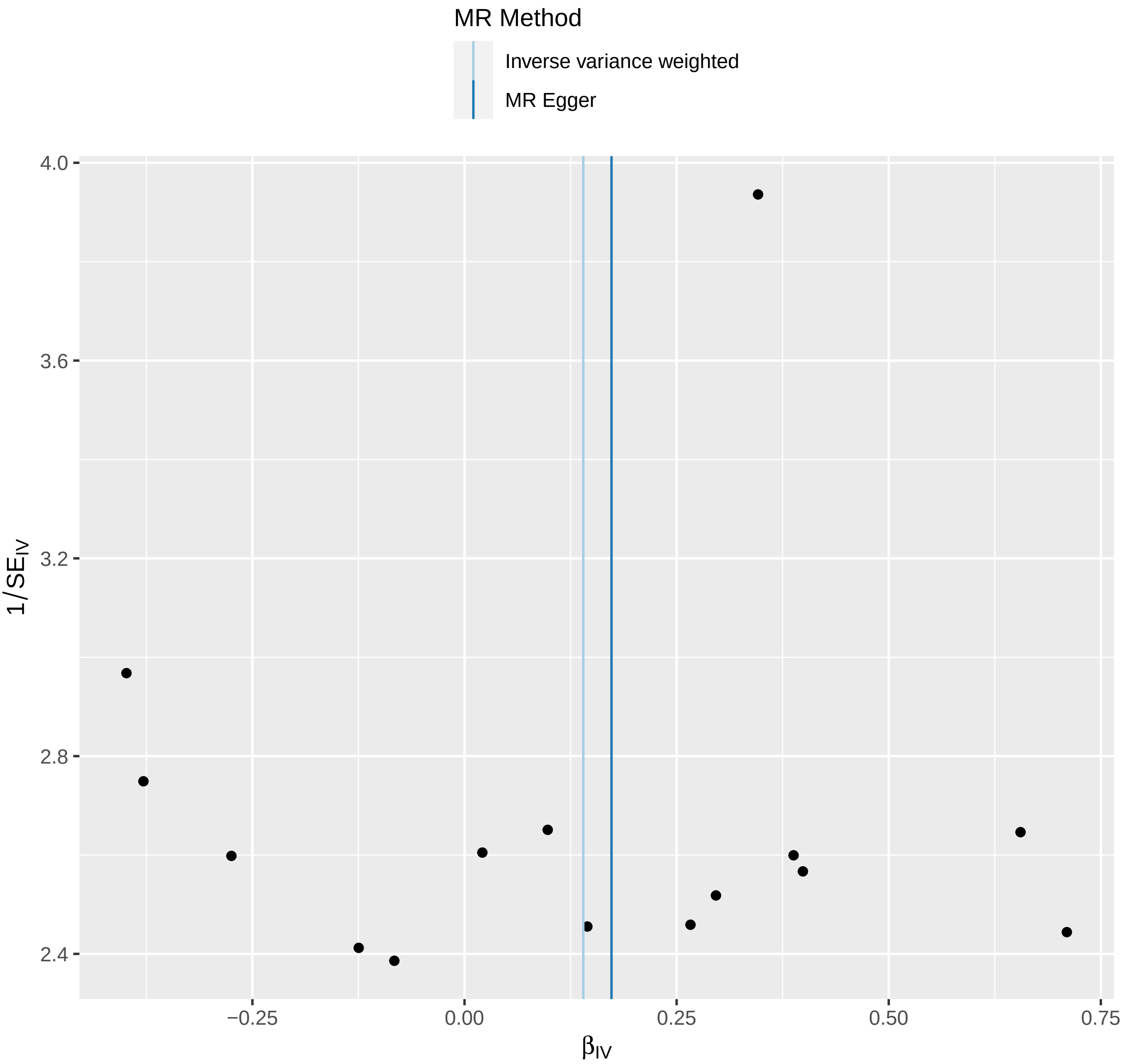

Batch 2−2617 : Gut microbiota abundance (genus Catenibacterium id.2153) on Histone acetyltransferase type B catalytic subunit with a threshold of 1e−05

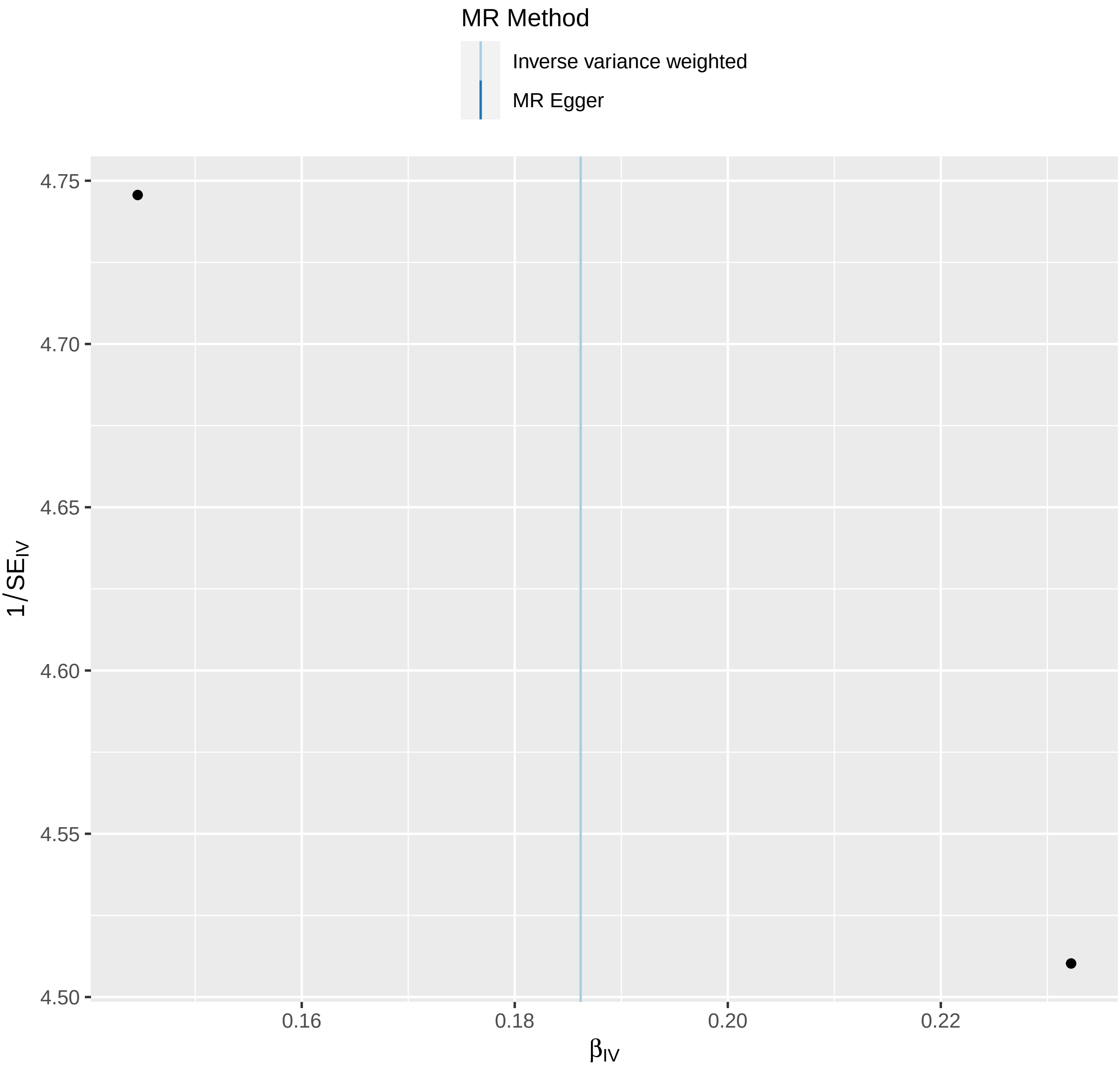

Batch 2−2618 : Gut microbiota abundance (genus Parasutterella id.2892) on Histone acetyltransferase type B catalytic subunit
with a threshold of 1e−05

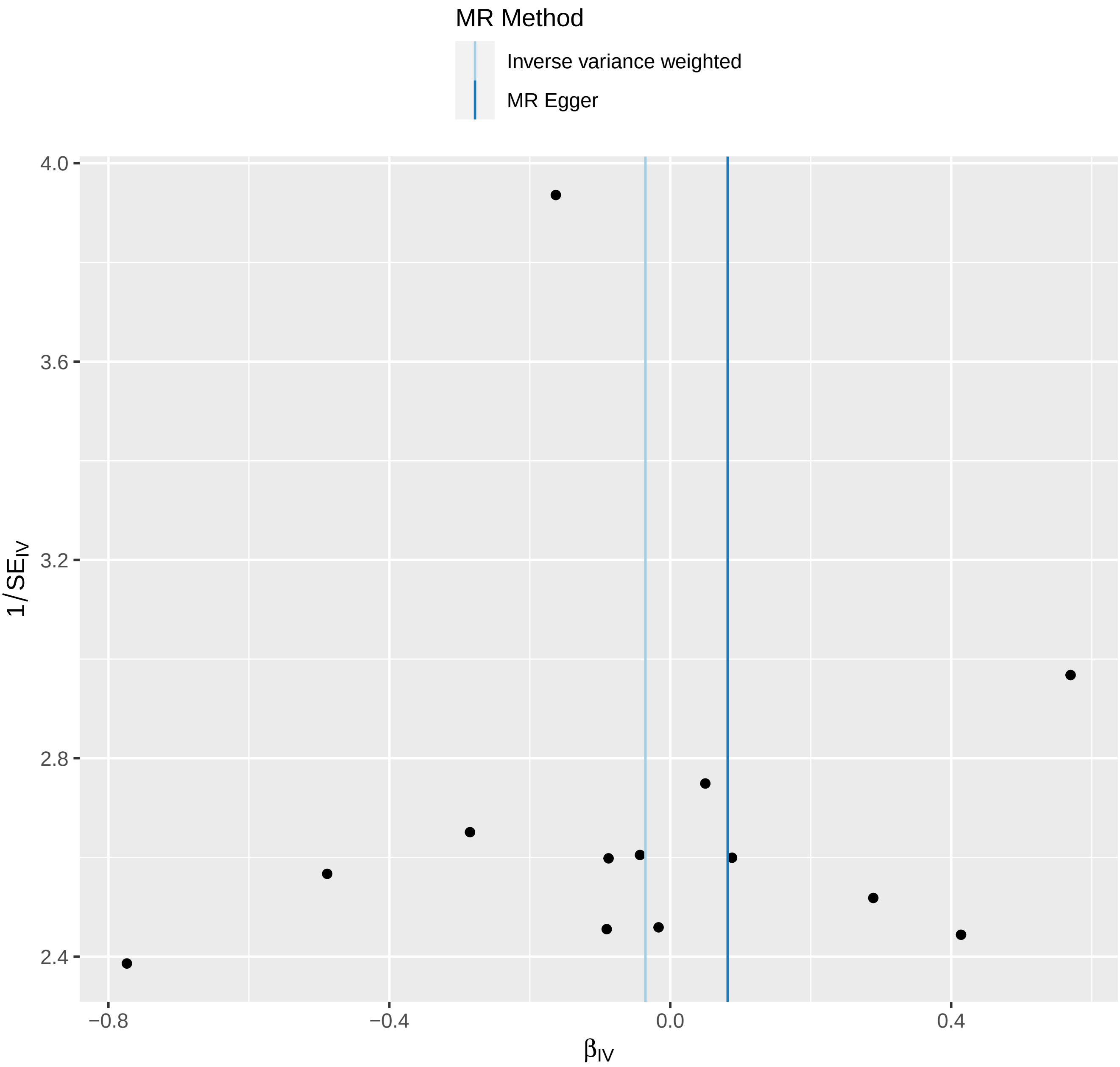

Batch 2−2619 : Gut microbiota abundance (genus Catenibacterium id.2153) on Hepatitis A virus cellular receptor 1
with a threshold of 1e−05

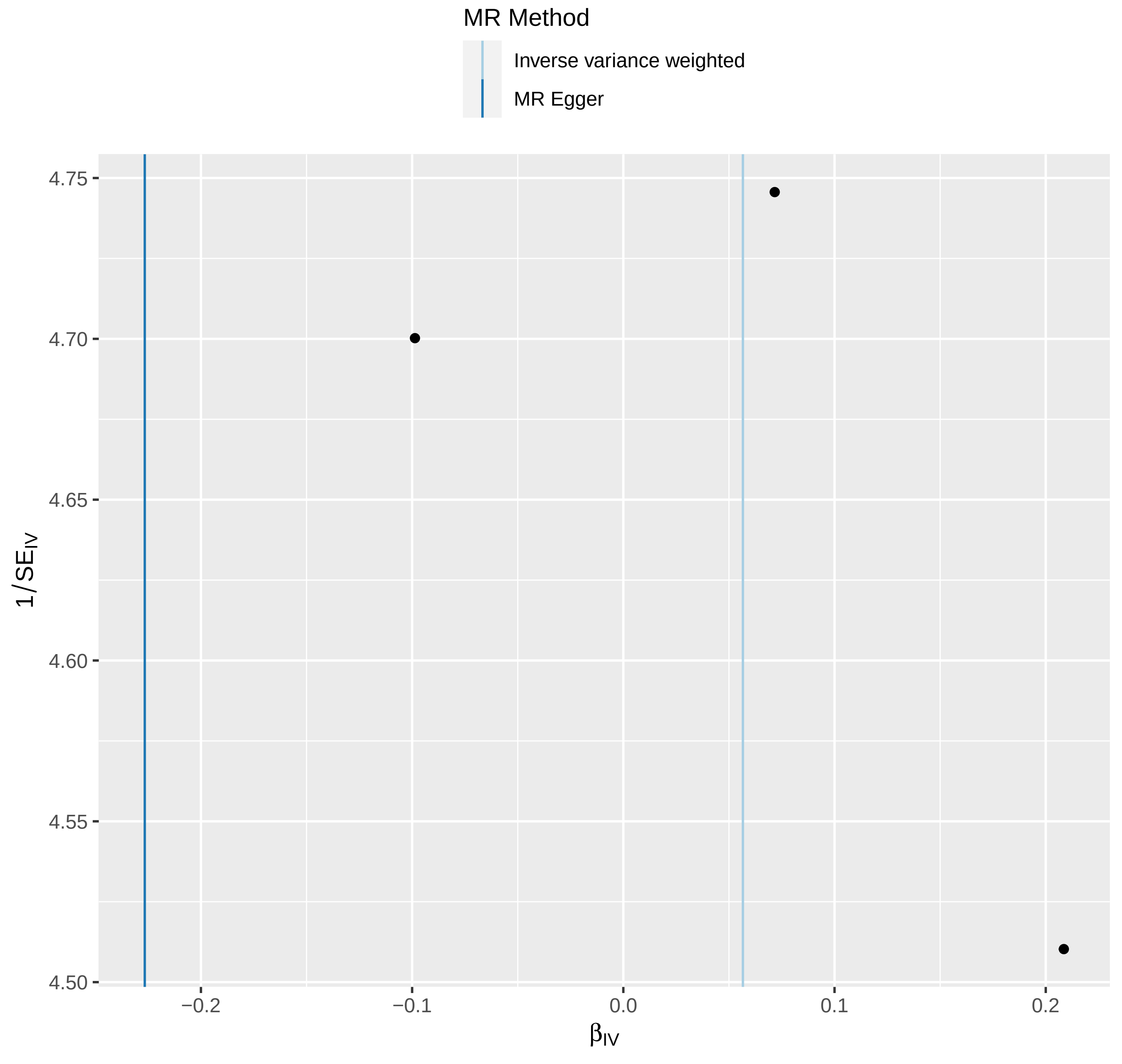

Batch 2−2620 : Gut microbiota abundance (genus Parasutterella id.2892) on Hepatitis A virus cellular receptor 1
with a threshold of 1e−05

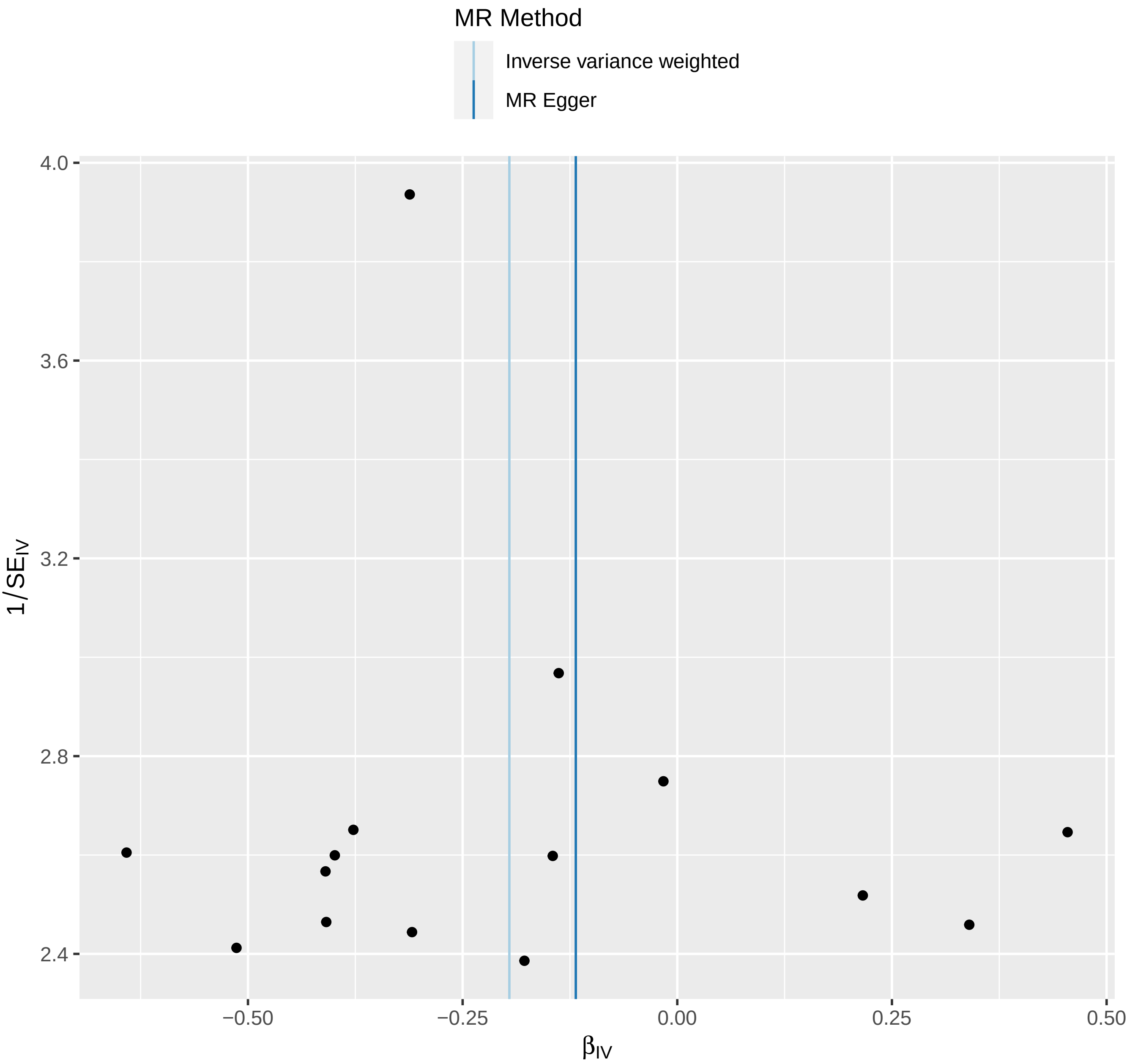

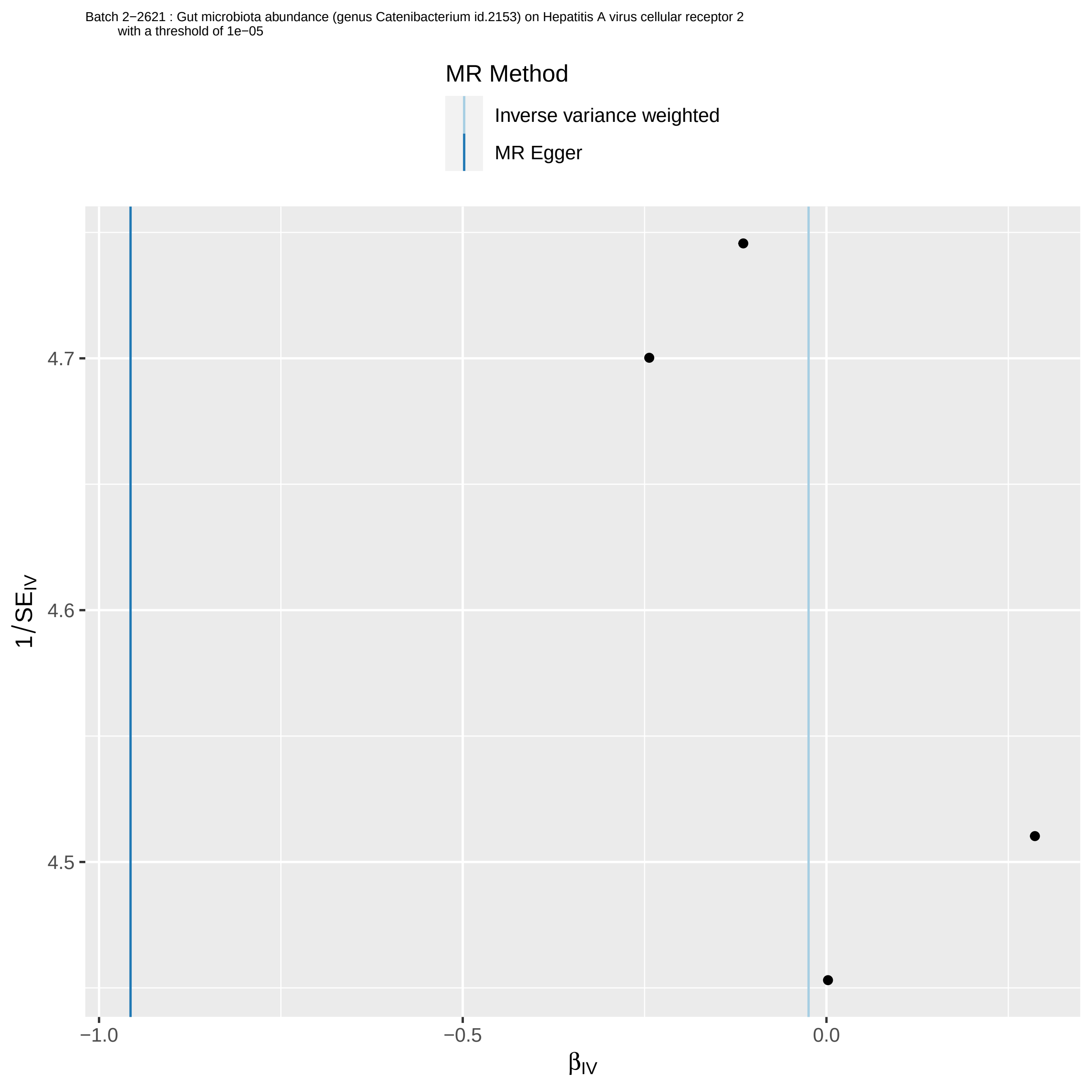
Batch 2−2621 : Gut microbiota abundance (genus Catenibacterium id.2153) on Hepatitis A virus cellular receptor 2
with a threshold of 1e−05
MR Method
Inverse variance weighted
MR Egger
4.7
4.6
4.5
1/SE_IV
−1.0
−0.5
0.0
β_IV

Batch 2−2622 : Gut microbiota abundance (genus Parasutterella id.2892) on Hepatitis A virus cellular receptor 2
with a threshold of 1e−05

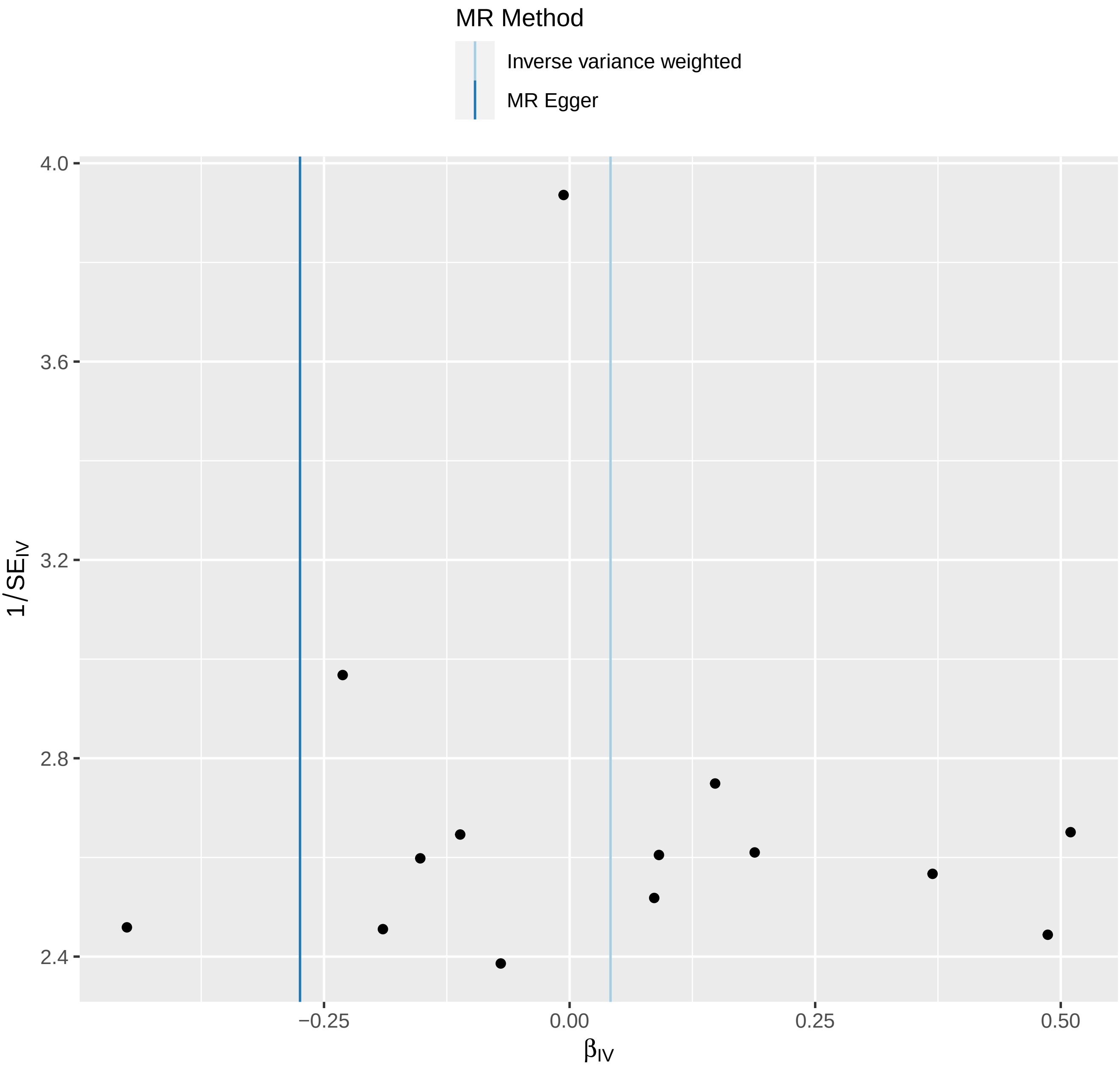

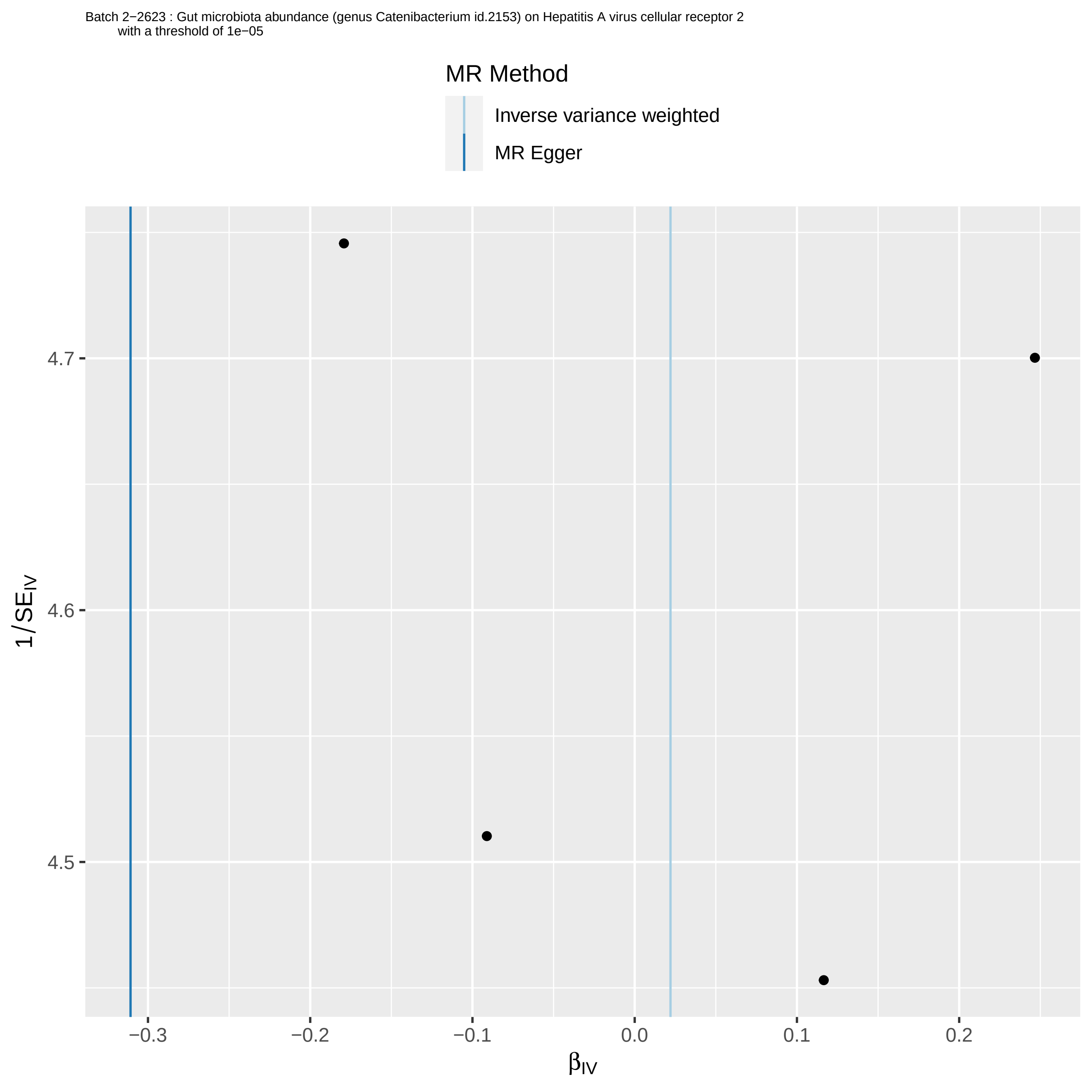
Batch 2−2623 : Gut microbiota abundance (genus Catenibacterium id.2153) on Hepatitis A virus cellular receptor 2
with a threshold of 1e−05
MR Method
Inverse variance weighted
MR Egger
4.7
4.6
4.5
1/SE$_{IV}$
−0.3
−0.2
−0.1
0.0
0.1
0.2
β_{IV}

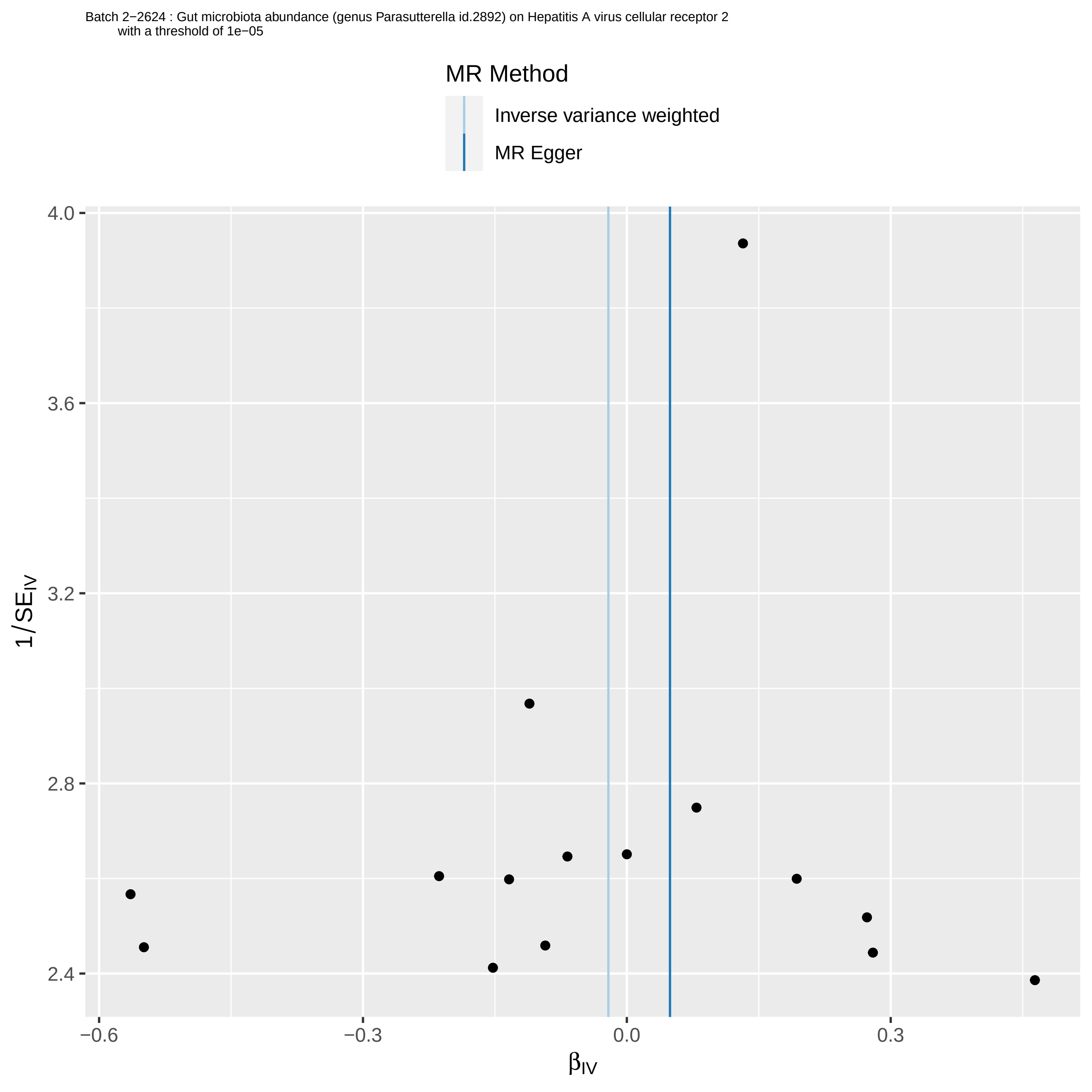

Batch 2−2624 : Gut microbiota abundance (genus Parasutterella id.2892) on Hepatitis A virus cellular receptor 2
with a threshold of 1e−05
MR Method
Inverse variance weighted
MR Egger
4.0
3.6
3.2
2.8
2.4
1/SE_IV
−0.6
−0.3
0.0
0.3
β_IV

Batch 2−2625 : Gut microbiota abundance (genus Catenibacterium id.2153) on Heparin−binding EGF−like growth factor with a threshold of 1e−05

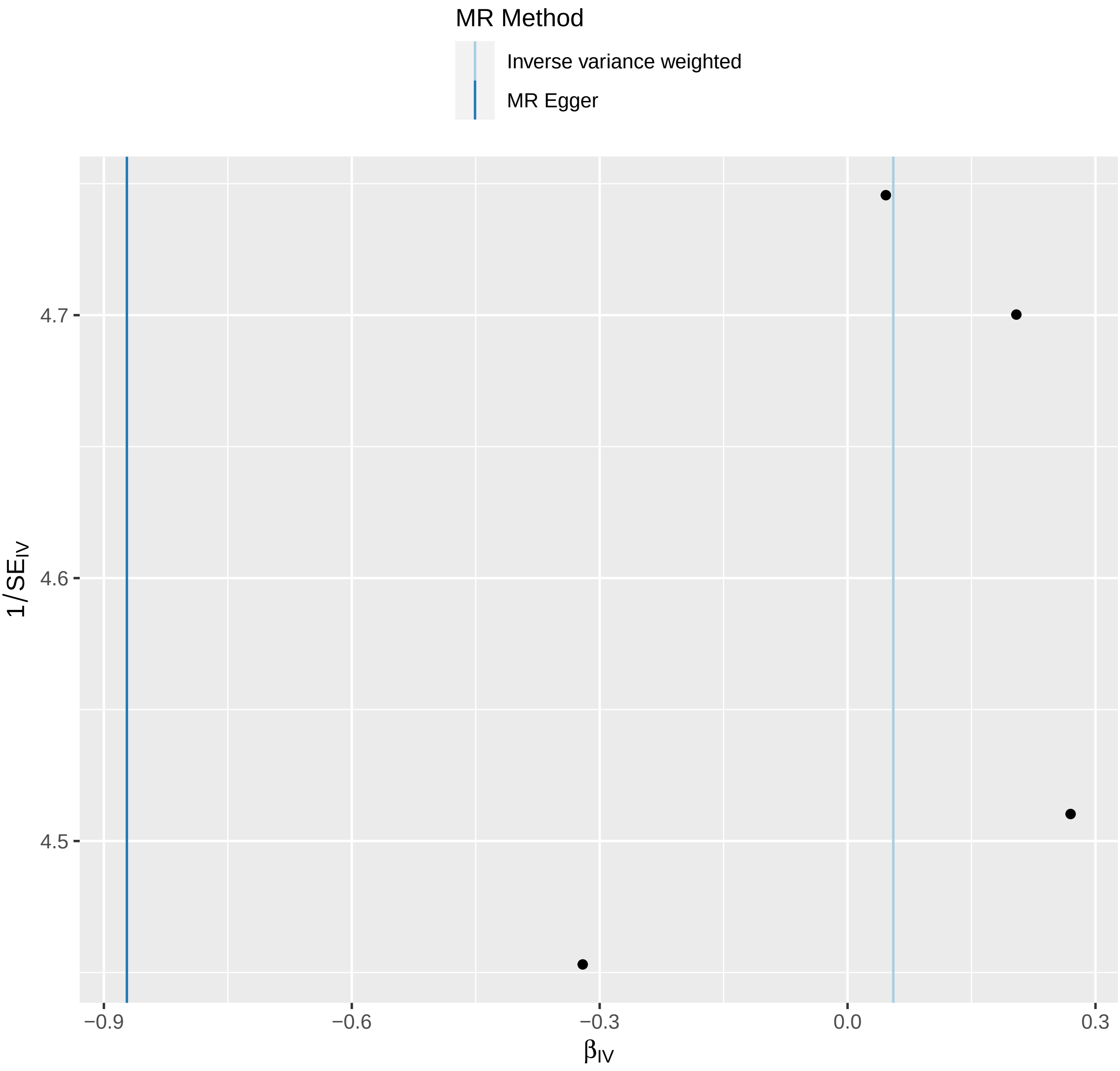

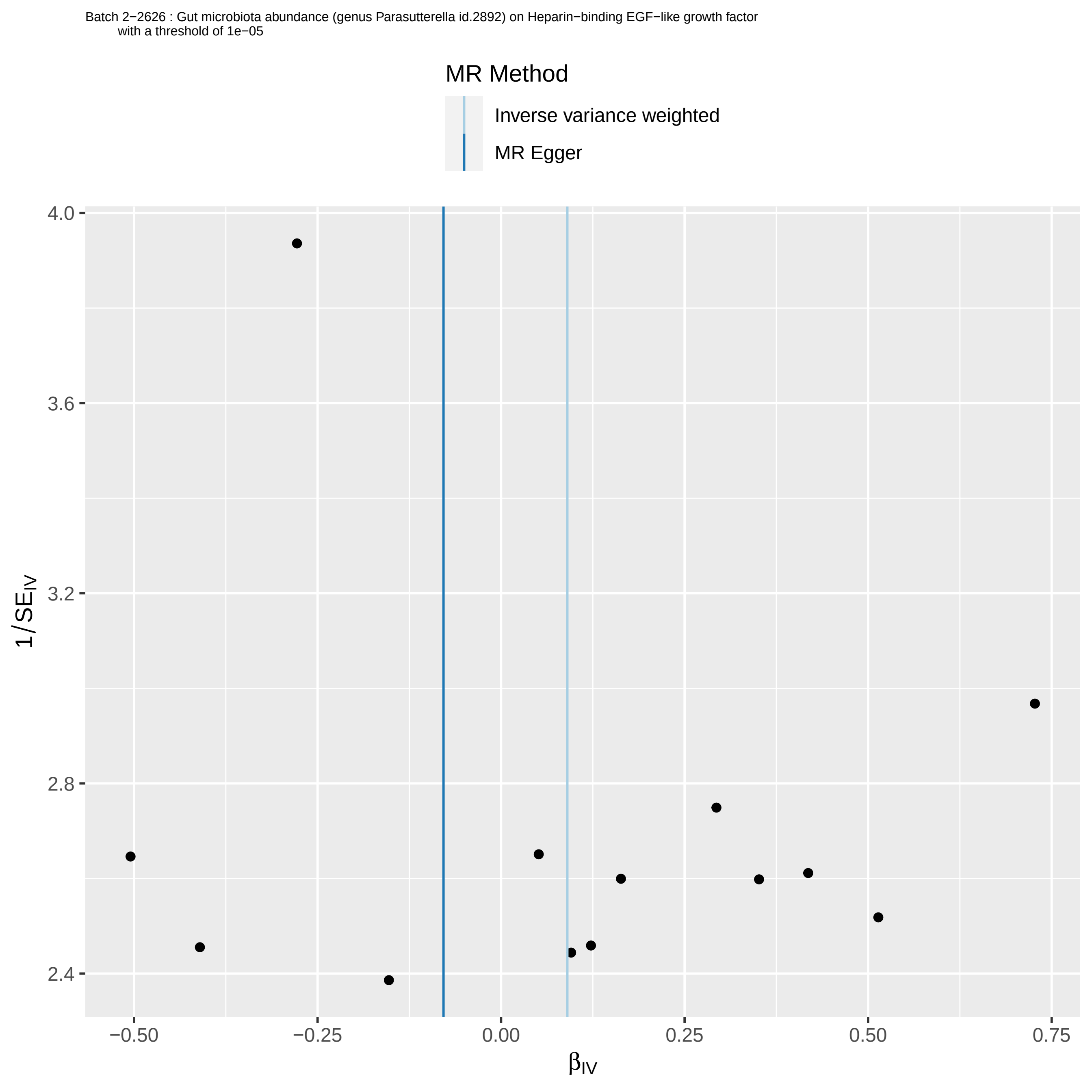
Batch 2−2626 : Gut microbiota abundance (genus Parasutterella id.2892) on Heparin−binding EGF−like growth factor
with a threshold of 1e−05
MR Method
Inverse variance weighted
MR Egger
4.0
3.6
3.2
2.8
2.4
1/SE_IV
−0.50
−0.25
0.00
0.25
0.50
0.75
β_IV

Batch 2−2627 : Gut microbiota abundance (genus Catenibacterium id.2153) on Heparin−binding EGF−like growth factor
with a threshold of 1e−05

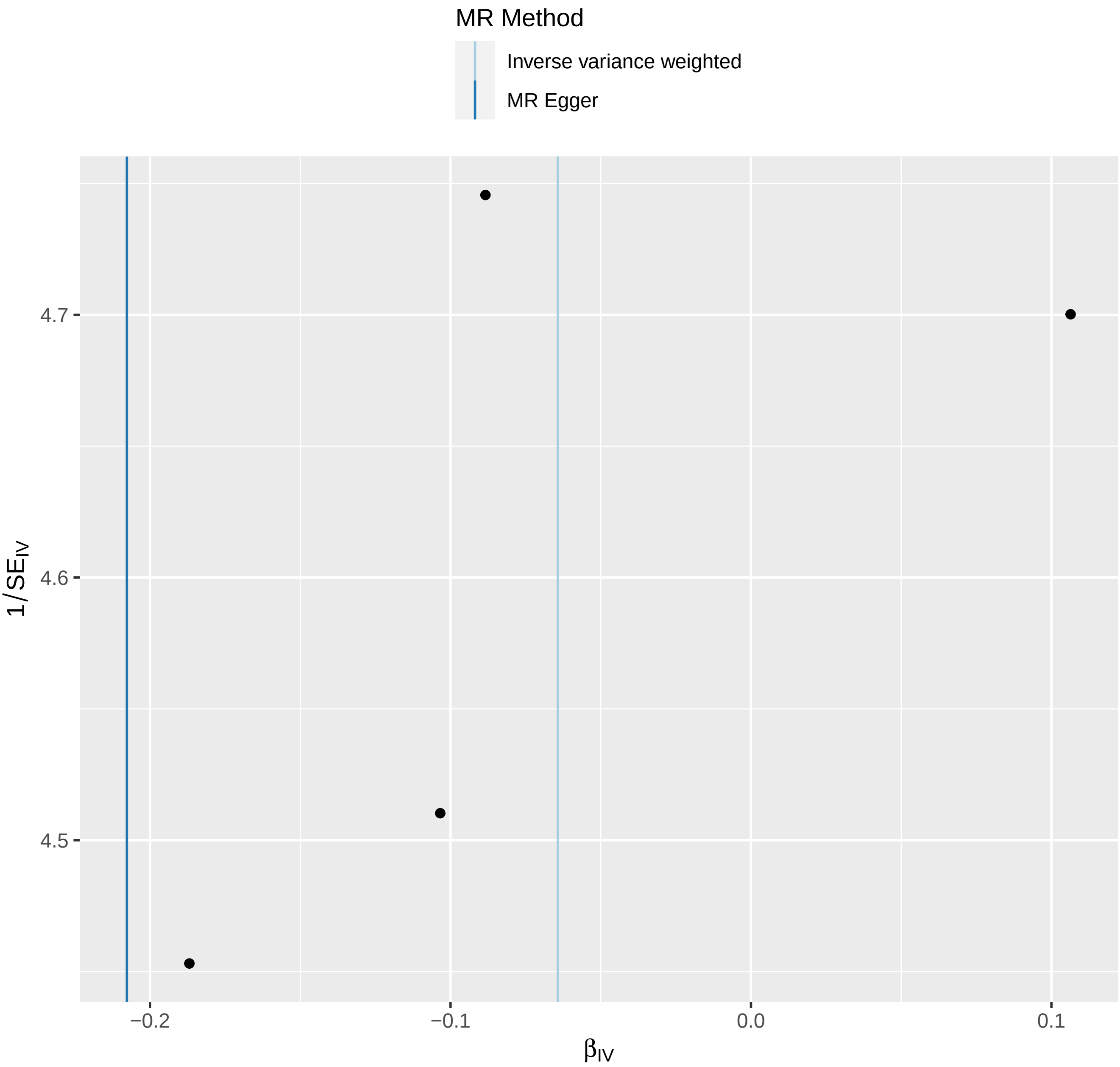

Batch 2−2628 : Gut microbiota abundance (genus Parasutterella id.2892) on Heparin−binding EGF−like growth factor
with a threshold of 1e−05

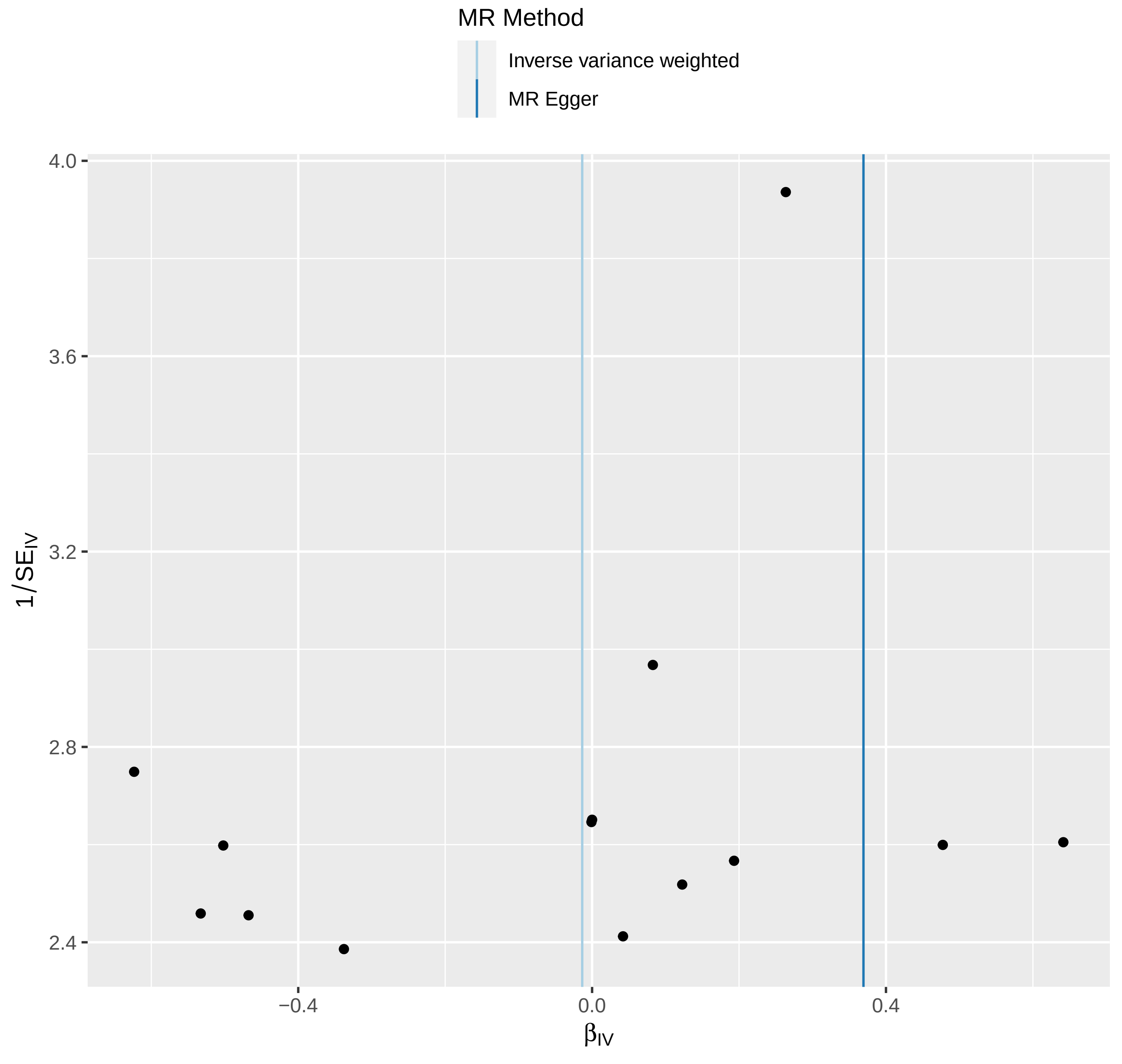

Batch 2−2629 : Gut microbiota abundance (genus Catenibacterium id.2153) on Hemoglobin subunit gamma−1
with a threshold of 1e−05

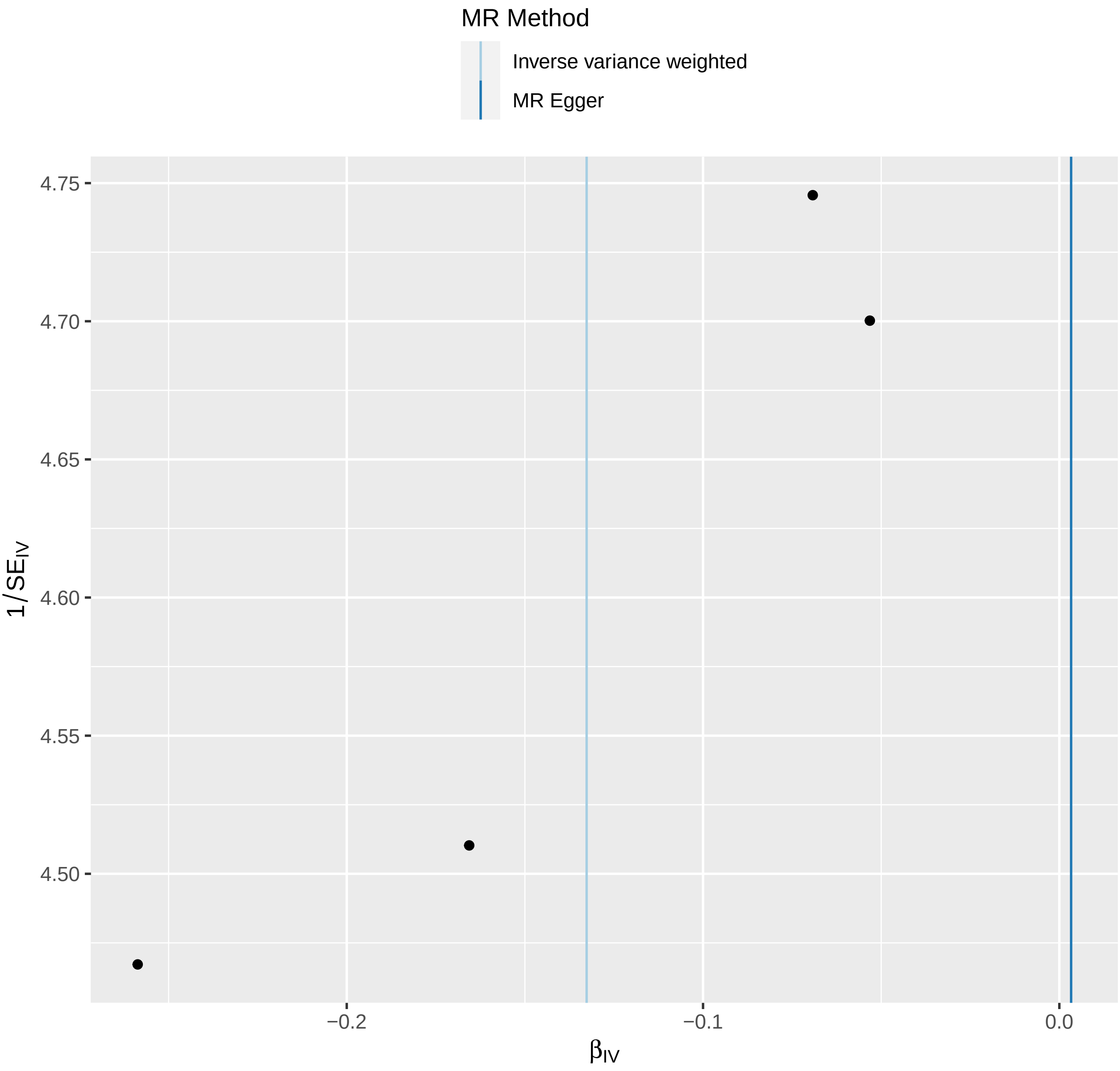

Batch 2−2630 : Gut microbiota abundance (genus Parasutterella id.2892) on Hemoglobin subunit gamma−1
with a threshold of 1e−05

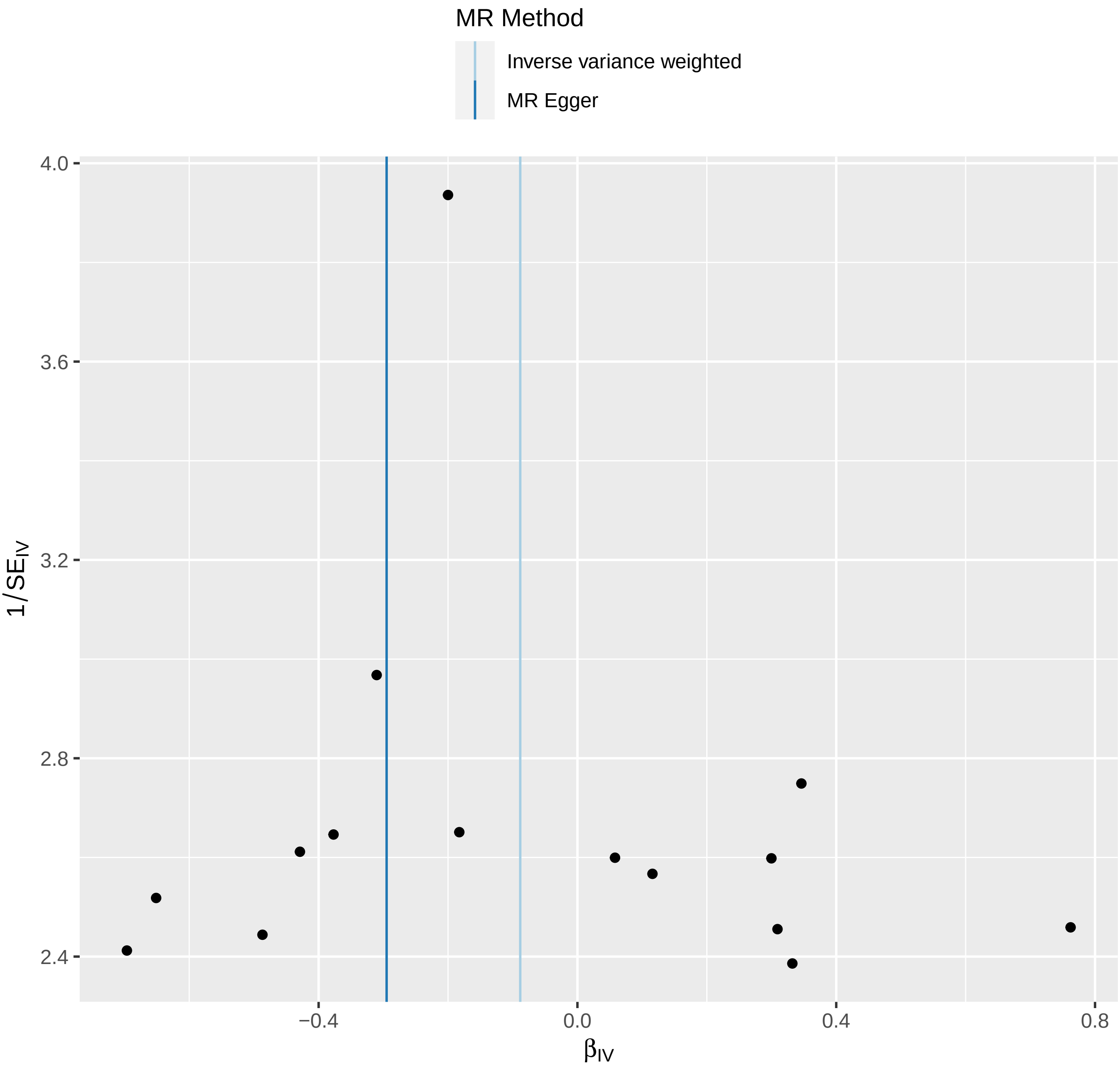

Batch 2−2631 : Gut microbiota abundance (genus Catenibacterium id.2153) on Hemoglobin subunit theta−1
with a threshold of 1e−05

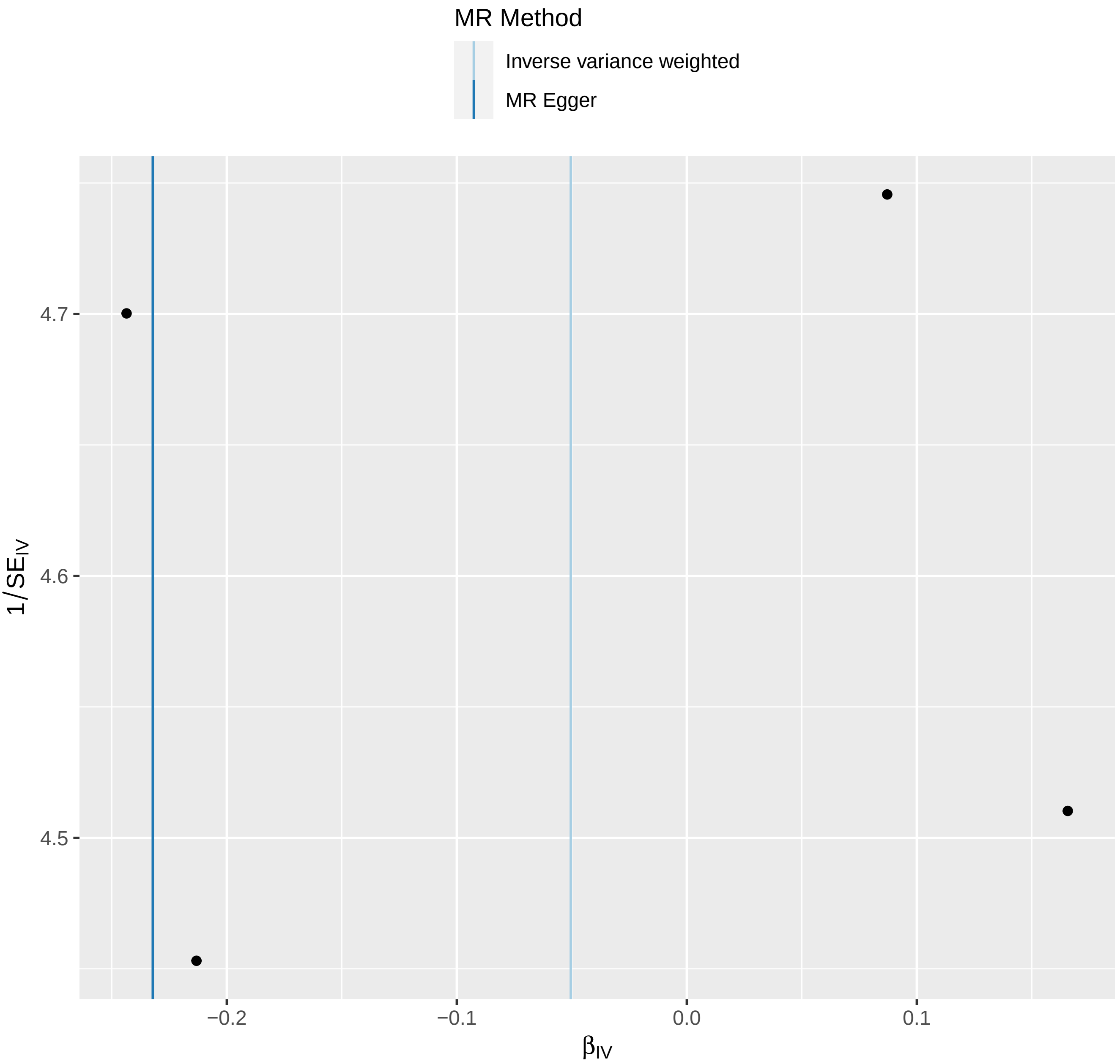

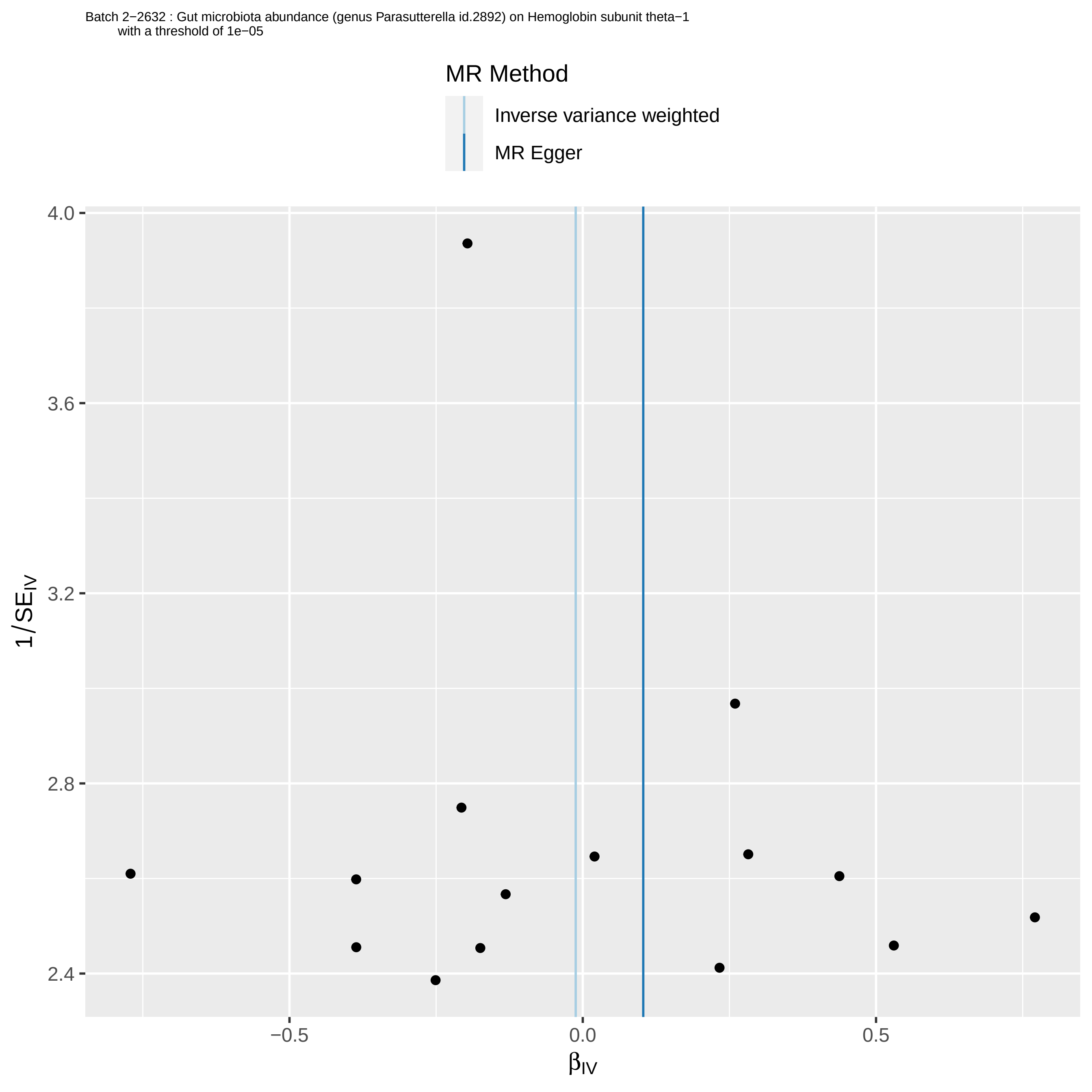

Batch 2−2632 : Gut microbiota abundance (genus Parasutterella id.2892) on Hemoglobin subunit theta−1
with a threshold of 1e−05
MR Method
Inverse variance weighted
MR Egger
4.0
3.6
3.2
2.8
2.4
1/SE$_{IV}$
−0.5
0.0
0.5
β$_{IV}$

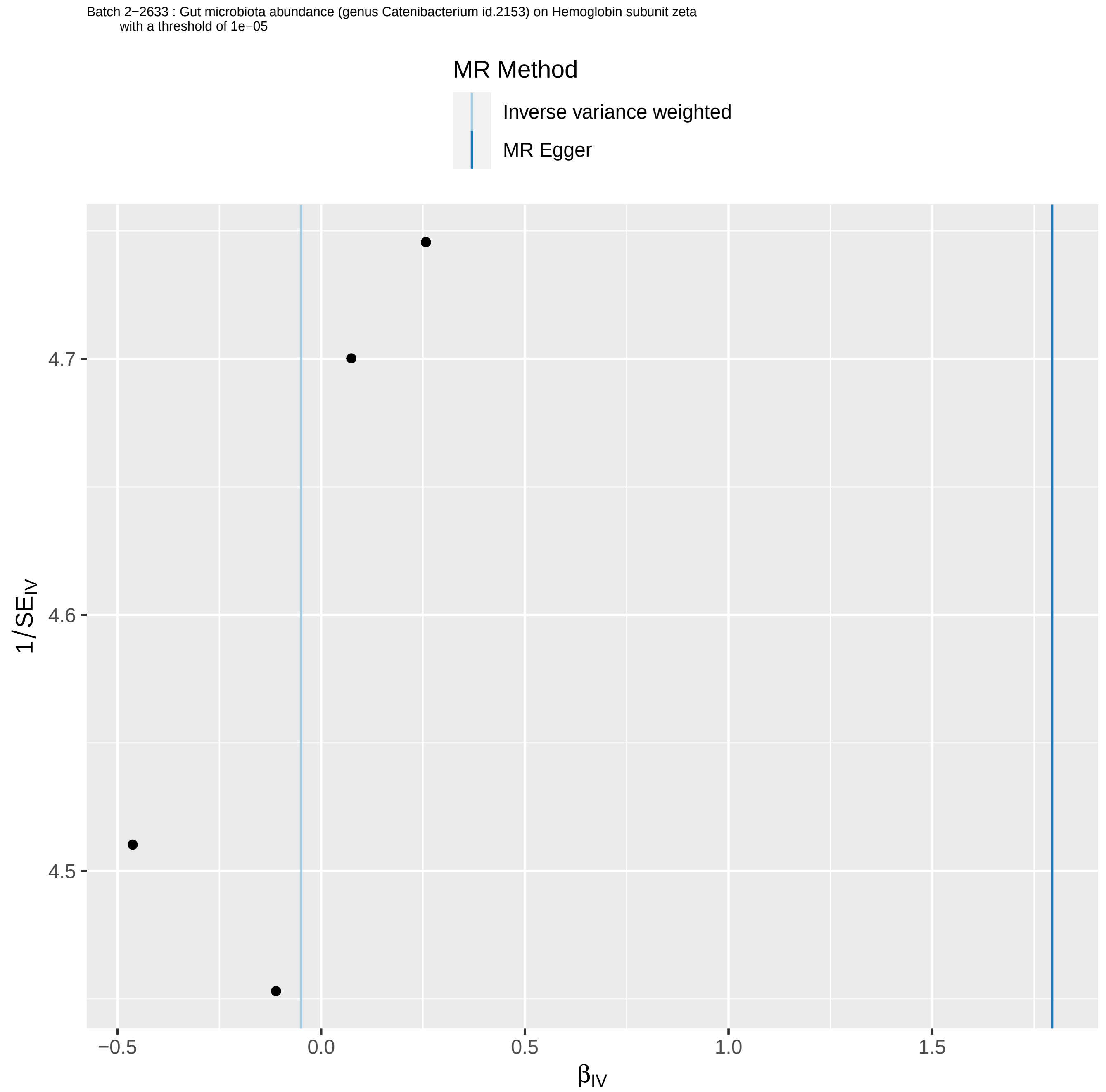
Batch 2−2633 : Gut microbiota abundance (genus Catenibacterium id.2153) on Hemoglobin subunit zeta
with a threshold of 1e−05
MR Method
Inverse variance weighted
MR Egger
4.7
4.6
4.5
1/SE_IV
−0.5
0.0
0.5
1.0
1.5
β_IV

Batch 2−2634 : Gut microbiota abundance (genus Parasutterella id.2892) on Hemoglobin subunit zeta
with a threshold of 1e−05

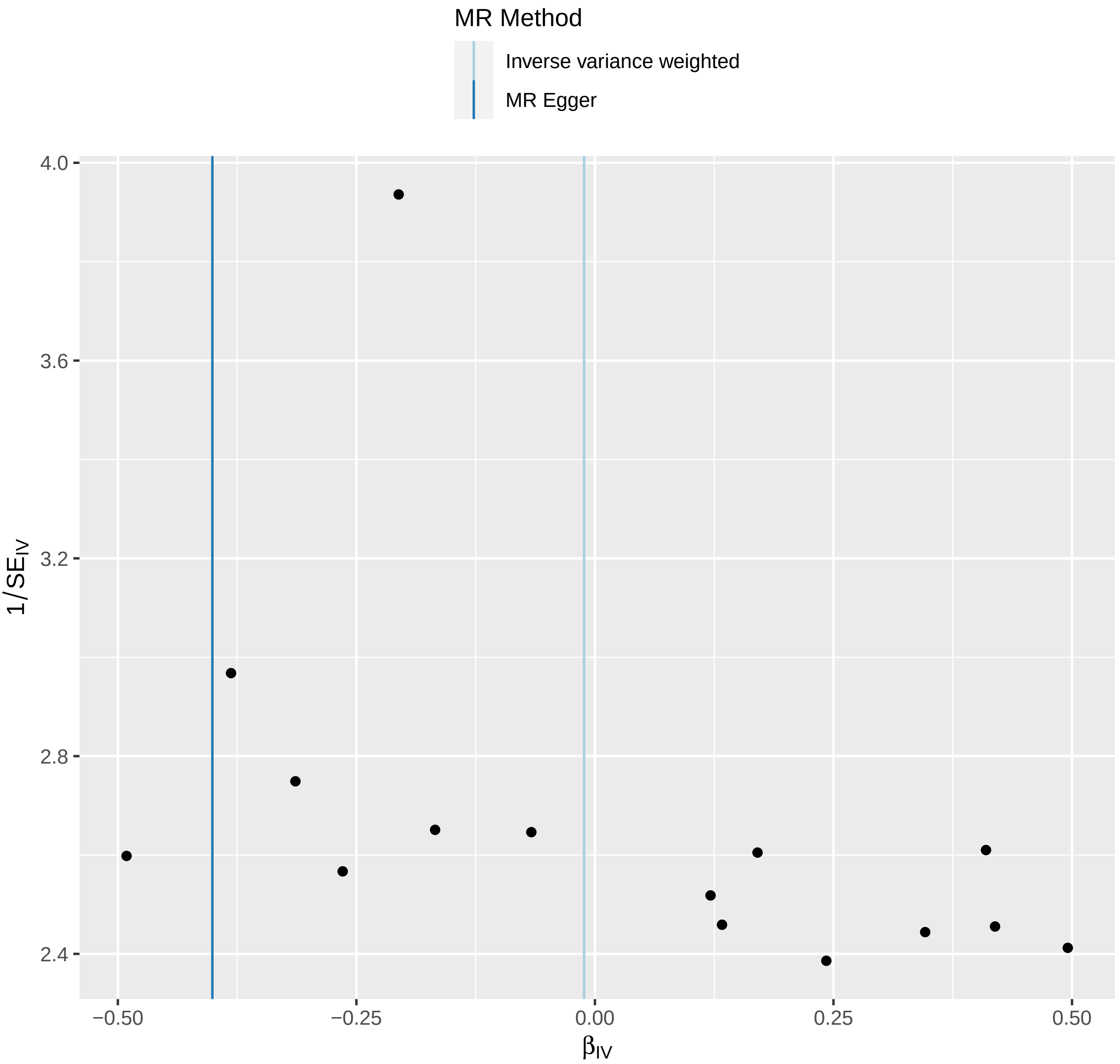

Batch 2−2635 : Gut microbiota abundance (genus Catenibacterium id.2153) on Hydroxycarboxylic acid receptor 2
with a threshold of 1e−05

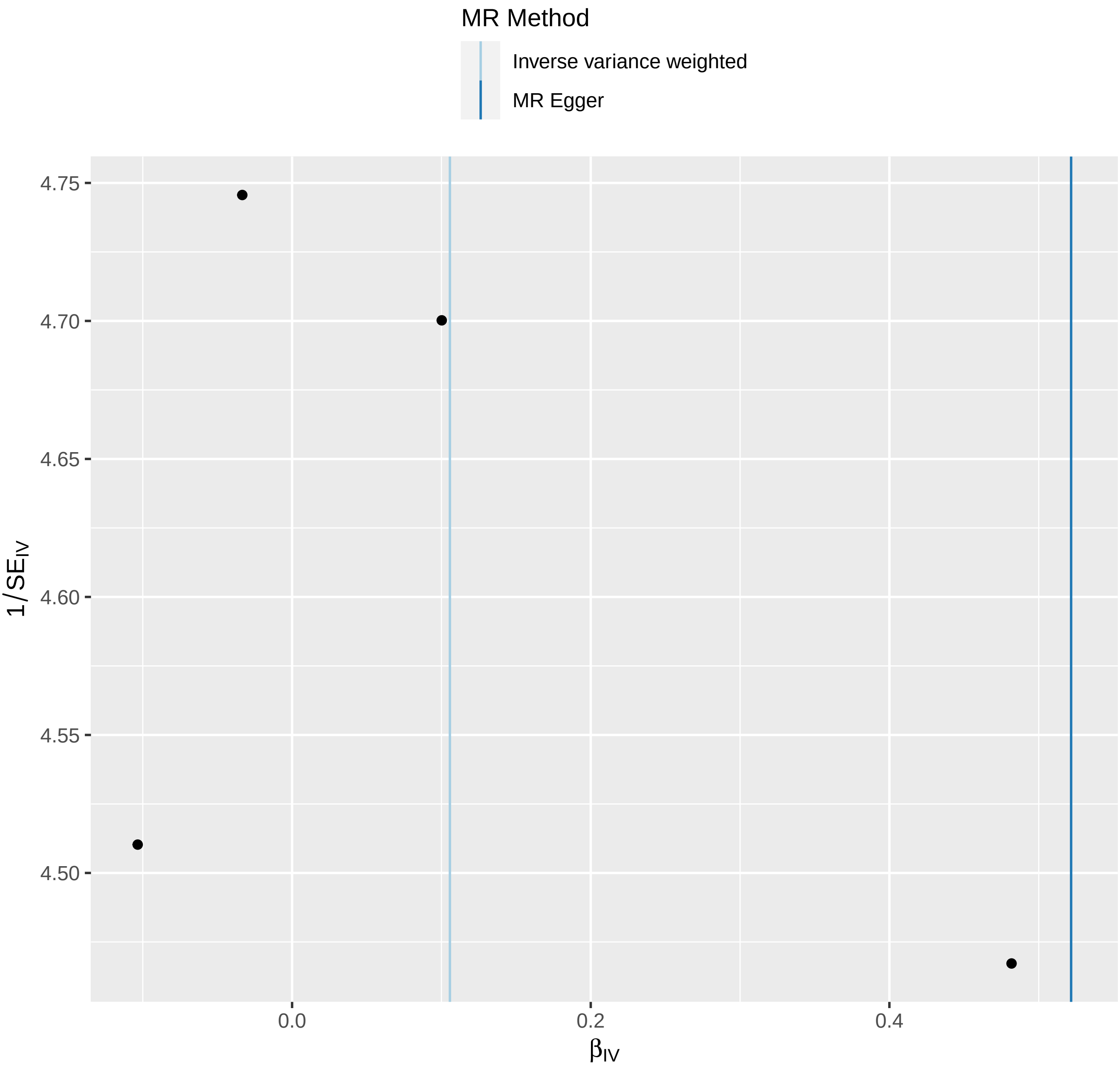

Batch 2−2636 : Gut microbiota abundance (genus Parasutterella id.2892) on Hydroxycarboxylic acid receptor 2
with a threshold of 1e−05

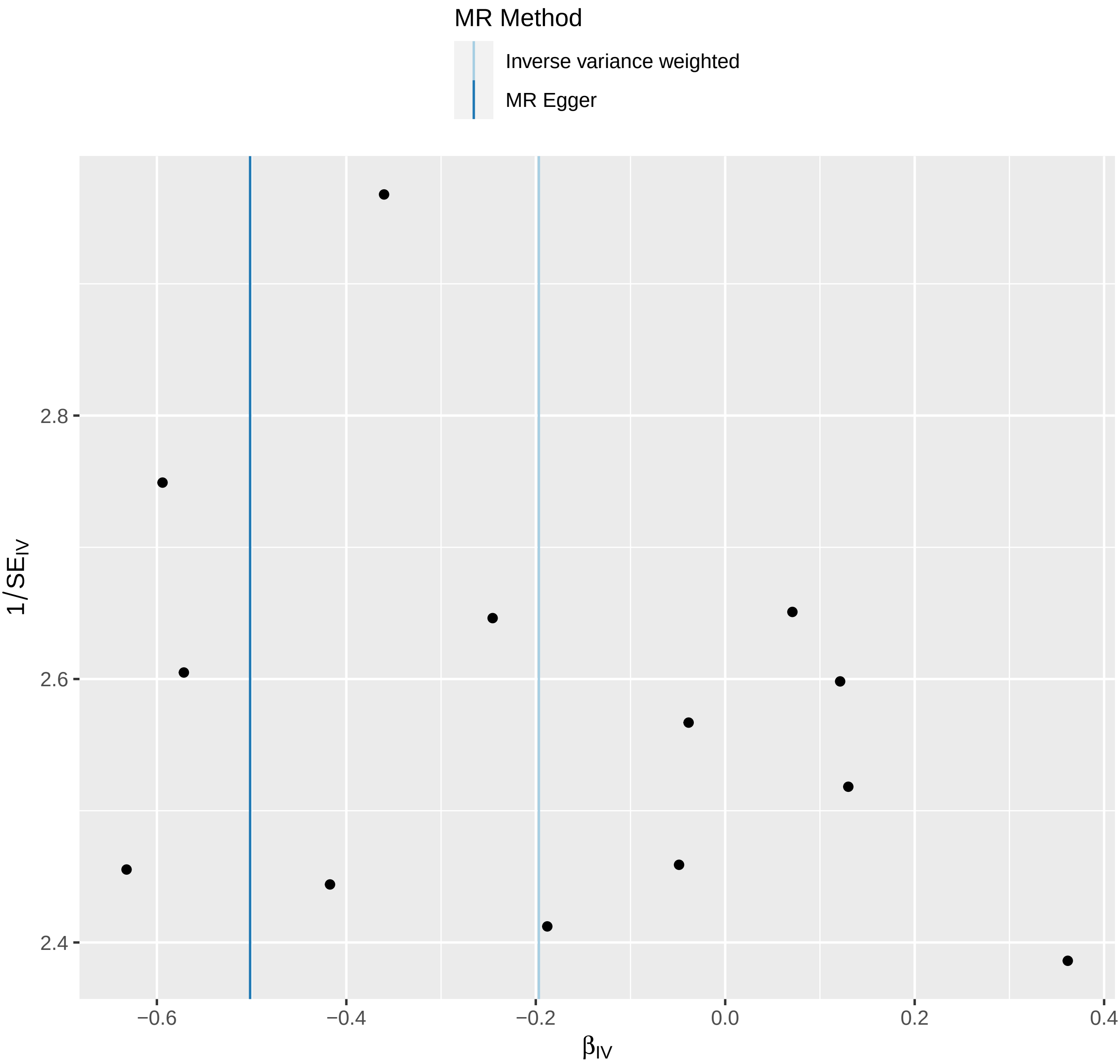

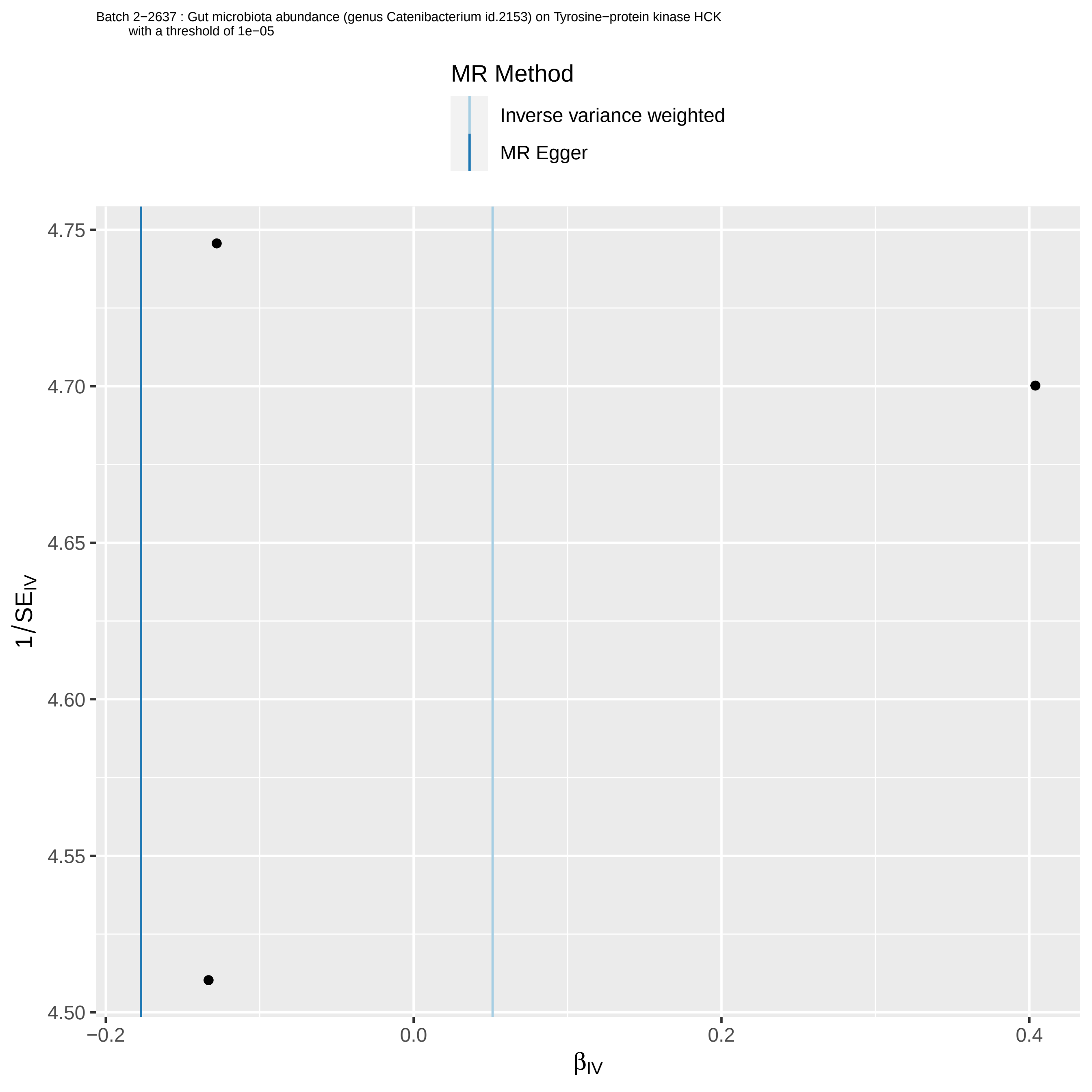
Batch 2−2637 : Gut microbiota abundance (genus Catenibacterium id.2153) on Tyrosine−protein kinase HCK
with a threshold of 1e−05
MR Method
Inverse variance weighted
MR Egger
4.75
4.70
4.65
4.60
4.55
4.50
1/SE_IV
−0.2
0.0
0.2
0.4
β_IV

Batch 2−2638 : Gut microbiota abundance (genus Parasutterella id.2892) on Tyrosine−protein kinase HCK
with a threshold of 1e−05

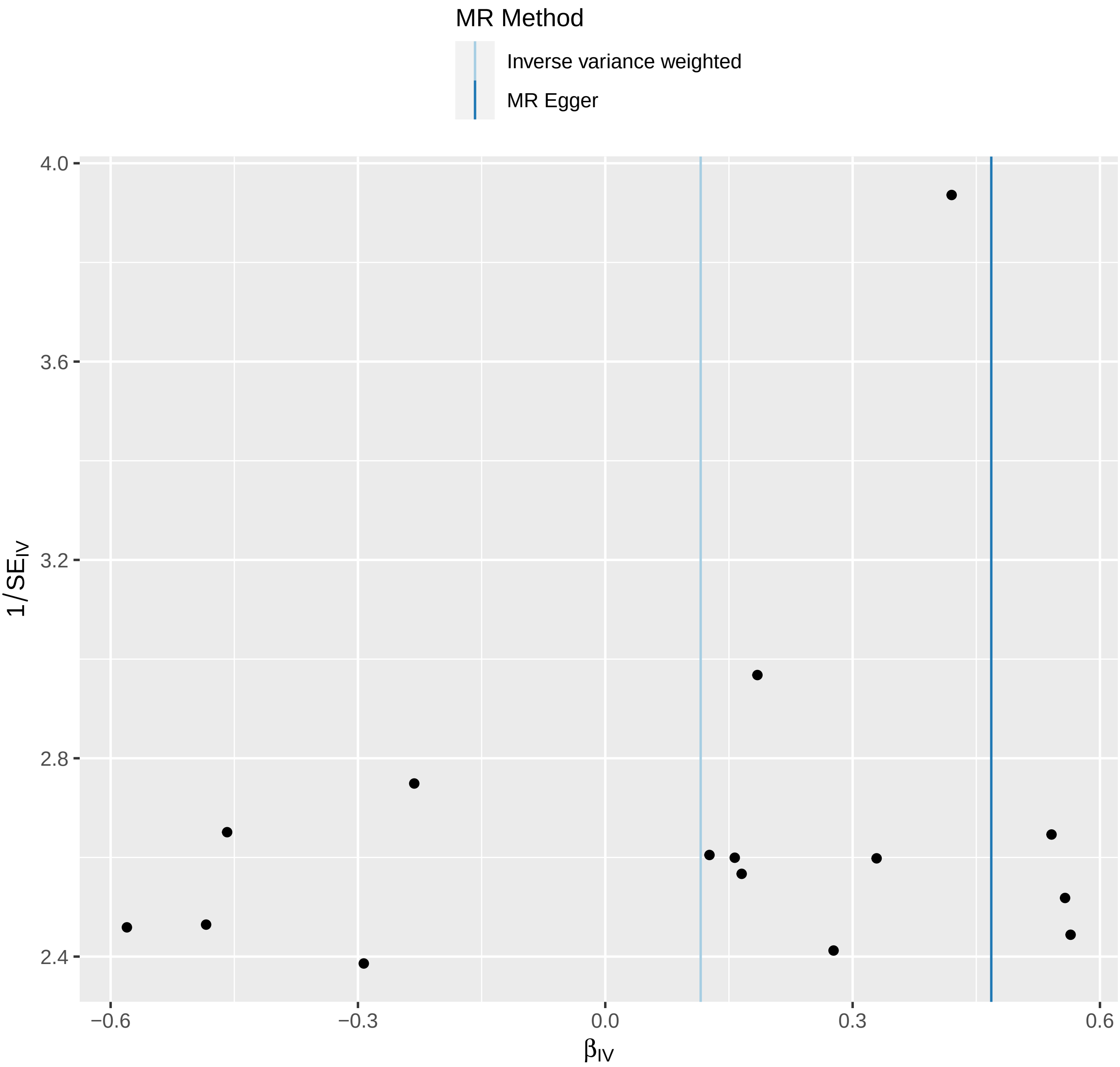

Batch 2−2639 : Gut microbiota abundance (genus Catenibacterium id.2153) on Histone deacetylase 8
with a threshold of 1e−05

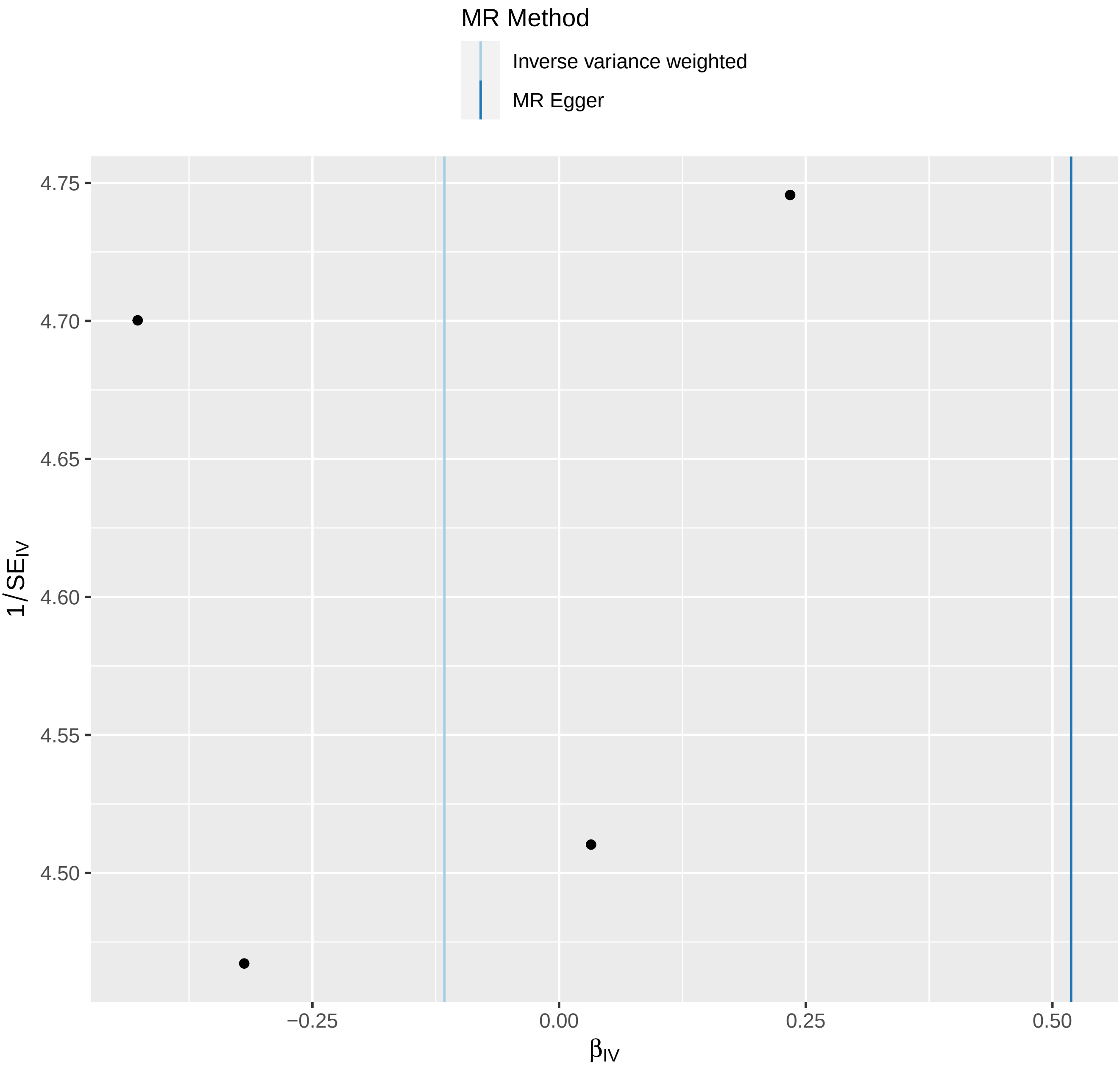

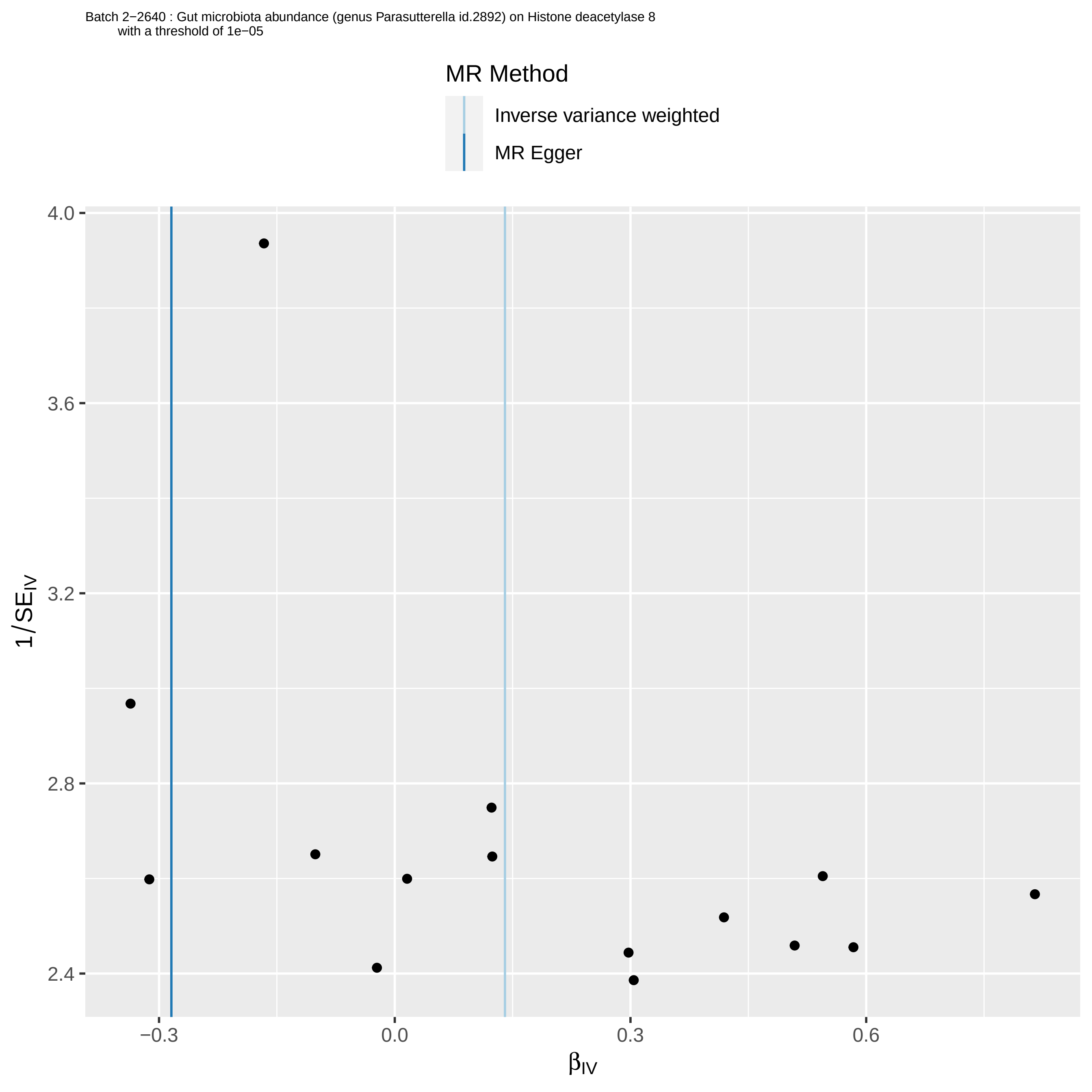
Batch 2−2640 : Gut microbiota abundance (genus Parasutterella id.2892) on Histone deacetylase 8
with a threshold of 1e−05
MR Method
Inverse variance weighted
MR Egger
4.0
3.6
3.2
2.8
2.4
1/SE$_{IV}$
−0.3
0.0
0.3
0.6
β_{IV}

Batch 2−2641 : Gut microbiota abundance (genus Catenibacterium id.2153) on Hepatoma−derived growth factor−related protein 2
with a threshold of 1e−05

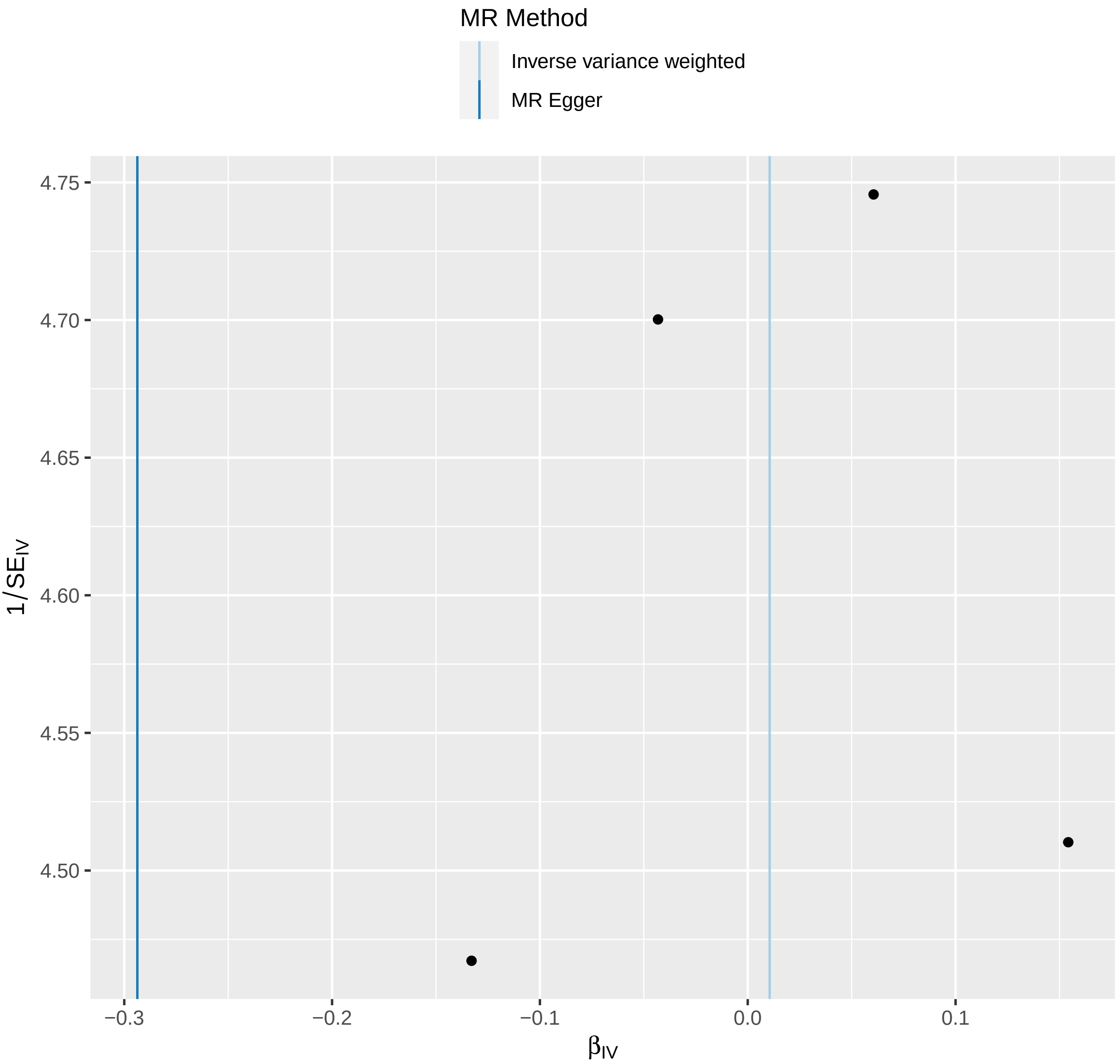

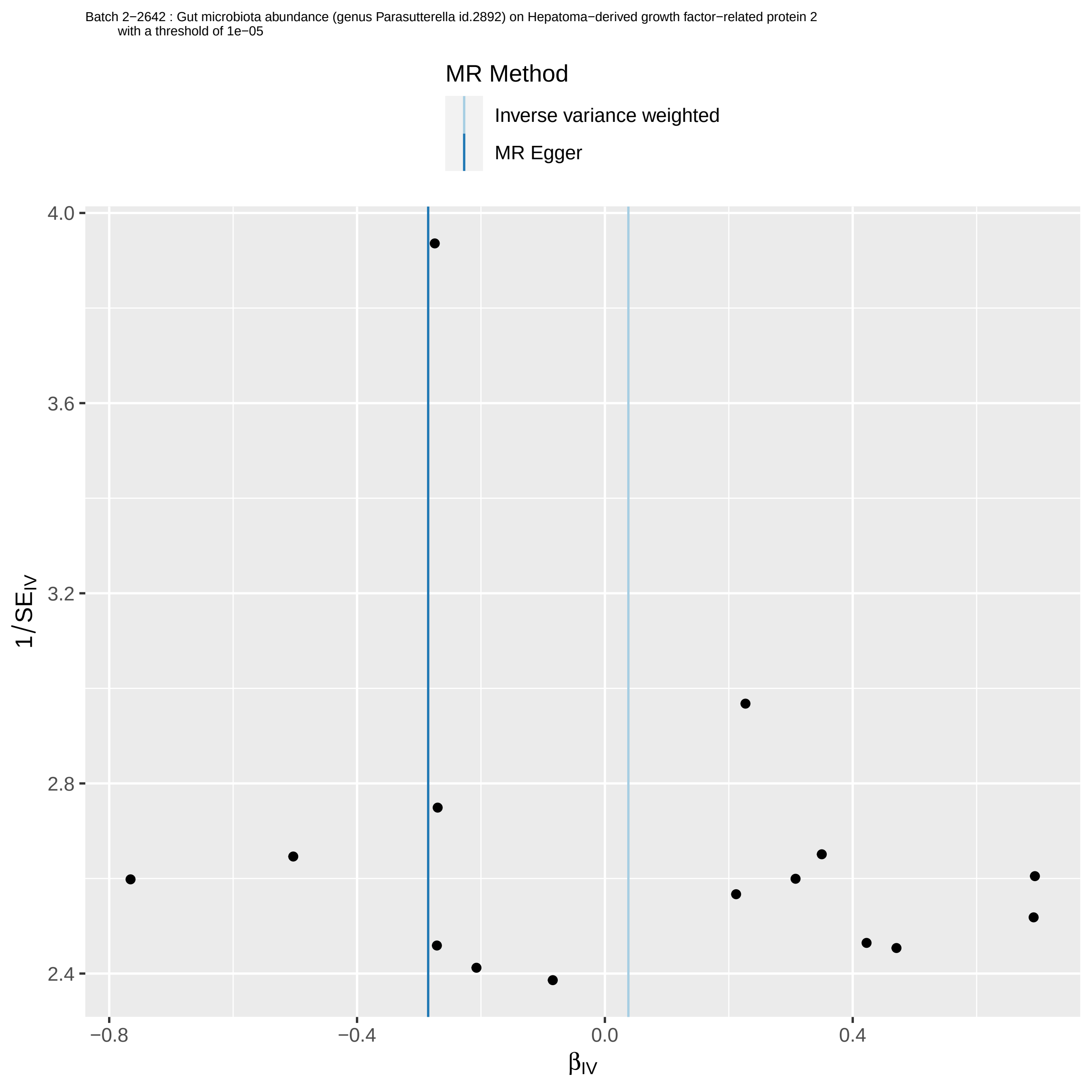
Batch 2−2642 : Gut microbiota abundance (genus Parasutterella id.2892) on Hepatoma−derived growth factor−related protein 2
with a threshold of 1e−05
MR Method
Inverse variance weighted
MR Egger
4.0
3.6
3.2
2.8
2.4
1/SE_IV
−0.8
−0.4
0.0
0.4
β_IV

Batch 2−2643 : Gut microbiota abundance (genus Catenibacterium id.2153) on Haloacid dehalogenase−like hydrolase domain−containing protein 2 with a threshold of 1e−05

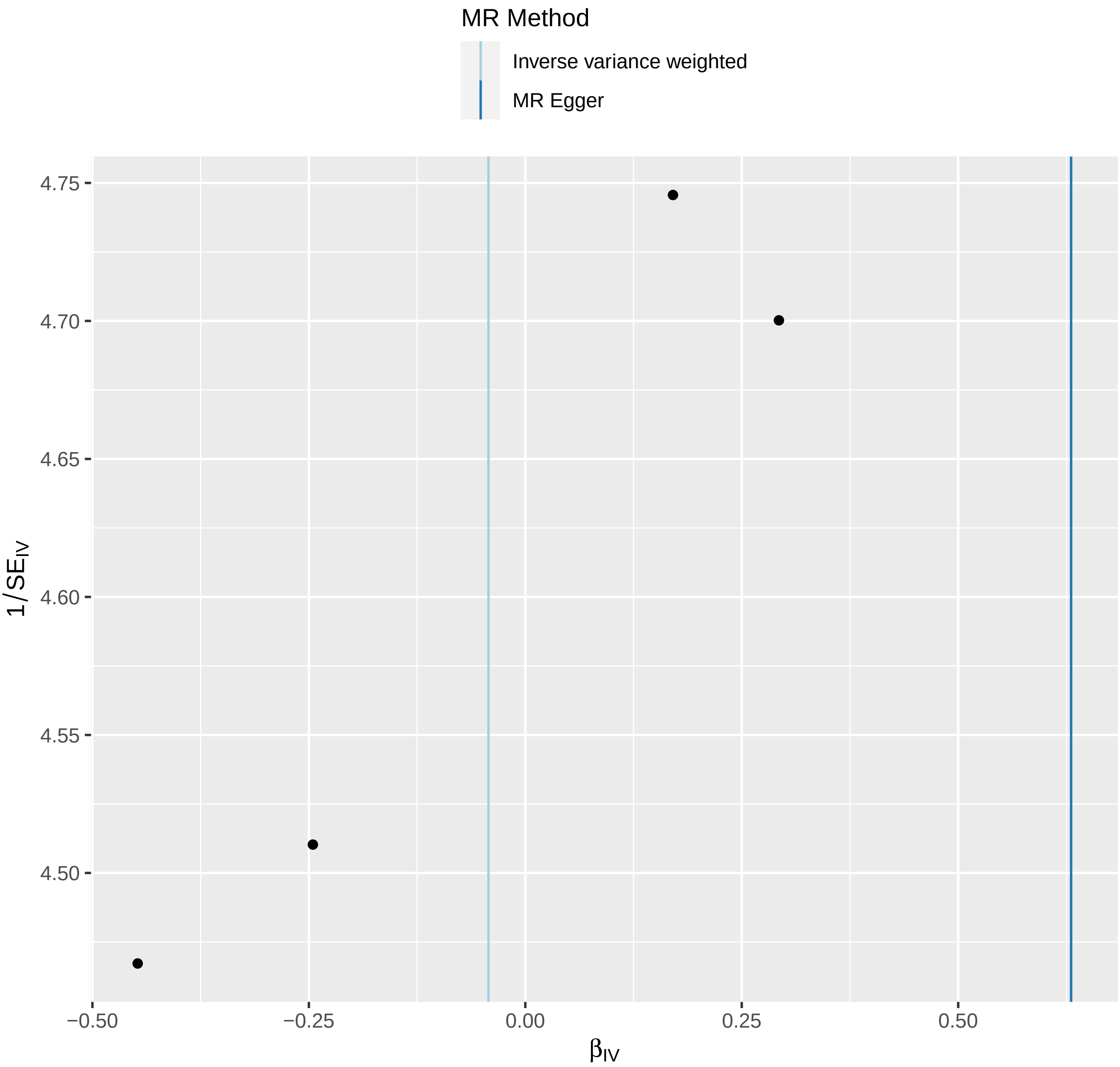

Batch 2−2644 : Gut microbiota abundance (genus Parasutterella id.2892) on Haloacid dehalogenase−like hydrolase domain−containing protein 2 with a threshold of 1e−05

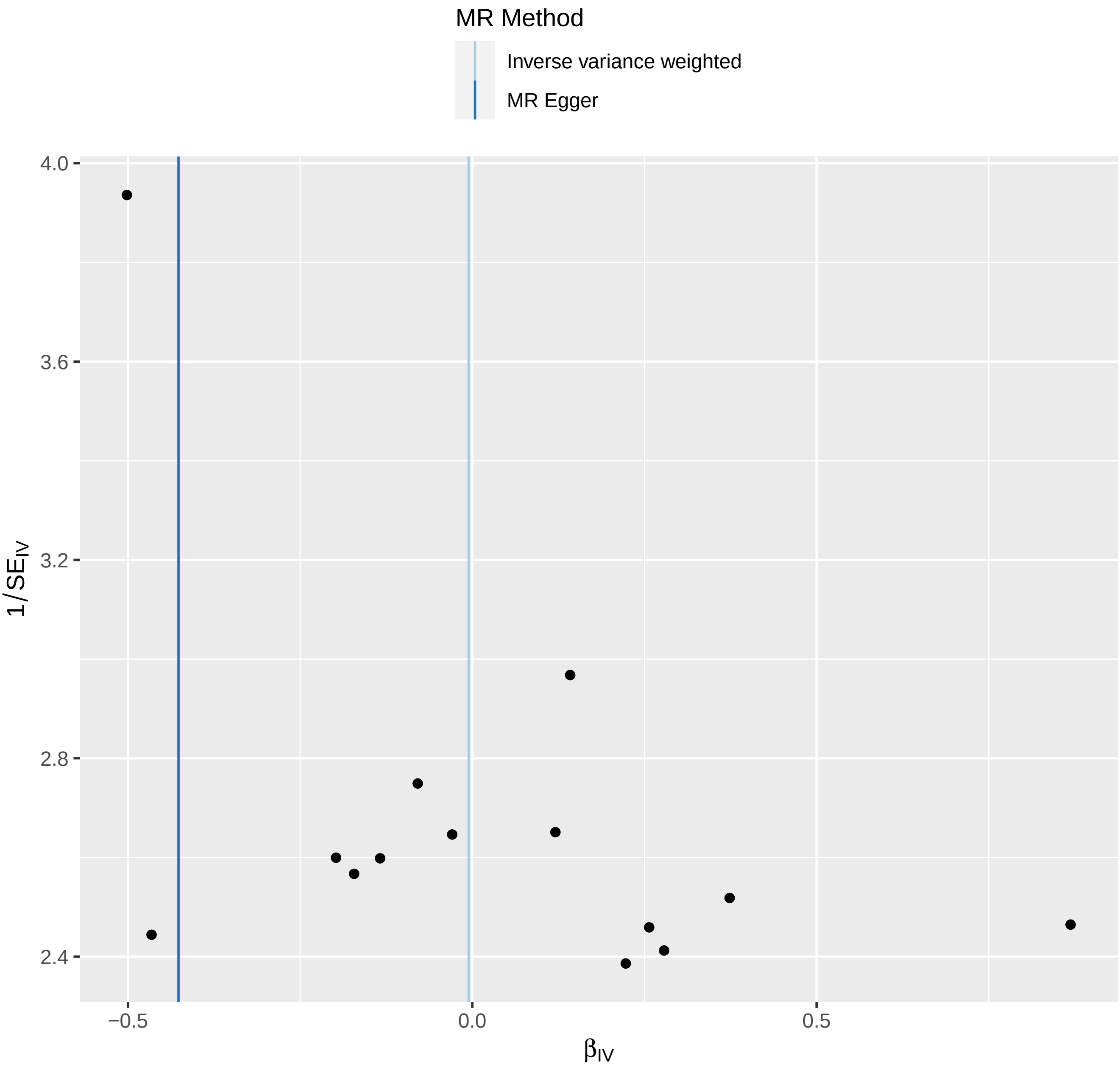

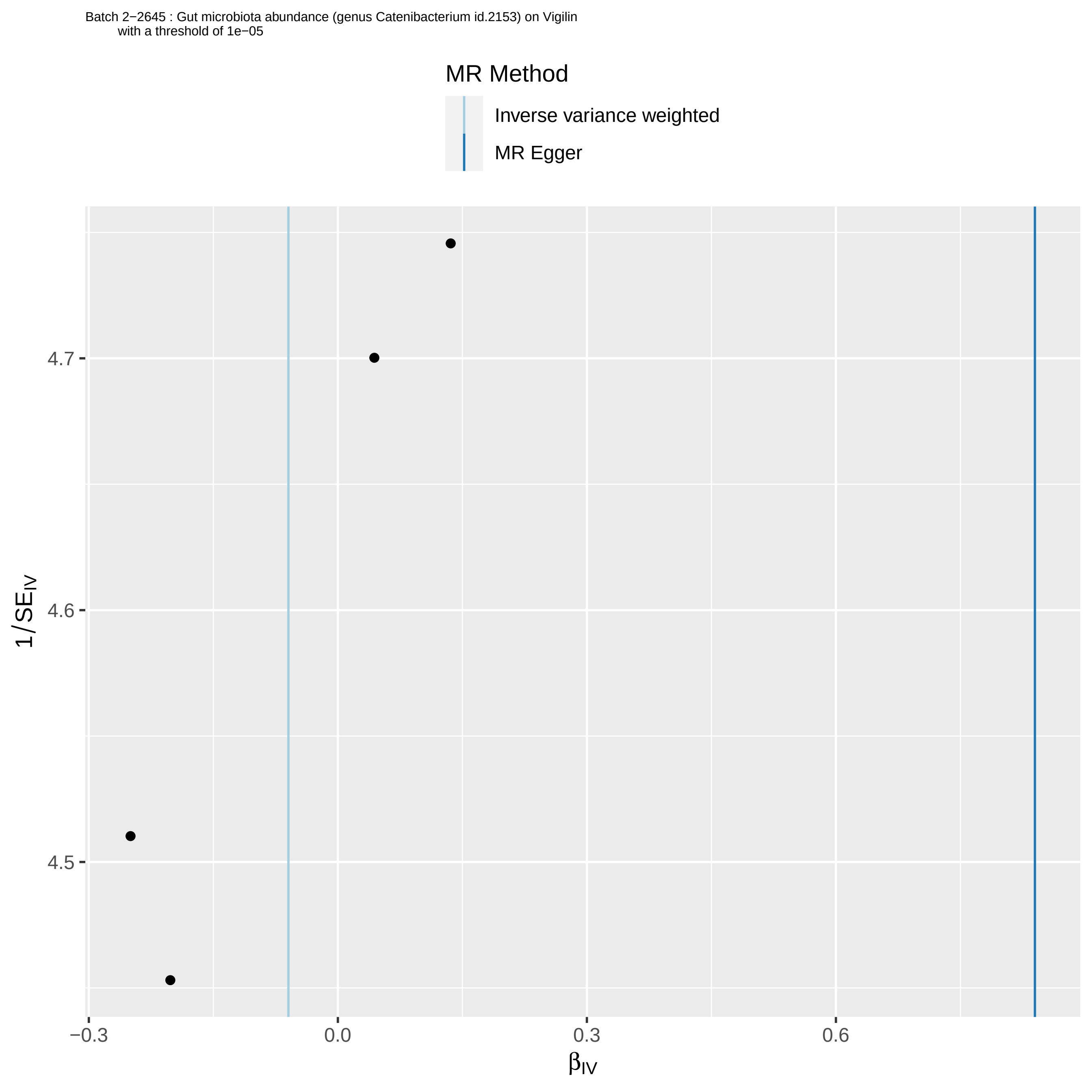

Batch 2−2645 : Gut microbiota abundance (genus Catenibacterium id.2153) on Vigilin
with a threshold of 1e−05
MR Method
Inverse variance weighted
MR Egger
4.7
4.6
4.5
1/SE_IV
−0.3
0.0
0.3
0.6
β_IV

Batch 2−2646 : Gut microbiota abundance (genus Parasutterella id.2892) on Vigilin
with a threshold of 1e−05

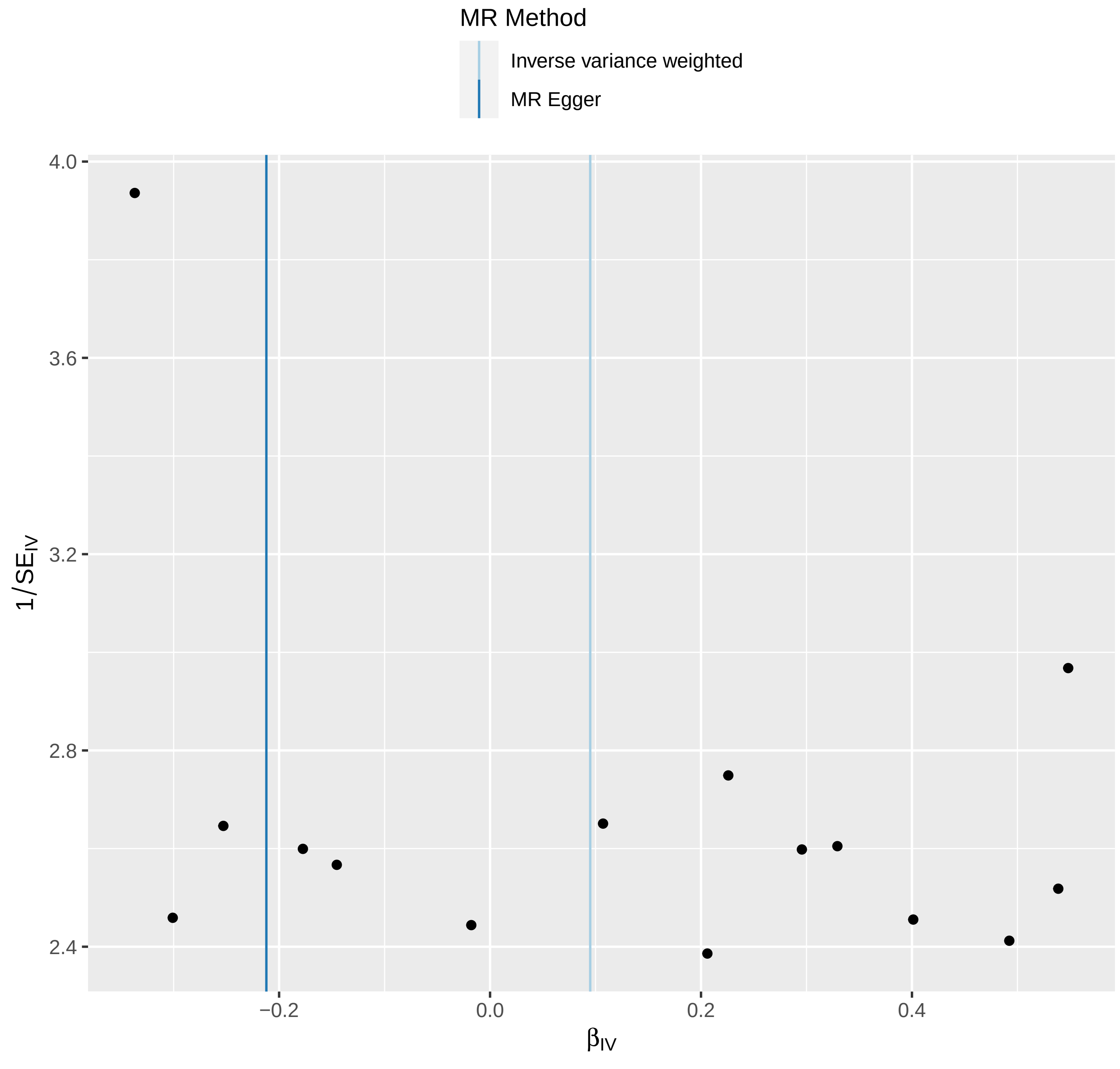

Batch 2−2647 : Gut microbiota abundance (genus Catenibacterium id.2153) on E3 ubiquitin−protein ligase HECW1
with a threshold of 1e−05

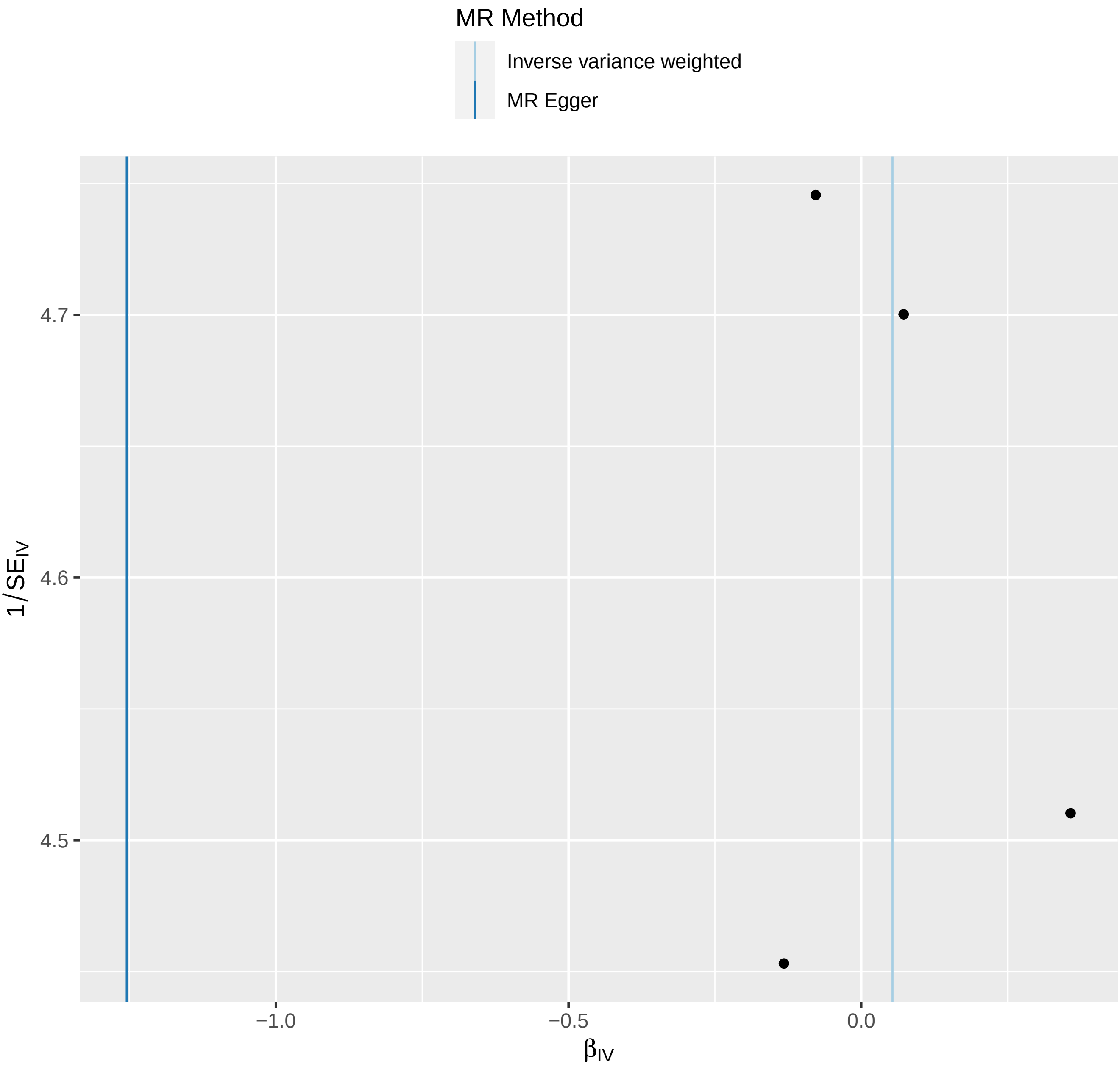

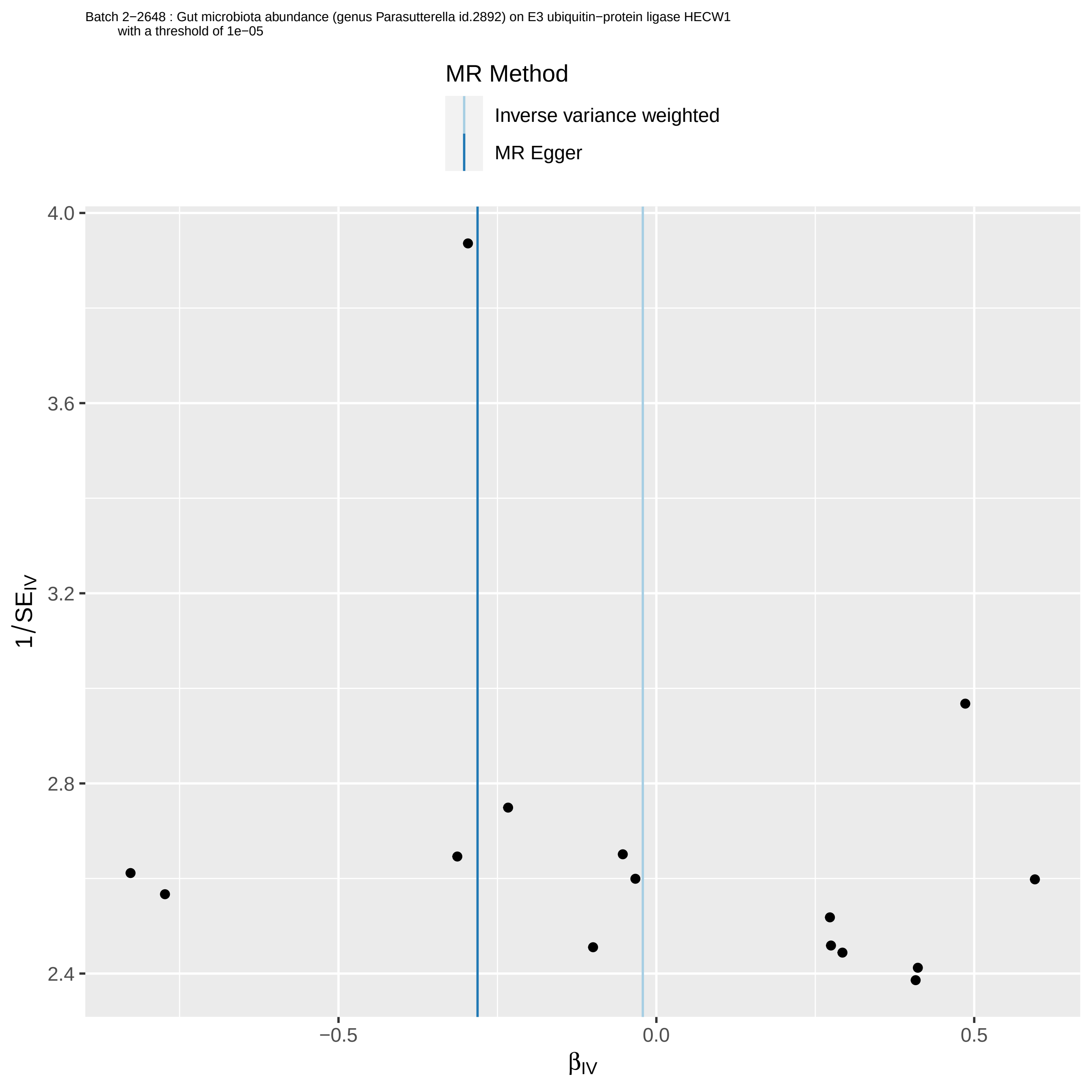
Batch 2−2648 : Gut microbiota abundance (genus Parasutterella id.2892) on E3 ubiquitin−protein ligase HECW1
with a threshold of 1e−05
MR Method
Inverse variance weighted
MR Egger
4.0
3.6
3.2
2.8
2.4
1/SE_IV
−0.5
0.0
0.5
β_IV

Batch 2−2649 : Gut microbiota abundance (genus Catenibacterium id.2153) on Protein HEG homolog 1
with a threshold of 1e−05

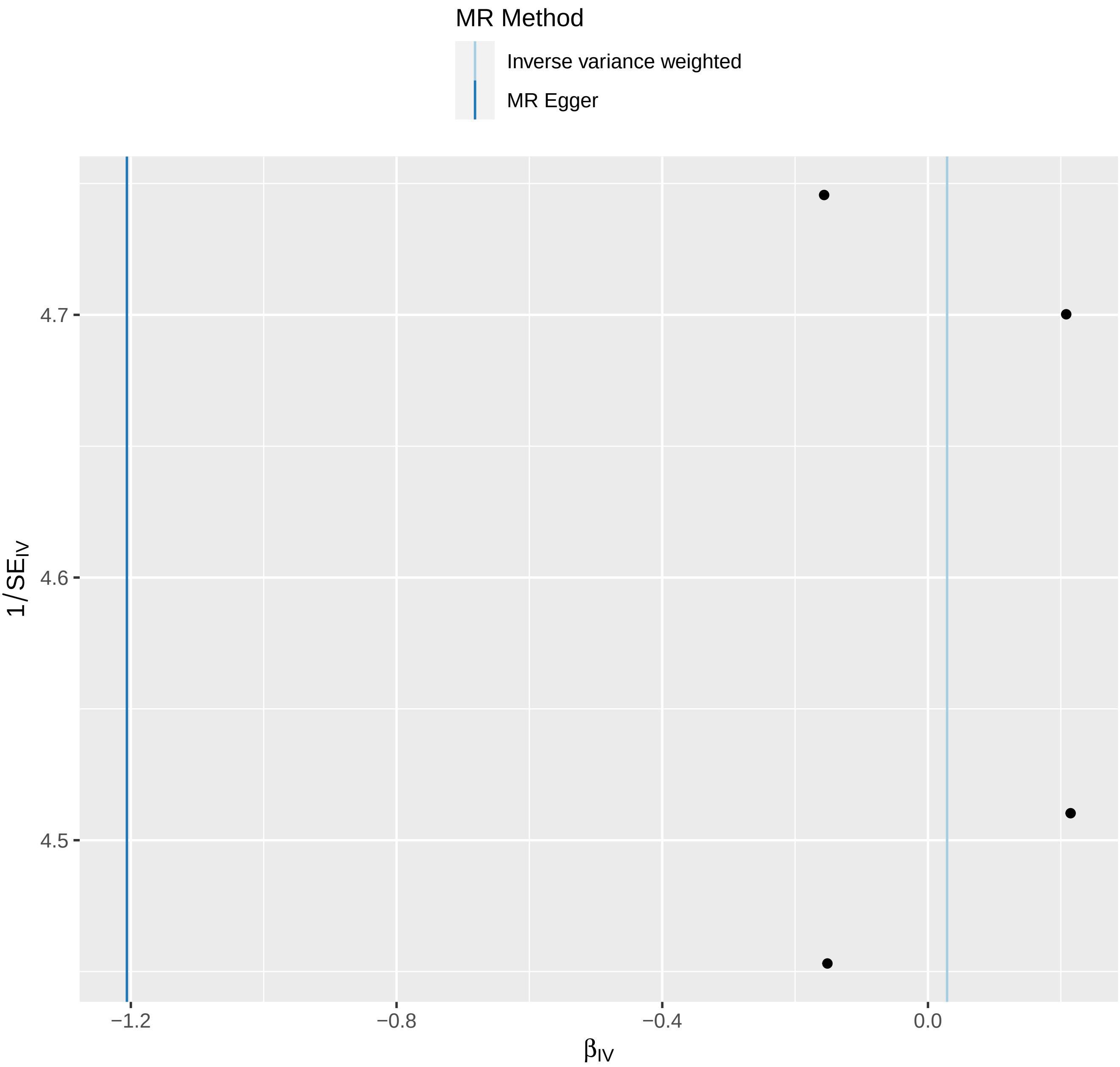

Batch 2−2650 : Gut microbiota abundance (genus Parasutterella id.2892) on Protein HEG homolog 1
with a threshold of 1e−05

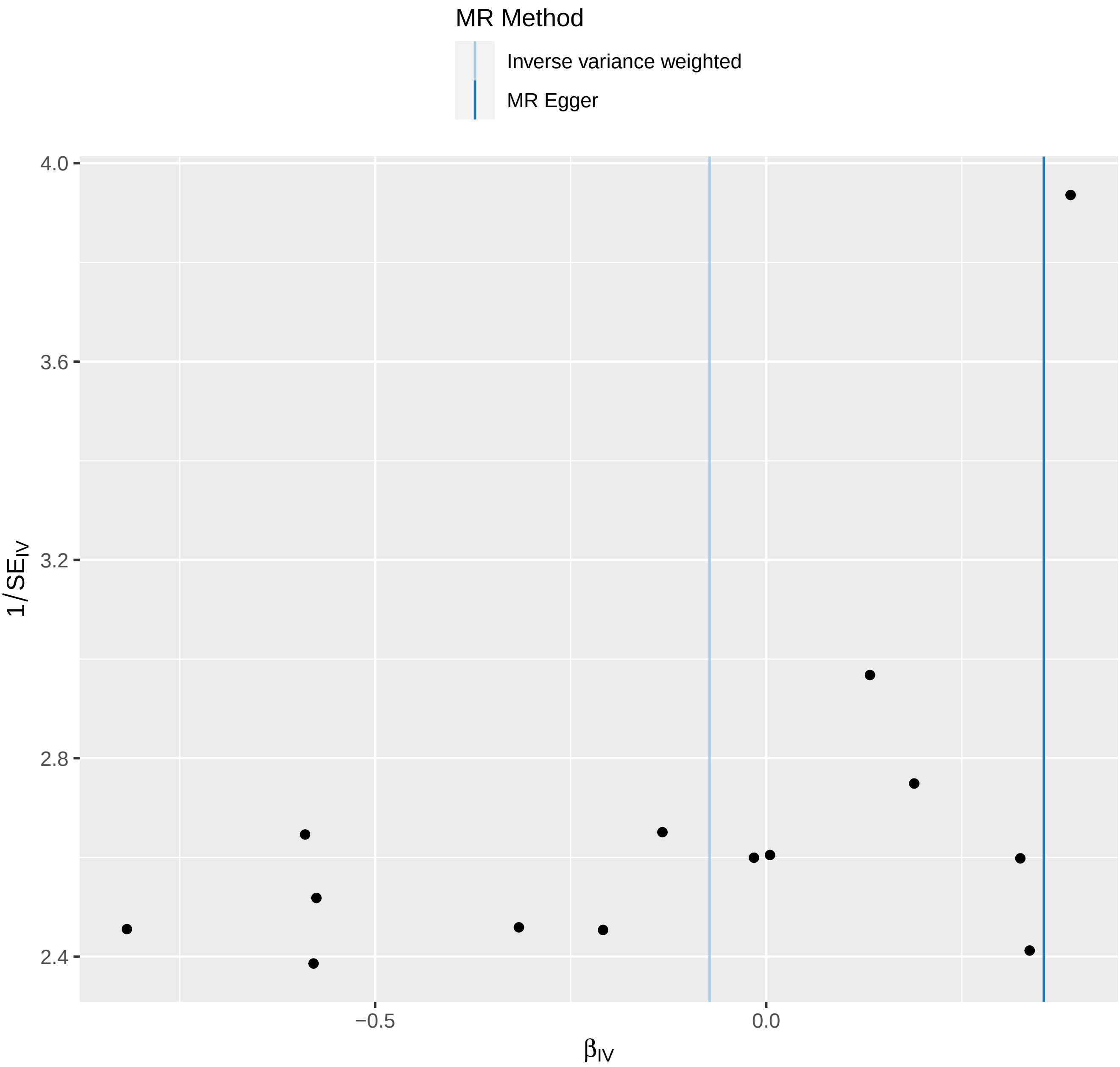

Batch 2−2651 : Gut microbiota abundance (genus Catenibacterium id.2153) on HEPACAM family member 2
with a threshold of 1e−05

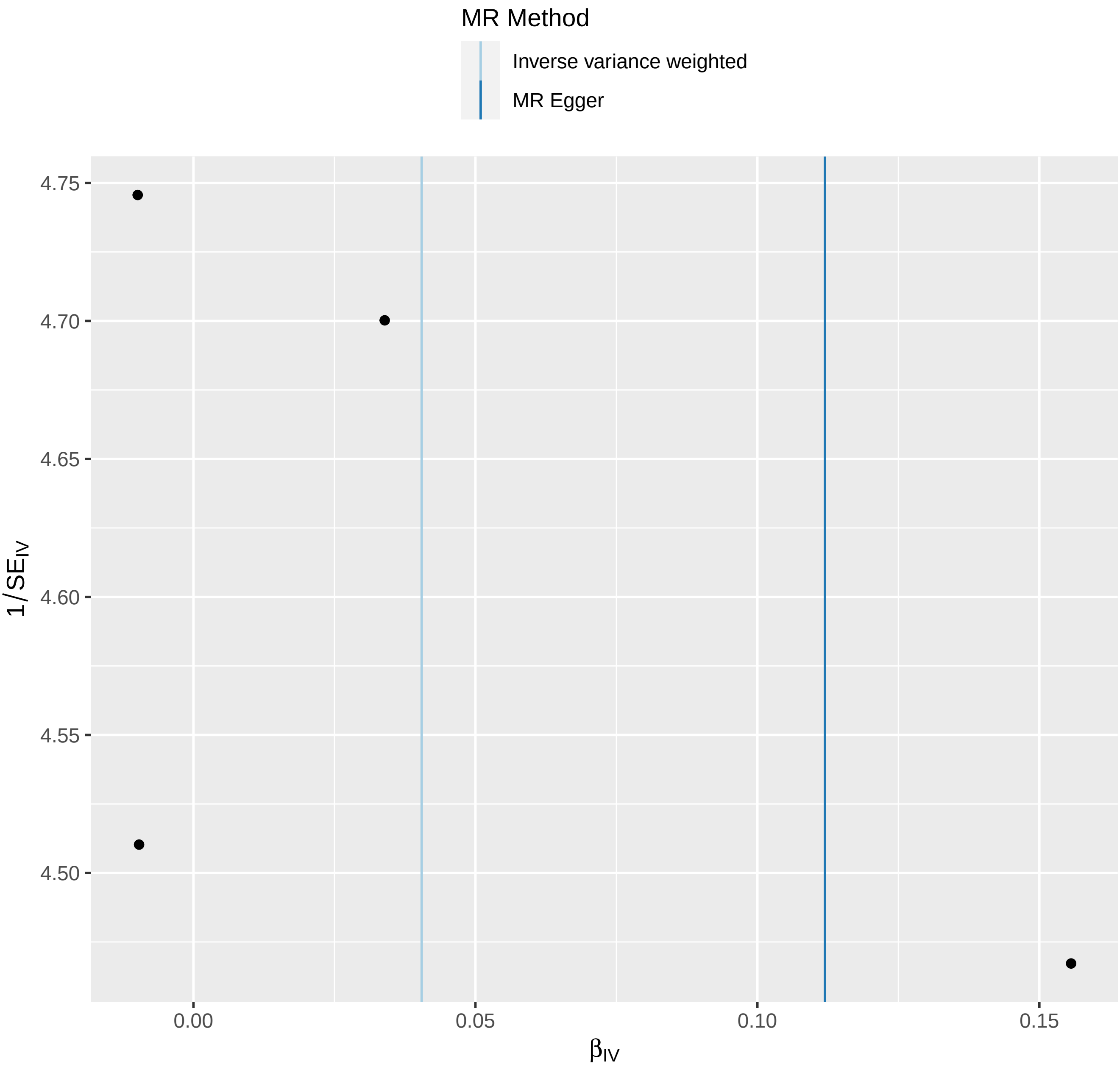

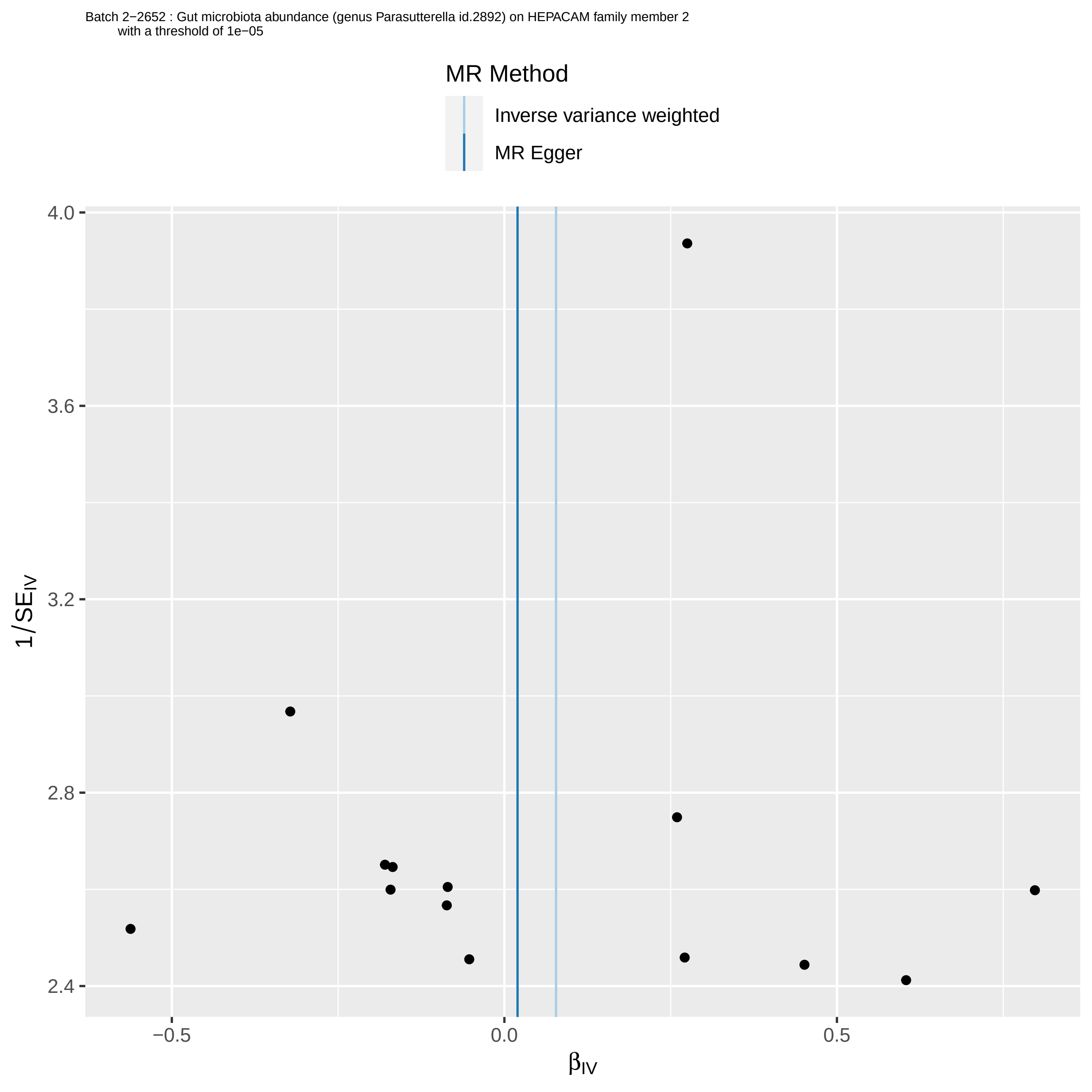
Batch 2−2652 : Gut microbiota abundance (genus Parasutterella id.2892) on HEPACAM family member 2
with a threshold of 1e−05
MR Method
Inverse variance weighted
MR Egger
4.0
3.6
3.2
2.8
2.4
1/SE_IV
−0.5
0.0
0.5
β_IV

Batch 2−2653 : Gut microbiota abundance (genus Catenibacterium id.2153) on HEPACAM family member 2
with a threshold of 1e−05

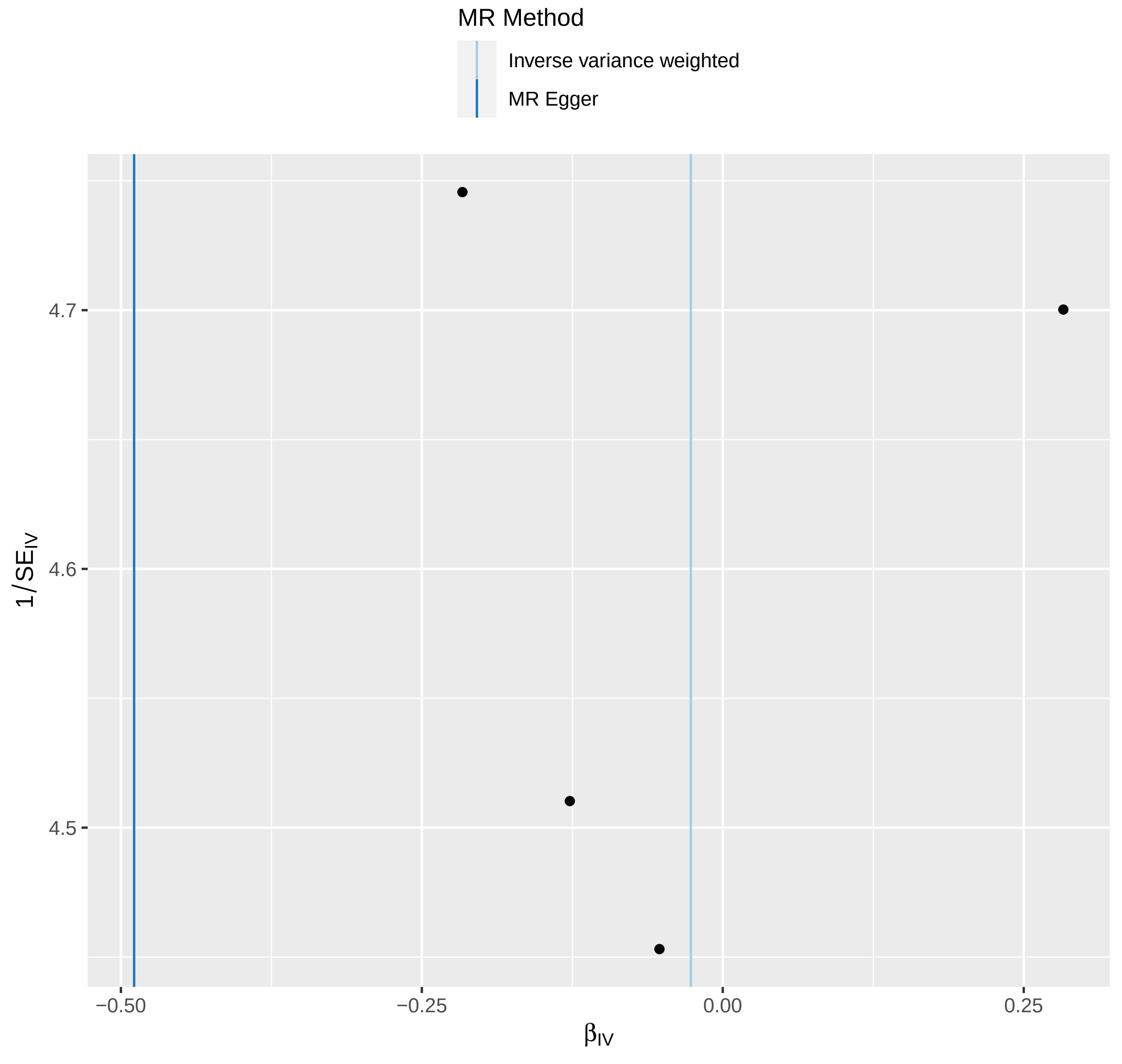

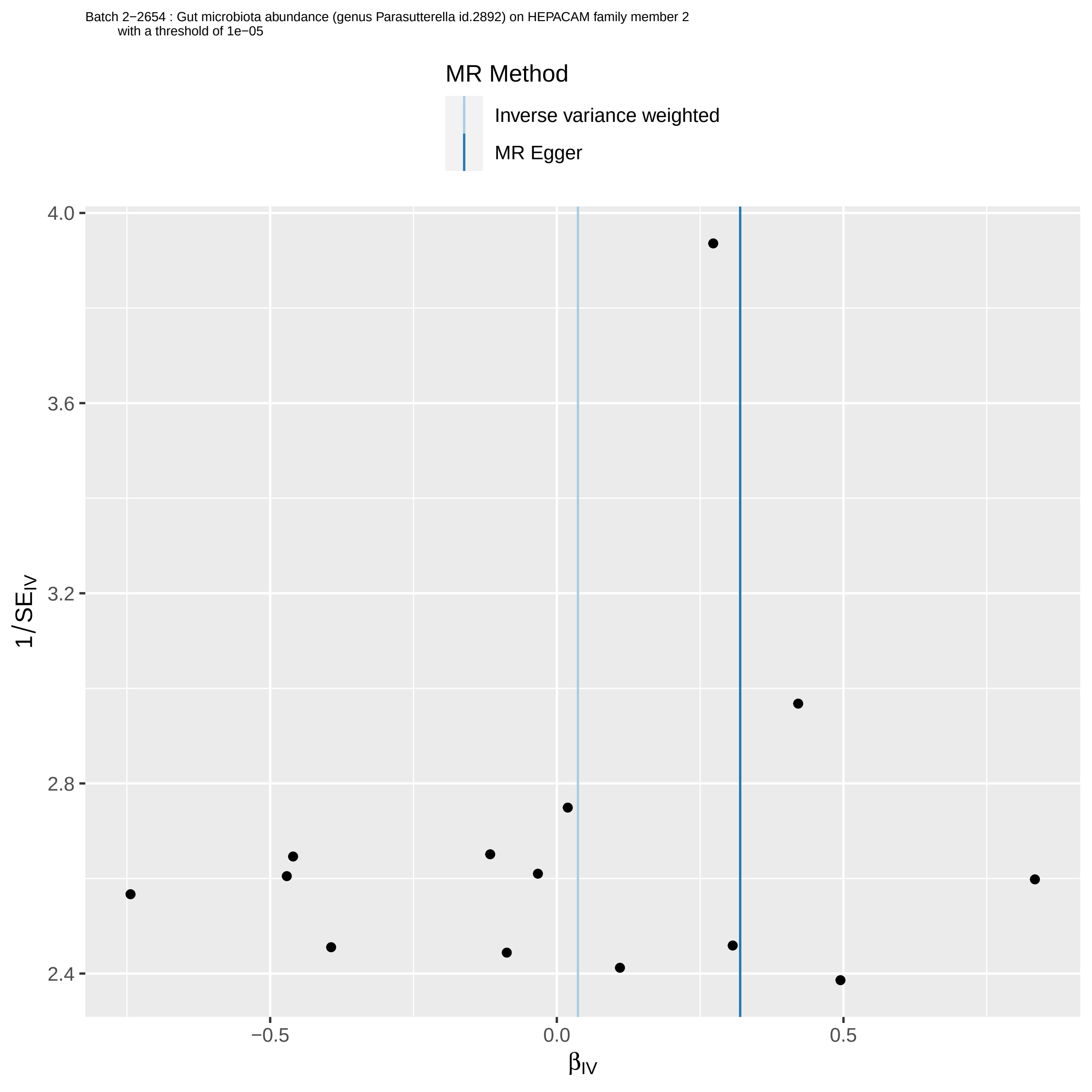
Batch 2−2654 : Gut microbiota abundance (genus Parasutterella id.2892) on HEPACAM family member 2
with a threshold of 1e−05
MR Method
Inverse variance weighted
MR Egger
4.0
3.6
3.2
2.8
2.4
1/SE_IV
−0.5
0.0
0.5
β_IV

Batch 2−2655 : Gut microbiota abundance (genus Catenibacterium id.2153) on Hephaestin−like protein 1
with a threshold of 1e−05

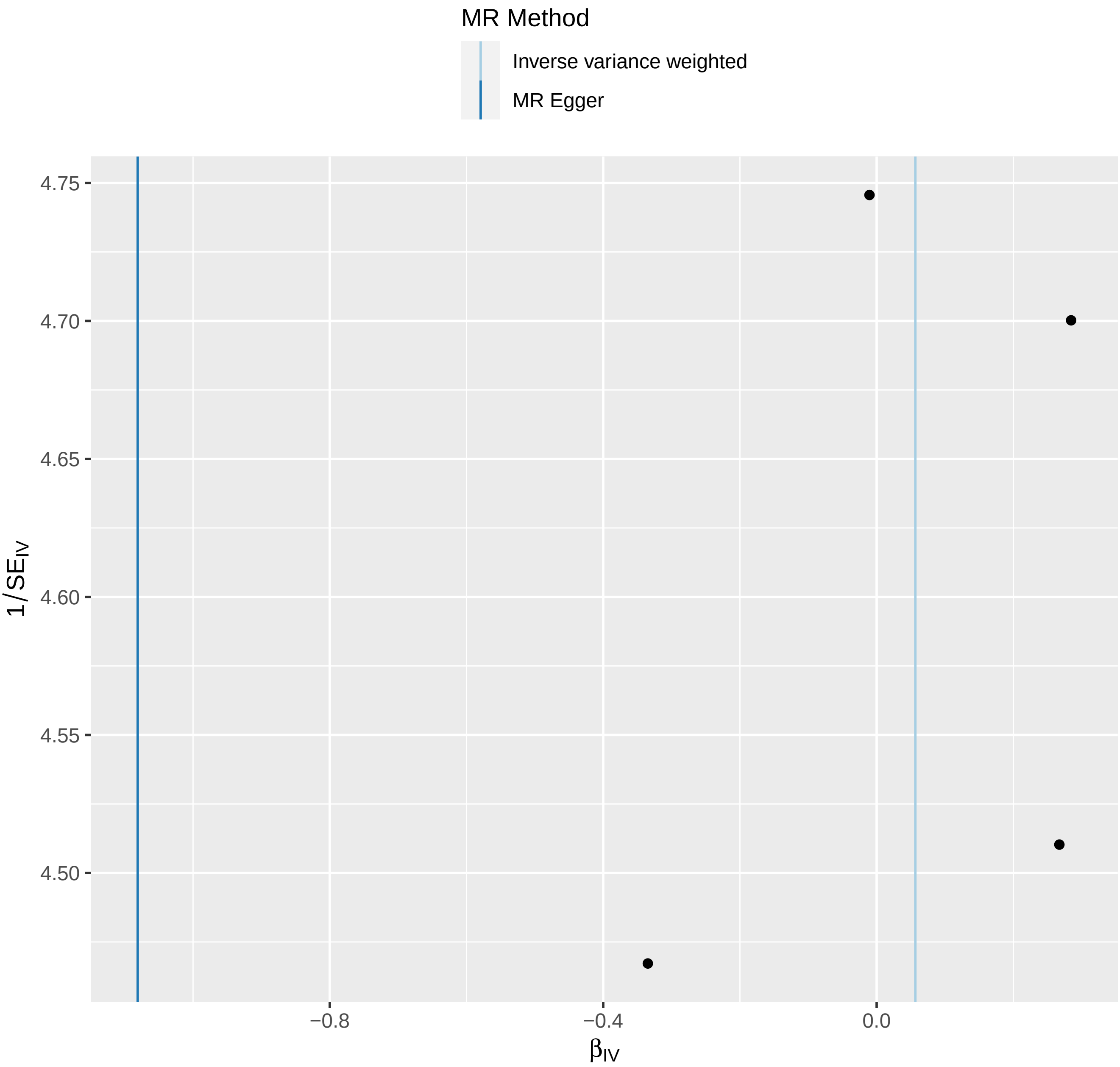

Batch 2−2656 : Gut microbiota abundance (genus Parasutterella id.2892) on Hephaestin−like protein 1
with a threshold of 1e−05

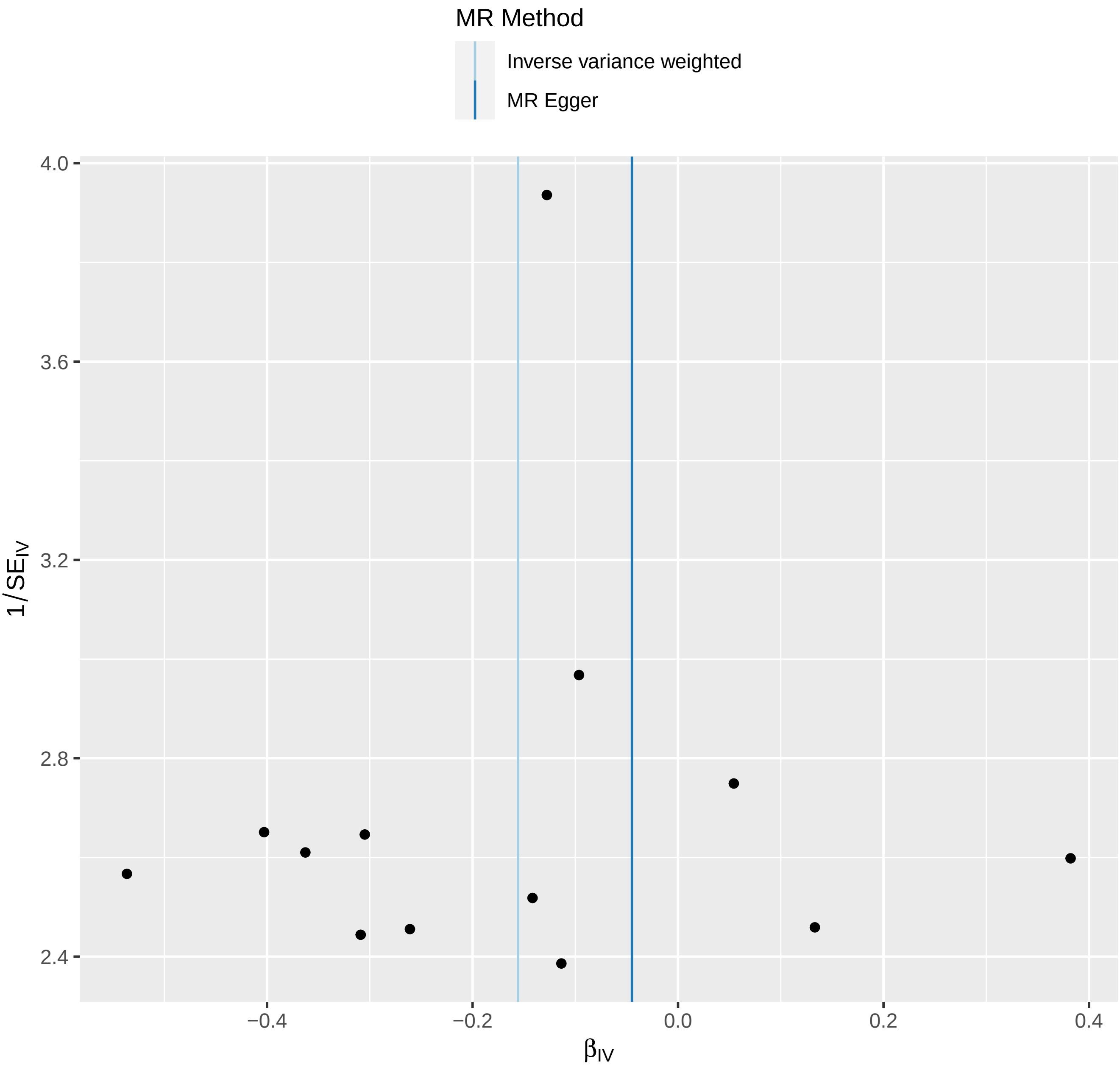

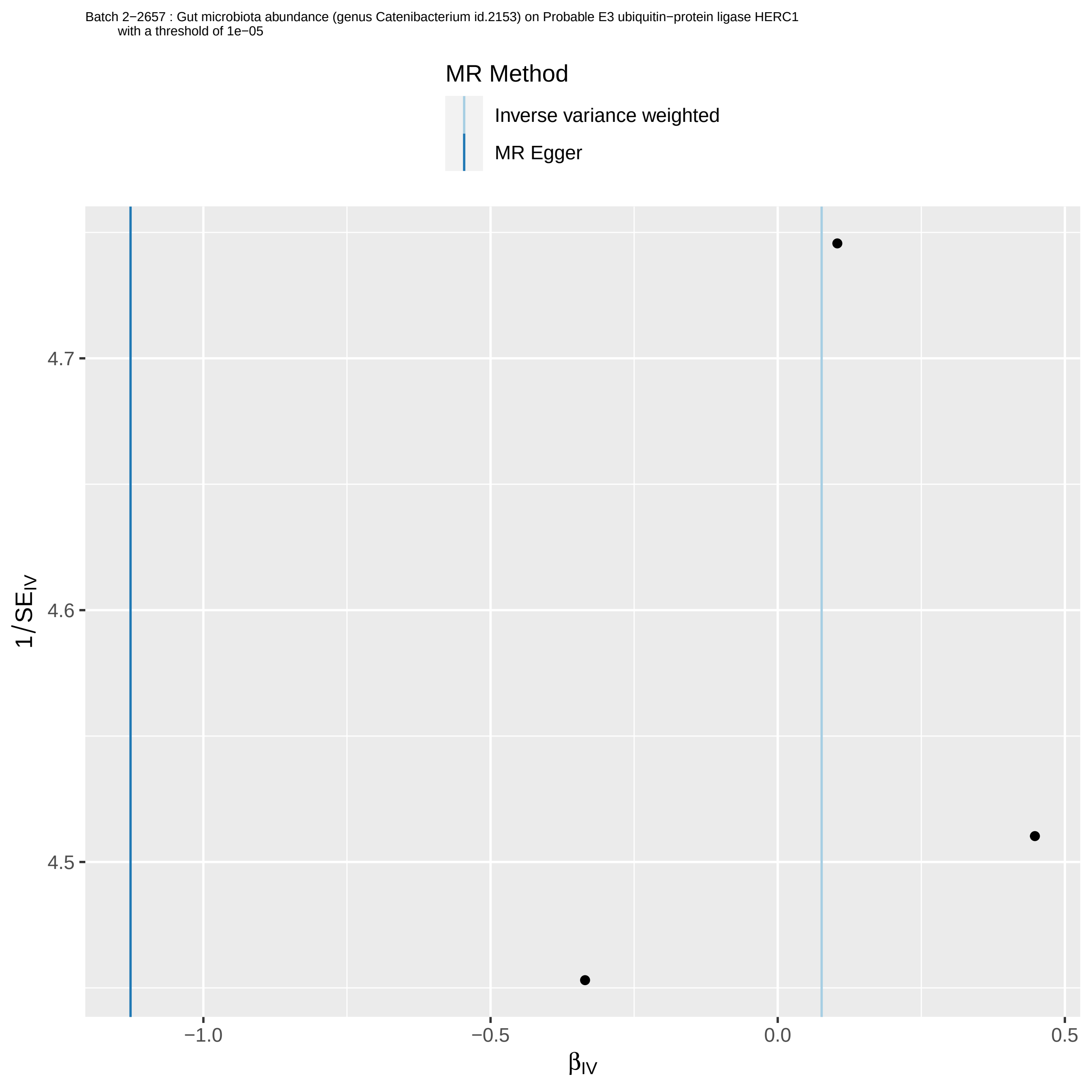
Batch 2−2657 : Gut microbiota abundance (genus Catenibacterium id.2153) on Probable E3 ubiquitin−protein ligase HERC1
with a threshold of 1e−05
MR Method
Inverse variance weighted
MR Egger
4.7
4.6
4.5
−1.0
−0.5
0.0
0.5
1/SE_IV
β_IV

Batch 2−2658 : Gut microbiota abundance (genus Parasutterella id.2892) on Probable E3 ubiquitin−protein ligase HERC1
with a threshold of 1e−05

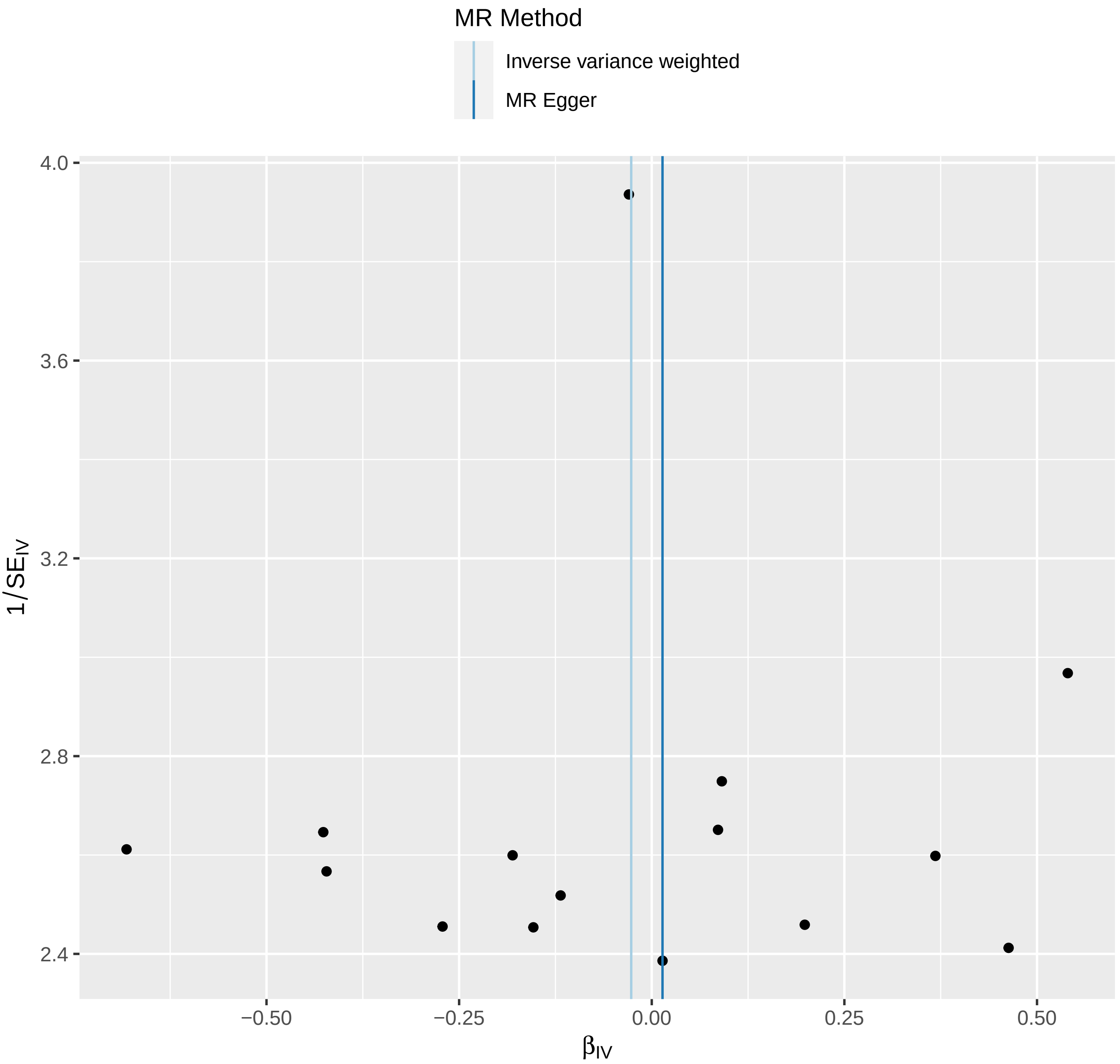

Batch 2−2659 : Gut microbiota abundance (genus Catenibacterium id.2153) on Probable E3 ubiquitin−protein ligase HERC4
with a threshold of 1e−05

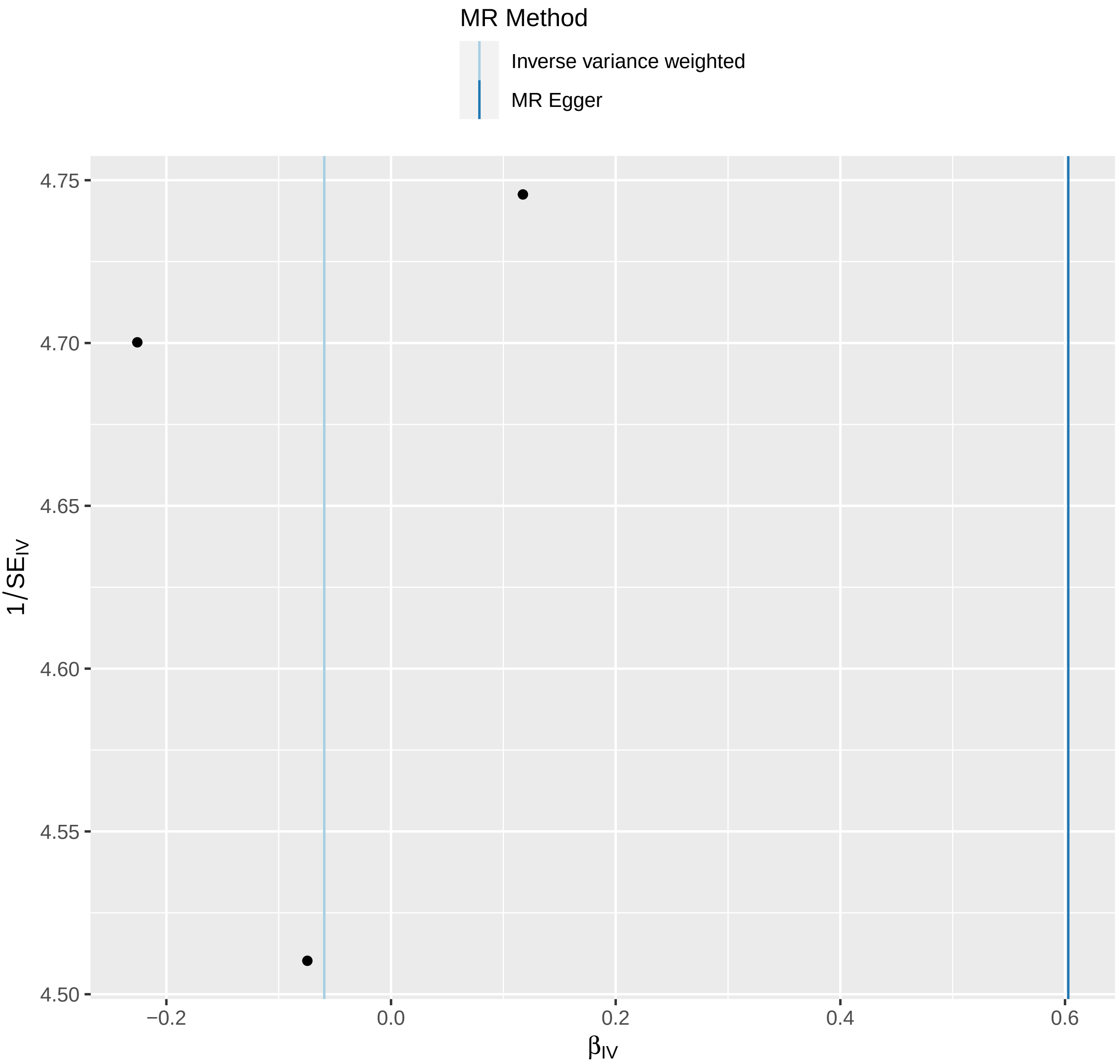

Batch 2−2660 : Gut microbiota abundance (genus Parasutterella id.2892) on Probable E3 ubiquitin−protein ligase HERC4
with a threshold of 1e−05

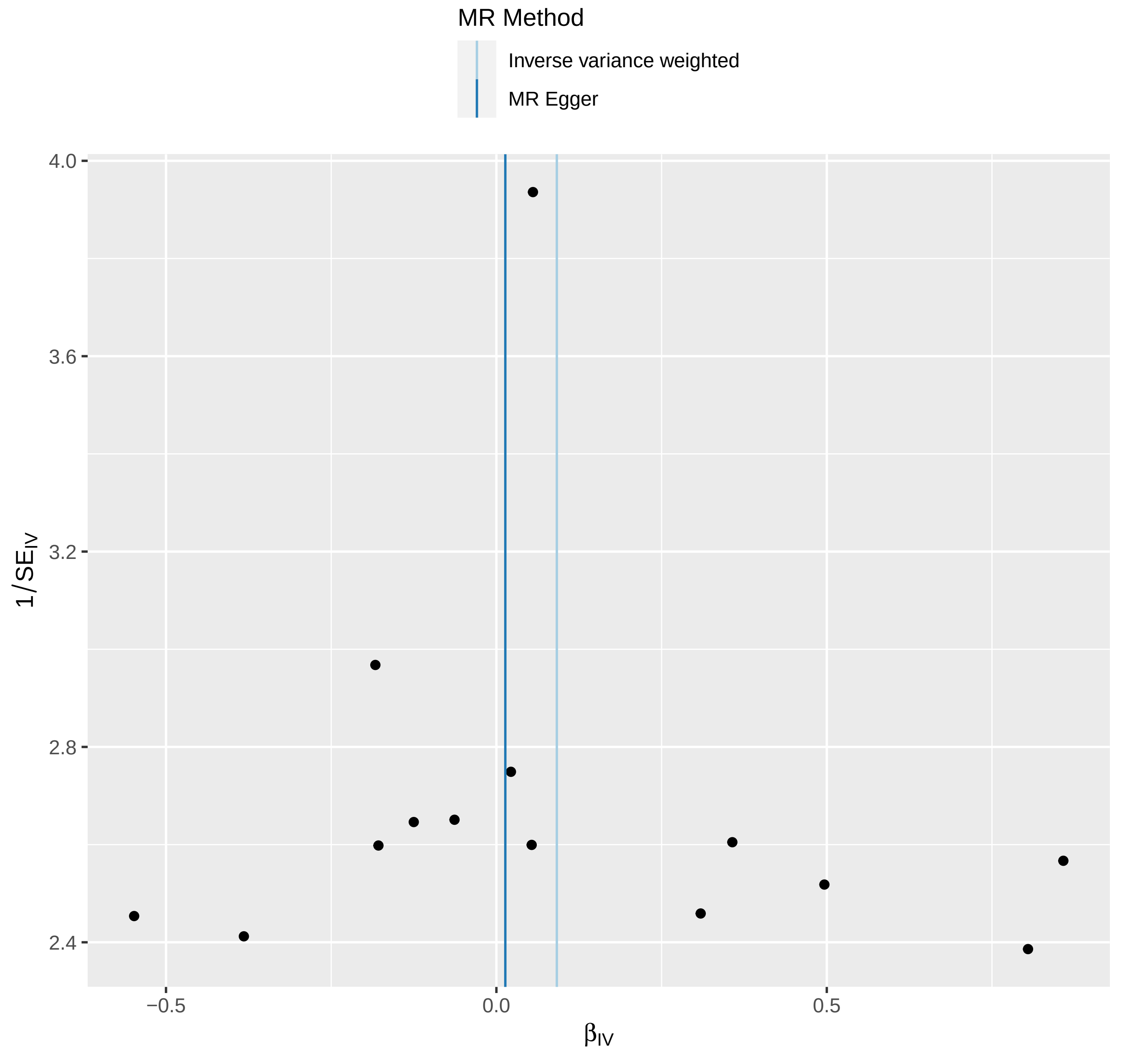

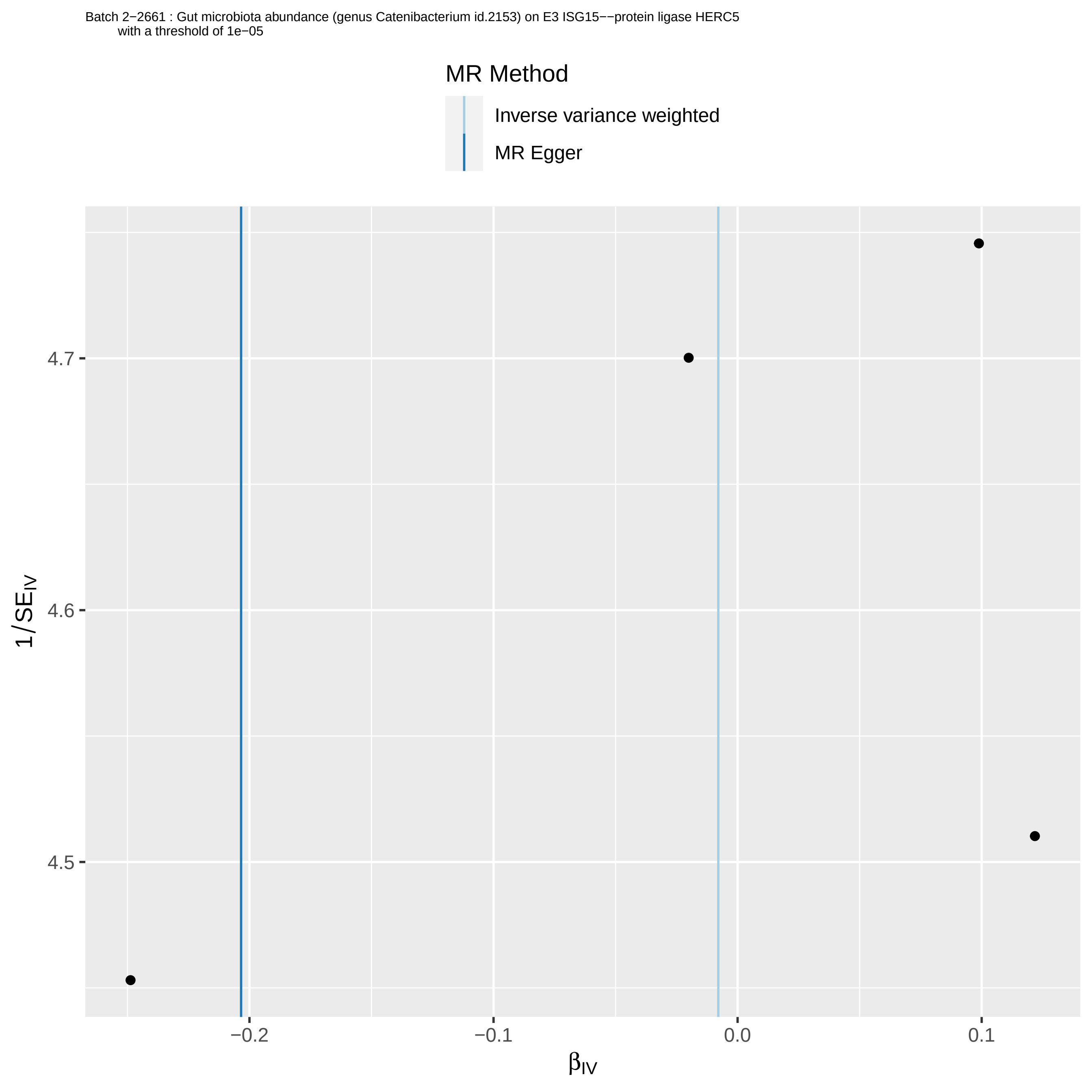
Batch 2−2661 : Gut microbiota abundance (genus Catenibacterium id.2153) on E3 ISG15−−protein ligase HERC5
with a threshold of 1e−05
MR Method
Inverse variance weighted
MR Egger
4.7
4.6
4.5
1/SE$_{IV}$
−0.2
−0.1
0.0
0.1
β$_{IV}$

Batch 2−2662 : Gut microbiota abundance (genus Parasutterella id.2892) on E3 ISG15−−protein ligase HERC5
with a threshold of 1e−05

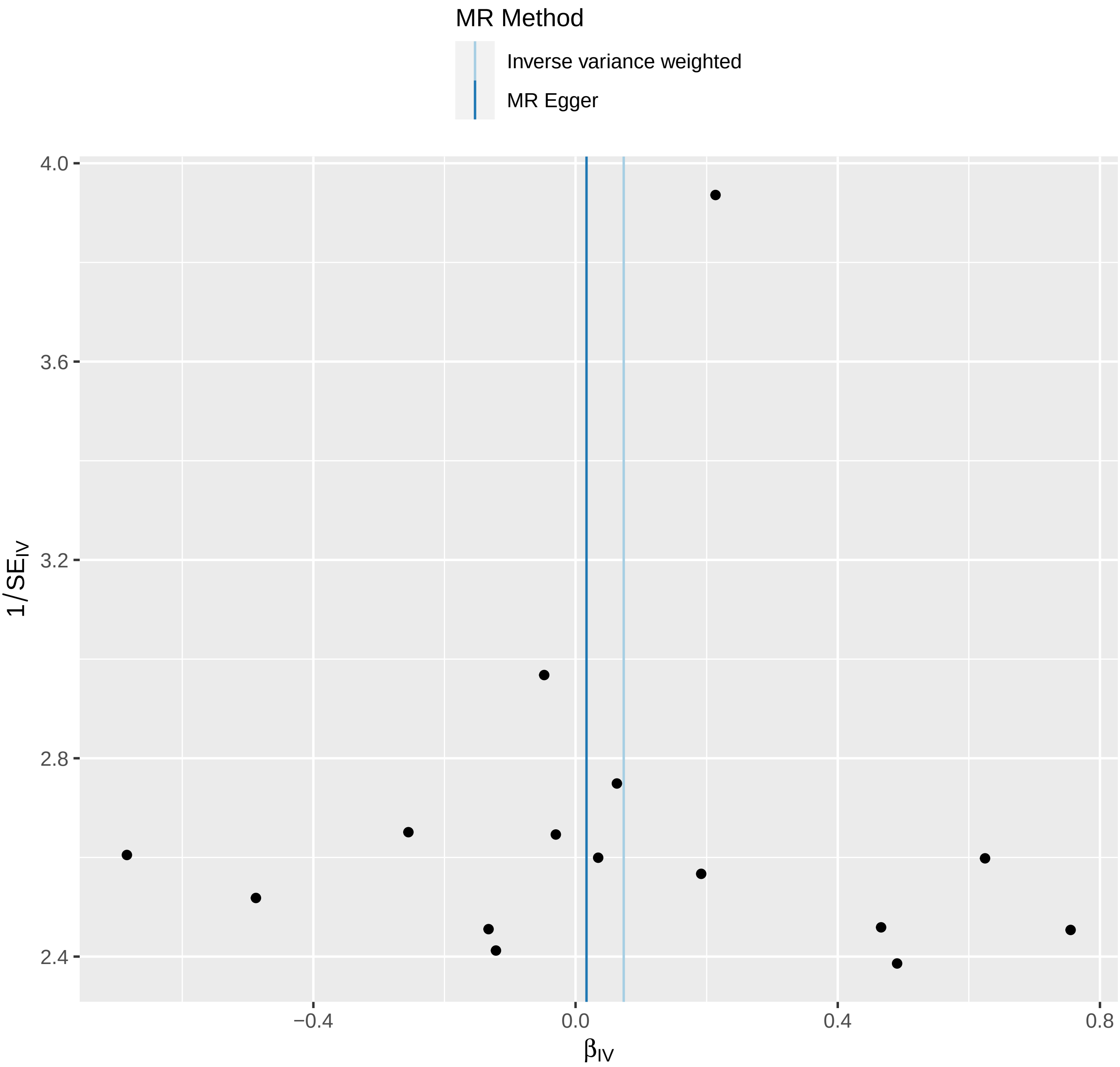

Batch 2−2663 : Gut microbiota abundance (genus Catenibacterium id.2153) on Hemojuvelin
with a threshold of 1e−05

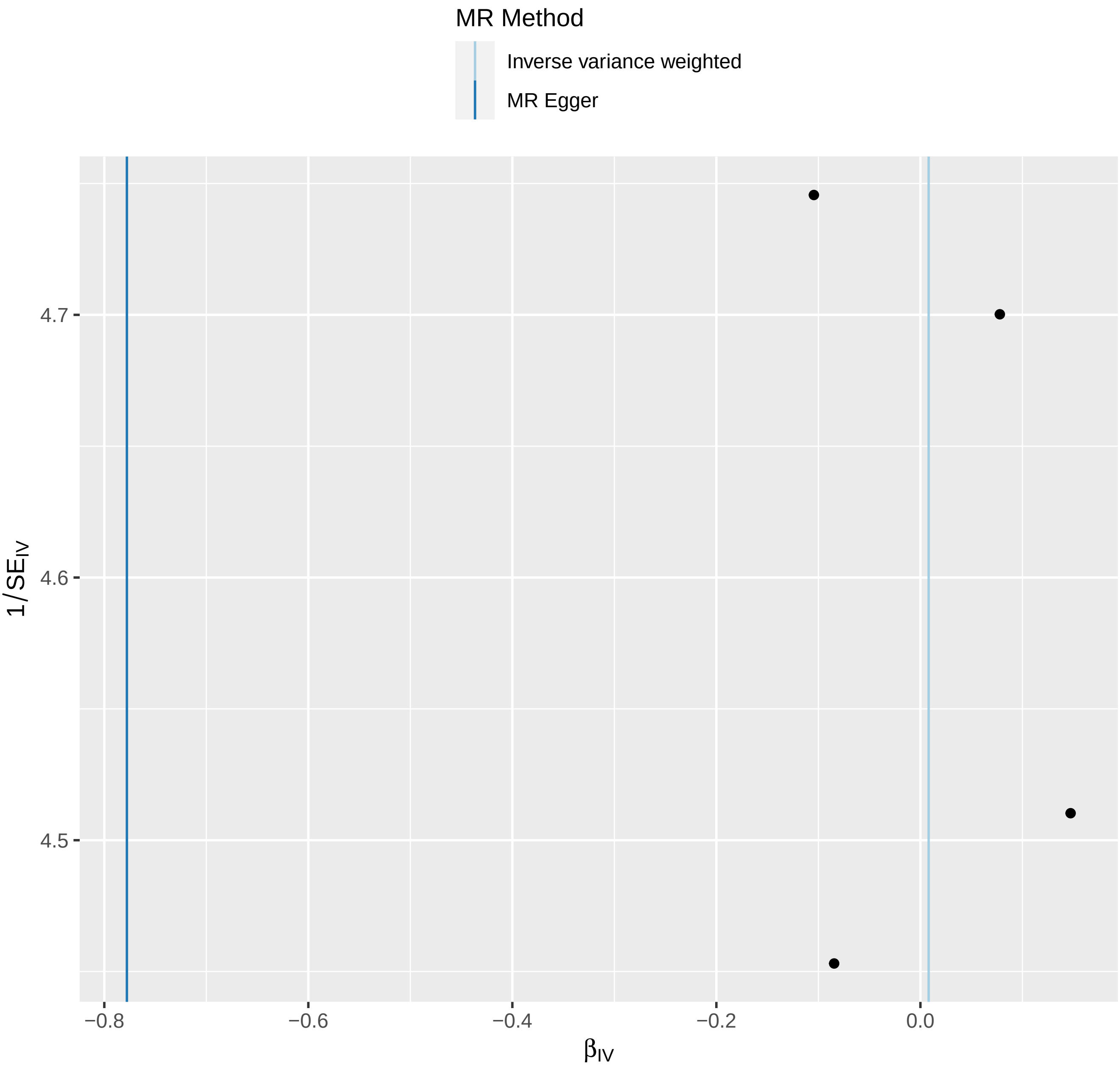

Batch 2−2664 : Gut microbiota abundance (genus Parasutterella id.2892) on Hemojuvelin
with a threshold of 1e−05

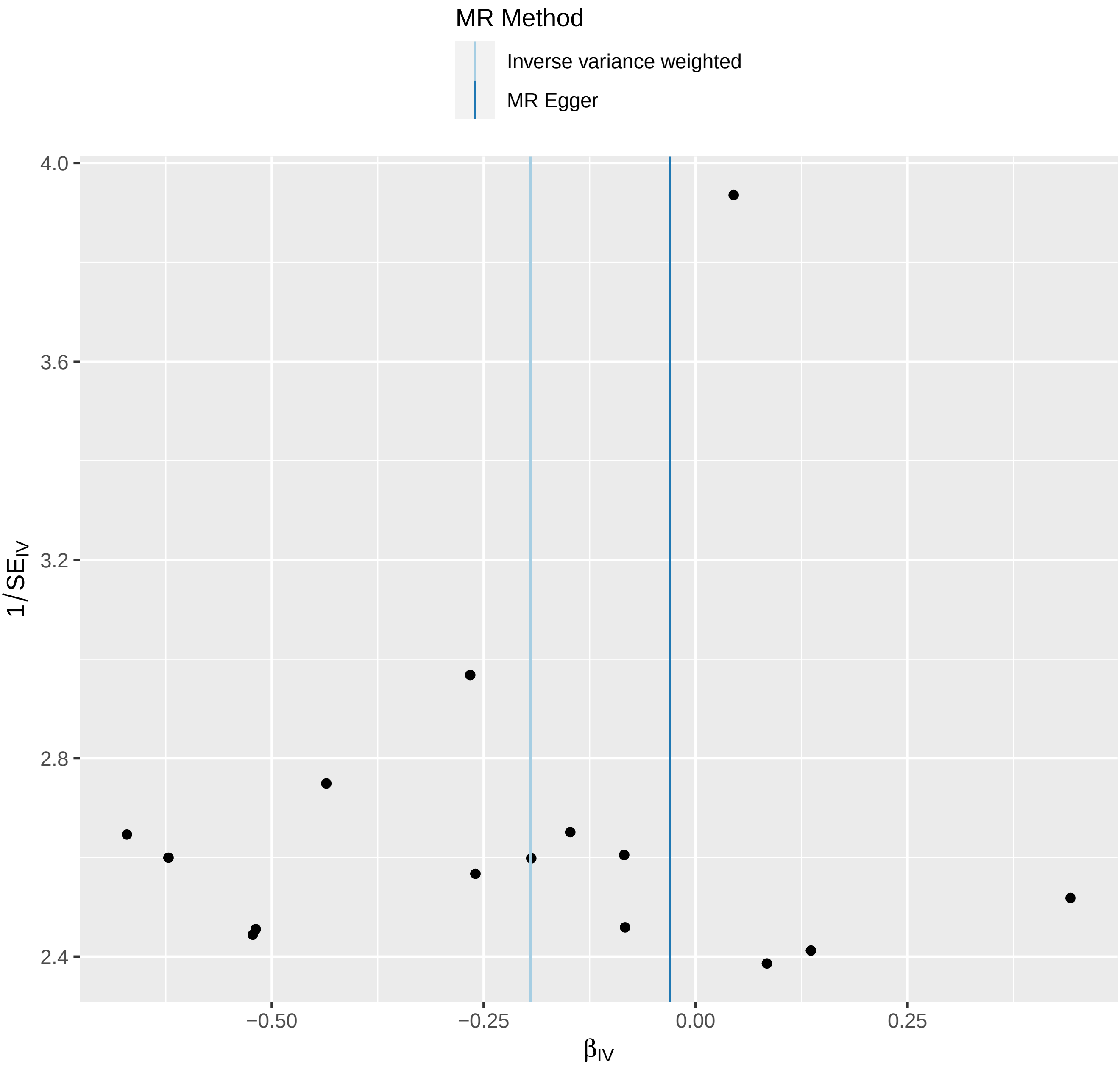

Batch 2−2665 : Gut microbiota abundance (genus Catenibacterium id.2153) on Homogentisate 1,2−dioxygenase
with a threshold of 1e−05

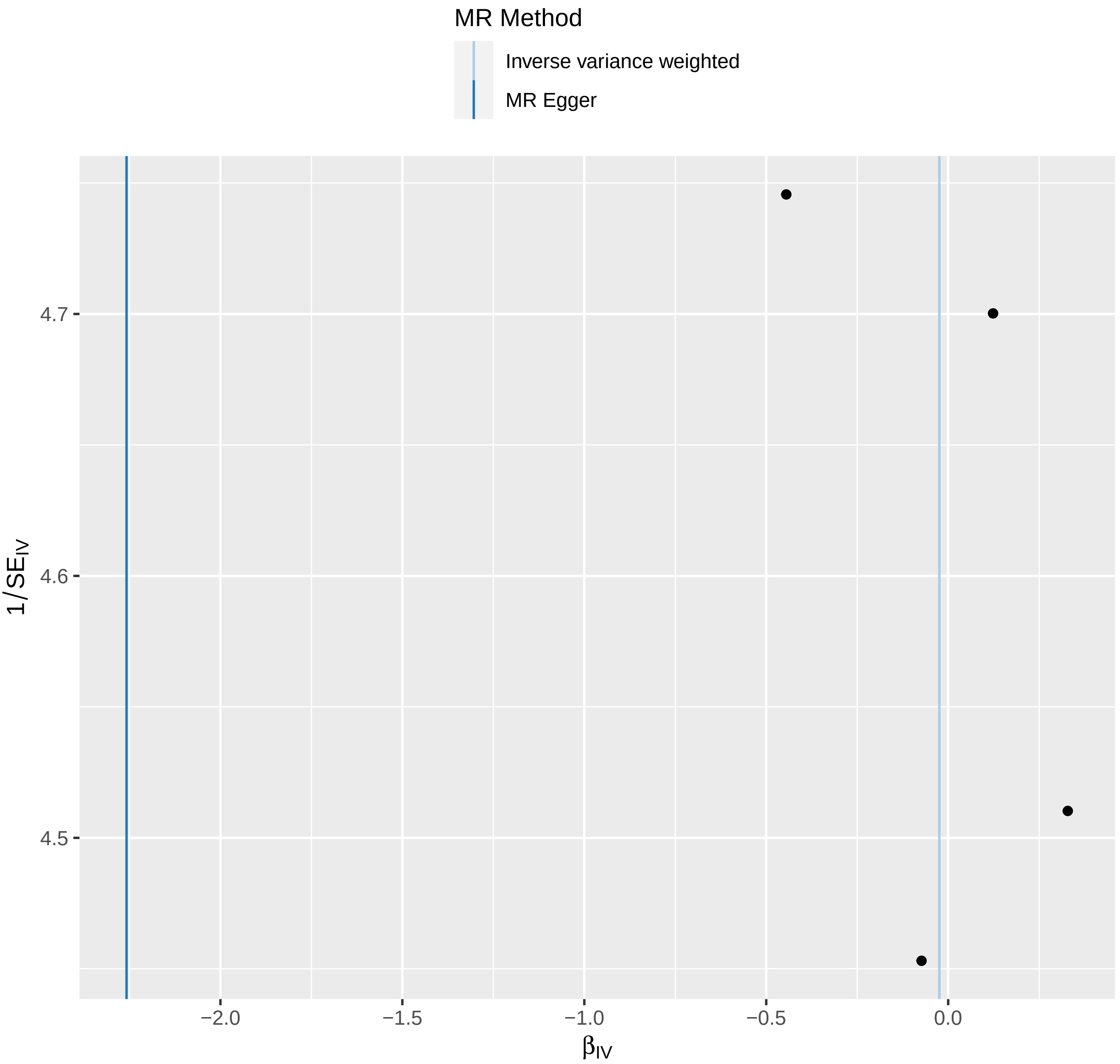

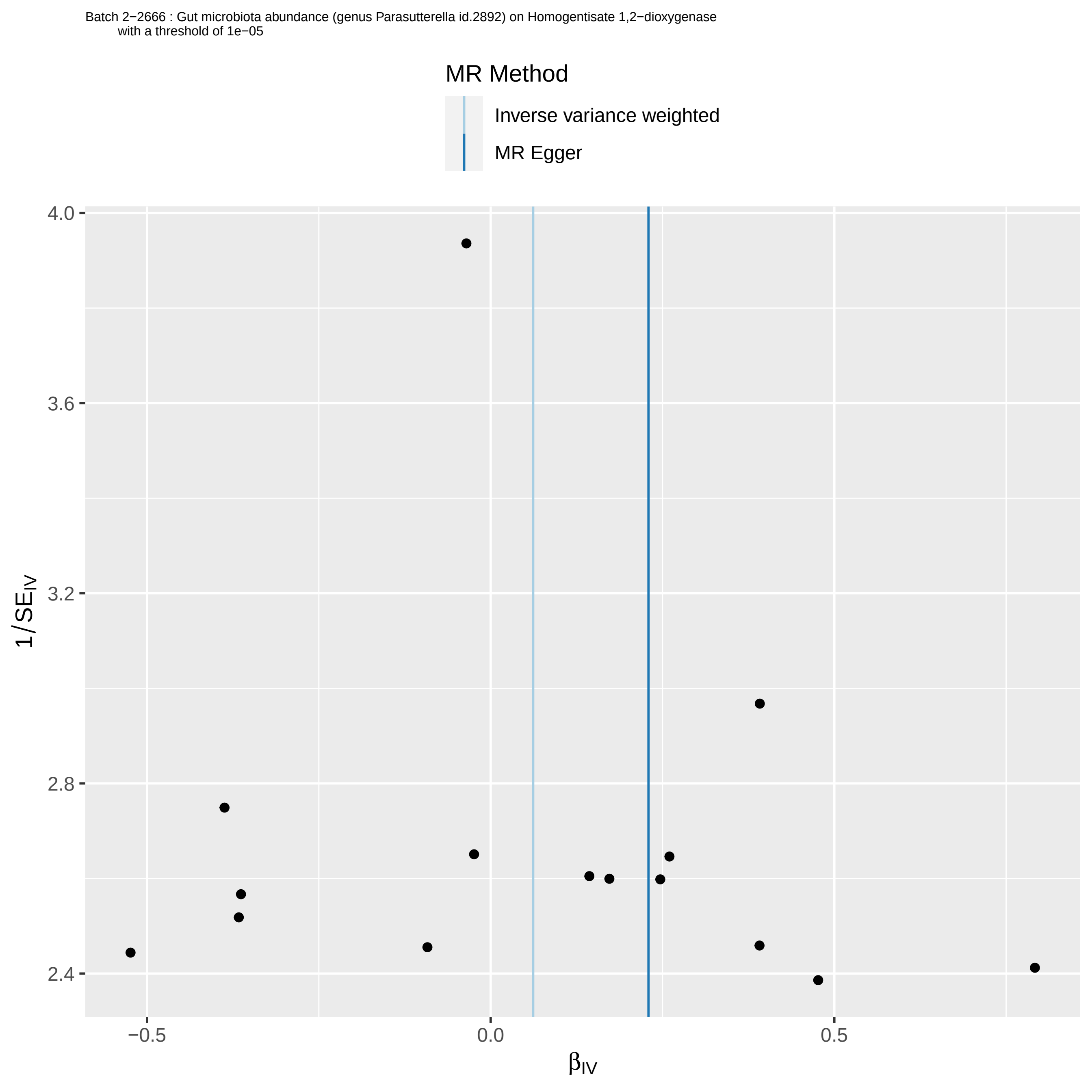
Batch 2−2666 : Gut microbiota abundance (genus Parasutterella id.2892) on Homogentisate 1,2−dioxygenase
with a threshold of 1e−05
MR Method
Inverse variance weighted
MR Egger
4.0
3.6
3.2
2.8
2.4
1/SE$_{IV}$
−0.5
0.0
0.5
β$_{IV}$

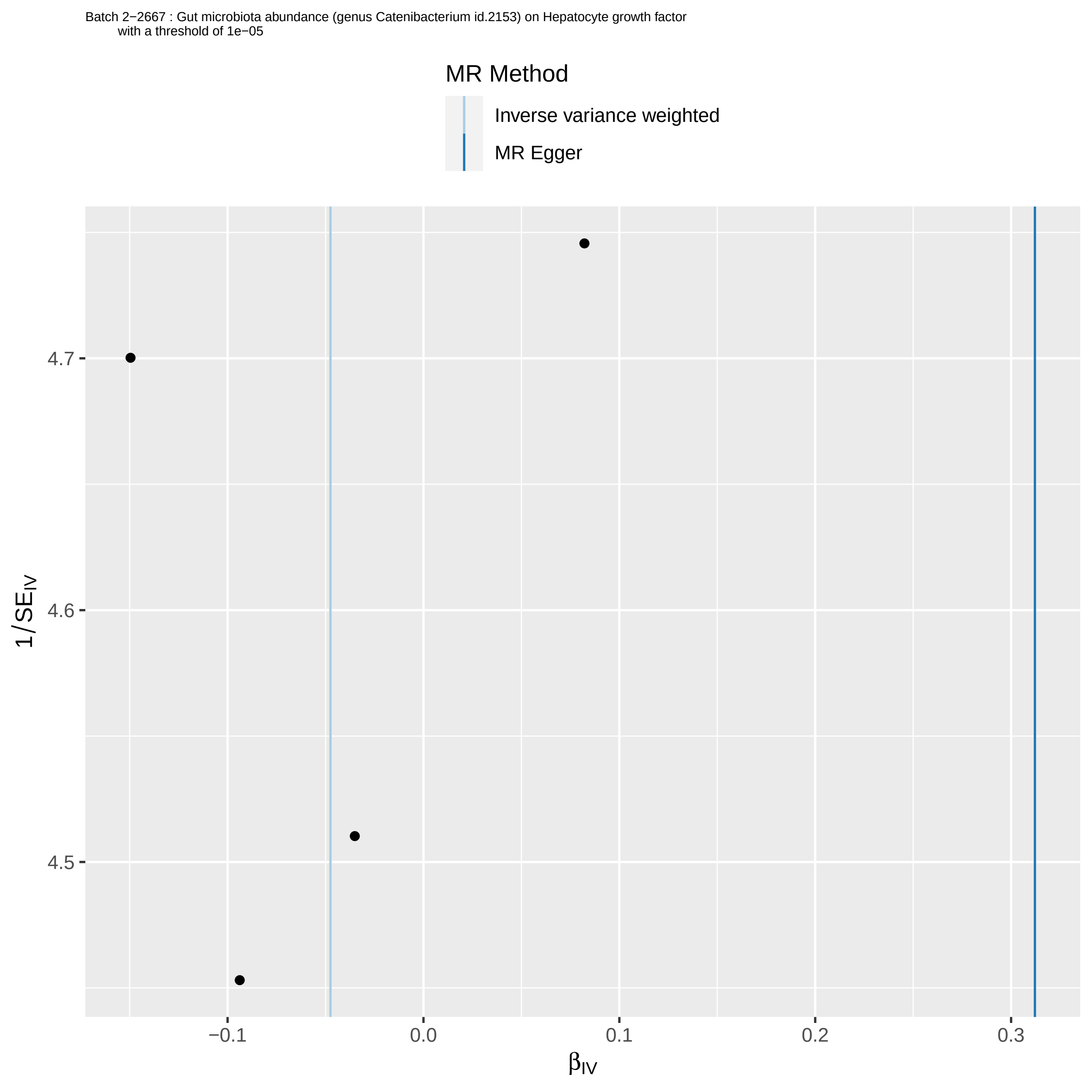
Batch 2−2667 : Gut microbiota abundance (genus Catenibacterium id.2153) on Hepatocyte growth factor
with a threshold of 1e−05
MR Method
Inverse variance weighted
MR Egger
4.7
4.6
4.5
1/SE_IV
−0.1
0.0
0.1
0.2
0.3
β_IV

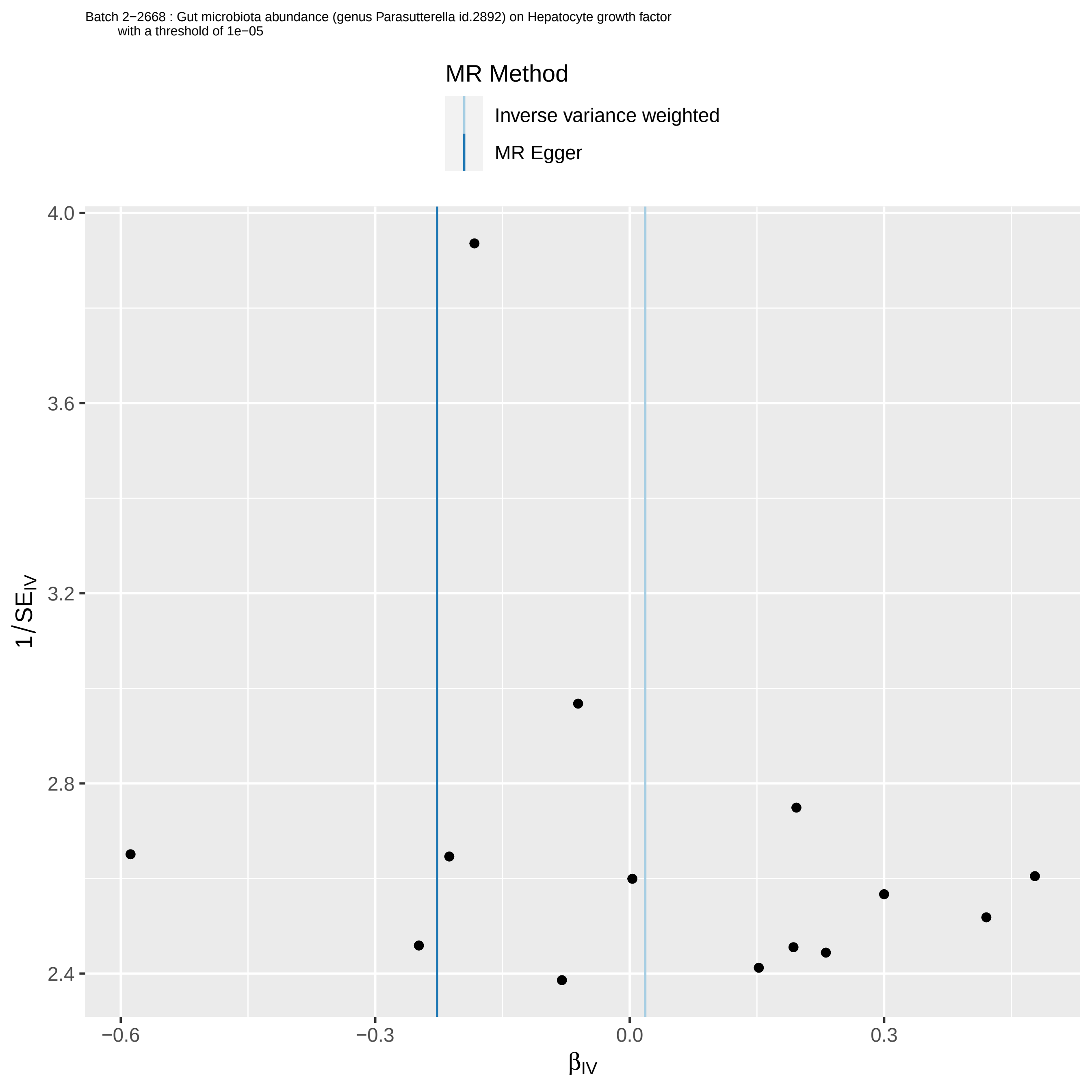

Batch 2−2668 : Gut microbiota abundance (genus Parasutterella id.2892) on Hepatocyte growth factor
with a threshold of 1e−05
MR Method
Inverse variance weighted
MR Egger
4.0
3.6
3.2
2.8
2.4
1/SE_IV
−0.6
−0.3
0.0
0.3
β_IV

Batch 2−2669 : Gut microbiota abundance (genus Catenibacterium id.2153) on Hepatocyte growth factor−regulated tyrosine kinase substrate with a threshold of 1e−05

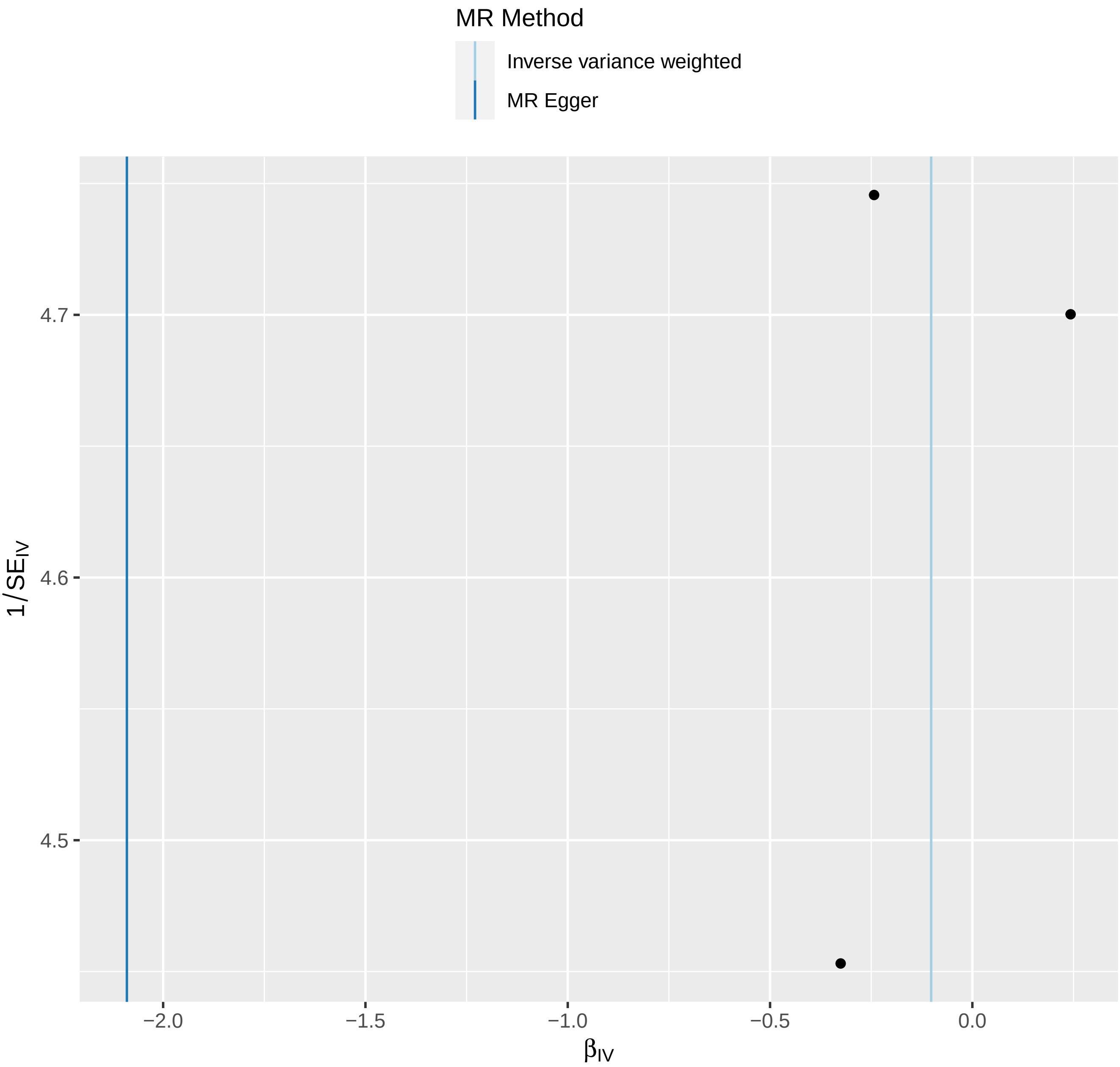

Batch 2−2670 : Gut microbiota abundance (genus Parasutterella id.2892) on Hepatocyte growth factor−regulated tyrosine kinase substrate
with a threshold of 1e−05

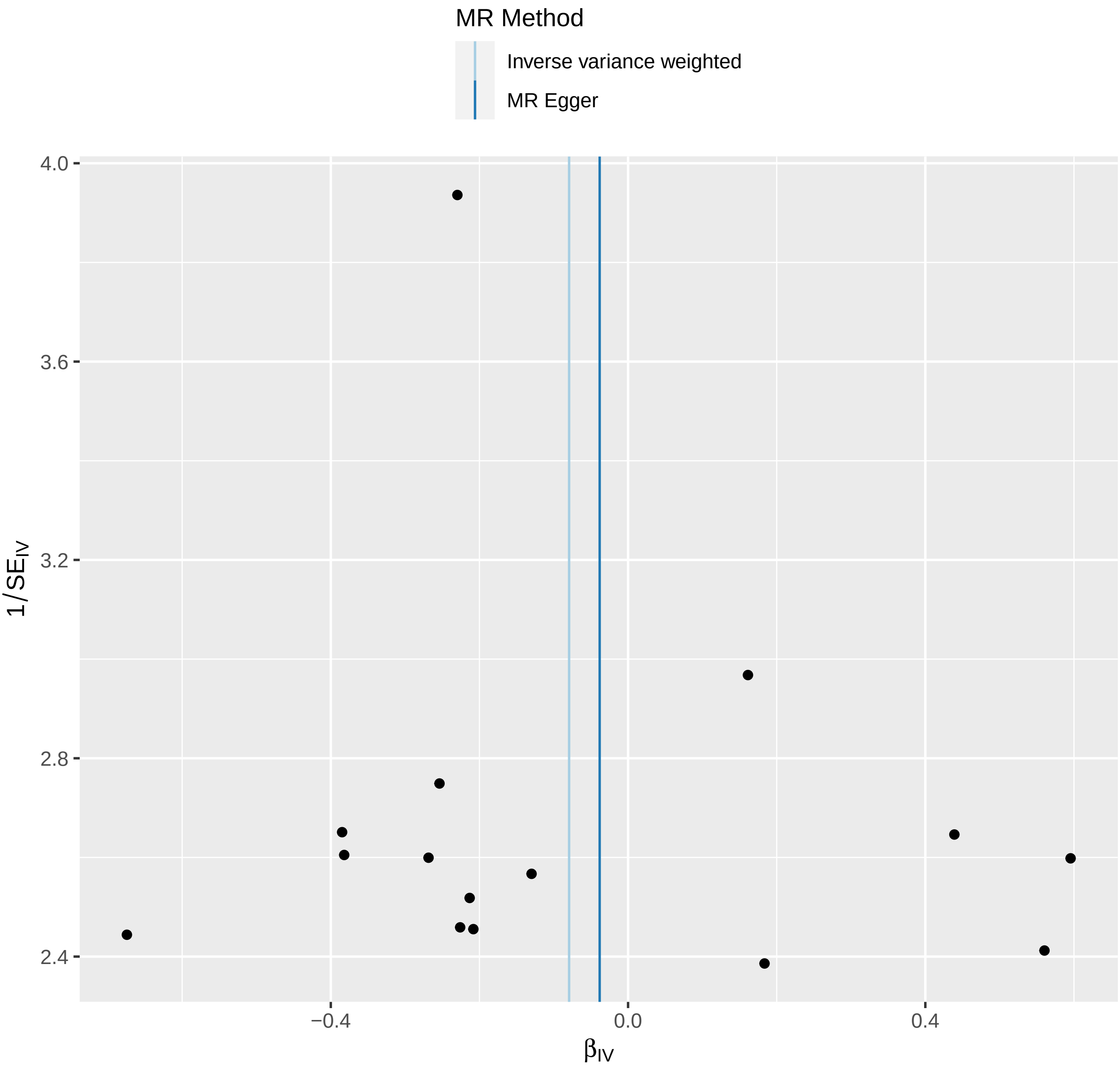

Batch 2−2671 : Gut microbiota abundance (genus Catenibacterium id.2153) on HERV−H LTR−associating protein 2
with a threshold of 1e−05

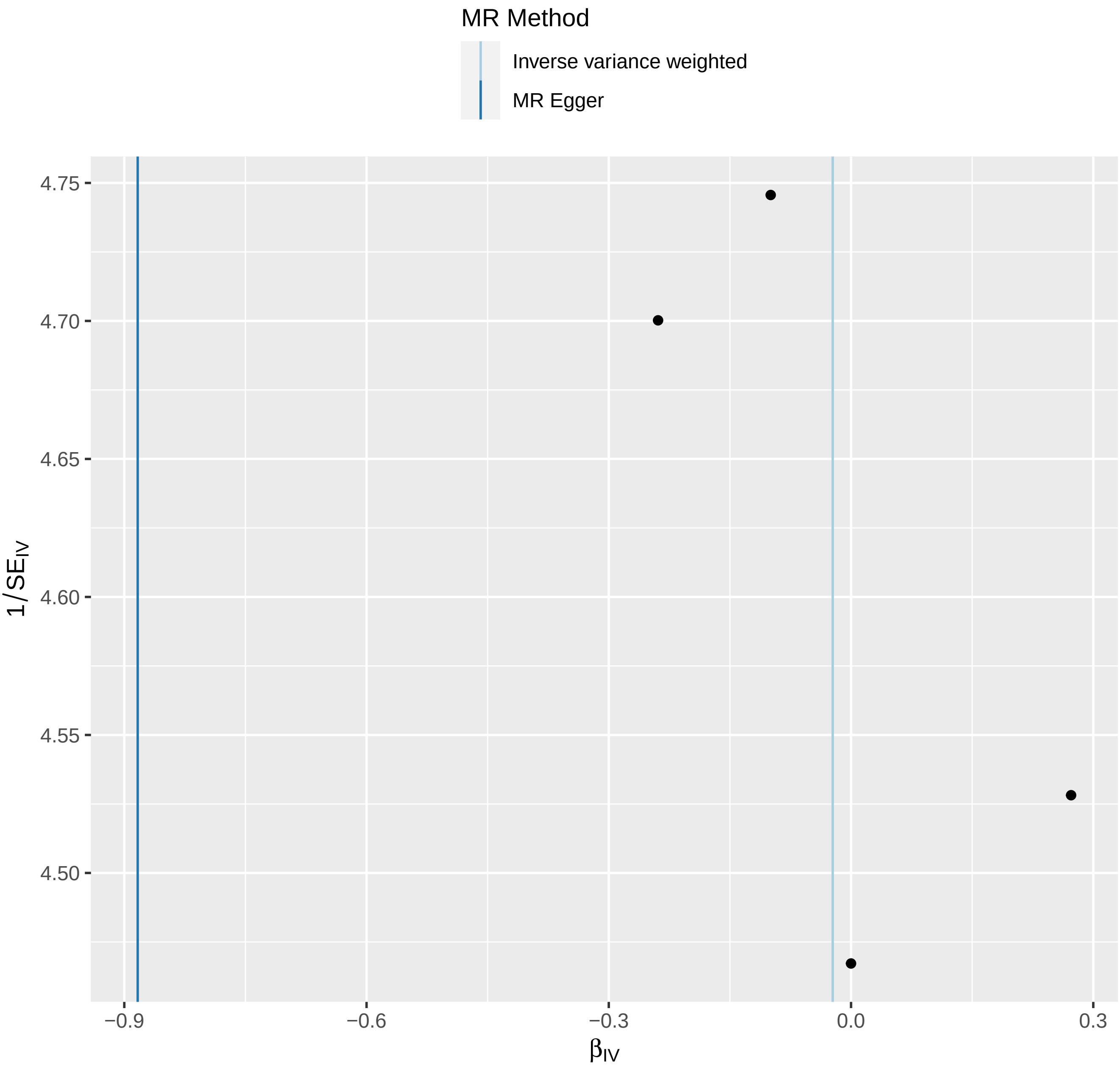

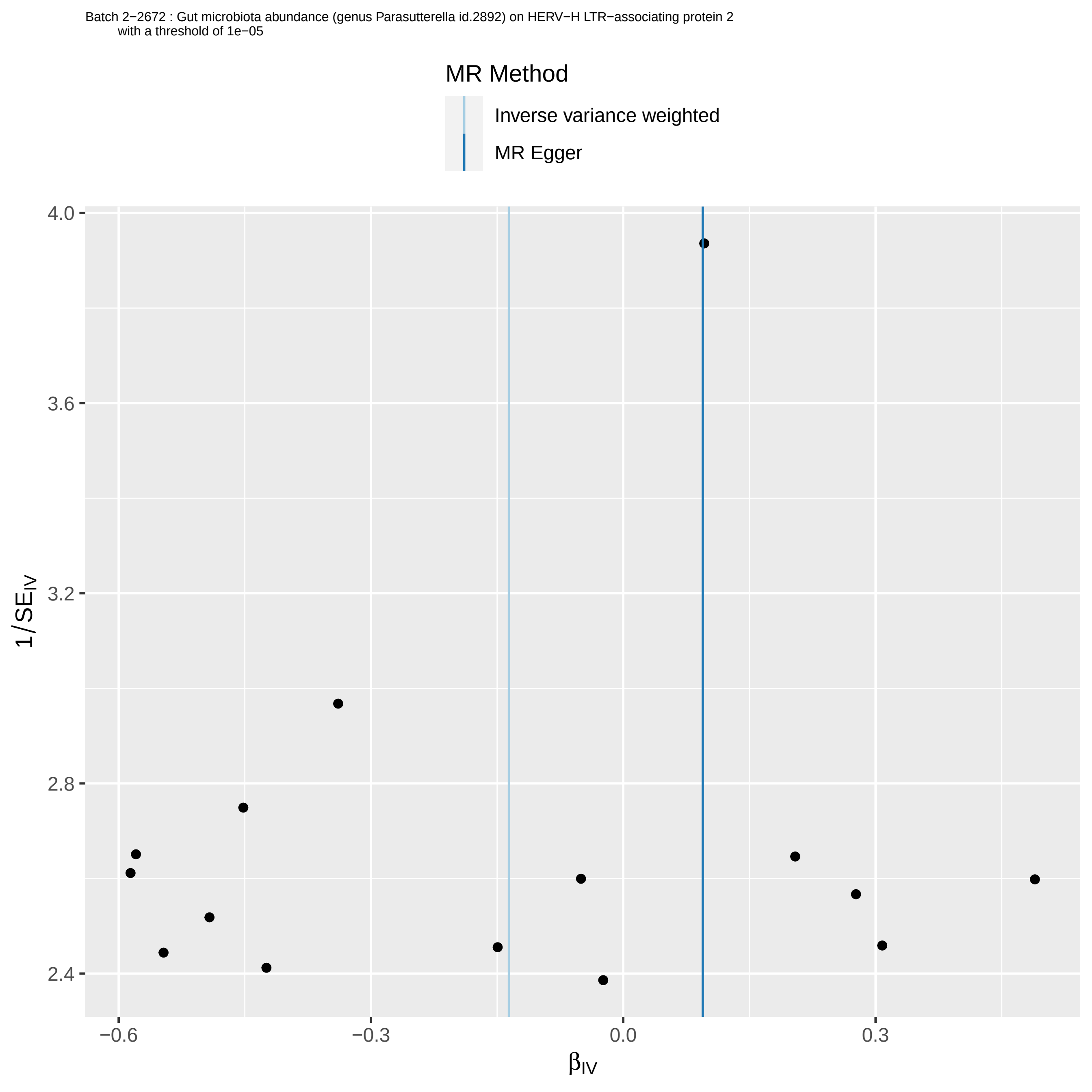

Batch 2−2672 : Gut microbiota abundance (genus Parasutterella id.2892) on HERV−H LTR−associating protein 2
with a threshold of 1e−05
MR Method
Inverse variance weighted
MR Egger
4.0
3.6
3.2
2.8
2.4
1/SE_IV
−0.6
−0.3
0.0
0.3
β_IV

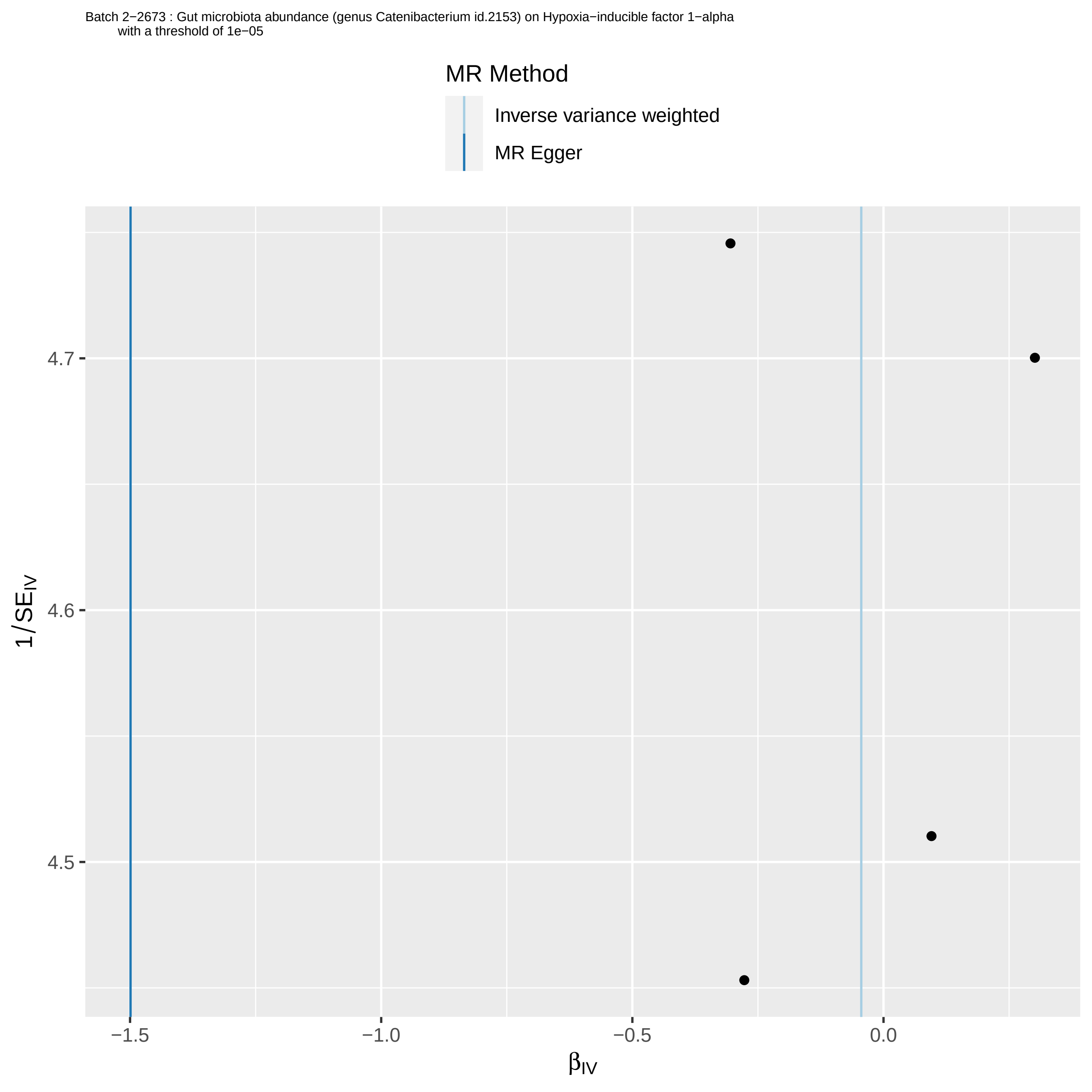
Batch 2−2673 : Gut microbiota abundance (genus Catenibacterium id.2153) on Hypoxia−inducible factor 1−alpha
with a threshold of 1e−05
MR Method
Inverse variance weighted
MR Egger
4.7
4.6
4.5
1/SE_IV
−1.5
−1.0
−0.5
0.0
β_IV

Batch 2−2674 : Gut microbiota abundance (genus Parasutterella id.2892) on Hypoxia−inducible factor 1−alpha
with a threshold of 1e−05

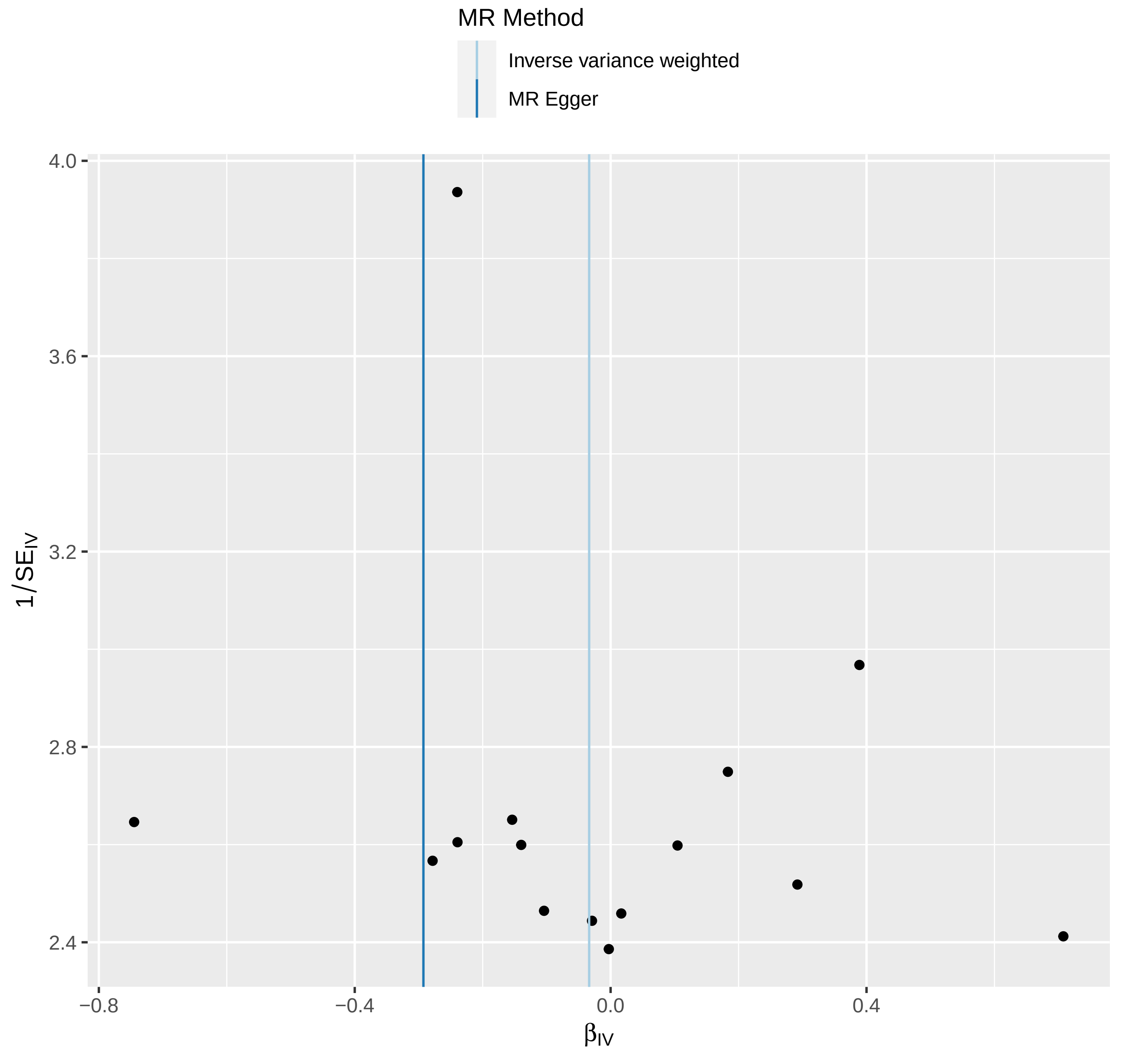

Batch 2−2675 : Gut microbiota abundance (genus Catenibacterium id.2153) on Histidine triad nucleotide−binding protein 1 with a threshold of 1e−05

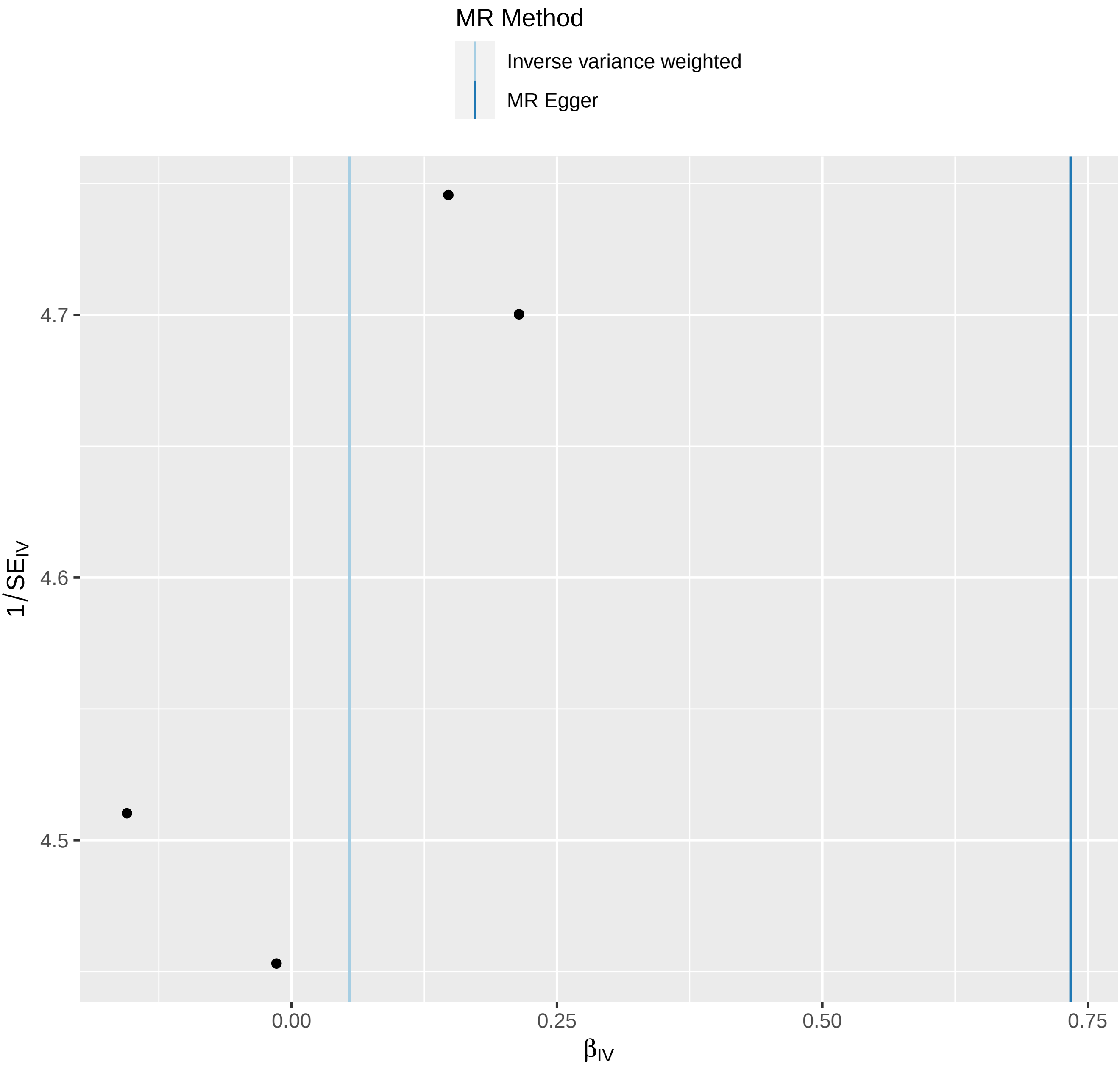

Batch 2−2676 : Gut microbiota abundance (genus Parasutterella id.2892) on Histidine triad nucleotide−binding protein 1 with a threshold of 1e−05

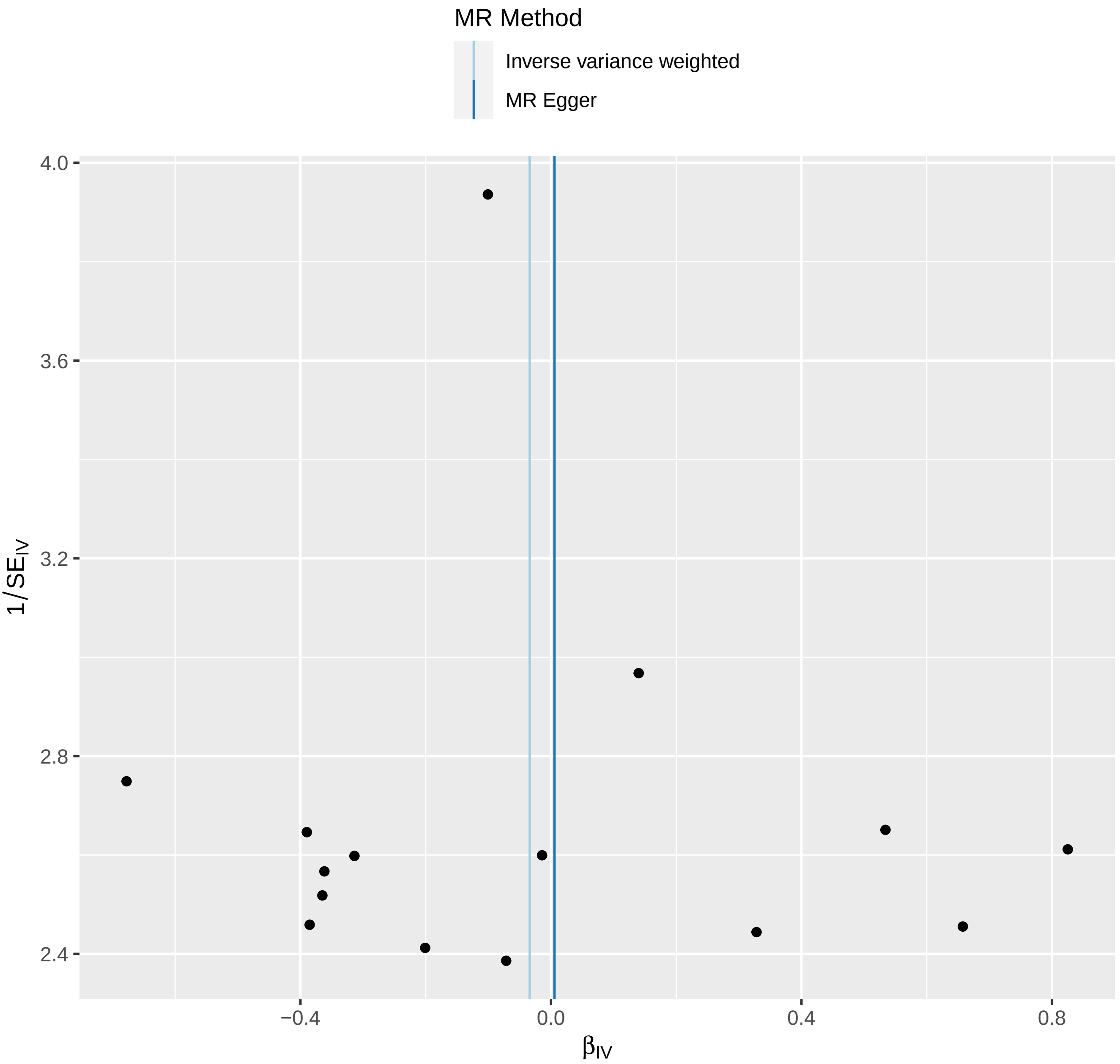

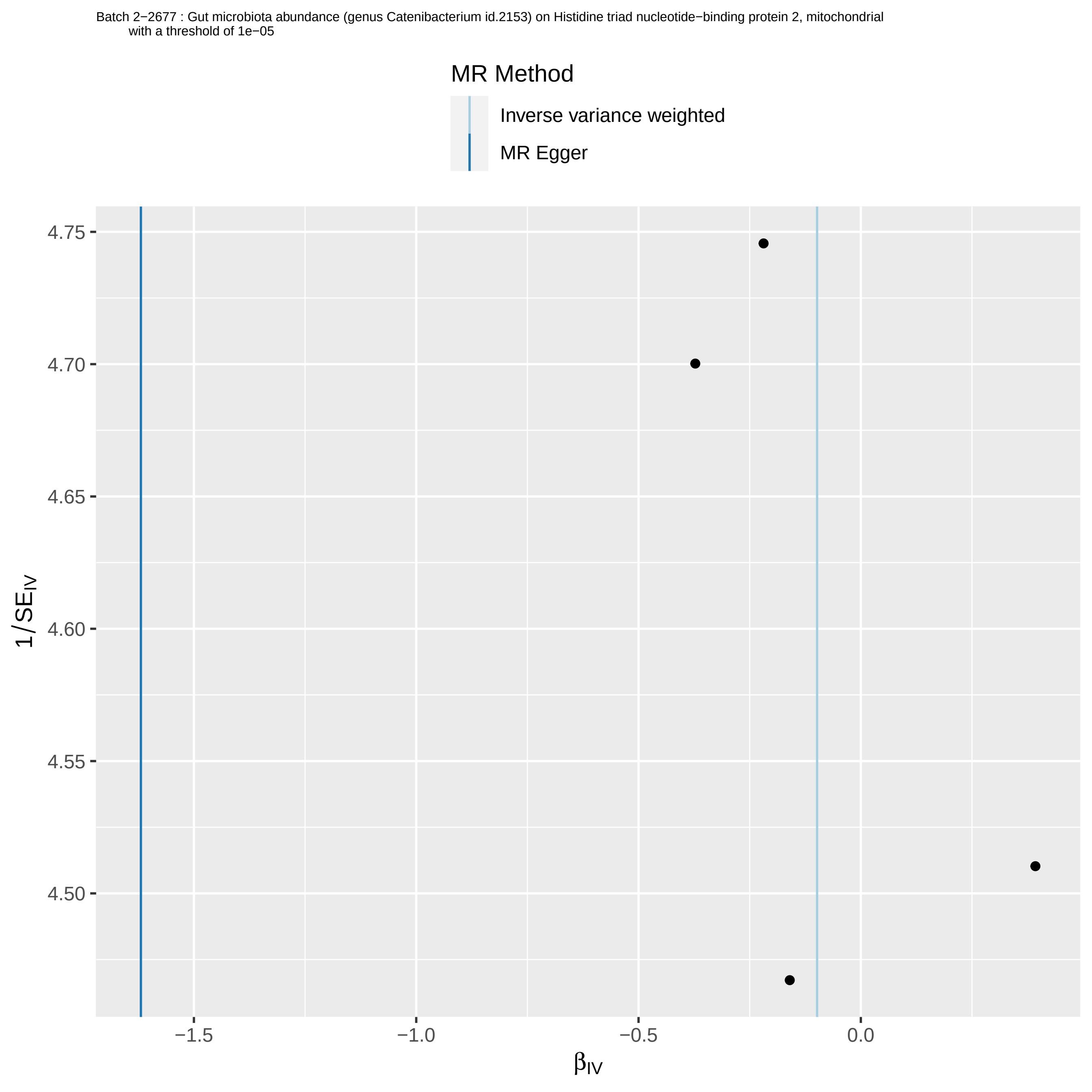
Batch 2−2677 : Gut microbiota abundance (genus Catenibacterium id.2153) on Histidine triad nucleotide−binding protein 2, mitochondrial
with a threshold of 1e−05
MR Method
Inverse variance weighted
MR Egger
4.75
4.70
4.65
4.60
4.55
4.50
1/SE_IV
−1.5
−1.0
−0.5
0.0
β_IV

Batch 2−2678 : Gut microbiota abundance (genus Parasutterella id.2892) on Histidine triad nucleotide−binding protein 2, mitochondrial
with a threshold of 1e−05

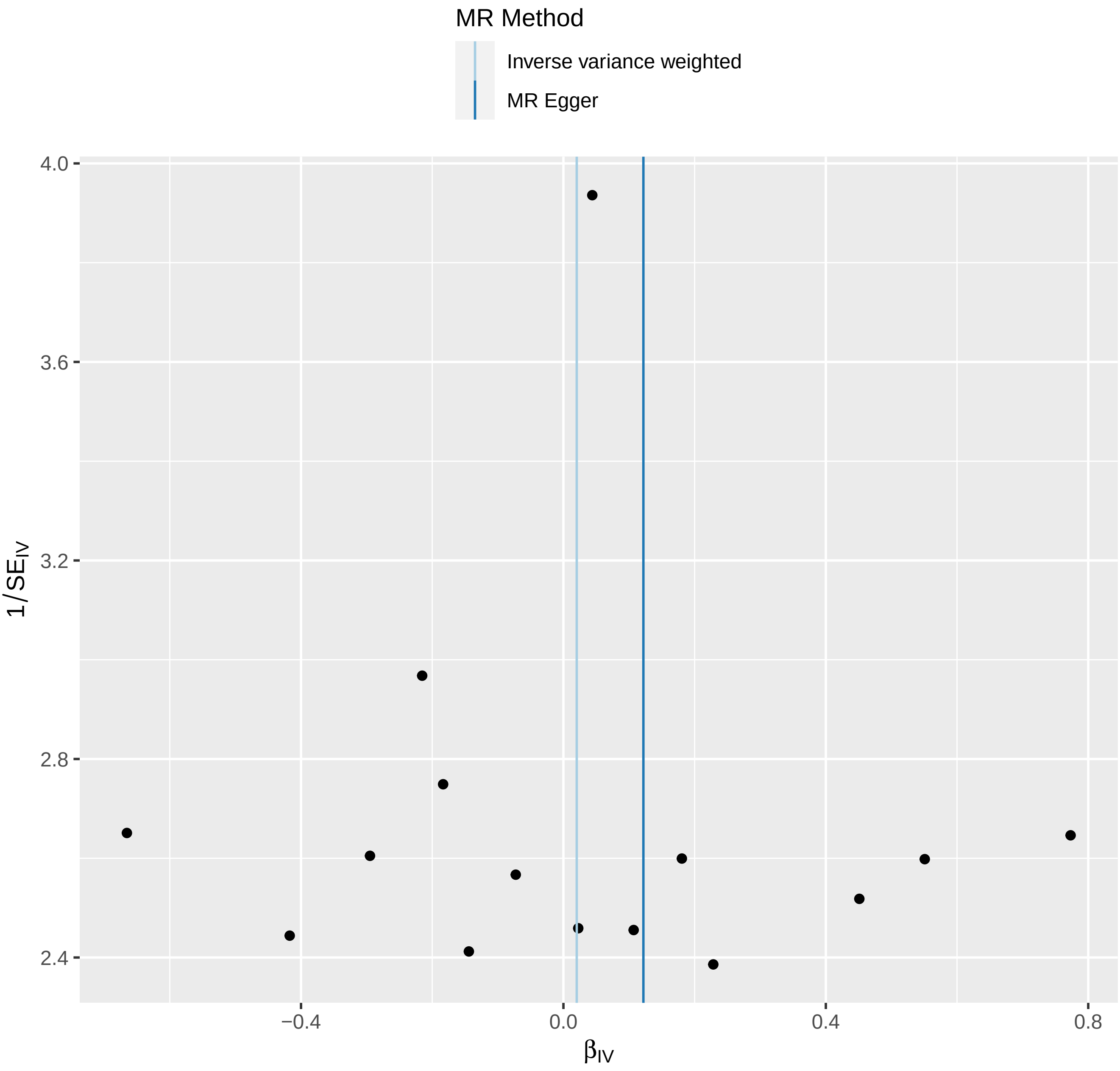

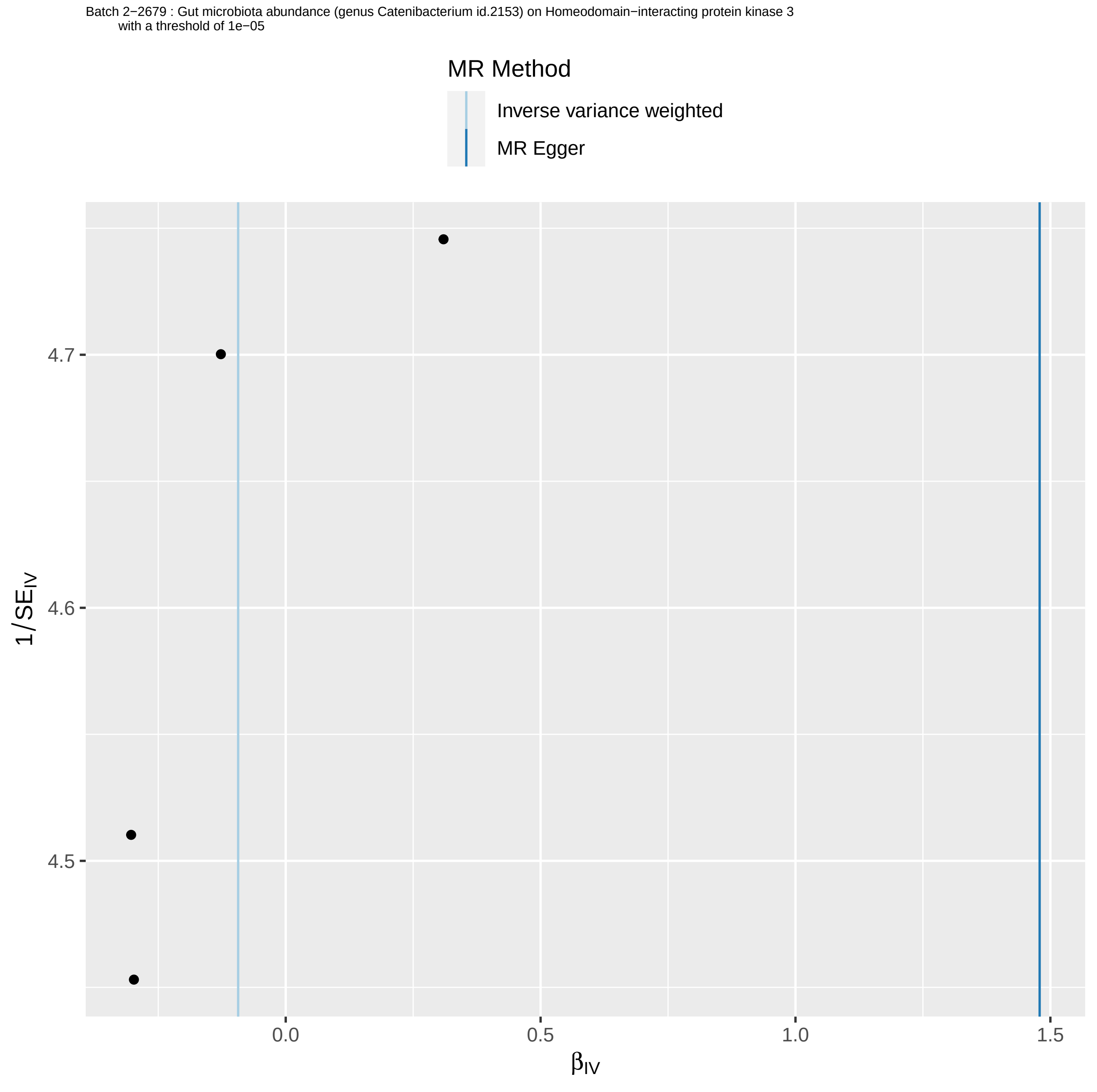
Batch 2−2679 : Gut microbiota abundance (genus Catenibacterium id.2153) on Homeodomain−interacting protein kinase 3
with a threshold of 1e−05
MR Method
Inverse variance weighted
MR Egger
4.7
4.6
4.5
1/SE_IV
0.0
0.5
1.0
1.5
β_IV

Batch 2−2680 : Gut microbiota abundance (genus Parasutterella id.2892) on Homeodomain−interacting protein kinase 3
with a threshold of 1e−05

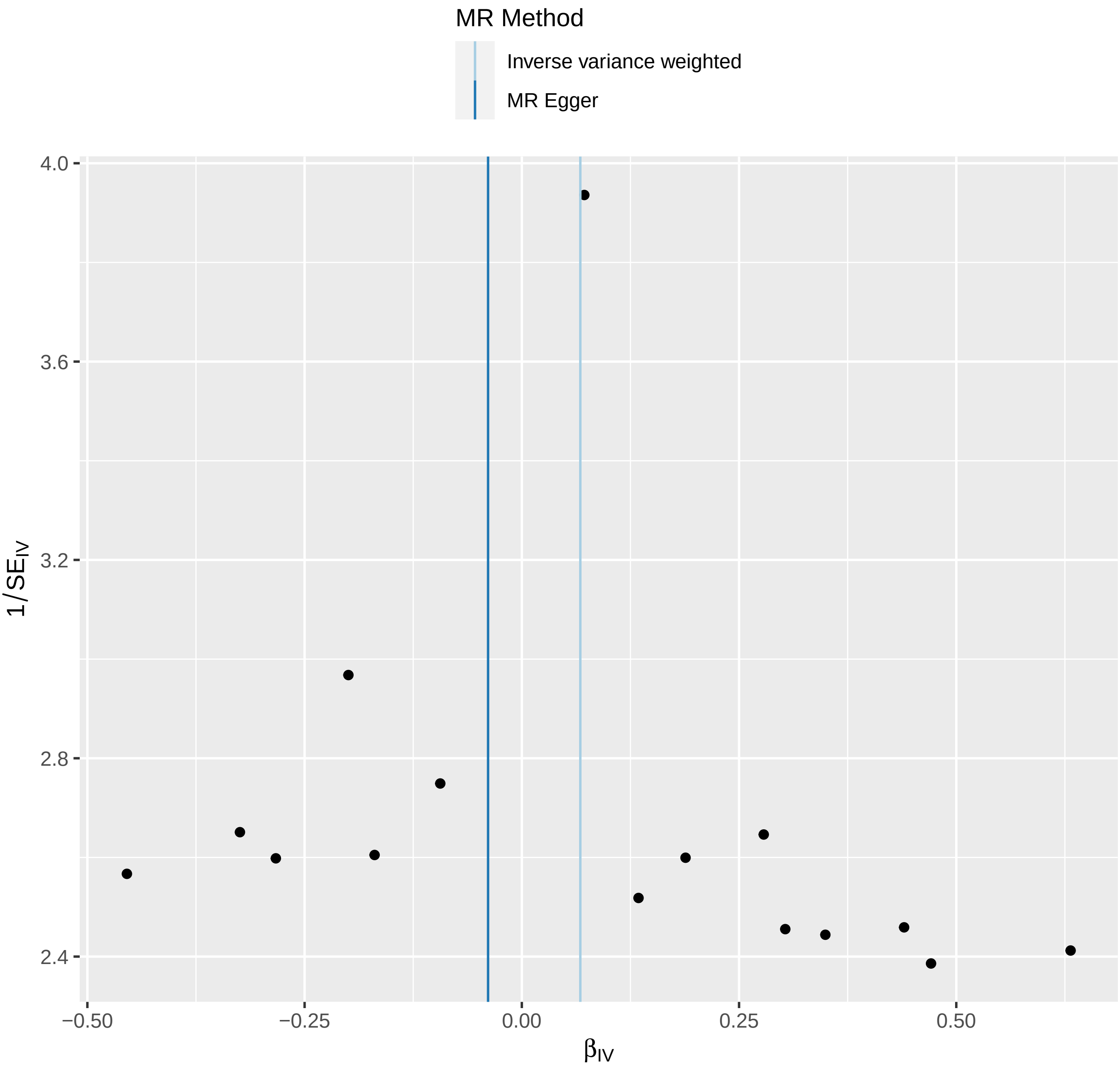

Batch 2−2681 : Gut microbiota abundance (genus Catenibacterium id.2153) on Histone H1.2
with a threshold of 1e−05

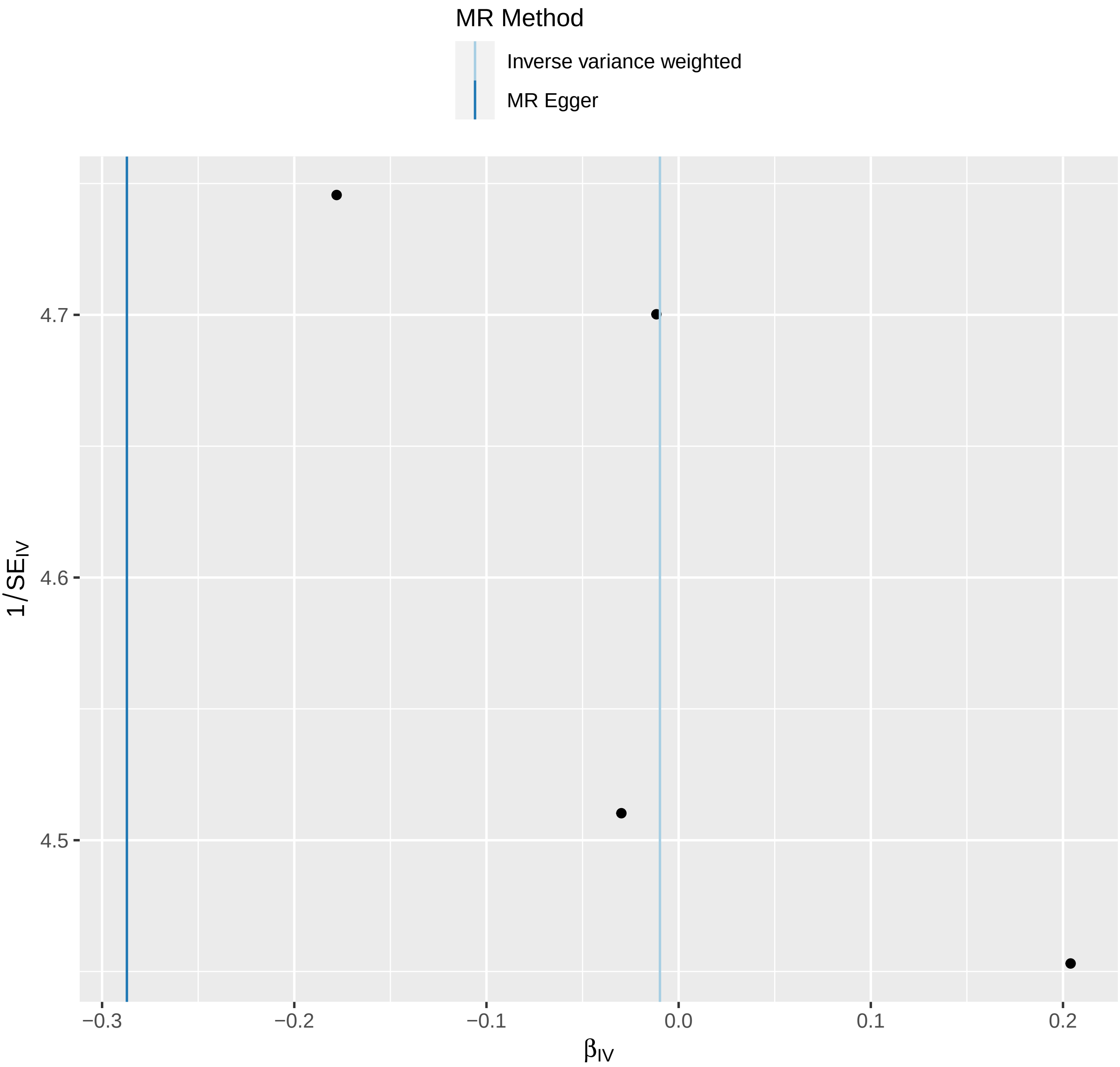

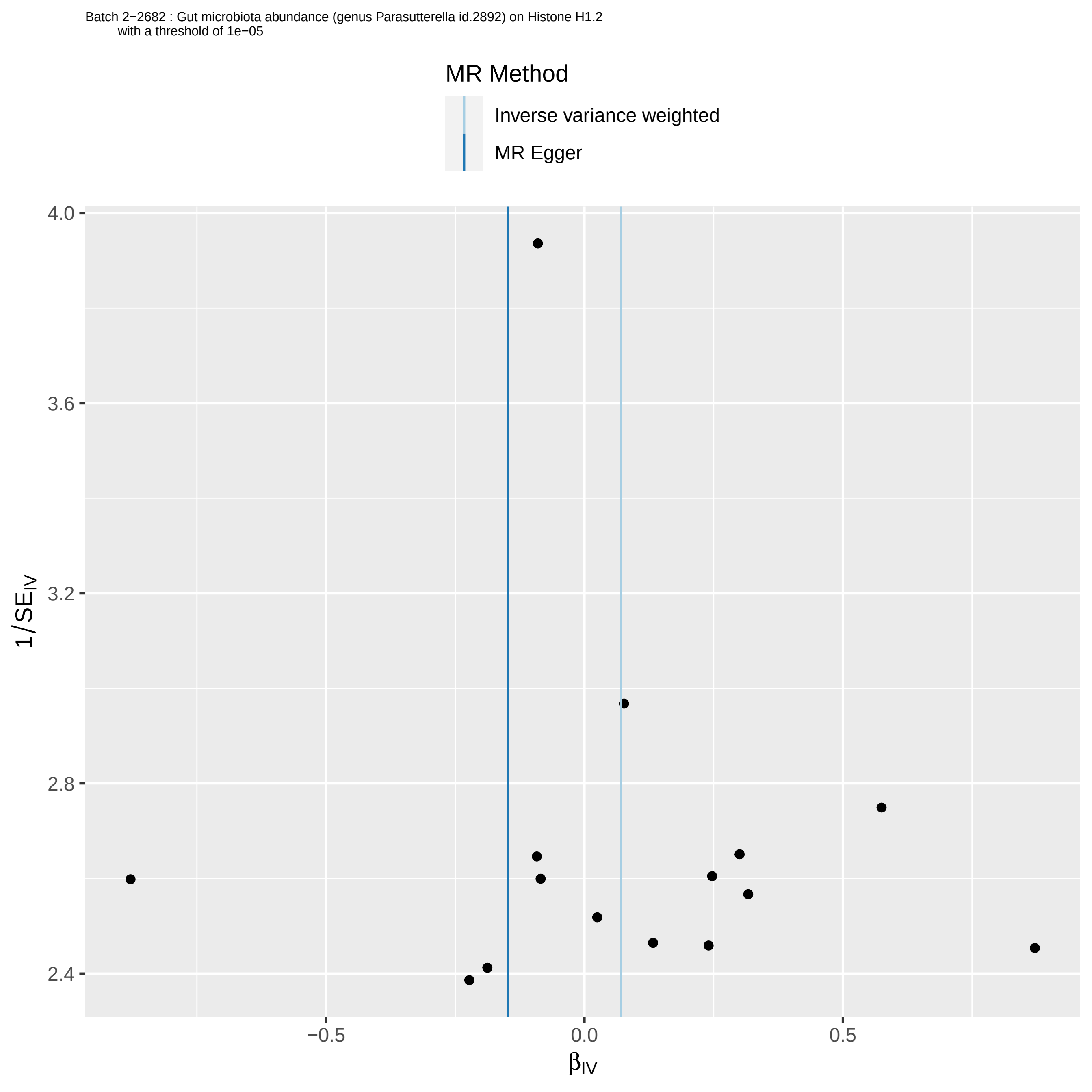
Batch 2−2682 : Gut microbiota abundance (genus Parasutterella id.2892) on Histone H1.2
with a threshold of 1e−05
MR Method
Inverse variance weighted
MR Egger
4.0
3.6
3.2
2.8
2.4
1/SE_IV
−0.5
0.0
0.5
β_IV

Batch 2−2683 : Gut microbiota abundance (genus Catenibacterium id.2153) on Histone H2A type 3
with a threshold of 1e−05

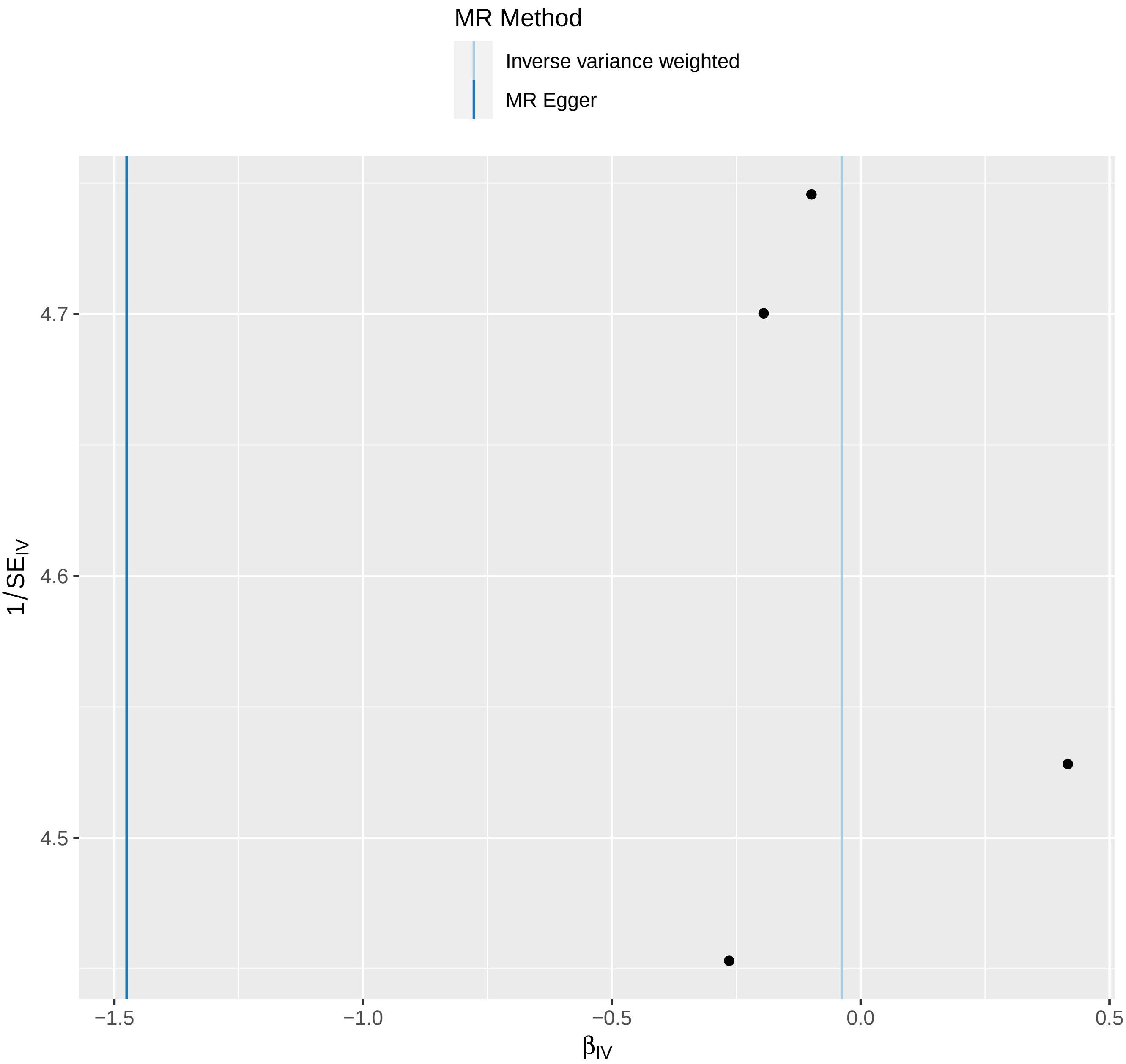

Batch 2−2684 : Gut microbiota abundance (genus Parasutterella id.2892) on Histone H2A type 3
with a threshold of 1e−05

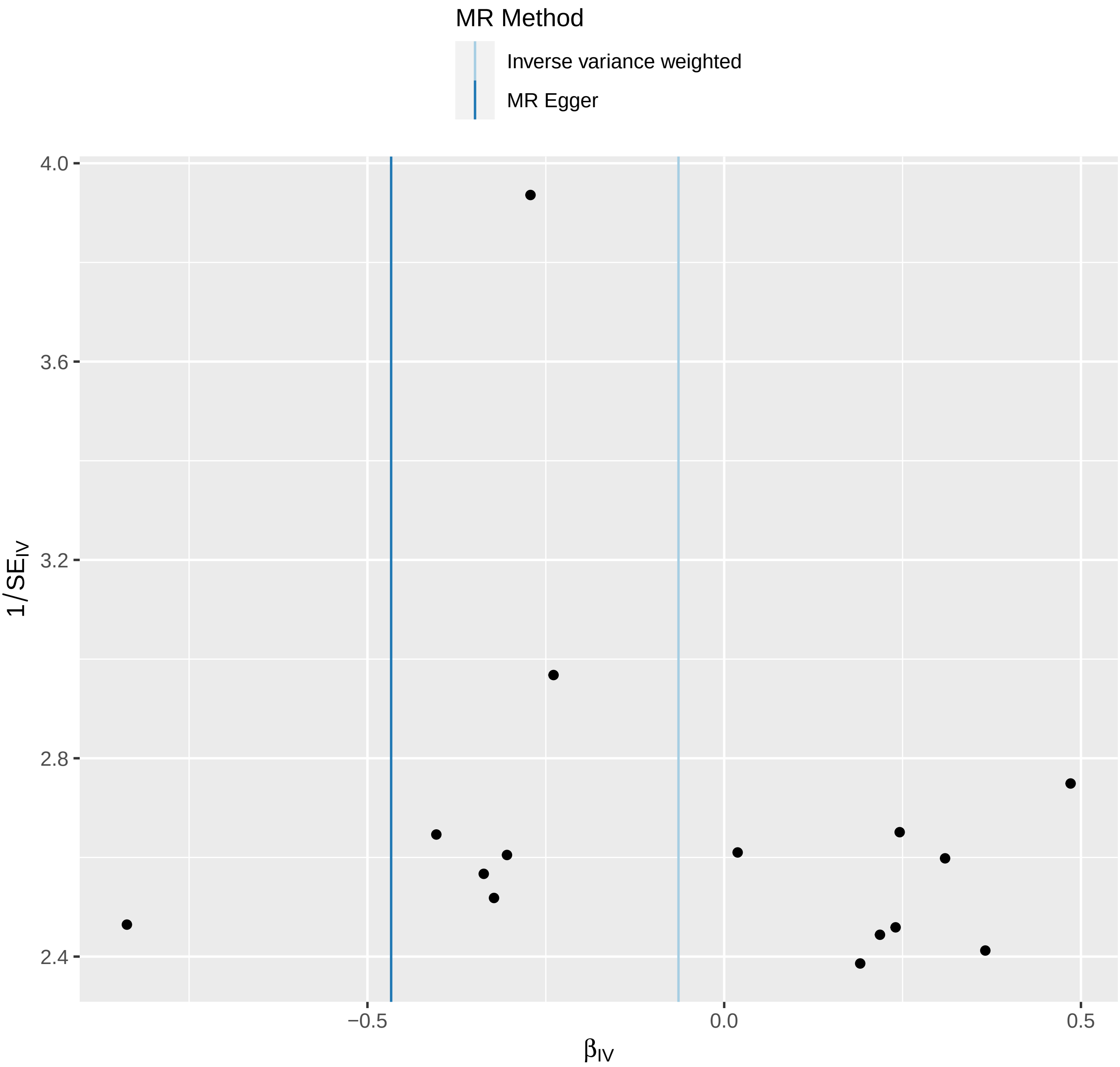

Batch 2−2685 : Gut microbiota abundance (genus Catenibacterium id.2153) on Hexokinase−1
with a threshold of 1e−05

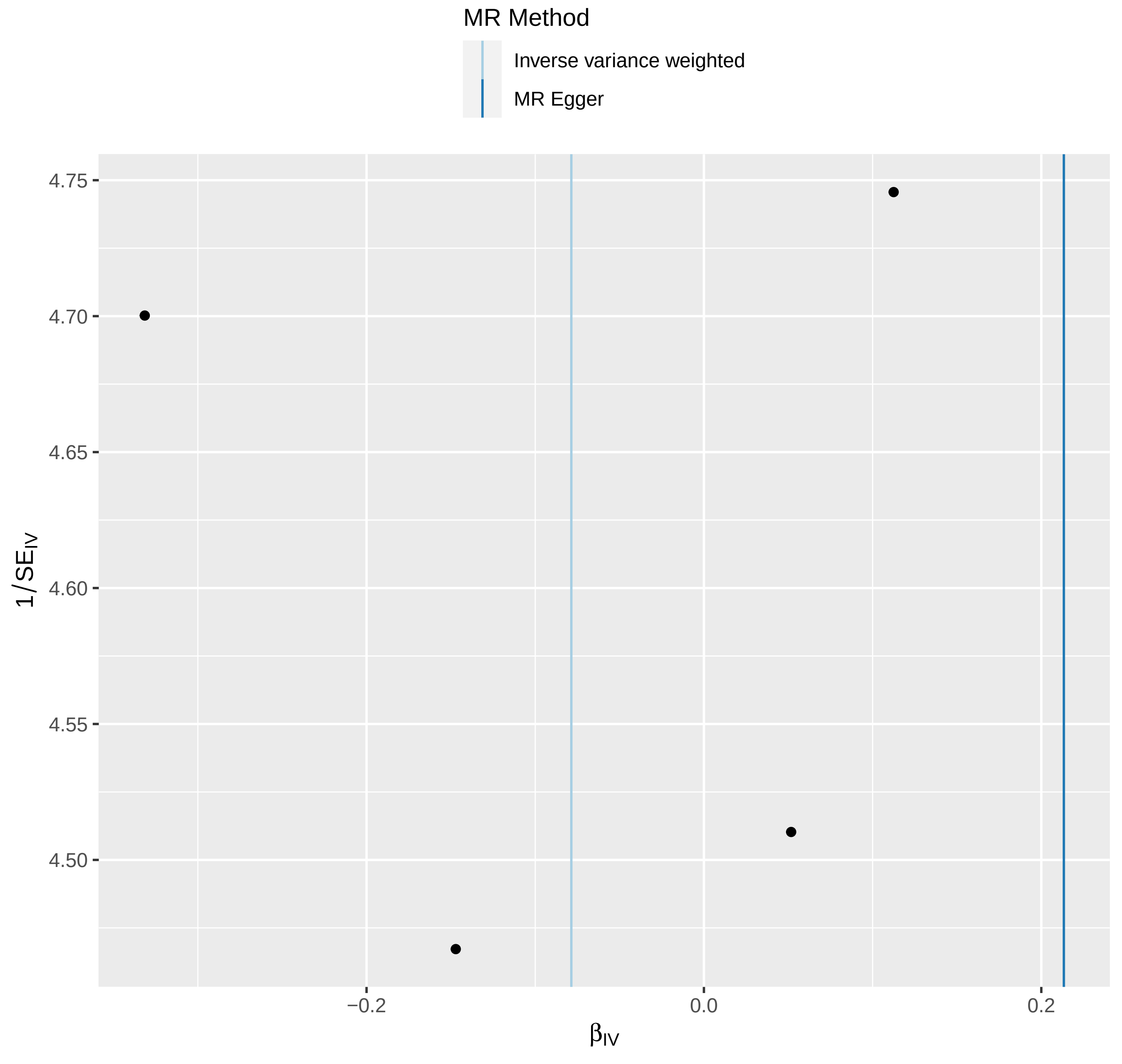

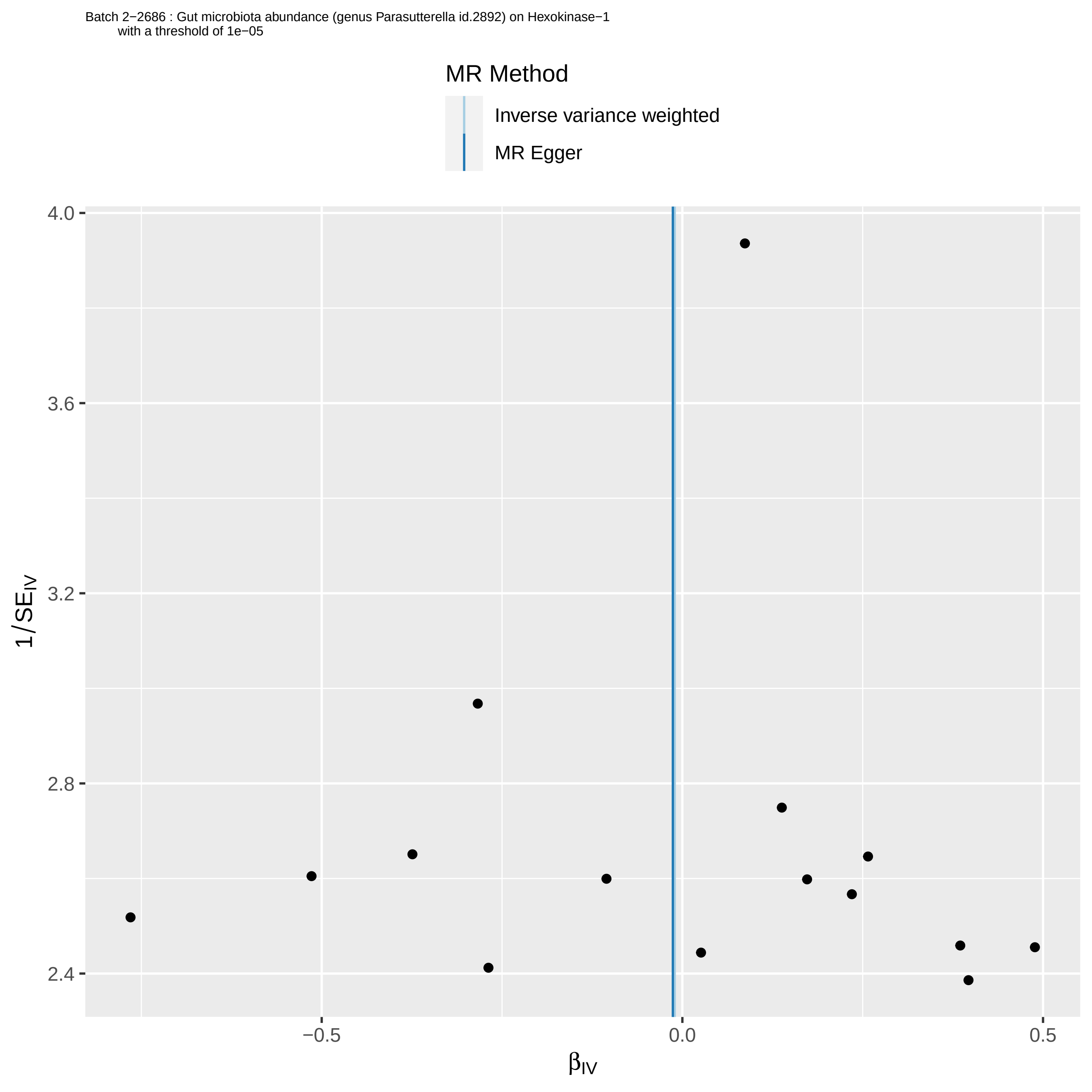
Batch 2−2686 : Gut microbiota abundance (genus Parasutterella id.2892) on Hexokinase−1
with a threshold of 1e−05
MR Method
Inverse variance weighted
MR Egger
4.0
3.6
3.2
2.8
2.4
1/SE_IV
−0.5
0.0
0.5
β_IV

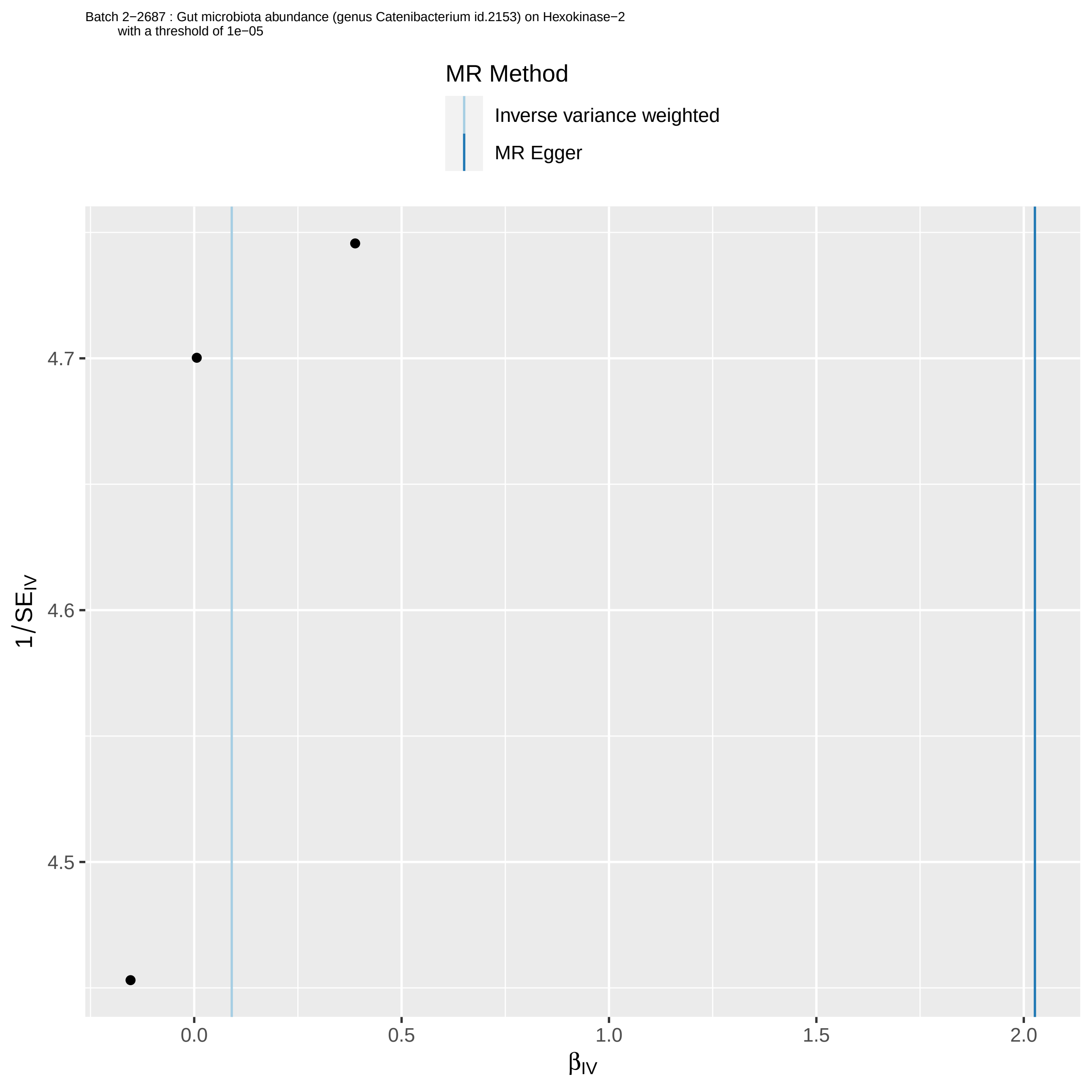
Batch 2−2687 : Gut microbiota abundance (genus Catenibacterium id.2153) on Hexokinase−2
with a threshold of 1e−05
MR Method
Inverse variance weighted
MR Egger
4.7
4.6
4.5
1/SE_IV
0.0
0.5
1.0
1.5
2.0
β_IV

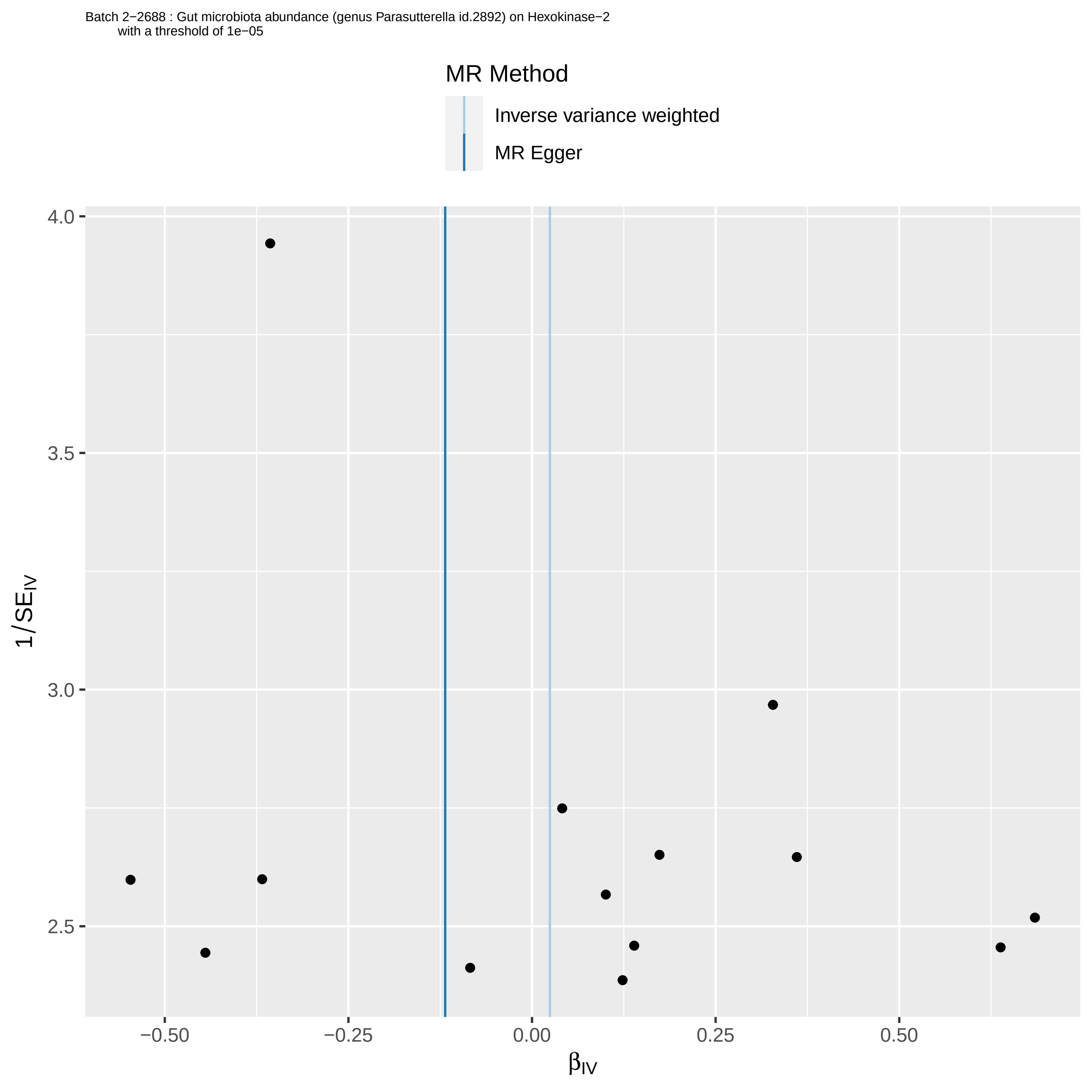

Batch 2−2688 : Gut microbiota abundance (genus Parasutterella id.2892) on Hexokinase−2
with a threshold of 1e−05
MR Method
Inverse variance weighted
MR Egger
4.0
3.5
3.0
2.5
1/SE_IV
−0.50
−0.25
0.00
0.25
0.50
β_IV

Batch 2−2689 : Gut microbiota abundance (genus Catenibacterium id.2153) on HLA class II histocompatibility antigen, DM alpha chain with a threshold of 1e−05

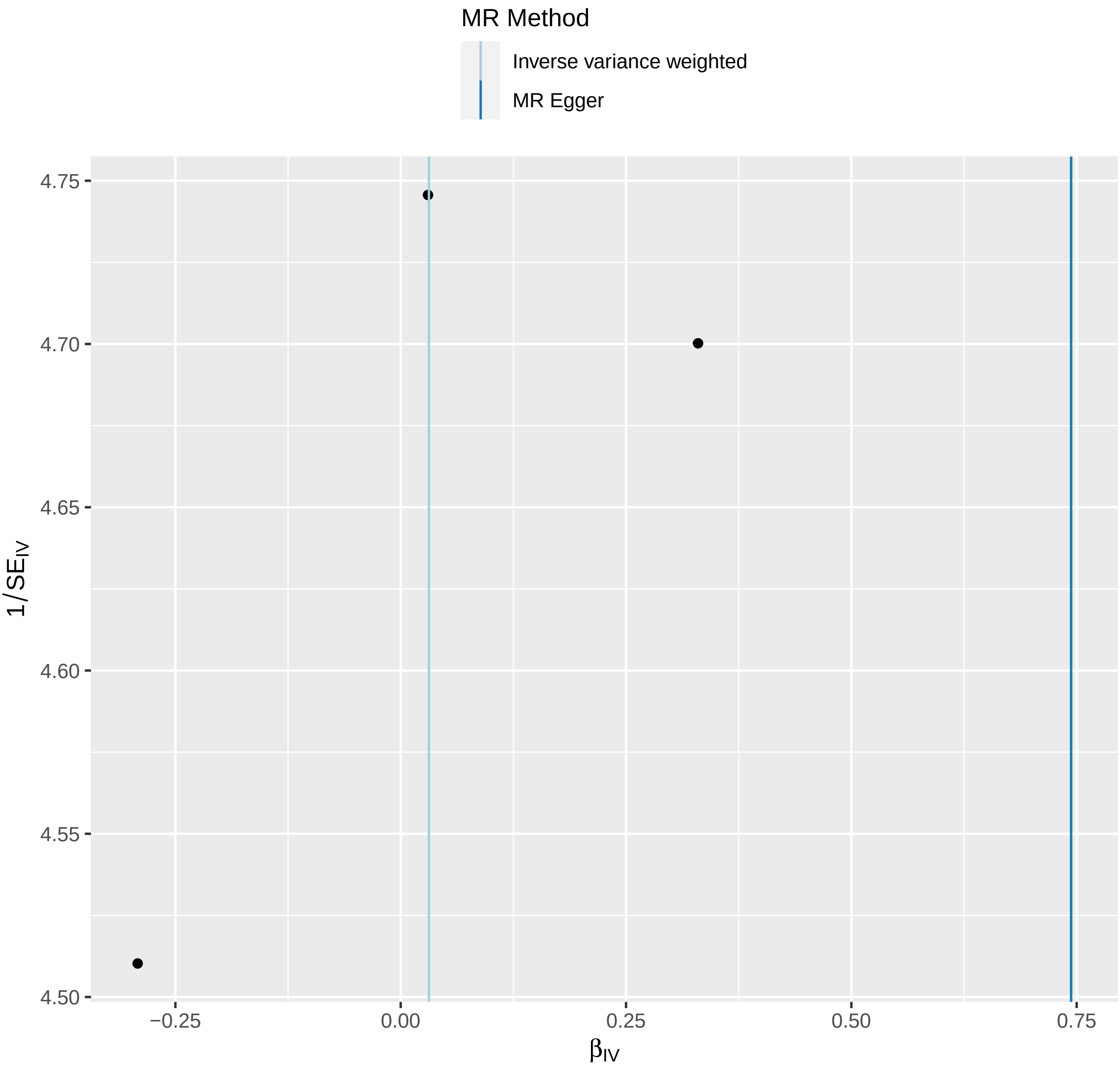

Batch 2−2690 : Gut microbiota abundance (genus Parasutterella id.2892) on HLA class II histocompatibility antigen, DM alpha chain
with a threshold of 1e−05

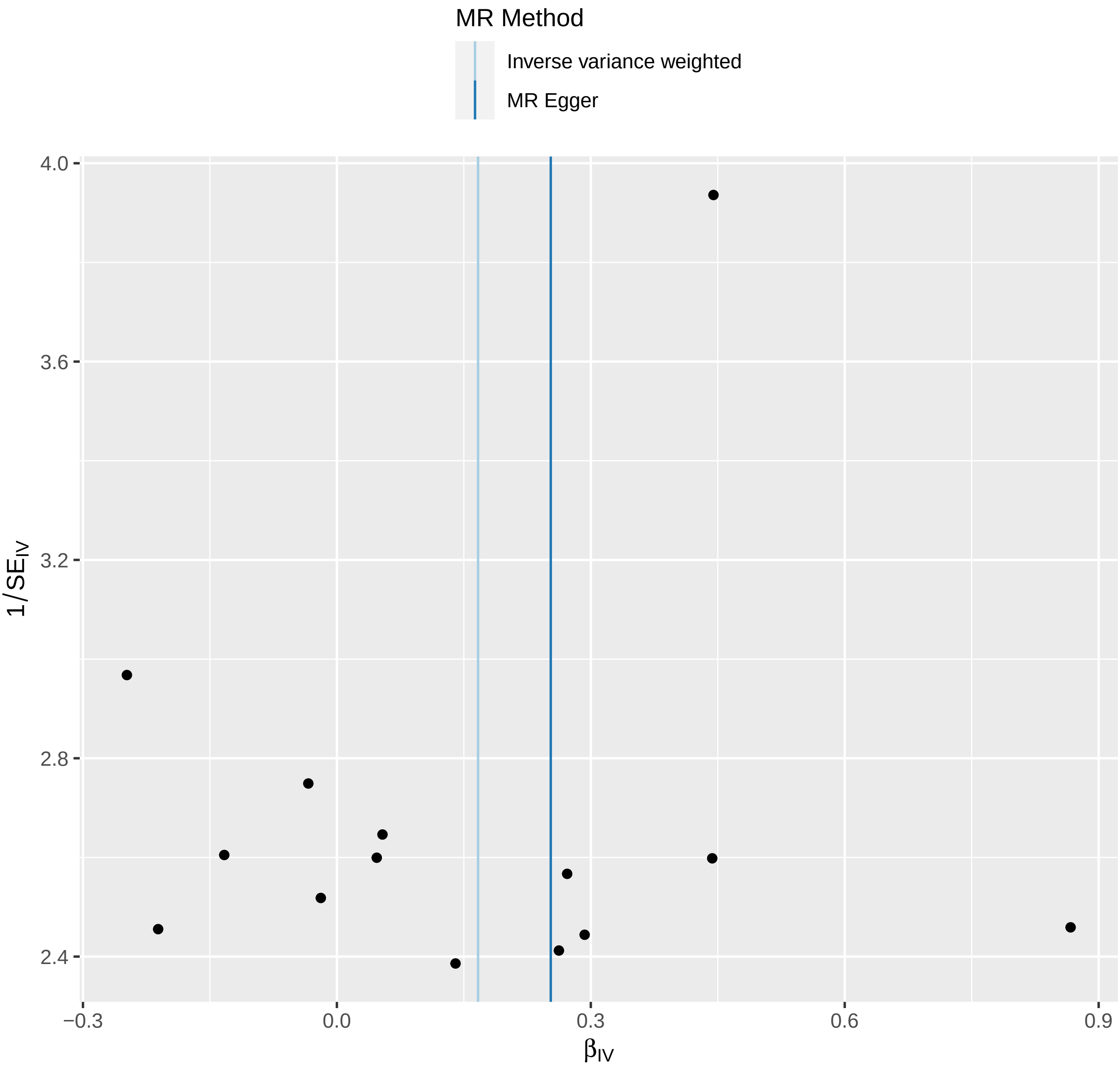

Batch 2−2691 : Gut microbiota abundance (genus Catenibacterium id.2153) on HLA class II histocompatibility antigen, DP beta 1 chain
with a threshold of 1e−05

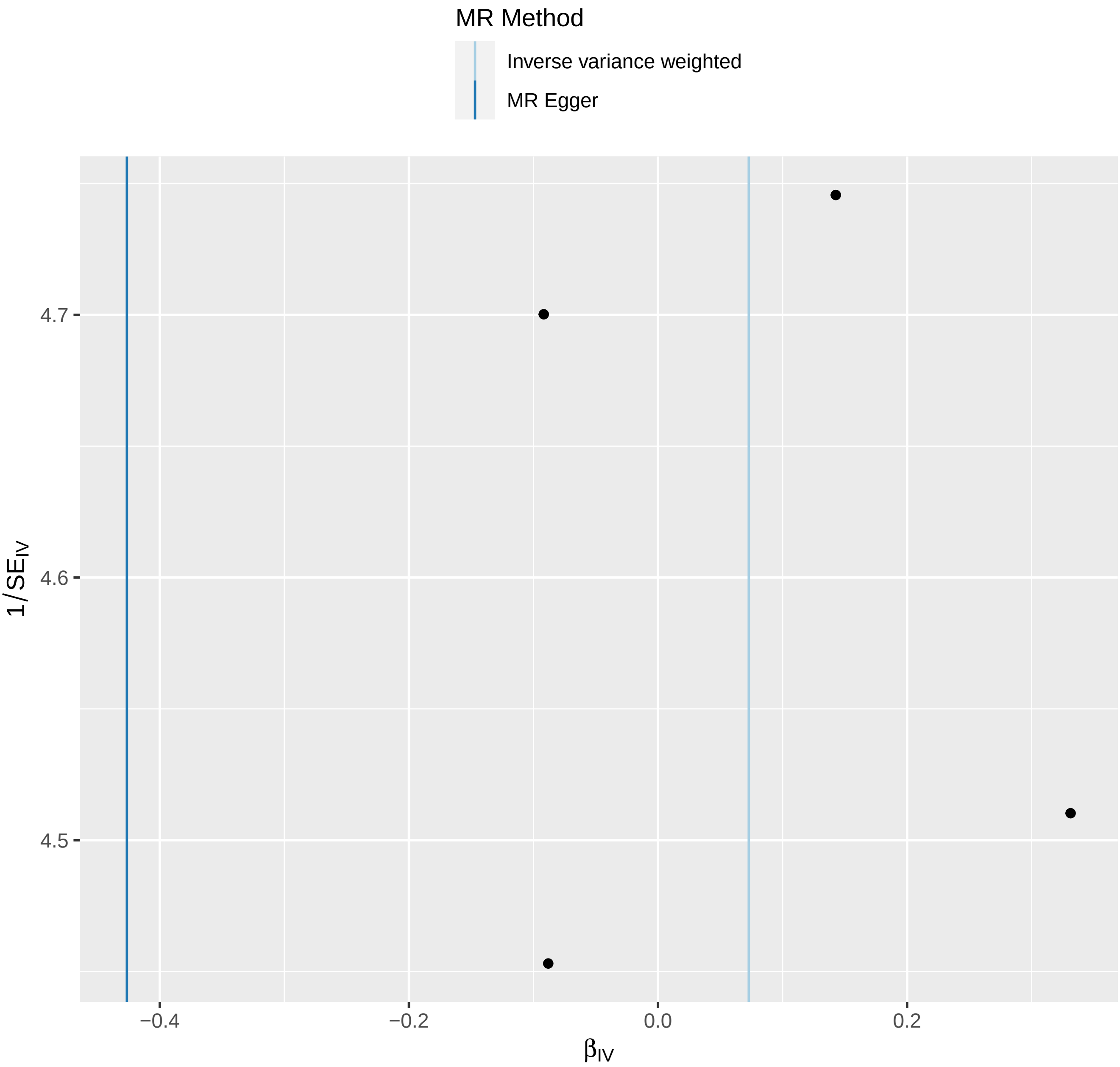

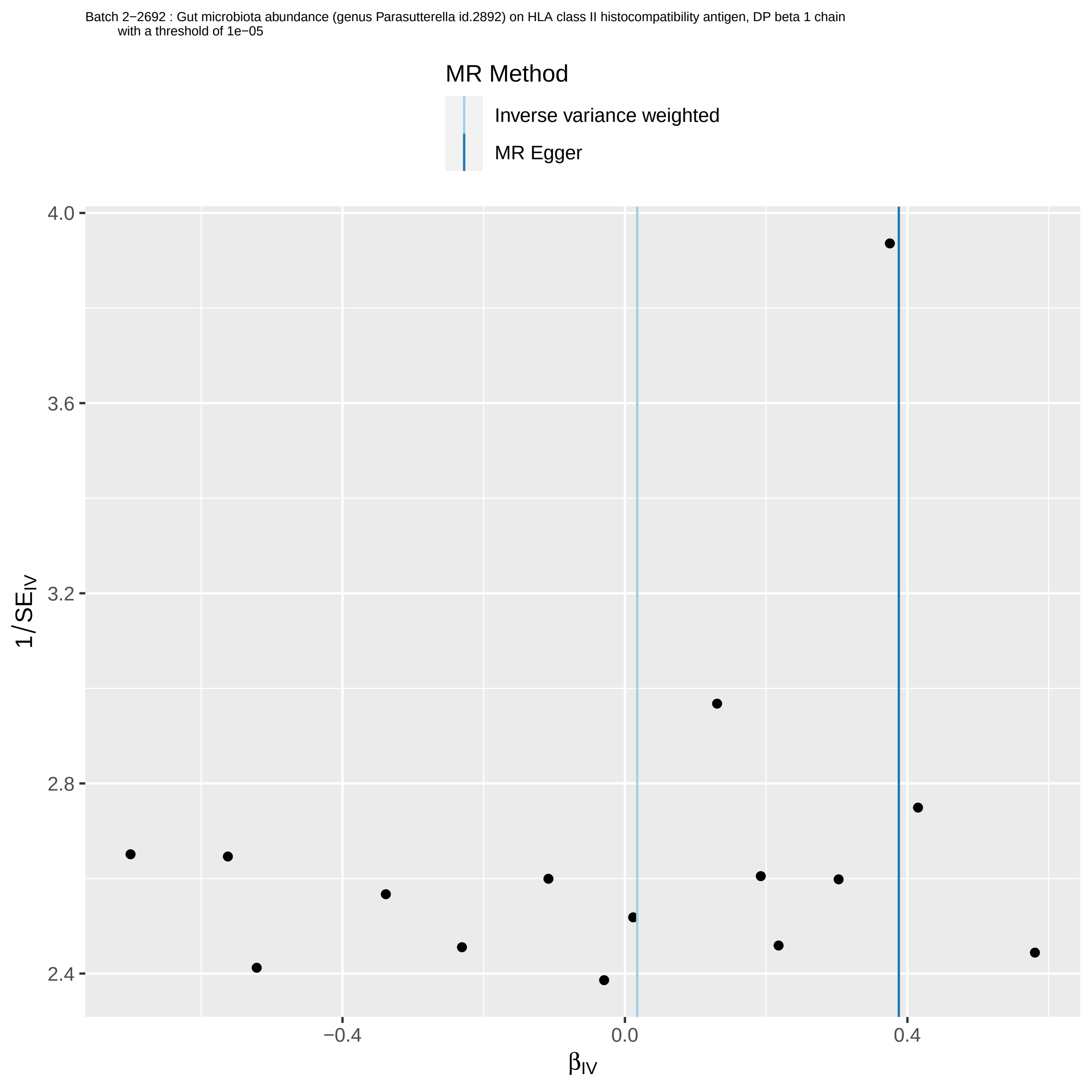
Batch 2−2692 : Gut microbiota abundance (genus Parasutterella id.2892) on HLA class II histocompatibility antigen, DP beta 1 chain
with a threshold of 1e−05
MR Method
Inverse variance weighted
MR Egger
4.0
3.6
3.2
2.8
2.4
1/SE$_{IV}$
−0.4
0.0
0.4
β_{IV}

Batch 2−2693 : Gut microbiota abundance (genus Catenibacterium id.2153) on HLA class II histocompatibility antigen, DQ alpha 2 chain with a threshold of 1e−05

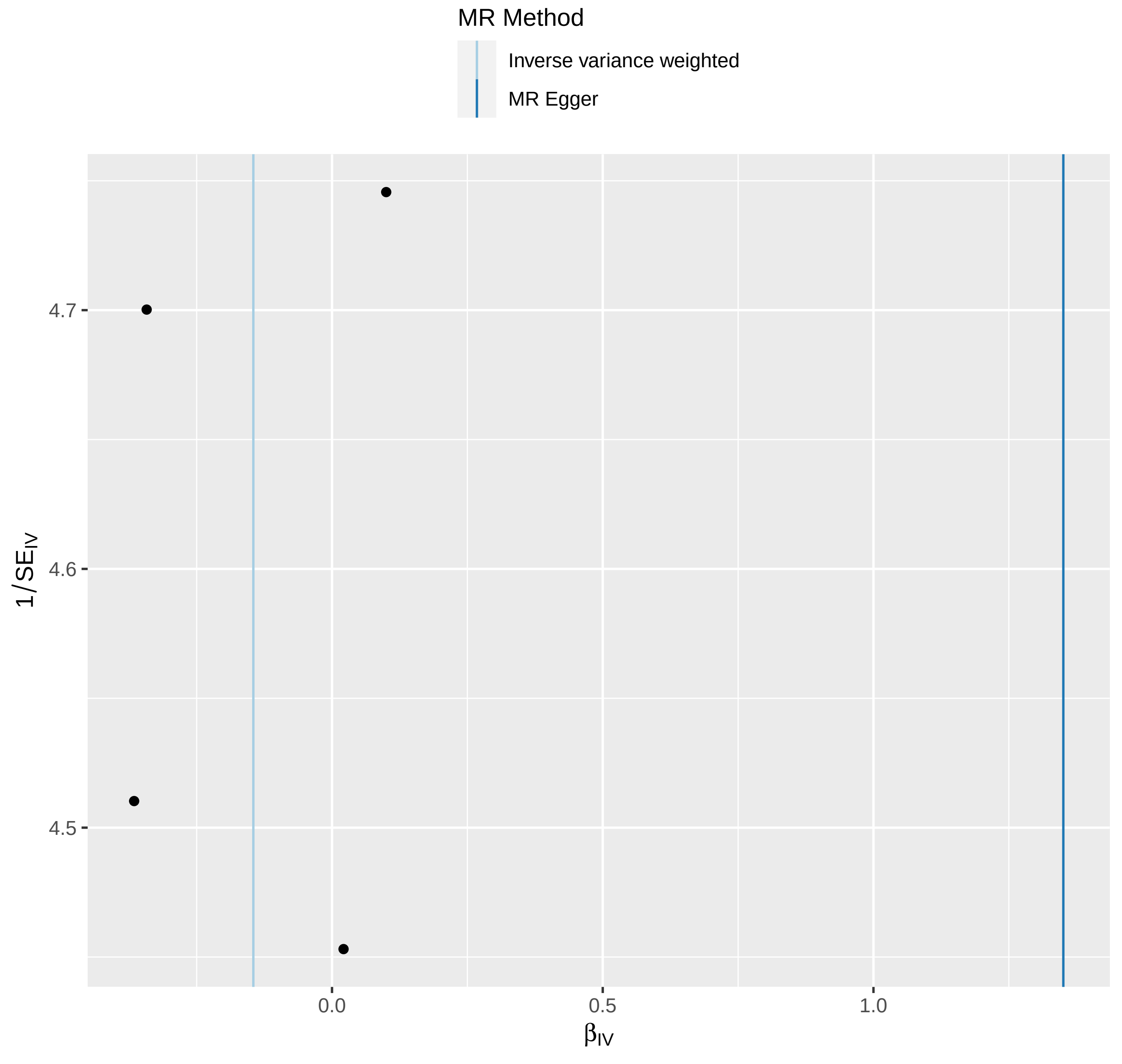

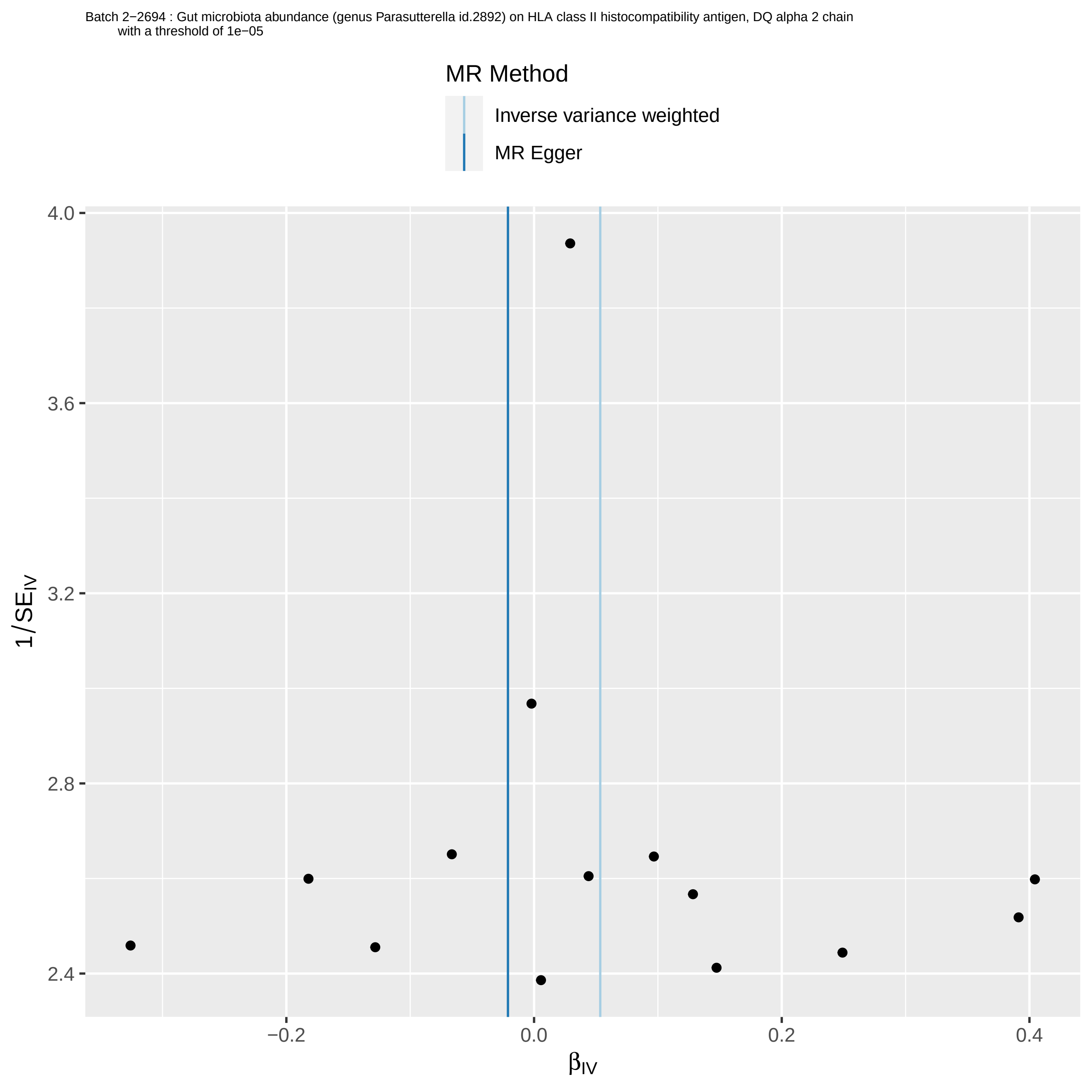

Batch 2−2694 : Gut microbiota abundance (genus Parasutterella id.2892) on HLA class II histocompatibility antigen, DQ alpha 2 chain
with a threshold of 1e−05
MR Method
Inverse variance weighted
MR Egger
4.0
3.6
3.2
2.8
2.4
1/SE_IV
−0.2
0.0
0.2
0.4
β_IV

Batch 2−2695 : Gut microbiota abundance (genus Catenibacterium id.2153) on Porphobilinogen deaminase
with a threshold of 1e−05

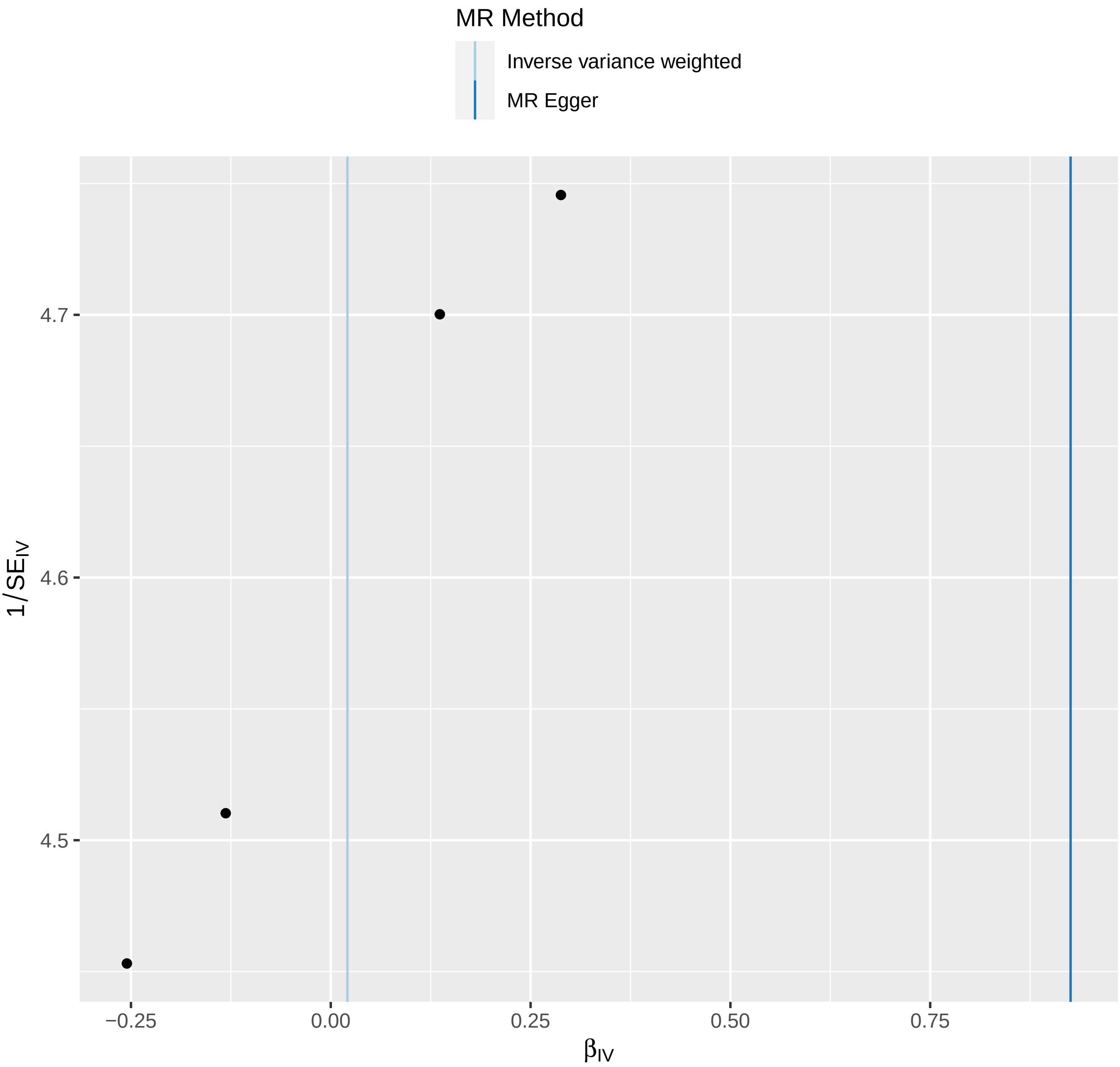

Batch 2−2696 : Gut microbiota abundance (genus Parasutterella id.2892) on Porphobilinogen deaminase with a threshold of 1e−05

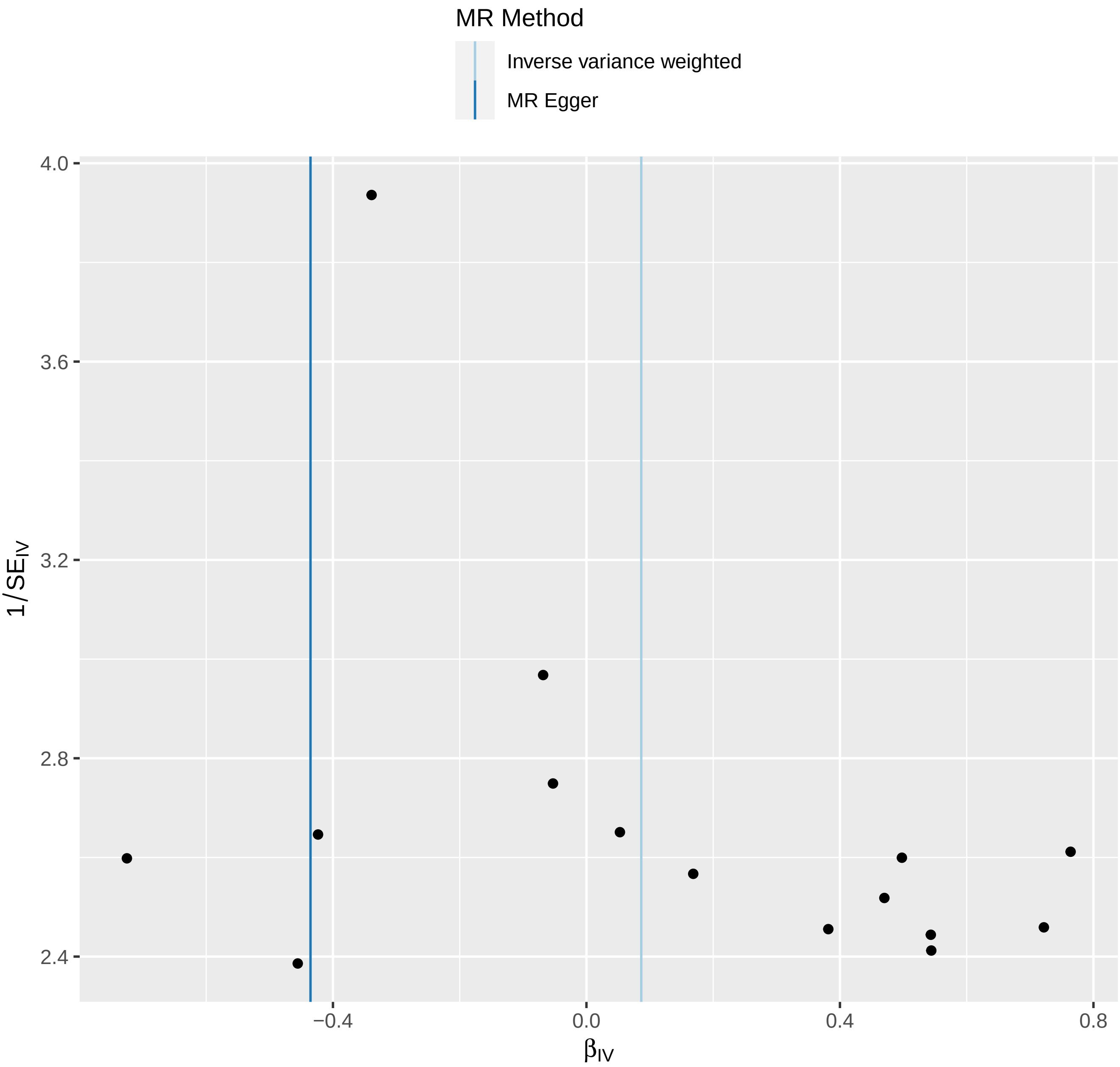

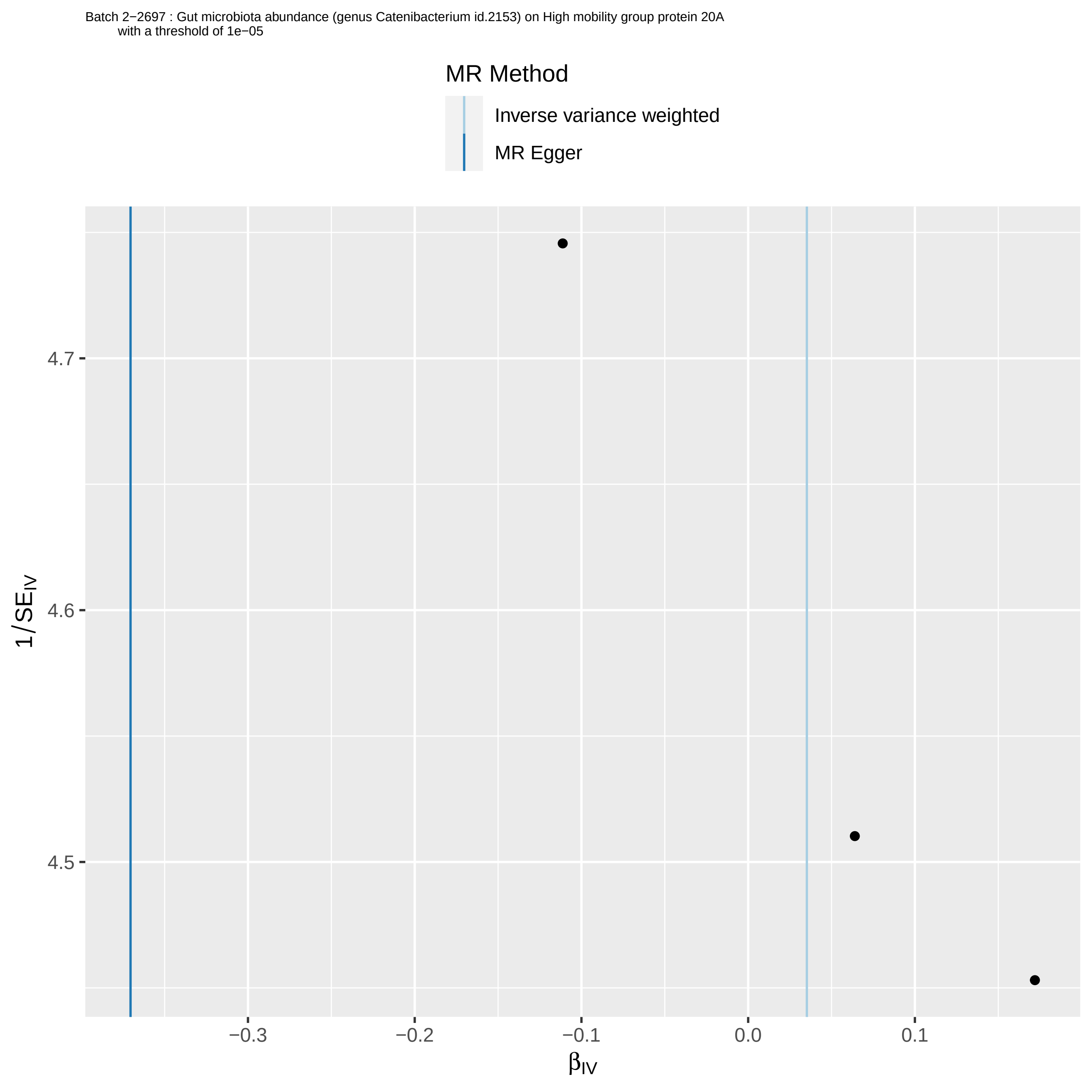

Batch 2−2697 : Gut microbiota abundance (genus Catenibacterium id.2153) on High mobility group protein 20A
with a threshold of 1e−05
MR Method
Inverse variance weighted
MR Egger
4.7
4.6
4.5
1/SE_IV
−0.3
−0.2
−0.1
0.0
0.1
β_IV

Batch 2−2698 : Gut microbiota abundance (genus Parasutterella id.2892) on High mobility group protein 20A
with a threshold of 1e−05

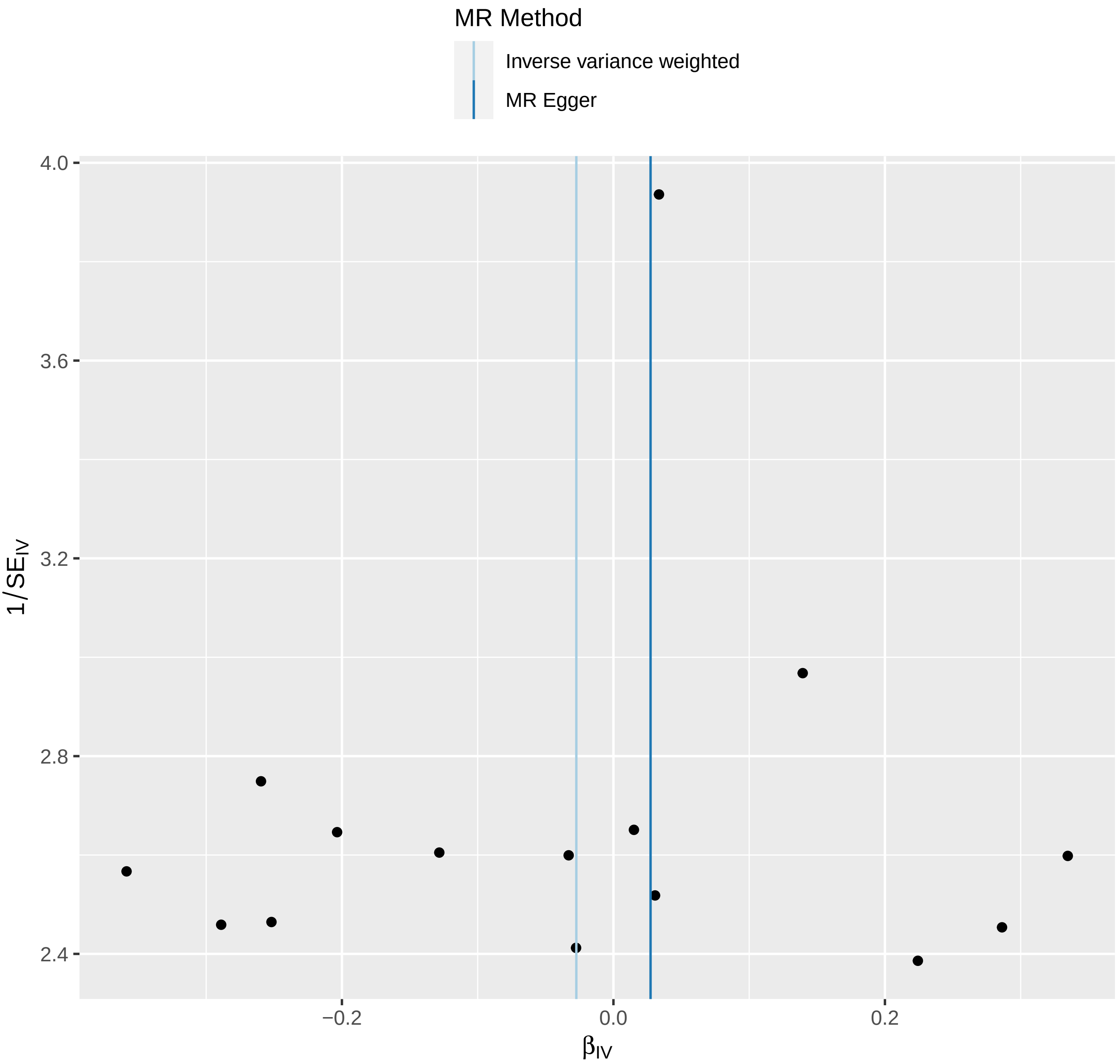

Batch 2−2699 : Gut microbiota abundance (genus Catenibacterium id.2153) on SWI/SNF−related matrix−associated actin−dependent regulator of chromatin subfamily E memb
with a threshold of 1e−05

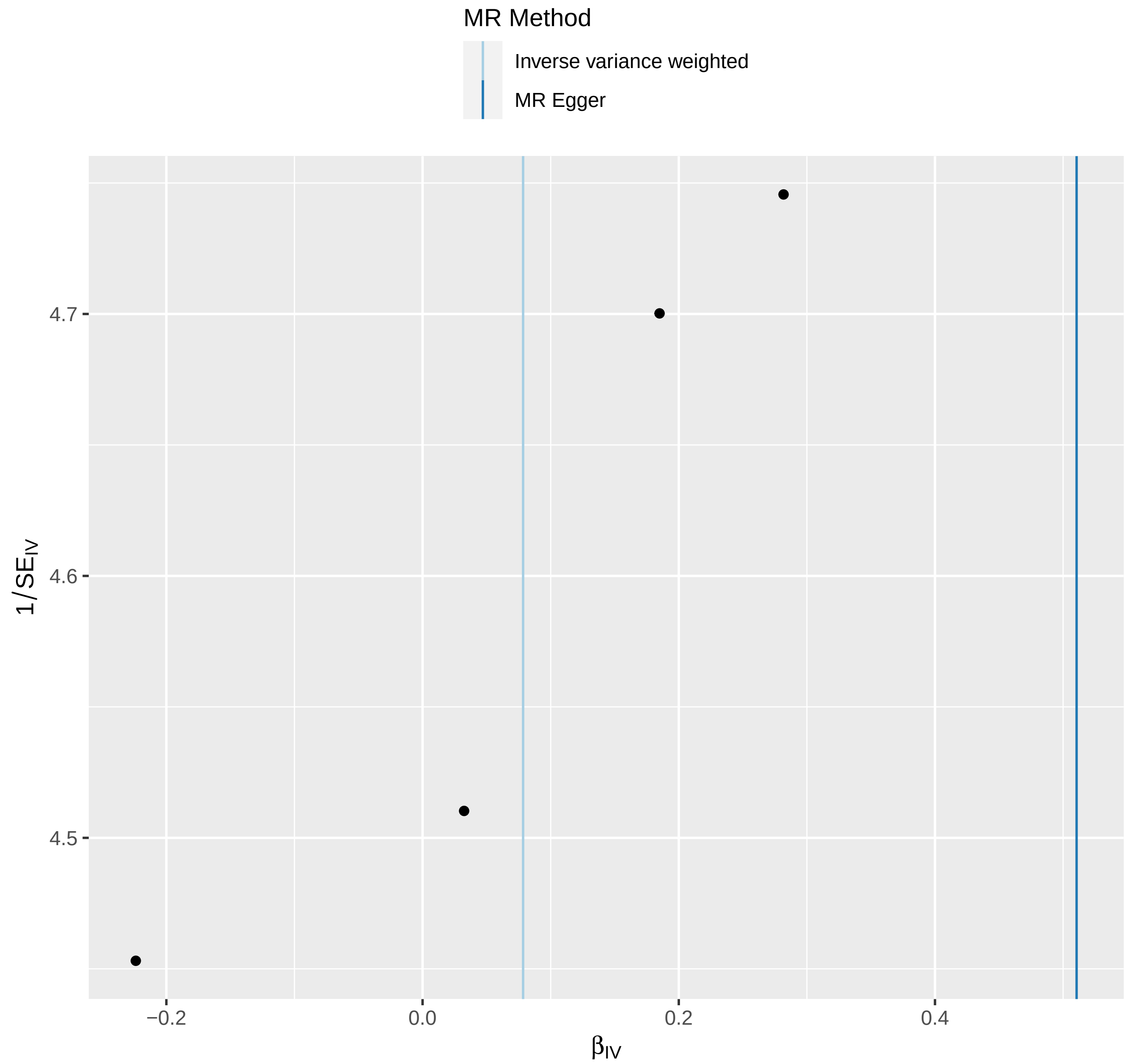

Batch 2−2700 : Gut microbiota abundance (genus Parasutterella id.2892) on SWI/SNF−related matrix−associated actin−dependent regulator of chromatin subfamily E member
with a threshold of 1e−05

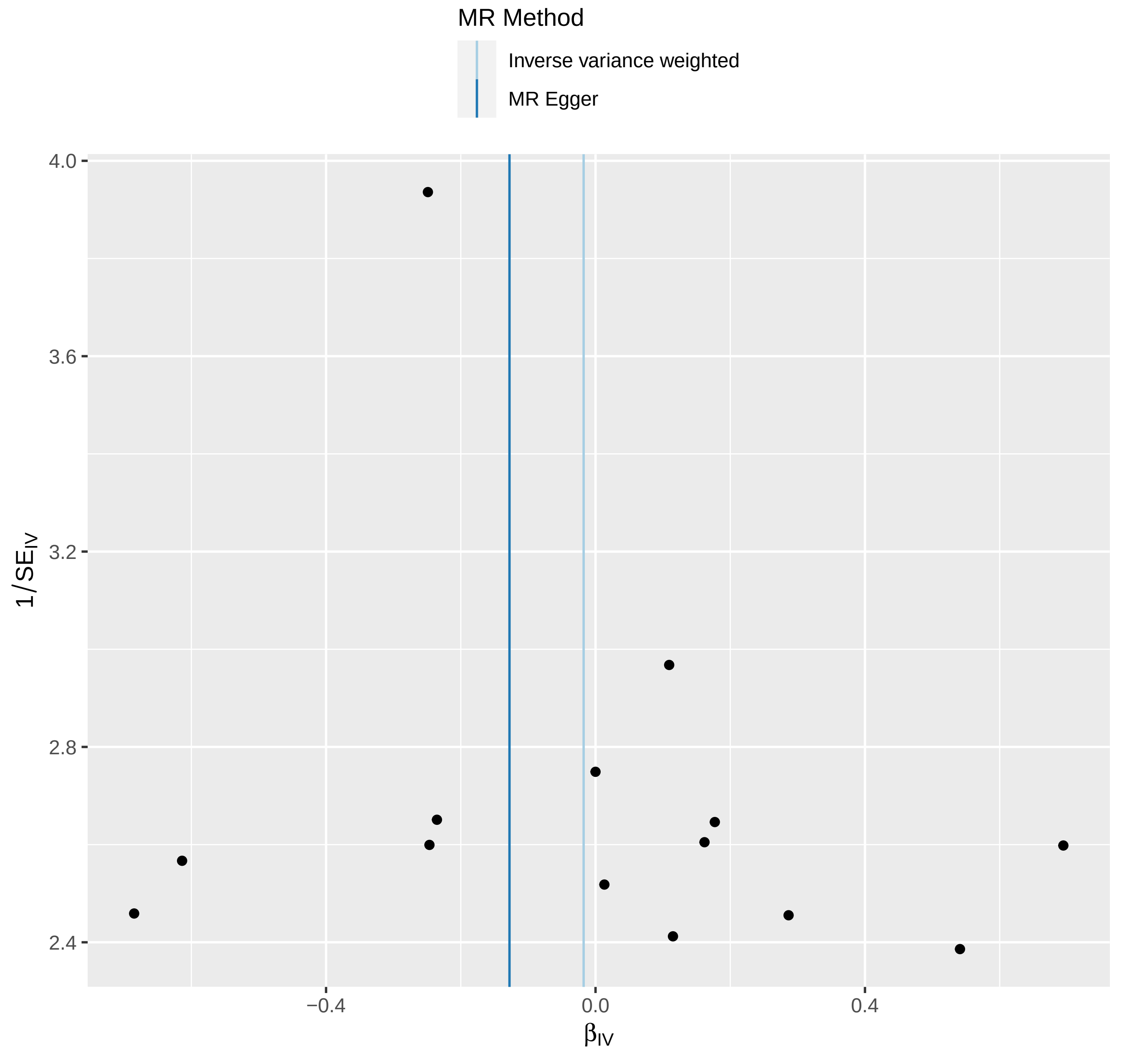

Batch 2−2701 : Gut microbiota abundance (genus Catenibacterium id.2153) on High mobility group protein B1
with a threshold of 1e−05

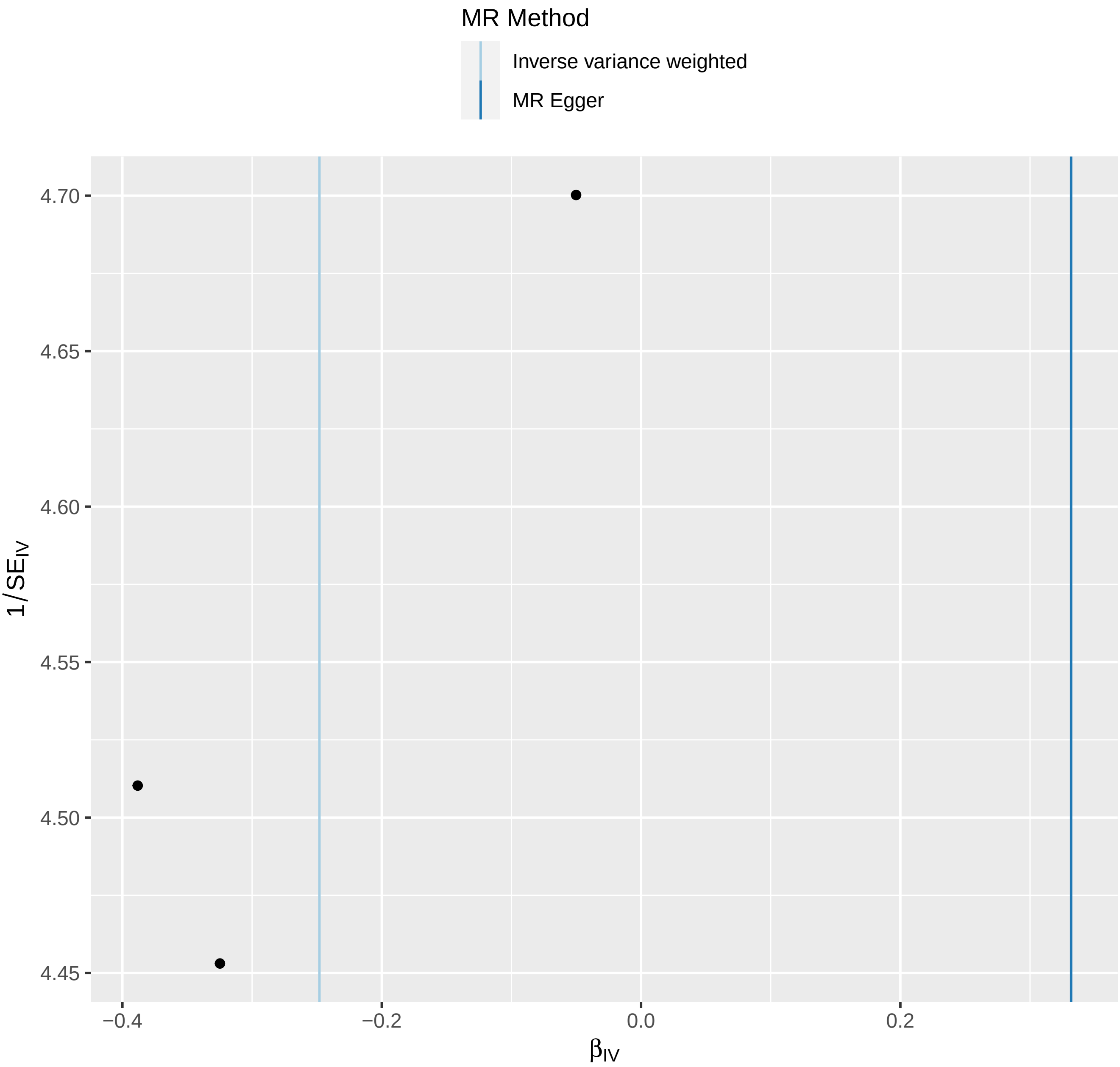

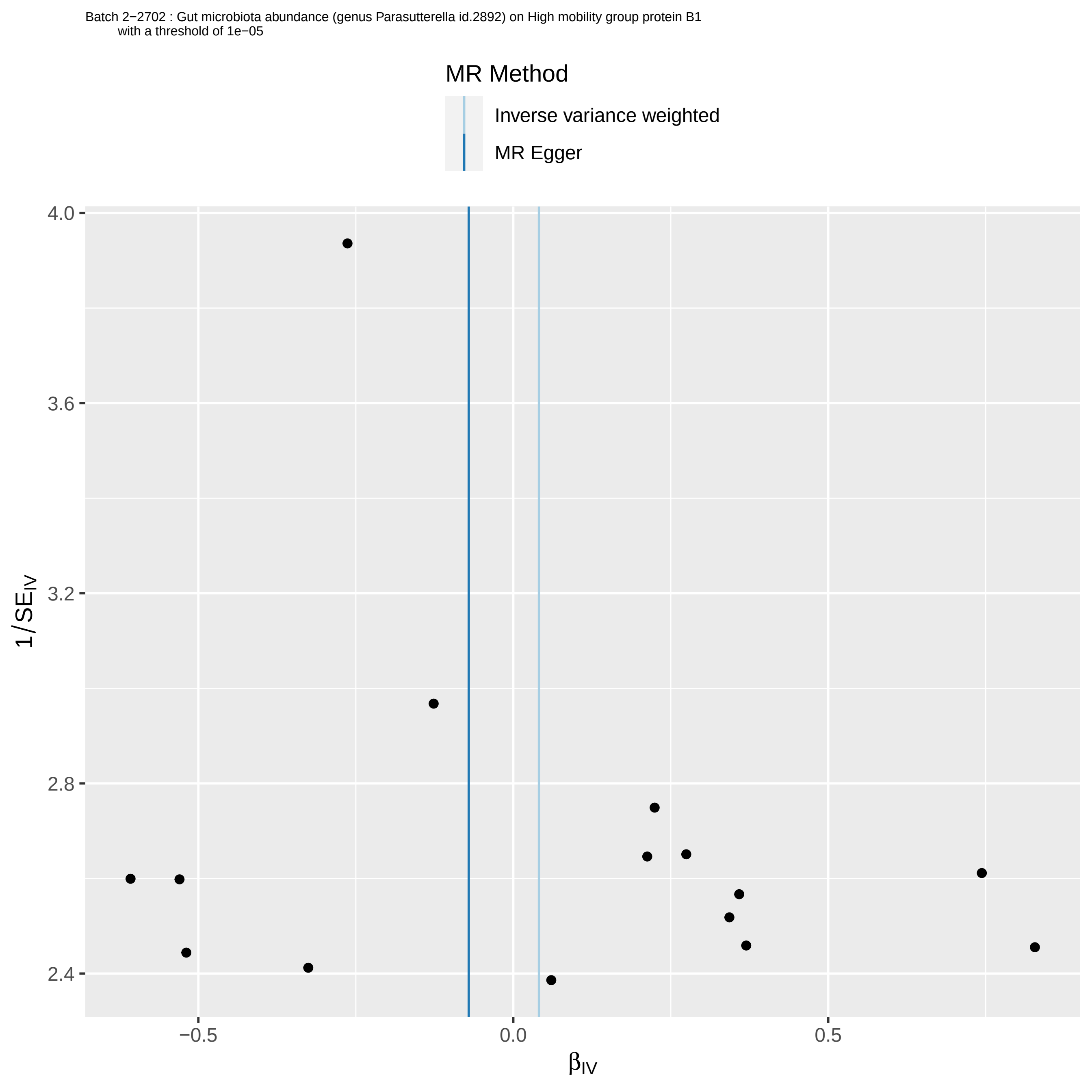
Batch 2−2702 : Gut microbiota abundance (genus Parasutterella id.2892) on High mobility group protein B1
with a threshold of 1e−05
MR Method
Inverse variance weighted
MR Egger
4.0
3.6
3.2
2.8
2.4
1/SE_IV
−0.5
0.0
0.5
β_IV

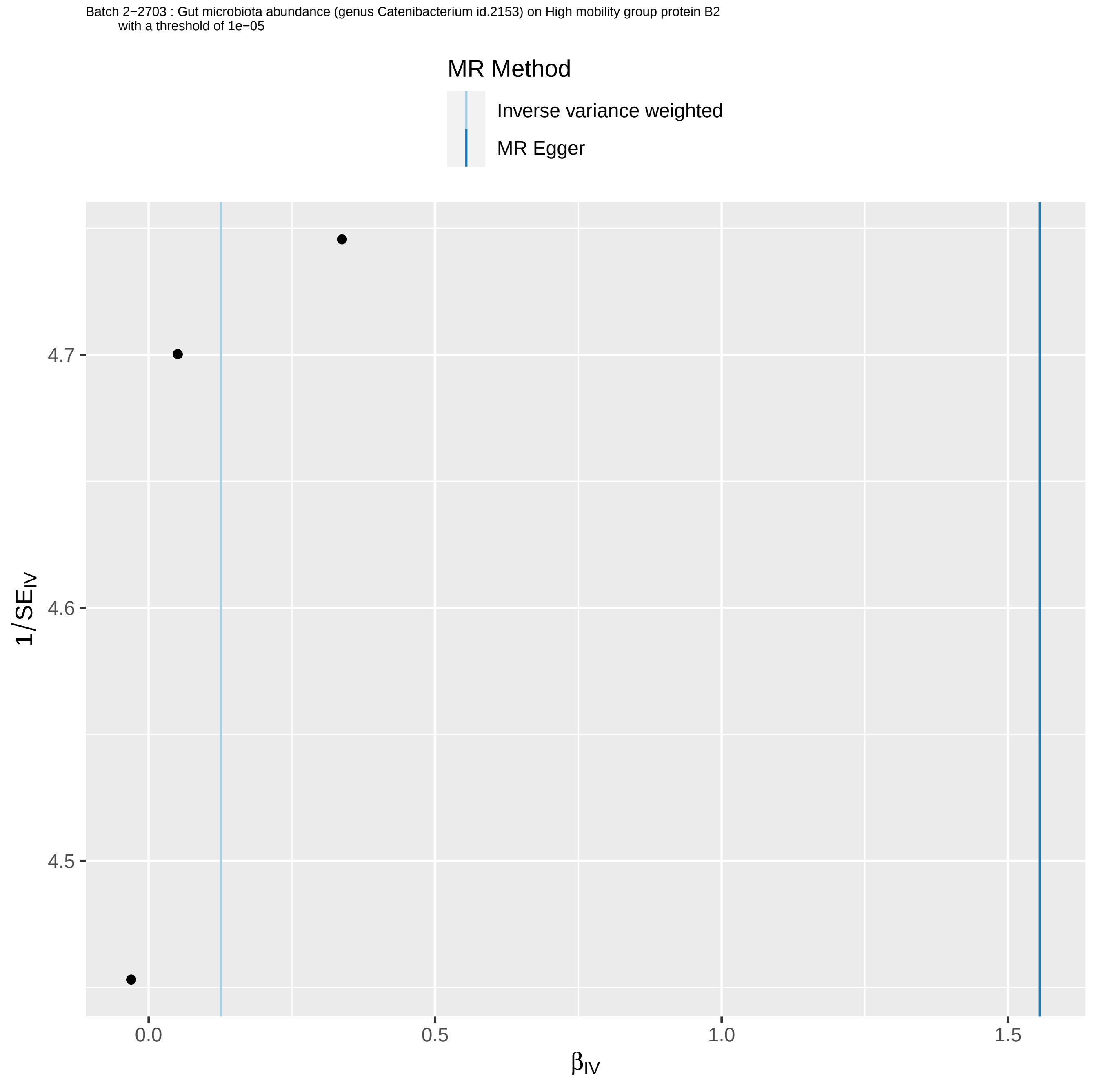
Batch 2−2703 : Gut microbiota abundance (genus Catenibacterium id.2153) on High mobility group protein B2
with a threshold of 1e−05
MR Method
Inverse variance weighted
MR Egger
4.7
4.6
4.5
1/SE_IV
0.0
0.5
1.0
1.5
β_IV

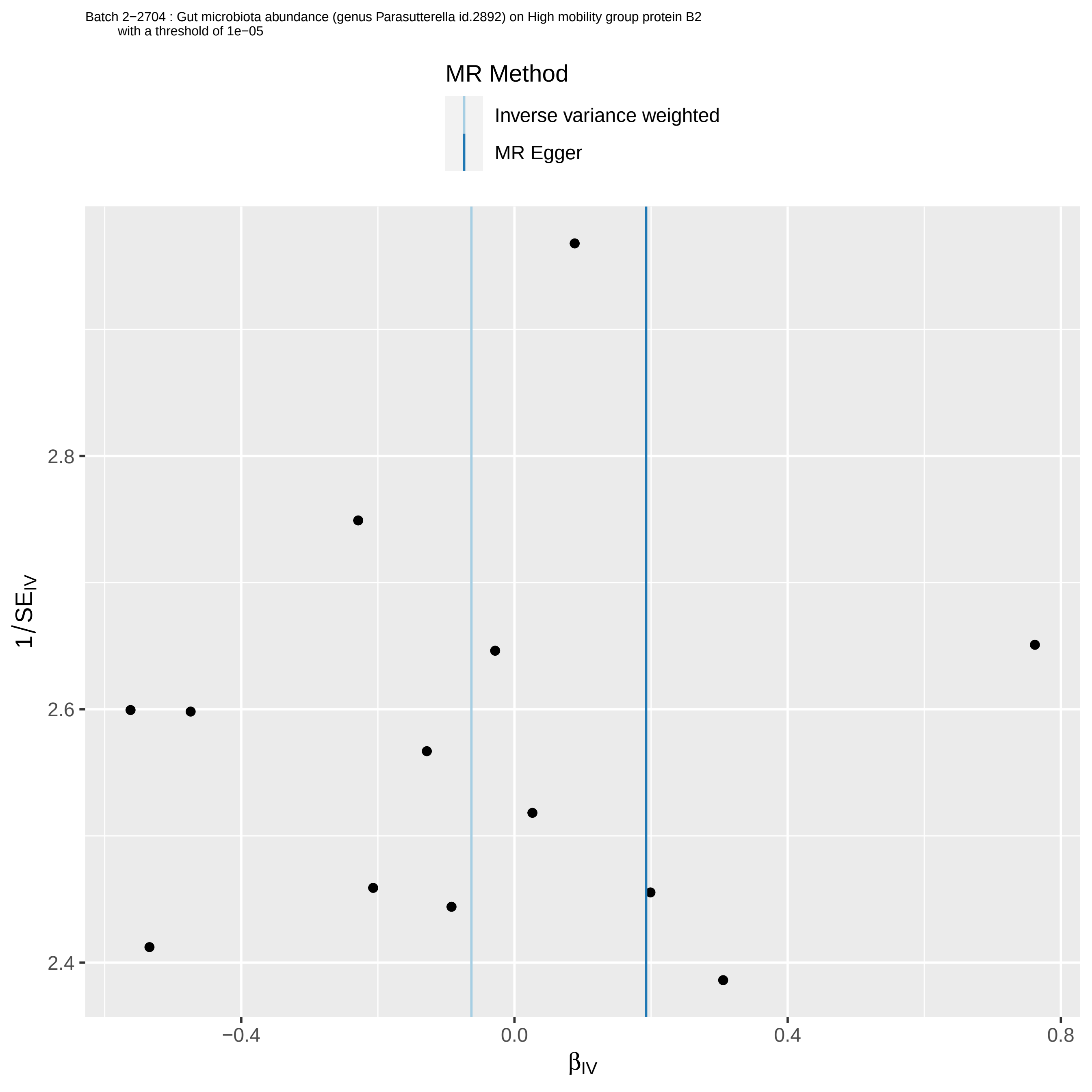
Batch 2−2704 : Gut microbiota abundance (genus Parasutterella id.2892) on High mobility group protein B2
with a threshold of 1e−05
MR Method
Inverse variance weighted
MR Egger
2.8
2.6
2.4
1/SE_IV
−0.4
0.0
0.4
0.8
β_IV

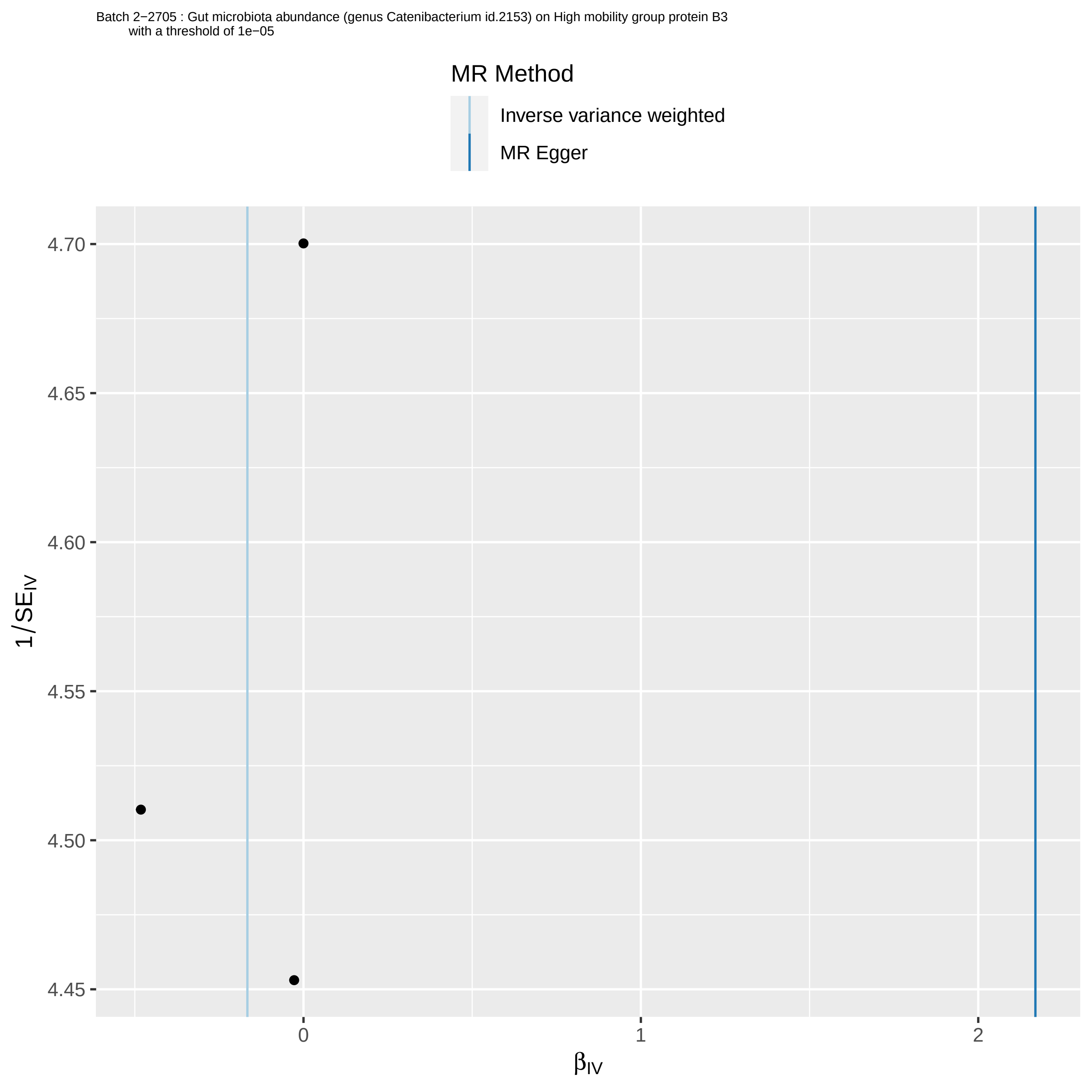
Batch 2−2705 : Gut microbiota abundance (genus Catenibacterium id.2153) on High mobility group protein B3
with a threshold of 1e−05
MR Method
Inverse variance weighted
MR Egger
4.70
4.65
4.60
4.55
4.50
4.45
$1/SE_{IV}$
0
1
2
β_{IV}

Batch 2−2706 : Gut microbiota abundance (genus Parasutterella id.2892) on High mobility group protein B3
with a threshold of 1e−05

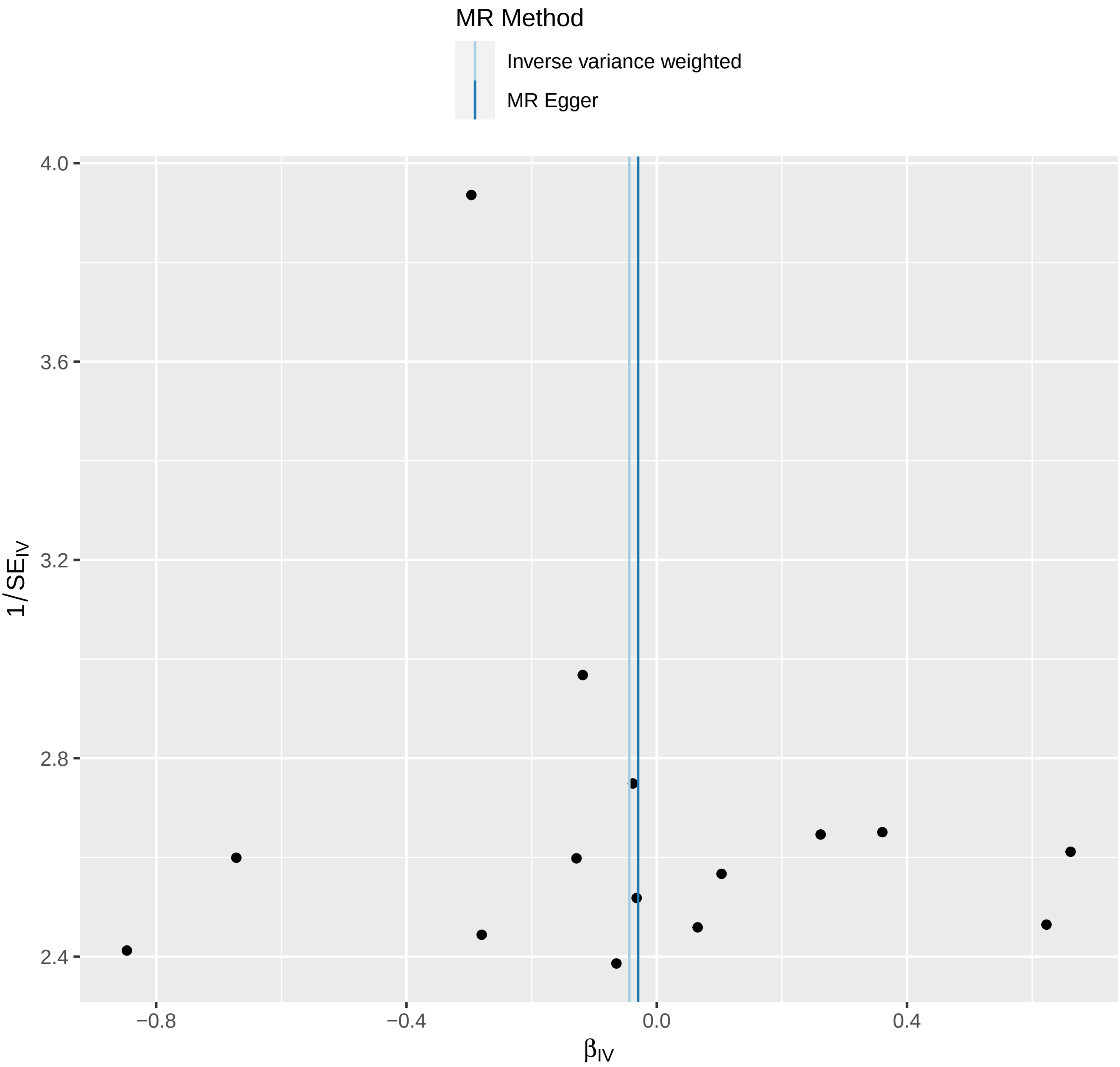

Batch 2−2707 : Gut microbiota abundance (genus Catenibacterium id.2153) on 3−hydroxy−3−methylglutaryl−coenzyme A reductase with a threshold of 1e−05

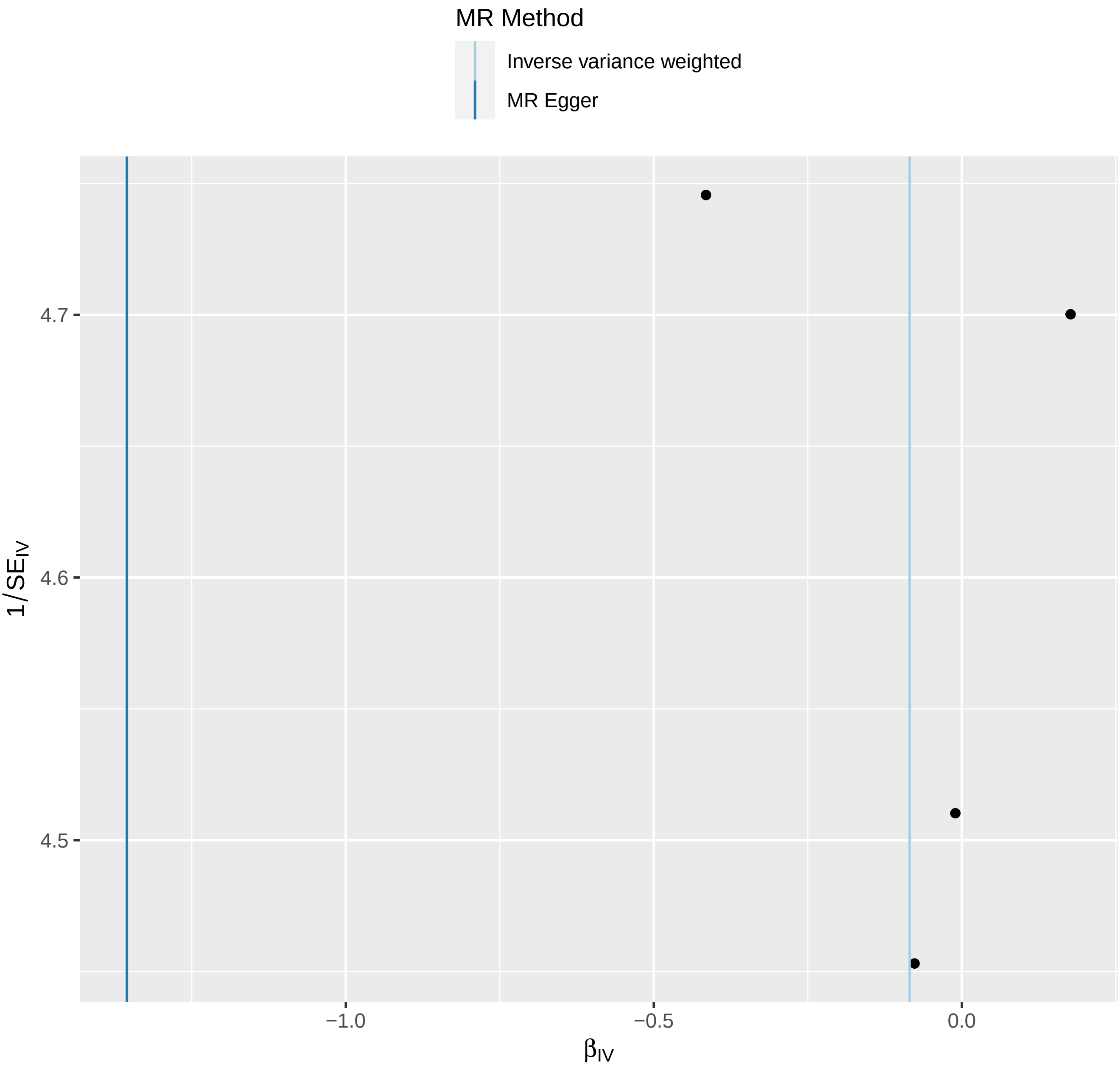

Batch 2−2708 : Gut microbiota abundance (genus Parasutterella id.2892) on 3−hydroxy−3−methylglutaryl−coenzyme A reductase with a threshold of 1e−05

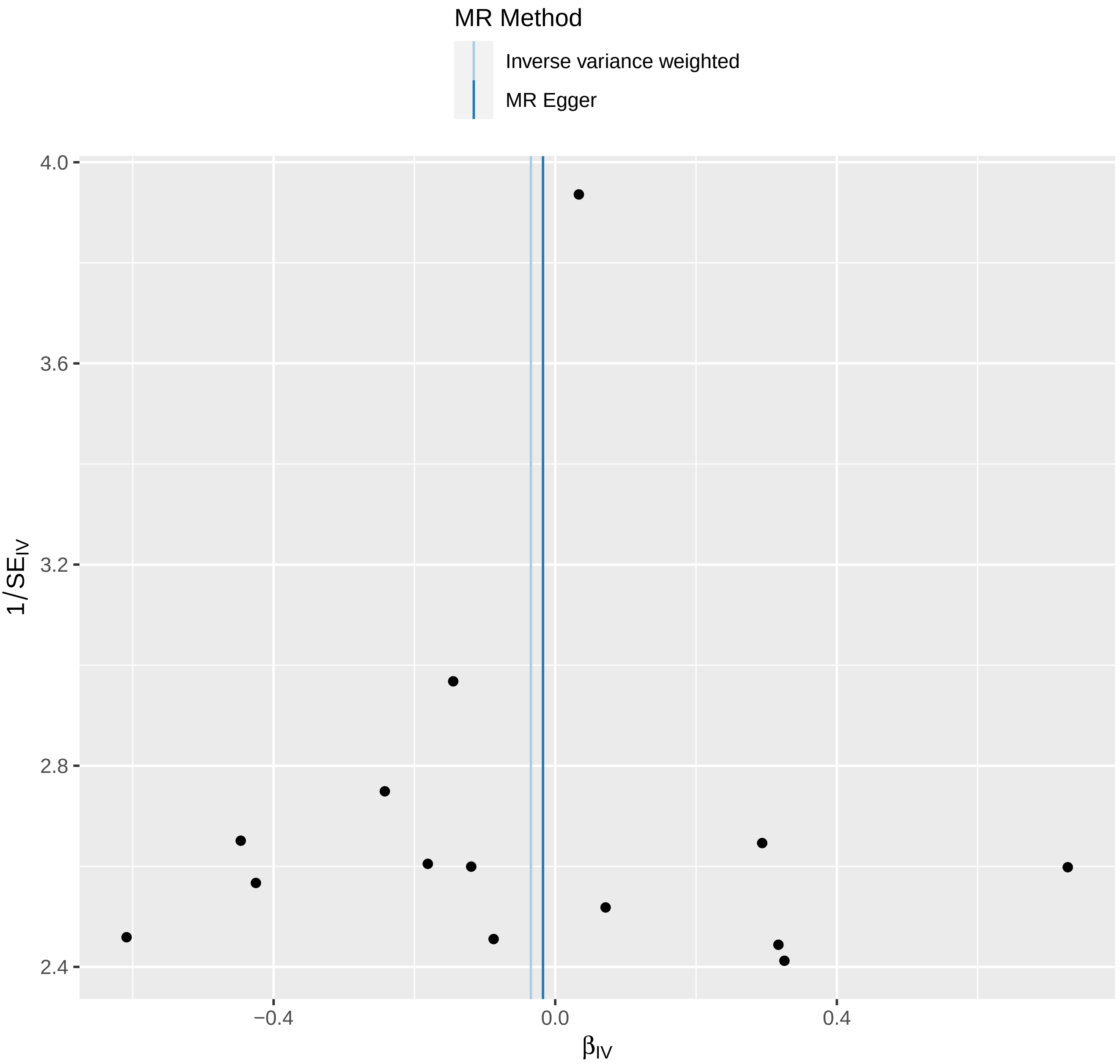

Batch 2−2709 : Gut microbiota abundance (genus Catenibacterium id.2153) on Hydroxymethylglutaryl−CoA synthase, cytoplasmic
with a threshold of 1e−05

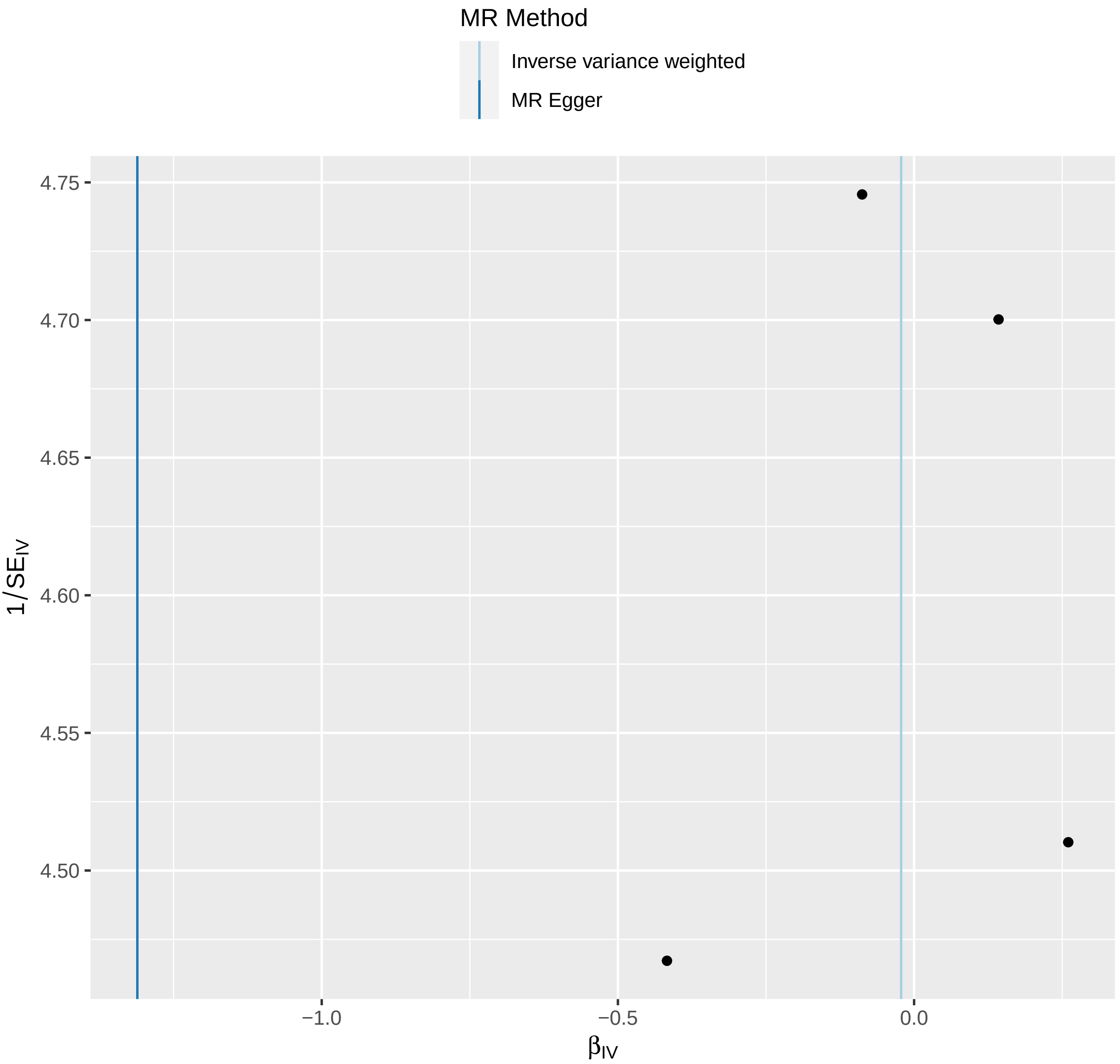

Batch 2−2710 : Gut microbiota abundance (genus Parasutterella id.2892) on Hydroxymethylglutaryl−CoA synthase, cytoplasmic
with a threshold of 1e−05

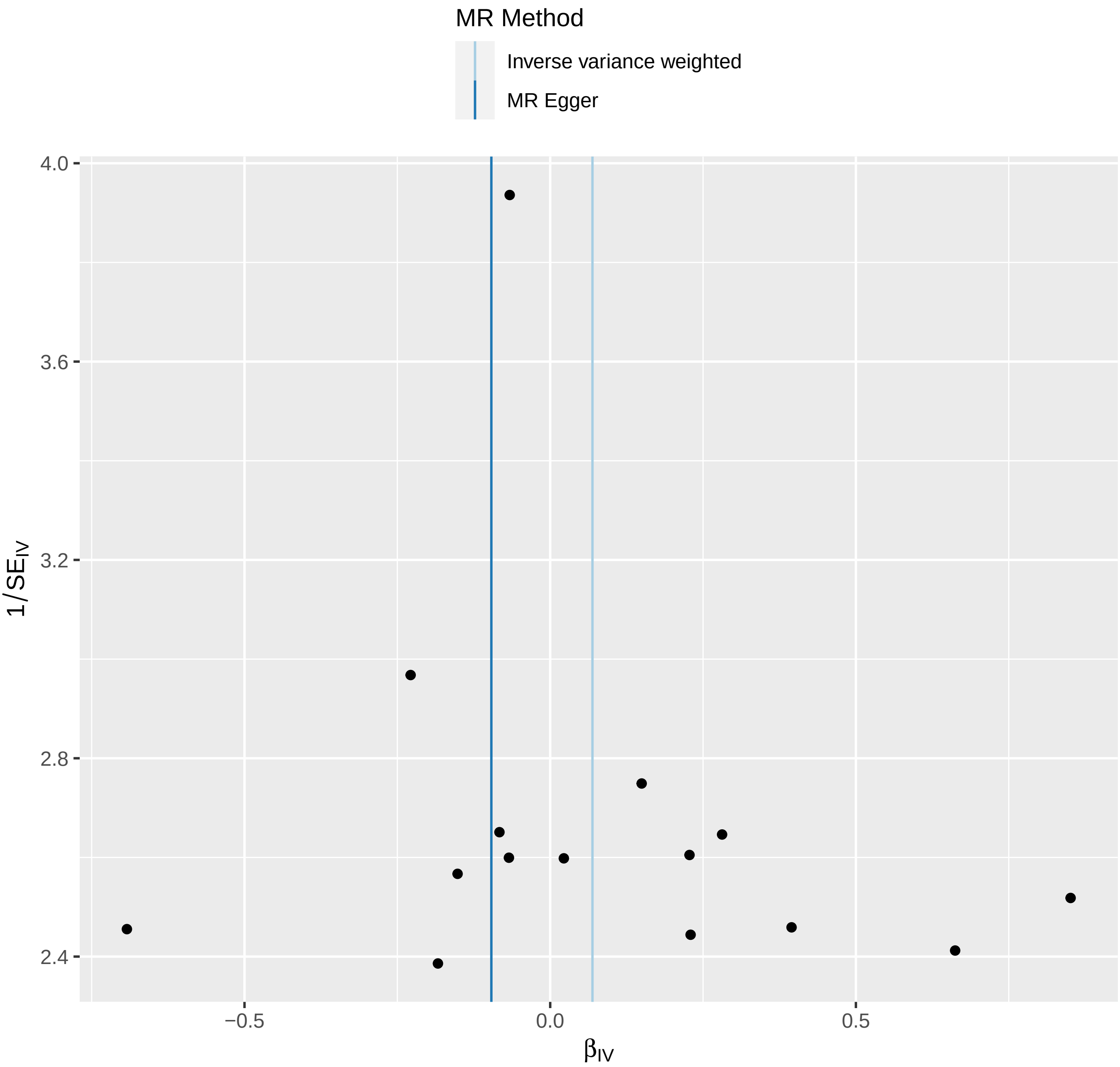

Batch 2−2711 : Gut microbiota abundance (genus Catenibacterium id.2153) on Hydroxymethylglutaryl−CoA synthase, mitochondrial
with a threshold of 1e−05

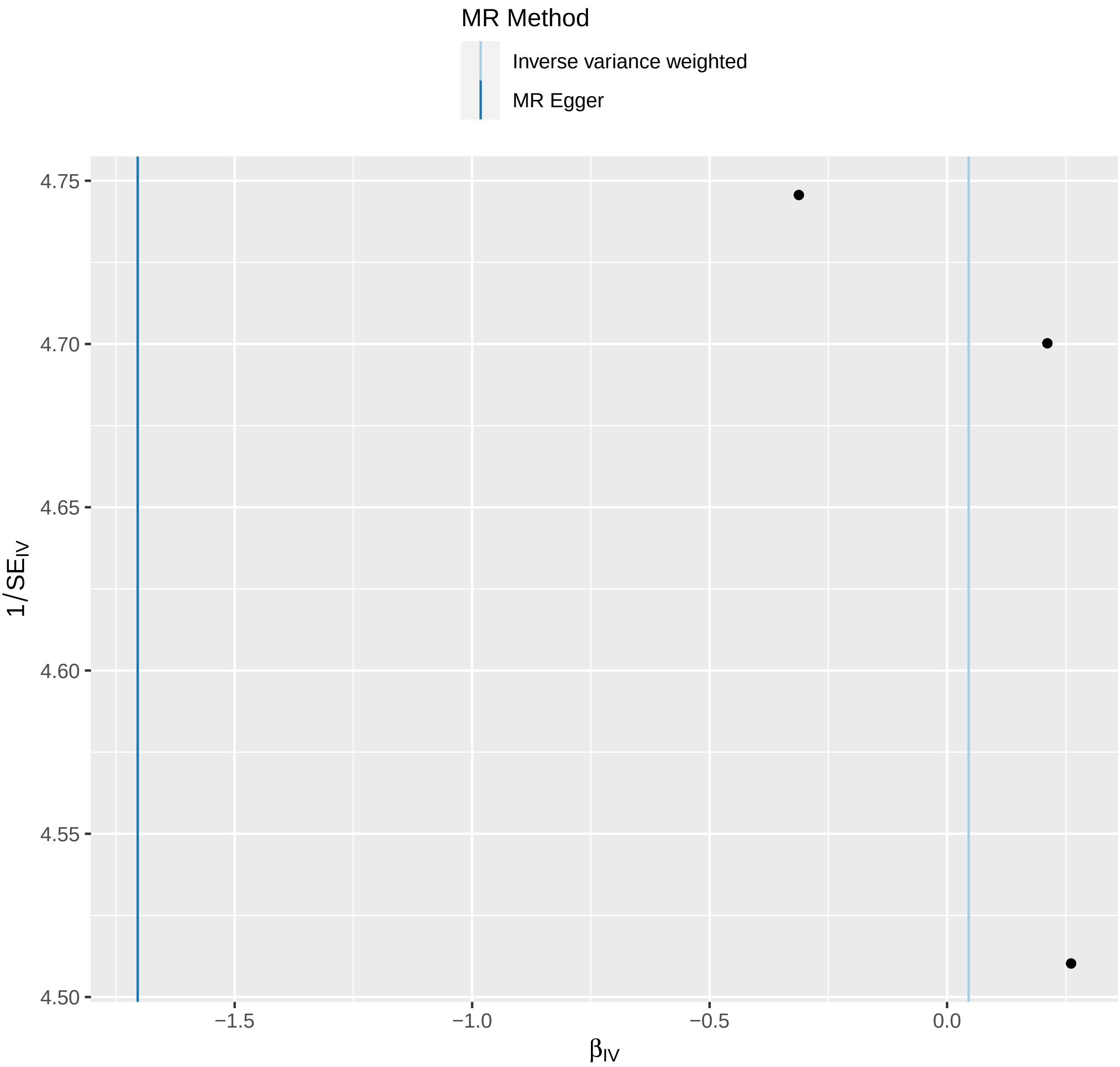

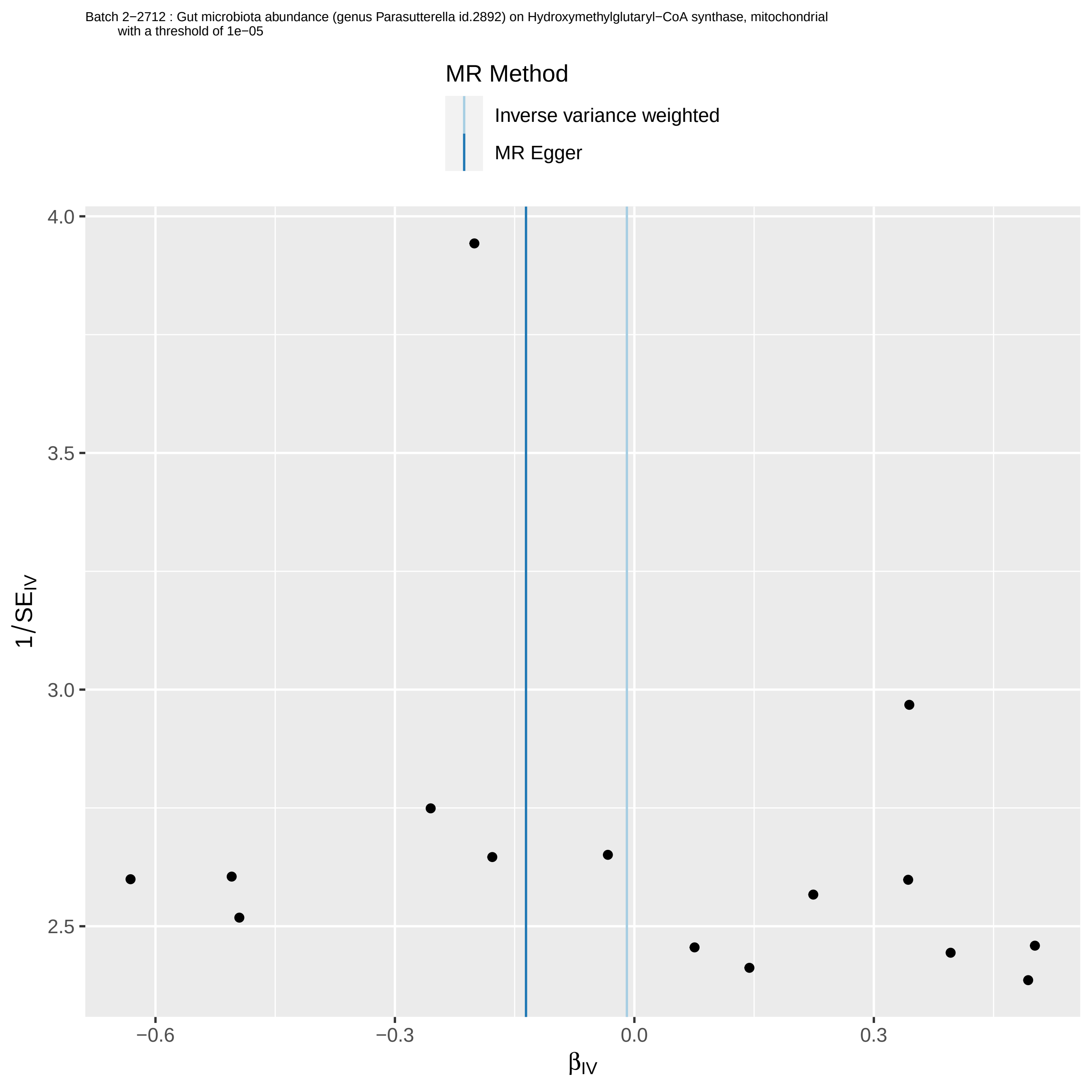
Batch 2−2712 : Gut microbiota abundance (genus Parasutterella id.2892) on Hydroxymethylglutaryl−CoA synthase, mitochondrial with a threshold of 1e−05
MR Method
Inverse variance weighted
MR Egger
4.0
3.5
3.0
2.5
$1/SE_{IV}$
−0.6
−0.3
0.0
0.3
β_{IV}

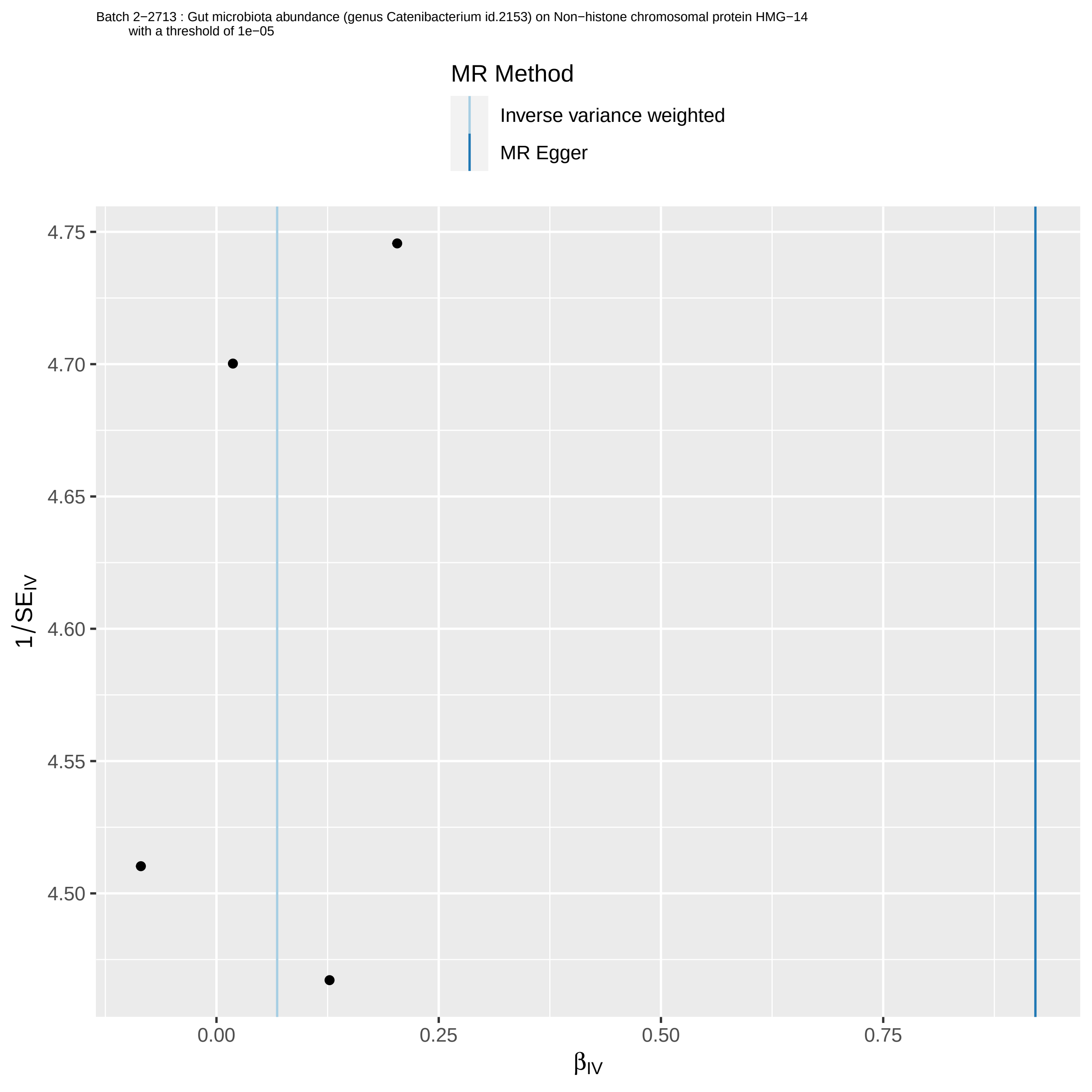

Batch 2−2713 : Gut microbiota abundance (genus Catenibacterium id.2153) on Non−histone chromosomal protein HMG−14
with a threshold of 1e−05
MR Method
Inverse variance weighted
MR Egger
4.75
4.70
4.65
4.60
4.55
4.50
1/SE_IV
0.00
0.25
0.50
0.75
β_IV

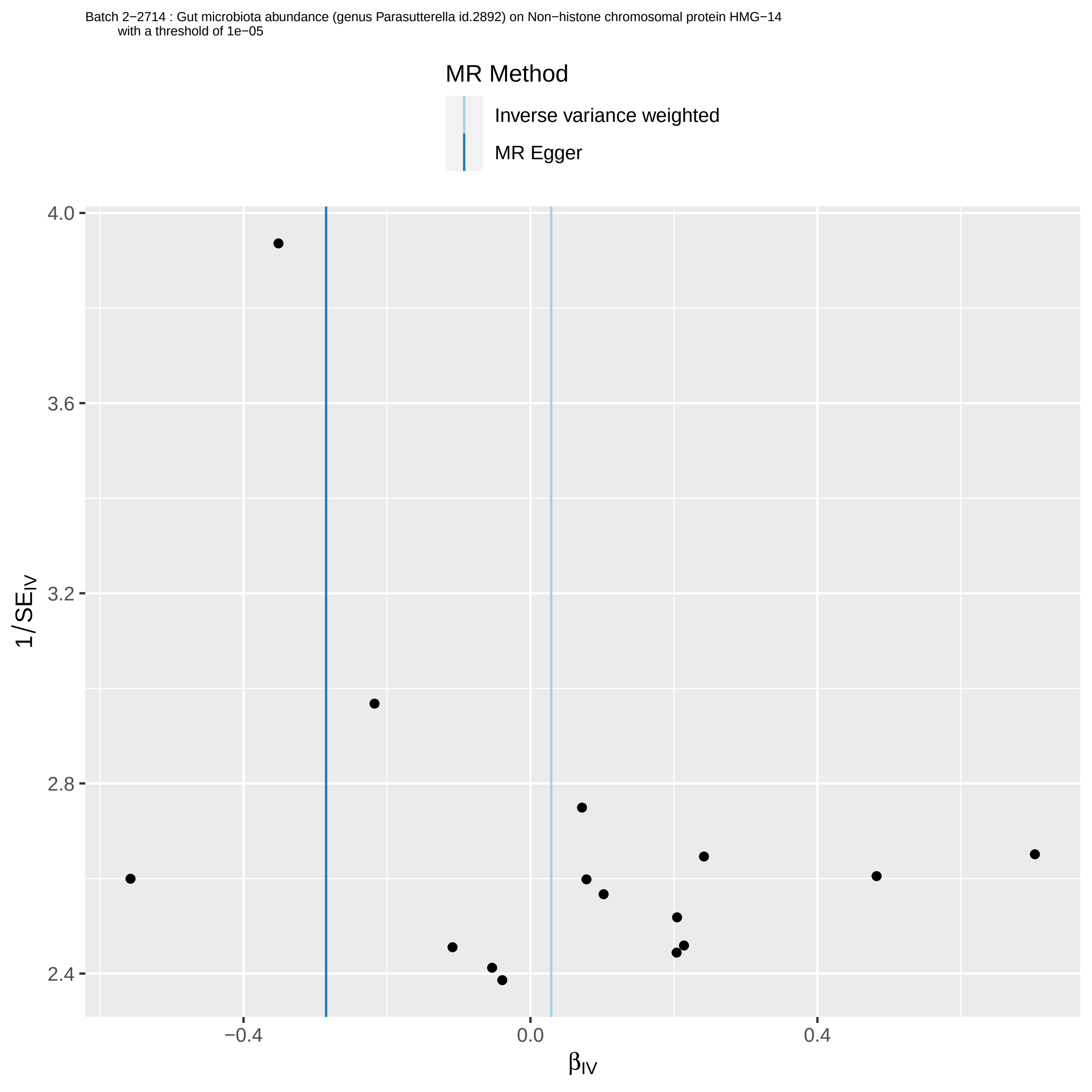

Batch 2−2714 : Gut microbiota abundance (genus Parasutterella id.2892) on Non−histone chromosomal protein HMG−14
with a threshold of 1e−05
MR Method
Inverse variance weighted
MR Egger
1/SE_IV
4.0
3.6
3.2
2.8
2.4
−0.4
0.0
0.4
β_IV

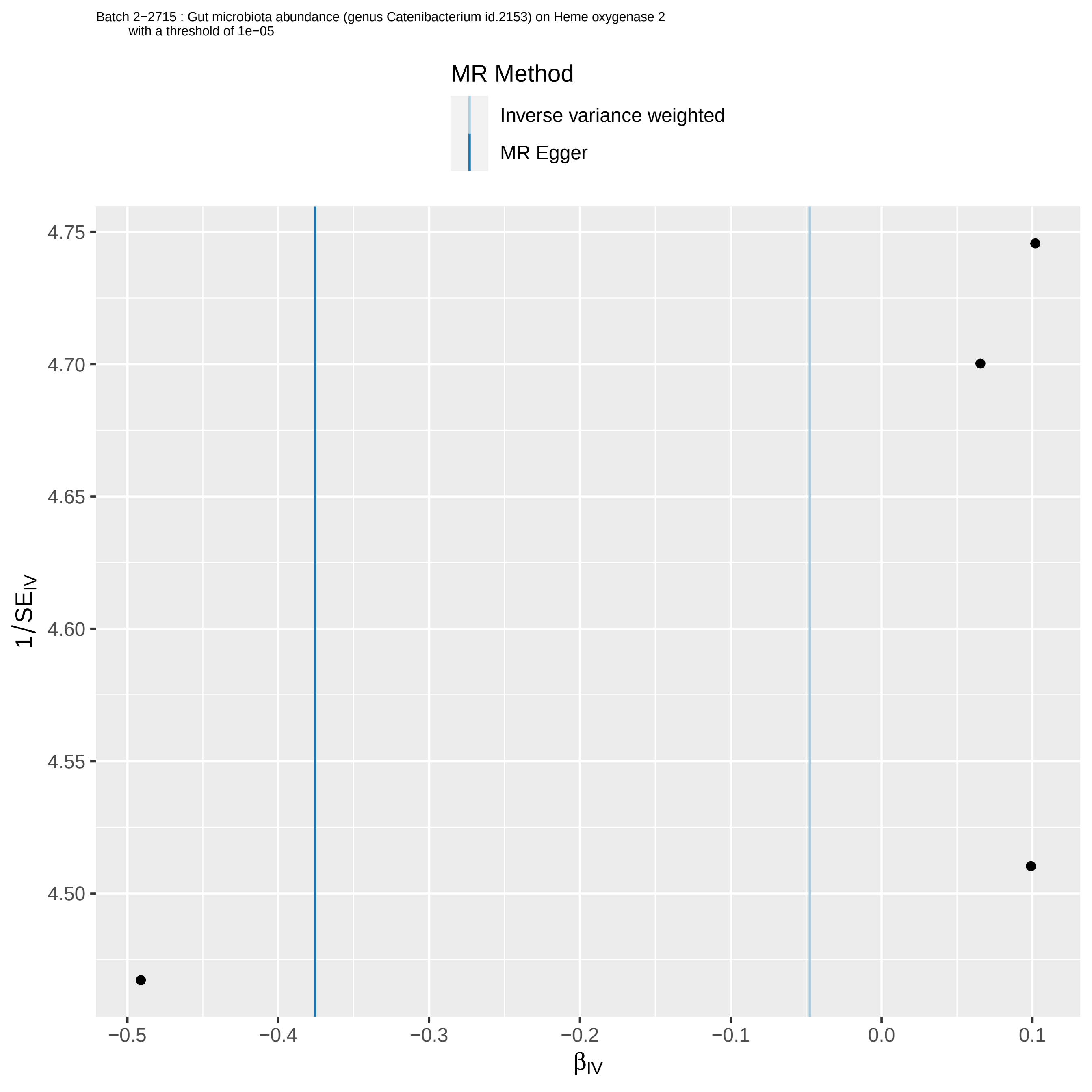

Batch 2−2715 : Gut microbiota abundance (genus Catenibacterium id.2153) on Heme oxygenase 2
with a threshold of 1e−05
MR Method
Inverse variance weighted
MR Egger
4.75
4.70
4.65
4.60
4.55
4.50
1/SE_IV
−0.5
−0.4
−0.3
−0.2
−0.1
0.0
0.1
β_IV

Batch 2−2716 : Gut microbiota abundance (genus Parasutterella id.2892) on Heme oxygenase 2
with a threshold of 1e−05

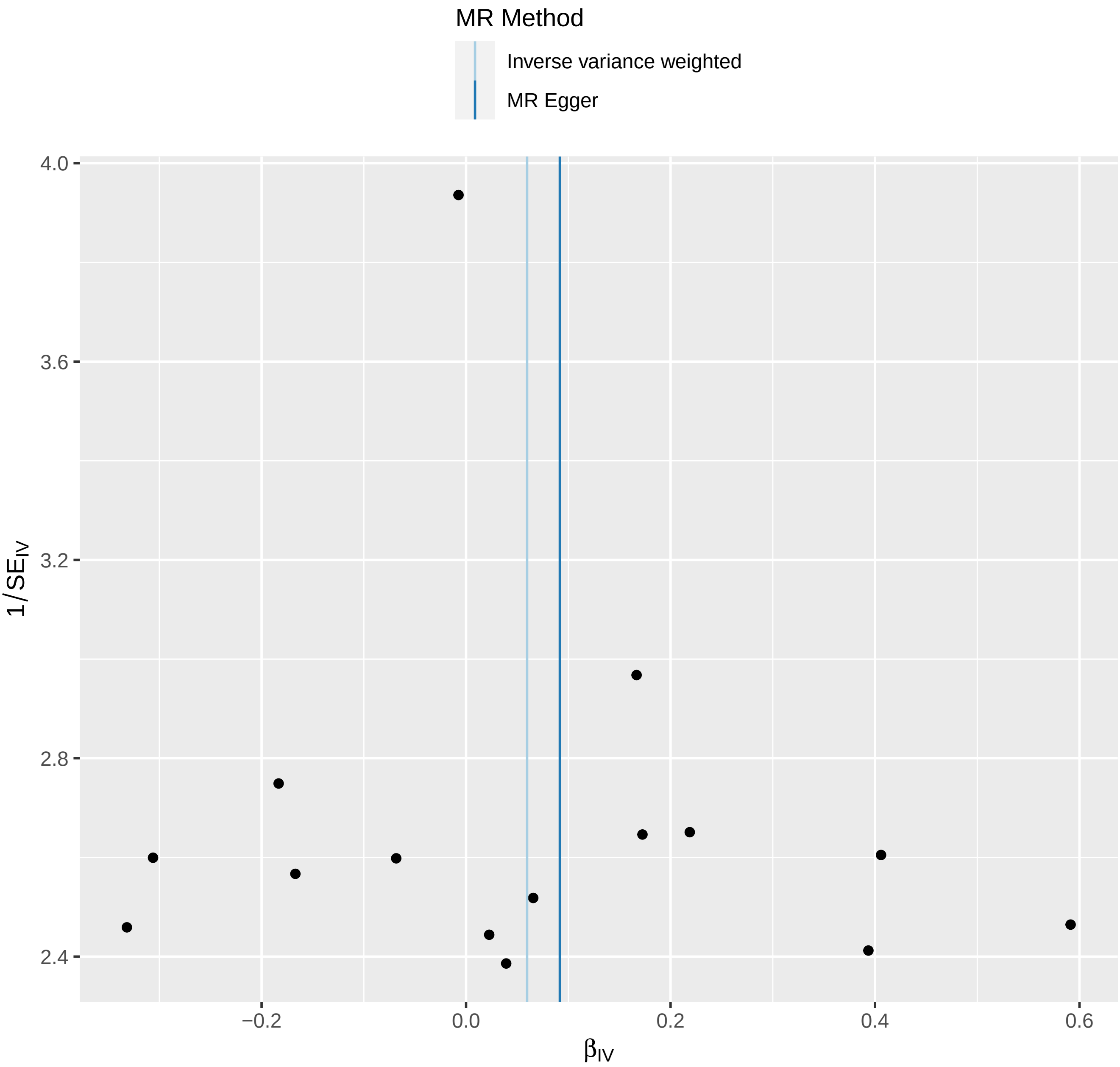

Batch 2−2717 : Gut microbiota abundance (genus Catenibacterium id.2153) on Hepatocyte nuclear factor 1−alpha
with a threshold of 1e−05

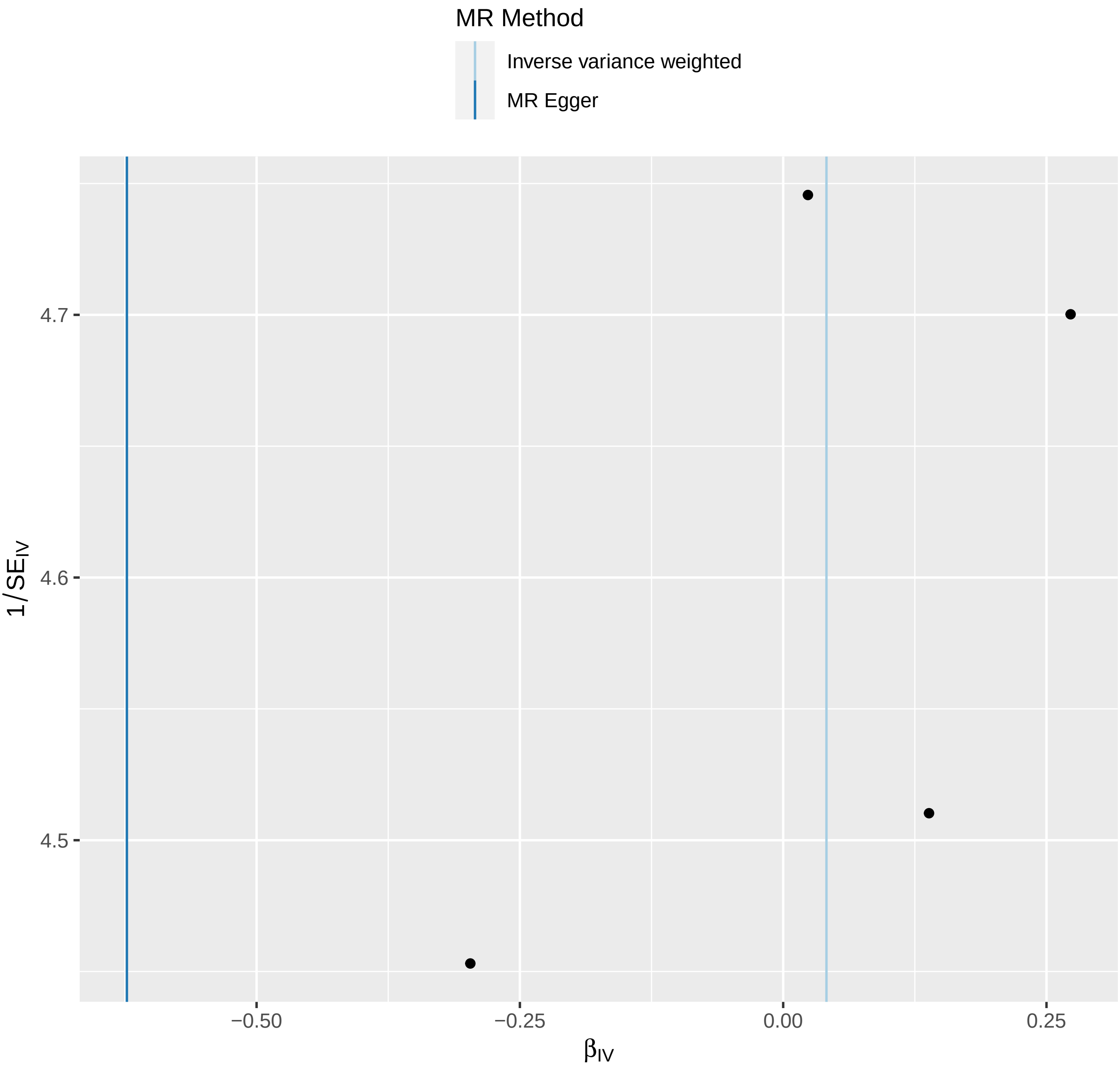

Batch 2−2718 : Gut microbiota abundance (genus Parasutterella id.2892) on Hepatocyte nuclear factor 1−alpha
with a threshold of 1e−05

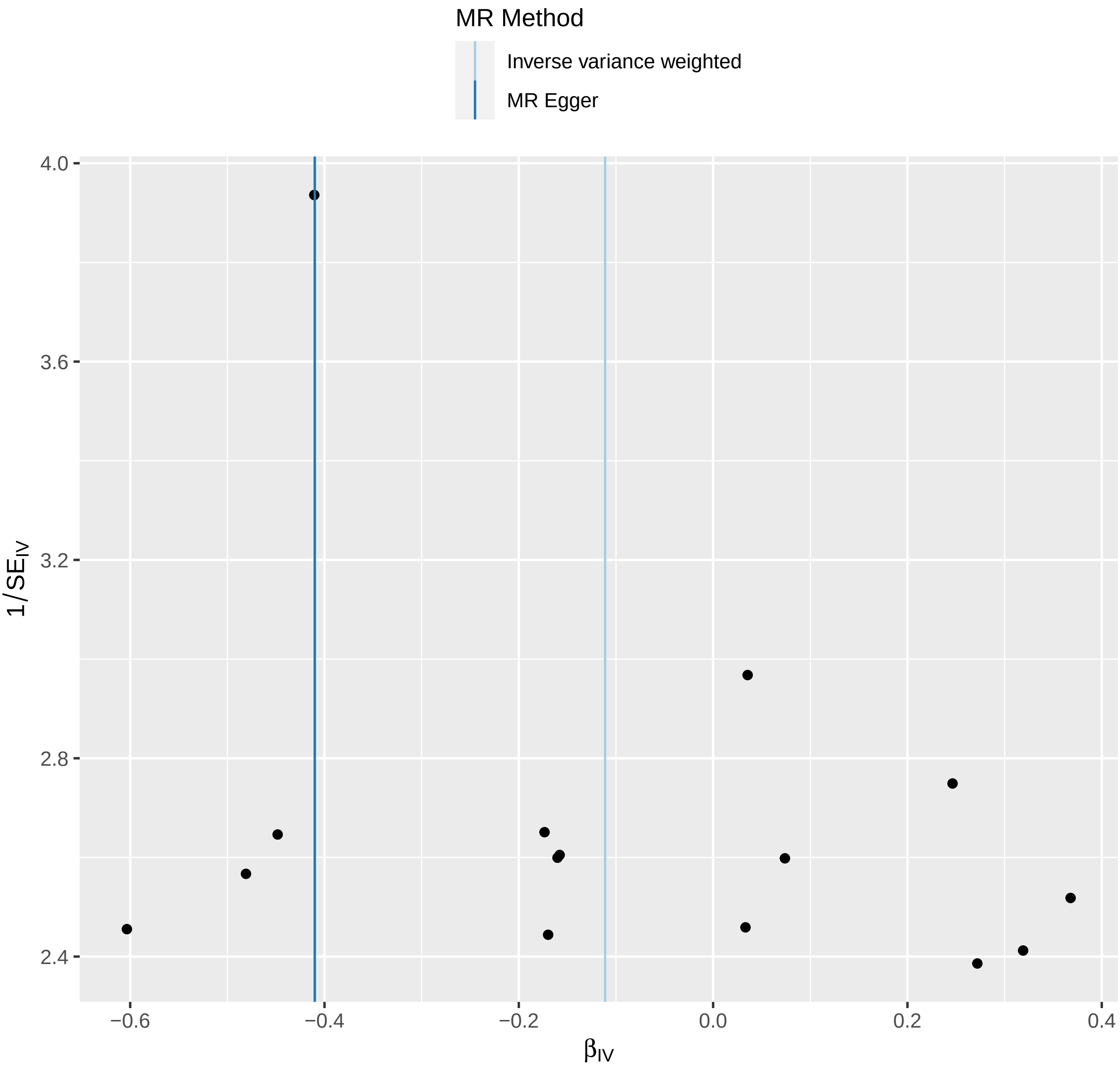

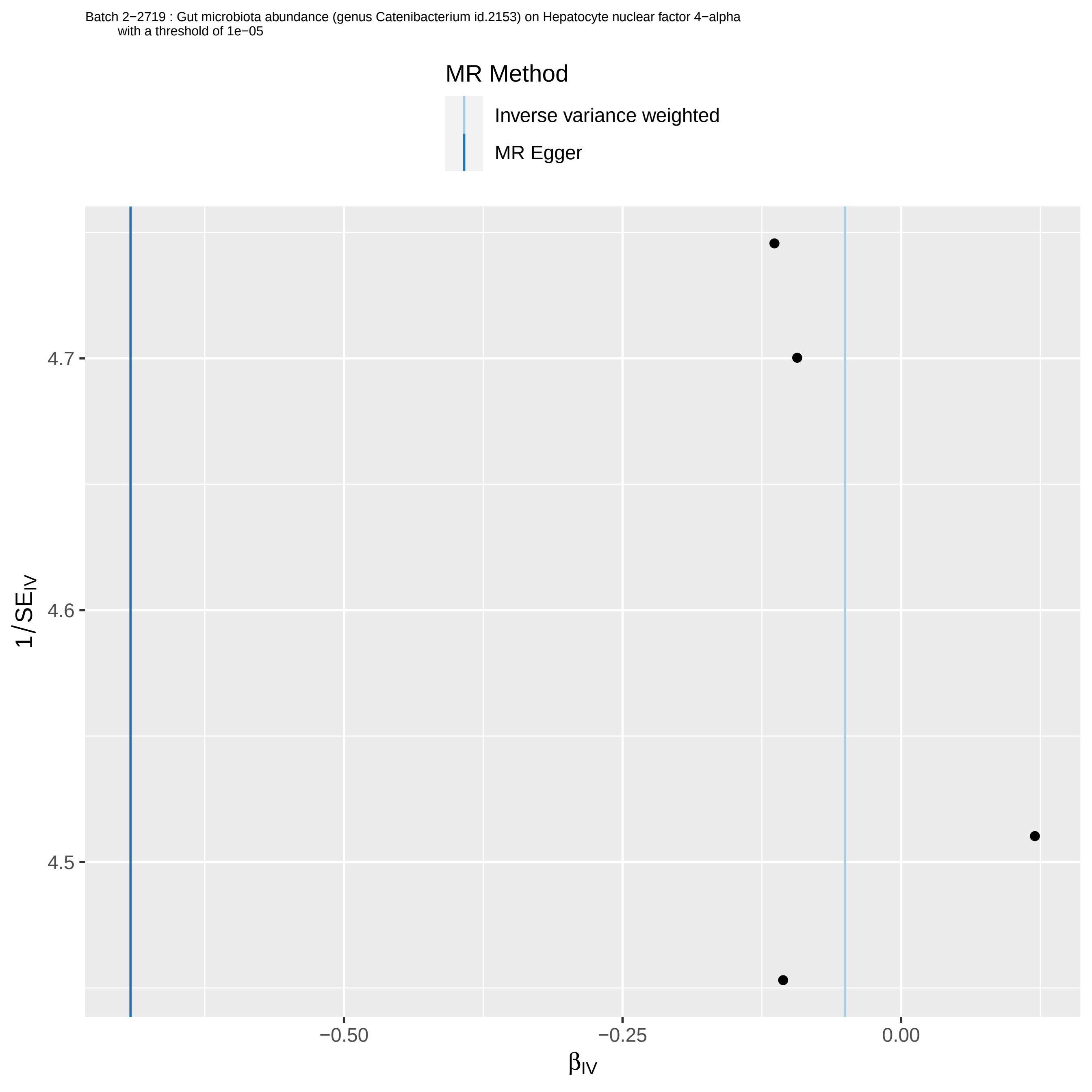
Batch 2−2719 : Gut microbiota abundance (genus Catenibacterium id.2153) on Hepatocyte nuclear factor 4−alpha
with a threshold of 1e−05
MR Method
Inverse variance weighted
MR Egger
4.7
4.6
4.5
1/SE_IV
−0.50
−0.25
0.00
β_IV

Batch 2−2720 : Gut microbiota abundance (genus Parasutterella id.2892) on Hepatocyte nuclear factor 4−alpha with a threshold of 1e−05

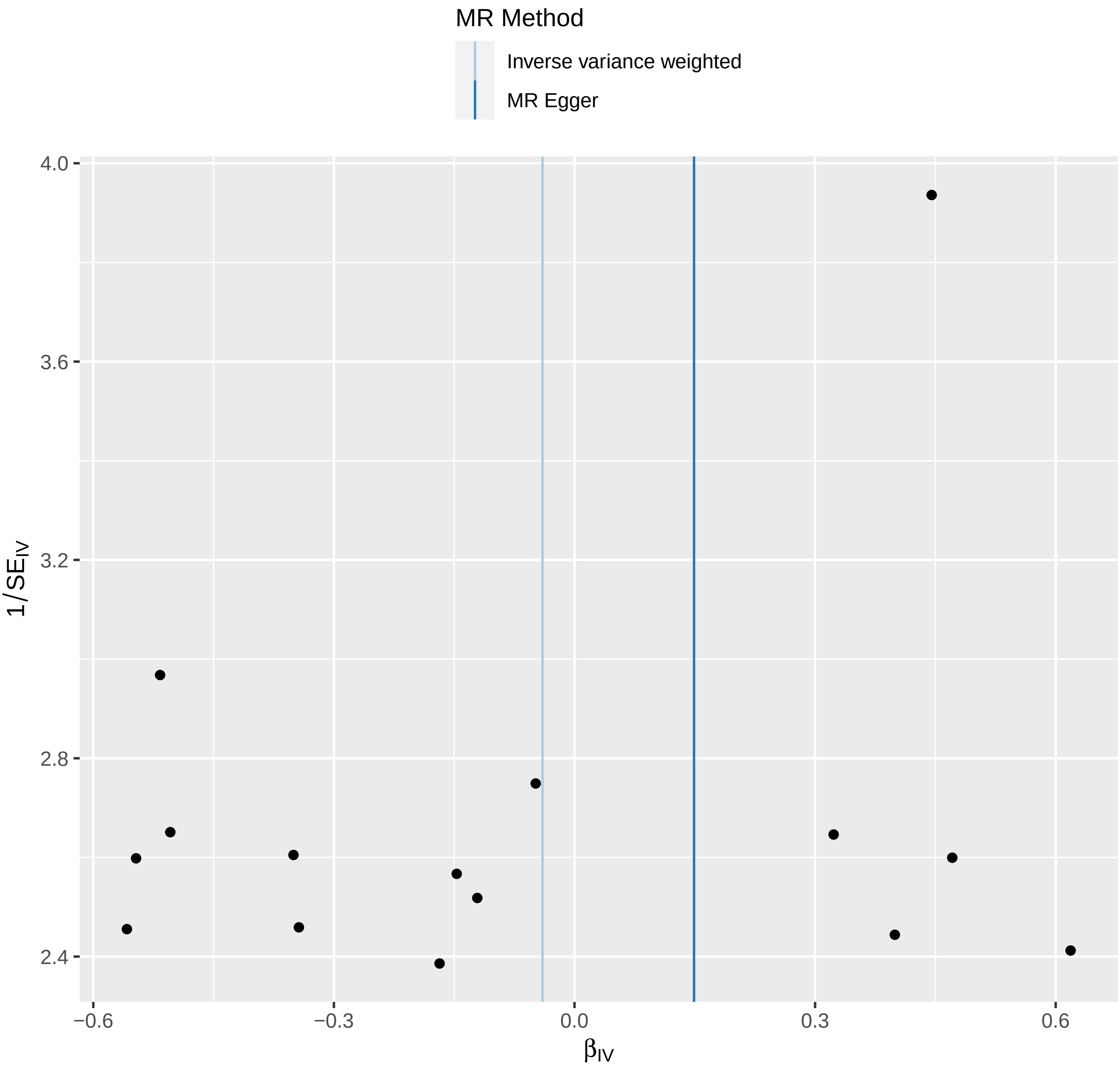

Batch 2−2721 : Gut microbiota abundance (genus Catenibacterium id.2153) on Heterogeneous nuclear ribonucleoproteins A2/B1
with a threshold of 1e−05

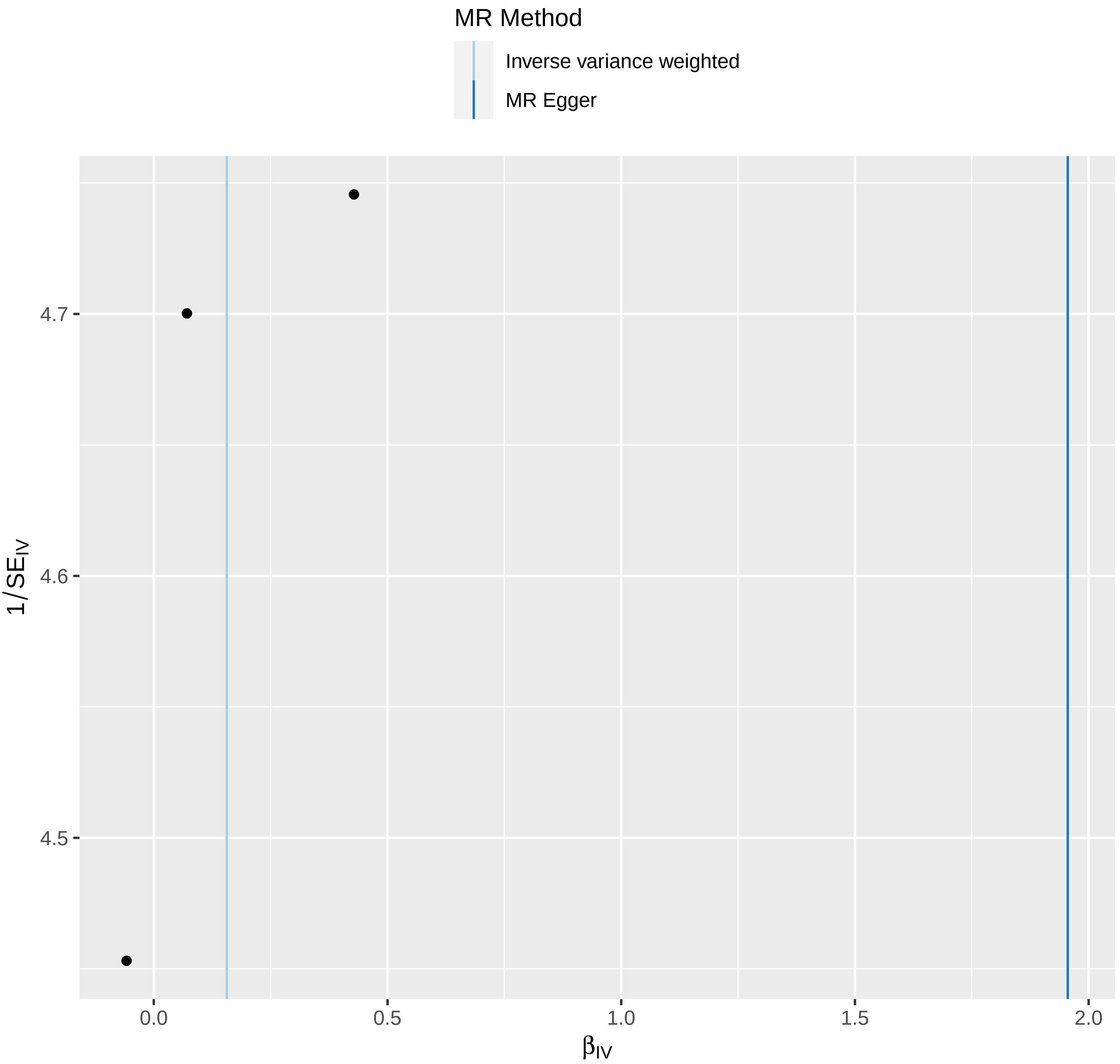

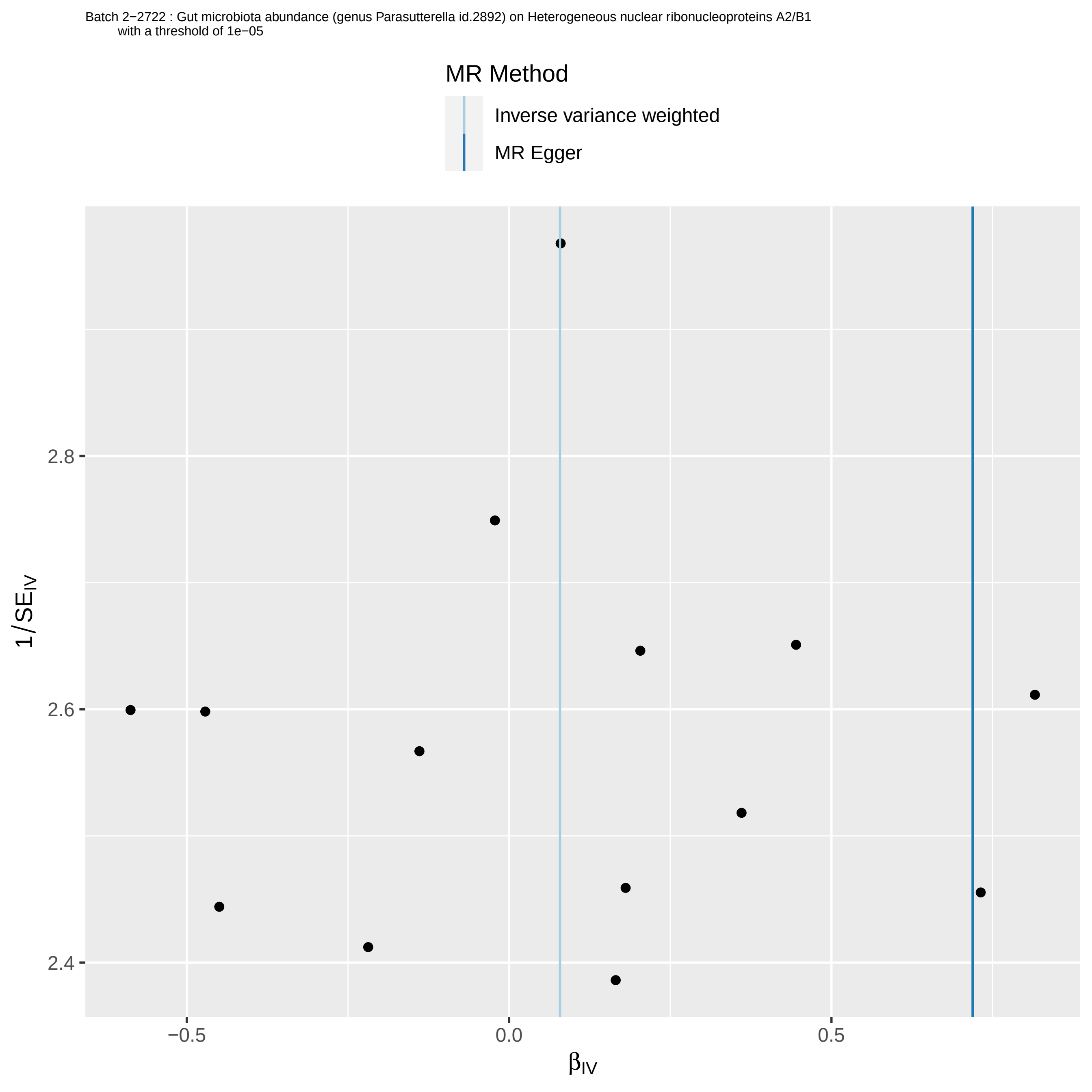

Batch 2−2722 : Gut microbiota abundance (genus Parasutterella id.2892) on Heterogeneous nuclear ribonucleoproteins A2/B1
with a threshold of 1e−05
MR Method
Inverse variance weighted
MR Egger
2.8
2.6
2.4
1/SE_IV
−0.5
0.0
0.5
β_IV

Batch 2−2723 : Gut microbiota abundance (genus Catenibacterium id.2153) on Heterogeneous nuclear ribonucleoprotein A/B with a threshold of 1e−05

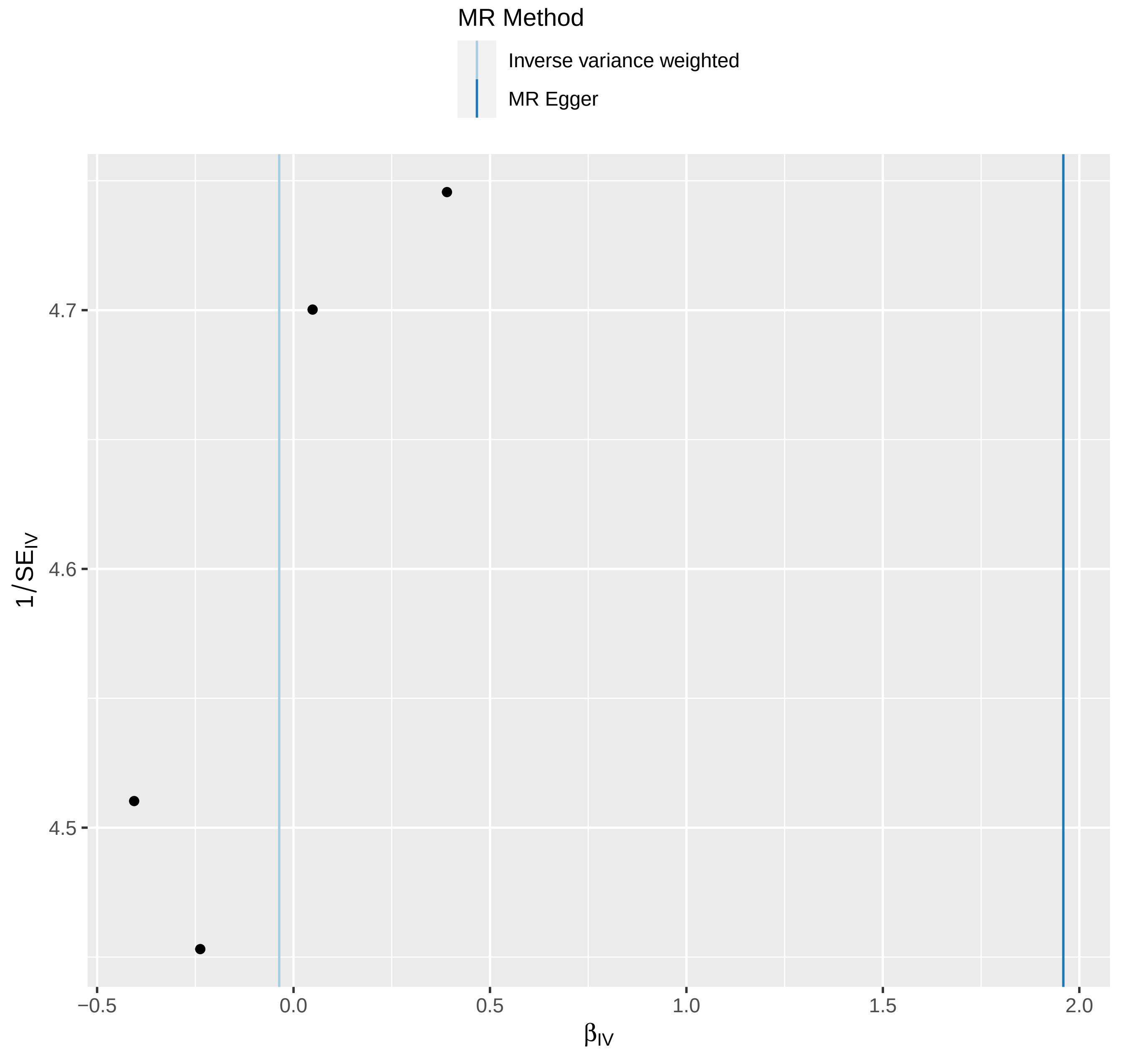

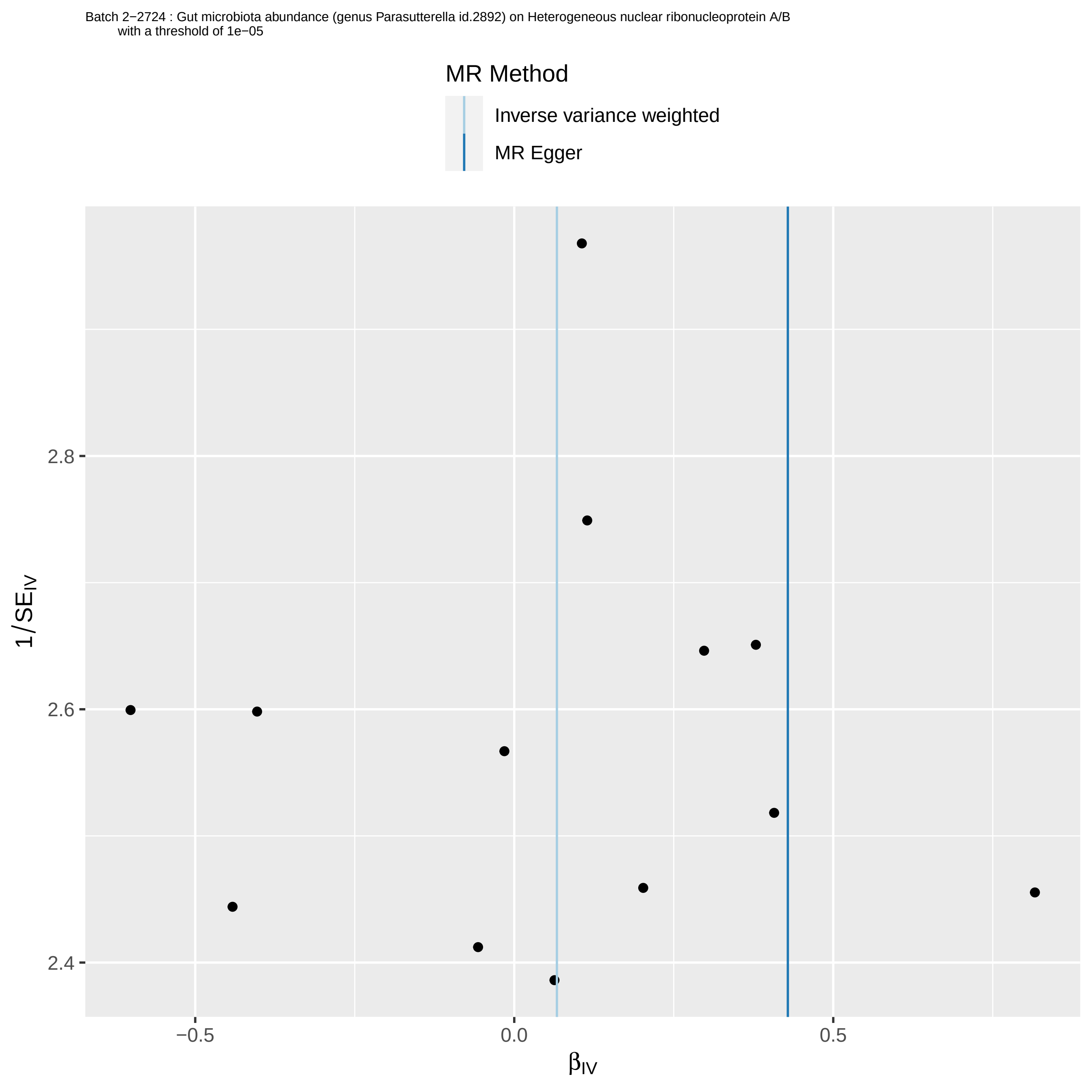
Batch 2−2724 : Gut microbiota abundance (genus Parasutterella id.2892) on Heterogeneous nuclear ribonucleoprotein A/B
with a threshold of 1e−05
MR Method
Inverse variance weighted
MR Egger
2.8
2.6
2.4
1/SE_IV
−0.5
0.0
0.5
β_IV

Batch 2−2725 : Gut microbiota abundance (genus Catenibacterium id.2153) on Heterogeneous nuclear ribonucleoprotein A/B with a threshold of 1e−05

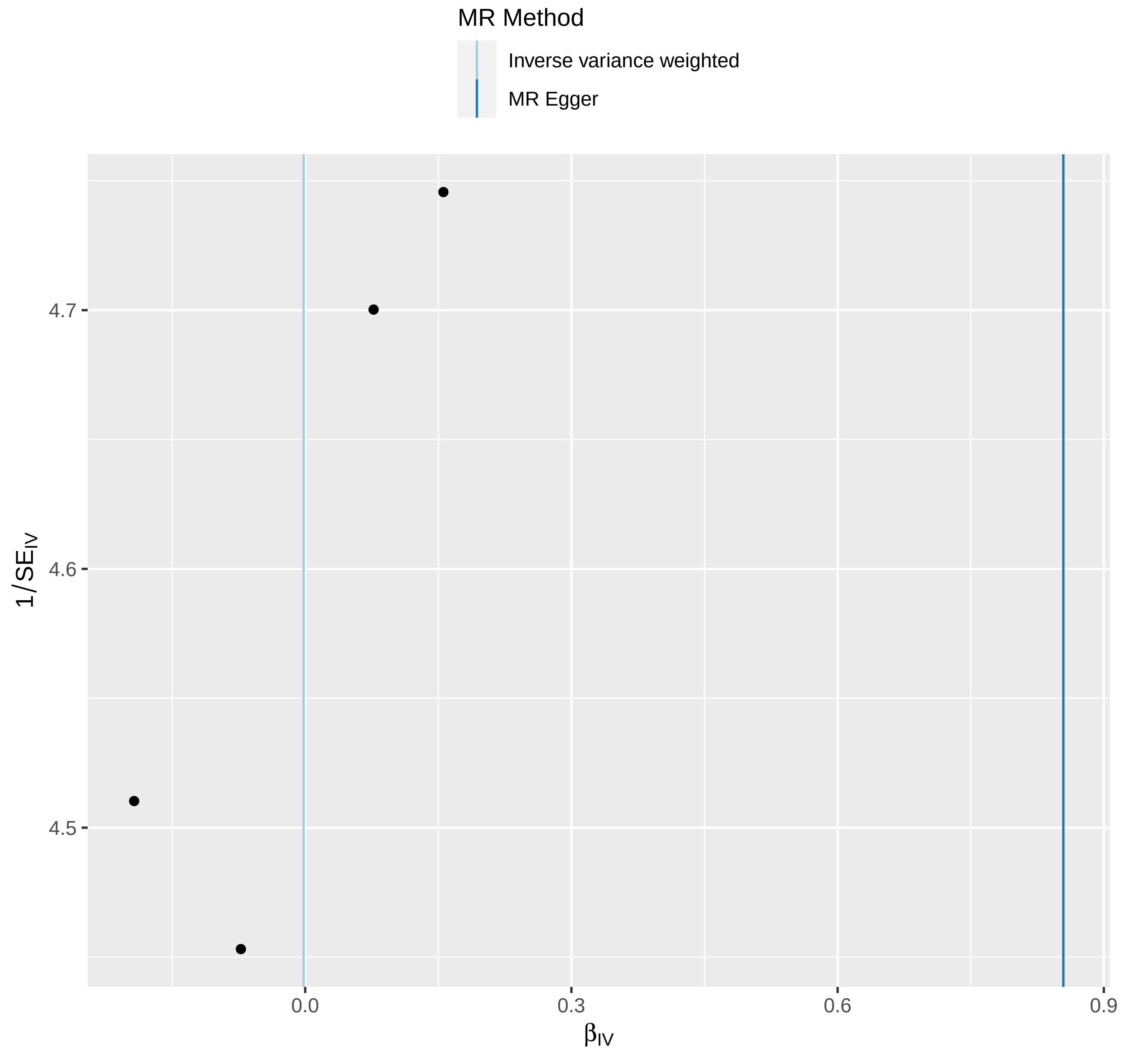

Batch 2−2726 : Gut microbiota abundance (genus Parasutterella id.2892) on Heterogeneous nuclear ribonucleoprotein A/B
with a threshold of 1e−05

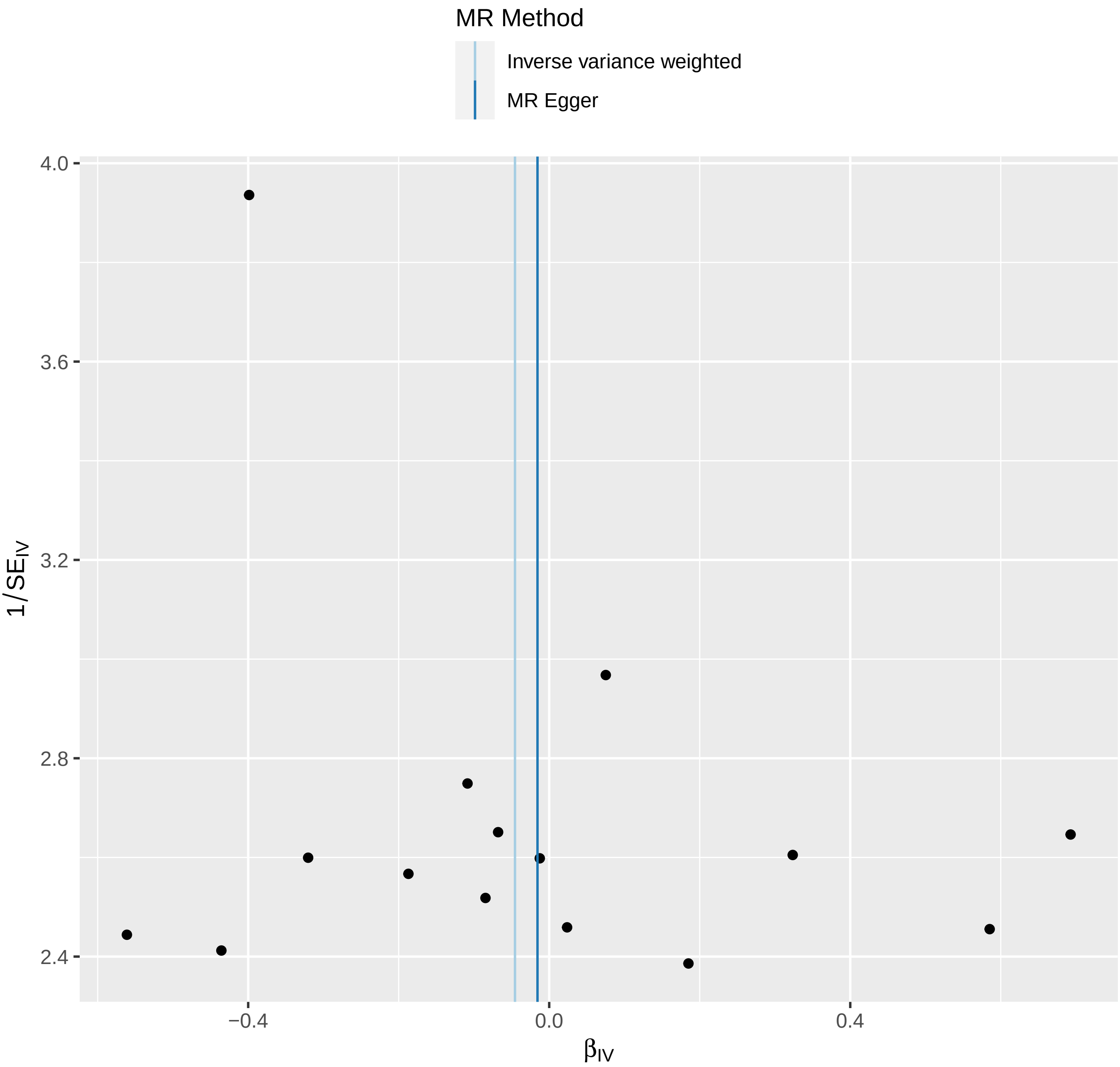

Batch 2−2727 : Gut microbiota abundance (genus Catenibacterium id.2153) on Heterogeneous nuclear ribonucleoproteins C1/C2
with a threshold of 1e−05

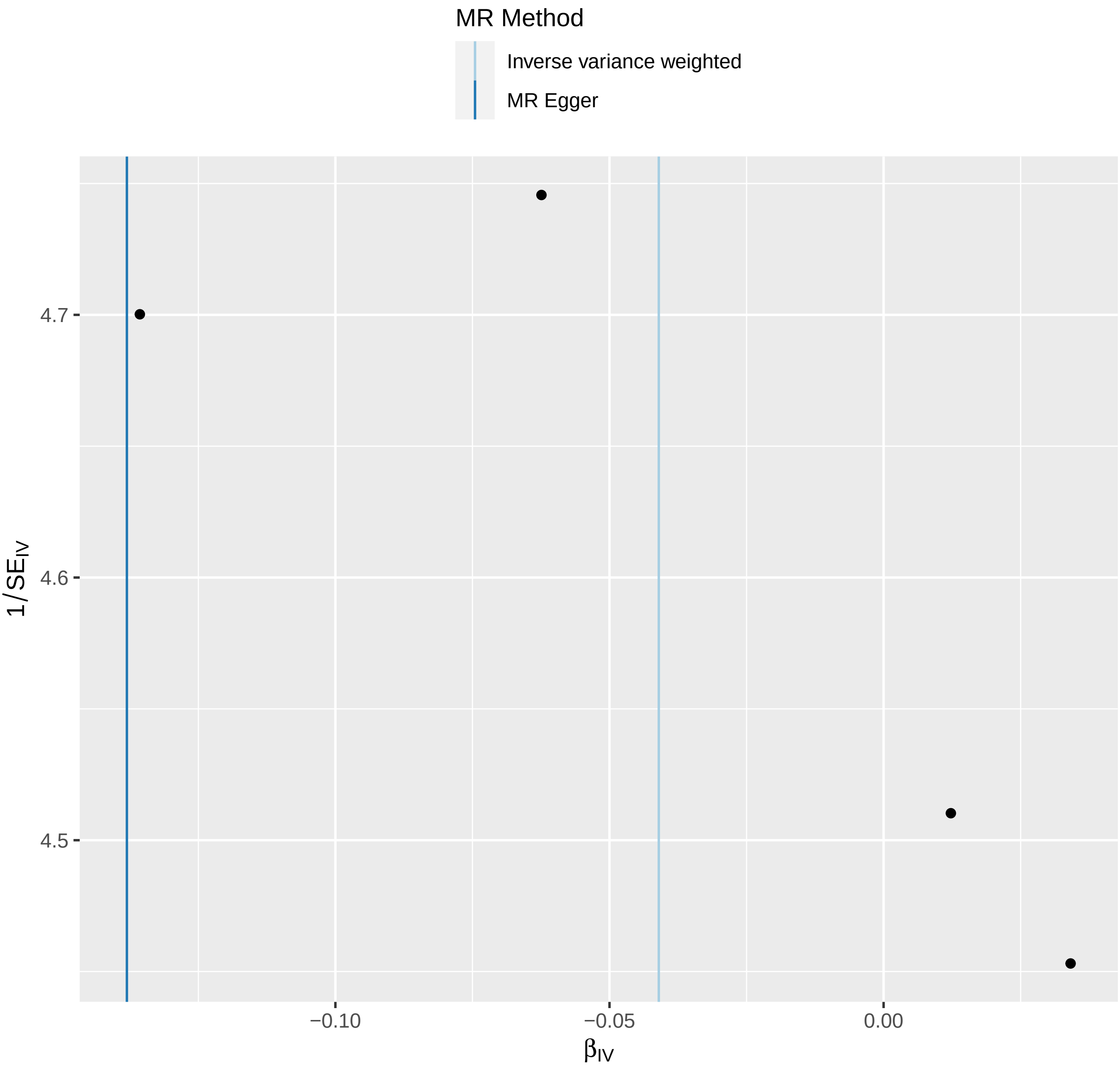

Batch 2−2728 : Gut microbiota abundance (genus Parasutterella id.2892) on Heterogeneous nuclear ribonucleoproteins C1/C2
with a threshold of 1e−05

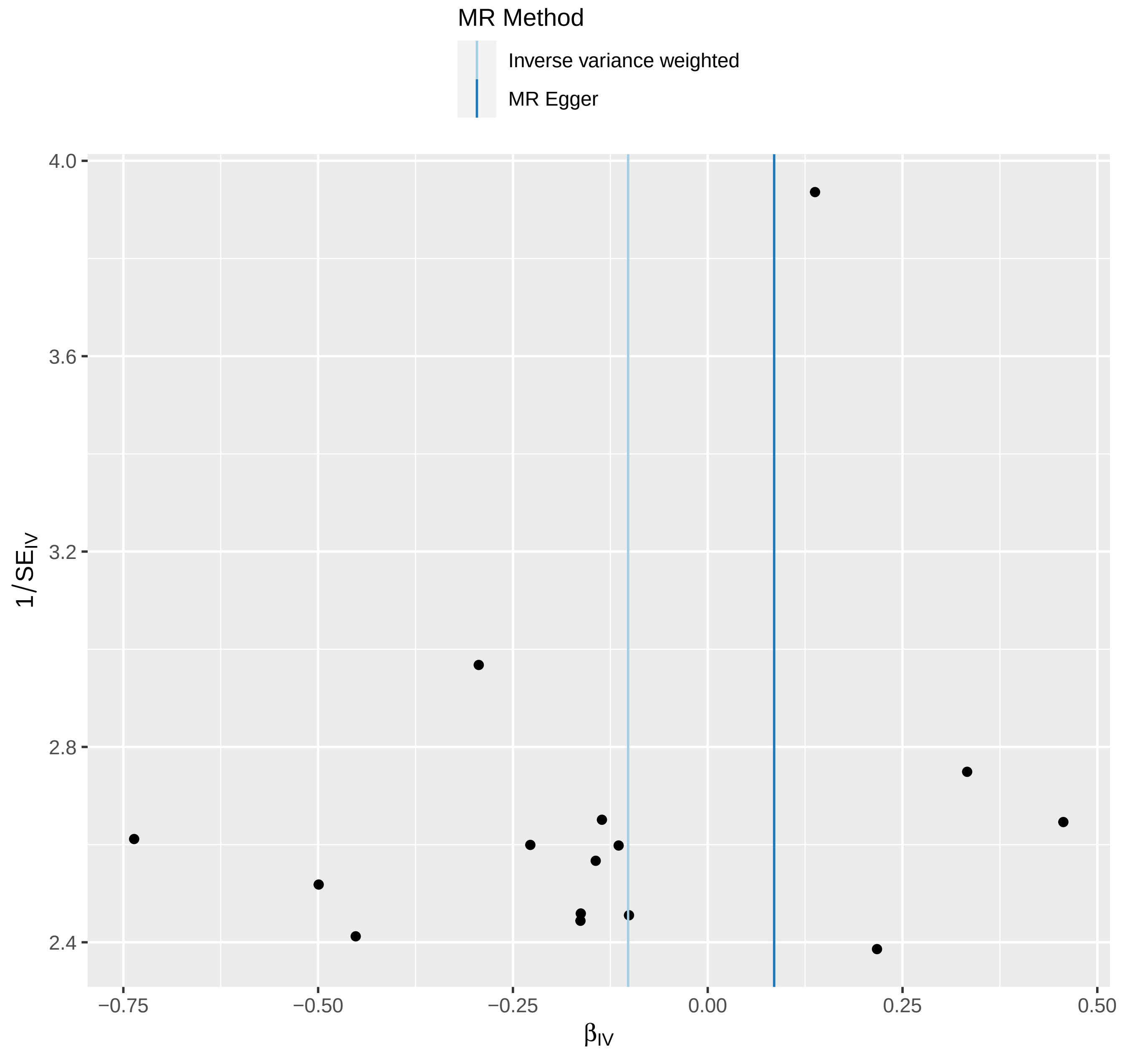

Batch 2−2729 : Gut microbiota abundance (genus Catenibacterium id.2153) on Heterogeneous nuclear ribonucleoprotein D−like
with a threshold of 1e−05

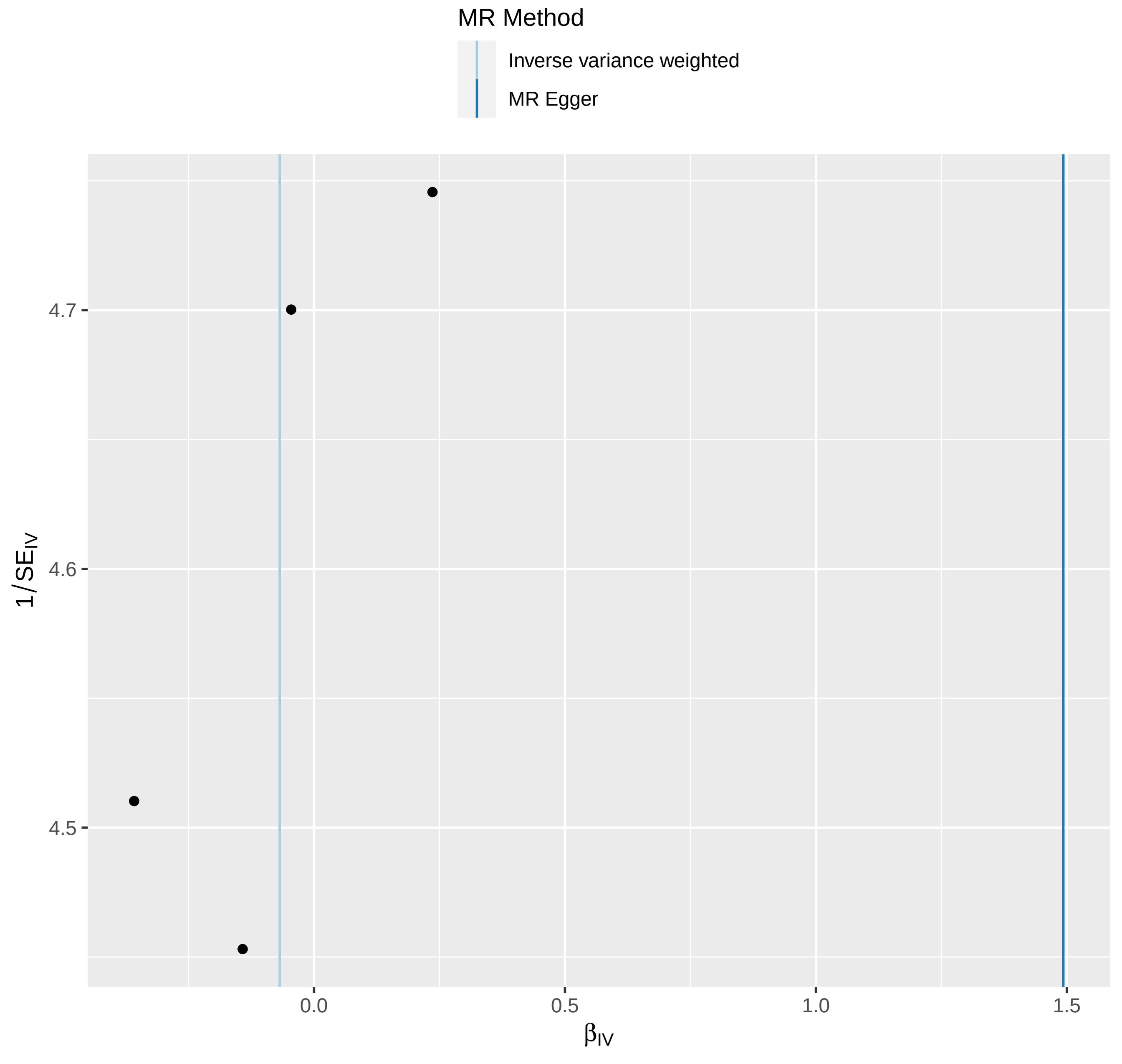

Batch 2−2730 : Gut microbiota abundance (genus Parasutterella id.2892) on Heterogeneous nuclear ribonucleoprotein D−like
with a threshold of 1e−05

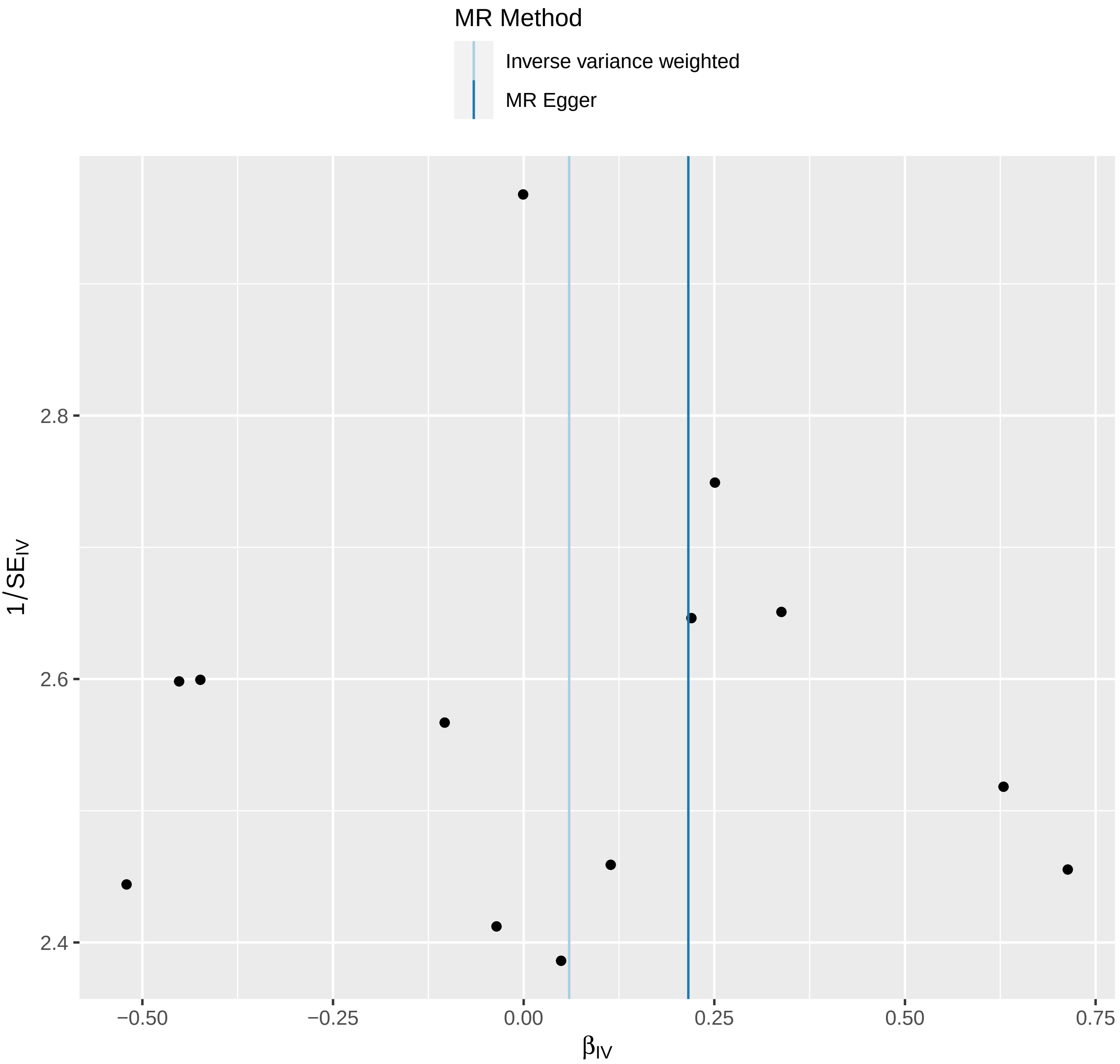

Batch 2−2731 : Gut microbiota abundance (genus Catenibacterium id.2153) on Heterogeneous nuclear ribonucleoprotein K with a threshold of 1e−05

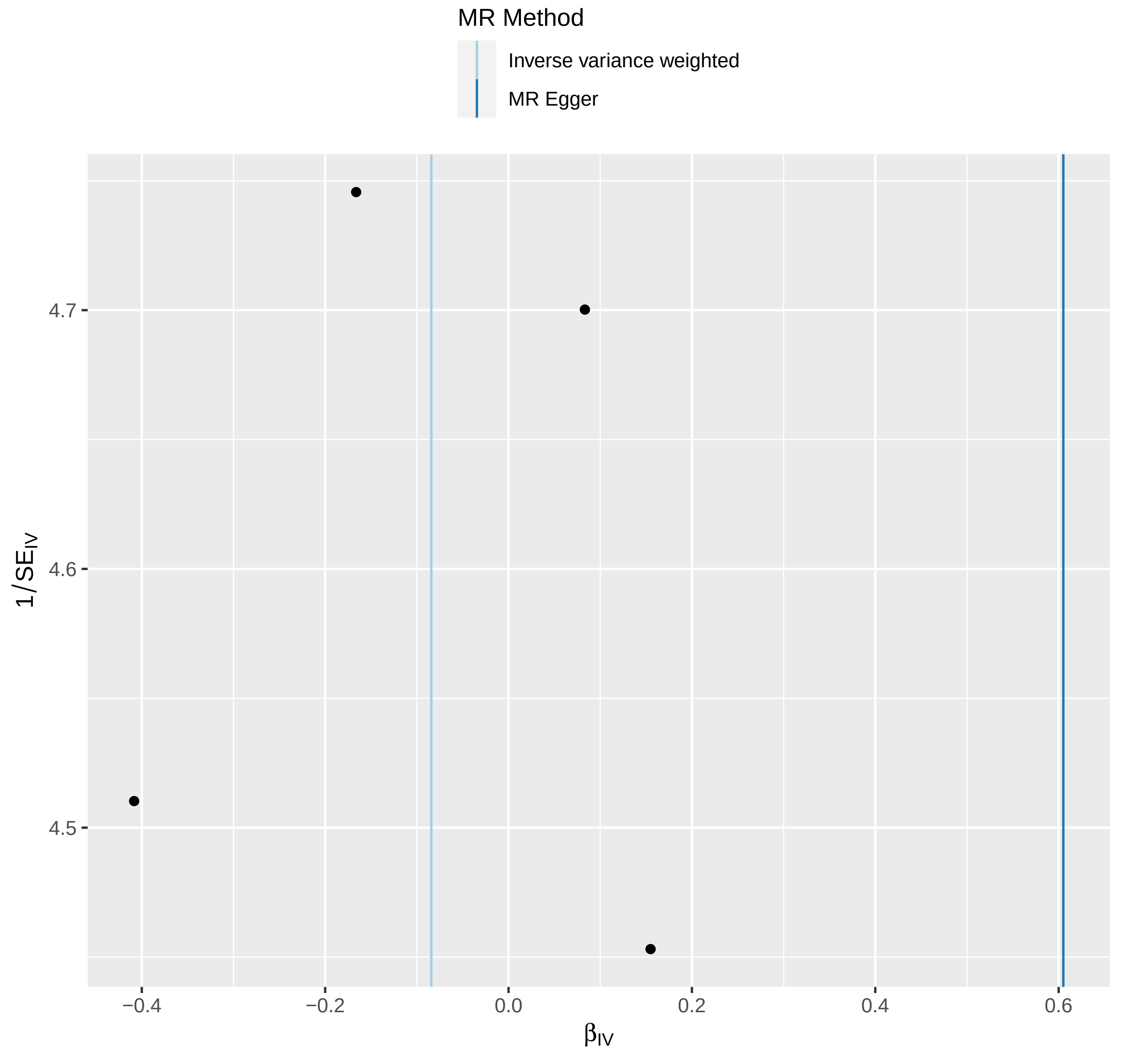

Batch 2−2732 : Gut microbiota abundance (genus Parasutterella id.2892) on Heterogeneous nuclear ribonucleoprotein K
with a threshold of 1e−05

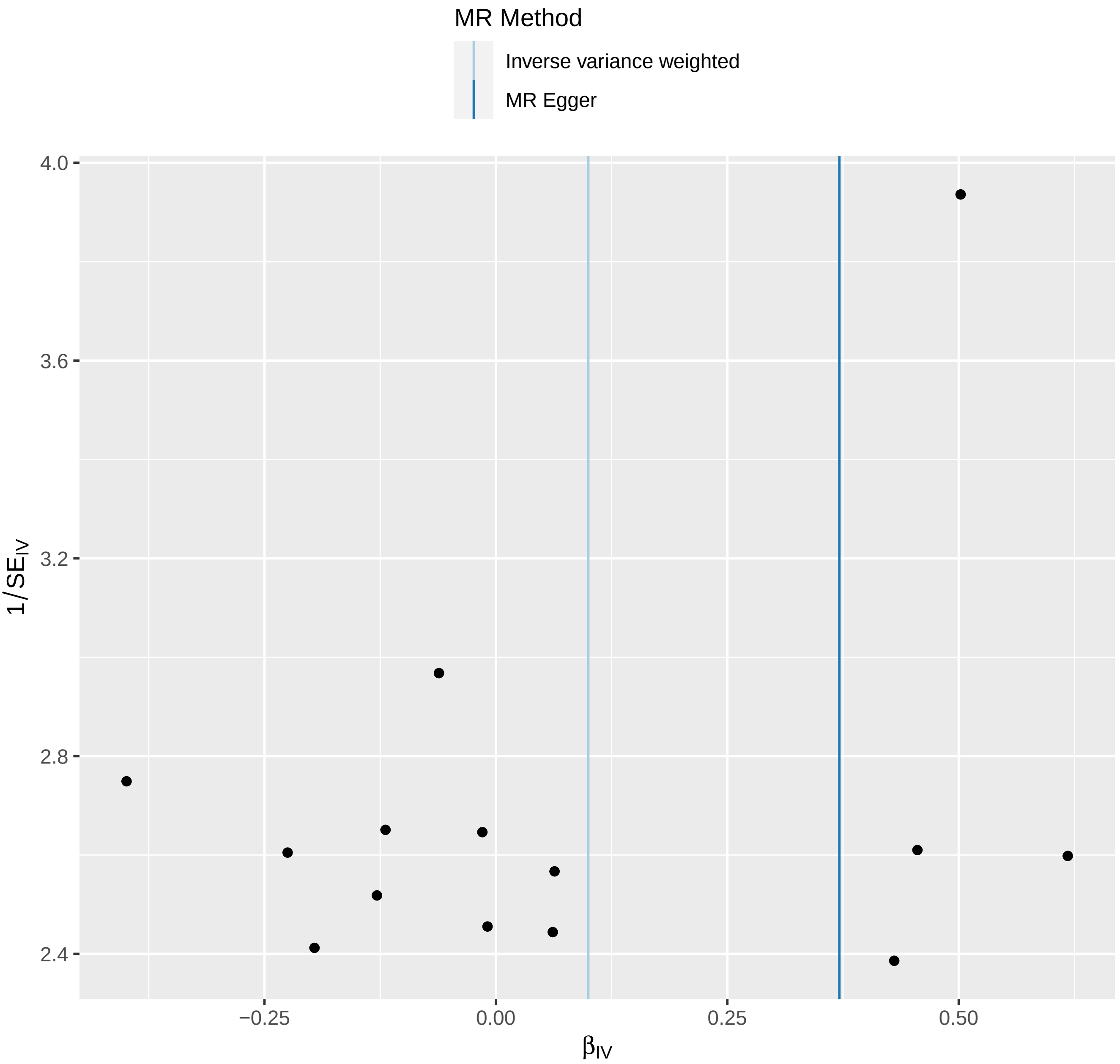

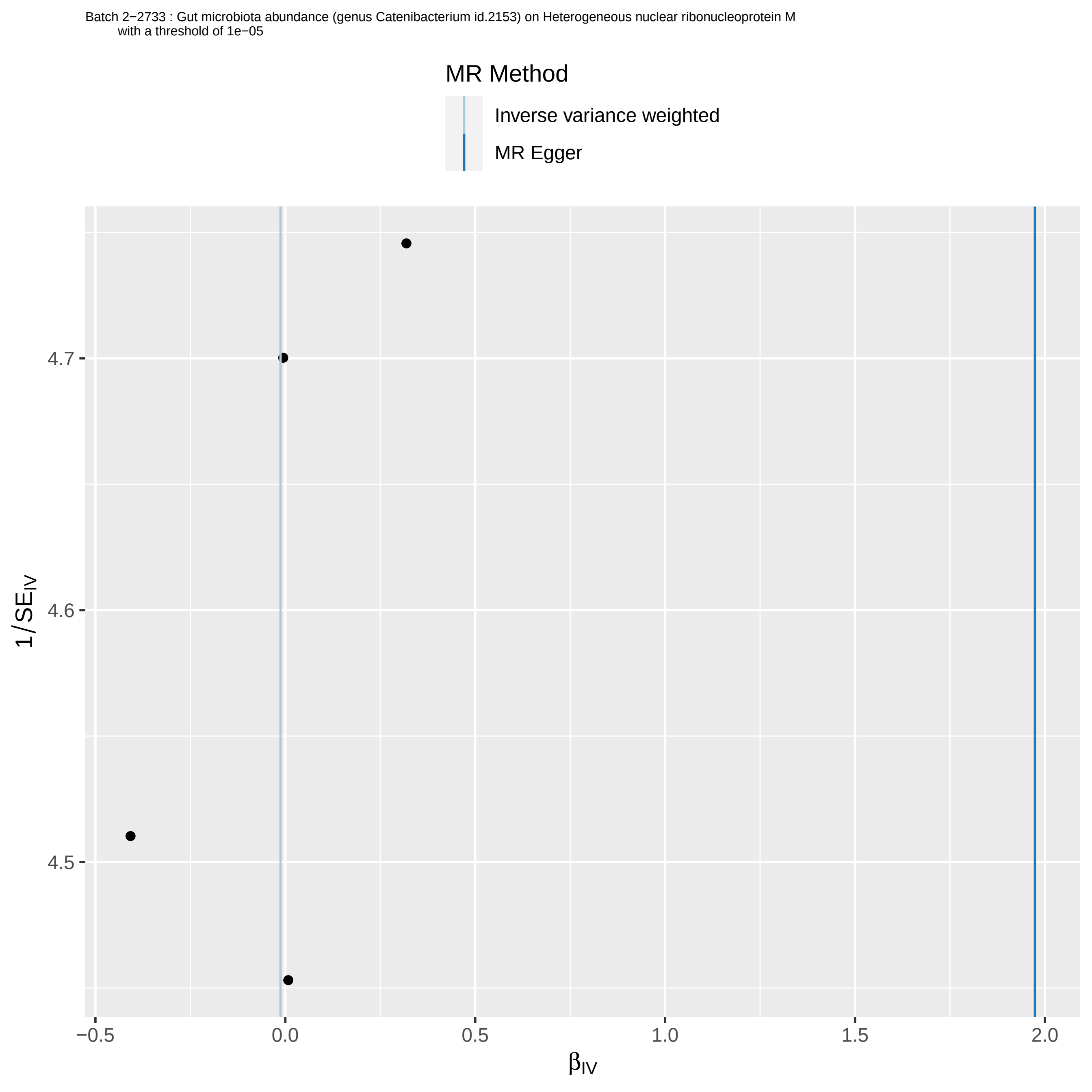
Batch 2−2733 : Gut microbiota abundance (genus Catenibacterium id.2153) on Heterogeneous nuclear ribonucleoprotein M
with a threshold of 1e−05
MR Method
Inverse variance weighted
MR Egger
4.7
4.6
4.5
1/SE_IV
−0.5
0.0
0.5
1.0
1.5
2.0
β_IV

Batch 2−2734 : Gut microbiota abundance (genus Parasutterella id.2892) on Heterogeneous nuclear ribonucleoprotein M
with a threshold of 1e−05

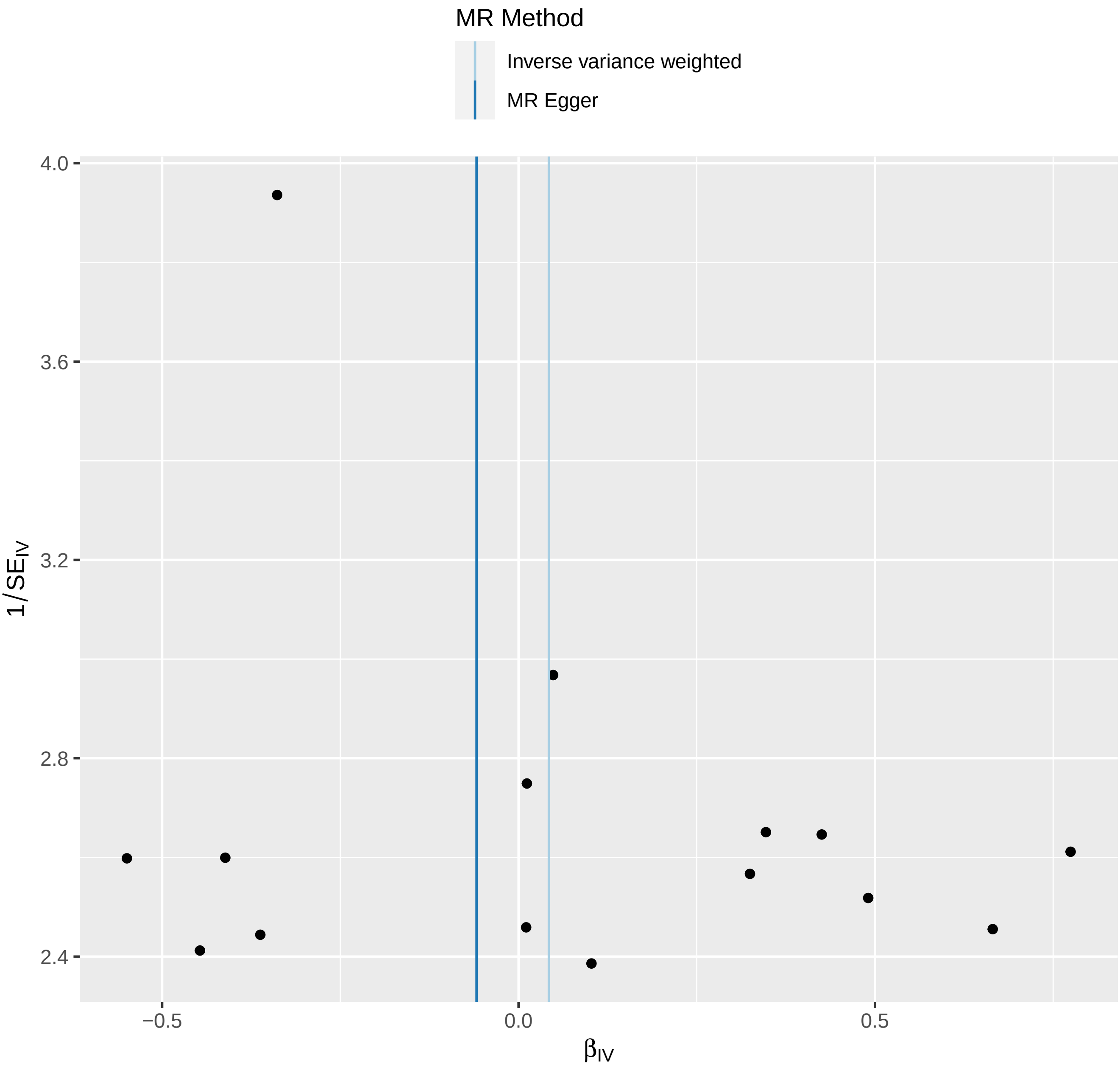

Batch 2−2735 : Gut microbiota abundance (genus Catenibacterium id.2153) on 4−hydroxy−2−oxoglutarate aldolase, mitochondrial
with a threshold of 1e−05

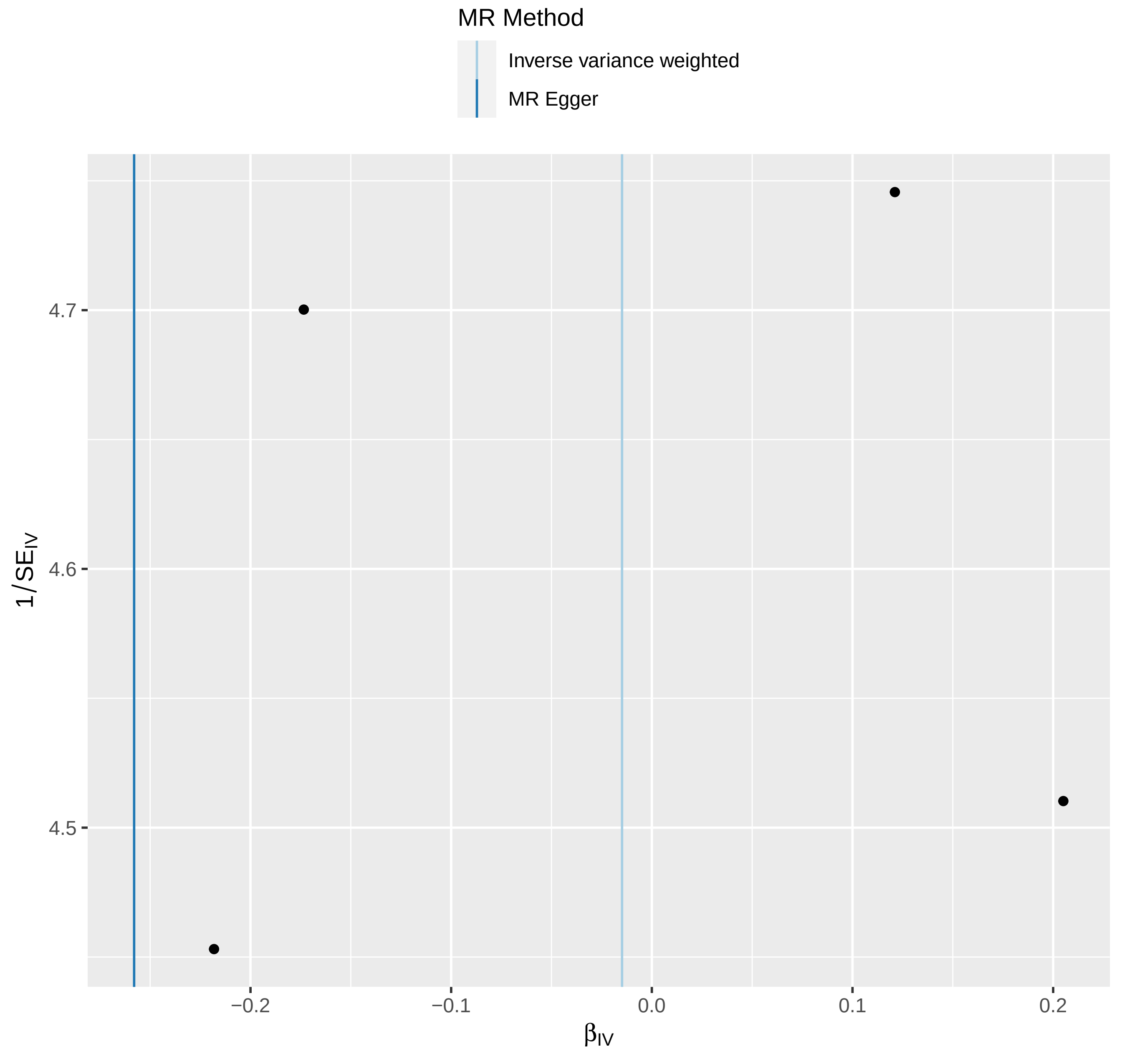

Batch 2−2736 : Gut microbiota abundance (genus Parasutterella id.2892) on 4−hydroxy−2−oxoglutarate aldolase, mitochondrial
with a threshold of 1e−05

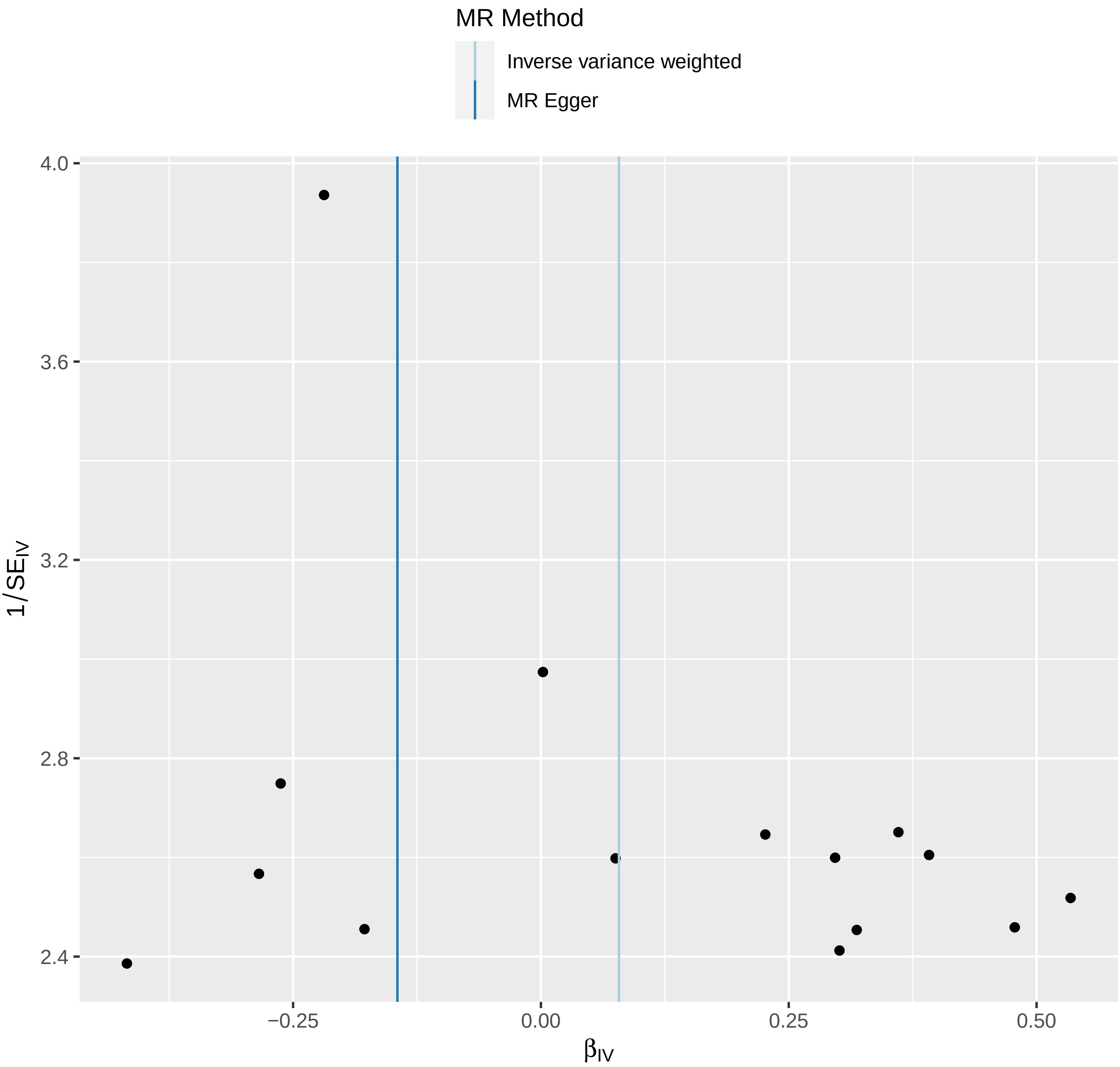

Batch 2−2737 : Gut microbiota abundance (genus Catenibacterium id.2153) on Haptoglobin
with a threshold of 1e−05

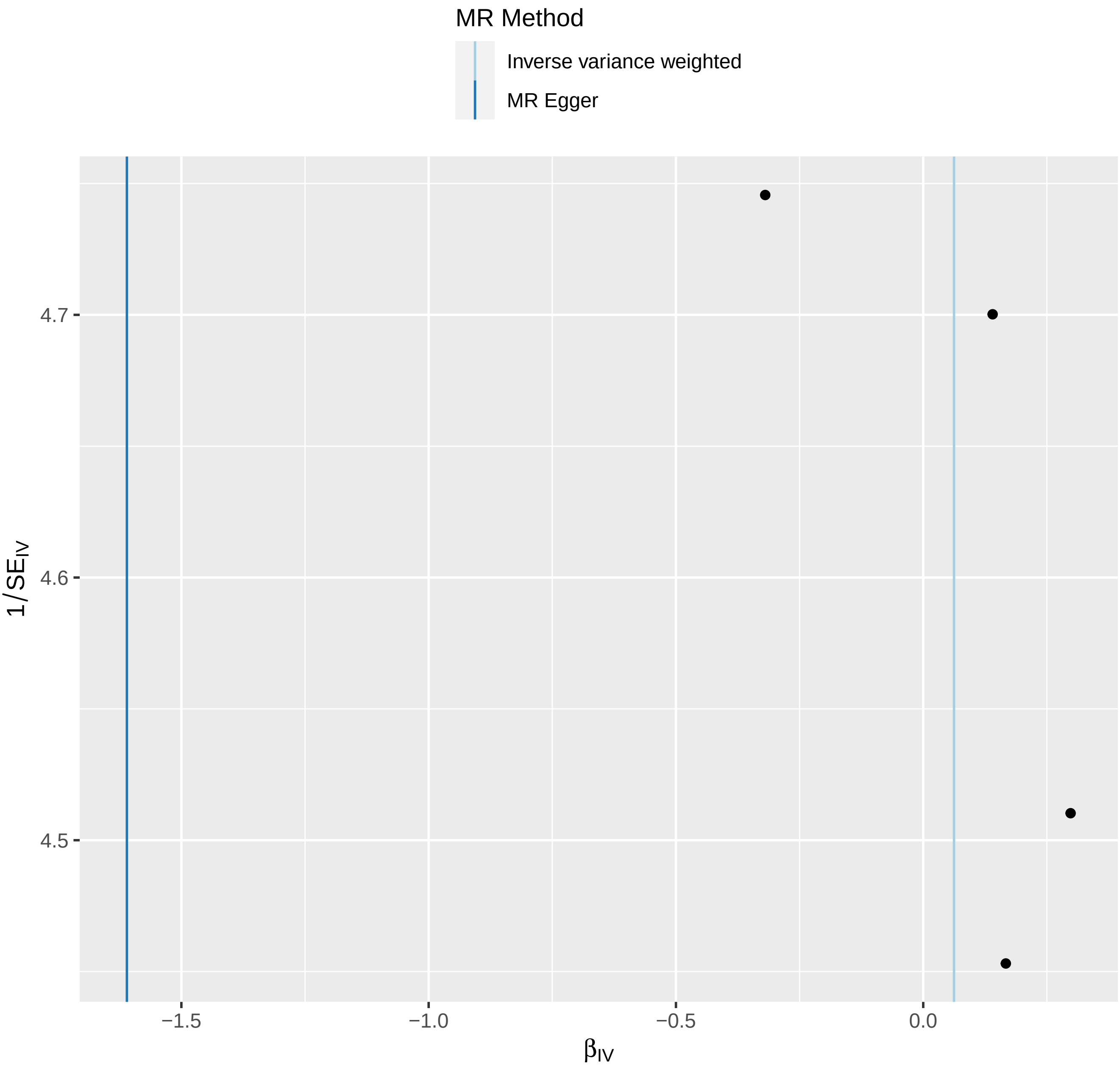

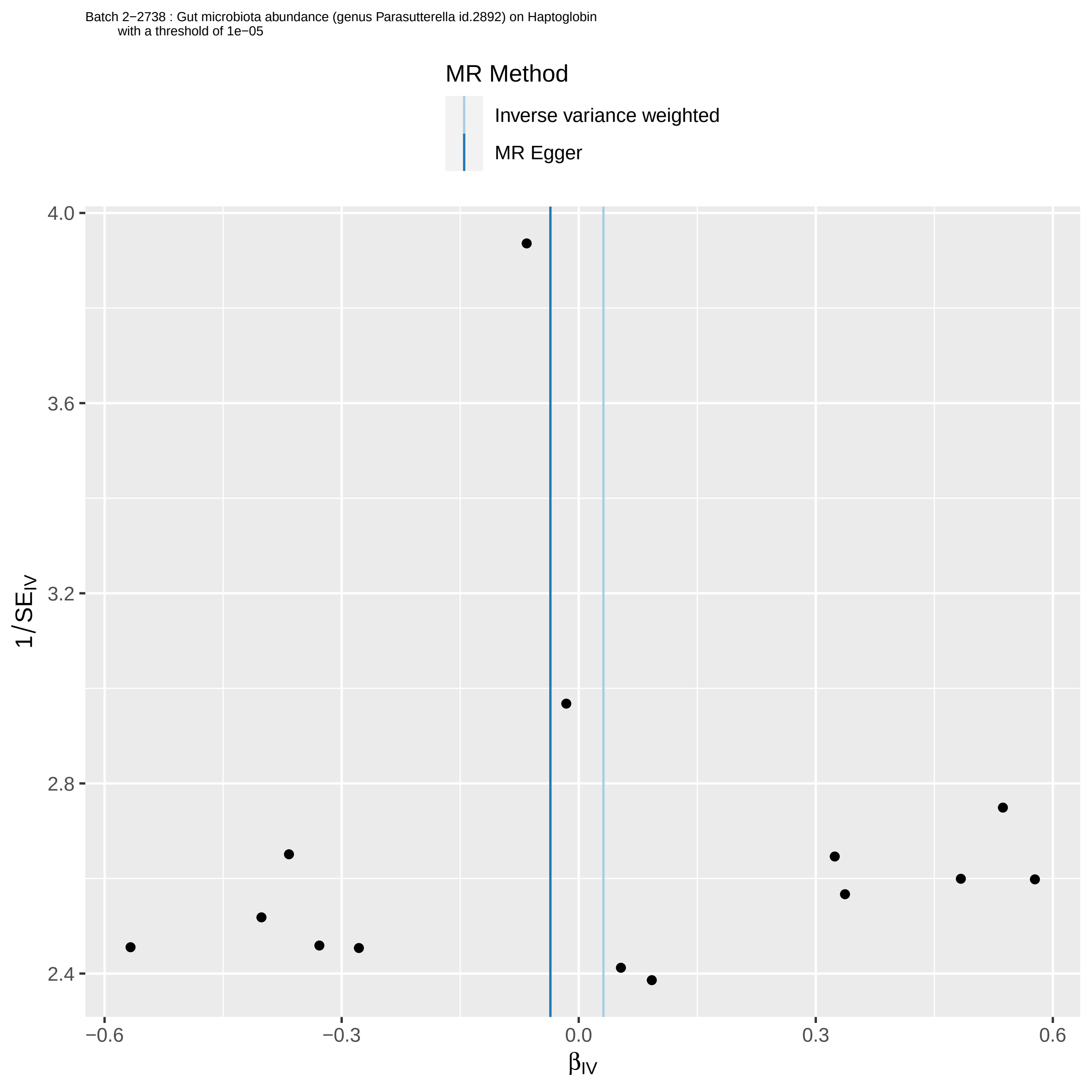
Batch 2−2738 : Gut microbiota abundance (genus Parasutterella id.2892) on Haptoglobin
with a threshold of 1e−05
MR Method
Inverse variance weighted
MR Egger
4.0
3.6
3.2
2.8
2.4
1/SE_IV
−0.6
−0.3
0.0
0.3
0.6
β_IV

Batch 2−2739 : Gut microbiota abundance (genus Catenibacterium id.2153) on 15−hydroxyprostaglandin dehydrogenase [NAD(+)] with a threshold of 1e−05

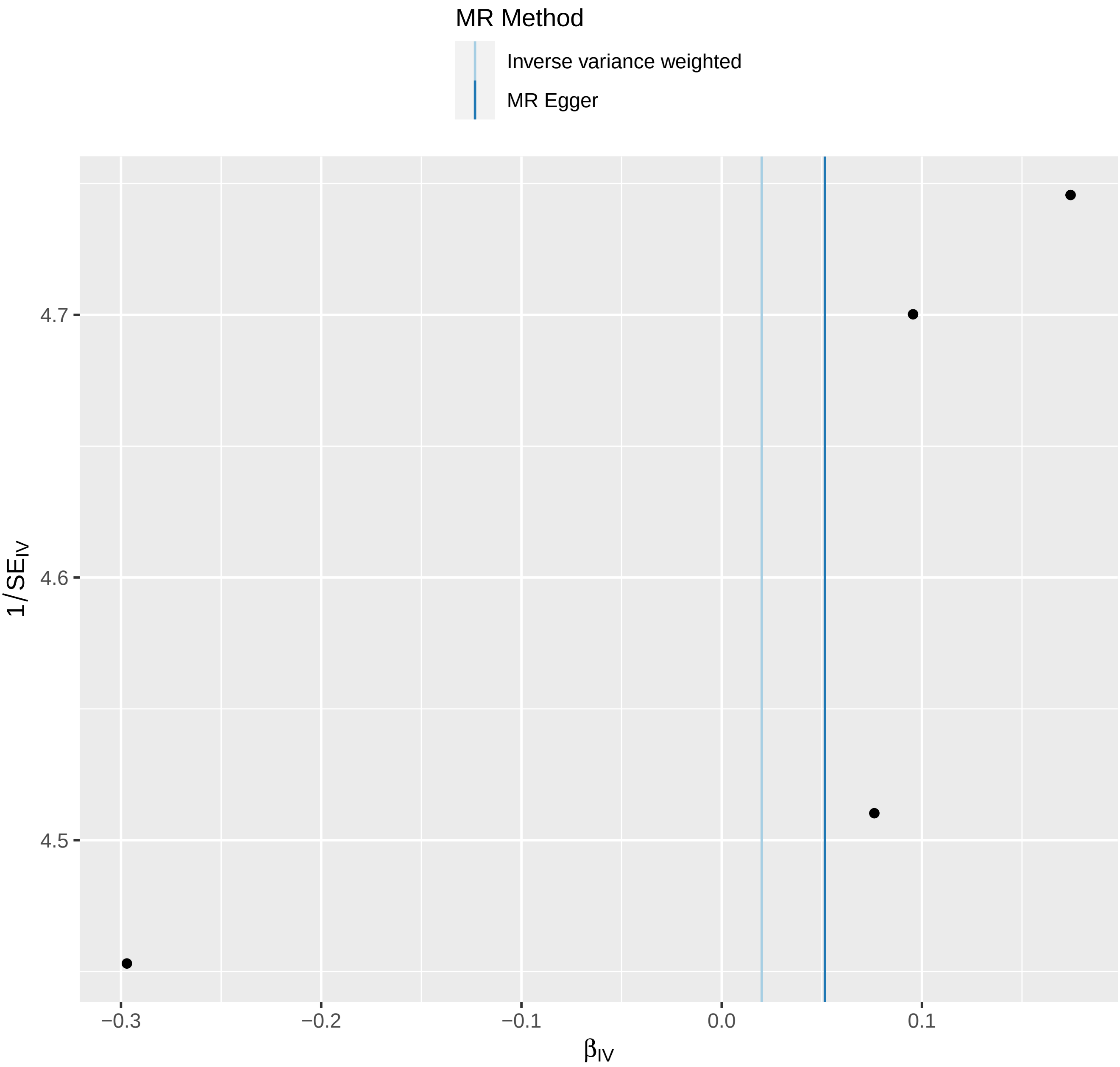

Batch 2−2740 : Gut microbiota abundance (genus Parasutterella id.2892) on 15−hydroxyprostaglandin dehydrogenase [NAD(+)] with a threshold of 1e−05

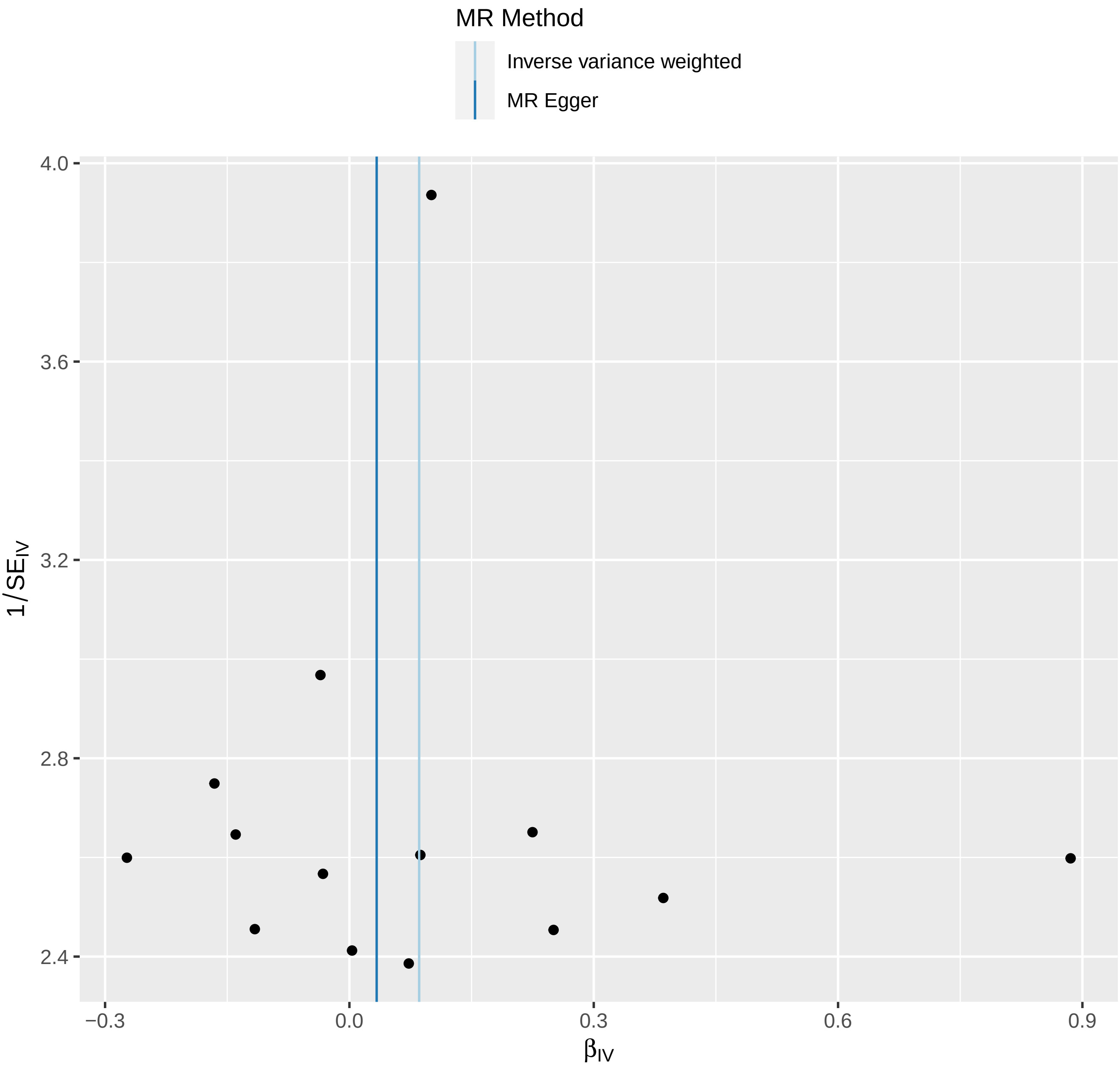

Batch 2−2741 : Gut microbiota abundance (genus Catenibacterium id.2153) on Hematopoietic prostaglandin D synthase
with a threshold of 1e−05

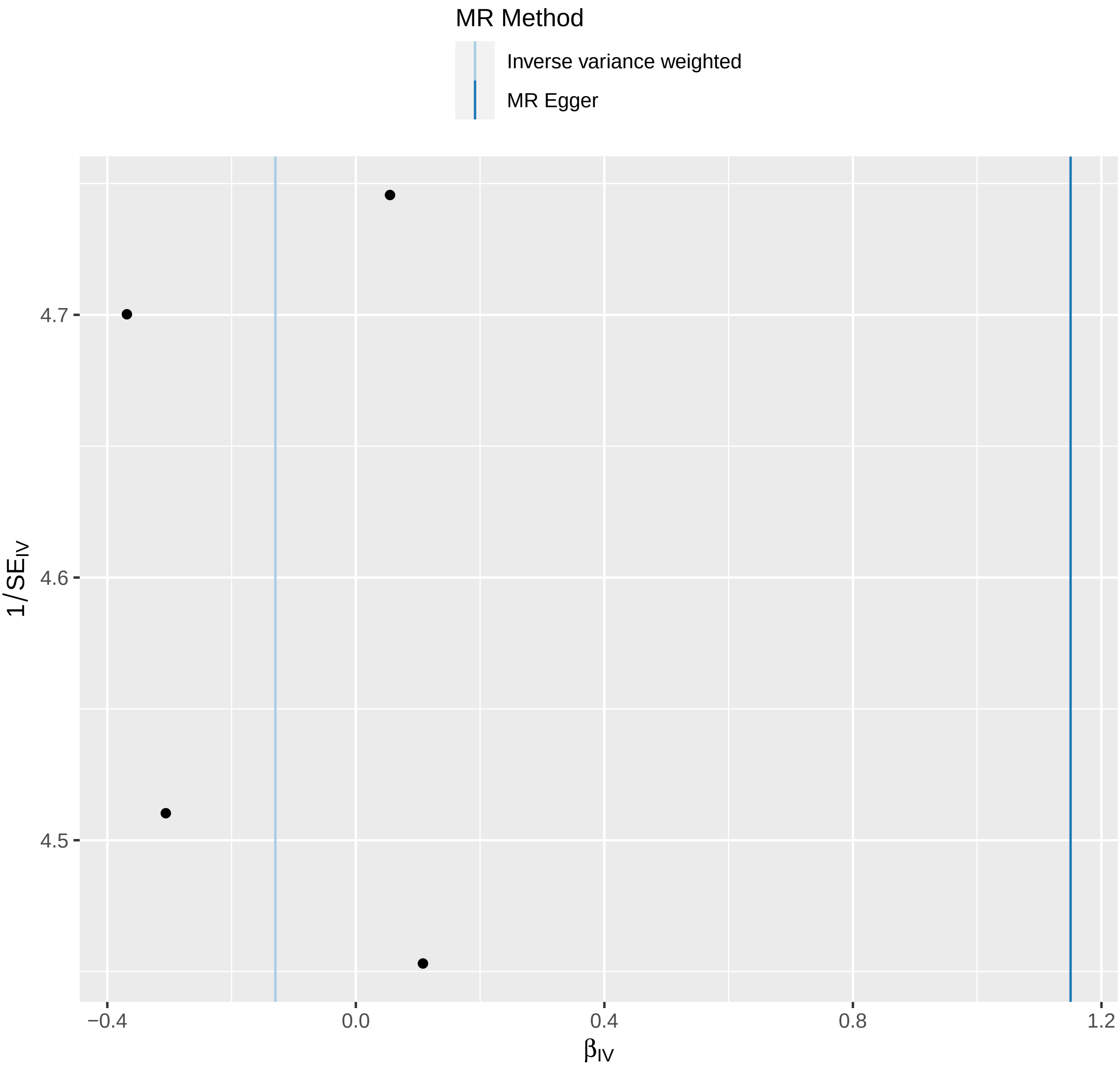

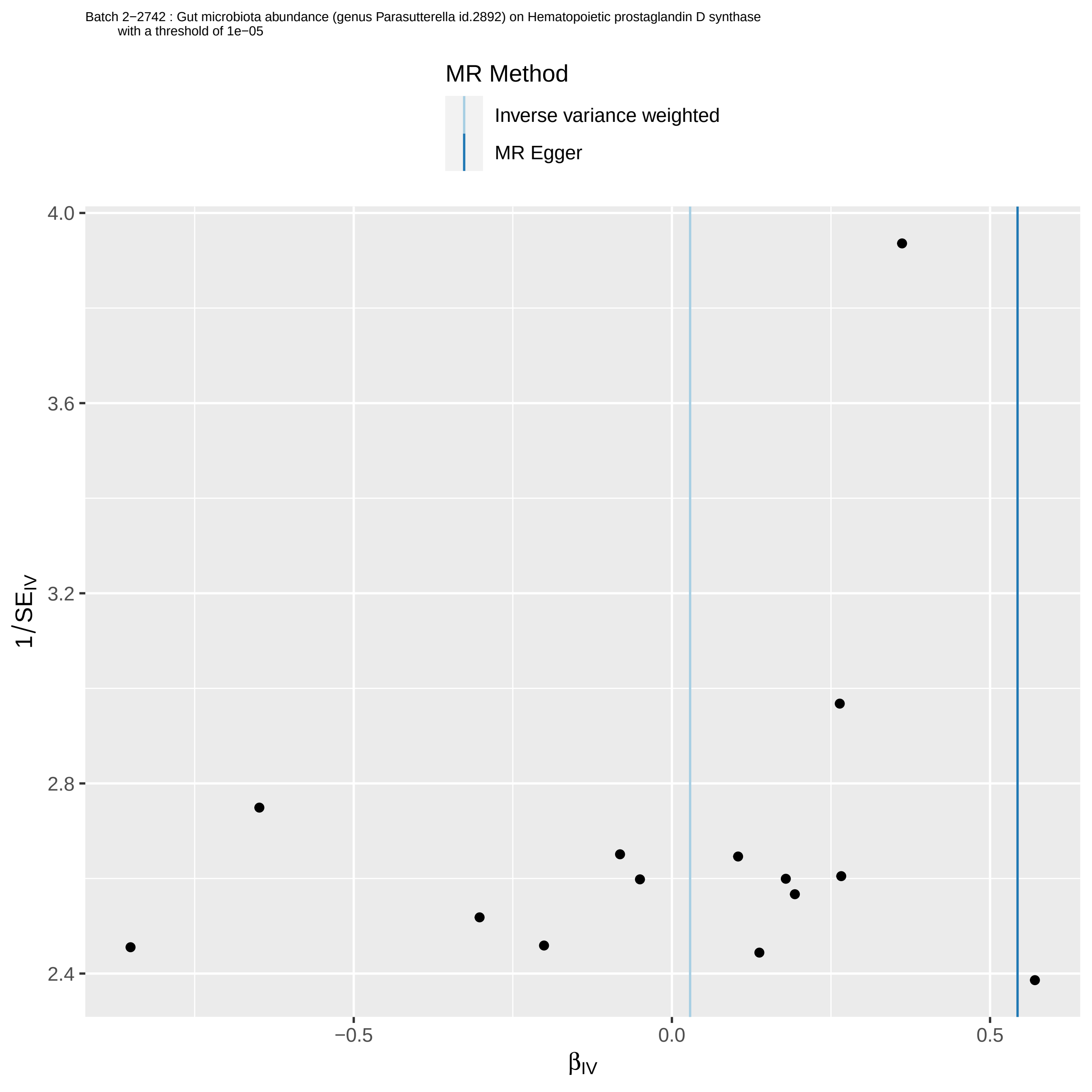

Batch 2−2742 : Gut microbiota abundance (genus Parasutterella id.2892) on Hematopoietic prostaglandin D synthase
with a threshold of 1e−05
MR Method
Inverse variance weighted
MR Egger
4.0
3.6
3.2
2.8
2.4
1/SE_IV
−0.5
0.0
0.5
β_IV

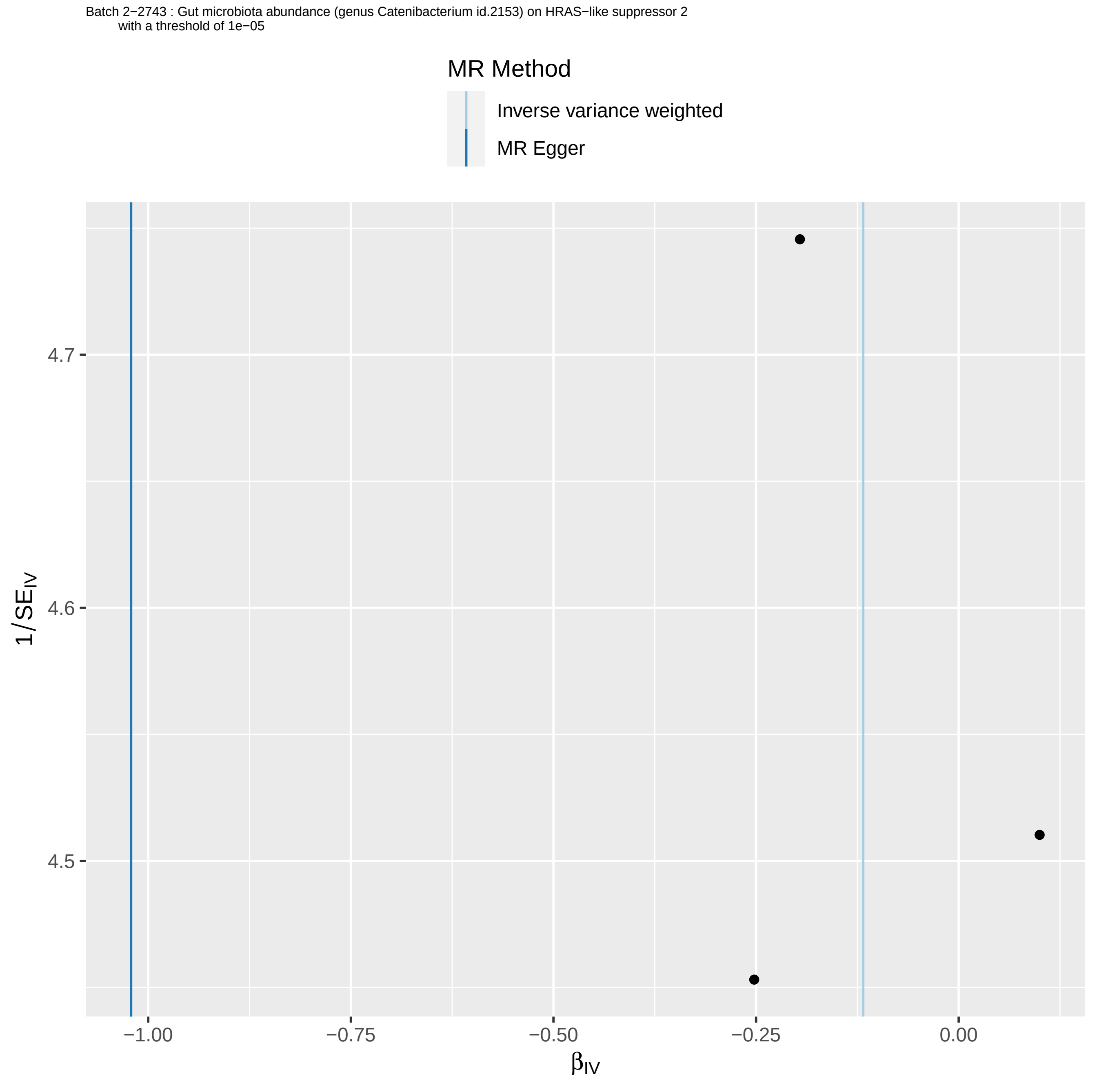

Batch 2−2743 : Gut microbiota abundance (genus Catenibacterium id.2153) on HRAS−like suppressor 2
with a threshold of 1e−05
MR Method
Inverse variance weighted
MR Egger
4.7
4.6
4.5
1/SE_IV
−1.00
−0.75
−0.50
−0.25
0.00
β_IV

Batch 2−2744 : Gut microbiota abundance (genus Parasutterella id.2892) on HRAS−like suppressor 2
with a threshold of 1e−05

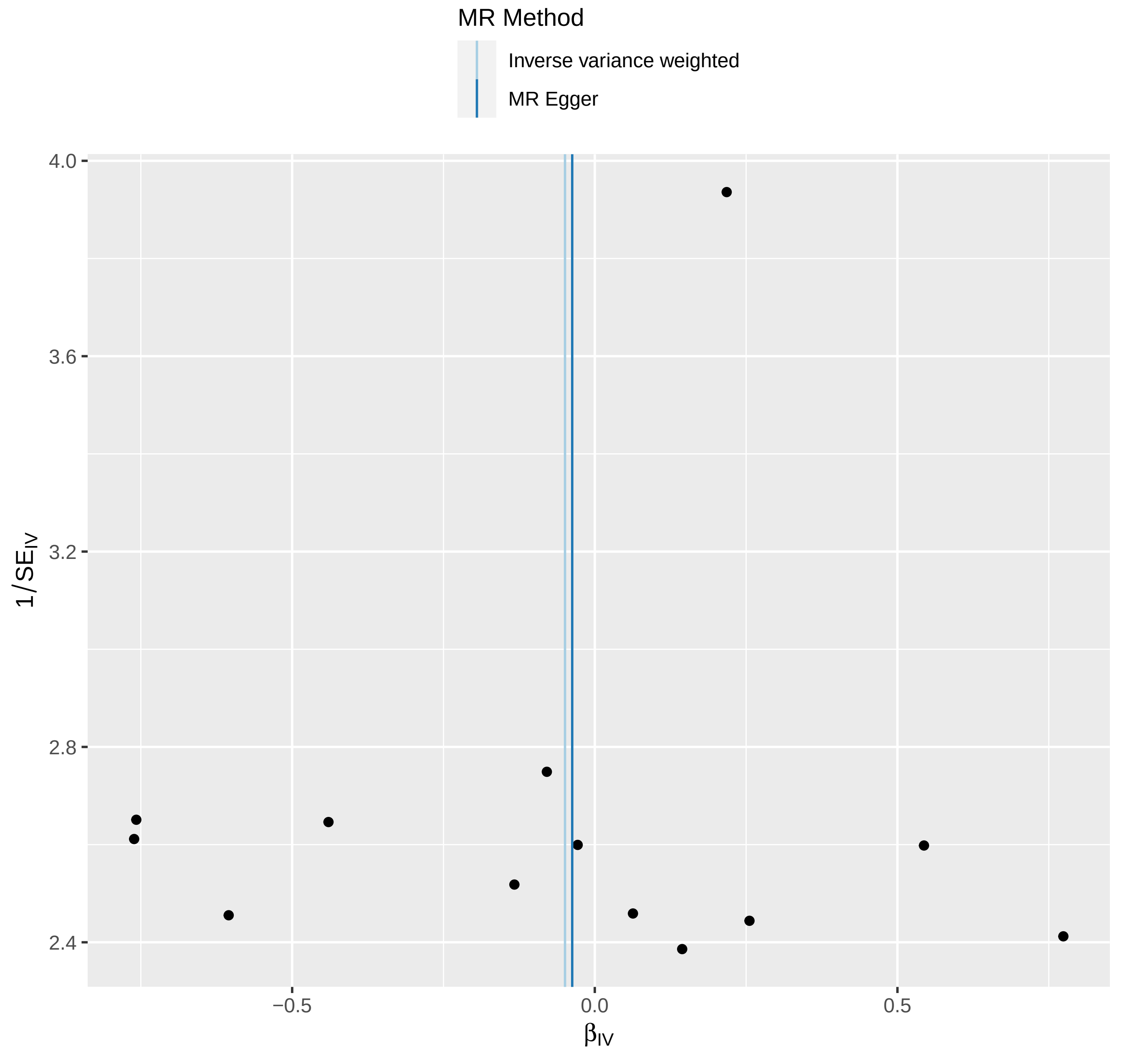

Batch 2−2745 : Gut microbiota abundance (genus Catenibacterium id.2153) on Activator of apoptosis harakiri
with a threshold of 1e−05

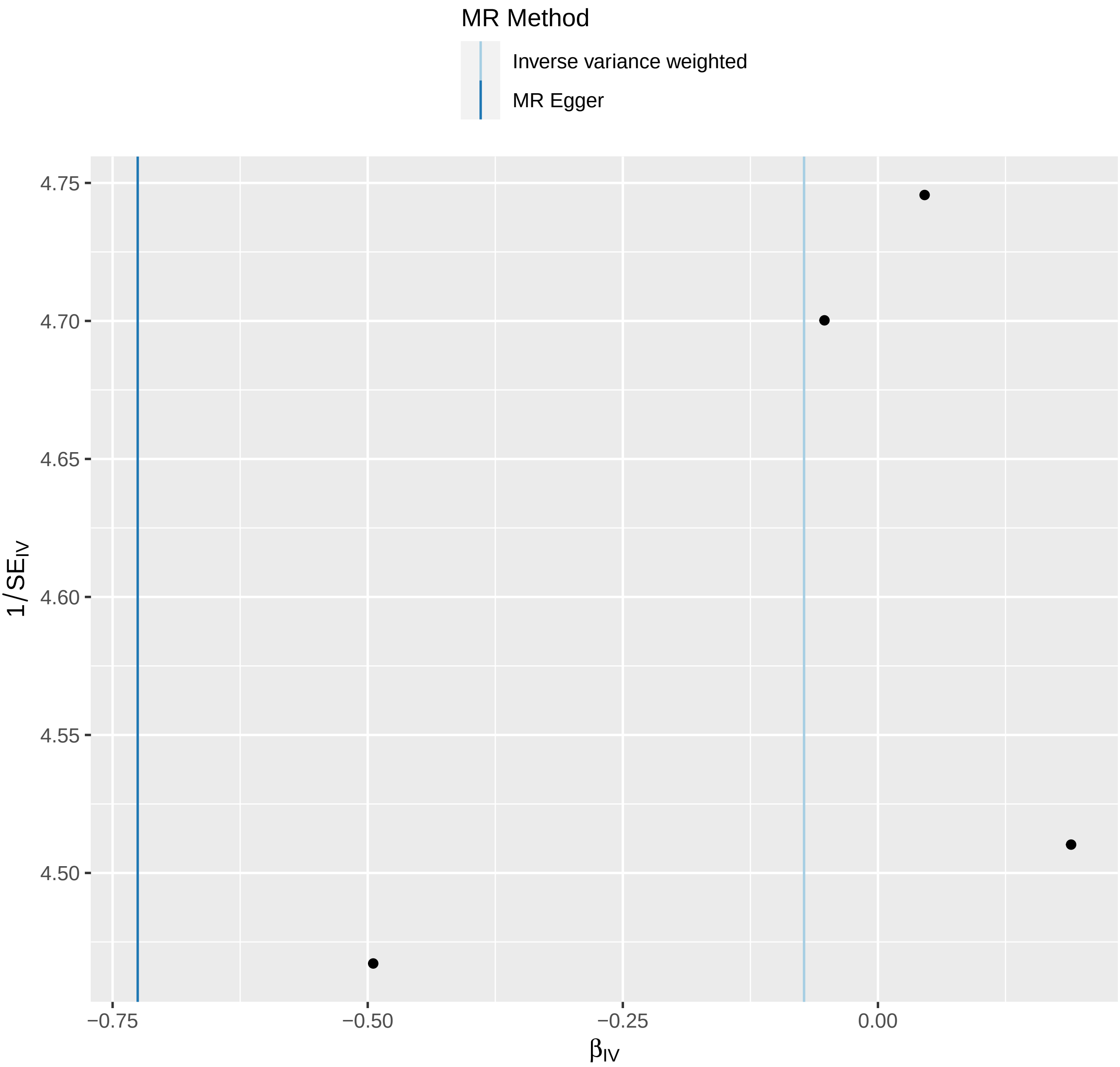

Batch 2−2746 : Gut microbiota abundance (genus Parasutterella id.2892) on Activator of apoptosis harakiri
with a threshold of 1e−05

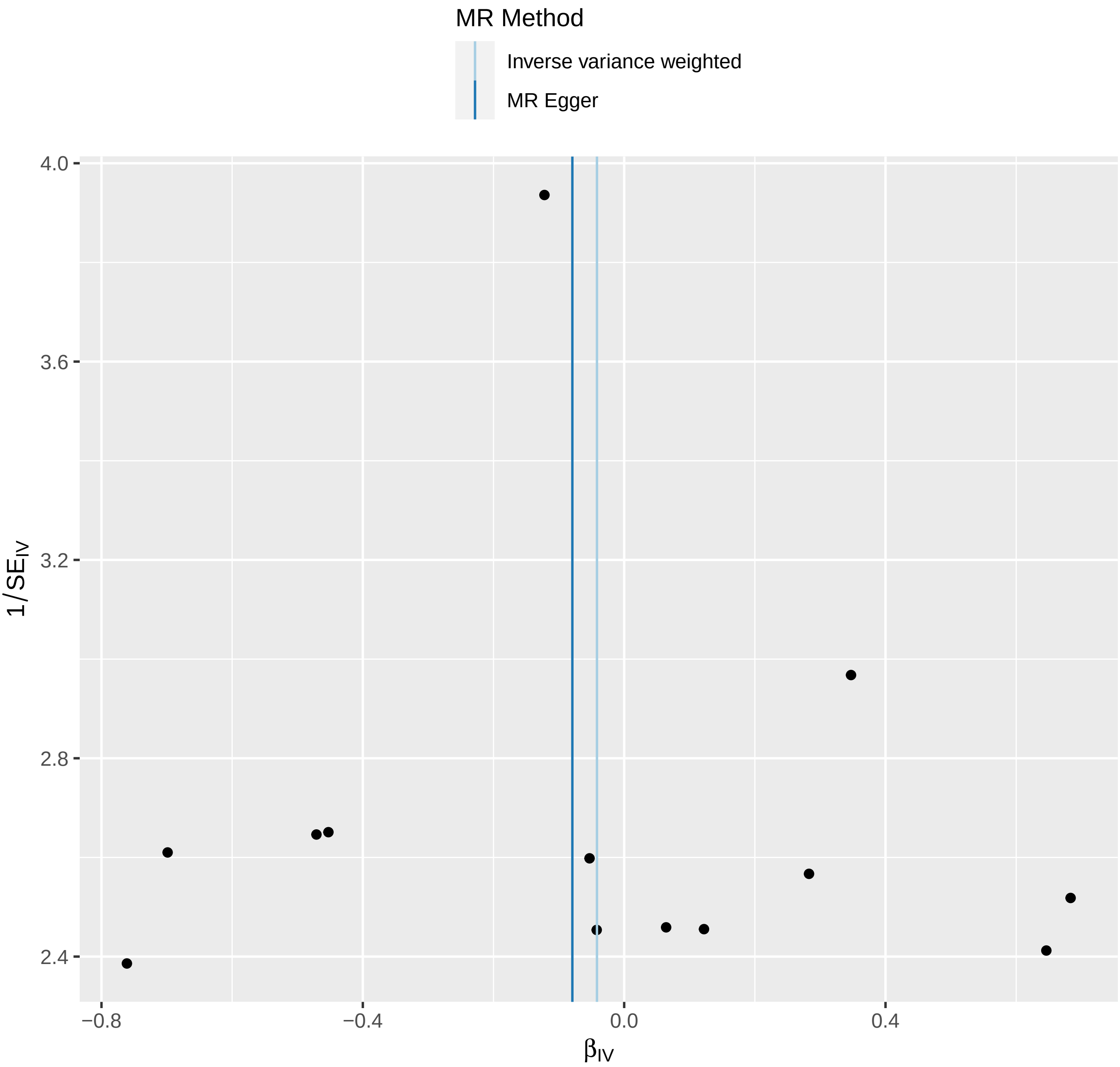

Batch 2−2747 : Gut microbiota abundance (genus Catenibacterium id.2153) on Ribonuclease UK114
with a threshold of 1e−05

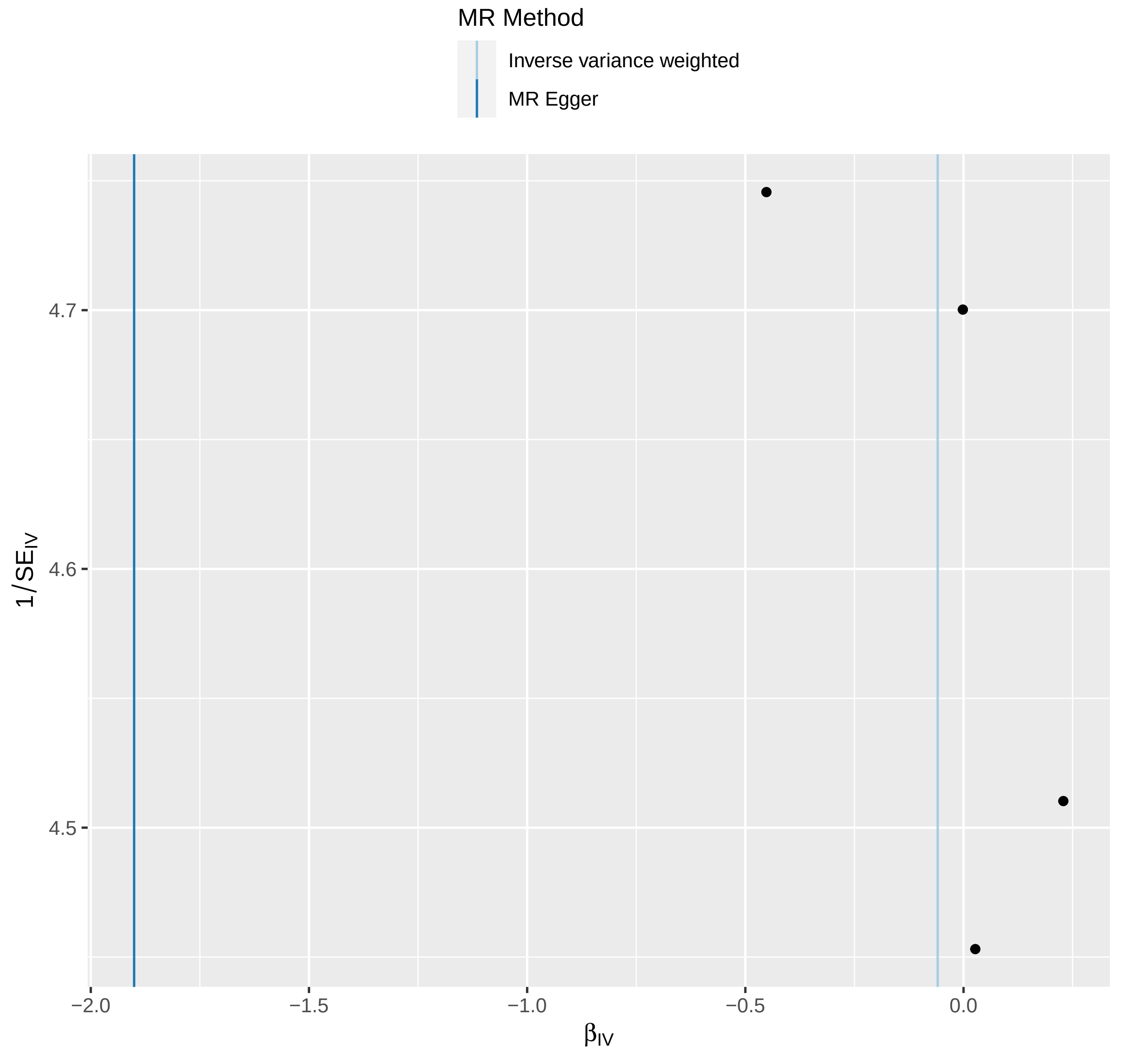

Batch 2−2748 : Gut microbiota abundance (genus Parasutterella id.2892) on Ribonuclease UK114
with a threshold of 1e−05

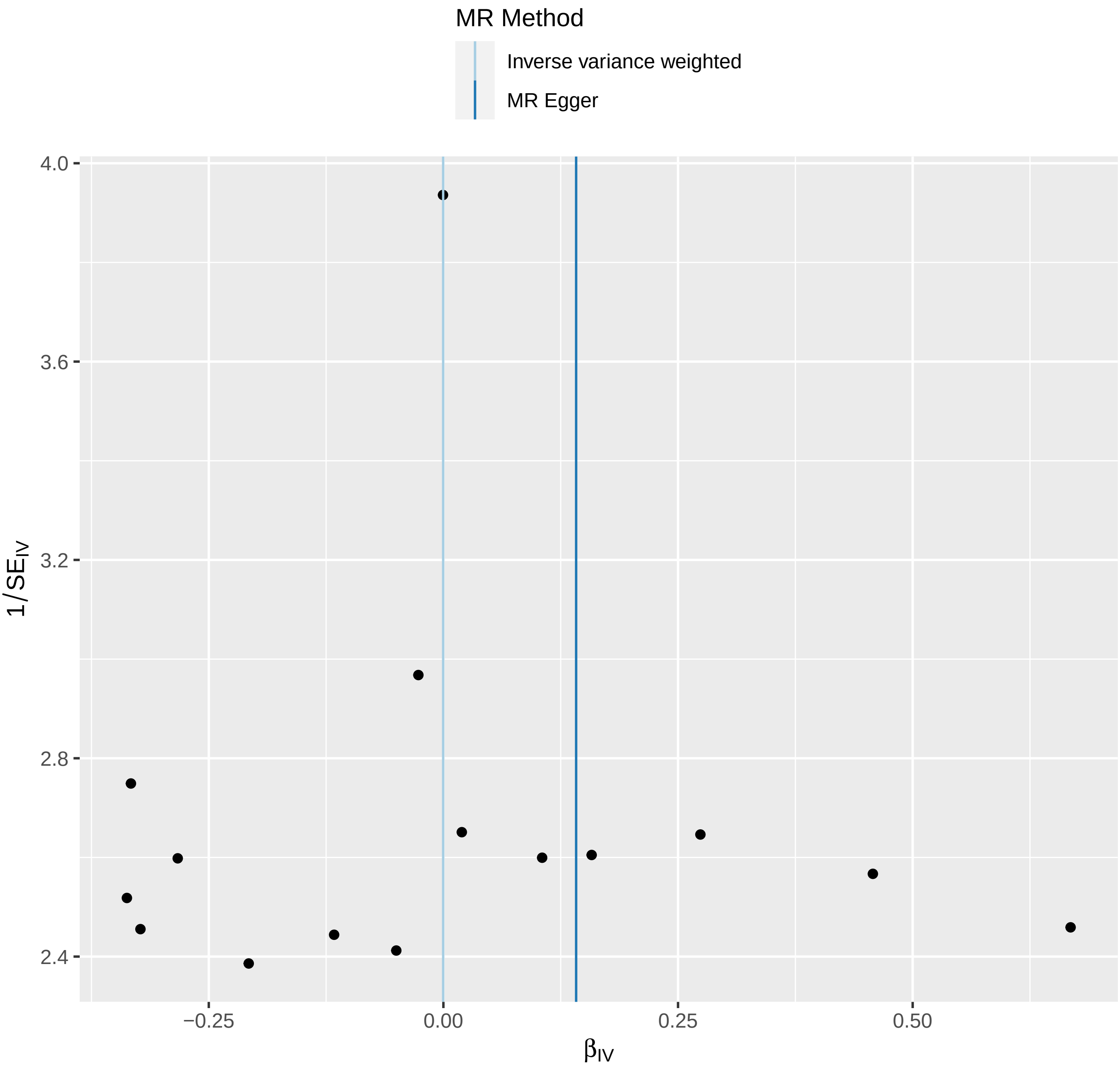

Batch 2−2749 : Gut microbiota abundance (genus Catenibacterium id.2153) on Heparan sulfate glucosamine 3−O−sulfotransferase 3A1 with a threshold of 1e−05

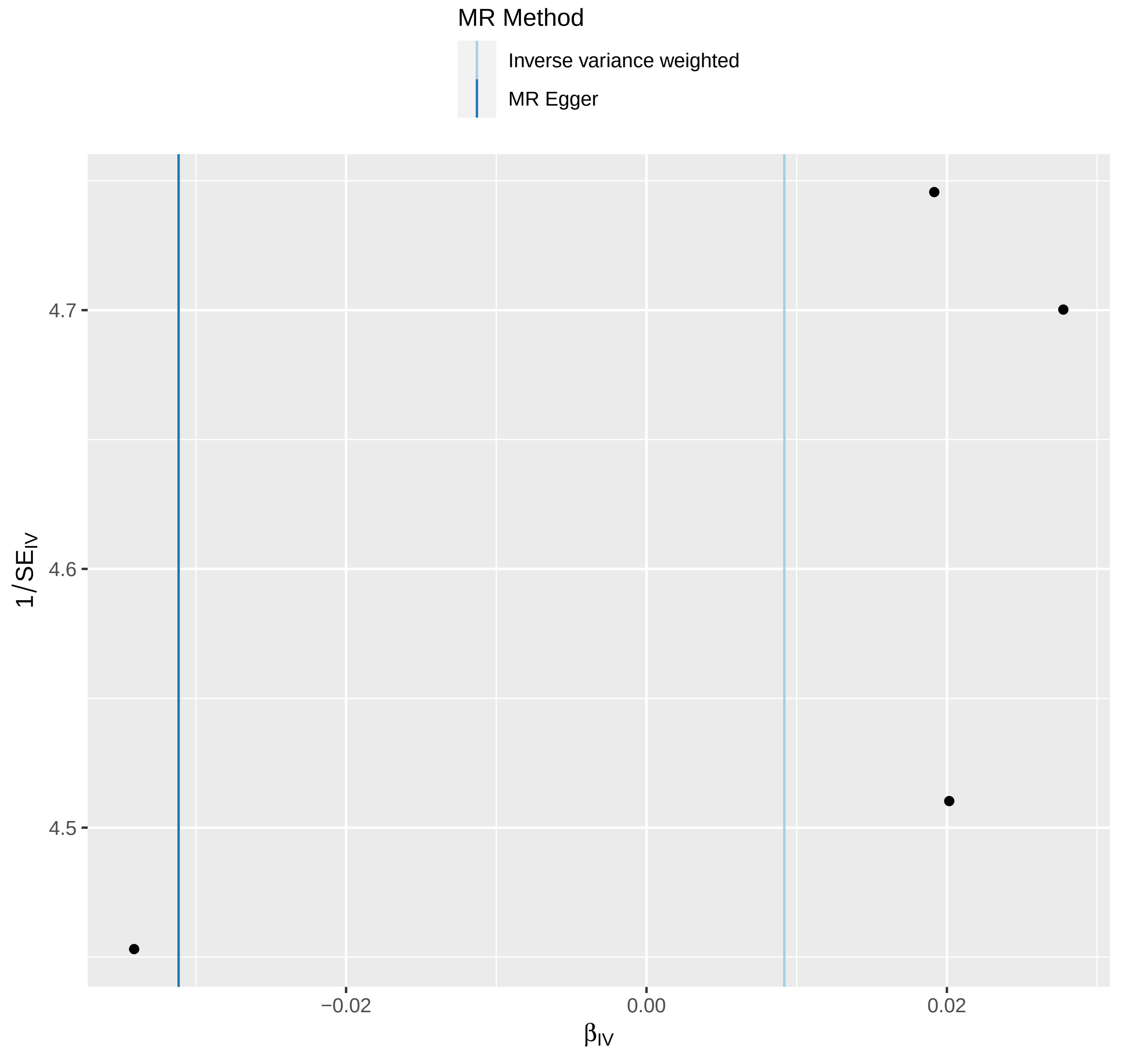

Batch 2−2750 : Gut microbiota abundance (genus Parasutterella id.2892) on Heparan sulfate glucosamine 3−O−sulfotransferase 3A1
with a threshold of 1e−05

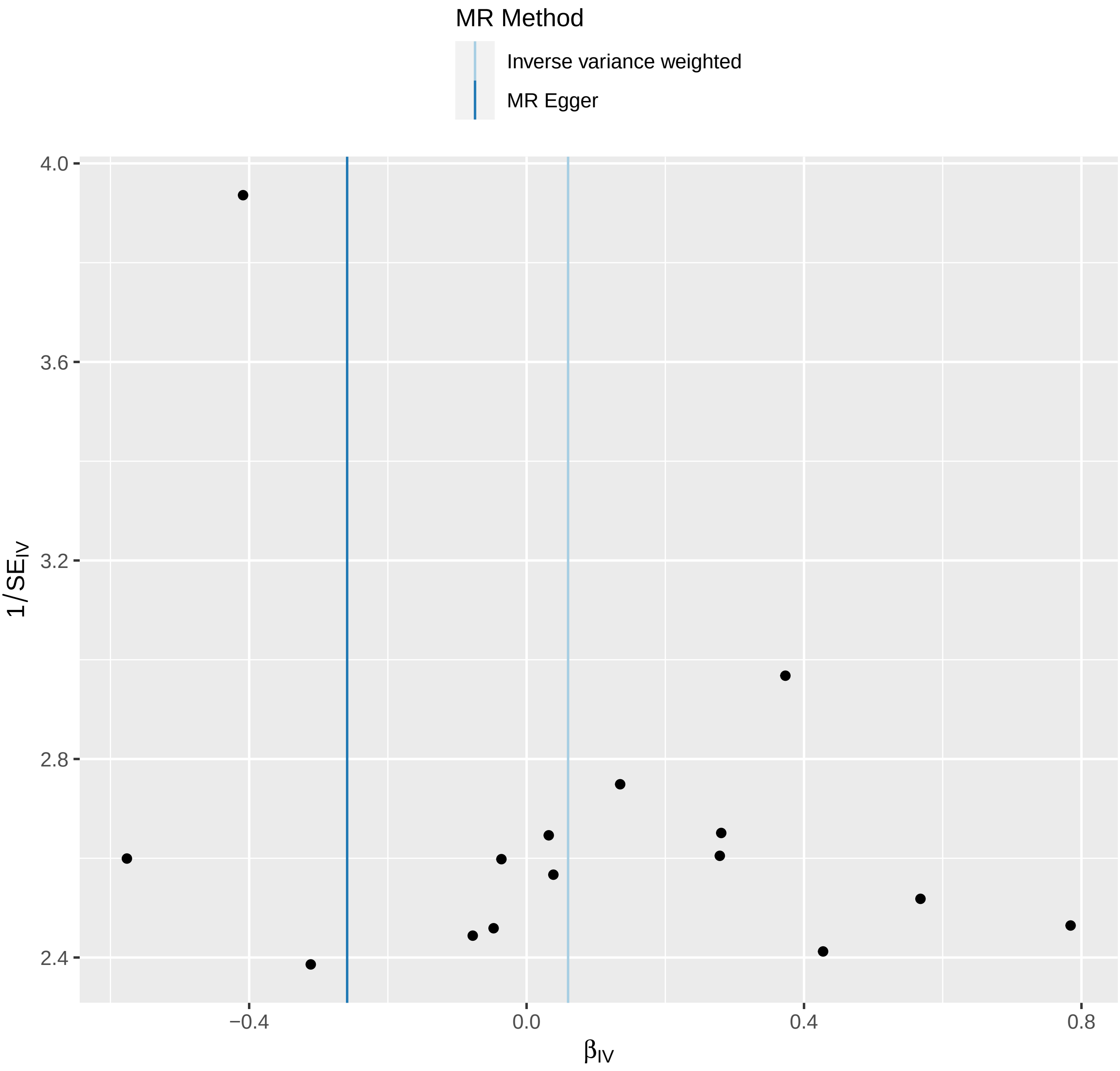

Batch 2−2751 : Gut microbiota abundance (genus Catenibacterium id.2153) on Heparan sulfate glucosamine 3−O−sulfotransferase 3B1 with a threshold of 1e−05

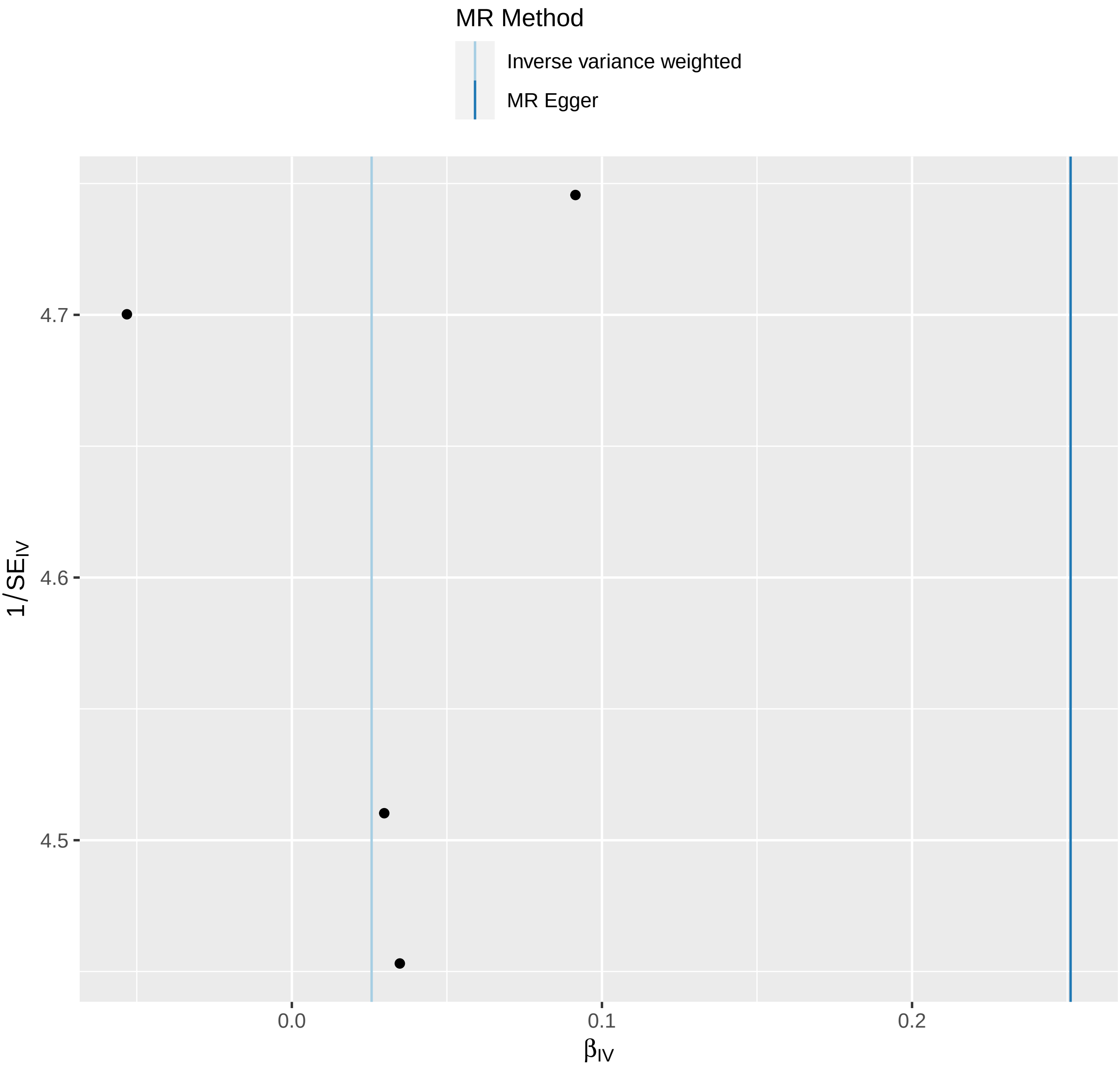

Batch 2−2752 : Gut microbiota abundance (genus Parasutterella id.2892) on Heparan sulfate glucosamine 3−O−sulfotransferase 3B1
with a threshold of 1e−05

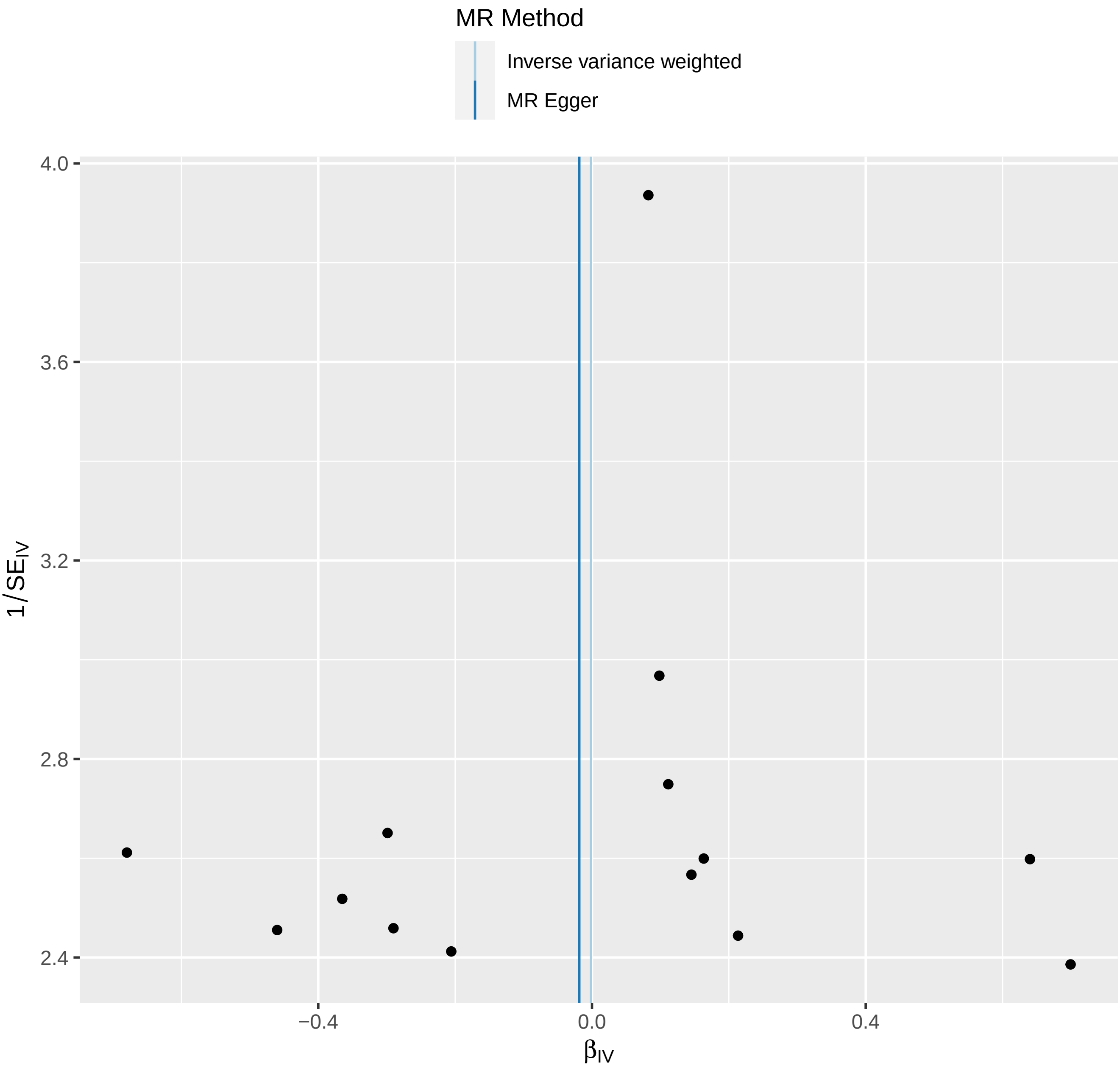

Batch 2−2753 : Gut microbiota abundance (genus Catenibacterium id.2153) on Heparan sulfate glucosamine 3−O−sulfotransferase 4
with a threshold of 1e−05

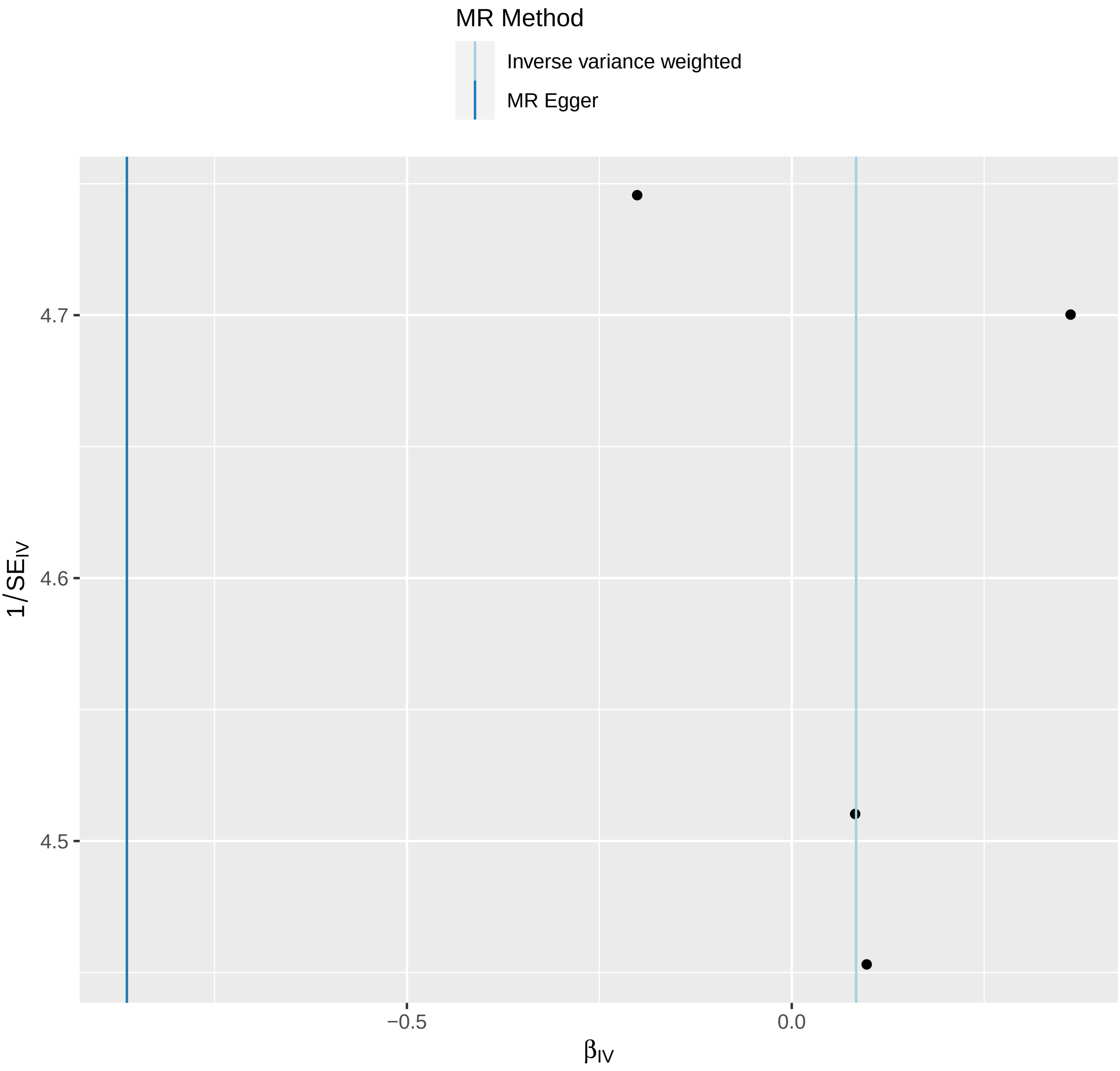

Batch 2−2754 : Gut microbiota abundance (genus Parasutterella id.2892) on Heparan sulfate glucosamine 3−O−sulfotransferase 4 with a threshold of 1e−05

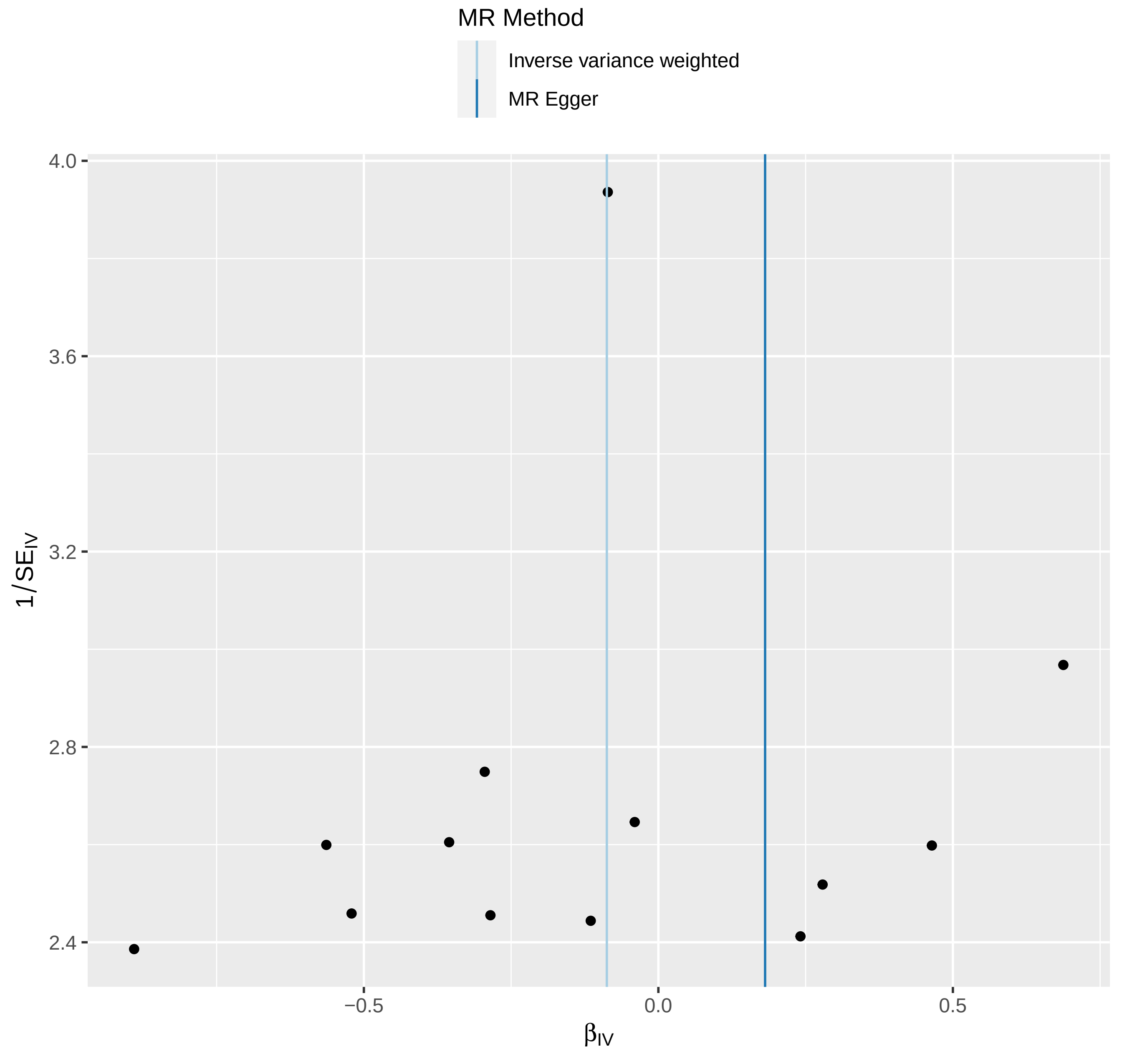

Batch 2−2755 : Gut microbiota abundance (genus Catenibacterium id.2153) on Heparan−sulfate 6−O−sulfotransferase 1
with a threshold of 1e−05

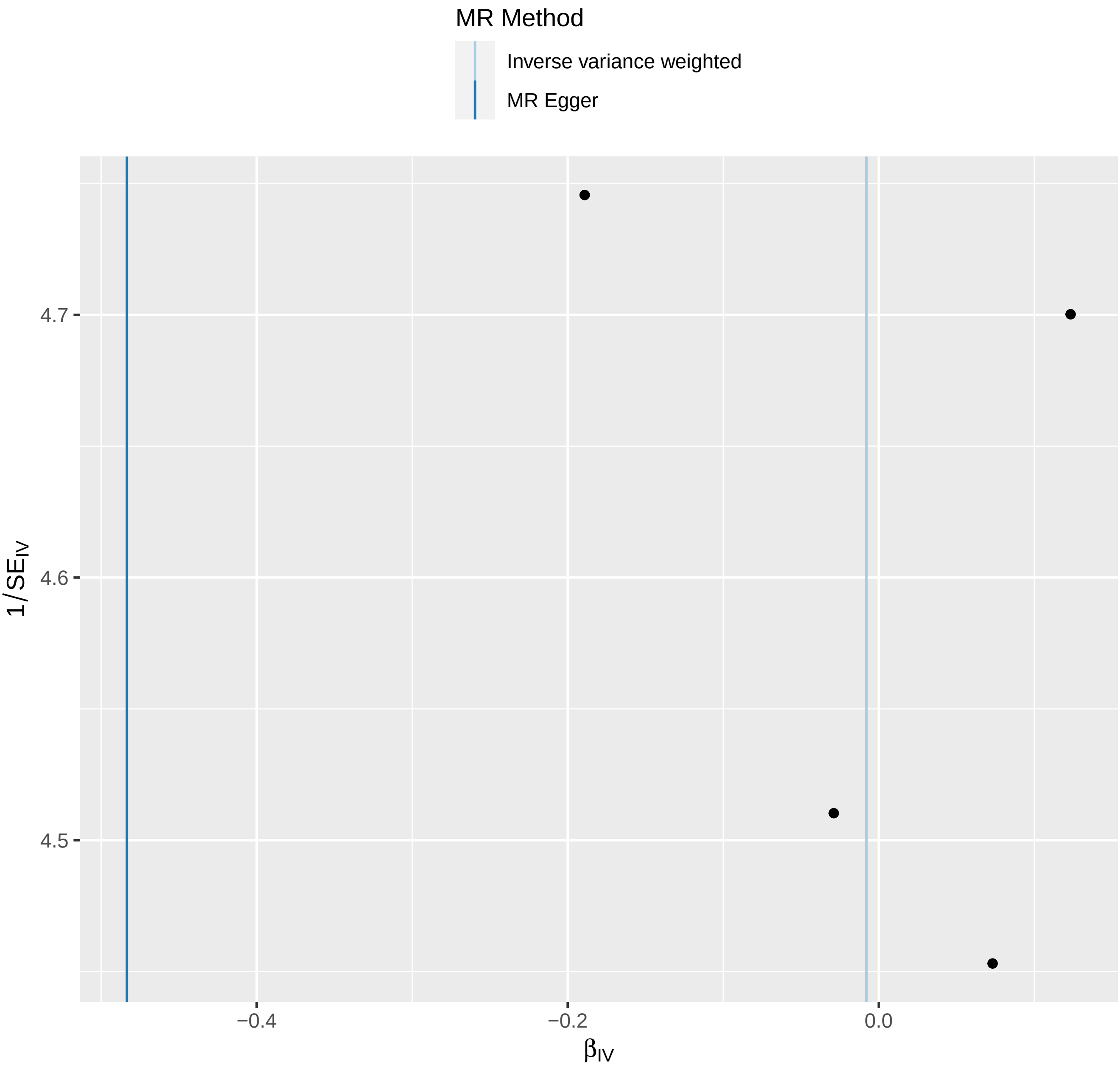

Batch 2−2756 : Gut microbiota abundance (genus Parasutterella id.2892) on Heparan−sulfate 6−O−sulfotransferase 1
with a threshold of 1e−05

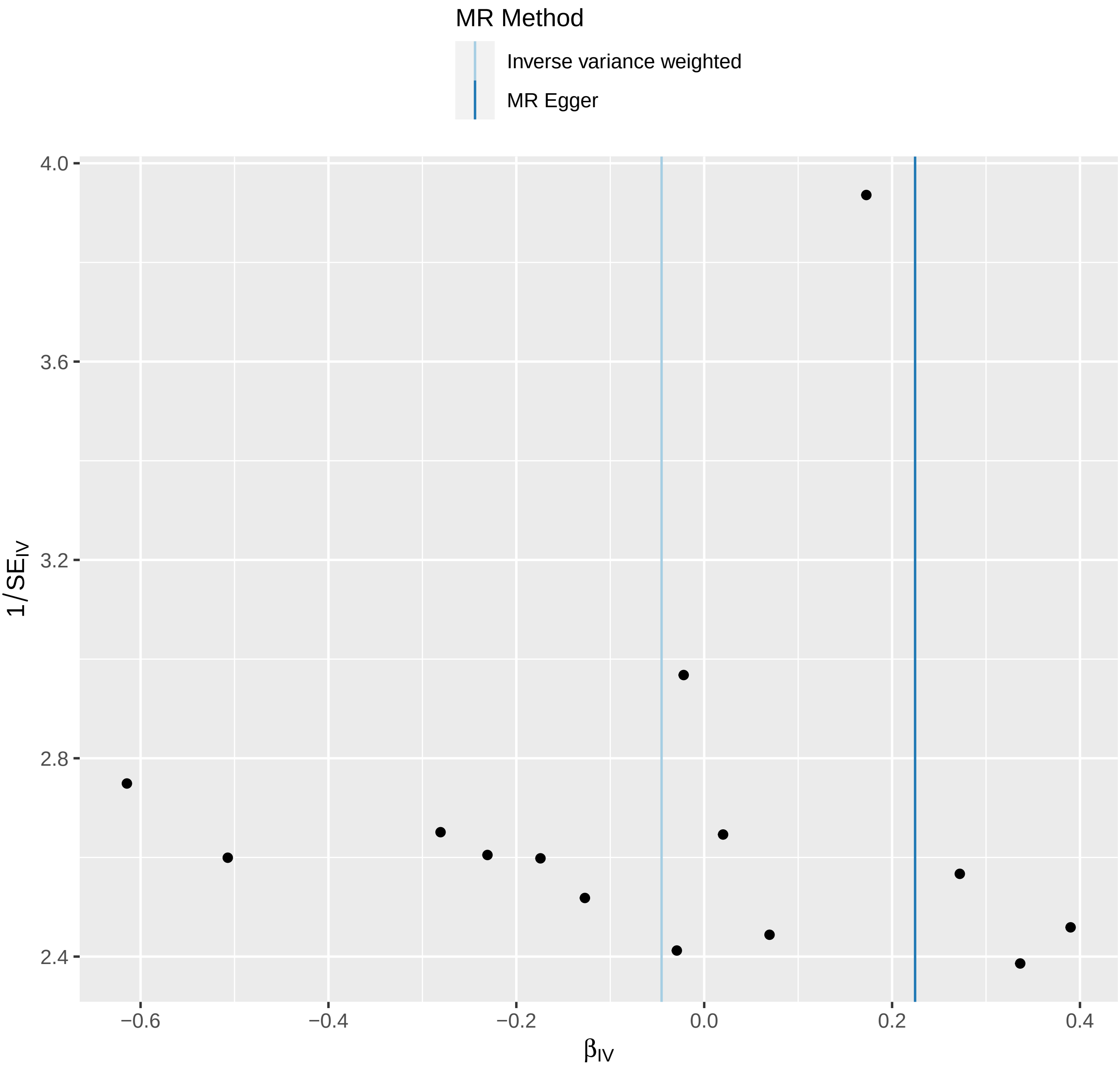

Batch 2−2757 : Gut microbiota abundance (genus Catenibacterium id.2153) on Heparan−sulfate 6−O−sulfotransferase 2
with a threshold of 1e−05

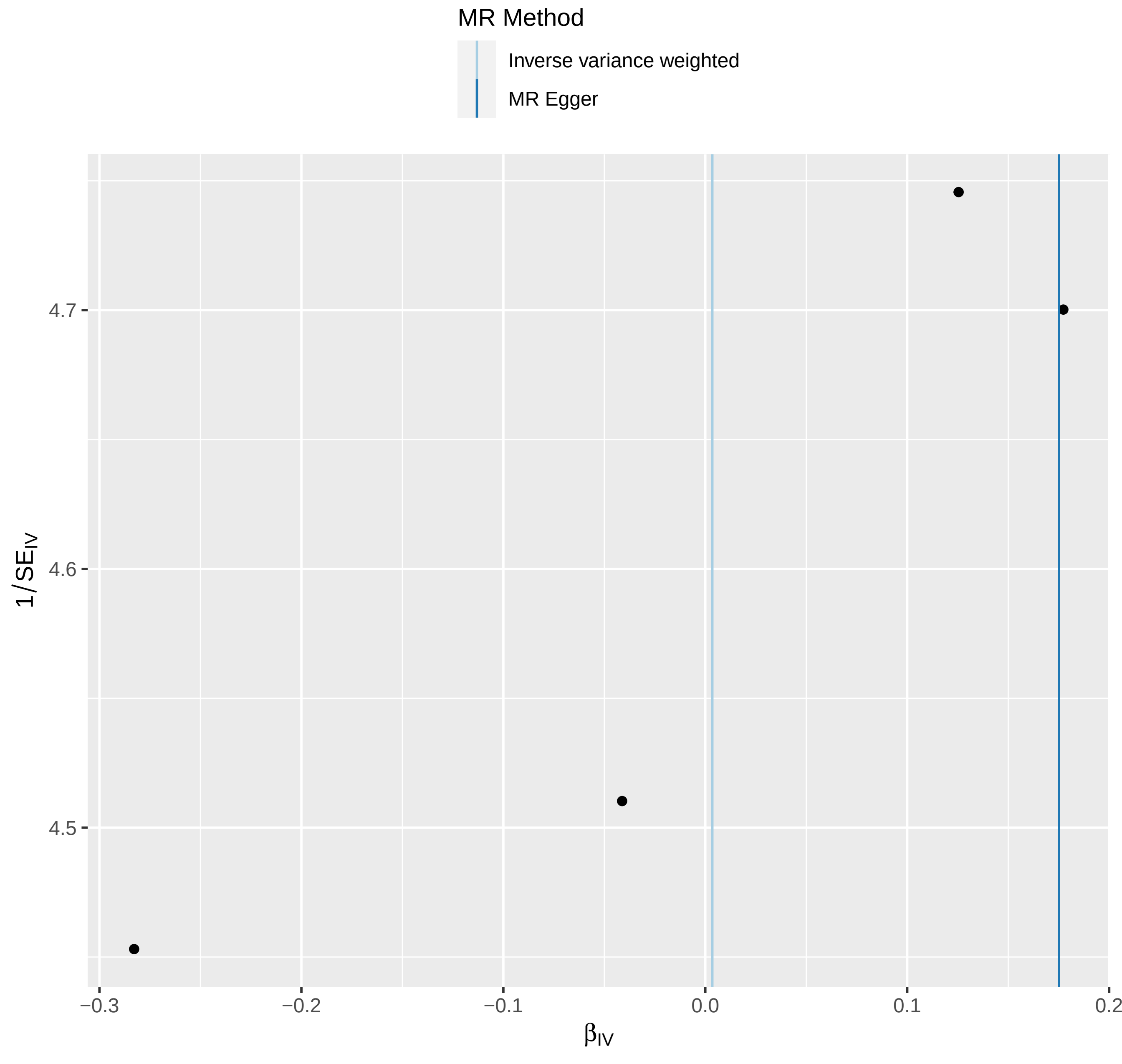

Batch 2−2758 : Gut microbiota abundance (genus Parasutterella id.2892) on Heparan−sulfate 6−O−sulfotransferase 2
with a threshold of 1e−05

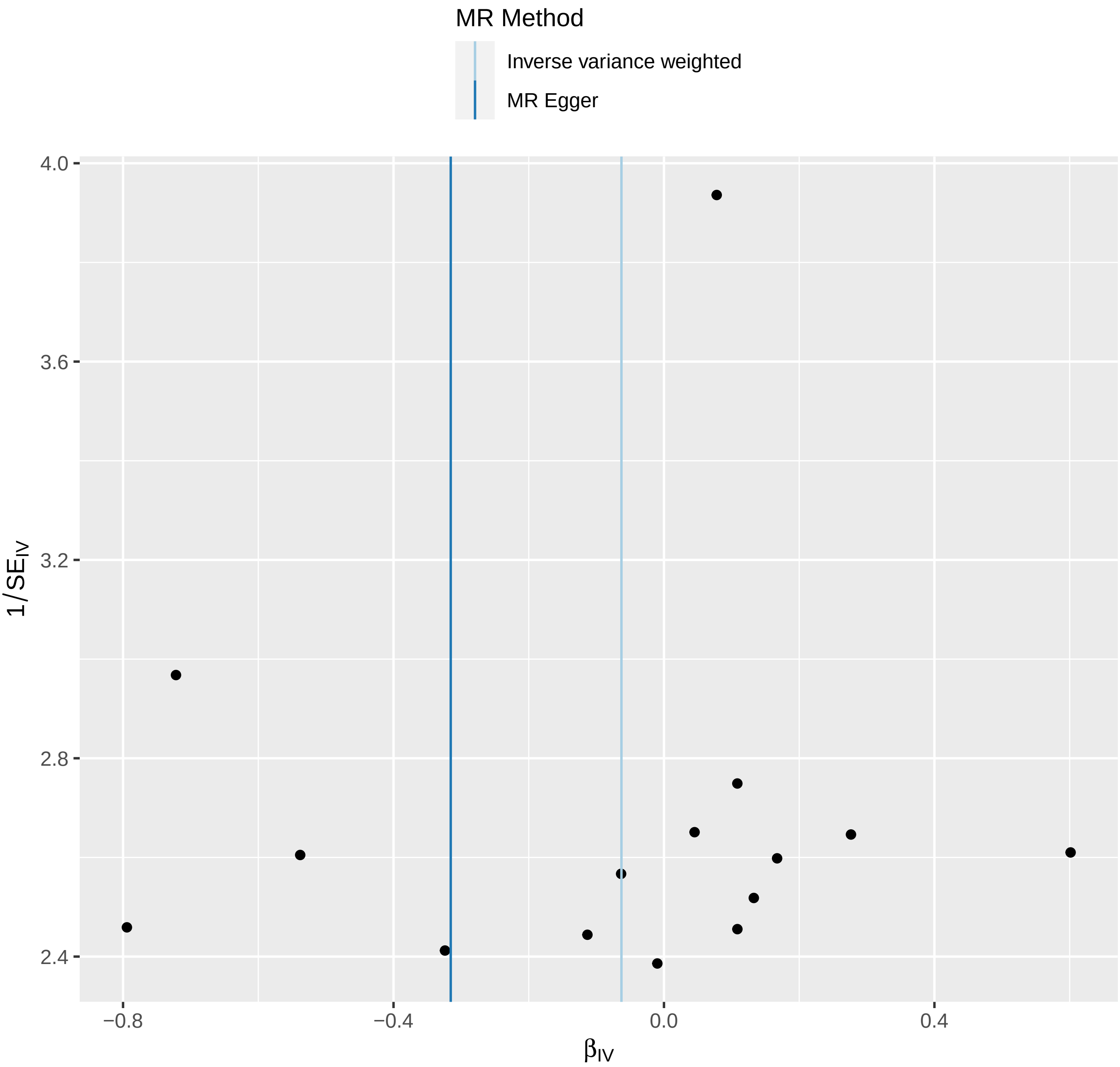

Batch 2−2759 : Gut microbiota abundance (genus Catenibacterium id.2153) on 3−hydroxyacyl−CoA dehydrogenase type−2
with a threshold of 1e−05

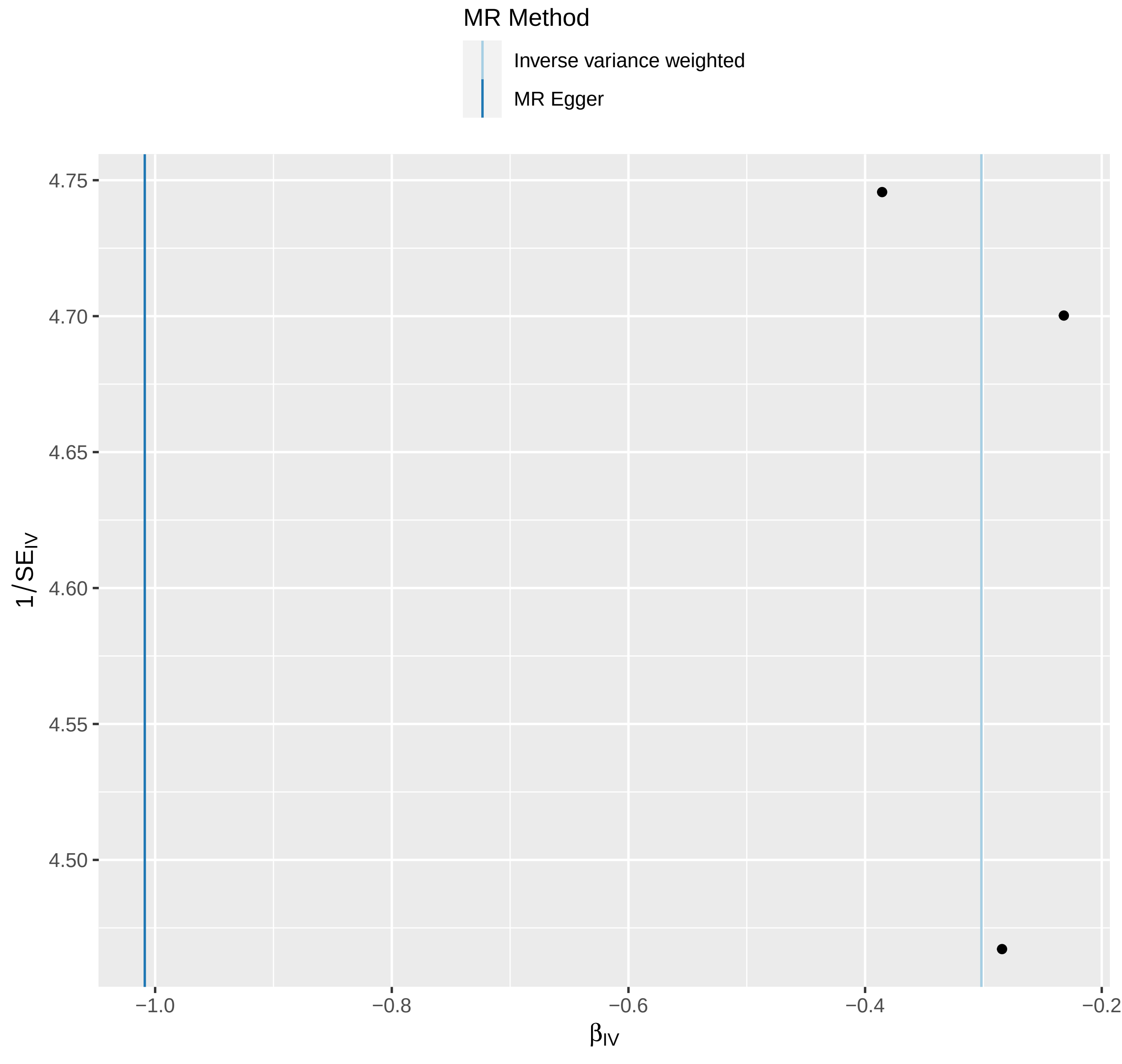

Batch 2−2760 : Gut microbiota abundance (genus Parasutterella id.2892) on 3−hydroxyacyl−CoA dehydrogenase type−2
with a threshold of 1e−05

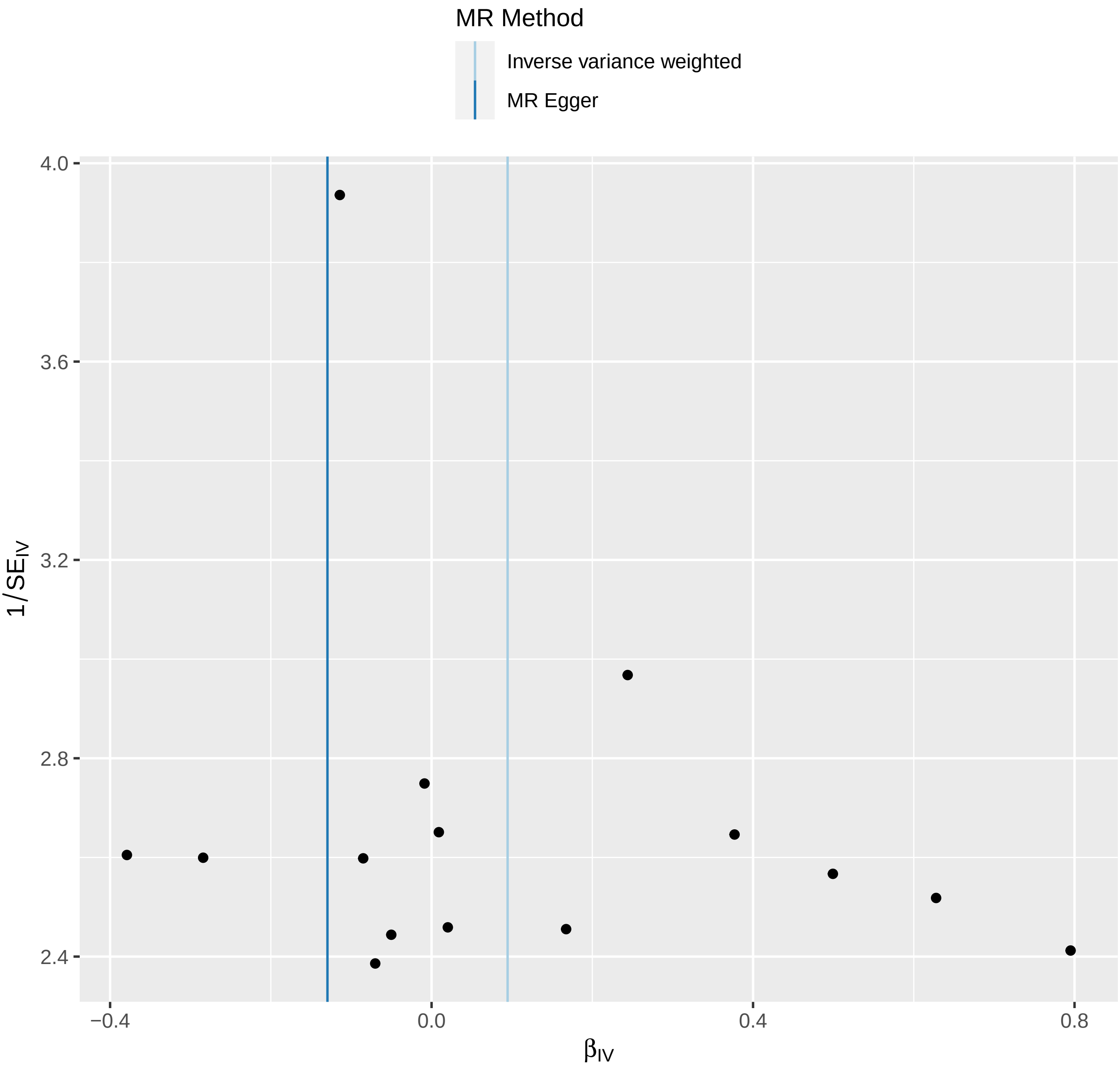

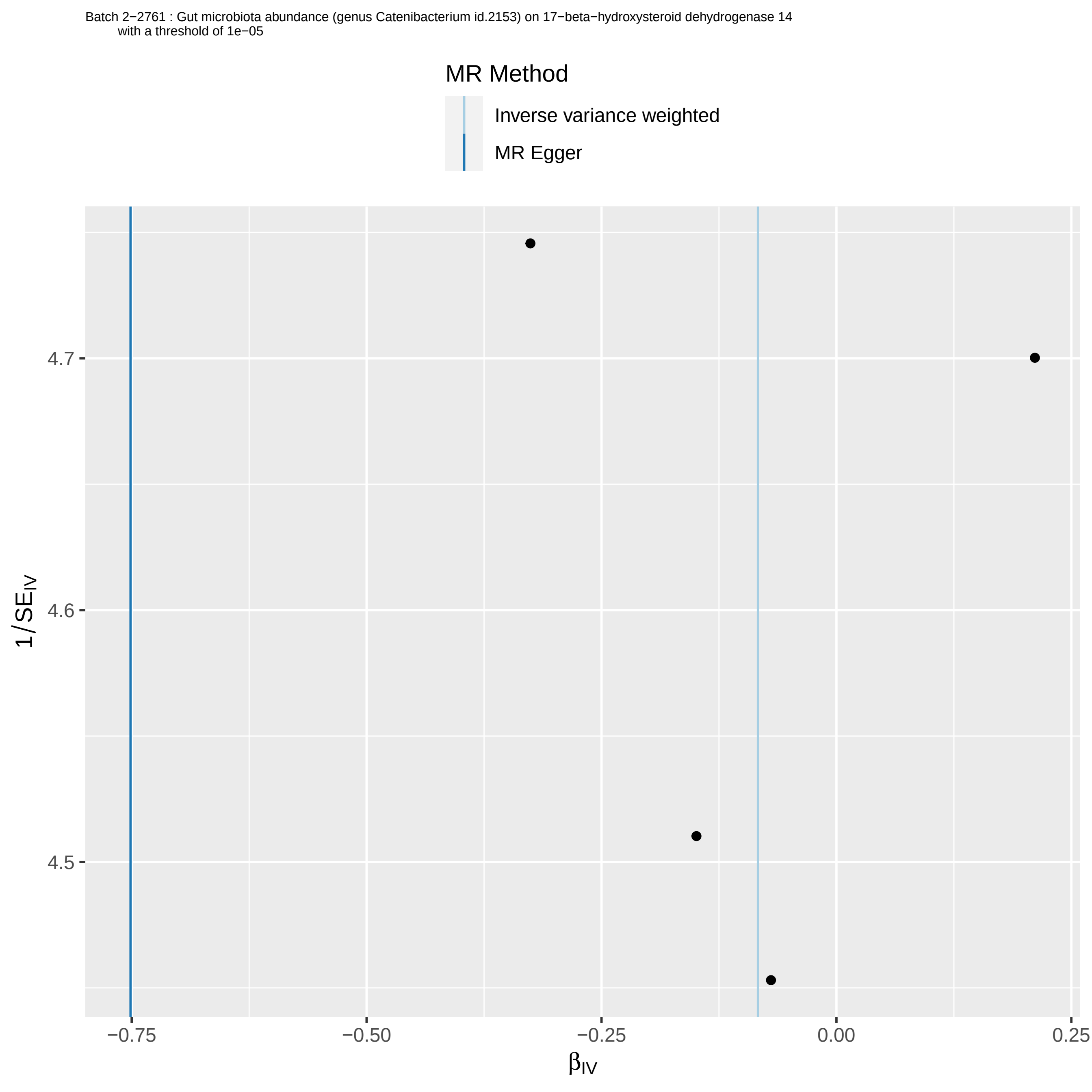

Batch 2−2761 : Gut microbiota abundance (genus Catenibacterium id.2153) on 17−beta−hydroxysteroid dehydrogenase 14
with a threshold of 1e−05
MR Method
Inverse variance weighted
MR Egger
4.7
4.6
4.5
1/SE_IV
−0.75
−0.50
−0.25
0.00
0.25
β_IV

Batch 2−2762 : Gut microbiota abundance (genus Parasutterella id.2892) on 17−beta−hydroxysteroid dehydrogenase 14
with a threshold of 1e−05

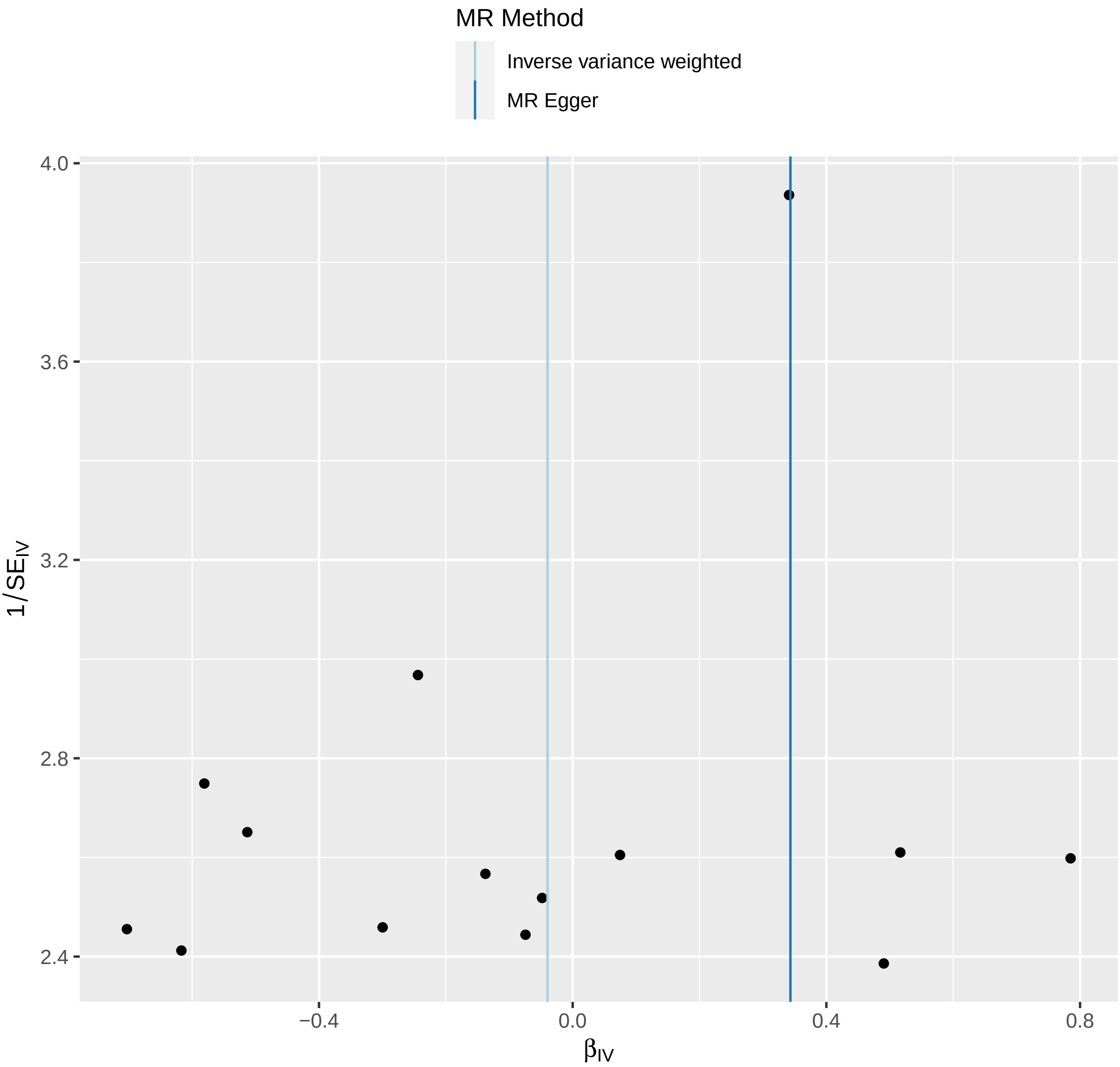

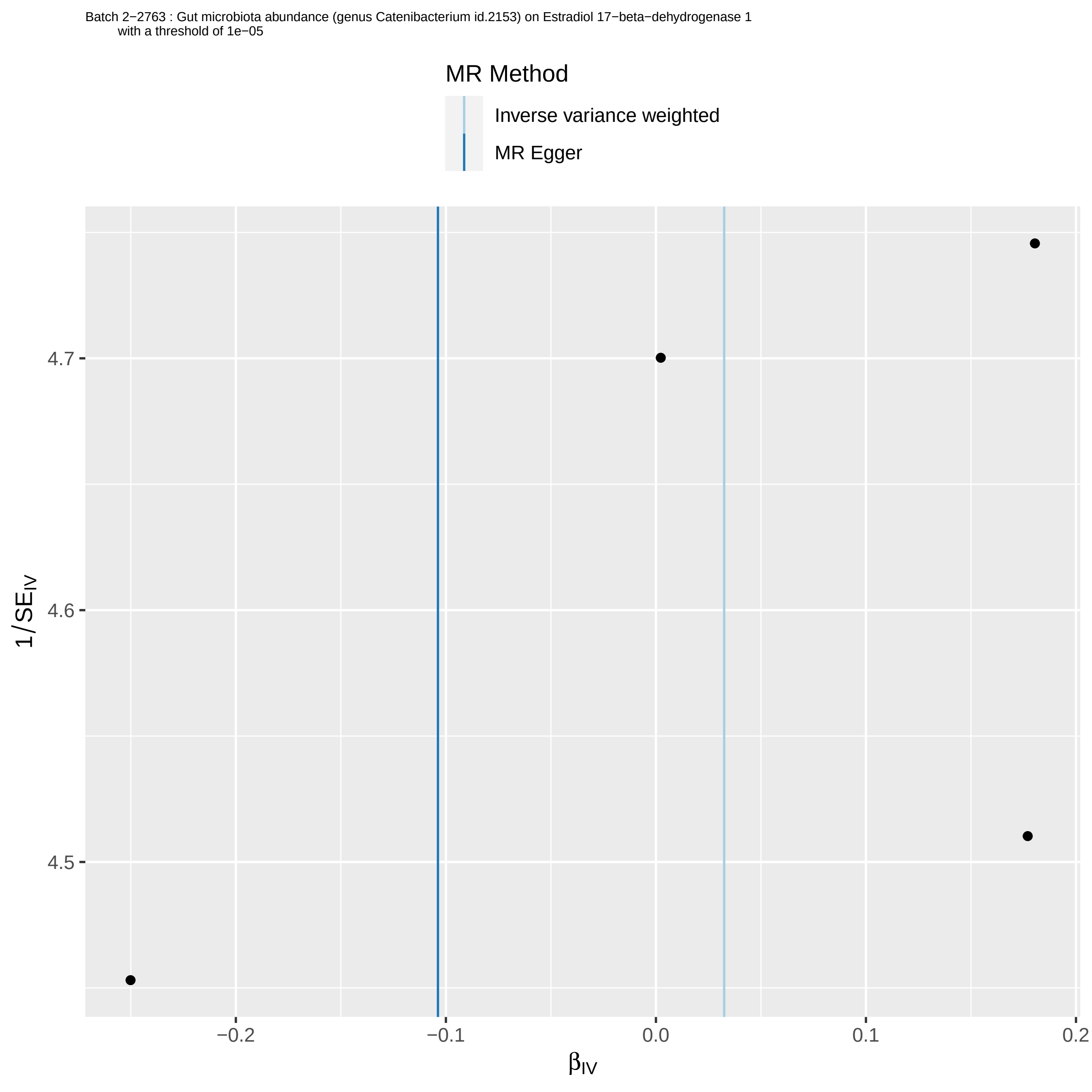
Batch 2−2763 : Gut microbiota abundance (genus Catenibacterium id.2153) on Estradiol 17−beta−dehydrogenase 1
with a threshold of 1e−05
MR Method
Inverse variance weighted
MR Egger
4.7
4.6
4.5
1/SE_IV
−0.2
−0.1
0.0
0.1
0.2
β_IV

Batch 2−2764 : Gut microbiota abundance (genus Parasutterella id.2892) on Estradiol 17−beta−dehydrogenase 1
with a threshold of 1e−05

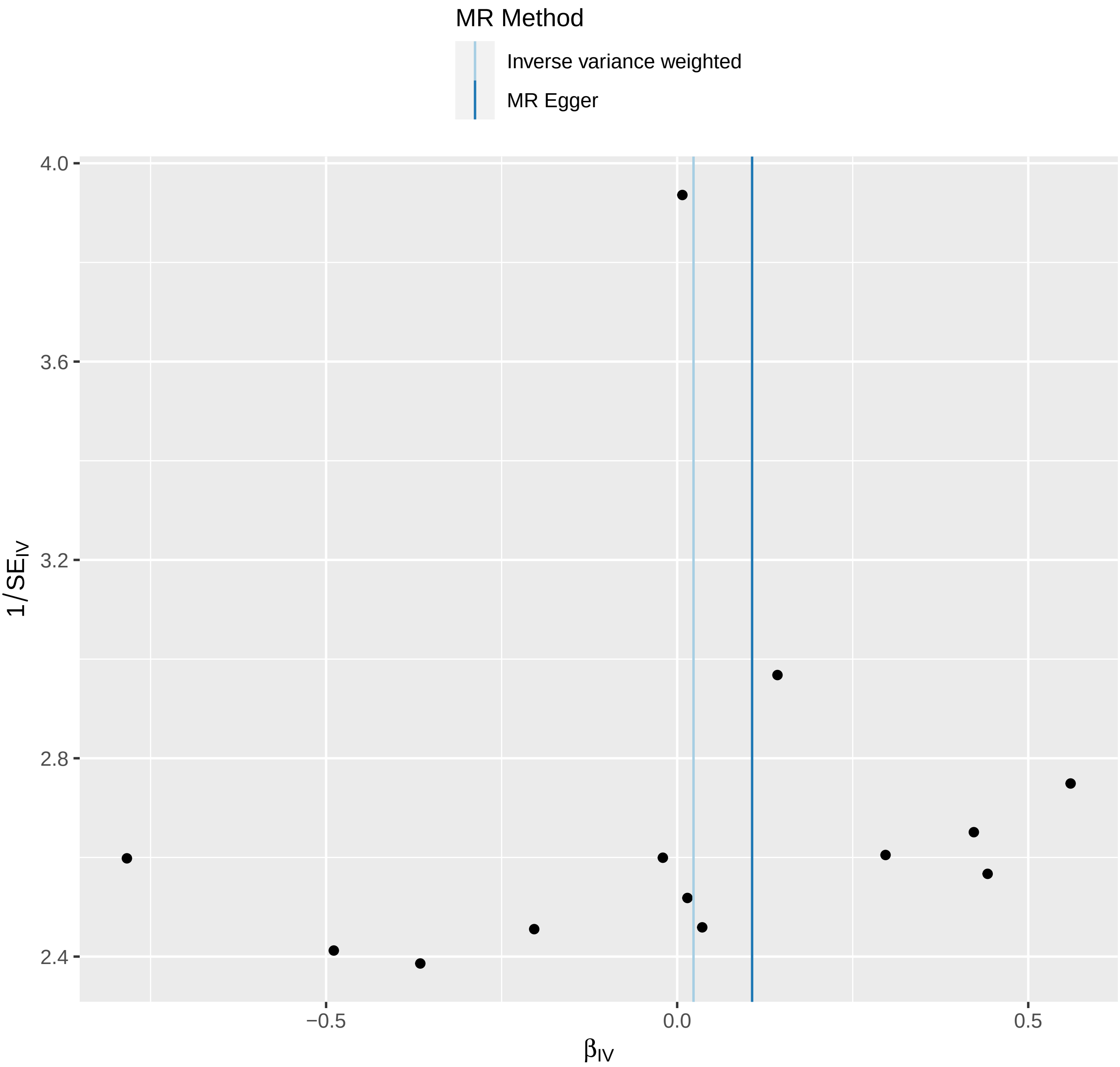

Batch 2−2765 : Gut microbiota abundance (genus Catenibacterium id.2153) on Estradiol 17−beta−dehydrogenase 2
with a threshold of 1e−05

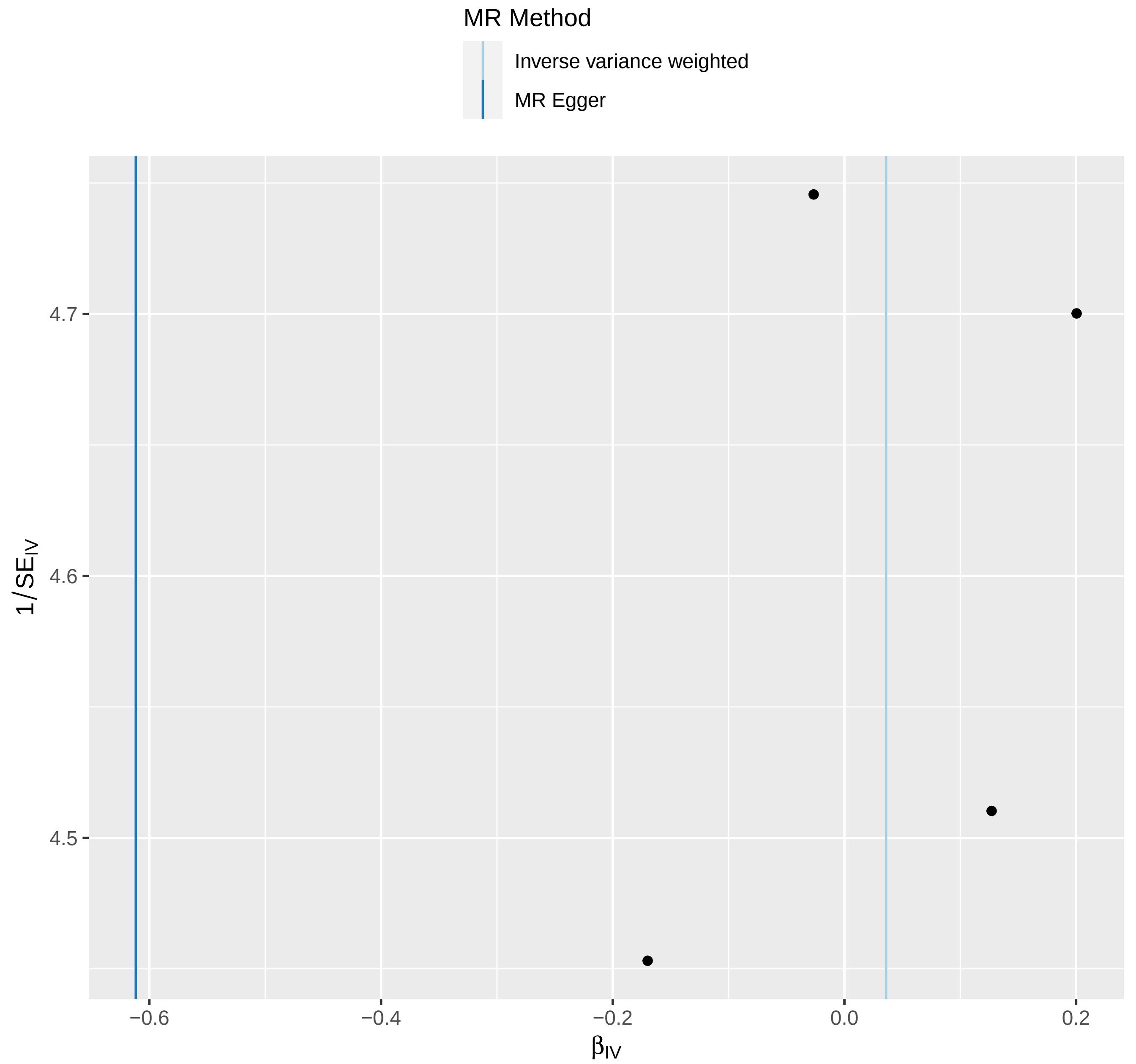

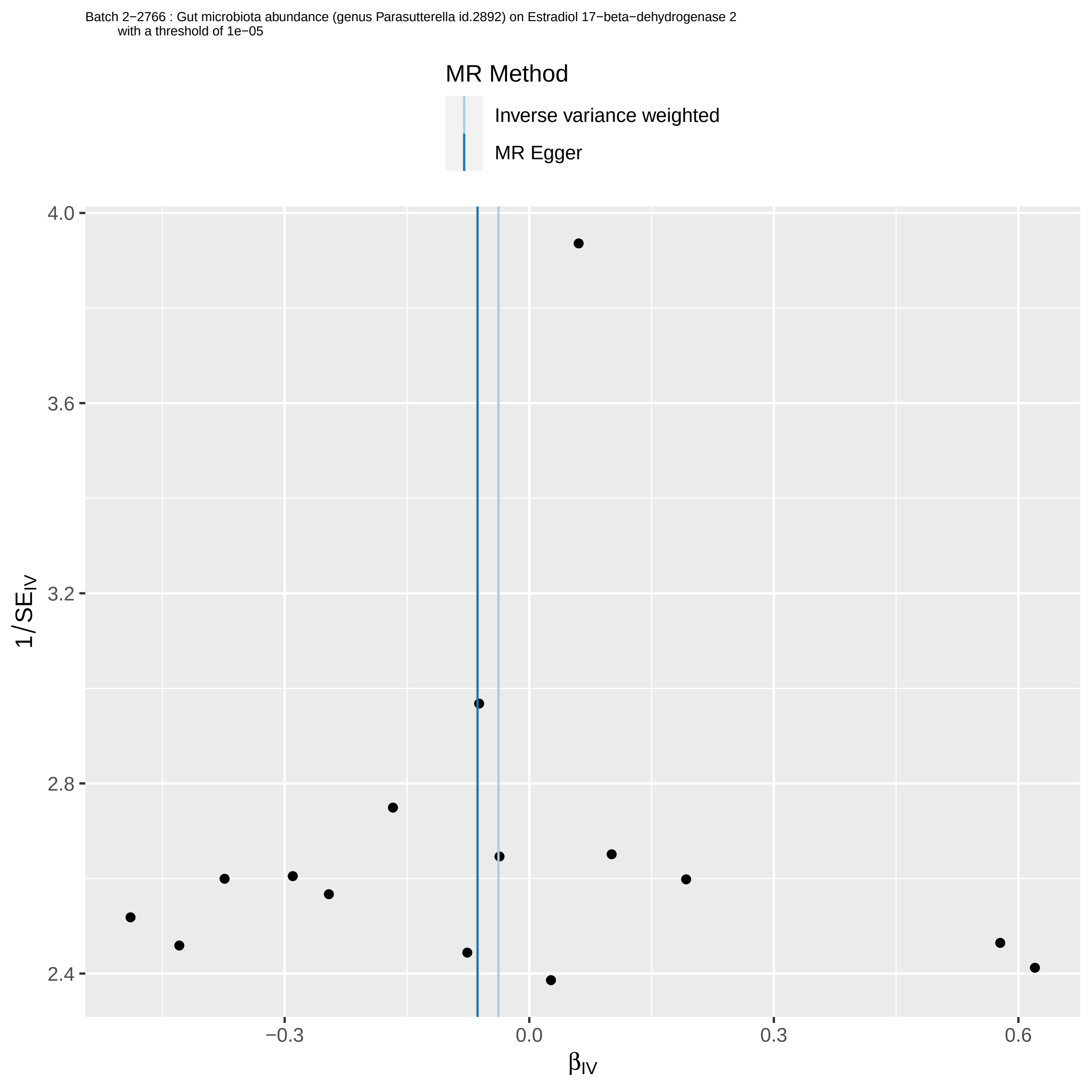
Batch 2−2766 : Gut microbiota abundance (genus Parasutterella id.2892) on Estradiol 17−beta−dehydrogenase 2
with a threshold of 1e−05
MR Method
Inverse variance weighted
MR Egger
4.0
3.6
3.2
2.8
2.4
1/SE_IV
−0.3
0.0
0.3
0.6
β_IV

Batch 2−2767 : Gut microbiota abundance (genus Catenibacterium id.2153) on Heat shock protein HSP 90−alpha/beta
with a threshold of 1e−05

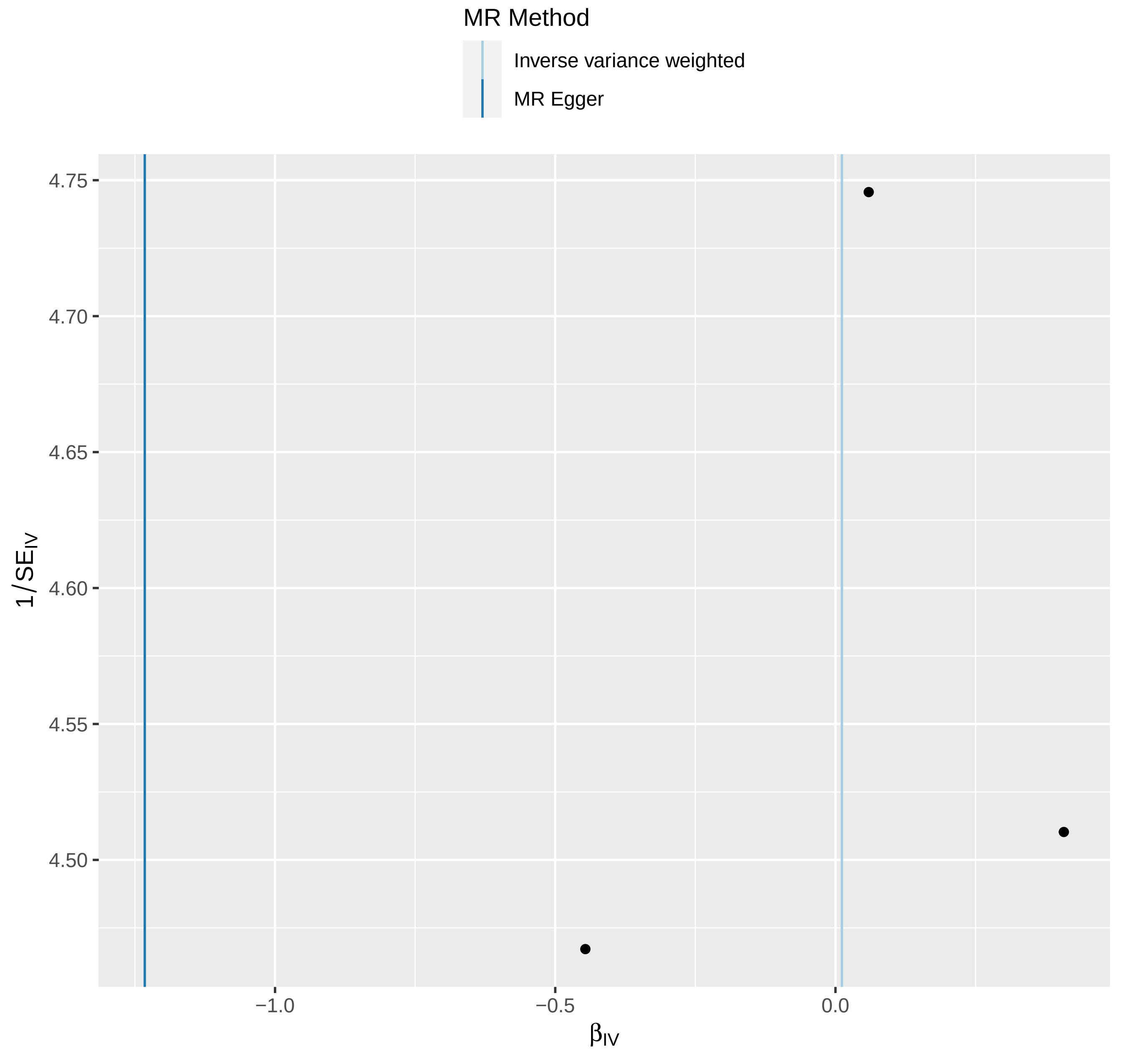

Batch 2−2768 : Gut microbiota abundance (genus Parasutterella id.2892) on Heat shock protein HSP 90−alpha/beta
with a threshold of 1e−05

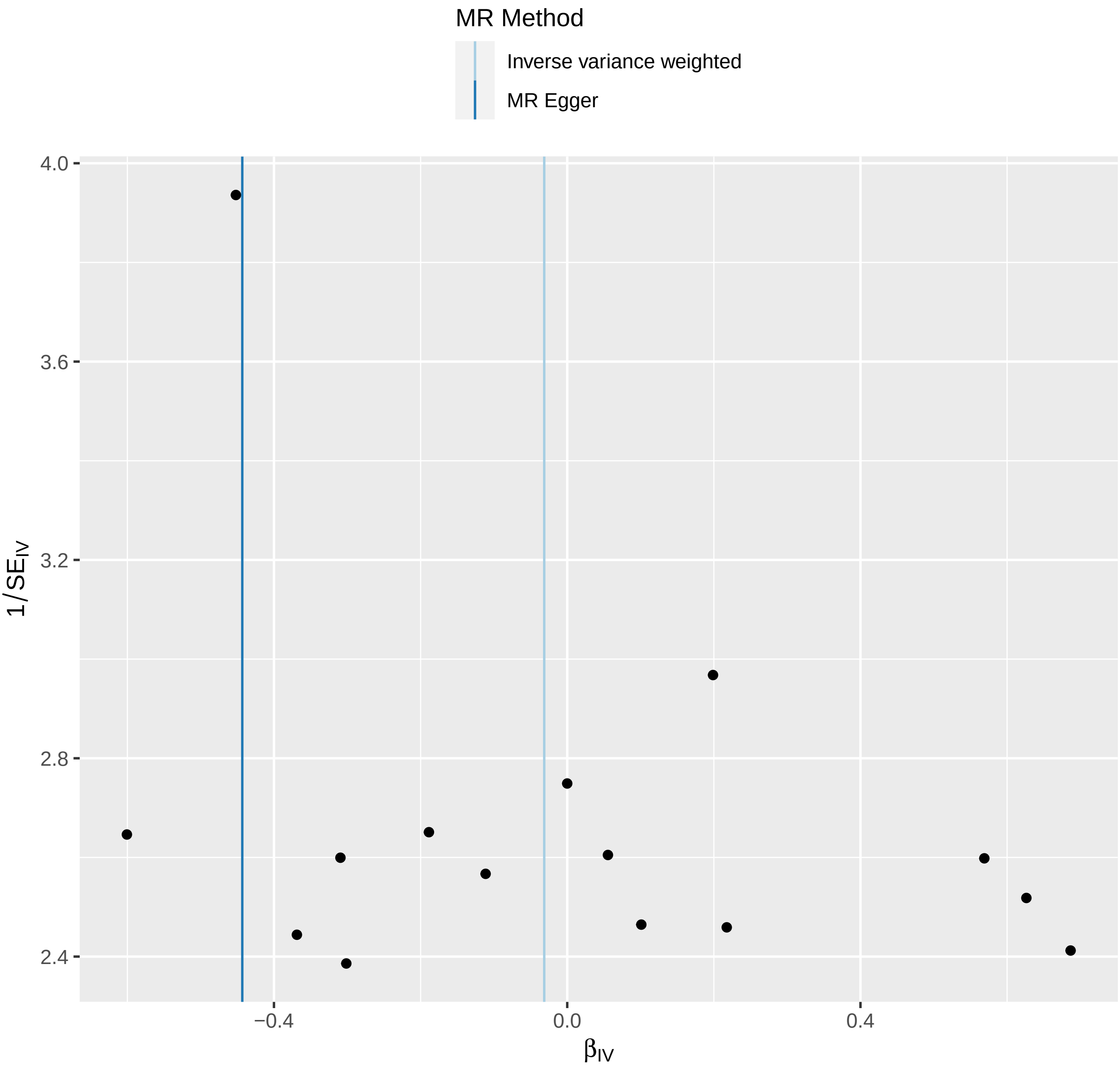

Batch 2−2769 : Gut microbiota abundance (genus Catenibacterium id.2153) on Heat shock protein HSP 90−beta
with a threshold of 1e−05

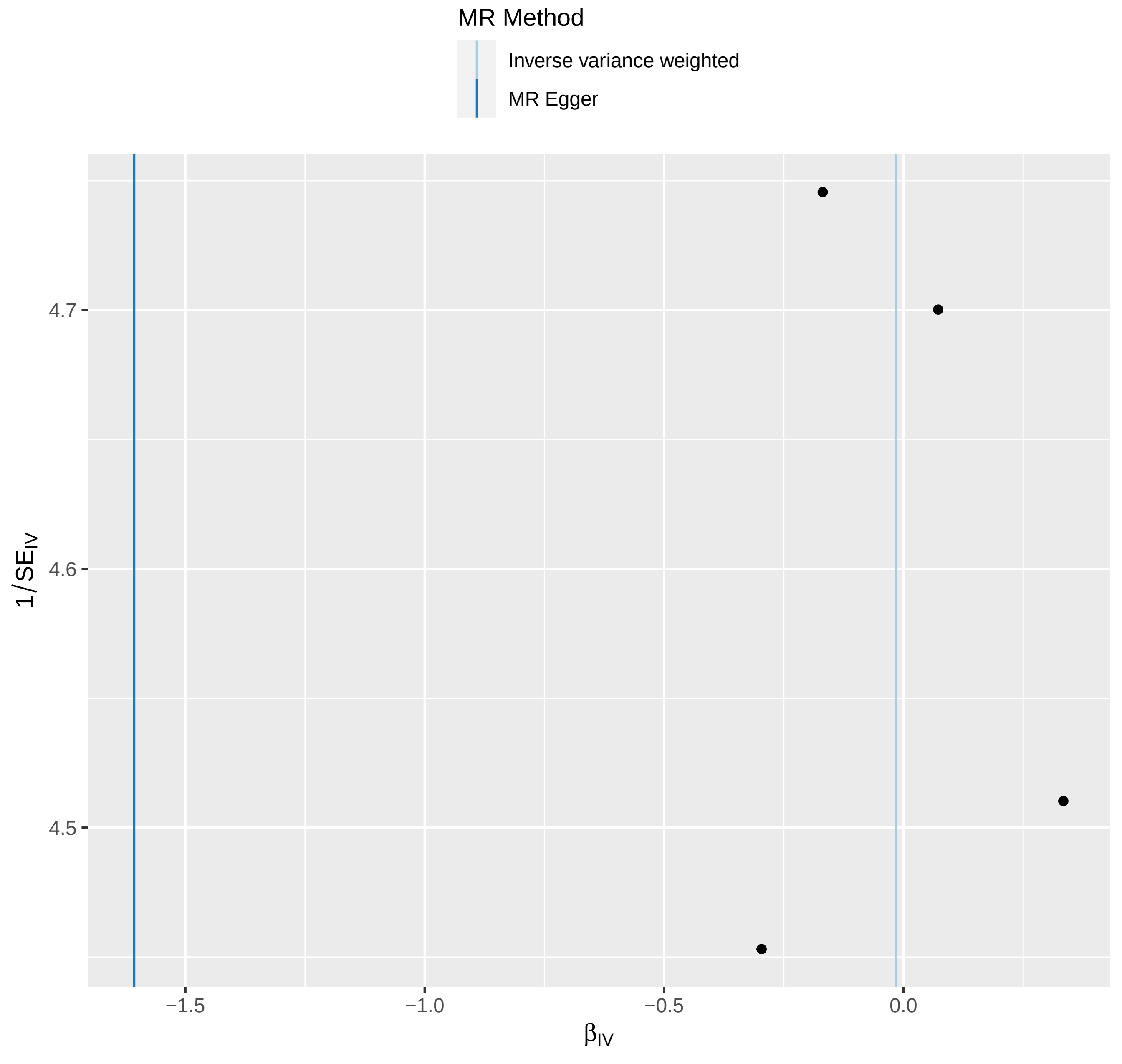

Batch 2−2770 : Gut microbiota abundance (genus Parasutterella id.2892) on Heat shock protein HSP 90−beta
with a threshold of 1e−05

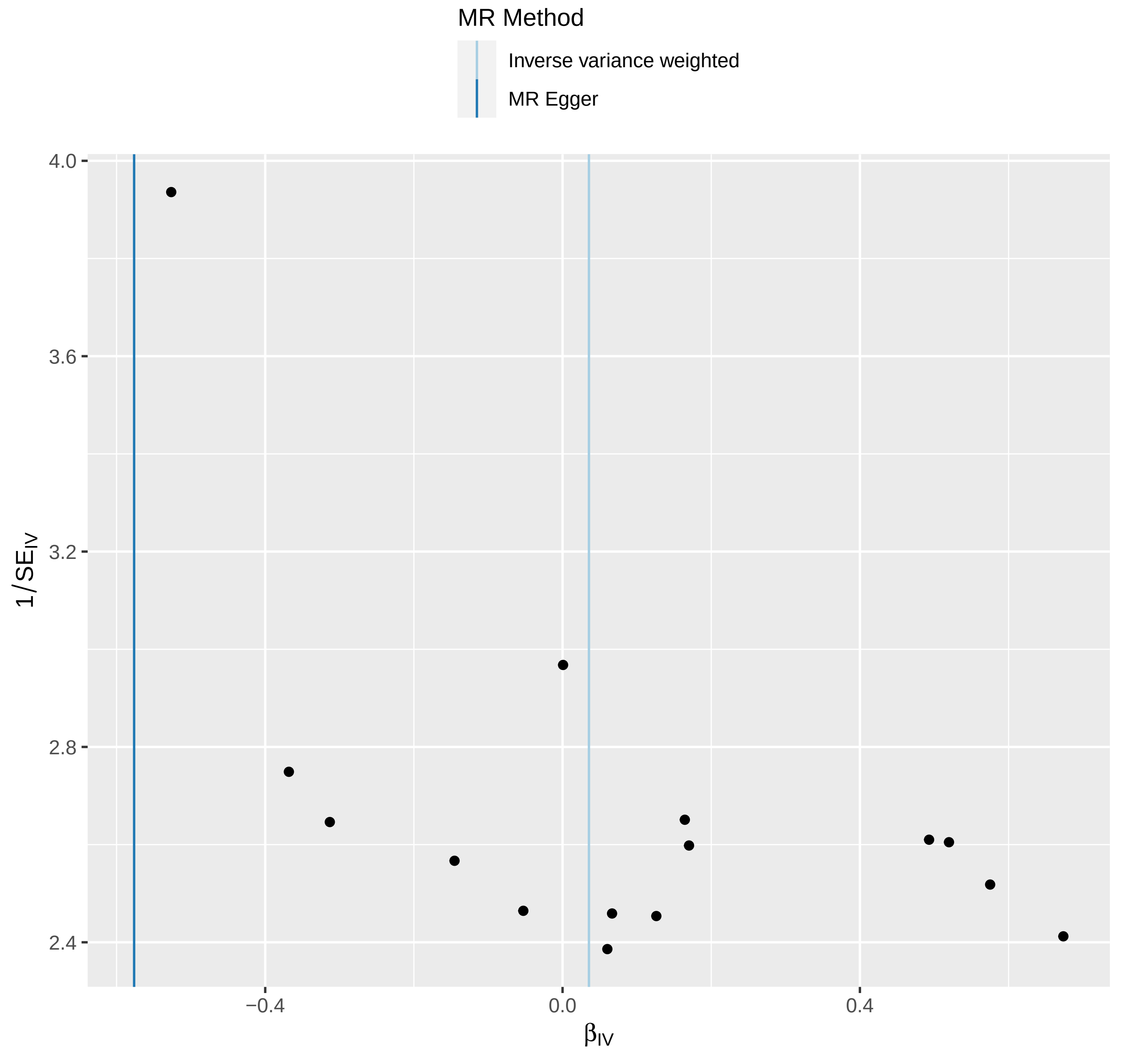

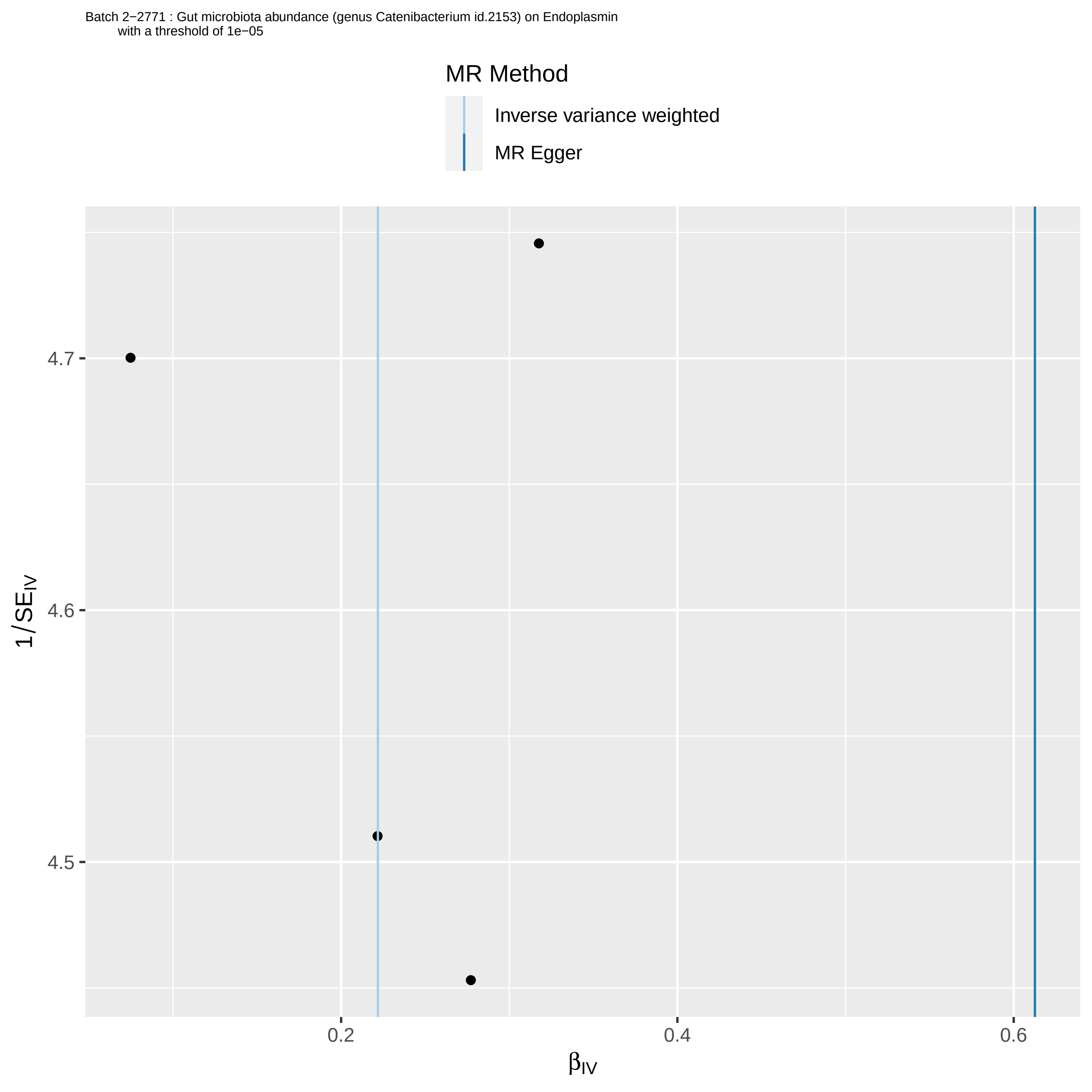
Batch 2−2771 : Gut microbiota abundance (genus Catenibacterium id.2153) on Endoplasmin
with a threshold of 1e−05
MR Method
Inverse variance weighted
MR Egger
4.7
4.6
4.5
$1/SE_{IV}$
0.2
0.4
0.6
β_{IV}

Batch 2−2772 : Gut microbiota abundance (genus Parasutterella id.2892) on Endoplasmin
with a threshold of 1e−05

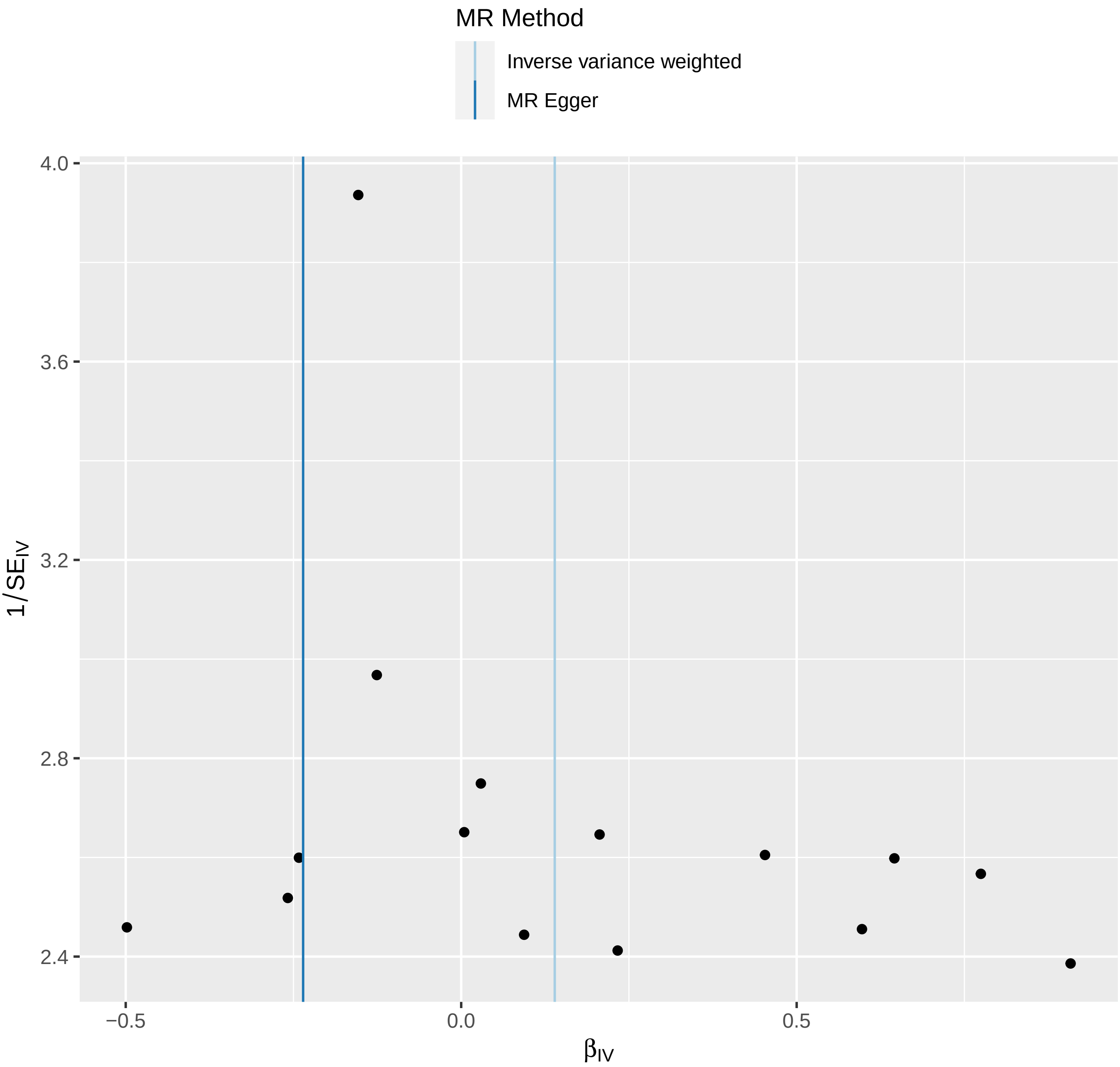

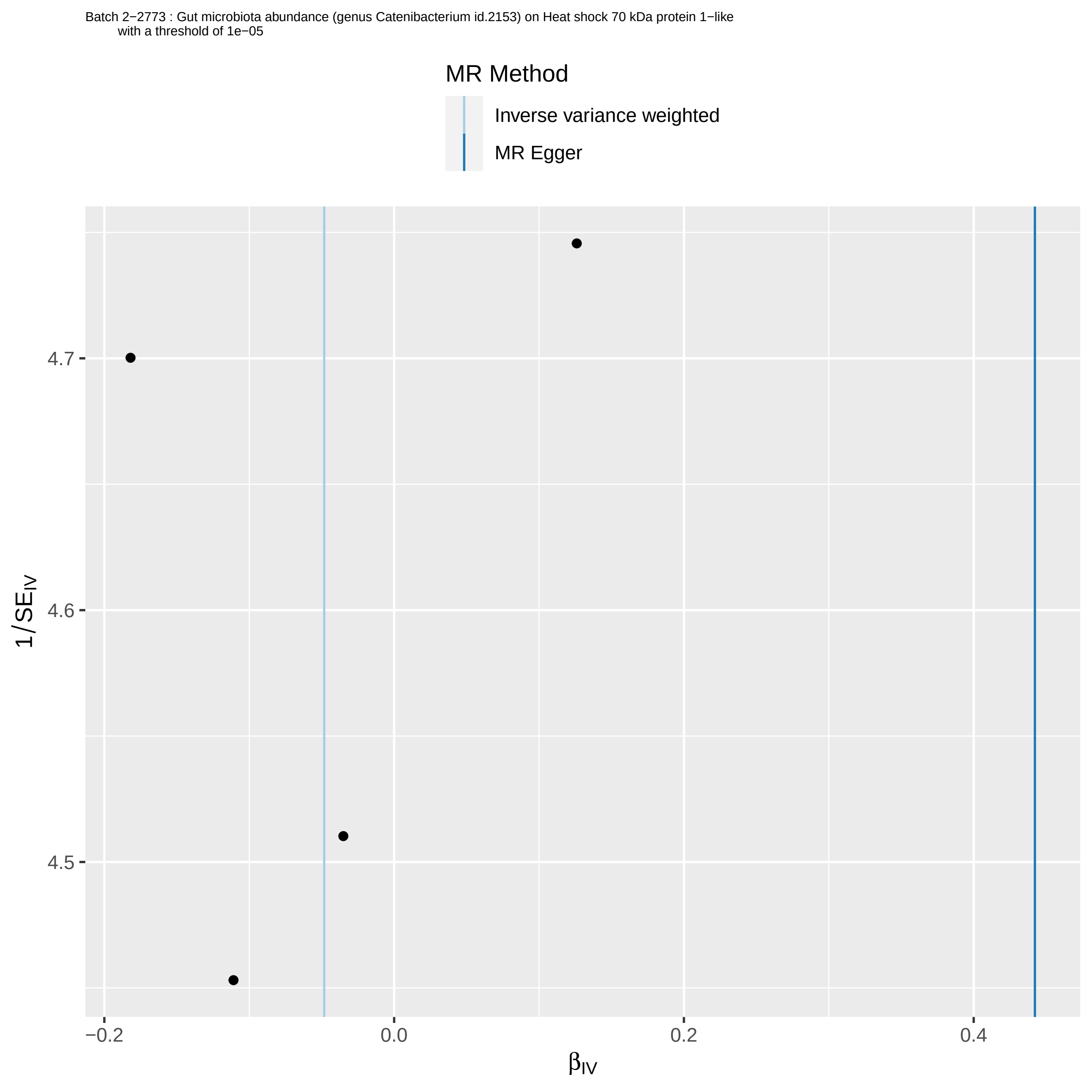

Batch 2−2773 : Gut microbiota abundance (genus Catenibacterium id.2153) on Heat shock 70 kDa protein 1−like
with a threshold of 1e−05
MR Method
Inverse variance weighted
MR Egger
4.7
4.6
4.5
1/SE_IV
−0.2
0.0
0.2
0.4
β_IV

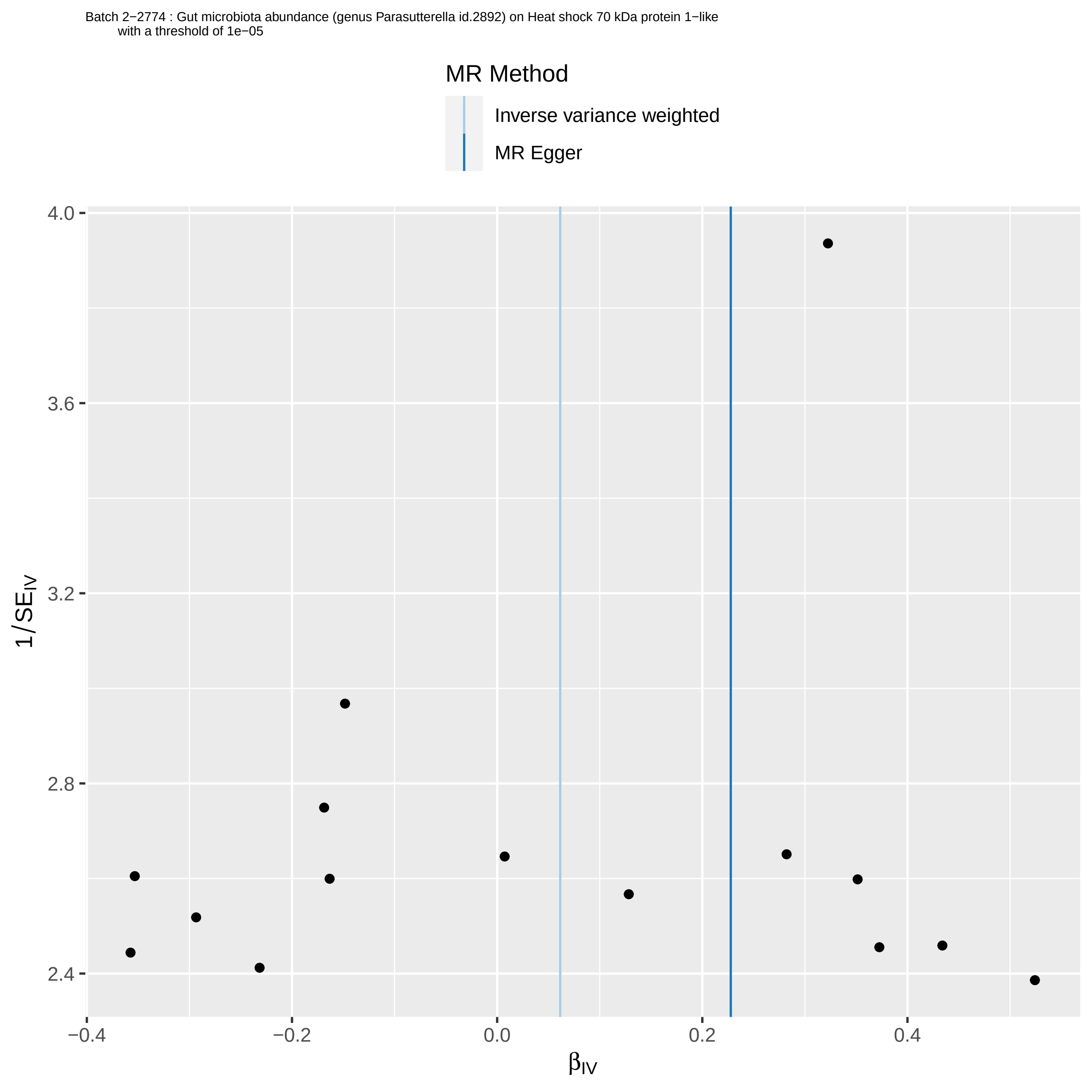
Batch 2−2774 : Gut microbiota abundance (genus Parasutterella id.2892) on Heat shock 70 kDa protein 1−like
with a threshold of 1e−05
MR Method
Inverse variance weighted
MR Egger
4.0
3.6
3.2
2.8
2.4
1/SE_IV
−0.4
−0.2
0.0
0.2
0.4
β_IV

Batch 2−2775 : Gut microbiota abundance (genus Catenibacterium id.2153) on Heat shock 70 kDa protein 6
with a threshold of 1e−05

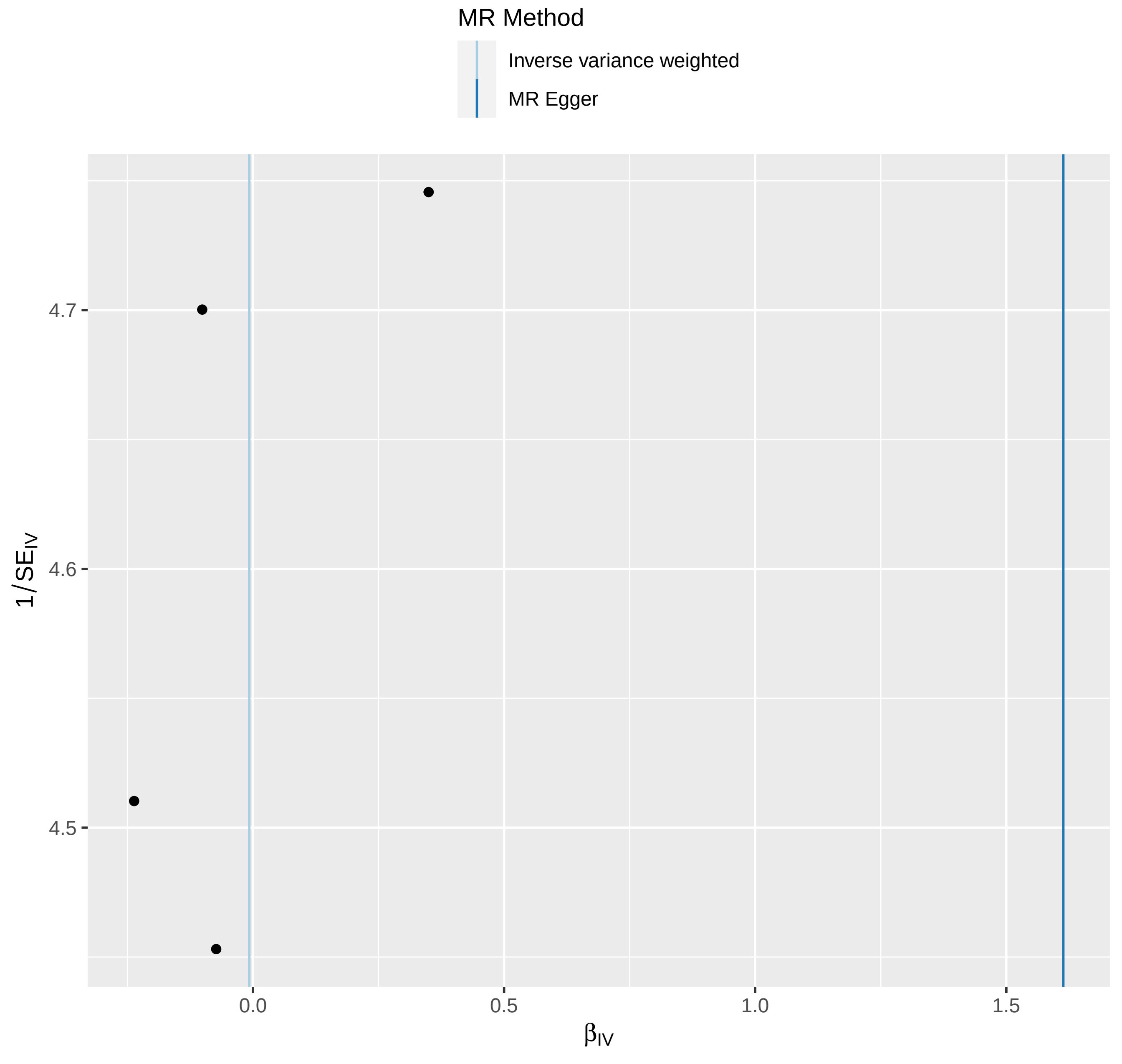

Batch 2−2776 : Gut microbiota abundance (genus Parasutterella id.2892) on Heat shock 70 kDa protein 6
with a threshold of 1e−05

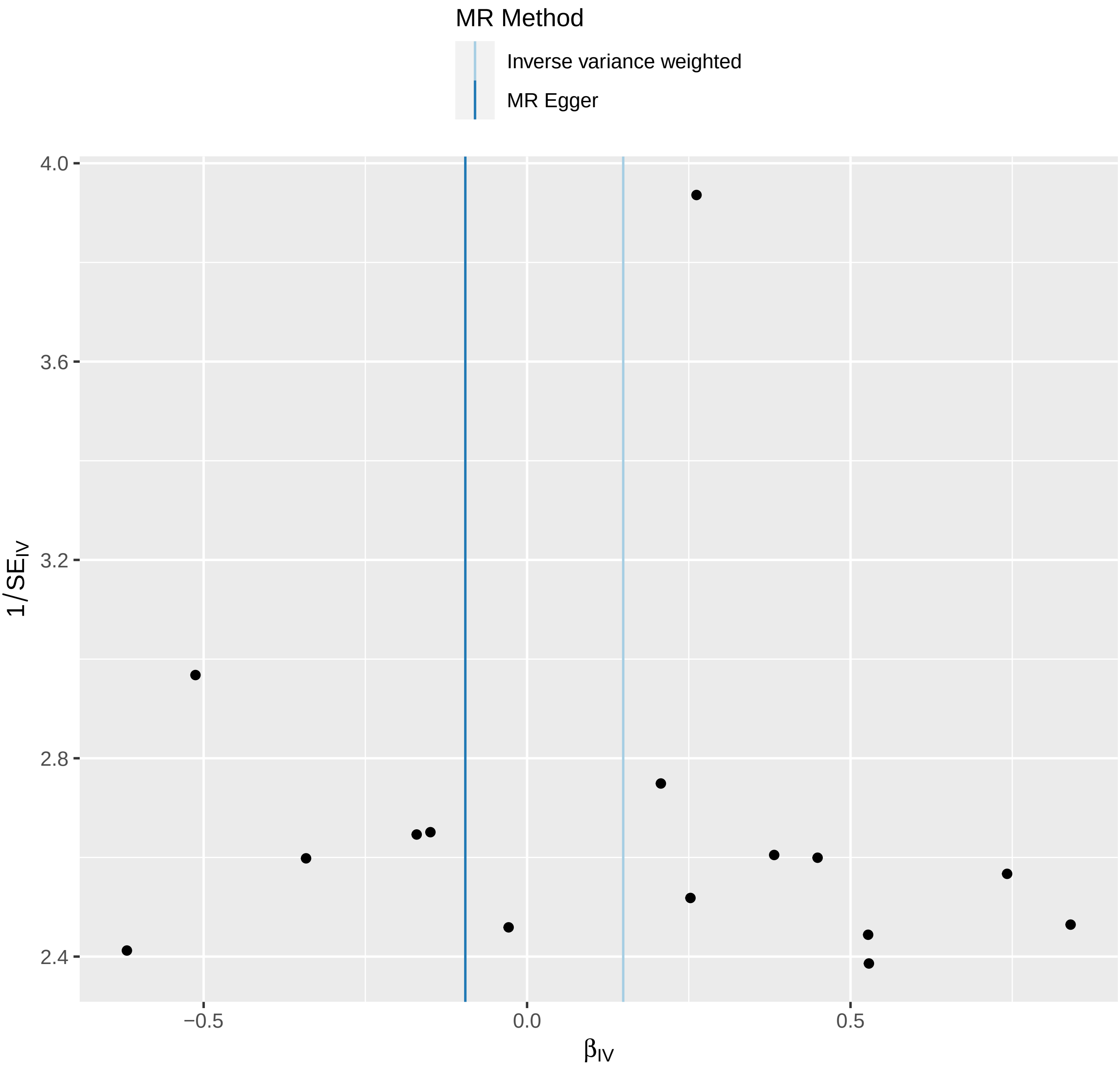

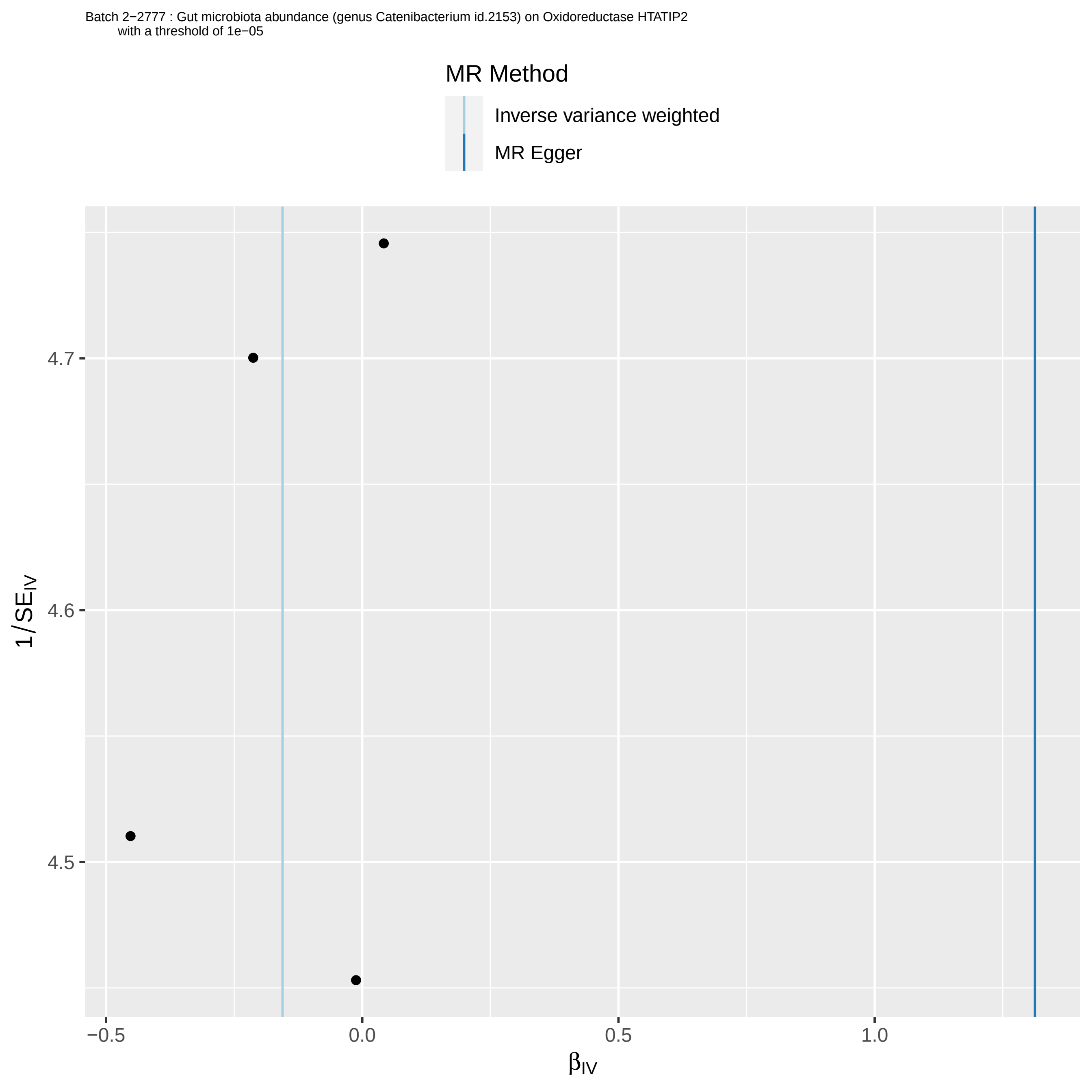
Batch 2−2777 : Gut microbiota abundance (genus Catenibacterium id.2153) on Oxidoreductase HTATIP2
with a threshold of 1e−05
MR Method
Inverse variance weighted
MR Egger
4.7
4.6
4.5
1/SE_IV
−0.5
0.0
0.5
1.0
β_IV

Batch 2−2778 : Gut microbiota abundance (genus Parasutterella id.2892) on Oxidoreductase HTATIP2
with a threshold of 1e−05

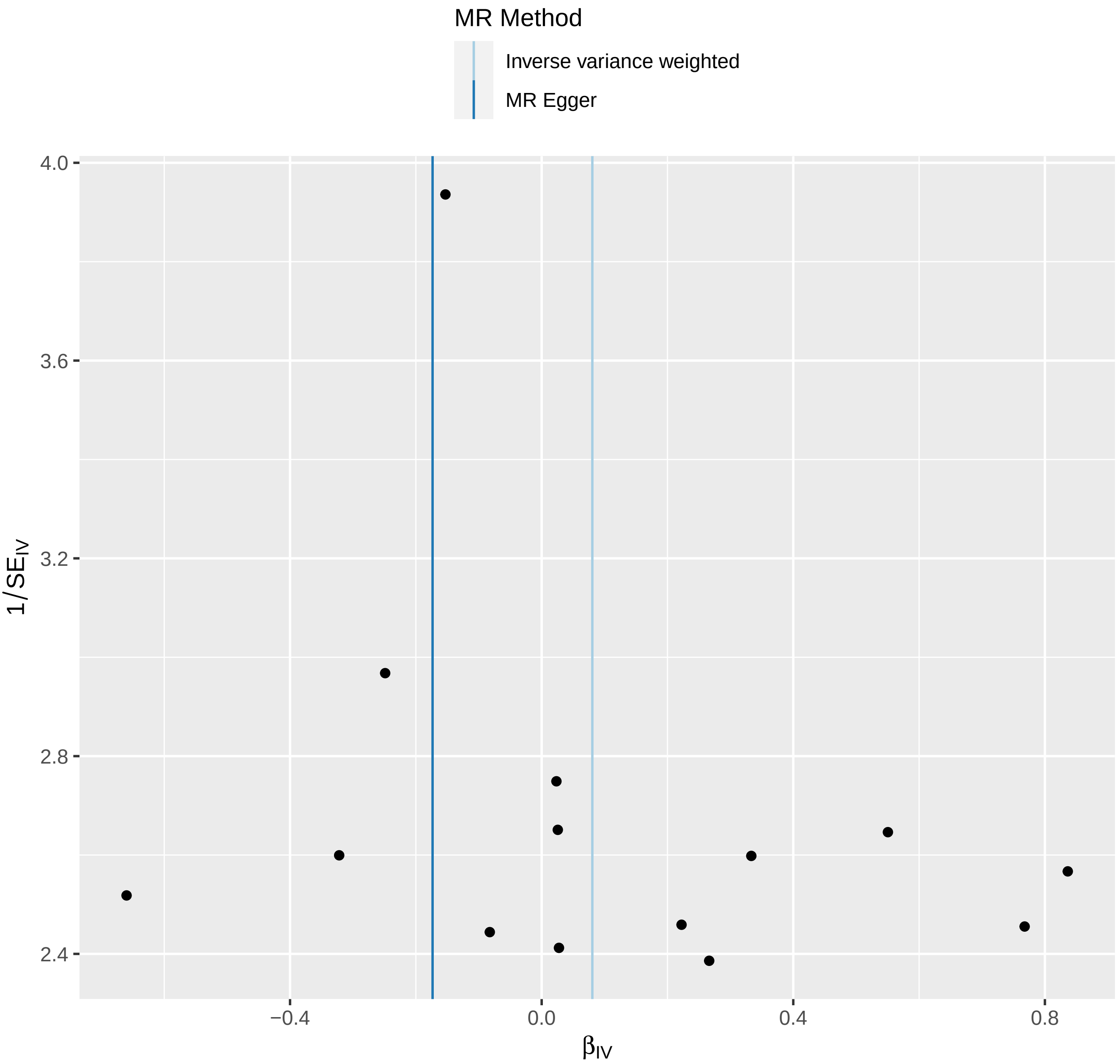

Batch 2−2779 : Gut microbiota abundance (genus Catenibacterium id.2153) on Oxidoreductase HTATIP2
with a threshold of 1e−05

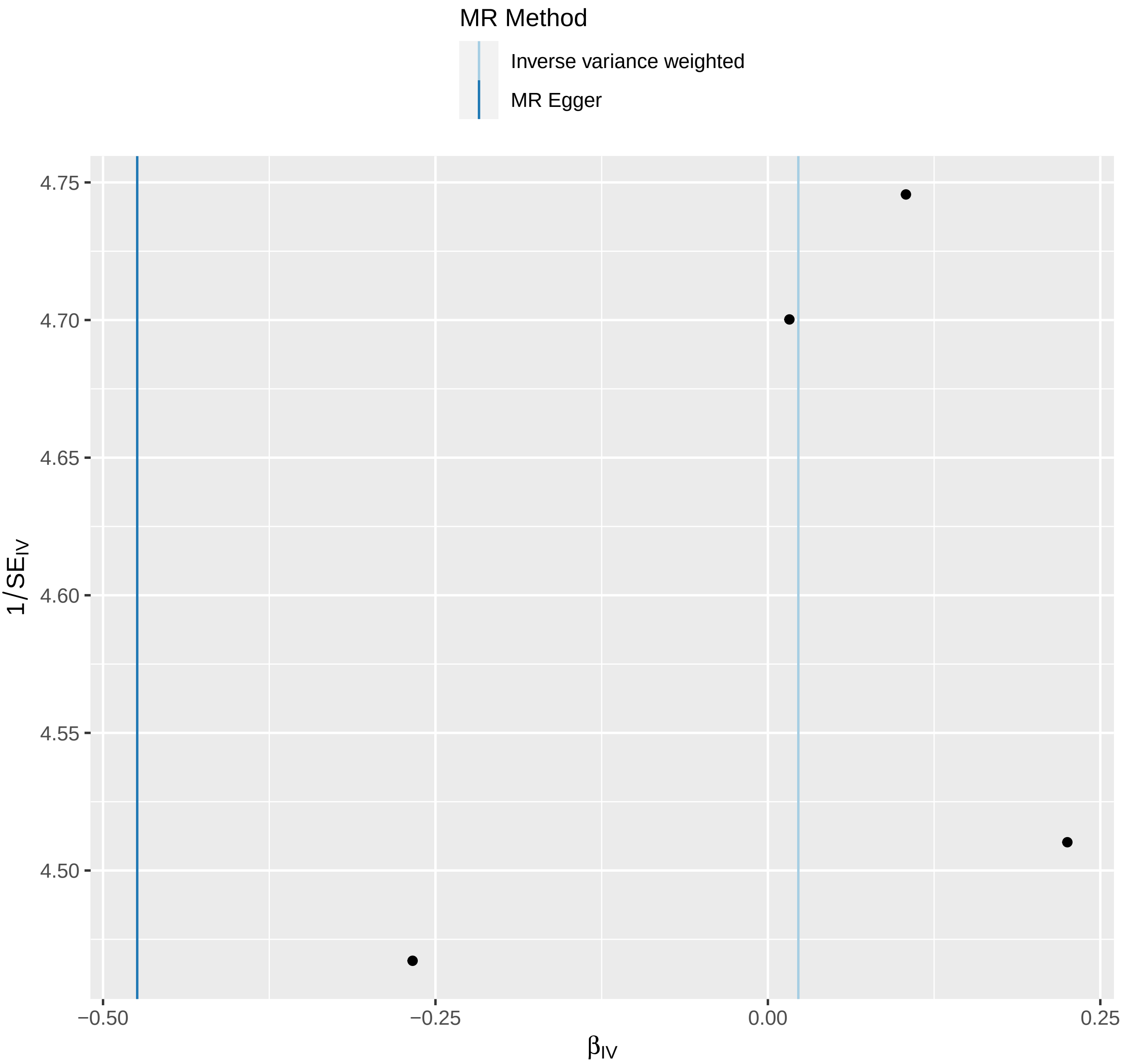

Batch 2−2780 : Gut microbiota abundance (genus Parasutterella id.2892) on Oxidoreductase HTATIP2
with a threshold of 1e−05

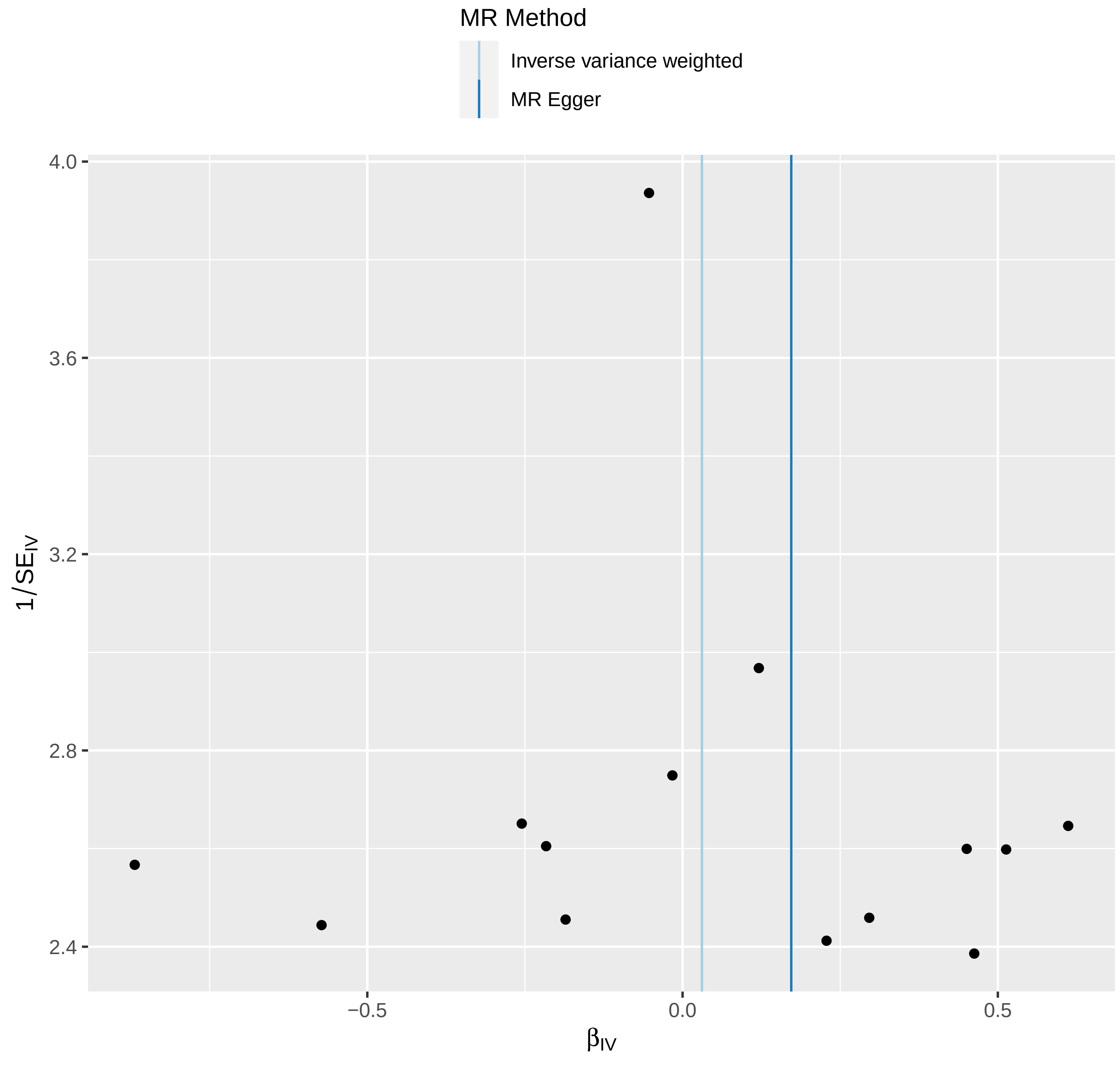

Batch 2−2781 : Gut microbiota abundance (genus Catenibacterium id.2153) on Histatin−1
with a threshold of 1e−05

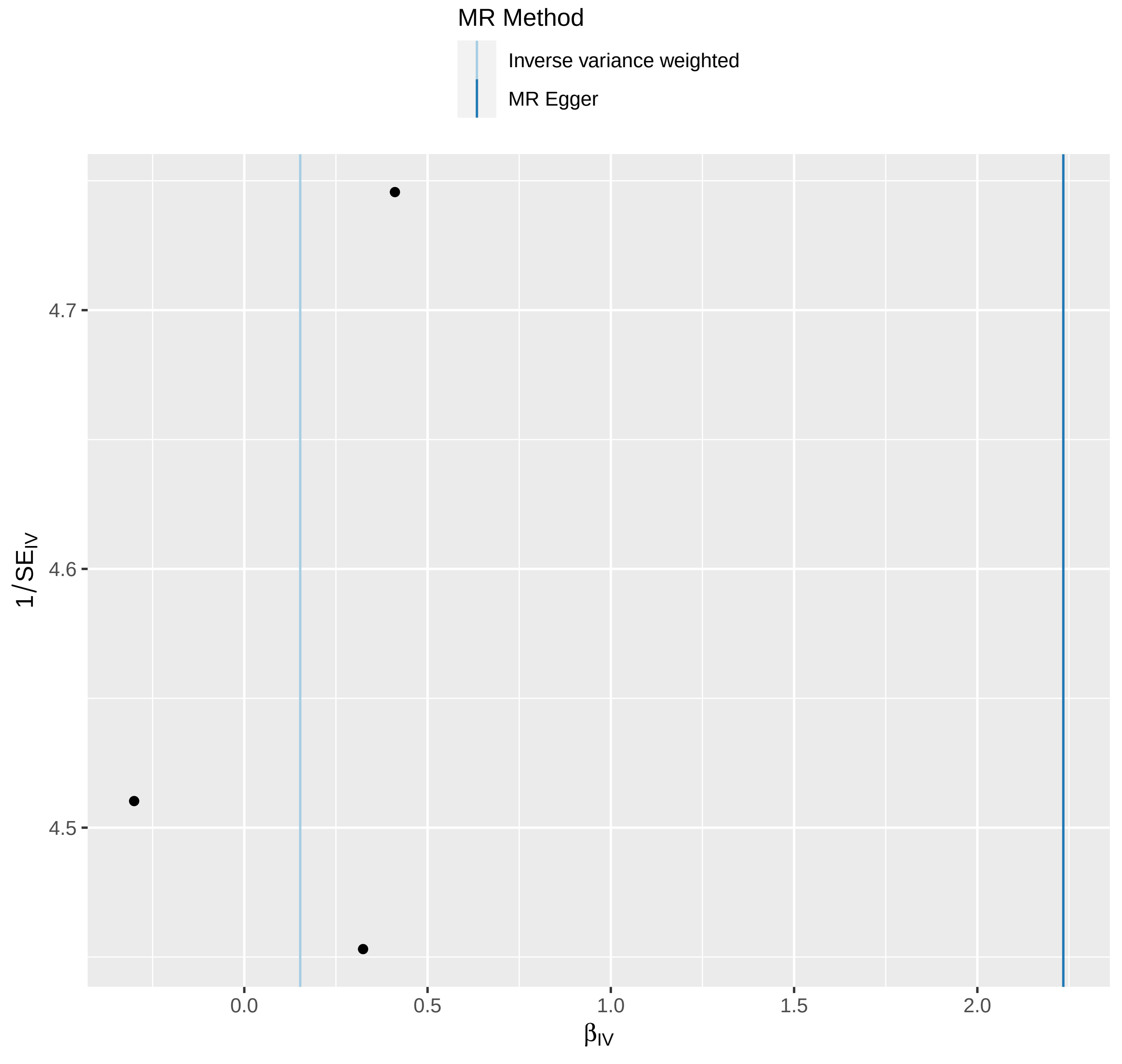

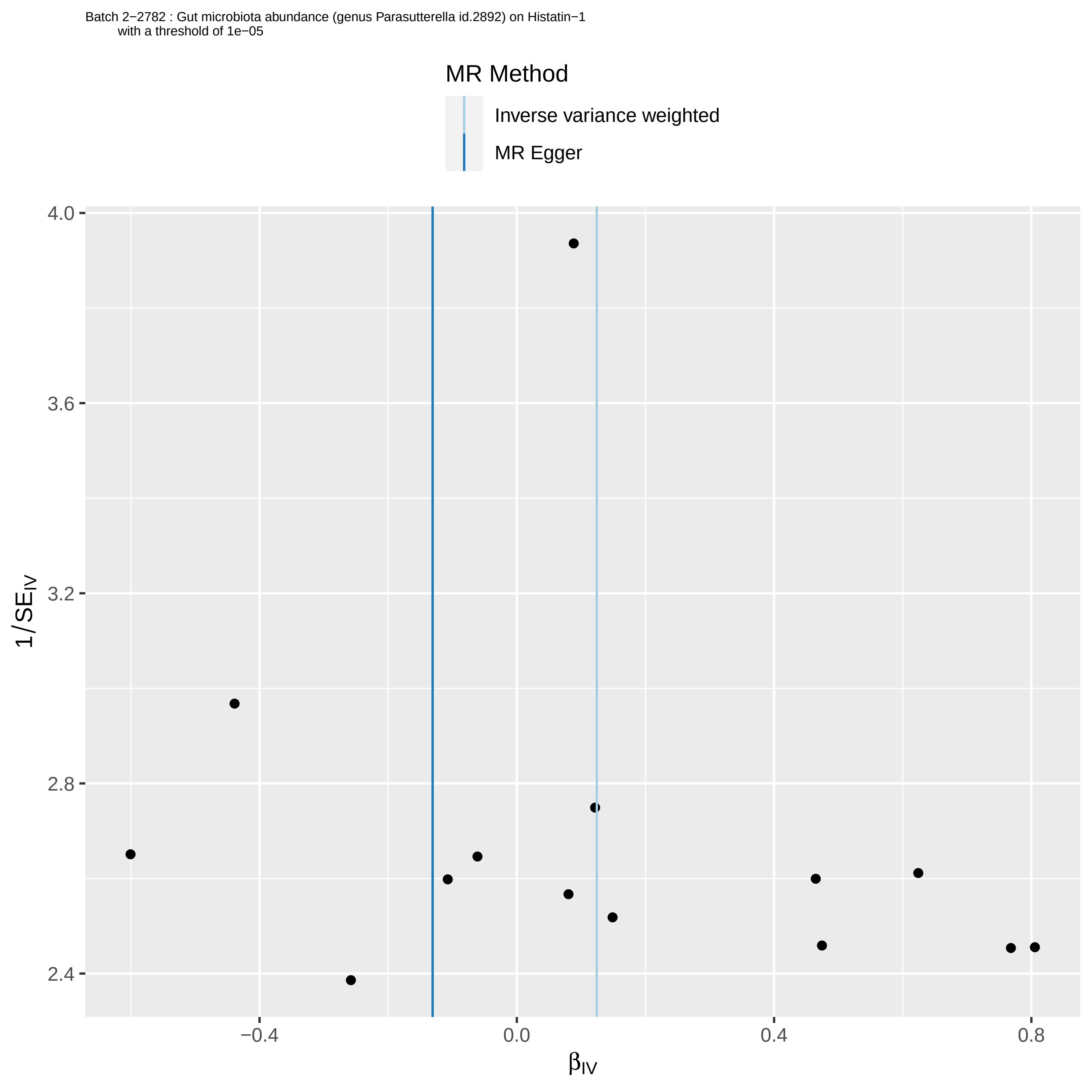

Batch 2−2782 : Gut microbiota abundance (genus Parasutterella id.2892) on Histatin−1
with a threshold of 1e−05
MR Method
Inverse variance weighted
MR Egger
4.0
3.6
3.2
2.8
2.4
1/SE_IV
−0.4
0.0
0.4
0.8
β_IV

Batch 2−2783 : Gut microbiota abundance (genus Catenibacterium id.2153) on Serine protease HTRA2, mitochondrial
with a threshold of 1e−05

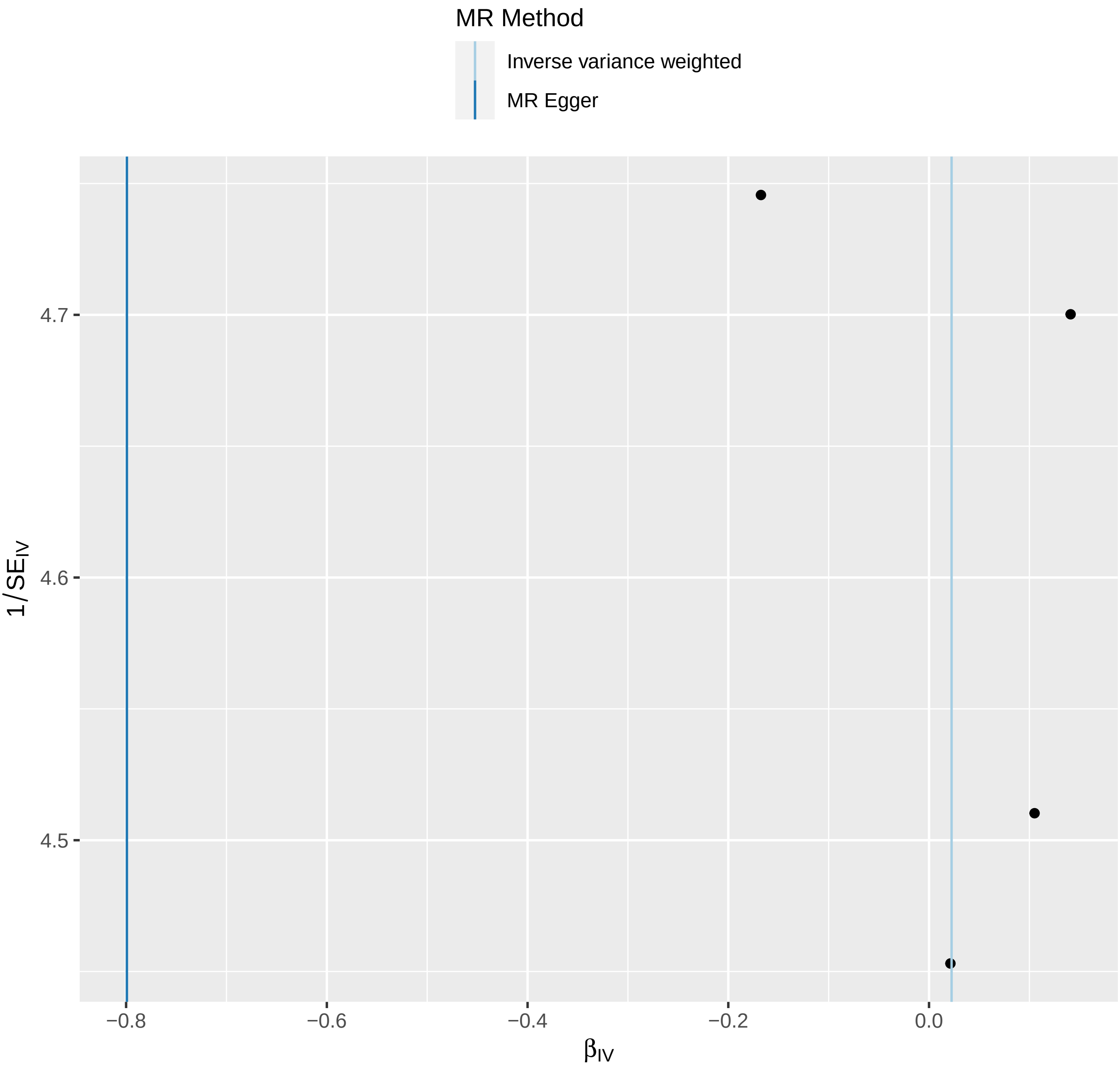

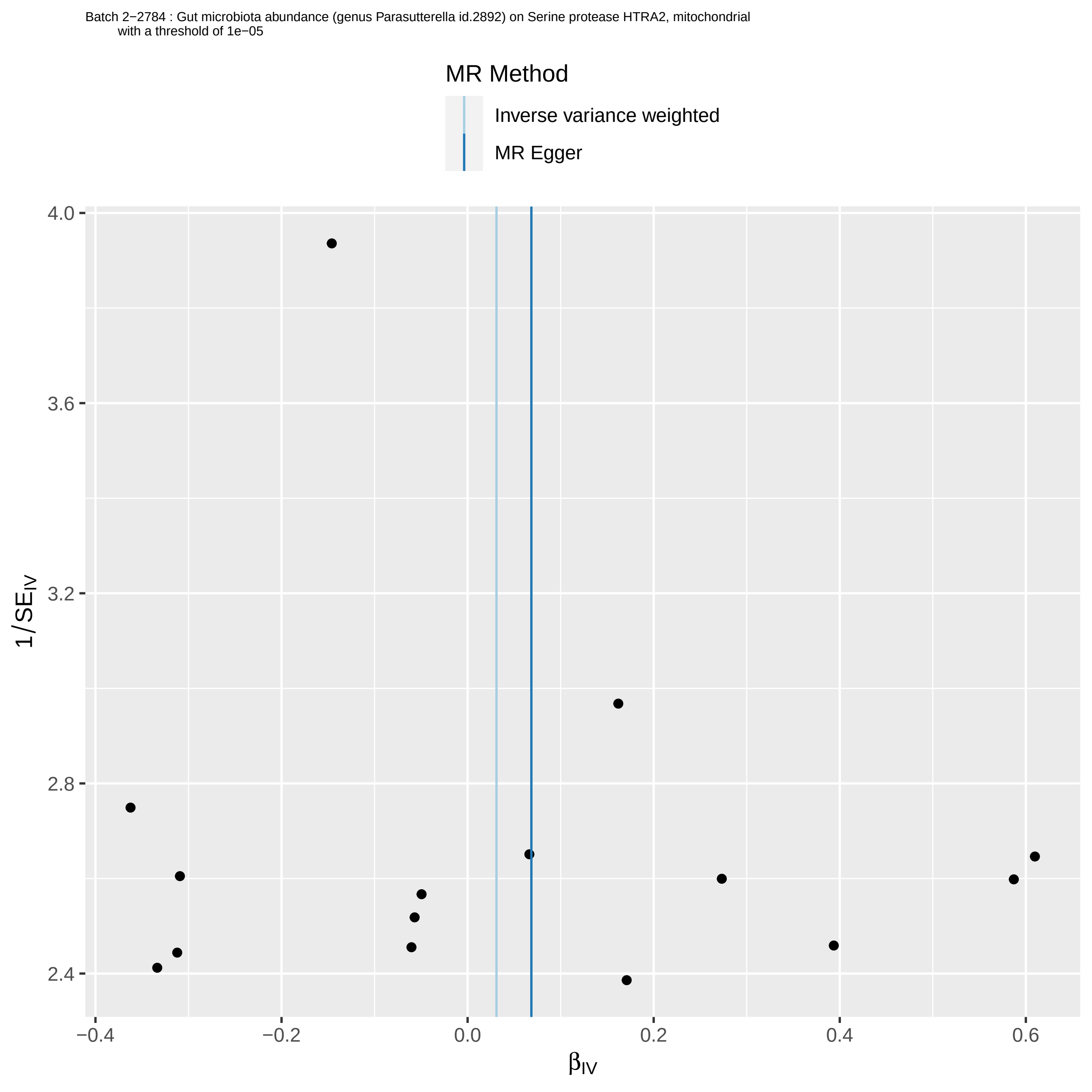

Batch 2−2784 : Gut microbiota abundance (genus Parasutterella id.2892) on Serine protease HTRA2, mitochondrial
with a threshold of 1e−05
MR Method
Inverse variance weighted
MR Egger
4.0
3.6
3.2
2.8
2.4
−0.4
−0.2
0.0
0.2
0.4
0.6
$1/SE_{IV}$
β_{IV}

Batch 2−2785 : Gut microbiota abundance (genus Catenibacterium id.2153) on Hyaluronidase−1
with a threshold of 1e−05

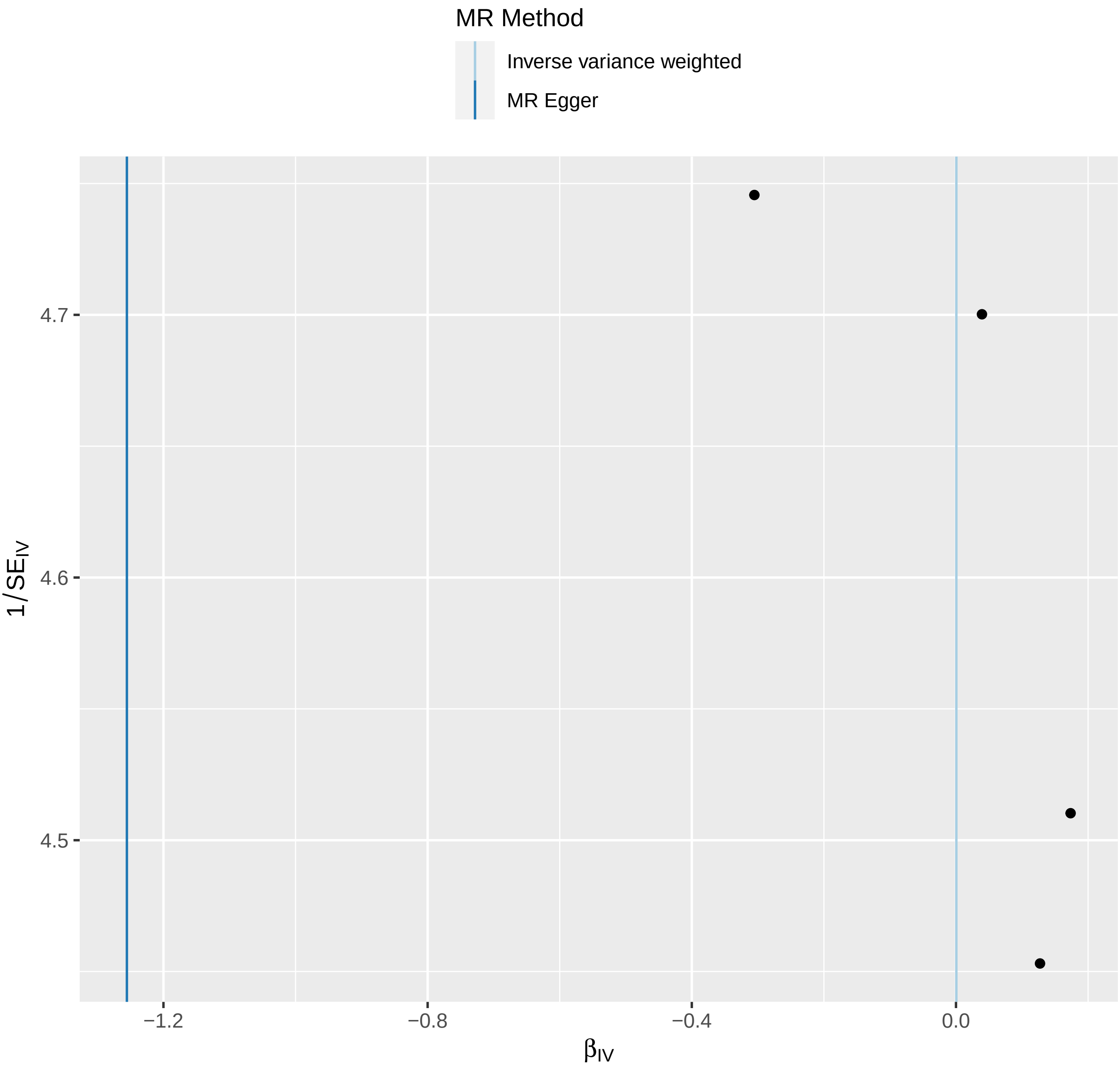

Batch 2−2786 : Gut microbiota abundance (genus Parasutterella id.2892) on Hyaluronidase−1
with a threshold of 1e−05

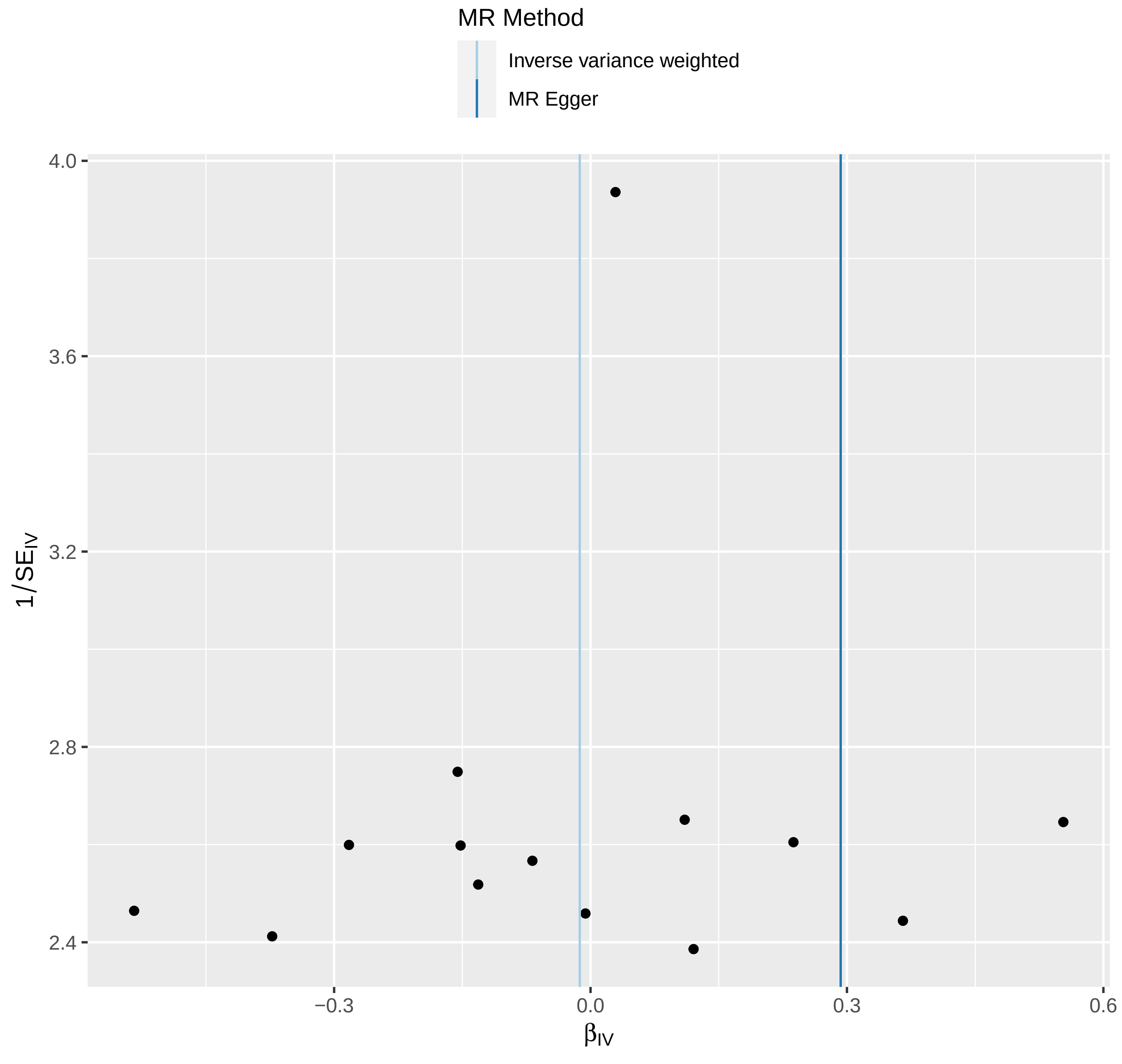

Batch 2−2787 : Gut microbiota abundance (genus Catenibacterium id.2153) on Isoleucine−−tRNA ligase, cytoplasmic
with a threshold of 1e−05

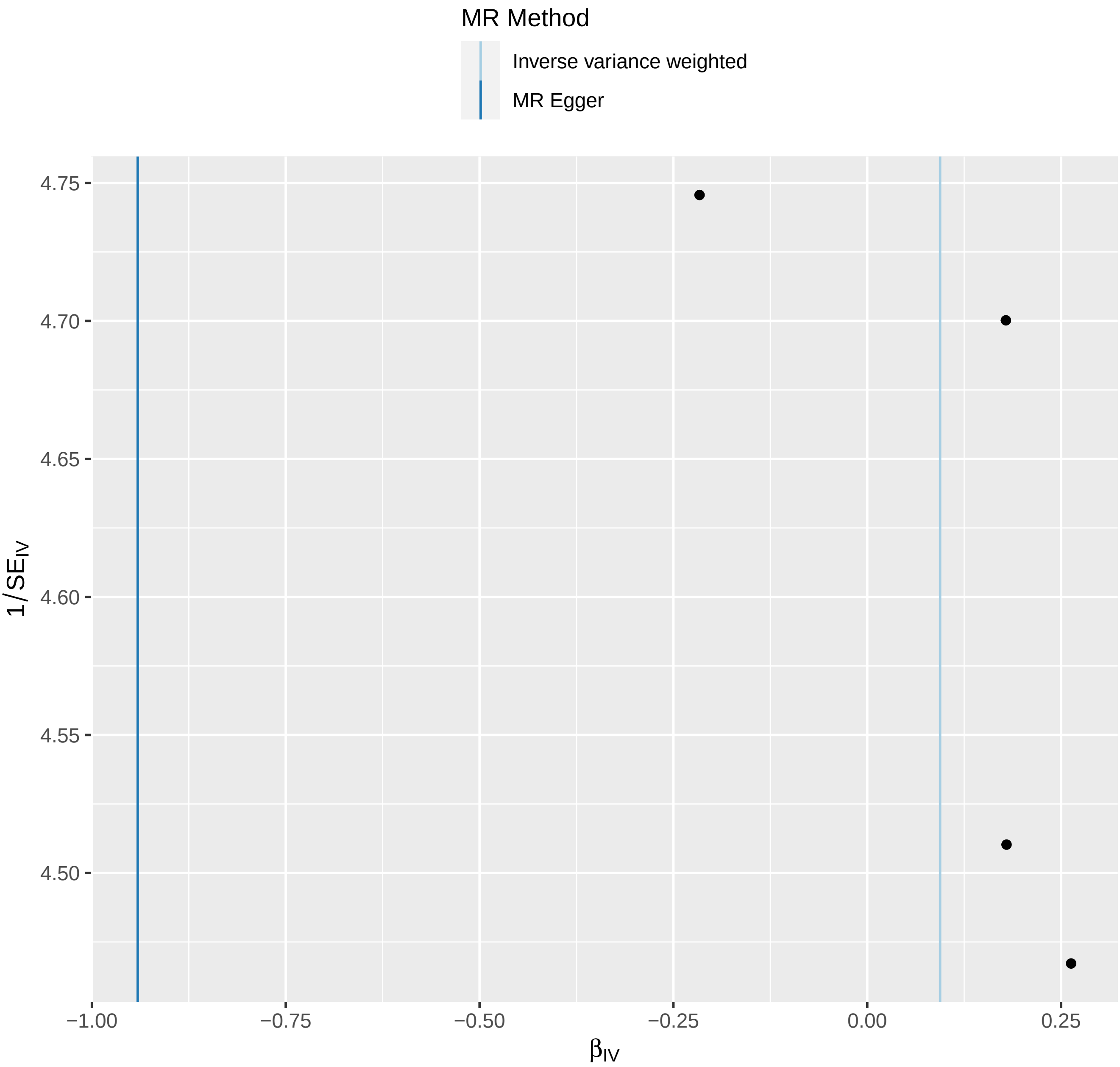

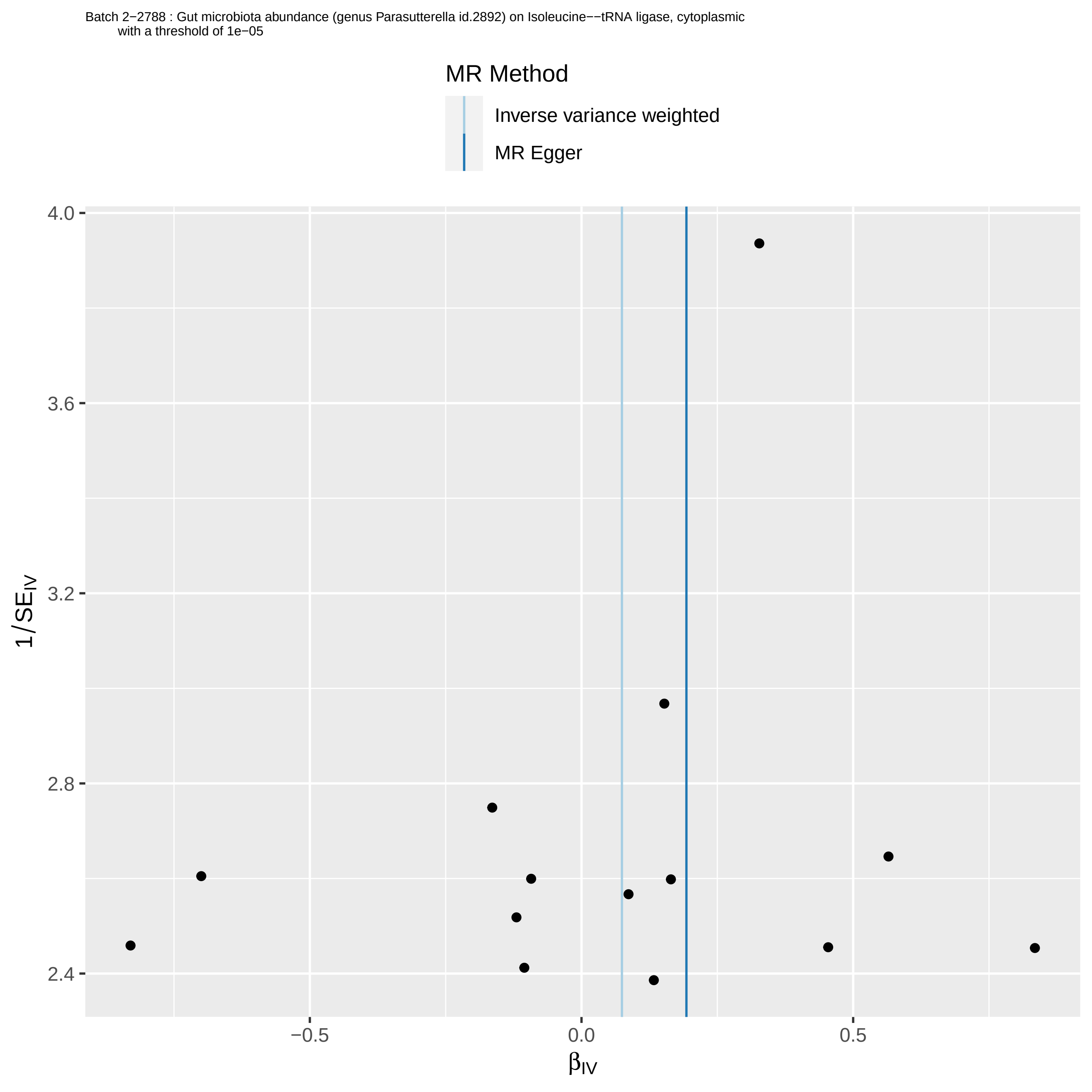
Batch 2−2788 : Gut microbiota abundance (genus Parasutterella id.2892) on Isoleucine−−tRNA ligase, cytoplasmic
with a threshold of 1e−05
MR Method
Inverse variance weighted
MR Egger
4.0
3.6
3.2
2.8
2.4
1/SE_IV
−0.5
0.0
0.5
β_IV

Batch 2−2789 : Gut microbiota abundance (genus Catenibacterium id.2153) on Bone sialoprotein 2
with a threshold of 1e−05

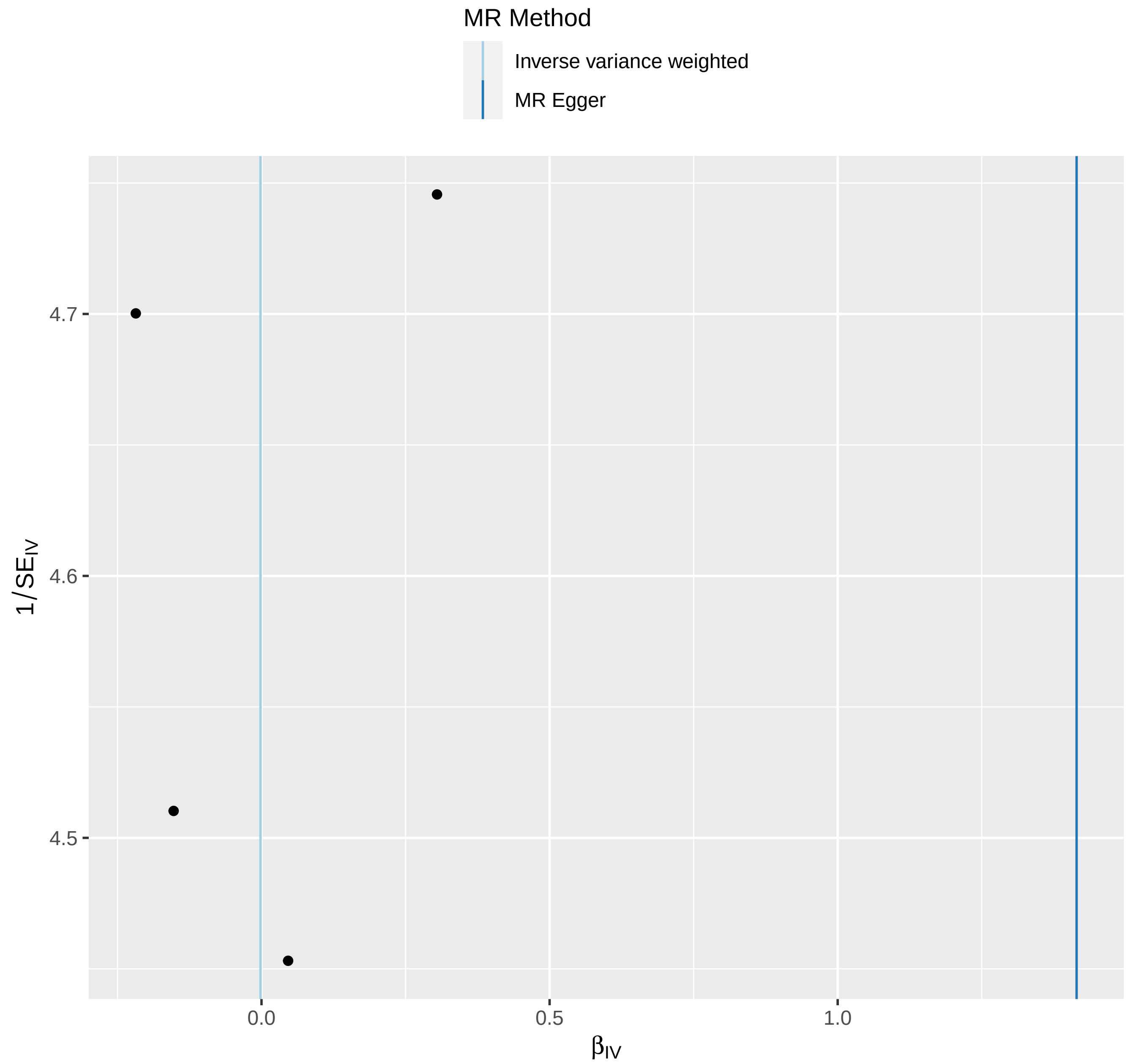

Batch 2−2790 : Gut microbiota abundance (genus Parasutterella id.2892) on Bone sialoprotein 2
with a threshold of 1e−05

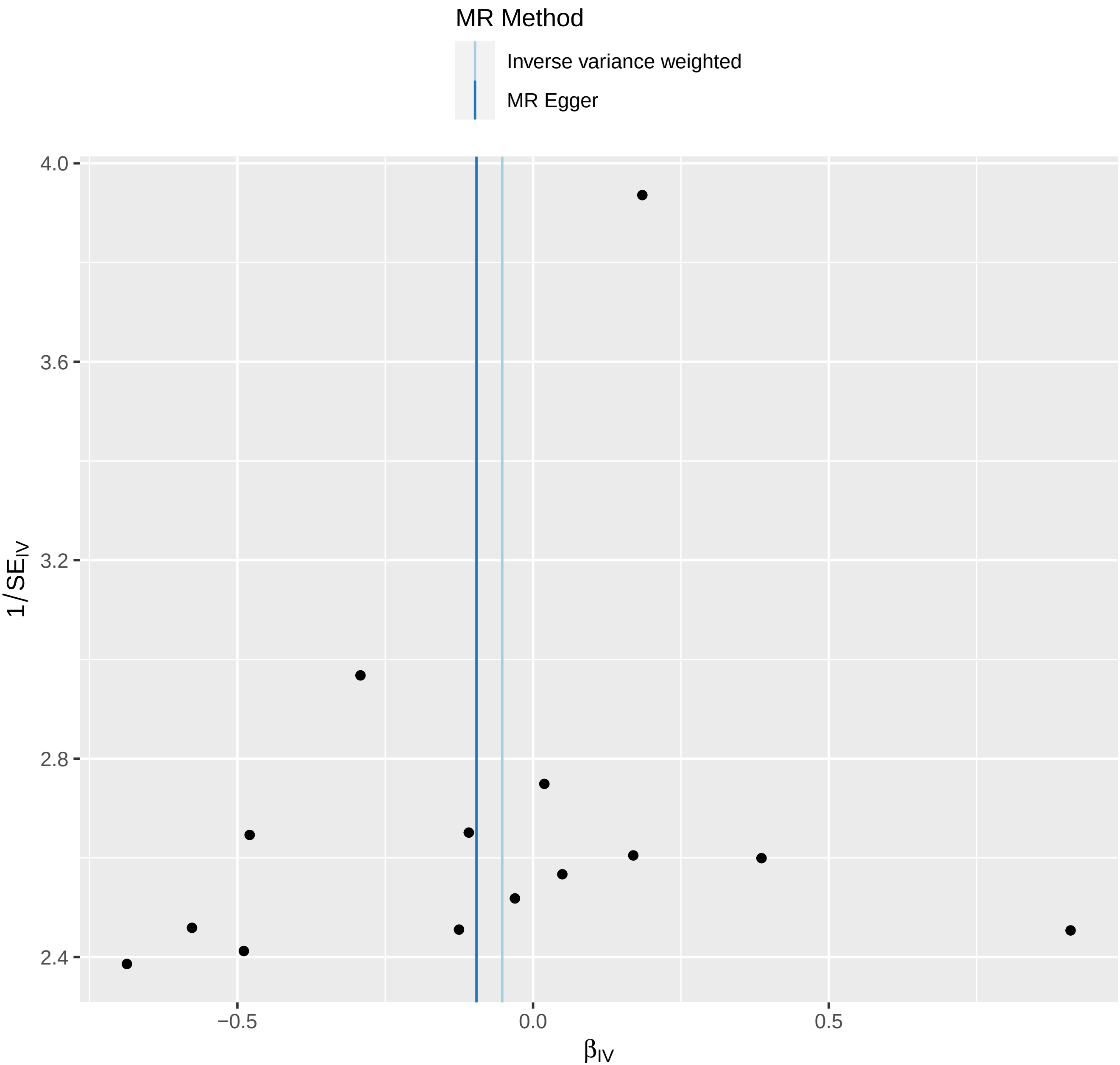

Batch 2−2791 : Gut microbiota abundance (genus Catenibacterium id.2153) on Bone sialoprotein 2
with a threshold of 1e−05

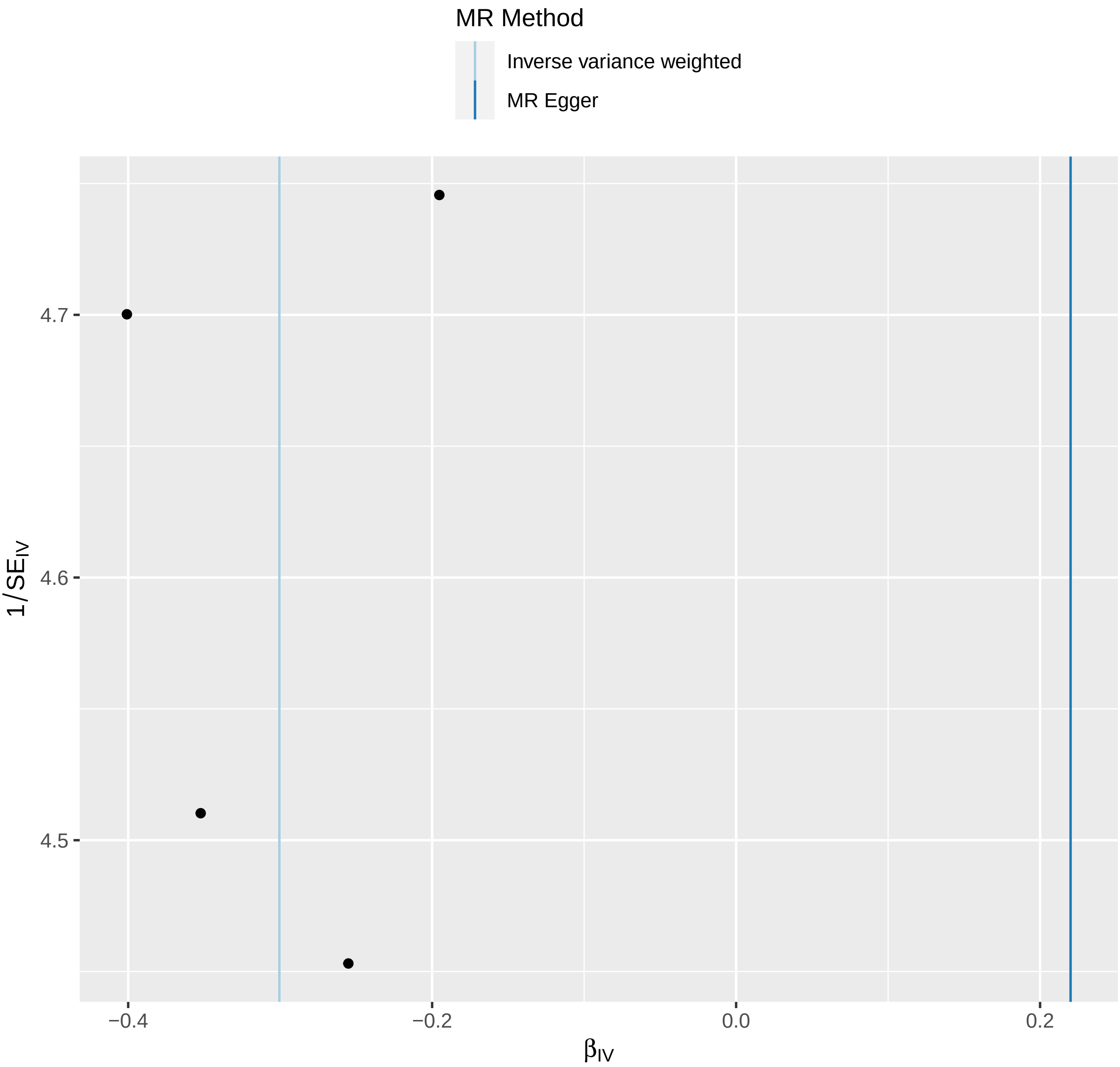

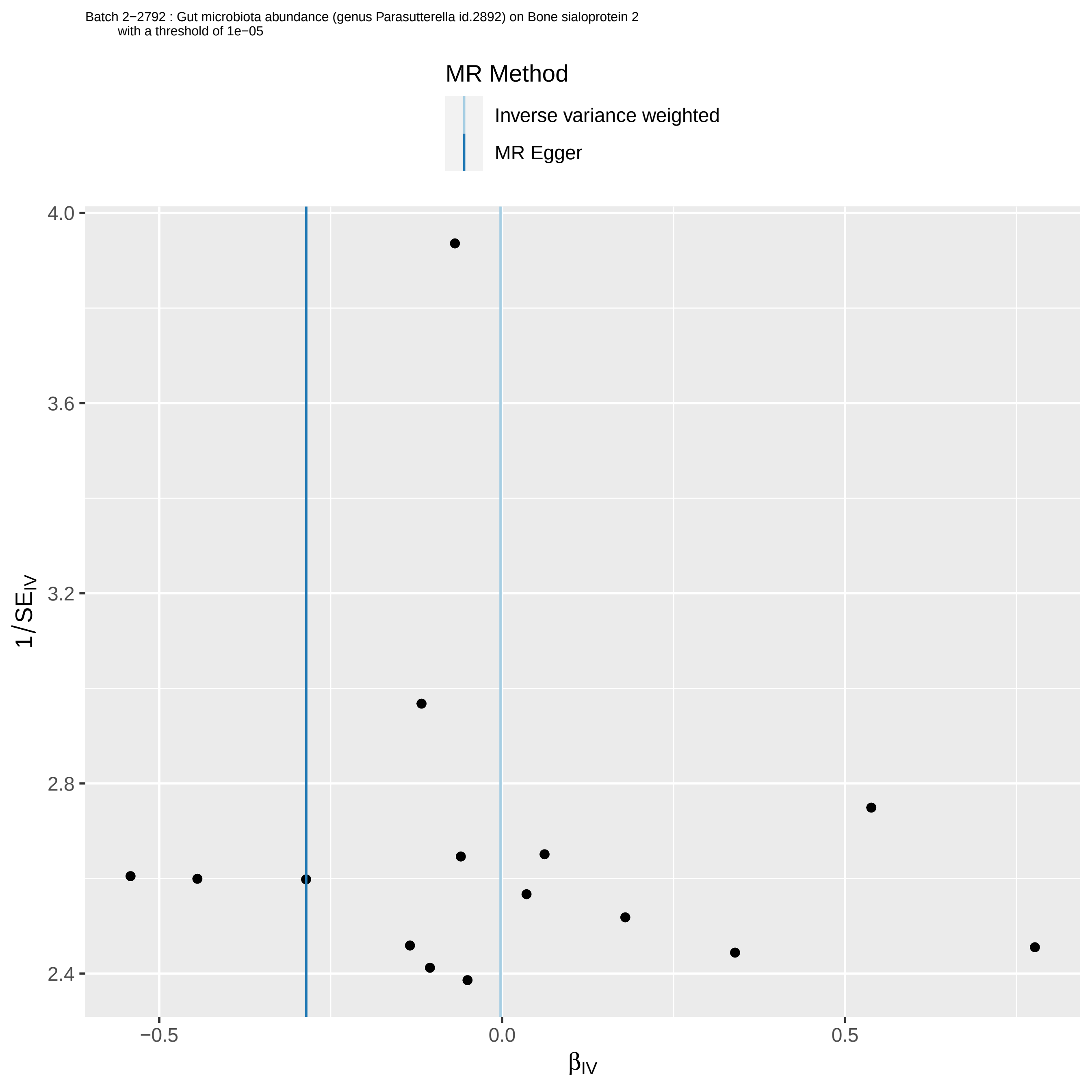
Batch 2−2792 : Gut microbiota abundance (genus Parasutterella id.2892) on Bone sialoprotein 2
with a threshold of 1e−05
MR Method
Inverse variance weighted
MR Egger
4.0
3.6
3.2
2.8
2.4
1/SE_IV
−0.5
0.0
0.5
β_IV

Batch 2−2793 : Gut microbiota abundance (genus Catenibacterium id.2153) on Intercellular adhesion molecule 1
with a threshold of 1e−05

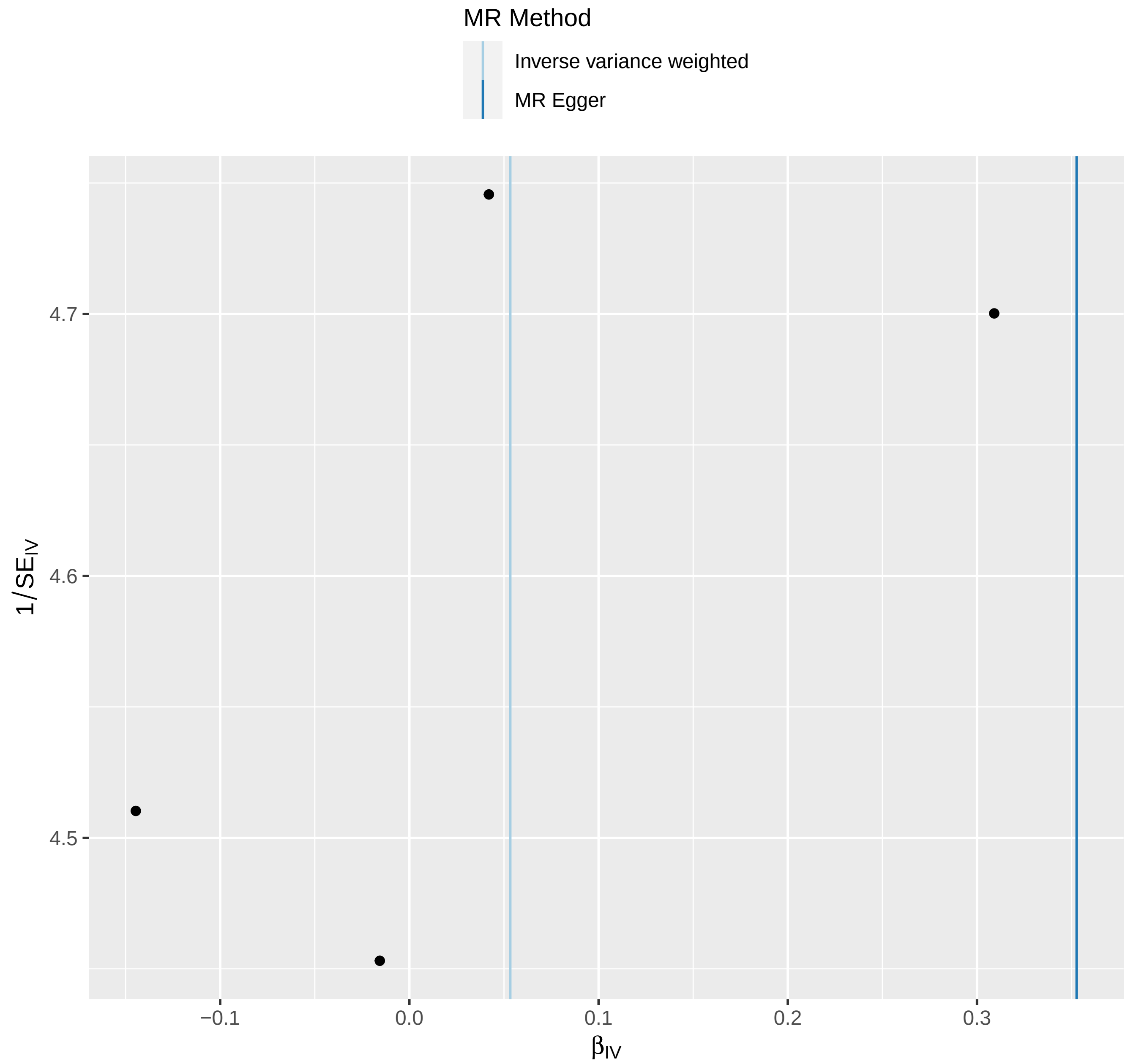

Batch 2−2794 : Gut microbiota abundance (genus Parasutterella id.2892) on Intercellular adhesion molecule 1
with a threshold of 1e−05

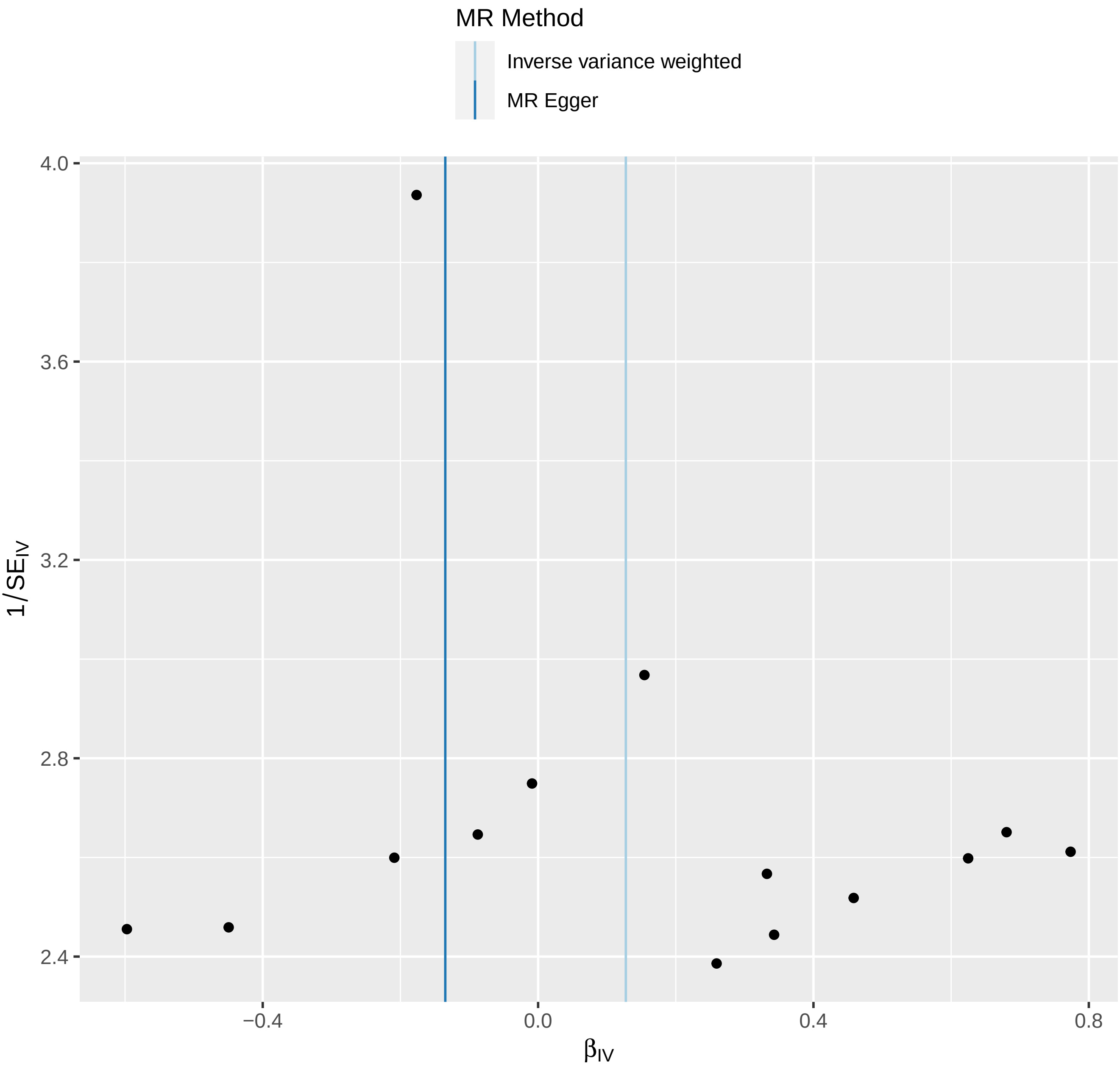

Batch 2−2795 : Gut microbiota abundance (genus Catenibacterium id.2153) on Intercellular adhesion molecule 2
with a threshold of 1e−05

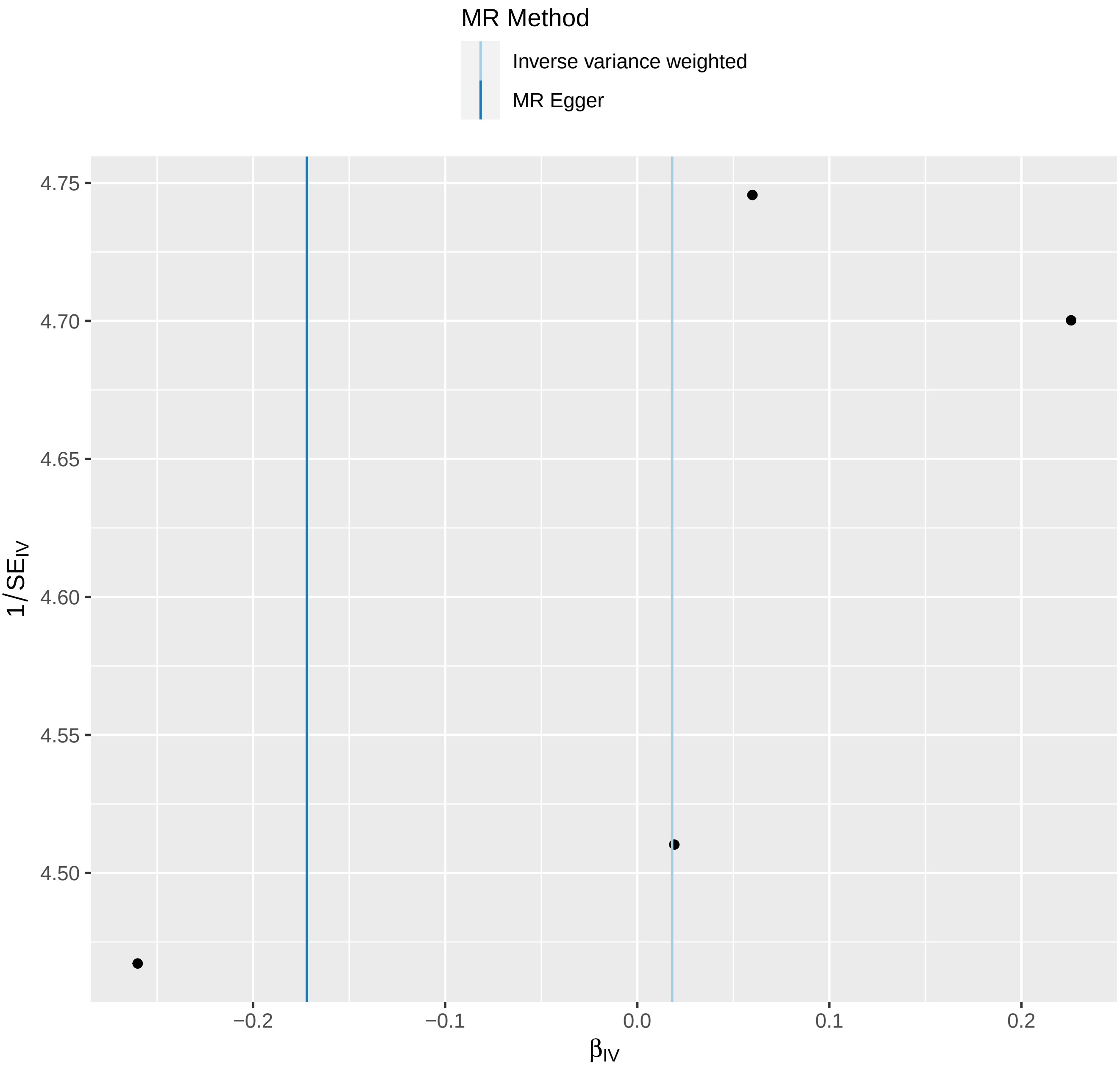

Batch 2−2796 : Gut microbiota abundance (genus Parasutterella id.2892) on Intercellular adhesion molecule 2
with a threshold of 1e−05

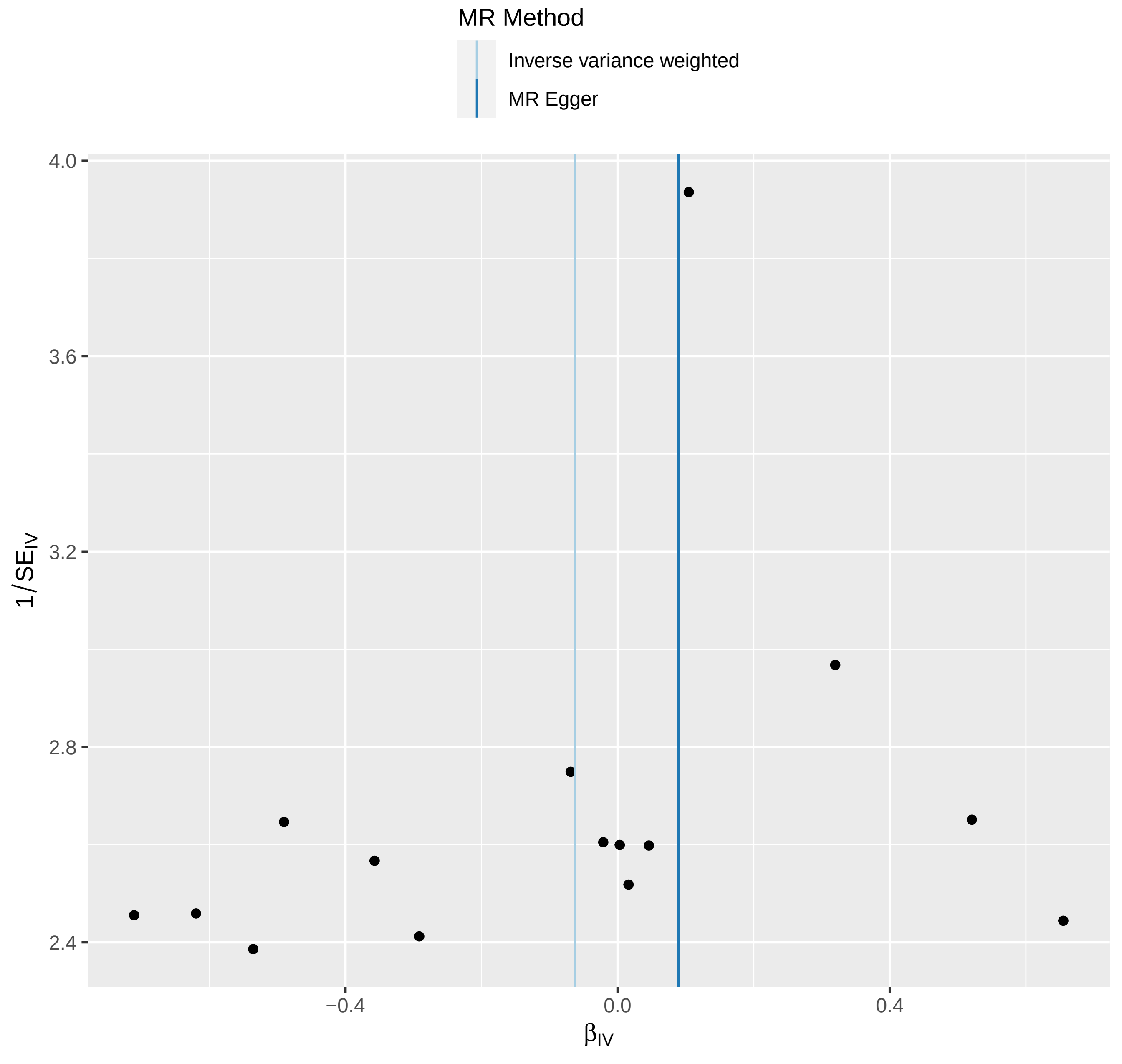

Batch 2−2797 : Gut microbiota abundance (genus Catenibacterium id.2153) on Intercellular adhesion molecule 3 with a threshold of 1e−05

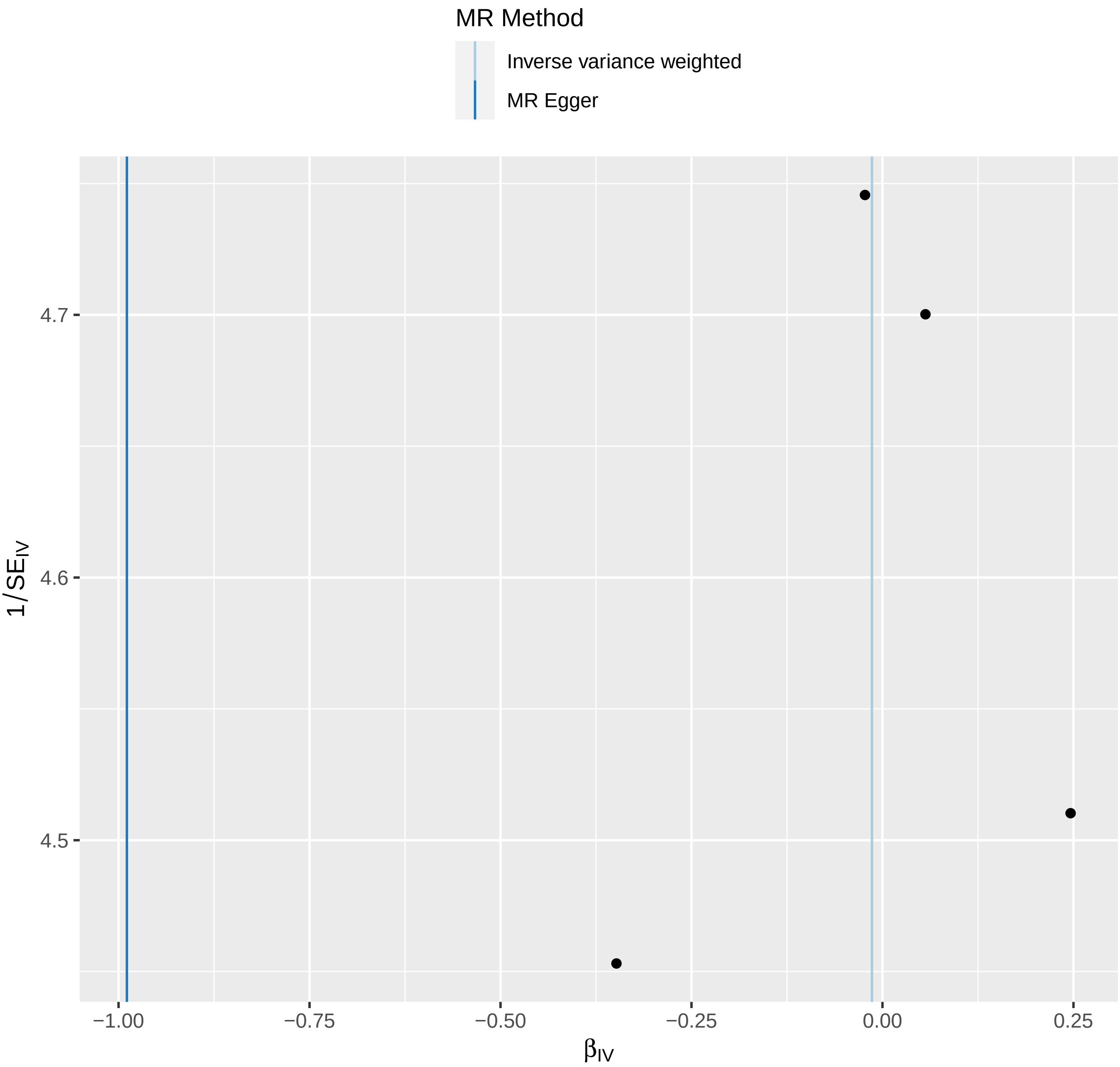

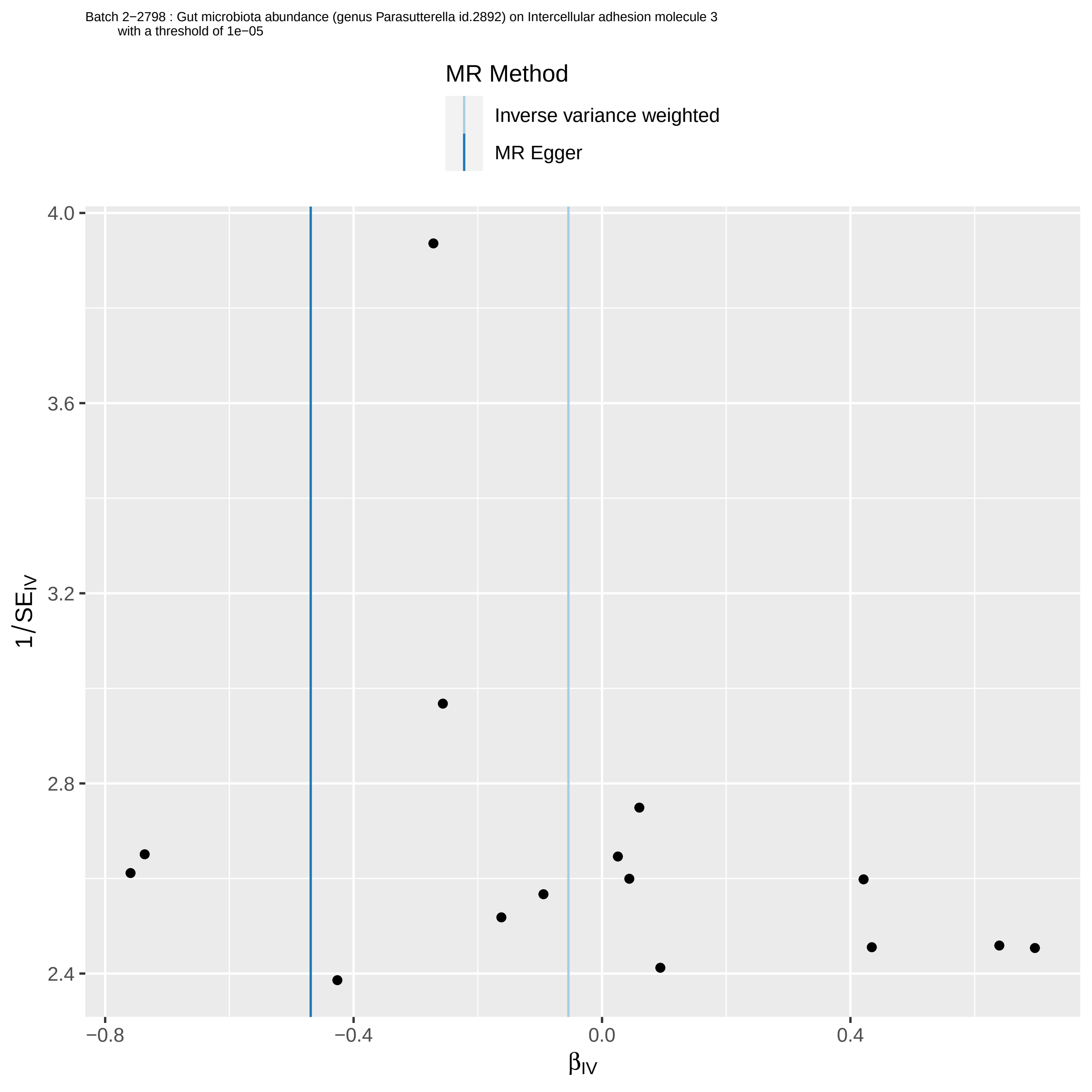

Batch 2−2798 : Gut microbiota abundance (genus Parasutterella id.2892) on Intercellular adhesion molecule 3
with a threshold of 1e−05
MR Method
Inverse variance weighted
MR Egger
4.0
3.6
3.2
2.8
2.4
1/SE_IV
−0.8
−0.4
0.0
0.4
β_IV

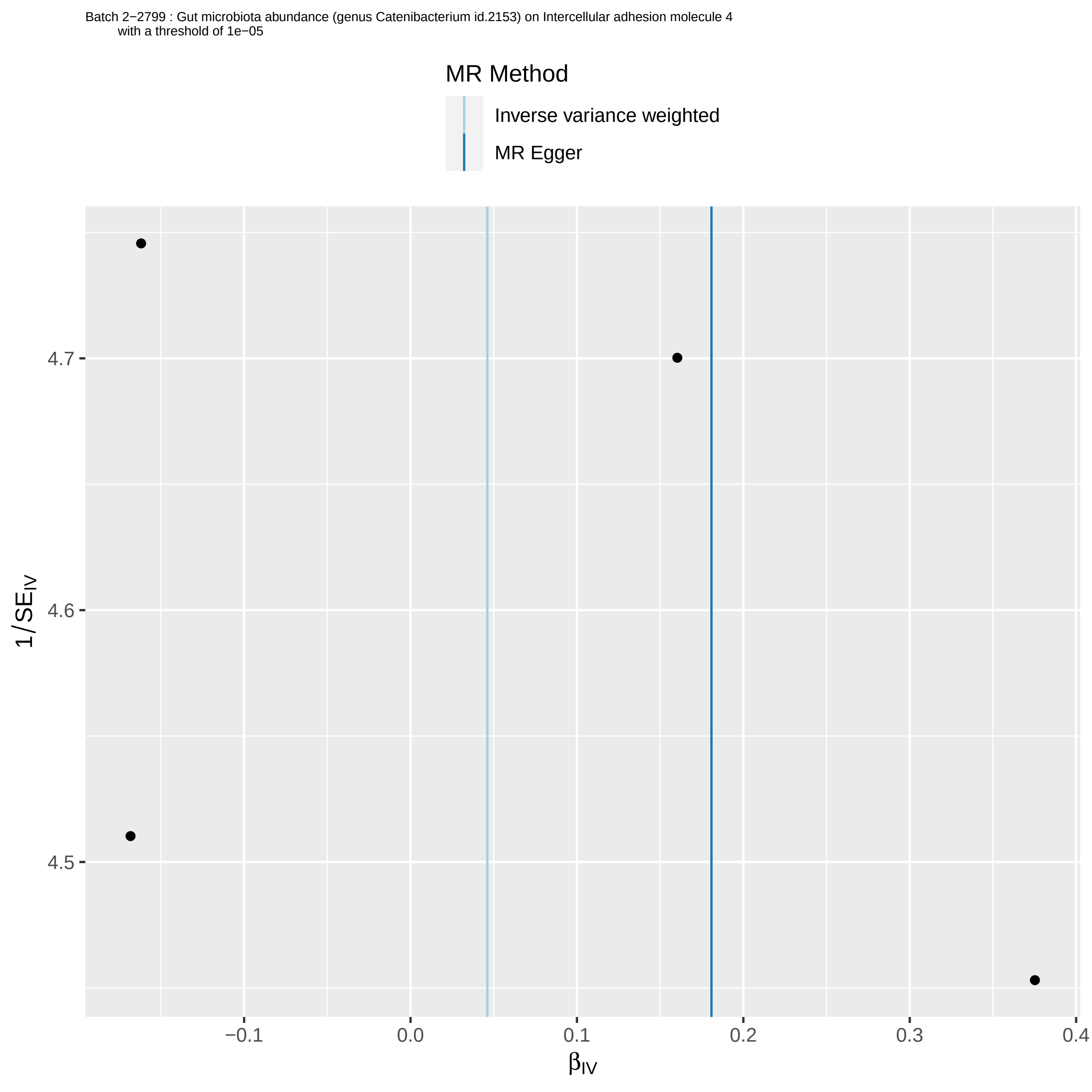

Batch 2−2799 : Gut microbiota abundance (genus Catenibacterium id.2153) on Intercellular adhesion molecule 4
with a threshold of 1e−05
MR Method
Inverse variance weighted
MR Egger
4.7
4.6
4.5
1/SE_IV
−0.1
0.0
0.1
0.2
0.3
0.4
β_IV

Batch 2−2800 : Gut microbiota abundance (genus Parasutterella id.2892) on Intercellular adhesion molecule 4
with a threshold of 1e−05

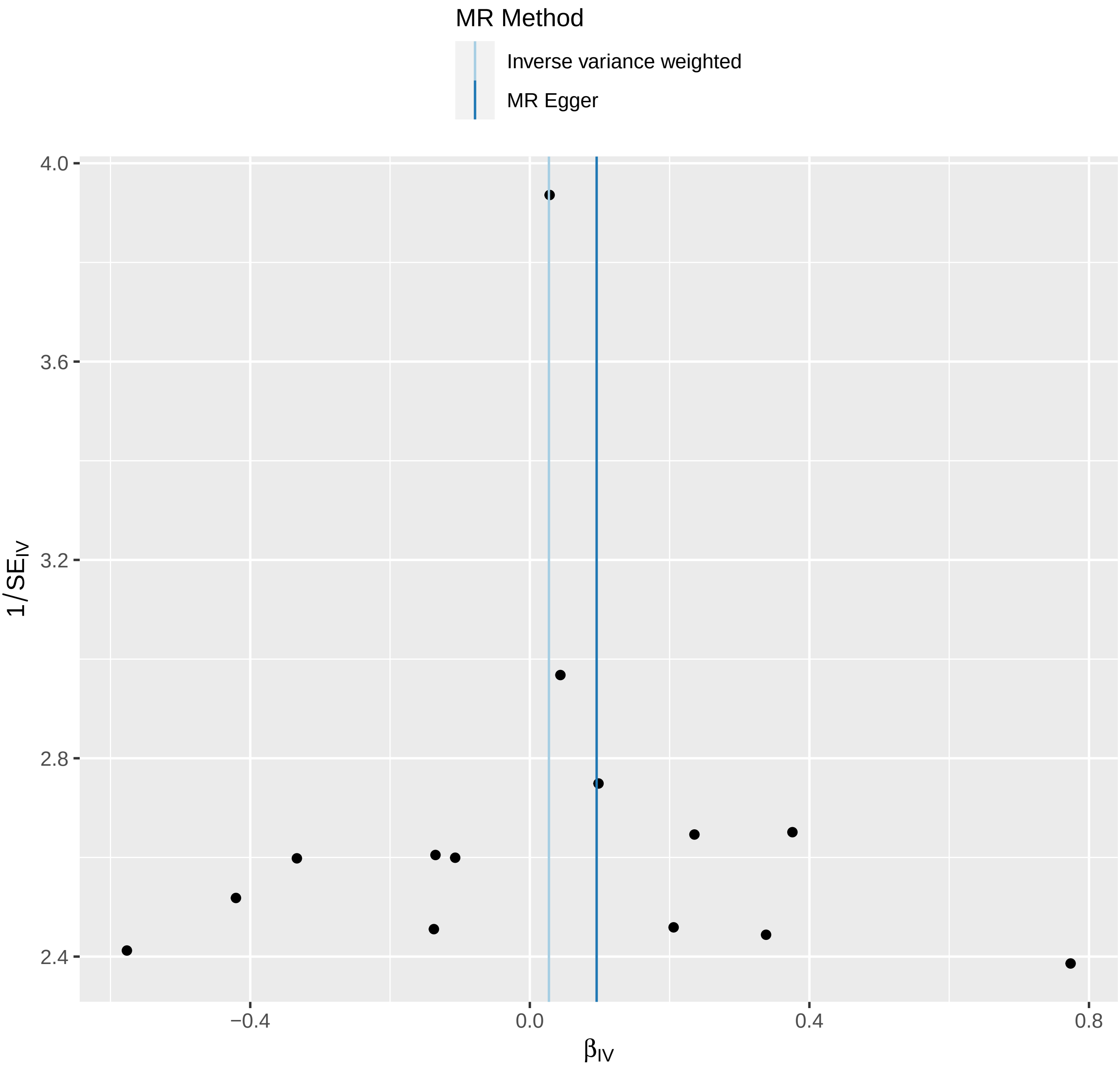

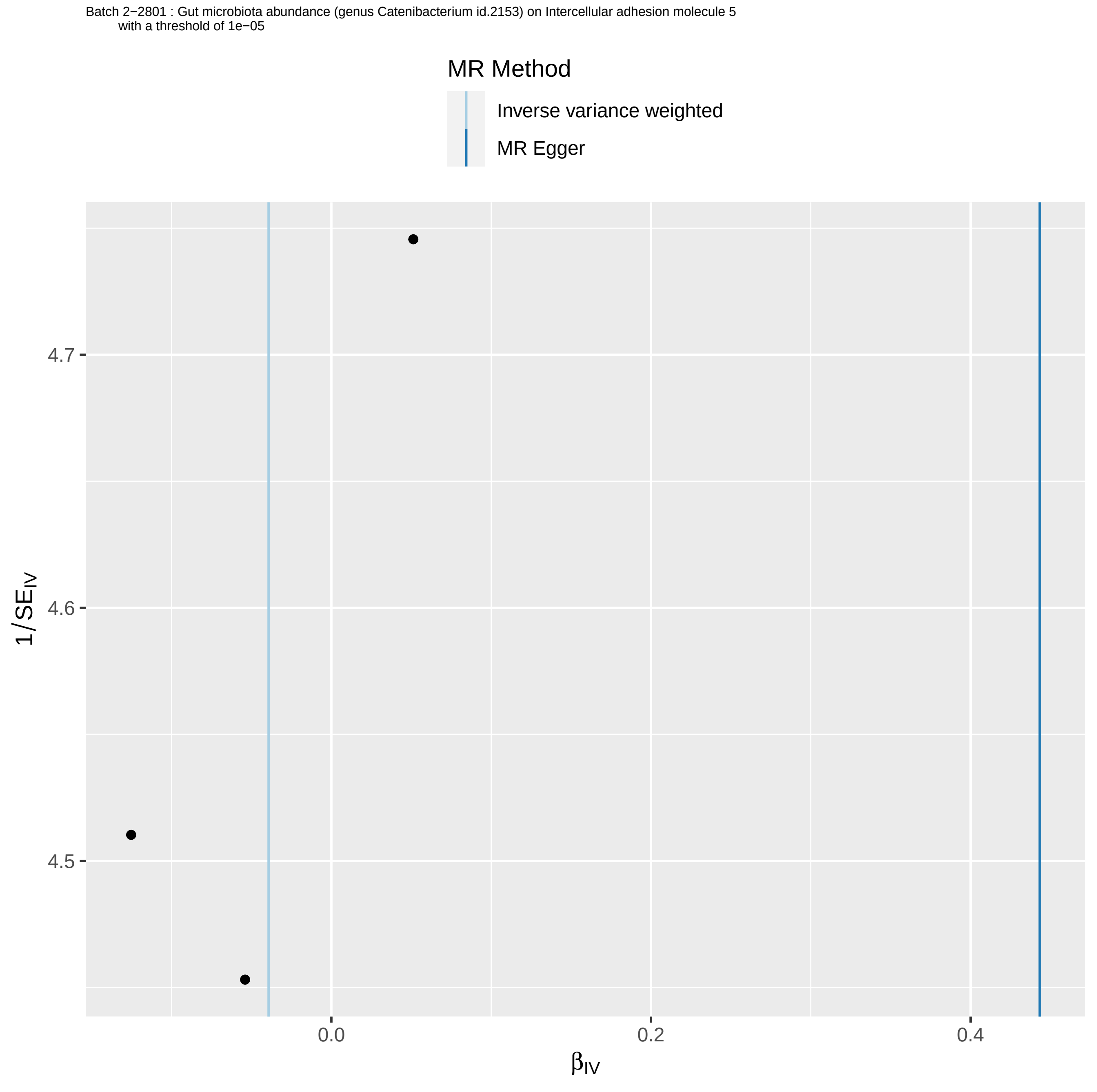
Batch 2−2801 : Gut microbiota abundance (genus Catenibacterium id.2153) on Intercellular adhesion molecule 5
with a threshold of 1e−05
MR Method
Inverse variance weighted
MR Egger
4.7
4.6
4.5
1/SE_IV
0.0
0.2
0.4
β_IV

Batch 2−2802 : Gut microbiota abundance (genus Parasutterella id.2892) on Intercellular adhesion molecule 5
with a threshold of 1e−05

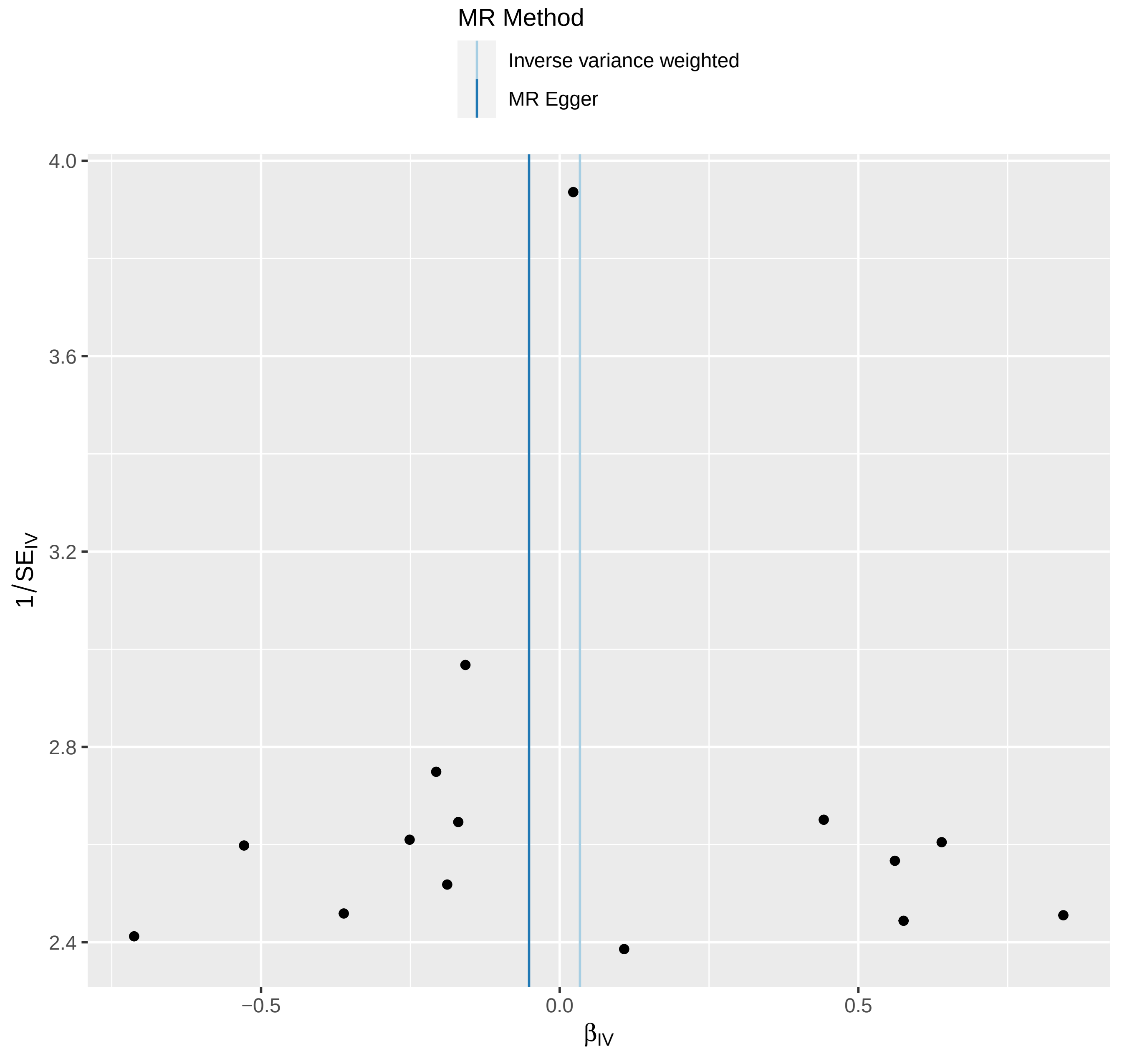

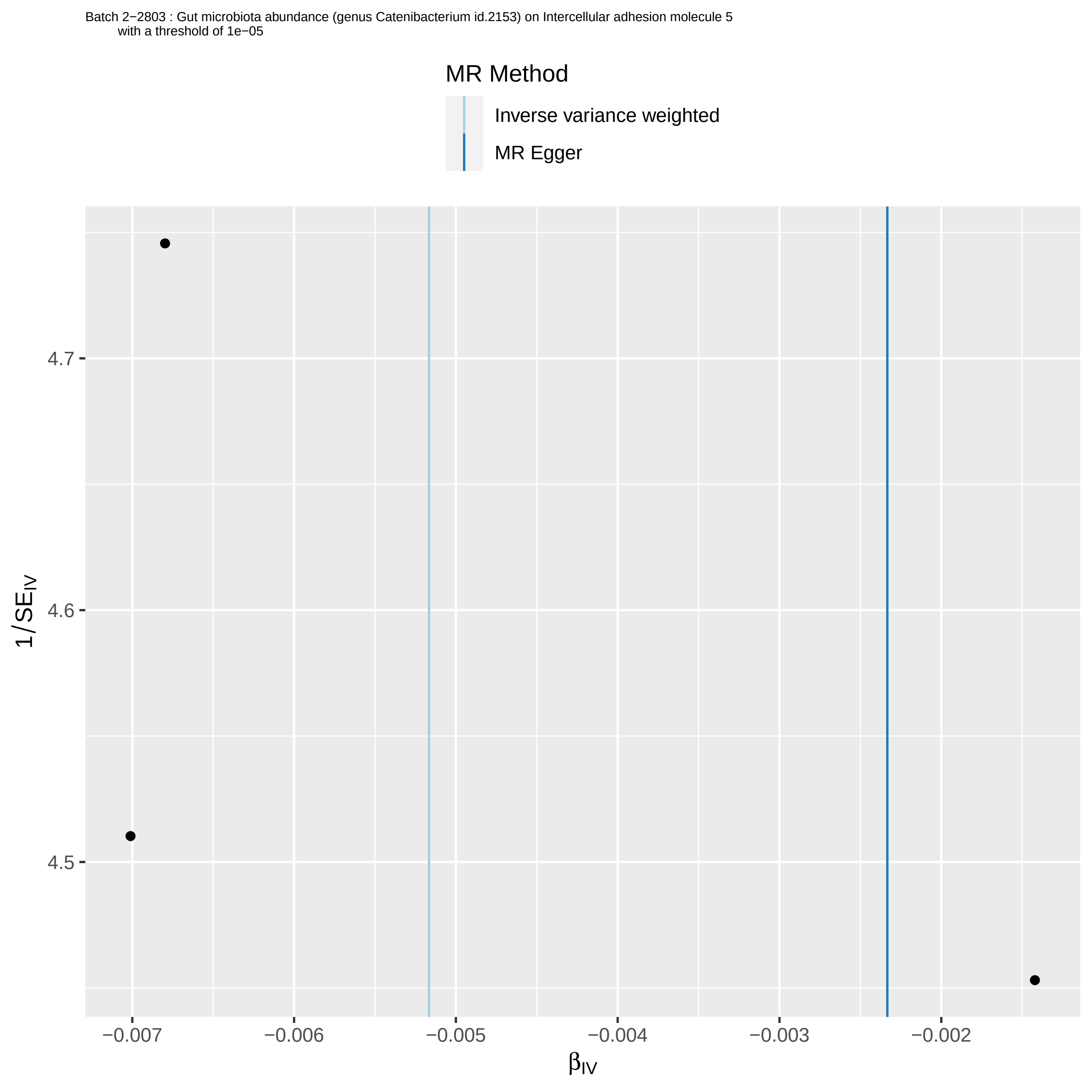
Batch 2−2803 : Gut microbiota abundance (genus Catenibacterium id.2153) on Intercellular adhesion molecule 5
with a threshold of 1e−05
MR Method
Inverse variance weighted
MR Egger
4.7
4.6
4.5
1/SE_IV
−0.007
−0.006
−0.005
−0.004
−0.003
−0.002
β_IV

Batch 2−2804 : Gut microbiota abundance (genus Parasutterella id.2892) on Intercellular adhesion molecule 5
with a threshold of 1e−05

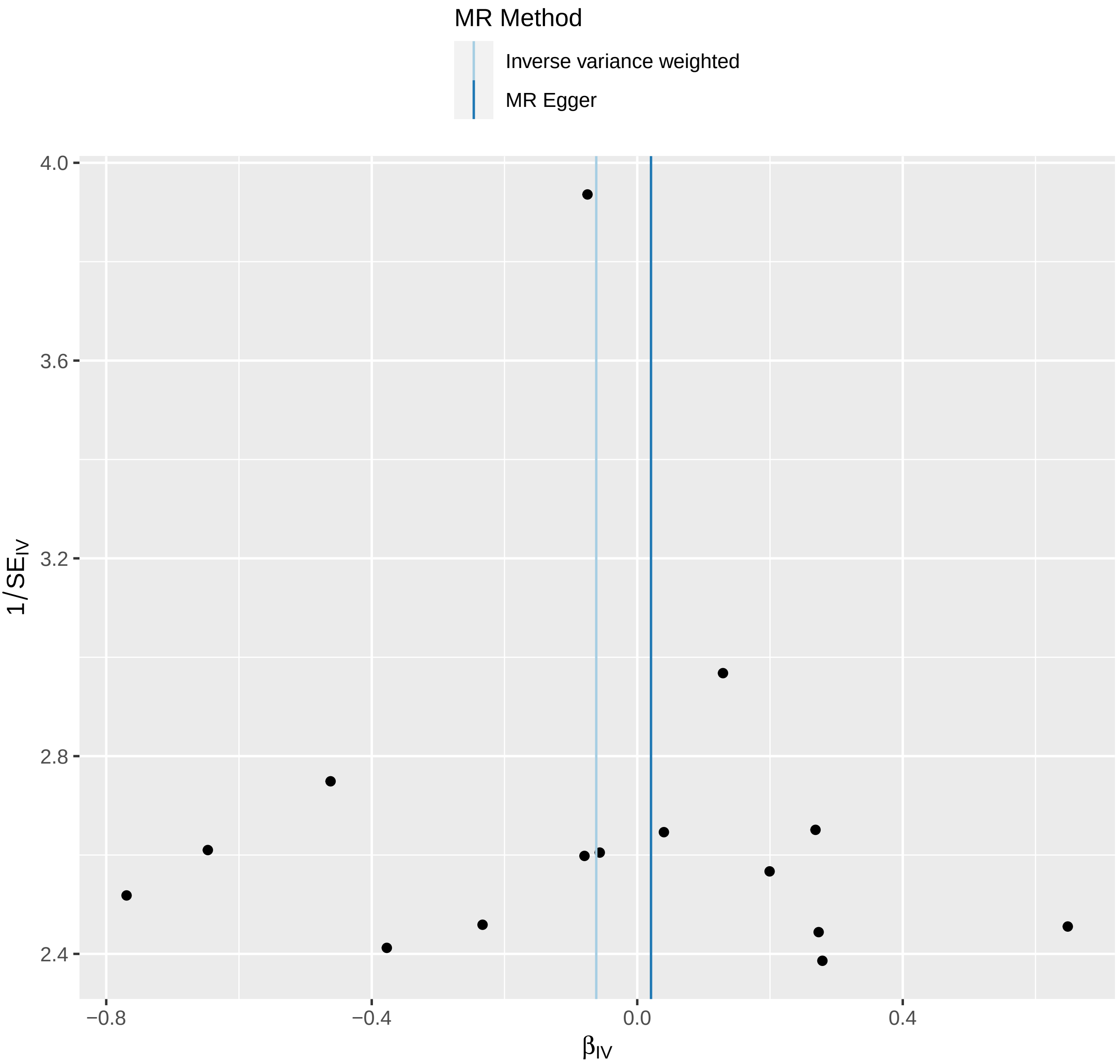

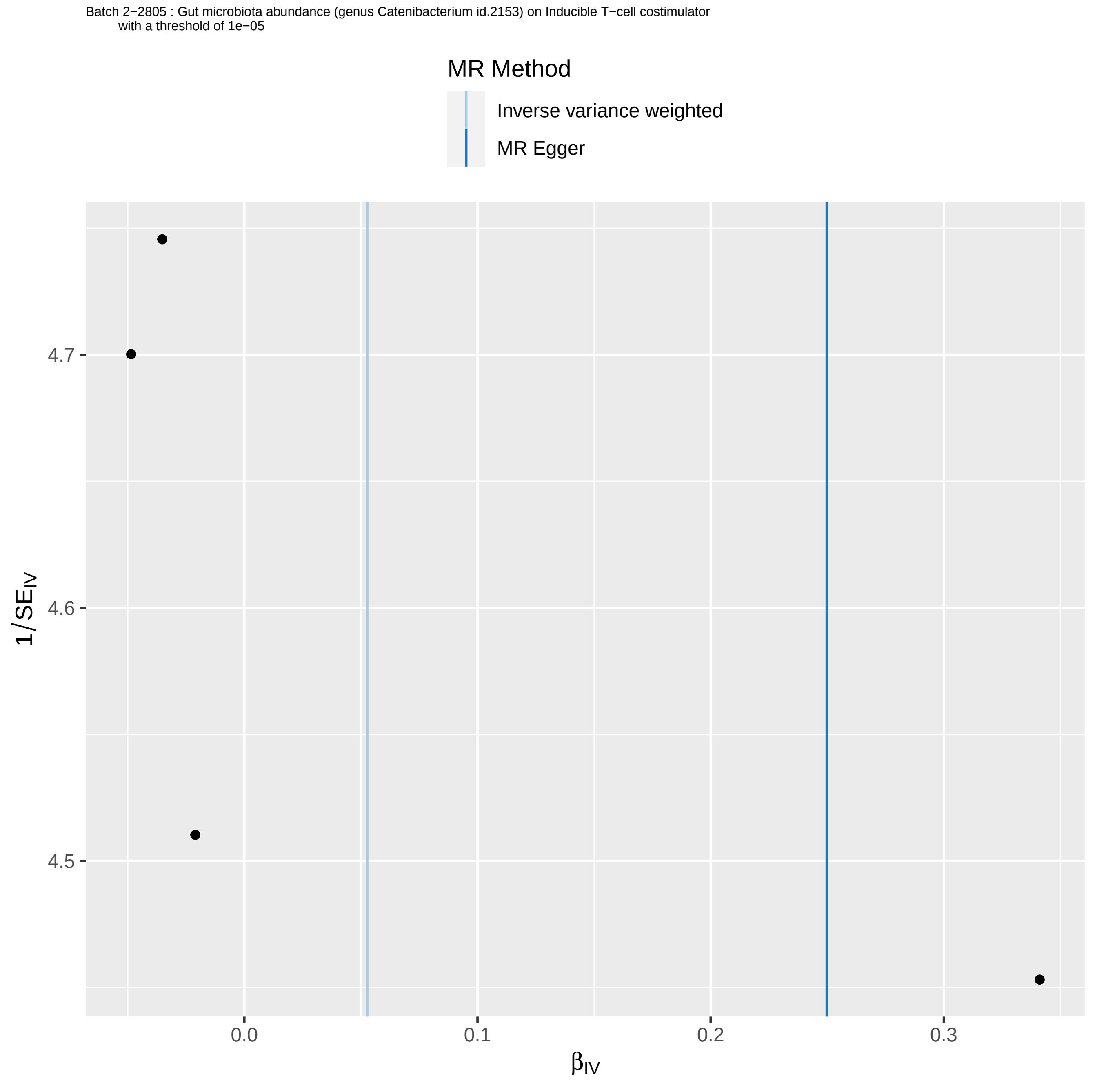
Batch 2−2805 : Gut microbiota abundance (genus Catenibacterium id.2153) on Inducible T−cell costimulator
with a threshold of 1e−05
MR Method
Inverse variance weighted
MR Egger
4.7
4.6
4.5
1/SE_IV
0.0
0.1
0.2
0.3
β_IV

Batch 2−2806 : Gut microbiota abundance (genus Parasutterella id.2892) on Inducible T−cell costimulator
with a threshold of 1e−05

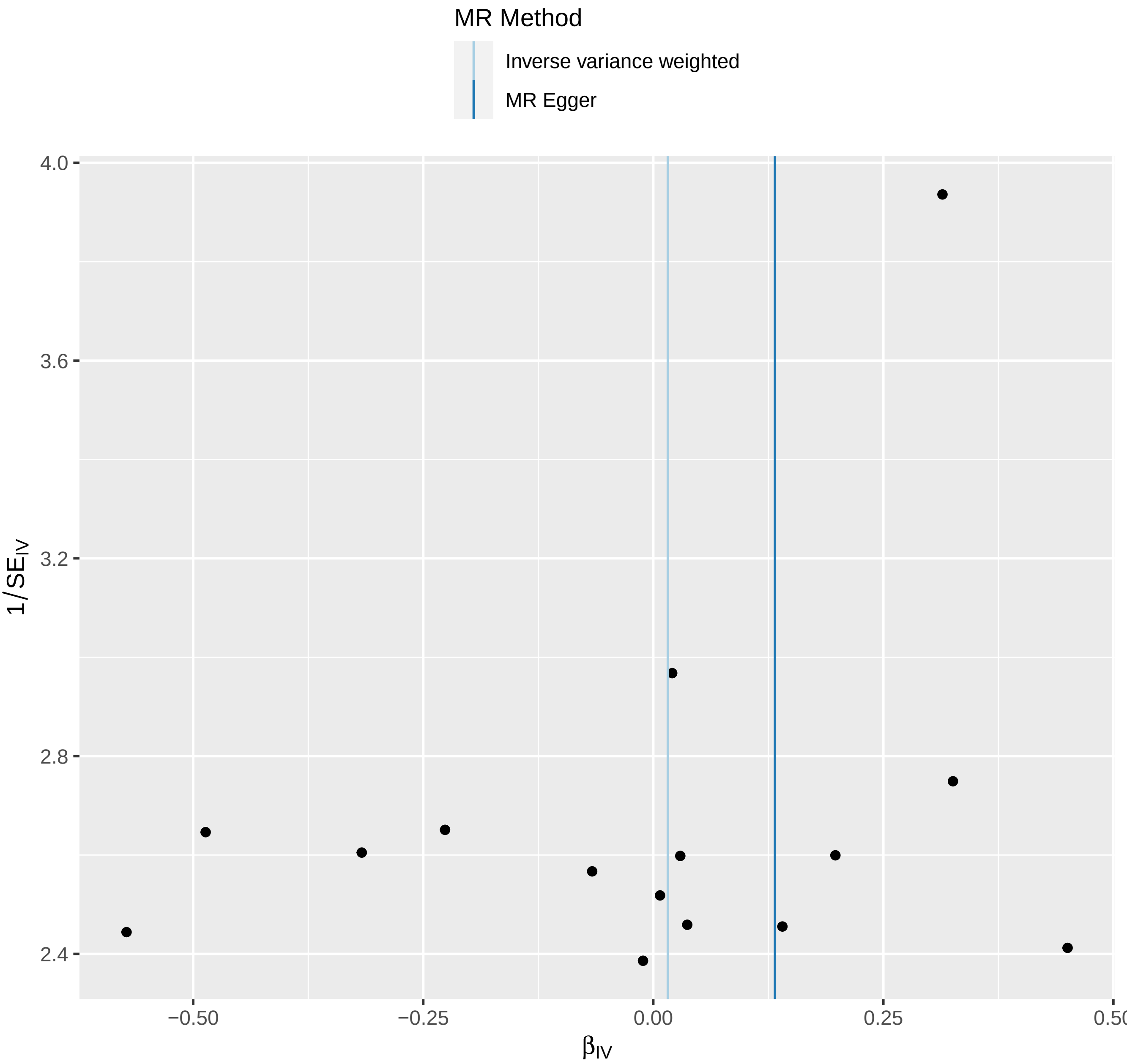

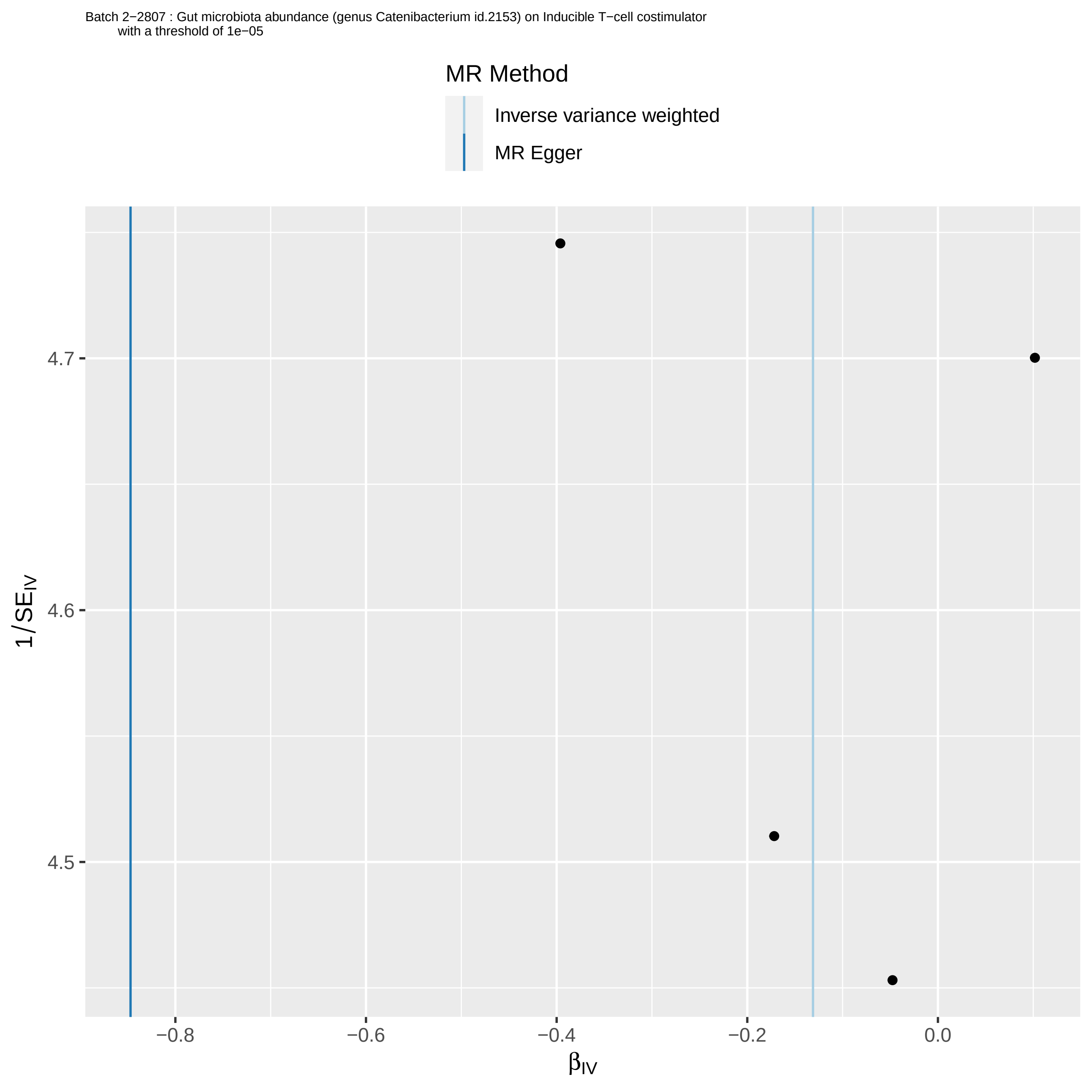
Batch 2−2807 : Gut microbiota abundance (genus Catenibacterium id.2153) on Inducible T−cell costimulator
with a threshold of 1e−05
MR Method
Inverse variance weighted
MR Egger
4.7
4.6
4.5
1/SE_IV
−0.8
−0.6
−0.4
−0.2
0.0
β_IV

Batch 2−2808 : Gut microbiota abundance (genus Parasutterella id.2892) on Inducible T−cell costimulator
with a threshold of 1e−05

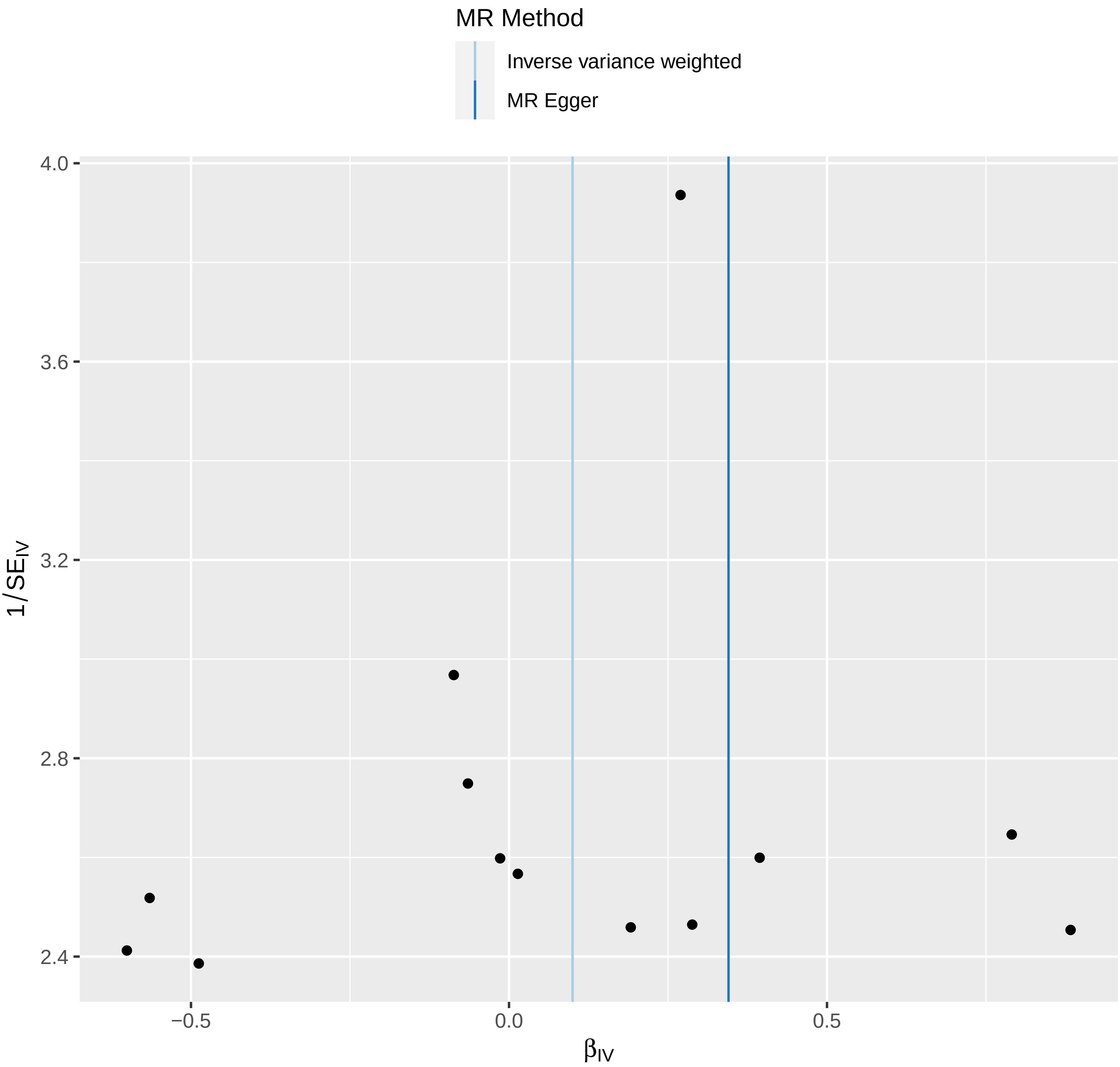

Batch 2−2809 : Gut microbiota abundance (genus Catenibacterium id.2153) on ICOS ligand
with a threshold of 1e−05

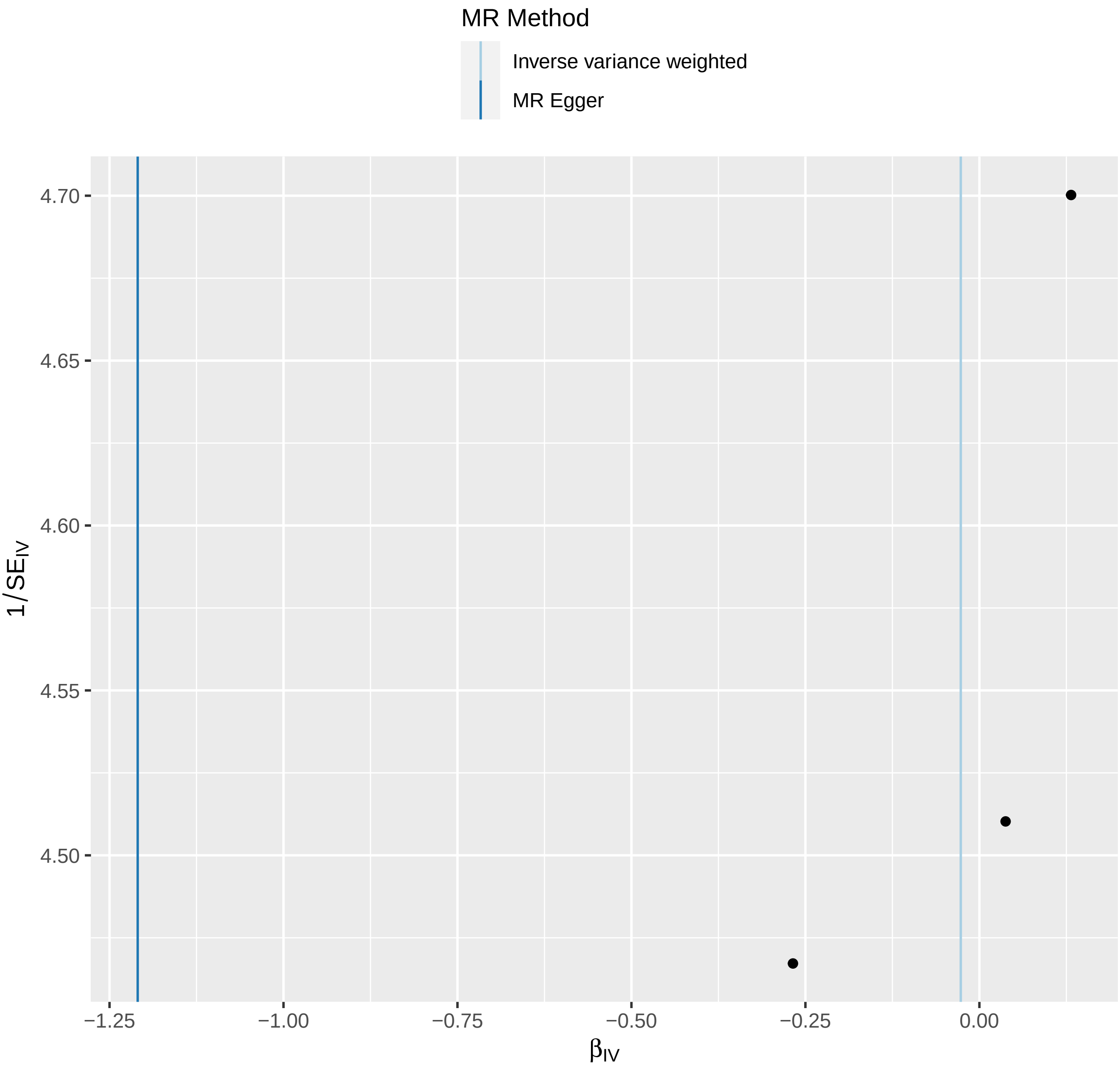

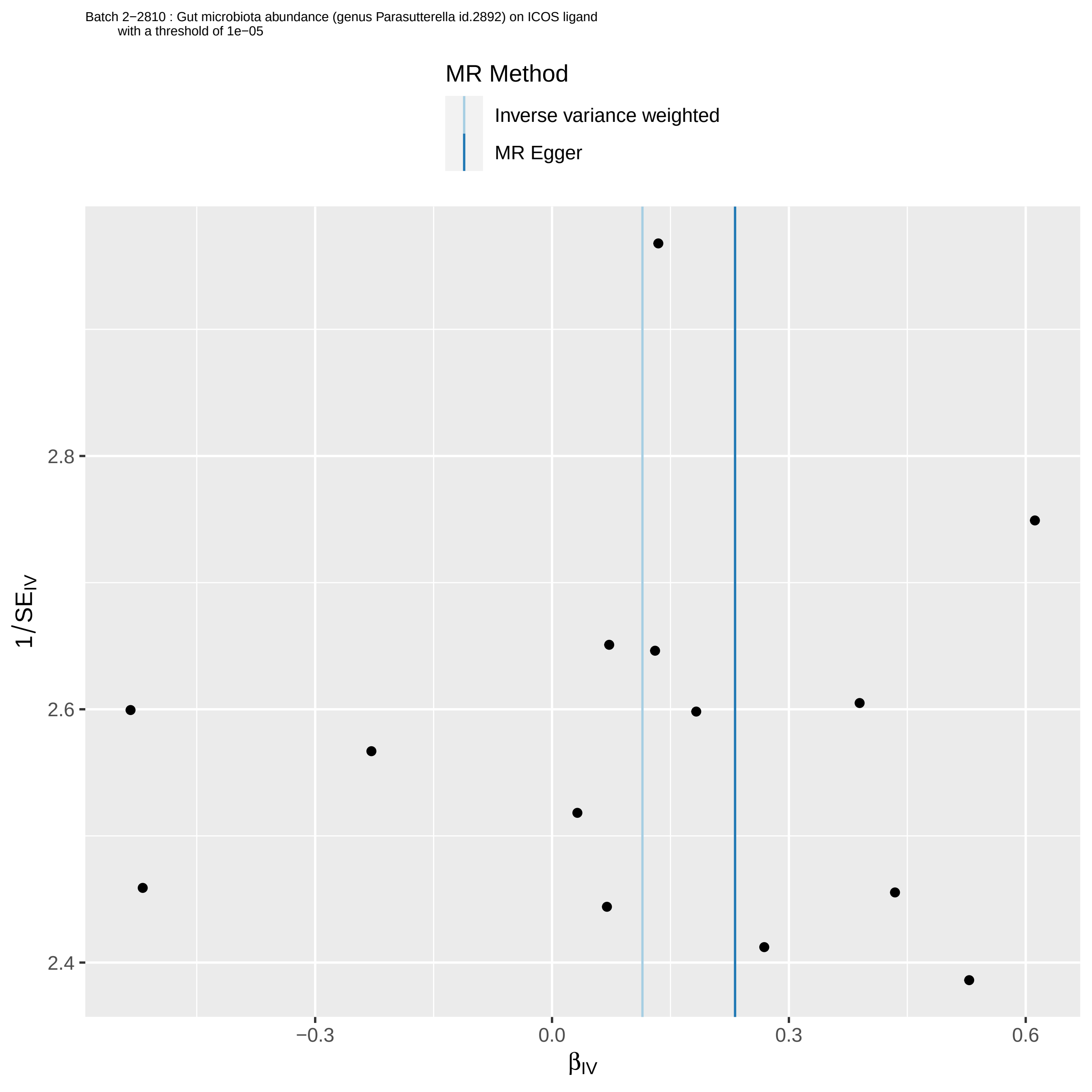
Batch 2−2810 : Gut microbiota abundance (genus Parasutterella id.2892) on ICOS ligand
with a threshold of 1e−05
MR Method
Inverse variance weighted
MR Egger
2.8
2.6
2.4
1/SE_IV
−0.3
0.0
0.3
0.6
β_IV

Batch 2−2811 : Gut microbiota abundance (genus Catenibacterium id.2153) on ICOS ligand
with a threshold of 1e−05

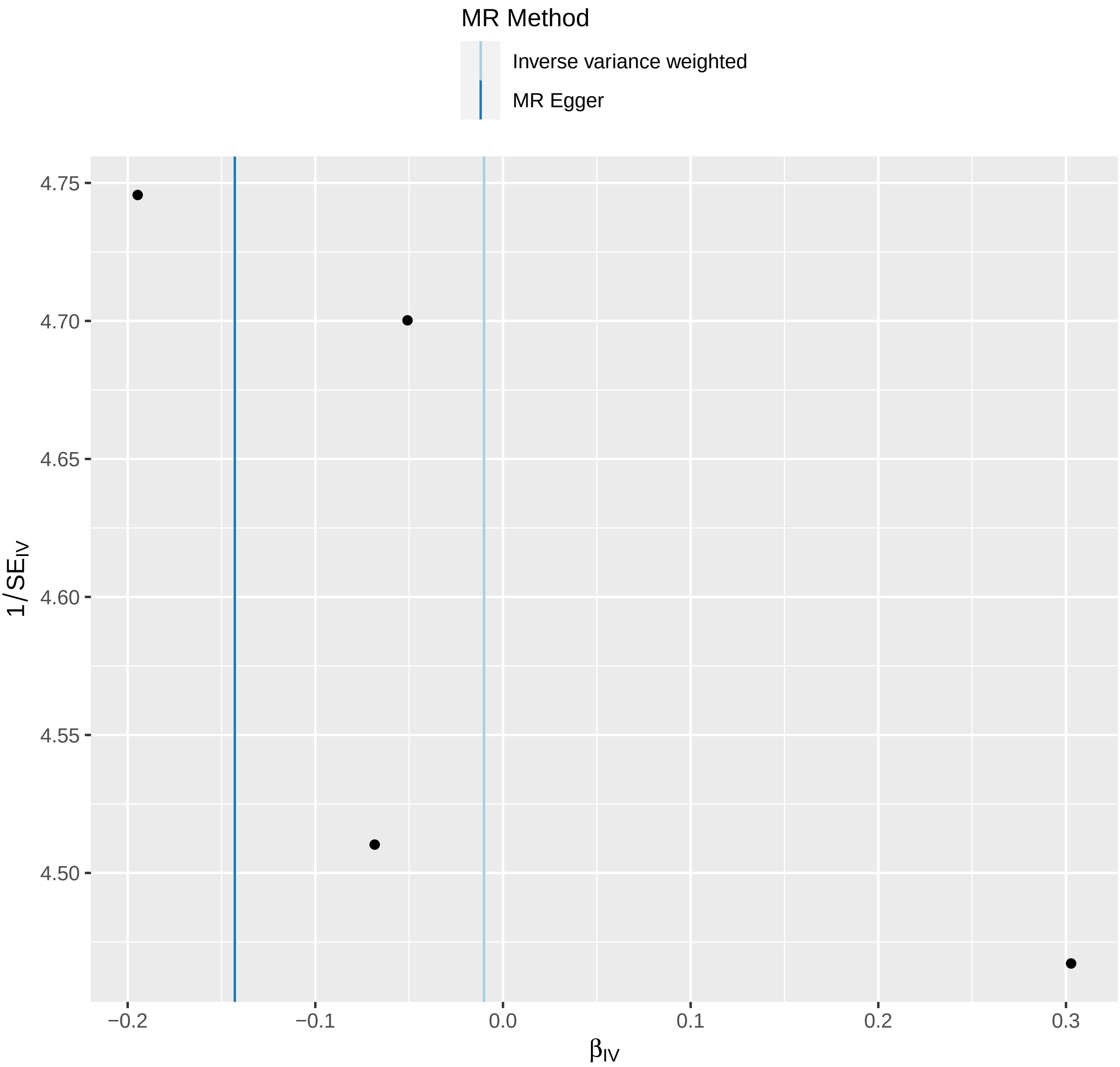

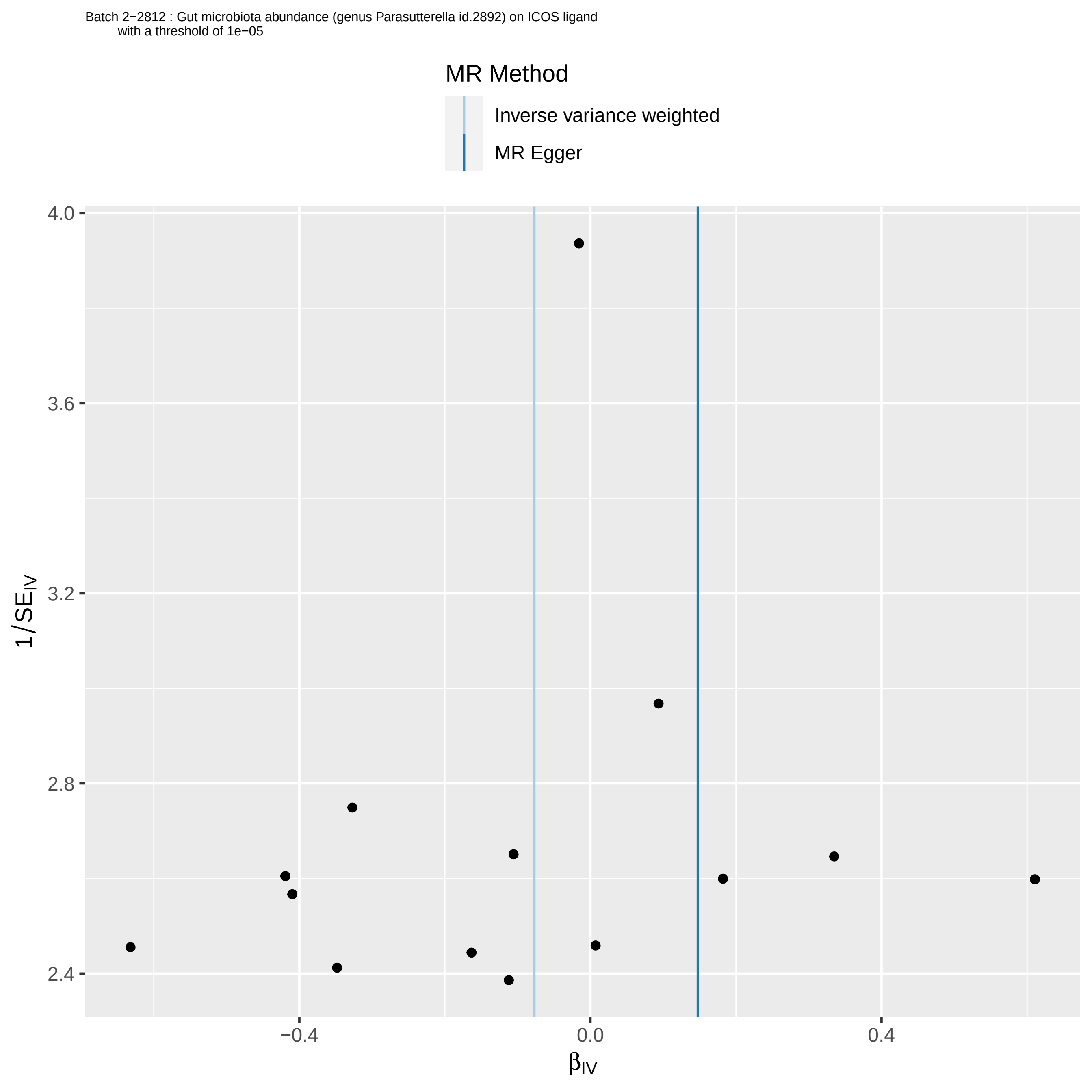

Batch 2−2812 : Gut microbiota abundance (genus Parasutterella id.2892) on ICOS ligand
with a threshold of 1e−05
MR Method
Inverse variance weighted
MR Egger
4.0
3.6
3.2
2.8
2.4
1/SE_IV
−0.4
0.0
0.4
β_IV

Batch 2−2813 : Gut microbiota abundance (genus Catenibacterium id.2153) on DNA−binding protein inhibitor ID−1
with a threshold of 1e−05

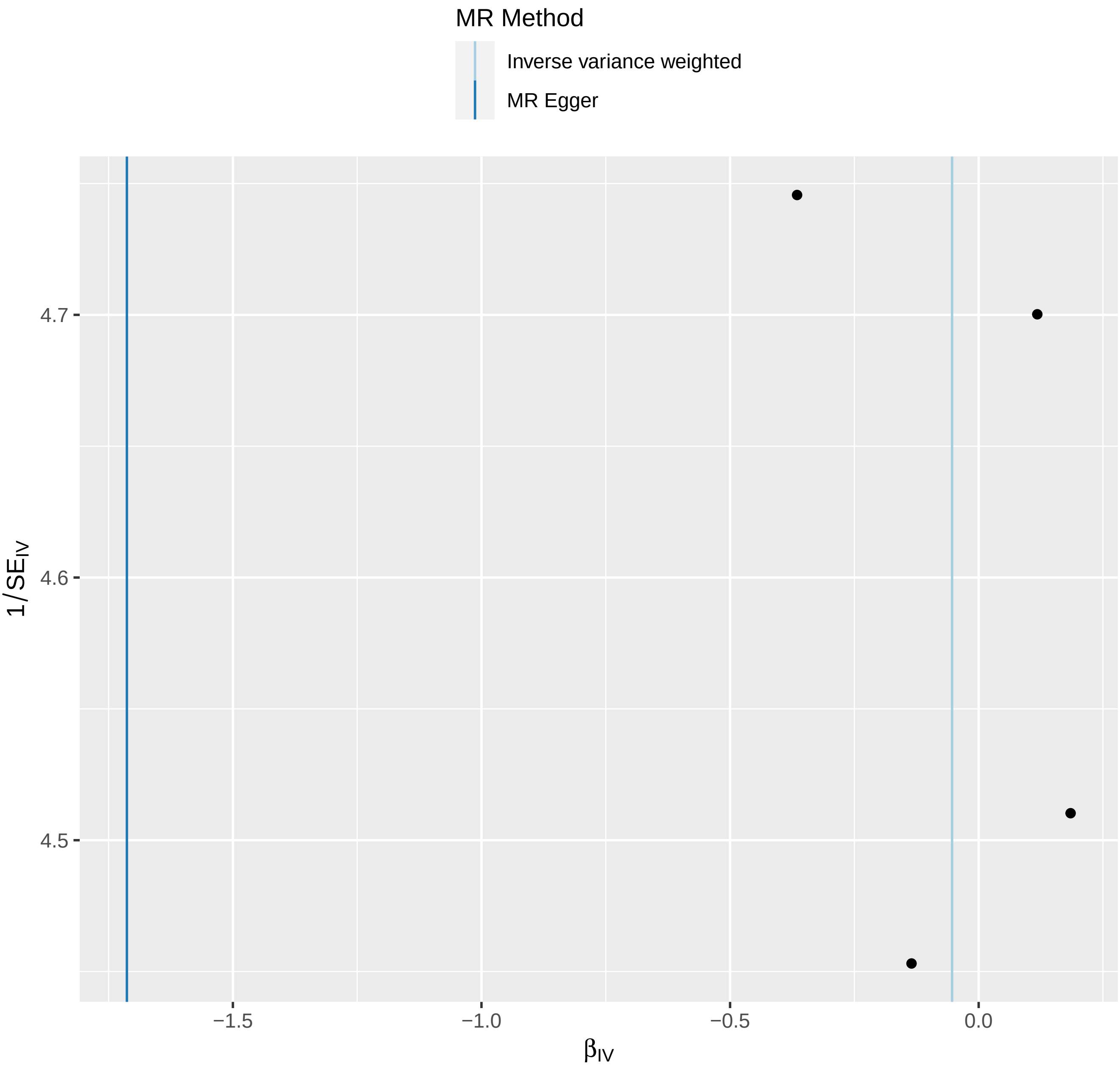

Batch 2−2814 : Gut microbiota abundance (genus Parasutterella id.2892) on DNA−binding protein inhibitor ID−1
with a threshold of 1e−05

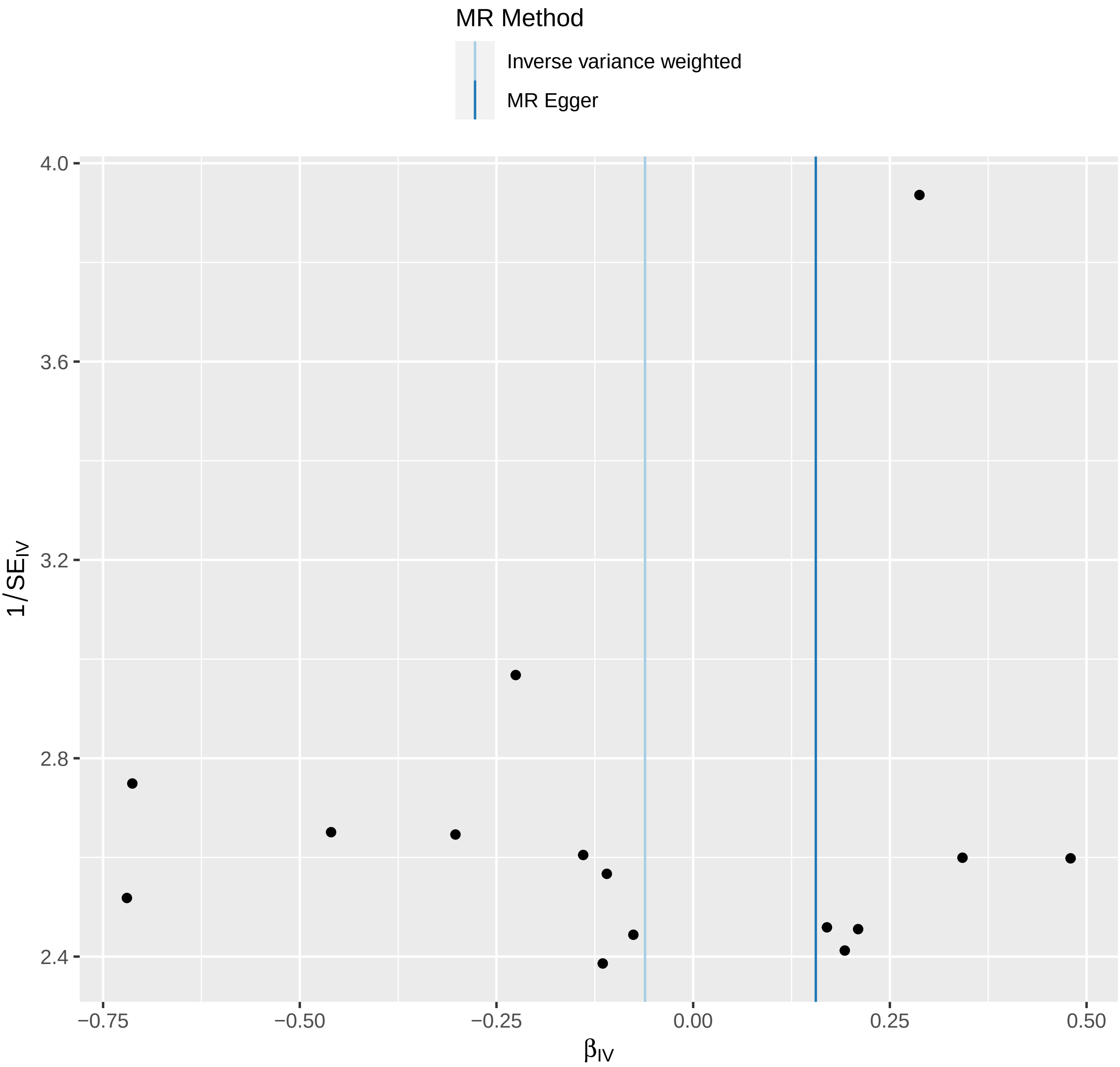

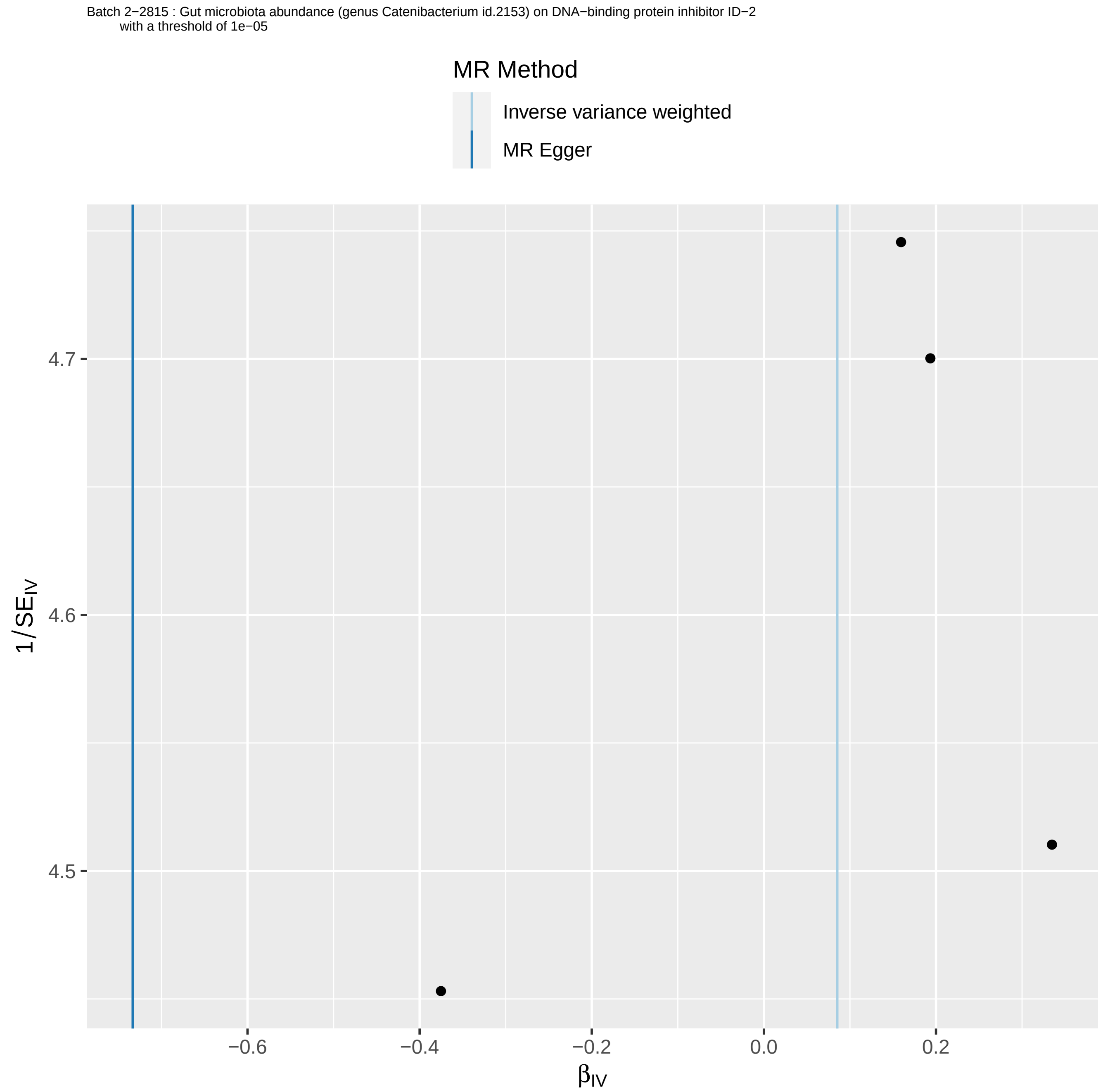

Batch 2−2815 : Gut microbiota abundance (genus Catenibacterium id.2153) on DNA−binding protein inhibitor ID−2
with a threshold of 1e−05
MR Method
Inverse variance weighted
MR Egger
4.7
4.6
4.5
1/SE_IV
−0.6
−0.4
−0.2
0.0
0.2
β_IV

Batch 2−2816 : Gut microbiota abundance (genus Parasutterella id.2892) on DNA−binding protein inhibitor ID−2
with a threshold of 1e−05

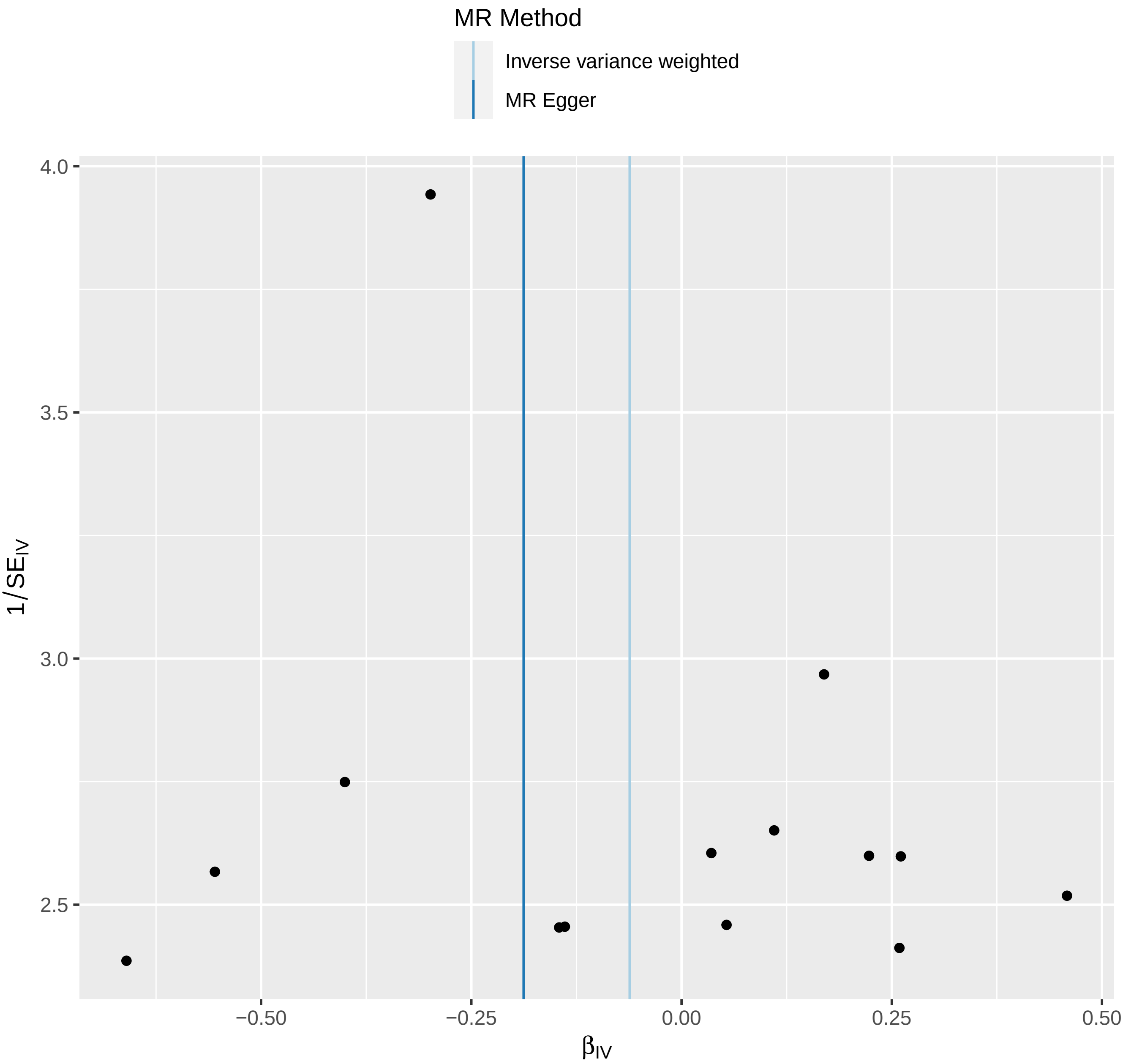

Batch 2−2817 : Gut microbiota abundance (genus Catenibacterium id.2153) on Insulin−degrading enzyme
with a threshold of 1e−05

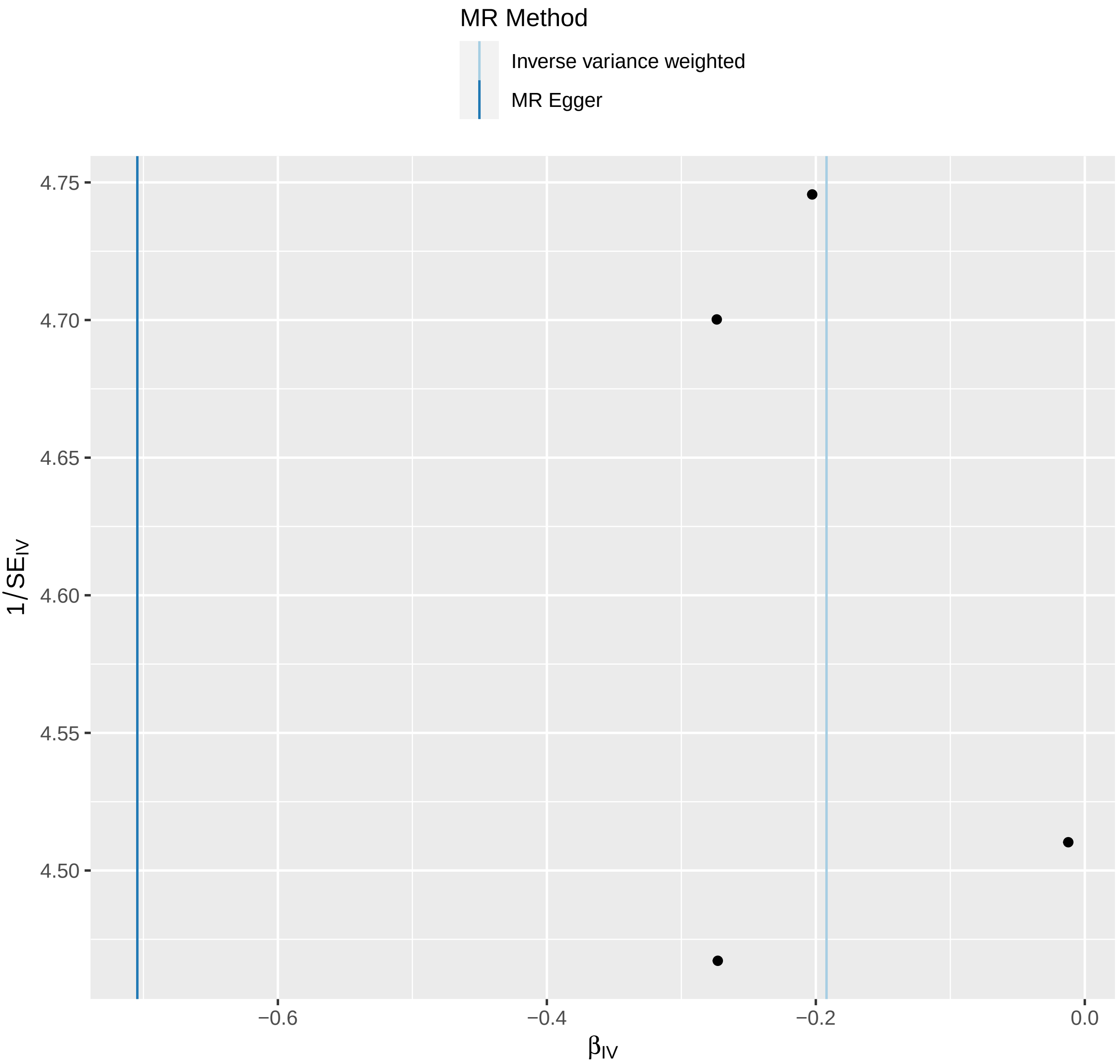

Batch 2−2818 : Gut microbiota abundance (genus Parasutterella id.2892) on Insulin−degrading enzyme with a threshold of 1e−05

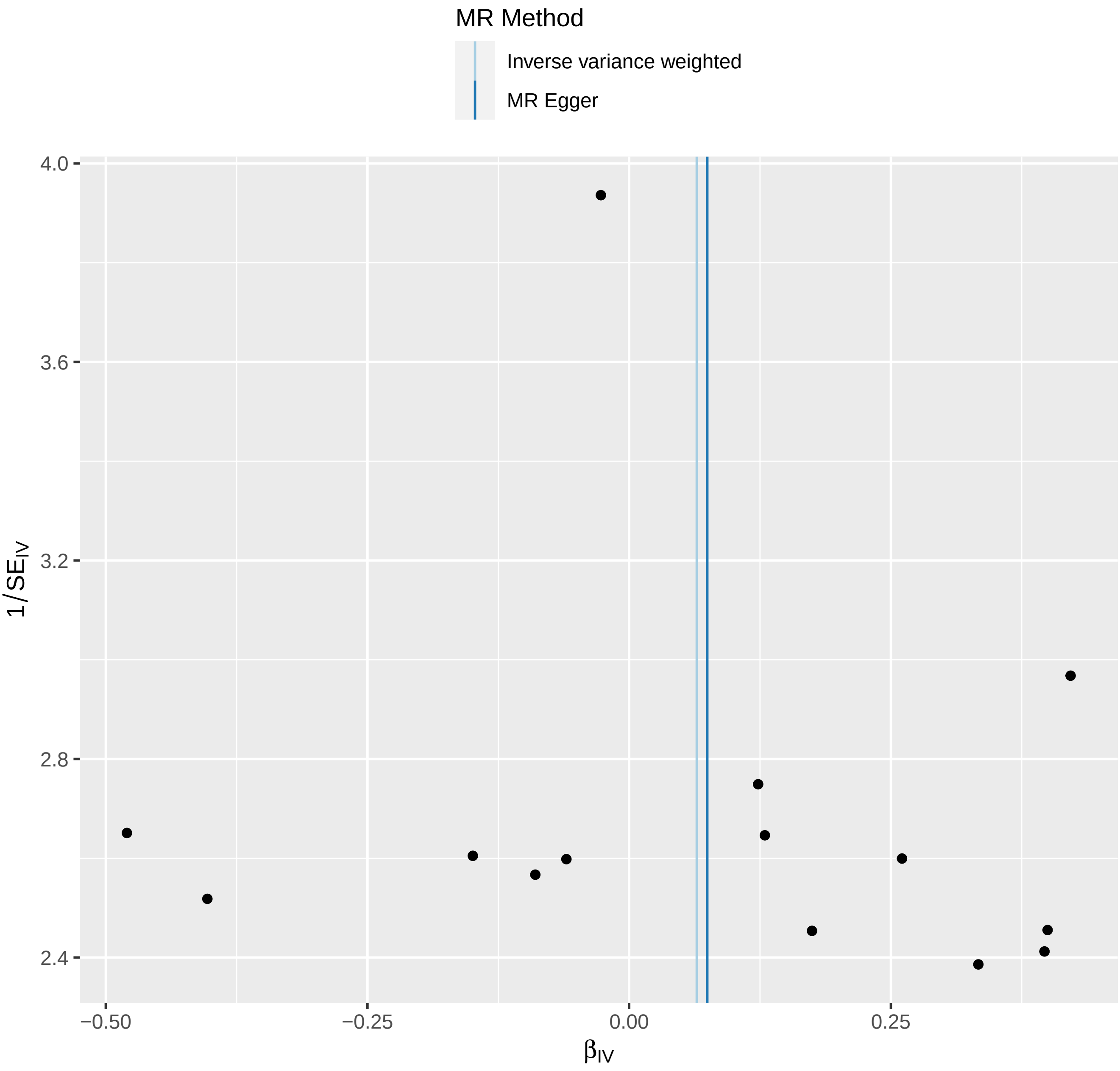

Batch 2−2819 : Gut microbiota abundance (genus Catenibacterium id.2153) on Indoleamine 2,3−dioxygenase 1
with a threshold of 1e−05

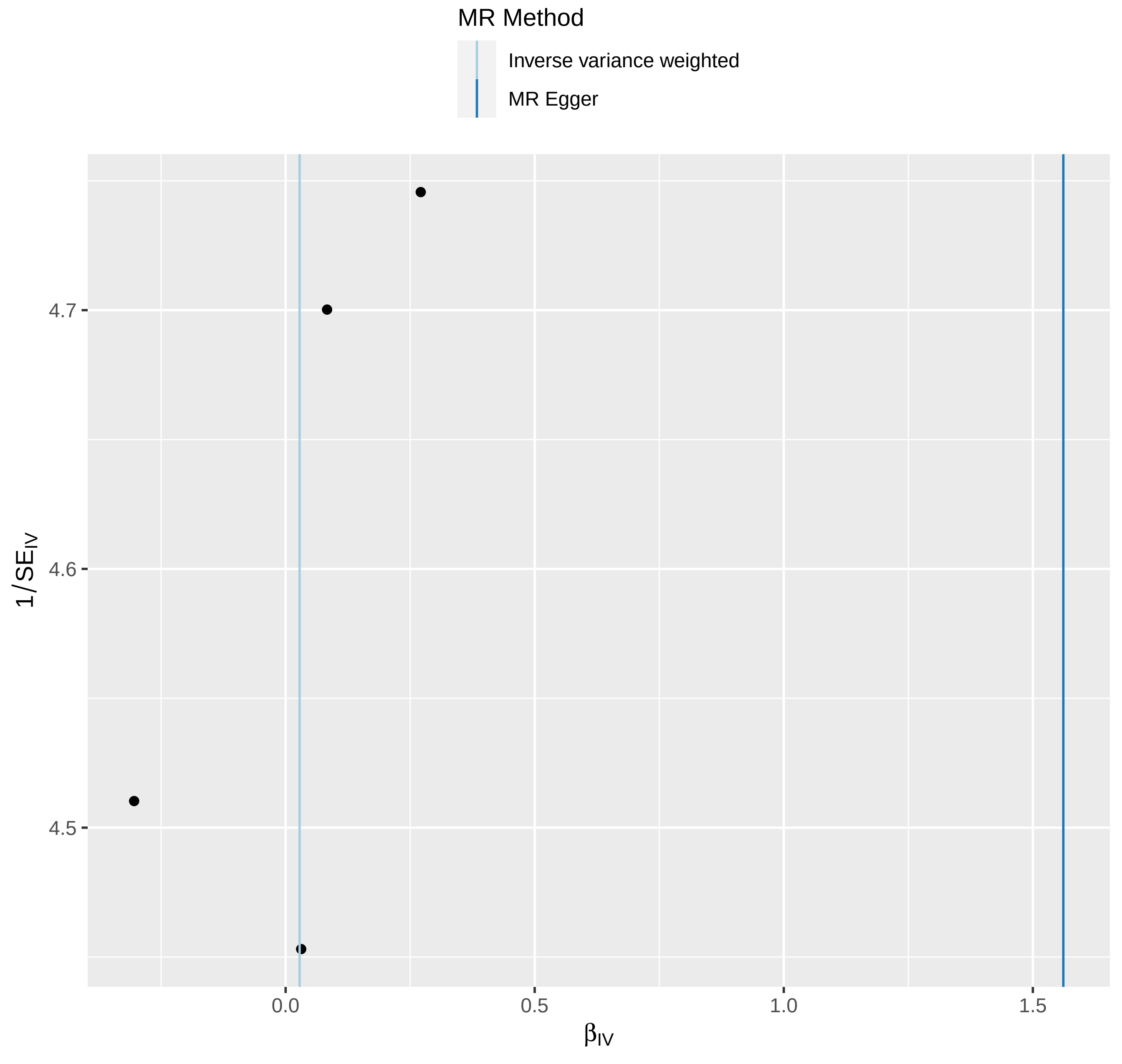

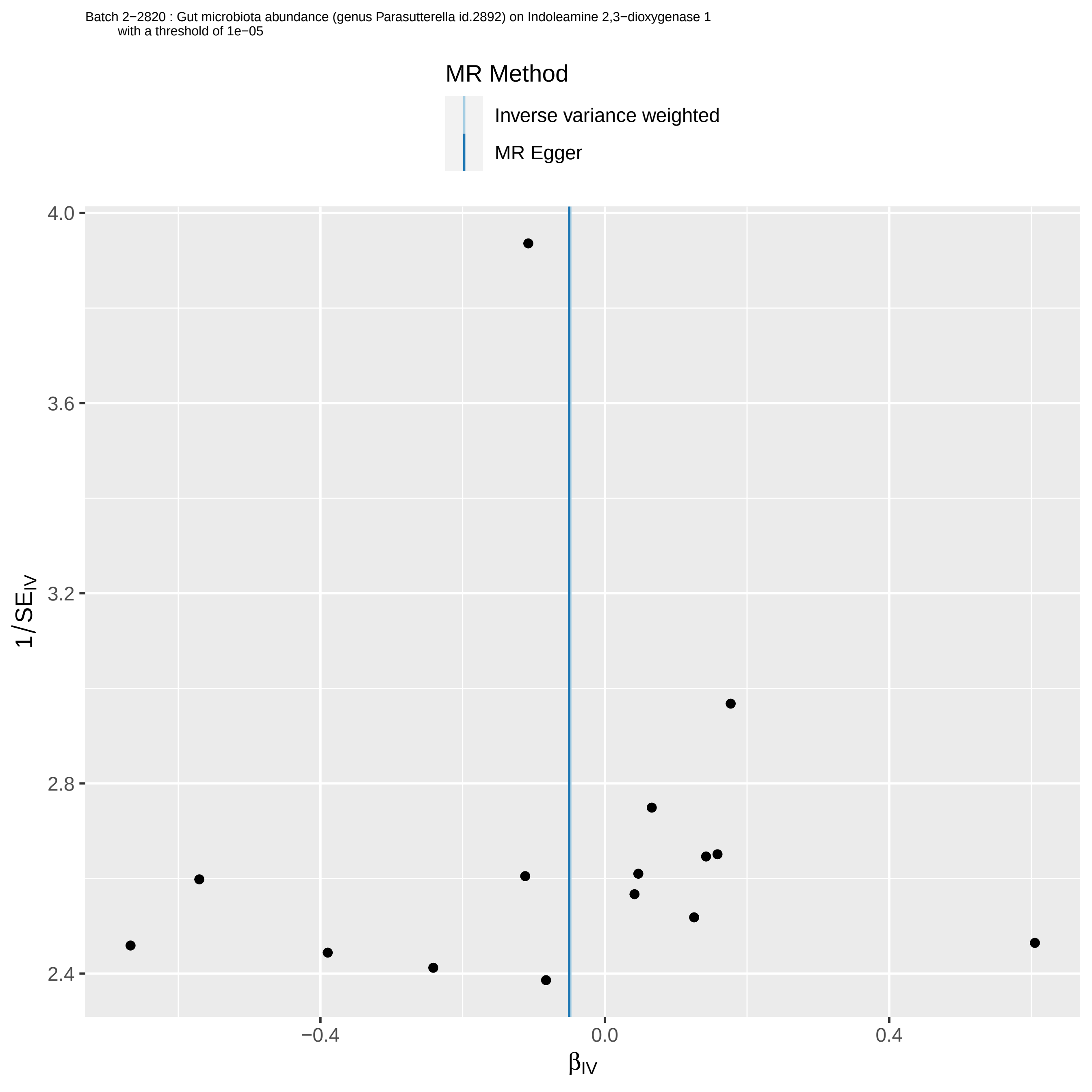
Batch 2−2820 : Gut microbiota abundance (genus Parasutterella id.2892) on Indoleamine 2,3−dioxygenase 1
with a threshold of 1e−05
MR Method
Inverse variance weighted
MR Egger
4.0
3.6
3.2
2.8
2.4
1/SE_IV
−0.4
0.0
0.4
β_IV

Batch 2−2821 : Gut microbiota abundance (genus Catenibacterium id.2153) on Alpha−L−iduronidase
with a threshold of 1e−05

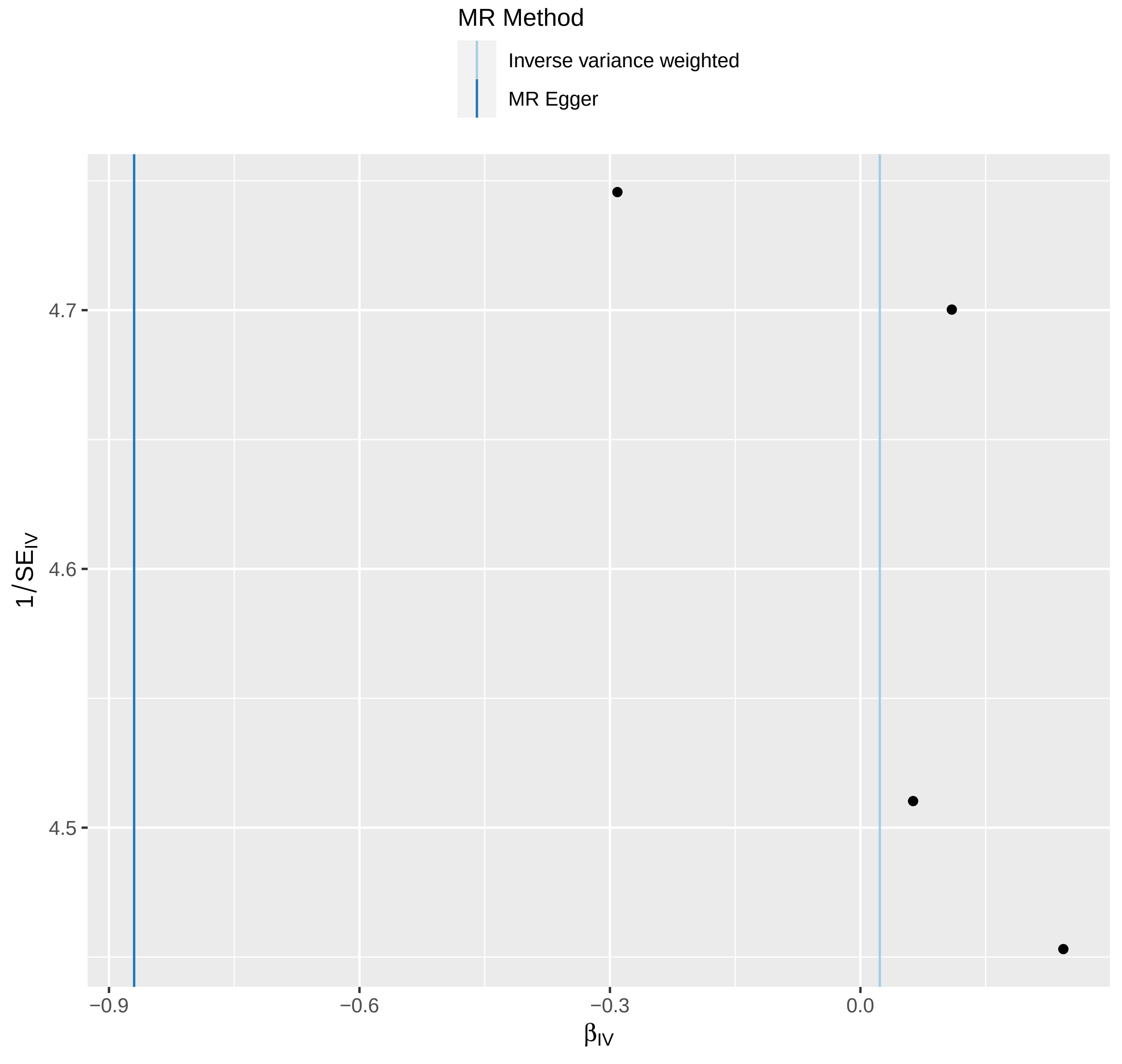

Batch 2−2822 : Gut microbiota abundance (genus Parasutterella id.2892) on Alpha−L−iduronidase
with a threshold of 1e−05

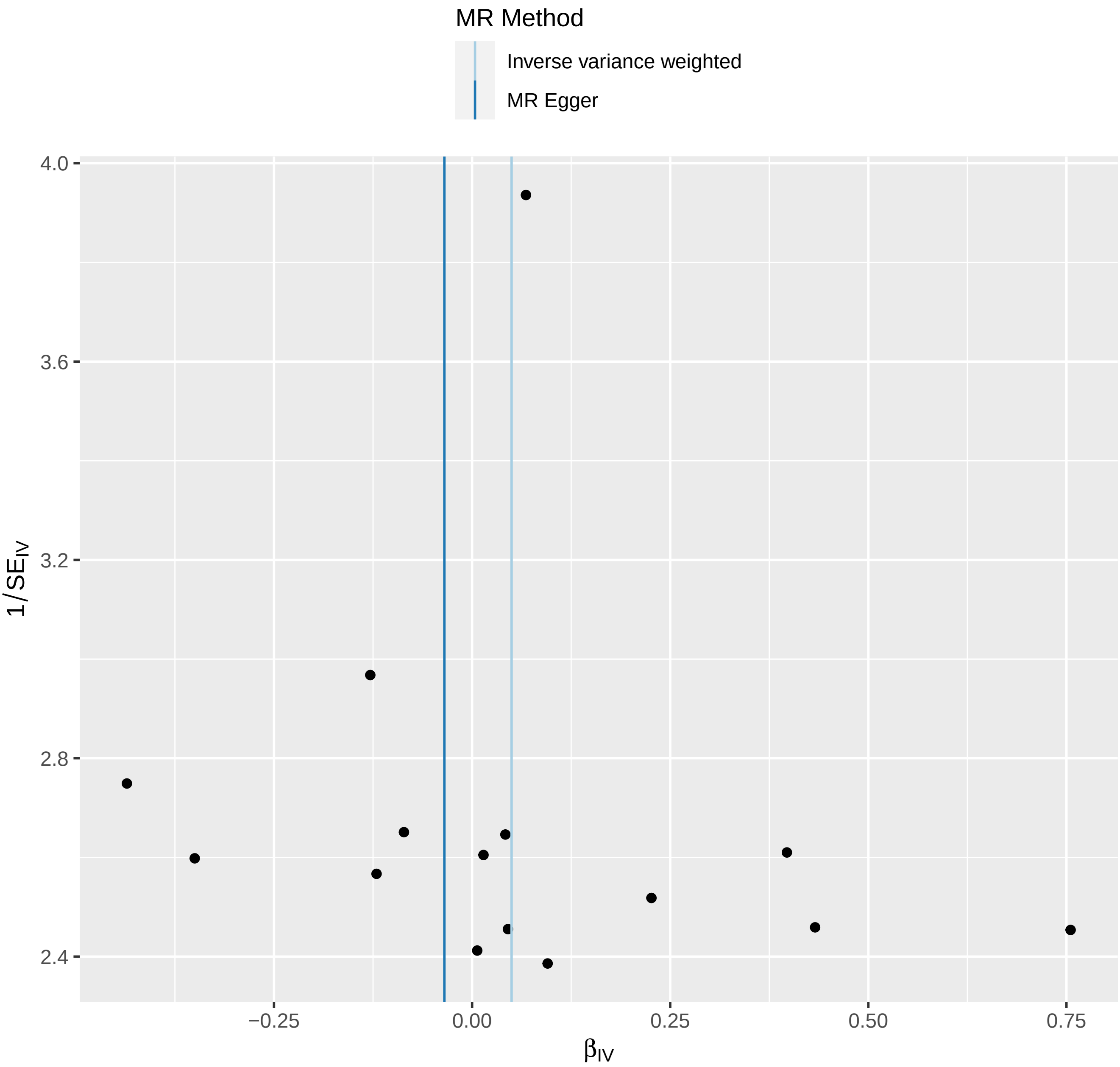

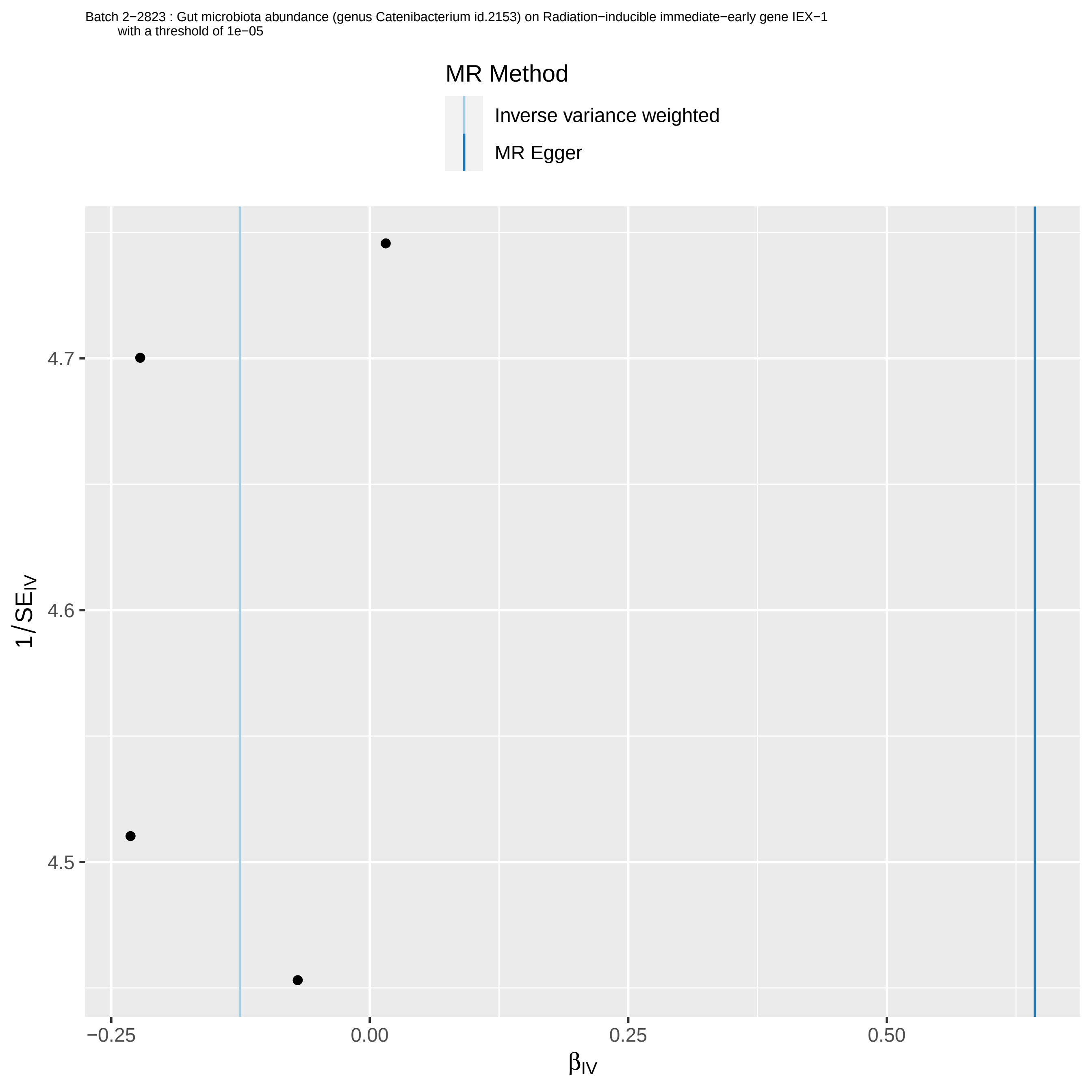

Batch 2−2823 : Gut microbiota abundance (genus Catenibacterium id.2153) on Radiation−inducible immediate−early gene IEX−1
with a threshold of 1e−05
MR Method
Inverse variance weighted
MR Egger
4.7
4.6
4.5
1/SE_IV
−0.25
0.00
0.25
0.50
β_IV

Batch 2−2824 : Gut microbiota abundance (genus Parasutterella id.2892) on Radiation−inducible immediate−early gene IEX−1
with a threshold of 1e−05

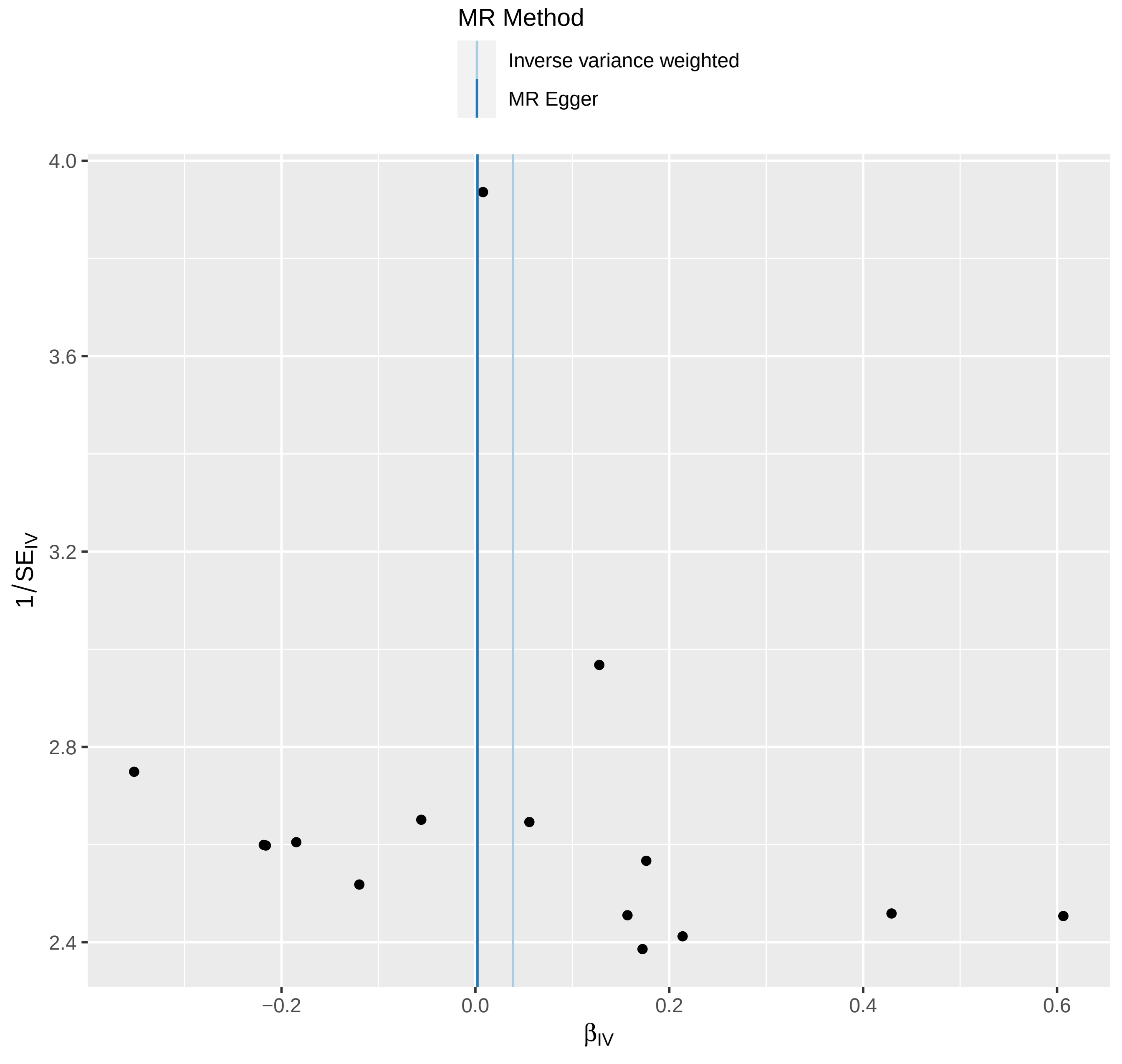

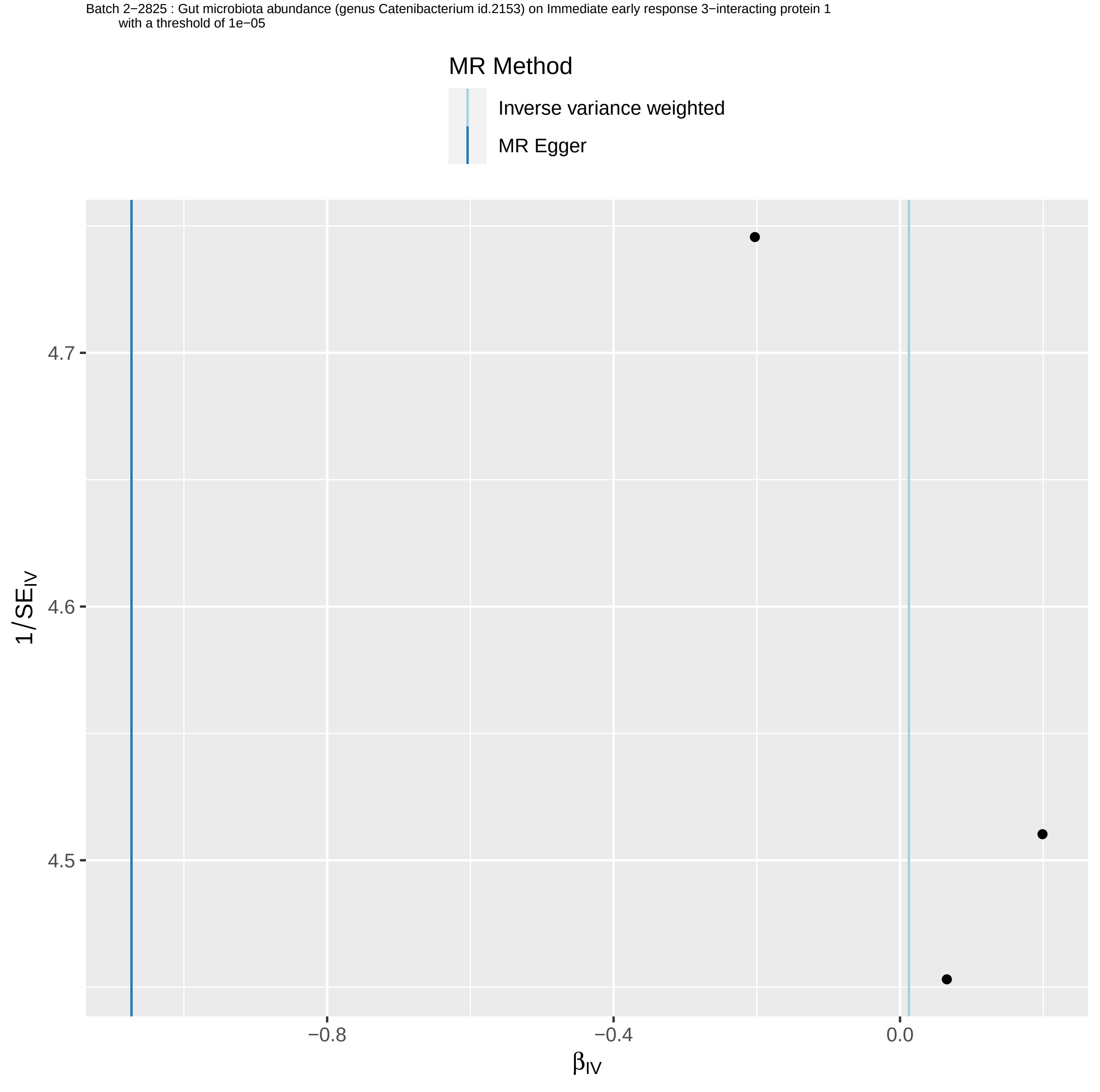
Batch 2−2825 : Gut microbiota abundance (genus Catenibacterium id.2153) on Immediate early response 3−interacting protein 1
with a threshold of 1e−05
MR Method
Inverse variance weighted
MR Egger
4.7
4.6
4.5
$1/SE_{IV}$
−0.8
−0.4
0.0
β_{IV}

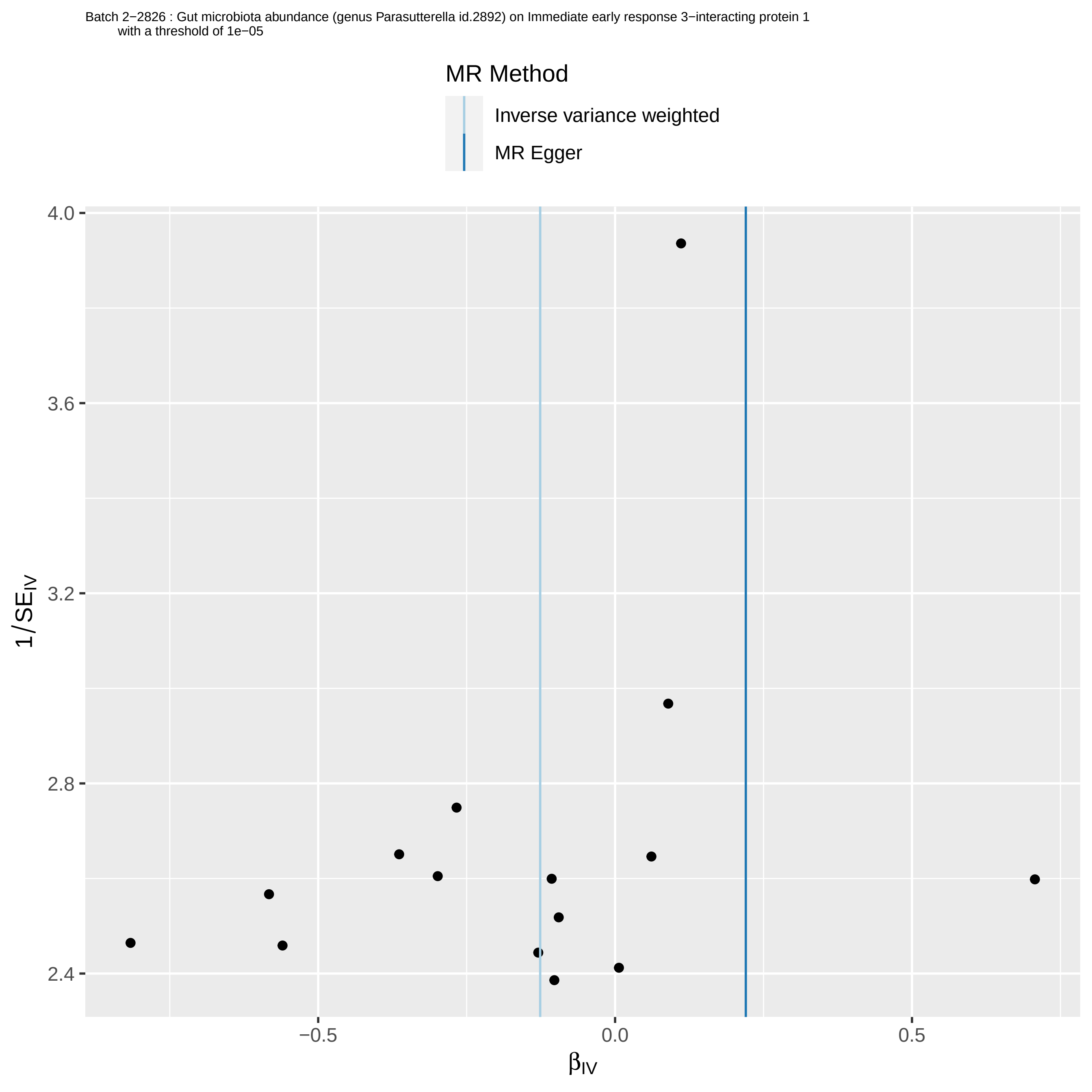
Batch 2−2826 : Gut microbiota abundance (genus Parasutterella id.2892) on Immediate early response 3−interacting protein 1
with a threshold of 1e−05
MR Method
Inverse variance weighted
MR Egger
4.0
3.6
3.2
2.8
2.4
1/SE_IV
−0.5
0.0
0.5
β_IV

Batch 2−2827 : Gut microbiota abundance (genus Catenibacterium id.2153) on Gamma−interferon−inducible protein 16
with a threshold of 1e−05

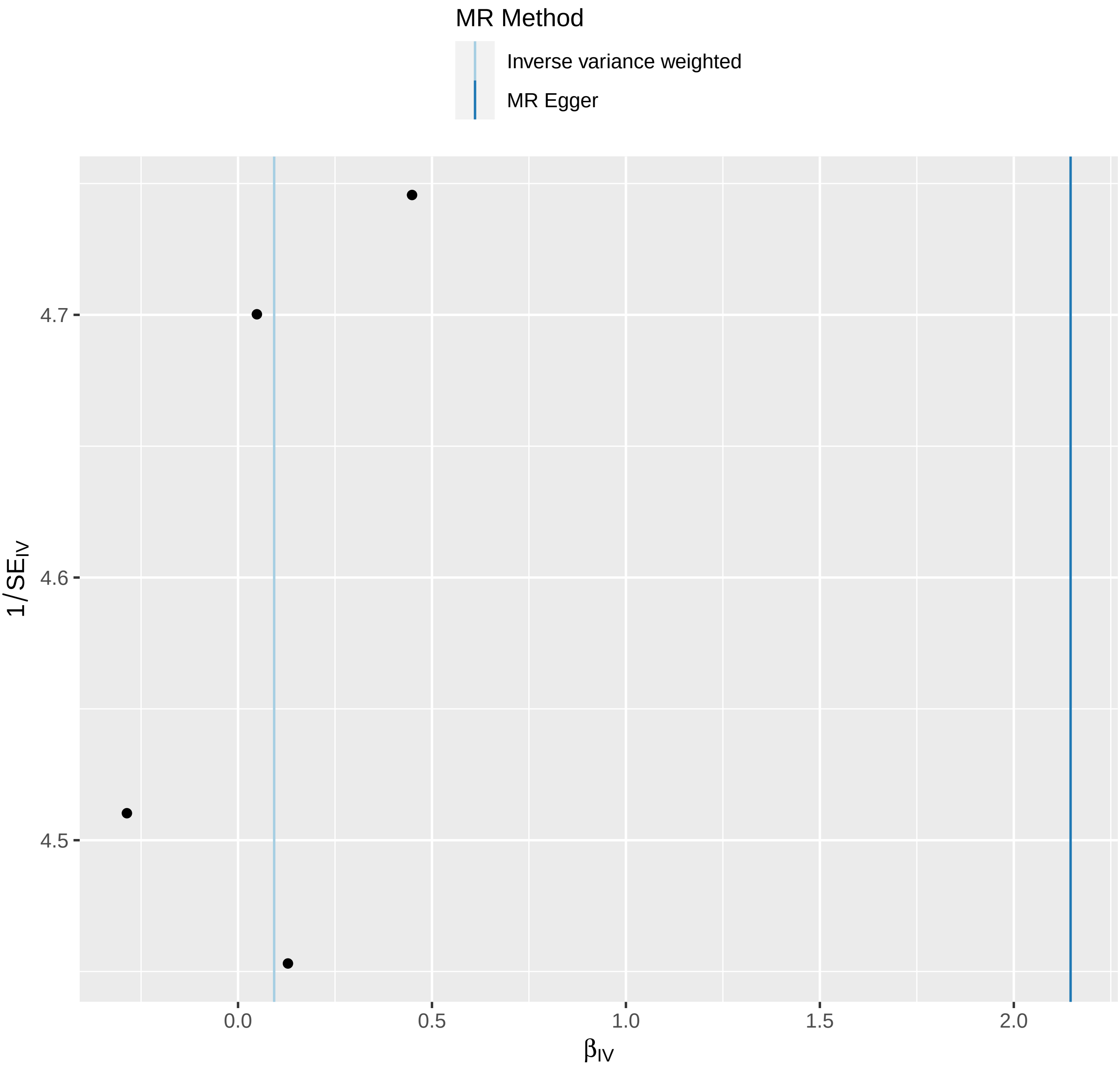

Batch 2−2828 : Gut microbiota abundance (genus Parasutterella id.2892) on Gamma−interferon−inducible protein 16
with a threshold of 1e−05

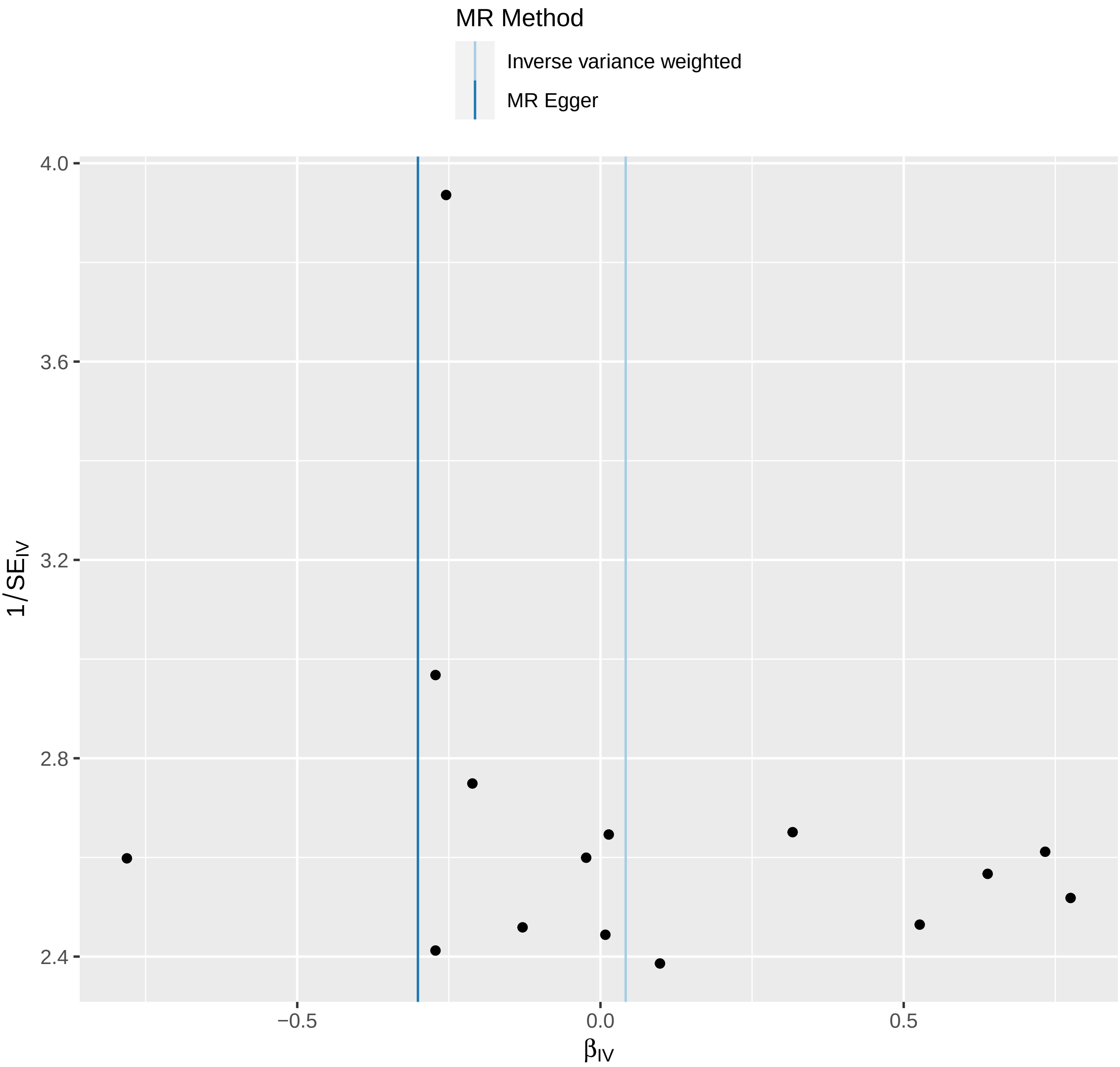

Batch 2−2829 : Gut microbiota abundance (genus Catenibacterium id.2153) on Gamma−interferon−inducible protein 16
with a threshold of 1e−05

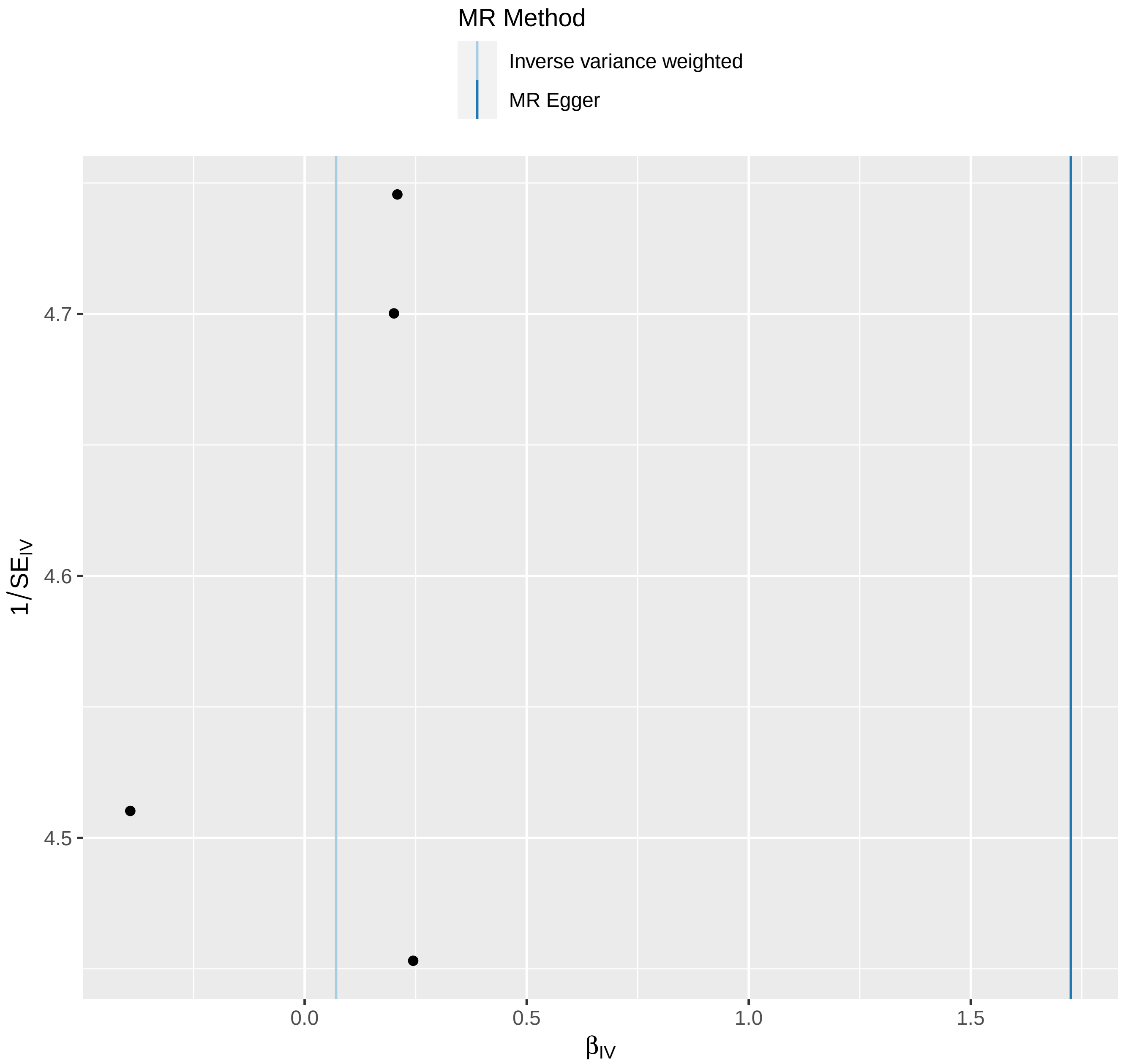

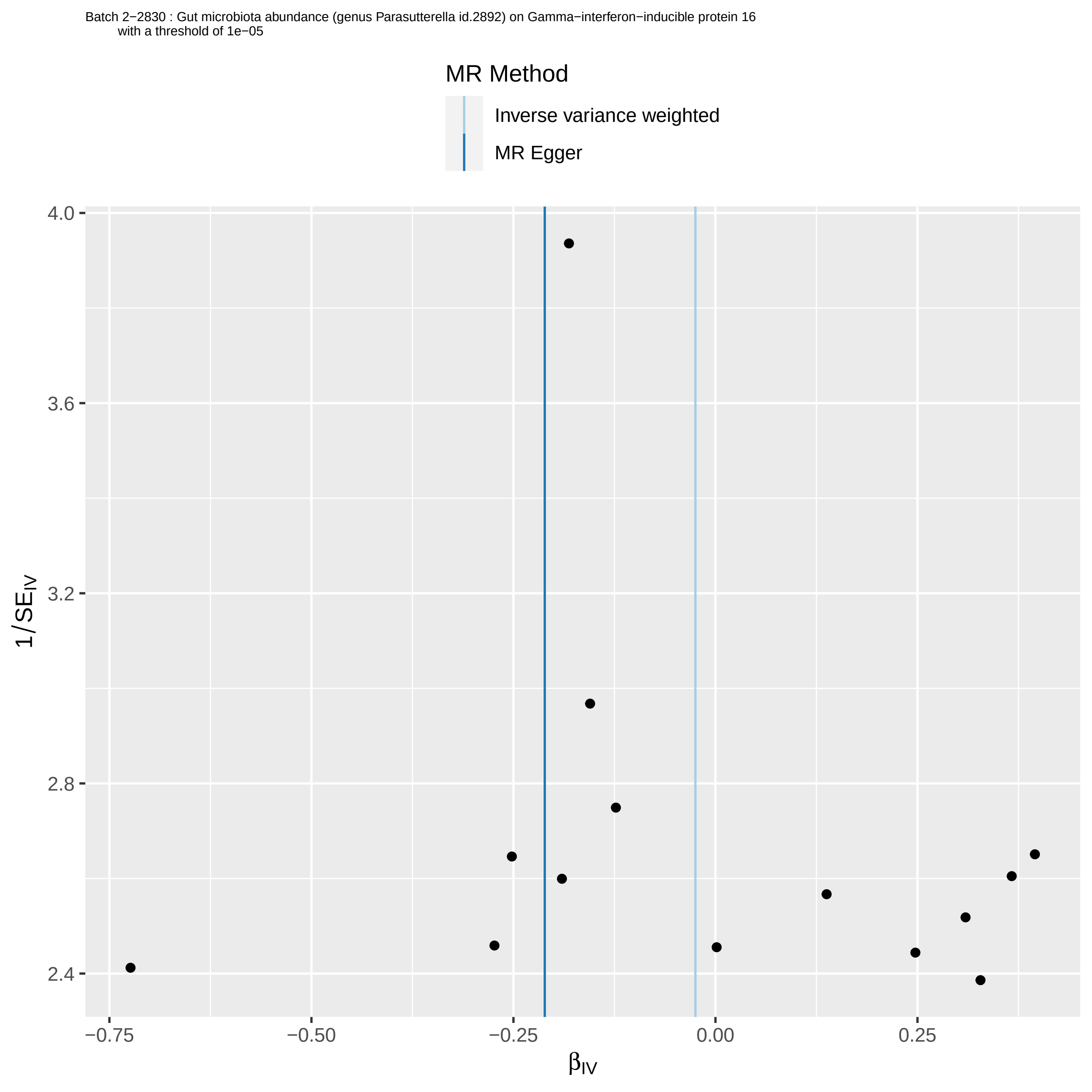

Batch 2−2830 : Gut microbiota abundance (genus Parasutterella id.2892) on Gamma−interferon−inducible protein 16
with a threshold of 1e−05
MR Method
Inverse variance weighted
MR Egger
4.0
3.6
3.2
2.8
2.4
1/SE_IV
−0.75
−0.50
−0.25
0.00
0.25
β_IV

Batch 2−2831 : Gut microbiota abundance (genus Catenibacterium id.2153) on Interferon−induced protein with tetratricopeptide repeats 2 with a threshold of 1e−05

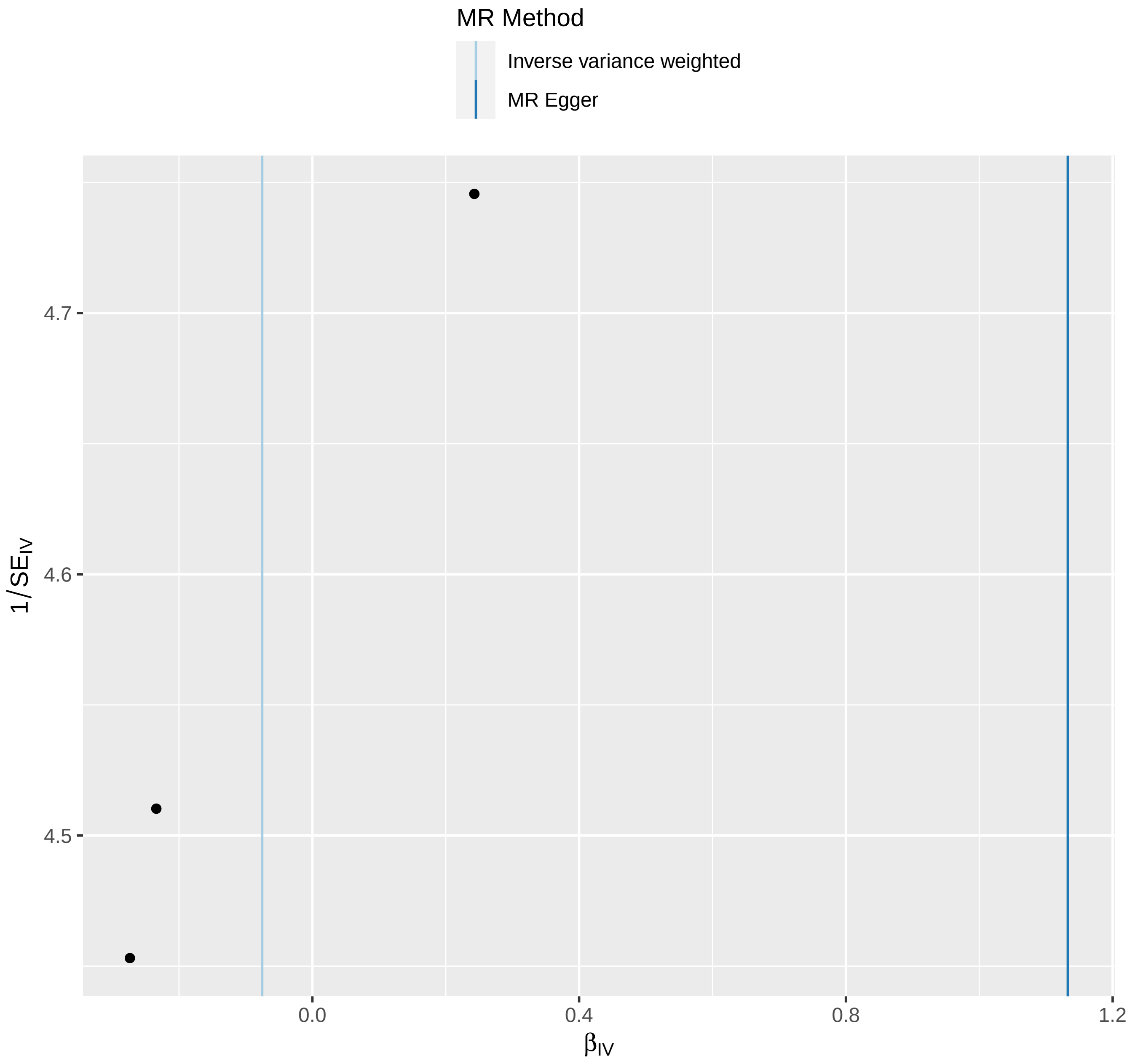

Batch 2−2832 : Gut microbiota abundance (genus Parasutterella id.2892) on Interferon−induced protein with tetratricopeptide repeats 2 with a threshold of 1e−05

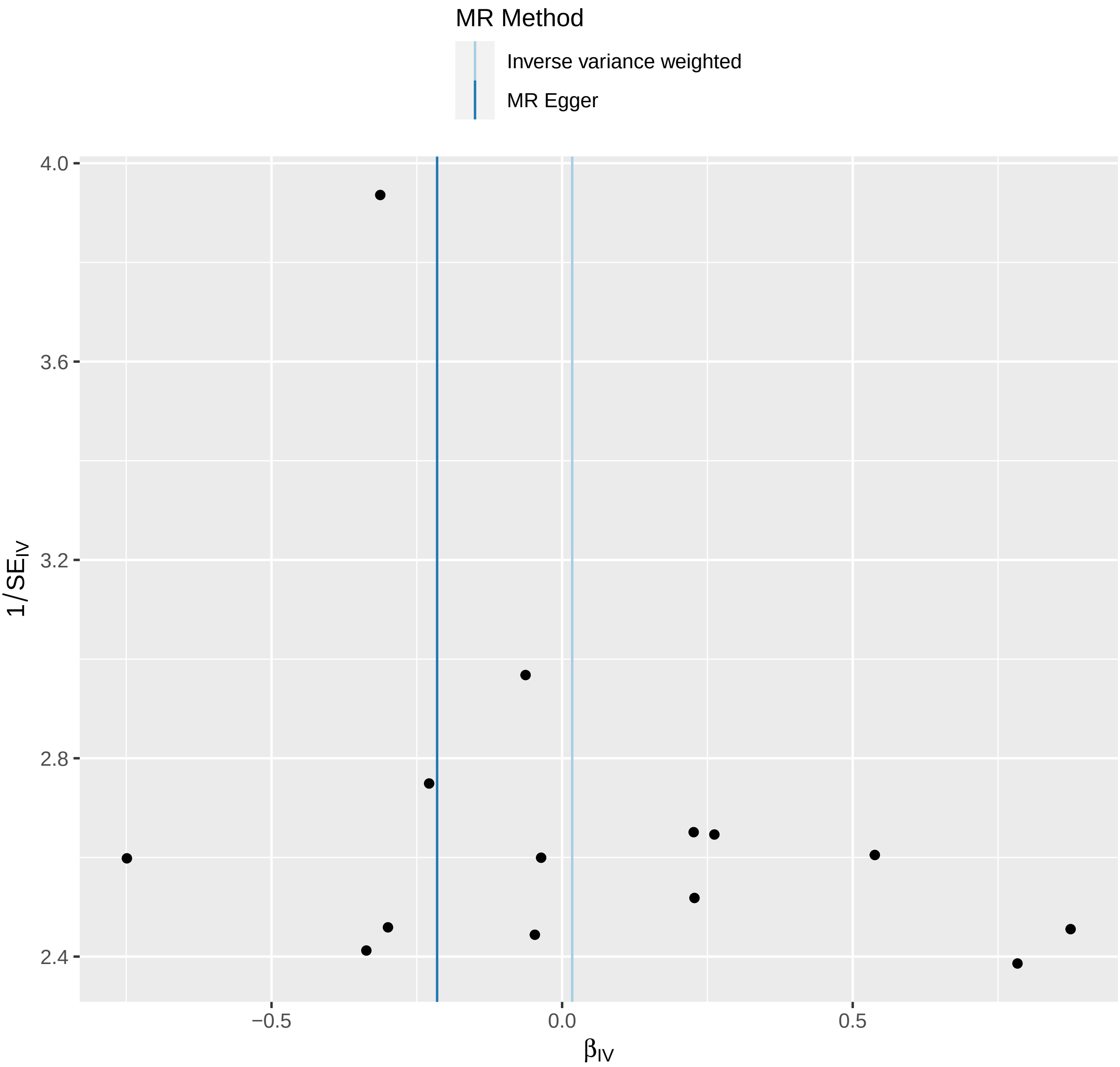

Batch 2−2833 : Gut microbiota abundance (genus Catenibacterium id.2153) on Interferon−induced protein with tetratricopeptide repeats 3 with a threshold of 1e−05

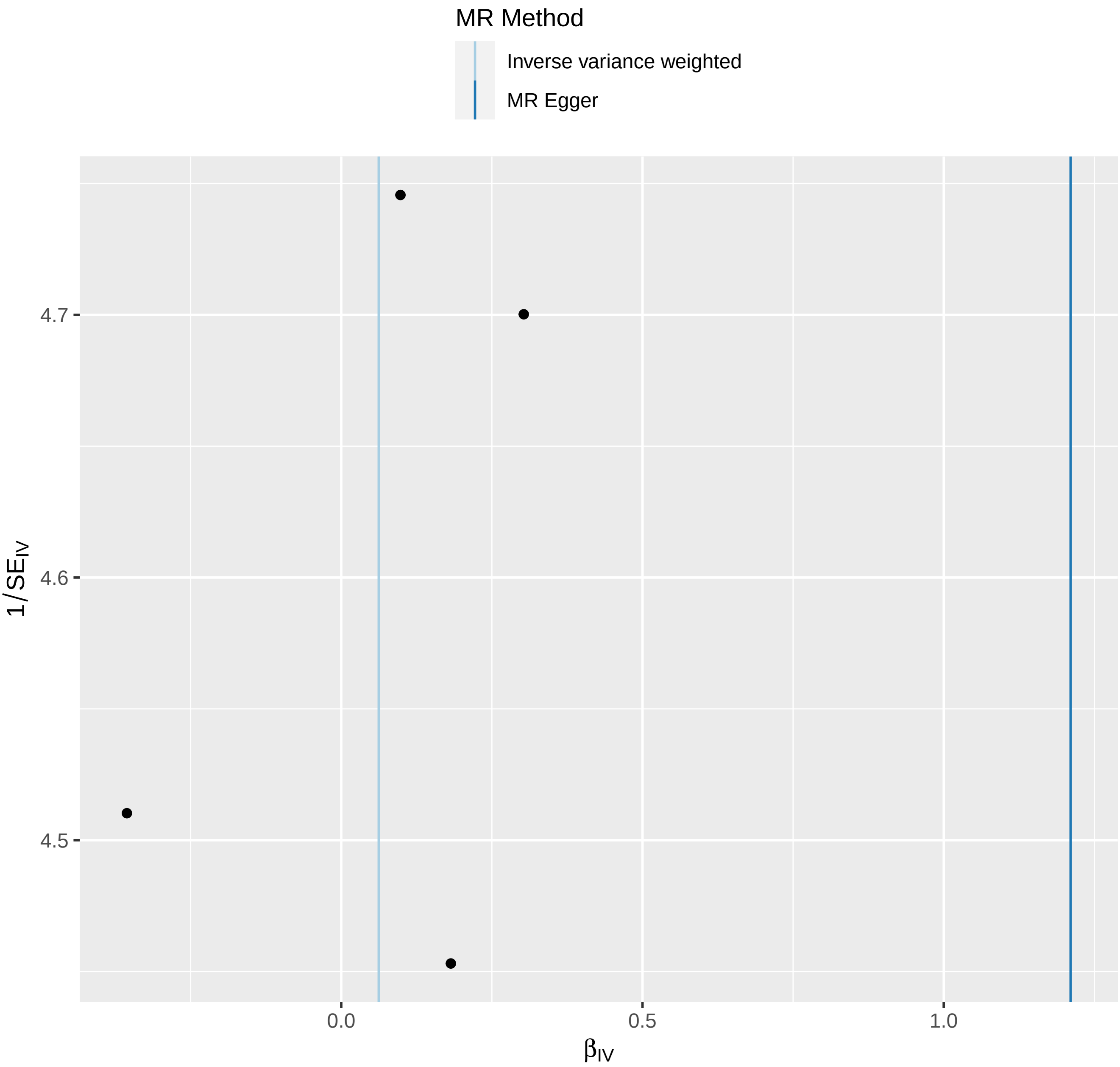

Batch 2−2834 : Gut microbiota abundance (genus Parasutterella id.2892) on Interferon−induced protein with tetratricopeptide repeats 3
with a threshold of 1e−05

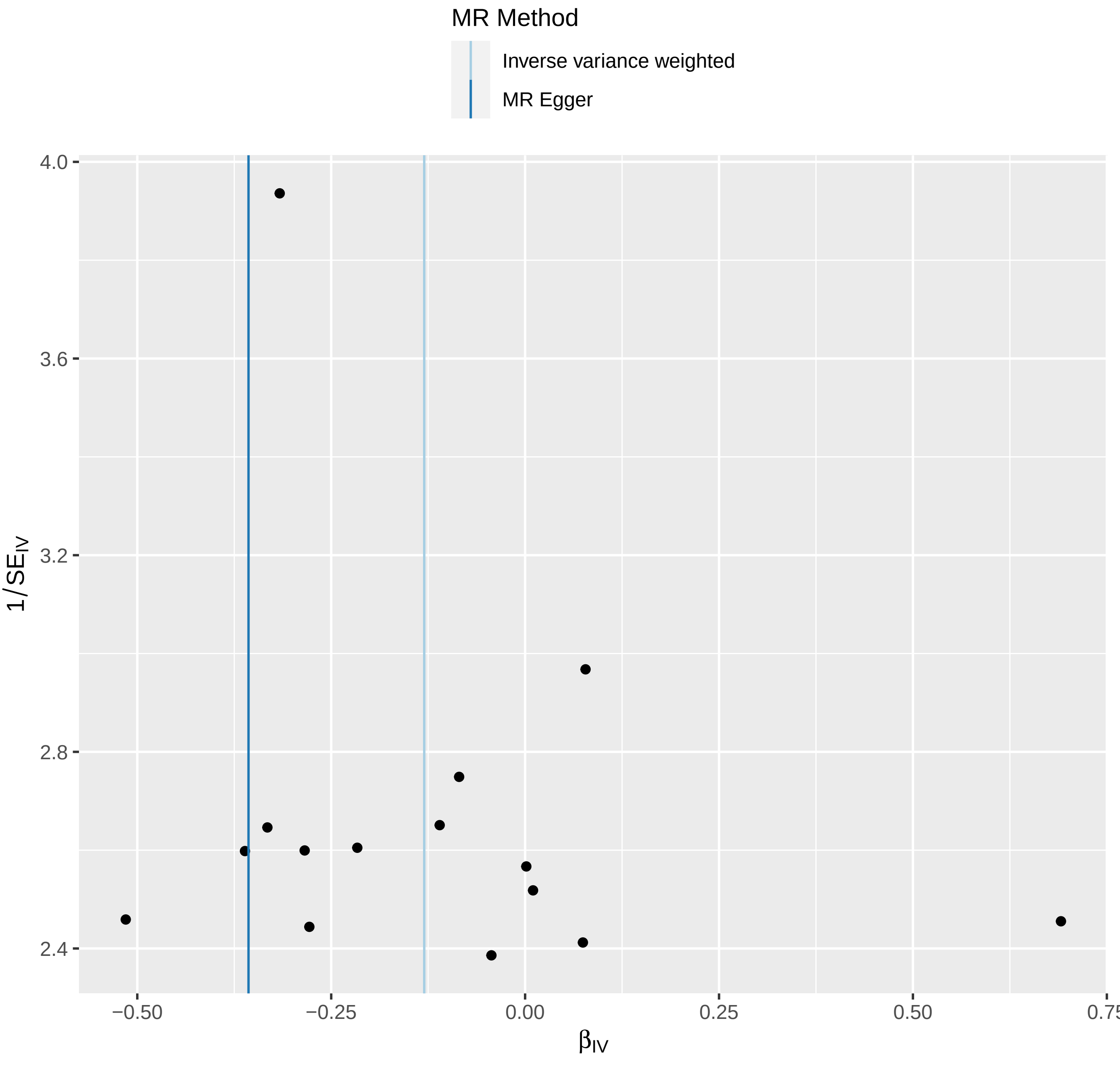

Batch 2−2835 : Gut microbiota abundance (genus Catenibacterium id.2153) on Interferon alpha−10
with a threshold of 1e−05

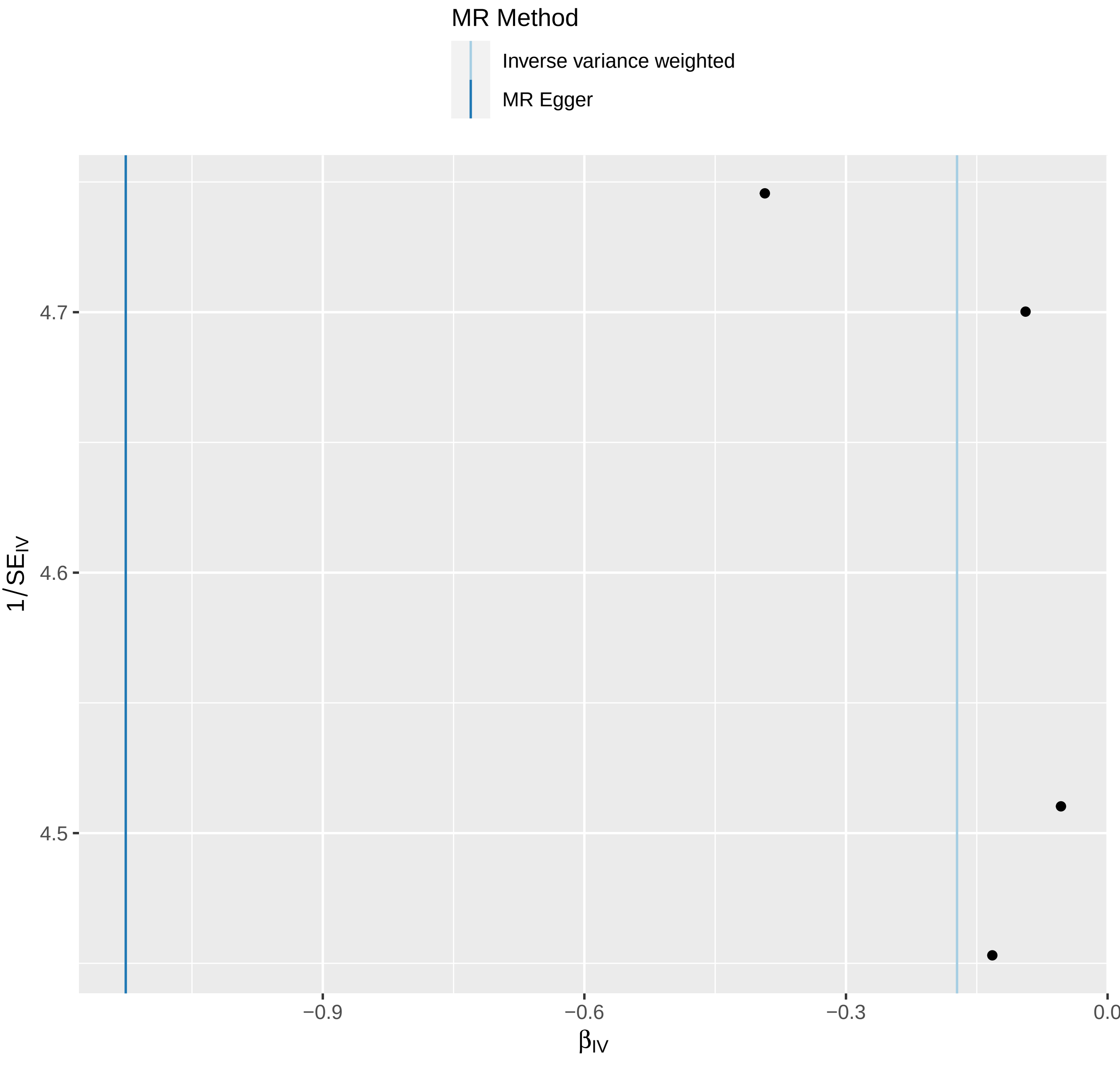

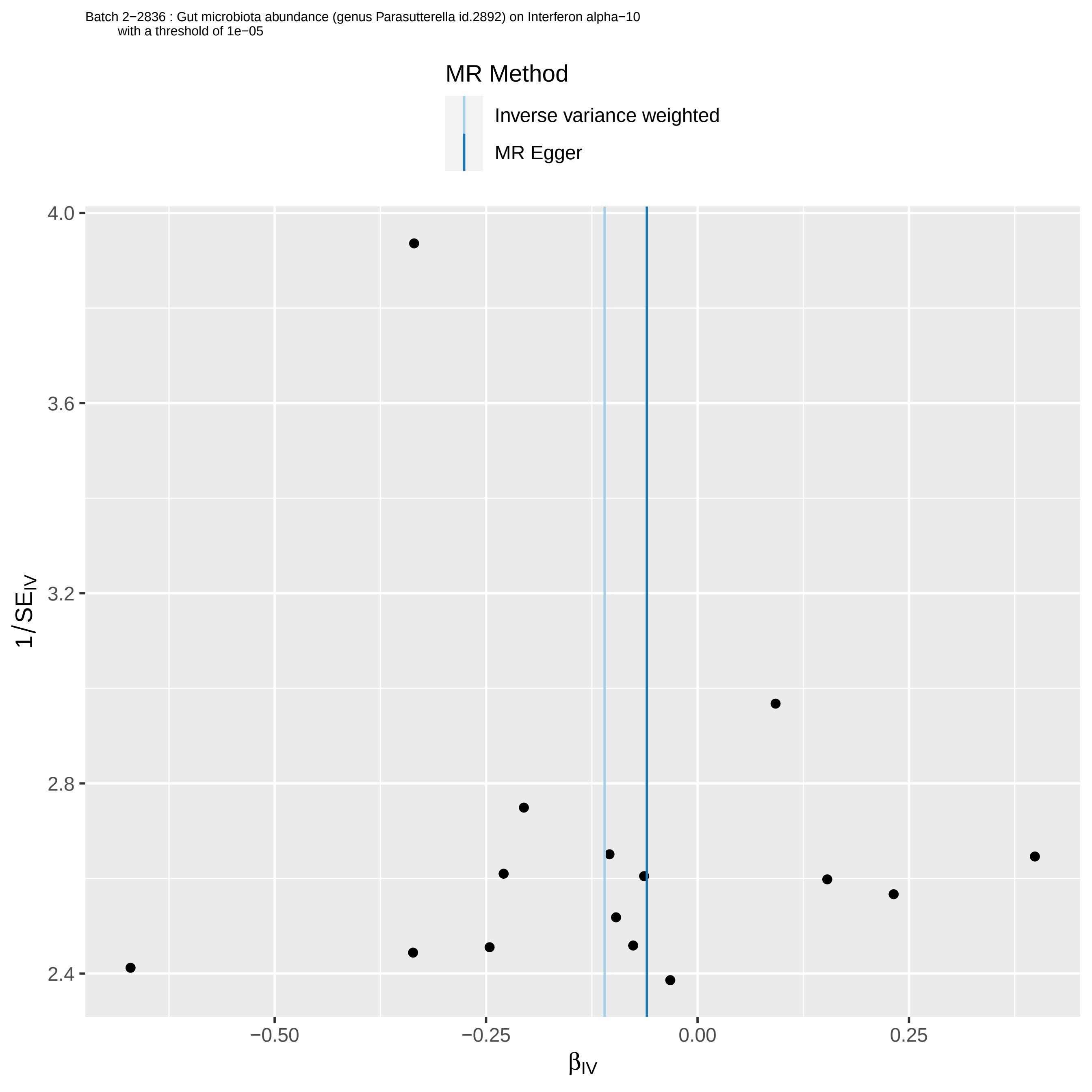

Batch 2−2836 : Gut microbiota abundance (genus Parasutterella id.2892) on Interferon alpha−10
with a threshold of 1e−05
MR Method
Inverse variance weighted
MR Egger
4.0
3.6
3.2
2.8
2.4
1/SE_IV
−0.50
−0.25
0.00
0.25
β_IV

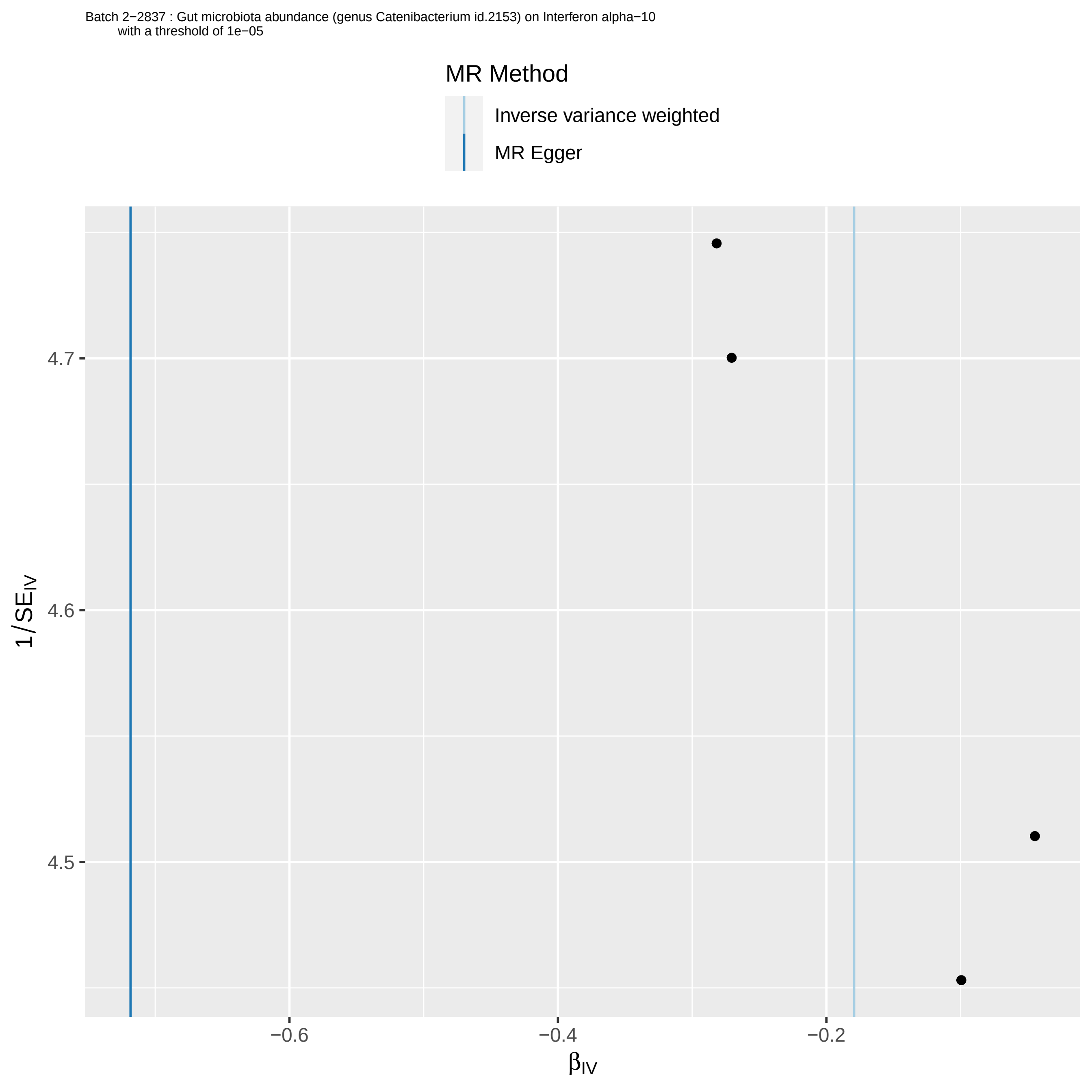

Batch 2−2837 : Gut microbiota abundance (genus Catenibacterium id.2153) on Interferon alpha−10
with a threshold of 1e−05
MR Method
Inverse variance weighted
MR Egger
4.7
4.6
4.5
1/SE_IV
−0.6
−0.4
−0.2
β_IV

Batch 2−2838 : Gut microbiota abundance (genus Parasutterella id.2892) on Interferon alpha−10
with a threshold of 1e−05

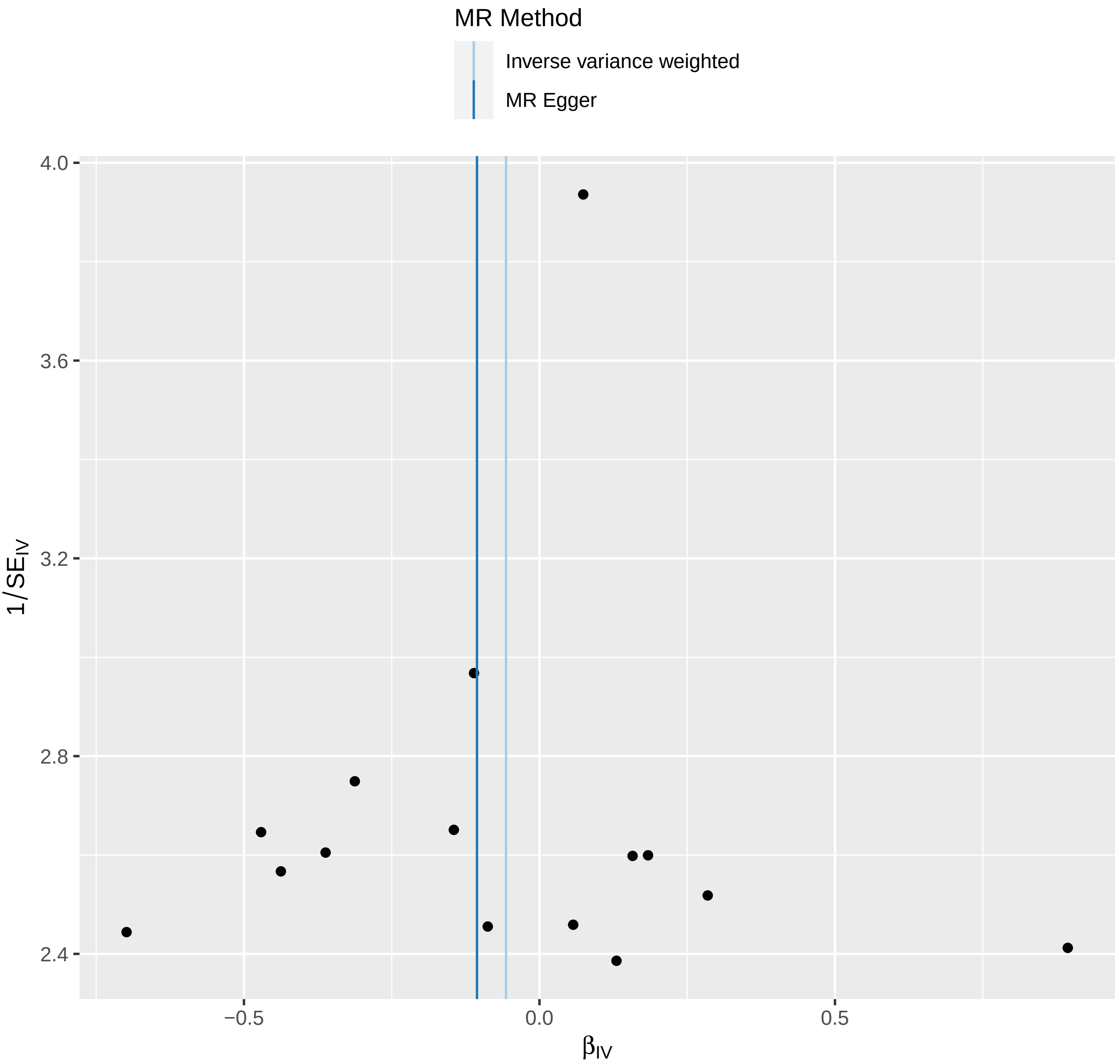

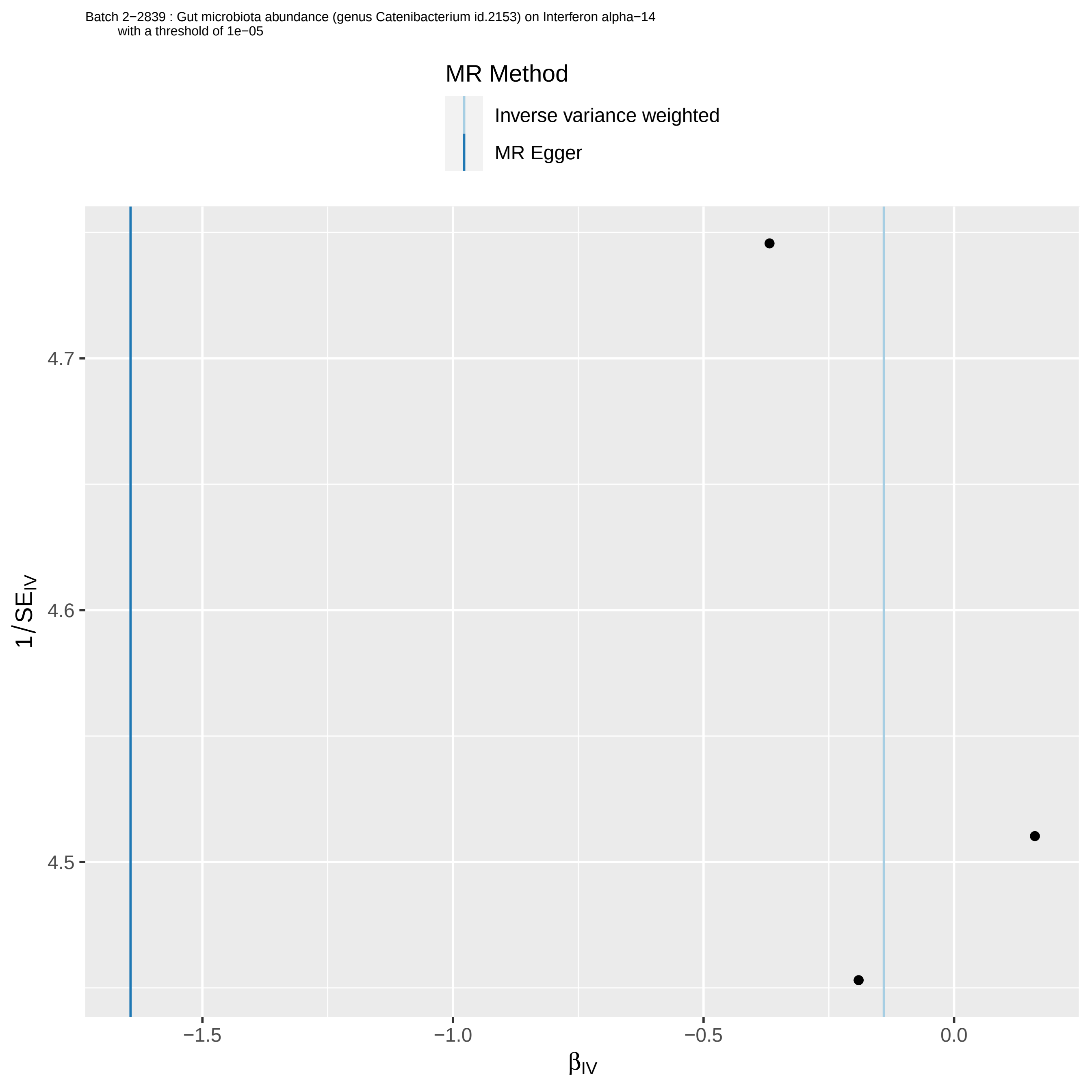
Batch 2−2839 : Gut microbiota abundance (genus Catenibacterium id.2153) on Interferon alpha−14
with a threshold of 1e−05
MR Method
Inverse variance weighted
MR Egger
4.7
4.6
4.5
1/SE_IV
−1.5
−1.0
−0.5
0.0
β_IV

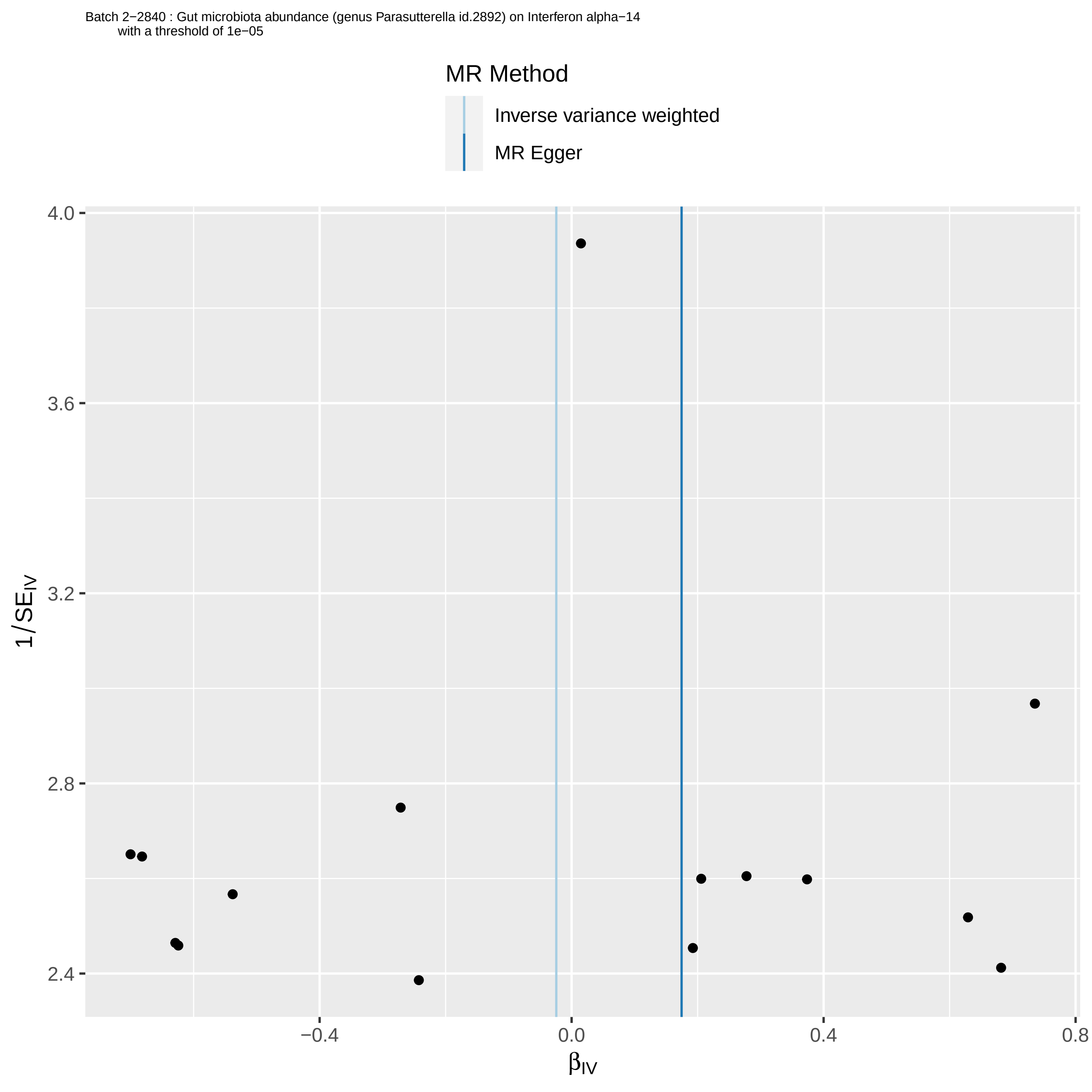
Batch 2−2840 : Gut microbiota abundance (genus Parasutterella id.2892) on Interferon alpha−14
with a threshold of 1e−05
MR Method
Inverse variance weighted
MR Egger
4.0
3.6
3.2
2.8
2.4
1/SE$_{IV}$
−0.4
0.0
0.4
0.8
β$_{IV}$

Batch 2−2841 : Gut microbiota abundance (genus Catenibacterium id.2153) on Interferon alpha−4
with a threshold of 1e−05

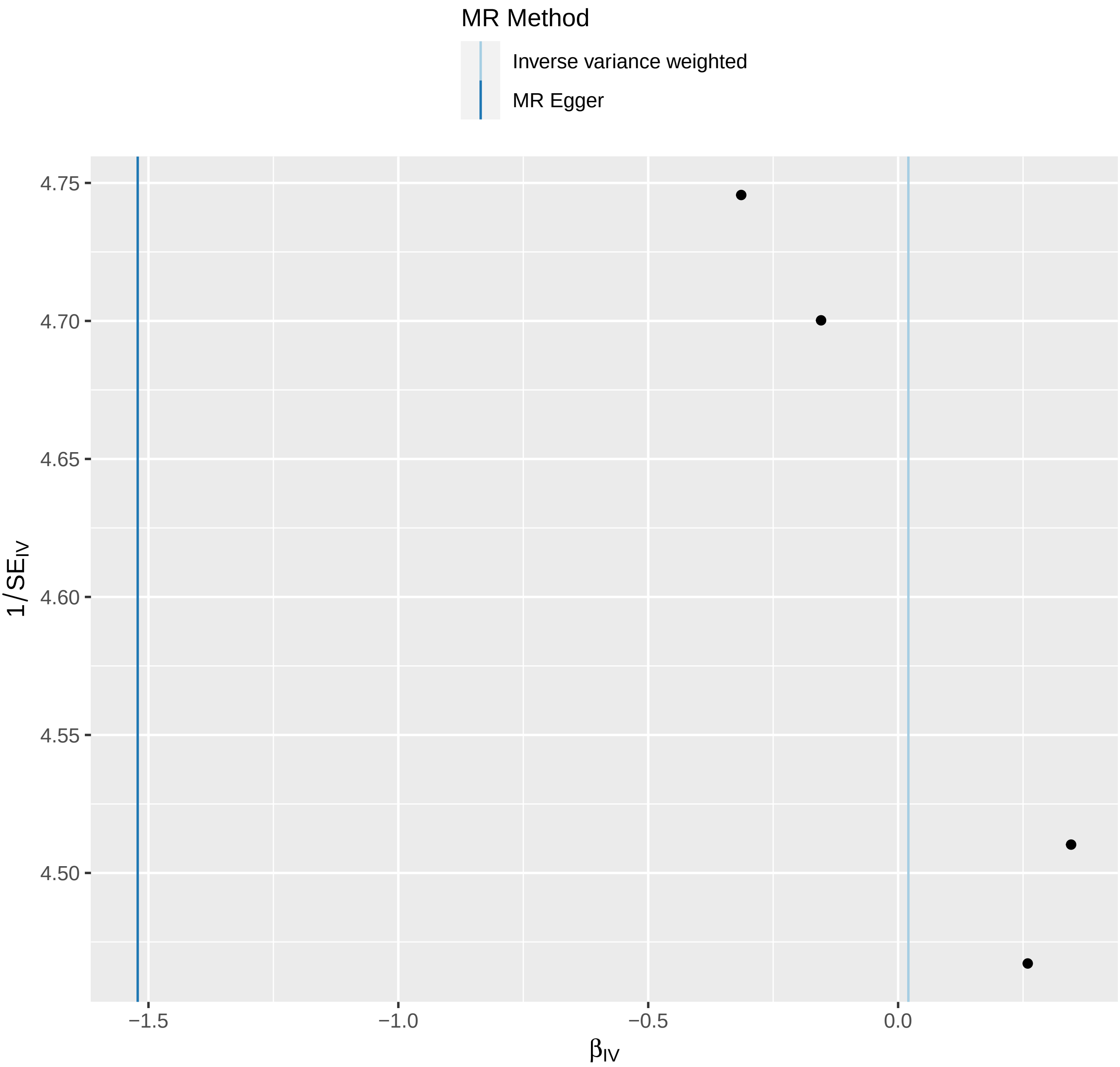

Batch 2−2842 : Gut microbiota abundance (genus Parasutterella id.2892) on Interferon alpha−4
with a threshold of 1e−05

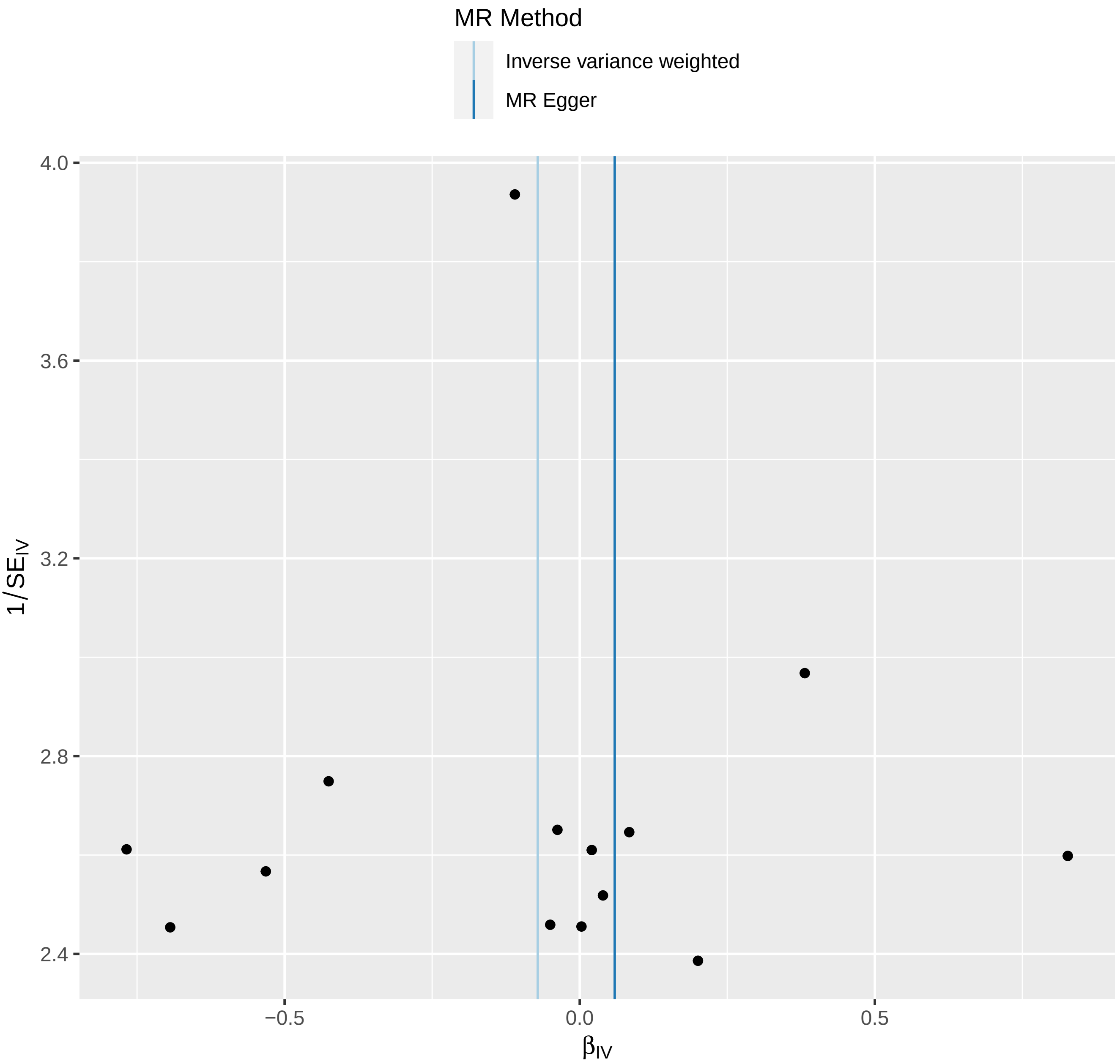

Batch 2−2843 : Gut microbiota abundance (genus Catenibacterium id.2153) on Interferon alpha−5
with a threshold of 1e−05

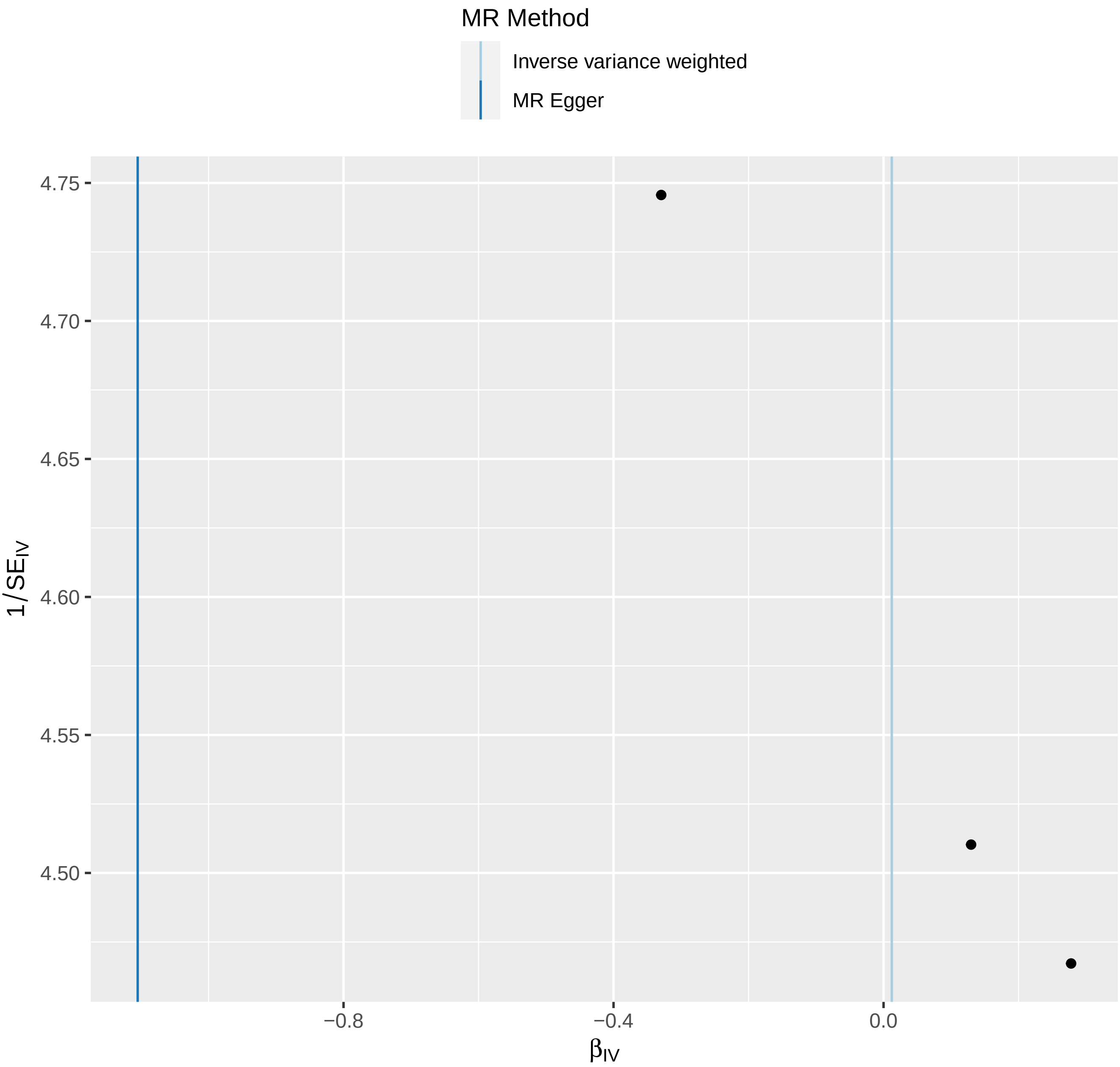

Batch 2−2844 : Gut microbiota abundance (genus Parasutterella id.2892) on Interferon alpha−5
with a threshold of 1e−05

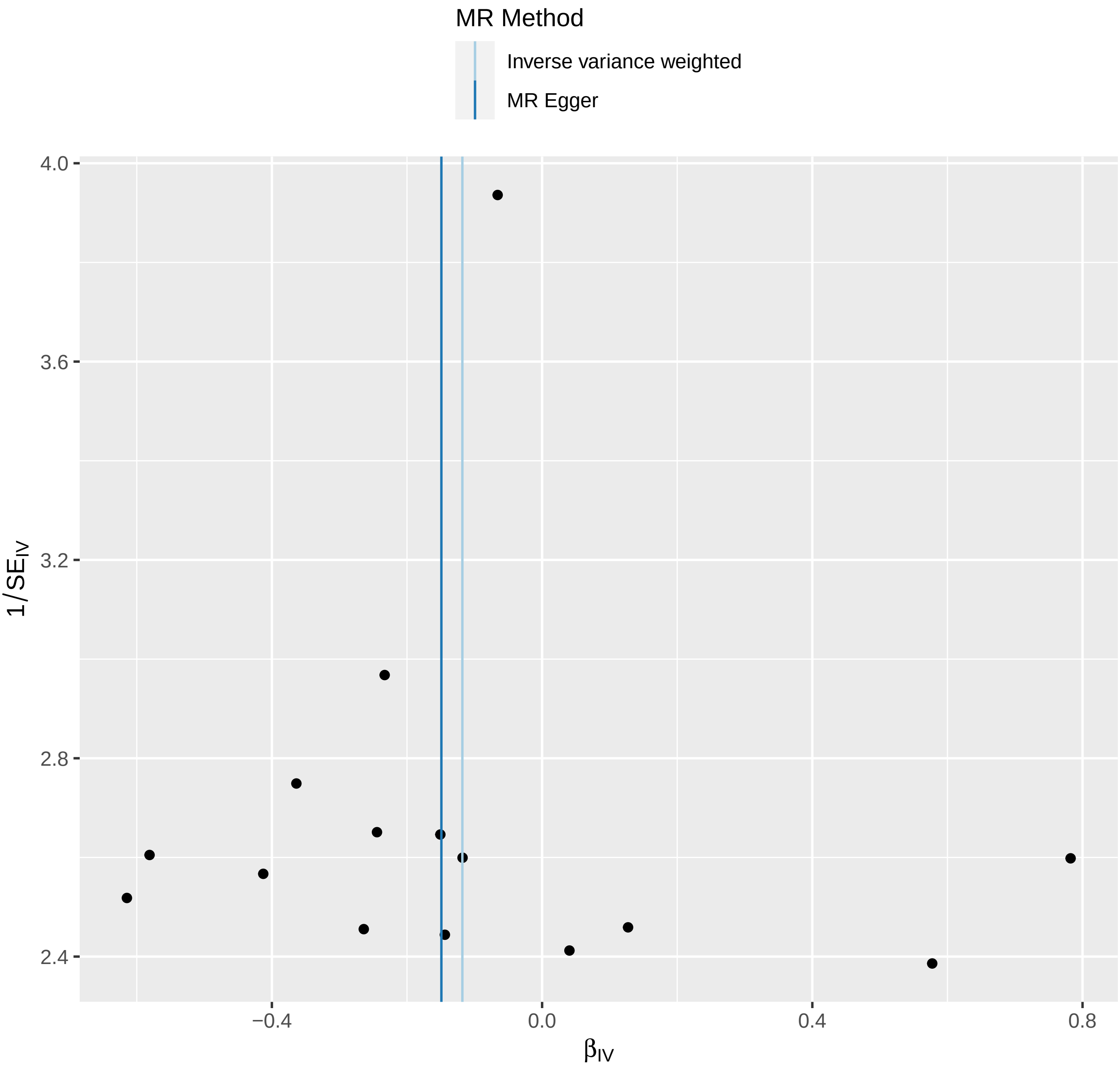

Batch 2−2845 : Gut microbiota abundance (genus Catenibacterium id.2153) on Interferon alpha−6
with a threshold of 1e−05

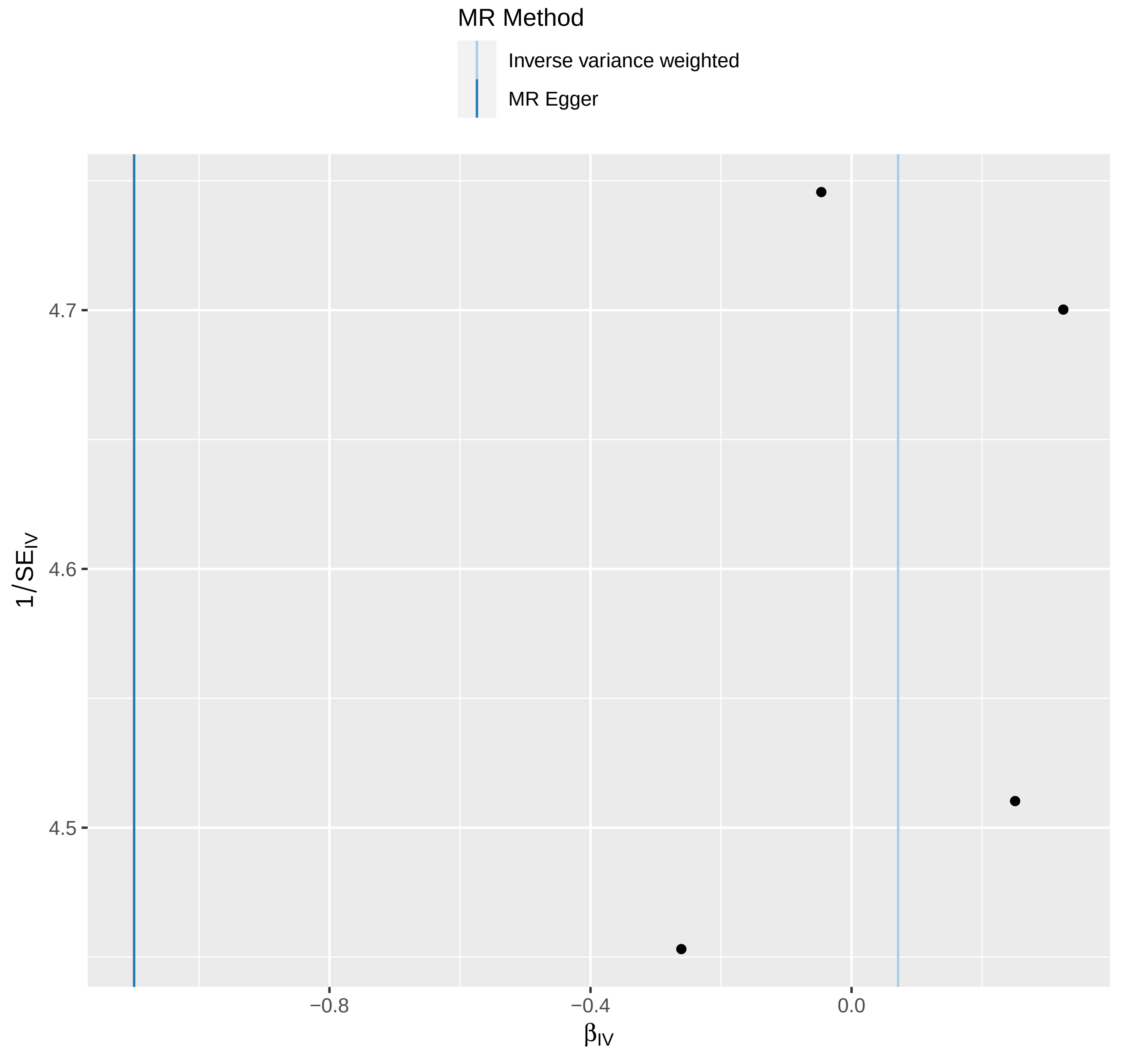

Batch 2−2846 : Gut microbiota abundance (genus Parasutterella id.2892) on Interferon alpha−6
with a threshold of 1e−05

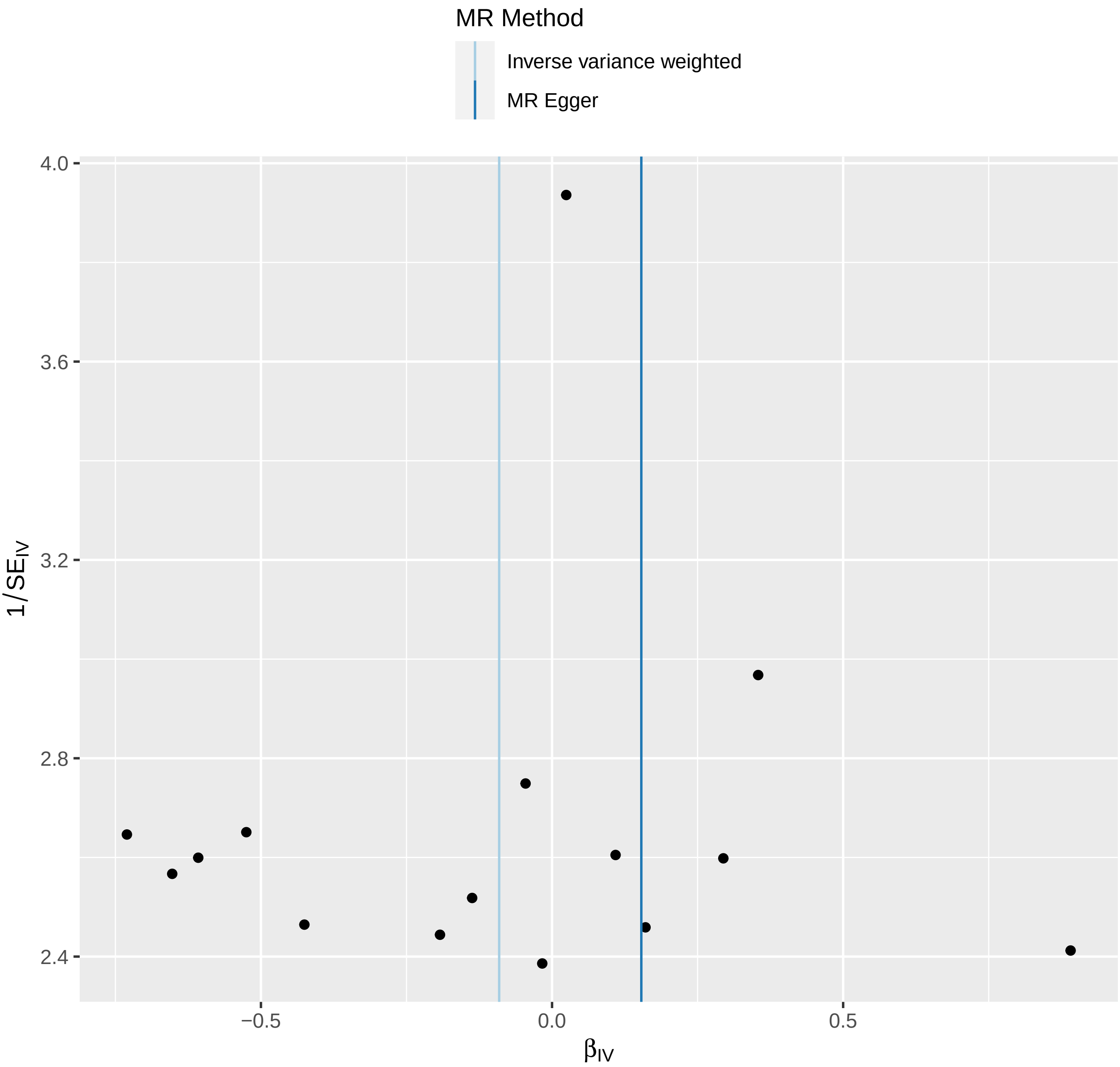

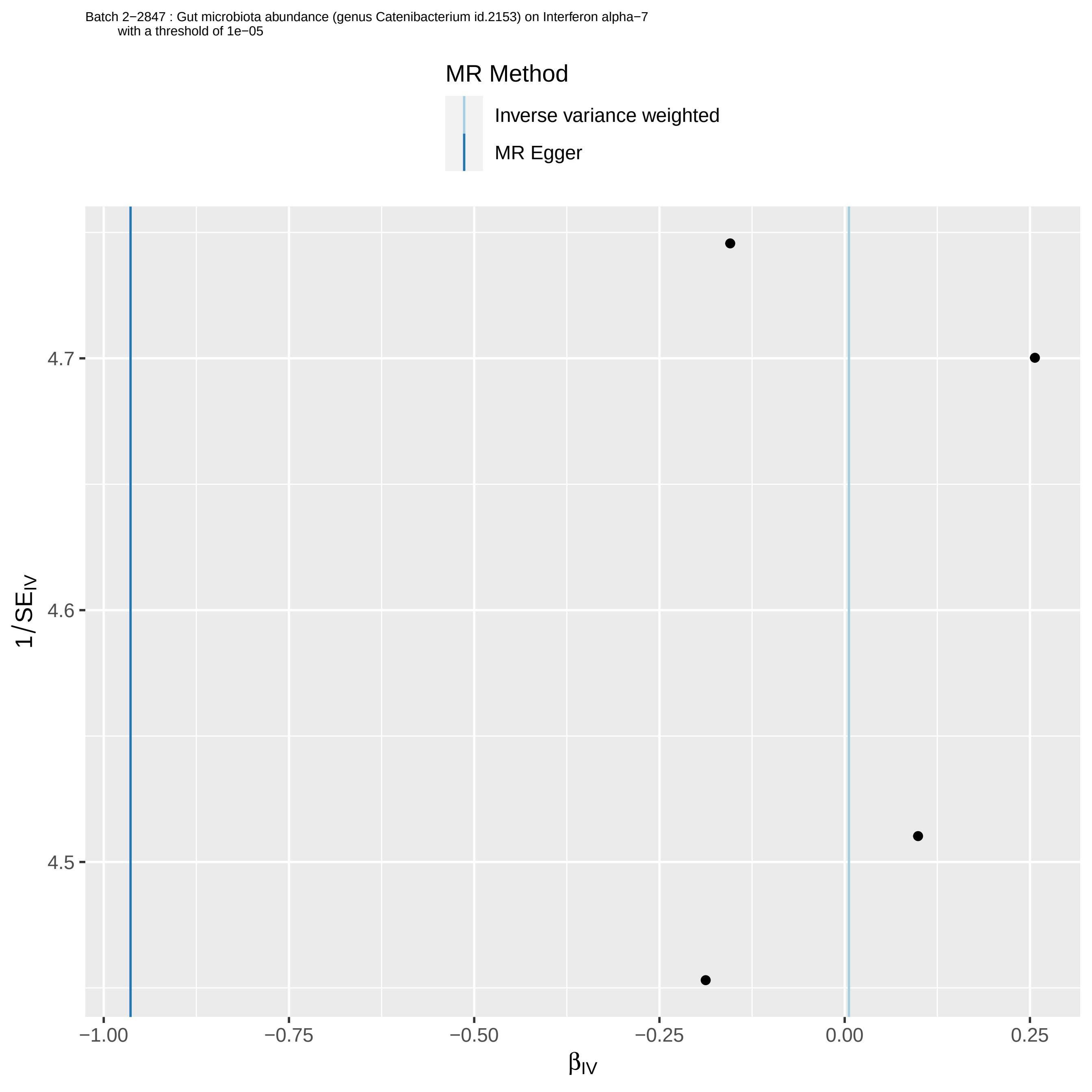
Batch 2−2847 : Gut microbiota abundance (genus Catenibacterium id.2153) on Interferon alpha−7
with a threshold of 1e−05
MR Method
Inverse variance weighted
MR Egger
4.7
4.6
4.5
1/SE_IV
−1.00
−0.75
−0.50
−0.25
0.00
0.25
β_IV

Batch 2−2848 : Gut microbiota abundance (genus Parasutterella id.2892) on Interferon alpha−7
with a threshold of 1e−05

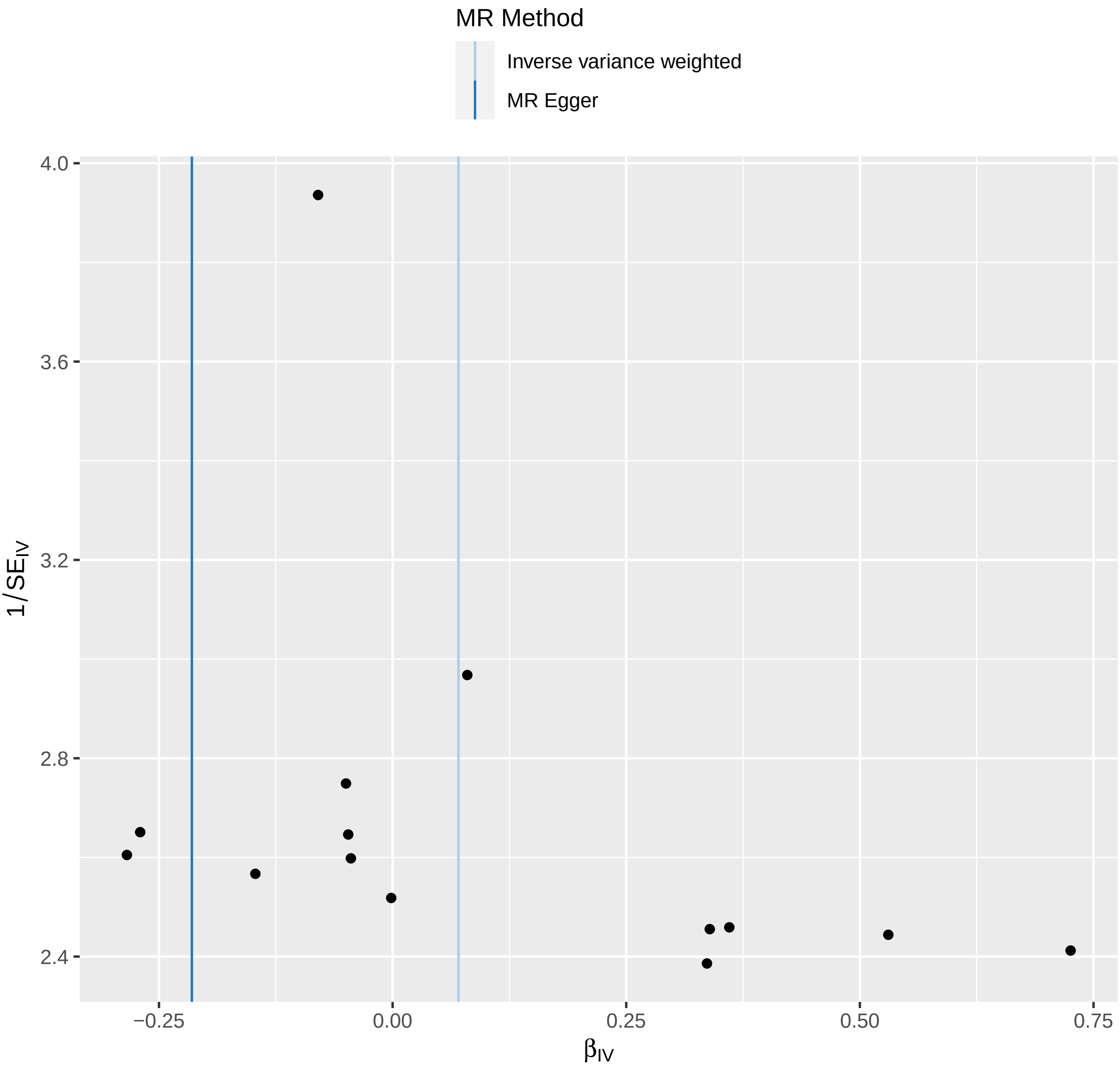

Batch 2−2849 : Gut microbiota abundance (genus Catenibacterium id.2153) on Interferon alpha−8
with a threshold of 1e−05

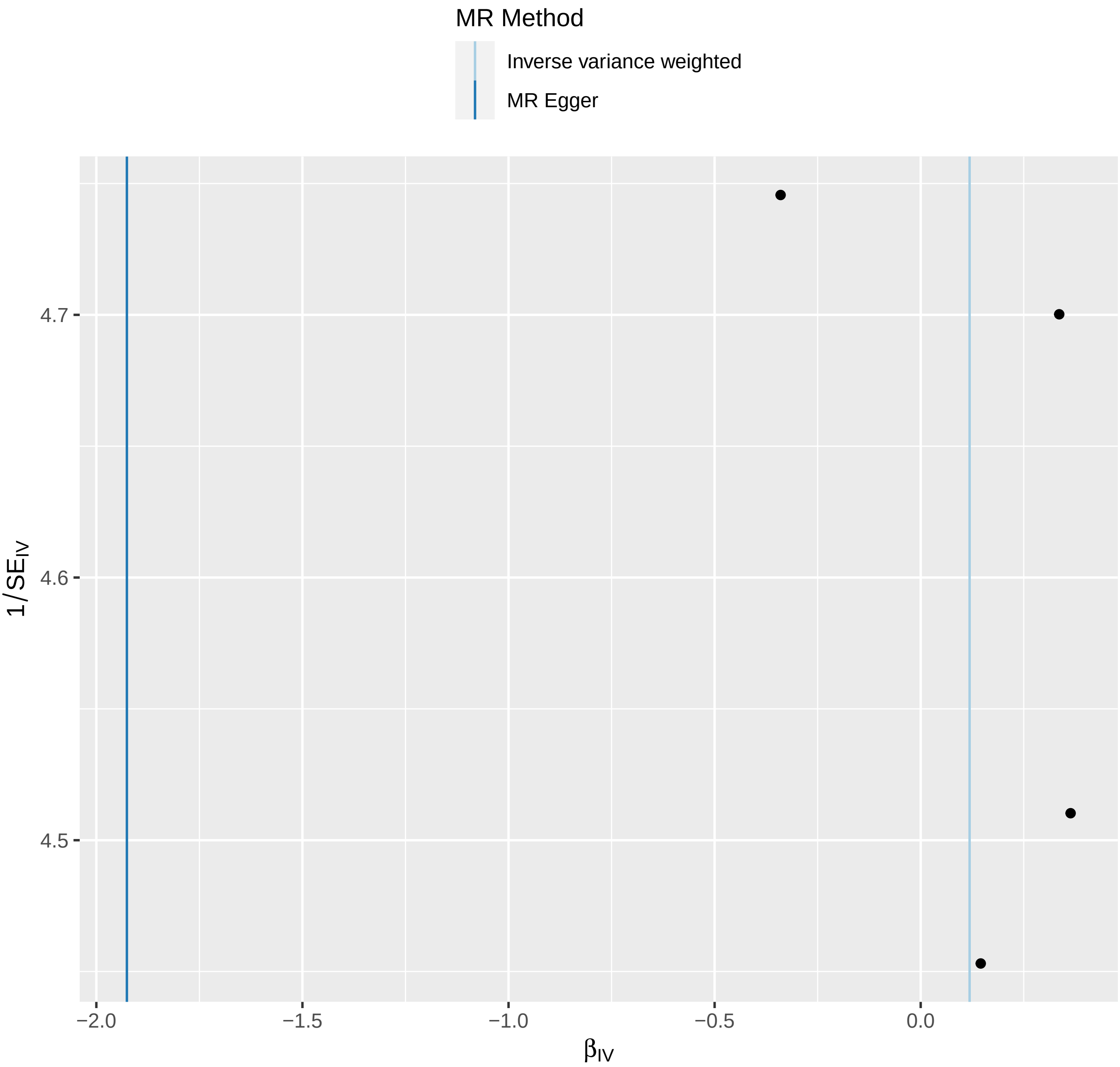

Batch 2−2850 : Gut microbiota abundance (genus Parasutterella id.2892) on Interferon alpha−8
with a threshold of 1e−05

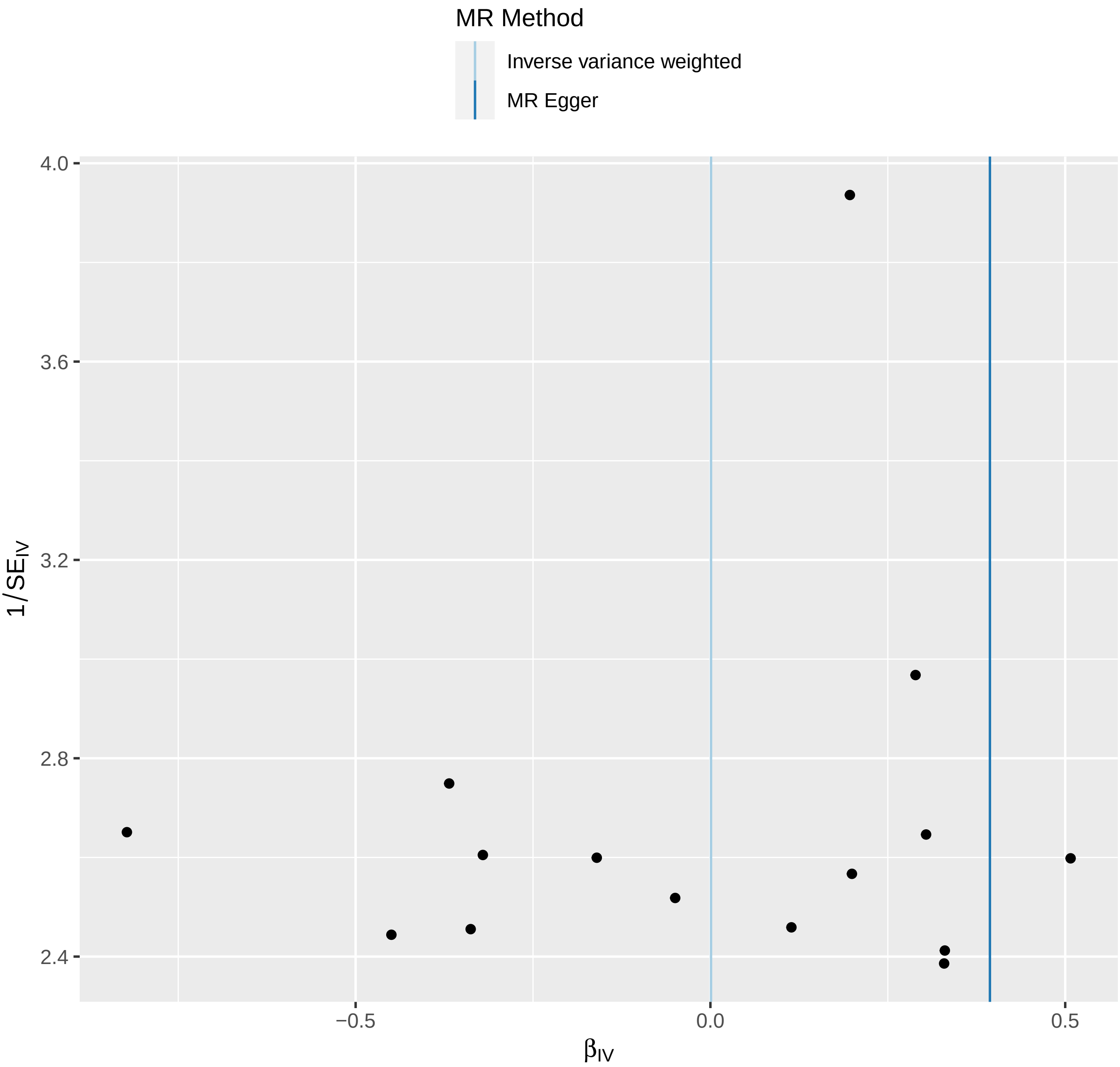

Batch 2−2851 : Gut microbiota abundance (genus Catenibacterium id.2153) on Interferon alpha/beta receptor 1
with a threshold of 1e−05

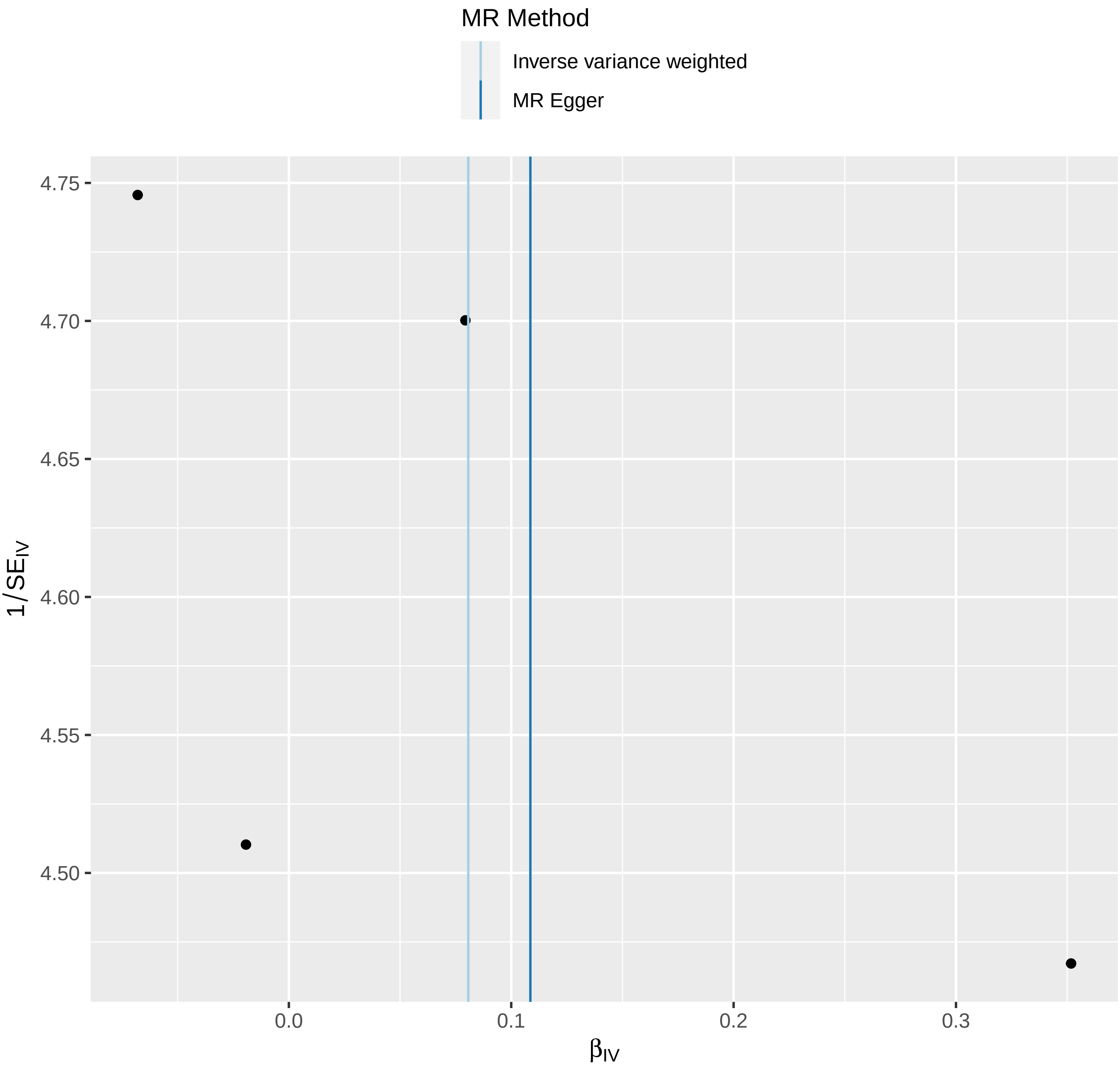

Batch 2−2852 : Gut microbiota abundance (genus Parasutterella id.2892) on Interferon alpha/beta receptor 1
with a threshold of 1e−05

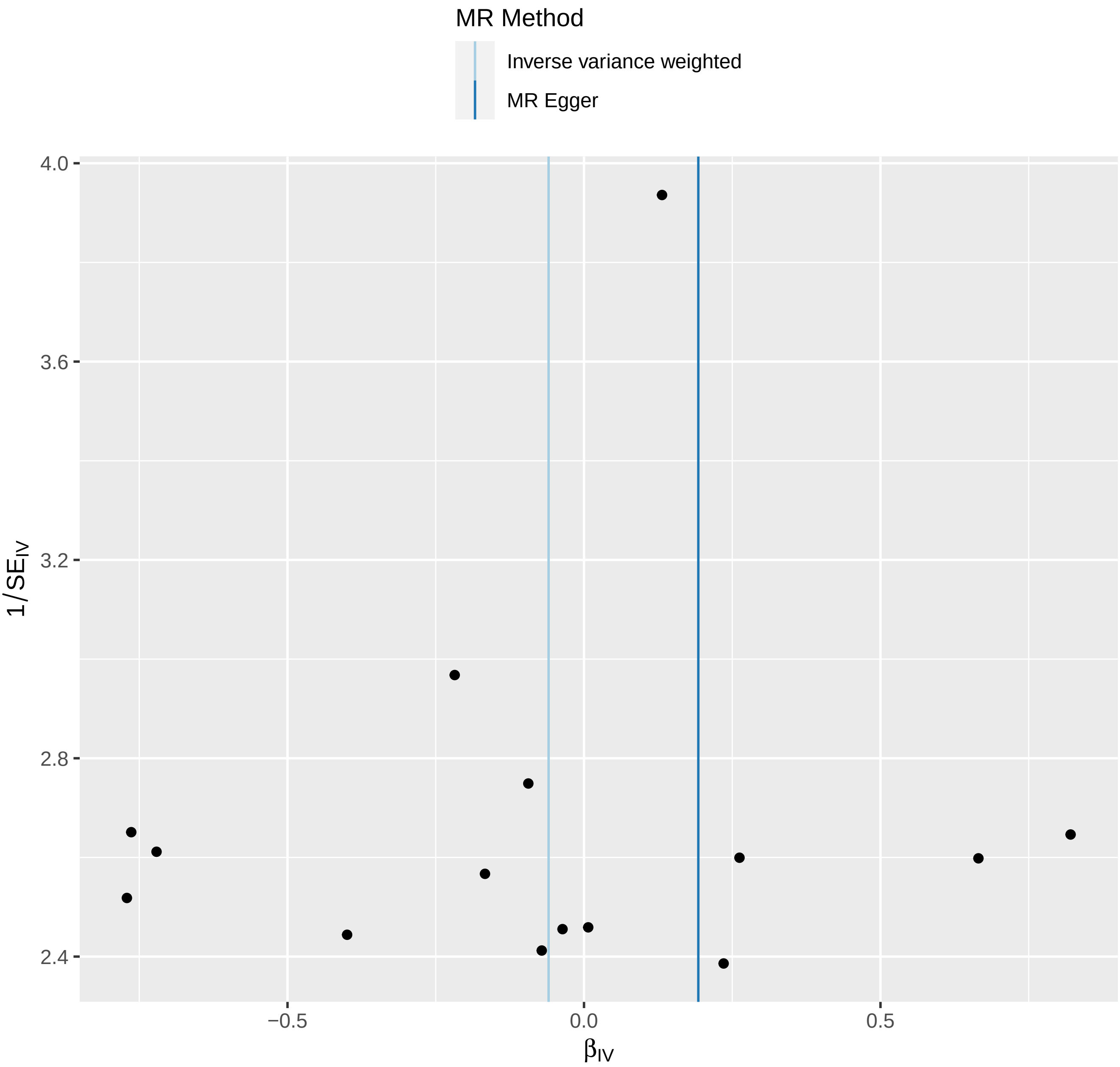

Batch 2−2853 : Gut microbiota abundance (genus Catenibacterium id.2153) on Interferon beta
with a threshold of 1e−05

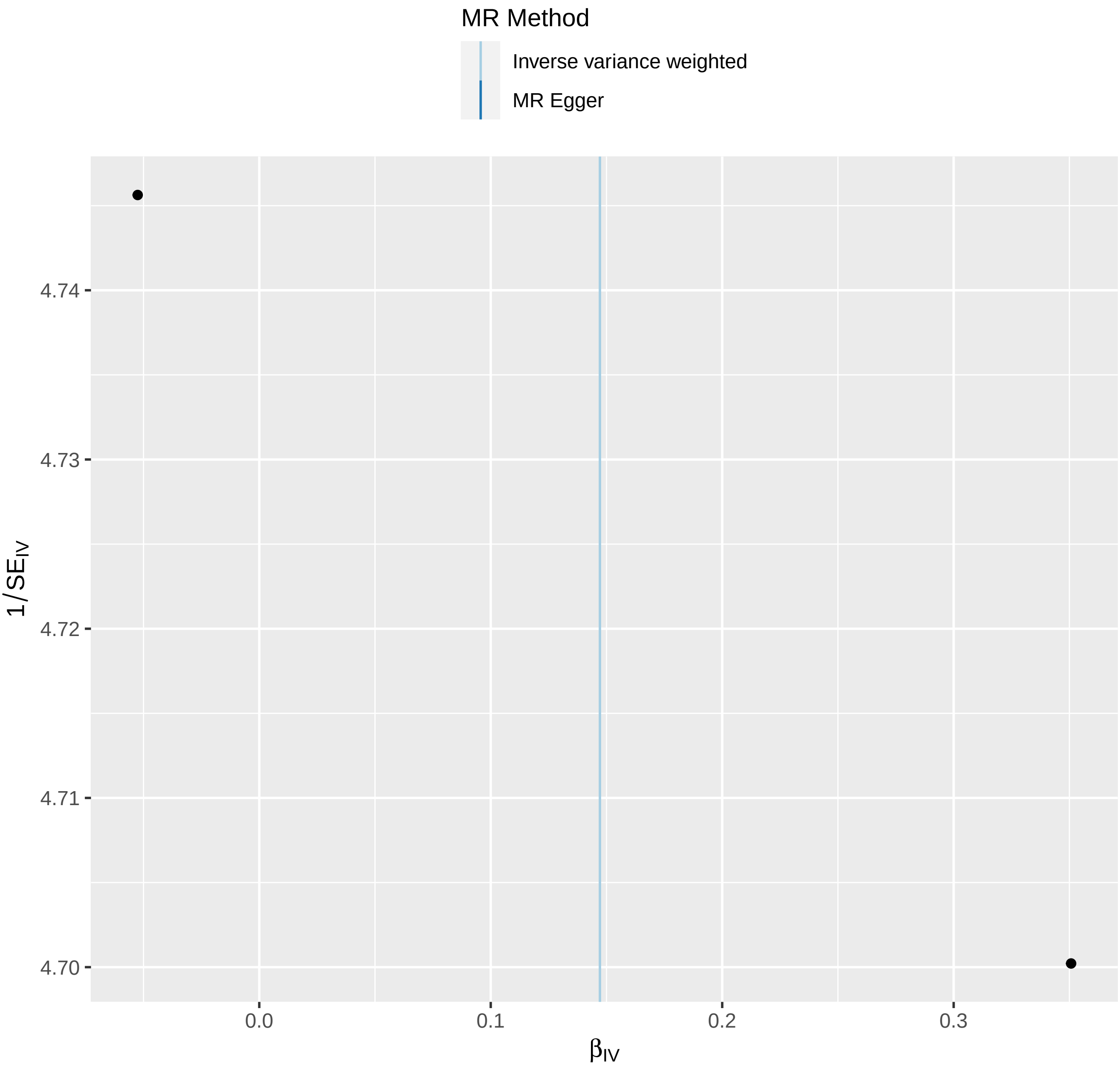

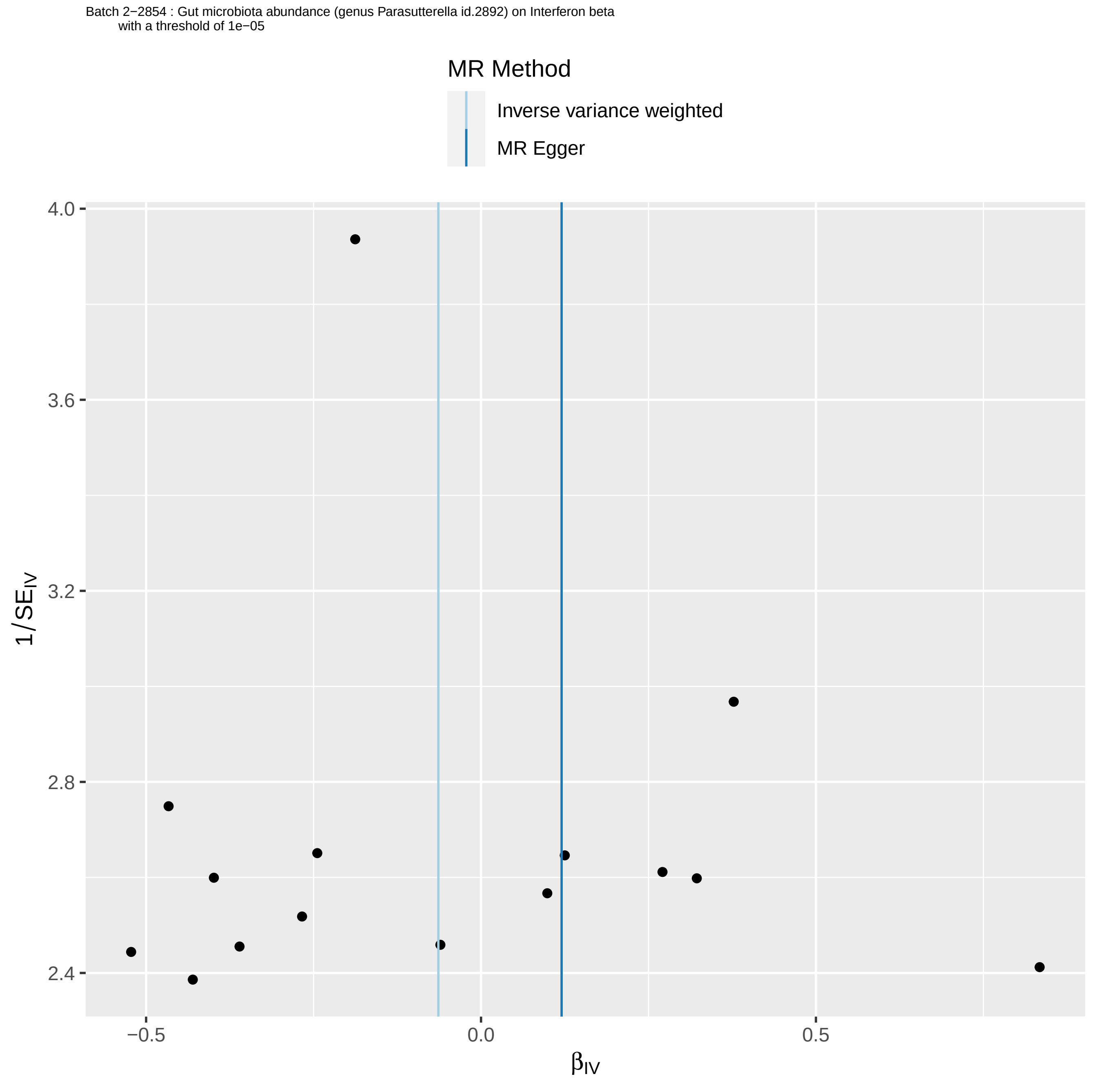

Batch 2−2854 : Gut microbiota abundance (genus Parasutterella id.2892) on Interferon beta
with a threshold of 1e−05
MR Method
Inverse variance weighted
MR Egger
4.0
3.6
3.2
2.8
2.4
1/SE$_{IV}$
−0.5
0.0
0.5
β$_{IV}$

Batch 2−2855 : Gut microbiota abundance (genus Catenibacterium id.2153) on Interferon gamma
with a threshold of 1e−05

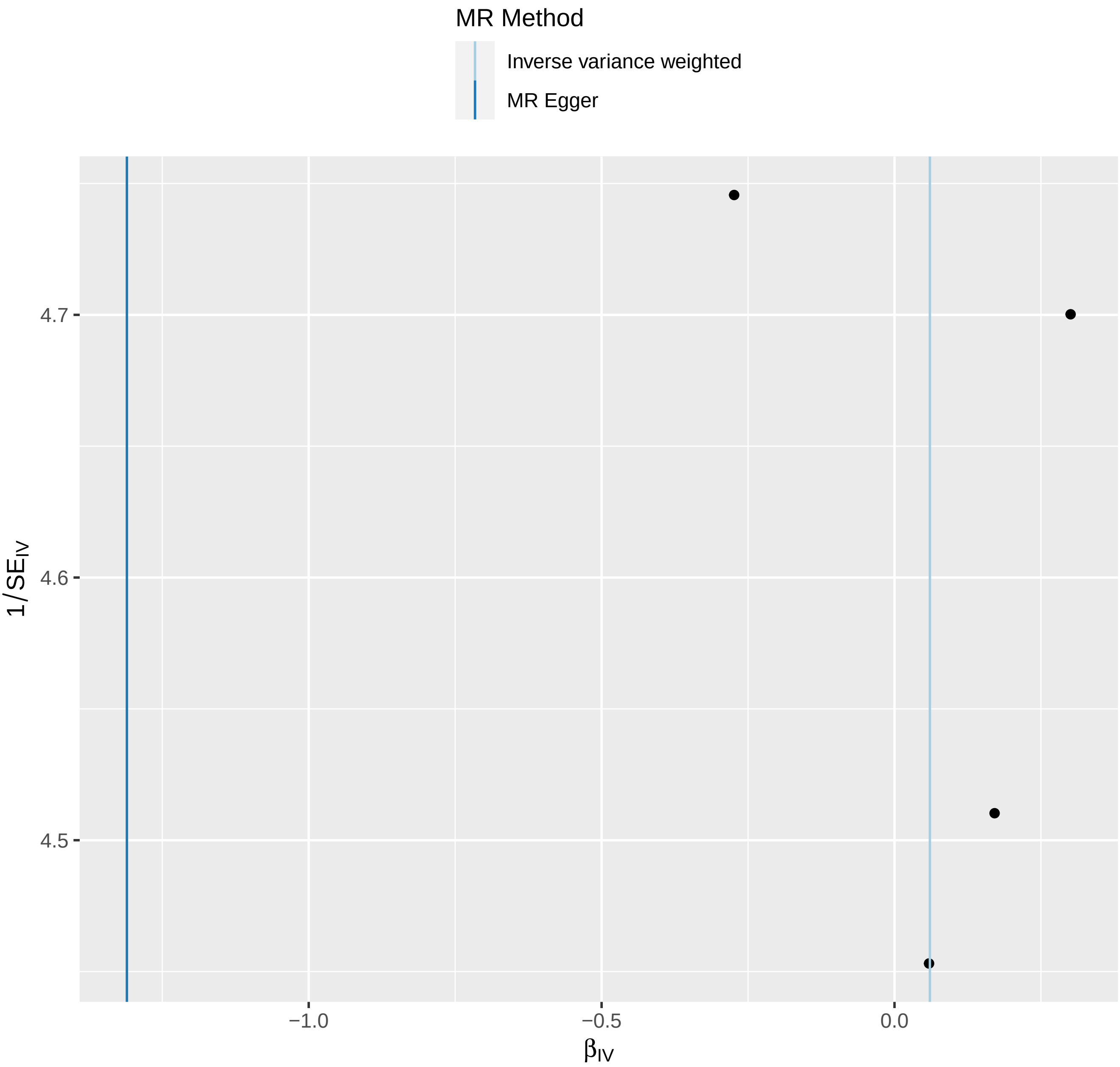

Batch 2−2856 : Gut microbiota abundance (genus Parasutterella id.2892) on Interferon gamma
with a threshold of 1e−05

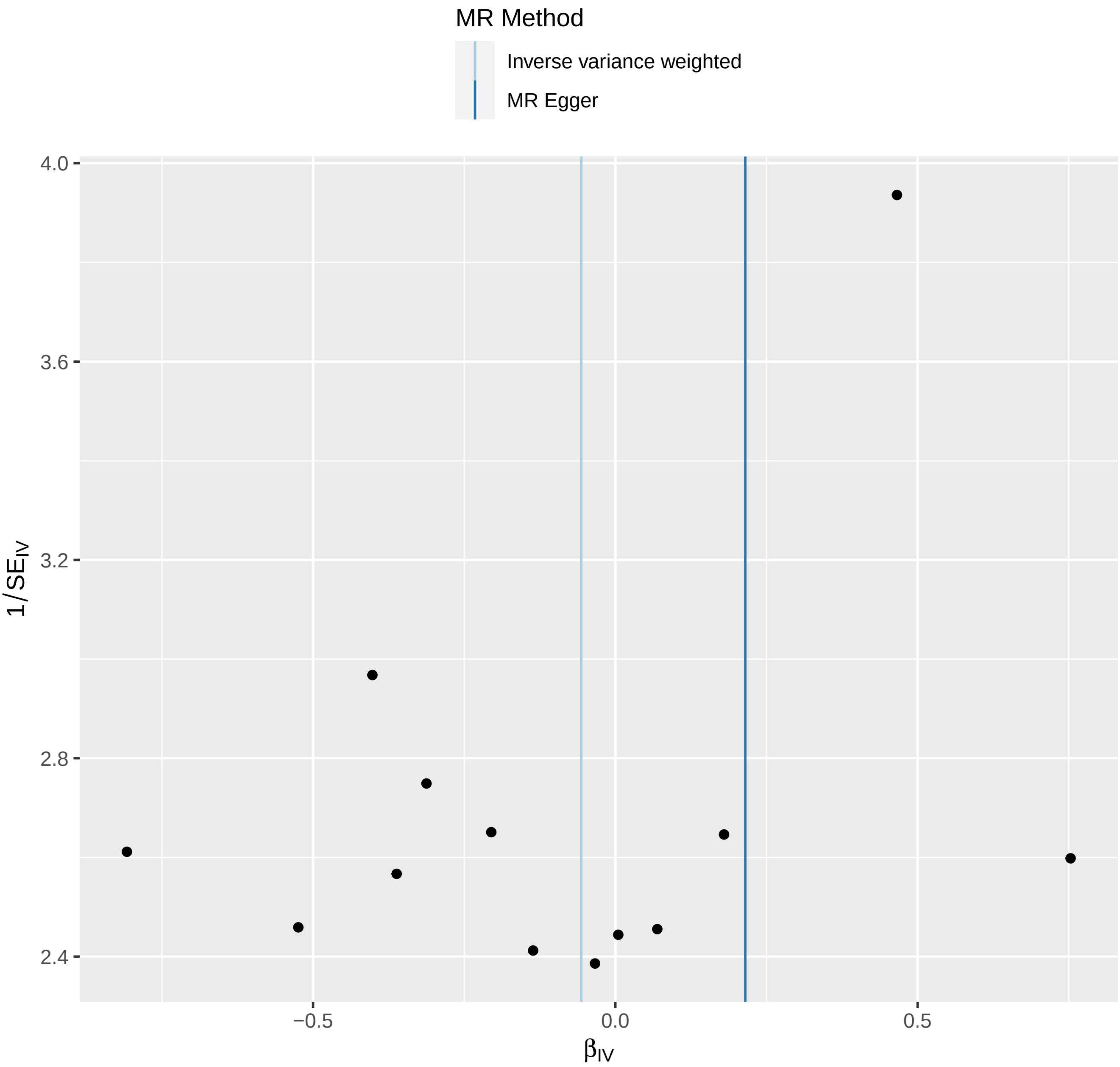

Batch 2−2857 : Gut microbiota abundance (genus Catenibacterium id.2153) on Interferon gamma
with a threshold of 1e−05

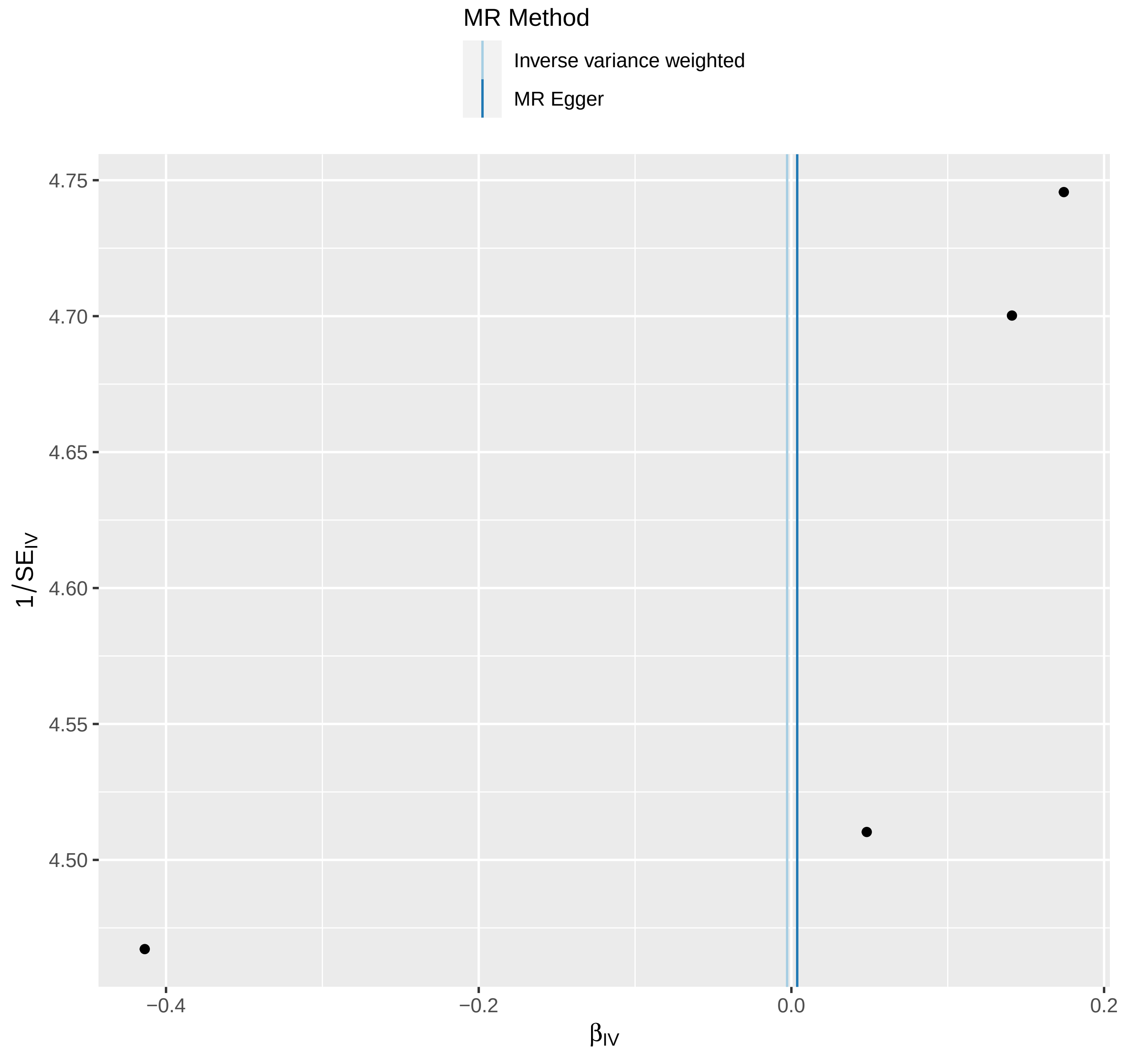

Batch 2−2858 : Gut microbiota abundance (genus Parasutterella id.2892) on Interferon gamma
with a threshold of 1e−05

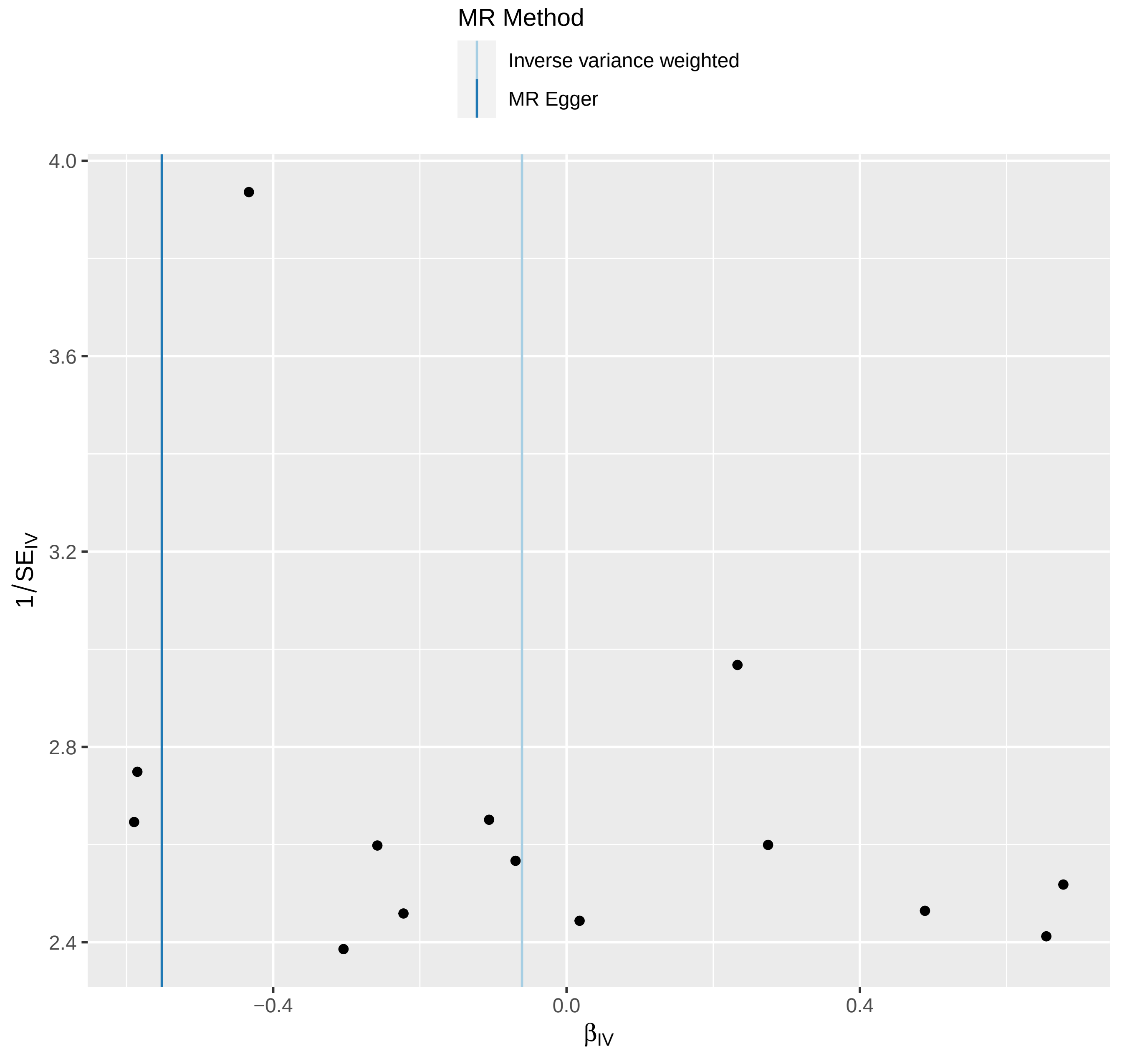

Batch 2−2859 : Gut microbiota abundance (genus Catenibacterium id.2153) on Interferon gamma receptor 1
with a threshold of 1e−05

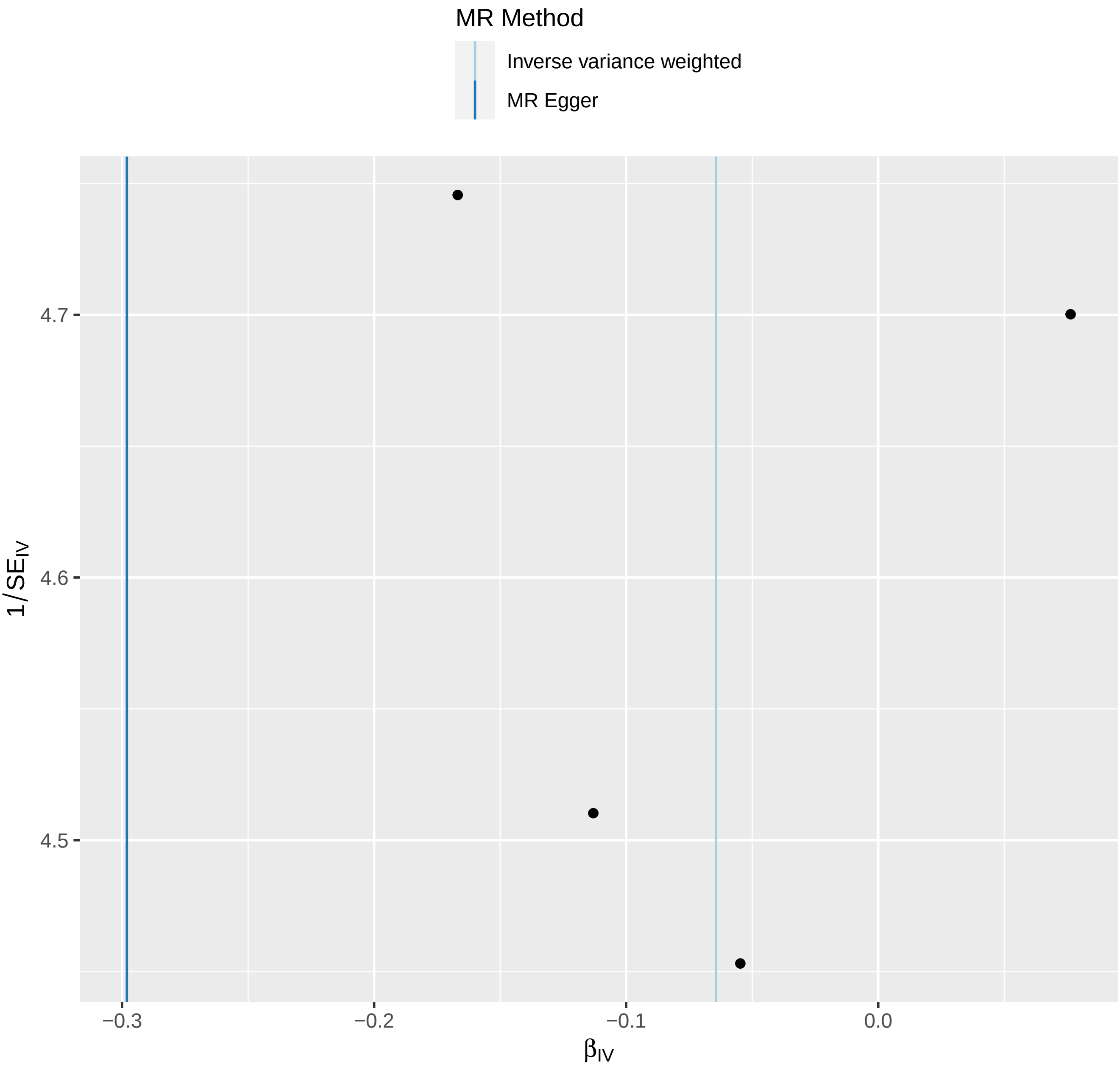

Batch 2−2860 : Gut microbiota abundance (genus Parasutterella id.2892) on Interferon gamma receptor 1
with a threshold of 1e−05

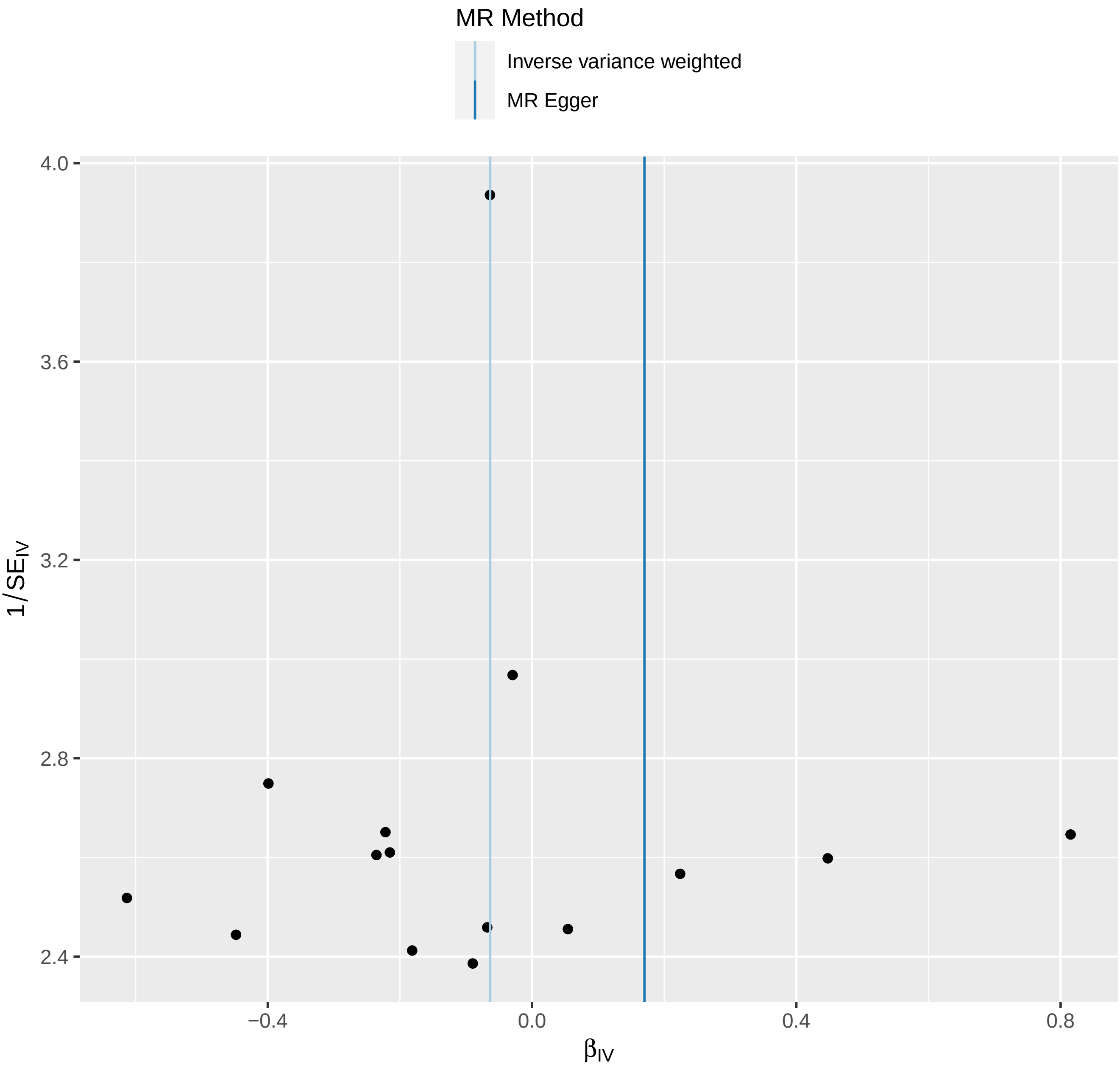

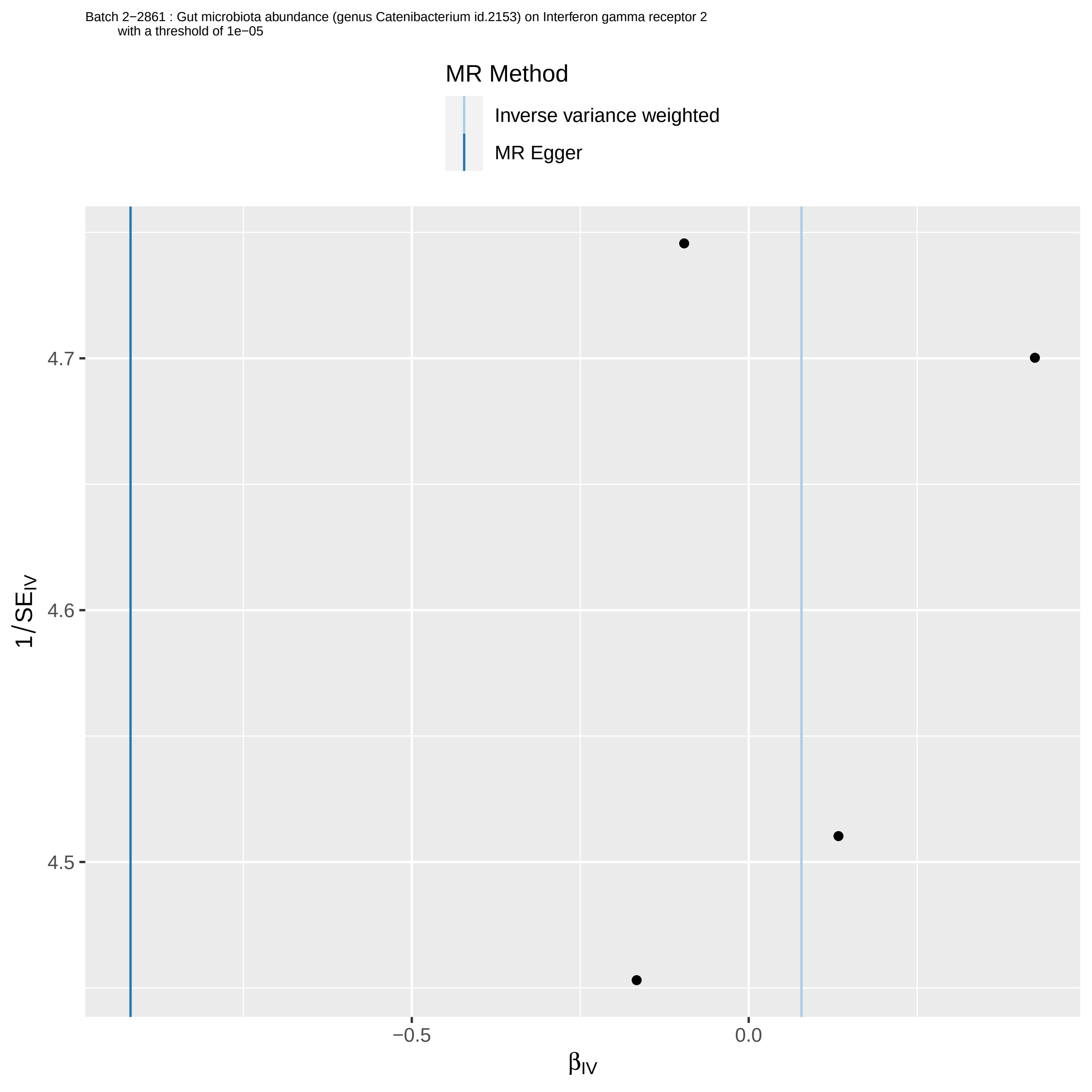

Batch 2−2861 : Gut microbiota abundance (genus Catenibacterium id.2153) on Interferon gamma receptor 2
with a threshold of 1e−05
MR Method
Inverse variance weighted
MR Egger
4.7
4.6
4.5
1/SE_IV
−0.5
0.0
β_IV

Batch 2−2862 : Gut microbiota abundance (genus Parasutterella id.2892) on Interferon gamma receptor 2
with a threshold of 1e−05

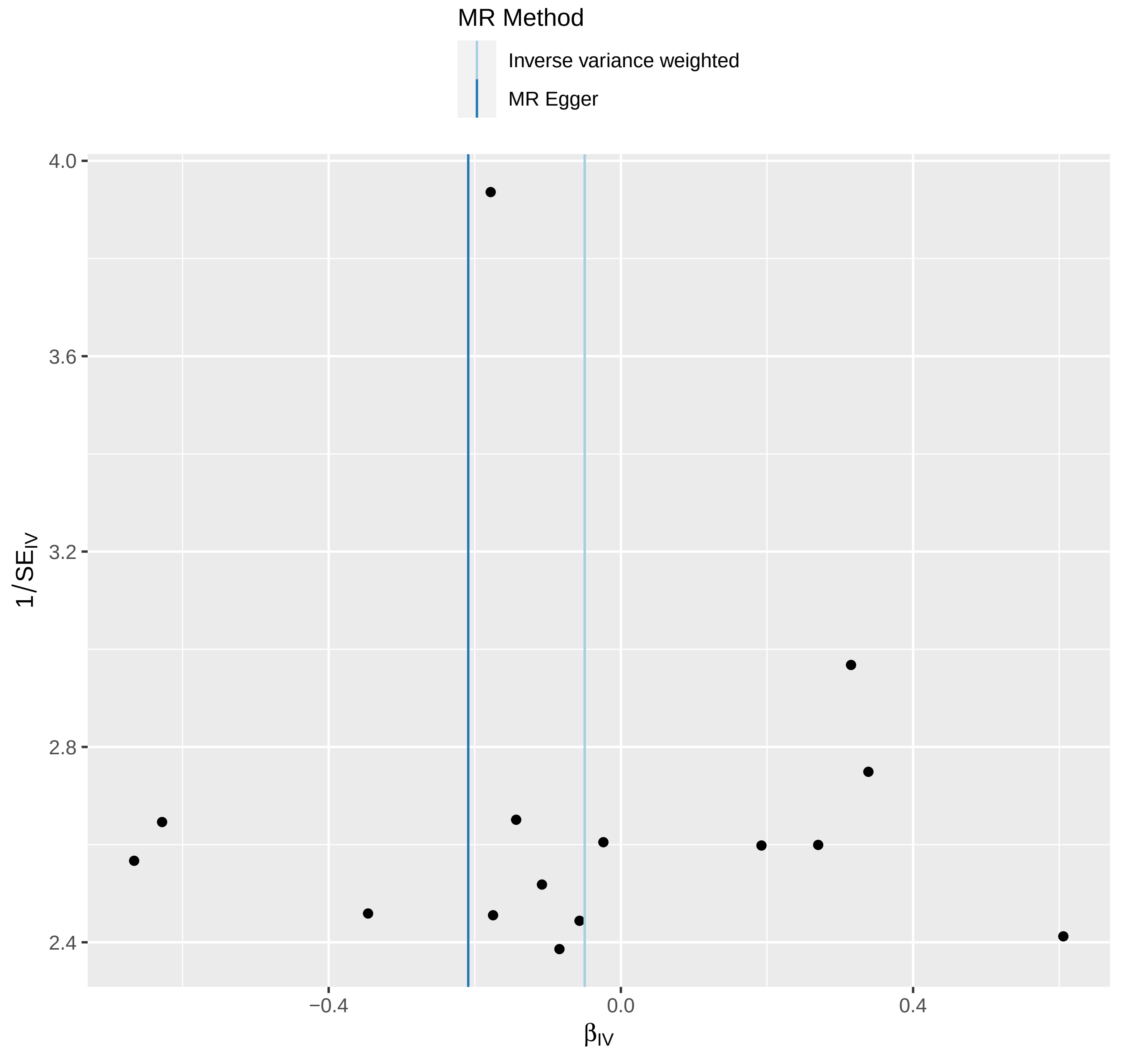

Batch 2−2863 : Gut microbiota abundance (genus Catenibacterium id.2153) on Interferon gamma receptor 2
with a threshold of 1e−05

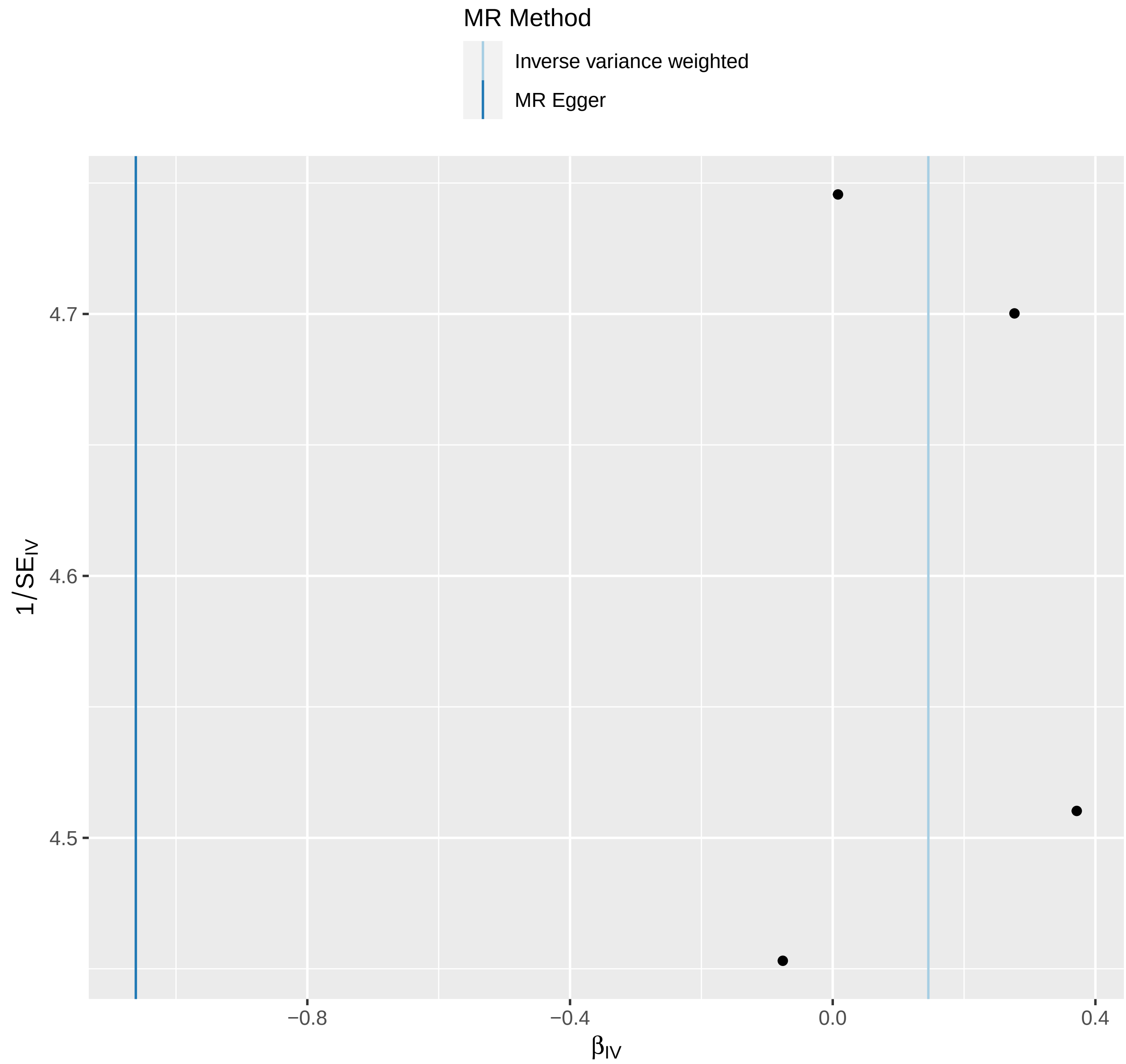

Batch 2−2864 : Gut microbiota abundance (genus Parasutterella id.2892) on Interferon gamma receptor 2
with a threshold of 1e−05

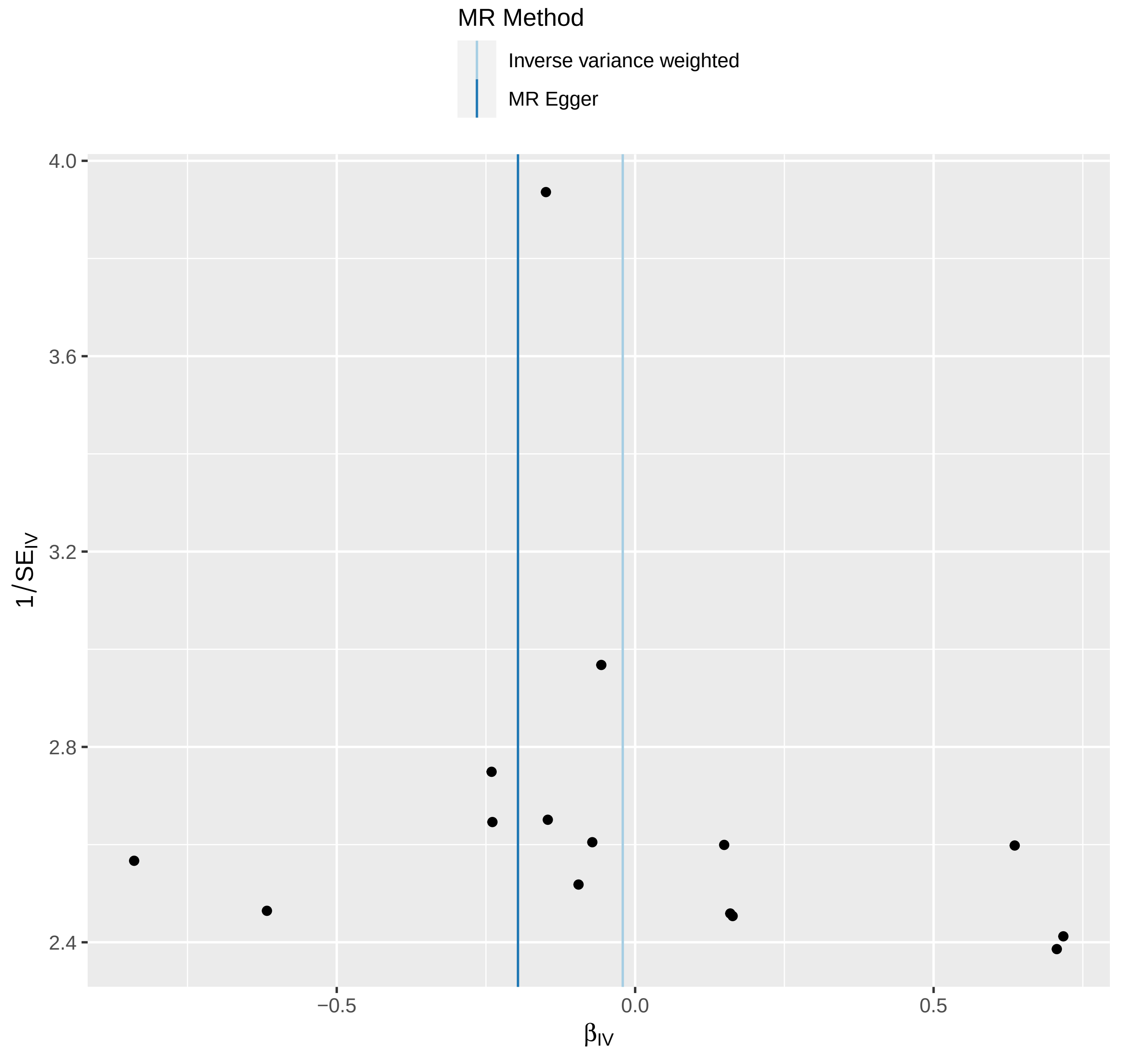

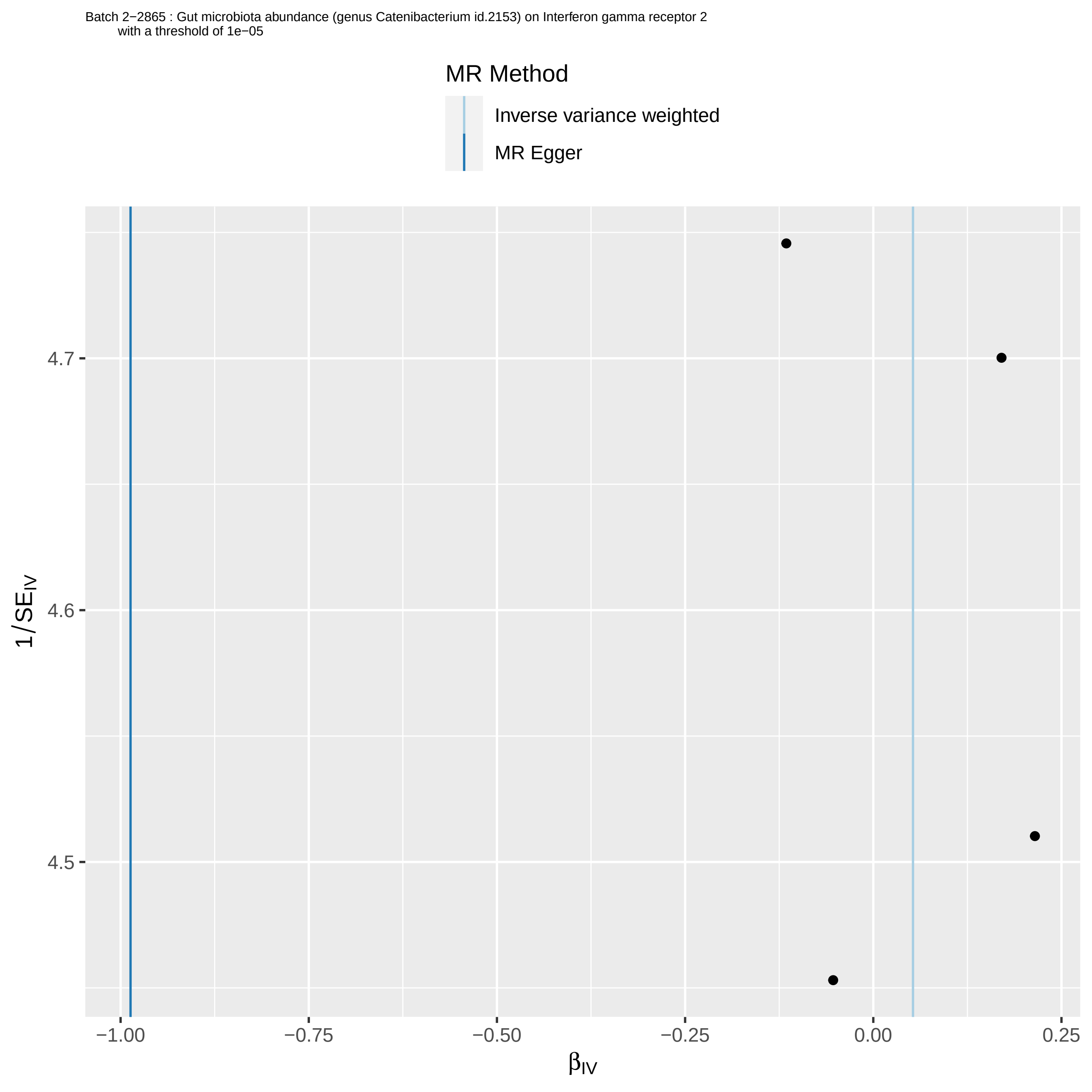
Batch 2−2865 : Gut microbiota abundance (genus Catenibacterium id.2153) on Interferon gamma receptor 2
with a threshold of 1e−05
MR Method
Inverse variance weighted
MR Egger
4.7
4.6
4.5
1/SE_IV
−1.00
−0.75
−0.50
−0.25
0.00
0.25
β_IV

Batch 2−2866 : Gut microbiota abundance (genus Parasutterella id.2892) on Interferon gamma receptor 2
with a threshold of 1e−05

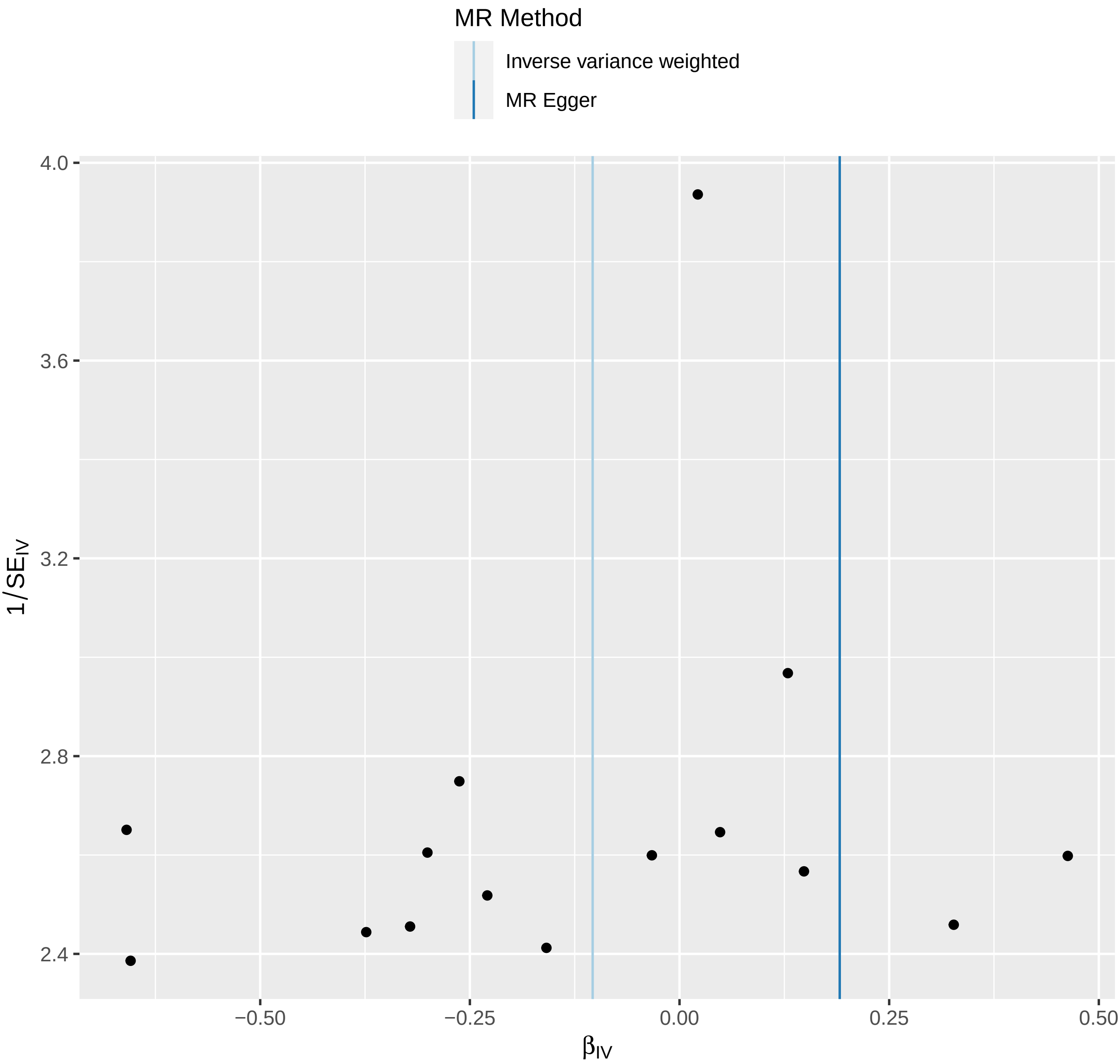

Batch 2−2867 : Gut microbiota abundance (genus Catenibacterium id.2153) on Interferon lambda−1
with a threshold of 1e−05

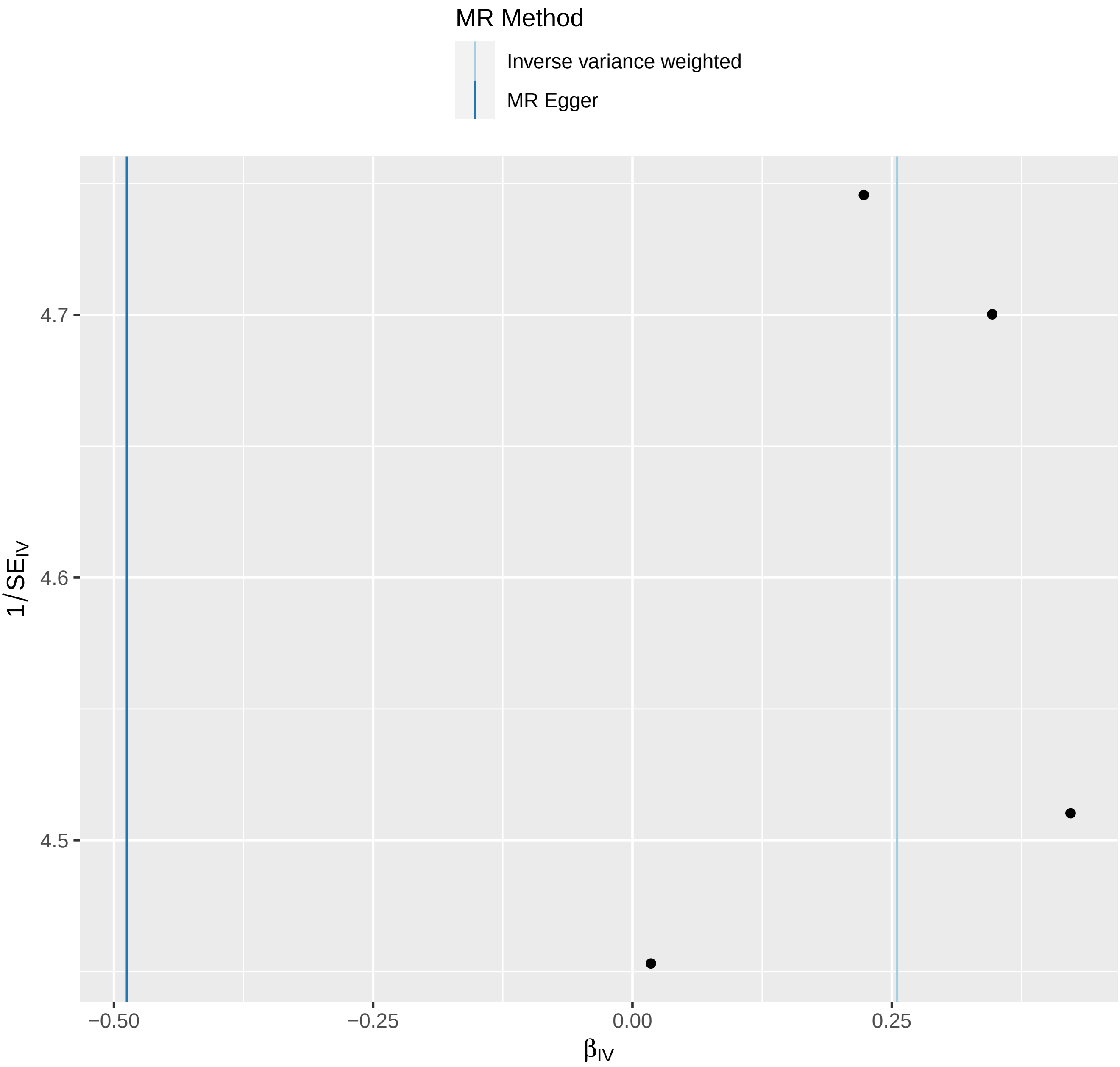

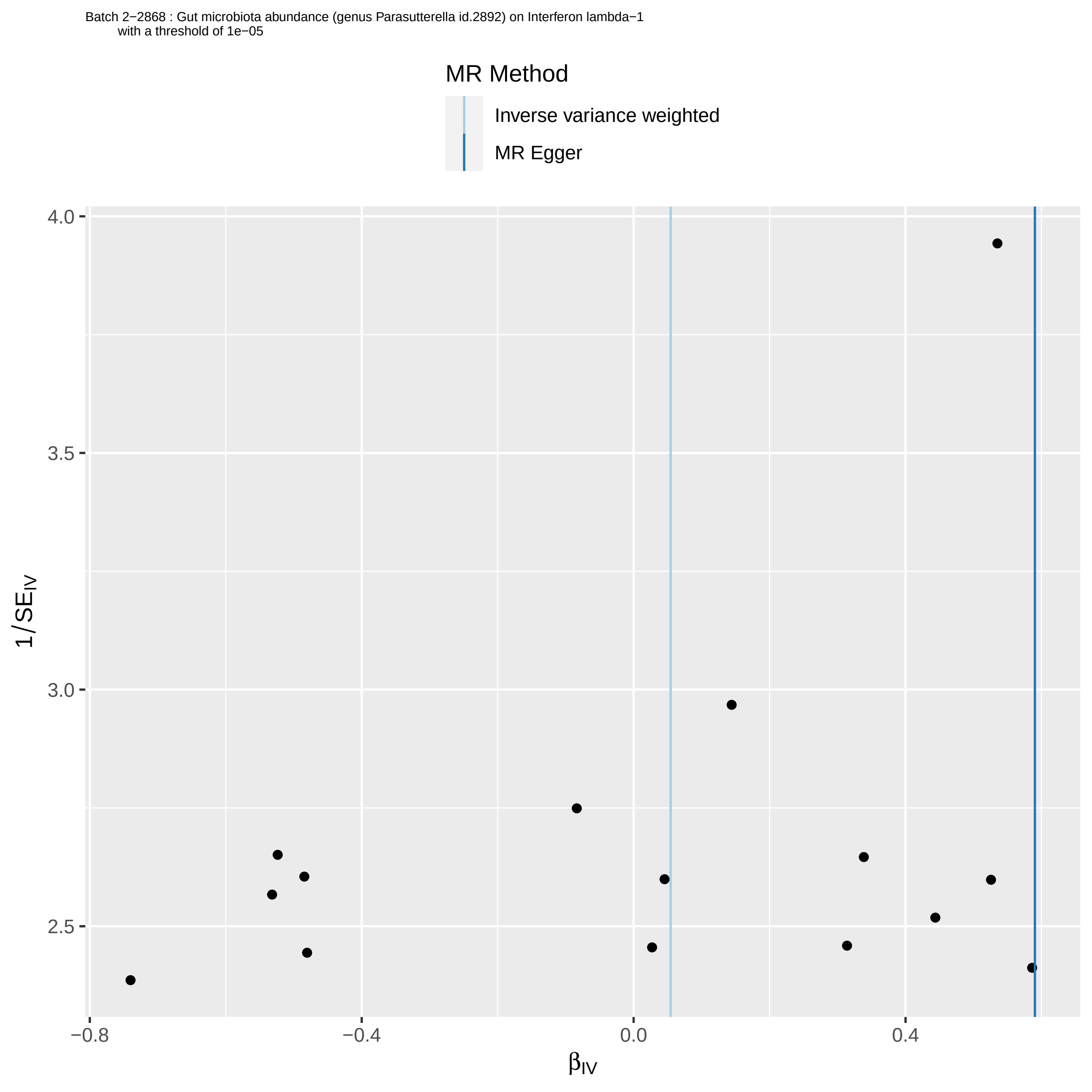

Batch 2−2868 : Gut microbiota abundance (genus Parasutterella id.2892) on Interferon lambda−1
with a threshold of 1e−05
MR Method
Inverse variance weighted
MR Egger
4.0
3.5
3.0
2.5
1/SE$_{IV}$
−0.8
−0.4
0.0
0.4
β$_{IV}$

Batch 2−2869 : Gut microbiota abundance (genus Catenibacterium id.2153) on Interferon lambda−1
with a threshold of 1e−05

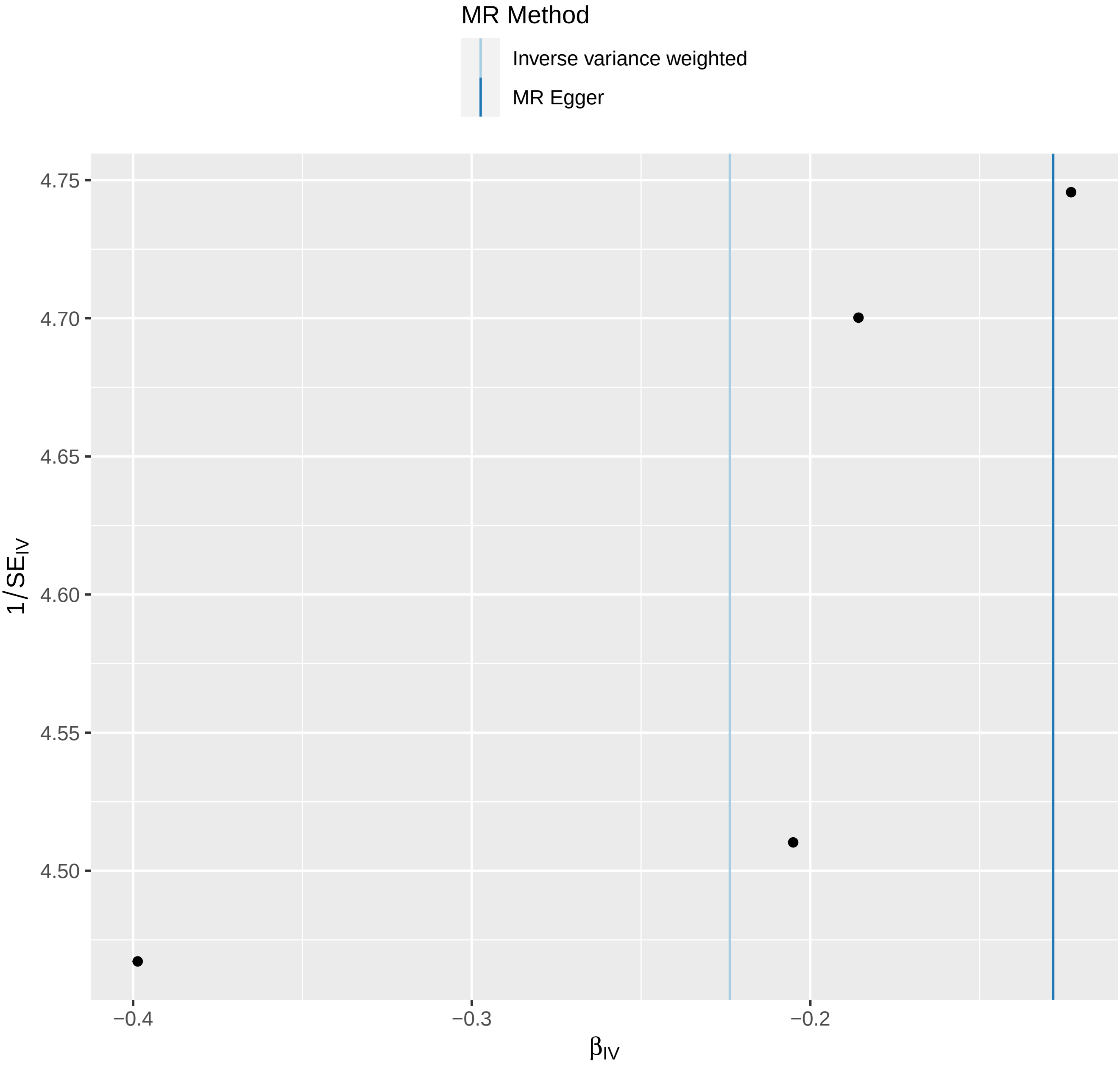

Batch 2−2870 : Gut microbiota abundance (genus Parasutterella id.2892) on Interferon lambda−1
with a threshold of 1e−05

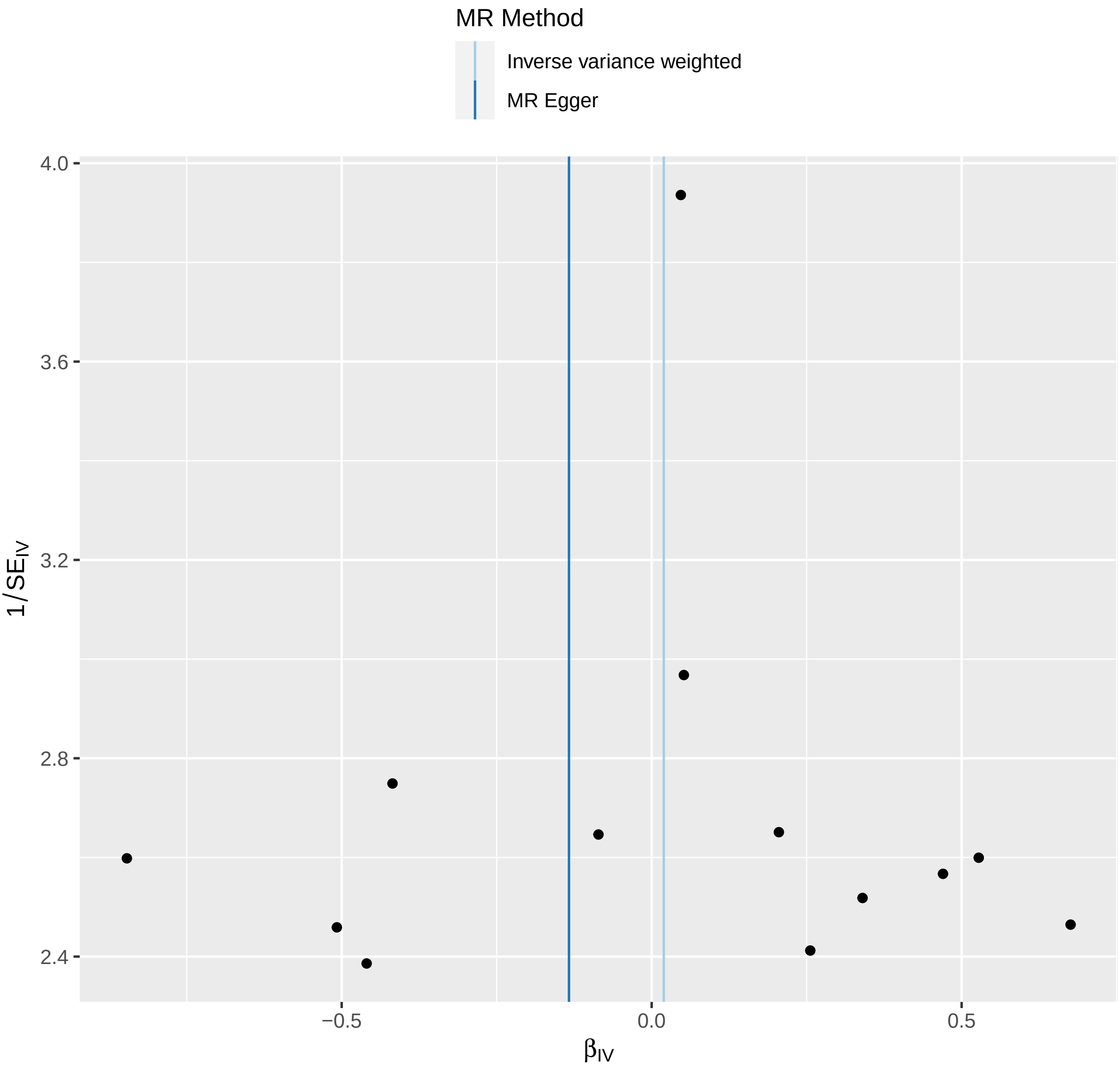

Batch 2−2871 : Gut microbiota abundance (genus Catenibacterium id.2153) on Interferon lambda−2
with a threshold of 1e−05

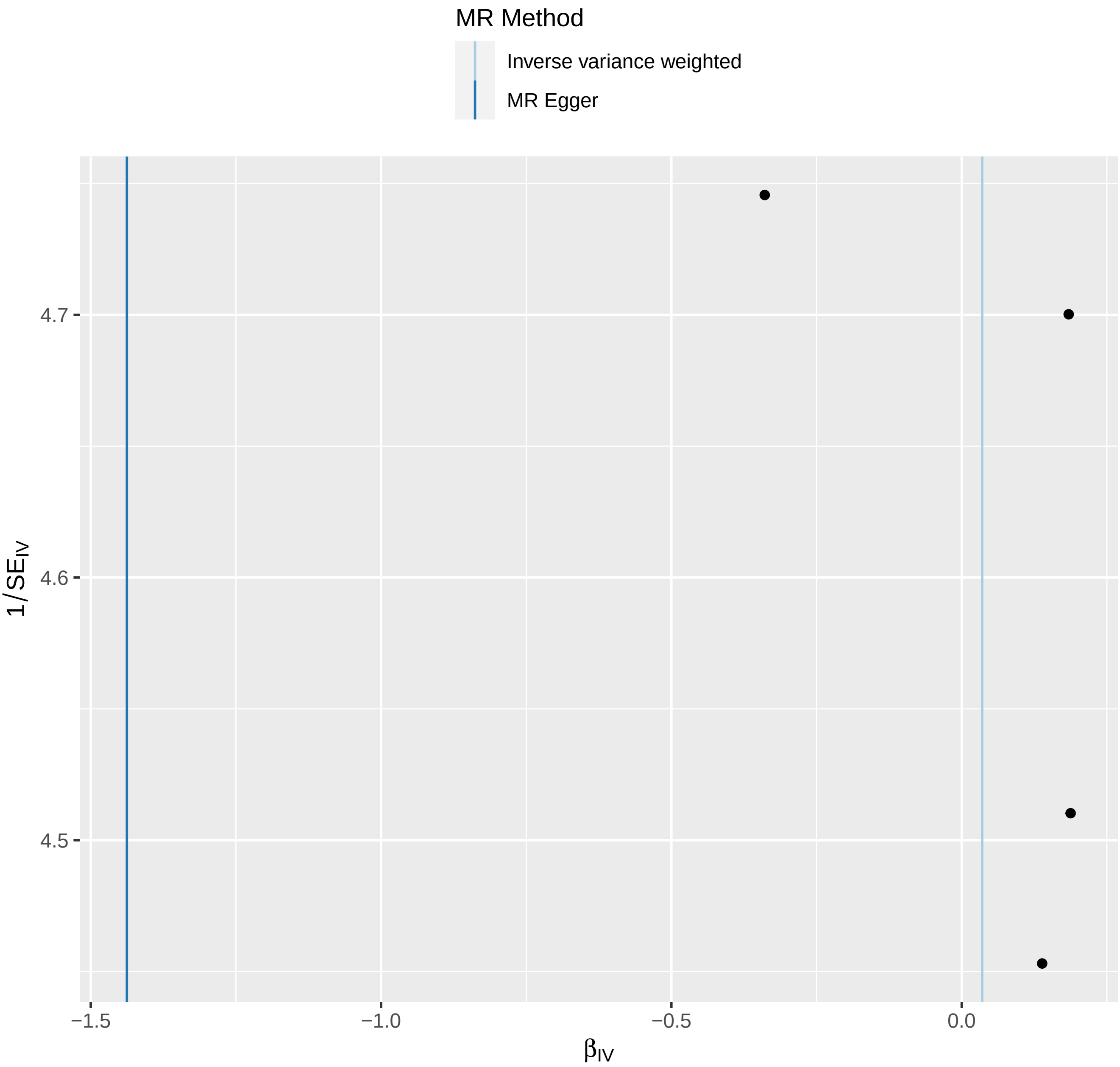

Batch 2−2872 : Gut microbiota abundance (genus Parasutterella id.2892) on Interferon lambda−2
with a threshold of 1e−05

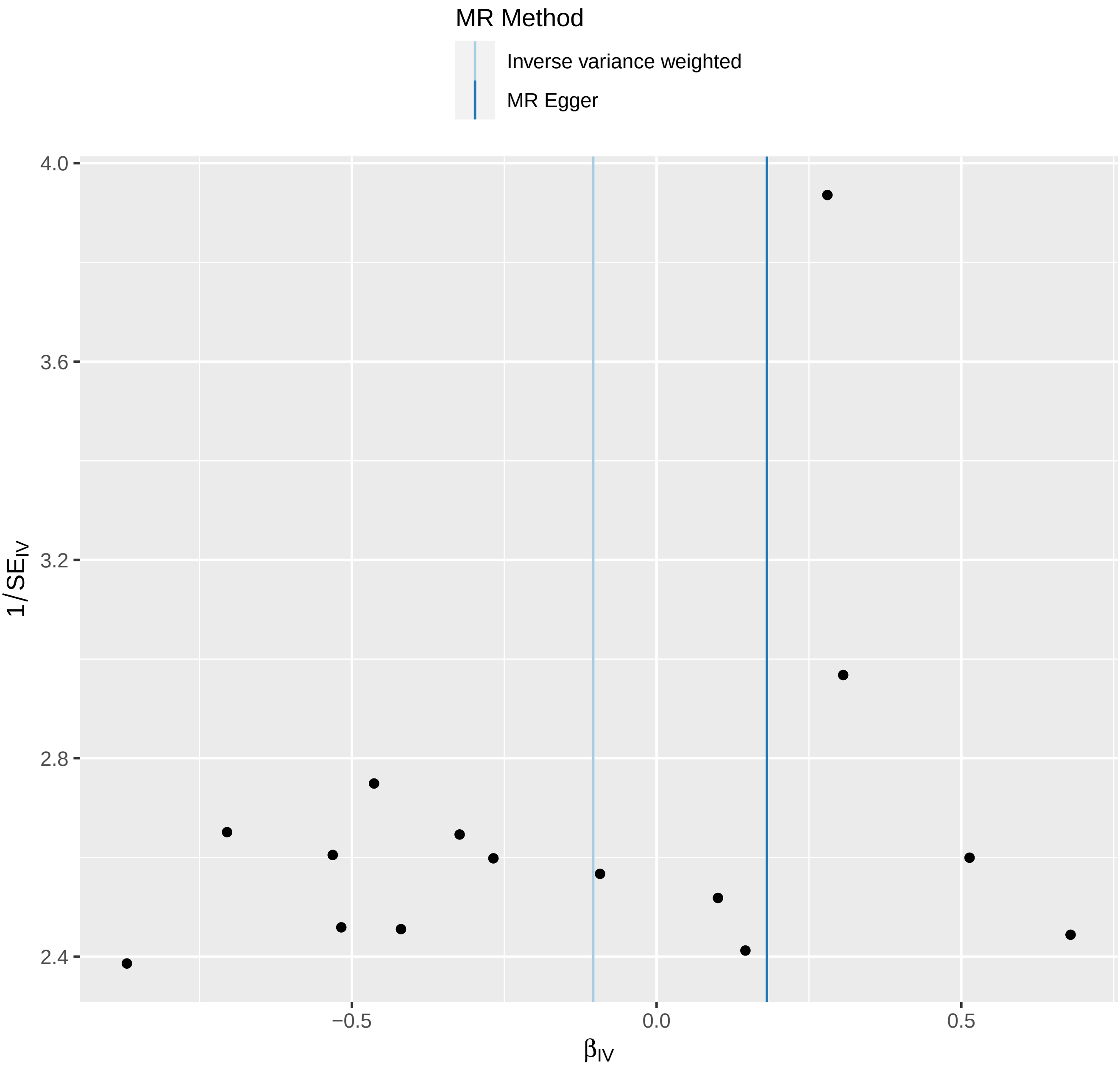

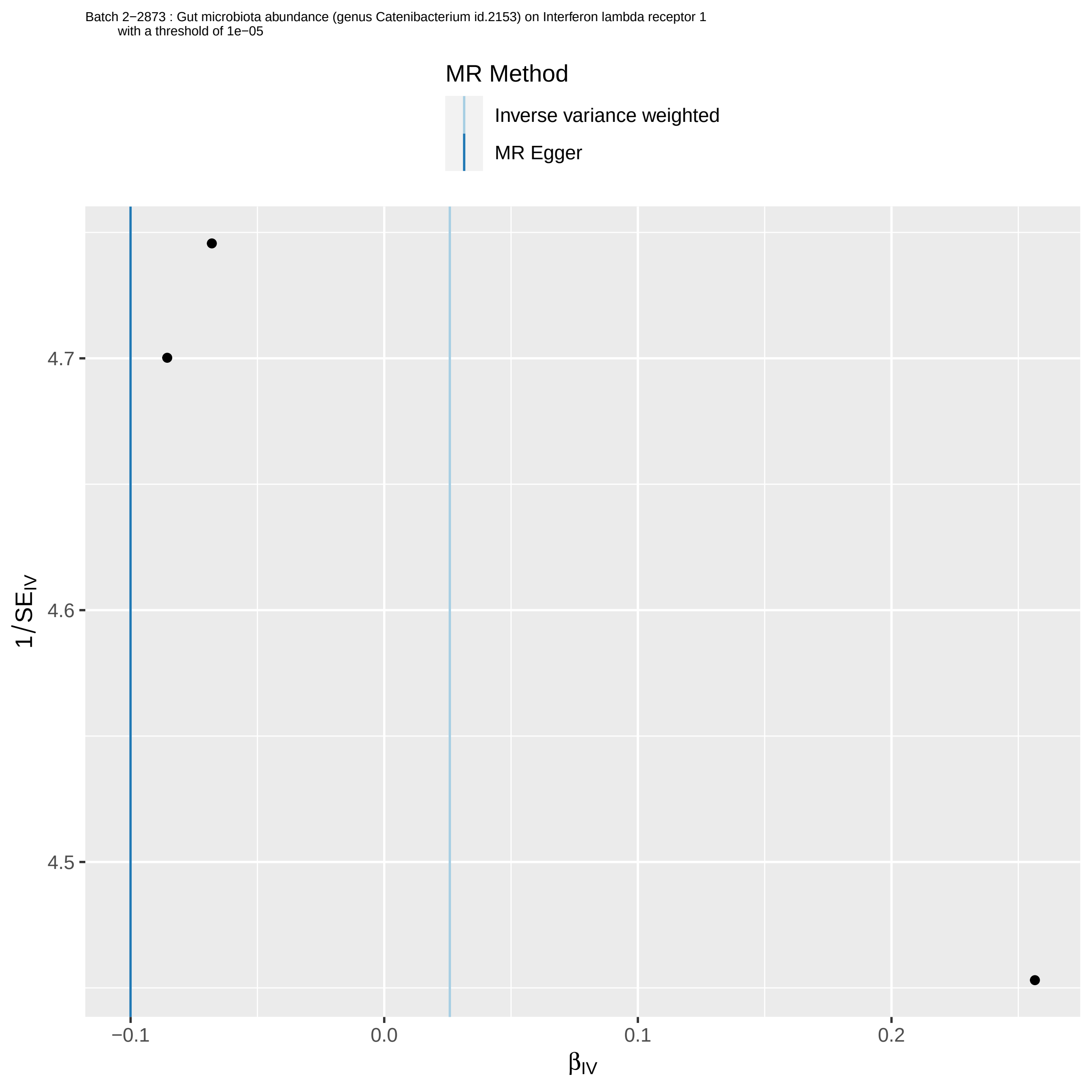

Batch 2−2873 : Gut microbiota abundance (genus Catenibacterium id.2153) on Interferon lambda receptor 1
with a threshold of 1e−05
MR Method
Inverse variance weighted
MR Egger
4.7
4.6
4.5
1/SE_IV
−0.1
0.0
0.1
0.2
β_IV

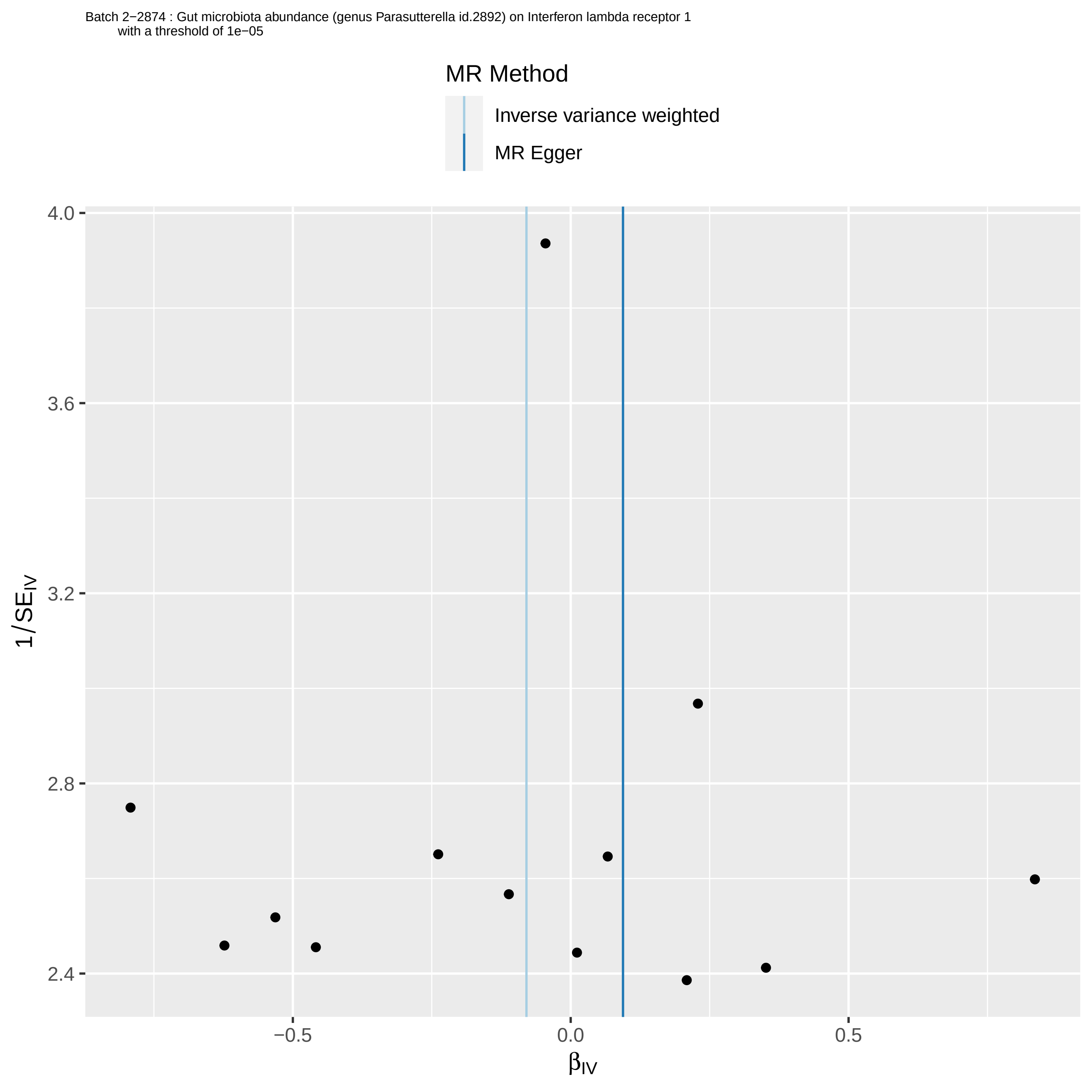

Batch 2−2874 : Gut microbiota abundance (genus Parasutterella id.2892) on Interferon lambda receptor 1
with a threshold of 1e−05
MR Method
Inverse variance weighted
MR Egger
4.0
3.6
3.2
2.8
2.4
−0.5
0.0
0.5
1/SE_IV
β_IV

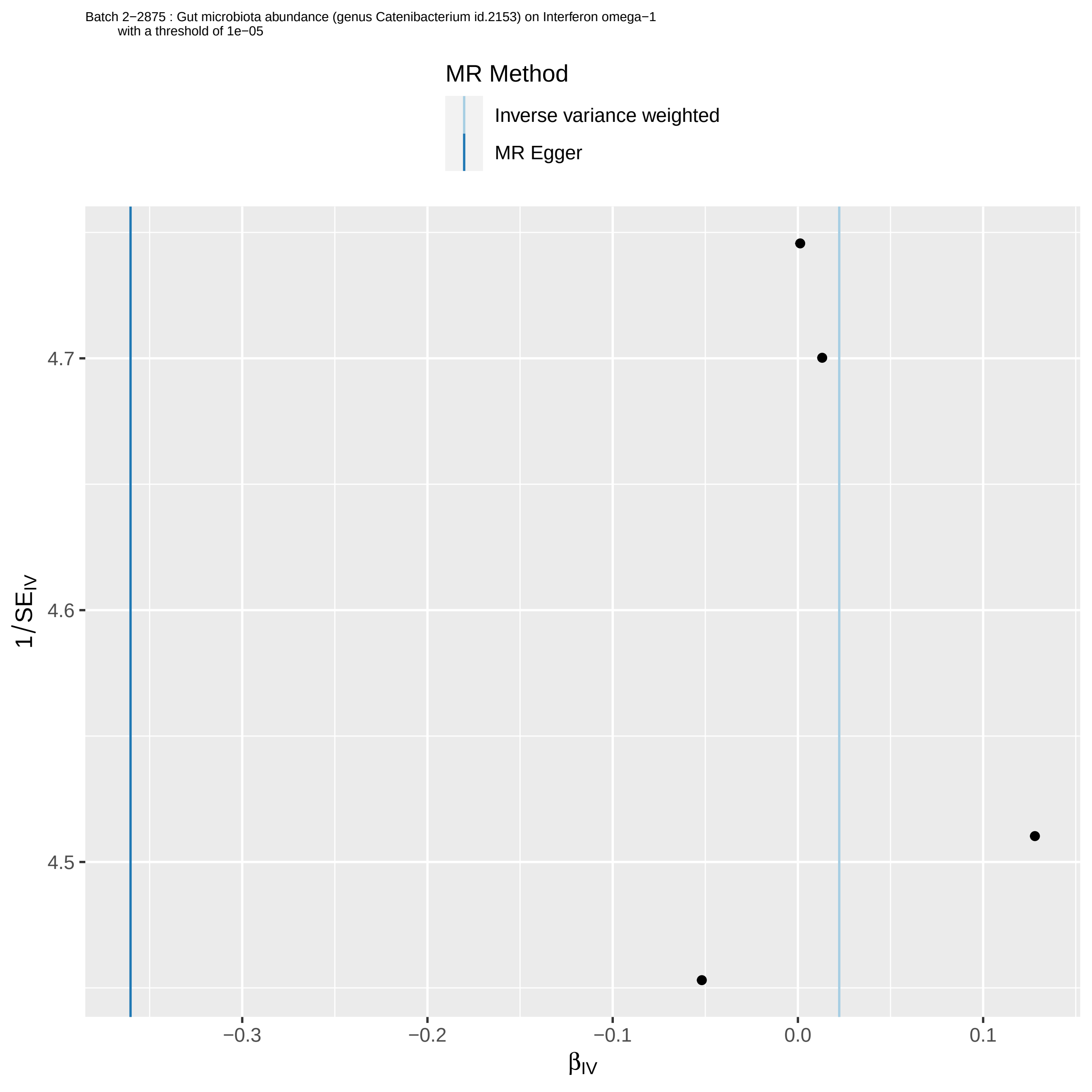

Batch 2−2875 : Gut microbiota abundance (genus Catenibacterium id.2153) on Interferon omega−1
with a threshold of 1e−05
MR Method
Inverse variance weighted
MR Egger
4.7
4.6
4.5
1/SE_IV
−0.3
−0.2
−0.1
0.0
0.1
β_IV

Batch 2−2876 : Gut microbiota abundance (genus Parasutterella id.2892) on Interferon omega−1
with a threshold of 1e−05

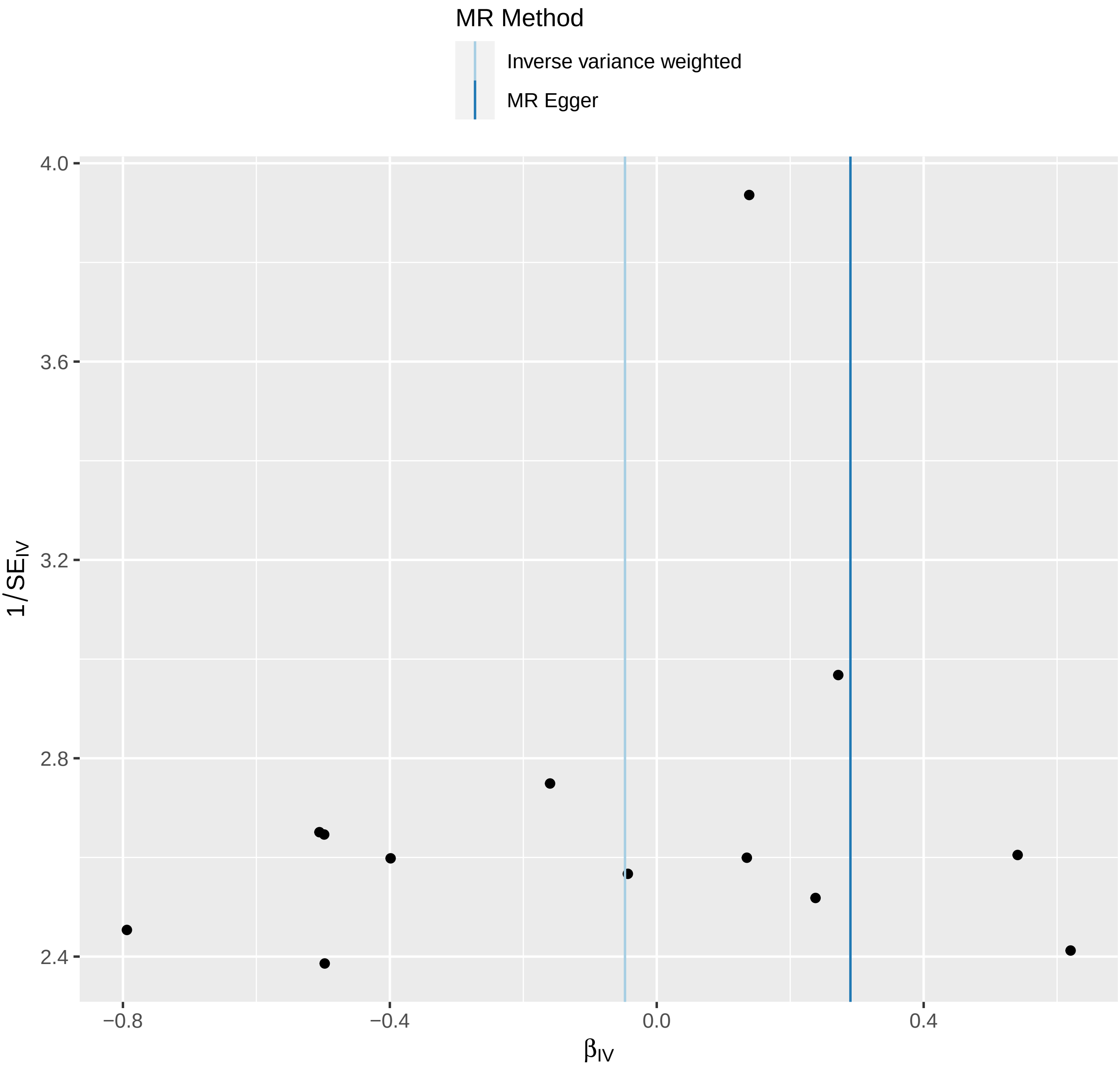

Batch 2−2877 : Gut microbiota abundance (genus Catenibacterium id.2153) on Immunoglobulin−binding protein 1
with a threshold of 1e−05

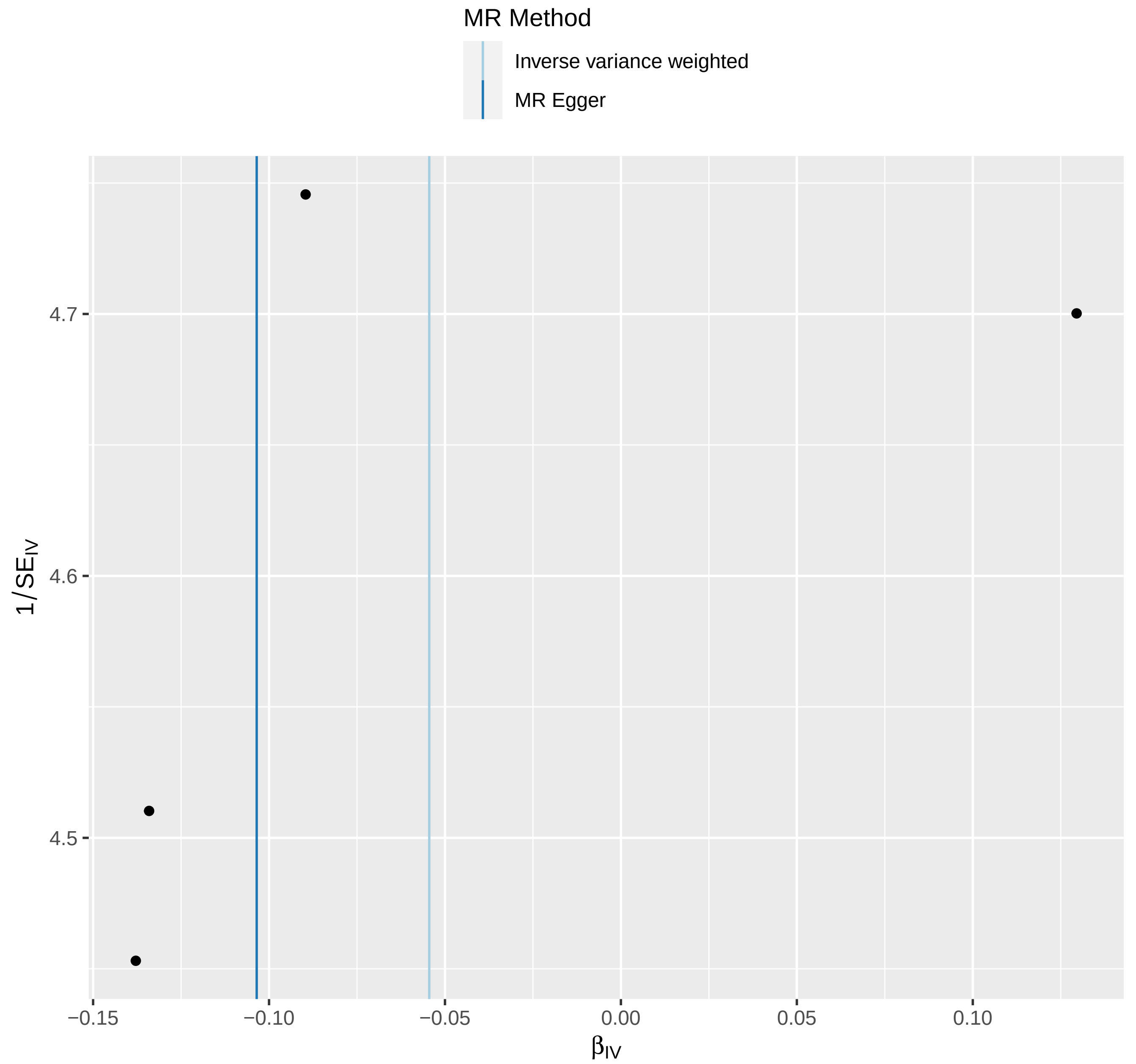

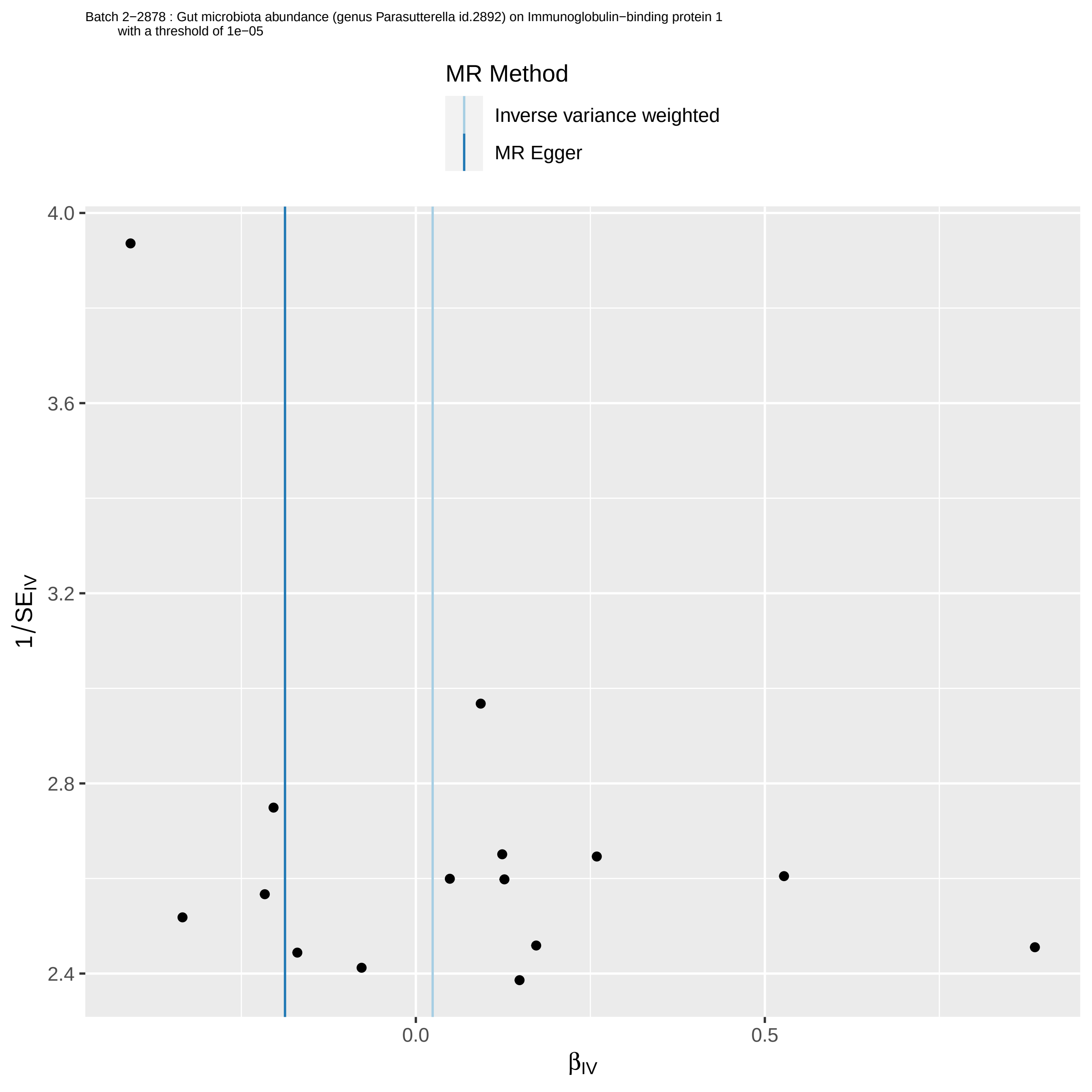

Batch 2−2878 : Gut microbiota abundance (genus Parasutterella id.2892) on Immunoglobulin−binding protein 1
with a threshold of 1e−05
MR Method
Inverse variance weighted
MR Egger
4.0
3.6
3.2
2.8
2.4
1/SE_IV
0.0
0.5
β_IV

Batch 2−2879 : Gut microbiota abundance (genus Catenibacterium id.2153) on Immunoglobulin superfamily DCC subclass member 3
with a threshold of 1e−05

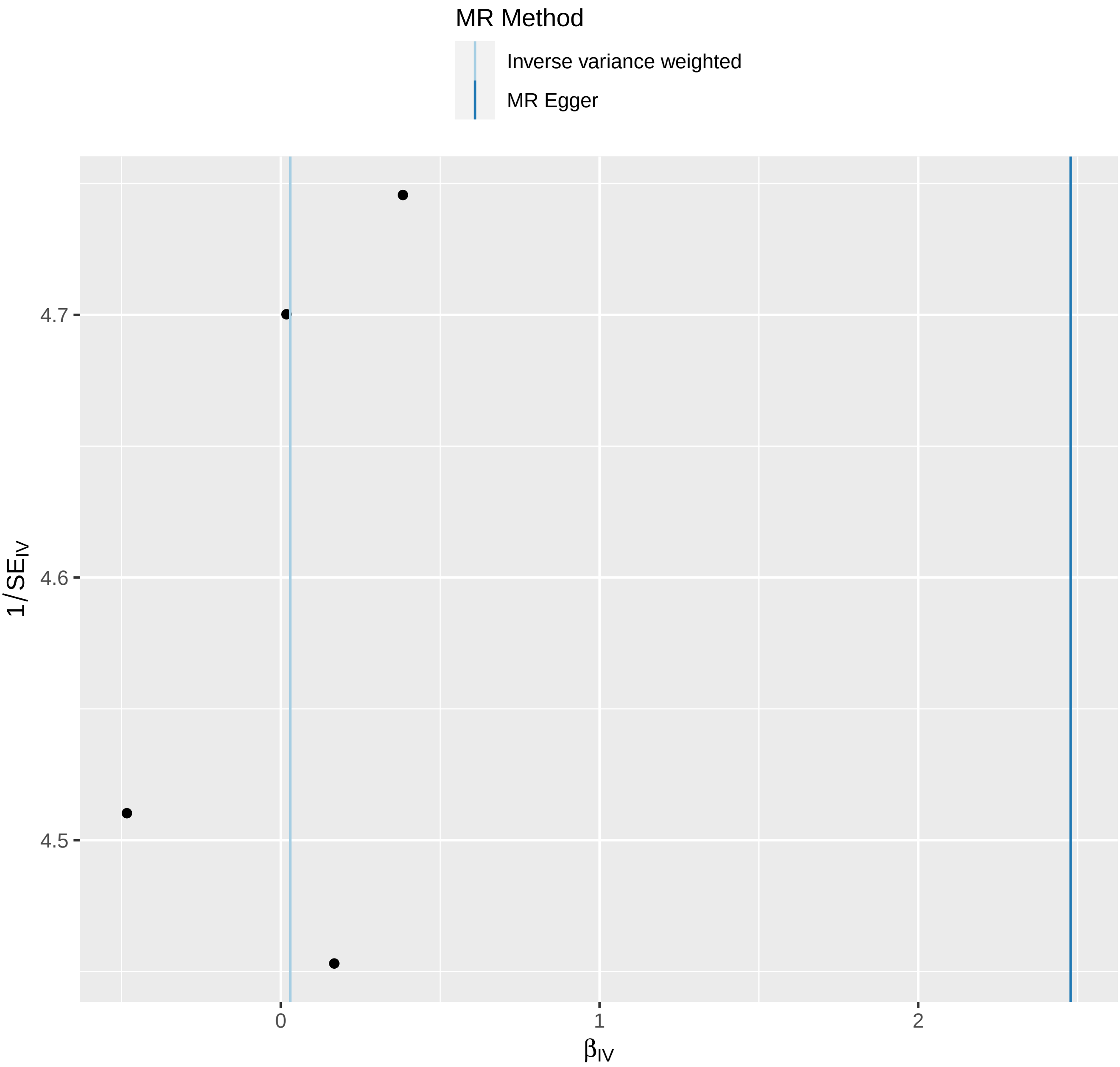

Batch 2−2880 : Gut microbiota abundance (genus Parasutterella id.2892) on Immunoglobulin superfamily DCC subclass member 3
with a threshold of 1e−05

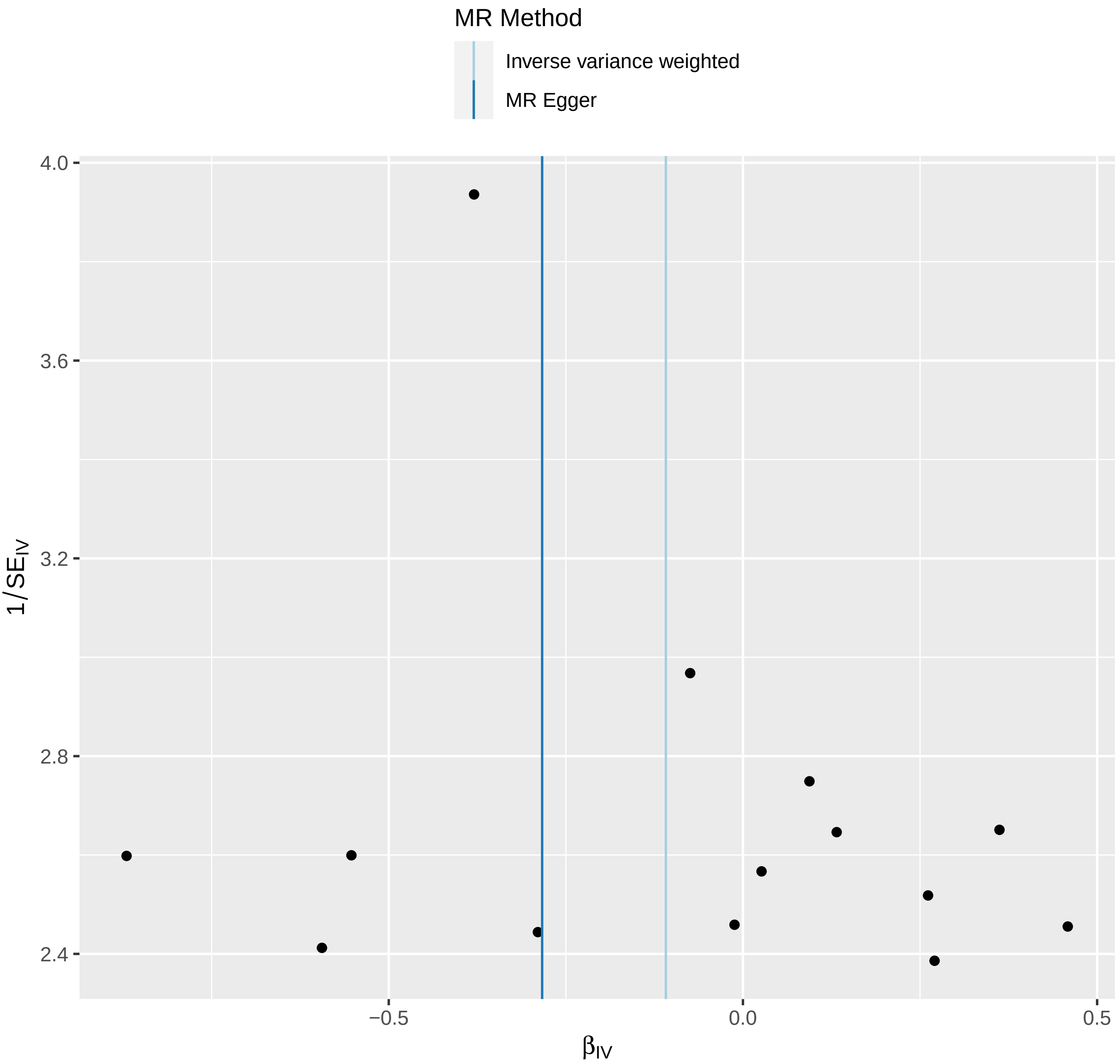

Batch 2−2881 : Gut microbiota abundance (genus Catenibacterium id.2153) on Immunoglobulin superfamily DCC subclass member 3
with a threshold of 1e−05

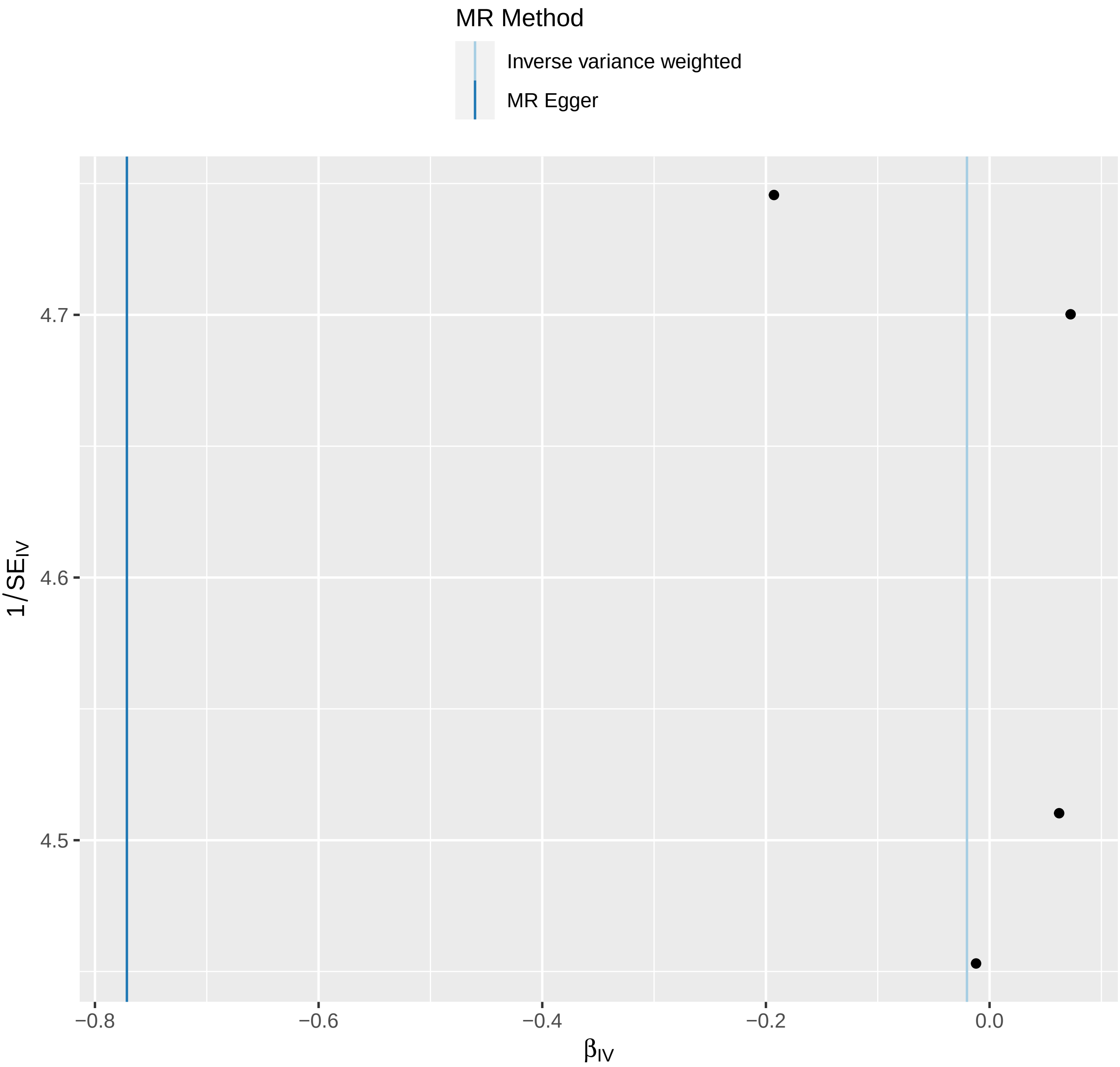

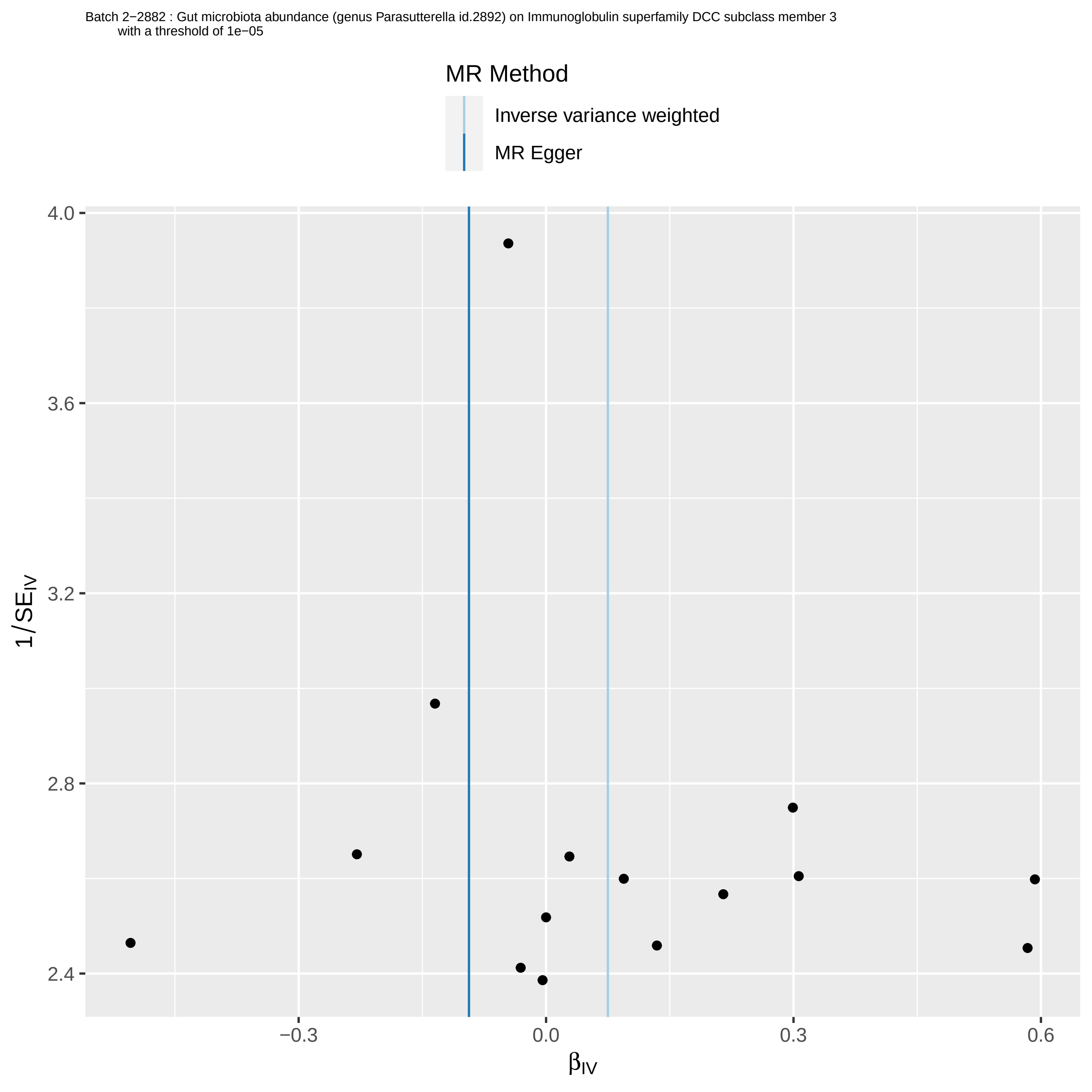

Batch 2−2882 : Gut microbiota abundance (genus Parasutterella id.2892) on Immunoglobulin superfamily DCC subclass member 3
with a threshold of 1e−05
MR Method
Inverse variance weighted
MR Egger
4.0
3.6
3.2
2.8
2.4
1/SE_IV
−0.3
0.0
0.3
0.6
β_IV

Batch 2−2883 : Gut microbiota abundance (genus Catenibacterium id.2153) on Immunoglobulin superfamily DCC subclass member 4 with a threshold of 1e−05

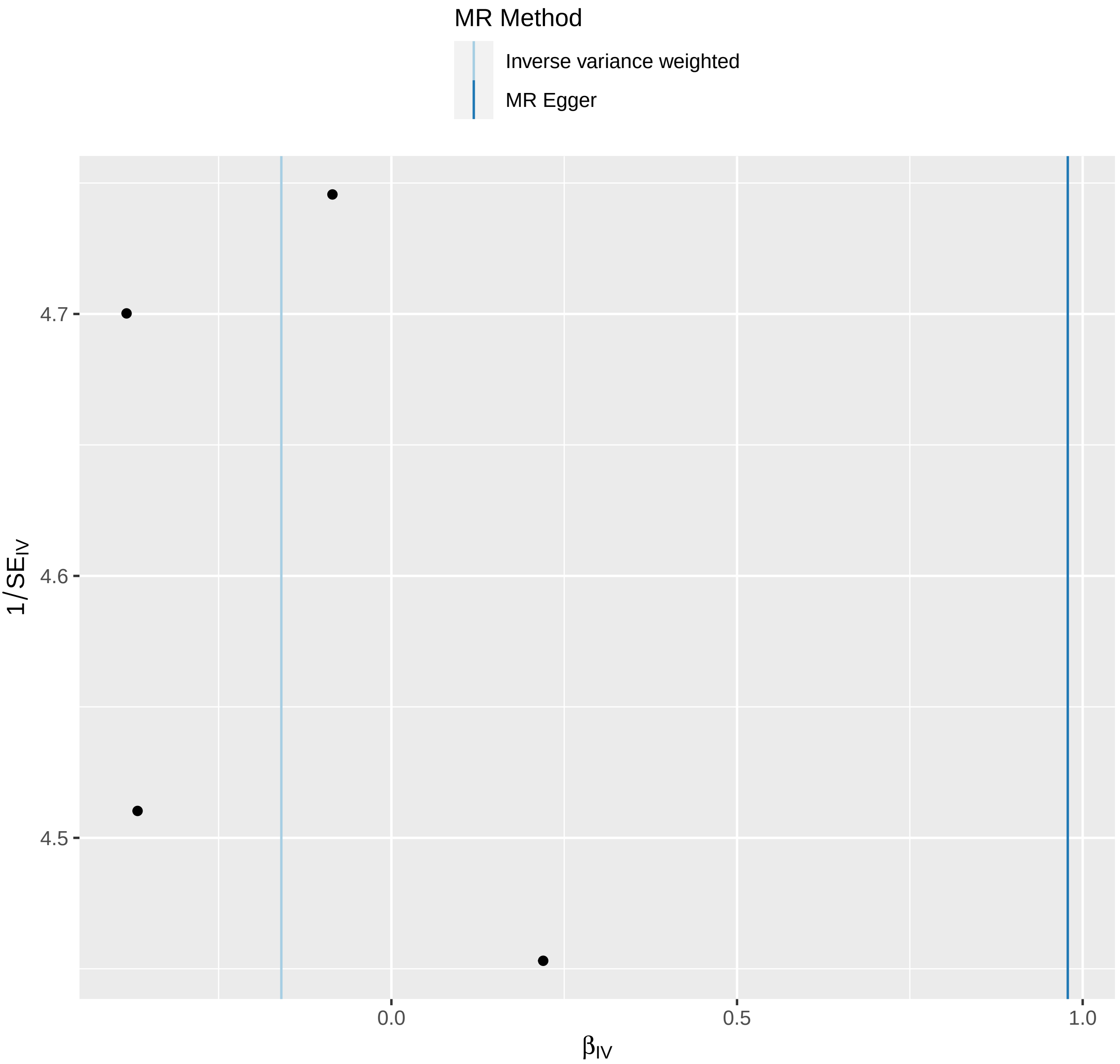

Batch 2−2884 : Gut microbiota abundance (genus Parasutterella id.2892) on Immunoglobulin superfamily DCC subclass member 4 with a threshold of 1e−05

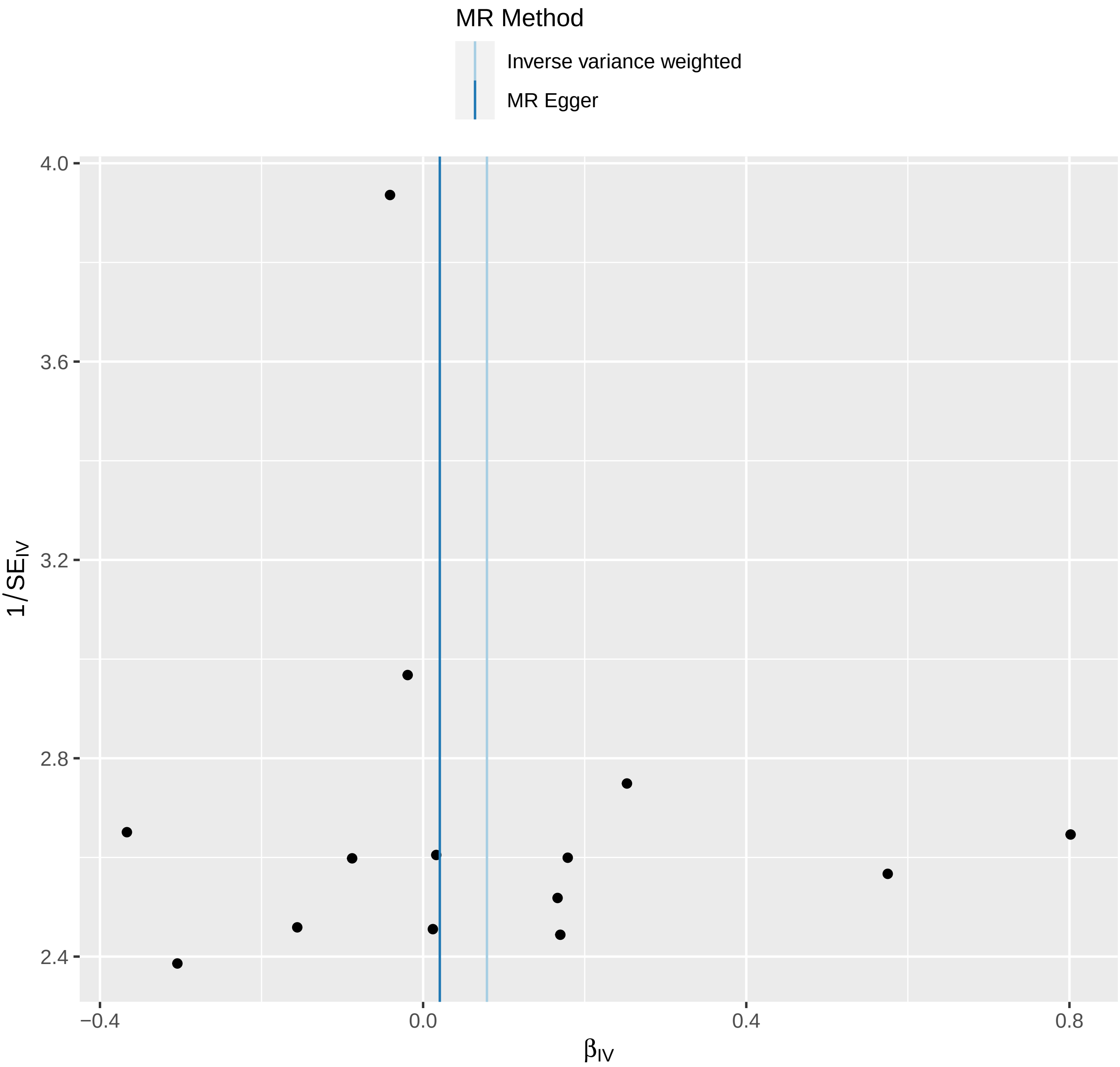

Batch 2−2885 : Gut microbiota abundance (genus Catenibacterium id.2153) on Insulin−like growth factor I
with a threshold of 1e−05

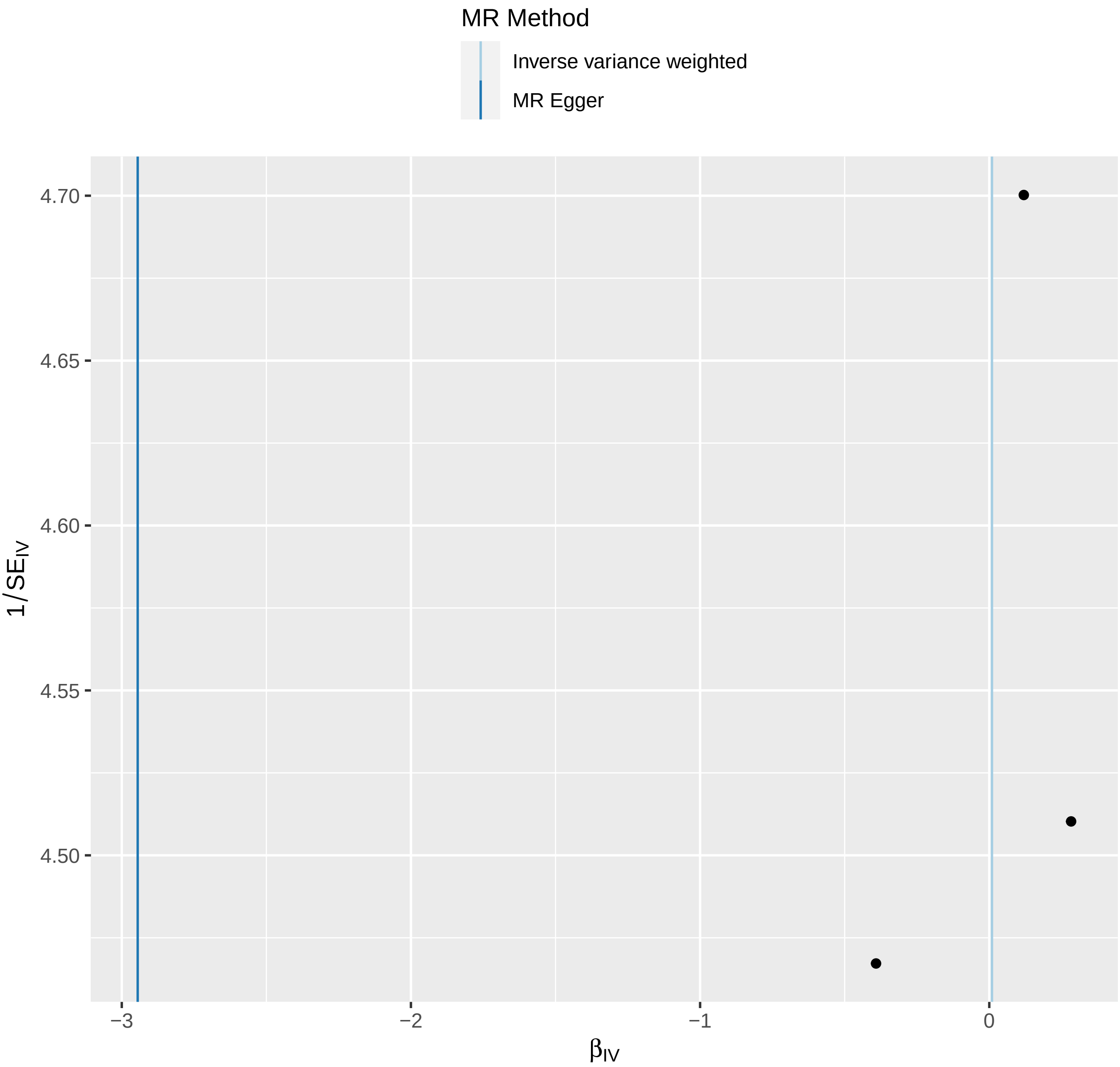

Batch 2−2886 : Gut microbiota abundance (genus Parasutterella id.2892) on Insulin−like growth factor I
with a threshold of 1e−05

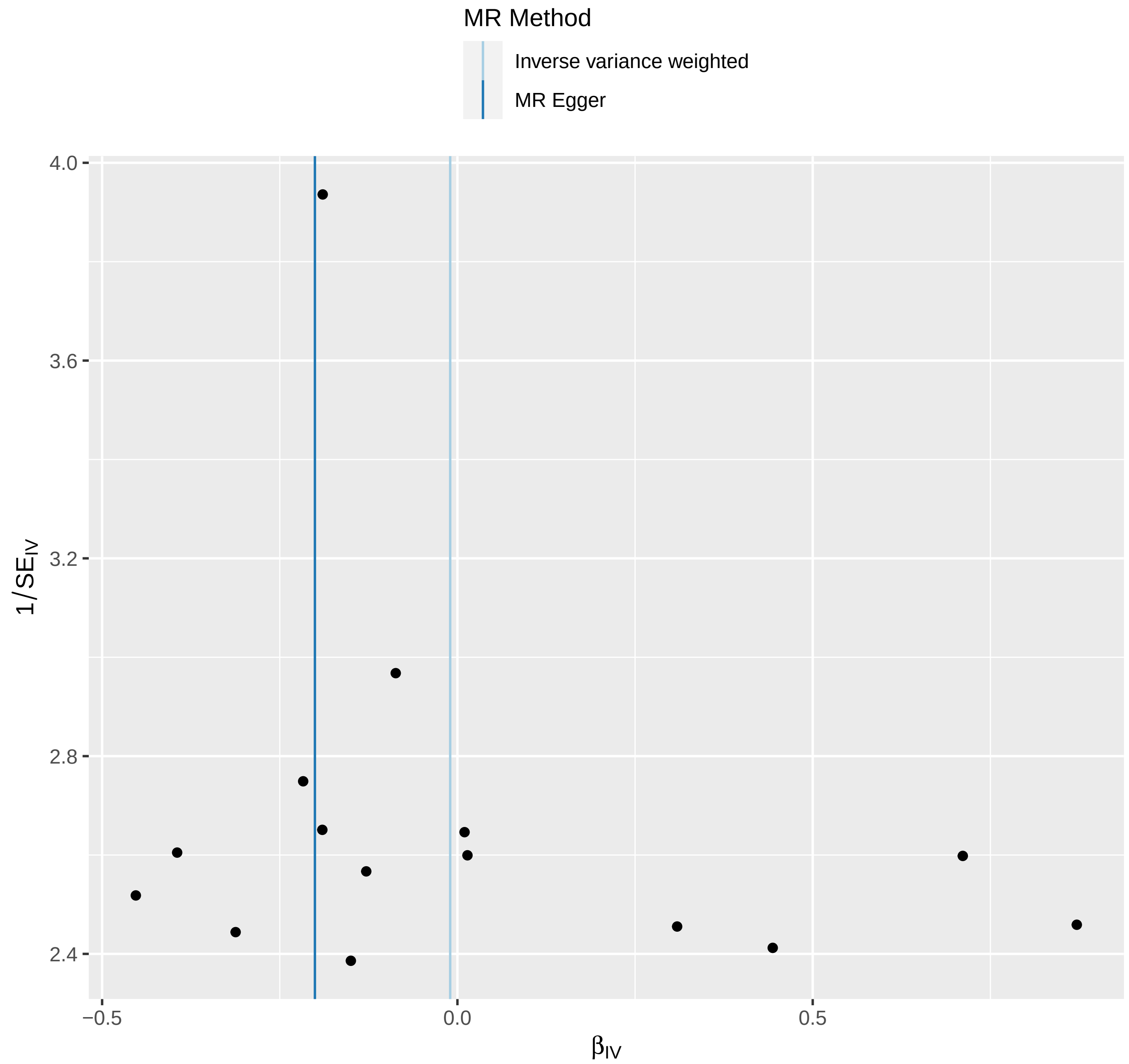

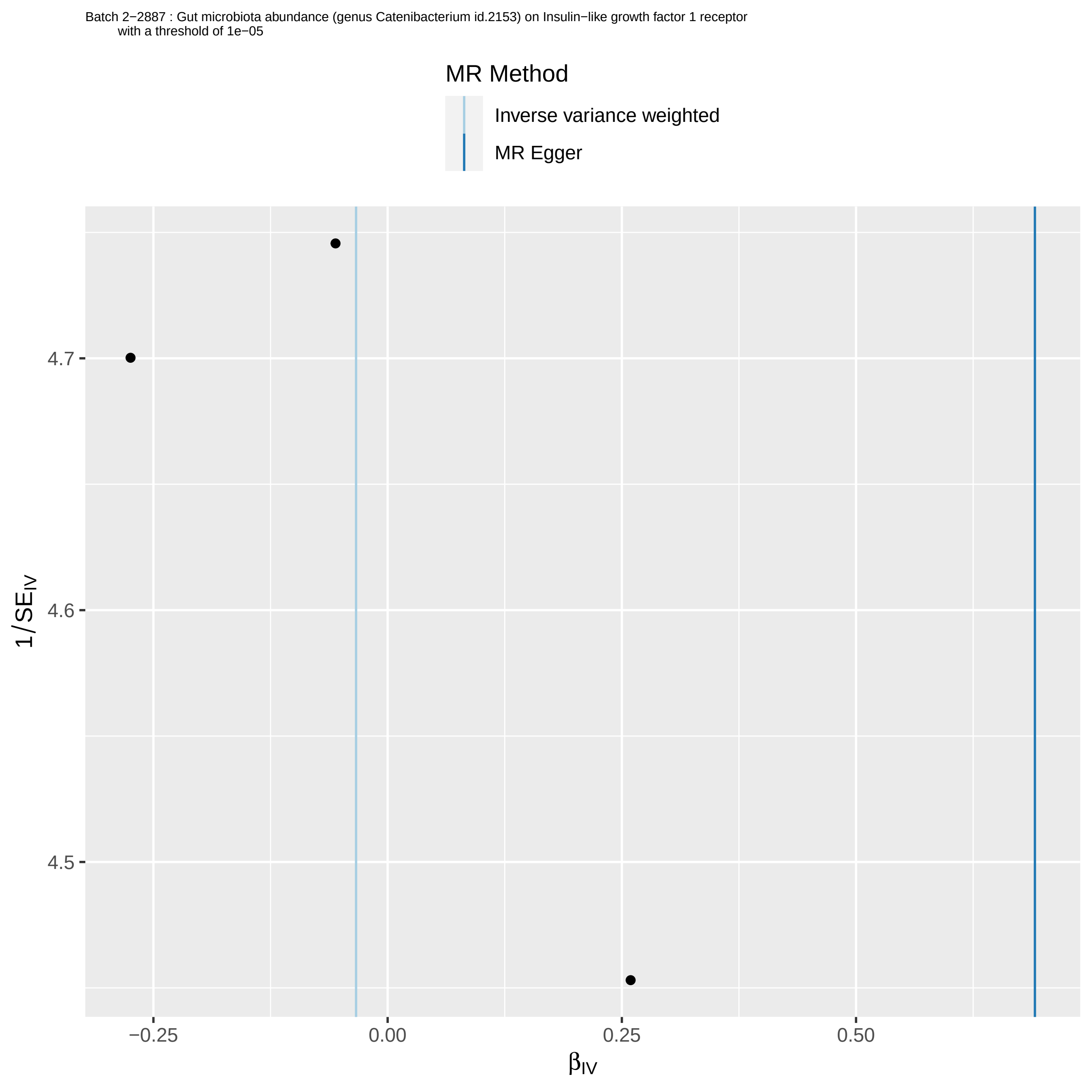
Batch 2−2887 : Gut microbiota abundance (genus Catenibacterium id.2153) on Insulin−like growth factor 1 receptor
with a threshold of 1e−05
MR Method
Inverse variance weighted
MR Egger
4.7
4.6
4.5
1/SE_IV
−0.25
0.00
0.25
0.50
β_IV

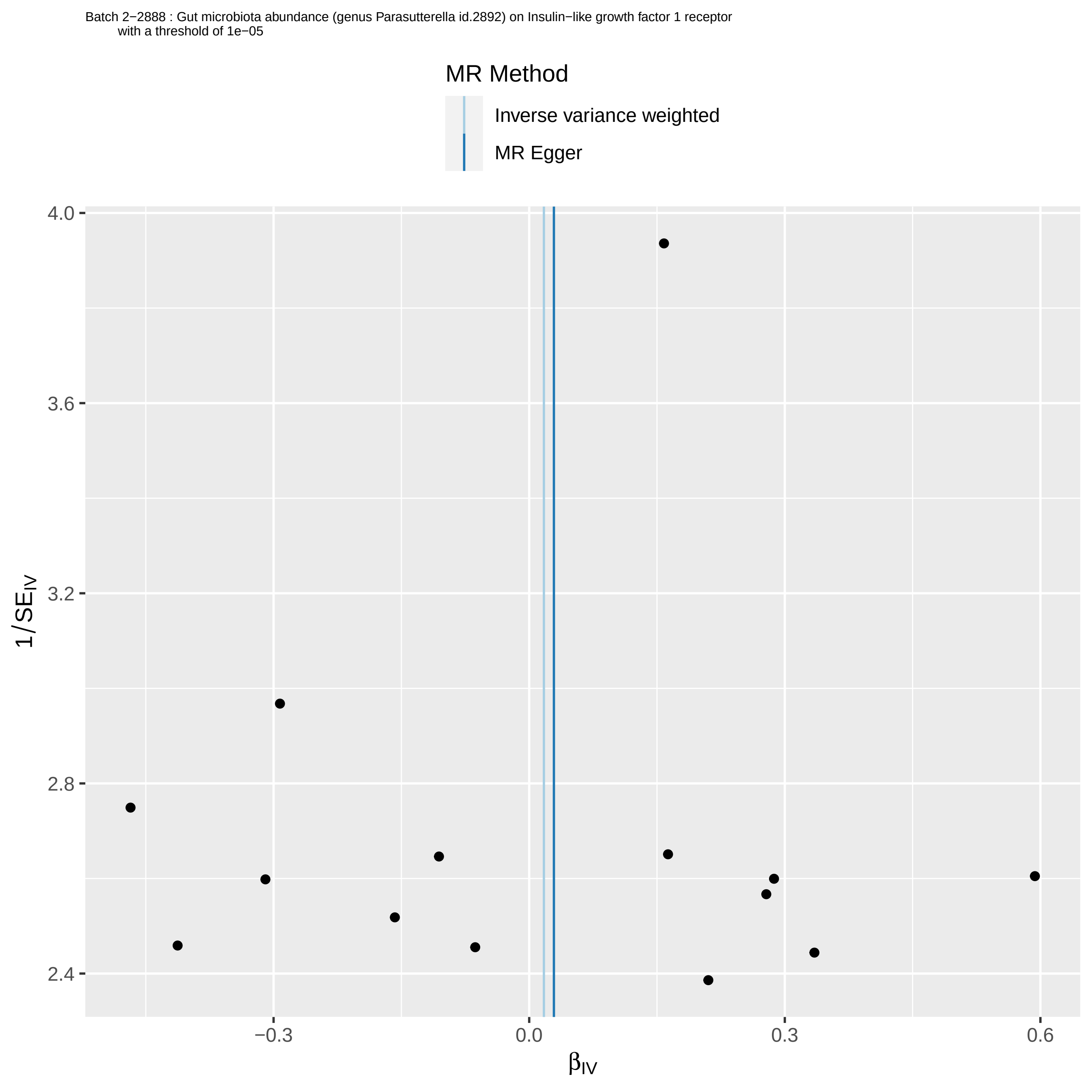
Batch 2−2888 : Gut microbiota abundance (genus Parasutterella id.2892) on Insulin−like growth factor 1 receptor
with a threshold of 1e−05
MR Method
Inverse variance weighted
MR Egger
4.0
3.6
3.2
2.8
2.4
1/SE_IV
−0.3
0.0
0.3
0.6
β_IV

Batch 2−2889 : Gut microbiota abundance (genus Catenibacterium id.2153) on Cation−independent mannose−6−phosphate receptor
with a threshold of 1e−05

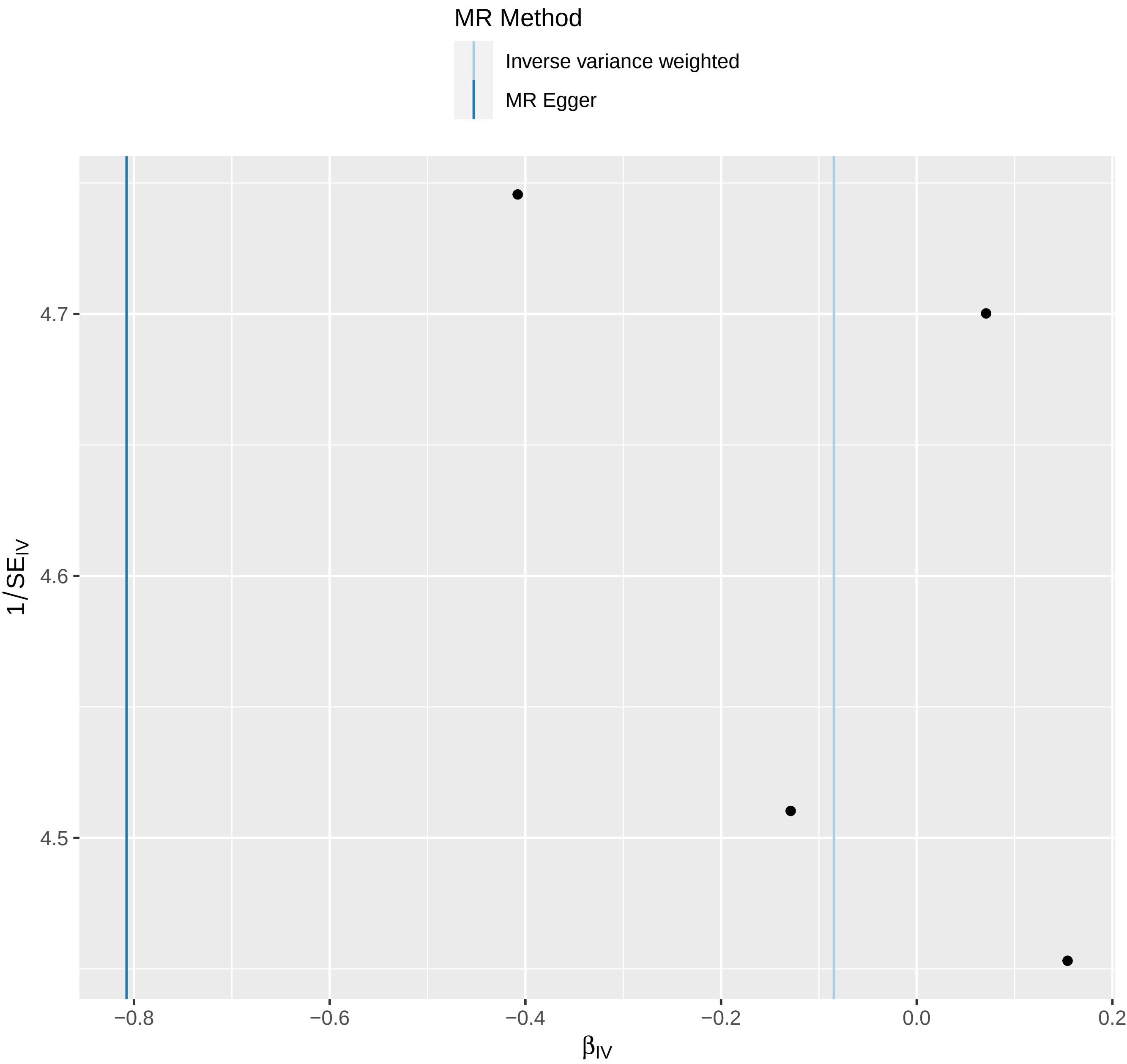

Batch 2−2890 : Gut microbiota abundance (genus Parasutterella id.2892) on Cation−independent mannose−6−phosphate receptor
with a threshold of 1e−05

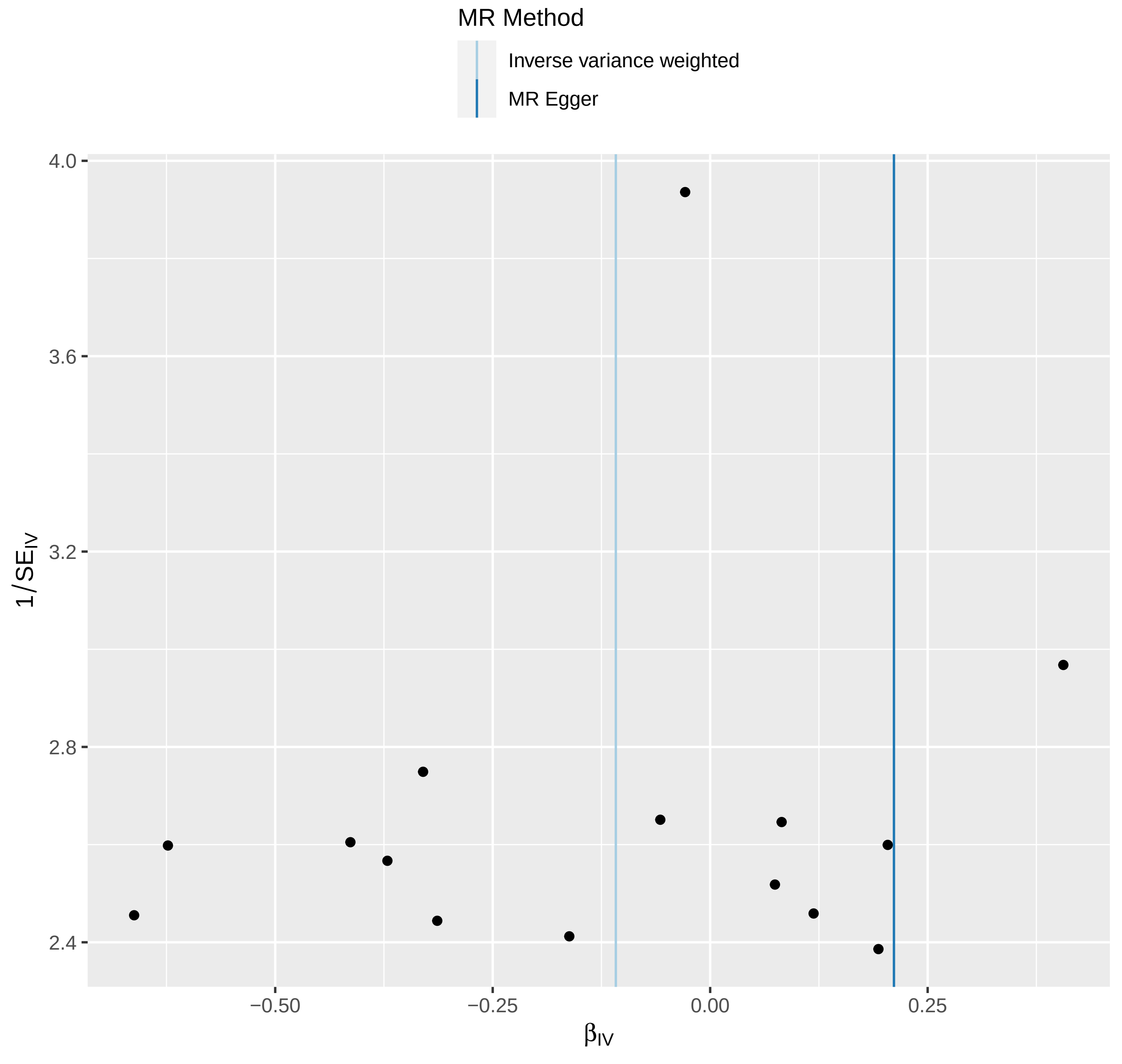

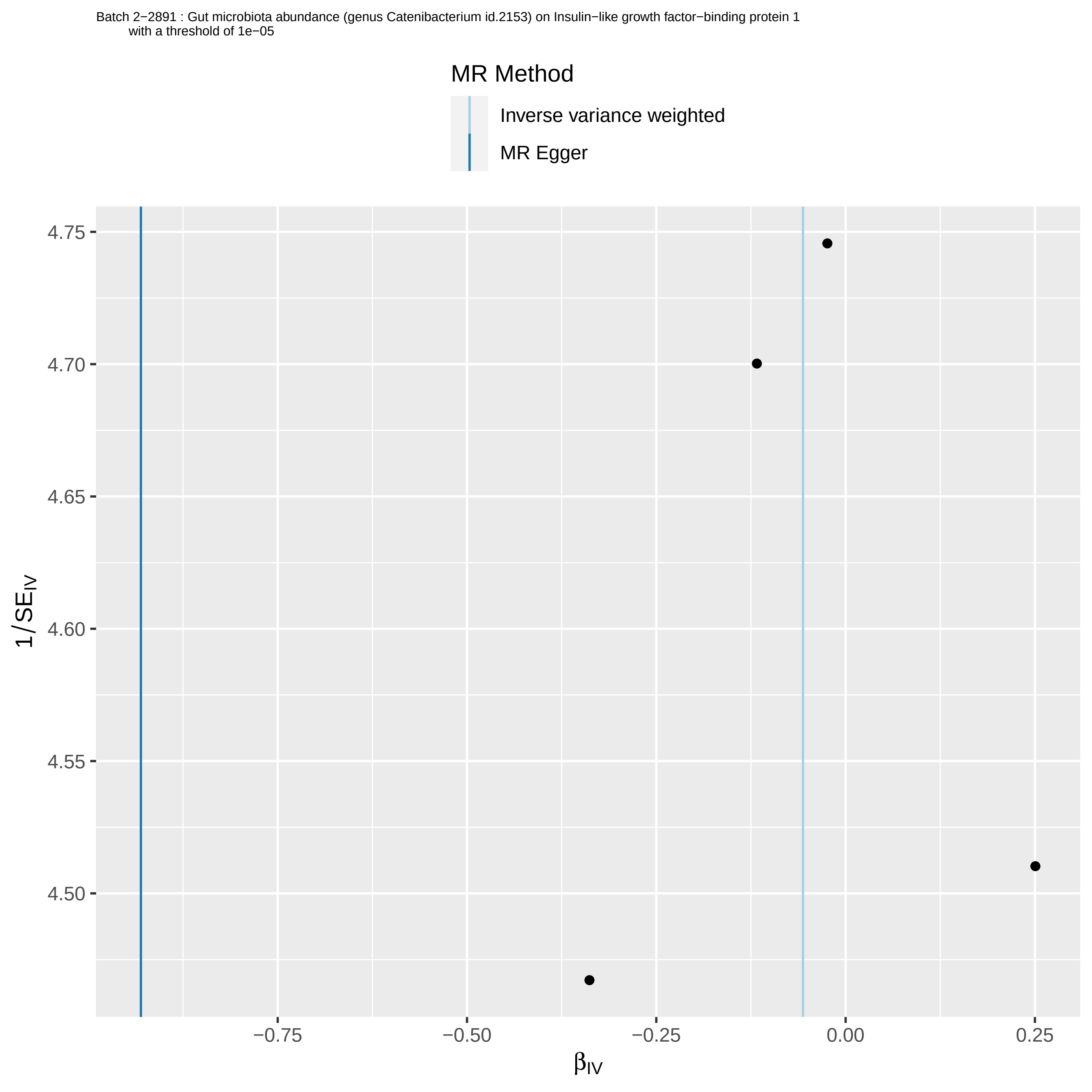

Batch 2−2891 : Gut microbiota abundance (genus Catenibacterium id.2153) on Insulin−like growth factor−binding protein 1
with a threshold of 1e−05
MR Method
Inverse variance weighted
MR Egger
4.75
4.70
4.65
4.60
4.55
4.50
1/SE_IV
−0.75
−0.50
−0.25
0.00
0.25
β_IV

Batch 2−2892 : Gut microbiota abundance (genus Parasutterella id.2892) on Insulin−like growth factor−binding protein 1
with a threshold of 1e−05

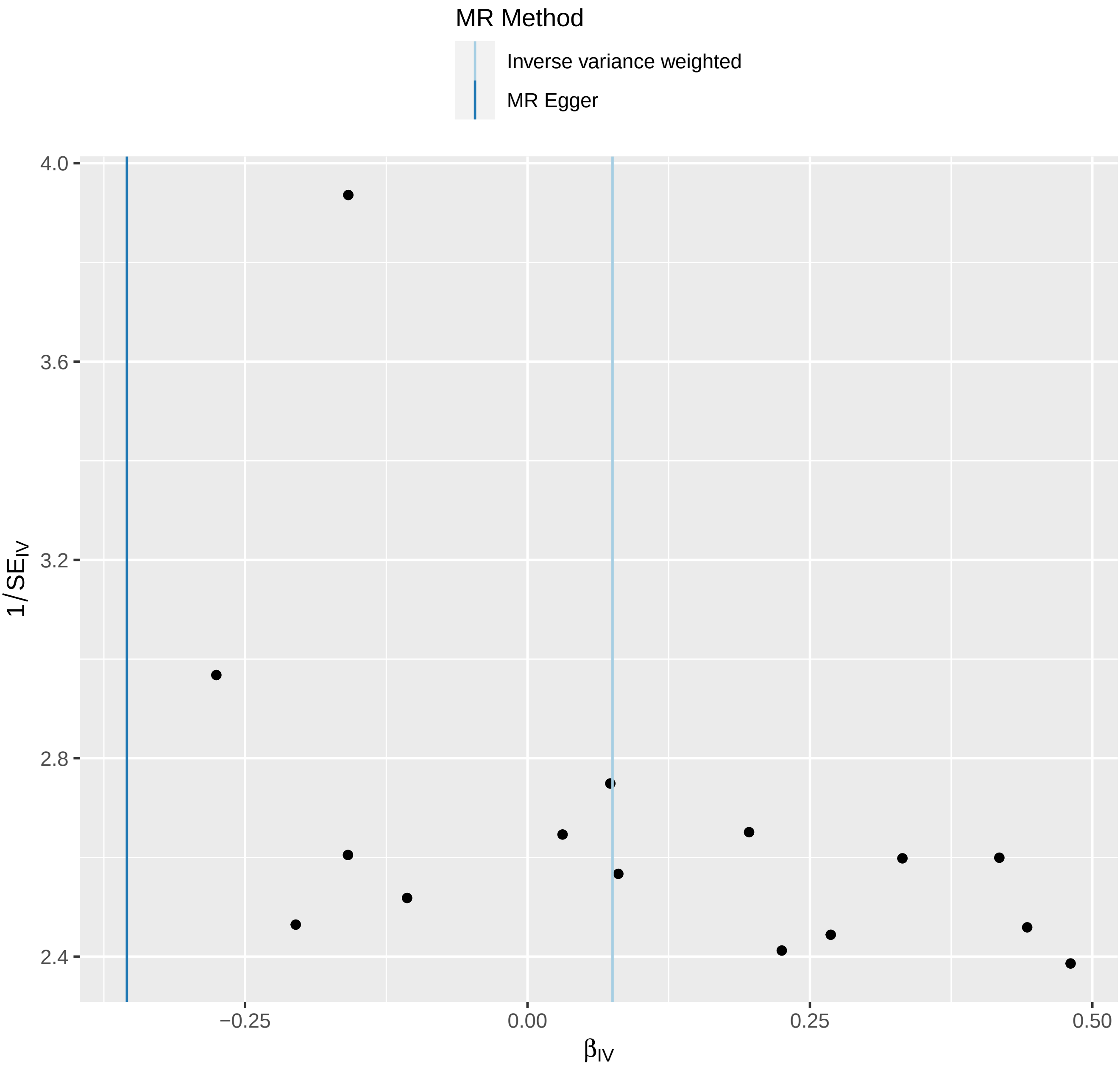

Batch 2−2893 : Gut microbiota abundance (genus Catenibacterium id.2153) on Insulin−like growth factor−binding protein 1
with a threshold of 1e−05

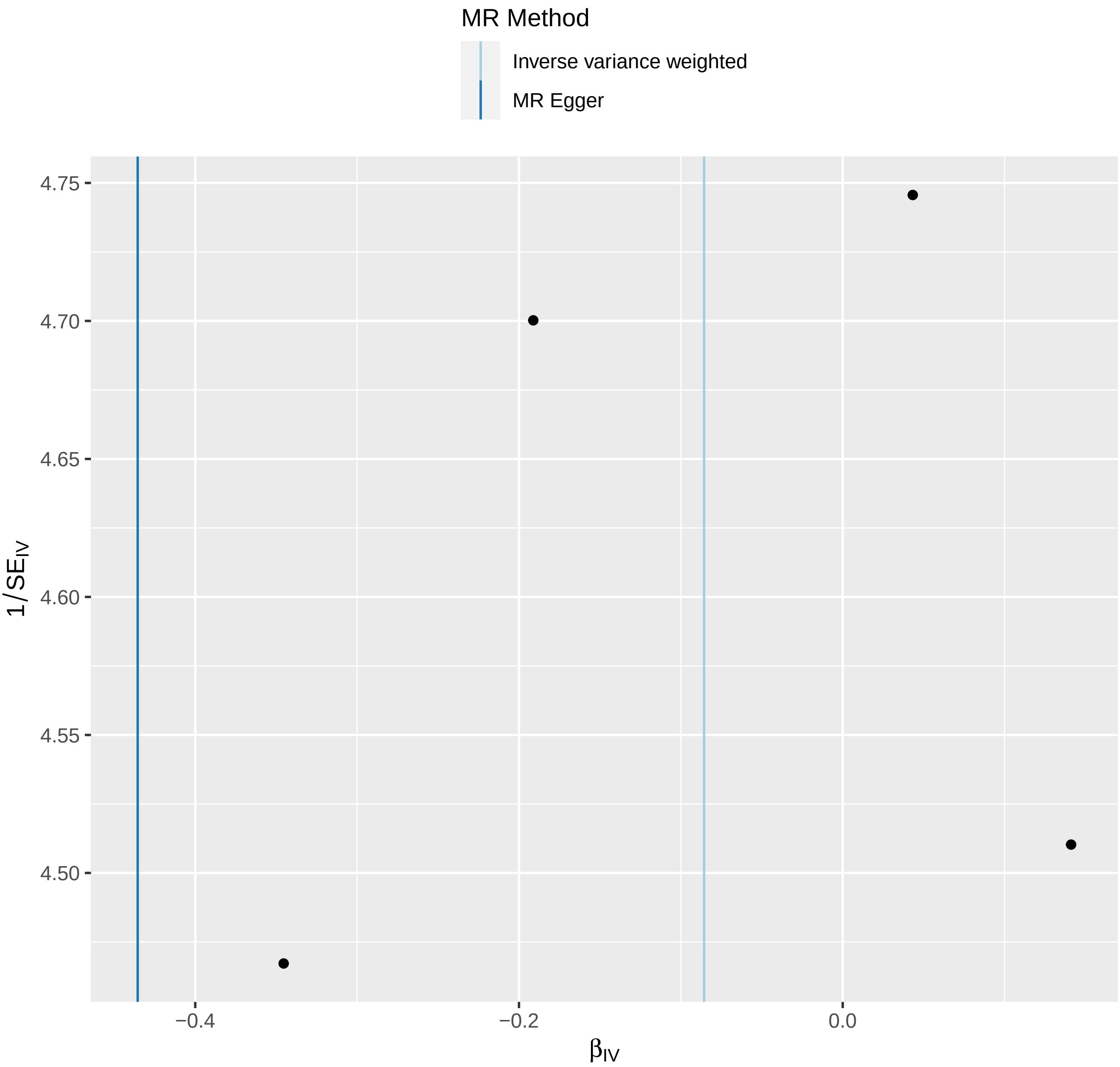

Batch 2−2894 : Gut microbiota abundance (genus Parasutterella id.2892) on Insulin−like growth factor−binding protein 1 with a threshold of 1e−05

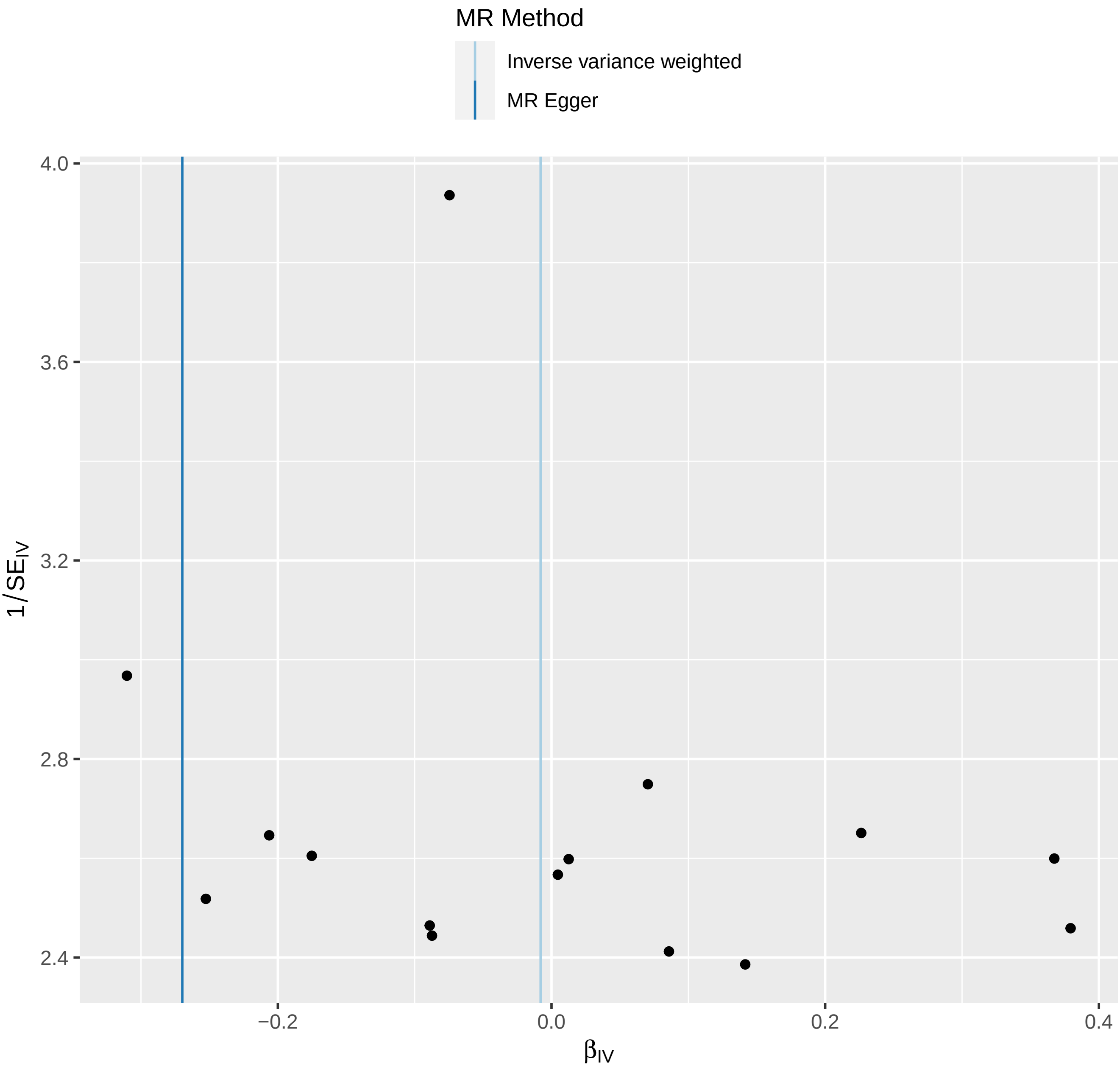

Batch 2−2895 : Gut microbiota abundance (genus Catenibacterium id.2153) on Insulin−like growth factor−binding protein 2
with a threshold of 1e−05

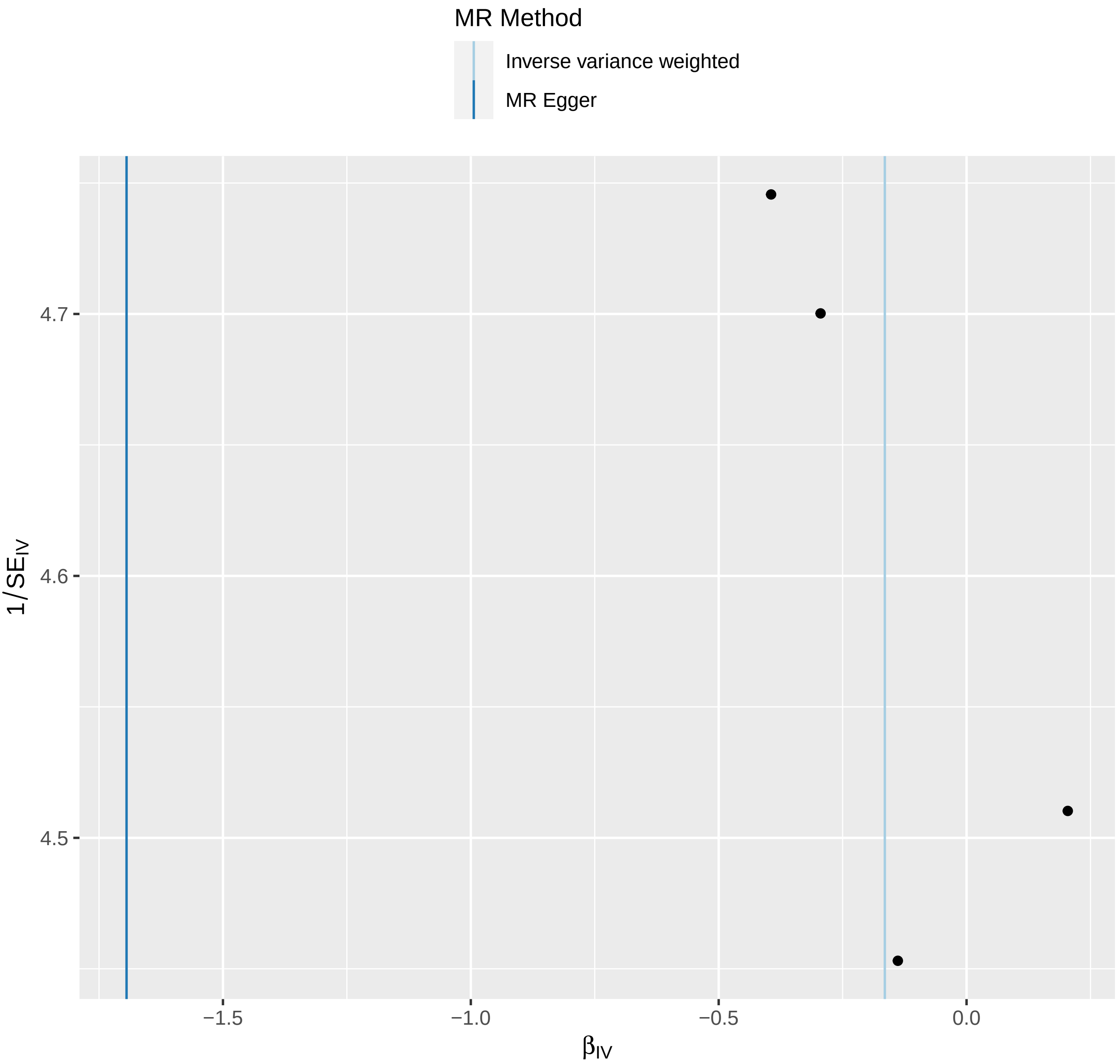

Batch 2−2896 : Gut microbiota abundance (genus Parasutterella id.2892) on Insulin−like growth factor−binding protein 2
with a threshold of 1e−05

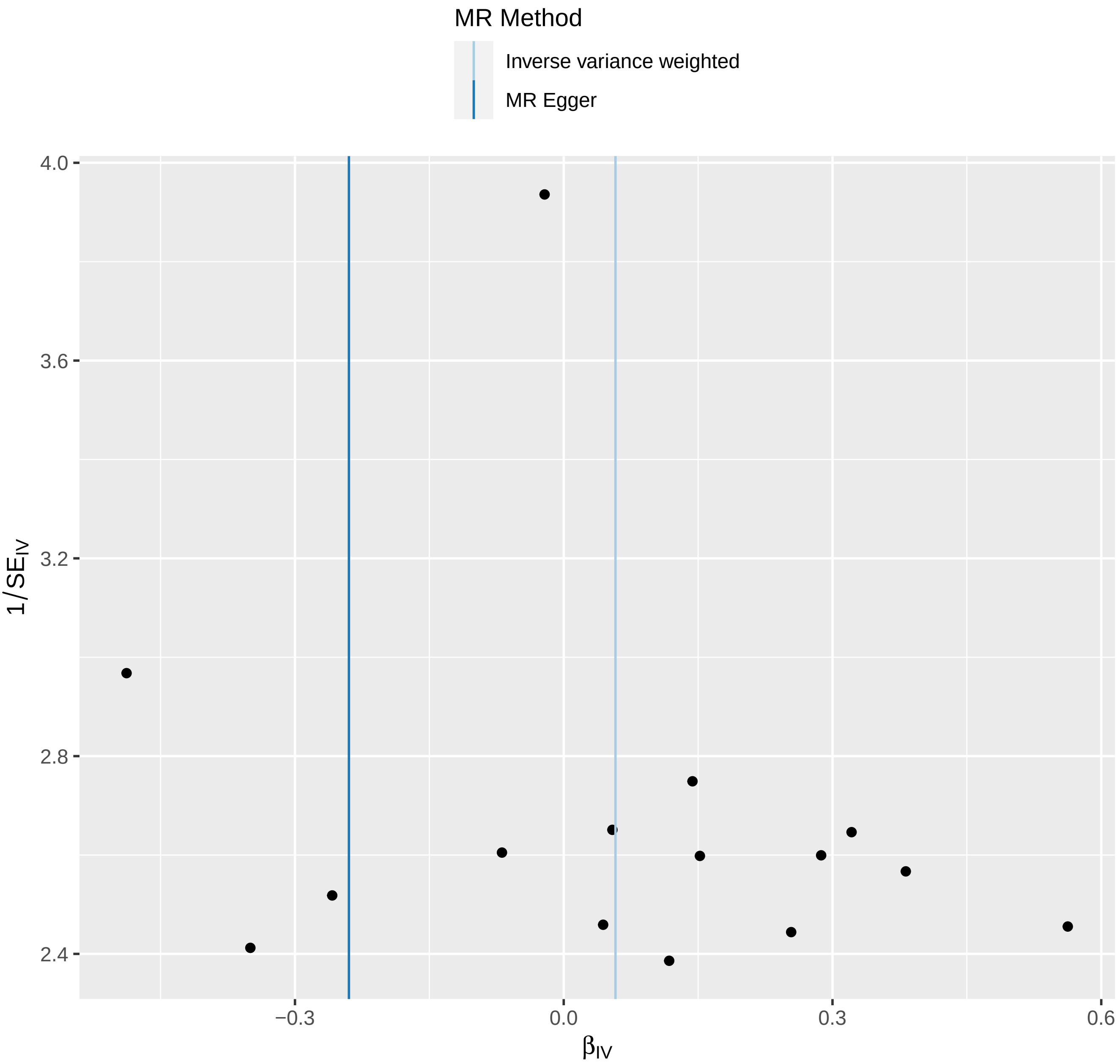

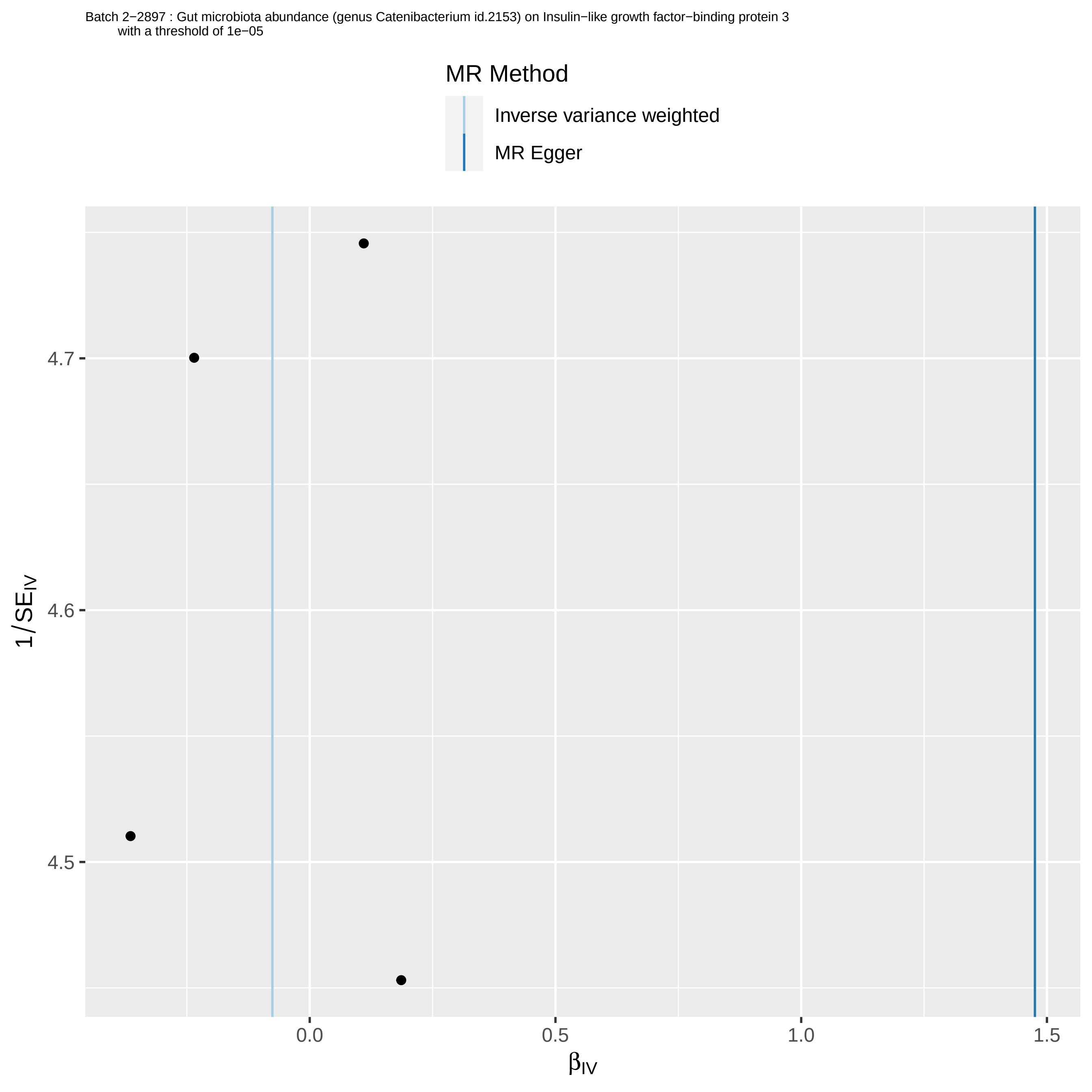

Batch 2−2897 : Gut microbiota abundance (genus Catenibacterium id.2153) on Insulin−like growth factor−binding protein 3
with a threshold of 1e−05
MR Method
Inverse variance weighted
MR Egger
4.7
4.6
4.5
$1/SE_{IV}$
0.0
0.5
1.0
1.5
β_{IV}

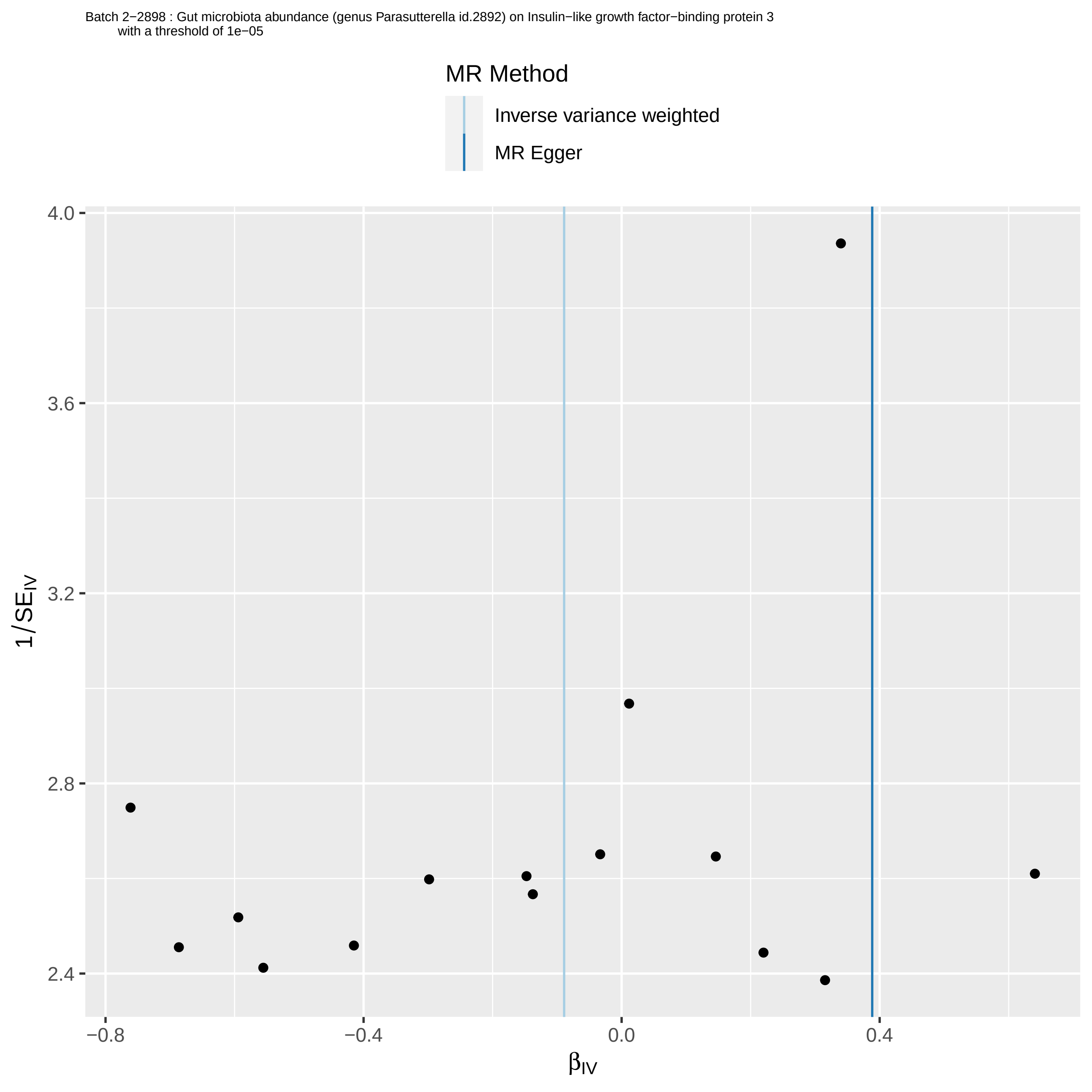
Batch 2−2898 : Gut microbiota abundance (genus Parasutterella id.2892) on Insulin−like growth factor−binding protein 3
with a threshold of 1e−05
MR Method
Inverse variance weighted
MR Egger
4.0
3.6
3.2
2.8
2.4
$1/SE_{IV}$
−0.8
−0.4
0.0
0.4
β_{IV}

Batch 2−2899 : Gut microbiota abundance (genus Catenibacterium id.2153) on Insulin−like growth factor−binding protein 6
with a threshold of 1e−05

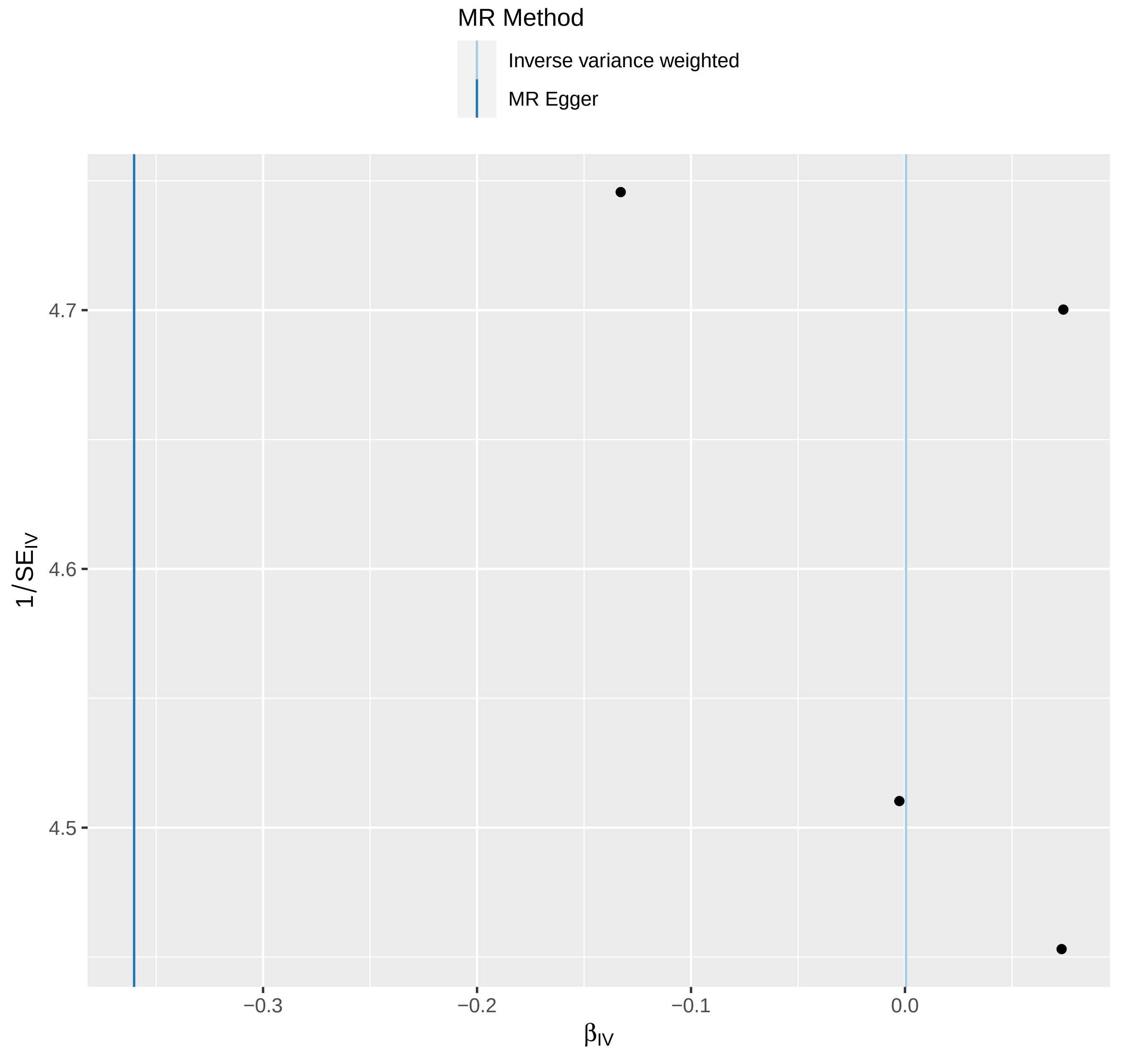

Batch 2−2900 : Gut microbiota abundance (genus Parasutterella id.2892) on Insulin−like growth factor−binding protein 6
with a threshold of 1e−05

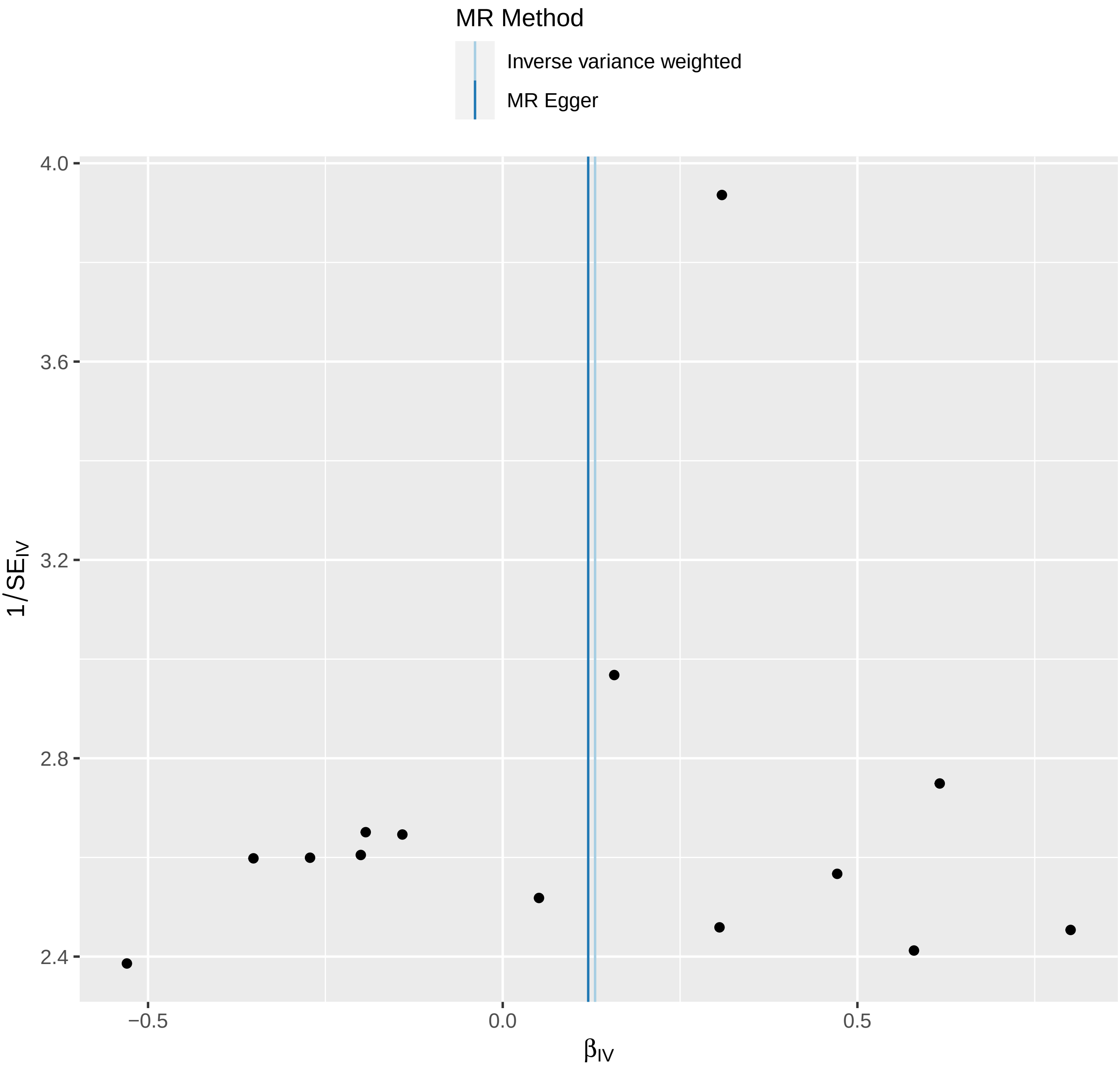

Batch 2−2901 : Gut microbiota abundance (genus Catenibacterium id.2153) on Insulin−like growth factor−binding protein 7
with a threshold of 1e−05

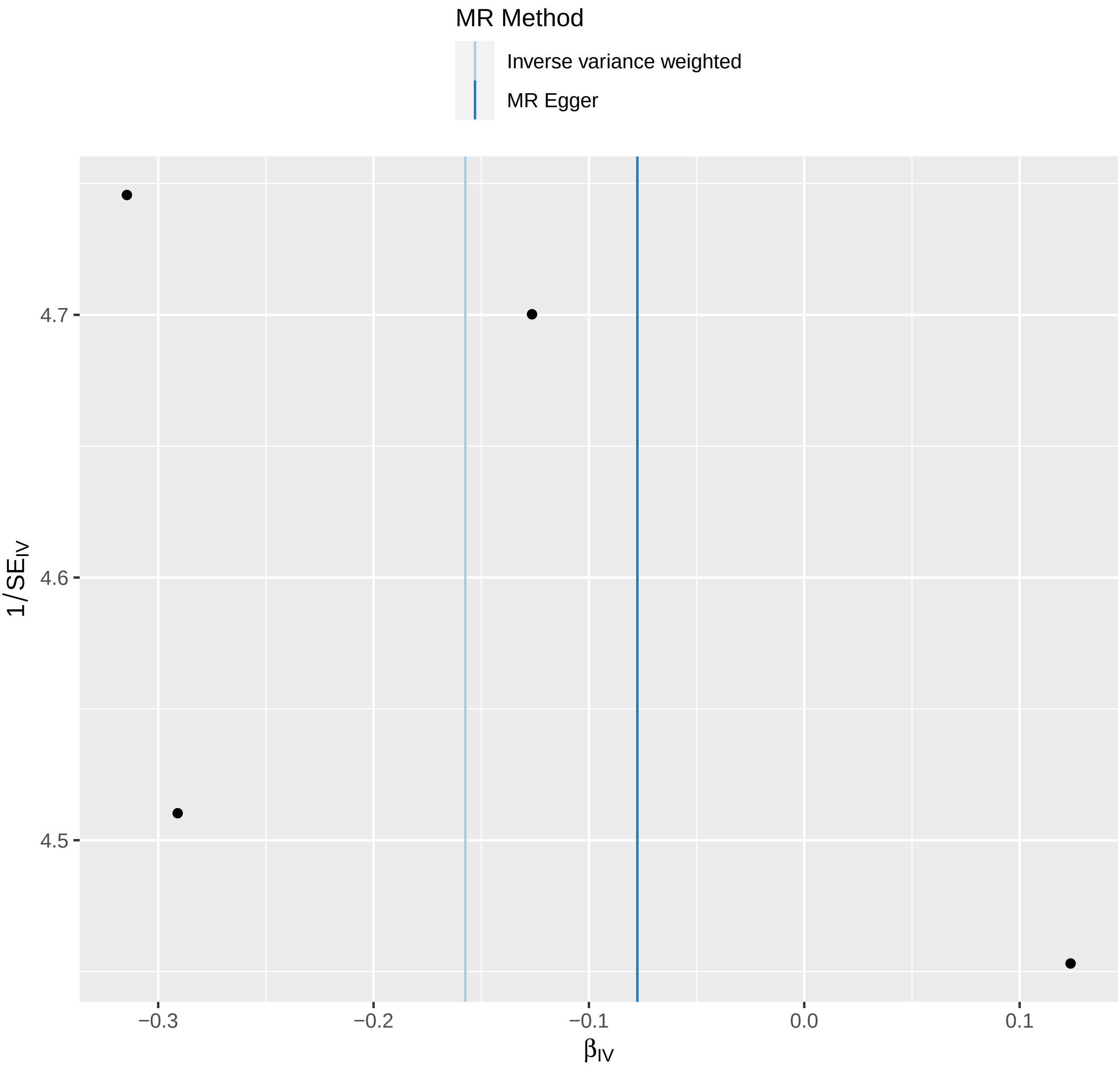

Batch 2−2902 : Gut microbiota abundance (genus Parasutterella id.2892) on Insulin−like growth factor−binding protein 7 with a threshold of 1e−05

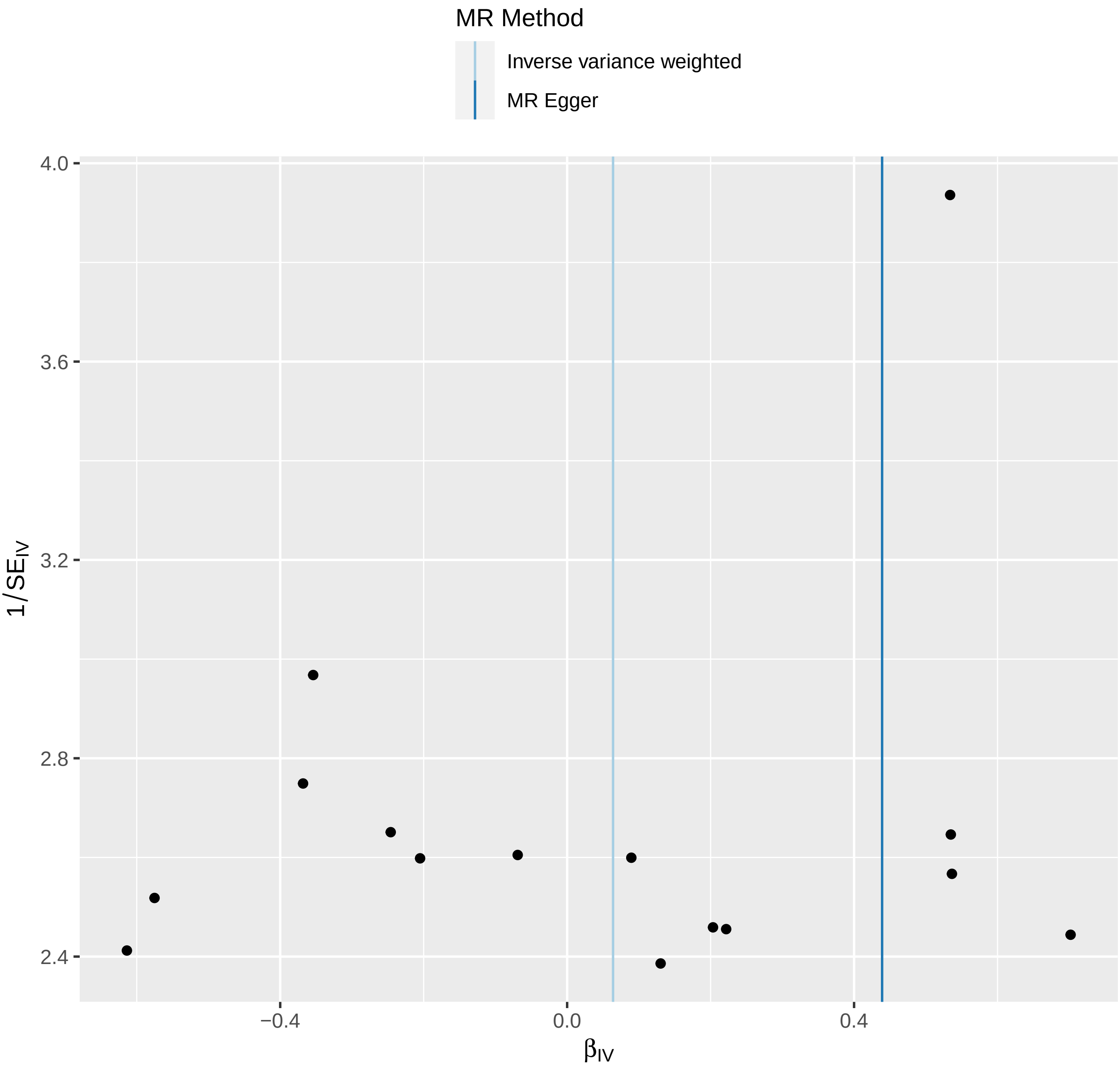

Batch 2−2903 : Gut microbiota abundance (genus Catenibacterium id.2153) on Insulin−like growth factor−binding protein−like 1
with a threshold of 1e−05

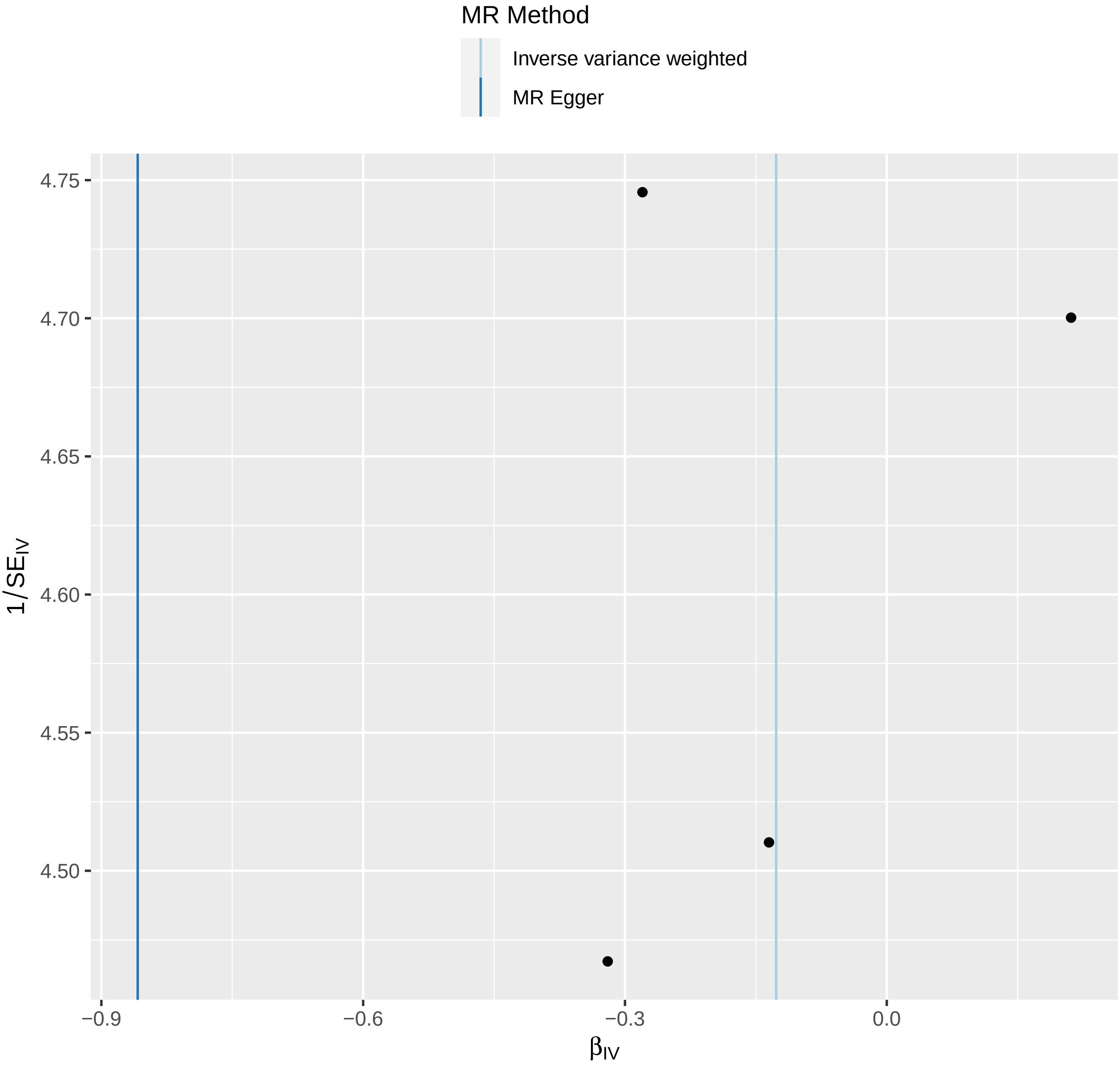

Batch 2−2904 : Gut microbiota abundance (genus Parasutterella id.2892) on Insulin−like growth factor−binding protein−like 1
with a threshold of 1e−05

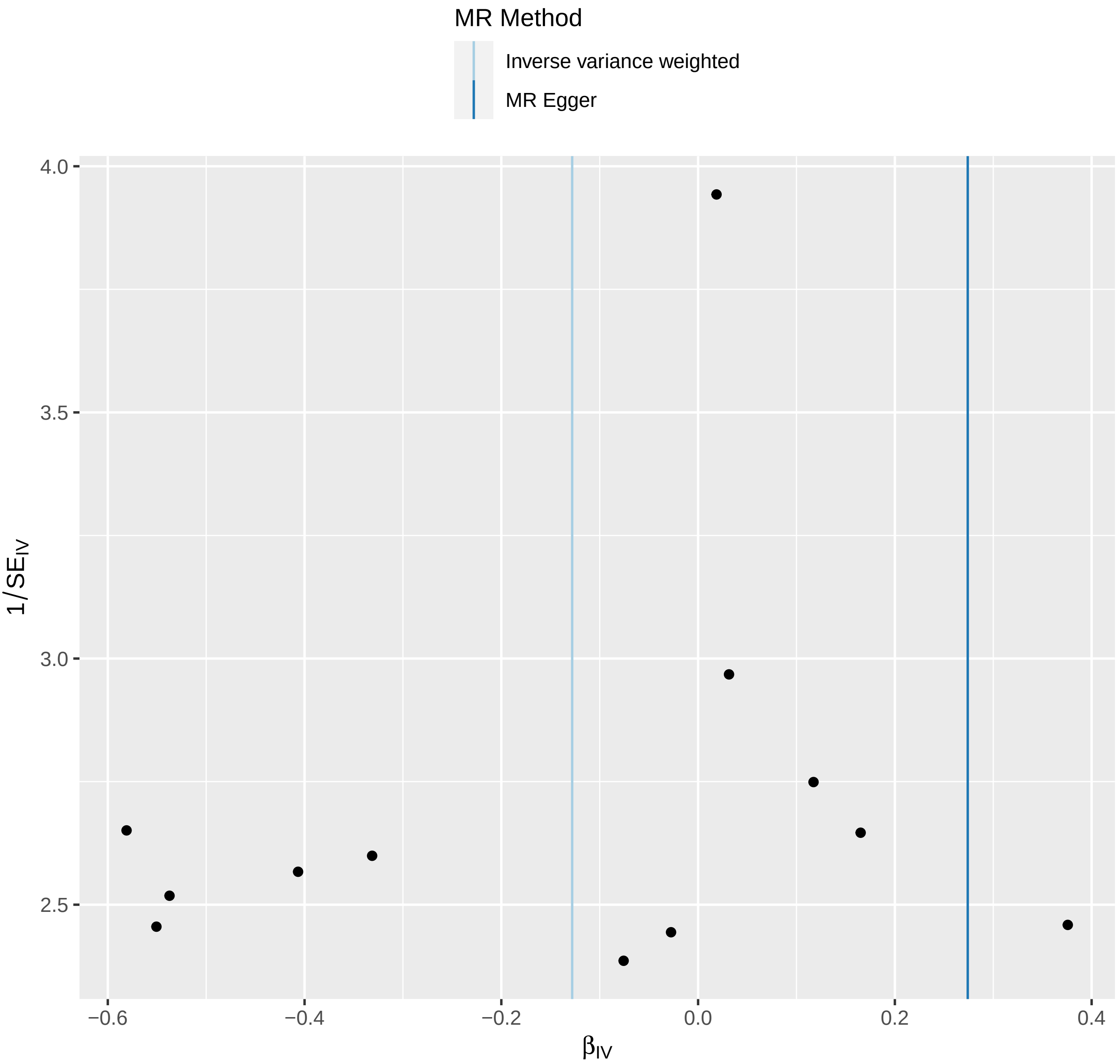

Batch 2−2905 : Gut microbiota abundance (genus Catenibacterium id.2153) on Insulin growth factor−like family member 3
with a threshold of 1e−05

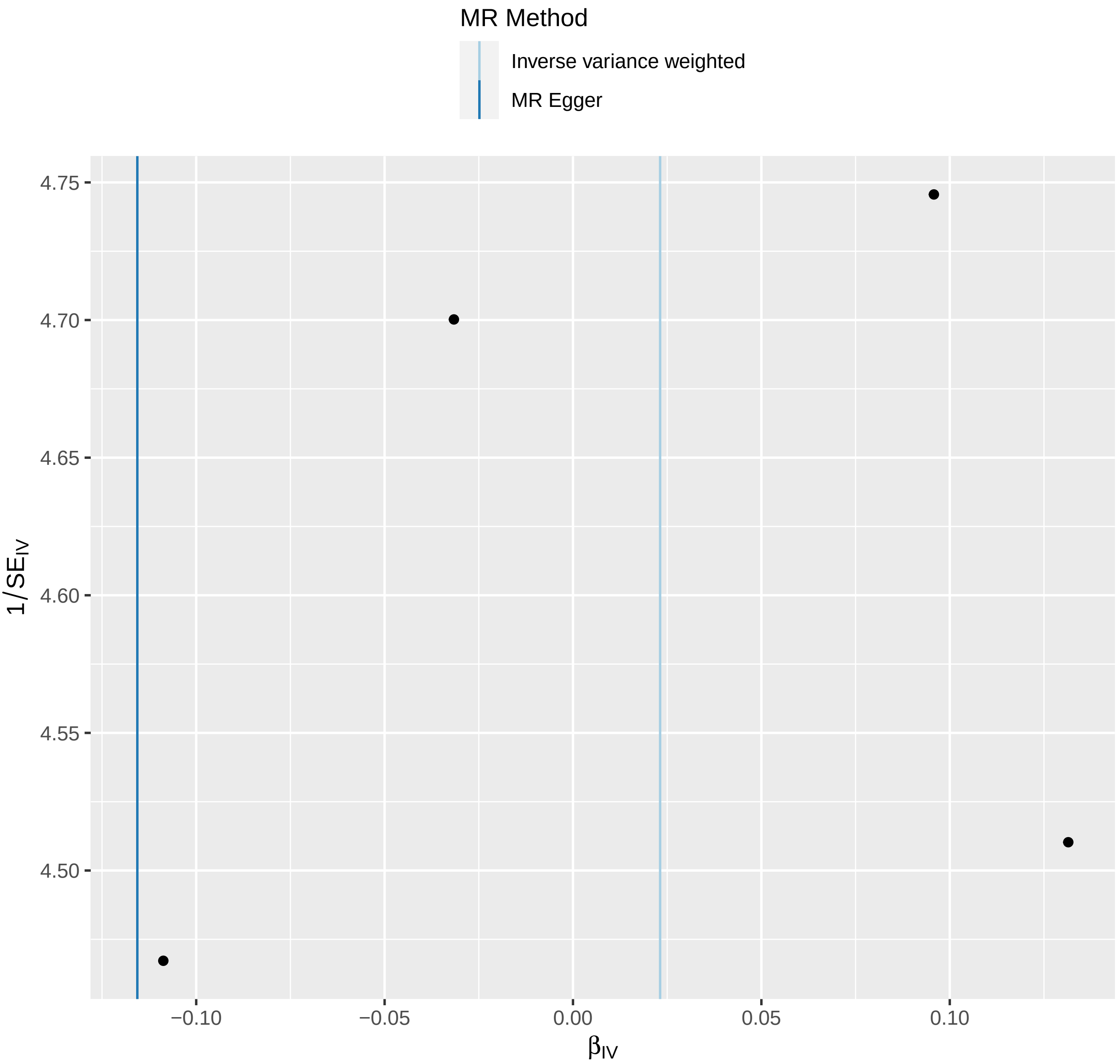

Batch 2−2906 : Gut microbiota abundance (genus Parasutterella id.2892) on Insulin growth factor−like family member 3
with a threshold of 1e−05

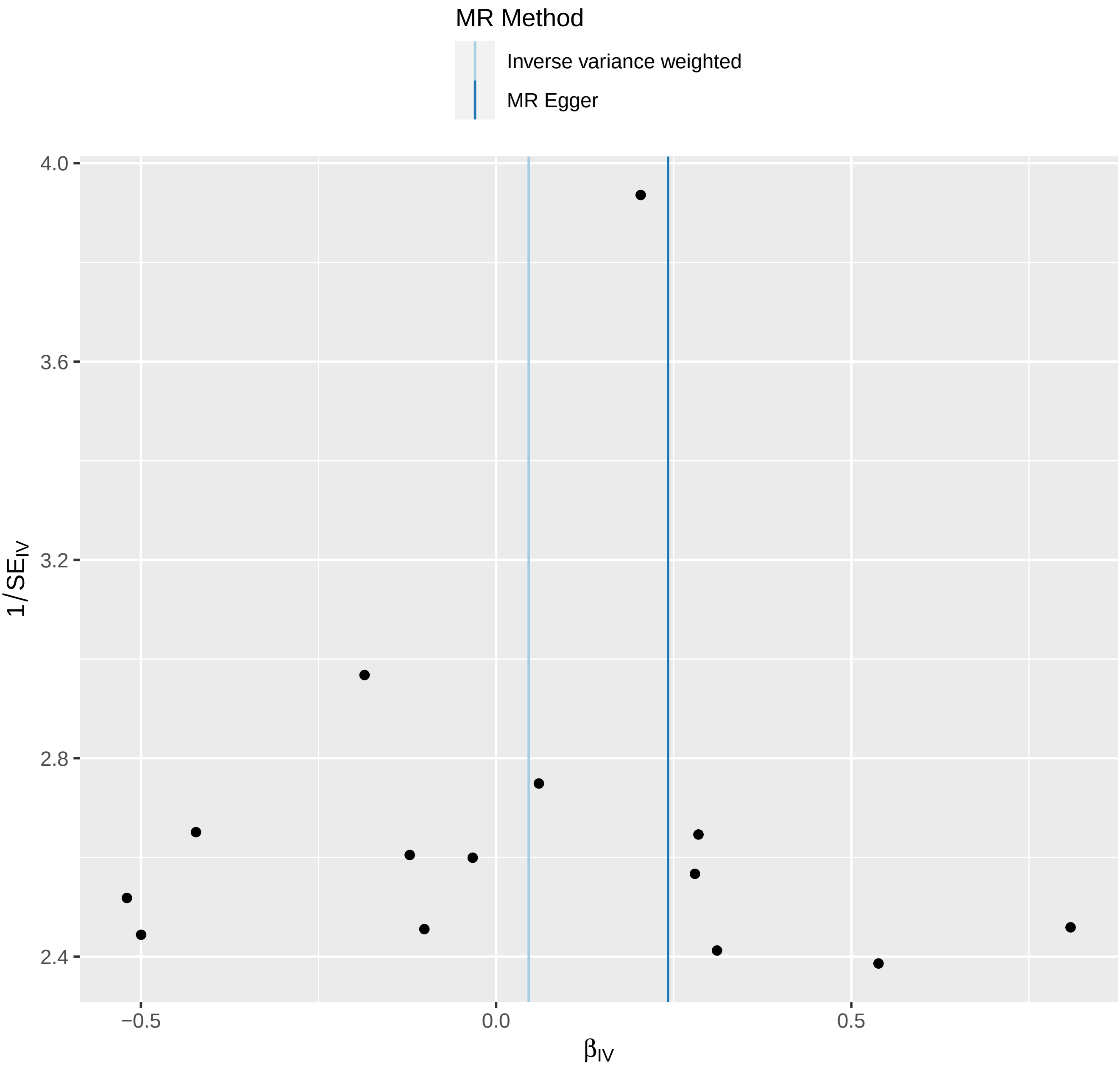

Batch 2−2907 : Gut microbiota abundance (genus Catenibacterium id.2153) on Insulin growth factor−like family member 4
with a threshold of 1e−05

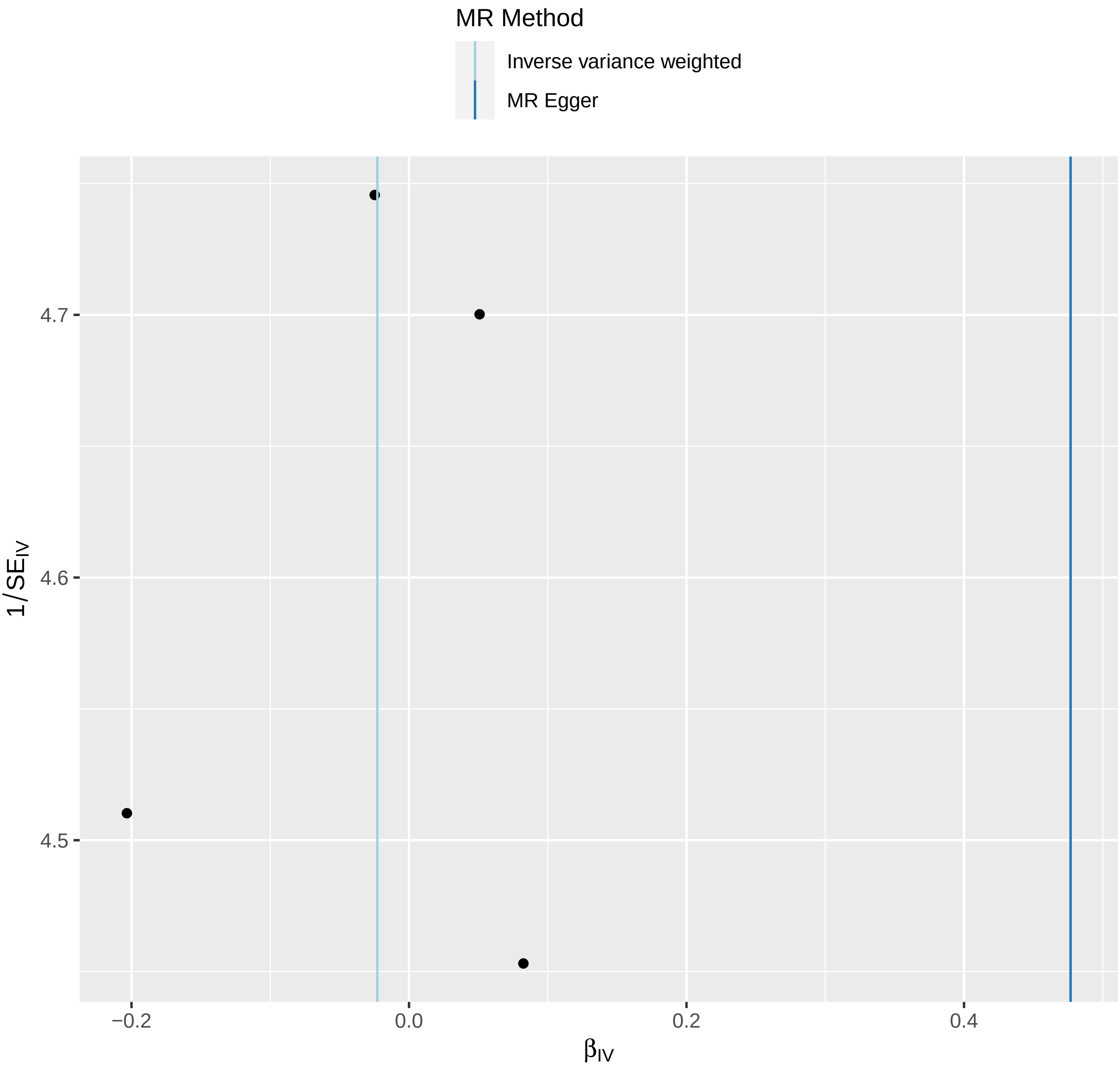

Batch 2−2908 : Gut microbiota abundance (genus Parasutterella id.2892) on Insulin growth factor−like family member 4
with a threshold of 1e−05

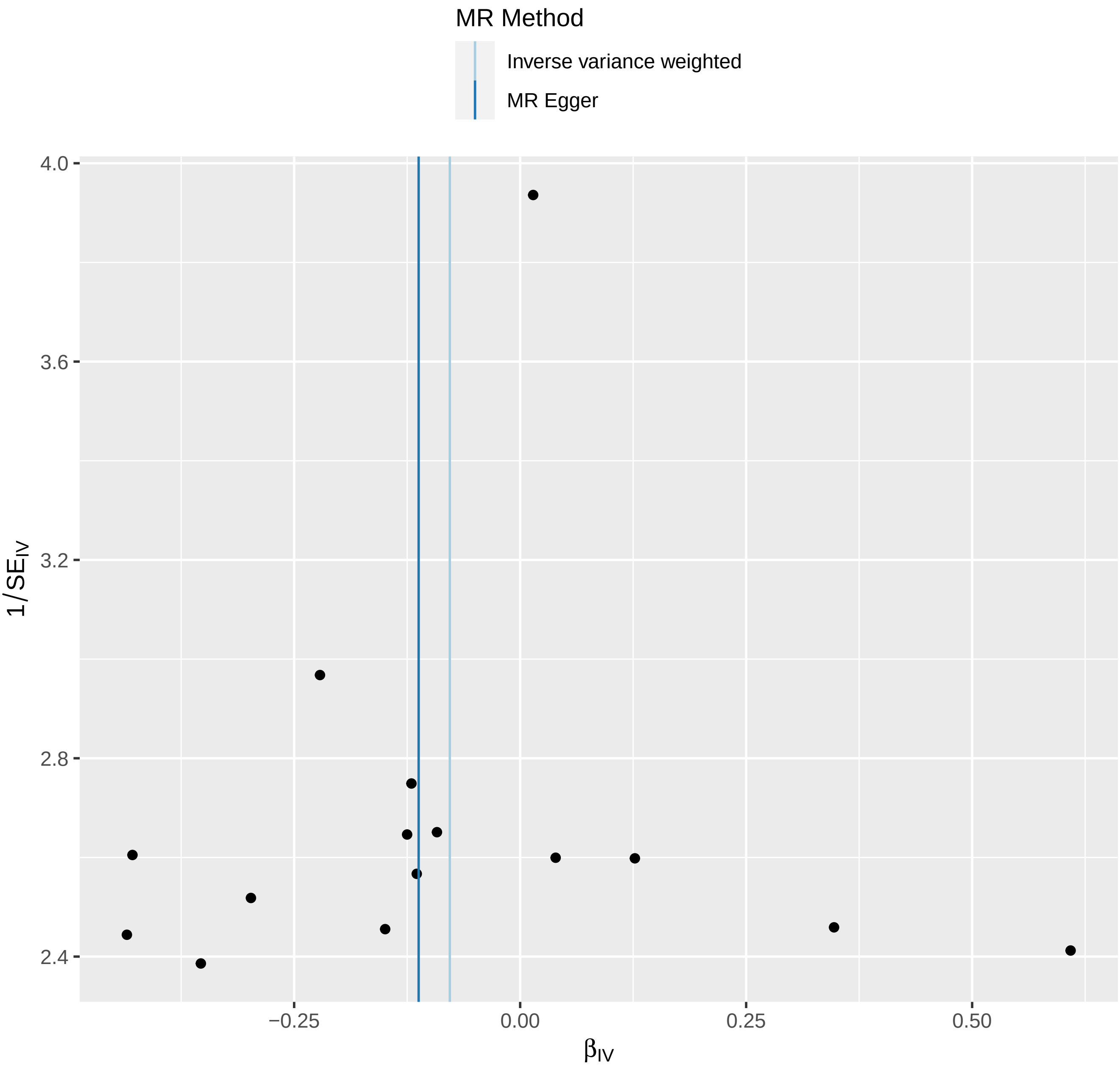

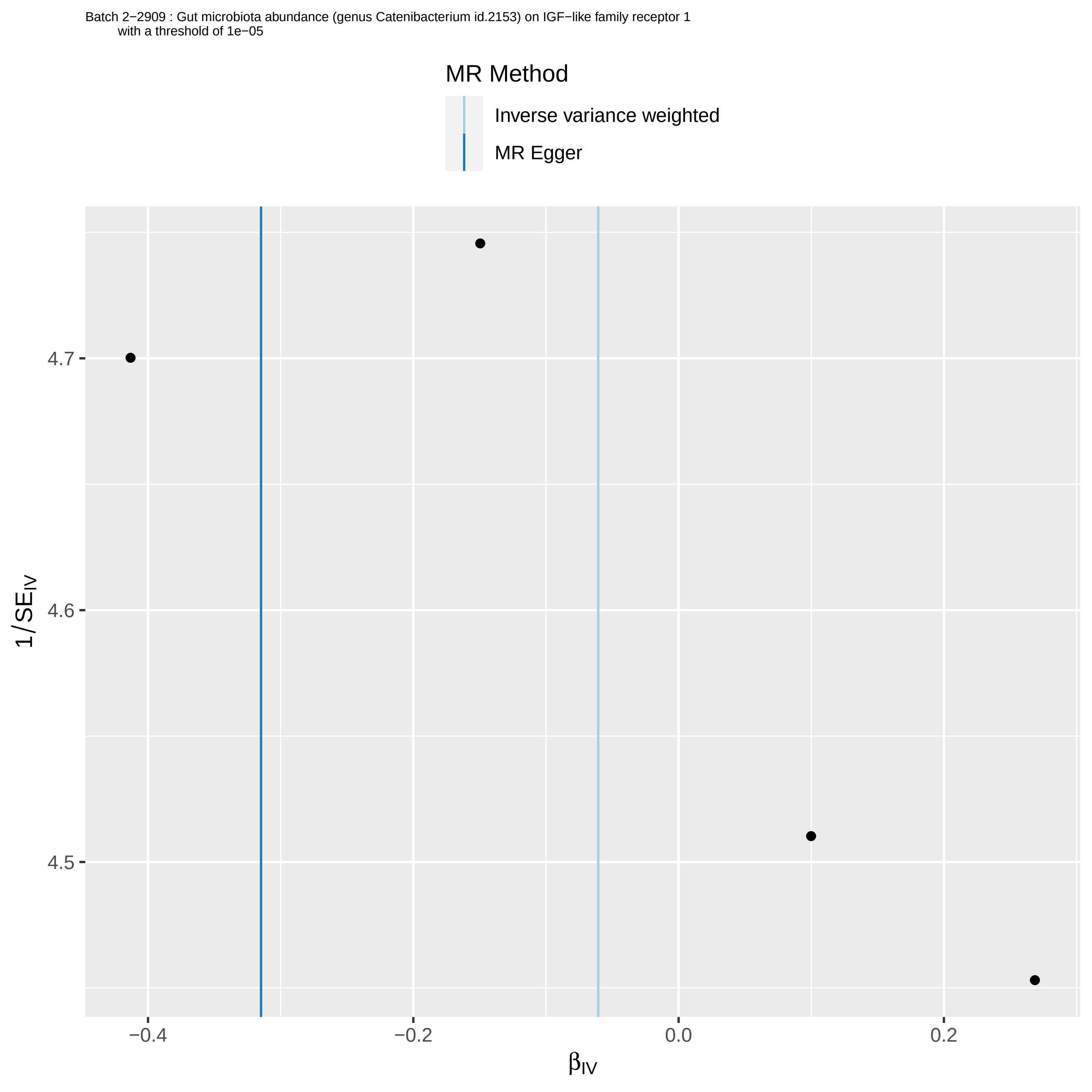

Batch 2−2909 : Gut microbiota abundance (genus Catenibacterium id.2153) on IGF−like family receptor 1
with a threshold of 1e−05
MR Method
Inverse variance weighted
MR Egger
4.7
4.6
4.5
1/SE_IV
−0.4
−0.2
0.0
0.2
β_IV

Batch 2−2910 : Gut microbiota abundance (genus Parasutterella id.2892) on IGF−like family receptor 1
with a threshold of 1e−05

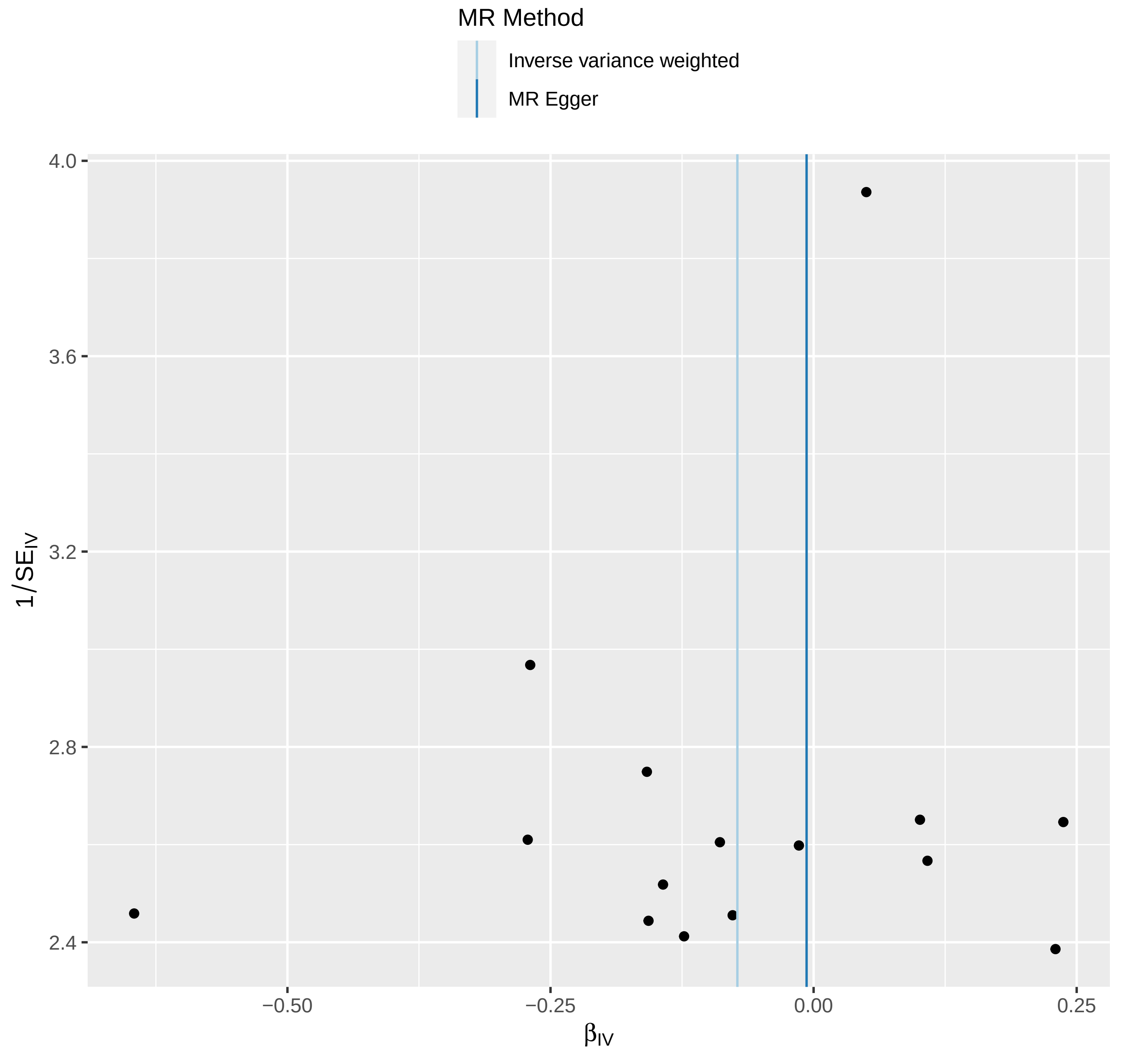

Batch 2−2911 : Gut microbiota abundance (genus Catenibacterium id.2153) on Immunoglobulin E
with a threshold of 1e−05

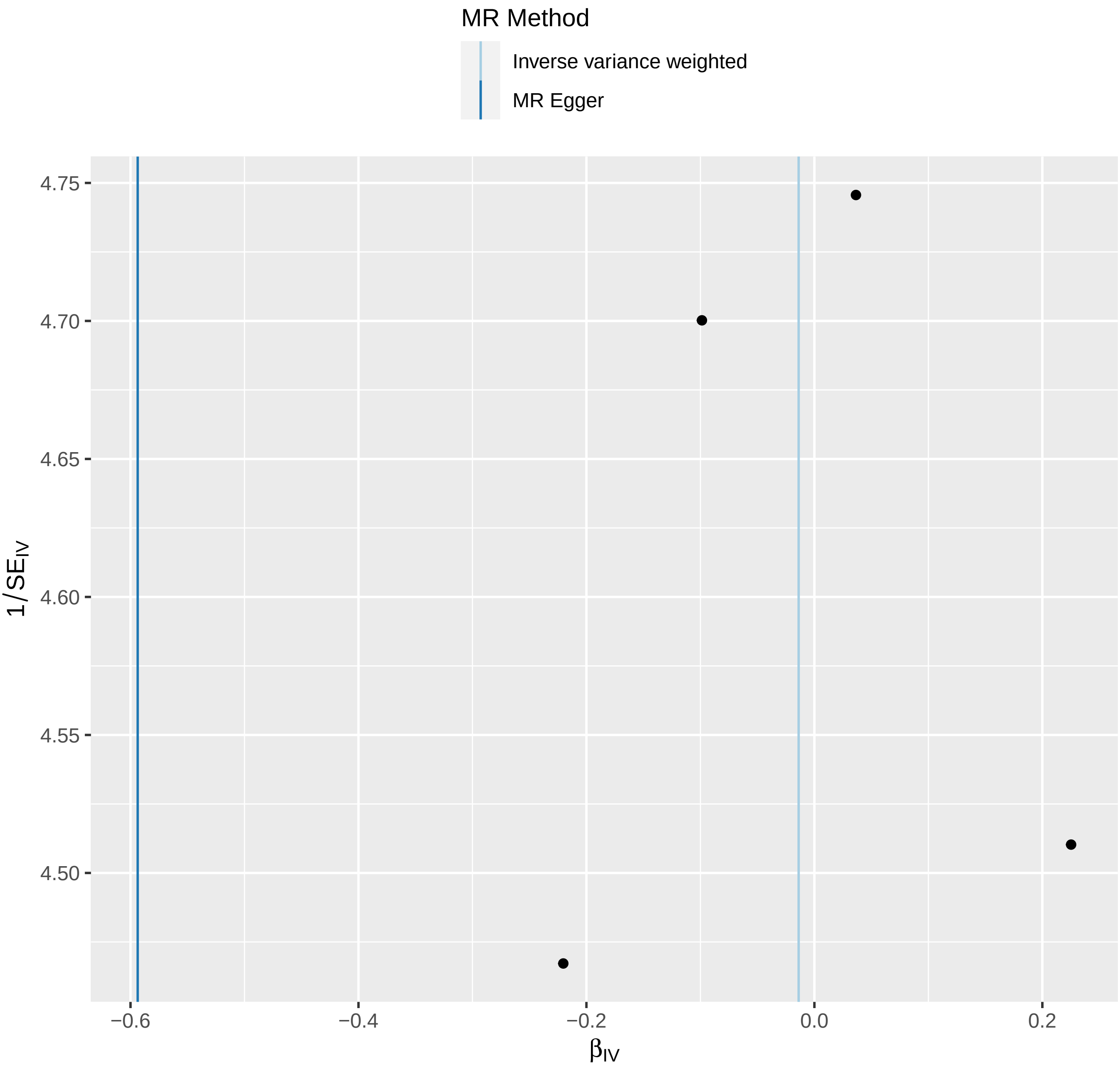

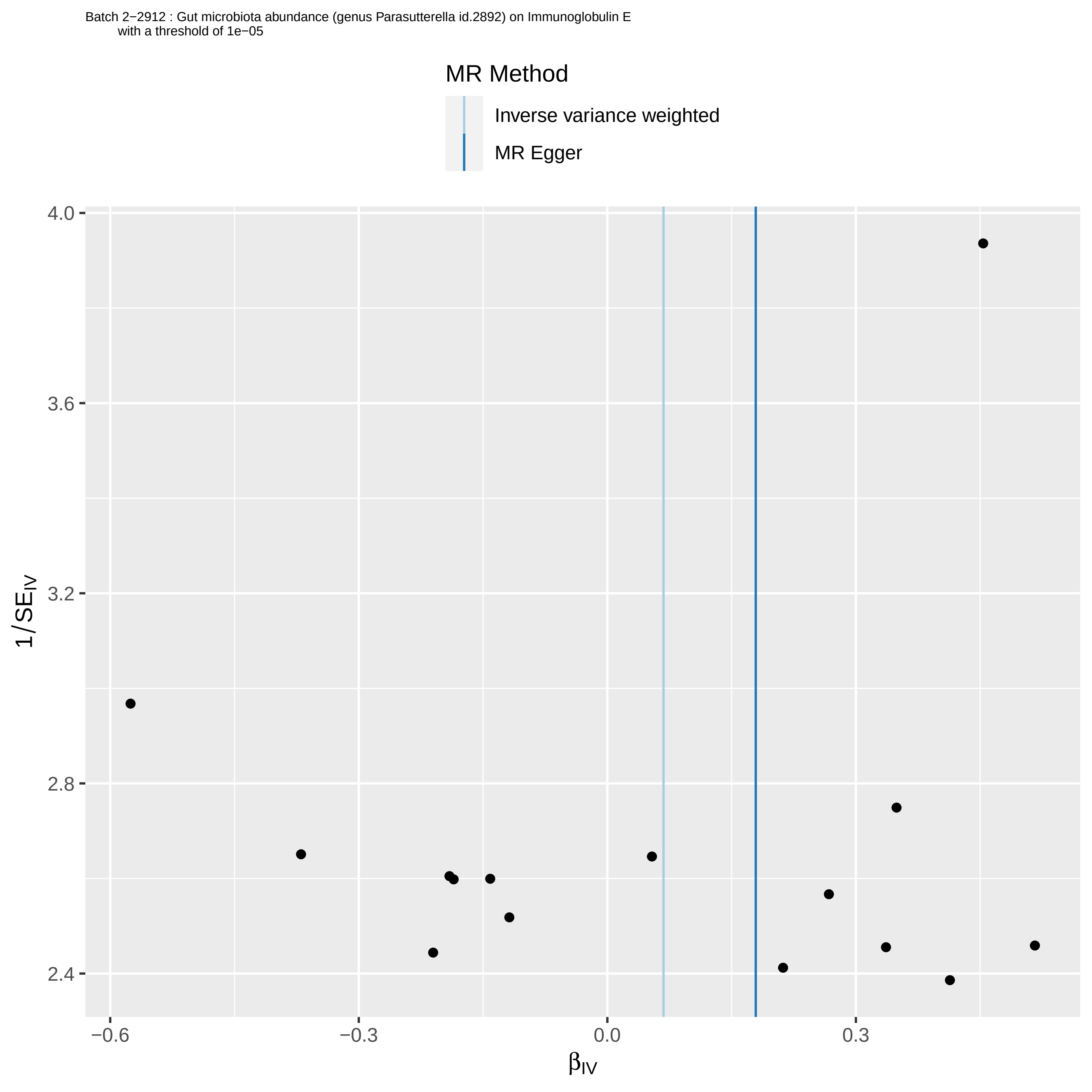

Batch 2−2912 : Gut microbiota abundance (genus Parasutterella id.2892) on Immunoglobulin E
with a threshold of 1e−05
MR Method
Inverse variance weighted
MR Egger
4.0
3.6
3.2
2.8
2.4
1/SE_IV
−0.6
−0.3
0.0
0.3
β_IV

Batch 2−2913 : Gut microbiota abundance (genus Catenibacterium id.2153) on Immunoglobulin J chain
with a threshold of 1e−05

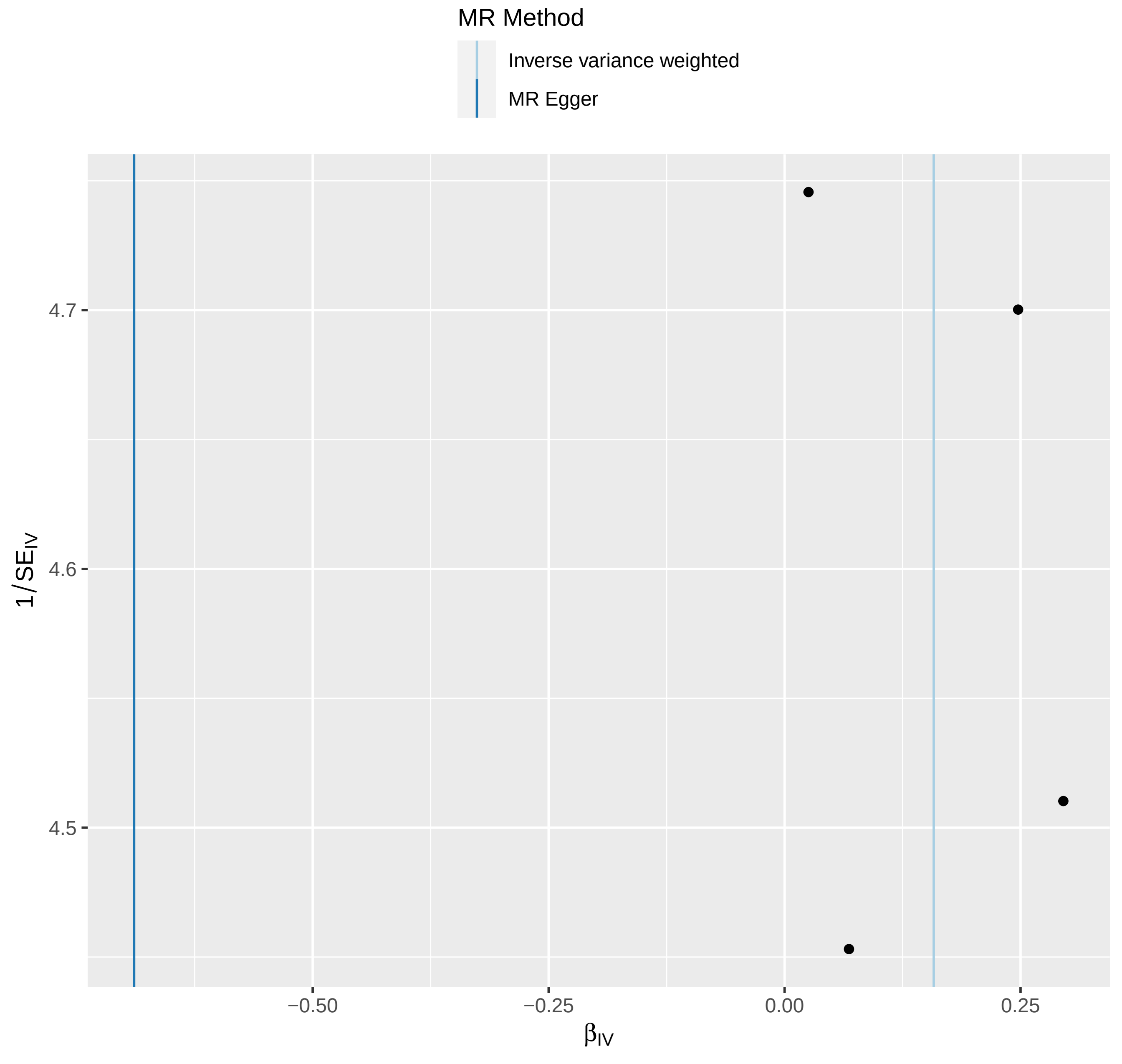

Batch 2−2914 : Gut microbiota abundance (genus Parasutterella id.2892) on Immunoglobulin J chain
with a threshold of 1e−05

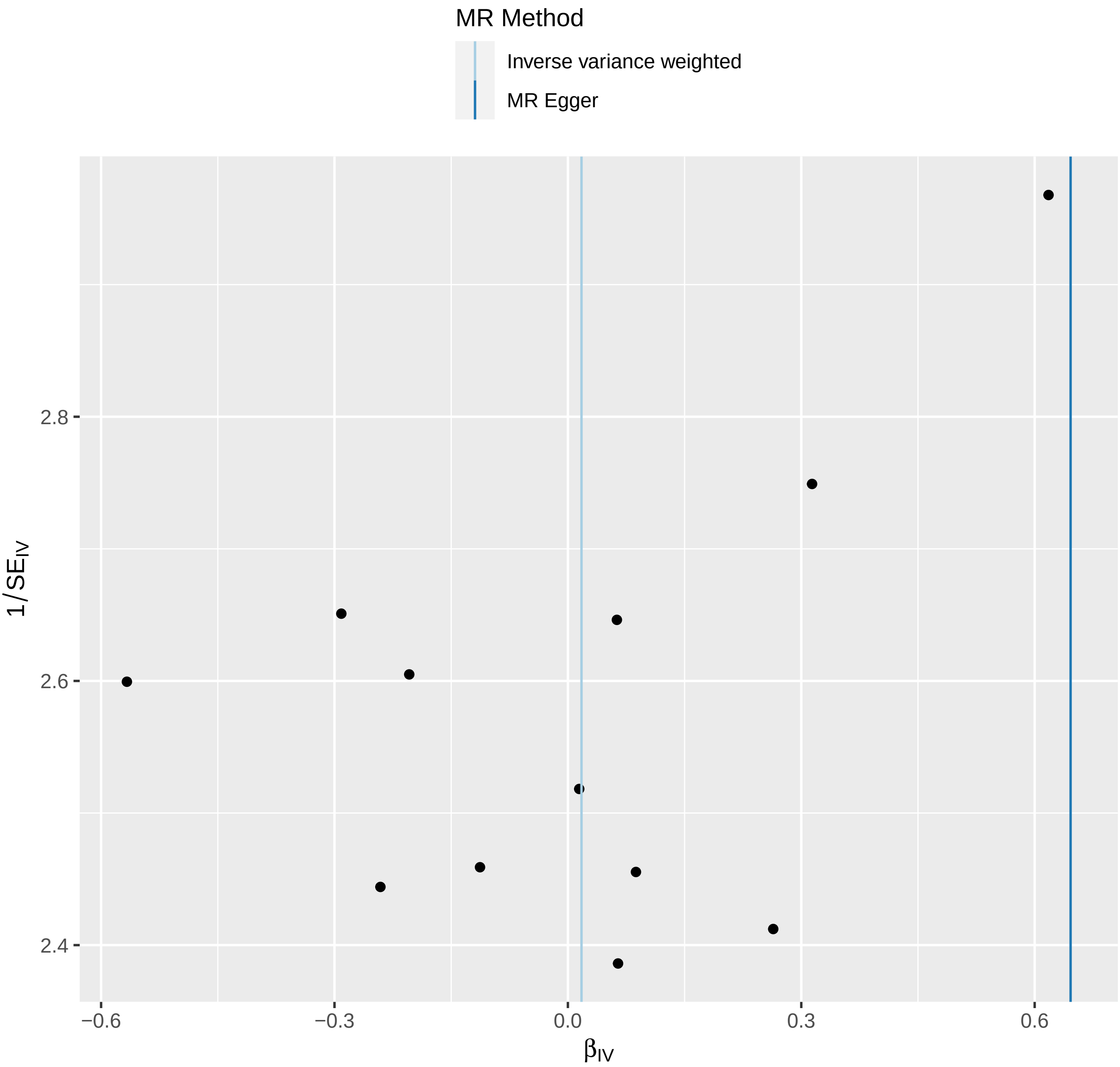

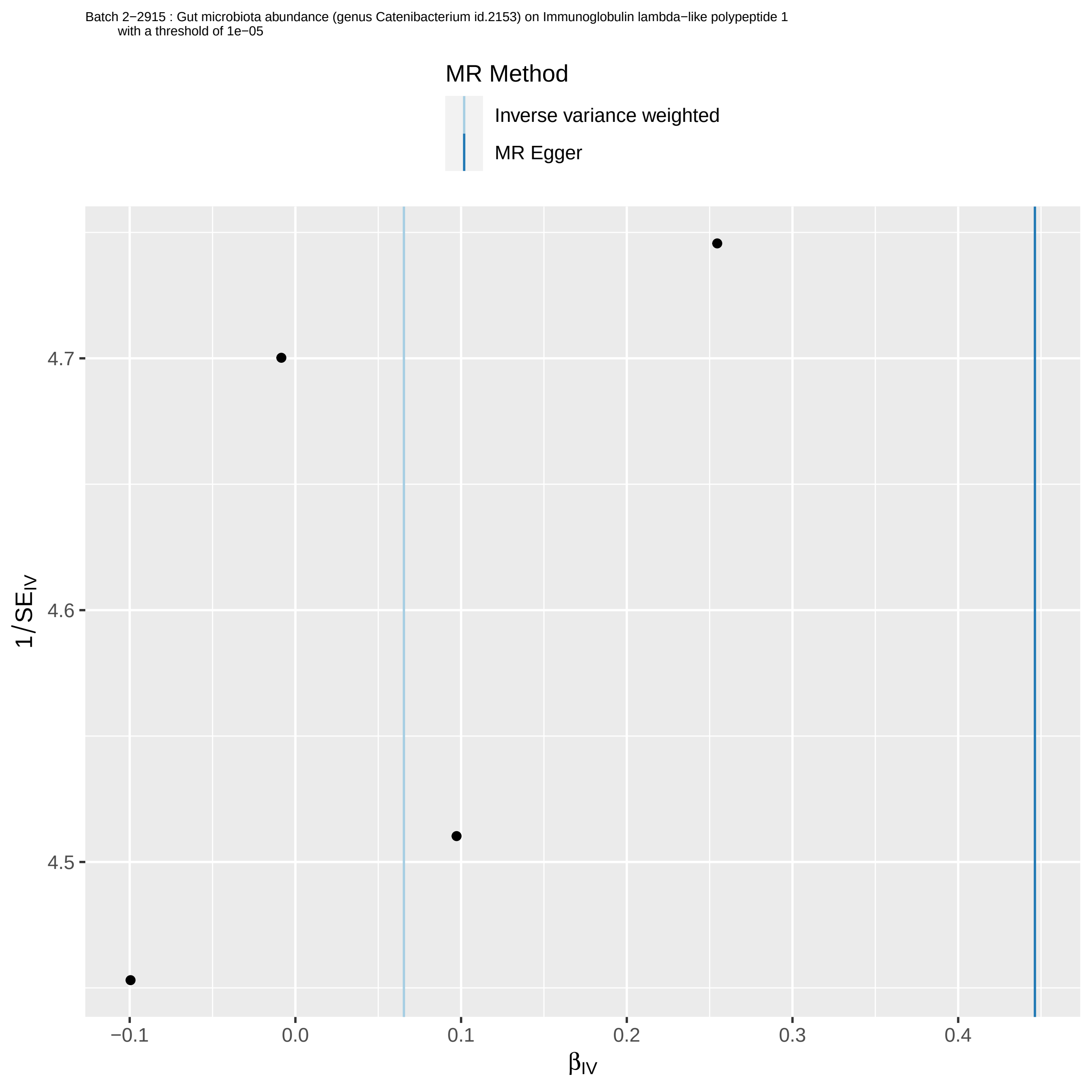

Batch 2−2915 : Gut microbiota abundance (genus Catenibacterium id.2153) on Immunoglobulin lambda−like polypeptide 1
with a threshold of 1e−05
MR Method
Inverse variance weighted
MR Egger
4.7
4.6
4.5
1/SE$_{IV}$
−0.1
0.0
0.1
0.2
0.3
0.4
β$_{IV}$

Batch 2−2916 : Gut microbiota abundance (genus Parasutterella id.2892) on Immunoglobulin lambda−like polypeptide 1
with a threshold of 1e−05

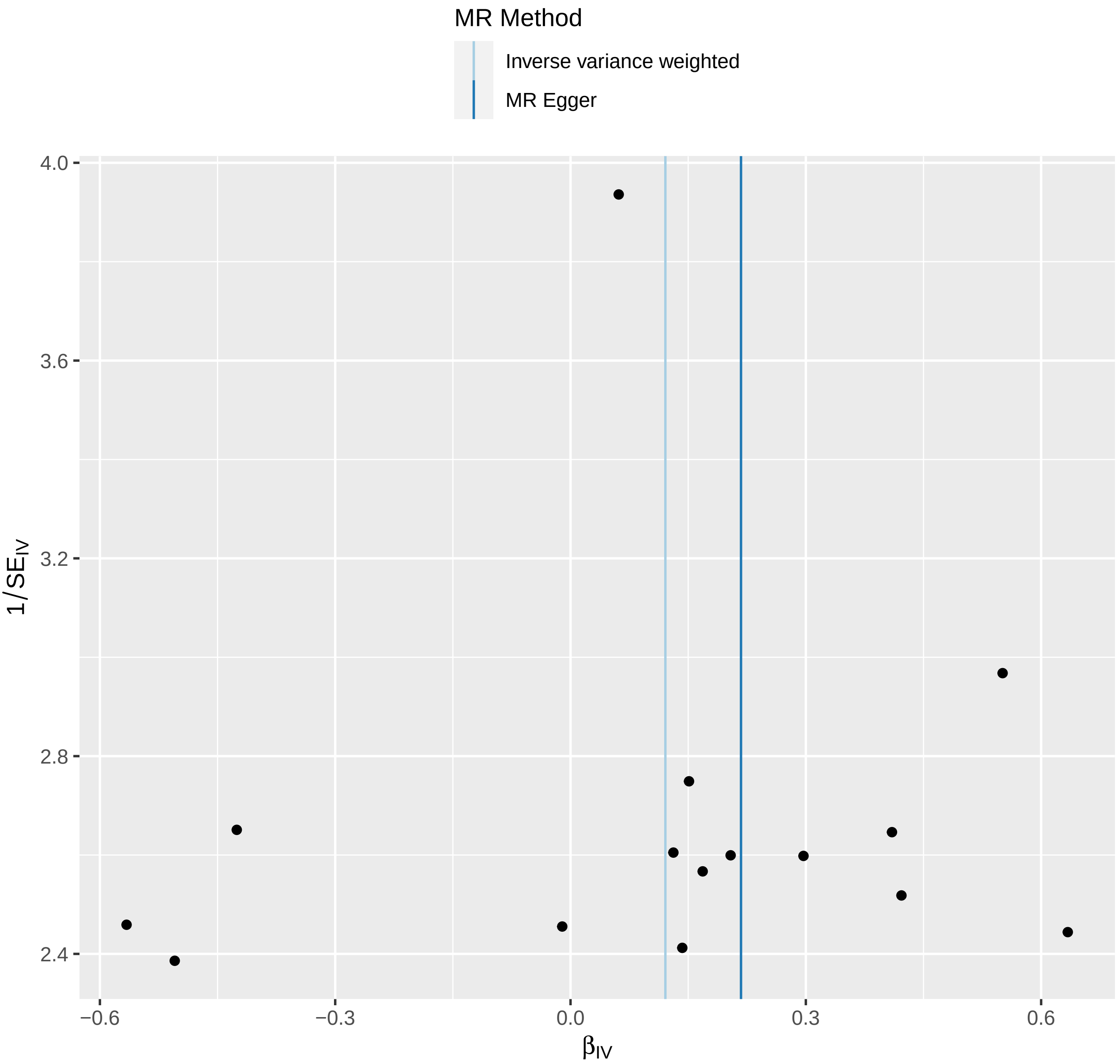

Batch 2−2917 : Gut microbiota abundance (genus Catenibacterium id.2153) on IgLON family member 5
with a threshold of 1e−05

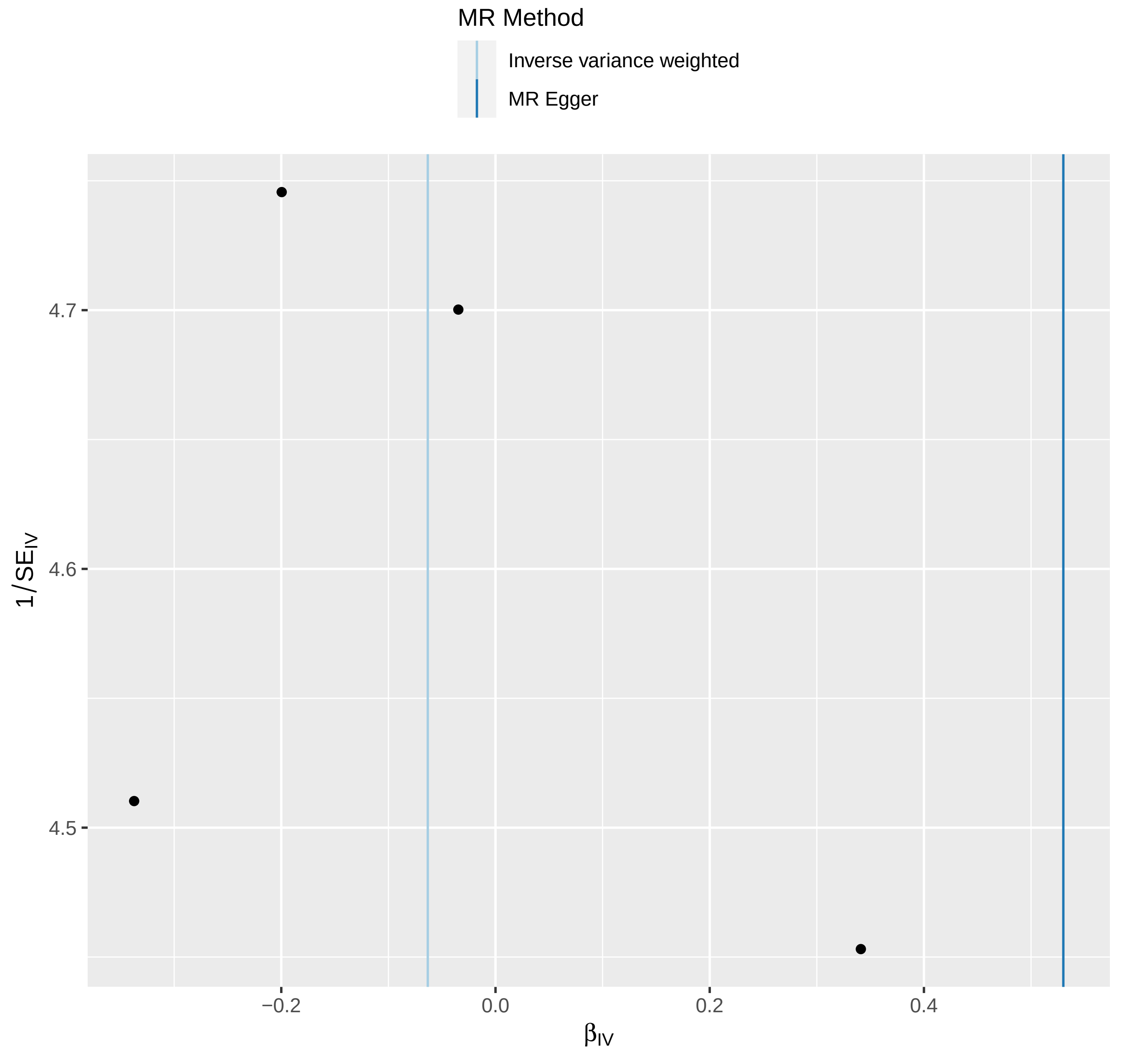

Batch 2−2918 : Gut microbiota abundance (genus Parasutterella id.2892) on IgLON family member 5
with a threshold of 1e−05

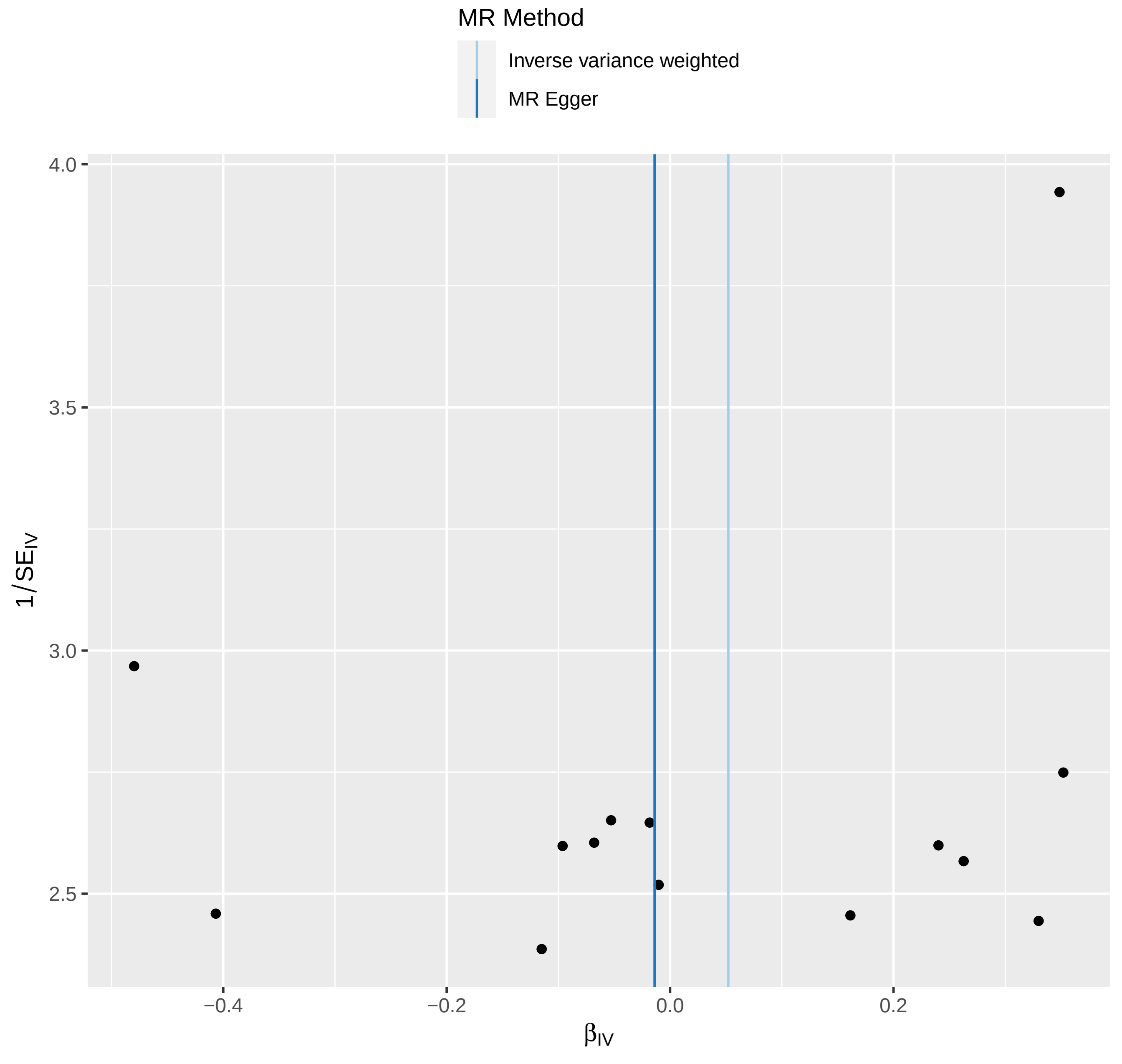

Batch 2−2919 : Gut microbiota abundance (genus Catenibacterium id.2153) on Immunoglobulin superfamily member 11
with a threshold of 1e−05

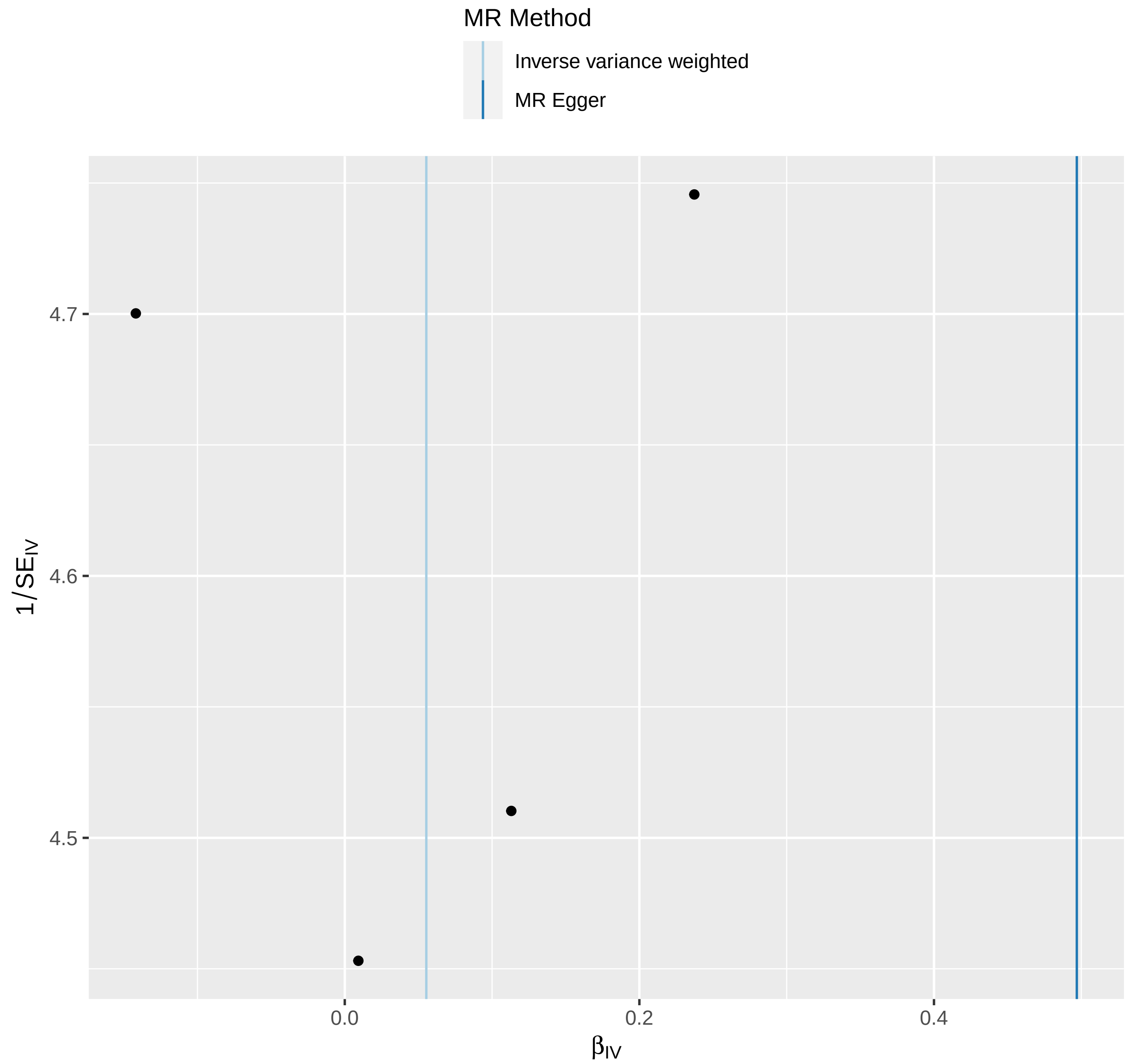

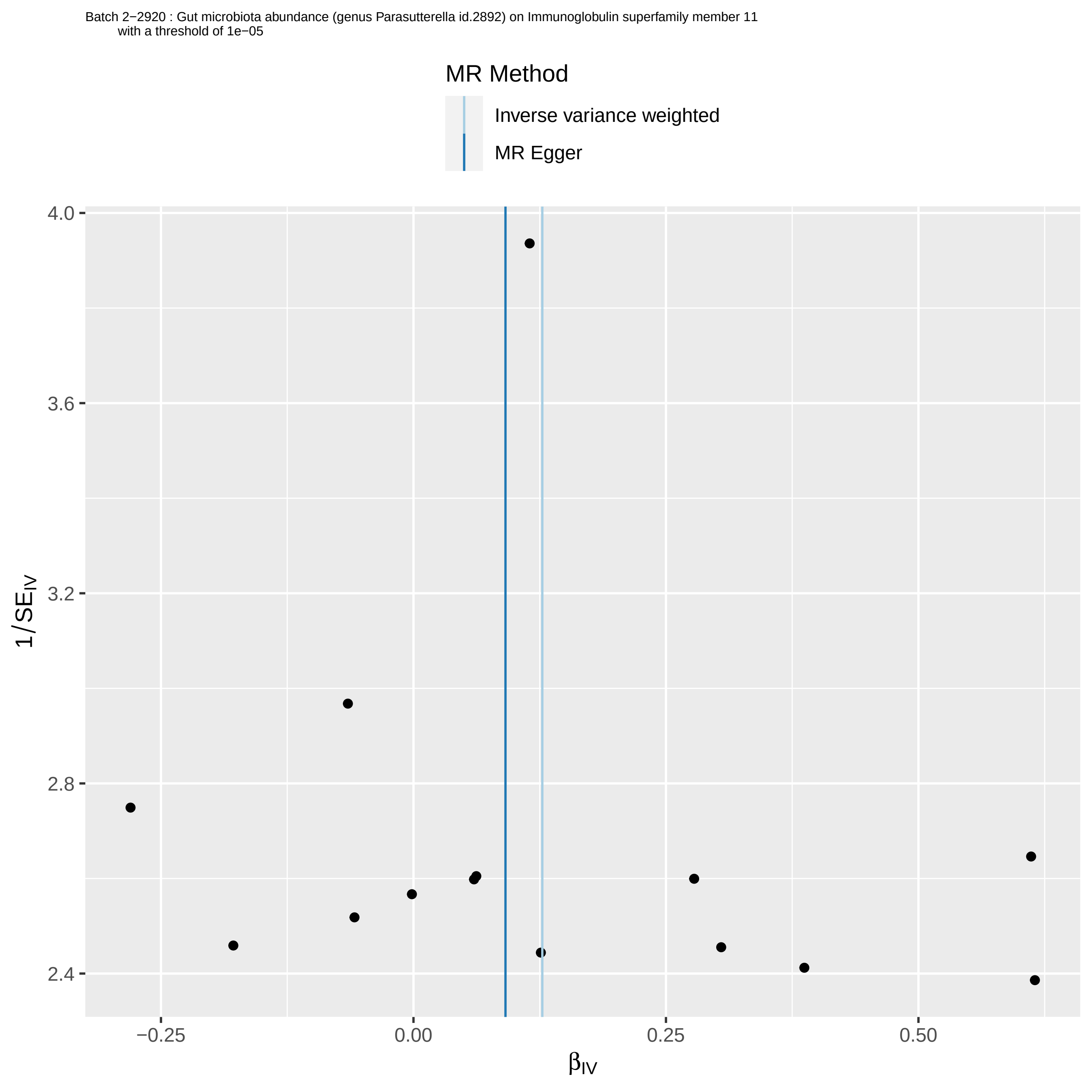
Batch 2−2920 : Gut microbiota abundance (genus Parasutterella id.2892) on Immunoglobulin superfamily member 11
with a threshold of 1e−05
MR Method
Inverse variance weighted
MR Egger
4.0
3.6
3.2
2.8
2.4
1/SE_IV
−0.25
0.00
0.25
0.50
β_IV

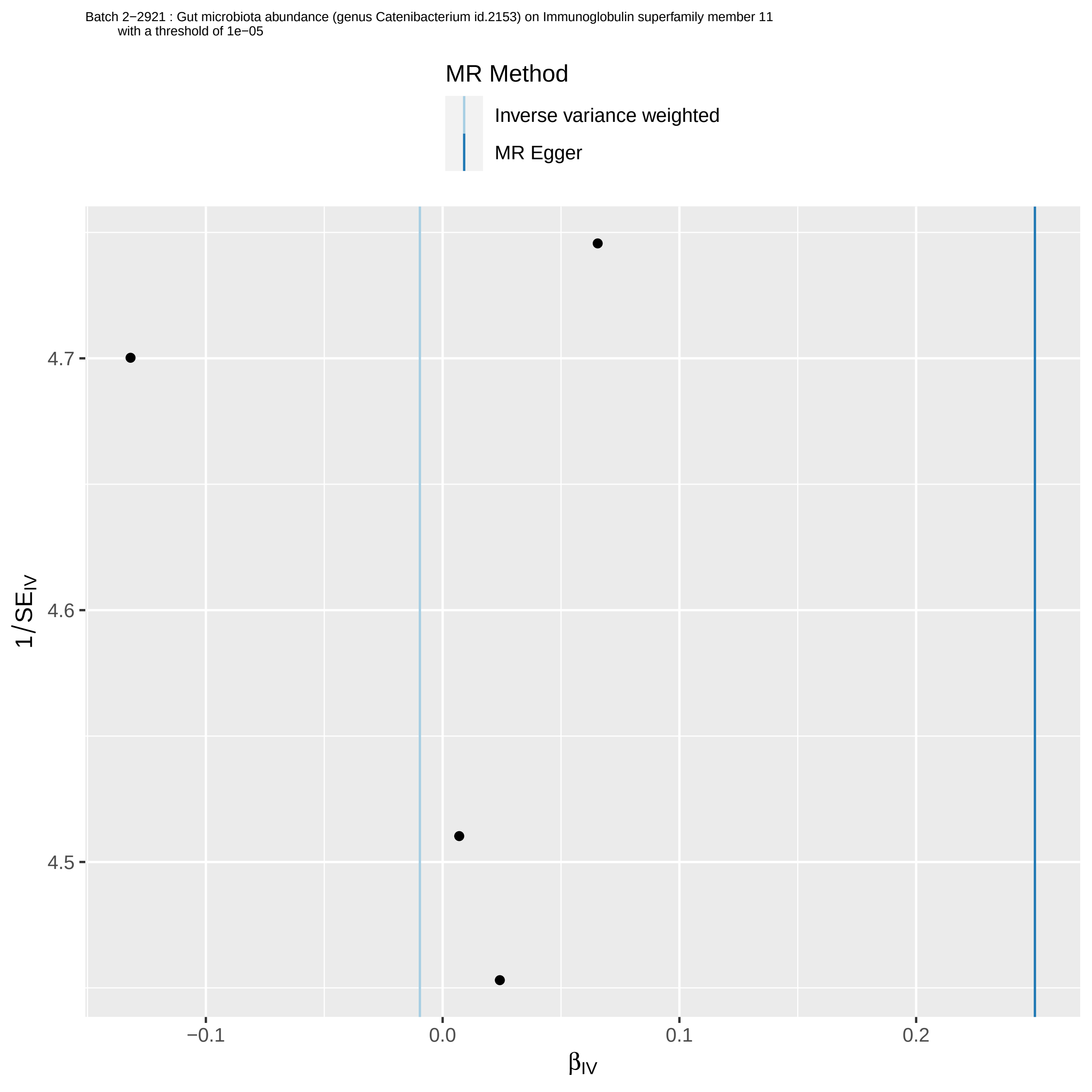

Batch 2−2921 : Gut microbiota abundance (genus Catenibacterium id.2153) on Immunoglobulin superfamily member 11
with a threshold of 1e−05
MR Method
Inverse variance weighted
MR Egger
4.7
4.6
4.5
1/SE_IV
−0.1
0.0
0.1
0.2
β_IV

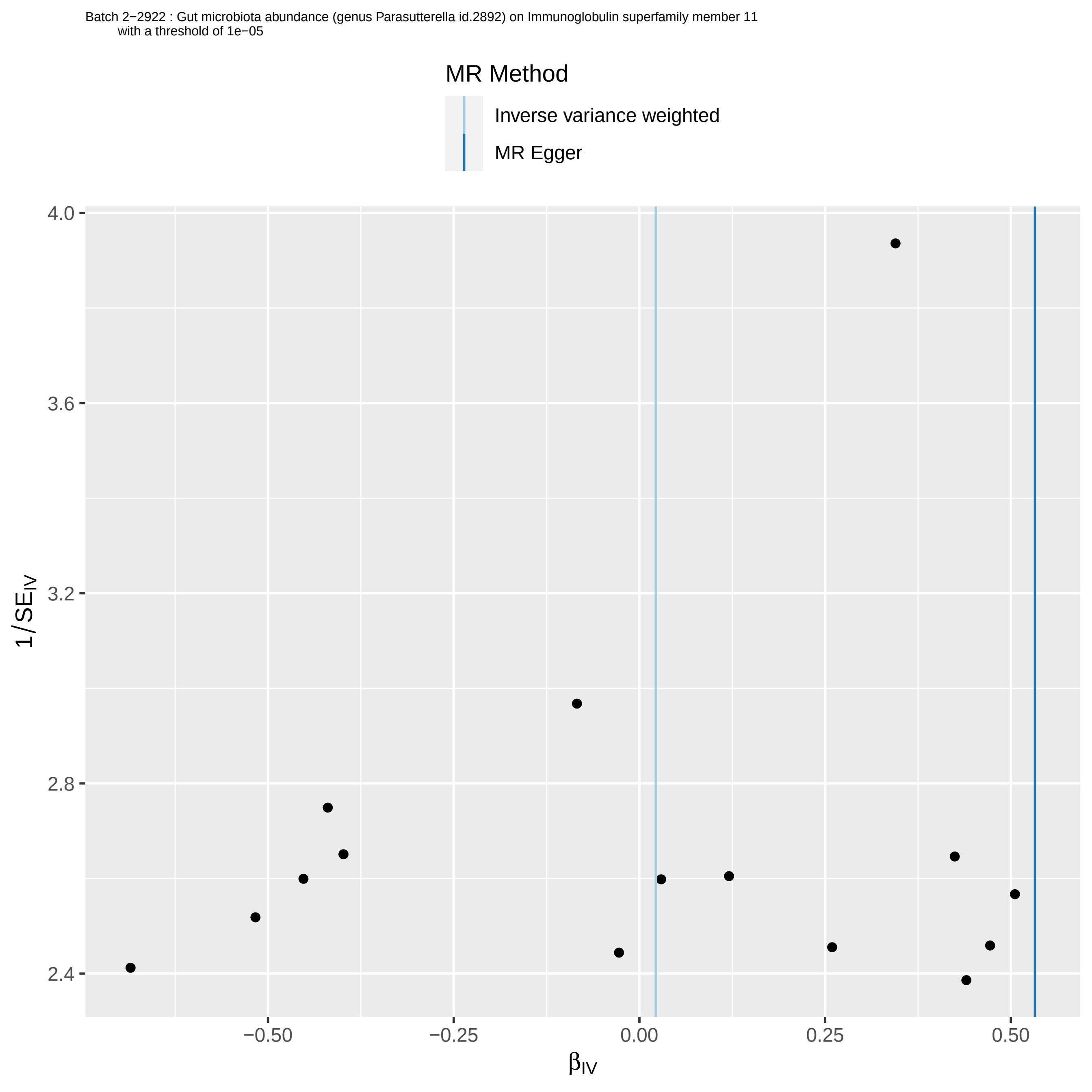

Batch 2−2922 : Gut microbiota abundance (genus Parasutterella id.2892) on Immunoglobulin superfamily member 11
with a threshold of 1e−05
MR Method
Inverse variance weighted
MR Egger
4.0
3.6
3.2
2.8
2.4
1/SE$_{IV}$
−0.50
−0.25
0.00
0.25
0.50
β$_{IV}$

Batch 2−2923 : Gut microbiota abundance (genus Catenibacterium id.2153) on Immunoglobulin superfamily member 8 with a threshold of 1e−05

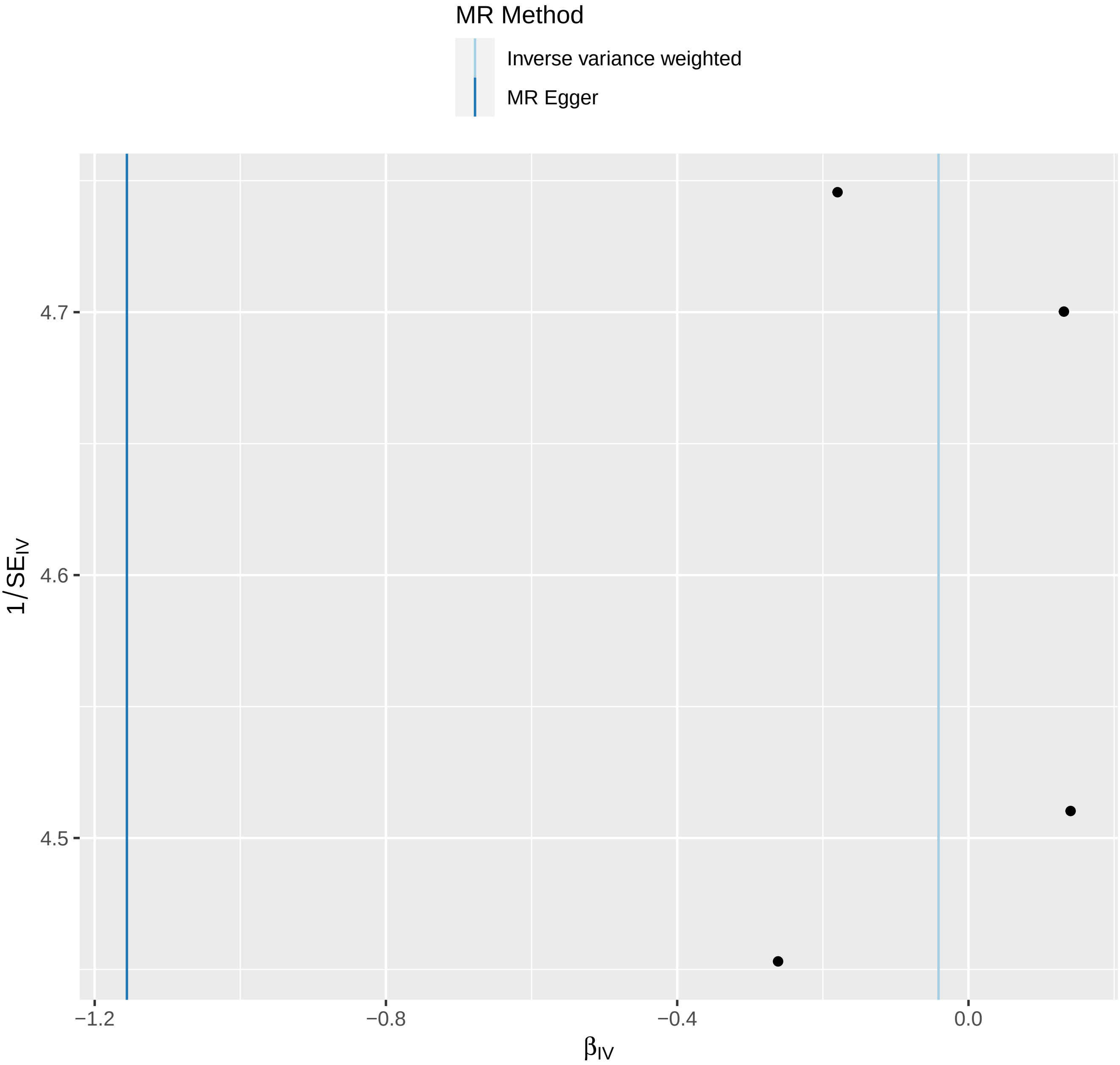

Batch 2−2924 : Gut microbiota abundance (genus Parasutterella id.2892) on Immunoglobulin superfamily member 8
with a threshold of 1e−05

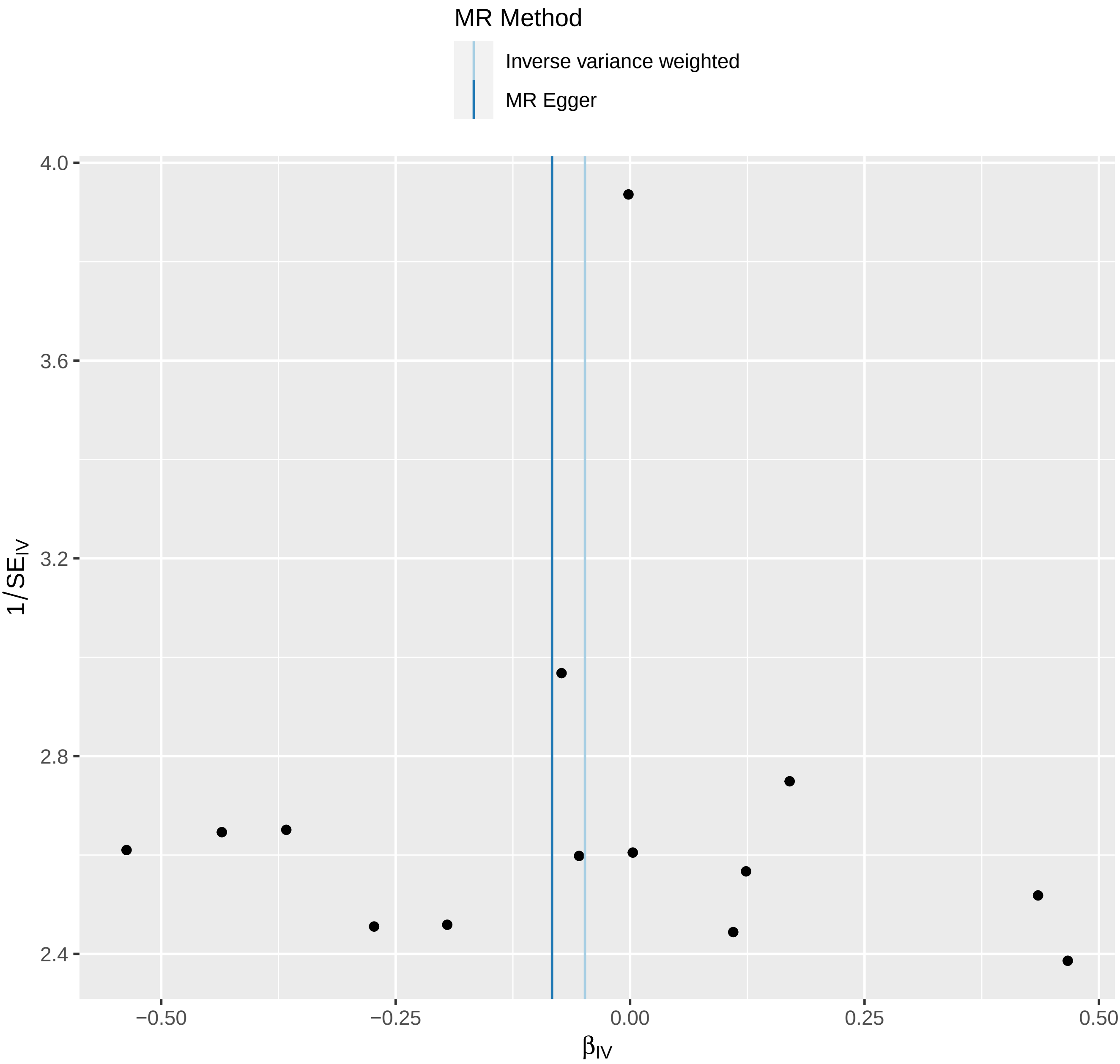

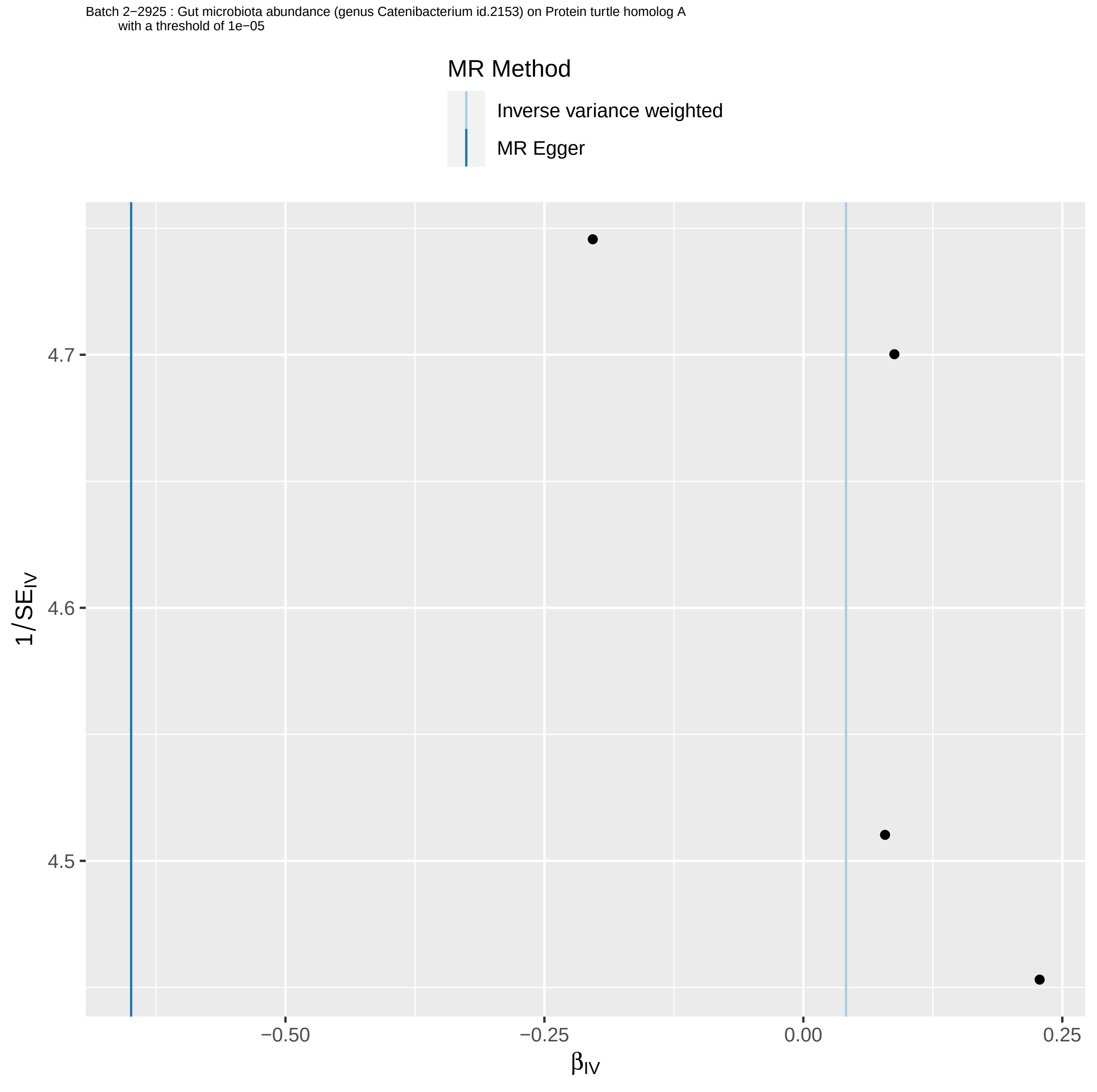

Batch 2−2925 : Gut microbiota abundance (genus Catenibacterium id.2153) on Protein turtle homolog A
with a threshold of 1e−05
MR Method
Inverse variance weighted
MR Egger
4.7
4.6
4.5
1/SE_IV
−0.50
−0.25
0.00
0.25
β_IV

Batch 2−2926 : Gut microbiota abundance (genus Parasutterella id.2892) on Protein turtle homolog A
with a threshold of 1e−05

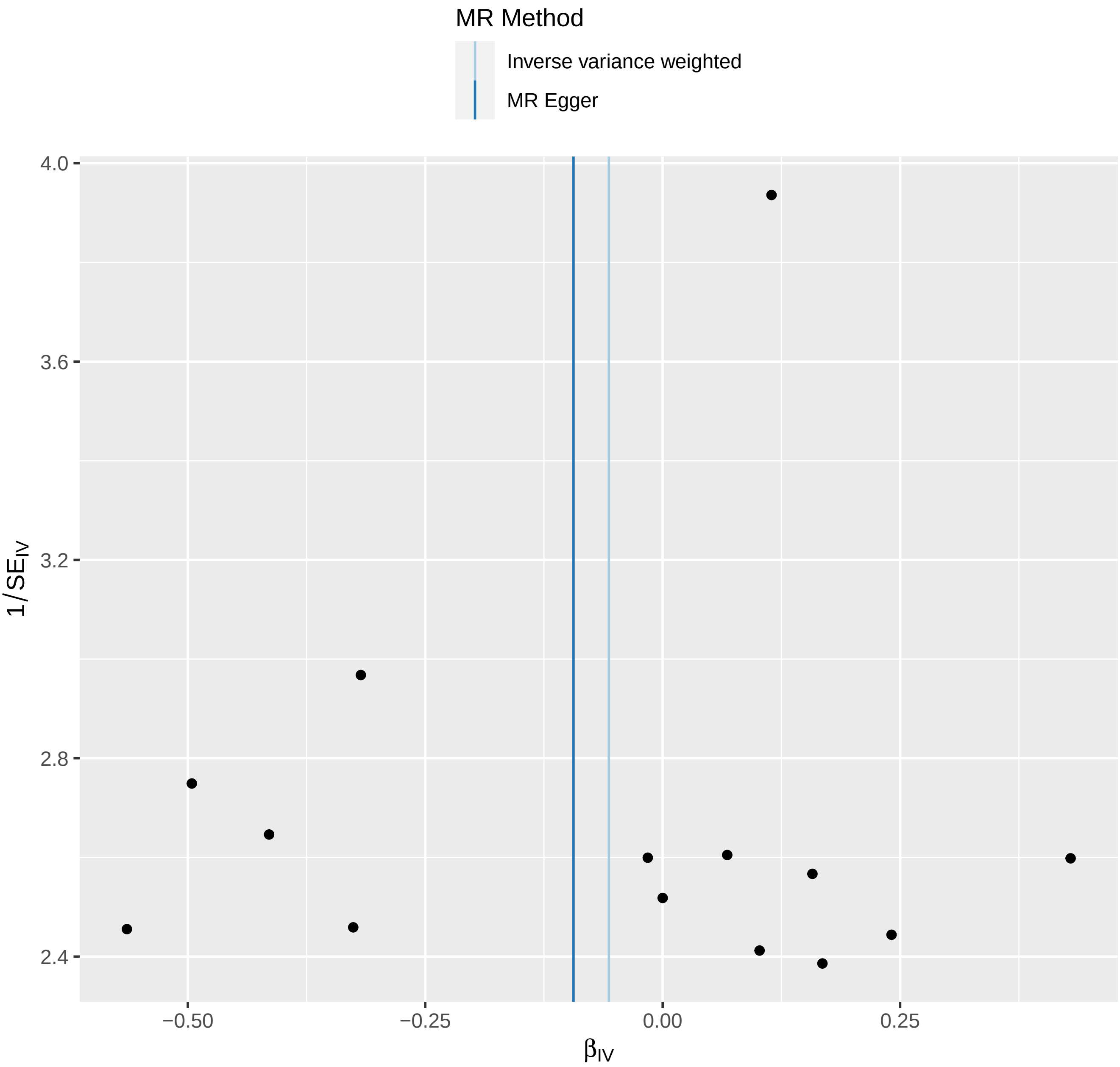

Batch 2−2927 : Gut microbiota abundance (genus Catenibacterium id.2153) on Interleukin−10
with a threshold of 1e−05

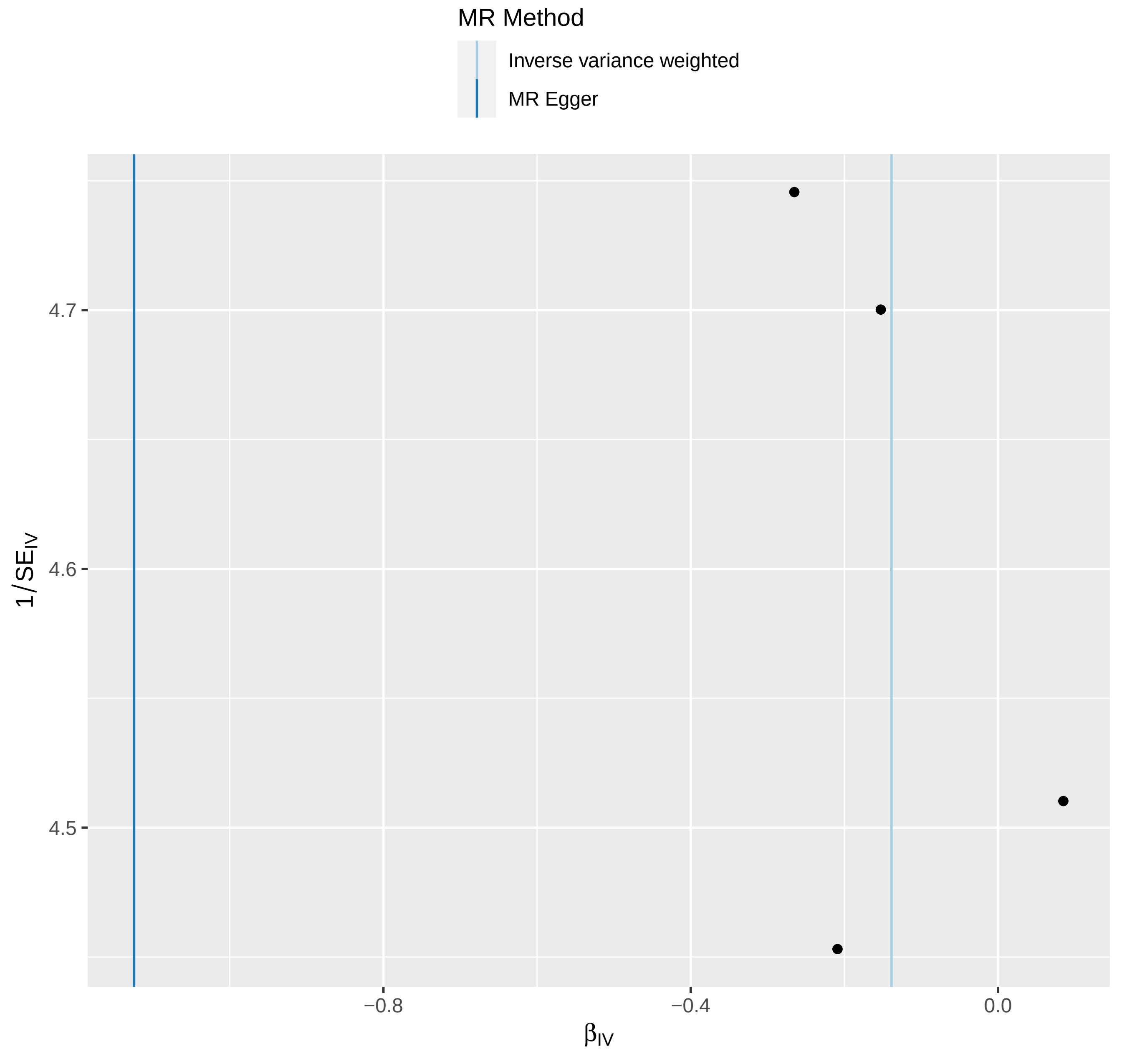

Batch 2−2928 : Gut microbiota abundance (genus Parasutterella id.2892) on Interleukin−10
with a threshold of 1e−05

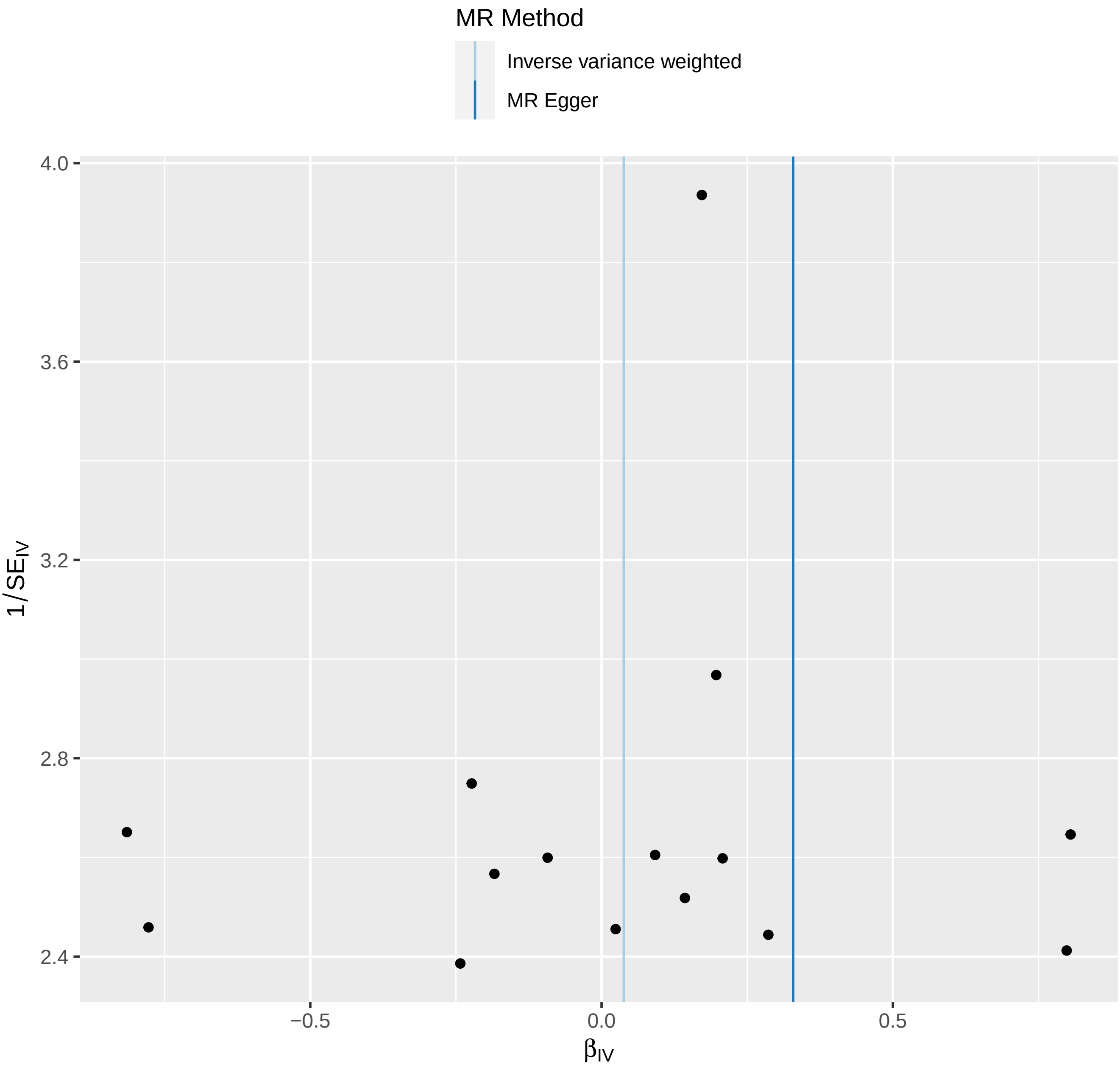

Batch 2−2929 : Gut microbiota abundance (genus Catenibacterium id.2153) on Interleukin−10 receptor subunit beta
with a threshold of 1e−05

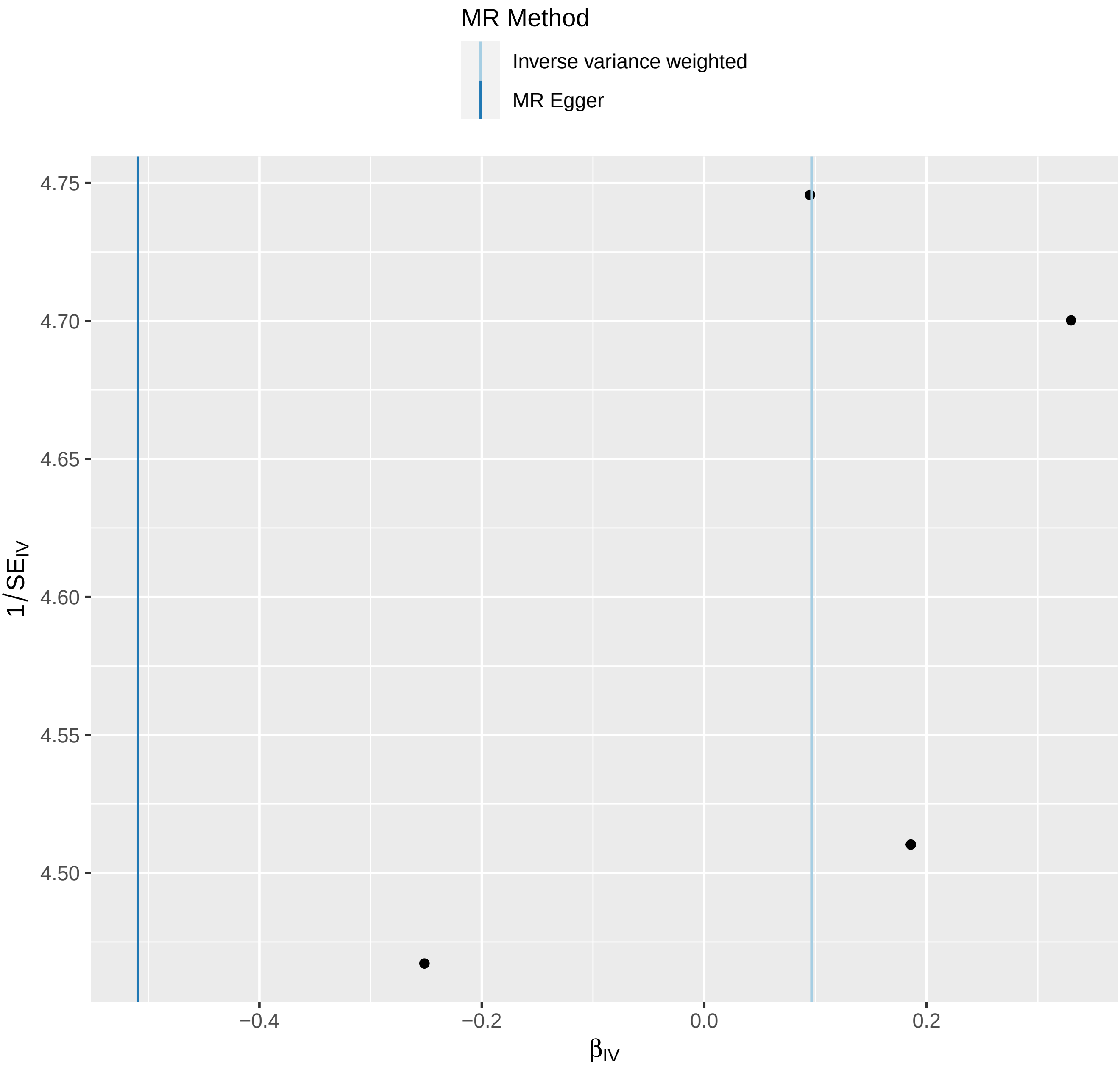

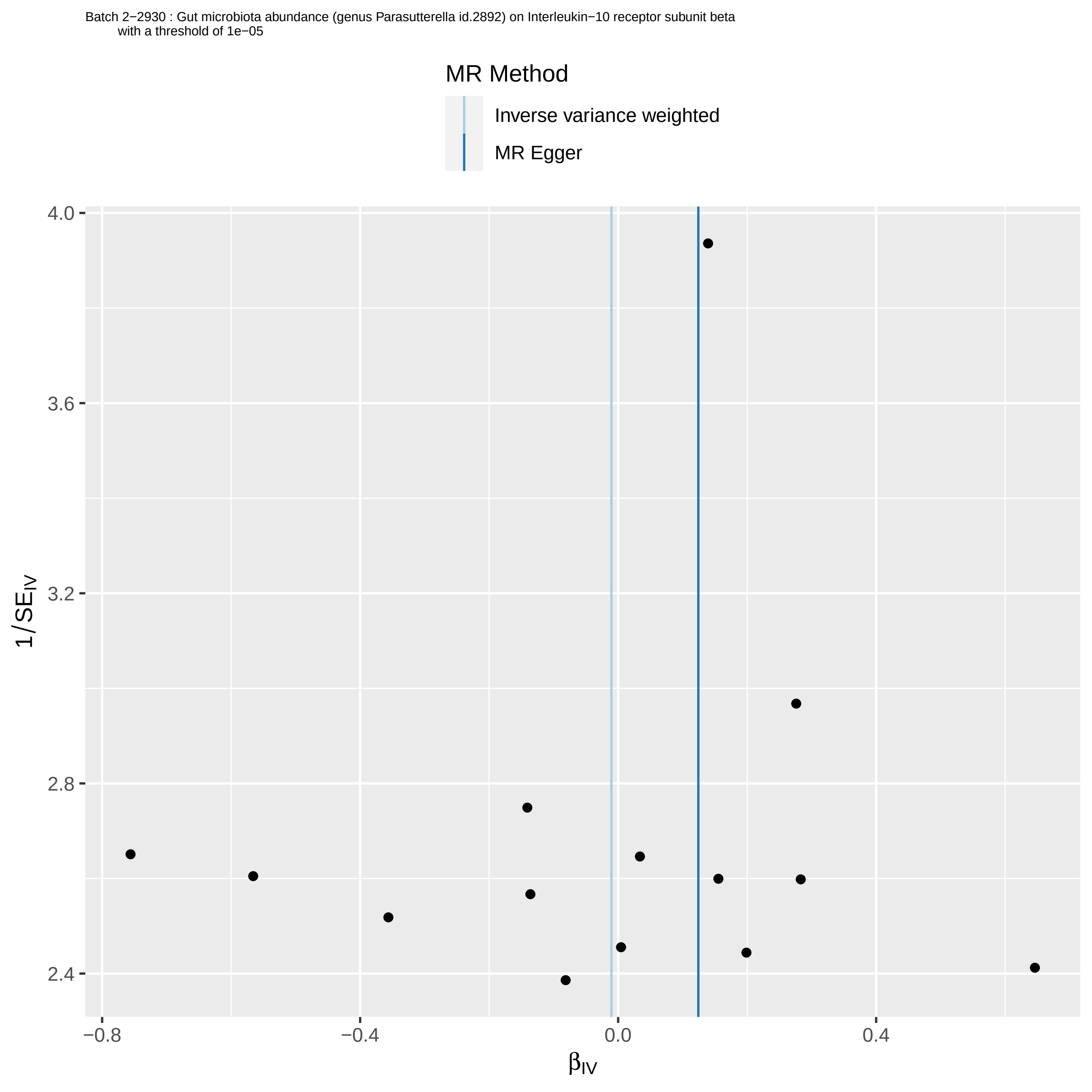
Batch 2−2930 : Gut microbiota abundance (genus Parasutterella id.2892) on Interleukin−10 receptor subunit beta
with a threshold of 1e−05
MR Method
Inverse variance weighted
MR Egger
4.0
3.6
3.2
2.8
2.4
1/SE$_{IV}$
−0.8
−0.4
0.0
0.4
β$_{IV}$

Batch 2−2931 : Gut microbiota abundance (genus Catenibacterium id.2153) on Interleukin−11
with a threshold of 1e−05

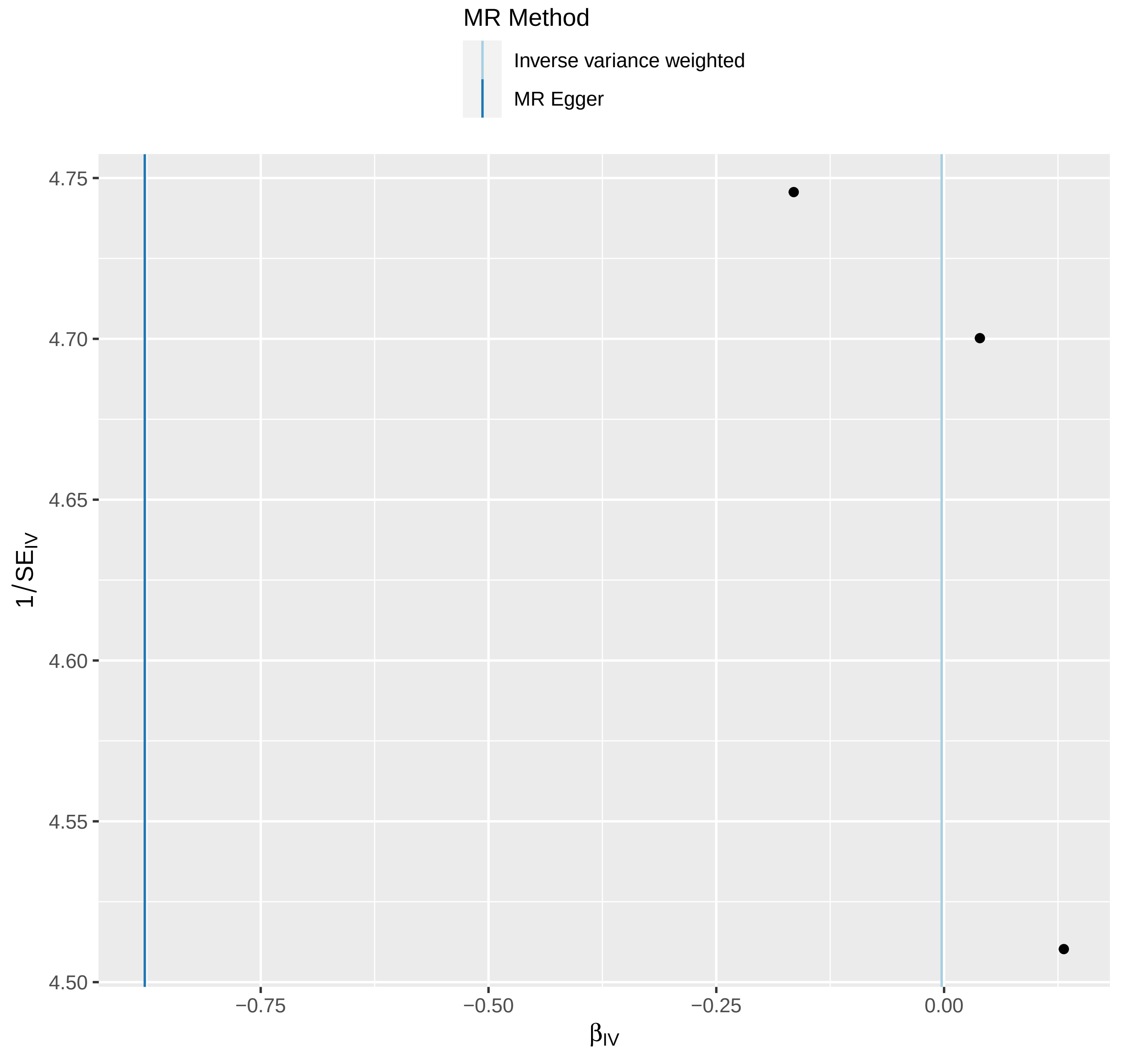

Batch 2−2932 : Gut microbiota abundance (genus Parasutterella id.2892) on Interleukin−11
with a threshold of 1e−05

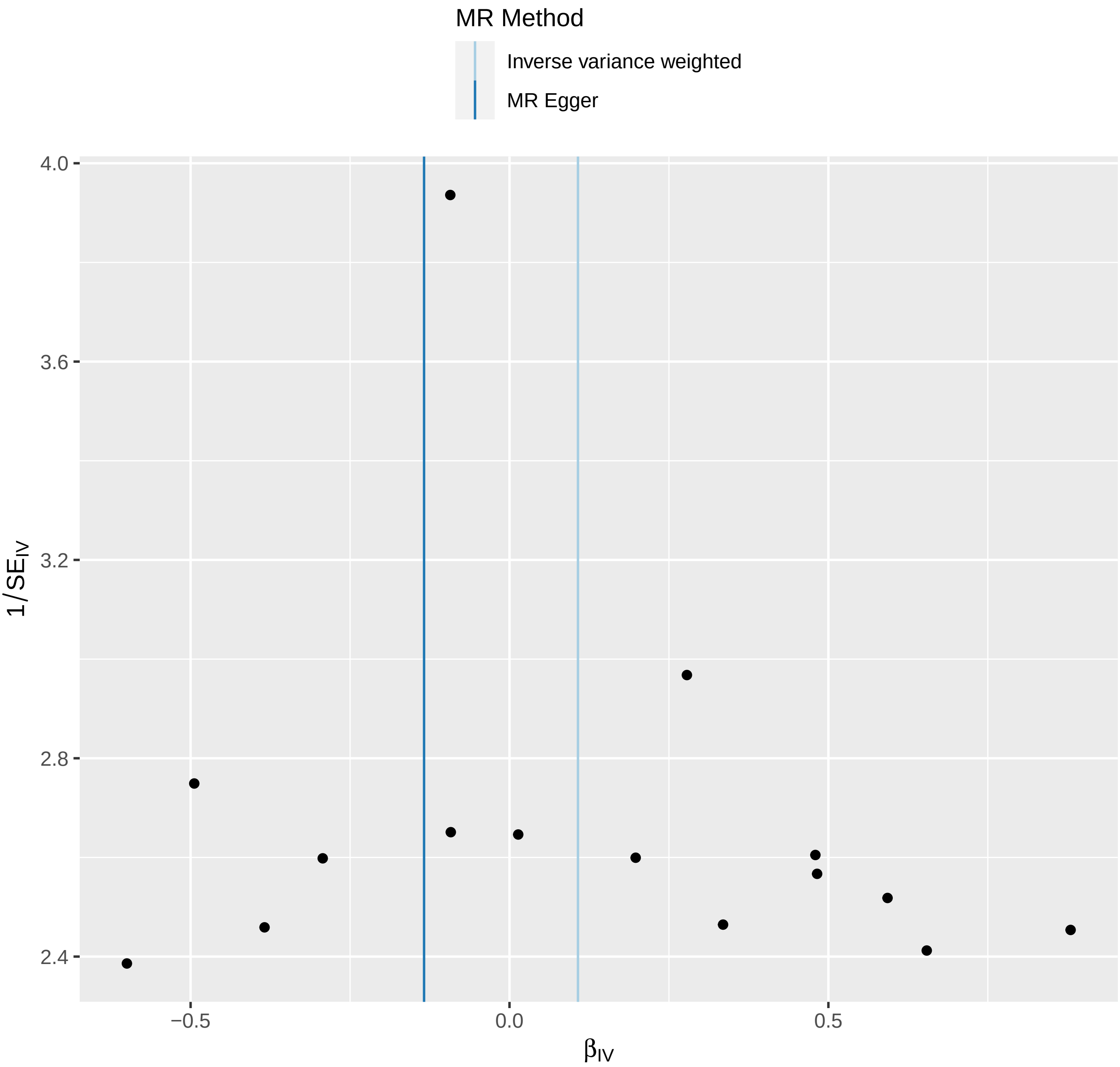

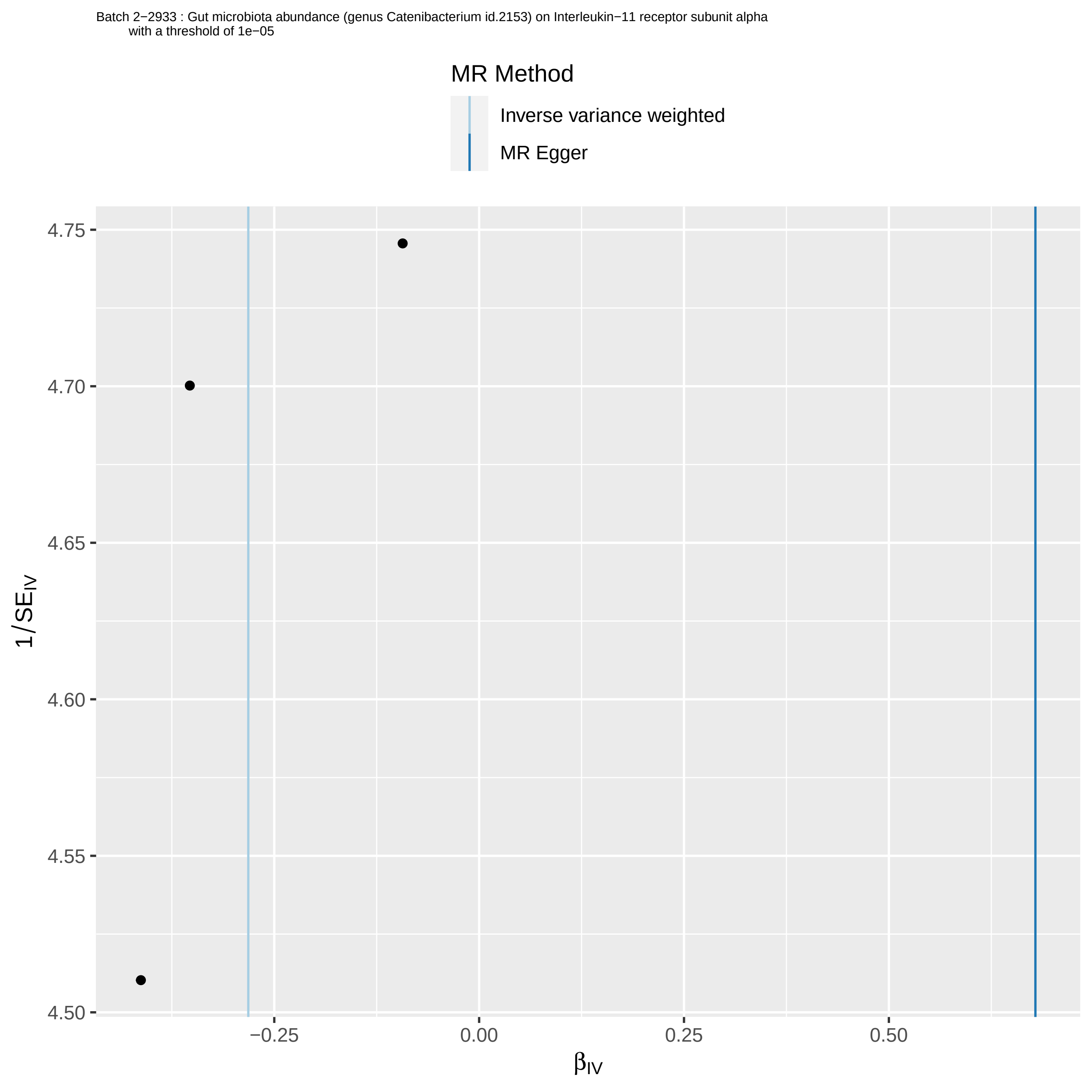

Batch 2−2933 : Gut microbiota abundance (genus Catenibacterium id.2153) on Interleukin−11 receptor subunit alpha
with a threshold of 1e−05
MR Method
Inverse variance weighted
MR Egger
4.75
4.70
4.65
4.60
4.55
4.50
1/SE_IV
−0.25
0.00
0.25
0.50
β_IV

Batch 2−2934 : Gut microbiota abundance (genus Parasutterella id.2892) on Interleukin−11 receptor subunit alpha
with a threshold of 1e−05

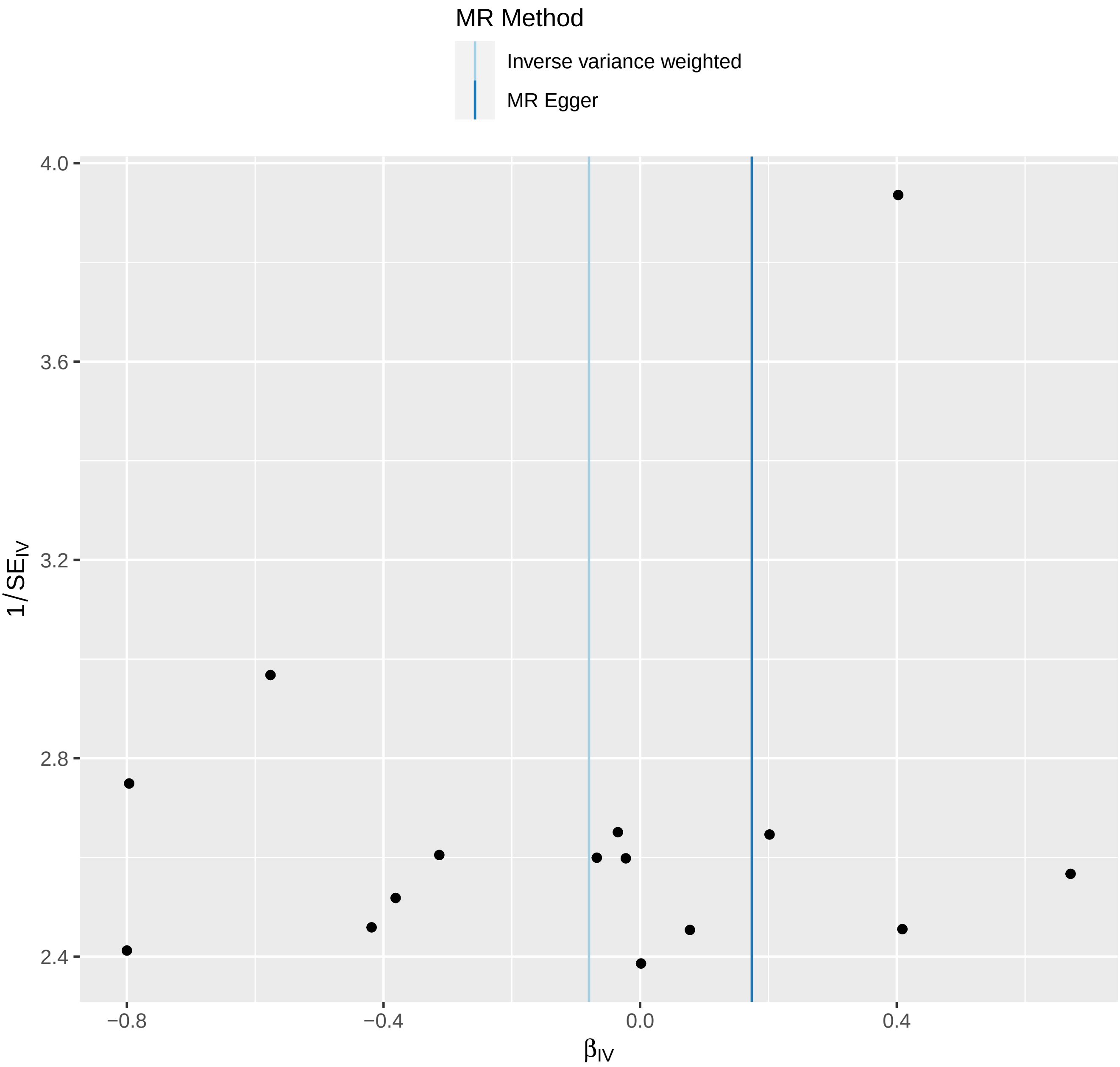

Batch 2−2935 : Gut microbiota abundance (genus Catenibacterium id.2153) on Interleukin−12
with a threshold of 1e−05

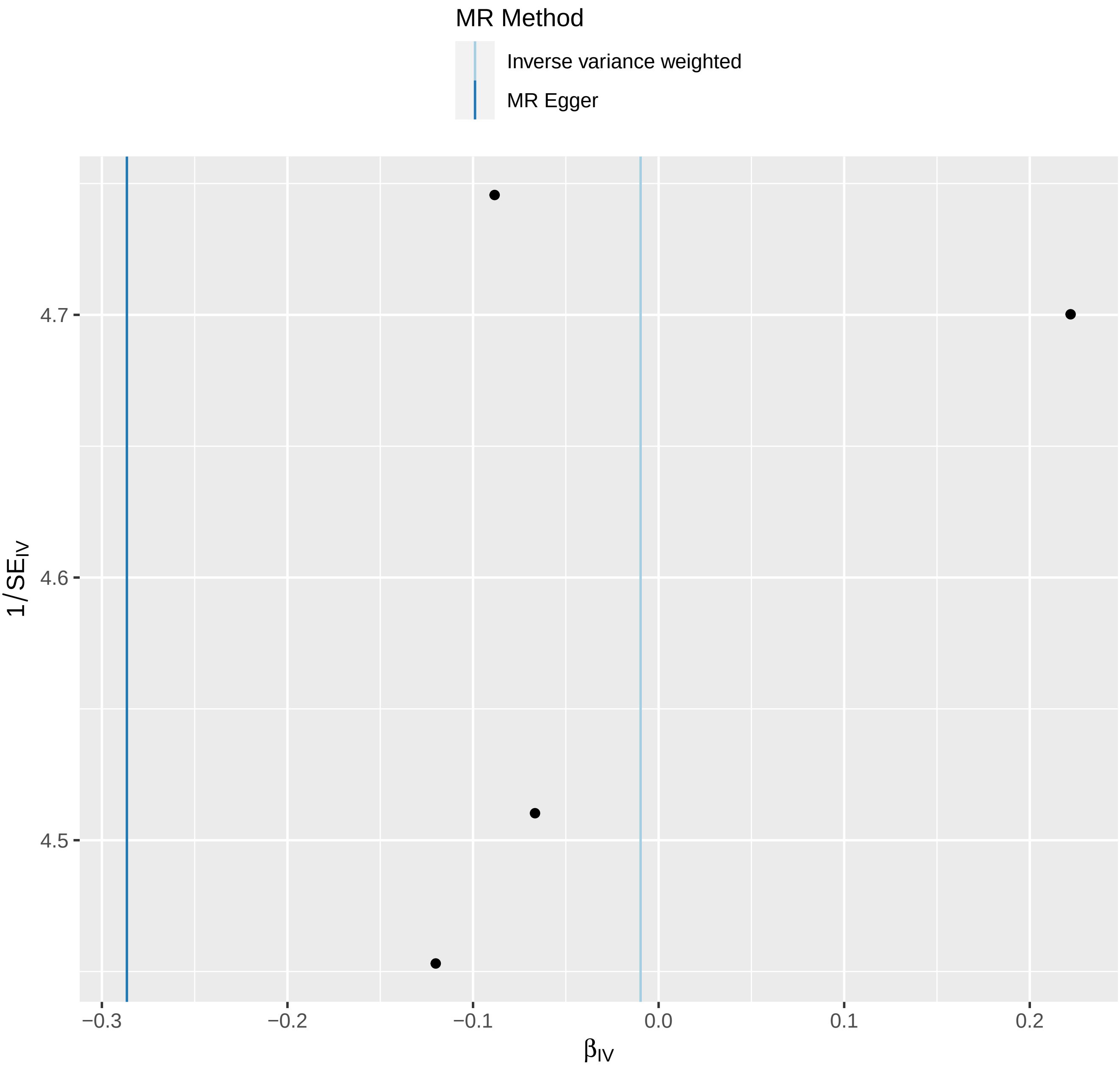

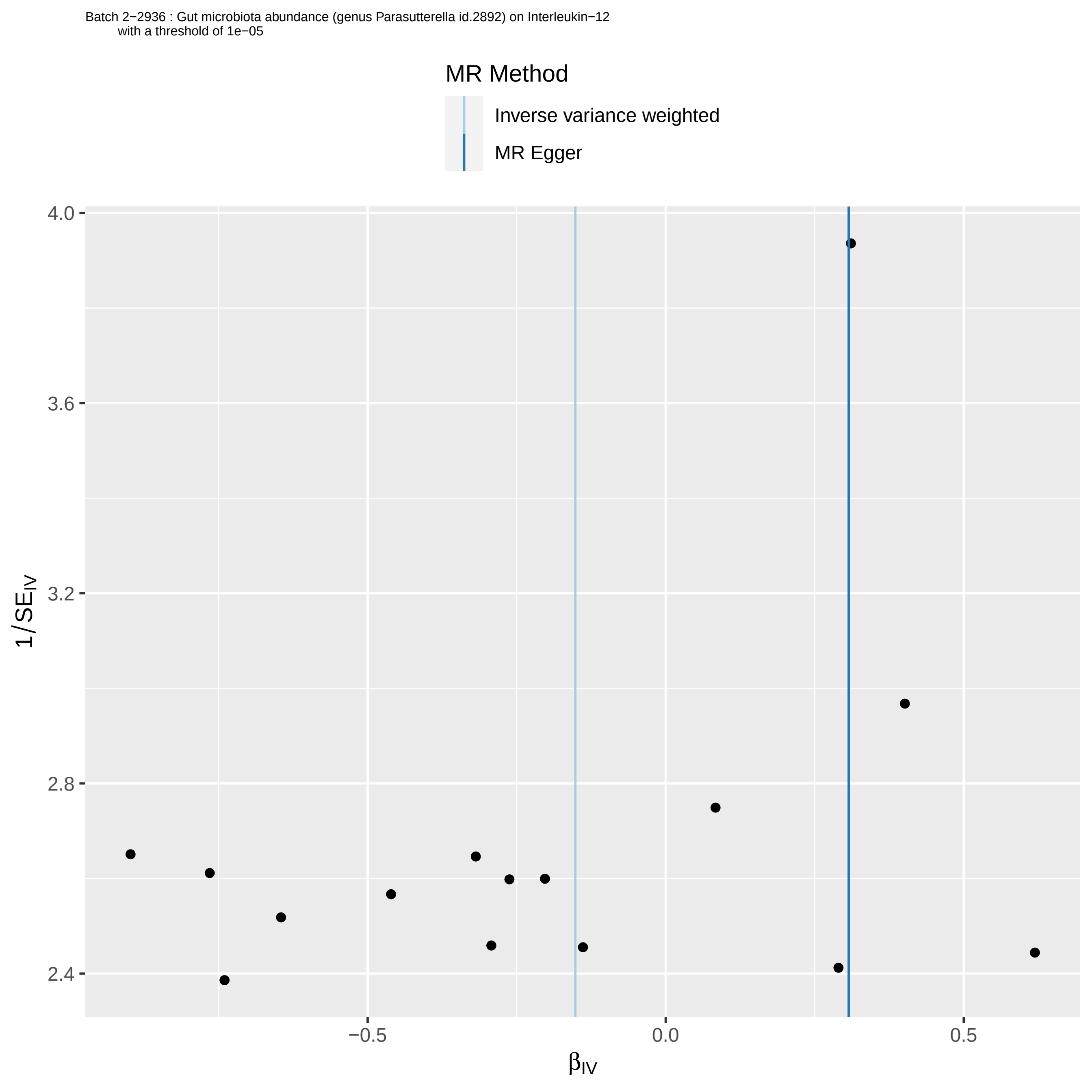

Batch 2−2936 : Gut microbiota abundance (genus Parasutterella id.2892) on Interleukin−12
with a threshold of 1e−05
MR Method
Inverse variance weighted
MR Egger
4.0
3.6
3.2
2.8
2.4
1/SE_IV
−0.5
0.0
0.5
β_IV

Batch 2−2937 : Gut microbiota abundance (genus Catenibacterium id.2153) on Interleukin−12
with a threshold of 1e−05

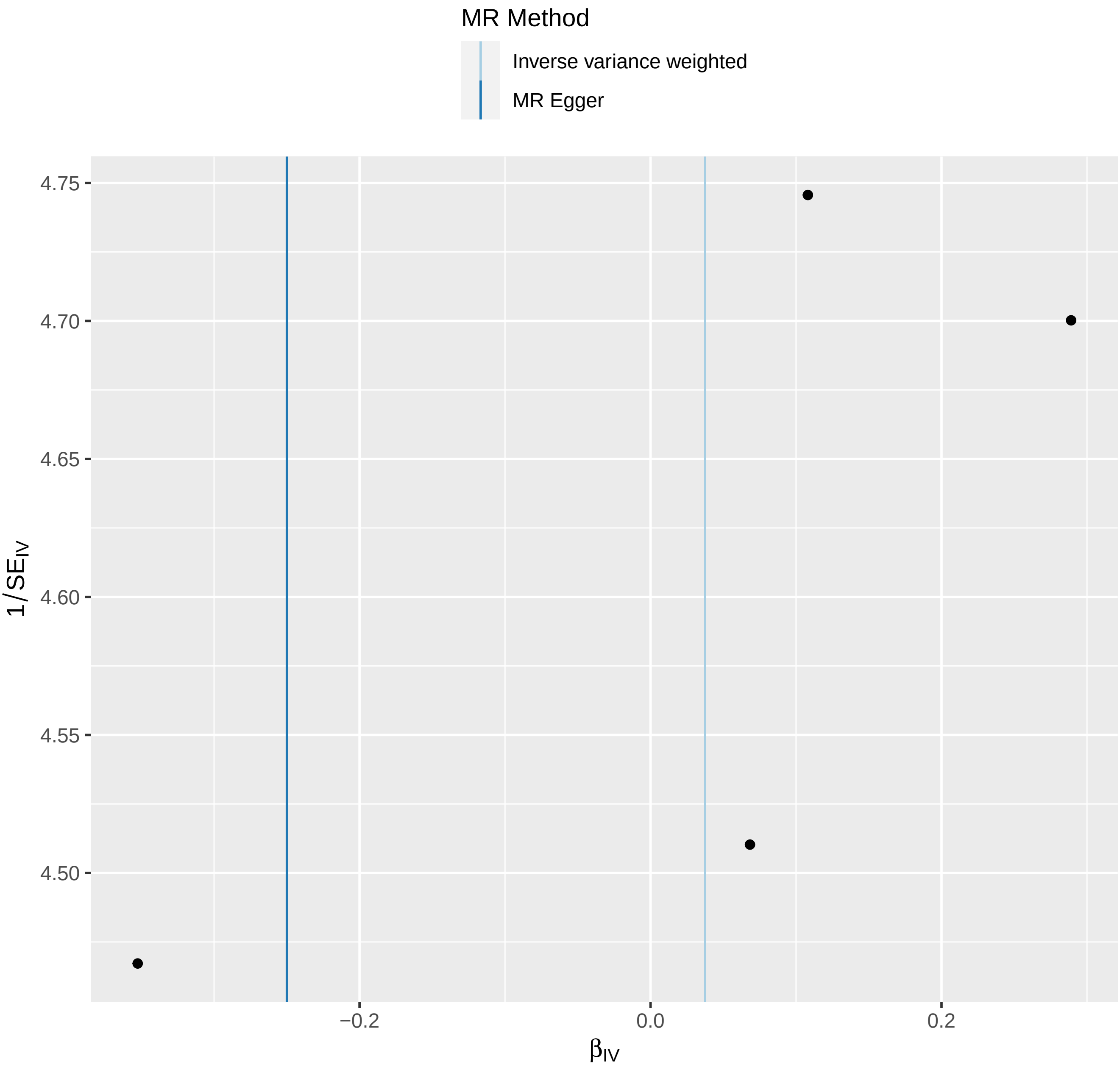

Batch 2−2938 : Gut microbiota abundance (genus Parasutterella id.2892) on Interleukin−12
with a threshold of 1e−05

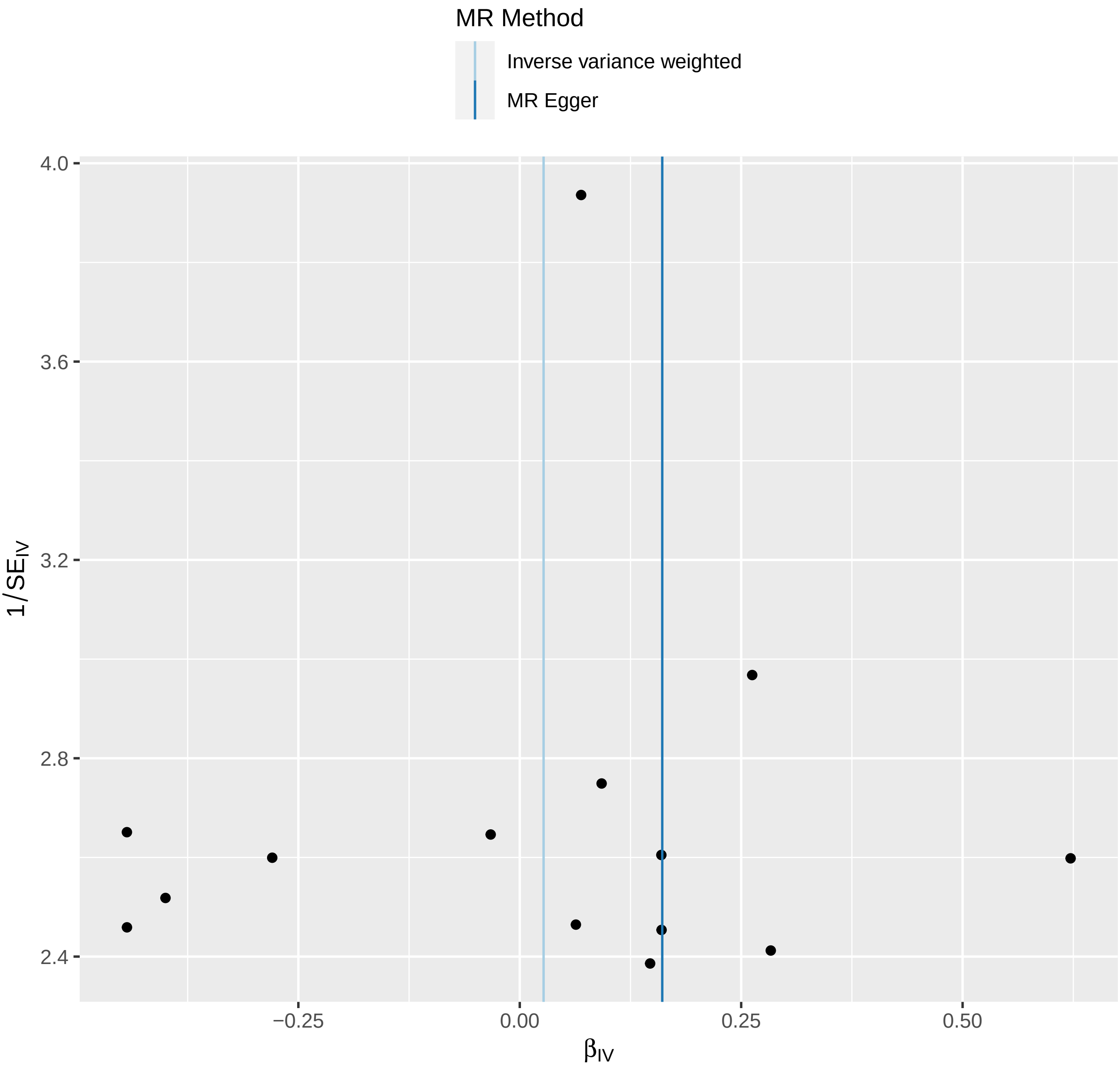

Batch 2−2939 : Gut microbiota abundance (genus Catenibacterium id.2153) on Interleukin−12
with a threshold of 1e−05

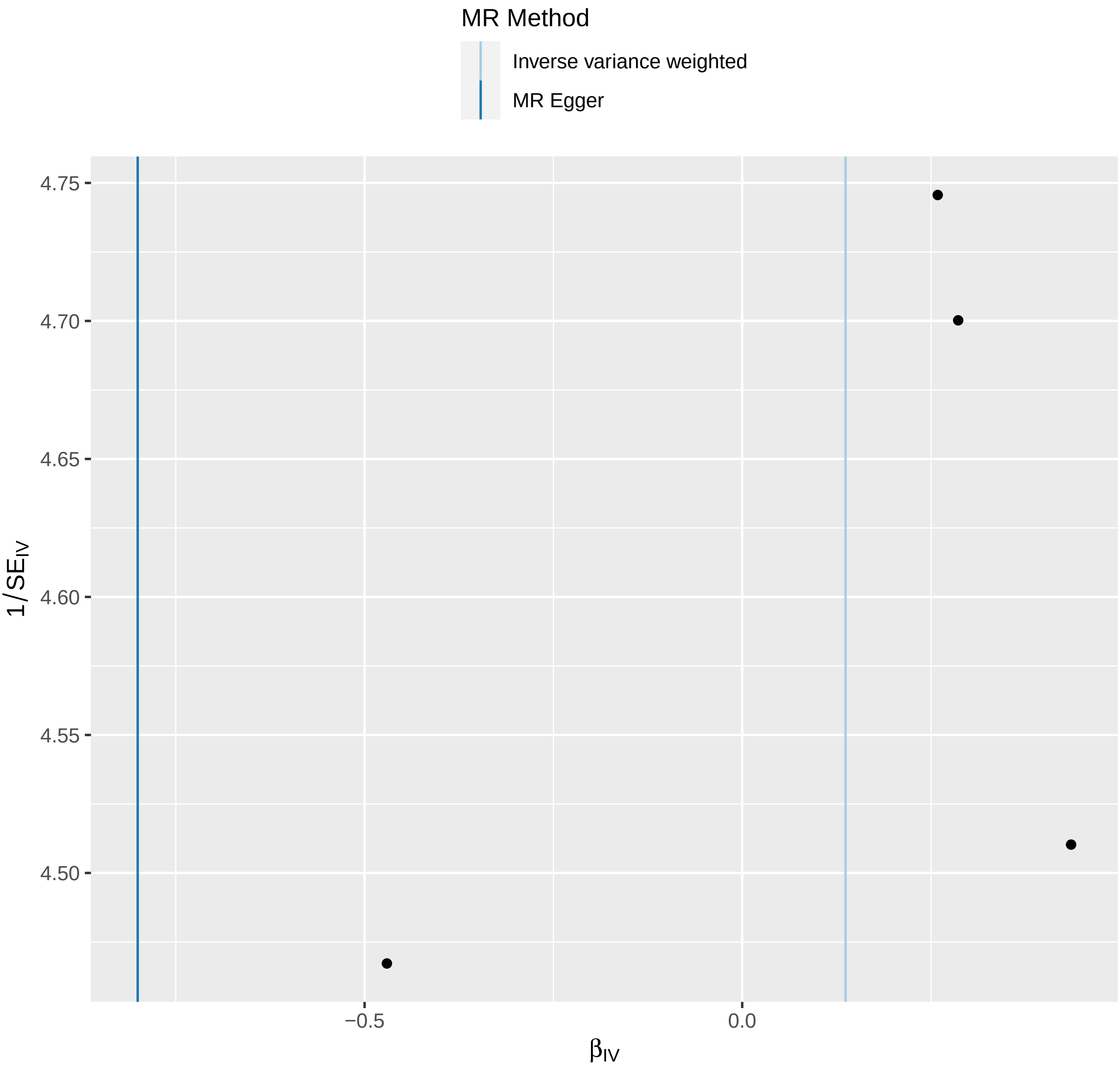

Batch 2−2940 : Gut microbiota abundance (genus Parasutterella id.2892) on Interleukin−12
with a threshold of 1e−05

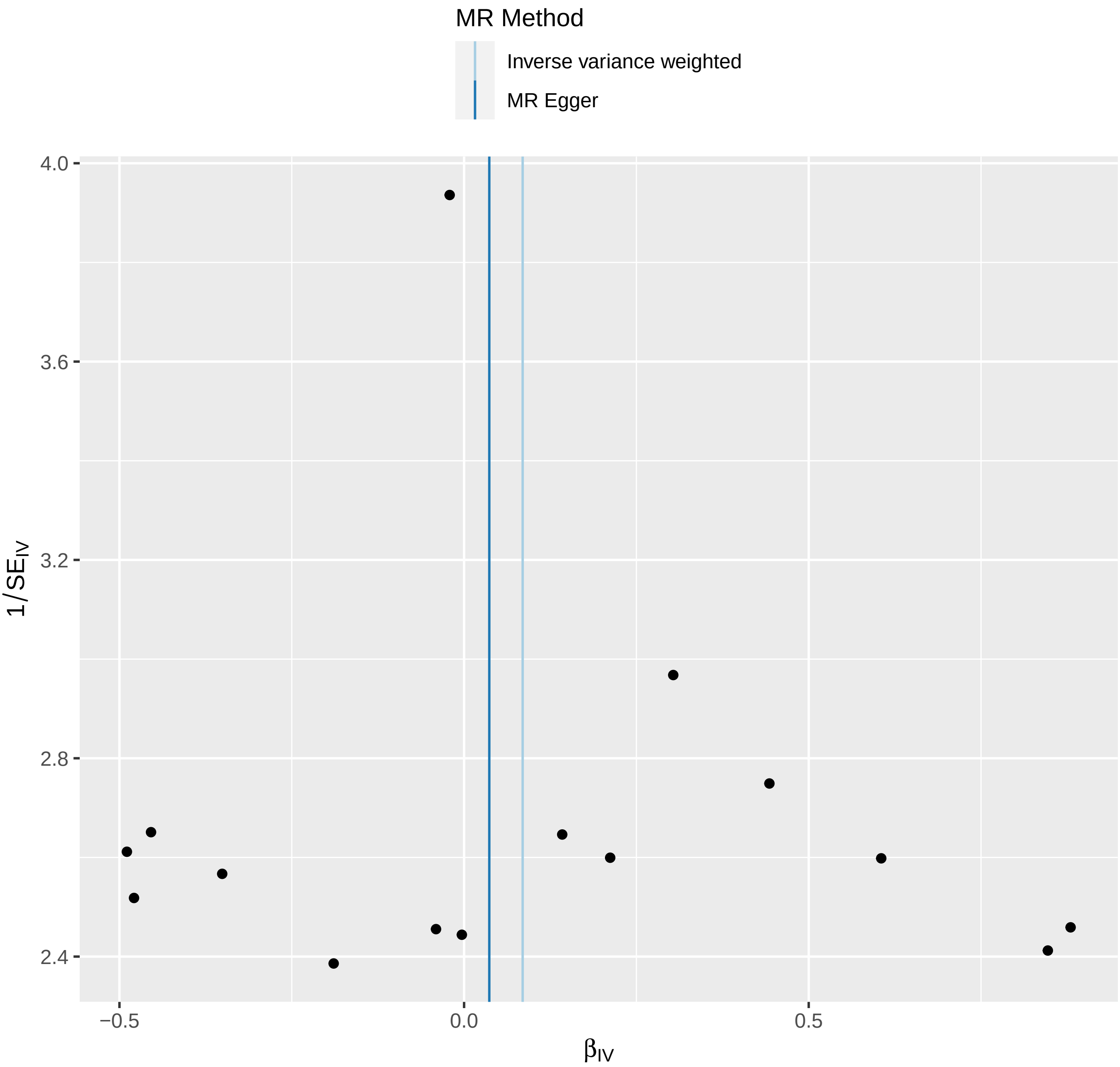

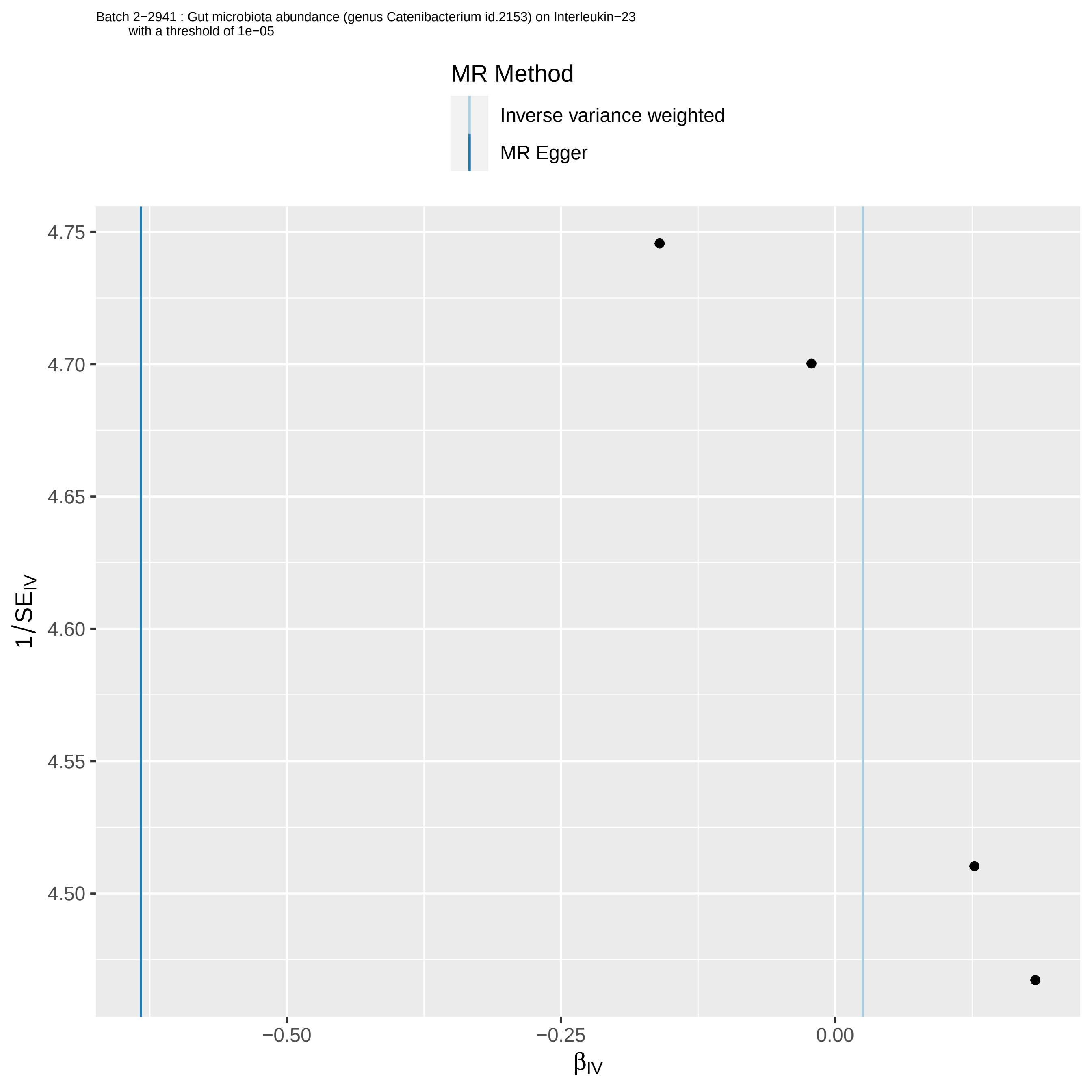

Batch 2−2941 : Gut microbiota abundance (genus Catenibacterium id.2153) on Interleukin−23
with a threshold of 1e−05
MR Method
Inverse variance weighted
MR Egger
4.75
4.70
4.65
4.60
4.55
4.50
1/SE_IV
−0.50
−0.25
0.00
β_IV

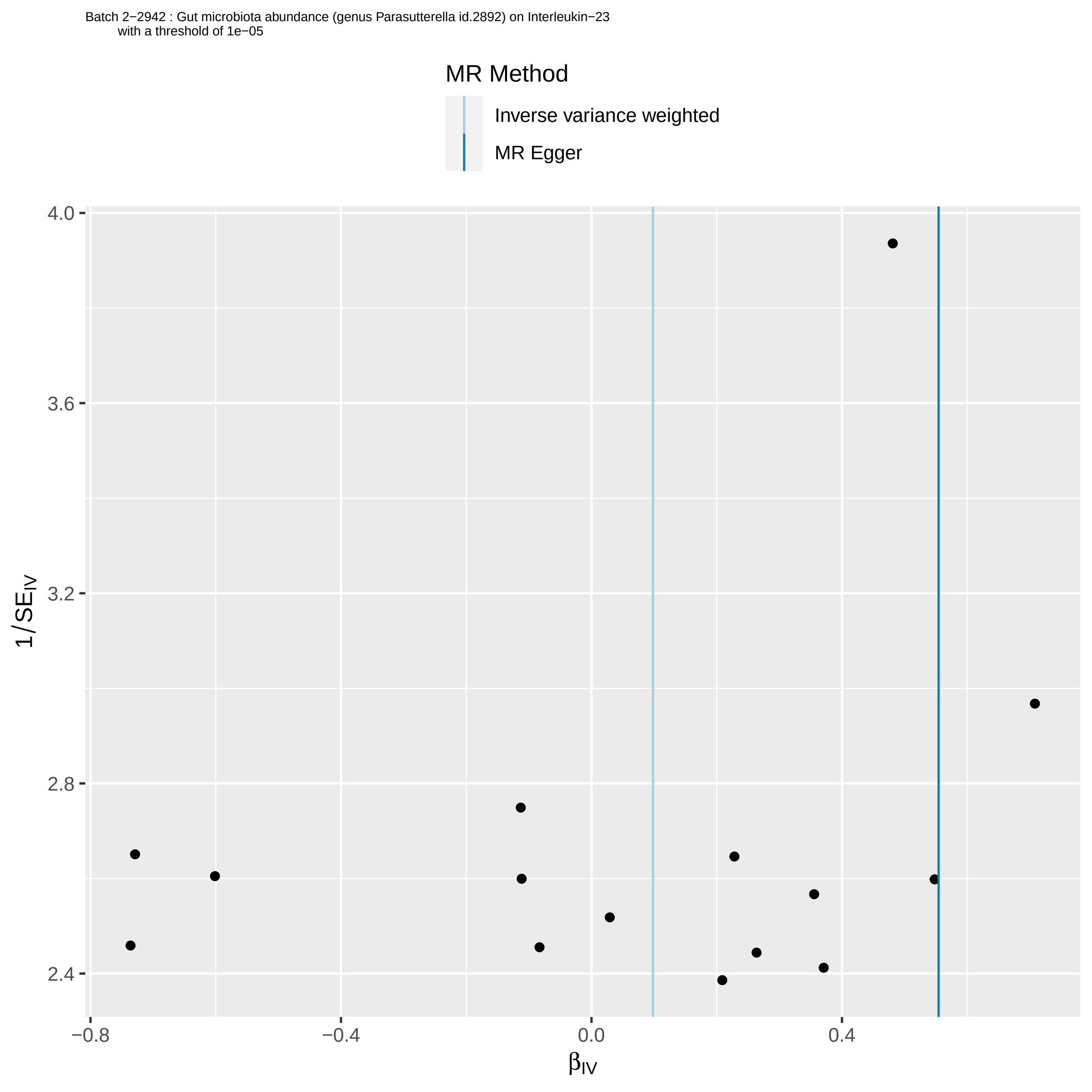

Batch 2−2942 : Gut microbiota abundance (genus Parasutterella id.2892) on Interleukin−23
with a threshold of 1e−05
MR Method
Inverse variance weighted
MR Egger
4.0
3.6
3.2
2.8
2.4
1/SE$_{IV}$
−0.8
−0.4
0.0
0.4
β$_{IV}$

Batch 2−2943 : Gut microbiota abundance (genus Catenibacterium id.2153) on Interleukin−23
with a threshold of 1e−05

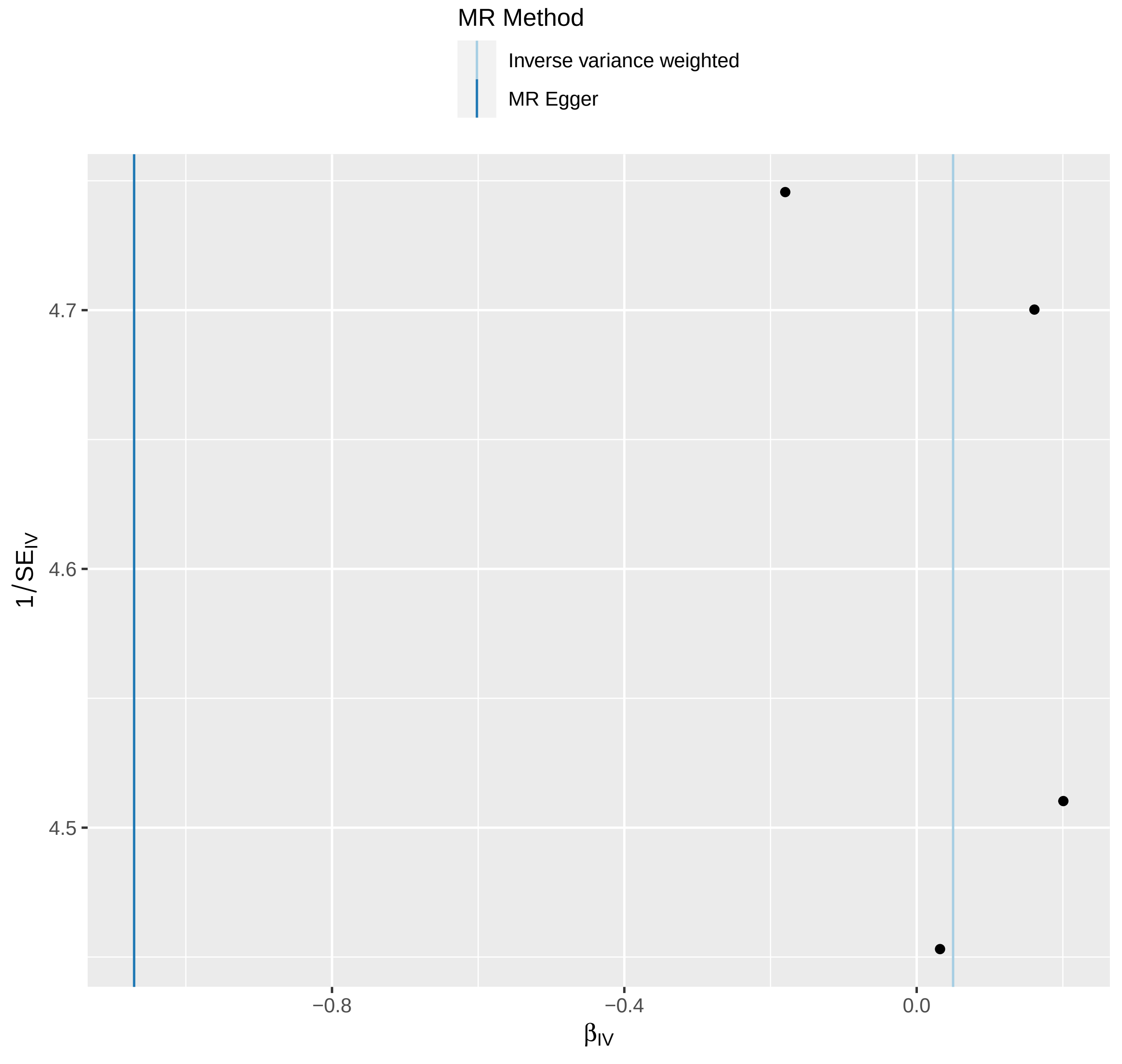

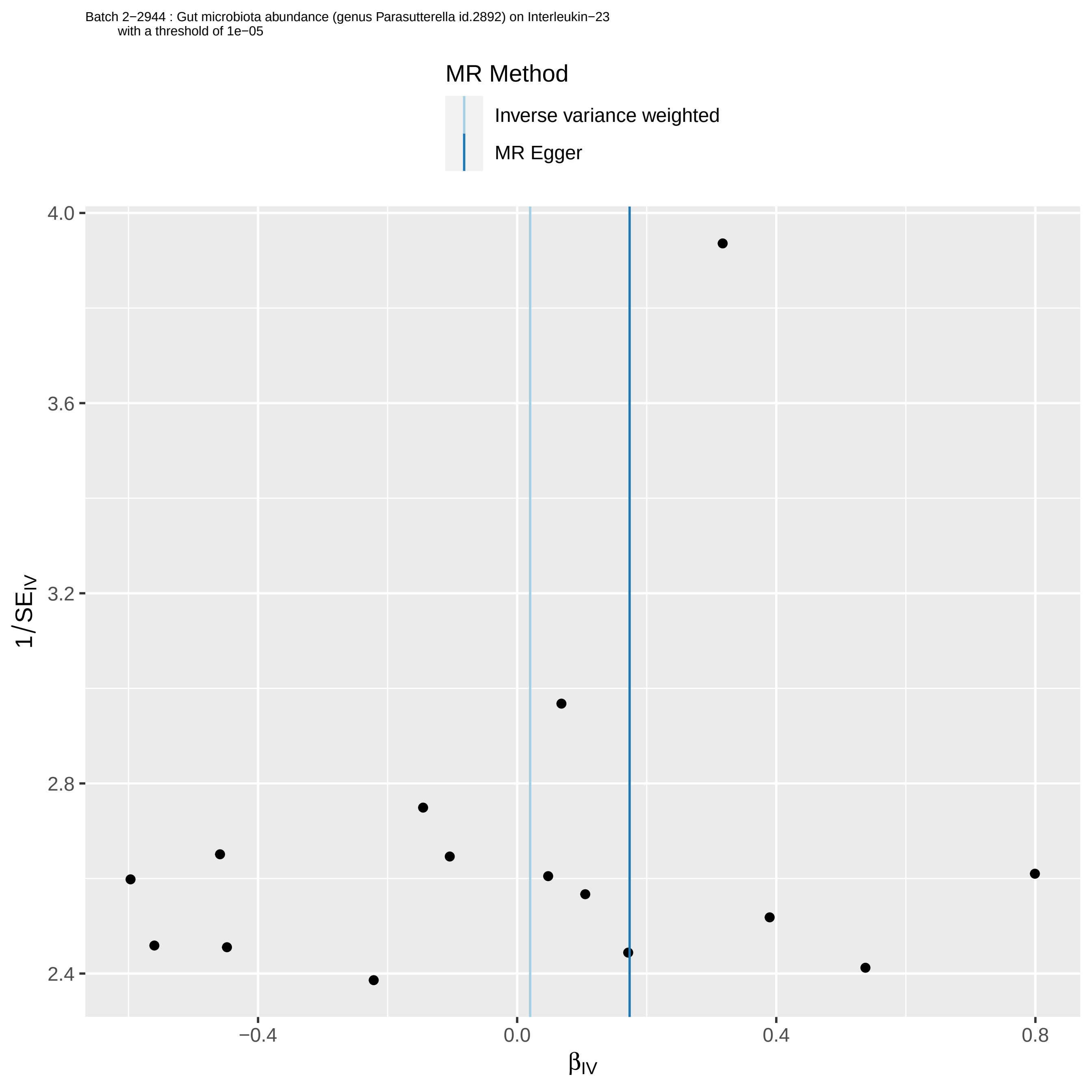

Batch 2−2944 : Gut microbiota abundance (genus Parasutterella id.2892) on Interleukin−23
with a threshold of 1e−05
MR Method
Inverse variance weighted
MR Egger
4.0
3.6
3.2
2.8
2.4
1/SE_IV
−0.4
0.0
0.4
0.8
β_IV

Batch 2−2945 : Gut microbiota abundance (genus Catenibacterium id.2153) on Interleukin−12 receptor subunit beta−1 with a threshold of 1e−05

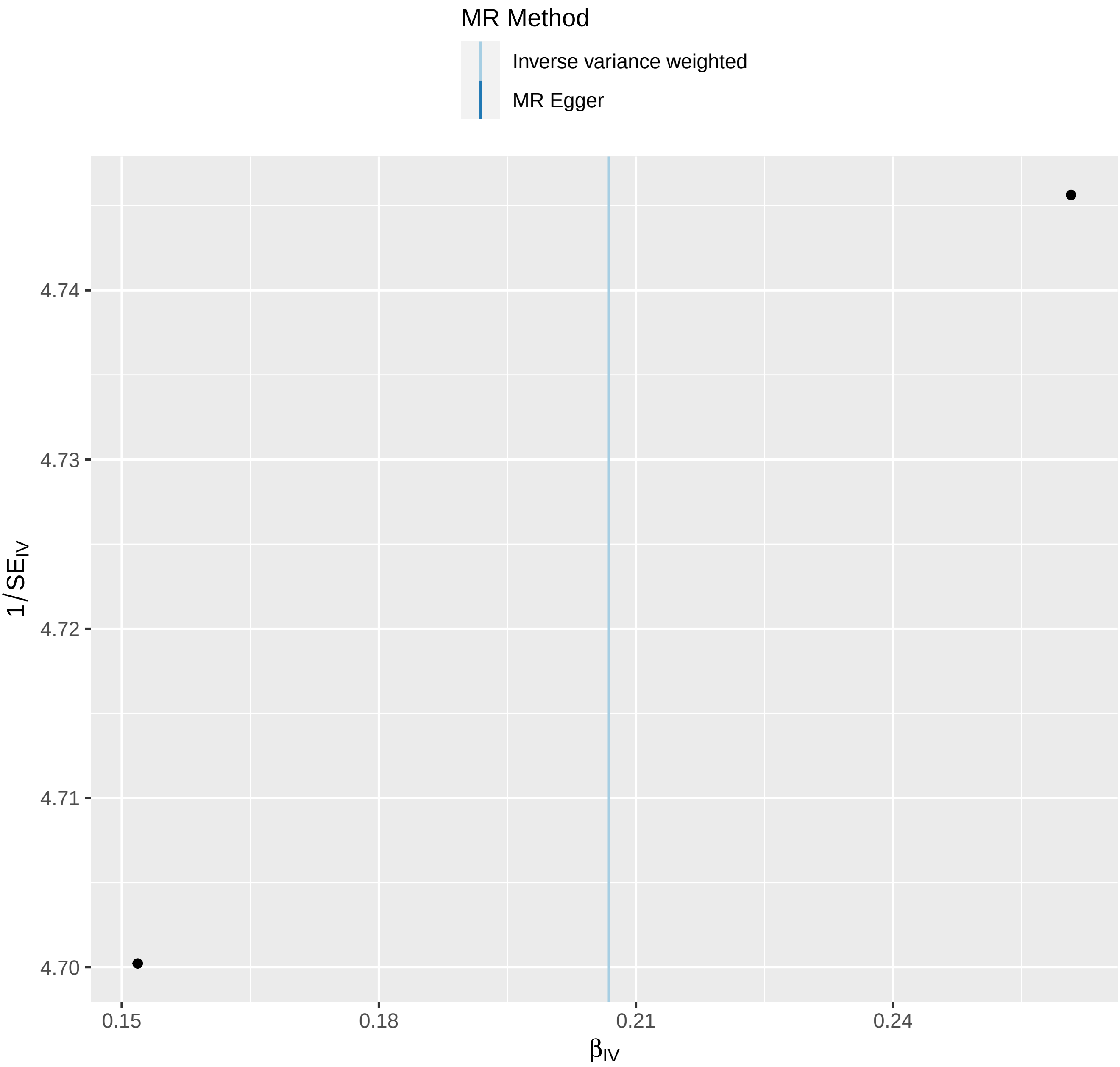

Batch 2−2946 : Gut microbiota abundance (genus Parasutterella id.2892) on Interleukin−12 receptor subunit beta−1
with a threshold of 1e−05

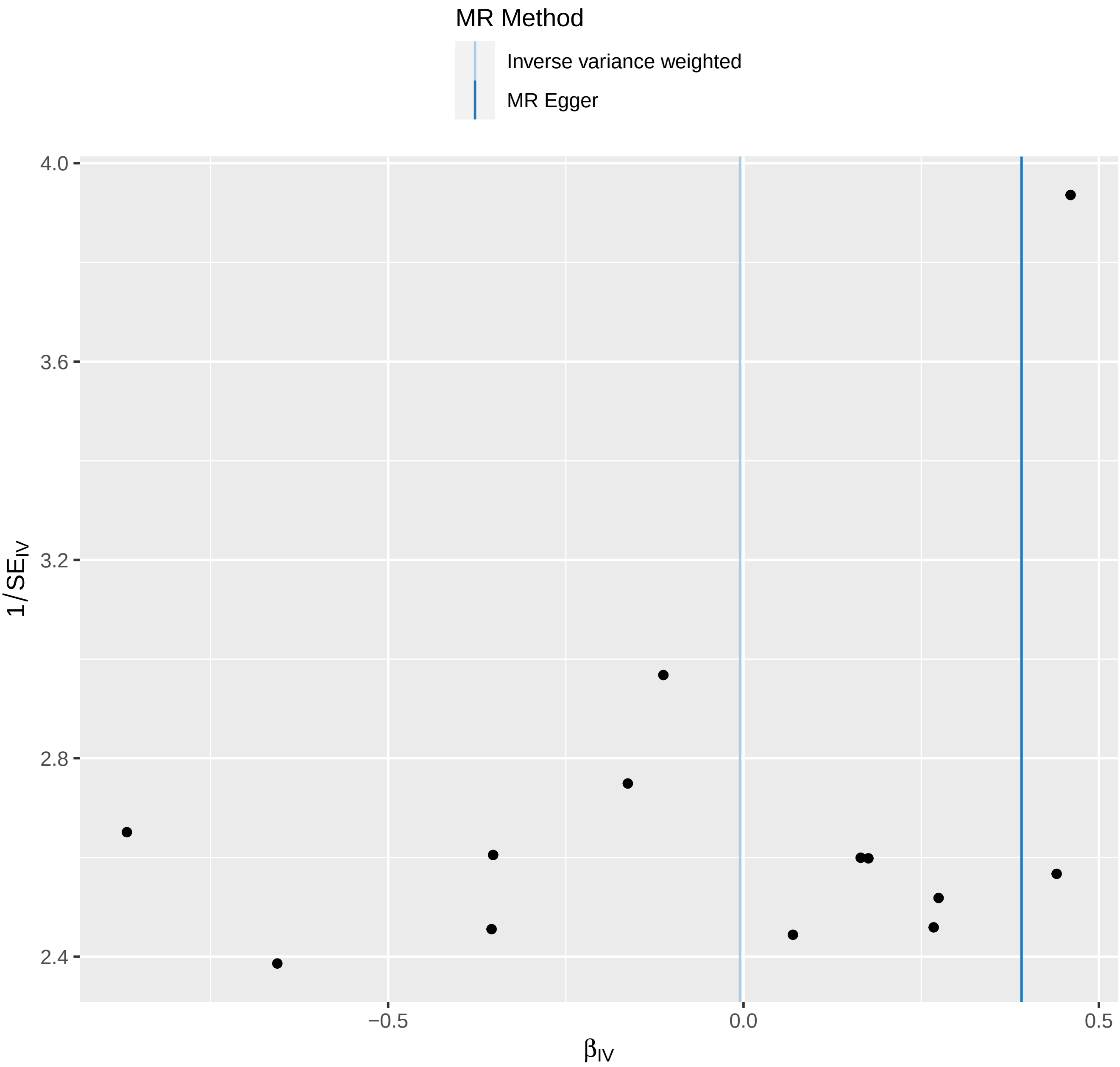

Batch 2−2947 : Gut microbiota abundance (genus Catenibacterium id.2153) on Interleukin−12 receptor subunit beta−2
with a threshold of 1e−05

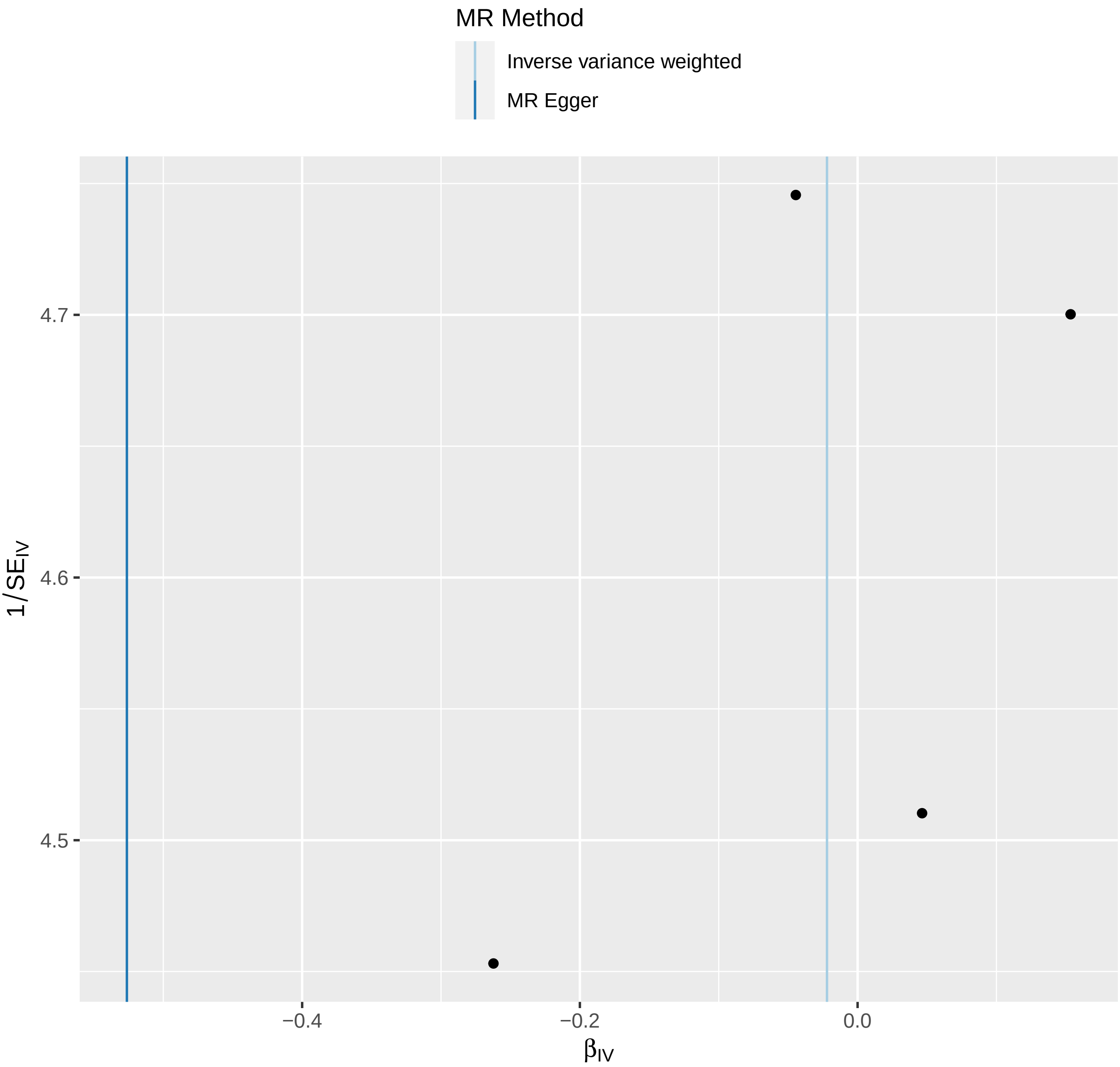

Batch 2−2948 : Gut microbiota abundance (genus Parasutterella id.2892) on Interleukin−12 receptor subunit beta−2
with a threshold of 1e−05

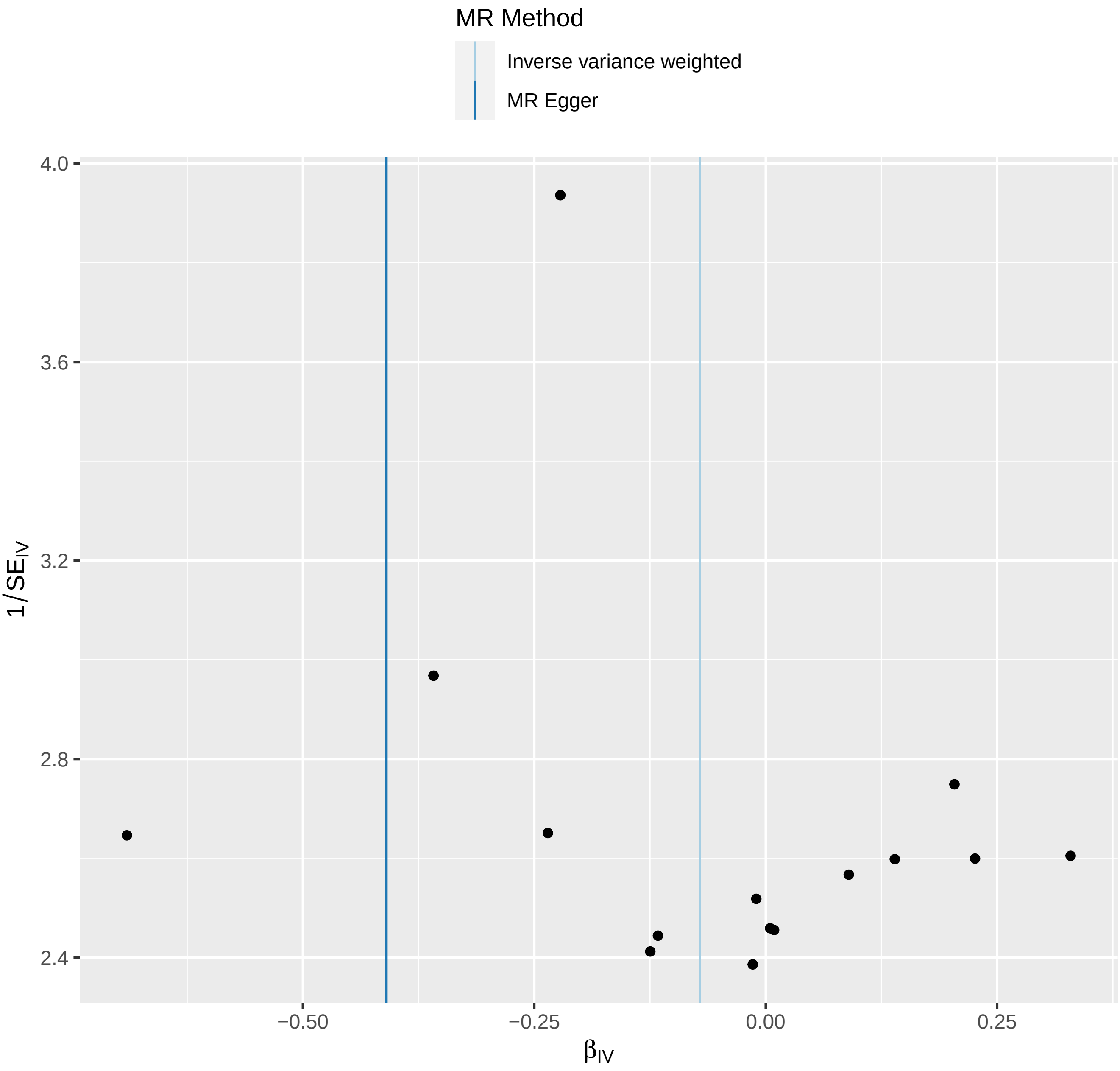

Batch 2−2949 : Gut microbiota abundance (genus Catenibacterium id.2153) on Interleukin−13
with a threshold of 1e−05

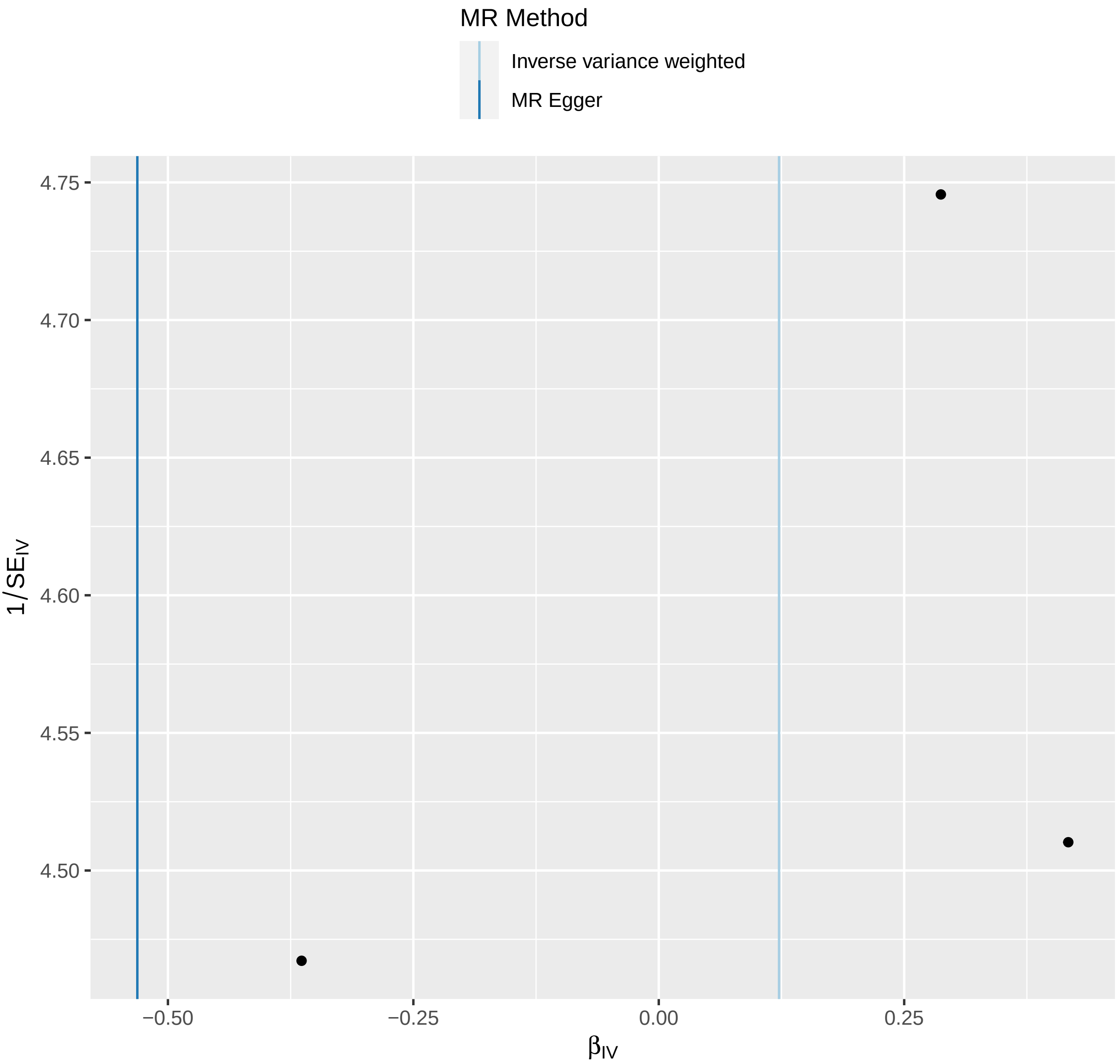

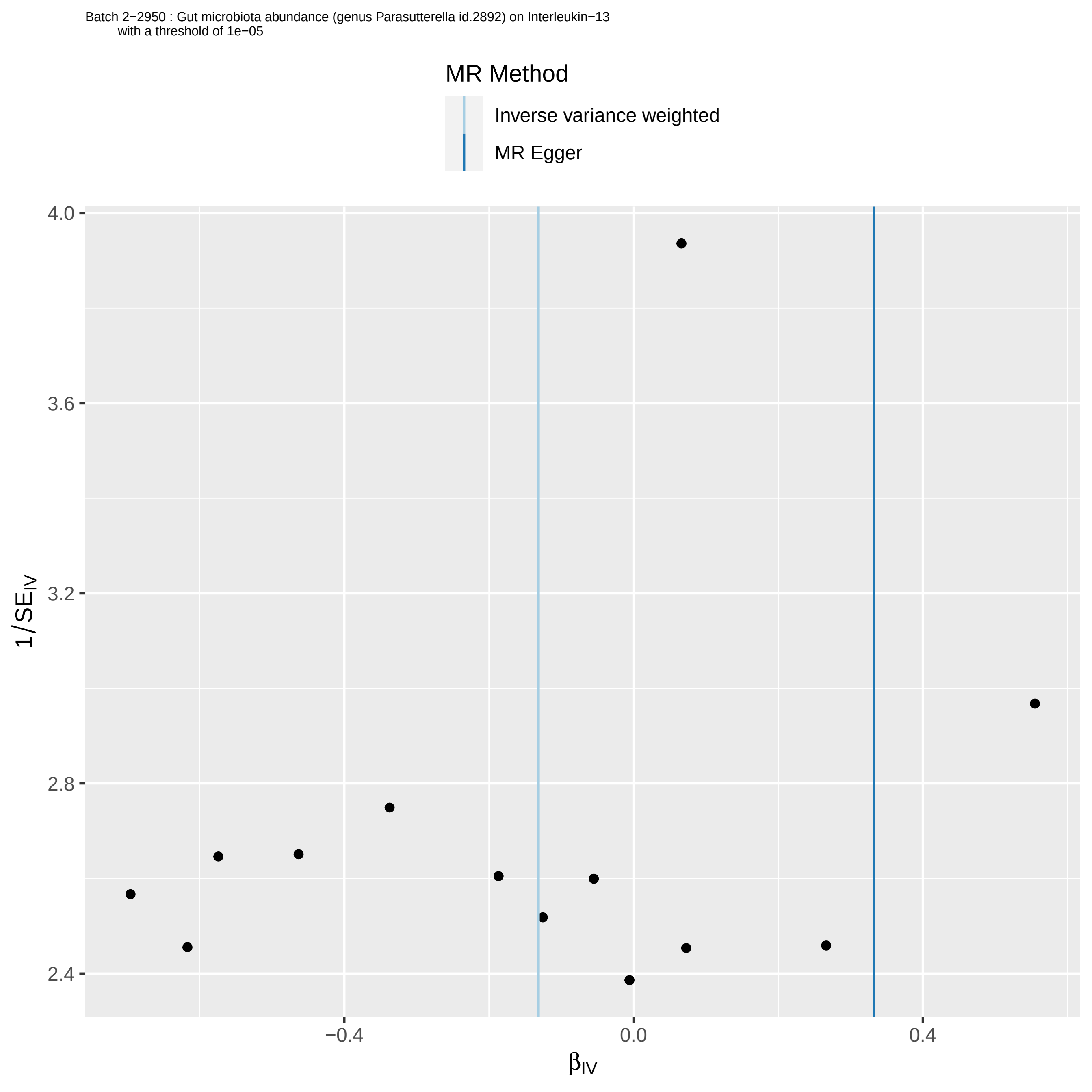

Batch 2−2950 : Gut microbiota abundance (genus Parasutterella id.2892) on Interleukin−13
with a threshold of 1e−05
MR Method
Inverse variance weighted
MR Egger
4.0
3.6
3.2
2.8
2.4
1/SE_IV
−0.4
0.0
0.4
β_IV

Batch 2−2951 : Gut microbiota abundance (genus Catenibacterium id.2153) on Interleukin−13 receptor subunit alpha−1 with a threshold of 1e−05

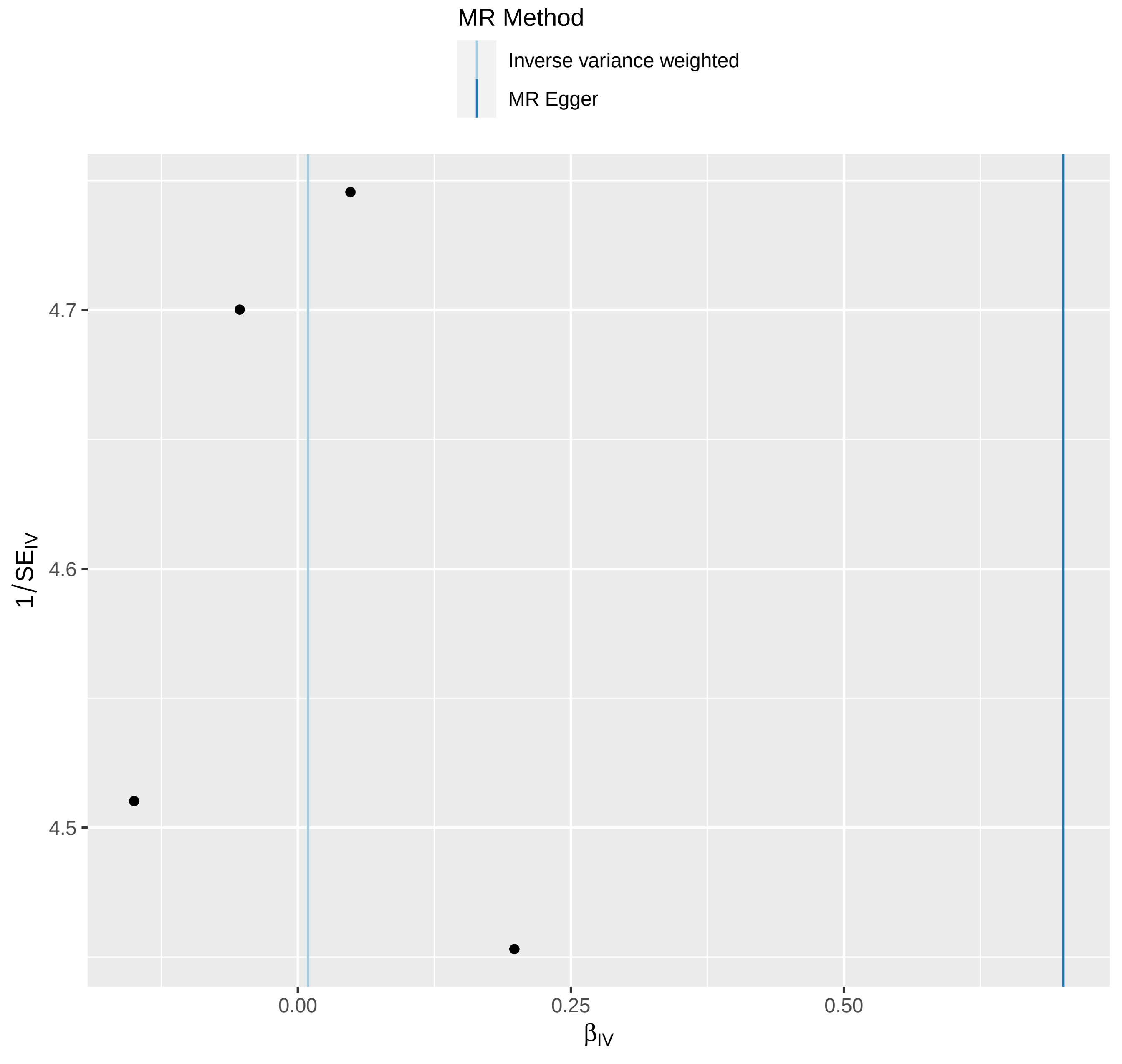

Batch 2−2952 : Gut microbiota abundance (genus Parasutterella id.2892) on Interleukin−13 receptor subunit alpha−1
with a threshold of 1e−05

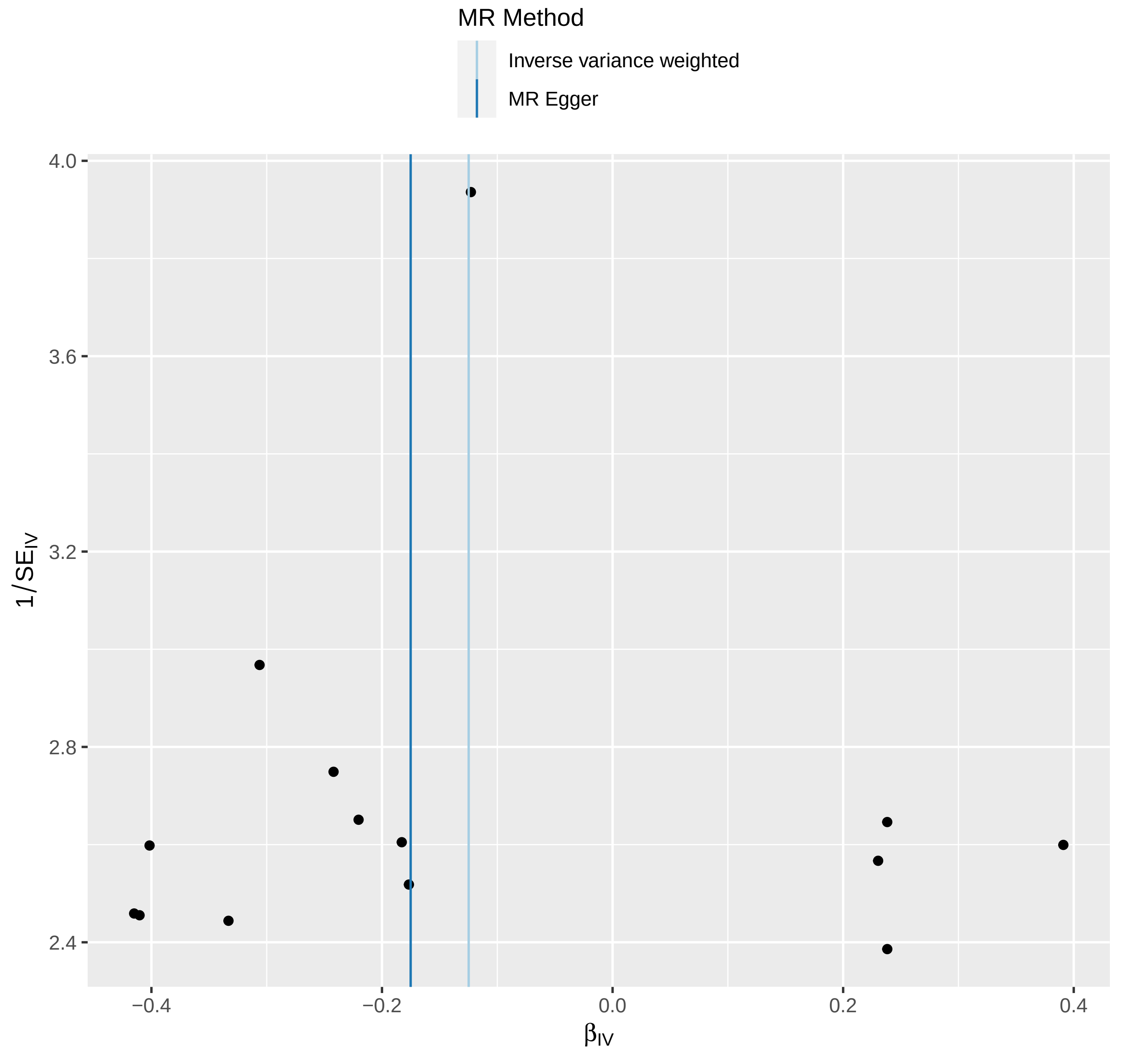

Batch 2−2953 : Gut microbiota abundance (genus Catenibacterium id.2153) on Interleukin−15 receptor subunit alpha with a threshold of 1e−05

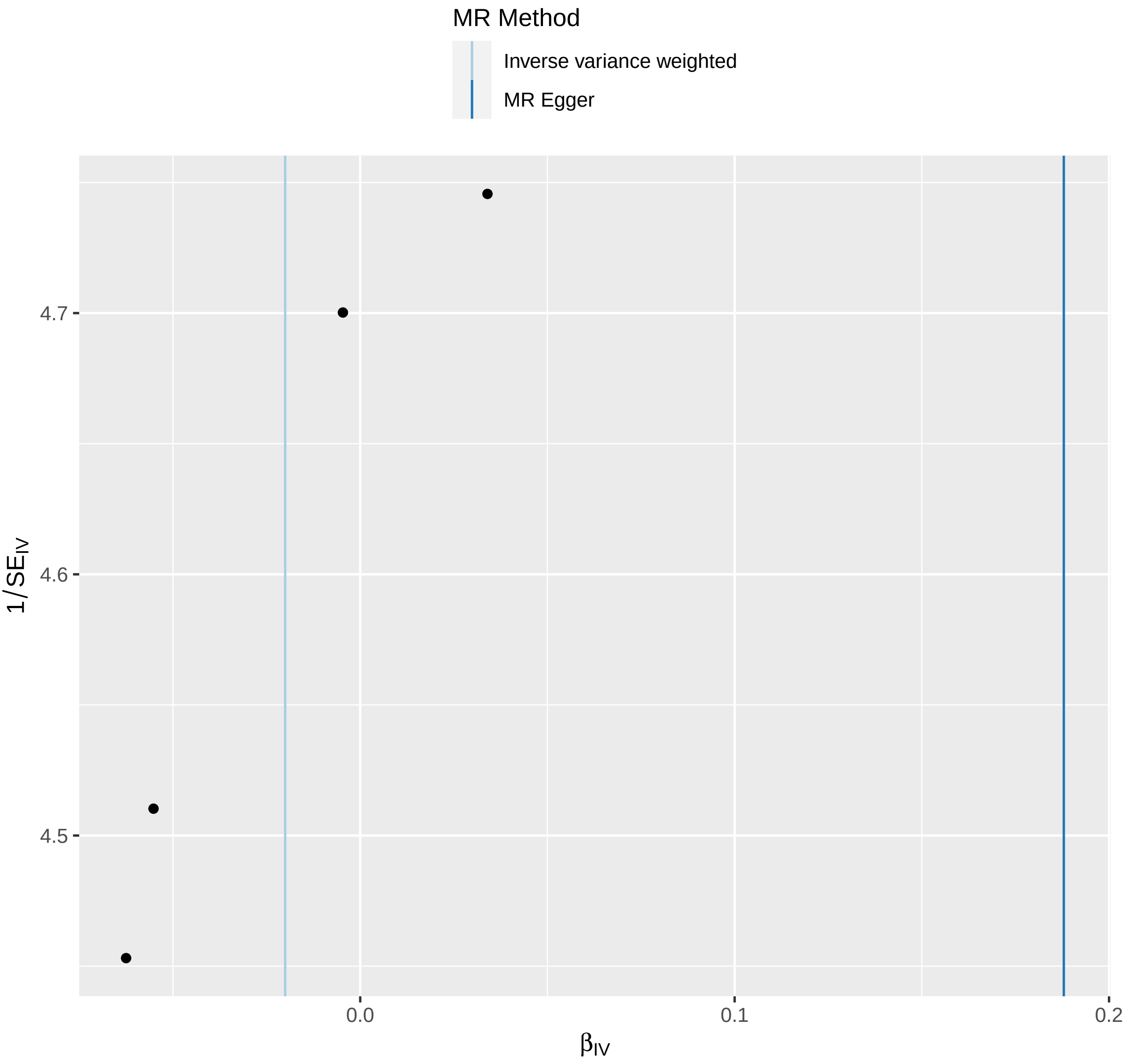

Batch 2−2954 : Gut microbiota abundance (genus Parasutterella id.2892) on Interleukin−15 receptor subunit alpha
with a threshold of 1e−05

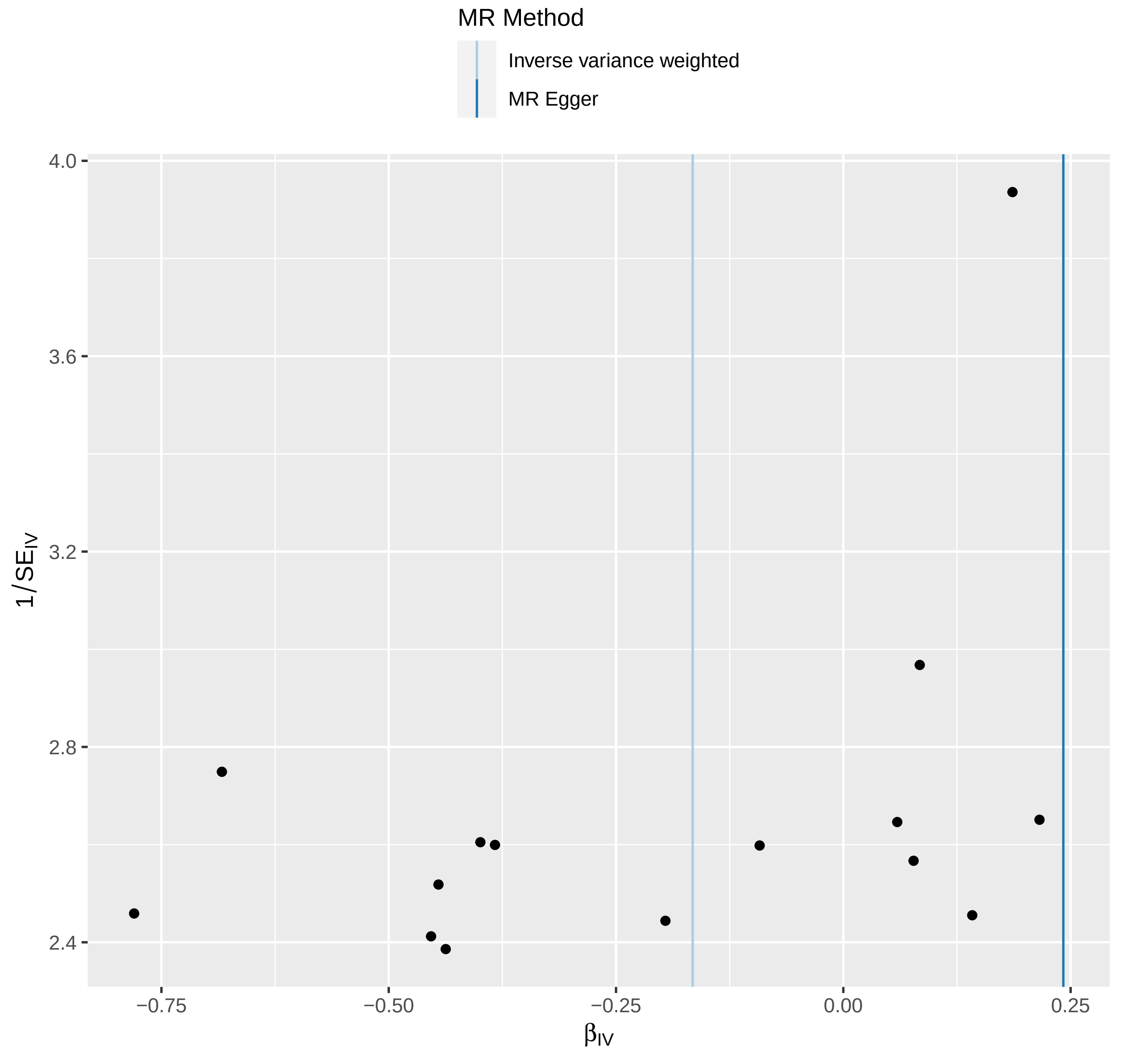

Batch 2−2955 : Gut microbiota abundance (genus Catenibacterium id.2153) on Interleukin−15 receptor subunit alpha
with a threshold of 1e−05

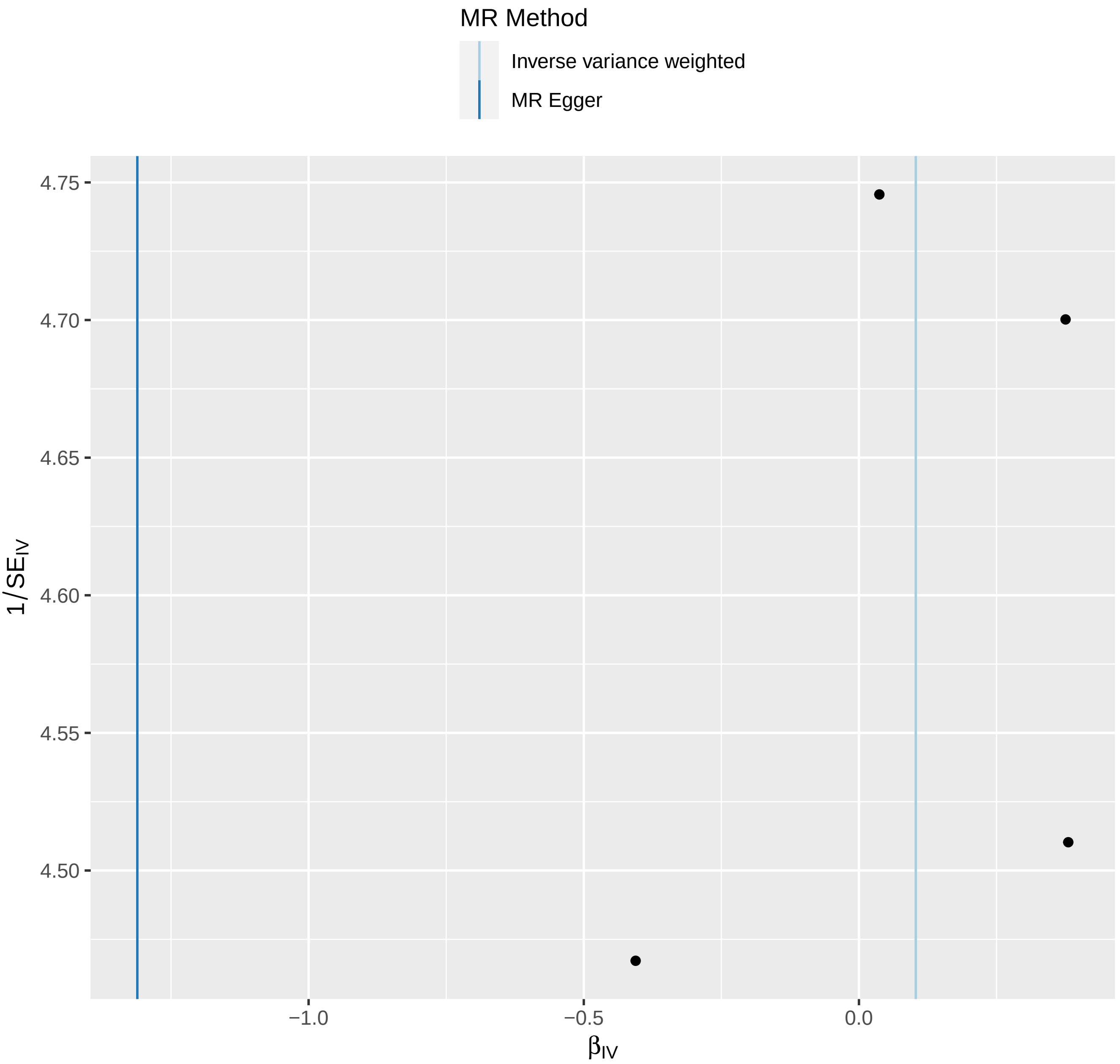

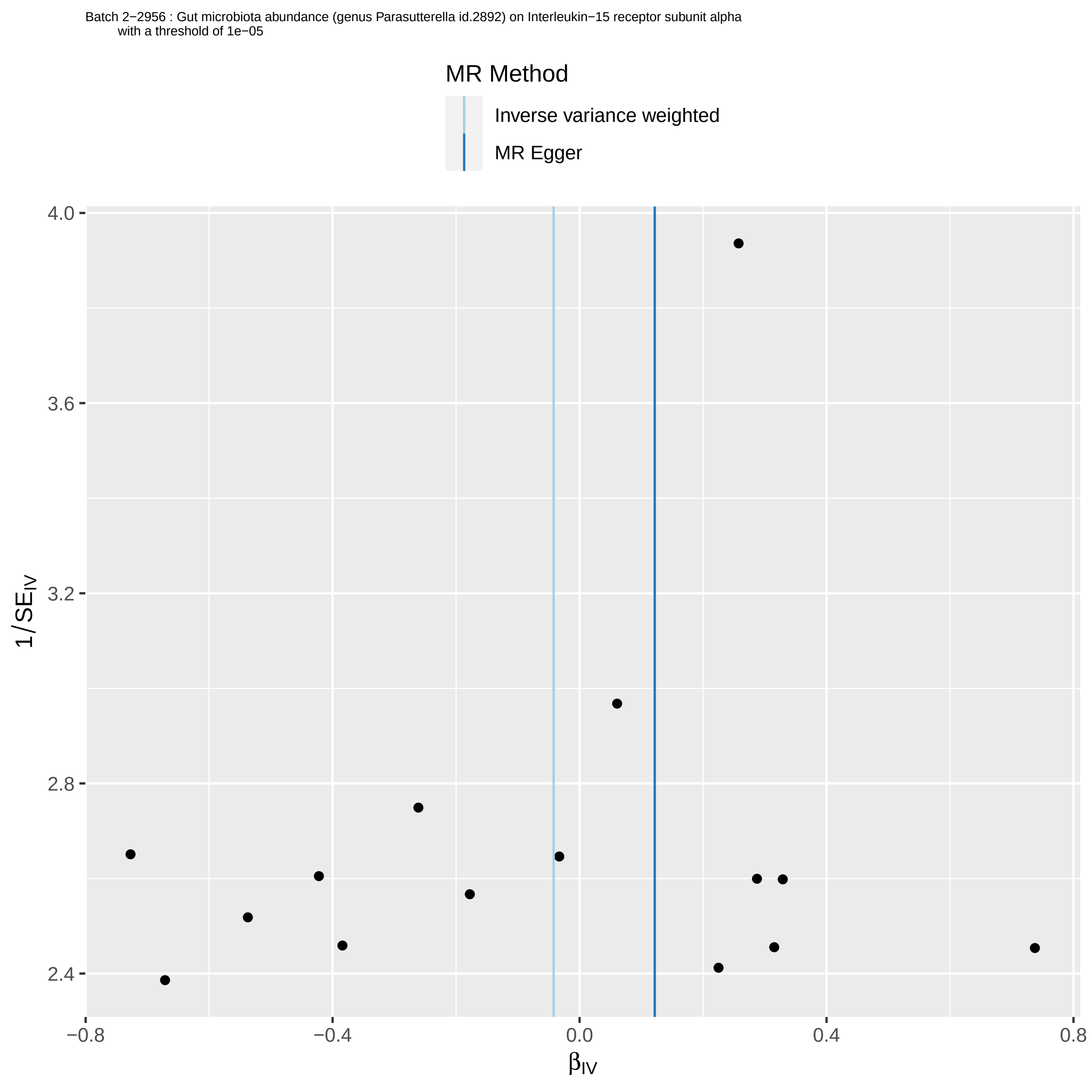
Batch 2−2956 : Gut microbiota abundance (genus Parasutterella id.2892) on Interleukin−15 receptor subunit alpha
with a threshold of 1e−05
MR Method
Inverse variance weighted
MR Egger
4.0
3.6
3.2
2.8
2.4
1/SE_IV
−0.8
−0.4
0.0
0.4
0.8
β_IV

Batch 2−2957 : Gut microbiota abundance (genus Catenibacterium id.2153) on Interleukin−16
with a threshold of 1e−05

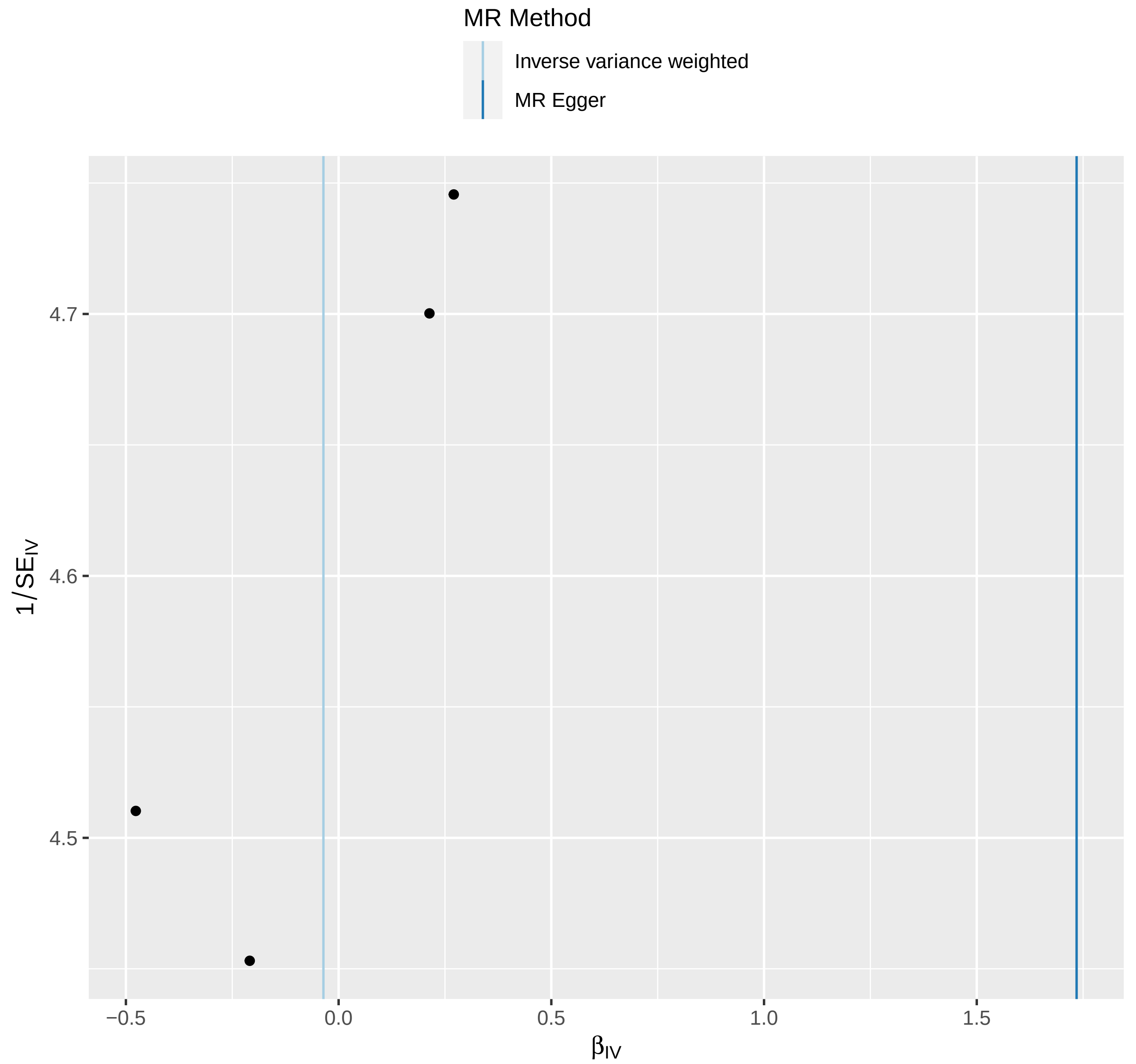

Batch 2−2958 : Gut microbiota abundance (genus Parasutterella id.2892) on Interleukin−16
with a threshold of 1e−05

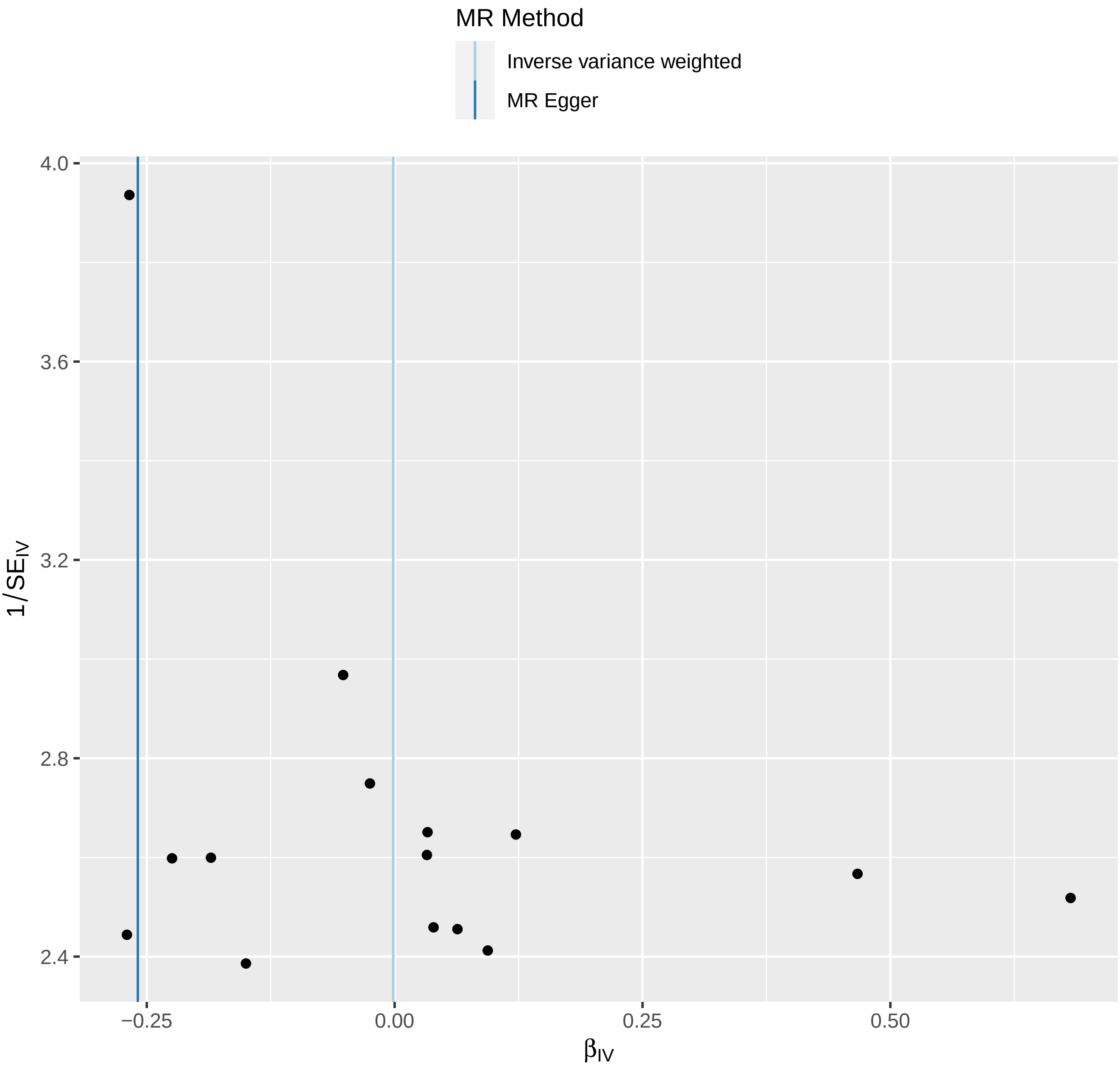

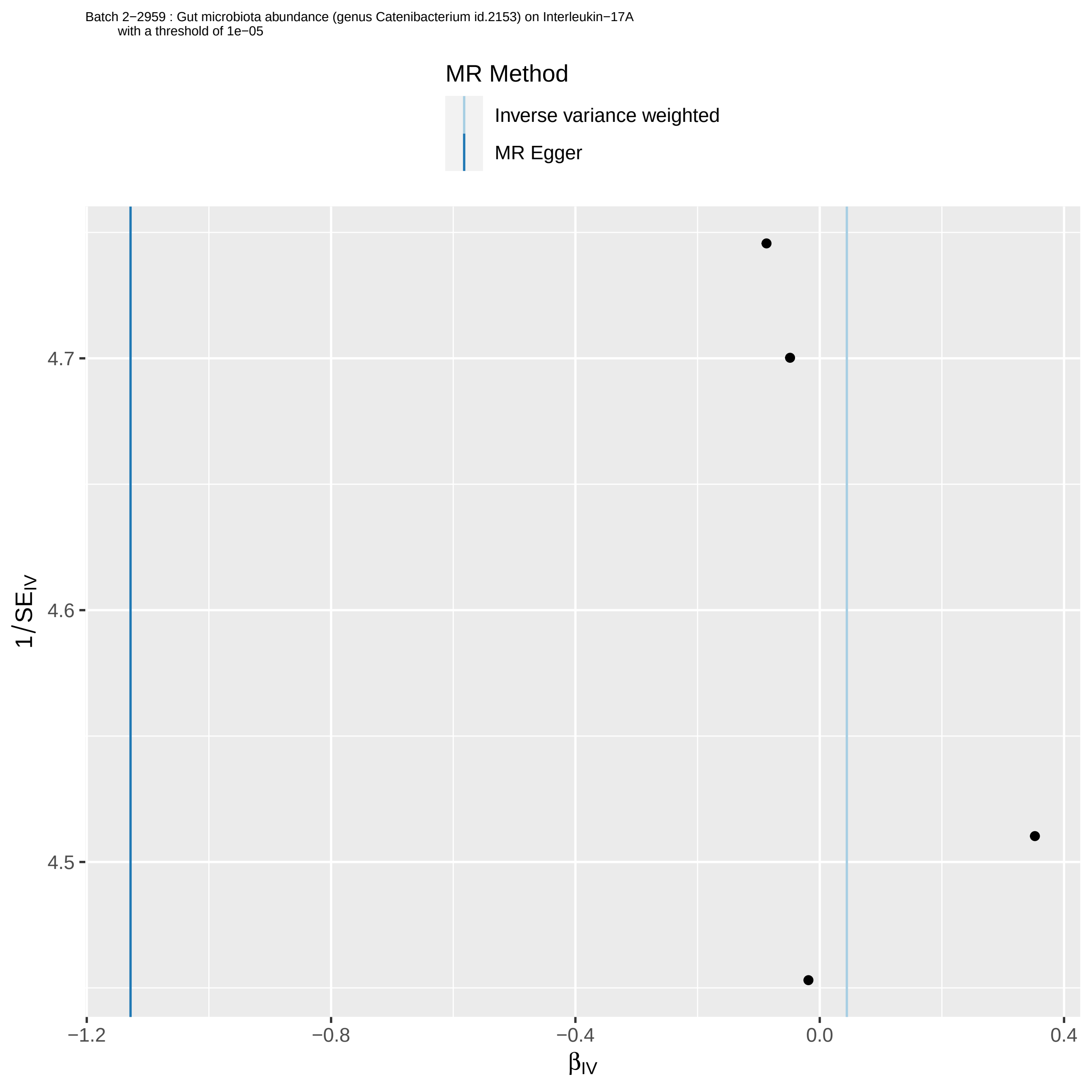

Batch 2−2959 : Gut microbiota abundance (genus Catenibacterium id.2153) on Interleukin−17A
with a threshold of 1e−05
MR Method
Inverse variance weighted
MR Egger
4.7
4.6
4.5
1/SE_IV
−1.2
−0.8
−0.4
0.0
0.4
β_IV

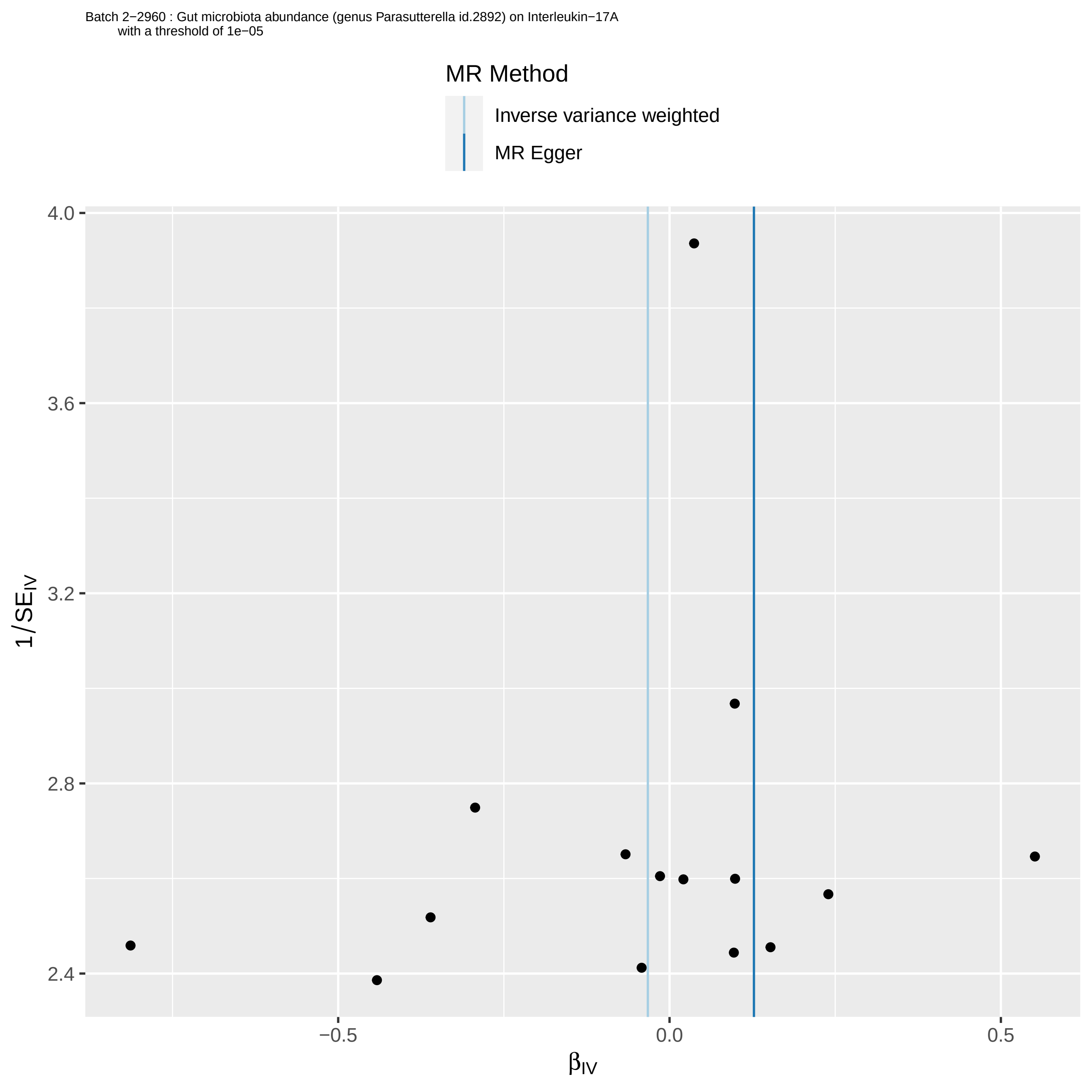
Batch 2−2960 : Gut microbiota abundance (genus Parasutterella id.2892) on Interleukin−17A
with a threshold of 1e−05
MR Method
Inverse variance weighted
MR Egger
4.0
3.6
3.2
2.8
2.4
1/SE$_{IV}$
−0.5
0.0
0.5
β_{IV}

Batch 2−2961 : Gut microbiota abundance (genus Catenibacterium id.2153) on Interleukin−17A
with a threshold of 1e−05

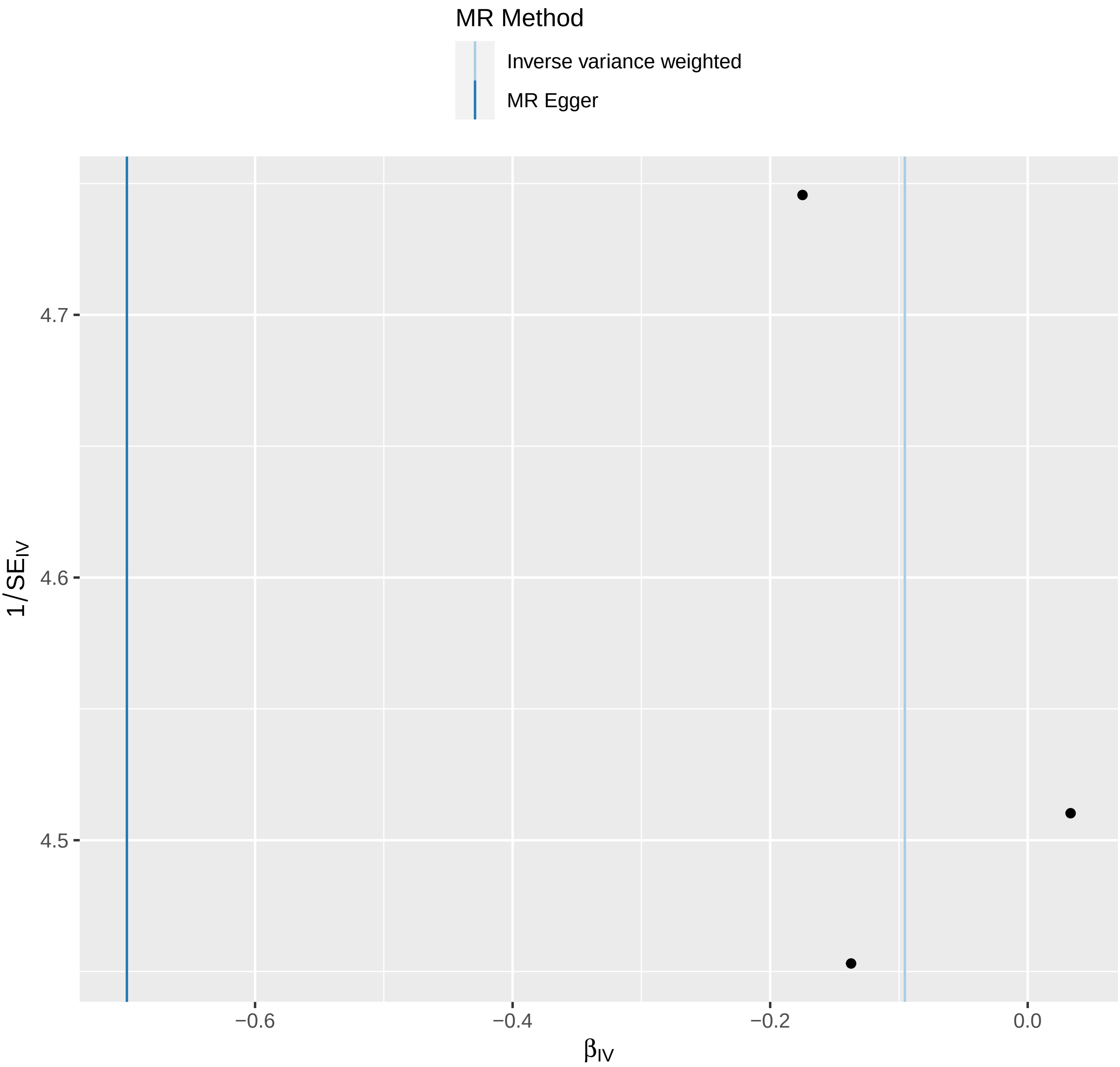

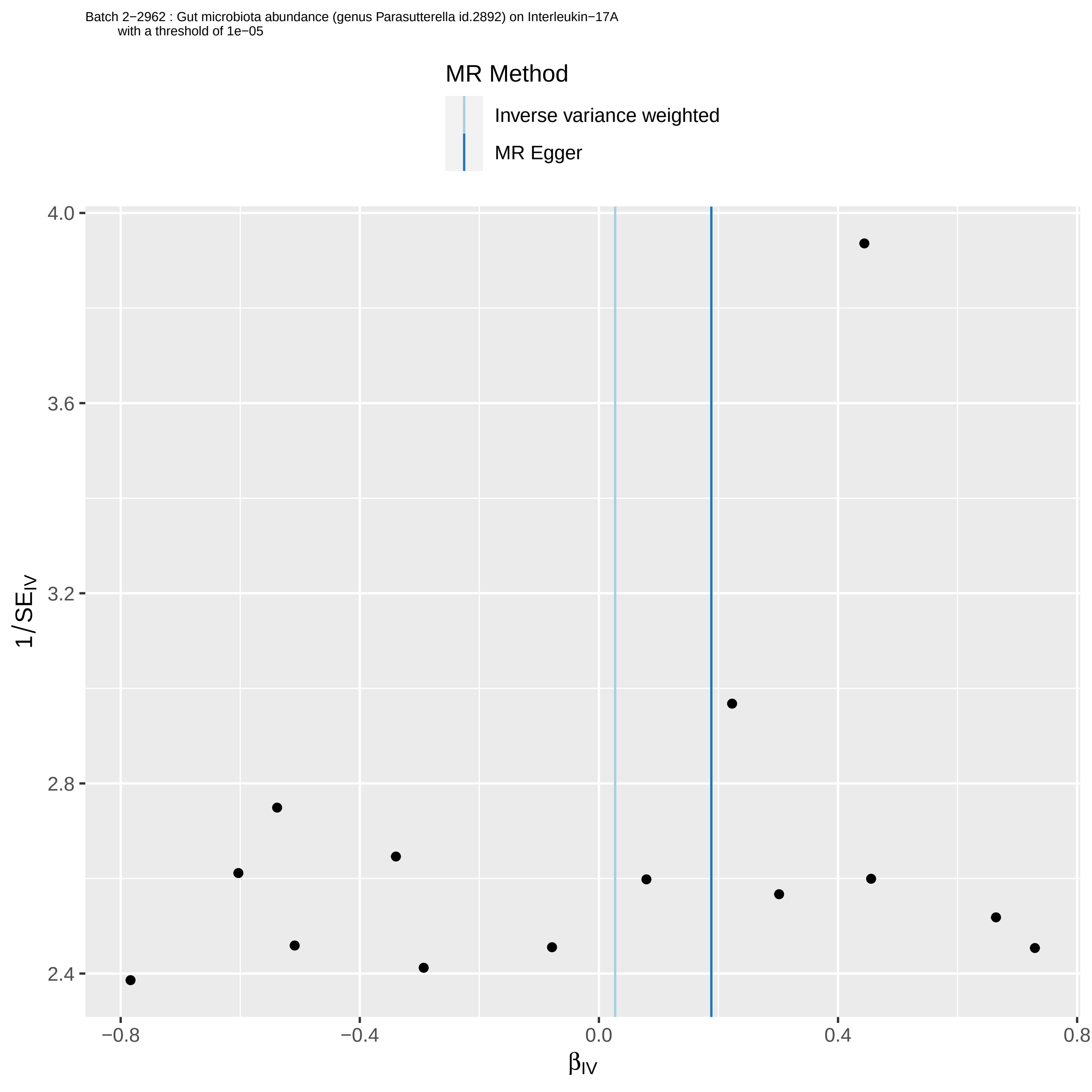
Batch 2−2962 : Gut microbiota abundance (genus Parasutterella id.2892) on Interleukin−17A
with a threshold of 1e−05
MR Method
Inverse variance weighted
MR Egger
4.0
3.6
3.2
2.8
2.4
1/SE_IV
−0.8
−0.4
0.0
0.4
0.8
β_IV

Batch 2−2963 : Gut microbiota abundance (genus Catenibacterium id.2153) on Interleukin−17A
with a threshold of 1e−05

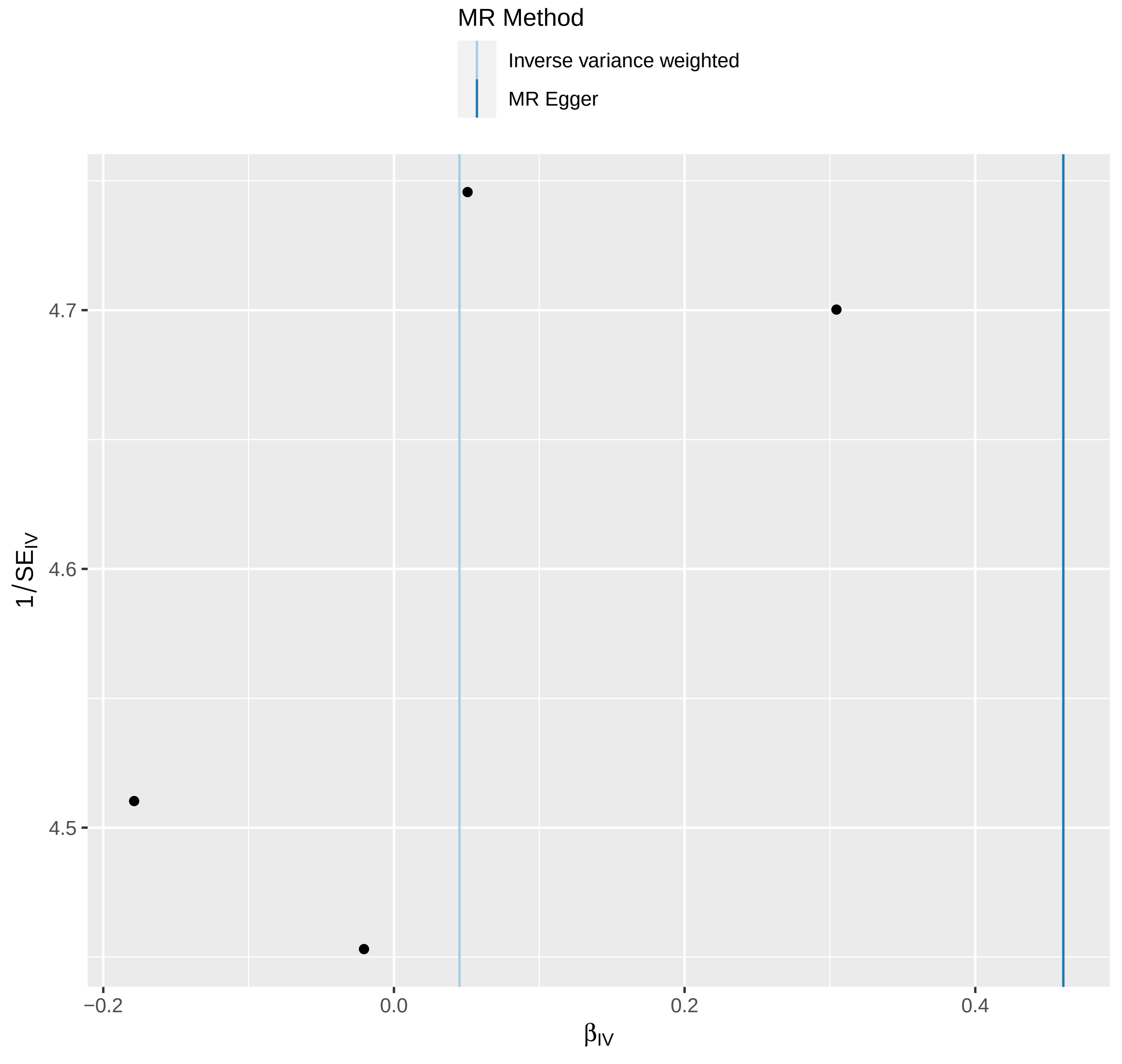

Batch 2−2964 : Gut microbiota abundance (genus Parasutterella id.2892) on Interleukin−17A
with a threshold of 1e−05

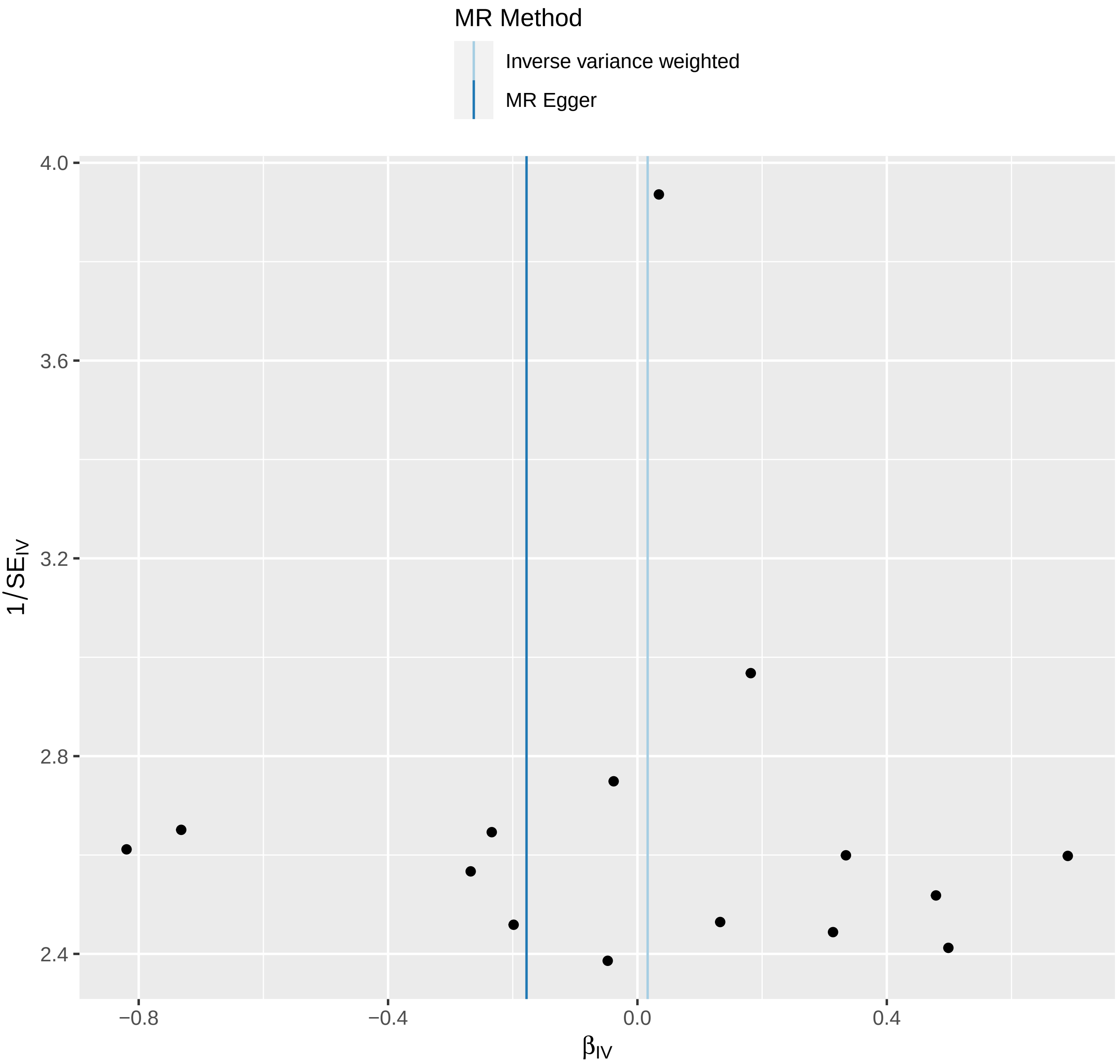

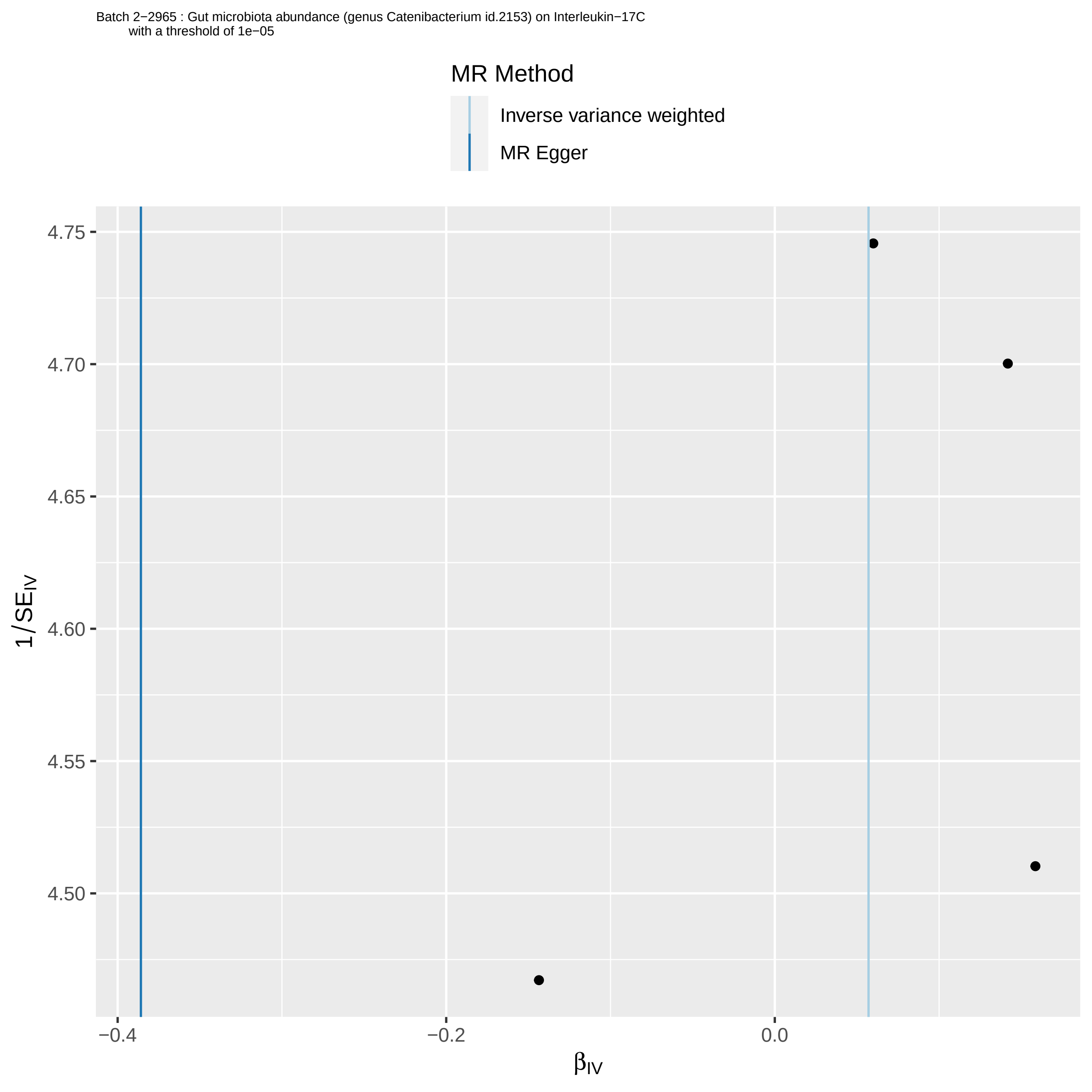
Batch 2−2965 : Gut microbiota abundance (genus Catenibacterium id.2153) on Interleukin−17C
with a threshold of 1e−05
MR Method
Inverse variance weighted
MR Egger
4.75
4.70
4.65
4.60
4.55
4.50
1/SE_IV
−0.4
−0.2
0.0
β_IV

Batch 2−2966 : Gut microbiota abundance (genus Parasutterella id.2892) on Interleukin−17C
with a threshold of 1e−05

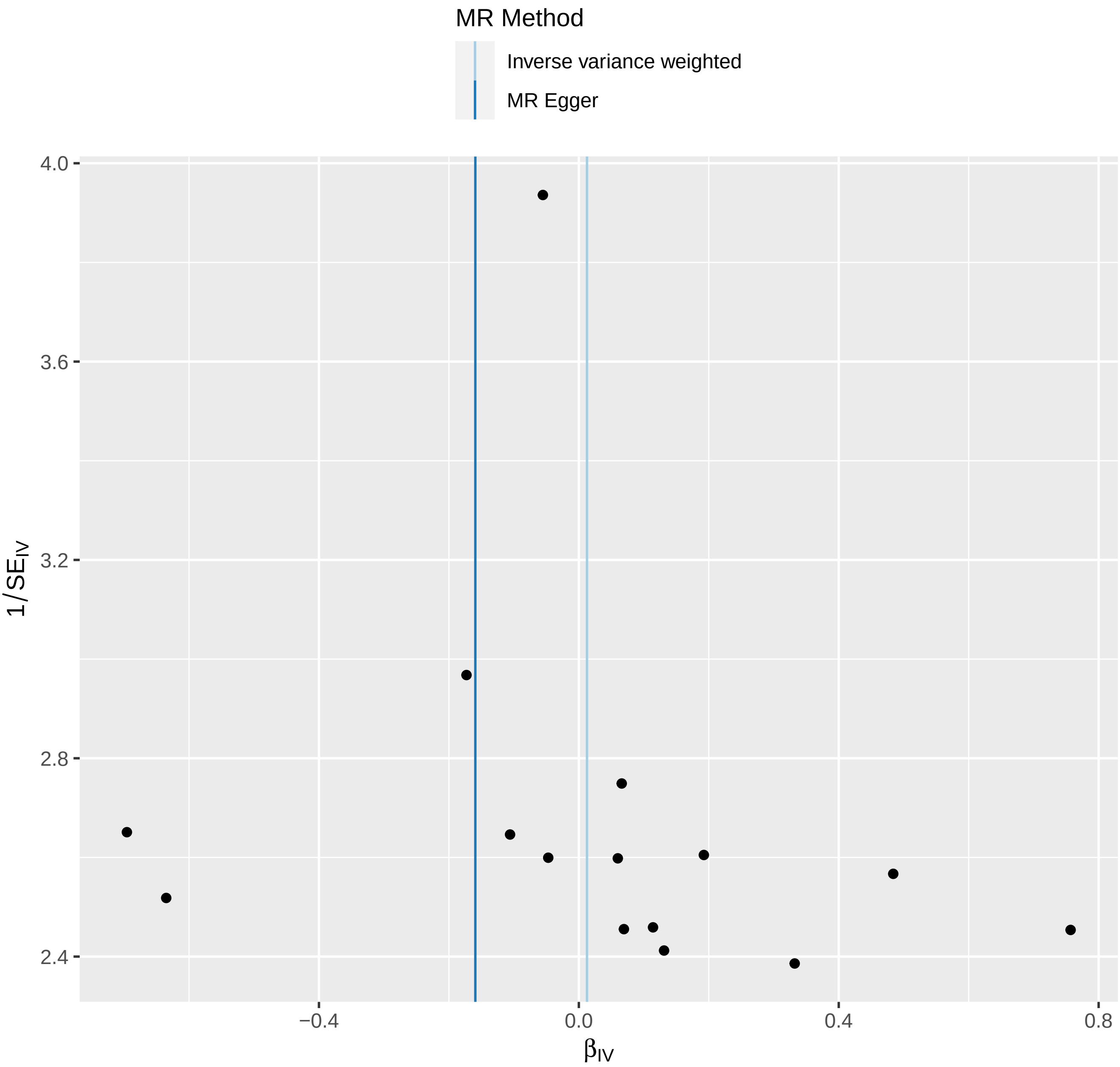

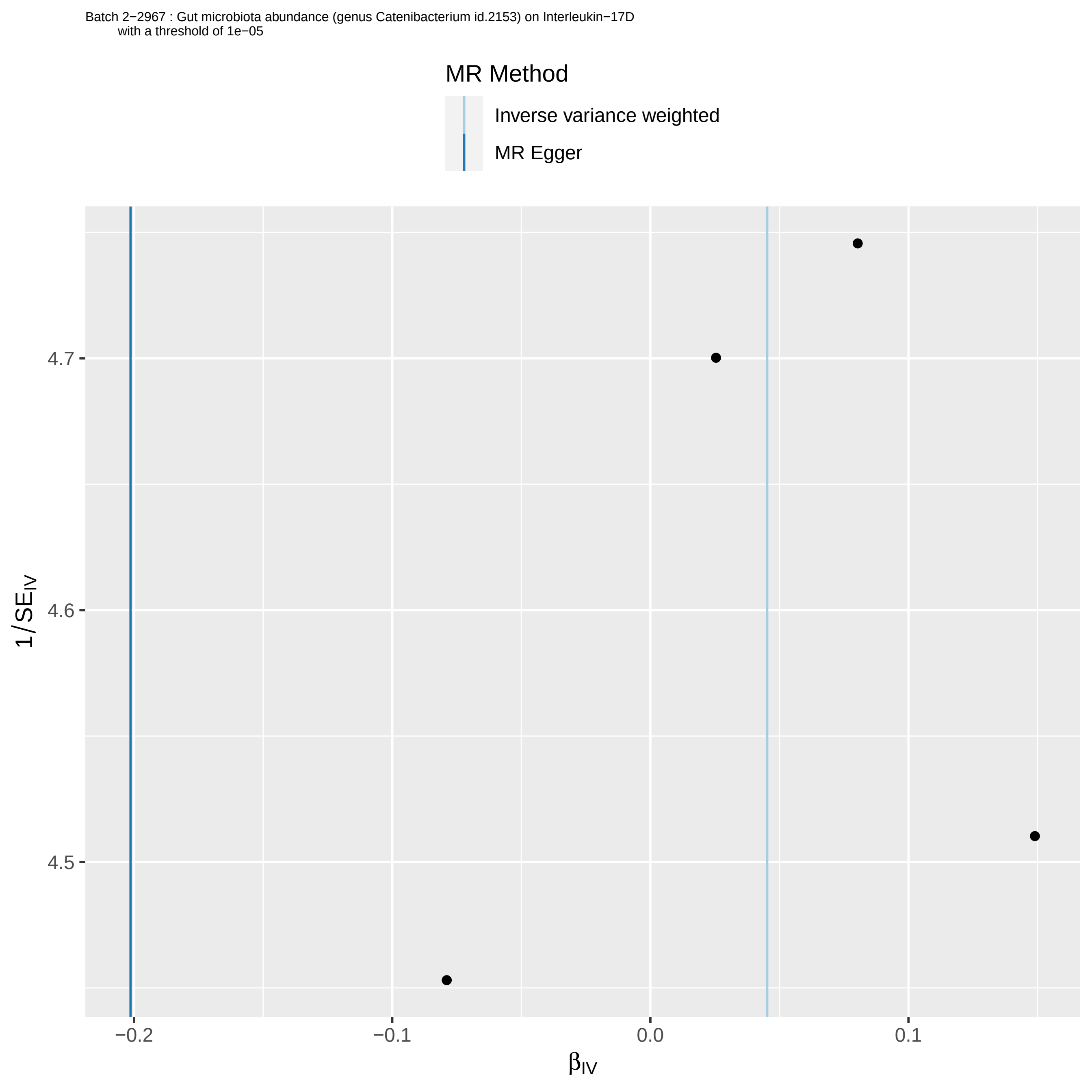

Batch 2−2967 : Gut microbiota abundance (genus Catenibacterium id.2153) on Interleukin−17D
with a threshold of 1e−05
MR Method
Inverse variance weighted
MR Egger
4.7
4.6
4.5
1/SE_IV
−0.2
−0.1
0.0
0.1
β_IV

Batch 2−2968 : Gut microbiota abundance (genus Parasutterella id.2892) on Interleukin−17D
with a threshold of 1e−05

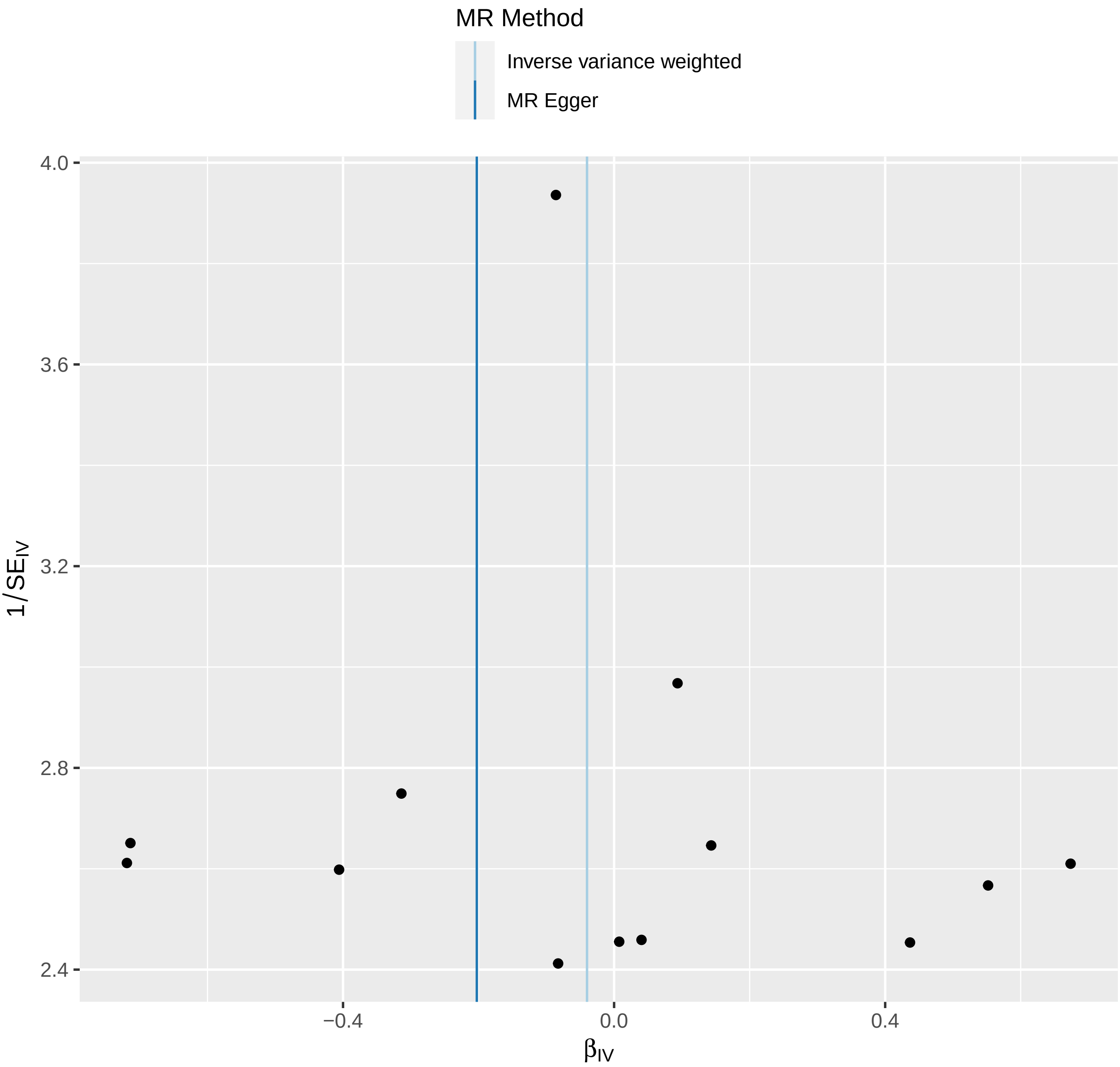

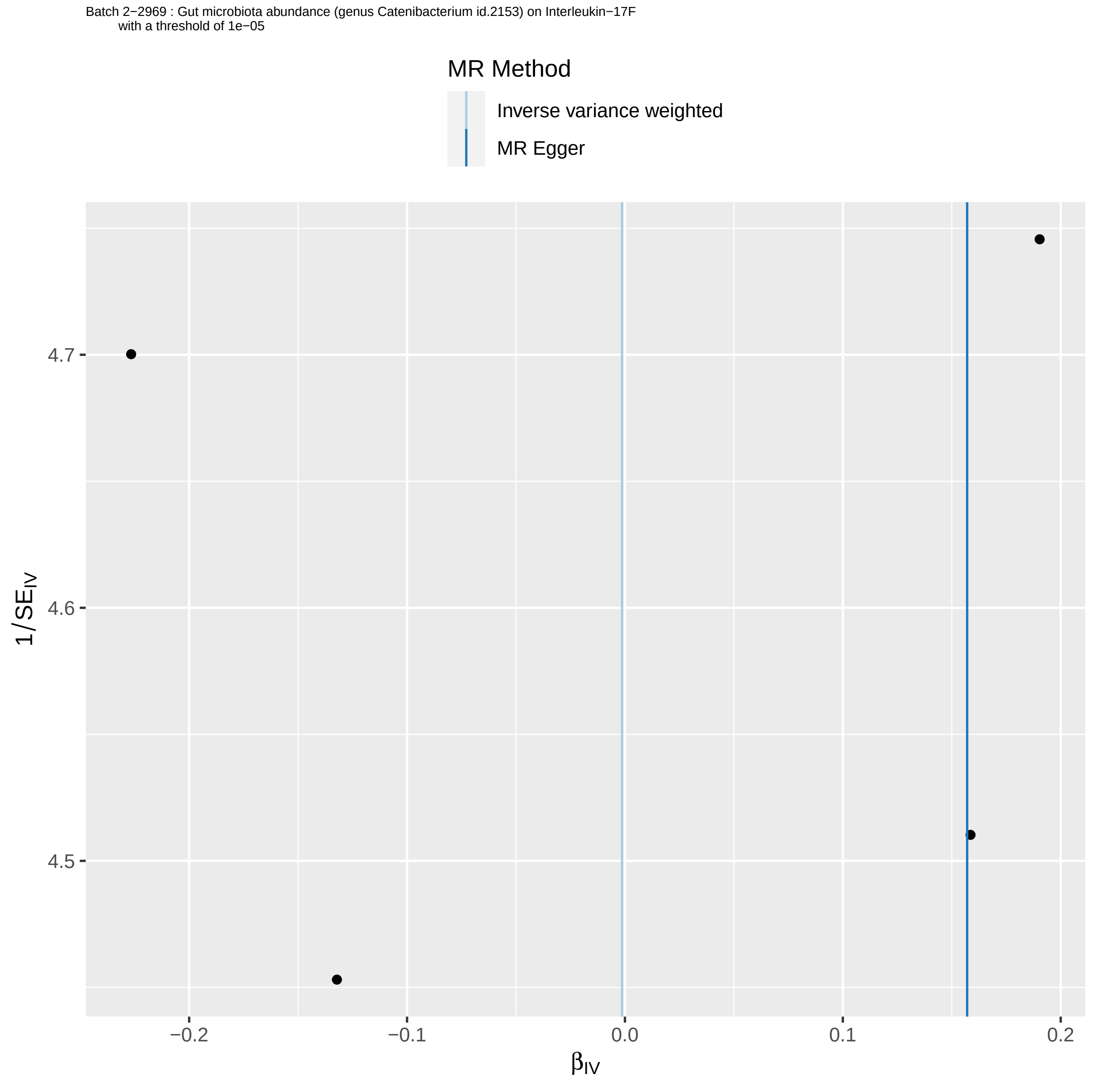
Batch 2−2969 : Gut microbiota abundance (genus Catenibacterium id.2153) on Interleukin−17F
with a threshold of 1e−05
MR Method
Inverse variance weighted
MR Egger
4.7
4.6
4.5
1/SE_IV
−0.2
−0.1
0.0
0.1
0.2
β_IV

Batch 2−2970 : Gut microbiota abundance (genus Parasutterella id.2892) on Interleukin−17F
with a threshold of 1e−05

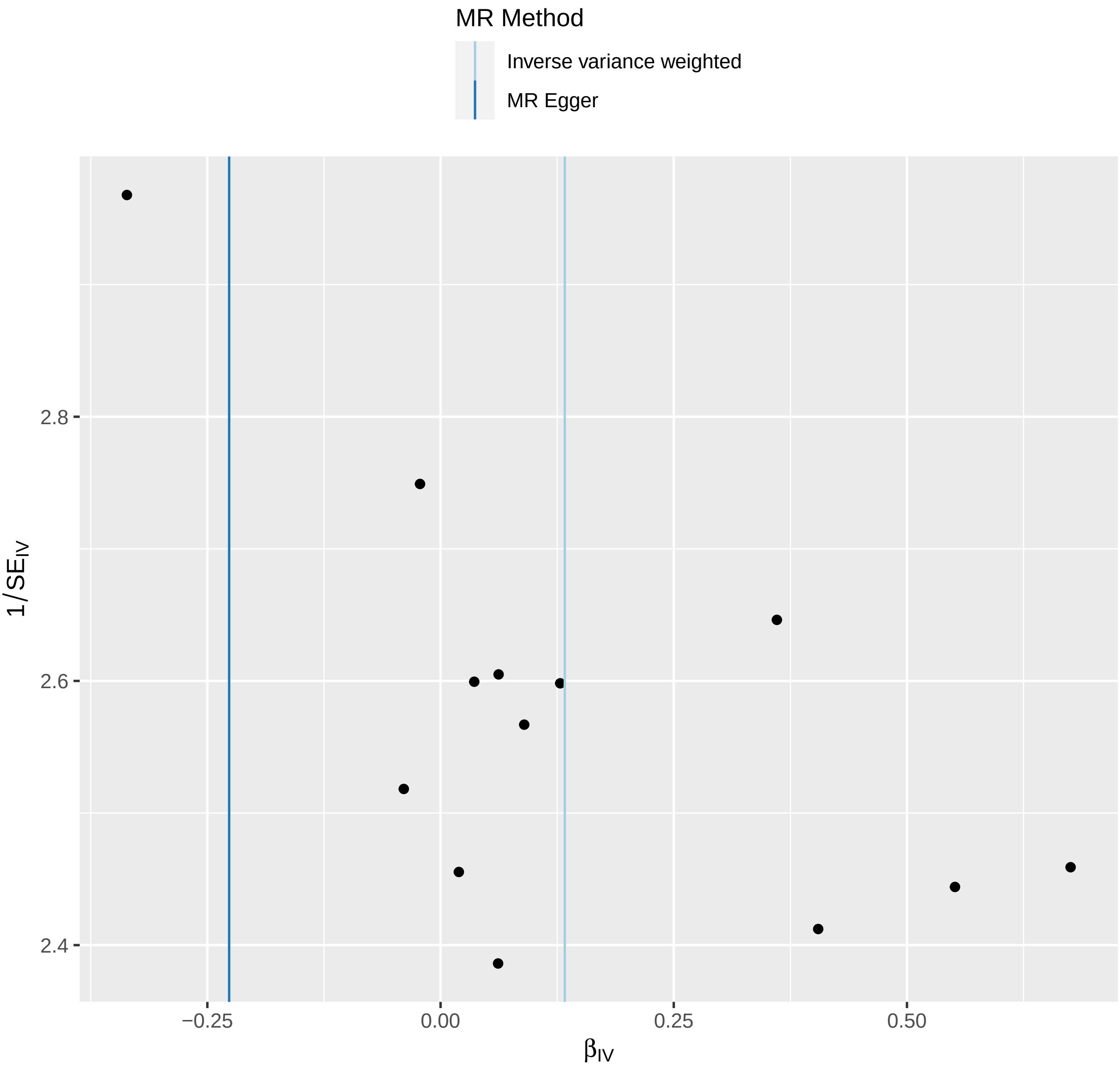

Batch 2−2971 : Gut microbiota abundance (genus Catenibacterium id.2153) on Interleukin−17 receptor A
with a threshold of 1e−05

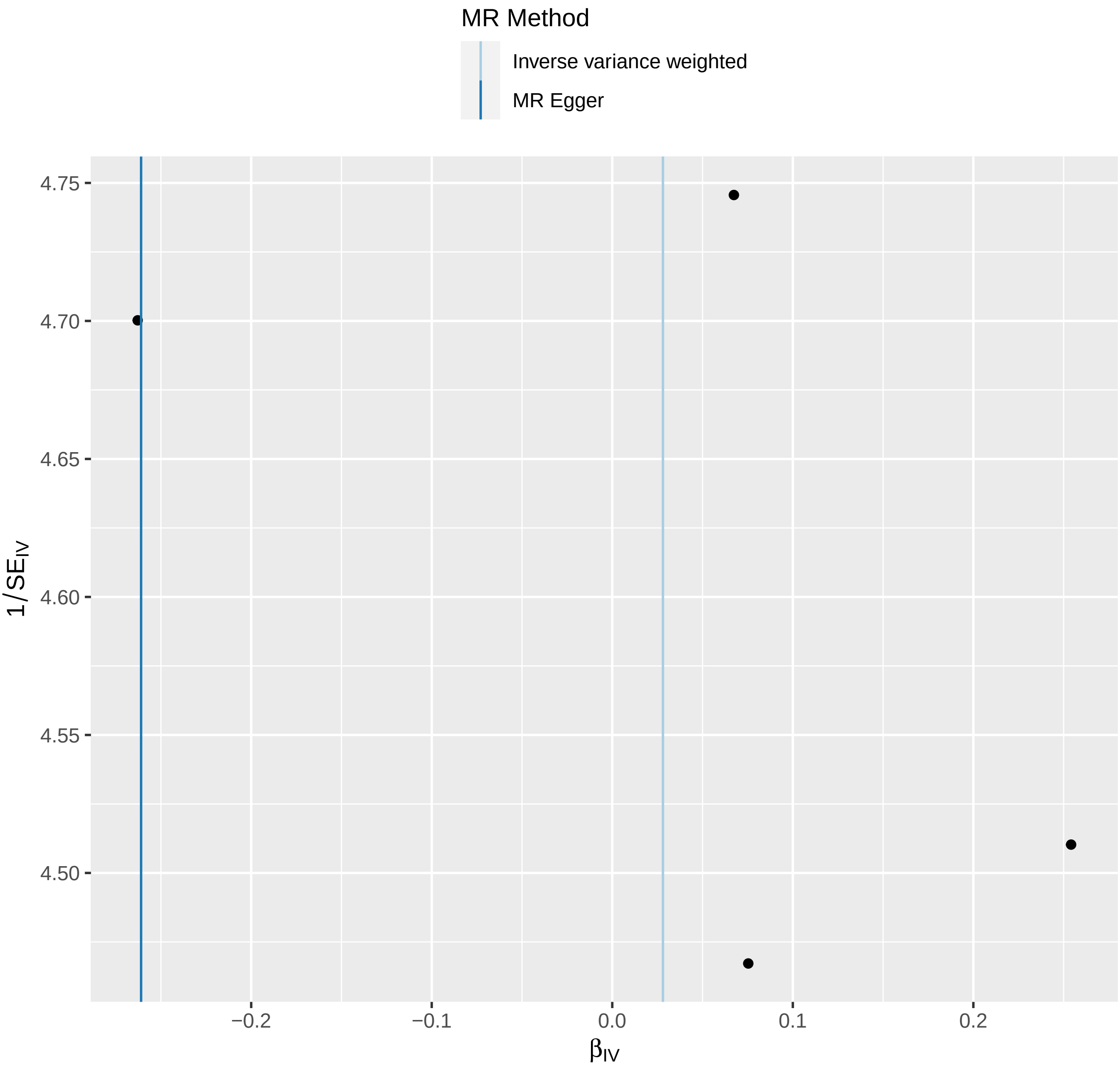

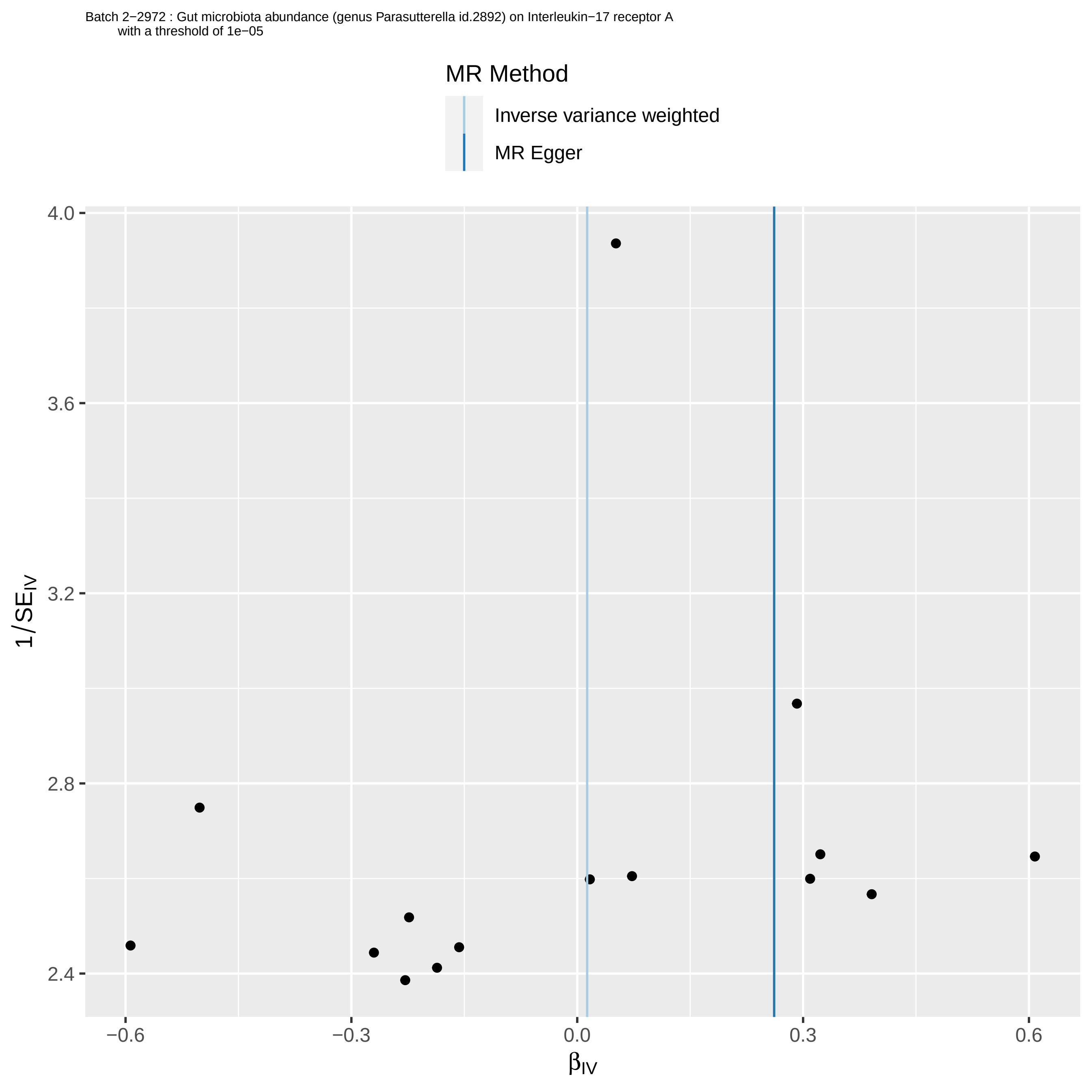
Batch 2−2972 : Gut microbiota abundance (genus Parasutterella id.2892) on Interleukin−17 receptor A
with a threshold of 1e−05
MR Method
Inverse variance weighted
MR Egger
4.0
3.6
3.2
2.8
2.4
1/SE$_{IV}$
−0.6
−0.3
0.0
0.3
0.6
β$_{IV}$

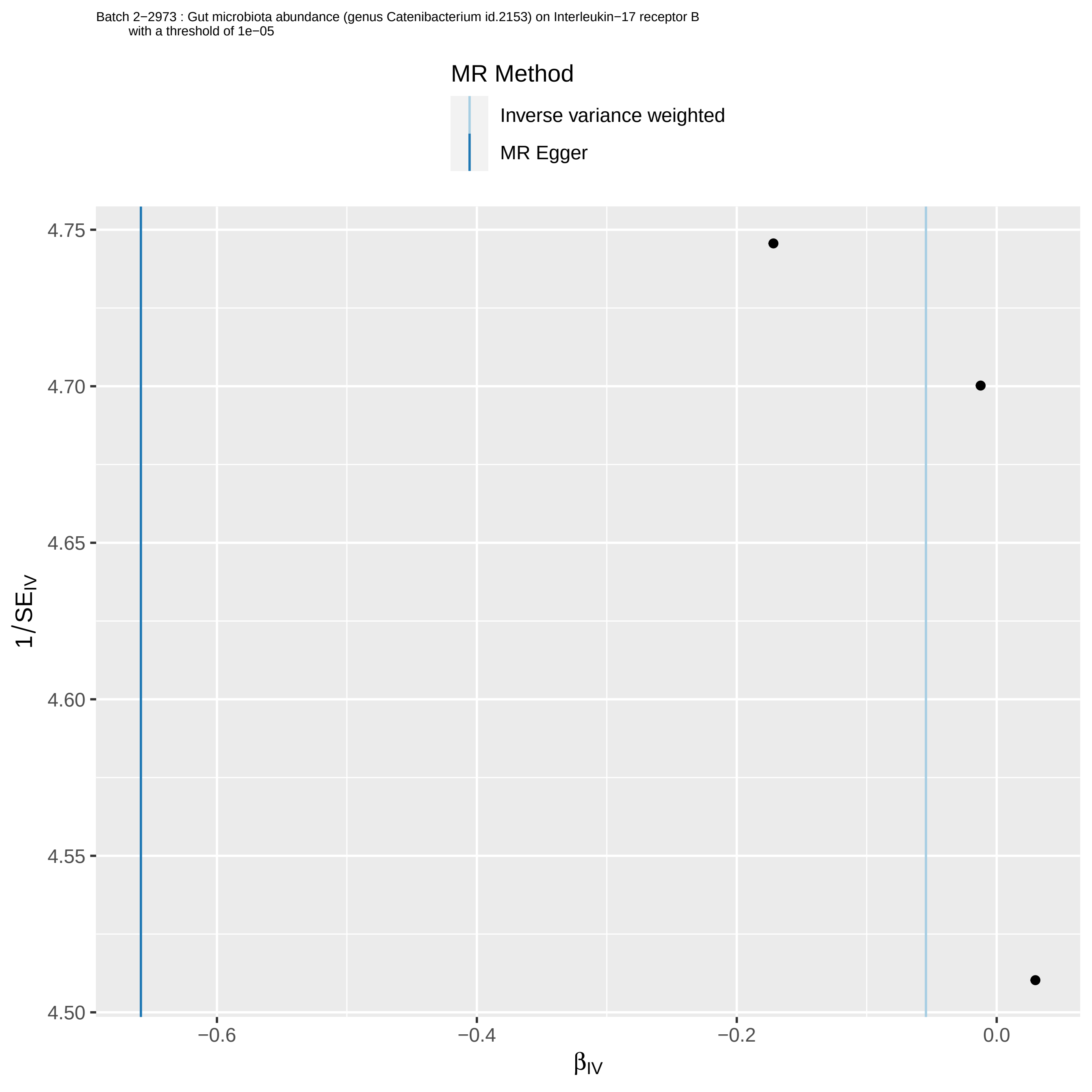
Batch 2−2973 : Gut microbiota abundance (genus Catenibacterium id.2153) on Interleukin−17 receptor B
with a threshold of 1e−05
MR Method
Inverse variance weighted
MR Egger
4.75
4.70
4.65
4.60
4.55
4.50
1/SE_IV
−0.6
−0.4
−0.2
0.0
β_IV

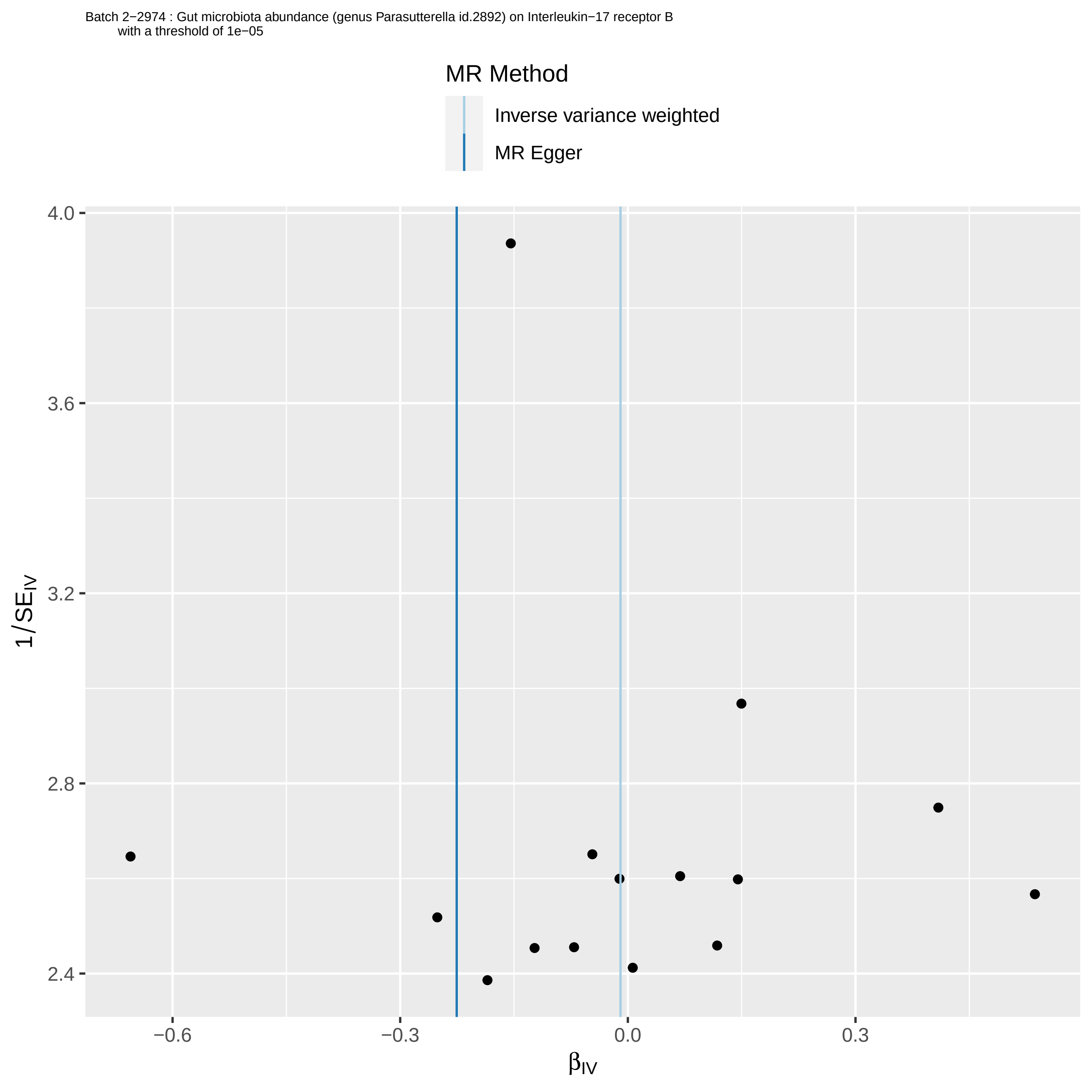

Batch 2−2974 : Gut microbiota abundance (genus Parasutterella id.2892) on Interleukin−17 receptor B
with a threshold of 1e−05
MR Method
Inverse variance weighted
MR Egger
4.0
3.6
3.2
2.8
2.4
1/SE_IV
−0.6
−0.3
0.0
0.3
β_IV

Batch 2−2975 : Gut microbiota abundance (genus Catenibacterium id.2153) on Interleukin−17 receptor C
with a threshold of 1e−05

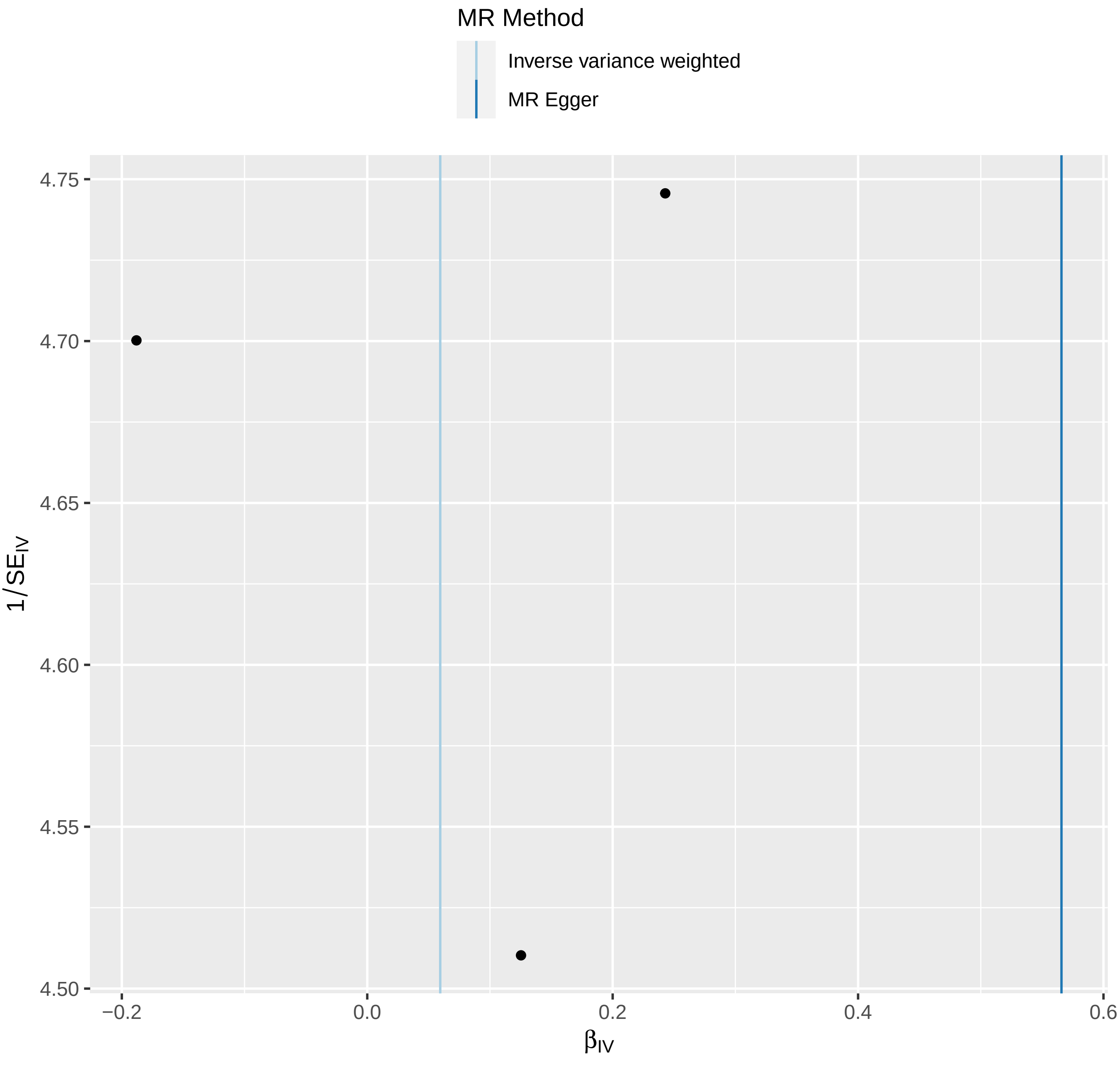

Batch 2−2976 : Gut microbiota abundance (genus Parasutterella id.2892) on Interleukin−17 receptor C
with a threshold of 1e−05

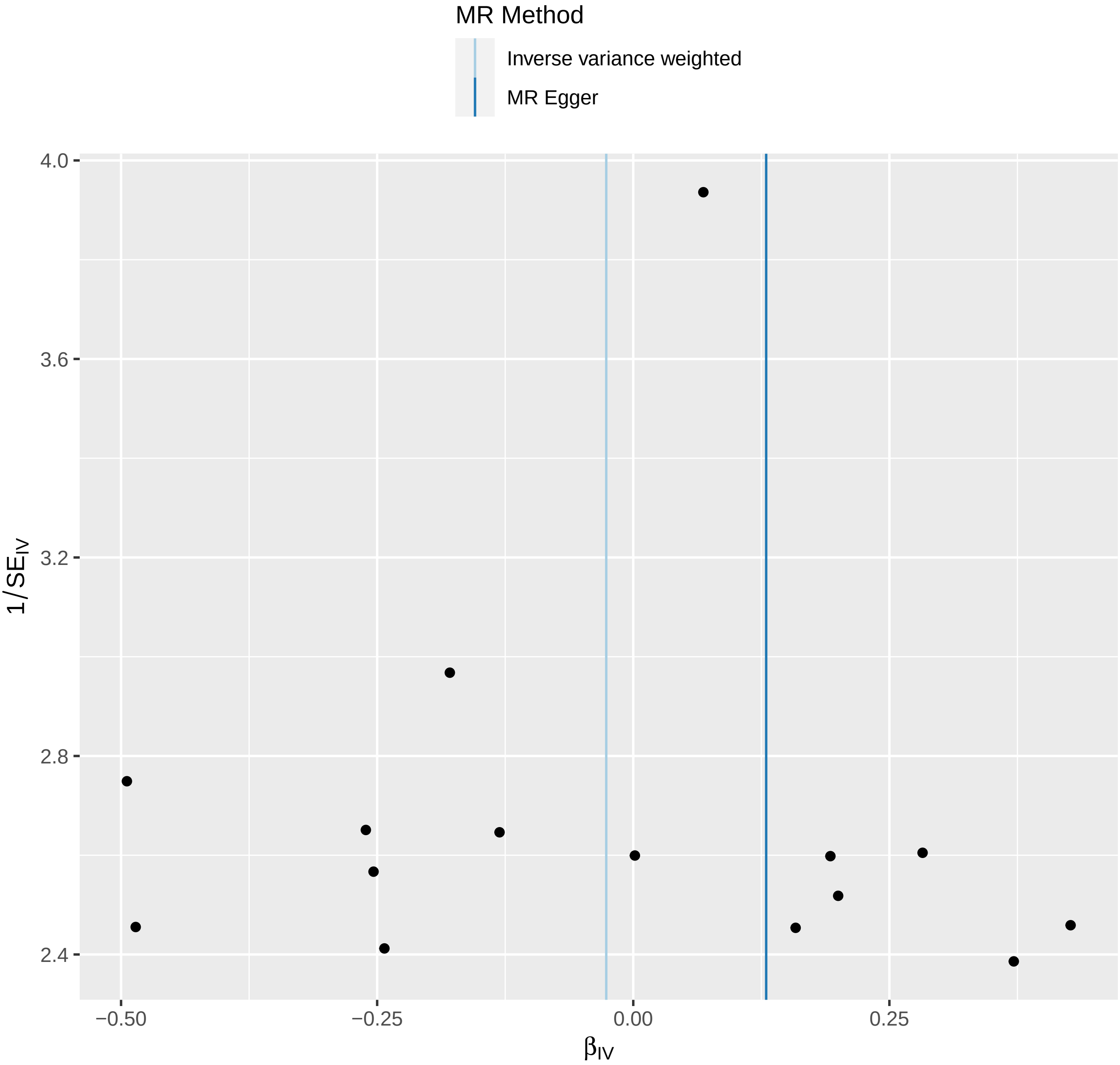

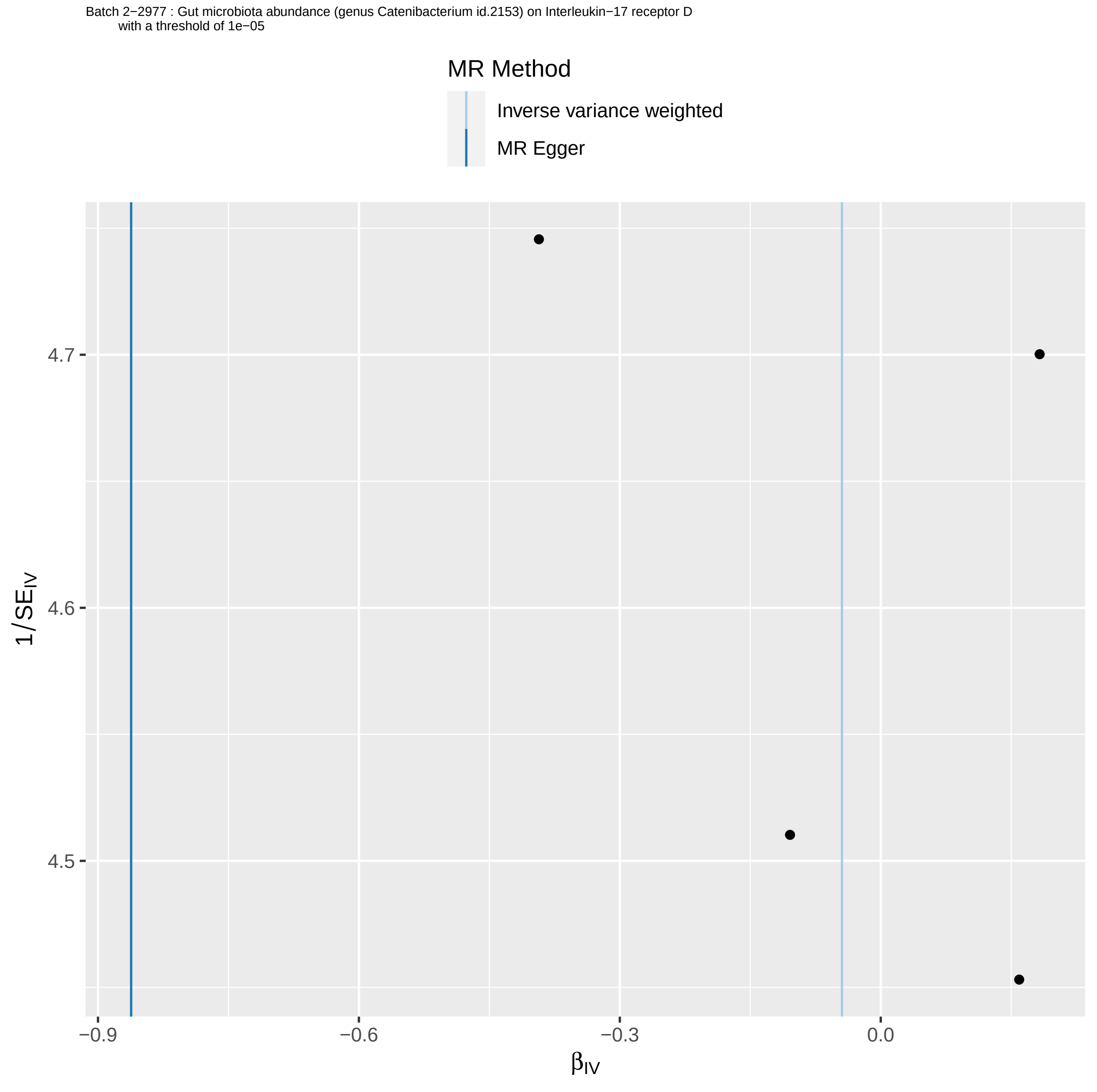
Batch 2−2977 : Gut microbiota abundance (genus Catenibacterium id.2153) on Interleukin−17 receptor D
with a threshold of 1e−05
MR Method
Inverse variance weighted
MR Egger
4.7
4.6
4.5
1/SE_IV
−0.9
−0.6
−0.3
0.0
β_IV

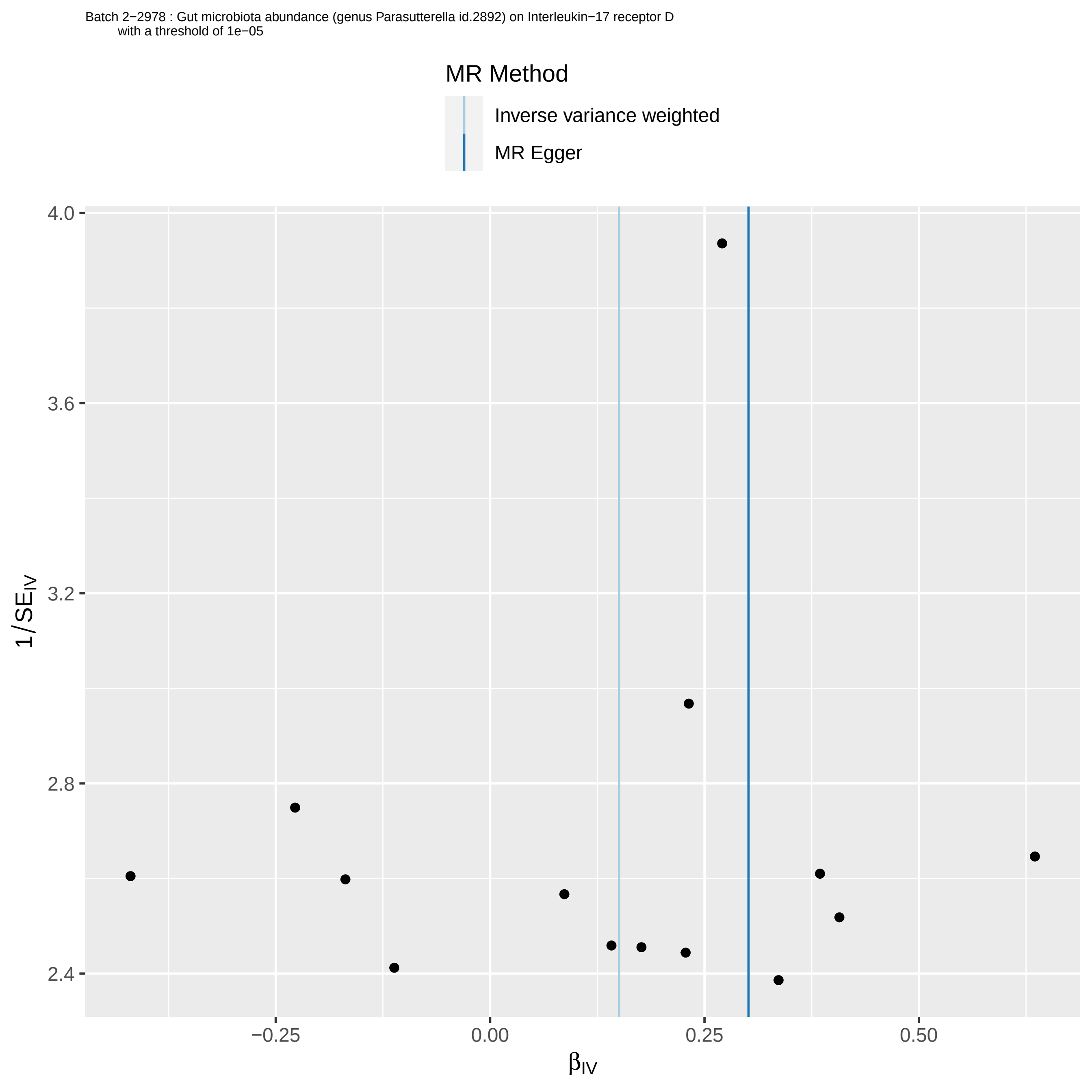
Batch 2−2978 : Gut microbiota abundance (genus Parasutterella id.2892) on Interleukin−17 receptor D
with a threshold of 1e−05
MR Method
Inverse variance weighted
MR Egger
4.0
3.6
3.2
2.8
2.4
1/SE$_{IV}$
−0.25
0.00
0.25
0.50
β_{IV}

Batch 2−2979 : Gut microbiota abundance (genus Catenibacterium id.2153) on Interleukin−18−binding protein
with a threshold of 1e−05

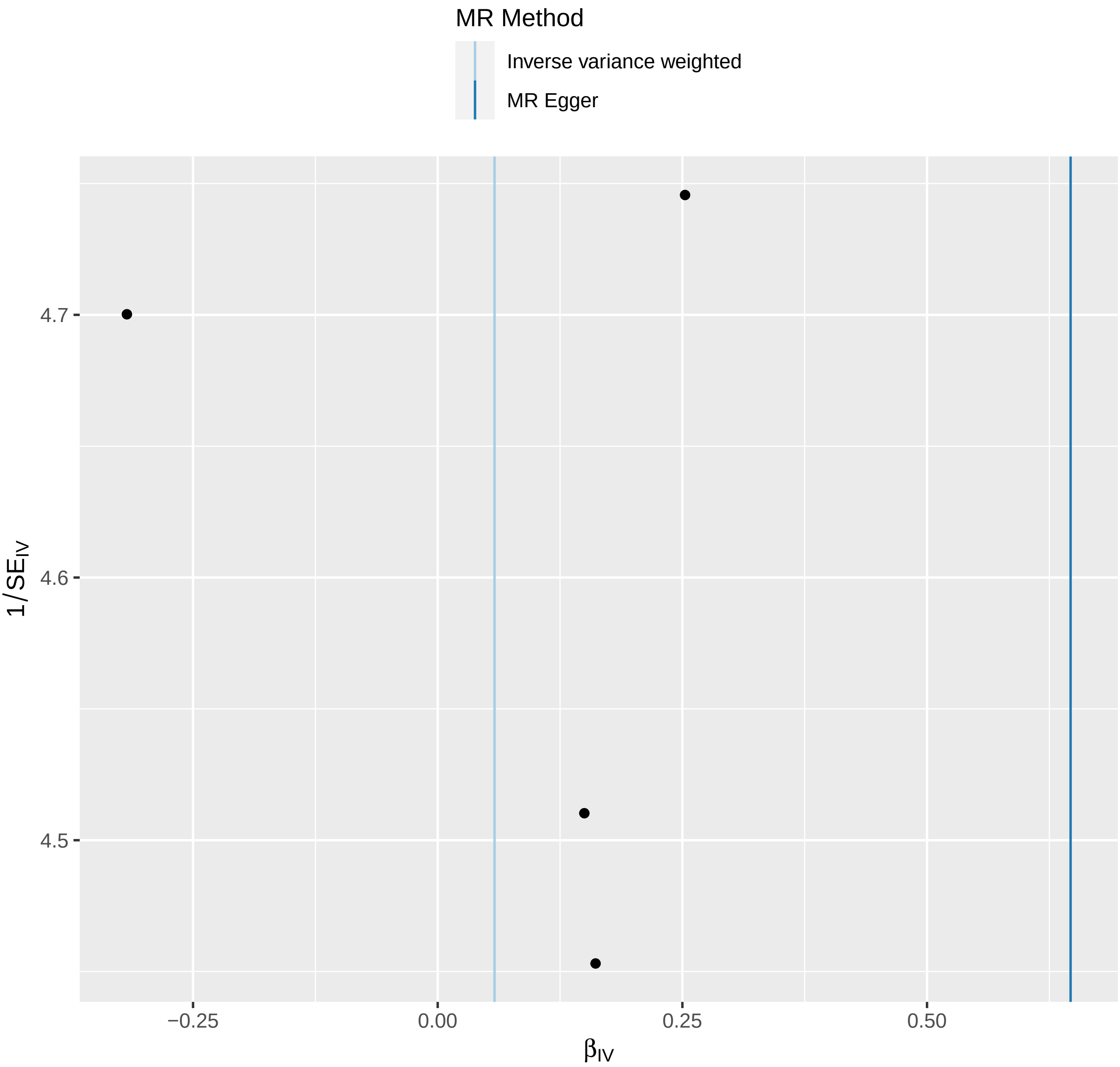

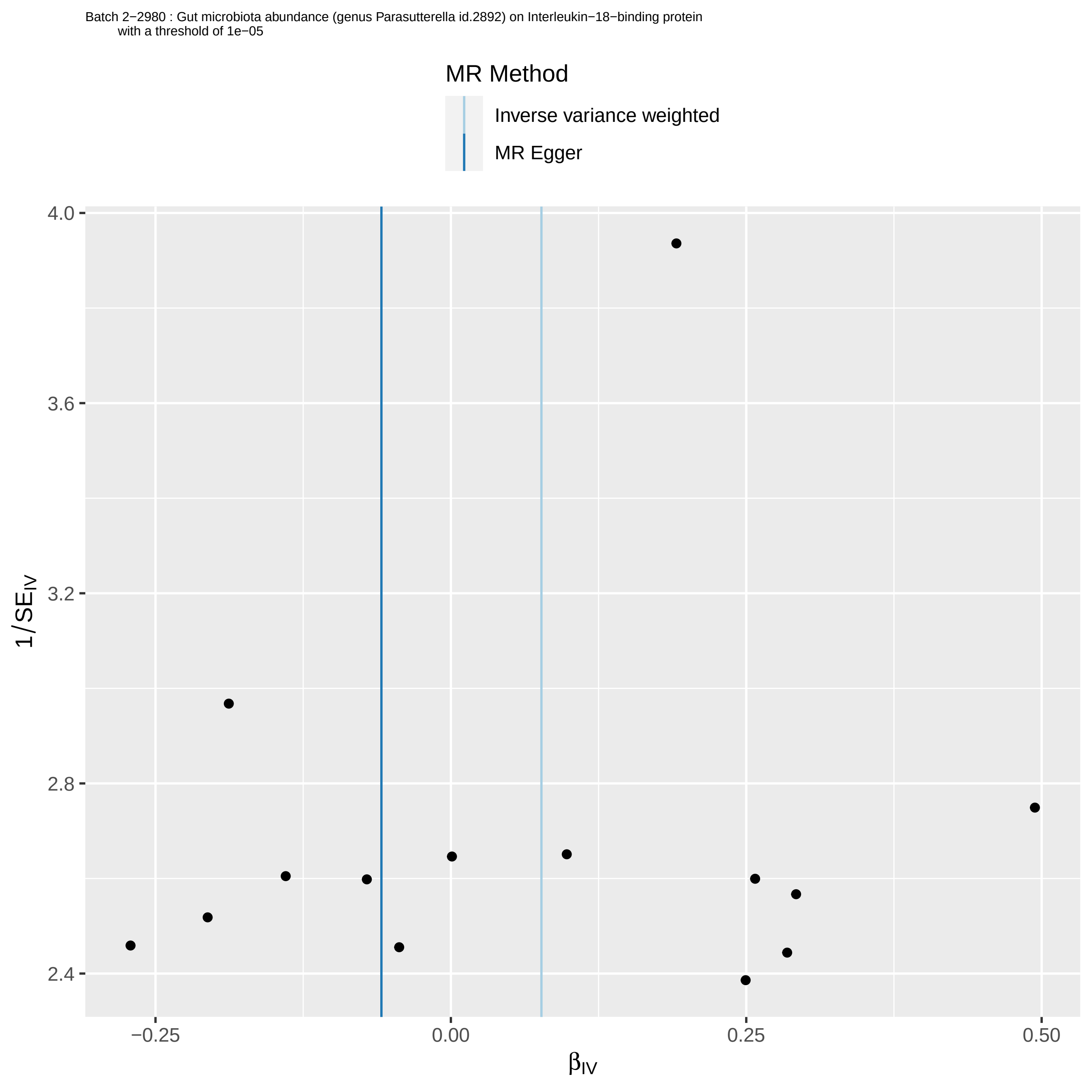

Batch 2−2980 : Gut microbiota abundance (genus Parasutterella id.2892) on Interleukin−18−binding protein
with a threshold of 1e−05
MR Method
Inverse variance weighted
MR Egger
4.0
3.6
3.2
2.8
2.4
1/SE_IV
−0.25
0.00
0.25
0.50
β_IV

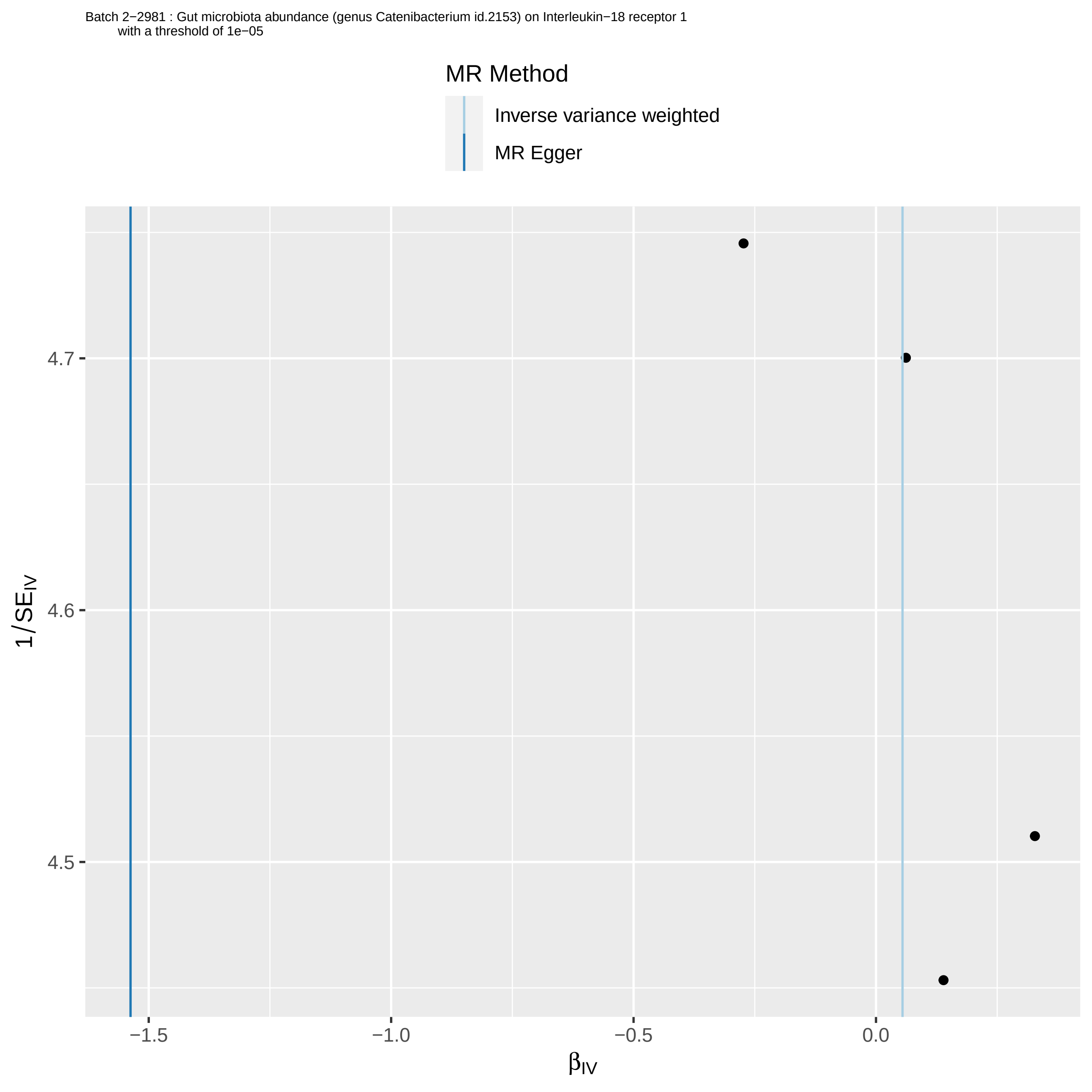
Batch 2−2981 : Gut microbiota abundance (genus Catenibacterium id.2153) on Interleukin−18 receptor 1
with a threshold of 1e−05
MR Method
Inverse variance weighted
MR Egger
4.7
4.6
4.5
1/SE_IV
−1.5
−1.0
−0.5
0.0
β_IV

Batch 2−2982 : Gut microbiota abundance (genus Parasutterella id.2892) on Interleukin−18 receptor 1
with a threshold of 1e−05

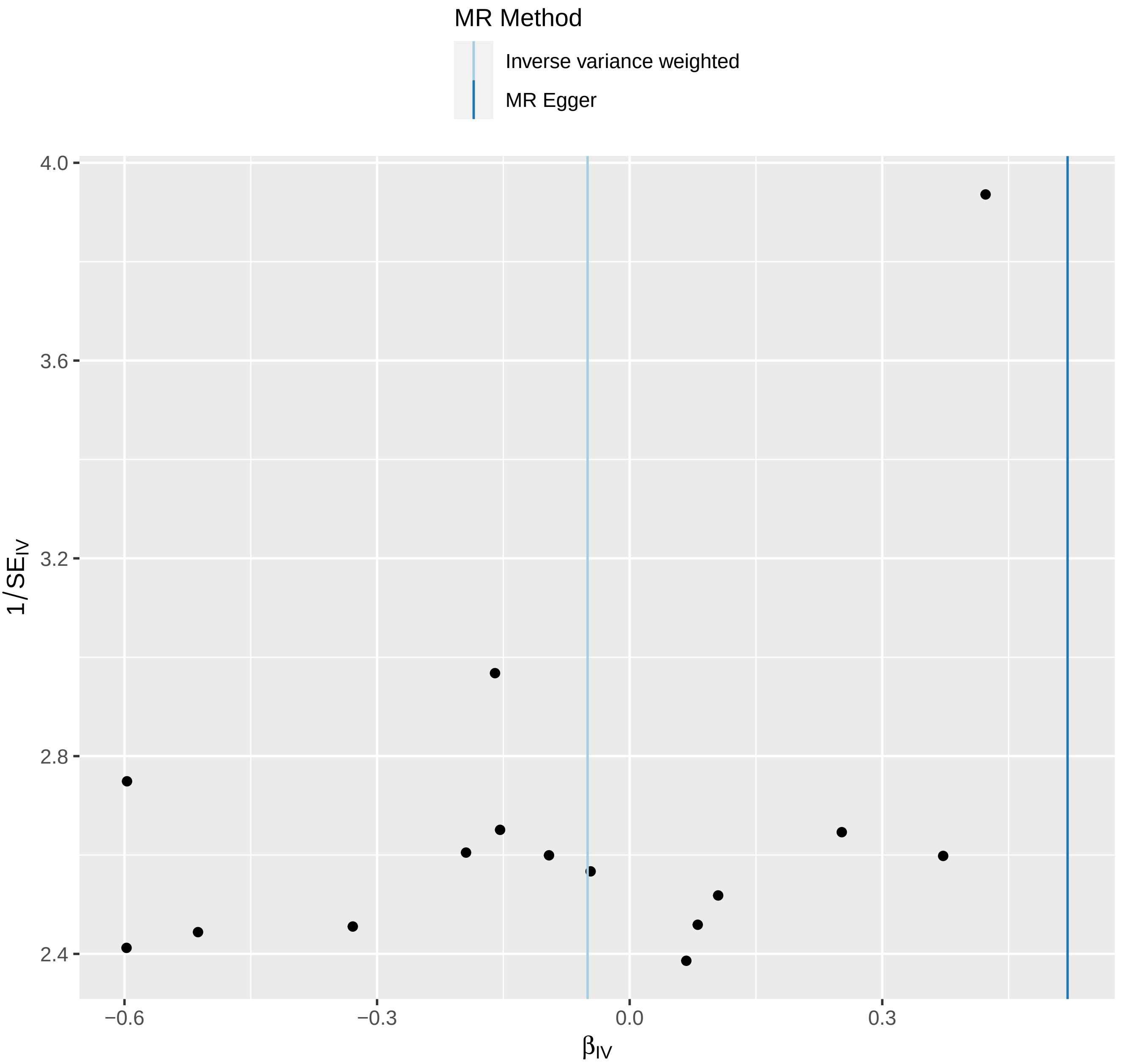

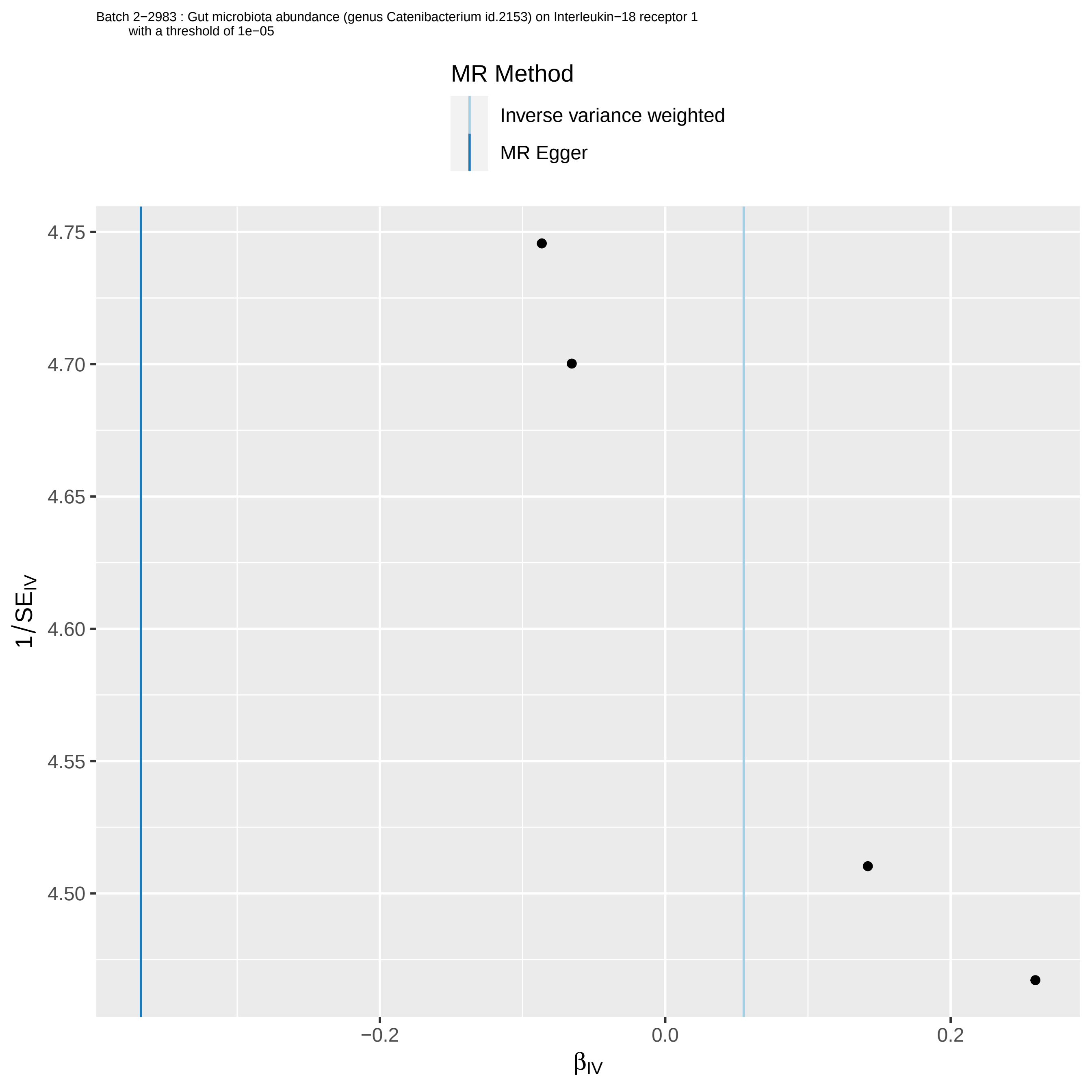
Batch 2−2983 : Gut microbiota abundance (genus Catenibacterium id.2153) on Interleukin−18 receptor 1
with a threshold of 1e−05
MR Method
Inverse variance weighted
MR Egger
4.75
4.70
4.65
4.60
4.55
4.50
1/SE_IV
−0.2
0.0
0.2
β_IV

Batch 2−2984 : Gut microbiota abundance (genus Parasutterella id.2892) on Interleukin−18 receptor 1
with a threshold of 1e−05

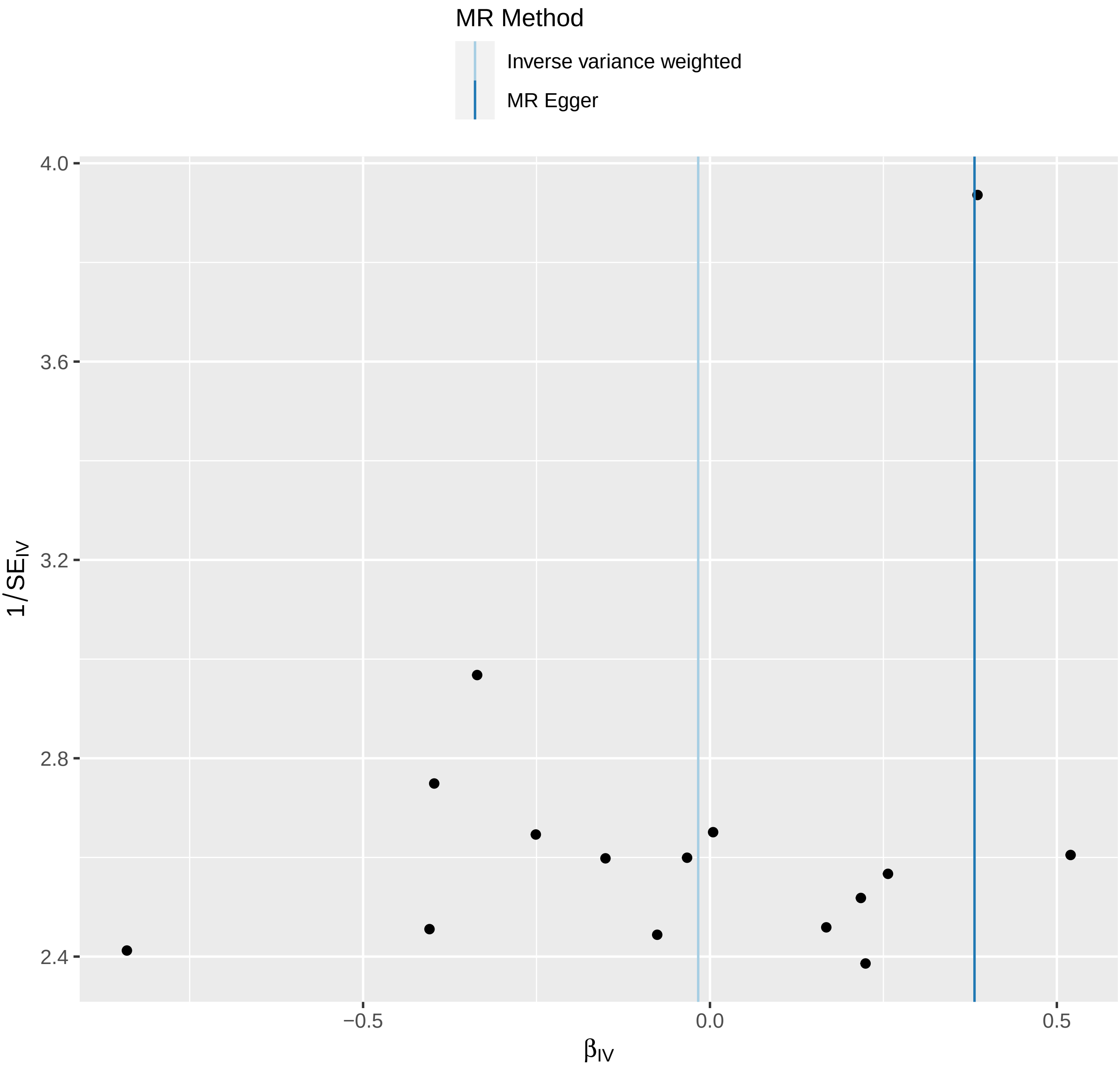

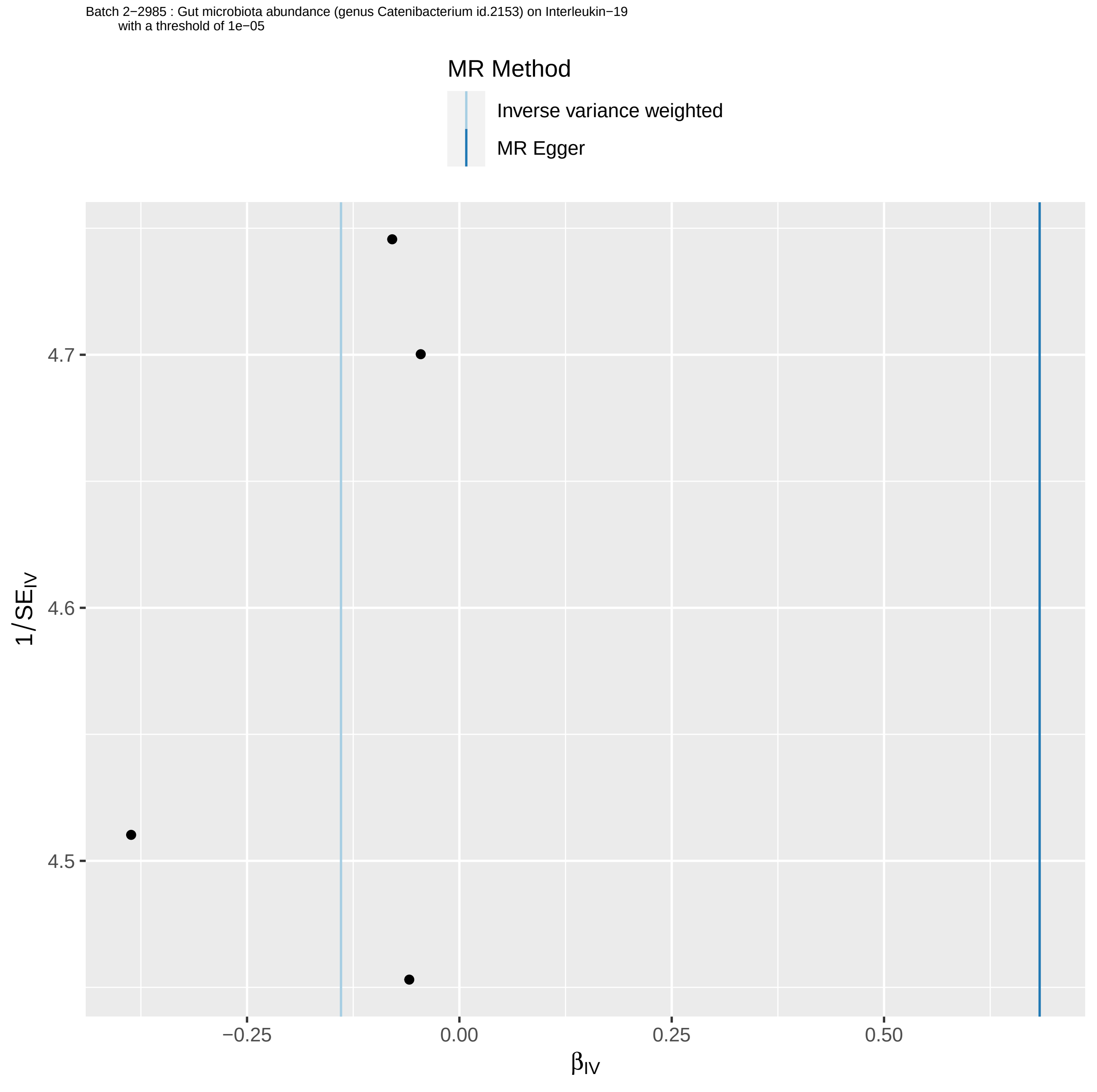

Batch 2−2985 : Gut microbiota abundance (genus Catenibacterium id.2153) on Interleukin−19
with a threshold of 1e−05
MR Method
Inverse variance weighted
MR Egger
4.7
4.6
4.5
1/SE_IV
−0.25
0.00
0.25
0.50
β_IV

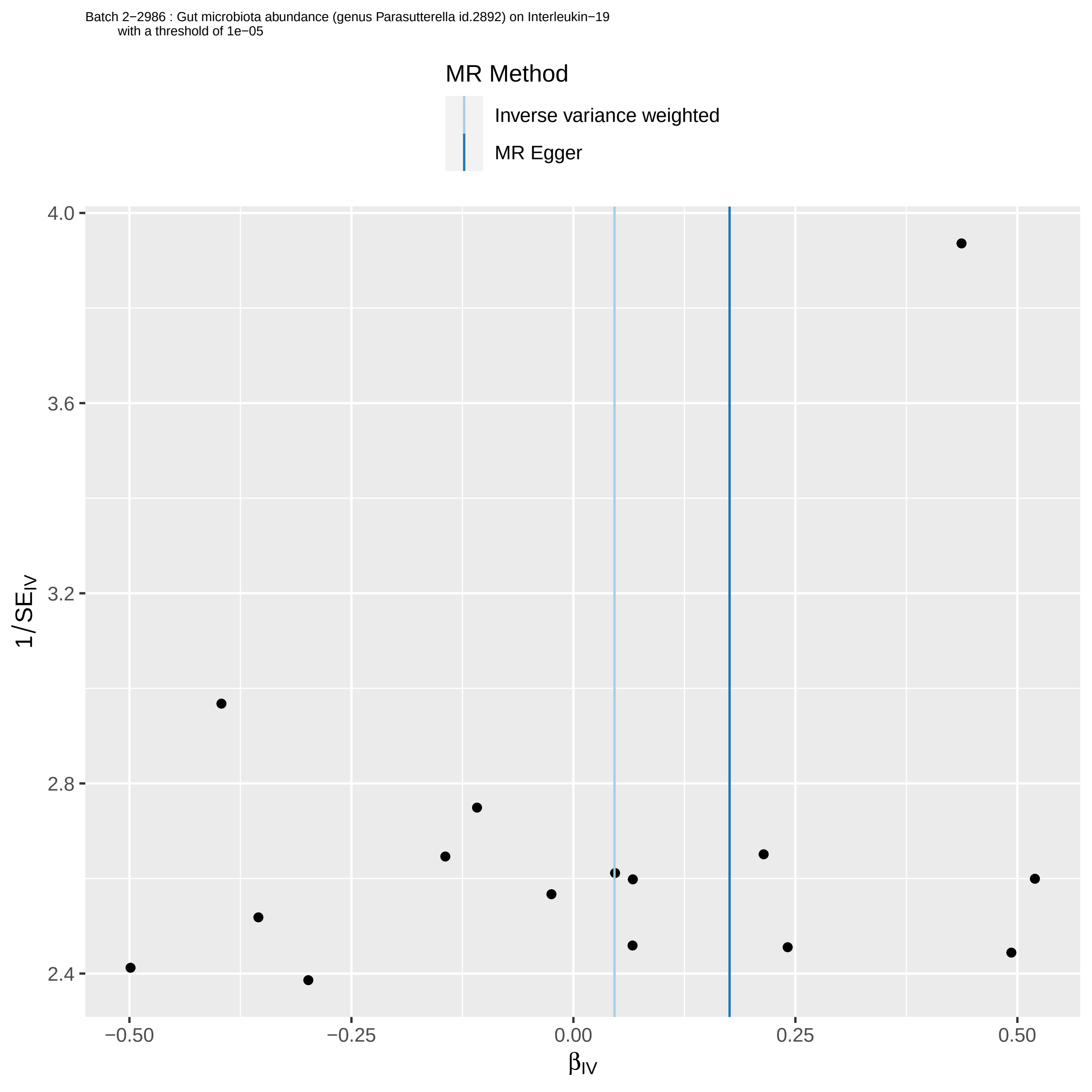
Batch 2−2986 : Gut microbiota abundance (genus Parasutterella id.2892) on Interleukin−19
with a threshold of 1e−05
MR Method
Inverse variance weighted
MR Egger
4.0
3.6
3.2
2.8
2.4
1/SE_IV
−0.50
−0.25
0.00
0.25
0.50
β_IV

Batch 2−2987 : Gut microbiota abundance (genus Catenibacterium id.2153) on Interleukin−1 alpha
with a threshold of 1e−05

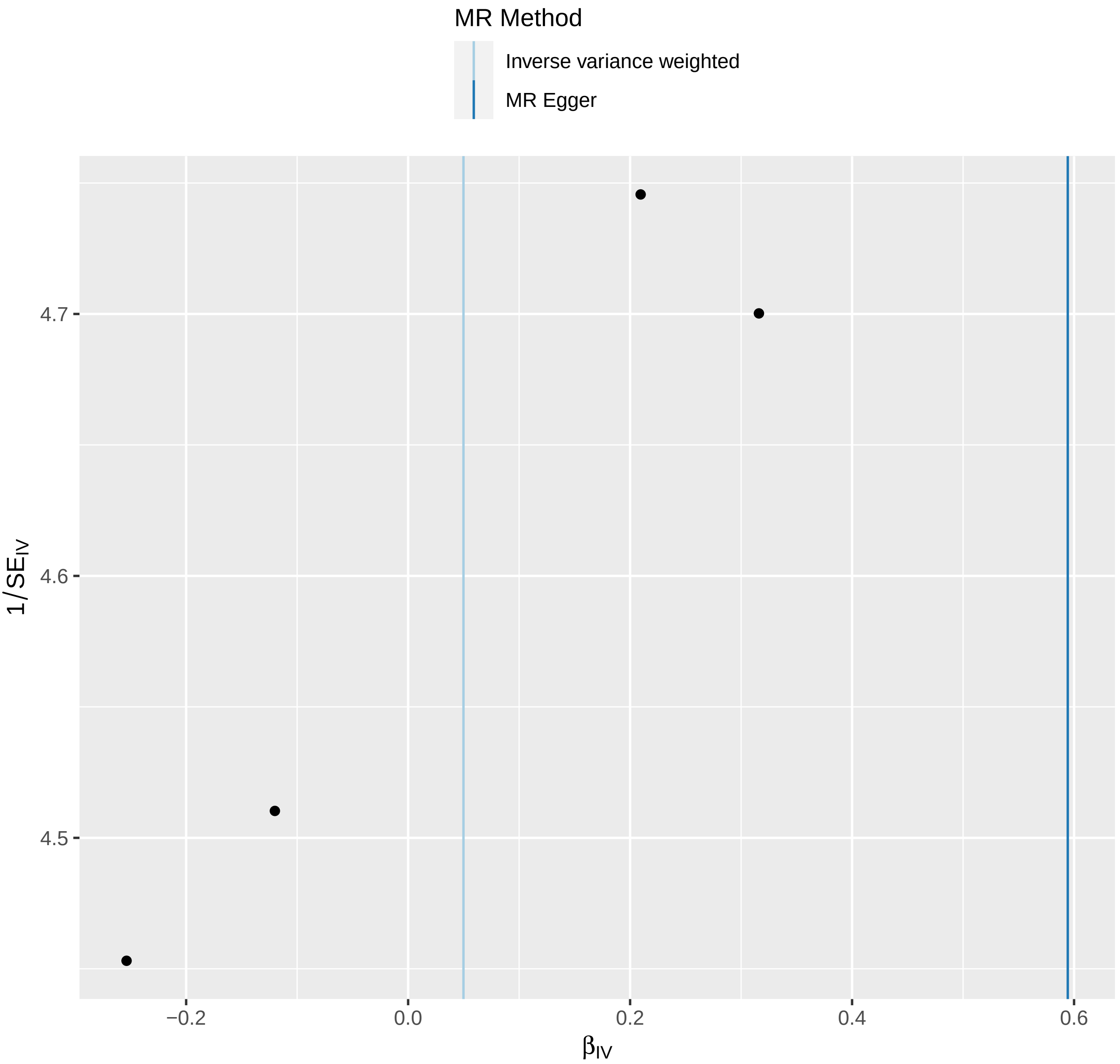

Batch 2−2988 : Gut microbiota abundance (genus Parasutterella id.2892) on Interleukin−1 alpha
with a threshold of 1e−05

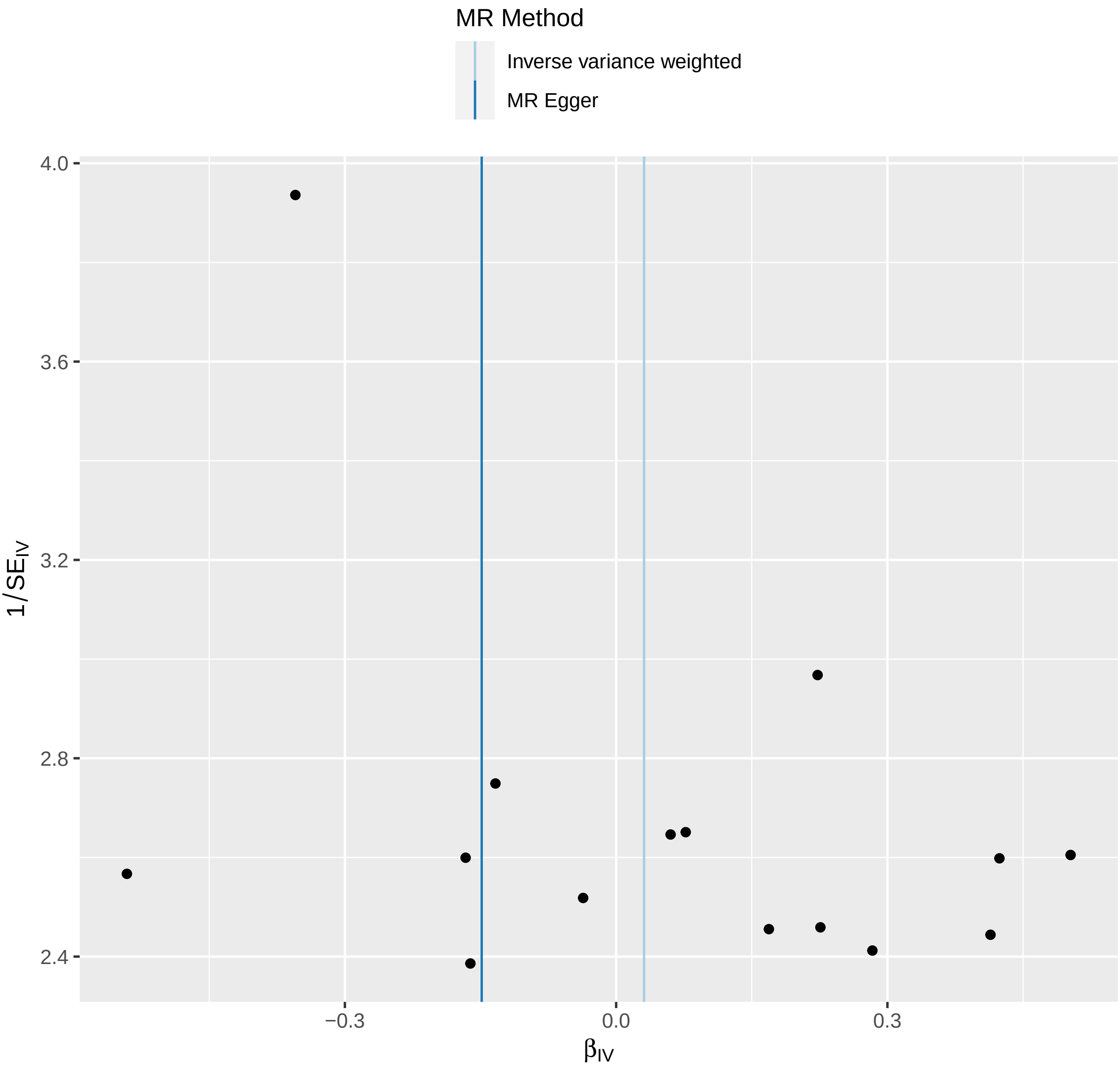

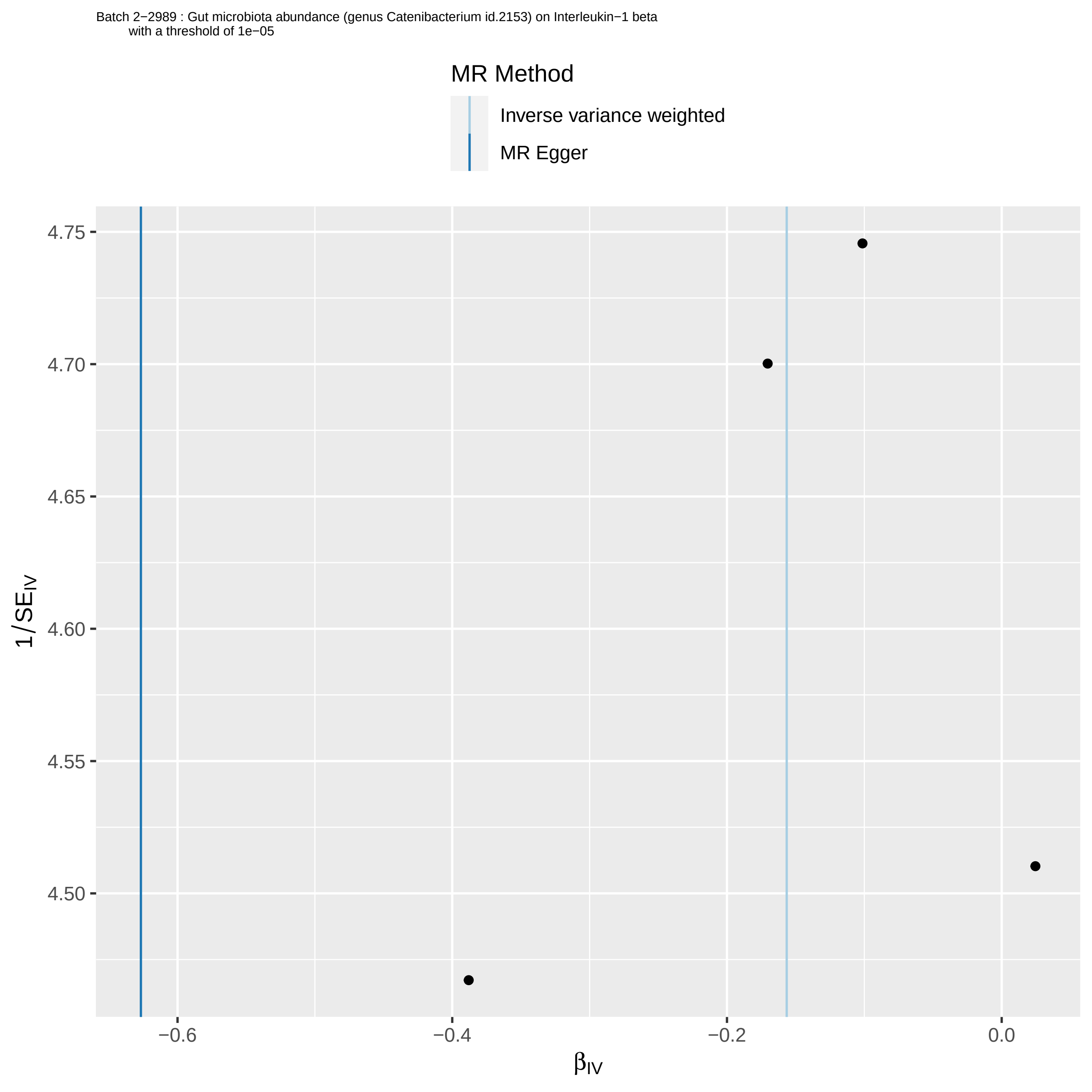
Batch 2−2989 : Gut microbiota abundance (genus Catenibacterium id.2153) on Interleukin−1 beta
with a threshold of 1e−05
MR Method
Inverse variance weighted
MR Egger
4.75
4.70
4.65
4.60
4.55
4.50
1/SE$_{IV}$
−0.6
−0.4
−0.2
0.0
β$_{IV}$

Batch 2−2990 : Gut microbiota abundance (genus Parasutterella id.2892) on Interleukin−1 beta
with a threshold of 1e−05

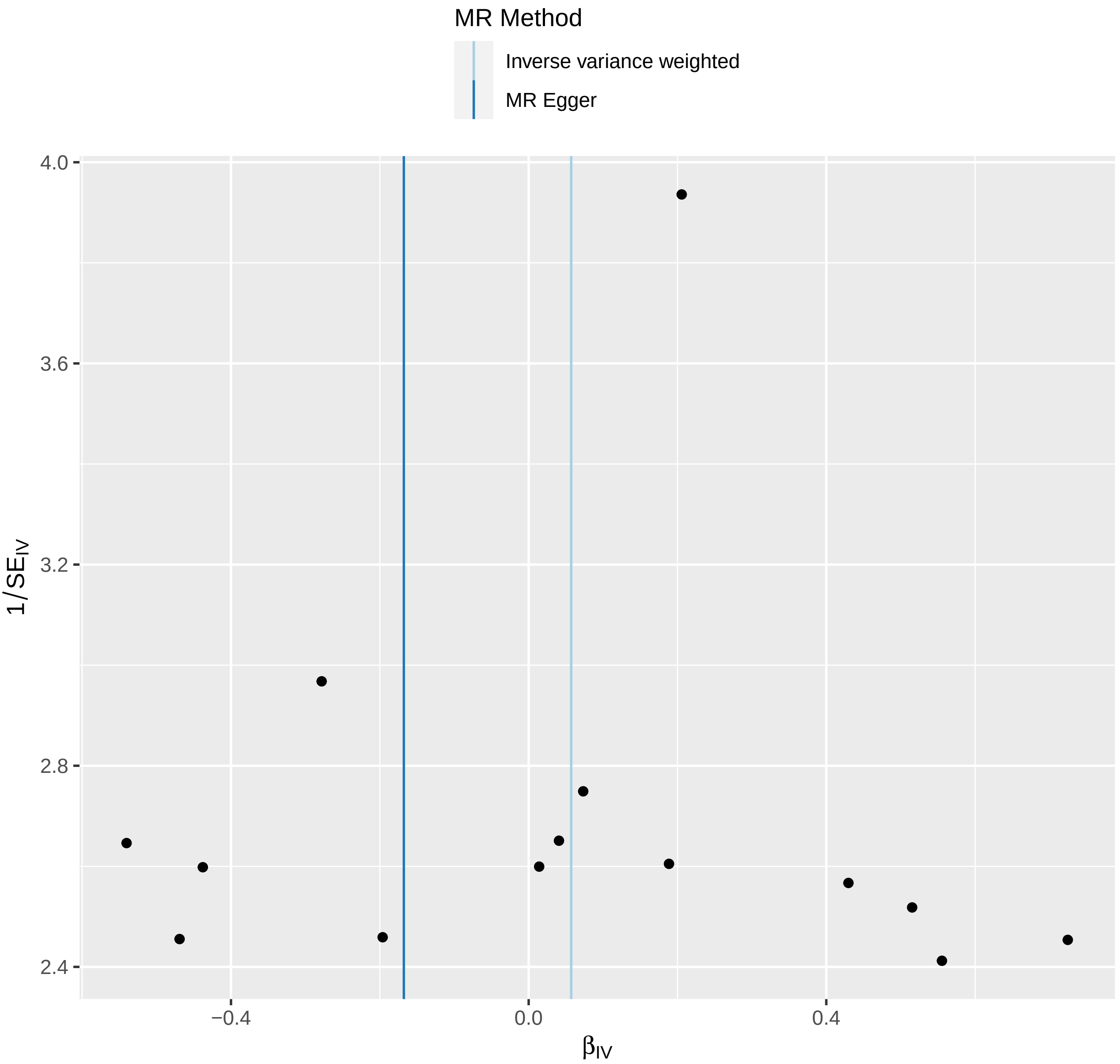

Batch 2−2991 : Gut microbiota abundance (genus Catenibacterium id.2153) on Interleukin−1 receptor type 1
with a threshold of 1e−05

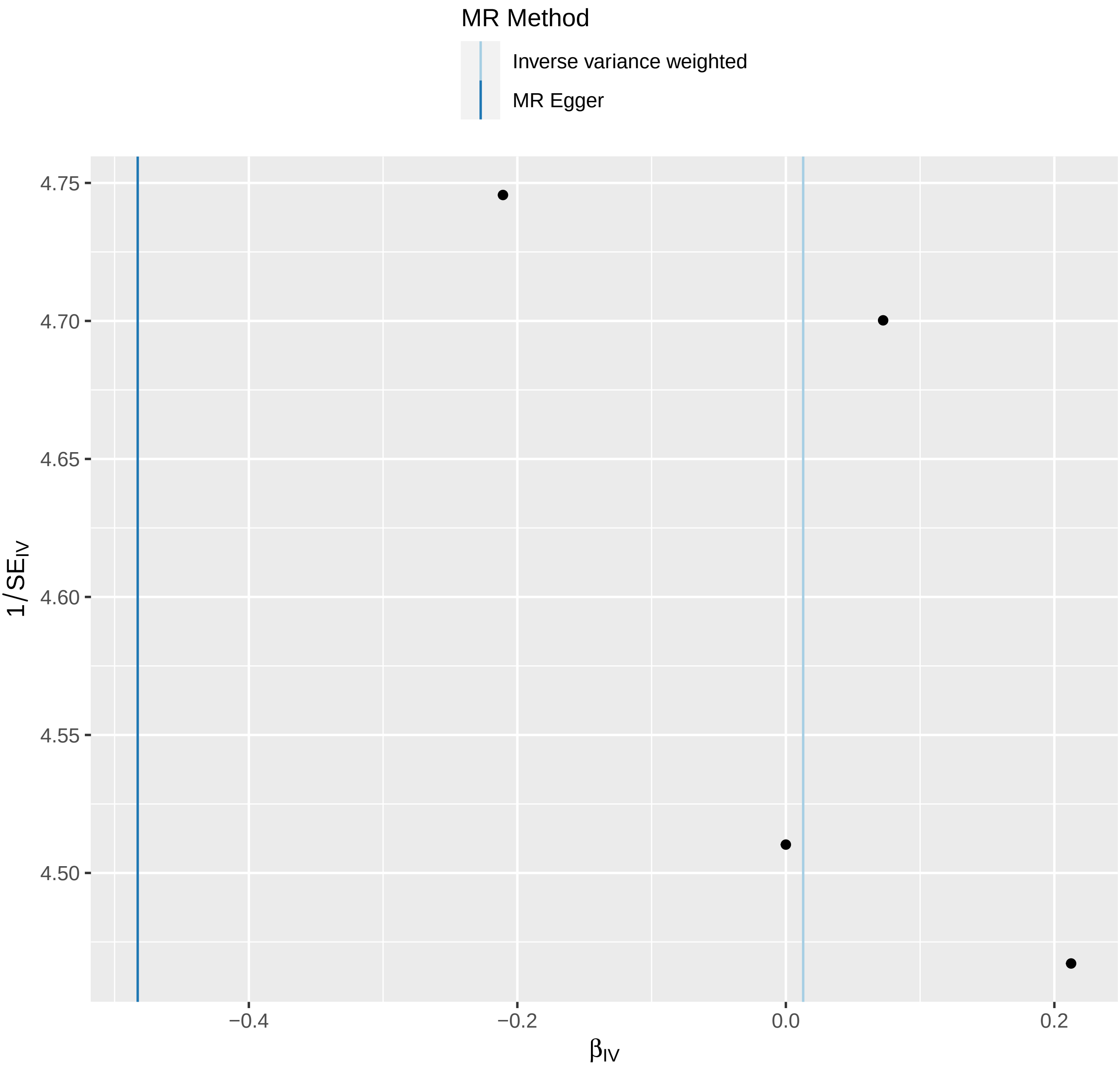

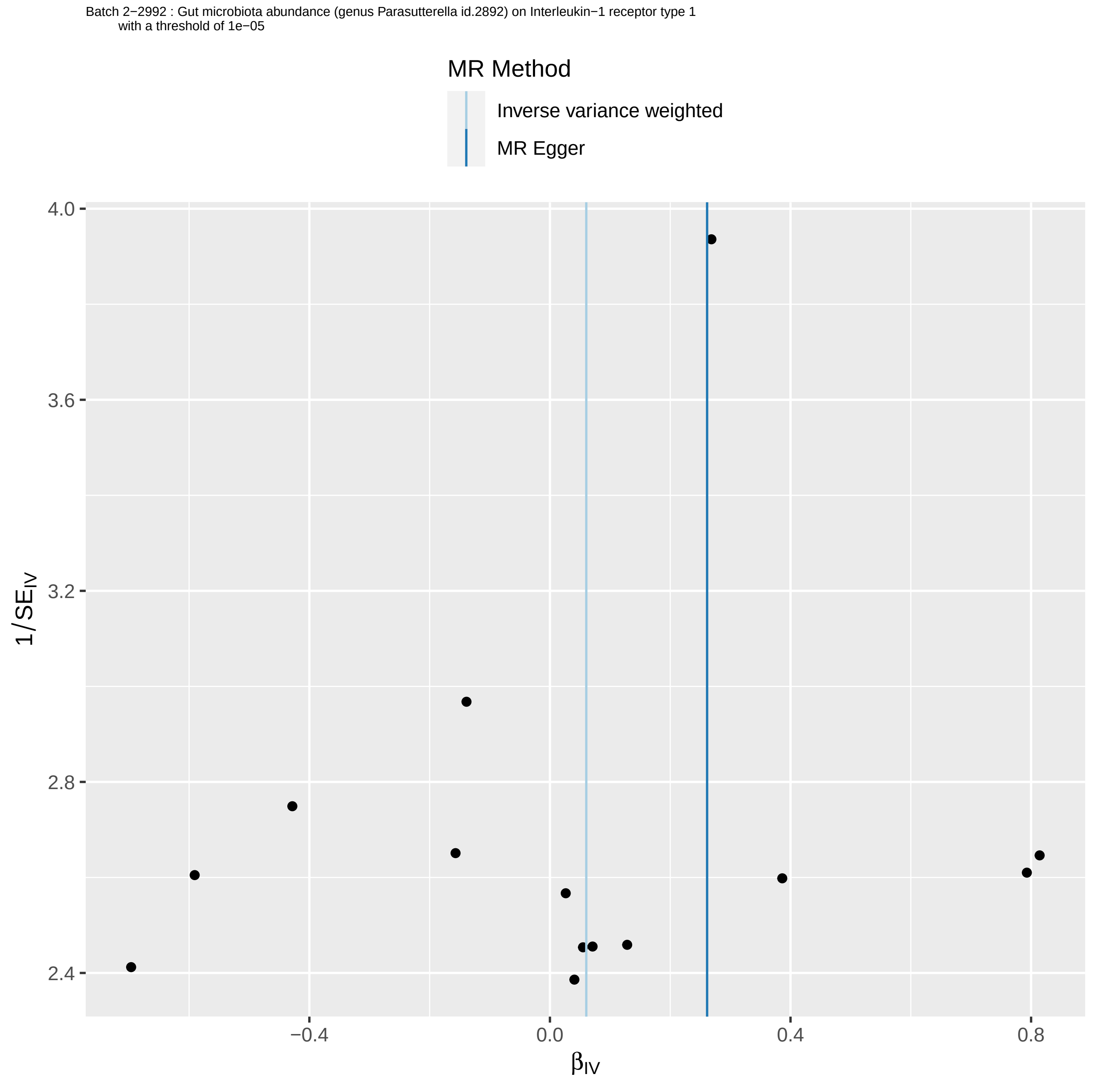
Batch 2−2992 : Gut microbiota abundance (genus Parasutterella id.2892) on Interleukin−1 receptor type 1
with a threshold of 1e−05
MR Method
Inverse variance weighted
MR Egger
4.0
3.6
3.2
2.8
2.4
1/SE_IV
−0.4
0.0
0.4
0.8
β_IV

Batch 2−2993 : Gut microbiota abundance (genus Catenibacterium id.2153) on Interleukin−1 receptor type 2
with a threshold of 1e−05

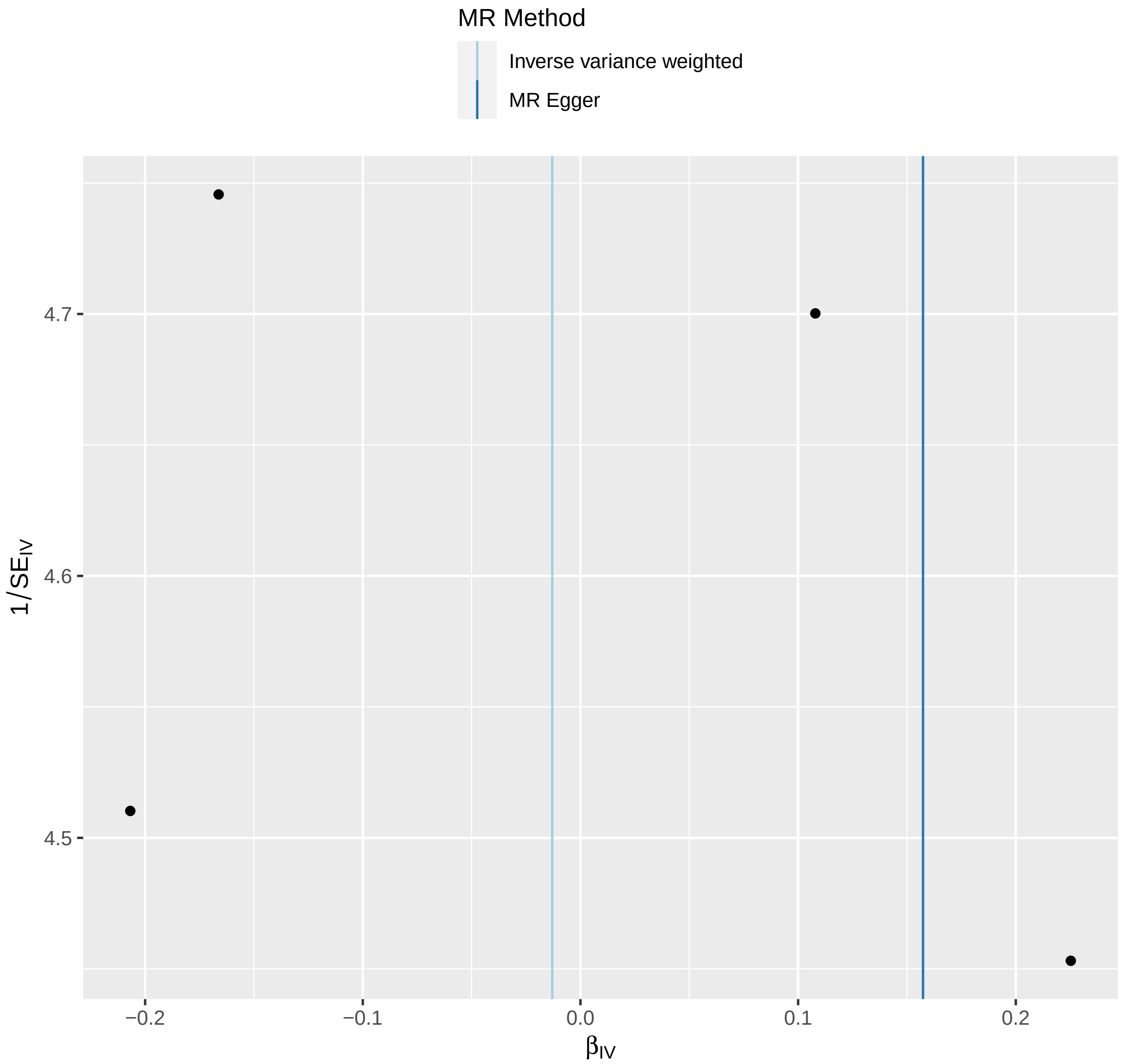

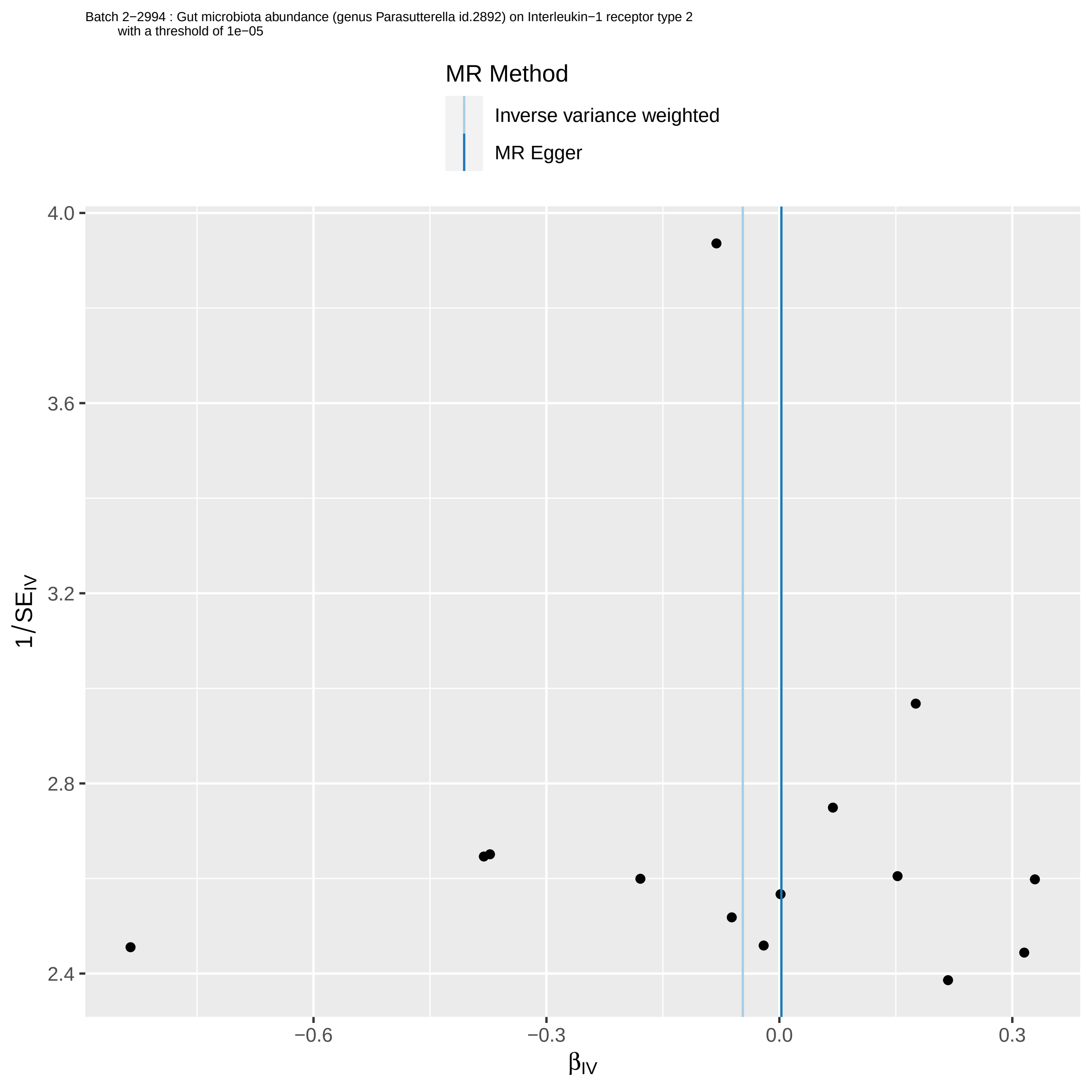

Batch 2−2994 : Gut microbiota abundance (genus Parasutterella id.2892) on Interleukin−1 receptor type 2
with a threshold of 1e−05
MR Method
Inverse variance weighted
MR Egger
4.0
3.6
3.2
2.8
2.4
1/SE_IV
−0.6
−0.3
0.0
0.3
β_IV

Batch 2−2995 : Gut microbiota abundance (genus Catenibacterium id.2153) on Interleukin−1 receptor type 2
with a threshold of 1e−05

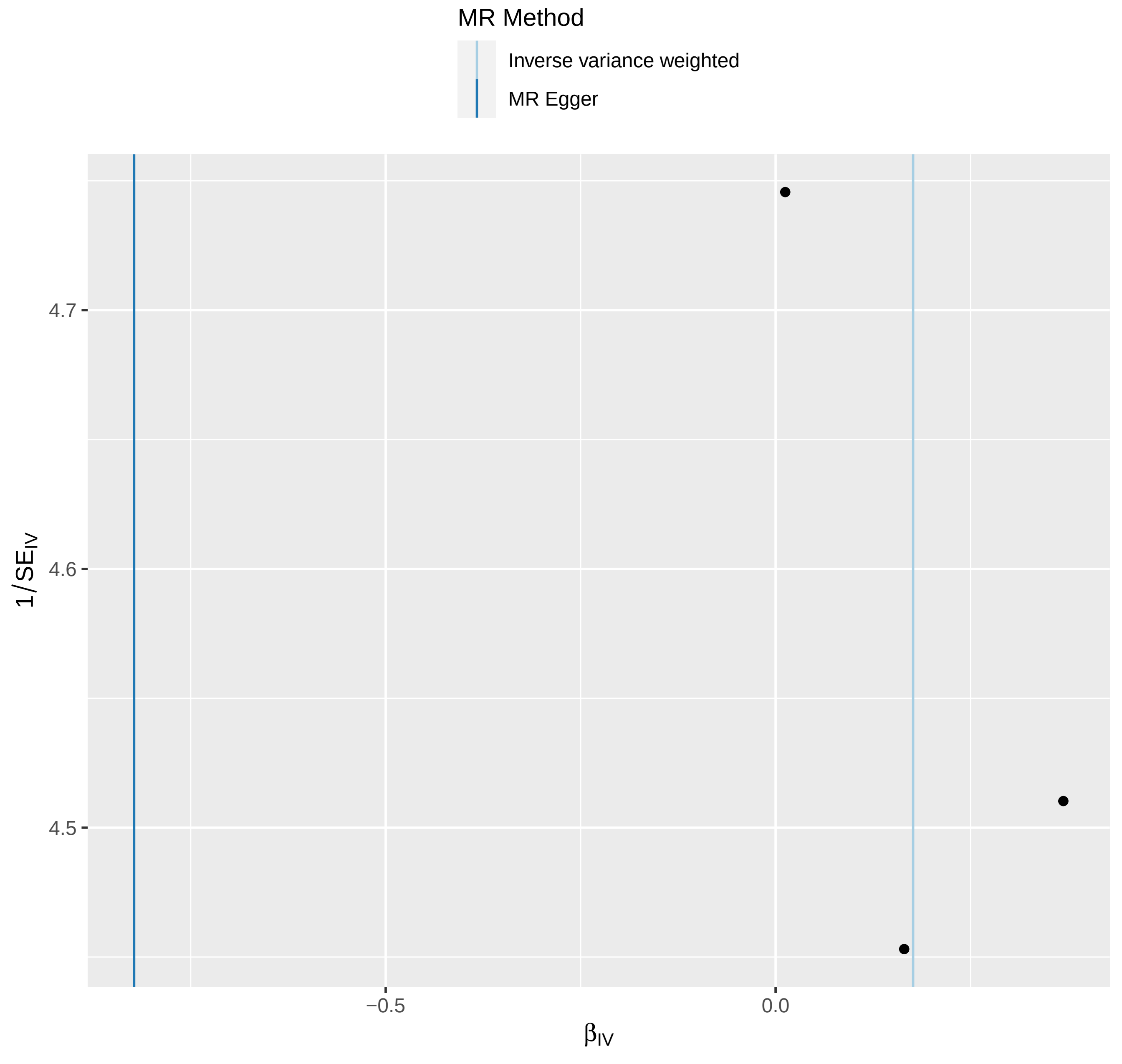

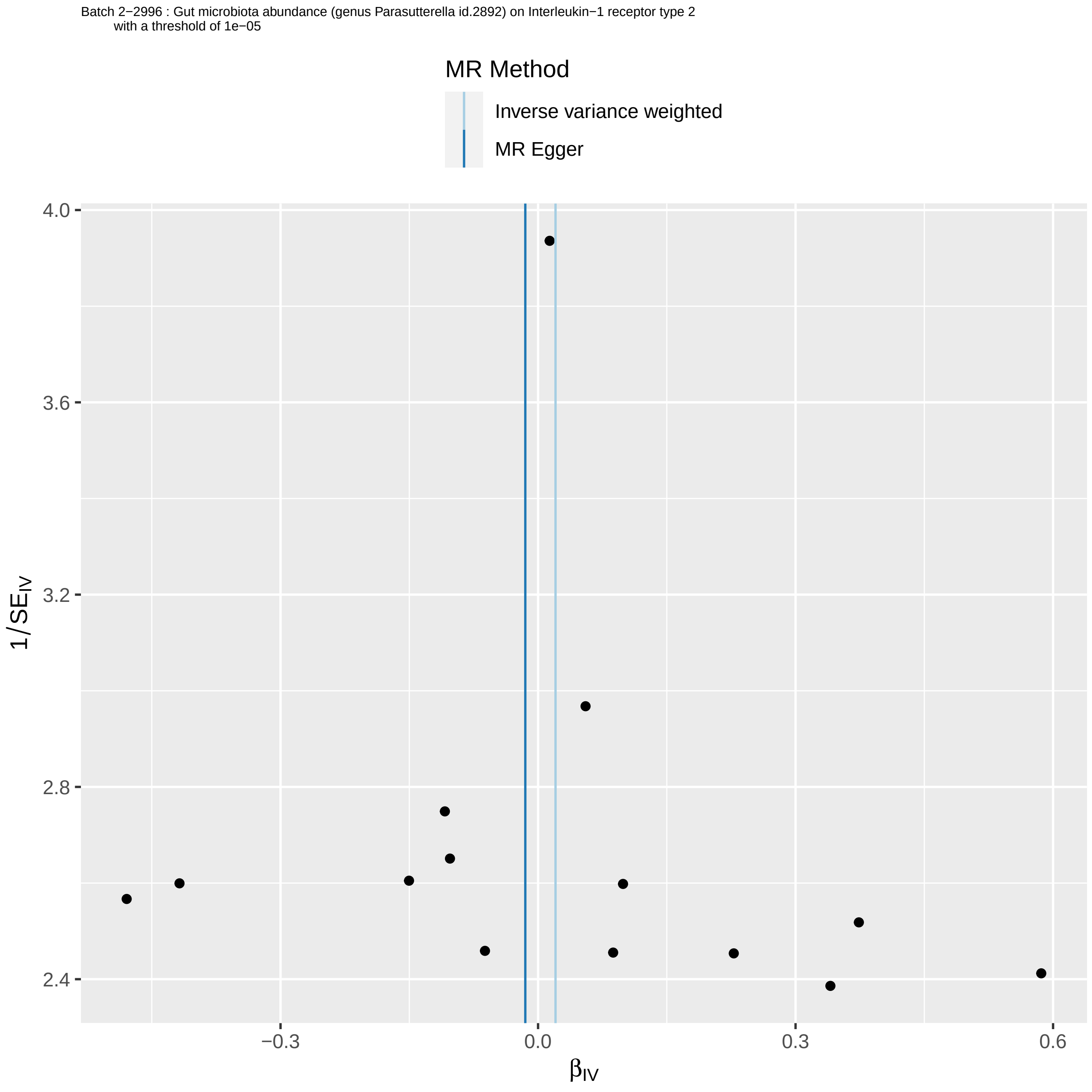
Batch 2−2996 : Gut microbiota abundance (genus Parasutterella id.2892) on Interleukin−1 receptor type 2
with a threshold of 1e−05
MR Method
Inverse variance weighted
MR Egger
4.0
3.6
3.2
2.8
2.4
1/SE_IV
−0.3
0.0
0.3
0.6
β_IV

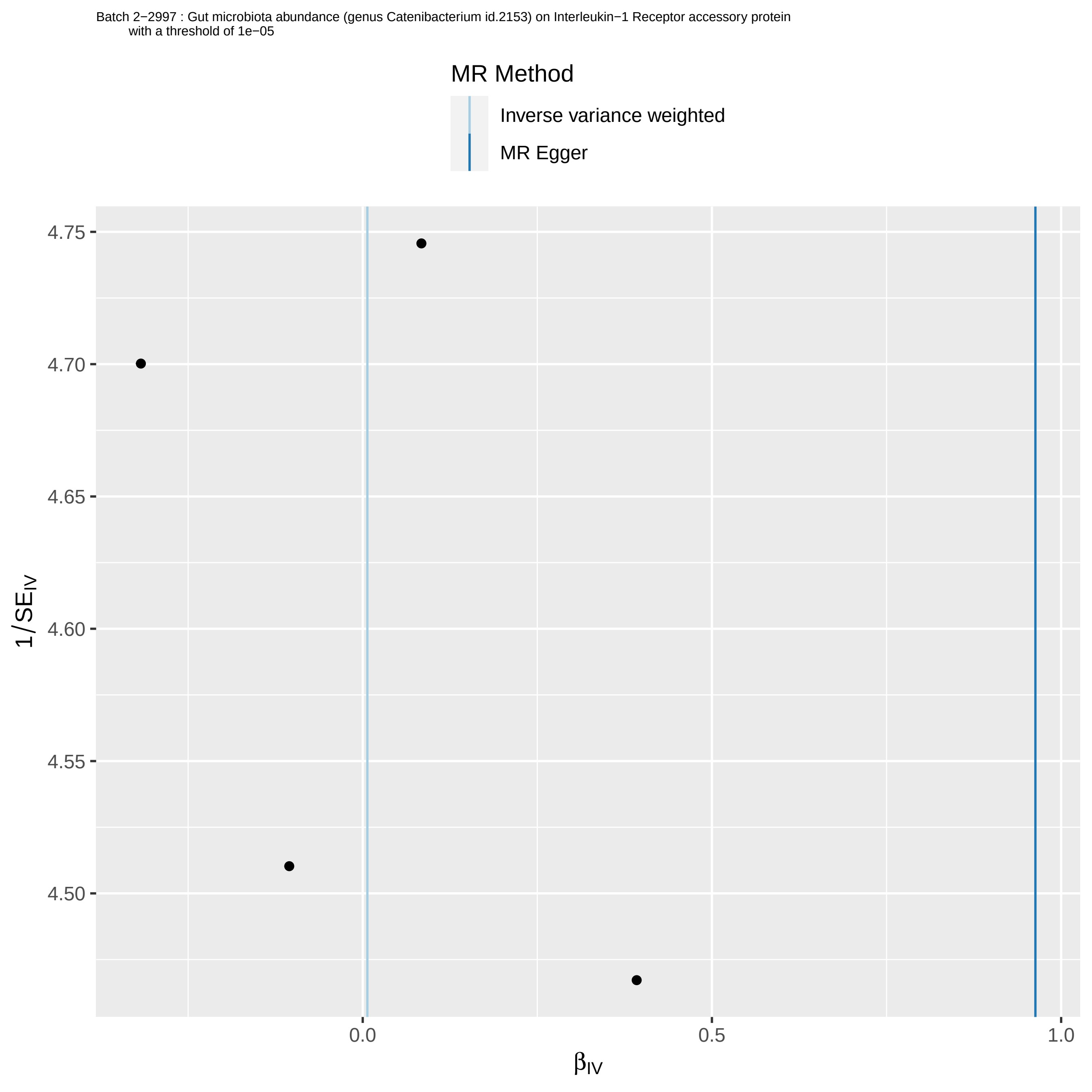

Batch 2−2997 : Gut microbiota abundance (genus Catenibacterium id.2153) on Interleukin−1 Receptor accessory protein
with a threshold of 1e−05
MR Method
Inverse variance weighted
MR Egger
4.75
4.70
4.65
4.60
4.55
4.50
1/SE$_{IV}$
0.0
0.5
1.0
β_{IV}

Batch 2−2998 : Gut microbiota abundance (genus Parasutterella id.2892) on Interleukin−1 Receptor accessory protein
with a threshold of 1e−05

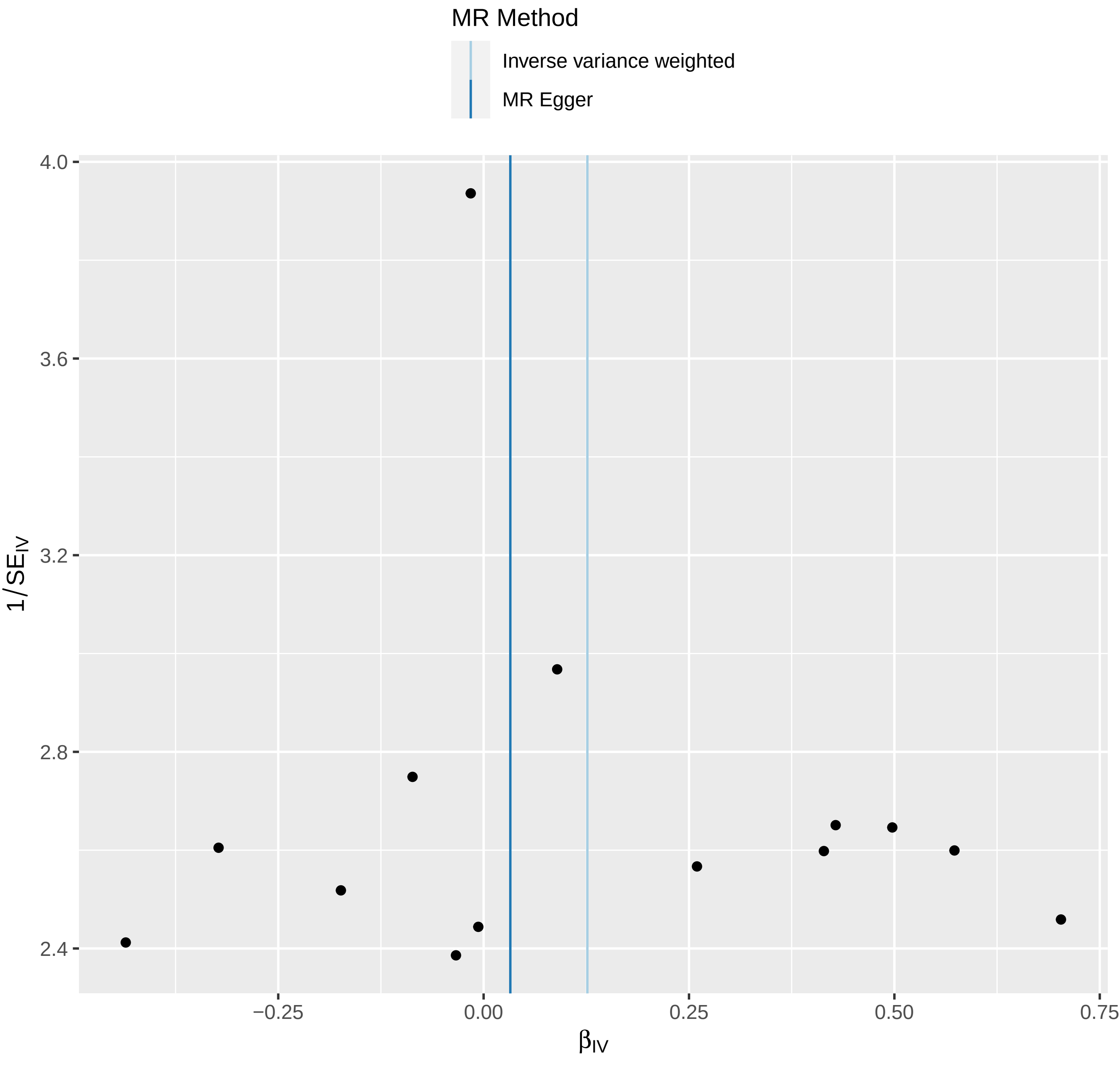

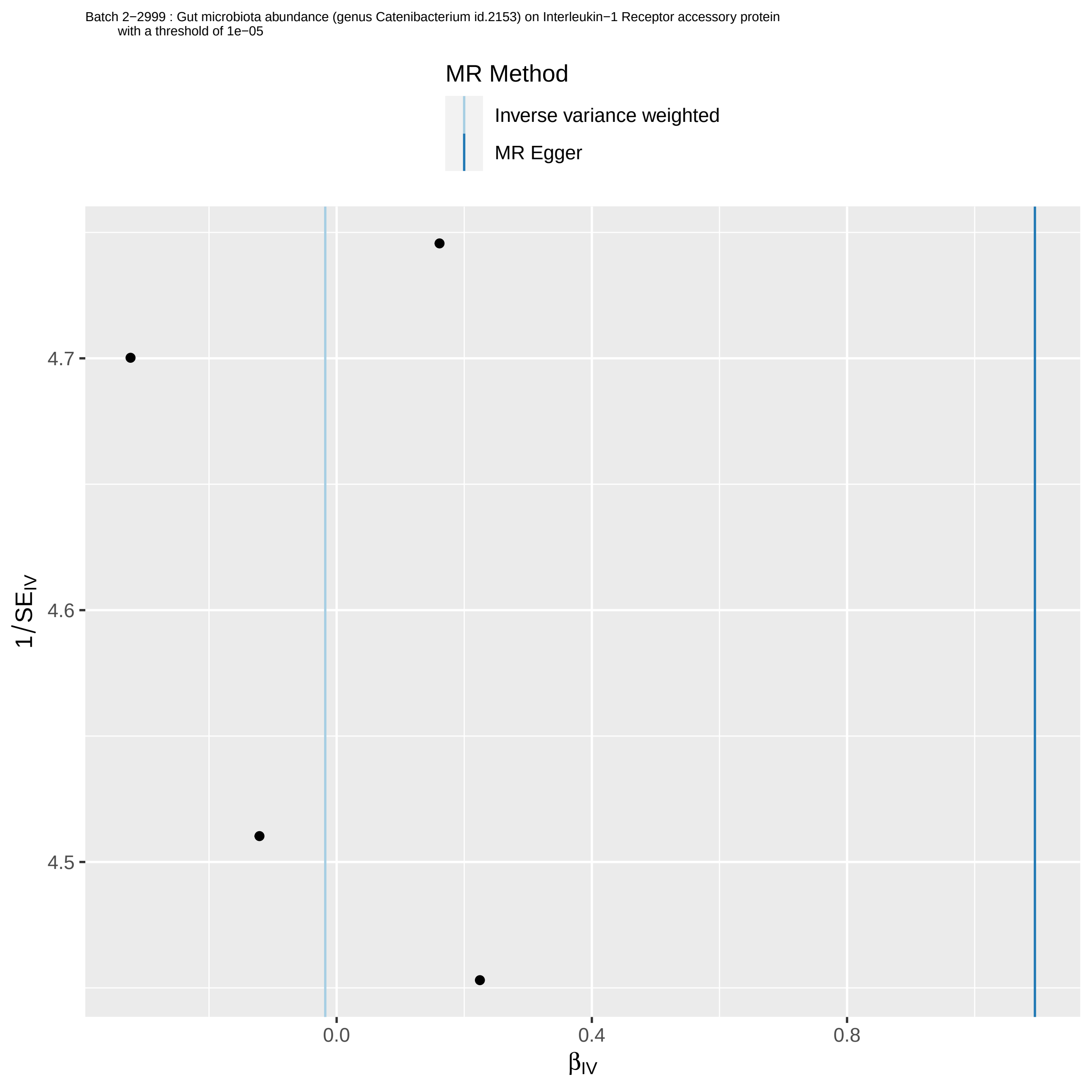
Batch 2−2999 : Gut microbiota abundance (genus Catenibacterium id.2153) on Interleukin−1 Receptor accessory protein
with a threshold of 1e−05
MR Method
Inverse variance weighted
MR Egger
4.7
4.6
4.5
1/SE_IV
0.0
0.4
0.8
β_IV

Batch 2−3000 : Gut microbiota abundance (genus Parasutterella id.2892) on Interleukin−1 Receptor accessory protein
with a threshold of 1e−05

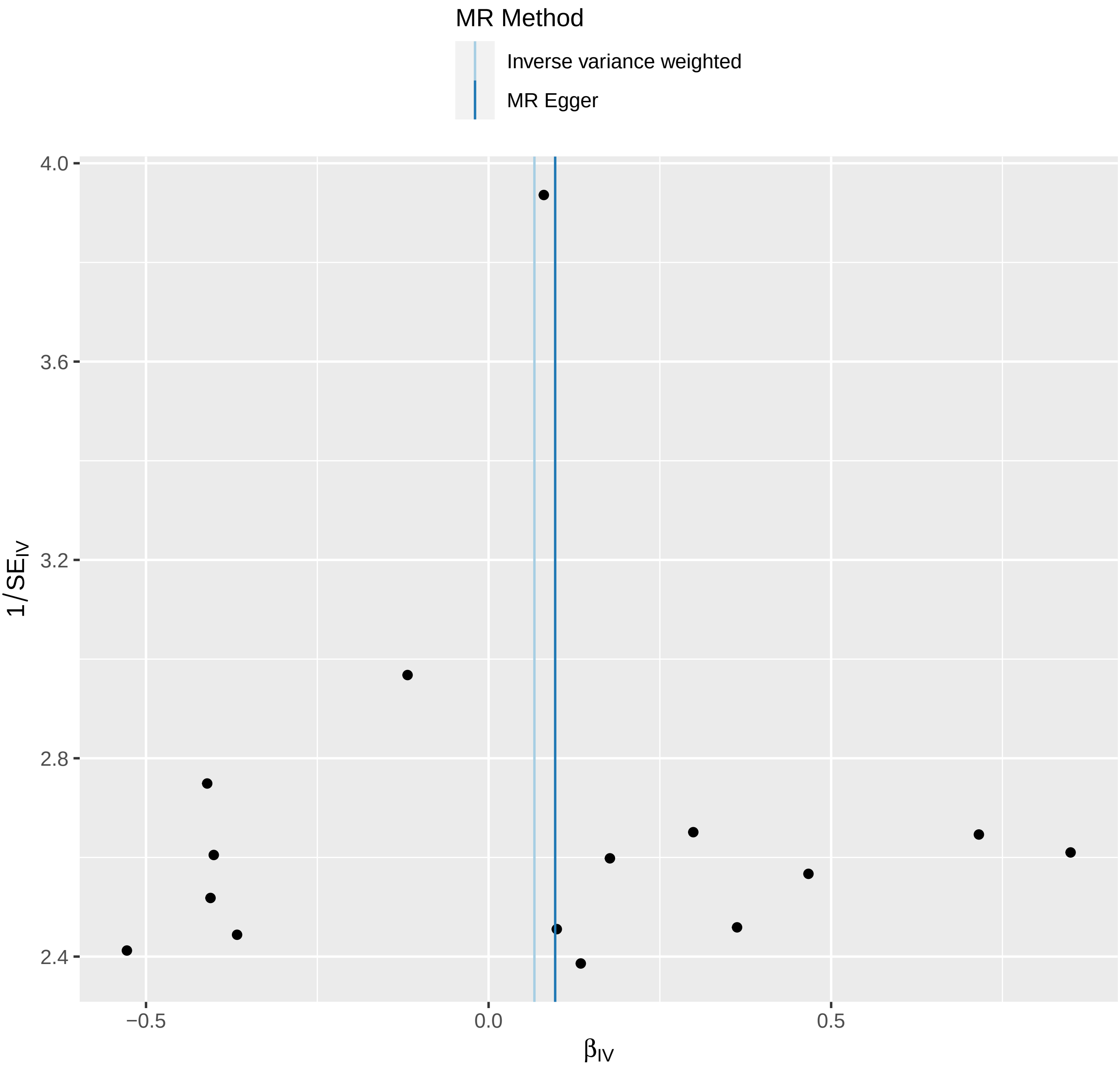

Batch 2−3001 : Gut microbiota abundance (genus Catenibacterium id.2153) on X−linked interleukin−1 receptor accessory protein−like 2
with a threshold of 1e−05

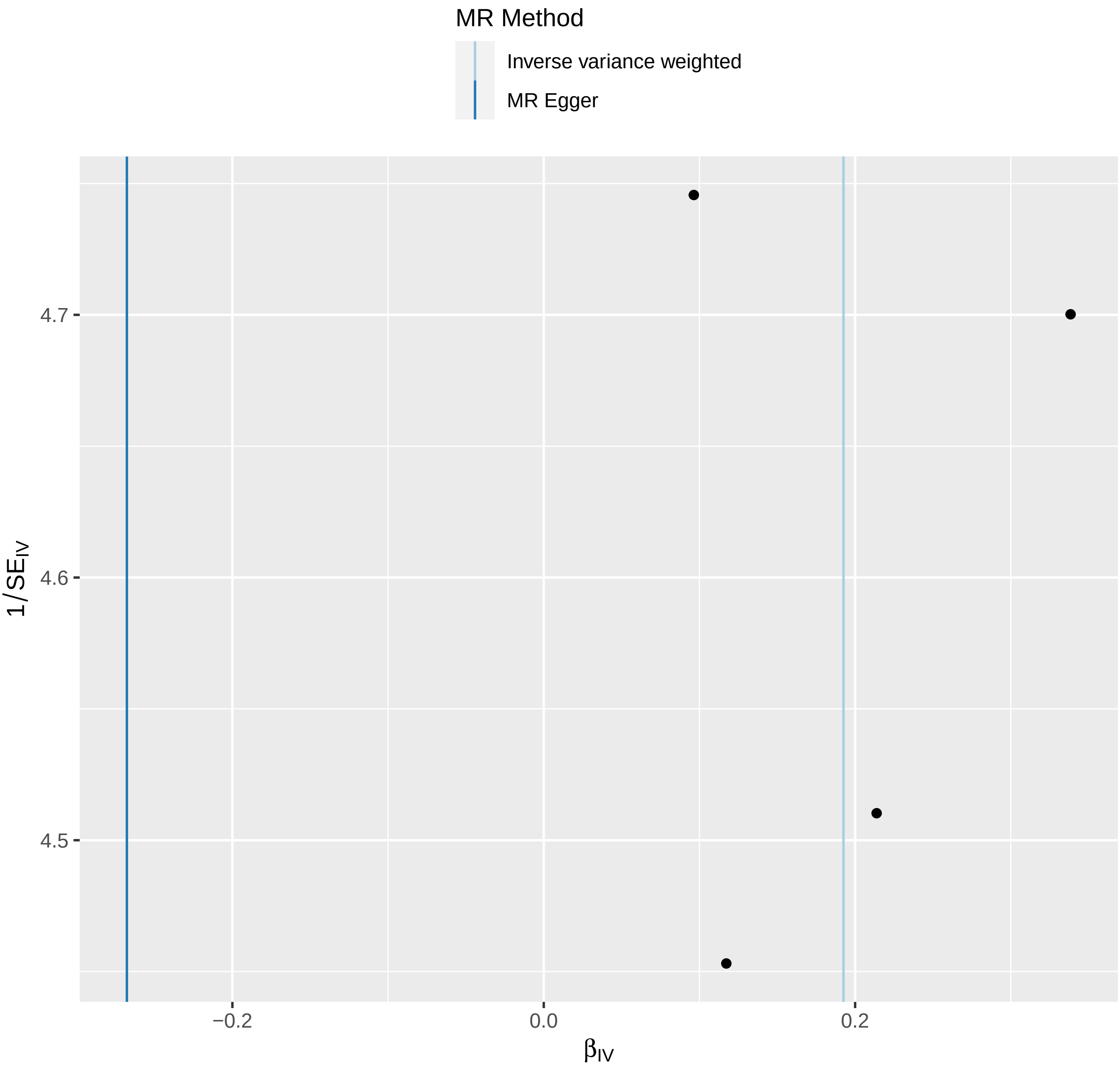

Batch 2−3002 : Gut microbiota abundance (genus Parasutterella id.2892) on X−linked interleukin−1 receptor accessory protein−like 2 with a threshold of 1e−05

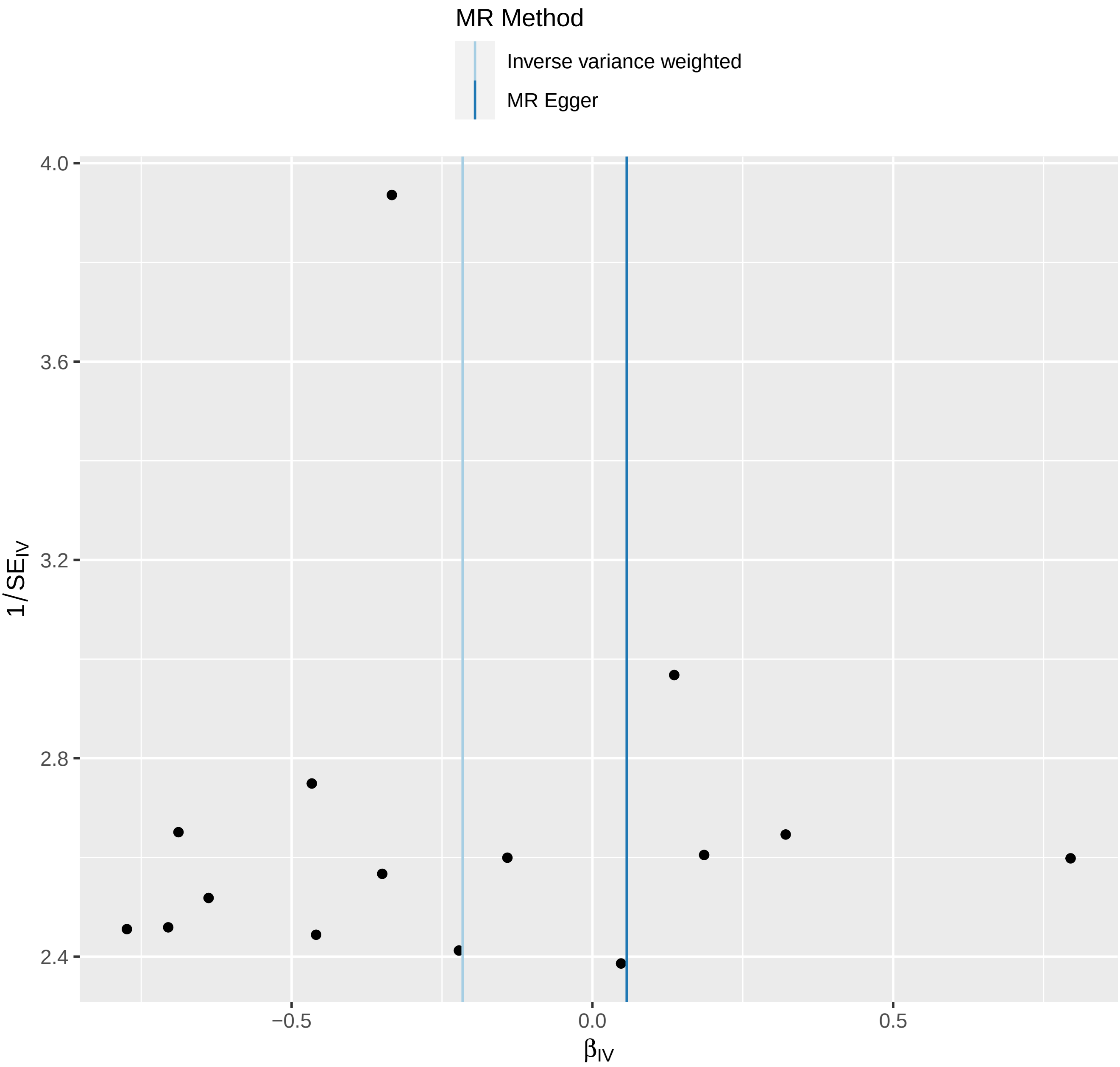

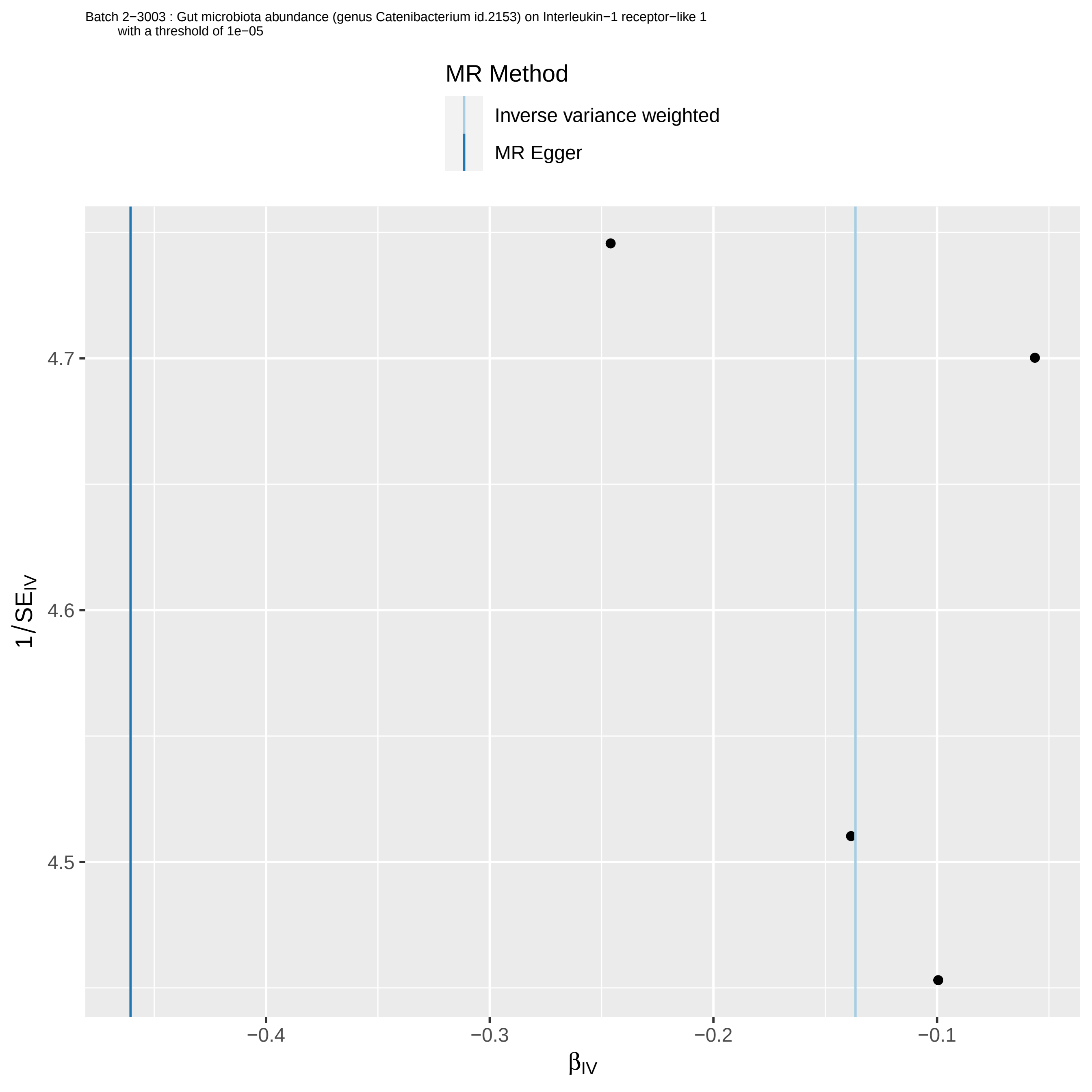
Batch 2−3003 : Gut microbiota abundance (genus Catenibacterium id.2153) on Interleukin−1 receptor−like 1
with a threshold of 1e−05
MR Method
Inverse variance weighted
MR Egger
4.7
4.6
4.5
1/SE_IV
−0.4
−0.3
−0.2
−0.1
β_IV

Batch 2−3004 : Gut microbiota abundance (genus Parasutterella id.2892) on Interleukin−1 receptor−like 1
with a threshold of 1e−05

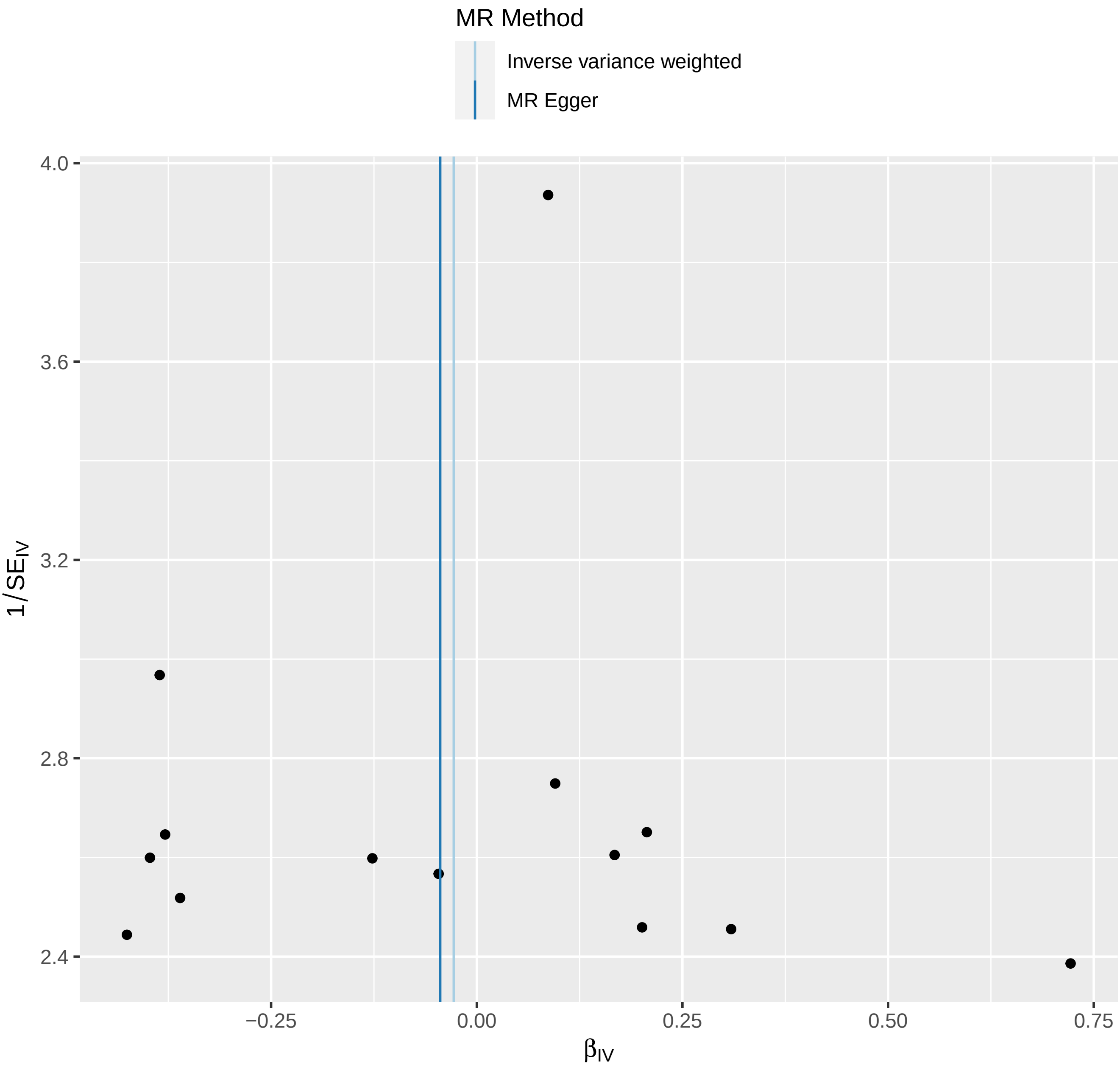

Batch 2−3005 : Gut microbiota abundance (genus Catenibacterium id.2153) on Interleukin−1 receptor−like 2
with a threshold of 1e−05

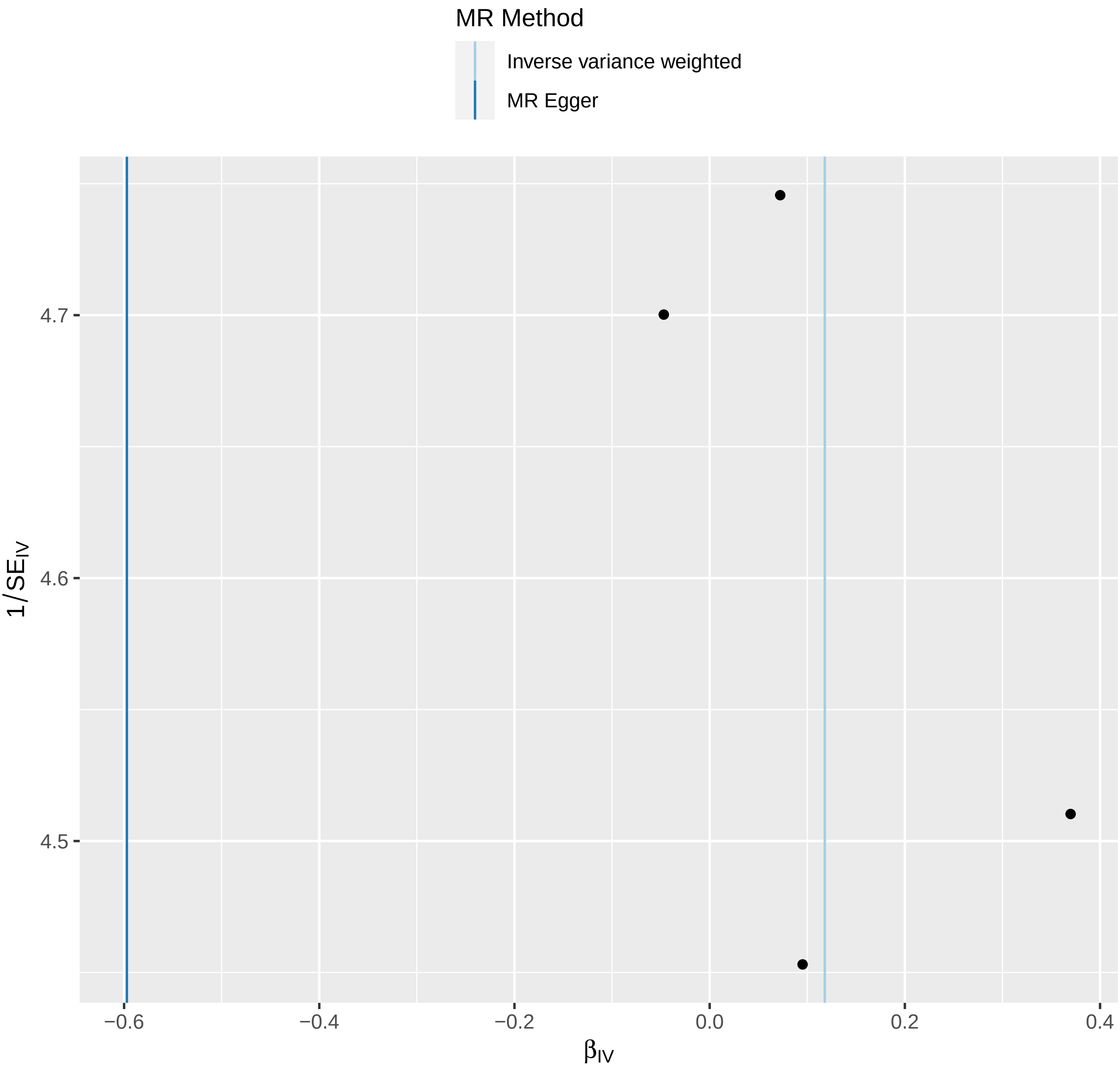

Batch 2−3006 : Gut microbiota abundance (genus Parasutterella id.2892) on Interleukin−1 receptor−like 2
with a threshold of 1e−05

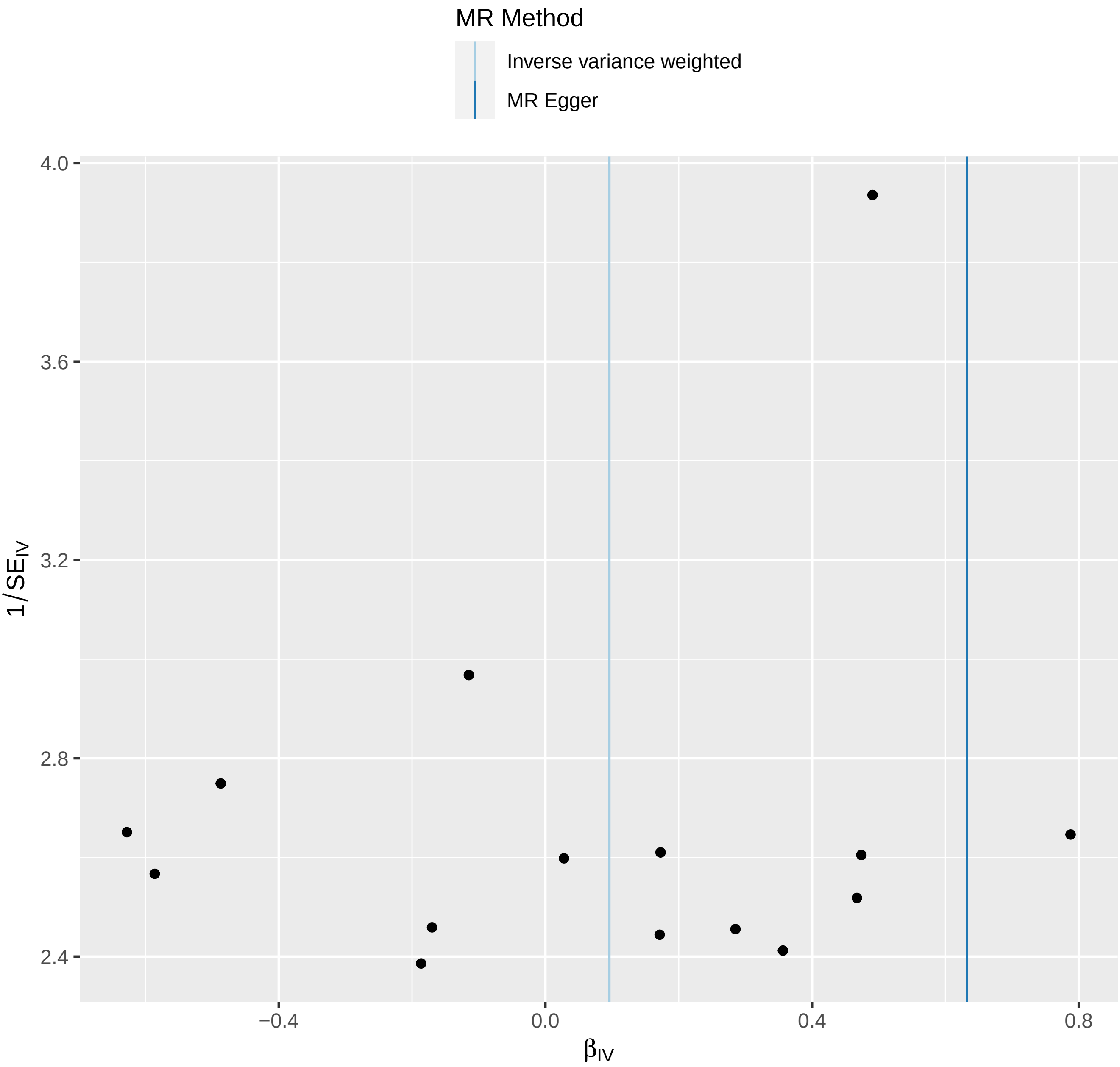

Batch 2−3007 : Gut microbiota abundance (genus Catenibacterium id.2153) on Interleukin−1 receptor antagonist protein with a threshold of 1e−05

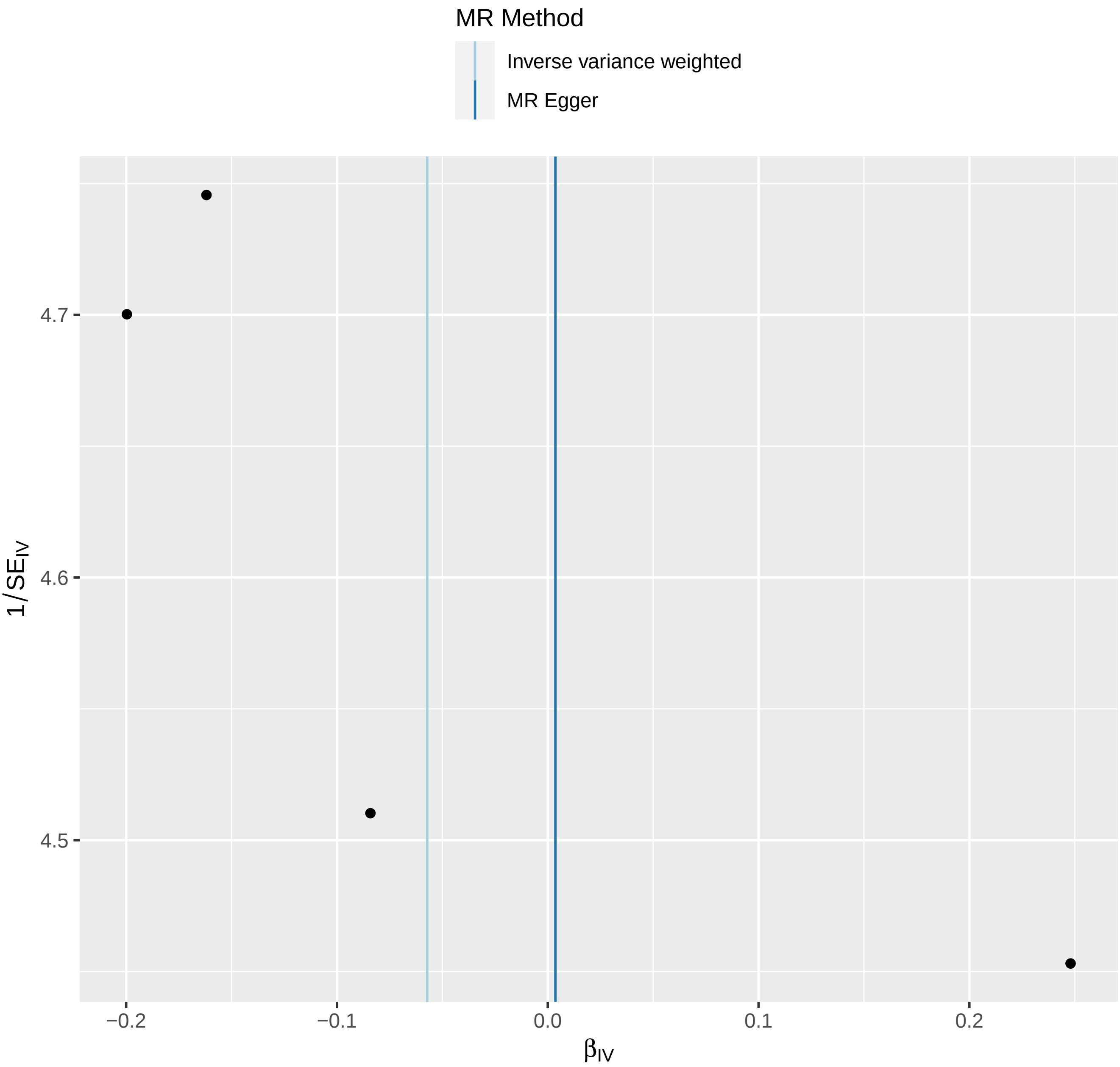

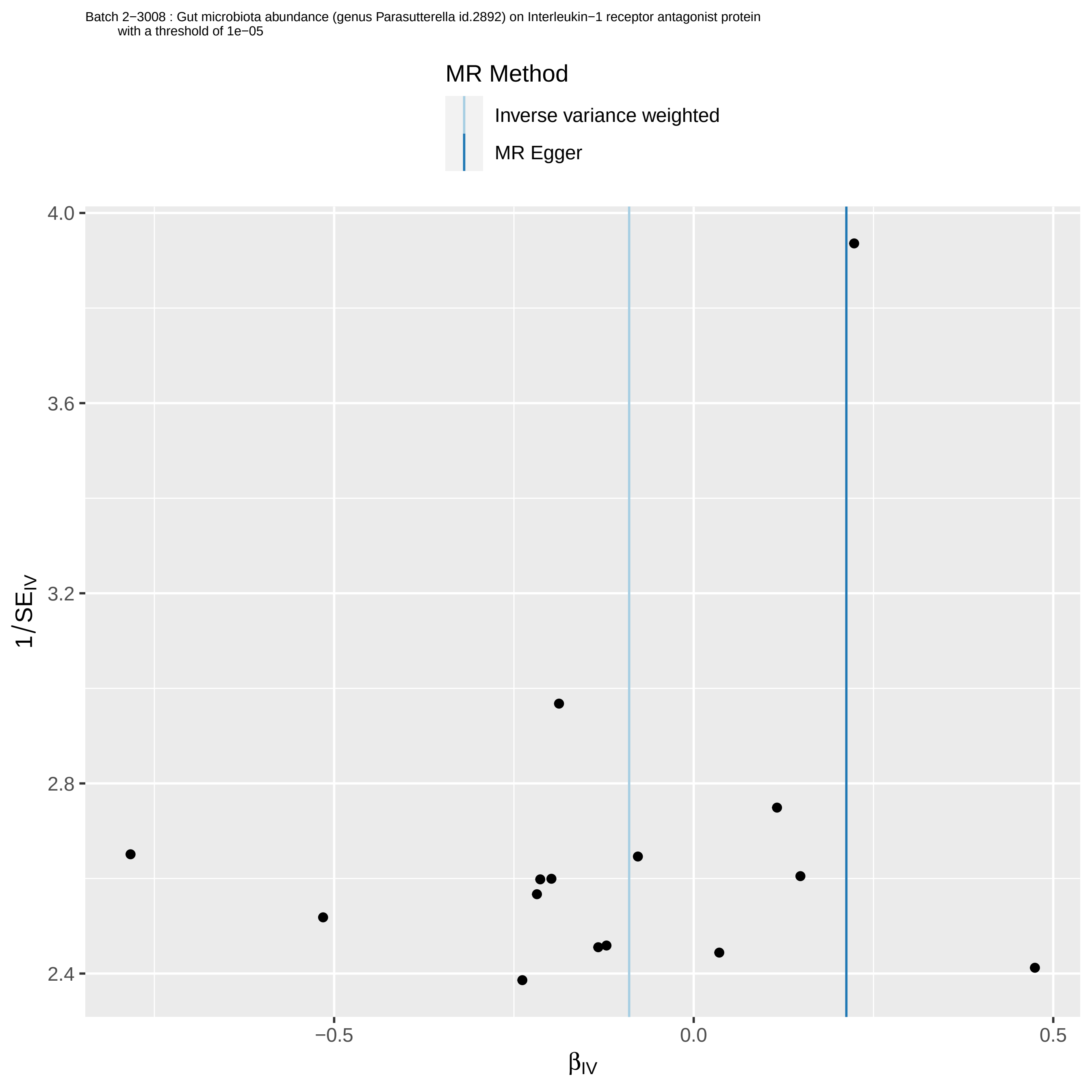

Batch 2−3008 : Gut microbiota abundance (genus Parasutterella id.2892) on Interleukin−1 receptor antagonist protein
with a threshold of 1e−05
MR Method
Inverse variance weighted
MR Egger
4.0
3.6
3.2
2.8
2.4
1/SE_IV
−0.5
0.0
0.5
β_IV

Batch 2−3009 : Gut microbiota abundance (genus Catenibacterium id.2153) on Interleukin−20
with a threshold of 1e−05

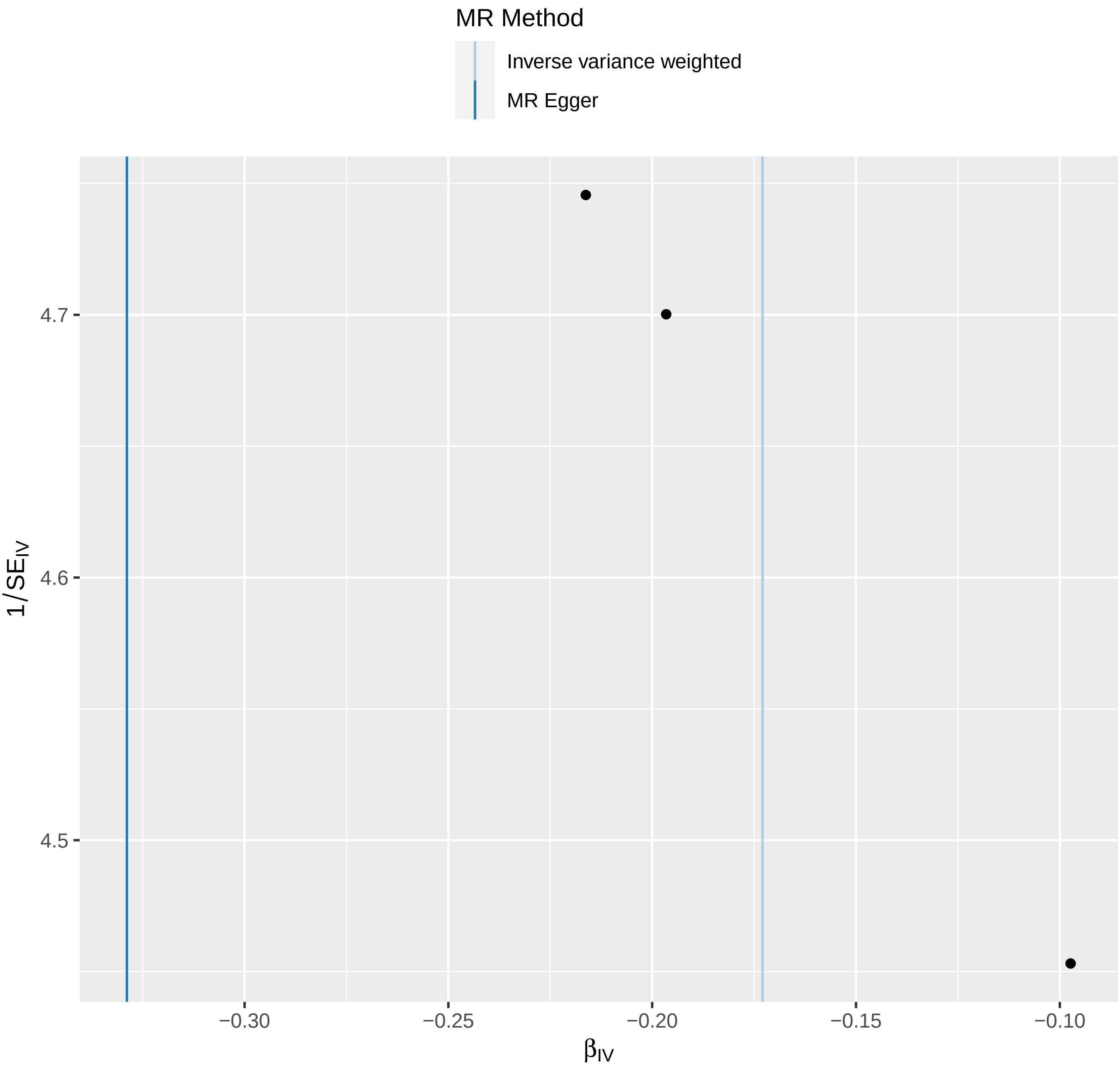

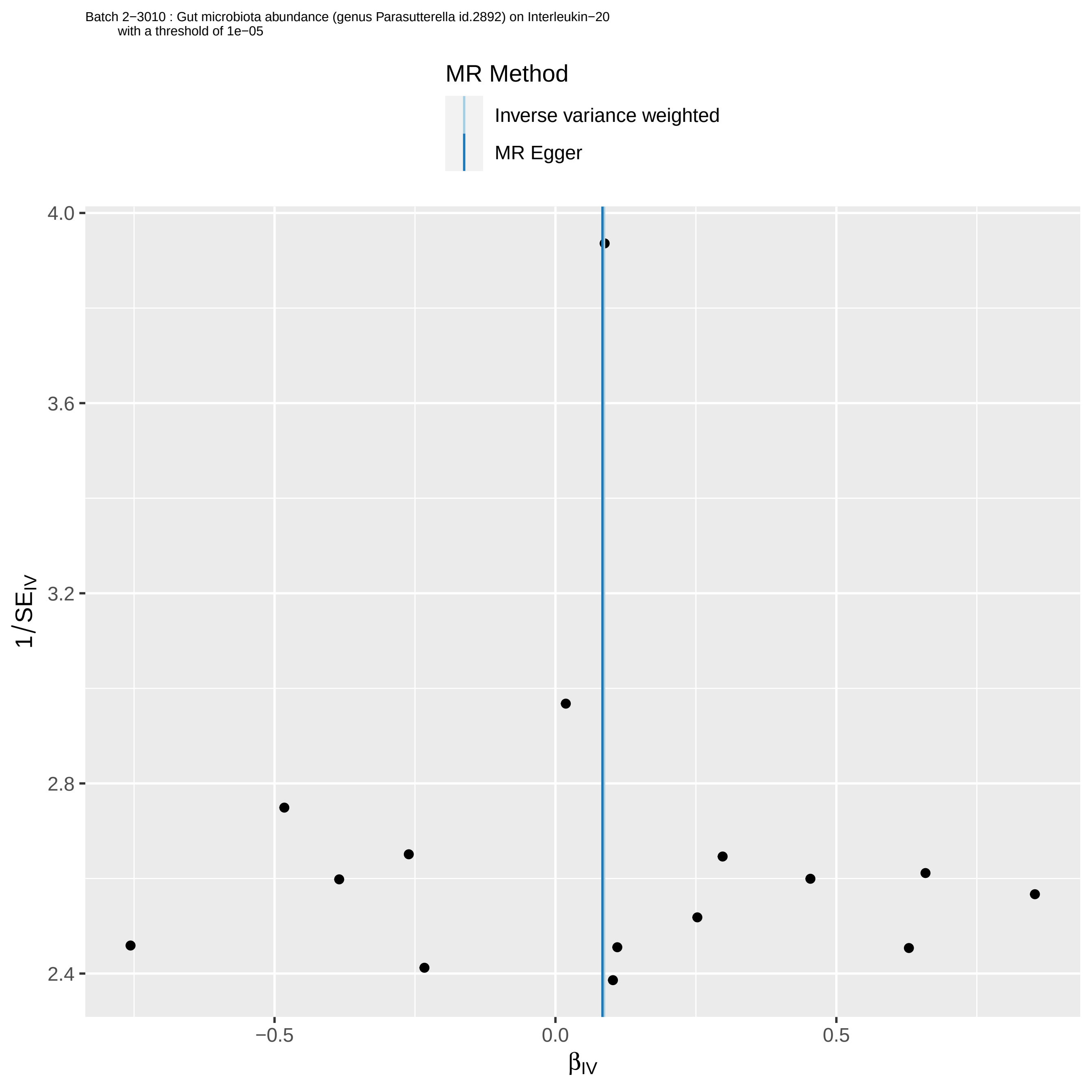
Batch 2−3010 : Gut microbiota abundance (genus Parasutterella id.2892) on Interleukin−20
with a threshold of 1e−05
MR Method
Inverse variance weighted
MR Egger
4.0
3.6
3.2
2.8
2.4
1/SE_IV
−0.5
0.0
0.5
β_IV

Batch 2−3011 : Gut microbiota abundance (genus Catenibacterium id.2153) on Interleukin−21
with a threshold of 1e−05

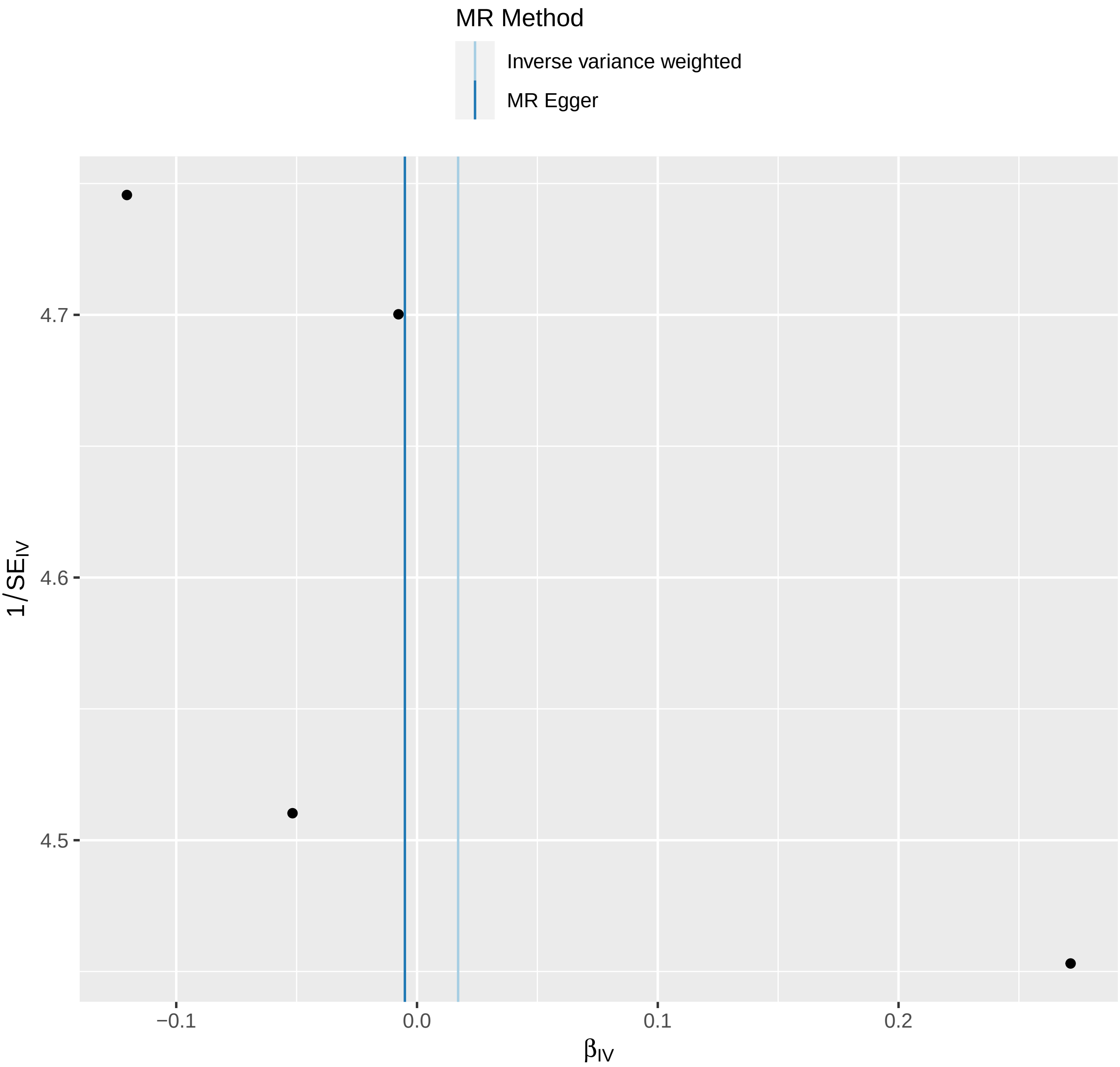

Batch 2−3012 : Gut microbiota abundance (genus Parasutterella id.2892) on Interleukin−21
with a threshold of 1e−05

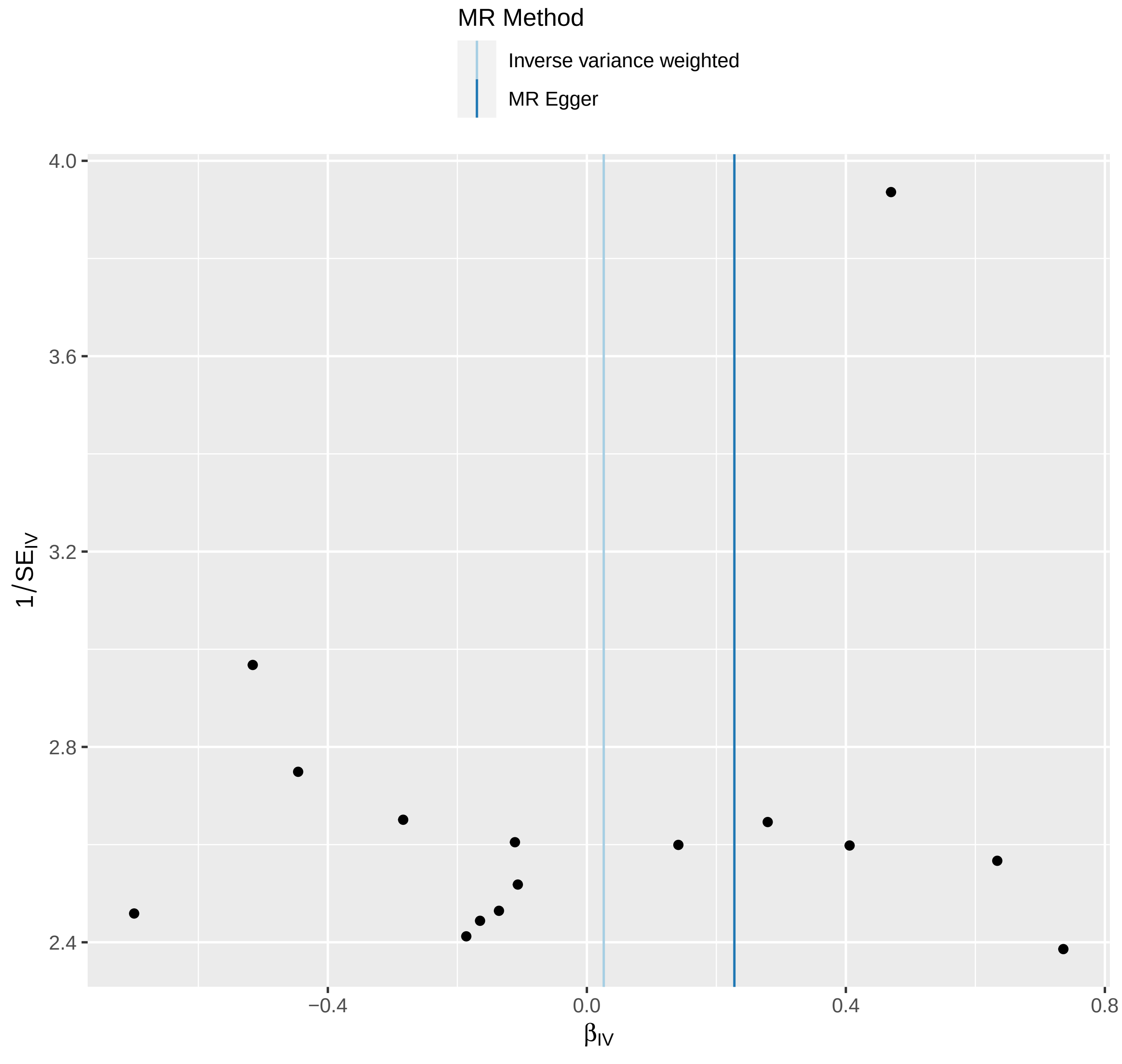

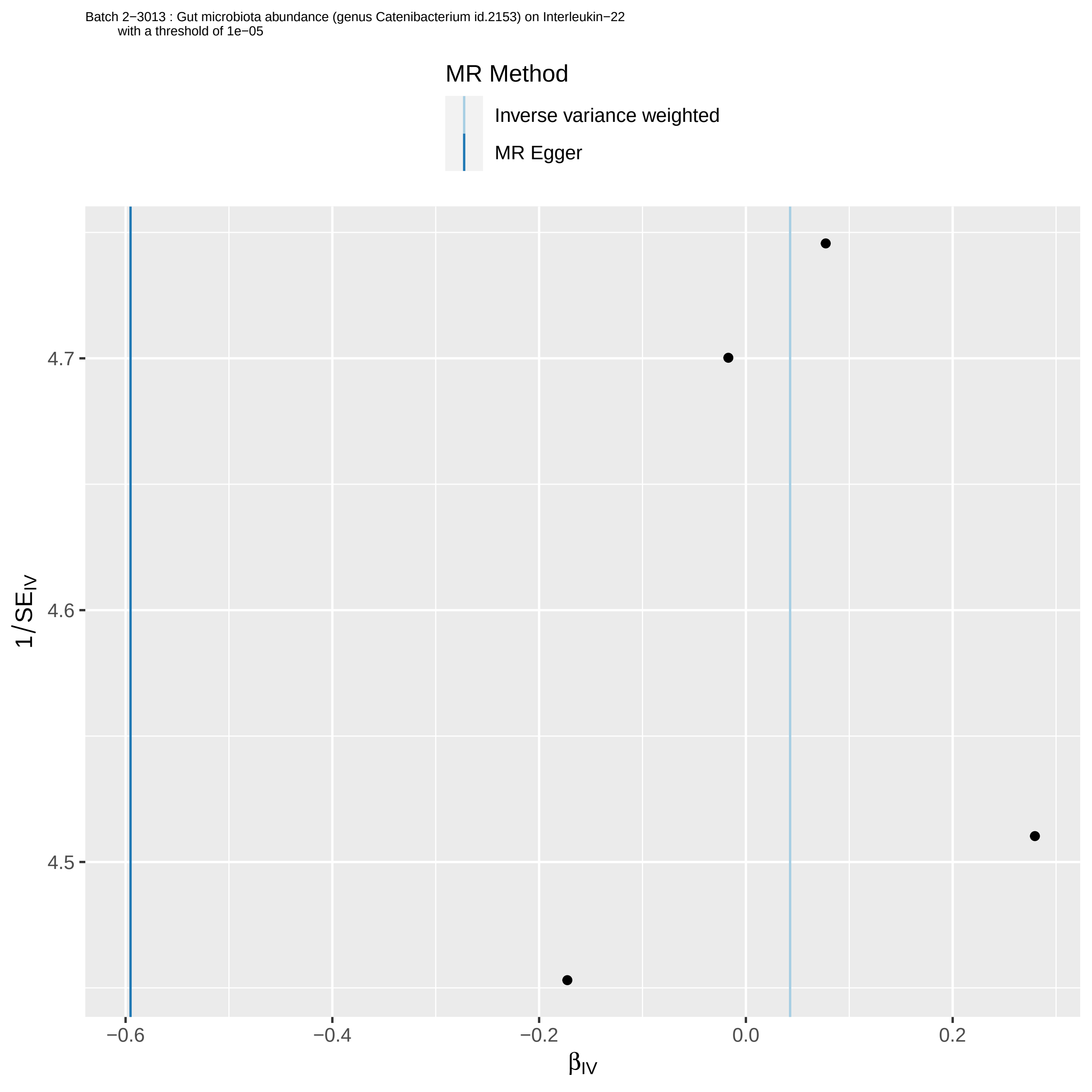
Batch 2−3013 : Gut microbiota abundance (genus Catenibacterium id.2153) on Interleukin−22
with a threshold of 1e−05
MR Method
Inverse variance weighted
MR Egger
4.7
4.6
4.5
1/SE_IV
−0.6
−0.4
−0.2
0.0
0.2
β_IV

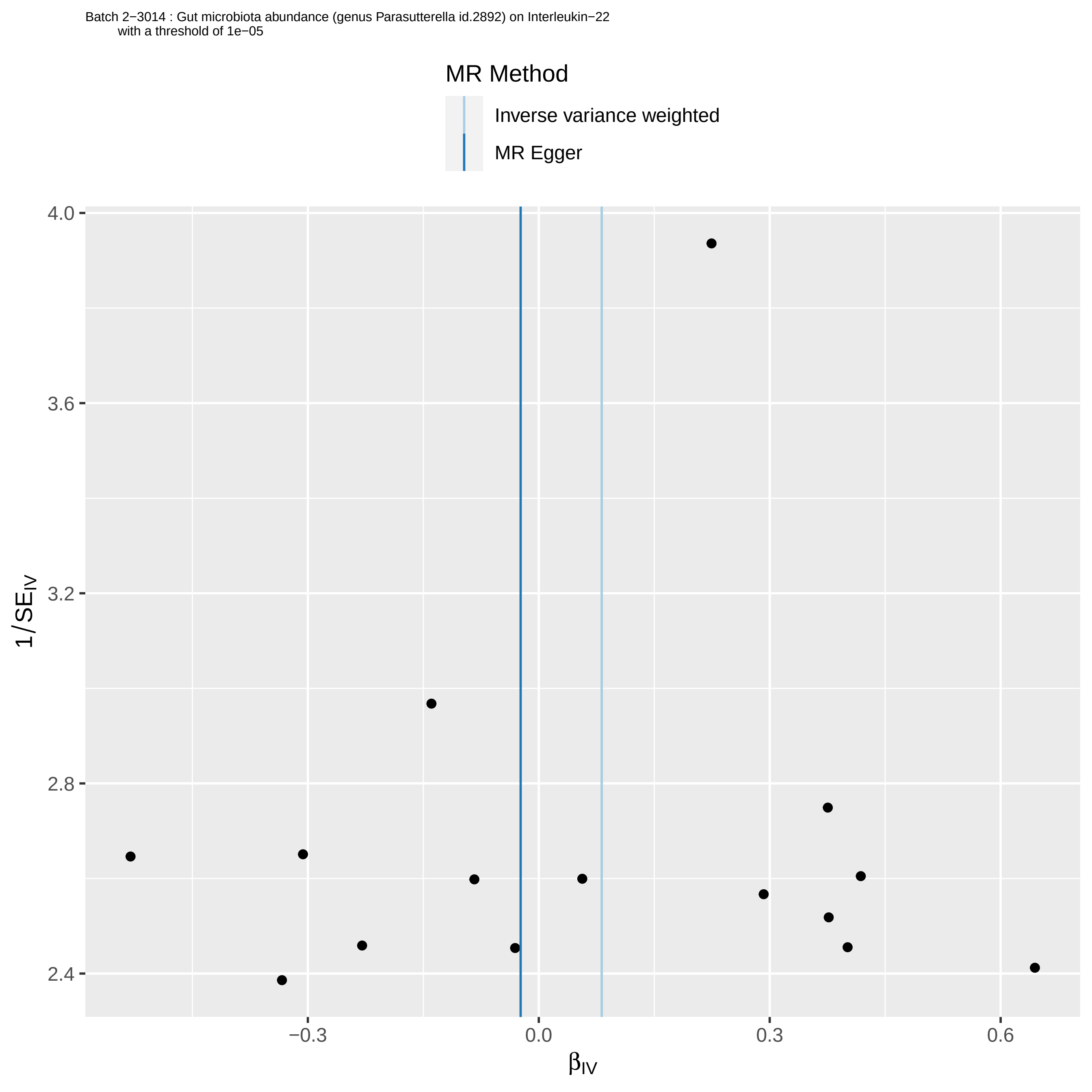

Batch 2−3014 : Gut microbiota abundance (genus Parasutterella id.2892) on Interleukin−22
with a threshold of 1e−05
MR Method
Inverse variance weighted
MR Egger
4.0
3.6
3.2
2.8
2.4
1/SE_IV
−0.3
0.0
0.3
0.6
β_IV

Batch 2−3015 : Gut microbiota abundance (genus Catenibacterium id.2153) on Interleukin−22 receptor subunit alpha−1
with a threshold of 1e−05

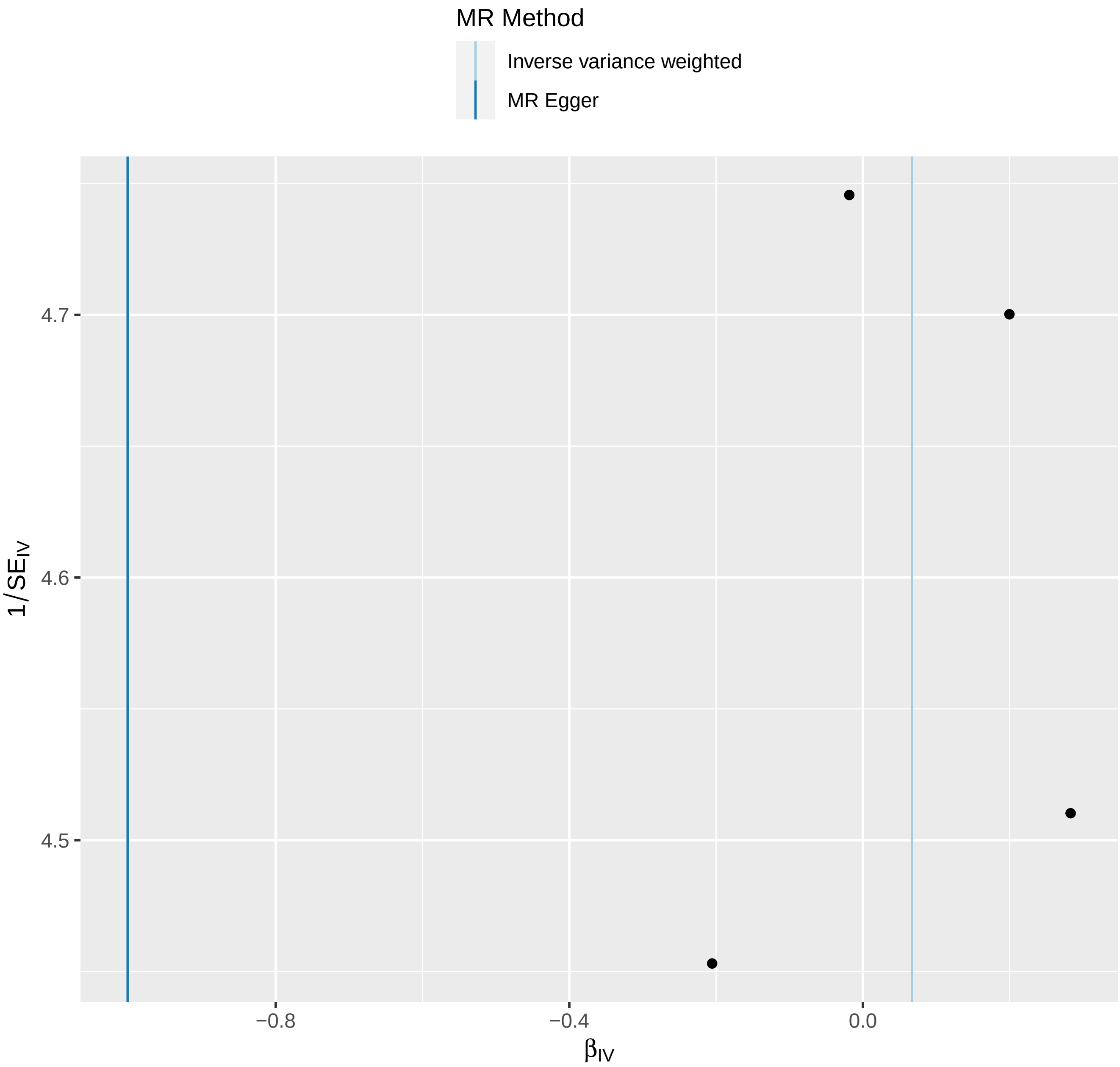

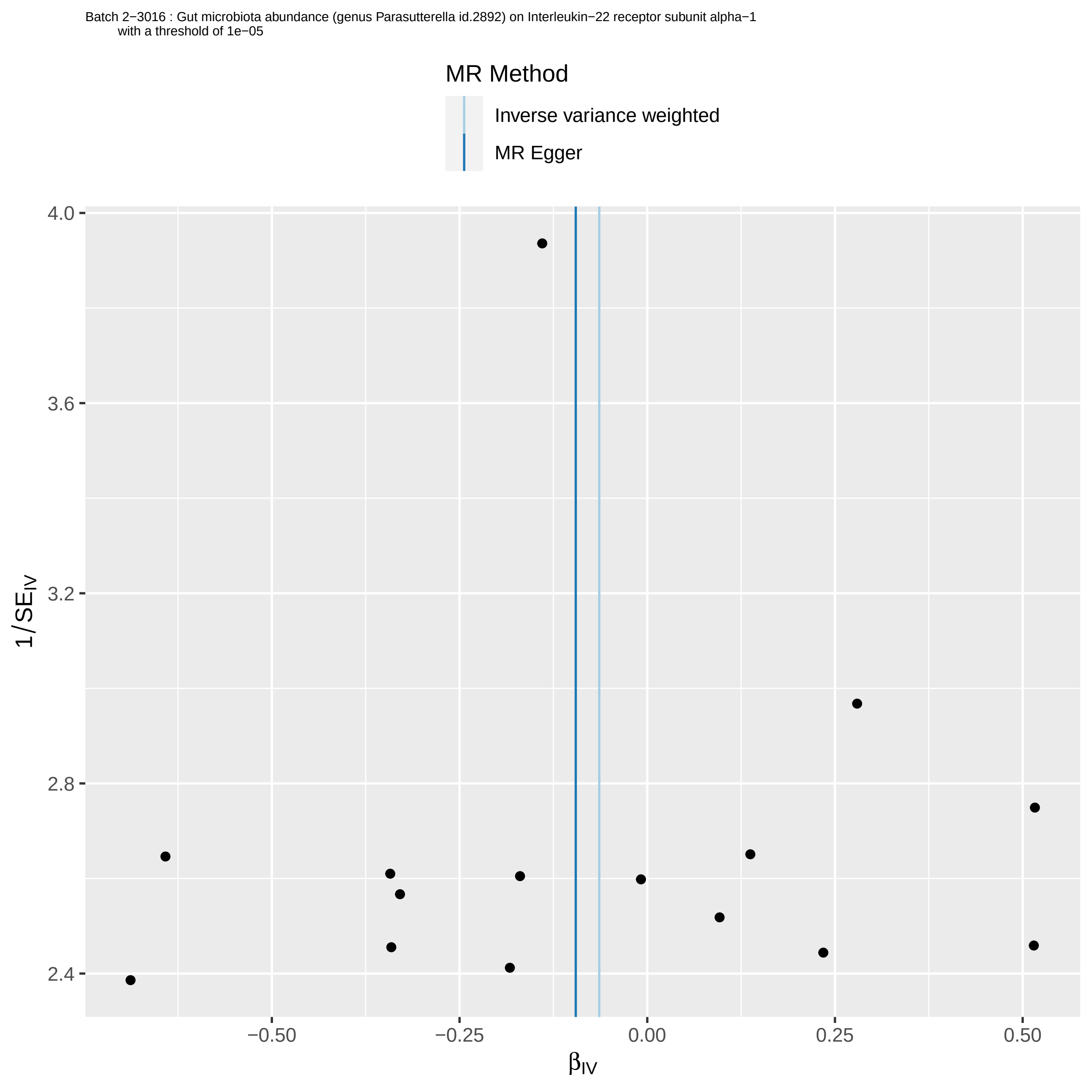
Batch 2−3016 : Gut microbiota abundance (genus Parasutterella id.2892) on Interleukin−22 receptor subunit alpha−1
with a threshold of 1e−05
MR Method
Inverse variance weighted
MR Egger
4.0
3.6
3.2
2.8
2.4
1/SE_IV
−0.50
−0.25
0.00
0.25
0.50
β_IV

Batch 2−3017 : Gut microbiota abundance (genus Catenibacterium id.2153) on Interleukin−22 receptor subunit alpha−1
with a threshold of 1e−05

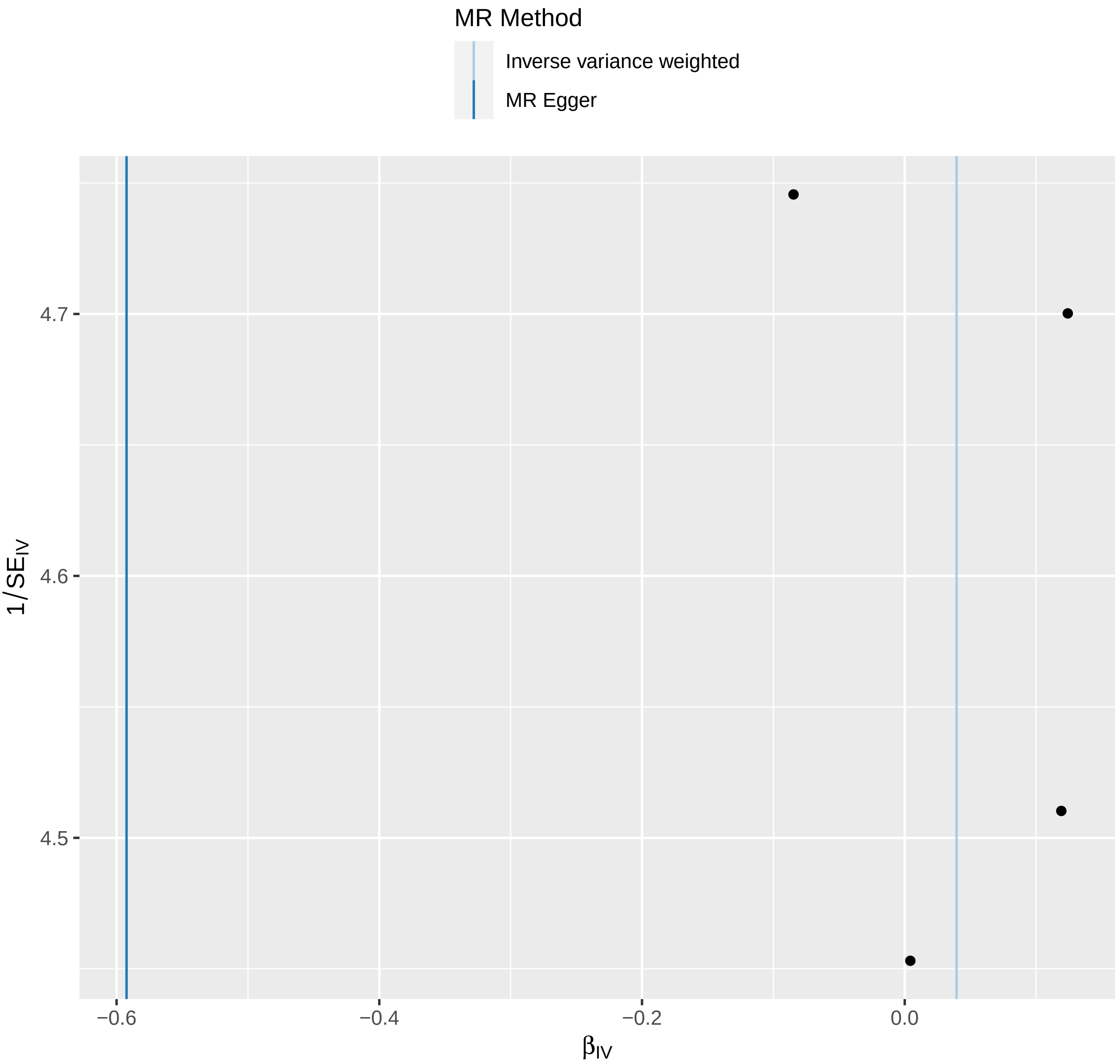

Batch 2−3018 : Gut microbiota abundance (genus Parasutterella id.2892) on Interleukin−22 receptor subunit alpha−1
with a threshold of 1e−05

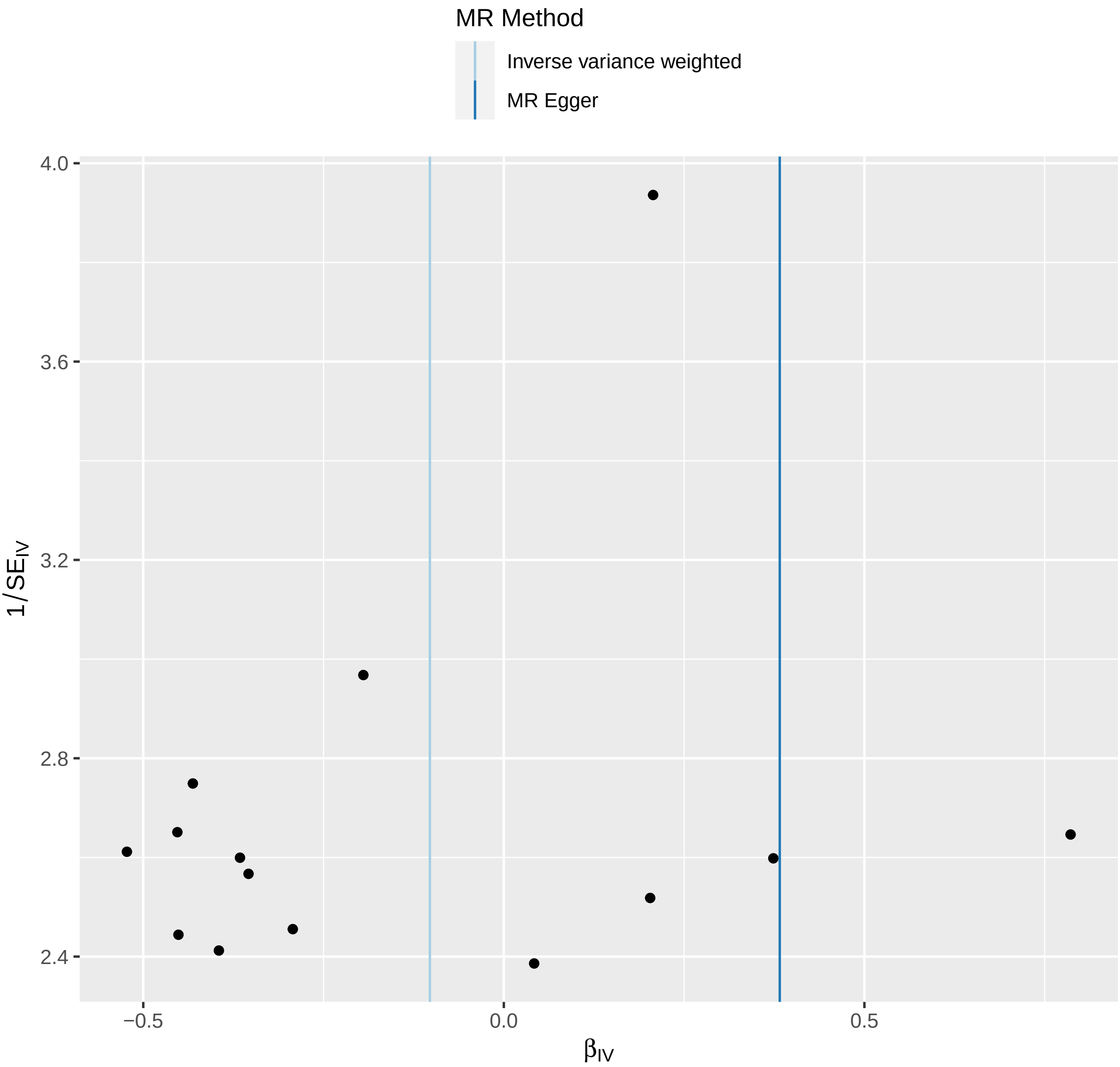

Batch 2−3019 : Gut microbiota abundance (genus Catenibacterium id.2153) on Interleukin−22 receptor subunit alpha−2
with a threshold of 1e−05

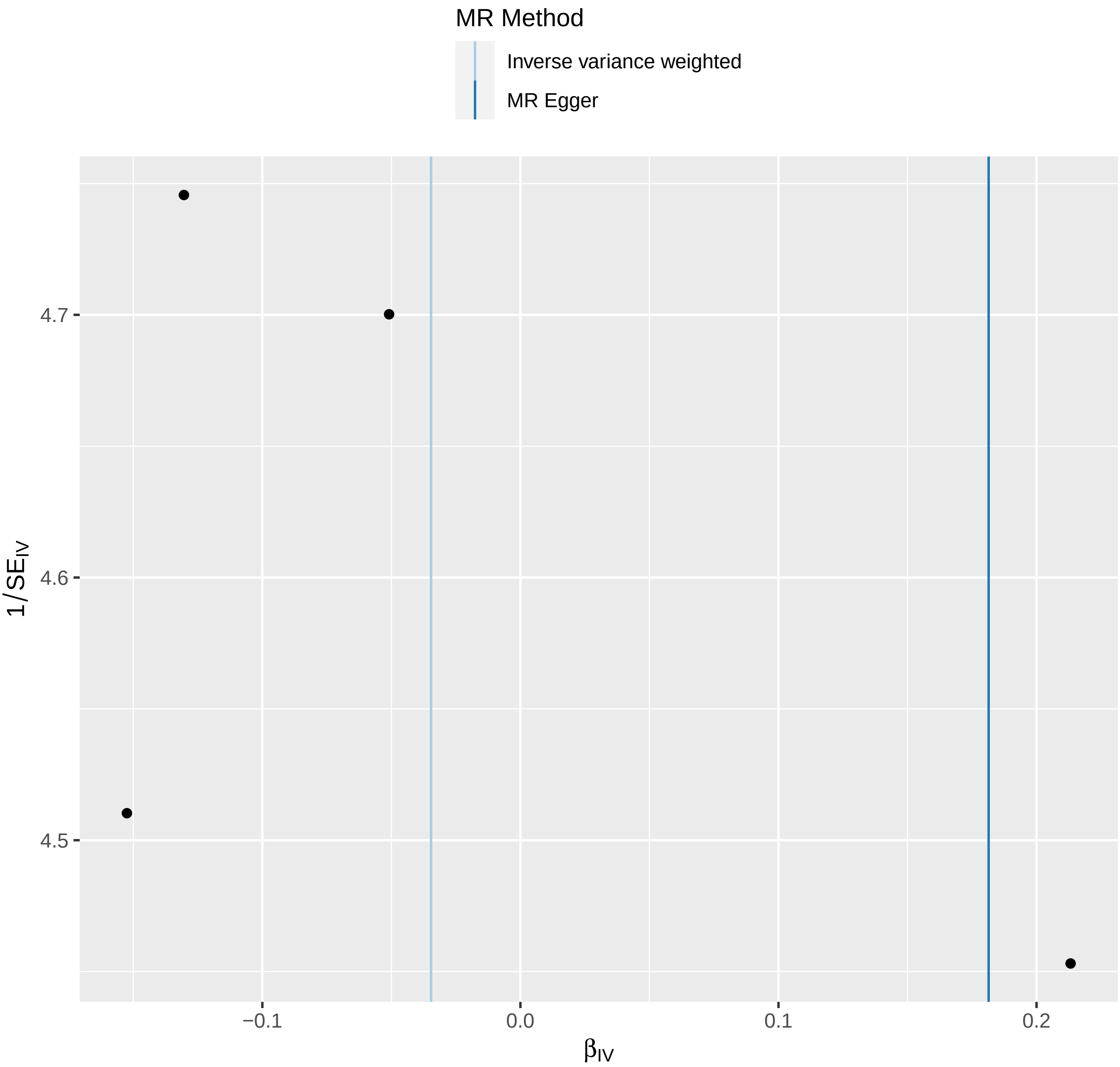

Batch 2−3020 : Gut microbiota abundance (genus Parasutterella id.2892) on Interleukin−22 receptor subunit alpha−2 with a threshold of 1e−05

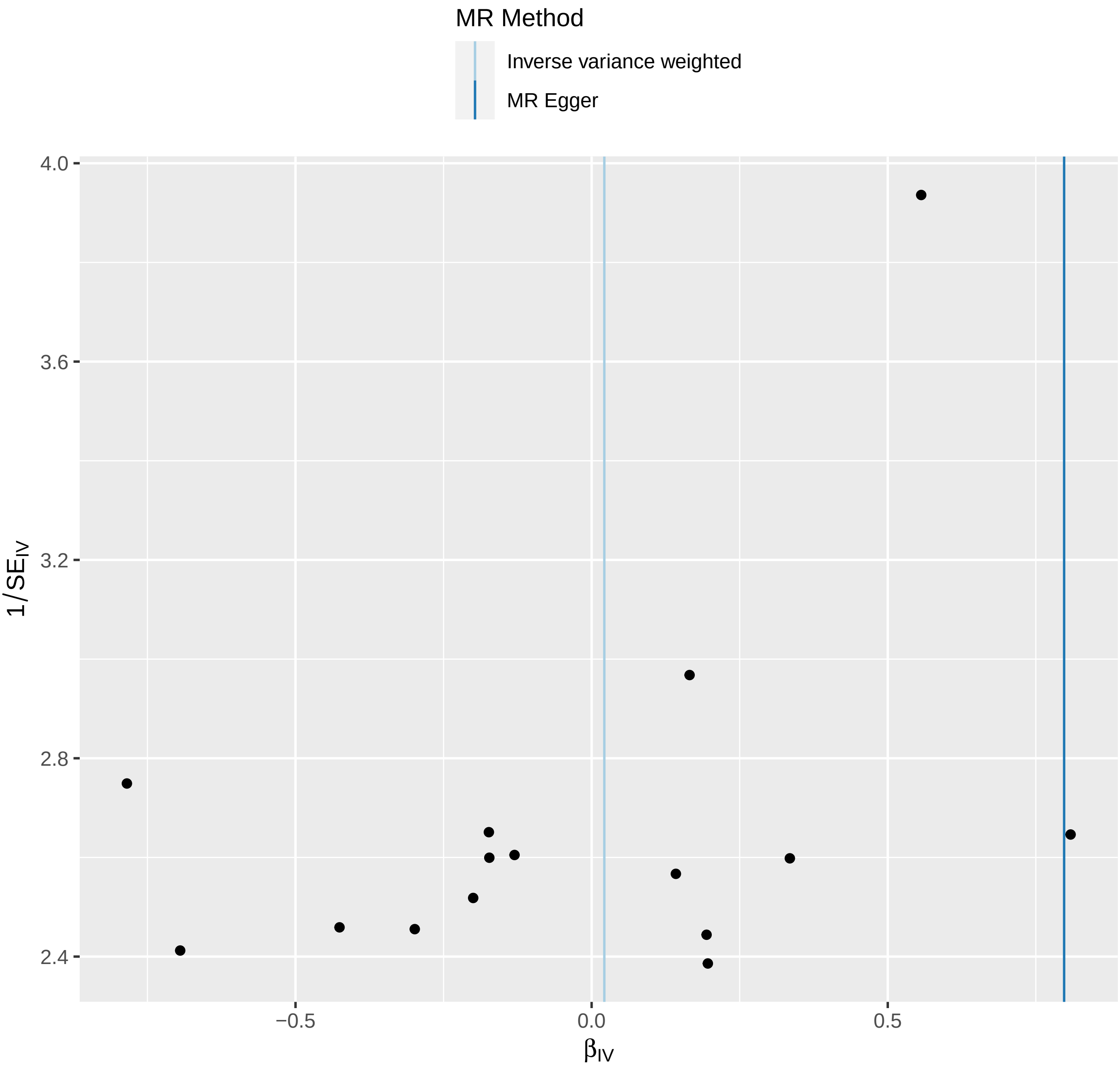

Batch 2−3021 : Gut microbiota abundance (genus Catenibacterium id.2153) on Interleukin−22 receptor subunit alpha−2 with a threshold of 1e−05

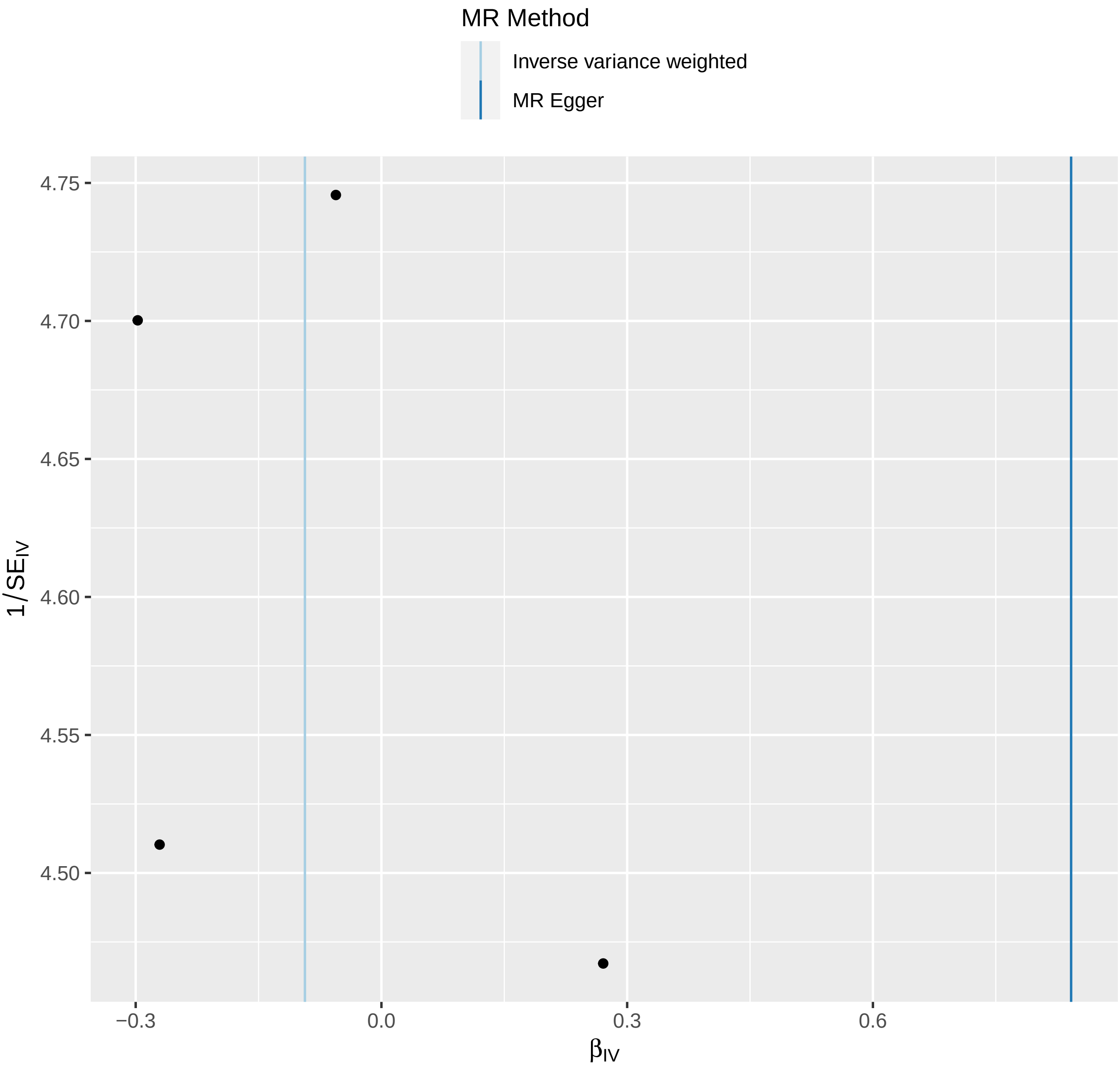

Batch 2−3022 : Gut microbiota abundance (genus Parasutterella id.2892) on Interleukin−22 receptor subunit alpha−2
with a threshold of 1e−05

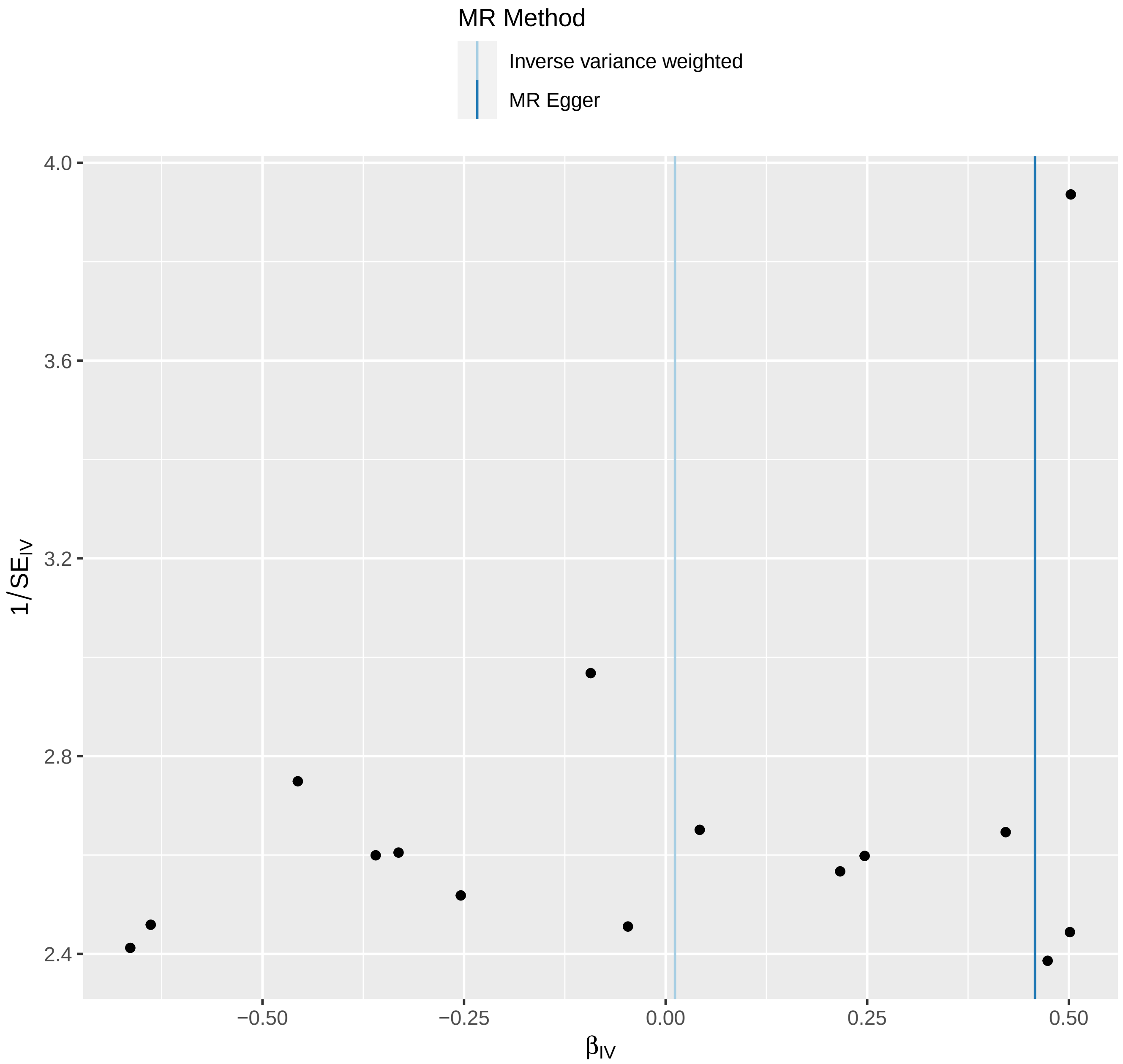

Batch 2−3023 : Gut microbiota abundance (genus Catenibacterium id.2153) on Interleukin−2
with a threshold of 1e−05

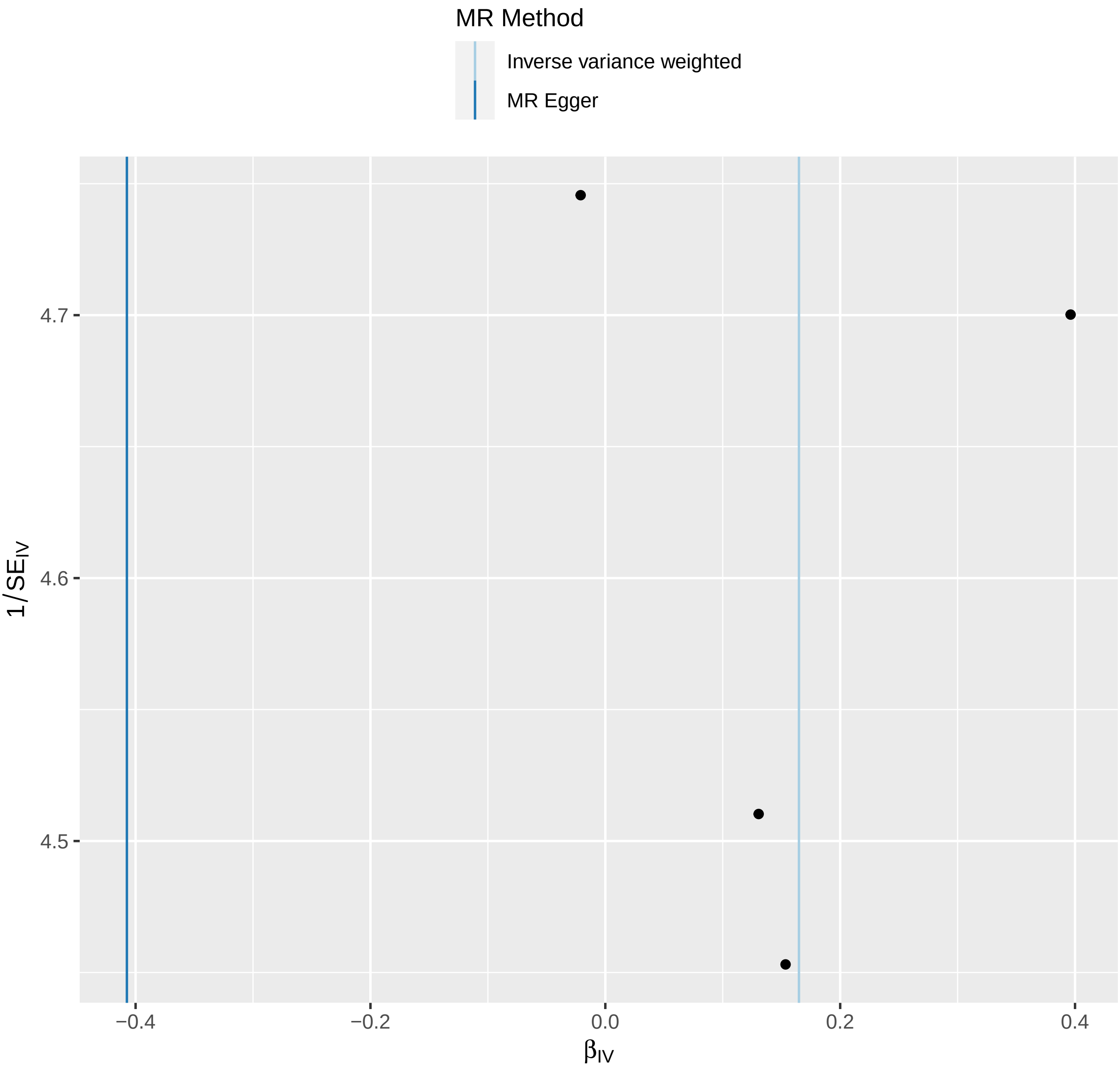

Batch 2−3024 : Gut microbiota abundance (genus Parasutterella id.2892) on Interleukin−2
with a threshold of 1e−05

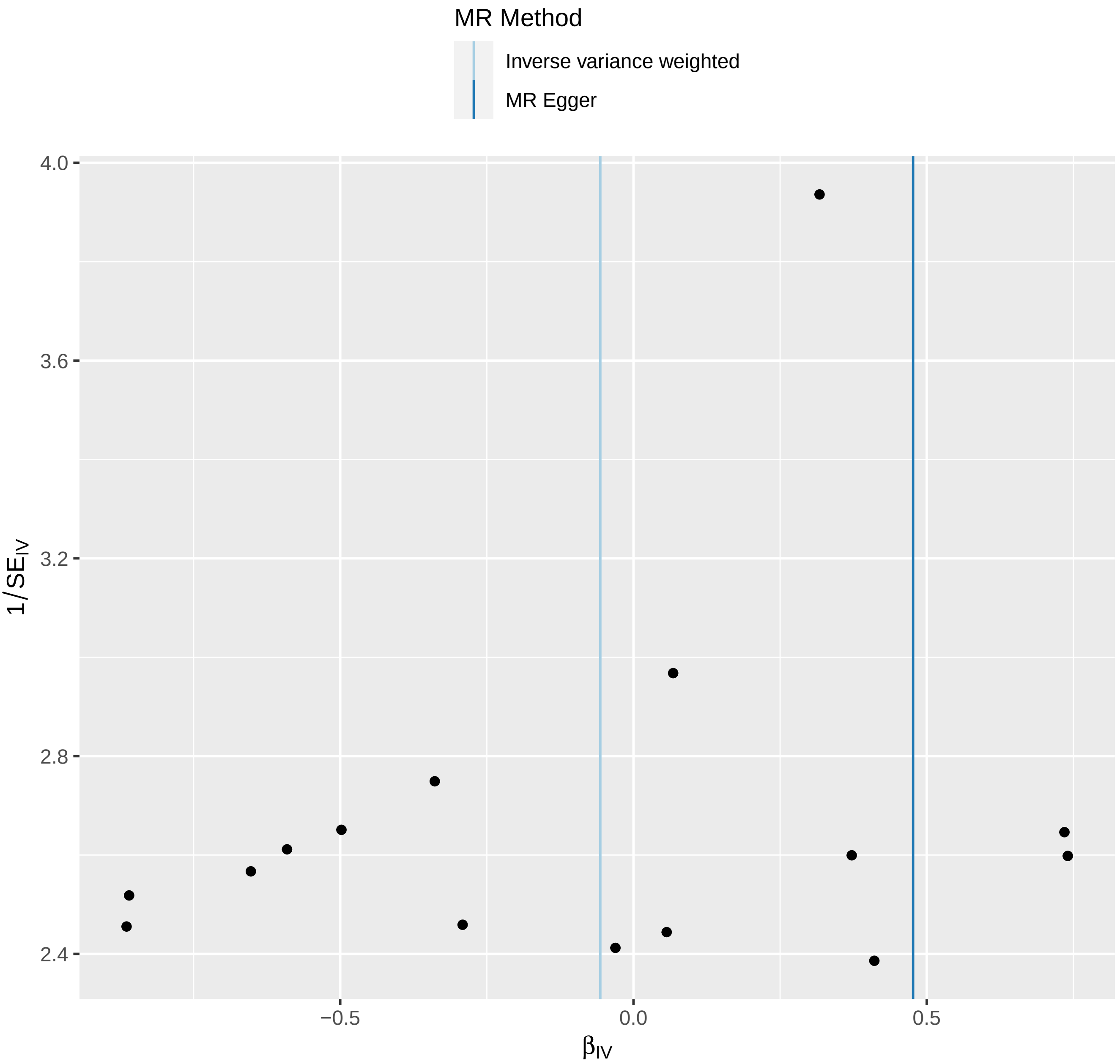

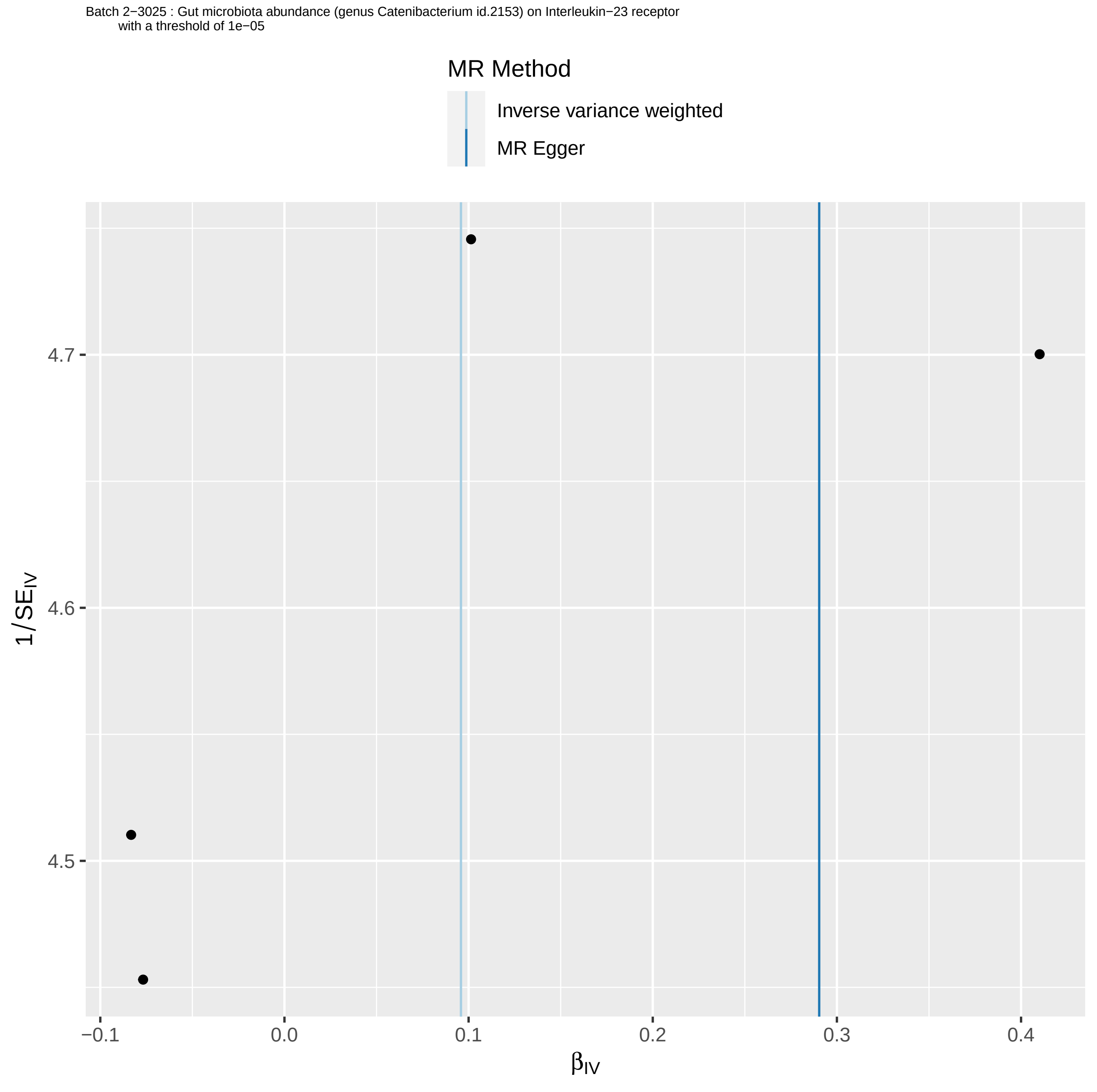
Batch 2−3025 : Gut microbiota abundance (genus Catenibacterium id.2153) on Interleukin−23 receptor
with a threshold of 1e−05
MR Method
Inverse variance weighted
MR Egger
4.7
4.6
4.5
1/SE_IV
−0.1
0.0
0.1
0.2
0.3
0.4
β_IV

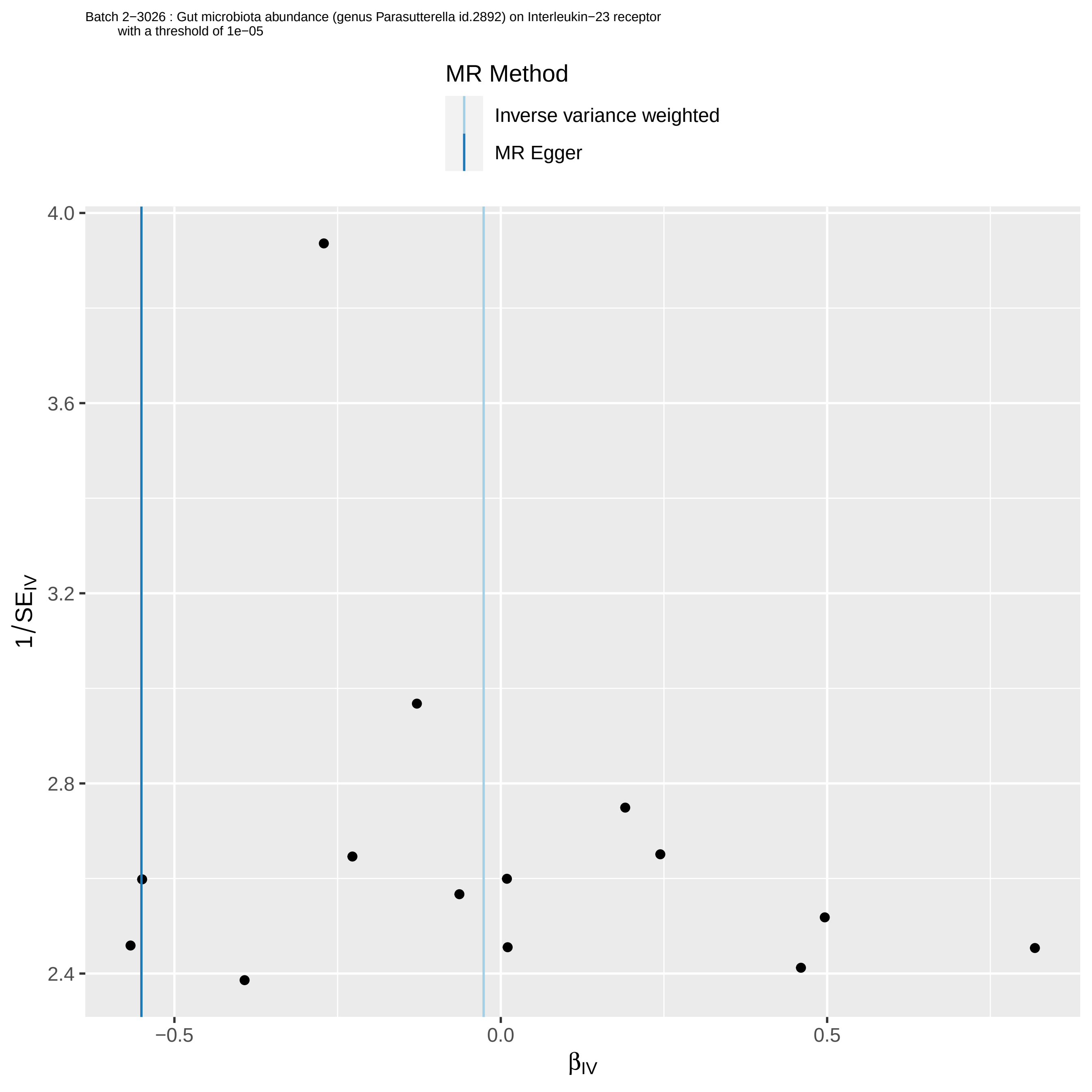

Batch 2−3026 : Gut microbiota abundance (genus Parasutterella id.2892) on Interleukin−23 receptor
with a threshold of 1e−05
MR Method
Inverse variance weighted
MR Egger
4.0
3.6
3.2
2.8
2.4
1/SE$_{IV}$
−0.5
0.0
0.5
β$_{IV}$

Batch 2−3027 : Gut microbiota abundance (genus Catenibacterium id.2153) on Interleukin−24
with a threshold of 1e−05

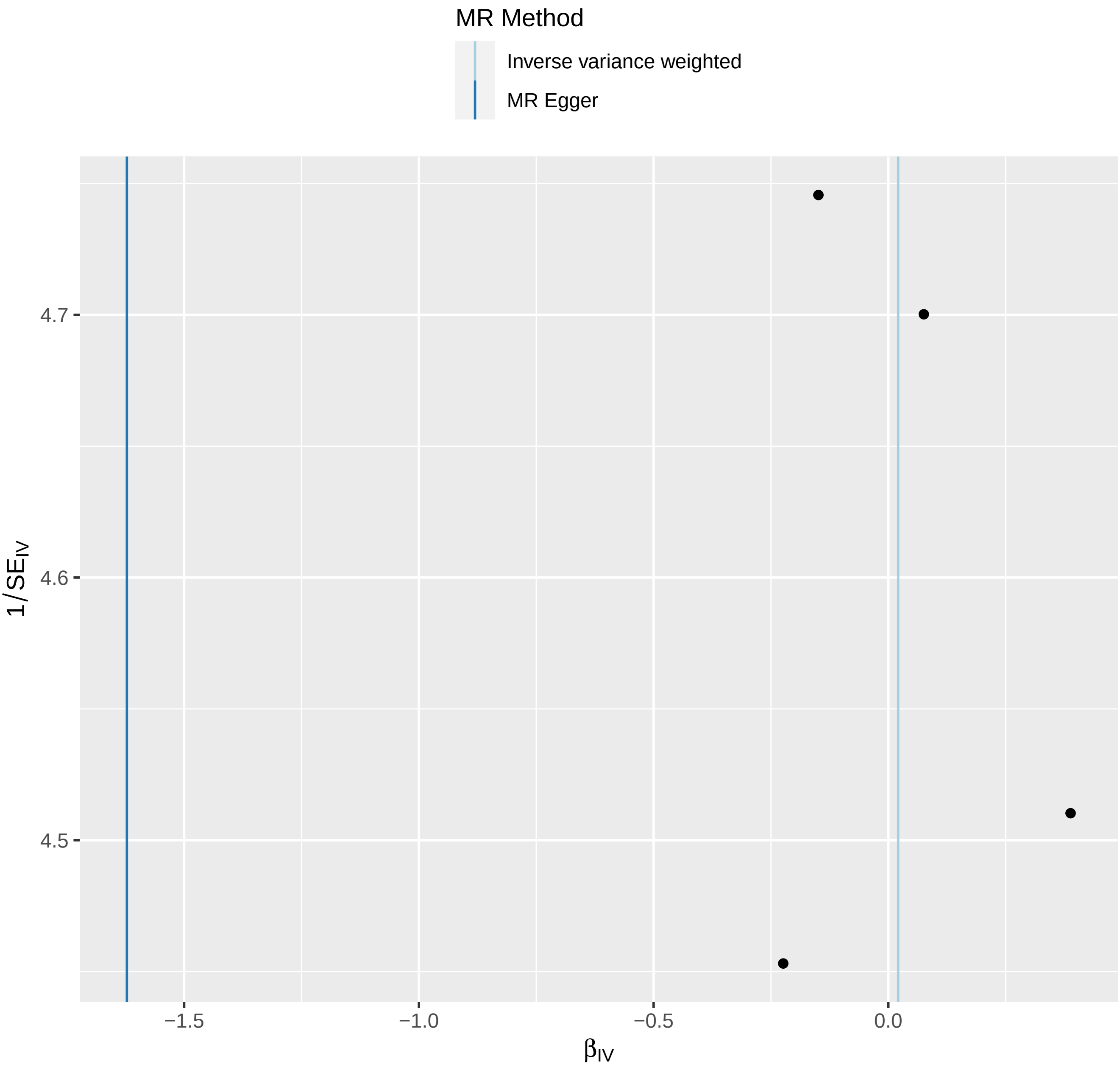

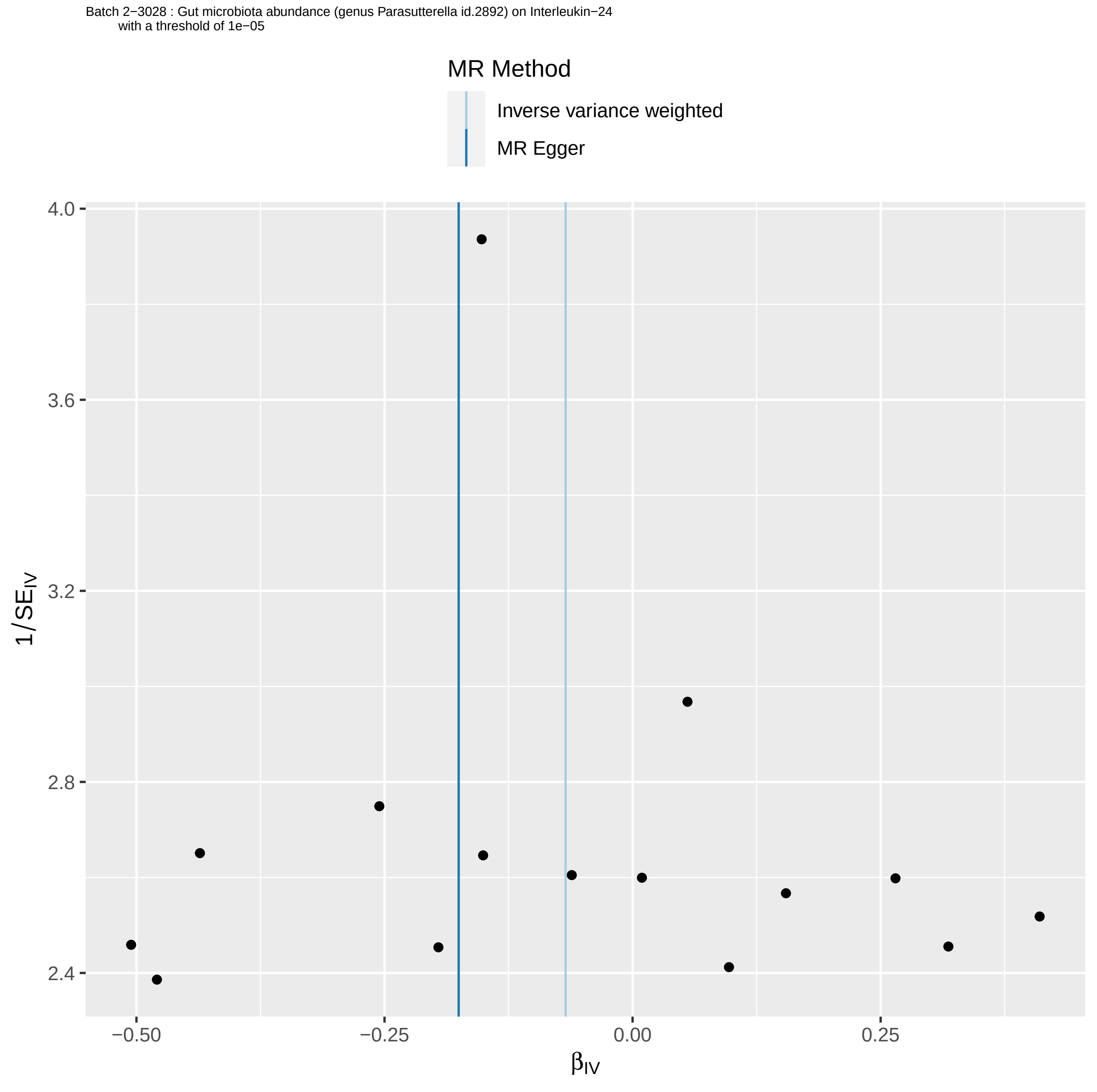
Batch 2−3028 : Gut microbiota abundance (genus Parasutterella id.2892) on Interleukin−24
with a threshold of 1e−05
MR Method
Inverse variance weighted
MR Egger
4.0
3.6
3.2
2.8
2.4
$1/SE_{IV}$
−0.50
−0.25
0.00
0.25
β_{IV}

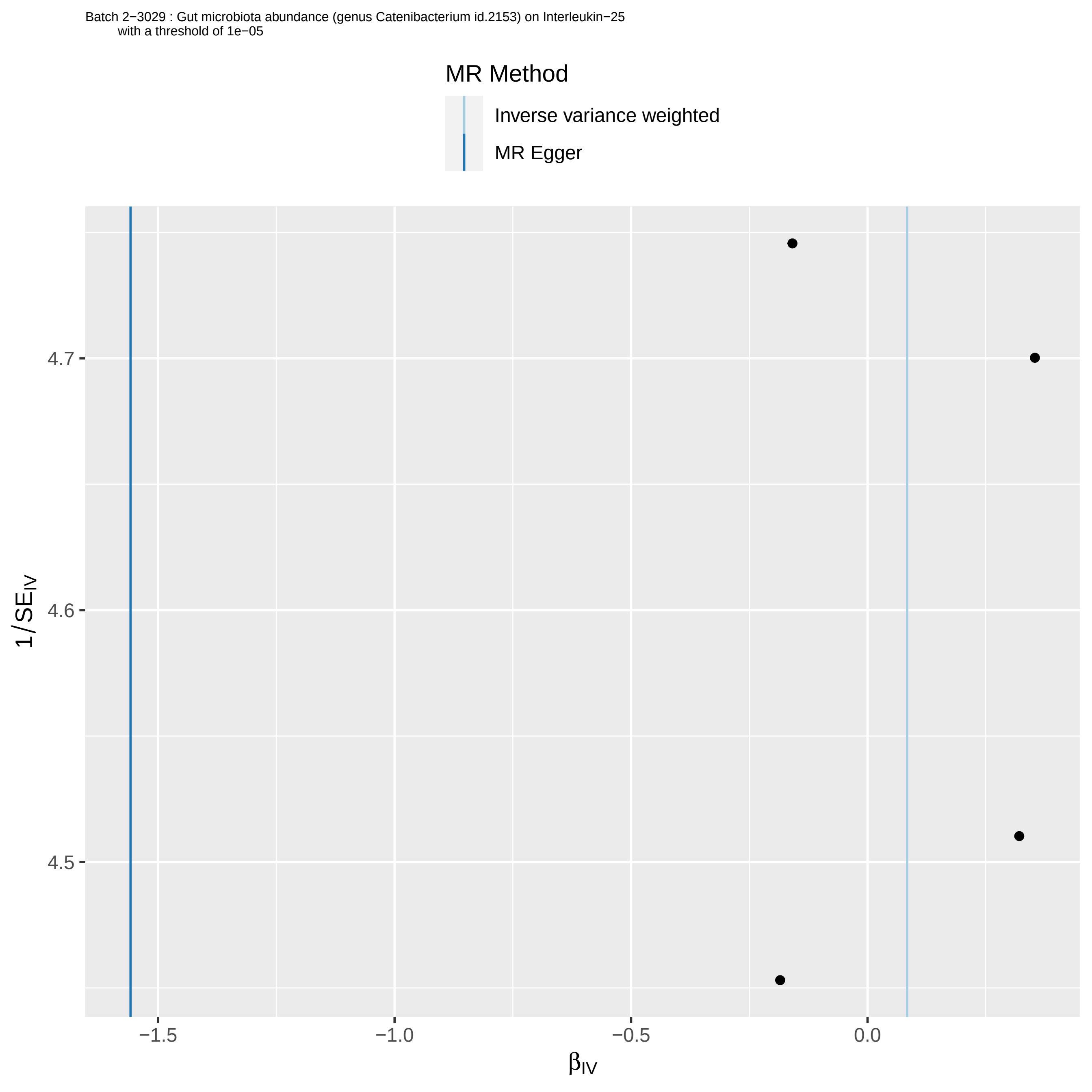
Batch 2−3029 : Gut microbiota abundance (genus Catenibacterium id.2153) on Interleukin−25
with a threshold of 1e−05
MR Method
Inverse variance weighted
MR Egger
4.7
4.6
4.5
1/SE_IV
−1.5
−1.0
−0.5
0.0
β_IV

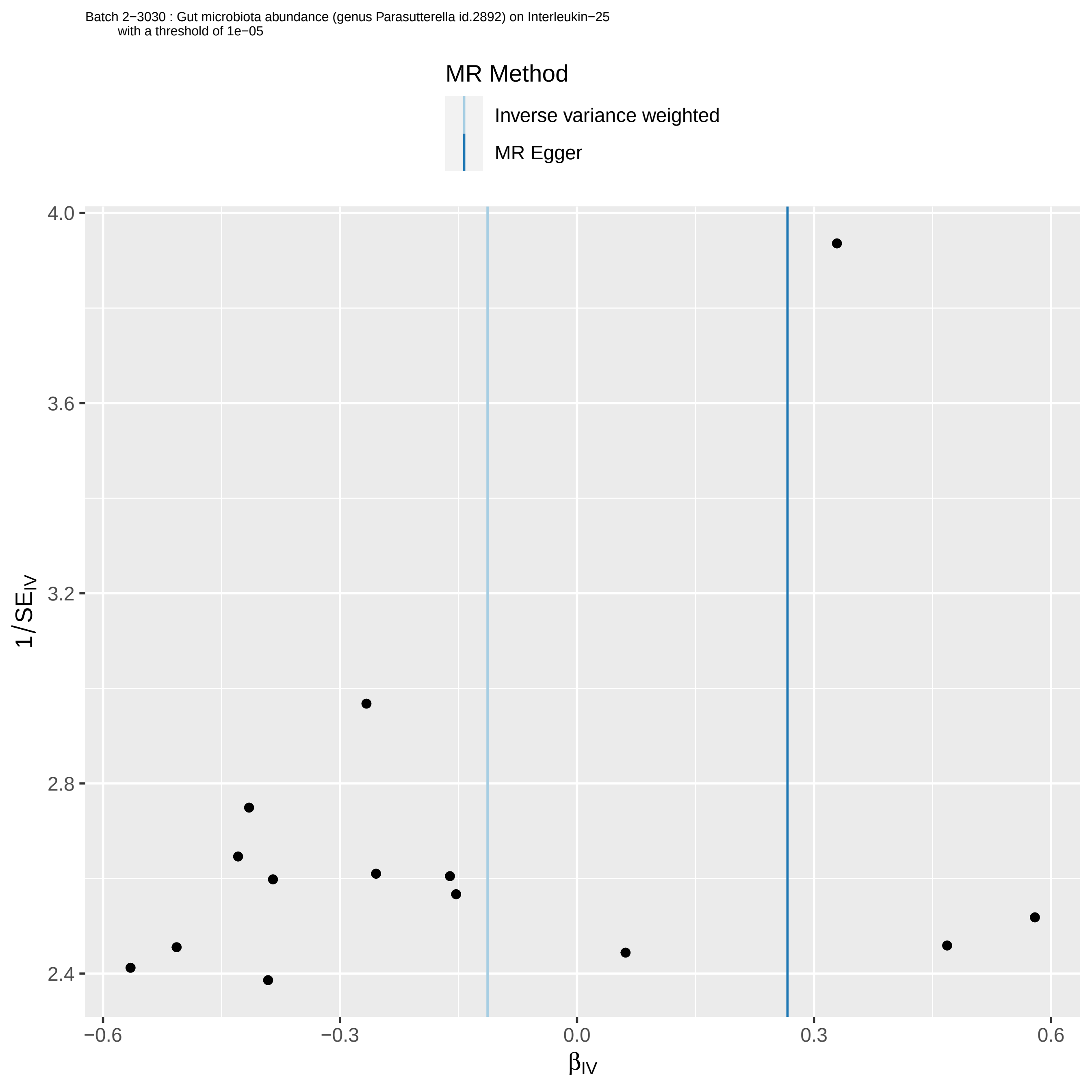

Batch 2−3030 : Gut microbiota abundance (genus Parasutterella id.2892) on Interleukin−25
with a threshold of 1e−05
MR Method
Inverse variance weighted
MR Egger
1/SE_IV
4.0
3.6
3.2
2.8
2.4
−0.6
−0.3
0.0
0.3
0.6
β_IV

Batch 2−3031 : Gut microbiota abundance (genus Catenibacterium id.2153) on Interleukin−27
with a threshold of 1e−05

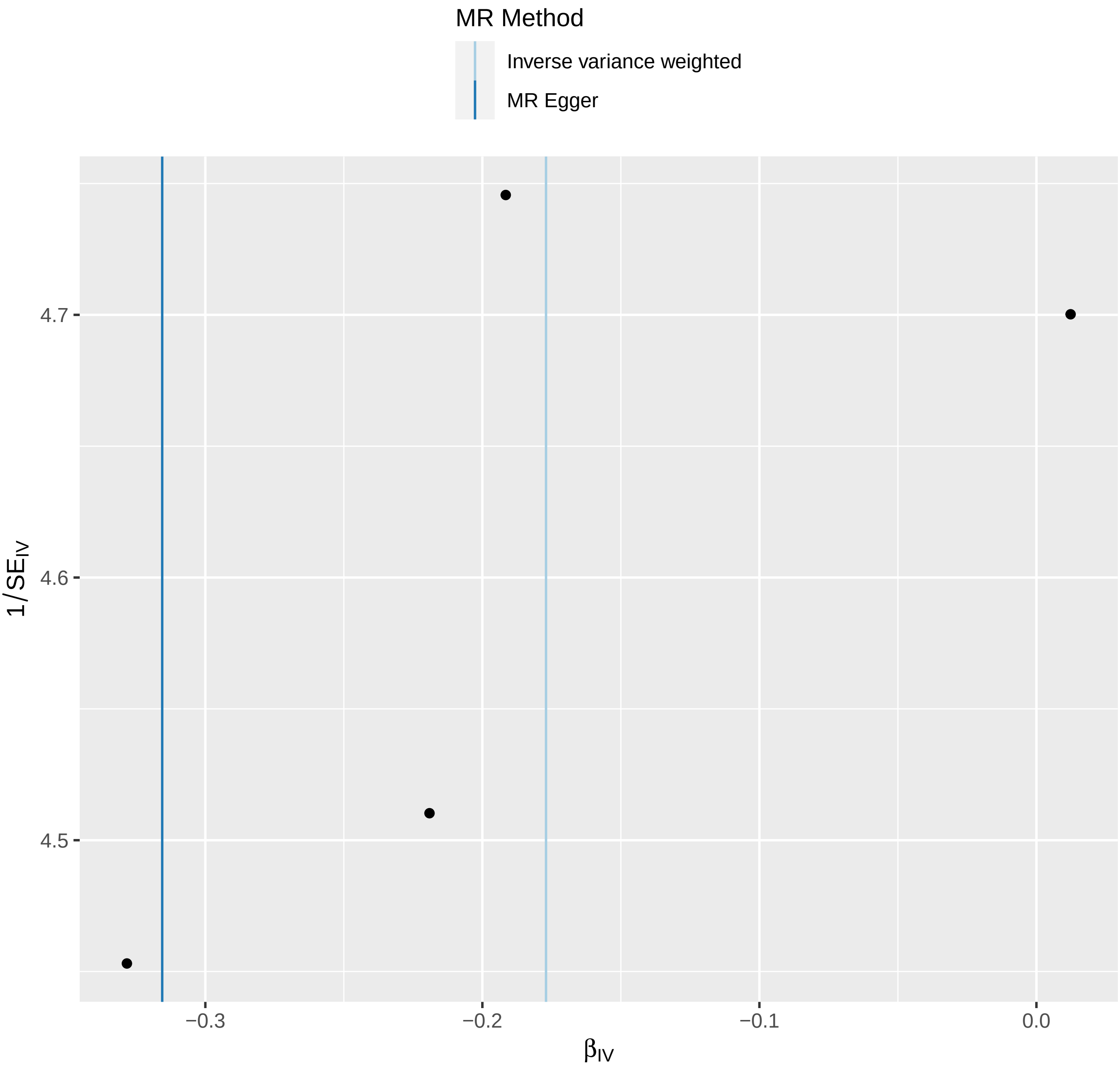

Batch 2−3032 : Gut microbiota abundance (genus Parasutterella id.2892) on Interleukin−27
with a threshold of 1e−05

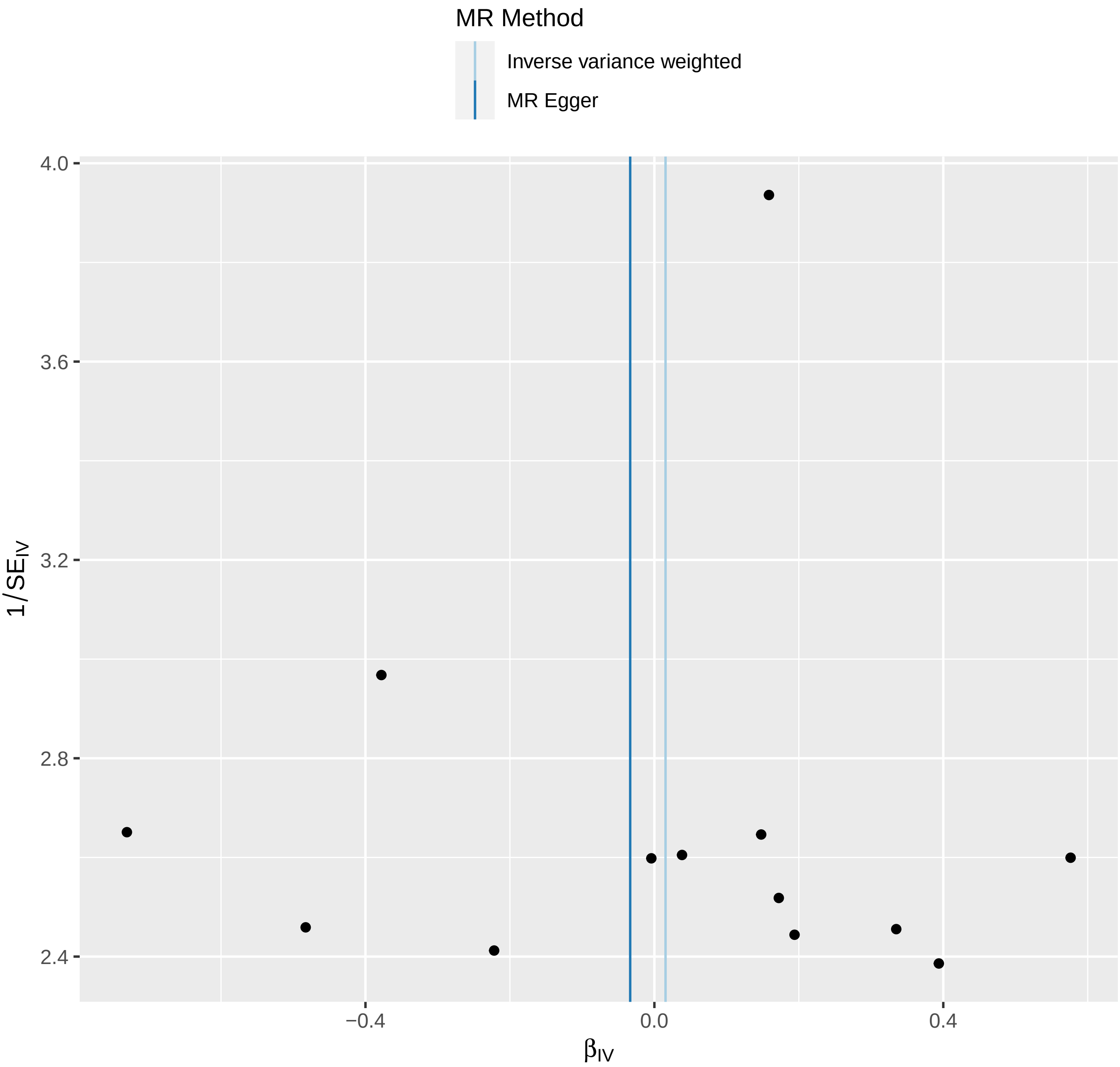

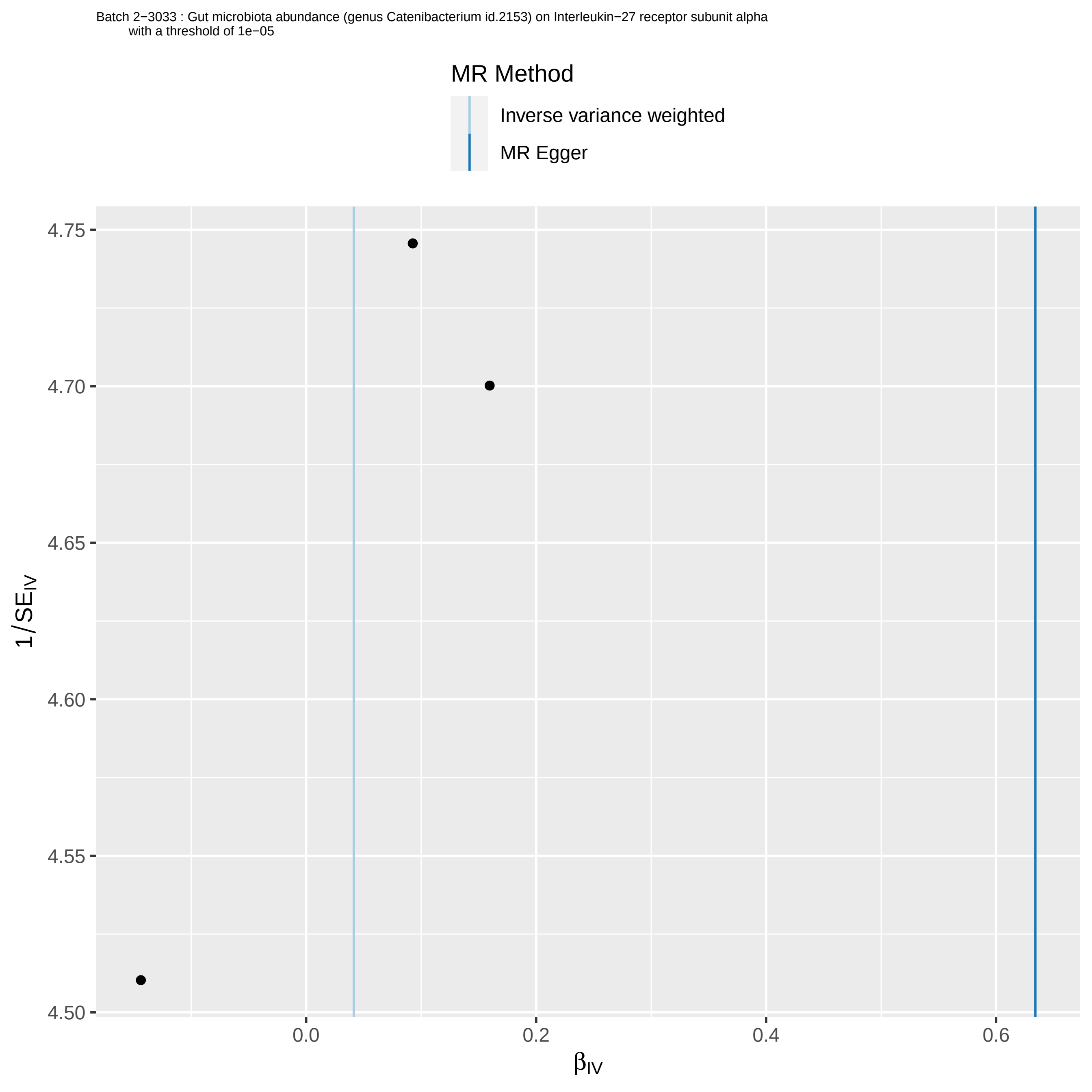

Batch 2−3033 : Gut microbiota abundance (genus Catenibacterium id.2153) on Interleukin−27 receptor subunit alpha
with a threshold of 1e−05
MR Method
Inverse variance weighted
MR Egger
4.75
4.70
4.65
4.60
4.55
4.50
1/SE_IV
0.0
0.2
0.4
0.6
β_IV

Batch 2−3034 : Gut microbiota abundance (genus Parasutterella id.2892) on Interleukin−27 receptor subunit alpha with a threshold of 1e−05

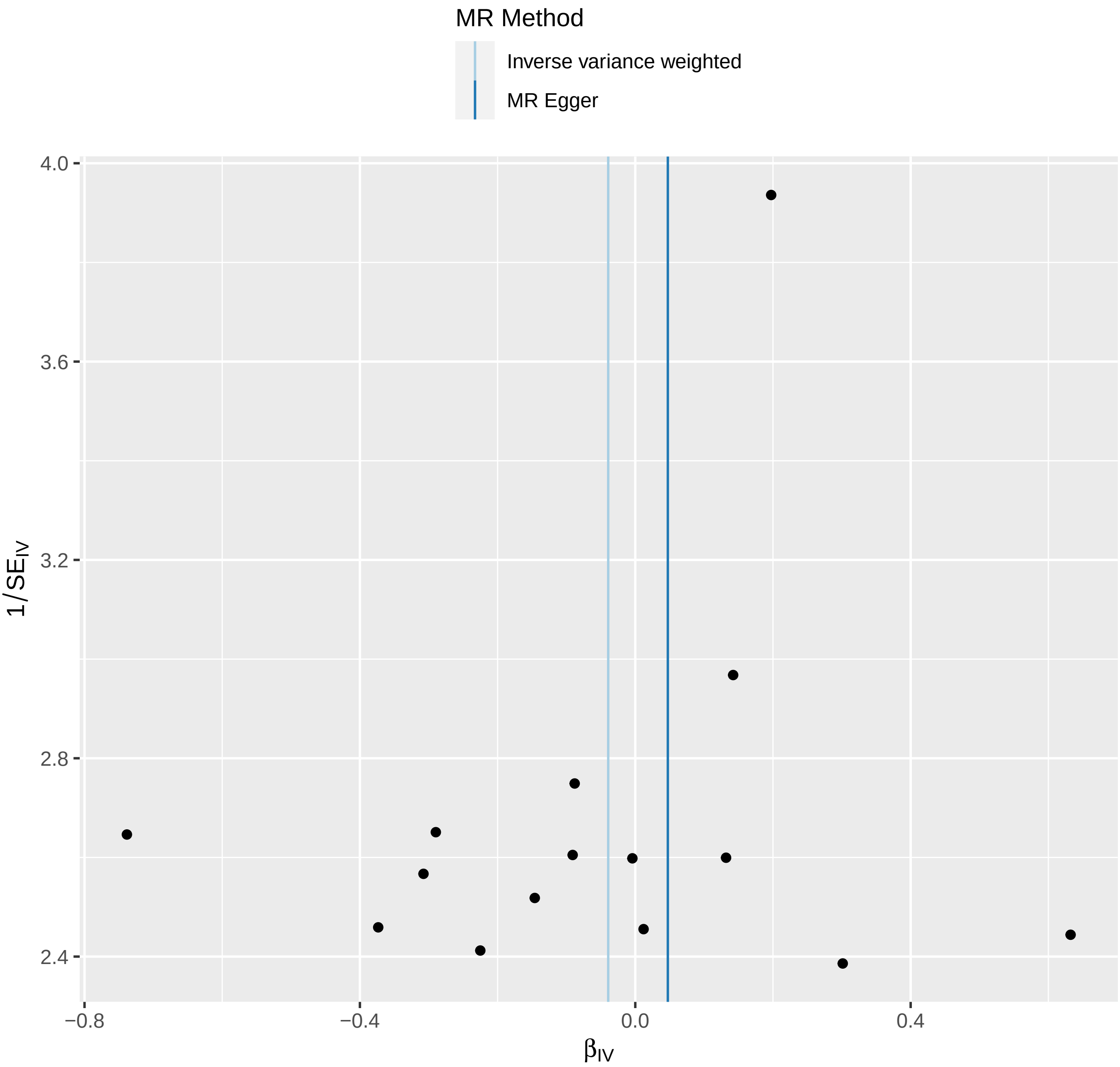

Batch 2−3035 : Gut microbiota abundance (genus Catenibacterium id.2153) on Interleukin−2 receptor subunit alpha
with a threshold of 1e−05

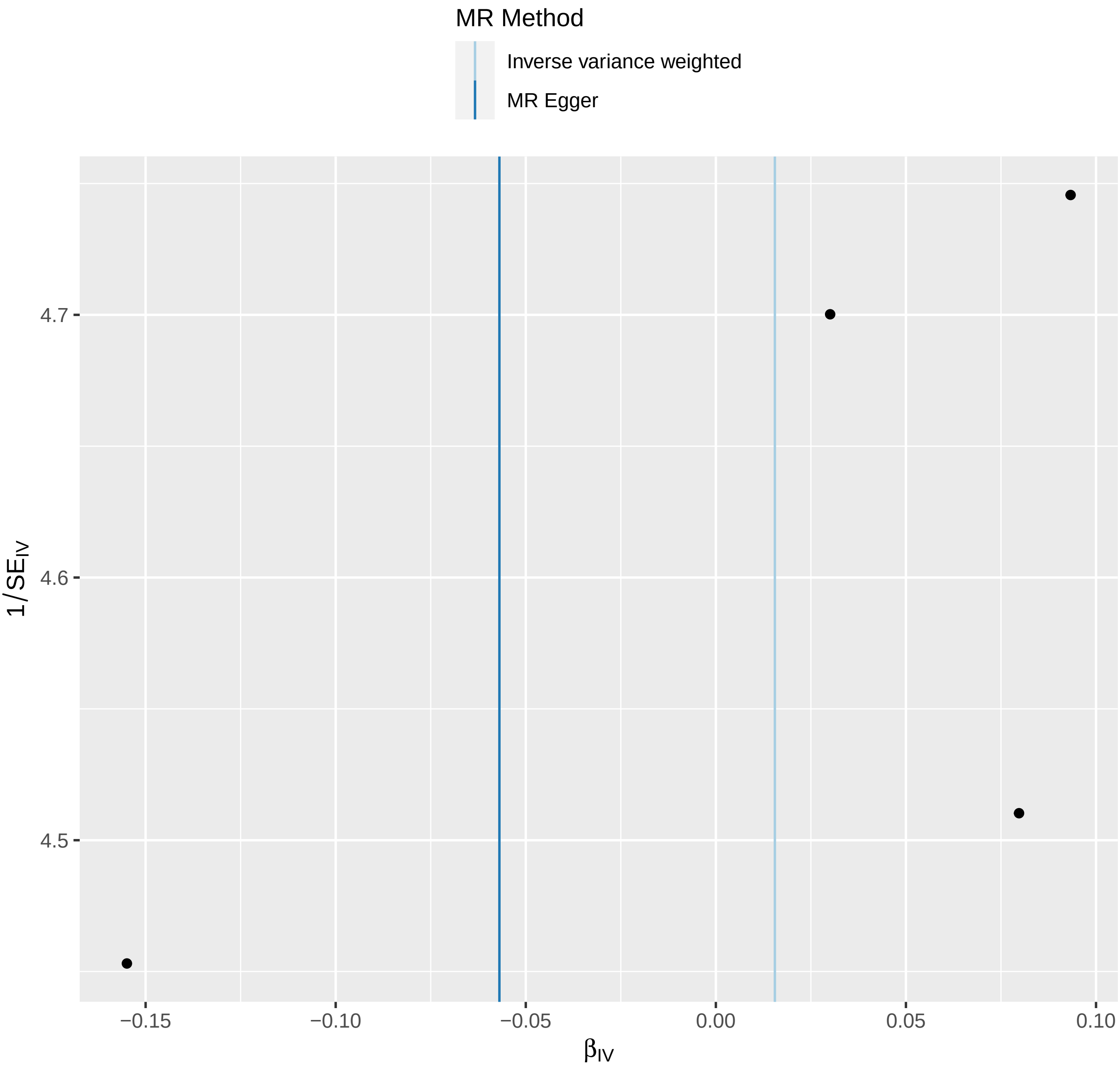

Batch 2−3036 : Gut microbiota abundance (genus Parasutterella id.2892) on Interleukin−2 receptor subunit alpha
with a threshold of 1e−05

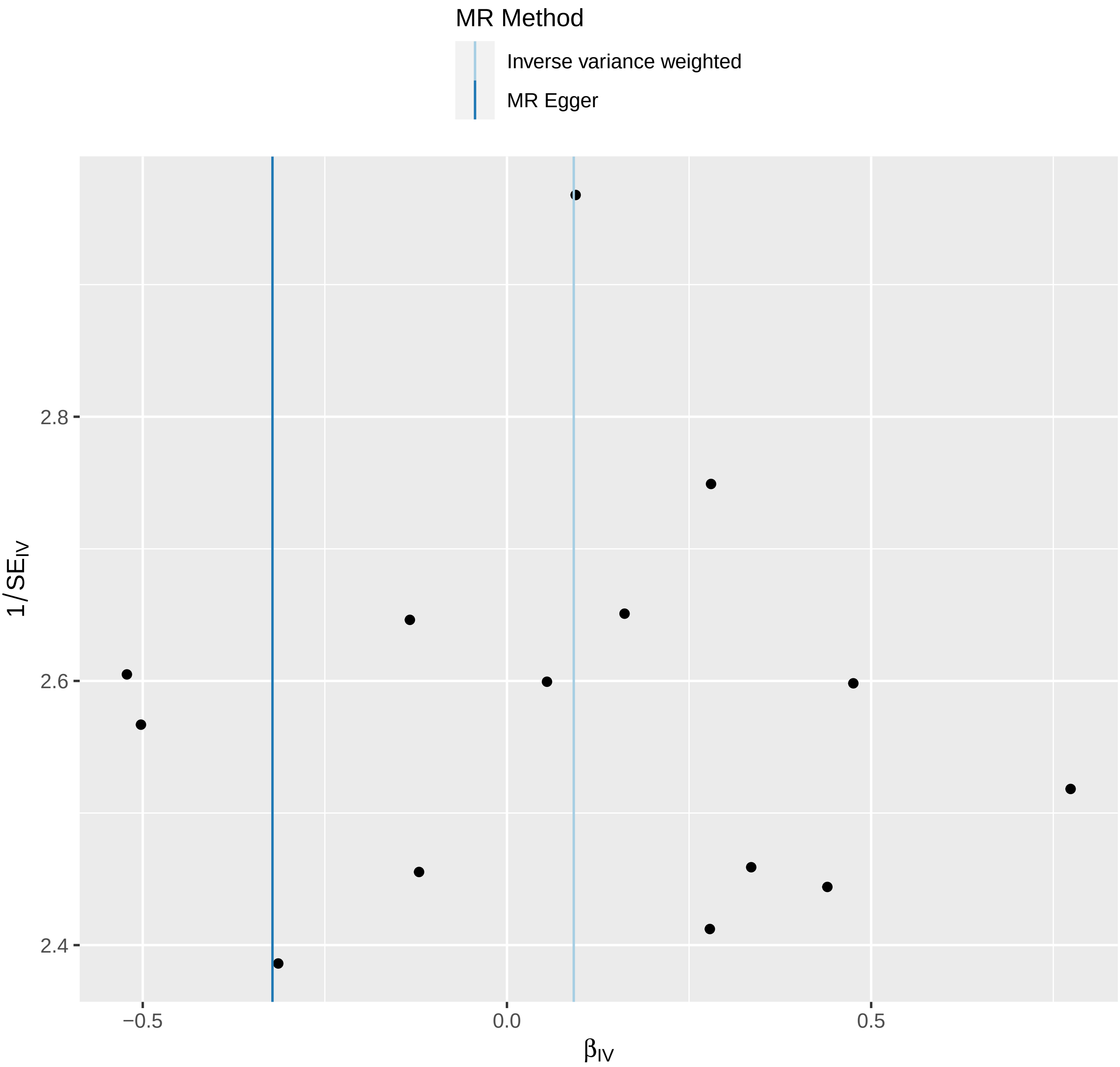

Batch 2−3037 : Gut microbiota abundance (genus Catenibacterium id.2153) on Interleukin−2 receptor subunit beta
with a threshold of 1e−05

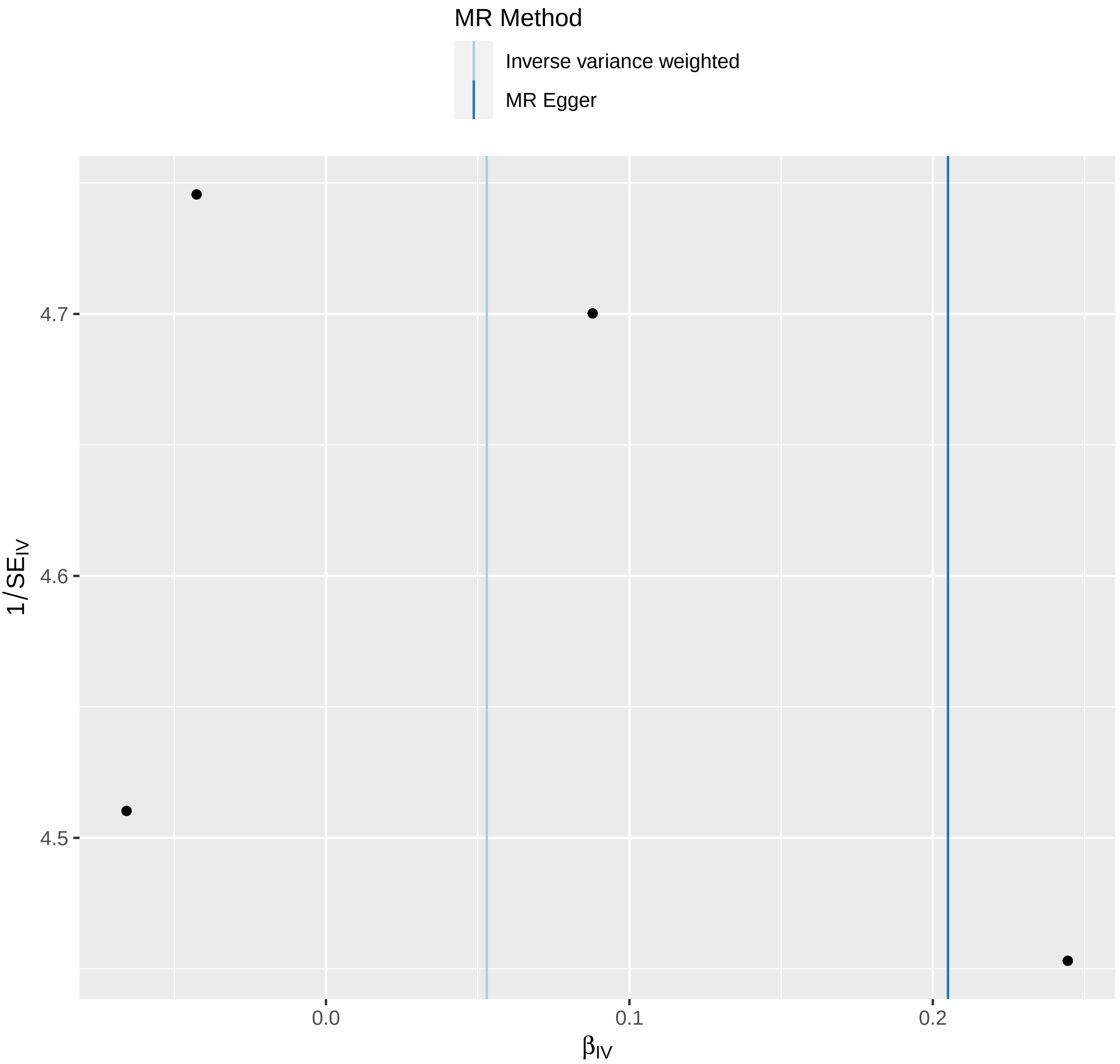

Batch 2−3038 : Gut microbiota abundance (genus Parasutterella id.2892) on Interleukin−2 receptor subunit beta
with a threshold of 1e−05

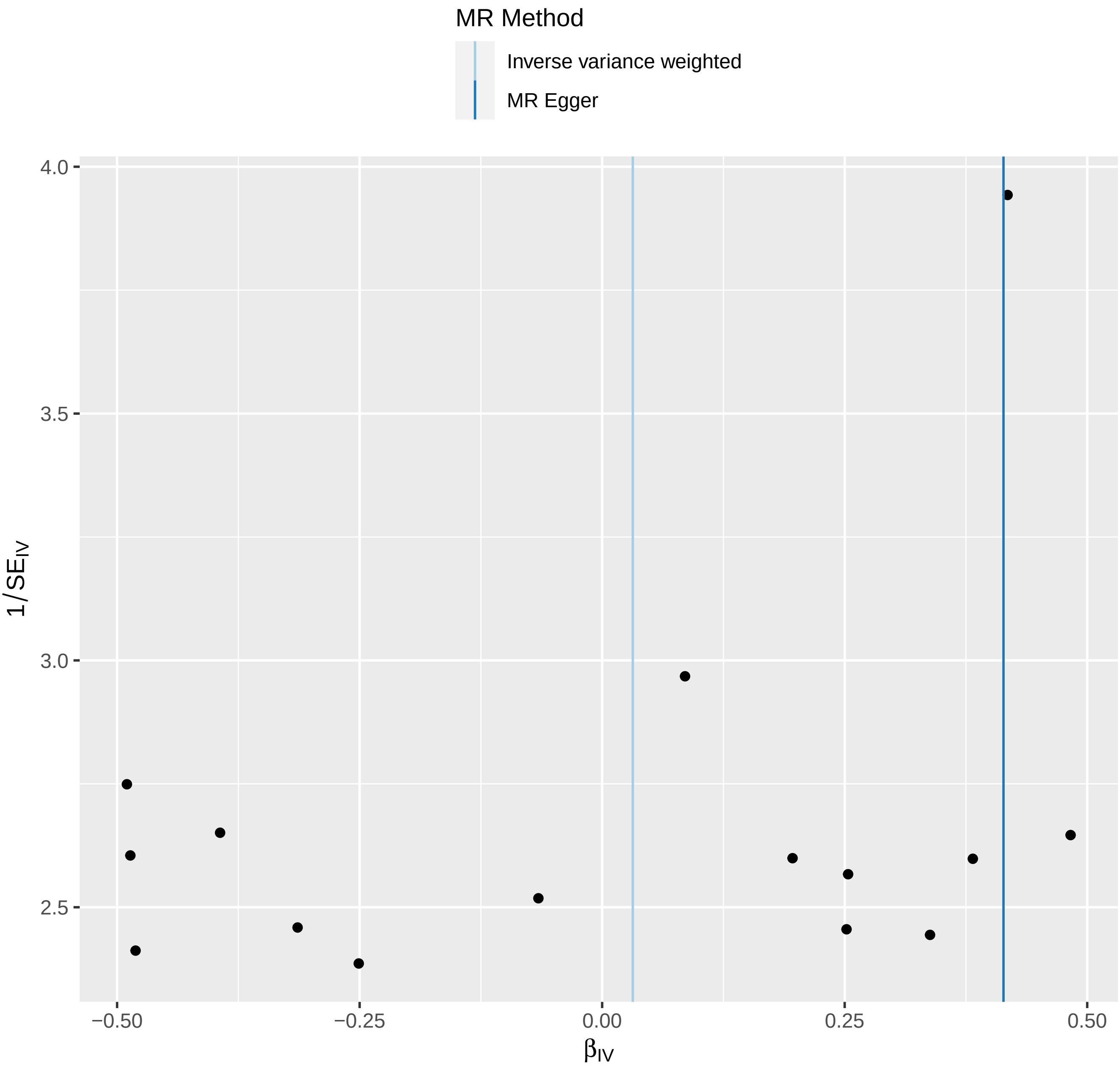

Batch 2−3039 : Gut microbiota abundance (genus Catenibacterium id.2153) on Cytokine receptor common subunit gamma
with a threshold of 1e−05

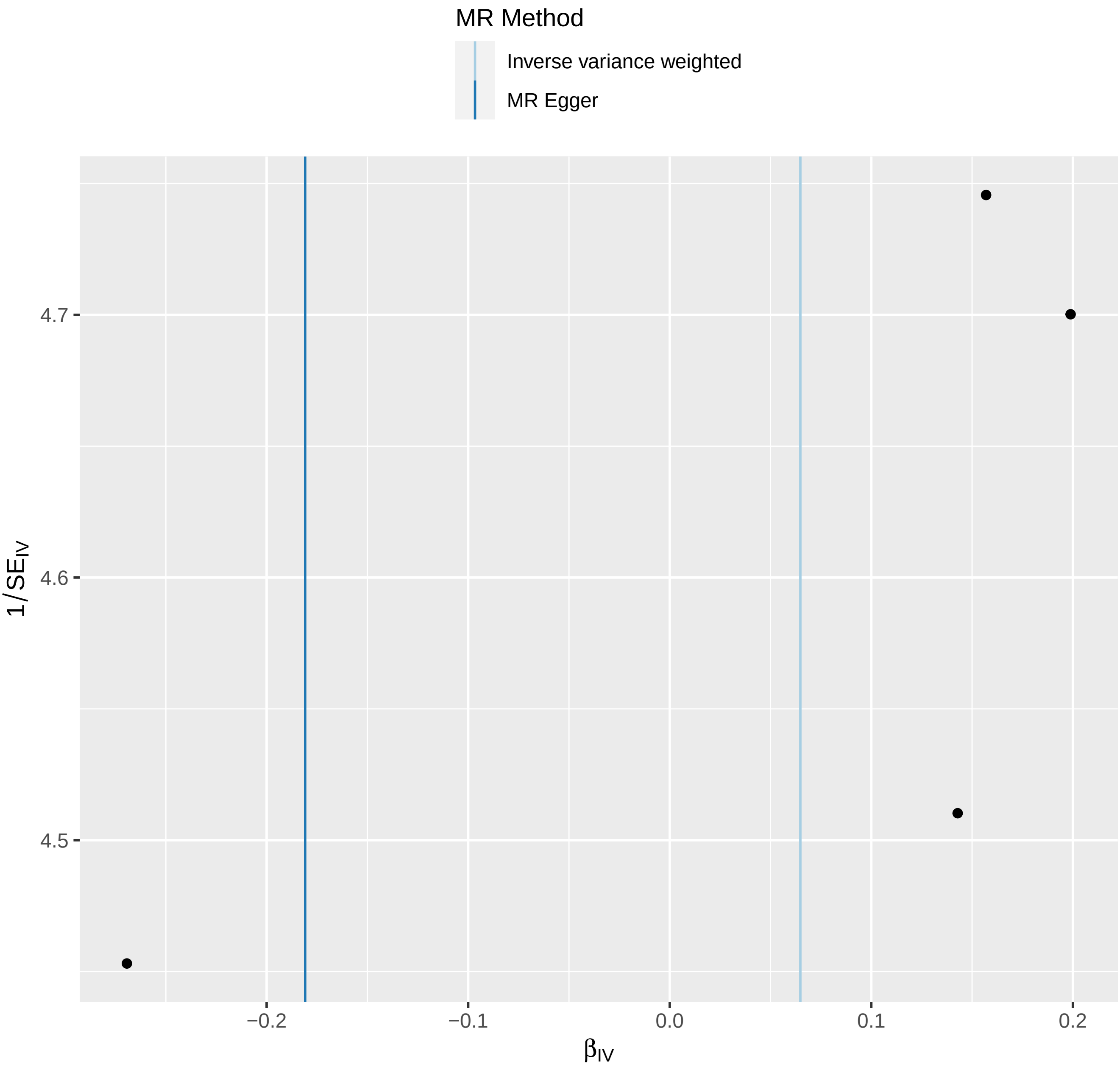

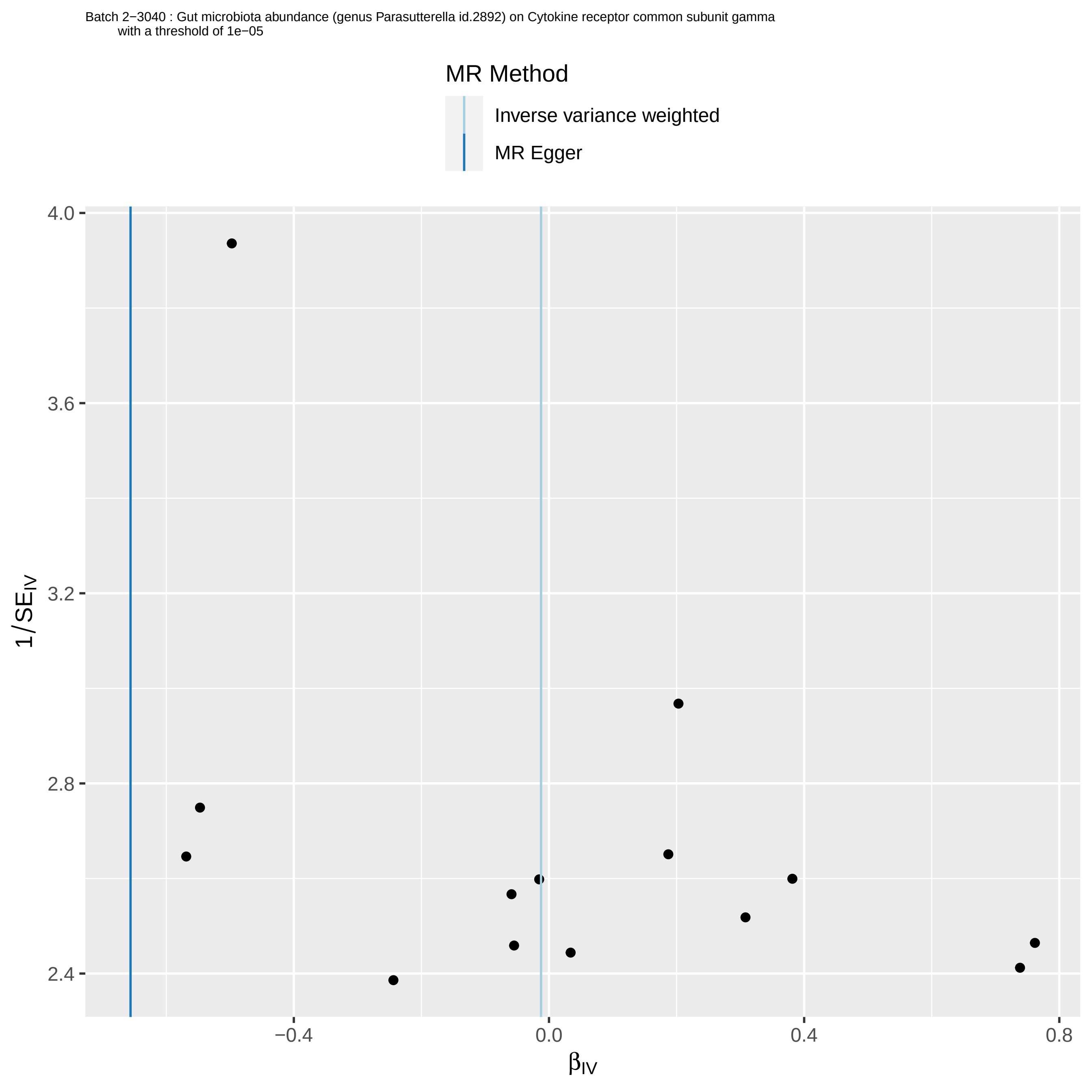

Batch 2−3040 : Gut microbiota abundance (genus Parasutterella id.2892) on Cytokine receptor common subunit gamma
with a threshold of 1e−05
MR Method
Inverse variance weighted
MR Egger
4.0
3.6
3.2
2.8
2.4
1/SE_IV
−0.4
0.0
0.4
0.8
β_IV

Batch 2−3041 : Gut microbiota abundance (genus Catenibacterium id.2153) on Interleukin−31
with a threshold of 1e−05

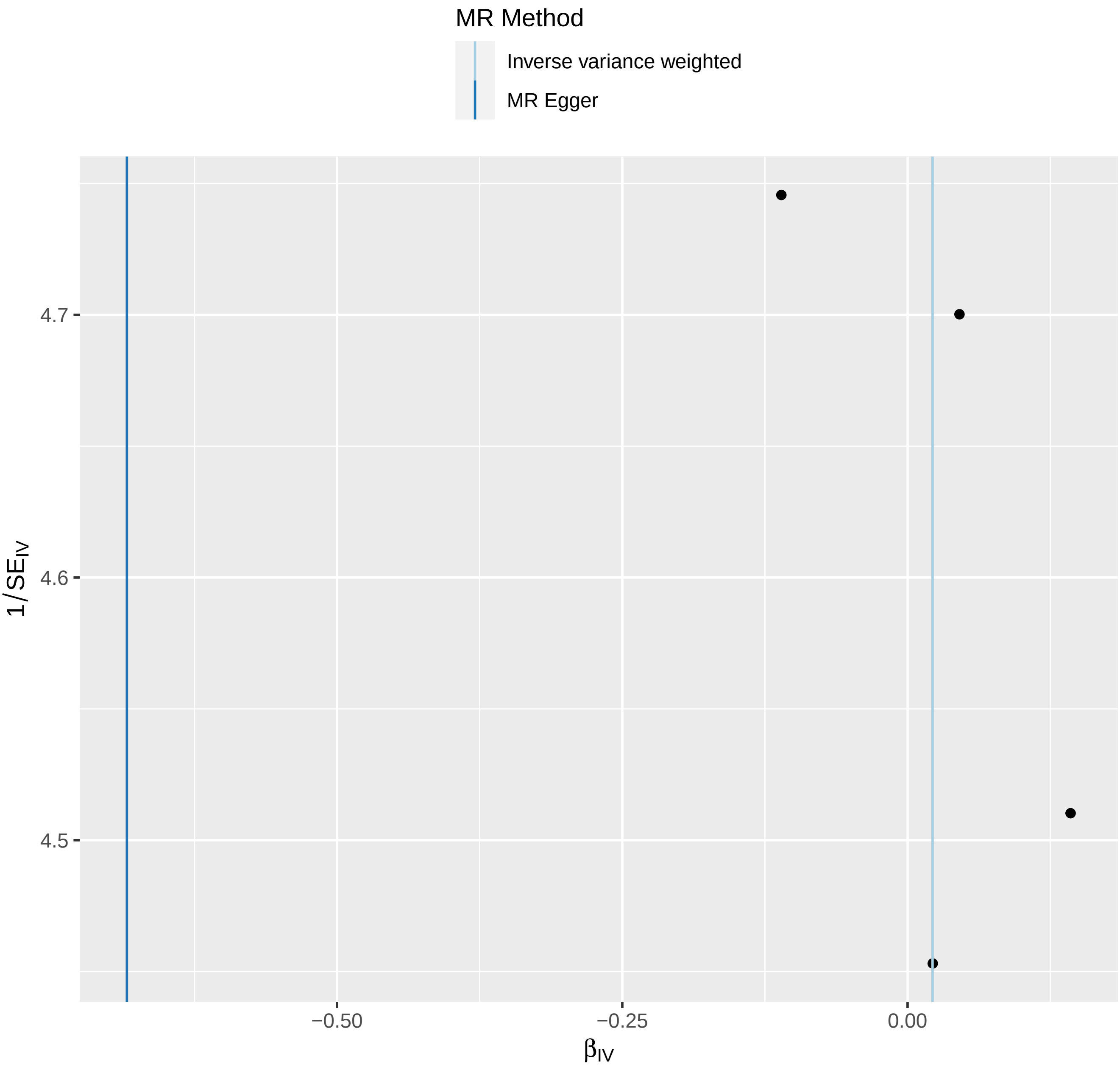

Batch 2−3042 : Gut microbiota abundance (genus Parasutterella id.2892) on Interleukin−31
with a threshold of 1e−05

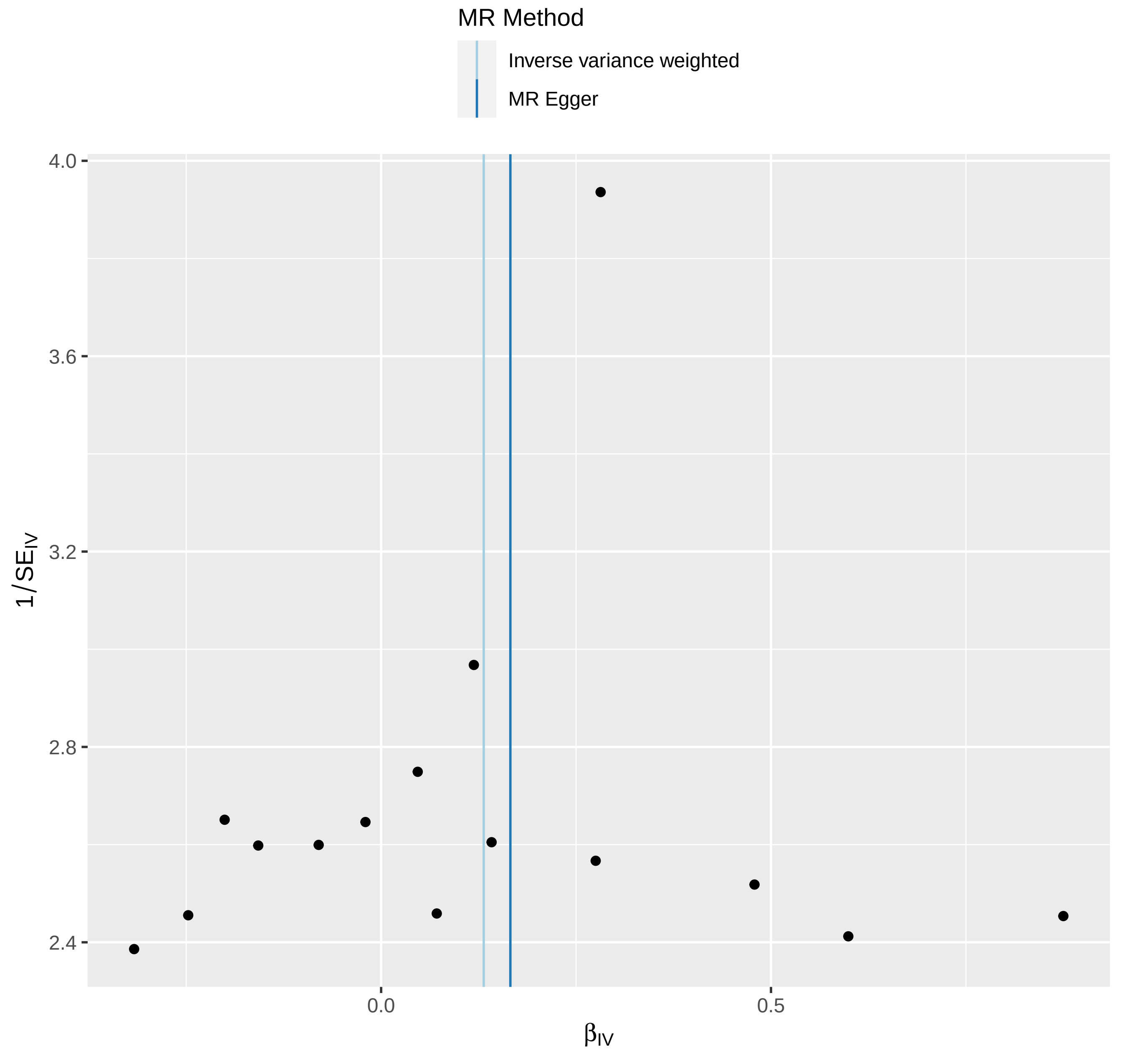

Batch 2−3043 : Gut microbiota abundance (genus Catenibacterium id.2153) on Interleukin−31 receptor subunit alpha
with a threshold of 1e−05

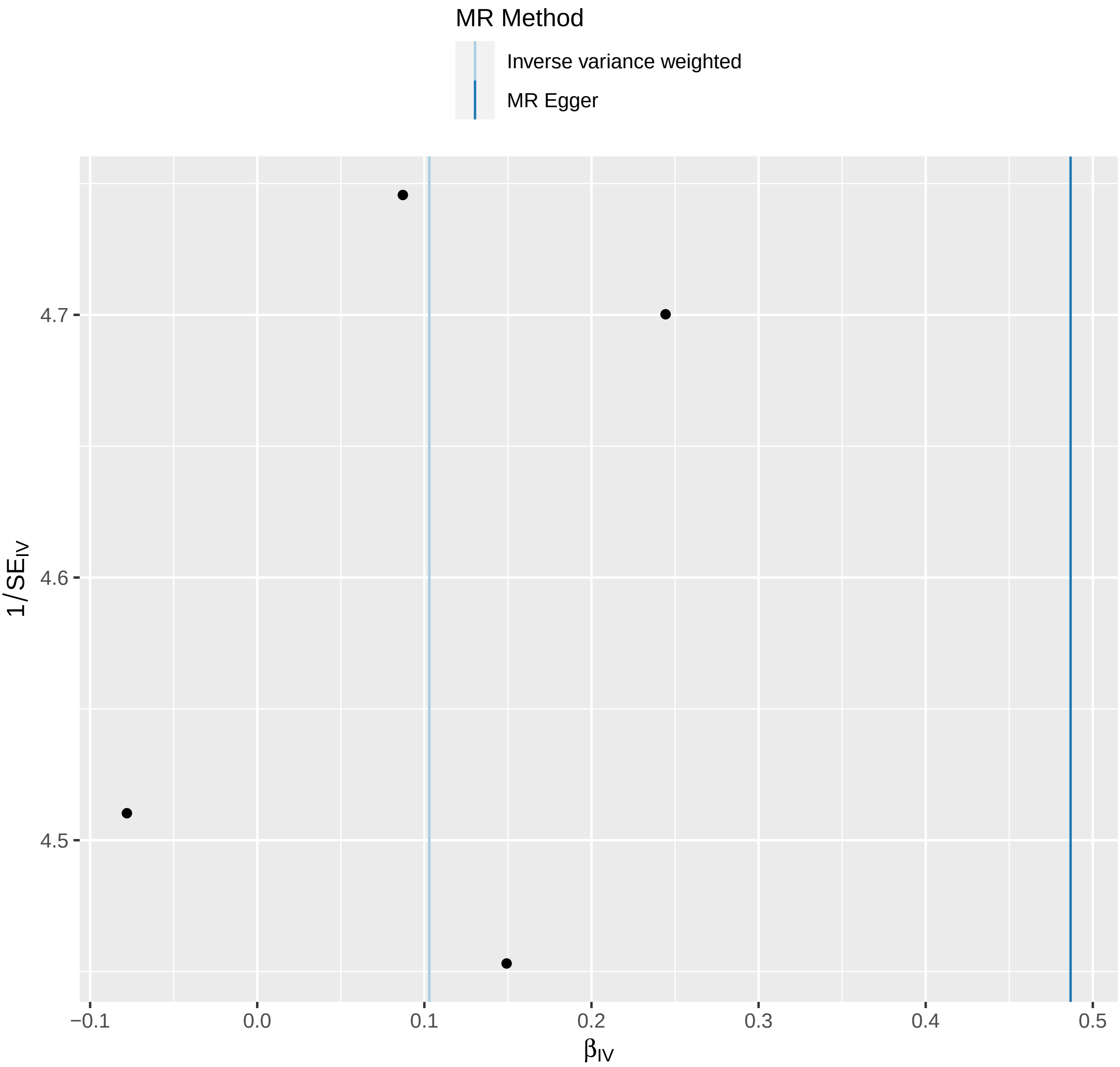

Batch 2−3044 : Gut microbiota abundance (genus Parasutterella id.2892) on Interleukin−31 receptor subunit alpha
with a threshold of 1e−05

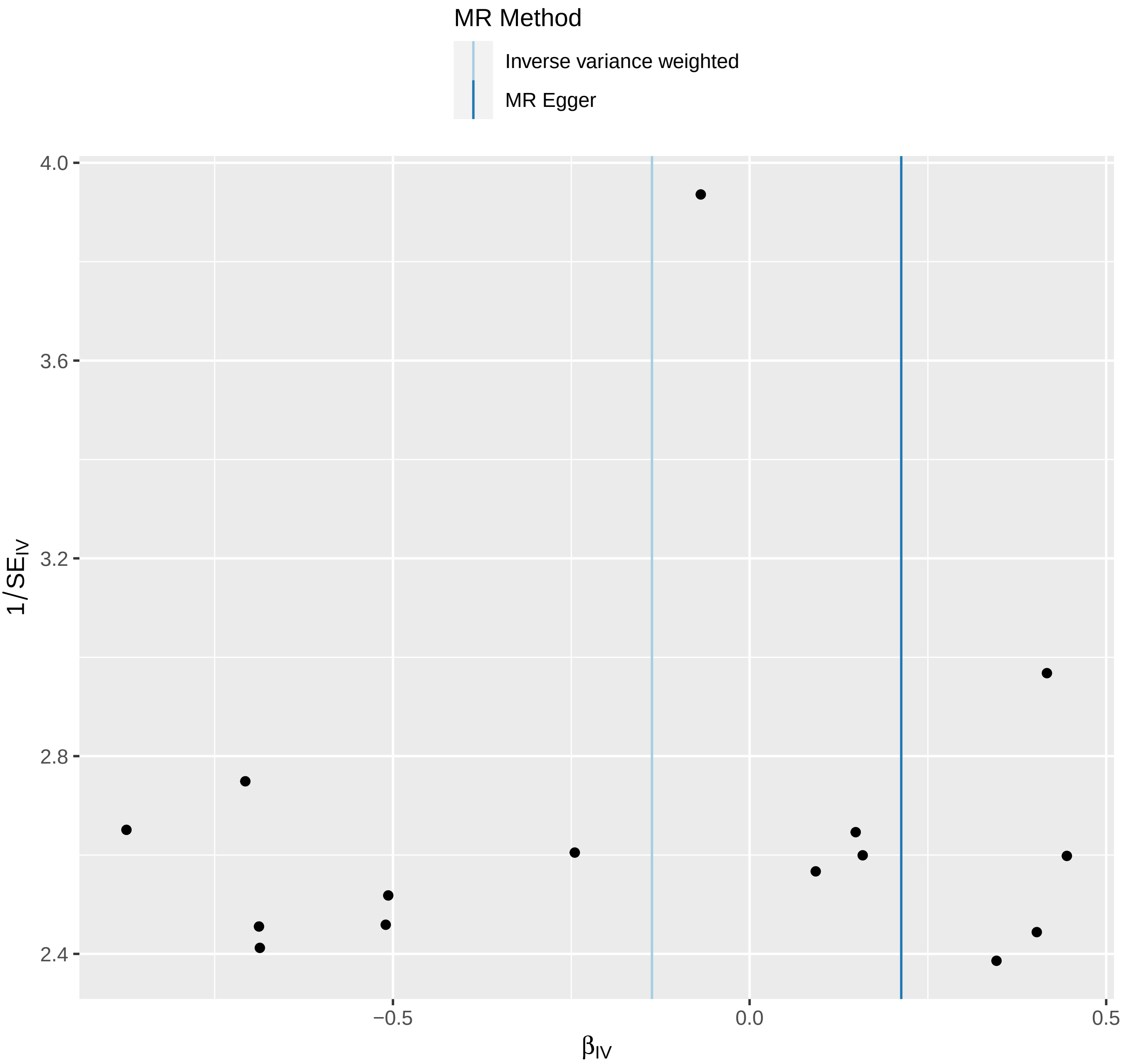

Batch 2−3045 : Gut microbiota abundance (genus Catenibacterium id.2153) on Interleukin−32
with a threshold of 1e−05

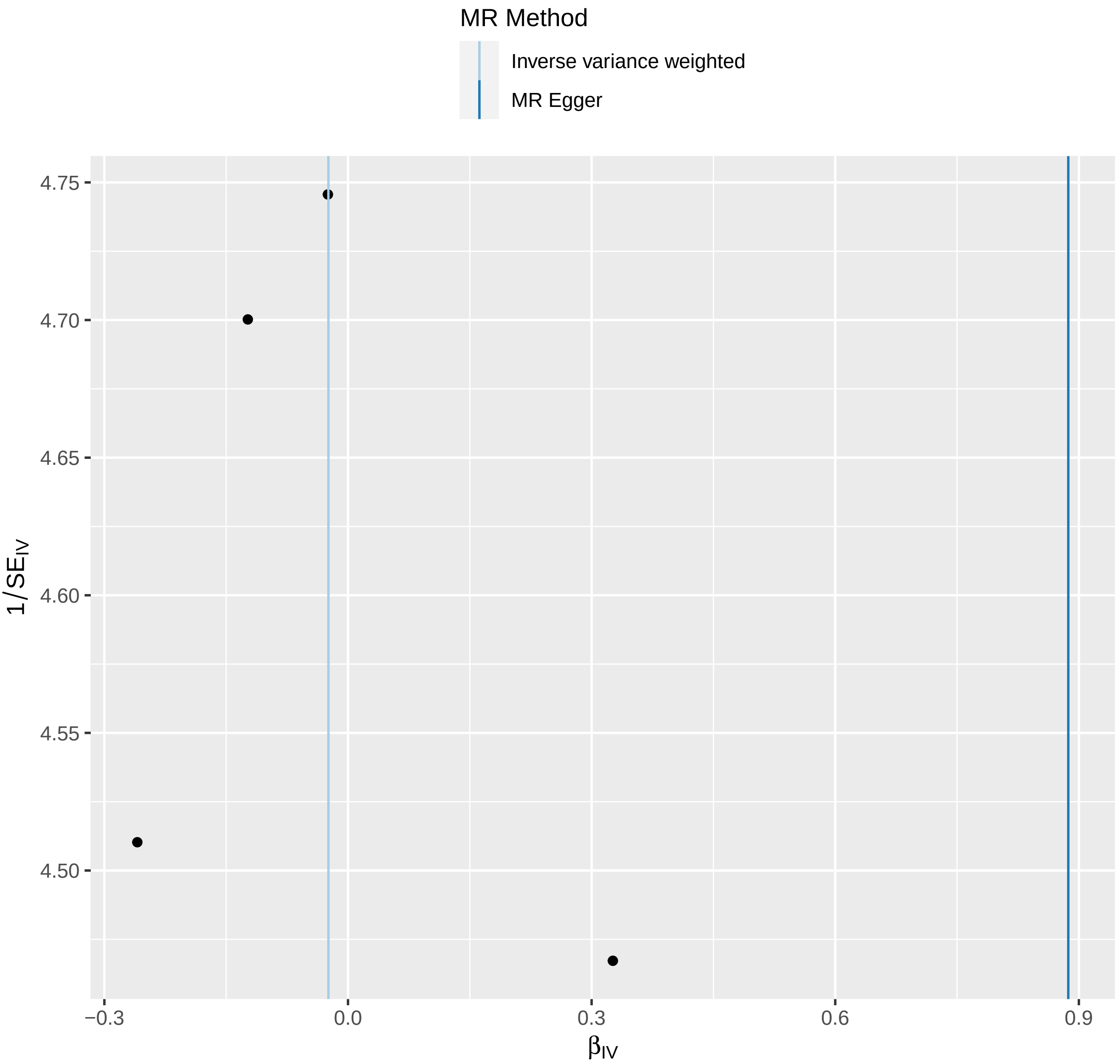

Batch 2−3046 : Gut microbiota abundance (genus Parasutterella id.2892) on Interleukin−32
with a threshold of 1e−05

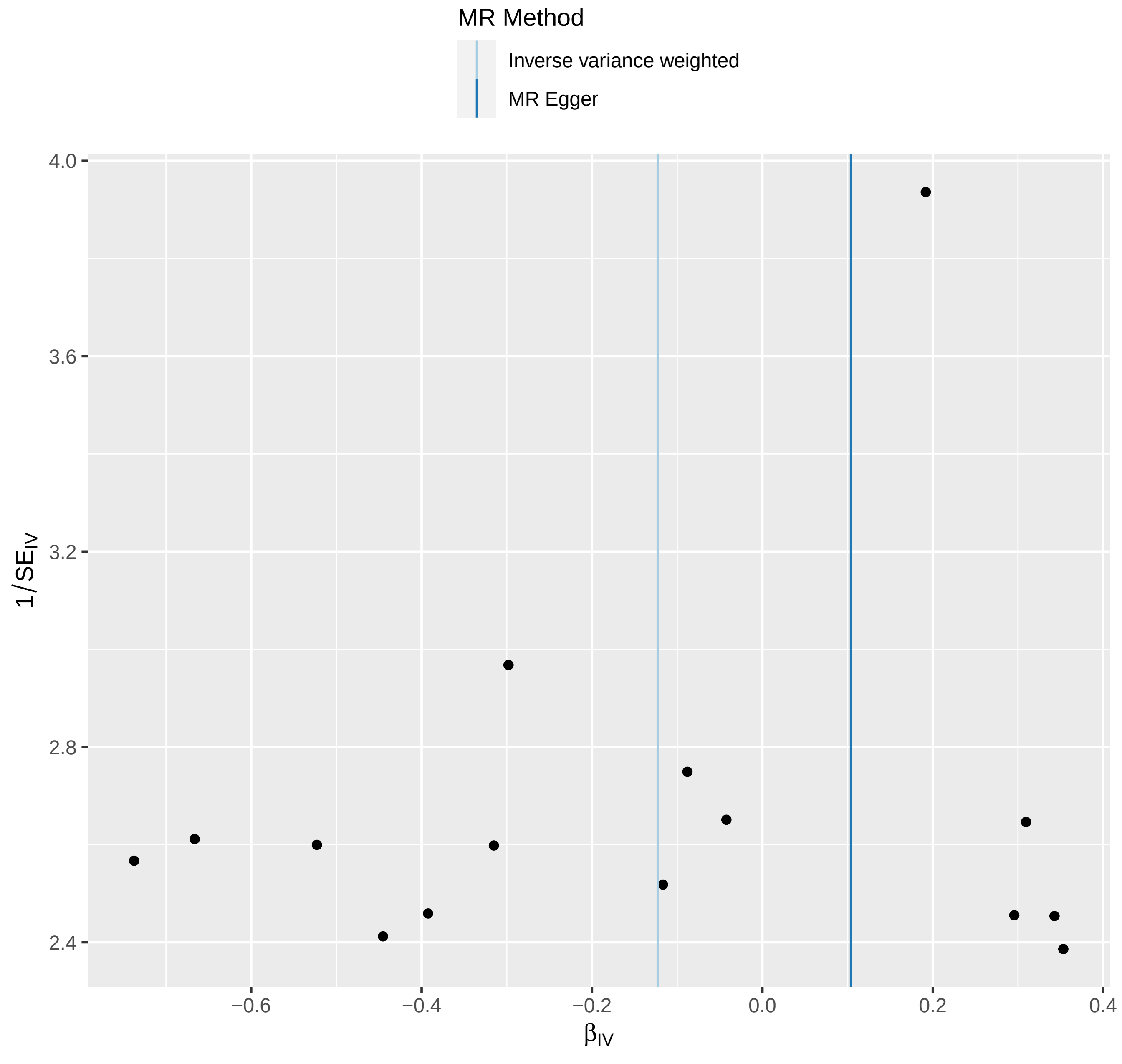

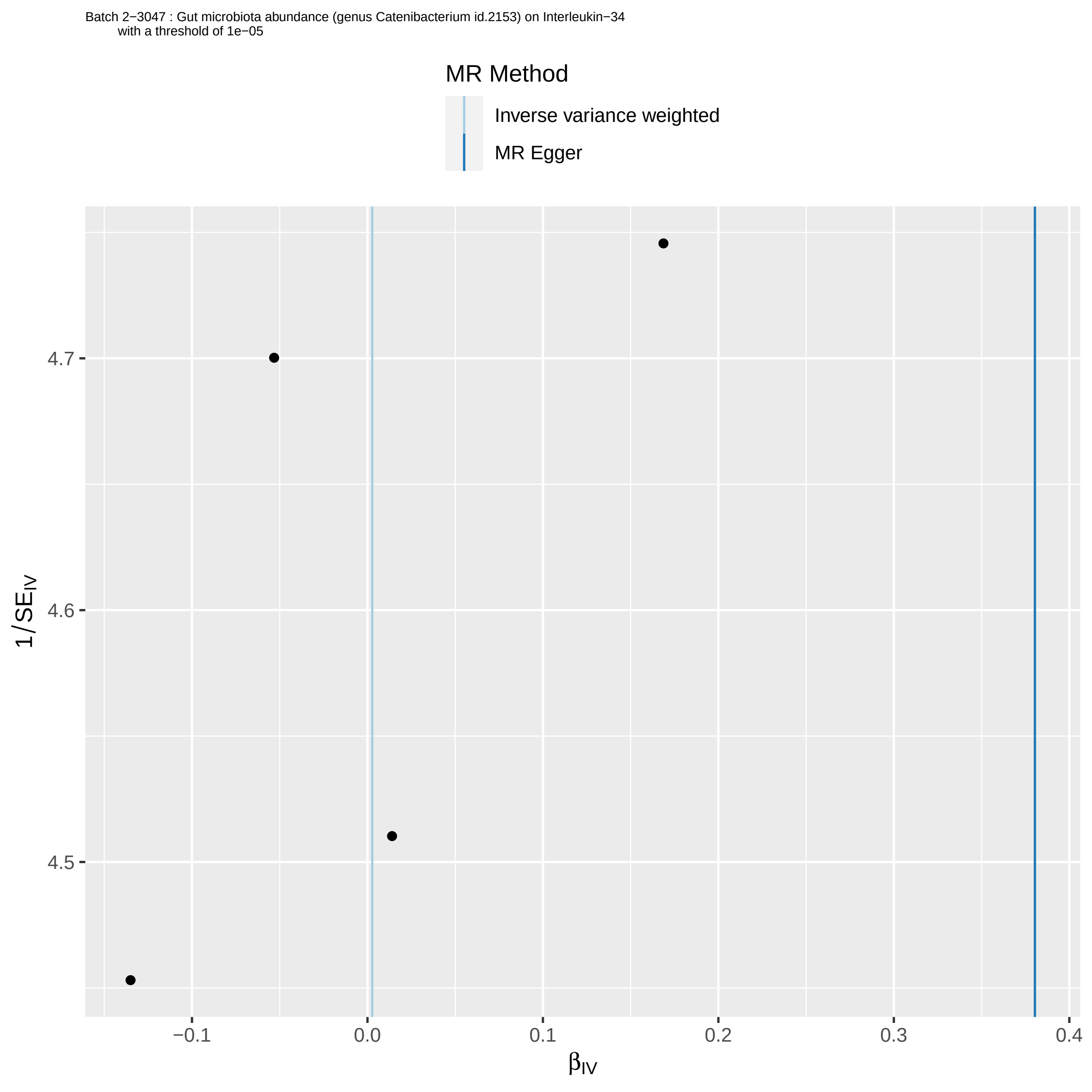

Batch 2−3047 : Gut microbiota abundance (genus Catenibacterium id.2153) on Interleukin−34
with a threshold of 1e−05
MR Method
Inverse variance weighted
MR Egger
4.7
4.6
4.5
1/SE$_{IV}$
−0.1
0.0
0.1
0.2
0.3
0.4
β$_{IV}$

Batch 2−3048 : Gut microbiota abundance (genus Parasutterella id.2892) on Interleukin−34
with a threshold of 1e−05

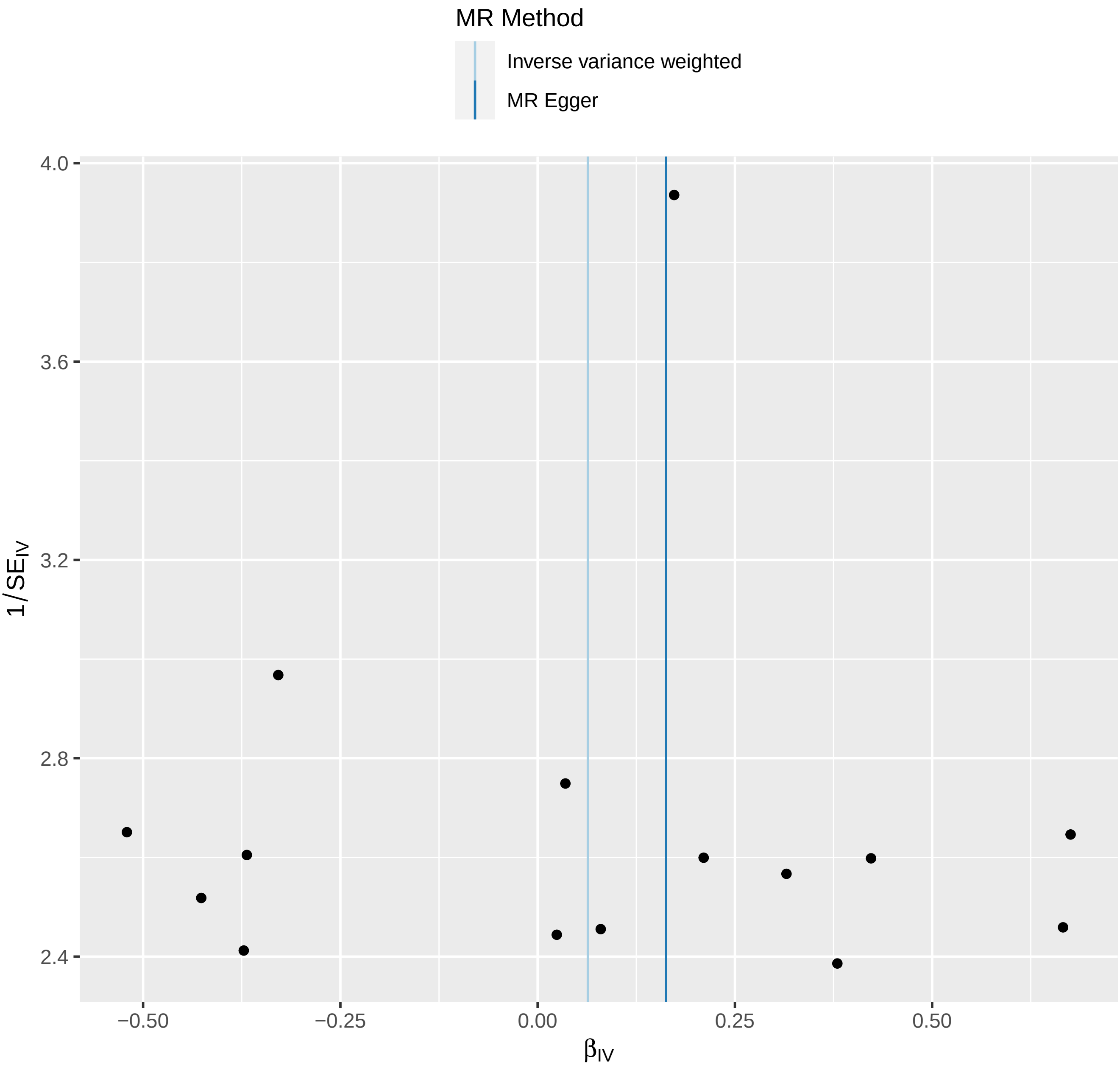

Batch 2−3049 : Gut microbiota abundance (genus Catenibacterium id.2153) on Interleukin−3
with a threshold of 1e−05

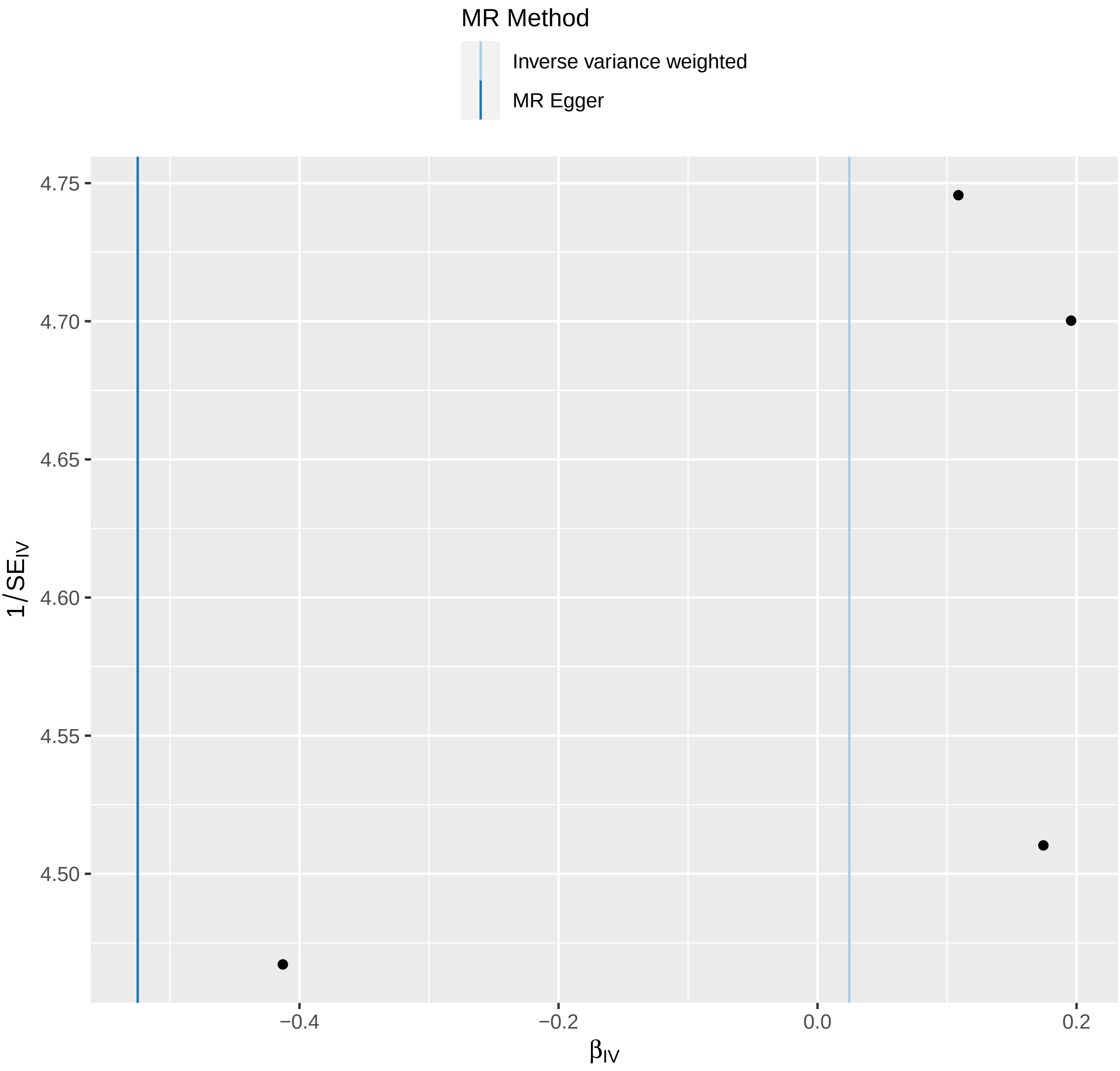

Batch 2−3050 : Gut microbiota abundance (genus Parasutterella id.2892) on Interleukin−3
with a threshold of 1e−05

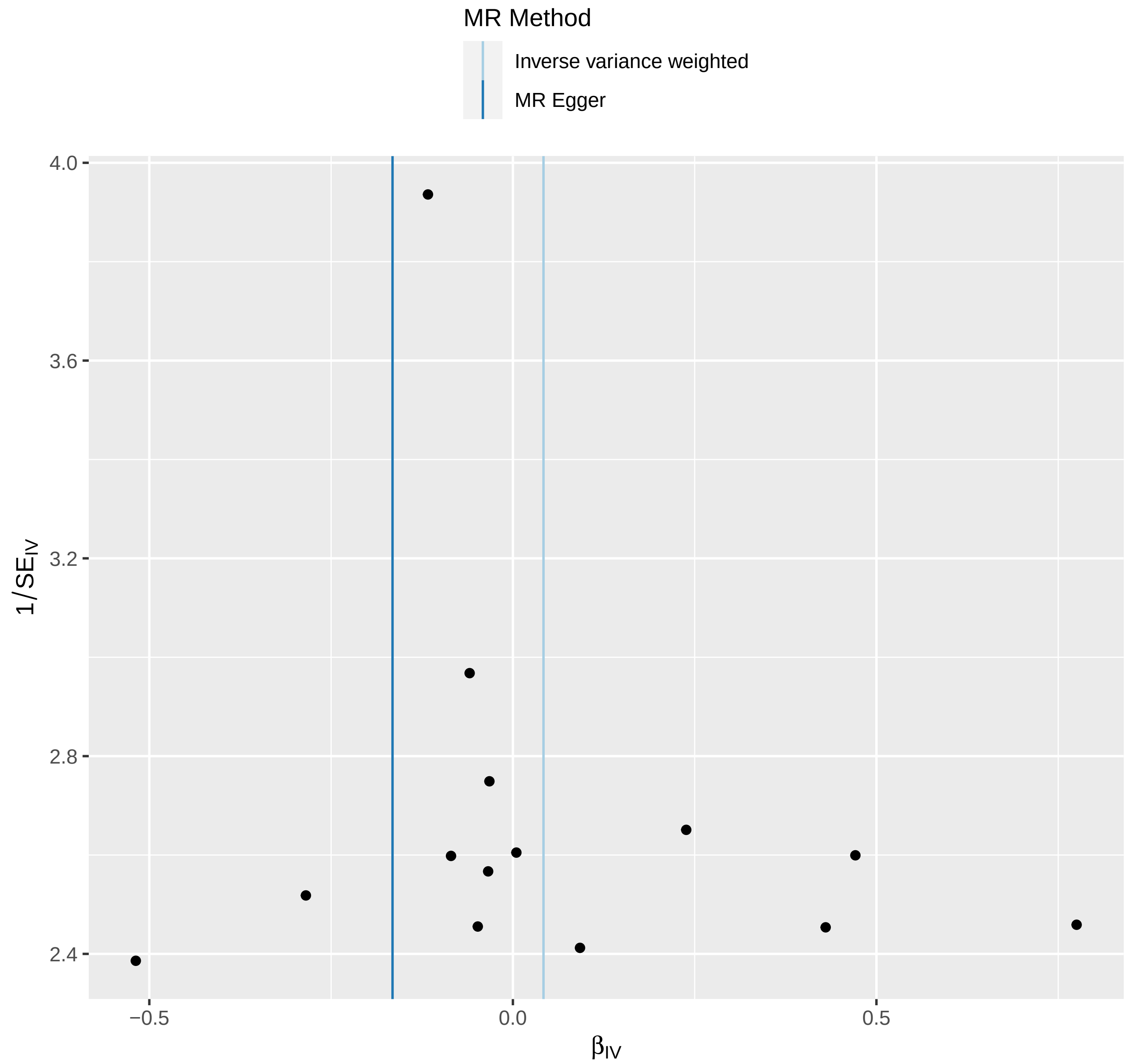

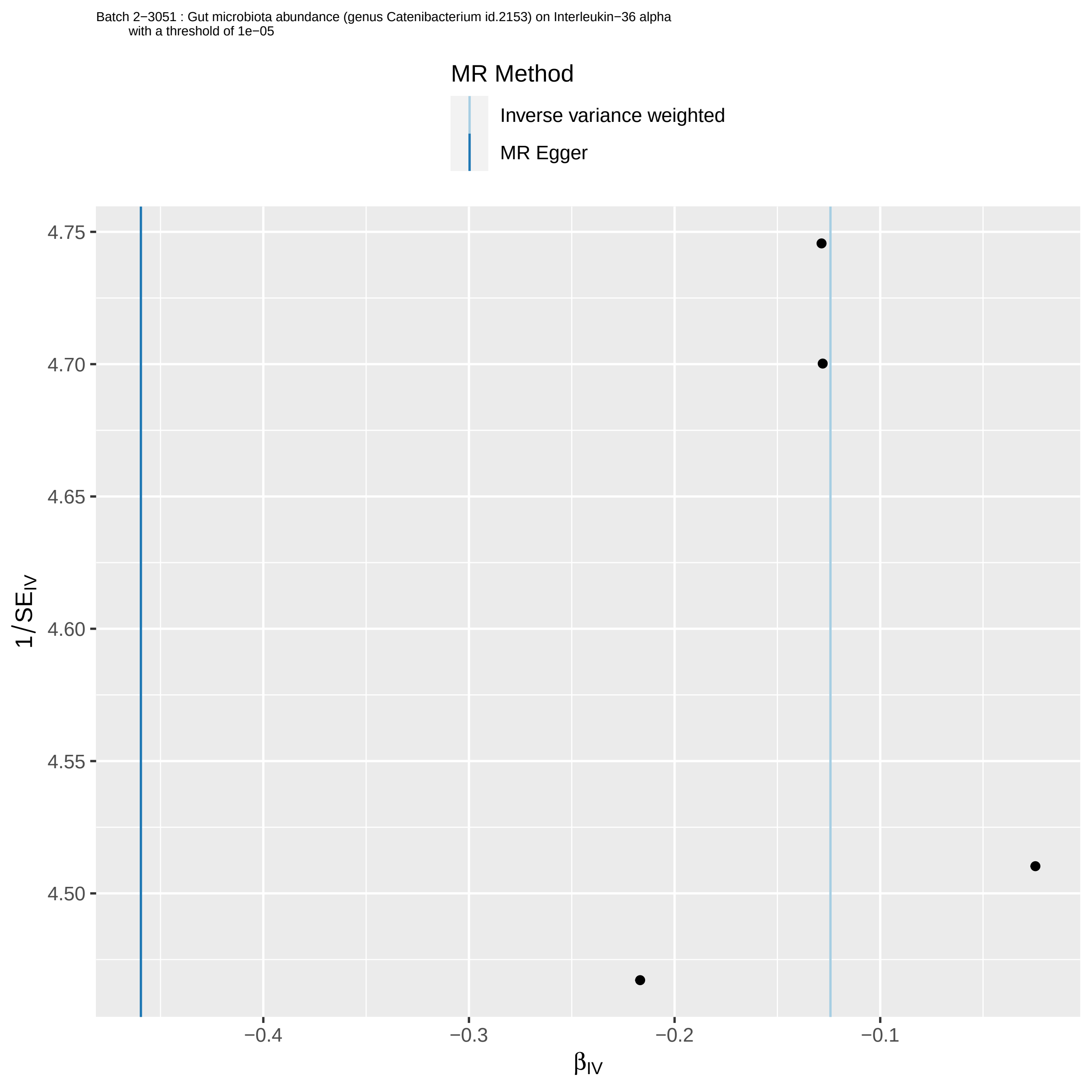

Batch 2−3051 : Gut microbiota abundance (genus Catenibacterium id.2153) on Interleukin−36 alpha
with a threshold of 1e−05
MR Method
Inverse variance weighted
MR Egger
4.75
4.70
4.65
4.60
4.55
4.50
1/SE_IV
−0.4
−0.3
−0.2
−0.1
β_IV

Batch 2−3052 : Gut microbiota abundance (genus Parasutterella id.2892) on Interleukin−36 alpha
with a threshold of 1e−05

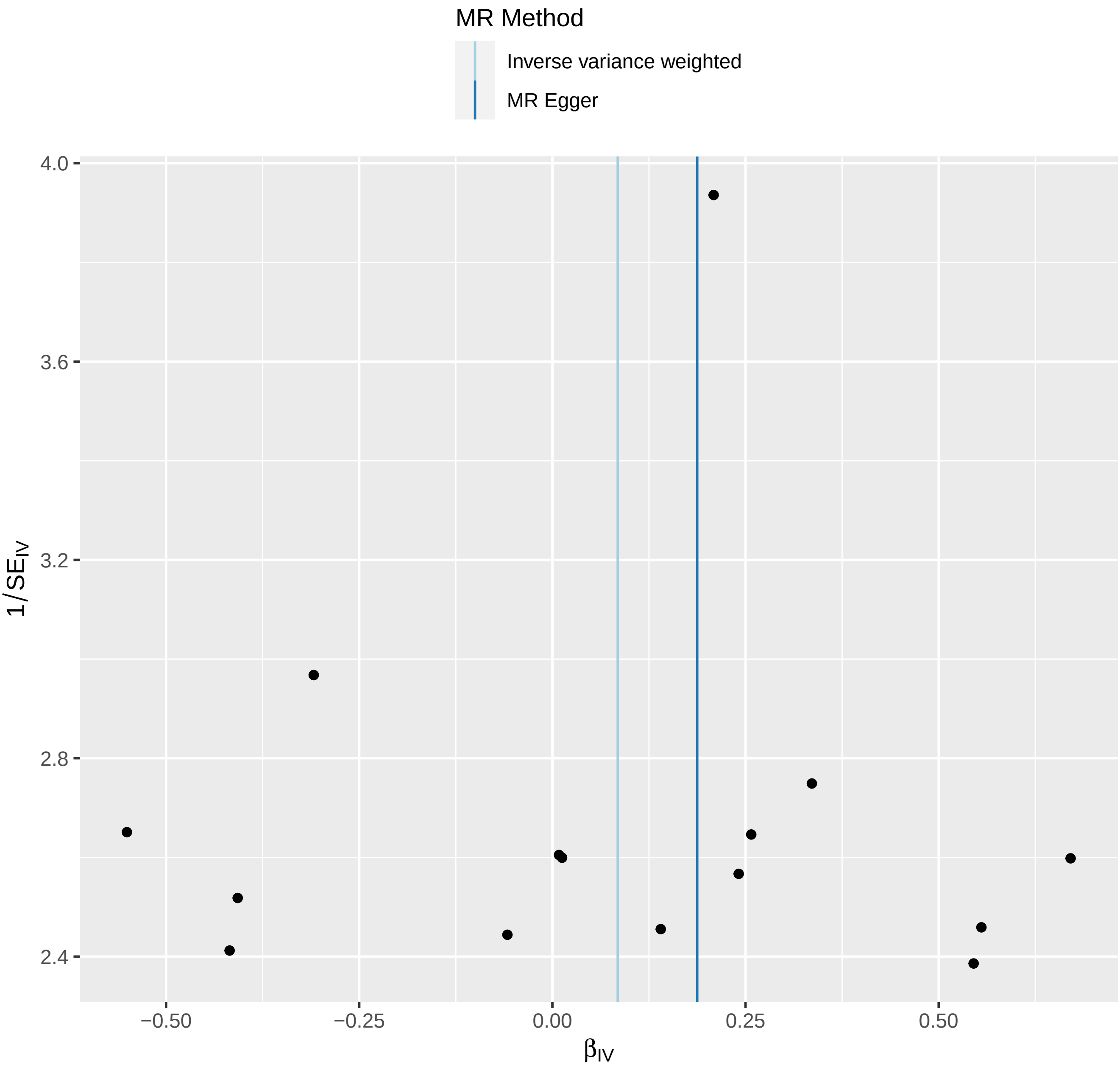

Batch 2−3053 : Gut microbiota abundance (genus Catenibacterium id.2153) on Interleukin−36 beta
with a threshold of 1e−05

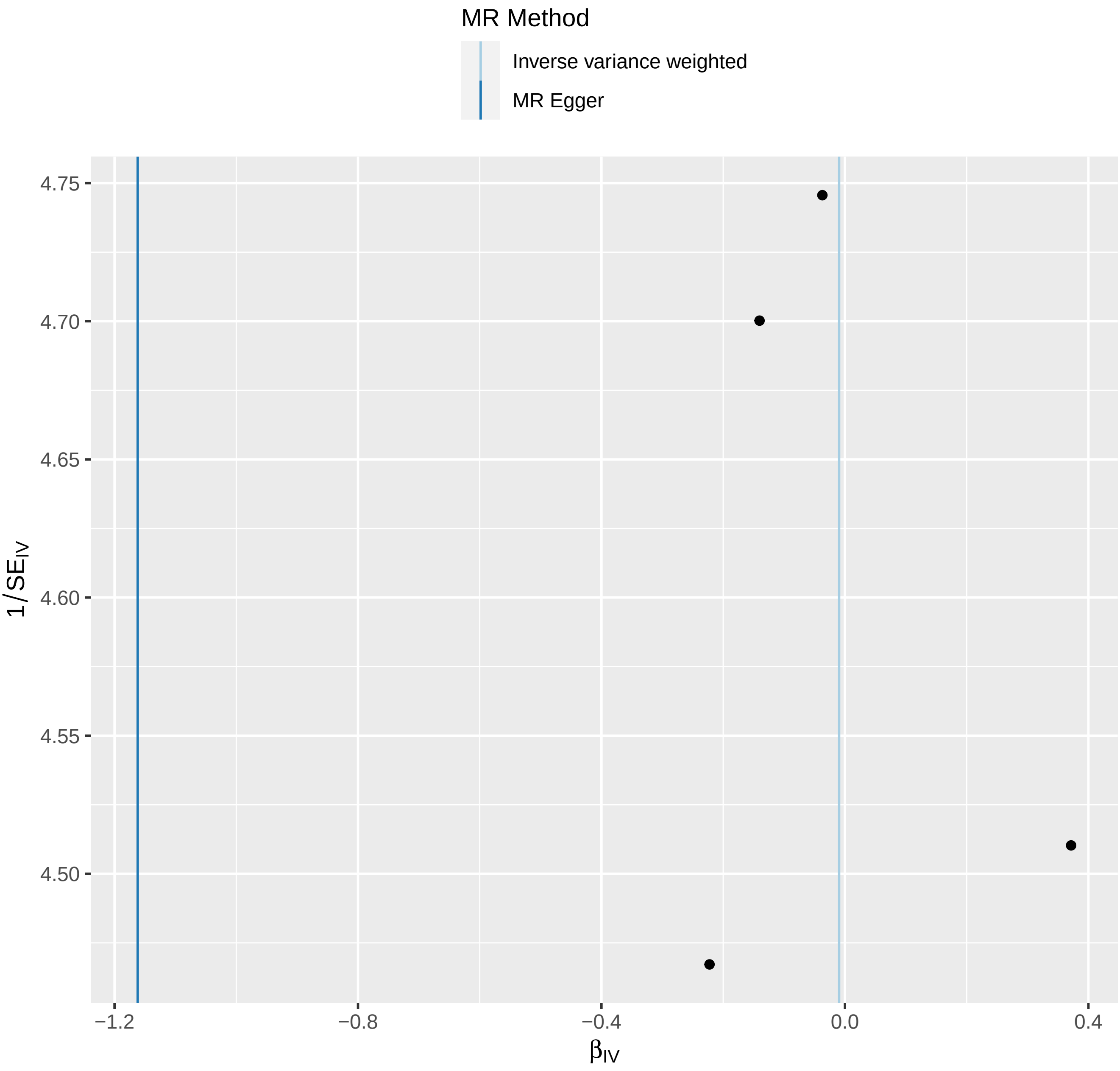

Batch 2−3054 : Gut microbiota abundance (genus Parasutterella id.2892) on Interleukin−36 beta
with a threshold of 1e−05

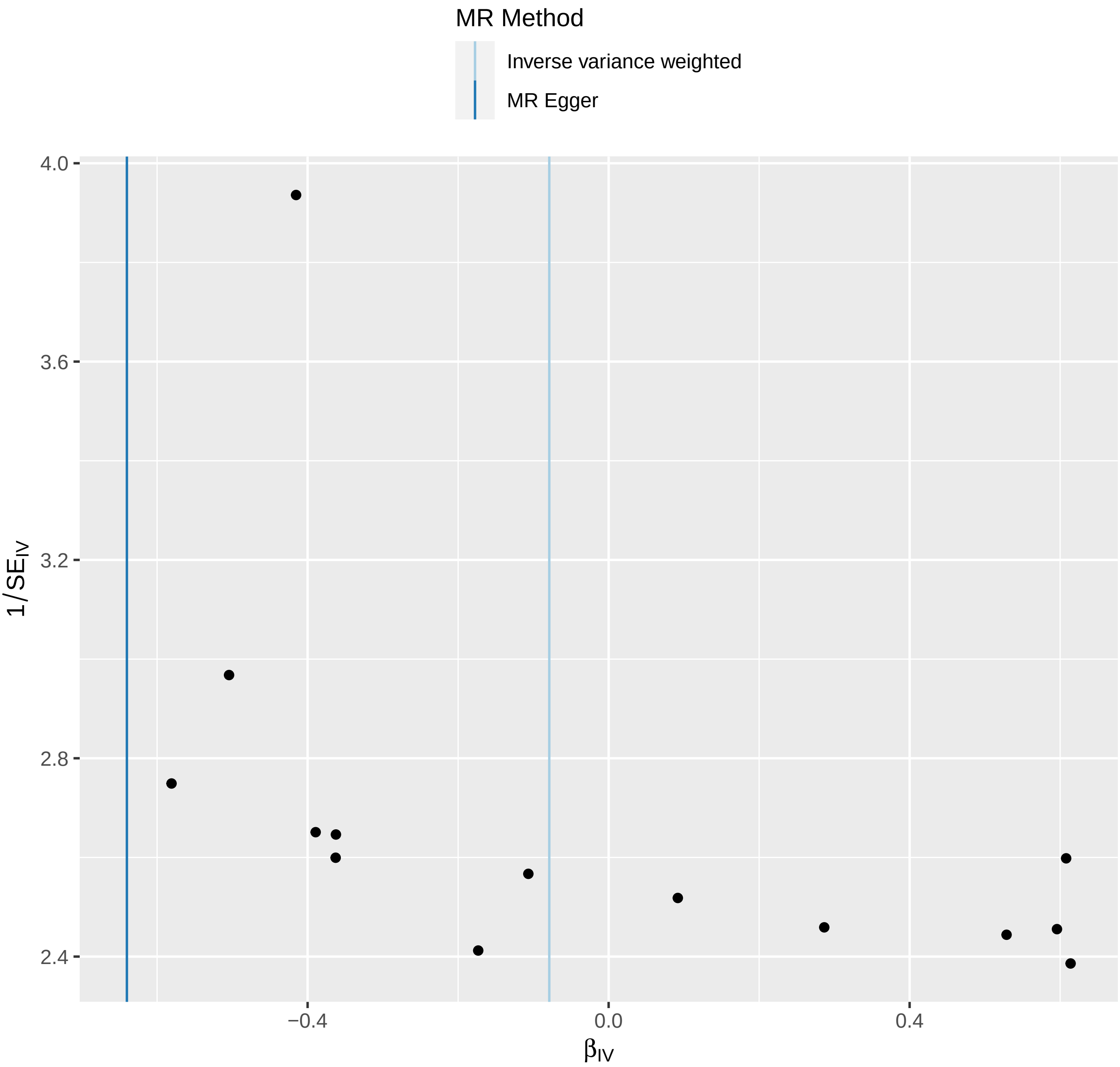

Batch 2−3055 : Gut microbiota abundance (genus Catenibacterium id.2153) on Interleukin−36 gamma
with a threshold of 1e−05

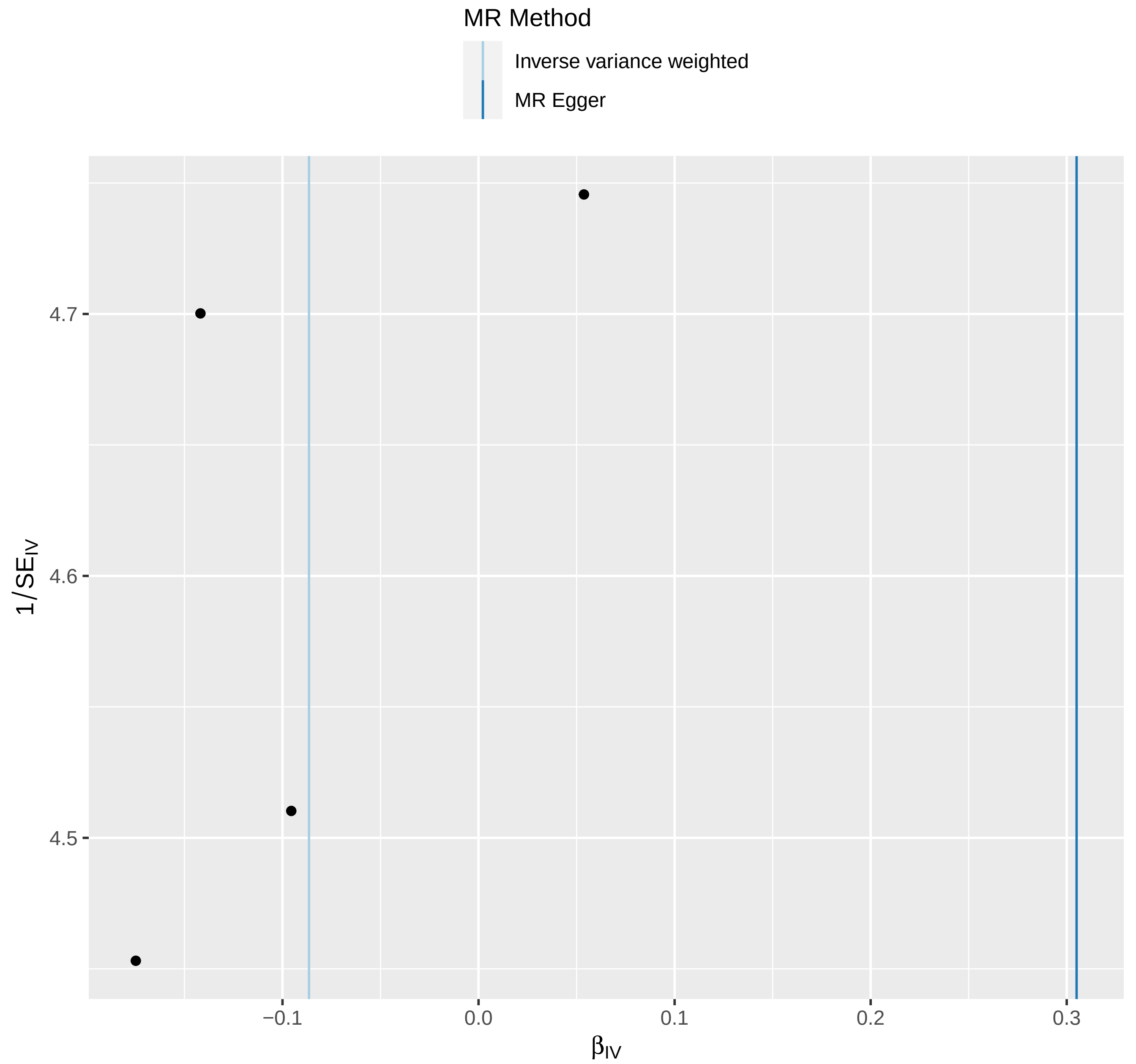

Batch 2−3056 : Gut microbiota abundance (genus Parasutterella id.2892) on Interleukin−36 gamma
with a threshold of 1e−05

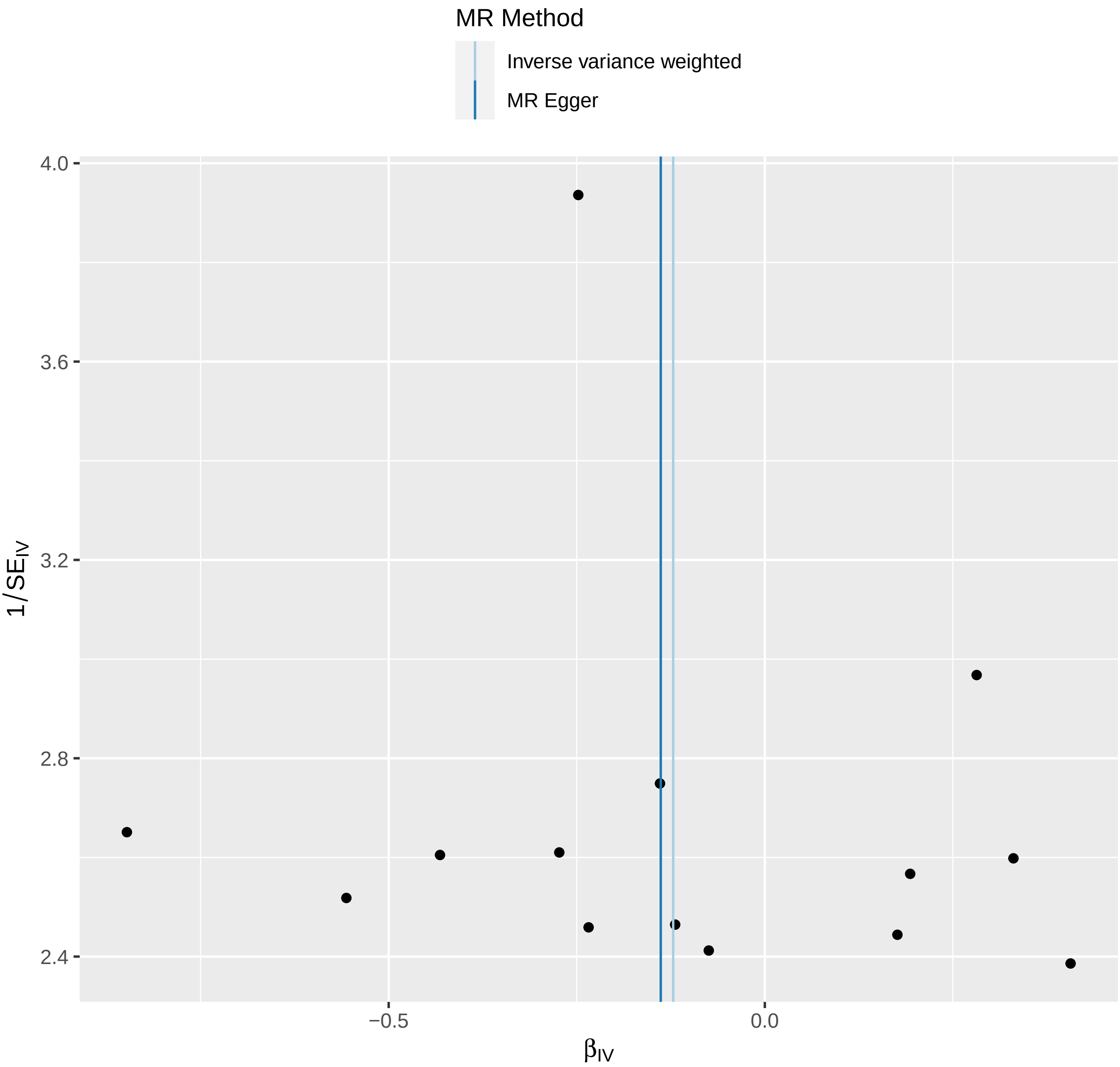

Batch 2−3057 : Gut microbiota abundance (genus Catenibacterium id.2153) on Interleukin−37
with a threshold of 1e−05

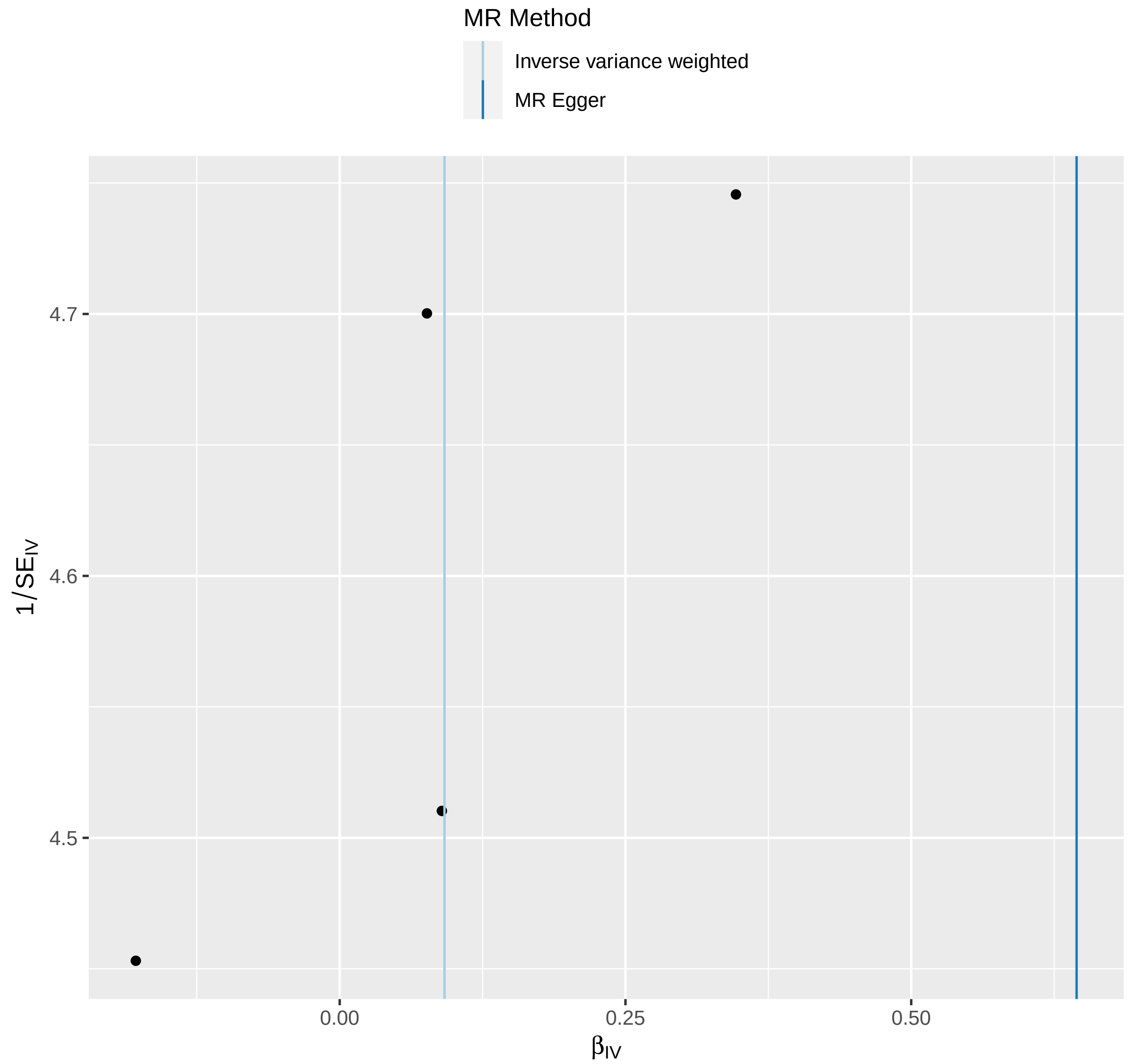

Batch 2−3058 : Gut microbiota abundance (genus Parasutterella id.2892) on Interleukin−37
with a threshold of 1e−05

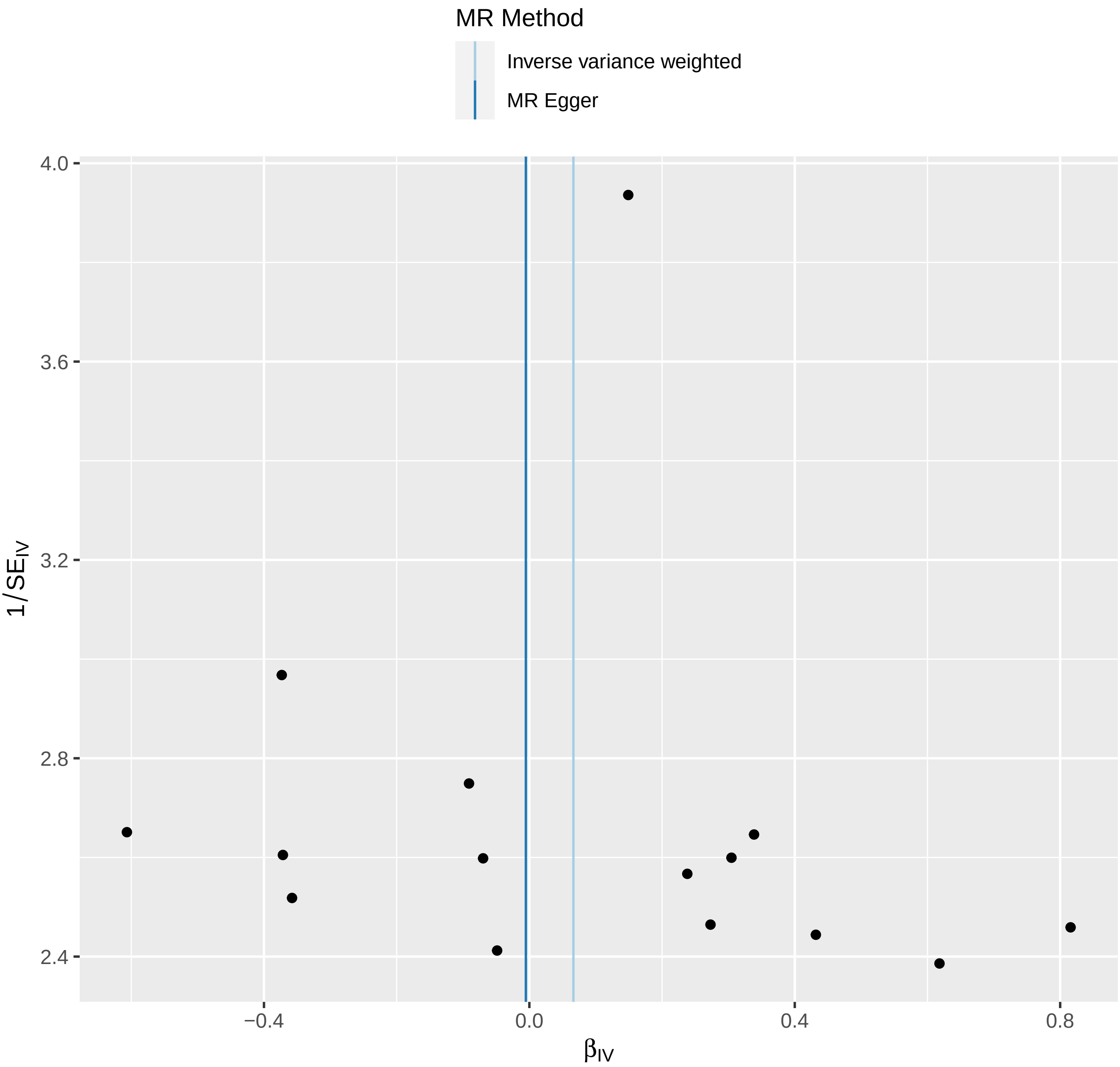

Batch 2−3059 : Gut microbiota abundance (genus Catenibacterium id.2153) on Interleukin−3 receptor subunit alpha with a threshold of 1e−05

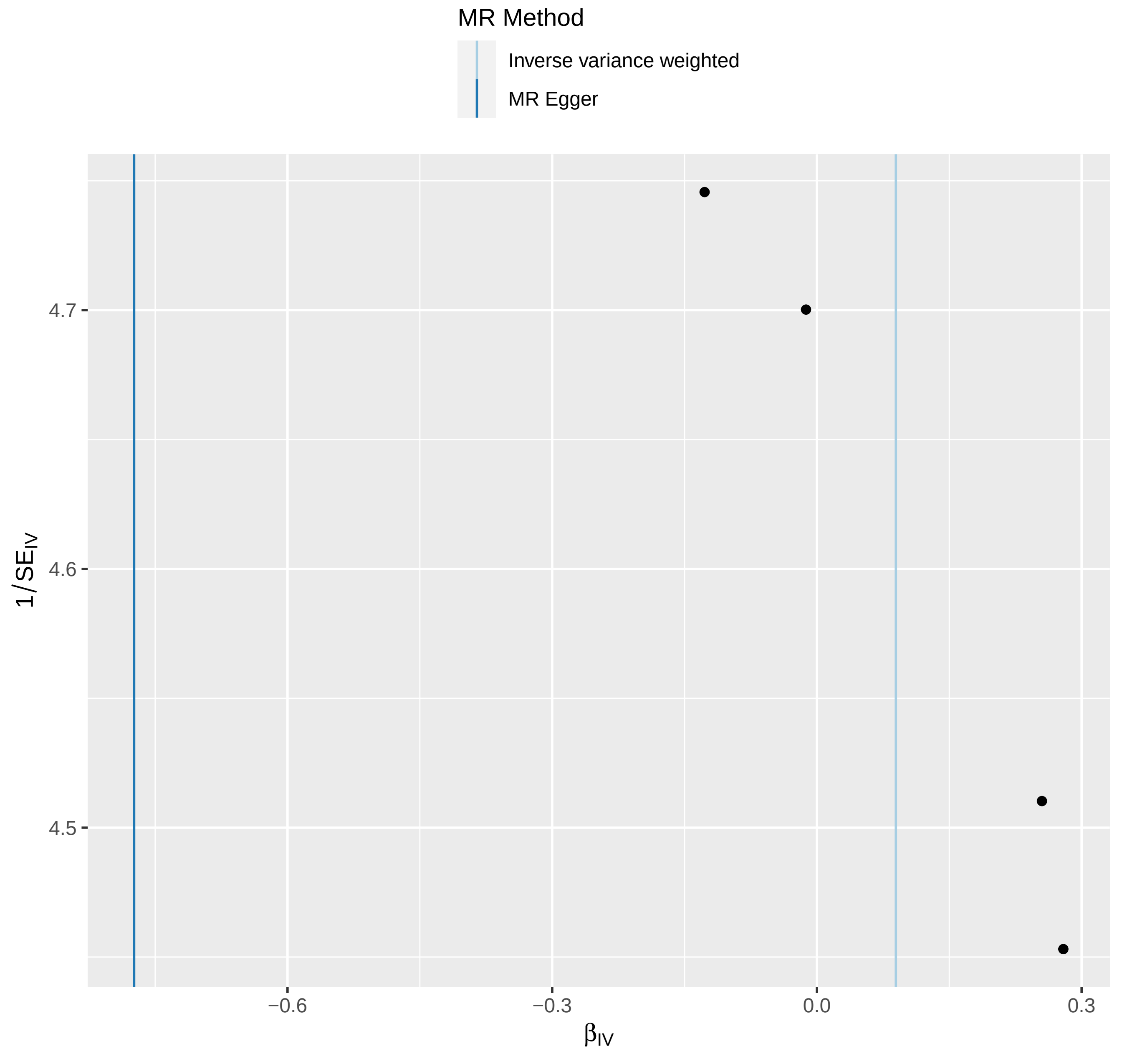

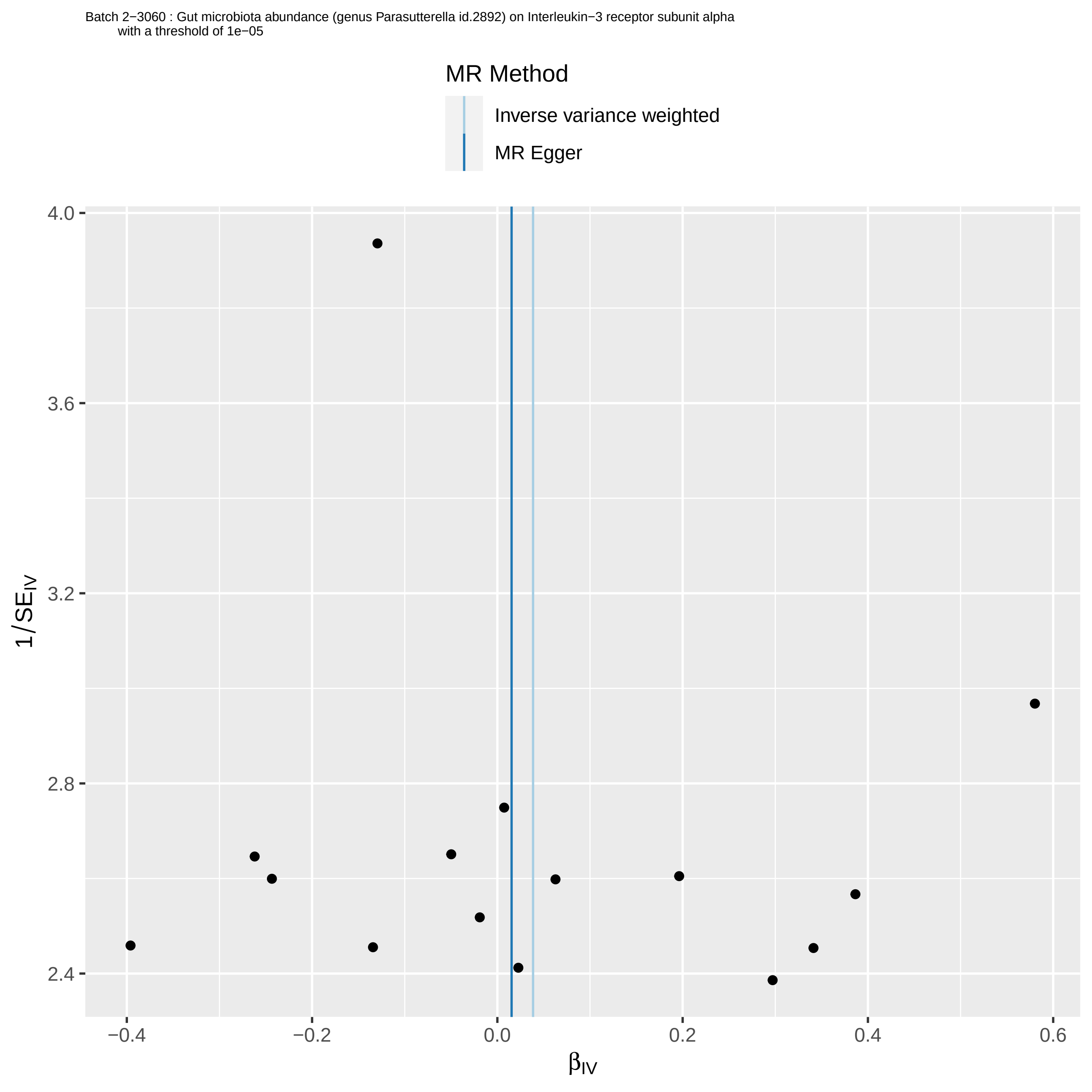

Batch 2−3060 : Gut microbiota abundance (genus Parasutterella id.2892) on Interleukin−3 receptor subunit alpha
with a threshold of 1e−05
MR Method
Inverse variance weighted
MR Egger
4.0
3.6
3.2
2.8
2.4
1/SE_IV
−0.4
−0.2
0.0
0.2
0.4
0.6
β_IV

Batch 2−3061 : Gut microbiota abundance (genus Catenibacterium id.2153) on Interleukin−3 receptor subunit alpha with a threshold of 1e−05

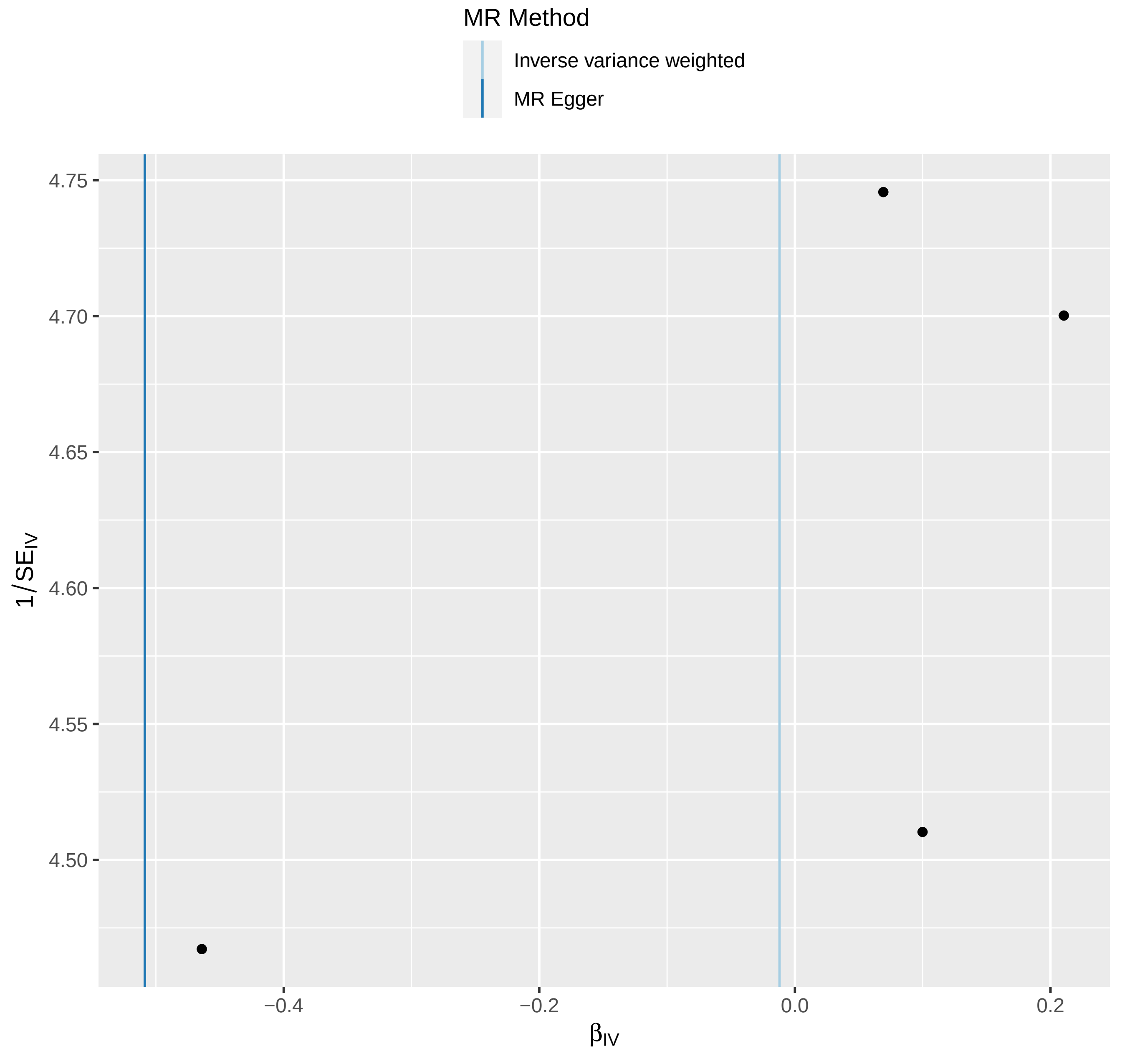

Batch 2−3062 : Gut microbiota abundance (genus Parasutterella id.2892) on Interleukin−3 receptor subunit alpha
with a threshold of 1e−05

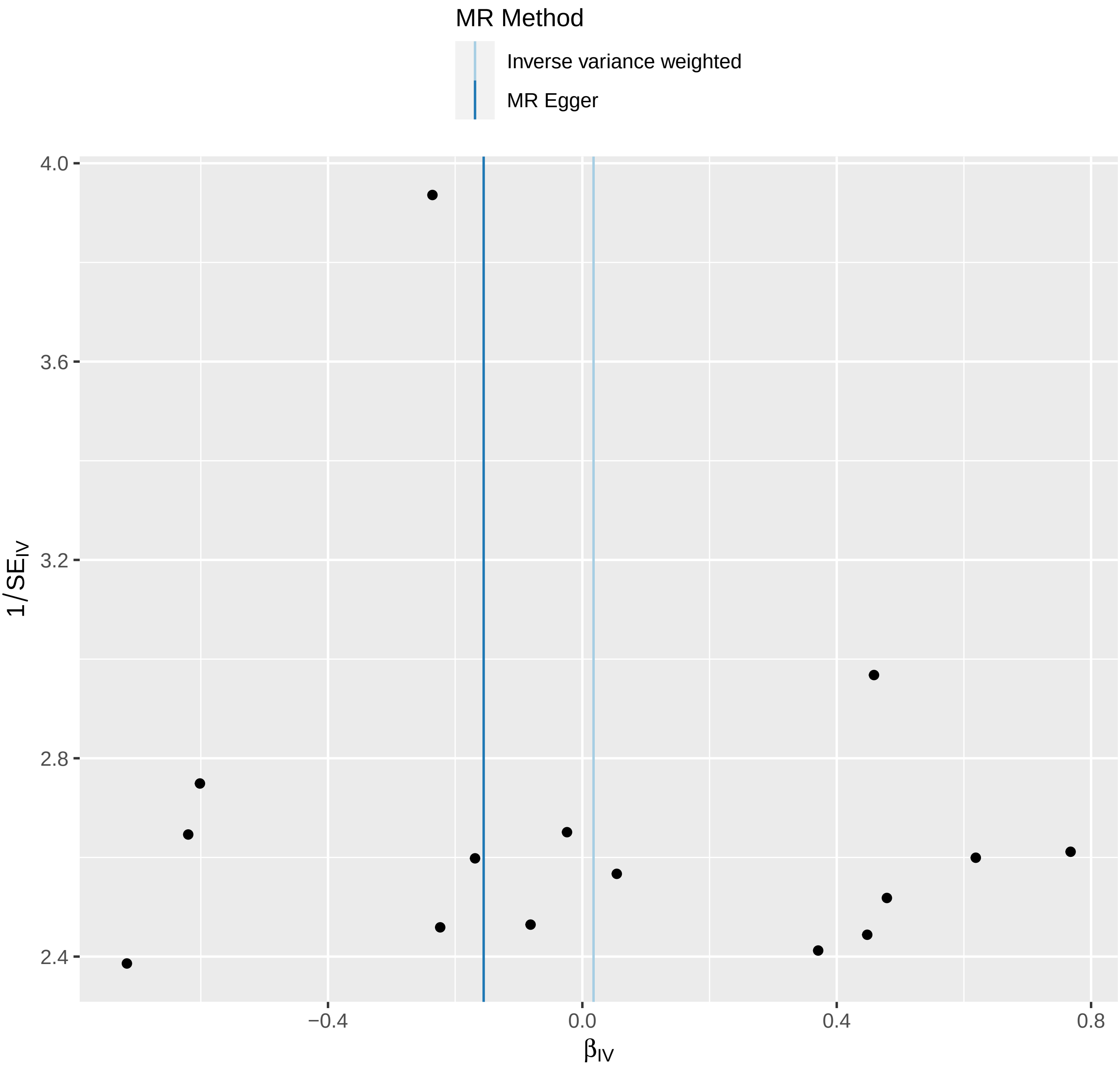

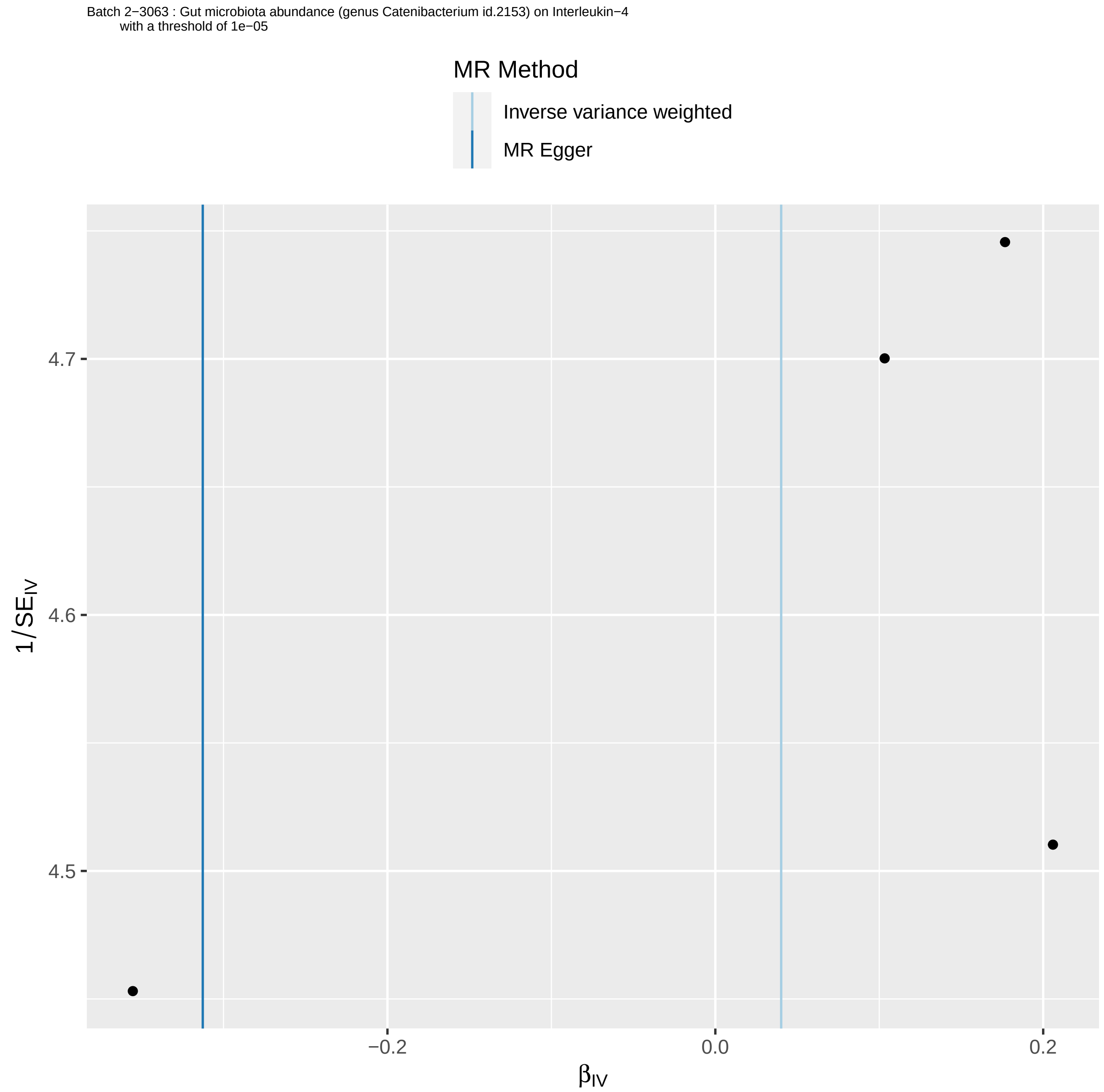
Batch 2−3063 : Gut microbiota abundance (genus Catenibacterium id.2153) on Interleukin−4
with a threshold of 1e−05
MR Method
Inverse variance weighted
MR Egger
4.7
4.6
4.5
1/SE_IV
−0.2
0.0
0.2
β_IV

Batch 2−3064 : Gut microbiota abundance (genus Parasutterella id.2892) on Interleukin−4
with a threshold of 1e−05

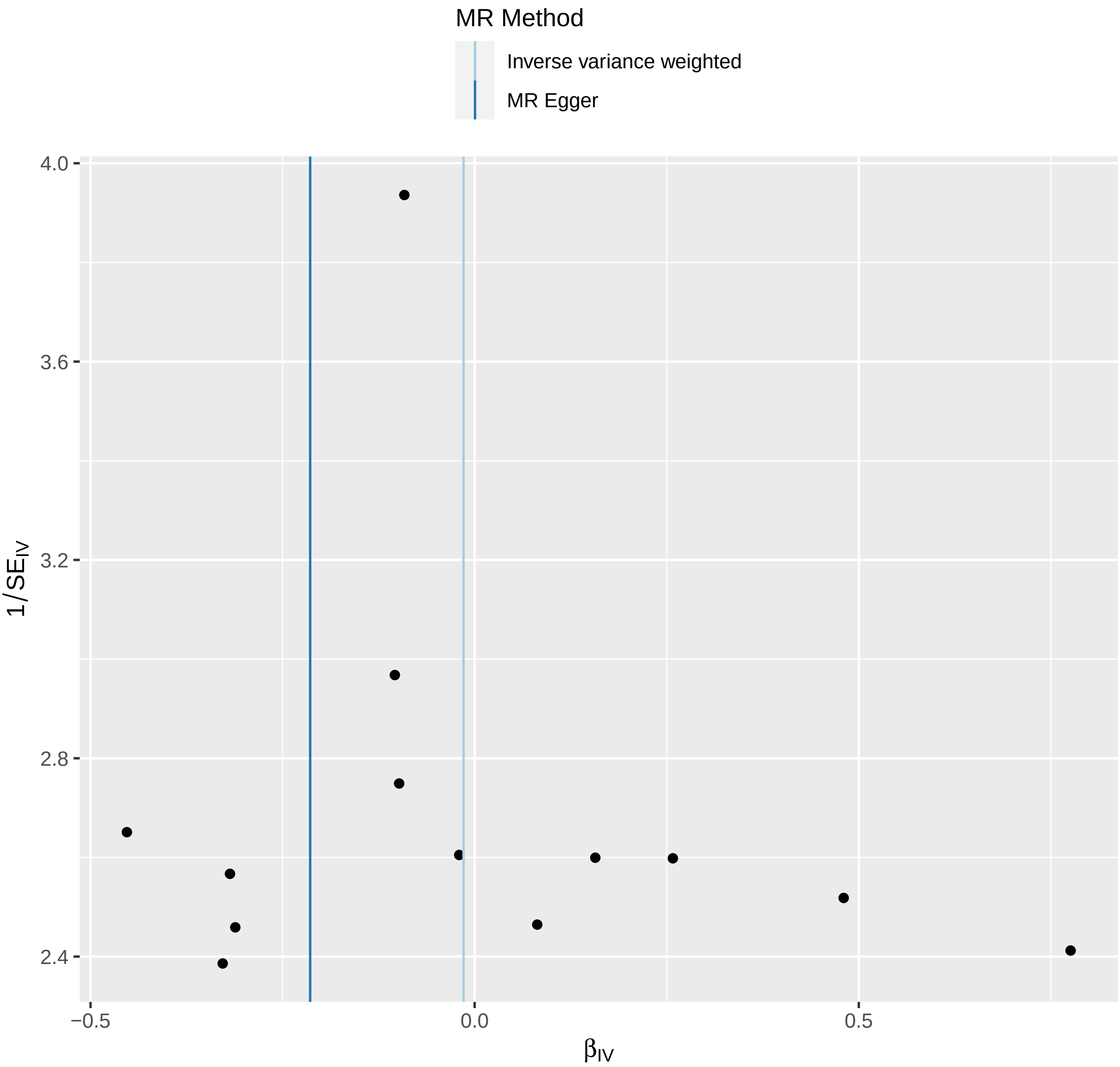

Batch 2−3065 : Gut microbiota abundance (genus Catenibacterium id.2153) on Interleukin−4 receptor subunit alpha
with a threshold of 1e−05

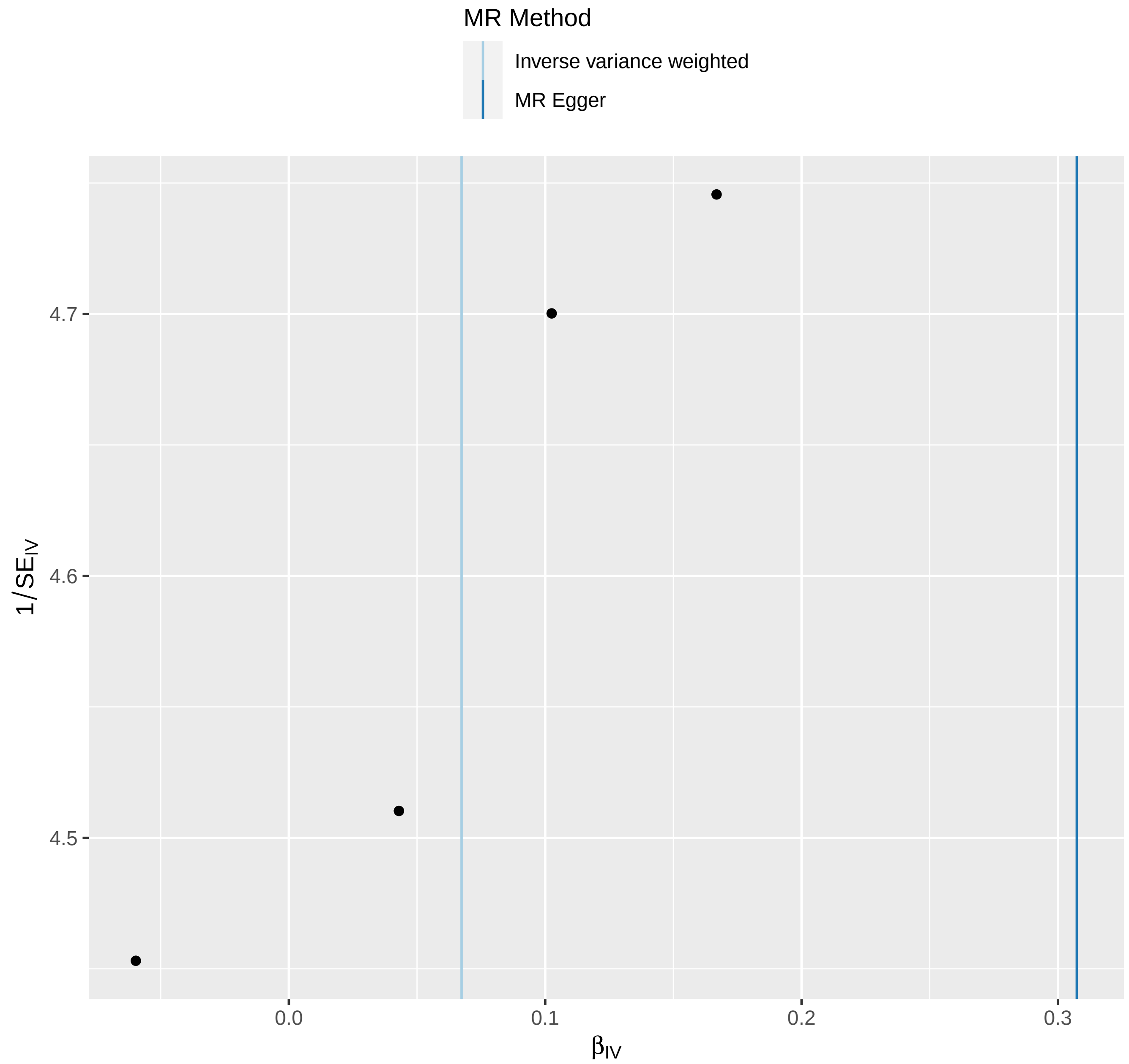

Batch 2−3066 : Gut microbiota abundance (genus Parasutterella id.2892) on Interleukin−4 receptor subunit alpha
with a threshold of 1e−05

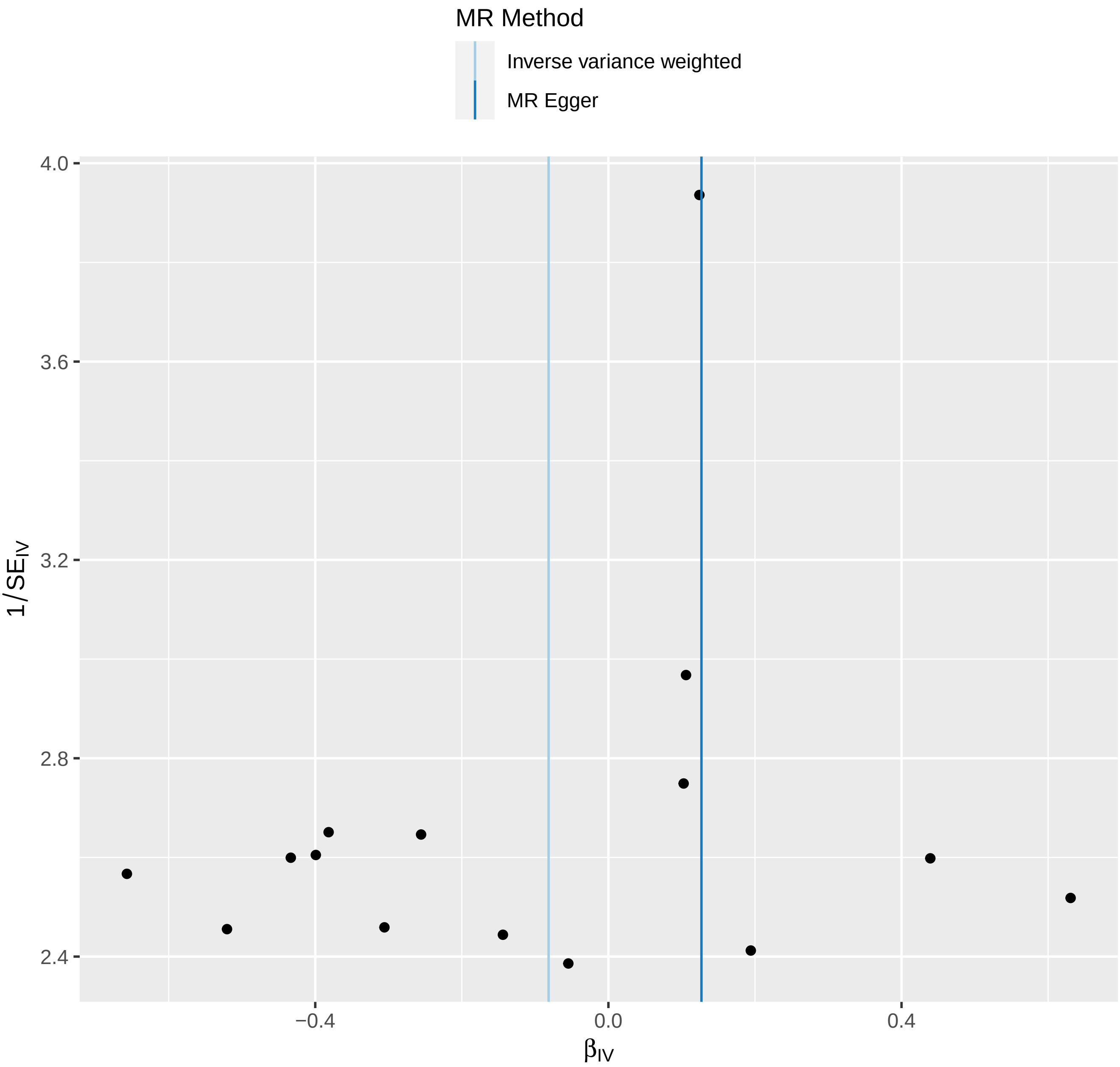

Batch 2−3067 : Gut microbiota abundance (genus Catenibacterium id.2153) on Interleukin−5
with a threshold of 1e−05

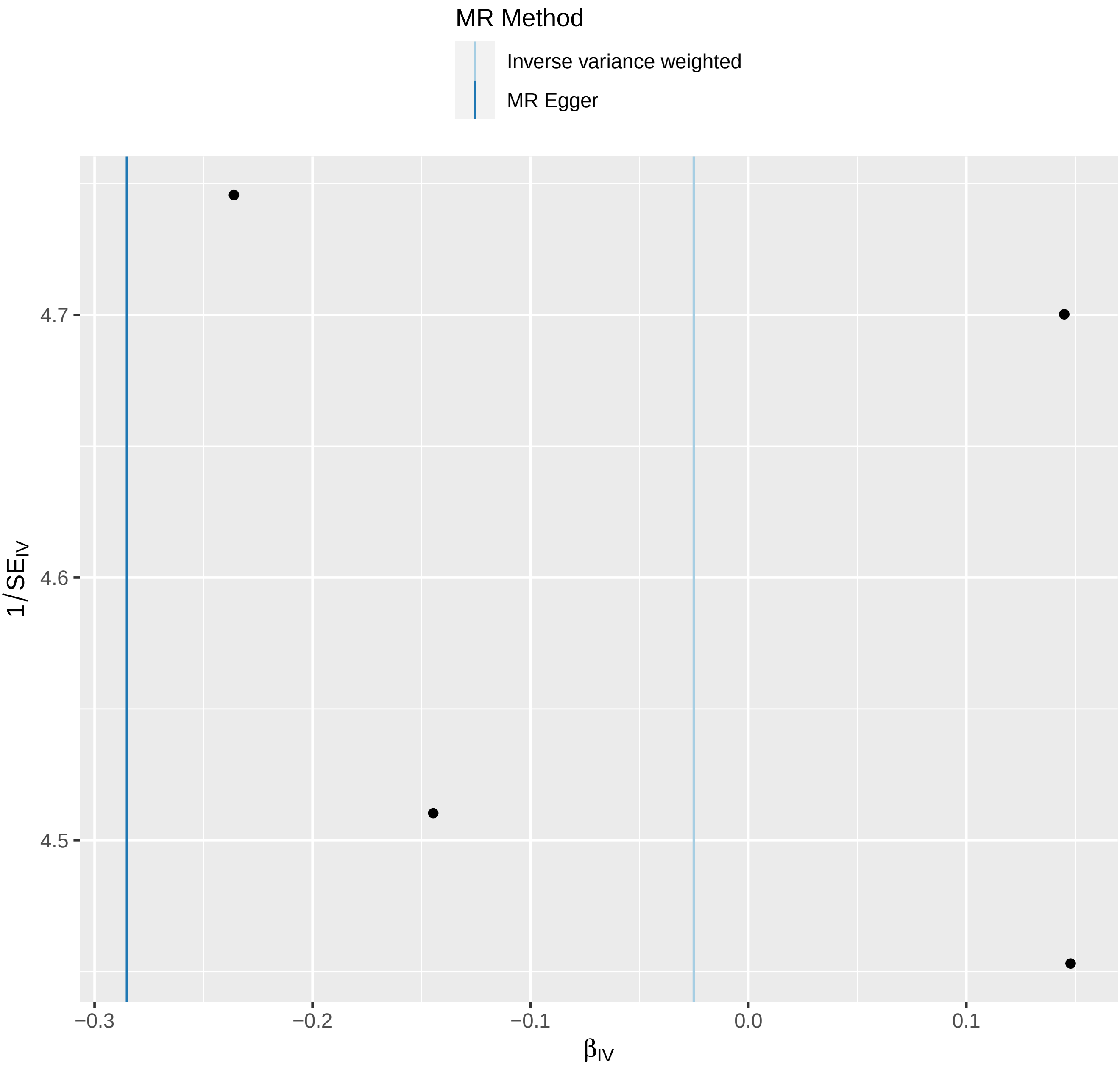

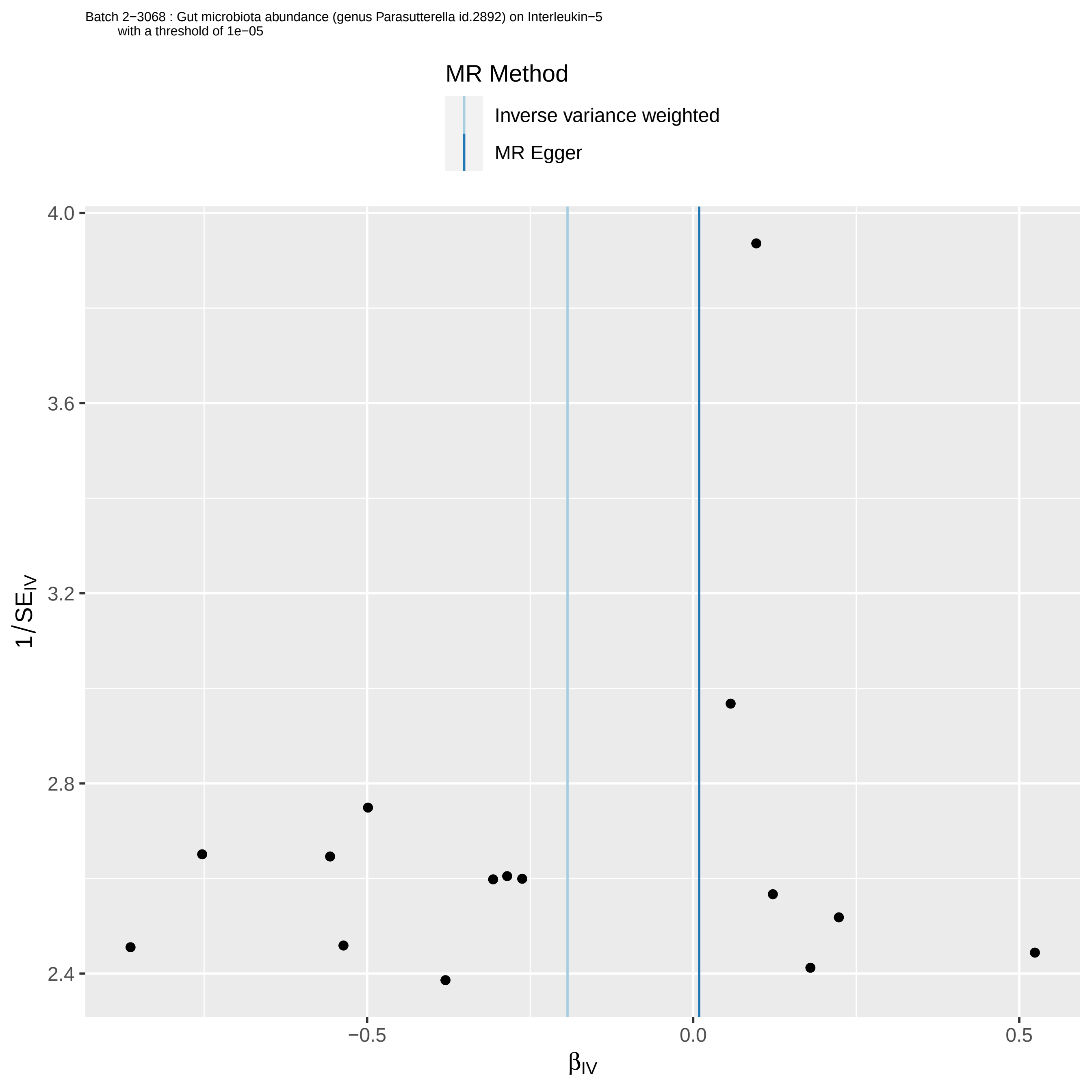
Batch 2−3068 : Gut microbiota abundance (genus Parasutterella id.2892) on Interleukin−5
with a threshold of 1e−05
MR Method
Inverse variance weighted
MR Egger
4.0
3.6
3.2
2.8
2.4
1/SE_IV
−0.5
0.0
0.5
β_IV

Batch 2−3069 : Gut microbiota abundance (genus Catenibacterium id.2153) on Interleukin−5
with a threshold of 1e−05

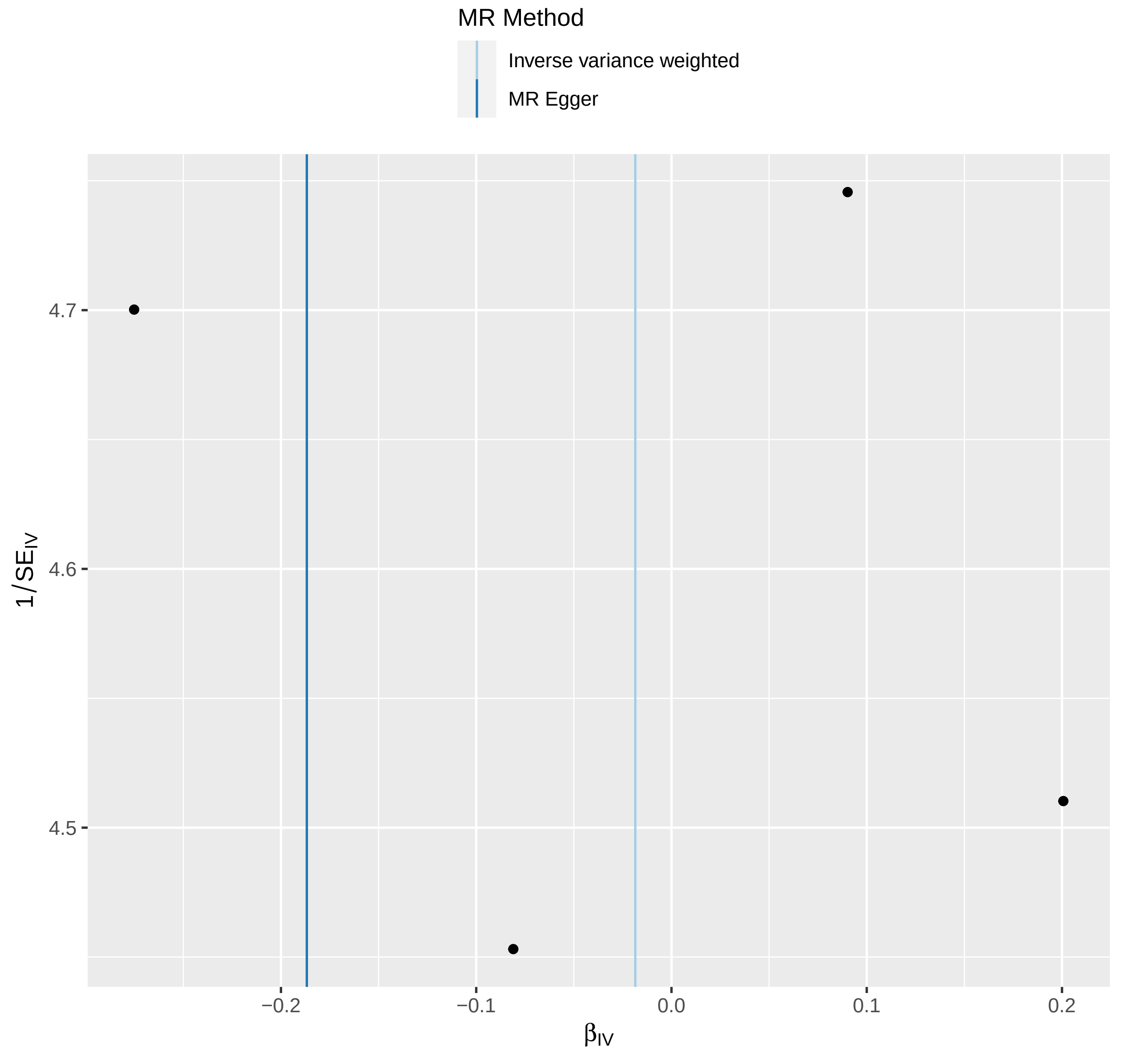

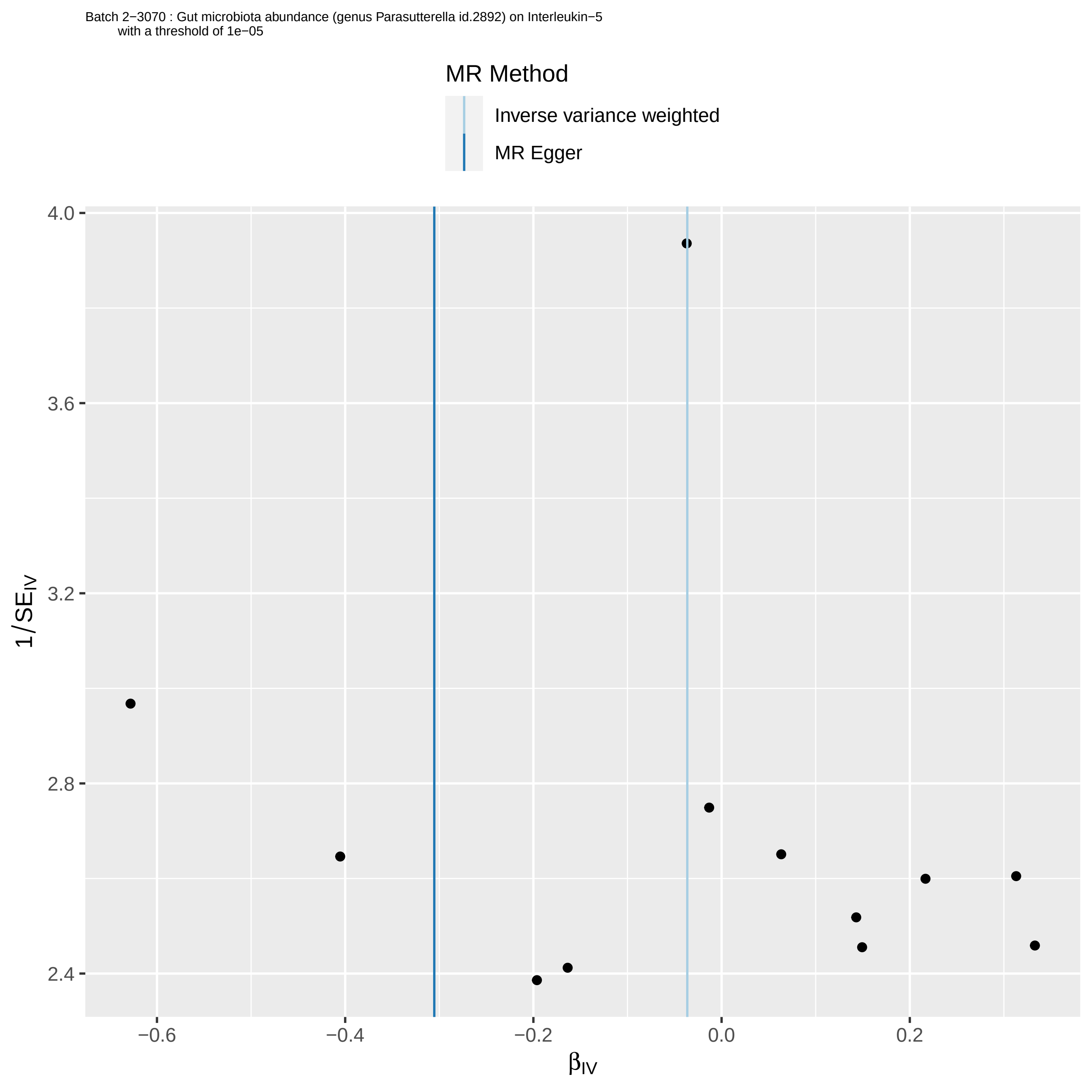

Batch 2−3070 : Gut microbiota abundance (genus Parasutterella id.2892) on Interleukin−5
with a threshold of 1e−05
MR Method
Inverse variance weighted
MR Egger
4.0
3.6
3.2
2.8
2.4
1/SE_IV
−0.6
−0.4
−0.2
0.0
0.2
β_IV

Batch 2−3071 : Gut microbiota abundance (genus Catenibacterium id.2153) on Interleukin−5 receptor subunit alpha
with a threshold of 1e−05

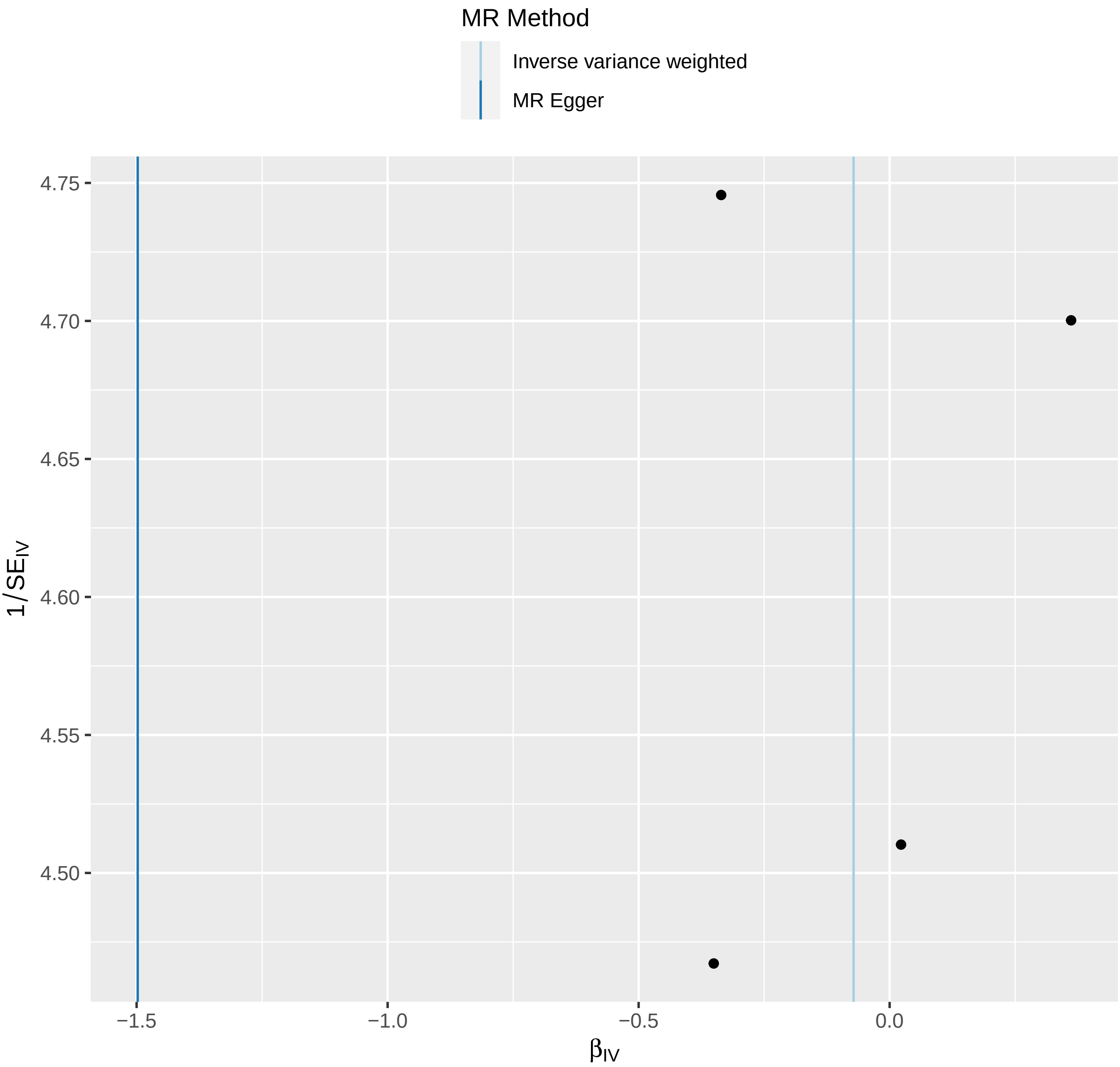

Batch 2−3072 : Gut microbiota abundance (genus Parasutterella id.2892) on Interleukin−5 receptor subunit alpha
with a threshold of 1e−05

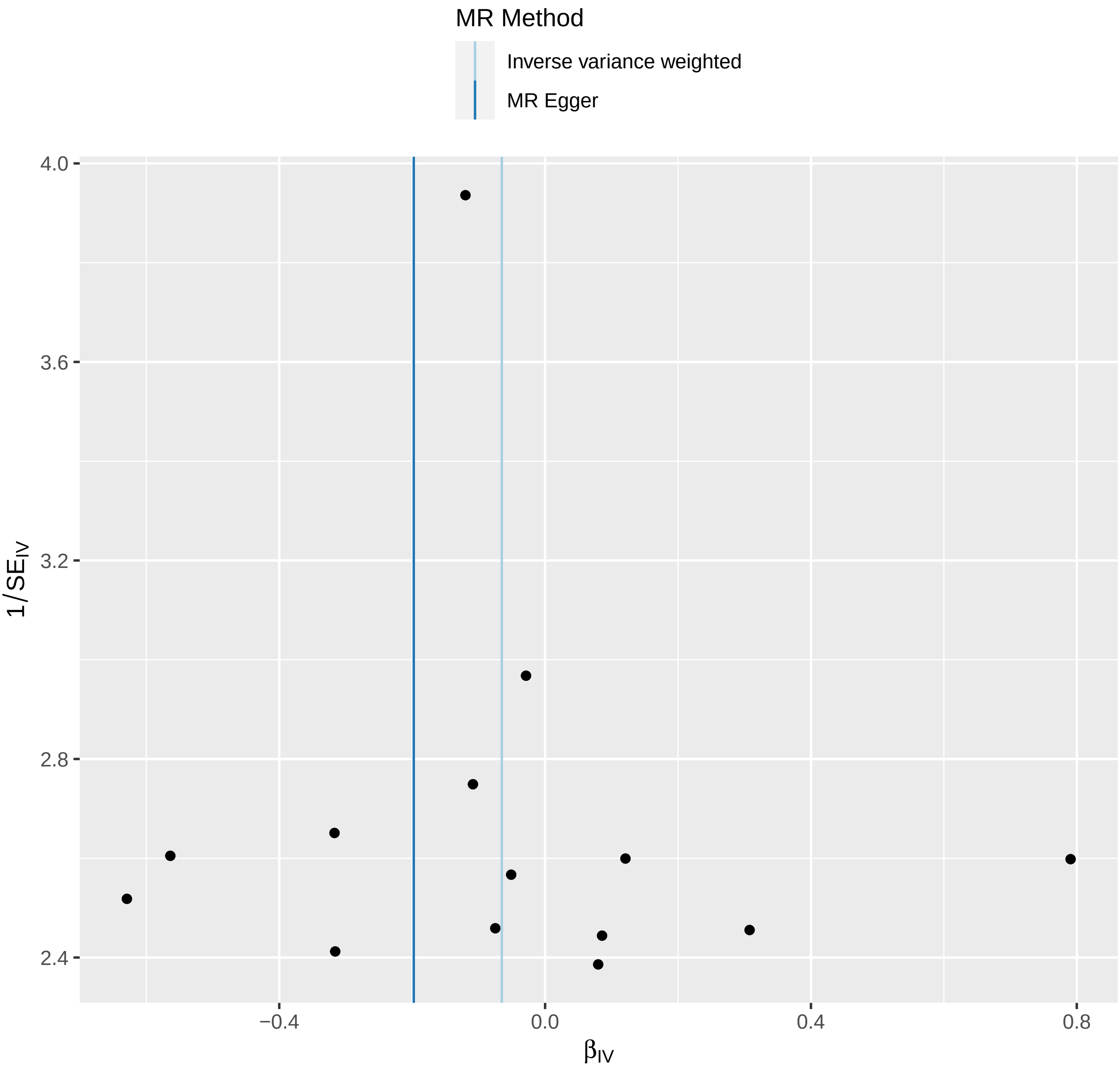

Batch 2−3073 : Gut microbiota abundance (genus Catenibacterium id.2153) on Interleukin−5 receptor subunit alpha
with a threshold of 1e−05

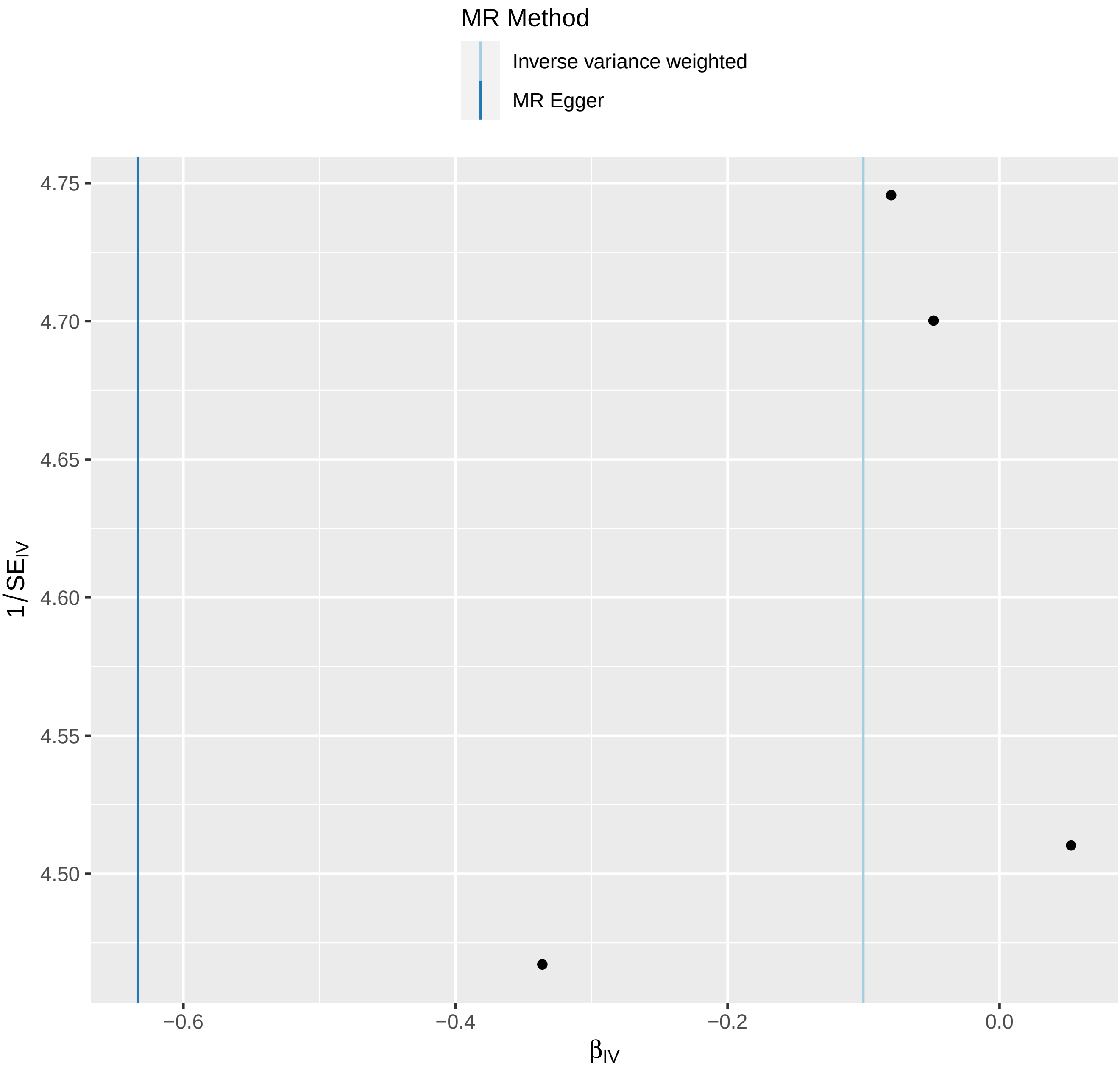

Batch 2−3074 : Gut microbiota abundance (genus Parasutterella id.2892) on Interleukin−5 receptor subunit alpha
with a threshold of 1e−05

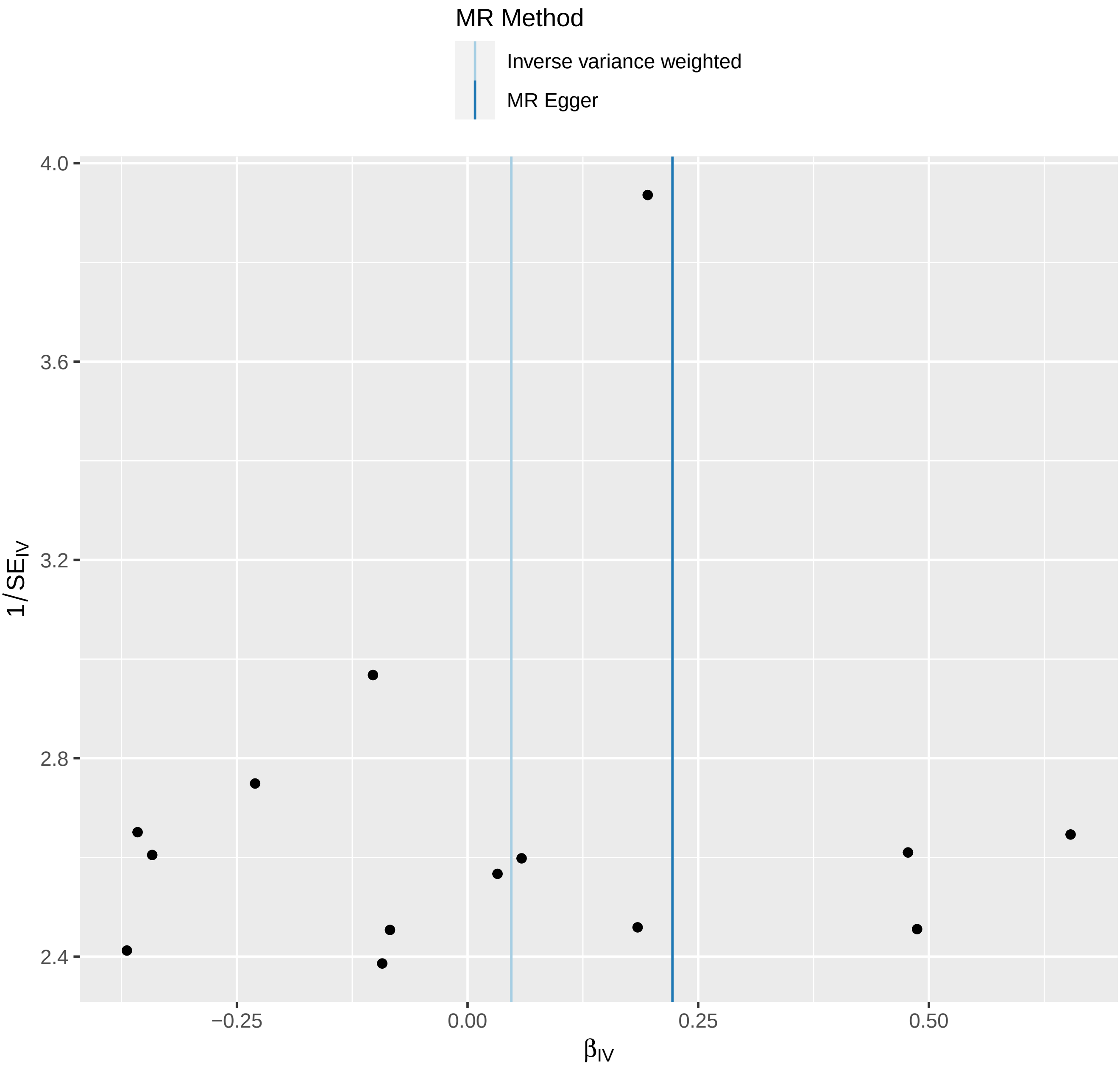

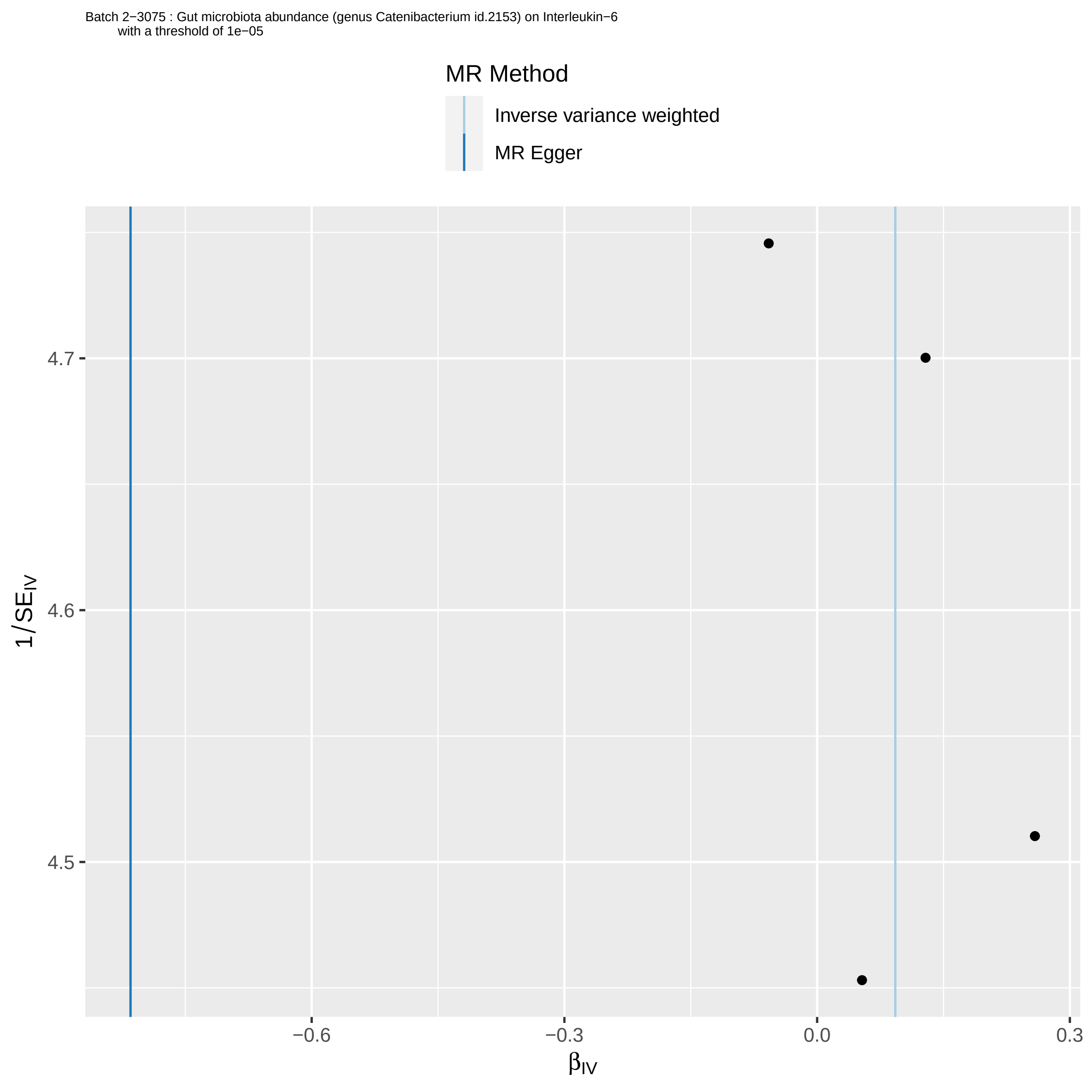
Batch 2−3075 : Gut microbiota abundance (genus Catenibacterium id.2153) on Interleukin−6
with a threshold of 1e−05
MR Method
Inverse variance weighted
MR Egger
4.7
4.6
4.5
1/SE_IV
−0.6
−0.3
0.0
0.3
β_IV

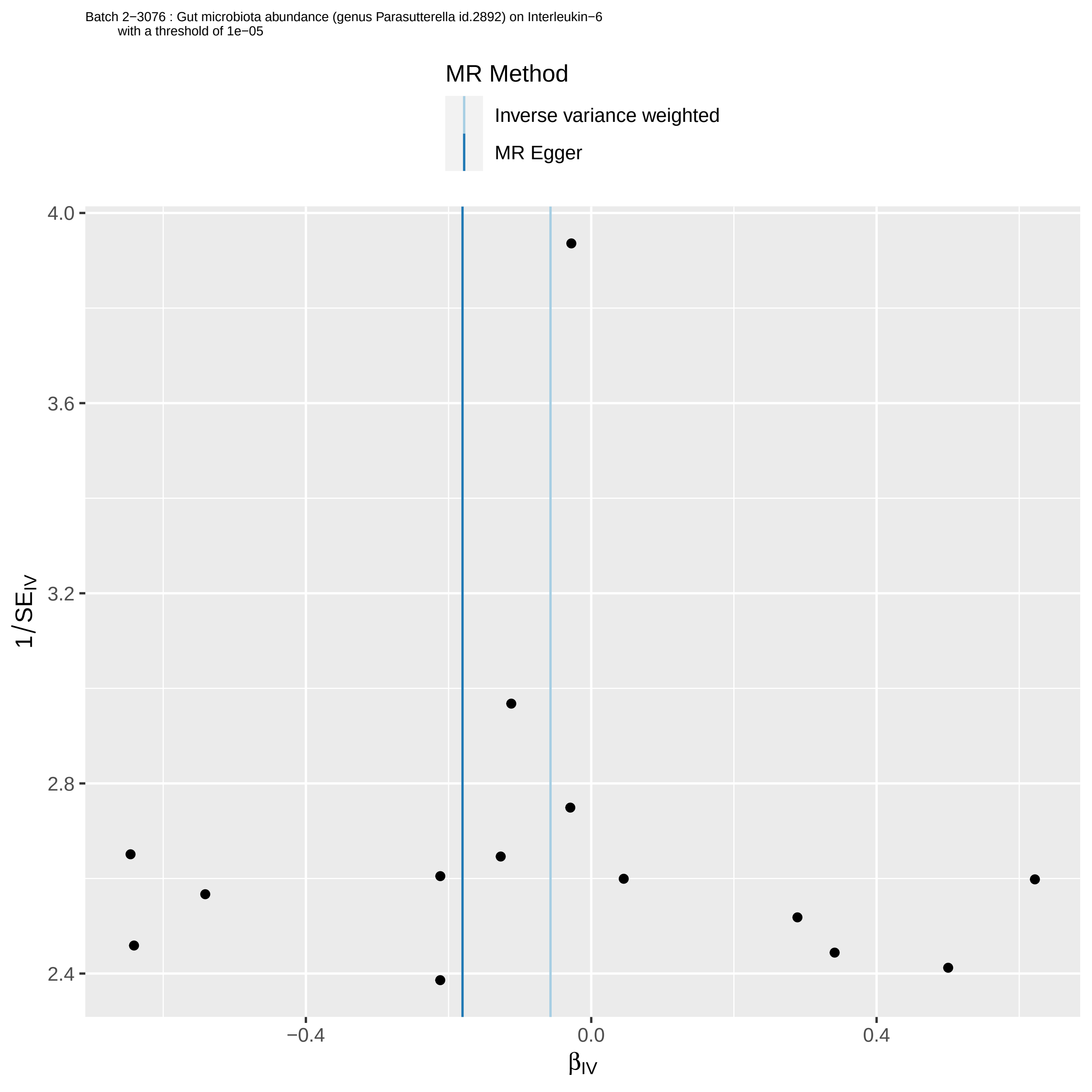

Batch 2−3076 : Gut microbiota abundance (genus Parasutterella id.2892) on Interleukin−6
with a threshold of 1e−05
MR Method
Inverse variance weighted
MR Egger
4.0
3.6
3.2
2.8
2.4
1/SE_IV
−0.4
0.0
0.4
β_IV

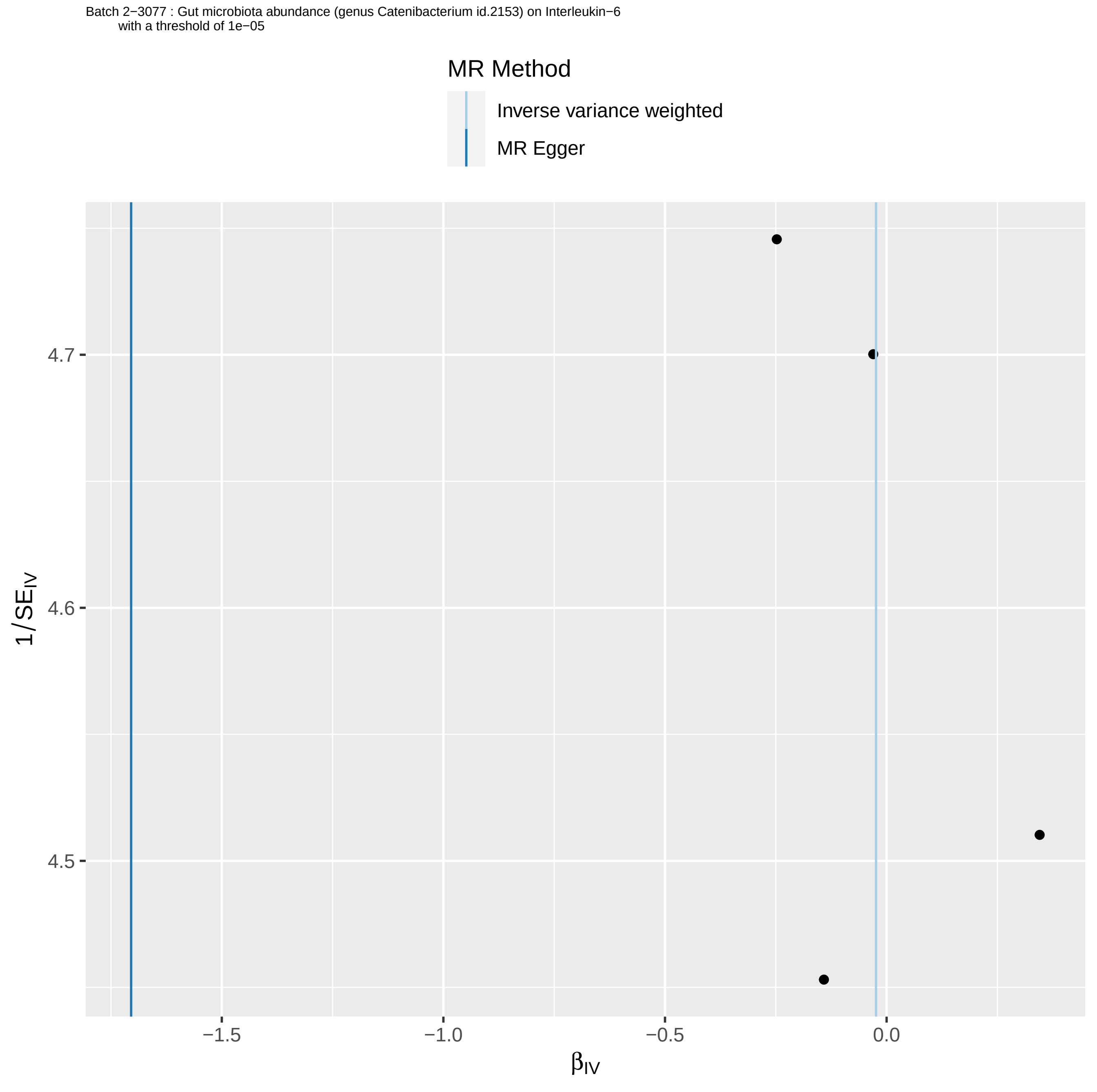
Batch 2−3077 : Gut microbiota abundance (genus Catenibacterium id.2153) on Interleukin−6
with a threshold of 1e−05
MR Method
Inverse variance weighted
MR Egger
4.7
4.6
4.5
1/SE_IV
−1.5
−1.0
−0.5
0.0
β_IV

Batch 2−3078 : Gut microbiota abundance (genus Parasutterella id.2892) on Interleukin−6
with a threshold of 1e−05

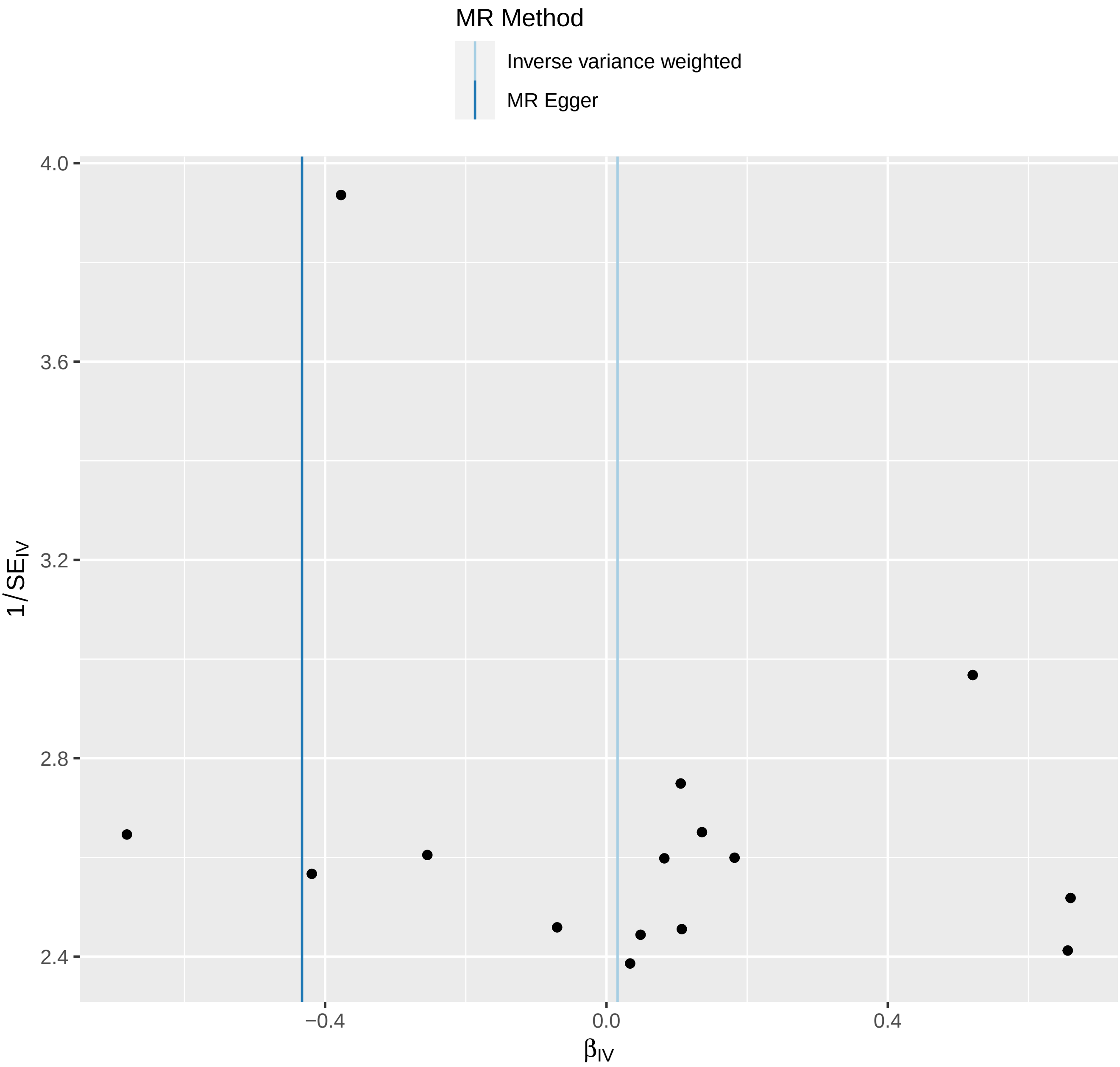

Batch 2−3079 : Gut microbiota abundance (genus Catenibacterium id.2153) on Interleukin−6 receptor subunit alpha
with a threshold of 1e−05

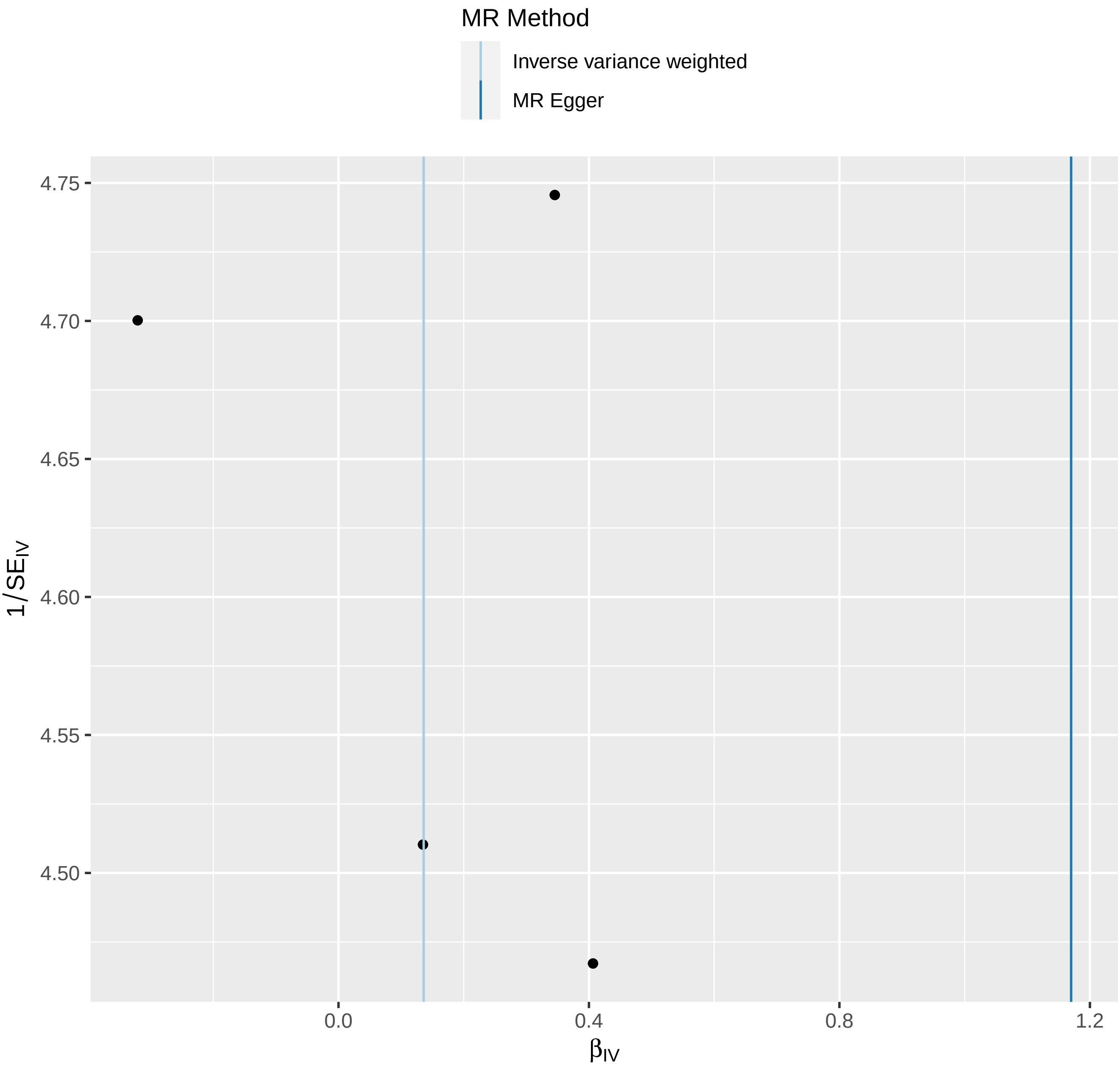

Batch 2−3080 : Gut microbiota abundance (genus Parasutterella id.2892) on Interleukin−6 receptor subunit alpha
with a threshold of 1e−05

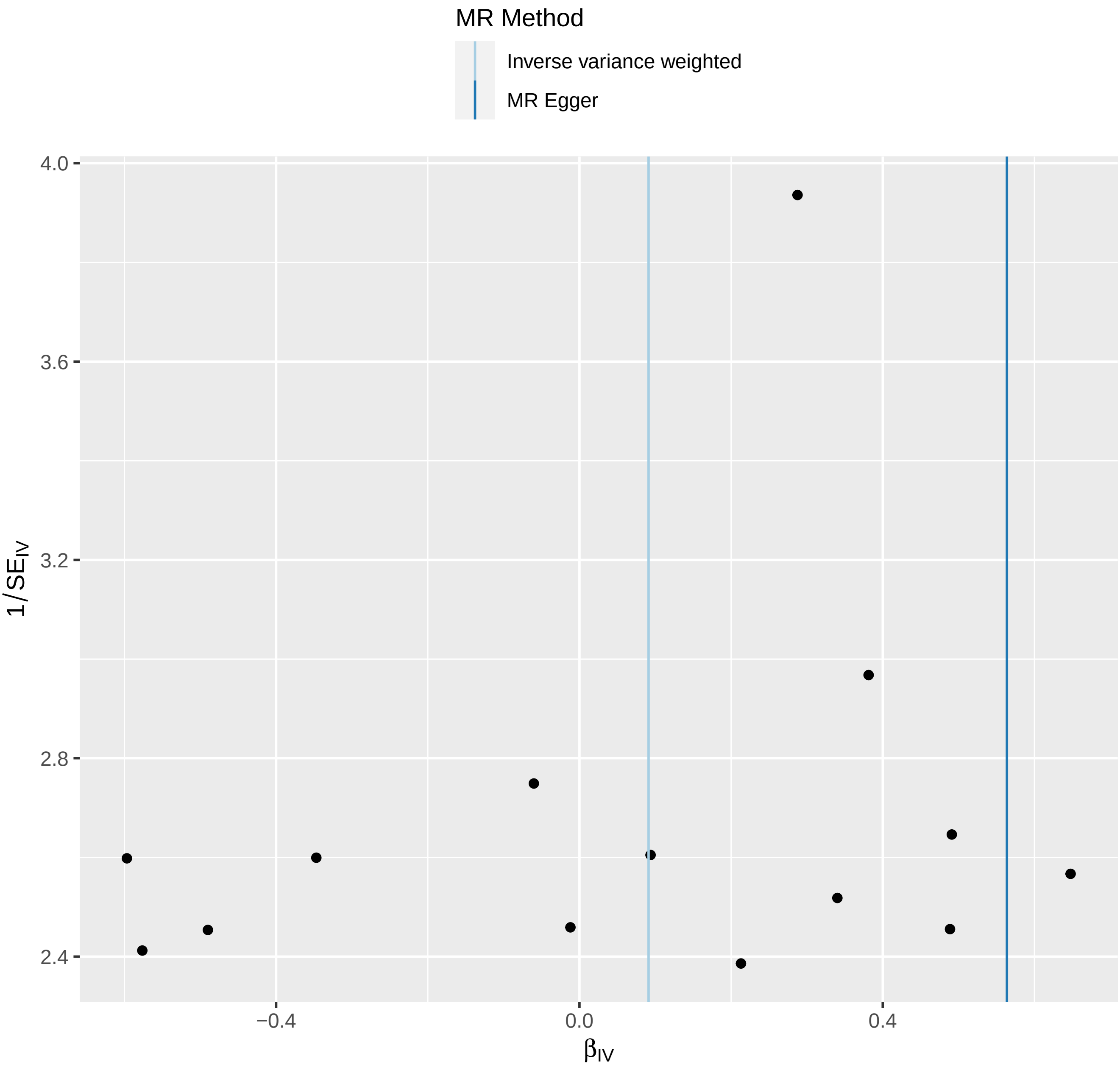

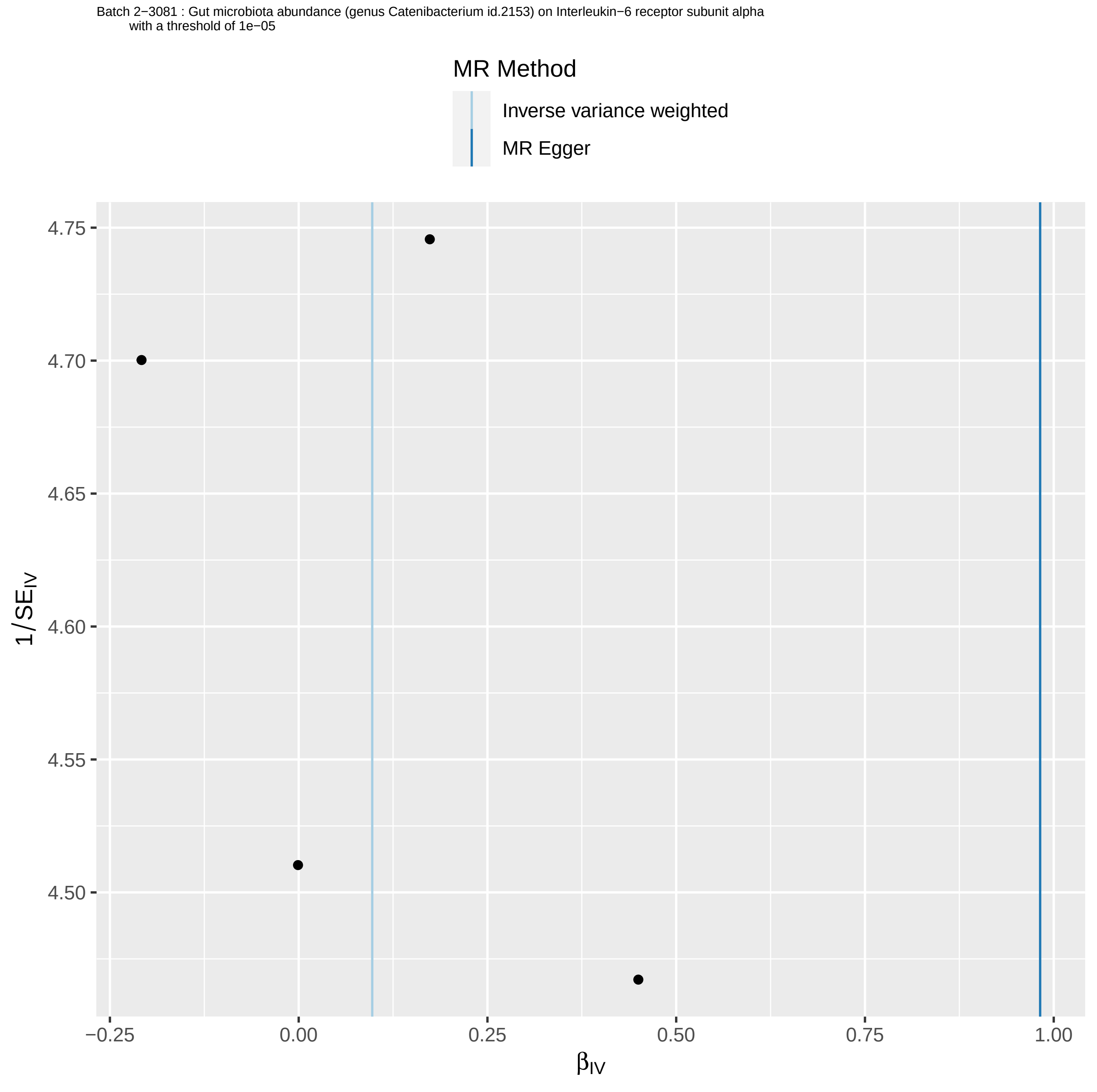
Batch 2−3081 : Gut microbiota abundance (genus Catenibacterium id.2153) on Interleukin−6 receptor subunit alpha
with a threshold of 1e−05
MR Method
Inverse variance weighted
MR Egger
4.75
4.70
4.65
4.60
4.55
4.50
1/SE_IV
−0.25
0.00
0.25
0.50
0.75
1.00
β_IV

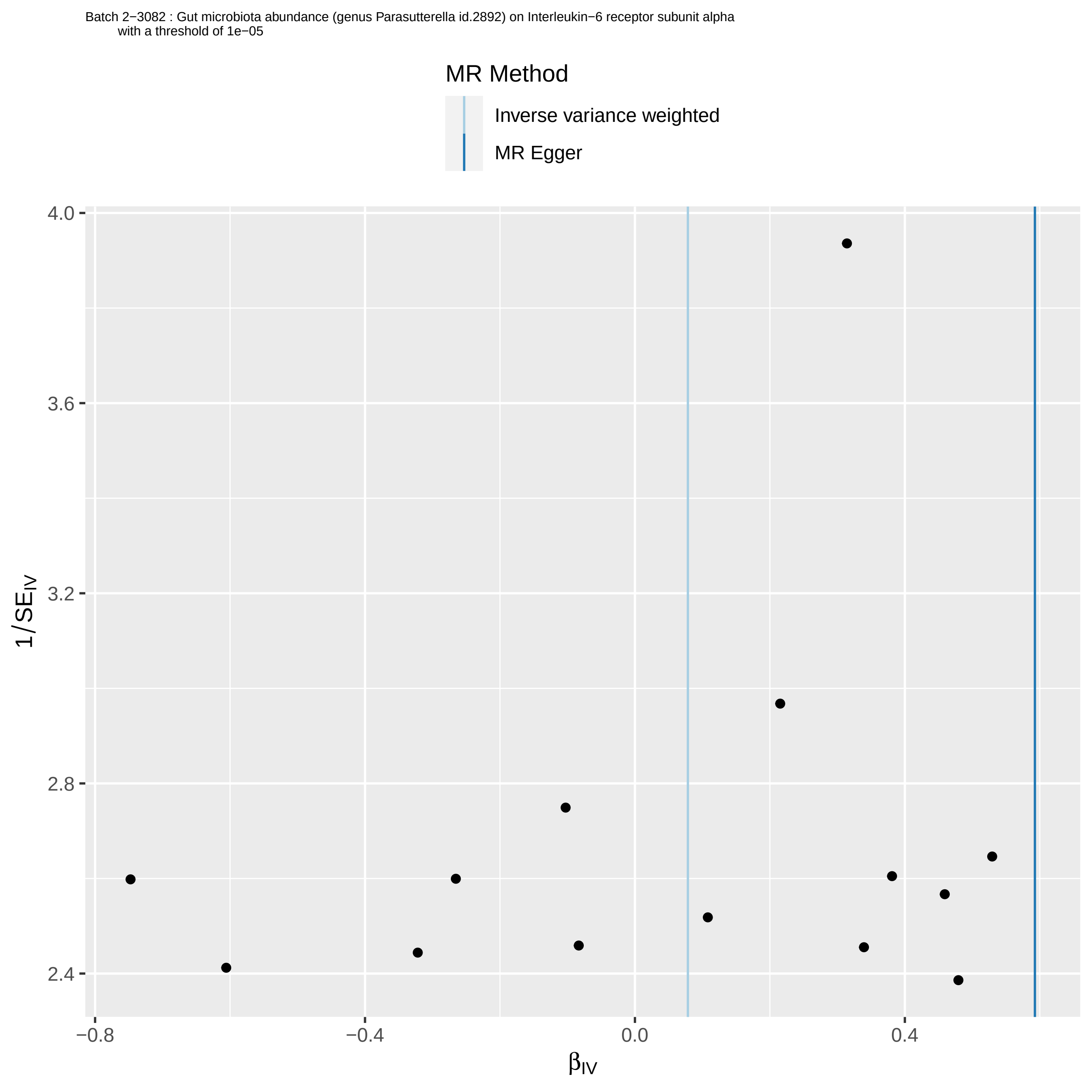
Batch 2−3082 : Gut microbiota abundance (genus Parasutterella id.2892) on Interleukin−6 receptor subunit alpha
with a threshold of 1e−05
MR Method
Inverse variance weighted
MR Egger
4.0
3.6
3.2
2.8
2.4
−0.8
−0.4
0.0
0.4
$1/SE_{IV}$
β_{IV}

Batch 2−3083 : Gut microbiota abundance (genus Catenibacterium id.2153) on Interleukin−6 receptor subunit beta
with a threshold of 1e−05

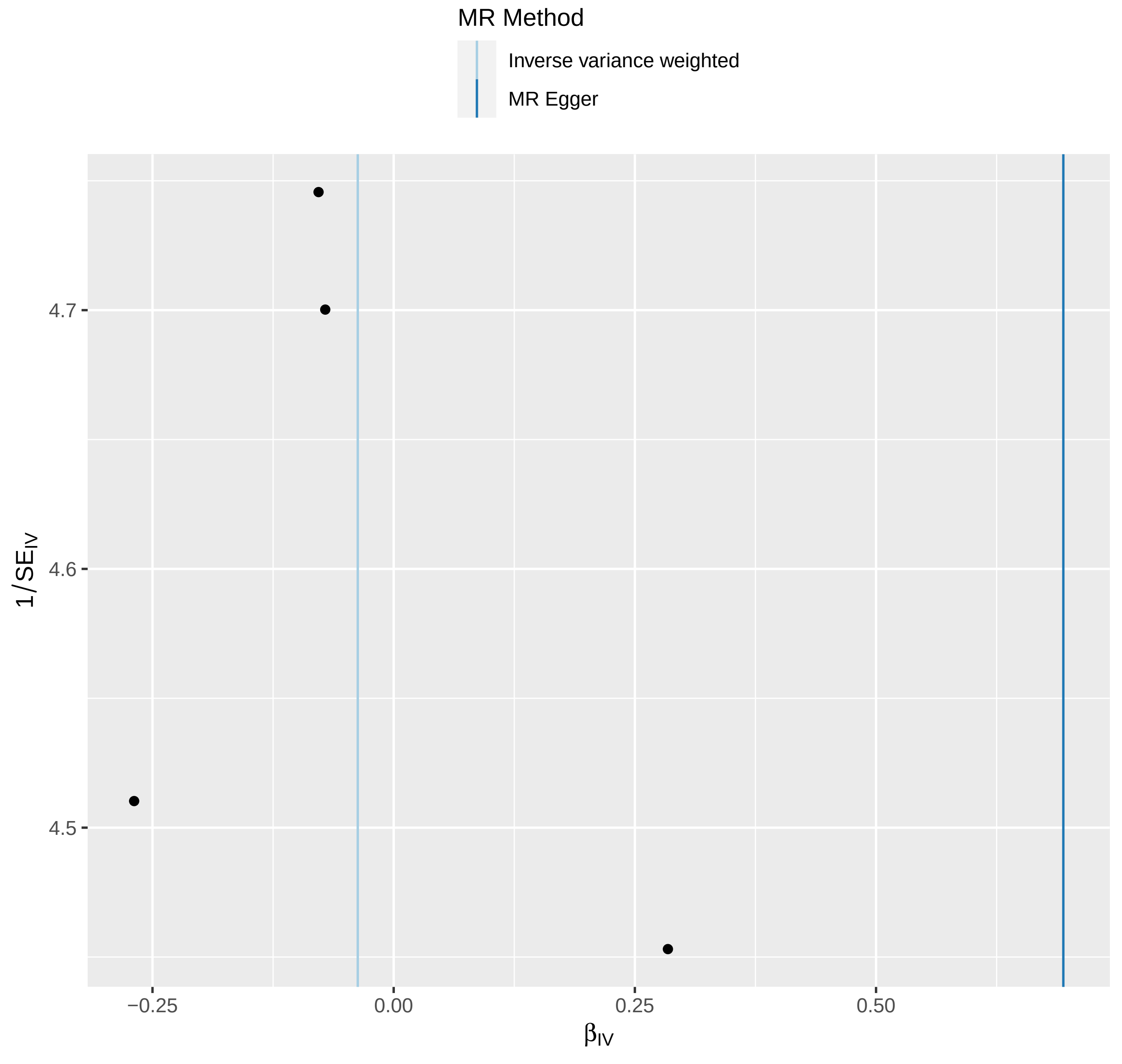

Batch 2−3084 : Gut microbiota abundance (genus Parasutterella id.2892) on Interleukin−6 receptor subunit beta
with a threshold of 1e−05

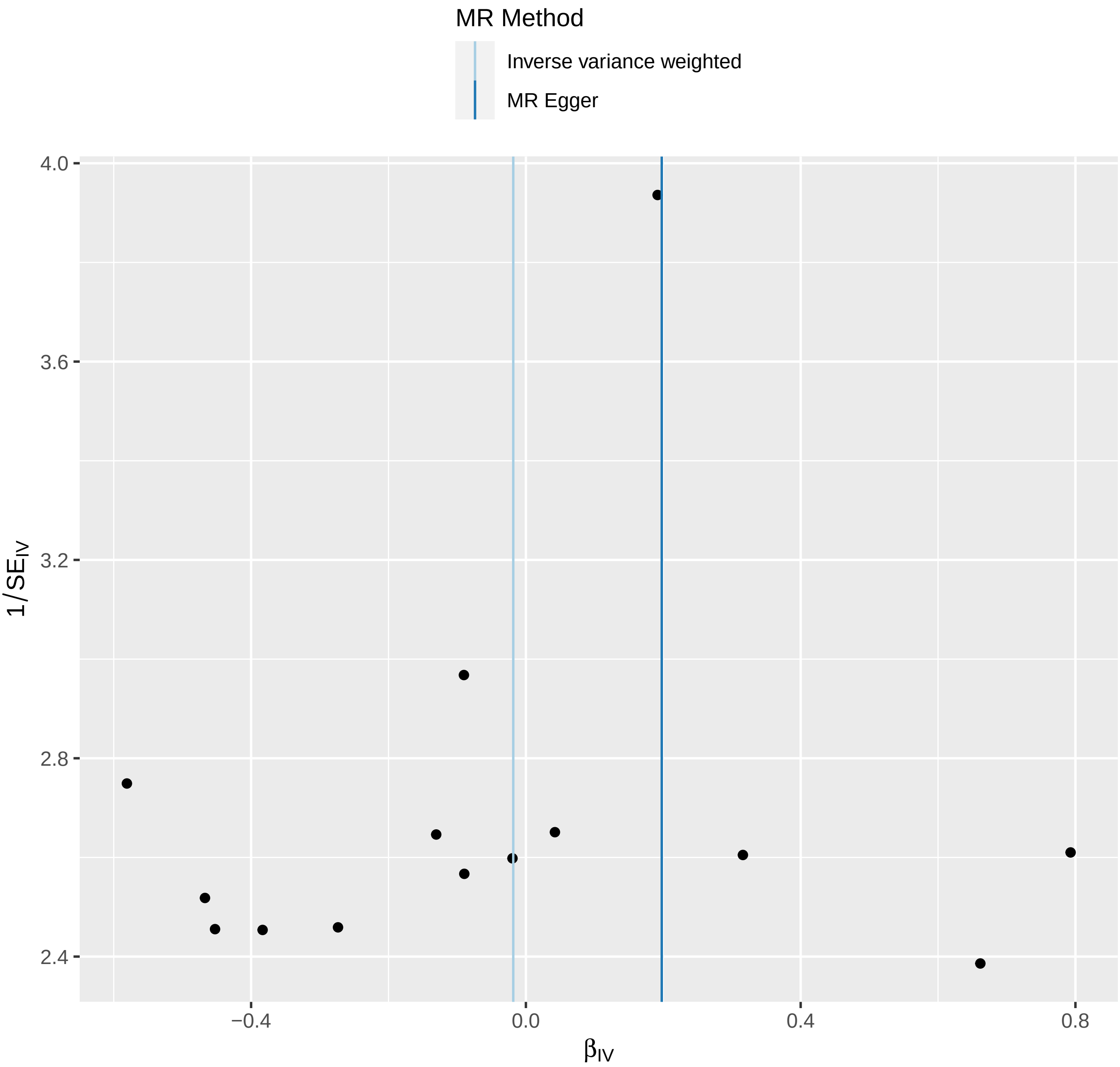

Batch 2−3085 : Gut microbiota abundance (genus Catenibacterium id.2153) on Interleukin−7
with a threshold of 1e−05

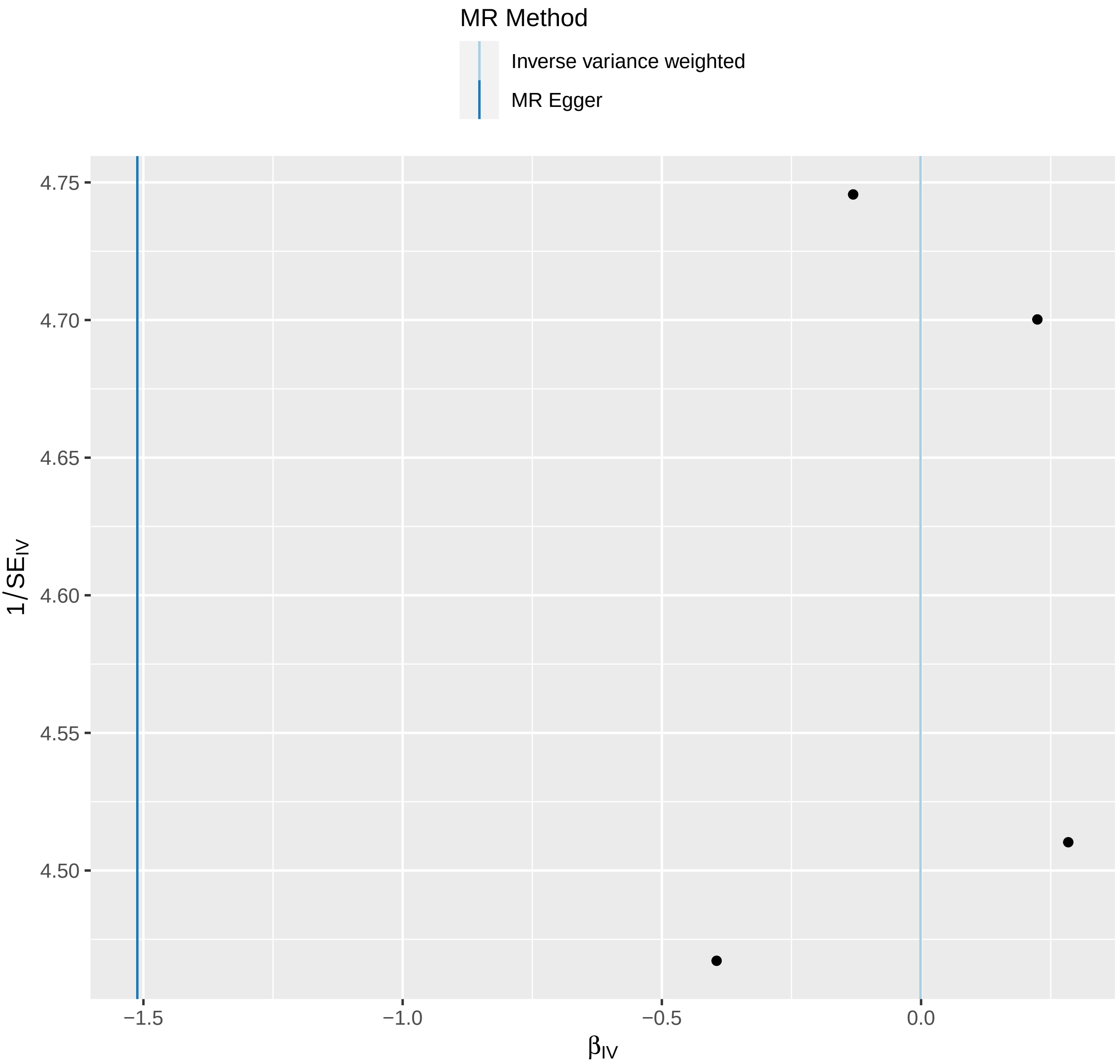

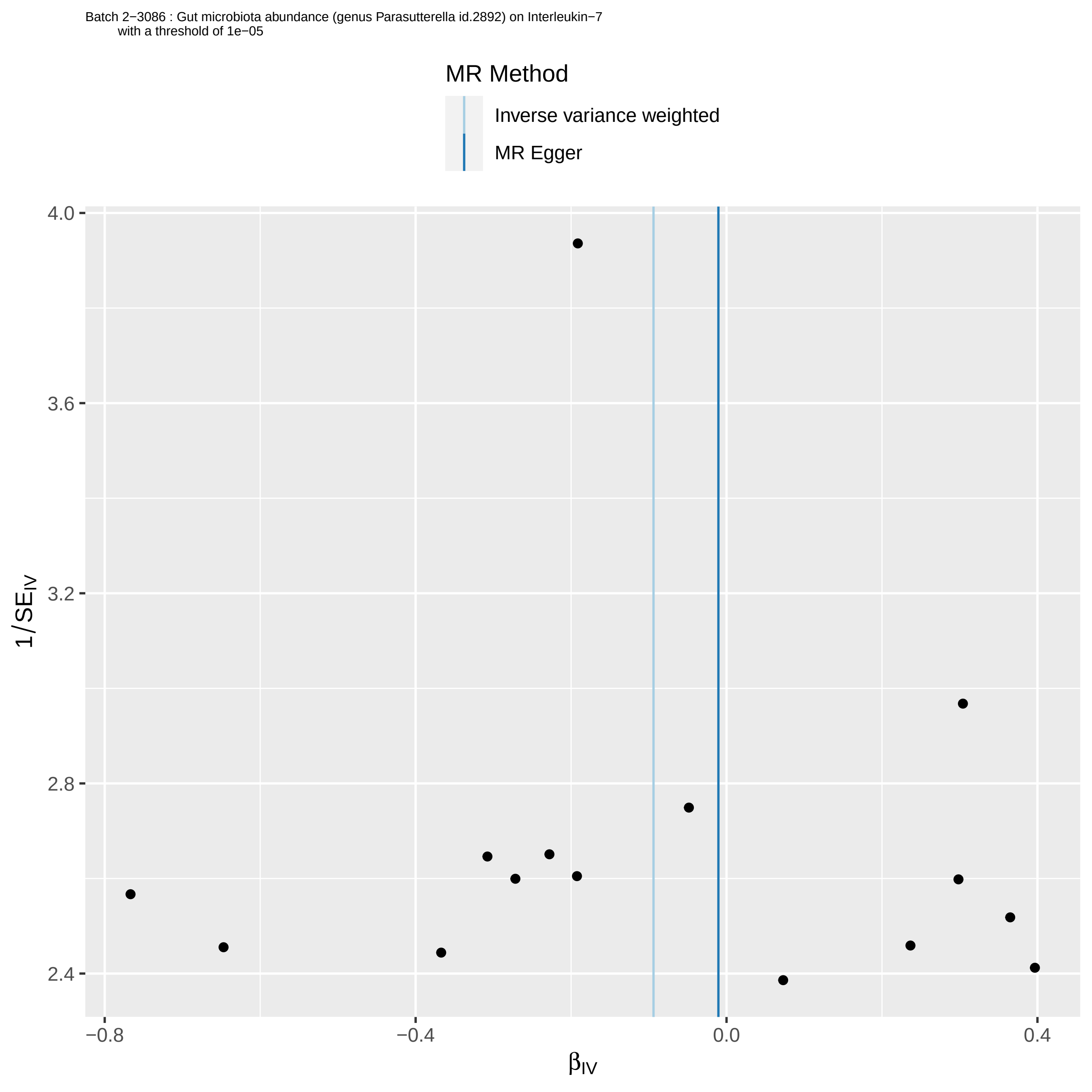

Batch 2−3086 : Gut microbiota abundance (genus Parasutterella id.2892) on Interleukin−7
with a threshold of 1e−05
MR Method
Inverse variance weighted
MR Egger
4.0
3.6
3.2
2.8
2.4
1/SE_IV
−0.8
−0.4
0.0
0.4
β_IV

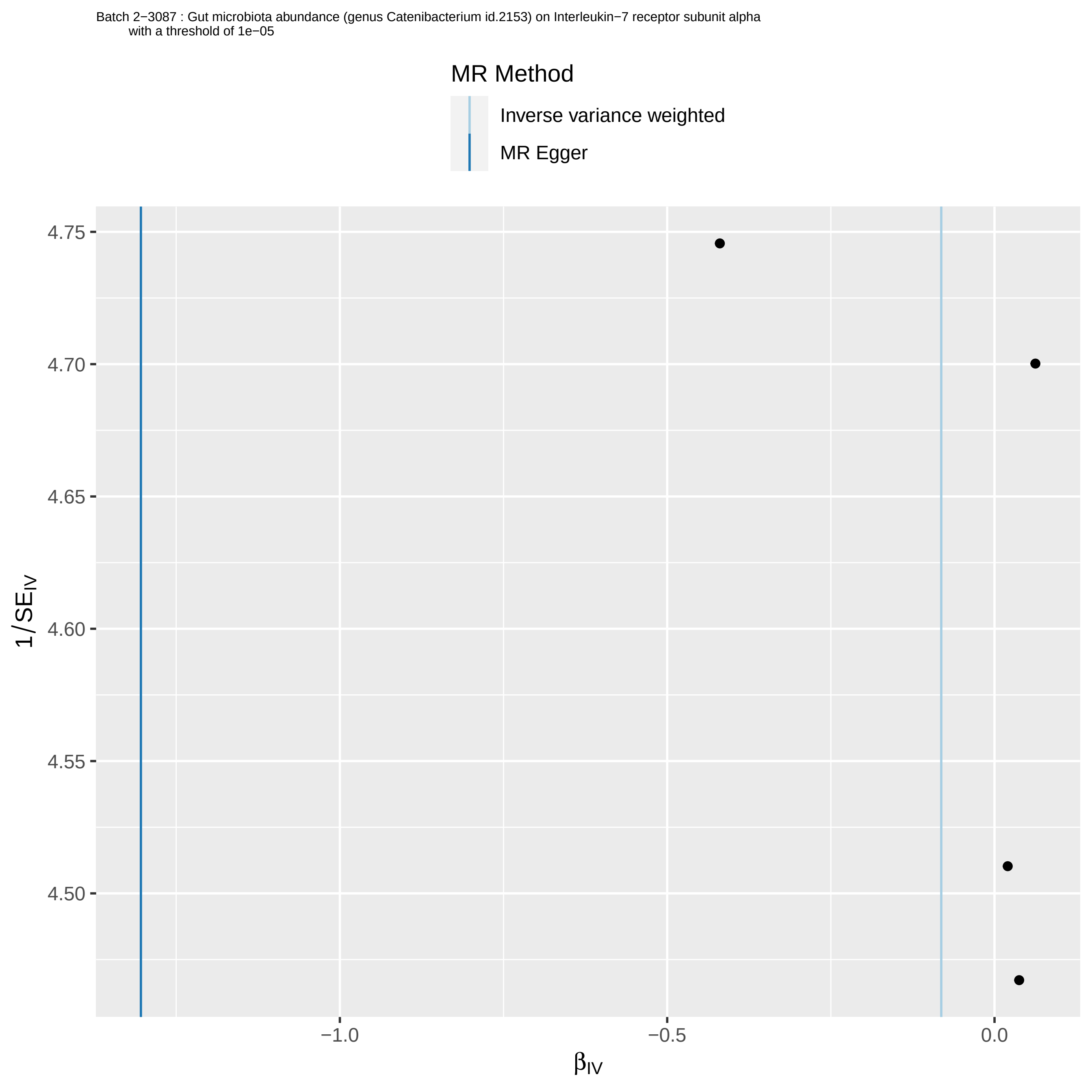
Batch 2−3087 : Gut microbiota abundance (genus Catenibacterium id.2153) on Interleukin−7 receptor subunit alpha
with a threshold of 1e−05
MR Method
Inverse variance weighted
MR Egger
4.75
4.70
4.65
4.60
4.55
4.50
1/SE_IV
−1.0
−0.5
0.0
β_IV

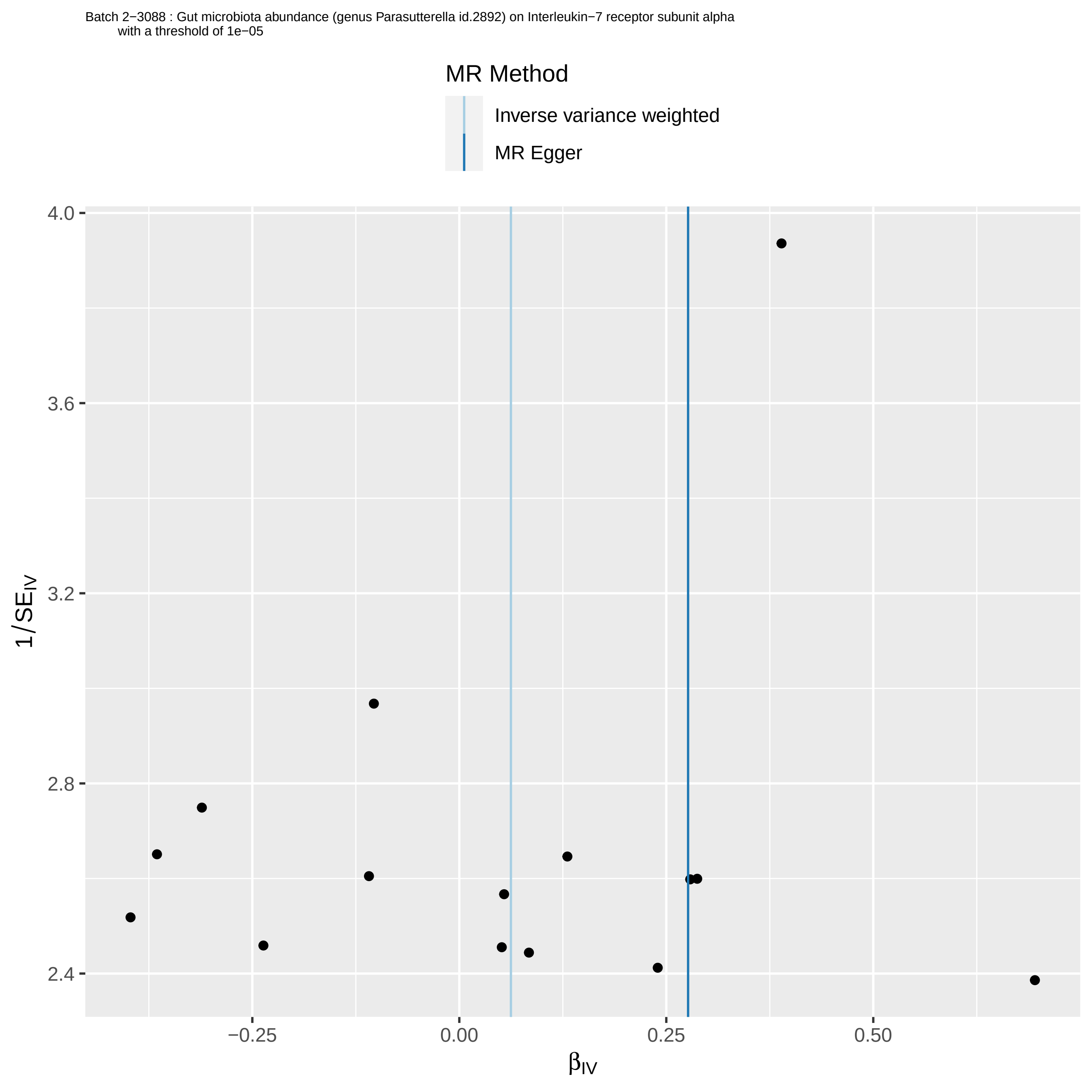

Batch 2−3088 : Gut microbiota abundance (genus Parasutterella id.2892) on Interleukin−7 receptor subunit alpha
with a threshold of 1e−05
MR Method
Inverse variance weighted
MR Egger
4.0
3.6
3.2
2.8
2.4
1/SE_IV
−0.25
0.00
0.25
0.50
β_IV

Batch 2−3089 : Gut microbiota abundance (genus Catenibacterium id.2153) on Interleukin−9
with a threshold of 1e−05

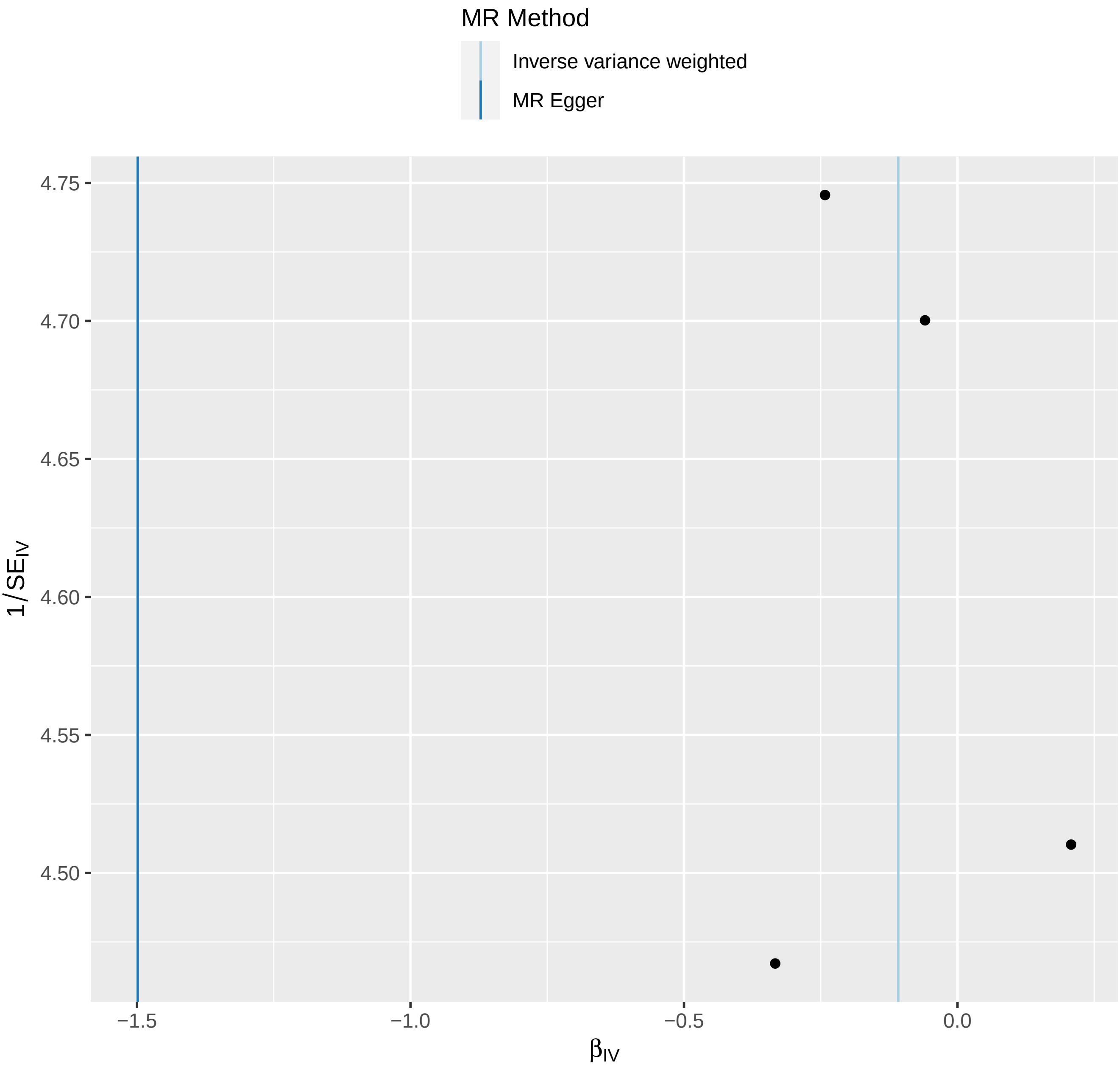

Batch 2−3090 : Gut microbiota abundance (genus Parasutterella id.2892) on Interleukin−9
with a threshold of 1e−05

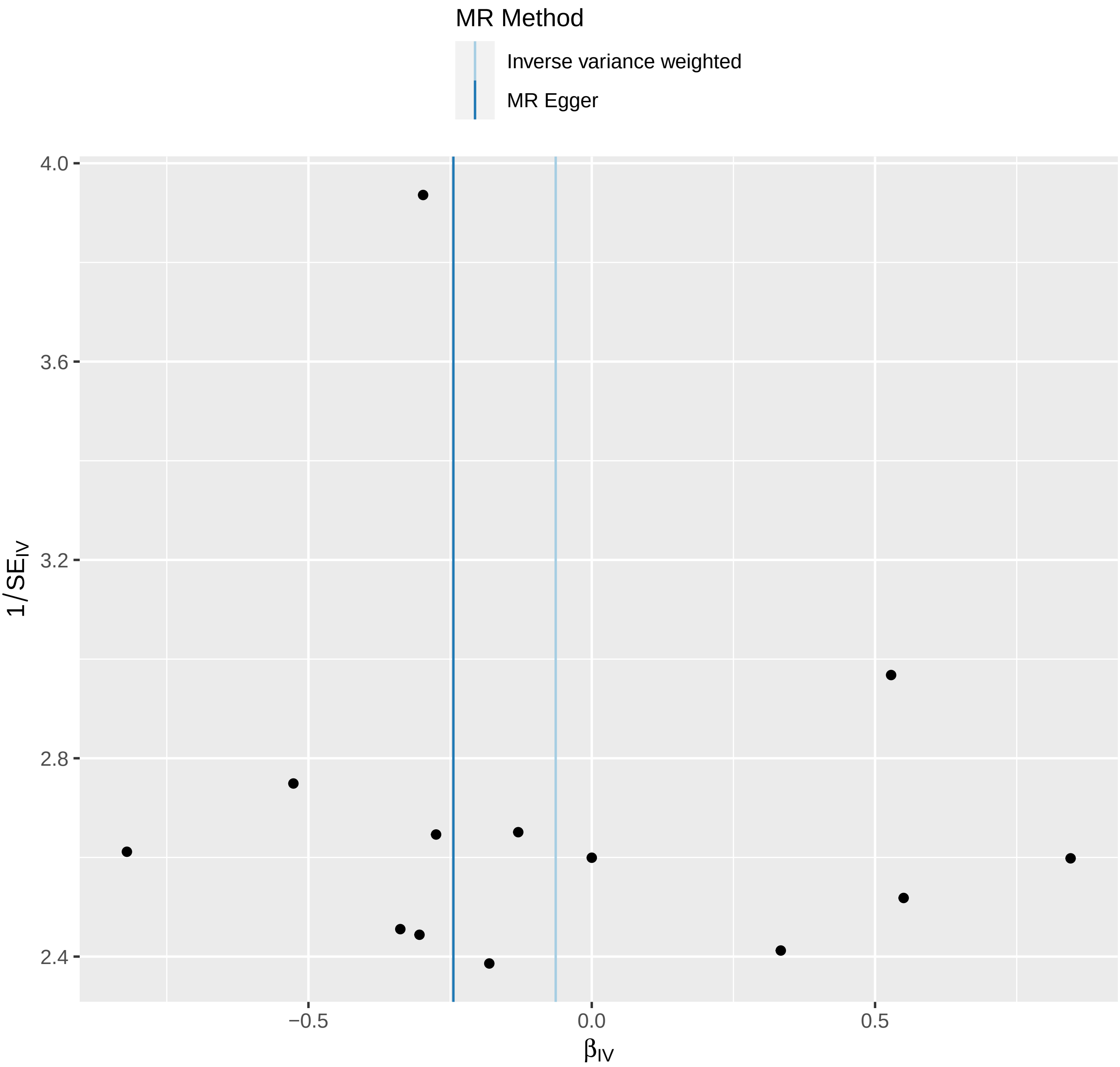

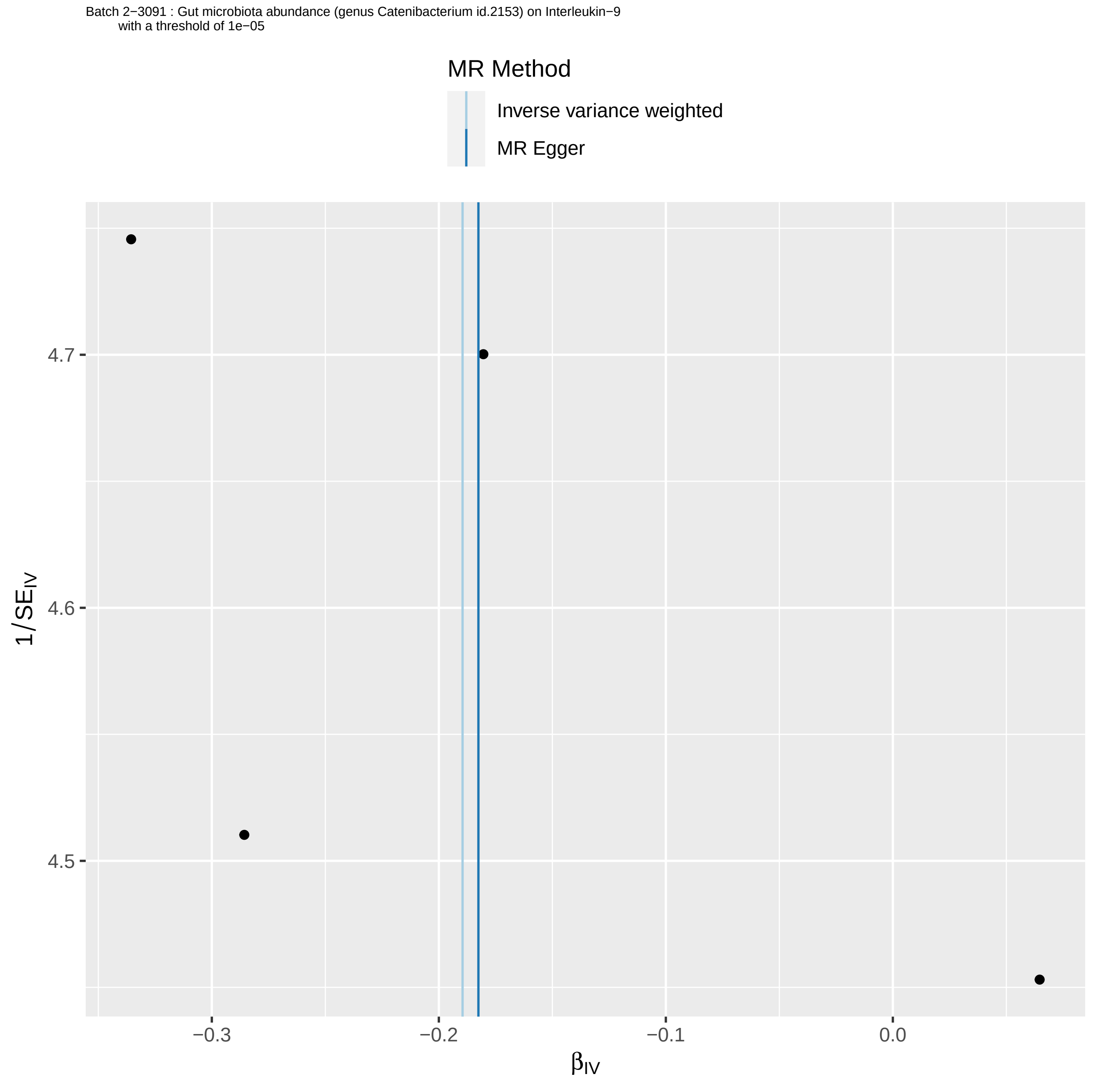
Batch 2−3091 : Gut microbiota abundance (genus Catenibacterium id.2153) on Interleukin−9
with a threshold of 1e−05
MR Method
Inverse variance weighted
MR Egger
4.7
4.6
4.5
1/SE_IV
−0.3
−0.2
−0.1
0.0
β_IV

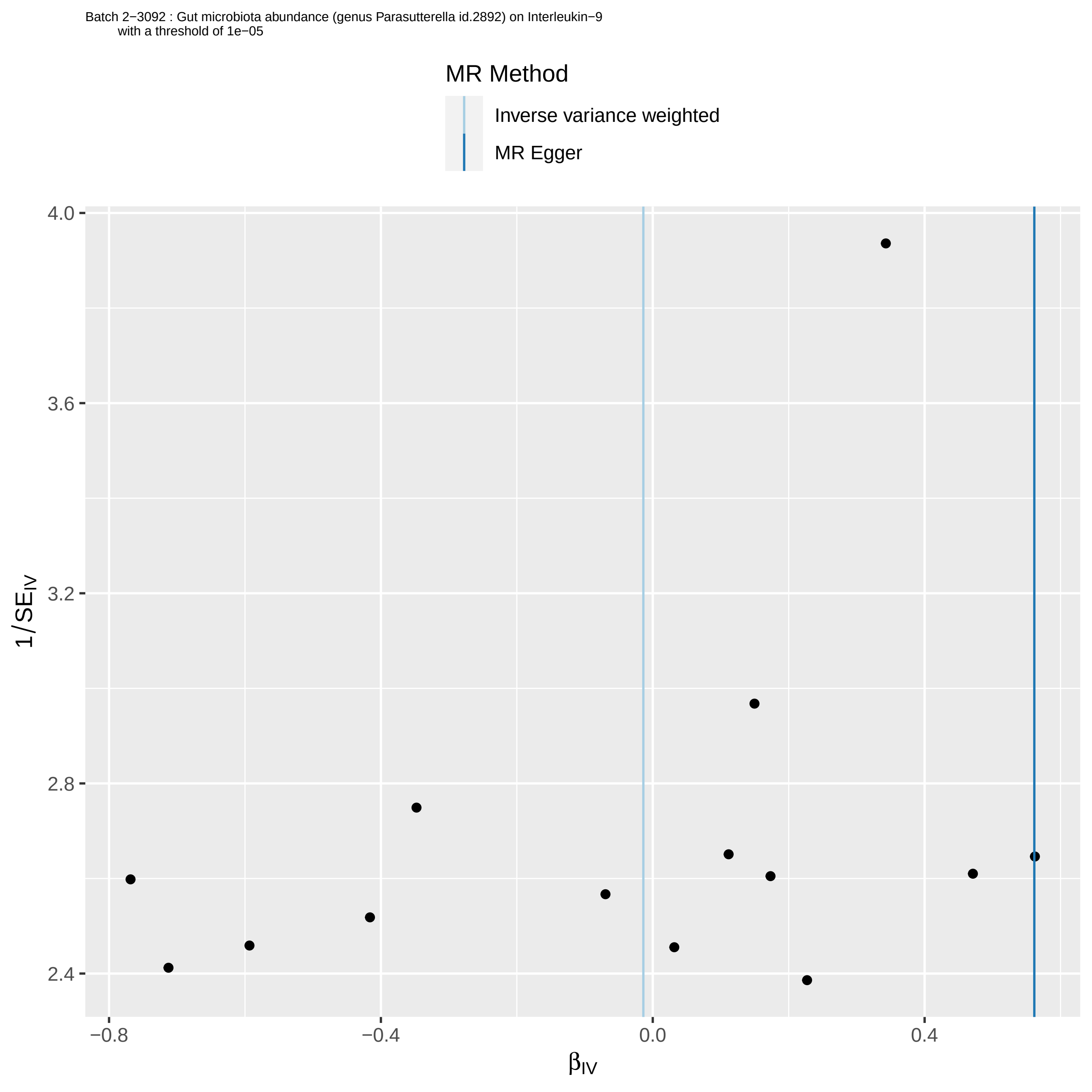

Batch 2−3092 : Gut microbiota abundance (genus Parasutterella id.2892) on Interleukin−9
with a threshold of 1e−05
MR Method
Inverse variance weighted
MR Egger
4.0
3.6
3.2
2.8
2.4
1/SE_IV
−0.8
−0.4
0.0
0.4
β_IV

Batch 2−3093 : Gut microbiota abundance (genus Catenibacterium id.2153) on Interleukin enhancer−binding factor 3
with a threshold of 1e−05

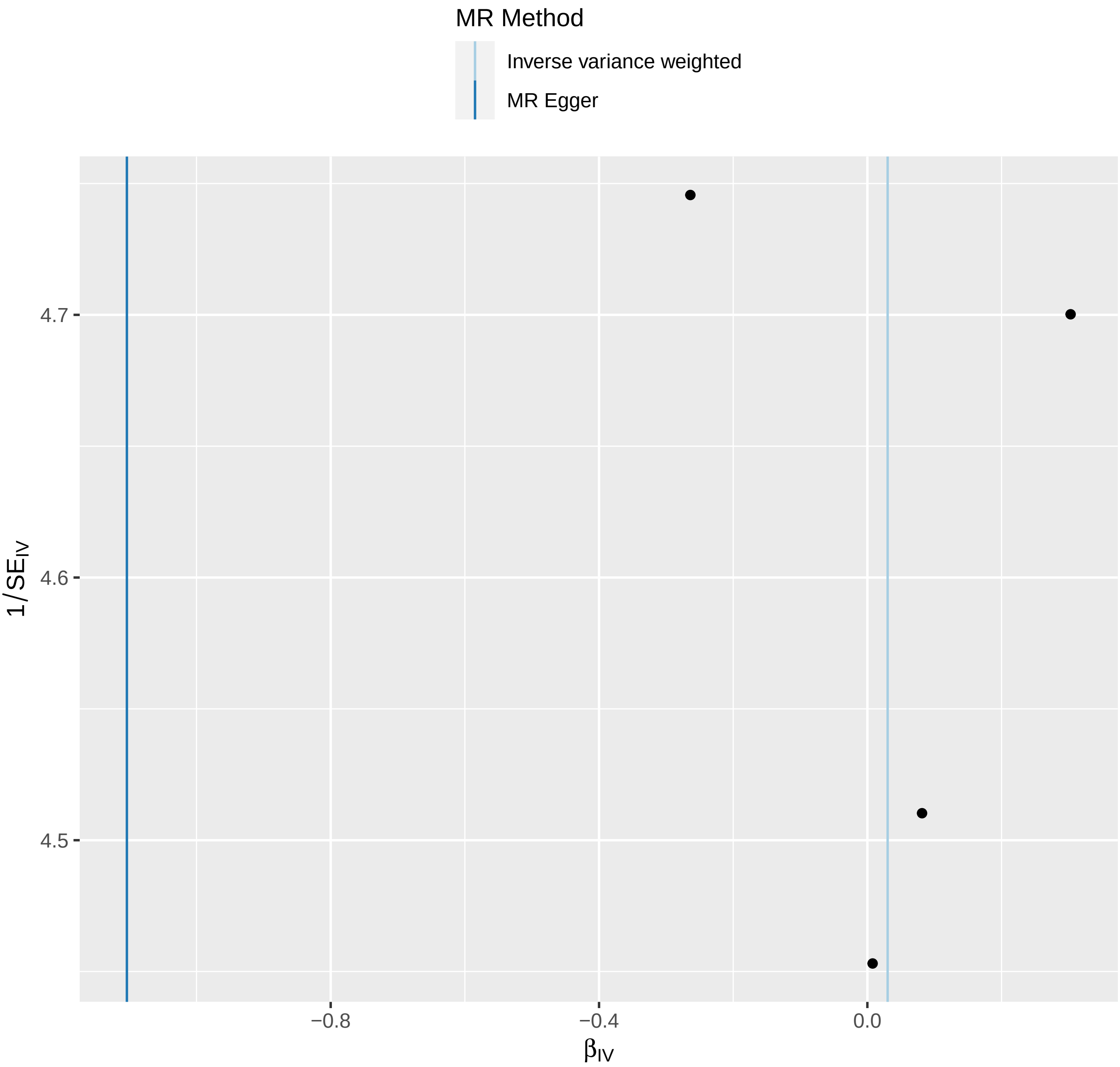

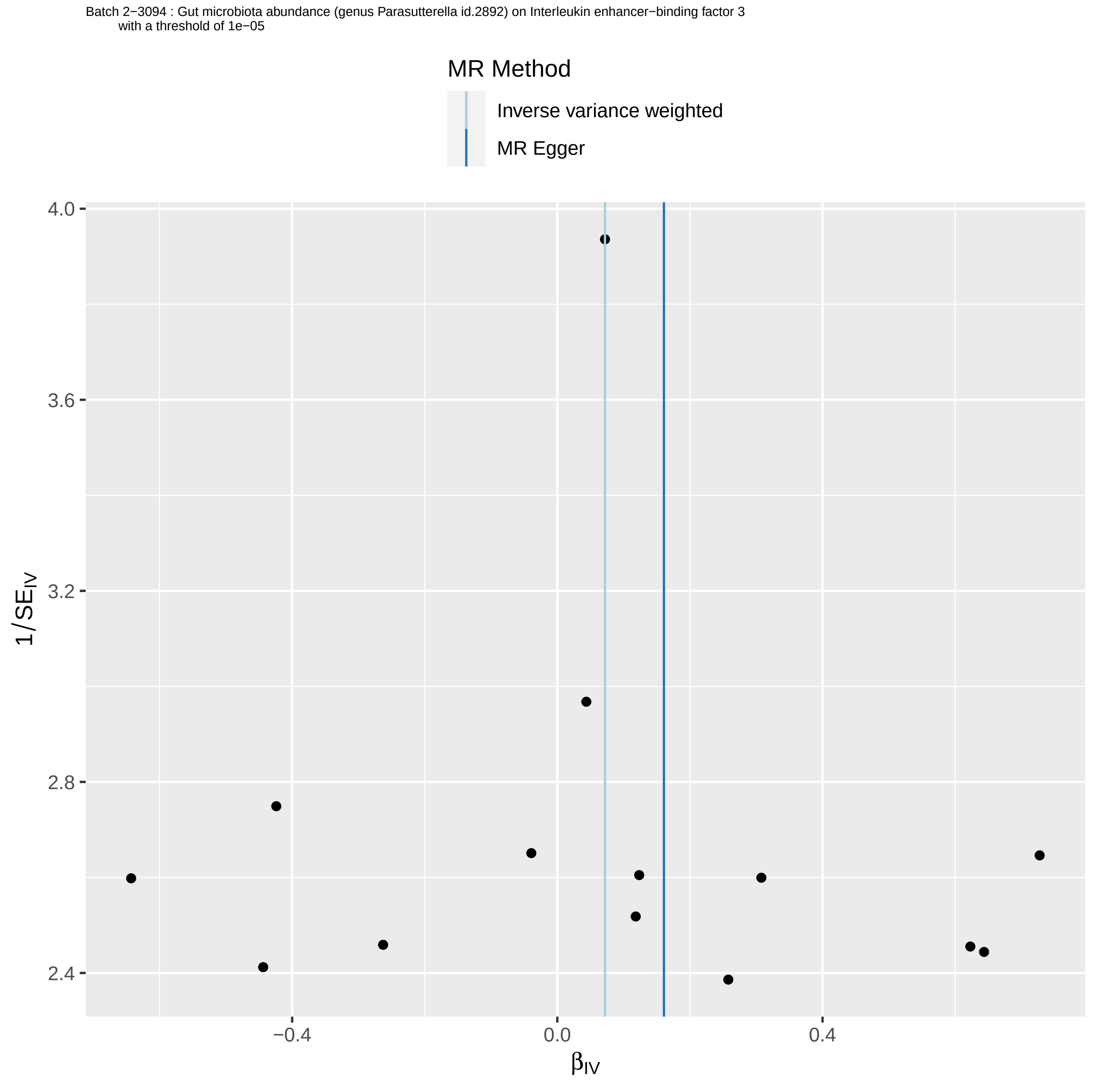

Batch 2−3094 : Gut microbiota abundance (genus Parasutterella id.2892) on Interleukin enhancer−binding factor 3
with a threshold of 1e−05
MR Method
Inverse variance weighted
MR Egger
4.0
3.6
3.2
2.8
2.4
1/SE_IV
−0.4
0.0
0.4
β_IV

Batch 2−3095 : Gut microbiota abundance (genus Catenibacterium id.2153) on Inositol monophosphatase 3 with a threshold of 1e−05

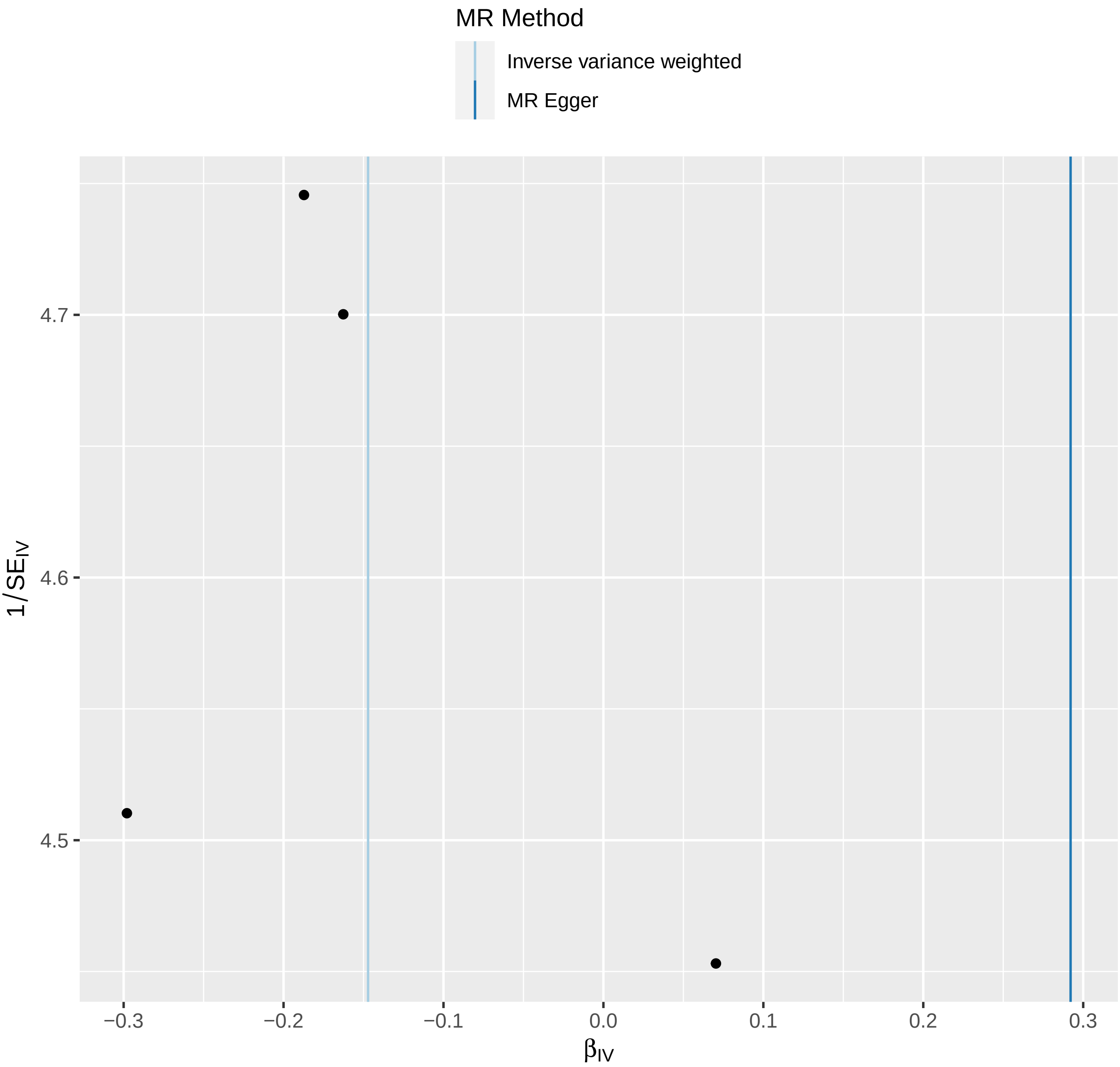

Batch 2−3096 : Gut microbiota abundance (genus Parasutterella id.2892) on Inositol monophosphatase 3
with a threshold of 1e−05

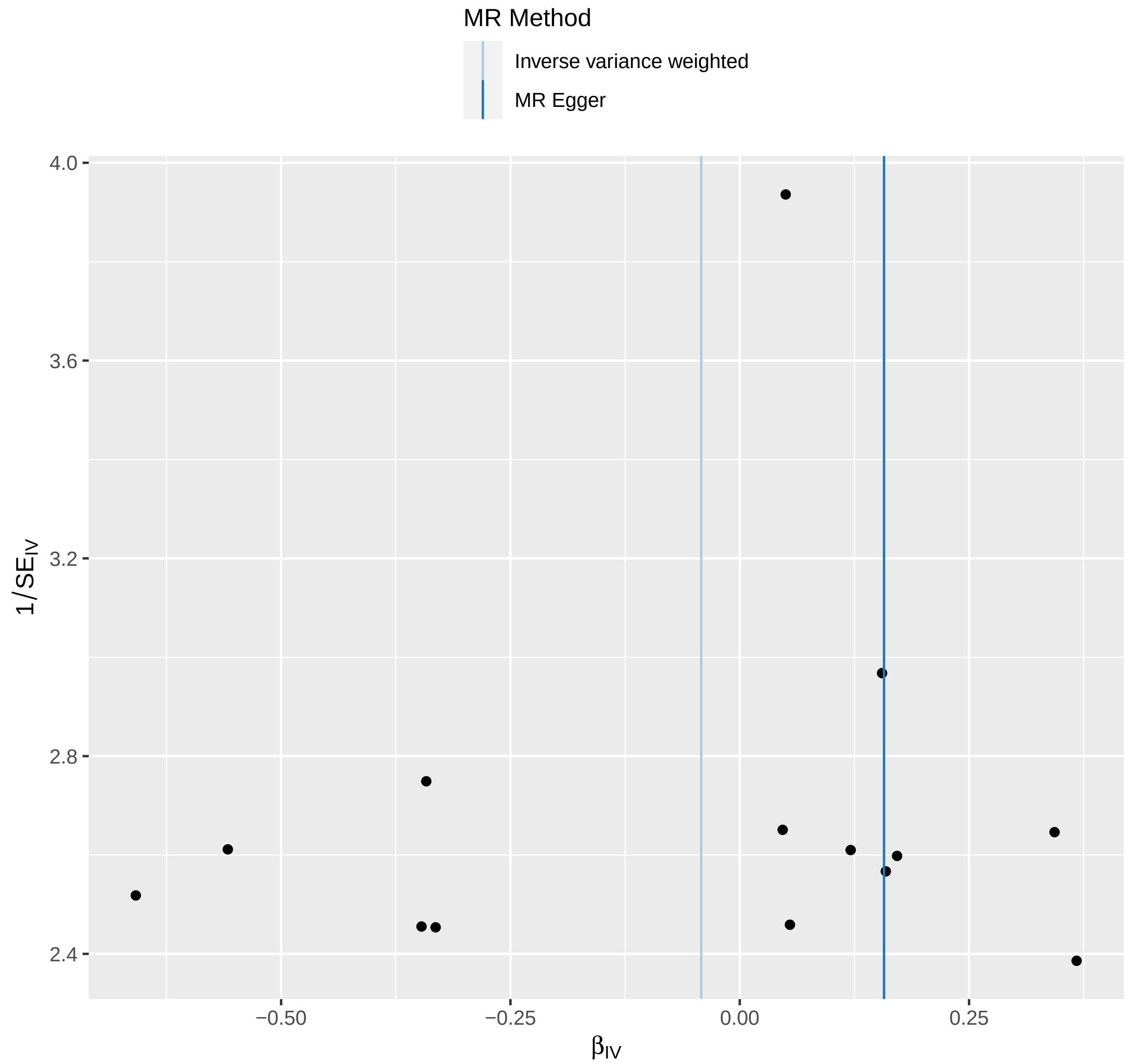

Batch 2−3097 : Gut microbiota abundance (genus Catenibacterium id.2153) on Inosine−5'−monophosphate dehydrogenase 1 with a threshold of 1e−05

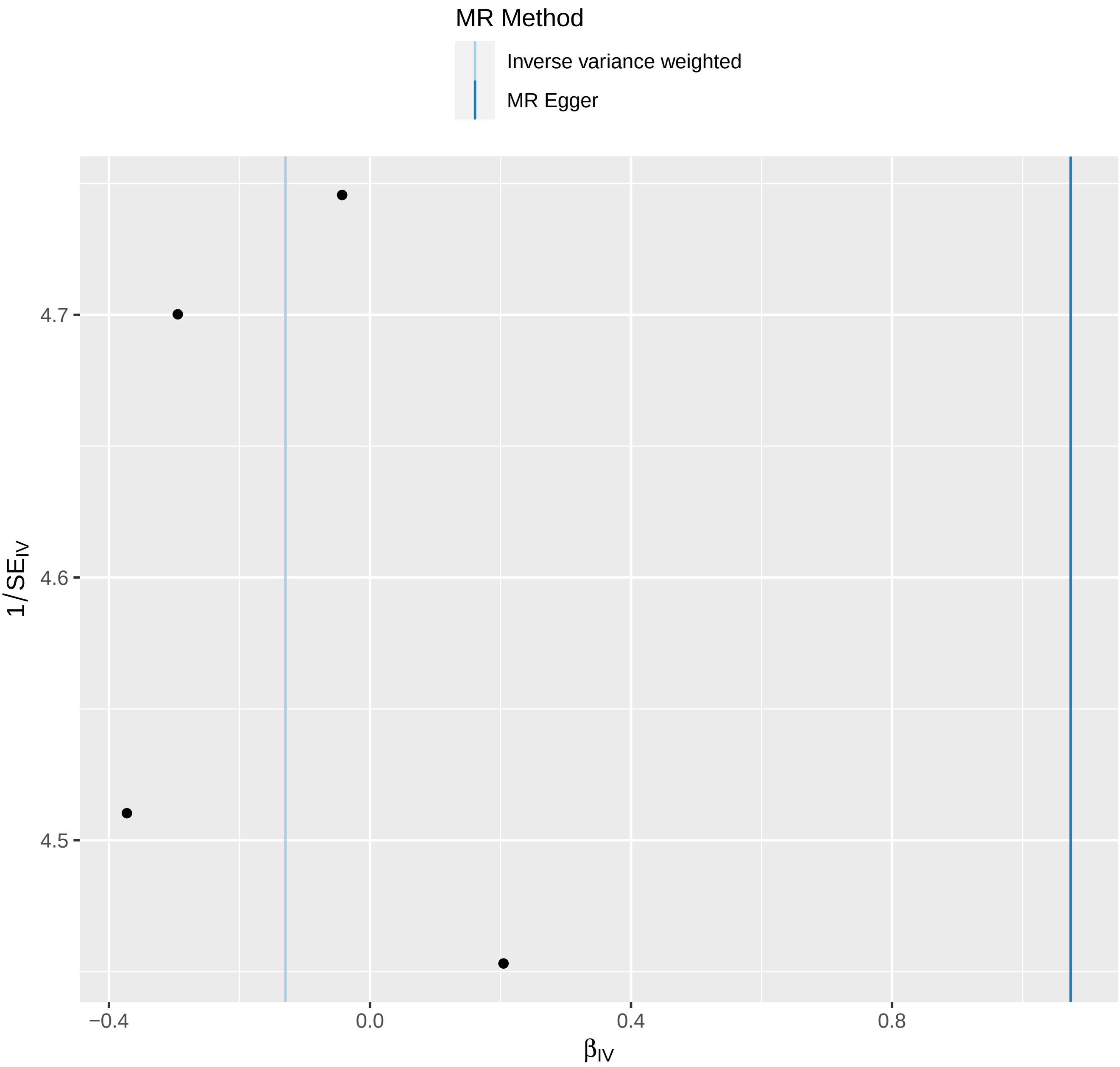

Batch 2−3098 : Gut microbiota abundance (genus Parasutterella id.2892) on Inosine−5'−monophosphate dehydrogenase 1
with a threshold of 1e−05

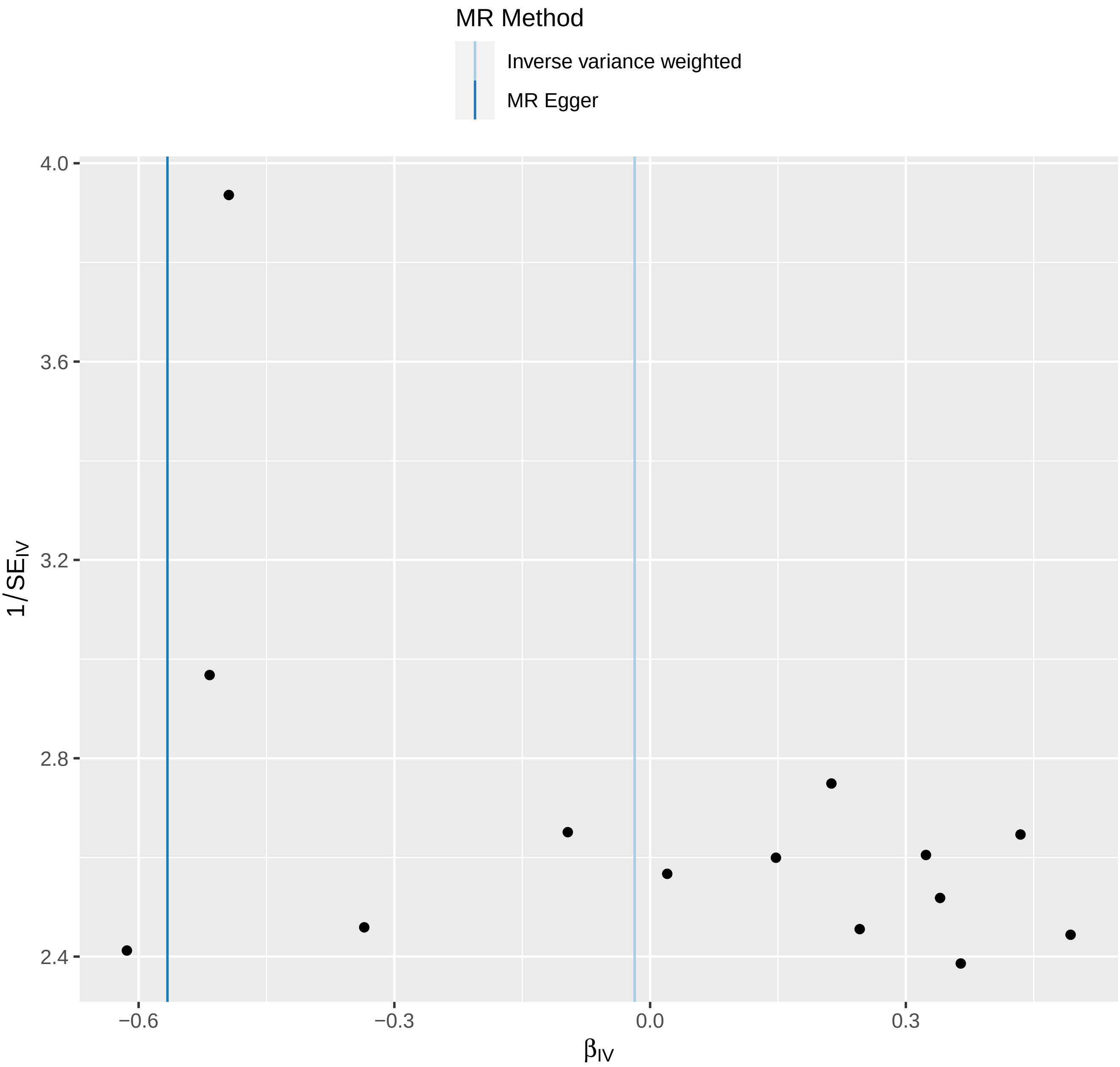

Batch 2−3099 : Gut microbiota abundance (genus Catenibacterium id.2153) on Inosine−5'−monophosphate dehydrogenase 2
with a threshold of 1e−05

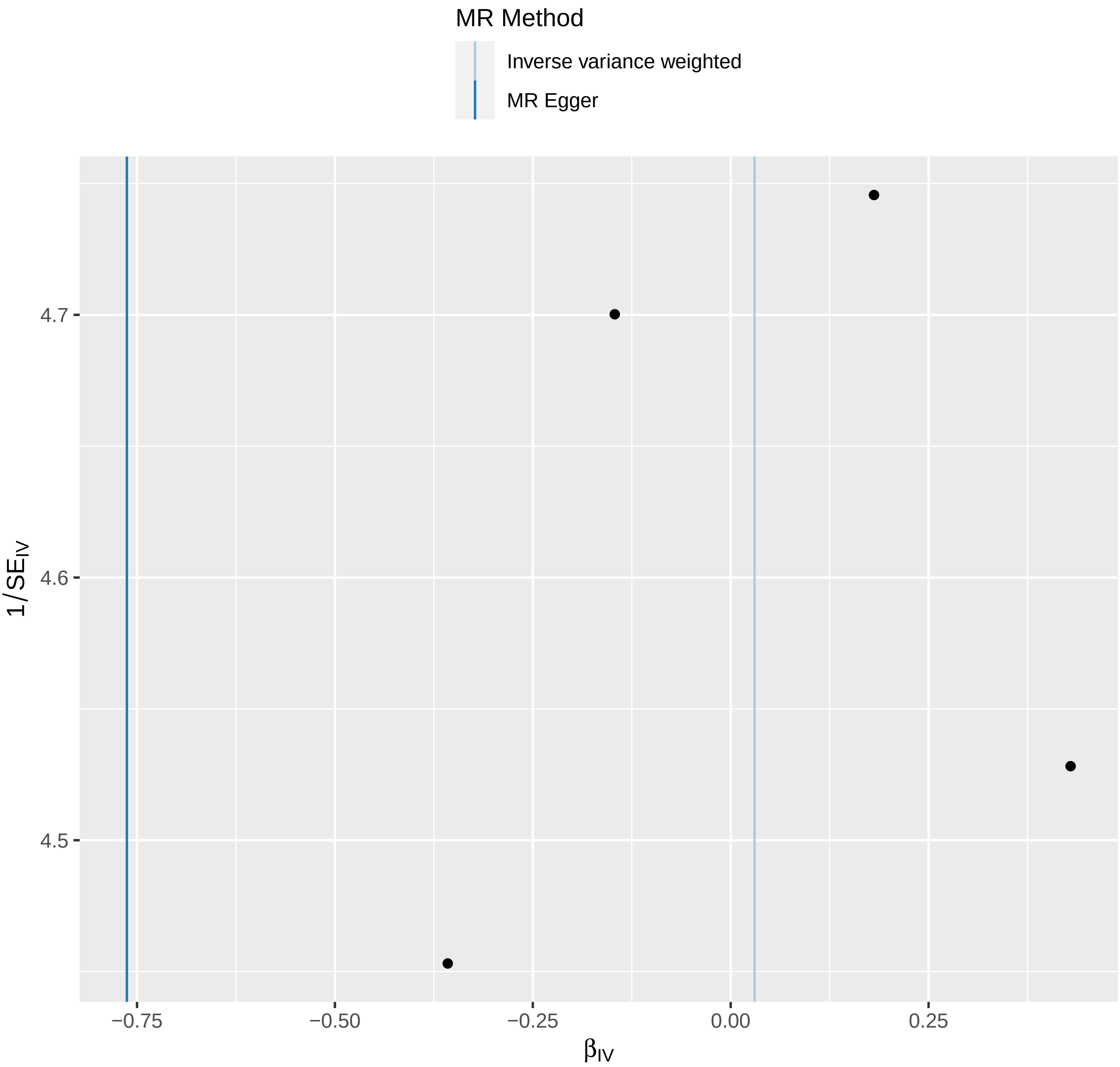

Batch 2−3100 : Gut microbiota abundance (genus Parasutterella id.2892) on Inosine−5'−monophosphate dehydrogenase 2
with a threshold of 1e−05

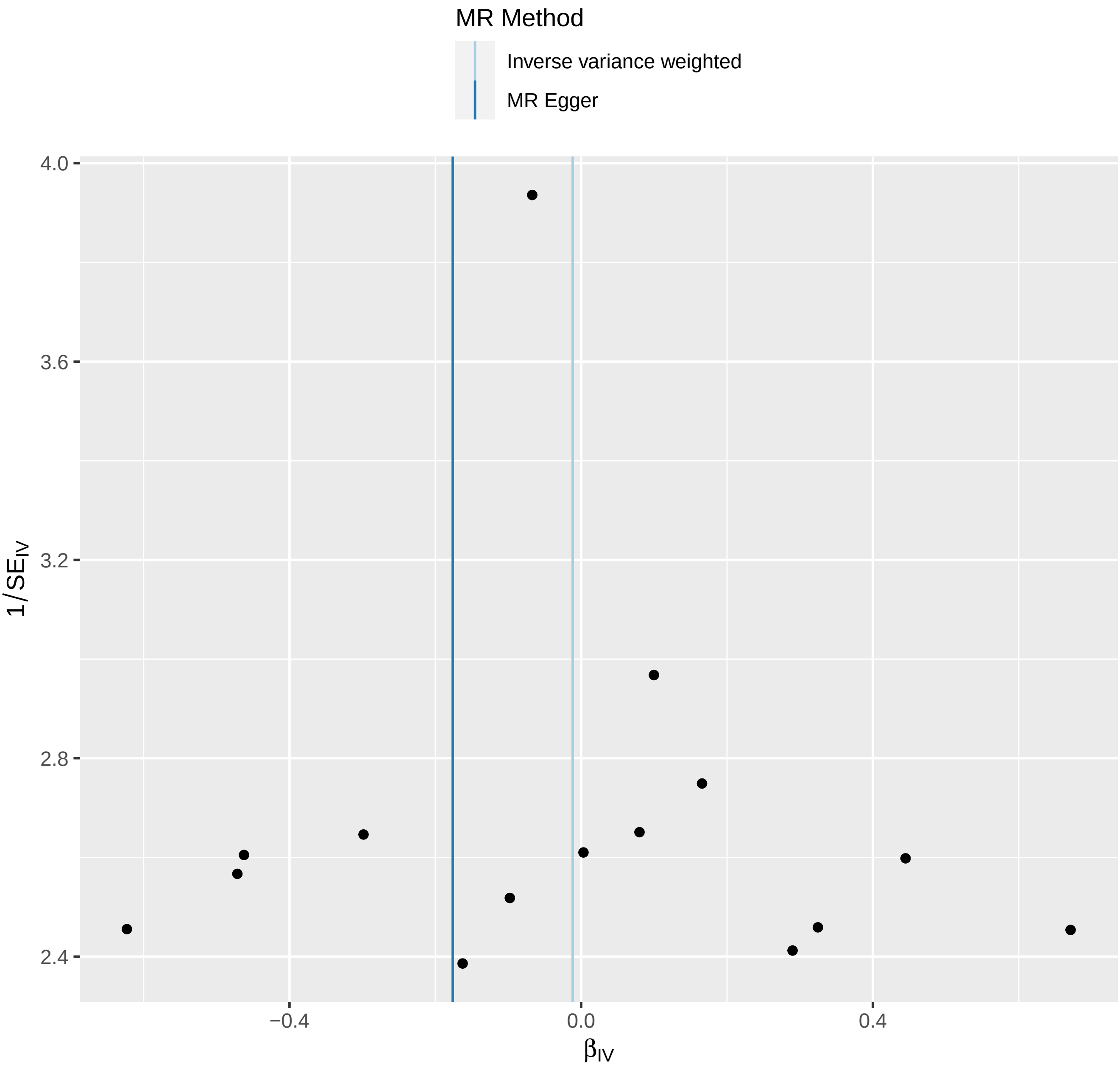

Batch 2−3101 : Gut microbiota abundance (genus Catenibacterium id.2153) on Alpha−internexin
with a threshold of 1e−05

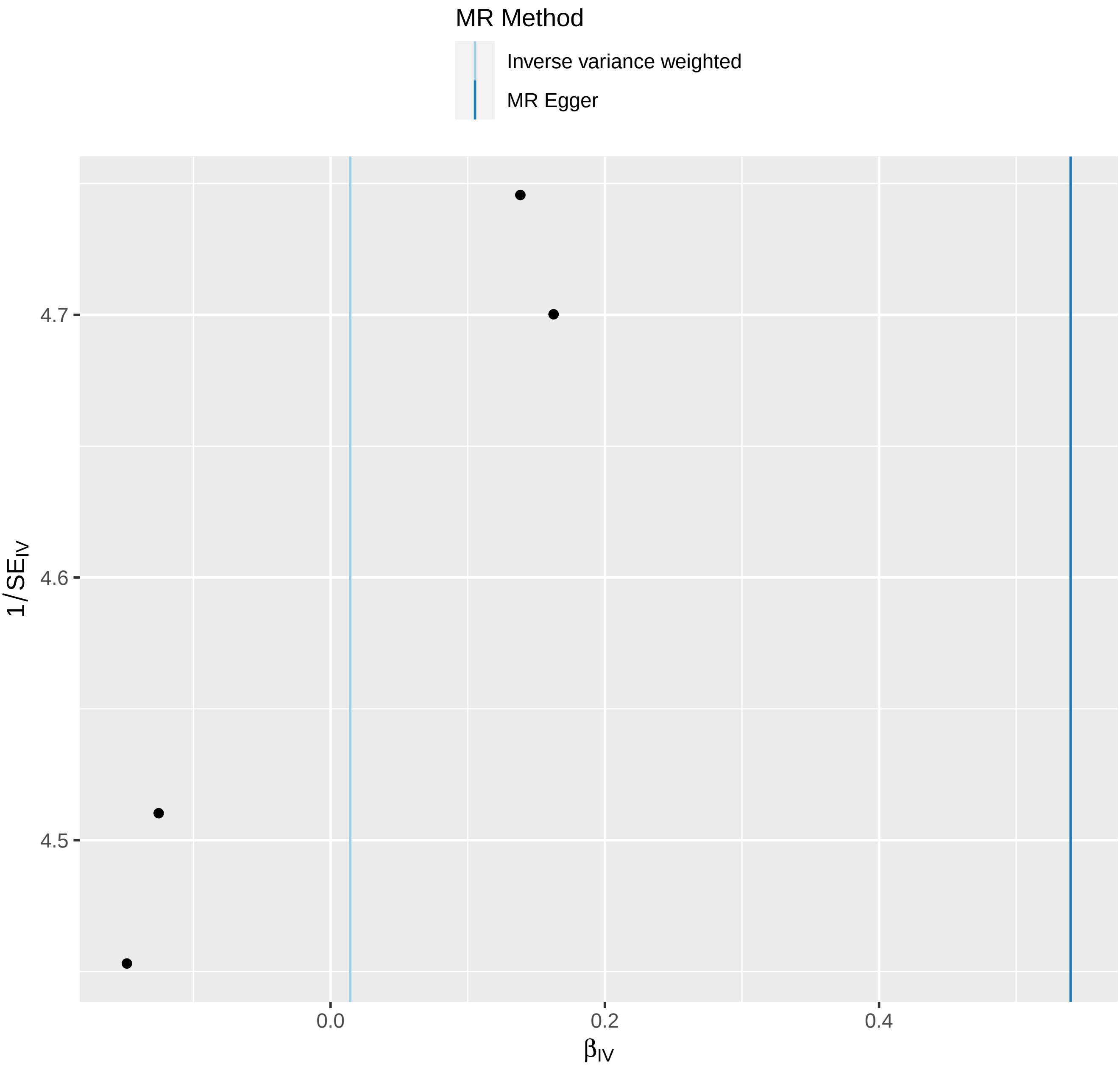

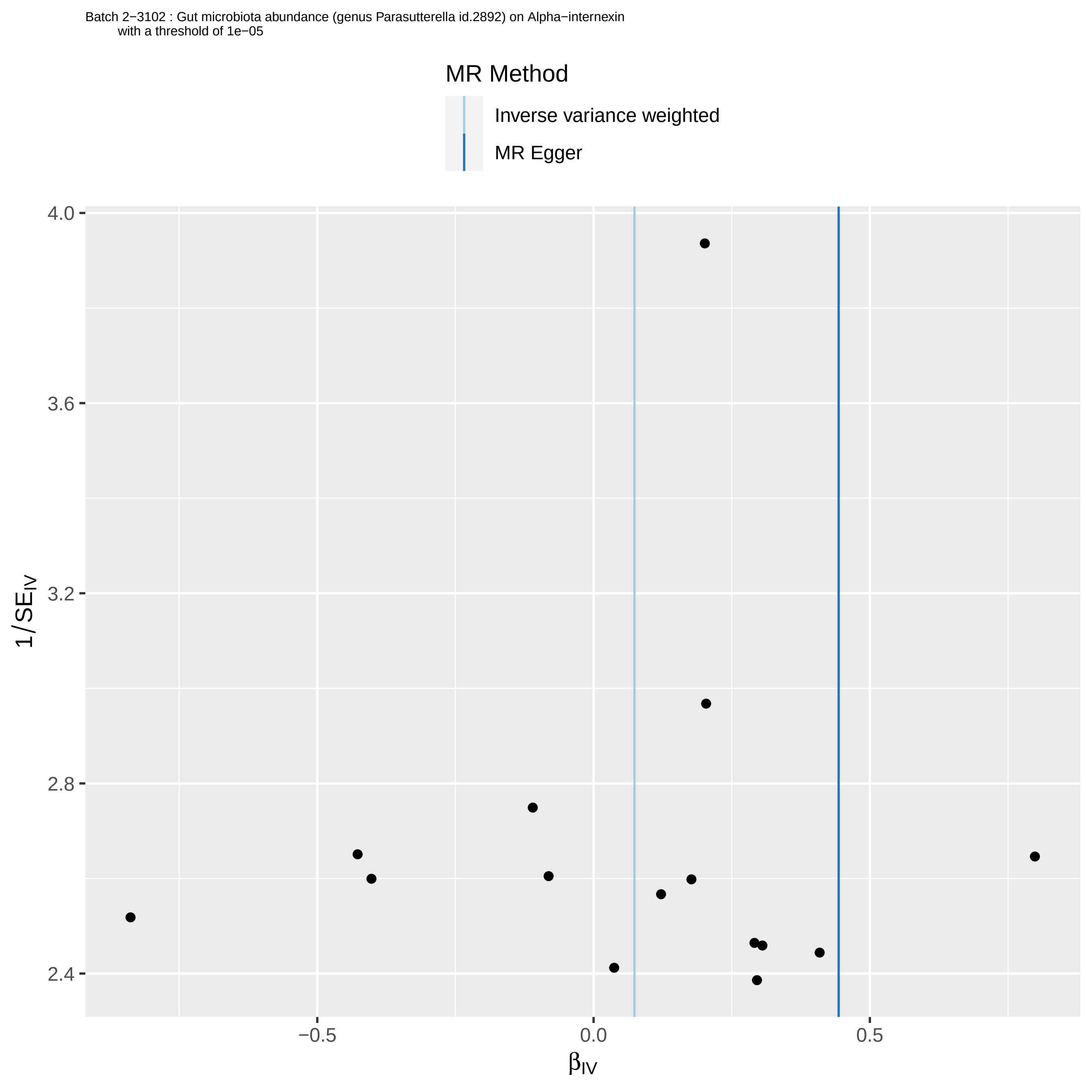
Batch 2−3102 : Gut microbiota abundance (genus Parasutterella id.2892) on Alpha−internexin
with a threshold of 1e−05
MR Method
Inverse variance weighted
MR Egger
4.0
3.6
3.2
2.8
2.4
$1/SE_{IV}$
−0.5
0.0
0.5
β_{IV}

Batch 2−3103 : Gut microbiota abundance (genus Catenibacterium id.2153) on Inhibitor of growth protein 1
with a threshold of 1e−05

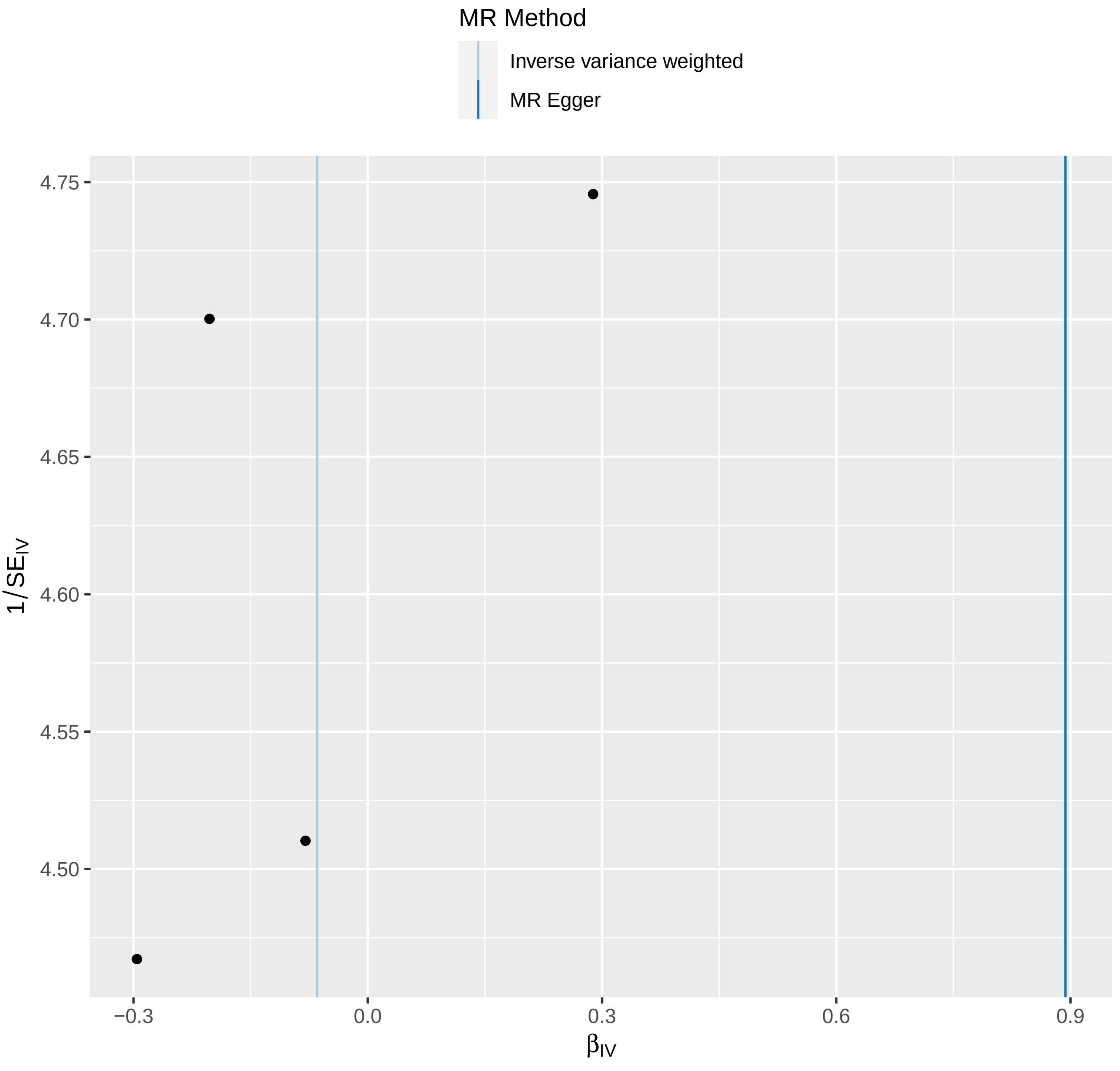

Batch 2−3104 : Gut microbiota abundance (genus Parasutterella id.2892) on Inhibitor of growth protein 1
with a threshold of 1e−05

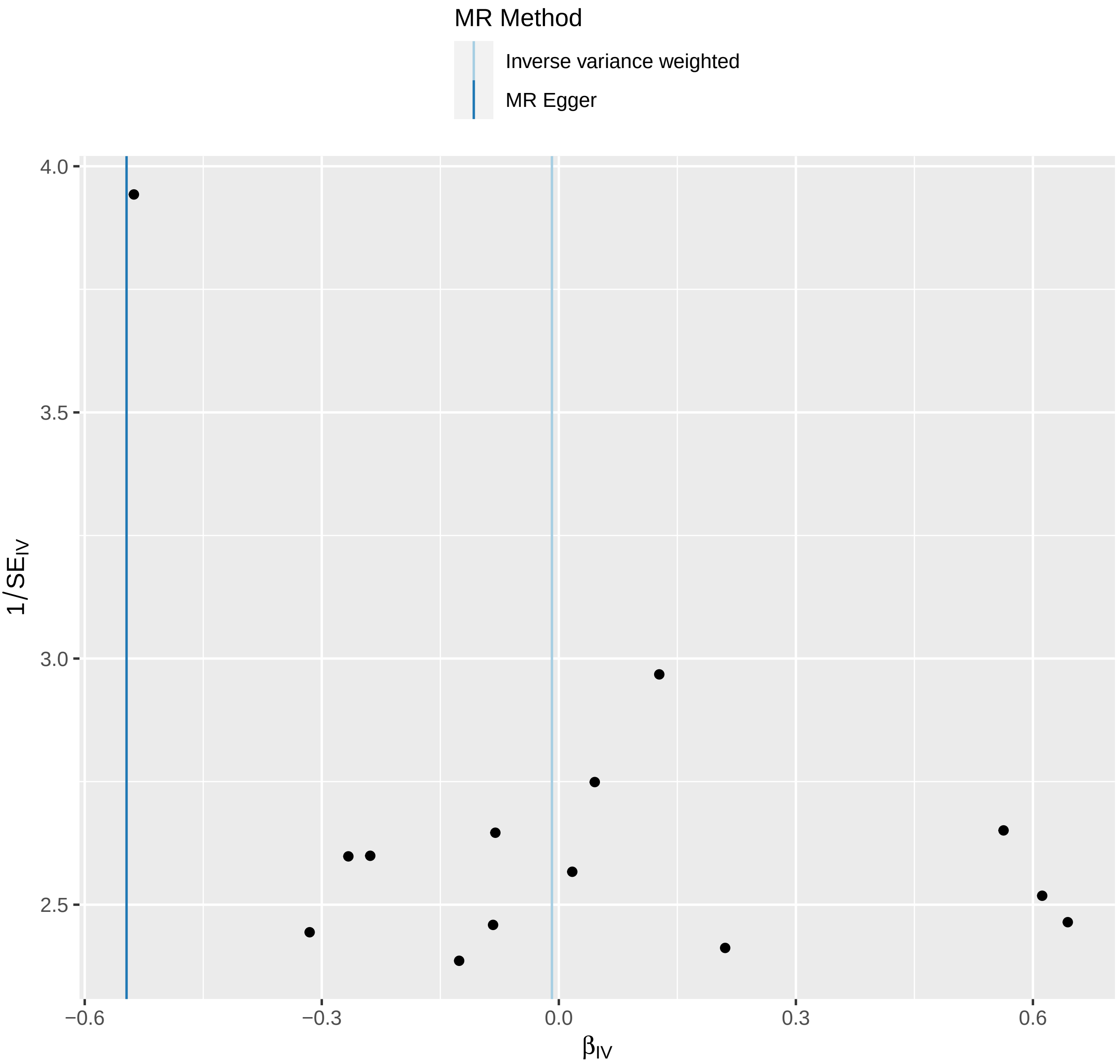

Batch 2−3105 : Gut microbiota abundance (genus Catenibacterium id.2153) on Inhibin beta A chain
with a threshold of 1e−05

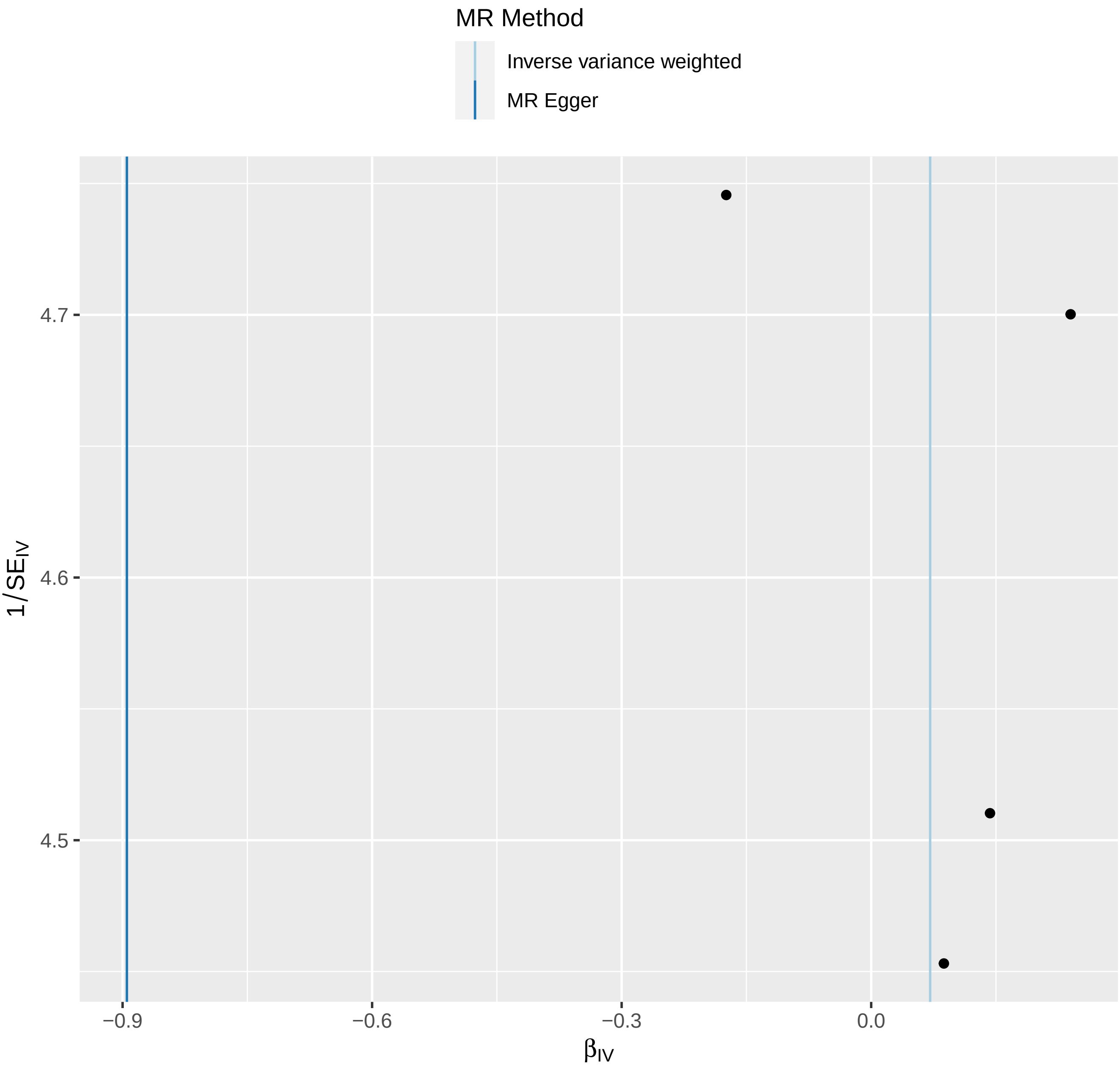

Batch 2−3106 : Gut microbiota abundance (genus Parasutterella id.2892) on Inhibin beta A chain
with a threshold of 1e−05

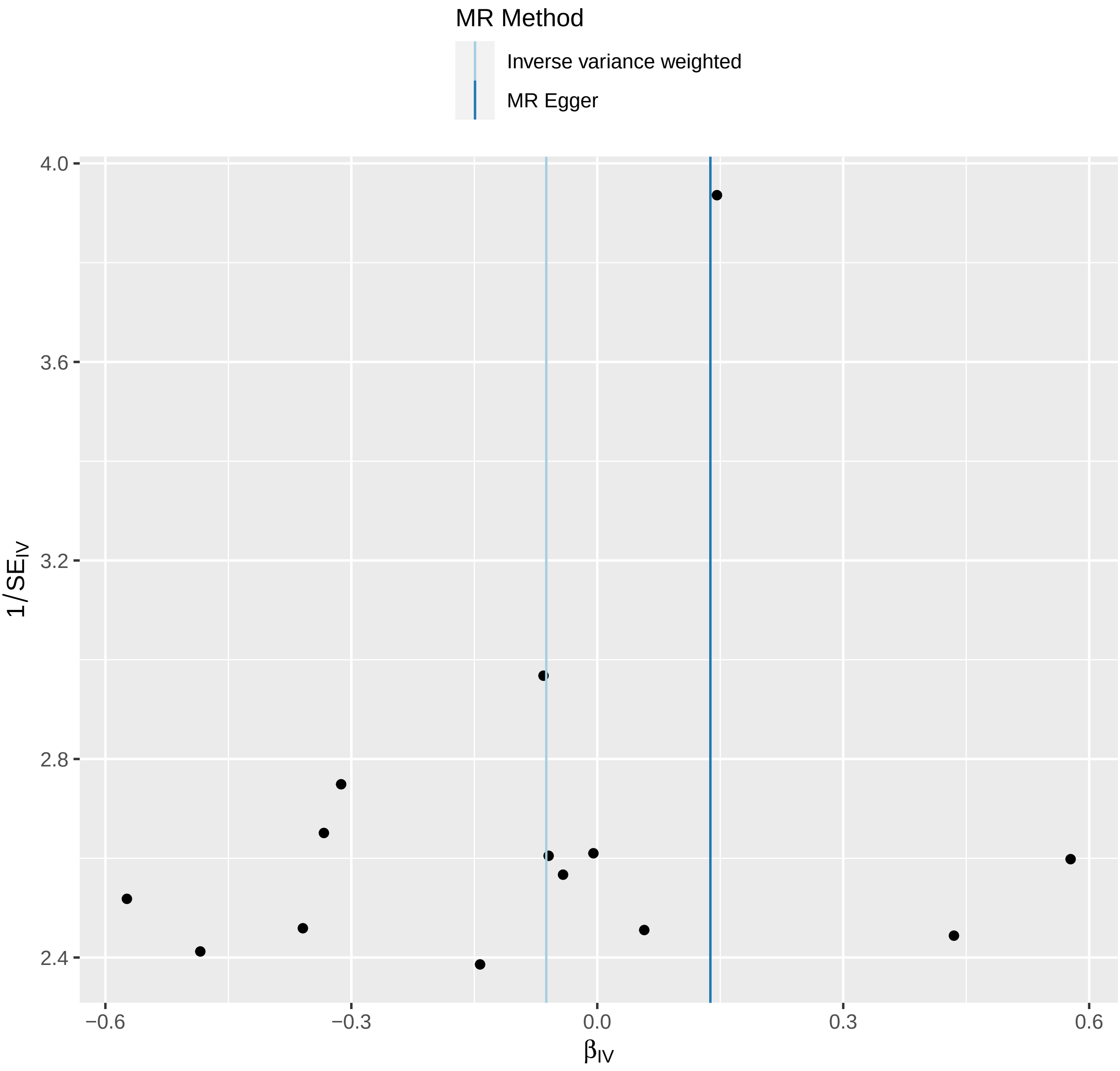

Batch 2−3107 : Gut microbiota abundance (genus Catenibacterium id.2153) on Inhibin beta A chain:Inhibin beta B chain heterodimer
with a threshold of 1e−05

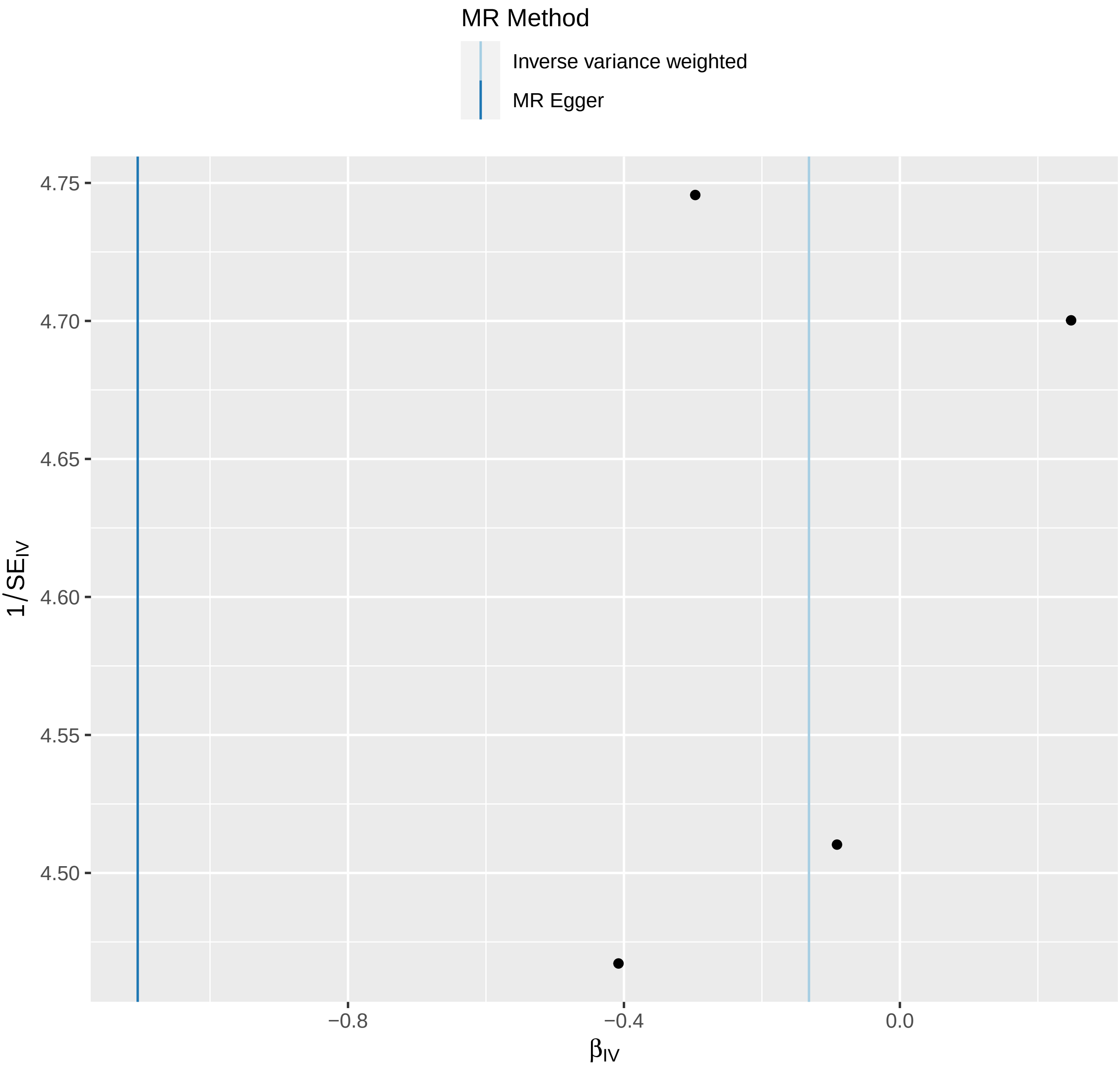

Batch 2−3108 : Gut microbiota abundance (genus Parasutterella id.2892) on Inhibin beta A chain:Inhibin beta B chain heterodimer
with a threshold of 1e−05

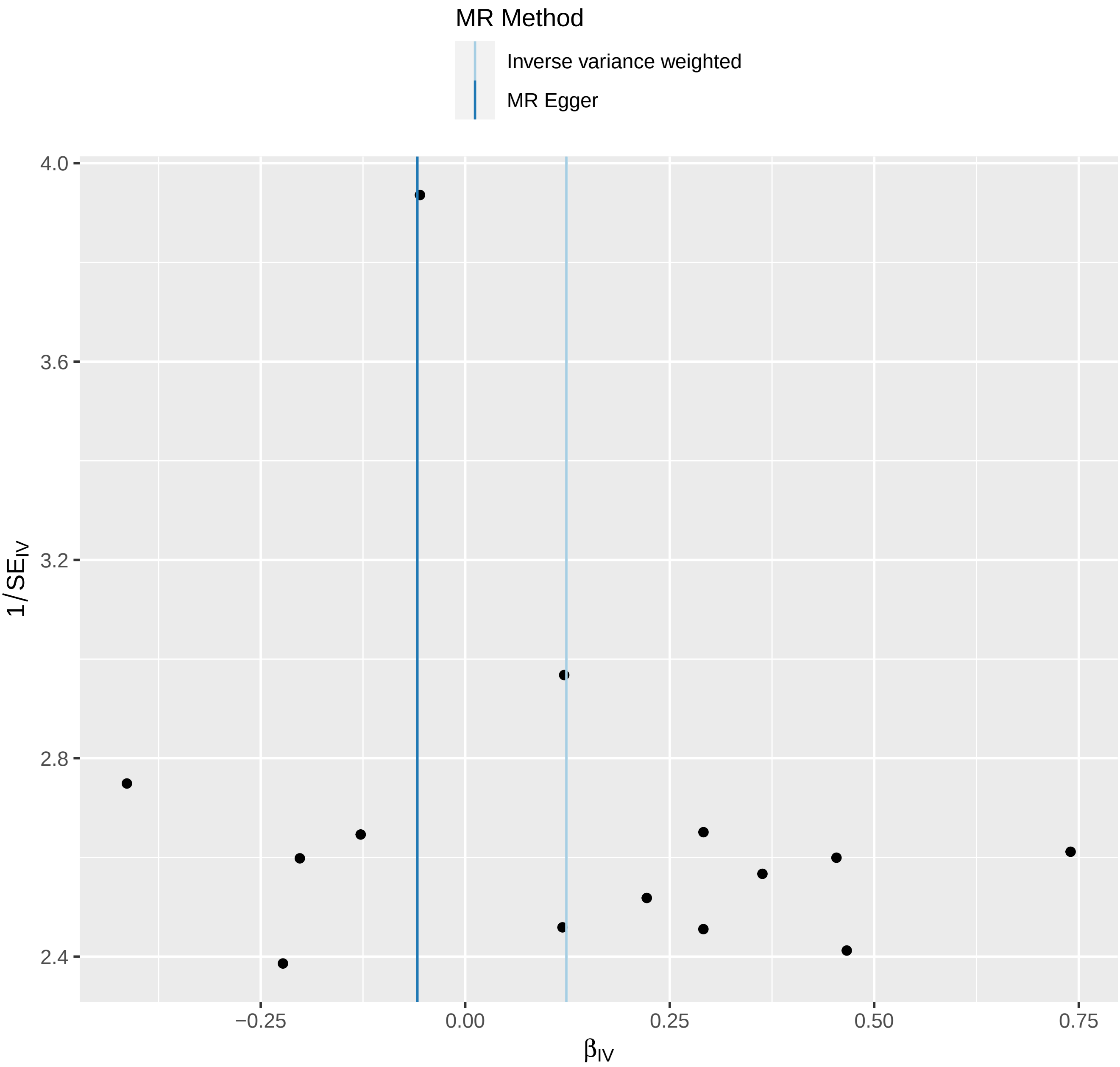

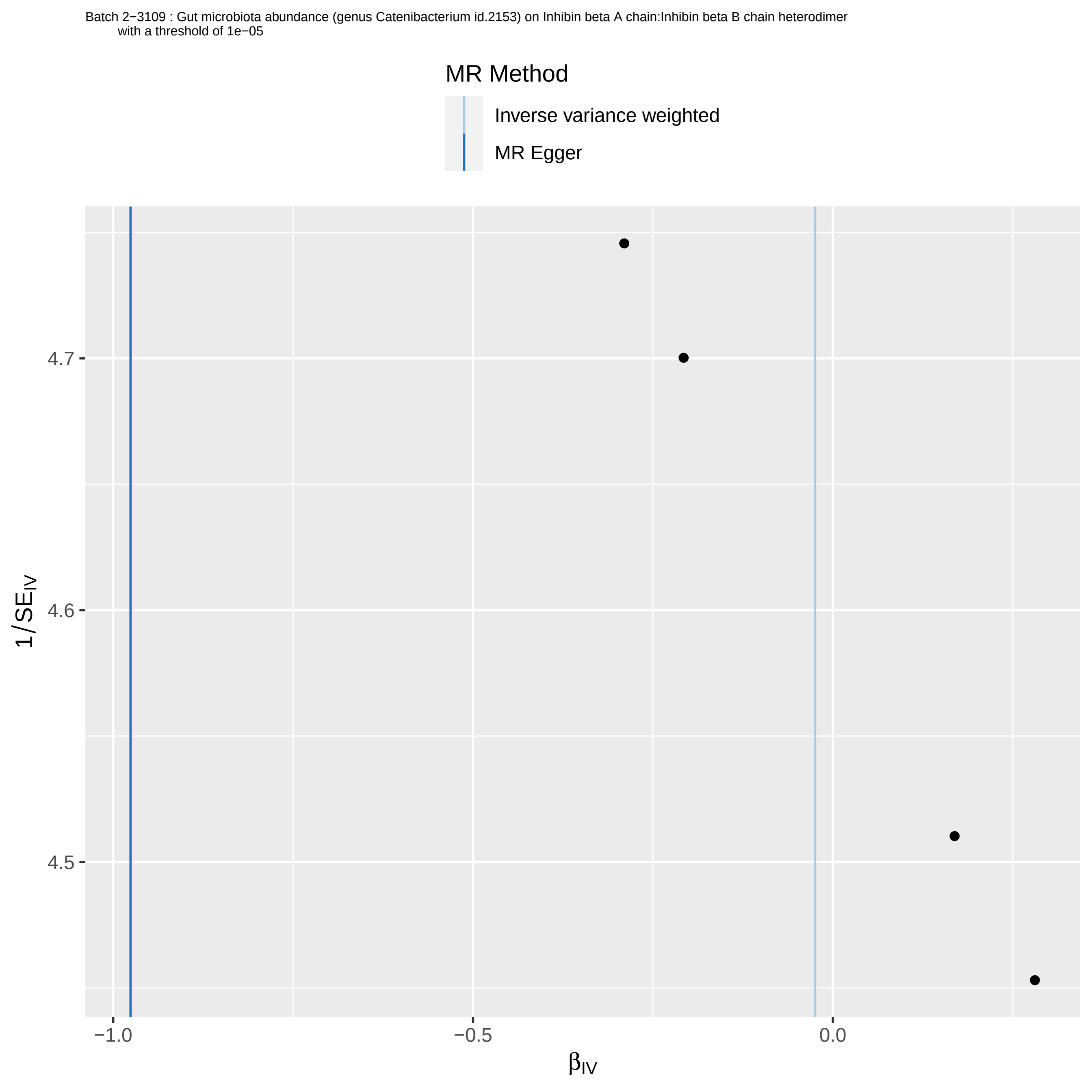

Batch 2−3109 : Gut microbiota abundance (genus Catenibacterium id.2153) on Inhibin beta A chain:Inhibin beta B chain heterodimer
with a threshold of 1e−05
MR Method
Inverse variance weighted
MR Egger
4.7
4.6
4.5
1/SE_IV
−1.0
−0.5
0.0
β_IV

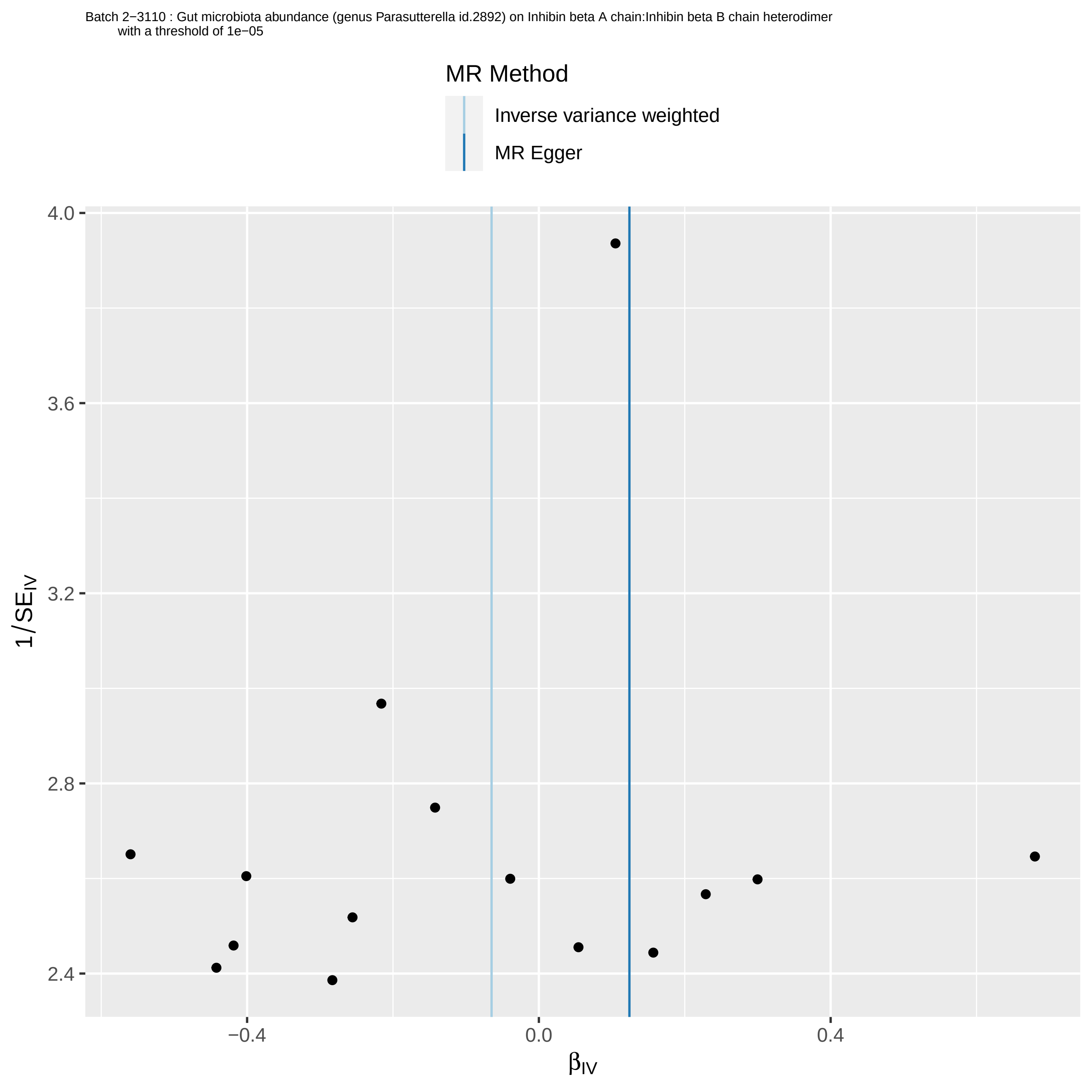
Batch 2−3110 : Gut microbiota abundance (genus Parasutterella id.2892) on Inhibin beta A chain:Inhibin beta B chain heterodimer
with a threshold of 1e−05
MR Method
Inverse variance weighted
MR Egger
4.0
3.6
3.2
2.8
2.4
−0.4
0.0
0.4
$1/SE_{IV}$
β_{IV}

Batch 2−3111 : Gut microbiota abundance (genus Catenibacterium id.2153) on Inhibin beta C chain
with a threshold of 1e−05

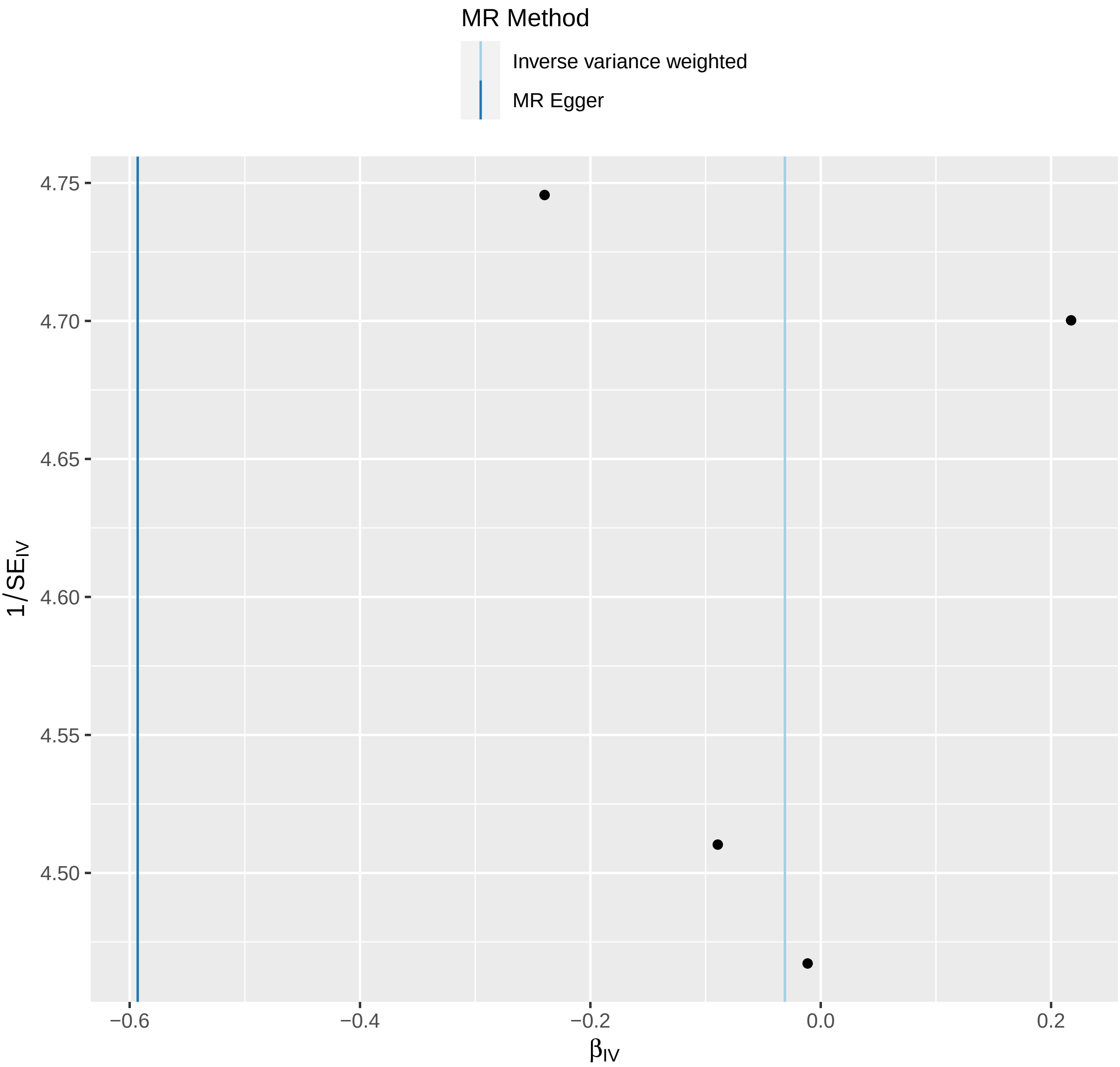

Batch 2−3112 : Gut microbiota abundance (genus Parasutterella id.2892) on Inhibin beta C chain
with a threshold of 1e−05

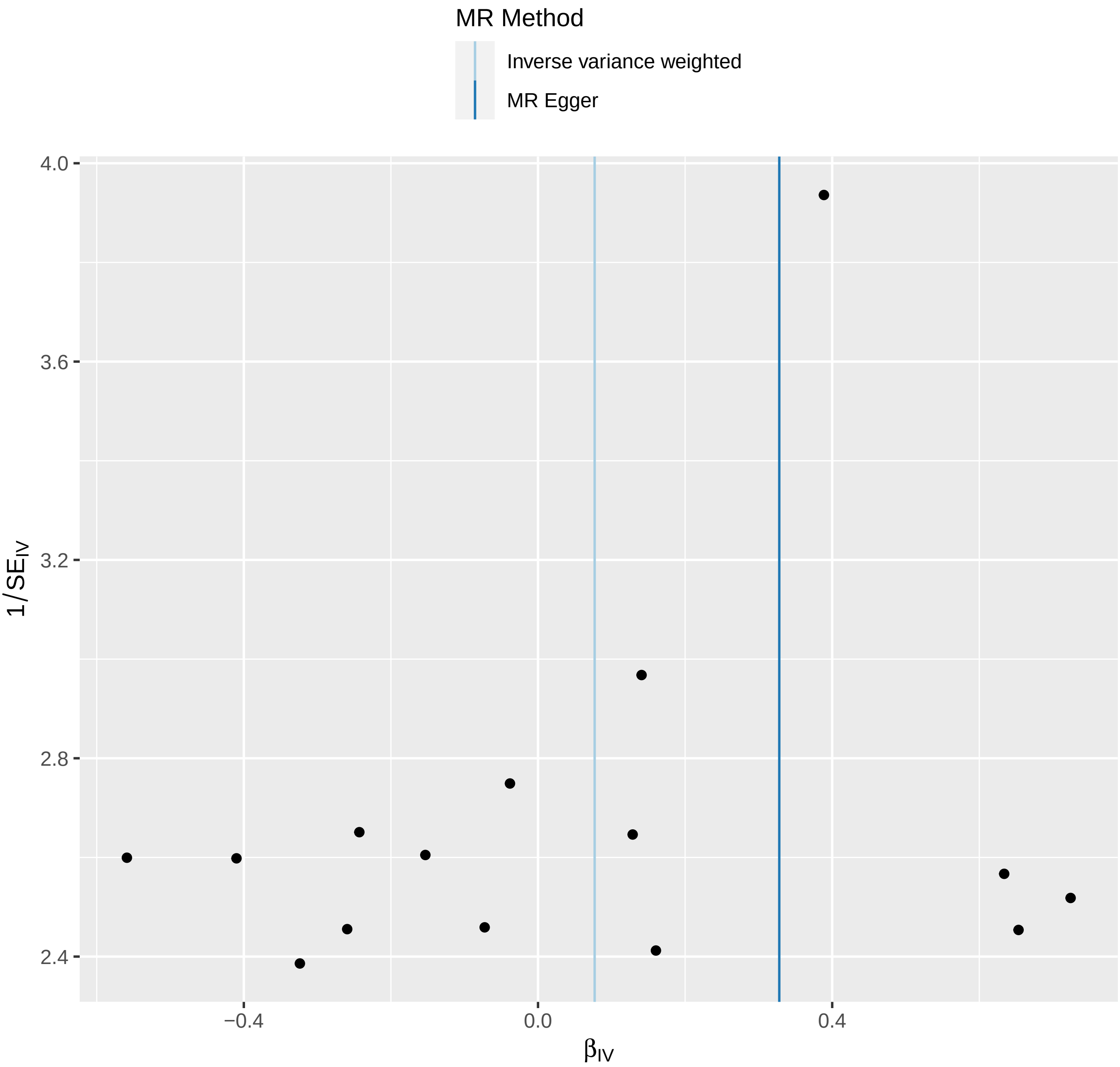

Batch 2−3113 : Gut microbiota abundance (genus Catenibacterium id.2153) on 72 kDa inositol polyphosphate 5−phosphatase
with a threshold of 1e−05

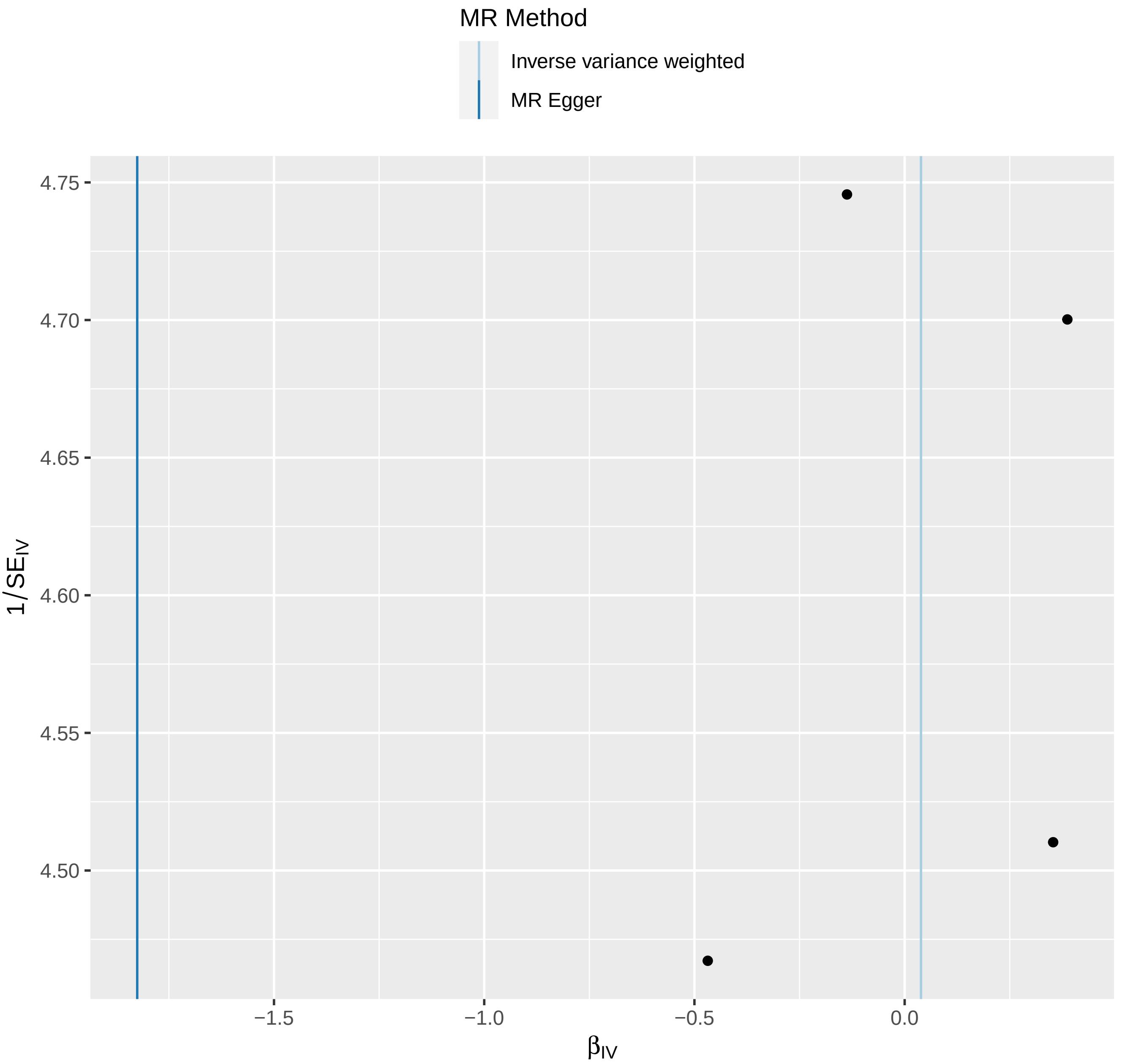

Batch 2−3114 : Gut microbiota abundance (genus Parasutterella id.2892) on 72 kDa inositol polyphosphate 5−phosphatase
with a threshold of 1e−05

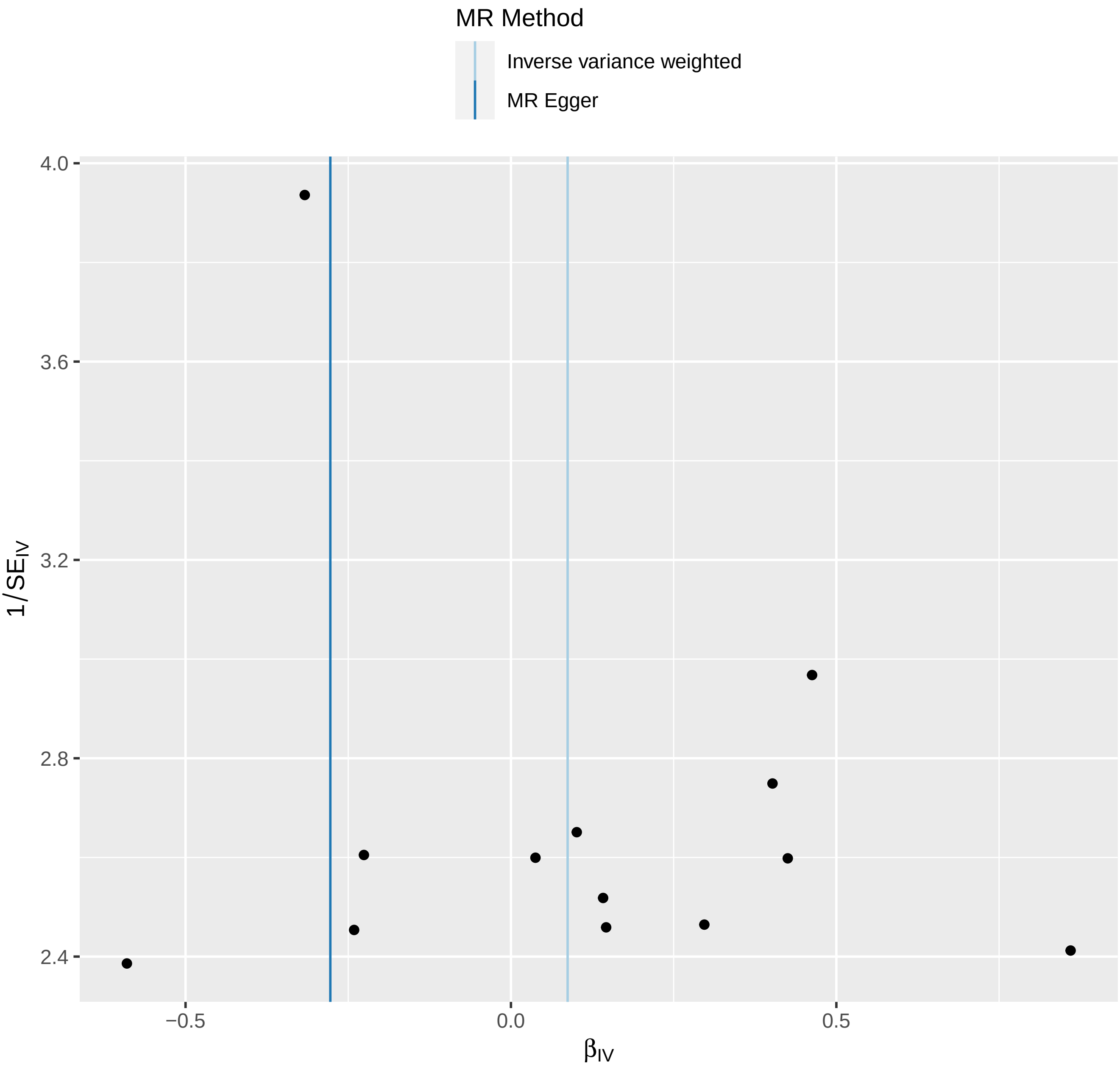

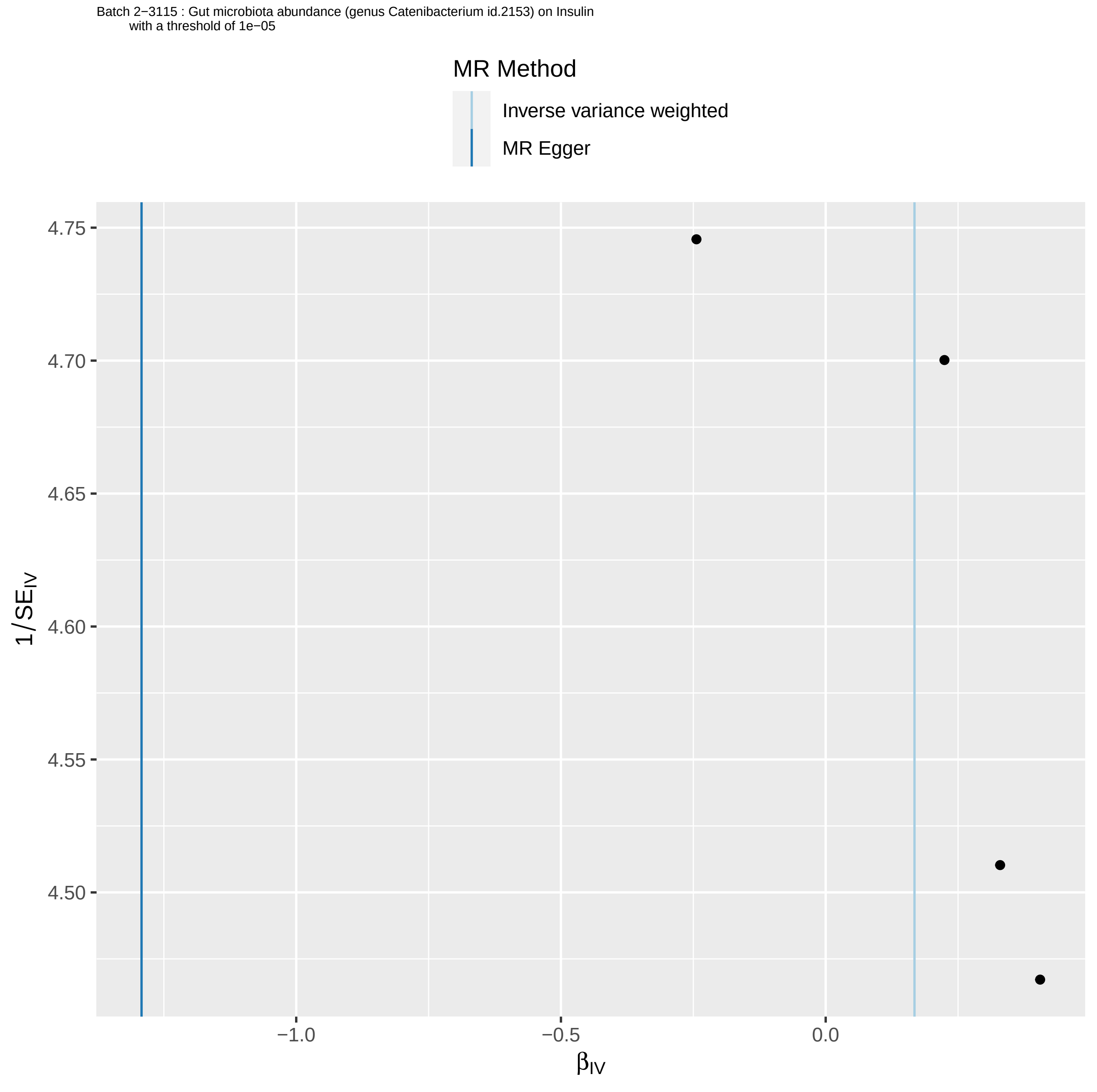
Batch 2−3115 : Gut microbiota abundance (genus Catenibacterium id.2153) on Insulin
with a threshold of 1e−05
MR Method
Inverse variance weighted
MR Egger
4.75
4.70
4.65
4.60
4.55
4.50
1/SE$_{IV}$
−1.0
−0.5
0.0
β$_{IV}$

Batch 2−3116 : Gut microbiota abundance (genus Parasutterella id.2892) on Insulin
with a threshold of 1e−05

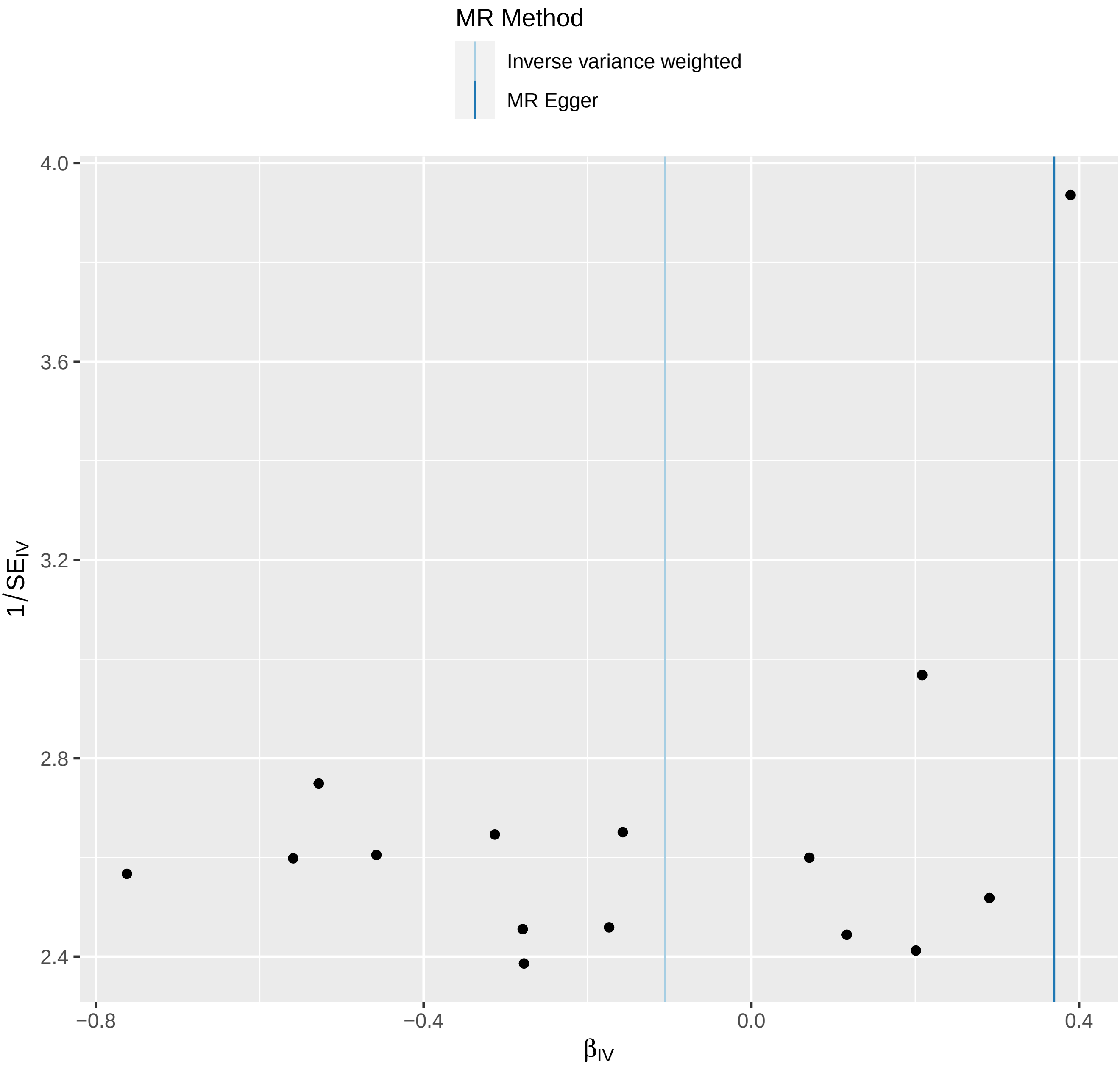

Batch 2−3117 : Gut microbiota abundance (genus Catenibacterium id.2153) on Insulin−induced gene 1 protein with a threshold of 1e−05

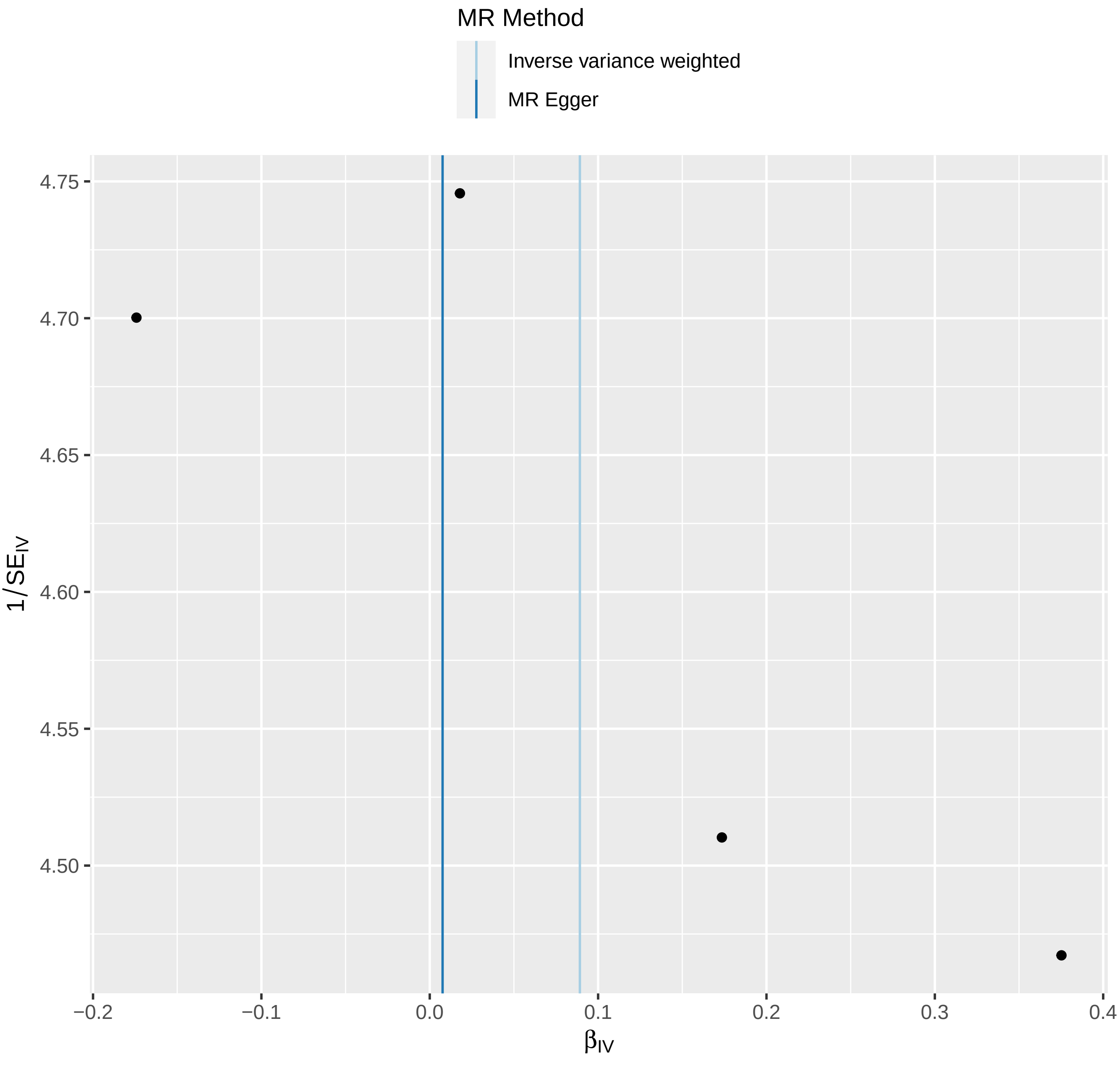

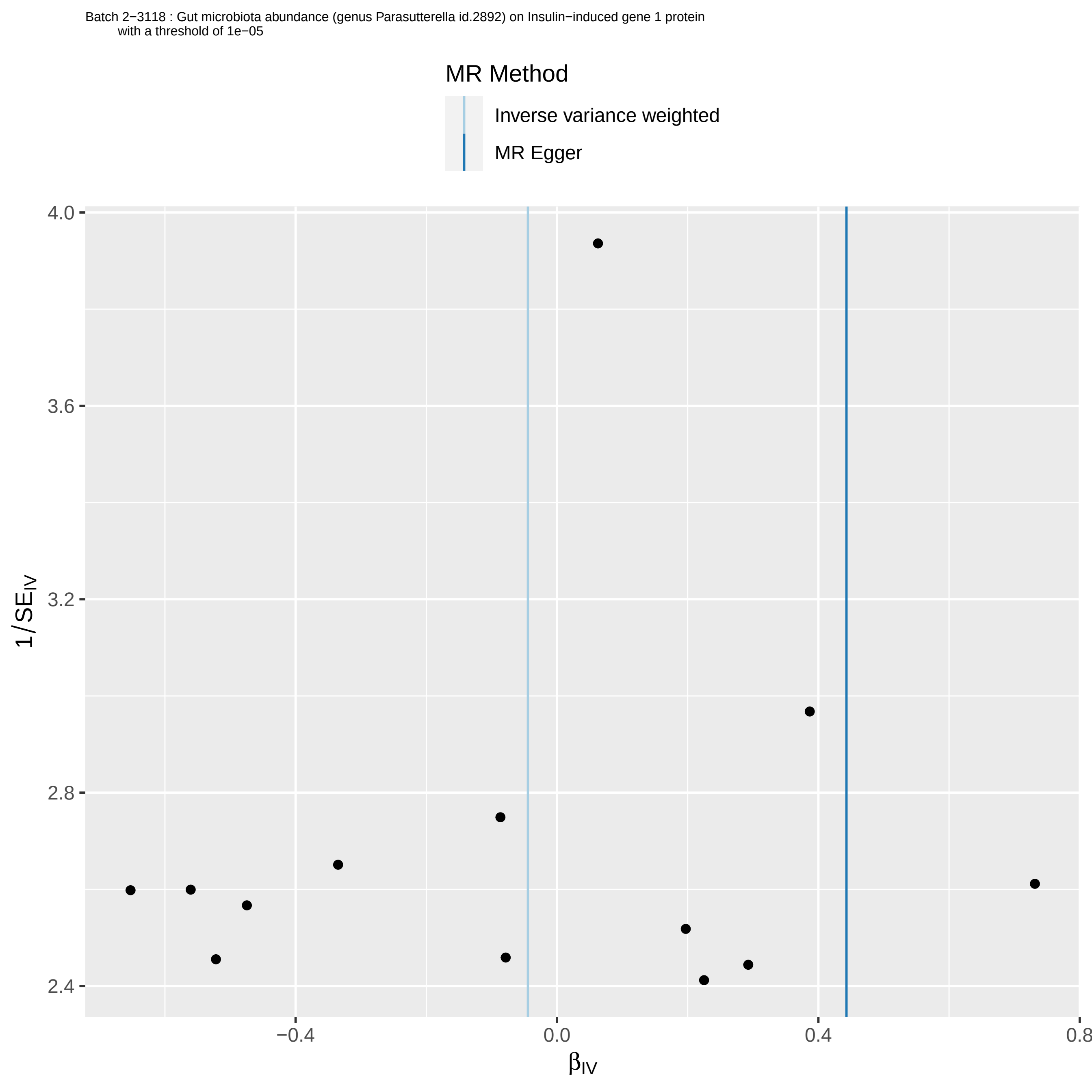

Batch 2−3118 : Gut microbiota abundance (genus Parasutterella id.2892) on Insulin−induced gene 1 protein
with a threshold of 1e−05
MR Method
Inverse variance weighted
MR Egger
4.0
3.6
3.2
2.8
2.4
1/SE_IV
−0.4
0.0
0.4
0.8
β_IV

Batch 2−3119 : Gut microbiota abundance (genus Catenibacterium id.2153) on Insulin−like 3
with a threshold of 1e−05

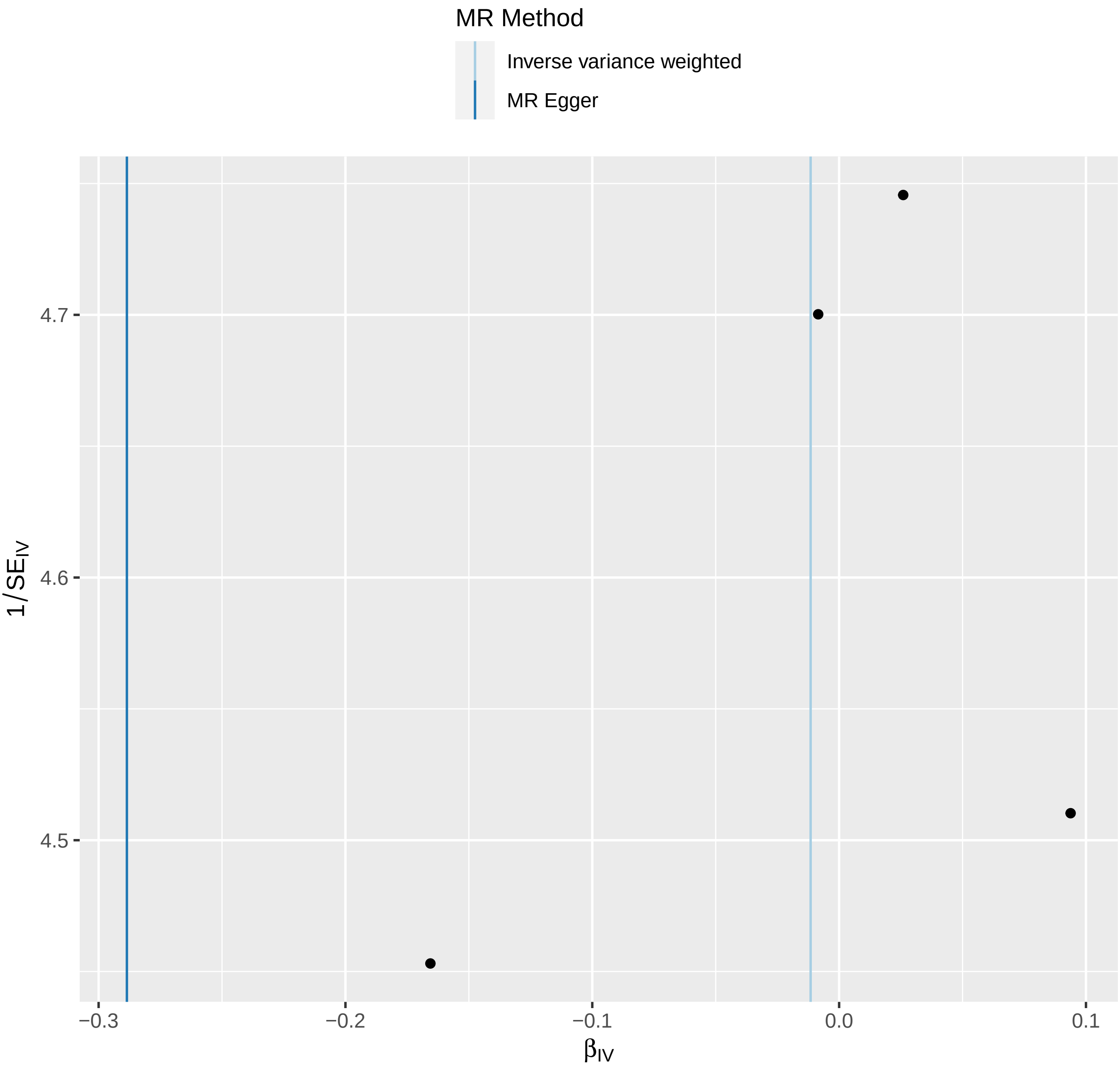

Batch 2−3120 : Gut microbiota abundance (genus Parasutterella id.2892) on Insulin−like 3
with a threshold of 1e−05

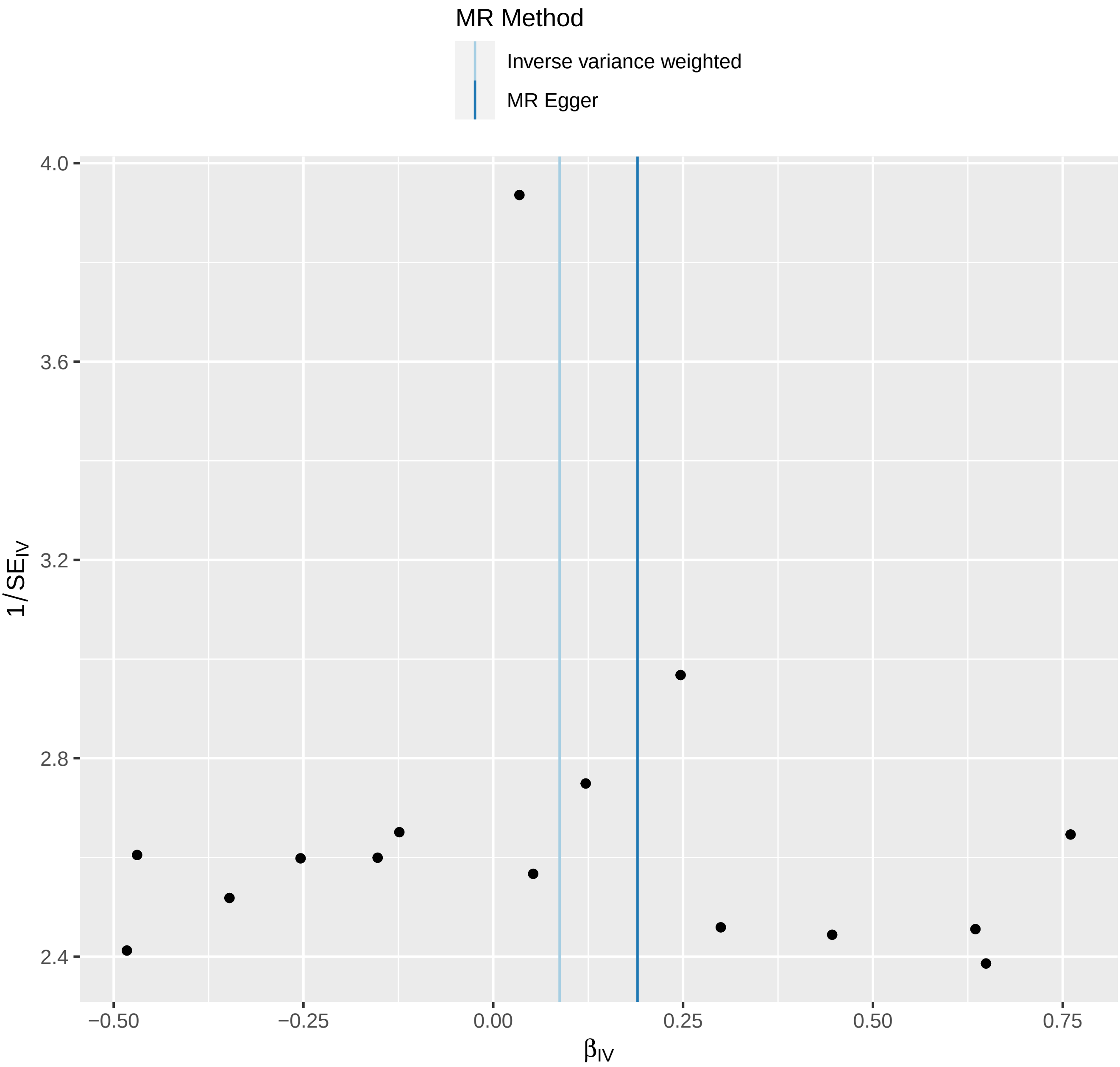

Batch 2−3121 : Gut microbiota abundance (genus Catenibacterium id.2153) on Early placenta insulin−like peptide with a threshold of 1e−05

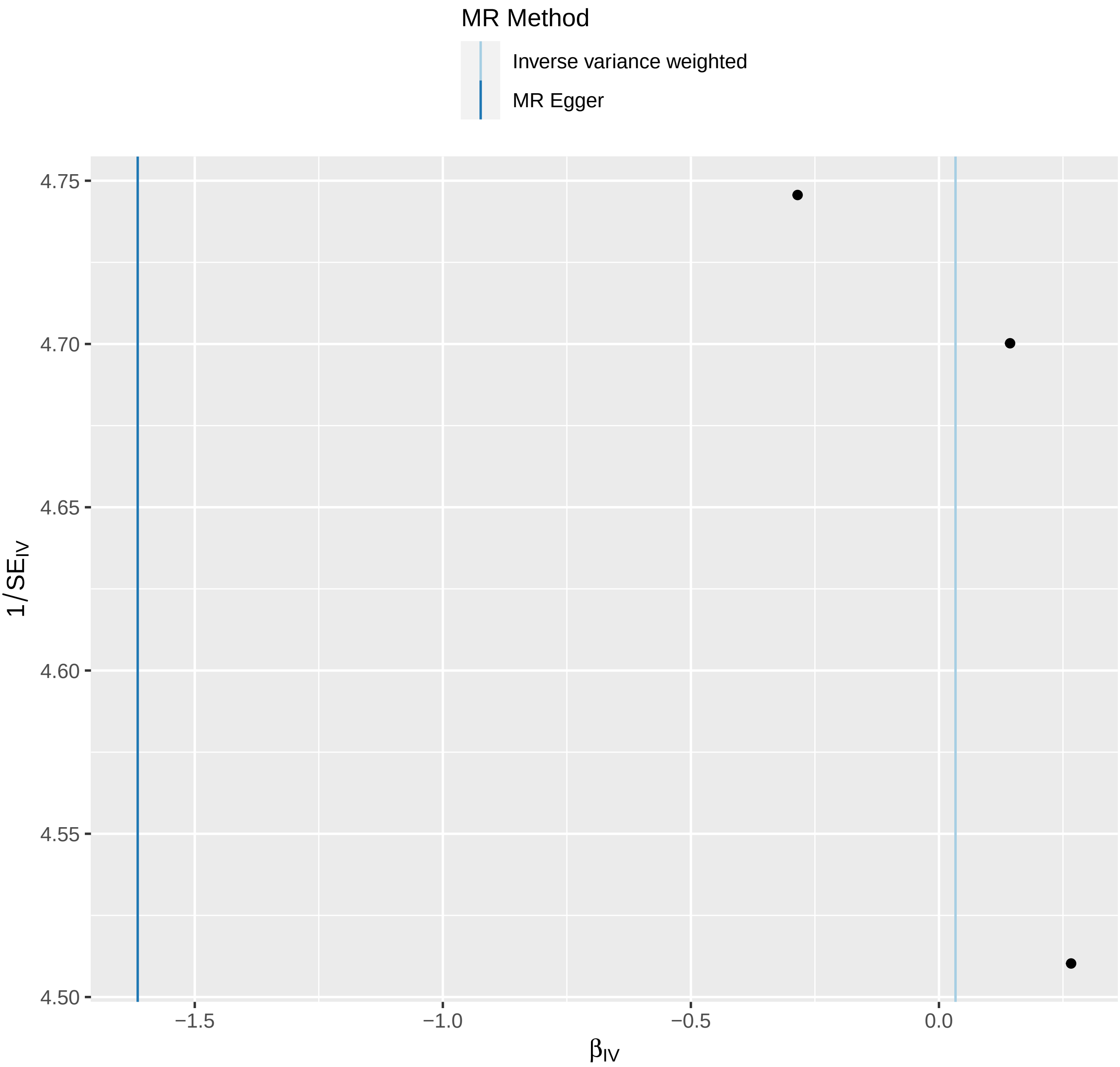

Batch 2−3122 : Gut microbiota abundance (genus Parasutterella id.2892) on Early placenta insulin−like peptide
with a threshold of 1e−05

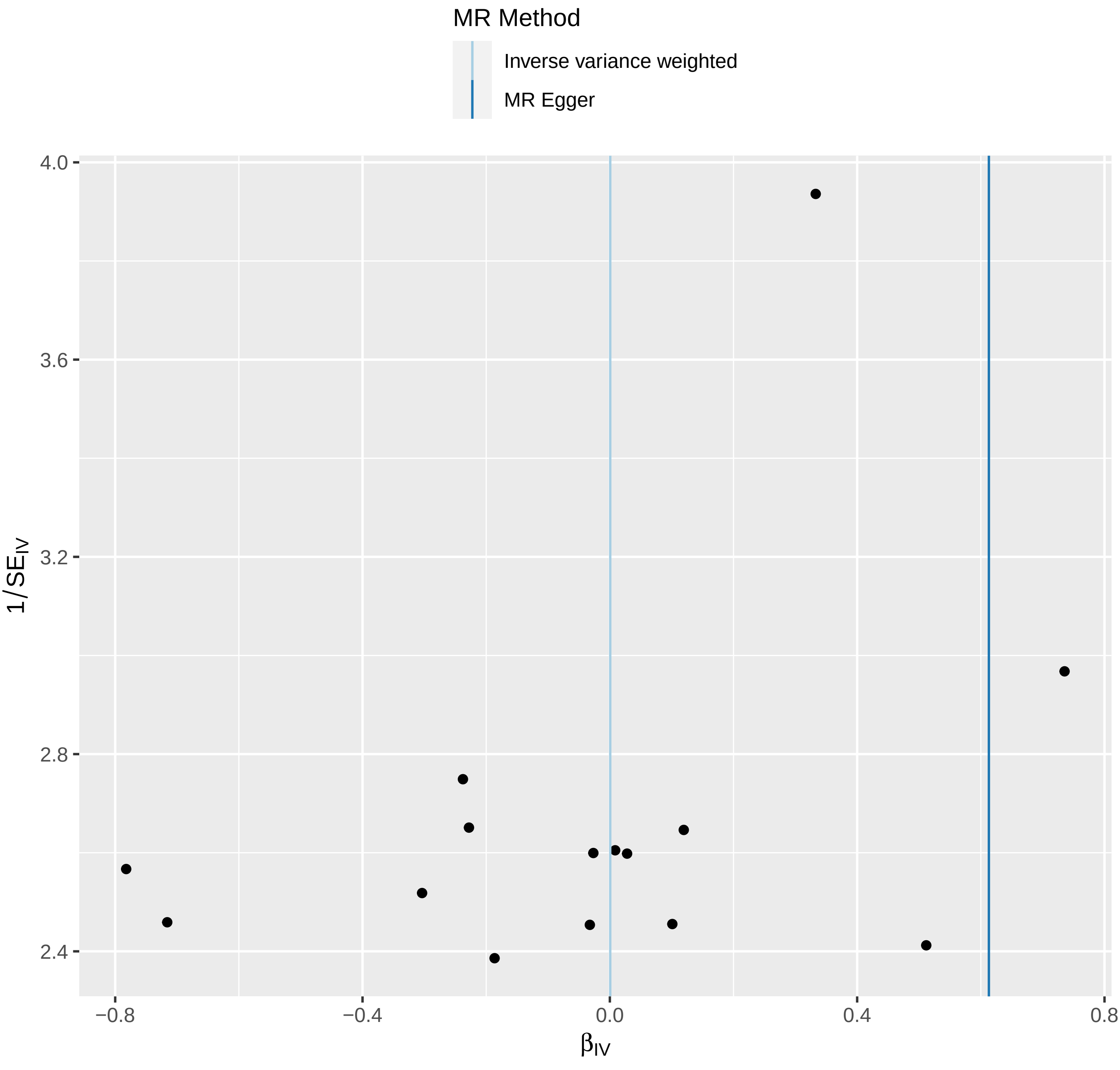

Batch 2−3123 : Gut microbiota abundance (genus Catenibacterium id.2153) on Insulin−like peptide INSL5
with a threshold of 1e−05

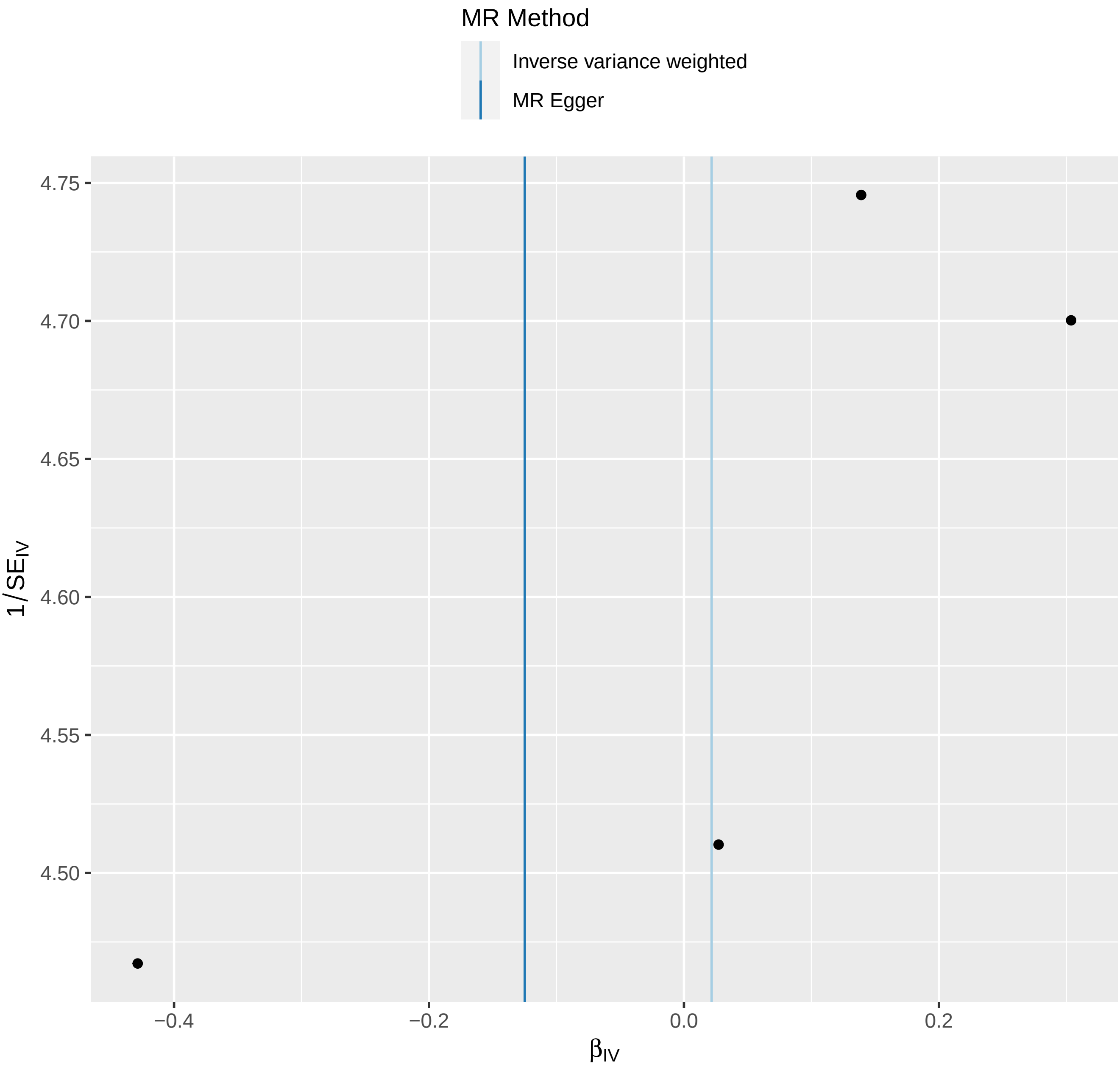

Batch 2−3124 : Gut microbiota abundance (genus Parasutterella id.2892) on Insulin−like peptide INSL5 with a threshold of 1e−05

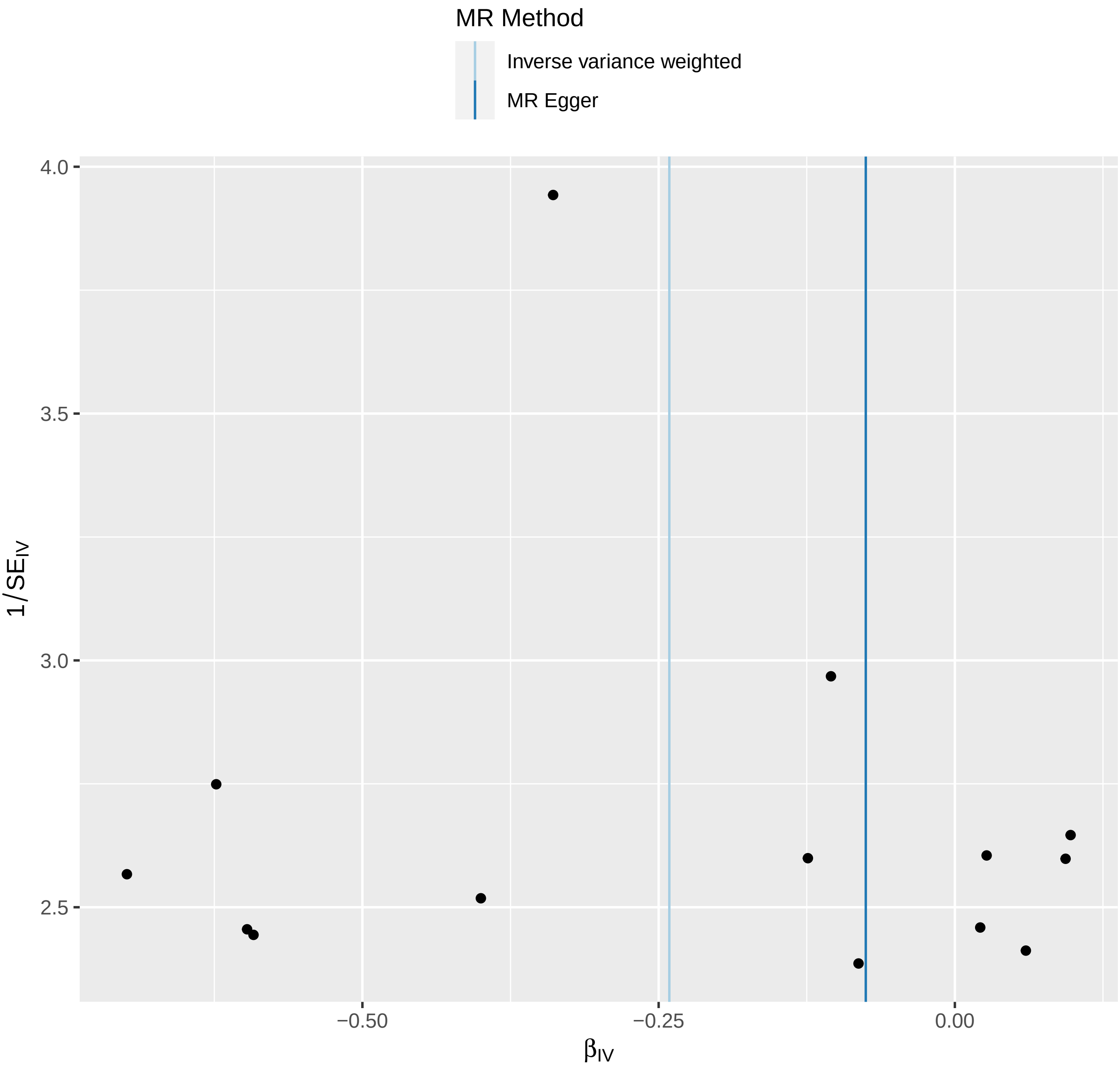

Batch 2−3125 : Gut microbiota abundance (genus Catenibacterium id.2153) on Insulin−like peptide INSL6
with a threshold of 1e−05

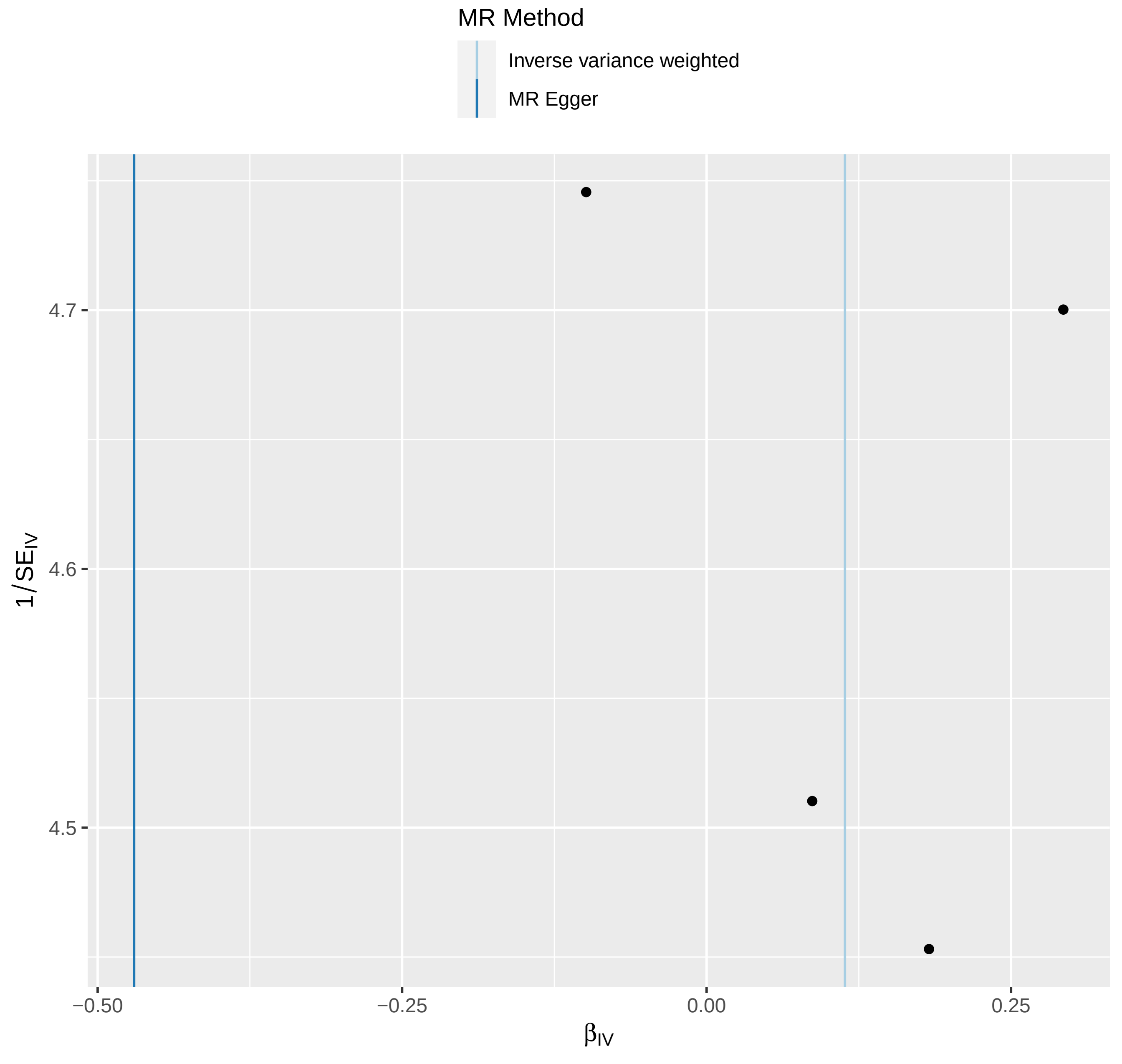

Batch 2−3126 : Gut microbiota abundance (genus Parasutterella id.2892) on Insulin−like peptide INSL6
with a threshold of 1e−05

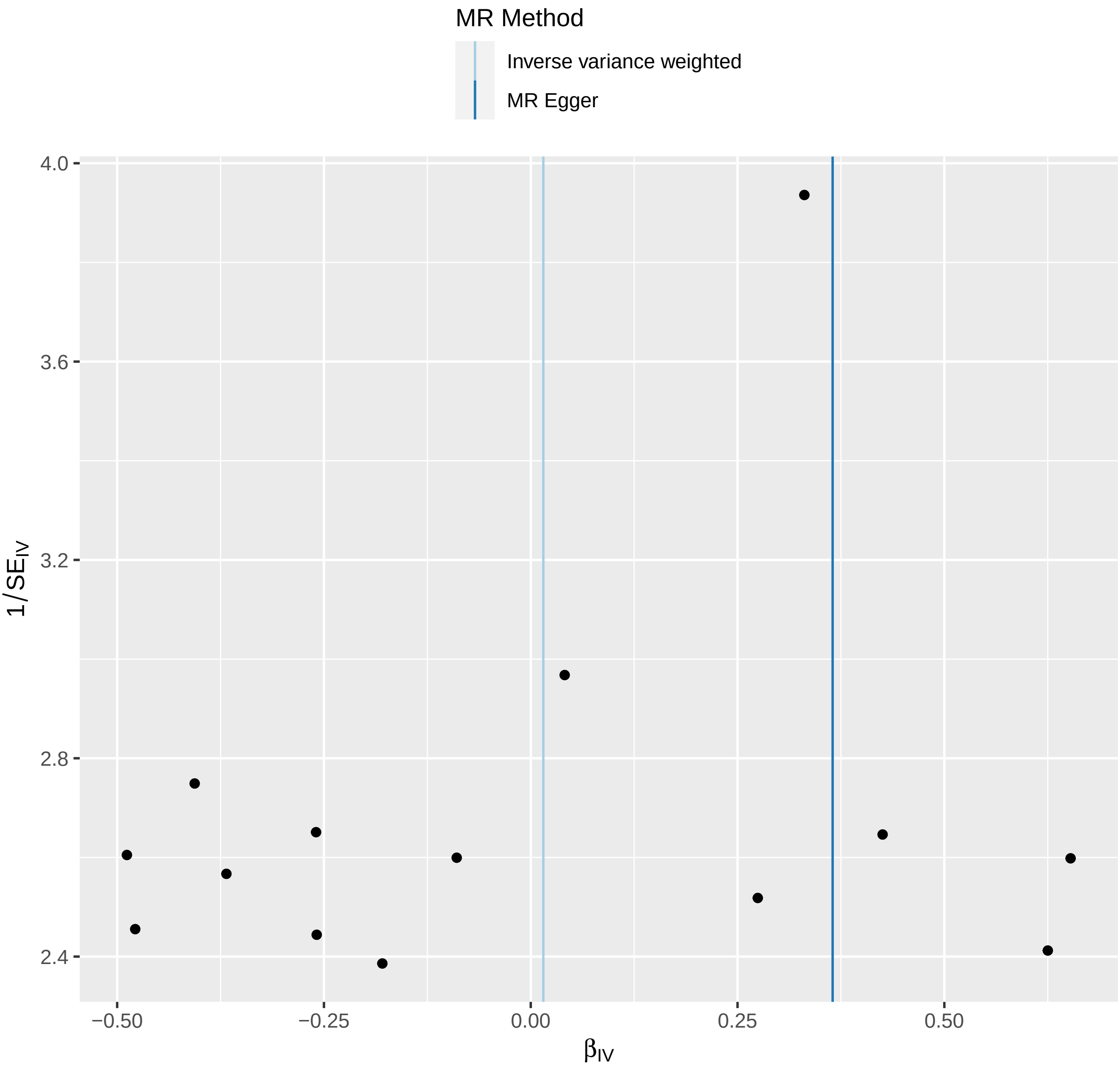

Batch 2−3127 : Gut microbiota abundance (genus Catenibacterium id.2153) on Insulin receptor
with a threshold of 1e−05

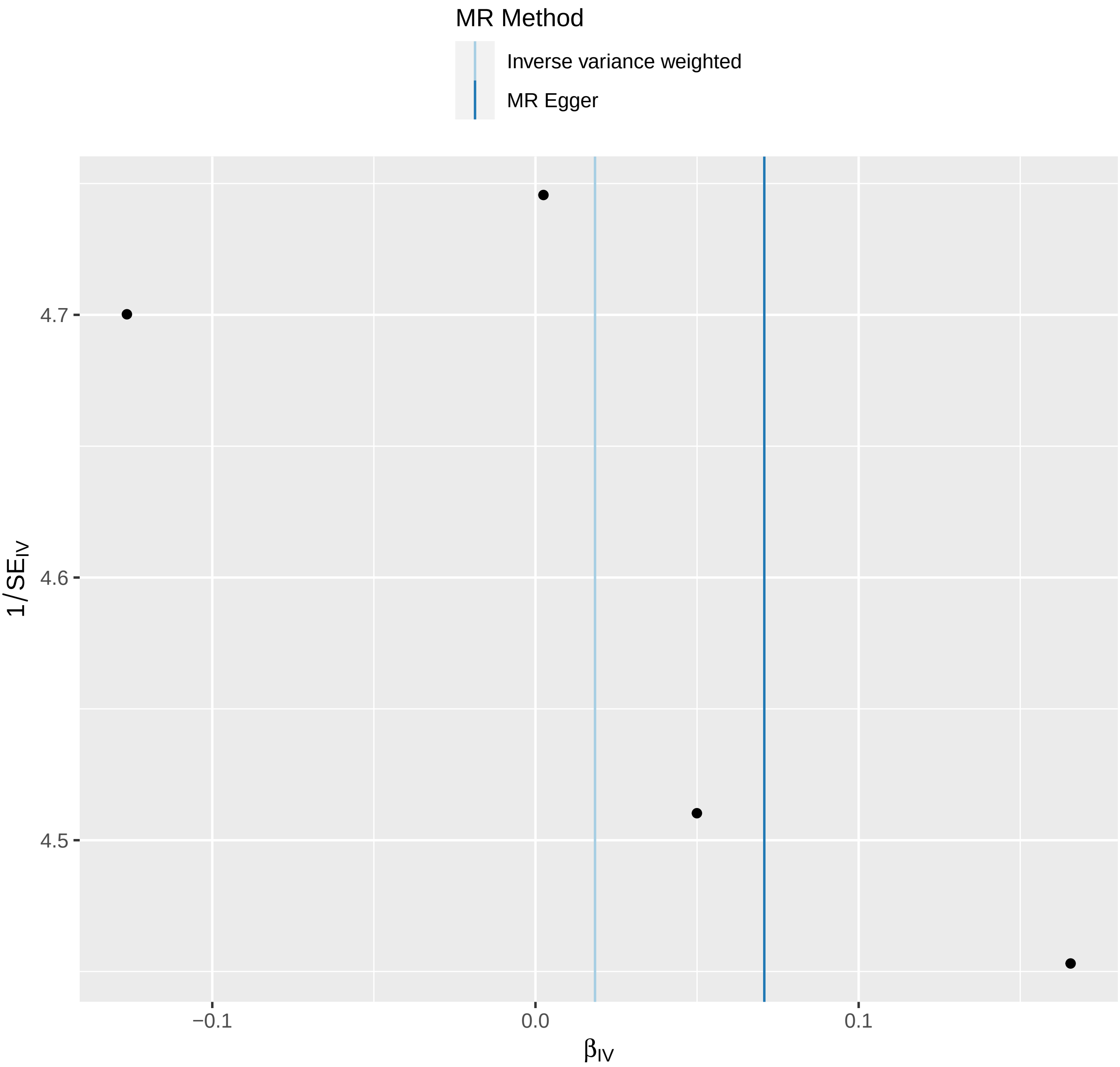

Batch 2−3128 : Gut microbiota abundance (genus Parasutterella id.2892) on Insulin receptor
with a threshold of 1e−05

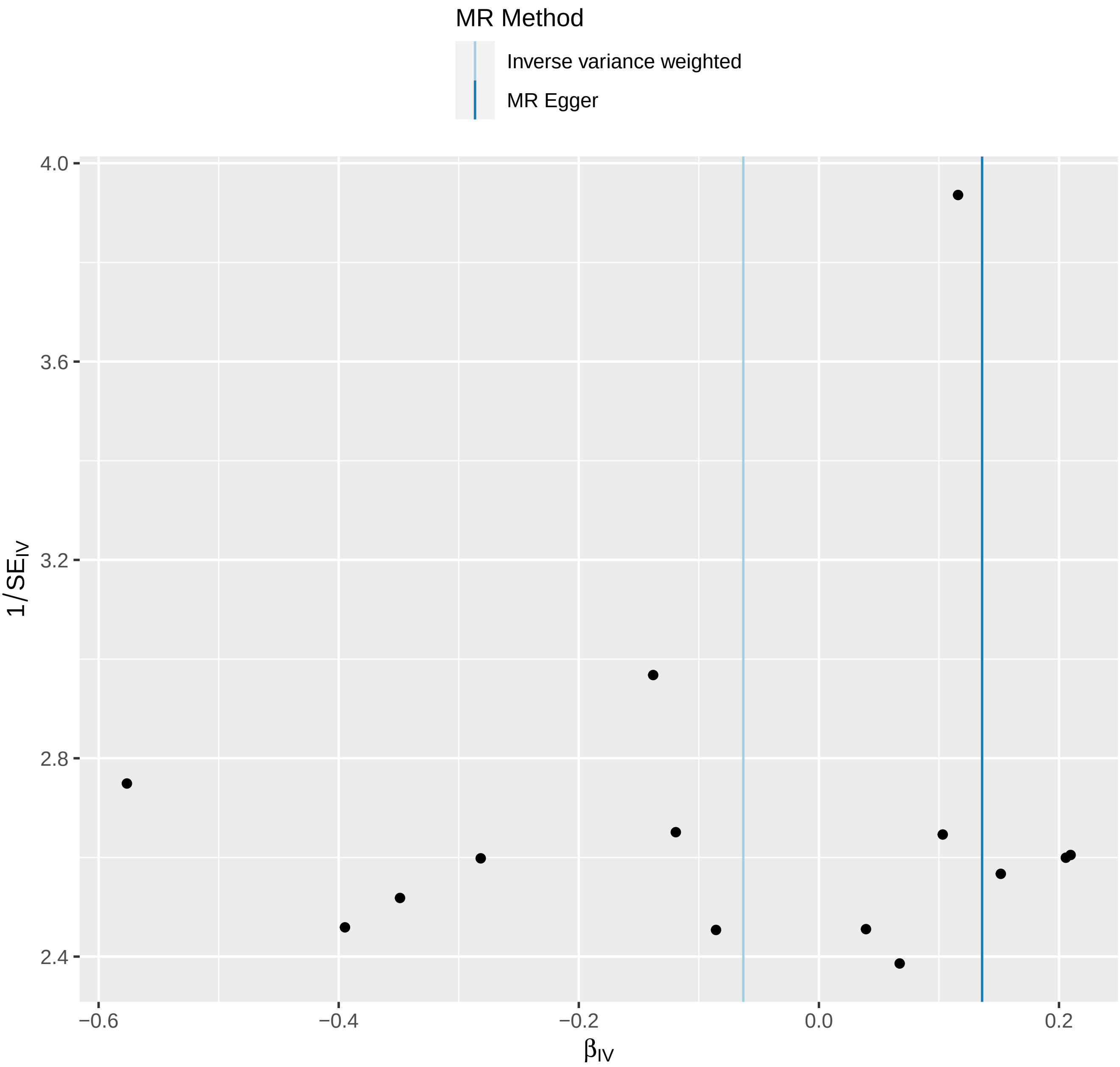

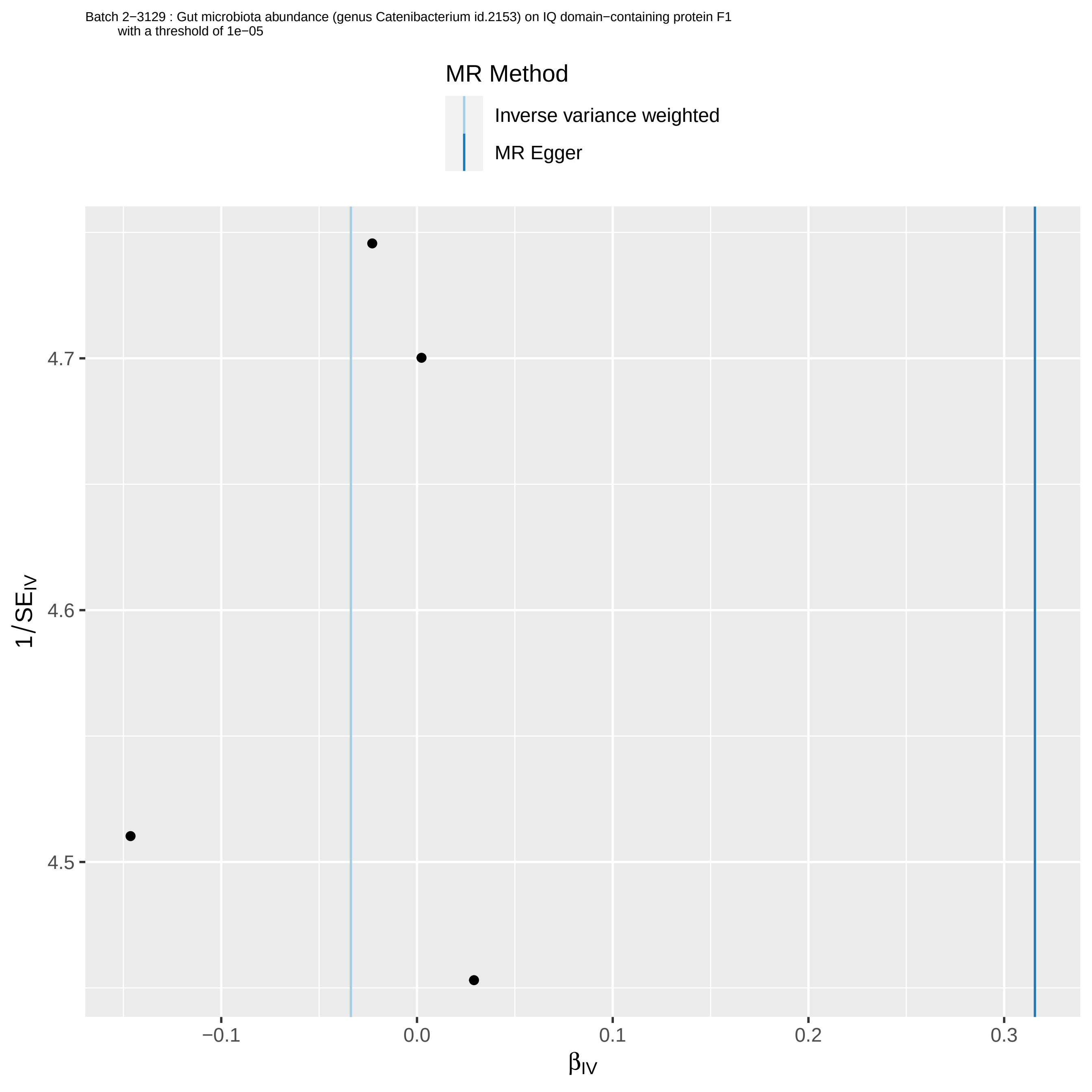
Batch 2−3129 : Gut microbiota abundance (genus Catenibacterium id.2153) on IQ domain−containing protein F1
with a threshold of 1e−05
MR Method
Inverse variance weighted
MR Egger
4.7
4.6
4.5
1/SE_IV
−0.1
0.0
0.1
0.2
0.3
β_IV

Batch 2−3130 : Gut microbiota abundance (genus Parasutterella id.2892) on IQ domain−containing protein F1
with a threshold of 1e−05

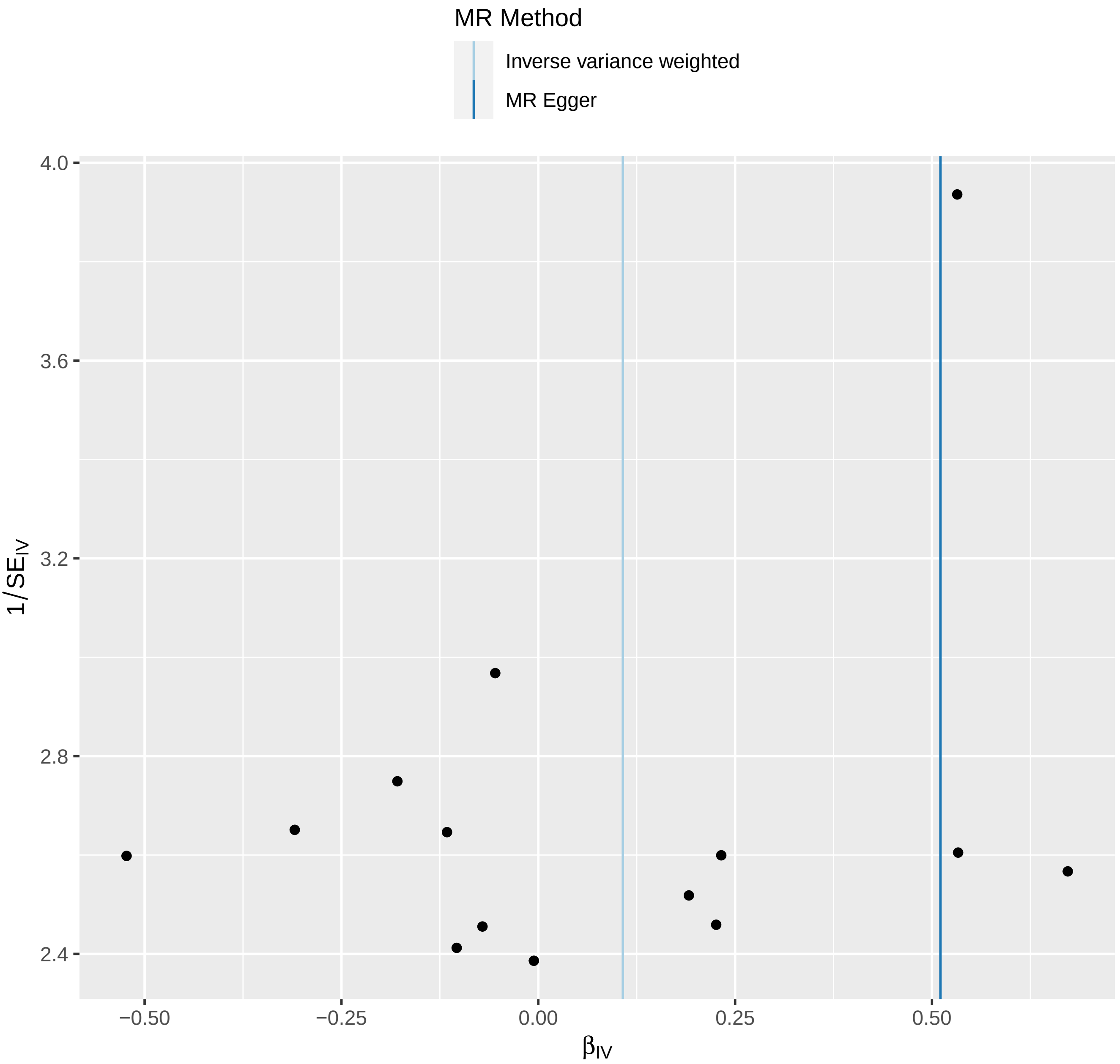

Batch 2−3131 : Gut microbiota abundance (genus Catenibacterium id.2153) on Interferon regulatory factor 1
with a threshold of 1e−05

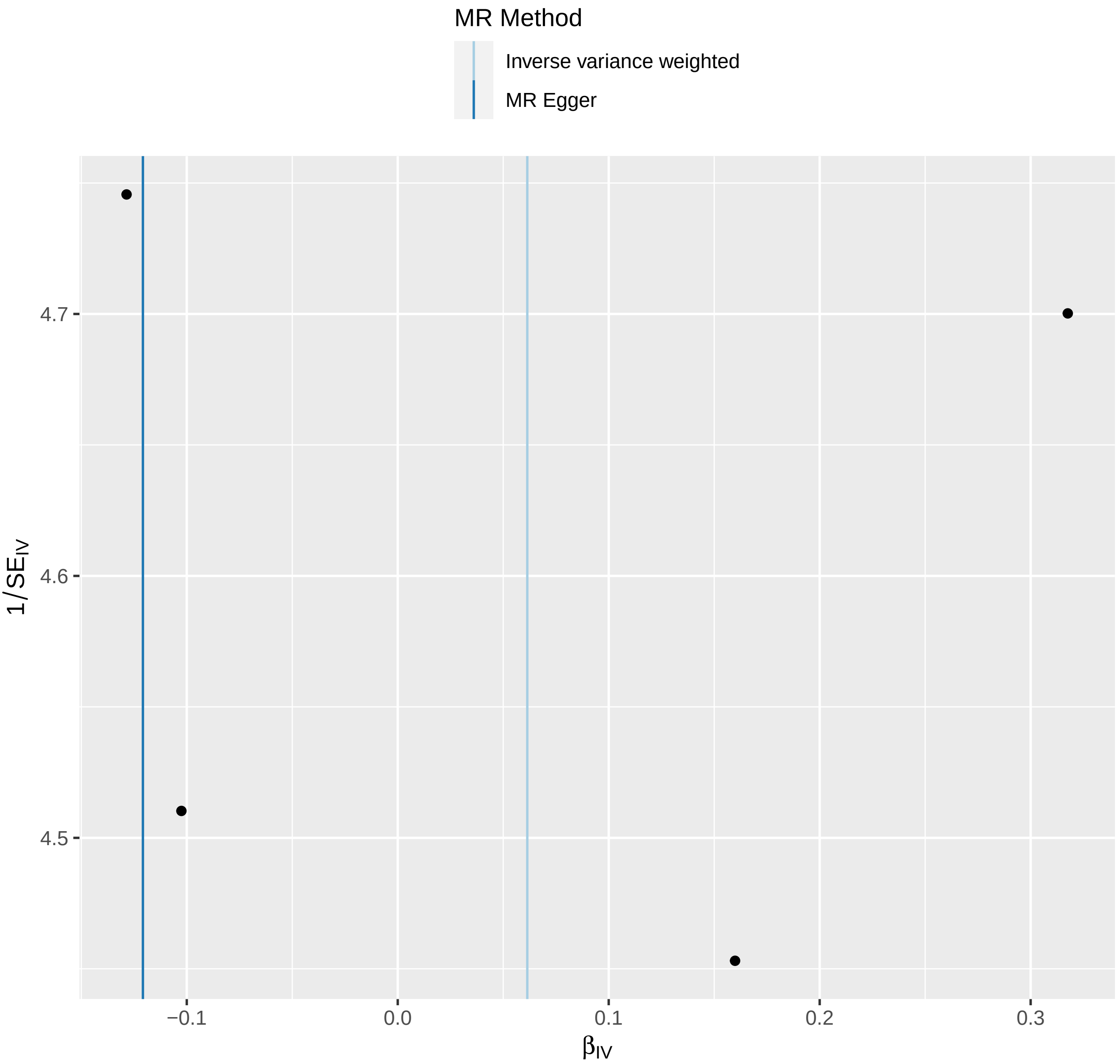

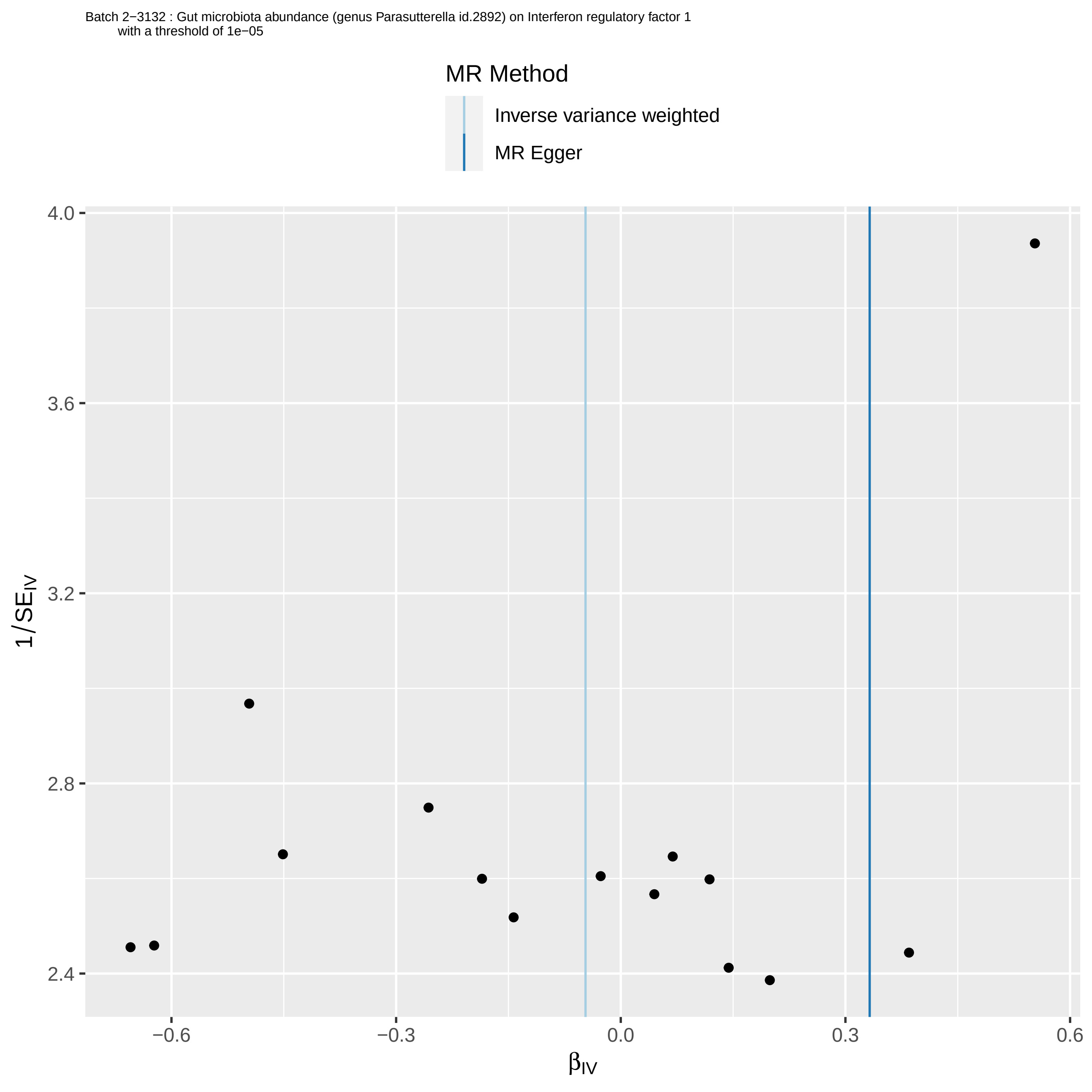

Batch 2−3132 : Gut microbiota abundance (genus Parasutterella id.2892) on Interferon regulatory factor 1
with a threshold of 1e−05
MR Method
Inverse variance weighted
MR Egger
1/SE_IV
4.0
3.6
3.2
2.8
2.4
−0.6
−0.3
0.0
0.3
0.6
β_IV

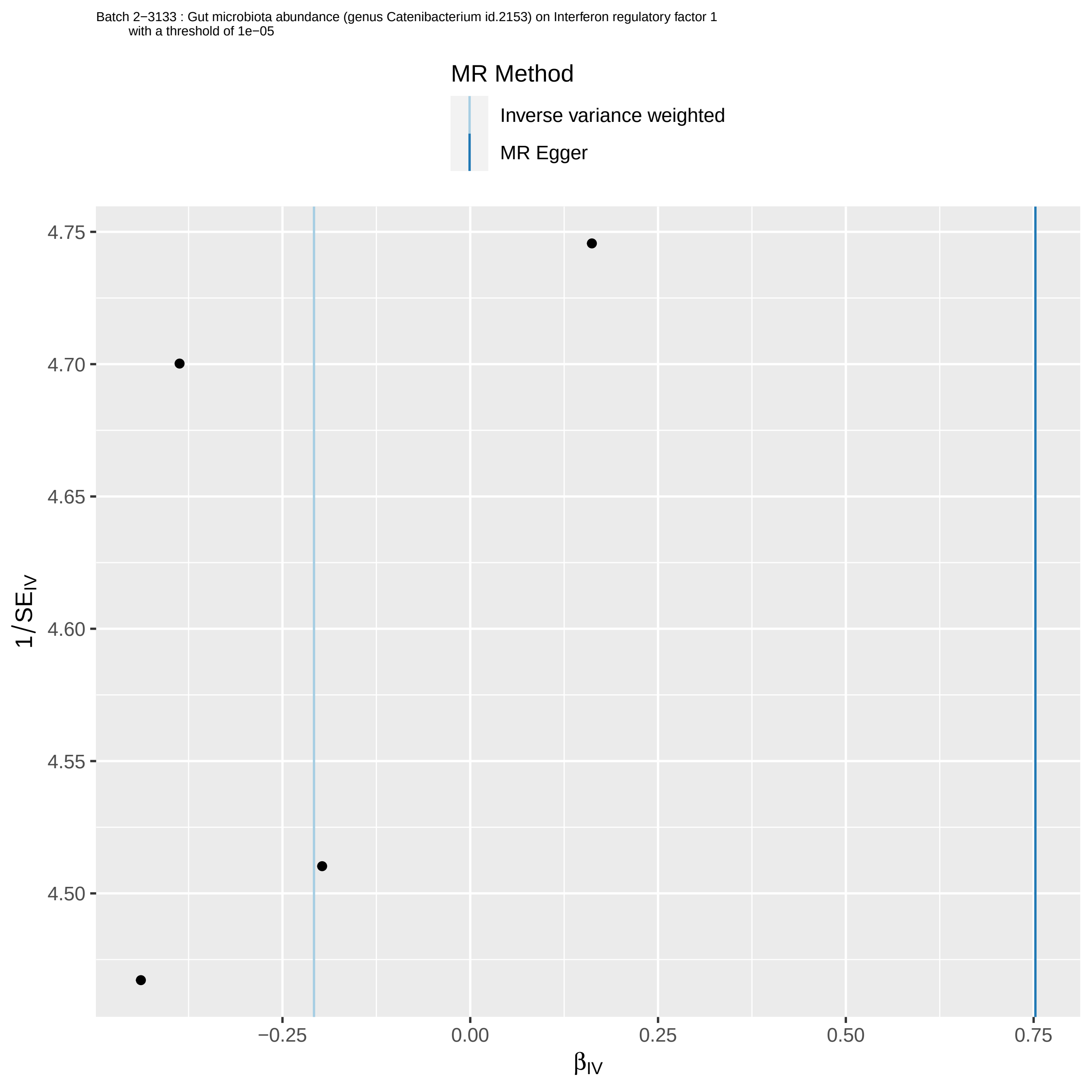
Batch 2−3133 : Gut microbiota abundance (genus Catenibacterium id.2153) on Interferon regulatory factor 1
with a threshold of 1e−05
MR Method
Inverse variance weighted
MR Egger
4.75
4.70
4.65
4.60
4.55
4.50
−0.25
0.00
0.25
0.50
0.75
1/SE_IV
β_IV

Batch 2−3134 : Gut microbiota abundance (genus Parasutterella id.2892) on Interferon regulatory factor 1
with a threshold of 1e−05

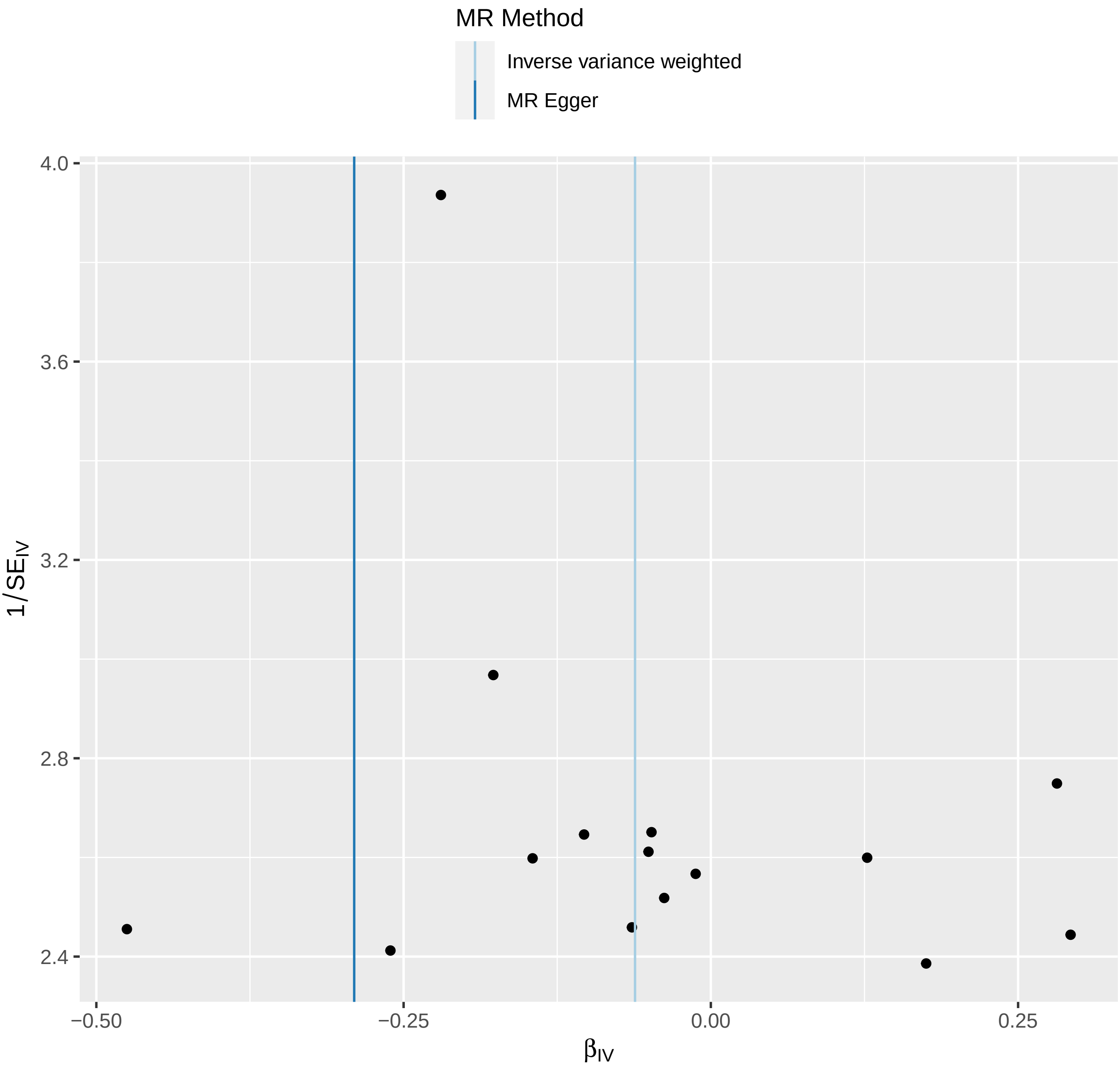

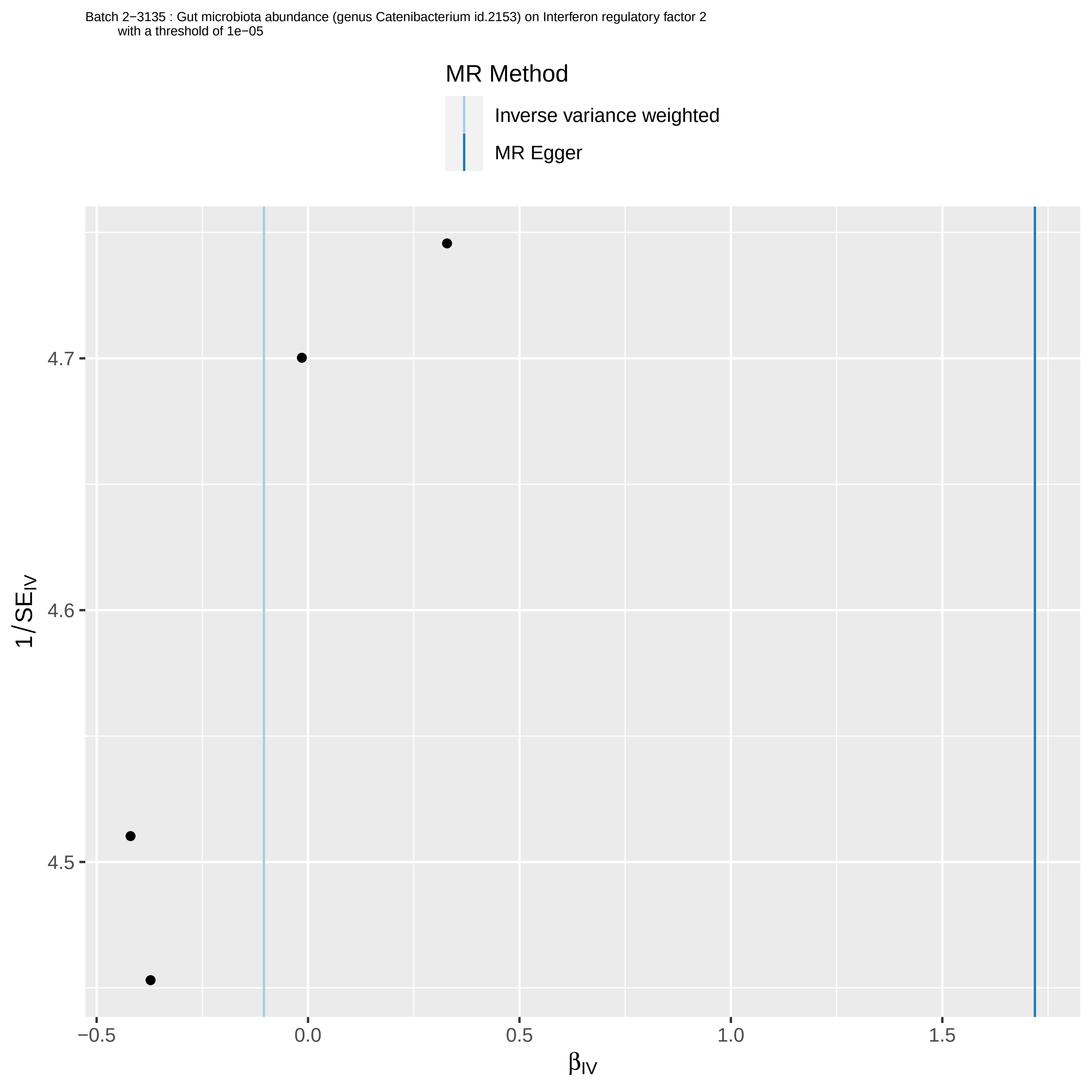

Batch 2−3135 : Gut microbiota abundance (genus Catenibacterium id.2153) on Interferon regulatory factor 2
with a threshold of 1e−05
MR Method
Inverse variance weighted
MR Egger
4.7
4.6
4.5
1/SE_IV
−0.5
0.0
0.5
1.0
1.5
β_IV

Batch 2−3136 : Gut microbiota abundance (genus Parasutterella id.2892) on Interferon regulatory factor 2
with a threshold of 1e−05

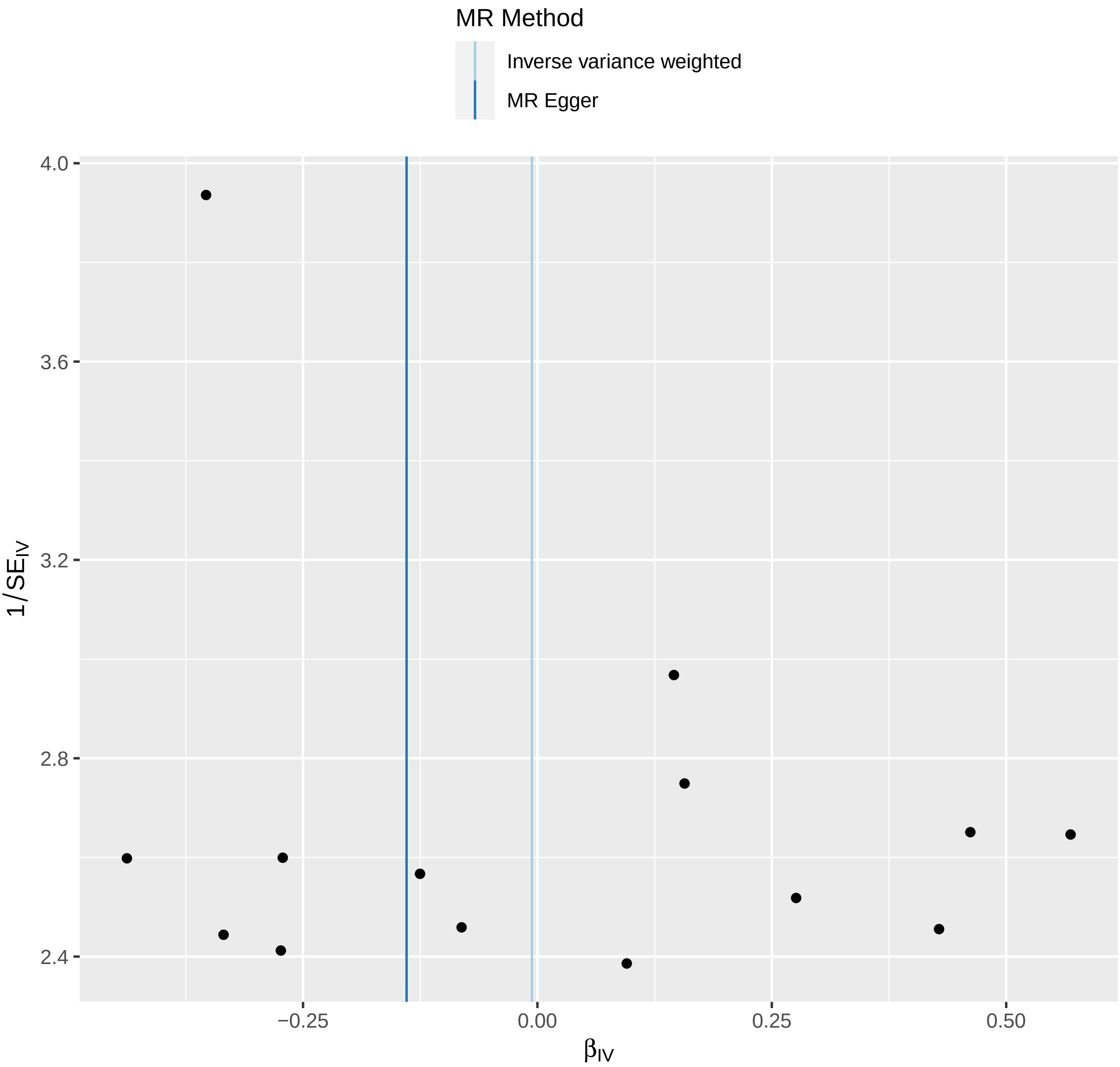

Batch 2−3137 : Gut microbiota abundance (genus Catenibacterium id.2153) on Interferon regulatory factor 4
with a threshold of 1e−05

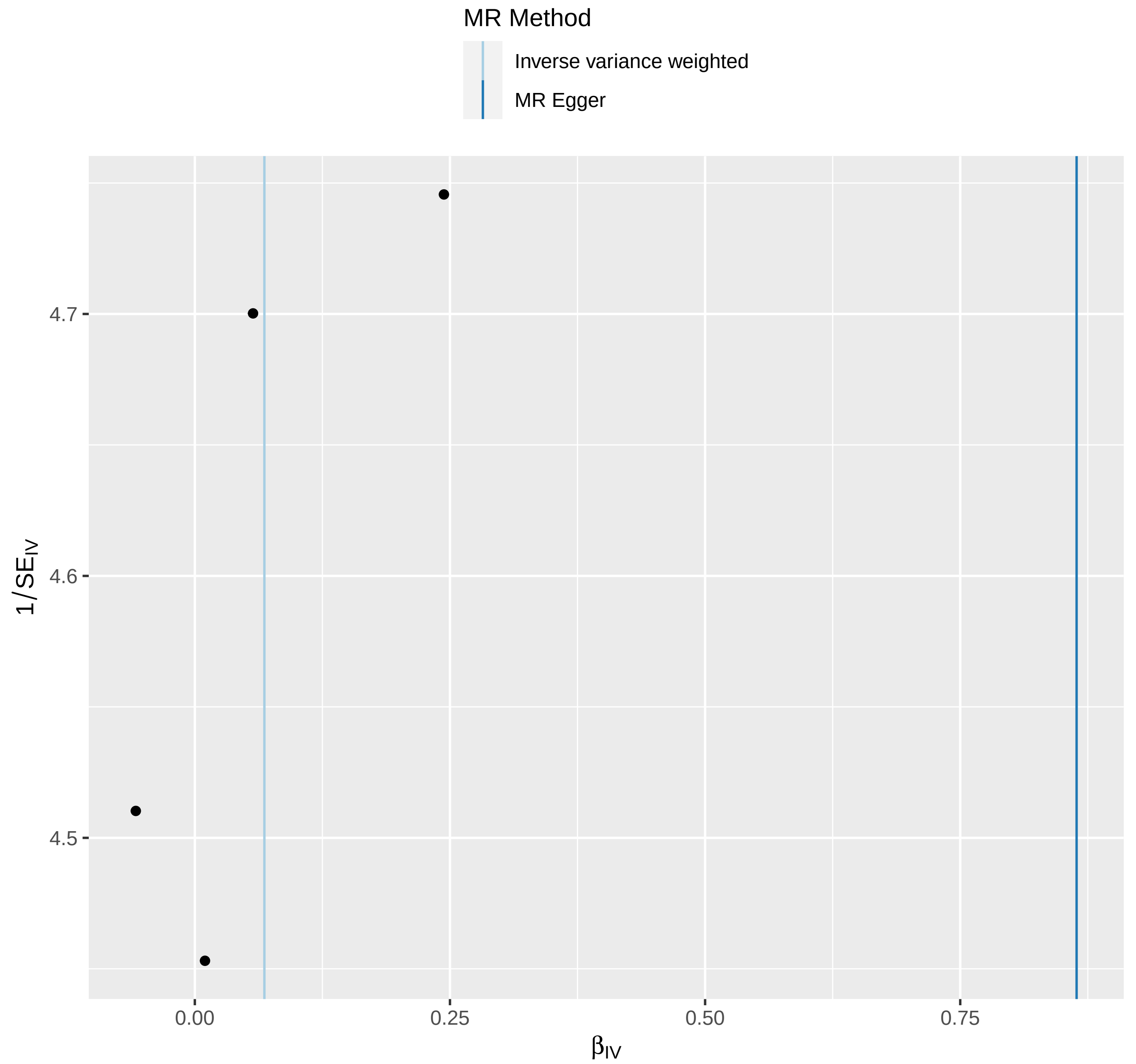

Batch 2−3138 : Gut microbiota abundance (genus Parasutterella id.2892) on Interferon regulatory factor 4
with a threshold of 1e−05

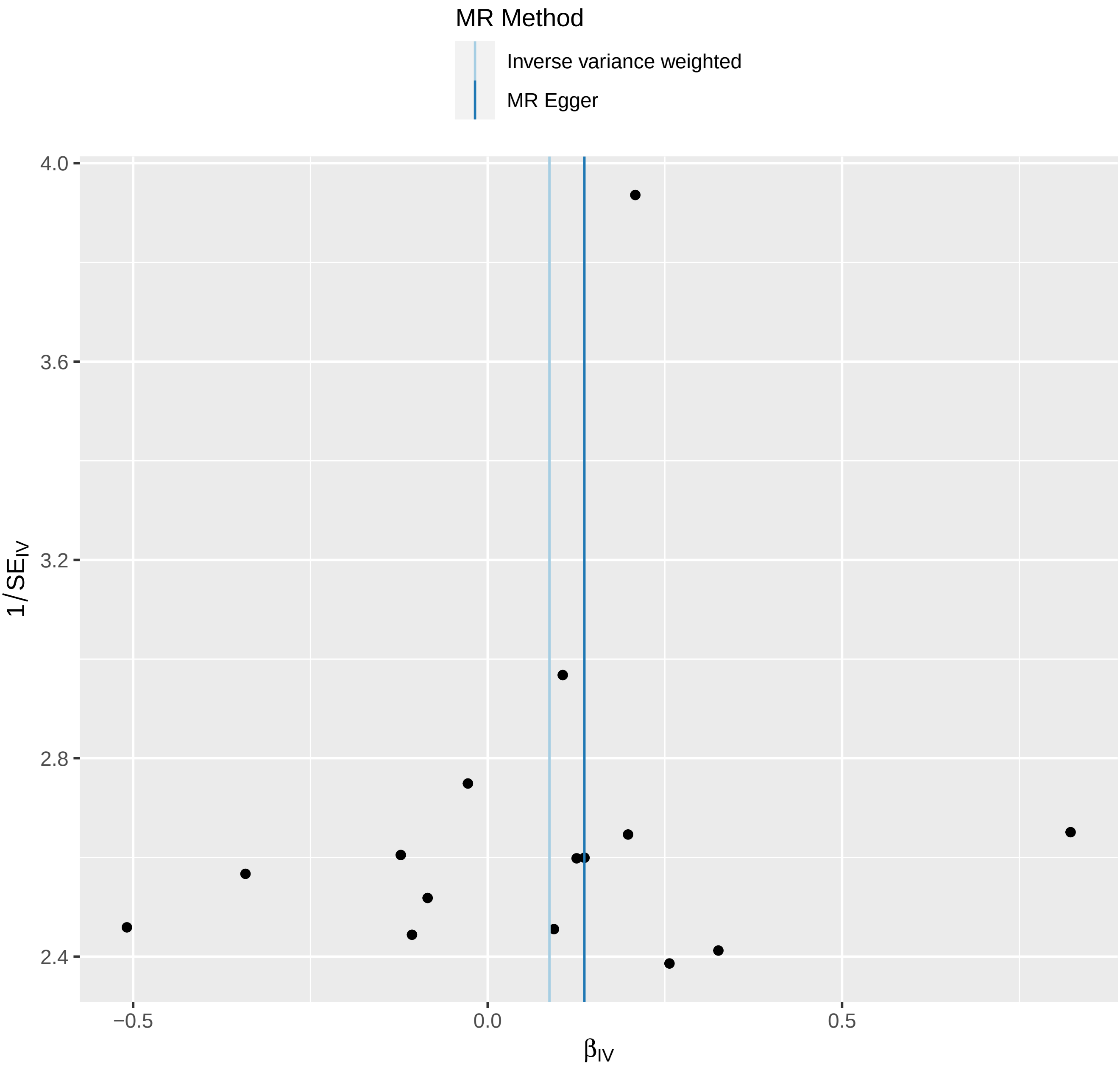

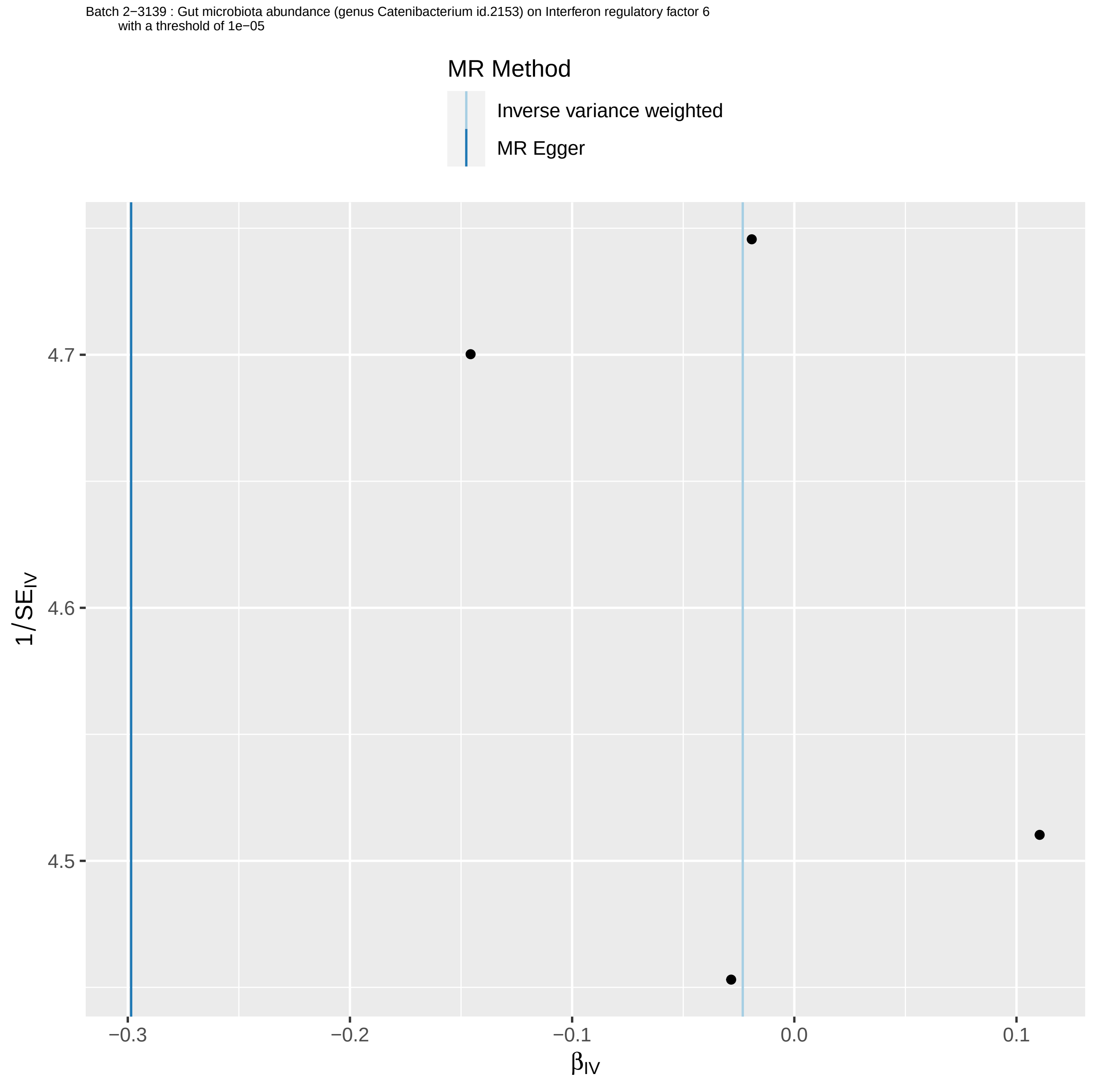

Batch 2−3139 : Gut microbiota abundance (genus Catenibacterium id.2153) on Interferon regulatory factor 6
with a threshold of 1e−05
MR Method
Inverse variance weighted
MR Egger
4.7
4.6
4.5
1/SE_IV
−0.3
−0.2
−0.1
0.0
0.1
β_IV

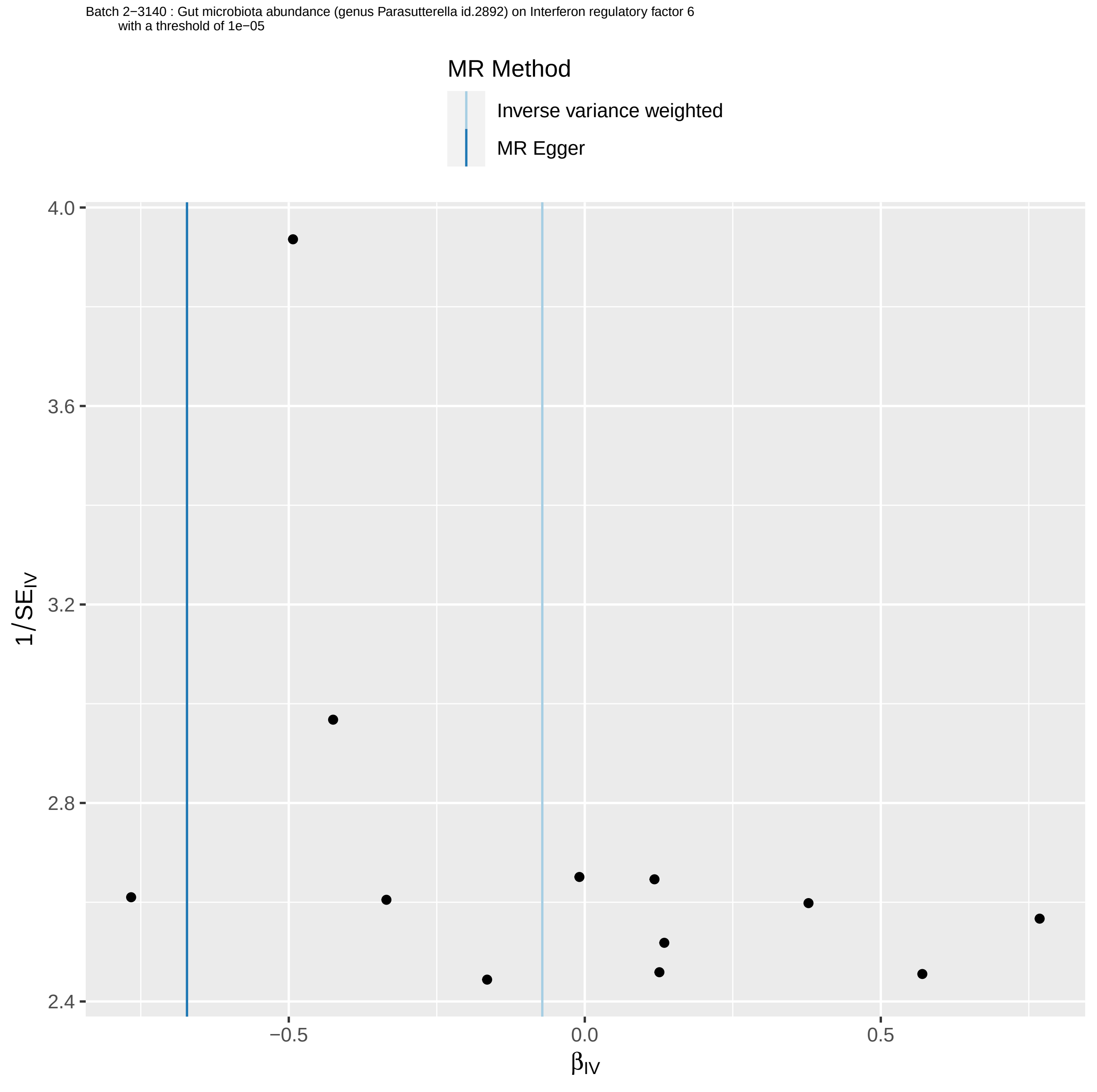
Batch 2−3140 : Gut microbiota abundance (genus Parasutterella id.2892) on Interferon regulatory factor 6
with a threshold of 1e−05
MR Method
Inverse variance weighted
MR Egger
4.0
3.6
3.2
2.8
2.4
1/SE_IV
−0.5
0.0
0.5
β_IV

Batch 2−3141 : Gut microbiota abundance (genus Catenibacterium id.2153) on Interferon regulatory factor 9
with a threshold of 1e−05

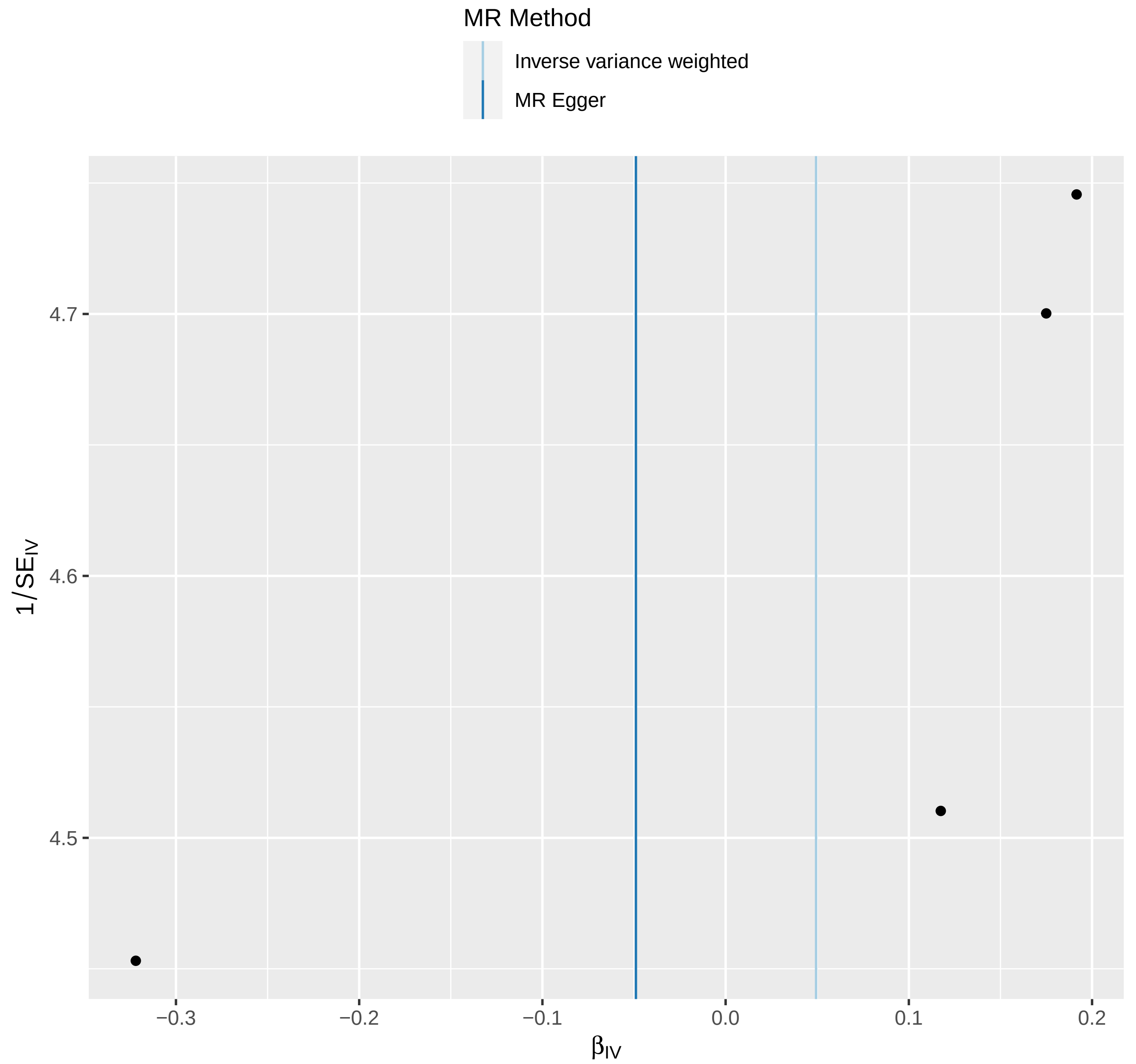

Batch 2−3142 : Gut microbiota abundance (genus Parasutterella id.2892) on Interferon regulatory factor 9
with a threshold of 1e−05

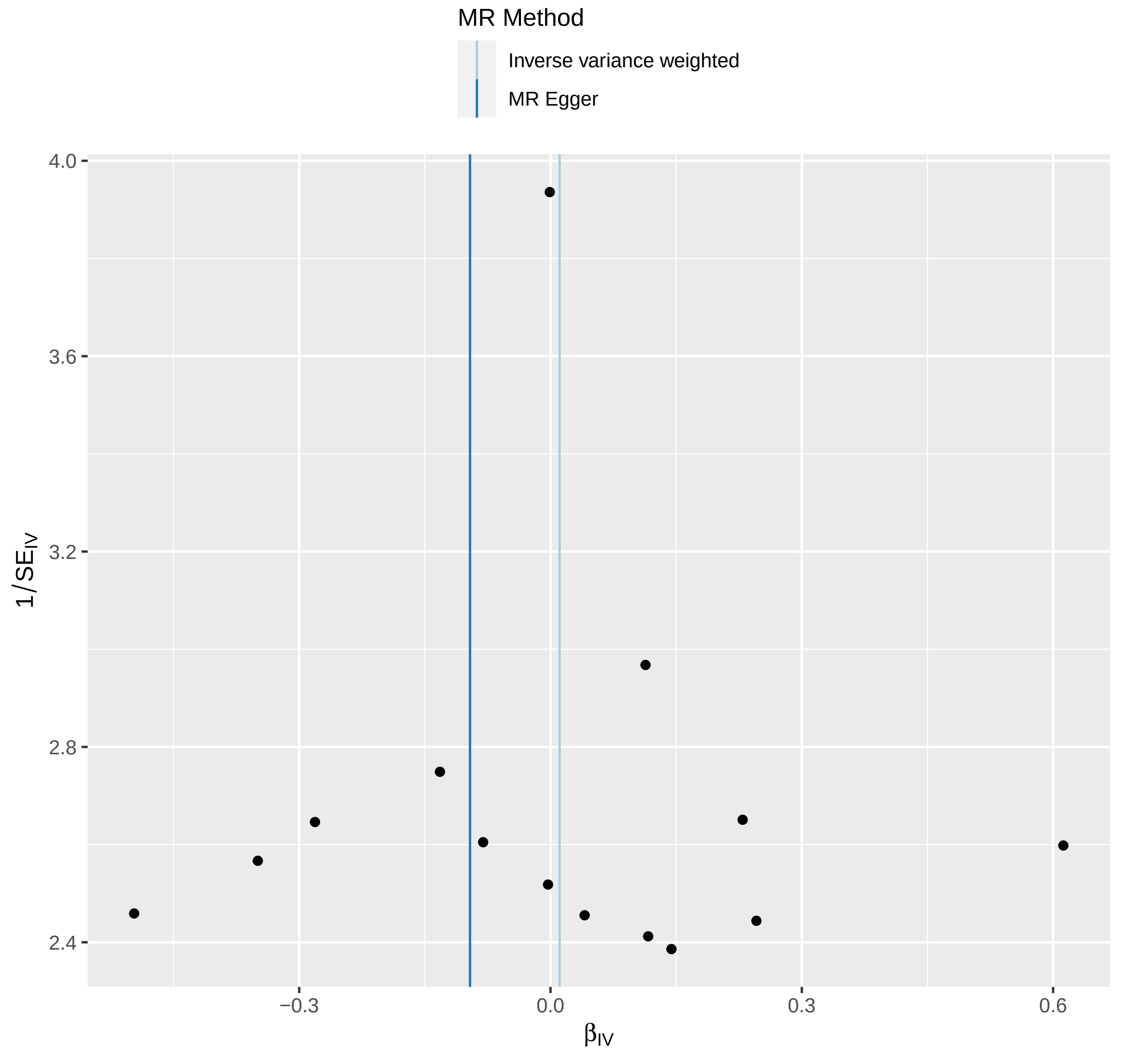

Batch 2−3143 : Gut microbiota abundance (genus Catenibacterium id.2153) on Iron−sulfur cluster assembly enzyme ISCU, mitochondrial with a threshold of 1e−05

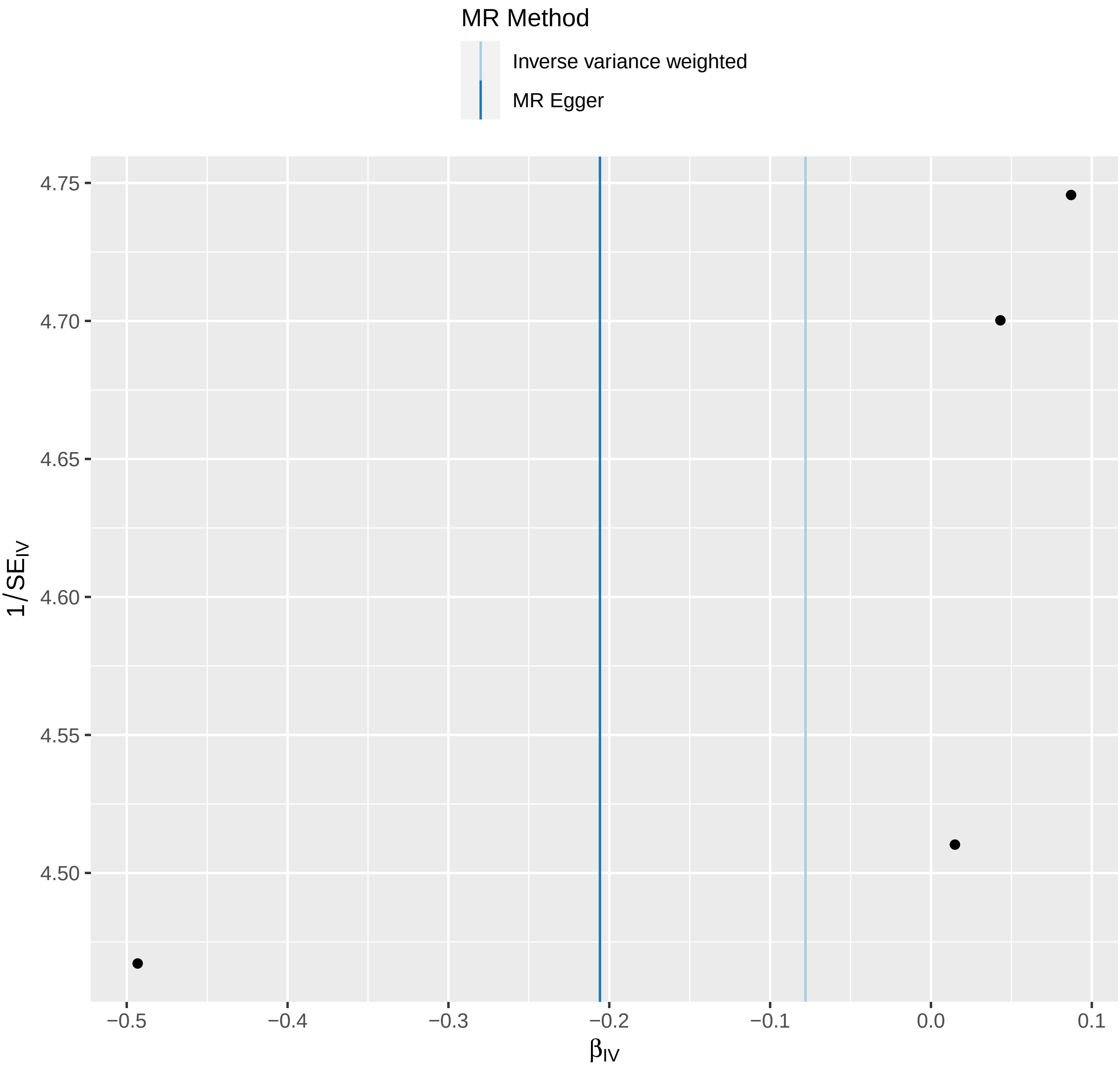

Batch 2−3144 : Gut microbiota abundance (genus Parasutterella id.2892) on Iron−sulfur cluster assembly enzyme ISCU, mitochondrial
with a threshold of 1e−05

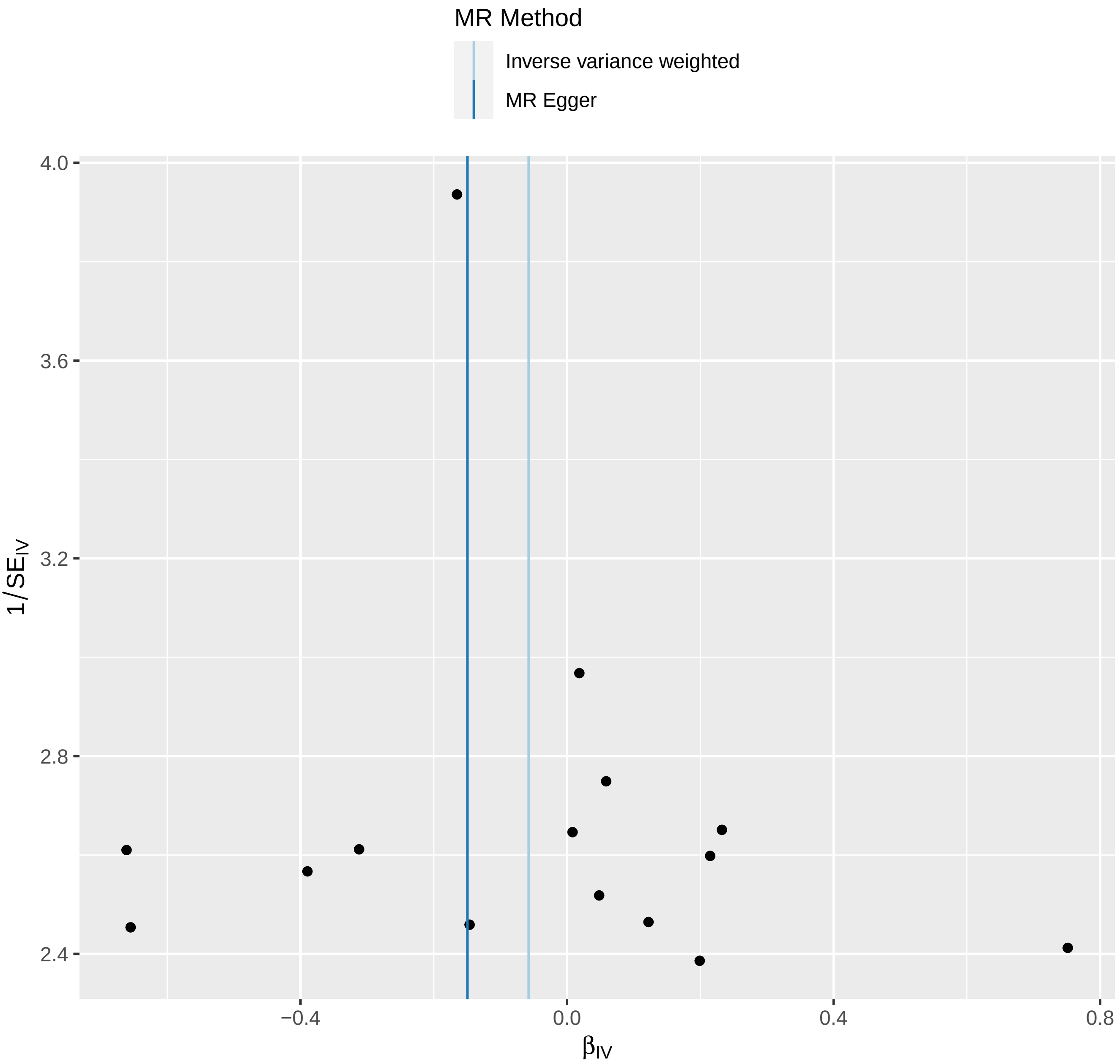

Batch 2−3145 : Gut microbiota abundance (genus Catenibacterium id.2153) on Ubiquitin−like protein ISG15
with a threshold of 1e−05

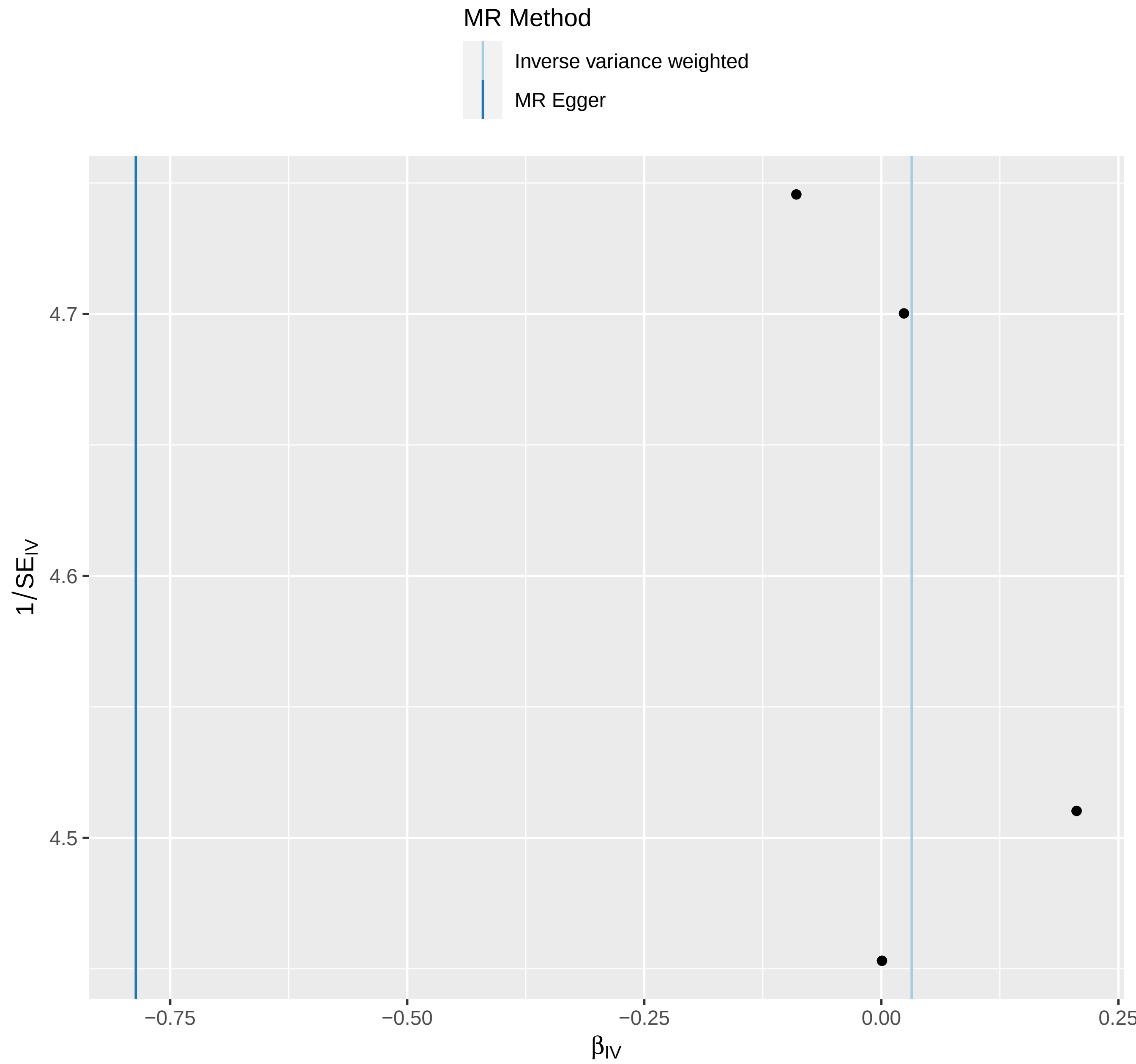

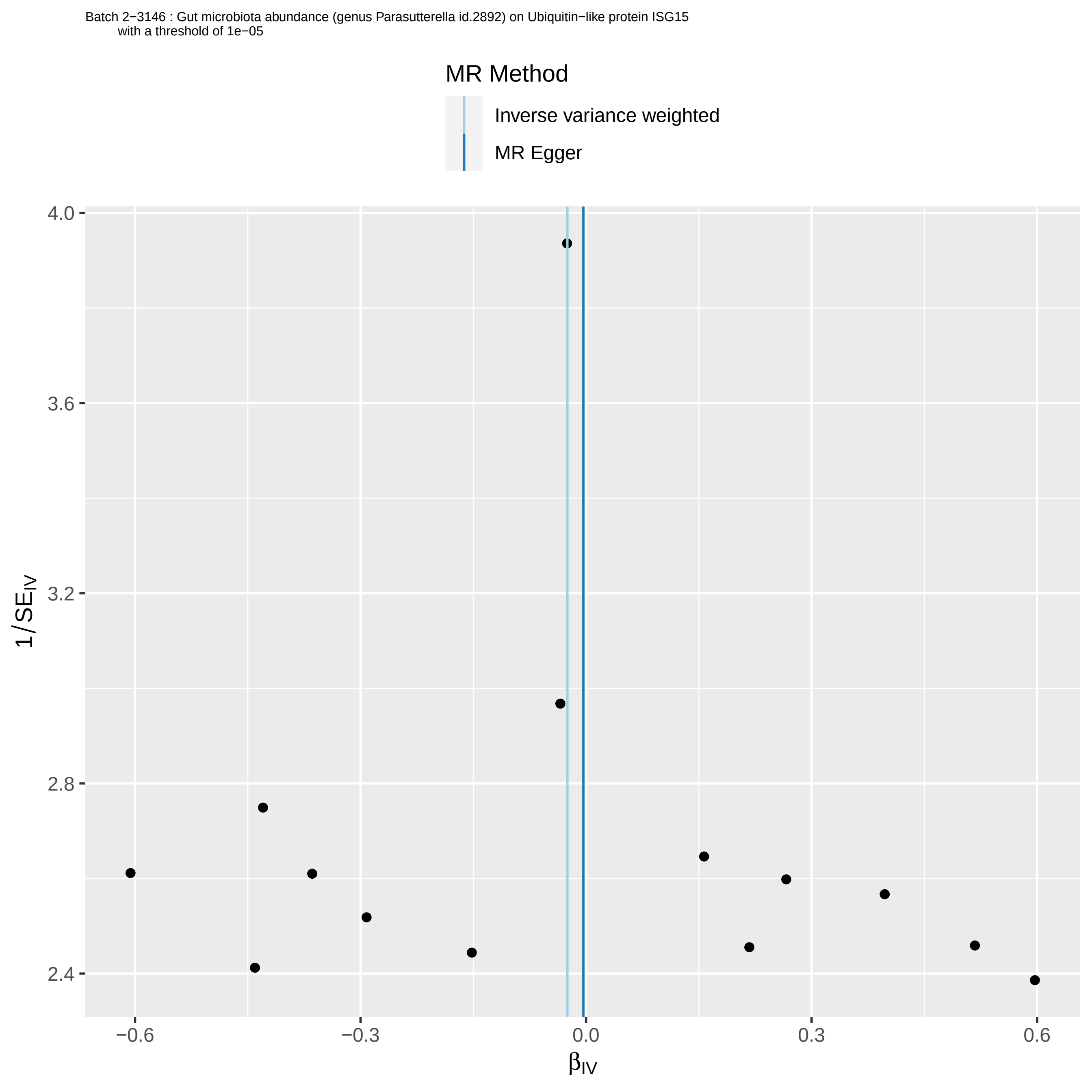
Batch 2−3146 : Gut microbiota abundance (genus Parasutterella id.2892) on Ubiquitin−like protein ISG15
with a threshold of 1e−05
MR Method
Inverse variance weighted
MR Egger
4.0
3.6
3.2
2.8
2.4
1/SE_IV
−0.6
−0.3
0.0
0.3
0.6
β_IV

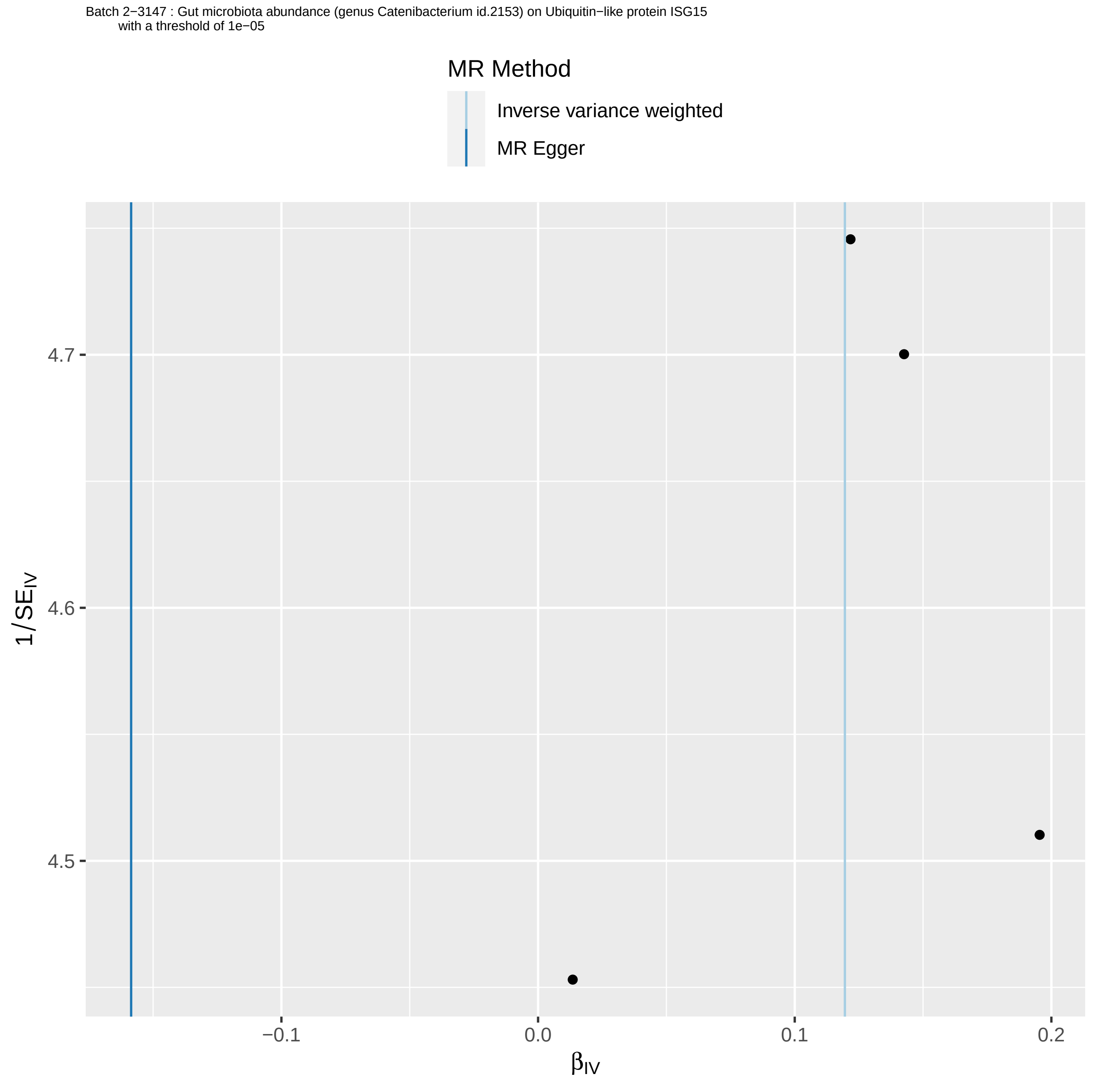
Batch 2−3147 : Gut microbiota abundance (genus Catenibacterium id.2153) on Ubiquitin−like protein ISG15
with a threshold of 1e−05
MR Method
Inverse variance weighted
MR Egger
4.7
4.6
4.5
1/SE_IV
−0.1
0.0
0.1
0.2
β_IV

Batch 2−3148 : Gut microbiota abundance (genus Parasutterella id.2892) on Ubiquitin−like protein ISG15
with a threshold of 1e−05

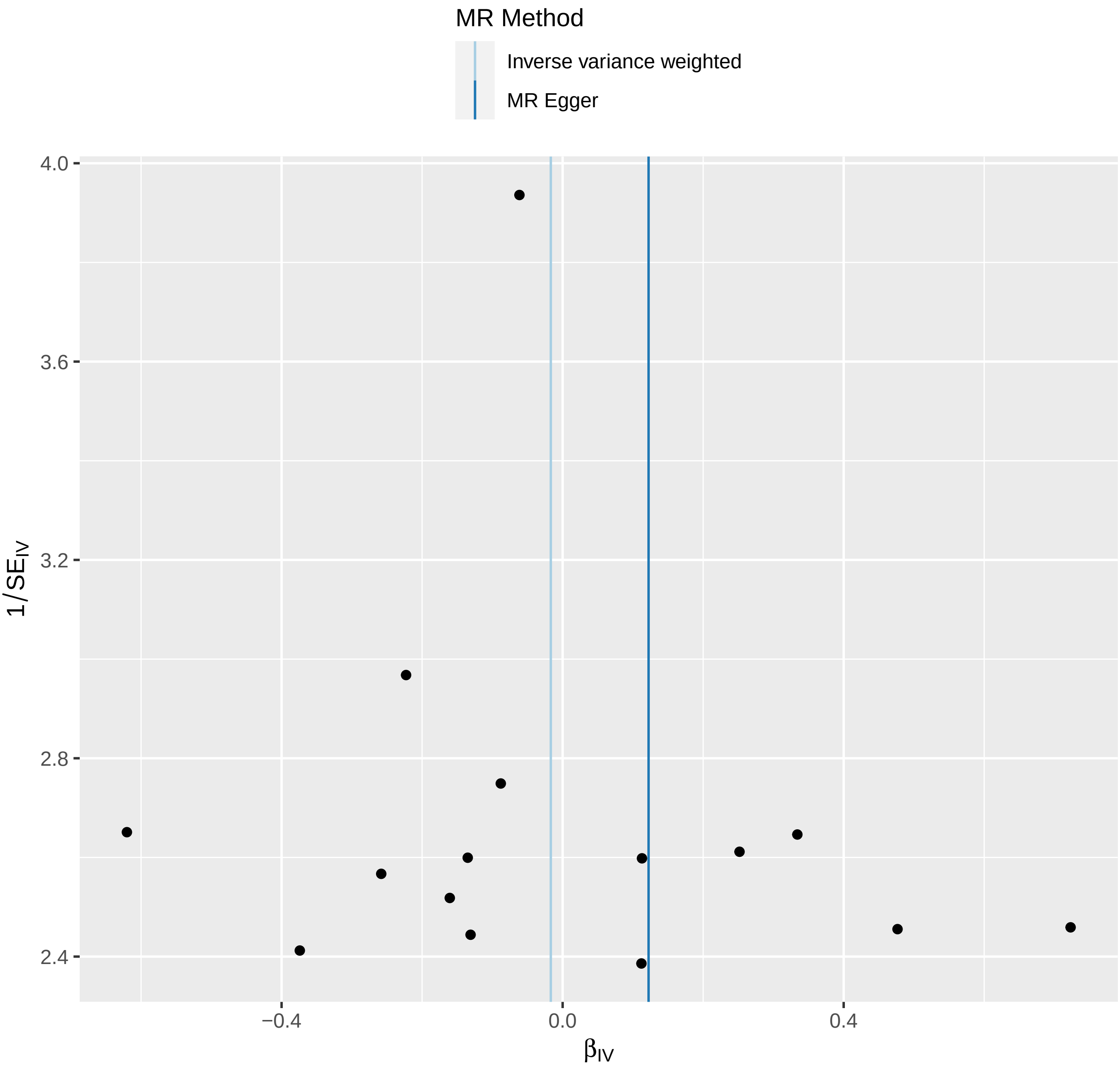

Batch 2−3149 : Gut microbiota abundance (genus Catenibacterium id.2153) on Insulin gene enhancer protein ISL−1
with a threshold of 1e−05

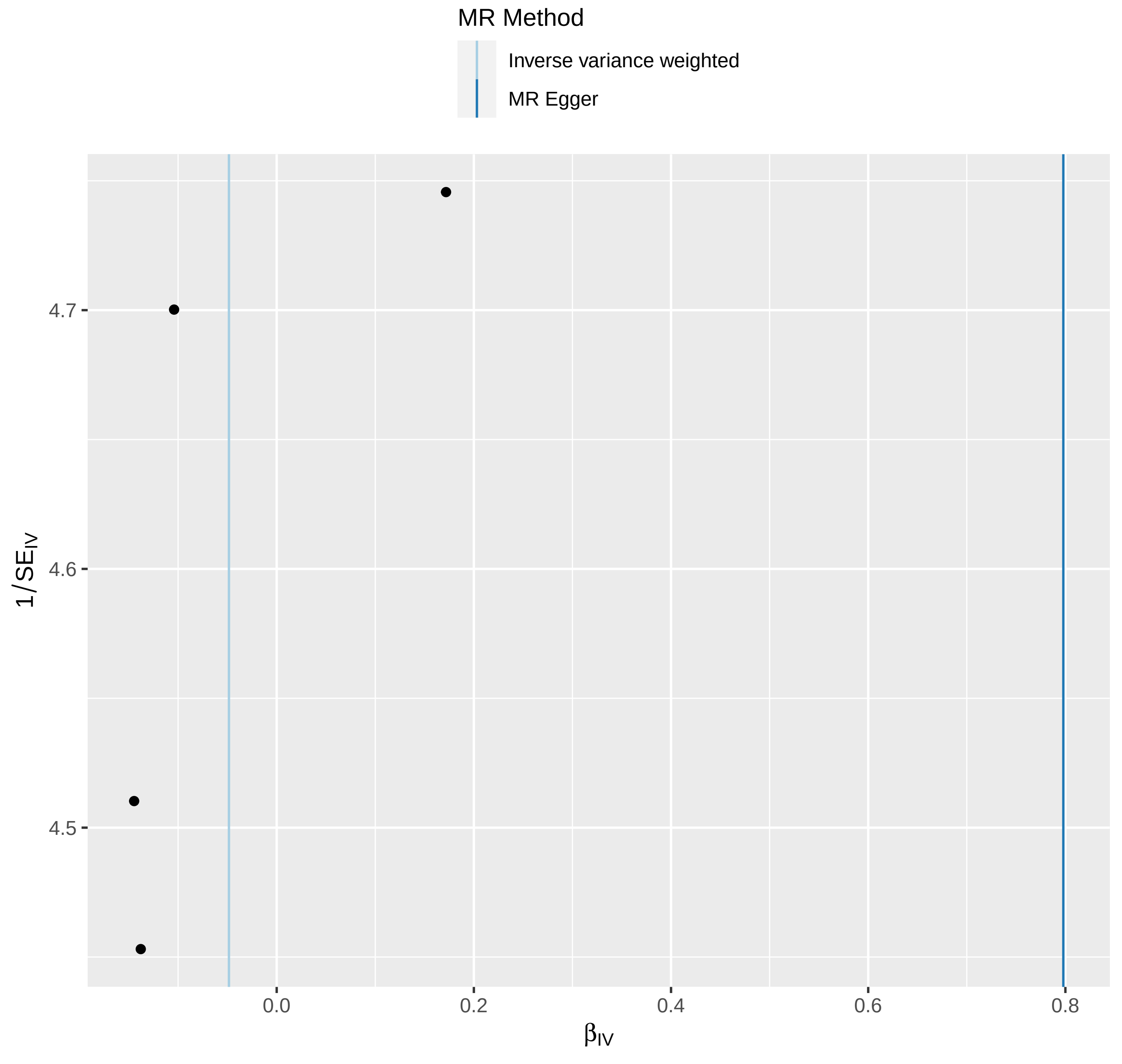

Batch 2−3150 : Gut microbiota abundance (genus Parasutterella id.2892) on Insulin gene enhancer protein ISL−1
with a threshold of 1e−05

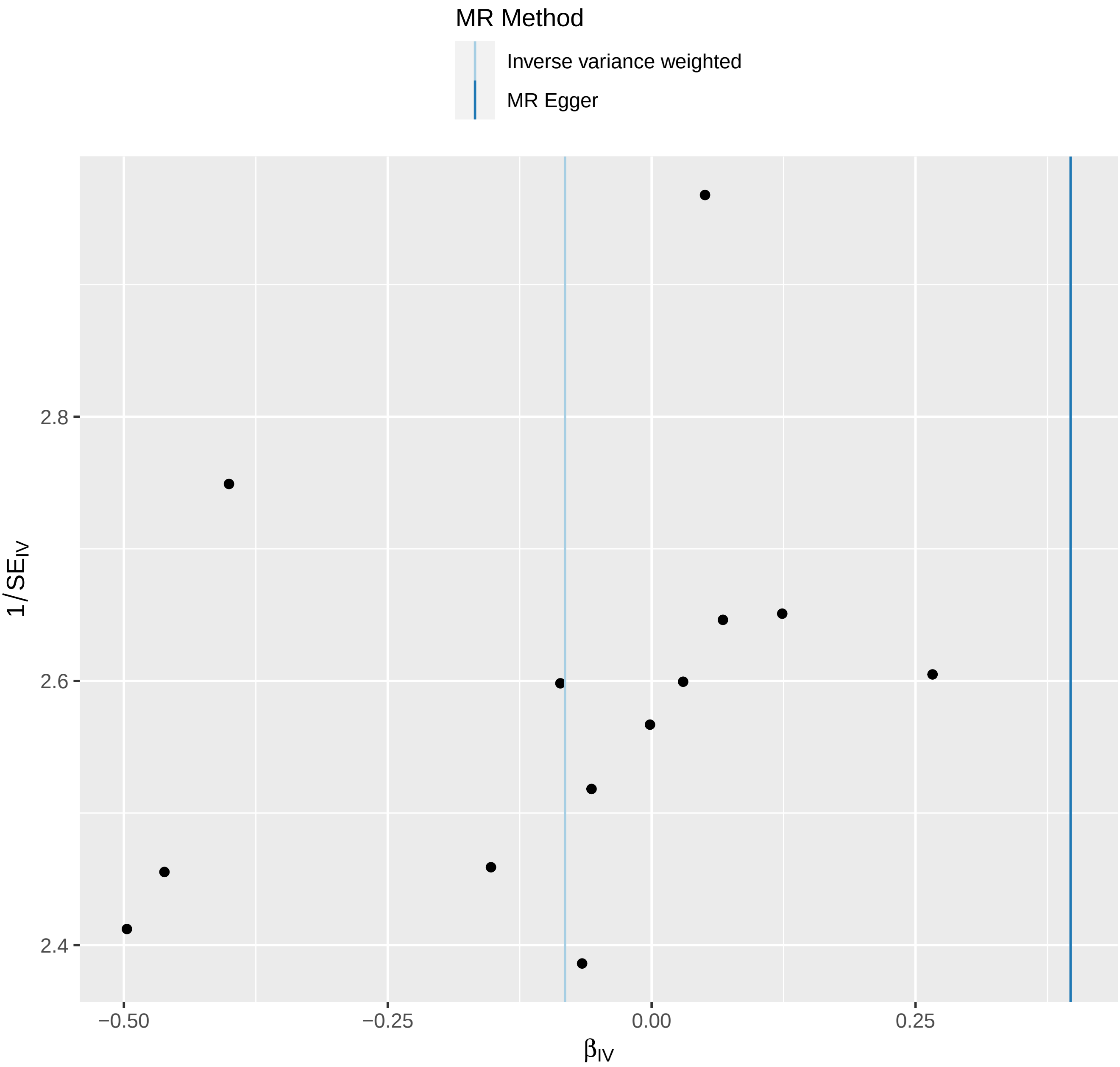

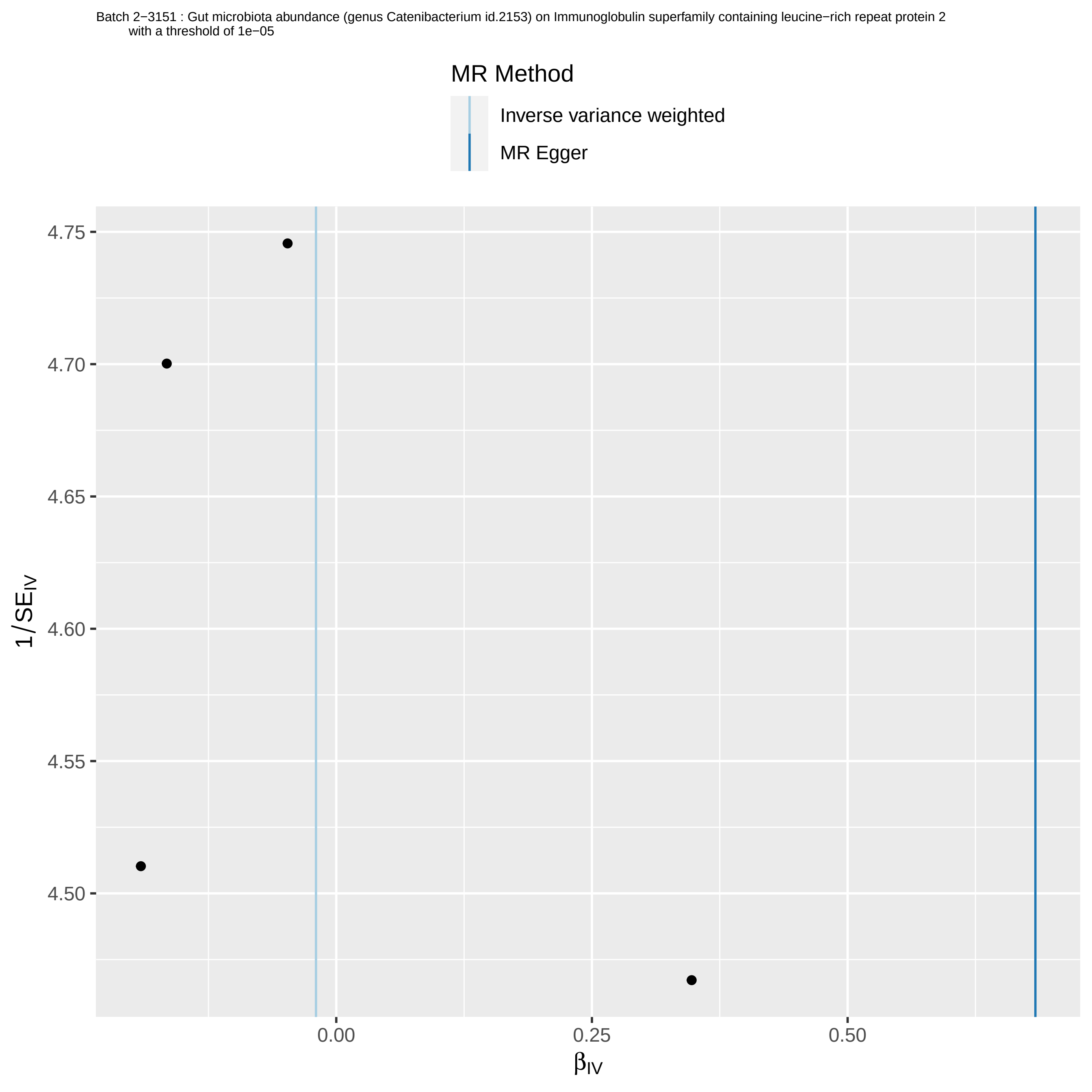

Batch 2−3151 : Gut microbiota abundance (genus Catenibacterium id.2153) on Immunoglobulin superfamily containing leucine−rich repeat protein 2
with a threshold of 1e−05
MR Method
Inverse variance weighted
MR Egger
4.75
4.70
4.65
4.60
4.55
4.50
1/SE_IV
0.00
0.25
0.50
β_IV

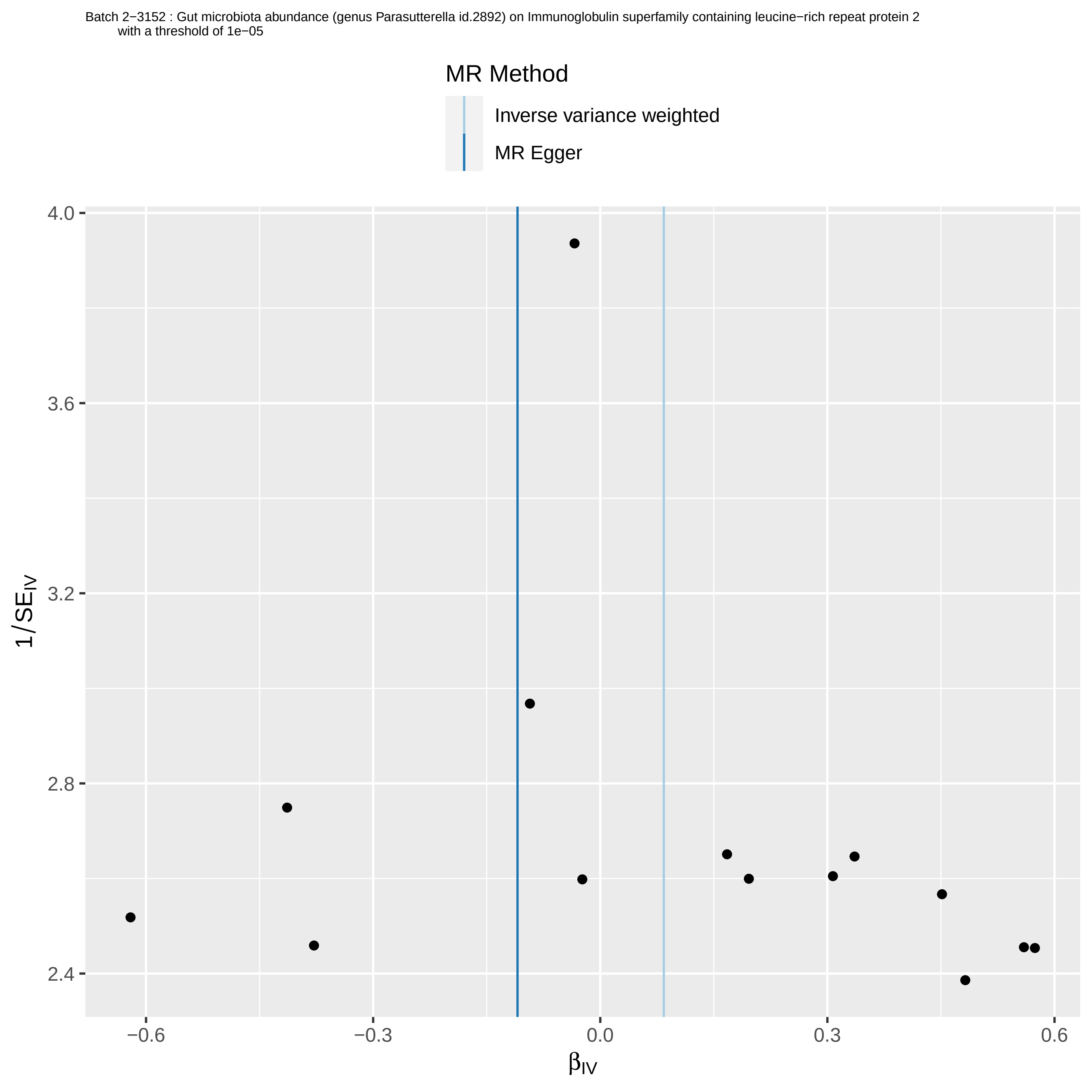

Batch 2−3152 : Gut microbiota abundance (genus Parasutterella id.2892) on Immunoglobulin superfamily containing leucine−rich repeat protein 2
with a threshold of 1e−05
MR Method
Inverse variance weighted
MR Egger
4.0
3.6
3.2
2.8
2.4
1/SE_IV
−0.6
−0.3
0.0
0.3
0.6
β_IV

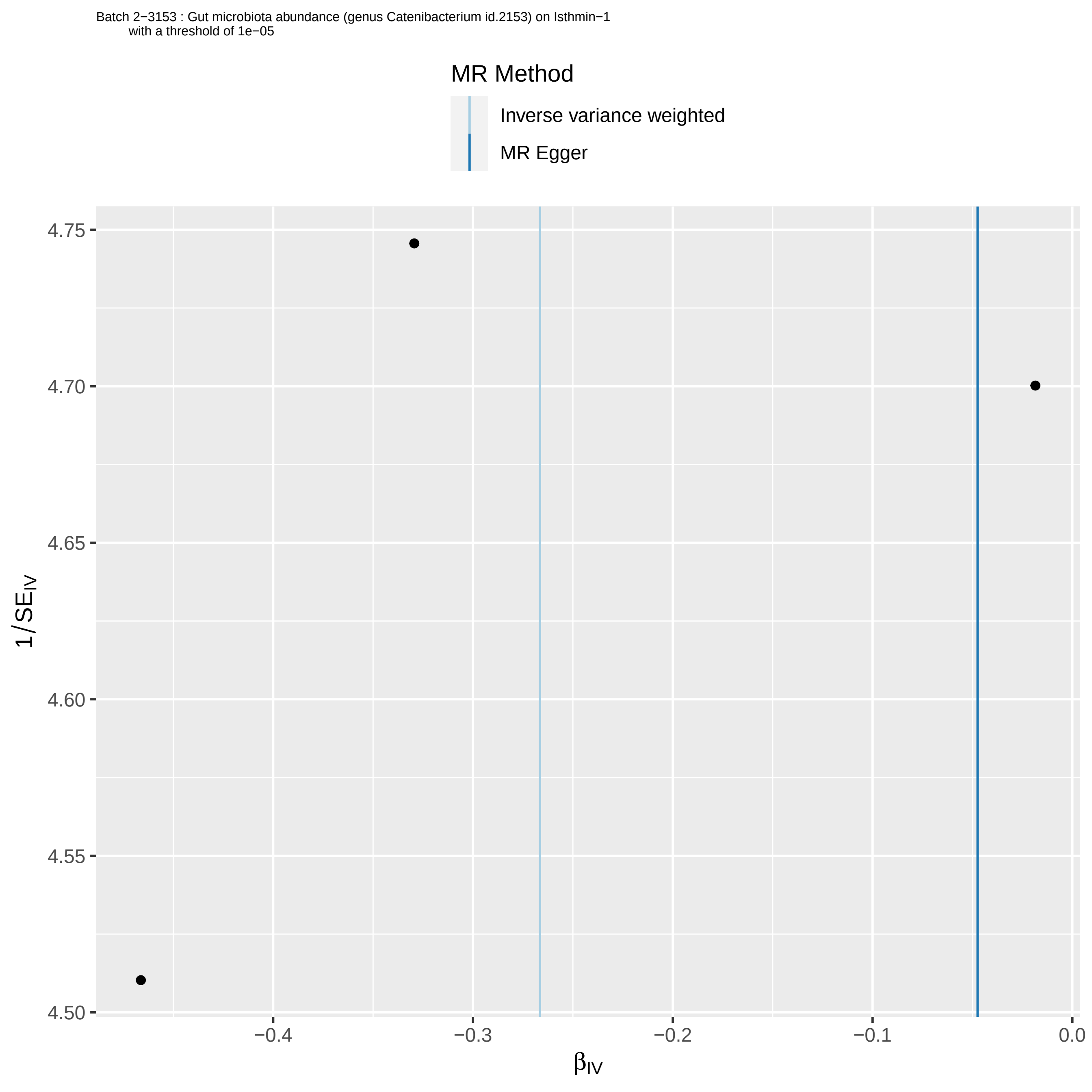
Batch 2−3153 : Gut microbiota abundance (genus Catenibacterium id.2153) on Isthmin−1
with a threshold of 1e−05
MR Method
Inverse variance weighted
MR Egger
4.75
4.70
4.65
4.60
4.55
4.50
1/SE_IV
−0.4
−0.3
−0.2
−0.1
0.0
β_IV

Batch 2−3154 : Gut microbiota abundance (genus Parasutterella id.2892) on Isthmin−1
with a threshold of 1e−05

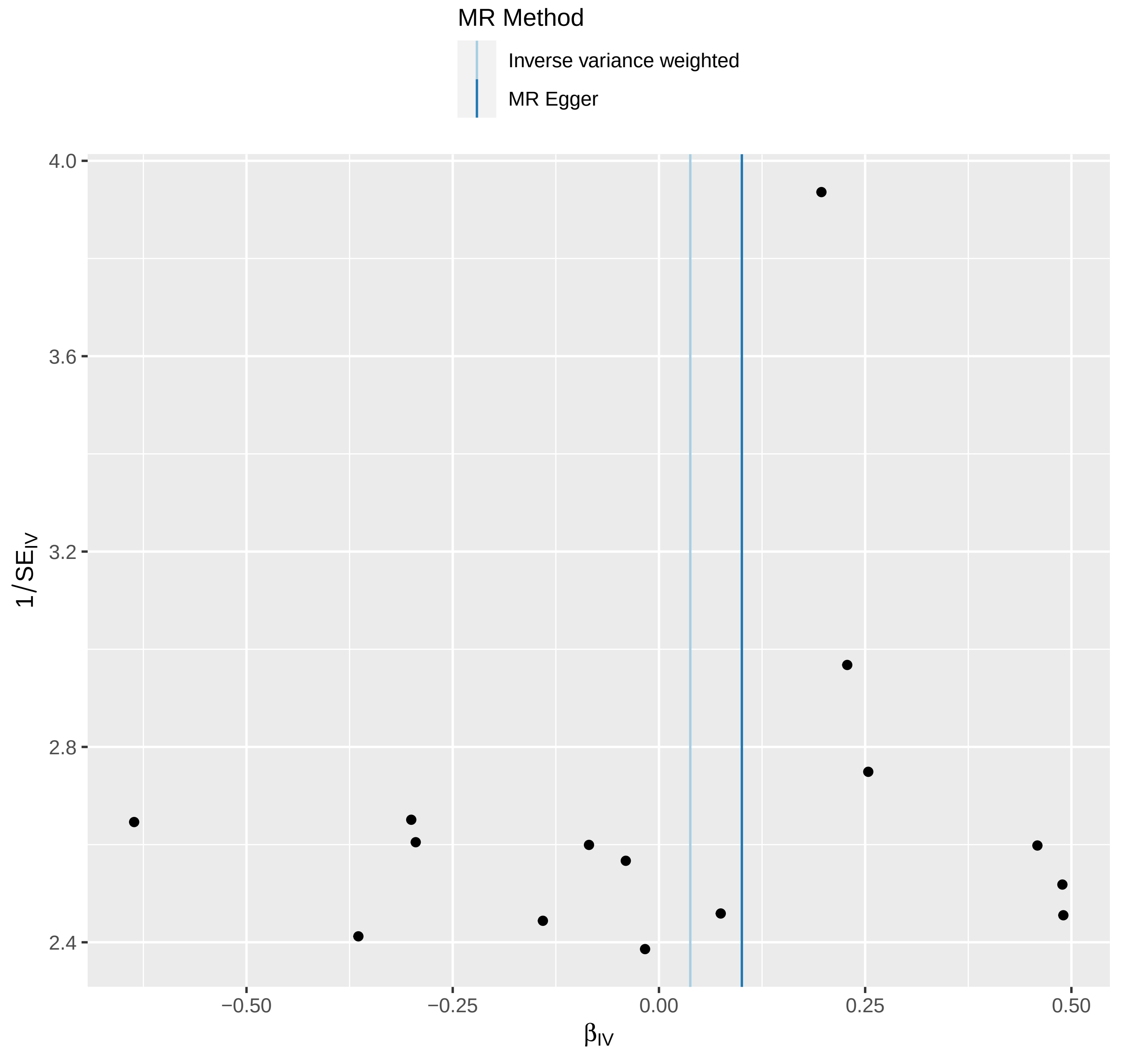

Batch 2−3155 : Gut microbiota abundance (genus Catenibacterium id.2153) on Isochorismatase domain−containing protein 1 with a threshold of 1e−05

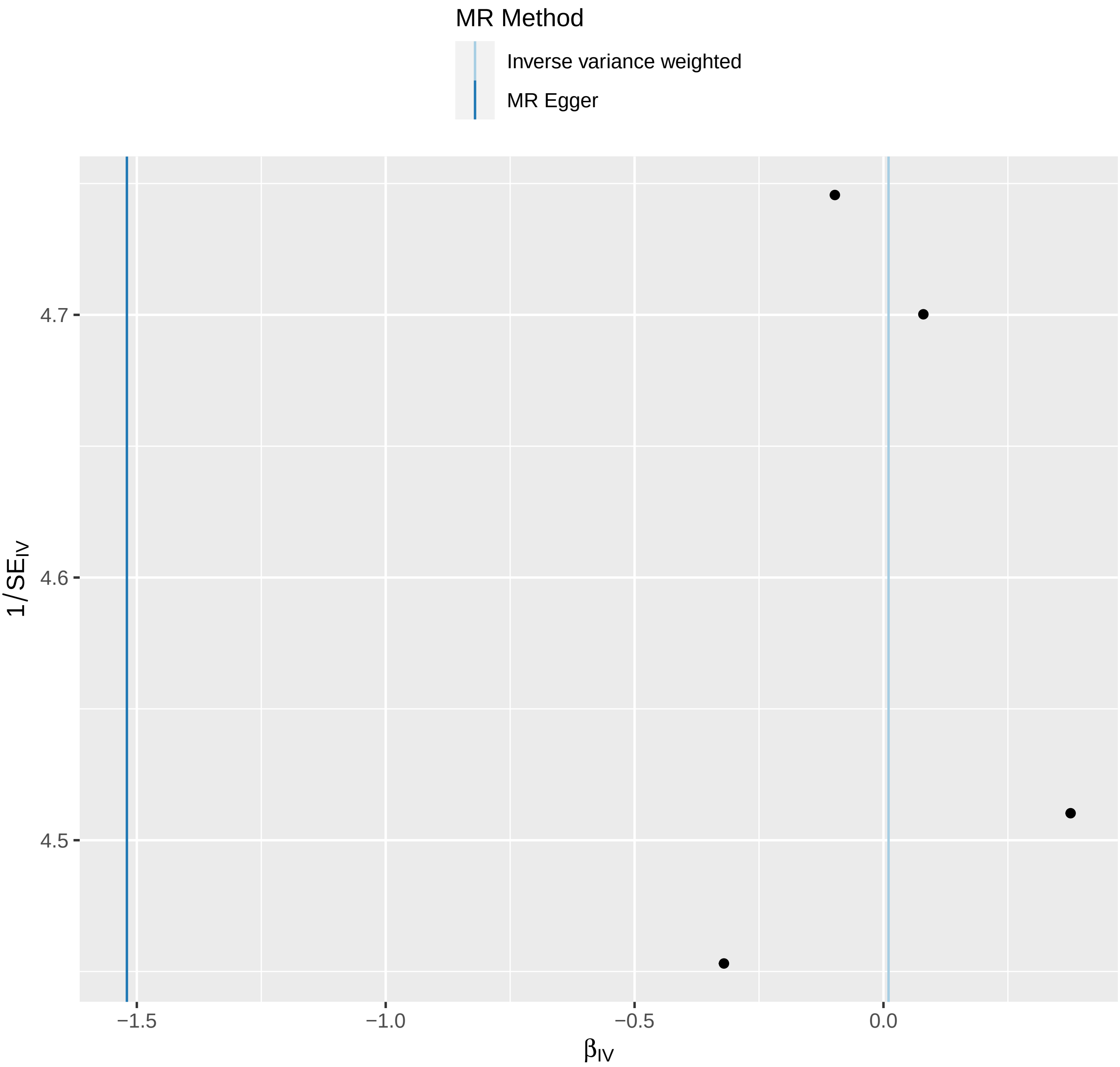

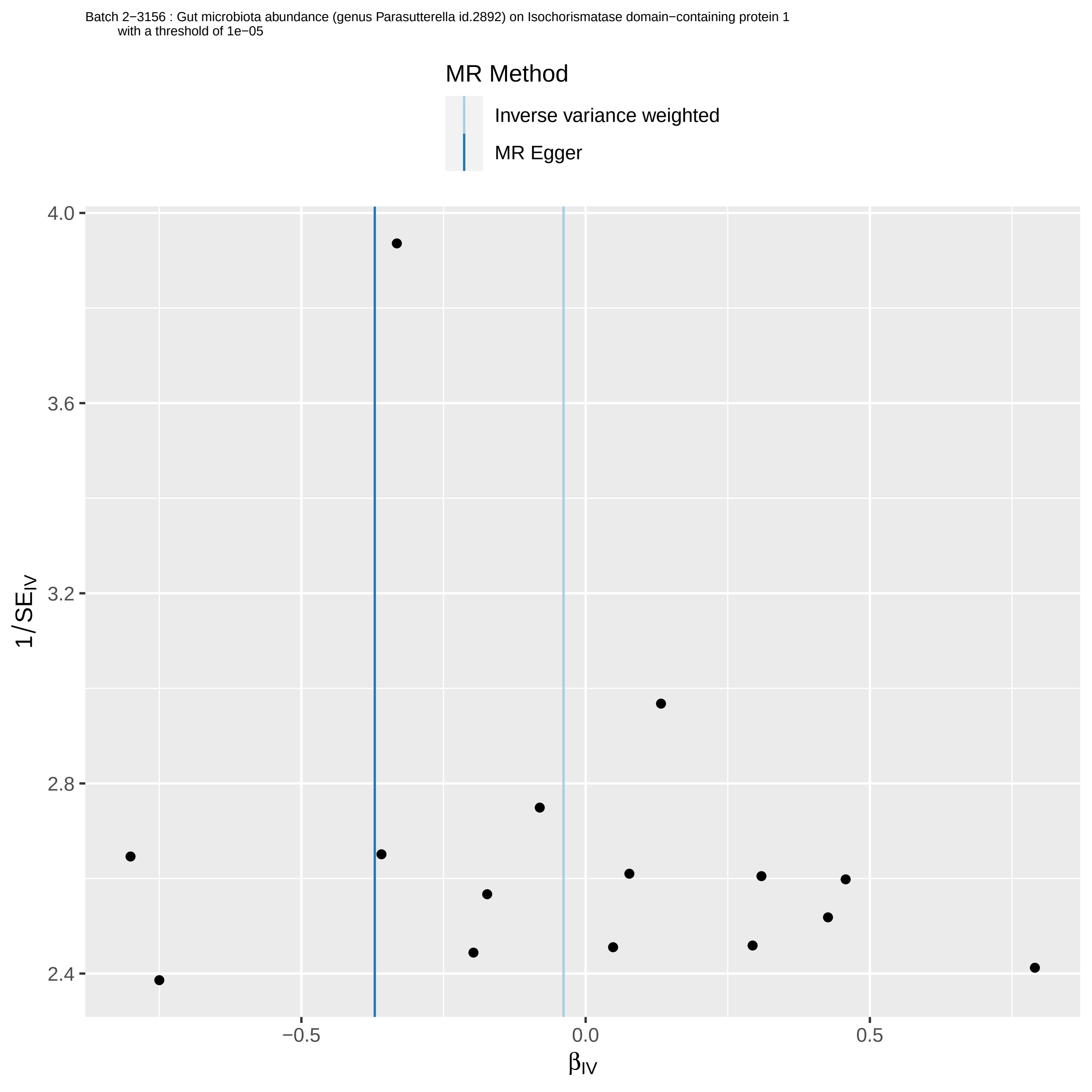
Batch 2−3156 : Gut microbiota abundance (genus Parasutterella id.2892) on Isochorismatase domain−containing protein 1
with a threshold of 1e−05
MR Method
Inverse variance weighted
MR Egger
4.0
3.6
3.2
2.8
2.4
1/SE$_{IV}$
−0.5
0.0
0.5
β_{IV}

Batch 2−3157 : Gut microbiota abundance (genus Catenibacterium id.2153) on IST1 homolog
with a threshold of 1e−05

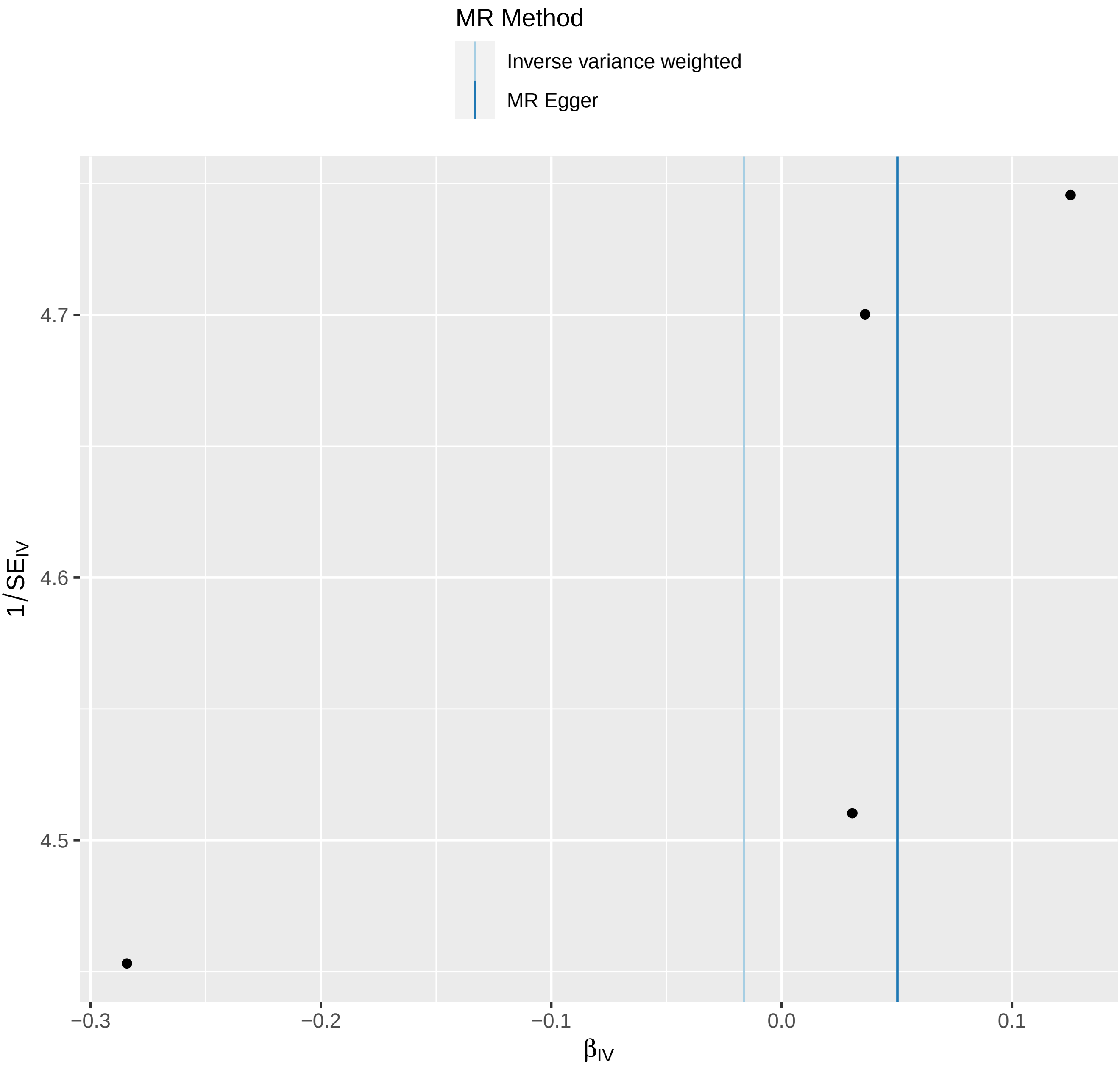

Batch 2−3158 : Gut microbiota abundance (genus Parasutterella id.2892) on IST1 homolog
with a threshold of 1e−05

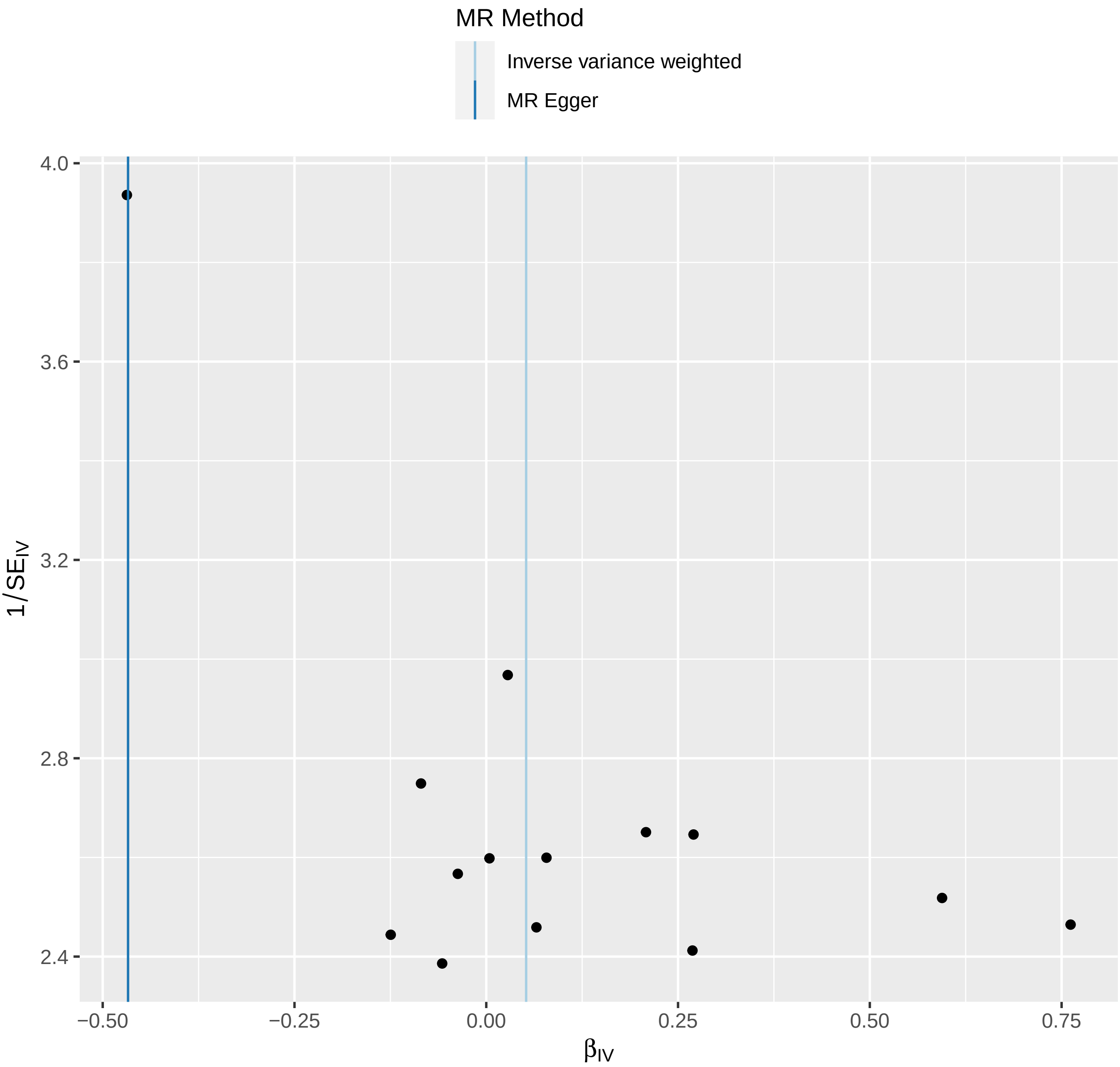

Batch 2−3159 : Gut microbiota abundance (genus Catenibacterium id.2153) on T−cell immunomodulatory protein
with a threshold of 1e−05

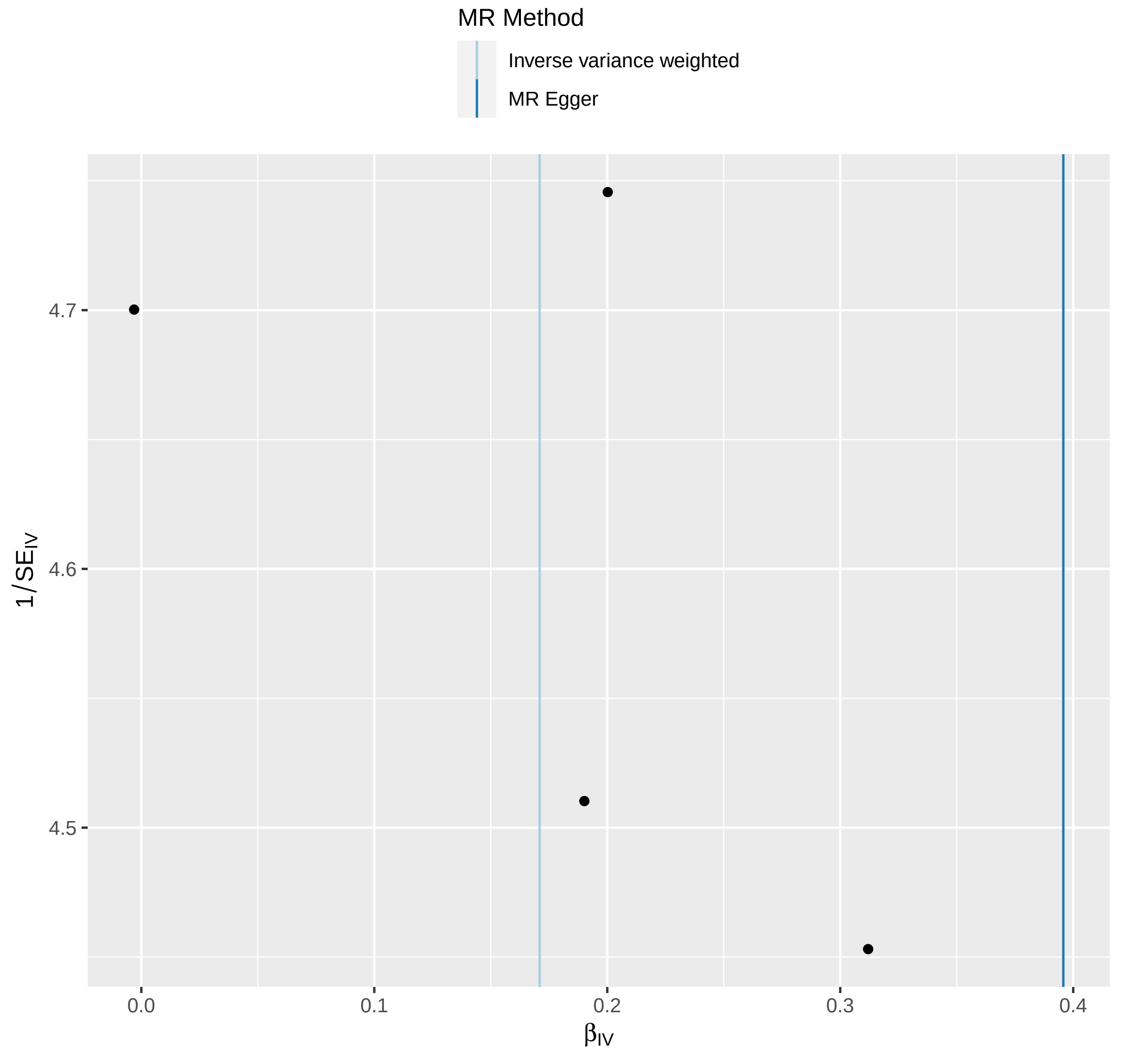

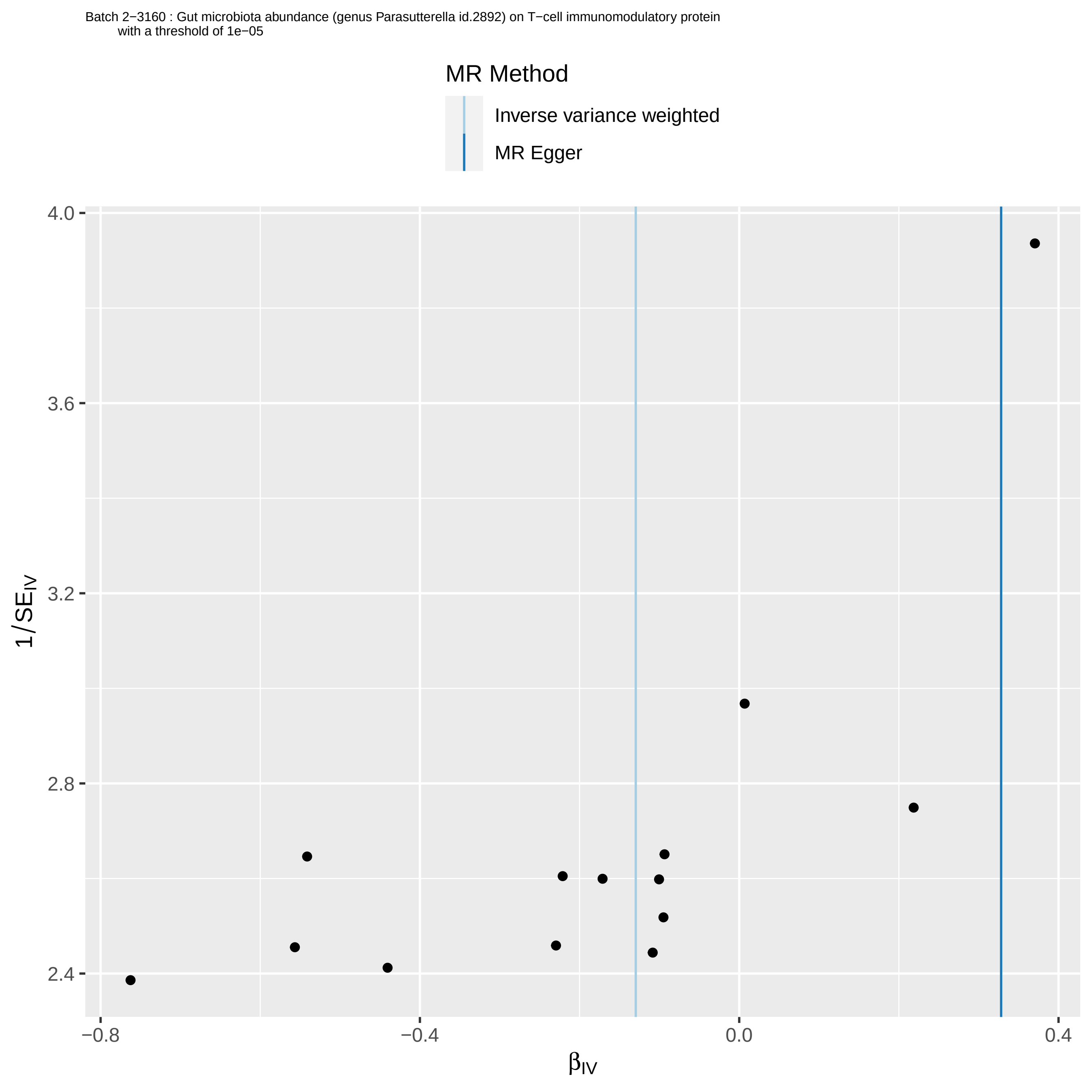
Batch 2−3160 : Gut microbiota abundance (genus Parasutterella id.2892) on T−cell immunomodulatory protein
with a threshold of 1e−05
MR Method
Inverse variance weighted
MR Egger
4.0
3.6
3.2
2.8
2.4
1/SE_IV
−0.8
−0.4
0.0
0.4
β_IV

Batch 2−3161 : Gut microbiota abundance (genus Catenibacterium id.2153) on Integrin alpha−I: beta−1 complex
with a threshold of 1e−05

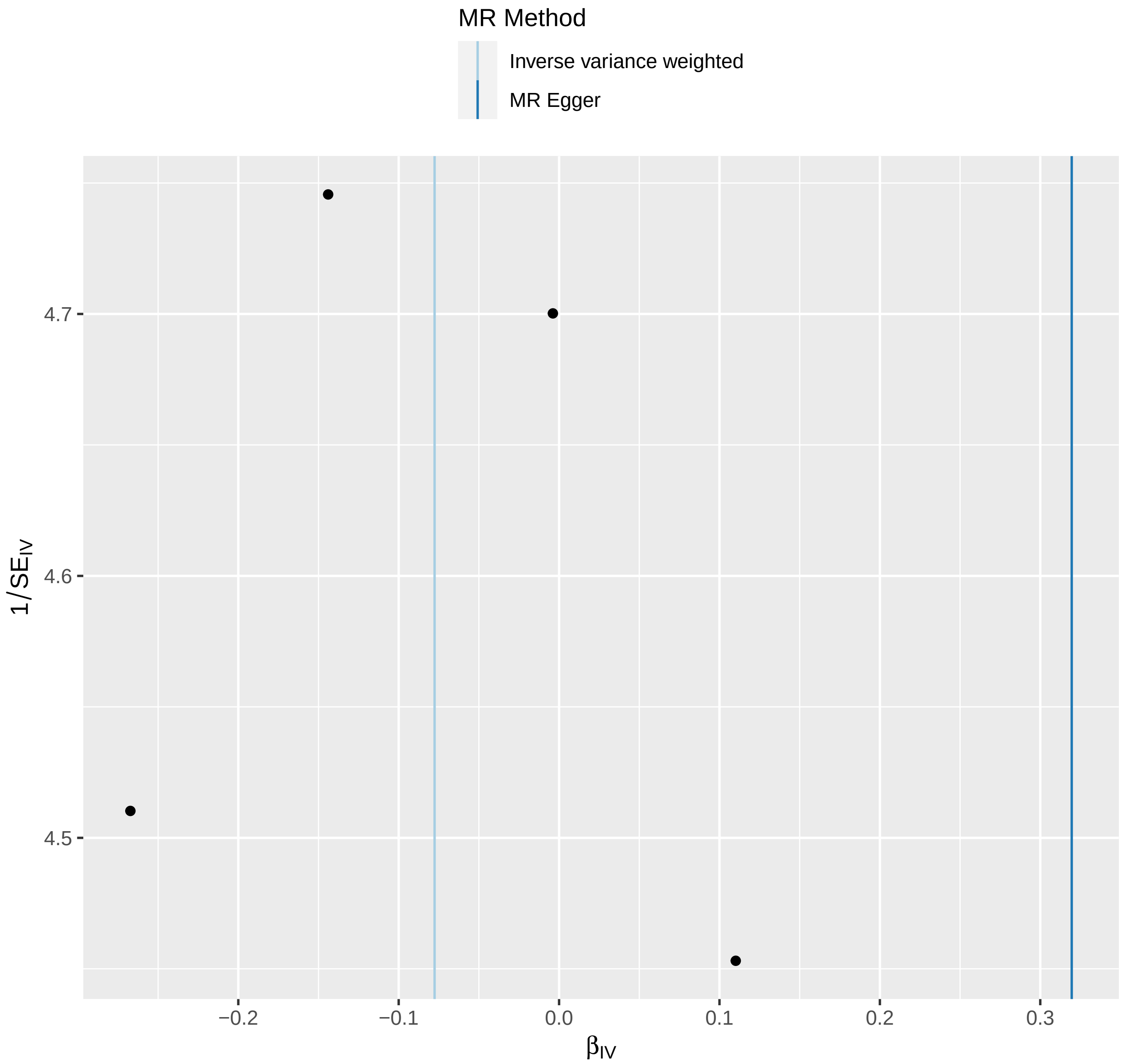

Batch 2−3162 : Gut microbiota abundance (genus Parasutterella id.2892) on Integrin alpha−I: beta−1 complex
with a threshold of 1e−05

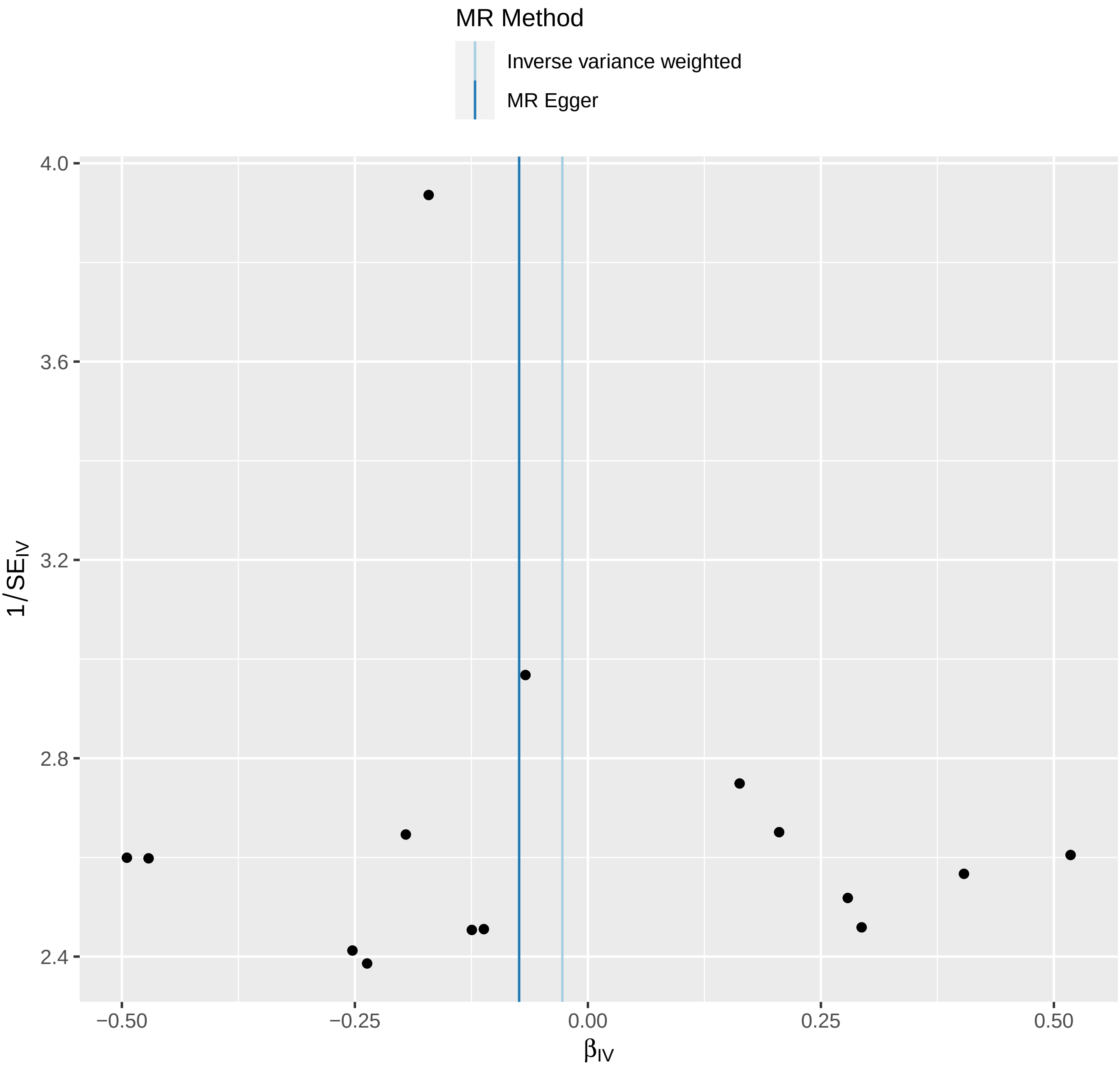

Batch 2−3163 : Gut microbiota abundance (genus Catenibacterium id.2153) on Integrin alpha−5
with a threshold of 1e−05

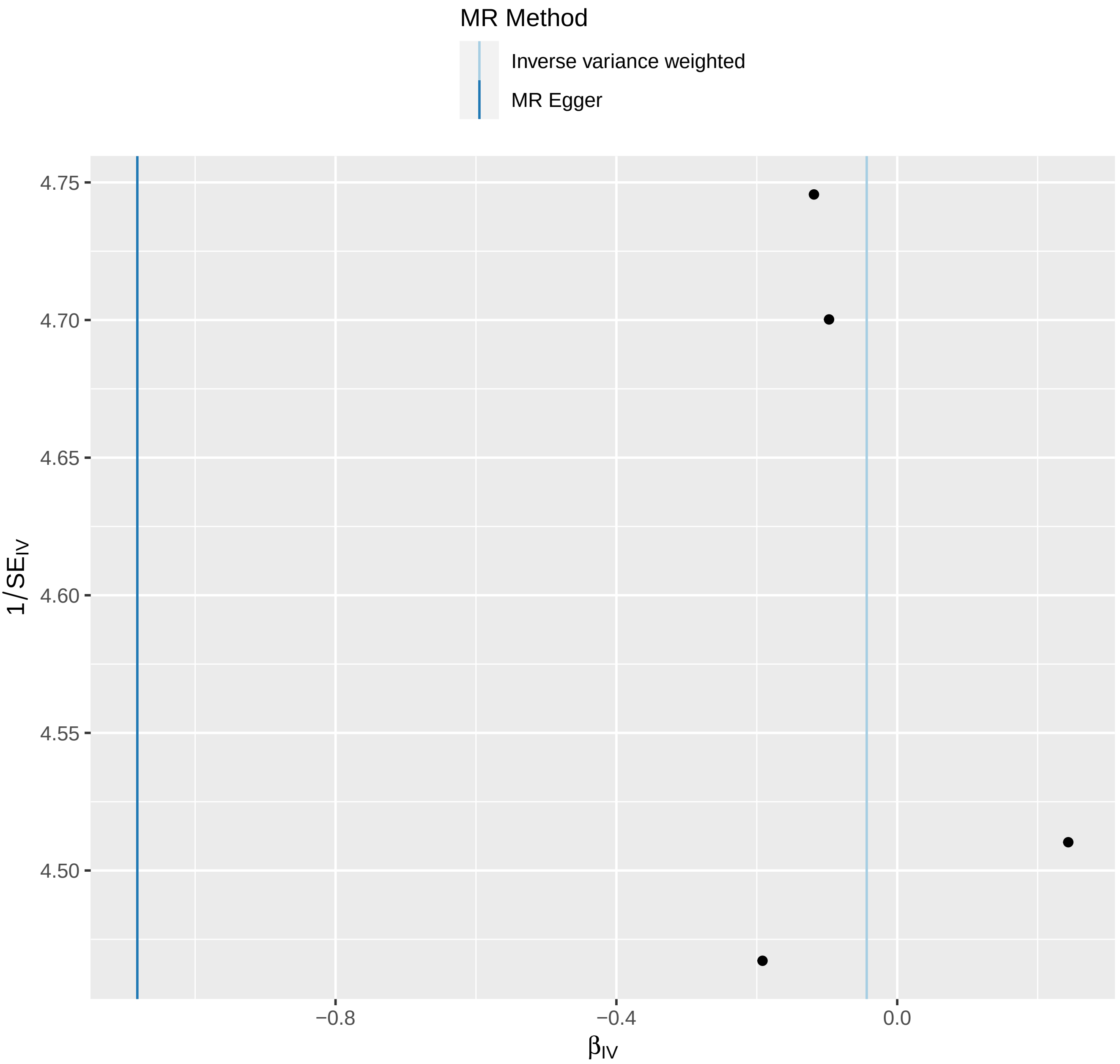

Batch 2−3164 : Gut microbiota abundance (genus Parasutterella id.2892) on Integrin alpha−5
with a threshold of 1e−05

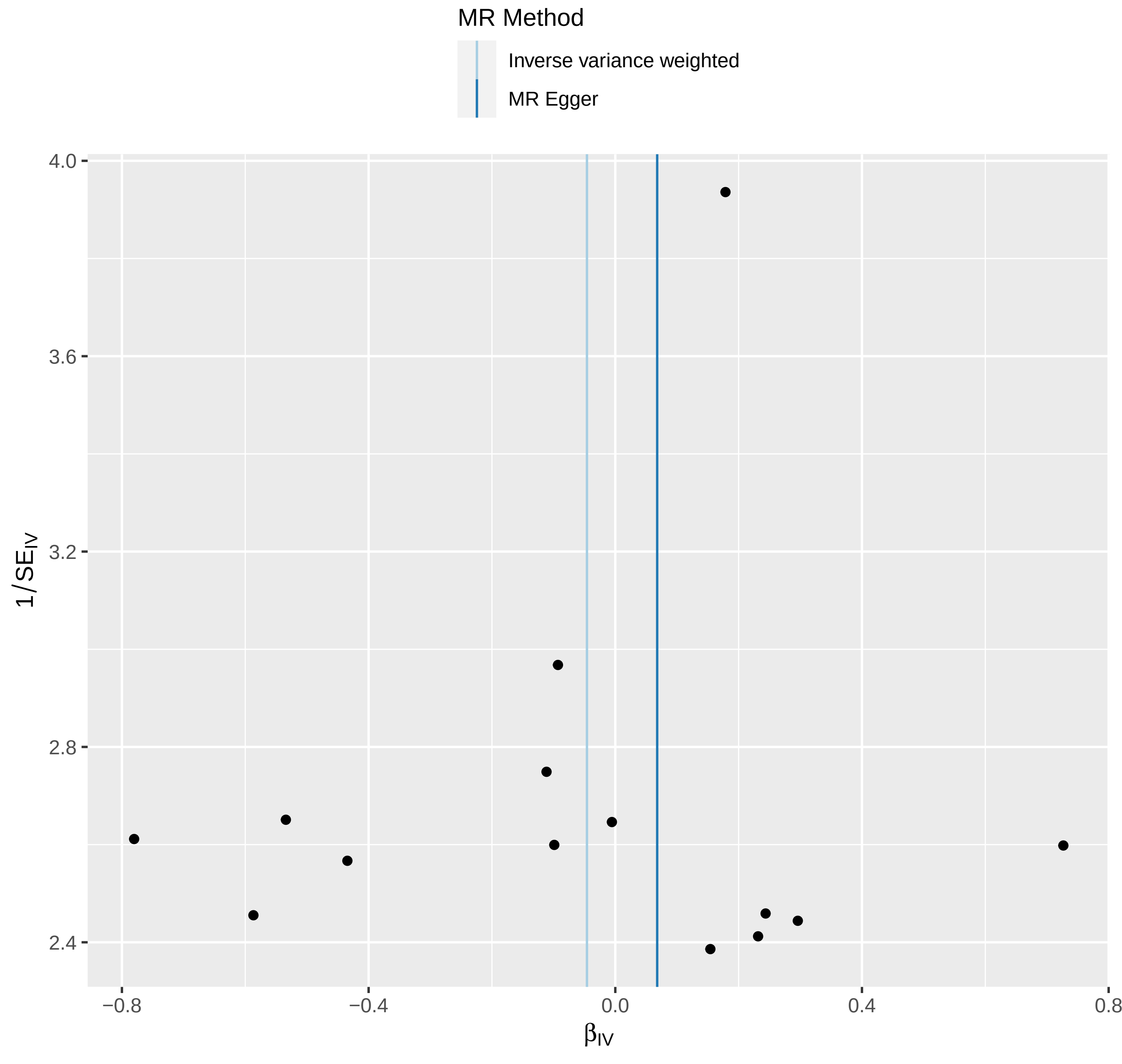

Batch 2−3165 : Gut microbiota abundance (genus Catenibacterium id.2153) on Integrin alpha−L
with a threshold of 1e−05

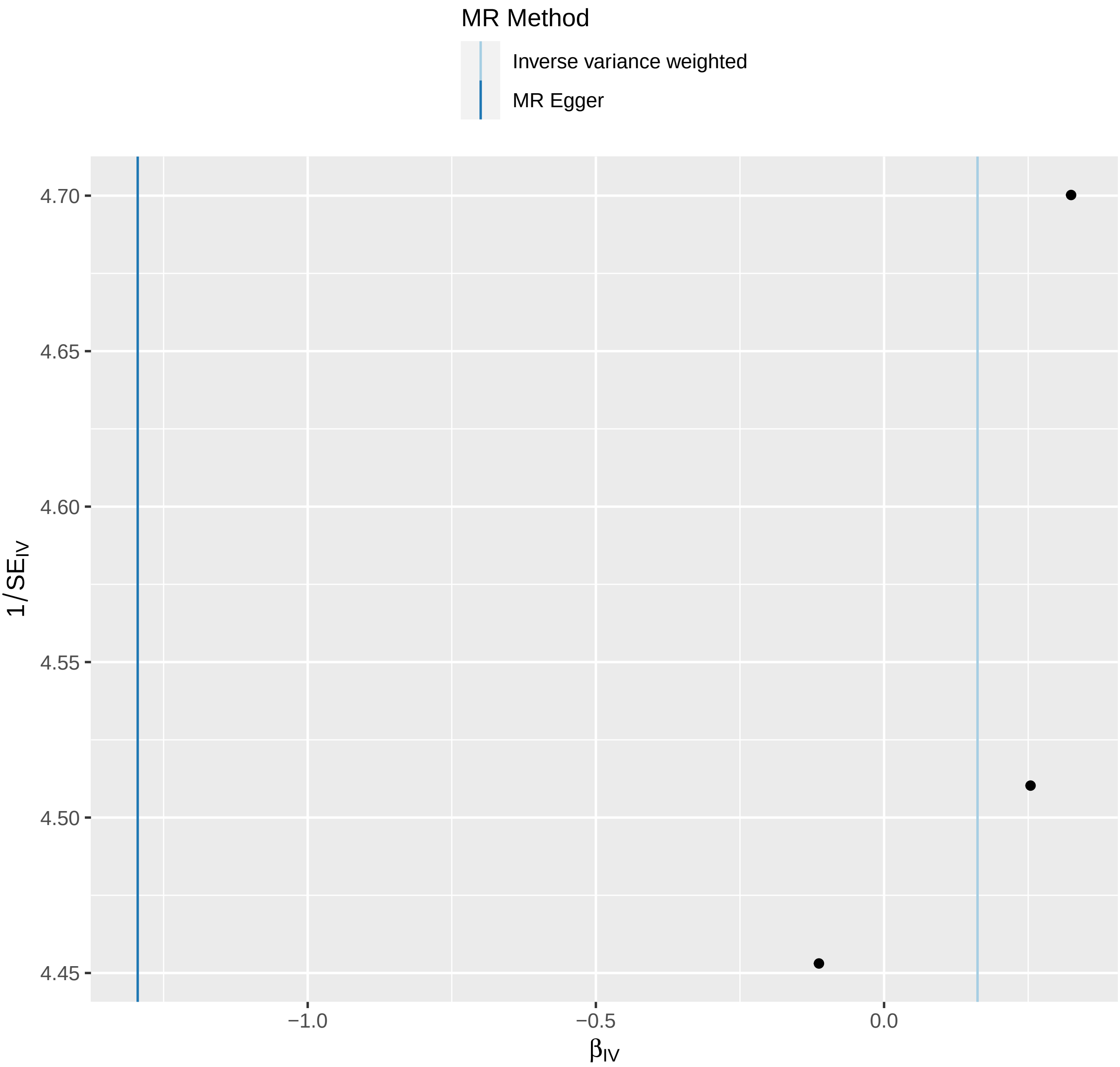

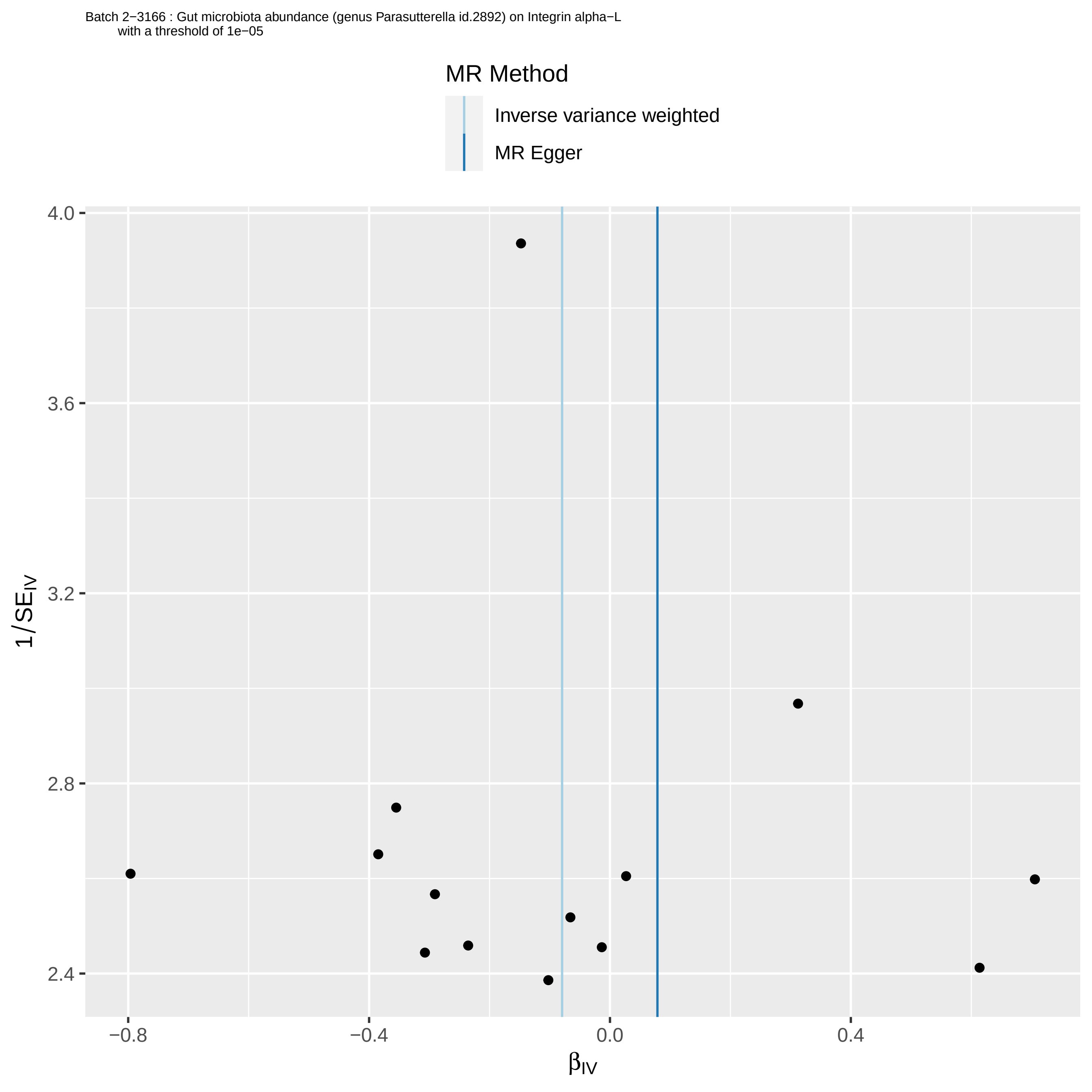
Batch 2−3166 : Gut microbiota abundance (genus Parasutterella id.2892) on Integrin alpha−L
with a threshold of 1e−05
MR Method
Inverse variance weighted
MR Egger
4.0
3.6
3.2
2.8
2.4
1/SE_IV
−0.8
−0.4
0.0
0.4
β_IV

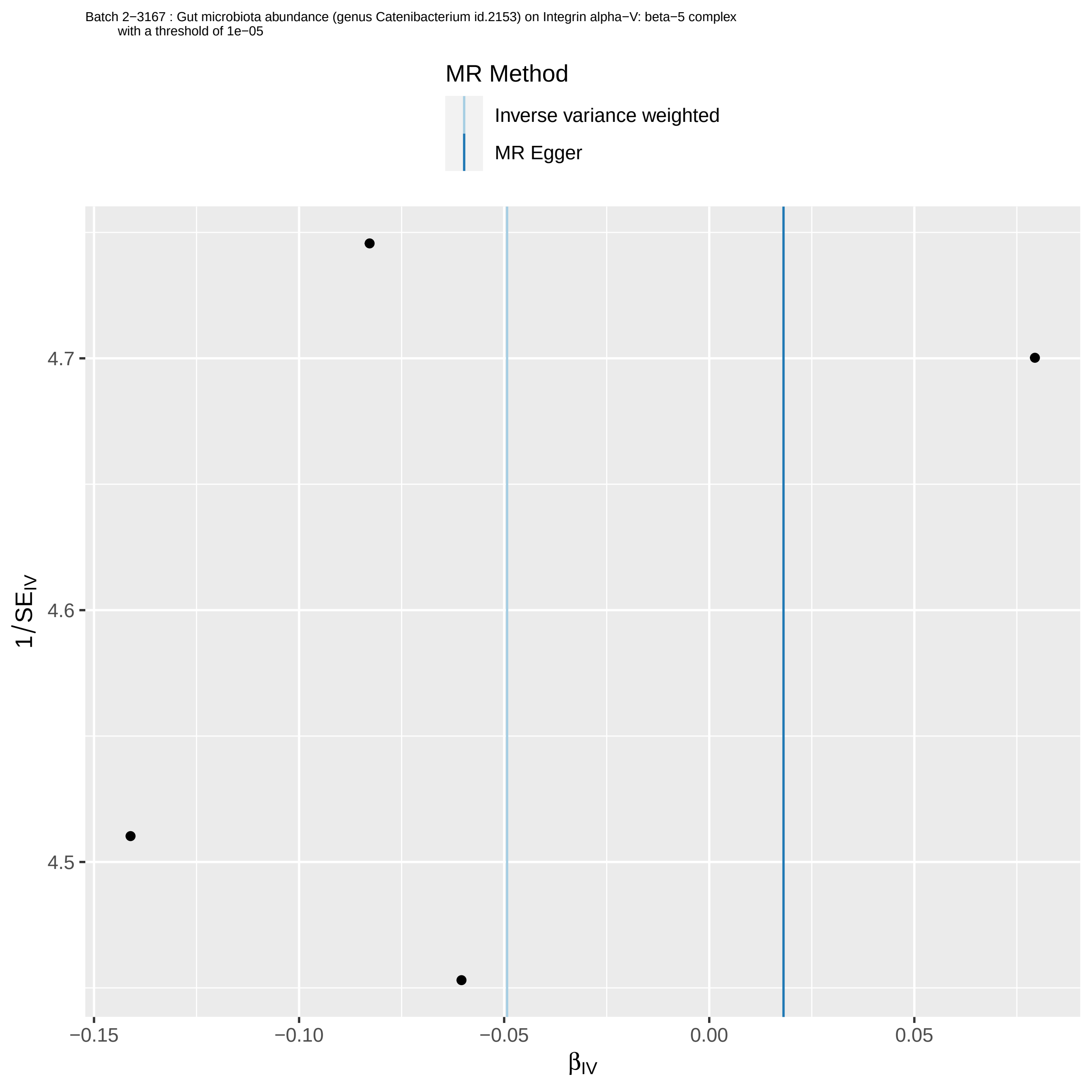
Batch 2−3167 : Gut microbiota abundance (genus Catenibacterium id.2153) on Integrin alpha−V: beta−5 complex
with a threshold of 1e−05
MR Method
Inverse variance weighted
MR Egger
4.7
4.6
4.5
1/SE_IV
−0.15
−0.10
−0.05
0.00
0.05
β_IV

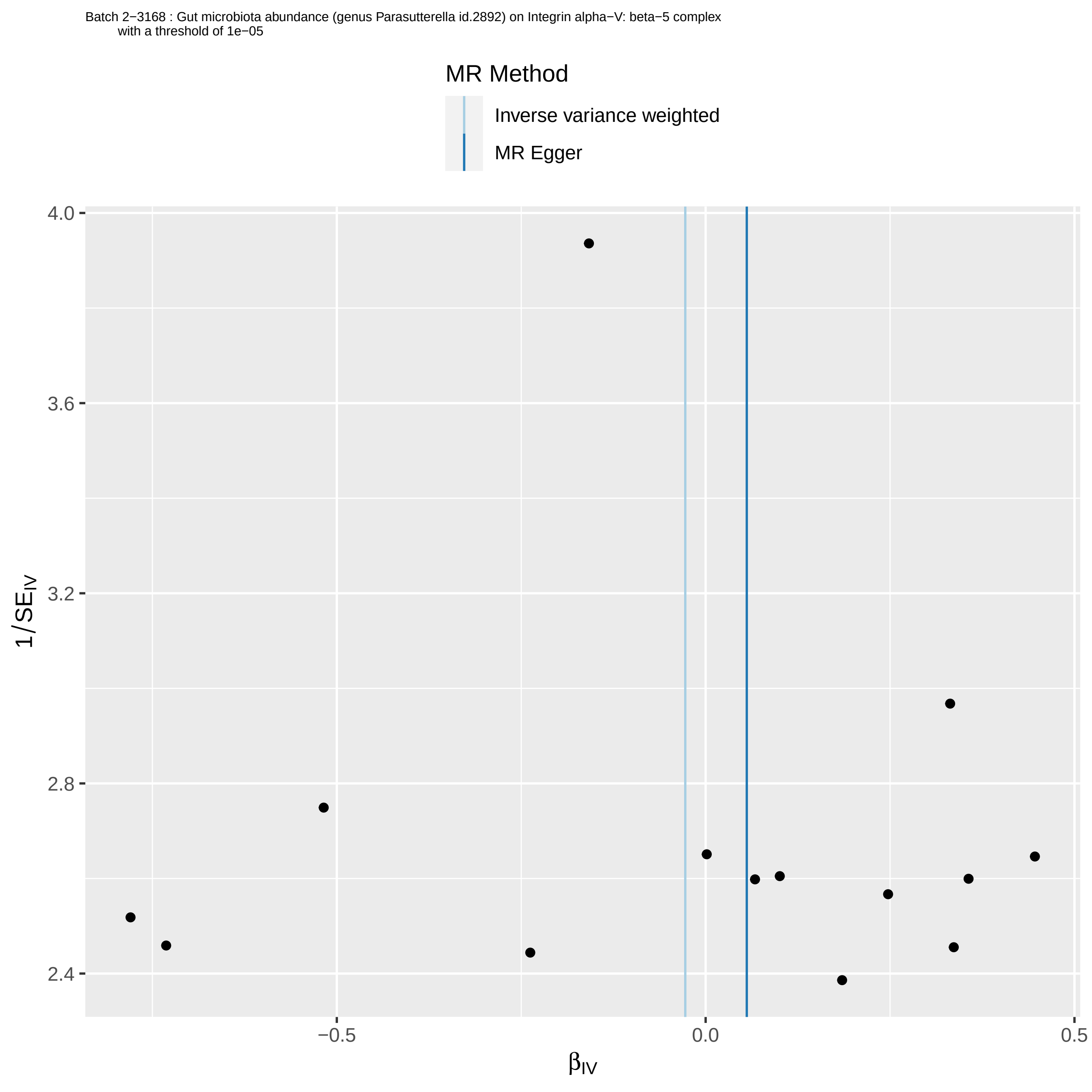

Batch 2−3168 : Gut microbiota abundance (genus Parasutterella id.2892) on Integrin alpha−V: beta−5 complex
with a threshold of 1e−05
MR Method
Inverse variance weighted
MR Egger
4.0
3.6
3.2
2.8
2.4
1/SE_IV
−0.5
0.0
0.5
β_IV

Batch 2−3169 : Gut microbiota abundance (genus Catenibacterium id.2153) on Integrin beta−7
with a threshold of 1e−05

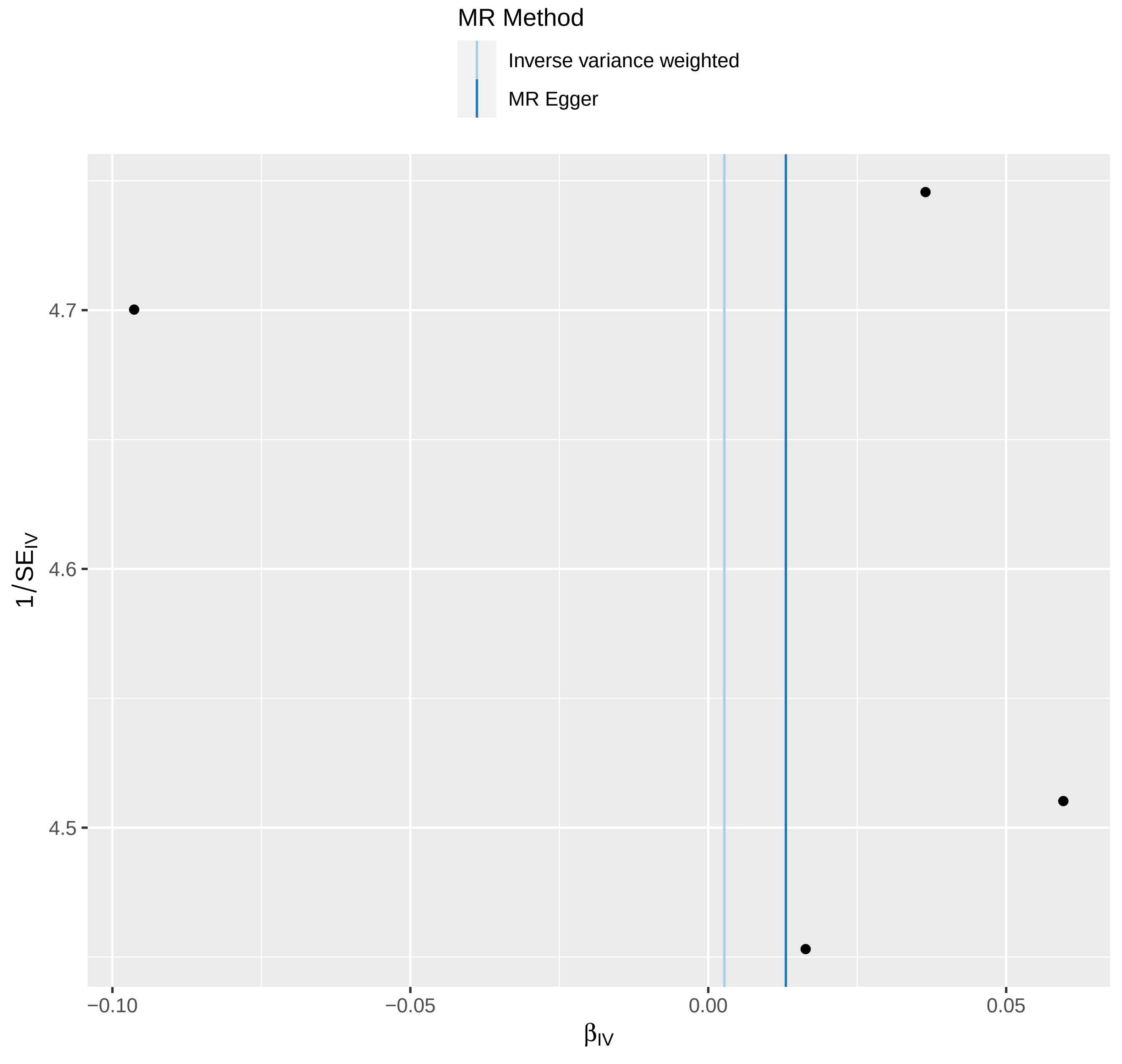

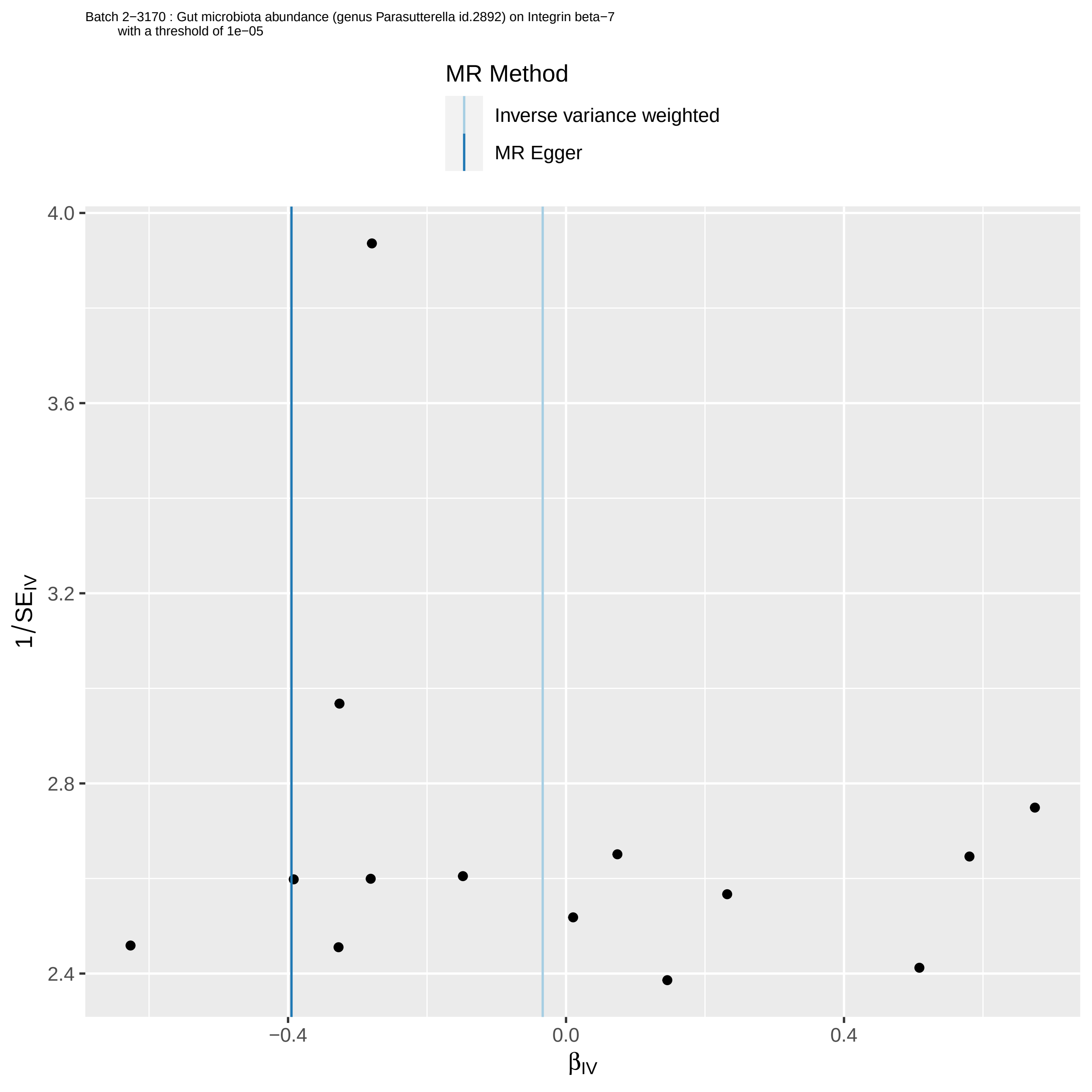

Batch 2−3170 : Gut microbiota abundance (genus Parasutterella id.2892) on Integrin beta−7
with a threshold of 1e−05
MR Method
Inverse variance weighted
MR Egger
4.0
3.6
3.2
2.8
2.4
1/SE_IV
−0.4
0.0
0.4
β_IV

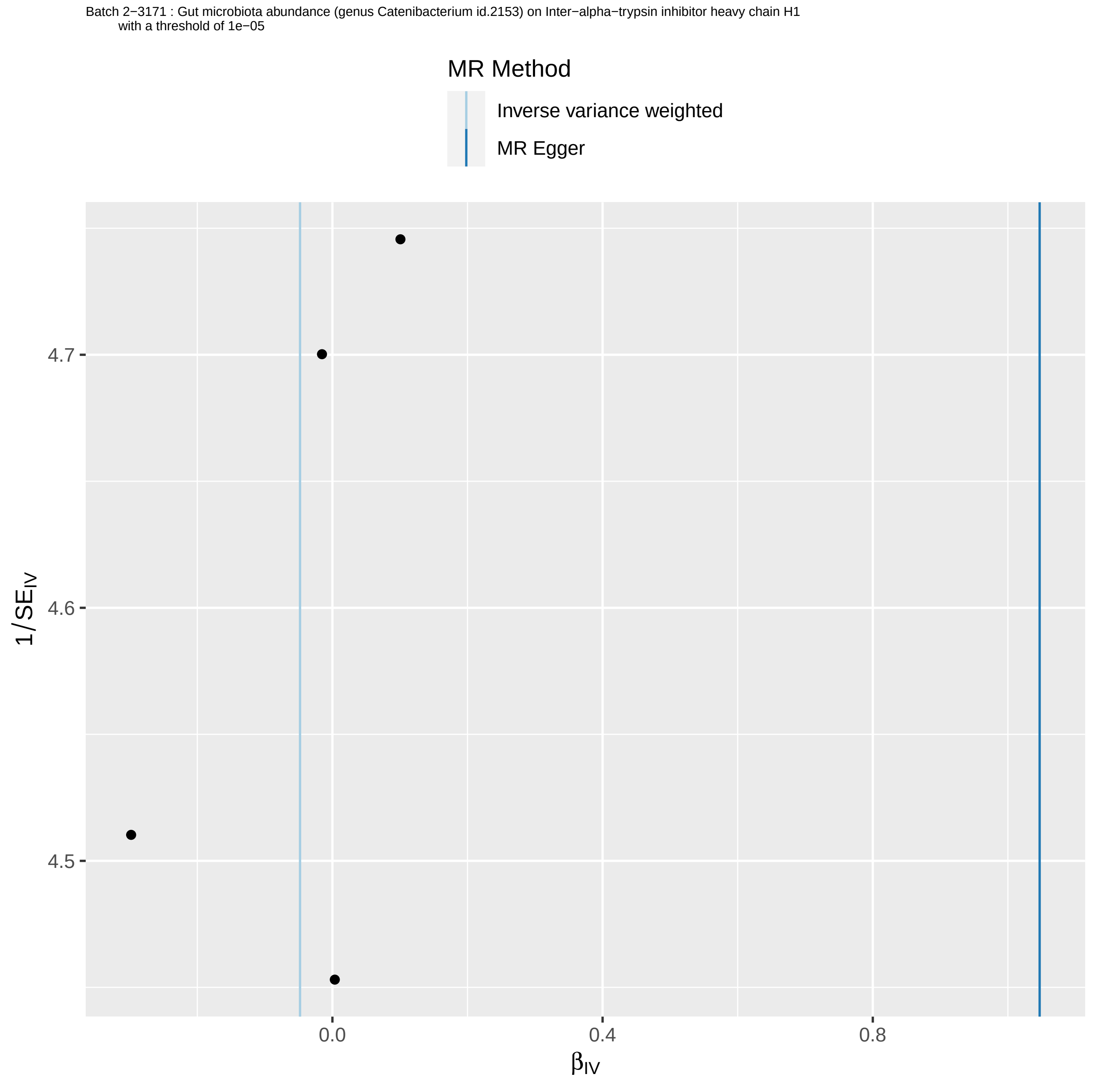
Batch 2−3171 : Gut microbiota abundance (genus Catenibacterium id.2153) on Inter−alpha−trypsin inhibitor heavy chain H1
with a threshold of 1e−05
MR Method
Inverse variance weighted
MR Egger
4.7
4.6
4.5
1/SE_IV
0.0
0.4
0.8
β_IV

Batch 2−3172 : Gut microbiota abundance (genus Parasutterella id.2892) on Inter−alpha−trypsin inhibitor heavy chain H1
with a threshold of 1e−05

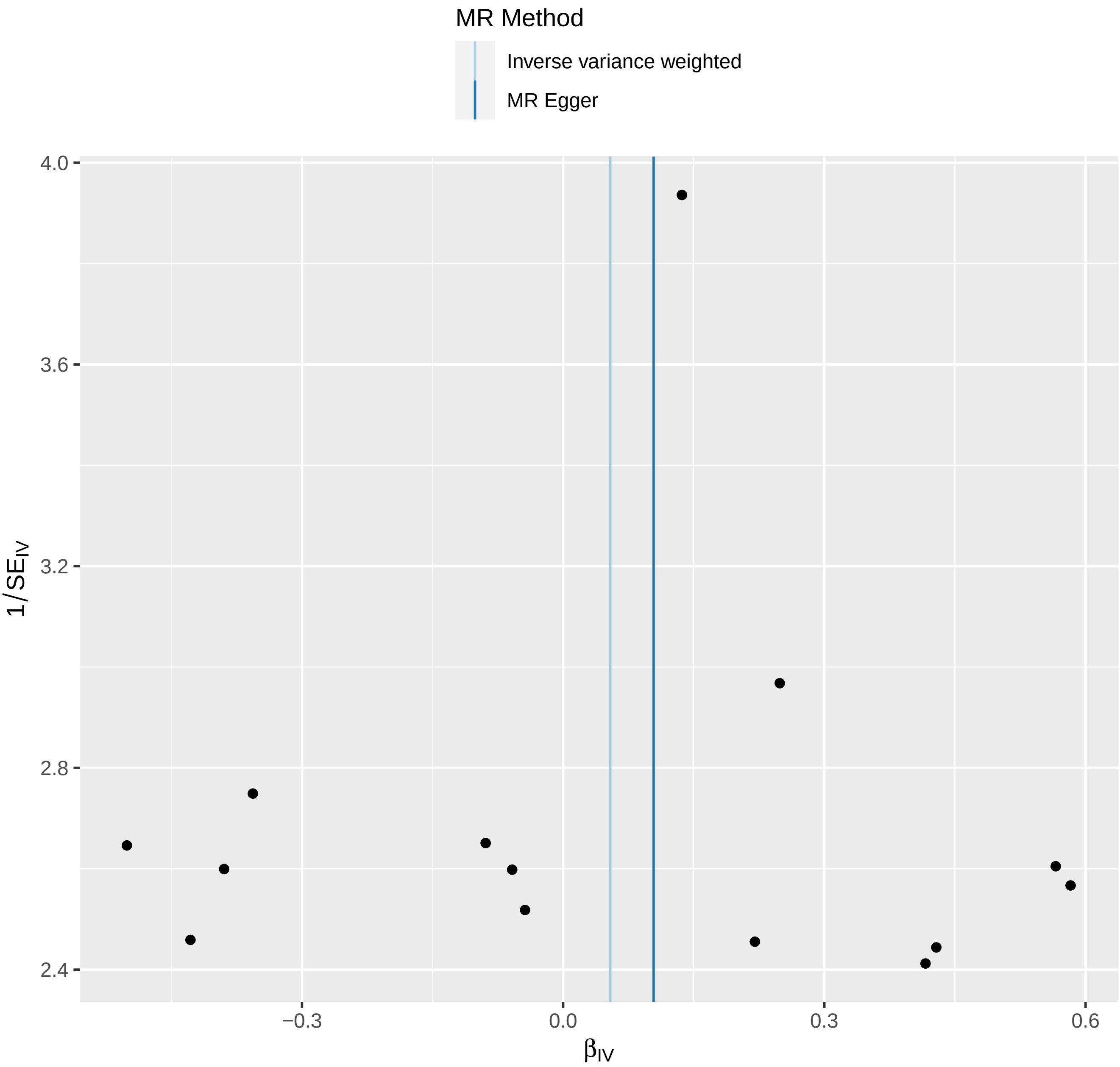

Batch 2−3173 : Gut microbiota abundance (genus Catenibacterium id.2153) on Inter−alpha−trypsin inhibitor heavy chain H5
with a threshold of 1e−05

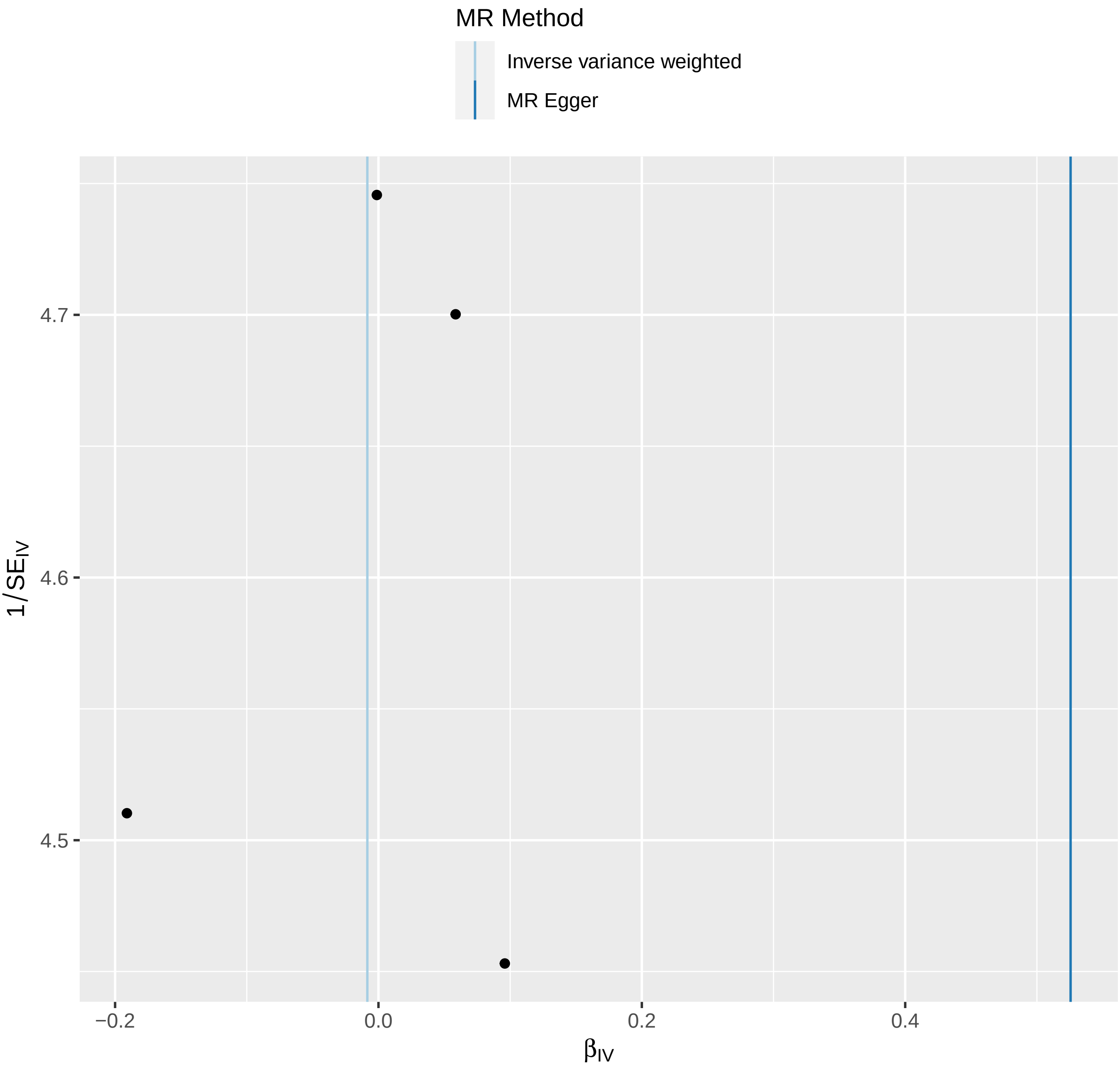

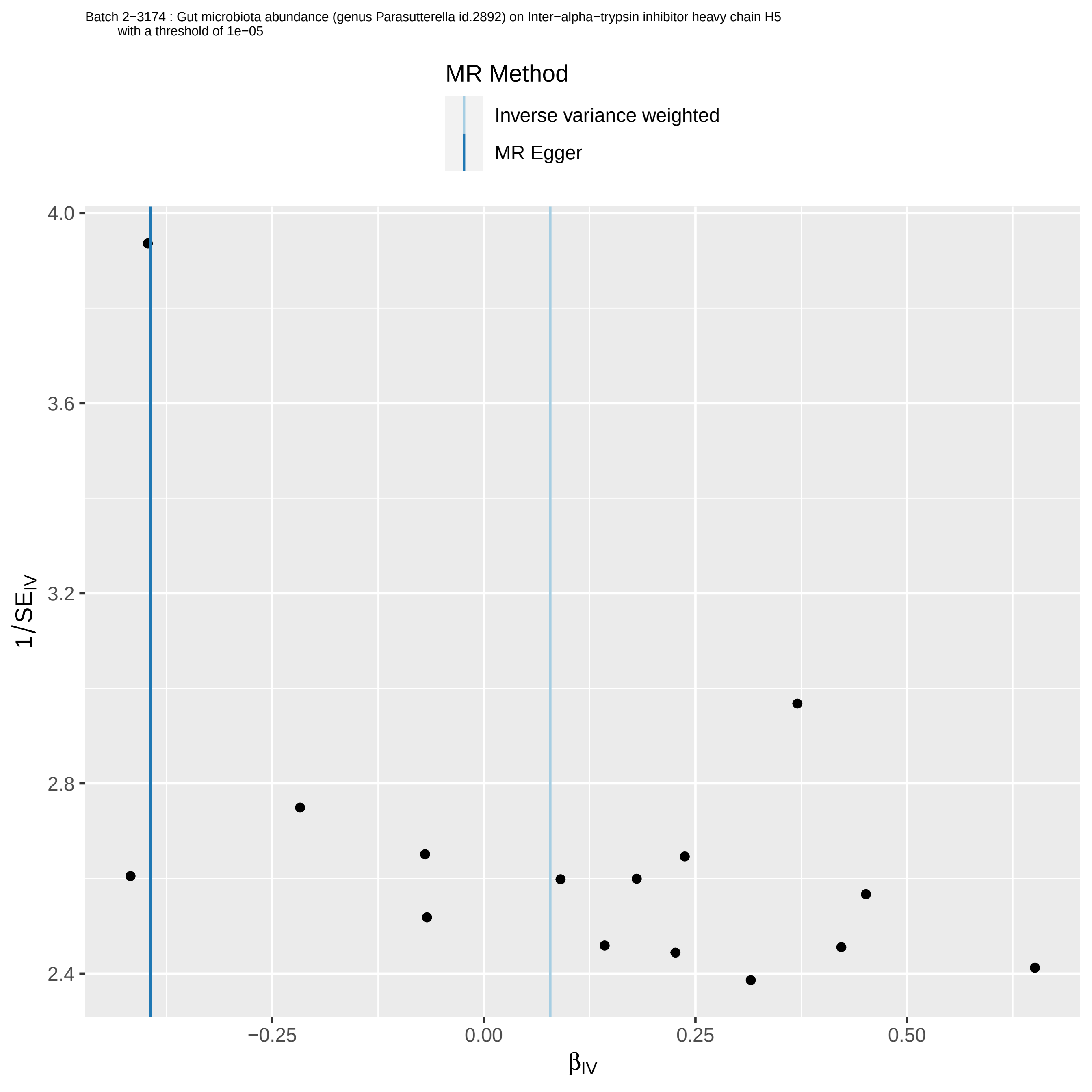

Batch 2−3174 : Gut microbiota abundance (genus Parasutterella id.2892) on Inter−alpha−trypsin inhibitor heavy chain H5
with a threshold of 1e−05
MR Method
Inverse variance weighted
MR Egger
4.0
3.6
3.2
2.8
2.4
1/SE_IV
−0.25
0.00
0.25
0.50
β_IV

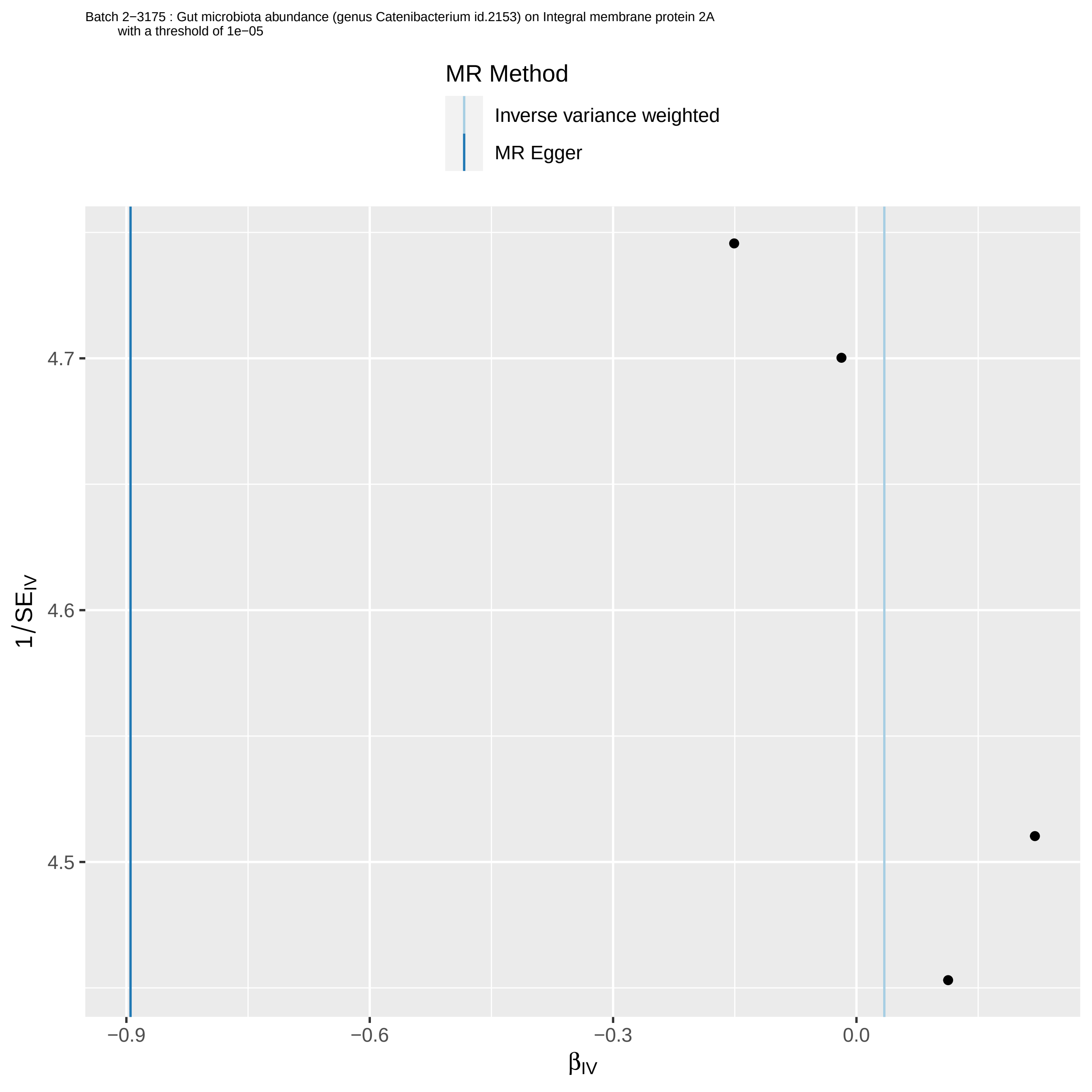
Batch 2−3175 : Gut microbiota abundance (genus Catenibacterium id.2153) on Integral membrane protein 2A
with a threshold of 1e−05
MR Method
Inverse variance weighted
MR Egger
4.7
4.6
4.5
1/SE_IV
−0.9
−0.6
−0.3
0.0
β_IV

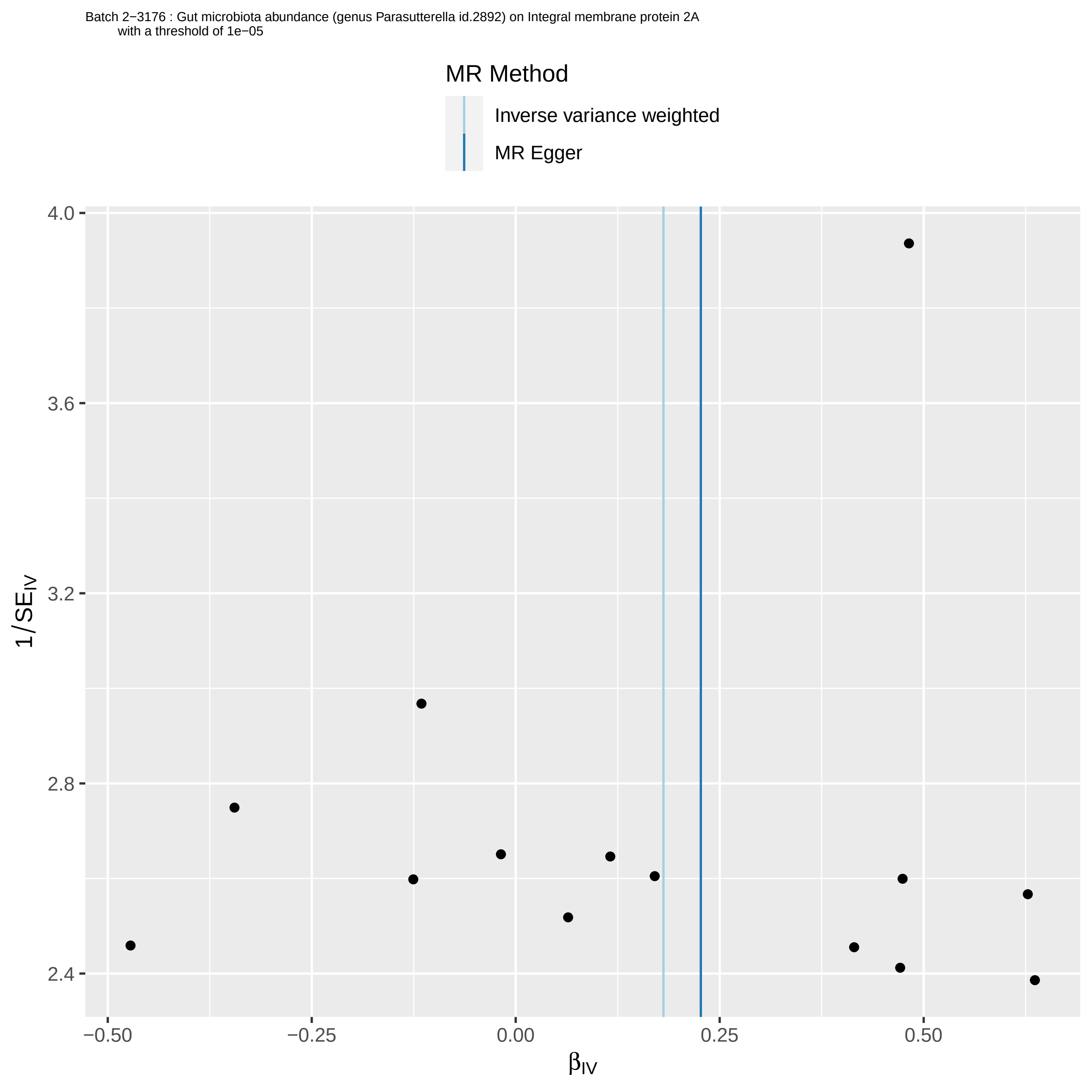
Batch 2−3176 : Gut microbiota abundance (genus Parasutterella id.2892) on Integral membrane protein 2A
with a threshold of 1e−05
MR Method
Inverse variance weighted
MR Egger
4.0
3.6
3.2
2.8
2.4
1/SE_IV
−0.50
−0.25
0.00
0.25
0.50
β_IV

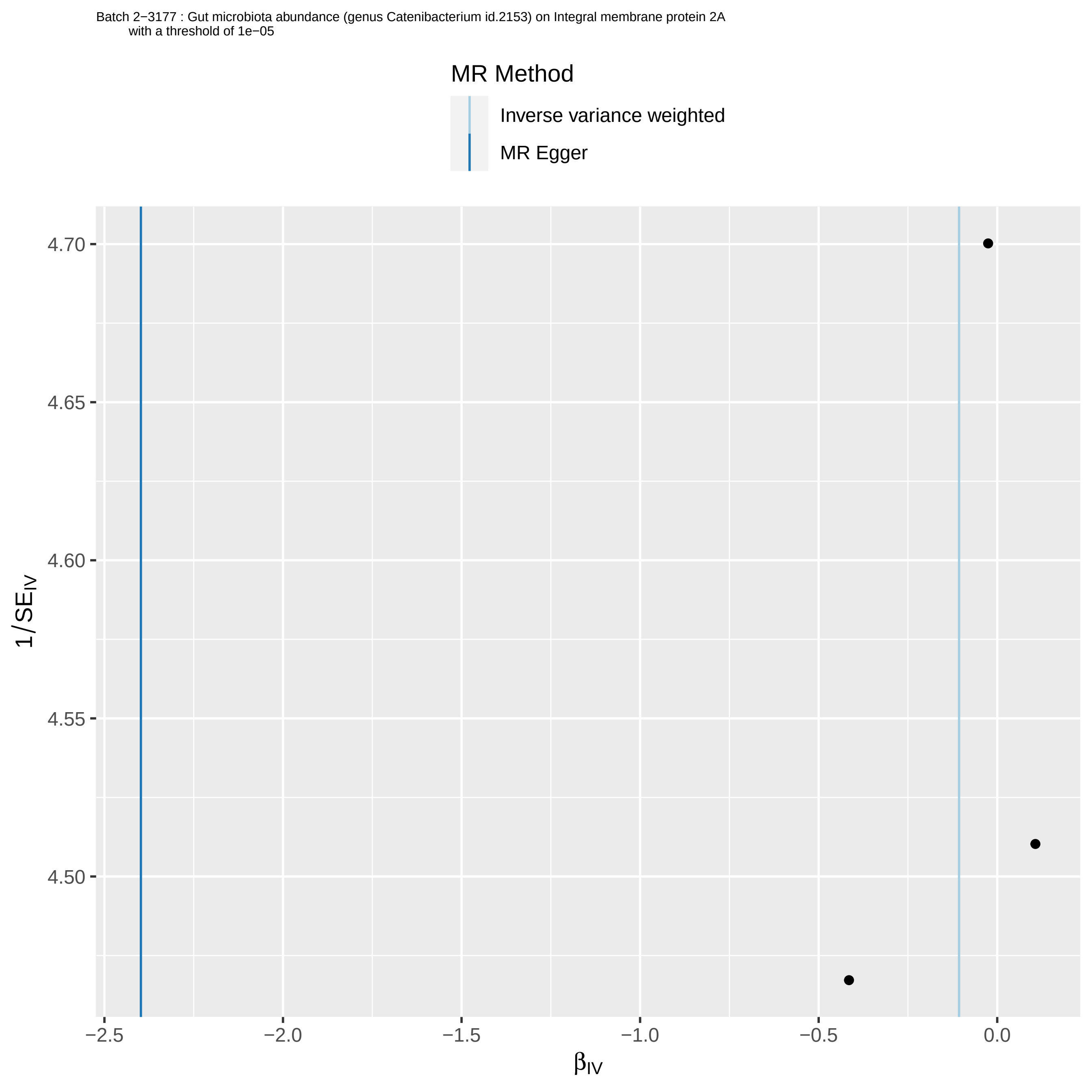

Batch 2−3177 : Gut microbiota abundance (genus Catenibacterium id.2153) on Integral membrane protein 2A
with a threshold of 1e−05
MR Method
Inverse variance weighted
MR Egger
4.70
4.65
4.60
4.55
4.50
1/SE_IV
−2.5
−2.0
−1.5
−1.0
−0.5
0.0
β_IV

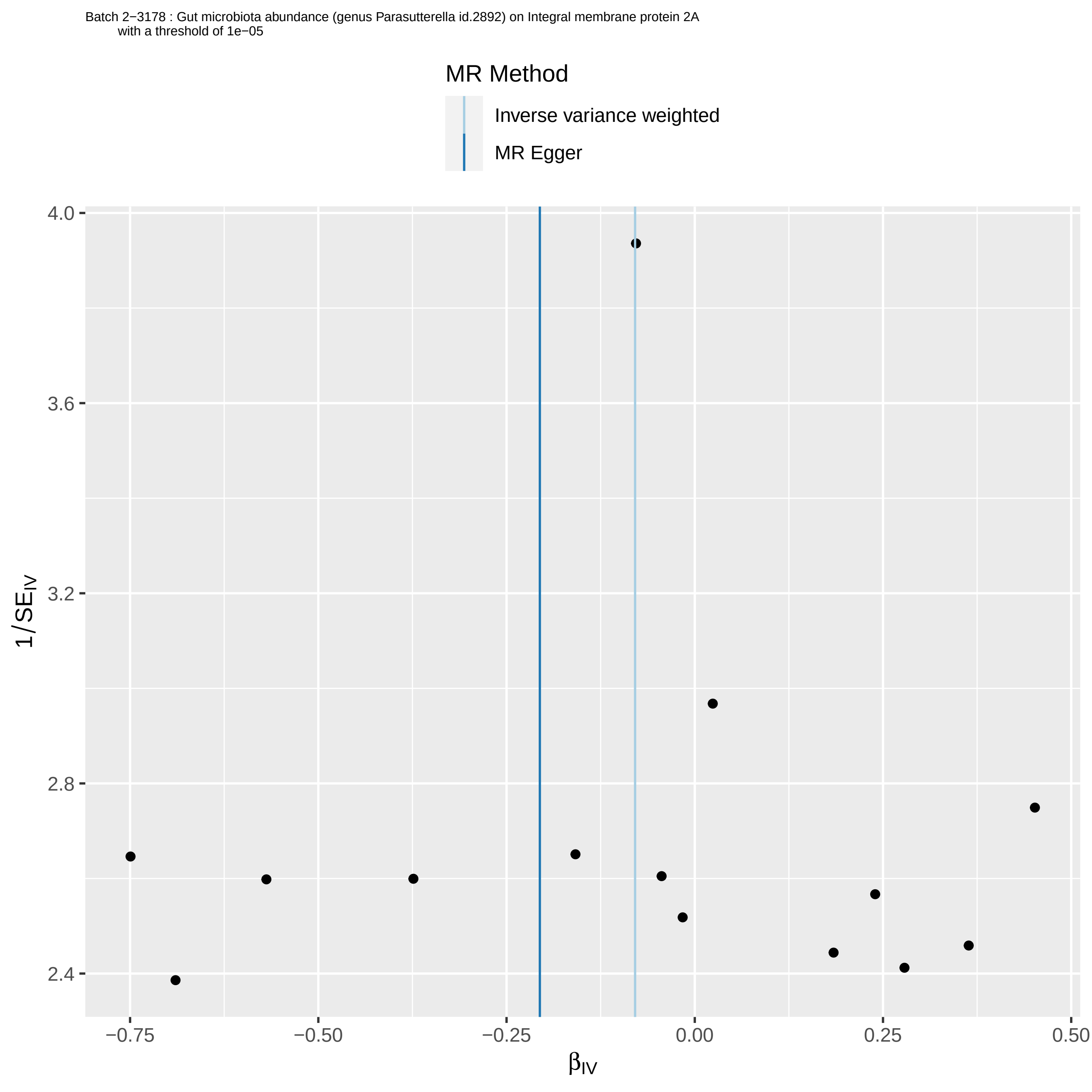
Batch 2−3178 : Gut microbiota abundance (genus Parasutterella id.2892) on Integral membrane protein 2A
with a threshold of 1e−05
MR Method
Inverse variance weighted
MR Egger
4.0
3.6
3.2
2.8
2.4
1/SE_IV
−0.75
−0.50
−0.25
0.00
0.25
0.50
β_IV

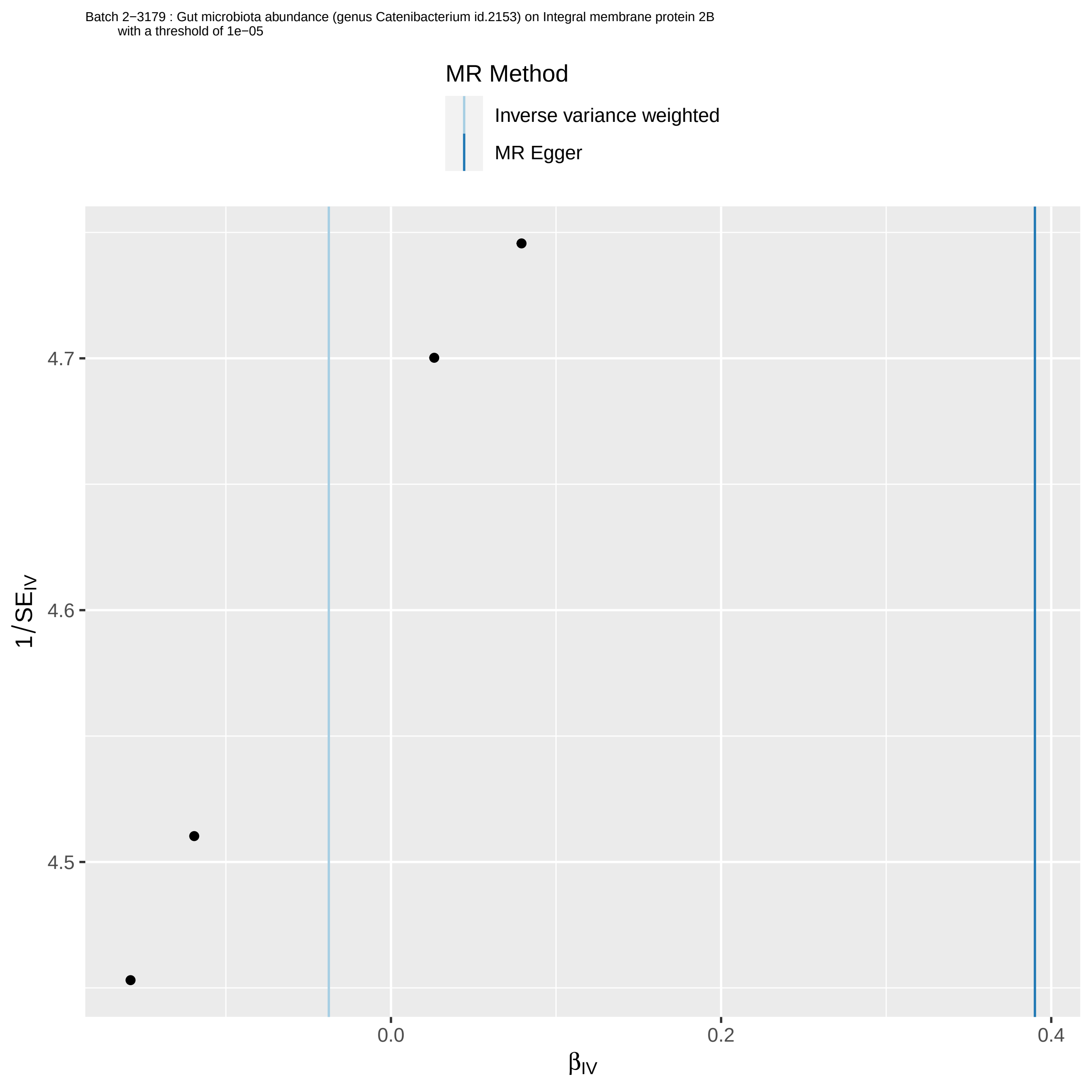
Batch 2−3179 : Gut microbiota abundance (genus Catenibacterium id.2153) on Integral membrane protein 2B
with a threshold of 1e−05
MR Method
Inverse variance weighted
MR Egger
4.7
4.6
4.5
1/SE$_{IV}$
0.0
0.2
0.4
β$_{IV}$

Batch 2−3180 : Gut microbiota abundance (genus Parasutterella id.2892) on Integral membrane protein 2B
with a threshold of 1e−05

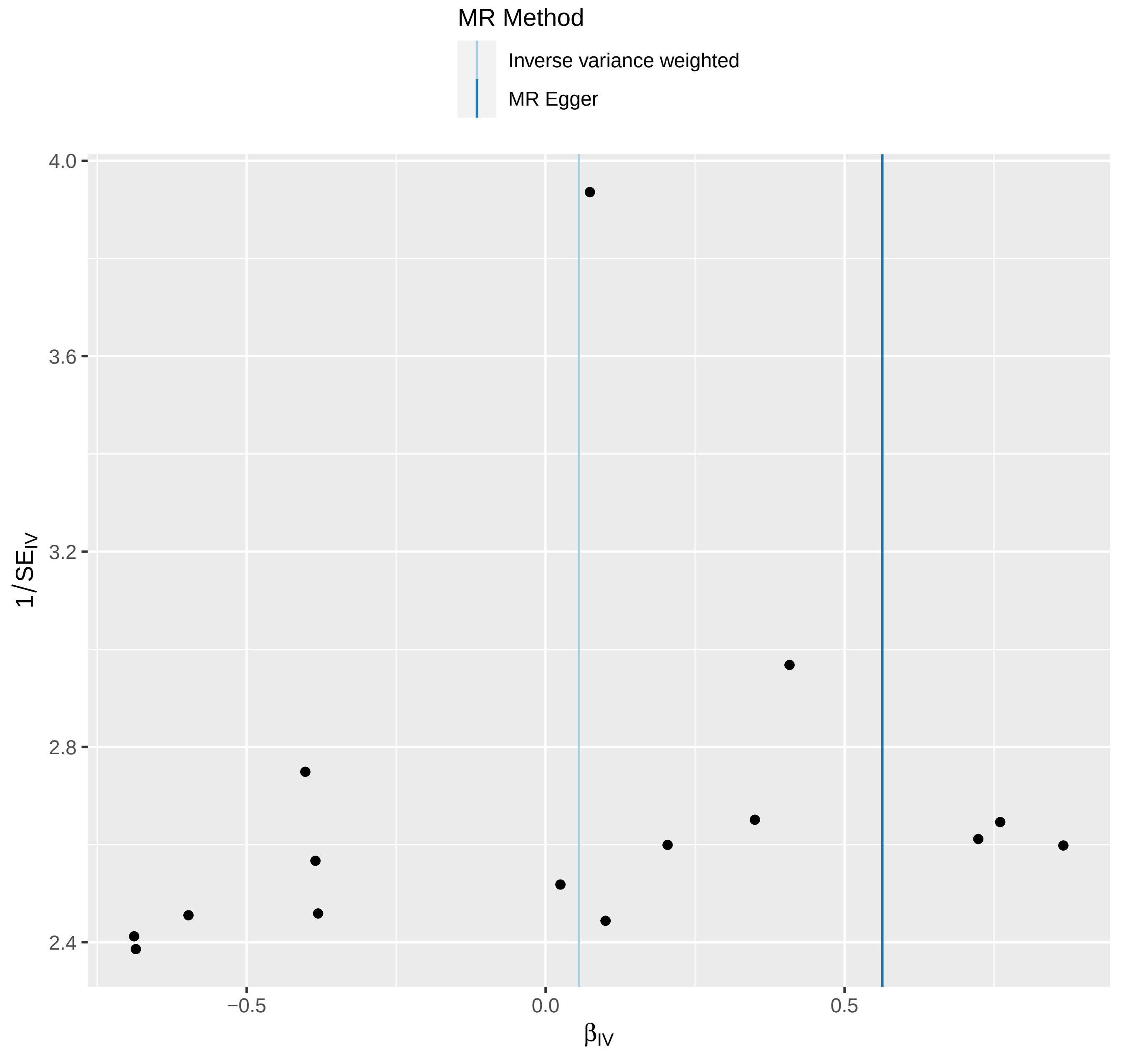

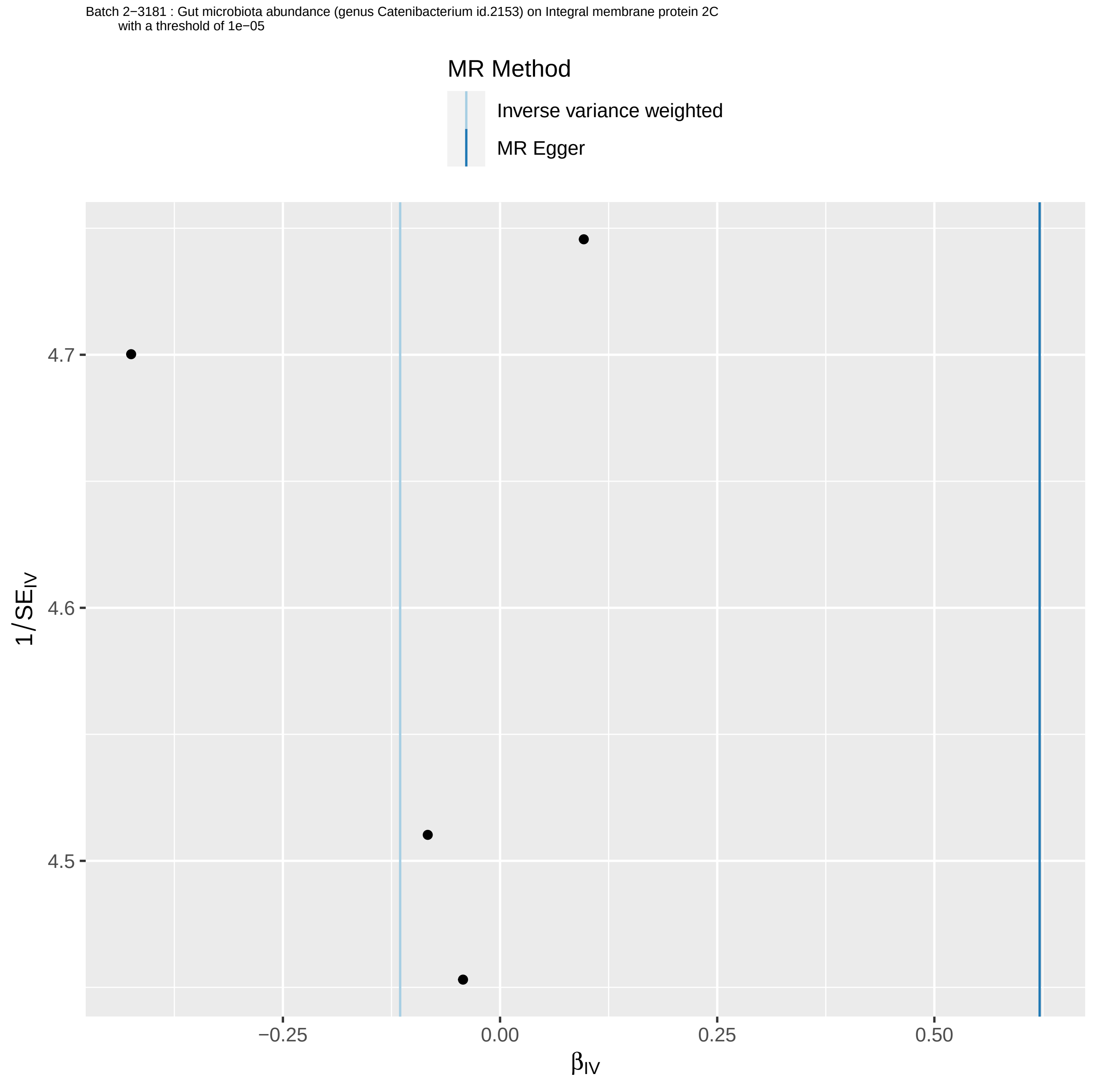

Batch 2−3181 : Gut microbiota abundance (genus Catenibacterium id.2153) on Integral membrane protein 2C
with a threshold of 1e−05
MR Method
Inverse variance weighted
MR Egger
4.7
4.6
4.5
1/SE_IV
−0.25
0.00
0.25
0.50
β_IV

Batch 2−3182 : Gut microbiota abundance (genus Parasutterella id.2892) on Integral membrane protein 2C
with a threshold of 1e−05

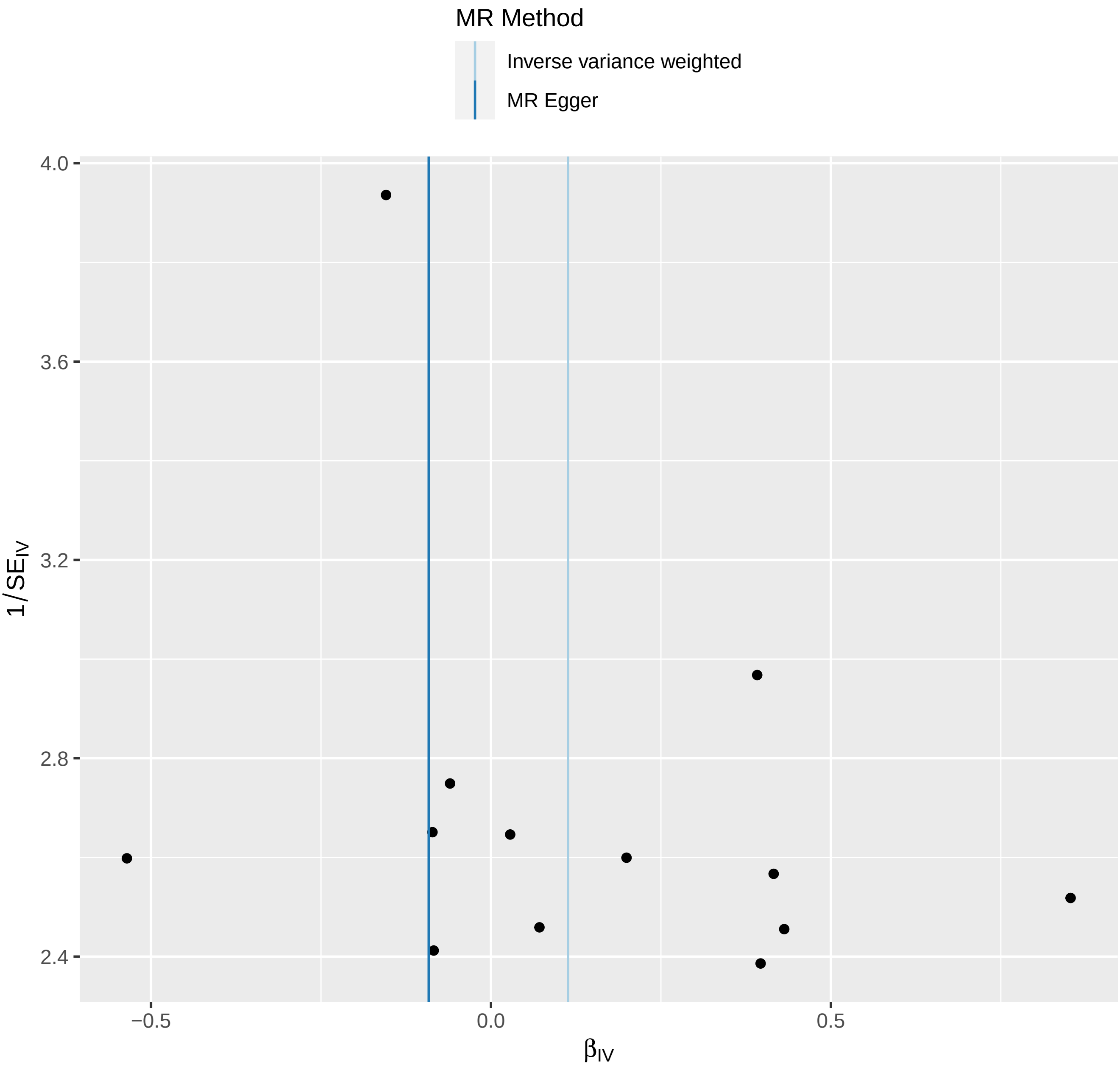

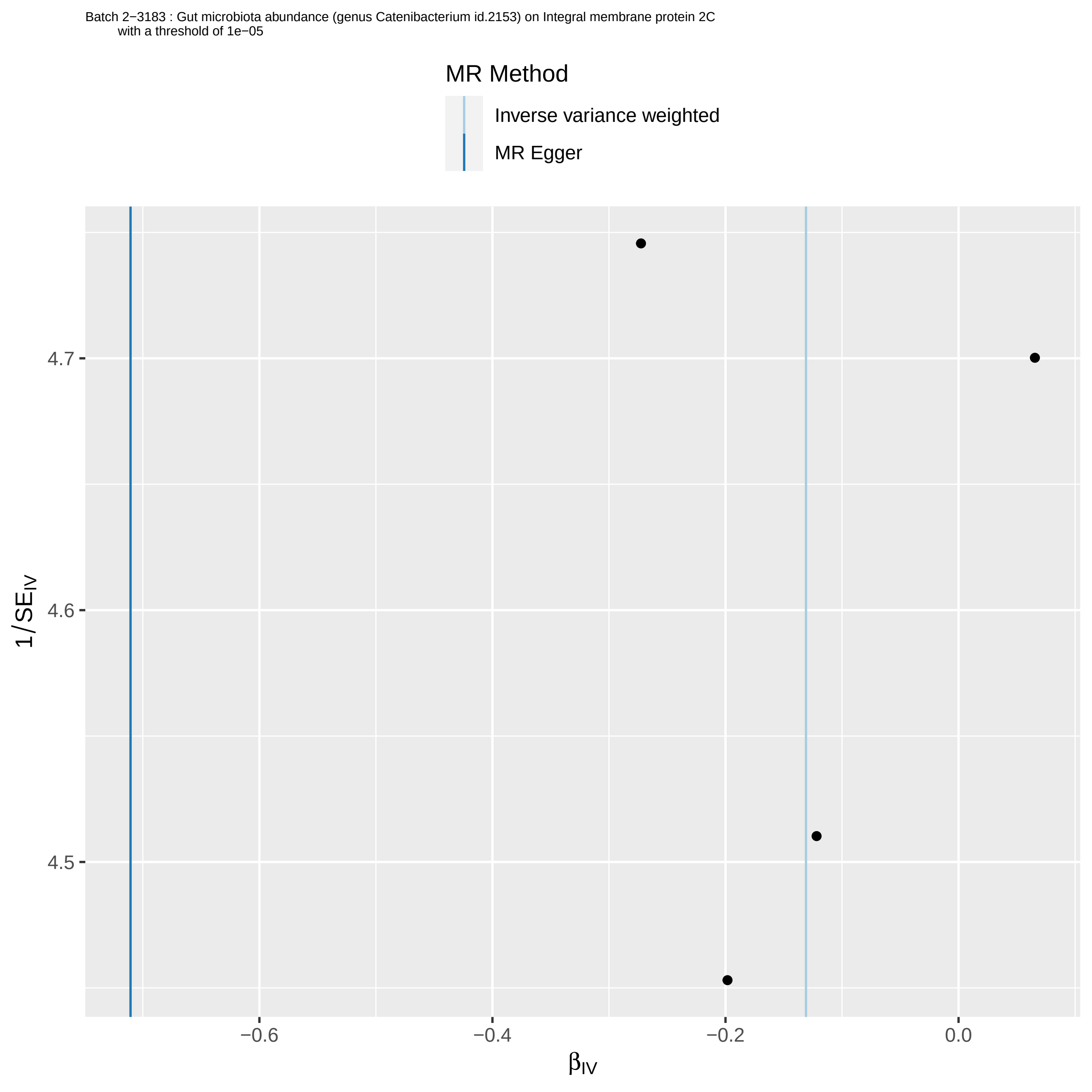

Batch 2−3183 : Gut microbiota abundance (genus Catenibacterium id.2153) on Integral membrane protein 2C
with a threshold of 1e−05
MR Method
Inverse variance weighted
MR Egger
4.7
4.6
4.5
1/SE$_{IV}$
−0.6
−0.4
−0.2
0.0
β_{IV}

Batch 2−3184 : Gut microbiota abundance (genus Parasutterella id.2892) on Integral membrane protein 2C
with a threshold of 1e−05

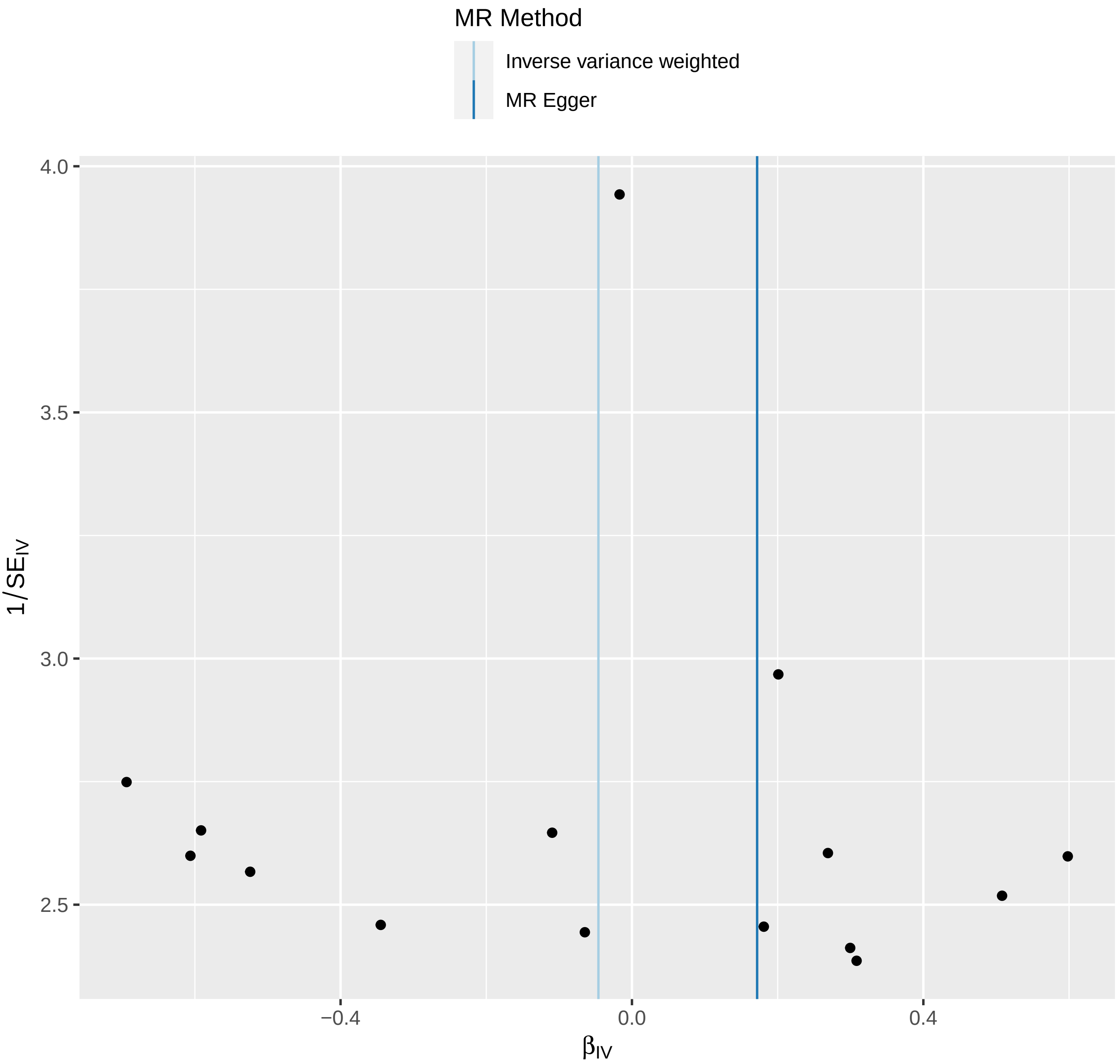

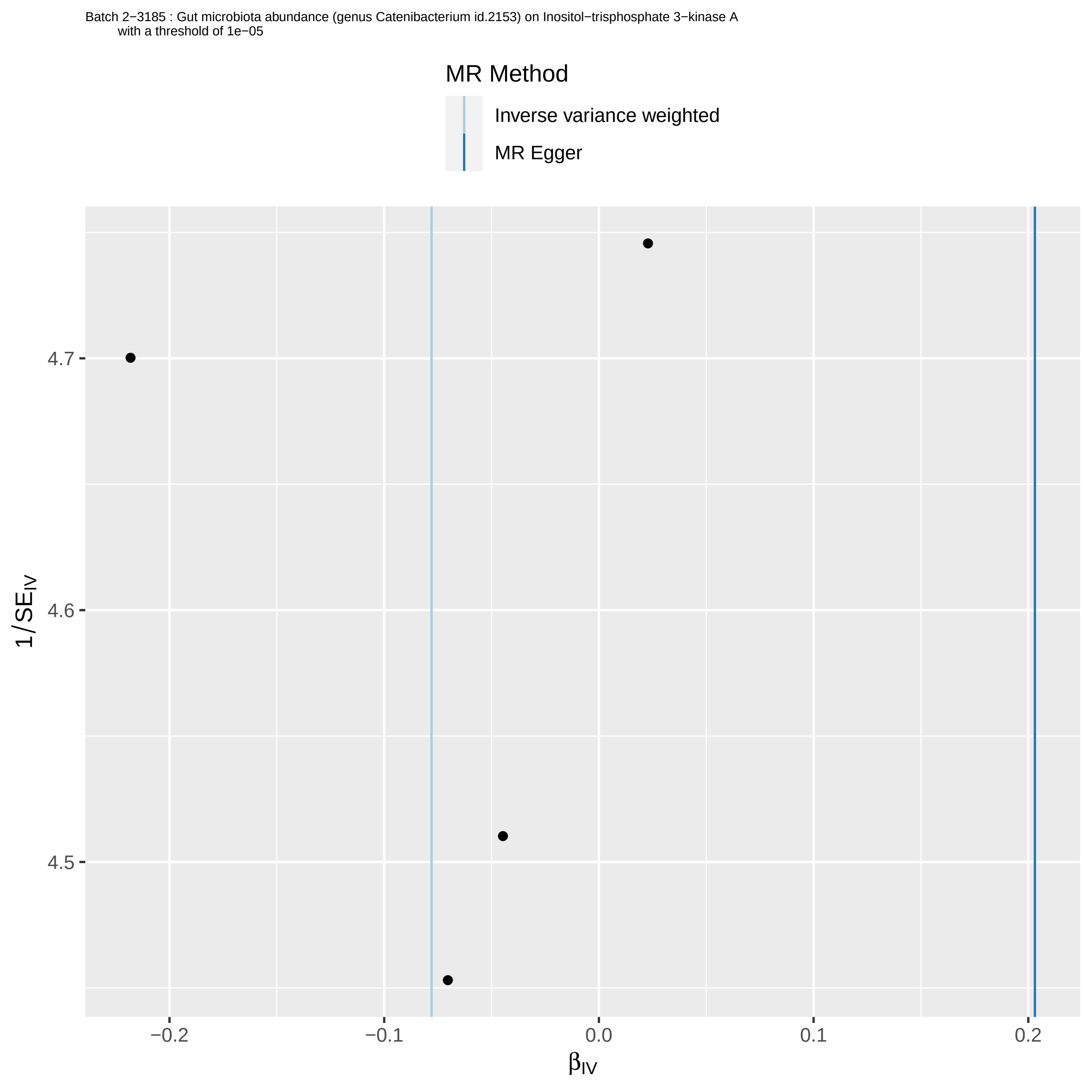
Batch 2−3185 : Gut microbiota abundance (genus Catenibacterium id.2153) on Inositol−trisphosphate 3−kinase A
with a threshold of 1e−05
MR Method
Inverse variance weighted
MR Egger
4.7
4.6
4.5
1/SE$_{IV}$
−0.2
−0.1
0.0
0.1
0.2
β$_{IV}$

Batch 2−3186 : Gut microbiota abundance (genus Parasutterella id.2892) on Inositol−trisphosphate 3−kinase A with a threshold of 1e−05

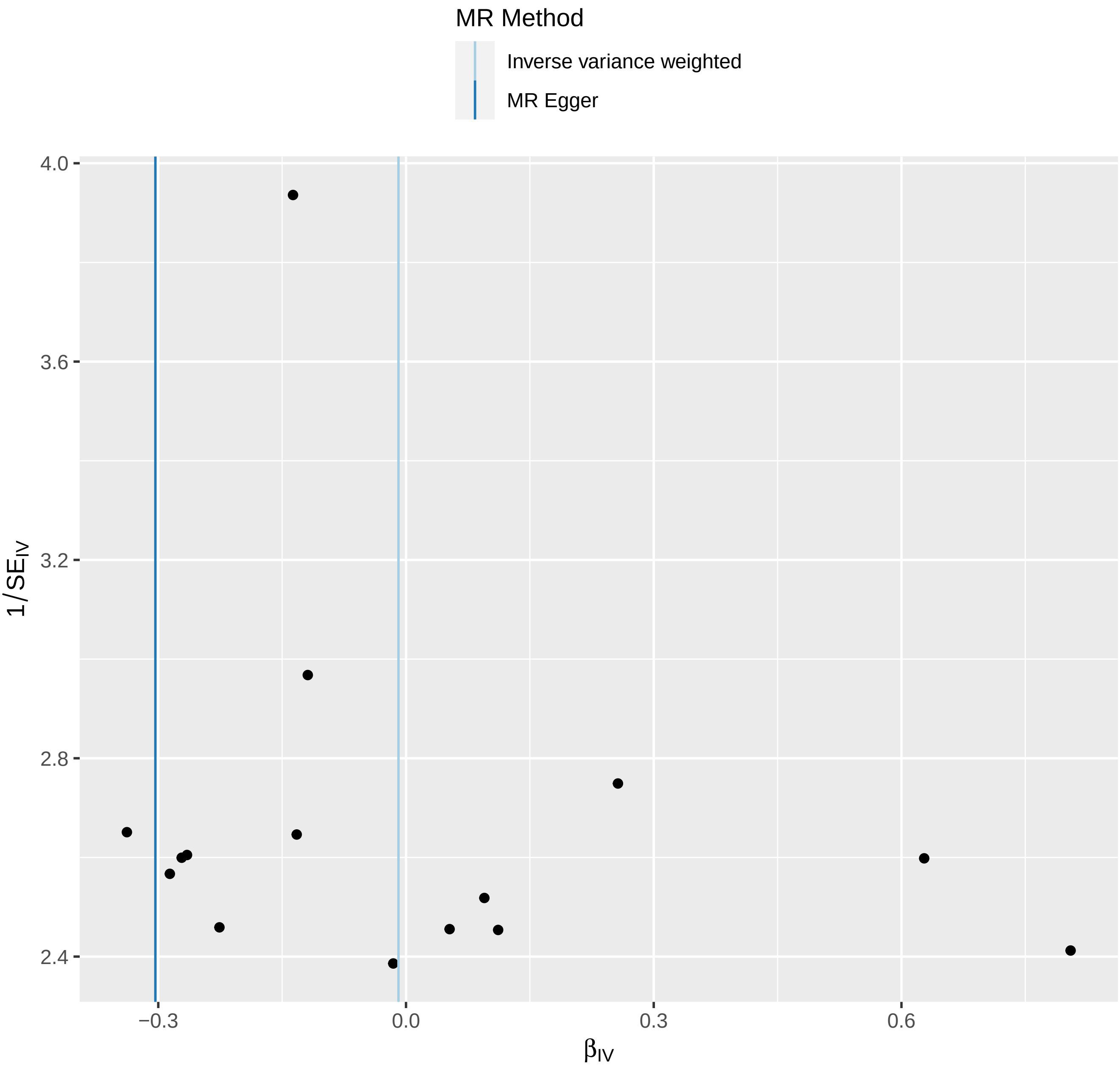

Batch 2−3187 : Gut microbiota abundance (genus Catenibacterium id.2153) on Inositol 1,4,5−trisphosphate receptor−interacting protein−like 1 with a threshold of 1e−05

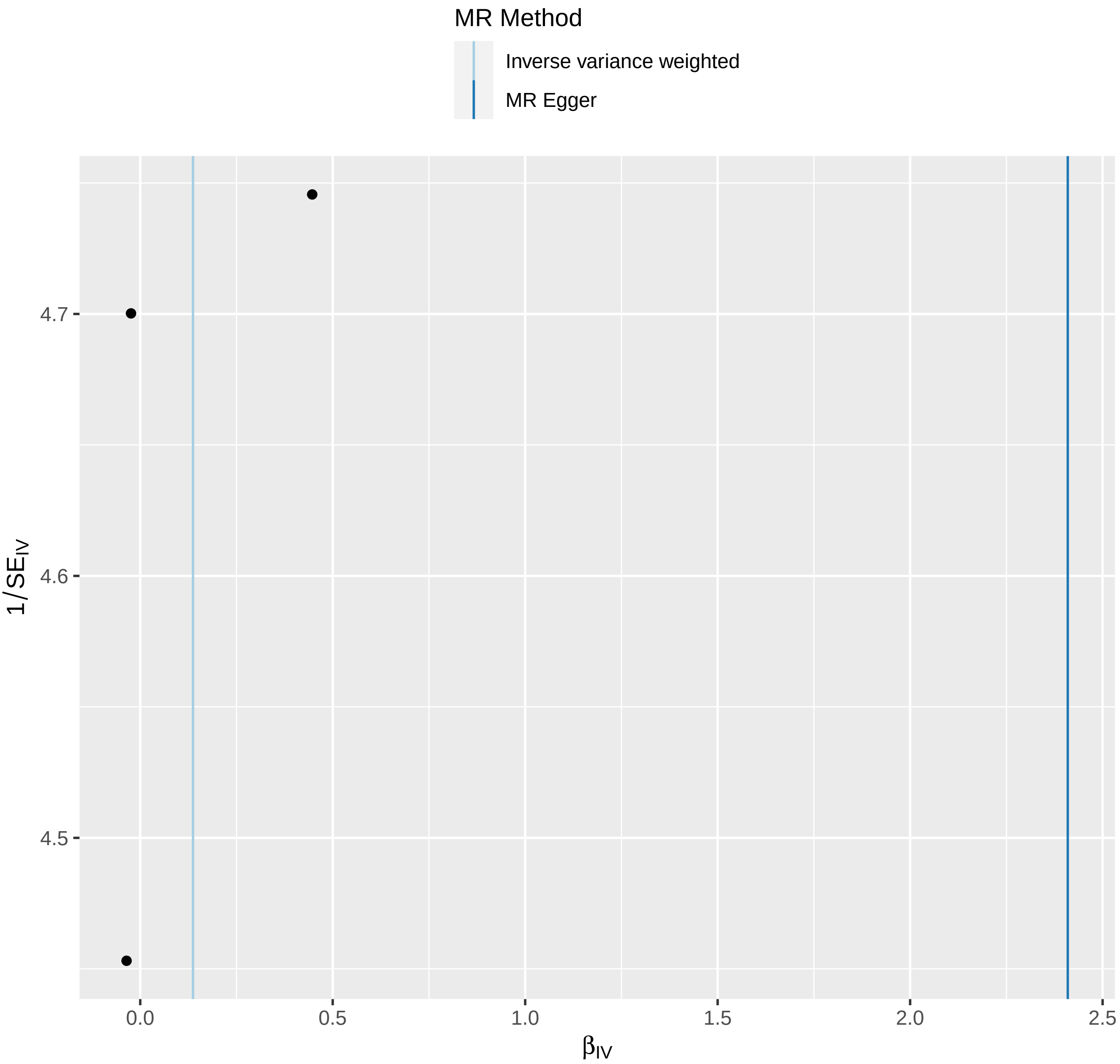

Batch 2−3188 : Gut microbiota abundance (genus Parasutterella id.2892) on Inositol 1,4,5−trisphosphate receptor−interacting protein−like 1
with a threshold of 1e−05

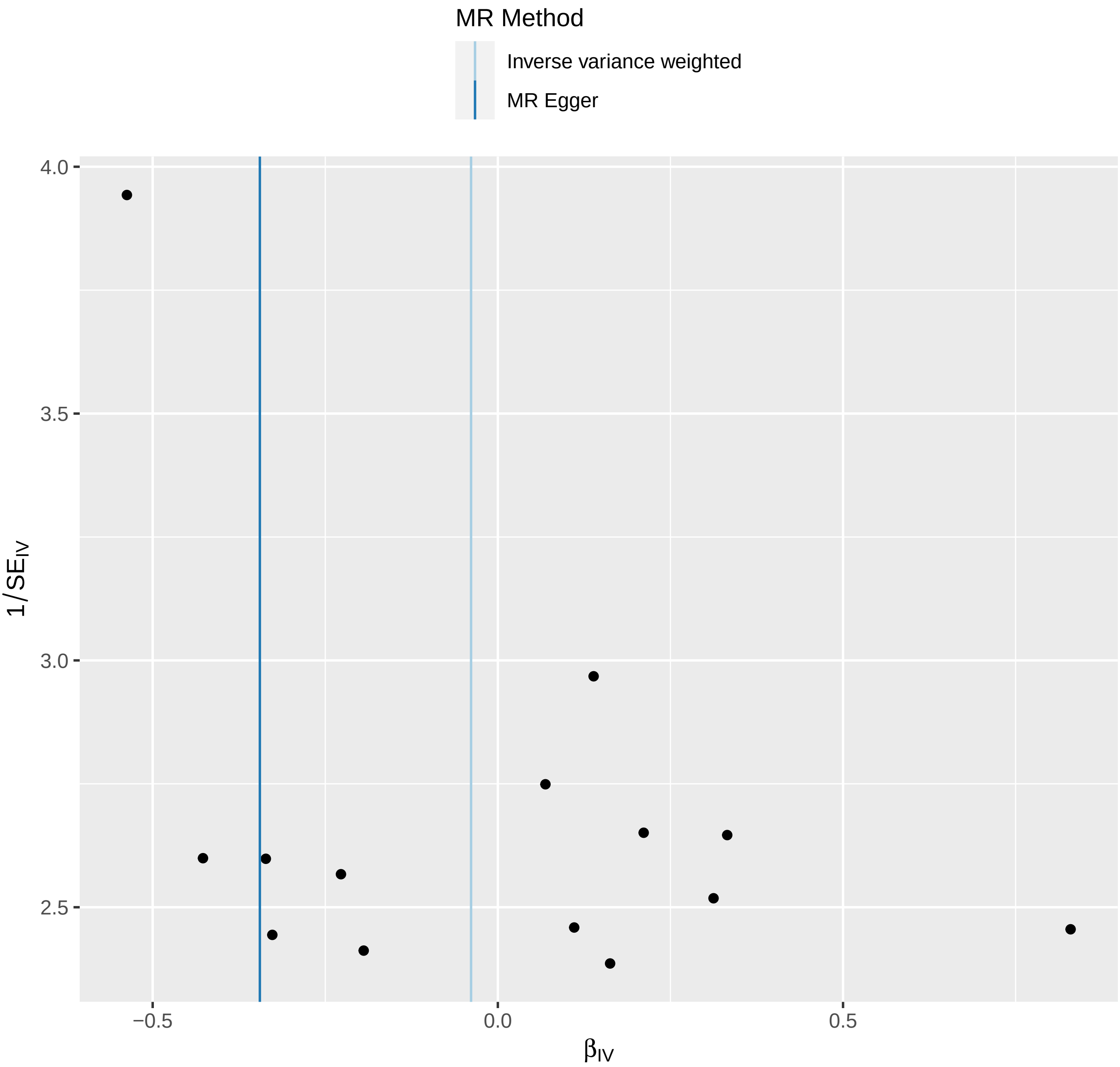

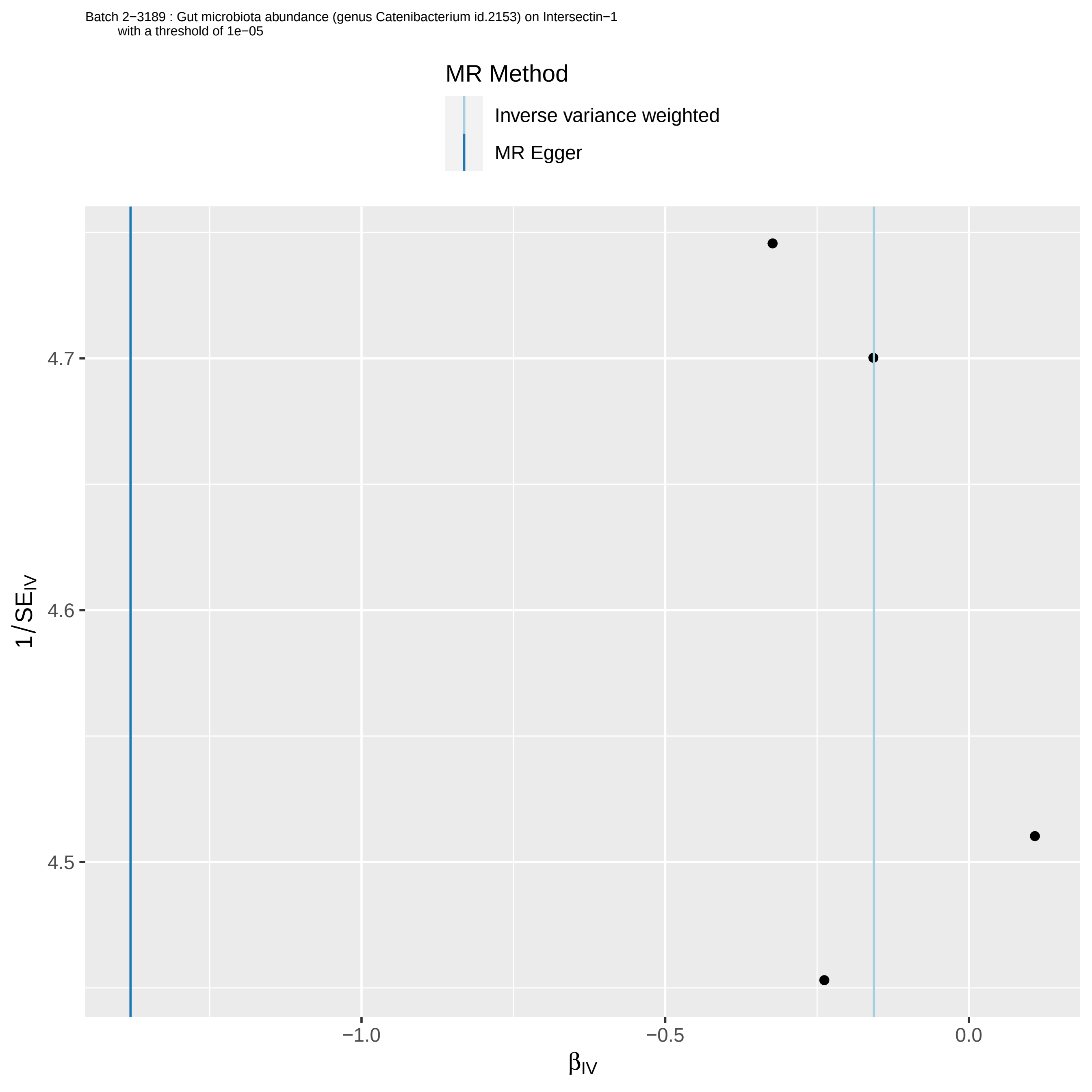
Batch 2−3189 : Gut microbiota abundance (genus Catenibacterium id.2153) on Intersectin−1
with a threshold of 1e−05
MR Method
Inverse variance weighted
MR Egger
4.7
4.6
4.5
1/SE$_{IV}$
−1.0
−0.5
0.0
β$_{IV}$

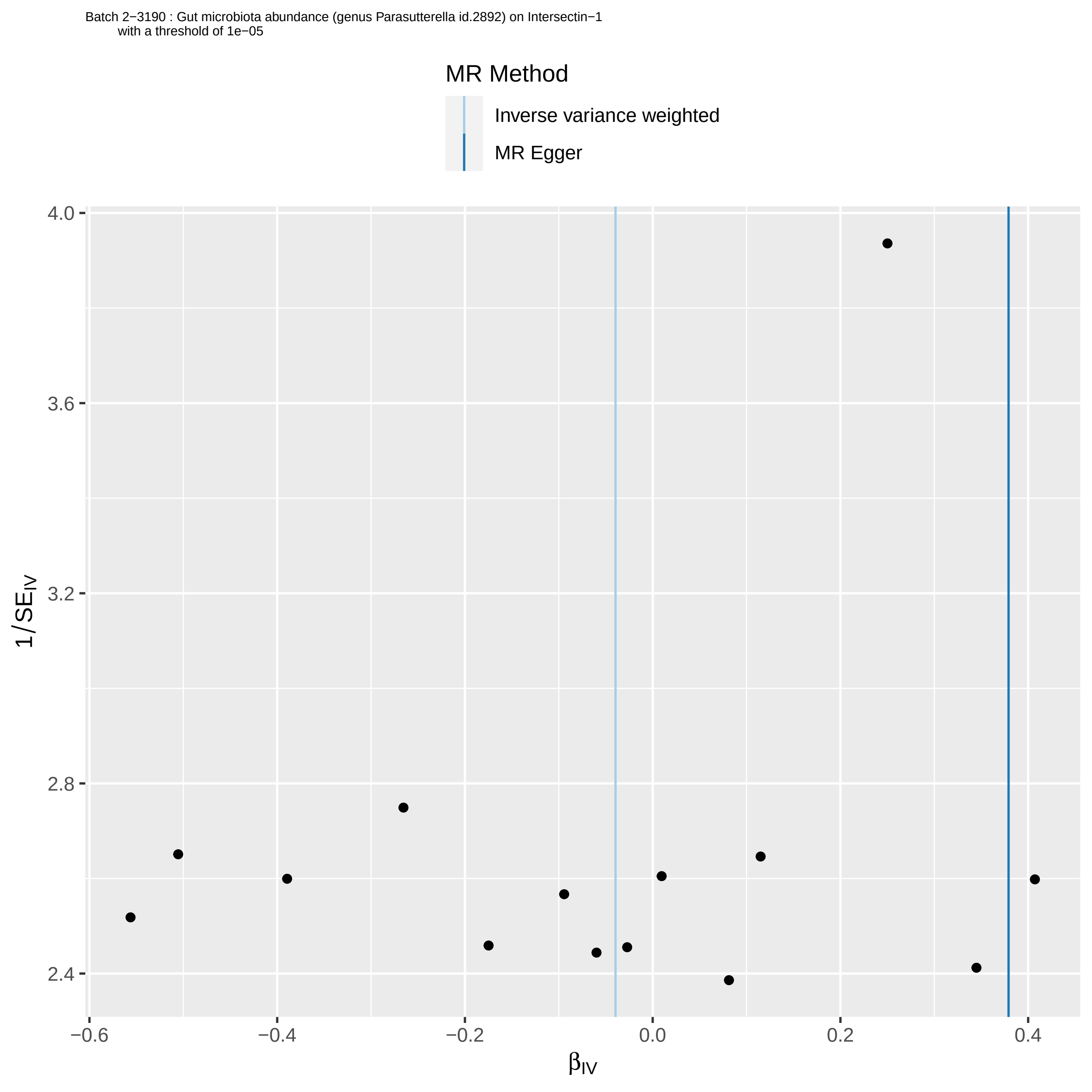

Batch 2−3190 : Gut microbiota abundance (genus Parasutterella id.2892) on Intersectin−1
with a threshold of 1e−05
MR Method
Inverse variance weighted
MR Egger
4.0
3.6
3.2
2.8
2.4
1/SE_IV
−0.6
−0.4
−0.2
0.0
0.2
0.4
β_IV

Batch 2−3191 : Gut microbiota abundance (genus Catenibacterium id.2153) on Protein jagged−1
with a threshold of 1e−05

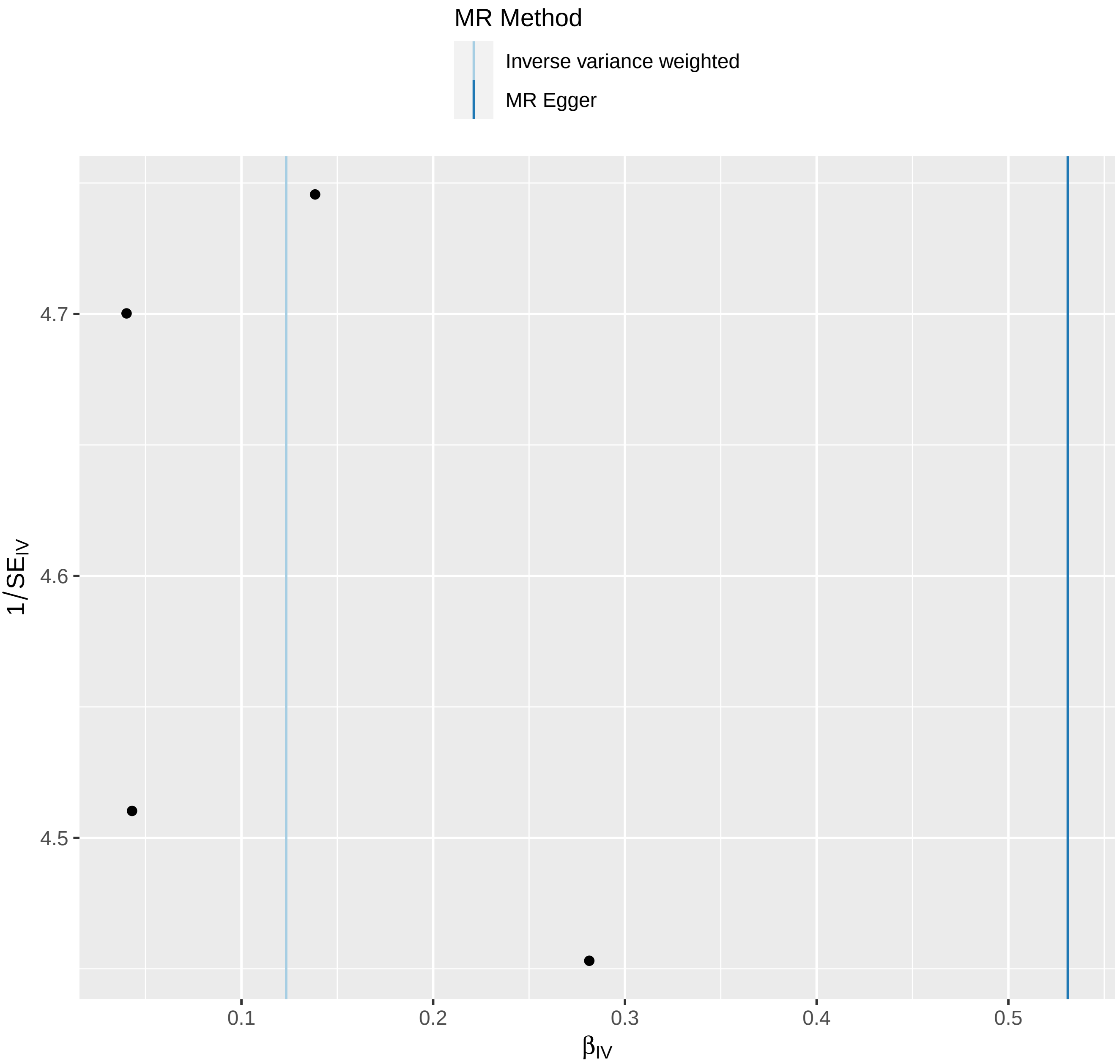

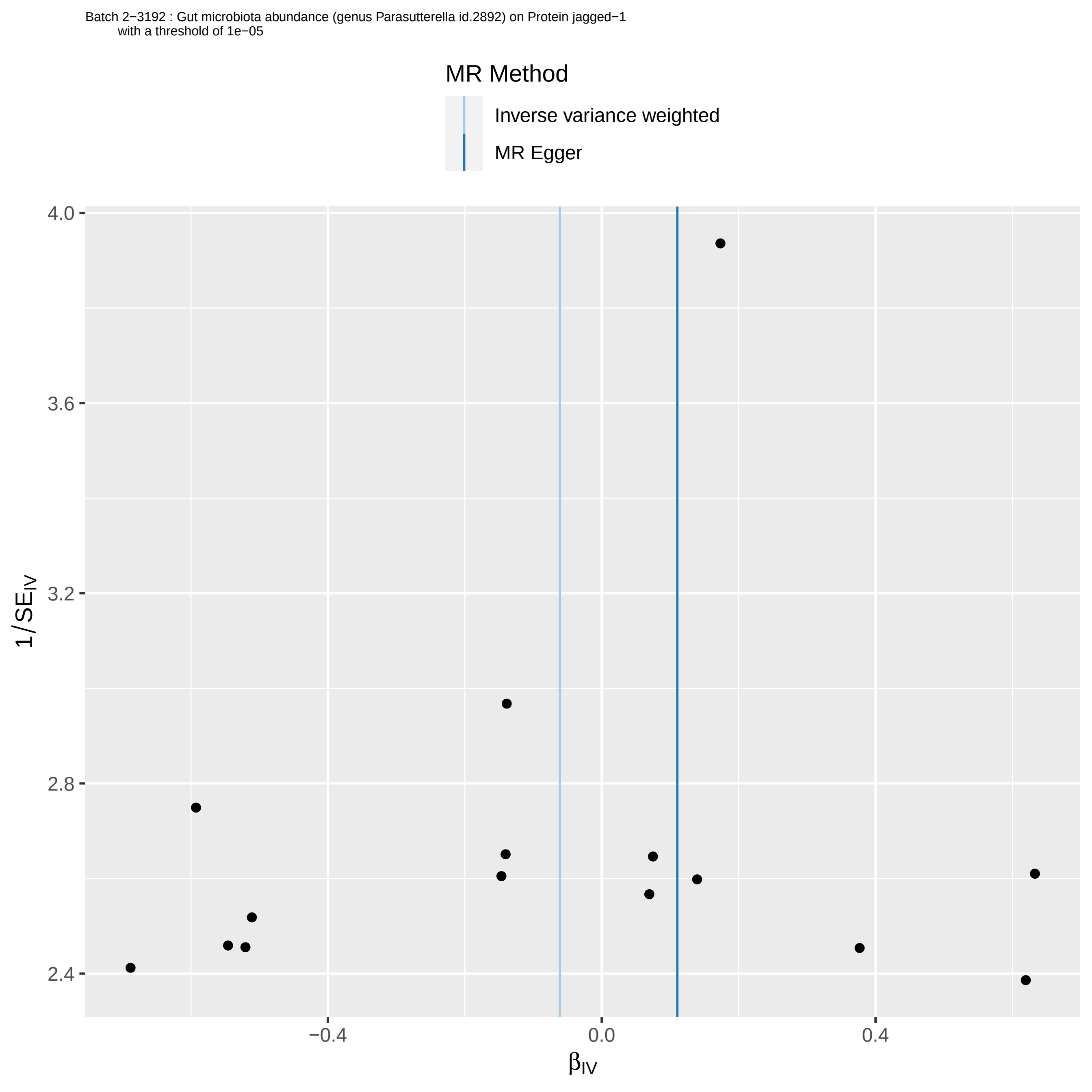

Batch 2−3192 : Gut microbiota abundance (genus Parasutterella id.2892) on Protein jagged−1
with a threshold of 1e−05
MR Method
Inverse variance weighted
MR Egger
4.0
3.6
3.2
2.8
2.4
1/SE_IV
−0.4
0.0
0.4
β_IV

Batch 2−3193 : Gut microbiota abundance (genus Catenibacterium id.2153) on Protein jagged−2
with a threshold of 1e−05

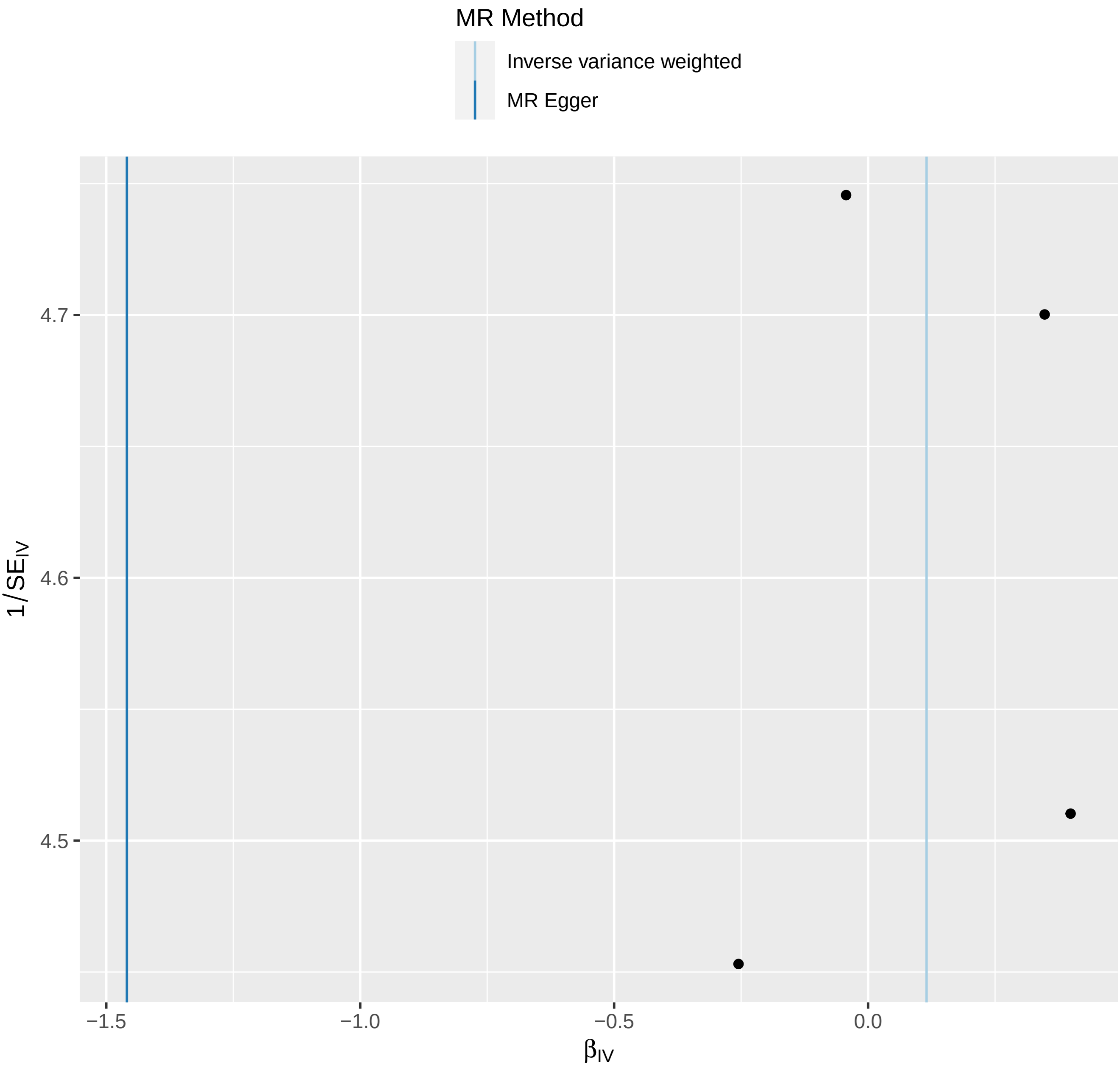

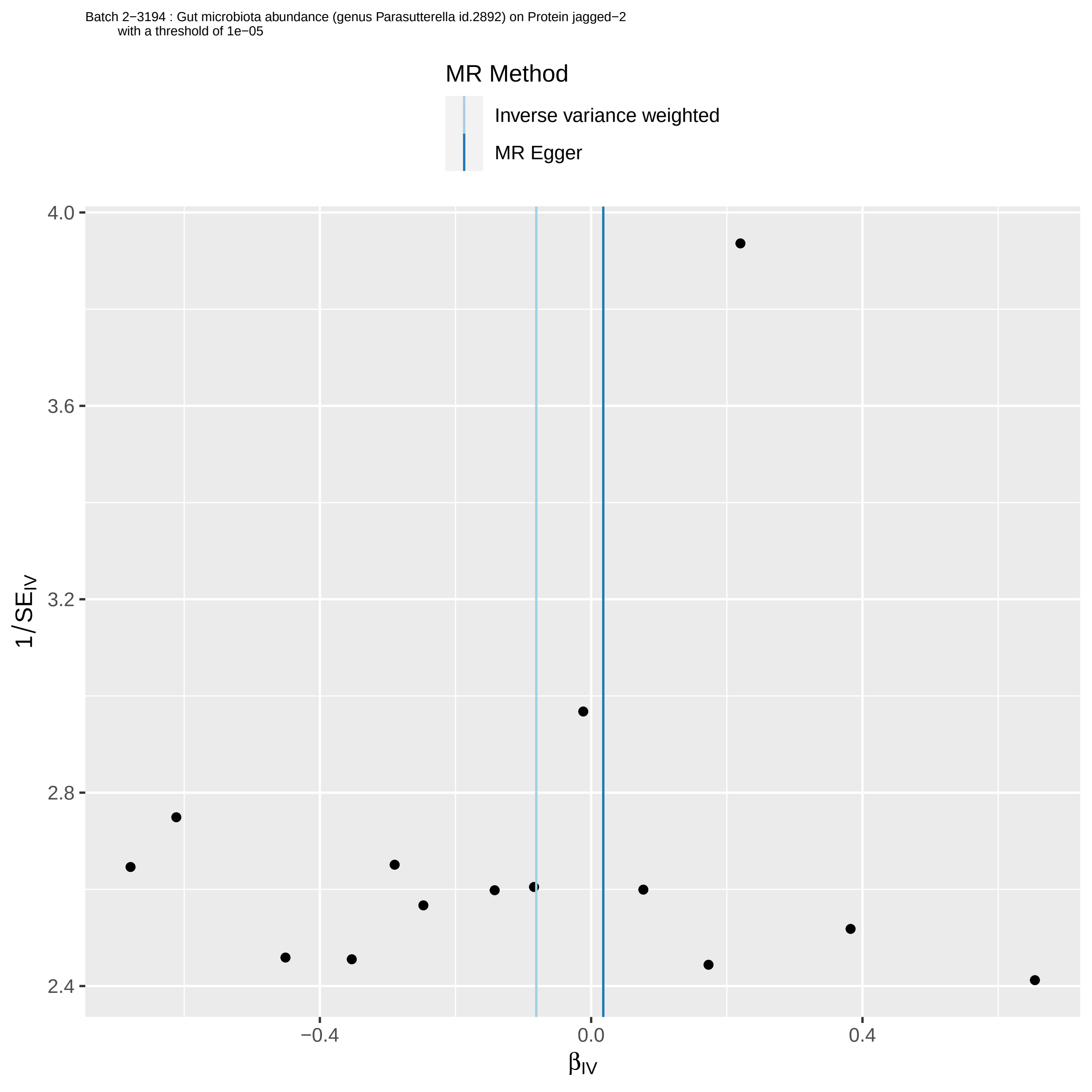

Batch 2−3194 : Gut microbiota abundance (genus Parasutterella id.2892) on Protein jagged−2
with a threshold of 1e−05
MR Method
Inverse variance weighted
MR Egger
4.0
3.6
3.2
2.8
2.4
1/SE_IV
−0.4
0.0
0.4
β_IV

Batch 2−3195 : Gut microbiota abundance (genus Catenibacterium id.2153) on Tyrosine−protein kinase JAK2
with a threshold of 1e−05

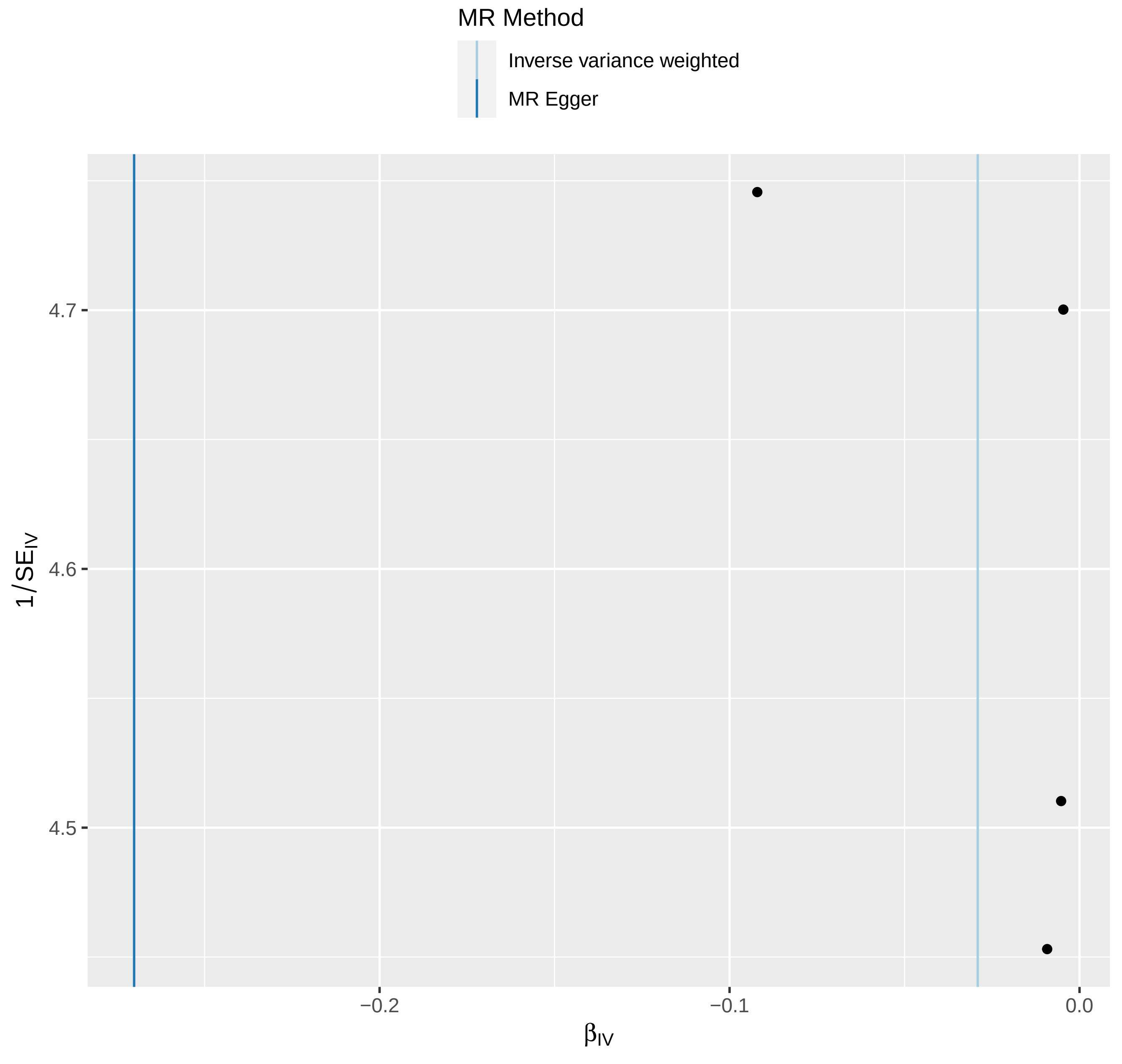

Batch 2−3196 : Gut microbiota abundance (genus Parasutterella id.2892) on Tyrosine−protein kinase JAK2
with a threshold of 1e−05

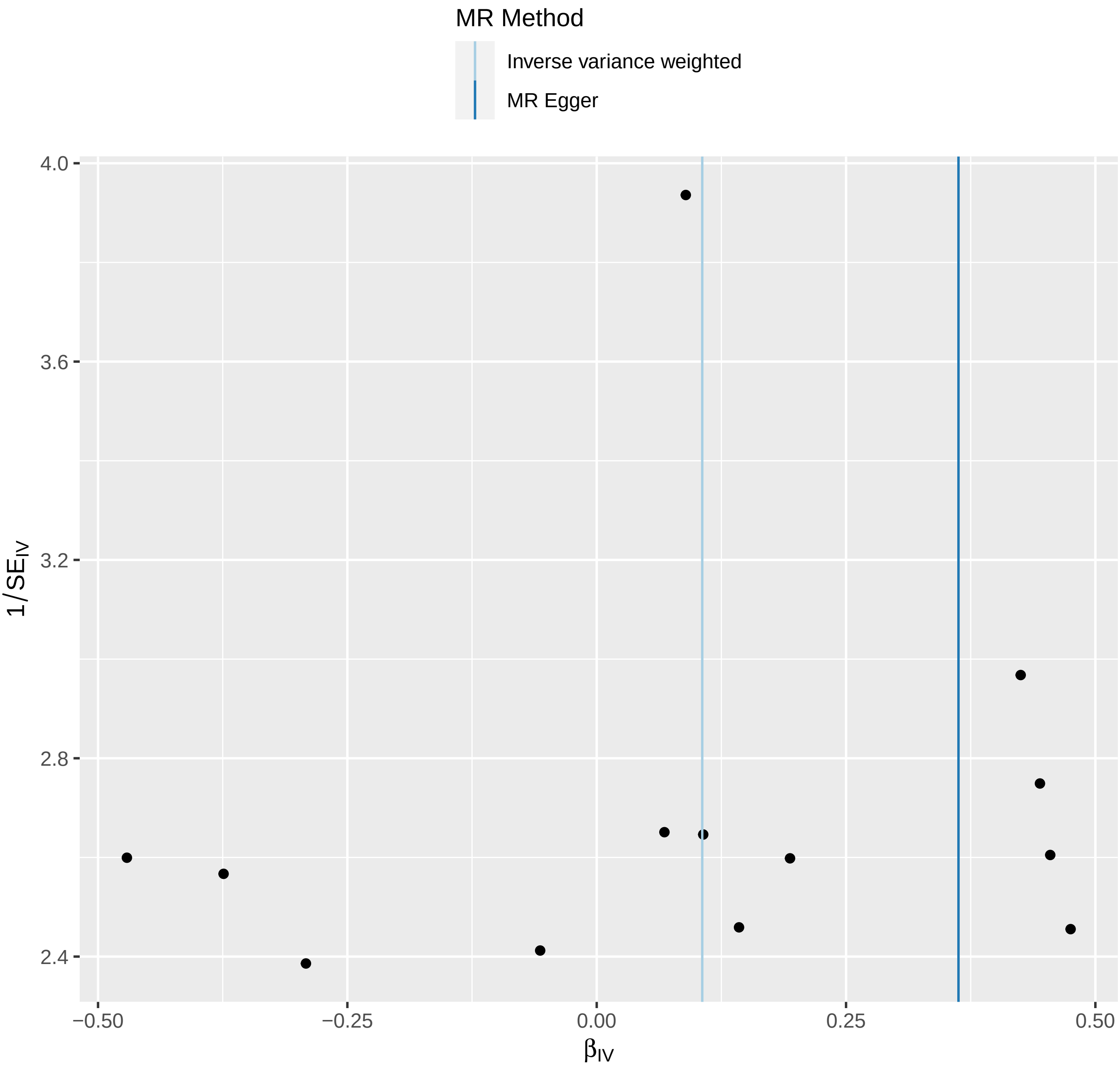

Batch 2−3197 : Gut microbiota abundance (genus Catenibacterium id.2153) on Janus kinase and microtubule−interacting protein 3
with a threshold of 1e−05

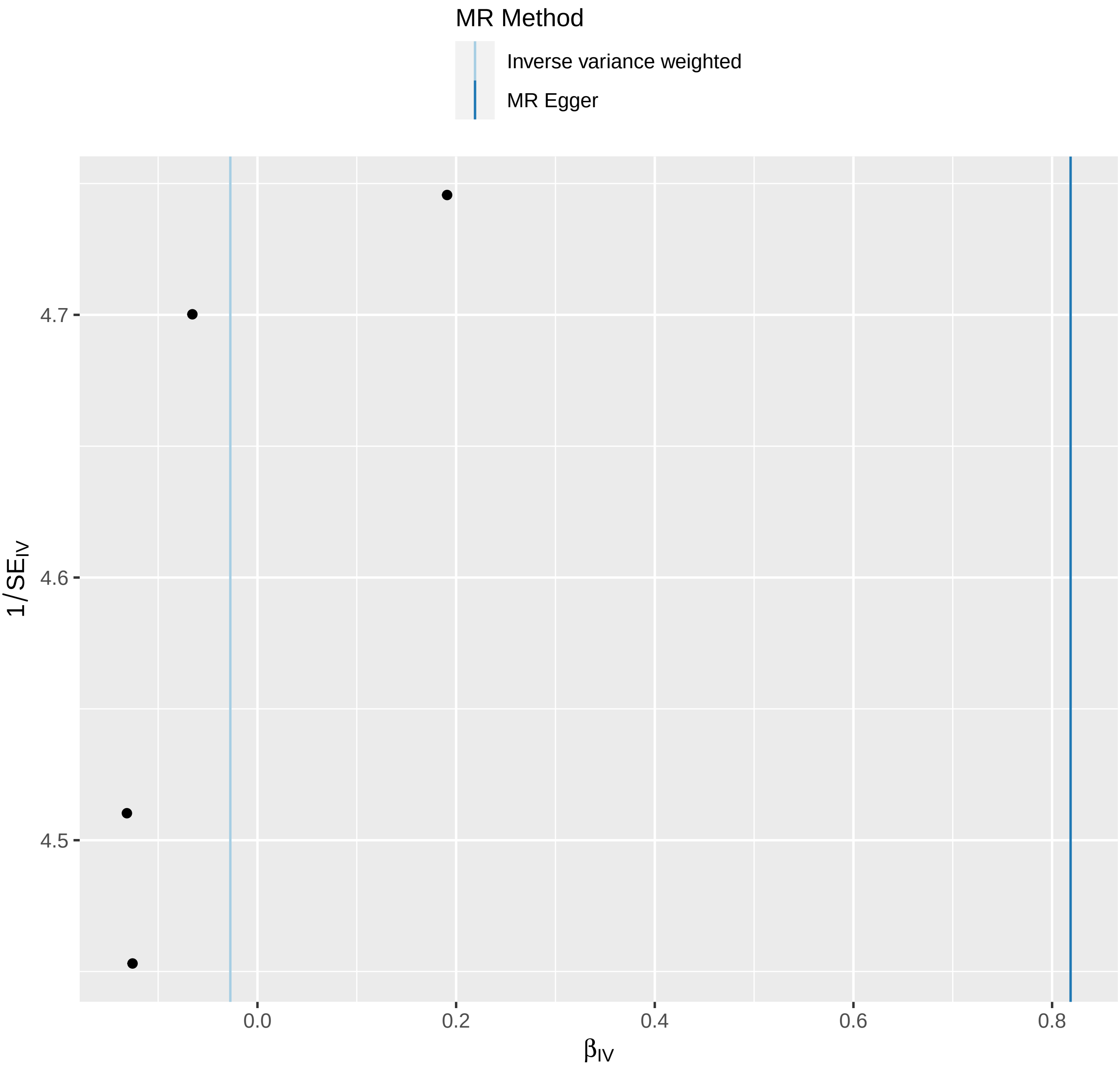

Batch 2−3198 : Gut microbiota abundance (genus Parasutterella id.2892) on Janus kinase and microtubule−interacting protein 3 with a threshold of 1e−05

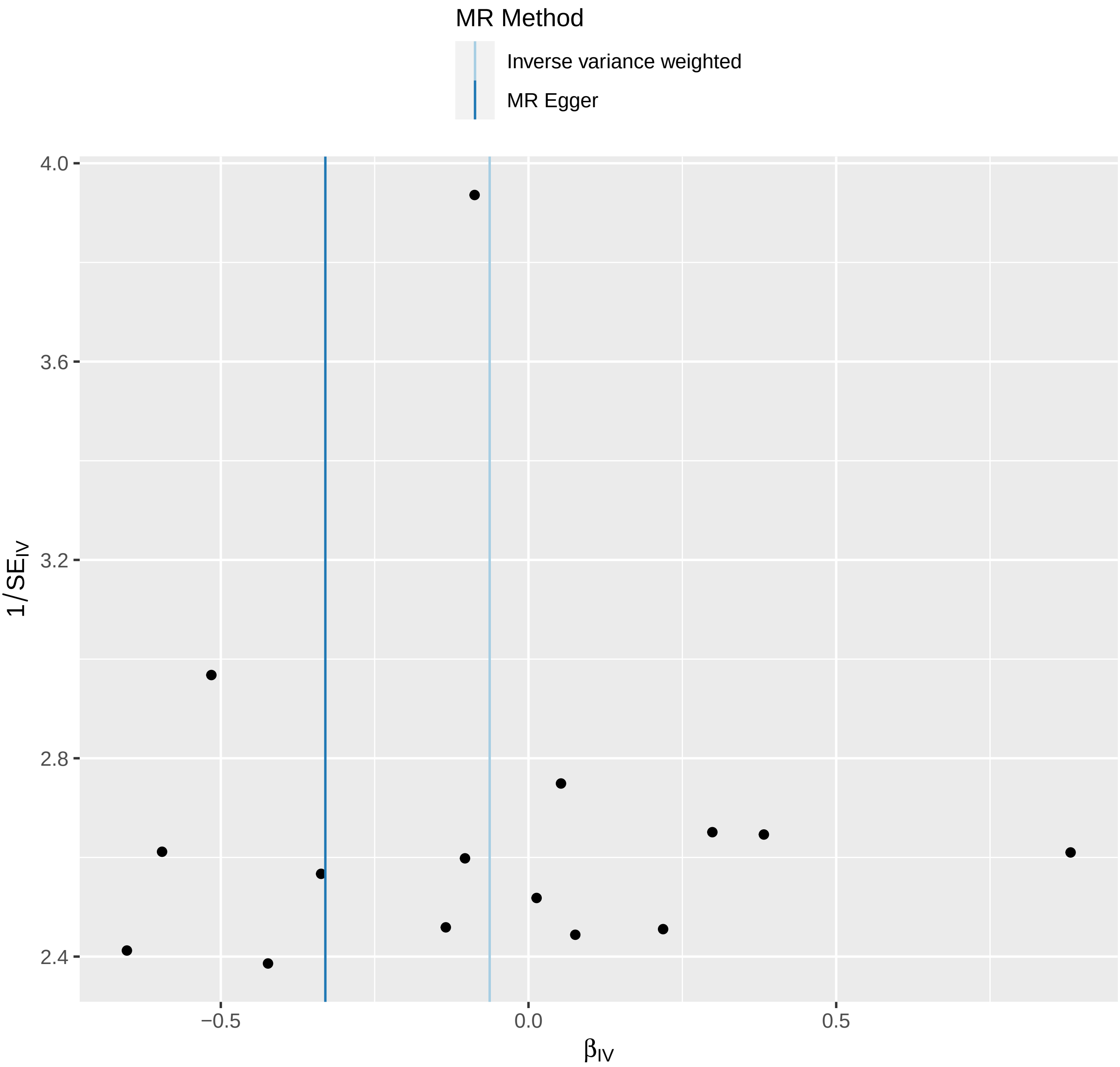

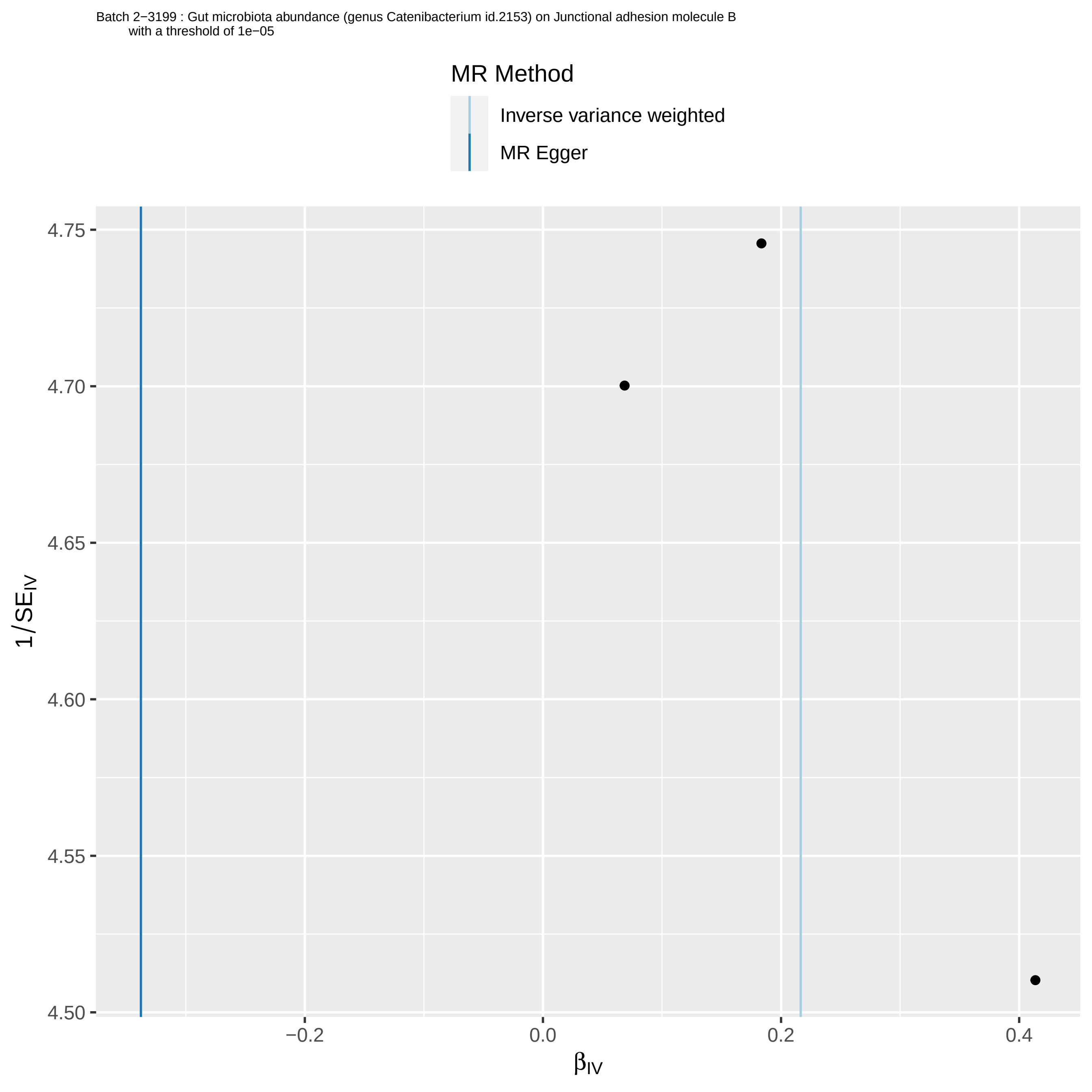

Batch 2−3199 : Gut microbiota abundance (genus Catenibacterium id.2153) on Junctional adhesion molecule B
with a threshold of 1e−05
MR Method
Inverse variance weighted
MR Egger
4.75
4.70
4.65
4.60
4.55
4.50
1/SE$_{IV}$
−0.2
0.0
0.2
0.4
β_{IV}

Batch 2−3200 : Gut microbiota abundance (genus Parasutterella id.2892) on Junctional adhesion molecule B
with a threshold of 1e−05

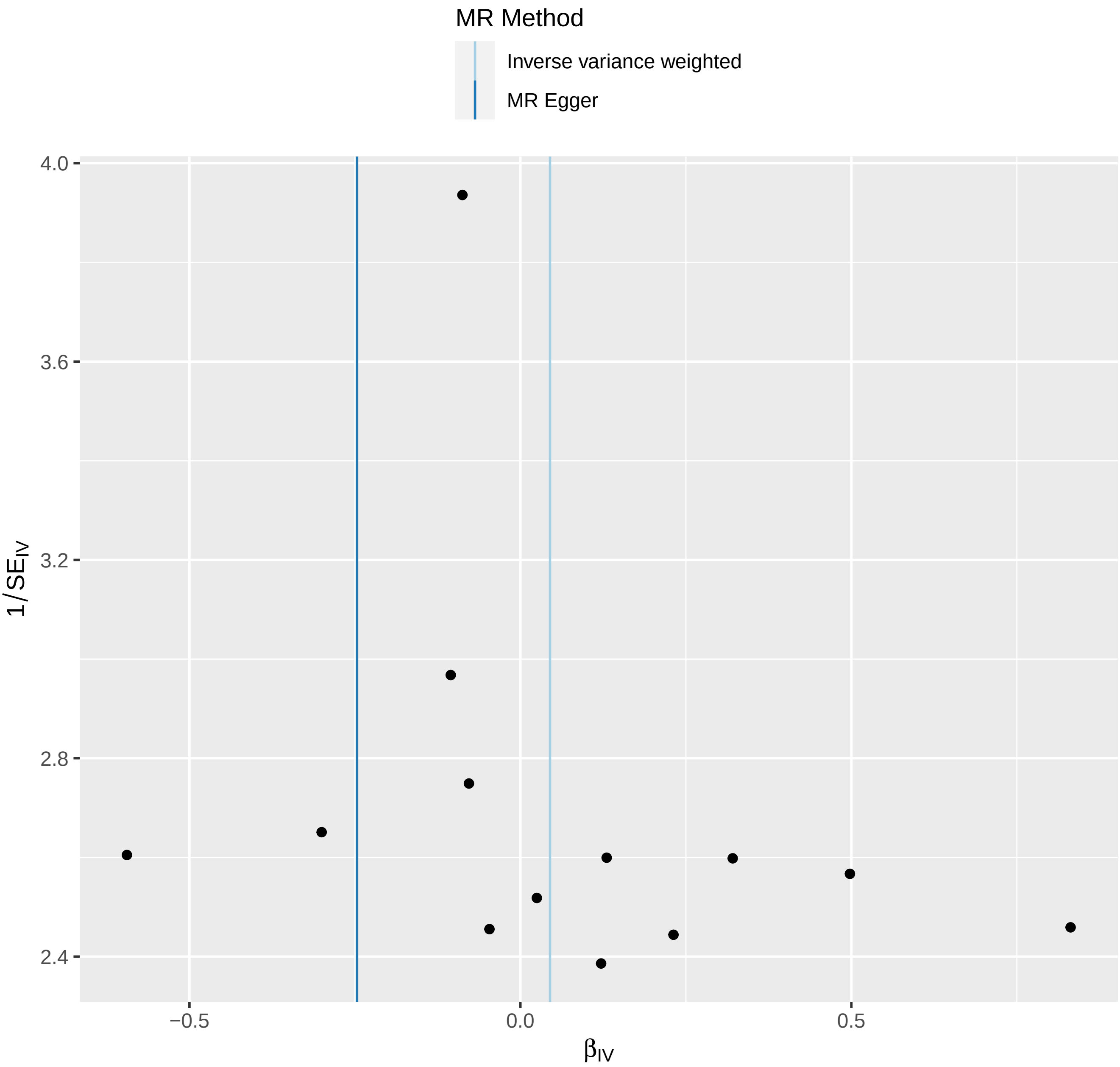

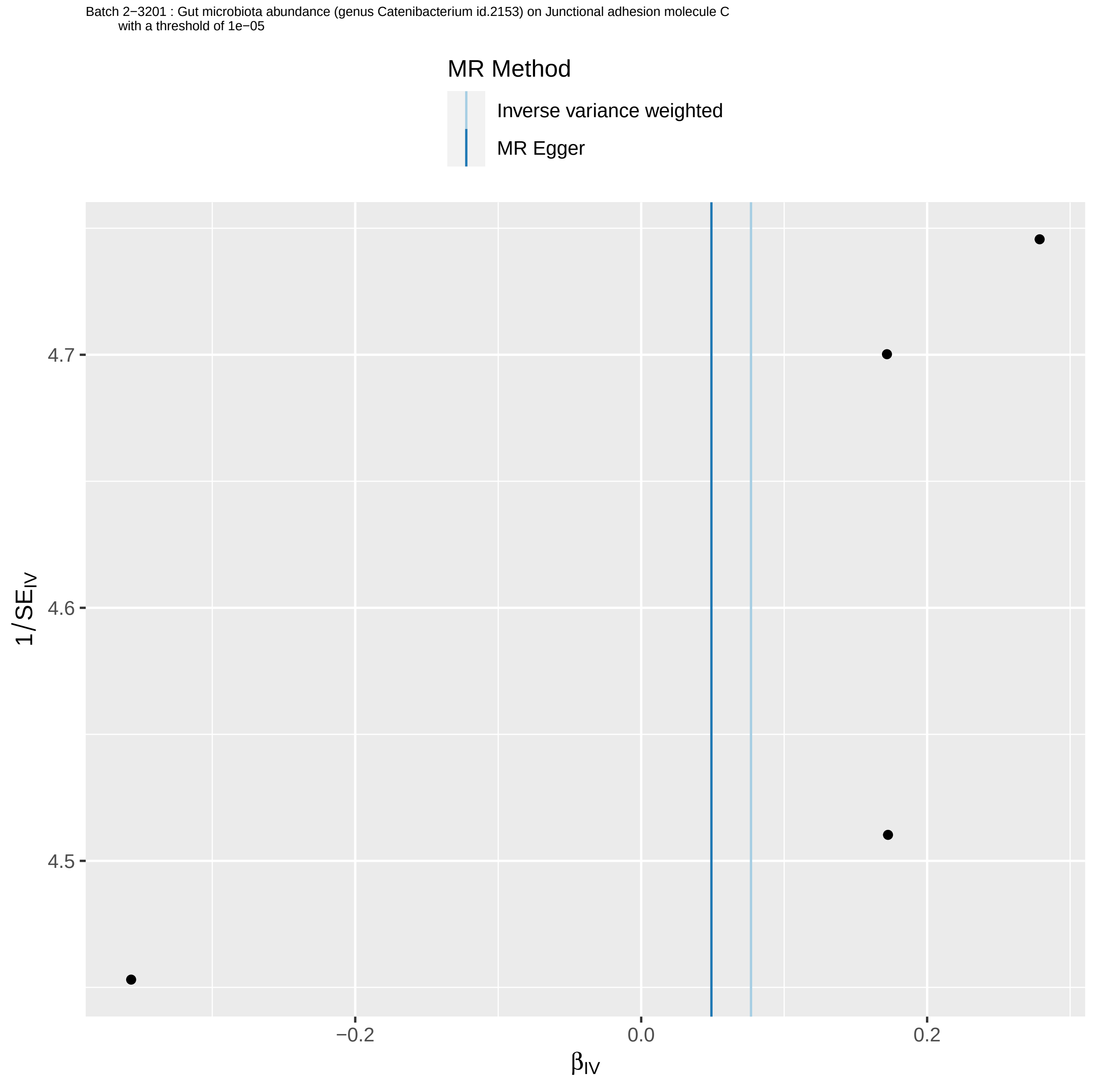

Batch 2−3201 : Gut microbiota abundance (genus Catenibacterium id.2153) on Junctional adhesion molecule C
with a threshold of 1e−05
MR Method
Inverse variance weighted
MR Egger
4.7
4.6
4.5
1/SE_IV
−0.2
0.0
0.2
β_IV

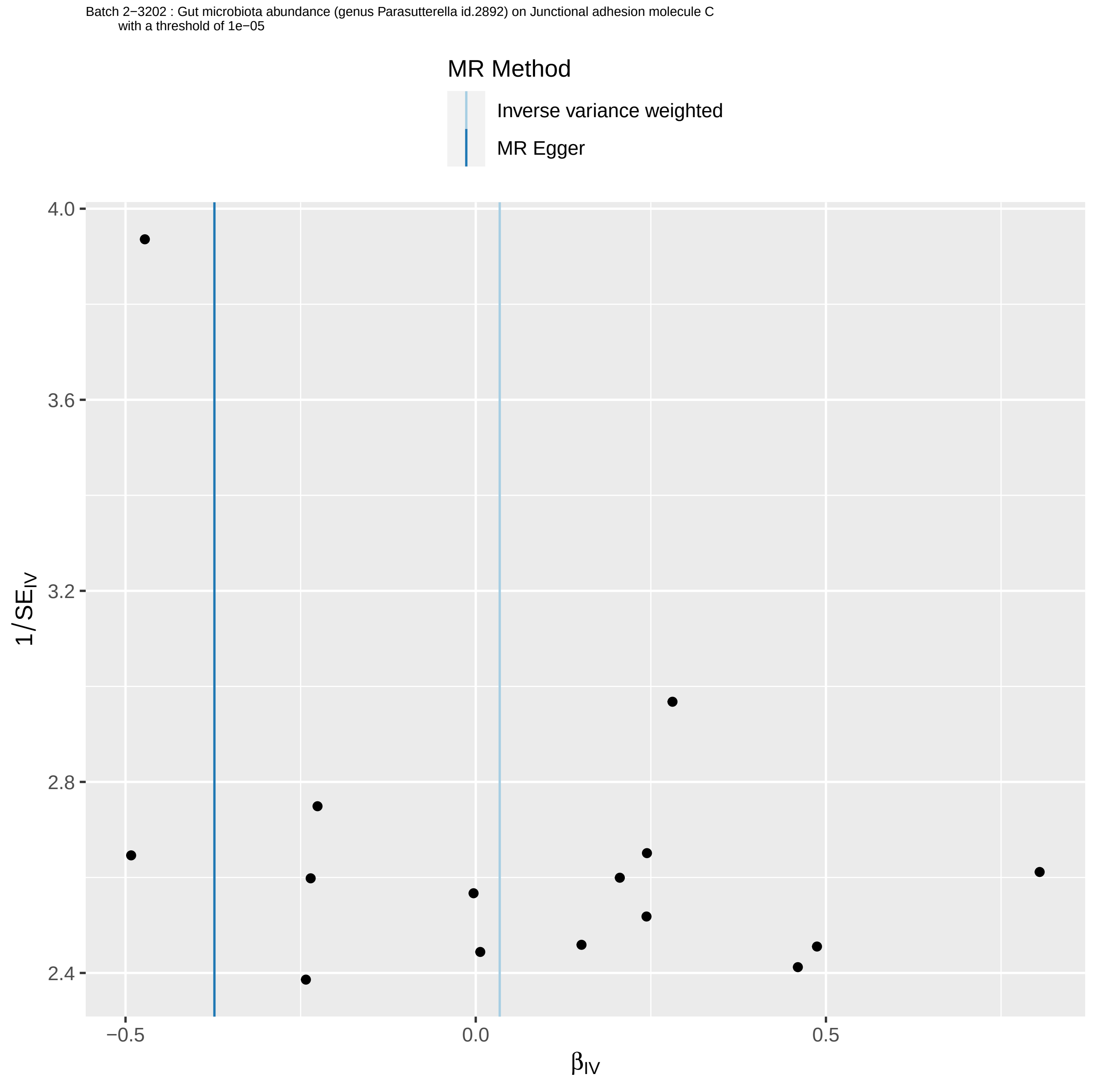
Batch 2−3202 : Gut microbiota abundance (genus Parasutterella id.2892) on Junctional adhesion molecule C
with a threshold of 1e−05
MR Method
Inverse variance weighted
MR Egger
4.0
3.6
3.2
2.8
2.4
1/SE_IV
−0.5
0.0
0.5
β_IV

Batch 2−3203 : Gut microbiota abundance (genus Catenibacterium id.2153) on Junctophilin−1
with a threshold of 1e−05

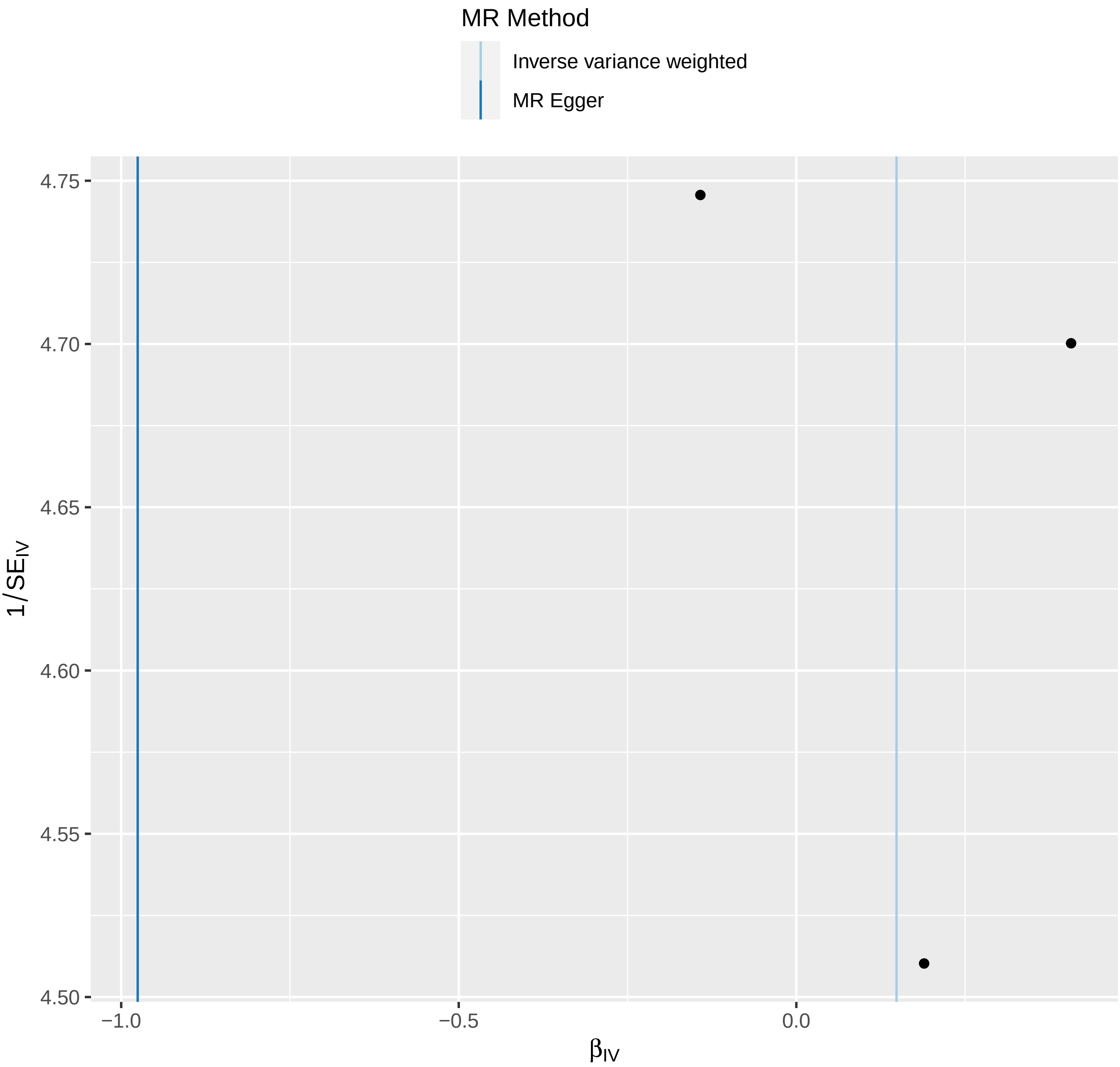

Batch 2−3204 : Gut microbiota abundance (genus Parasutterella id.2892) on Junctophilin−1
with a threshold of 1e−05

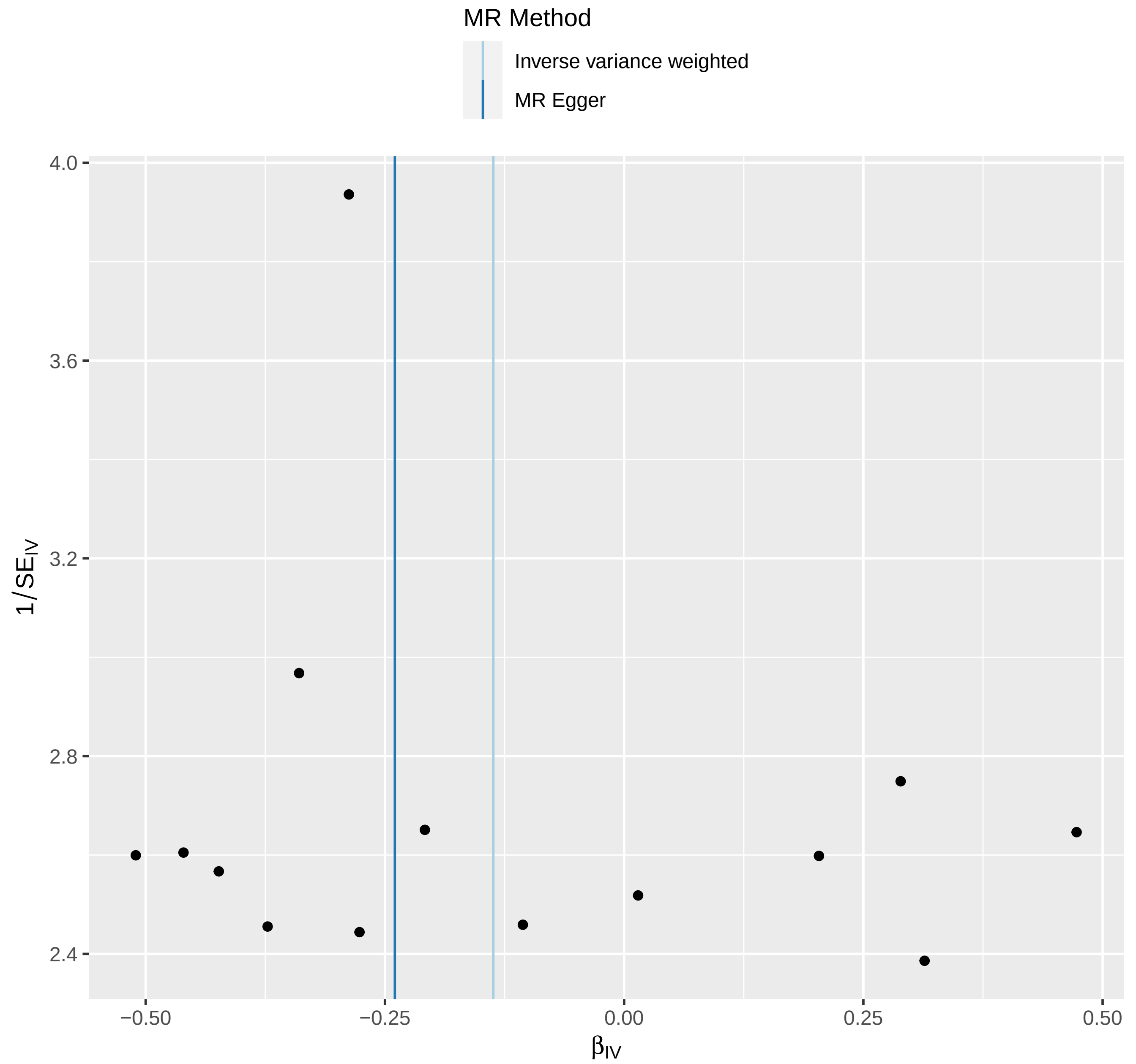

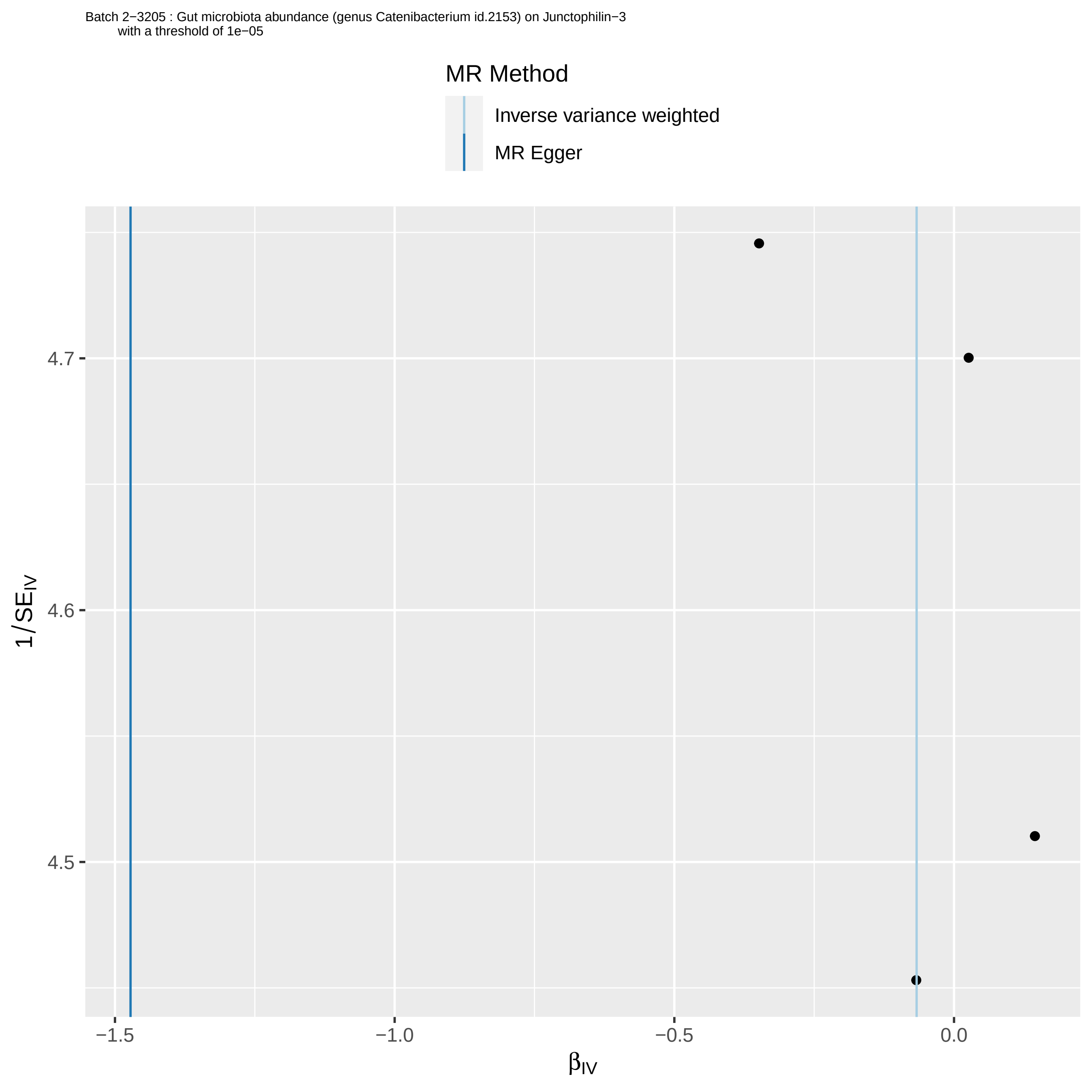

Batch 2−3205 : Gut microbiota abundance (genus Catenibacterium id.2153) on Junctophilin−3
with a threshold of 1e−05
MR Method
Inverse variance weighted
MR Egger
4.7
4.6
4.5
1/SE_IV
−1.5
−1.0
−0.5
0.0
β_IV

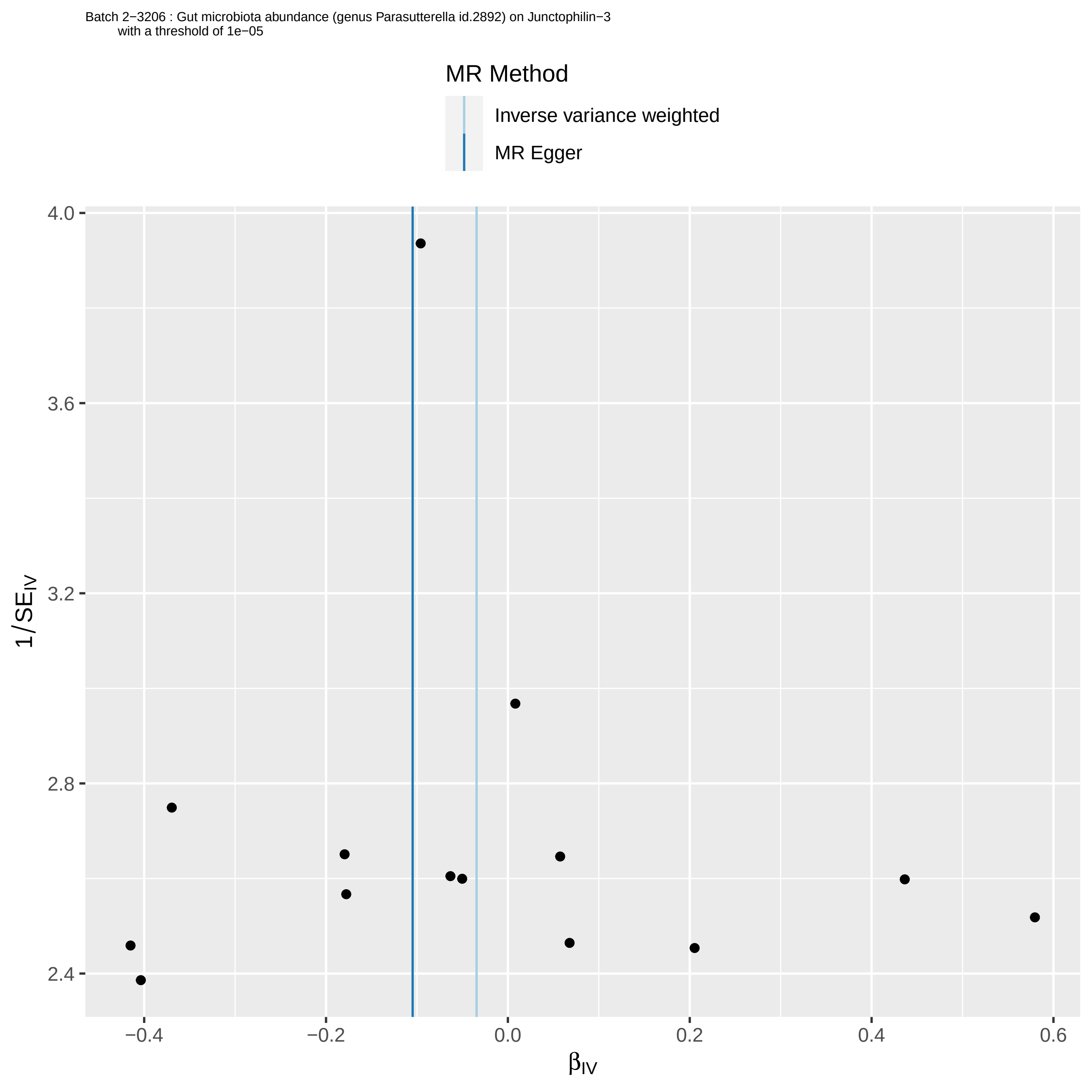

Batch 2−3206 : Gut microbiota abundance (genus Parasutterella id.2892) on Junctophilin−3
with a threshold of 1e−05
MR Method
Inverse variance weighted
MR Egger
4.0
3.6
3.2
2.8
2.4
1/SE_IV
−0.4
−0.2
0.0
0.2
0.4
0.6
β_IV

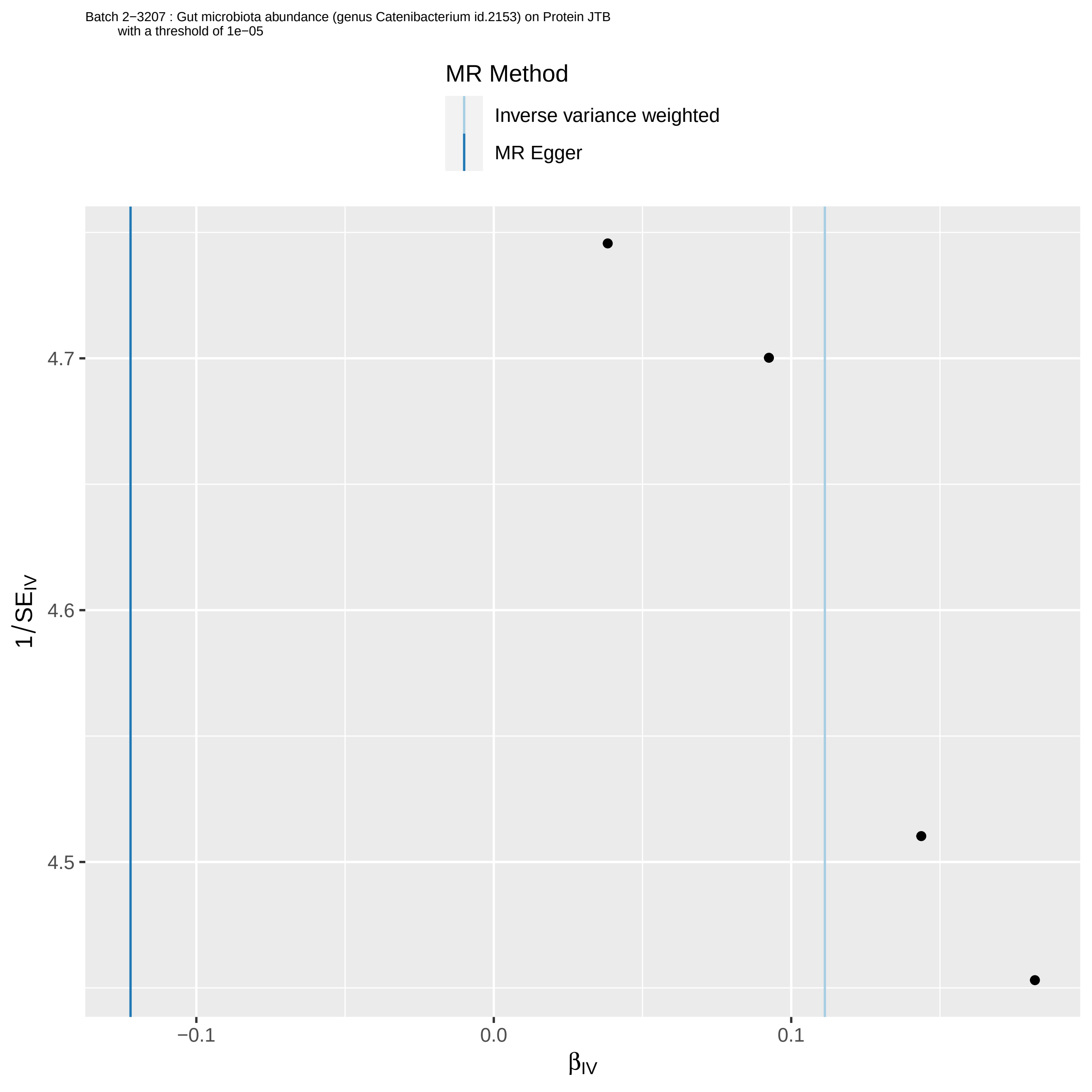

Batch 2−3207 : Gut microbiota abundance (genus Catenibacterium id.2153) on Protein JTB
with a threshold of 1e−05
MR Method
Inverse variance weighted
MR Egger
4.7
4.6
4.5
1/SE_IV
−0.1
0.0
0.1
β_IV

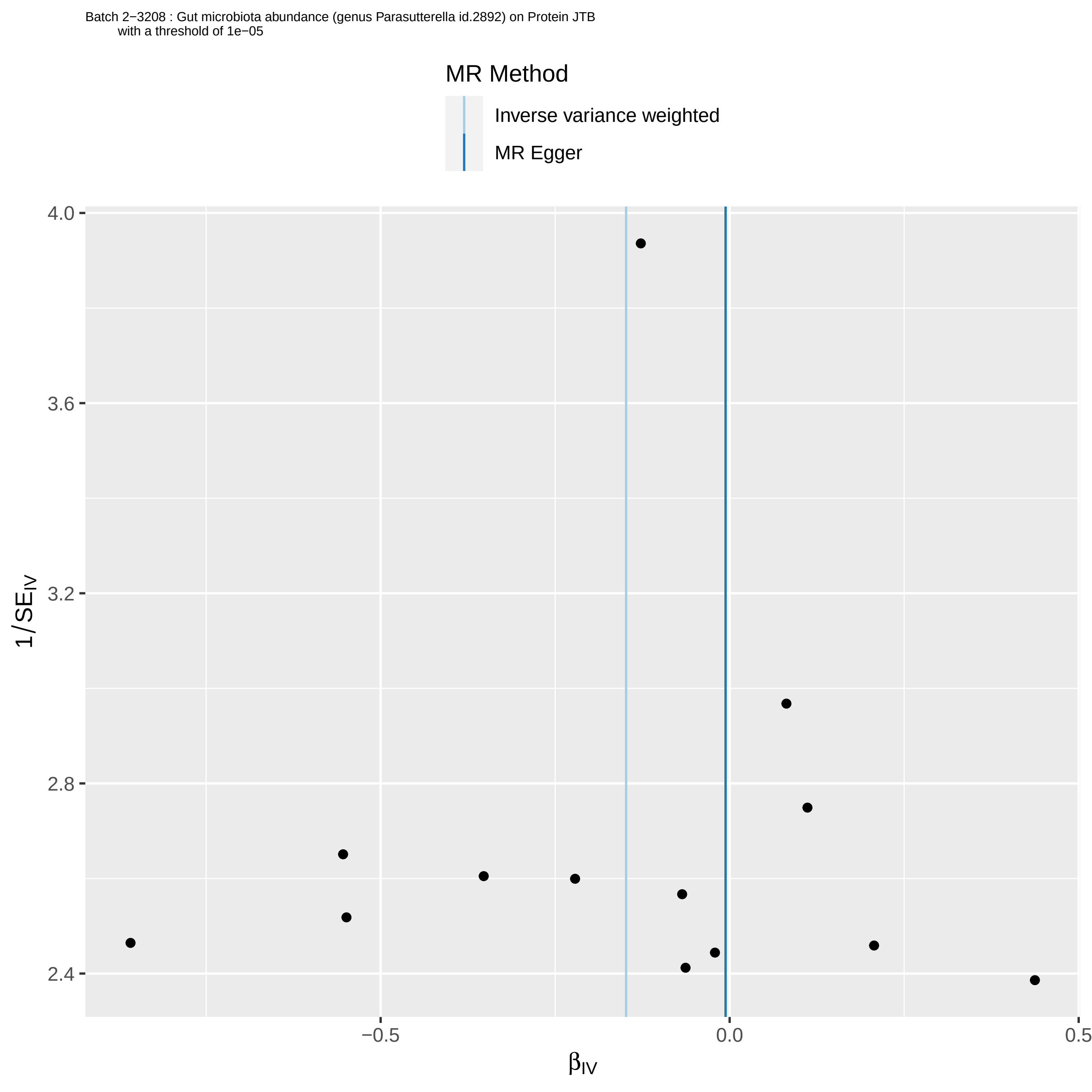
Batch 2−3208 : Gut microbiota abundance (genus Parasutterella id.2892) on Protein JTB
with a threshold of 1e−05
MR Method
Inverse variance weighted
MR Egger
4.0
3.6
3.2
2.8
2.4
1/SE_IV
−0.5
0.0
0.5
β_IV

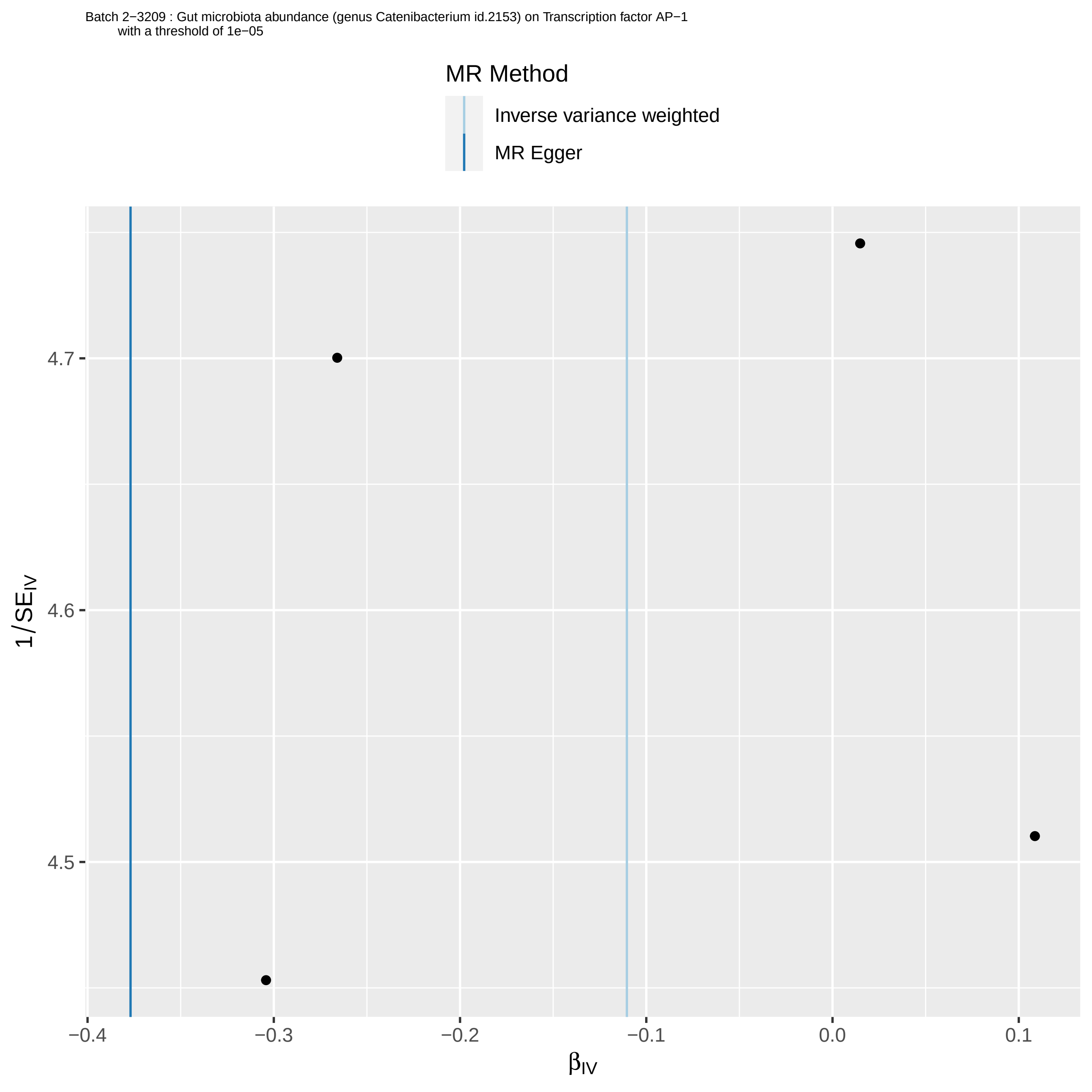

Batch 2−3209 : Gut microbiota abundance (genus Catenibacterium id.2153) on Transcription factor AP−1
with a threshold of 1e−05
MR Method
Inverse variance weighted
MR Egger
4.7
4.6
4.5
1/SE_IV
−0.4
−0.3
−0.2
−0.1
0.0
0.1
β_IV

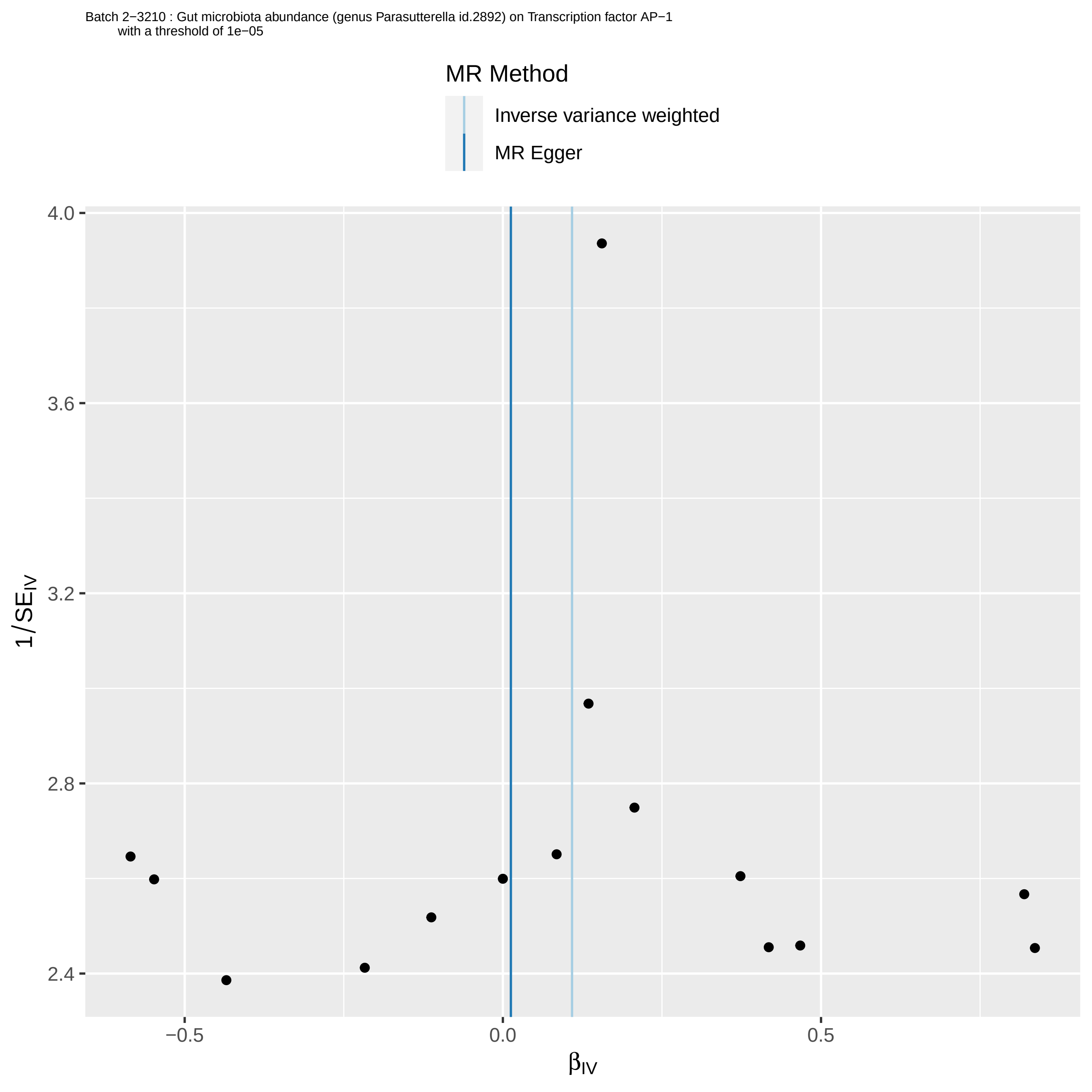

Batch 2−3210 : Gut microbiota abundance (genus Parasutterella id.2892) on Transcription factor AP−1
with a threshold of 1e−05
MR Method
Inverse variance weighted
MR Egger
4.0
3.6
3.2
2.8
2.4
1/SE_IV
−0.5
0.0
0.5
β_IV

Batch 2−3211 : Gut microbiota abundance (genus Catenibacterium id.2153) on Anosmin−1
with a threshold of 1e−05

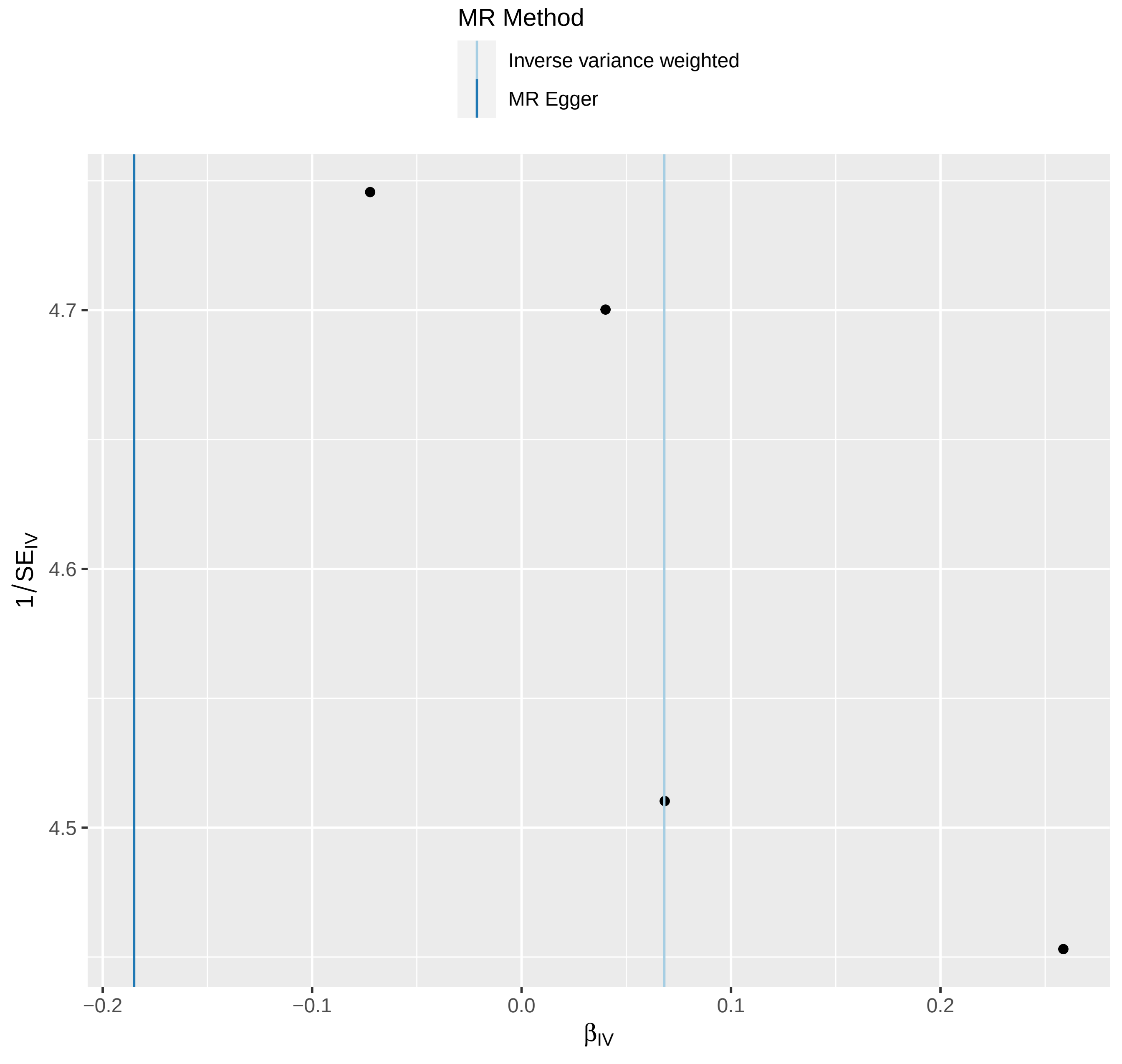

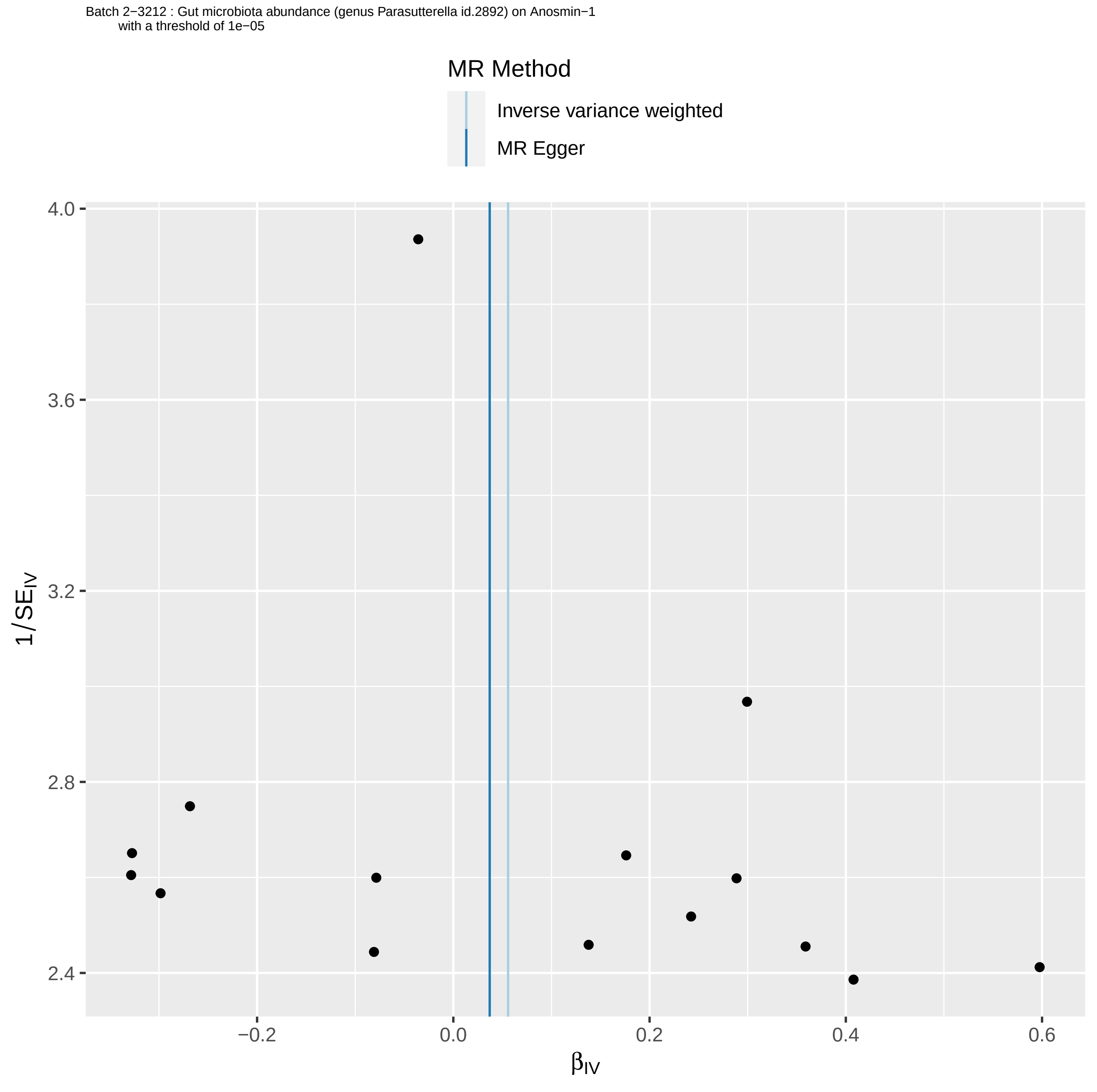
Batch 2−3212 : Gut microbiota abundance (genus Parasutterella id.2892) on Anosmin−1
with a threshold of 1e−05
MR Method
Inverse variance weighted
MR Egger
4.0
3.6
3.2
2.8
2.4
1/SE_IV
−0.2
0.0
0.2
0.4
0.6
β_IV

Batch 2−3213 : Gut microbiota abundance (genus Catenibacterium id.2153) on Histone acetyltransferase KAT6A
with a threshold of 1e−05

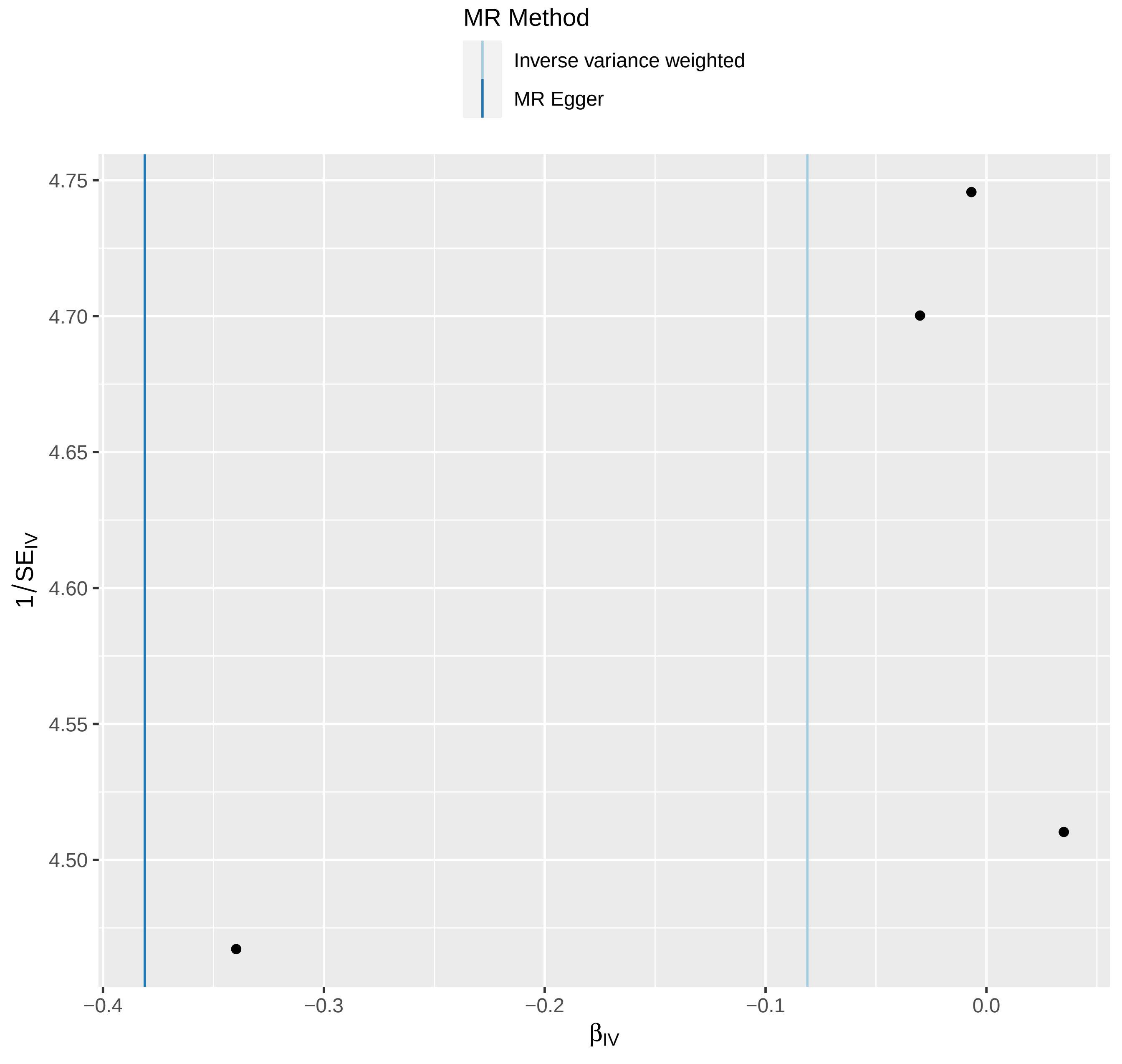

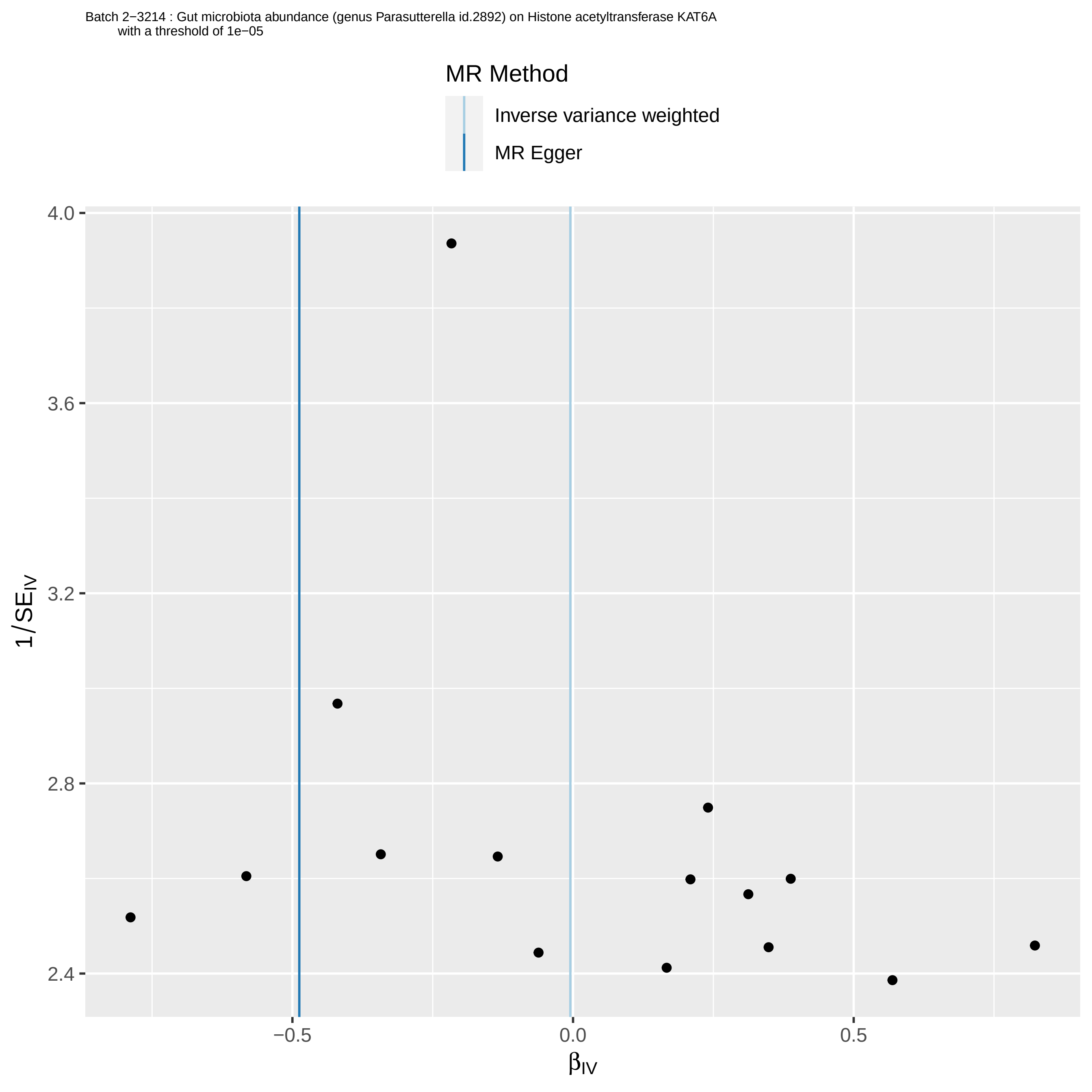
Batch 2−3214 : Gut microbiota abundance (genus Parasutterella id.2892) on Histone acetyltransferase KAT6A
with a threshold of 1e−05
MR Method
Inverse variance weighted
MR Egger
4.0
3.6
3.2
2.8
2.4
1/SE_IV
−0.5
0.0
0.5
β_IV

Batch 2−3215 : Gut microbiota abundance (genus Catenibacterium id.2153) on Potassium voltage−gated channel subfamily A member 10 with a threshold of 1e−05

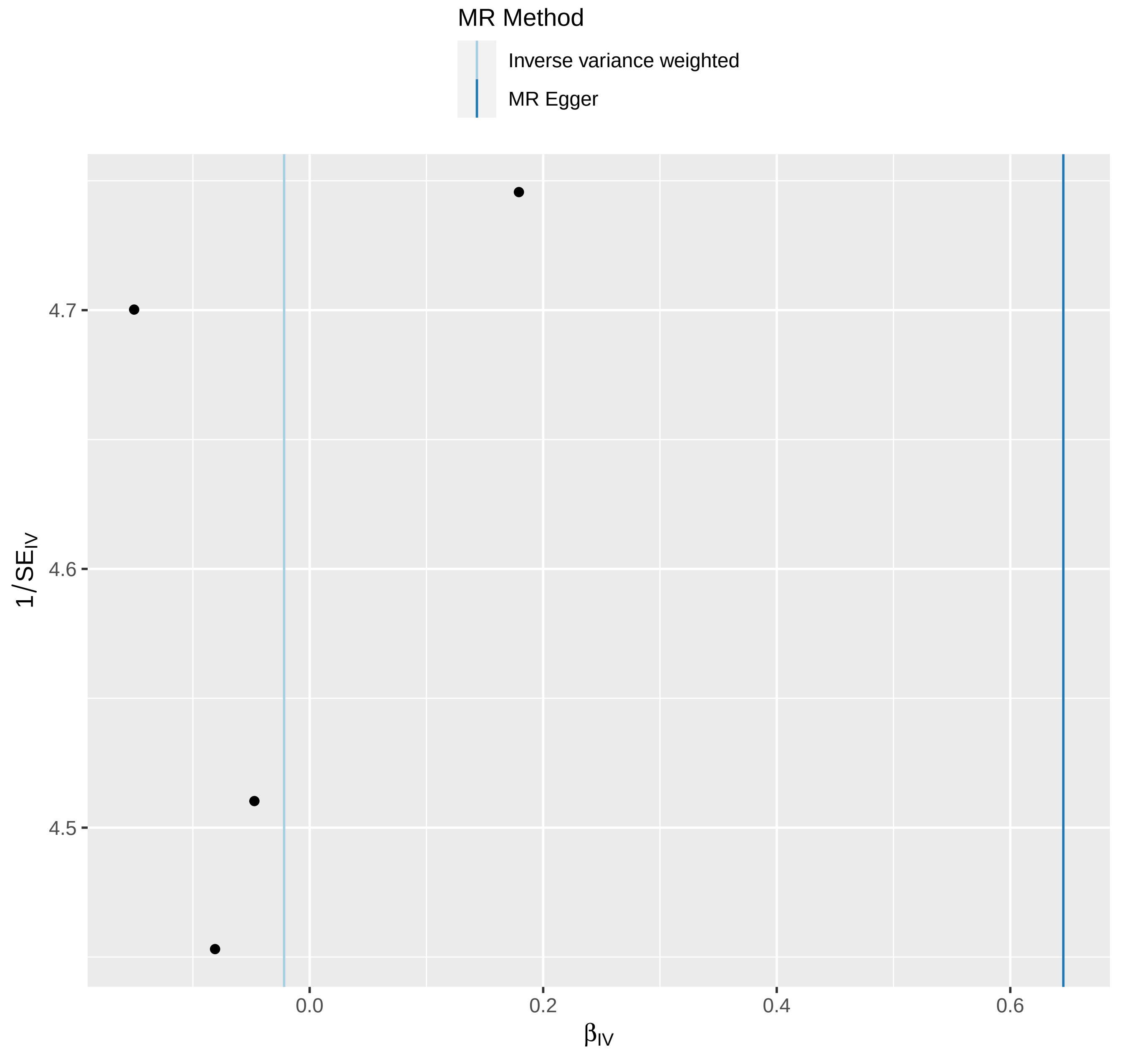

Batch 2−3216 : Gut microbiota abundance (genus Parasutterella id.2892) on Potassium voltage−gated channel subfamily A member 10
with a threshold of 1e−05

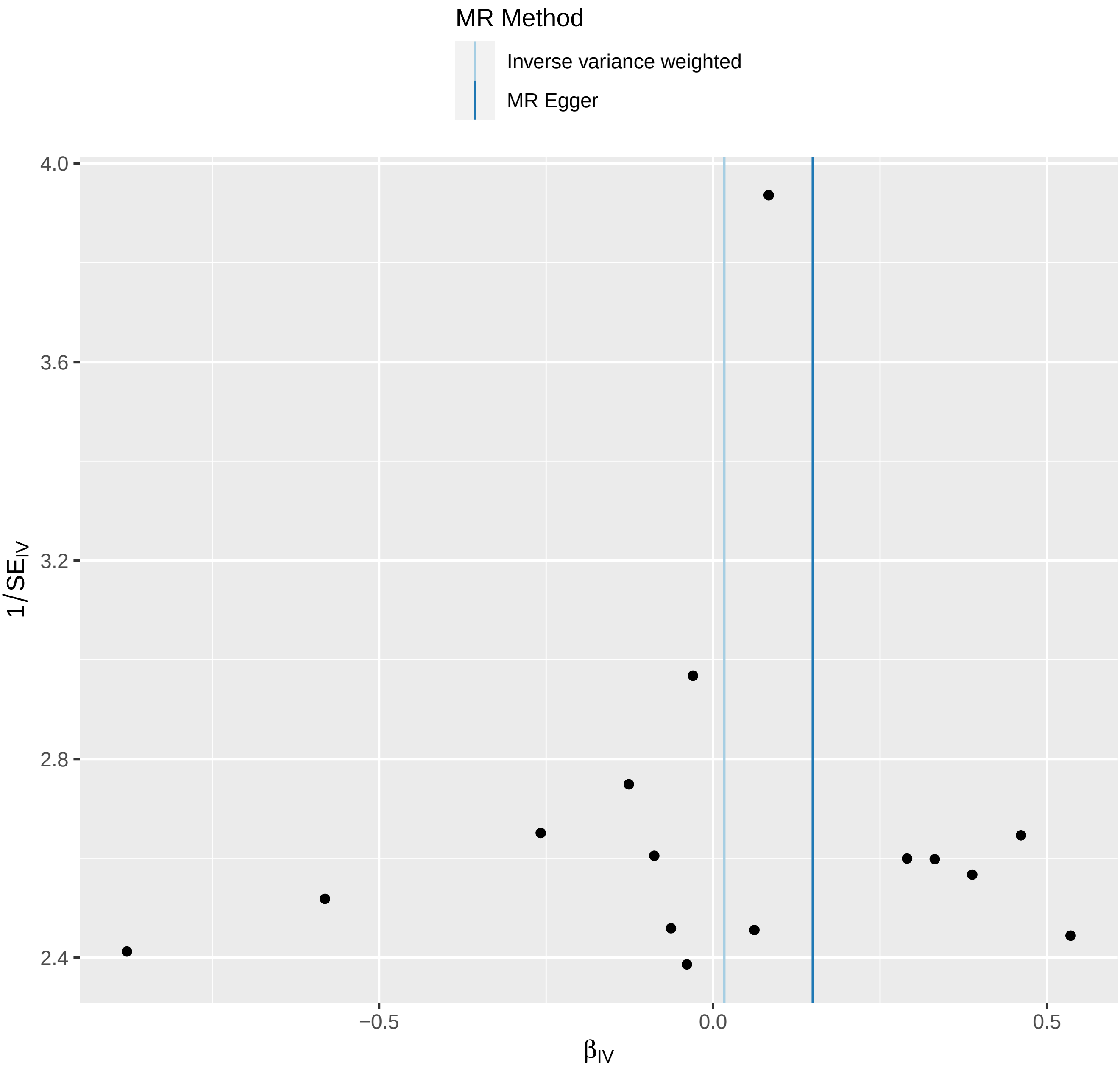

Batch 2−3217 : Gut microbiota abundance (genus Catenibacterium id.2153) on Voltage−gated potassium channel subunit beta−2 with a threshold of 1e−05

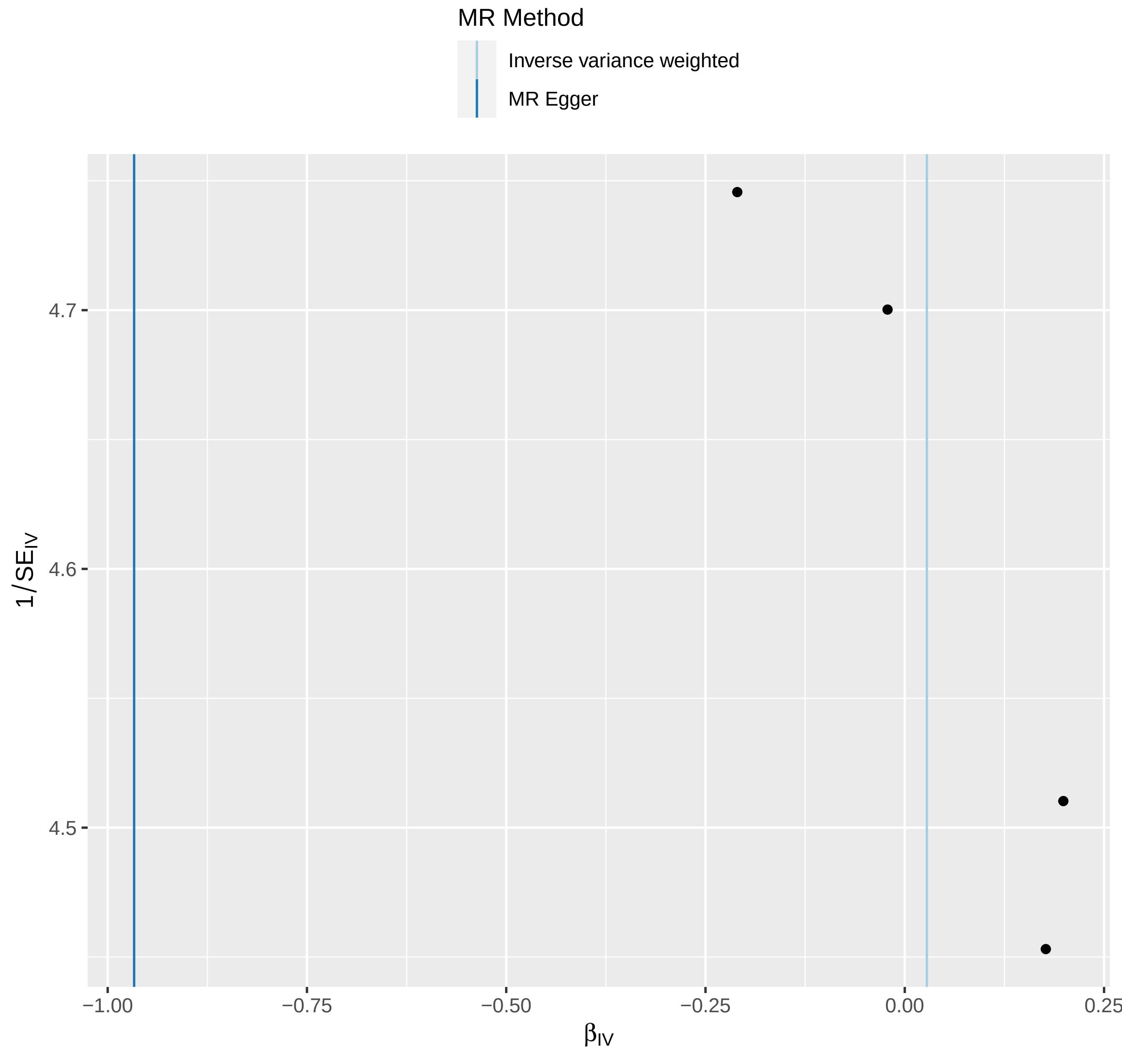

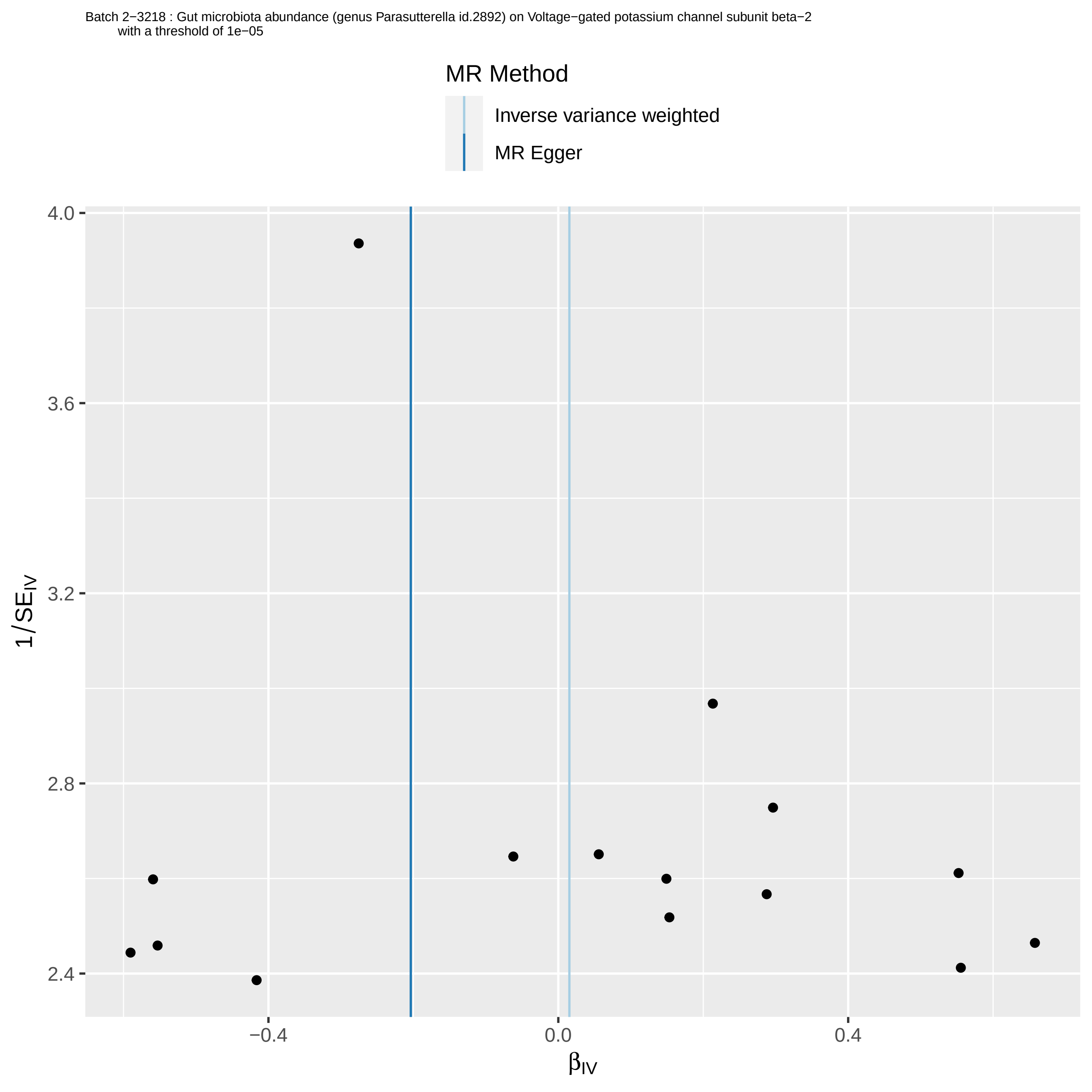
Batch 2−3218 : Gut microbiota abundance (genus Parasutterella id.2892) on Voltage−gated potassium channel subunit beta−2
with a threshold of 1e−05
MR Method
Inverse variance weighted
MR Egger
4.0
3.6
3.2
2.8
2.4
1/SE_IV
−0.4
0.0
0.4
β_IV

Batch 2−3219 : Gut microbiota abundance (genus Catenibacterium id.2153) on Potassium voltage−gated channel subfamily E member 2 with a threshold of 1e−05

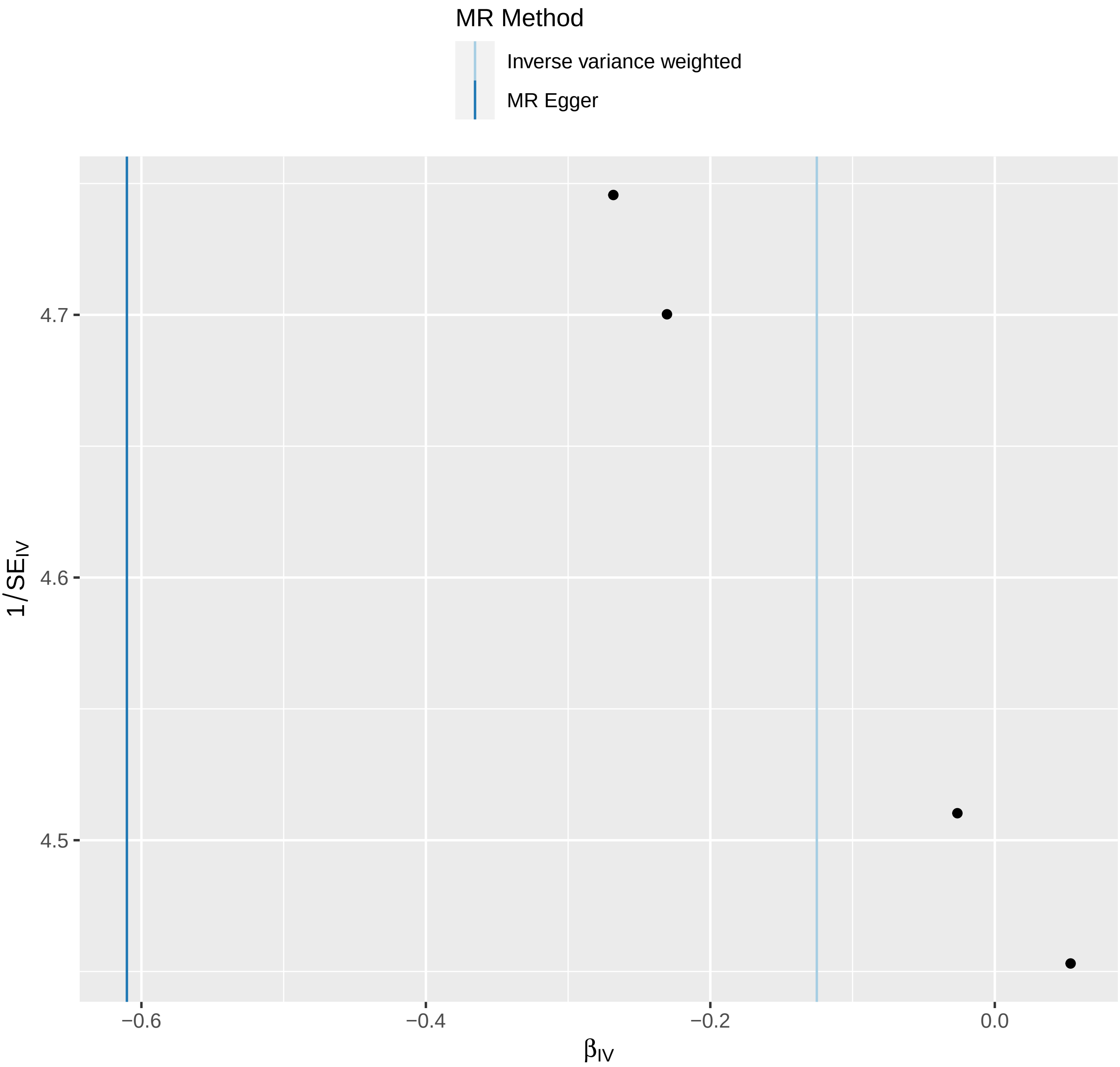

Batch 2−3220 : Gut microbiota abundance (genus Parasutterella id.2892) on Potassium voltage−gated channel subfamily E member 2
with a threshold of 1e−05

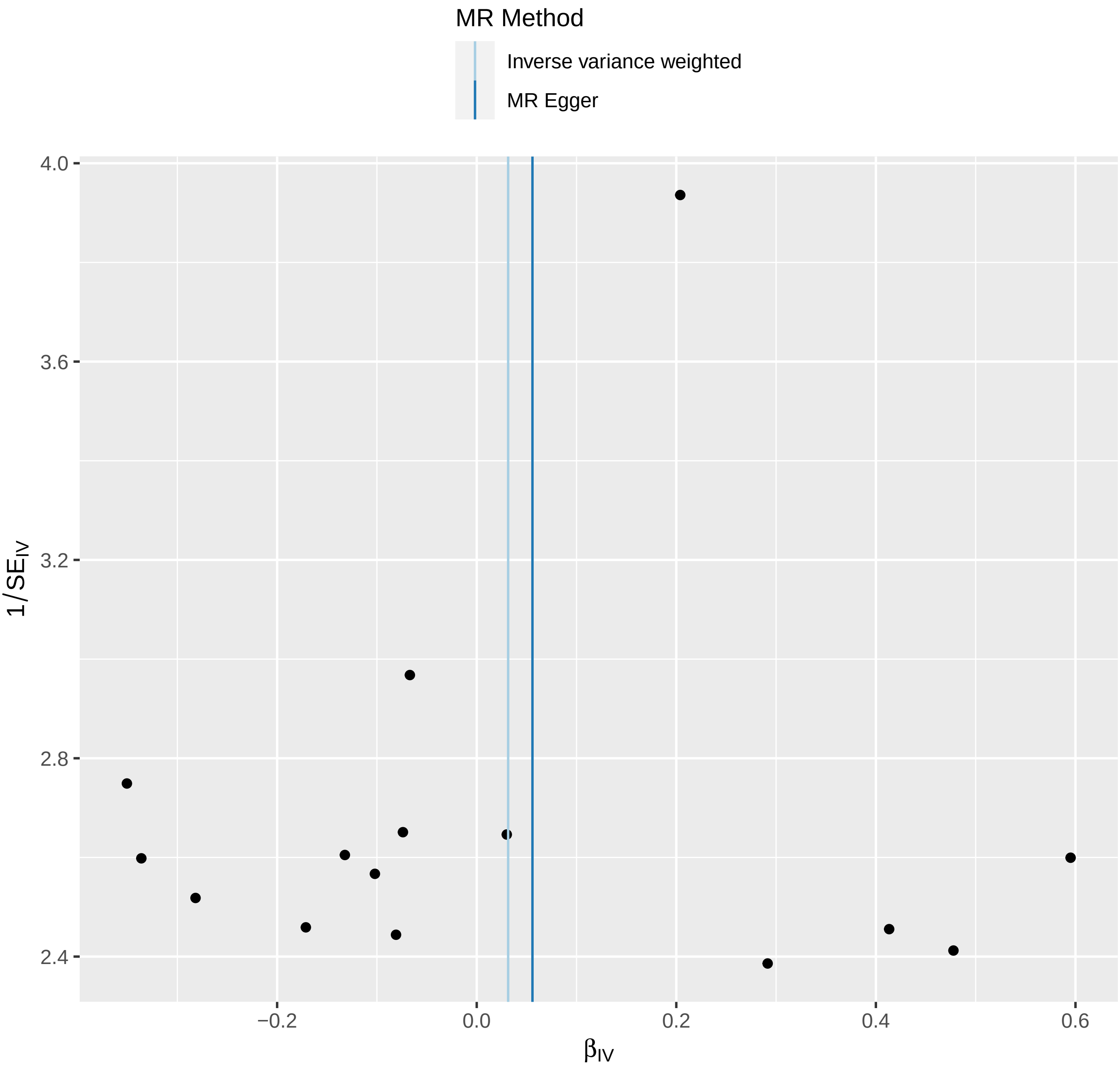

Batch 2−3221 : Gut microbiota abundance (genus Catenibacterium id.2153) on Potassium voltage−gated channel subfamily E member 3
with a threshold of 1e−05

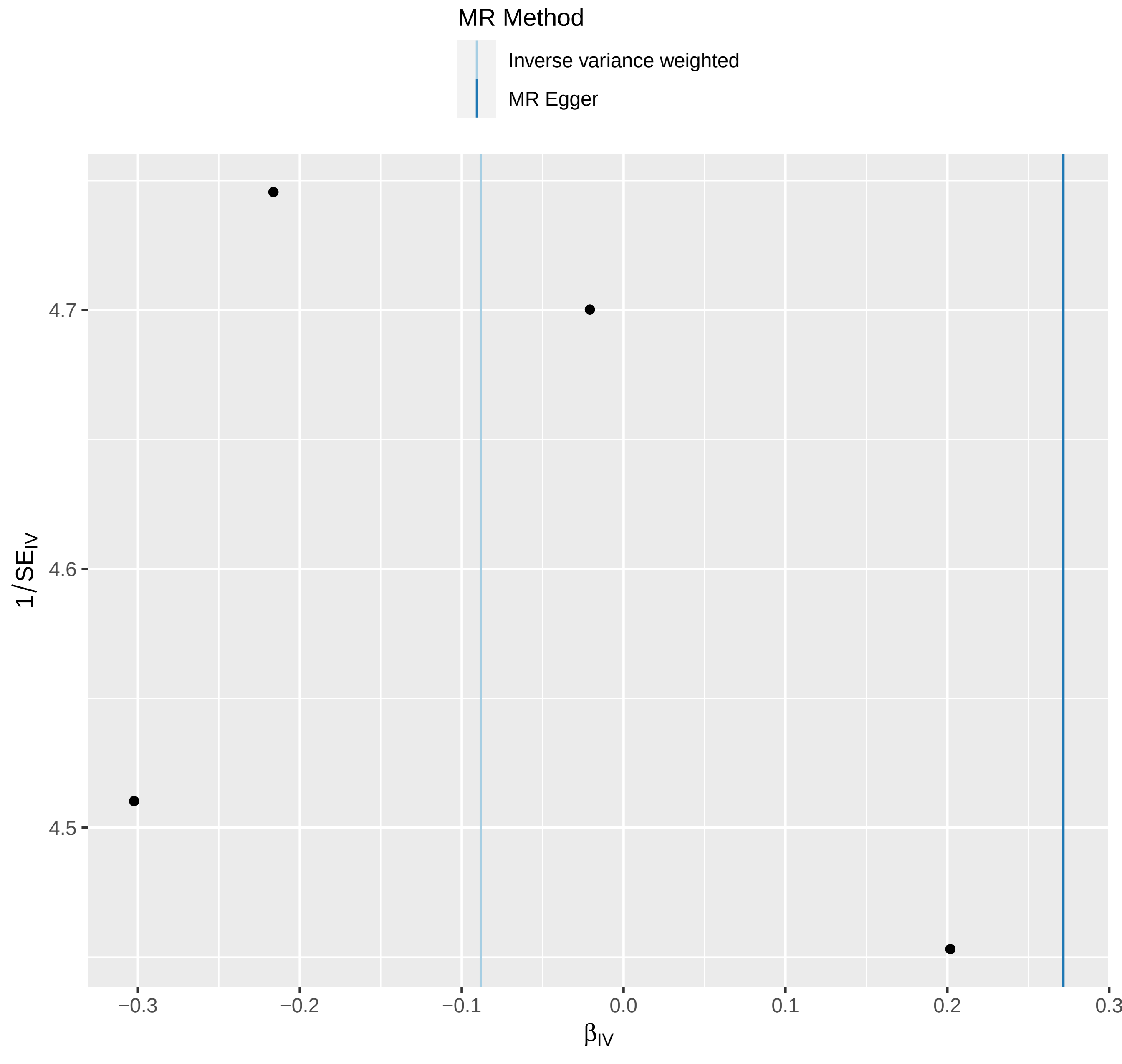

Batch 2−3222 : Gut microbiota abundance (genus Parasutterella id.2892) on Potassium voltage−gated channel subfamily E member 3 with a threshold of 1e−05

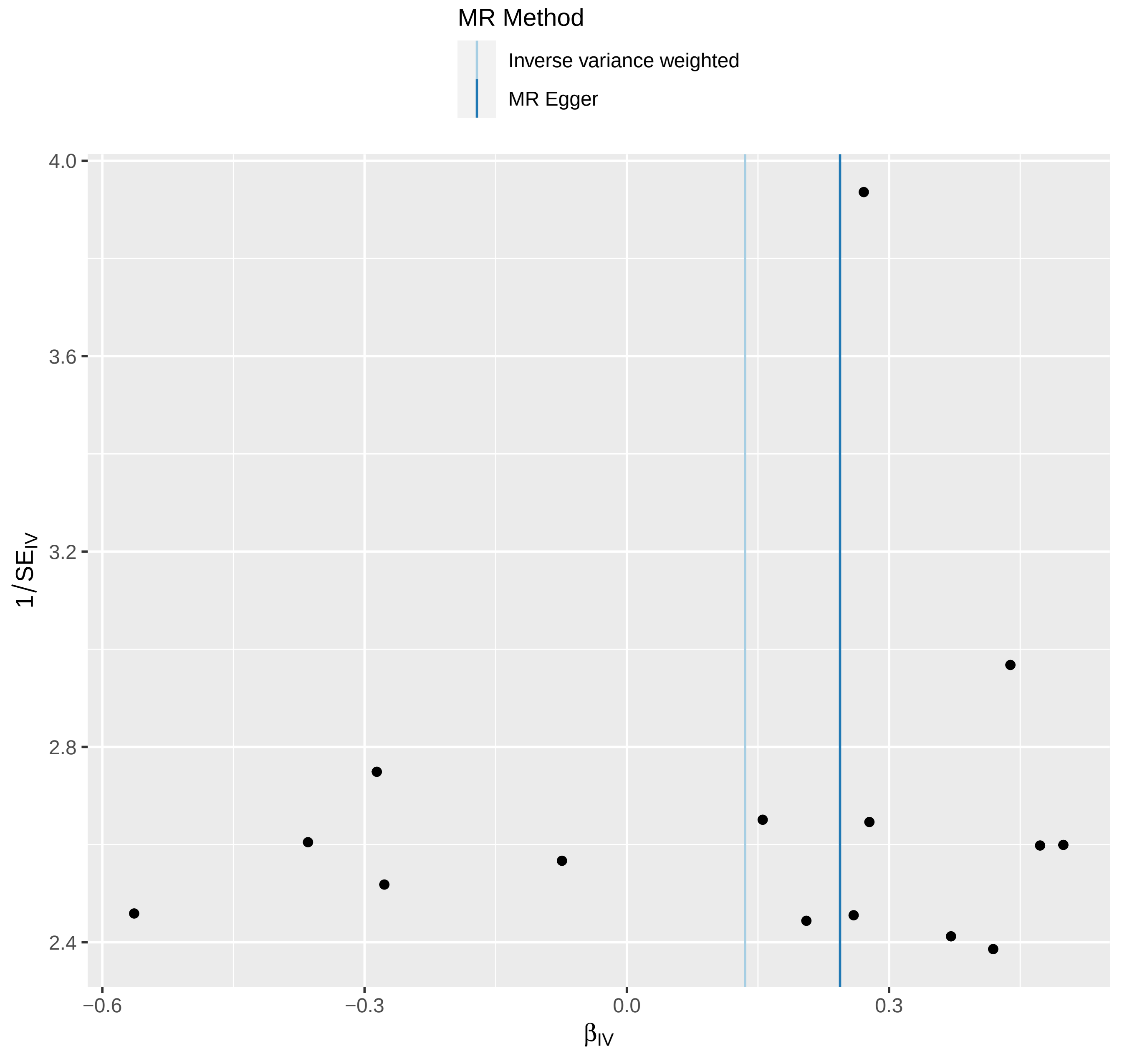

Batch 2−3223 : Gut microbiota abundance (genus Catenibacterium id.2153) on Potassium voltage−gated channel subfamily E regulatory beta subunit 5 with a threshold of 1e−05

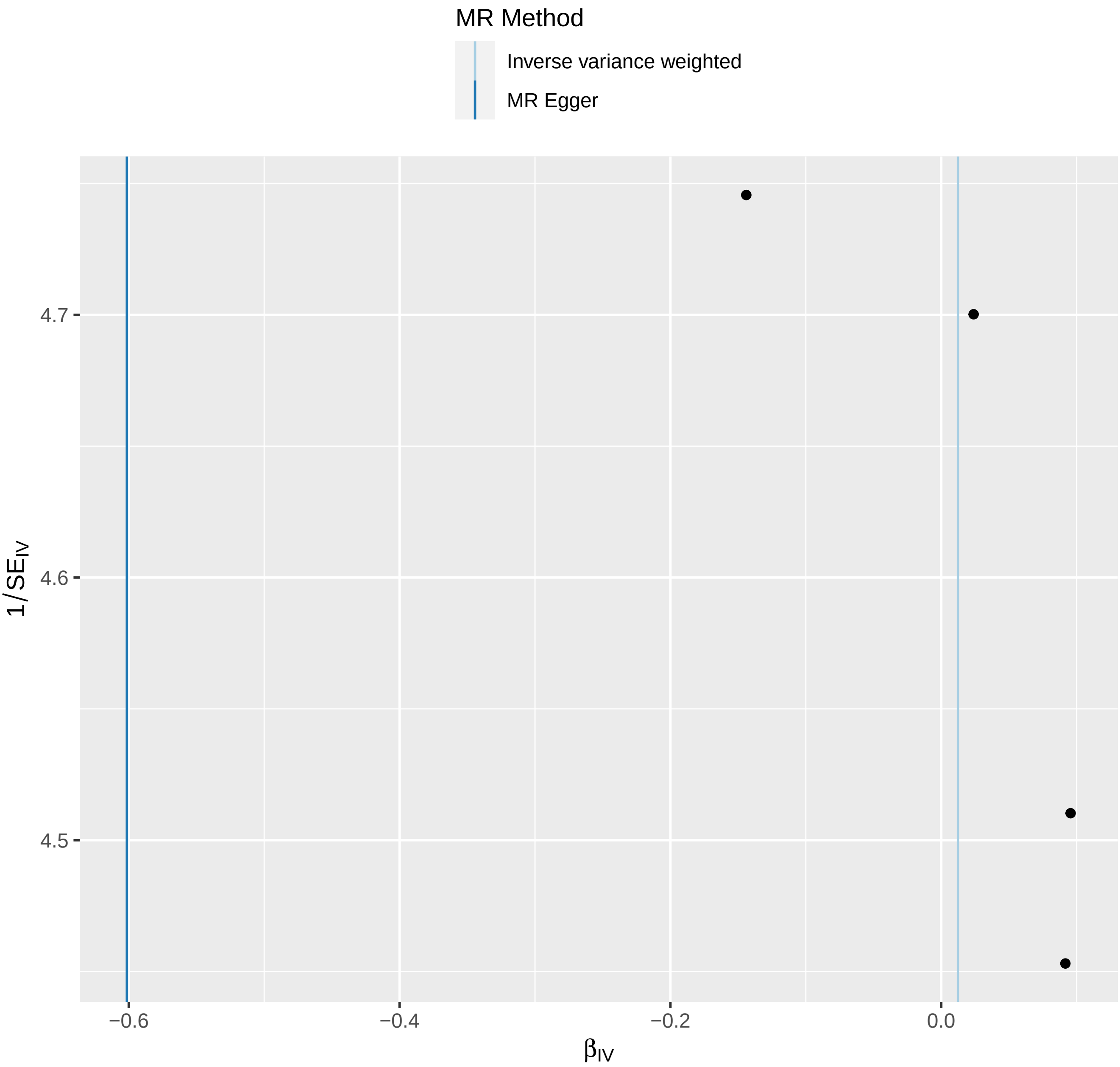

Batch 2−3224 : Gut microbiota abundance (genus Parasutterella id.2892) on Potassium voltage−gated channel subfamily E regulatory beta subunit 5
with a threshold of 1e−05

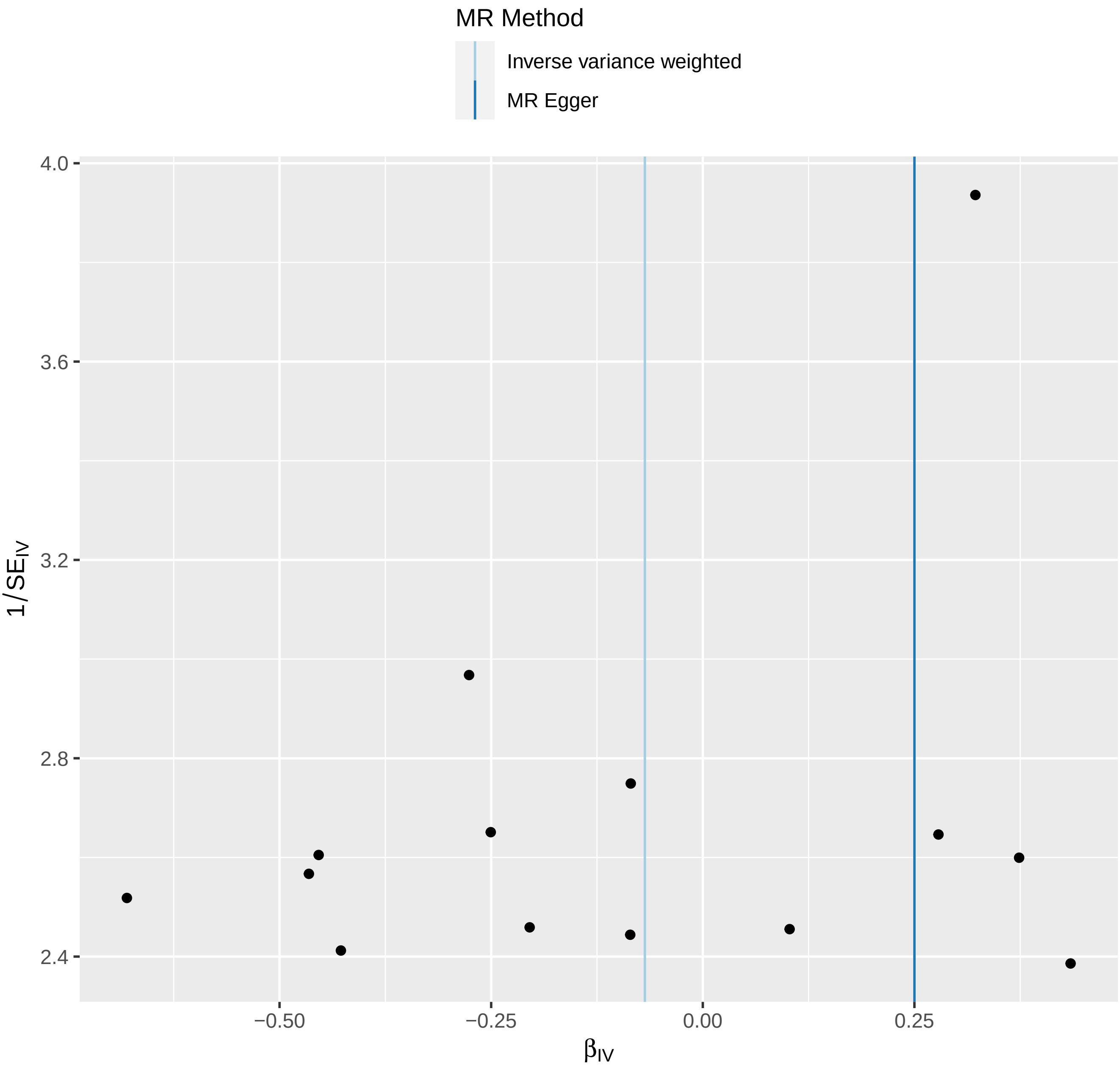

Batch 2−3225 : Gut microbiota abundance (genus Catenibacterium id.2153) on Potassium voltage−gated channel subfamily E regulatory beta subunit 5 with a threshold of 1e−05

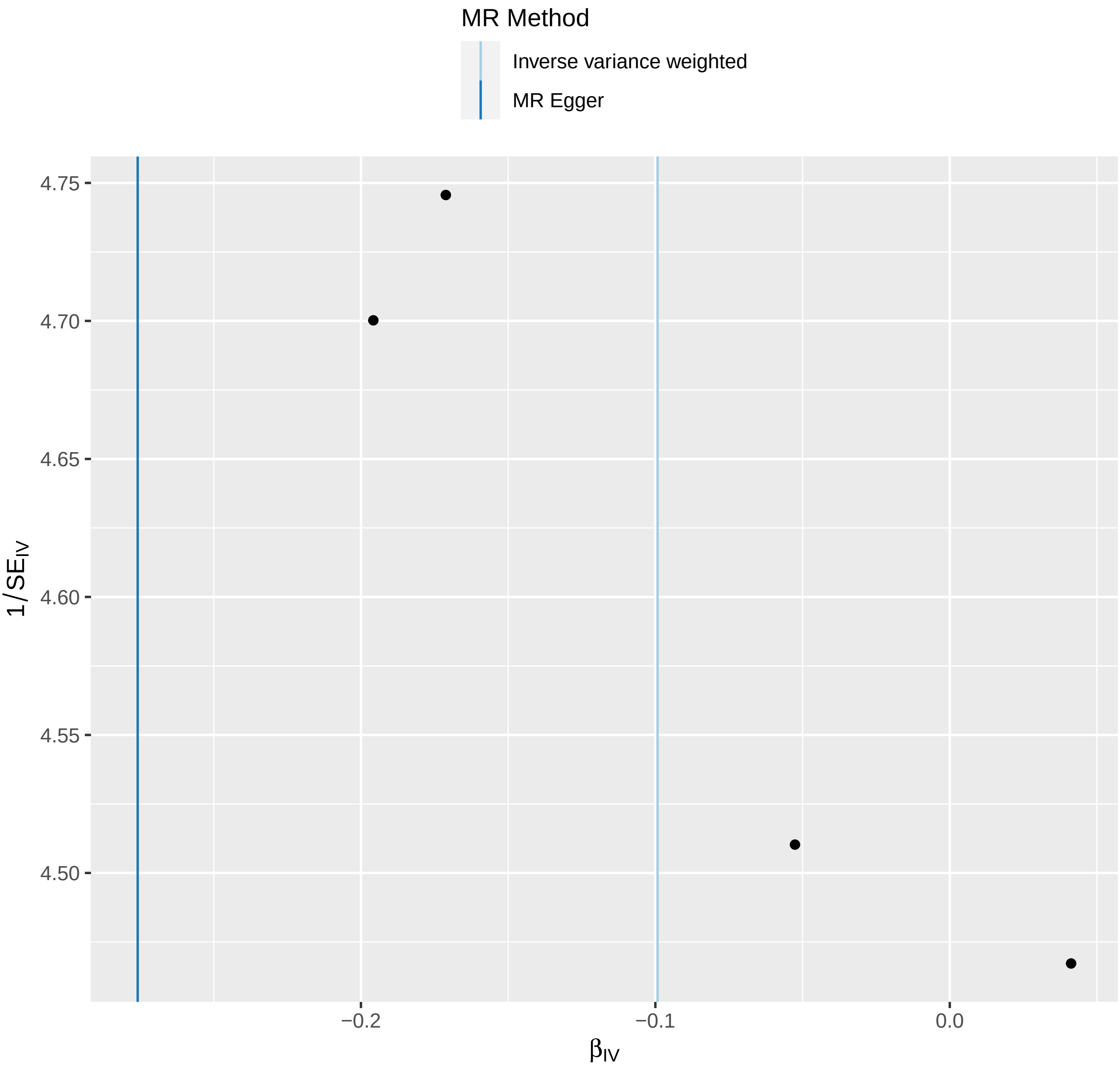

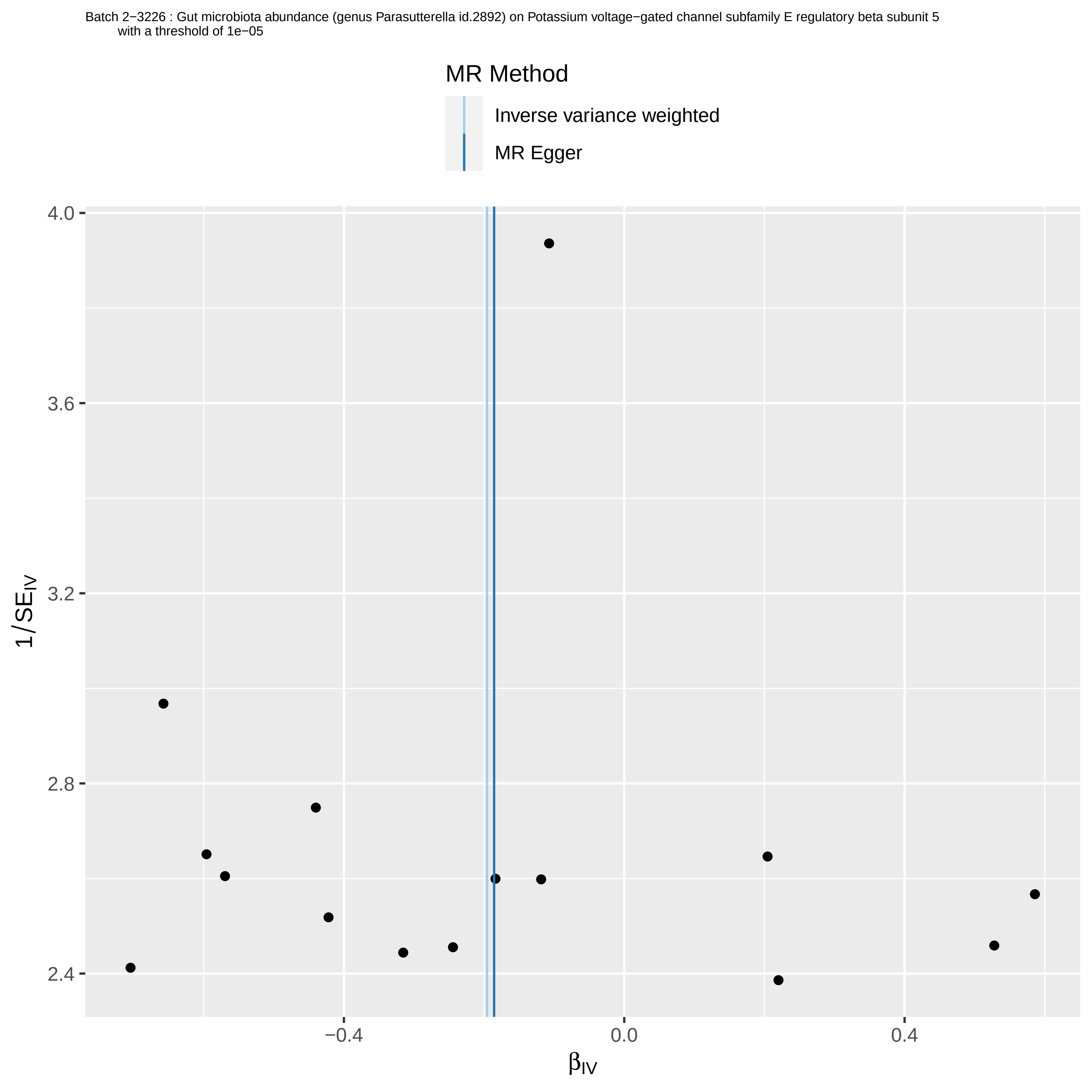

Batch 2−3226 : Gut microbiota abundance (genus Parasutterella id.2892) on Potassium voltage−gated channel subfamily E regulatory beta subunit 5
with a threshold of 1e−05
MR Method
Inverse variance weighted
MR Egger
4.0
3.6
3.2
2.8
2.4
1/SE_IV
−0.4
0.0
0.4
β_IV

Batch 2−3227 : Gut microbiota abundance (genus Catenibacterium id.2153) on Potassium voltage−gated channel subfamily F member 1 with a threshold of 1e−05

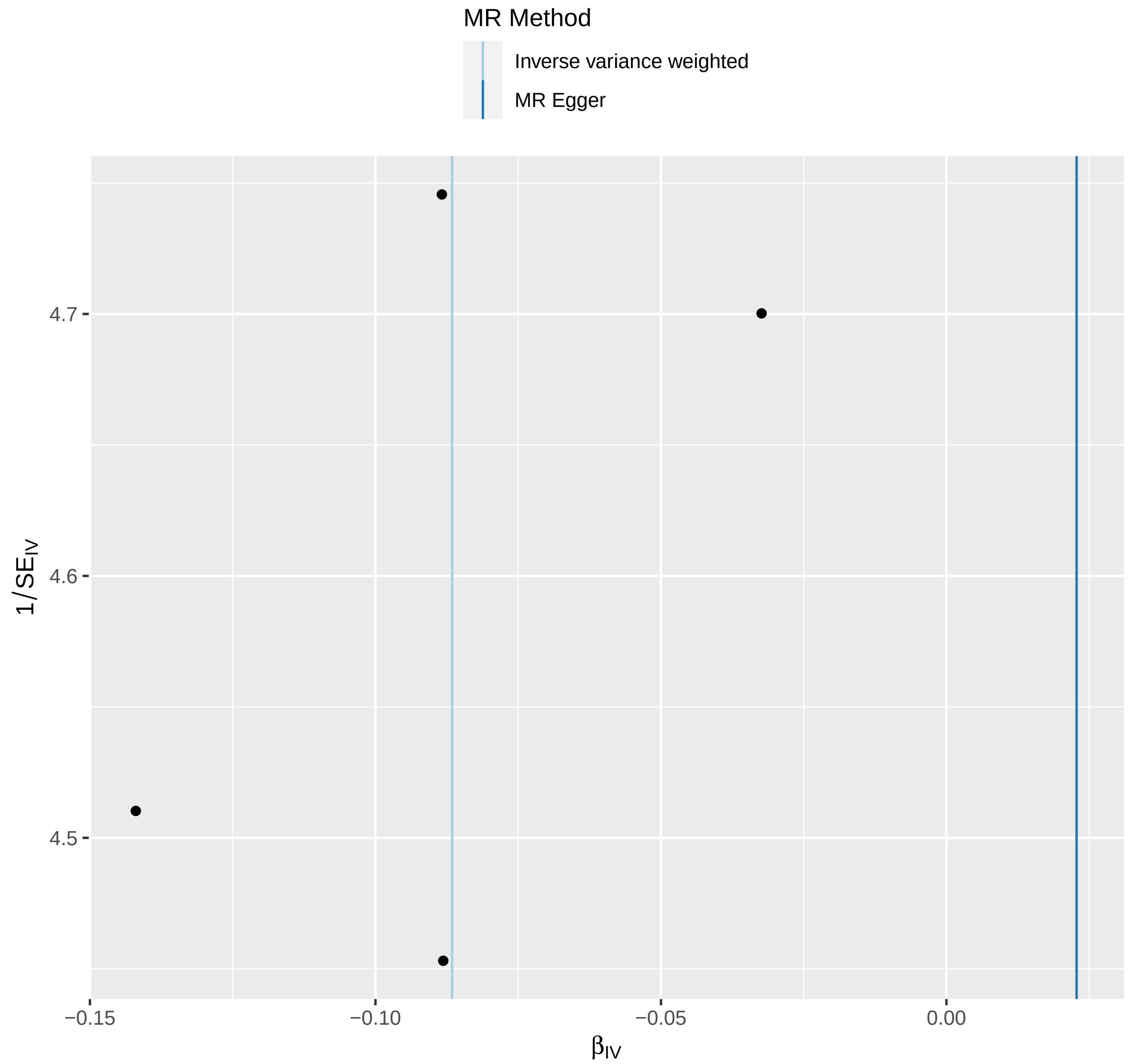

Batch 2−3228 : Gut microbiota abundance (genus Parasutterella id.2892) on Potassium voltage−gated channel subfamily F member 1
with a threshold of 1e−05

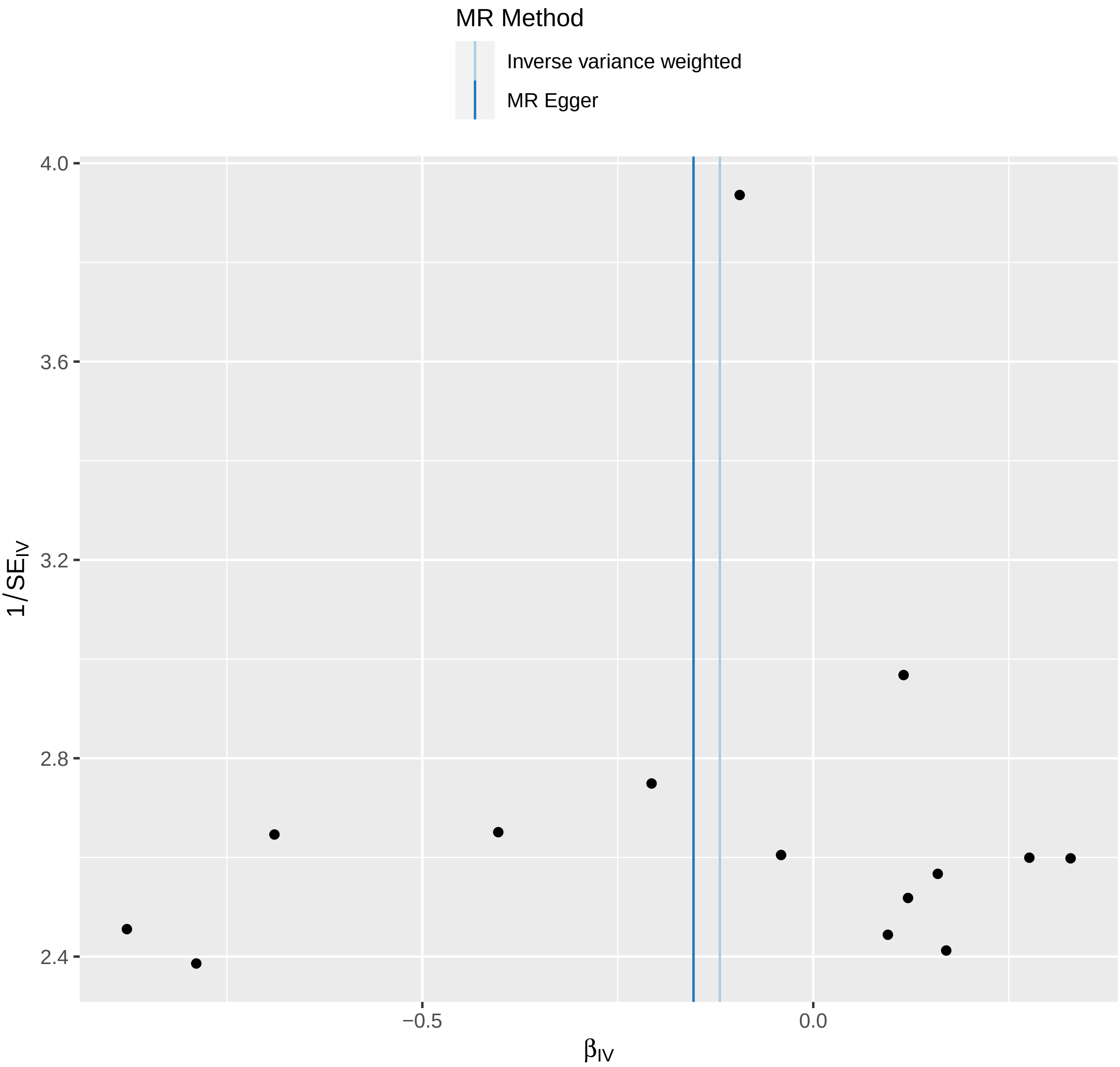

Batch 2−3229 : Gut microbiota abundance (genus Catenibacterium id.2153) on Potassium voltage−gated channel subfamily G member 4
with a threshold of 1e−05

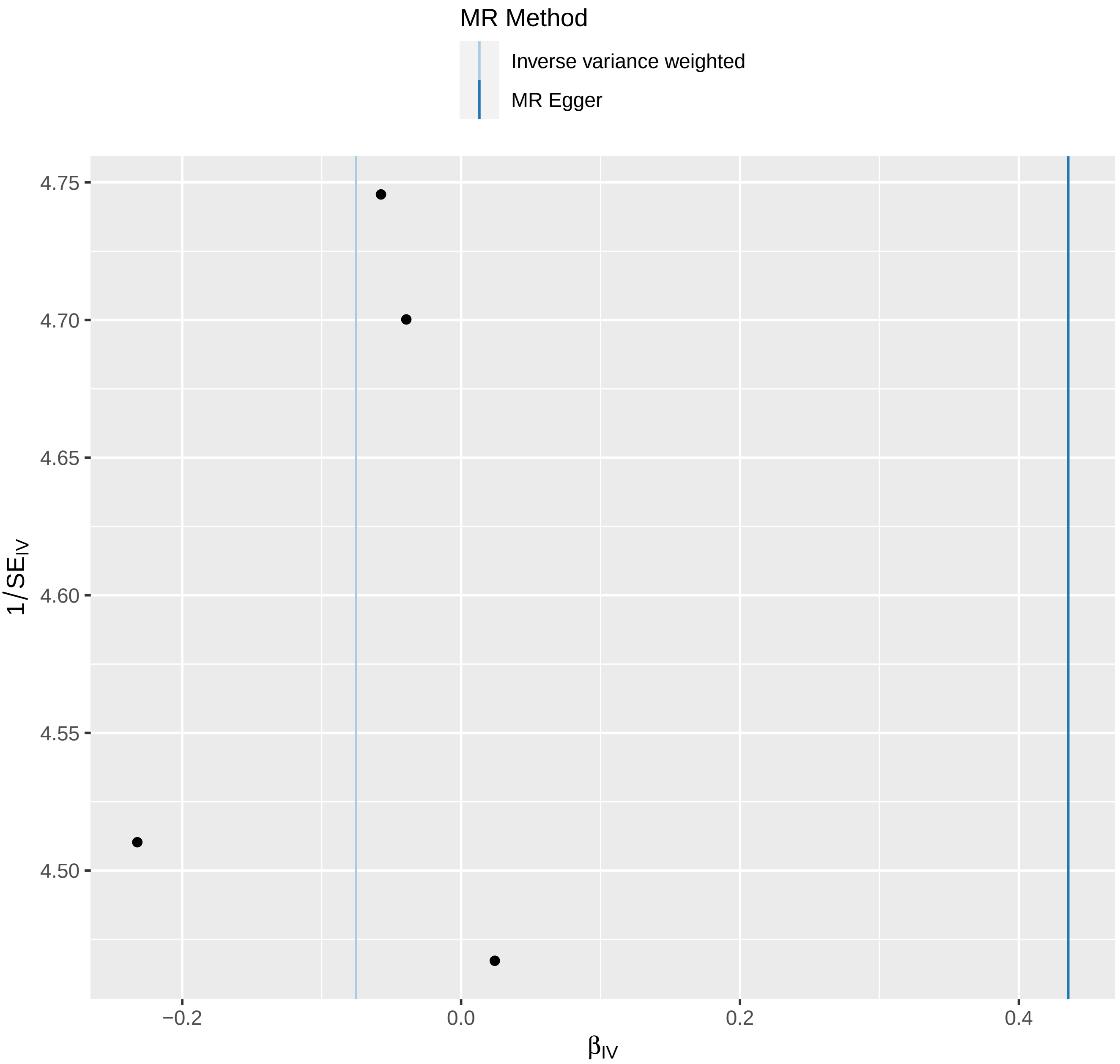

Batch 2−3230 : Gut microbiota abundance (genus Parasutterella id.2892) on Potassium voltage−gated channel subfamily G member 4
with a threshold of 1e−05

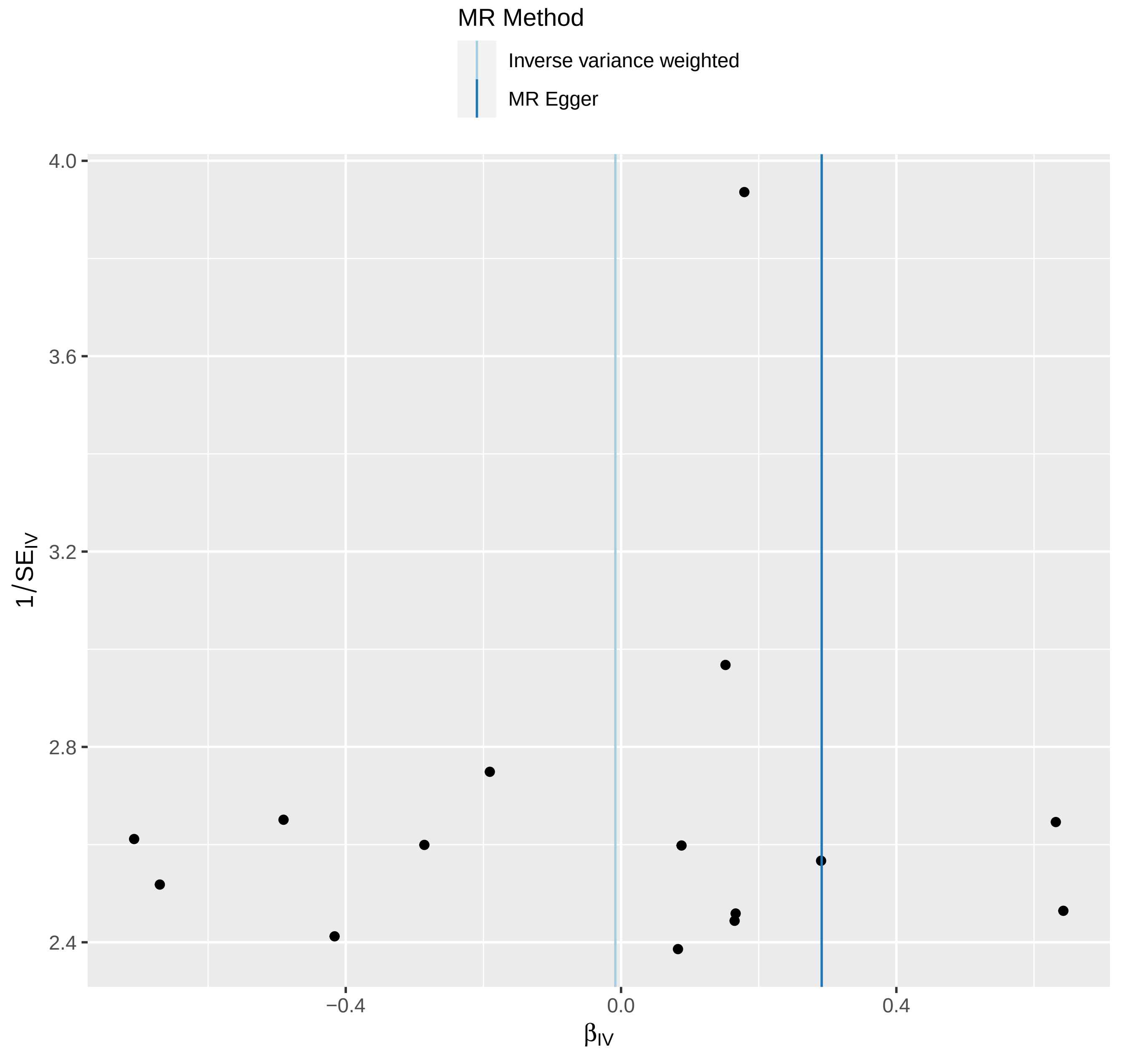

Batch 2−3231 : Gut microbiota abundance (genus Catenibacterium id.2153) on Kv channel−interacting protein 1
with a threshold of 1e−05

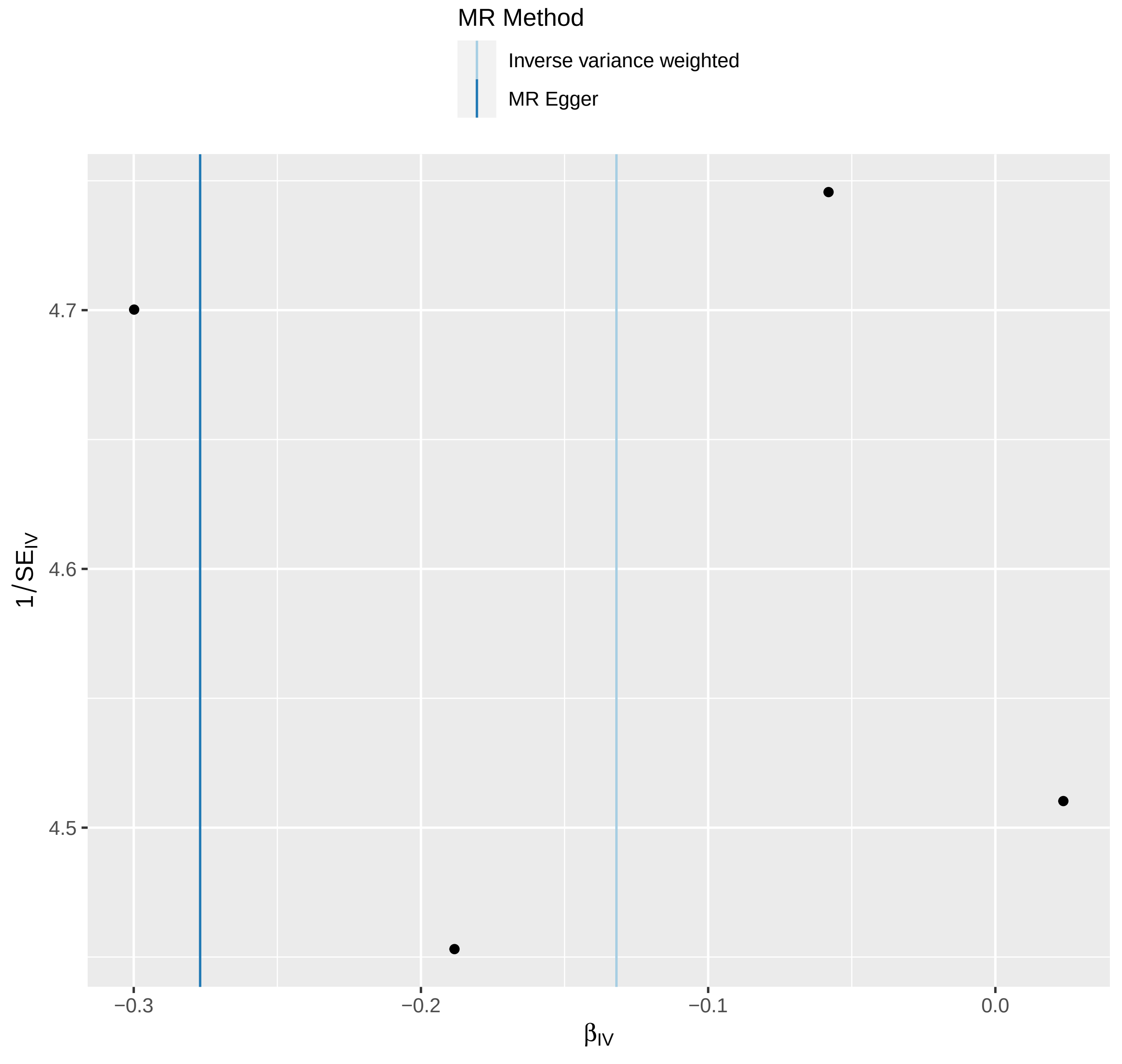

Batch 2−3232 : Gut microbiota abundance (genus Parasutterella id.2892) on Kv channel−interacting protein 1
with a threshold of 1e−05

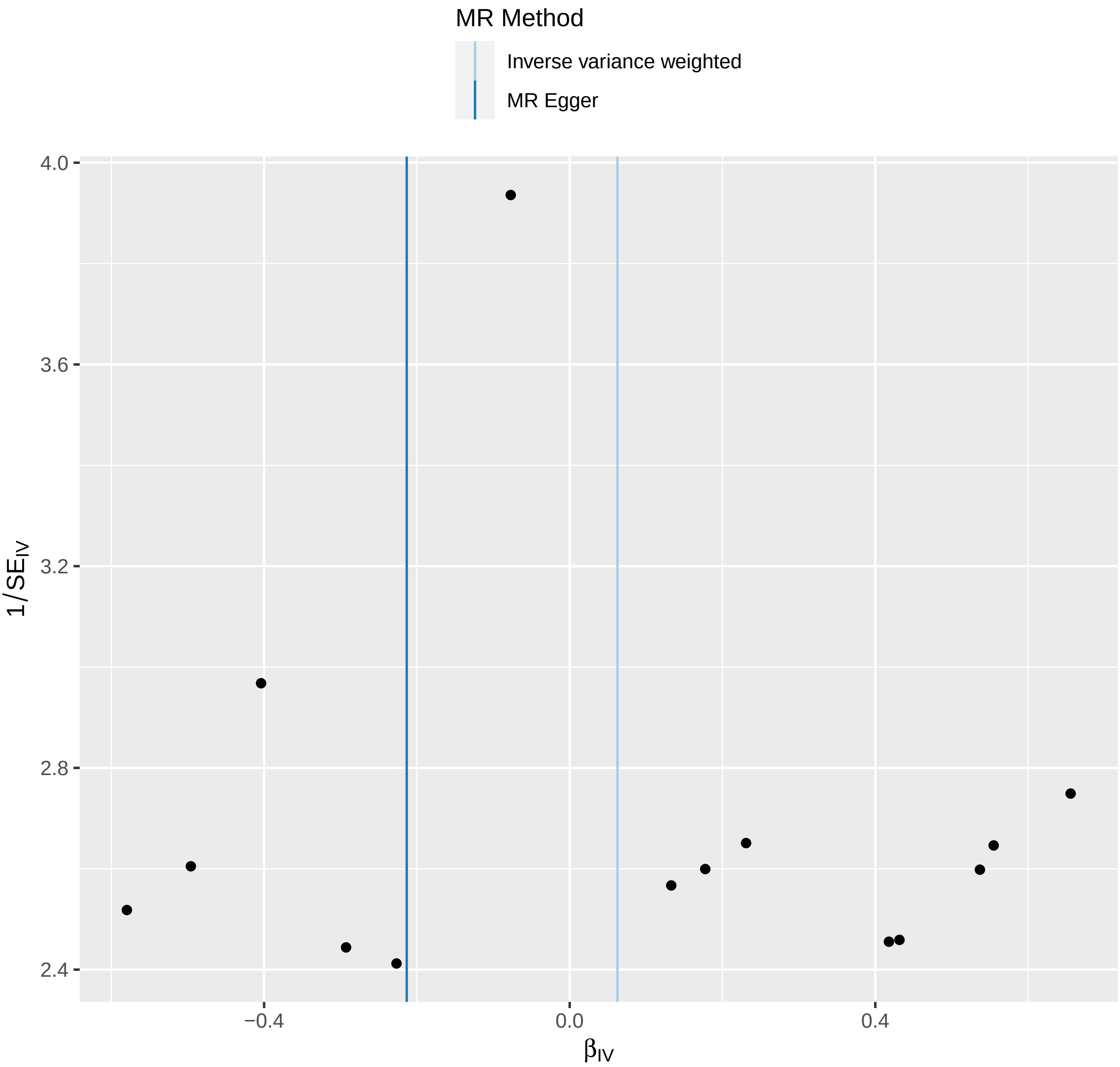

Batch 2−3233 : Gut microbiota abundance (genus Catenibacterium id.2153) on Calsenilin
with a threshold of 1e−05

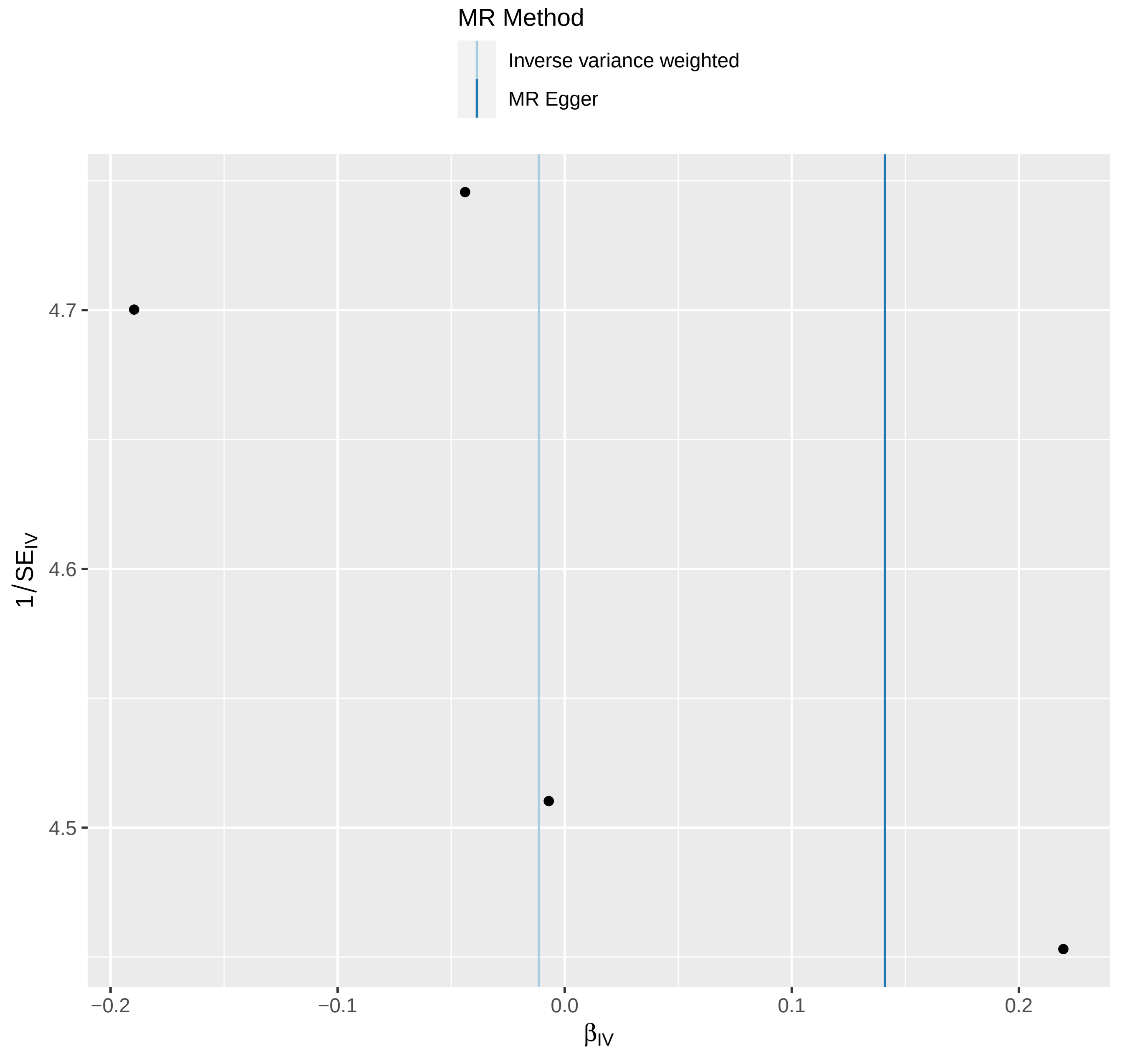

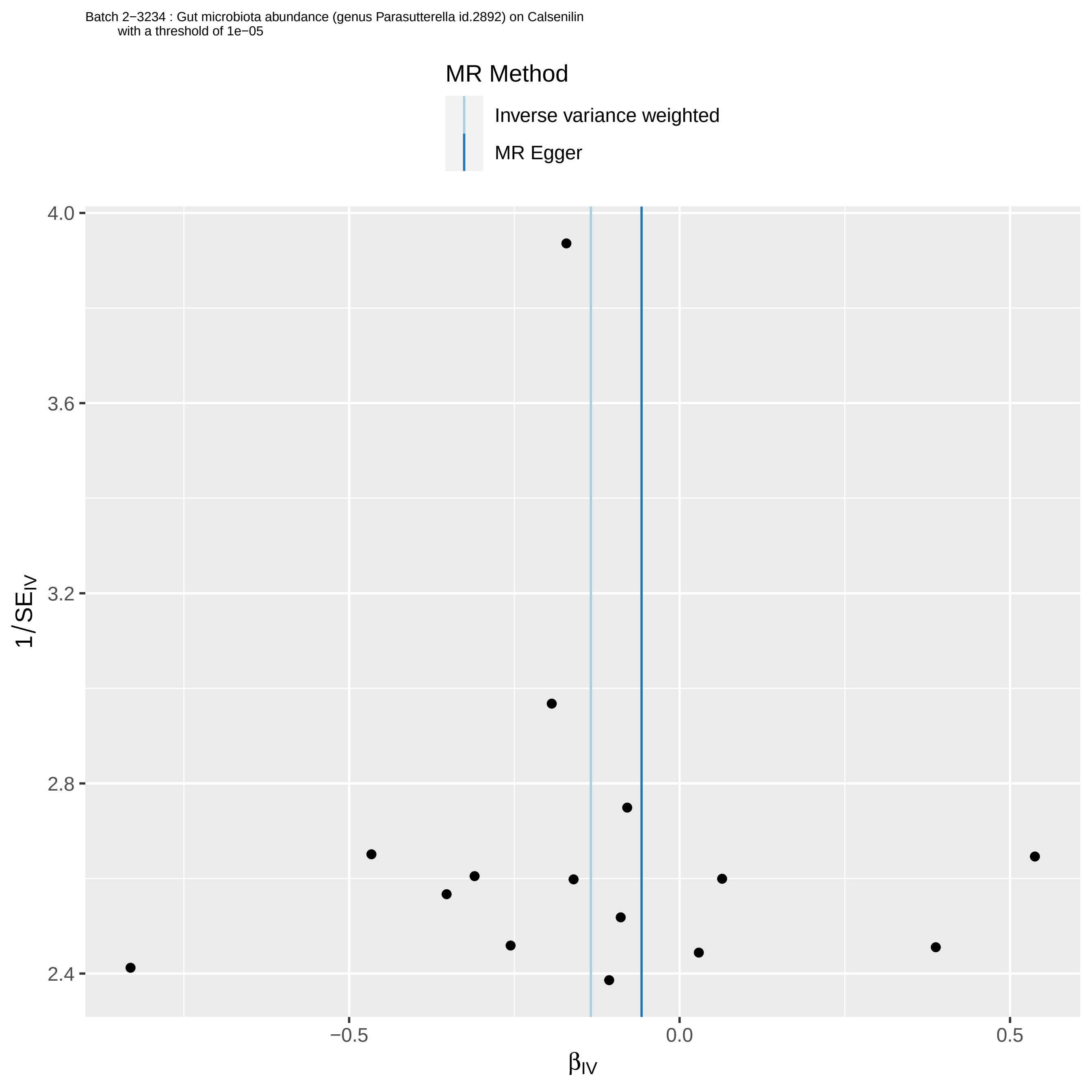
Batch 2−3234 : Gut microbiota abundance (genus Parasutterella id.2892) on Calsenilin
with a threshold of 1e−05
MR Method
Inverse variance weighted
MR Egger
4.0
3.6
3.2
2.8
2.4
1/SE_IV
−0.5
0.0
0.5
β_IV

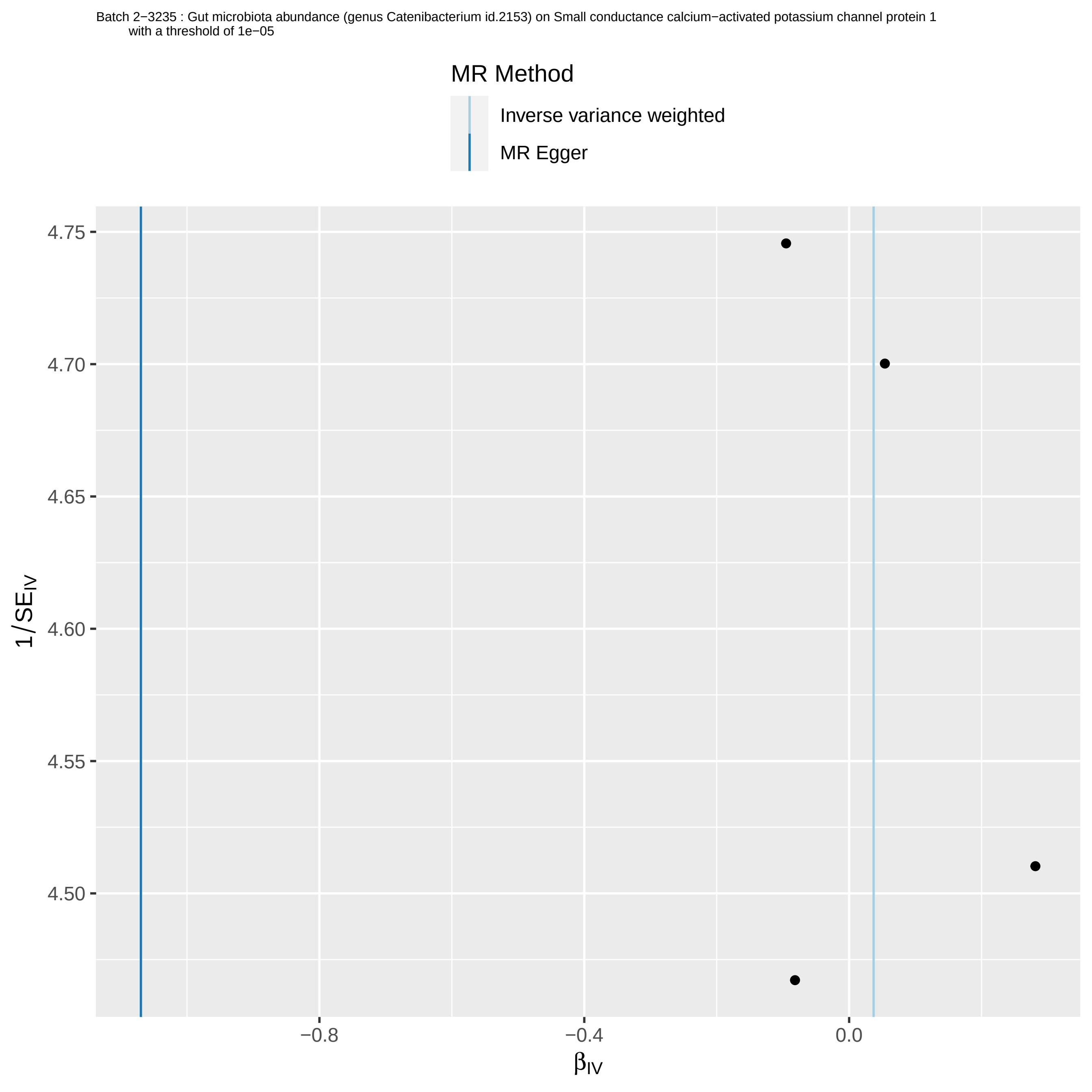
Batch 2−3235 : Gut microbiota abundance (genus Catenibacterium id.2153) on Small conductance calcium−activated potassium channel protein 1
with a threshold of 1e−05
MR Method
Inverse variance weighted
MR Egger
4.75
4.70
4.65
4.60
4.55
4.50
1/SE_IV
−0.8
−0.4
0.0
β_IV

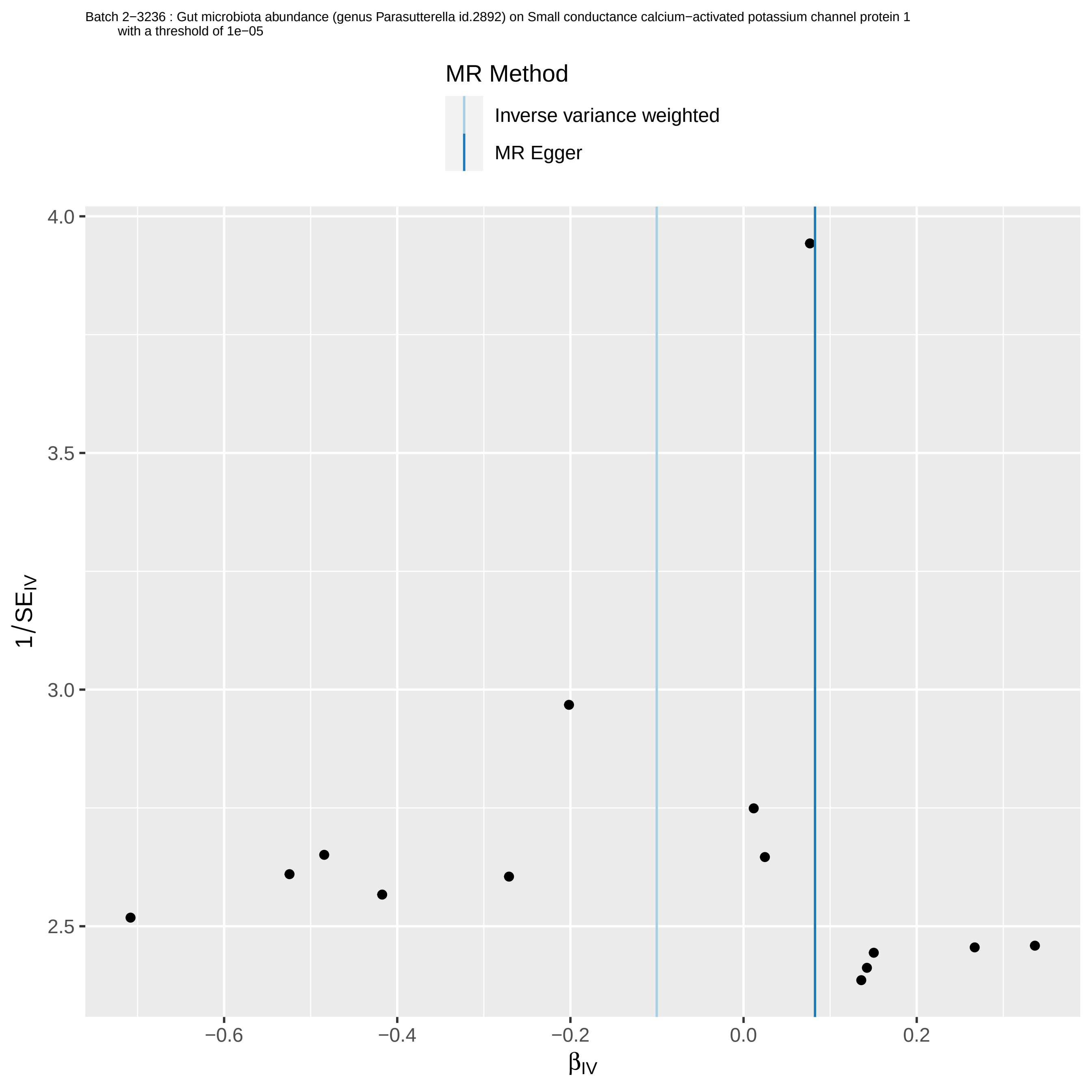

Batch 2−3236 : Gut microbiota abundance (genus Parasutterella id.2892) on Small conductance calcium−activated potassium channel protein 1
with a threshold of 1e−05
MR Method
Inverse variance weighted
MR Egger
4.0
3.5
3.0
2.5
1/SE_IV
−0.6
−0.4
−0.2
0.0
0.2
β_IV

Batch 2−3237 : Gut microbiota abundance (genus Catenibacterium id.2153) on Keratinocyte−associated transmembrane protein 2 with a threshold of 1e−05

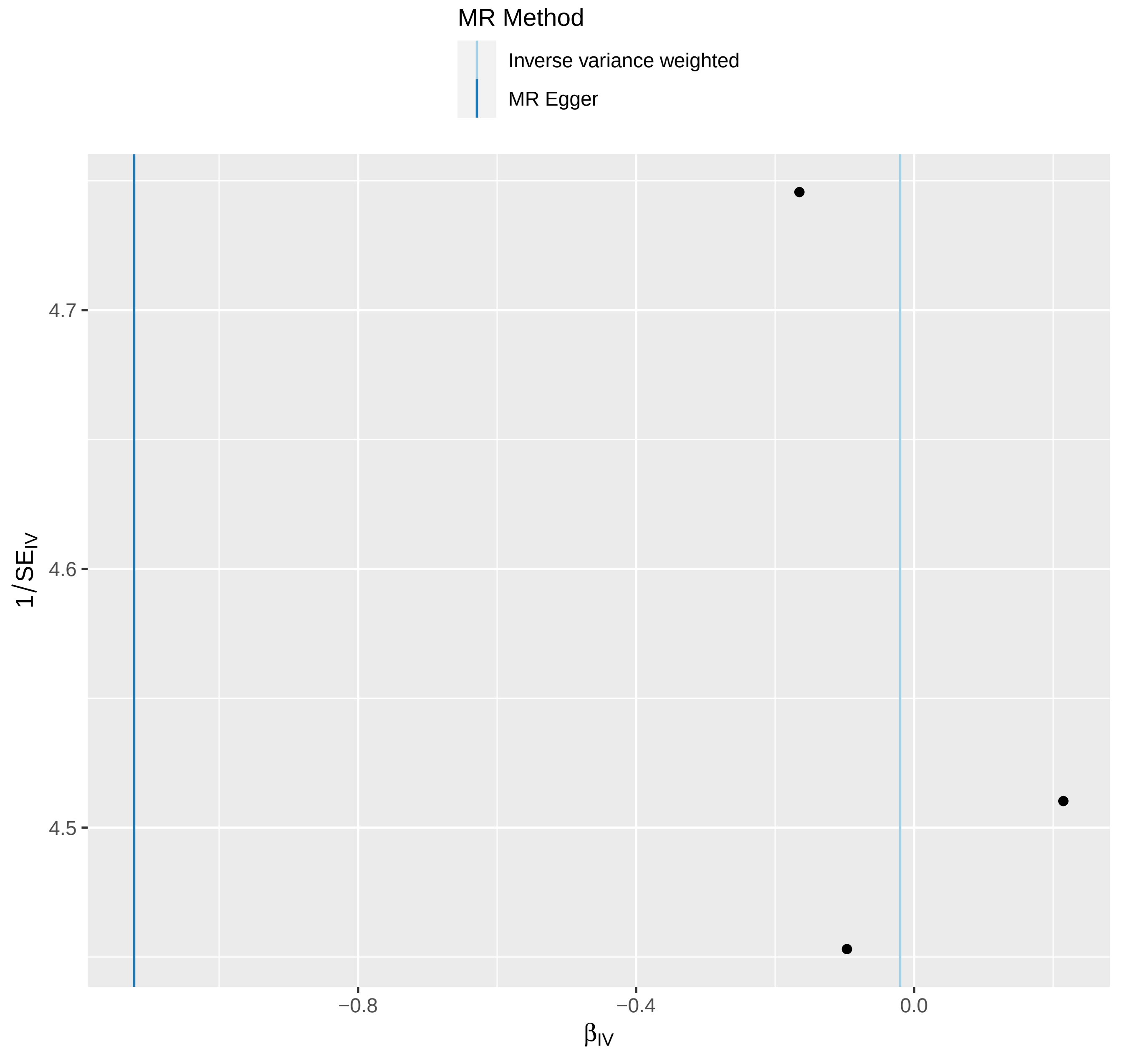

Batch 2−3238 : Gut microbiota abundance (genus Parasutterella id.2892) on Keratinocyte−associated transmembrane protein 2 with a threshold of 1e−05

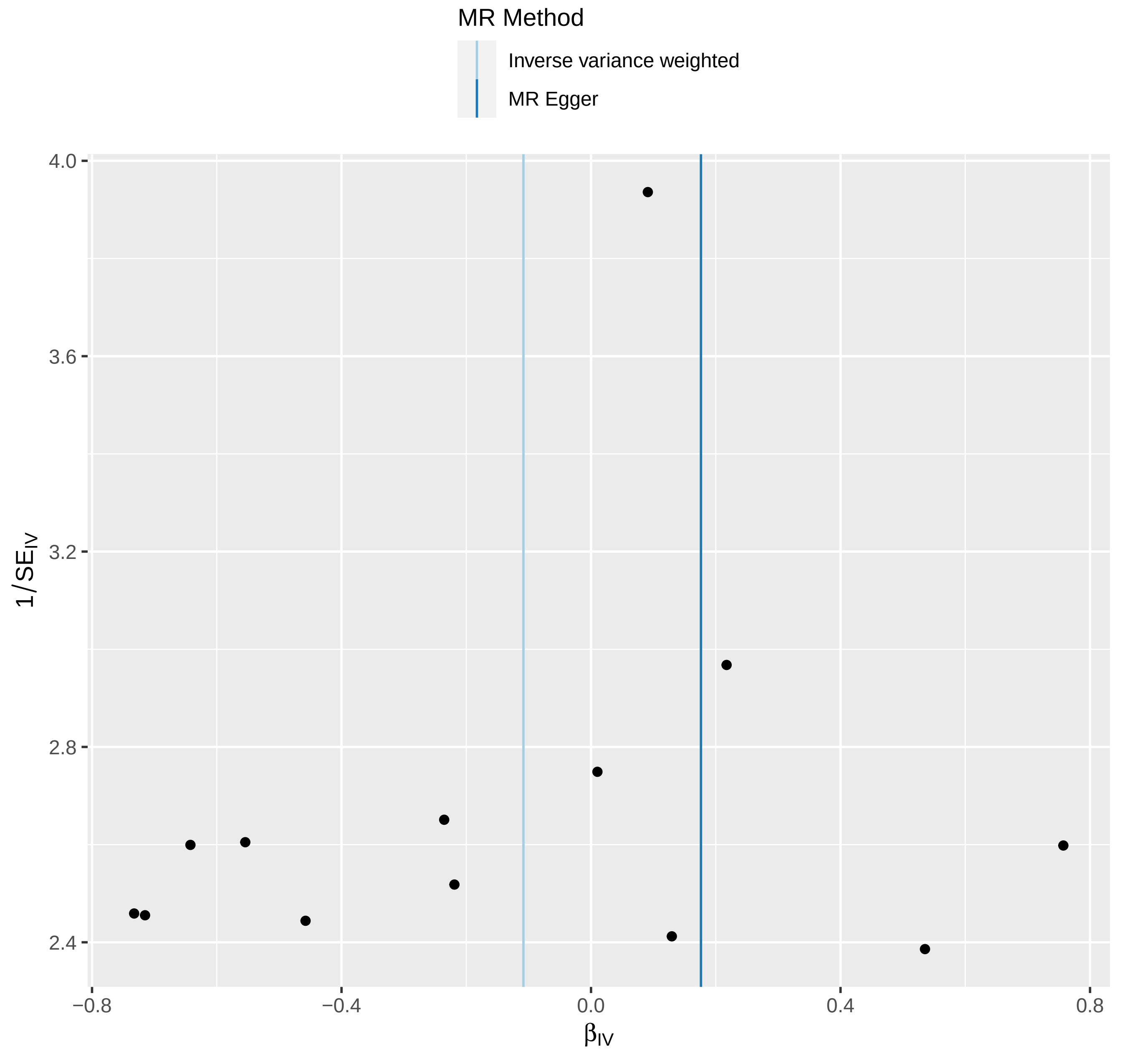

Batch 2−3239 : Gut microbiota abundance (genus Catenibacterium id.2153) on BTB/POZ domain−containing protein KCTD5
with a threshold of 1e−05

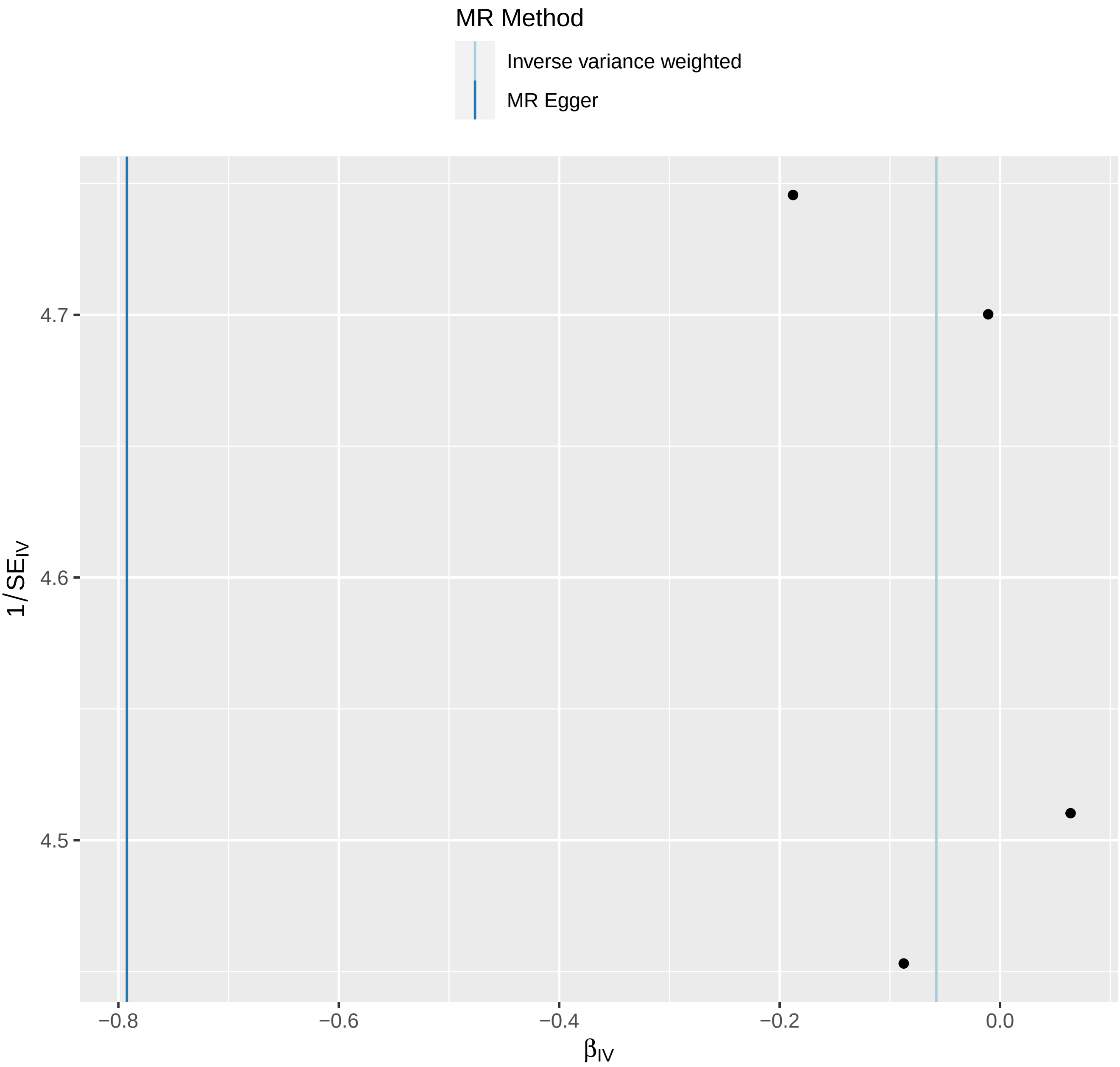

Batch 2−3240 : Gut microbiota abundance (genus Parasutterella id.2892) on BTB/POZ domain−containing protein KCTD5 with a threshold of 1e−05

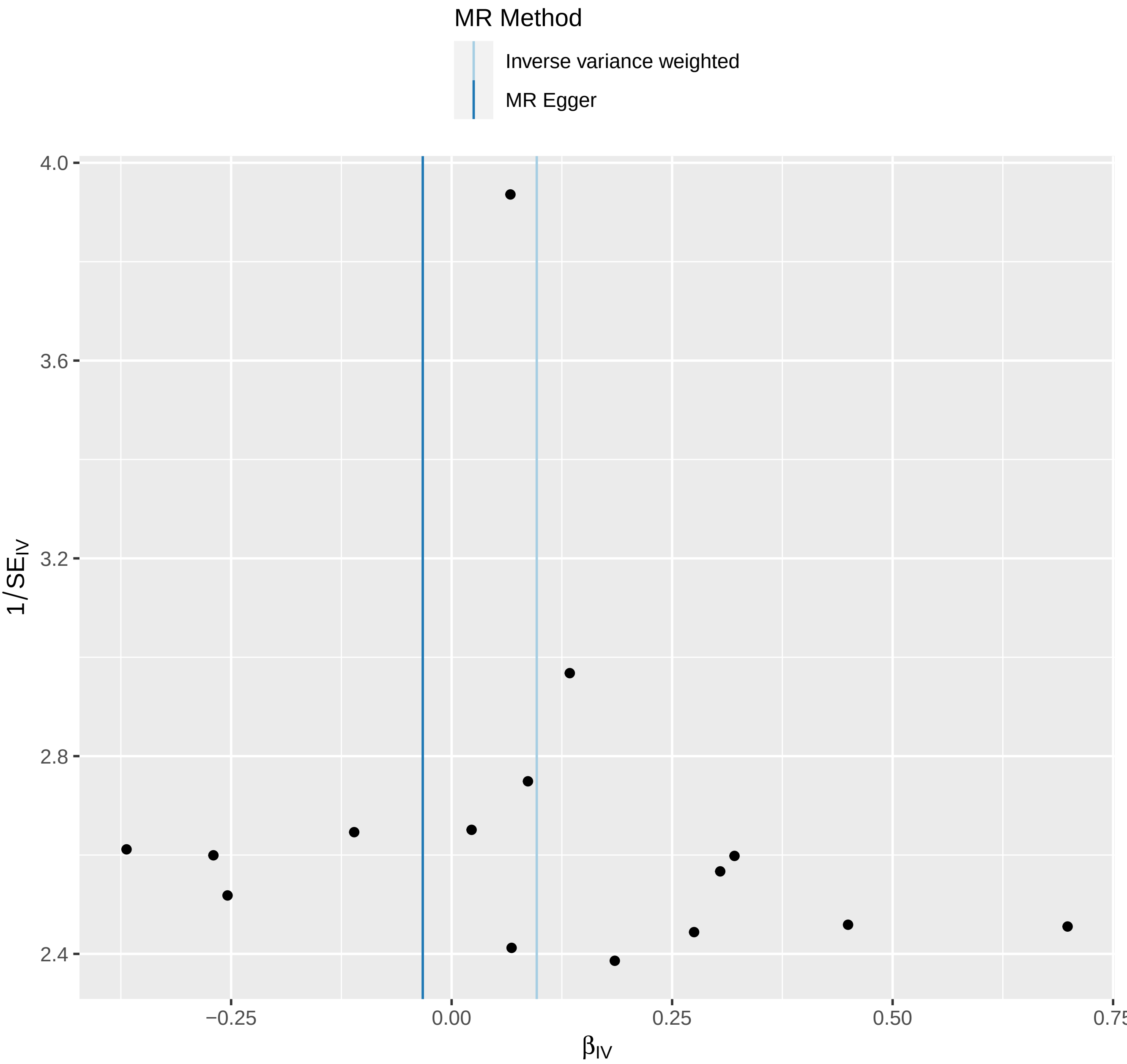

Batch 2−3241 : Gut microbiota abundance (genus Catenibacterium id.2153) on KDEL motif−containing protein 2 with a threshold of 1e−05

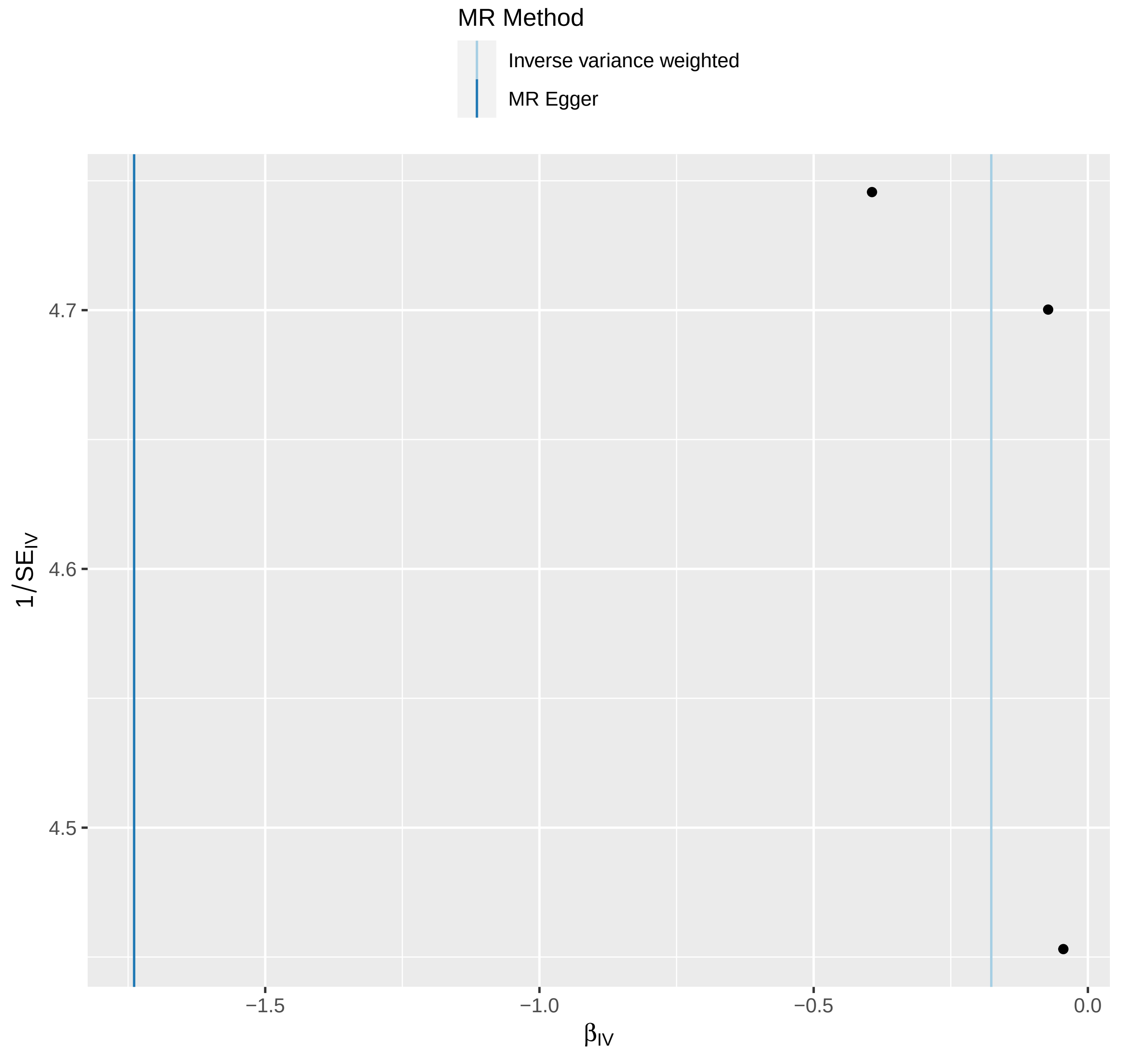

Batch 2−3242 : Gut microbiota abundance (genus Parasutterella id.2892) on KDEL motif−containing protein 2
with a threshold of 1e−05

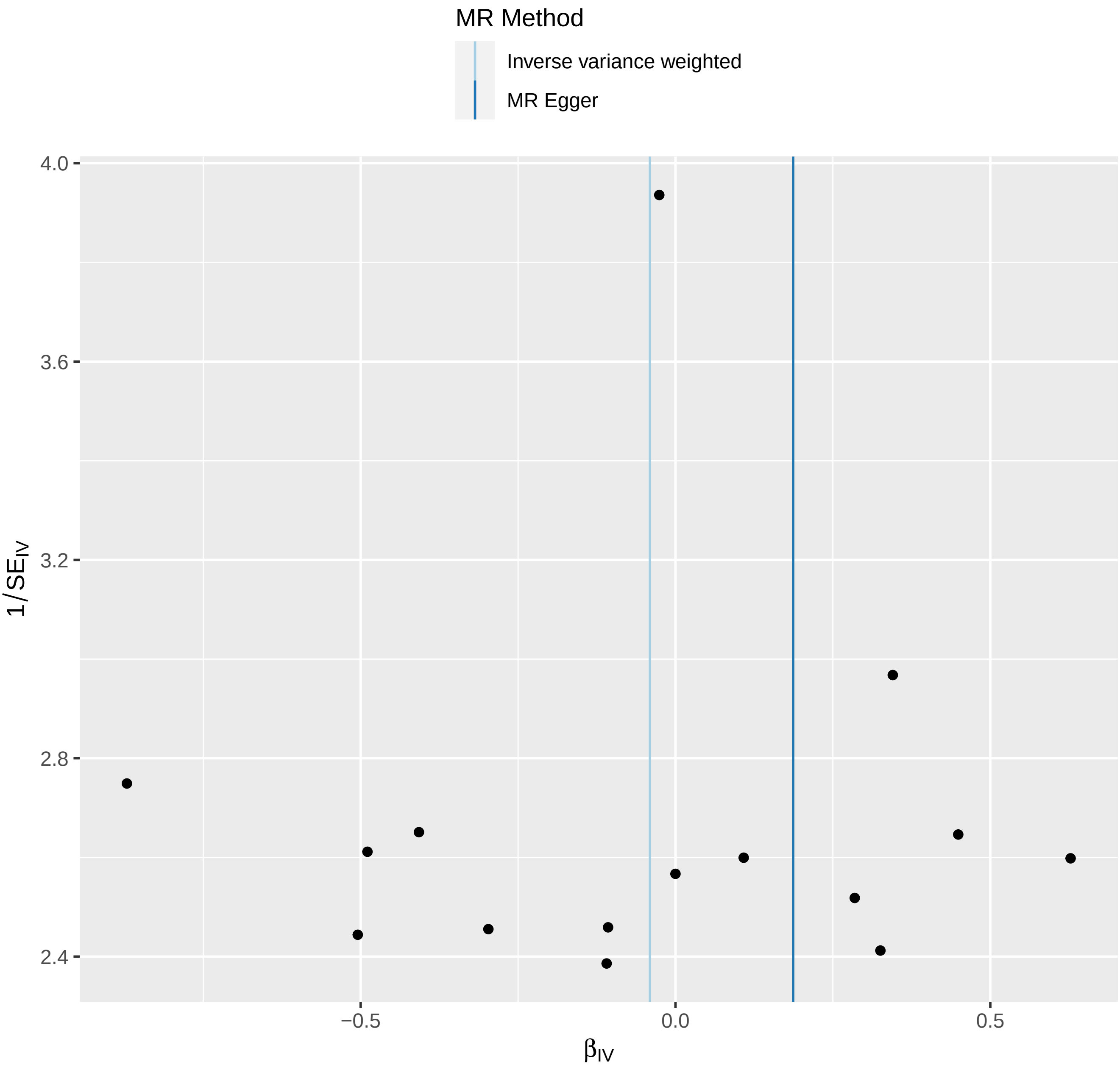

Batch 2−3243 : Gut microbiota abundance (genus Catenibacterium id.2153) on Vascular endothelial growth factor receptor 2 with a threshold of 1e−05

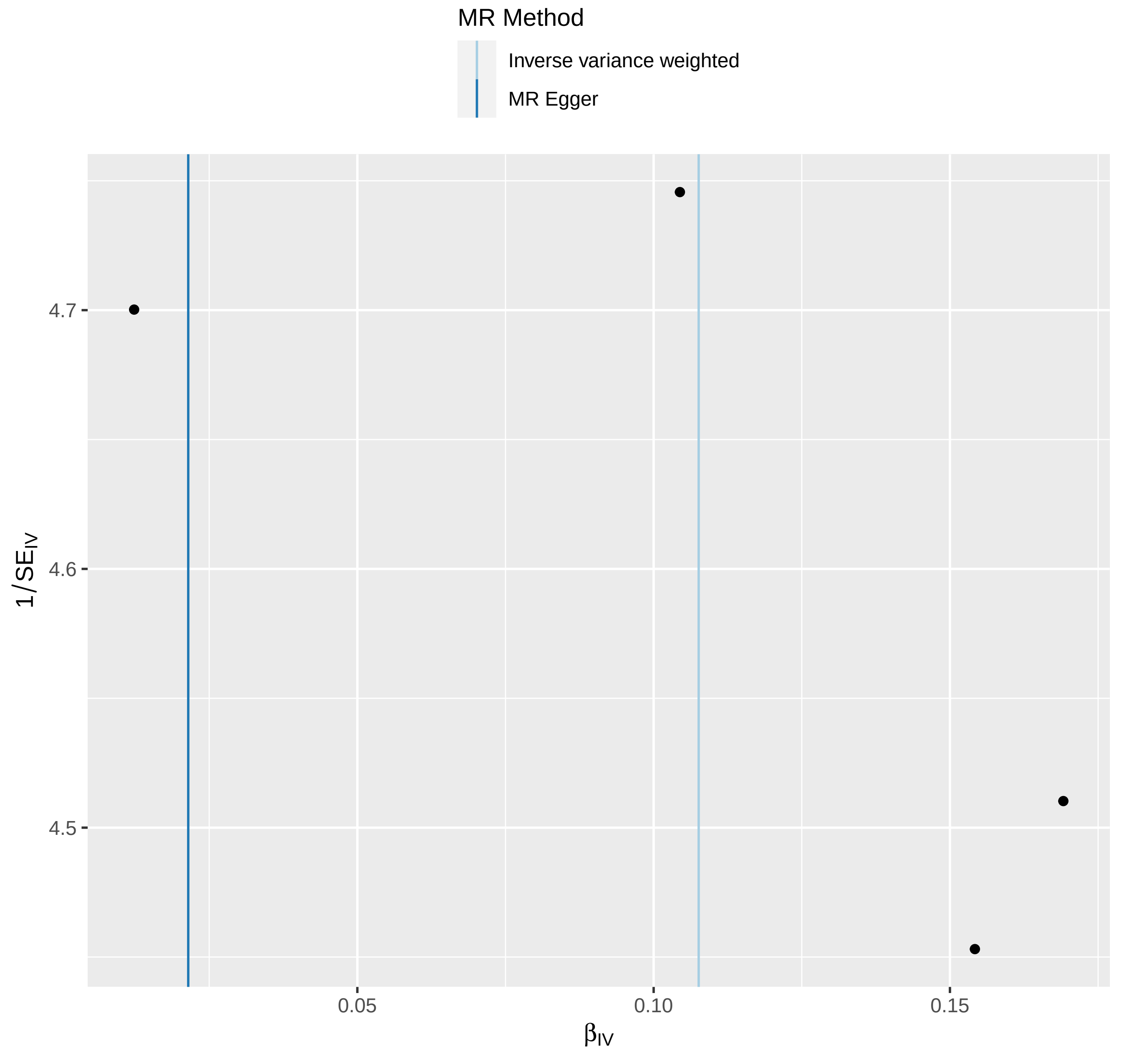

Batch 2−3244 : Gut microbiota abundance (genus Parasutterella id.2892) on Vascular endothelial growth factor receptor 2 with a threshold of 1e−05

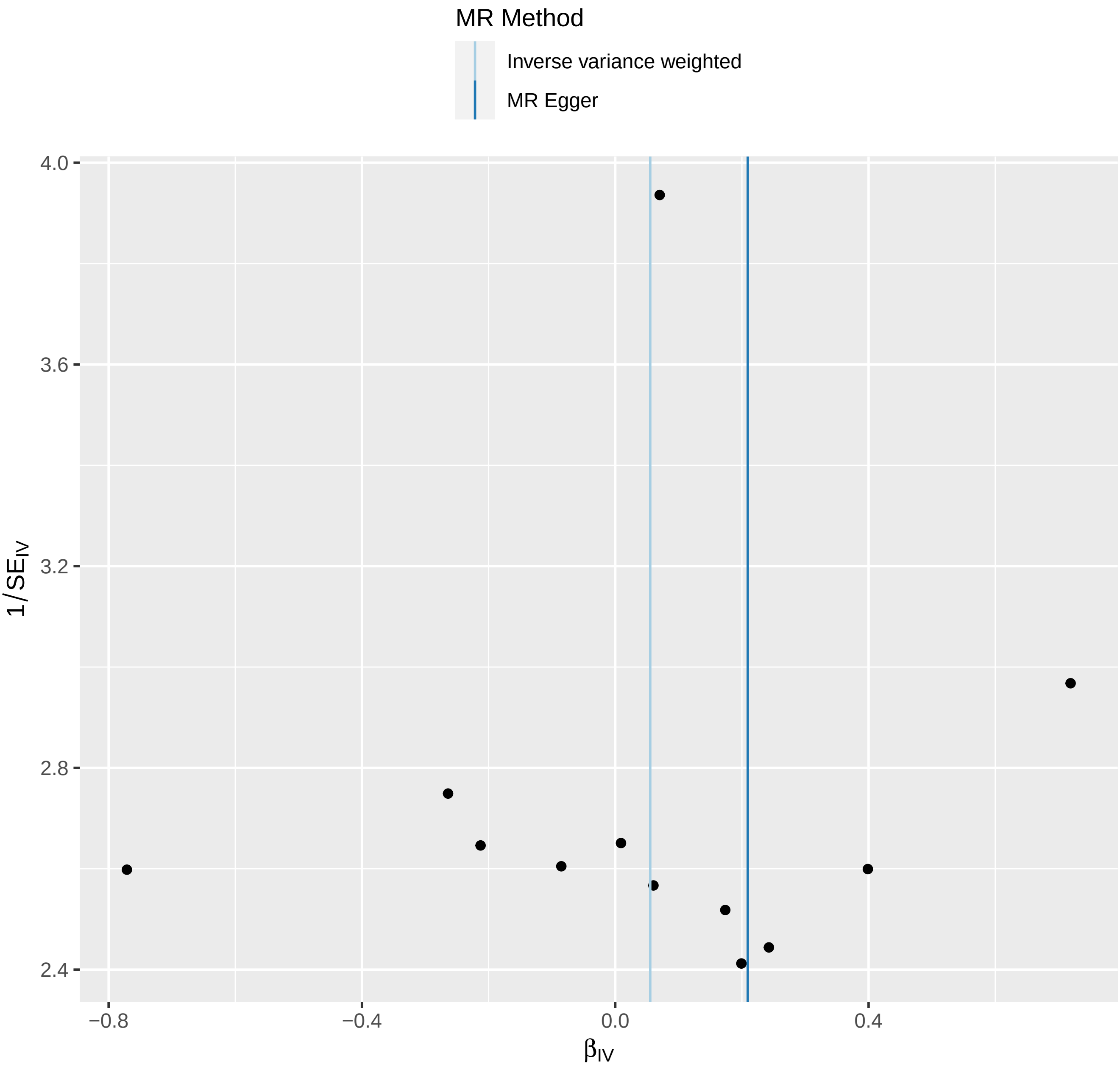

Batch 2−3245 : Gut microbiota abundance (genus Catenibacterium id.2153) on Kell blood group glycoprotein
with a threshold of 1e−05

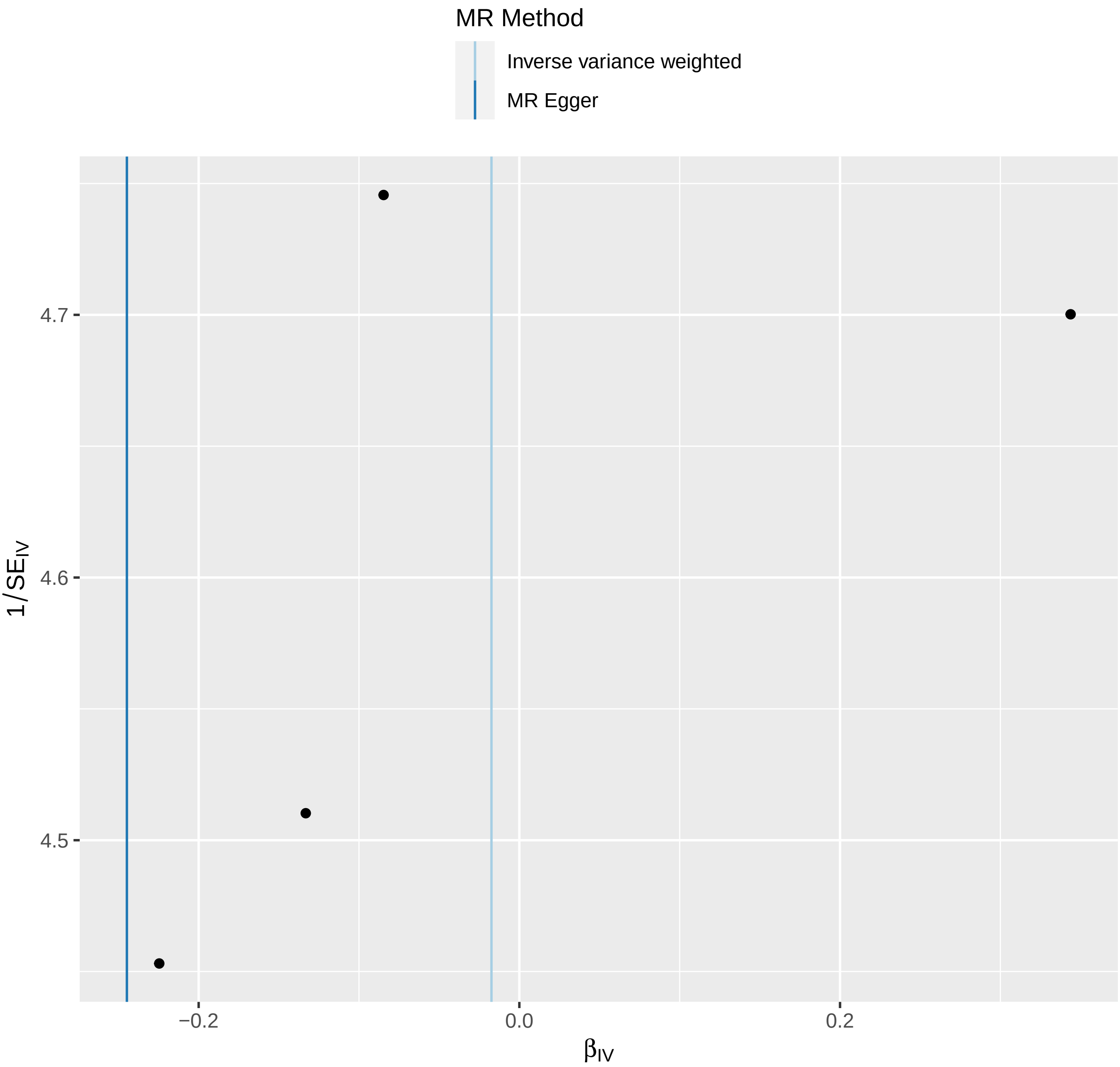

Batch 2−3246 : Gut microbiota abundance (genus Parasutterella id.2892) on Kell blood group glycoprotein
with a threshold of 1e−05

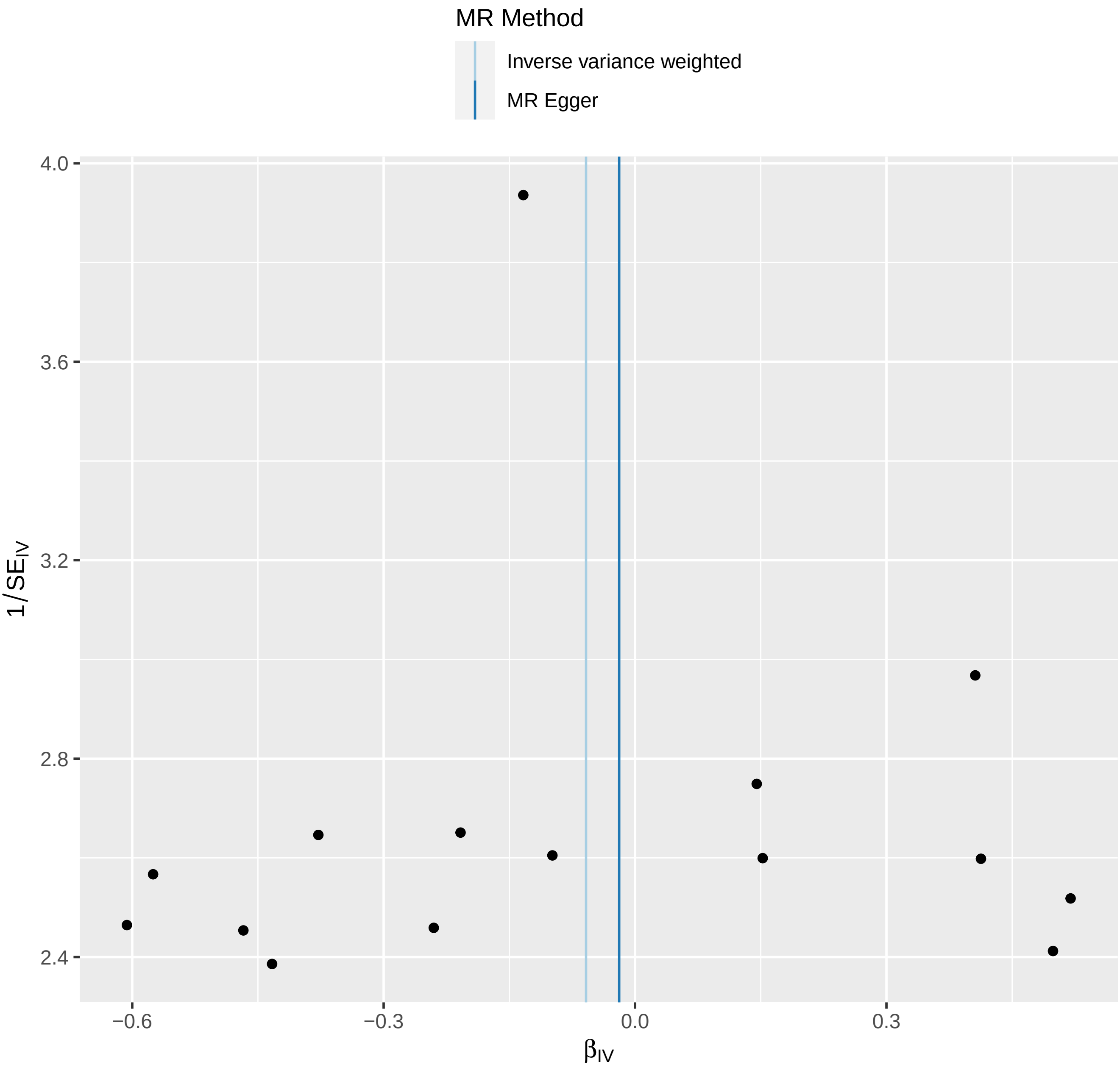

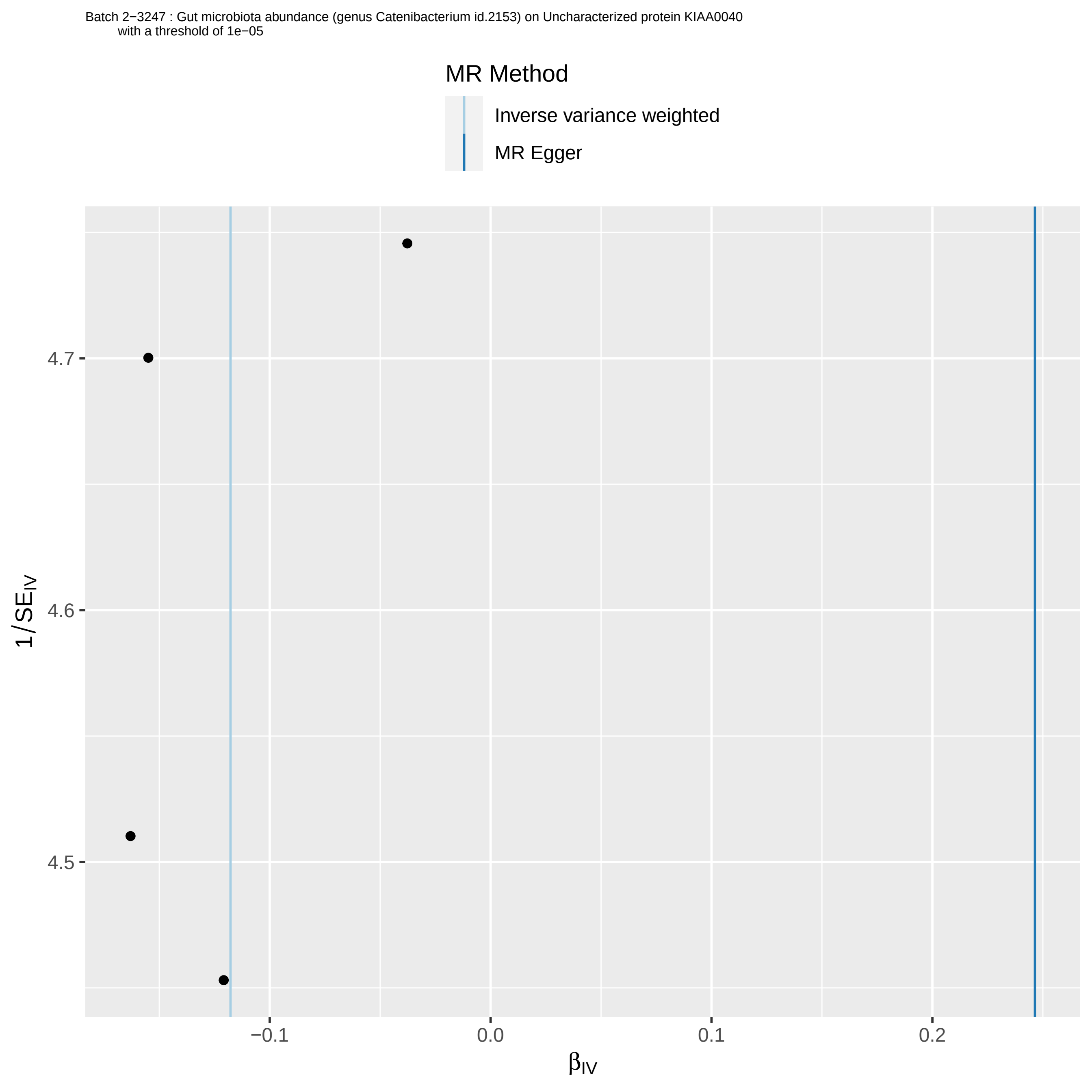

Batch 2−3247 : Gut microbiota abundance (genus Catenibacterium id.2153) on Uncharacterized protein KIAA0040
with a threshold of 1e−05
MR Method
Inverse variance weighted
MR Egger
4.7
4.6
4.5
1/SE_IV
−0.1
0.0
0.1
0.2
β_IV

Batch 2−3248 : Gut microbiota abundance (genus Parasutterella id.2892) on Uncharacterized protein KIAA0040
with a threshold of 1e−05

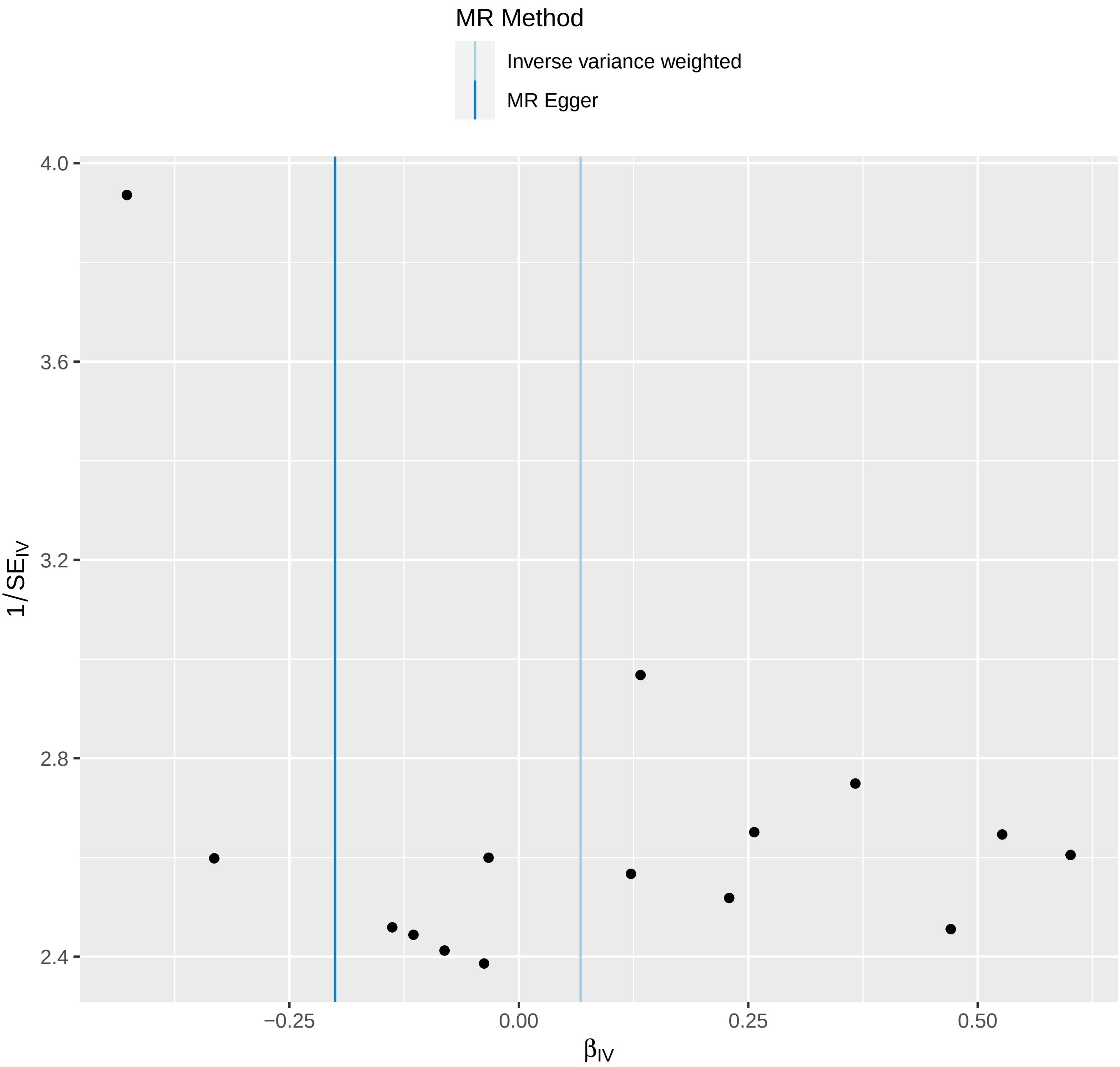

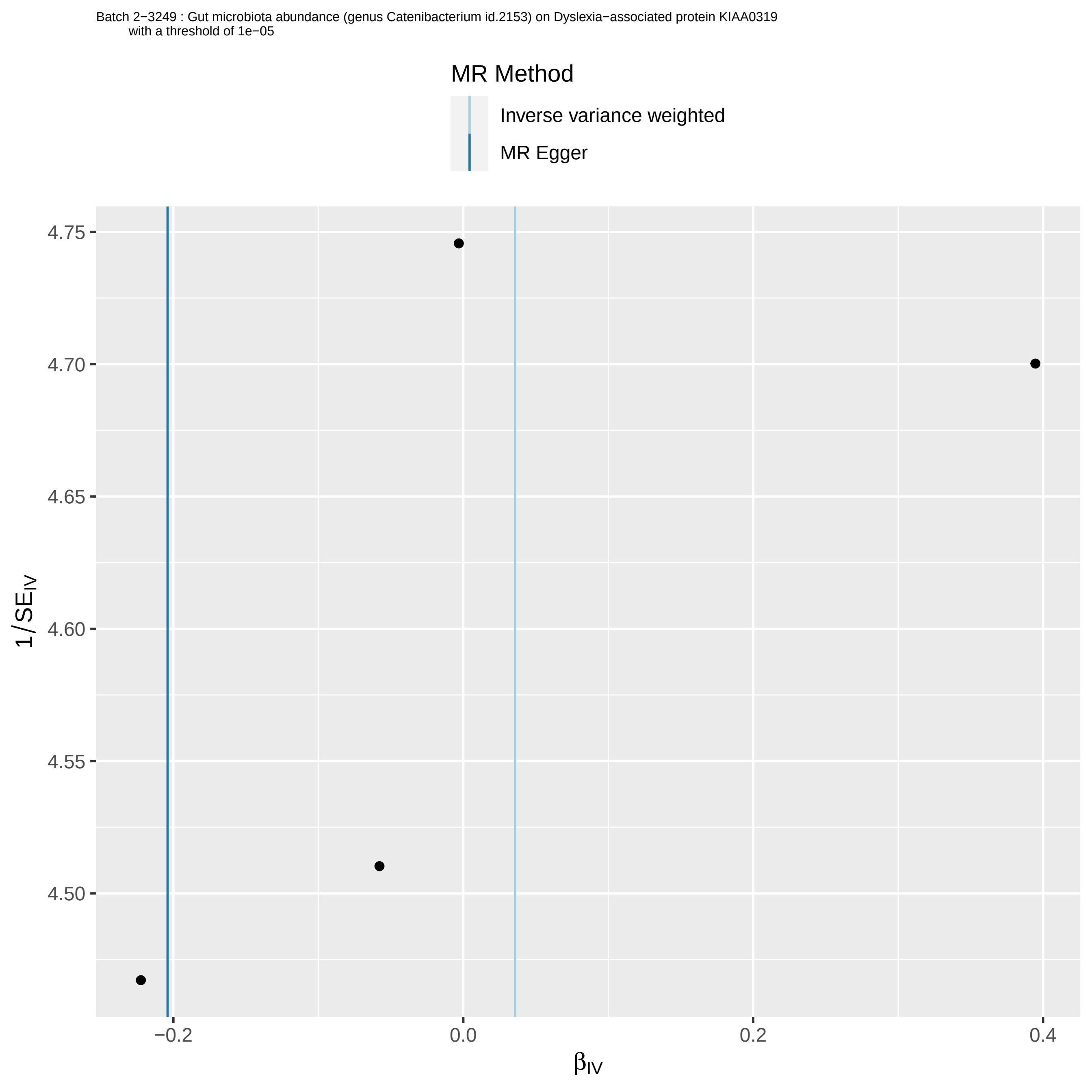

Batch 2−3249 : Gut microbiota abundance (genus Catenibacterium id.2153) on Dyslexia−associated protein KIAA0319
with a threshold of 1e−05
MR Method
Inverse variance weighted
MR Egger
4.75
4.70
4.65
4.60
4.55
4.50
1/SE_IV
−0.2
0.0
0.2
0.4
β_IV

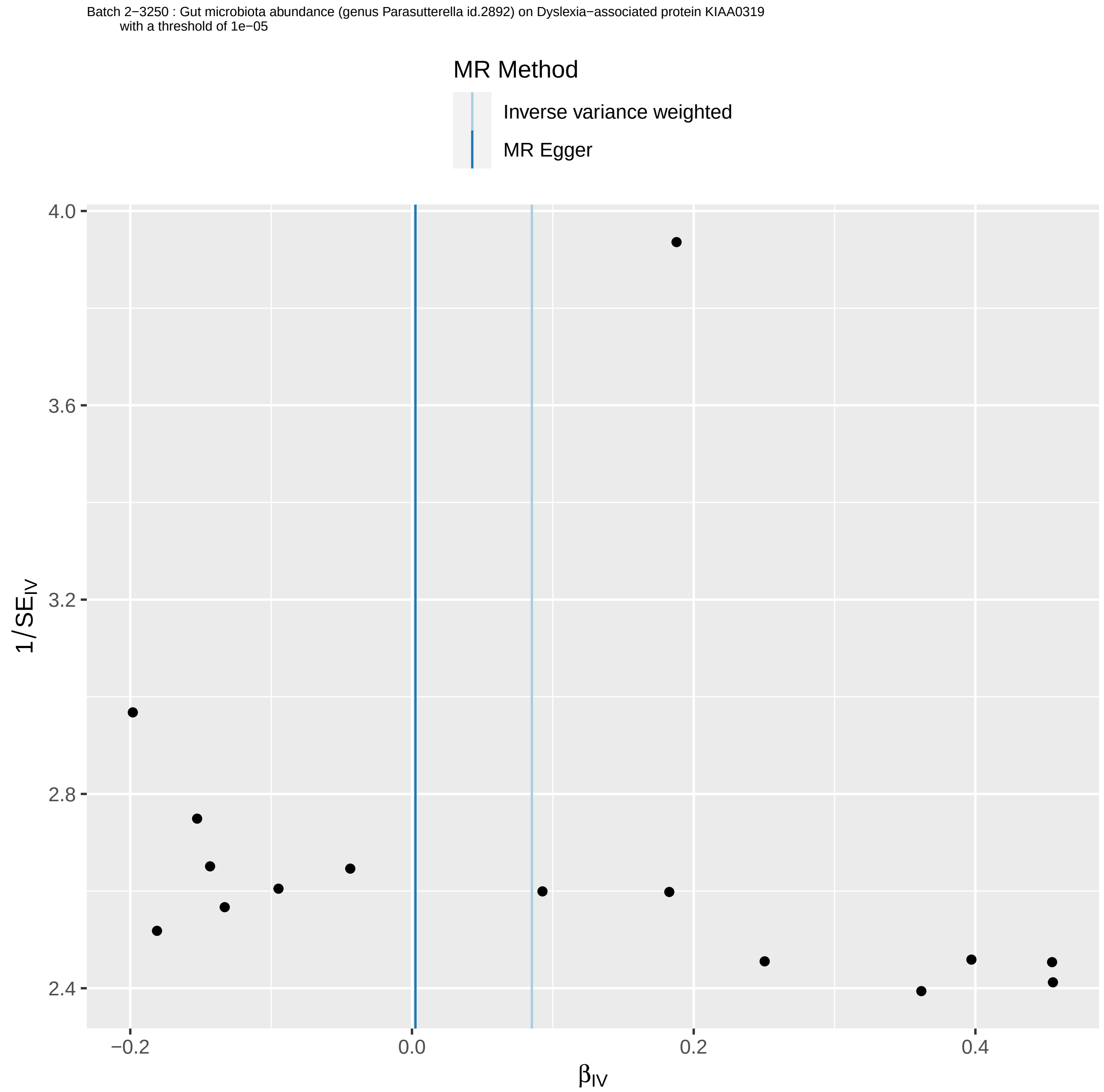

Batch 2−3250 : Gut microbiota abundance (genus Parasutterella id.2892) on Dyslexia−associated protein KIAA0319
with a threshold of 1e−05
MR Method
Inverse variance weighted
MR Egger
4.0
3.6
3.2
2.8
2.4
1/SE_IV
−0.2
0.0
0.2
0.4
β_IV

Batch 2−3251 : Gut microbiota abundance (genus Catenibacterium id.2153) on Dyslexia−associated protein KIAA0319−like protein
with a threshold of 1e−05

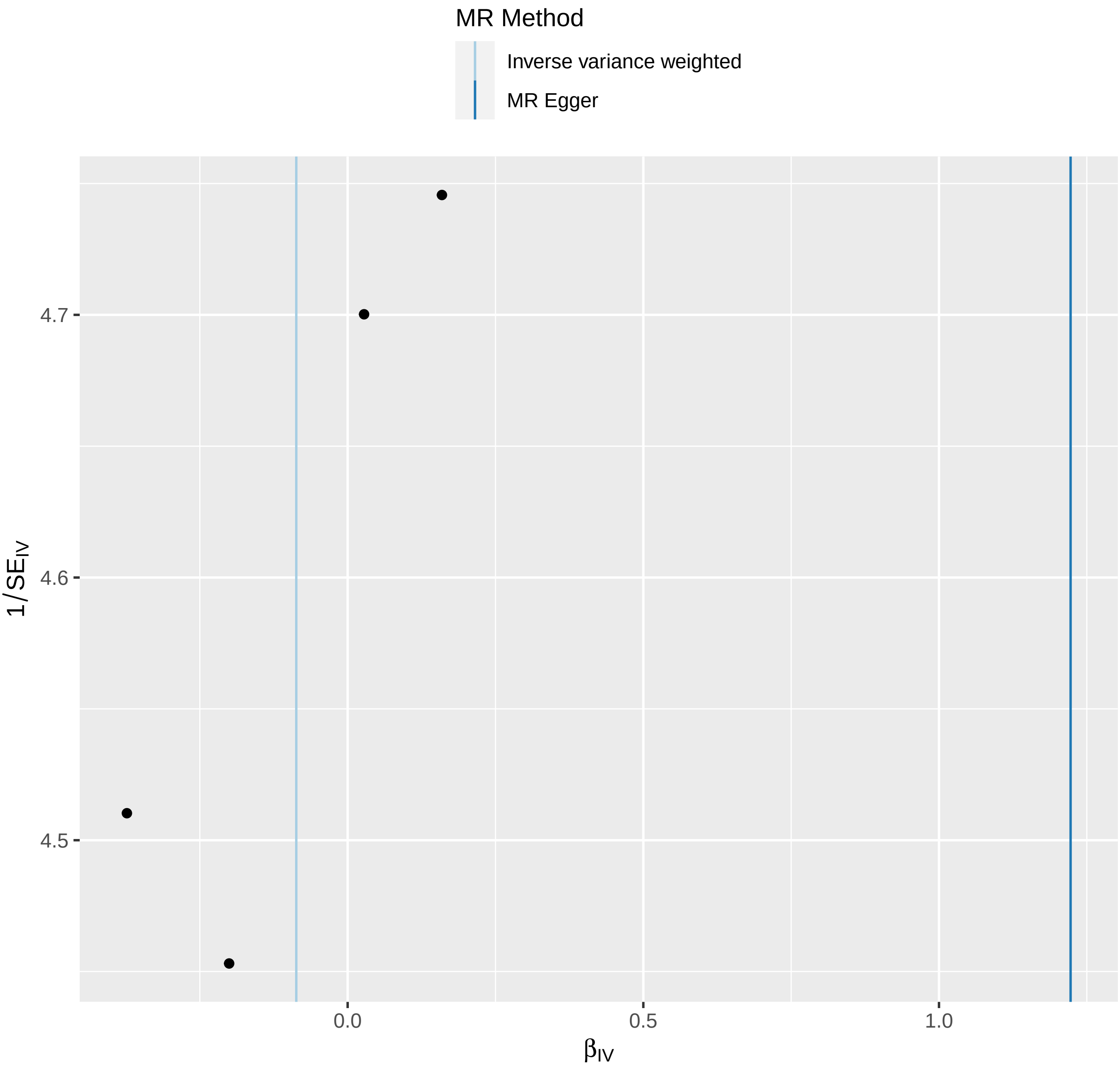

Batch 2−3252 : Gut microbiota abundance (genus Parasutterella id.2892) on Dyslexia−associated protein KIAA0319−like protein with a threshold of 1e−05

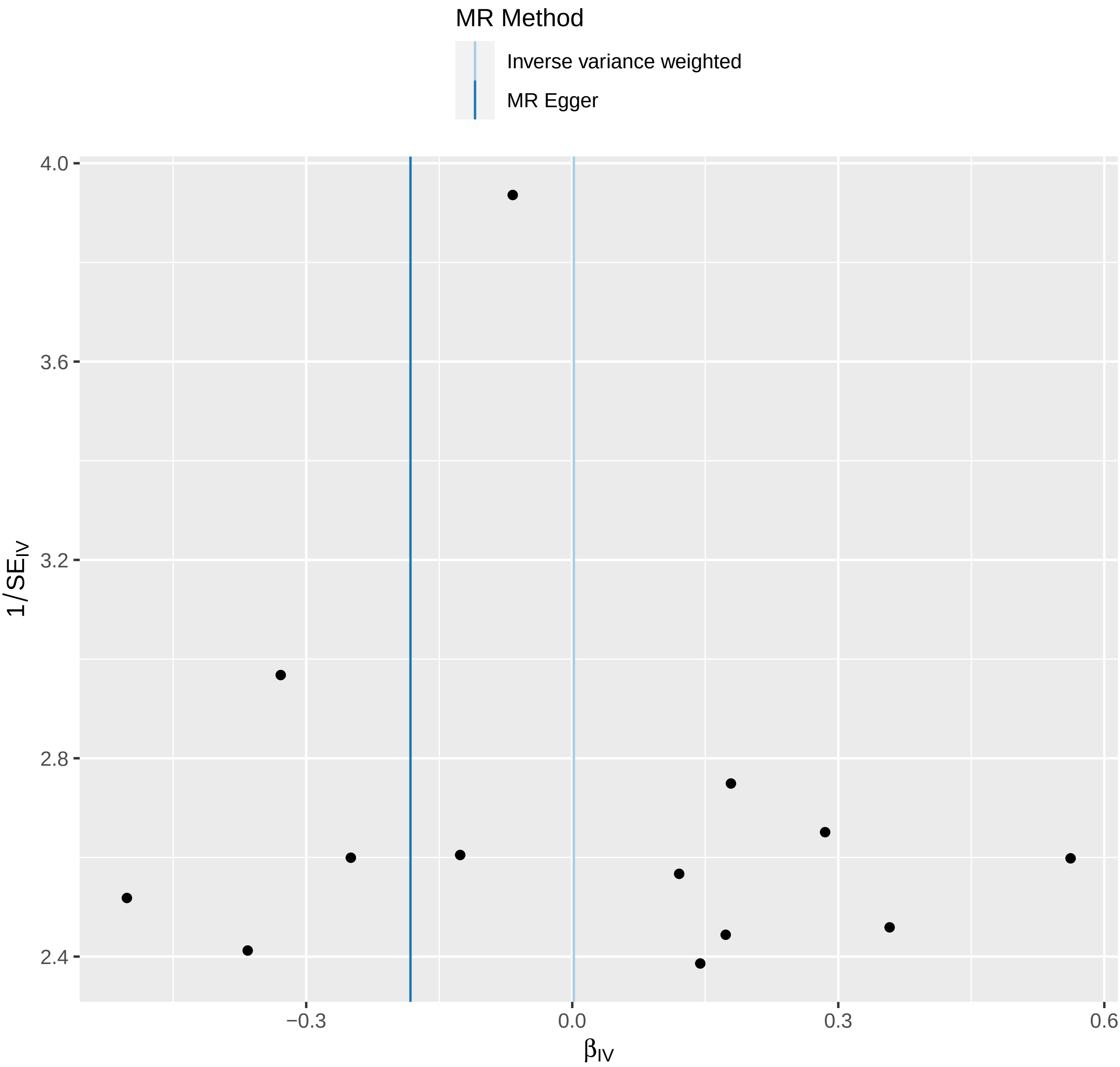

Batch 2−3253 : Gut microbiota abundance (genus Catenibacterium id.2153) on UPF0258 protein KIAA1024
with a threshold of 1e−05

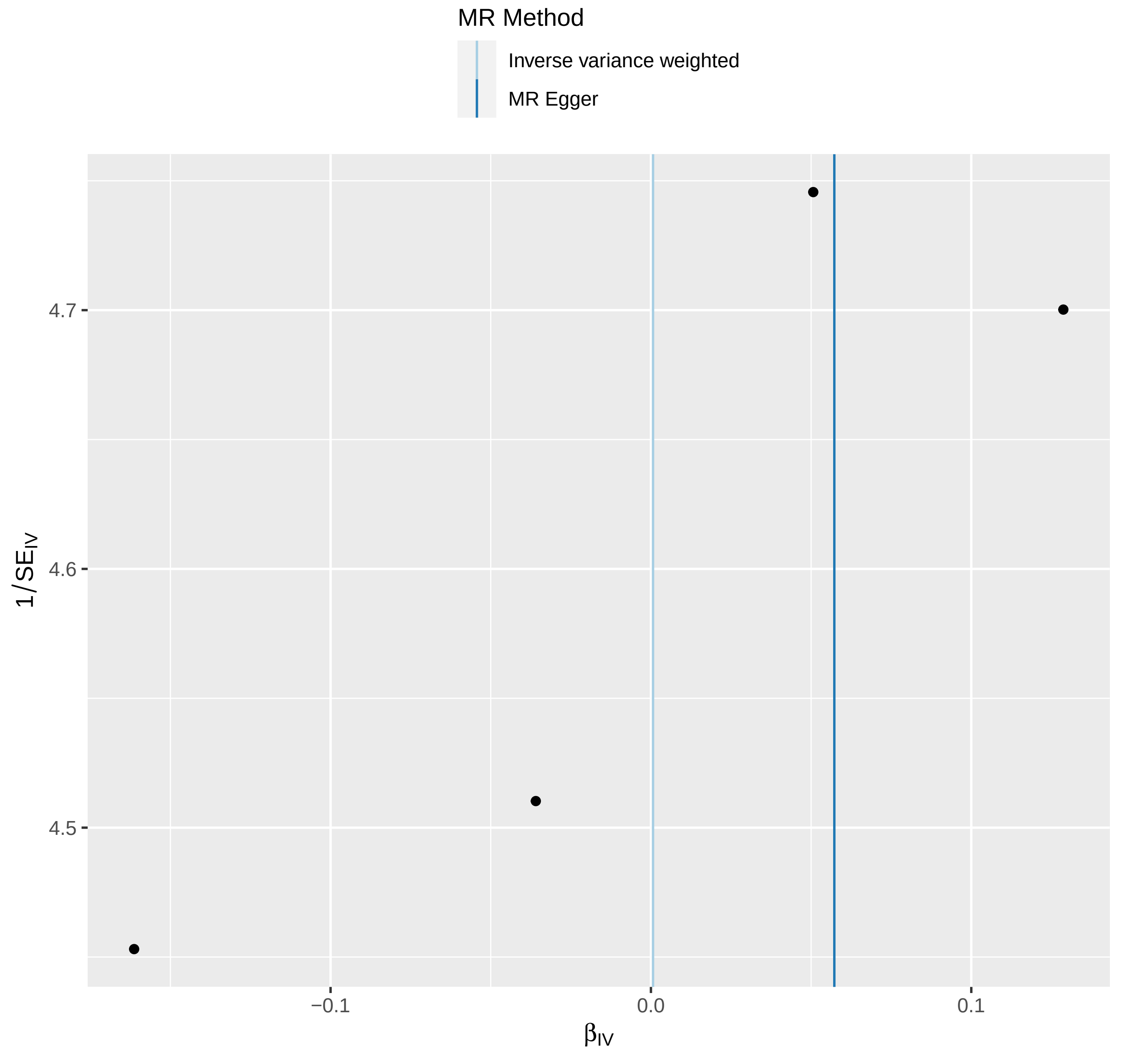

Batch 2−3254 : Gut microbiota abundance (genus Parasutterella id.2892) on UPF0258 protein KIAA1024
with a threshold of 1e−05

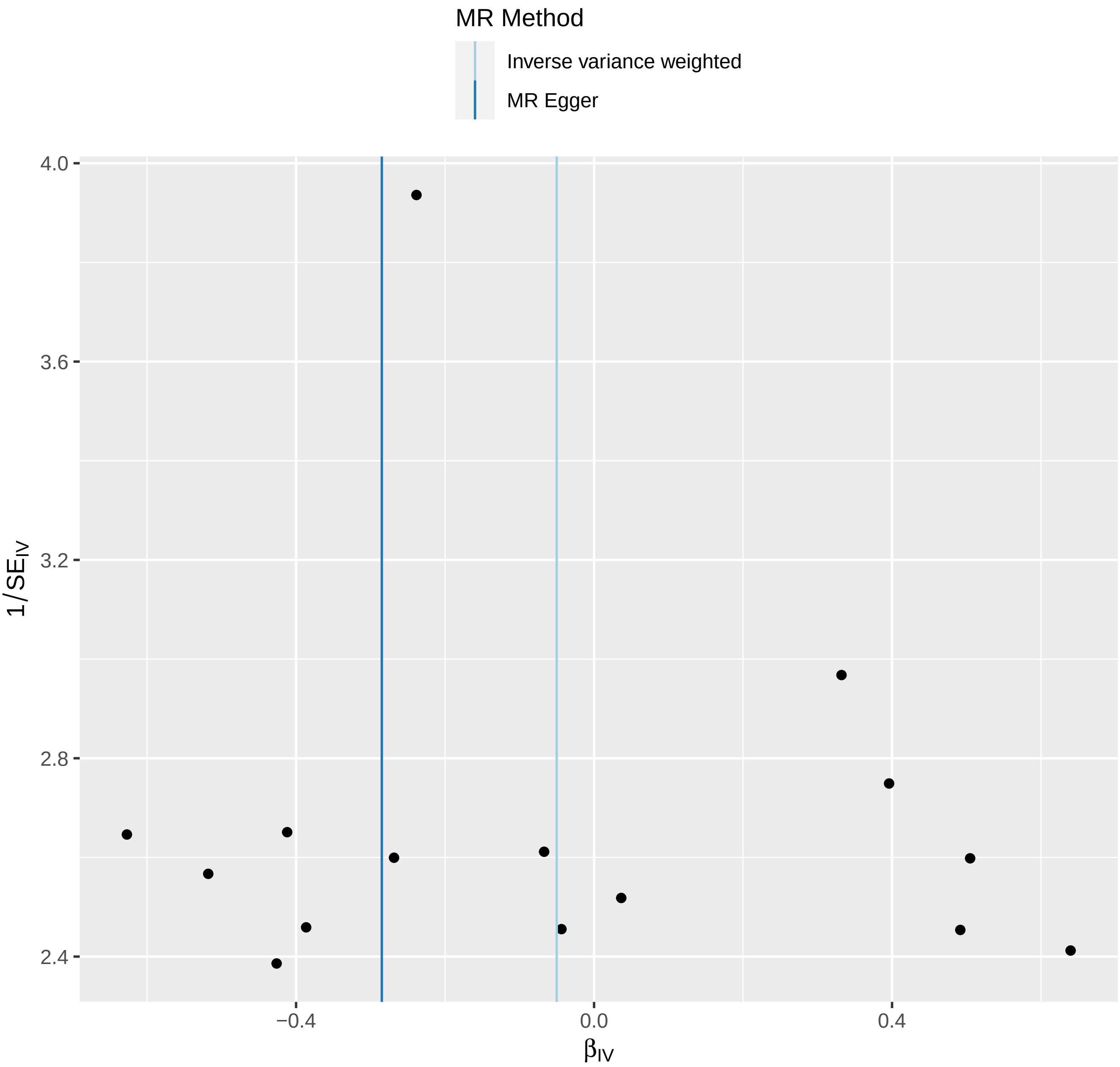

Batch 2−3255 : Gut microbiota abundance (genus Catenibacterium id.2153) on Uncharacterized family 31 glucosidase KIAA1161
with a threshold of 1e−05

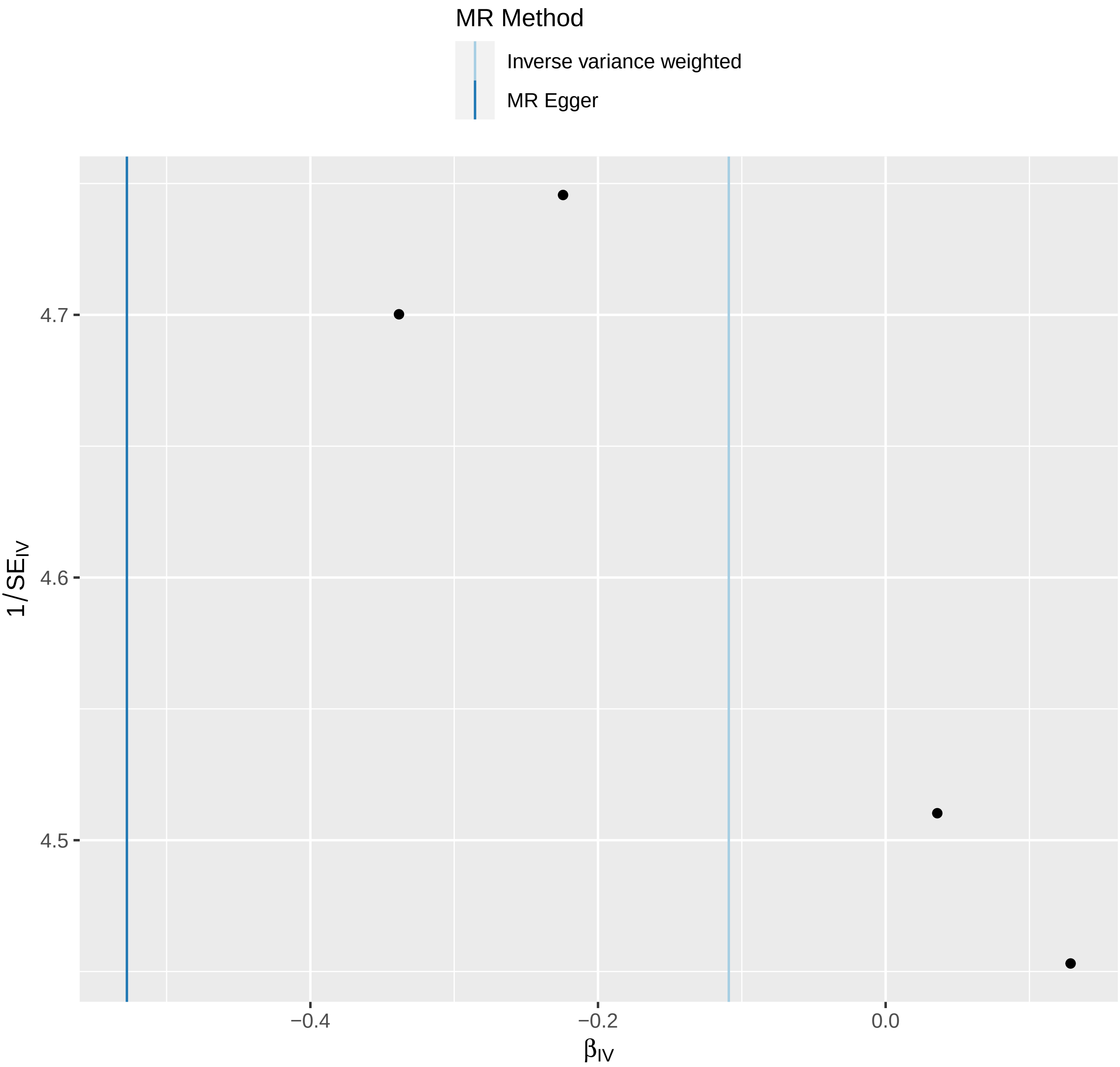

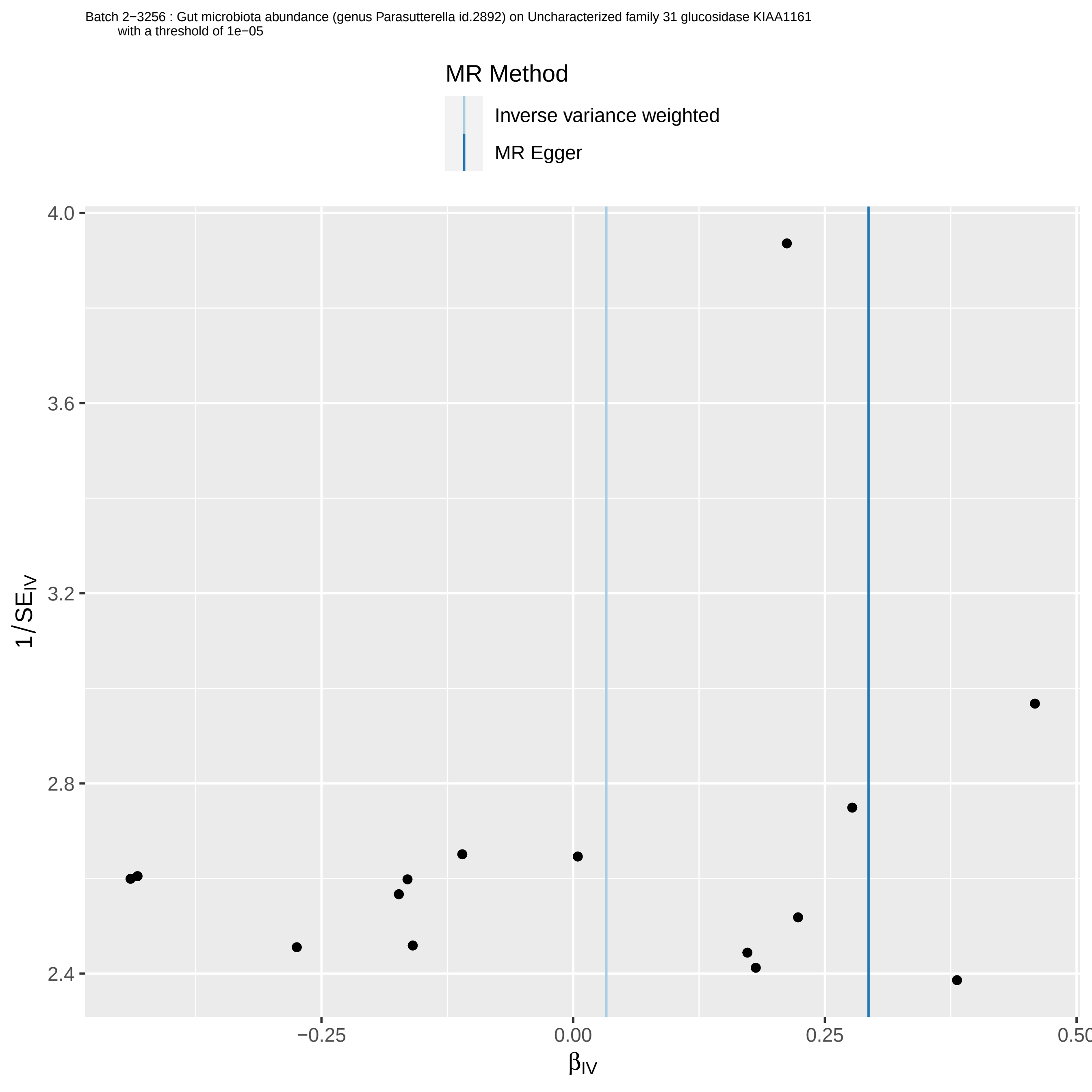

Batch 2−3256 : Gut microbiota abundance (genus Parasutterella id.2892) on Uncharacterized family 31 glucosidase KIAA1161
with a threshold of 1e−05
MR Method
Inverse variance weighted
MR Egger
4.0
3.6
3.2
2.8
2.4
1/SE_IV
−0.25
0.00
0.25
0.50
β_IV

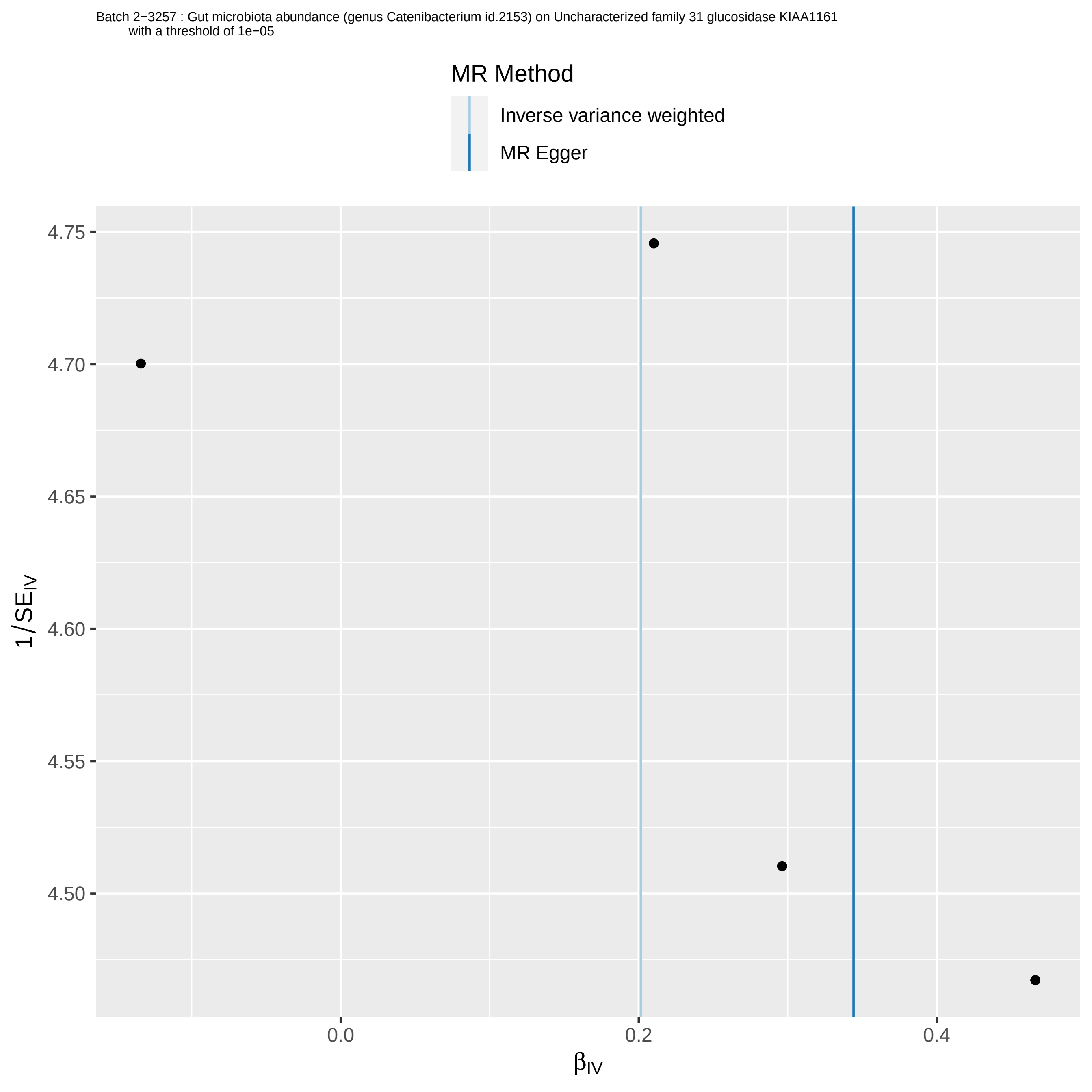

Batch 2−3257 : Gut microbiota abundance (genus Catenibacterium id.2153) on Uncharacterized family 31 glucosidase KIAA1161
with a threshold of 1e−05
MR Method
Inverse variance weighted
MR Egger
4.75
4.70
4.65
4.60
4.55
4.50
1/SE_IV
0.0
0.2
0.4
β_IV

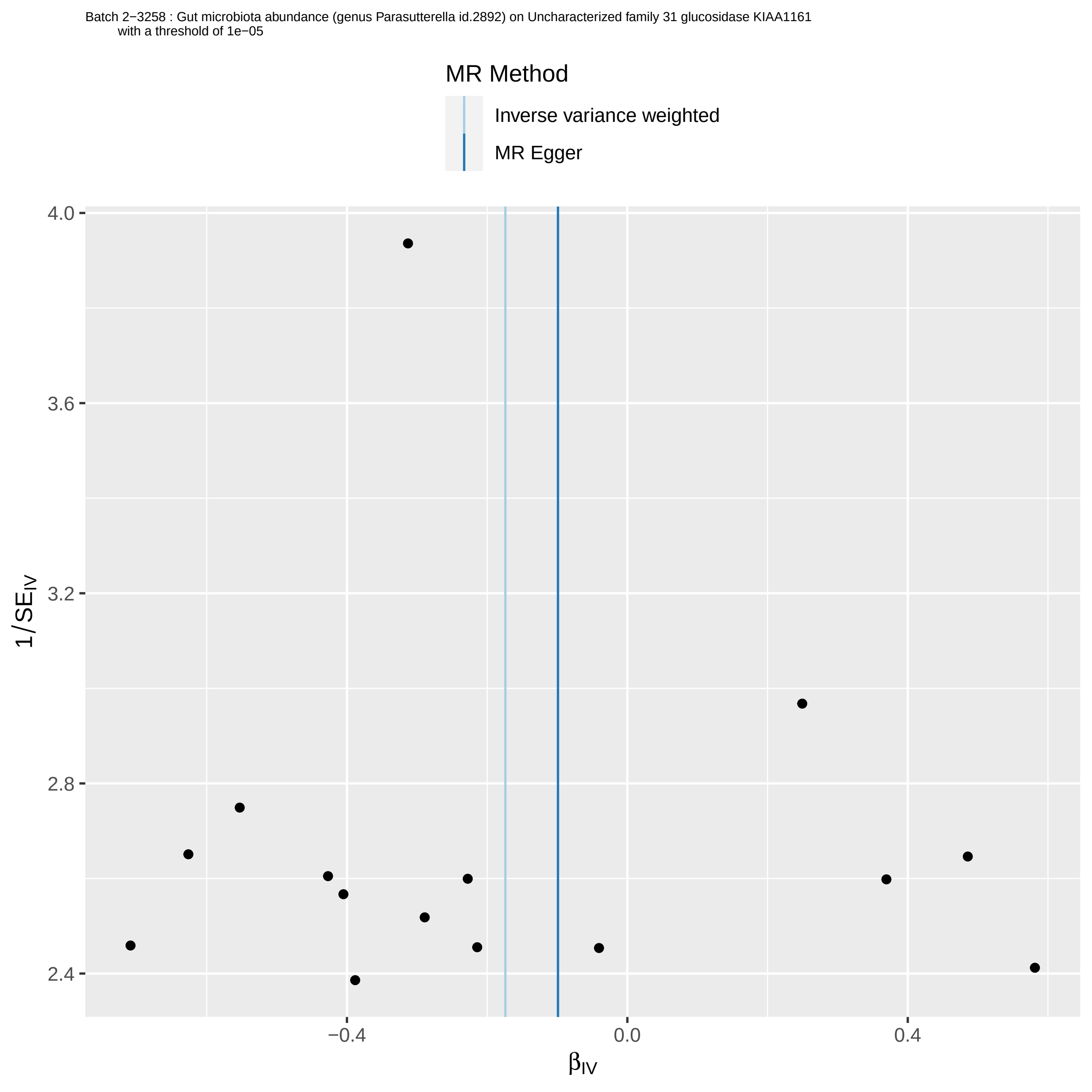
Batch 2−3258 : Gut microbiota abundance (genus Parasutterella id.2892) on Uncharacterized family 31 glucosidase KIAA1161
with a threshold of 1e−05
MR Method
Inverse variance weighted
MR Egger
4.0
3.6
3.2
2.8
2.4
1/SE_IV
−0.4
0.0
0.4
β_IV

Batch 2−3259 : Gut microbiota abundance (genus Catenibacterium id.2153) on UPF0577 protein KIAA1324
with a threshold of 1e−05

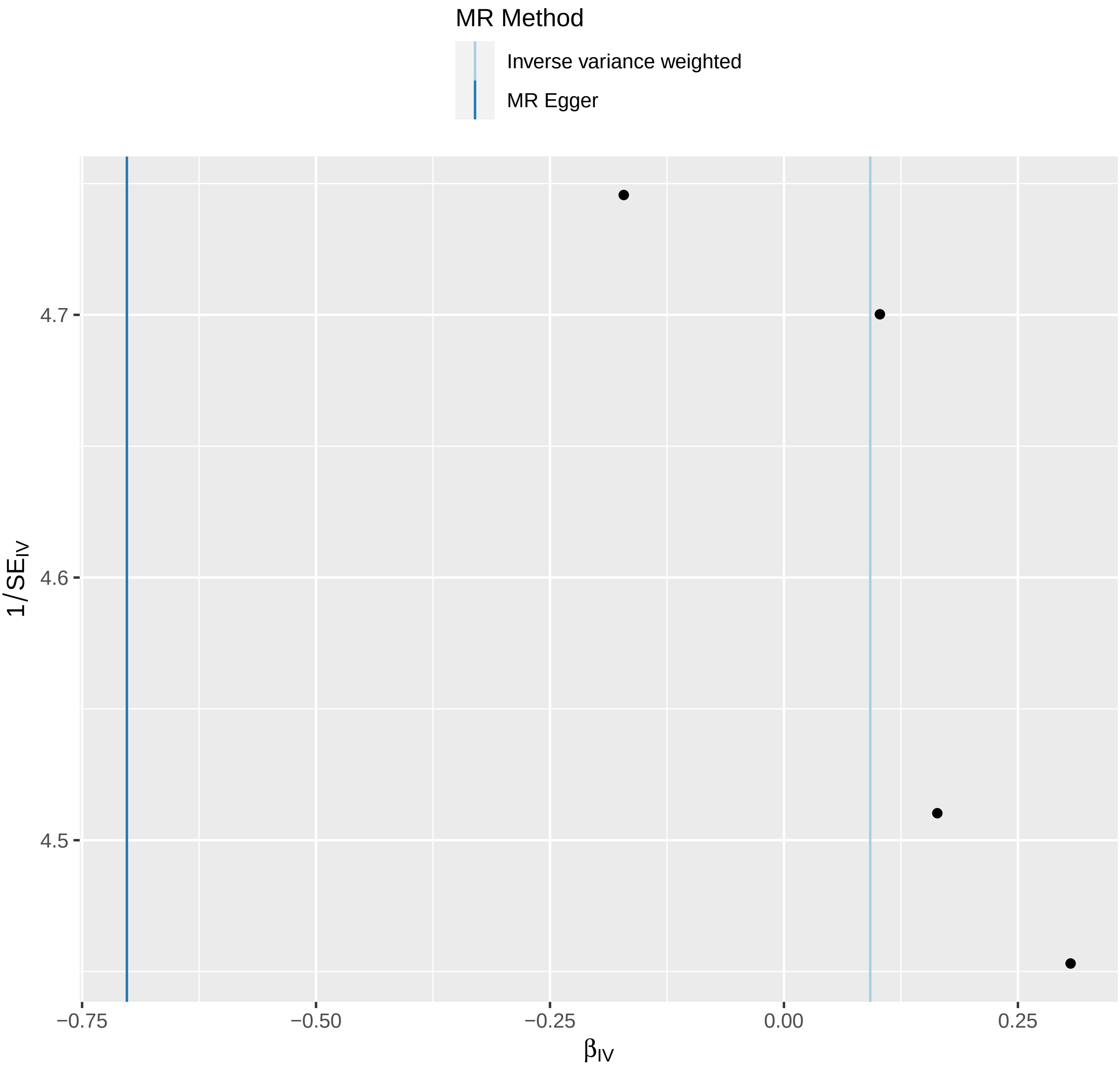

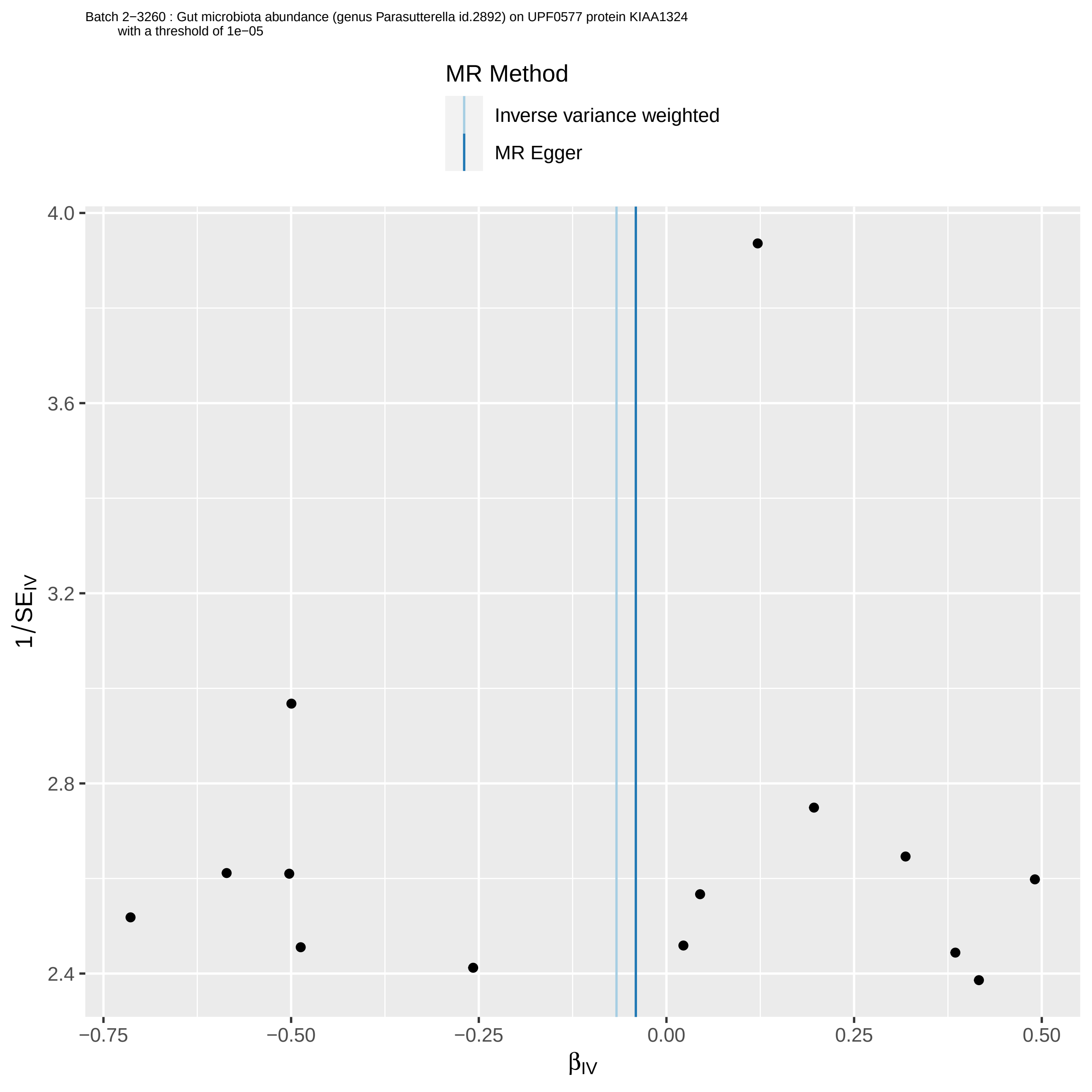

Batch 2−3260 : Gut microbiota abundance (genus Parasutterella id.2892) on UPF0577 protein KIAA1324
with a threshold of 1e−05
MR Method
Inverse variance weighted
MR Egger
4.0
3.6
3.2
2.8
2.4
1/SE$_{IV}$
−0.75
−0.50
−0.25
0.00
0.25
0.50
β$_{IV}$

Batch 2−3261 : Gut microbiota abundance (genus Catenibacterium id.2153) on UPF0577 protein KIAA1324
with a threshold of 1e−05

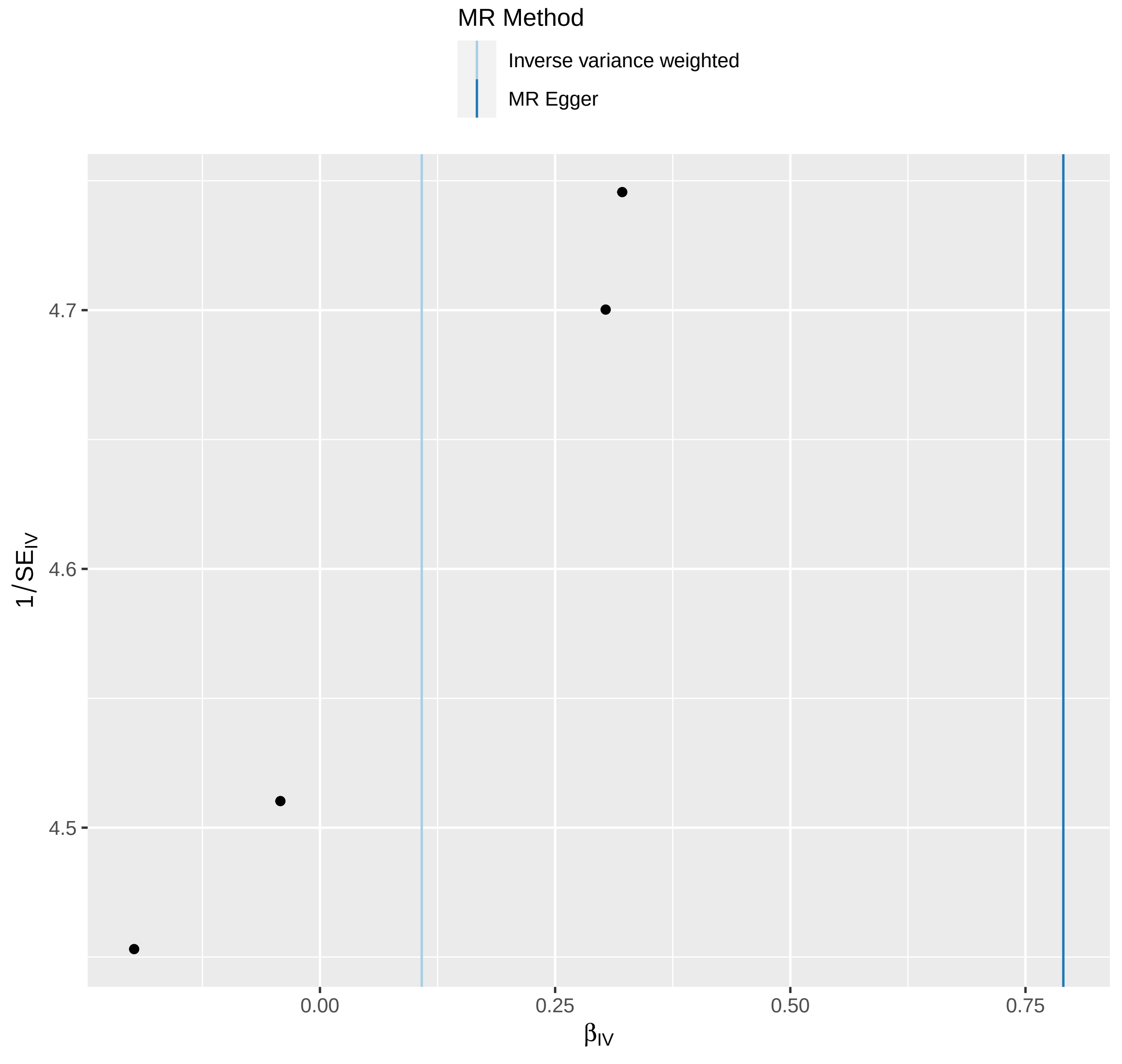

Batch 2−3262 : Gut microbiota abundance (genus Parasutterella id.2892) on UPF0577 protein KIAA1324
with a threshold of 1e−05

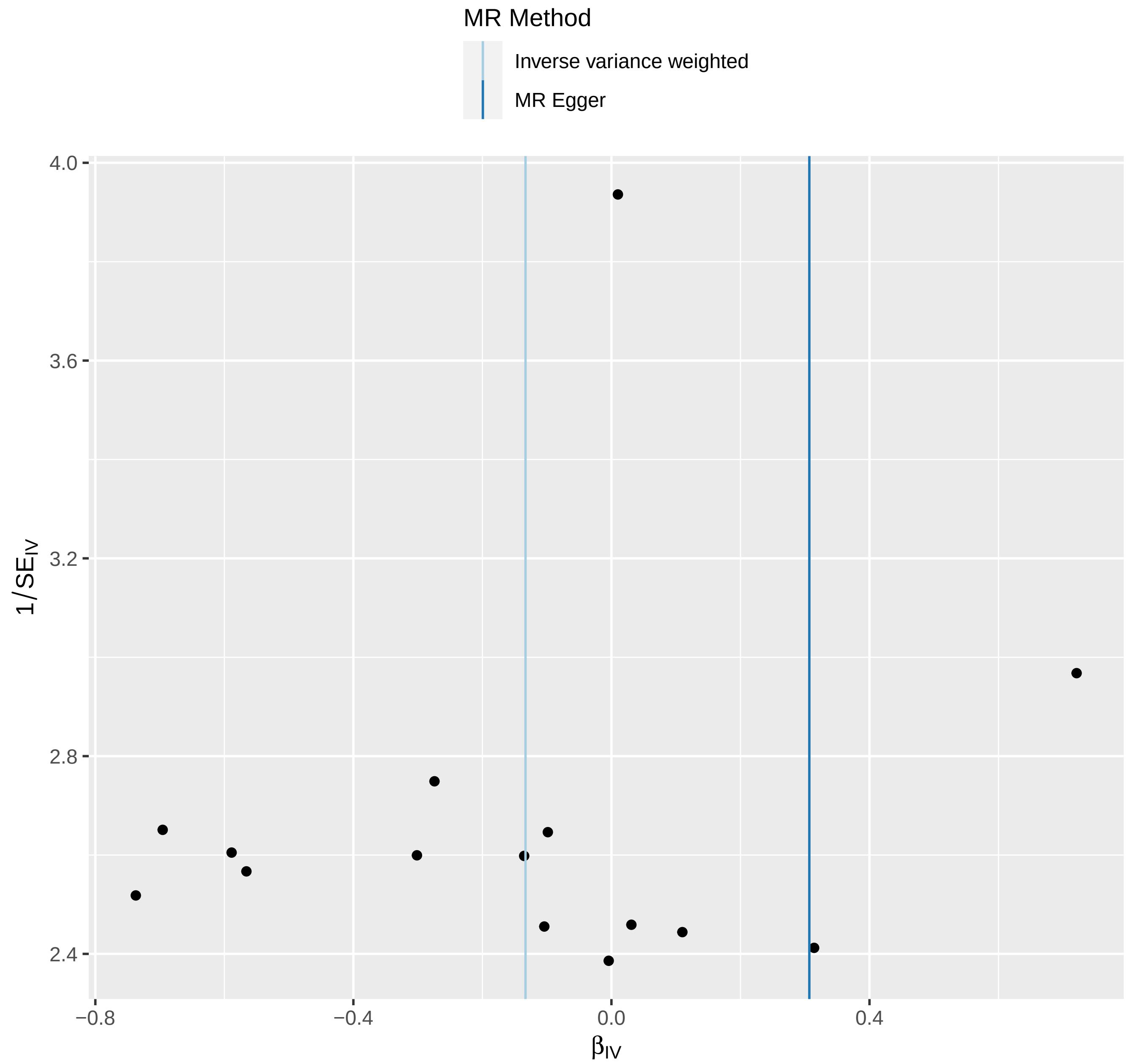

Batch 2−3263 : Gut microbiota abundance (genus Catenibacterium id.2153) on UPF0577 protein KIAA1324−like
with a threshold of 1e−05

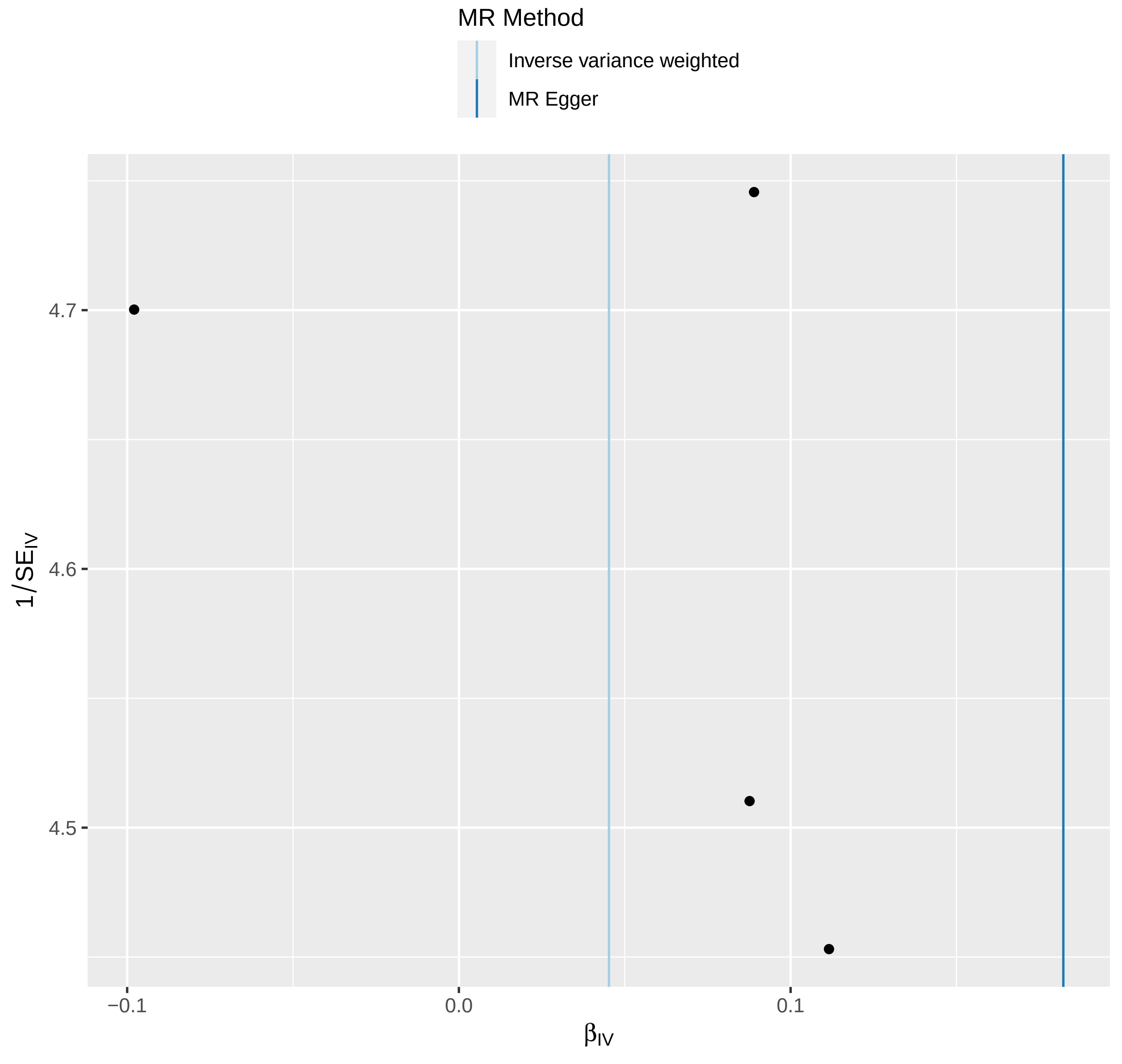

Batch 2−3264 : Gut microbiota abundance (genus Parasutterella id.2892) on UPF0577 protein KIAA1324−like
with a threshold of 1e−05

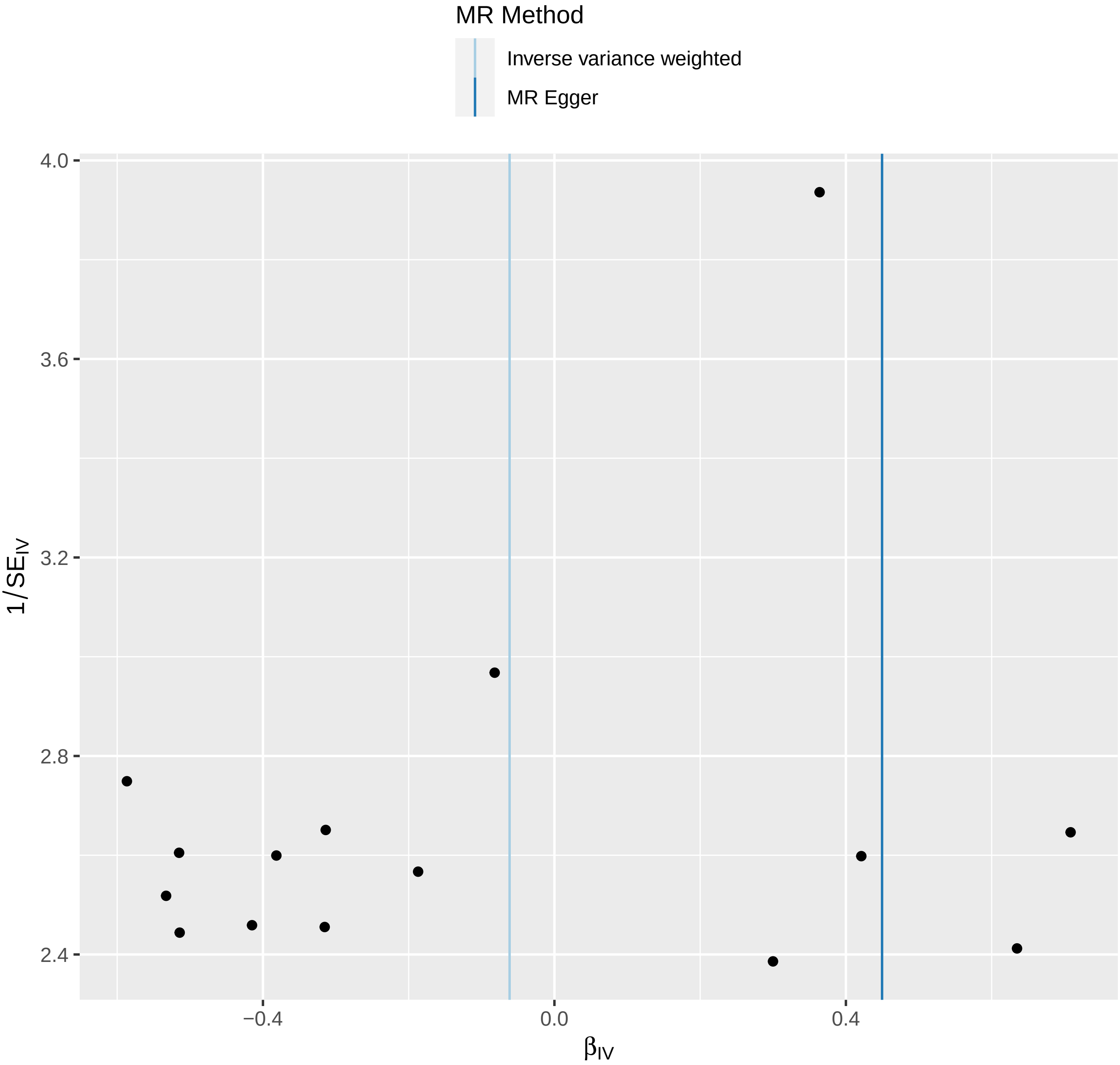

Batch 2−3265 : Gut microbiota abundance (genus Catenibacterium id.2153) on Uncharacterized protein KIAA1467
with a threshold of 1e−05

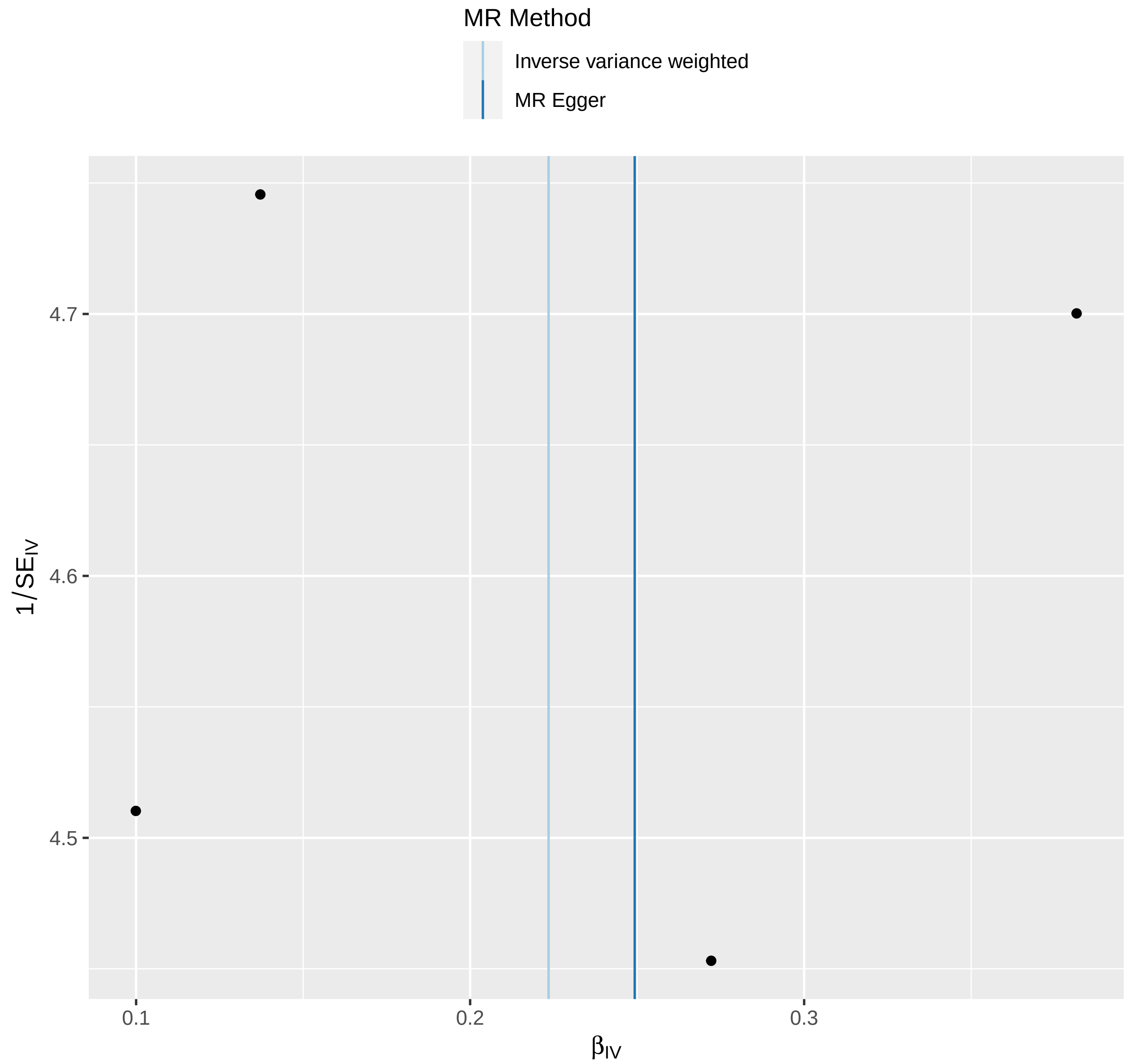

Batch 2−3266 : Gut microbiota abundance (genus Parasutterella id.2892) on Uncharacterized protein KIAA1467
with a threshold of 1e−05

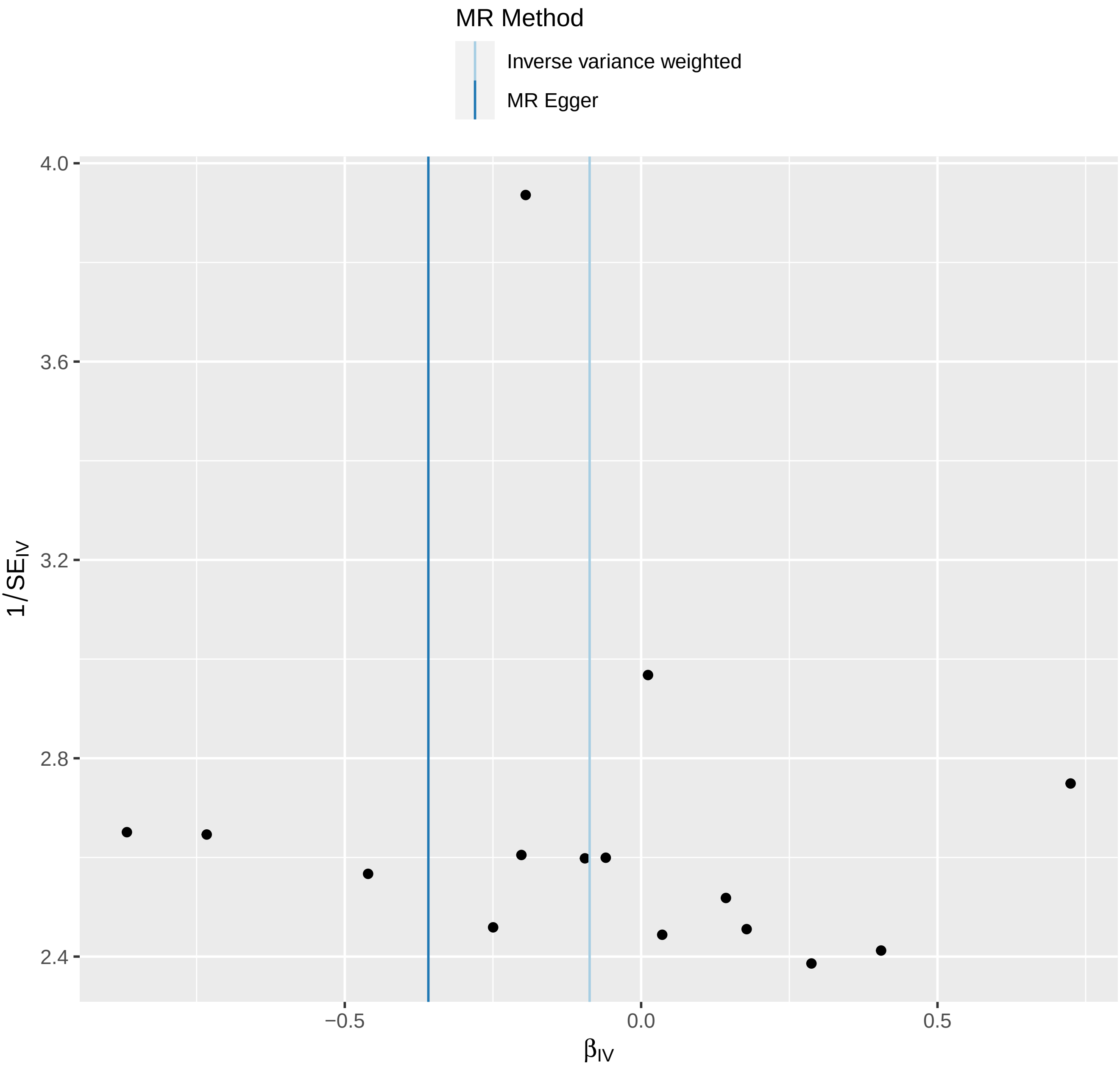

Batch 2−3267 : Gut microbiota abundance (genus Catenibacterium id.2153) on Uncharacterized protein KIAA2013
with a threshold of 1e−05

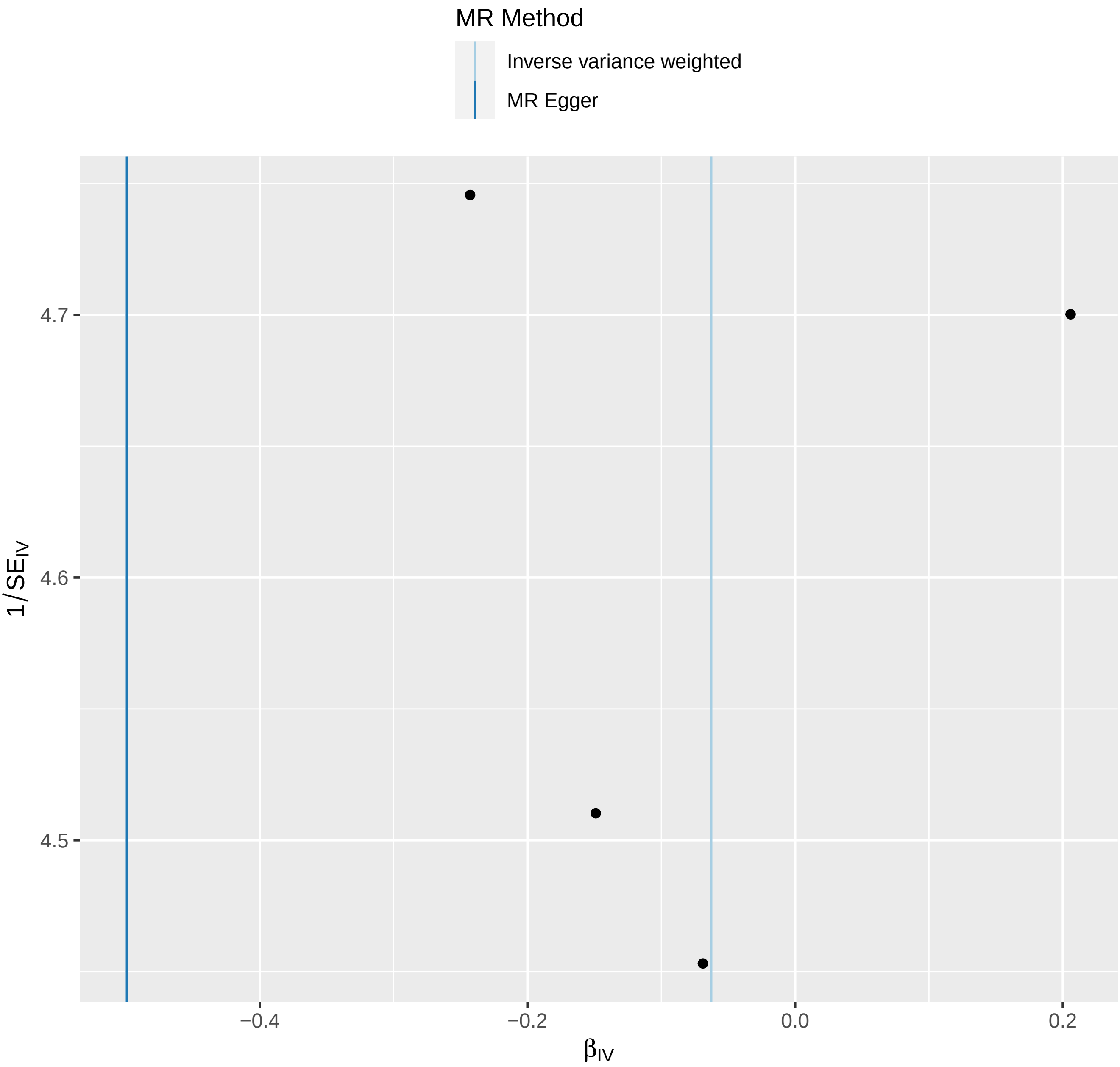

Batch 2−3268 : Gut microbiota abundance (genus Parasutterella id.2892) on Uncharacterized protein KIAA2013
with a threshold of 1e−05

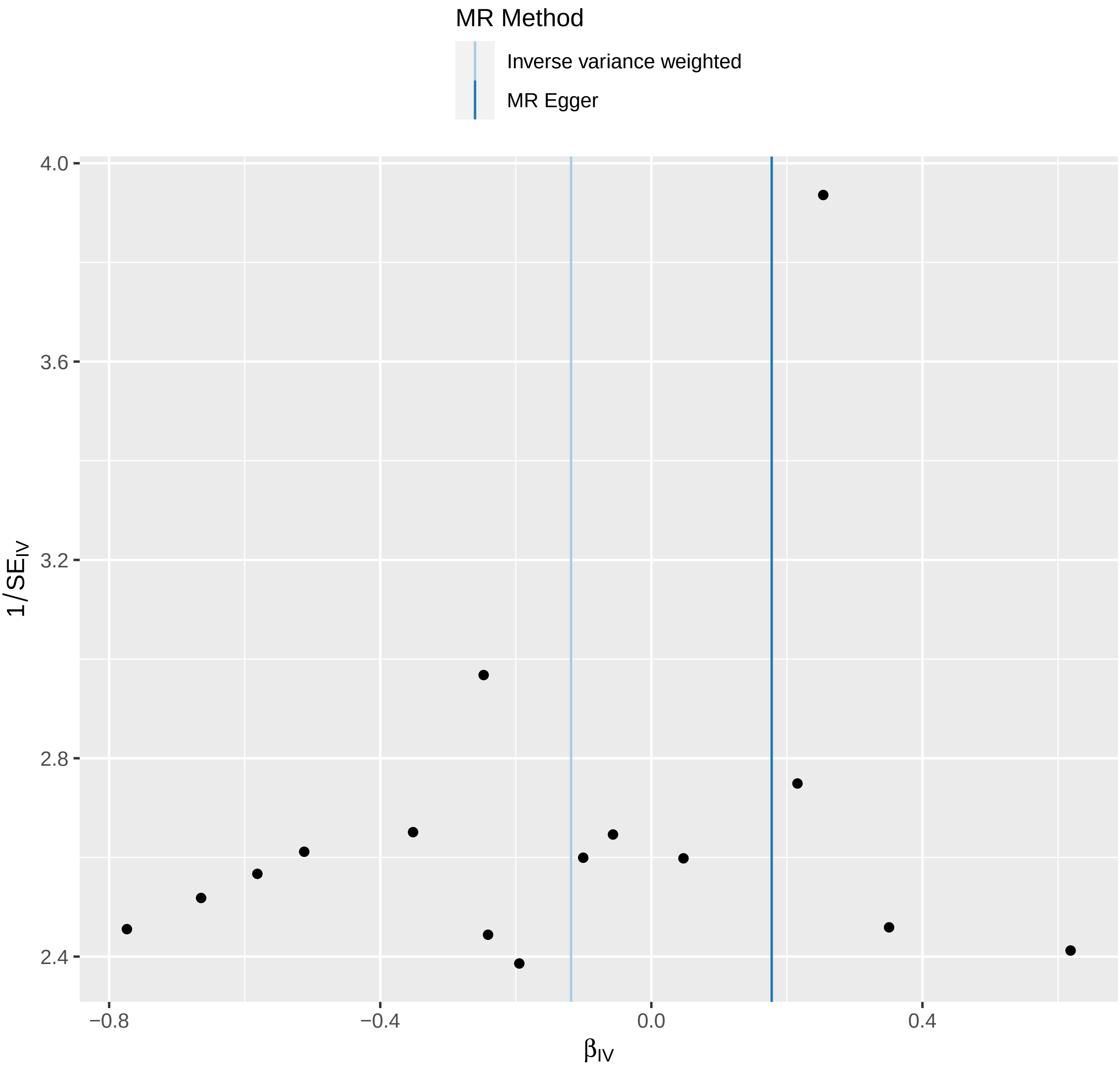

Batch 2−3269 : Gut microbiota abundance (genus Catenibacterium id.2153) on Kinesin−like protein KIF16B
with a threshold of 1e−05

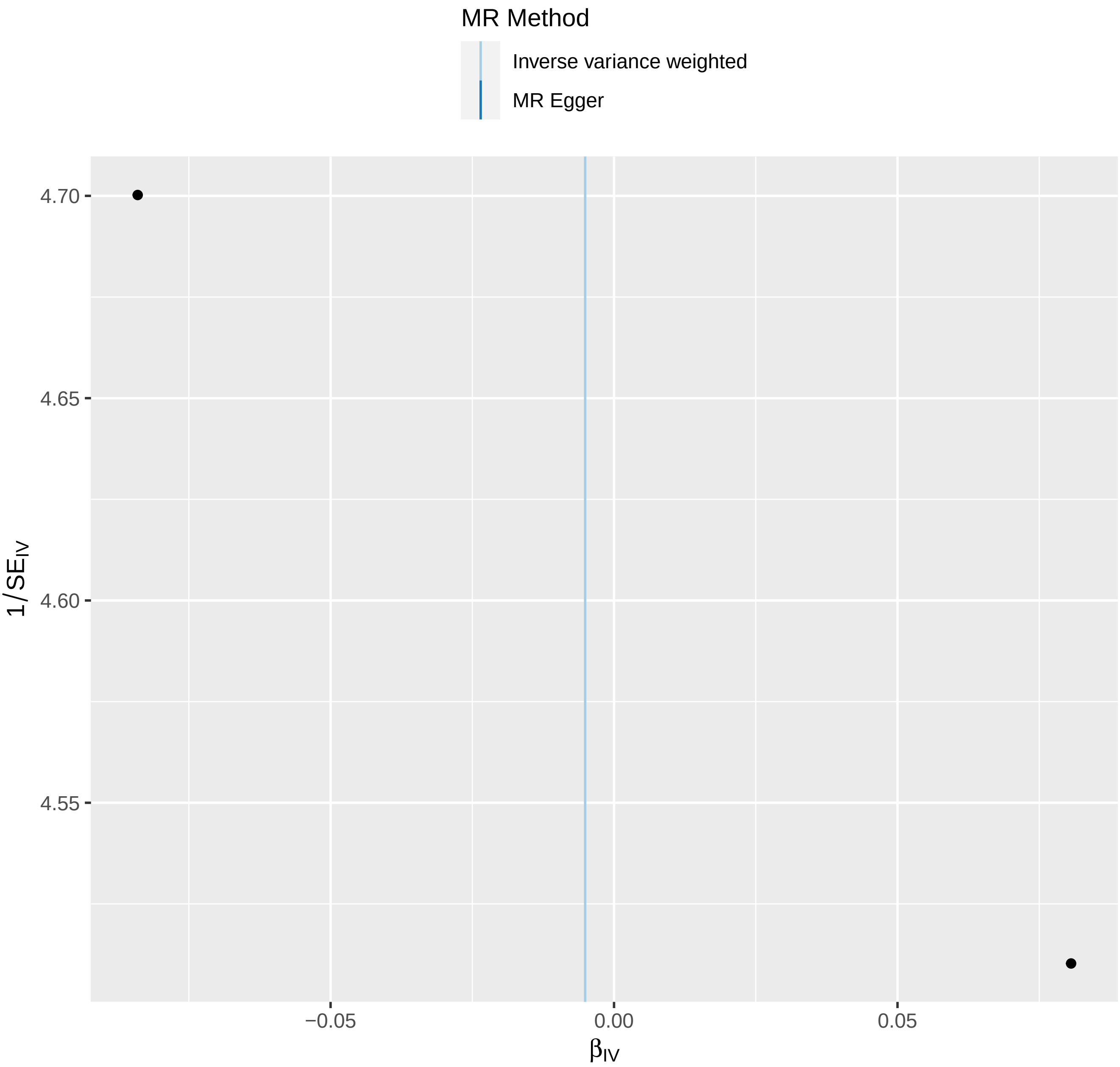

Batch 2−3270 : Gut microbiota abundance (genus Parasutterella id.2892) on Kinesin−like protein KIF16B
with a threshold of 1e−05

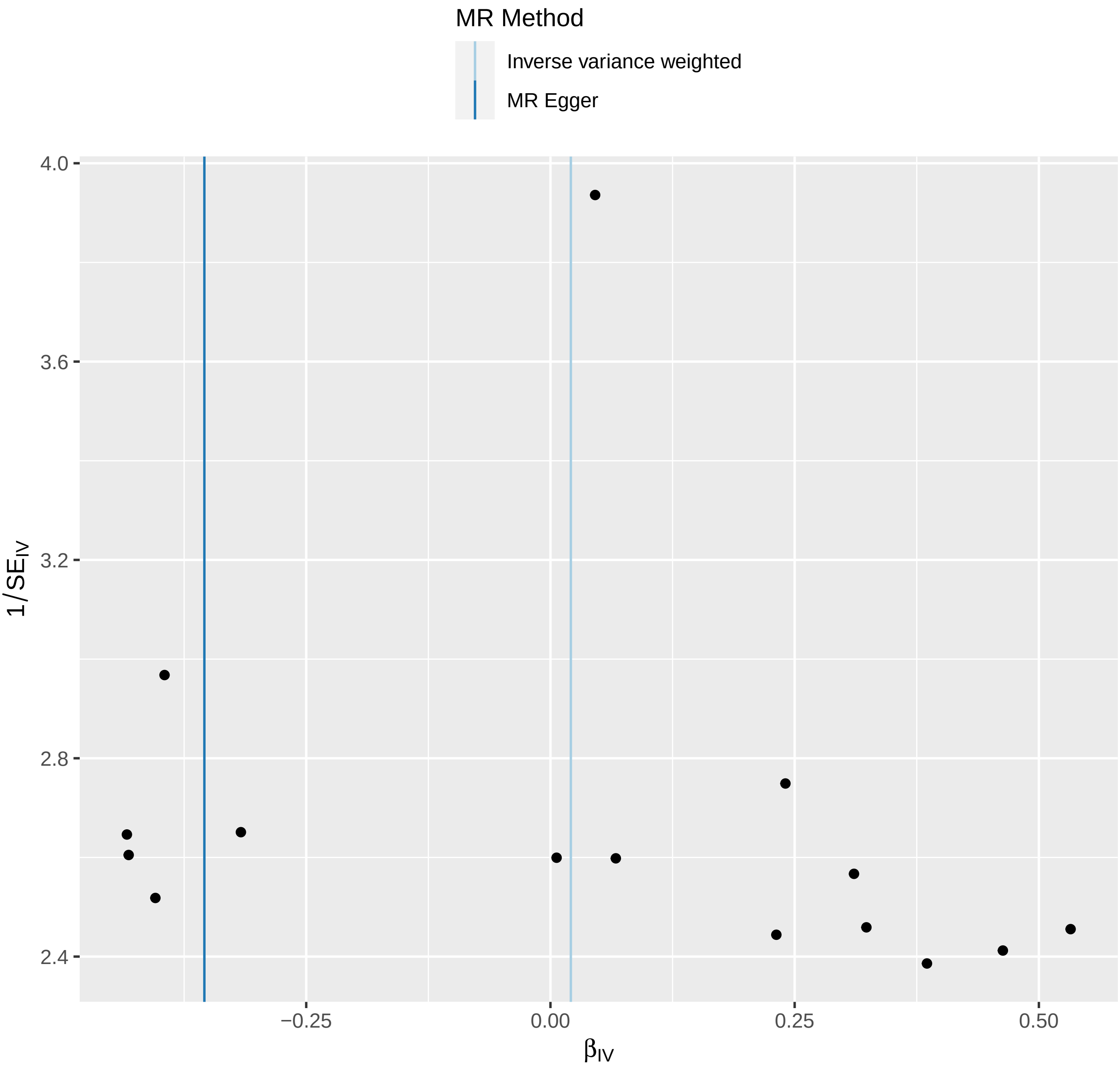

Batch 2−3271 : Gut microbiota abundance (genus Catenibacterium id.2153) on Kinesin−like protein KIF1C
with a threshold of 1e−05

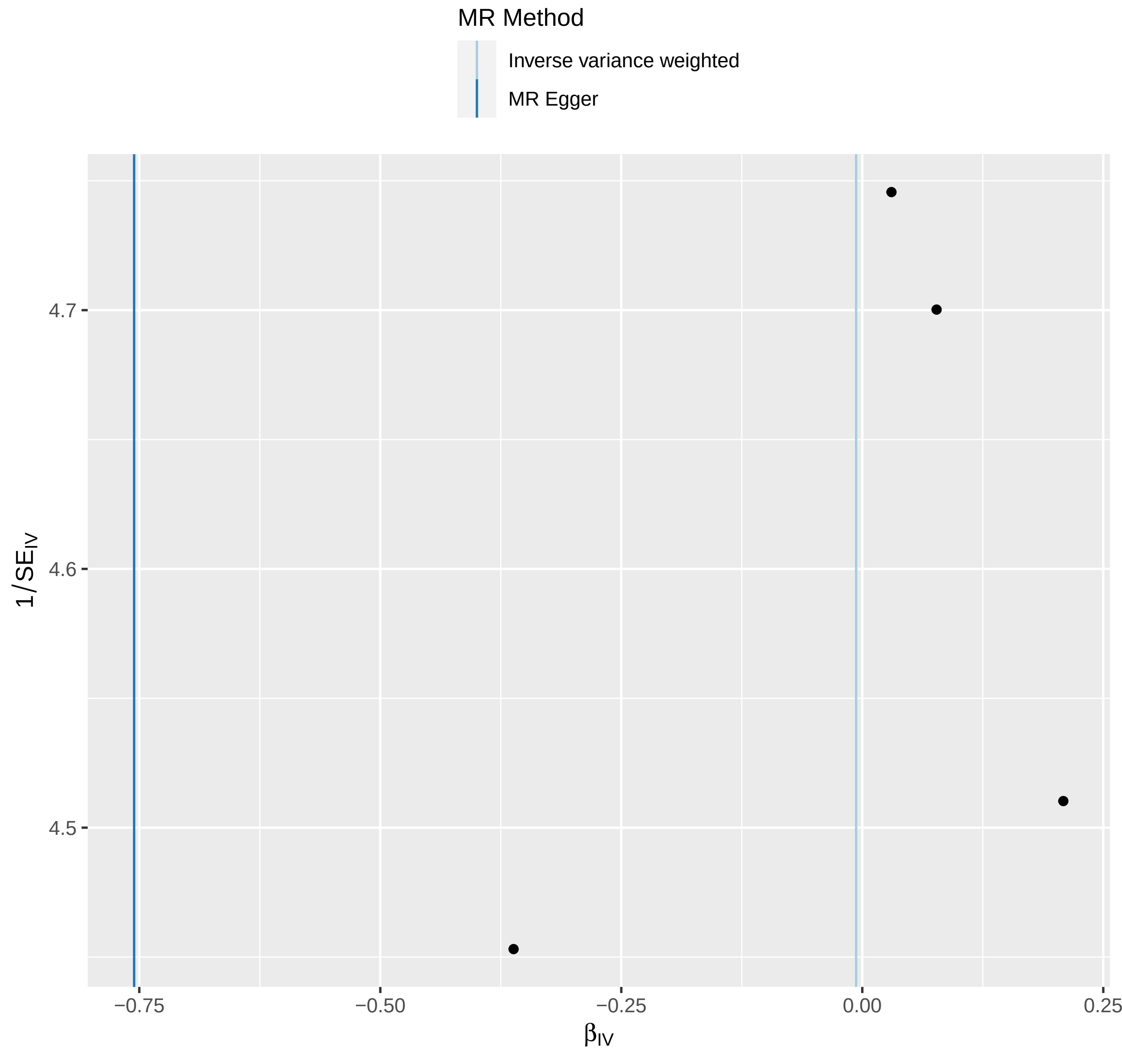

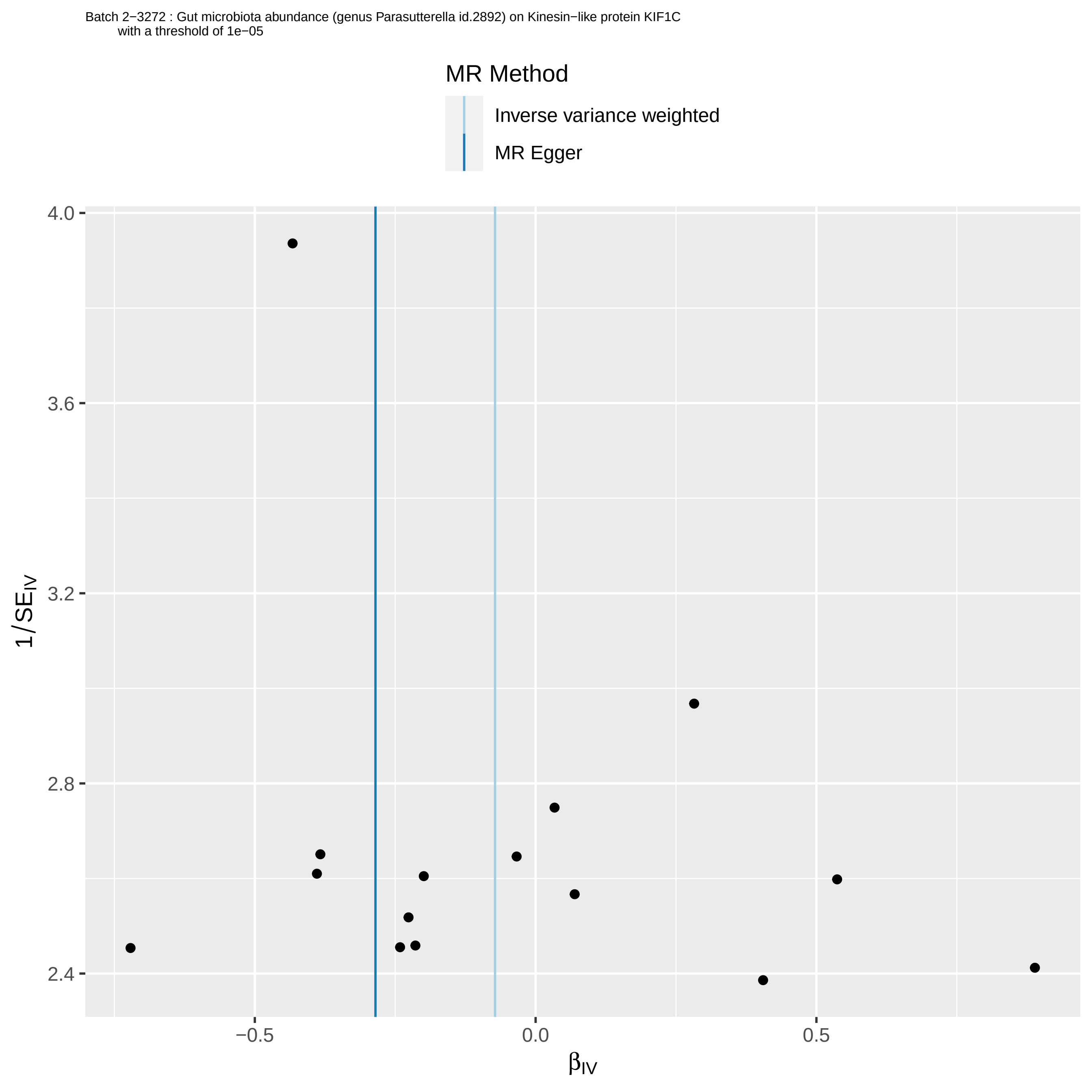
Batch 2−3272 : Gut microbiota abundance (genus Parasutterella id.2892) on Kinesin−like protein KIF1C
with a threshold of 1e−05
MR Method
Inverse variance weighted
MR Egger
4.0
3.6
3.2
2.8
2.4
1/SE_IV
−0.5
0.0
0.5
β_IV

Batch 2−3273 : Gut microbiota abundance (genus Catenibacterium id.2153) on Kinesin−like protein KIF23
with a threshold of 1e−05

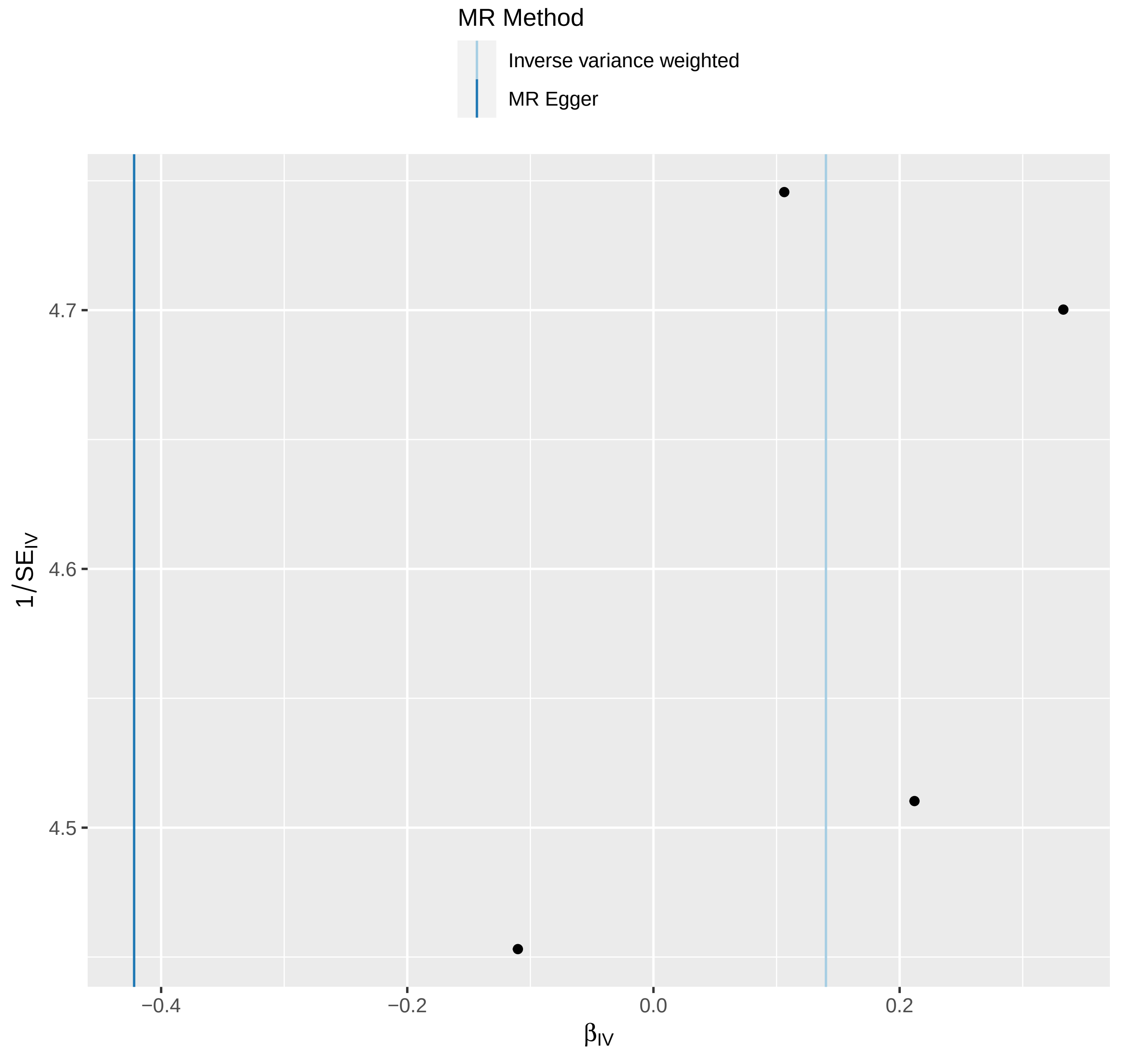

Batch 2−3274 : Gut microbiota abundance (genus Parasutterella id.2892) on Kinesin−like protein KIF23
with a threshold of 1e−05

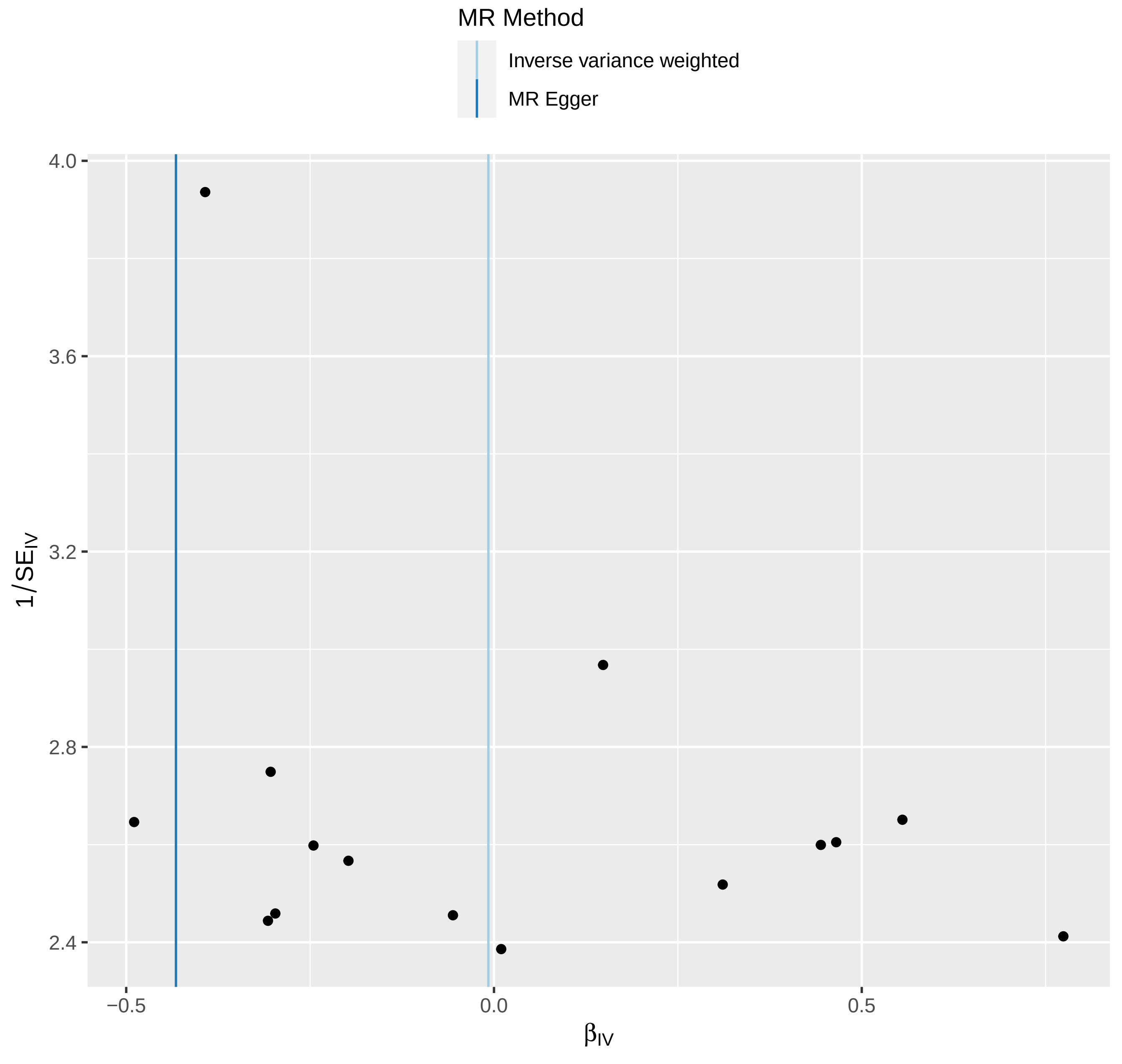

Batch 2−3275 : Gut microbiota abundance (genus Catenibacterium id.2153) on DNA/RNA−binding protein KIN17 with a threshold of 1e−05

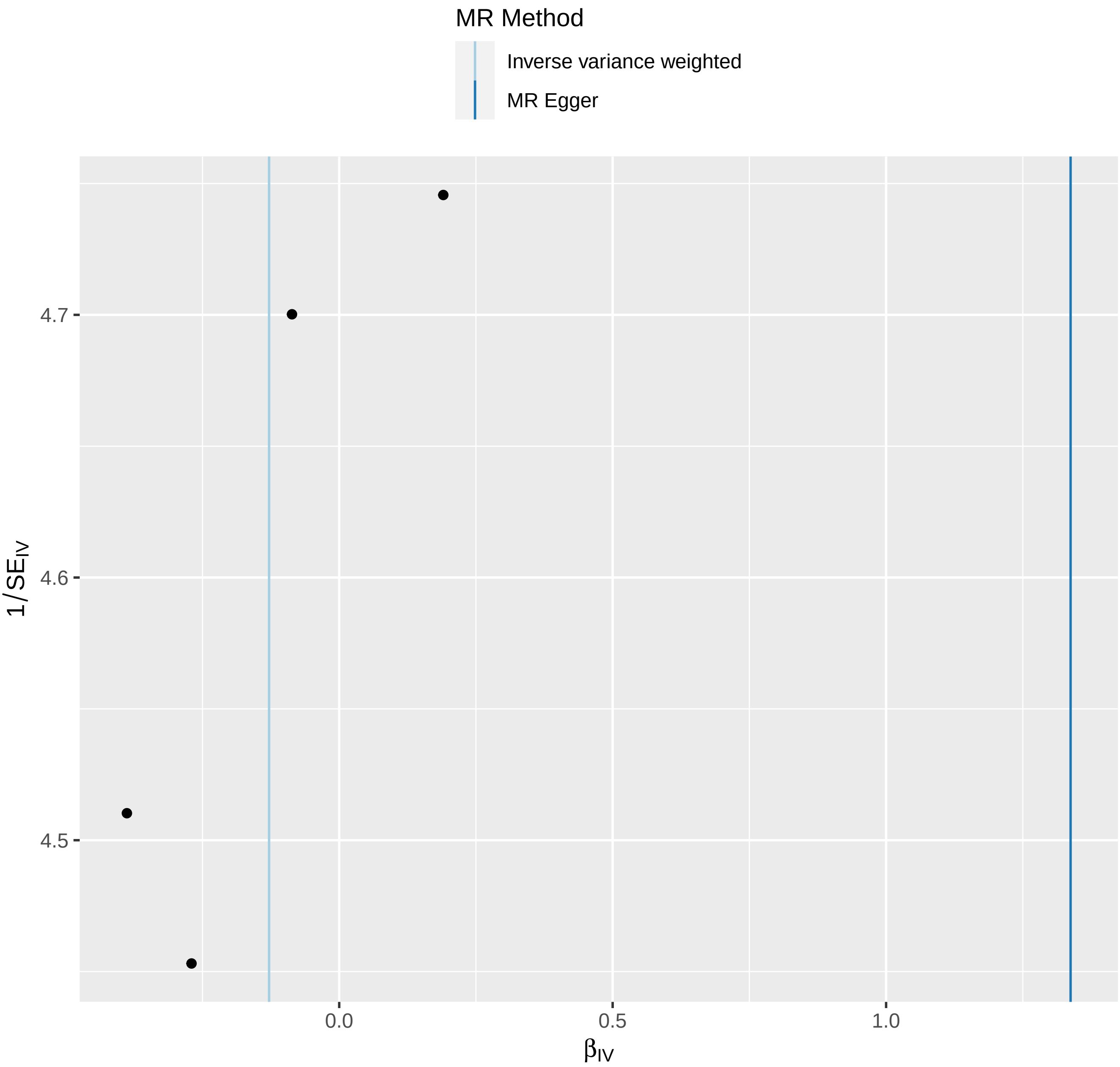

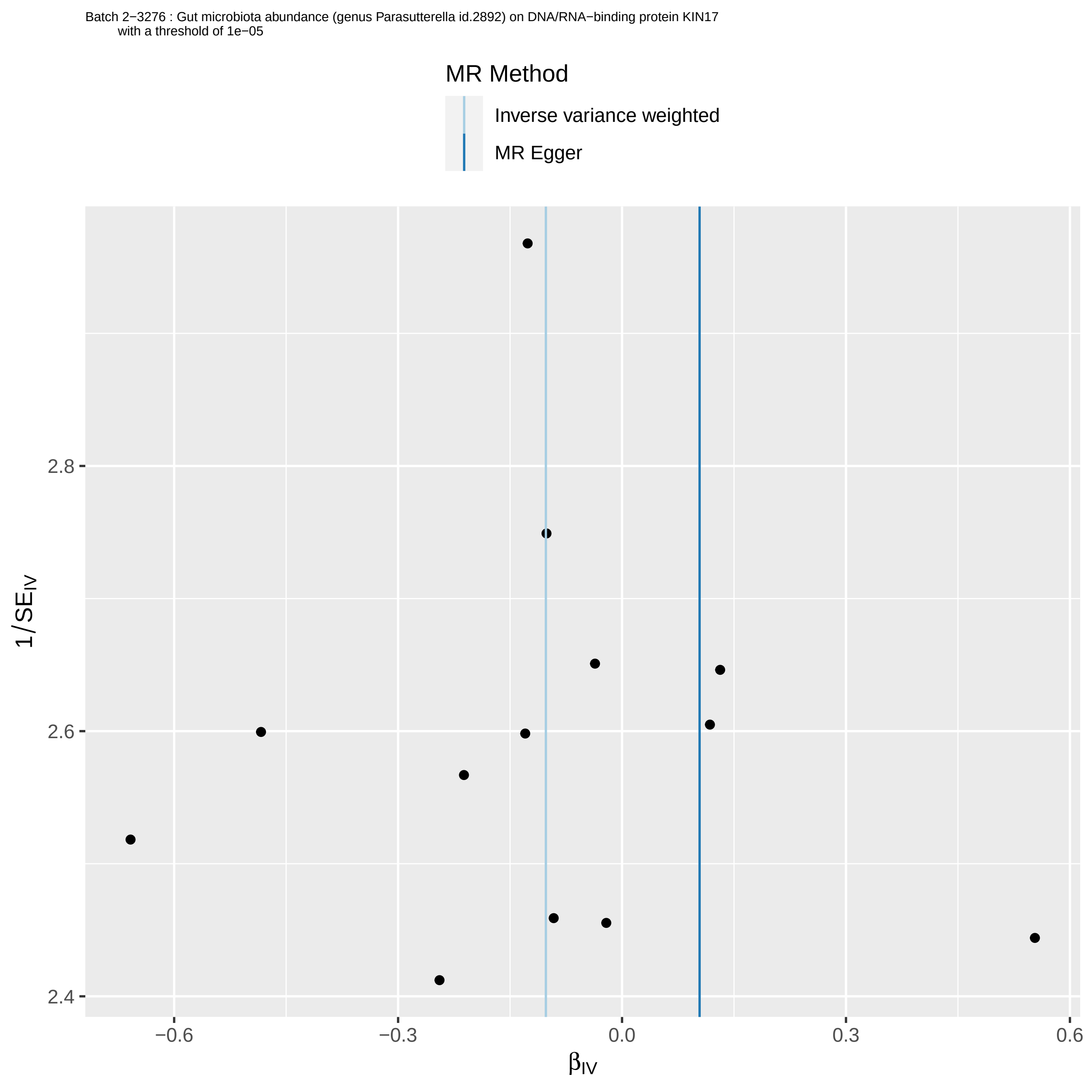

Batch 2−3276 : Gut microbiota abundance (genus Parasutterella id.2892) on DNA/RNA−binding protein KIN17
with a threshold of 1e−05
MR Method
Inverse variance weighted
MR Egger
2.8
2.6
2.4
1/SE_IV
−0.6
−0.3
0.0
0.3
0.6
β_IV

Batch 2−3277 : Gut microbiota abundance (genus Catenibacterium id.2153) on DNA/RNA−binding protein KIN17
with a threshold of 1e−05

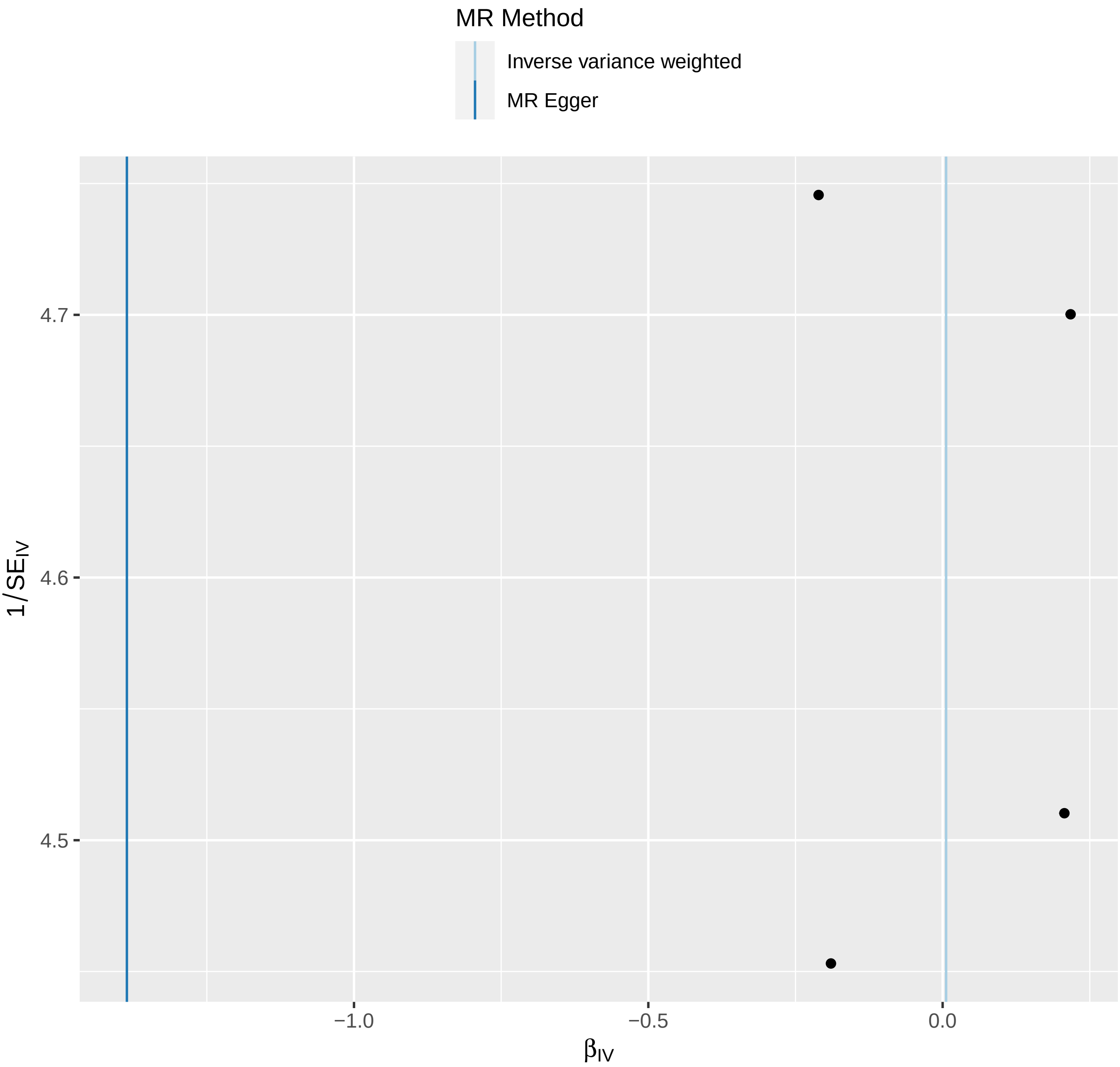

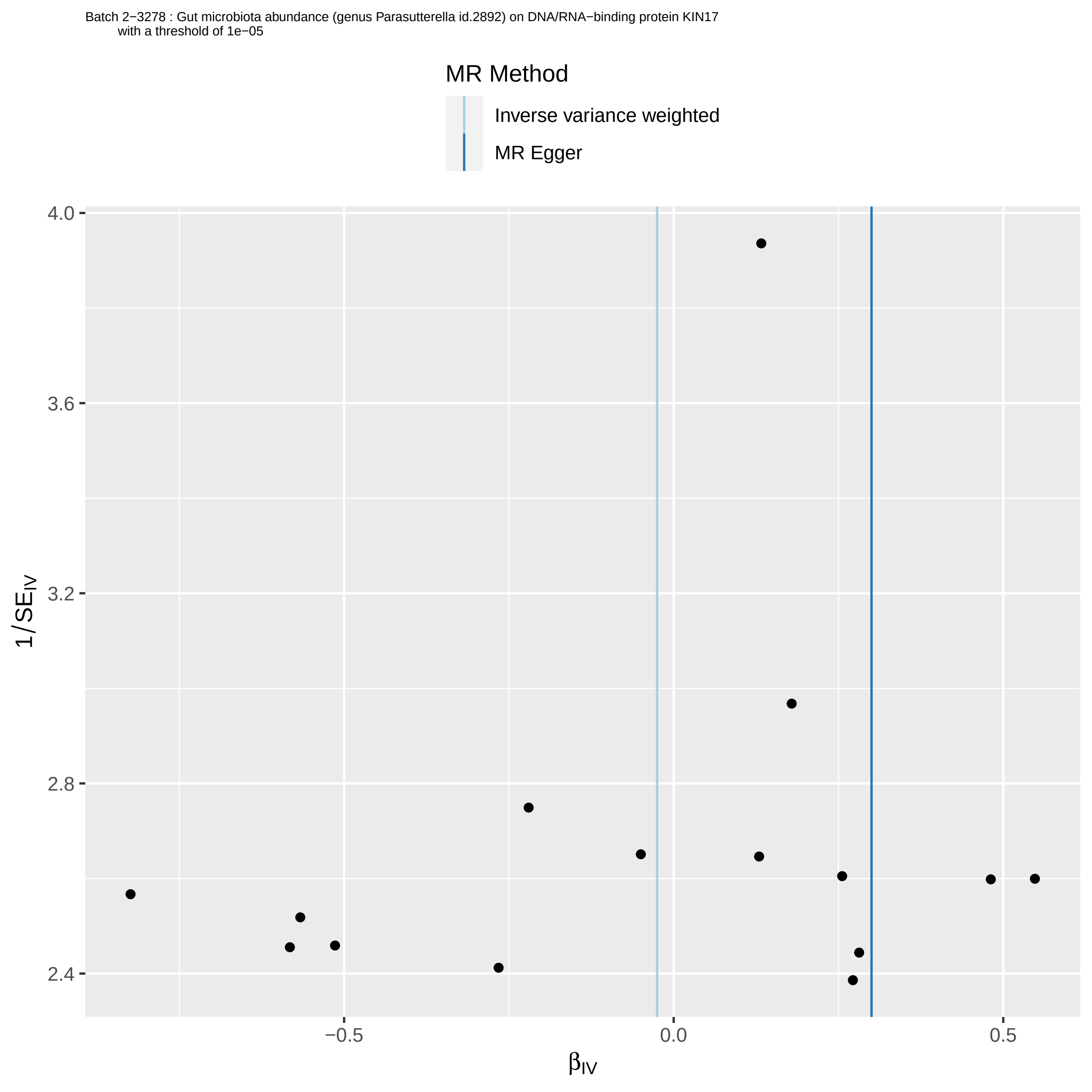

Batch 2−3278 : Gut microbiota abundance (genus Parasutterella id.2892) on DNA/RNA−binding protein KIN17
with a threshold of 1e−05
MR Method
Inverse variance weighted
MR Egger
4.0
3.6
3.2
2.8
2.4
1/SE_IV
−0.5
0.0
0.5
β_IV

Batch 2−3279 : Gut microbiota abundance (genus Catenibacterium id.2153) on Killer cell immunoglobulin−like receptor 2DL2 with a threshold of 1e−05

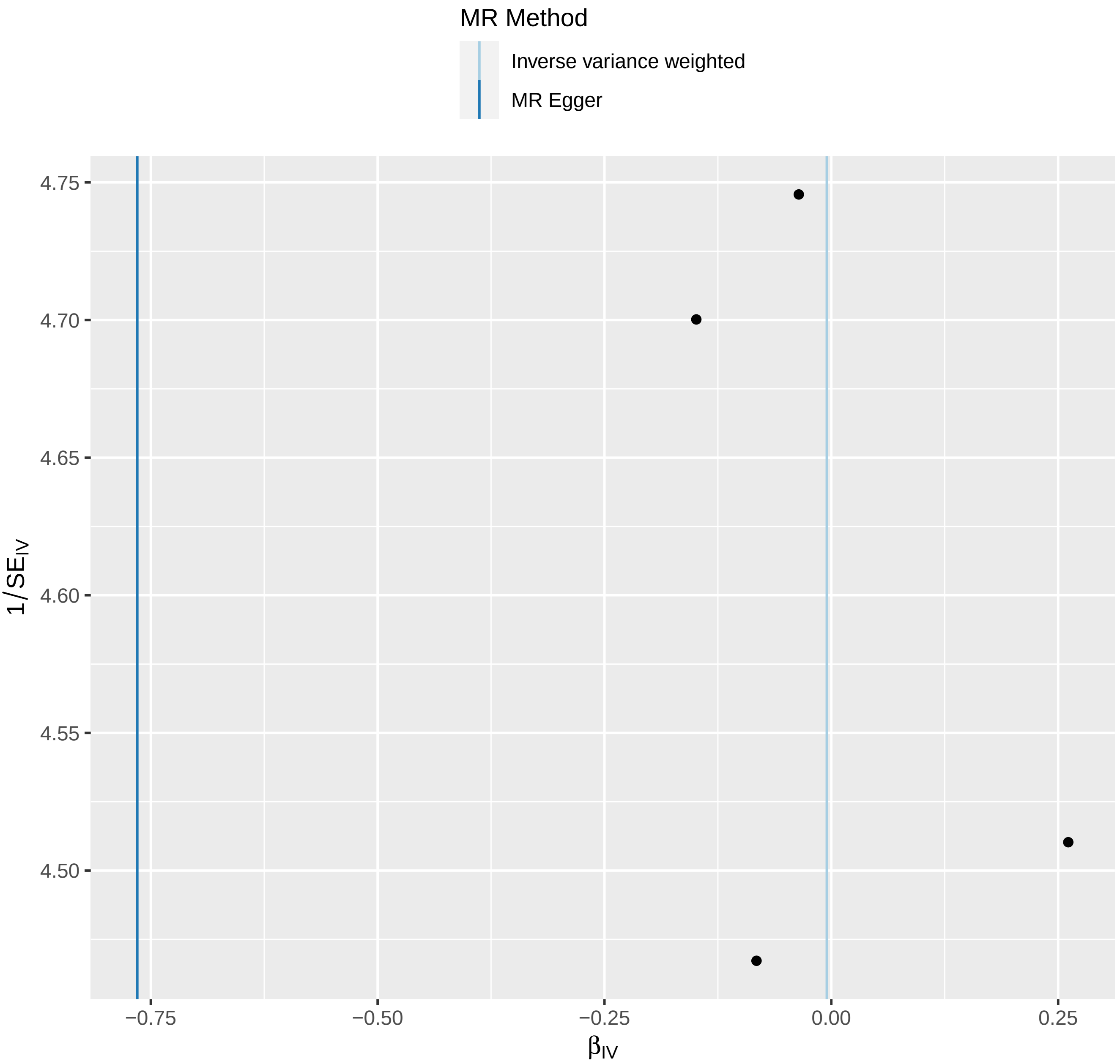

Batch 2−3280 : Gut microbiota abundance (genus Parasutterella id.2892) on Killer cell immunoglobulin−like receptor 2DL2
with a threshold of 1e−05

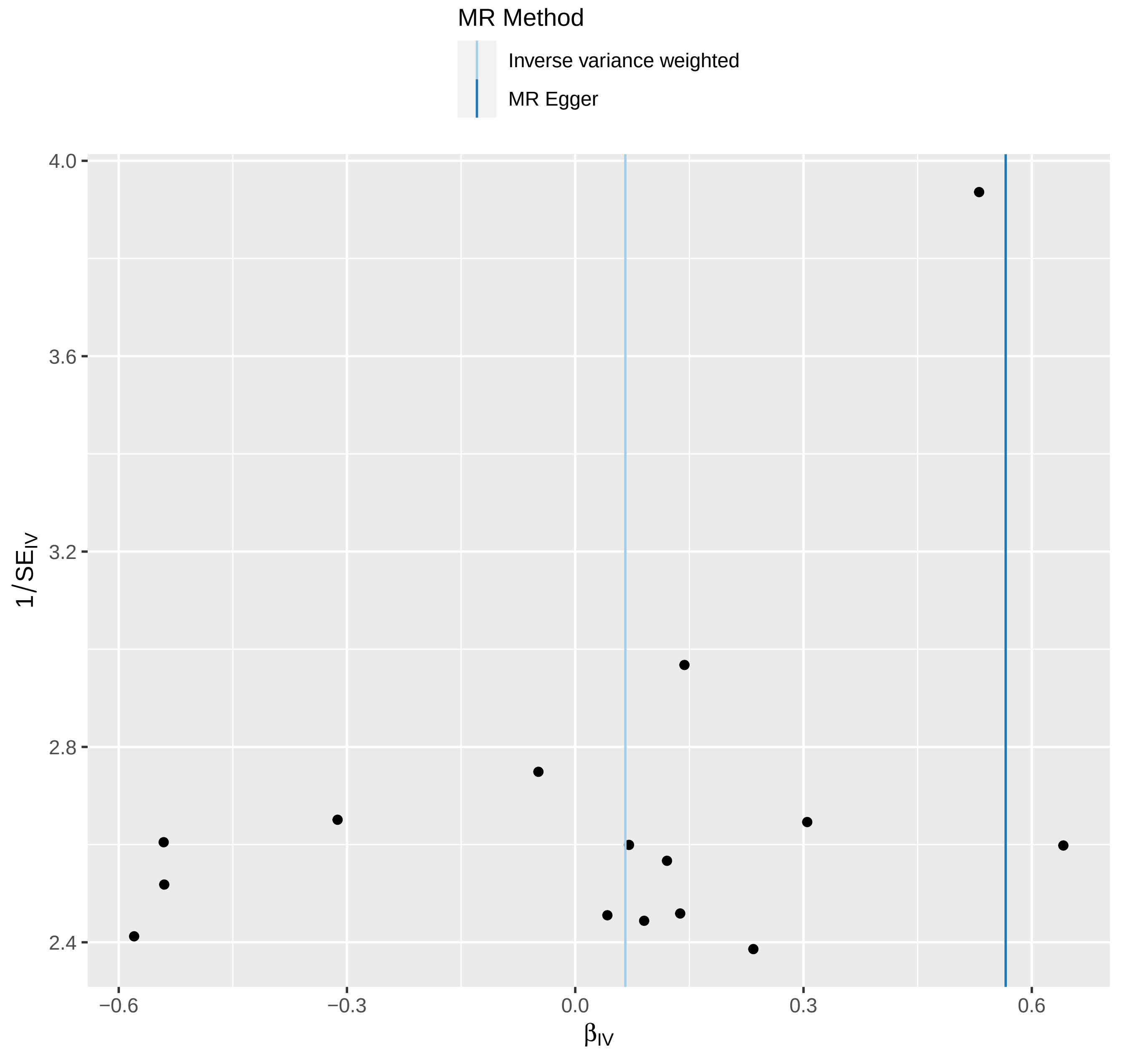

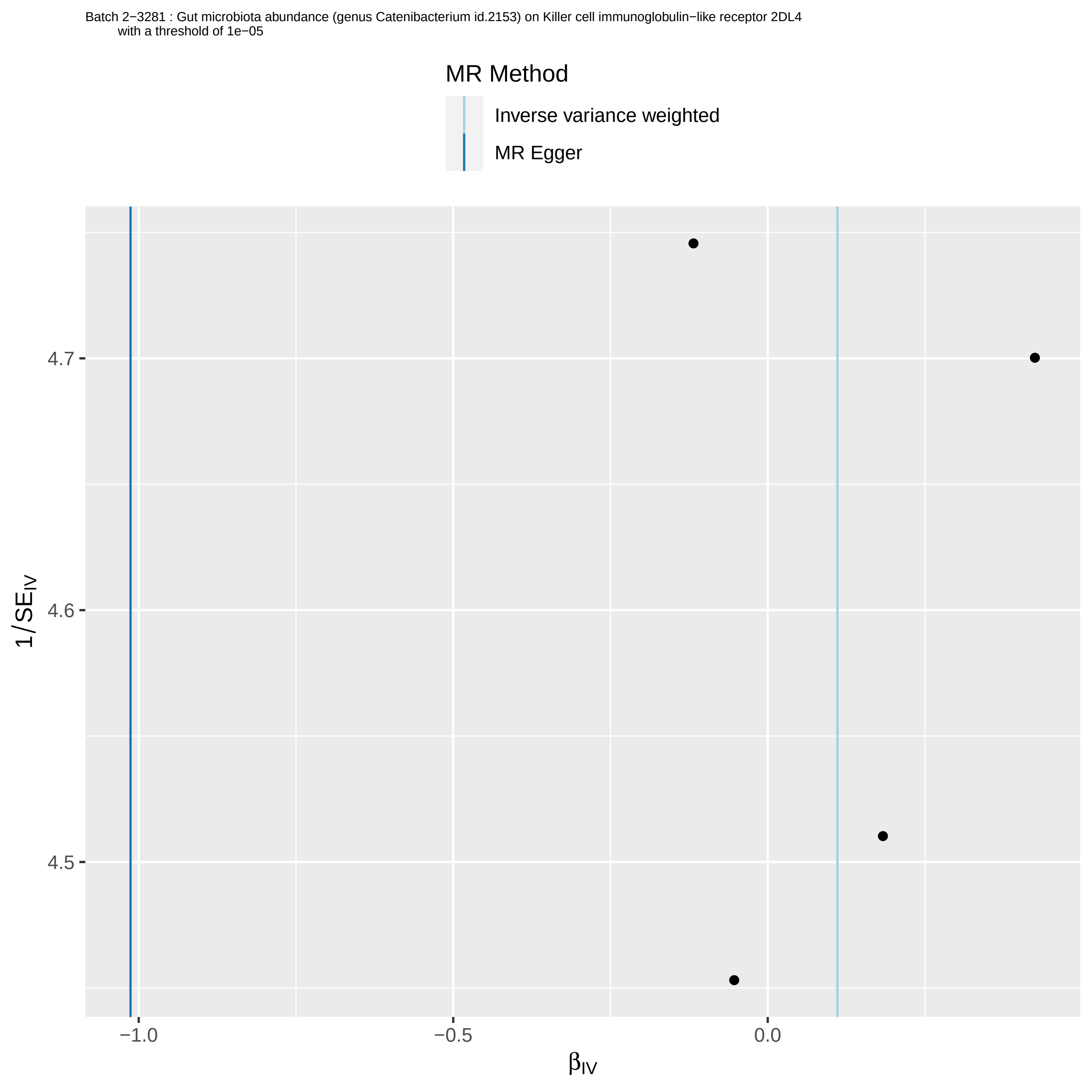

Batch 2−3281 : Gut microbiota abundance (genus Catenibacterium id.2153) on Killer cell immunoglobulin−like receptor 2DL4
with a threshold of 1e−05
MR Method
Inverse variance weighted
MR Egger
4.7
4.6
4.5
1/SE_IV
−1.0
−0.5
0.0
β_IV

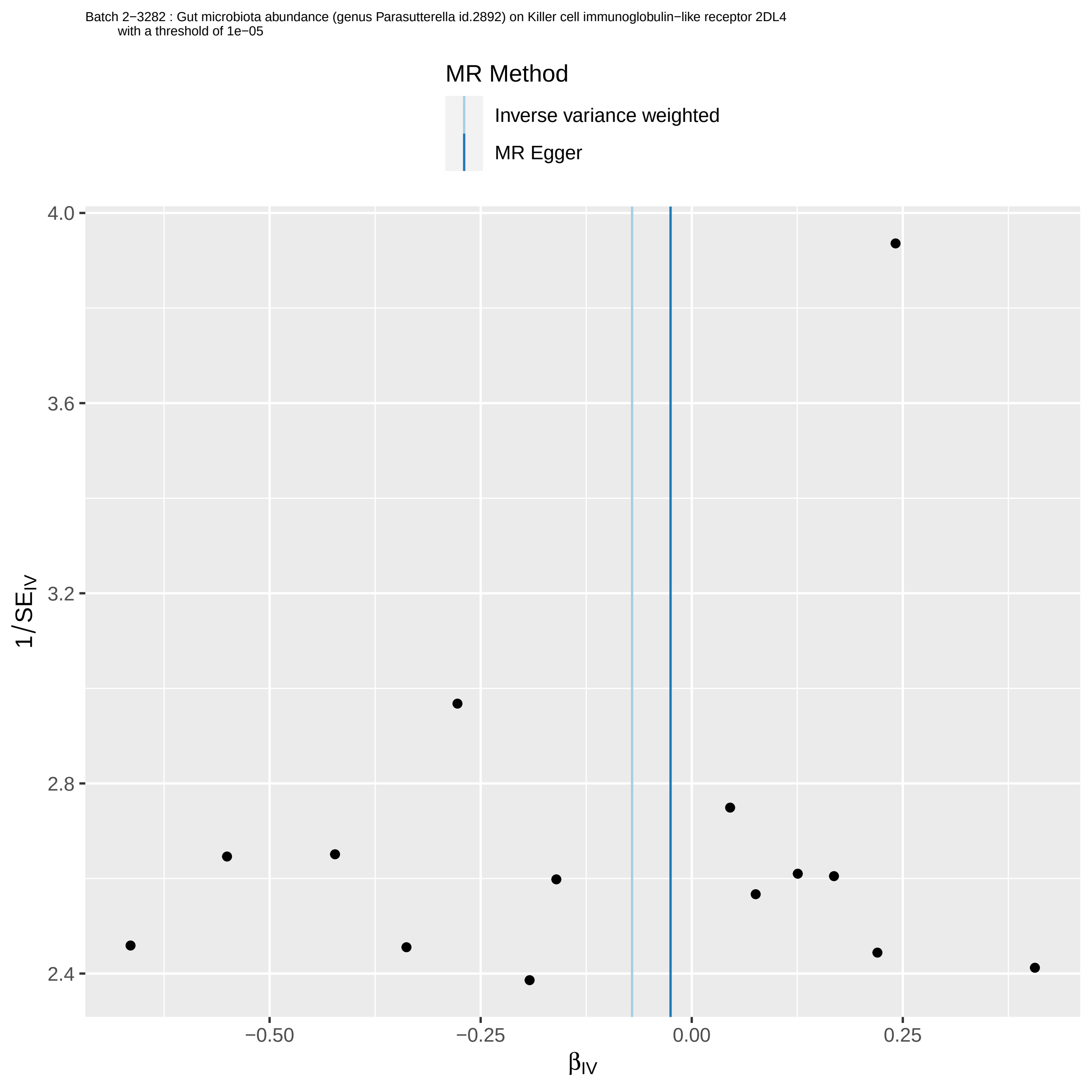

Batch 2−3282 : Gut microbiota abundance (genus Parasutterella id.2892) on Killer cell immunoglobulin−like receptor 2DL4
with a threshold of 1e−05
MR Method
Inverse variance weighted
MR Egger
4.0
3.6
3.2
2.8
2.4
1/SE_IV
−0.50
−0.25
0.00
0.25
β_IV

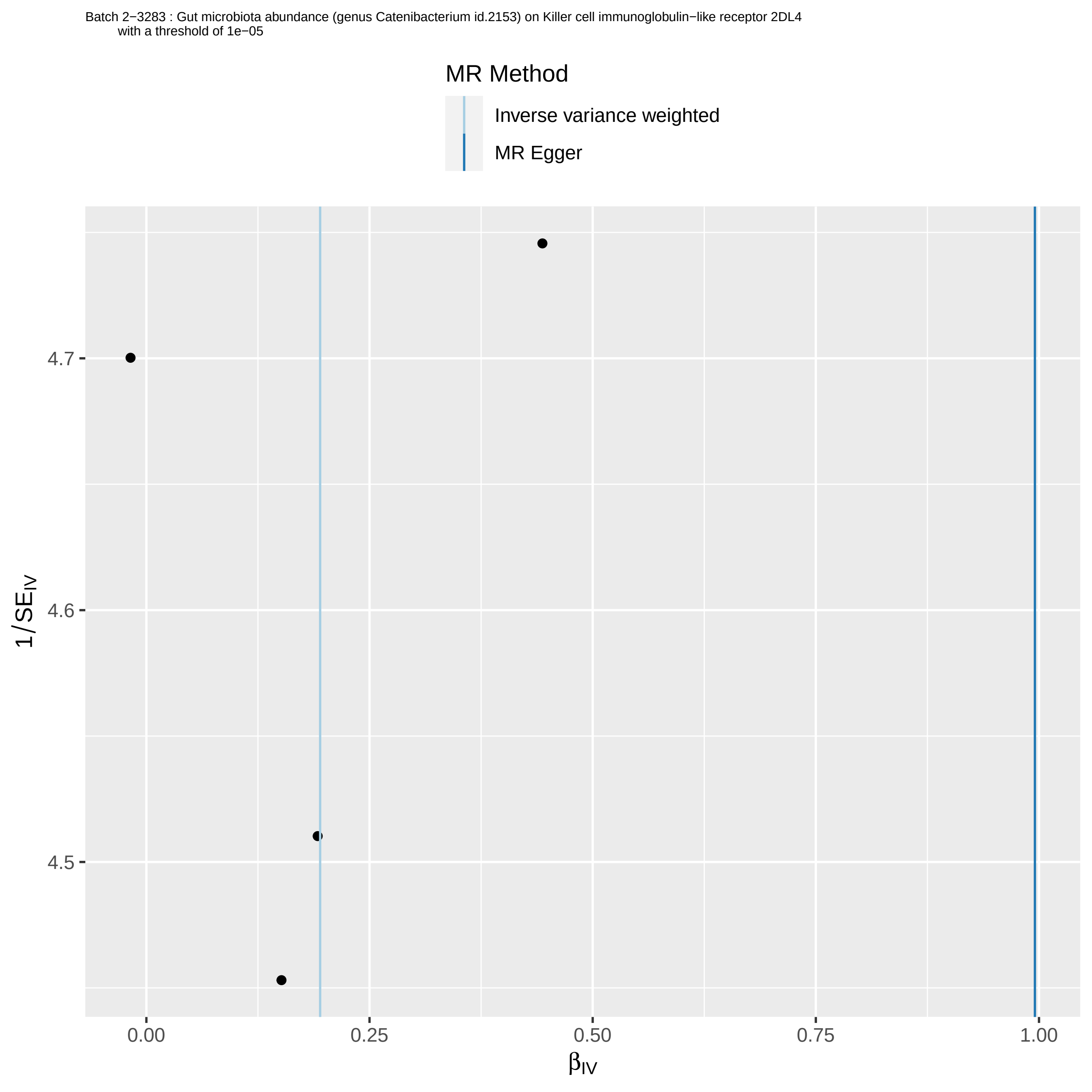

Batch 2−3283 : Gut microbiota abundance (genus Catenibacterium id.2153) on Killer cell immunoglobulin−like receptor 2DL4
with a threshold of 1e−05
MR Method
Inverse variance weighted
MR Egger
4.7
4.6
4.5
1/SE$_{IV}$
0.00
0.25
0.50
0.75
1.00
β_{IV}

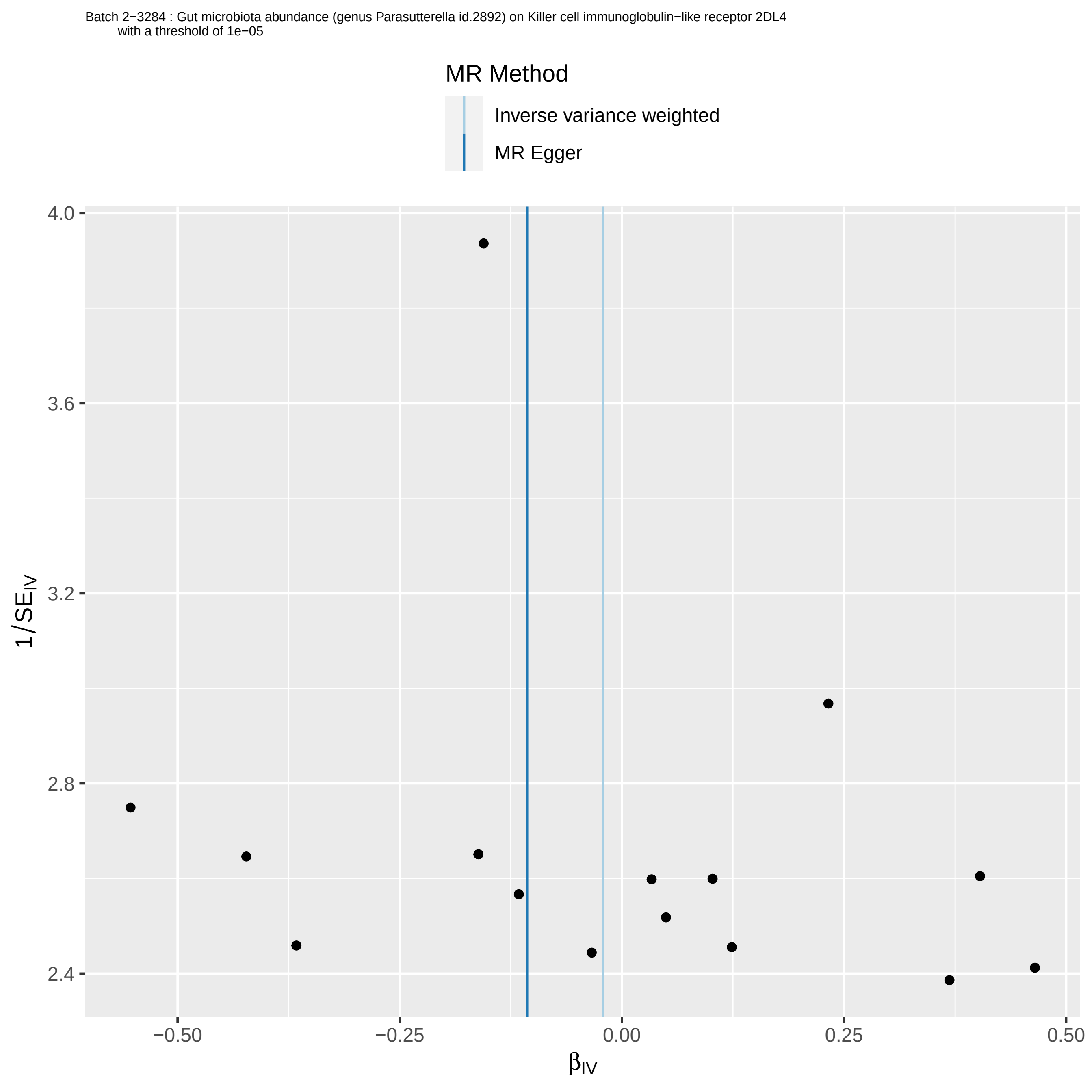
Batch 2−3284 : Gut microbiota abundance (genus Parasutterella id.2892) on Killer cell immunoglobulin−like receptor 2DL4
with a threshold of 1e−05
MR Method
Inverse variance weighted
MR Egger
4.0
3.6
3.2
2.8
2.4
1/SE$_{IV}$
−0.50
−0.25
0.00
0.25
0.50
β_{IV}

Batch 2−3285 : Gut microbiota abundance (genus Catenibacterium id.2153) on Killer cell immunoglobulin−like receptor 2DL5A with a threshold of 1e−05

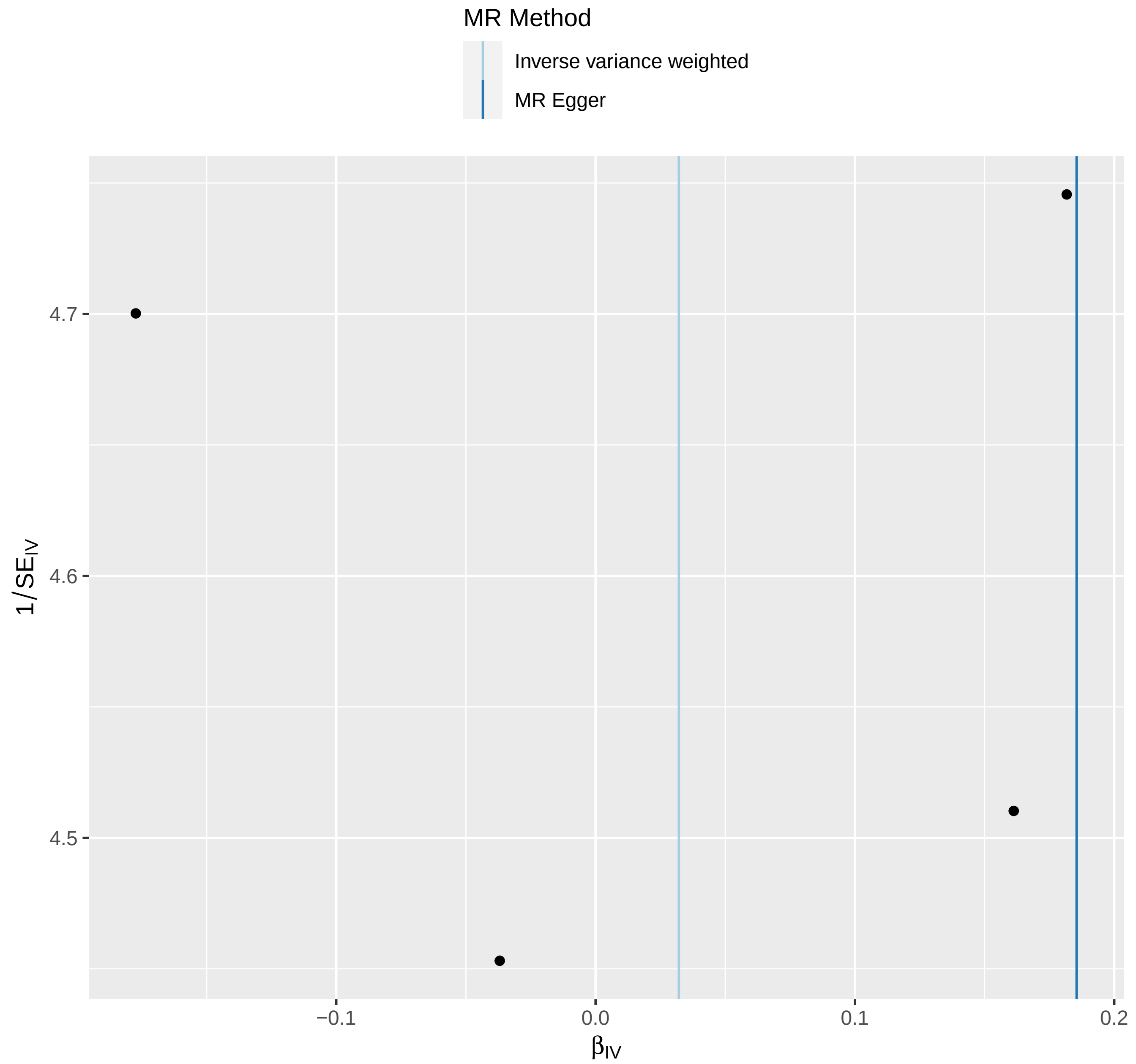

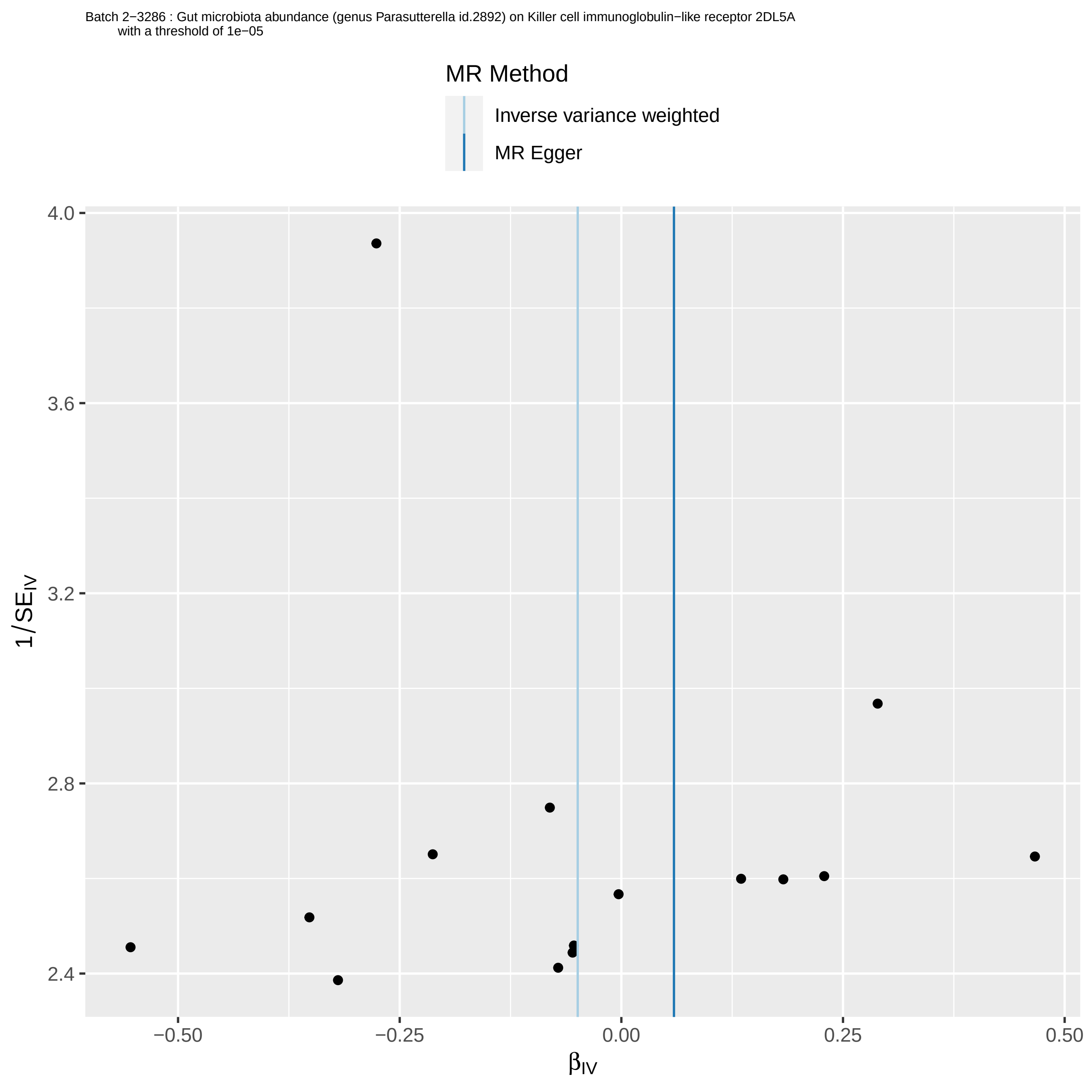

Batch 2−3286 : Gut microbiota abundance (genus Parasutterella id.2892) on Killer cell immunoglobulin−like receptor 2DL5A
with a threshold of 1e−05
MR Method
Inverse variance weighted
MR Egger
4.0
3.6
3.2
2.8
2.4
1/SE_IV
−0.50
−0.25
0.00
0.25
0.50
β_IV

Batch 2−3287 : Gut microbiota abundance (genus Catenibacterium id.2153) on Killer cell immunoglobulin−like receptor 2DL5A
with a threshold of 1e−05

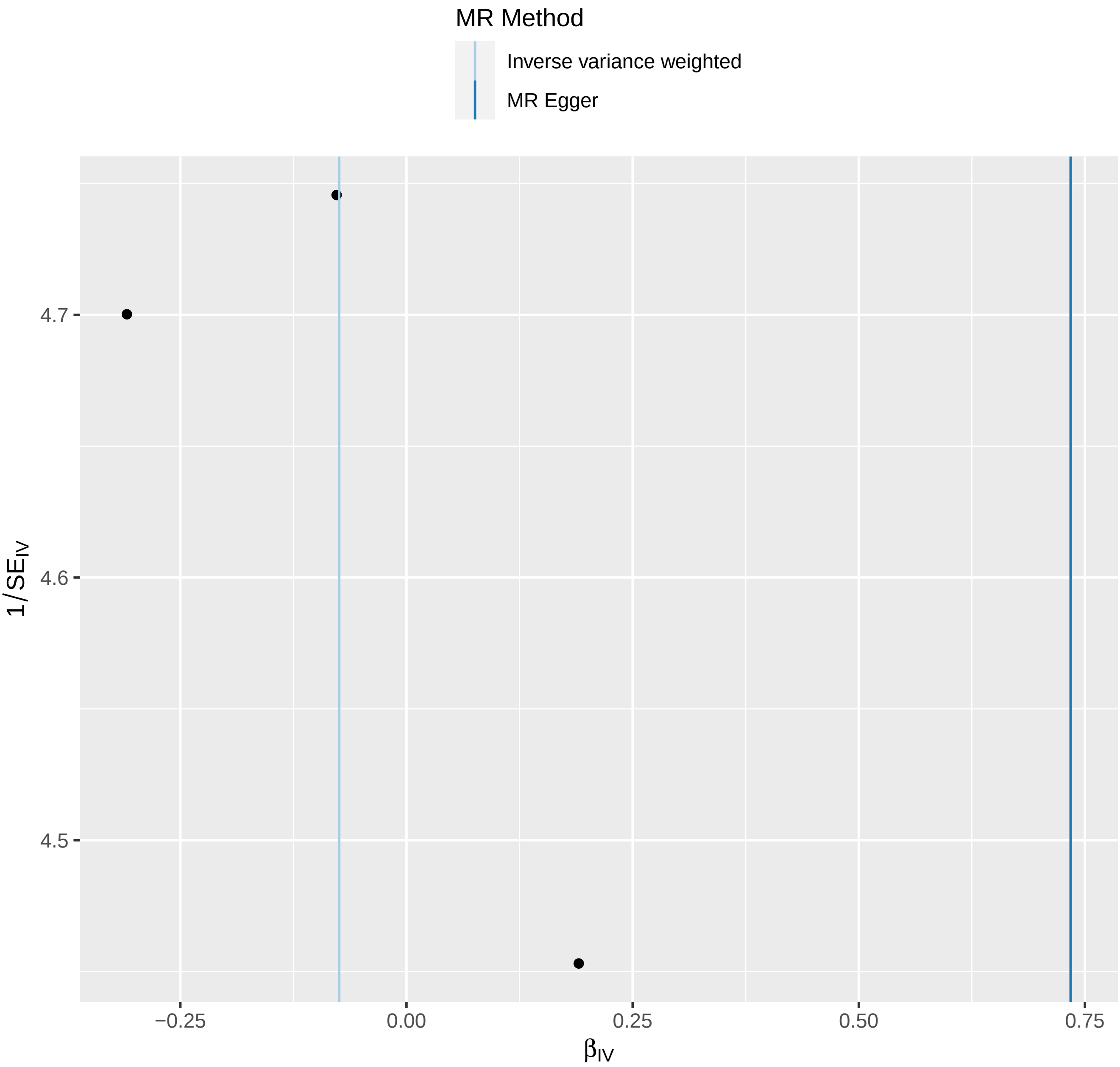

Batch 2−3288 : Gut microbiota abundance (genus Parasutterella id.2892) on Killer cell immunoglobulin−like receptor 2DL5A
with a threshold of 1e−05

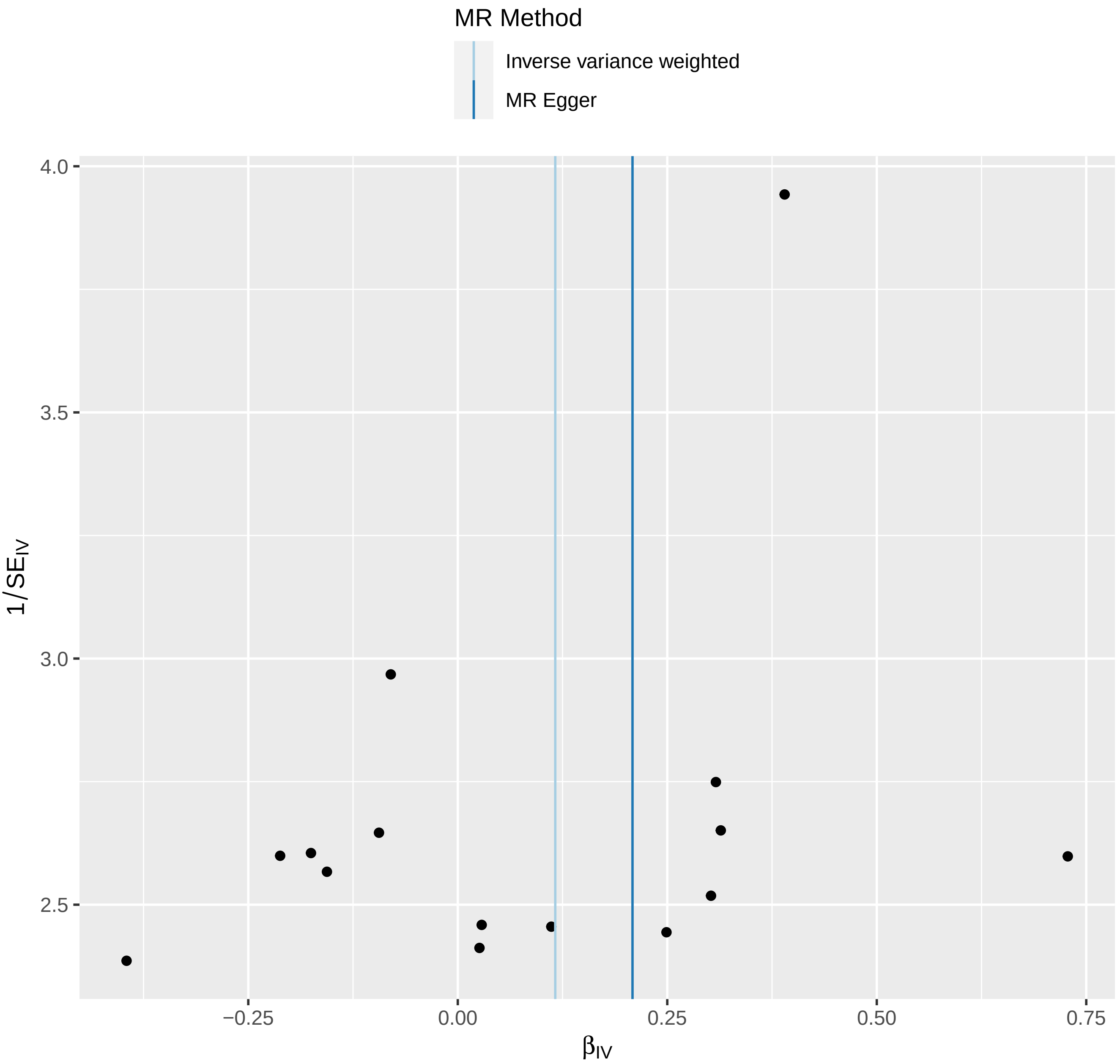

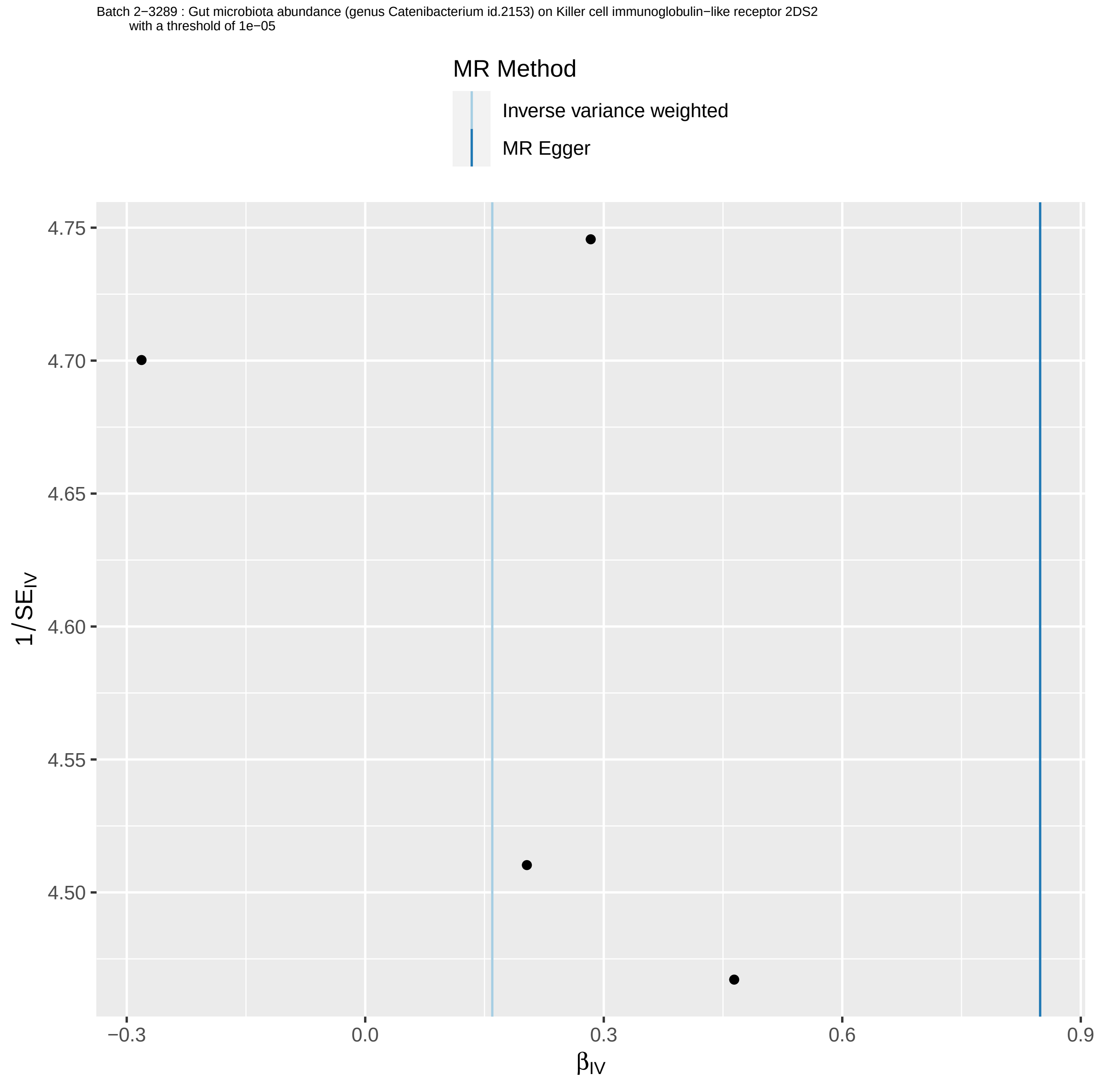
Batch 2−3289 : Gut microbiota abundance (genus Catenibacterium id.2153) on Killer cell immunoglobulin−like receptor 2DS2
with a threshold of 1e−05
MR Method
Inverse variance weighted
MR Egger
4.75
4.70
4.65
4.60
4.55
4.50
1/SE_IV
−0.3
0.0
0.3
0.6
0.9
β_IV

Batch 2−3290 : Gut microbiota abundance (genus Parasutterella id.2892) on Killer cell immunoglobulin−like receptor 2DS2
with a threshold of 1e−05

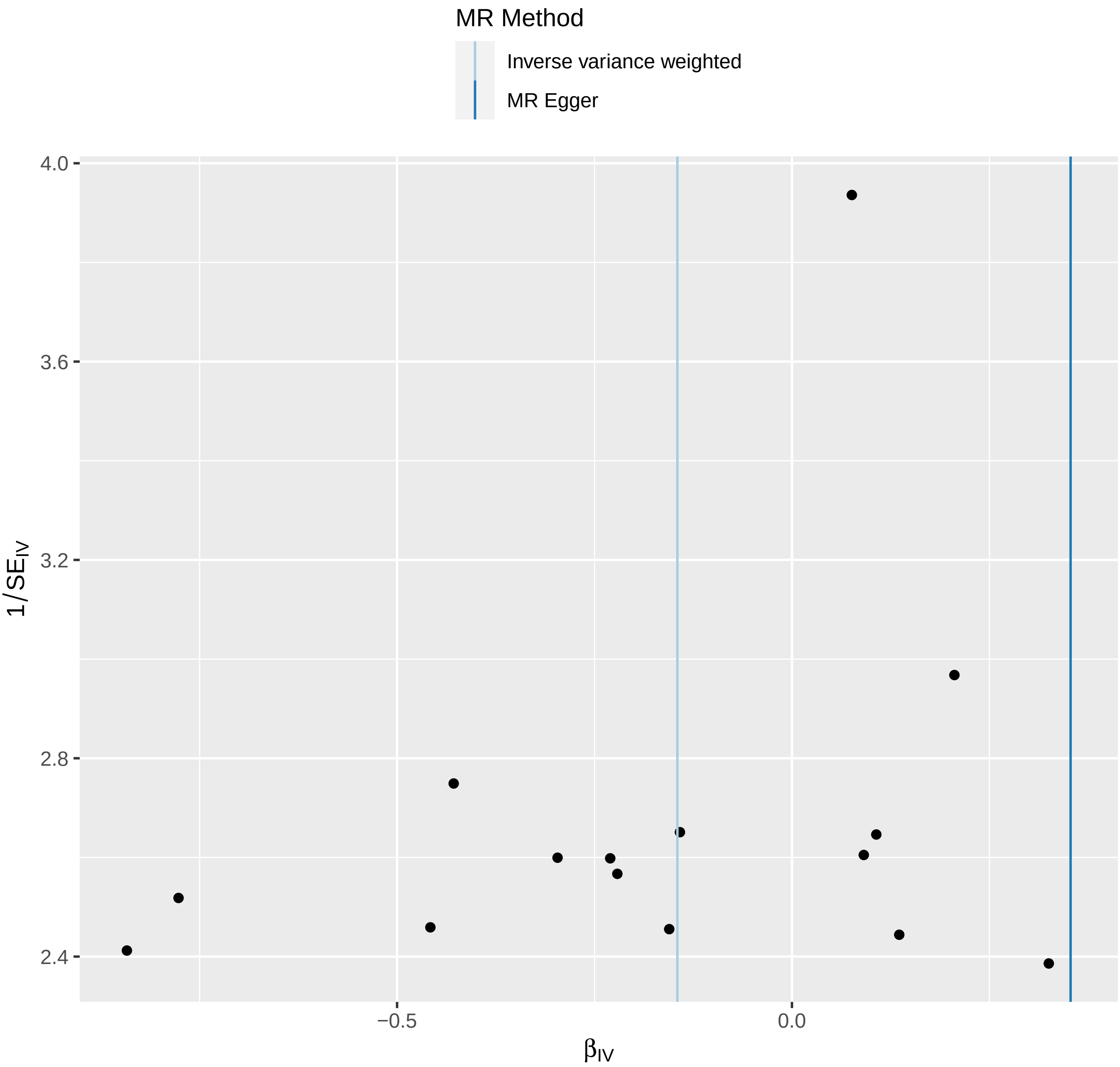

Batch 2−3291 : Gut microbiota abundance (genus Catenibacterium id.2153) on Killer cell immunoglobulin−like receptor 3DL2
with a threshold of 1e−05

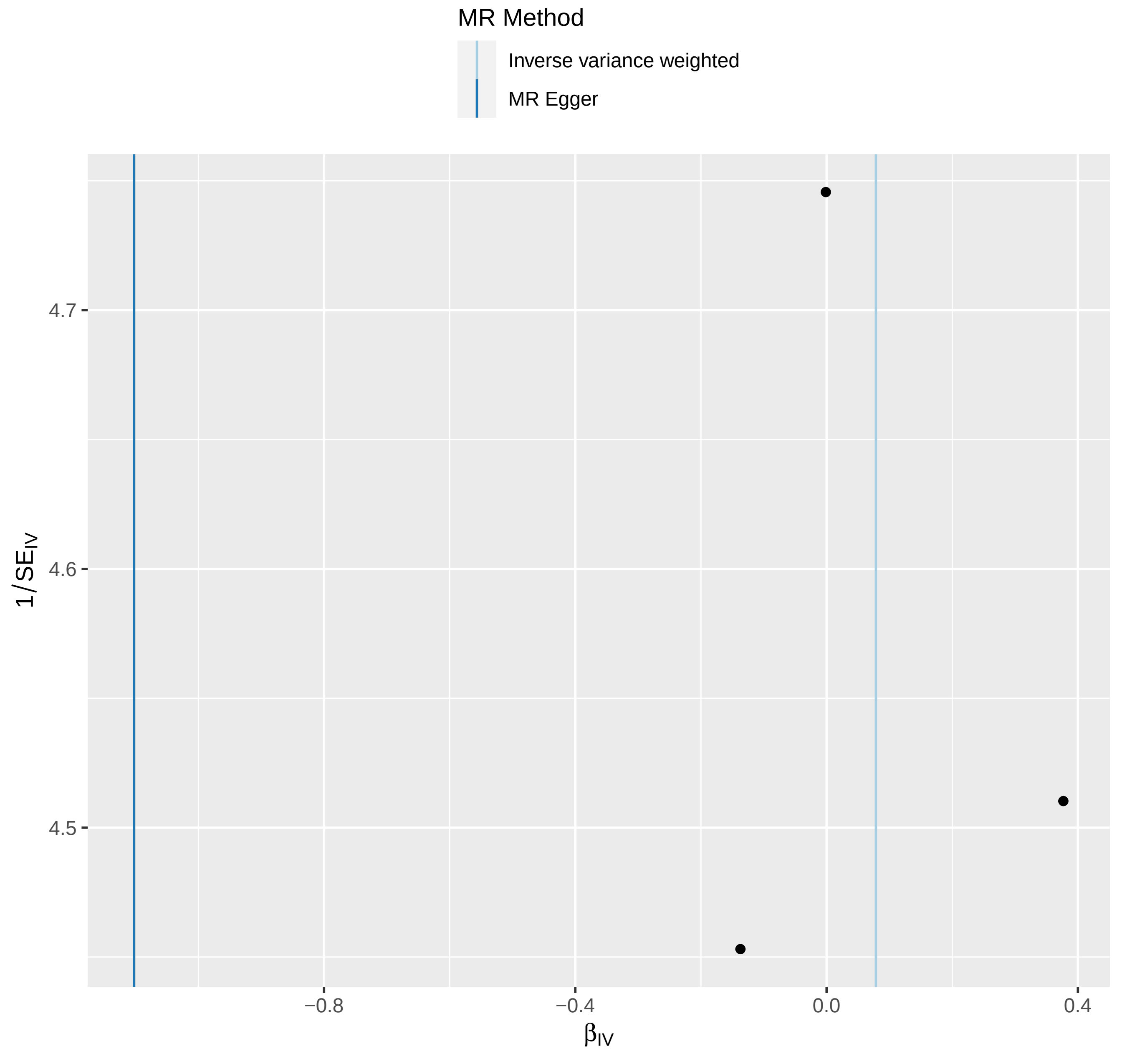

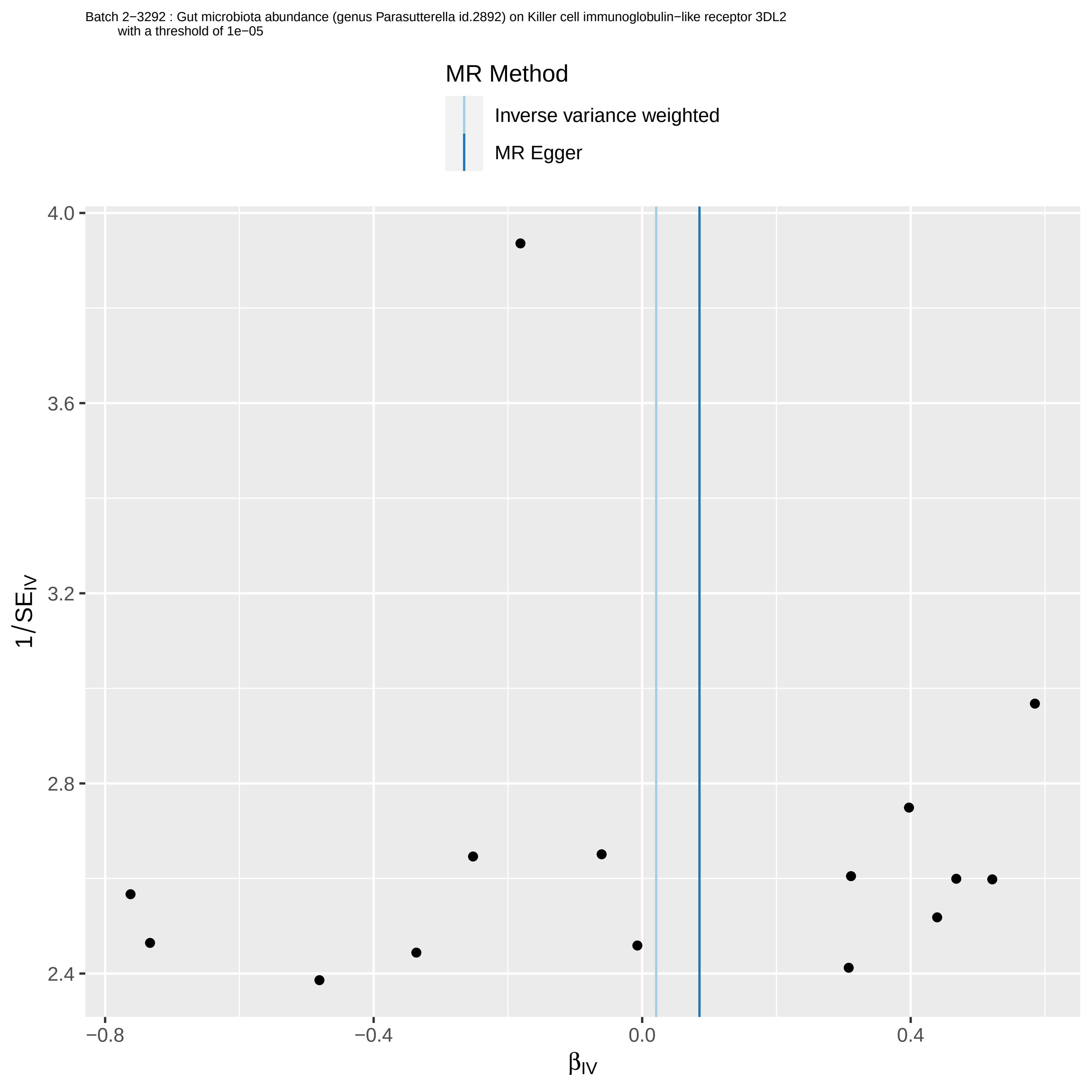

Batch 2−3292 : Gut microbiota abundance (genus Parasutterella id.2892) on Killer cell immunoglobulin−like receptor 3DL2
with a threshold of 1e−05
MR Method
Inverse variance weighted
MR Egger
4.0
3.6
3.2
2.8
2.4
1/SE_IV
−0.8
−0.4
0.0
0.4
β_IV

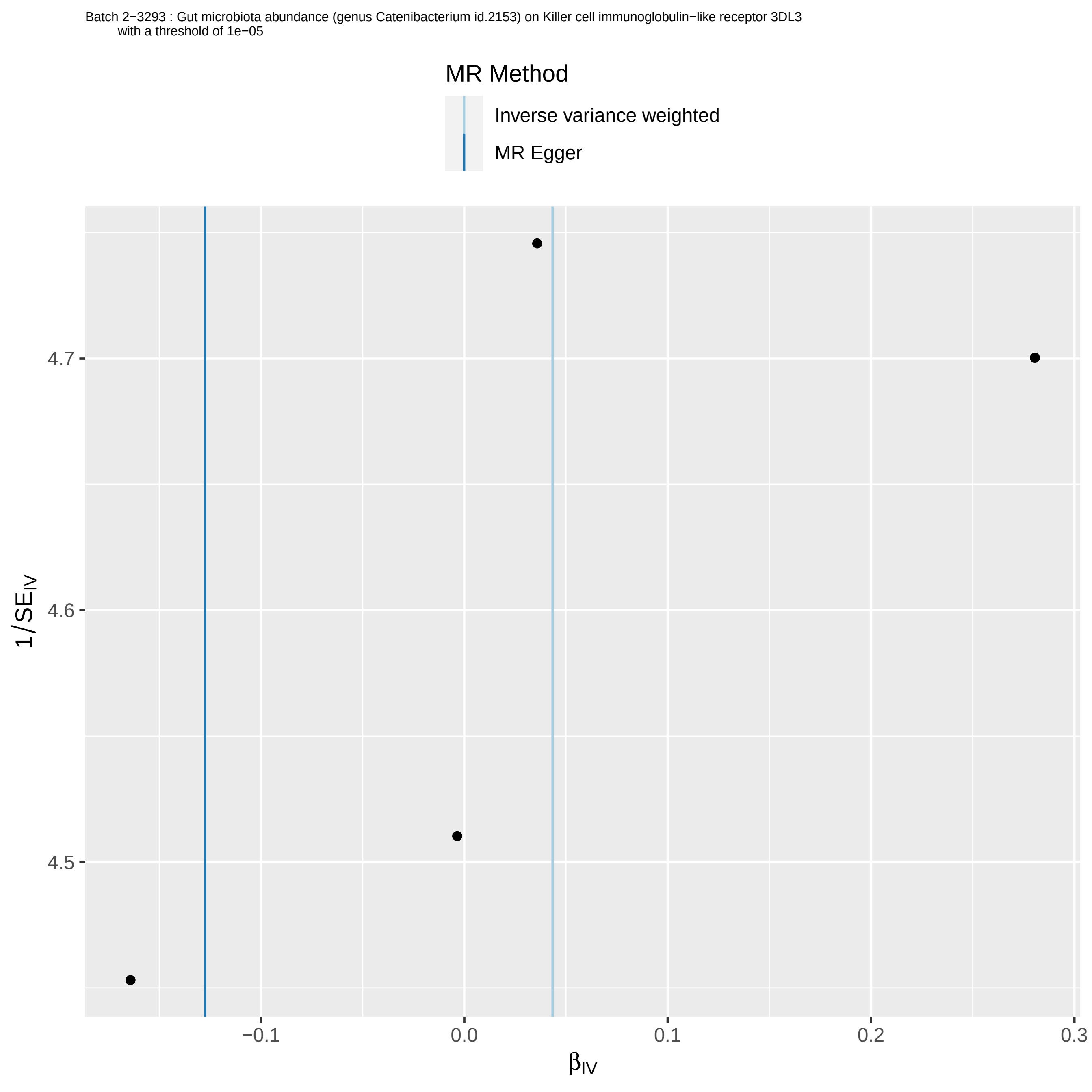

Batch 2−3293 : Gut microbiota abundance (genus Catenibacterium id.2153) on Killer cell immunoglobulin−like receptor 3DL3
with a threshold of 1e−05
MR Method
Inverse variance weighted
MR Egger
4.7
4.6
4.5
1/SE_IV
−0.1
0.0
0.1
0.2
0.3
β_IV

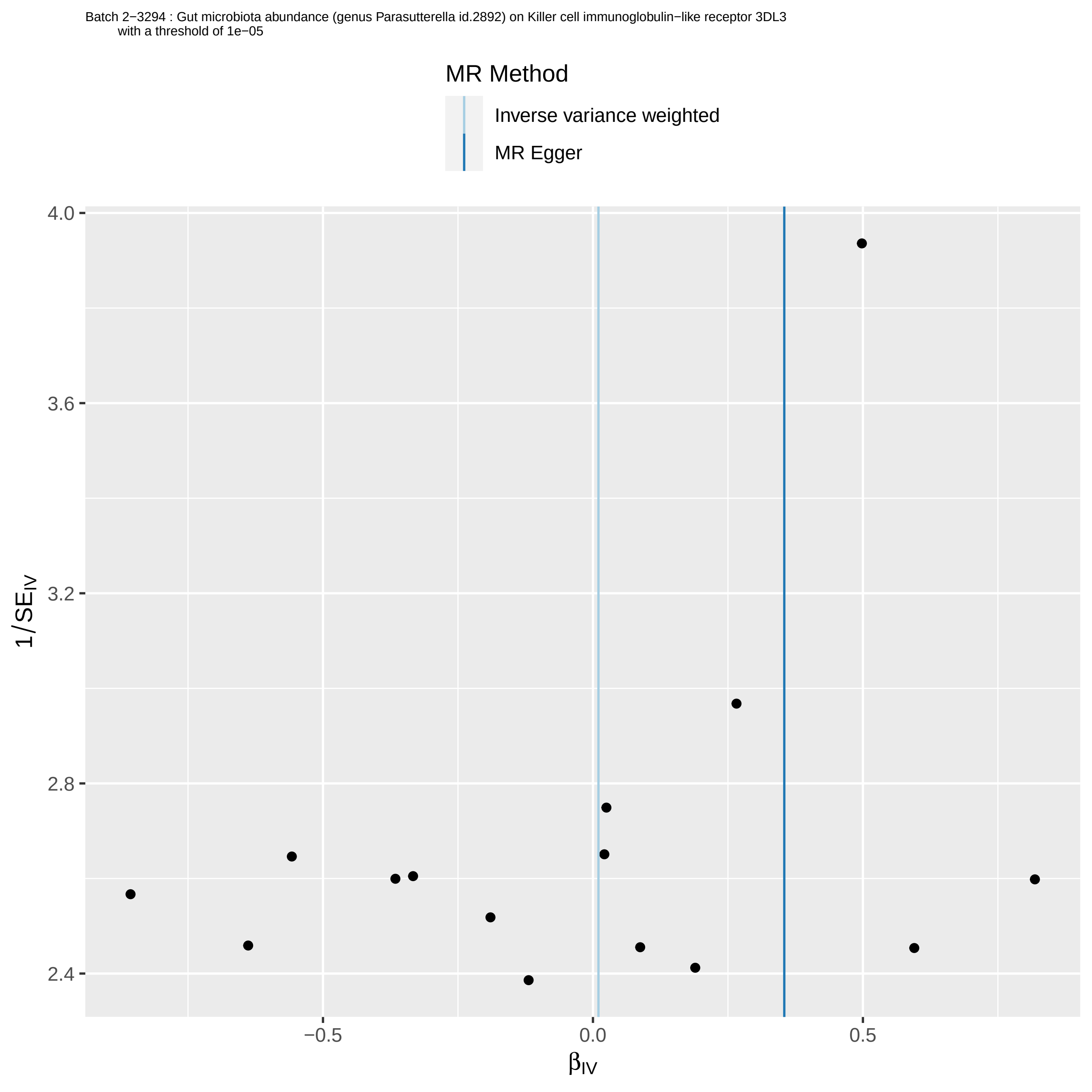

Batch 2−3294 : Gut microbiota abundance (genus Parasutterella id.2892) on Killer cell immunoglobulin−like receptor 3DL3
with a threshold of 1e−05
MR Method
Inverse variance weighted
MR Egger
4.0
3.6
3.2
2.8
2.4
1/SE_IV
−0.5
0.0
0.5
β_IV

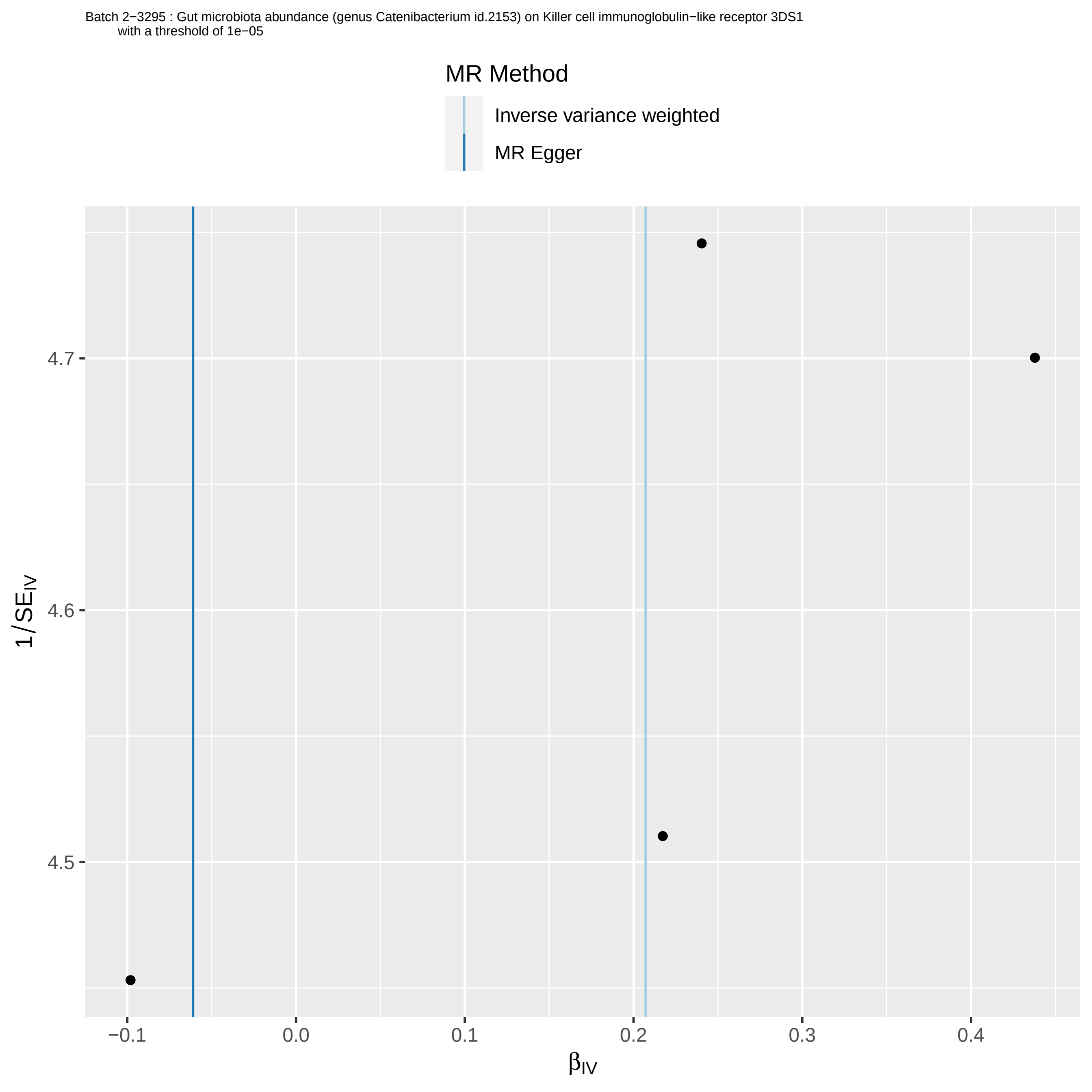

Batch 2−3295 : Gut microbiota abundance (genus Catenibacterium id.2153) on Killer cell immunoglobulin−like receptor 3DS1
with a threshold of 1e−05
MR Method
Inverse variance weighted
MR Egger
4.7
4.6
4.5
1/SE_IV
−0.1
0.0
0.1
0.2
0.3
0.4
β_IV

Batch 2−3296 : Gut microbiota abundance (genus Parasutterella id.2892) on Killer cell immunoglobulin−like receptor 3DS1
with a threshold of 1e−05

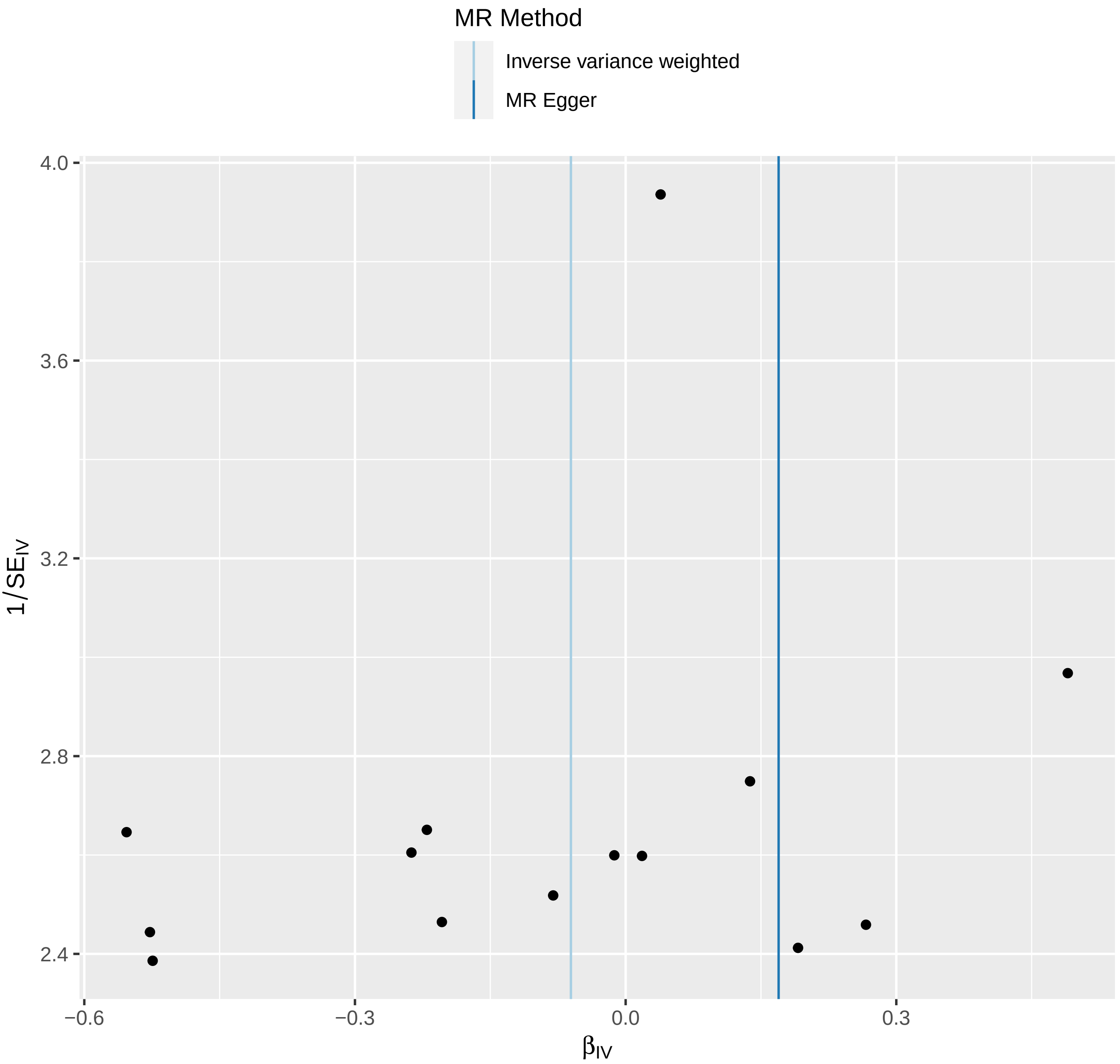

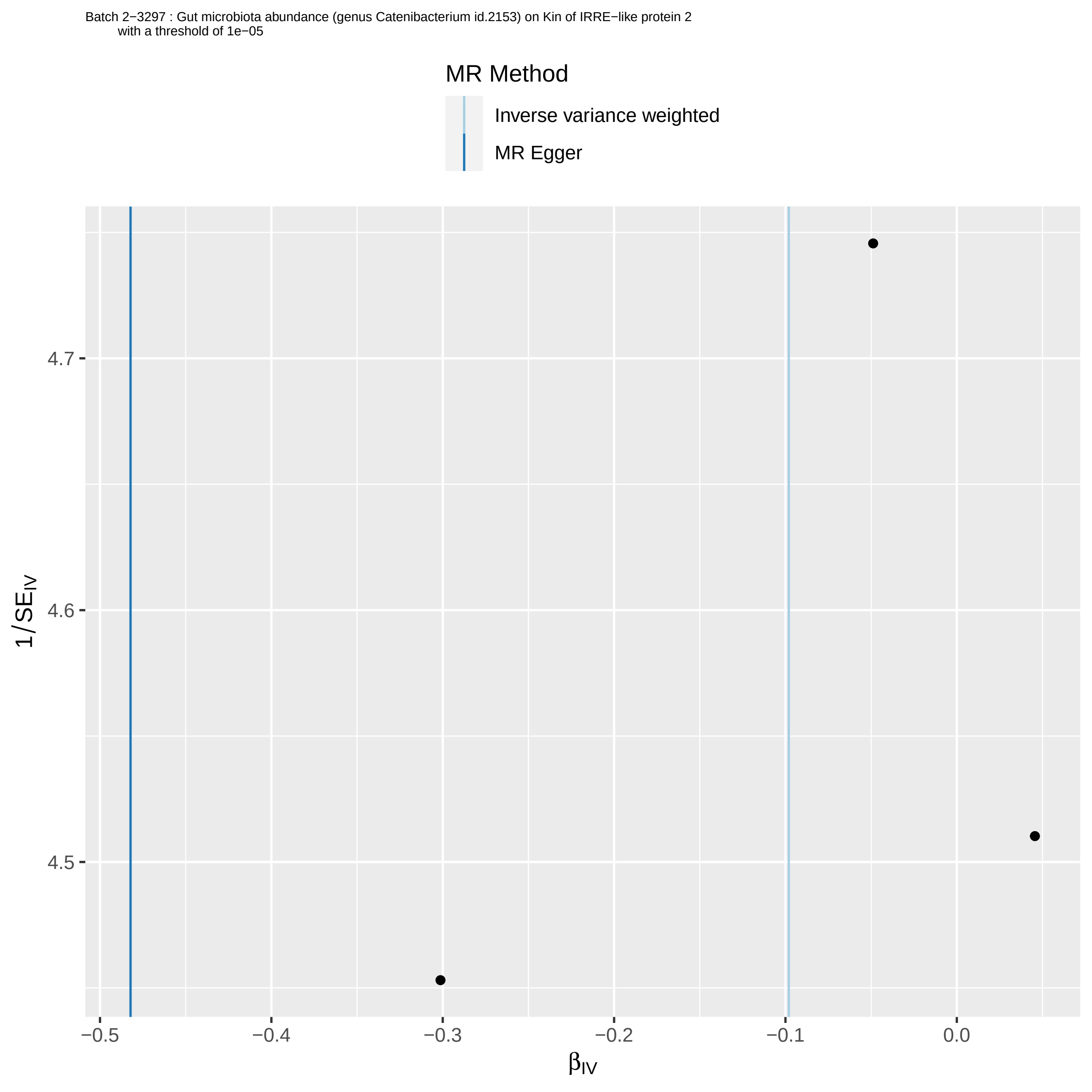

Batch 2−3297 : Gut microbiota abundance (genus Catenibacterium id.2153) on Kin of IRRE−like protein 2
with a threshold of 1e−05
MR Method
Inverse variance weighted
MR Egger
4.7
4.6
4.5
1/SE_IV
−0.5
−0.4
−0.3
−0.2
−0.1
0.0
β_IV

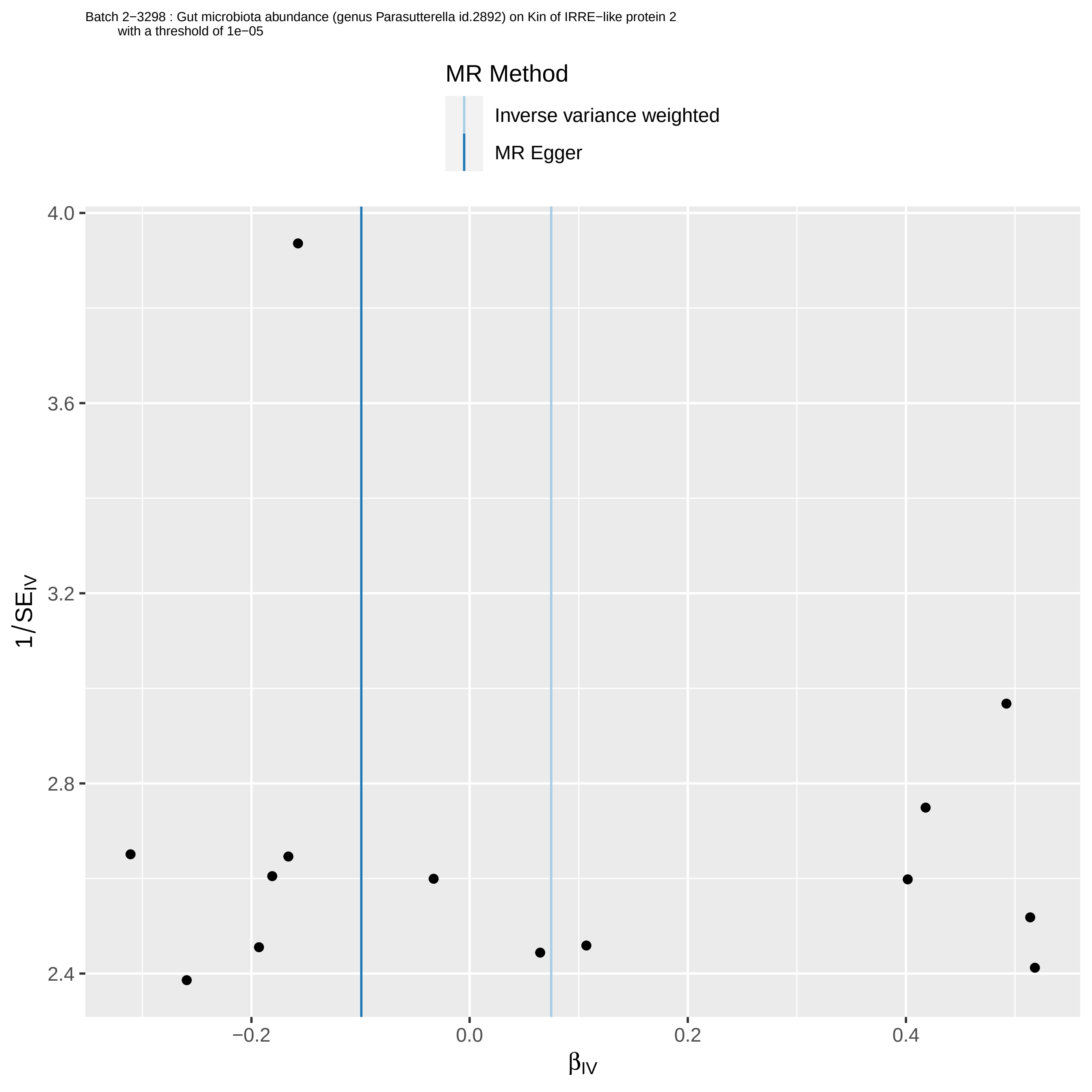
Batch 2−3298 : Gut microbiota abundance (genus Parasutterella id.2892) on Kin of IRRE−like protein 2
with a threshold of 1e−05
MR Method
Inverse variance weighted
MR Egger
4.0
3.6
3.2
2.8
2.4
1/SE_IV
−0.2
0.0
0.2
0.4
β_IV

Batch 2−3299 : Gut microbiota abundance (genus Catenibacterium id.2153) on Kin of IRRE−like protein 2
with a threshold of 1e−05

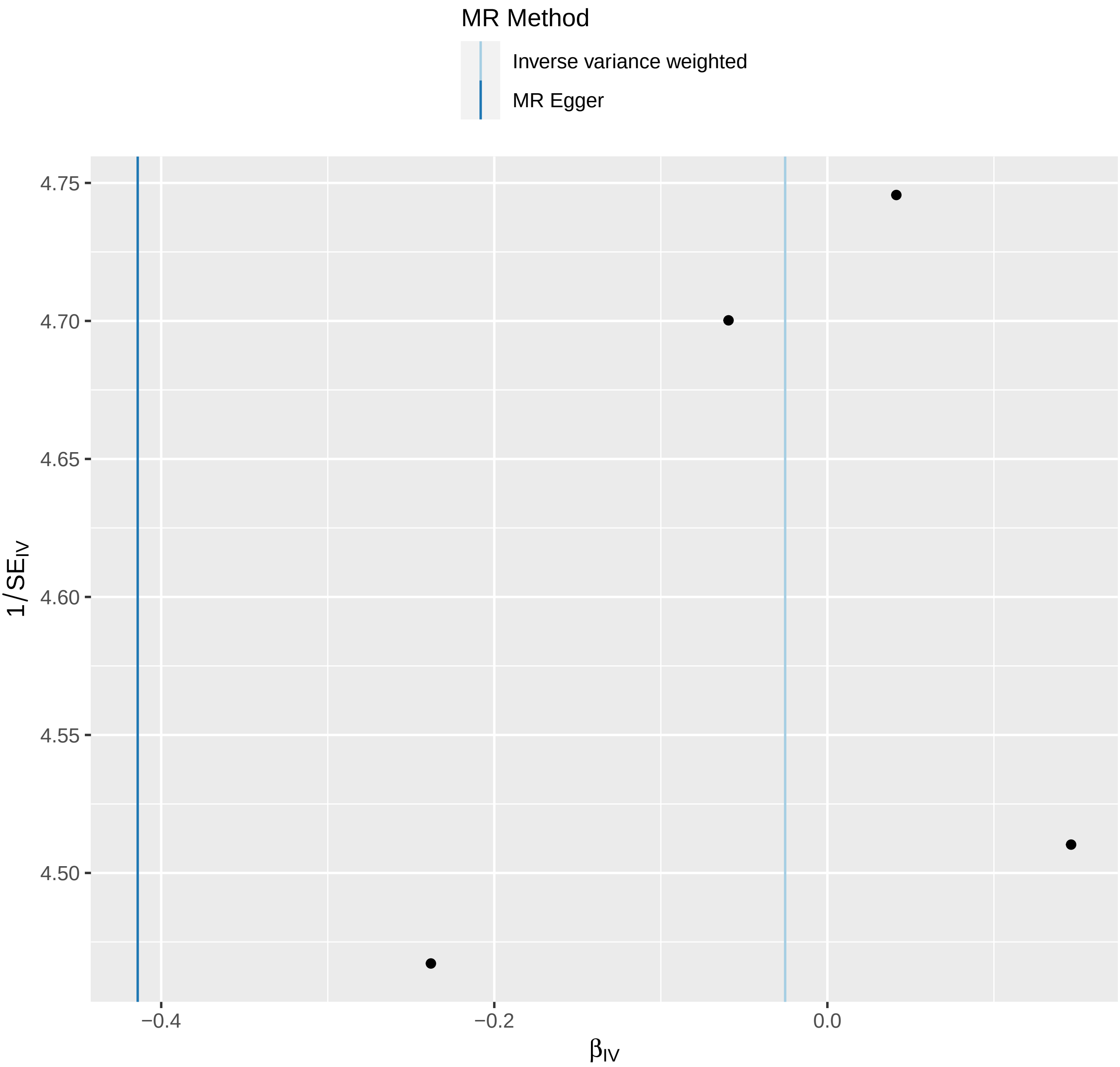

Batch 2−3300 : Gut microbiota abundance (genus Parasutterella id.2892) on Kin of IRRE−like protein 2
with a threshold of 1e−05

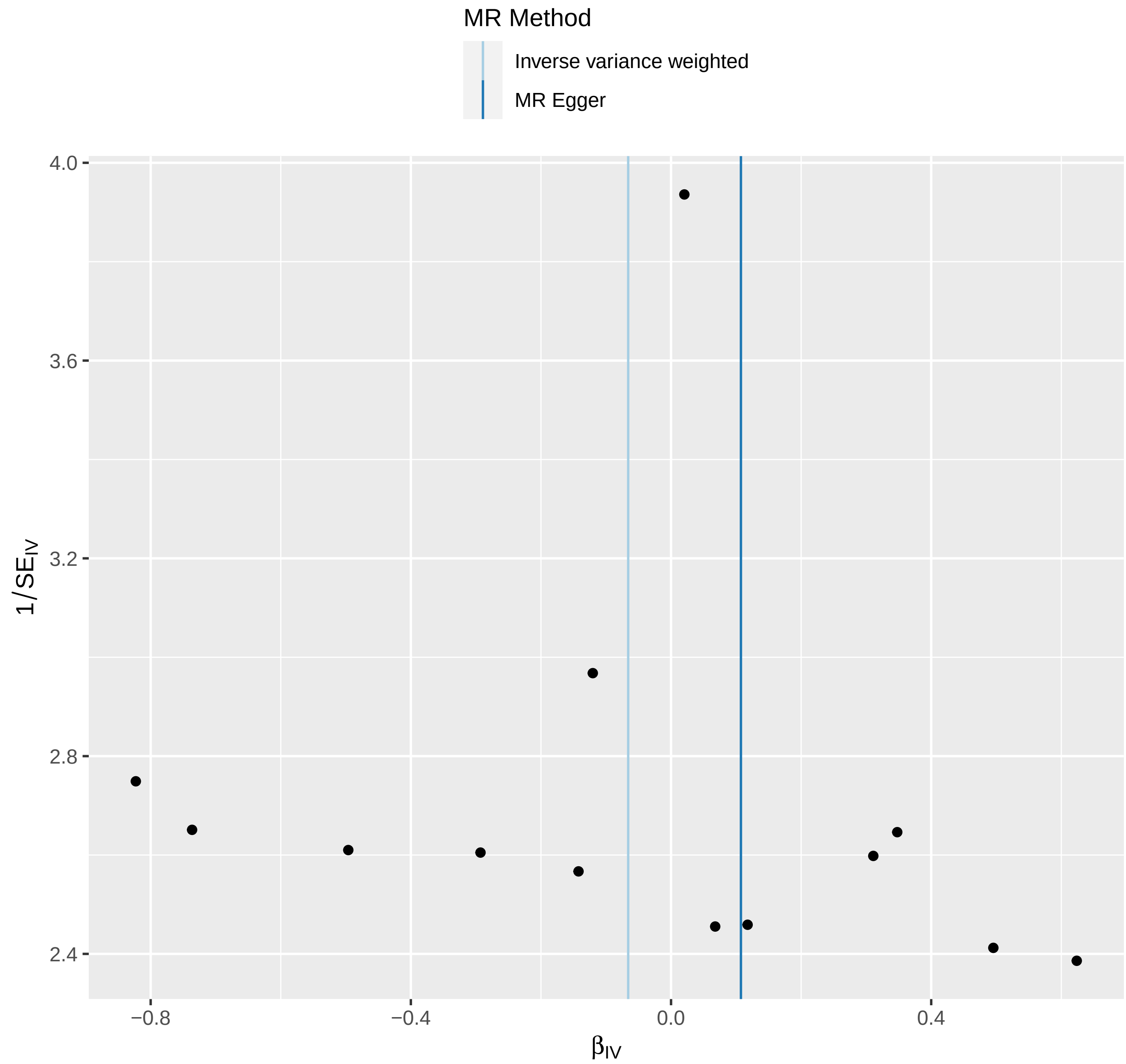

Batch 2−3301 : Gut microbiota abundance (genus Catenibacterium id.2153) on Kin of IRRE−like protein 3
with a threshold of 1e−05

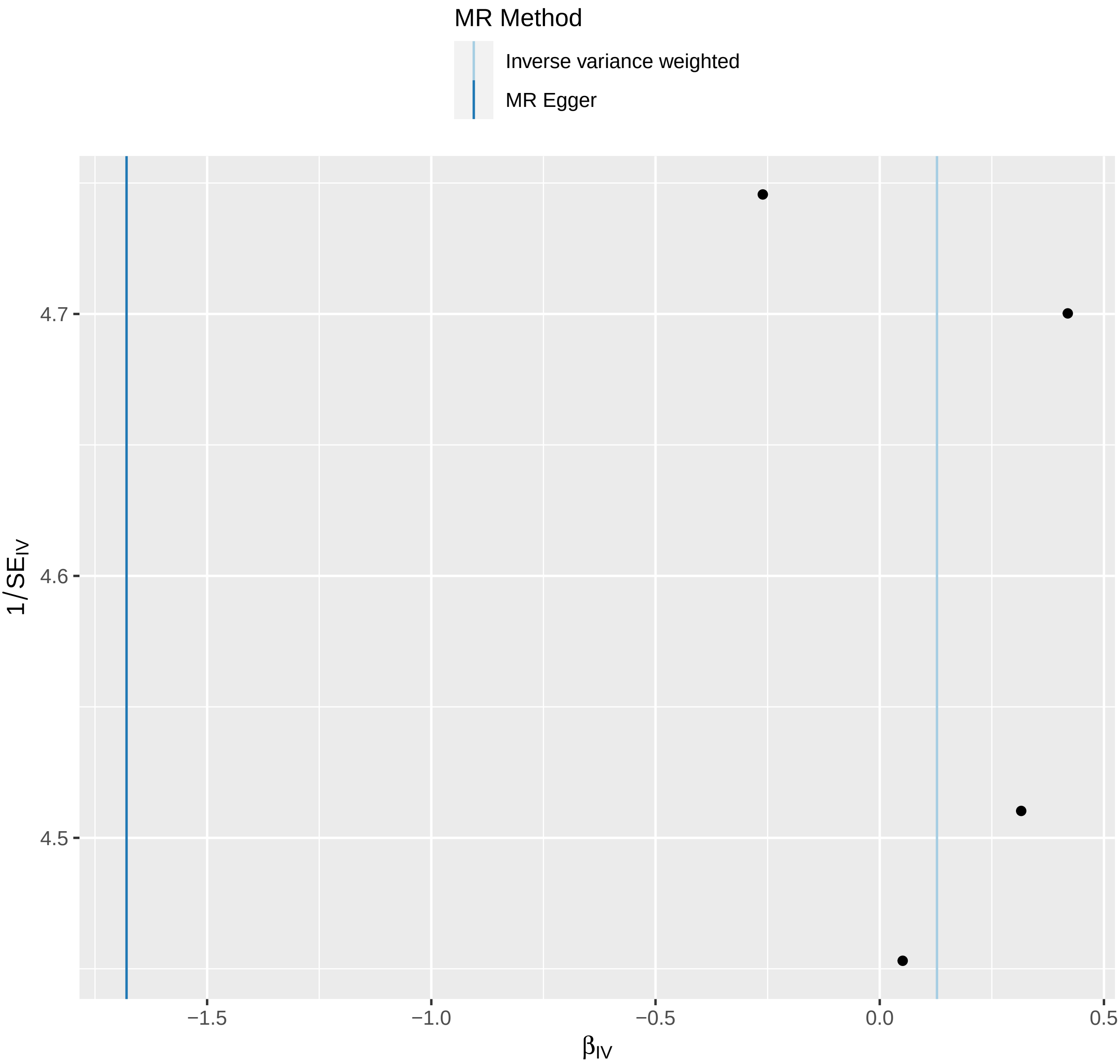

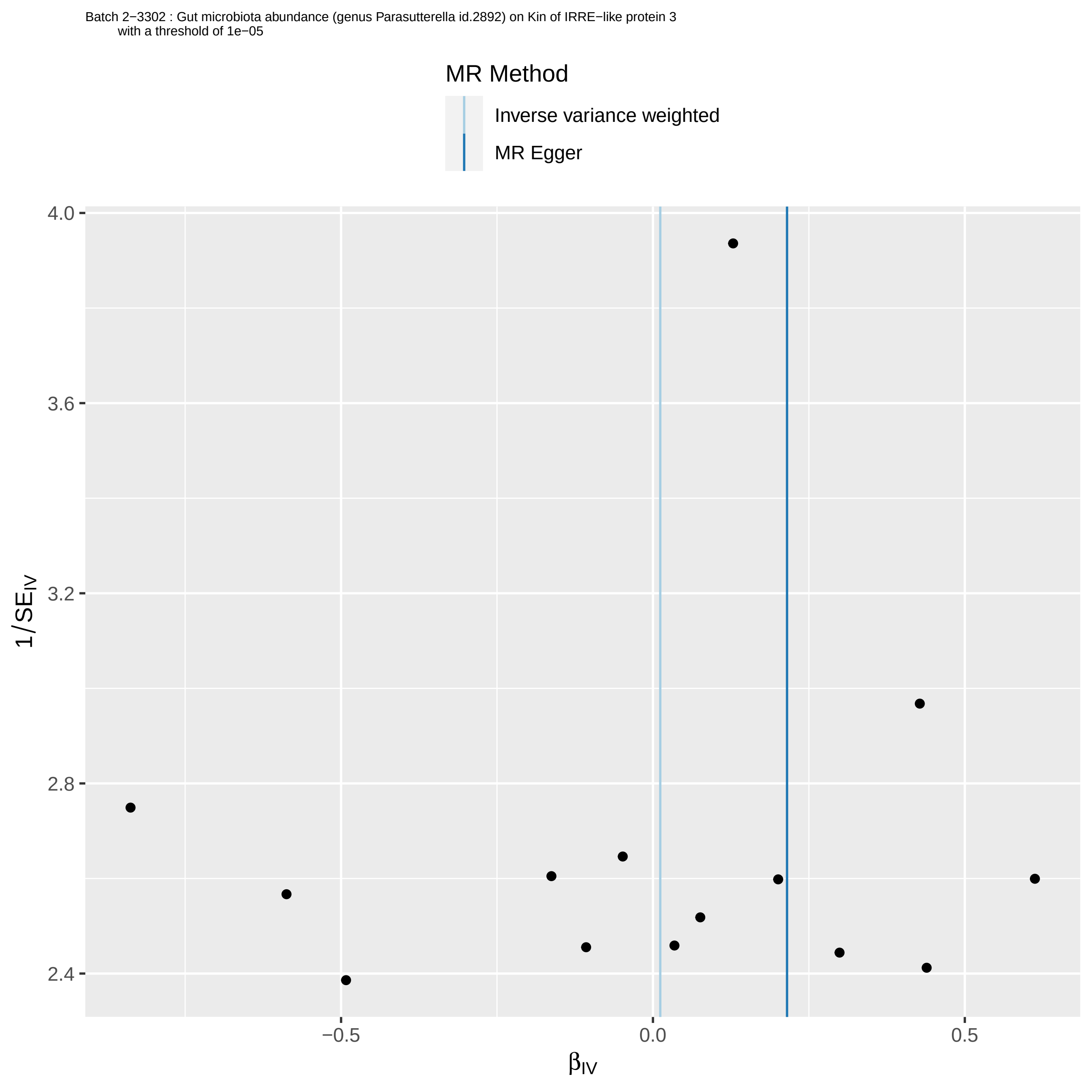

Batch 2−3302 : Gut microbiota abundance (genus Parasutterella id.2892) on Kin of IRRE−like protein 3
with a threshold of 1e−05
MR Method
Inverse variance weighted
MR Egger
4.0
3.6
3.2
2.8
2.4
1/SE_IV
−0.5
0.0
0.5
β_IV

Batch 2−3303 : Gut microbiota abundance (genus Catenibacterium id.2153) on Mast/stem cell growth factor receptor Kit with a threshold of 1e−05

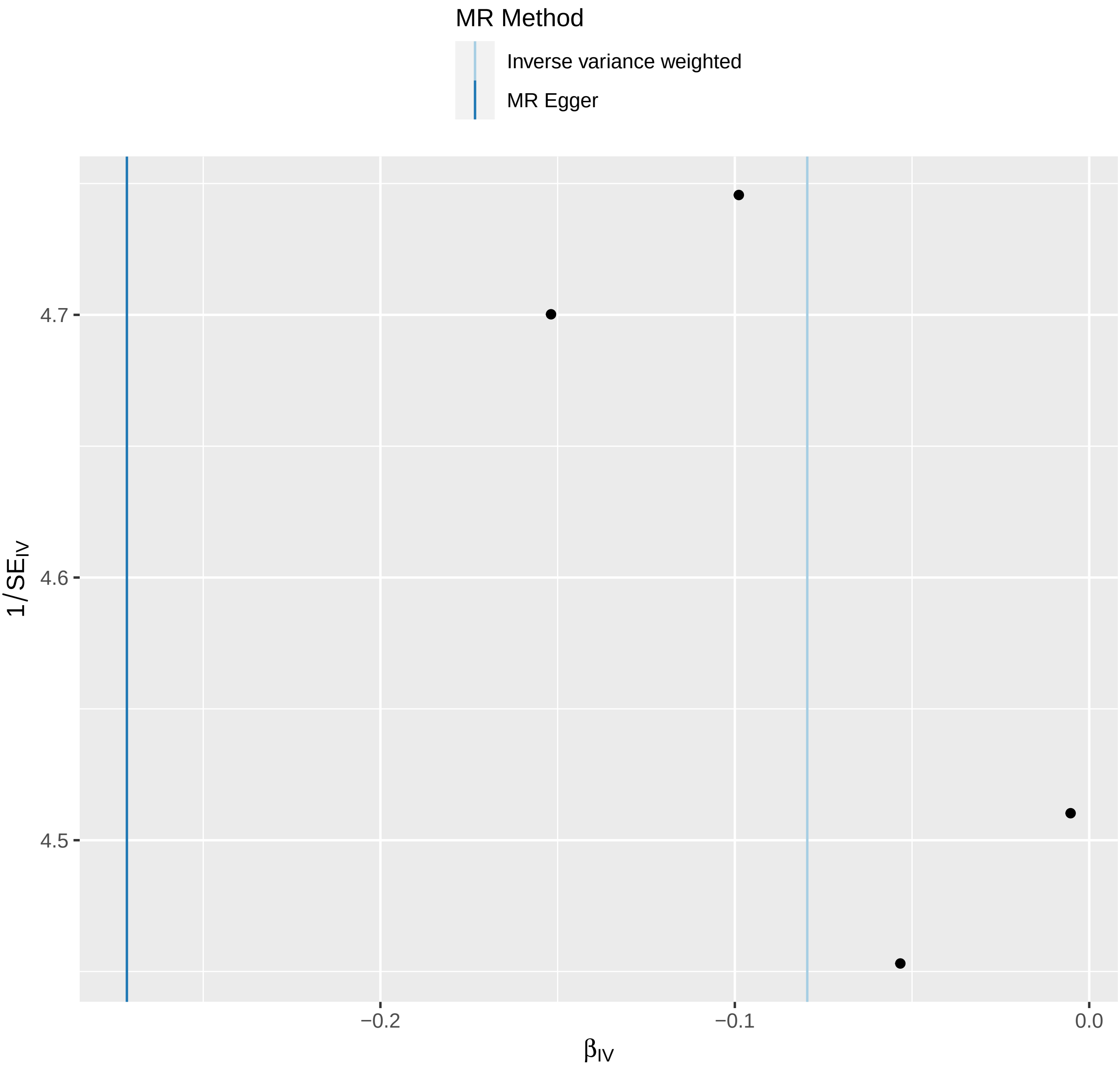

Batch 2−3304 : Gut microbiota abundance (genus Parasutterella id.2892) on Mast/stem cell growth factor receptor Kit
with a threshold of 1e−05

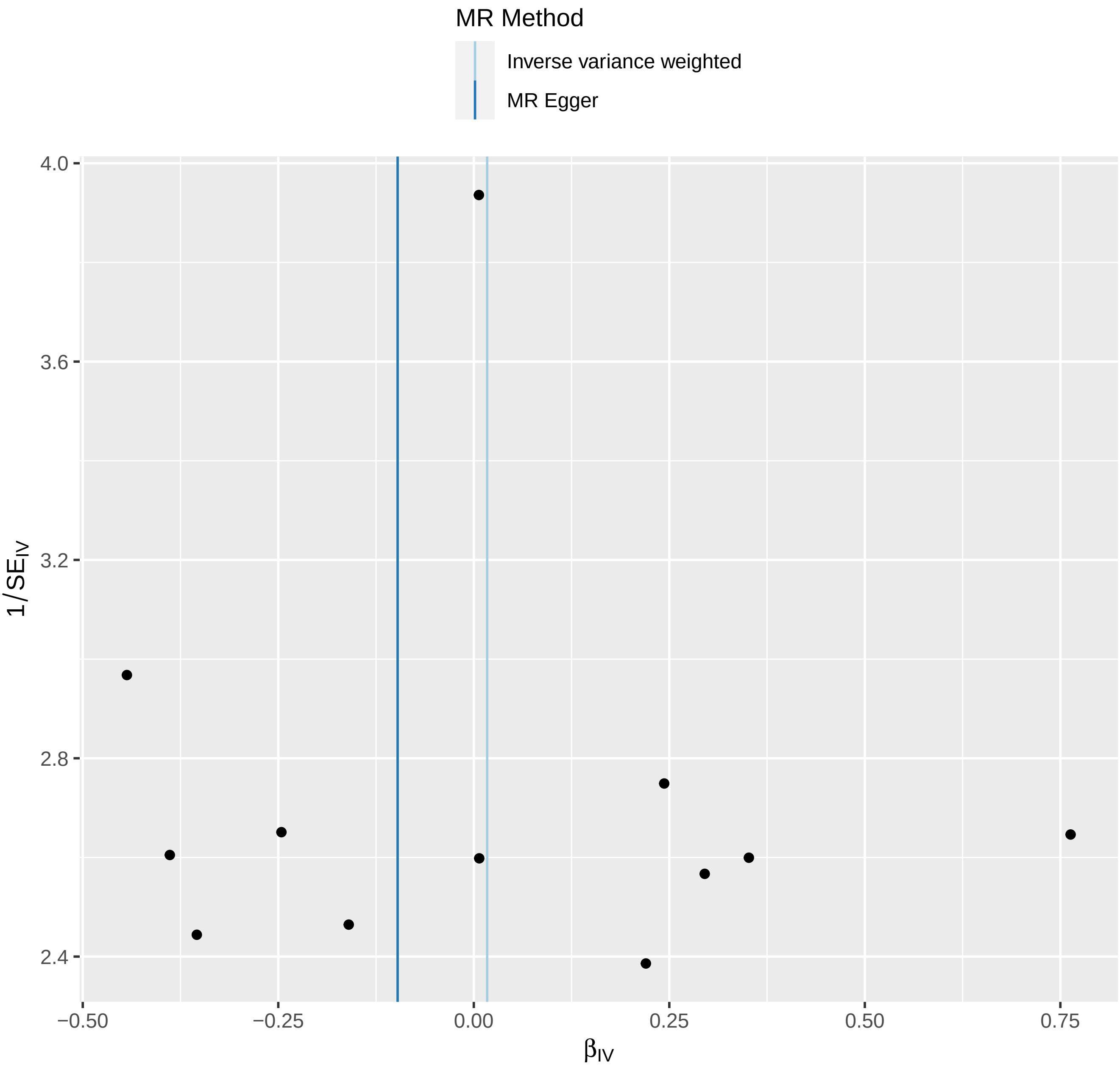

Batch 2−3305 : Gut microbiota abundance (genus Catenibacterium id.2153) on Kit ligand
with a threshold of 1e−05

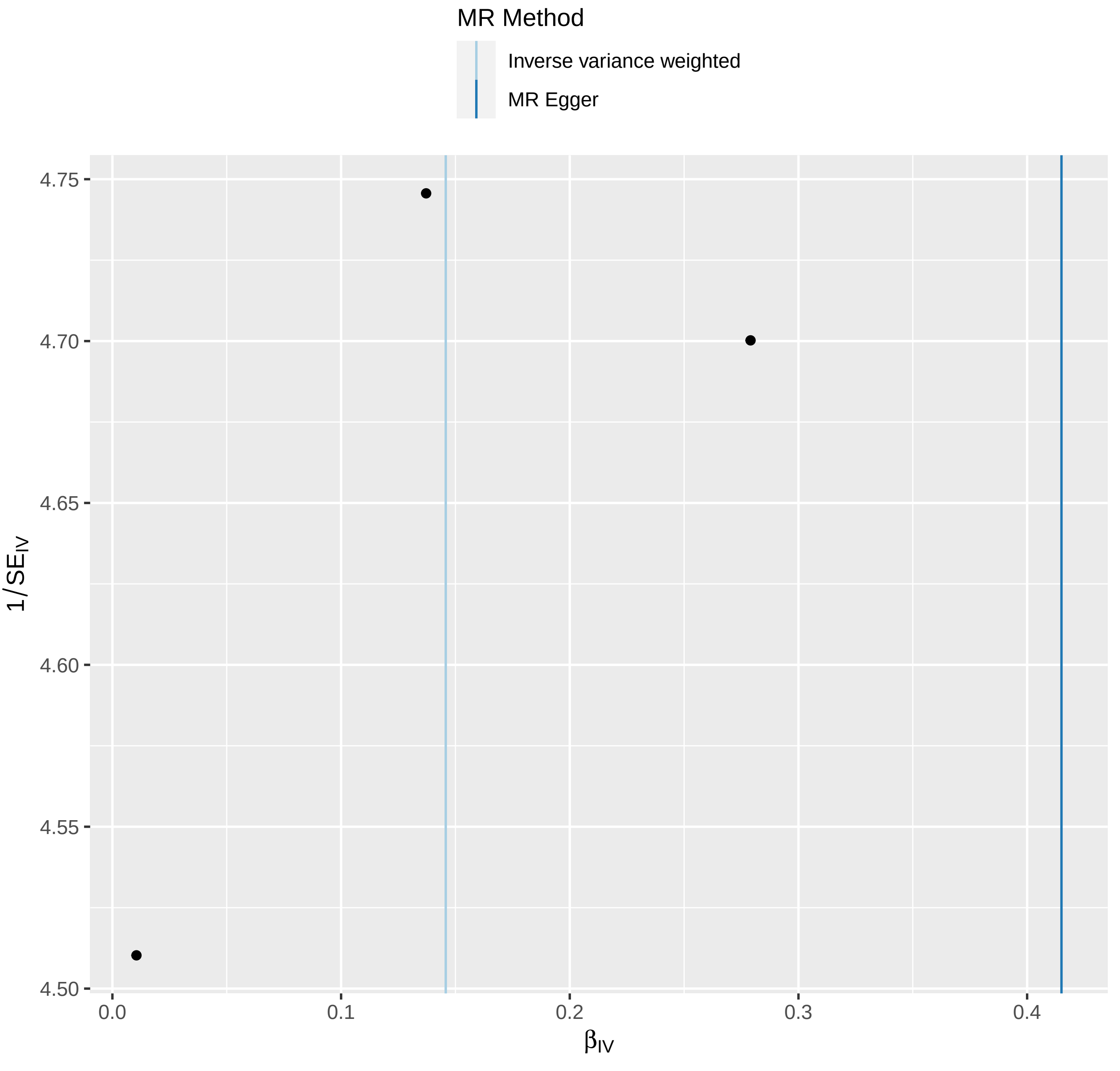

Batch 2−3306 : Gut microbiota abundance (genus Parasutterella id.2892) on Kit ligand
with a threshold of 1e−05

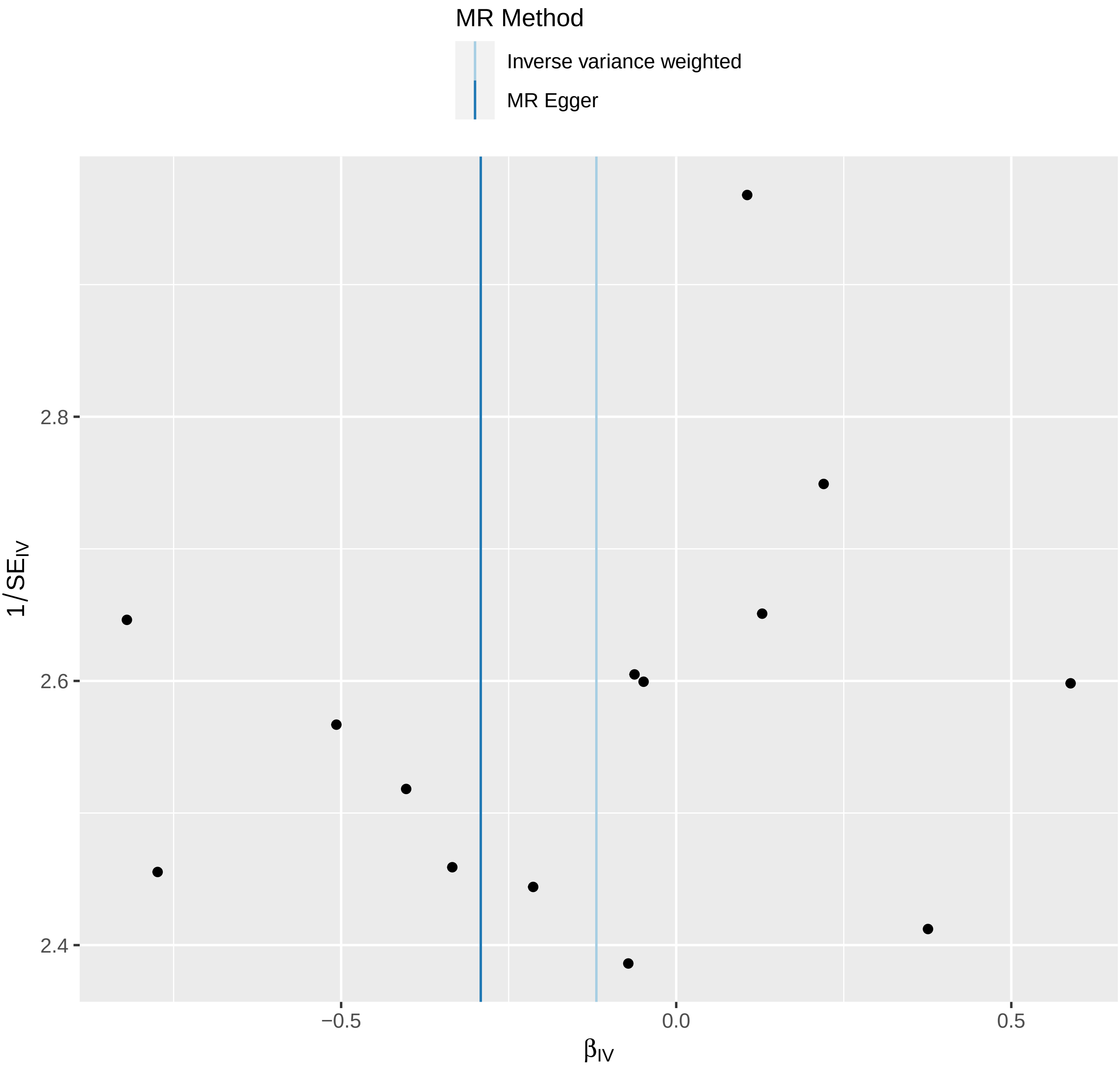

Batch 2−3307 : Gut microbiota abundance (genus Catenibacterium id.2153) on Kelch−like protein 12
with a threshold of 1e−05

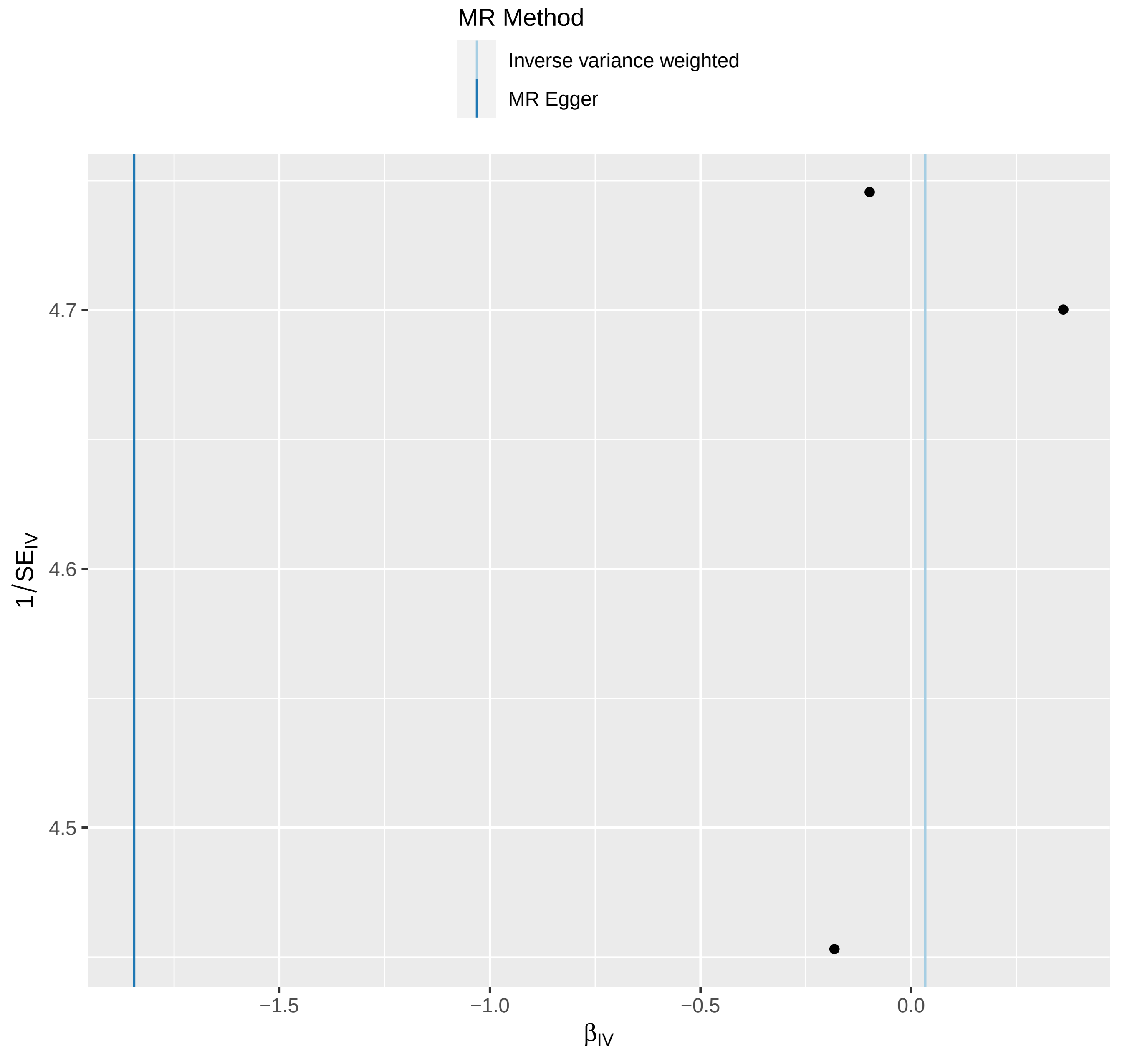

Batch 2−3308 : Gut microbiota abundance (genus Parasutterella id.2892) on Kelch−like protein 12
with a threshold of 1e−05

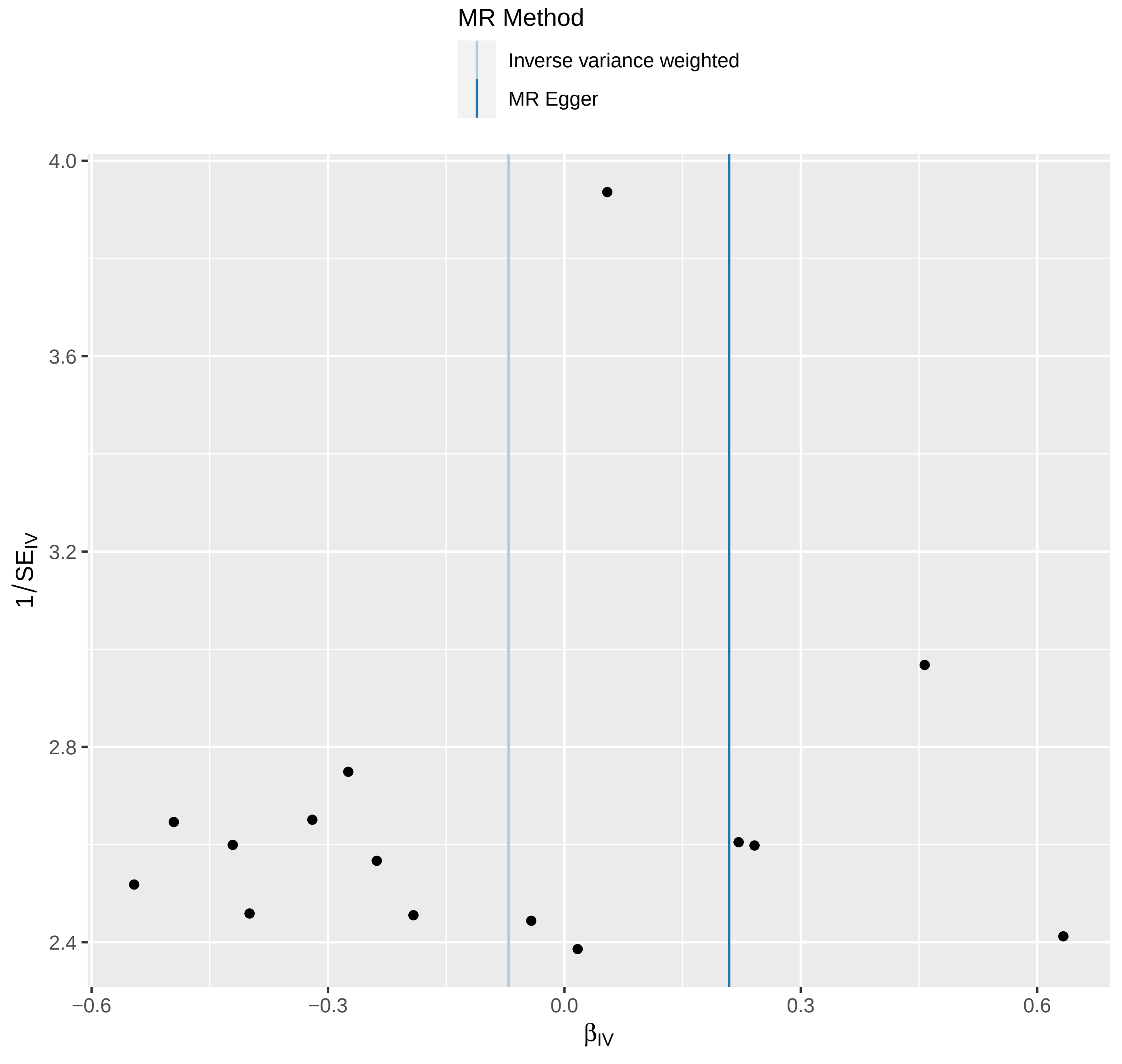

Batch 2−3309 : Gut microbiota abundance (genus Catenibacterium id.2153) on Kelch−like protein 13 with a threshold of 1e−05

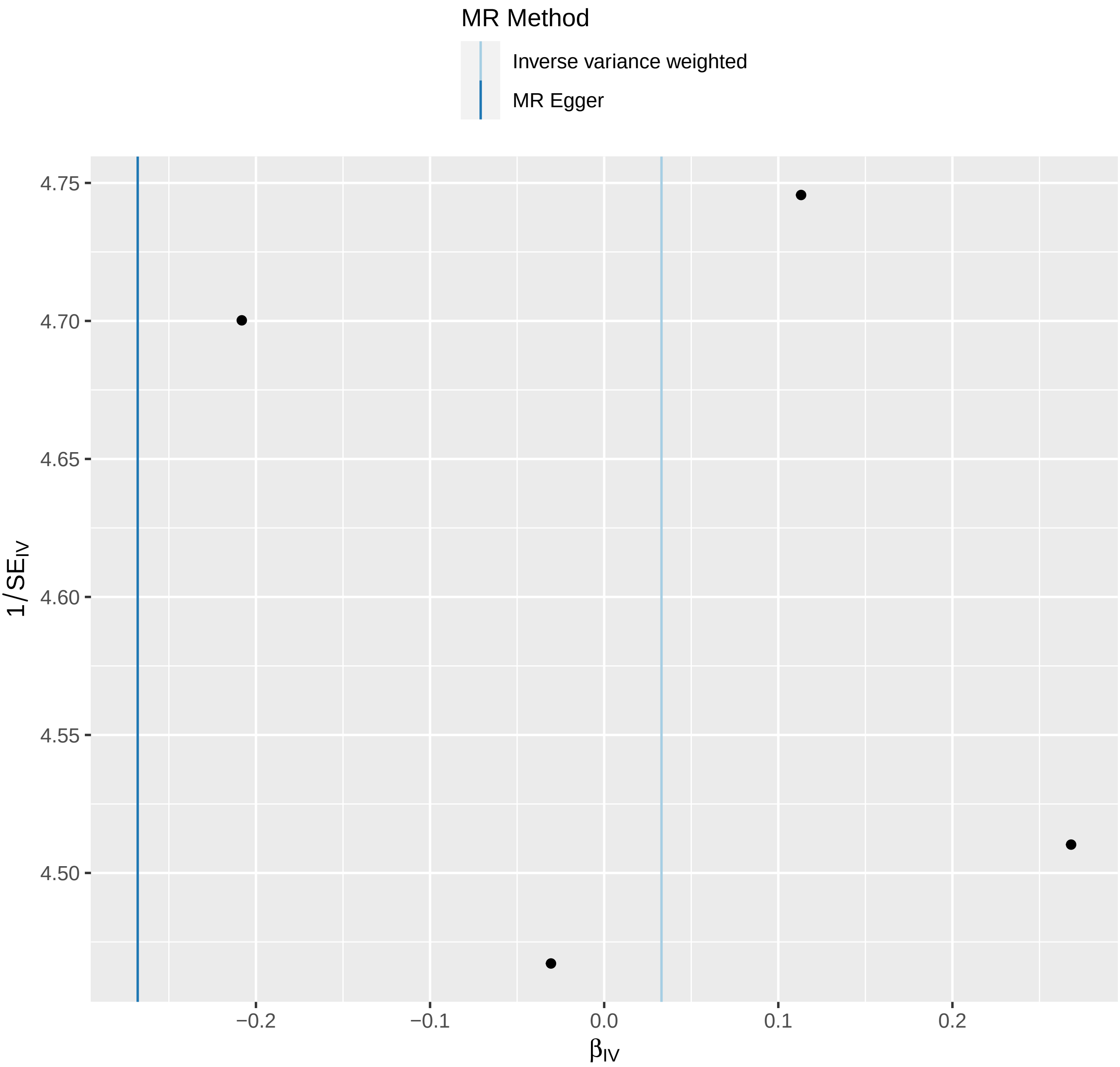

Batch 2−3310 : Gut microbiota abundance (genus Parasutterella id.2892) on Kelch−like protein 13
with a threshold of 1e−05

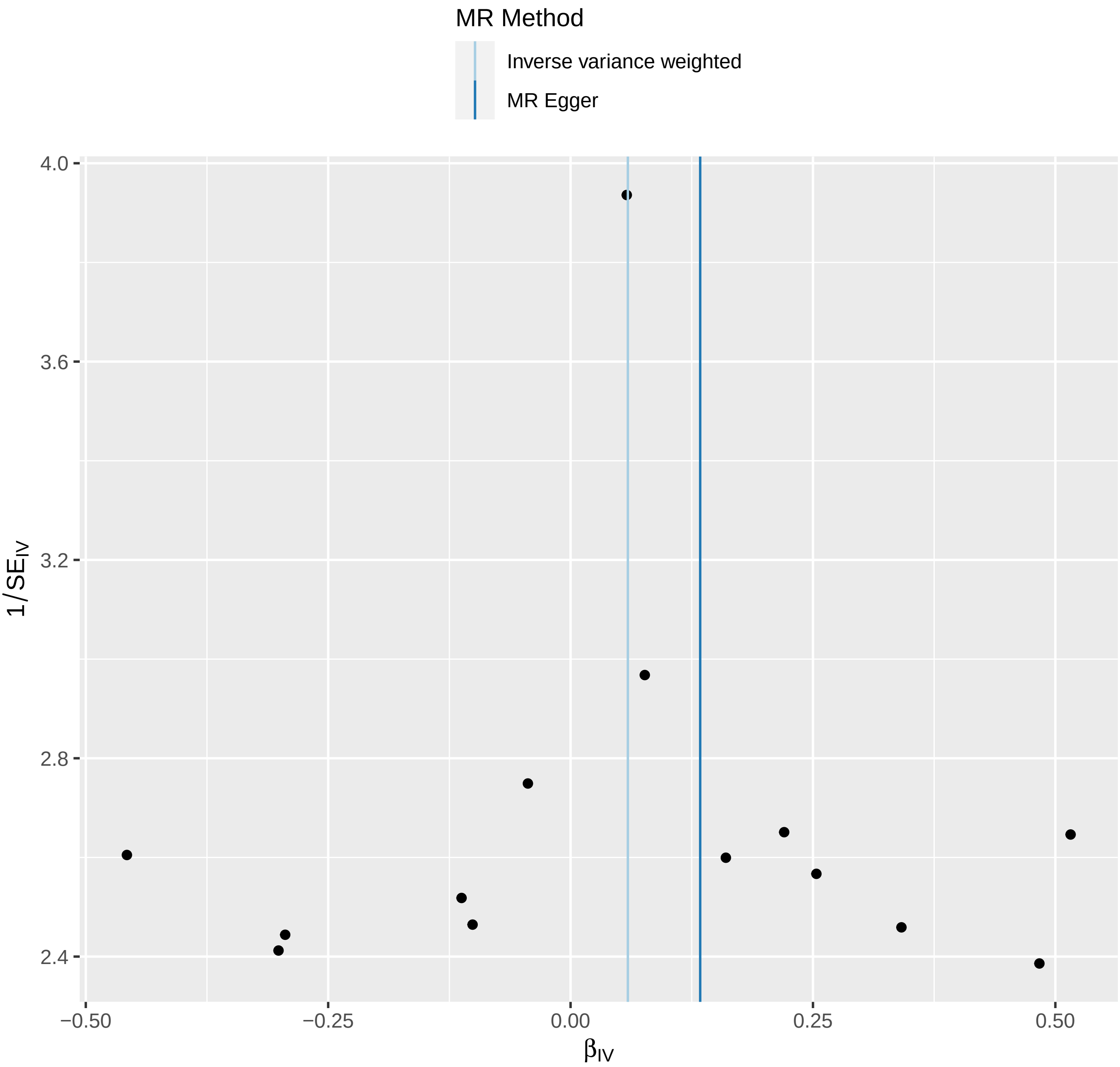

Batch 2−3311 : Gut microbiota abundance (genus Catenibacterium id.2153) on Kallikrein−11
with a threshold of 1e−05

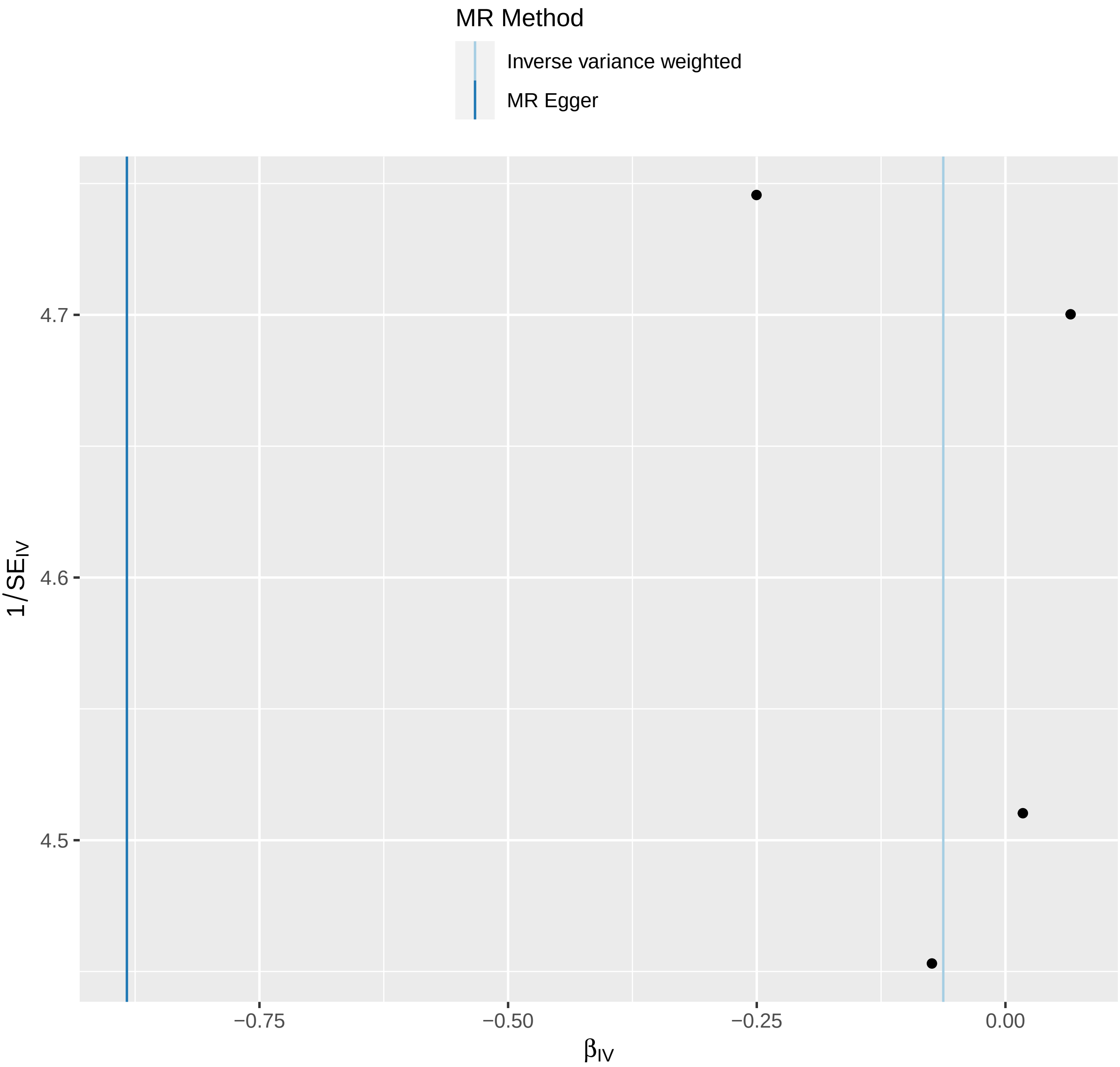

Batch 2−3312 : Gut microbiota abundance (genus Parasutterella id.2892) on Kallikrein−11
with a threshold of 1e−05

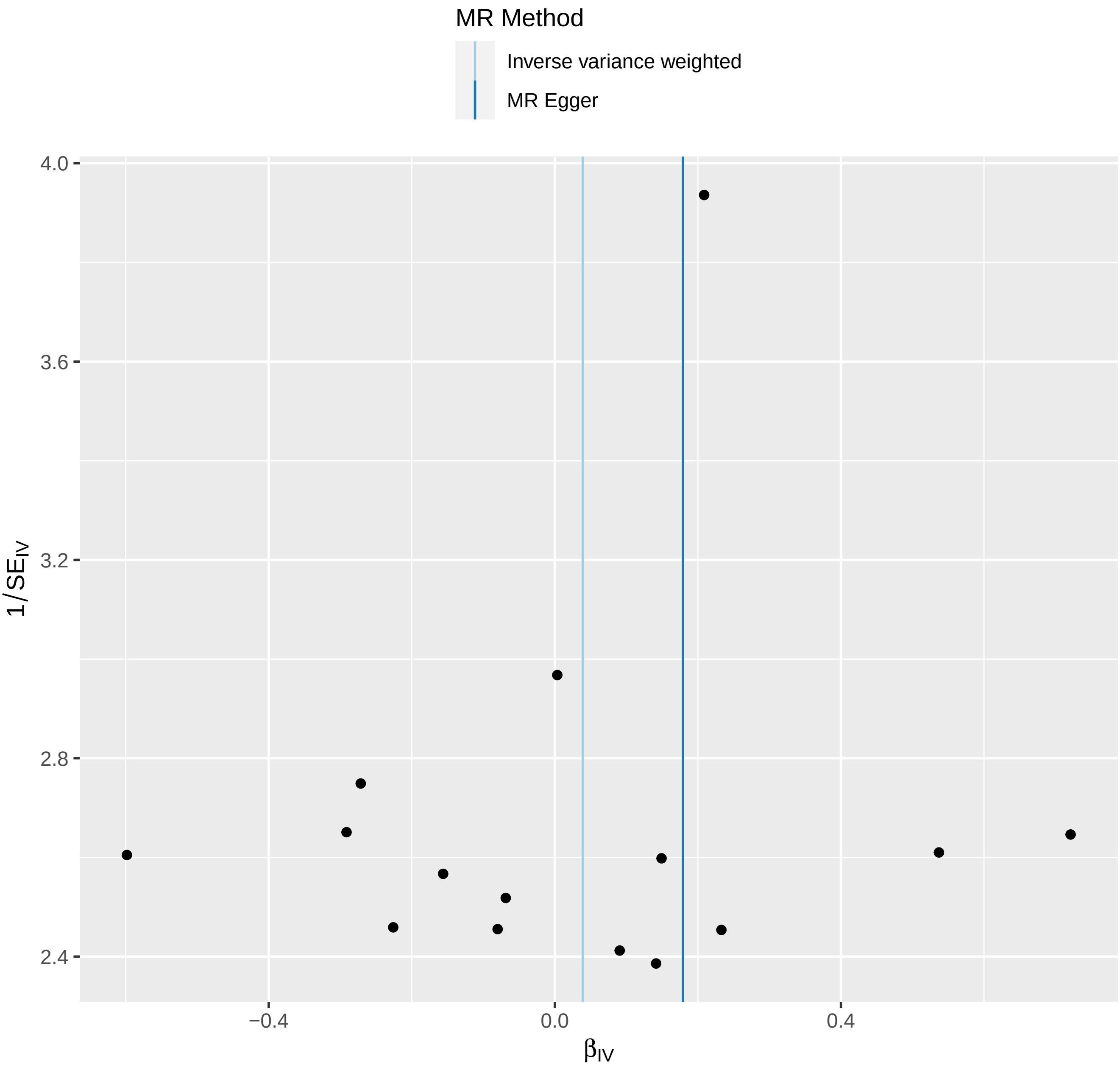

Batch 2−3313 : Gut microbiota abundance (genus Catenibacterium id.2153) on Kallikrein−12
with a threshold of 1e−05

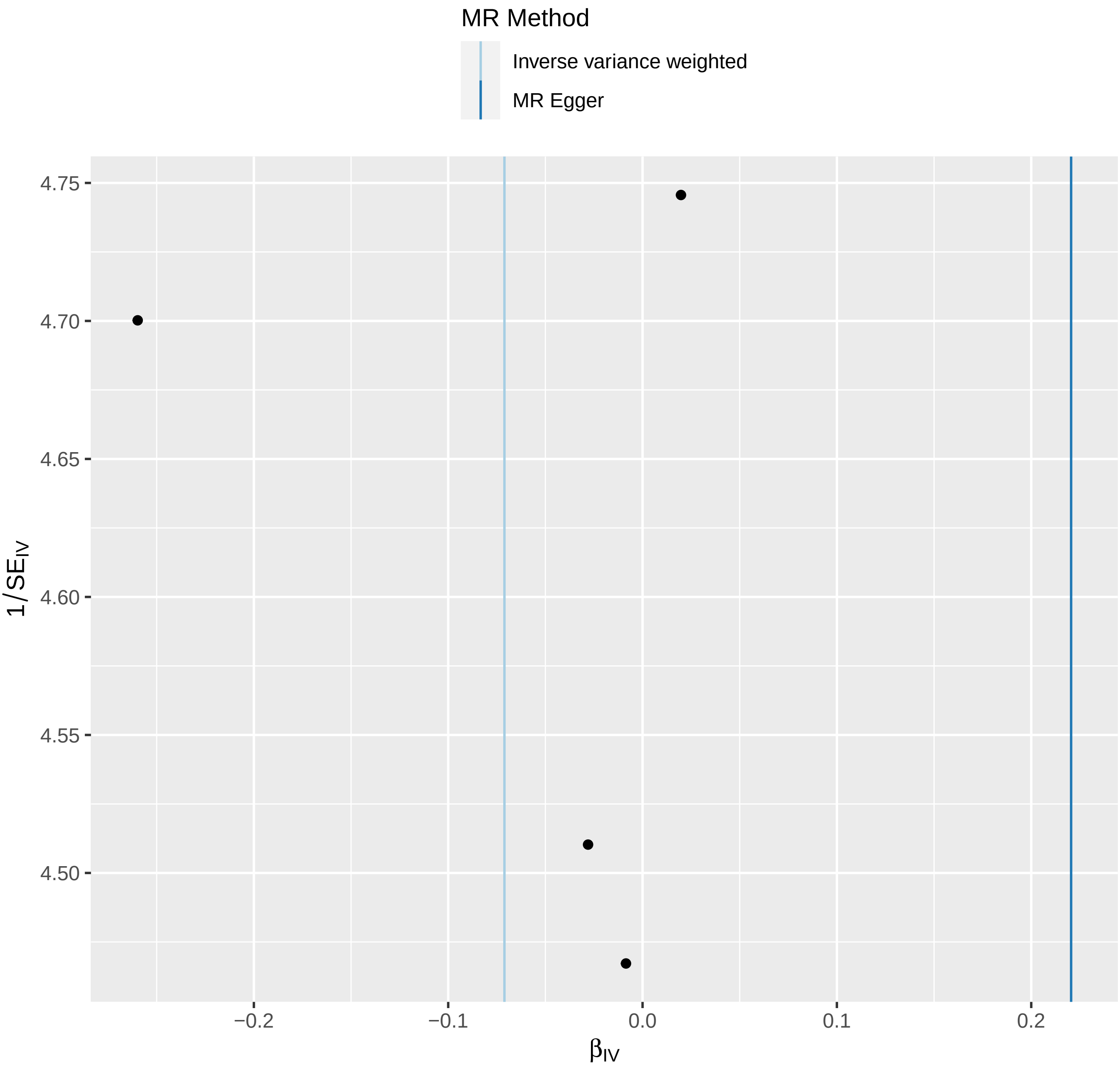

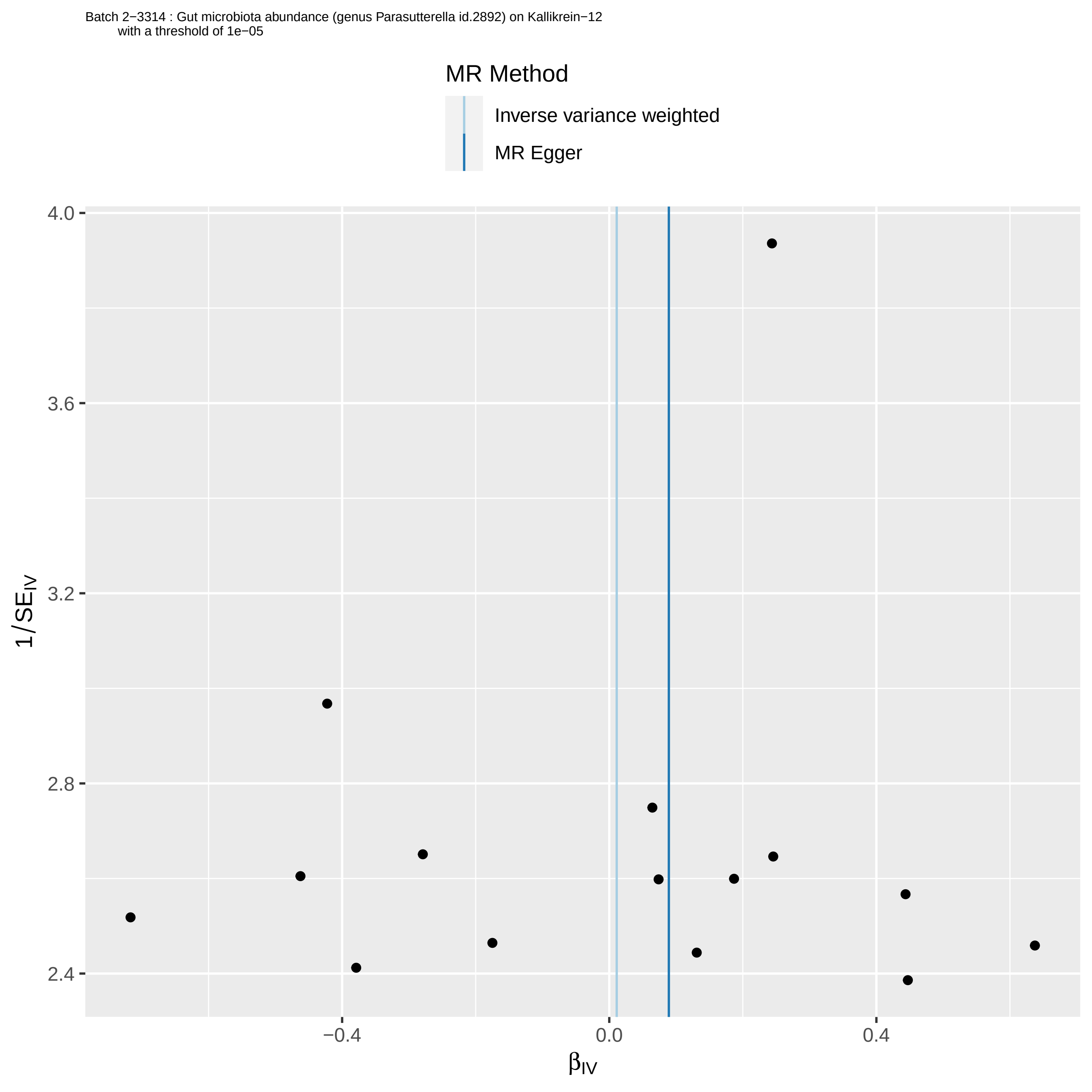

Batch 2−3314 : Gut microbiota abundance (genus Parasutterella id.2892) on Kallikrein−12
with a threshold of 1e−05
MR Method
Inverse variance weighted
MR Egger
4.0
3.6
3.2
2.8
2.4
1/SE_IV
−0.4
0.0
0.4
β_IV

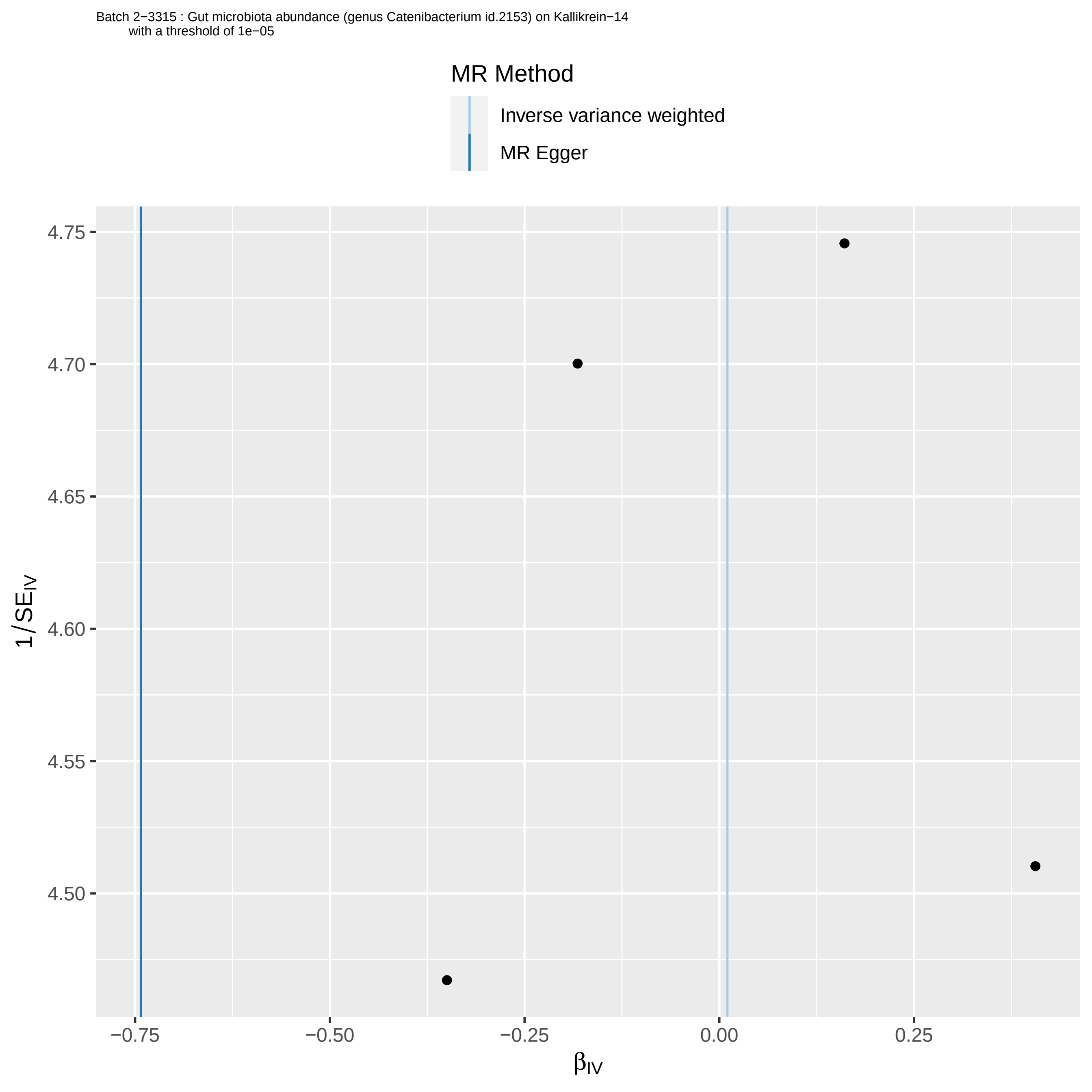

Batch 2−3315 : Gut microbiota abundance (genus Catenibacterium id.2153) on Kallikrein−14
with a threshold of 1e−05
MR Method
Inverse variance weighted
MR Egger
4.75
4.70
4.65
4.60
4.55
4.50
1/SE_IV
−0.75
−0.50
−0.25
0.00
0.25
β_IV

Batch 2−3316 : Gut microbiota abundance (genus Parasutterella id.2892) on Kallikrein−14
with a threshold of 1e−05

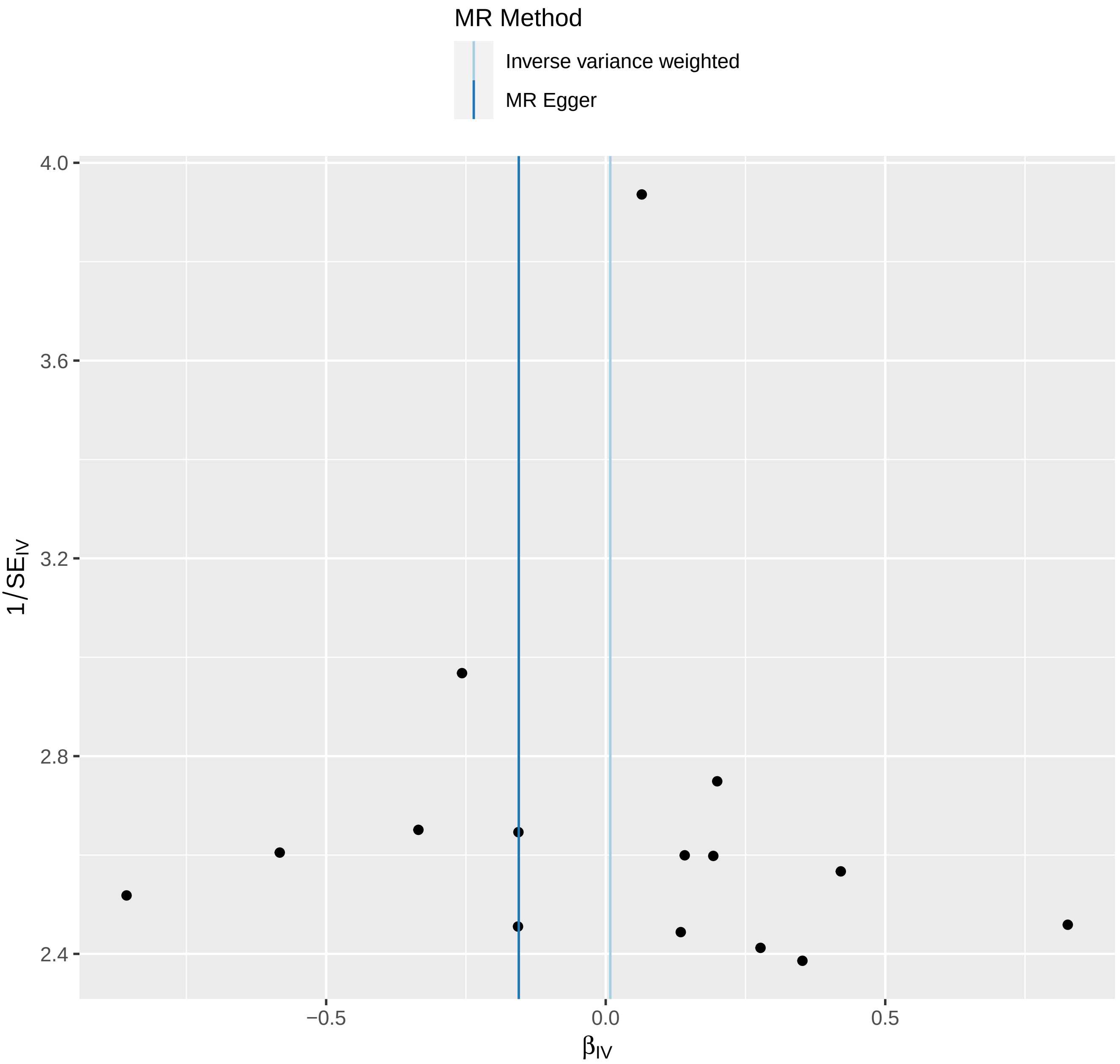

Batch 2−3317 : Gut microbiota abundance (genus Catenibacterium id.2153) on Kallikrein−14
with a threshold of 1e−05

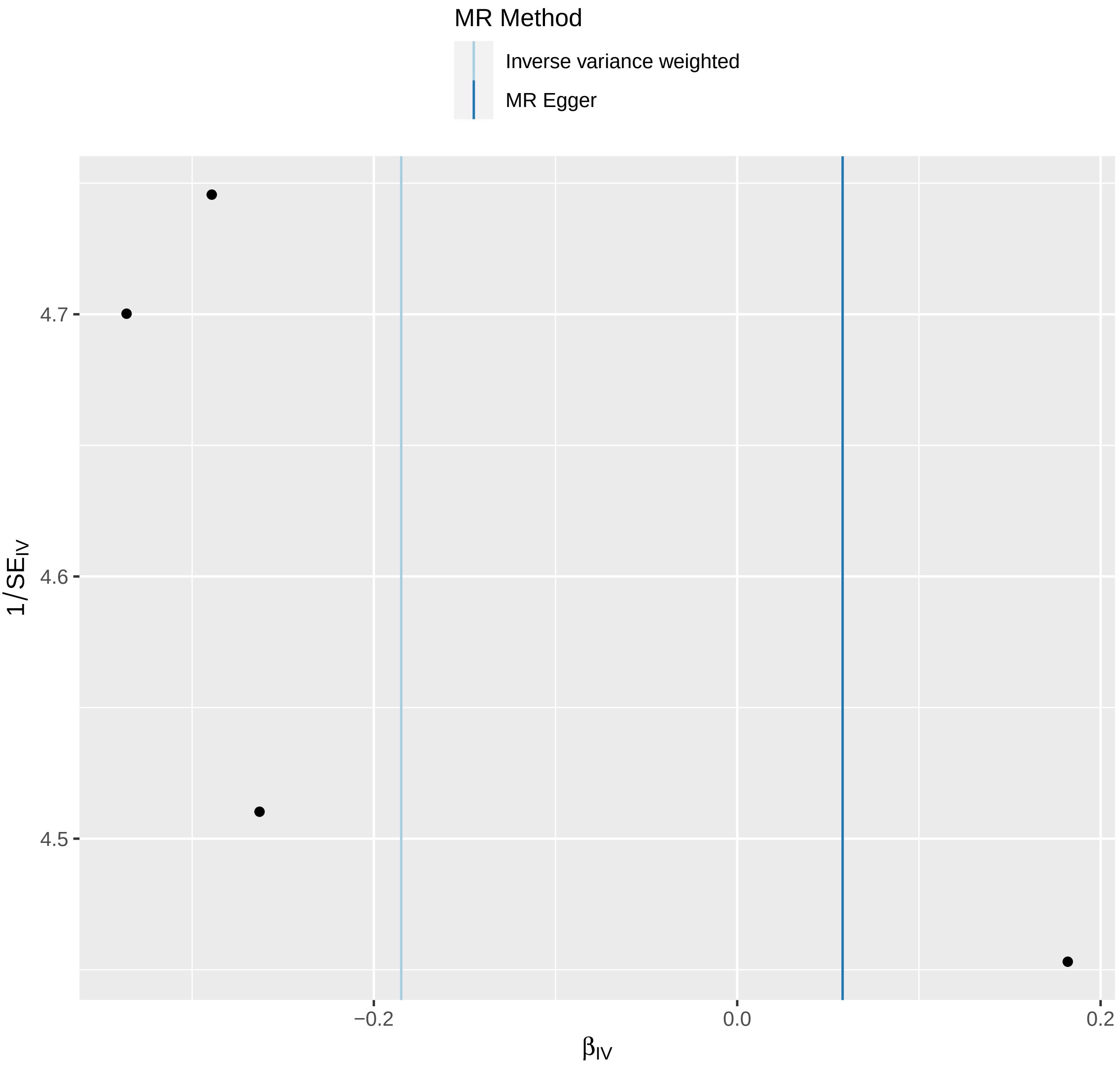

Batch 2−3318 : Gut microbiota abundance (genus Parasutterella id.2892) on Kallikrein−14
with a threshold of 1e−05

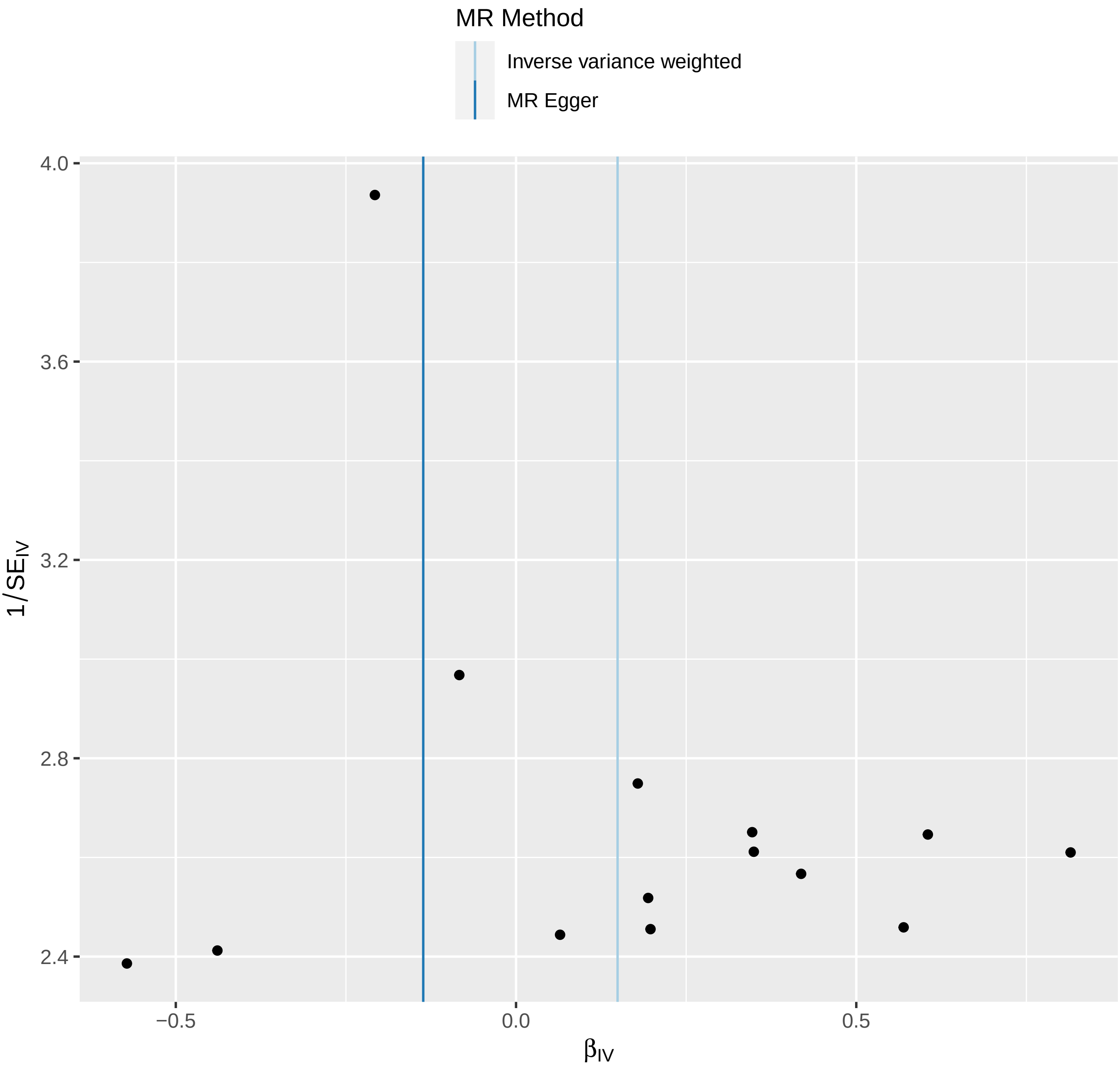

Batch 2−3319 : Gut microbiota abundance (genus Catenibacterium id.2153) on Kallikrein−15
with a threshold of 1e−05

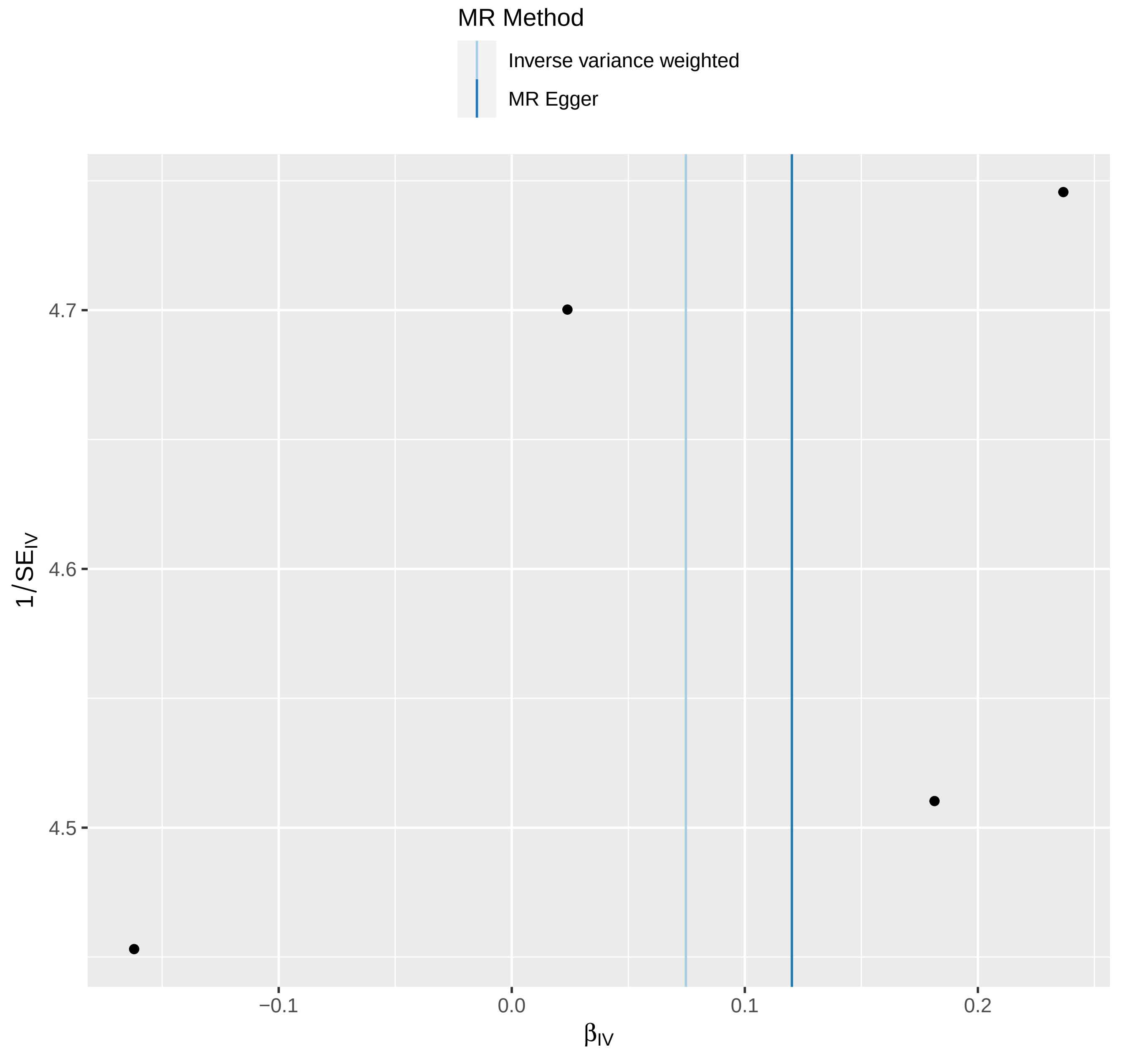

Batch 2−3320 : Gut microbiota abundance (genus Parasutterella id.2892) on Kallikrein−15
with a threshold of 1e−05

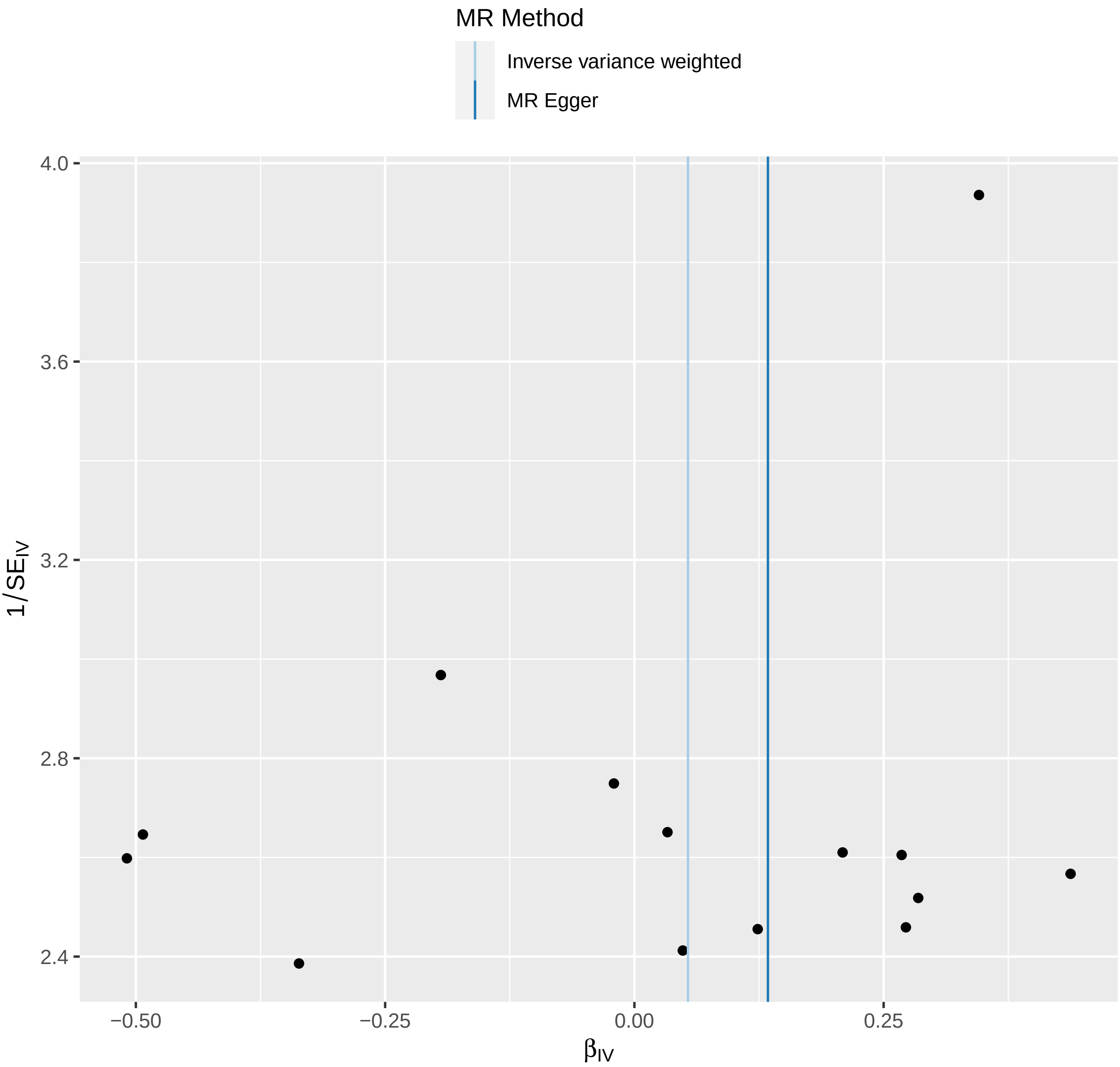

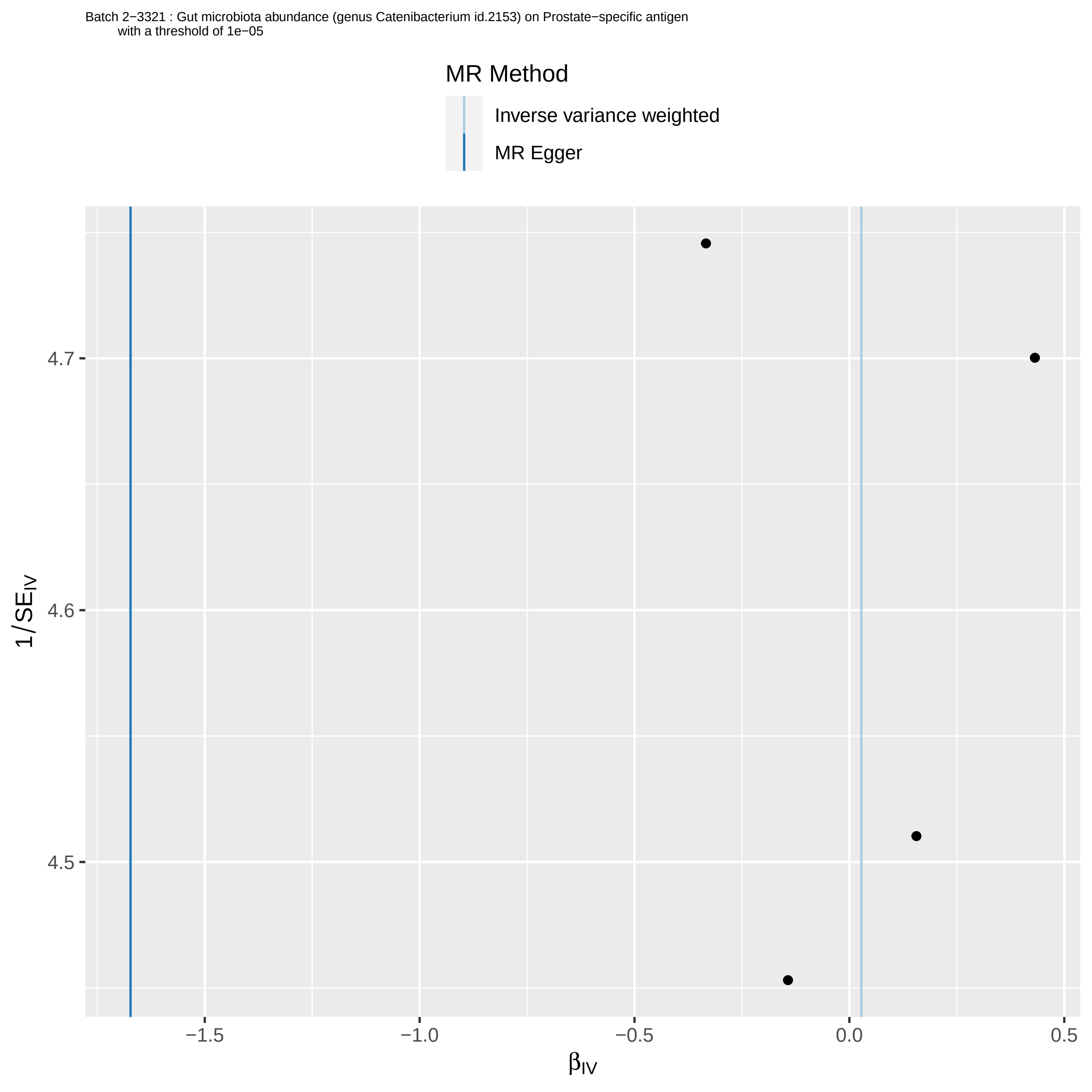
Batch 2−3321 : Gut microbiota abundance (genus Catenibacterium id.2153) on Prostate−specific antigen
with a threshold of 1e−05
MR Method
Inverse variance weighted
MR Egger
4.7
4.6
4.5
1/SE_IV
−1.5
−1.0
−0.5
0.0
0.5
β_IV

Batch 2−3322 : Gut microbiota abundance (genus Parasutterella id.2892) on Prostate−specific antigen
with a threshold of 1e−05

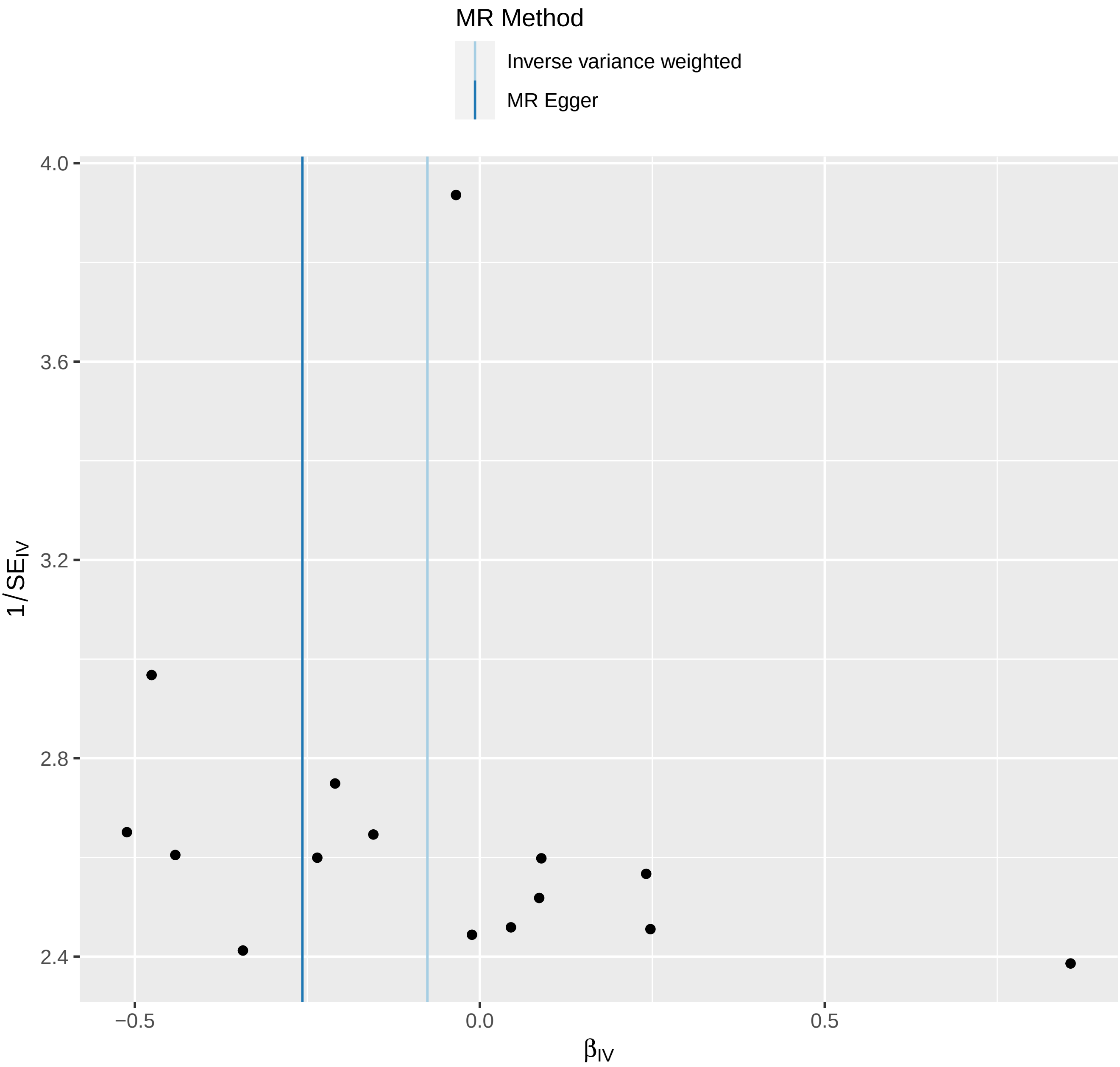

Batch 2−3323 : Gut microbiota abundance (genus Catenibacterium id.2153) on Kallikrein−4
with a threshold of 1e−05

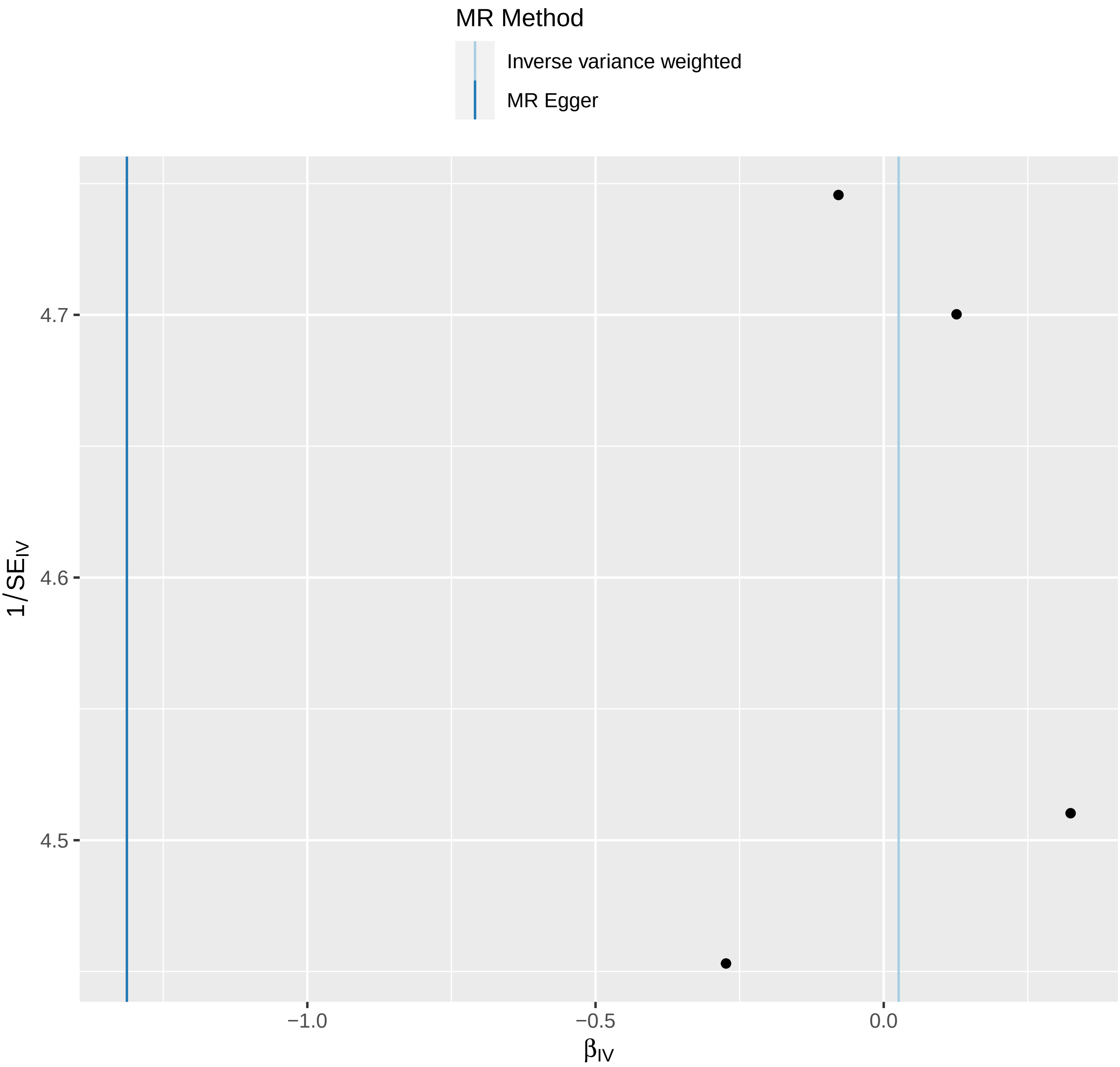

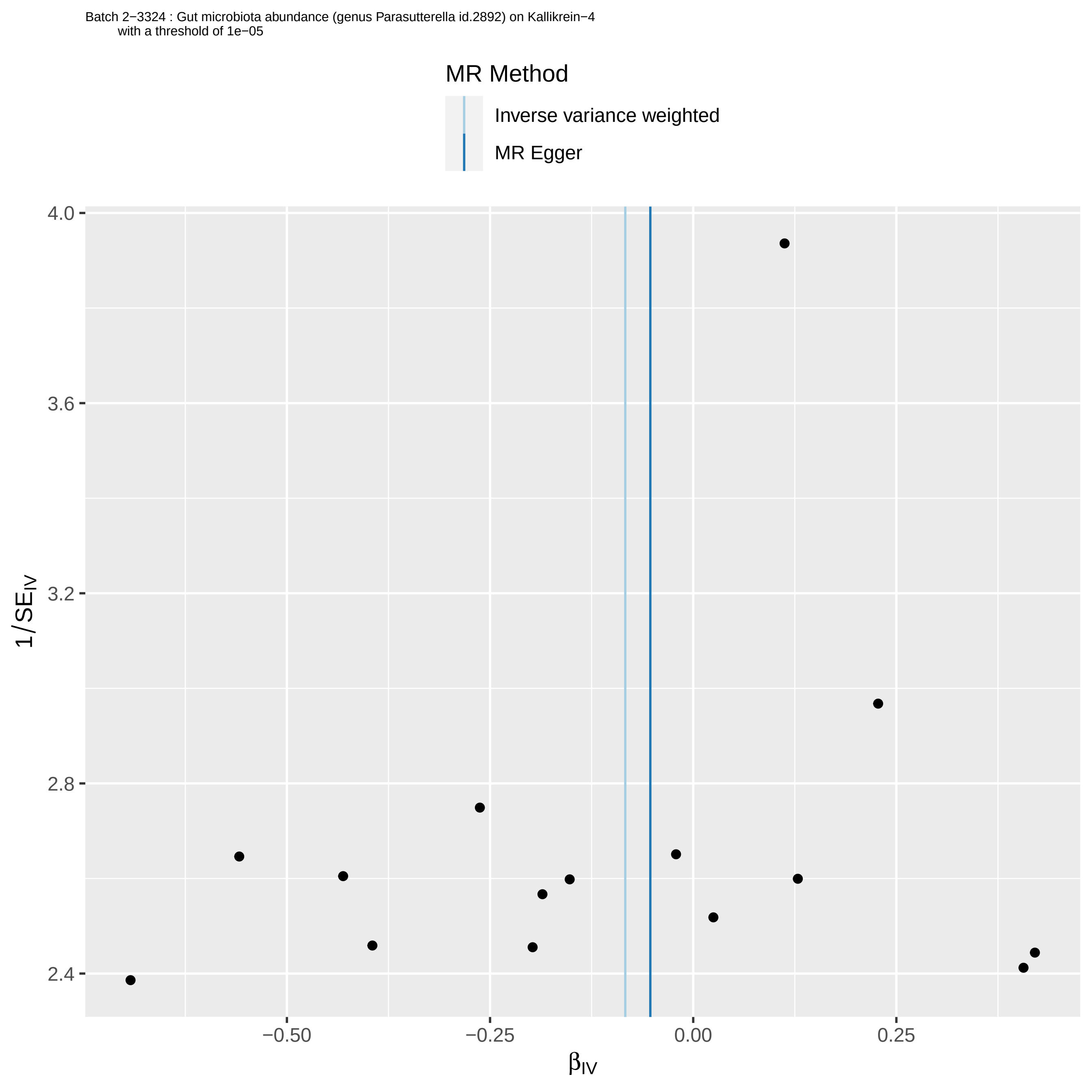

Batch 2−3324 : Gut microbiota abundance (genus Parasutterella id.2892) on Kallikrein−4
with a threshold of 1e−05
MR Method
Inverse variance weighted
MR Egger
4.0
3.6
3.2
2.8
2.4
1/SE_IV
−0.50
−0.25
0.00
0.25
β_IV

Batch 2−3325 : Gut microbiota abundance (genus Catenibacterium id.2153) on Kallikrein−5
with a threshold of 1e−05

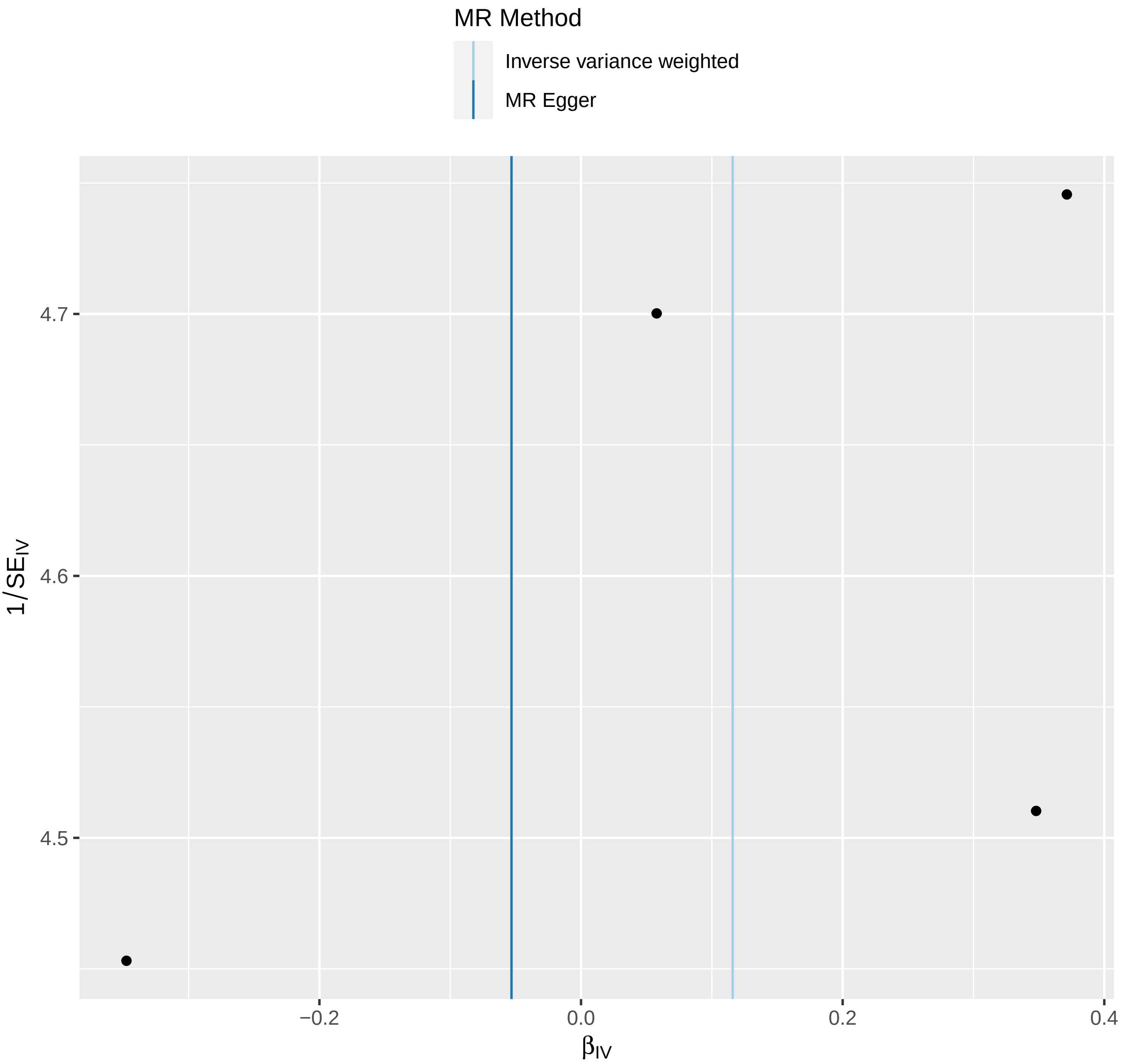

Batch 2−3326 : Gut microbiota abundance (genus Parasutterella id.2892) on Kallikrein−5
with a threshold of 1e−05

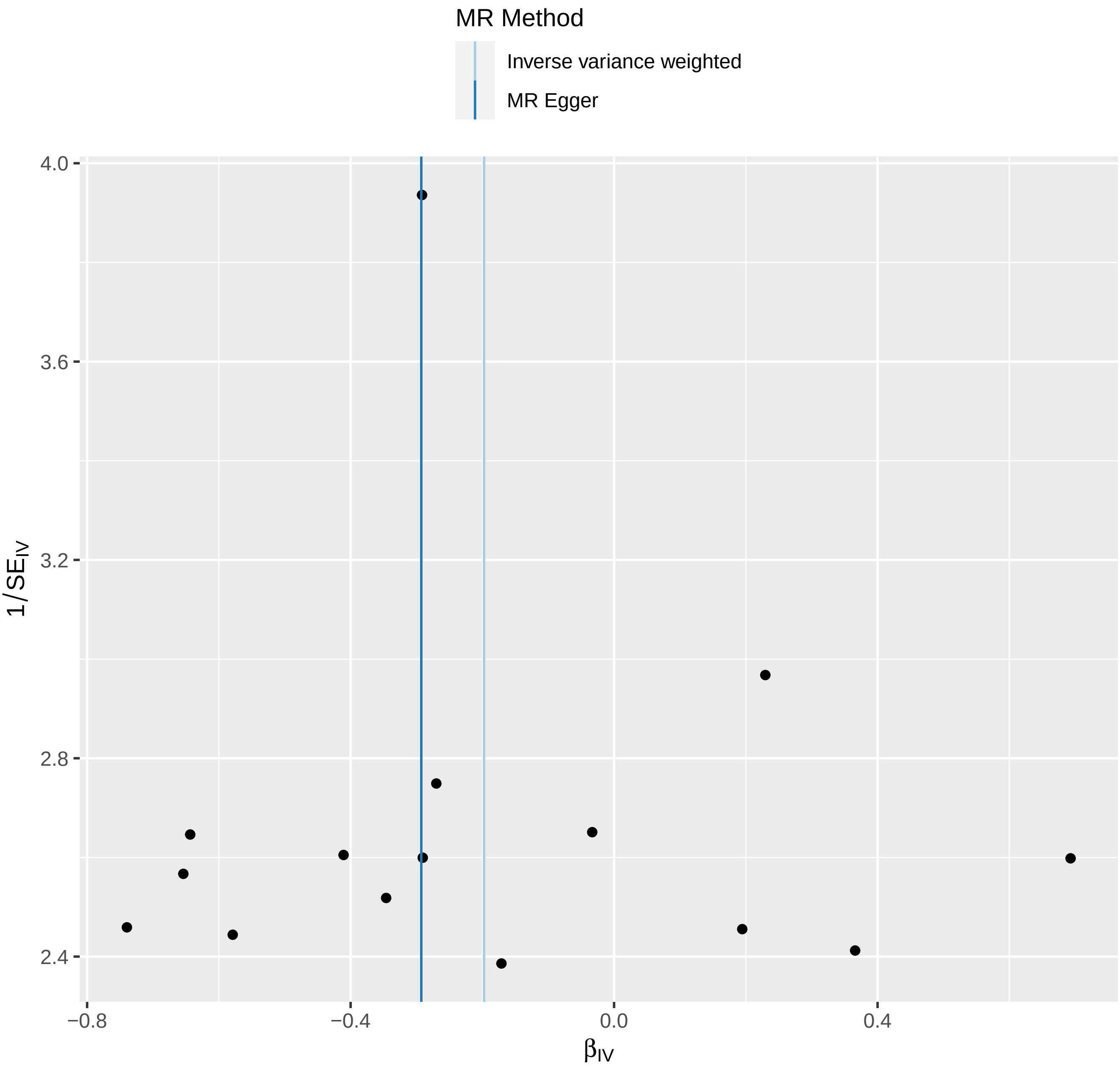

Batch 2−3327 : Gut microbiota abundance (genus Catenibacterium id.2153) on Kallikrein−5
with a threshold of 1e−05

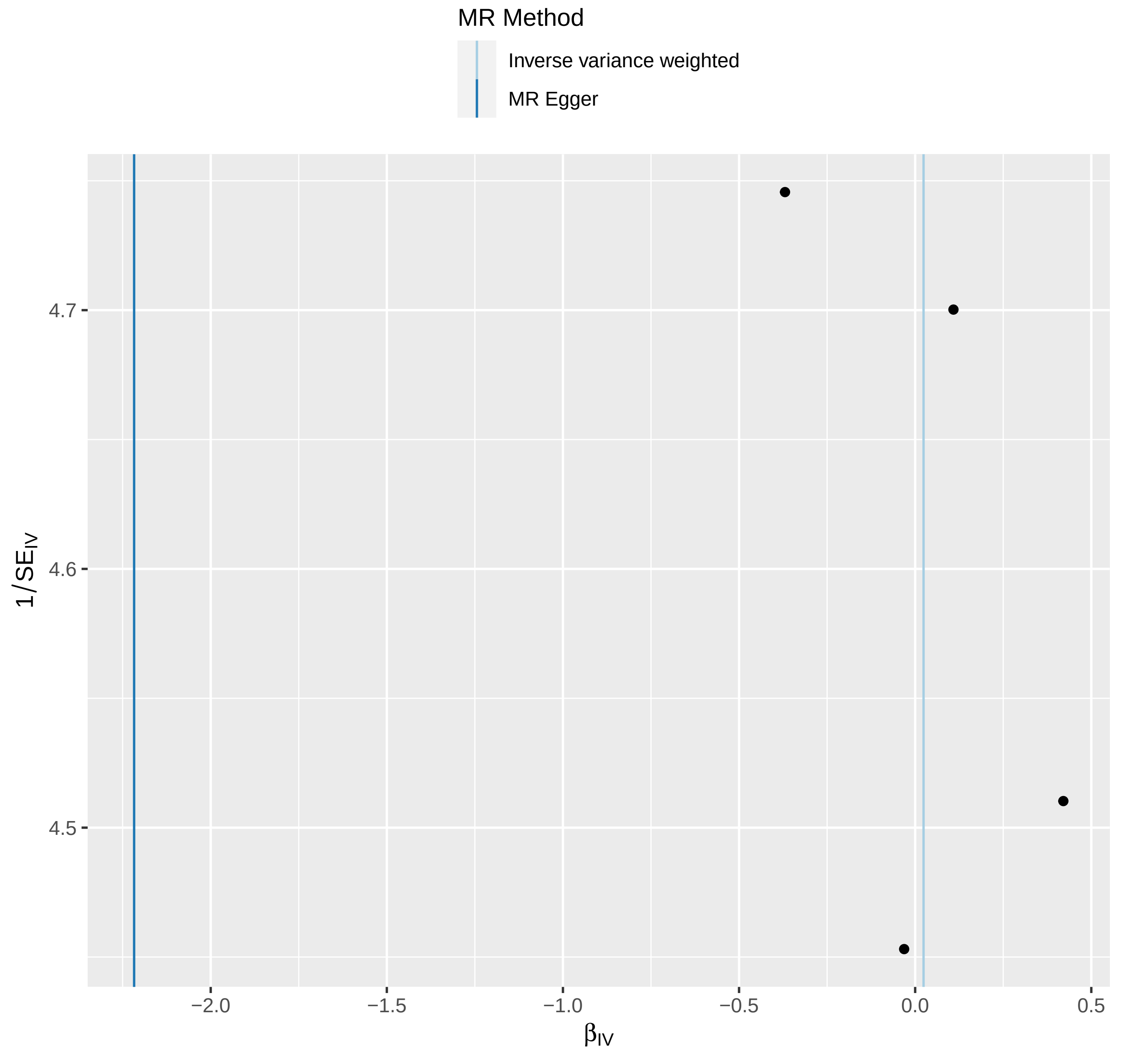

Batch 2−3328 : Gut microbiota abundance (genus Parasutterella id.2892) on Kallikrein−5
with a threshold of 1e−05

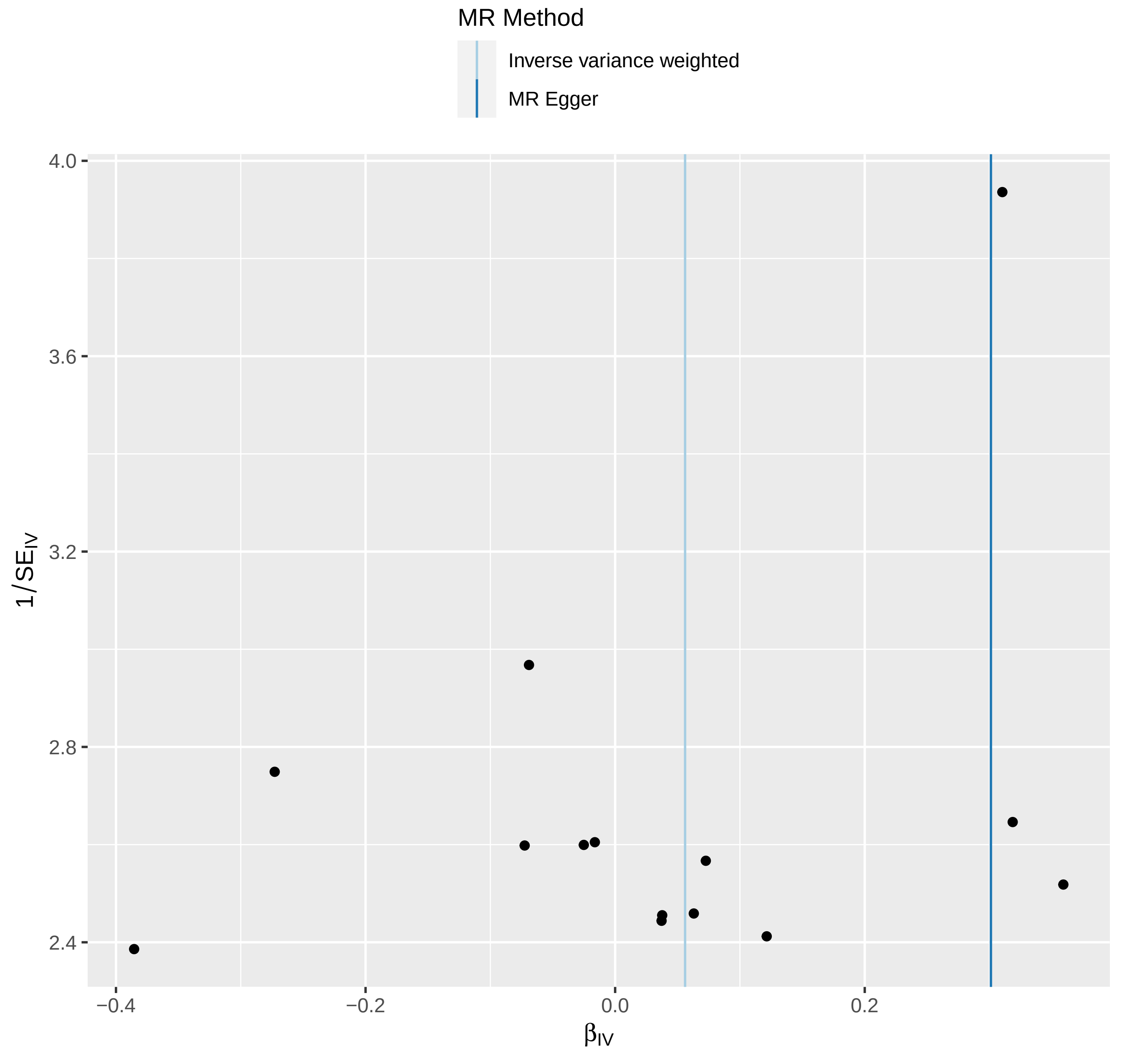

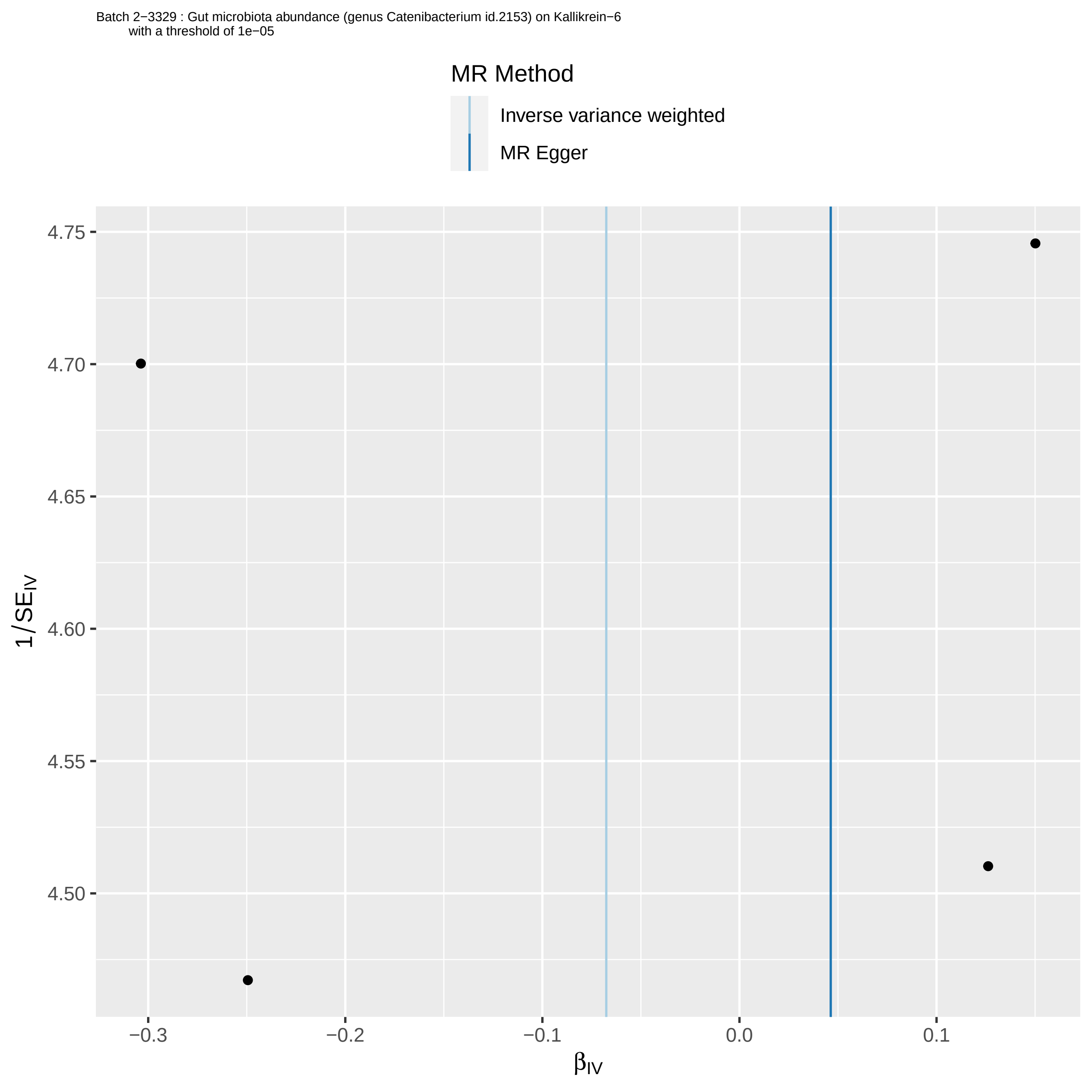
Batch 2−3329 : Gut microbiota abundance (genus Catenibacterium id.2153) on Kallikrein−6
with a threshold of 1e−05
MR Method
Inverse variance weighted
MR Egger
4.75
4.70
4.65
4.60
4.55
4.50
1/SE_IV
−0.3
−0.2
−0.1
0.0
0.1
β_IV

Batch 2−3330 : Gut microbiota abundance (genus Parasutterella id.2892) on Kallikrein−6
with a threshold of 1e−05

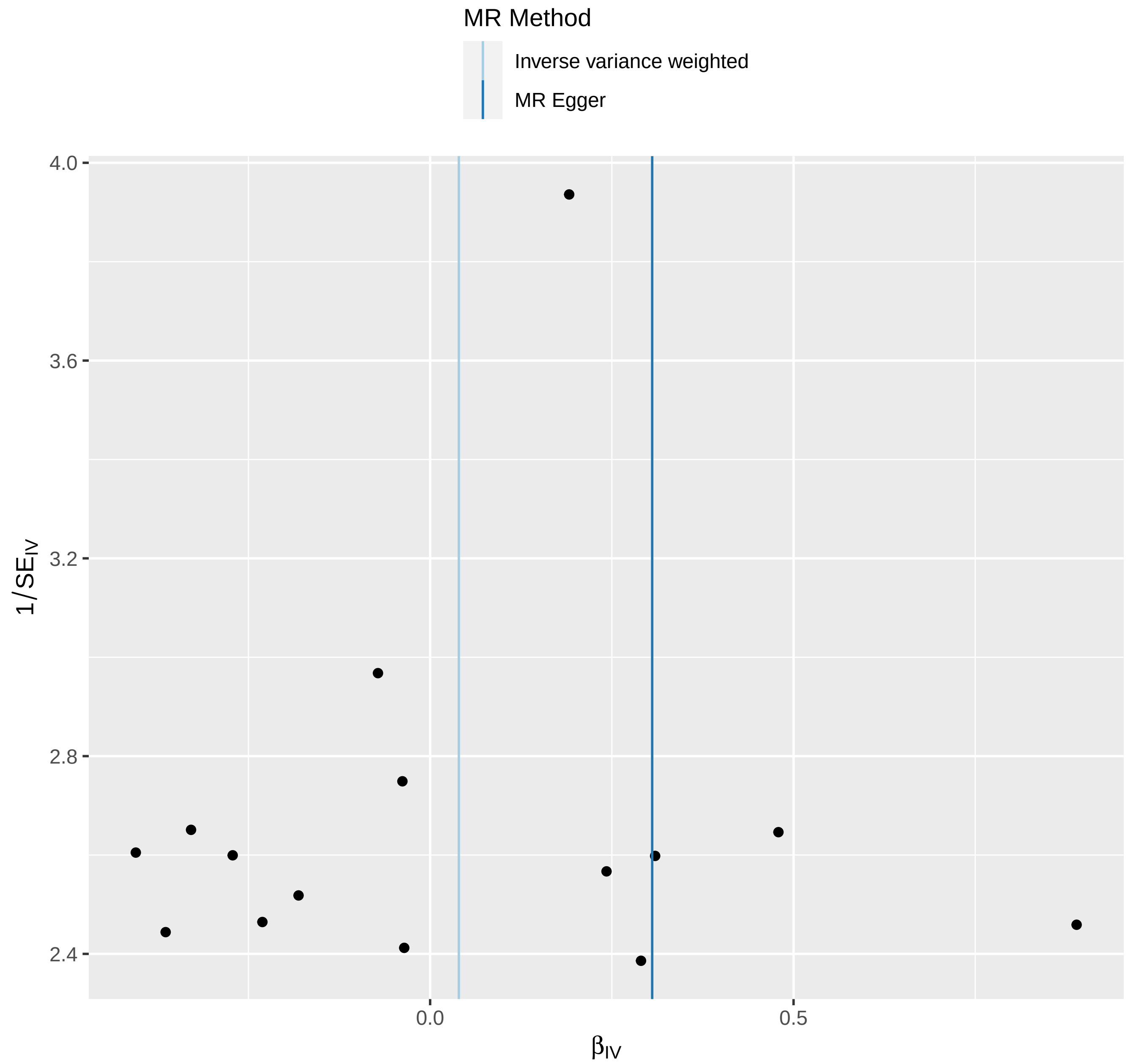

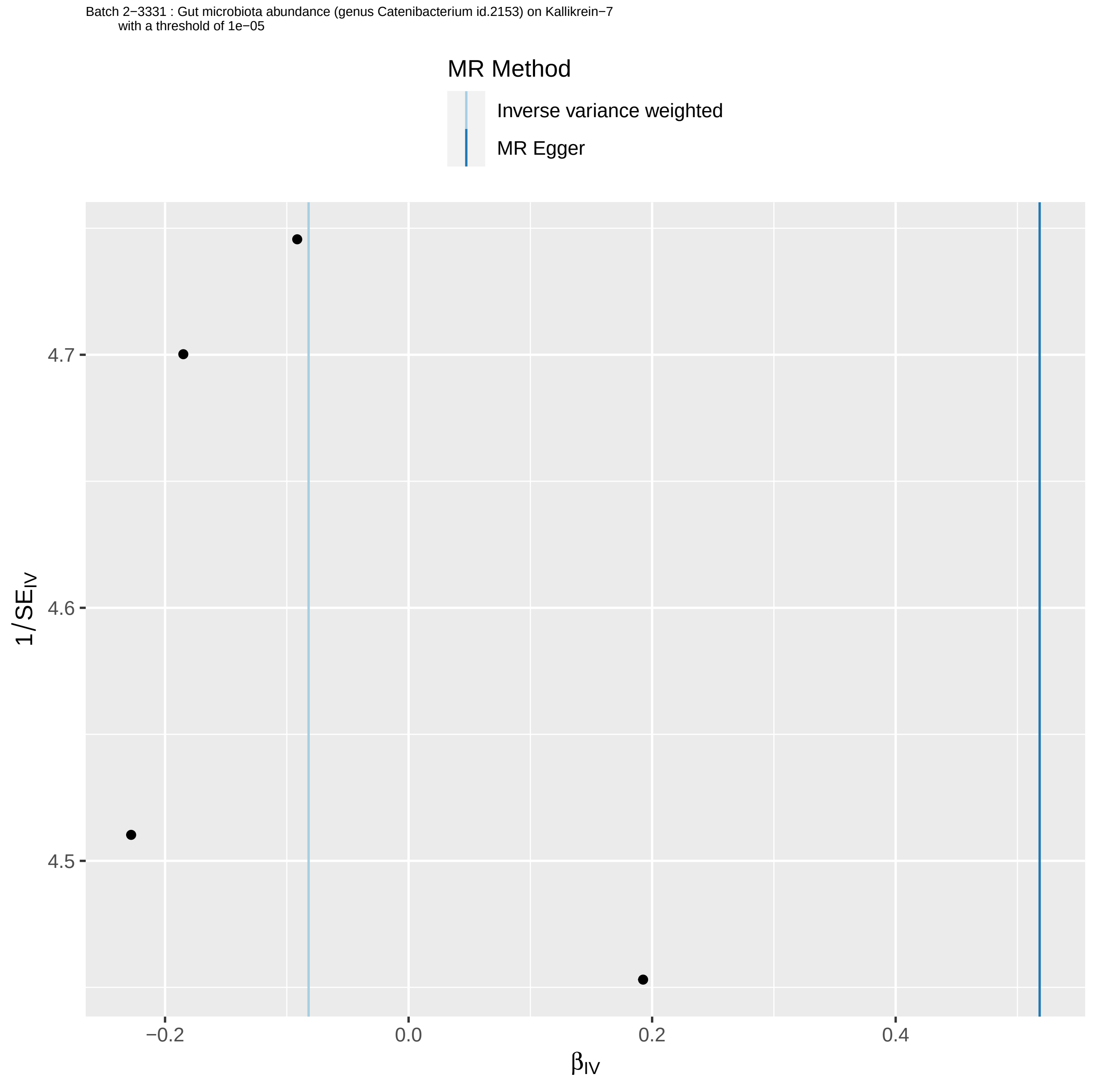
Batch 2−3331 : Gut microbiota abundance (genus Catenibacterium id.2153) on Kallikrein−7
with a threshold of 1e−05
MR Method
Inverse variance weighted
MR Egger
4.7
4.6
4.5
1/SE_IV
−0.2
0.0
0.2
0.4
β_IV

Batch 2−3332 : Gut microbiota abundance (genus Parasutterella id.2892) on Kallikrein−7
with a threshold of 1e−05

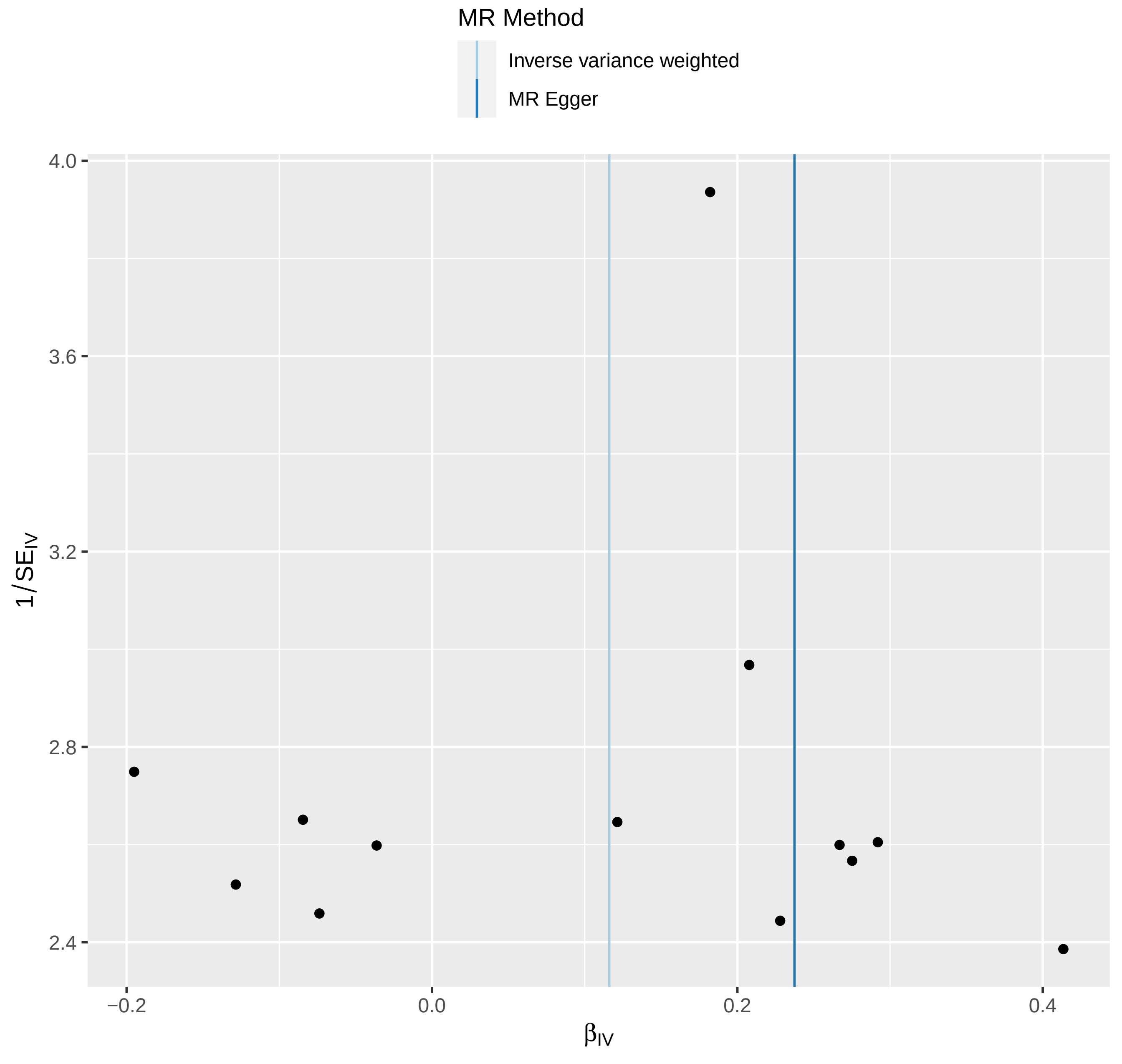

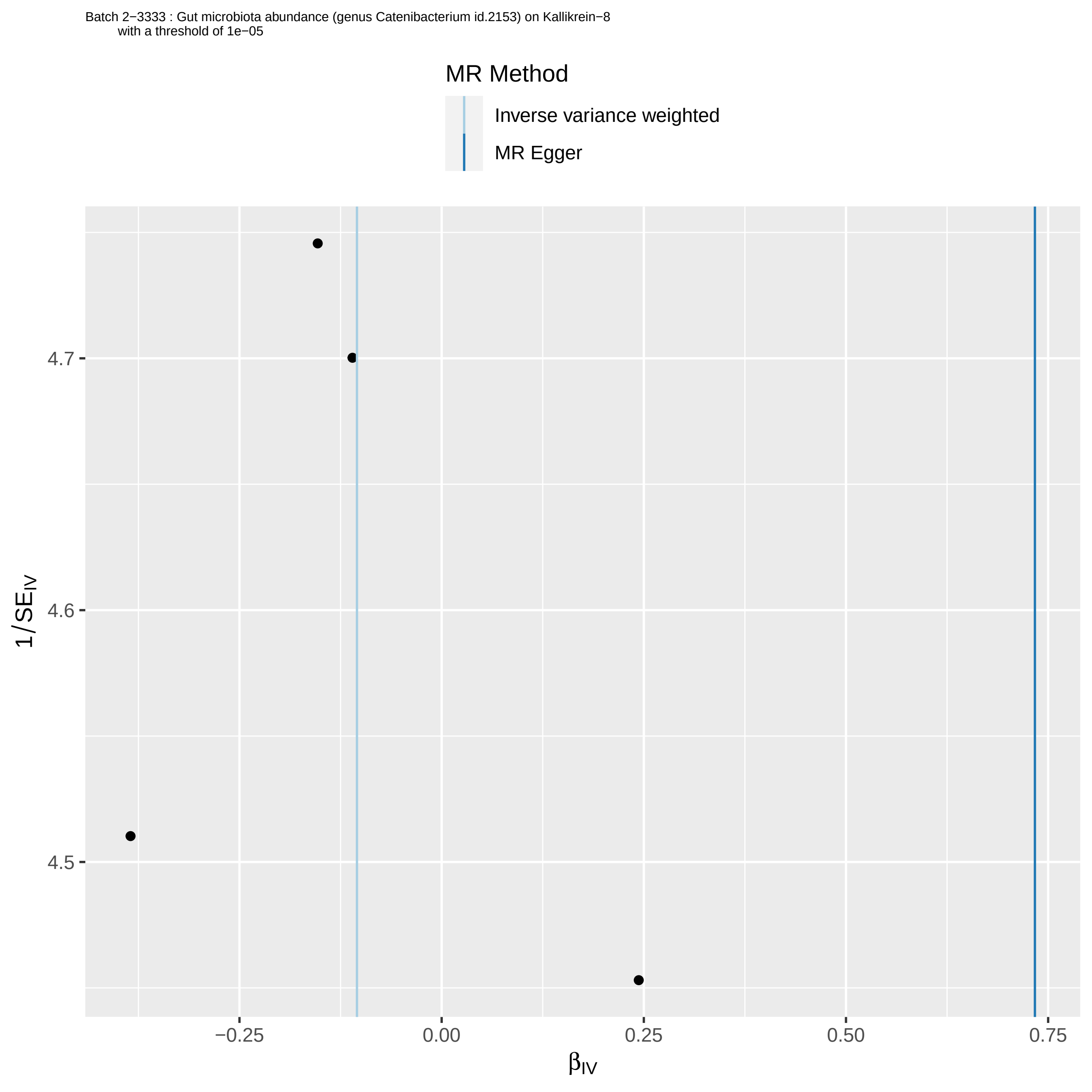
Batch 2−3333 : Gut microbiota abundance (genus Catenibacterium id.2153) on Kallikrein−8
with a threshold of 1e−05
MR Method
Inverse variance weighted
MR Egger
4.7
4.6
4.5
1/SE_IV
−0.25
0.00
0.25
0.50
0.75
β_IV

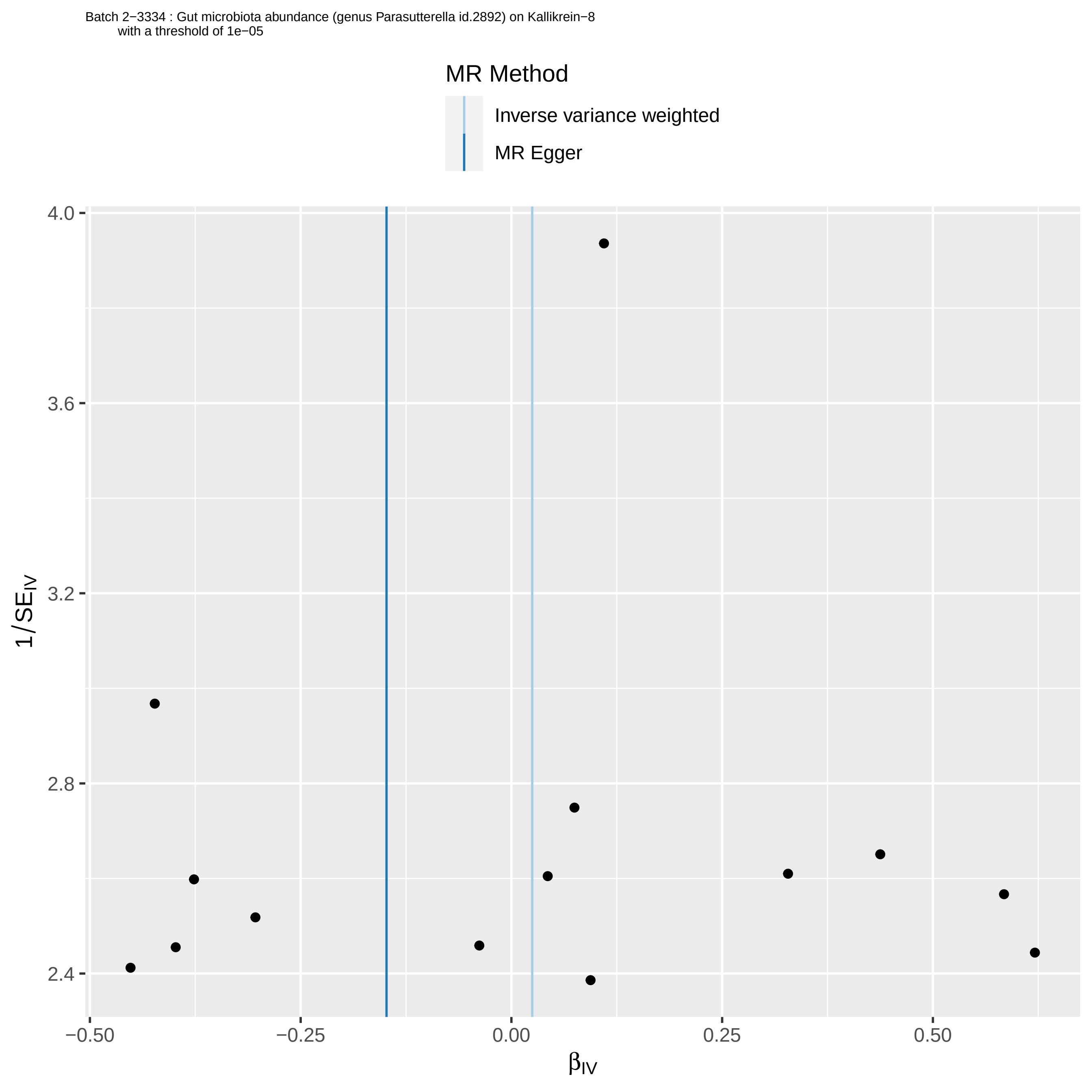

Batch 2−3334 : Gut microbiota abundance (genus Parasutterella id.2892) on Kallikrein−8
with a threshold of 1e−05
MR Method
Inverse variance weighted
MR Egger
4.0
3.6
3.2
2.8
2.4
1/SE_IV
−0.50
−0.25
0.00
0.25
0.50
β_IV

Batch 2−3335 : Gut microbiota abundance (genus Catenibacterium id.2153) on Kallikrein−8
with a threshold of 1e−05

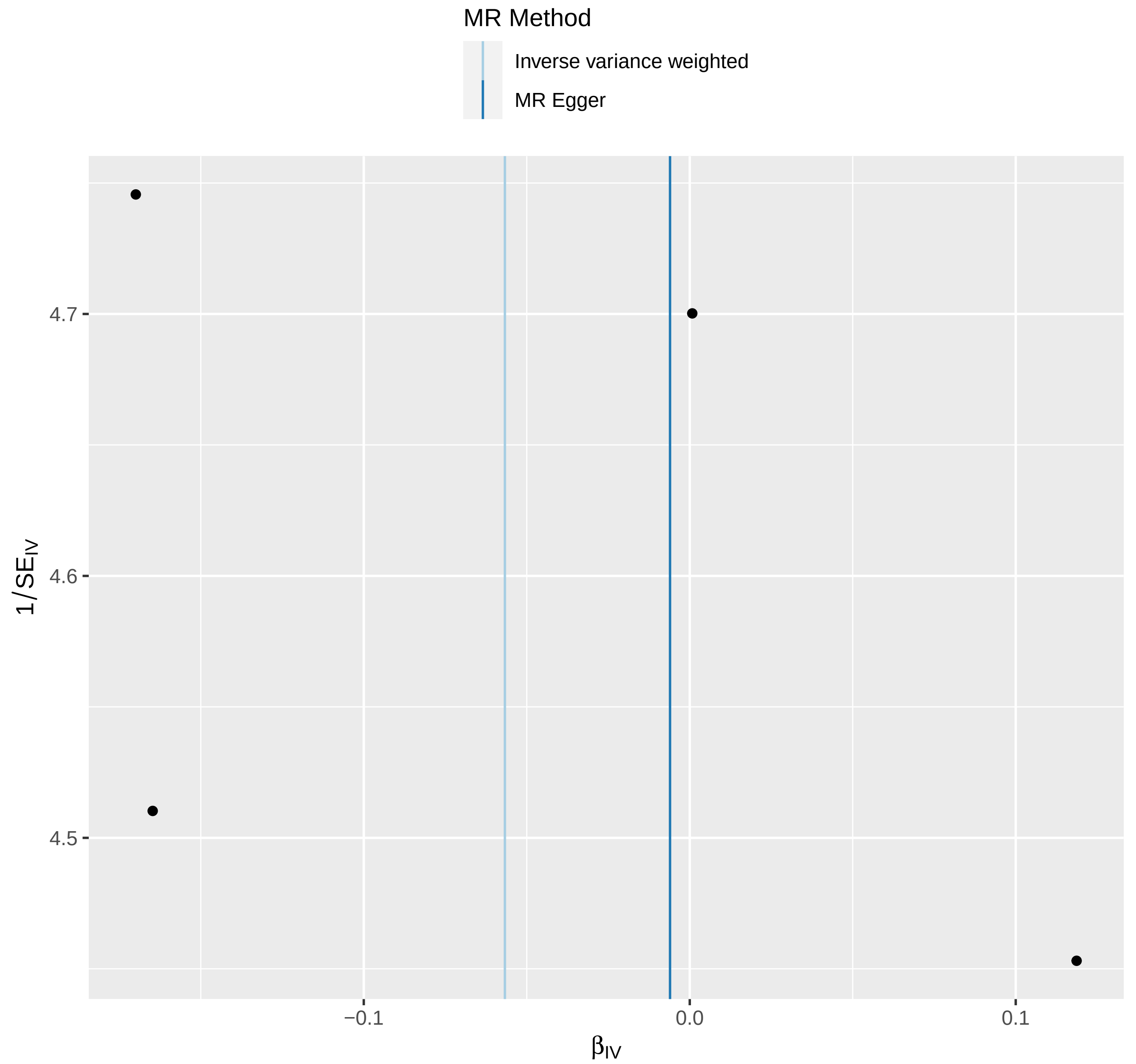

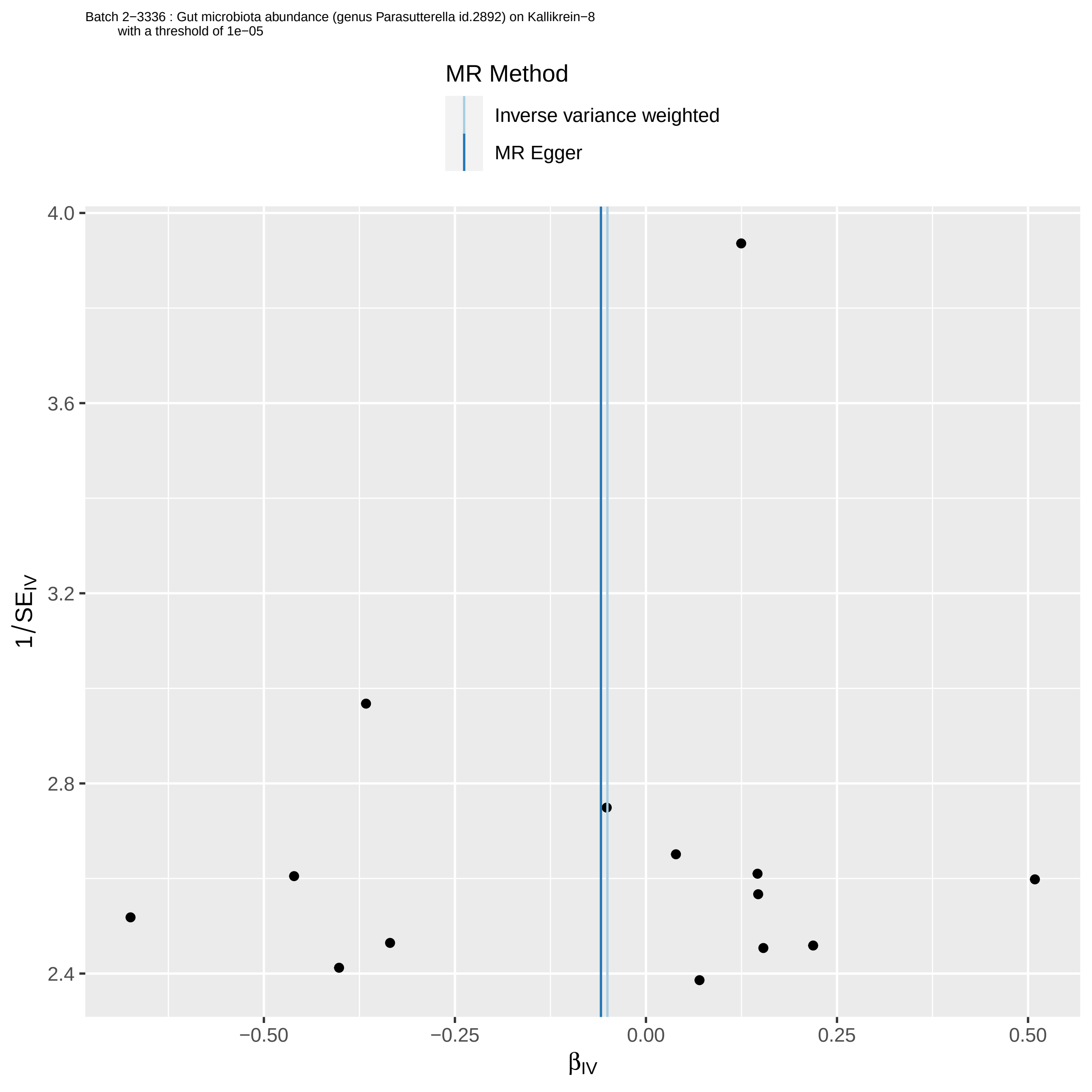

Batch 2−3336 : Gut microbiota abundance (genus Parasutterella id.2892) on Kallikrein−8
with a threshold of 1e−05
MR Method
Inverse variance weighted
MR Egger
4.0
3.6
3.2
2.8
2.4
1/SE_IV
−0.50
−0.25
0.00
0.25
0.50
β_IV

Batch 2−3337 : Gut microbiota abundance (genus Catenibacterium id.2153) on NKG2−A/NKG2−B type II integral membrane protein with a threshold of 1e−05

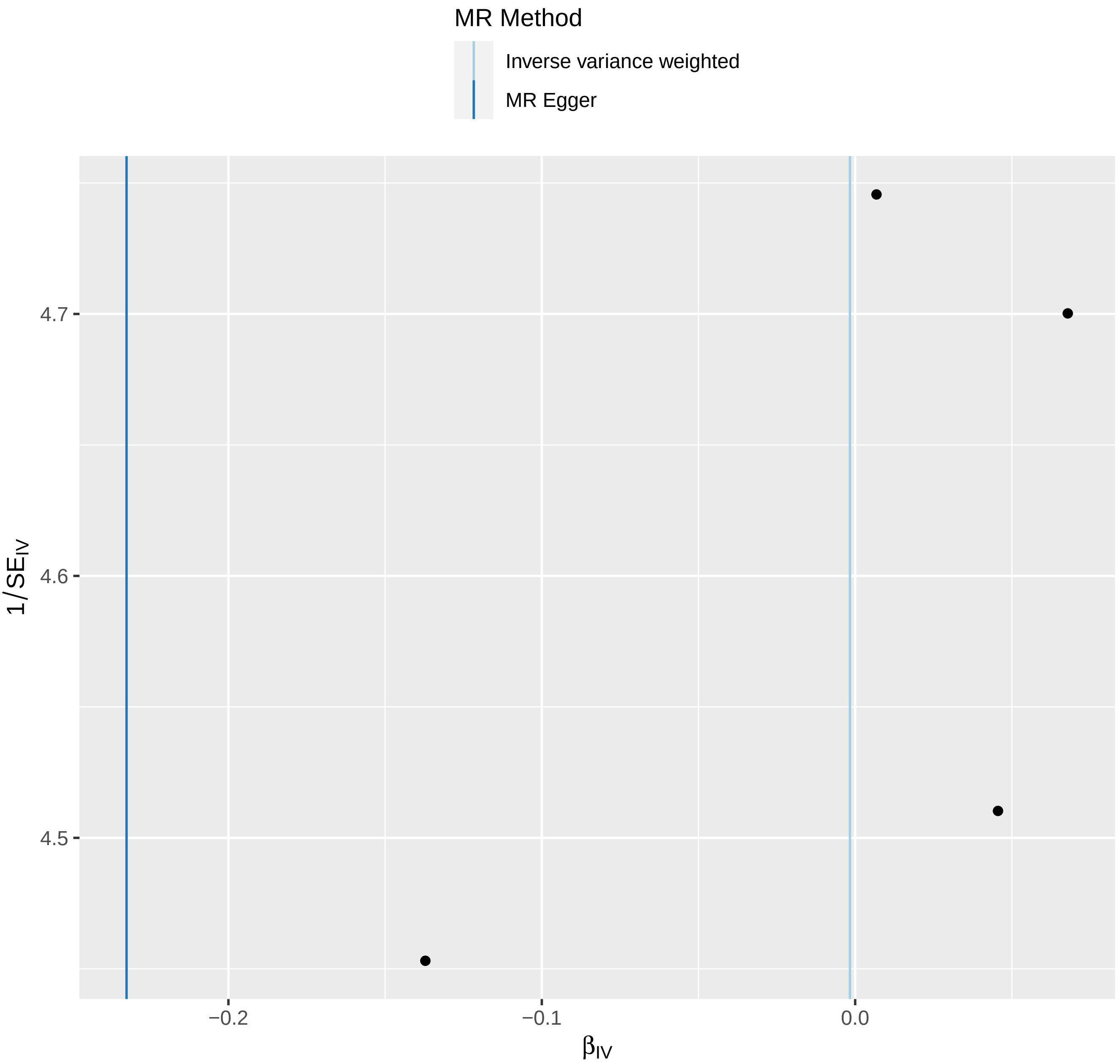

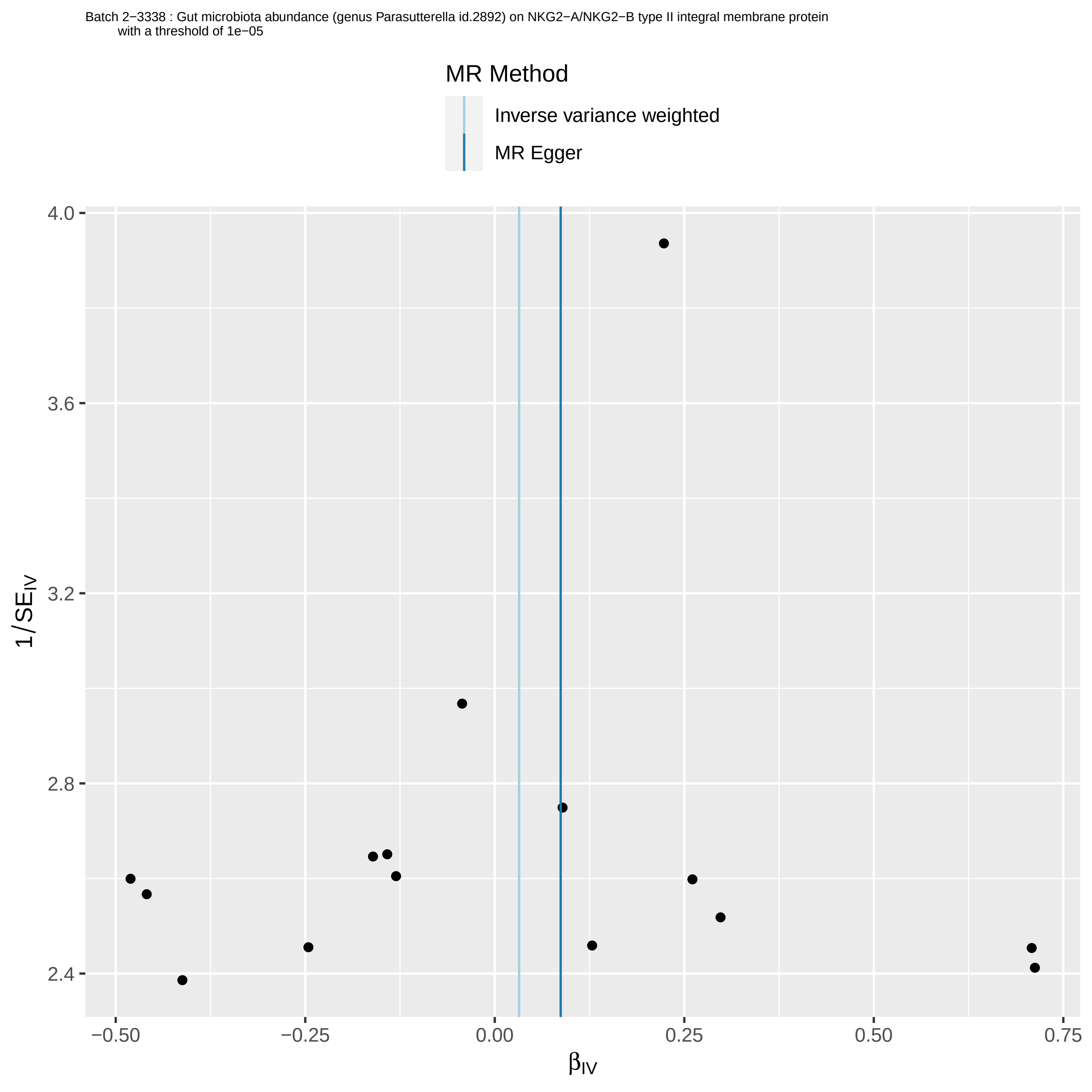
Batch 2−3338 : Gut microbiota abundance (genus Parasutterella id.2892) on NKG2−A/NKG2−B type II integral membrane protein
with a threshold of 1e−05
MR Method
Inverse variance weighted
MR Egger
4.0
3.6
3.2
2.8
2.4
1/SE_IV
−0.50
−0.25
0.00
0.25
0.50
0.75
β_IV

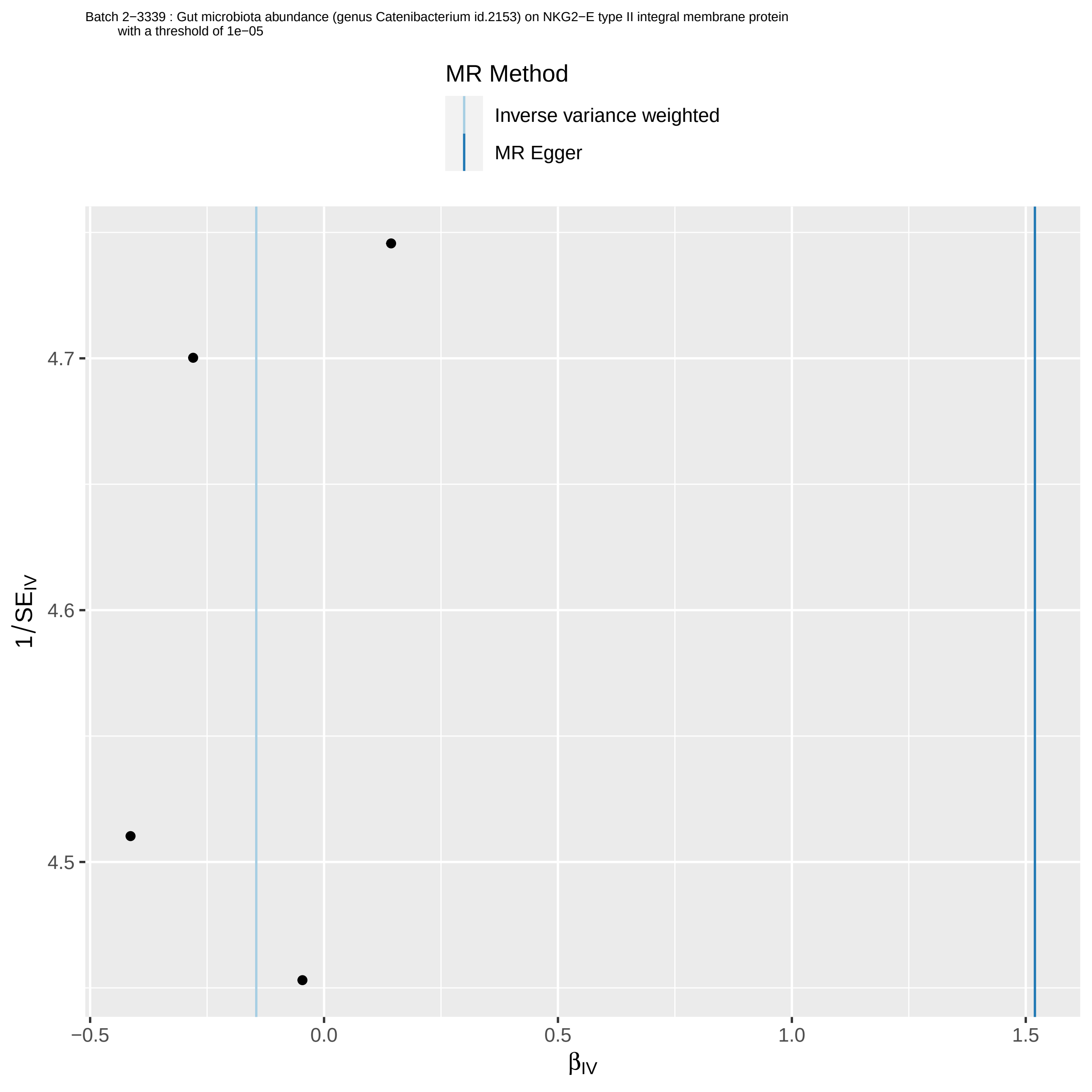

Batch 2−3339 : Gut microbiota abundance (genus Catenibacterium id.2153) on NKG2−E type II integral membrane protein
with a threshold of 1e−05
MR Method
Inverse variance weighted
MR Egger
4.7
4.6
4.5
1/SE_IV
−0.5
0.0
0.5
1.0
1.5
β_IV

Batch 2−3340 : Gut microbiota abundance (genus Parasutterella id.2892) on NKG2−E type II integral membrane protein
with a threshold of 1e−05

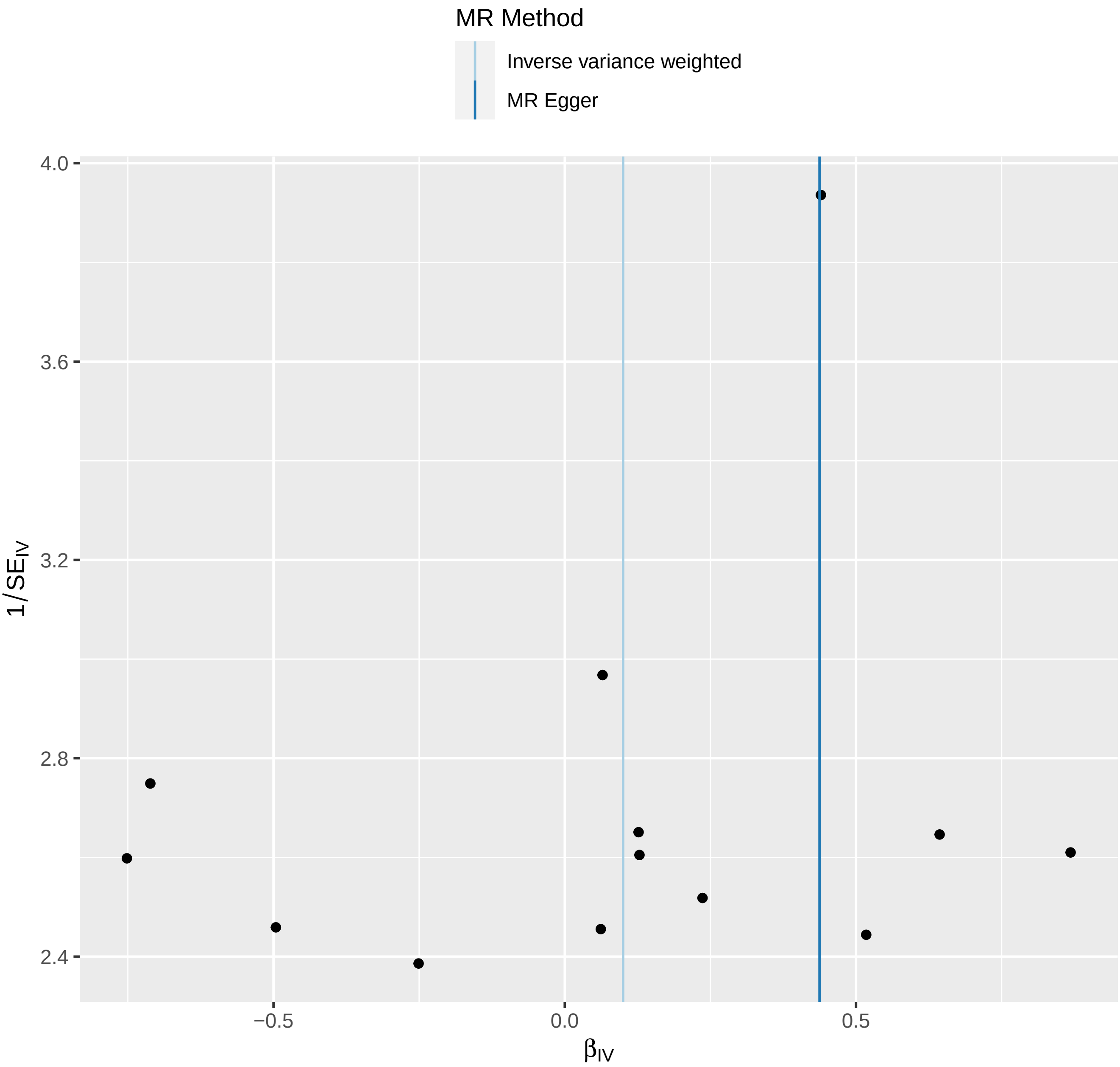

Batch 2−3341 : Gut microbiota abundance (genus Catenibacterium id.2153) on NKG2−E type II integral membrane protein
with a threshold of 1e−05

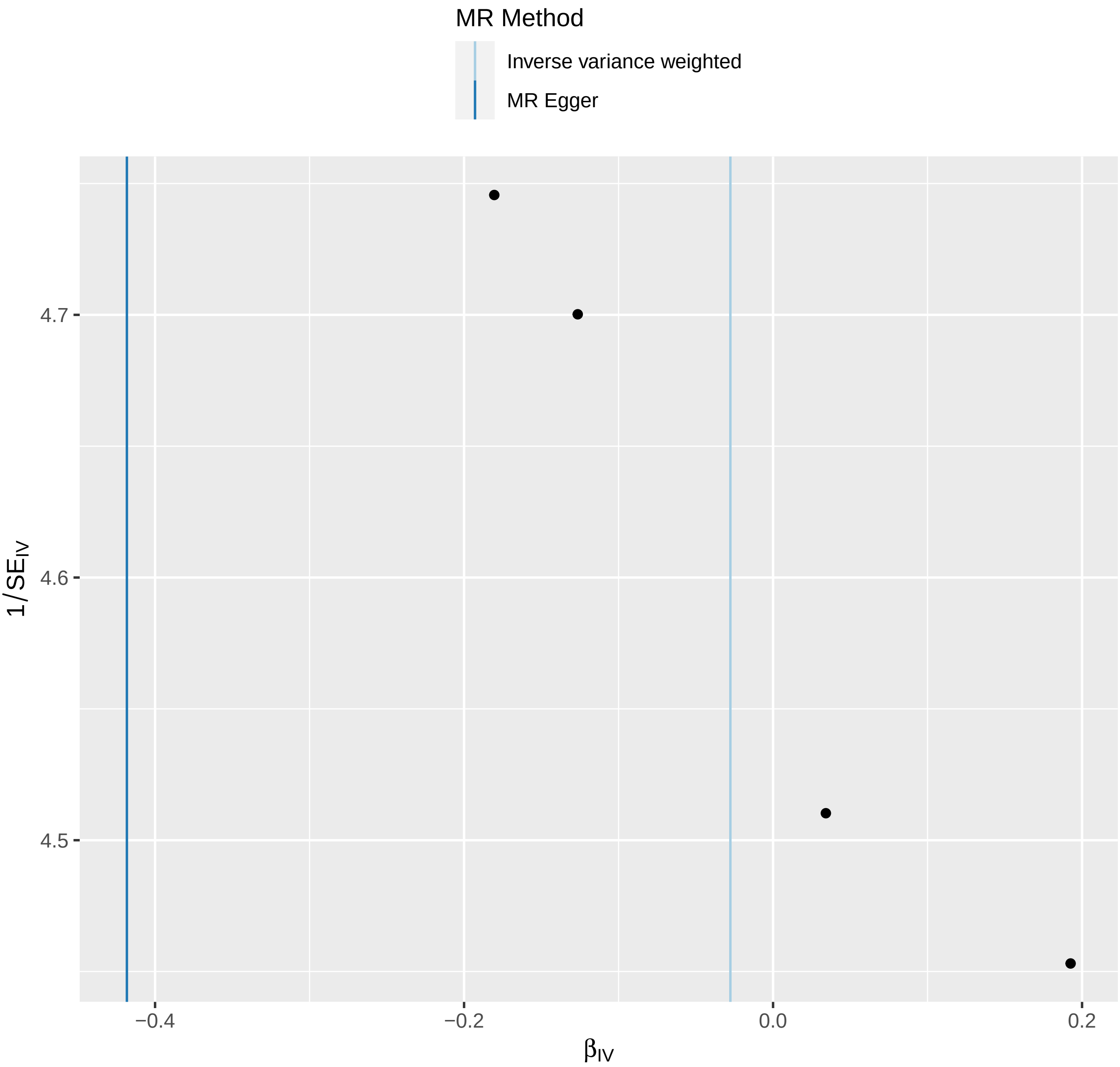

Batch 2−3342 : Gut microbiota abundance (genus Parasutterella id.2892) on NKG2−E type II integral membrane protein
with a threshold of 1e−05

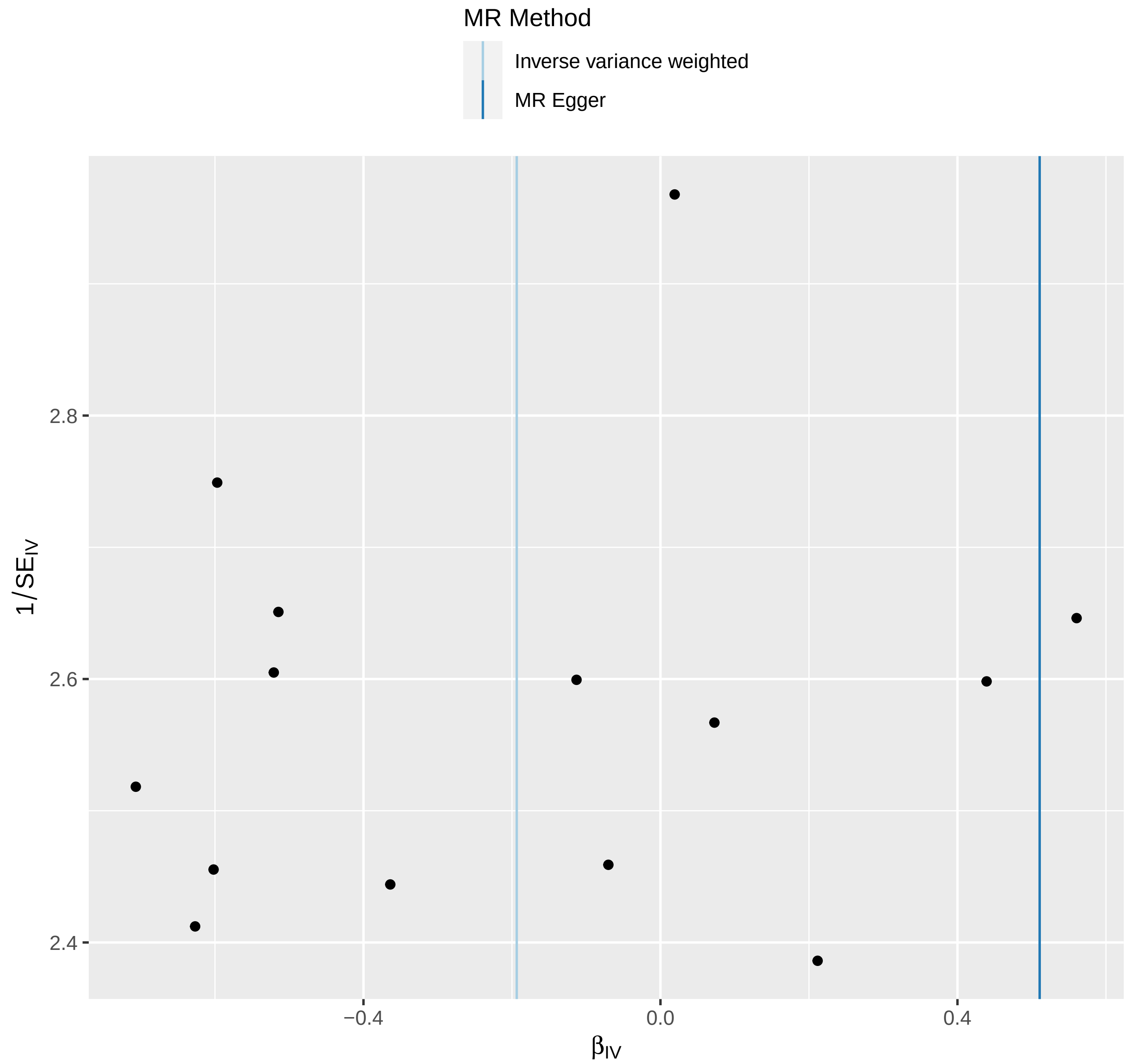

Batch 2−3343 : Gut microbiota abundance (genus Catenibacterium id.2153) on Killer cell lectin−like receptor subfamily F member 1 with a threshold of 1e−05

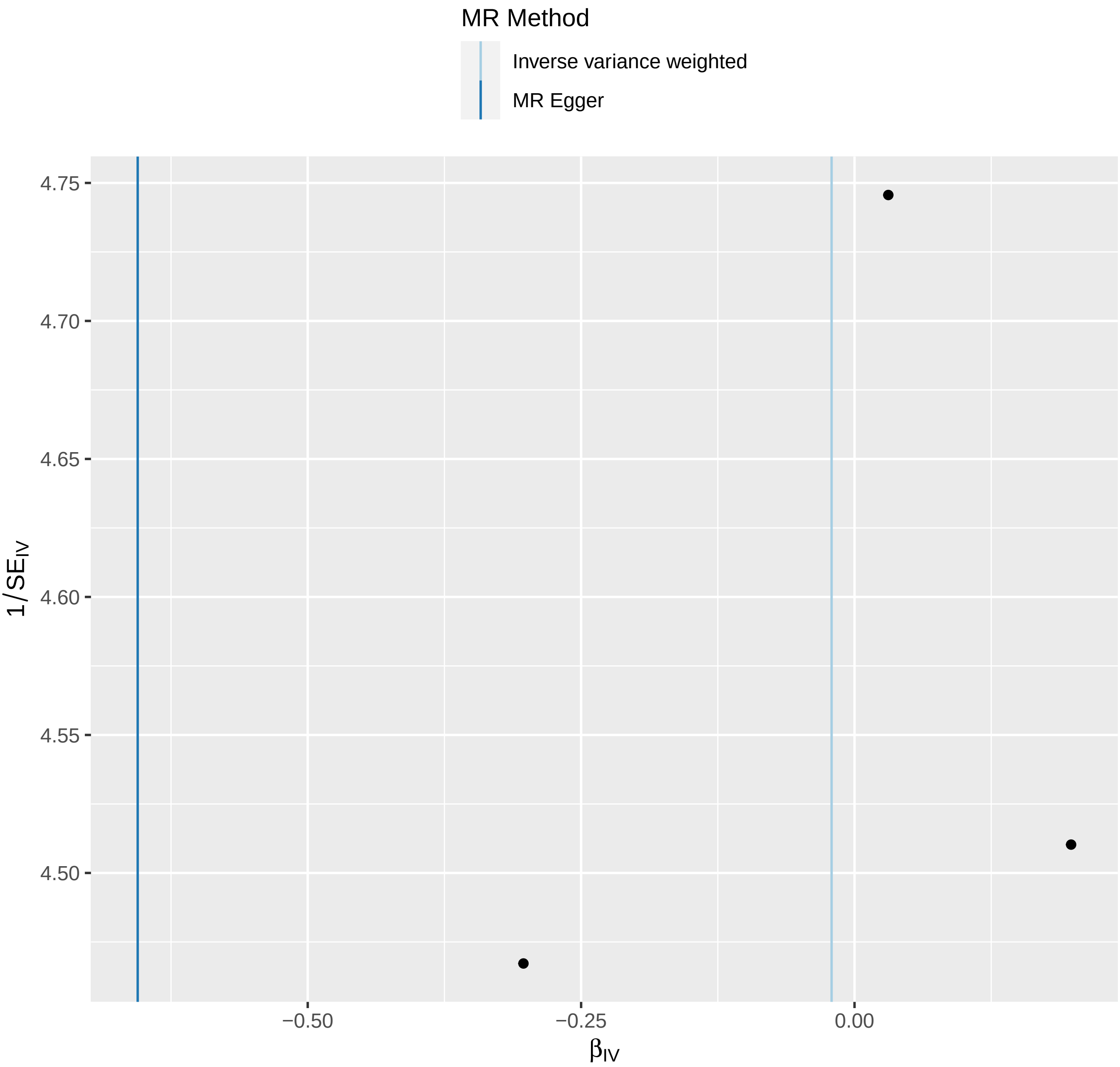

Batch 2−3344 : Gut microbiota abundance (genus Parasutterella id.2892) on Killer cell lectin−like receptor subfamily F member 1 with a threshold of 1e−05

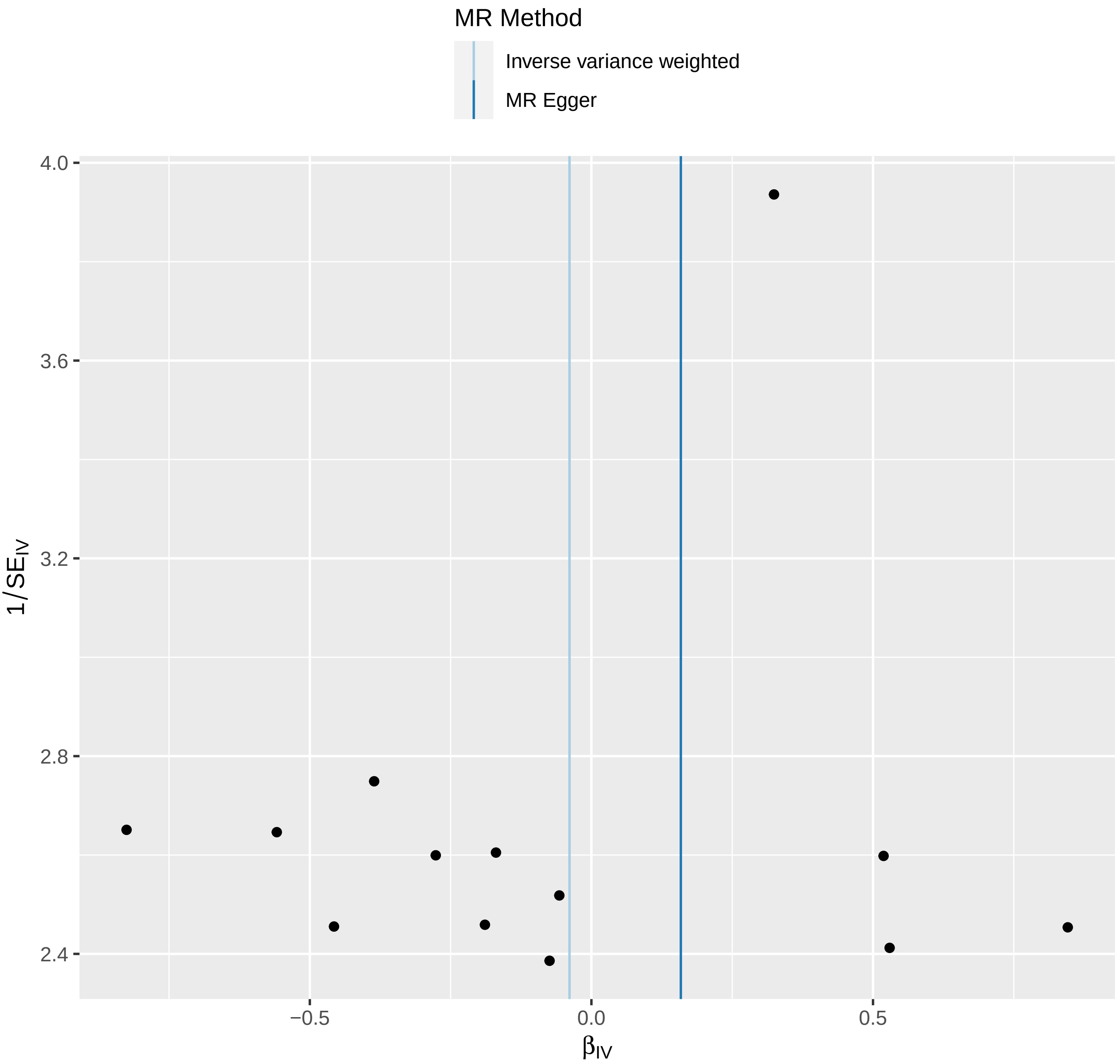

Batch 2−3345 : Gut microbiota abundance (genus Catenibacterium id.2153) on Killer cell lectin−like receptor subfamily F member 1 with a threshold of 1e−05

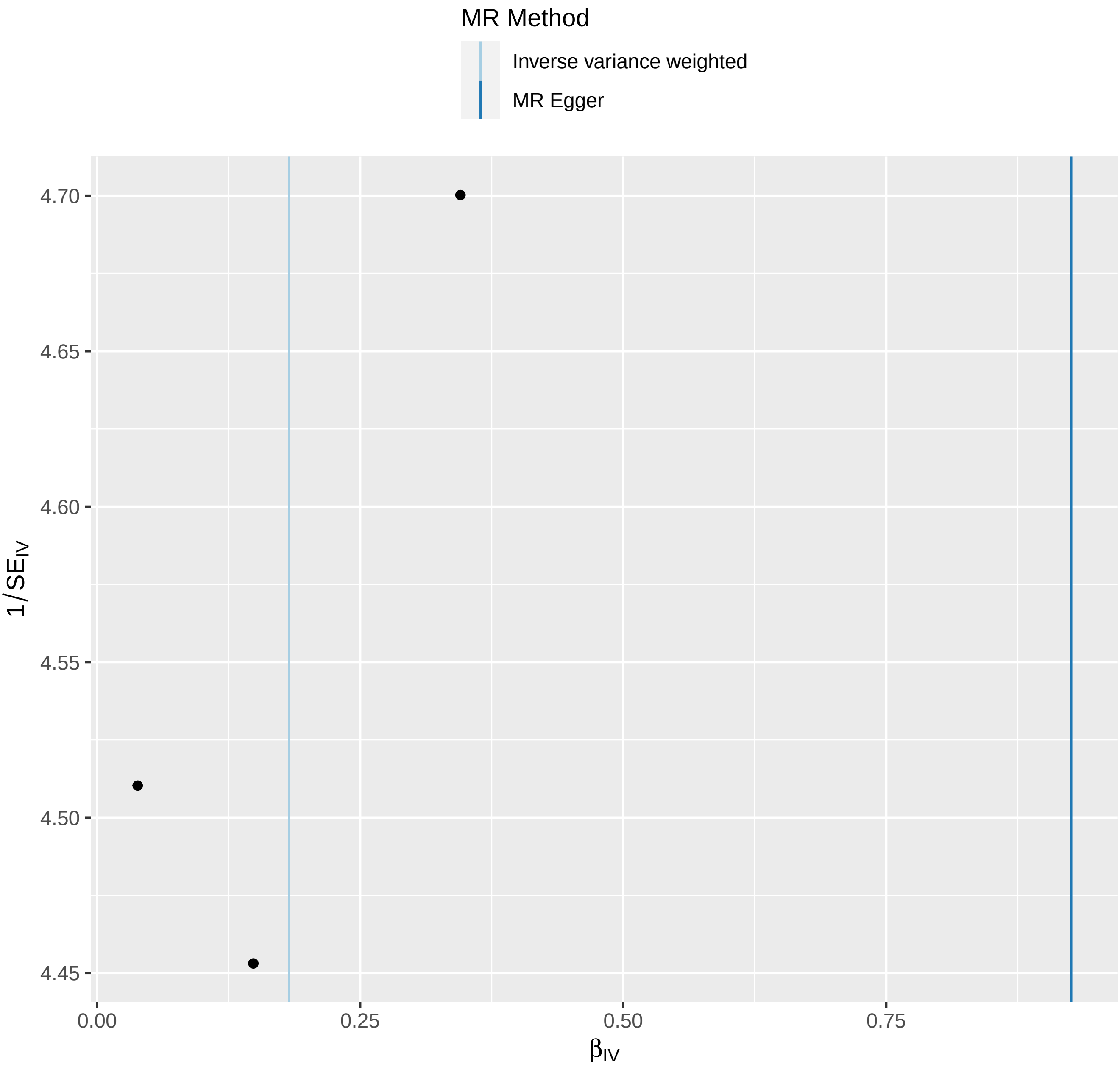

Batch 2−3346 : Gut microbiota abundance (genus Parasutterella id.2892) on Killer cell lectin−like receptor subfamily F member 1 with a threshold of 1e−05

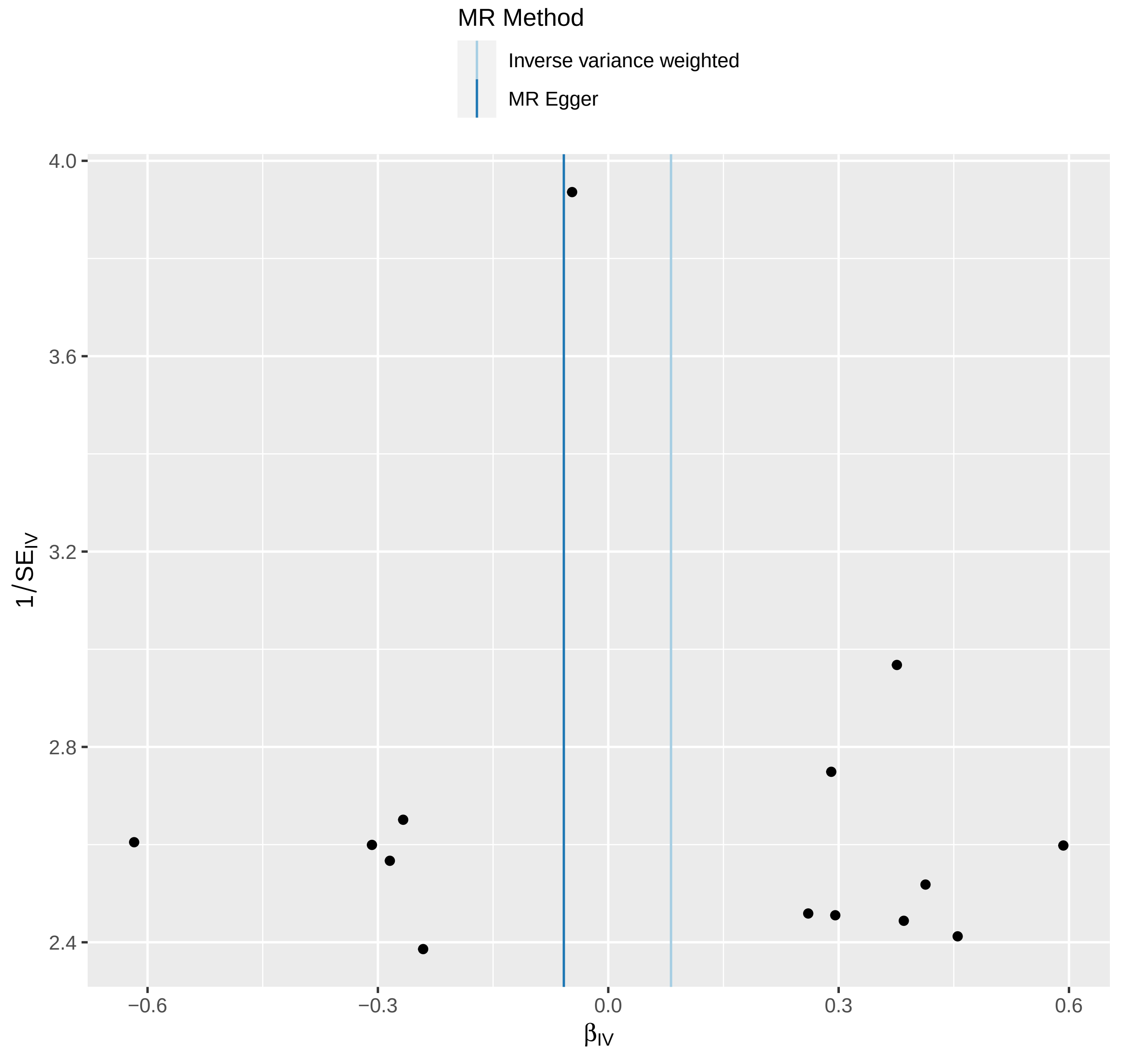

Batch 2−3347 : Gut microbiota abundance (genus Catenibacterium id.2153) on NKG2−D type II integral membrane protein with a threshold of 1e−05

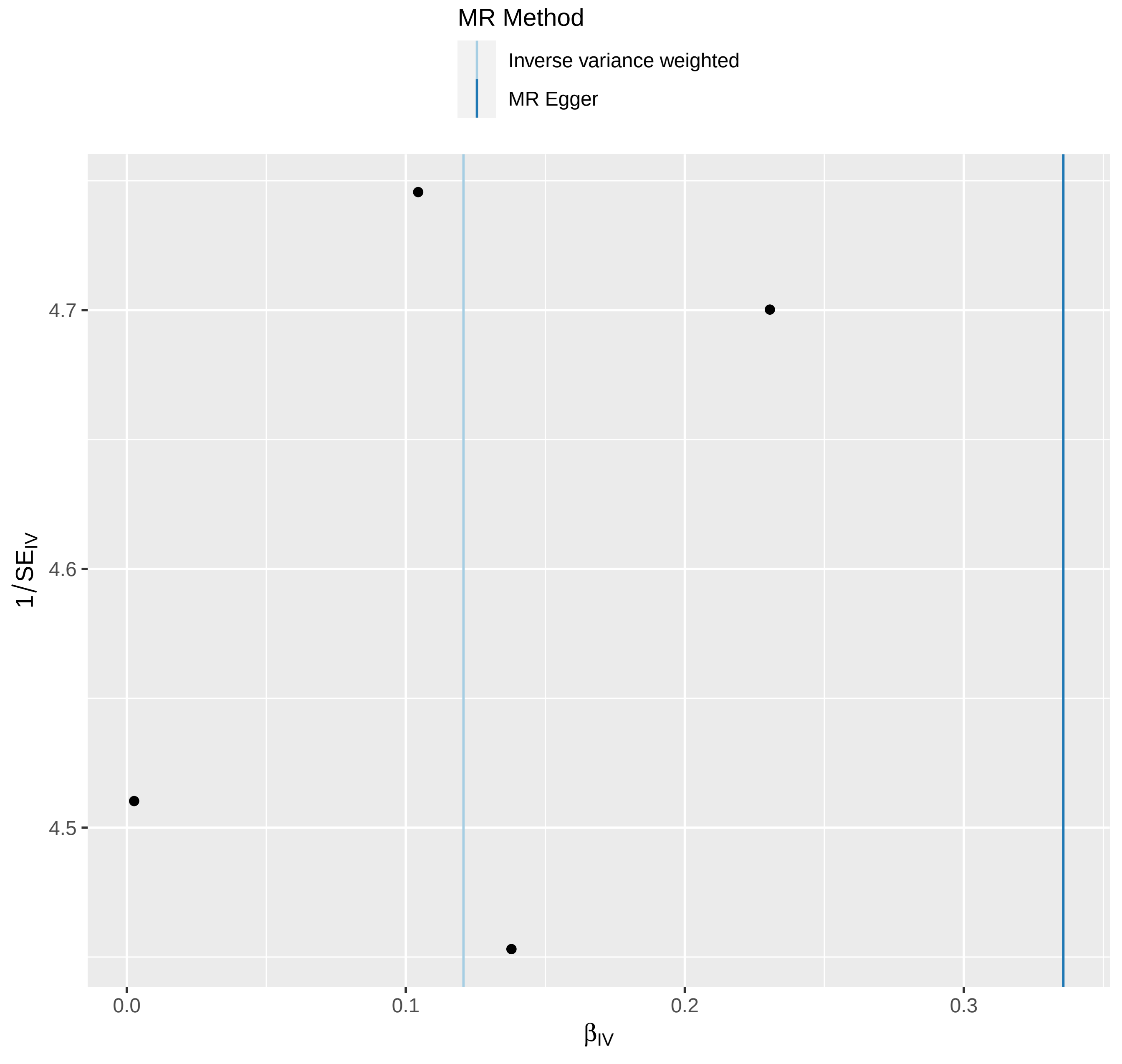

Batch 2−3348 : Gut microbiota abundance (genus Parasutterella id.2892) on NKG2−D type II integral membrane protein
with a threshold of 1e−05

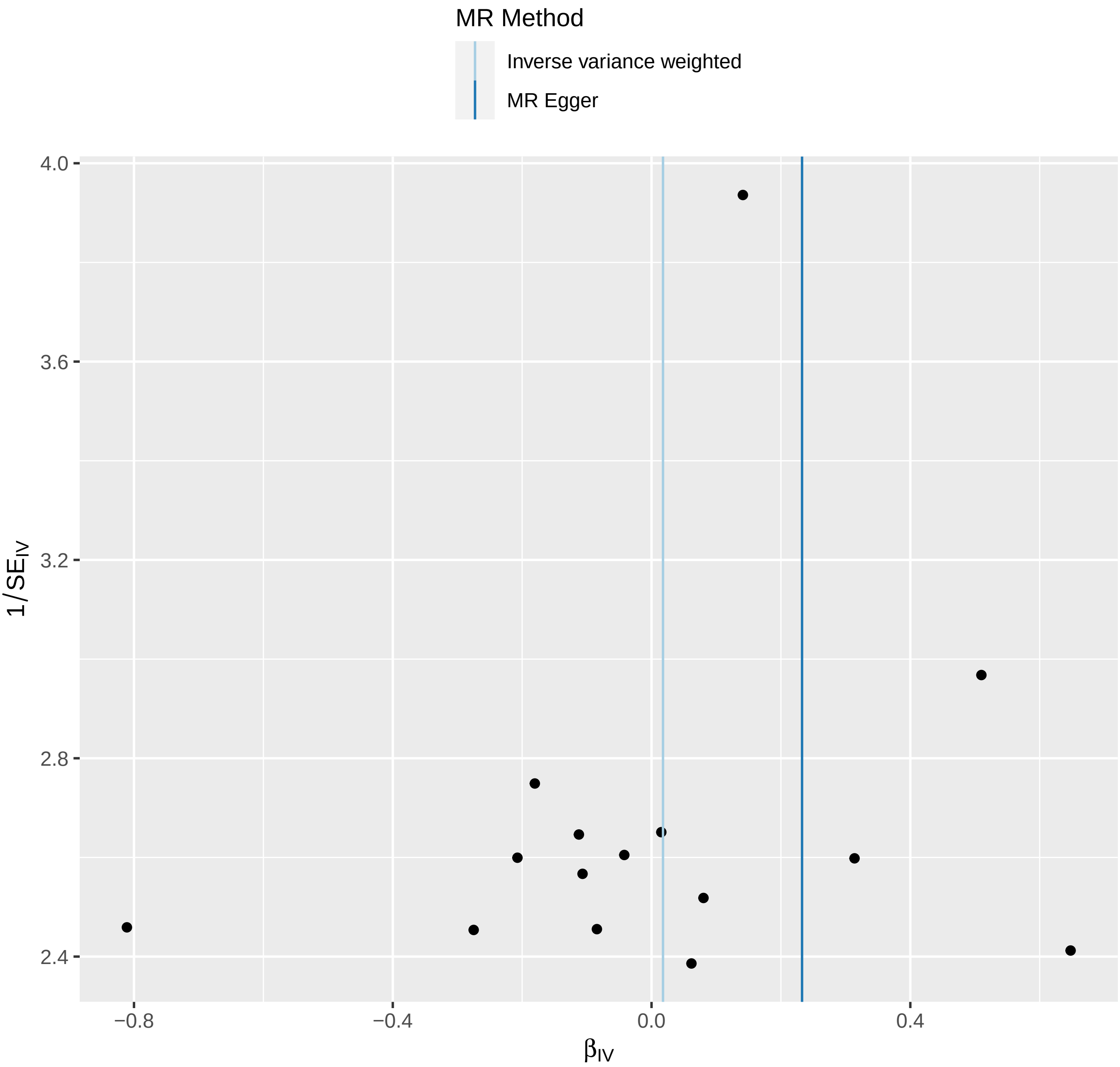

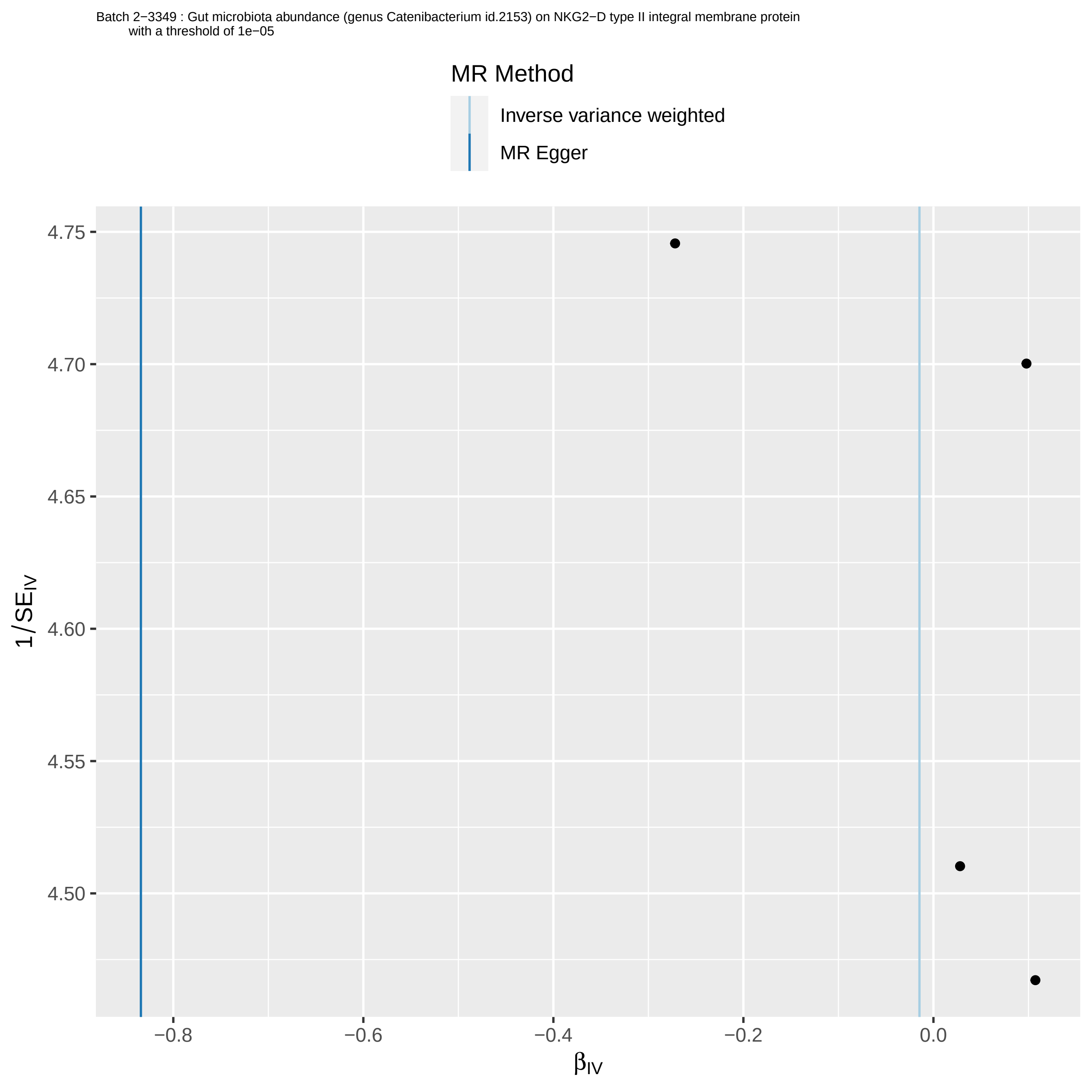

Batch 2−3349 : Gut microbiota abundance (genus Catenibacterium id.2153) on NKG2−D type II integral membrane protein
with a threshold of 1e−05
MR Method
Inverse variance weighted
MR Egger
4.75
4.70
4.65
4.60
4.55
4.50
1/SE_IV
−0.8
−0.6
−0.4
−0.2
0.0
β_IV

Batch 2−3350 : Gut microbiota abundance (genus Parasutterella id.2892) on NKG2−D type II integral membrane protein with a threshold of 1e−05

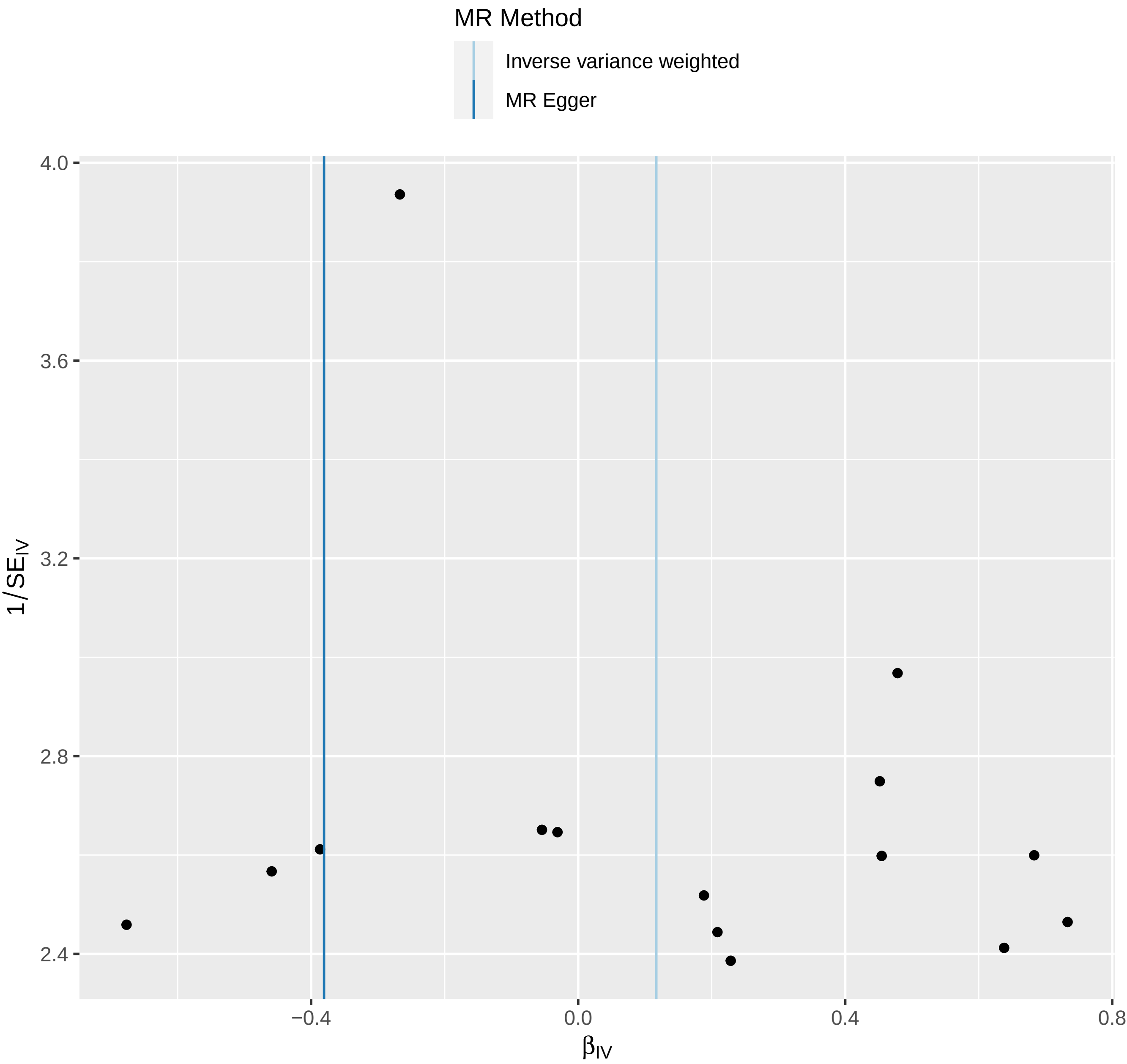

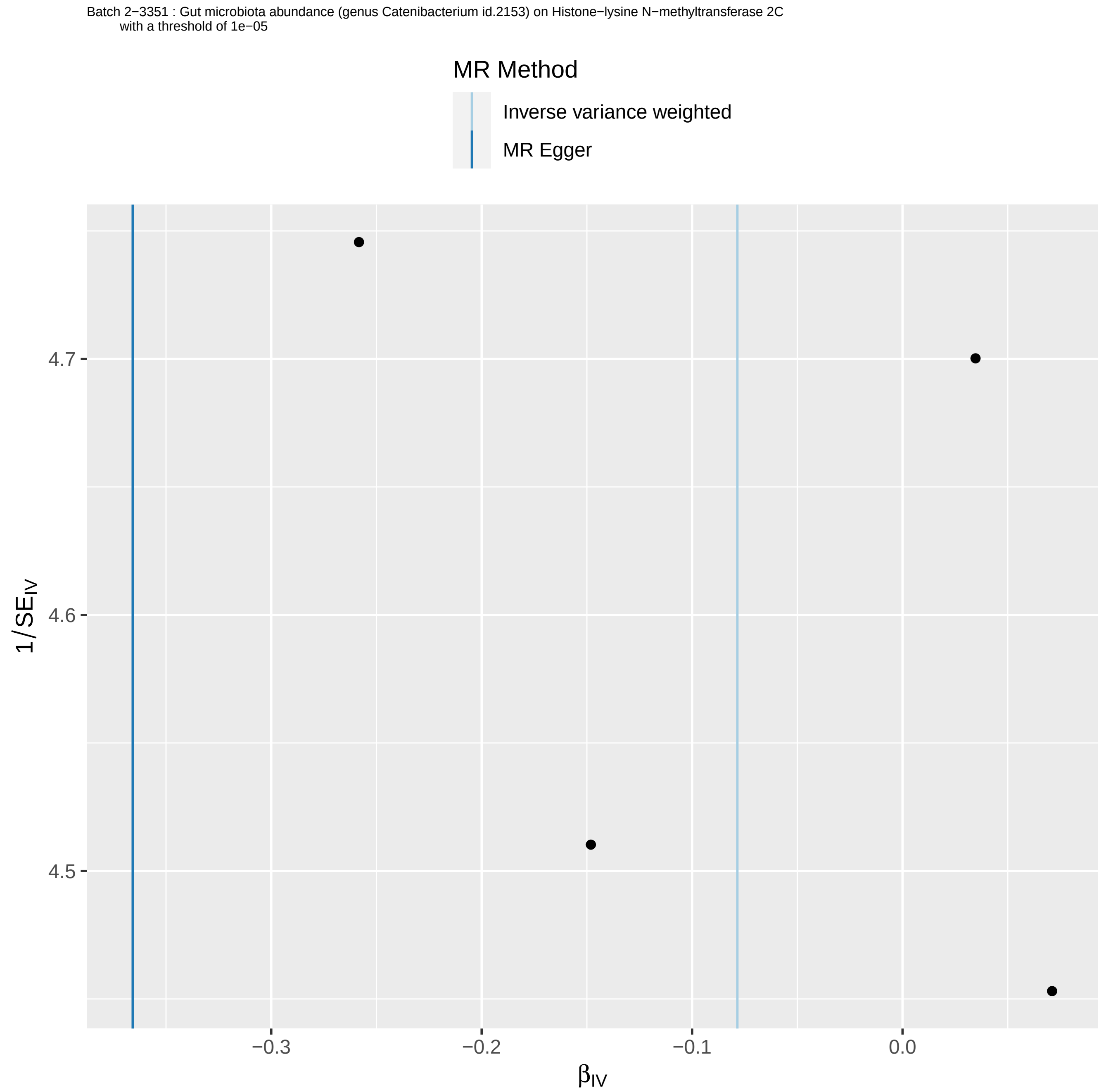

Batch 2−3351 : Gut microbiota abundance (genus Catenibacterium id.2153) on Histone−lysine N−methyltransferase 2C
with a threshold of 1e−05
MR Method
Inverse variance weighted
MR Egger
4.7
4.6
4.5
1/SE_IV
−0.3
−0.2
−0.1
0.0
β_IV

Batch 2−3352 : Gut microbiota abundance (genus Parasutterella id.2892) on Histone−lysine N−methyltransferase 2C with a threshold of 1e−05

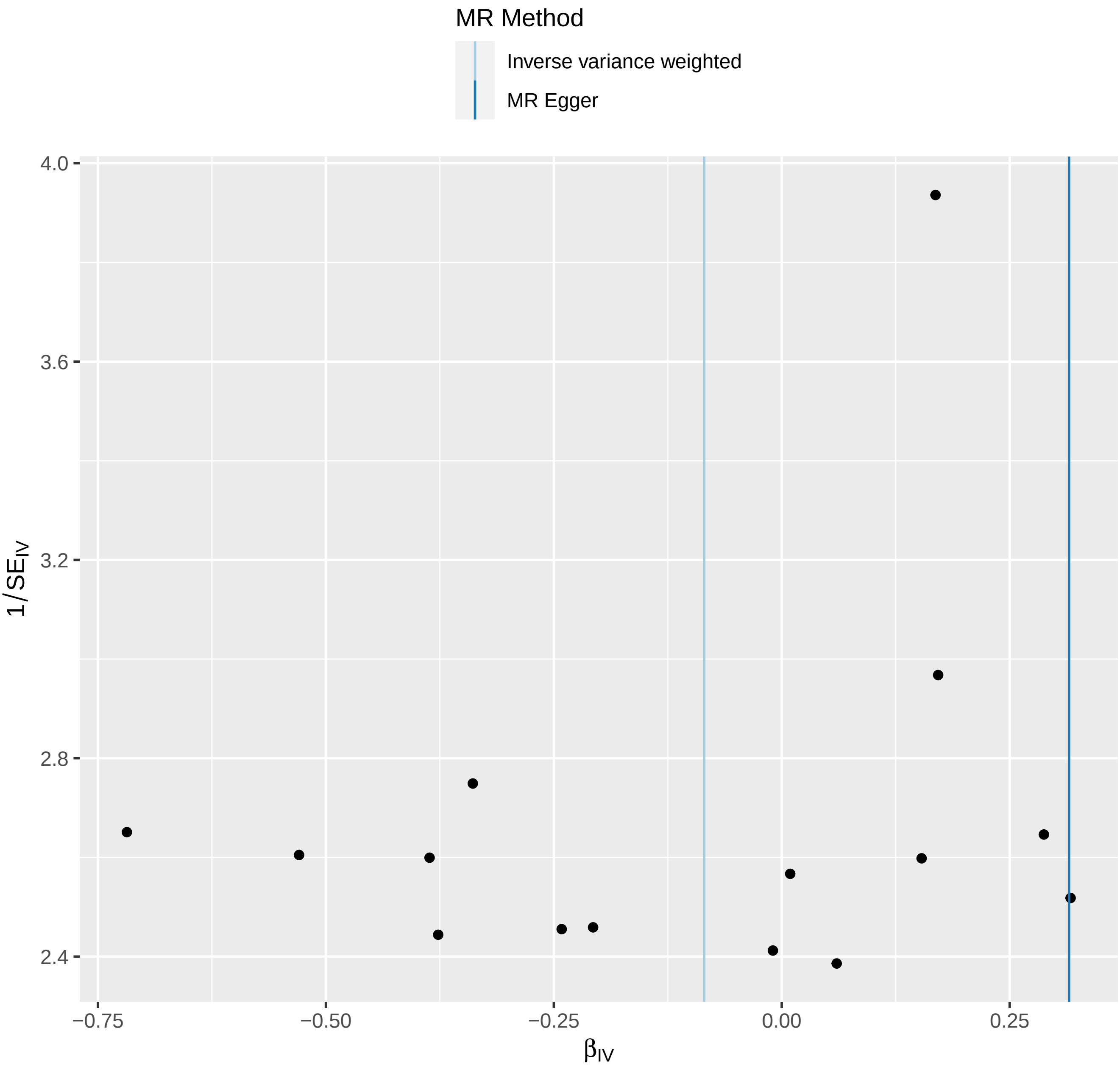

Batch 2−3353 : Gut microbiota abundance (genus Catenibacterium id.2153) on Importin subunit alpha−1
with a threshold of 1e−05

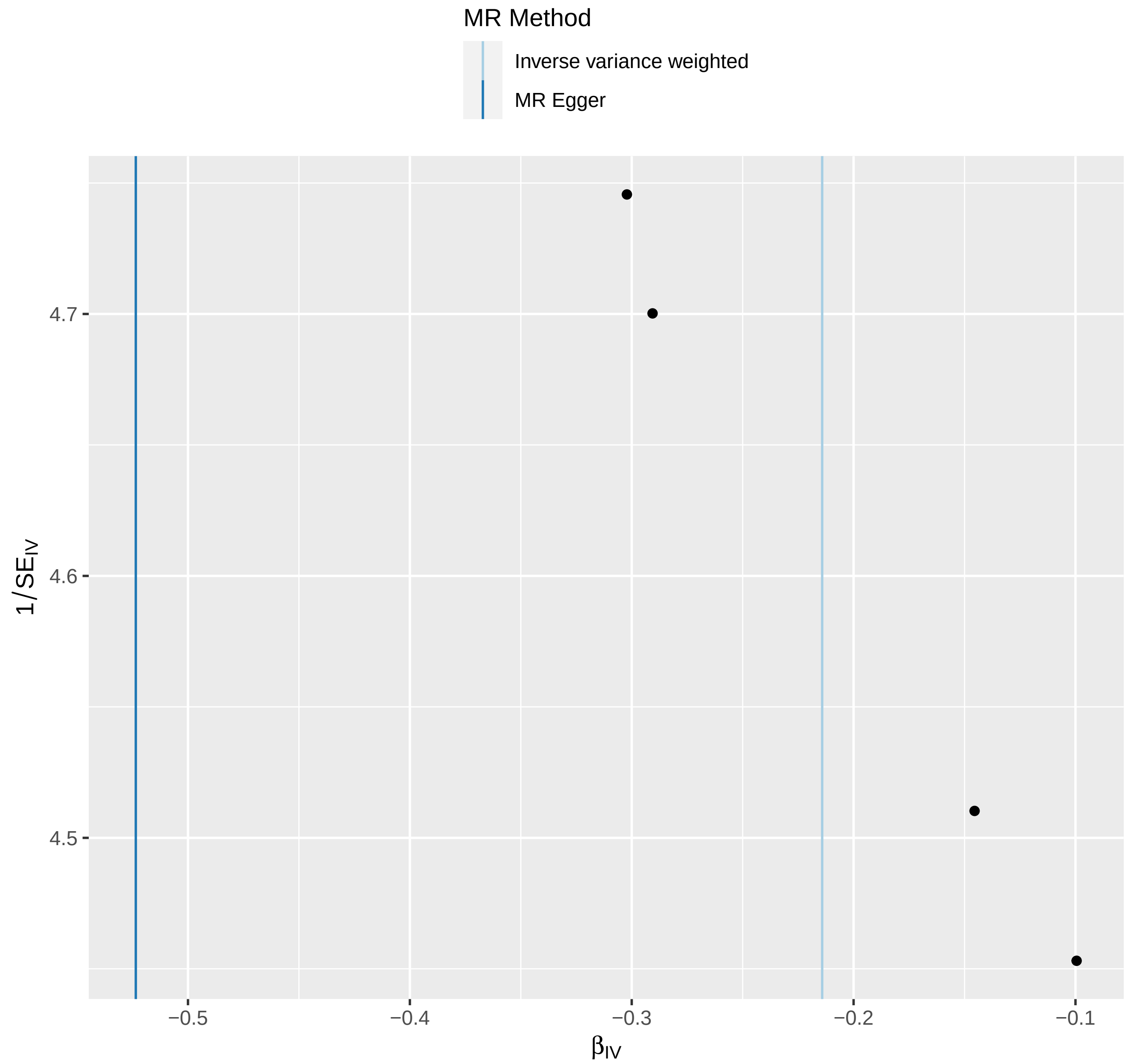

Batch 2−3354 : Gut microbiota abundance (genus Parasutterella id.2892) on Importin subunit alpha−1
with a threshold of 1e−05

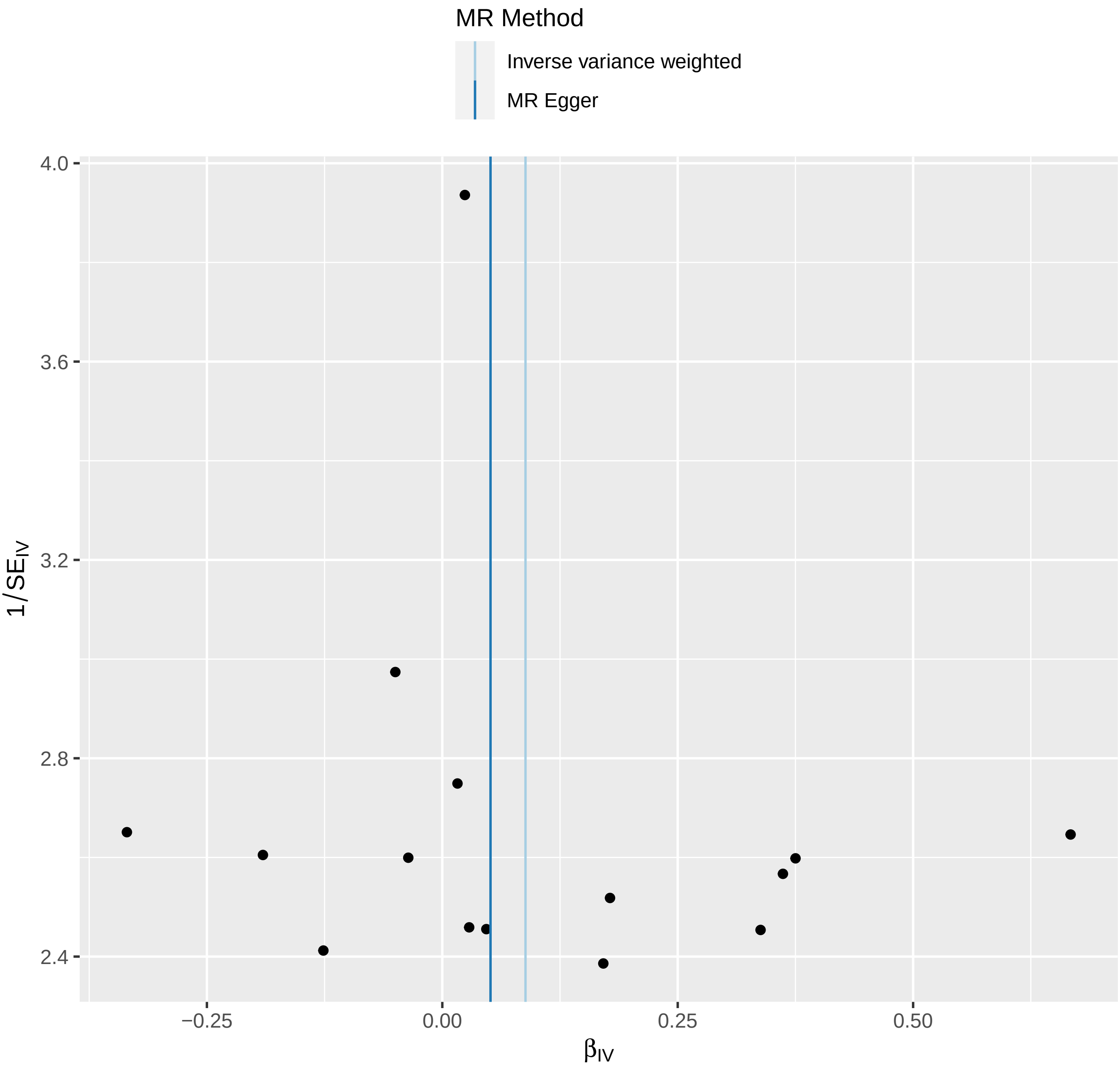

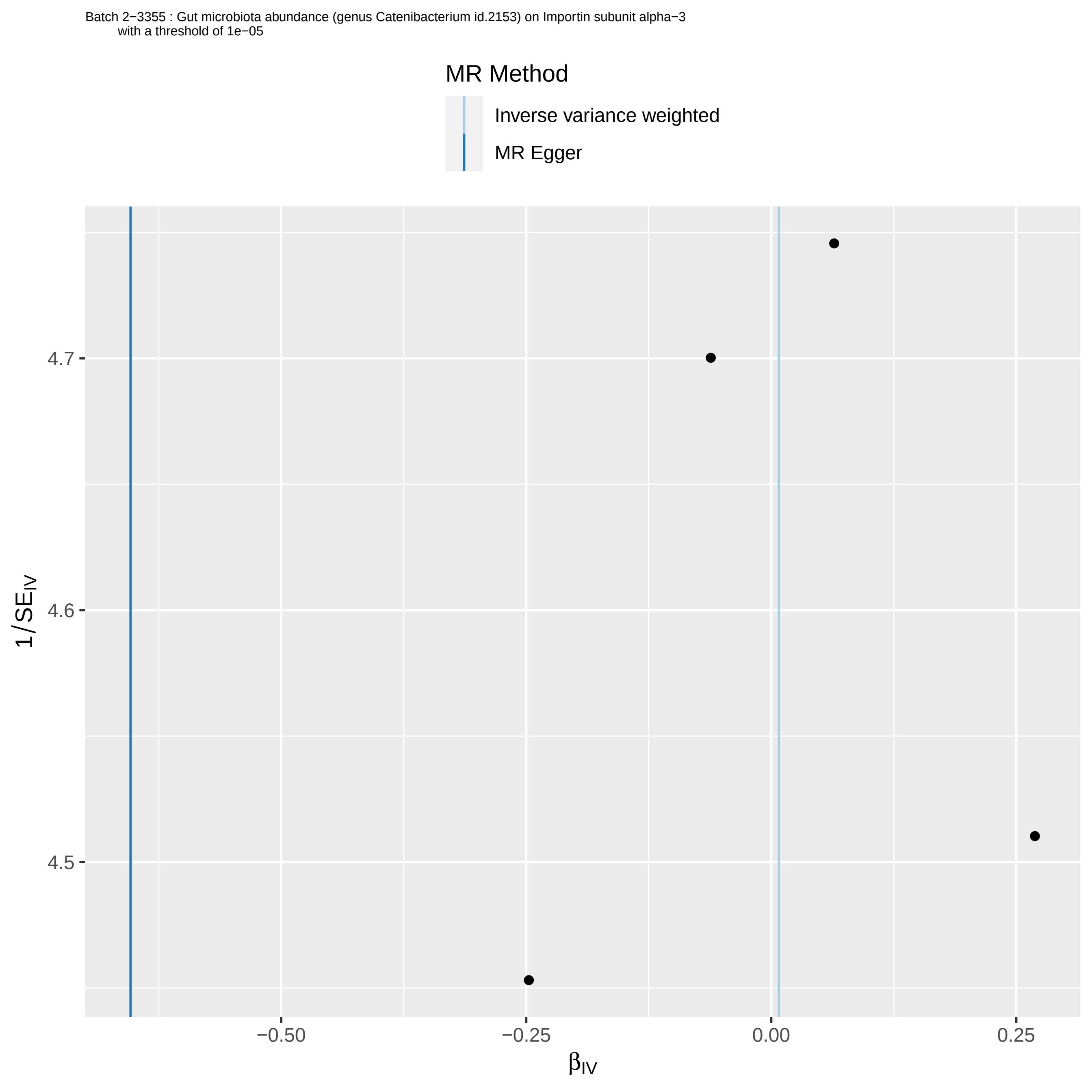

Batch 2−3355 : Gut microbiota abundance (genus Catenibacterium id.2153) on Importin subunit alpha−3
with a threshold of 1e−05
MR Method
Inverse variance weighted
MR Egger
4.7
4.6
4.5
1/SE_IV
−0.50
−0.25
0.00
0.25
β_IV

Batch 2−3356 : Gut microbiota abundance (genus Parasutterella id.2892) on Importin subunit alpha−3
with a threshold of 1e−05

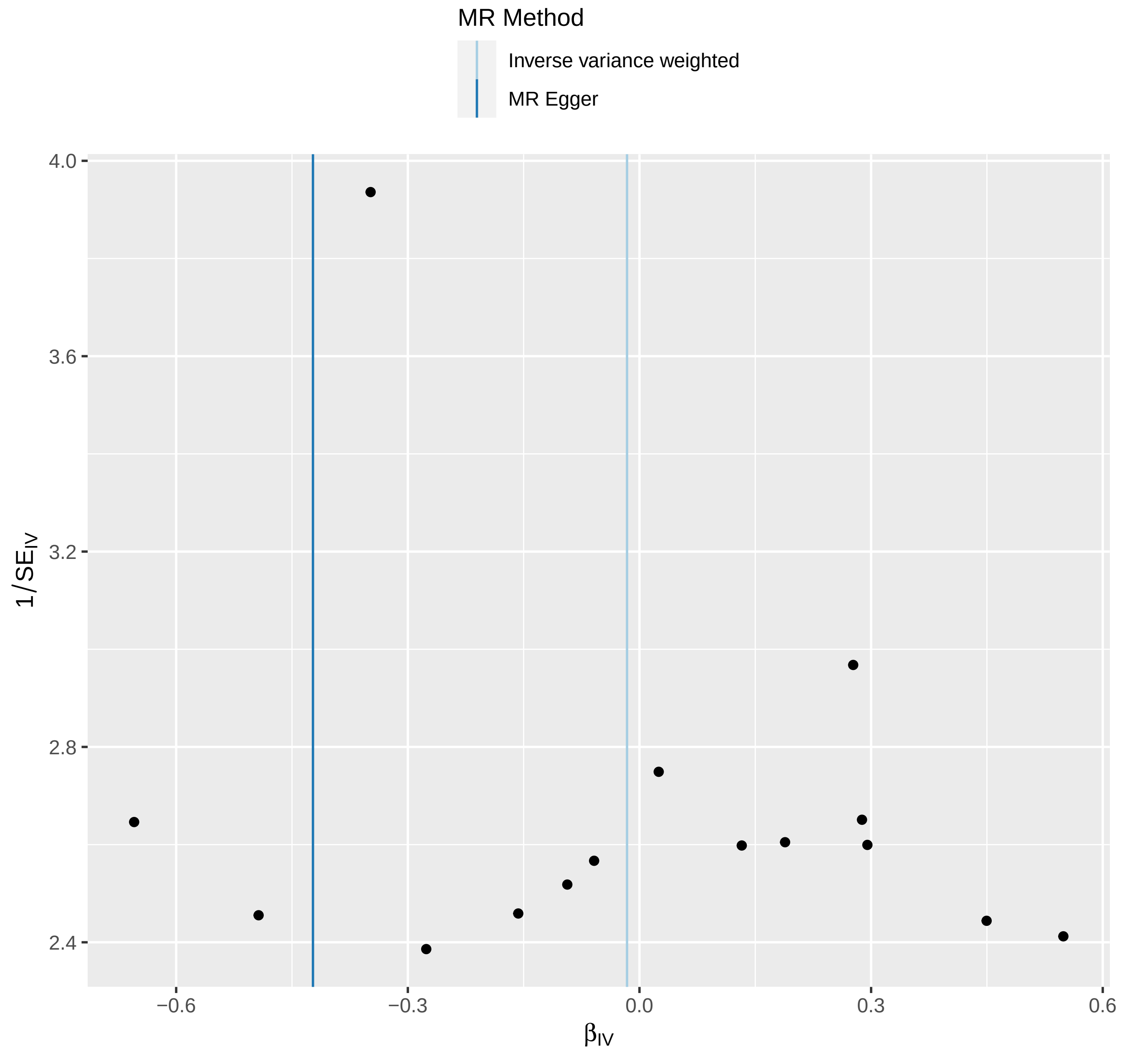

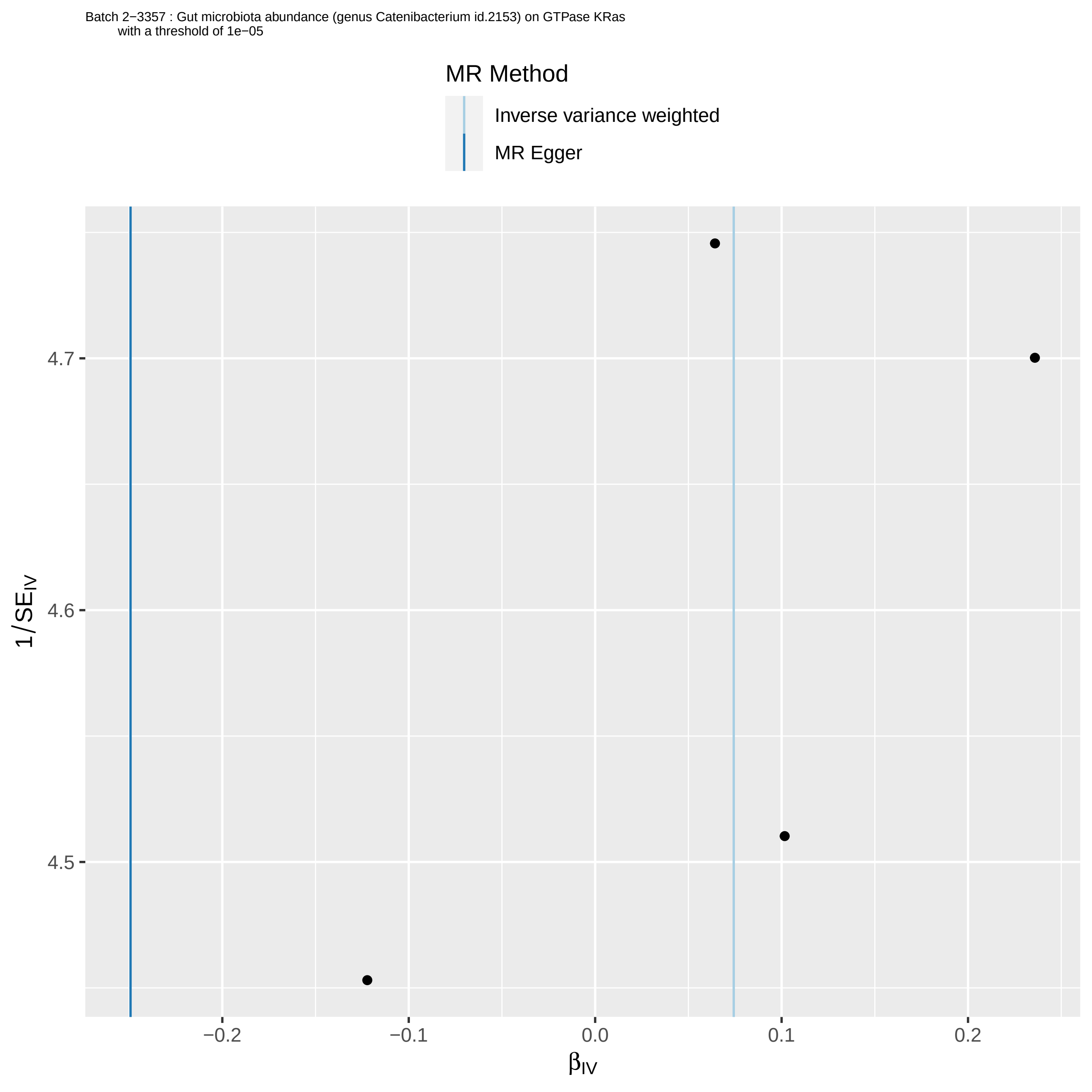
Batch 2−3357 : Gut microbiota abundance (genus Catenibacterium id.2153) on GTPase KRas
with a threshold of 1e−05
MR Method
Inverse variance weighted
MR Egger
4.7
4.6
4.5
1/SE$_{IV}$
−0.2
−0.1
0.0
0.1
0.2
β_{IV}

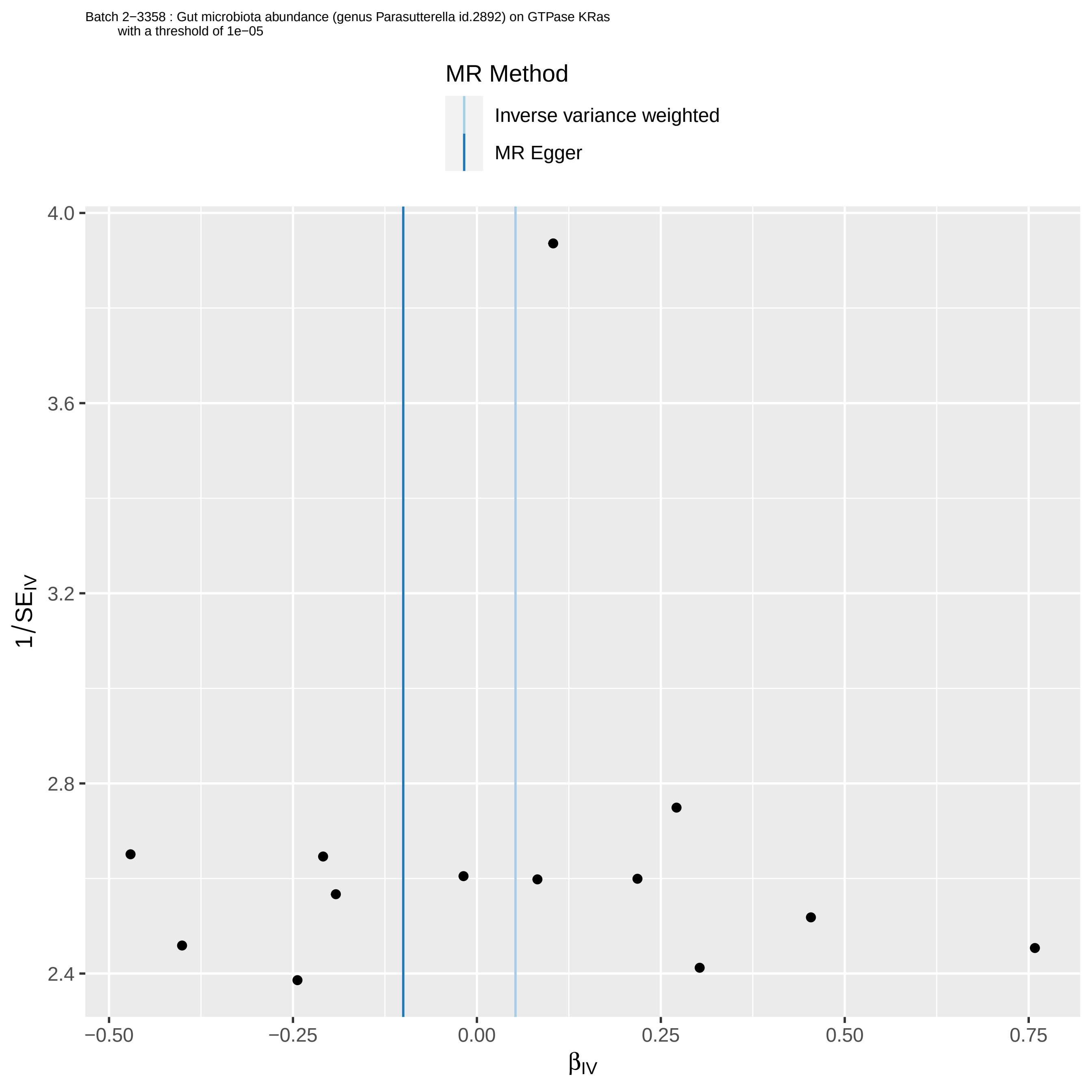

Batch 2−3358 : Gut microbiota abundance (genus Parasutterella id.2892) on GTPase KRas
with a threshold of 1e−05
MR Method
Inverse variance weighted
MR Egger
4.0
3.6
3.2
2.8
2.4
1/SE_IV
−0.50
−0.25
0.00
0.25
0.50
0.75
β_IV

Batch 2−3359 : Gut microbiota abundance (genus Catenibacterium id.2153) on Kremen protein 1
with a threshold of 1e−05

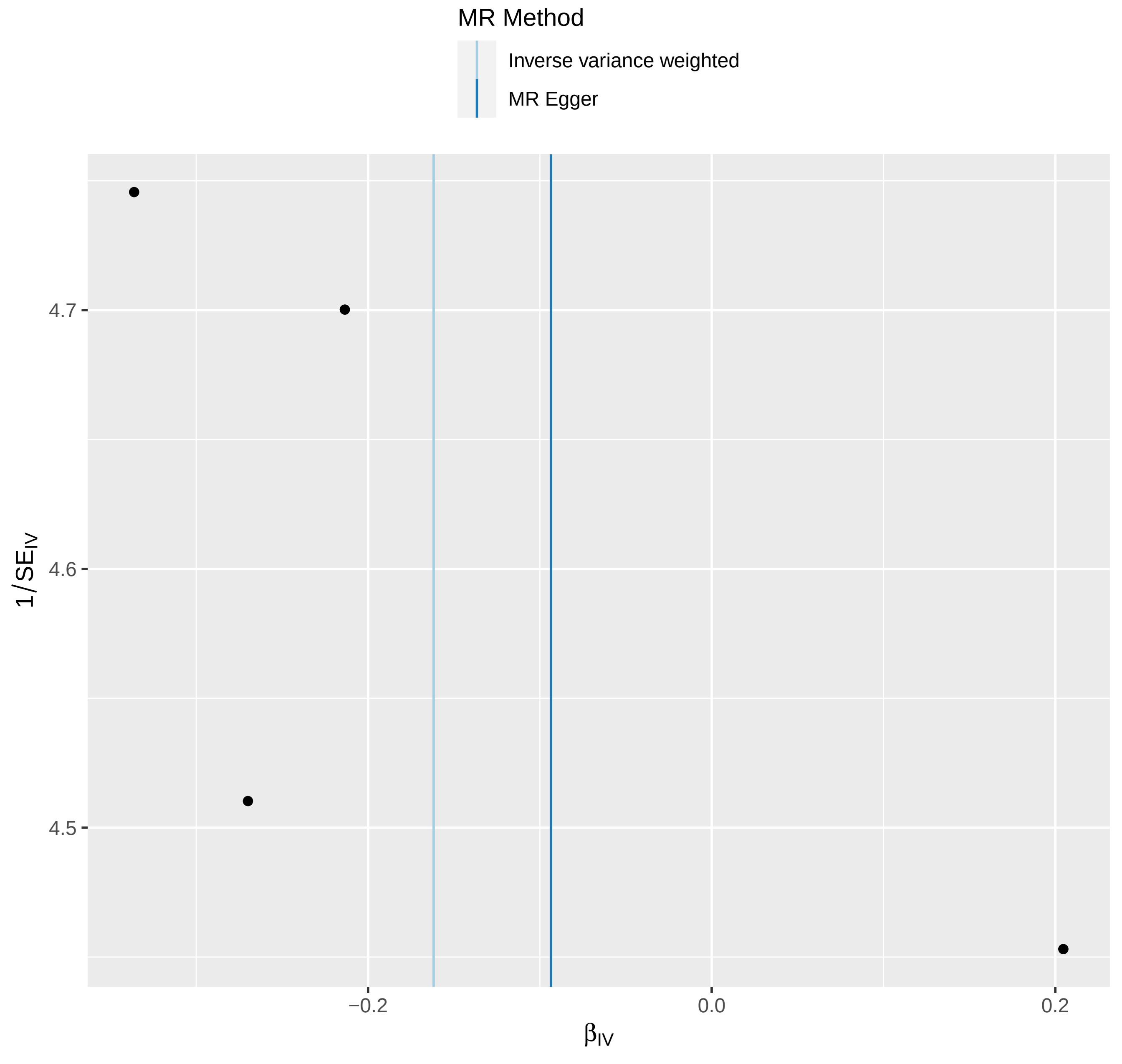

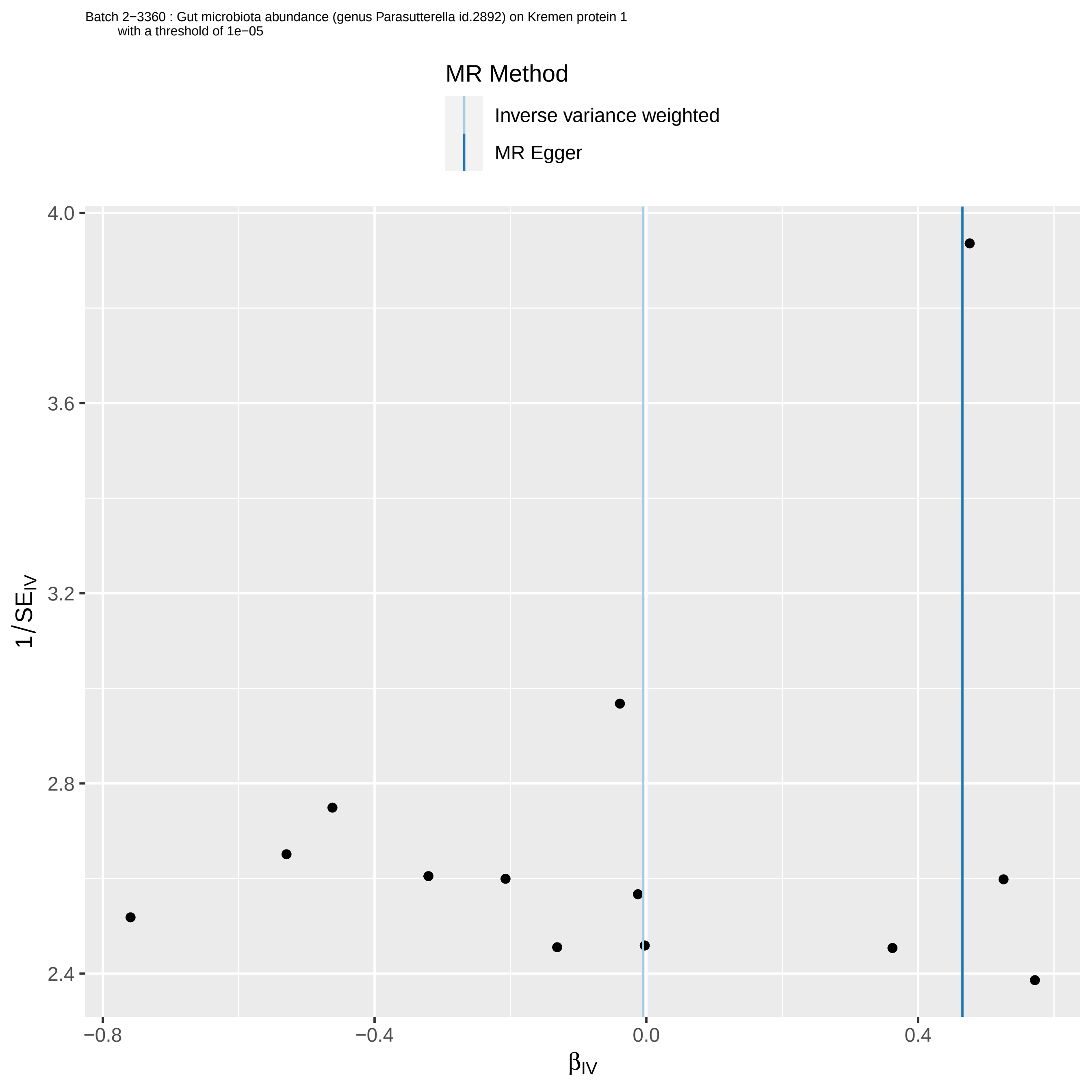

Batch 2−3360 : Gut microbiota abundance (genus Parasutterella id.2892) on Kremen protein 1
with a threshold of 1e−05
MR Method
Inverse variance weighted
MR Egger
4.0
3.6
3.2
2.8
2.4
1/SE_IV
−0.8
−0.4
0.0
0.4
β_IV

Batch 2−3361 : Gut microbiota abundance (genus Catenibacterium id.2153) on Kremen protein 2
with a threshold of 1e−05

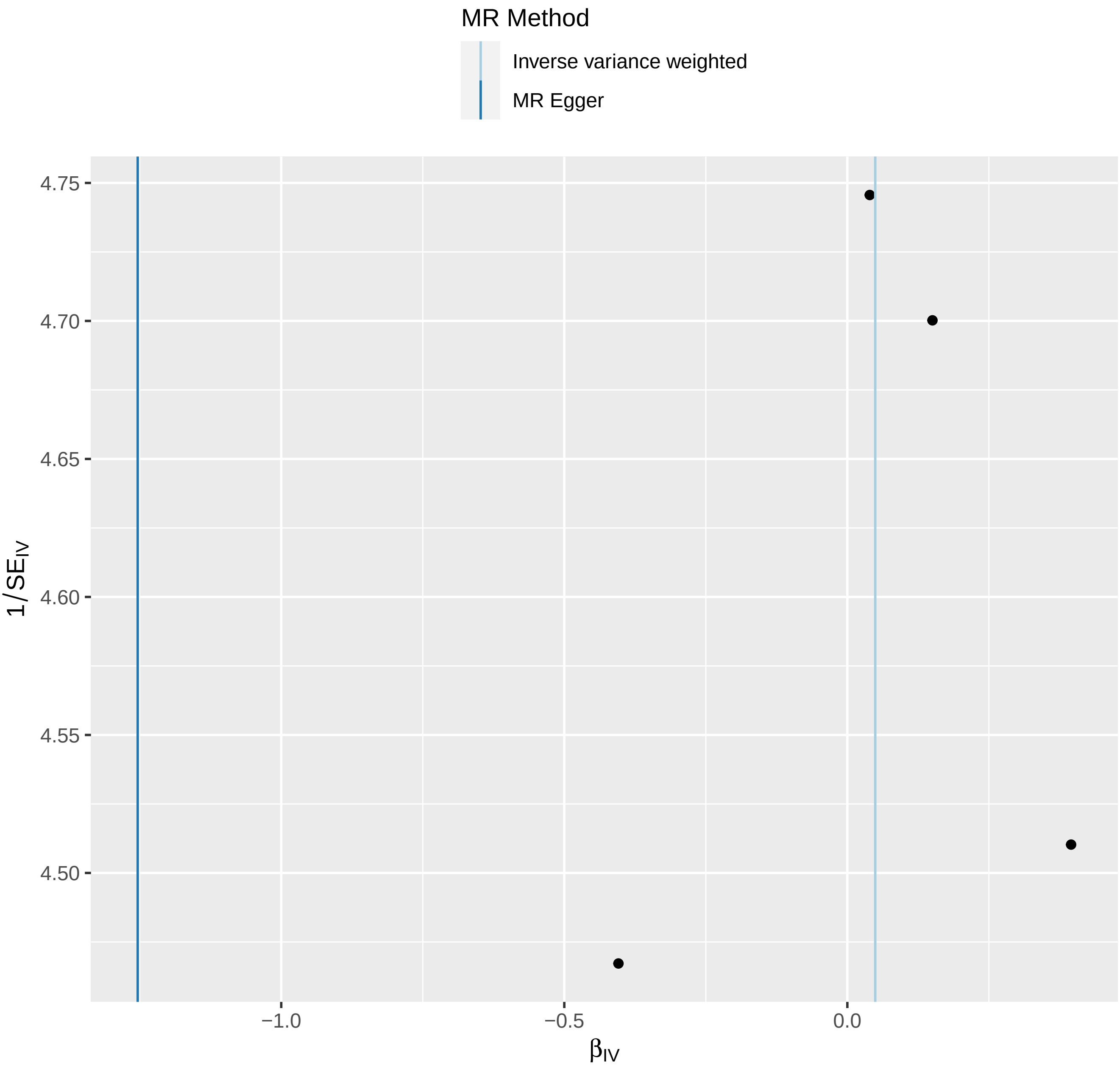

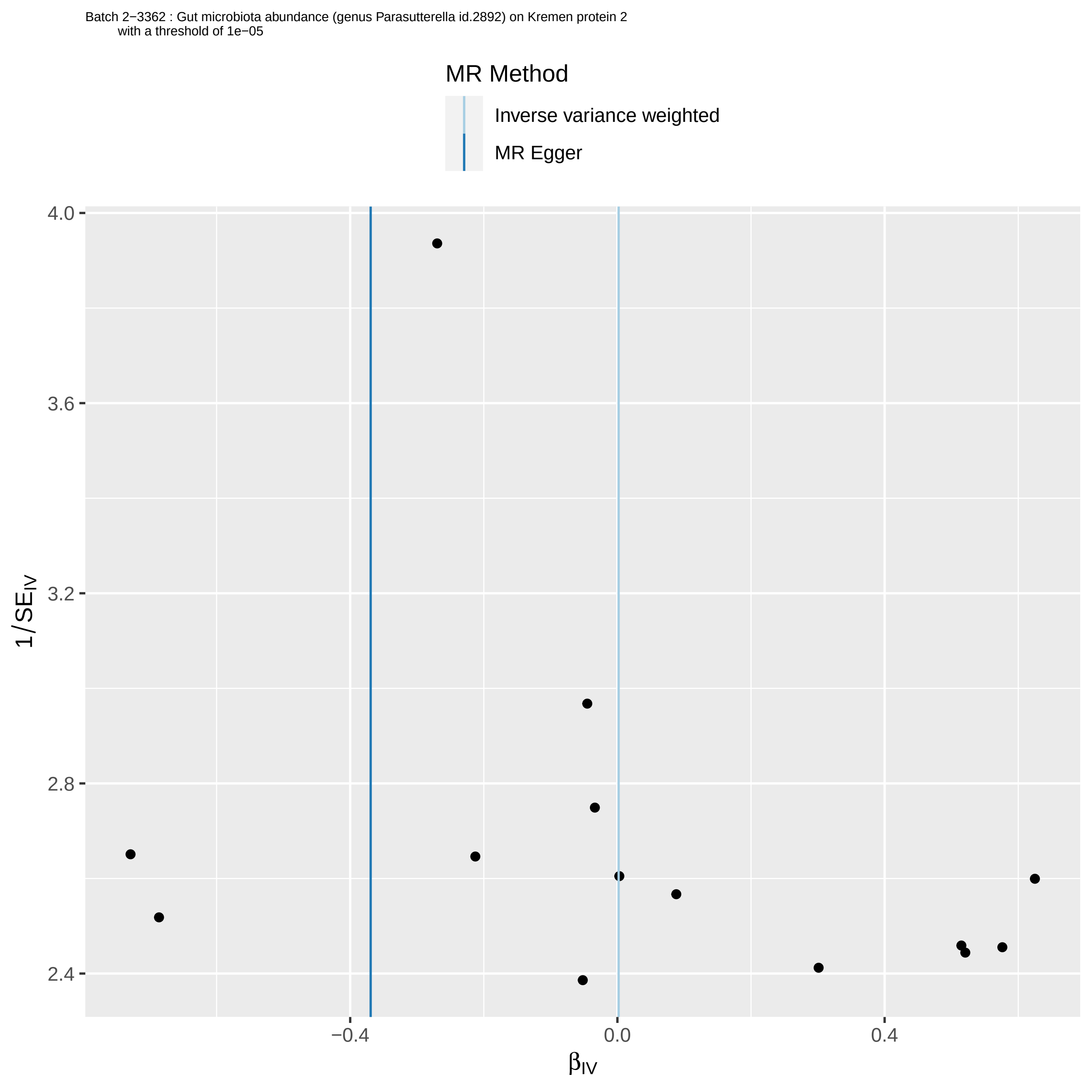

Batch 2−3362 : Gut microbiota abundance (genus Parasutterella id.2892) on Kremen protein 2
with a threshold of 1e−05
MR Method
Inverse variance weighted
MR Egger
4.0
3.6
3.2
2.8
2.4
1/SE$_{IV}$
−0.4
0.0
0.4
β_{IV}

Batch 2−3363 : Gut microbiota abundance (genus Catenibacterium id.2153) on Keratin, type I cytoskeletal 17
with a threshold of 1e−05

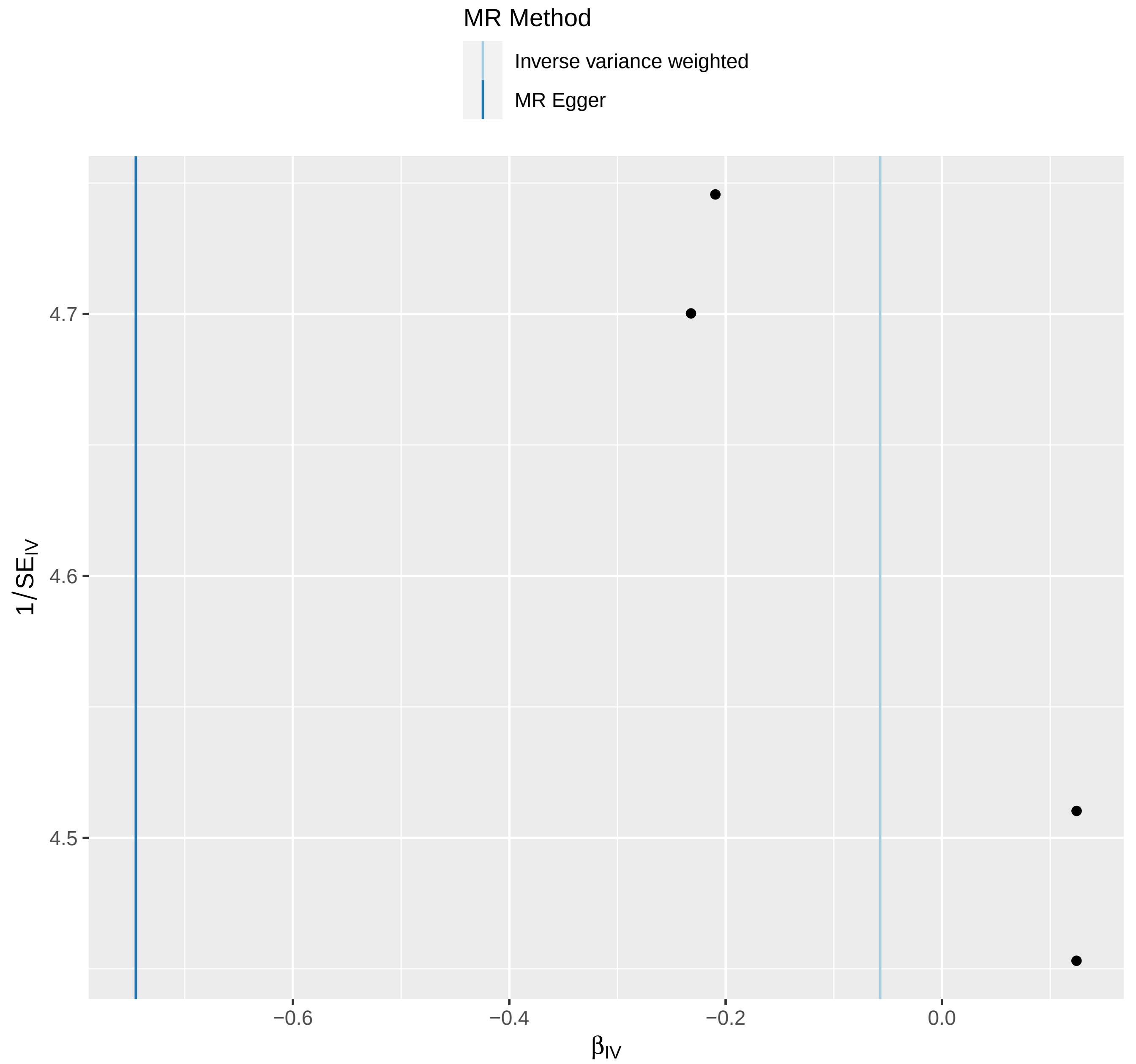

Batch 2−3364 : Gut microbiota abundance (genus Parasutterella id.2892) on Keratin, type I cytoskeletal 17
with a threshold of 1e−05

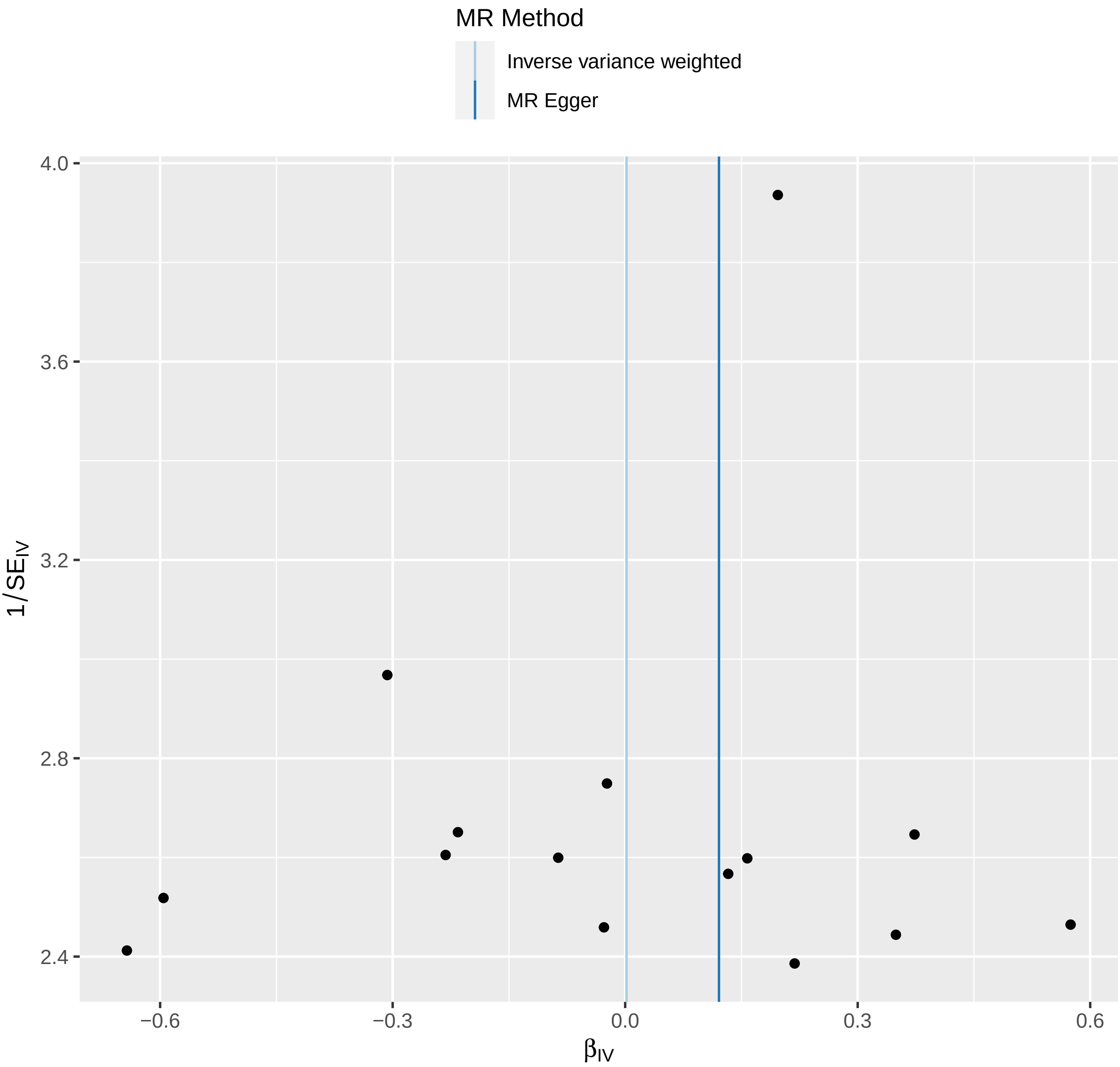

Batch 2−3365 : Gut microbiota abundance (genus Catenibacterium id.2153) on Keratin, type I cytoskeletal 18
with a threshold of 1e−05

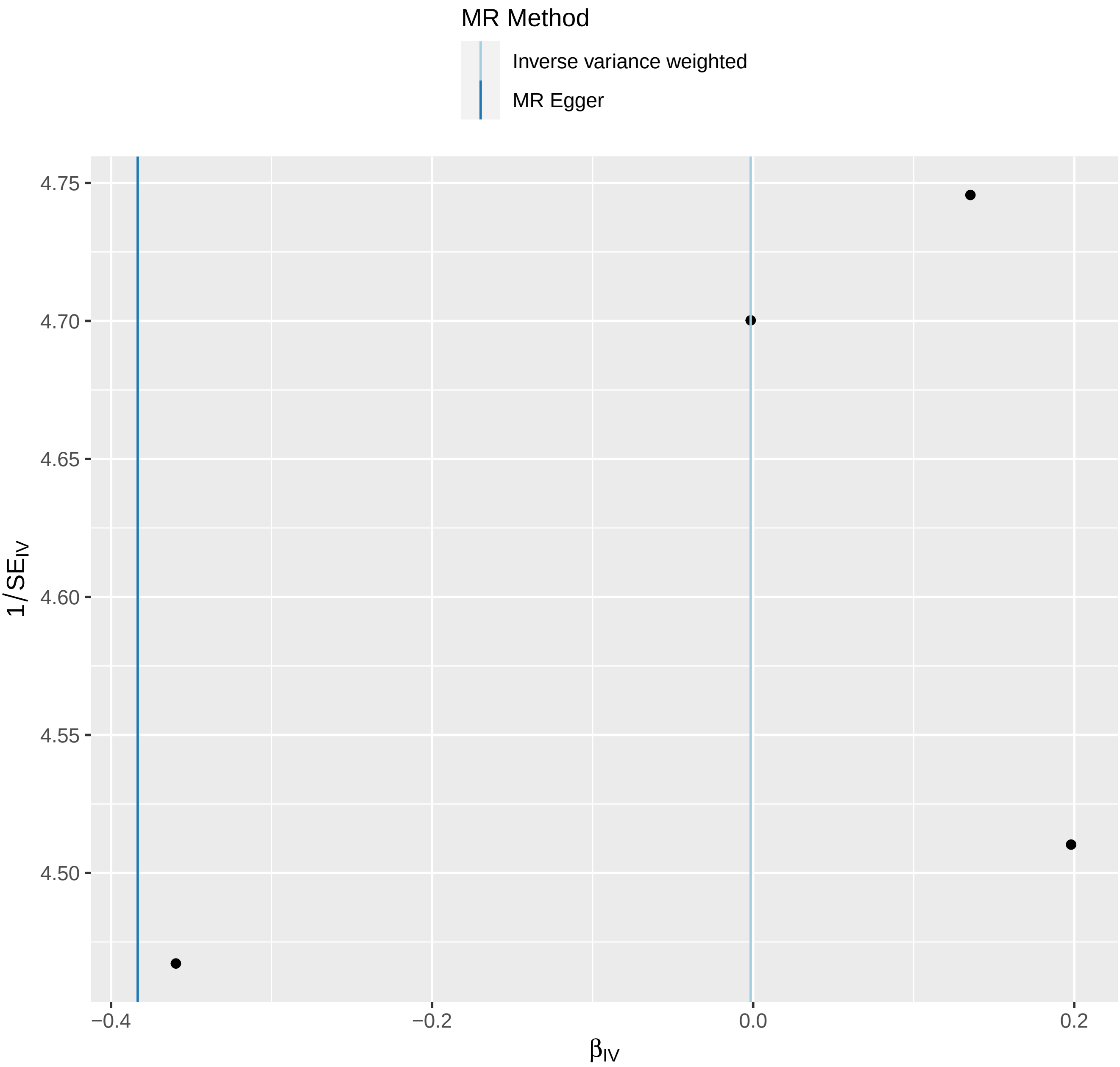

Batch 2−3366 : Gut microbiota abundance (genus Parasutterella id.2892) on Keratin, type I cytoskeletal 18
with a threshold of 1e−05

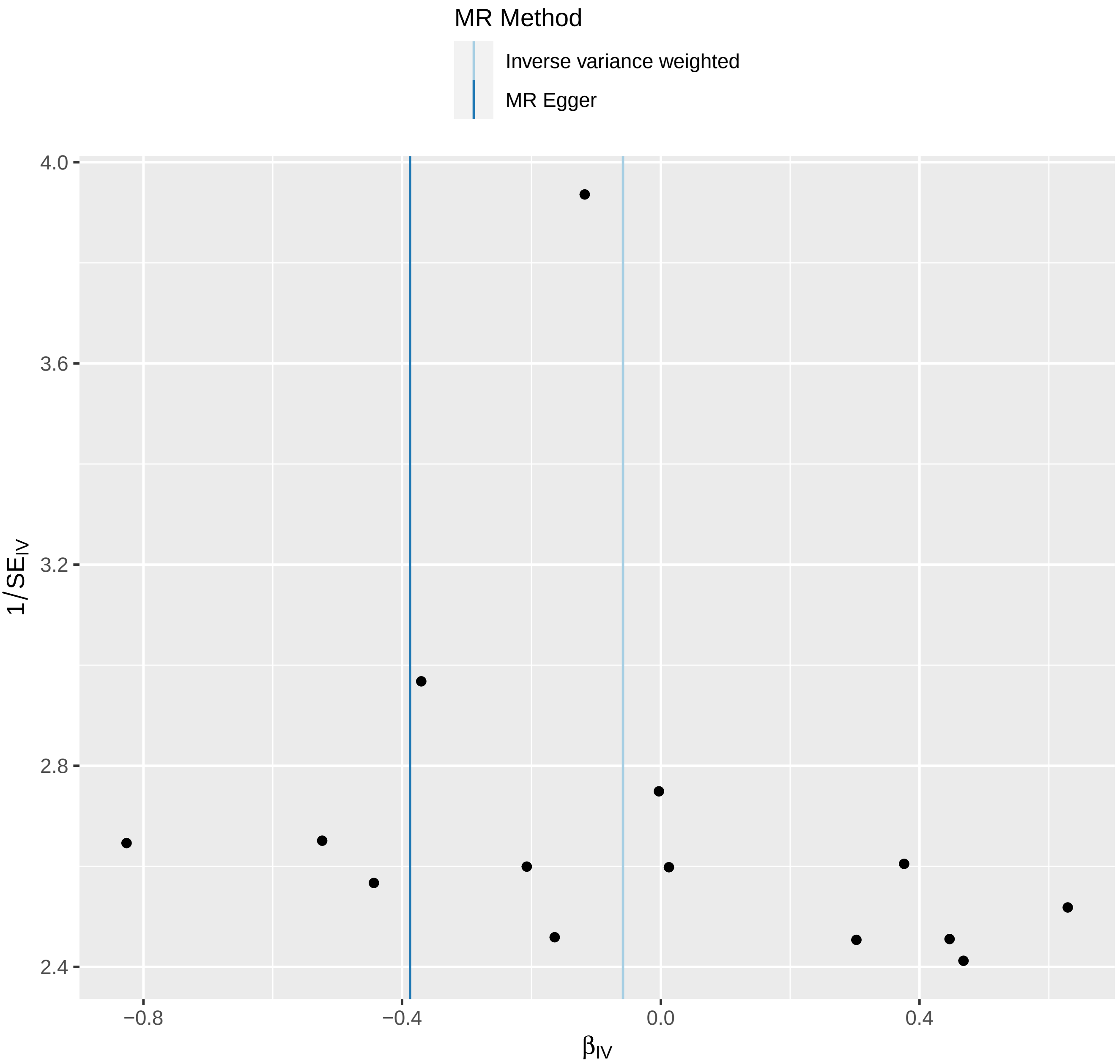

Batch 2−3367 : Gut microbiota abundance (genus Catenibacterium id.2153) on Keratin, type I cytoskeletal 20
with a threshold of 1e−05

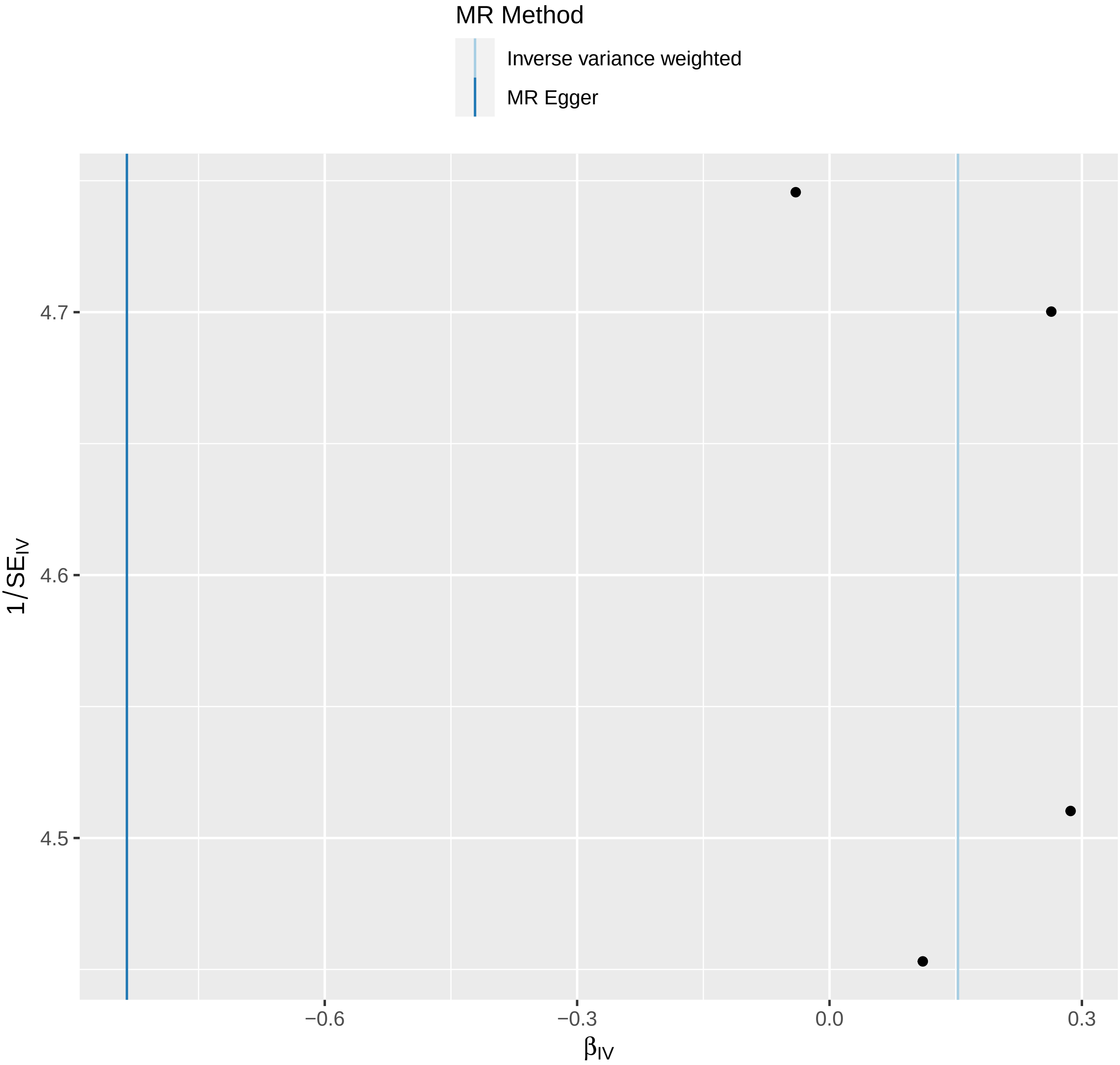

Batch 2−3368 : Gut microbiota abundance (genus Parasutterella id.2892) on Keratin, type I cytoskeletal 20
with a threshold of 1e−05

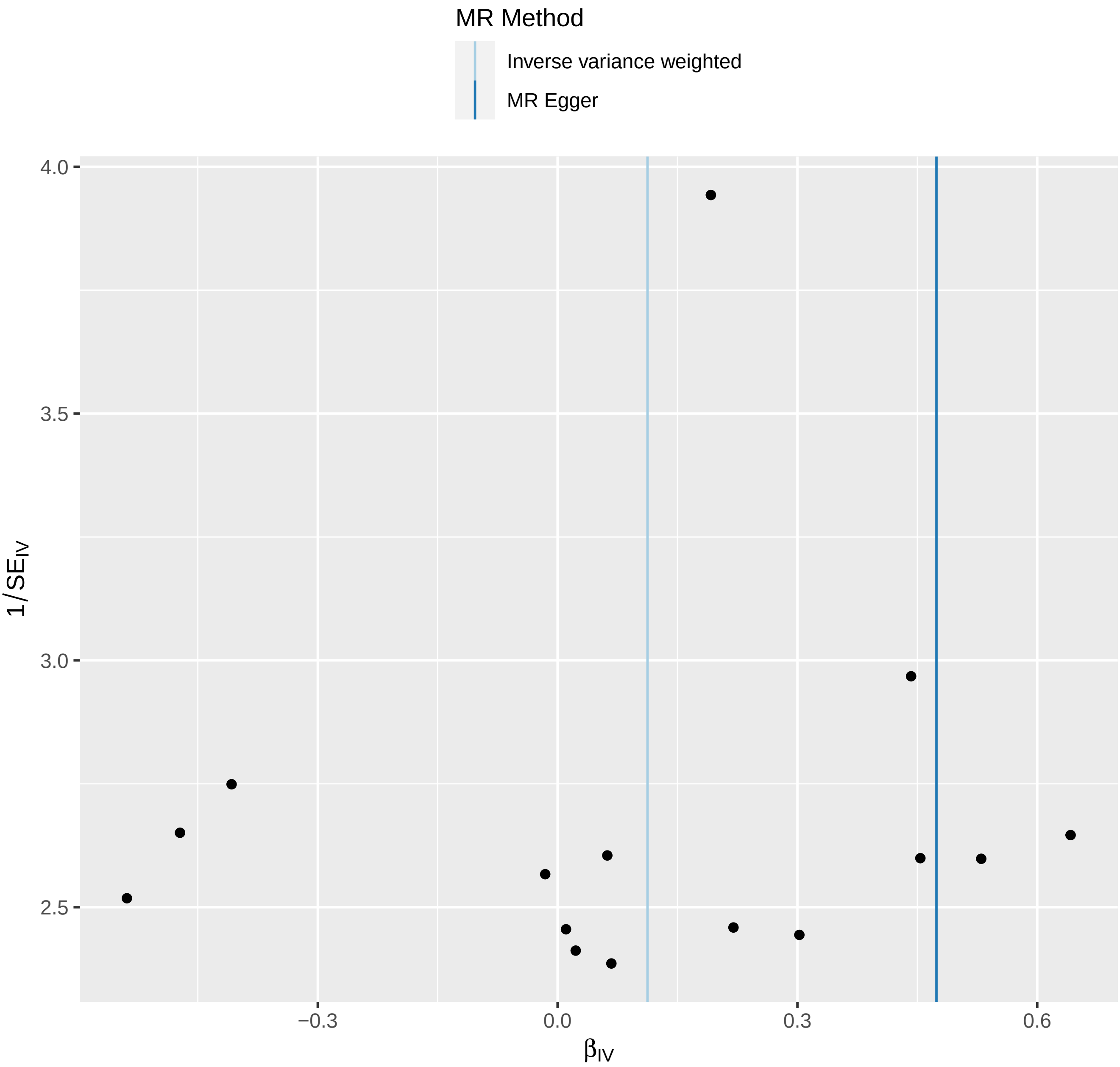

Batch 2−3369 : Gut microbiota abundance (genus Catenibacterium id.2153) on Keratin, type II cytoskeletal 5
with a threshold of 1e−05

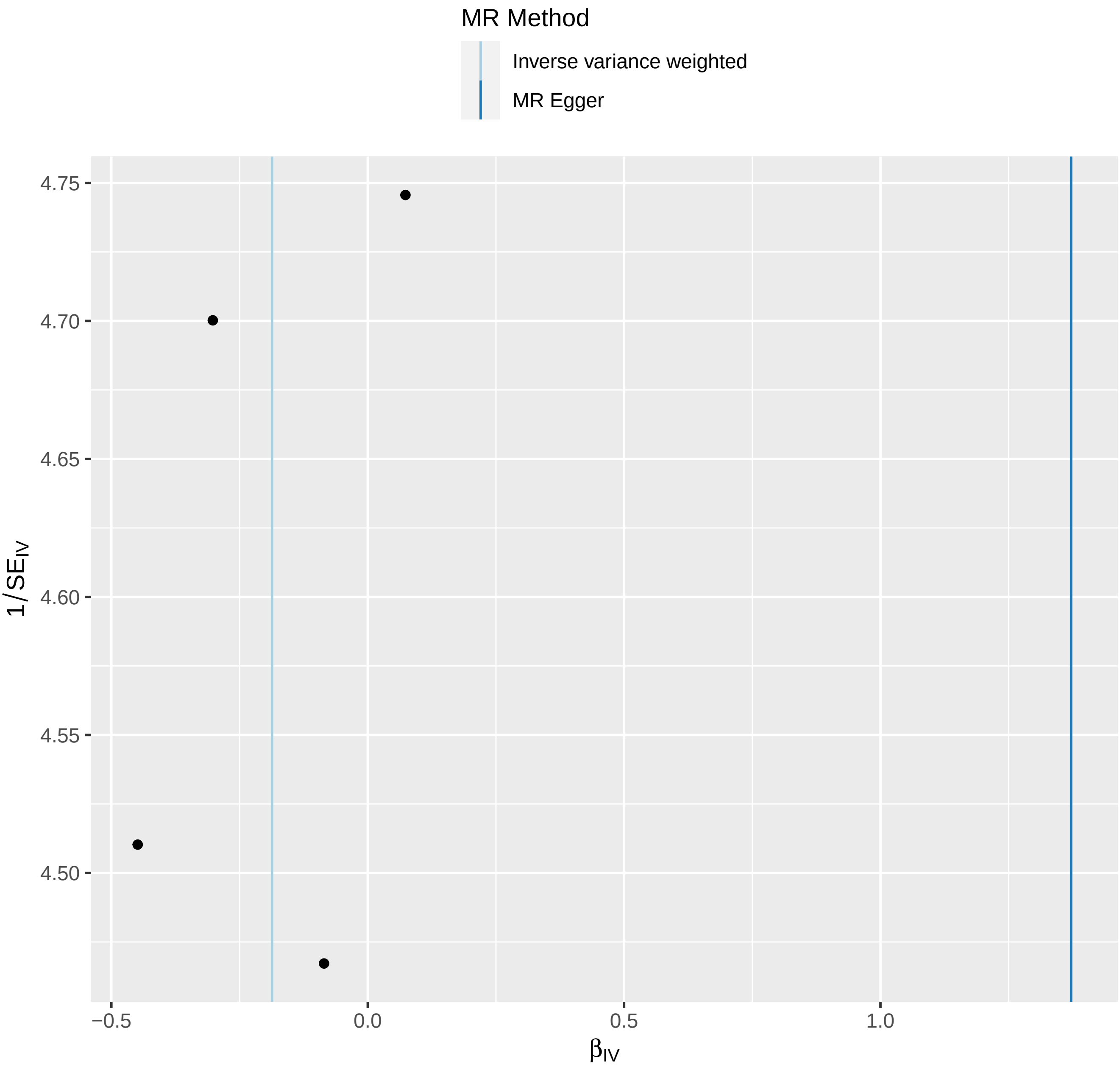

Batch 2−3370 : Gut microbiota abundance (genus Parasutterella id.2892) on Keratin, type II cytoskeletal 5
with a threshold of 1e−05

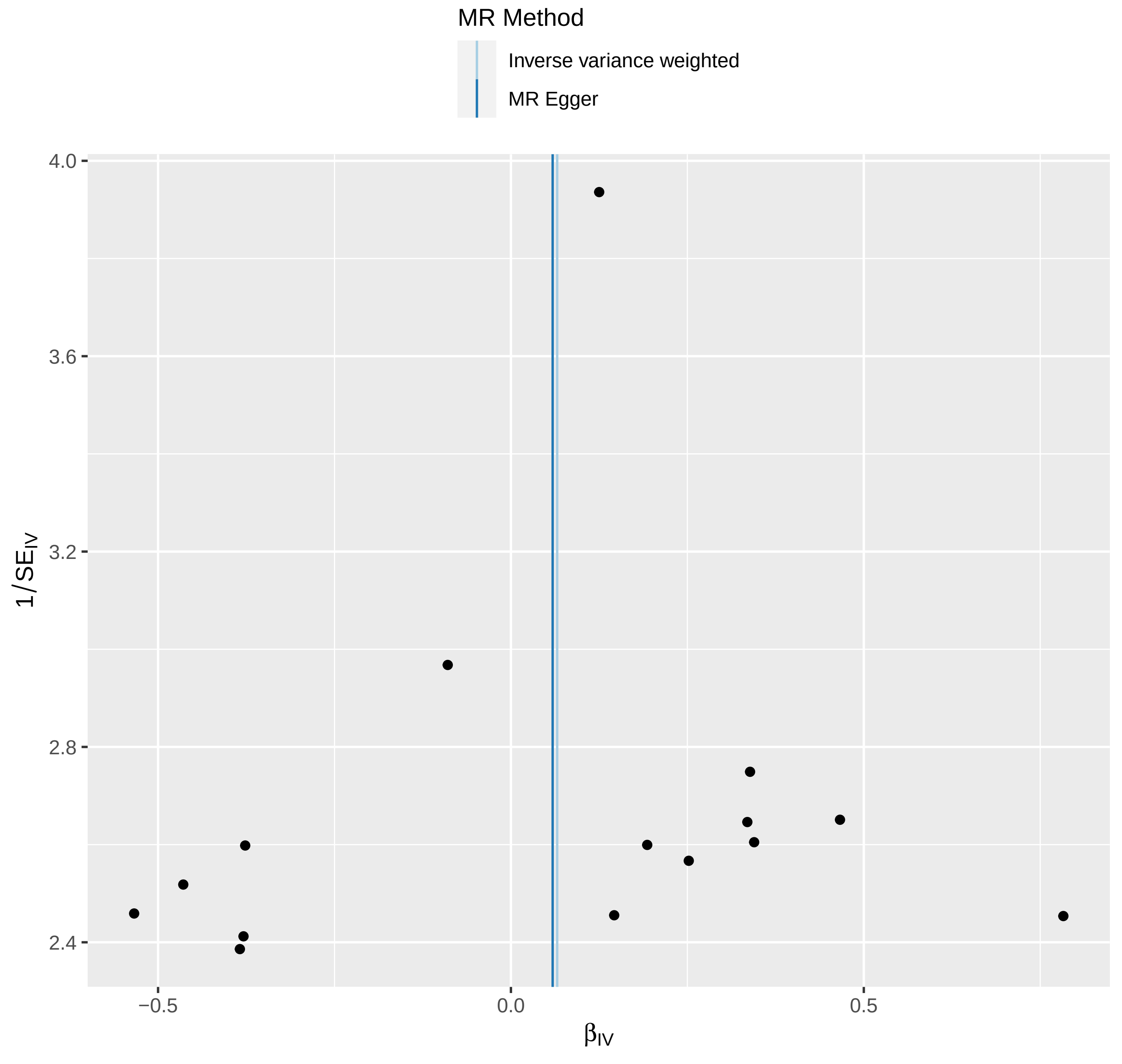

Batch 2−3371 : Gut microbiota abundance (genus Catenibacterium id.2153) on Keratin, type II cytoskeletal 7
with a threshold of 1e−05

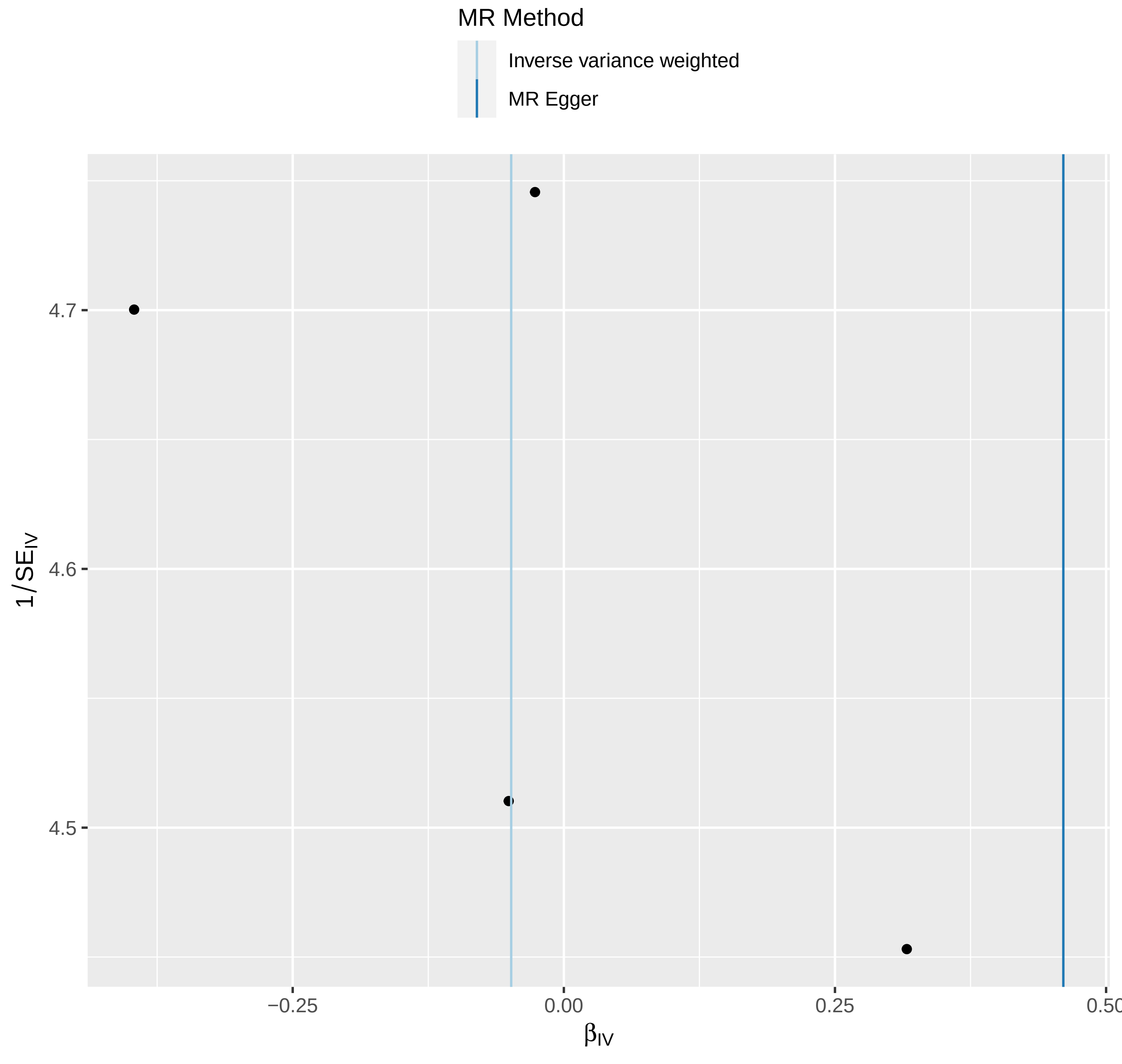

Batch 2−3372 : Gut microbiota abundance (genus Parasutterella id.2892) on Keratin, type II cytoskeletal 7
with a threshold of 1e−05

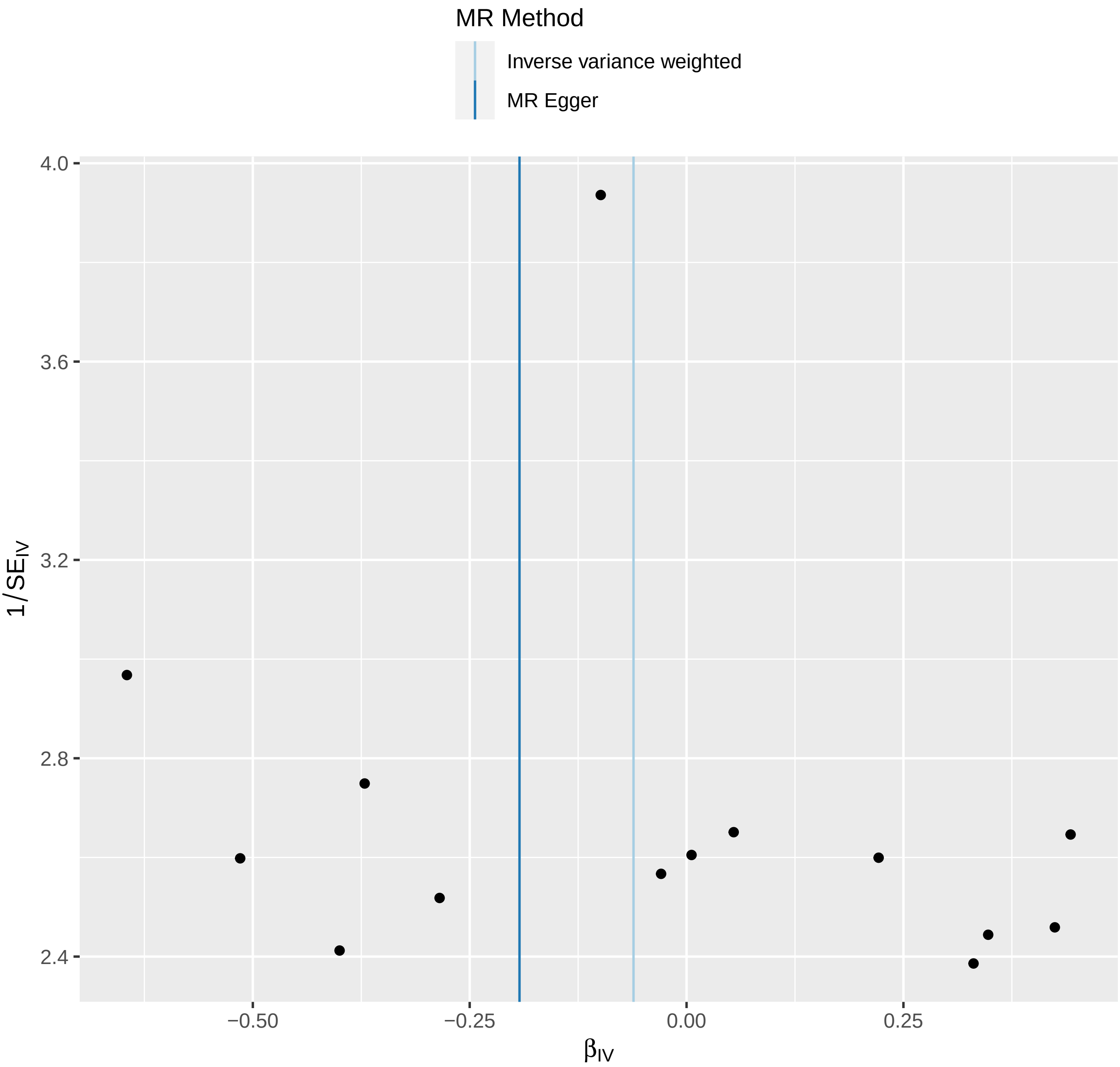

Batch 2−3373 : Gut microbiota abundance (genus Catenibacterium id.2153) on Keratin−associated protein 2−4
with a threshold of 1e−05

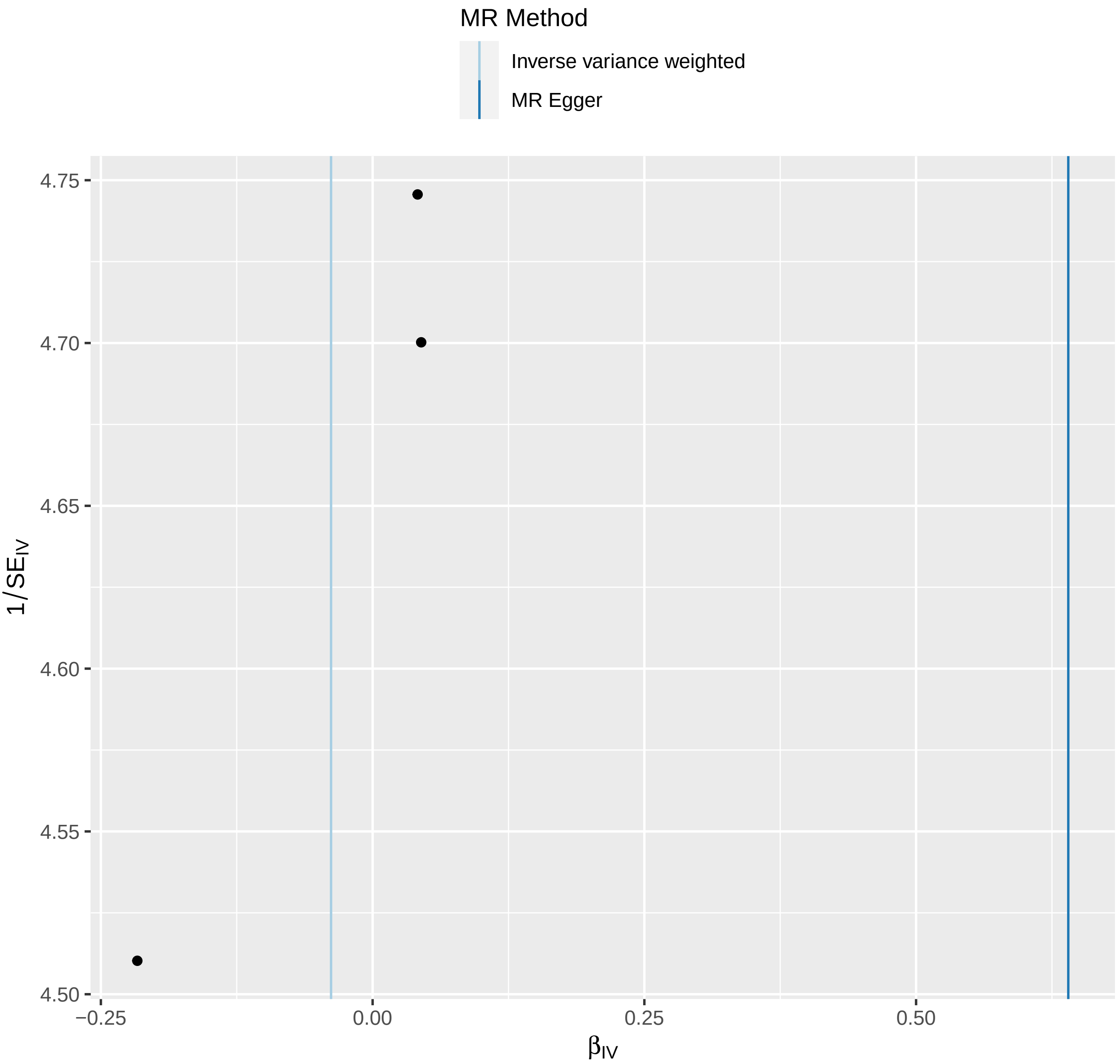

Batch 2−3374 : Gut microbiota abundance (genus Parasutterella id.2892) on Keratin−associated protein 2−4
with a threshold of 1e−05

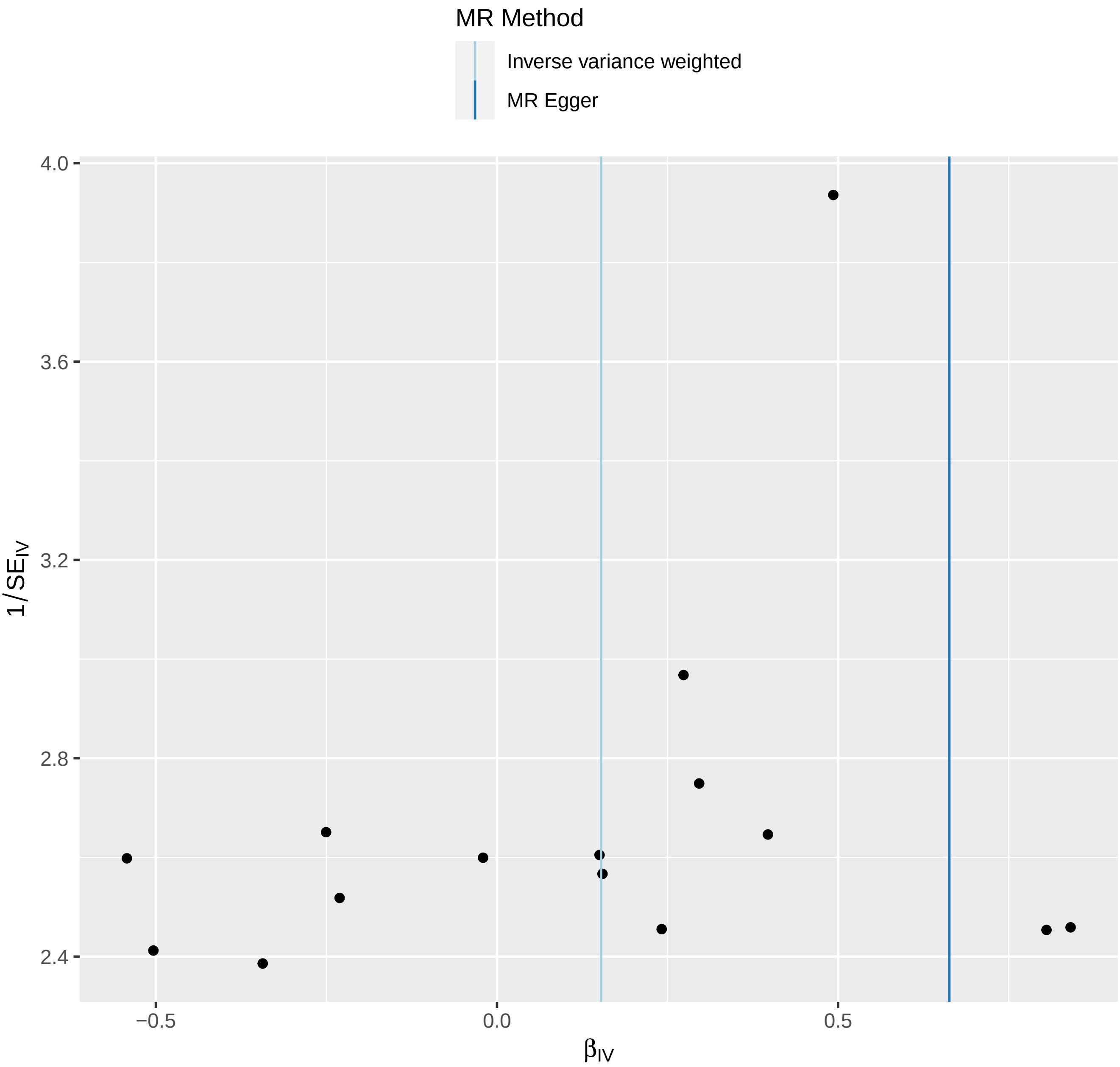

Batch 2−3375 : Gut microbiota abundance (genus Catenibacterium id.2153) on Keratinocyte differentiation−associated protein
with a threshold of 1e−05

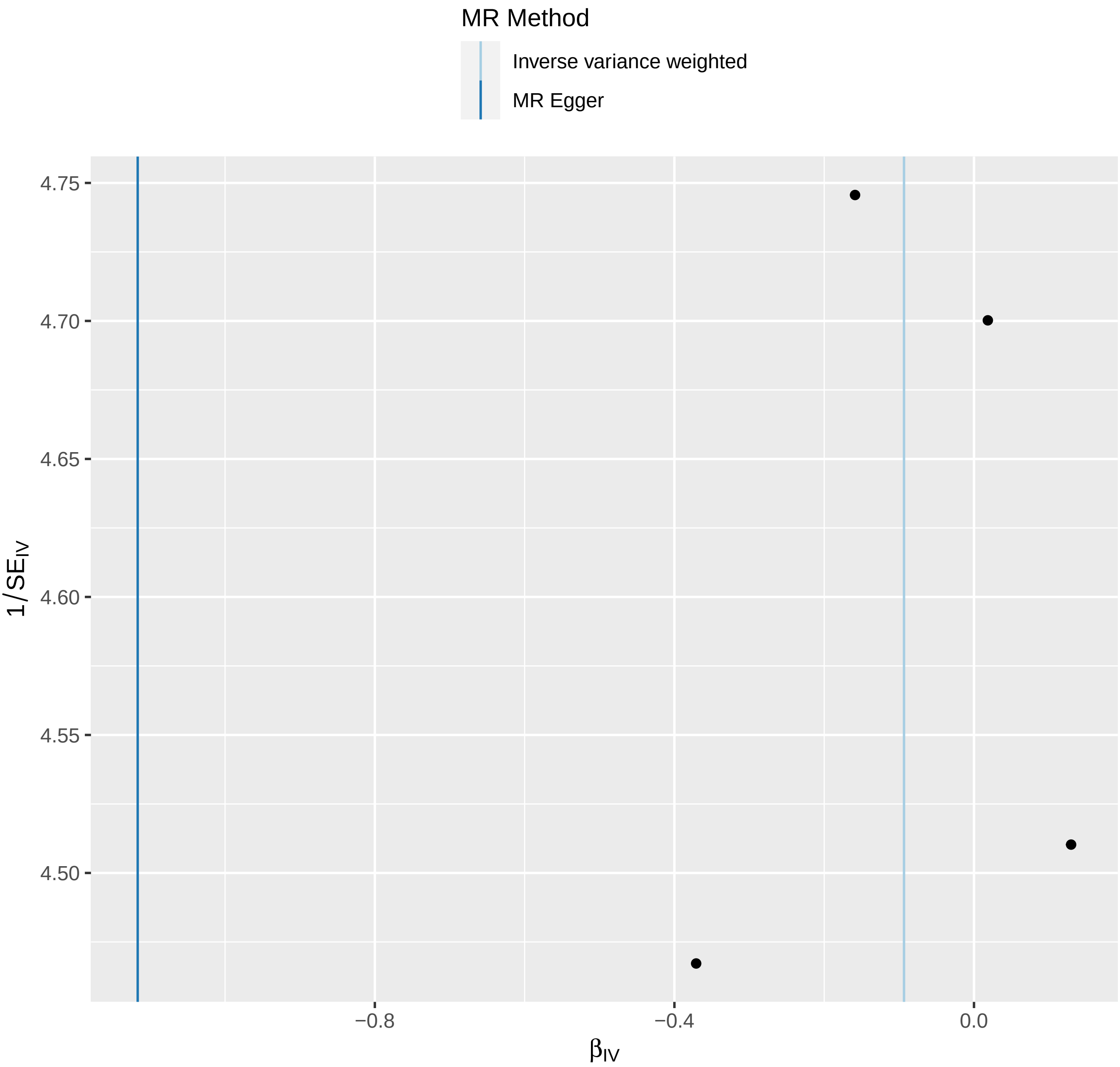

Batch 2−3376 : Gut microbiota abundance (genus Parasutterella id.2892) on Keratinocyte differentiation−associated protein
with a threshold of 1e−05

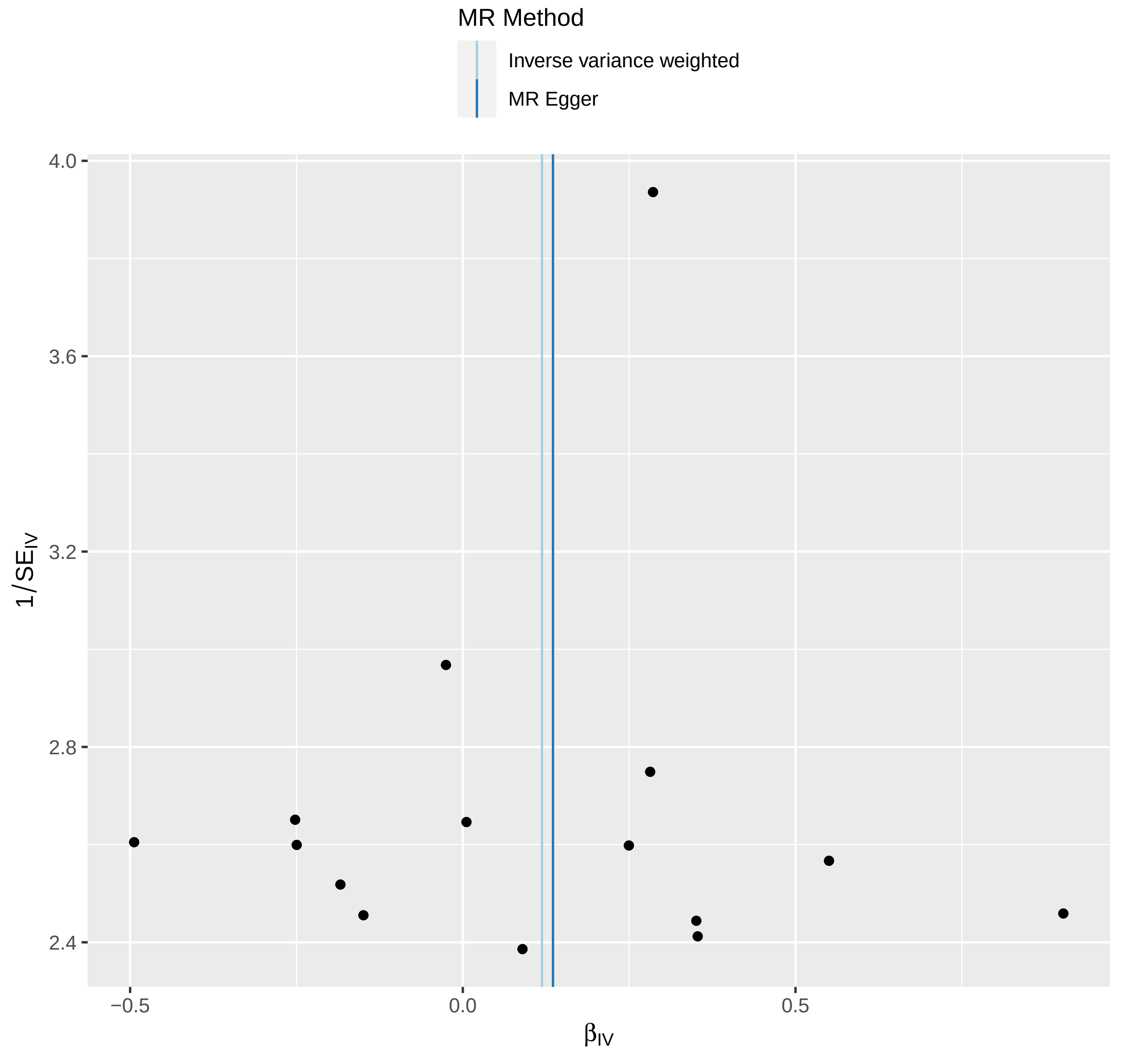

Batch 2−3377 : Gut microbiota abundance (genus Catenibacterium id.2153) on Kynureninase
with a threshold of 1e−05

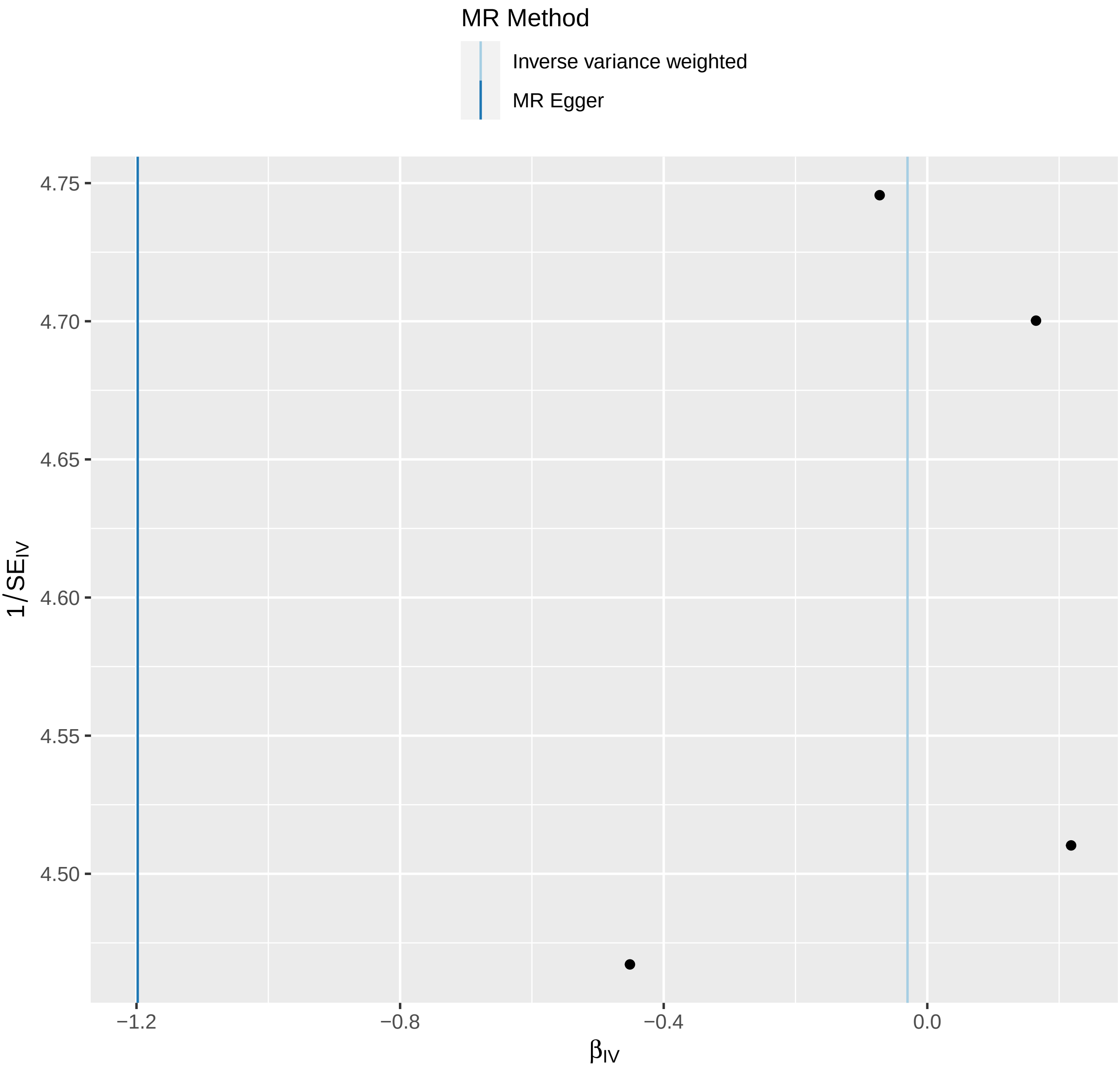

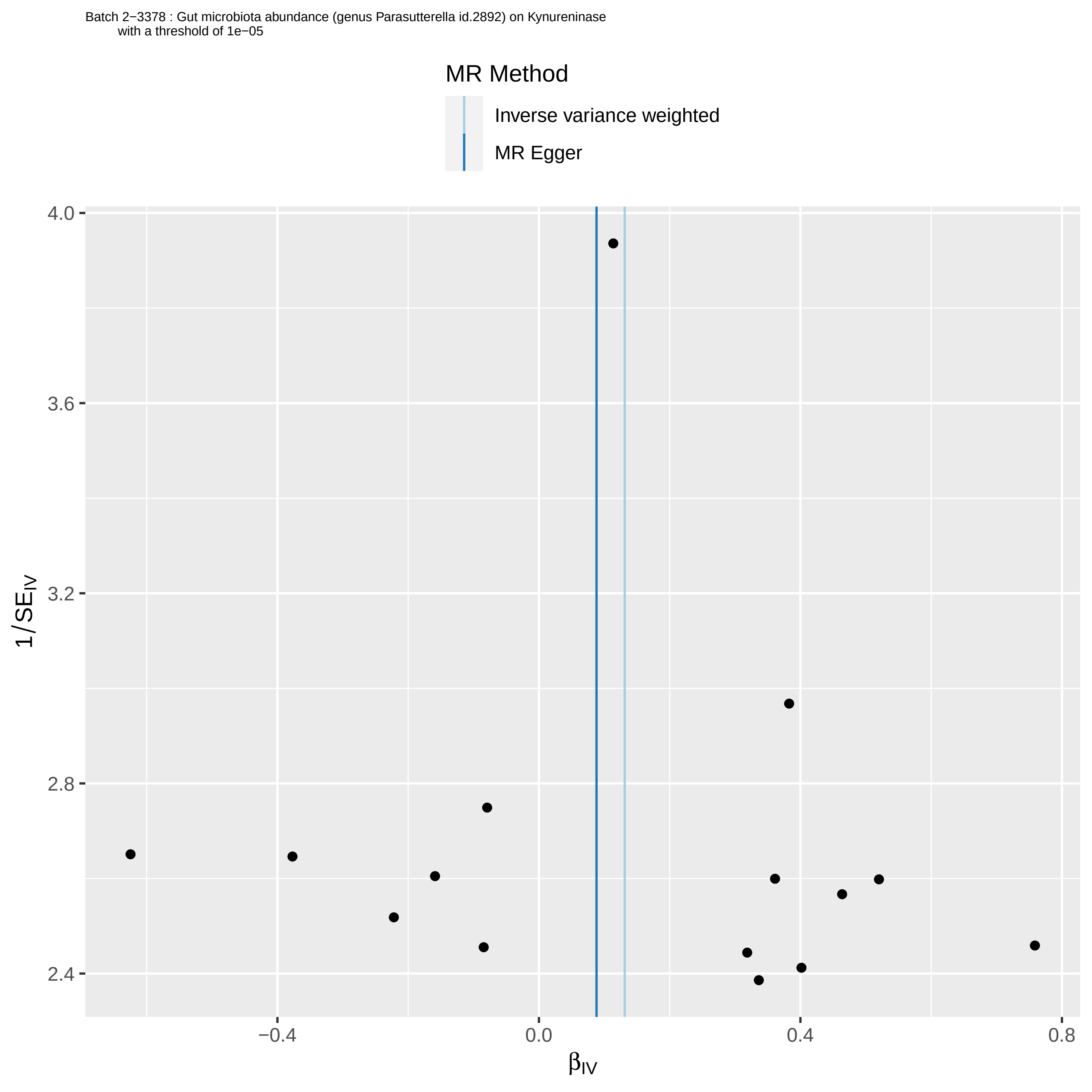
Batch 2−3378 : Gut microbiota abundance (genus Parasutterella id.2892) on Kynureninase
with a threshold of 1e−05
MR Method
Inverse variance weighted
MR Egger
4.0
3.6
3.2
2.8
2.4
1/SE$_{IV}$
−0.4
0.0
0.4
0.8
β_{IV}

Batch 2−3379 : Gut microbiota abundance (genus Catenibacterium id.2153) on Neural cell adhesion molecule L1
with a threshold of 1e−05

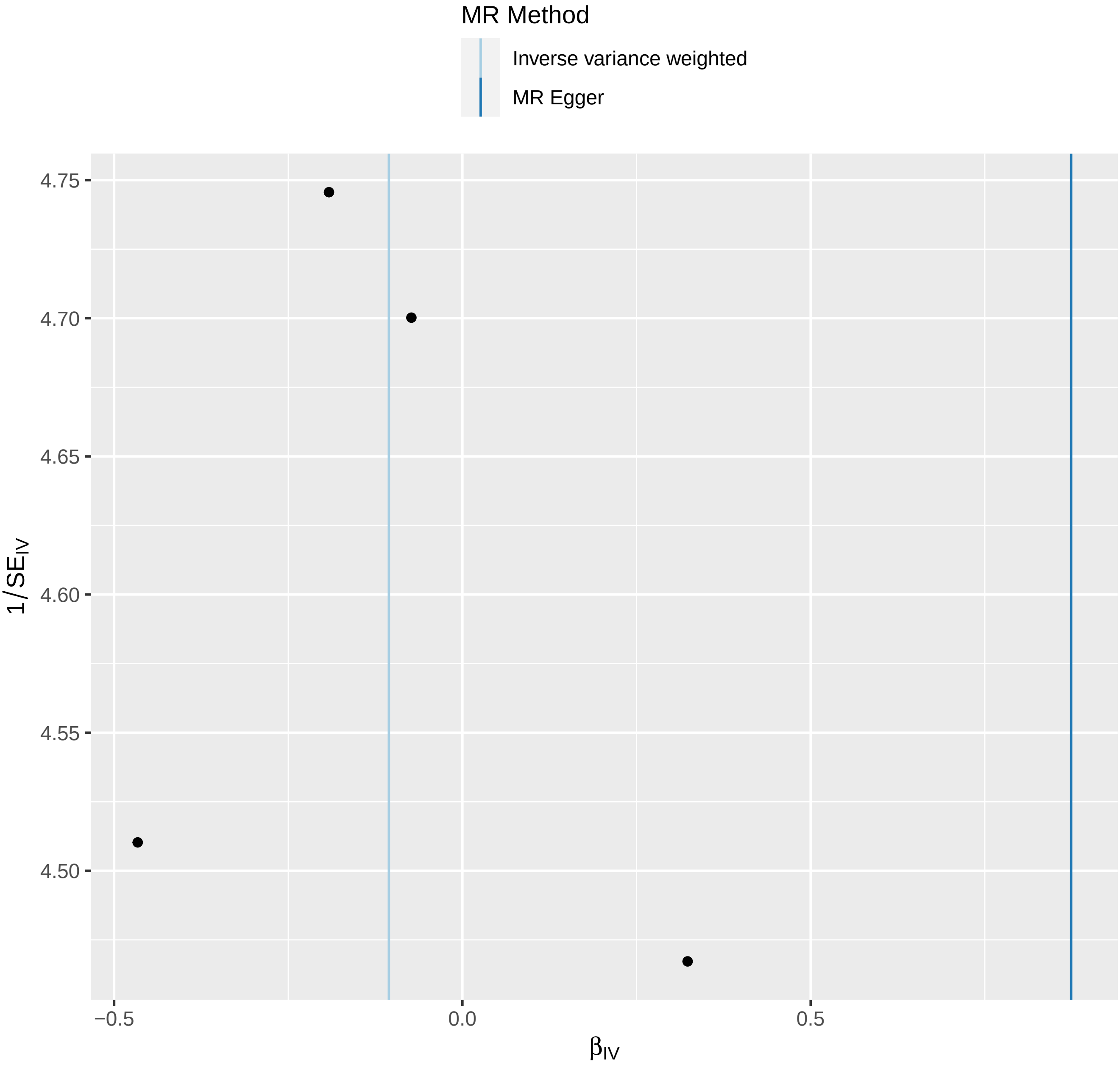

Batch 2−3380 : Gut microbiota abundance (genus Parasutterella id.2892) on Neural cell adhesion molecule L1
with a threshold of 1e−05

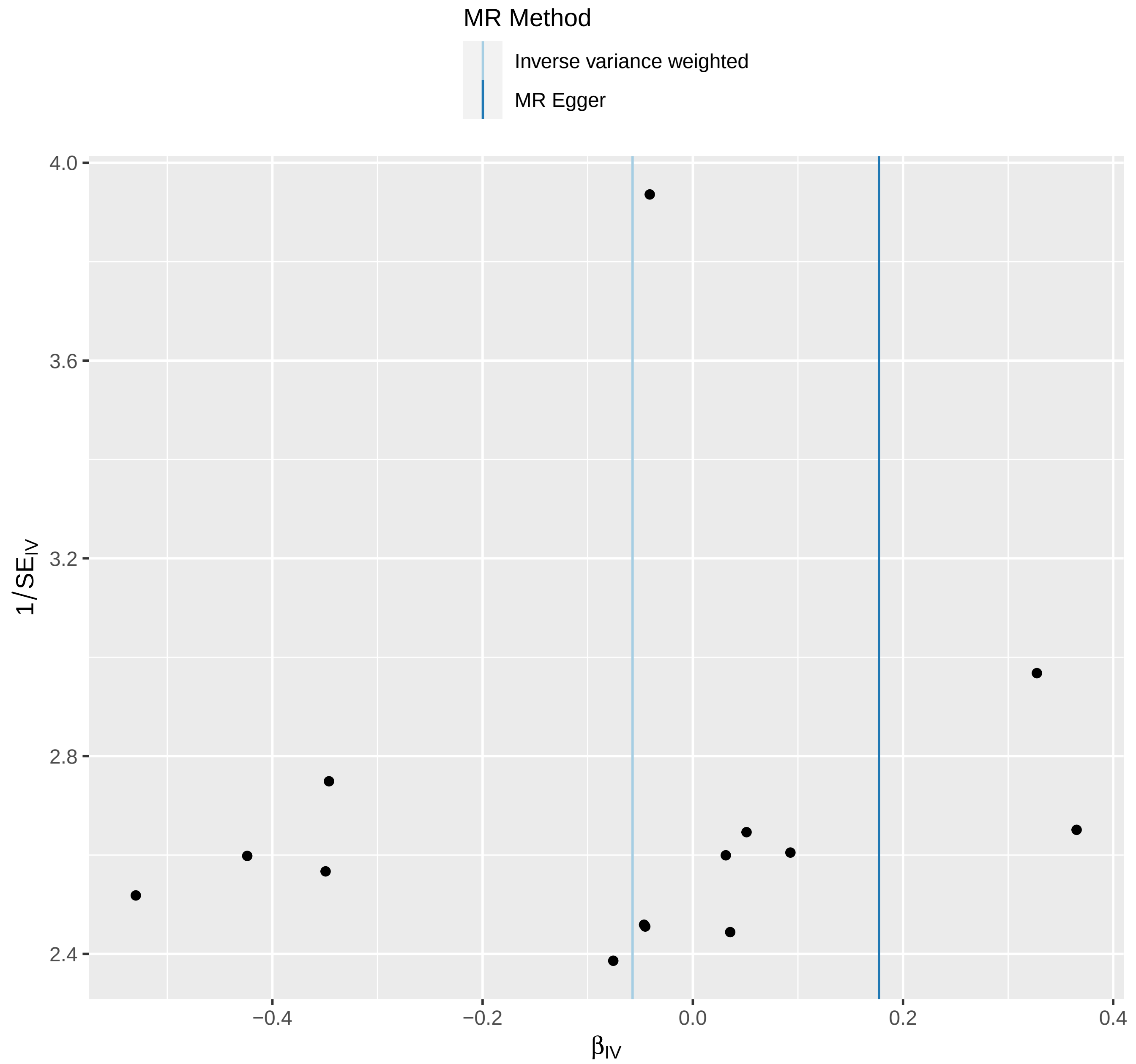

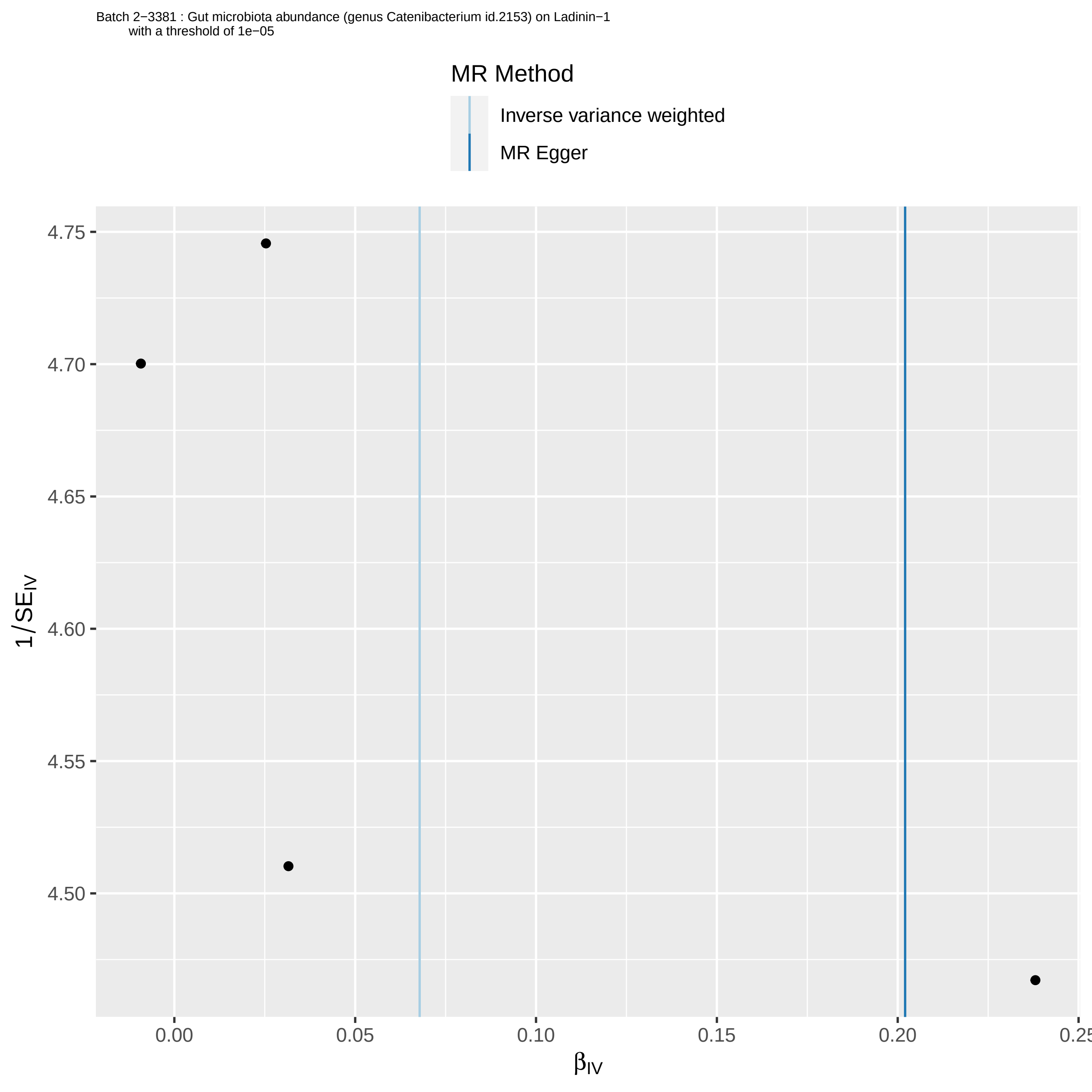
Batch 2−3381 : Gut microbiota abundance (genus Catenibacterium id.2153) on Ladinin−1
with a threshold of 1e−05
MR Method
Inverse variance weighted
MR Egger
4.75
4.70
4.65
4.60
4.55
4.50
1/SE_IV
0.00
0.05
0.10
0.15
0.20
0.25
β_IV

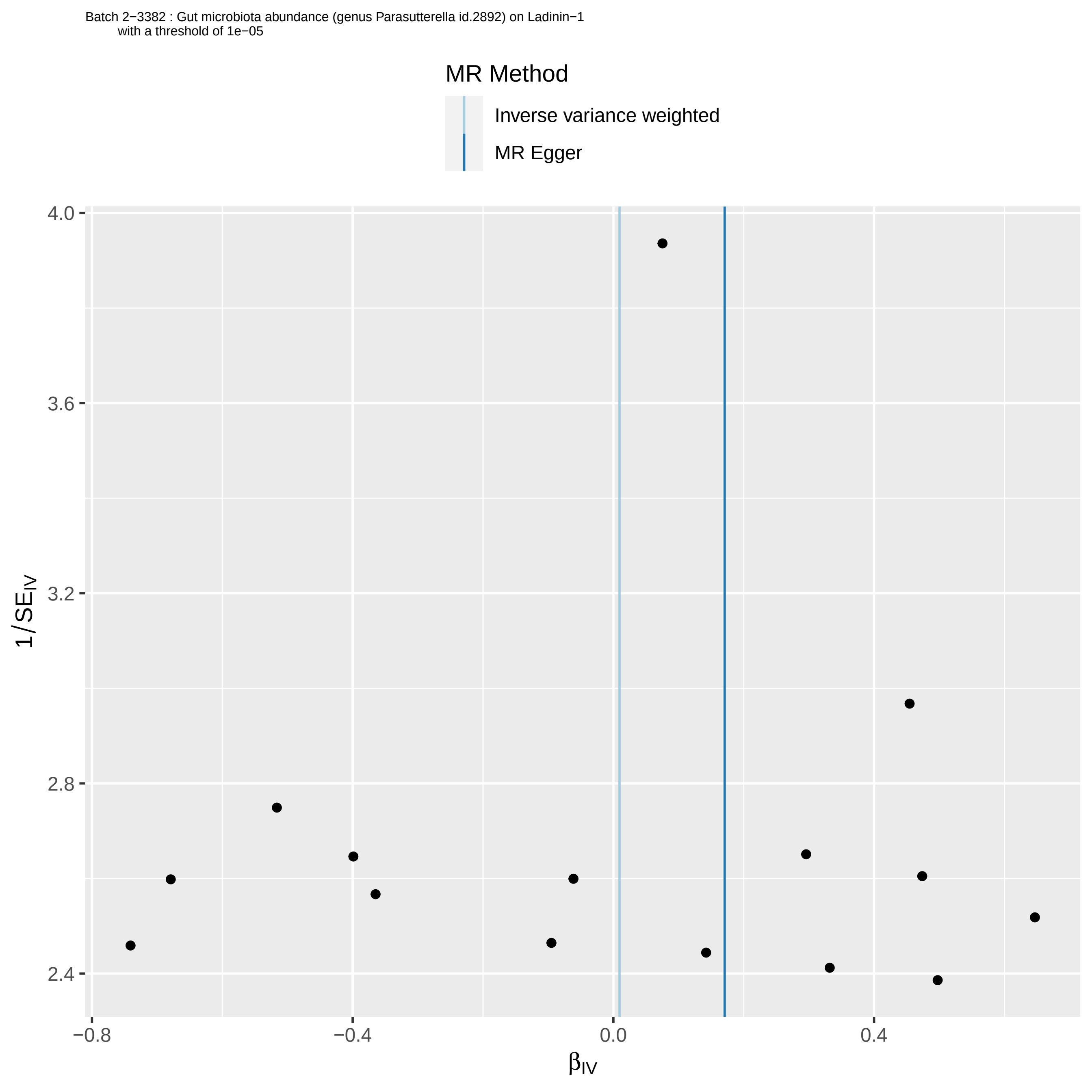

Batch 2−3382 : Gut microbiota abundance (genus Parasutterella id.2892) on Ladinin−1
with a threshold of 1e−05
MR Method
Inverse variance weighted
MR Egger
4.0
3.6
3.2
2.8
2.4
1/SE_IV
−0.8
−0.4
0.0
0.4
β_IV

Batch 2−3383 : Gut microbiota abundance (genus Catenibacterium id.2153) on Lymphocyte activation gene 3 protein with a threshold of 1e−05

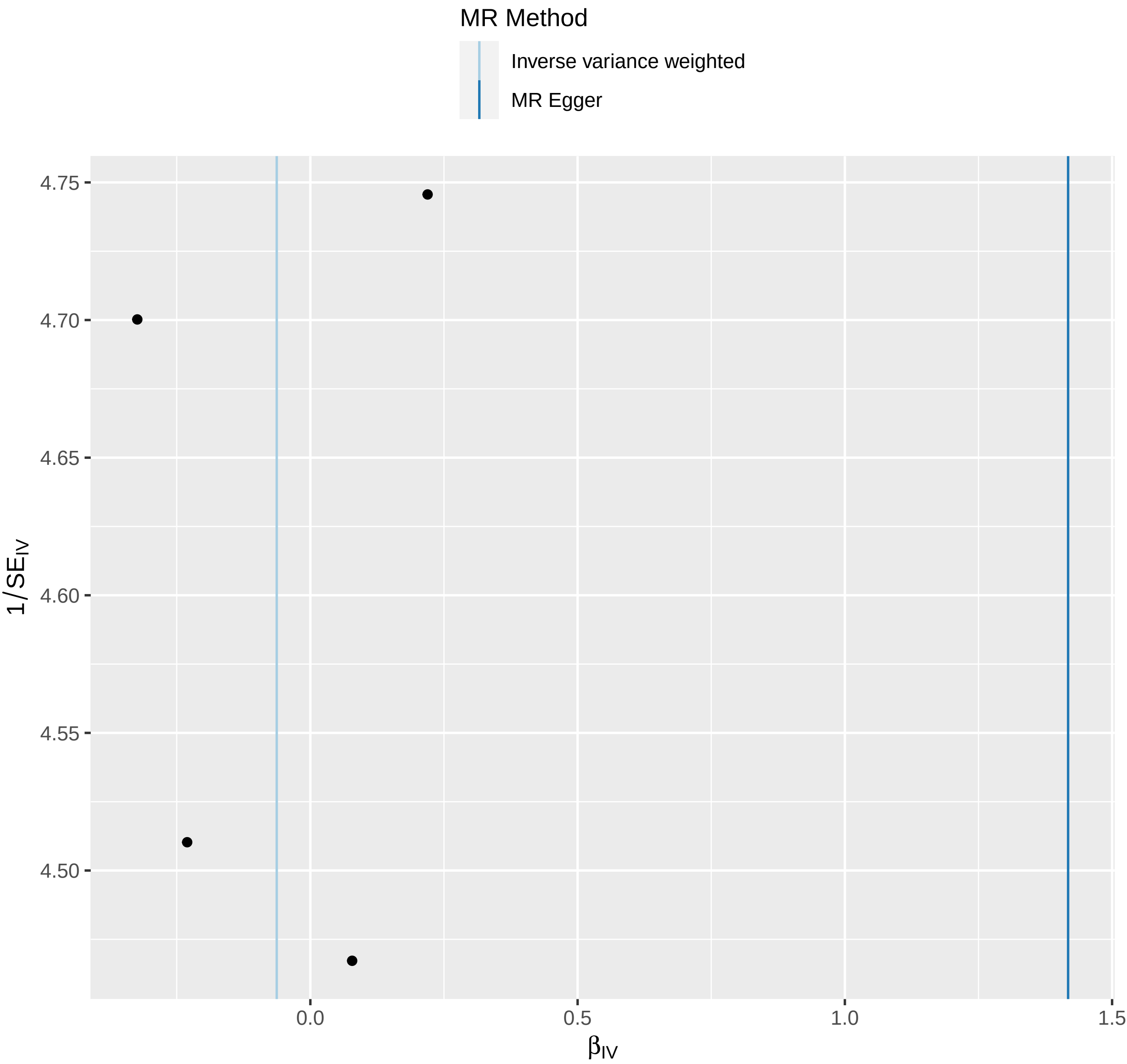

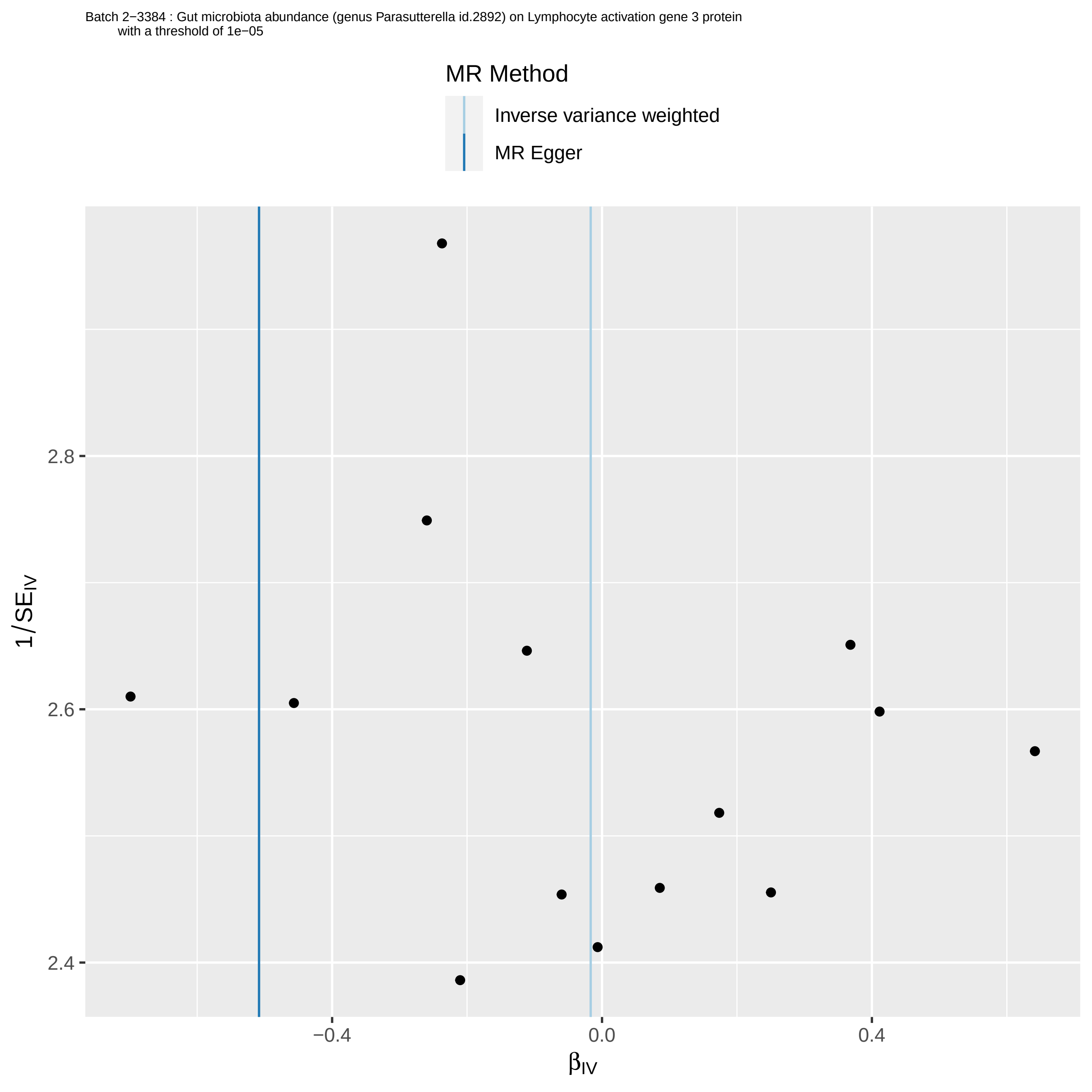

Batch 2−3384 : Gut microbiota abundance (genus Parasutterella id.2892) on Lymphocyte activation gene 3 protein
with a threshold of 1e−05
MR Method
Inverse variance weighted
MR Egger
2.8
2.6
2.4
1/SE_IV
−0.4
0.0
0.4
β_IV

Batch 2−3385 : Gut microbiota abundance (genus Catenibacterium id.2153) on Lymphocyte activation gene 3 protein
with a threshold of 1e−05

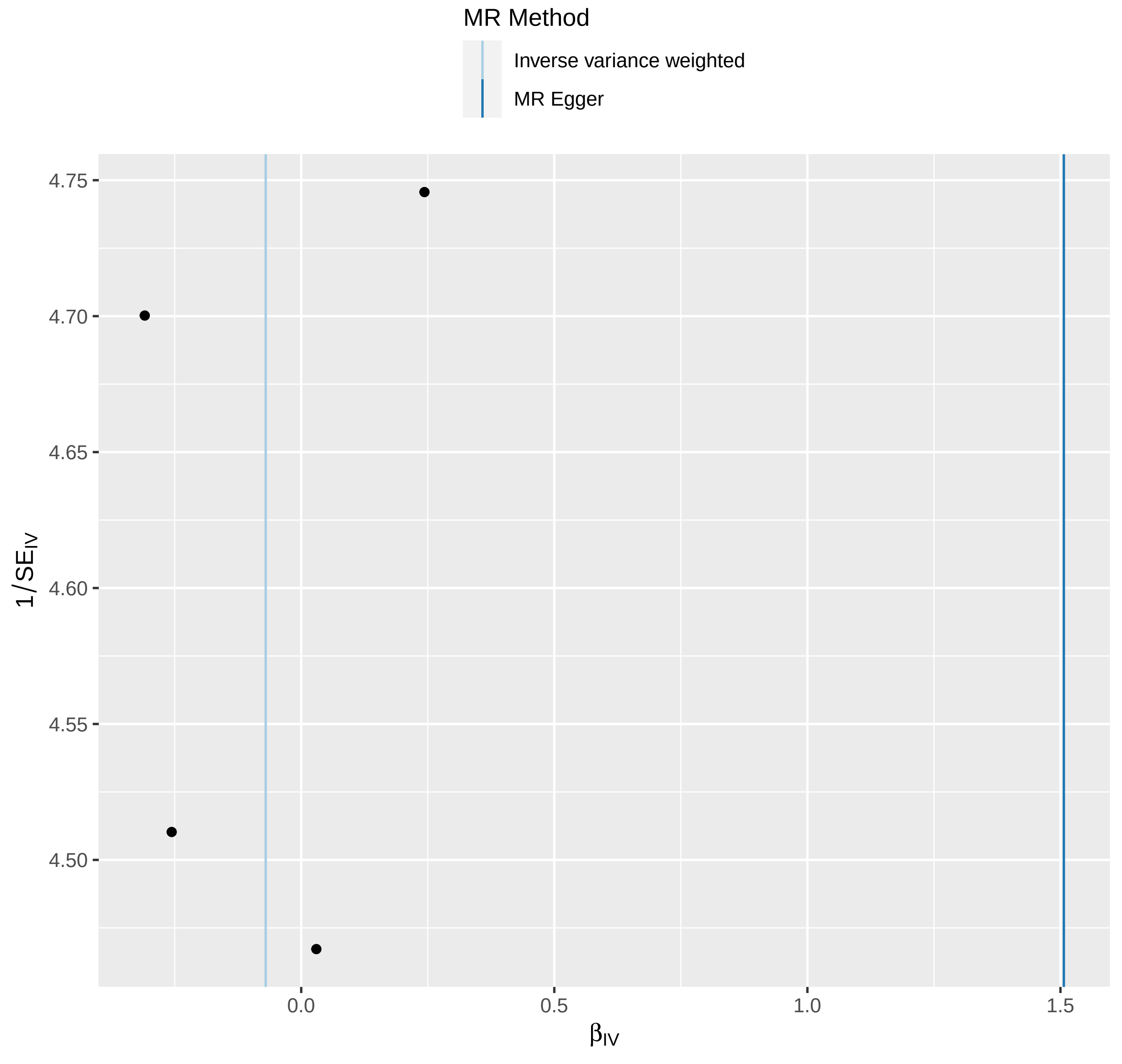

Batch 2−3386 : Gut microbiota abundance (genus Parasutterella id.2892) on Lymphocyte activation gene 3 protein
with a threshold of 1e−05

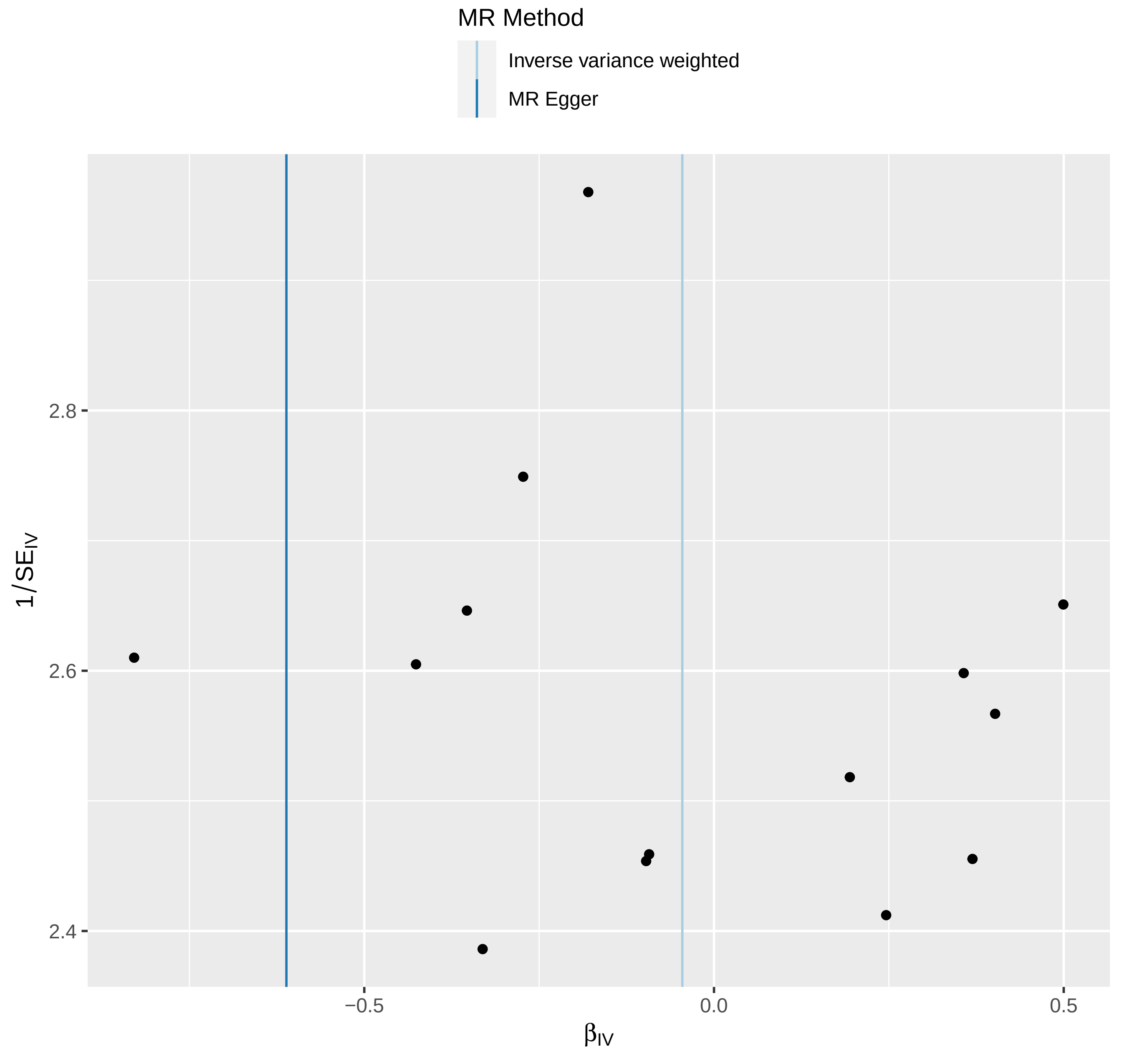

Batch 2−3387 : Gut microbiota abundance (genus Catenibacterium id.2153) on Leukocyte−associated immunoglobulin−like receptor 1 with a threshold of 1e−05

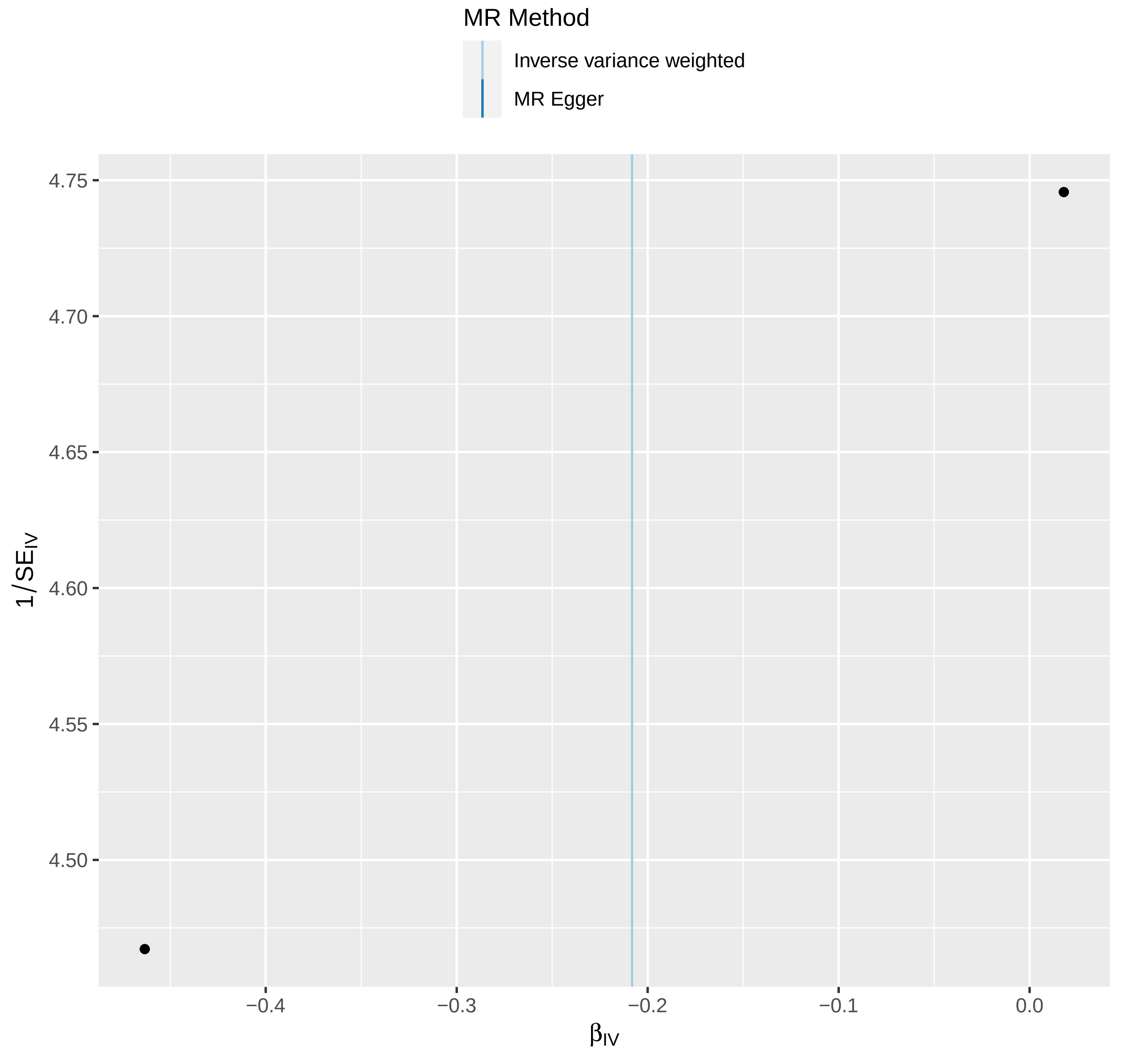

Batch 2−3388 : Gut microbiota abundance (genus Parasutterella id.2892) on Leukocyte−associated immunoglobulin−like receptor 1 with a threshold of 1e−05

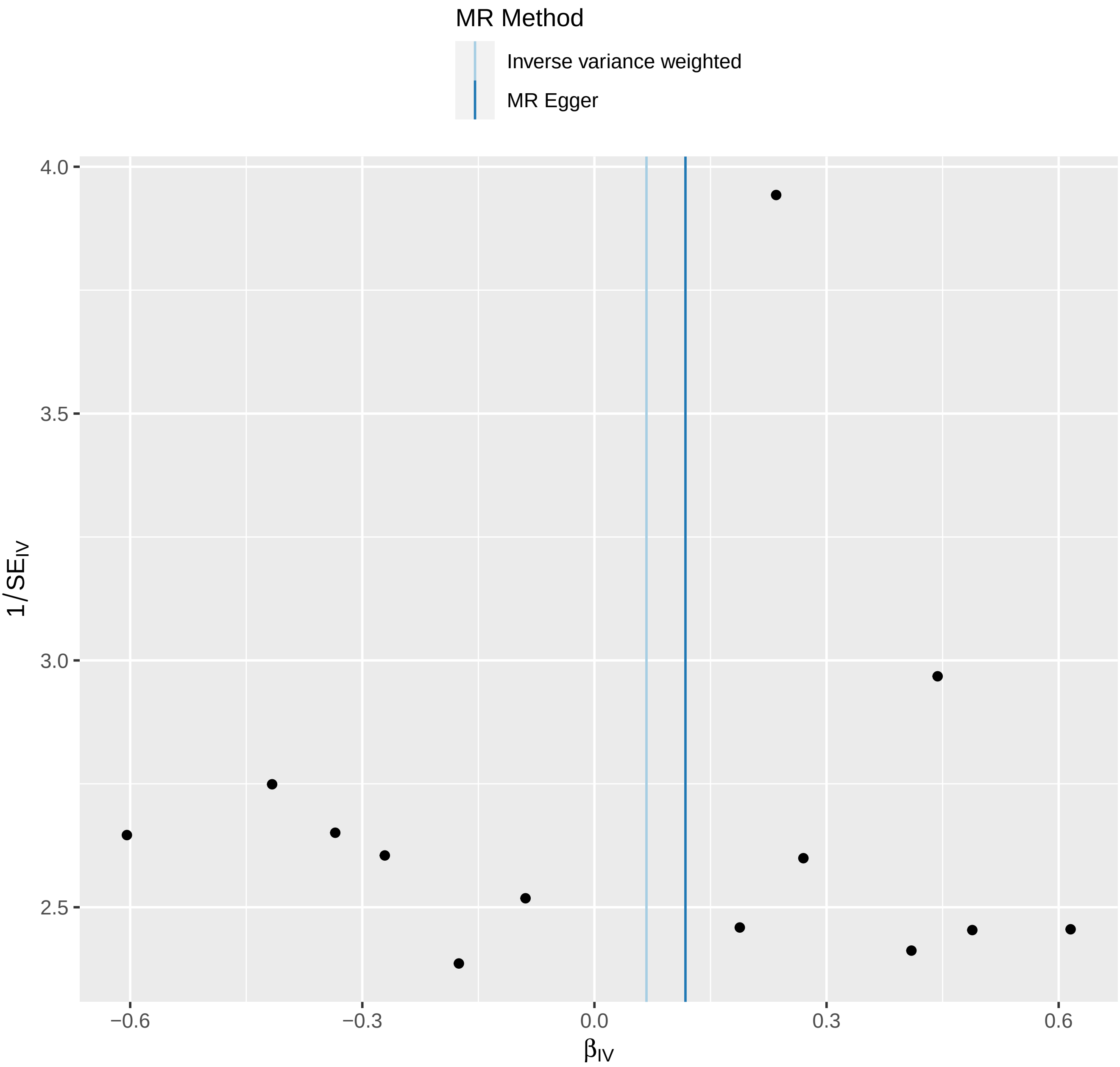

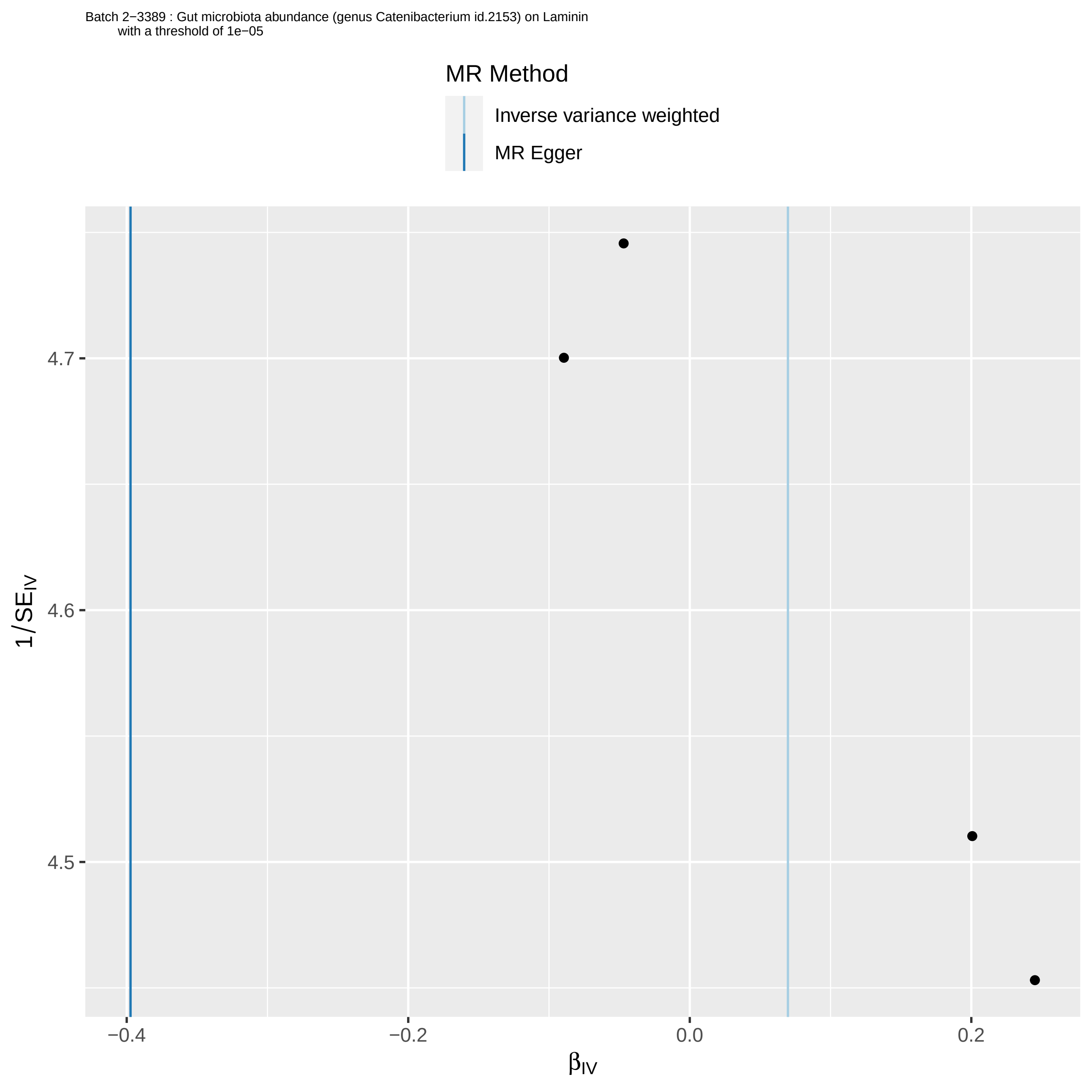
Batch 2−3389 : Gut microbiota abundance (genus Catenibacterium id.2153) on Laminin
with a threshold of 1e−05
MR Method
Inverse variance weighted
MR Egger
4.7
4.6
4.5
1/SE_IV
−0.4
−0.2
0.0
0.2
β_IV

Batch 2−3390 : Gut microbiota abundance (genus Parasutterella id.2892) on Laminin
with a threshold of 1e−05

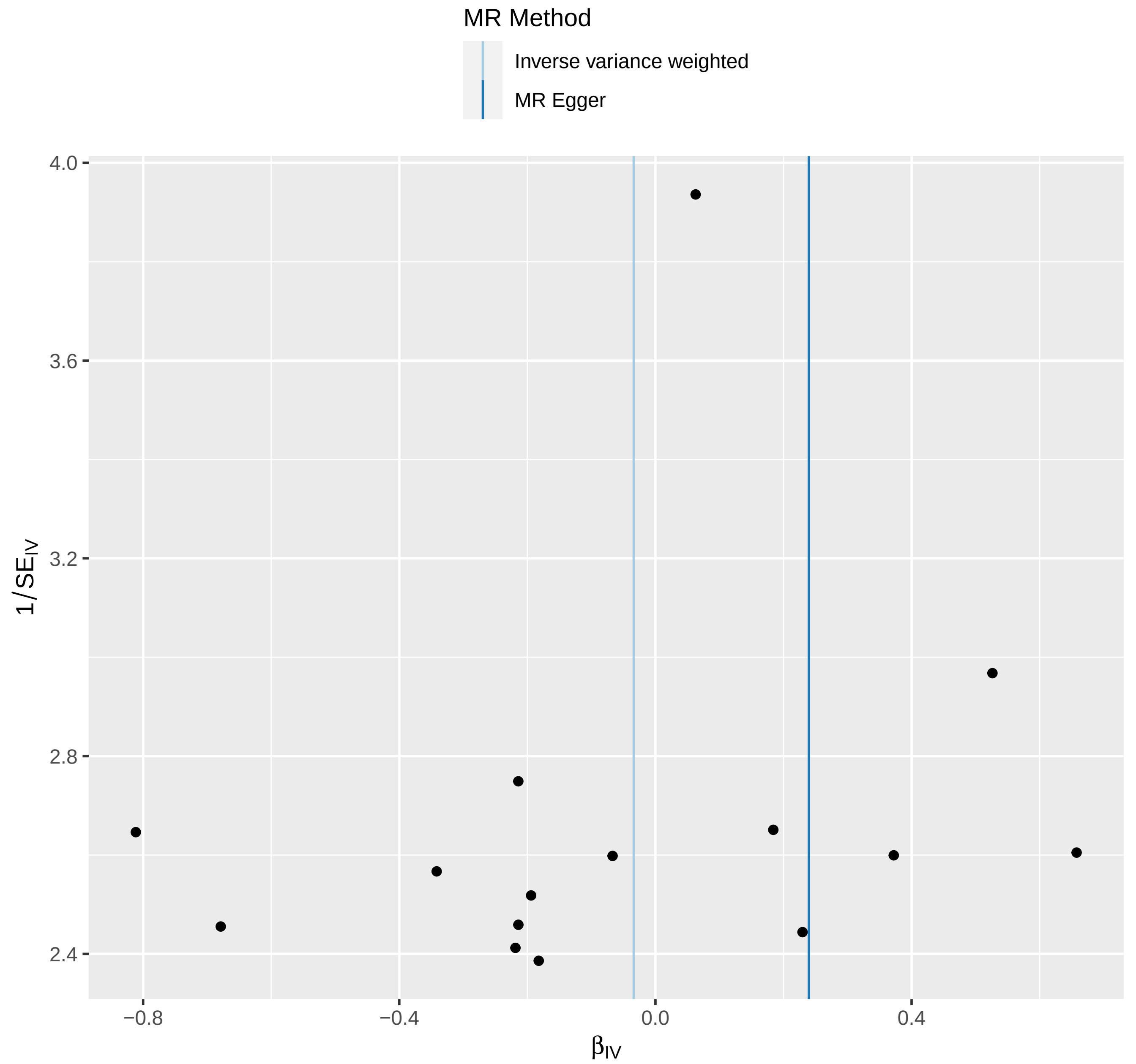

Batch 2−3391 : Gut microbiota abundance (genus Catenibacterium id.2153) on Laminin subunit alpha−4
with a threshold of 1e−05

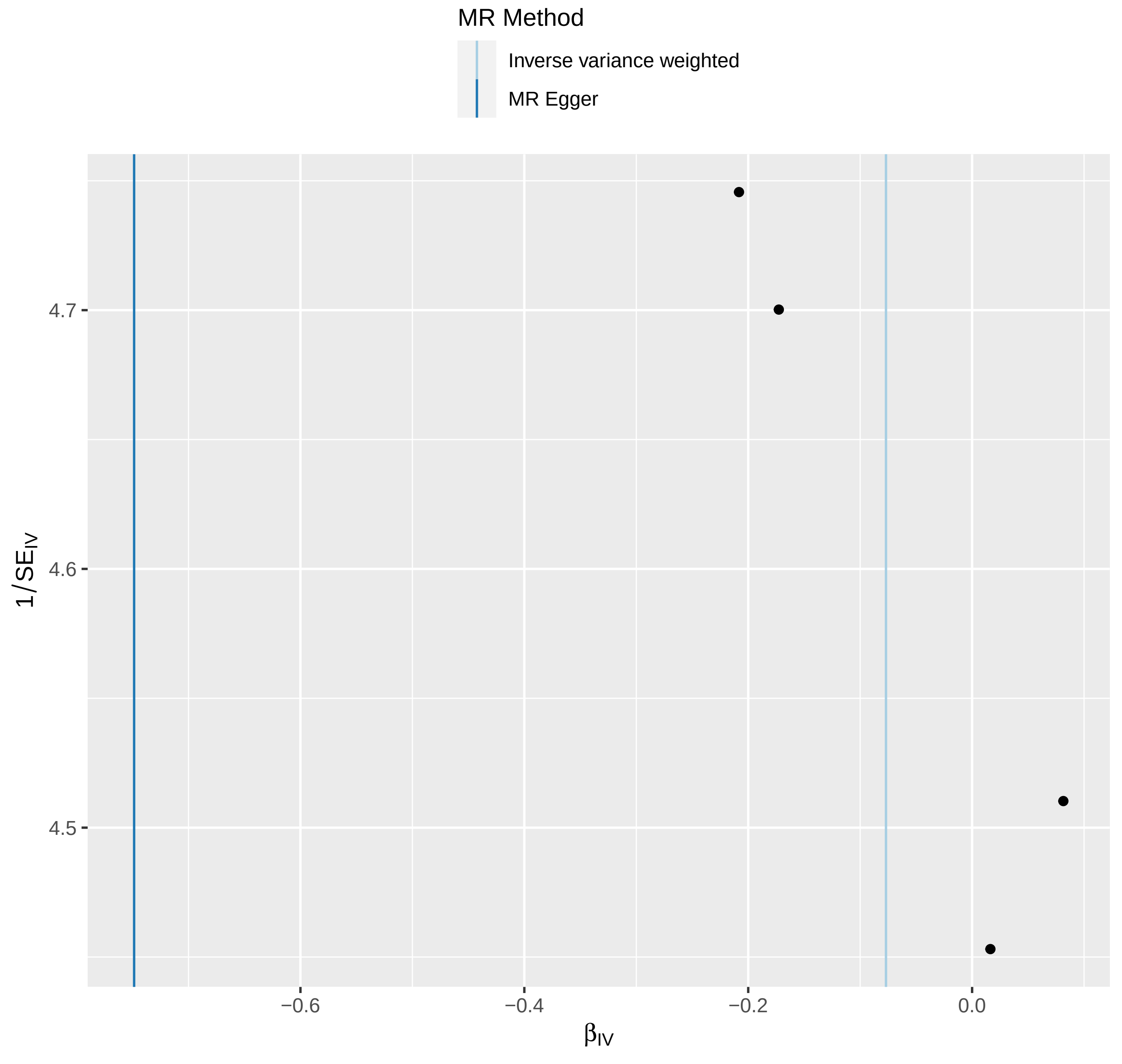

Batch 2−3392 : Gut microbiota abundance (genus Parasutterella id.2892) on Laminin subunit alpha−4
with a threshold of 1e−05

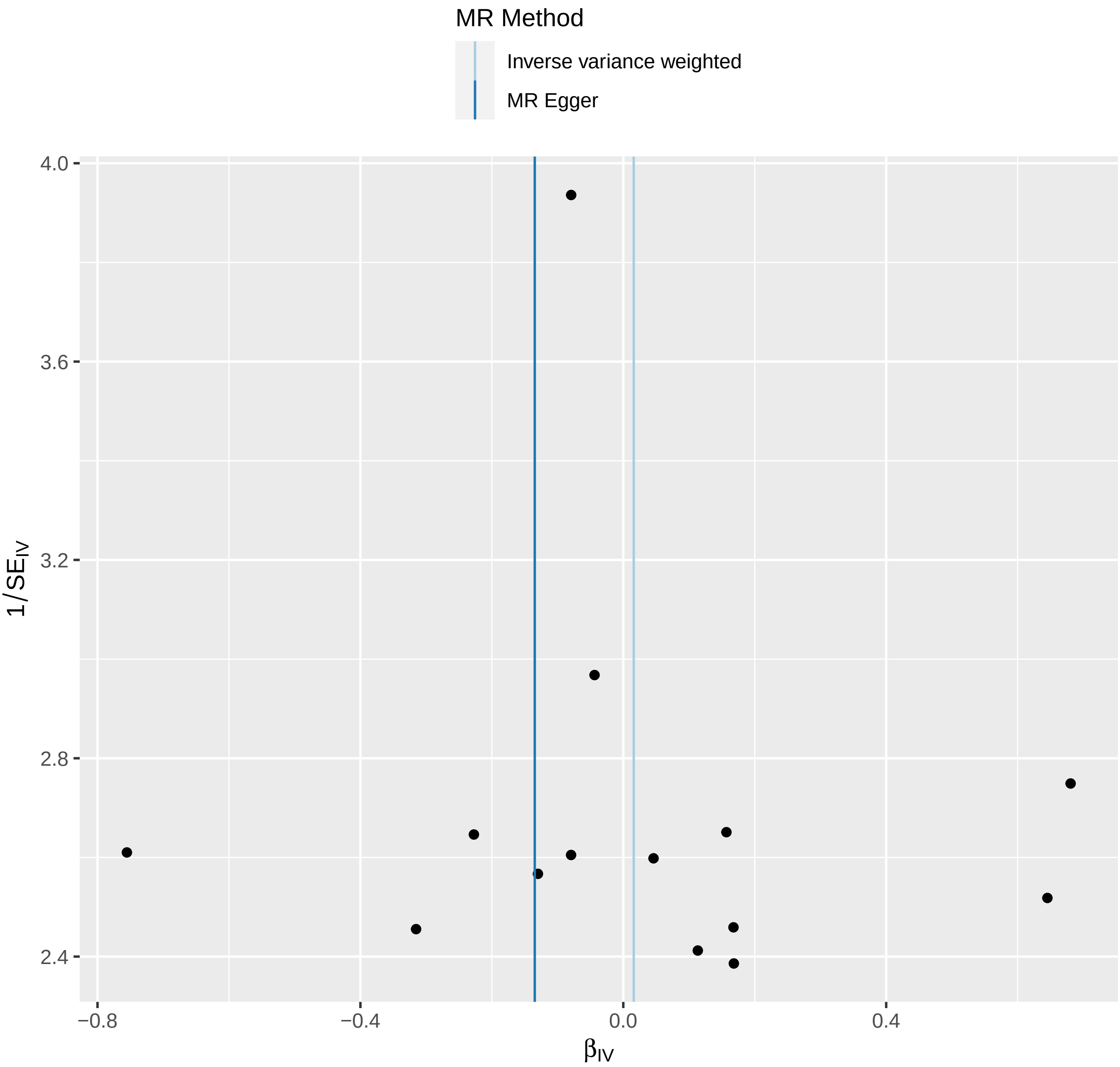

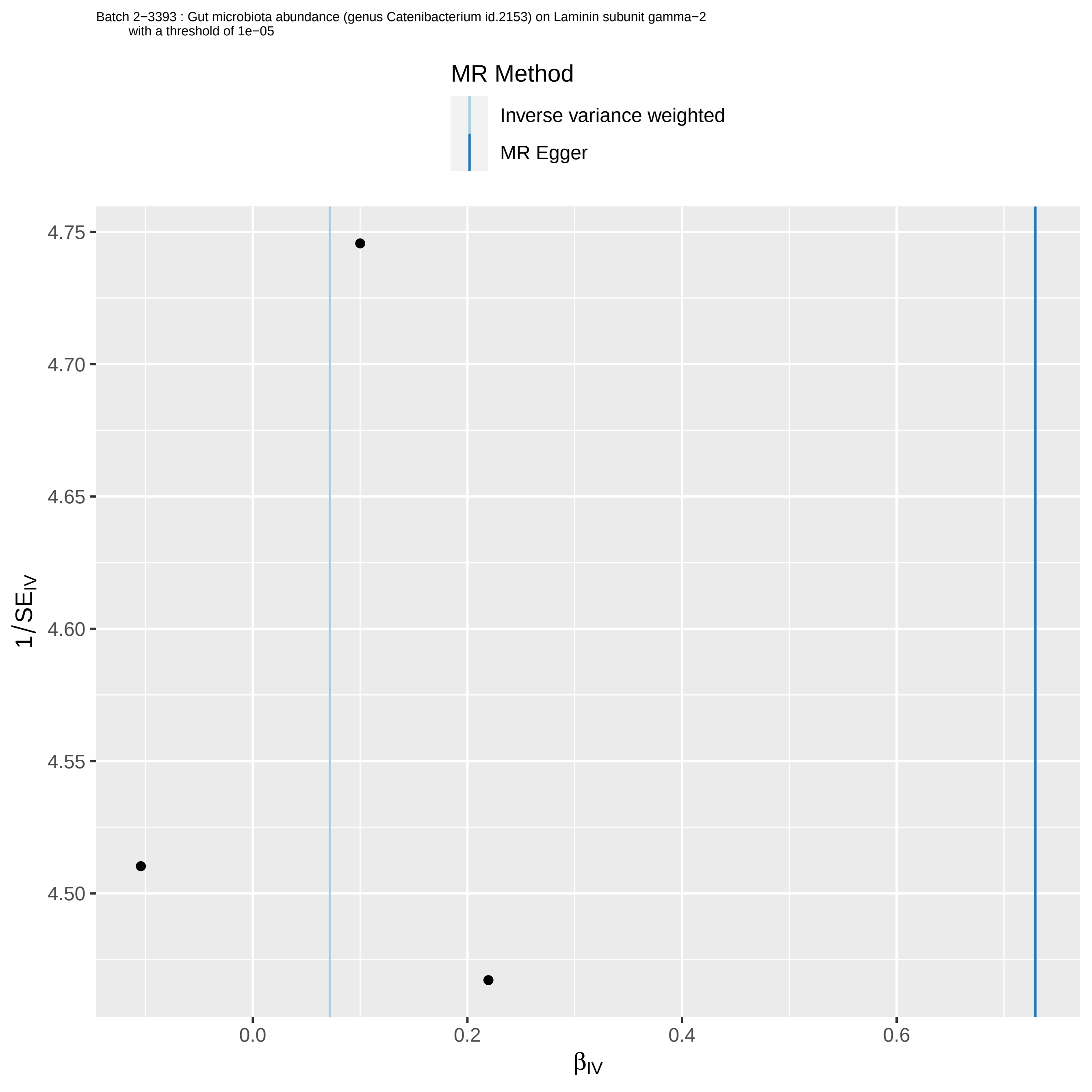
Batch 2−3393 : Gut microbiota abundance (genus Catenibacterium id.2153) on Laminin subunit gamma−2
with a threshold of 1e−05
MR Method
Inverse variance weighted
MR Egger
4.75
4.70
4.65
4.60
4.55
4.50
1/SE_IV
0.0
0.2
0.4
0.6
β_IV

Batch 2−3394 : Gut microbiota abundance (genus Parasutterella id.2892) on Laminin subunit gamma−2
with a threshold of 1e−05

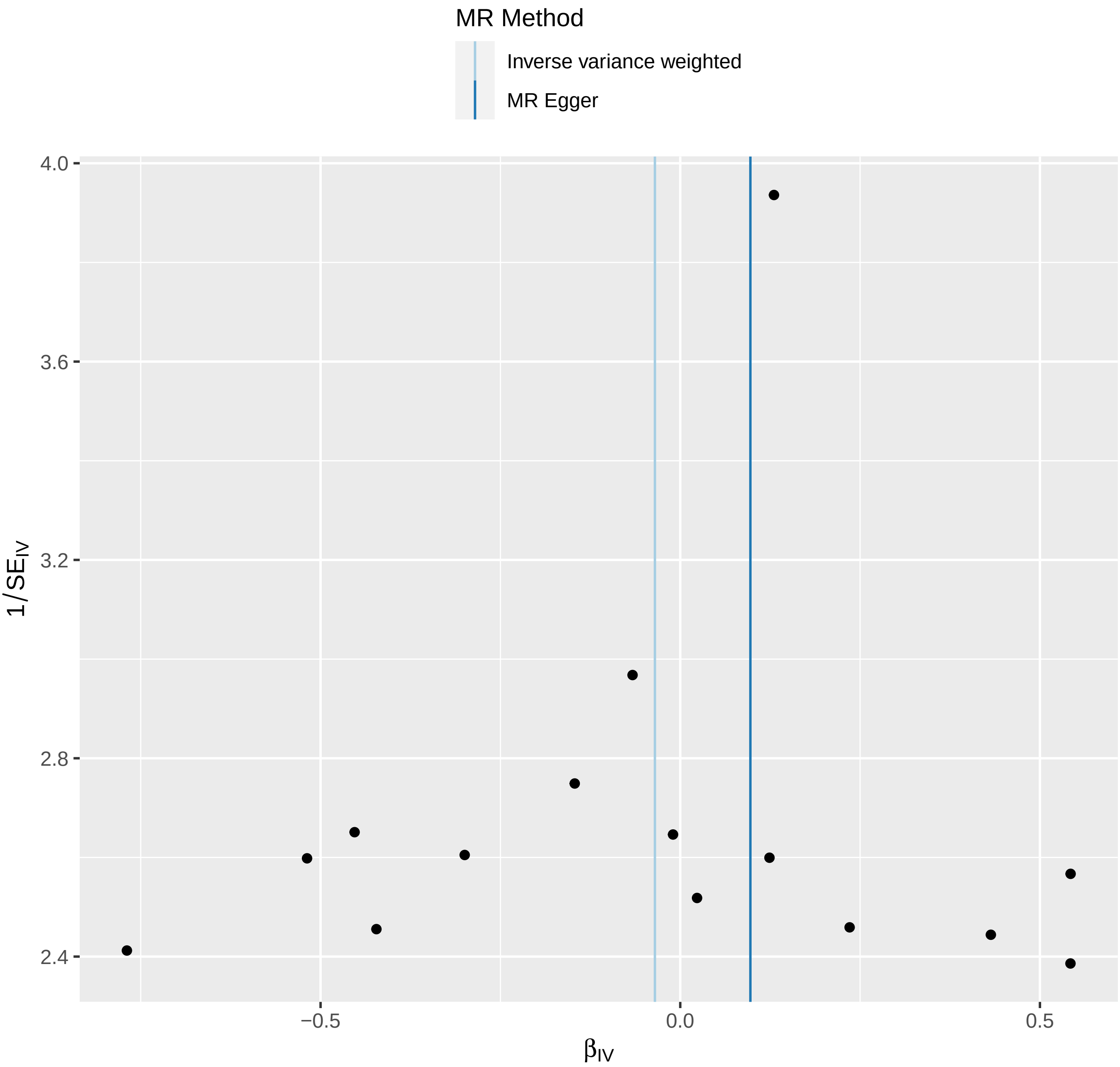

Batch 2−3395 : Gut microbiota abundance (genus Catenibacterium id.2153) on Lysosome−associated membrane glycoprotein 3
with a threshold of 1e−05

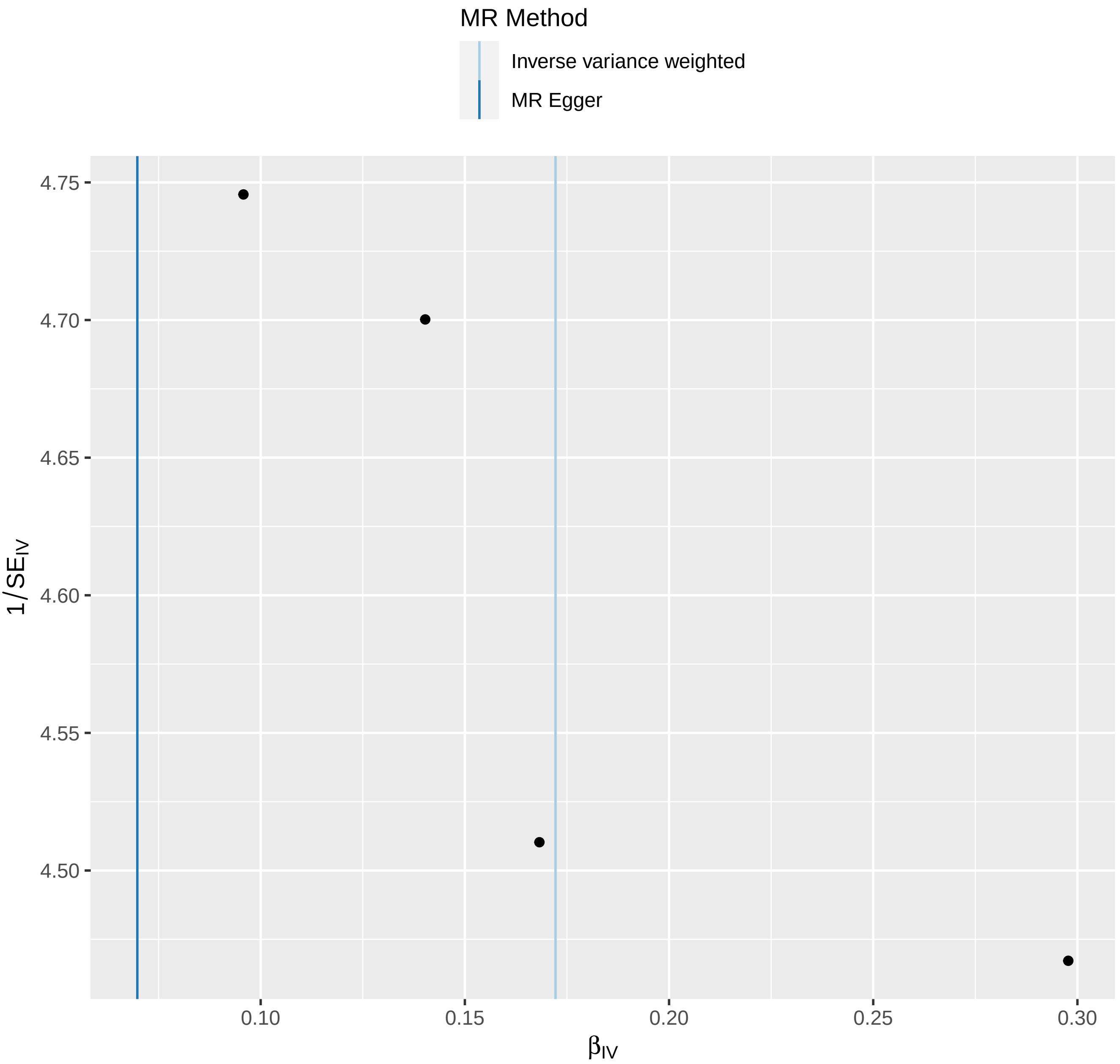

Batch 2−3396 : Gut microbiota abundance (genus Parasutterella id.2892) on Lysosome−associated membrane glycoprotein 3
with a threshold of 1e−05

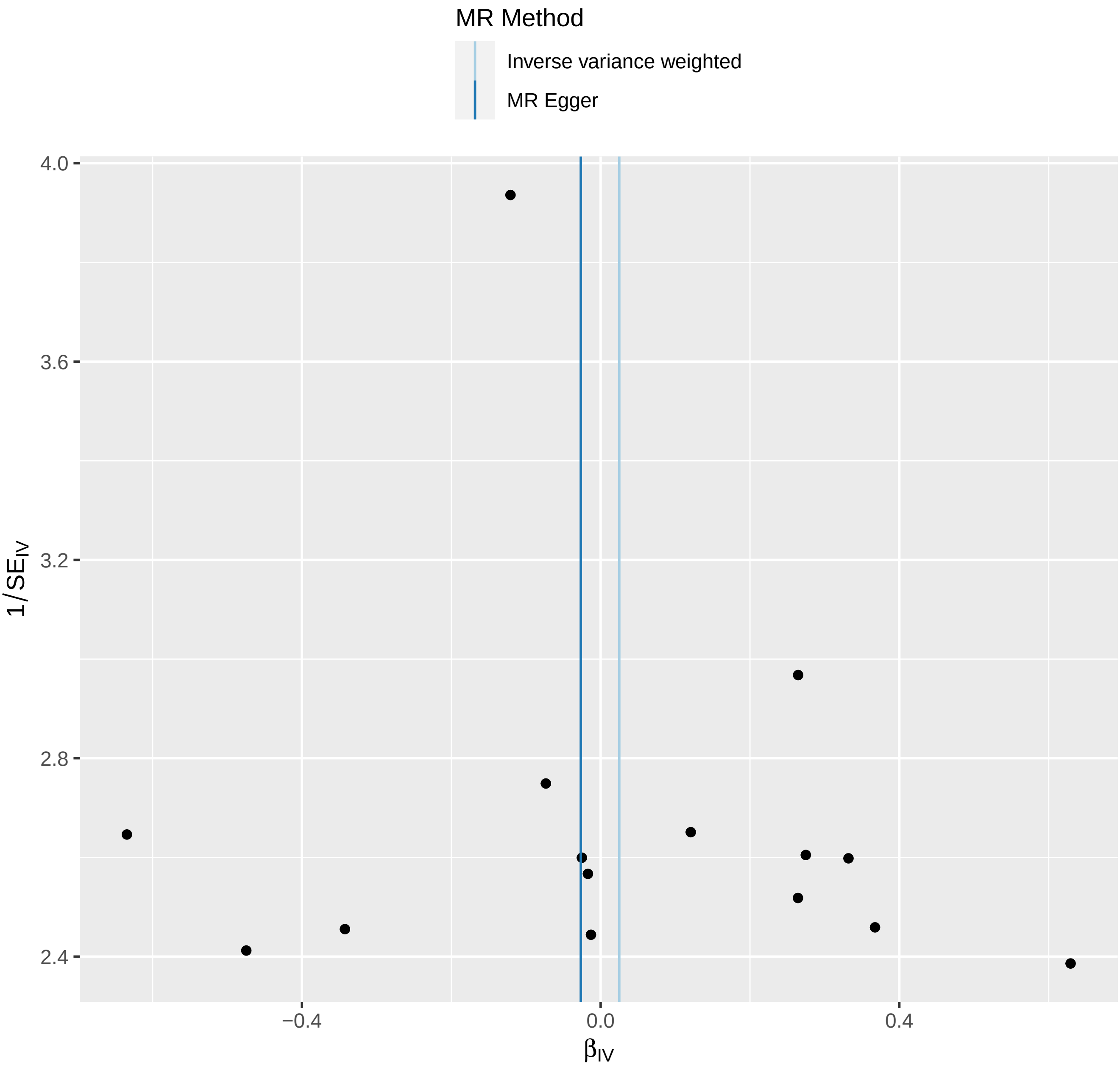

Batch 2−3397 : Gut microbiota abundance (genus Catenibacterium id.2153) on Ragulator complex protein LAMTOR3
with a threshold of 1e−05

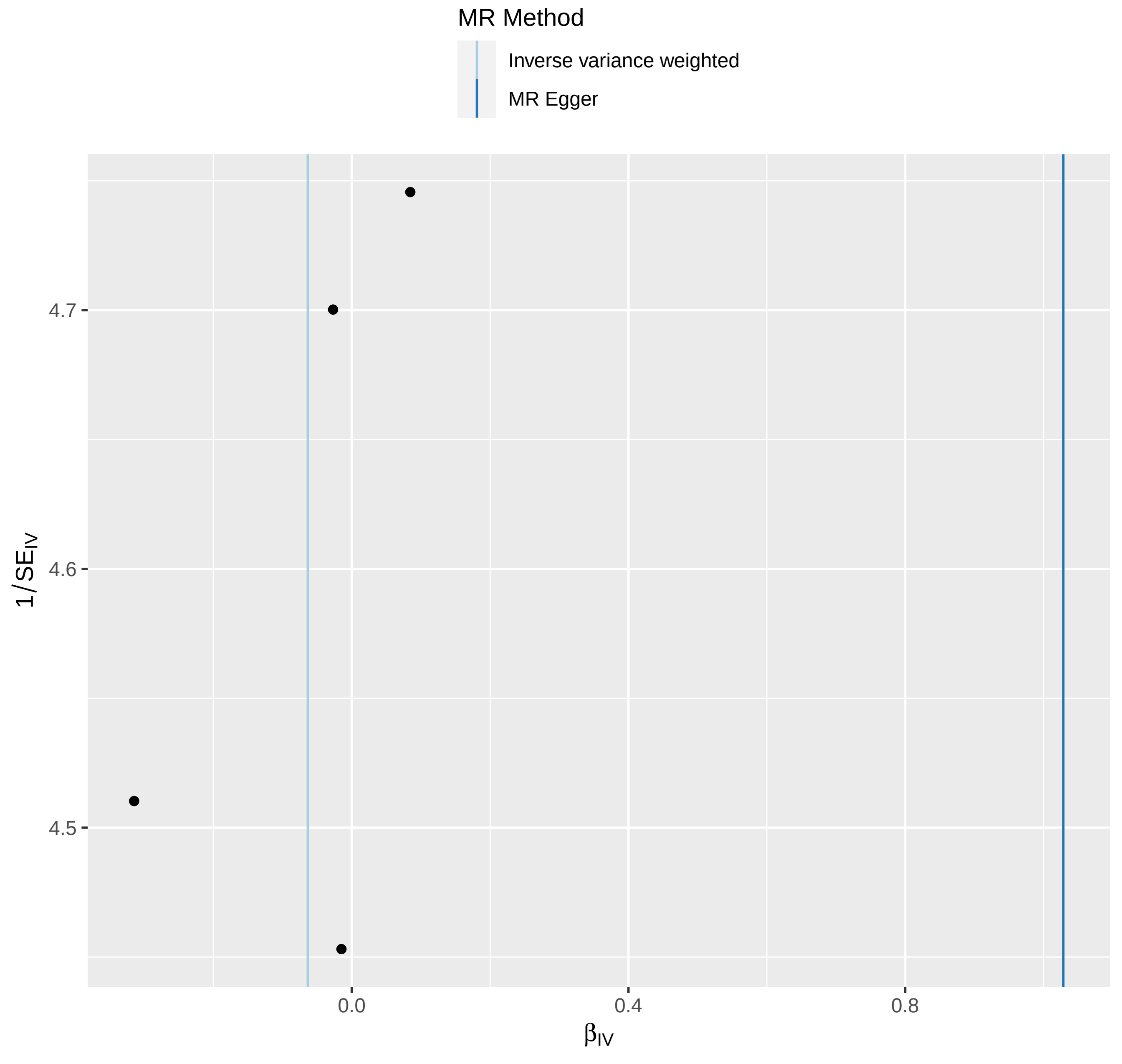

Batch 2−3398 : Gut microbiota abundance (genus Parasutterella id.2892) on Ragulator complex protein LAMTOR3
with a threshold of 1e−05

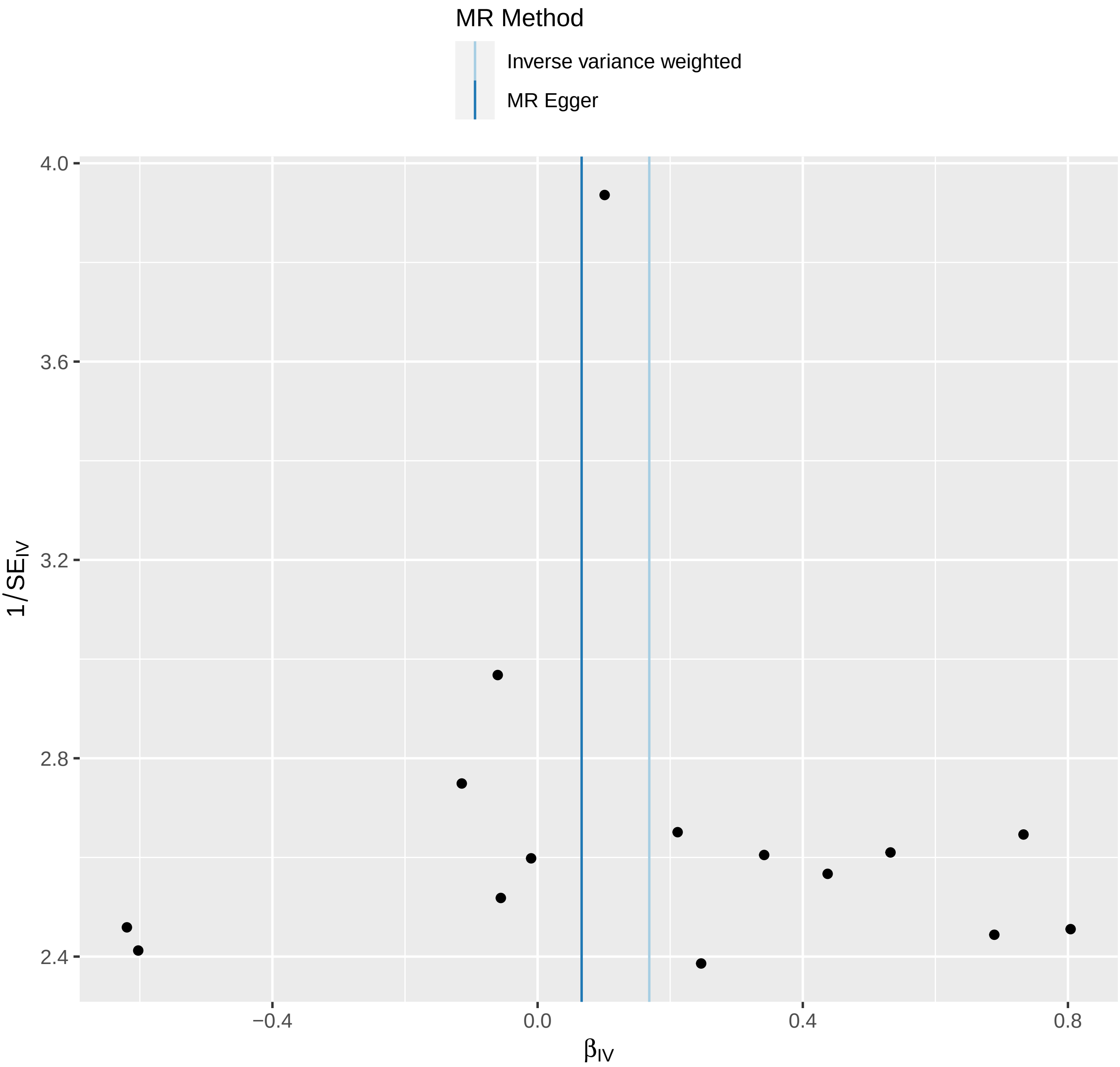

Batch 2−3399 : Gut microbiota abundance (genus Catenibacterium id.2153) on Glycosyltransferase−like protein LARGE1
with a threshold of 1e−05

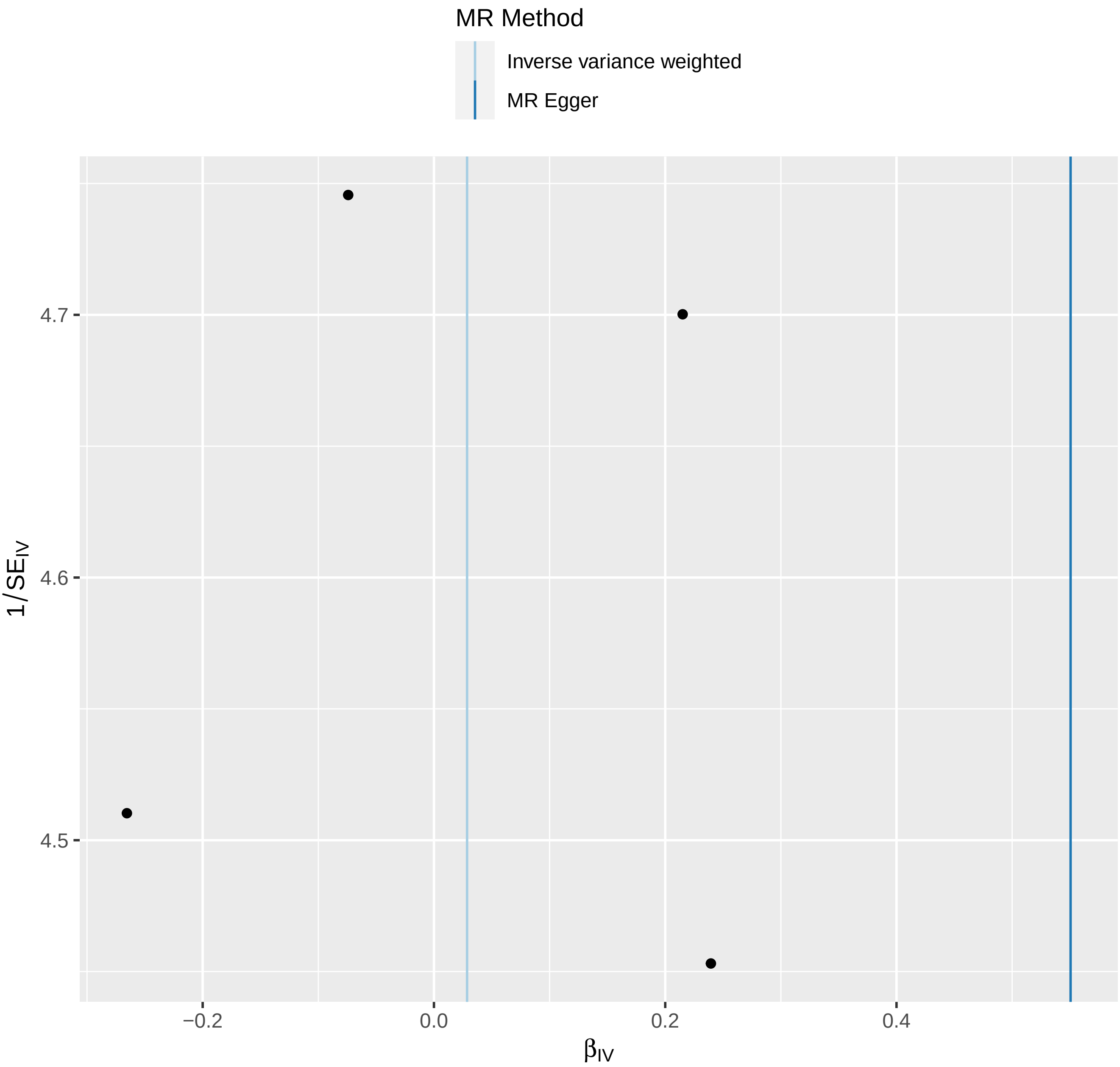

Batch 2−3400 : Gut microbiota abundance (genus Parasutterella id.2892) on Glycosyltransferase−like protein LARGE1
with a threshold of 1e−05

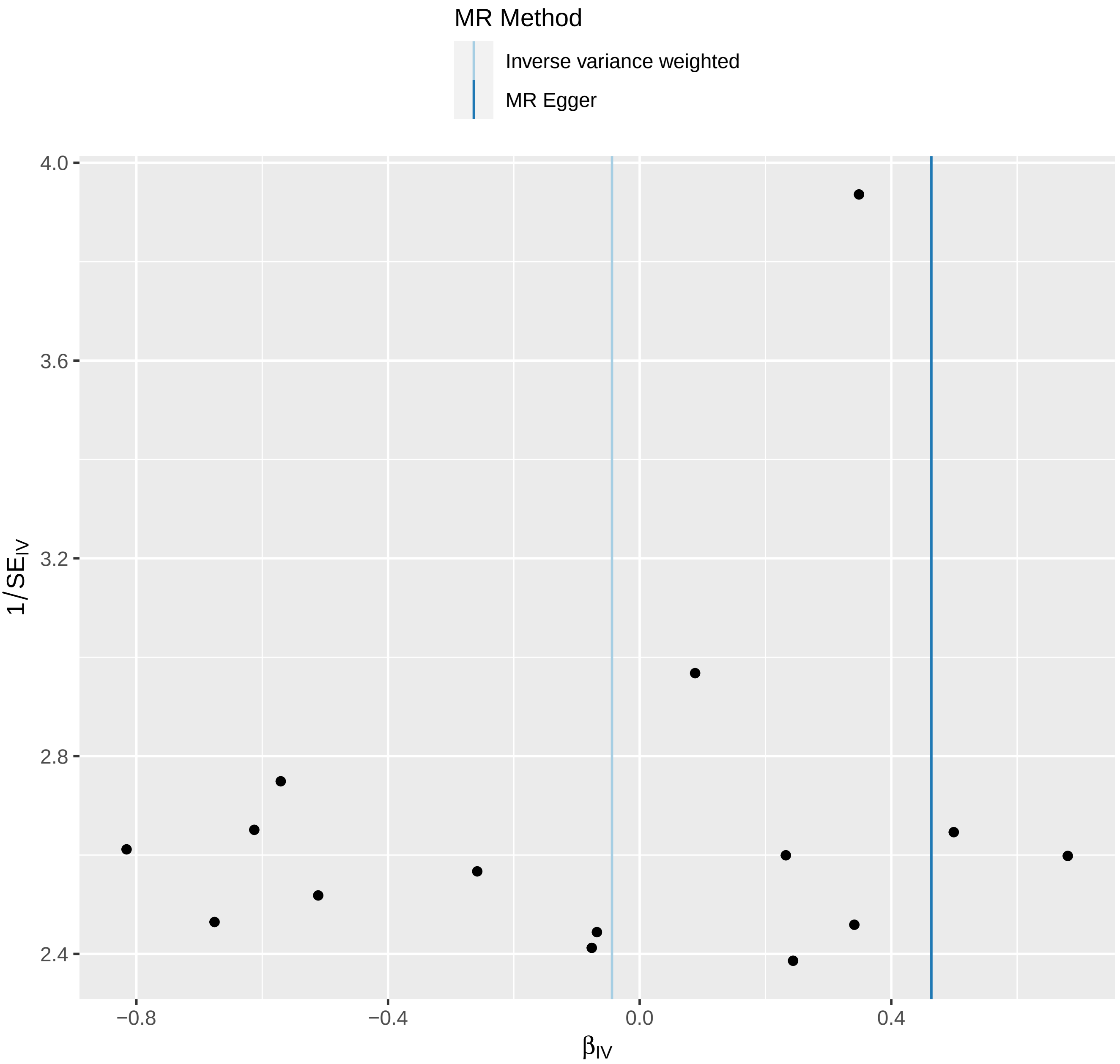

Batch 2−3401 : Gut microbiota abundance (genus Catenibacterium id.2153) on Linker for activation of T−cells family member 1
with a threshold of 1e−05

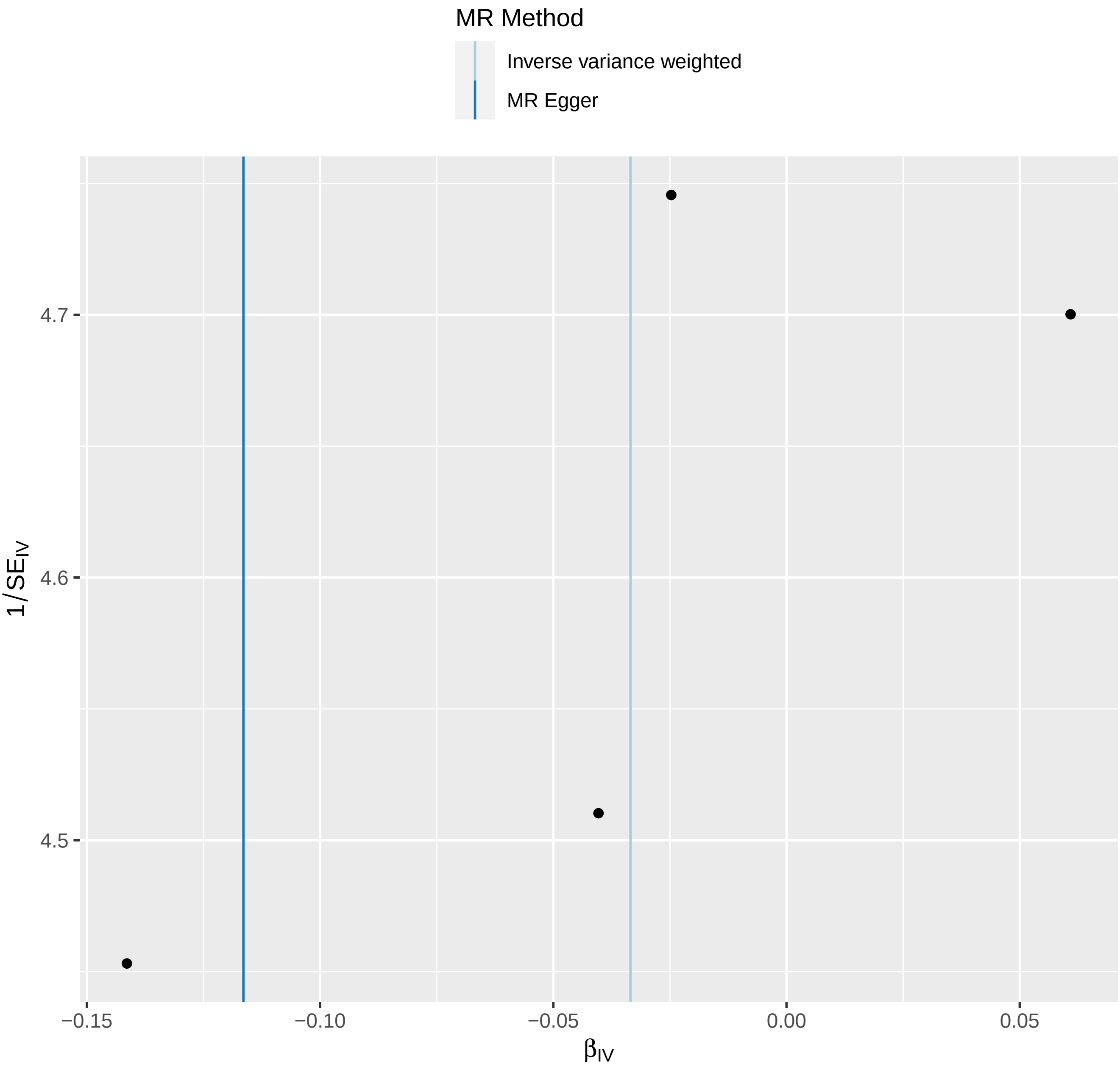

Batch 2−3402 : Gut microbiota abundance (genus Parasutterella id.2892) on Linker for activation of T−cells family member 1
with a threshold of 1e−05

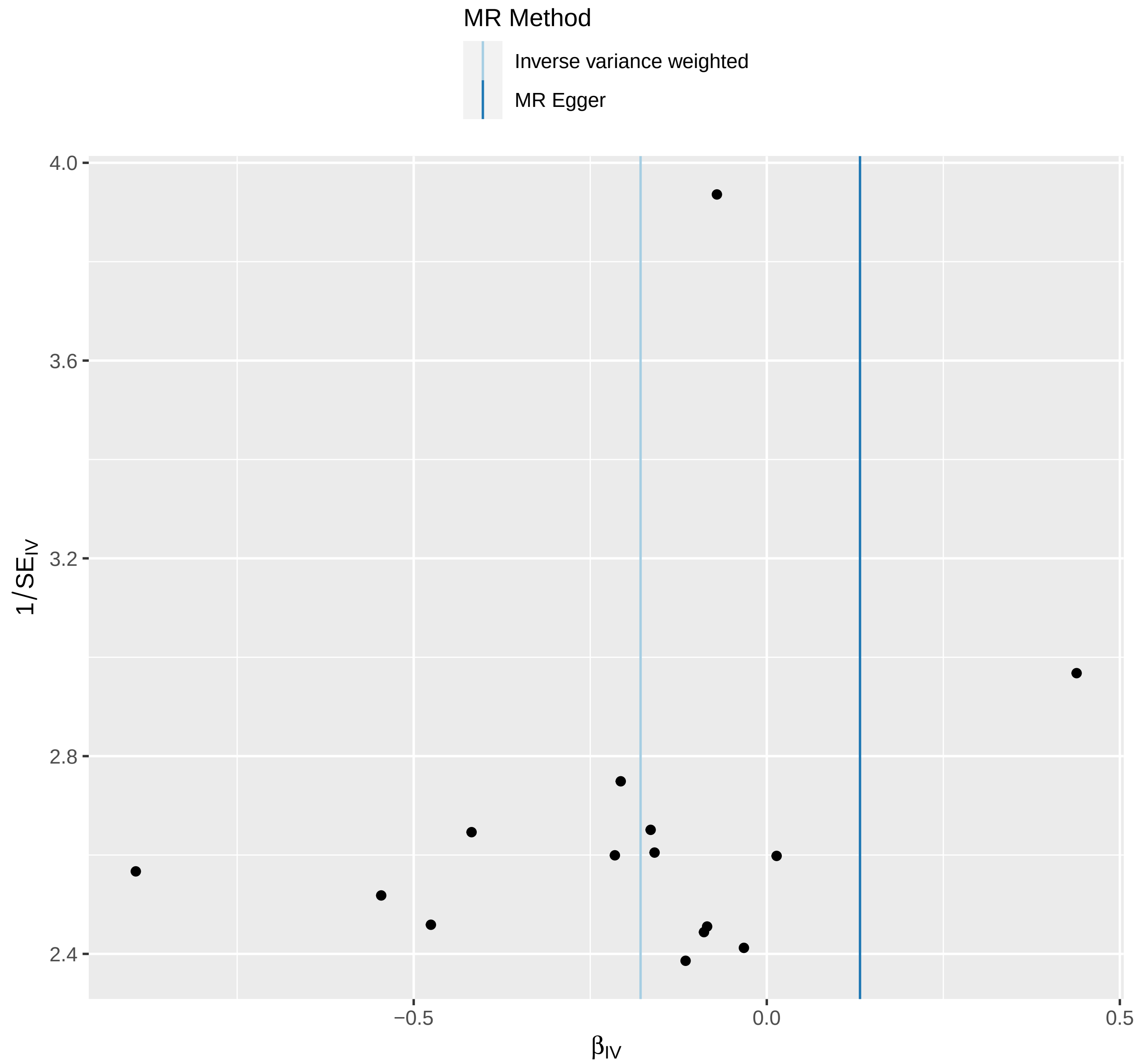

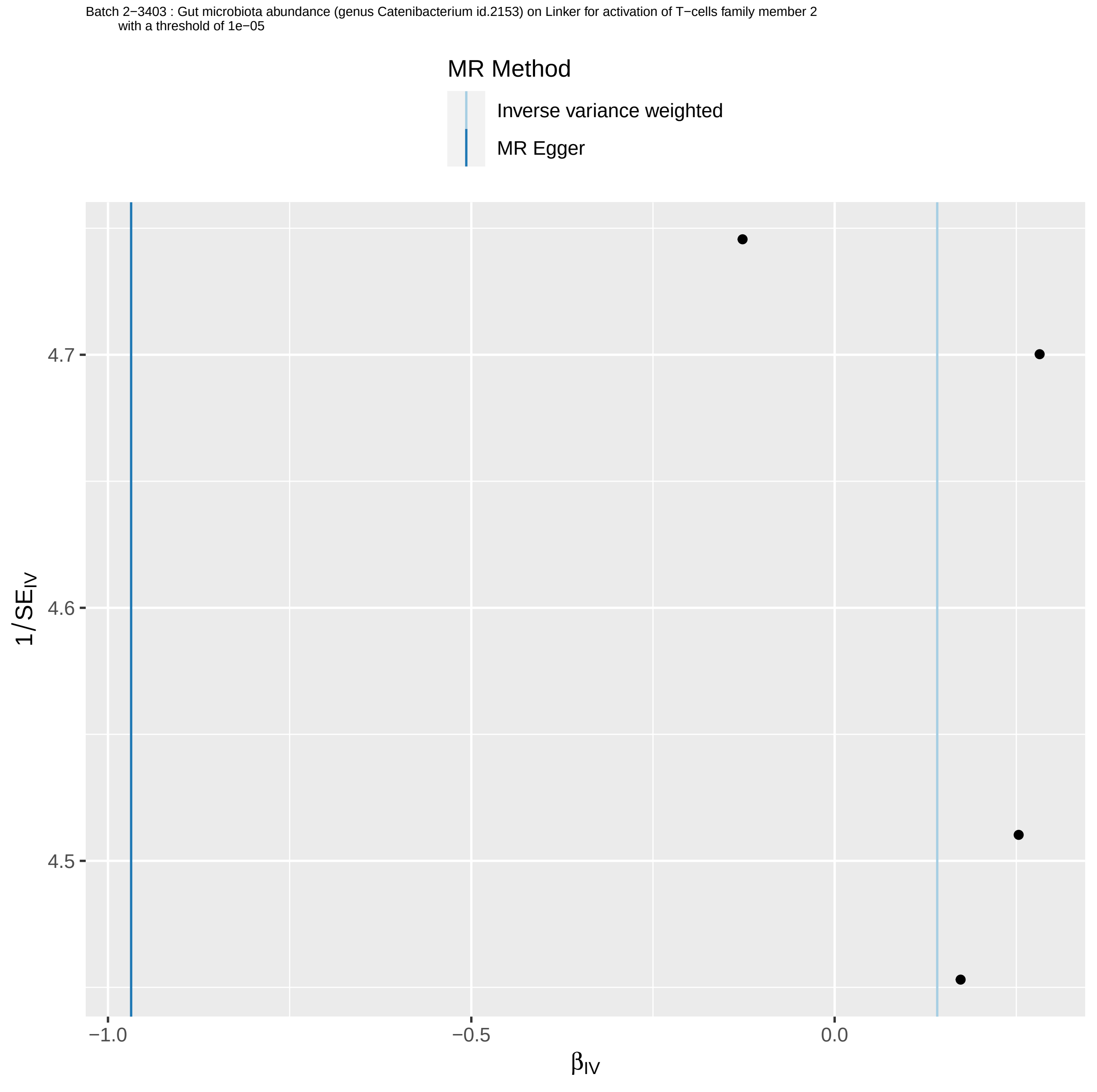
Batch 2−3403 : Gut microbiota abundance (genus Catenibacterium id.2153) on Linker for activation of T−cells family member 2
with a threshold of 1e−05
MR Method
Inverse variance weighted
MR Egger
4.7
4.6
4.5
1/SE_IV
−1.0
−0.5
0.0
β_IV

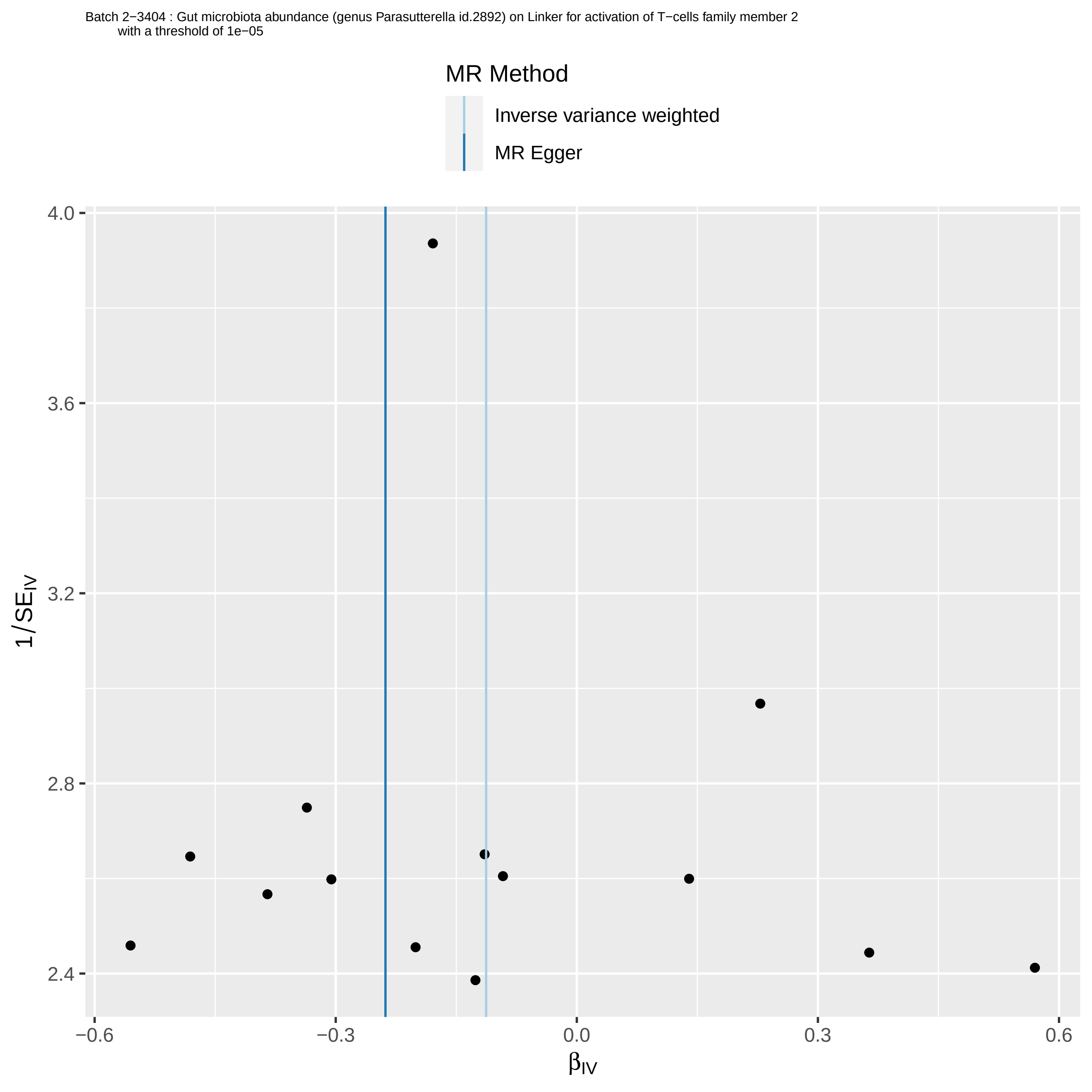
Batch 2−3404 : Gut microbiota abundance (genus Parasutterella id.2892) on Linker for activation of T−cells family member 2
with a threshold of 1e−05
MR Method
Inverse variance weighted
MR Egger
4.0
3.6
3.2
2.8
2.4
1/SE_IV
−0.6
−0.3
0.0
0.3
0.6
β_IV

Batch 2−3405 : Gut microbiota abundance (genus Catenibacterium id.2153) on Layilin
with a threshold of 1e−05

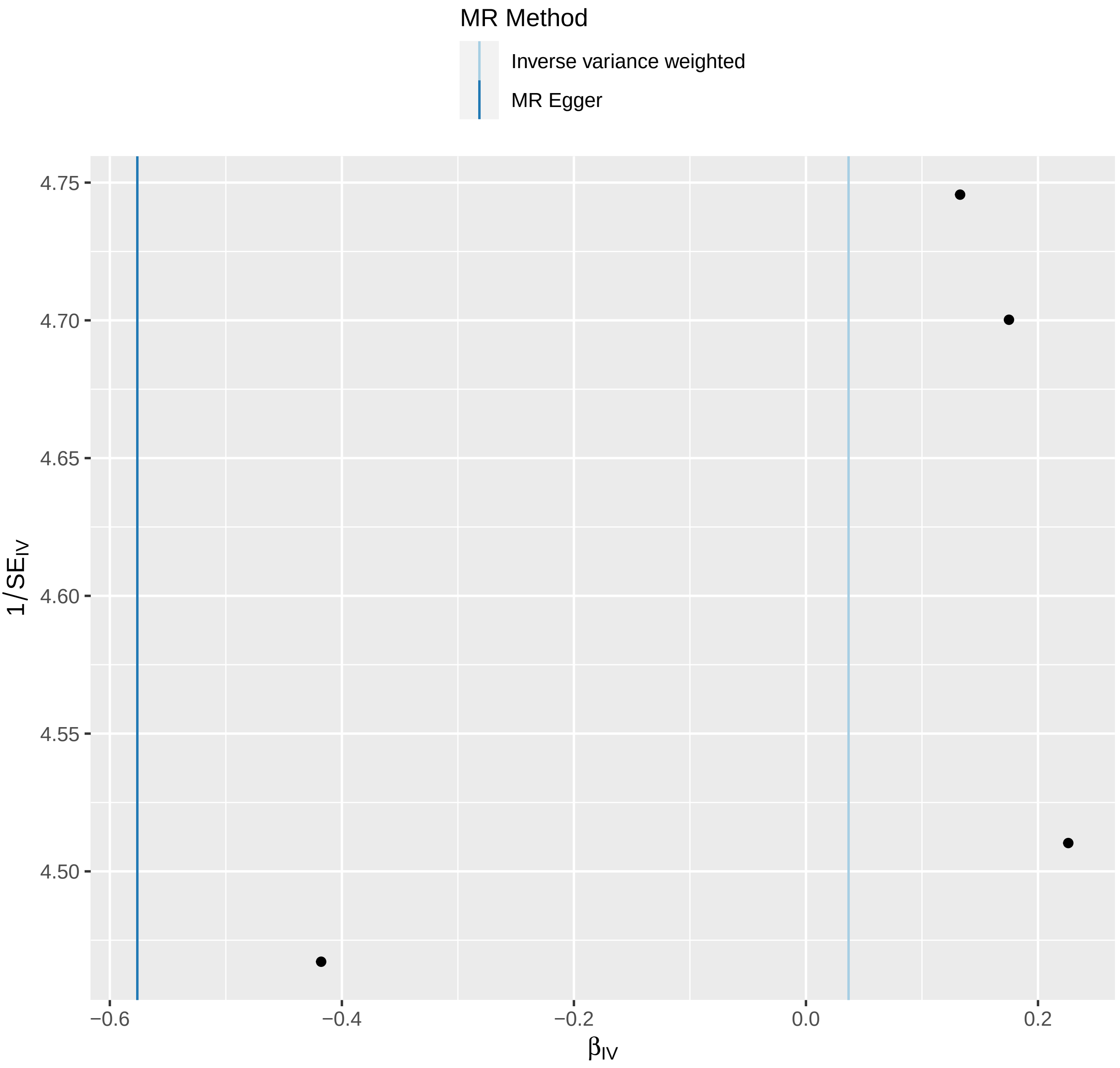

Batch 2−3406 : Gut microbiota abundance (genus Parasutterella id.2892) on Layilin
with a threshold of 1e−05

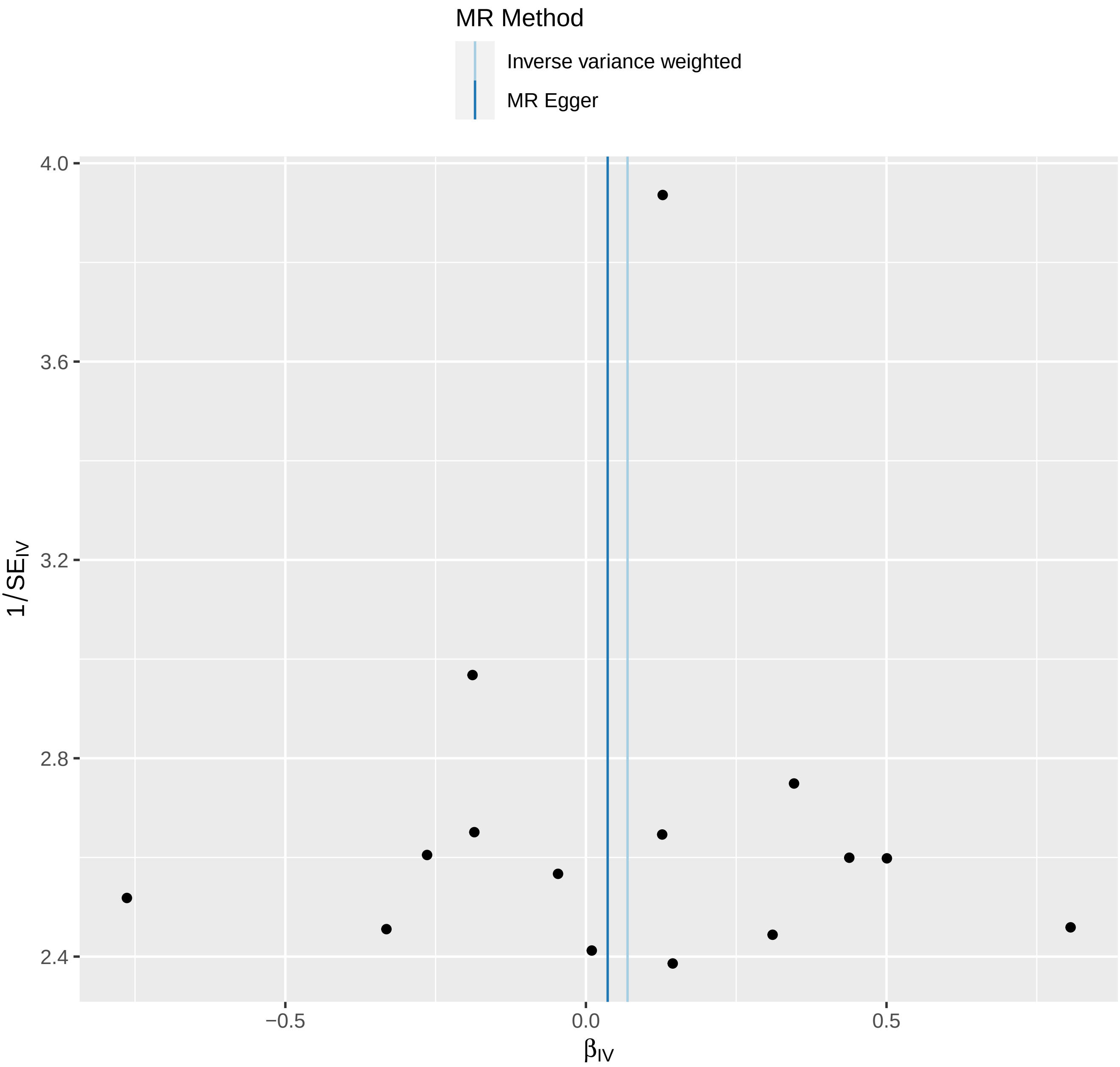

Batch 2−3407 : Gut microbiota abundance (genus Catenibacterium id.2153) on Tyrosine−protein kinase Lck
with a threshold of 1e−05

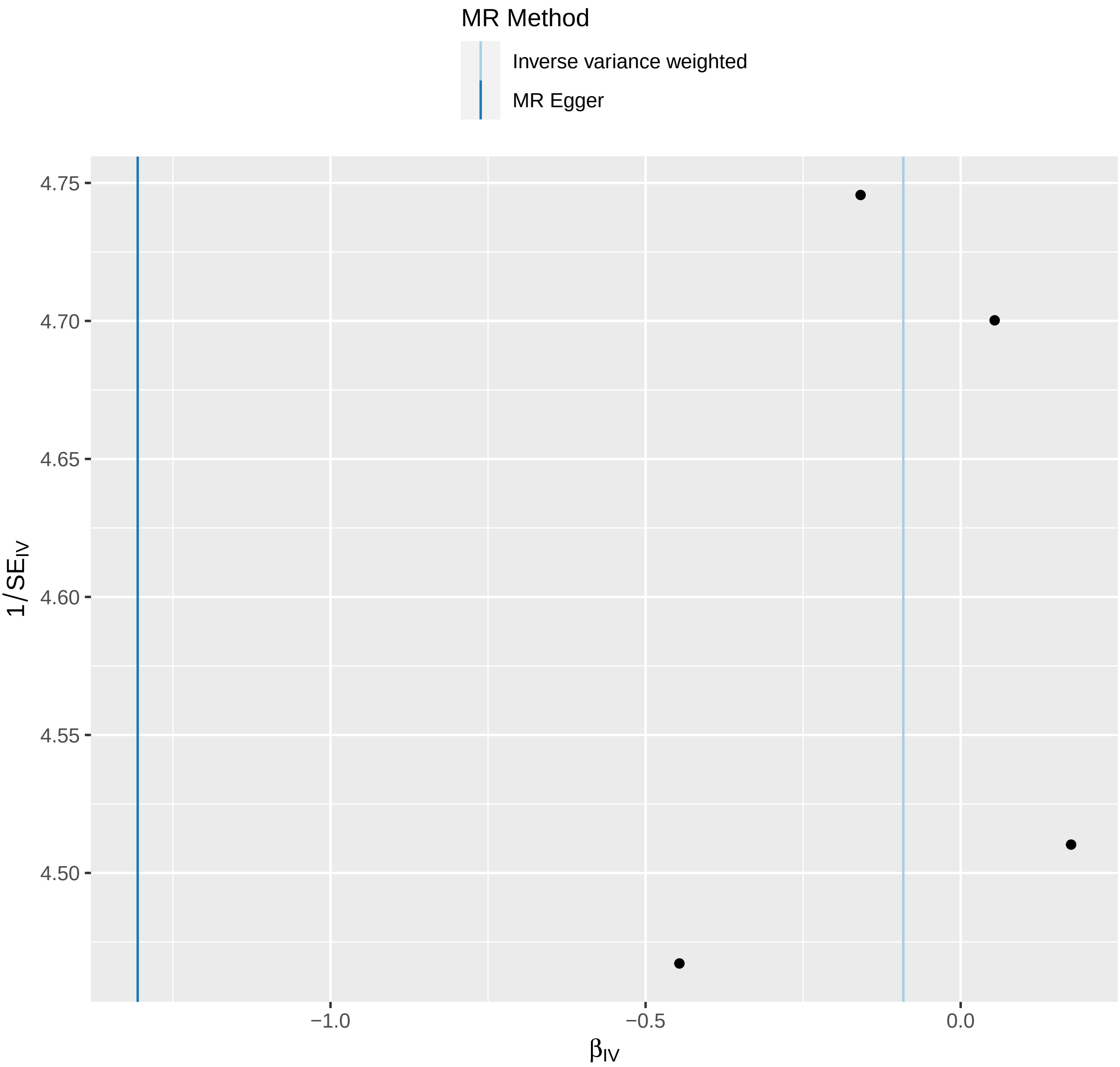

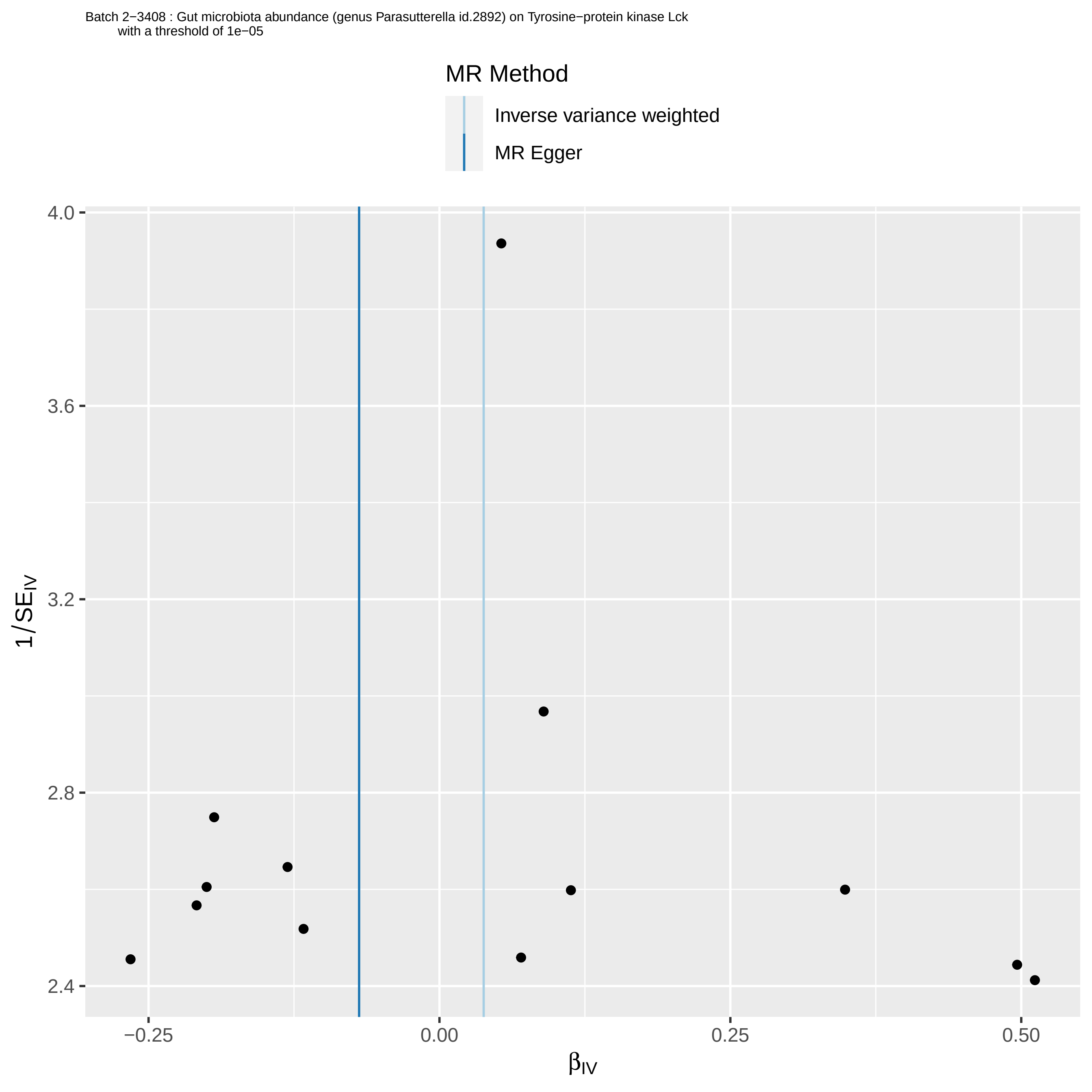

Batch 2−3408 : Gut microbiota abundance (genus Parasutterella id.2892) on Tyrosine−protein kinase Lck
with a threshold of 1e−05
MR Method
Inverse variance weighted
MR Egger
4.0
3.6
3.2
2.8
2.4
1/SE_IV
−0.25
0.00
0.25
0.50
β_IV

Batch 2−3409 : Gut microbiota abundance (genus Catenibacterium id.2153) on Tyrosine−protein kinase Lck
with a threshold of 1e−05

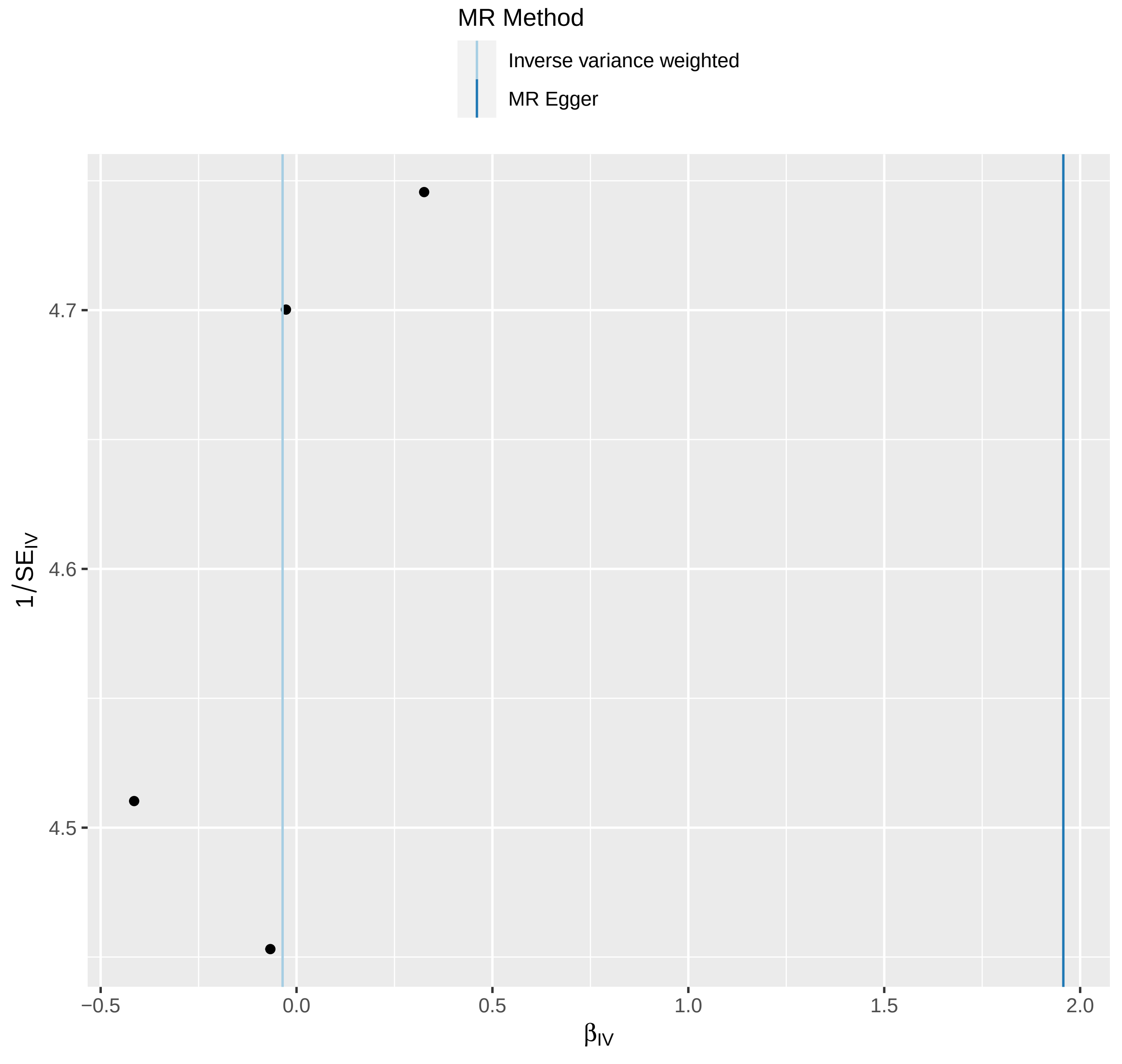

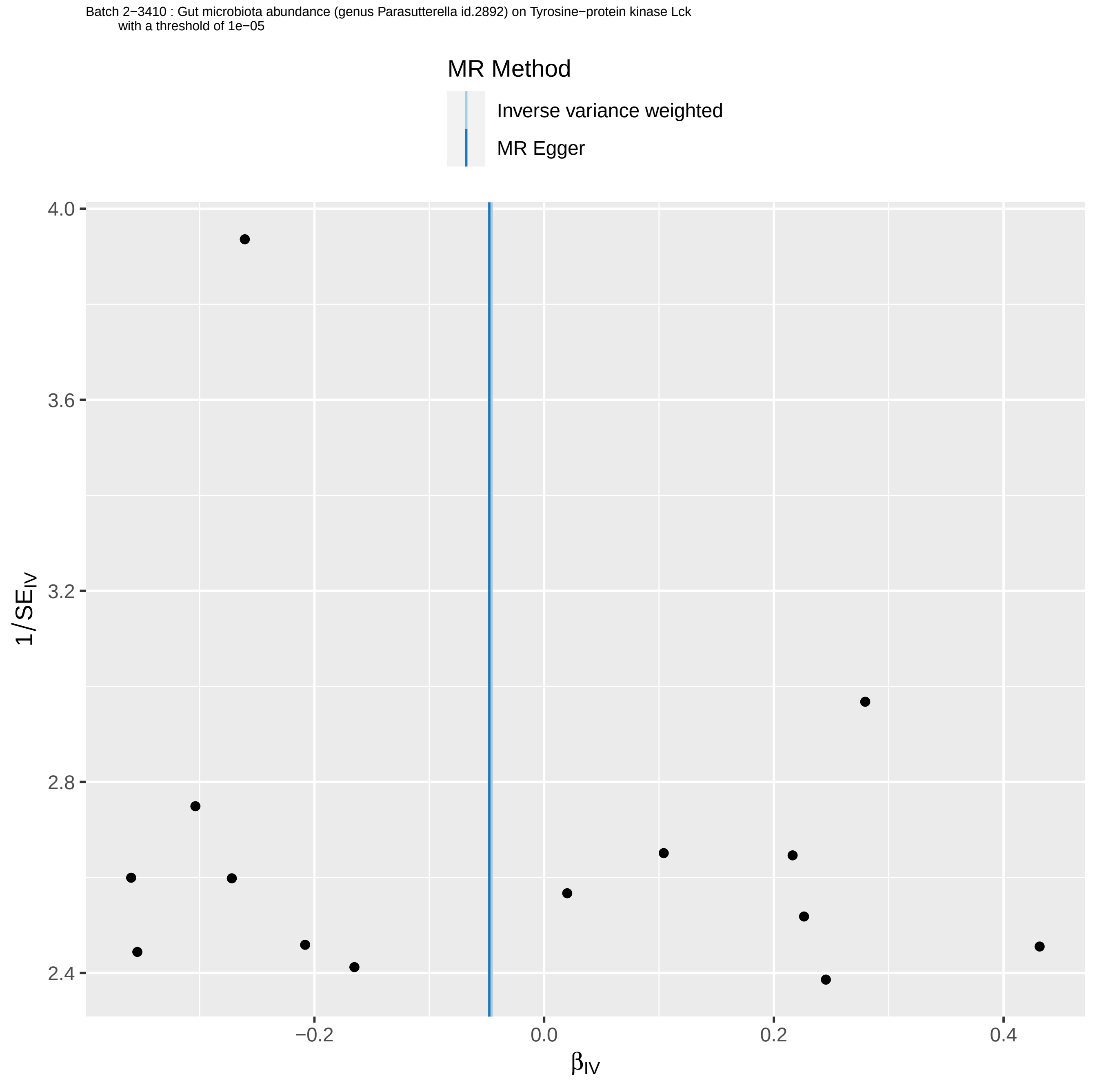

Batch 2−3410 : Gut microbiota abundance (genus Parasutterella id.2892) on Tyrosine−protein kinase Lck
with a threshold of 1e−05
MR Method
Inverse variance weighted
MR Egger
4.0
3.6
3.2
2.8
2.4
1/SE_IV
−0.2
0.0
0.2
0.4
β_IV

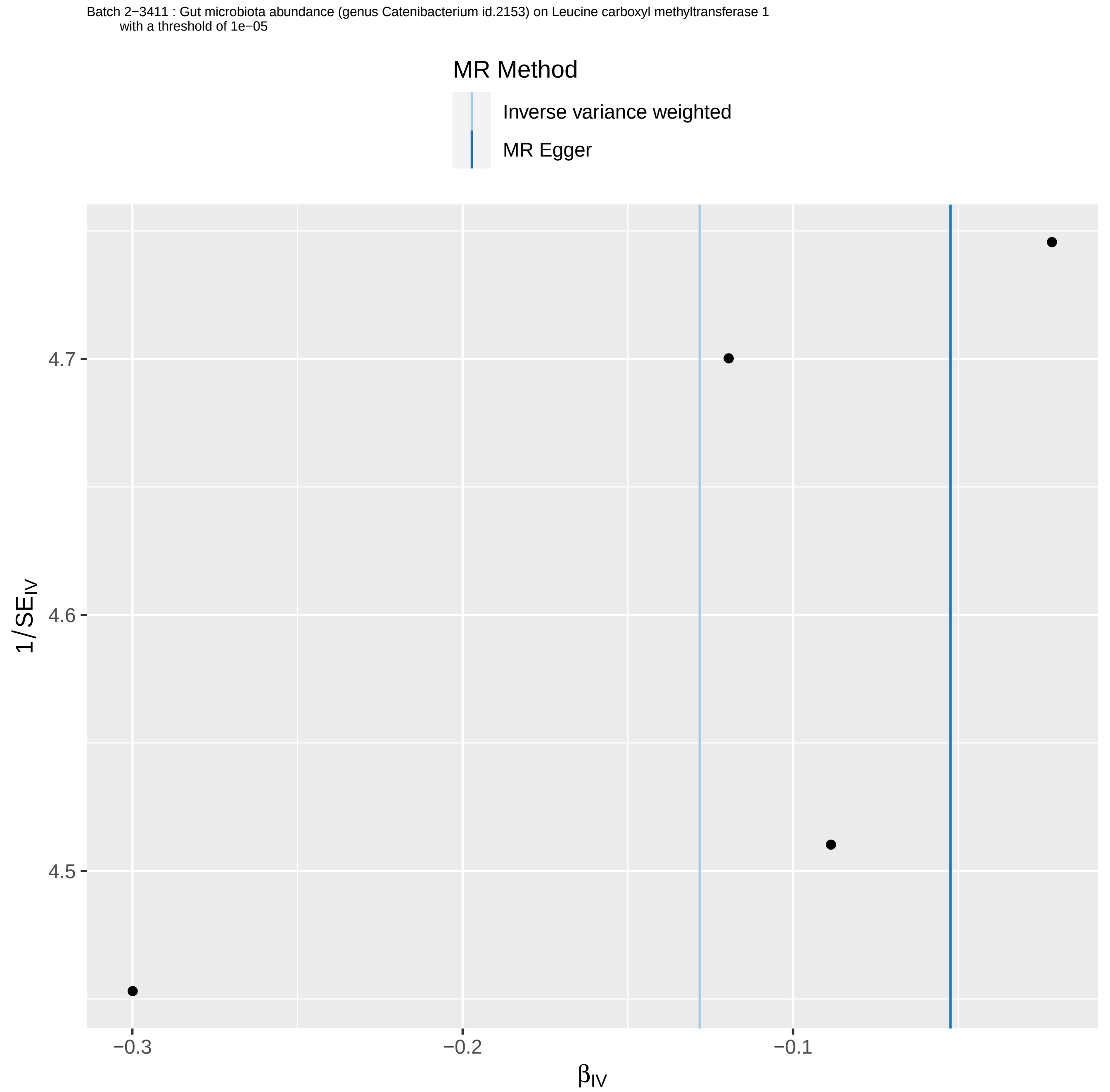

Batch 2−3411 : Gut microbiota abundance (genus Catenibacterium id.2153) on Leucine carboxyl methyltransferase 1
with a threshold of 1e−05
MR Method
Inverse variance weighted
MR Egger
4.7
4.6
4.5
1/SE_IV
−0.3
−0.2
−0.1
β_IV

Batch 2−3412 : Gut microbiota abundance (genus Parasutterella id.2892) on Leucine carboxyl methyltransferase 1
with a threshold of 1e−05

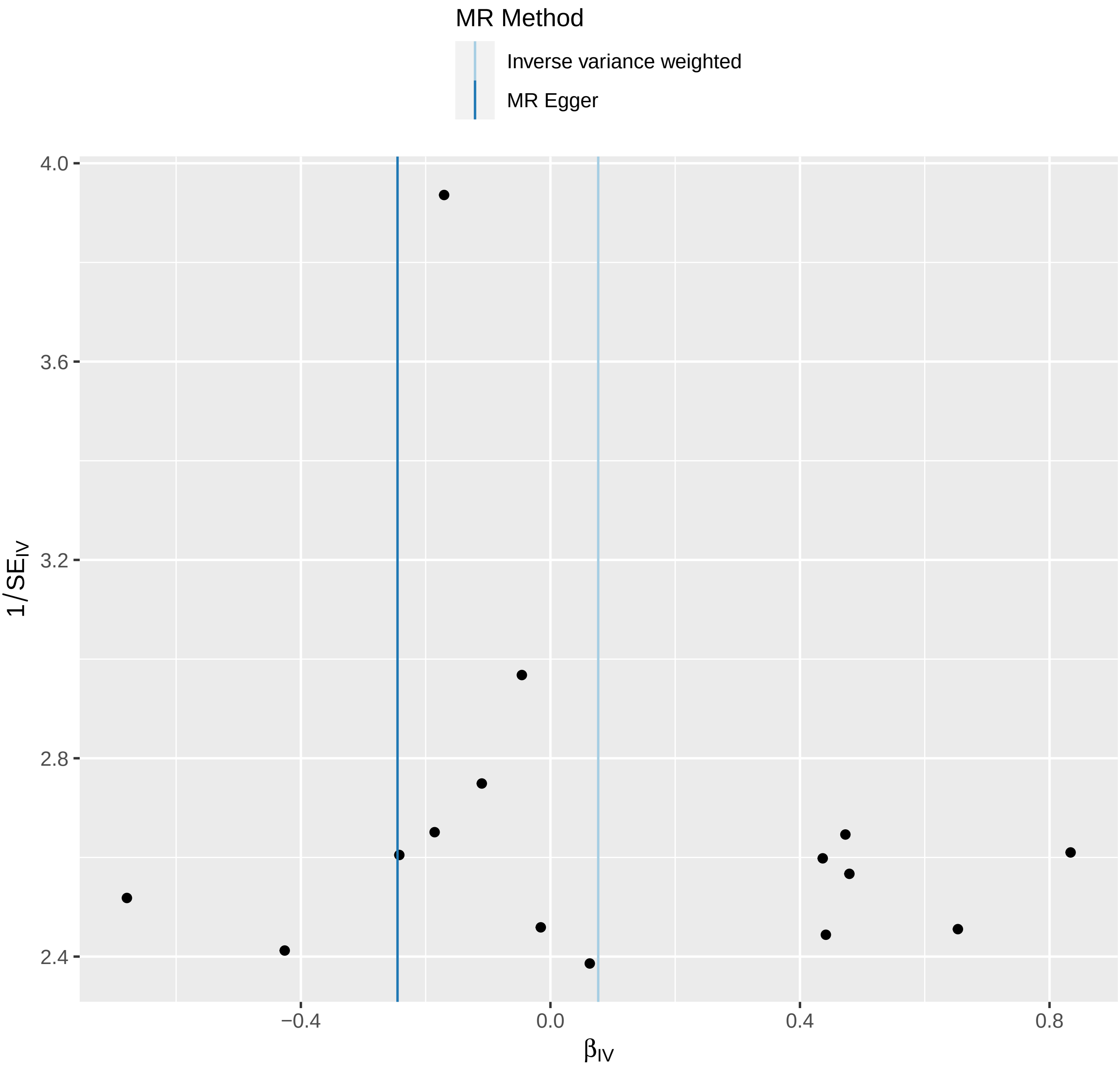

Batch 2−3413 : Gut microbiota abundance (genus Catenibacterium id.2153) on Epididymal−specific lipocalin−10
with a threshold of 1e−05

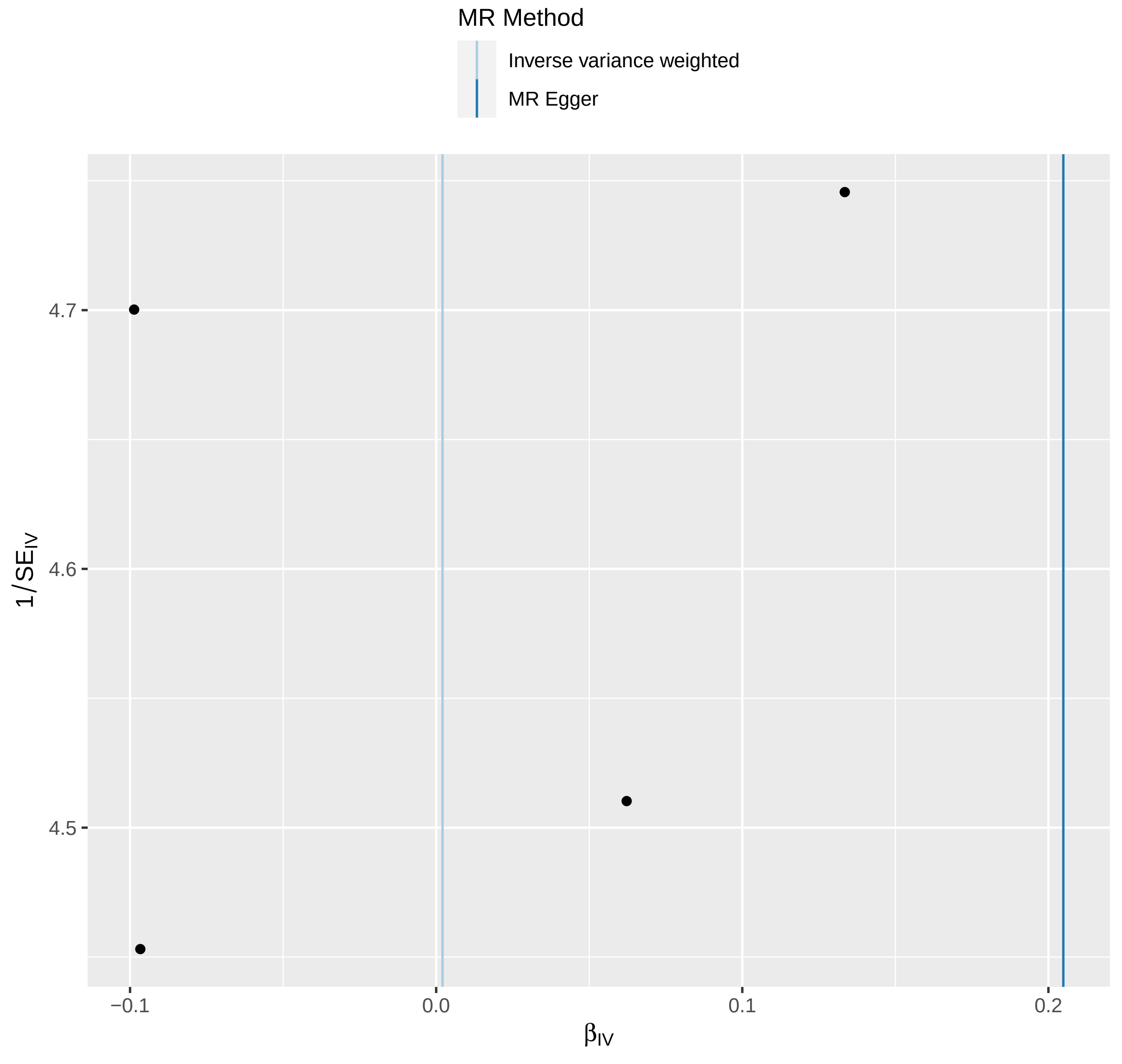

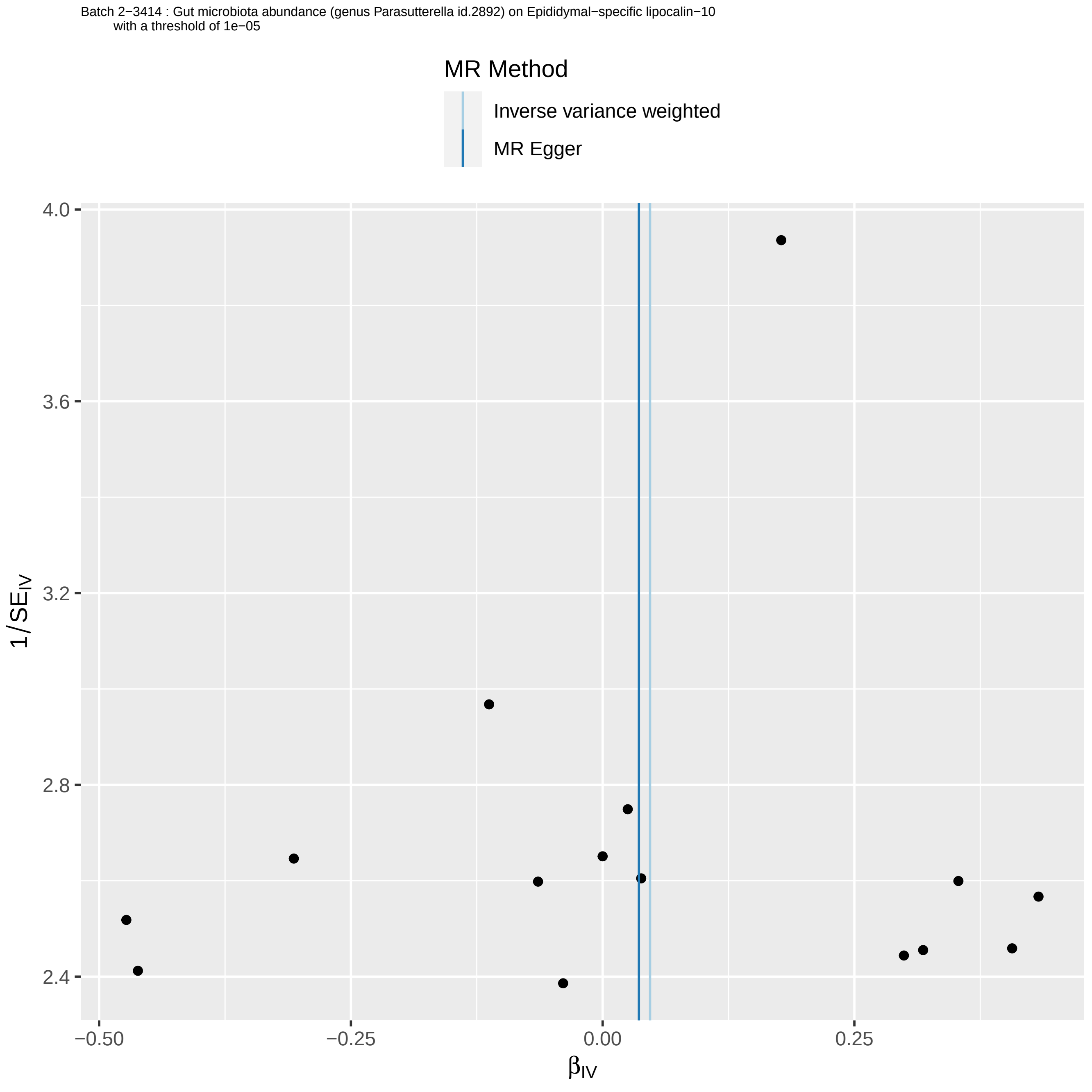
Batch 2−3414 : Gut microbiota abundance (genus Parasutterella id.2892) on Epididymal−specific lipocalin−10
with a threshold of 1e−05
MR Method
Inverse variance weighted
MR Egger
4.0
3.6
3.2
2.8
2.4
1/SE_IV
−0.50
−0.25
0.00
0.25
β_IV

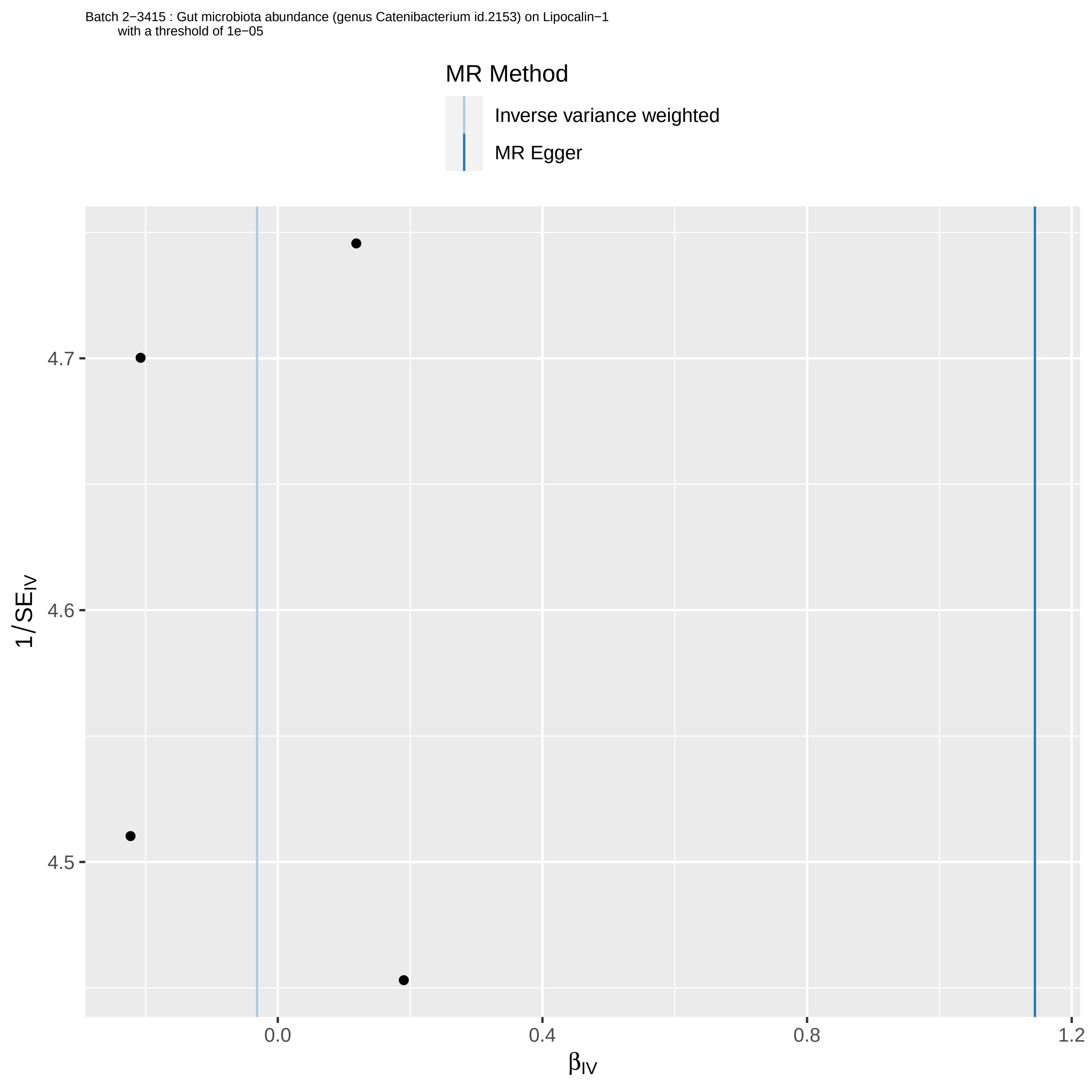

Batch 2−3415 : Gut microbiota abundance (genus Catenibacterium id.2153) on Lipocalin−1
with a threshold of 1e−05
MR Method
Inverse variance weighted
MR Egger
4.7
4.6
4.5
$1/SE_{IV}$
0.0
0.4
0.8
1.2
β_{IV}

Batch 2−3416 : Gut microbiota abundance (genus Parasutterella id.2892) on Lipocalin−1
with a threshold of 1e−05

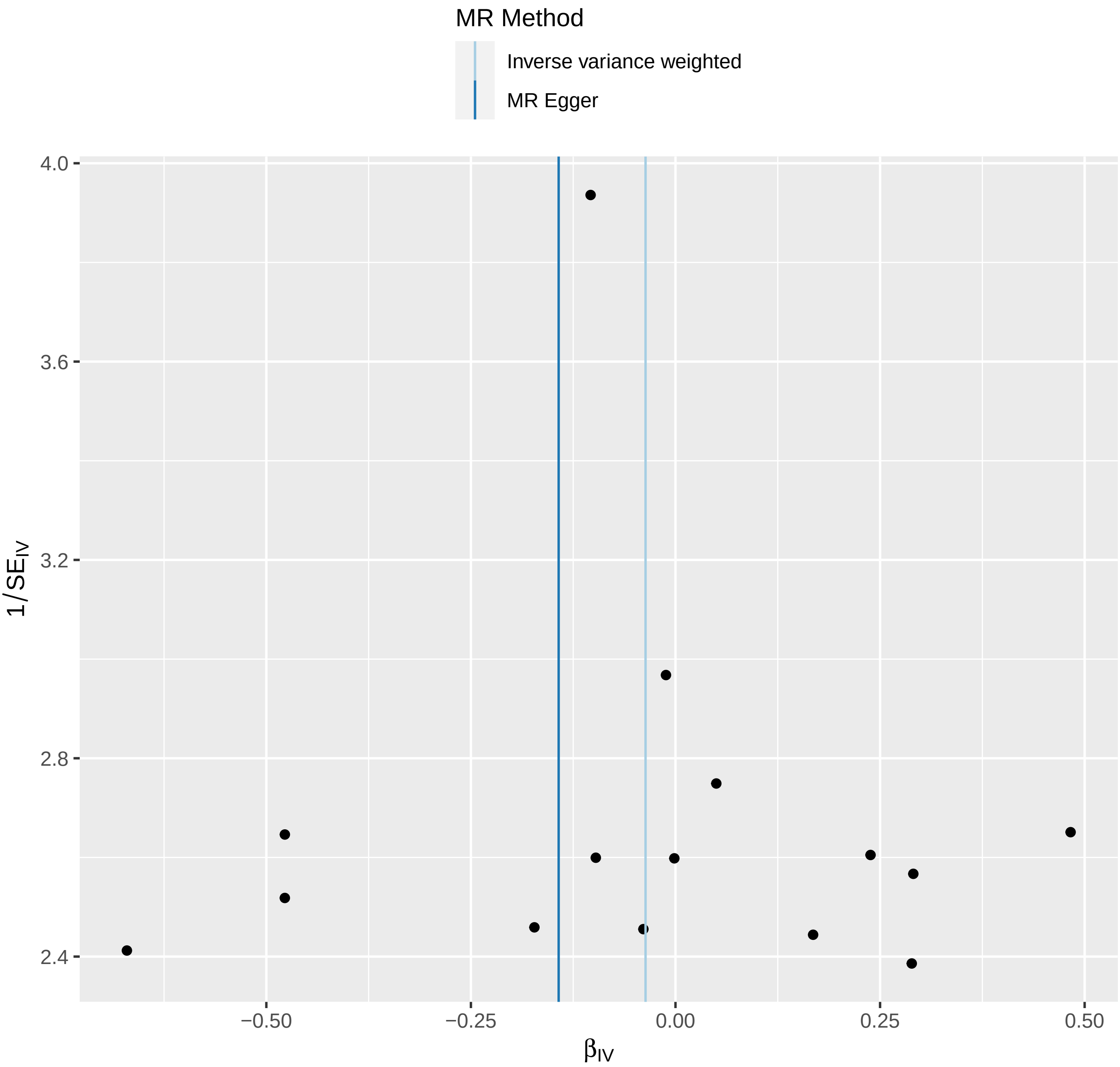

Batch 2−3417 : Gut microbiota abundance (genus Catenibacterium id.2153) on Neutrophil gelatinase−associated lipocalin with a threshold of 1e−05

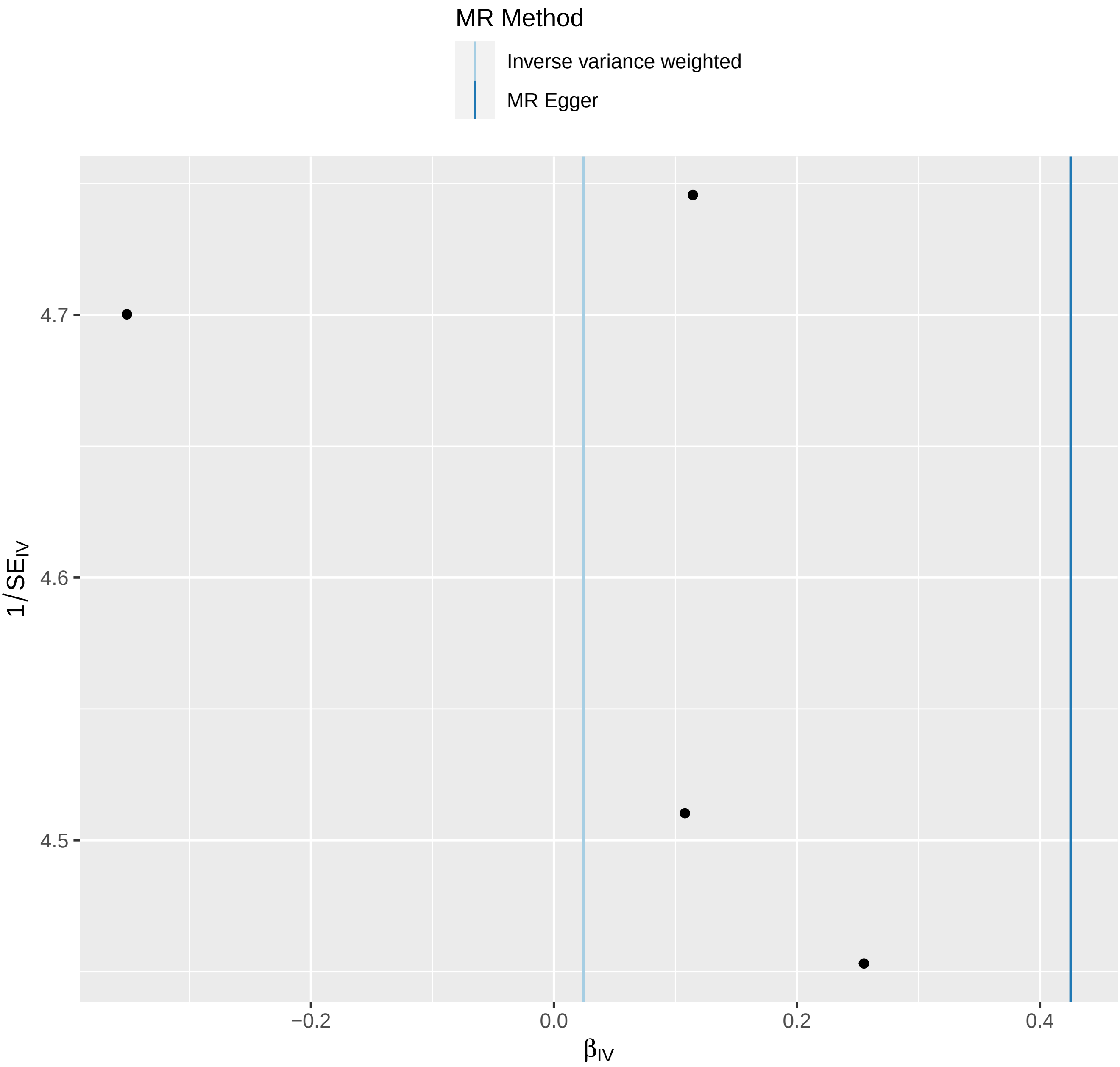

Batch 2−3418 : Gut microbiota abundance (genus Parasutterella id.2892) on Neutrophil gelatinase−associated lipocalin
with a threshold of 1e−05

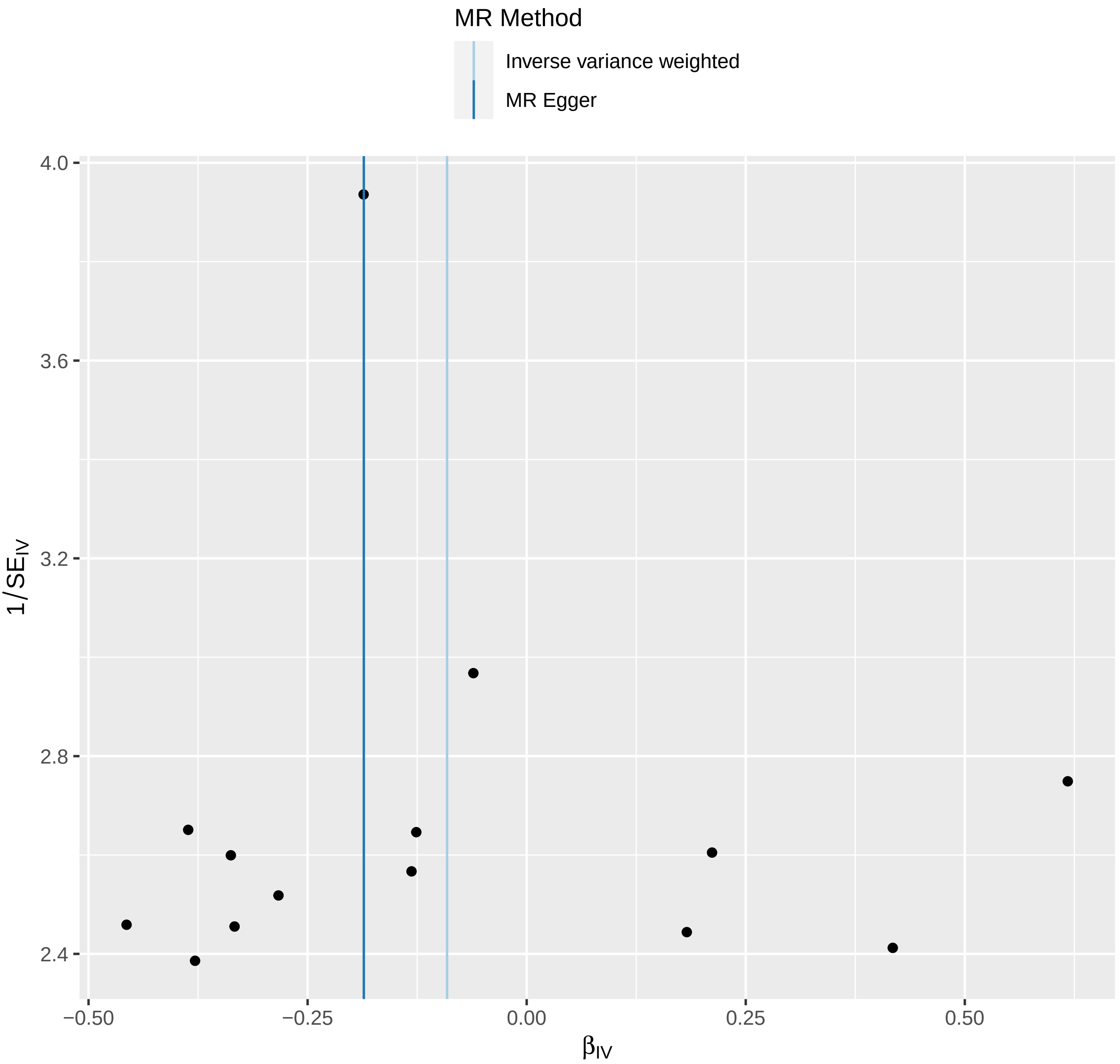

Batch 2−3419 : Gut microbiota abundance (genus Catenibacterium id.2153) on Epididymal−specific lipocalin−8
with a threshold of 1e−05

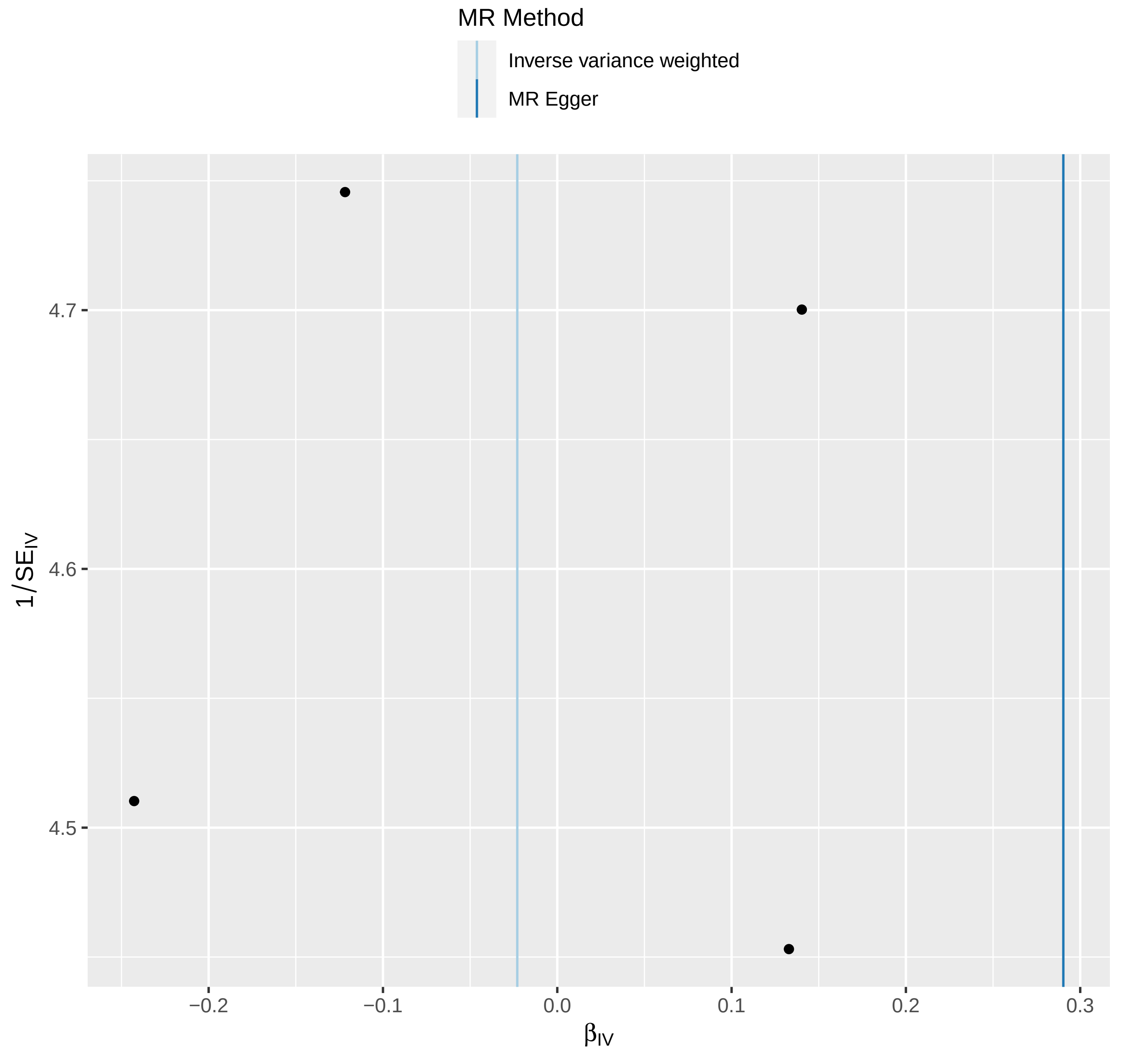

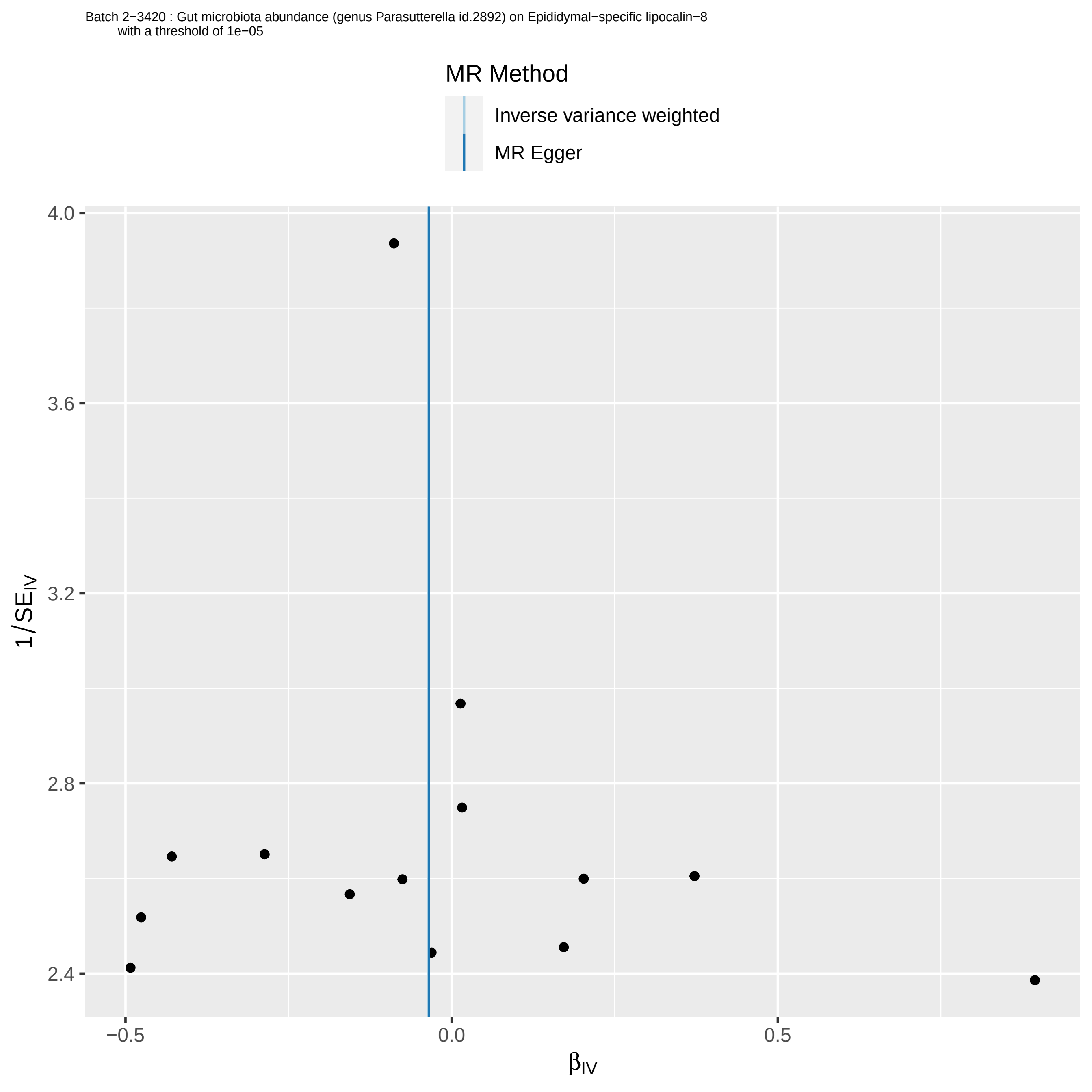

Batch 2−3420 : Gut microbiota abundance (genus Parasutterella id.2892) on Epididymal−specific lipocalin−8
with a threshold of 1e−05
MR Method
Inverse variance weighted
MR Egger
4.0
3.6
3.2
2.8
2.4
1/SE_IV
−0.5
0.0
0.5
β_IV

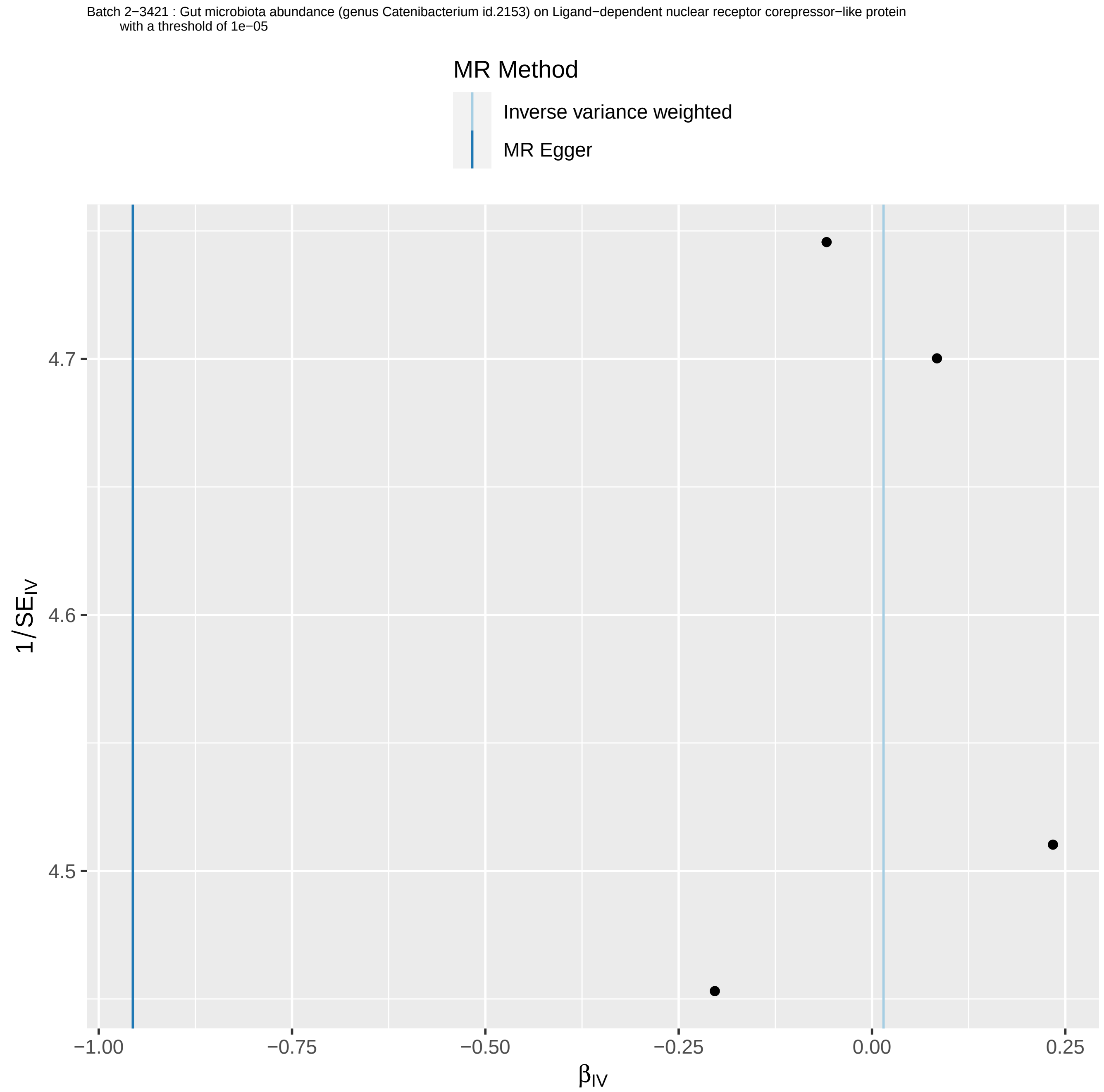
Batch 2−3421 : Gut microbiota abundance (genus Catenibacterium id.2153) on Ligand−dependent nuclear receptor corepressor−like protein
with a threshold of 1e−05
MR Method
Inverse variance weighted
MR Egger
4.7
4.6
4.5
1/SE$_{IV}$
−1.00
−0.75
−0.50
−0.25
0.00
0.25
β$_{IV}$

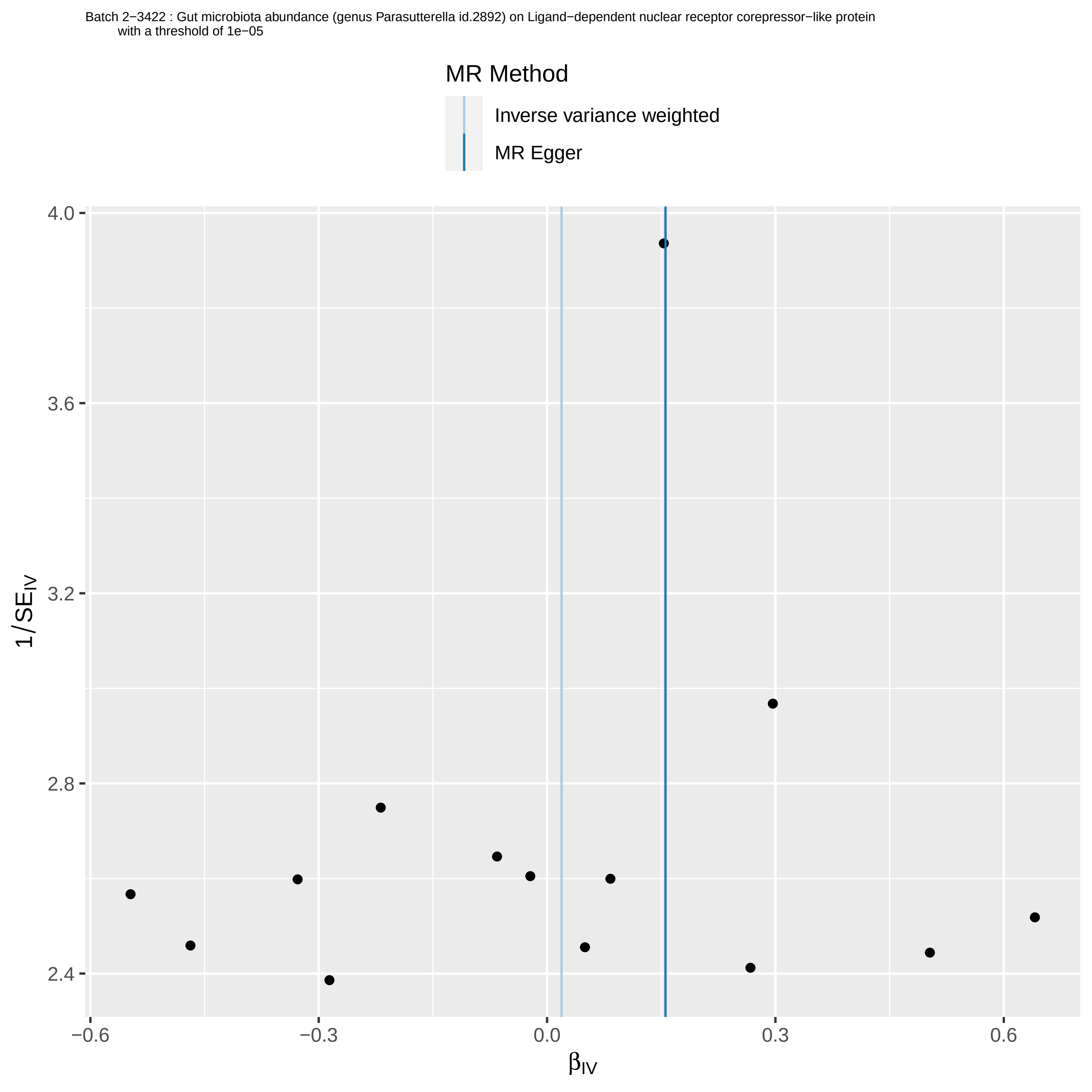
Batch 2−3422 : Gut microbiota abundance (genus Parasutterella id.2892) on Ligand−dependent nuclear receptor corepressor−like protein
with a threshold of 1e−05
MR Method
Inverse variance weighted
MR Egger
4.0
3.6
3.2
2.8
2.4
1/SE_IV
−0.6
−0.3
0.0
0.3
0.6
β_IV

Batch 2−3423 : Gut microbiota abundance (genus Catenibacterium id.2153) on Plastin−2
with a threshold of 1e−05

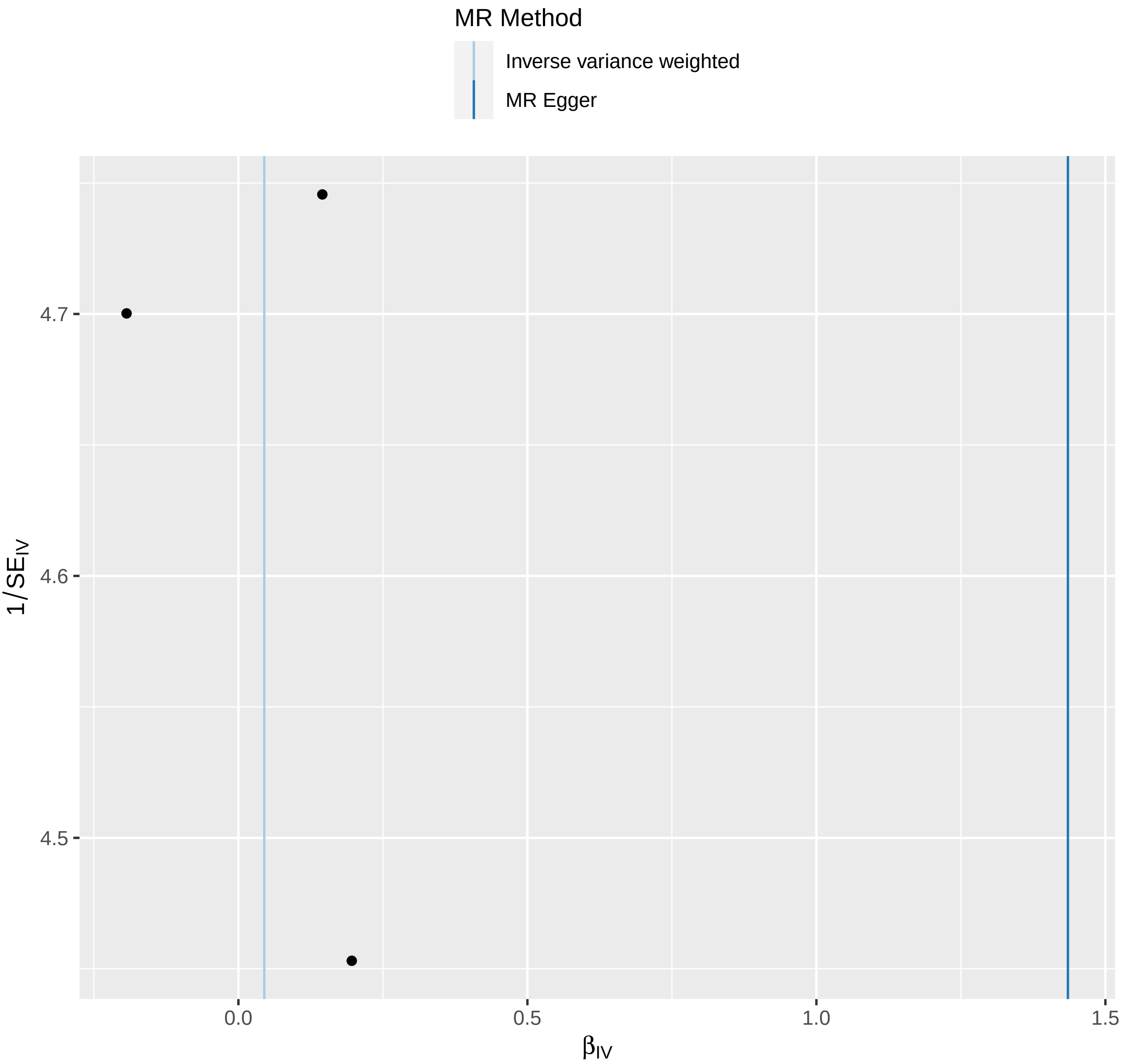

Batch 2−3424 : Gut microbiota abundance (genus Parasutterella id.2892) on Plastin−2
with a threshold of 1e−05

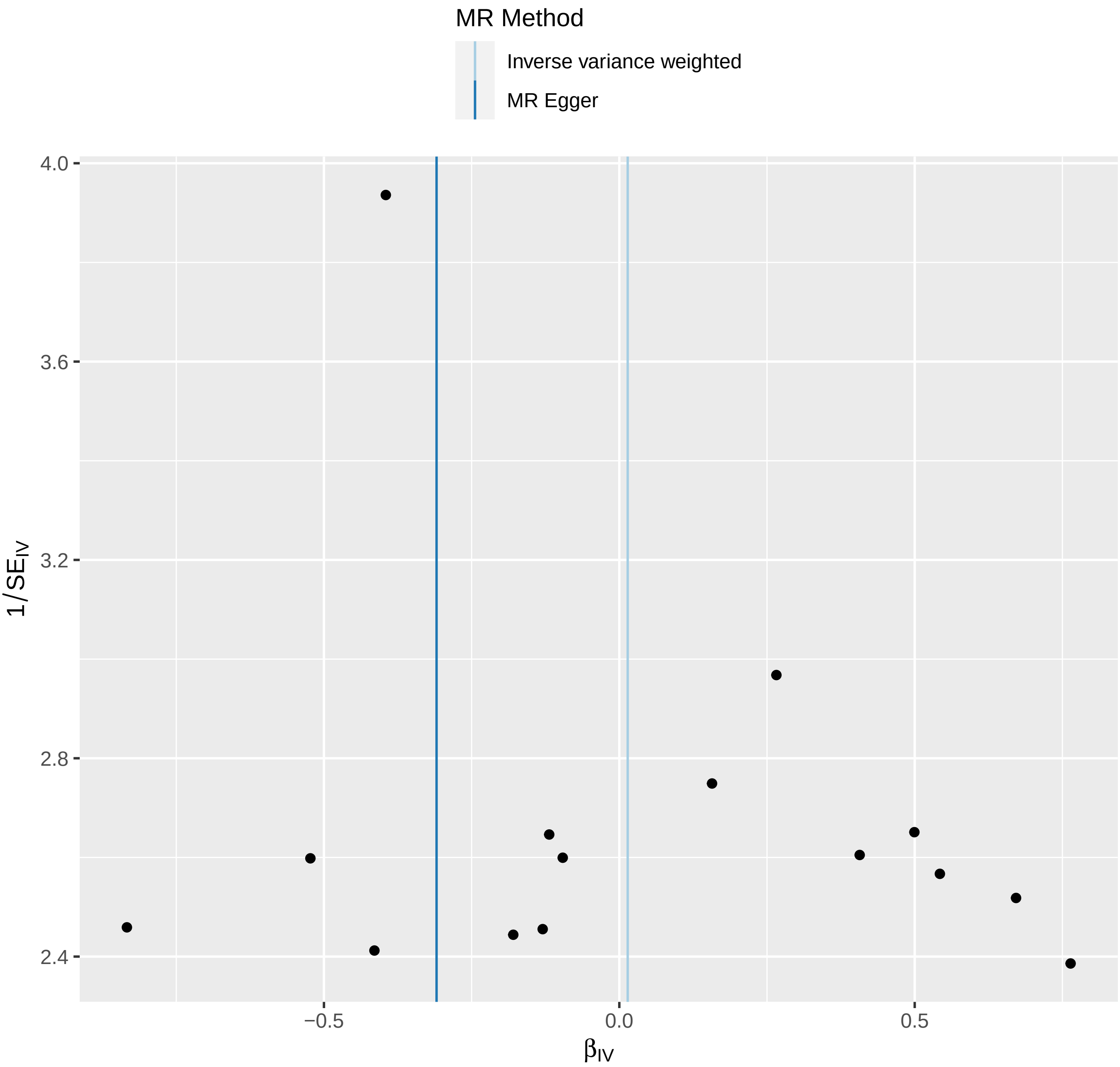

Batch 2−3425 : Gut microbiota abundance (genus Catenibacterium id.2153) on Lactase−phlorizin hydrolase
with a threshold of 1e−05

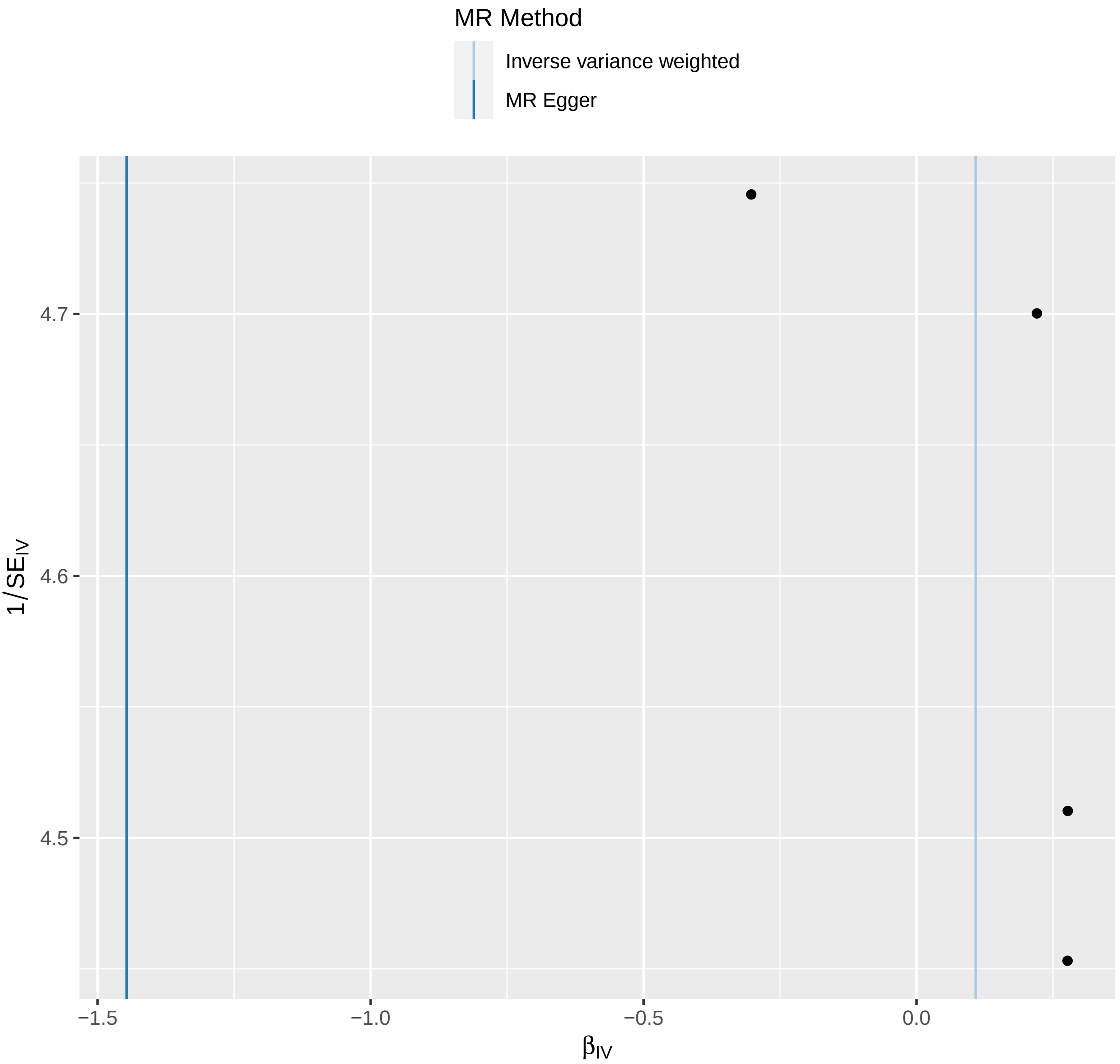

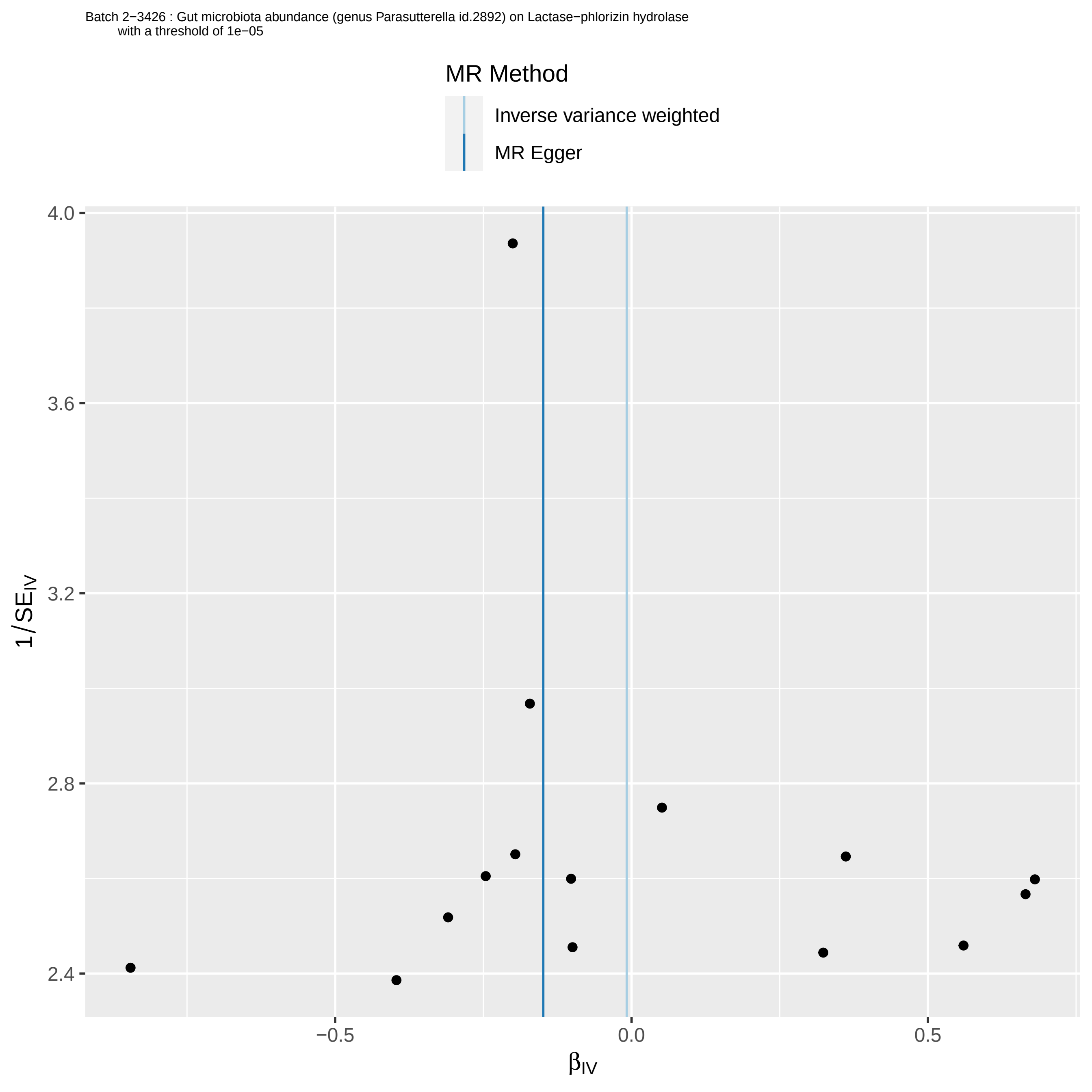

Batch 2−3426 : Gut microbiota abundance (genus Parasutterella id.2892) on Lactase−phlorizin hydrolase
with a threshold of 1e−05
MR Method
Inverse variance weighted
MR Egger
4.0
3.6
3.2
2.8
2.4
1/SE_IV
−0.5
0.0
0.5
β_IV

Batch 2−3427 : Gut microbiota abundance (genus Catenibacterium id.2153) on Lactase−like protein
with a threshold of 1e−05

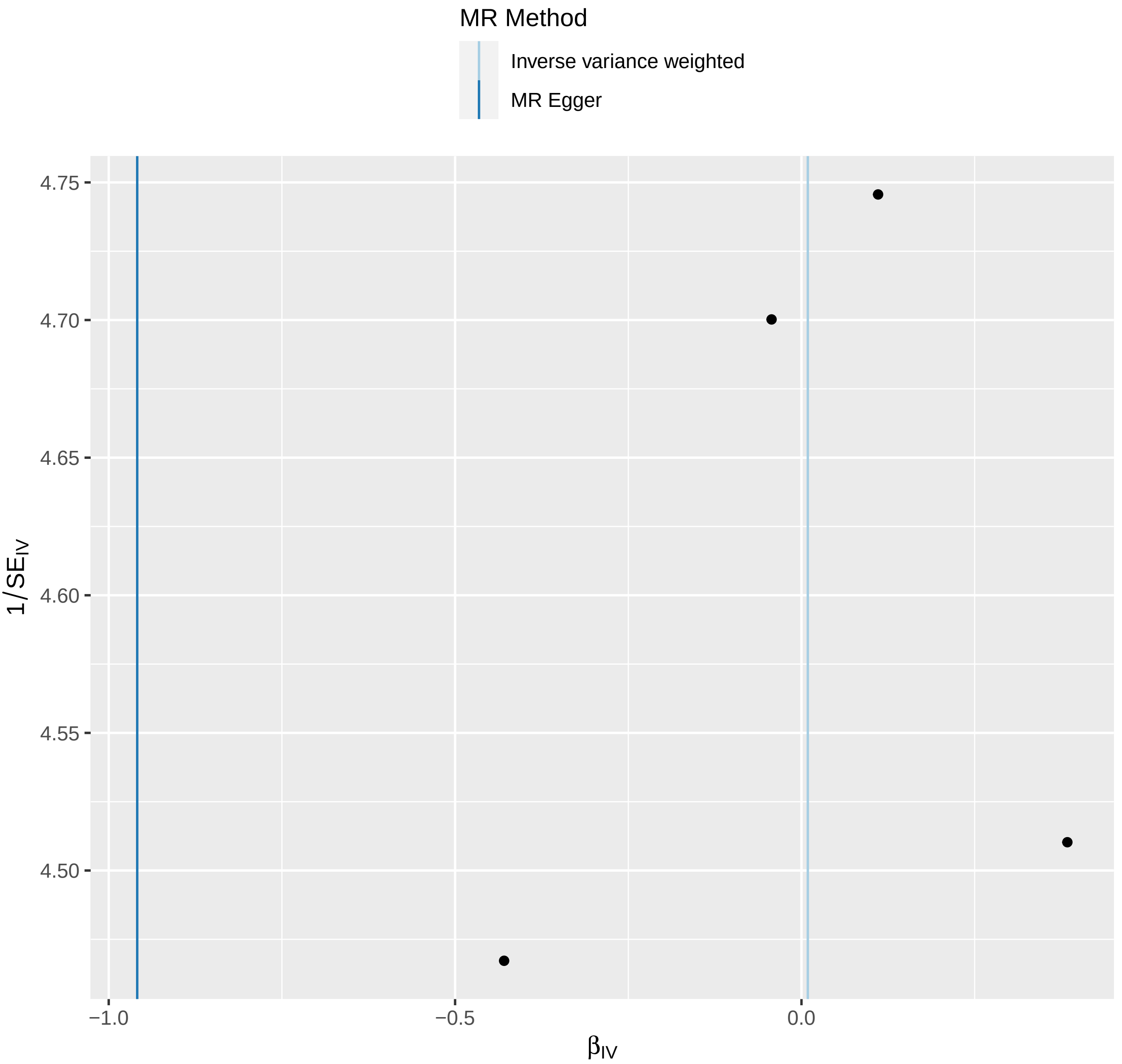

Batch 2−3428 : Gut microbiota abundance (genus Parasutterella id.2892) on Lactase−like protein
with a threshold of 1e−05

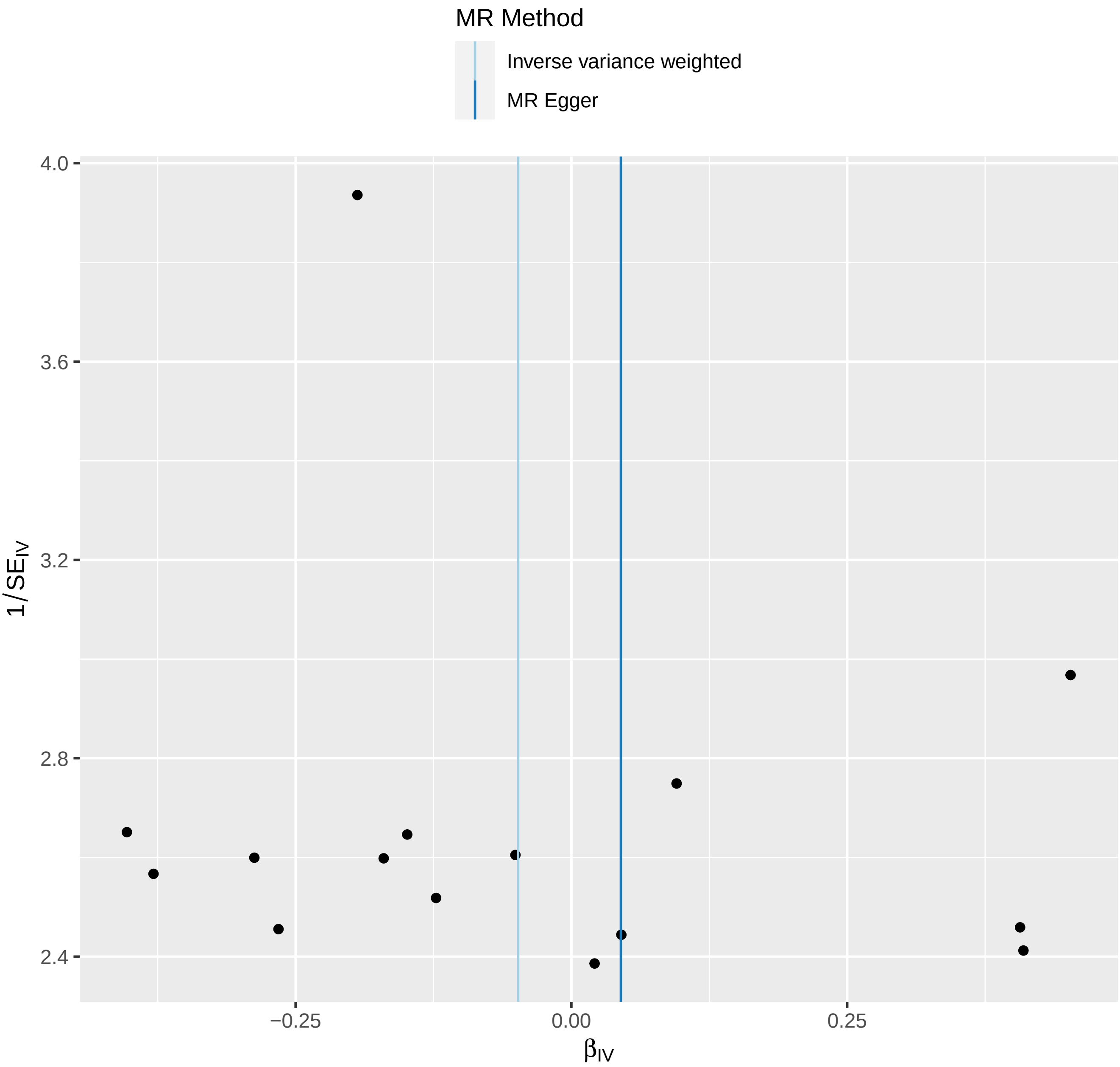

Batch 2−3429 : Gut microbiota abundance (genus Catenibacterium id.2153) on L−lactate dehydrogenase B chain
with a threshold of 1e−05

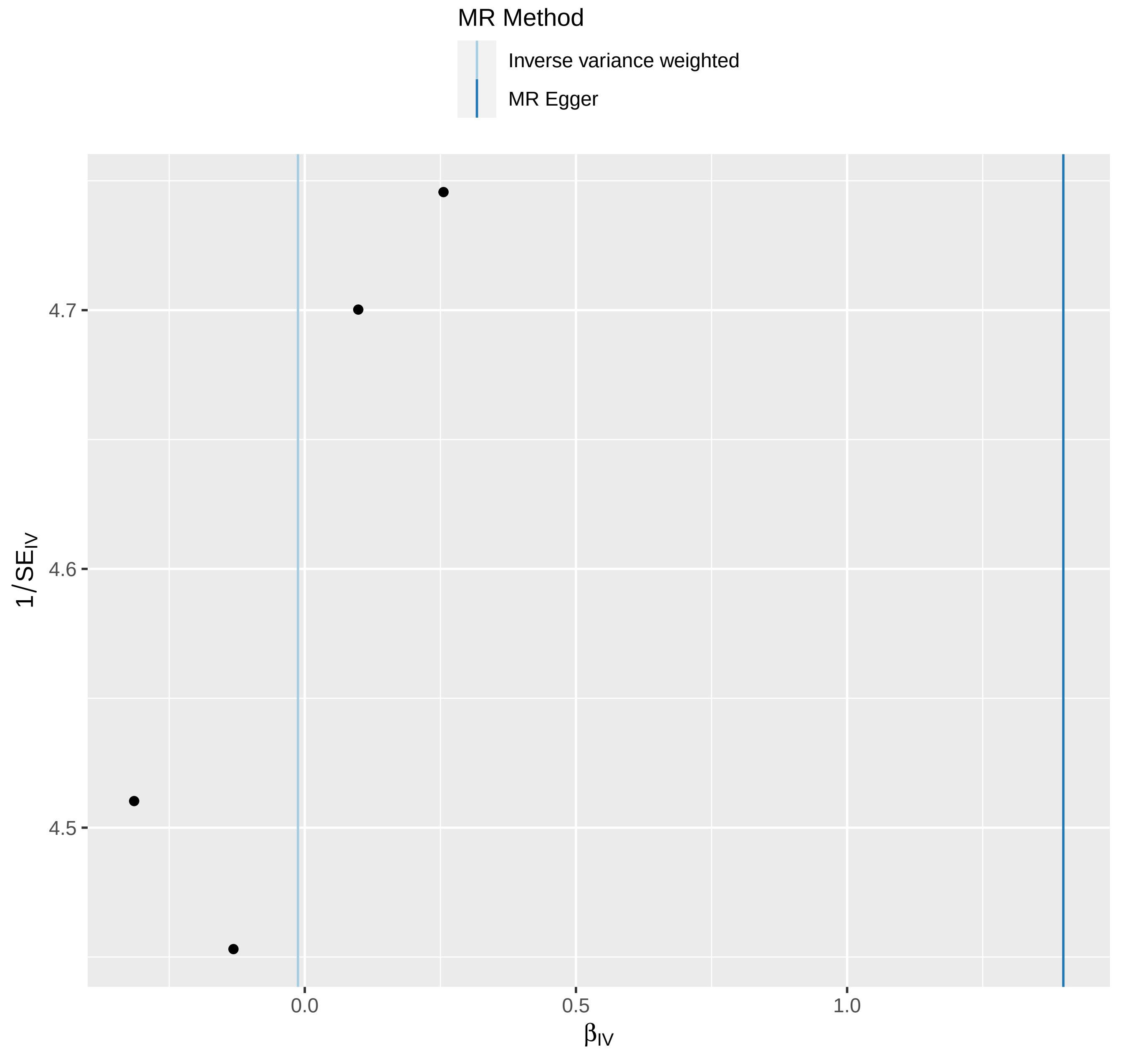

Batch 2−3430 : Gut microbiota abundance (genus Parasutterella id.2892) on L−lactate dehydrogenase B chain with a threshold of 1e−05

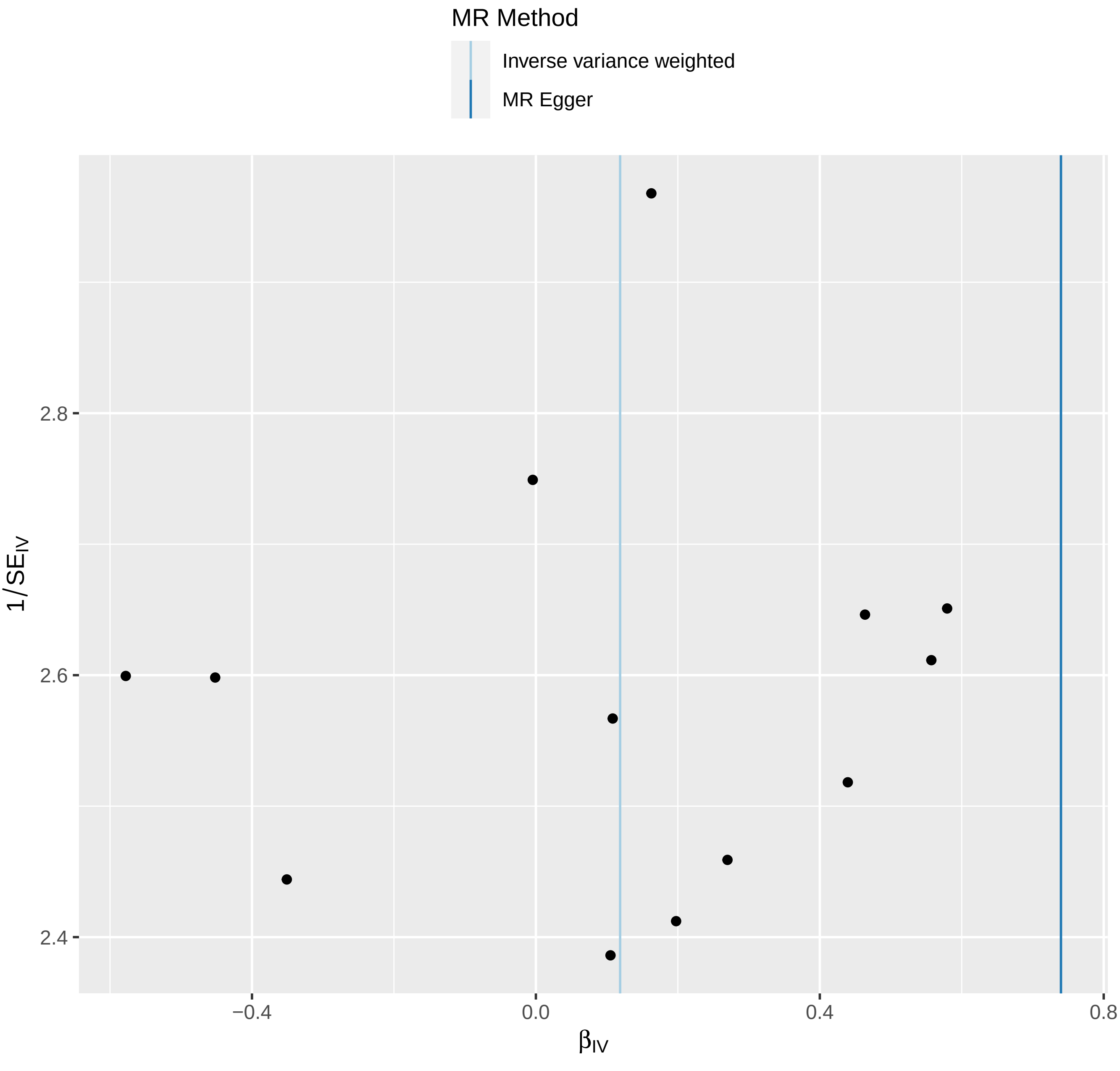

Batch 2−3431 : Gut microbiota abundance (genus Catenibacterium id.2153) on L−lactate dehydrogenase C chain
with a threshold of 1e−05

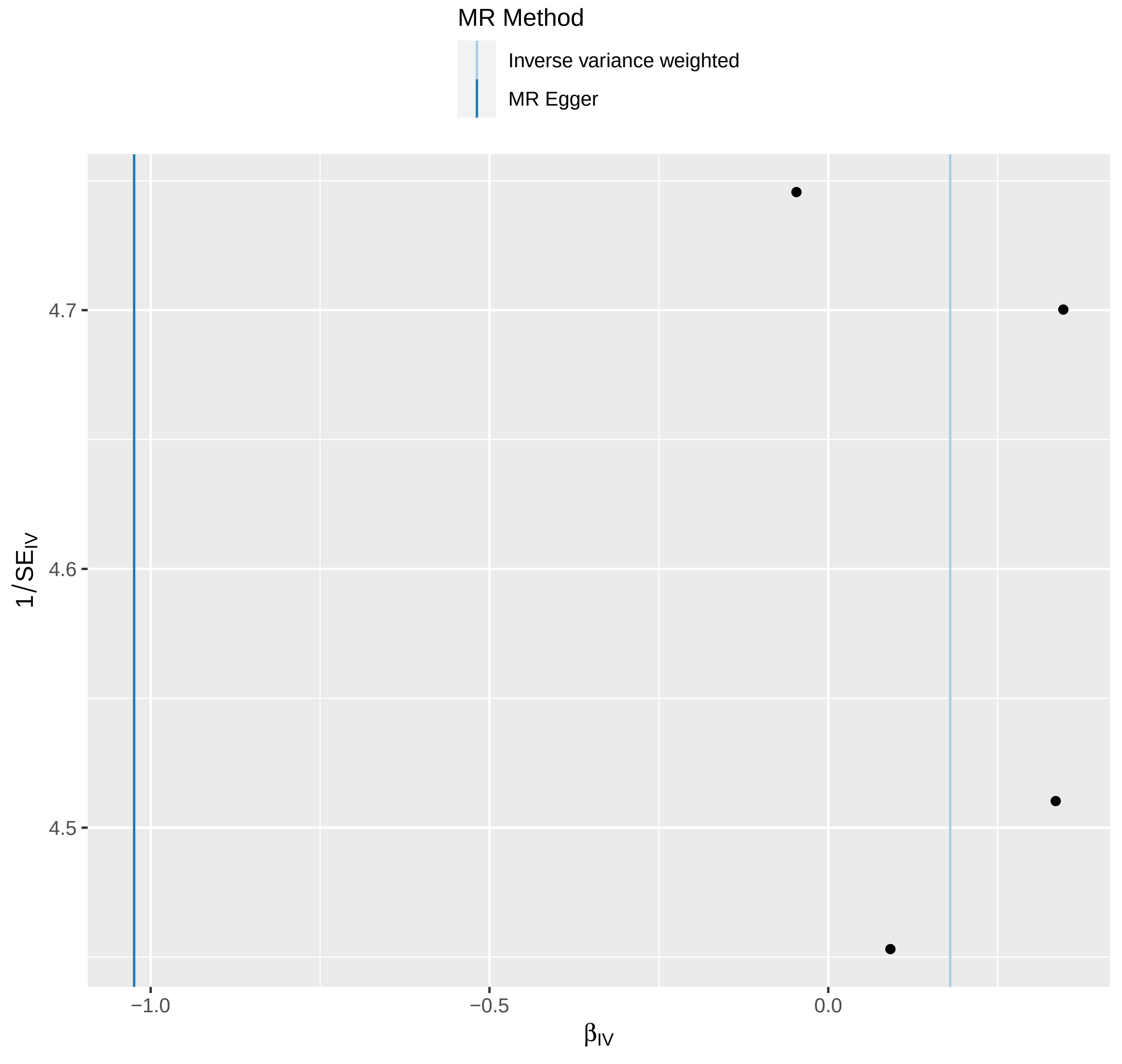

Batch 2−3432 : Gut microbiota abundance (genus Parasutterella id.2892) on L−lactate dehydrogenase C chain
with a threshold of 1e−05

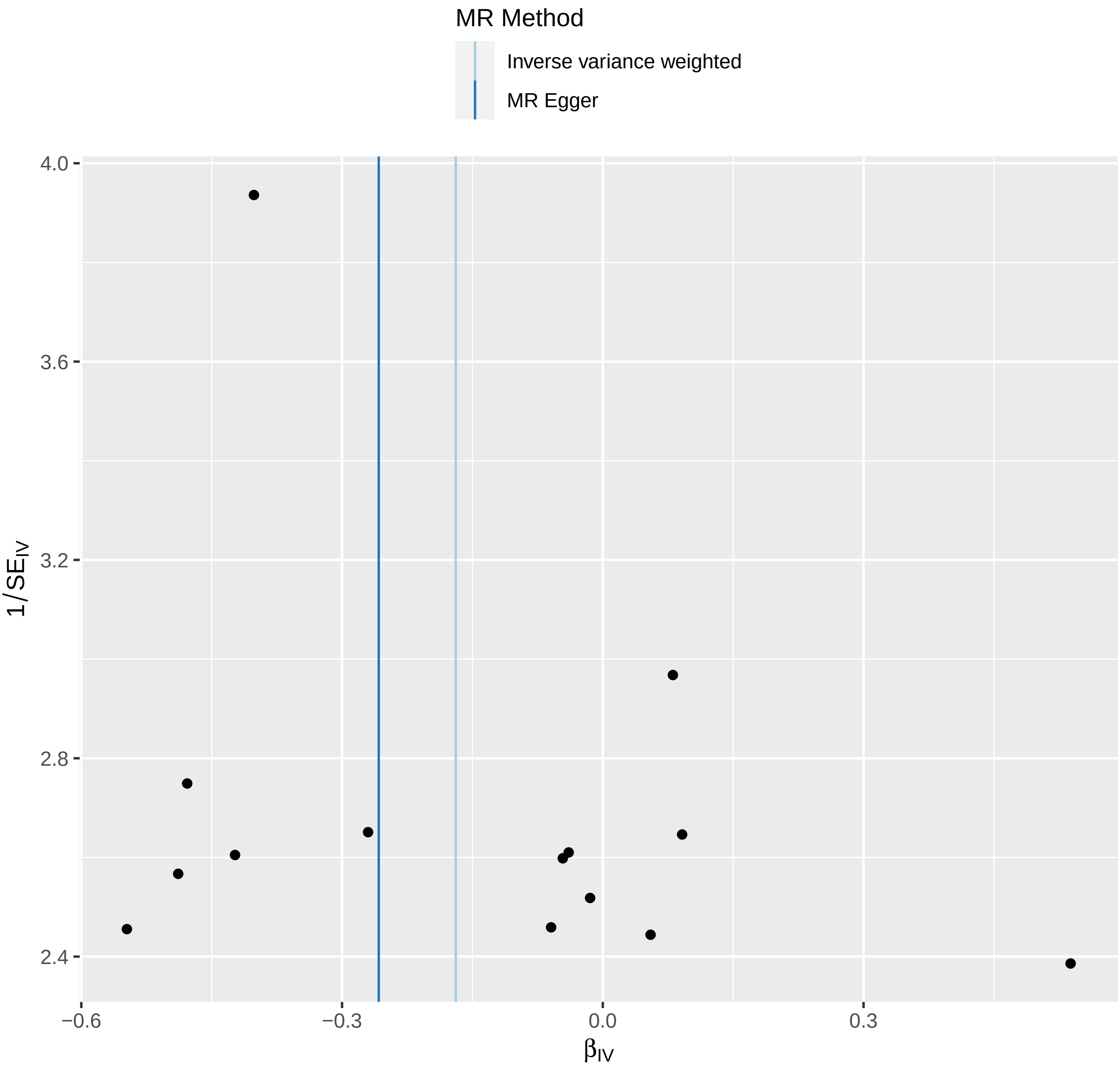

Batch 2−3433 : Gut microbiota abundance (genus Catenibacterium id.2153) on Low−density lipoprotein receptor
with a threshold of 1e−05

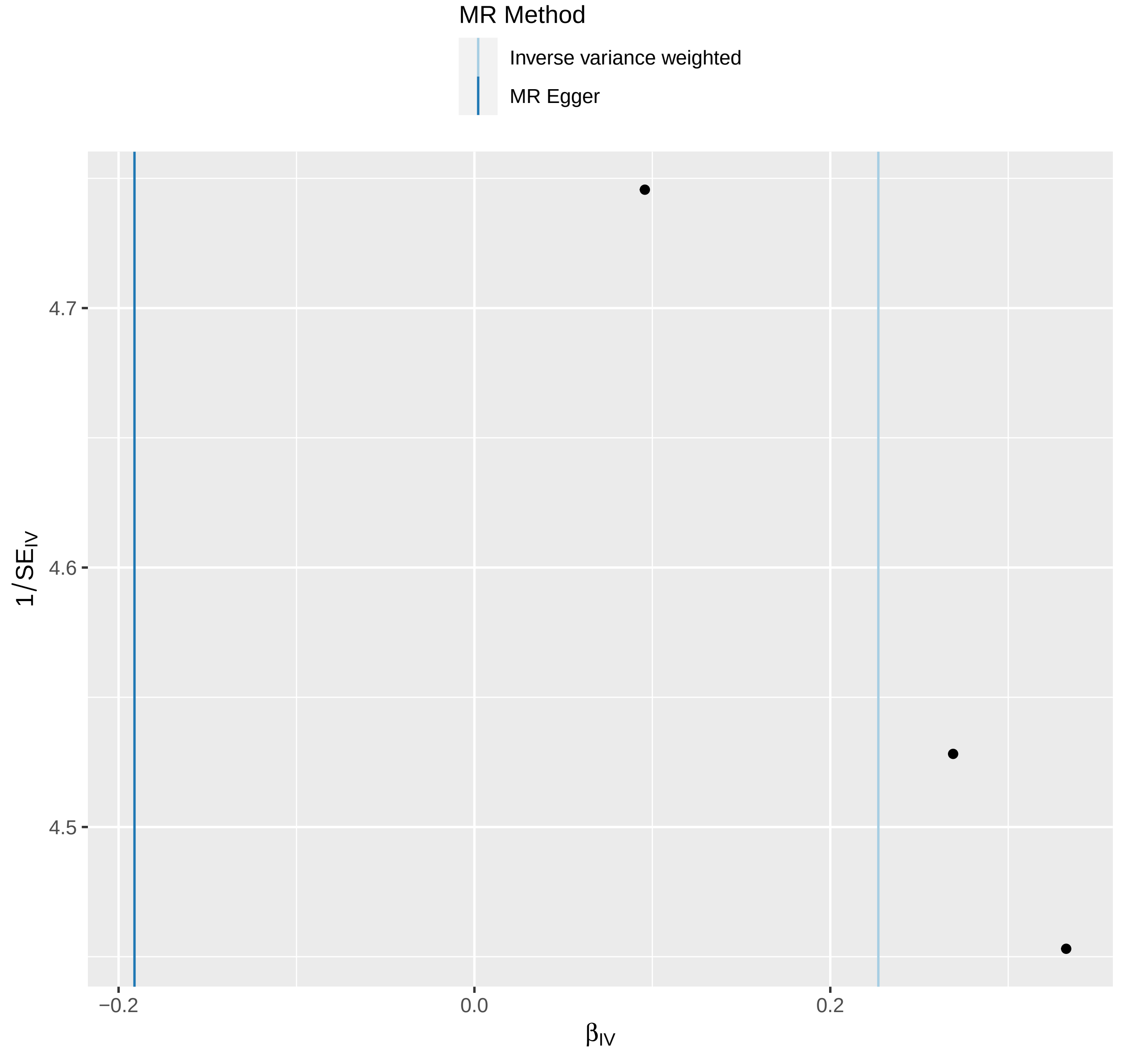

Batch 2−3434 : Gut microbiota abundance (genus Parasutterella id.2892) on Low−density lipoprotein receptor
with a threshold of 1e−05

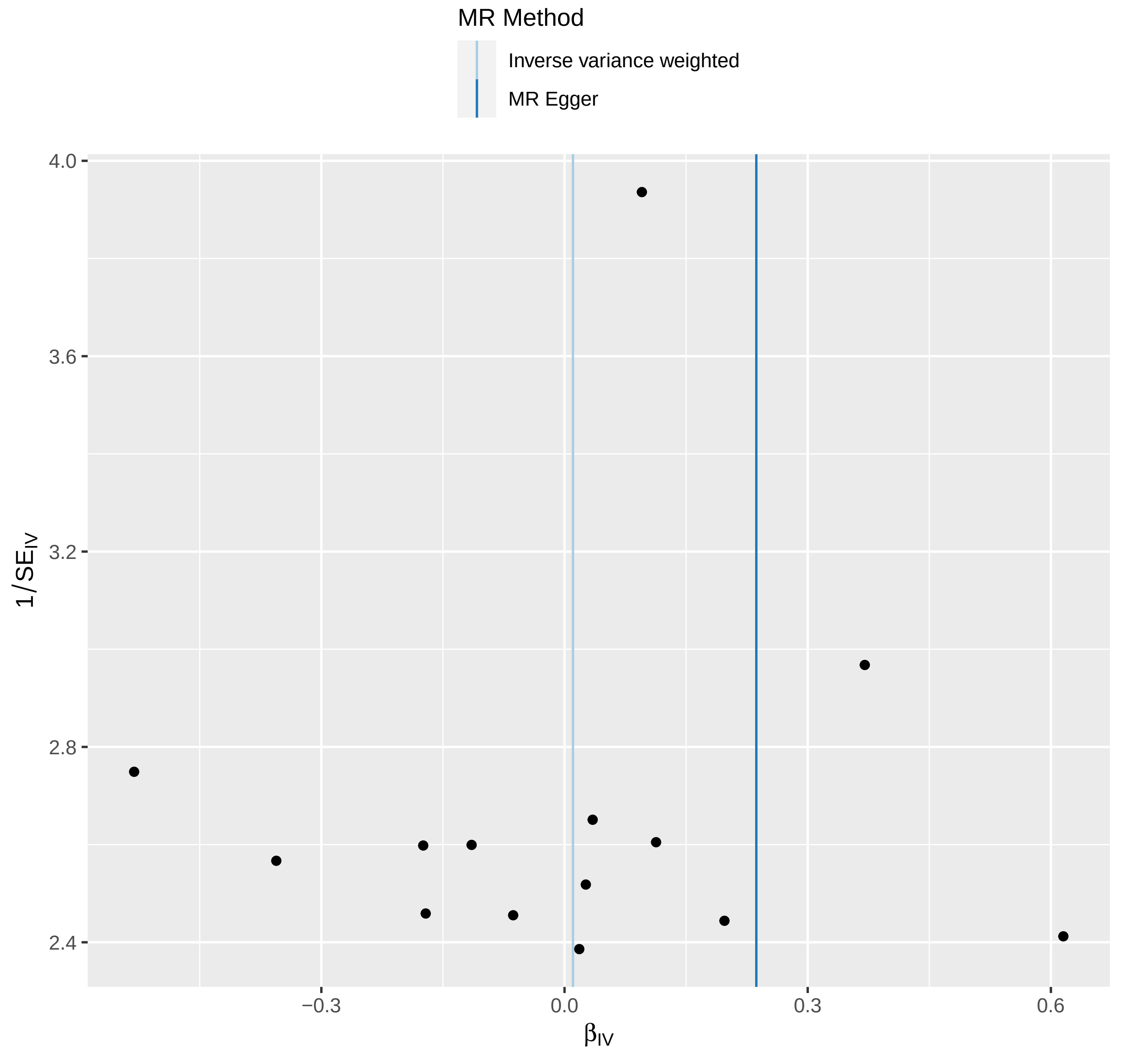

Batch 2−3435 : Gut microbiota abundance (genus Catenibacterium id.2153) on Protein LDOC1
with a threshold of 1e−05

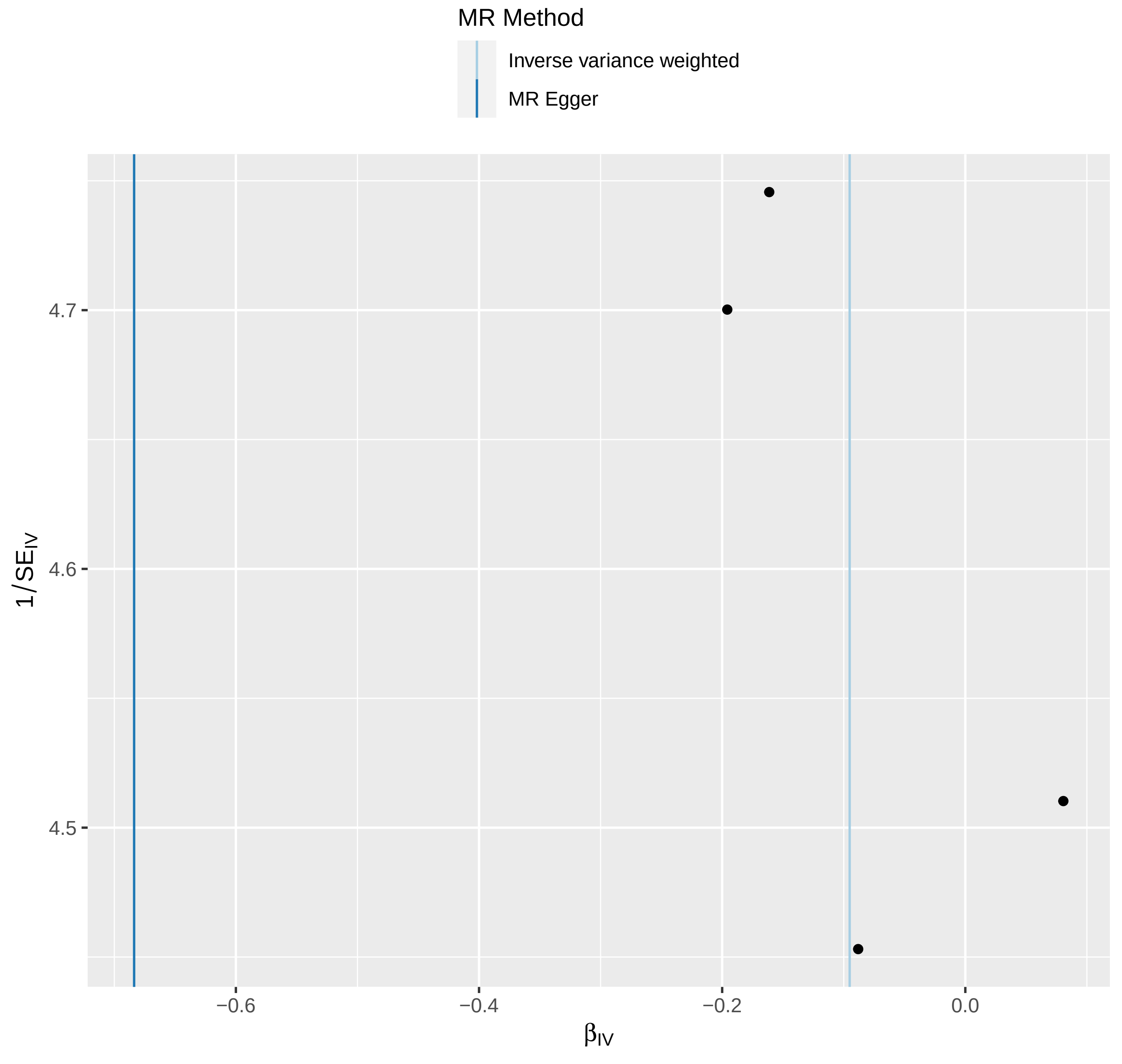

Batch 2−3436 : Gut microbiota abundance (genus Parasutterella id.2892) on Protein LDOC1
with a threshold of 1e−05

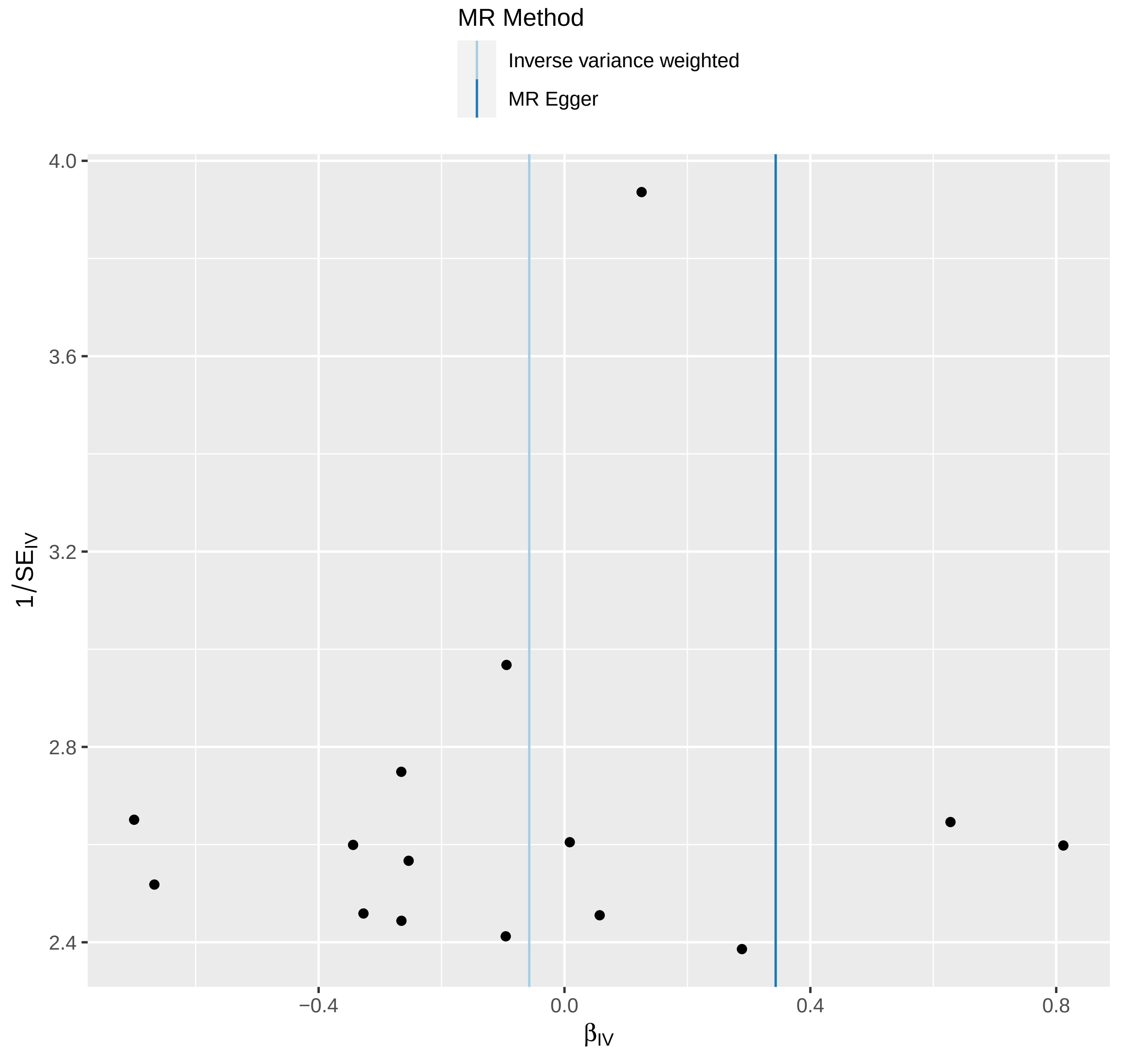

Batch 2−3437 : Gut microbiota abundance (genus Catenibacterium id.2153) on Leukocyte cell−derived chemotaxin 1 with a threshold of 1e−05

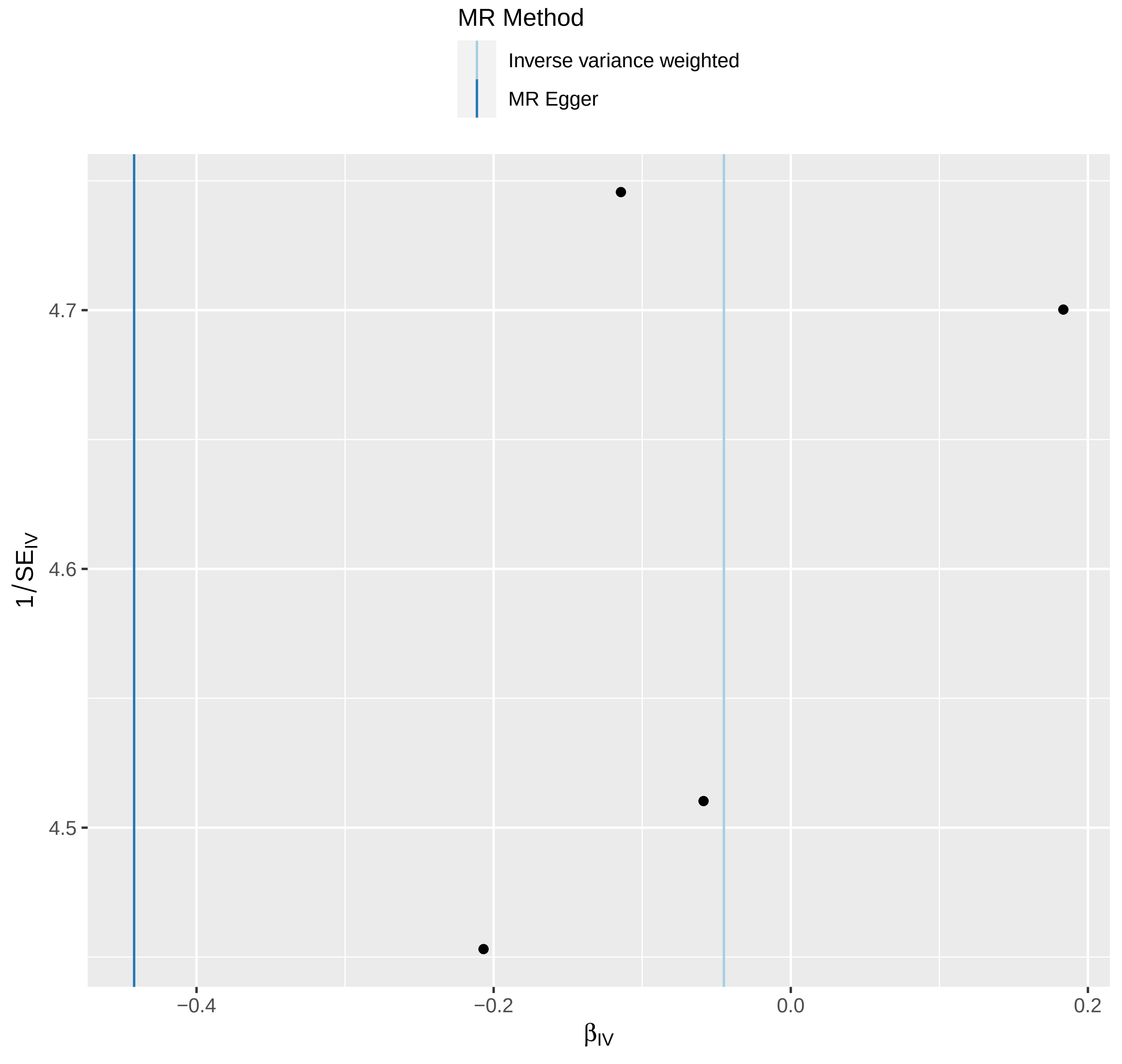

Batch 2−3438 : Gut microbiota abundance (genus Parasutterella id.2892) on Leukocyte cell−derived chemotaxin 1
with a threshold of 1e−05

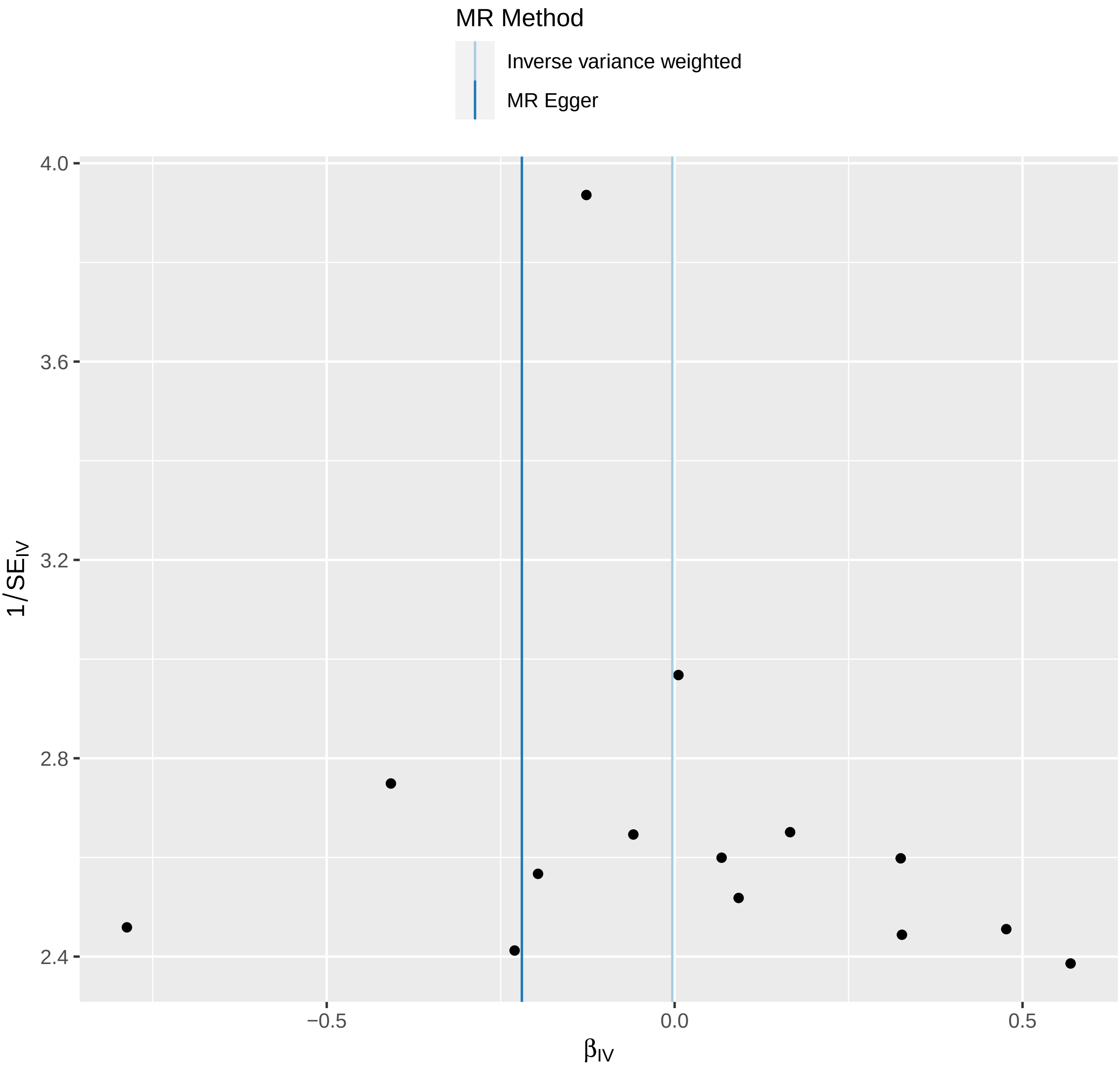

Batch 2−3439 : Gut microbiota abundance (genus Catenibacterium id.2153) on LEM domain−containing protein 1
with a threshold of 1e−05

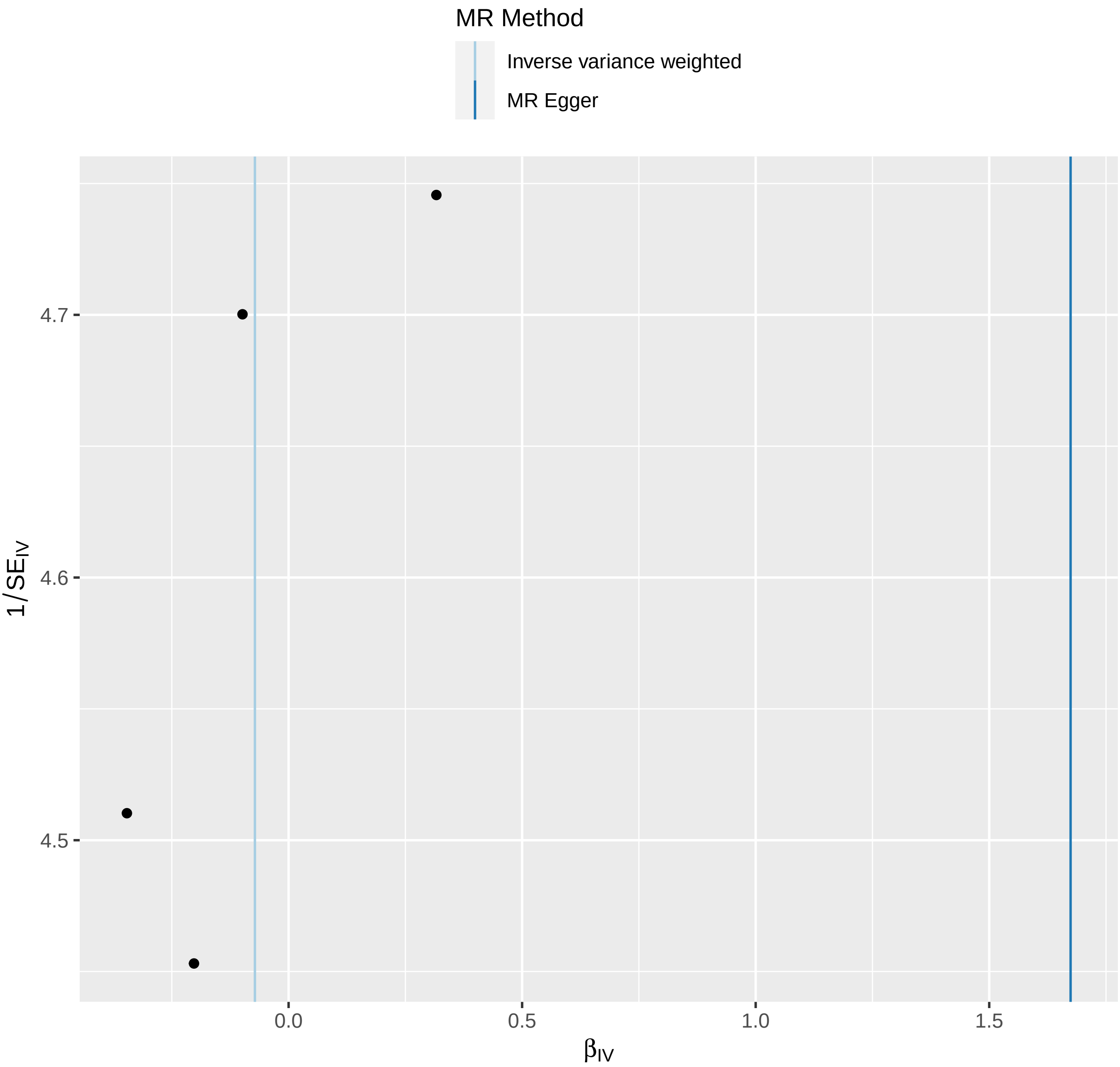

Batch 2−3440 : Gut microbiota abundance (genus Parasutterella id.2892) on LEM domain−containing protein 1 with a threshold of 1e−05

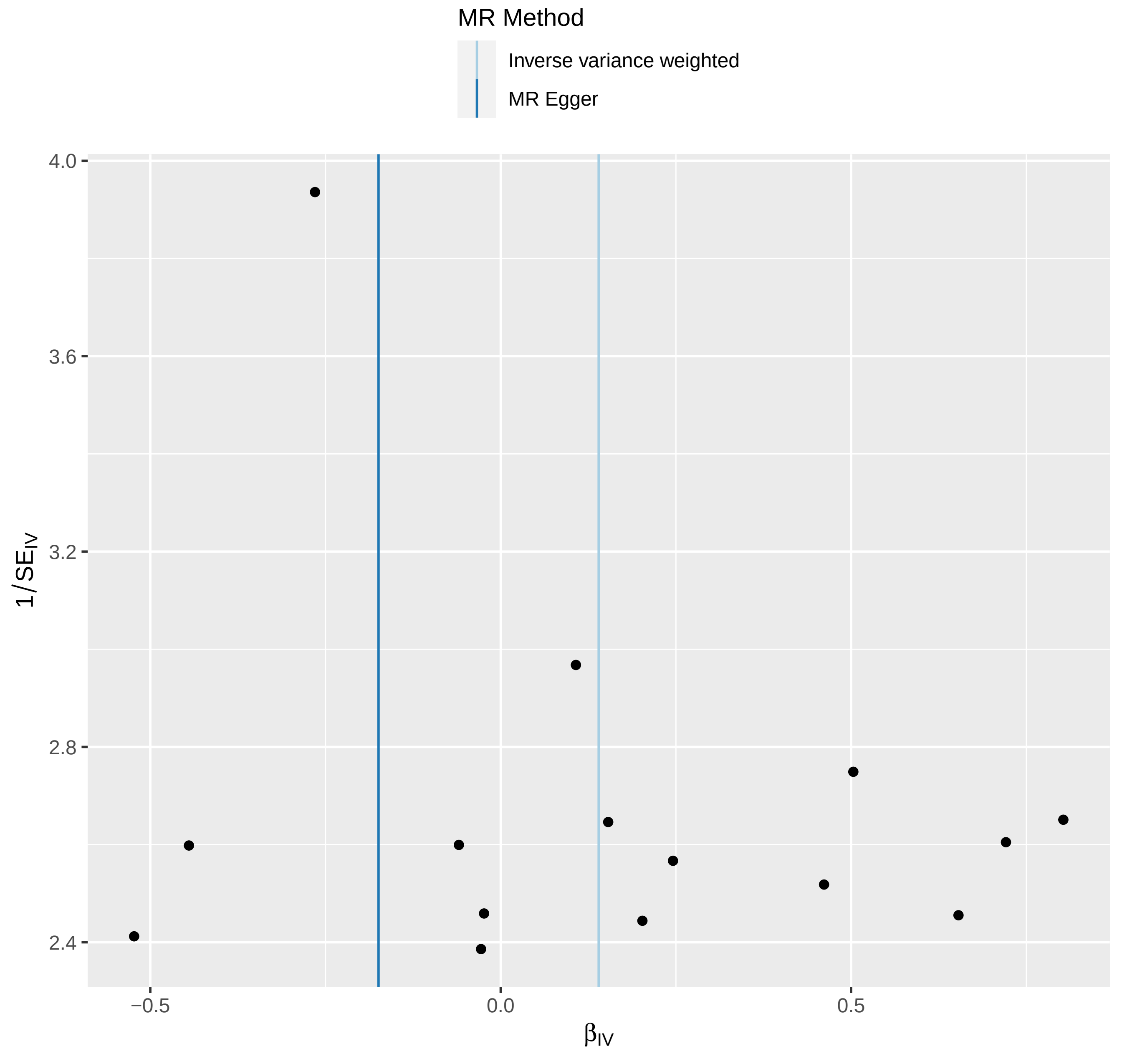

Batch 2−3441 : Gut microbiota abundance (genus Catenibacterium id.2153) on Leptin
with a threshold of 1e−05

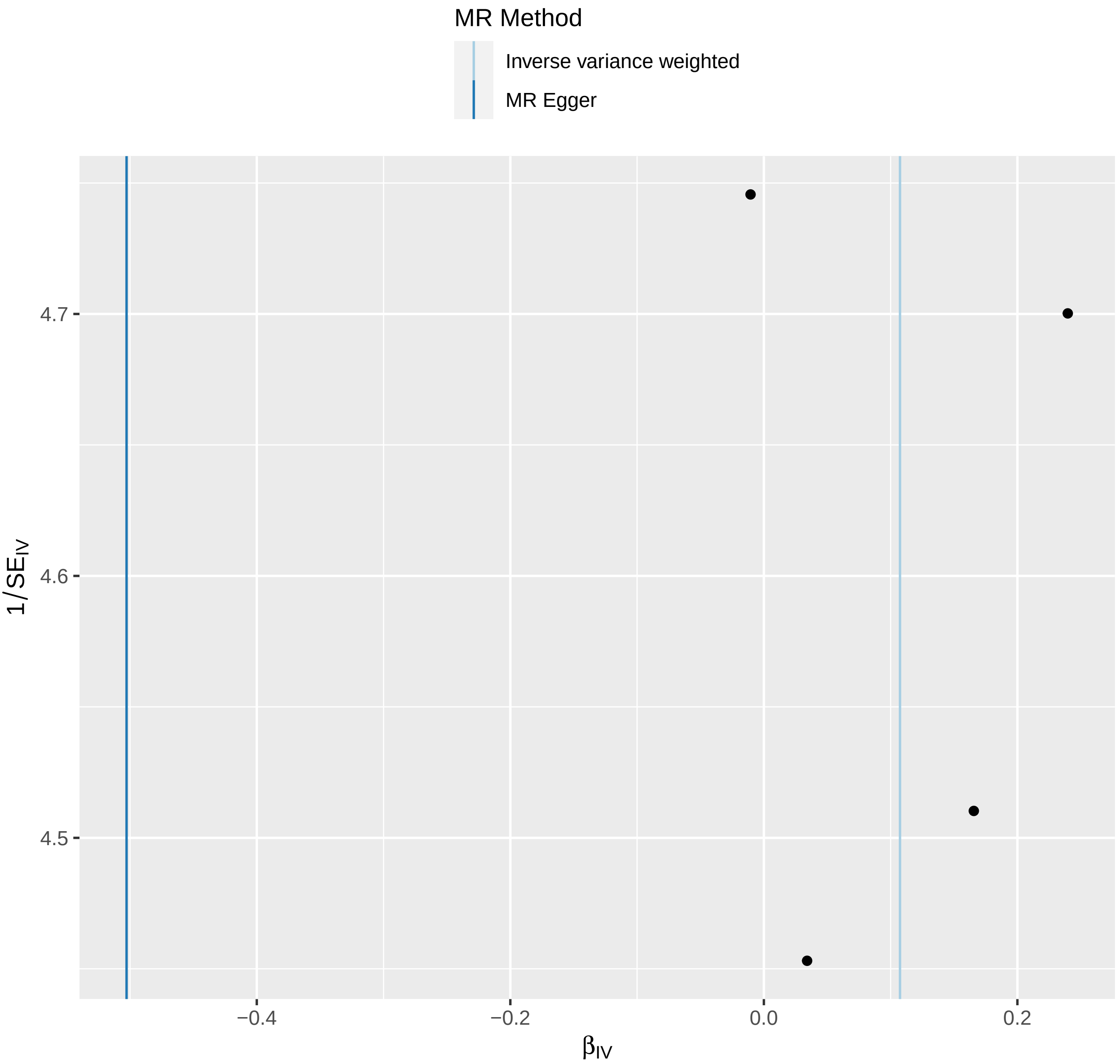

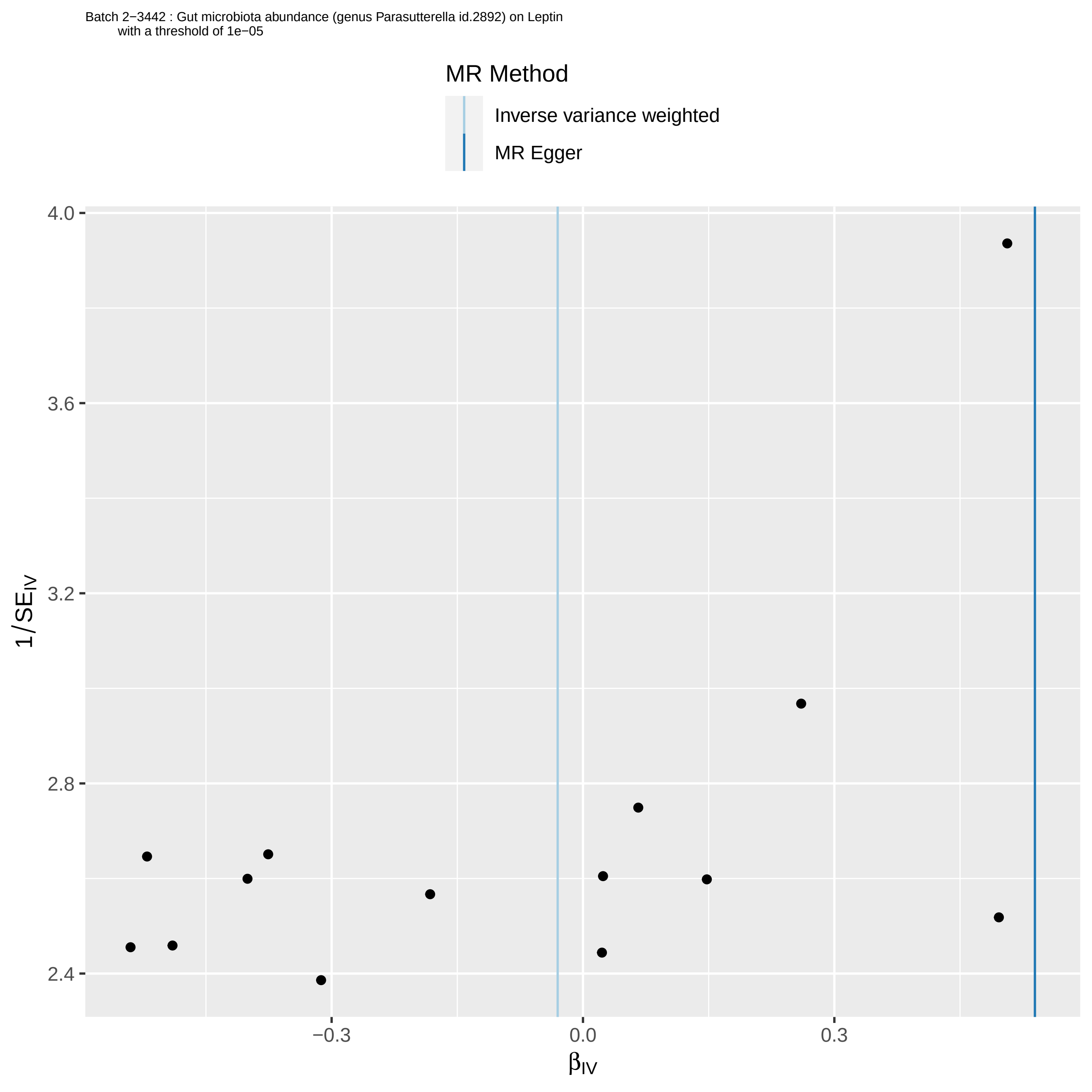

Batch 2−3442 : Gut microbiota abundance (genus Parasutterella id.2892) on Leptin
with a threshold of 1e−05
MR Method
Inverse variance weighted
MR Egger
4.0
3.6
3.2
2.8
2.4
1/SE_IV
−0.3
0.0
0.3
β_IV

Batch 2−3443 : Gut microbiota abundance (genus Catenibacterium id.2153) on Leptin
with a threshold of 1e−05

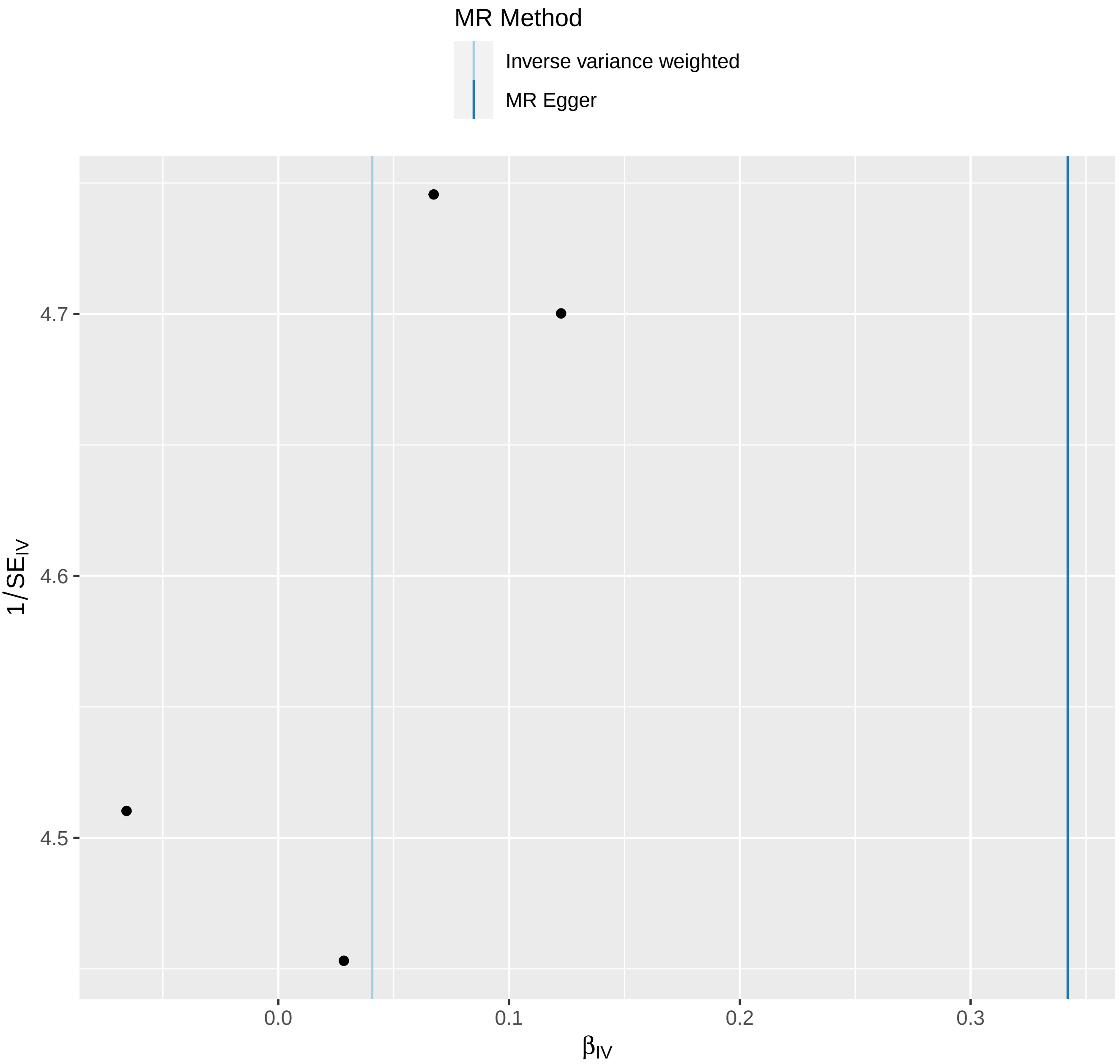

Batch 2−3444 : Gut microbiota abundance (genus Parasutterella id.2892) on Leptin
with a threshold of 1e−05

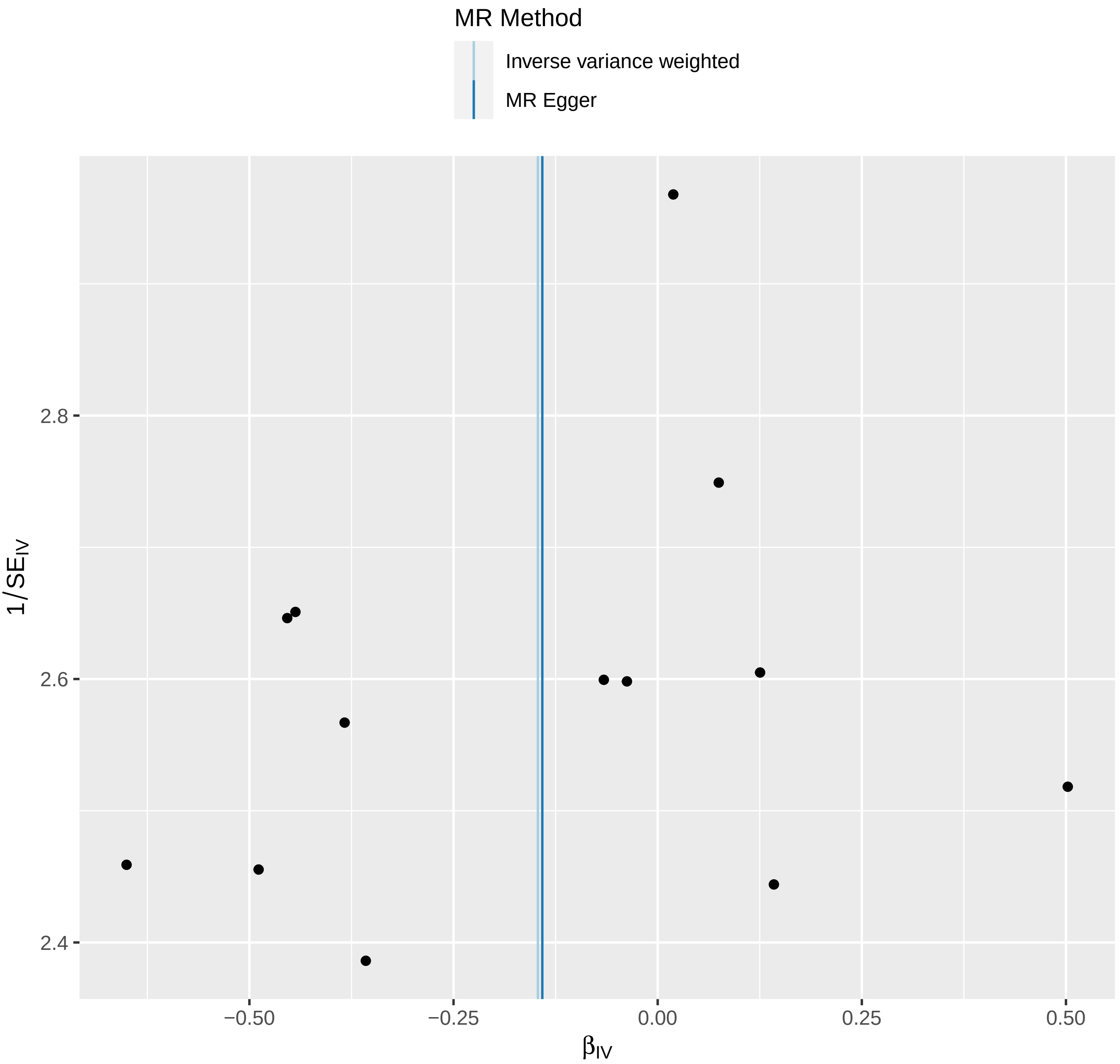

Batch 2−3445 : Gut microbiota abundance (genus Catenibacterium id.2153) on Leptin
with a threshold of 1e−05

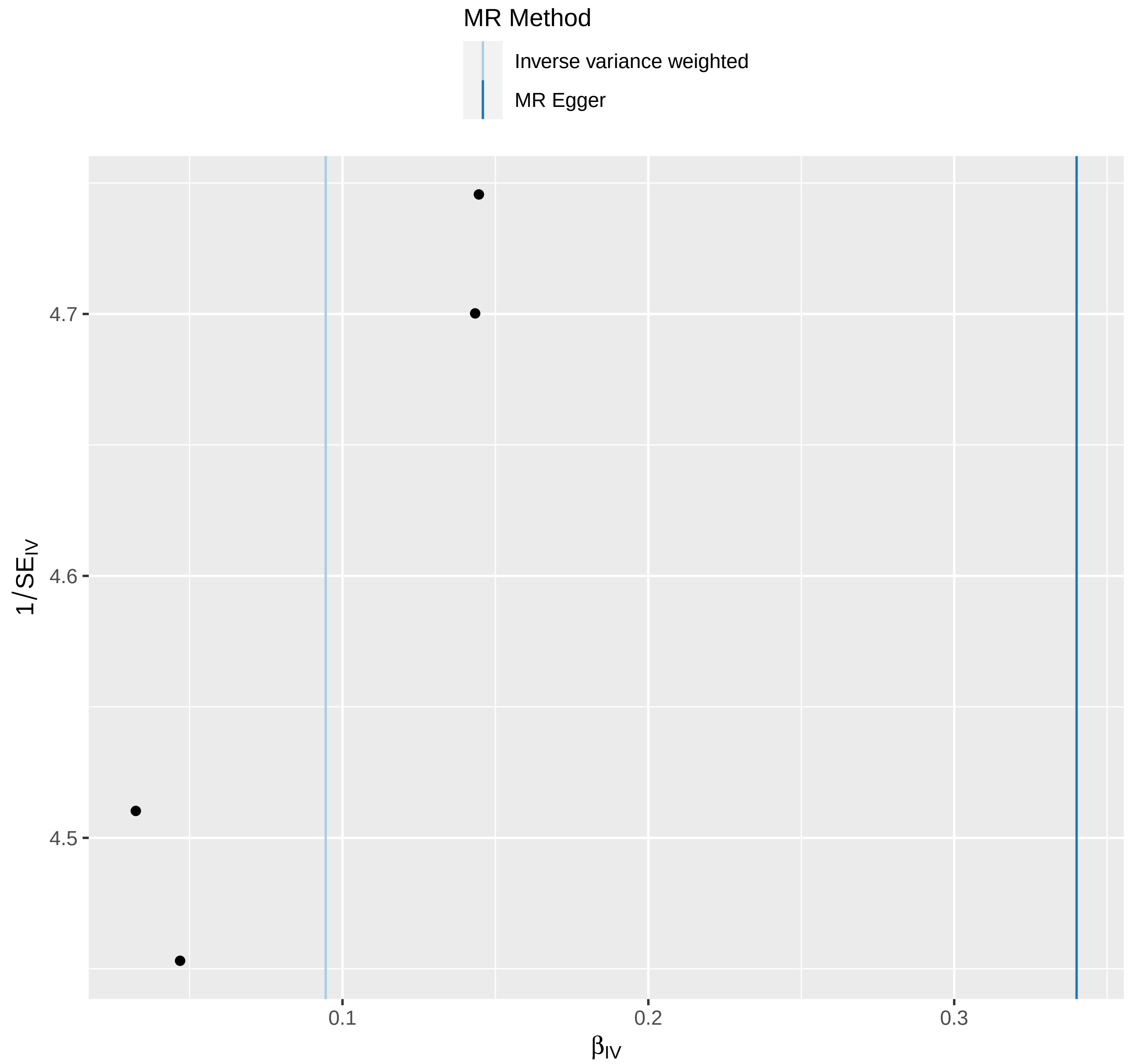

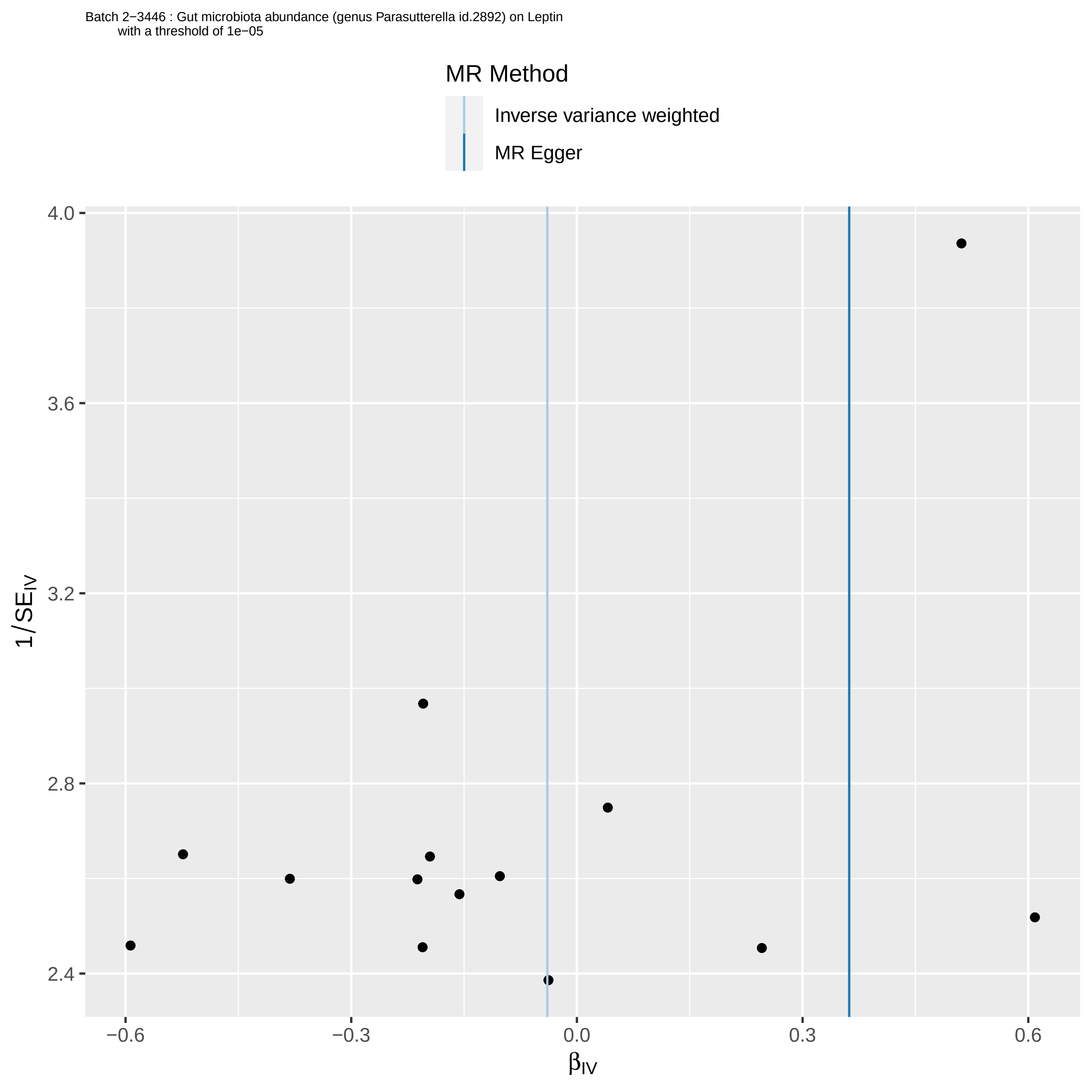
Batch 2−3446 : Gut microbiota abundance (genus Parasutterella id.2892) on Leptin
with a threshold of 1e−05
MR Method
Inverse variance weighted
MR Egger
4.0
3.6
3.2
2.8
2.4
1/SE$_{IV}$
−0.6
−0.3
0.0
0.3
0.6
β$_{IV}$

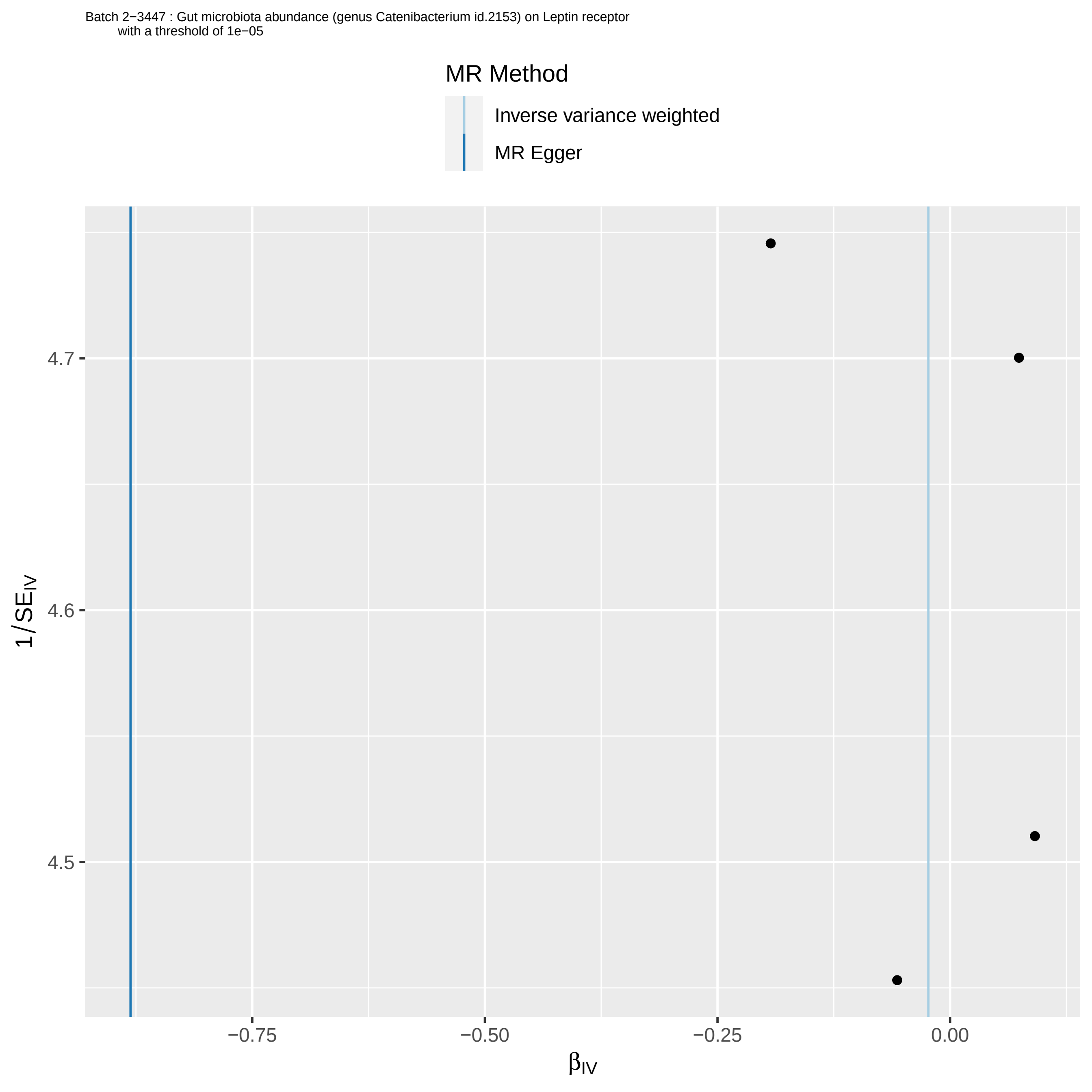

Batch 2−3447 : Gut microbiota abundance (genus Catenibacterium id.2153) on Leptin receptor
with a threshold of 1e−05
MR Method
Inverse variance weighted
MR Egger
4.7
4.6
4.5
1/SE_IV
−0.75
−0.50
−0.25
0.00
β_IV

Batch 2−3448 : Gut microbiota abundance (genus Parasutterella id.2892) on Leptin receptor
with a threshold of 1e−05

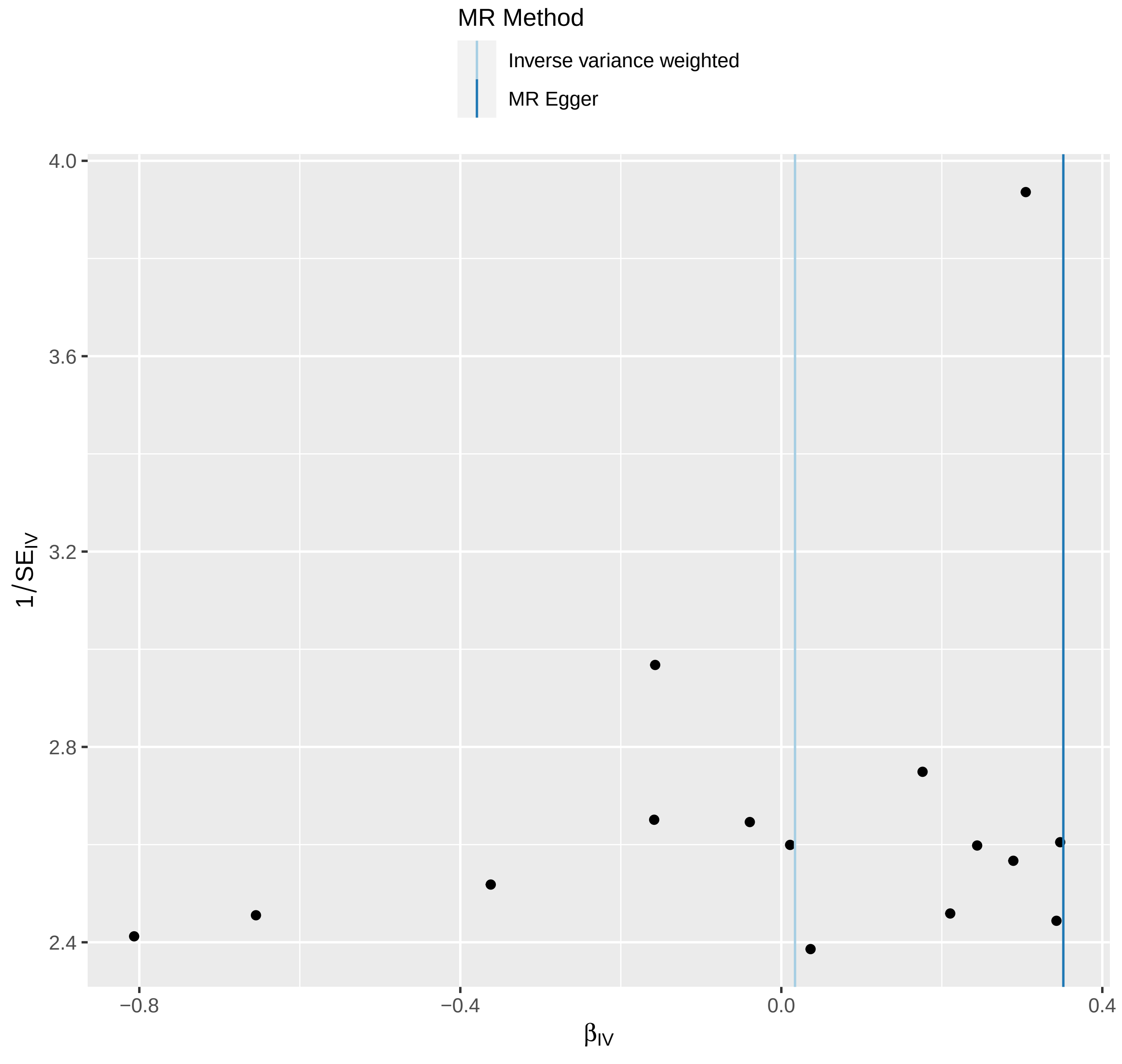

Batch 2−3449 : Gut microbiota abundance (genus Catenibacterium id.2153) on Leptin receptor
with a threshold of 1e−05

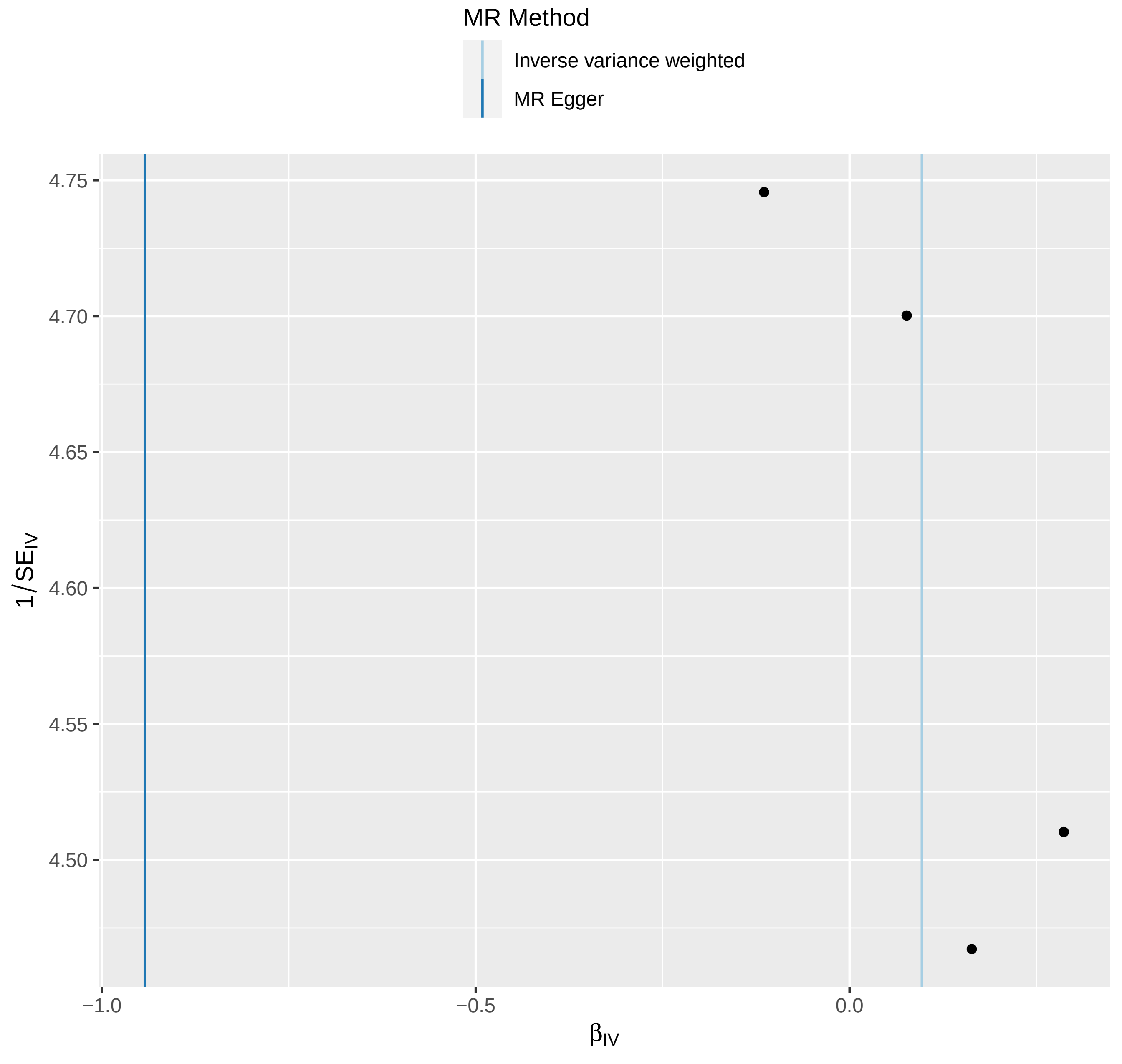

Batch 2−3450 : Gut microbiota abundance (genus Parasutterella id.2892) on Leptin receptor
with a threshold of 1e−05

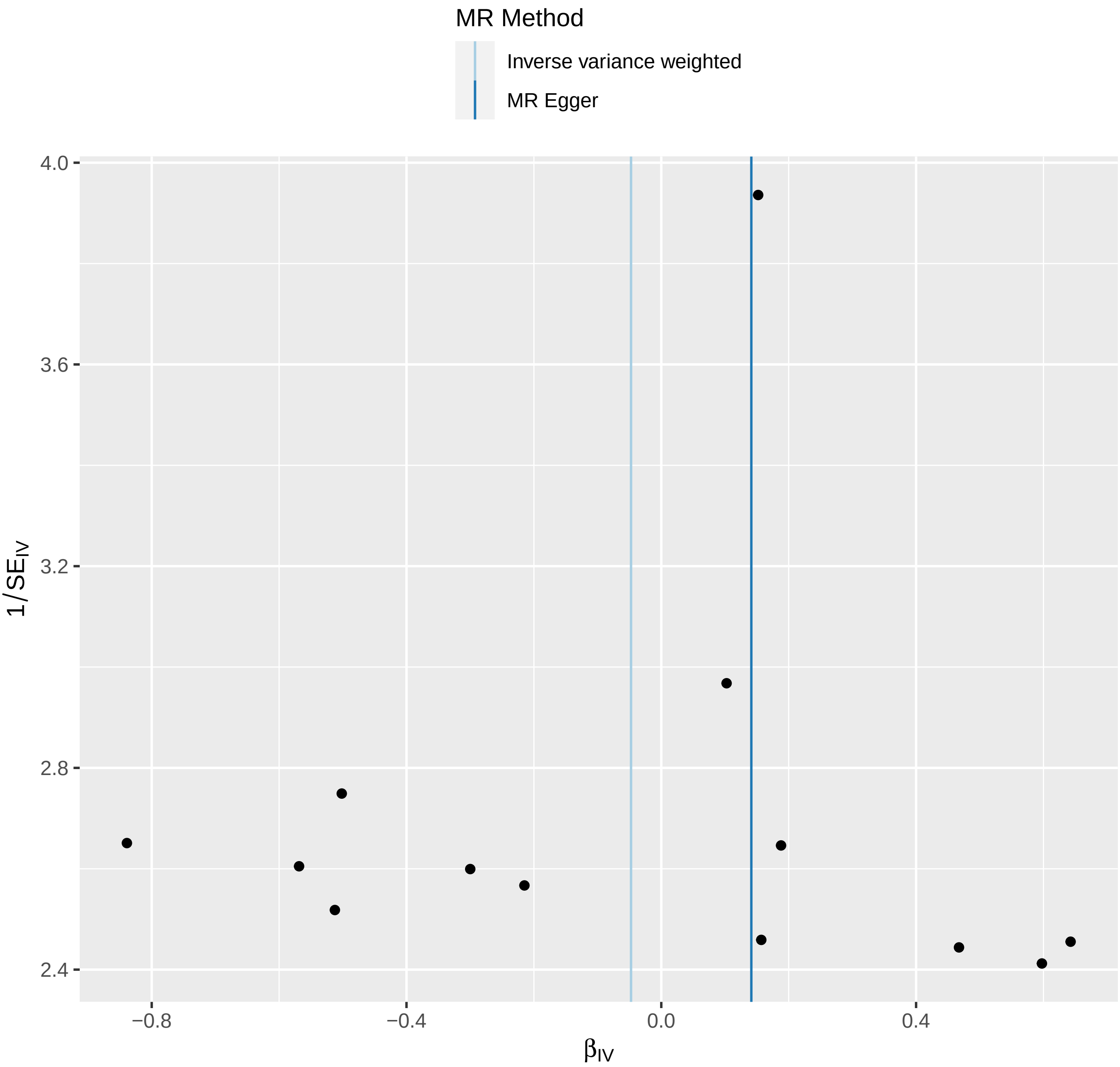

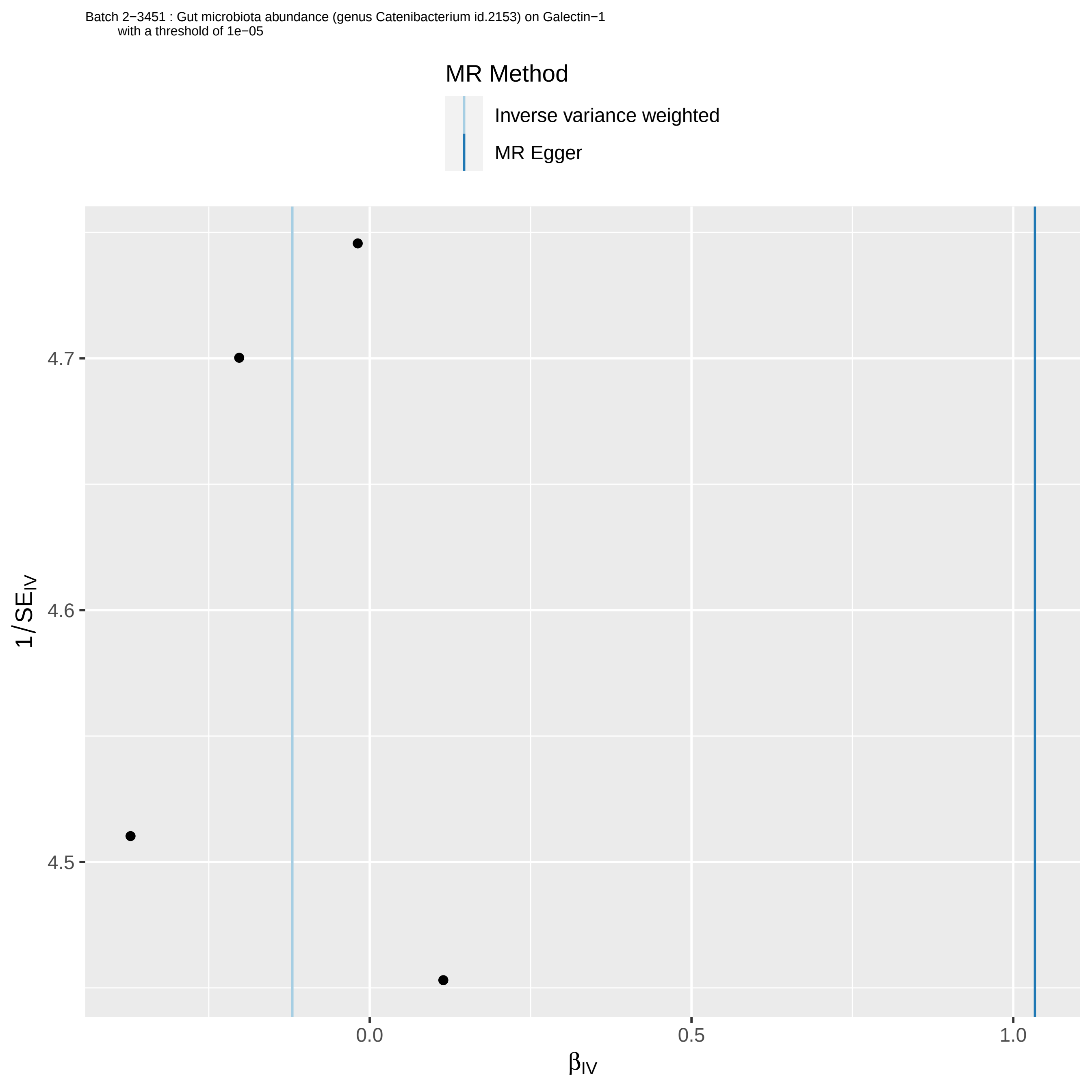

Batch 2−3451 : Gut microbiota abundance (genus Catenibacterium id.2153) on Galectin−1
with a threshold of 1e−05
MR Method
Inverse variance weighted
MR Egger
4.7
4.6
4.5
0.0
0.5
1.0
1/SE_IV
β_IV

Batch 2−3452 : Gut microbiota abundance (genus Parasutterella id.2892) on Galectin−1
with a threshold of 1e−05

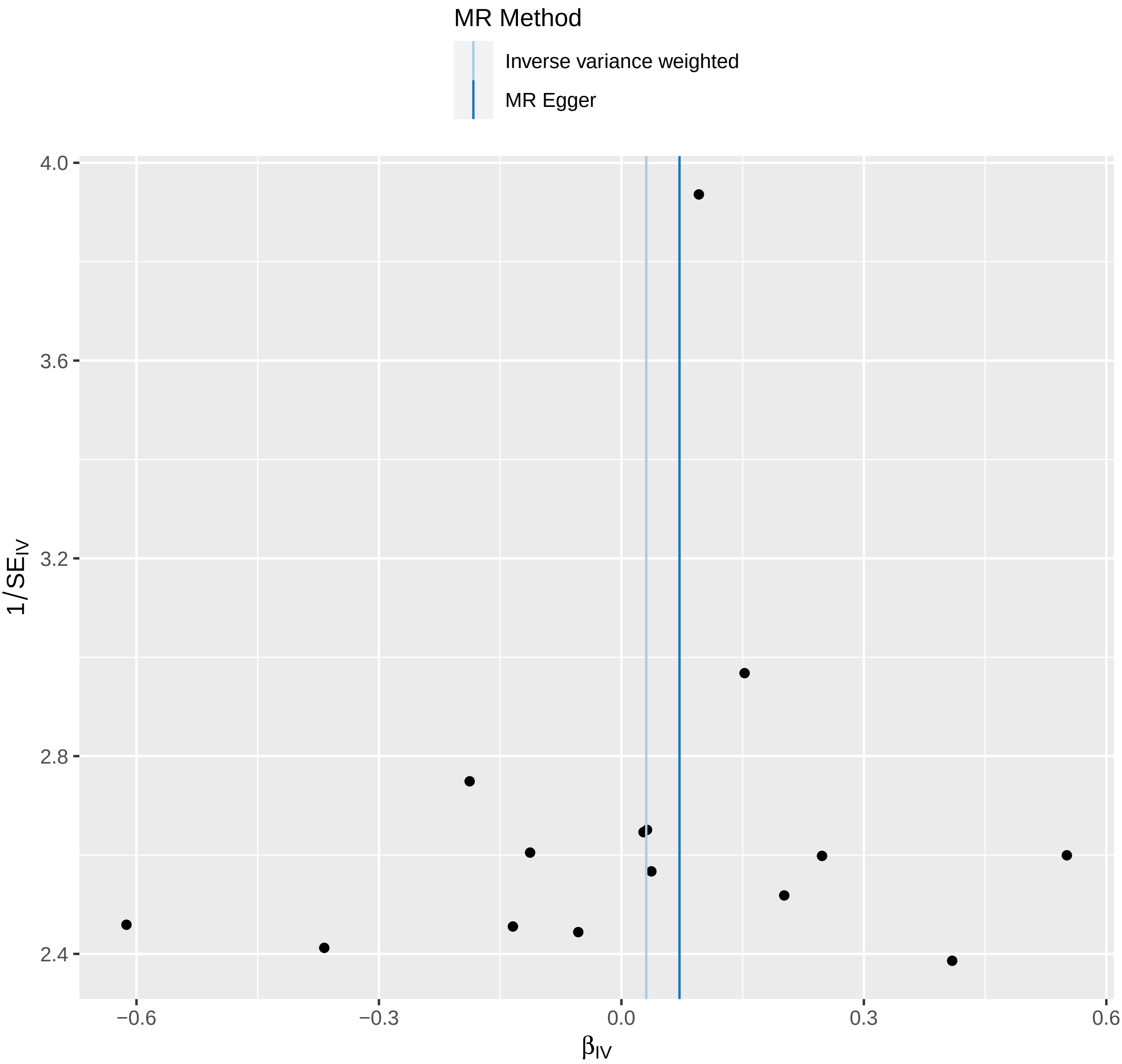

Batch 2−3453 : Gut microbiota abundance (genus Catenibacterium id.2153) on Galectin−2
with a threshold of 1e−05

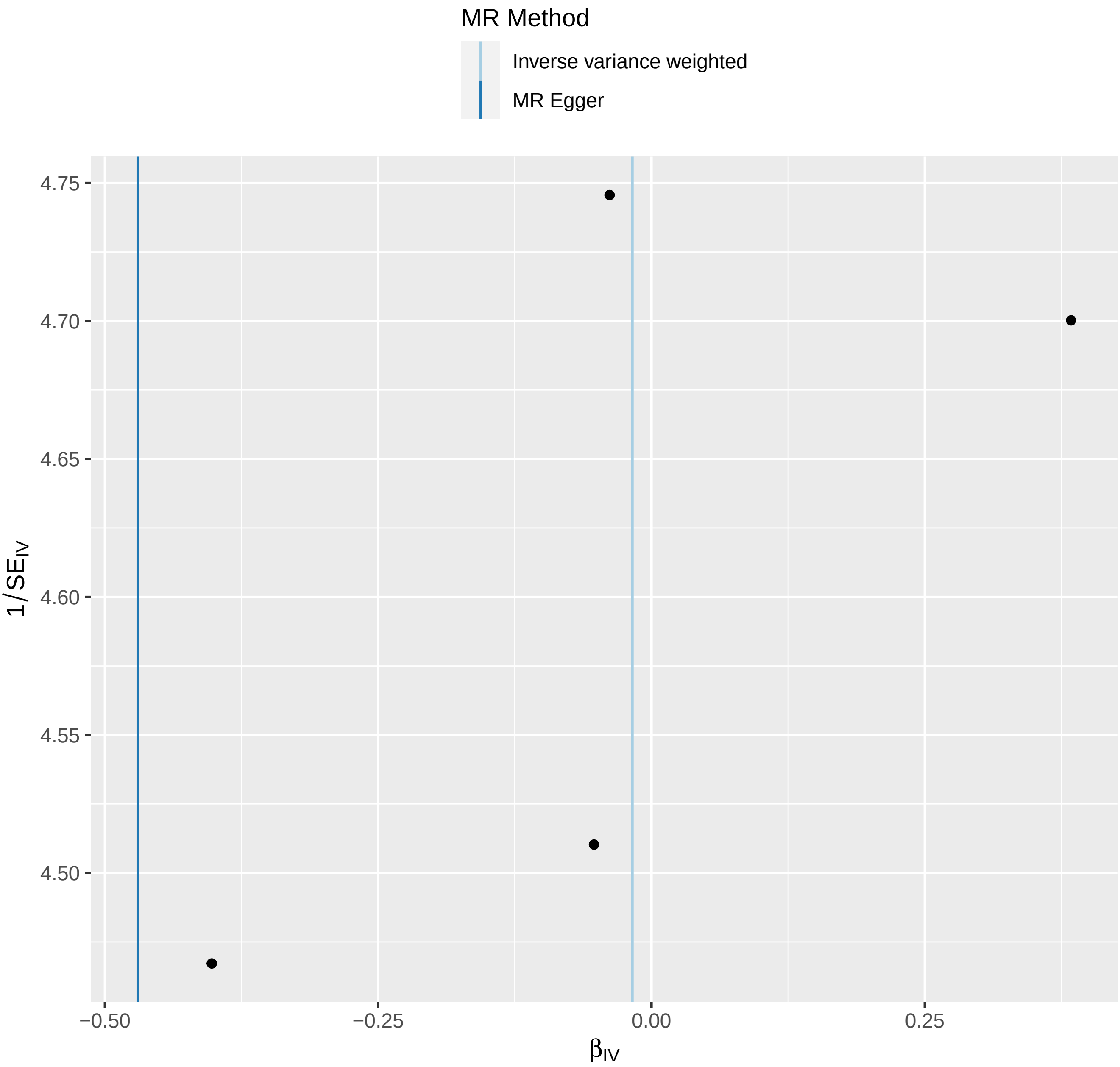

Batch 2−3454 : Gut microbiota abundance (genus Parasutterella id.2892) on Galectin−2
with a threshold of 1e−05

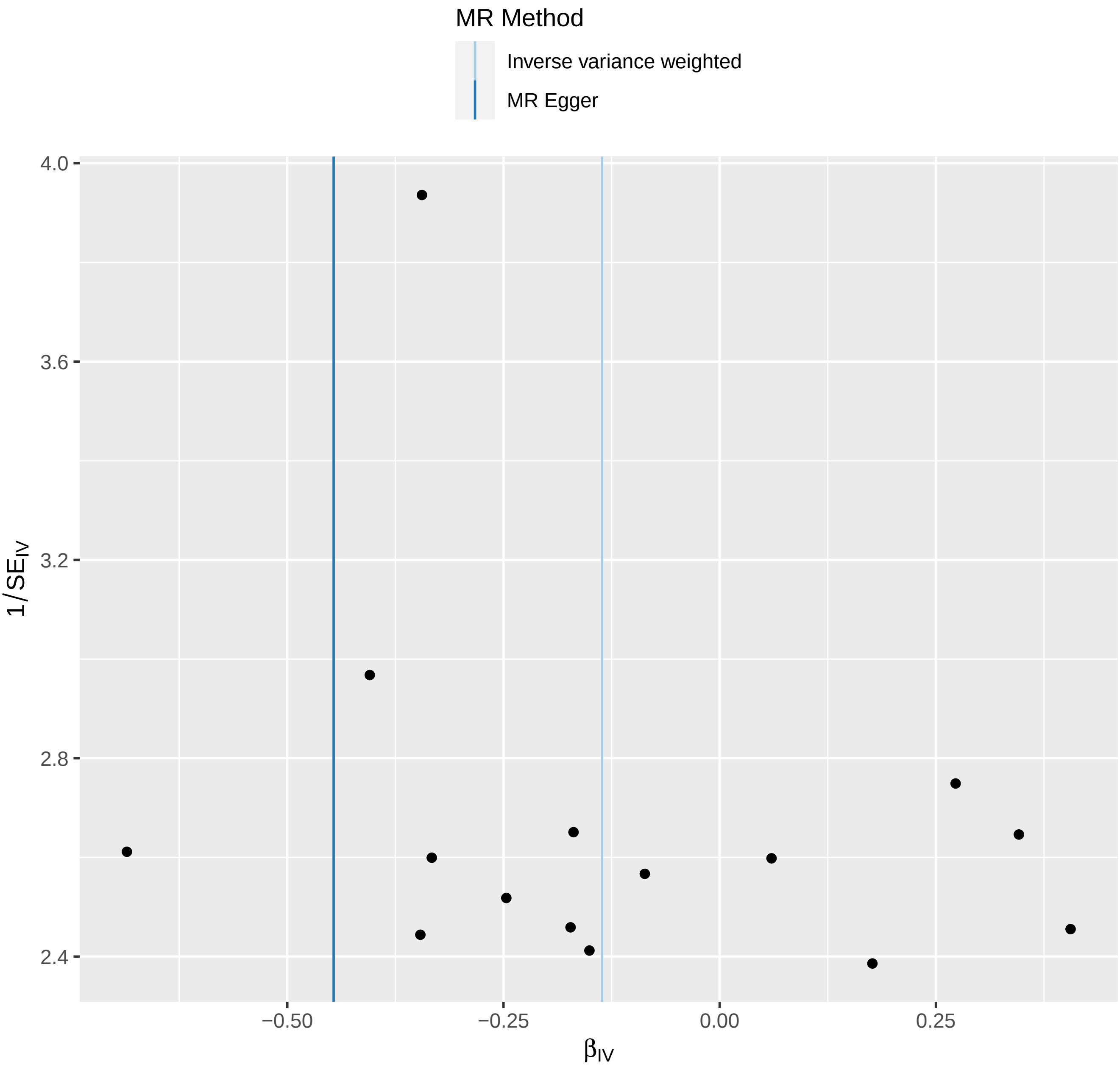

Batch 2−3455 : Gut microbiota abundance (genus Catenibacterium id.2153) on Galectin−4
with a threshold of 1e−05

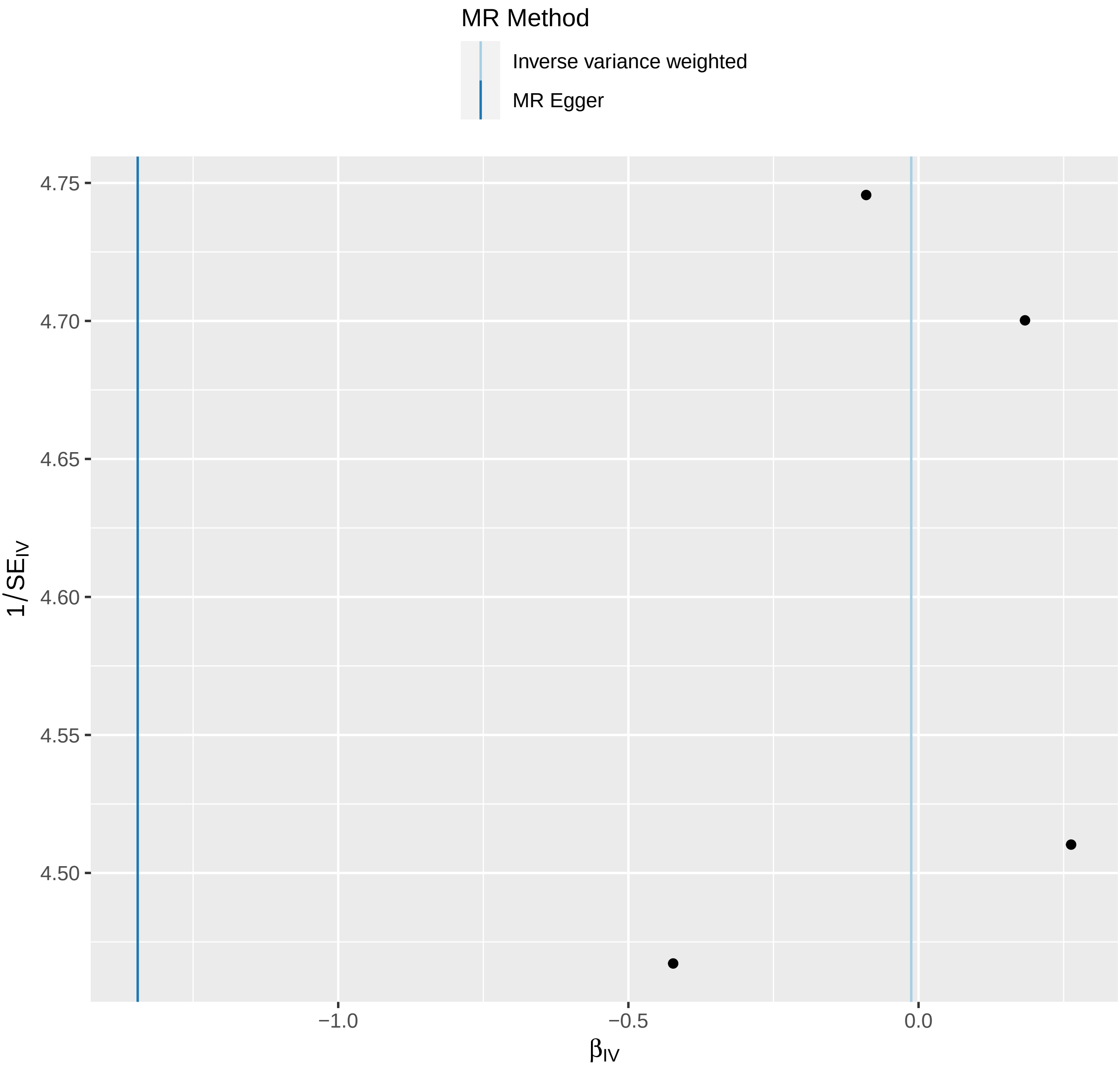

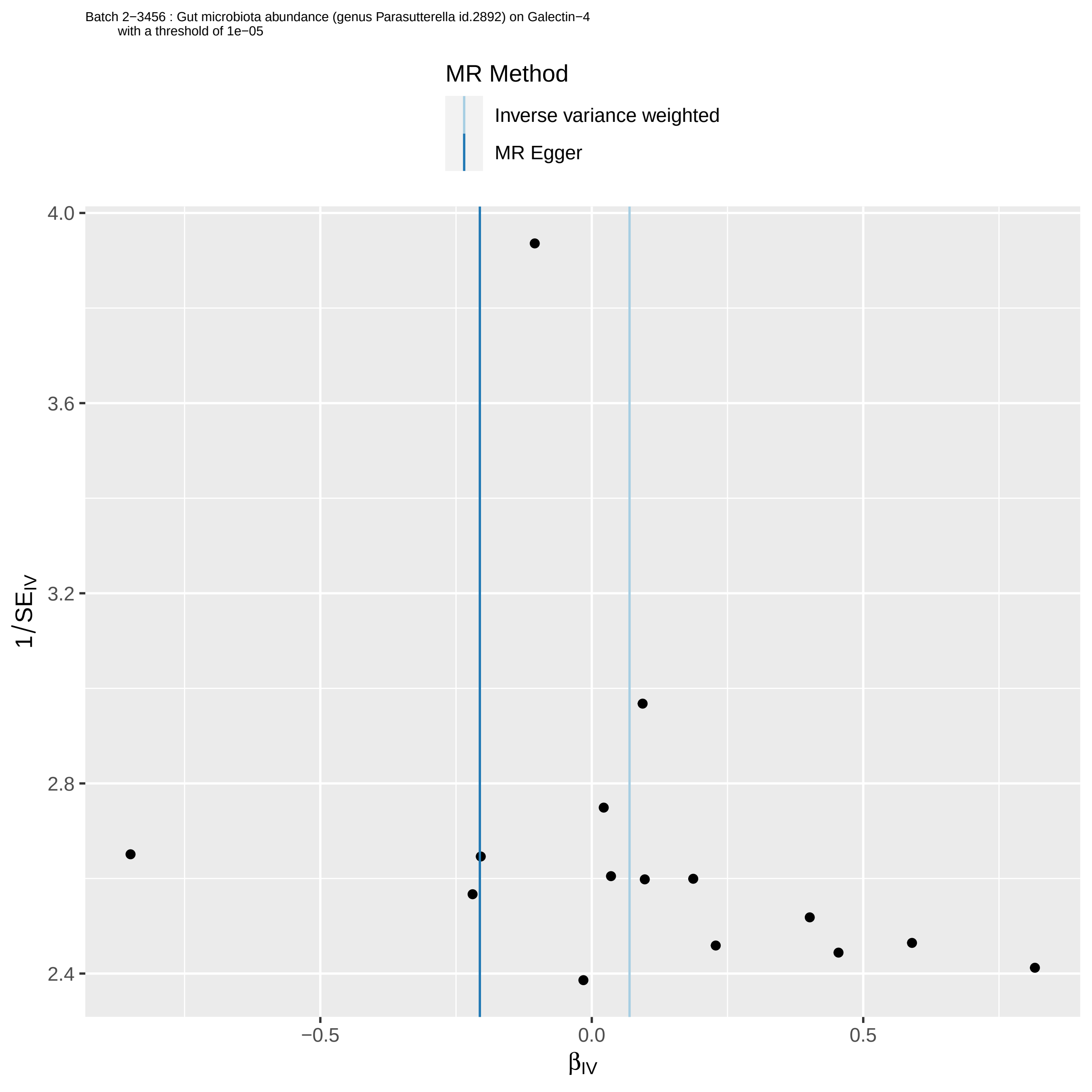

Batch 2−3456 : Gut microbiota abundance (genus Parasutterella id.2892) on Galectin−4
with a threshold of 1e−05
MR Method
Inverse variance weighted
MR Egger
4.0
3.6
3.2
2.8
2.4
1/SE_IV
−0.5
0.0
0.5
β_IV

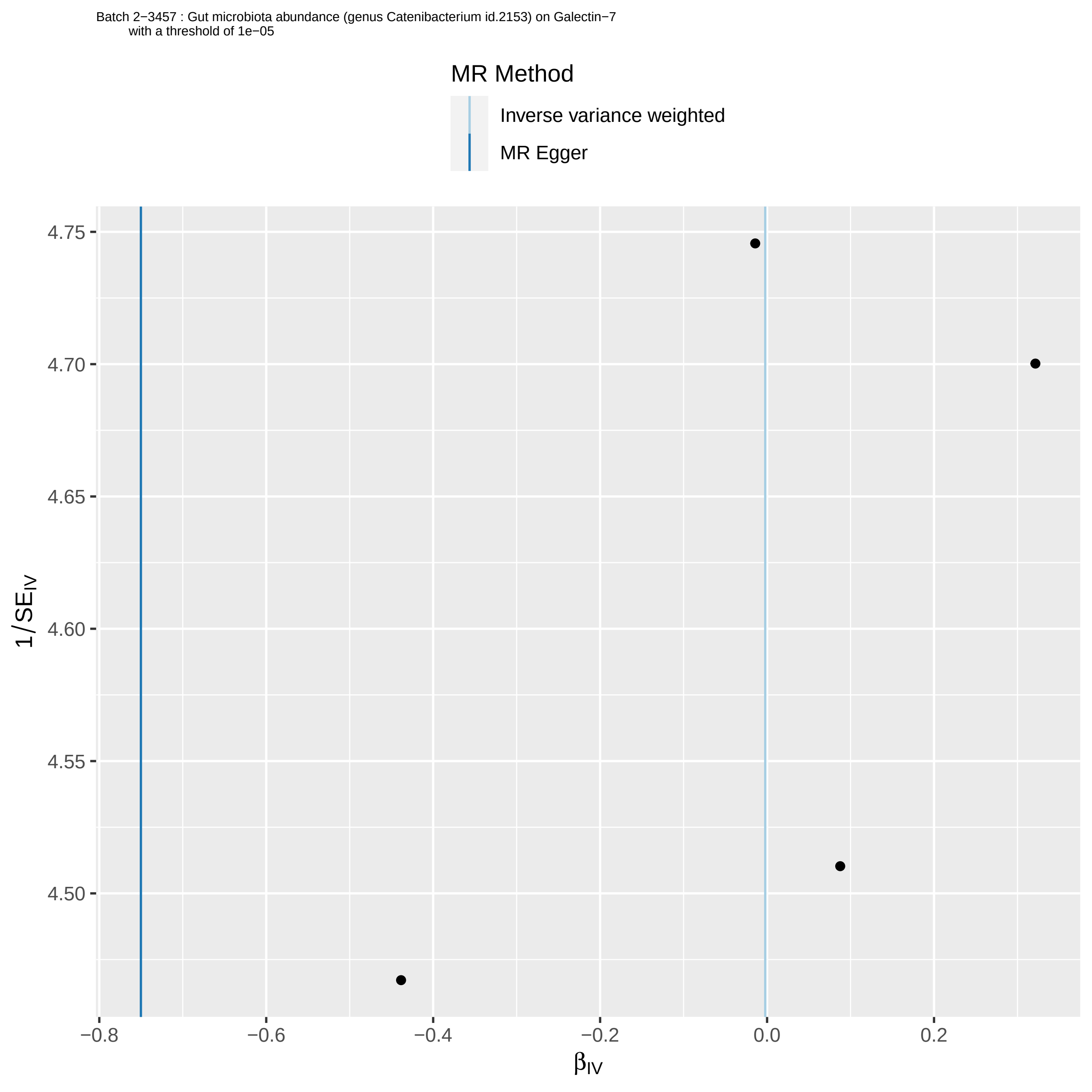

Batch 2−3457 : Gut microbiota abundance (genus Catenibacterium id.2153) on Galectin−7
with a threshold of 1e−05
MR Method
Inverse variance weighted
MR Egger
4.75
4.70
4.65
4.60
4.55
4.50
1/SE_IV
−0.8
−0.6
−0.4
−0.2
0.0
0.2
β_IV

Batch 2−3458 : Gut microbiota abundance (genus Parasutterella id.2892) on Galectin−7
with a threshold of 1e−05

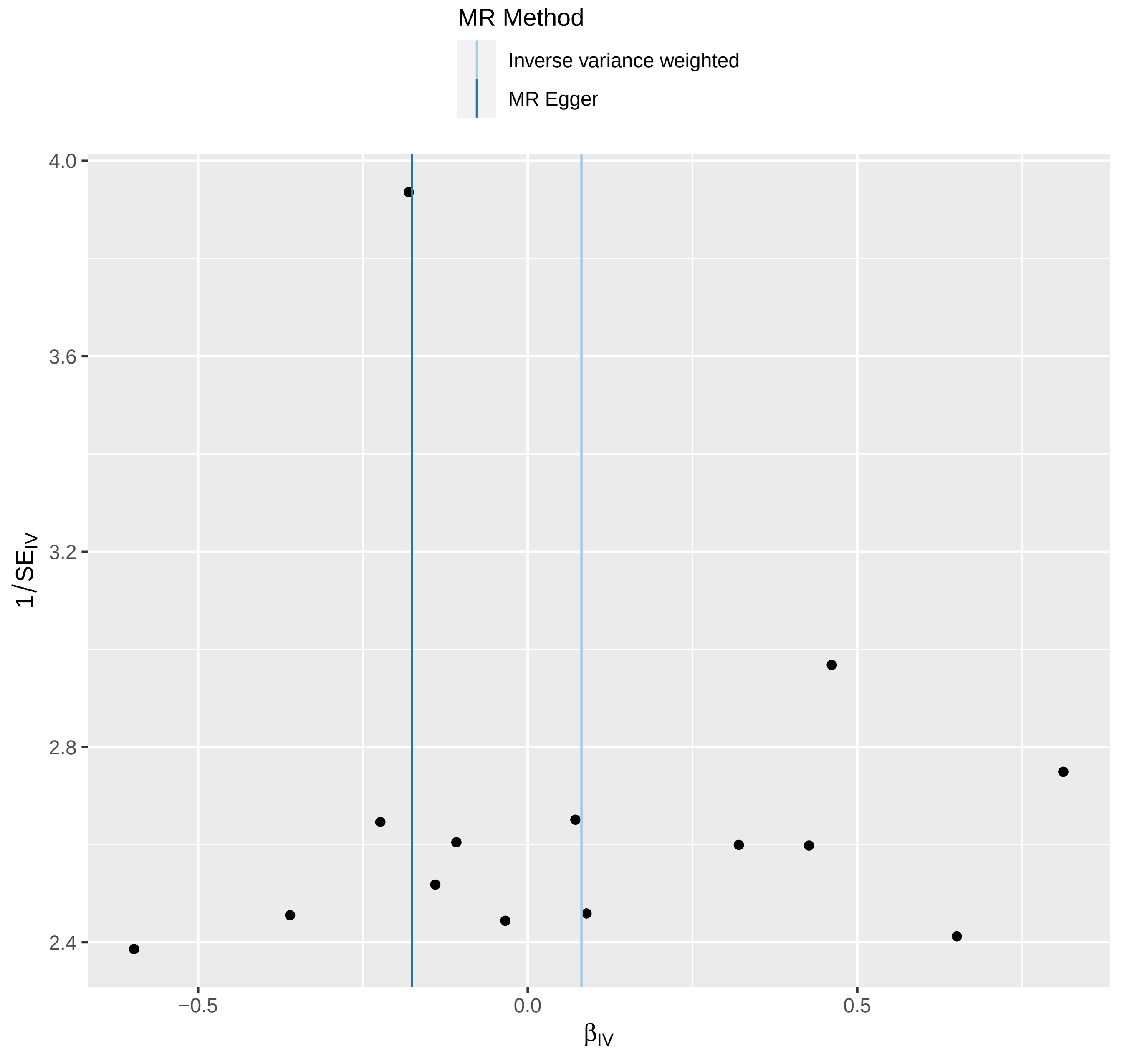

Batch 2−3459 : Gut microbiota abundance (genus Catenibacterium id.2153) on Galectin−7
with a threshold of 1e−05

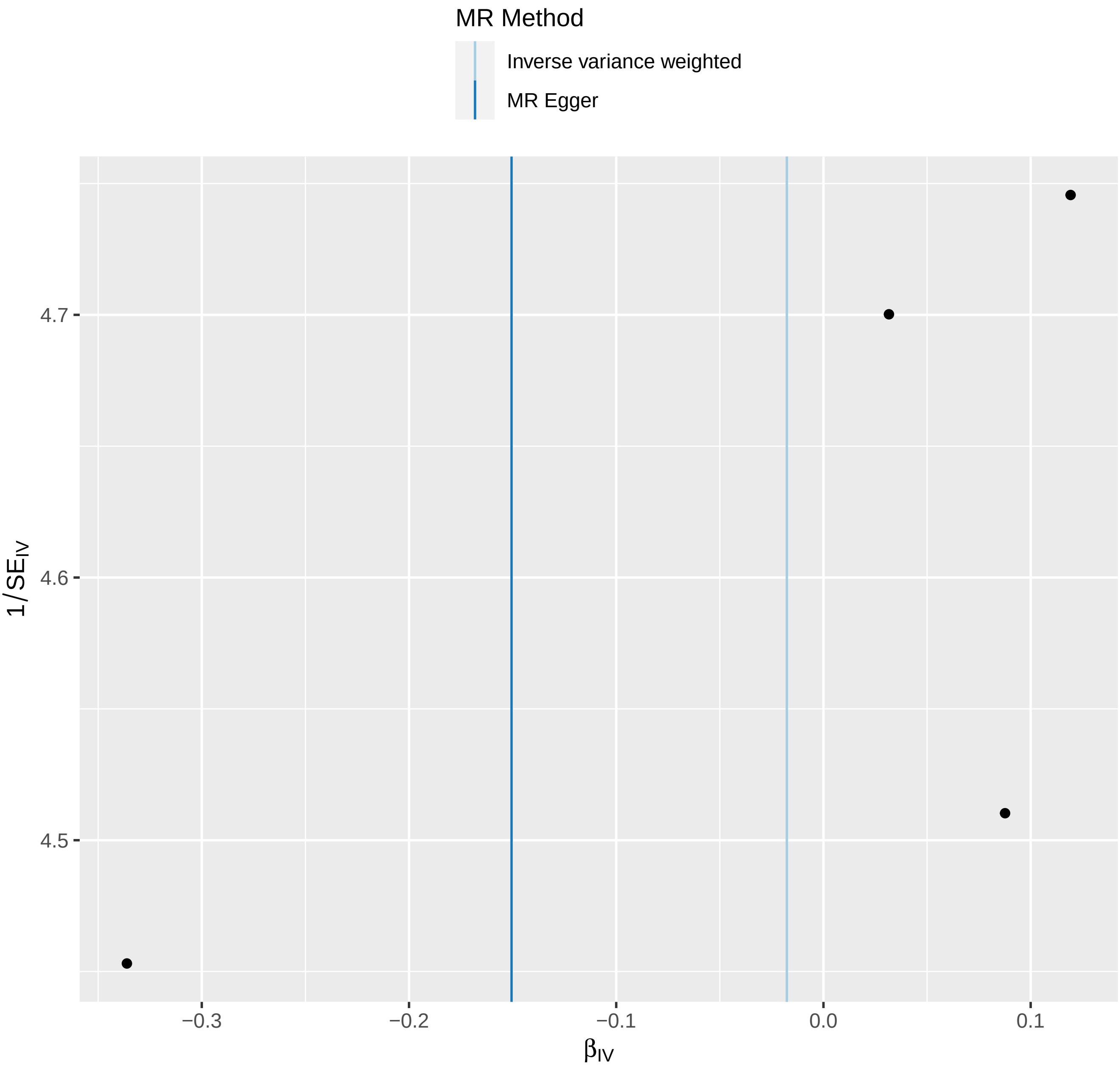

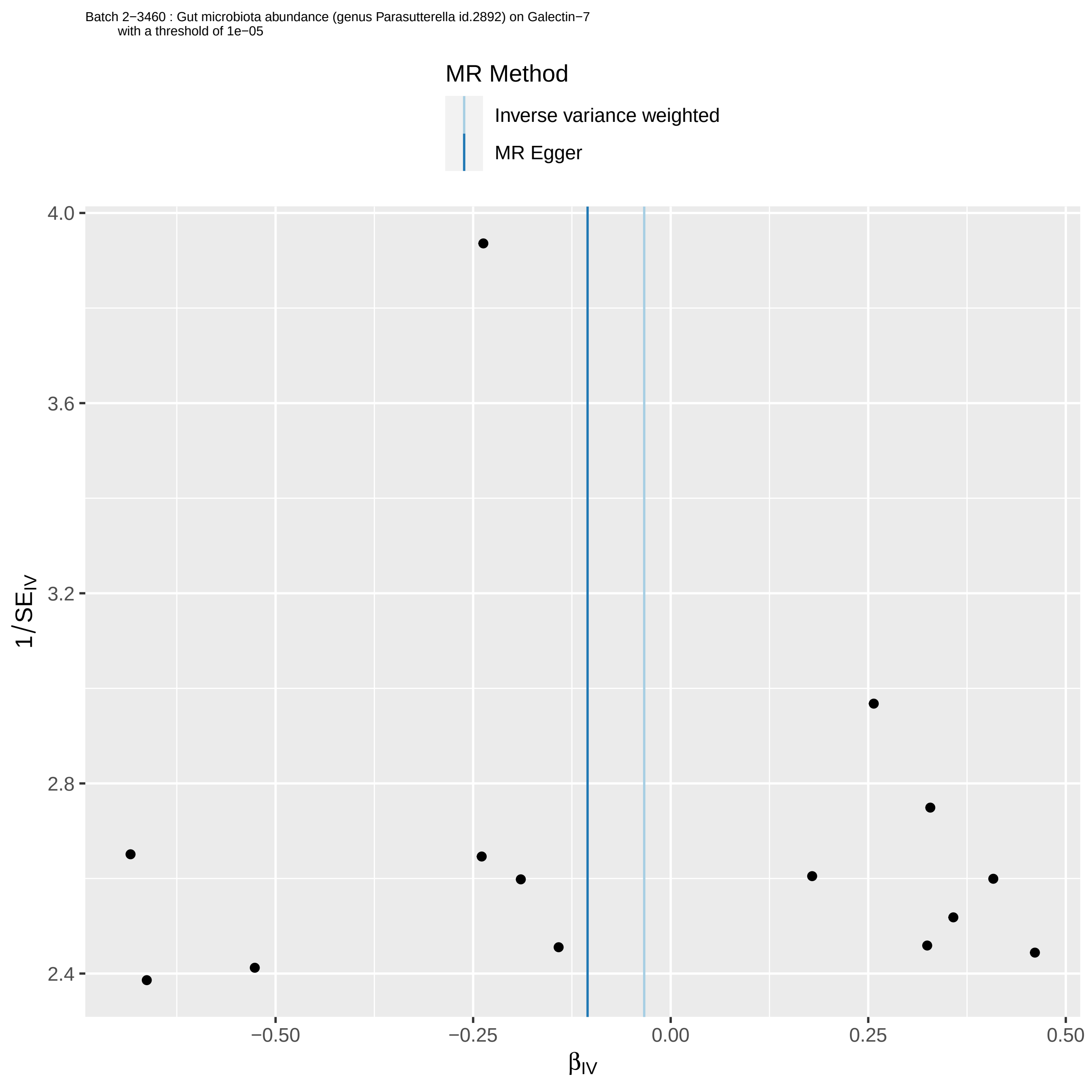

Batch 2−3460 : Gut microbiota abundance (genus Parasutterella id.2892) on Galectin−7
with a threshold of 1e−05
MR Method
Inverse variance weighted
MR Egger
4.0
3.6
3.2
2.8
2.4
1/SE_IV
−0.50
−0.25
0.00
0.25
0.50
β_IV

Batch 2−3461 : Gut microbiota abundance (genus Catenibacterium id.2153) on Galectin−8
with a threshold of 1e−05

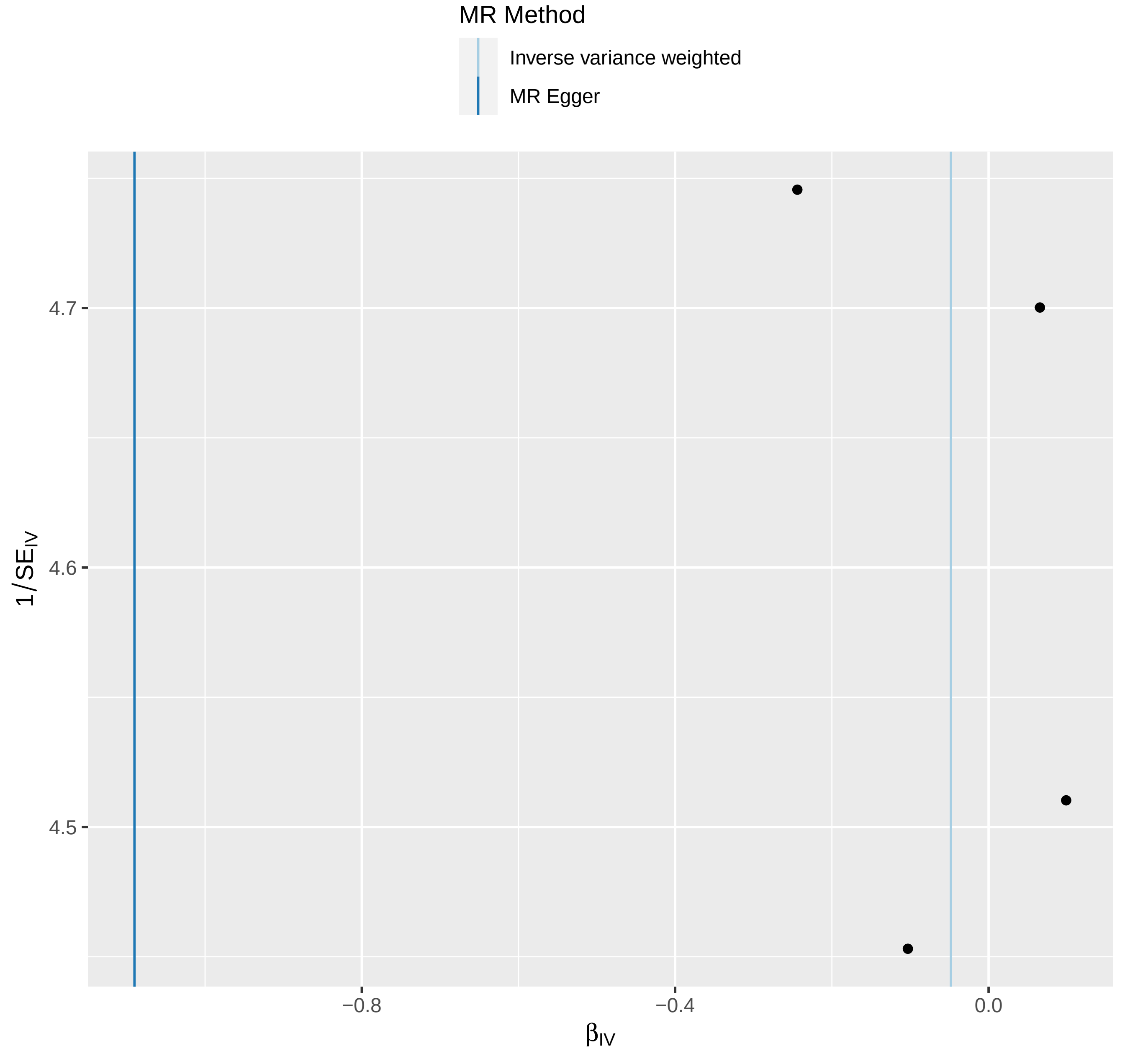

Batch 2−3462 : Gut microbiota abundance (genus Parasutterella id.2892) on Galectin−8
with a threshold of 1e−05

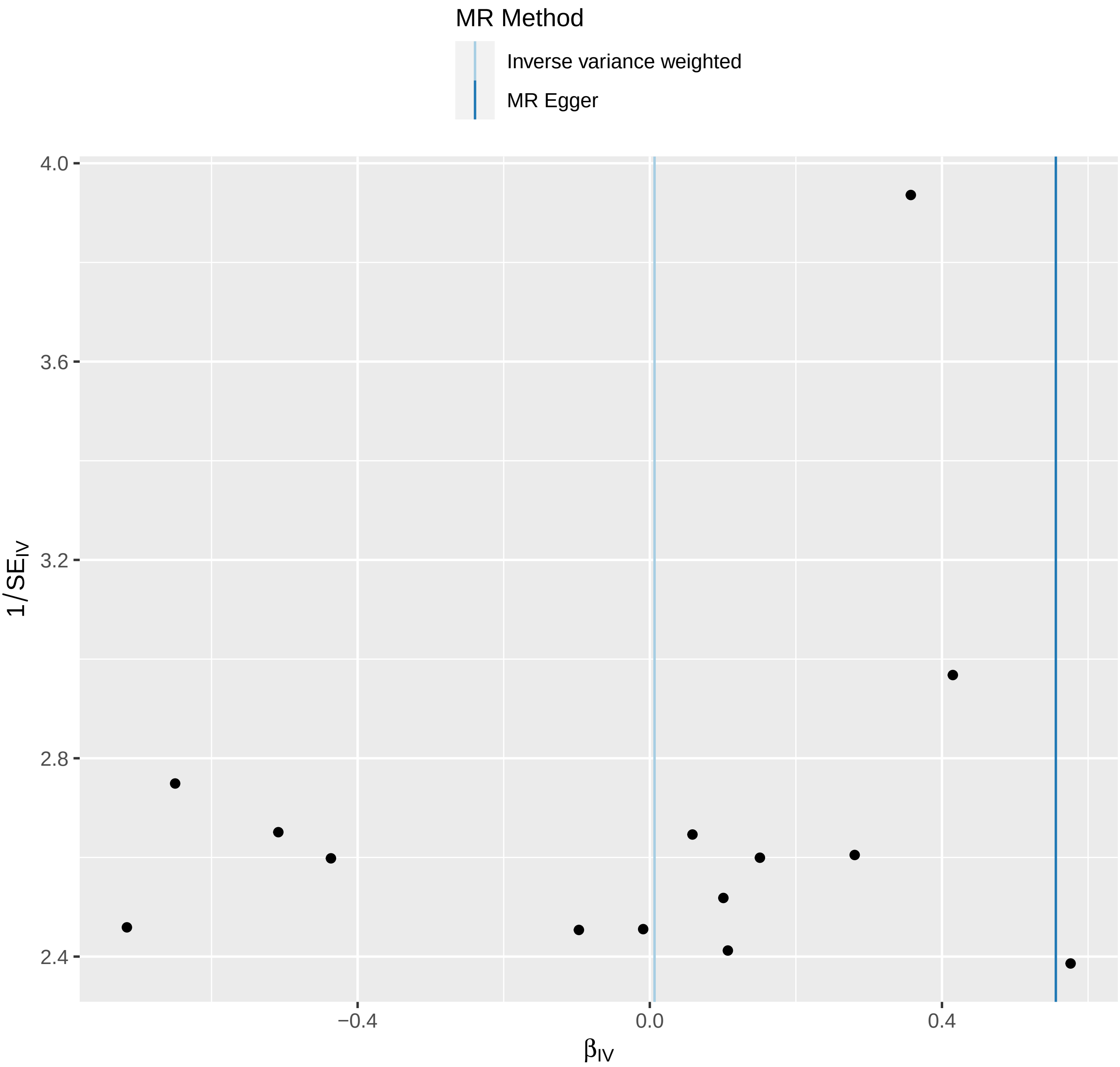

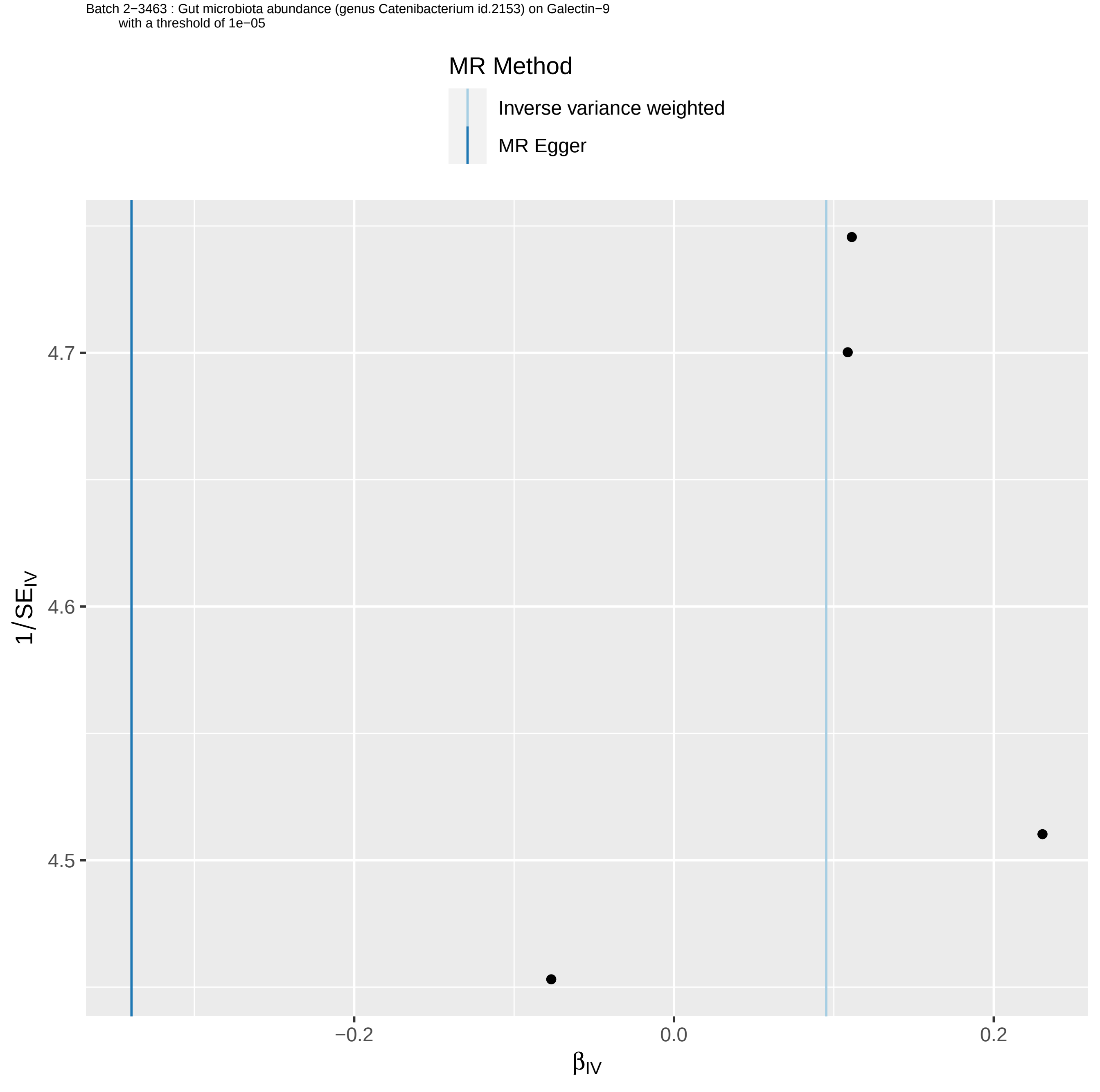

Batch 2−3463 : Gut microbiota abundance (genus Catenibacterium id.2153) on Galectin−9
with a threshold of 1e−05
MR Method
Inverse variance weighted
MR Egger
4.7
4.6
4.5
1/SE$_{IV}$
−0.2
0.0
0.2
β_{IV}

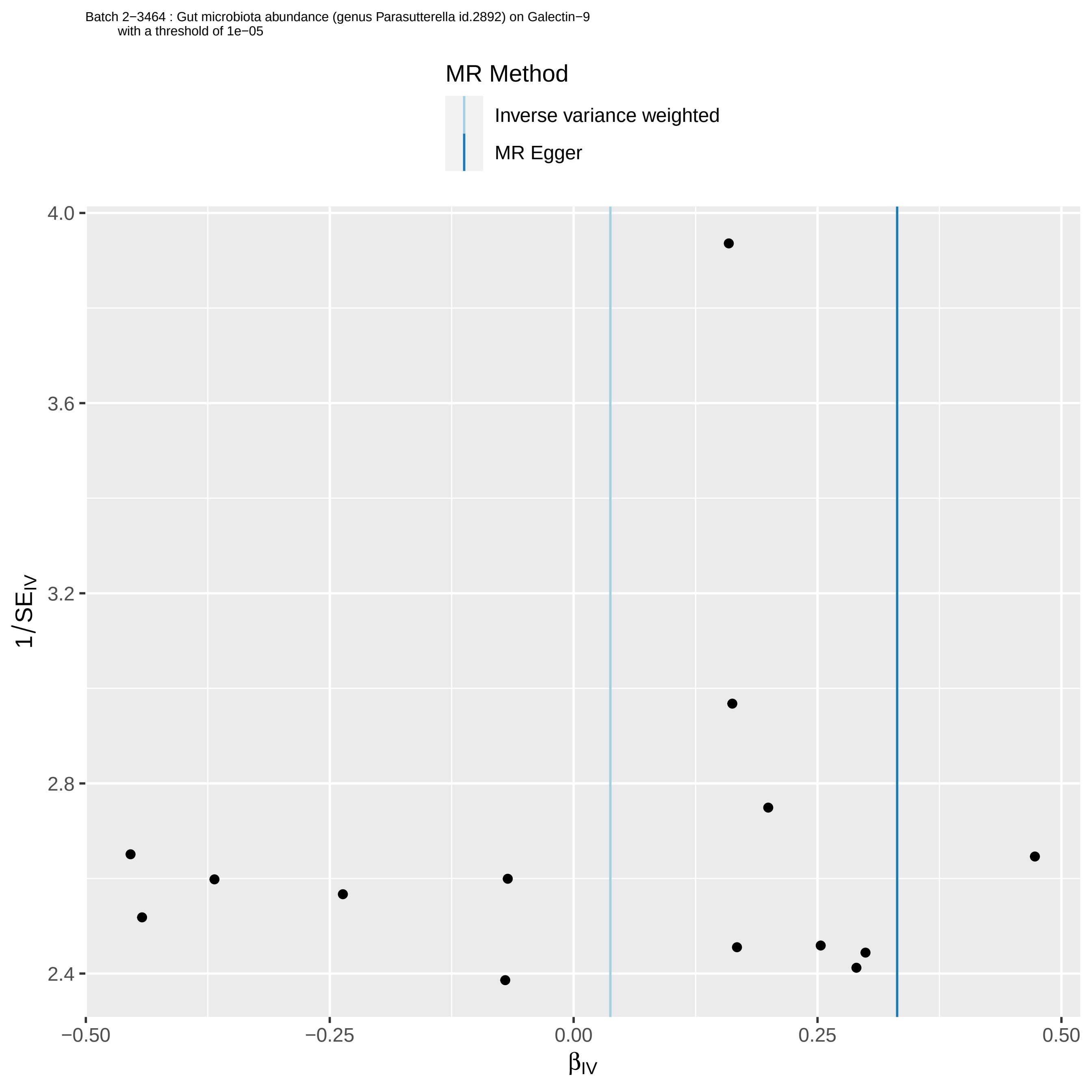

Batch 2−3464 : Gut microbiota abundance (genus Parasutterella id.2892) on Galectin−9
with a threshold of 1e−05
MR Method
Inverse variance weighted
MR Egger
4.0
3.6
3.2
2.8
2.4
1/SE_IV
−0.50
−0.25
0.00
0.25
0.50
β_IV

Batch 2−3465 : Gut microbiota abundance (genus Catenibacterium id.2153) on Leucine−rich repeat LGI family member 3 with a threshold of 1e−05

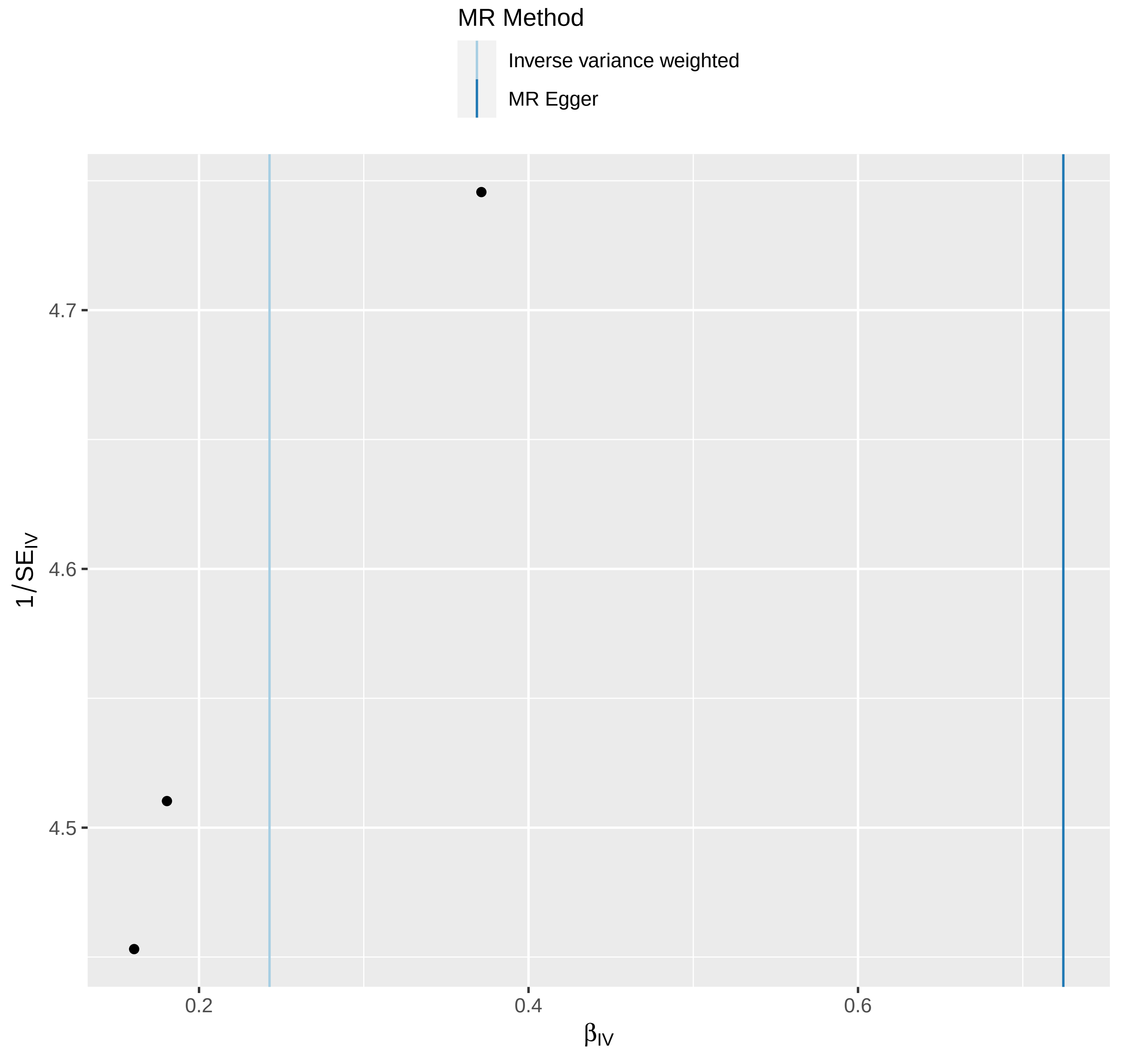

Batch 2−3466 : Gut microbiota abundance (genus Parasutterella id.2892) on Leucine−rich repeat LGI family member 3
with a threshold of 1e−05

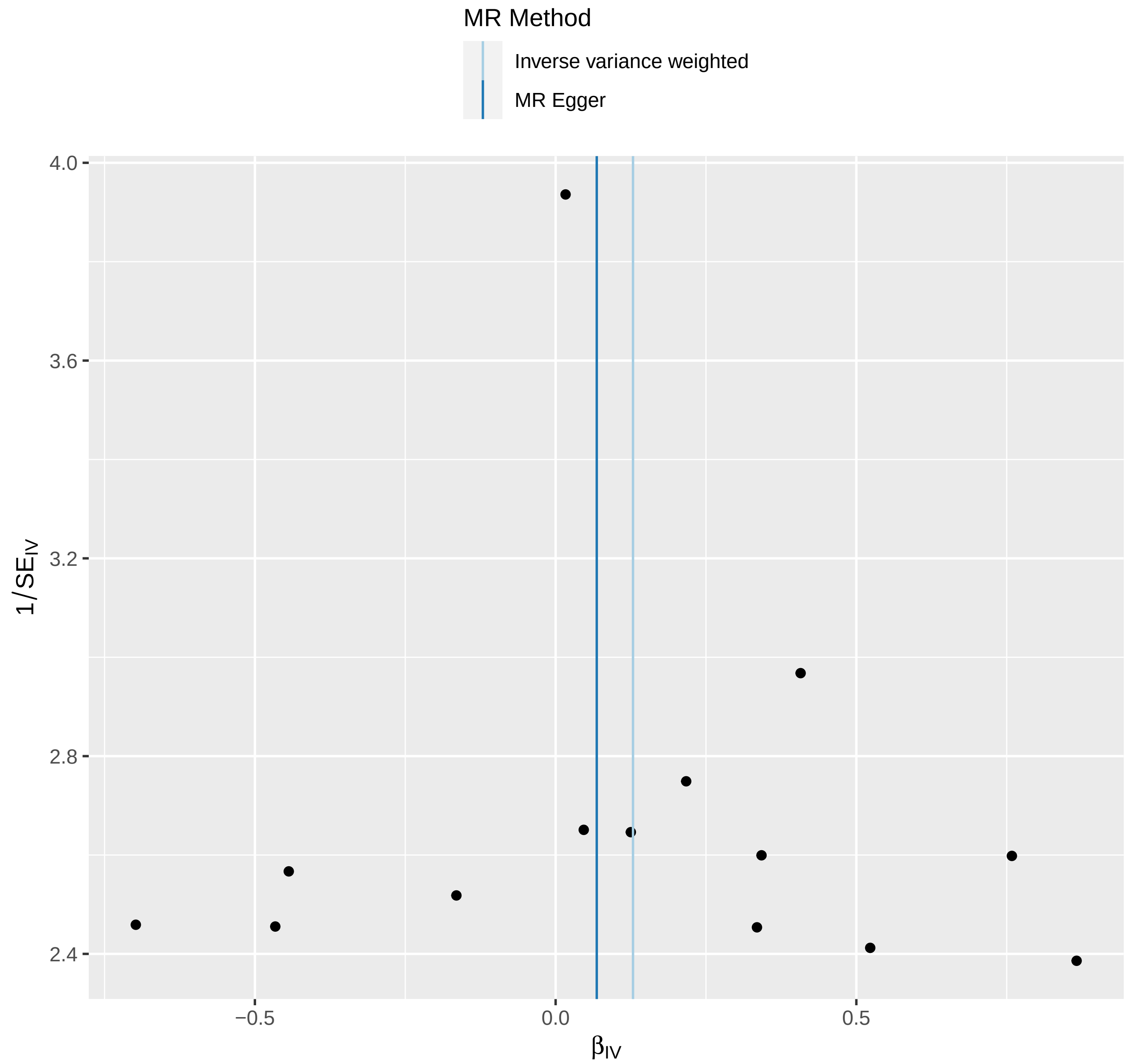

Batch 2−3467 : Gut microbiota abundance (genus Catenibacterium id.2153) on Legumain
with a threshold of 1e−05

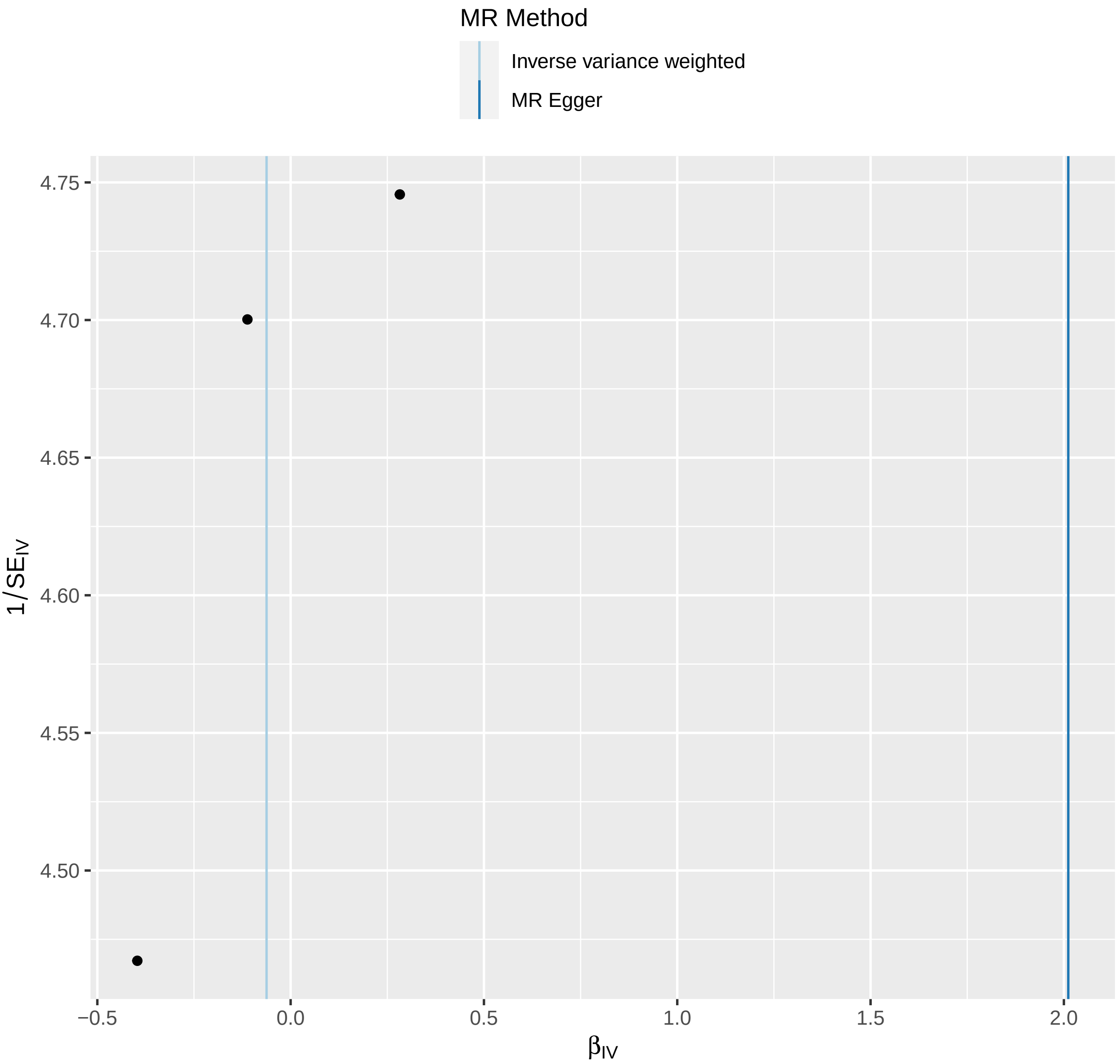

Batch 2−3468 : Gut microbiota abundance (genus Parasutterella id.2892) on Legumain
with a threshold of 1e−05

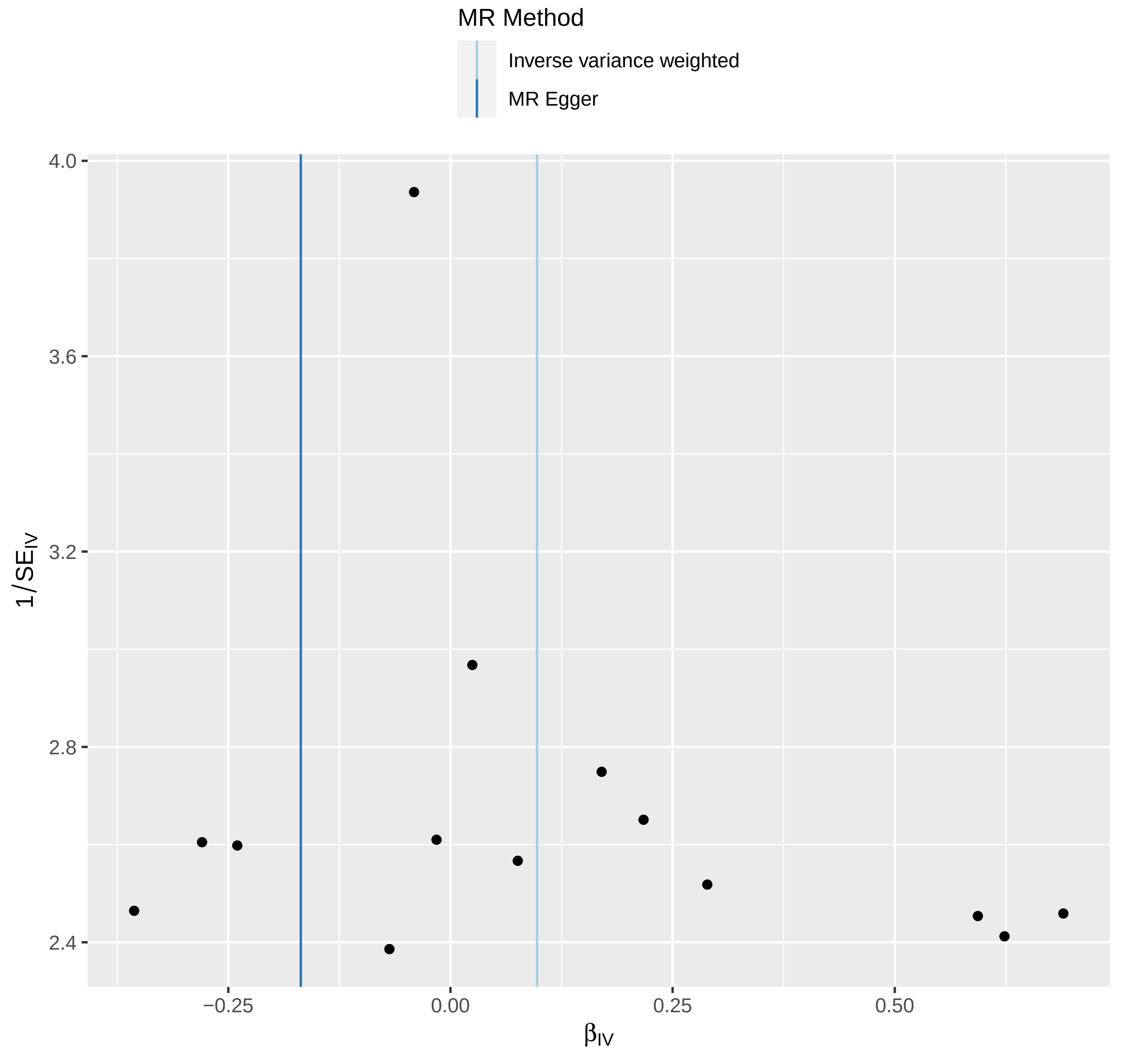

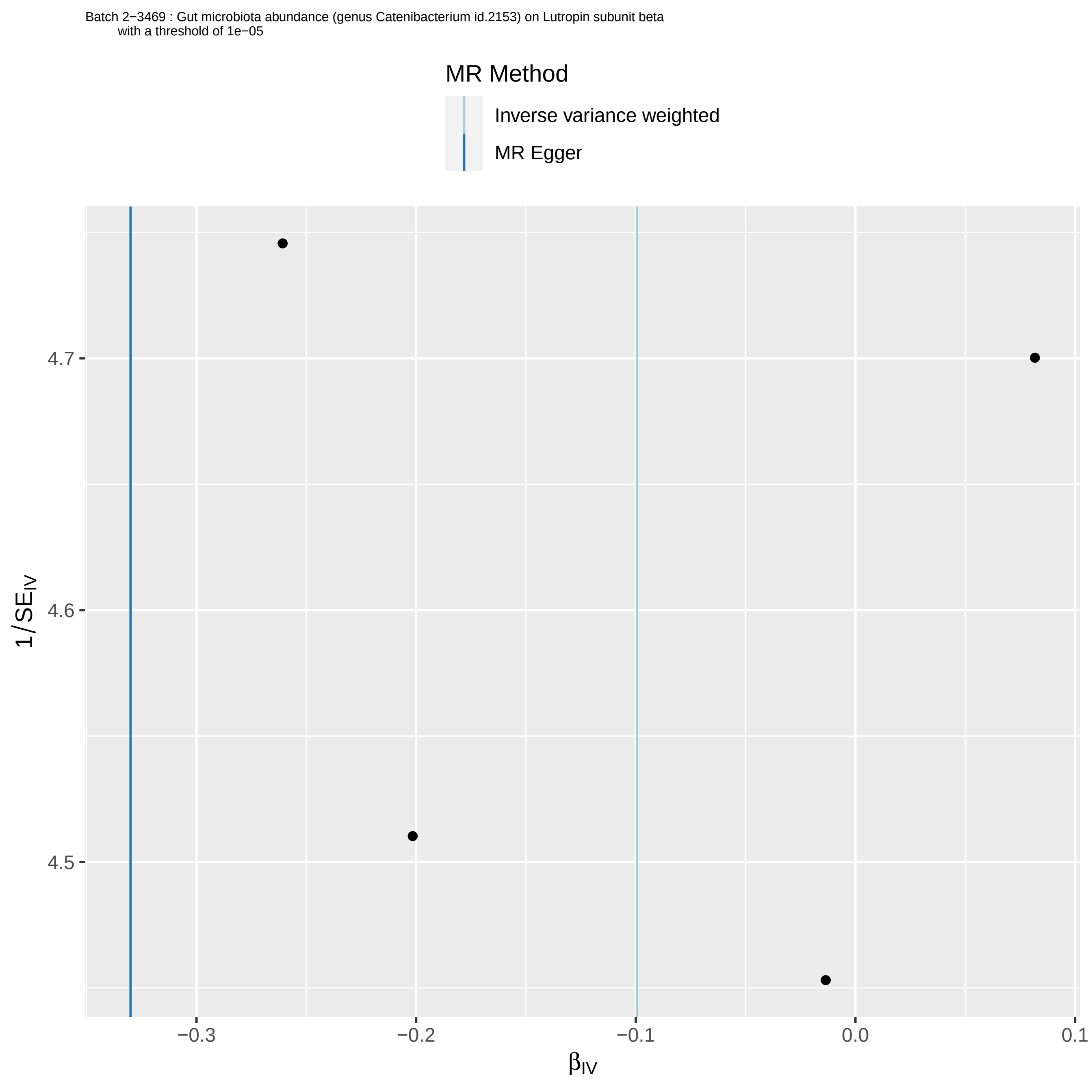

Batch 2−3469 : Gut microbiota abundance (genus Catenibacterium id.2153) on Lutropin subunit beta
with a threshold of 1e−05
MR Method
Inverse variance weighted
MR Egger
4.7
4.6
4.5
1/SE_IV
−0.3
−0.2
−0.1
0.0
0.1
β_IV

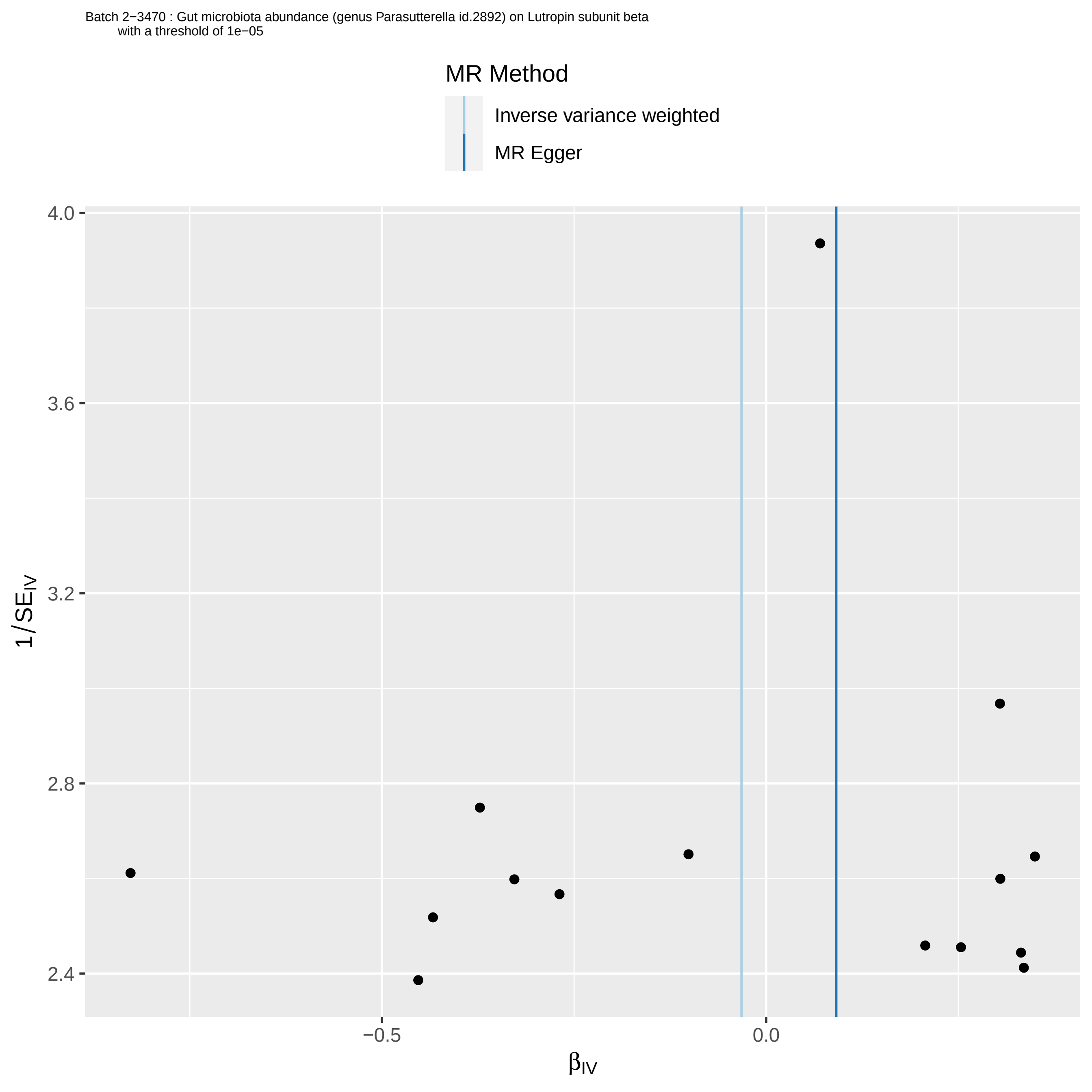
Batch 2−3470 : Gut microbiota abundance (genus Parasutterella id.2892) on Lutropin subunit beta
with a threshold of 1e−05
MR Method
Inverse variance weighted
MR Egger
4.0
3.6
3.2
2.8
2.4
$1/SE_{IV}$
−0.5
0.0
β_{IV}

Batch 2−3471 : Gut microbiota abundance (genus Catenibacterium id.2153) on Leukemia inhibitory factor
with a threshold of 1e−05

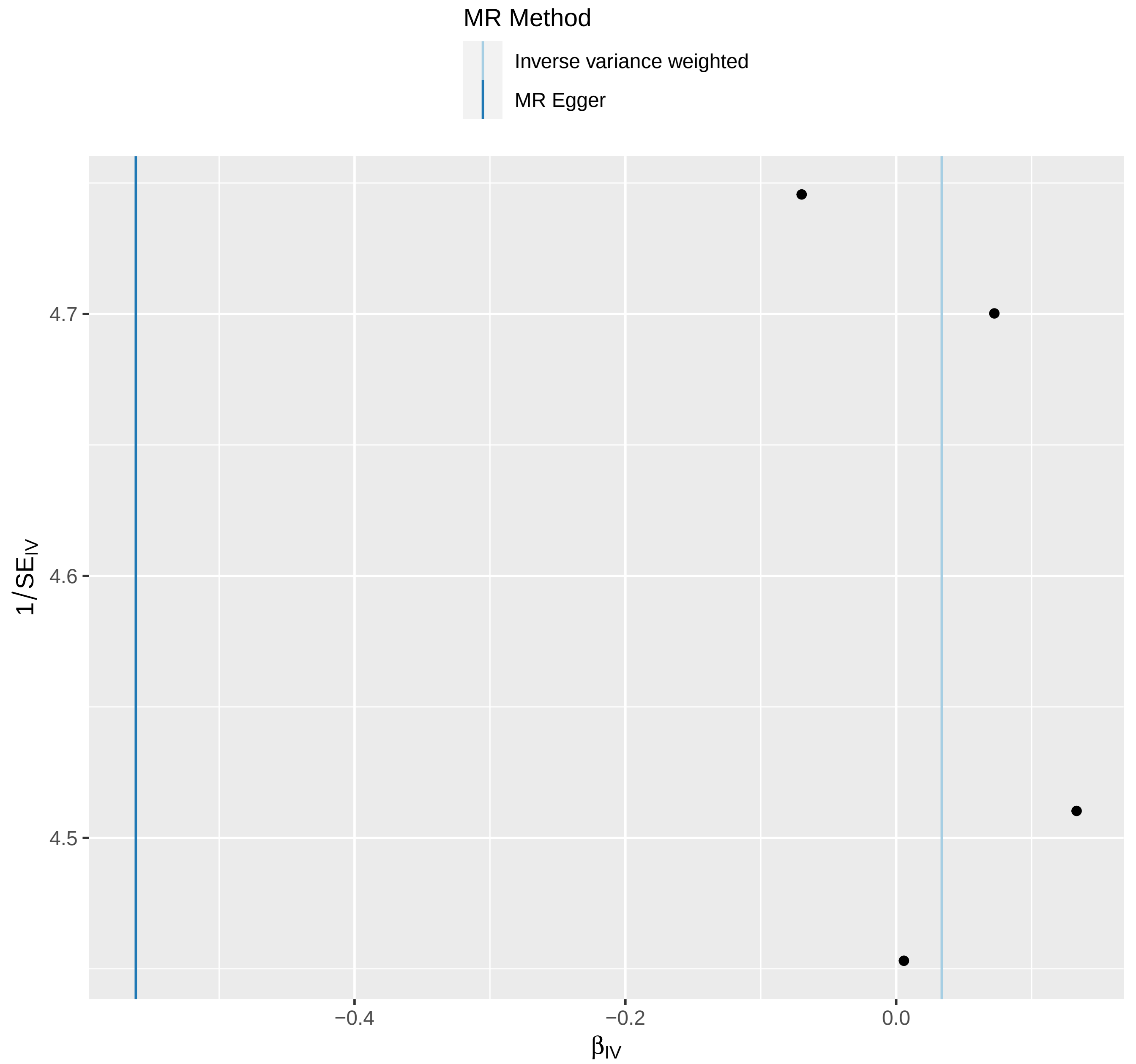

Batch 2−3472 : Gut microbiota abundance (genus Parasutterella id.2892) on Leukemia inhibitory factor
with a threshold of 1e−05

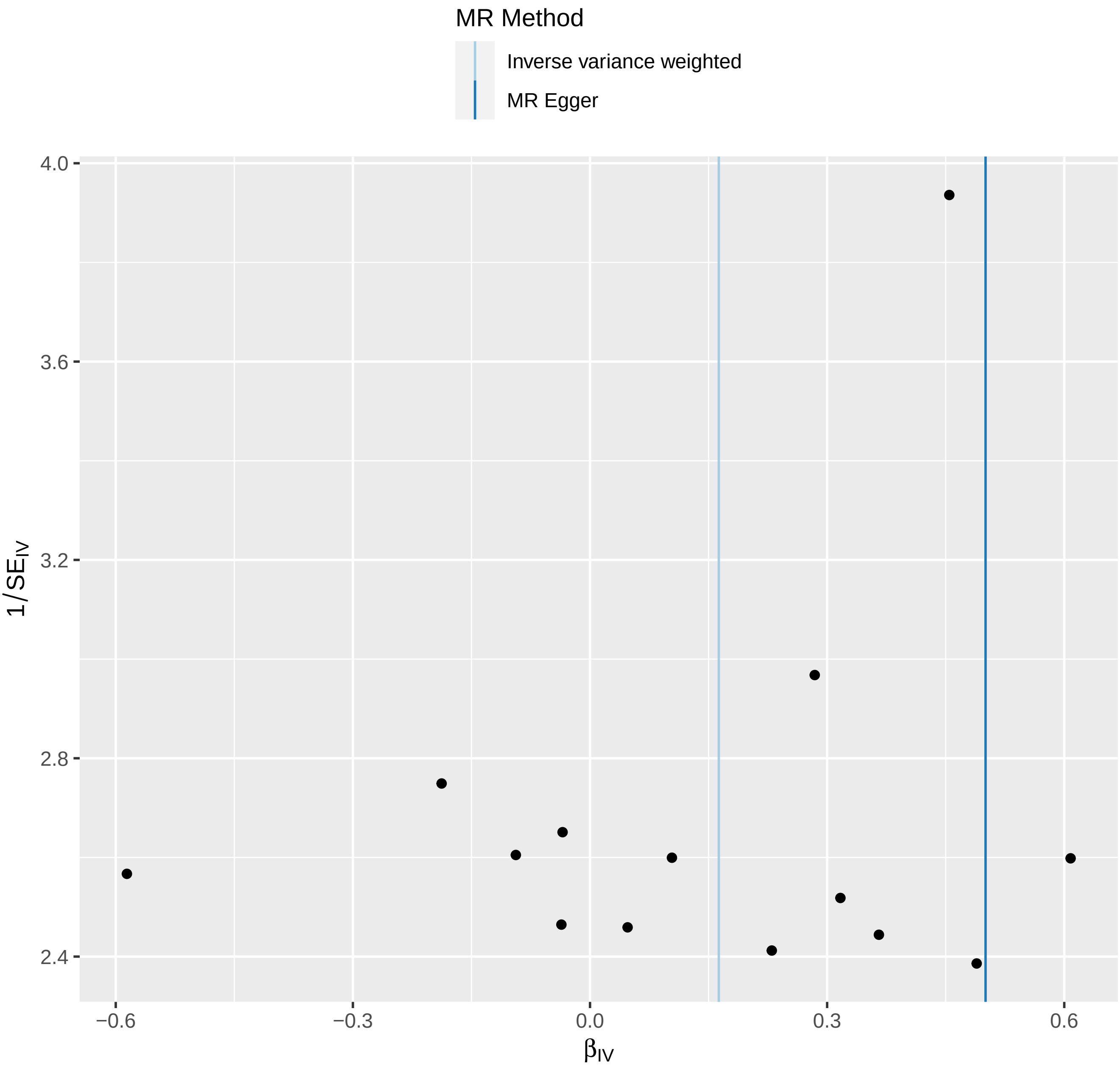

Batch 2−3473 : Gut microbiota abundance (genus Catenibacterium id.2153) on Leukemia inhibitory factor receptor
with a threshold of 1e−05

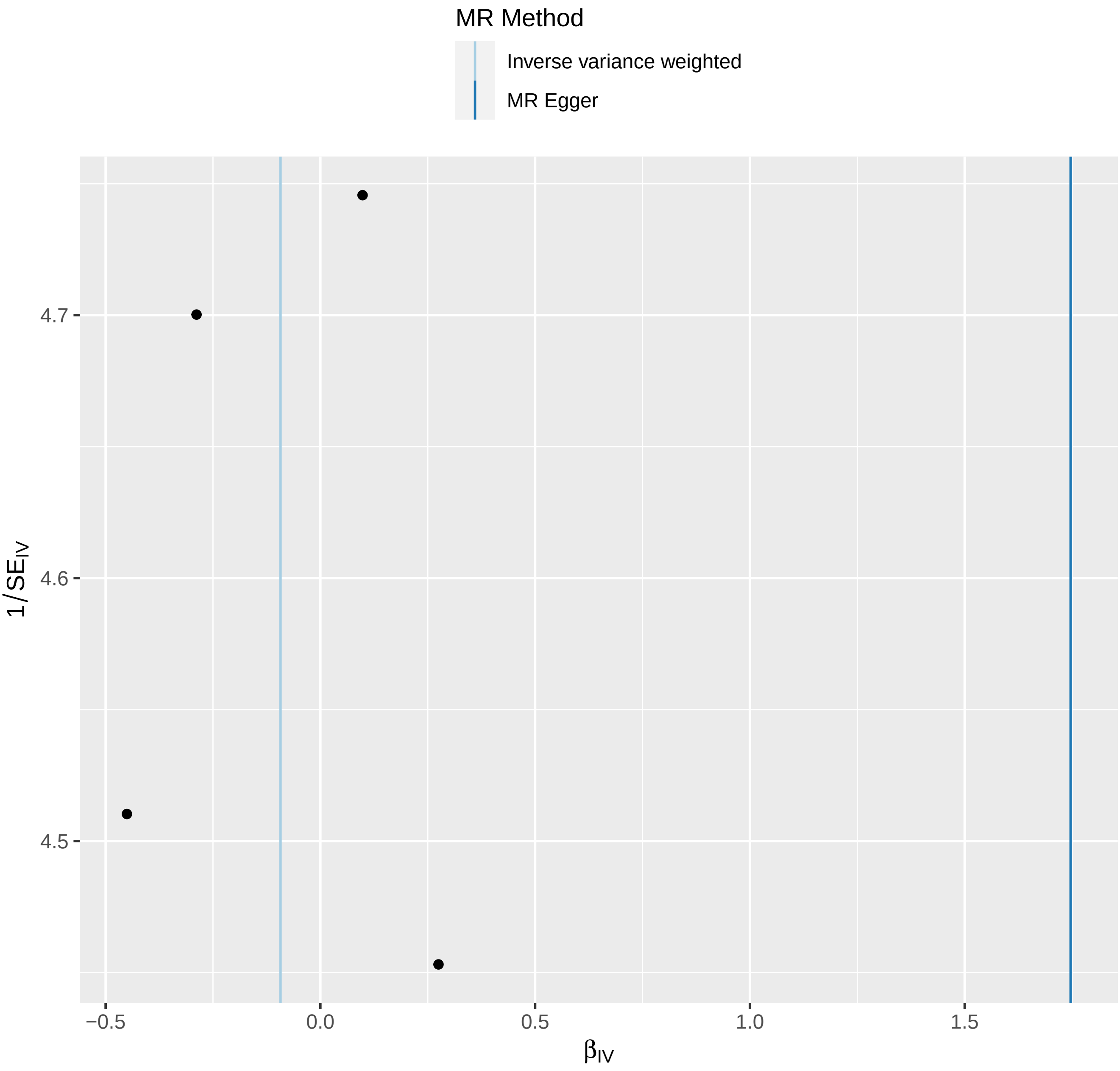

Batch 2−3474 : Gut microbiota abundance (genus Parasutterella id.2892) on Leukemia inhibitory factor receptor with a threshold of 1e−05

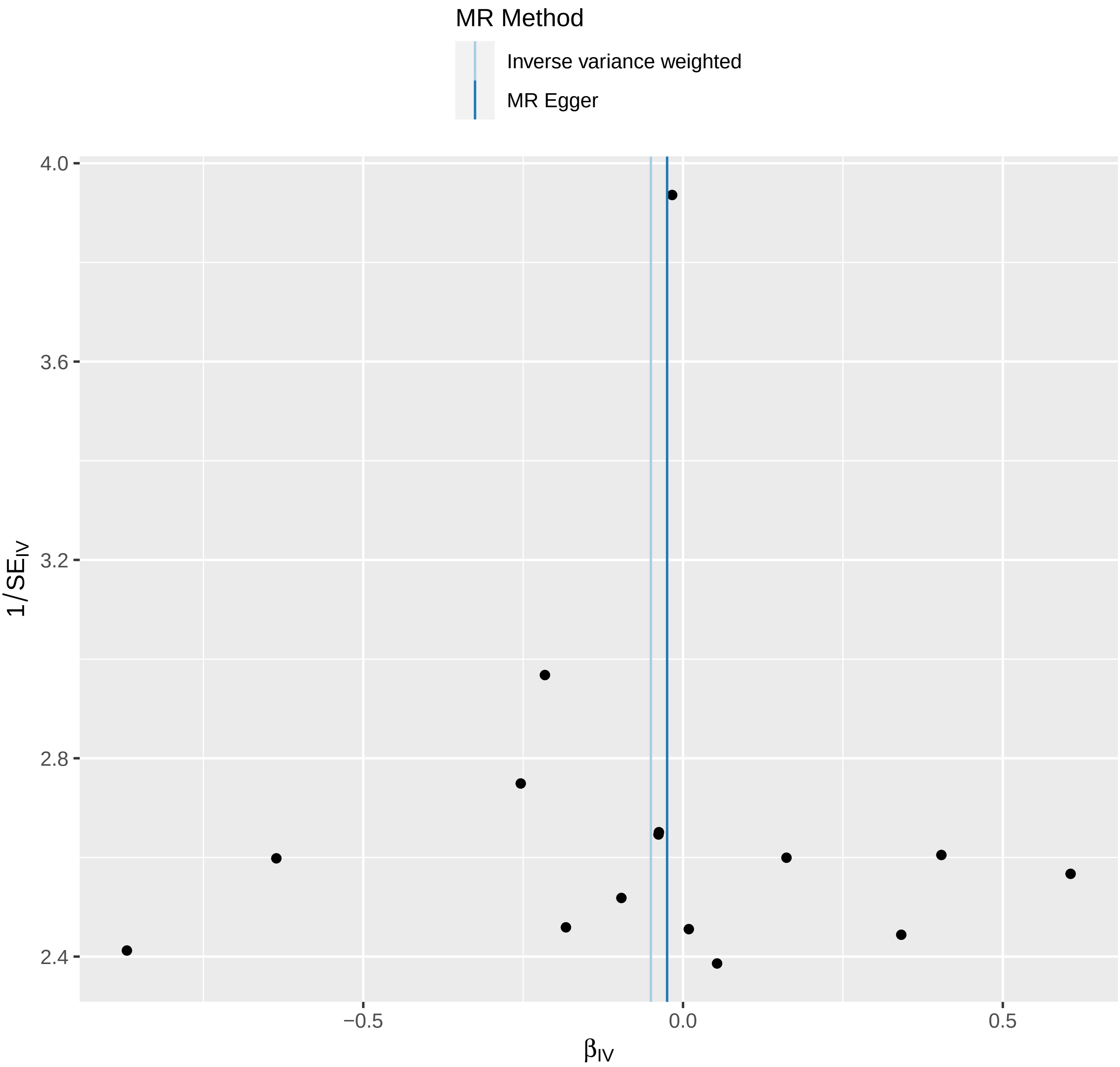

Batch 2−3475 : Gut microbiota abundance (genus Catenibacterium id.2153) on Leukocyte immunoglobulin−like receptor subfamily A member 4 with a threshold of 1e−05

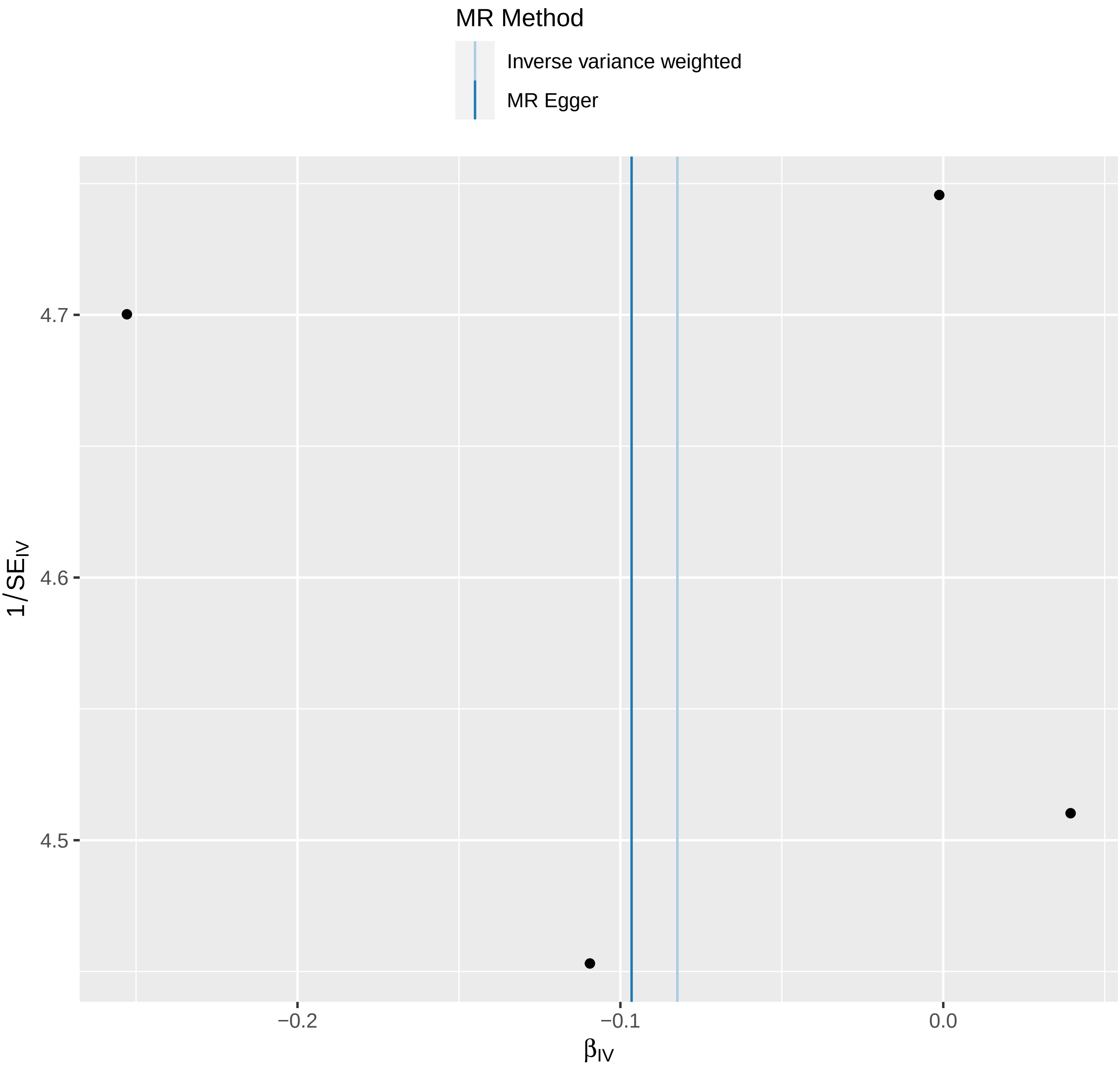

Batch 2−3476 : Gut microbiota abundance (genus Parasutterella id.2892) on Leukocyte immunoglobulin−like receptor subfamily A member 4 with a threshold of 1e−05

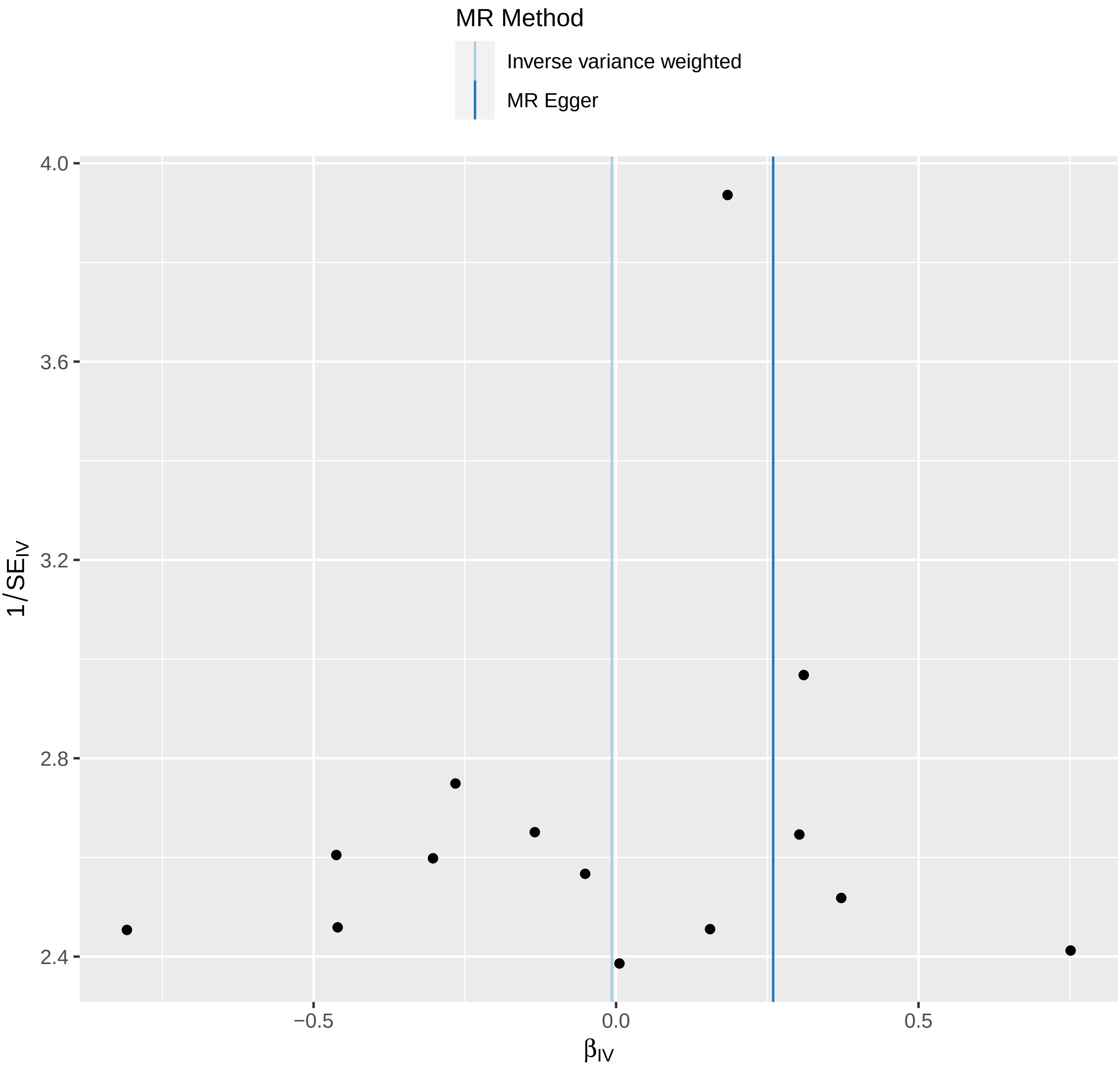

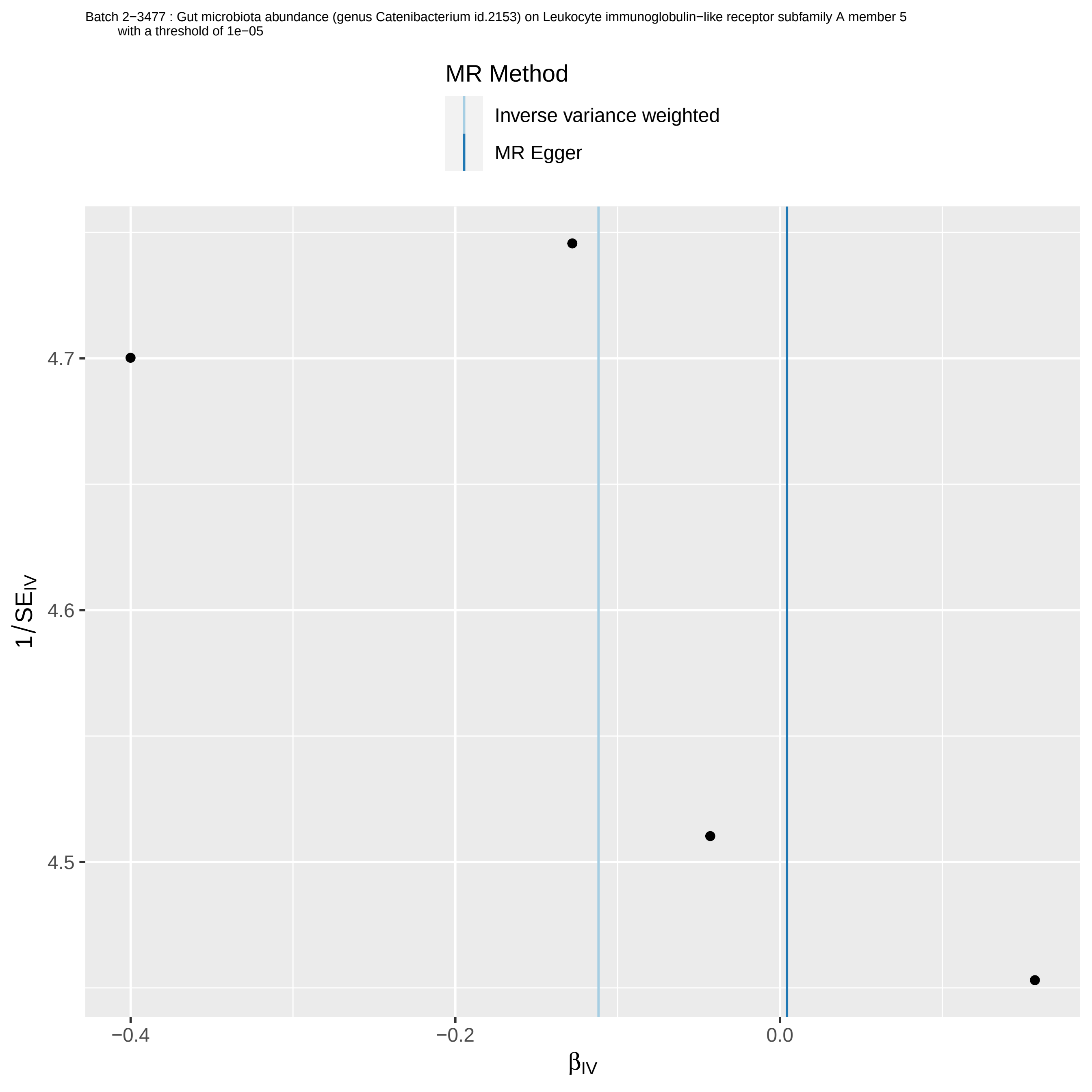
Batch 2−3477 : Gut microbiota abundance (genus Catenibacterium id.2153) on Leukocyte immunoglobulin−like receptor subfamily A member 5
with a threshold of 1e−05
MR Method
Inverse variance weighted
MR Egger
4.7
4.6
4.5
1/SE_IV
−0.4
−0.2
0.0
β_IV

Batch 2−3478 : Gut microbiota abundance (genus Parasutterella id.2892) on Leukocyte immunoglobulin−like receptor subfamily A member 5
with a threshold of 1e−05

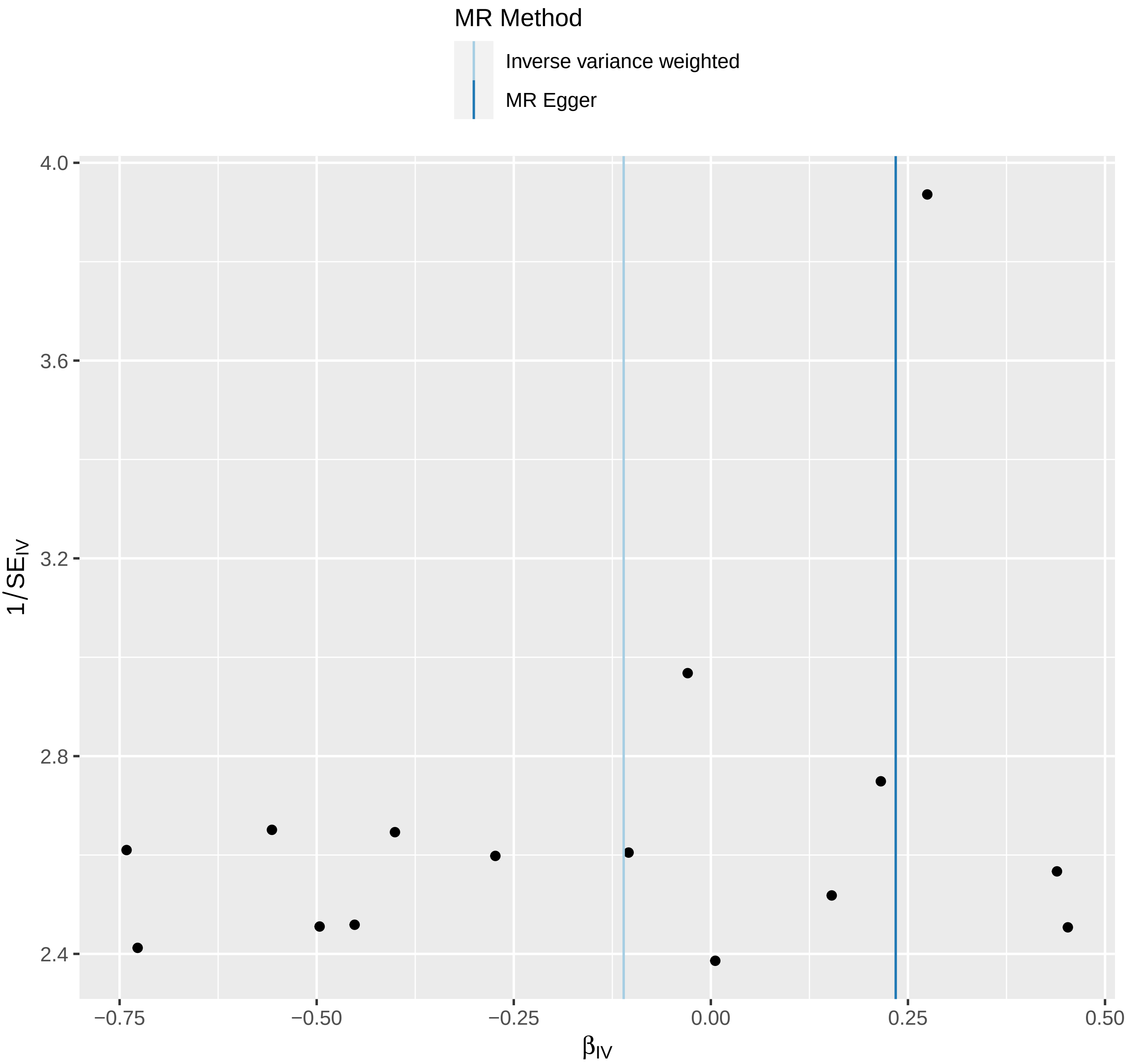

Batch 2−3479 : Gut microbiota abundance (genus Catenibacterium id.2153) on Leukocyte immunoglobulin−like receptor subfamily A member 5 with a threshold of 1e−05

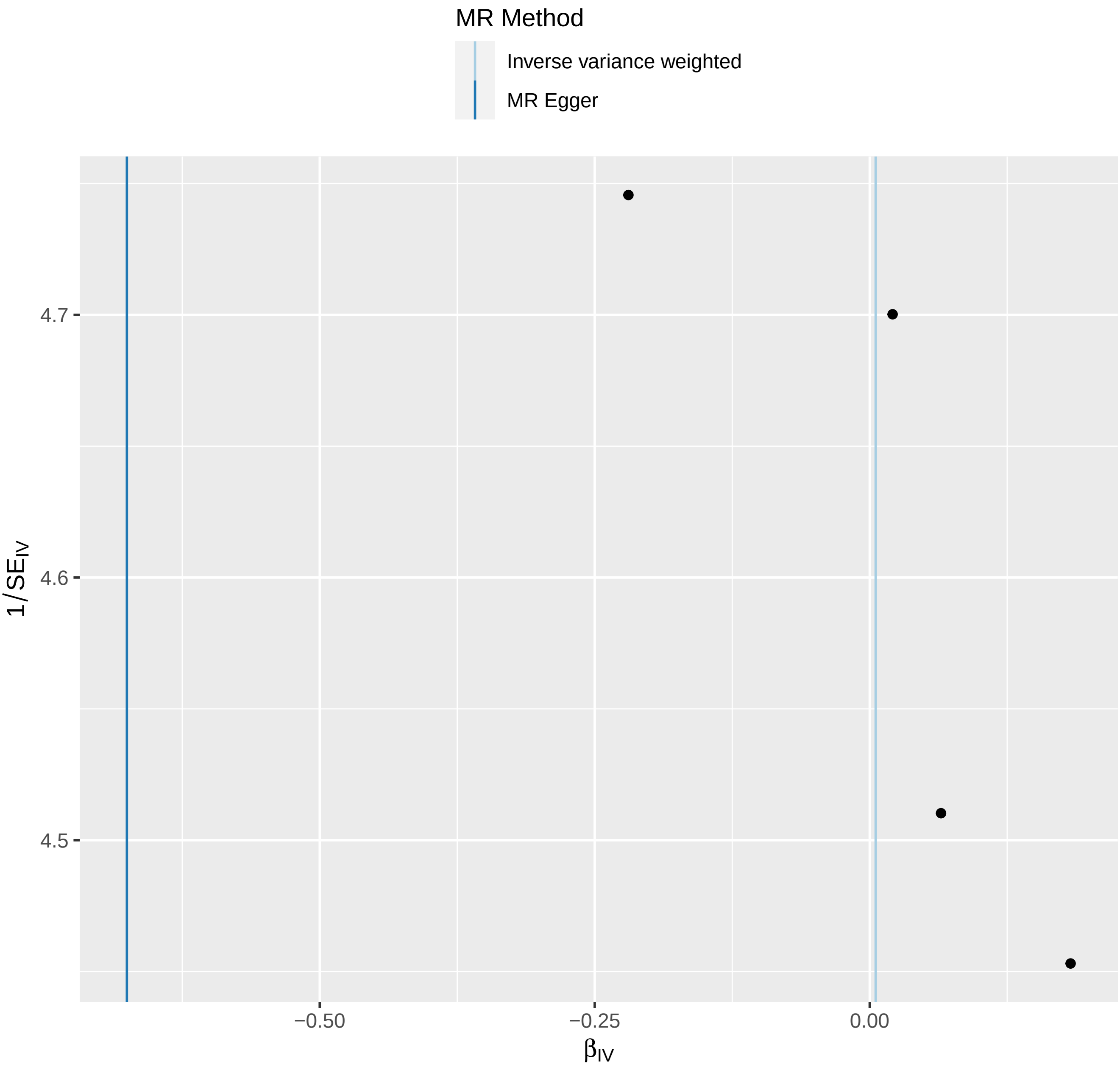

Batch 2−3480 : Gut microbiota abundance (genus Parasutterella id.2892) on Leukocyte immunoglobulin−like receptor subfamily A member 5
with a threshold of 1e−05

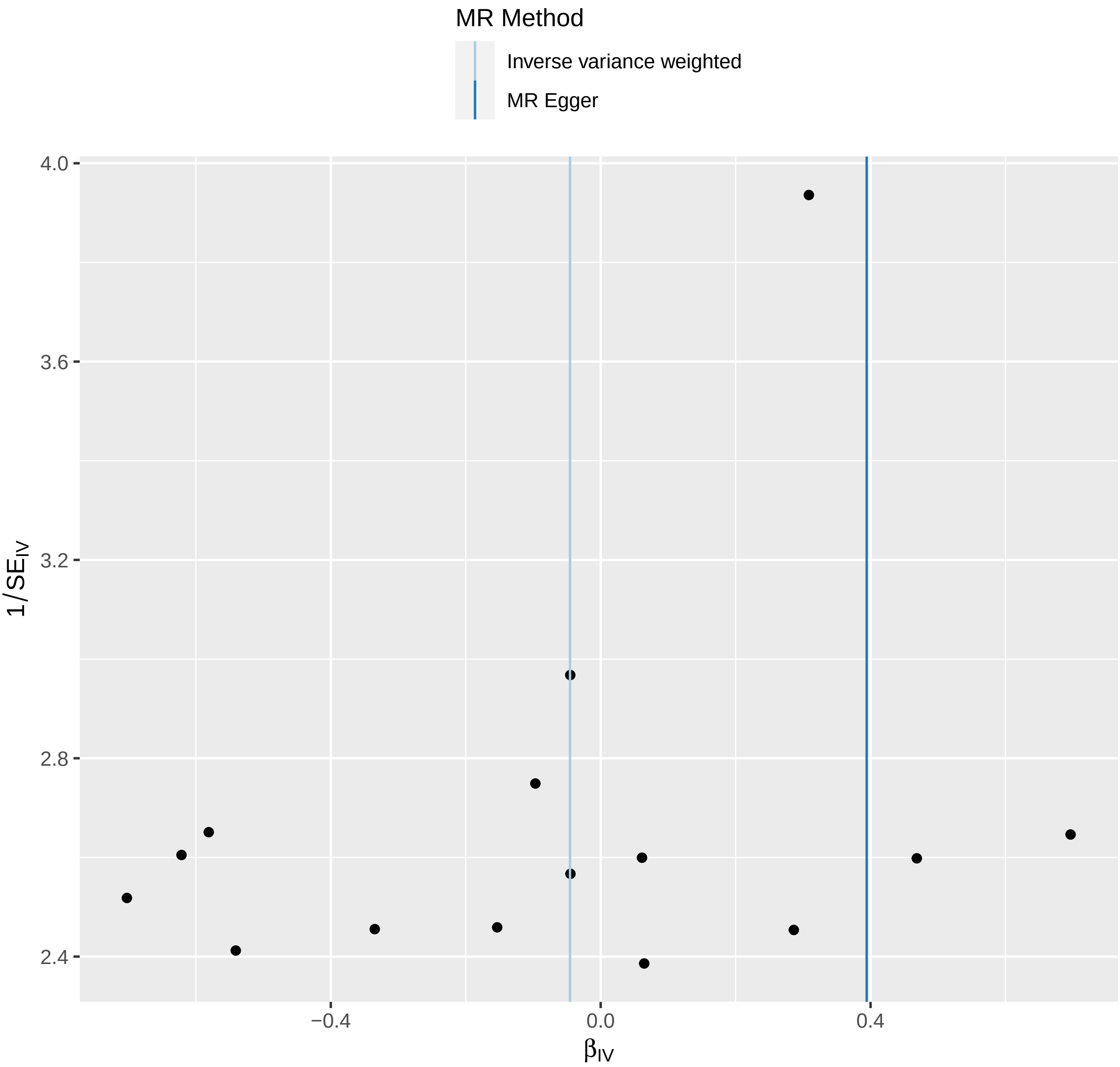

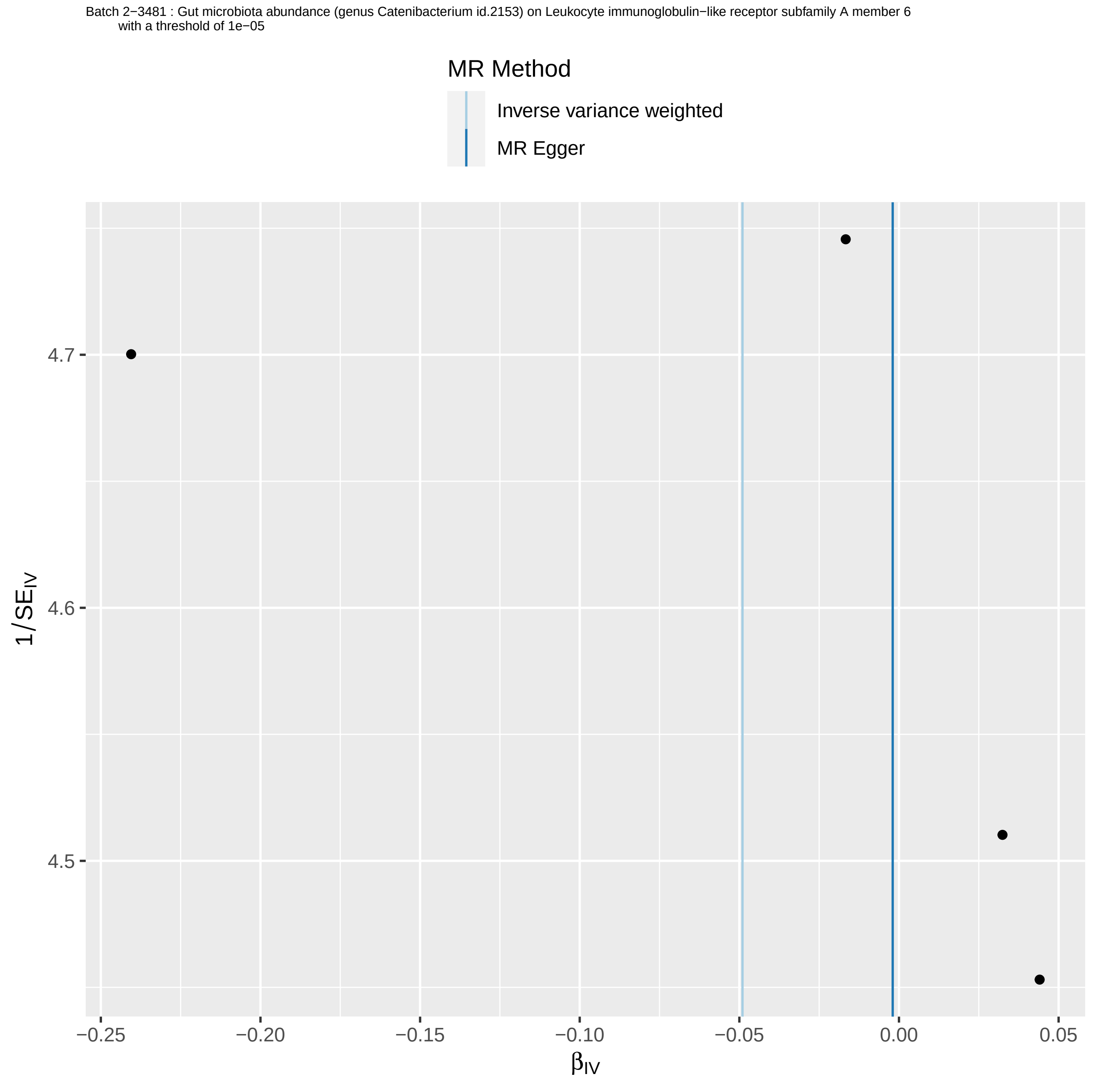

Batch 2−3481 : Gut microbiota abundance (genus Catenibacterium id.2153) on Leukocyte immunoglobulin−like receptor subfamily A member 6
with a threshold of 1e−05
MR Method
Inverse variance weighted
MR Egger
4.7
4.6
4.5
1/SE_IV
−0.25
−0.20
−0.15
−0.10
−0.05
0.00
0.05
β_IV

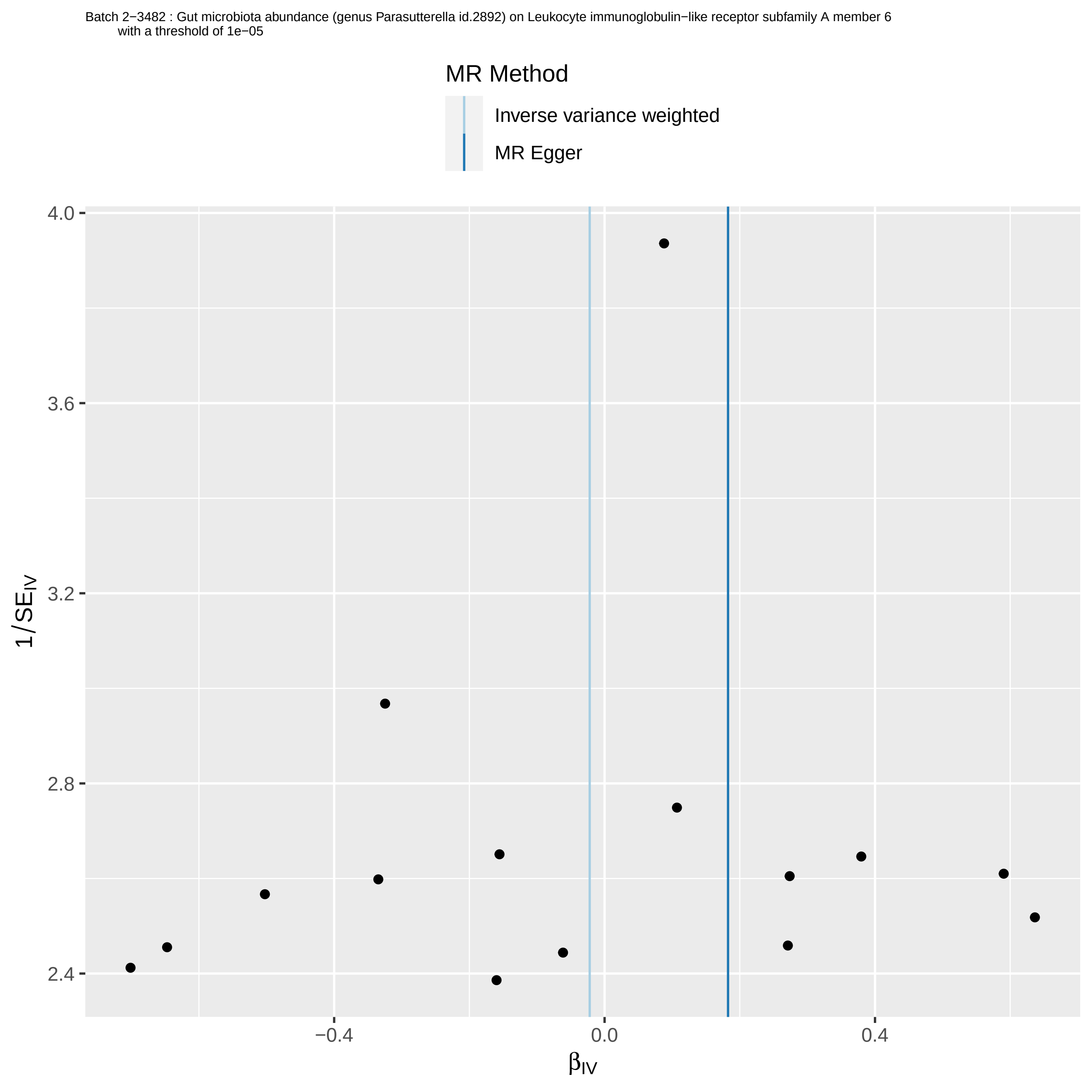

Batch 2−3482 : Gut microbiota abundance (genus Parasutterella id.2892) on Leukocyte immunoglobulin−like receptor subfamily A member 6
with a threshold of 1e−05
MR Method
Inverse variance weighted
MR Egger
4.0
3.6
3.2
2.8
2.4
1/SE_IV
−0.4
0.0
0.4
β_IV

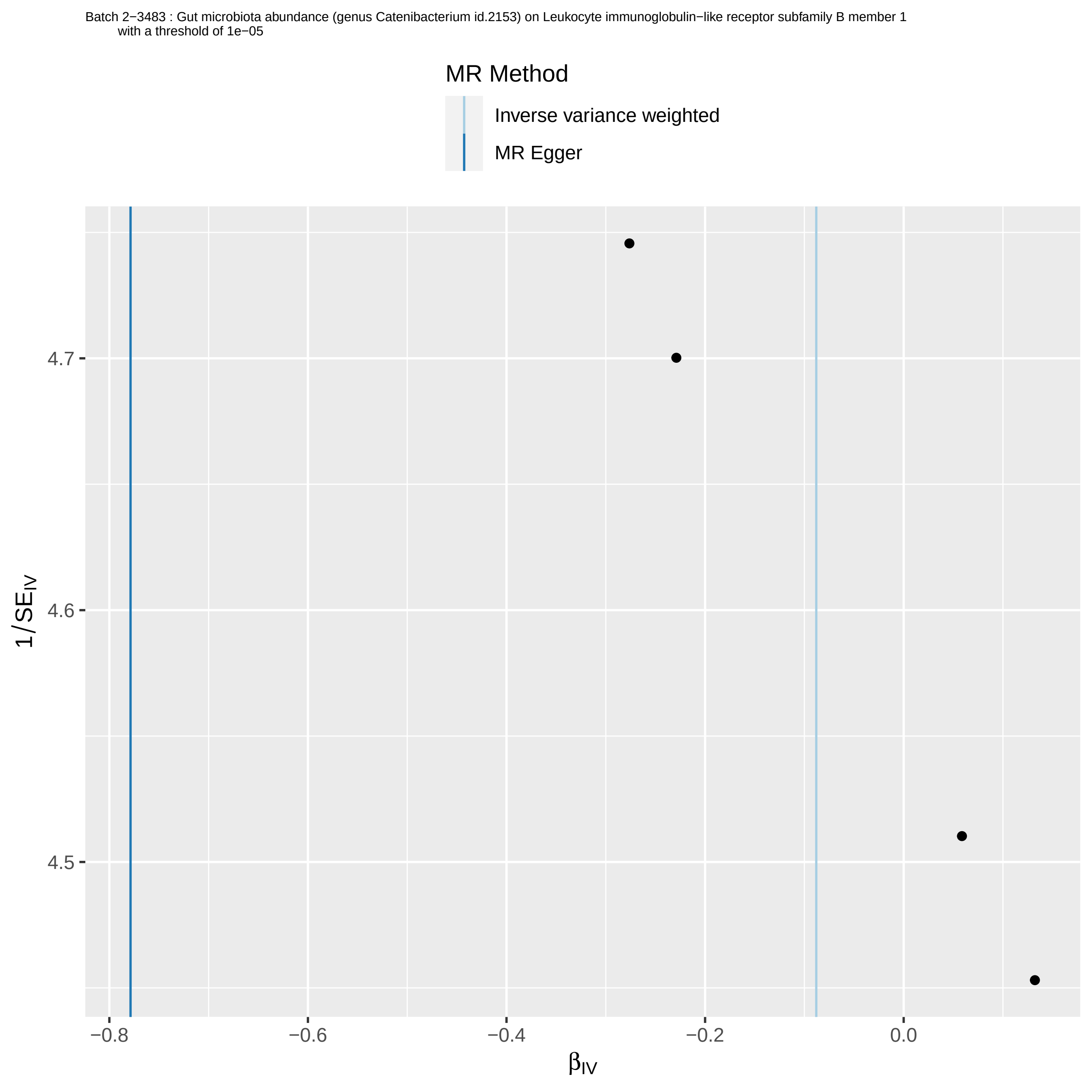
Batch 2−3483 : Gut microbiota abundance (genus Catenibacterium id.2153) on Leukocyte immunoglobulin−like receptor subfamily B member 1
with a threshold of 1e−05
MR Method
Inverse variance weighted
MR Egger
4.7
4.6
4.5
−0.8
−0.6
−0.4
−0.2
0.0
$1/SE_{IV}$
β_{IV}

Batch 2−3484 : Gut microbiota abundance (genus Parasutterella id.2892) on Leukocyte immunoglobulin−like receptor subfamily B member 1 with a threshold of 1e−05

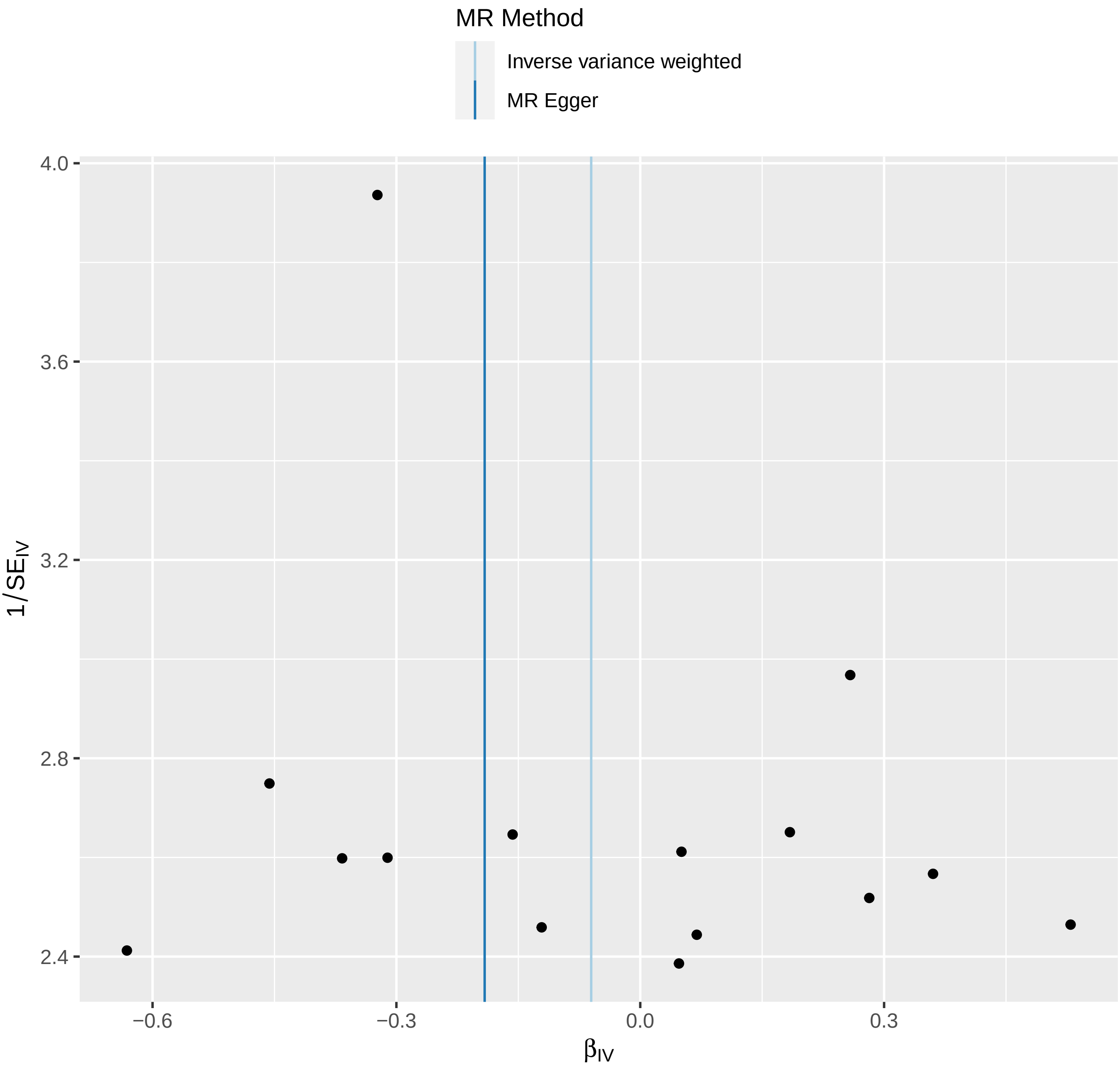

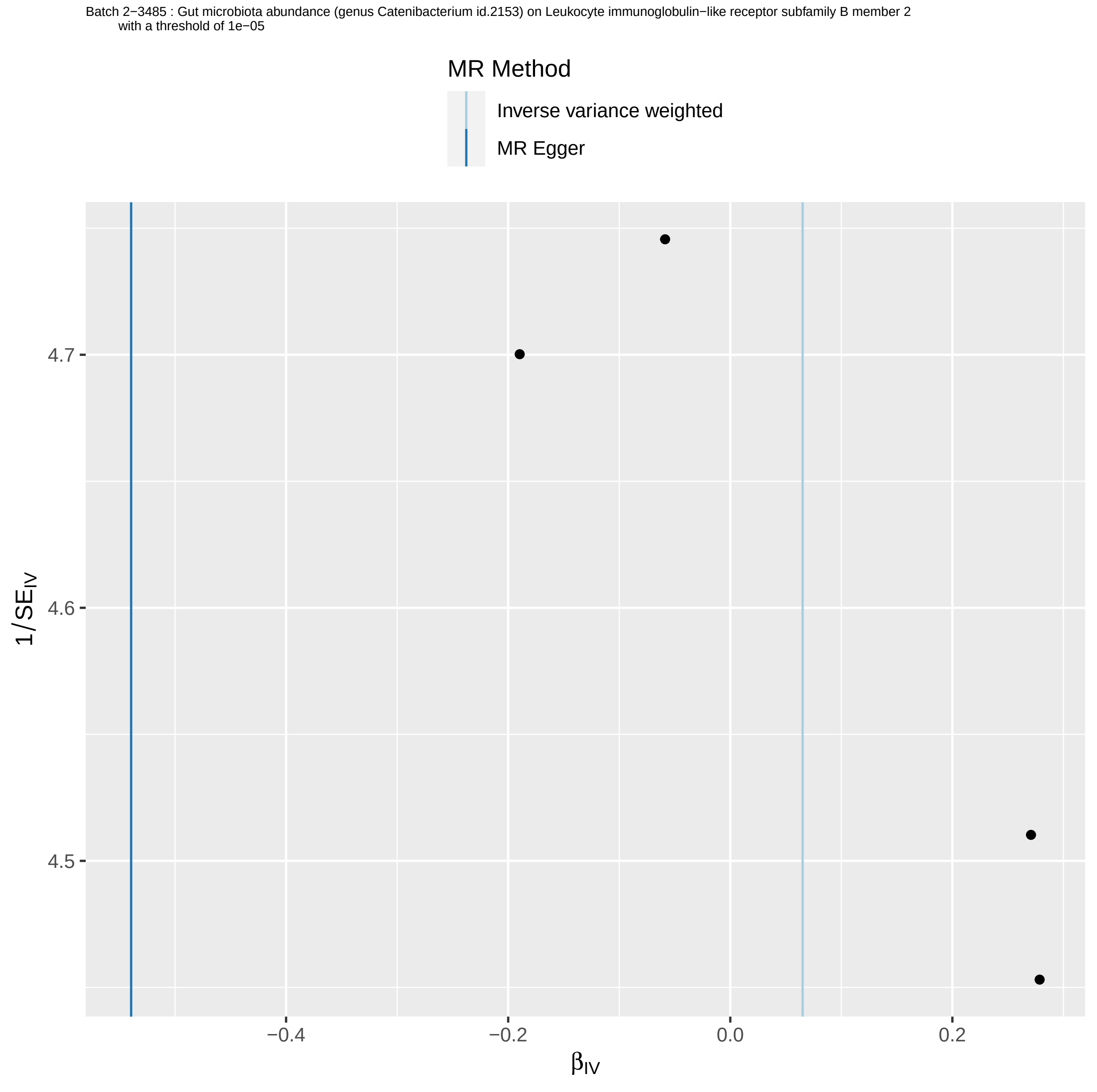

Batch 2−3485 : Gut microbiota abundance (genus Catenibacterium id.2153) on Leukocyte immunoglobulin−like receptor subfamily B member 2 with a threshold of 1e−05
MR Method
Inverse variance weighted
MR Egger
4.7
4.6
4.5
1/SE_IV
−0.4
−0.2
0.0
0.2
β_IV

Batch 2−3486 : Gut microbiota abundance (genus Parasutterella id.2892) on Leukocyte immunoglobulin−like receptor subfamily B member 2
with a threshold of 1e−05

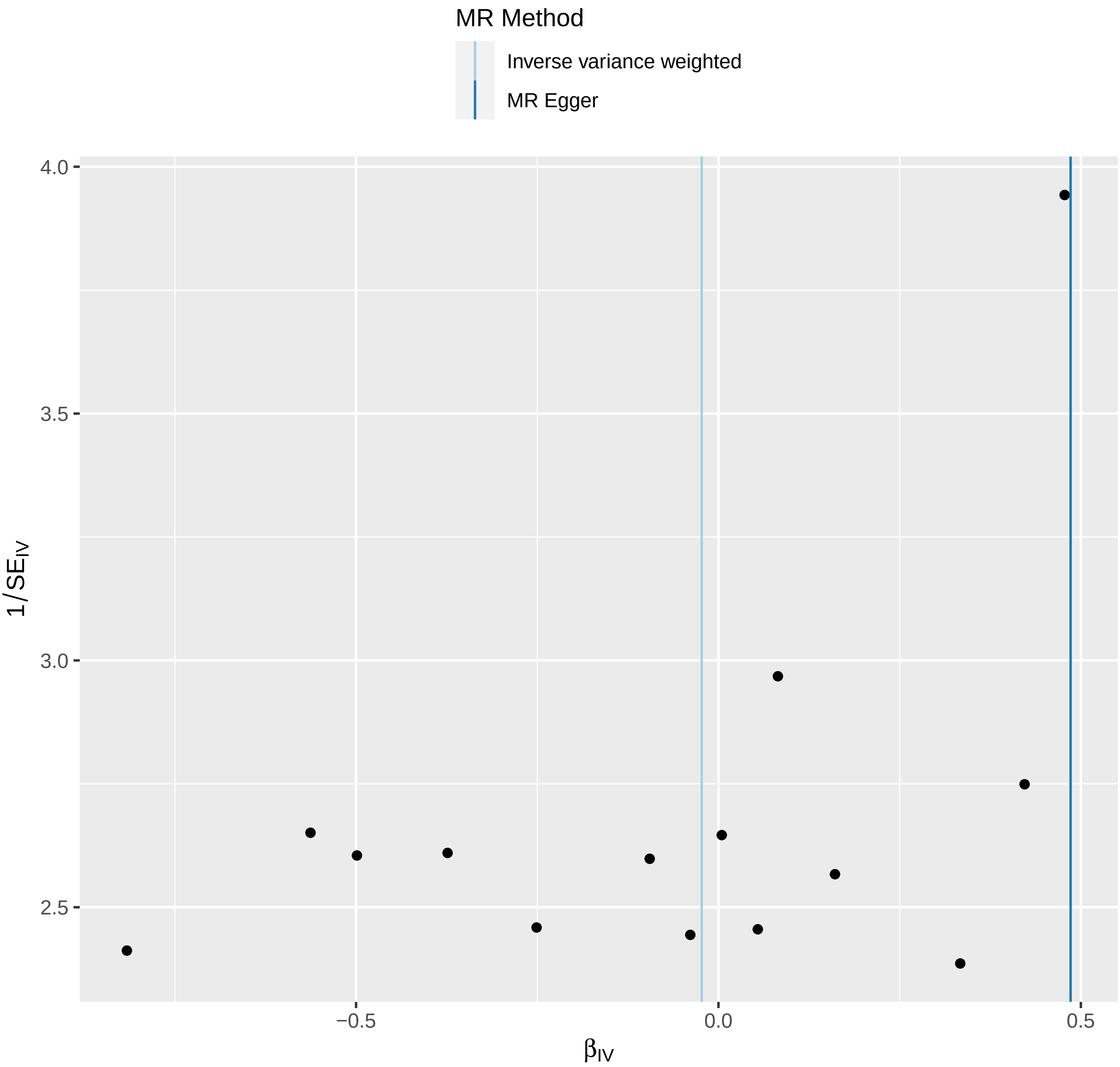

Batch 2−3487 : Gut microbiota abundance (genus Catenibacterium id.2153) on Leukocyte immunoglobulin−like receptor subfamily B member 2 with a threshold of 1e−05

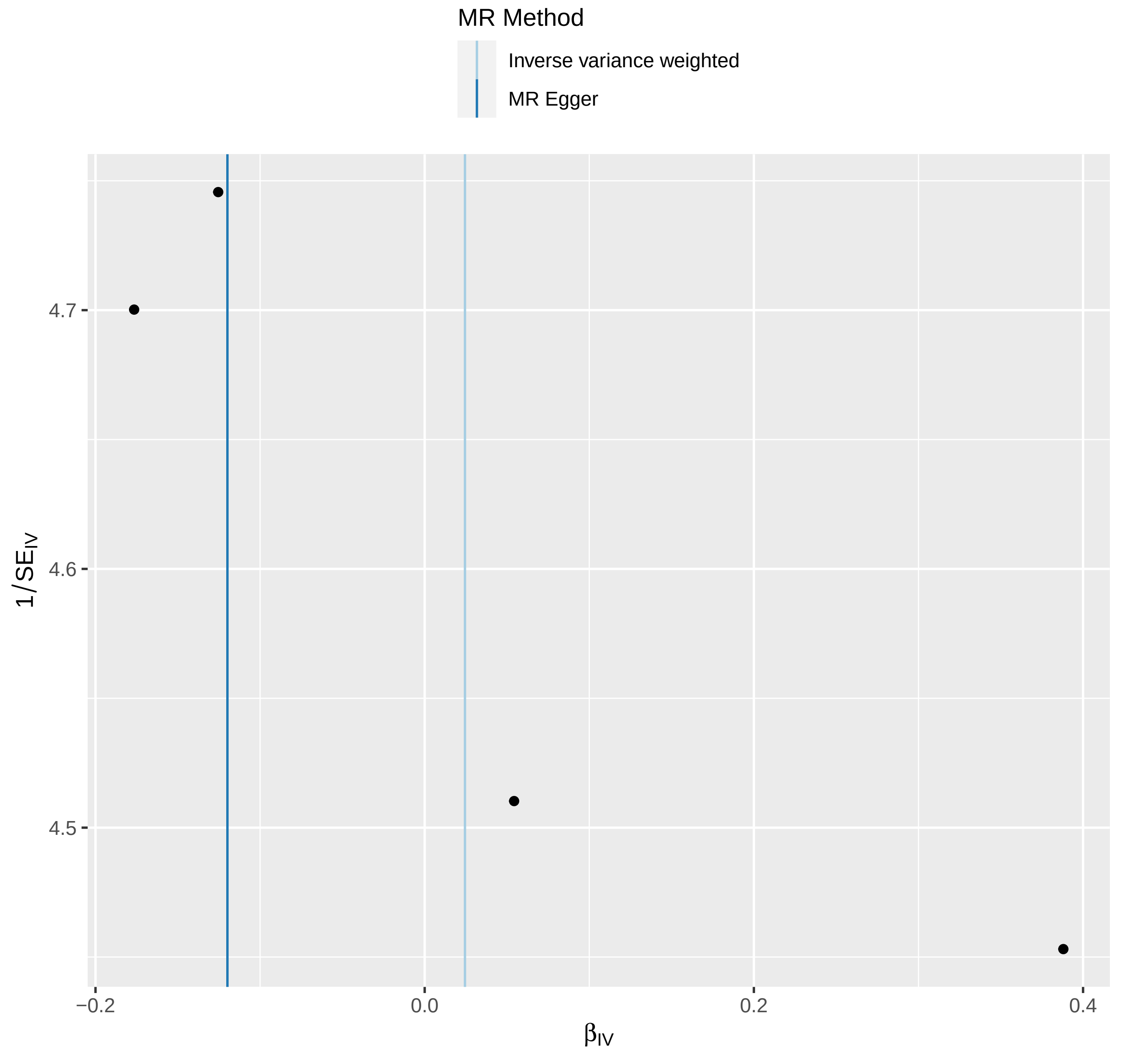

Batch 2−3488 : Gut microbiota abundance (genus Parasutterella id.2892) on Leukocyte immunoglobulin−like receptor subfamily B member 2
with a threshold of 1e−05

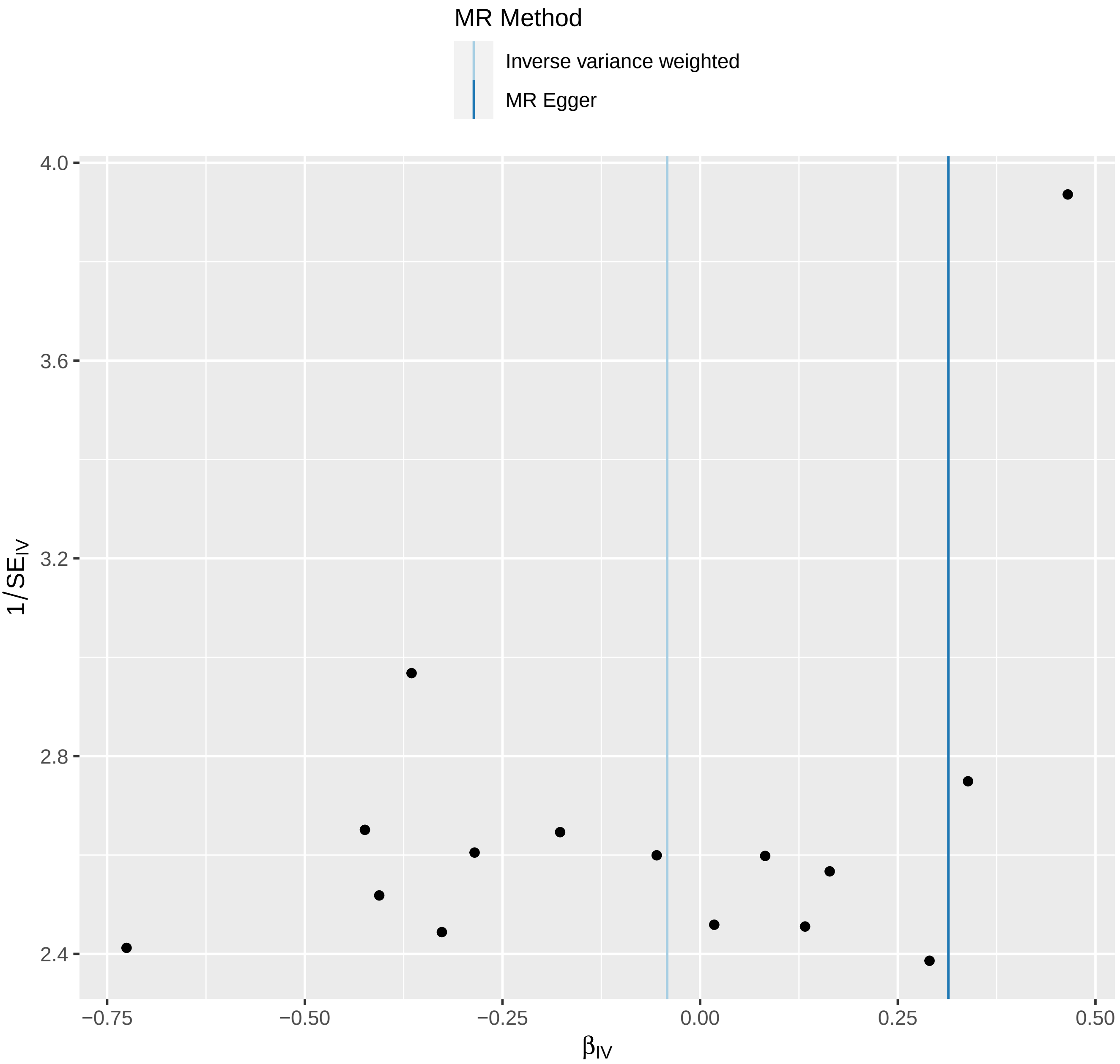

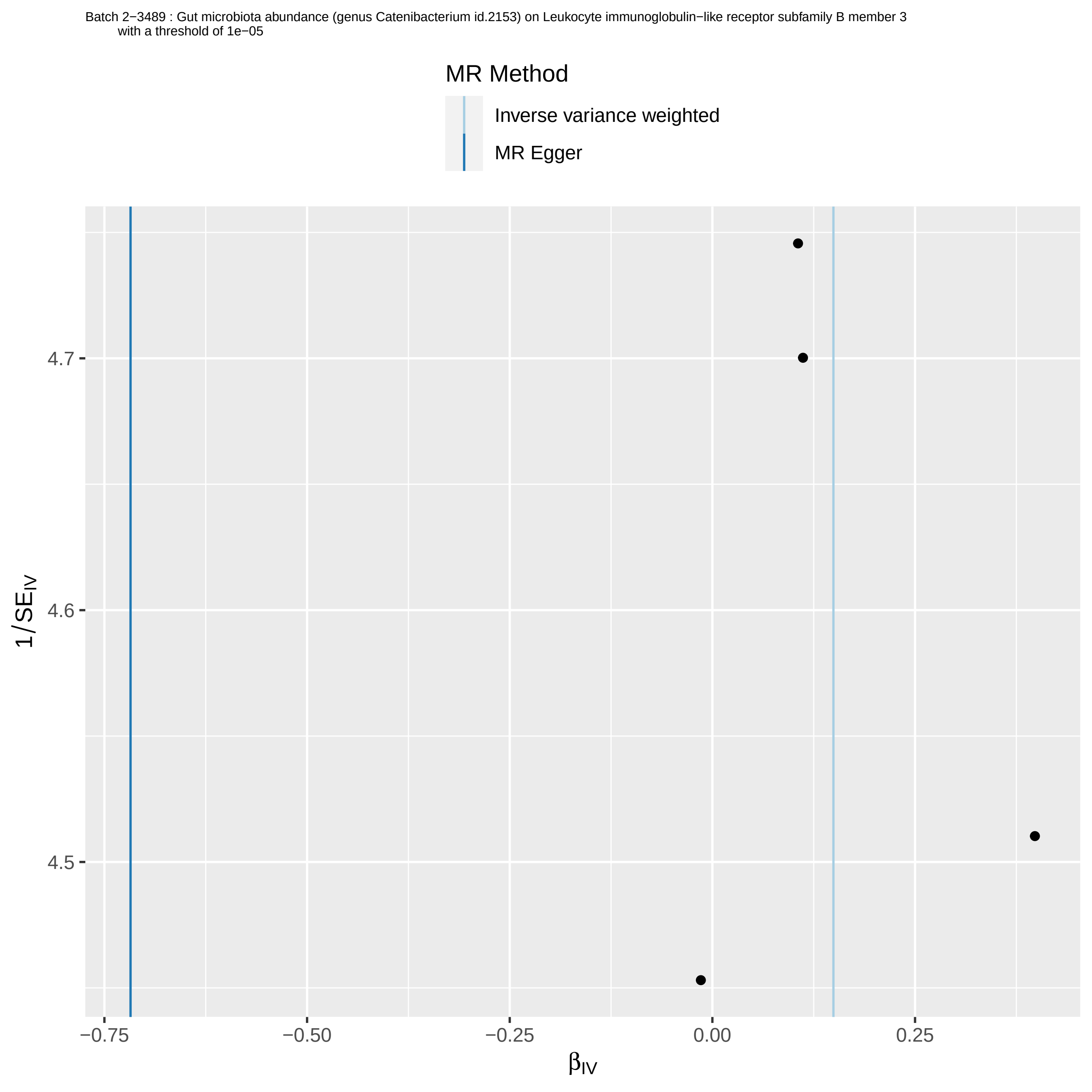

Batch 2−3489 : Gut microbiota abundance (genus Catenibacterium id.2153) on Leukocyte immunoglobulin−like receptor subfamily B member 3
with a threshold of 1e−05
MR Method
Inverse variance weighted
MR Egger
4.7
4.6
4.5
1/SE_IV
−0.75
−0.50
−0.25
0.00
0.25
β_IV

Batch 2−3490 : Gut microbiota abundance (genus Parasutterella id.2892) on Leukocyte immunoglobulin−like receptor subfamily B member 3 with a threshold of 1e−05

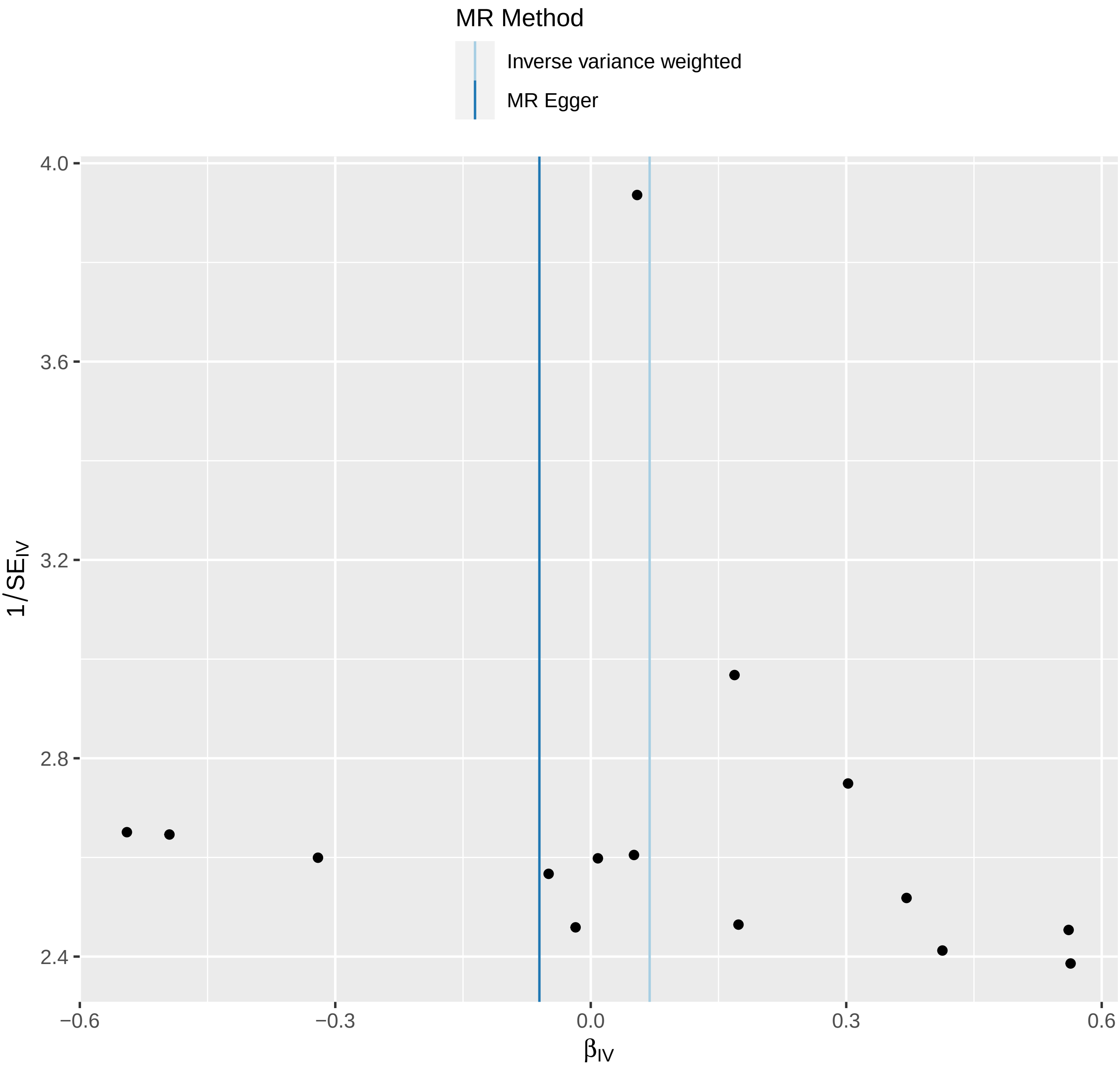

Batch 2−3491 : Gut microbiota abundance (genus Catenibacterium id.2153) on Leukocyte immunoglobulin−like receptor subfamily B member 4
with a threshold of 1e−05

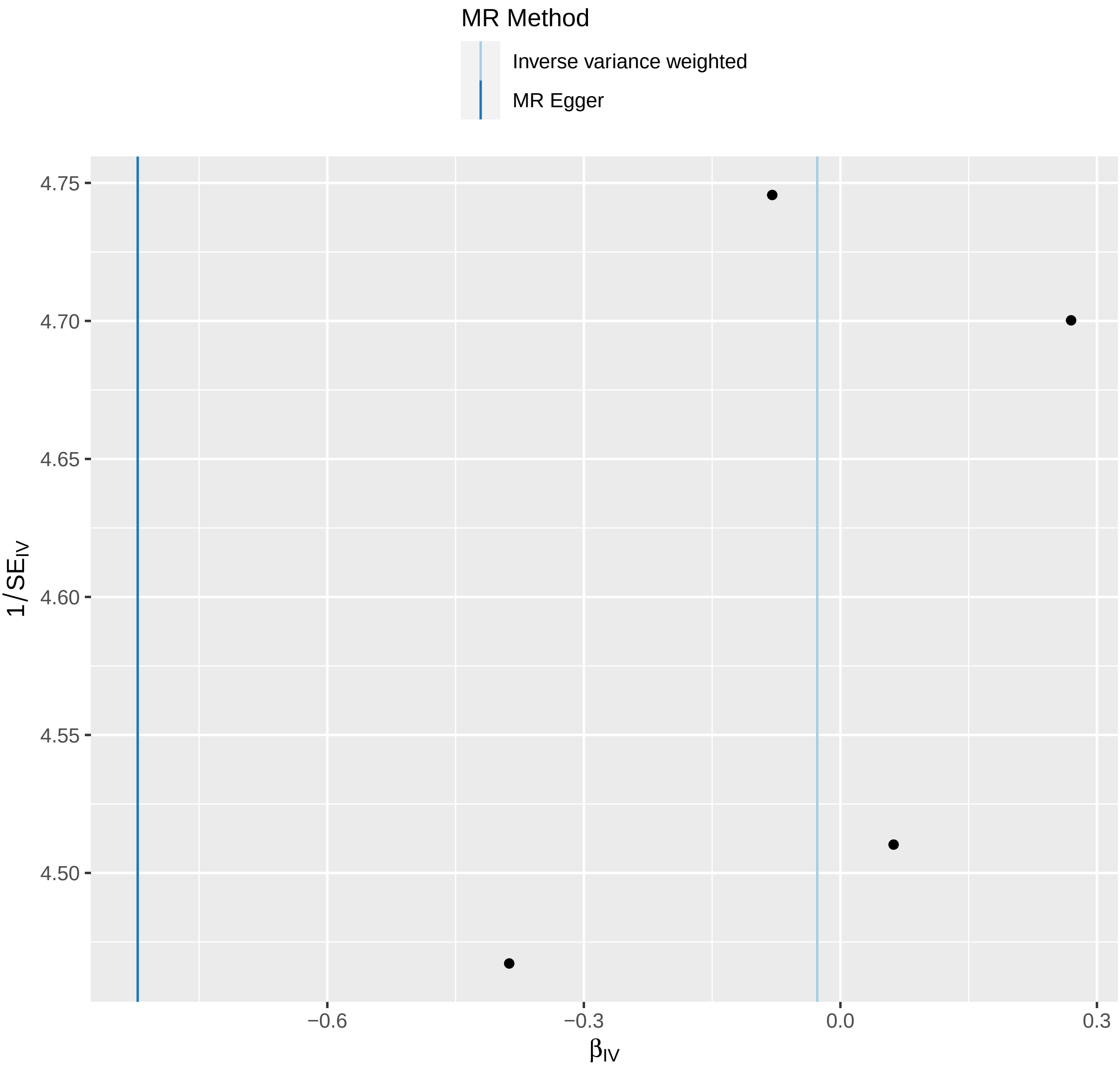

Batch 2−3492 : Gut microbiota abundance (genus Parasutterella id.2892) on Leukocyte immunoglobulin−like receptor subfamily B member 4 with a threshold of 1e−05

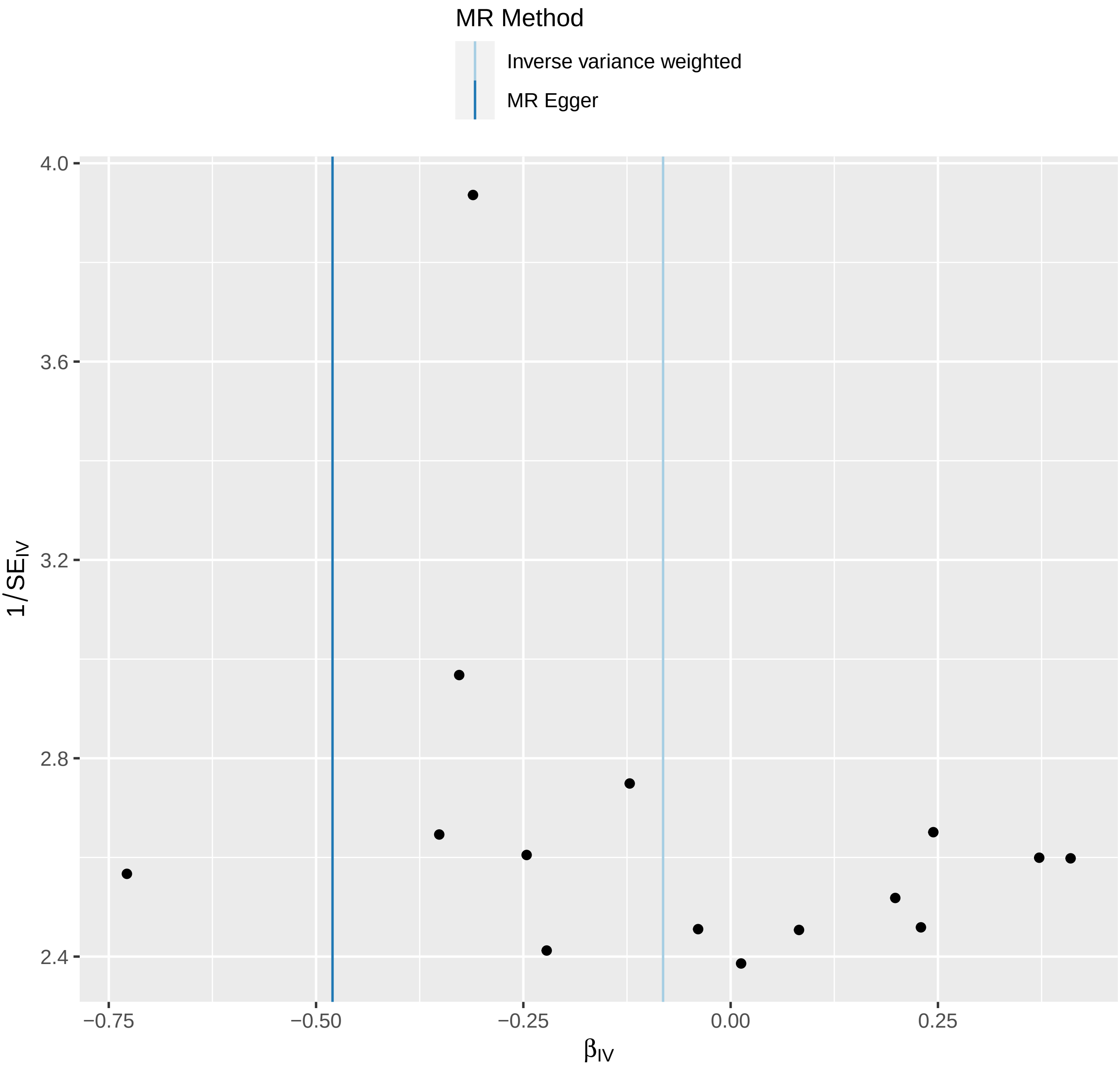

Batch 2−3493 : Gut microbiota abundance (genus Catenibacterium id.2153) on Leukocyte immunoglobulin−like receptor subfamily B member 5 with a threshold of 1e−05

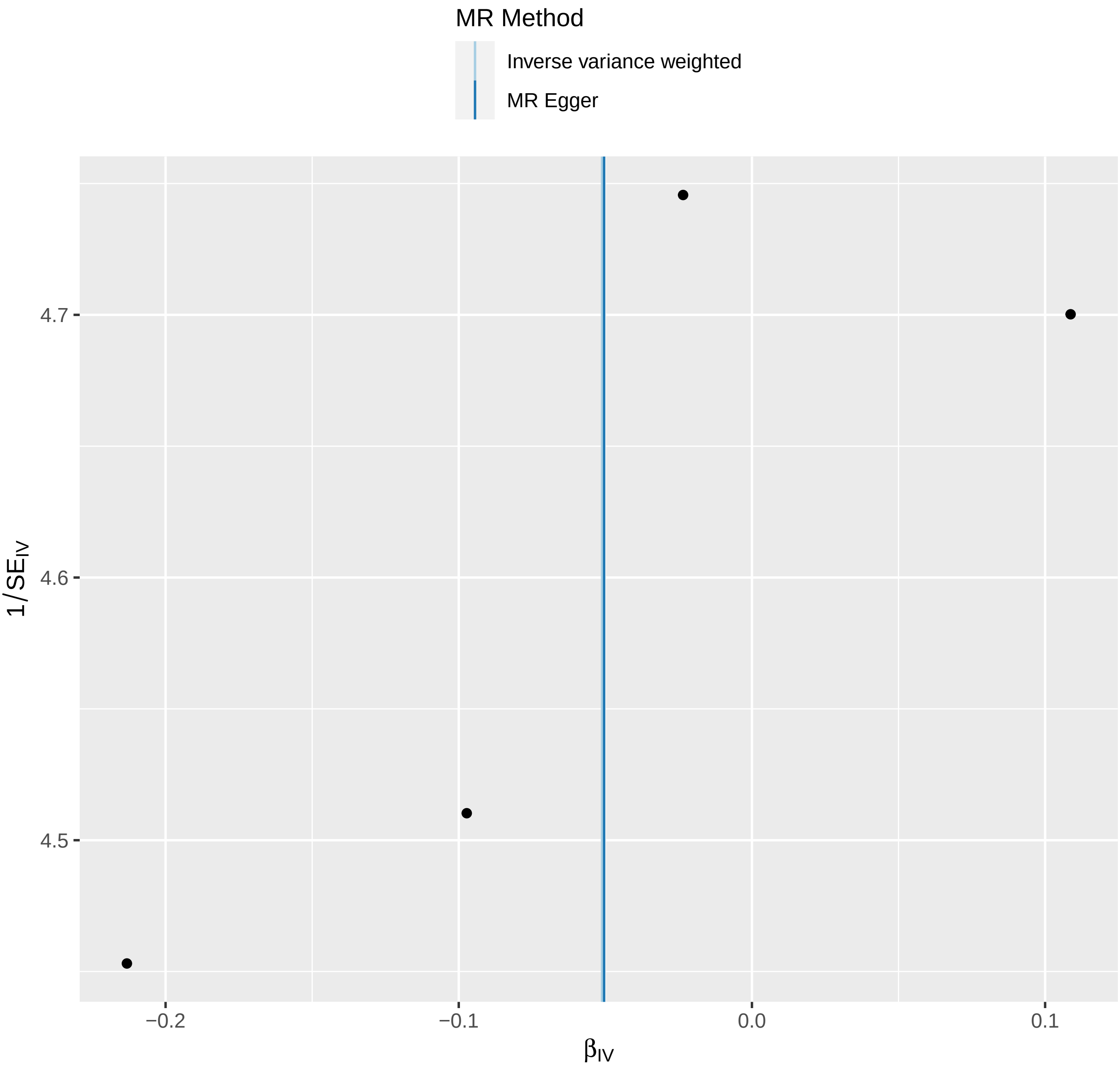

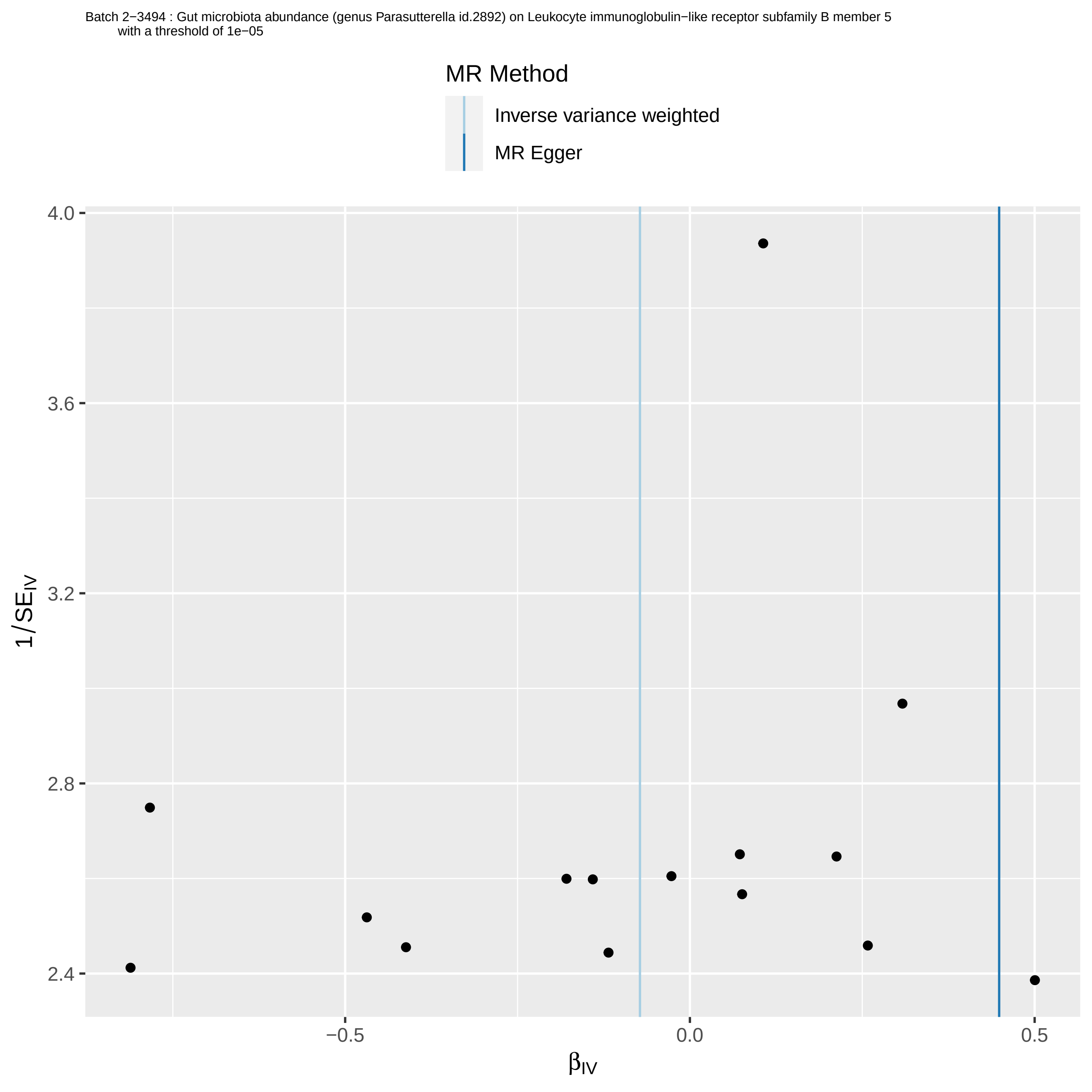
Batch 2−3494 : Gut microbiota abundance (genus Parasutterella id.2892) on Leukocyte immunoglobulin−like receptor subfamily B member 5
with a threshold of 1e−05
MR Method
Inverse variance weighted
MR Egger
4.0
3.6
3.2
2.8
2.4
1/SE_IV
−0.5
0.0
0.5
β_IV

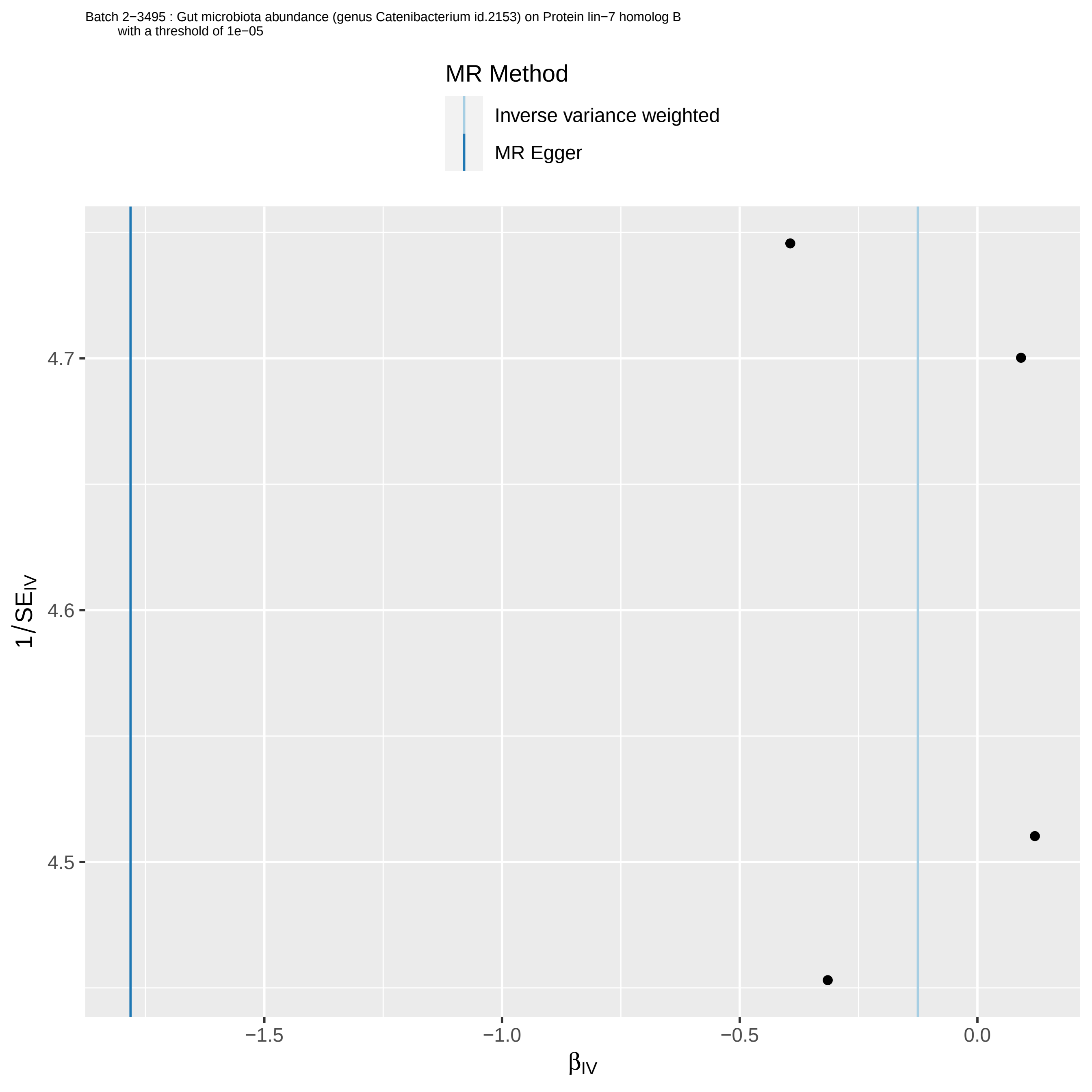

Batch 2−3495 : Gut microbiota abundance (genus Catenibacterium id.2153) on Protein lin−7 homolog B
with a threshold of 1e−05
MR Method
Inverse variance weighted
MR Egger
4.7
4.6
4.5
$1/SE_{IV}$
−1.5
−1.0
−0.5
0.0
β_{IV}

Batch 2−3496 : Gut microbiota abundance (genus Parasutterella id.2892) on Protein lin−7 homolog B
with a threshold of 1e−05

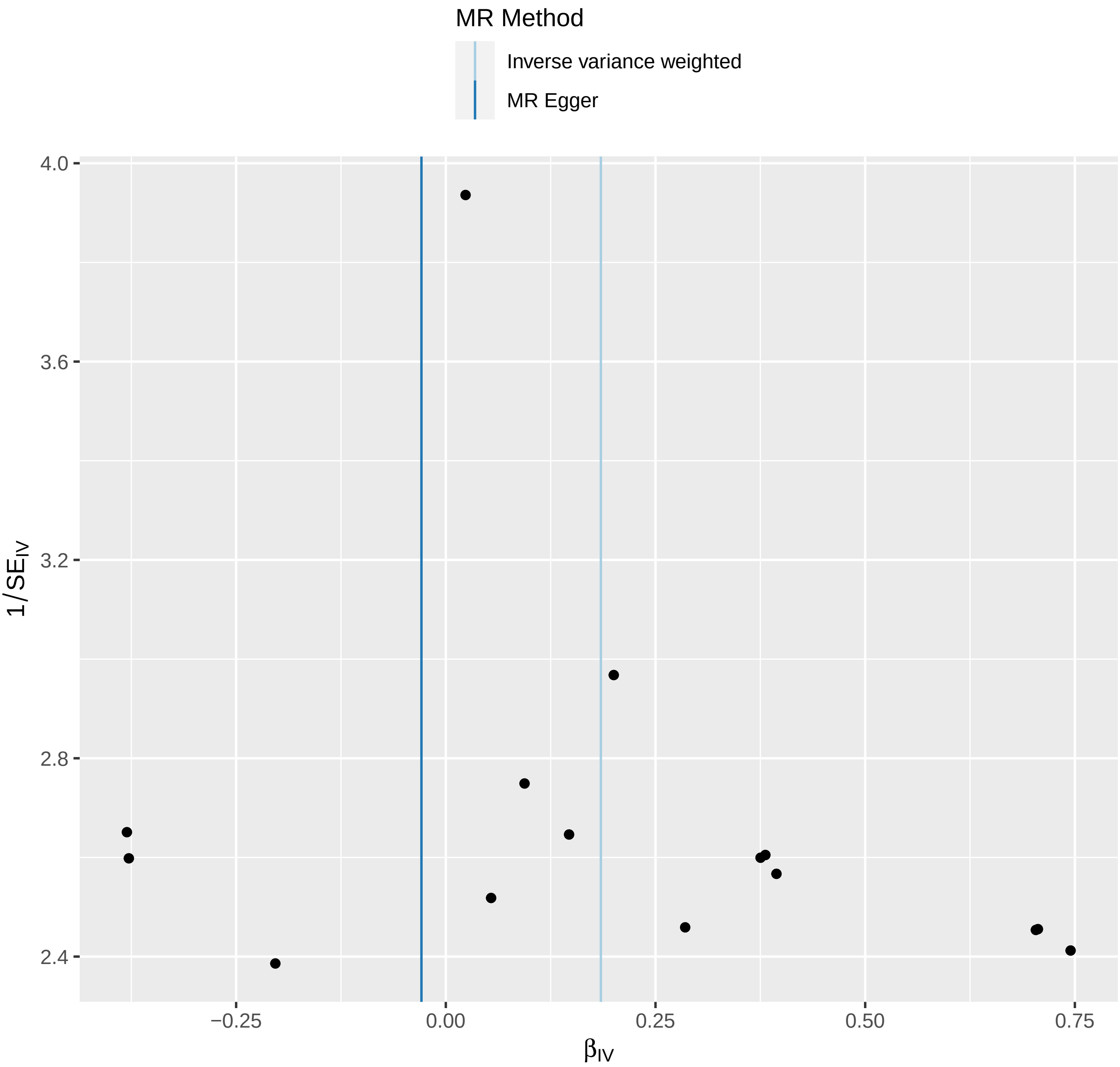

Batch 2−3497 : Gut microbiota abundance (genus Catenibacterium id.2153) on Leucine−rich repeat and immunoglobulin−like domain−containing nogo receptor−interacting pro
with a threshold of 1e−05

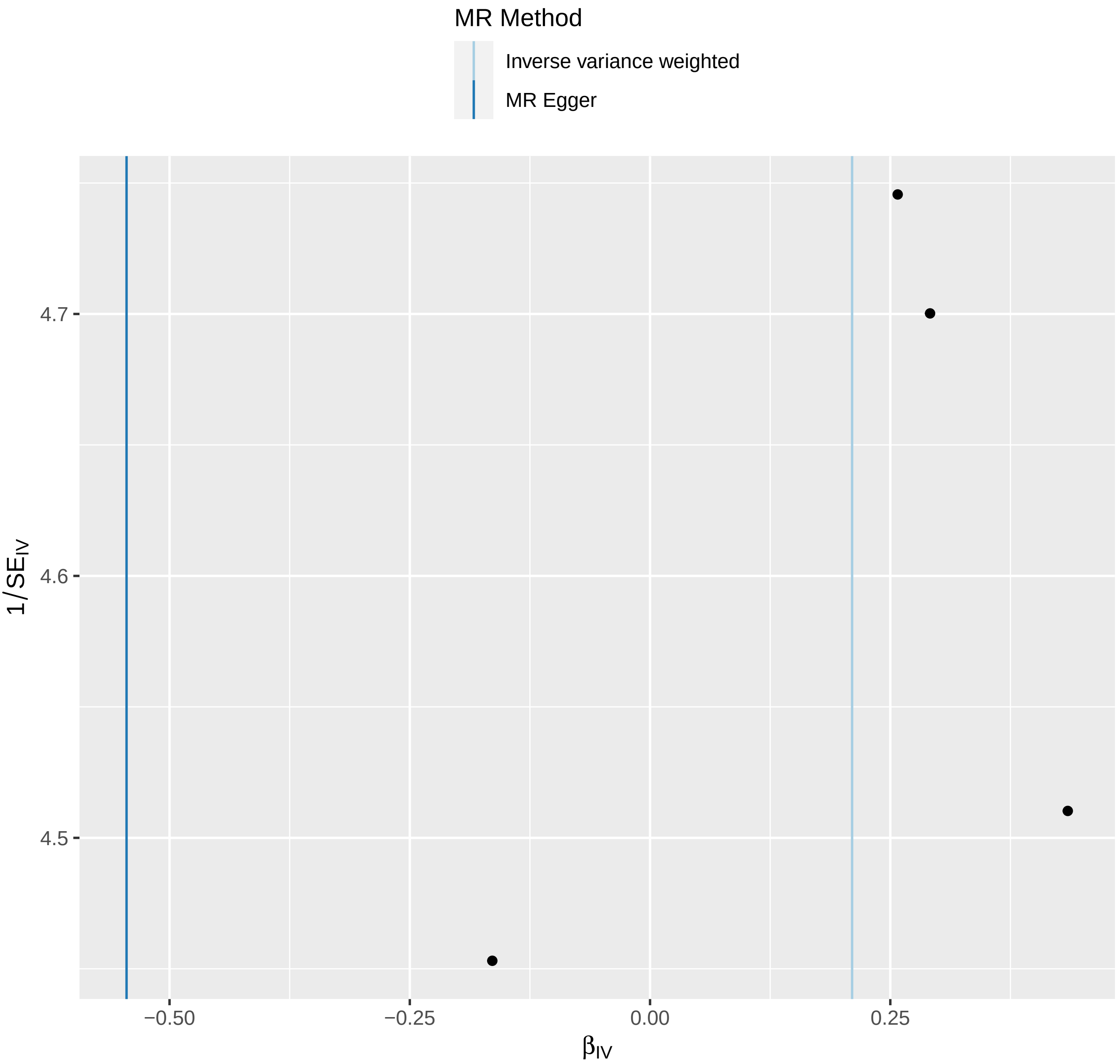

Batch 2−3498 : Gut microbiota abundance (genus Parasutterella id.2892) on Leucine−rich repeat and immunoglobulin−like domain−containing nogo receptor−interacting protei
with a threshold of 1e−05

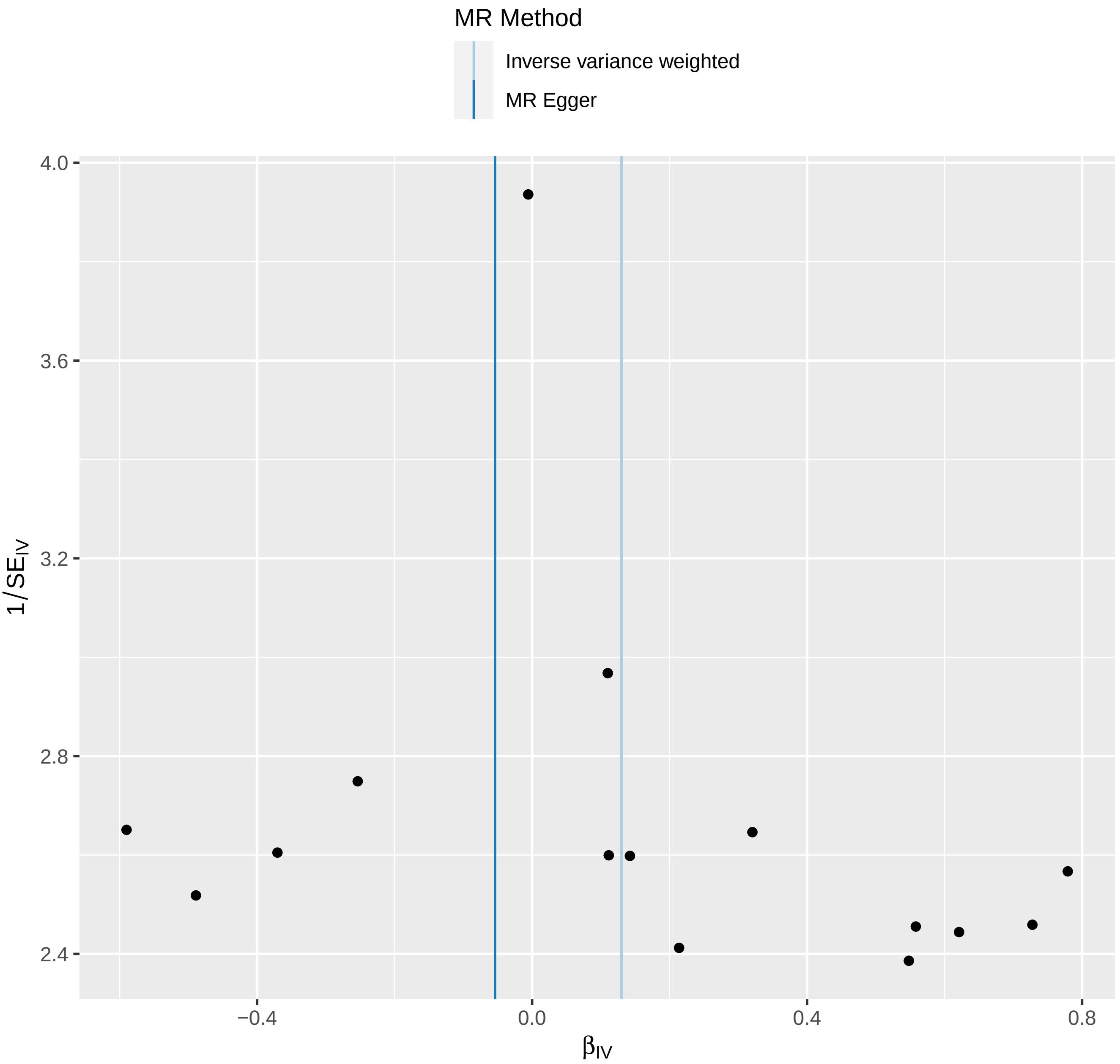

Batch 2−3499 : Gut microbiota abundance (genus Catenibacterium id.2153) on Leucine−rich repeat and immunoglobulin−like domain−containing nogo receptor−interacting pro
with a threshold of 1e−05

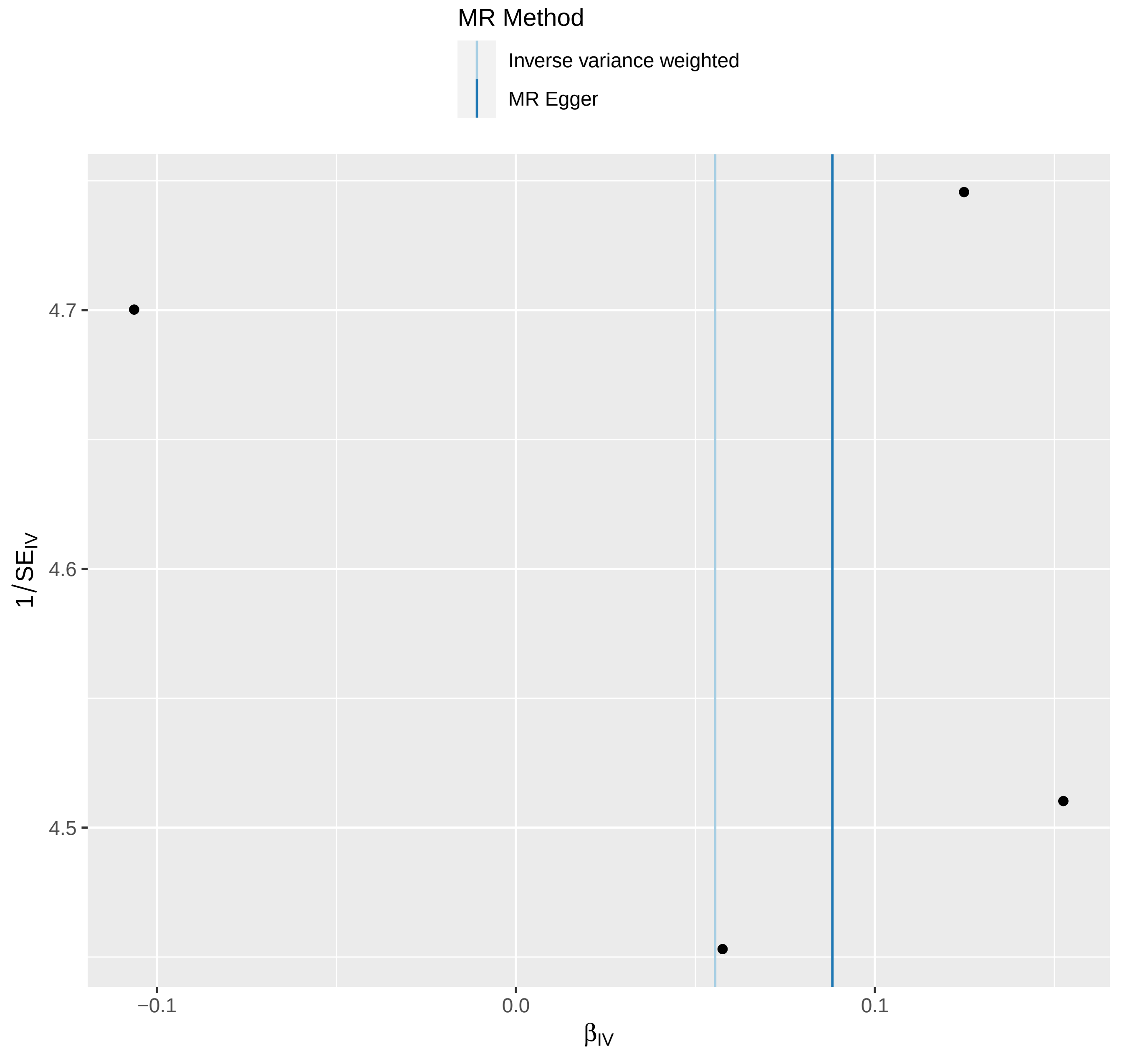

Batch 2−3500 : Gut microbiota abundance (genus Parasutterella id.2892) on Leucine−rich repeat and immunoglobulin−like domain−containing nogo receptor−interacting protei
with a threshold of 1e−05

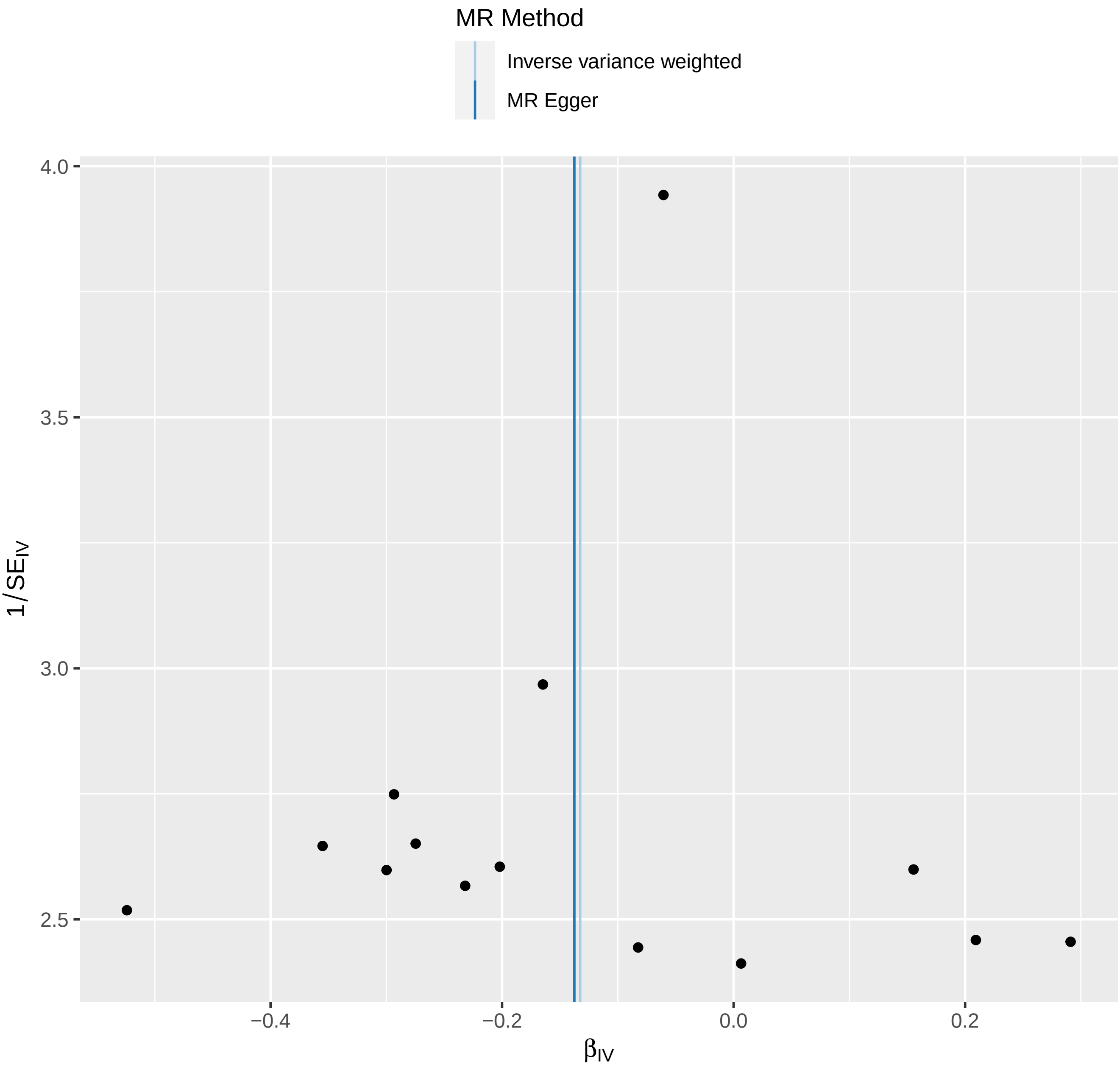

Batch 2−3501 : Gut microbiota abundance (genus Catenibacterium id.2153) on Gastric triacylglycerol lipase
with a threshold of 1e−05

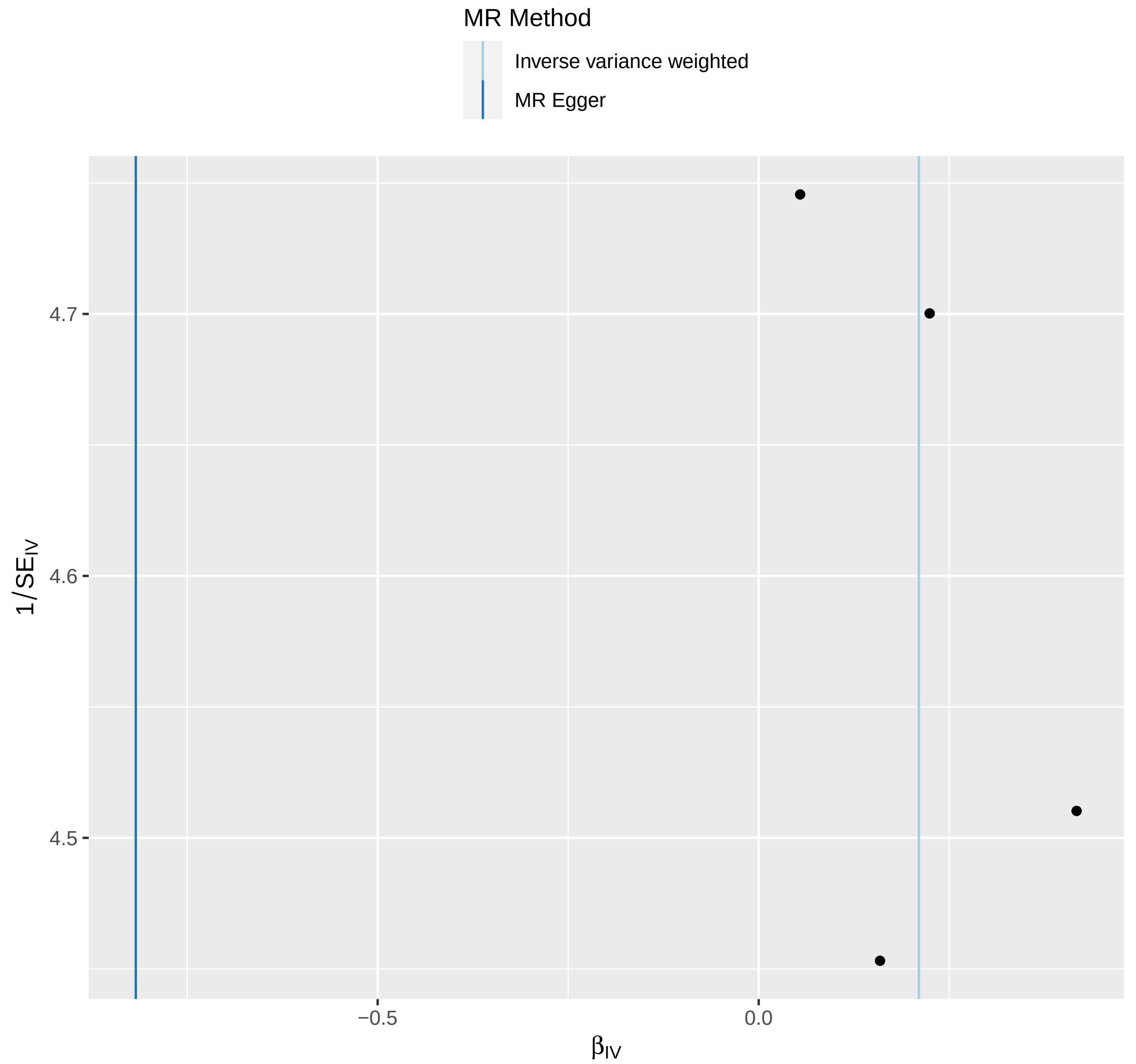

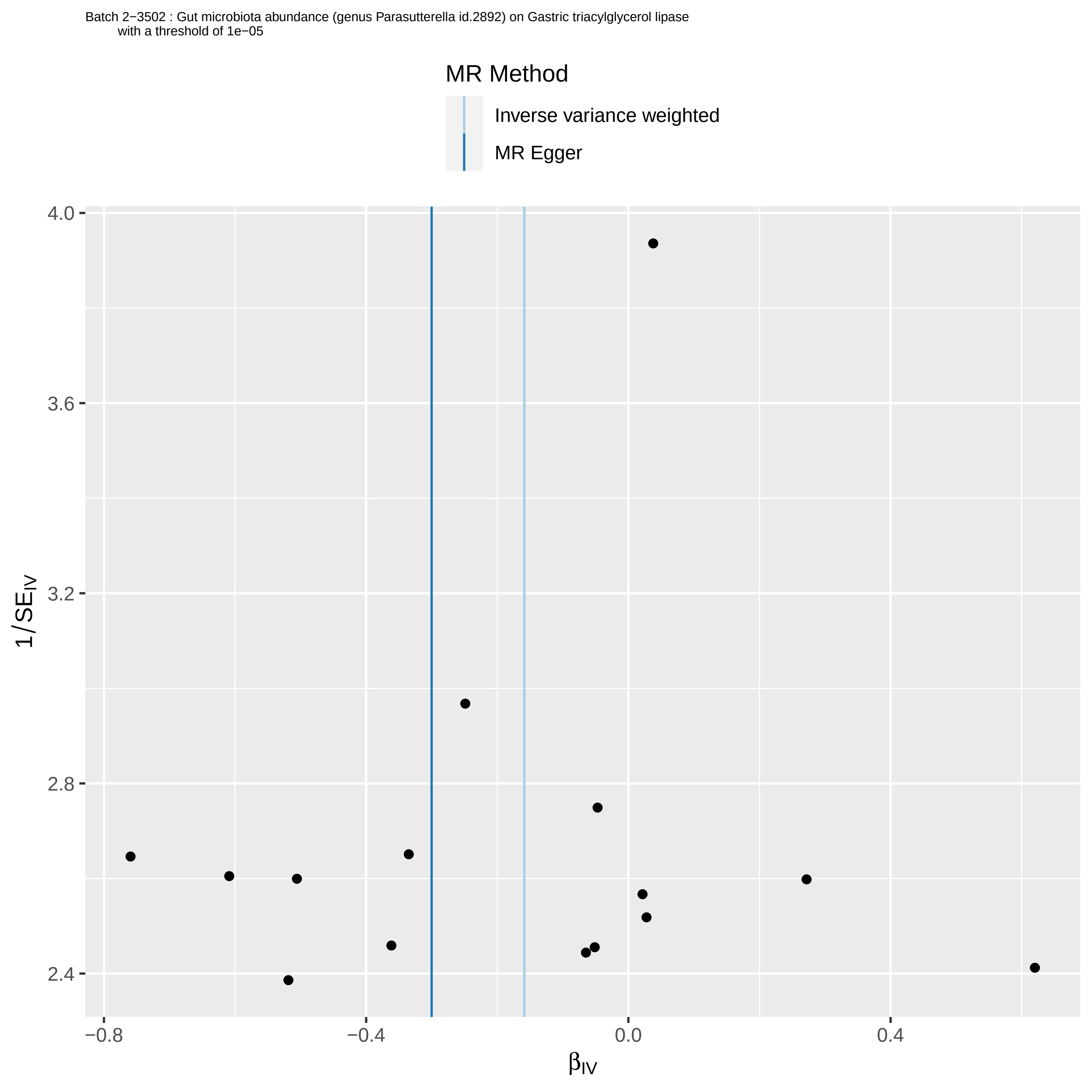

Batch 2−3502 : Gut microbiota abundance (genus Parasutterella id.2892) on Gastric triacylglycerol lipase
with a threshold of 1e−05
MR Method
Inverse variance weighted
MR Egger
4.0
3.6
3.2
2.8
2.4
1/SE_IV
−0.8
−0.4
0.0
0.4
β_IV

Batch 2−3503 : Gut microbiota abundance (genus Catenibacterium id.2153) on Lipase member K
with a threshold of 1e−05

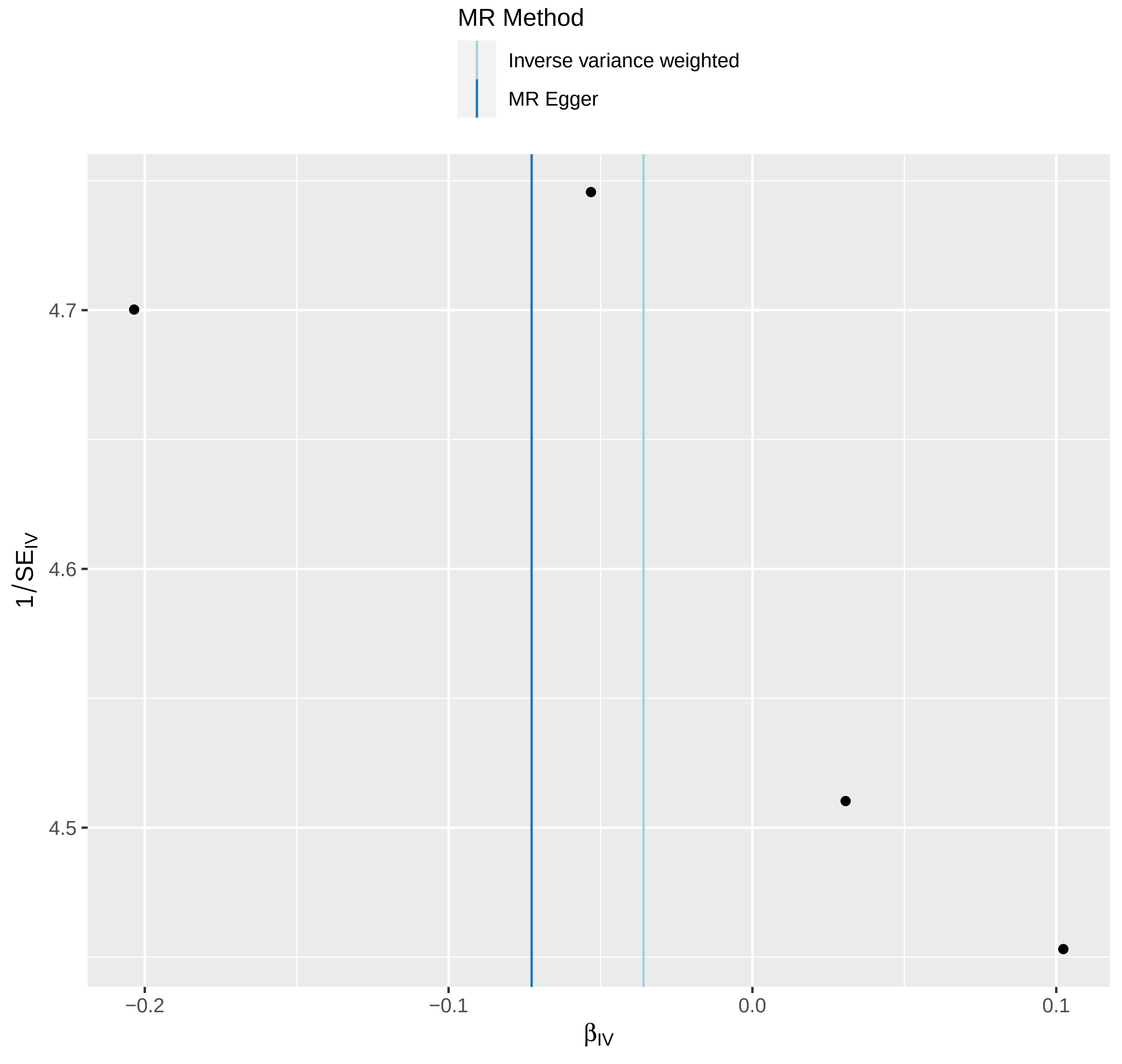

Batch 2−3504 : Gut microbiota abundance (genus Parasutterella id.2892) on Lipase member K
with a threshold of 1e−05

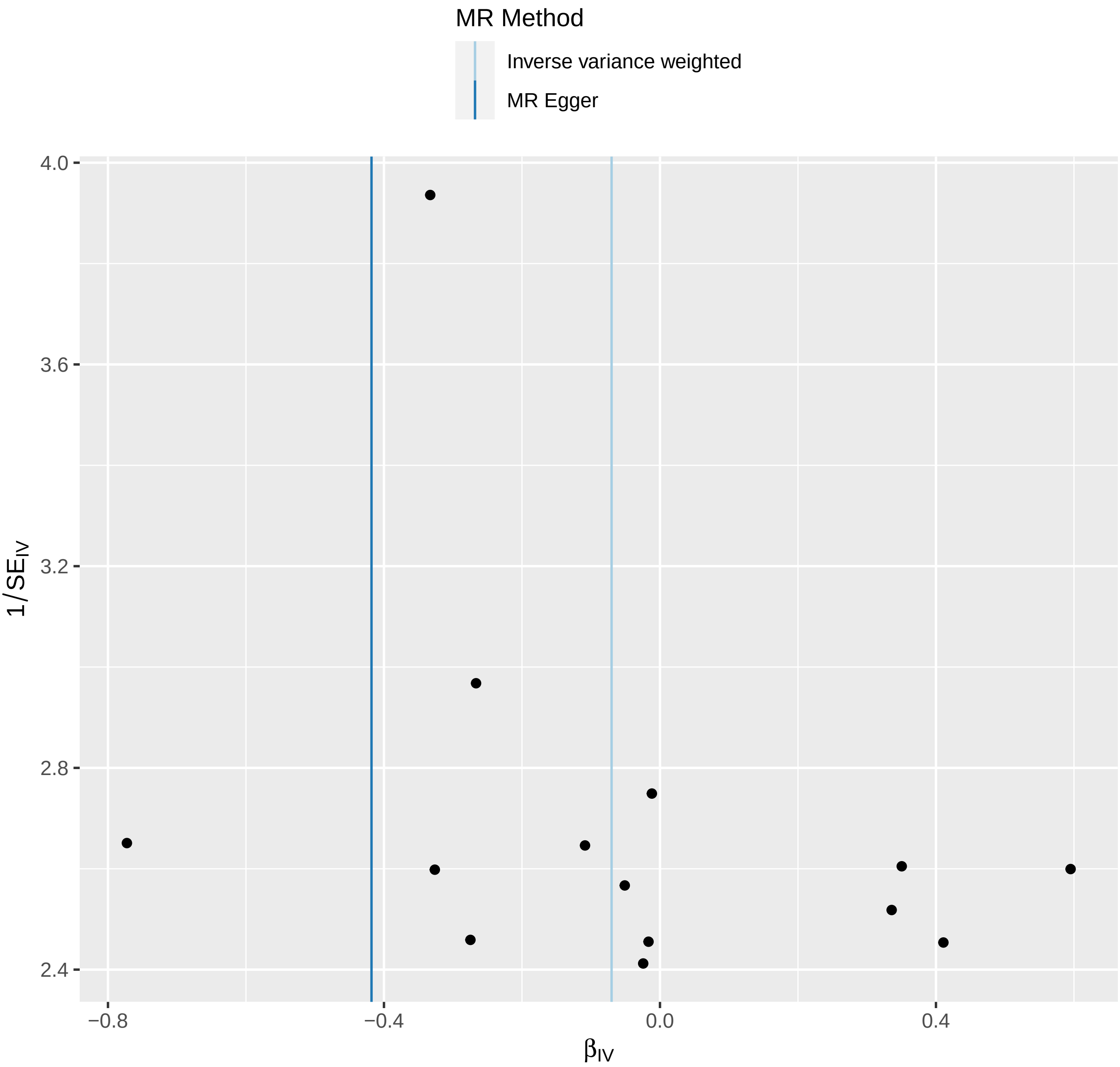

Batch 2−3505 : Gut microbiota abundance (genus Catenibacterium id.2153) on Lipase member N
with a threshold of 1e−05

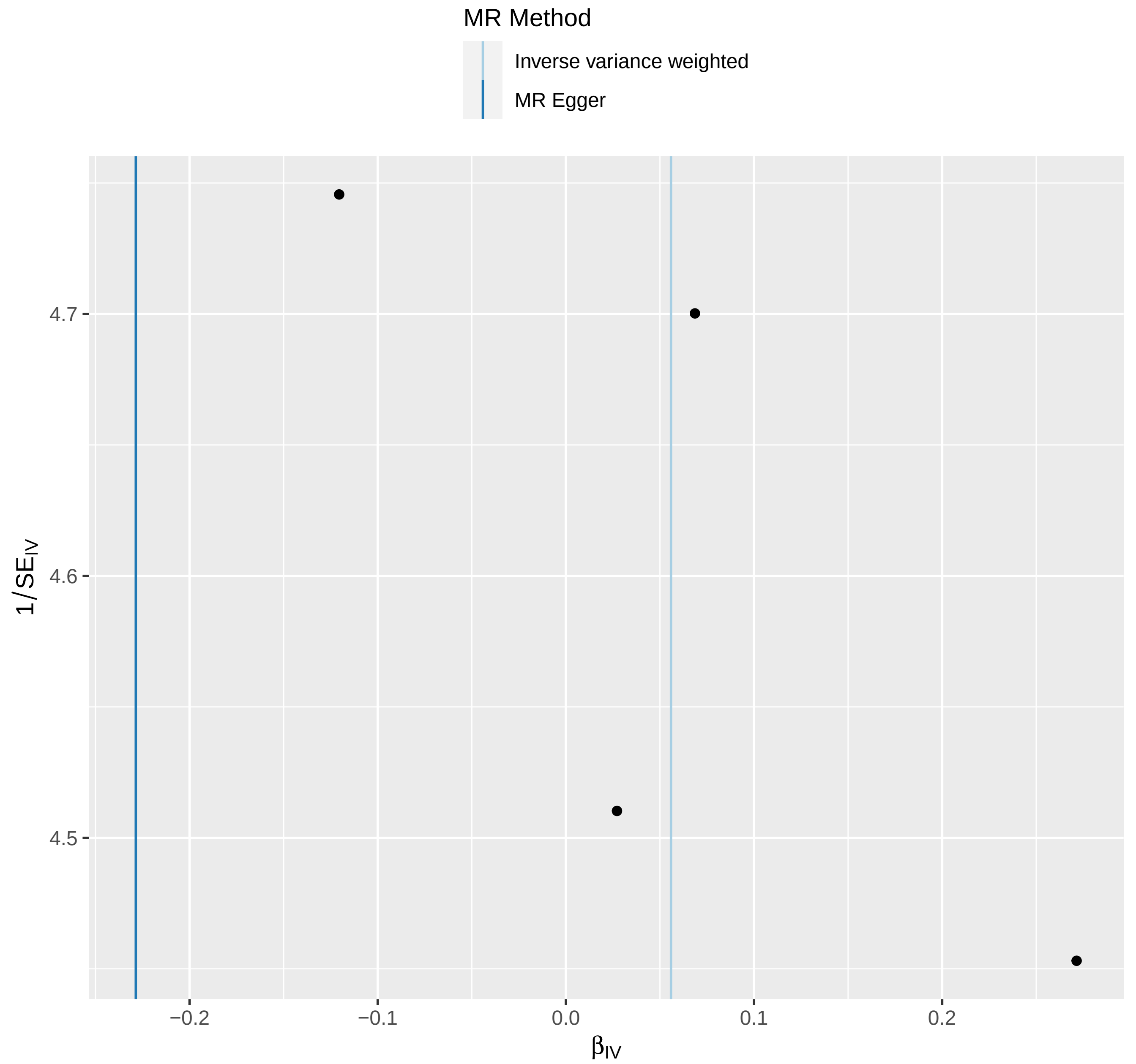

Batch 2−3506 : Gut microbiota abundance (genus Parasutterella id.2892) on Lipase member N
with a threshold of 1e−05

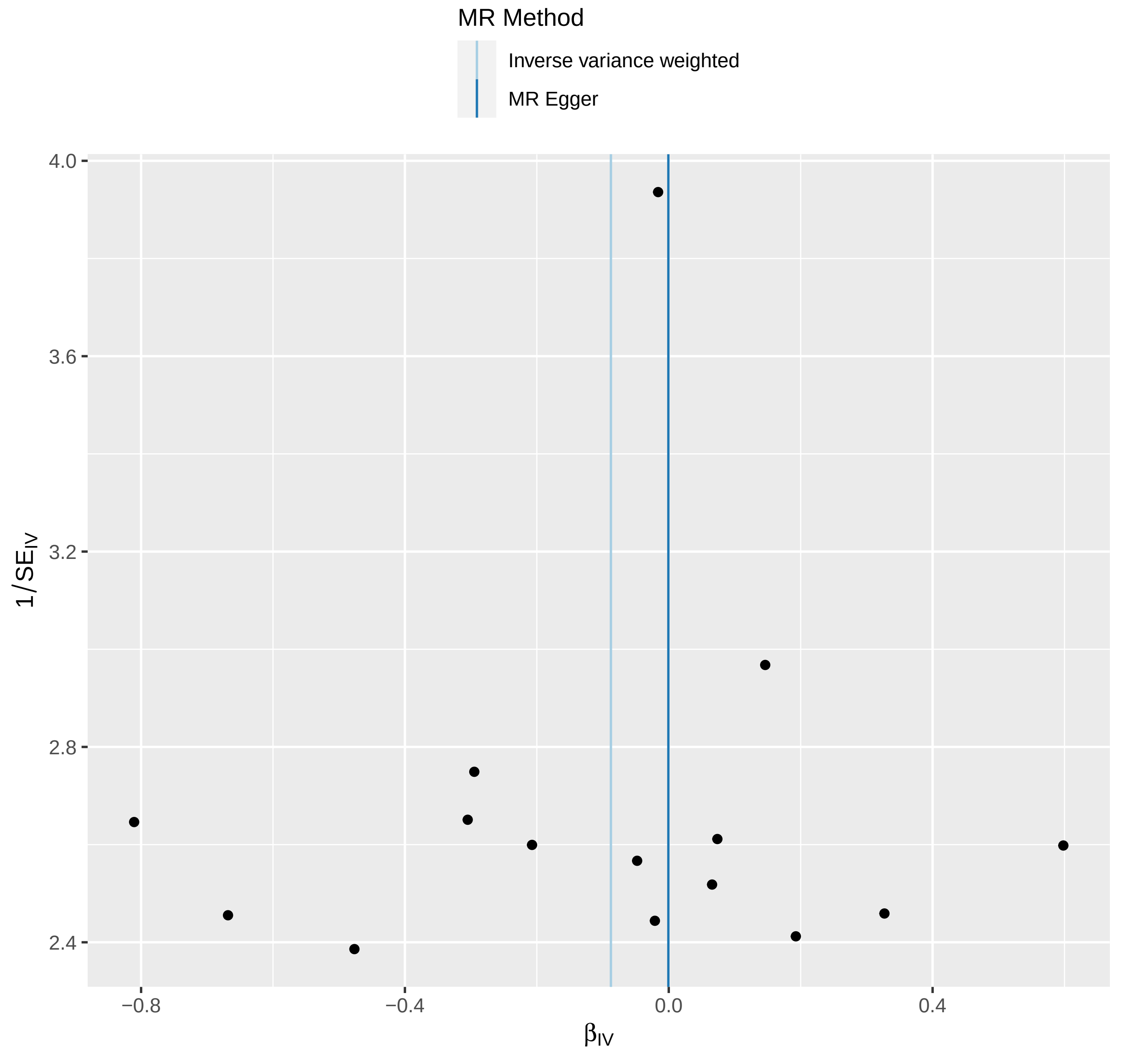

Batch 2−3507 : Gut microbiota abundance (genus Catenibacterium id.2153) on Vesicular integral−membrane protein VIP36
with a threshold of 1e−05

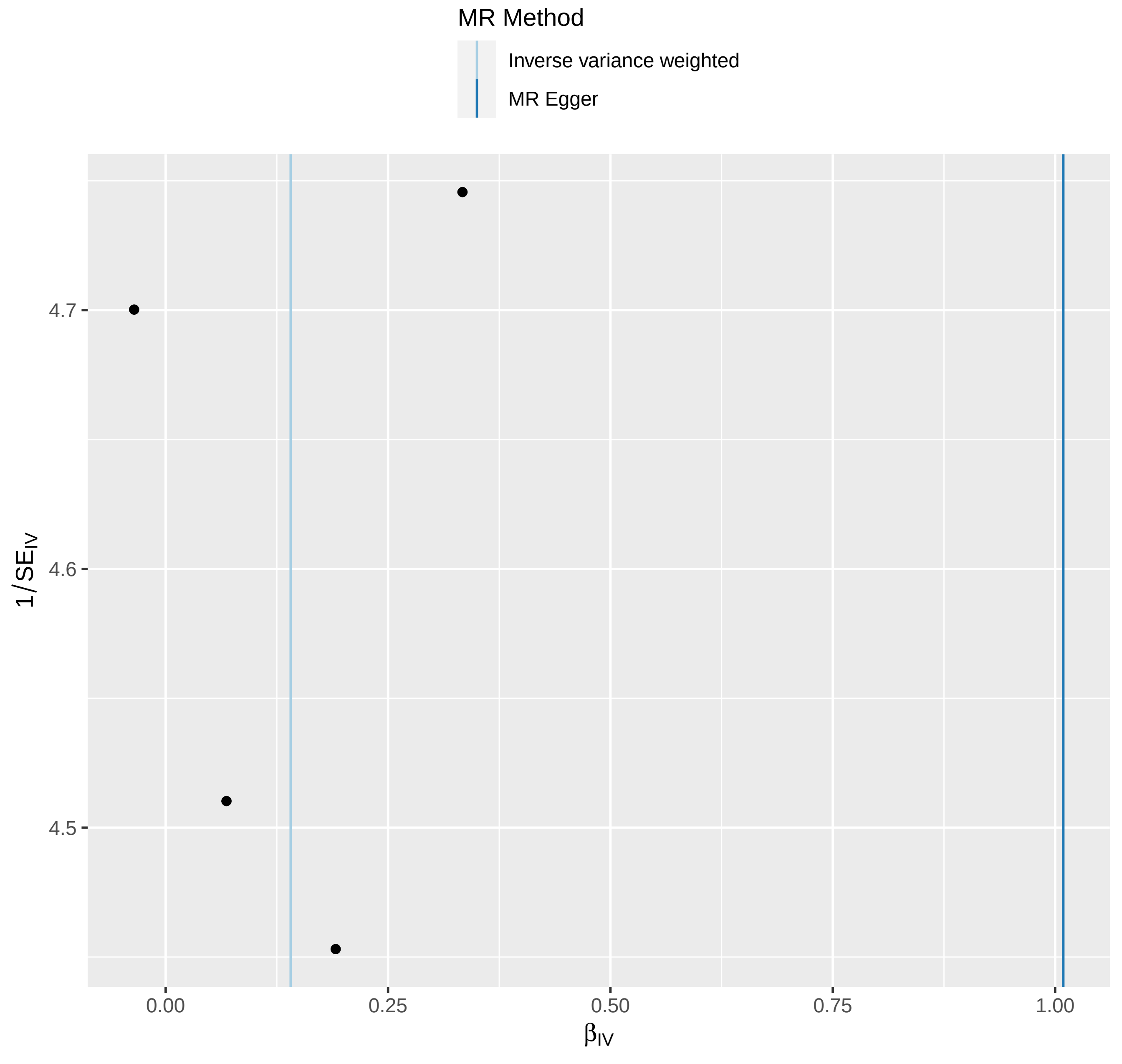

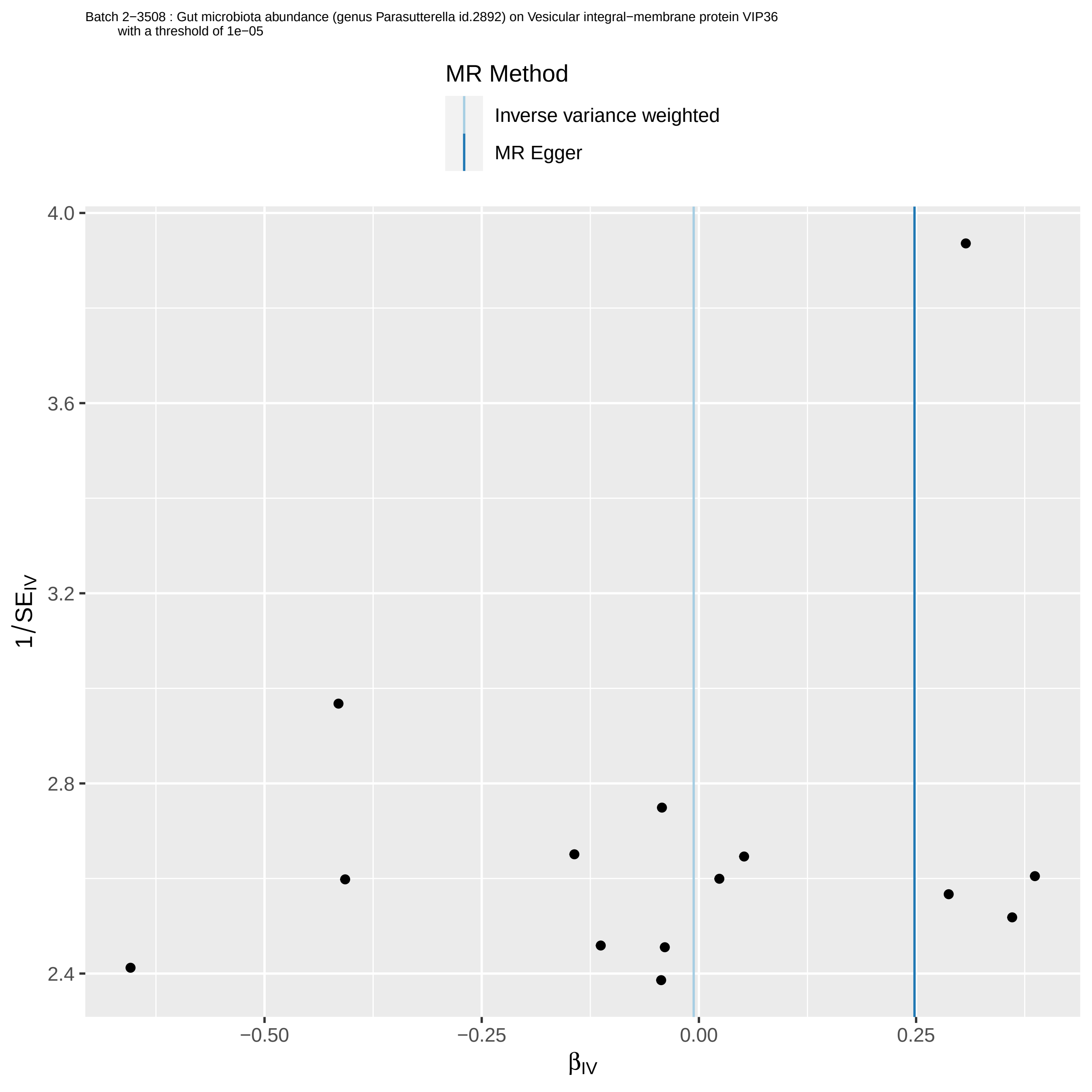

Batch 2−3508 : Gut microbiota abundance (genus Parasutterella id.2892) on Vesicular integral−membrane protein VIP36
with a threshold of 1e−05
MR Method
Inverse variance weighted
MR Egger
4.0
3.6
3.2
2.8
2.4
1/SE_IV
−0.50
−0.25
0.00
0.25
β_IV

Batch 2−3509 : Gut microbiota abundance (genus Catenibacterium id.2153) on Vesicular integral−membrane protein VIP36 with a threshold of 1e−05

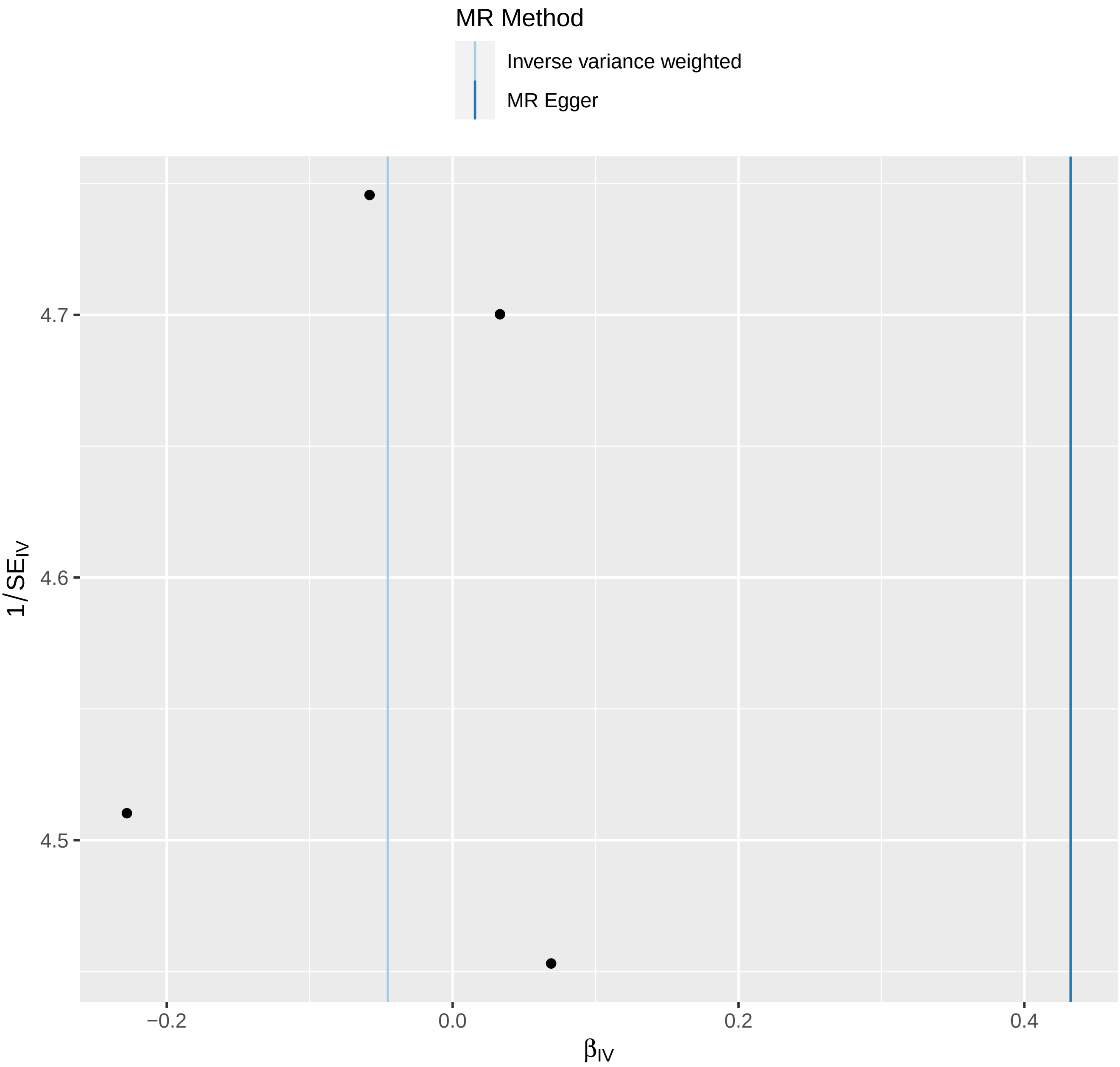

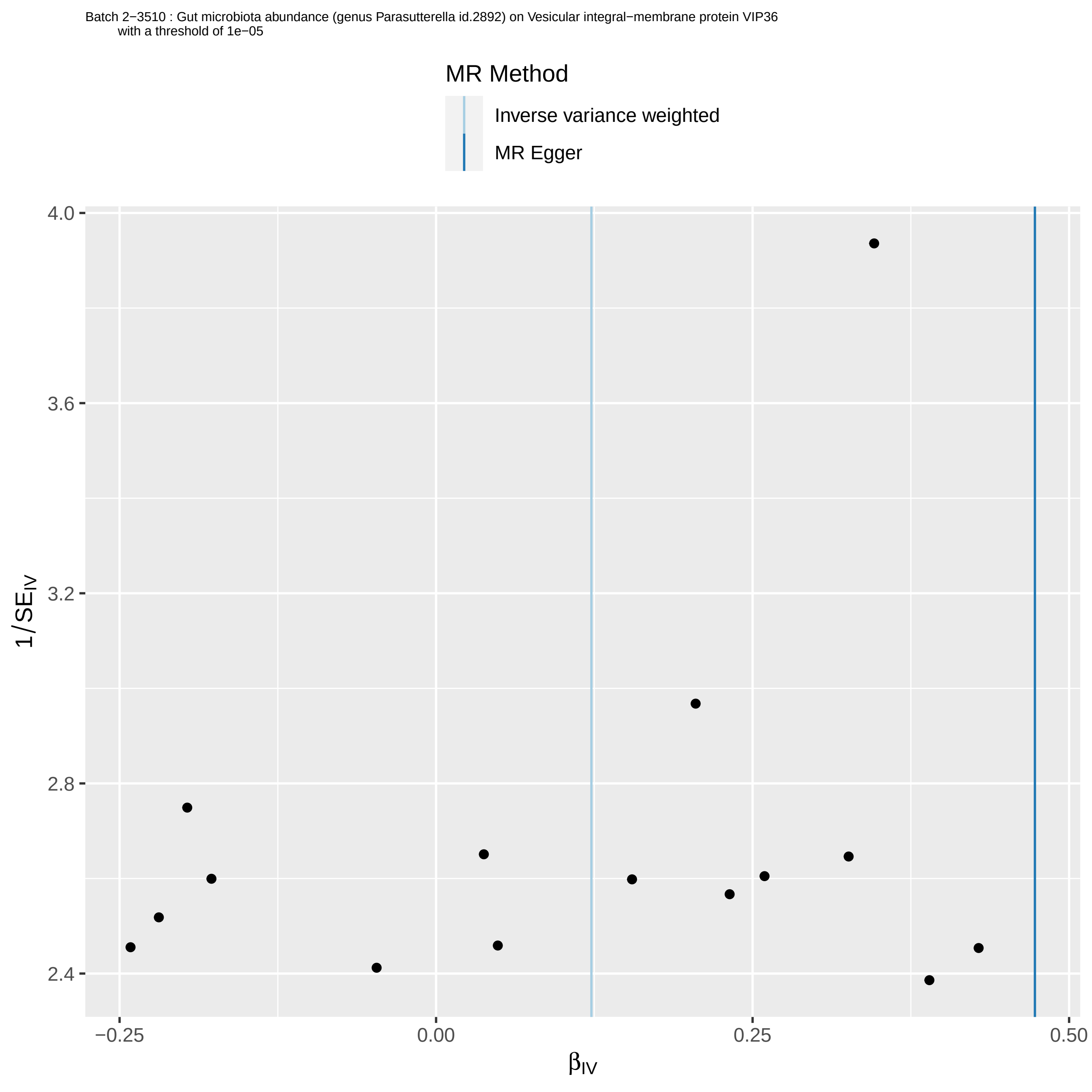
Batch 2−3510 : Gut microbiota abundance (genus Parasutterella id.2892) on Vesicular integral−membrane protein VIP36
with a threshold of 1e−05
MR Method
Inverse variance weighted
MR Egger
4.0
3.6
3.2
2.8
2.4
1/SE_IV
−0.25
0.00
0.25
0.50
β_IV

Batch 2−3511 : Gut microbiota abundance (genus Catenibacterium id.2153) on VIP36−like protein
with a threshold of 1e−05

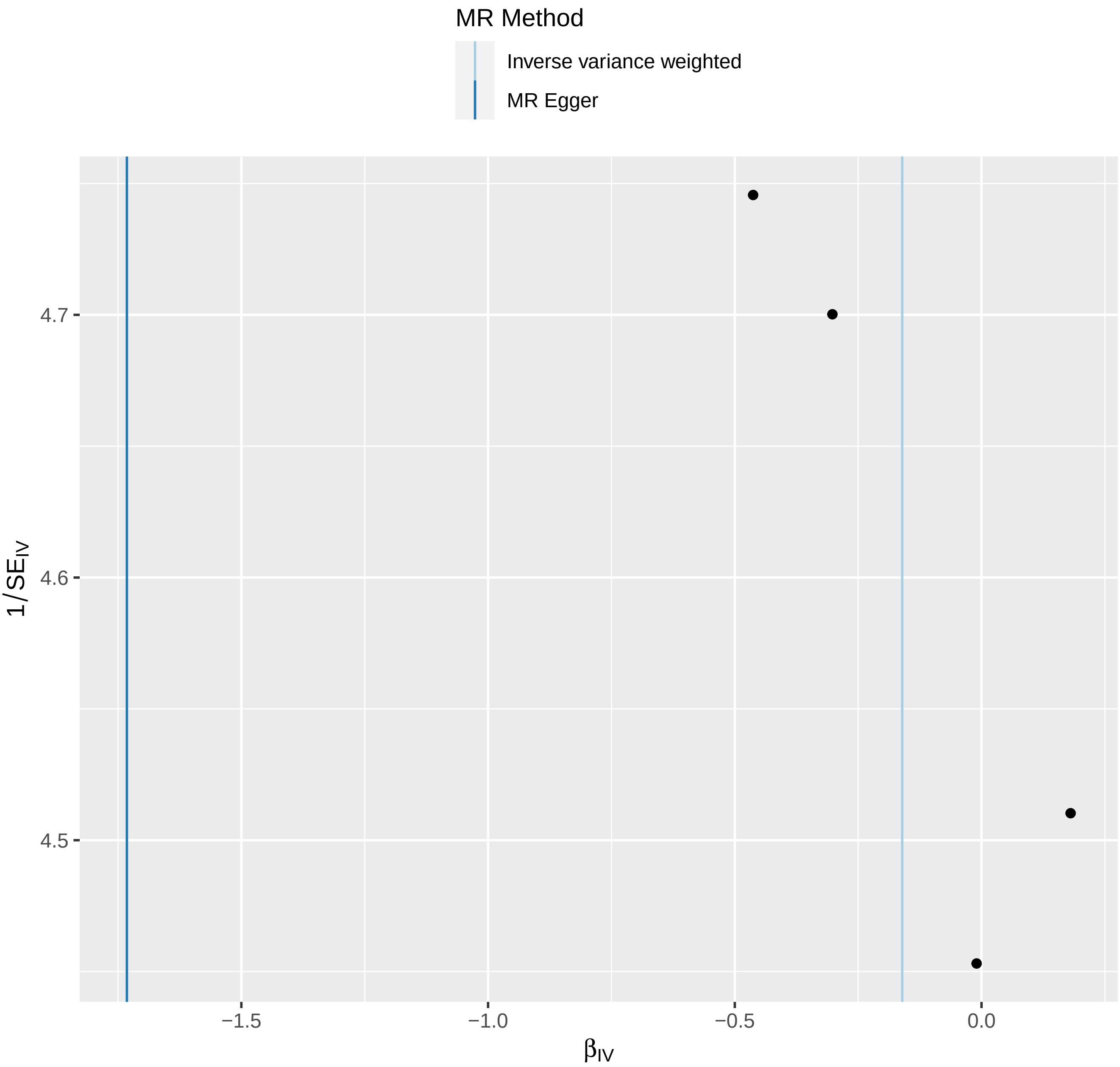

Batch 2−3512 : Gut microbiota abundance (genus Parasutterella id.2892) on VIP36−like protein
with a threshold of 1e−05

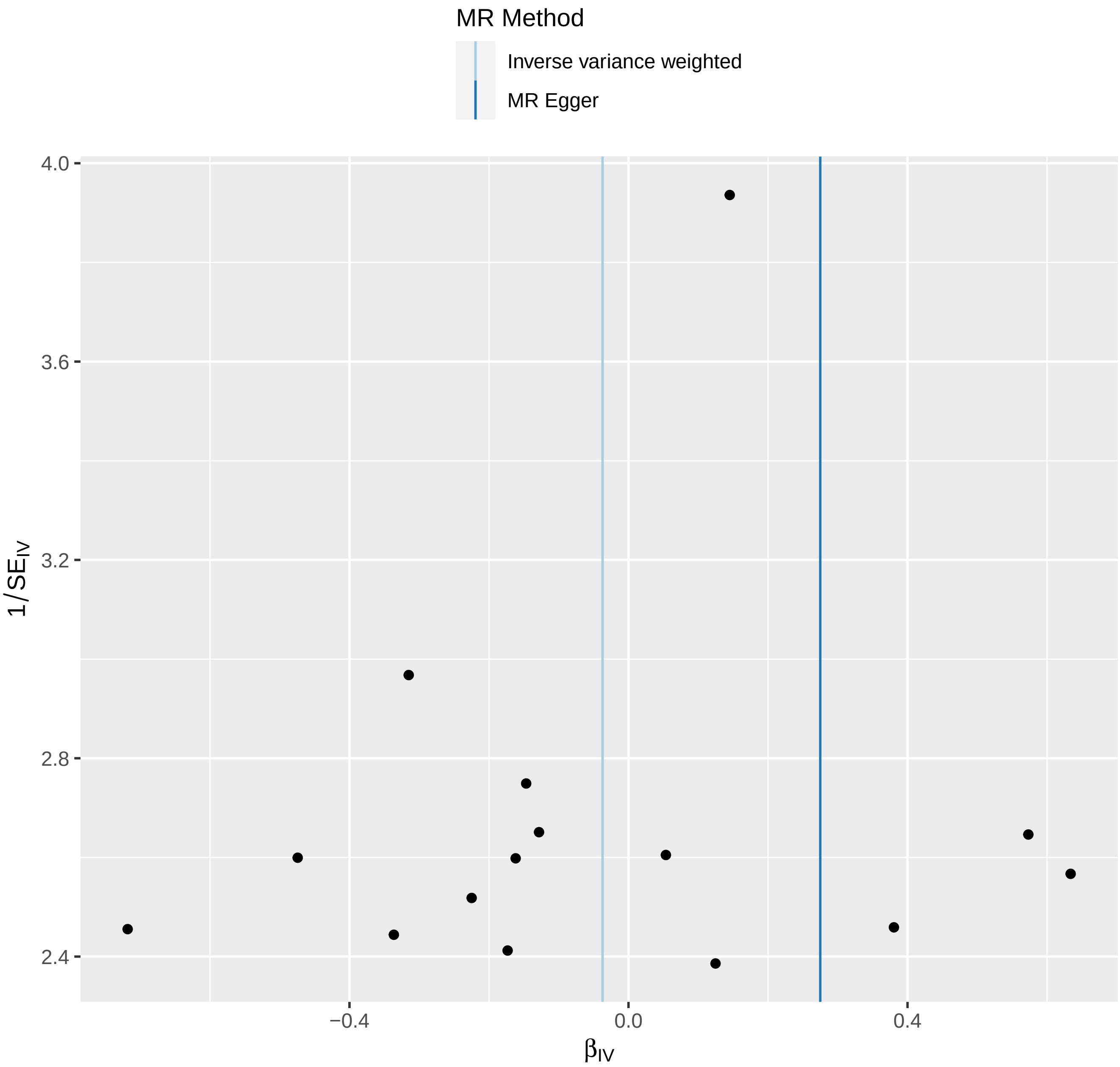

Batch 2−3513 : Gut microbiota abundance (genus Catenibacterium id.2153) on LIM and cysteine−rich domains protein 1
with a threshold of 1e−05

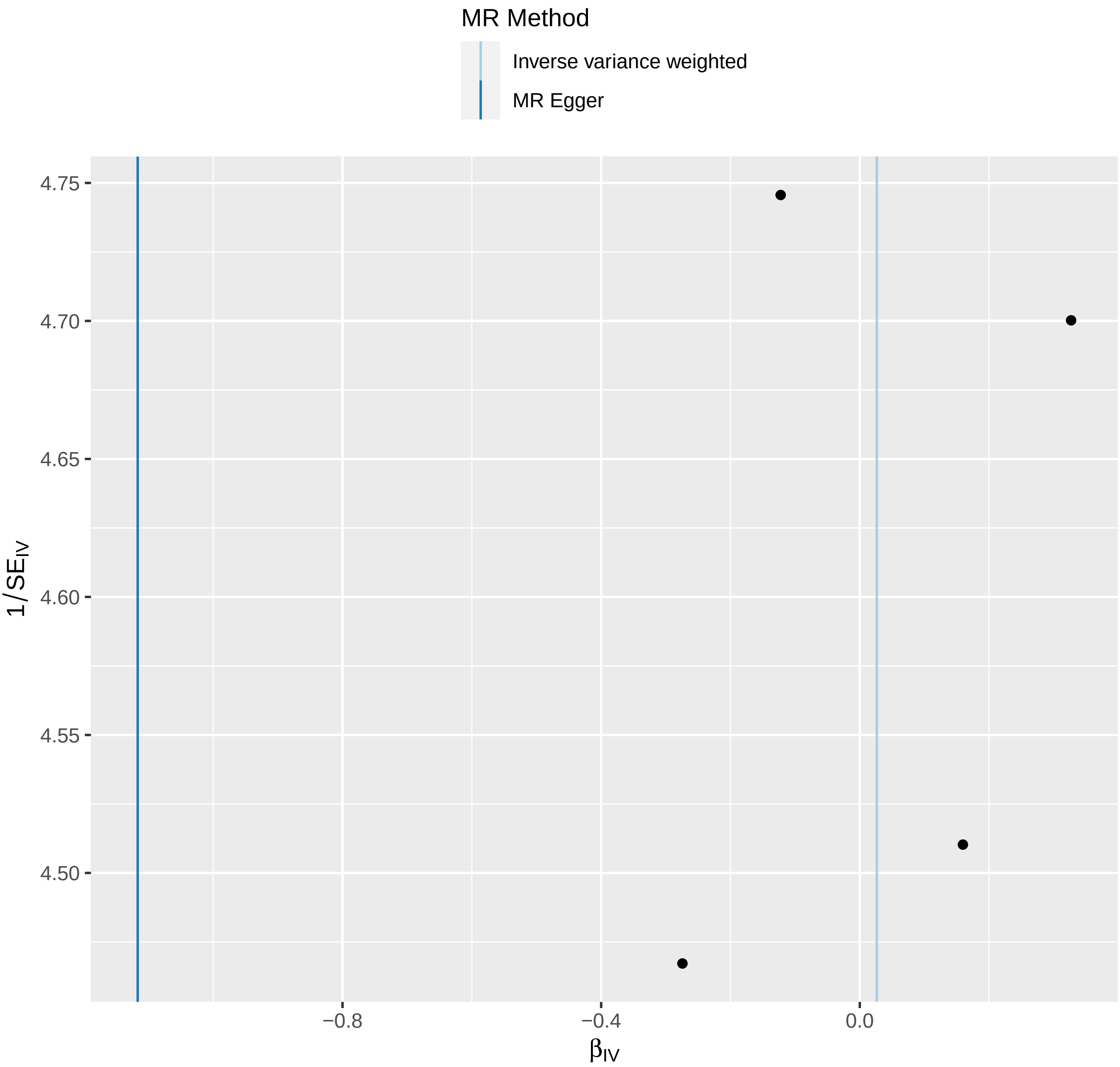

Batch 2−3514 : Gut microbiota abundance (genus Parasutterella id.2892) on LIM and cysteine−rich domains protein 1 with a threshold of 1e−05

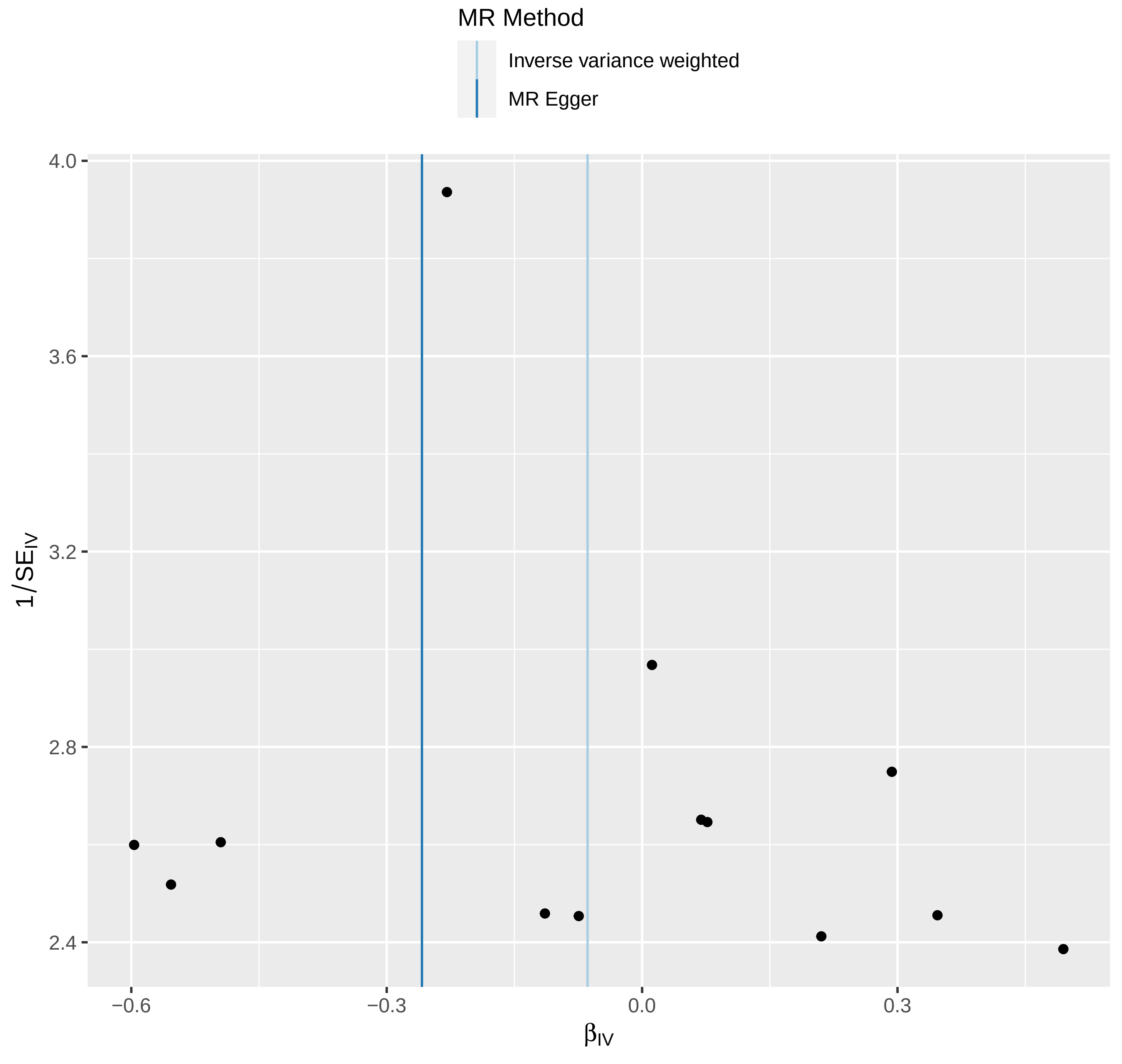

Batch 2−3515 : Gut microbiota abundance (genus Catenibacterium id.2153) on Lamin−B1
with a threshold of 1e−05

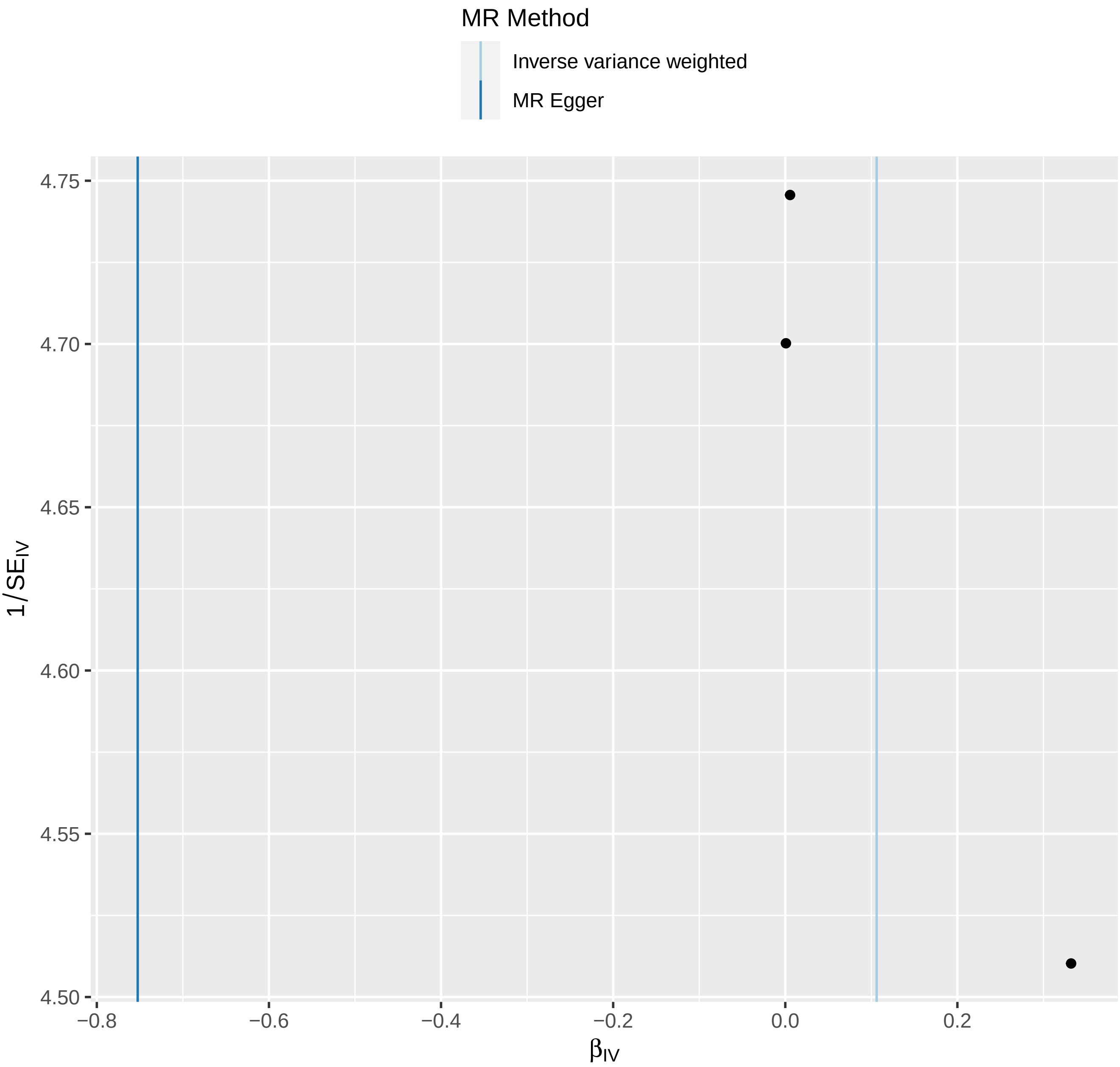

Batch 2−3516 : Gut microbiota abundance (genus Parasutterella id.2892) on Lamin−B1
with a threshold of 1e−05

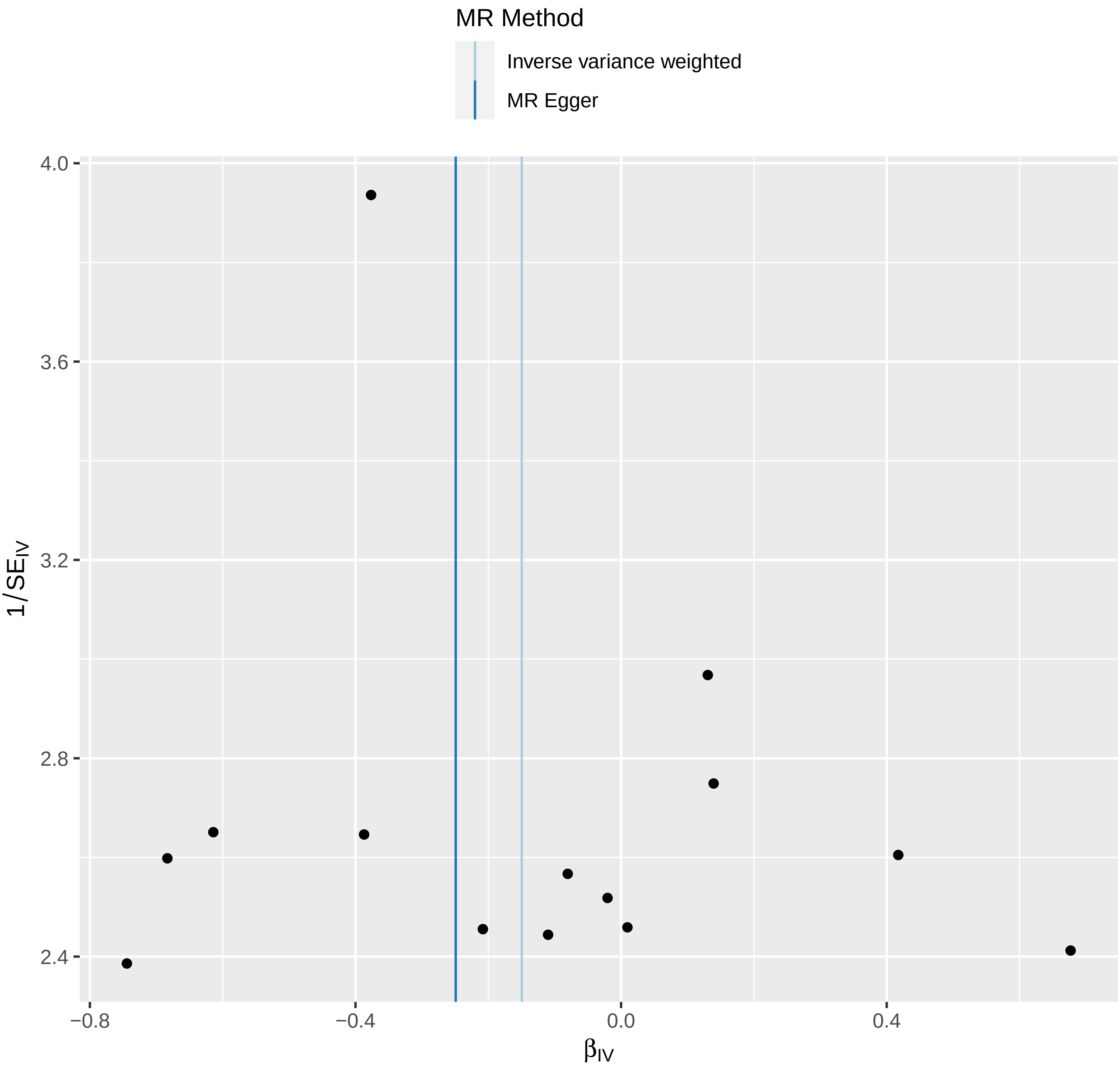

Batch 2−3517 : Gut microbiota abundance (genus Catenibacterium id.2153) on Leiomodin−1
with a threshold of 1e−05

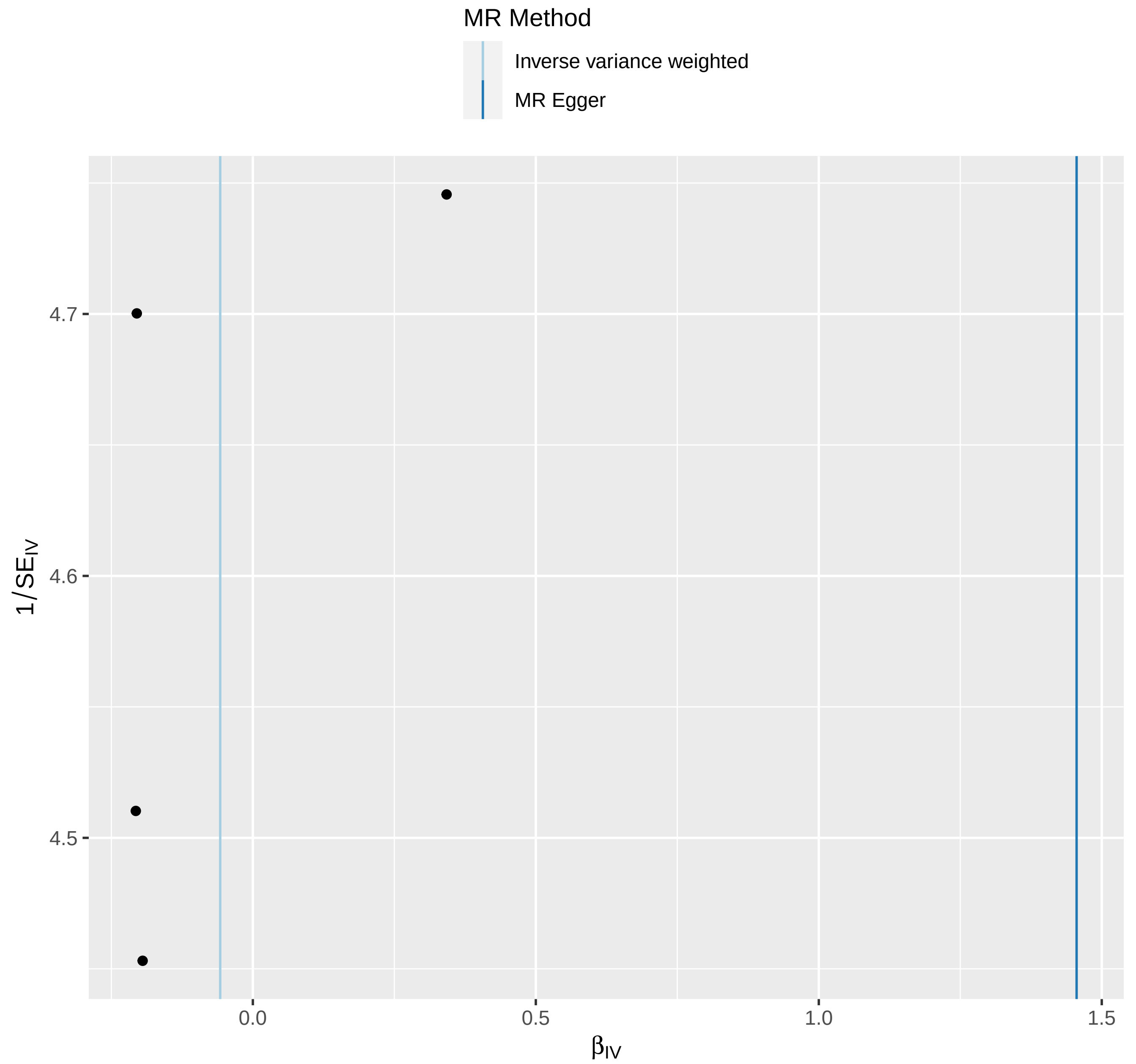

Batch 2−3518 : Gut microbiota abundance (genus Parasutterella id.2892) on Leiomodin−1
with a threshold of 1e−05

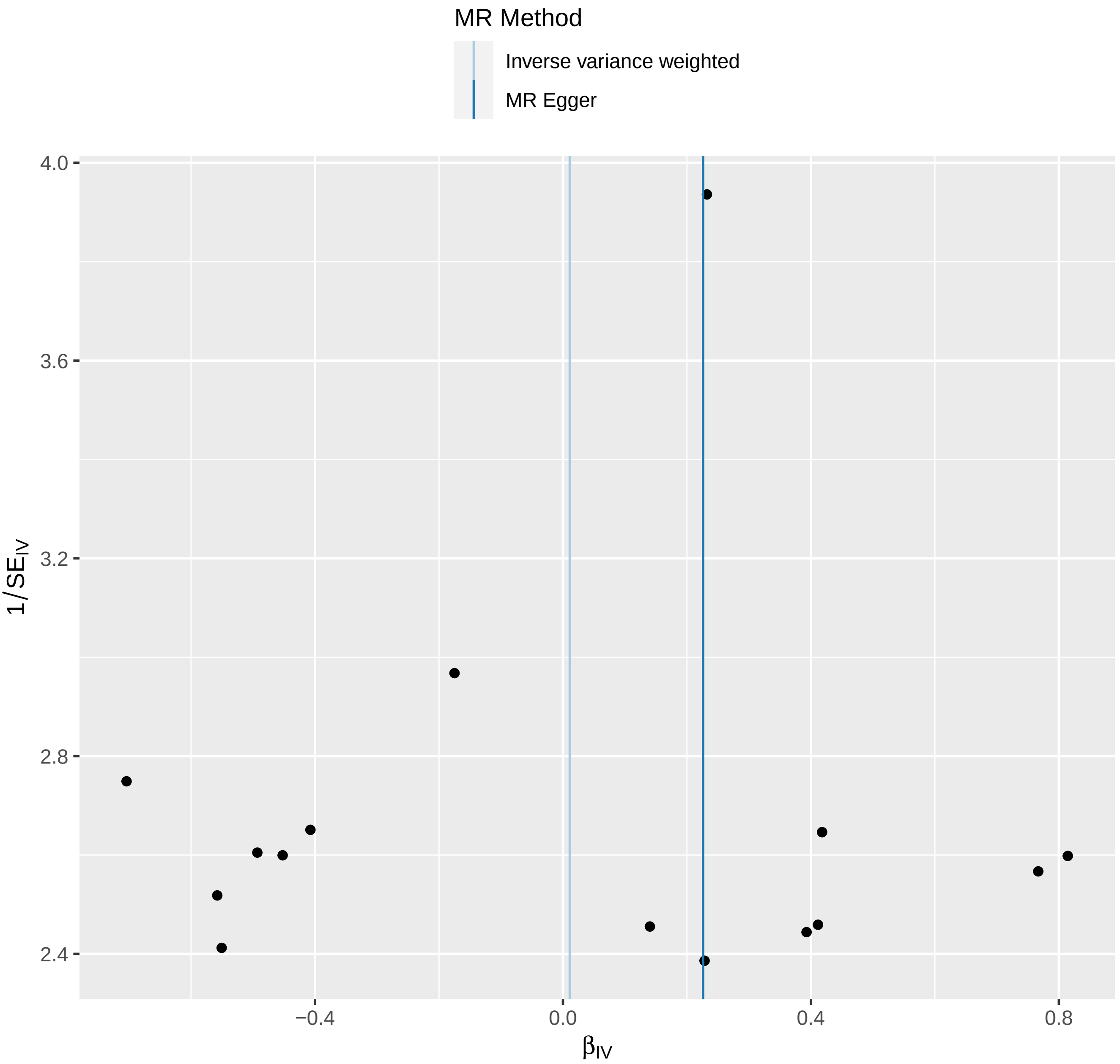

Batch 2−3519 : Gut microbiota abundance (genus Catenibacterium id.2153) on Ig Kappa chain V−I region HK102− like
with a threshold of 1e−05

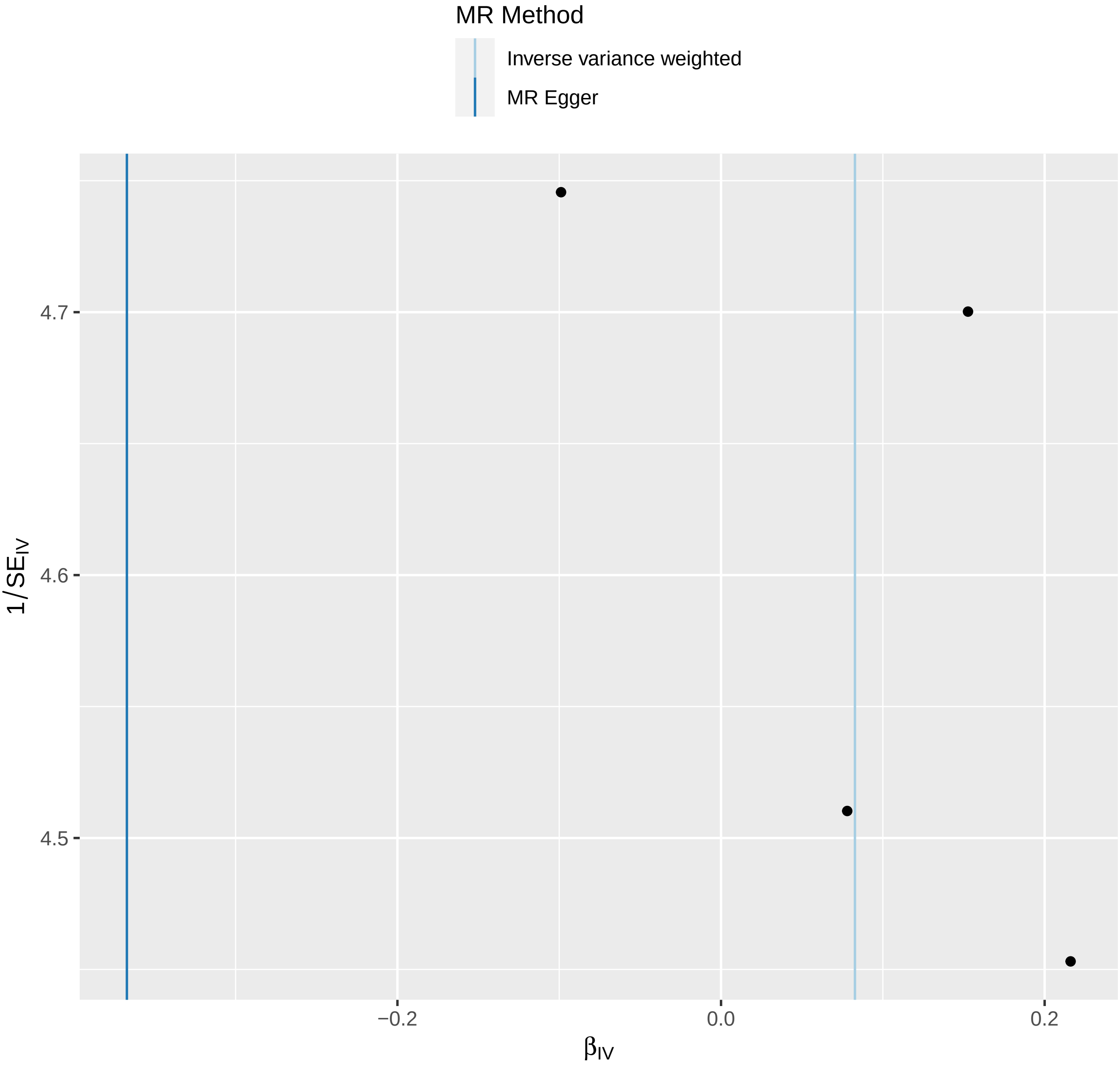

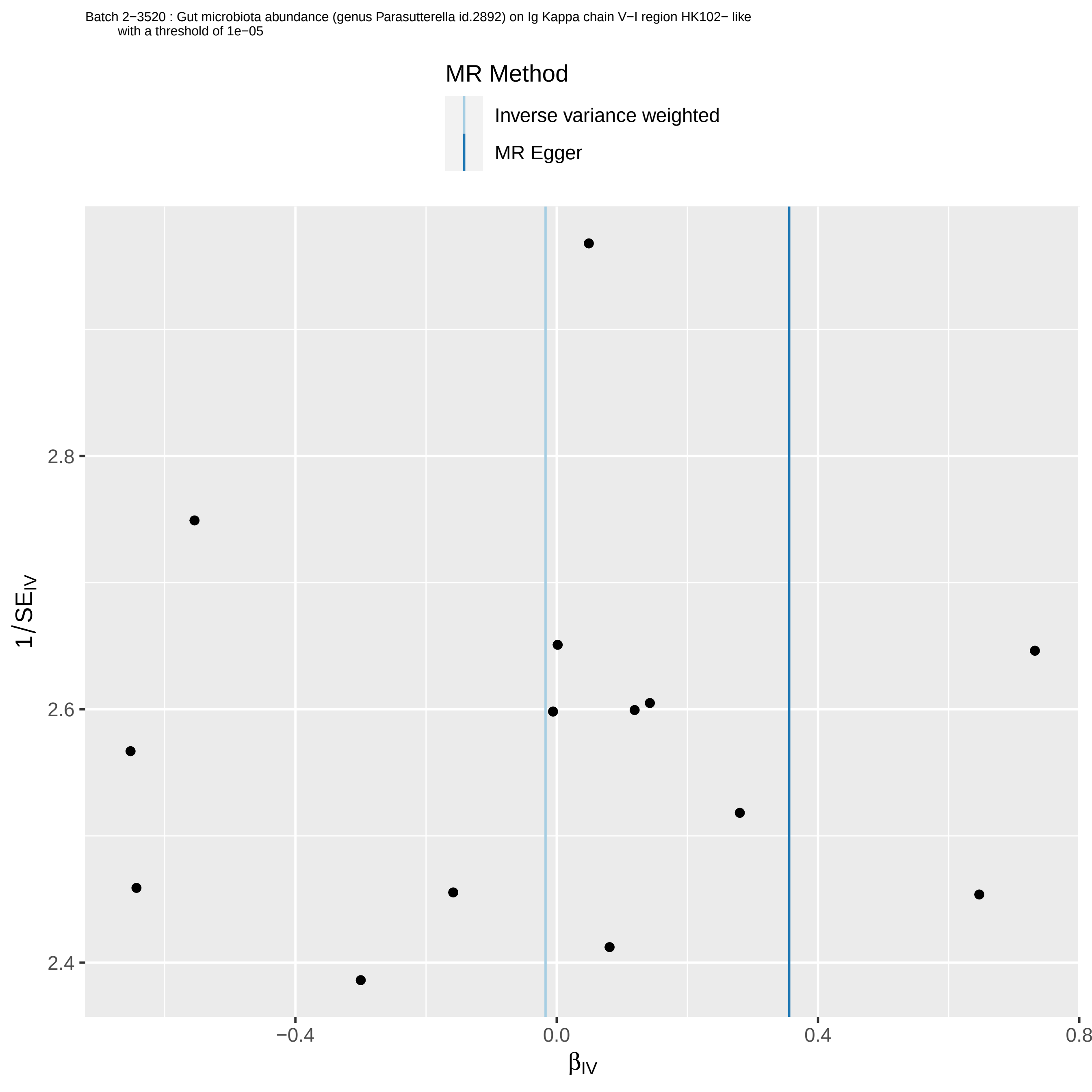

Batch 2−3520 : Gut microbiota abundance (genus Parasutterella id.2892) on Ig Kappa chain V−I region HK102− like
with a threshold of 1e−05
MR Method
Inverse variance weighted
MR Egger
2.8
2.6
2.4
1/SE_IV
−0.4
0.0
0.4
0.8
β_IV

Batch 2−3521 : Gut microbiota abundance (genus Catenibacterium id.2153) on Lon protease homolog, mitochondrial
with a threshold of 1e−05

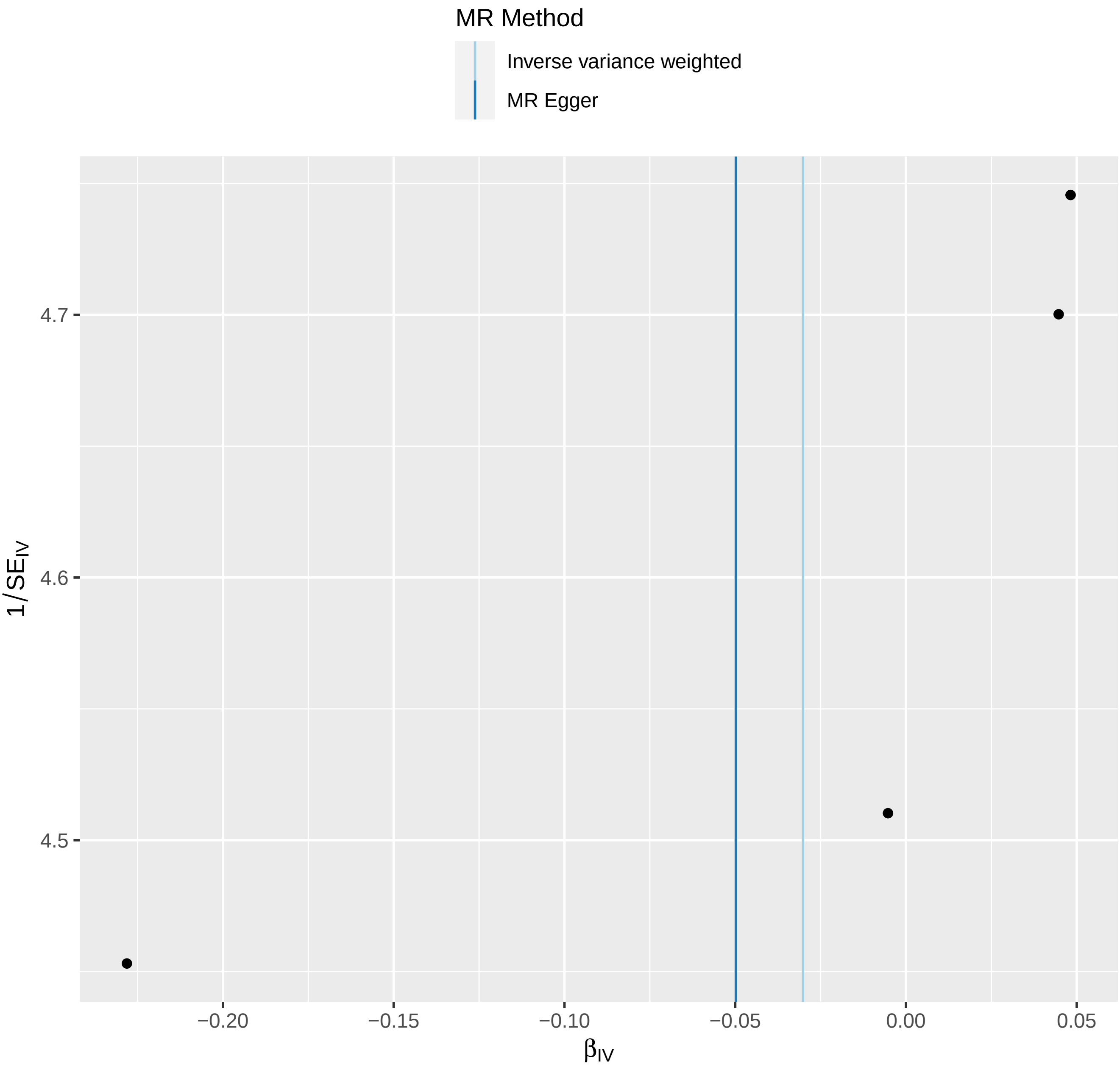

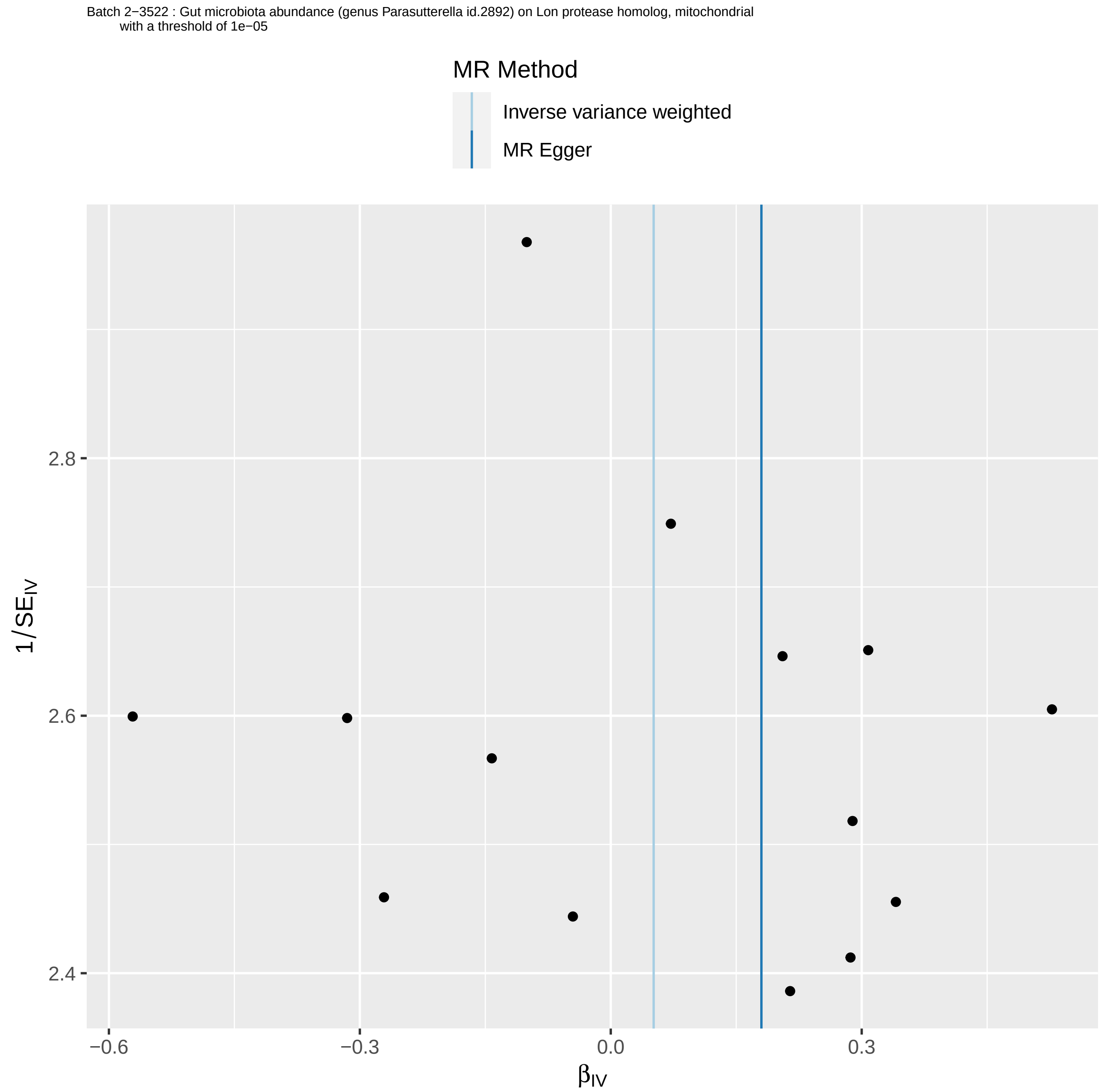
Batch 2−3522 : Gut microbiota abundance (genus Parasutterella id.2892) on Lon protease homolog, mitochondrial
with a threshold of 1e−05
MR Method
Inverse variance weighted
MR Egger
2.8
2.6
2.4
1/SE_IV
−0.6
−0.3
0.0
0.3
β_IV

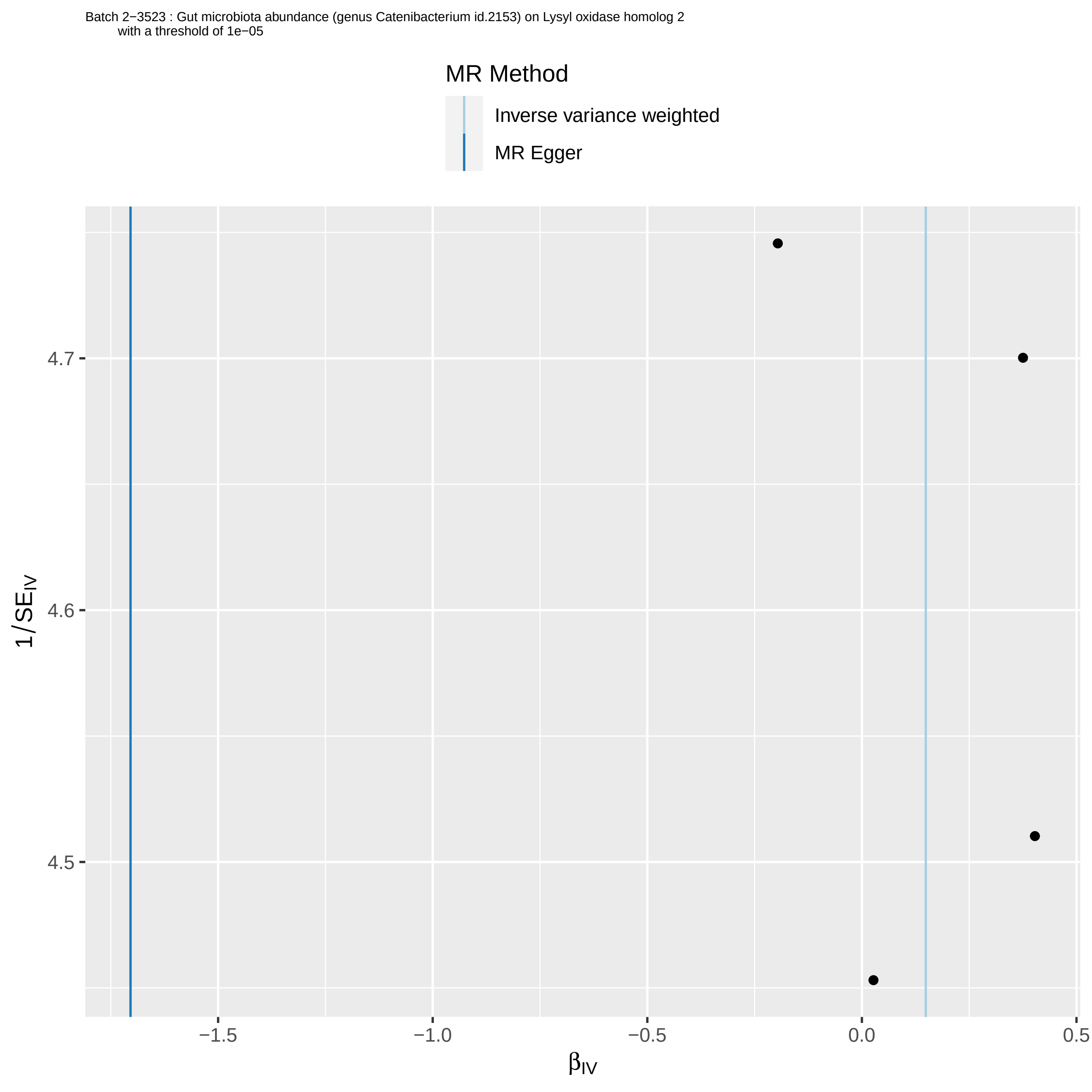

Batch 2−3523 : Gut microbiota abundance (genus Catenibacterium id.2153) on Lysyl oxidase homolog 2
with a threshold of 1e−05
MR Method
Inverse variance weighted
MR Egger
4.7
4.6
4.5
1/SE_IV
−1.5
−1.0
−0.5
0.0
0.5
β_IV

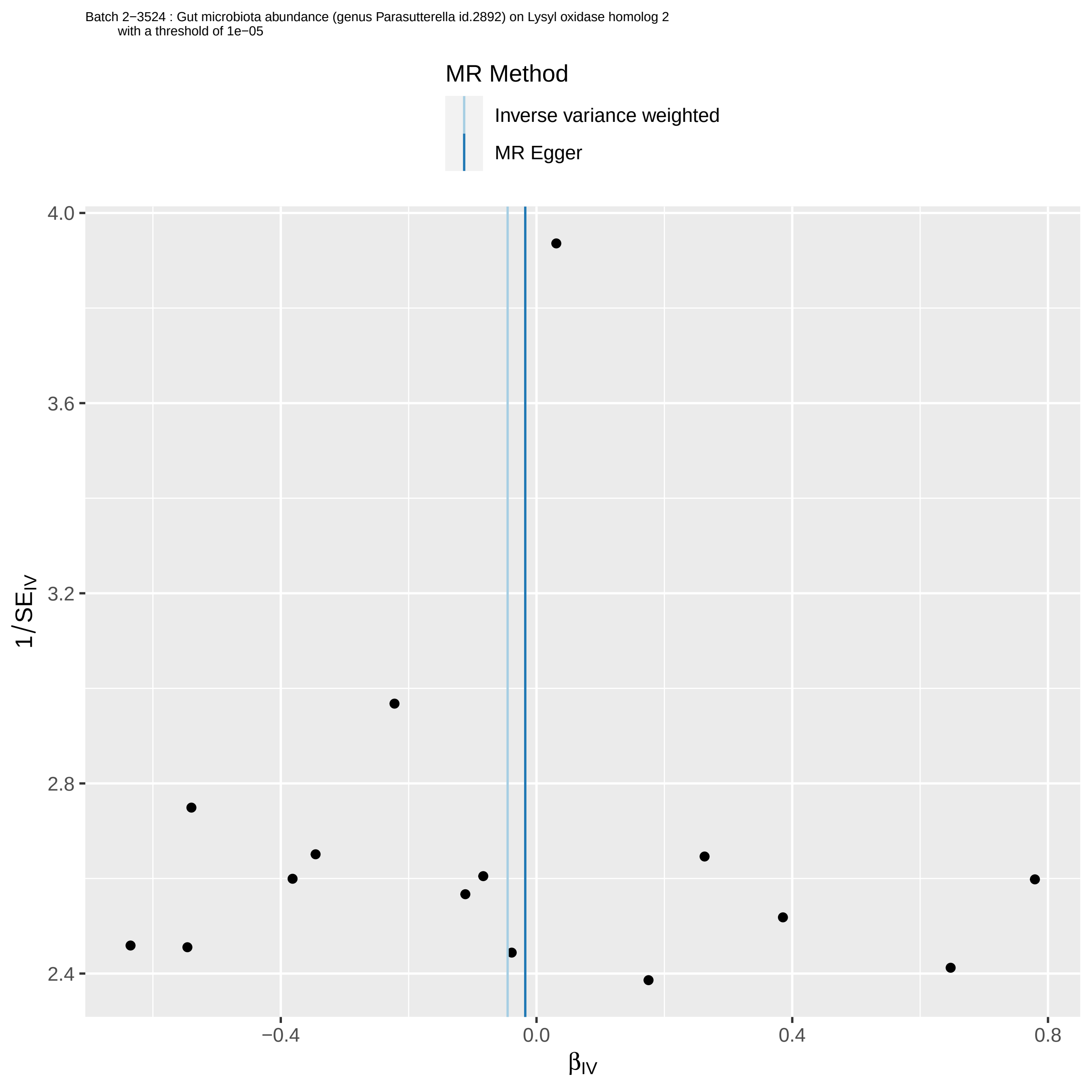

Batch 2−3524 : Gut microbiota abundance (genus Parasutterella id.2892) on Lysyl oxidase homolog 2
with a threshold of 1e−05
MR Method
Inverse variance weighted
MR Egger
4.0
3.6
3.2
2.8
2.4
1/SE_IV
−0.4
0.0
0.4
0.8
β_IV

Batch 2−3525 : Gut microbiota abundance (genus Catenibacterium id.2153) on Putative apolipoprotein(a)−like protein 2
with a threshold of 1e−05

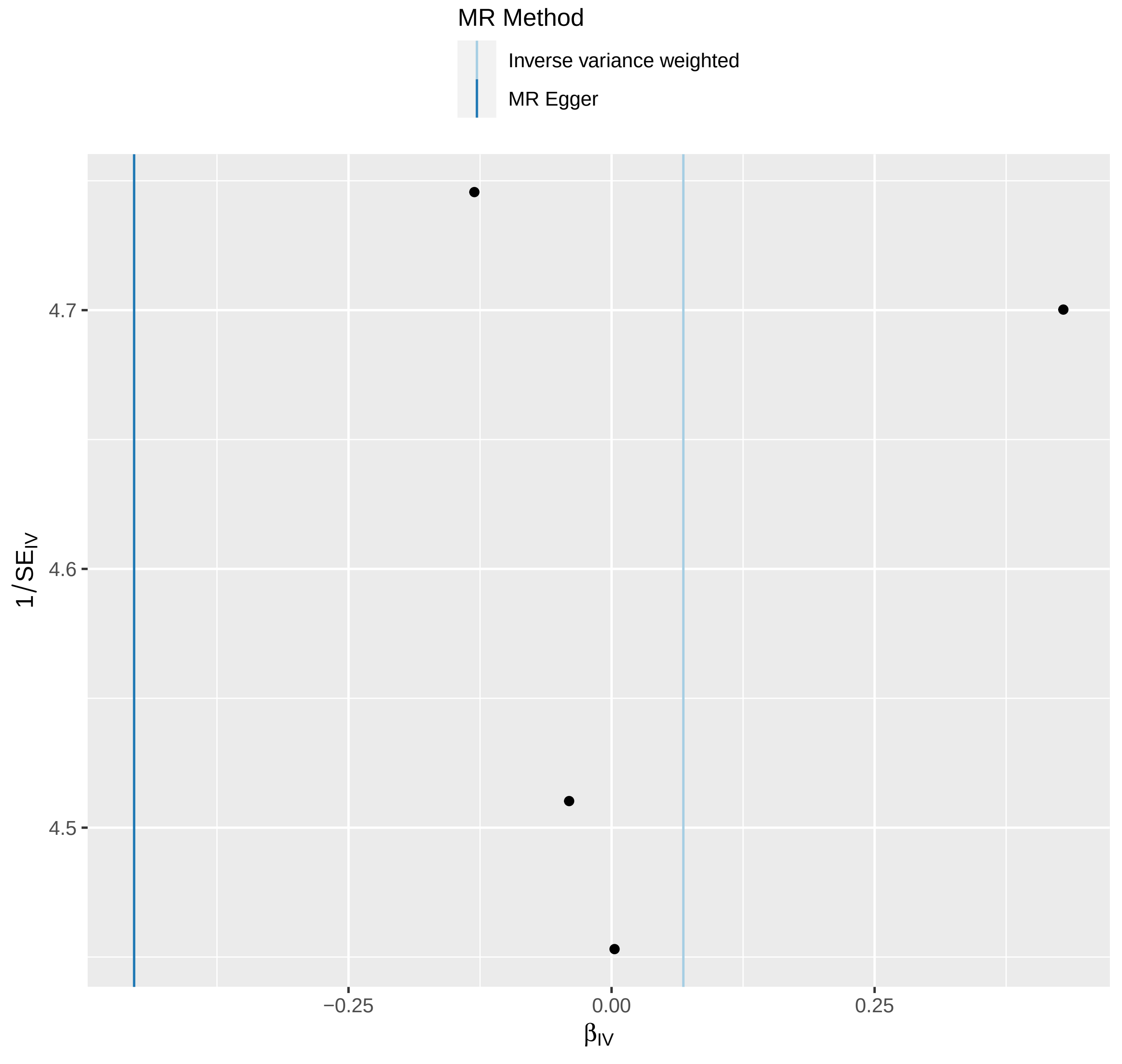

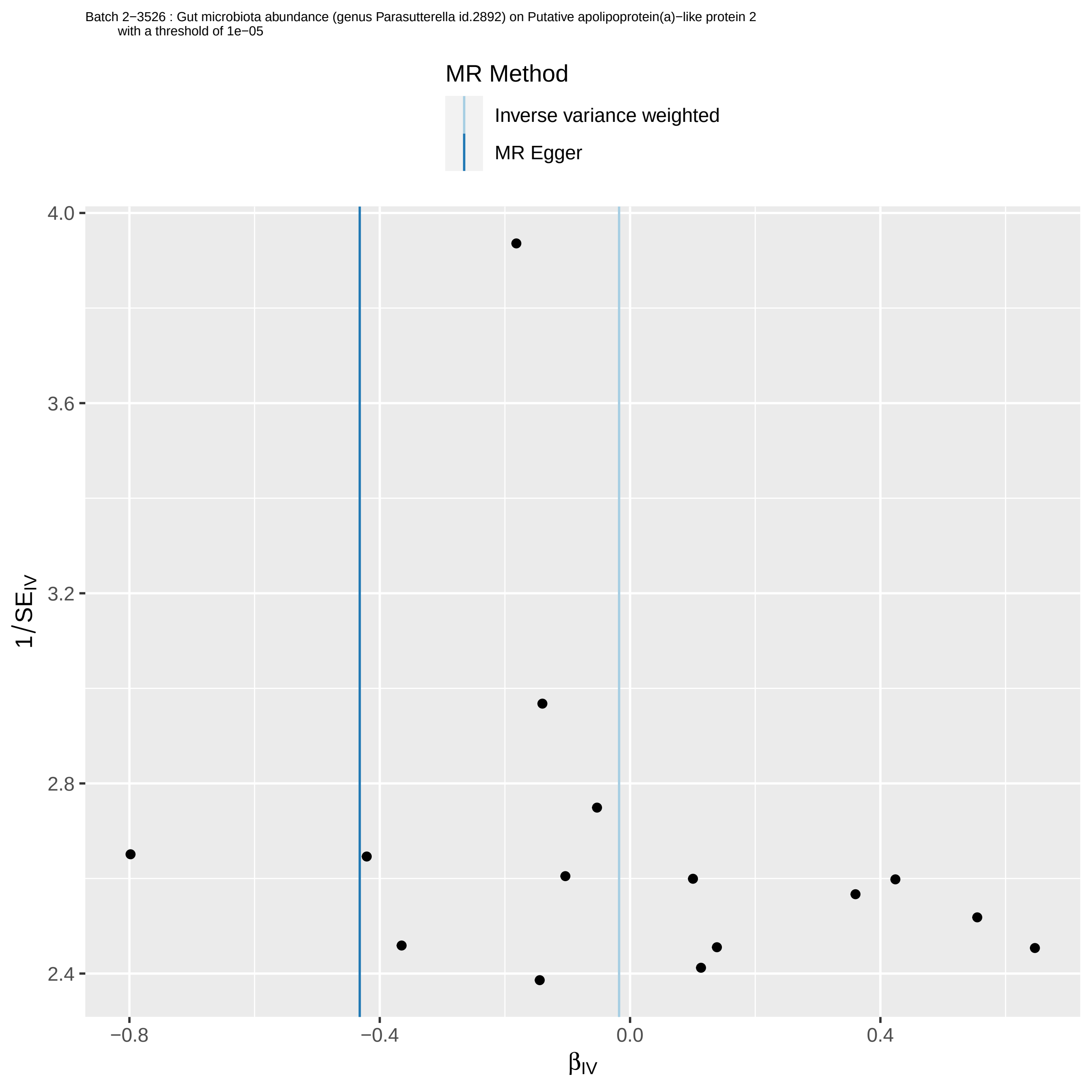
Batch 2−3526 : Gut microbiota abundance (genus Parasutterella id.2892) on Putative apolipoprotein(a)−like protein 2
with a threshold of 1e−05
MR Method
Inverse variance weighted
MR Egger
4.0
3.6
3.2
2.8
2.4
1/SE_IV
−0.8
−0.4
0.0
0.4
β_IV

Batch 2−3527 : Gut microbiota abundance (genus Catenibacterium id.2153) on Lysophosphatidylcholine acyltransferase 2
with a threshold of 1e−05

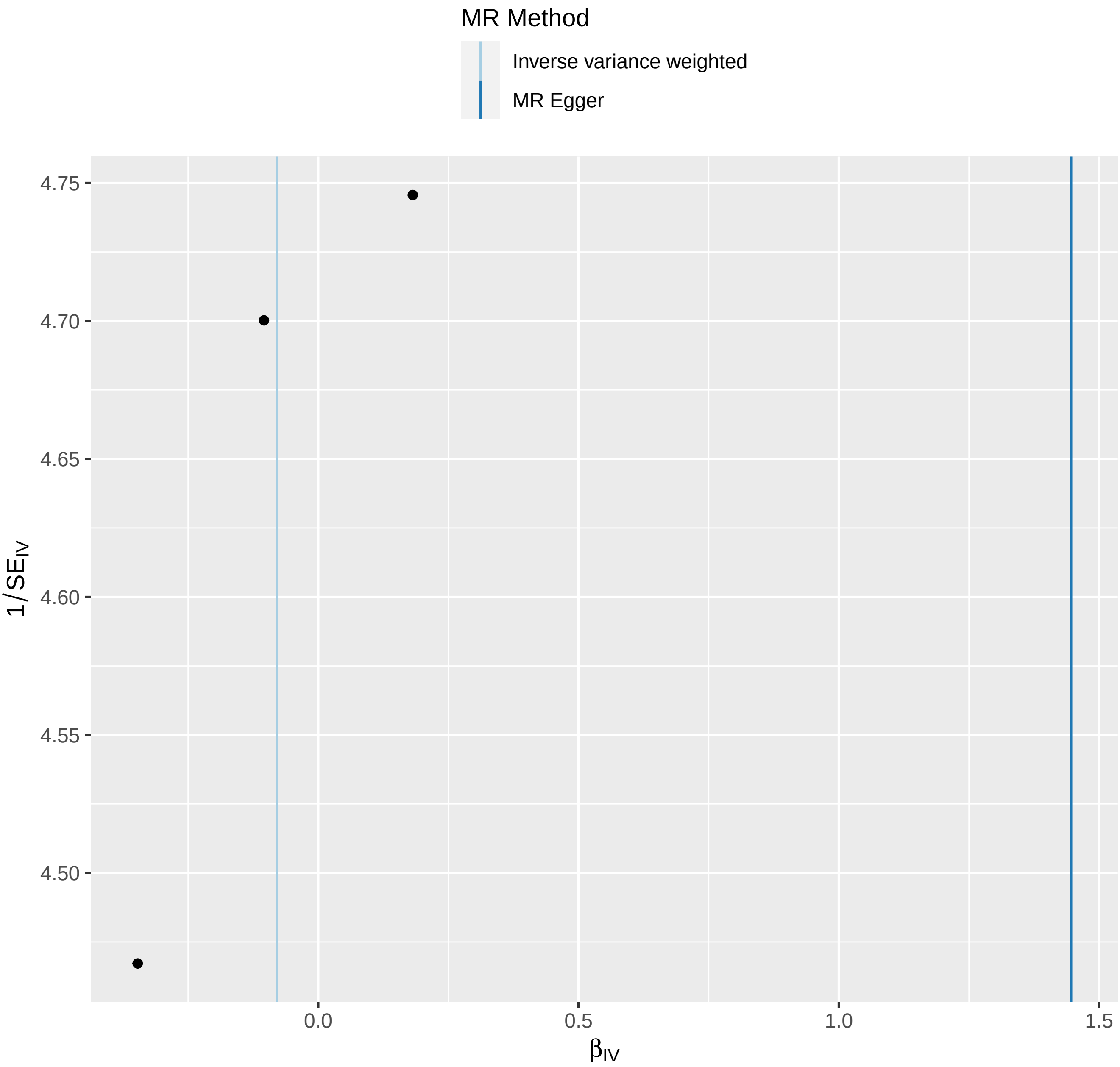

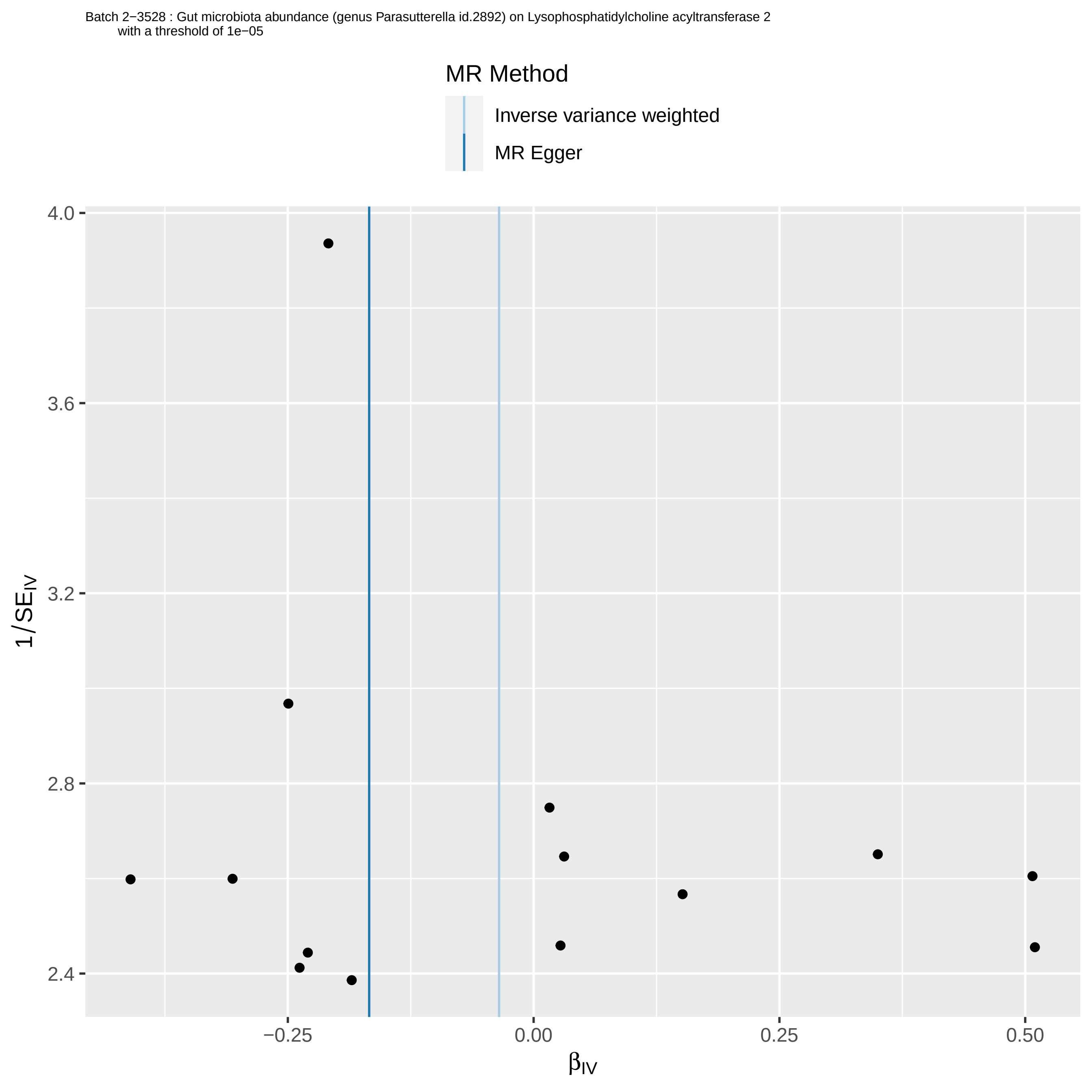
Batch 2−3528 : Gut microbiota abundance (genus Parasutterella id.2892) on Lysophosphatidylcholine acyltransferase 2
with a threshold of 1e−05
MR Method
Inverse variance weighted
MR Egger
4.0
3.6
3.2
2.8
2.4
$1/SE_{IV}$
−0.25
0.00
0.25
0.50
β_{IV}

Batch 2−3529 : Gut microbiota abundance (genus Catenibacterium id.2153) on Lactoperoxidase
with a threshold of 1e−05

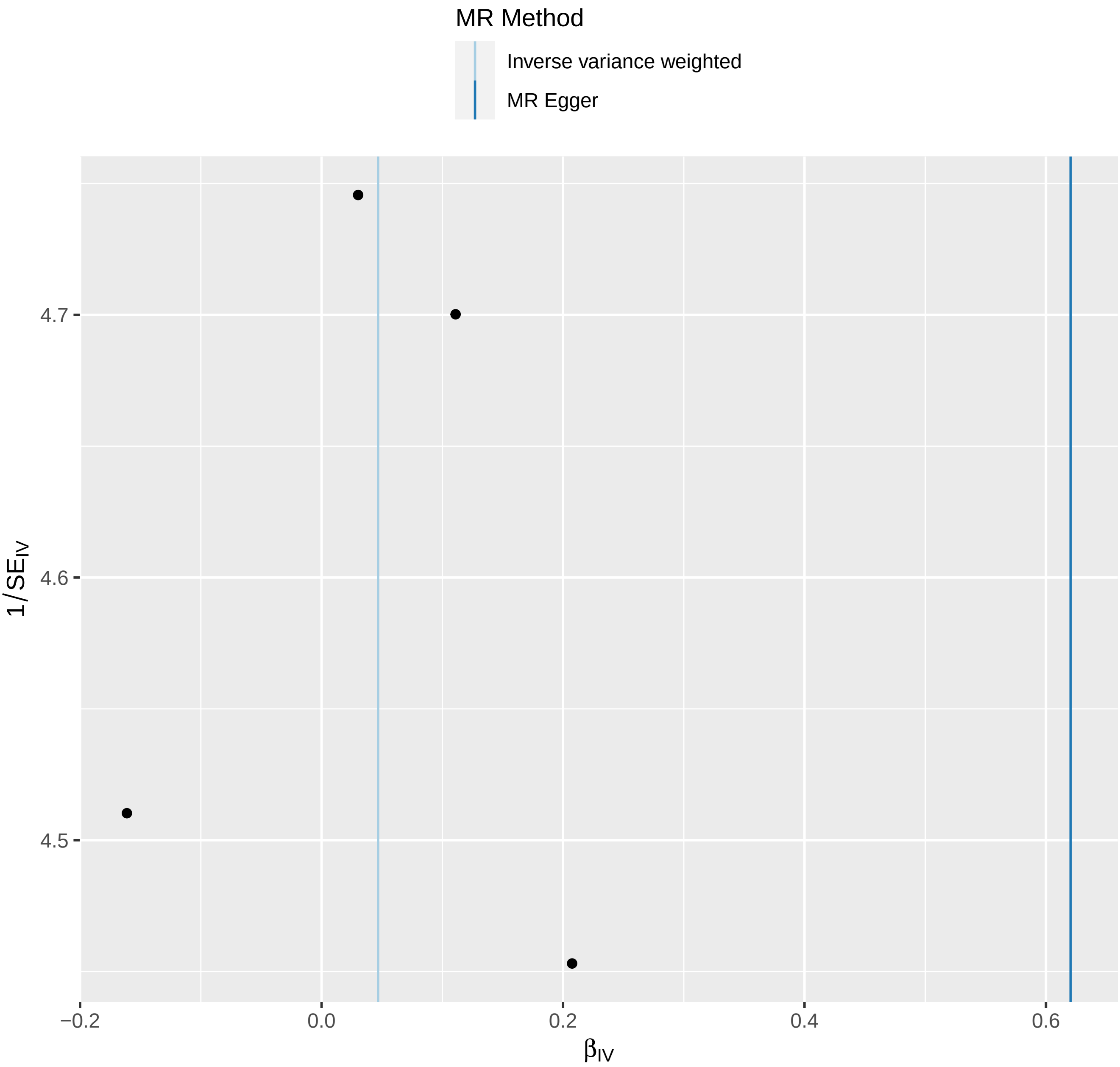

Batch 2−3530 : Gut microbiota abundance (genus Parasutterella id.2892) on Lactoperoxidase
with a threshold of 1e−05

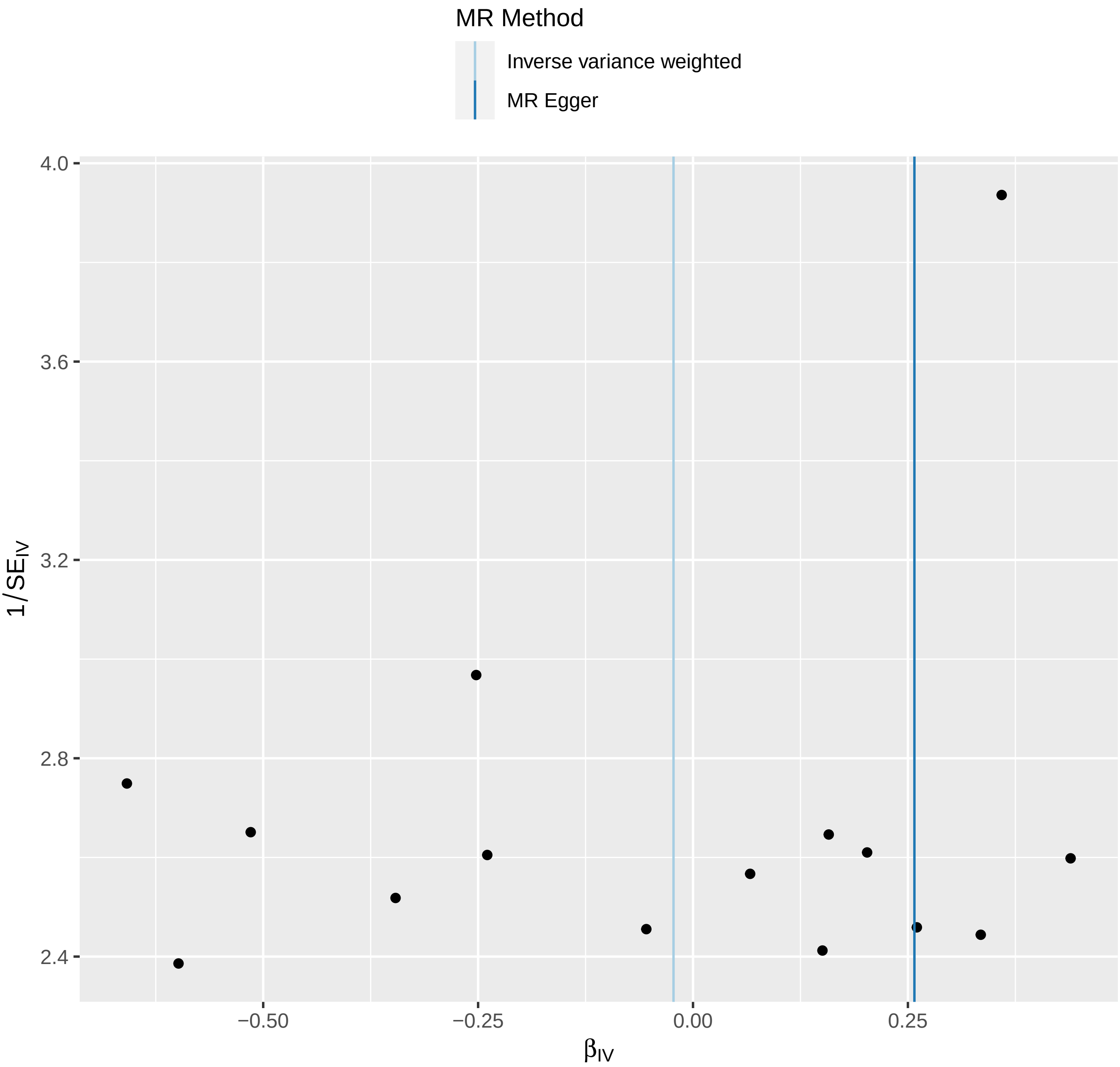

Batch 2−3531 : Gut microbiota abundance (genus Catenibacterium id.2153) on Leucine−rich repeat and calponin homology domain−containing protein 4 with a threshold of 1e−05

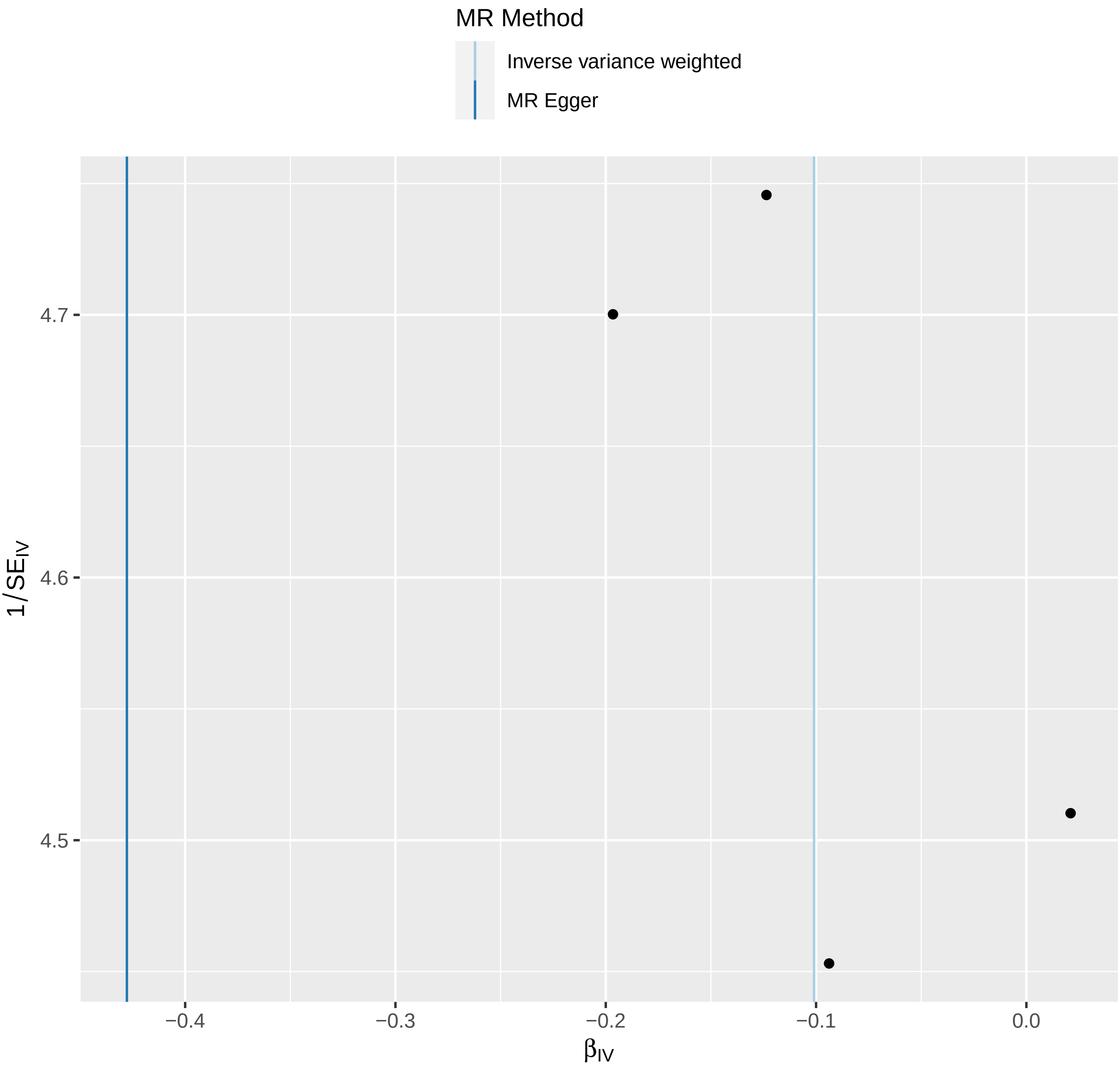

Batch 2−3532 : Gut microbiota abundance (genus Parasutterella id.2892) on Leucine−rich repeat and calponin homology domain−containing protein 4 with a threshold of 1e−05

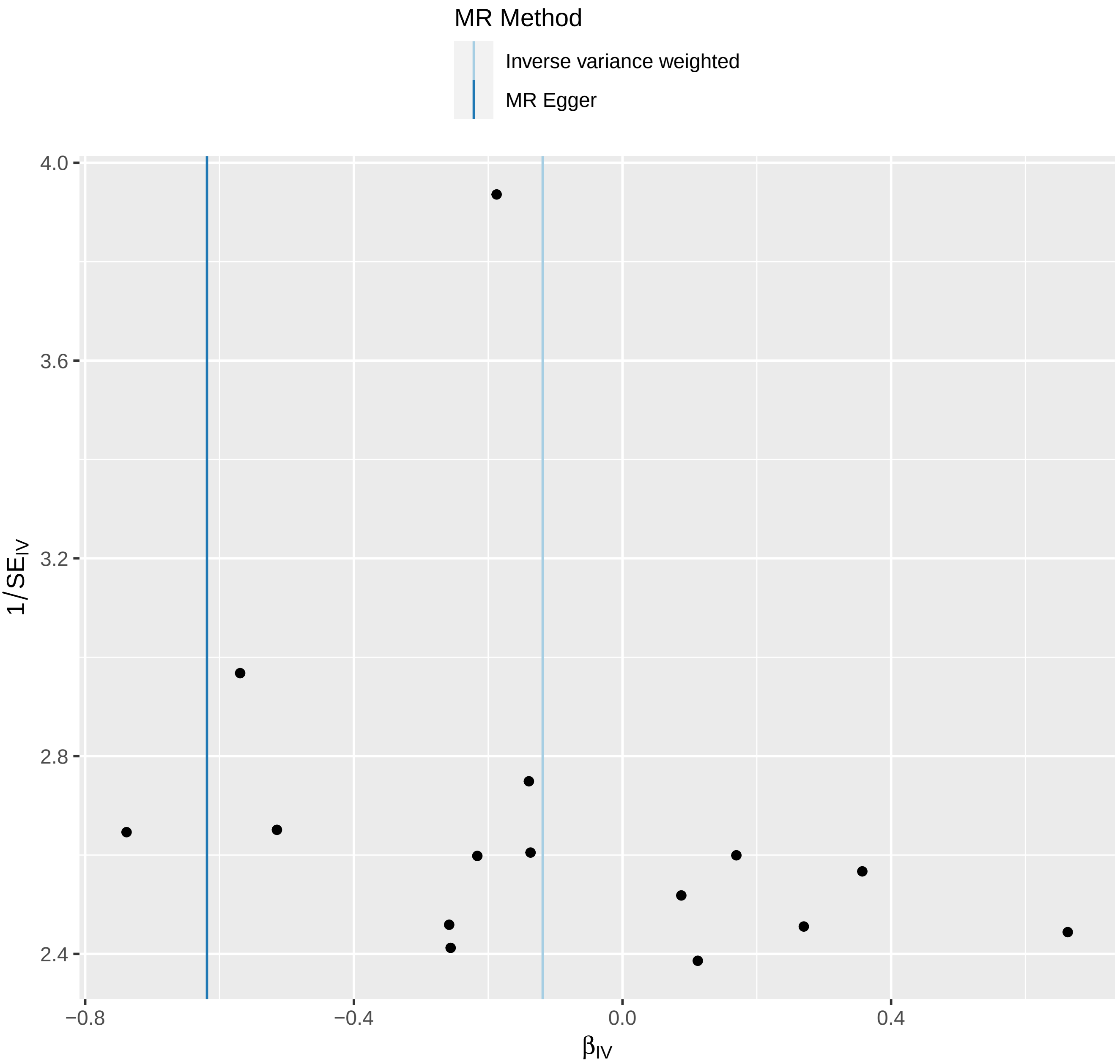

Batch 2−3533 : Gut microbiota abundance (genus Catenibacterium id.2153) on Leucine−rich repeat and calponin homology domain−containing protein 4 with a threshold of 1e−05

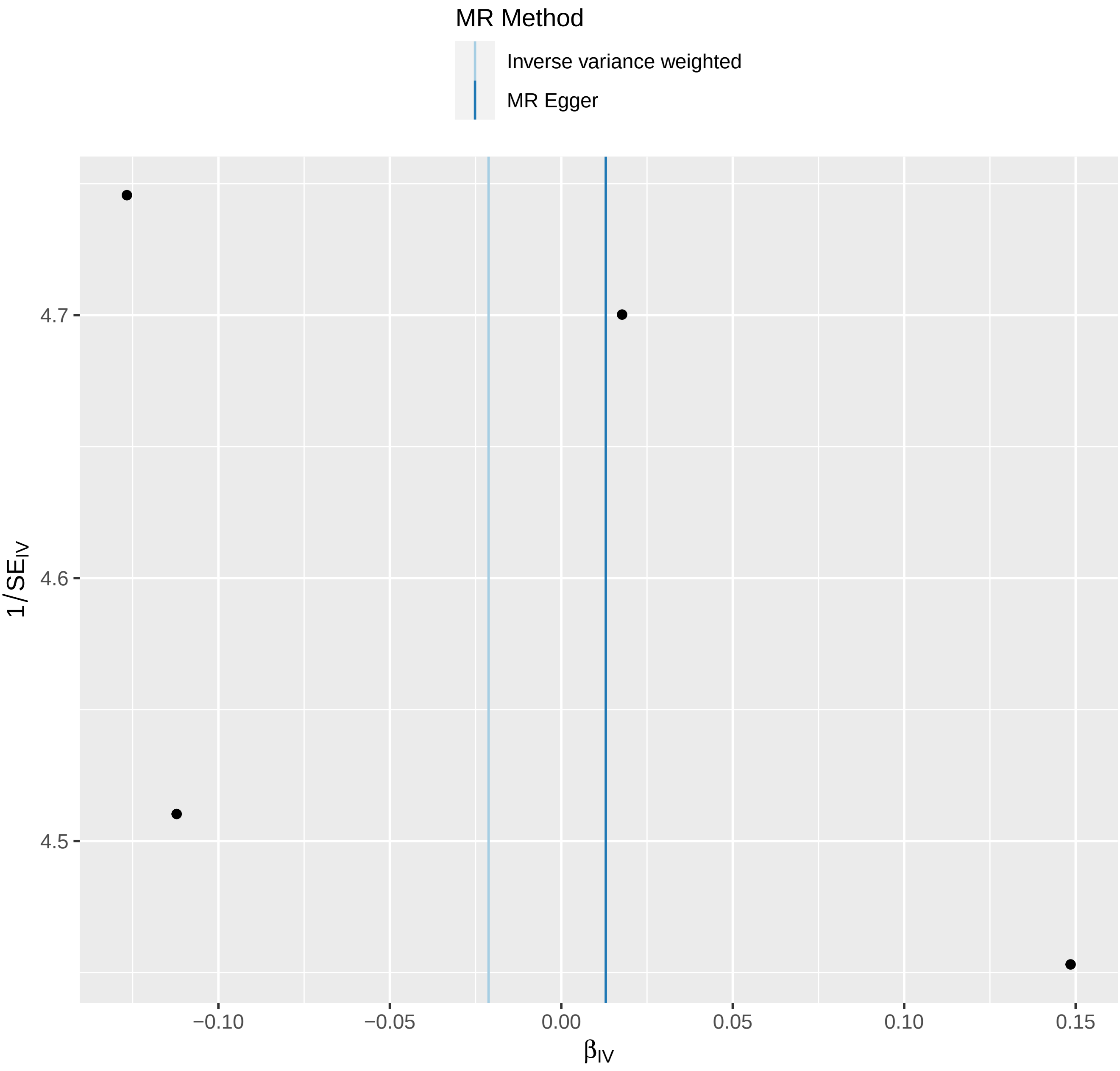

Batch 2−3534 : Gut microbiota abundance (genus Parasutterella id.2892) on Leucine−rich repeat and calponin homology domain−containing protein 4 with a threshold of 1e−05

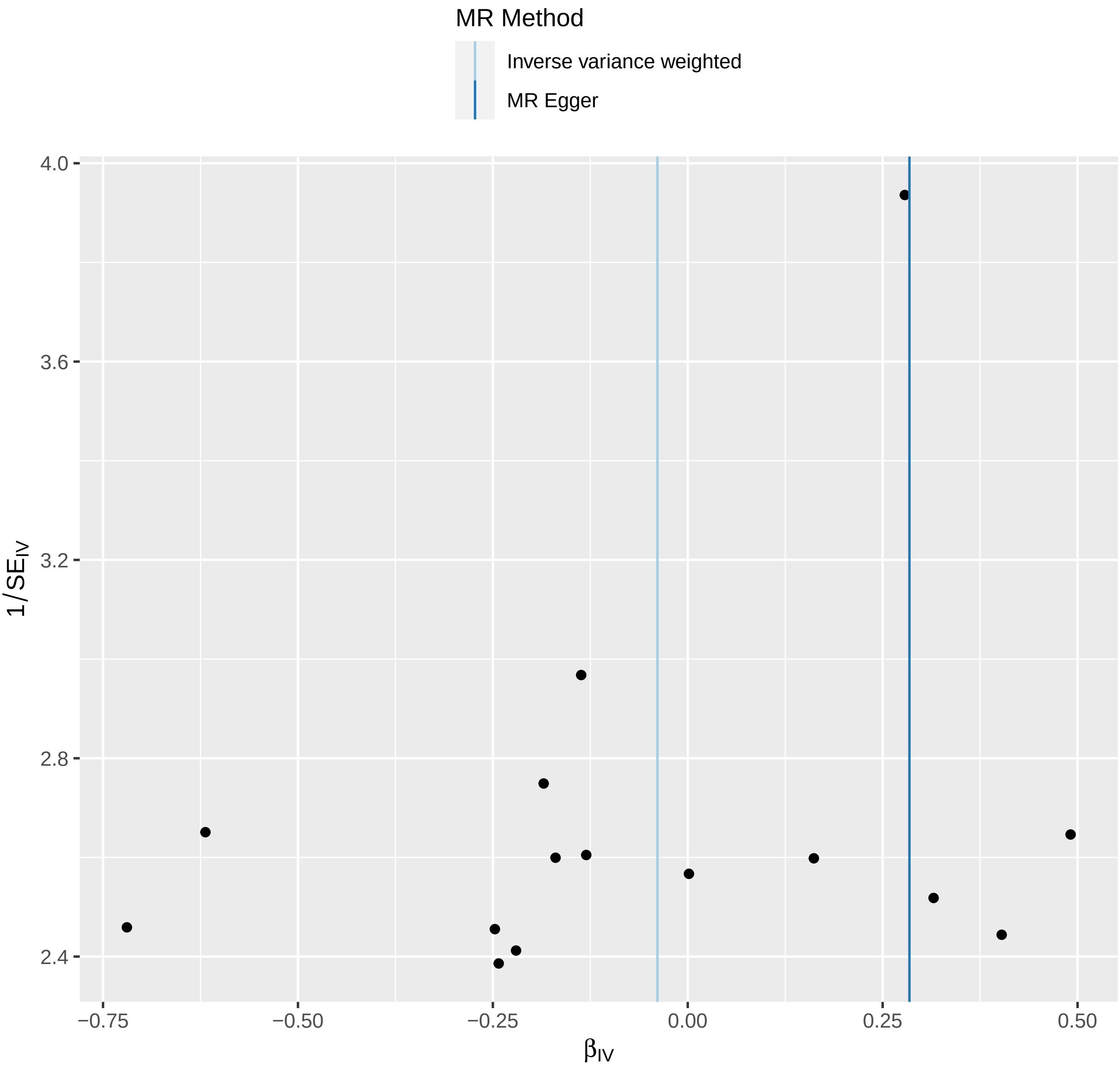

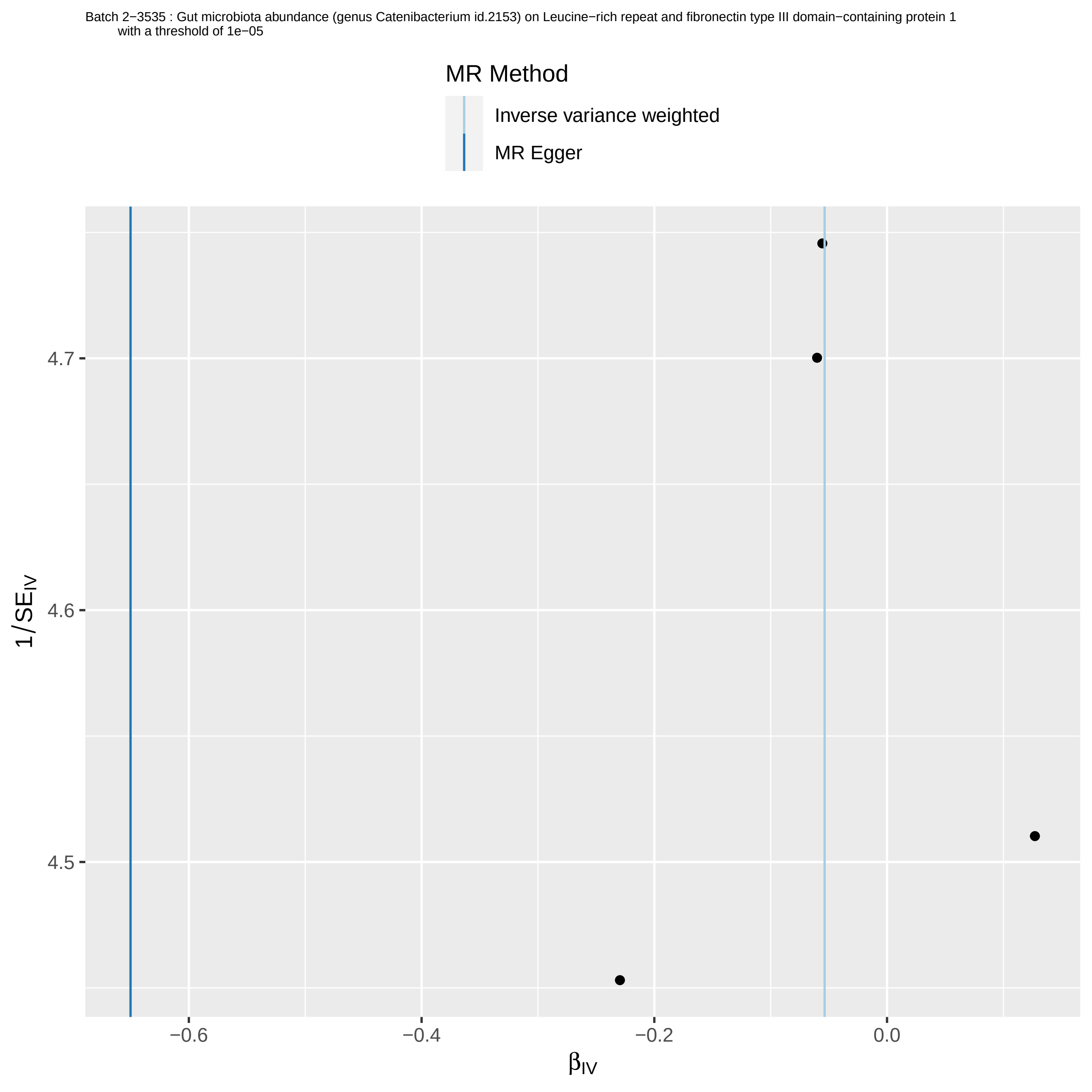

Batch 2−3535 : Gut microbiota abundance (genus Catenibacterium id.2153) on Leucine−rich repeat and fibronectin type III domain−containing protein 1
with a threshold of 1e−05
MR Method
Inverse variance weighted
MR Egger
4.7
4.6
4.5
1/SE_IV
−0.6
−0.4
−0.2
0.0
β_IV

Batch 2−3536 : Gut microbiota abundance (genus Parasutterella id.2892) on Leucine−rich repeat and fibronectin type III domain−containing protein 1 with a threshold of 1e−05

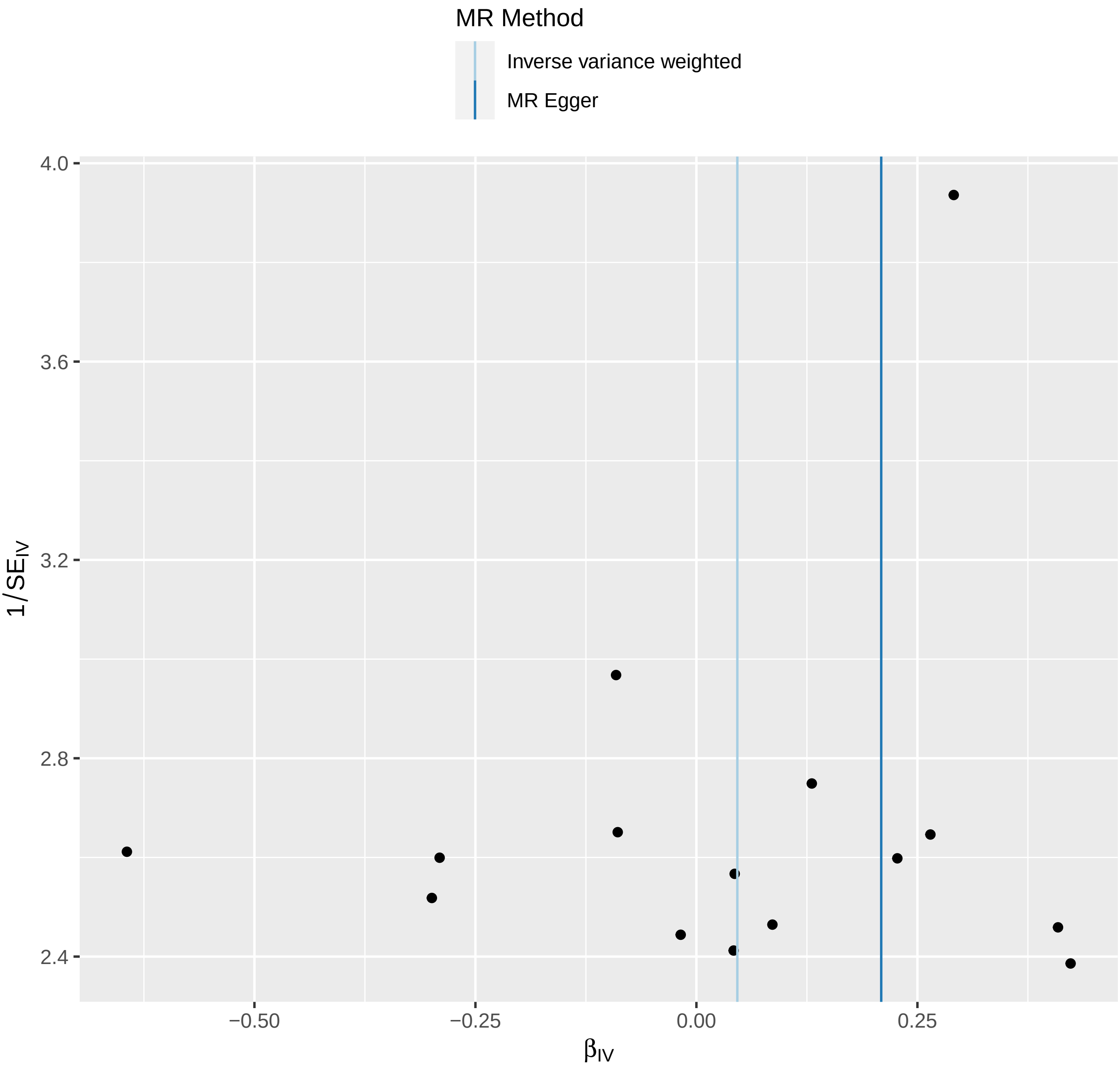

Batch 2−3537 : Gut microbiota abundance (genus Catenibacterium id.2153) on Leucine−rich repeat and fibronectin type−III domain−containing protein 2 with a threshold of 1e−05

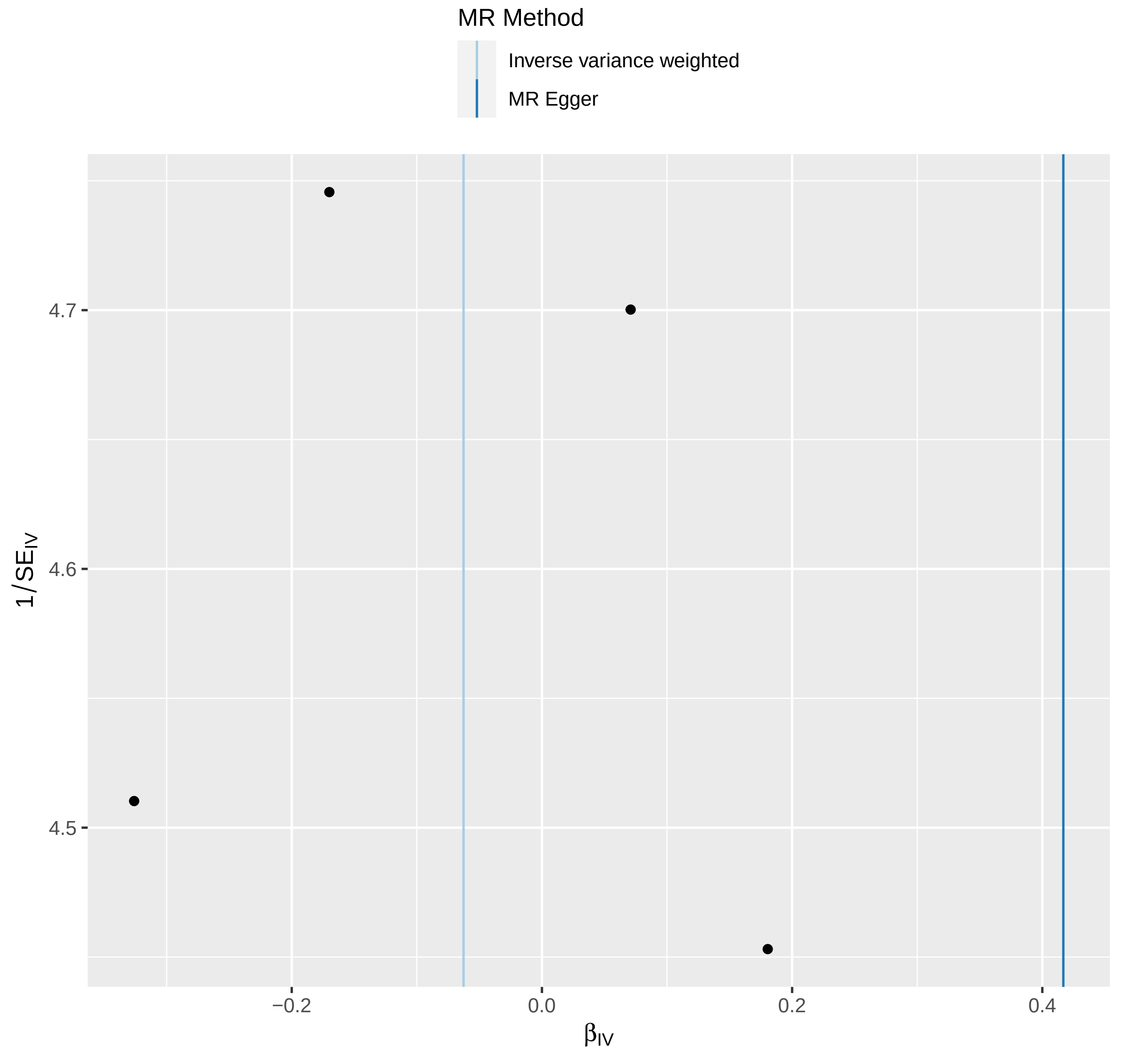

Batch 2−3538 : Gut microbiota abundance (genus Parasutterella id.2892) on Leucine−rich repeat and fibronectin type−III domain−containing protein 2 with a threshold of 1e−05

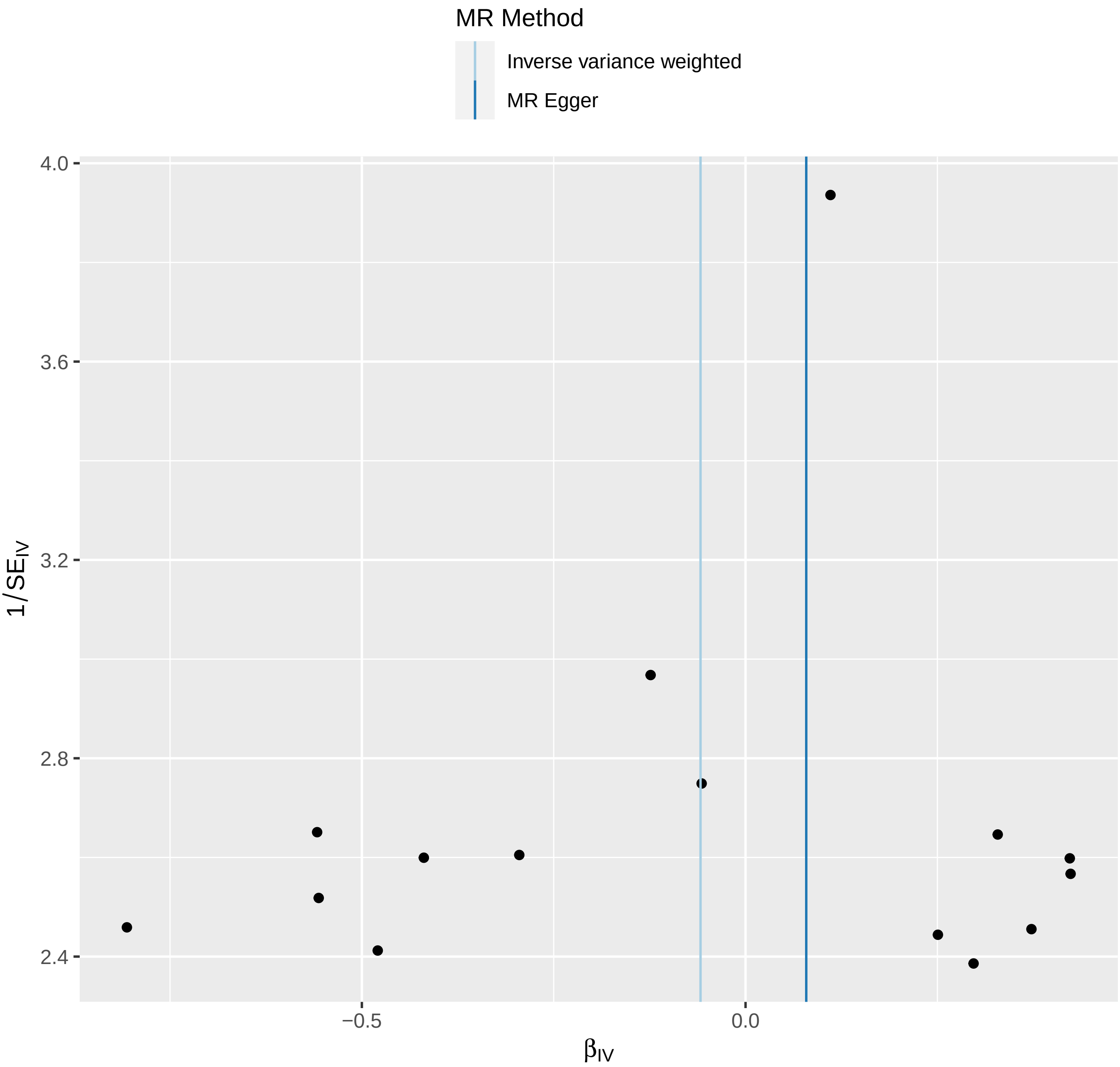

Batch 2−3539 : Gut microbiota abundance (genus Catenibacterium id.2153) on Leucine−rich repeats and immunoglobulin−like domains protein 3 with a threshold of 1e−05

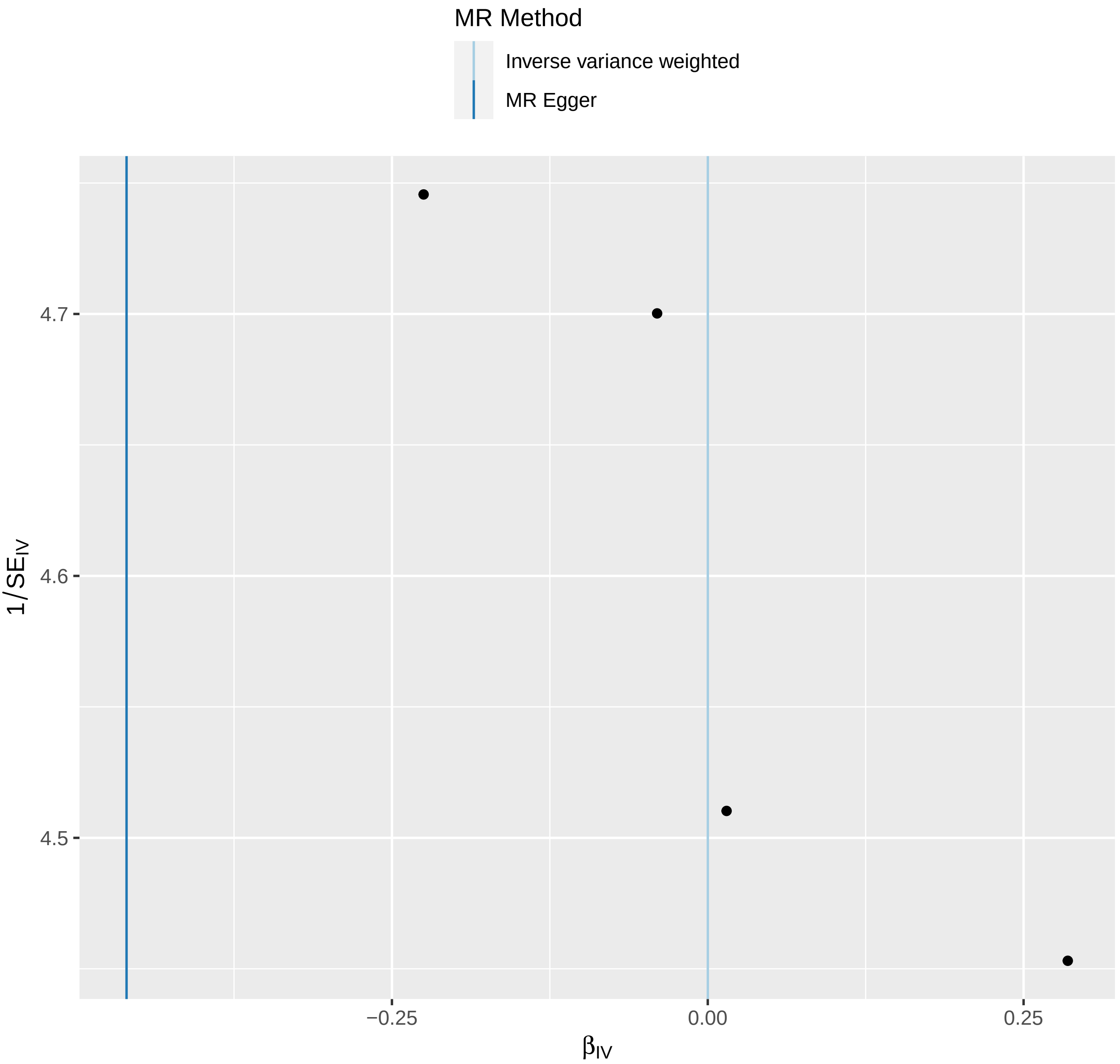

Batch 2−3540 : Gut microbiota abundance (genus Parasutterella id.2892) on Leucine−rich repeats and immunoglobulin−like domains protein 3 with a threshold of 1e−05

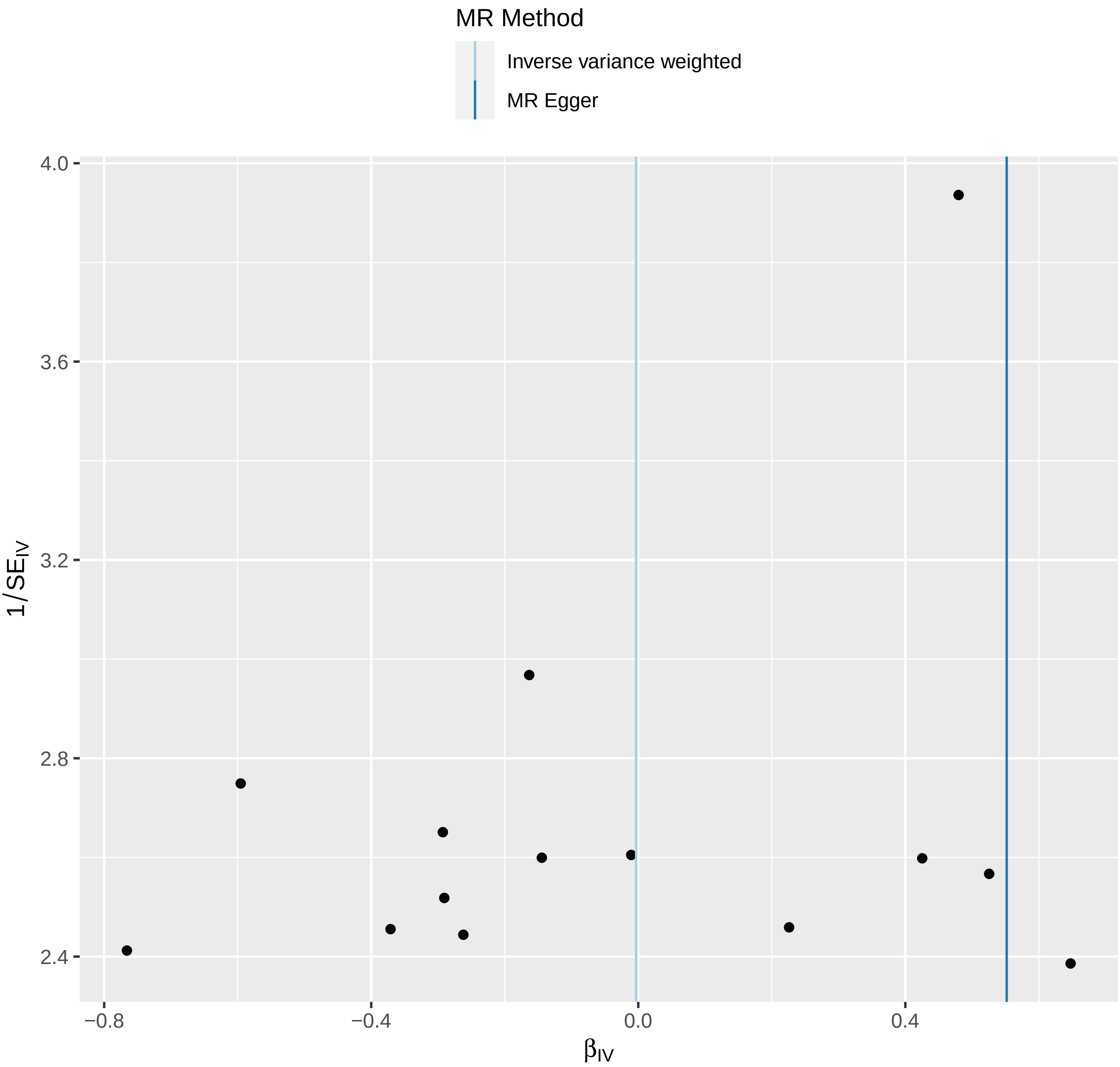

Batch 2−3541 : Gut microbiota abundance (genus Catenibacterium id.2153) on Leucine−rich repeat, immunoglobulin−like domain and transmembrane domain−containing prote
with a threshold of 1e−05

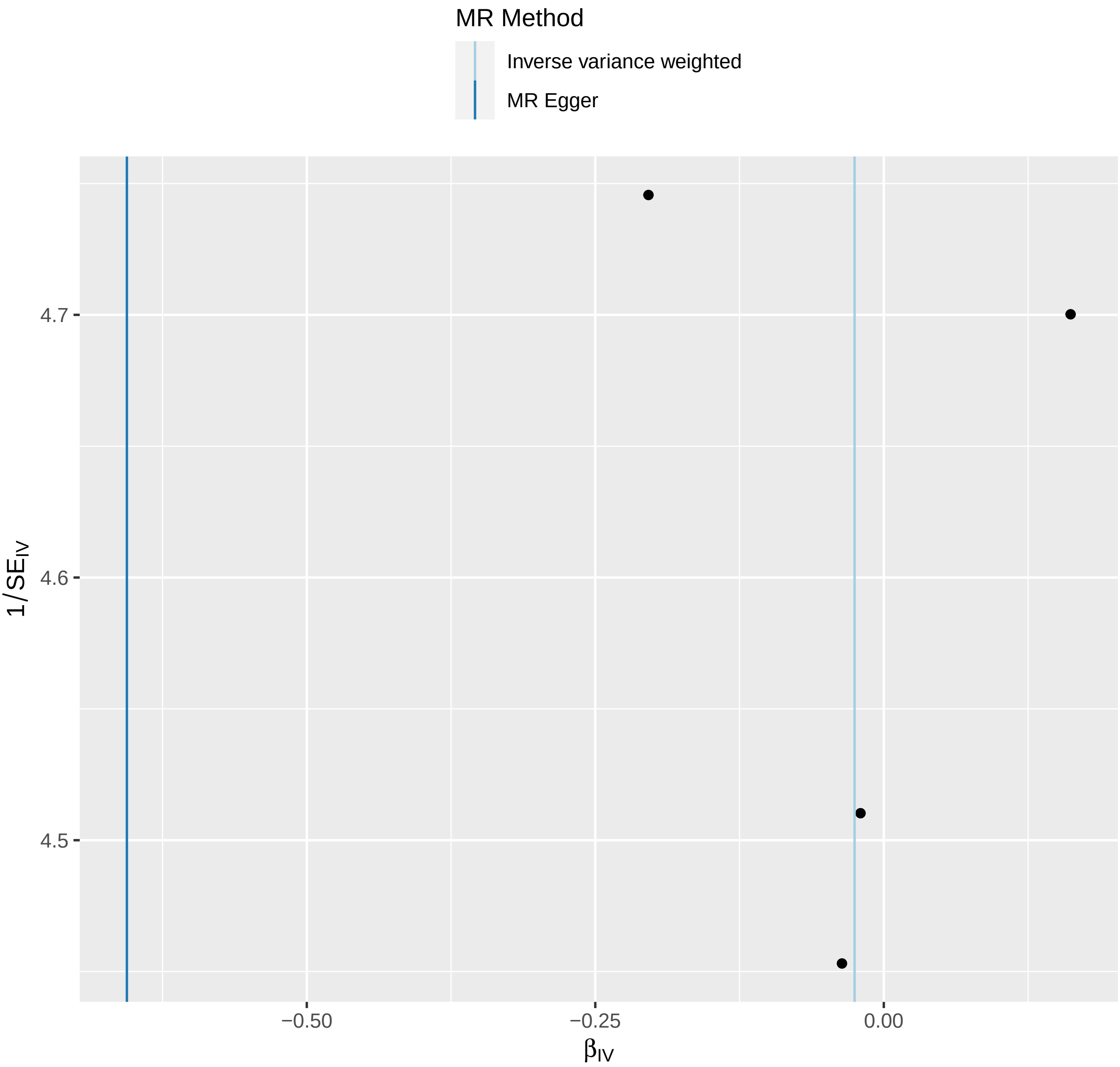

Batch 2−3542 : Gut microbiota abundance (genus Parasutterella id.2892) on Leucine−rich repeat, immunoglobulin−like domain and transmembrane domain−containing protein with a threshold of 1e−05

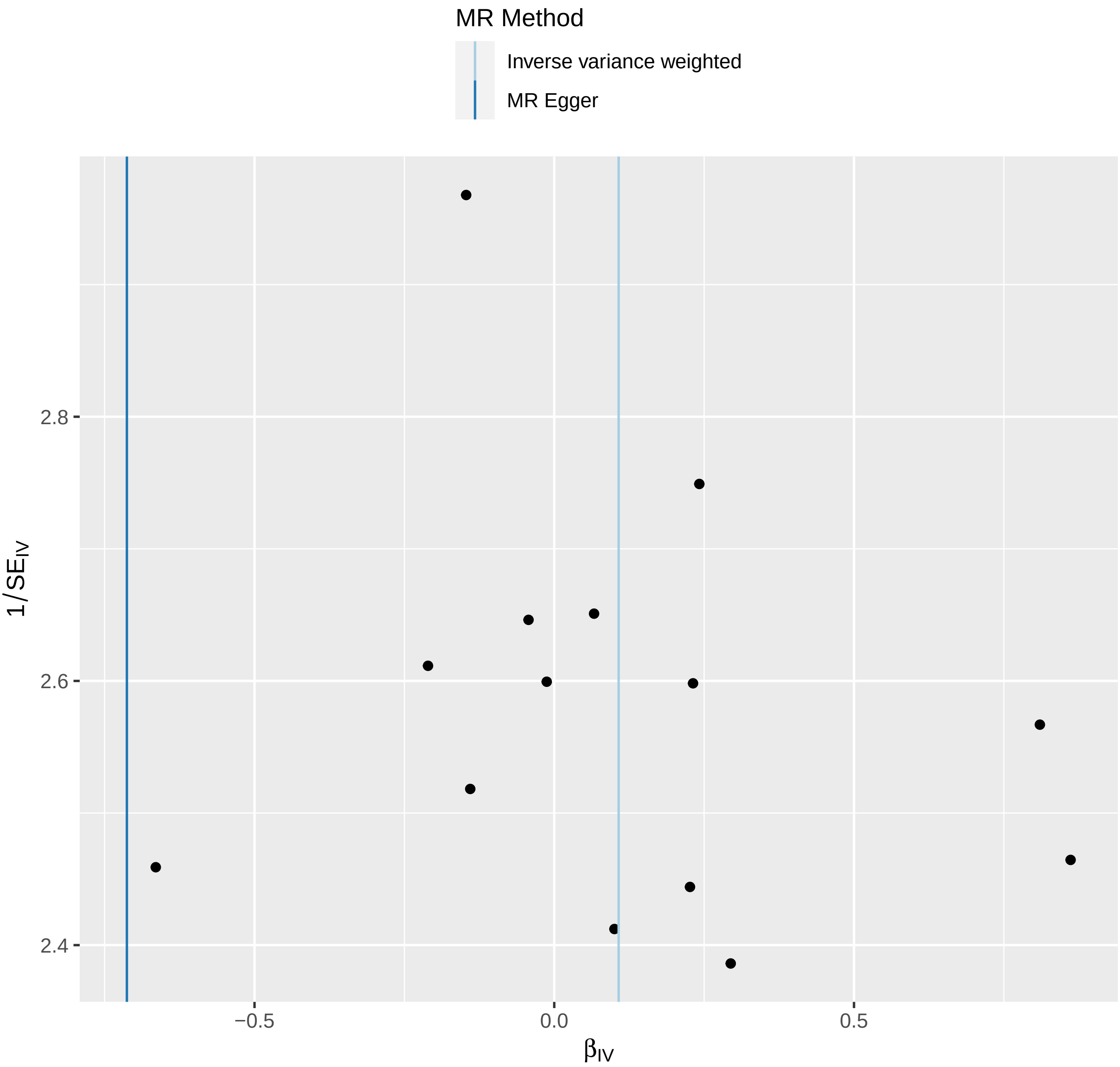

Batch 2−3543 : Gut microbiota abundance (genus Catenibacterium id.2153) on Leucine−rich repeat, immunoglobulin−like domain and transmembrane domain−containing prote
with a threshold of 1e−05

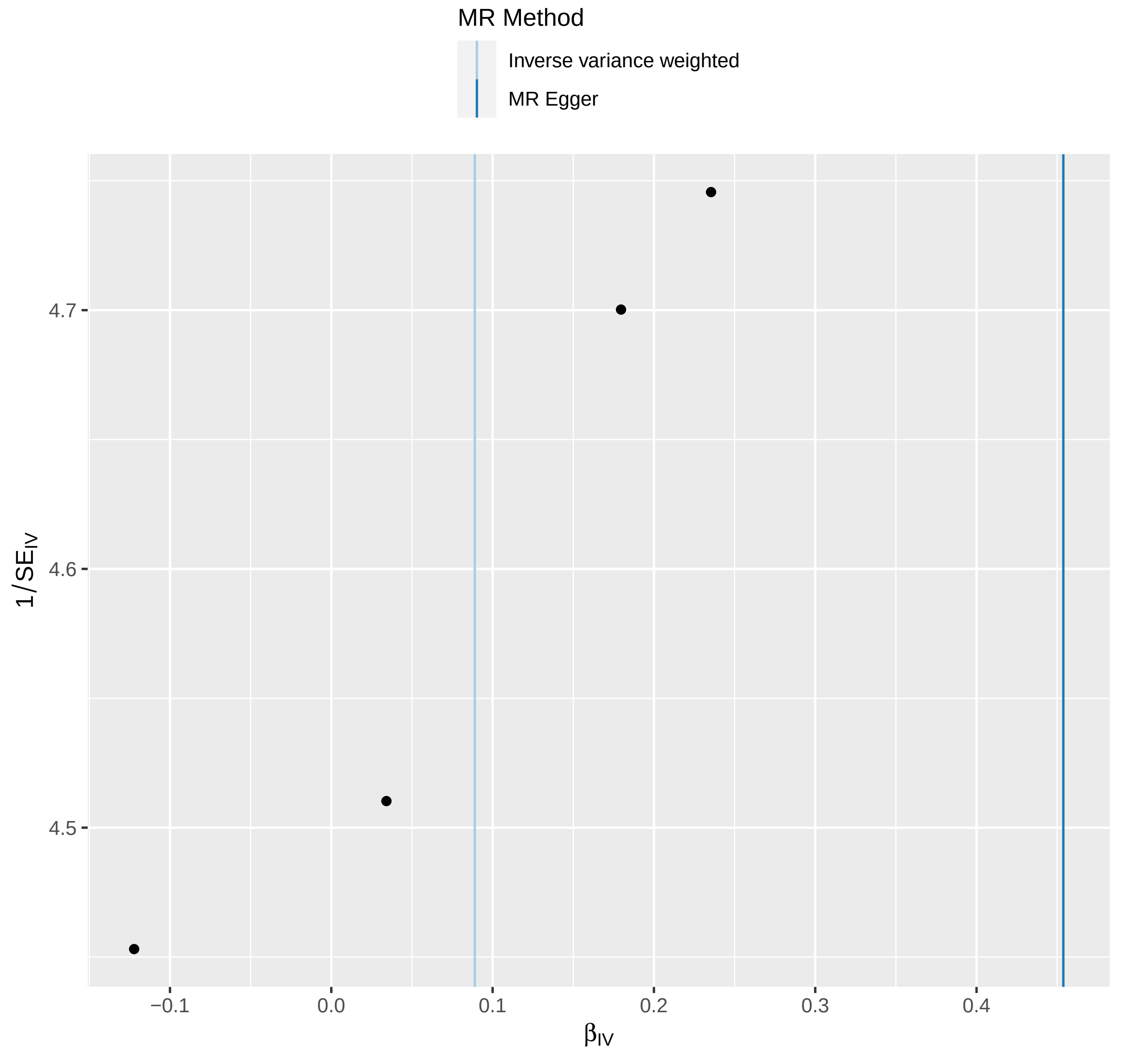

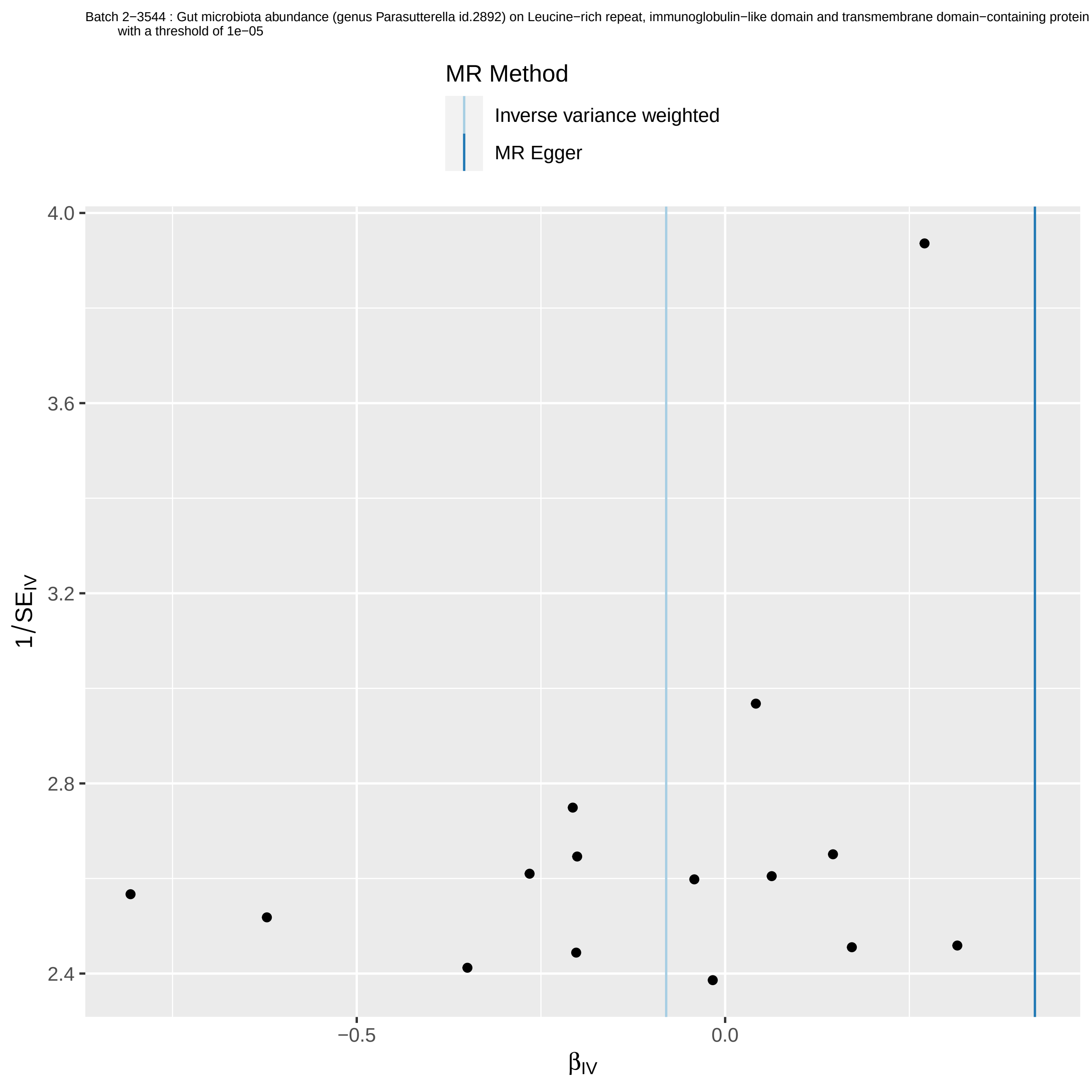

Batch 2−3544 : Gut microbiota abundance (genus Parasutterella id.2892) on Leucine−rich repeat, immunoglobulin−like domain and transmembrane domain−containing protein
with a threshold of 1e−05
MR Method
Inverse variance weighted
MR Egger
4.0
3.6
3.2
2.8
2.4
1/SE$_{IV}$
−0.5
0.0
β$_{IV}$

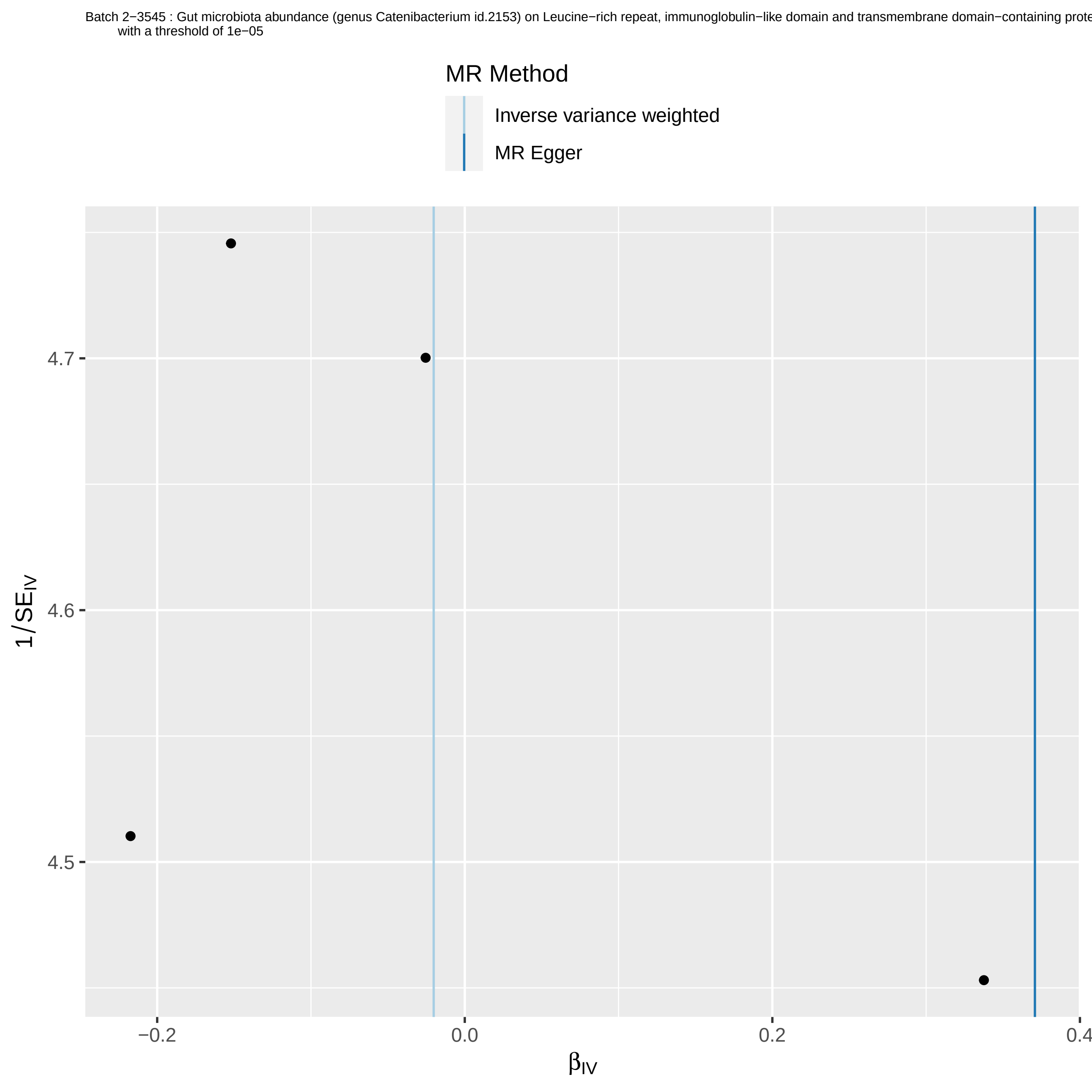

Batch 2−3545 : Gut microbiota abundance (genus Catenibacterium id.2153) on Leucine−rich repeat, immunoglobulin−like domain and transmembrane domain−containing prote
with a threshold of 1e−05
MR Method
Inverse variance weighted
MR Egger
4.7
4.6
4.5
1/SE_IV
−0.2
0.0
0.2
0.4
β_IV

Batch 2−3546 : Gut microbiota abundance (genus Parasutterella id.2892) on Leucine−rich repeat, immunoglobulin−like domain and transmembrane domain−containing protein
with a threshold of 1e−05

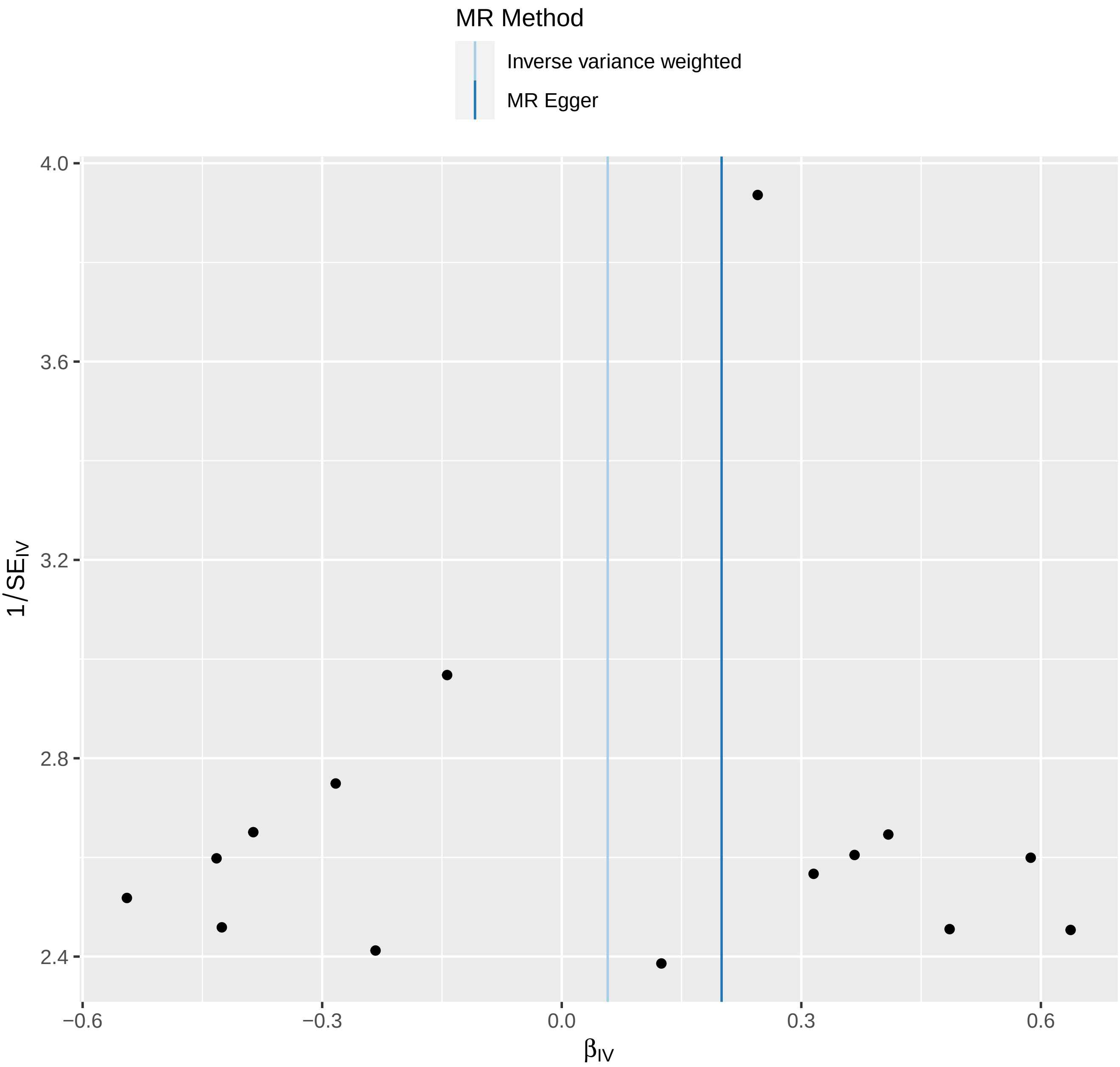

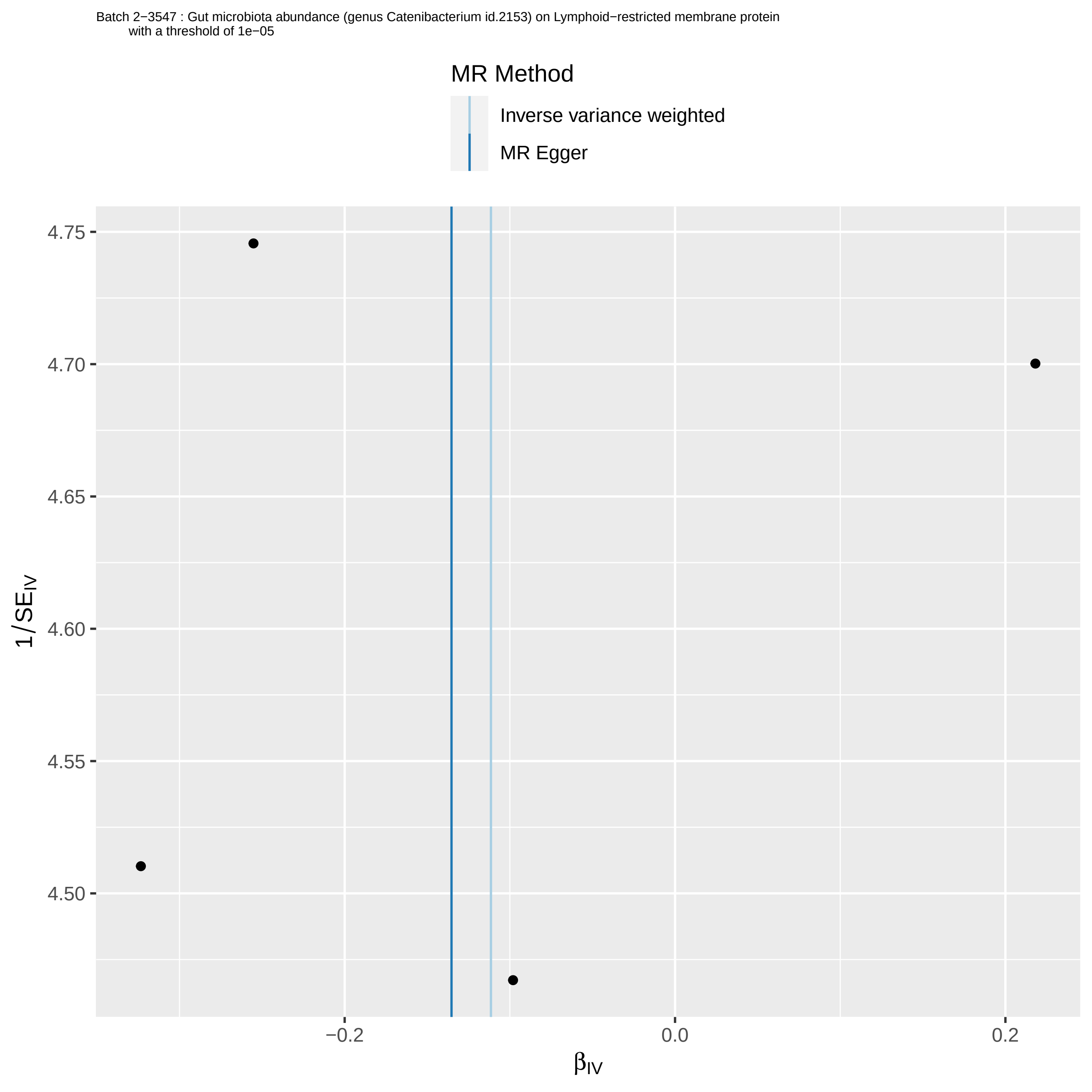

Batch 2−3547 : Gut microbiota abundance (genus Catenibacterium id.2153) on Lymphoid−restricted membrane protein
with a threshold of 1e−05
MR Method
Inverse variance weighted
MR Egger
4.75
4.70
4.65
4.60
4.55
4.50
1/SE$_{IV}$
−0.2
0.0
0.2
β$_{IV}$

Batch 2−3548 : Gut microbiota abundance (genus Parasutterella id.2892) on Lymphoid−restricted membrane protein
with a threshold of 1e−05

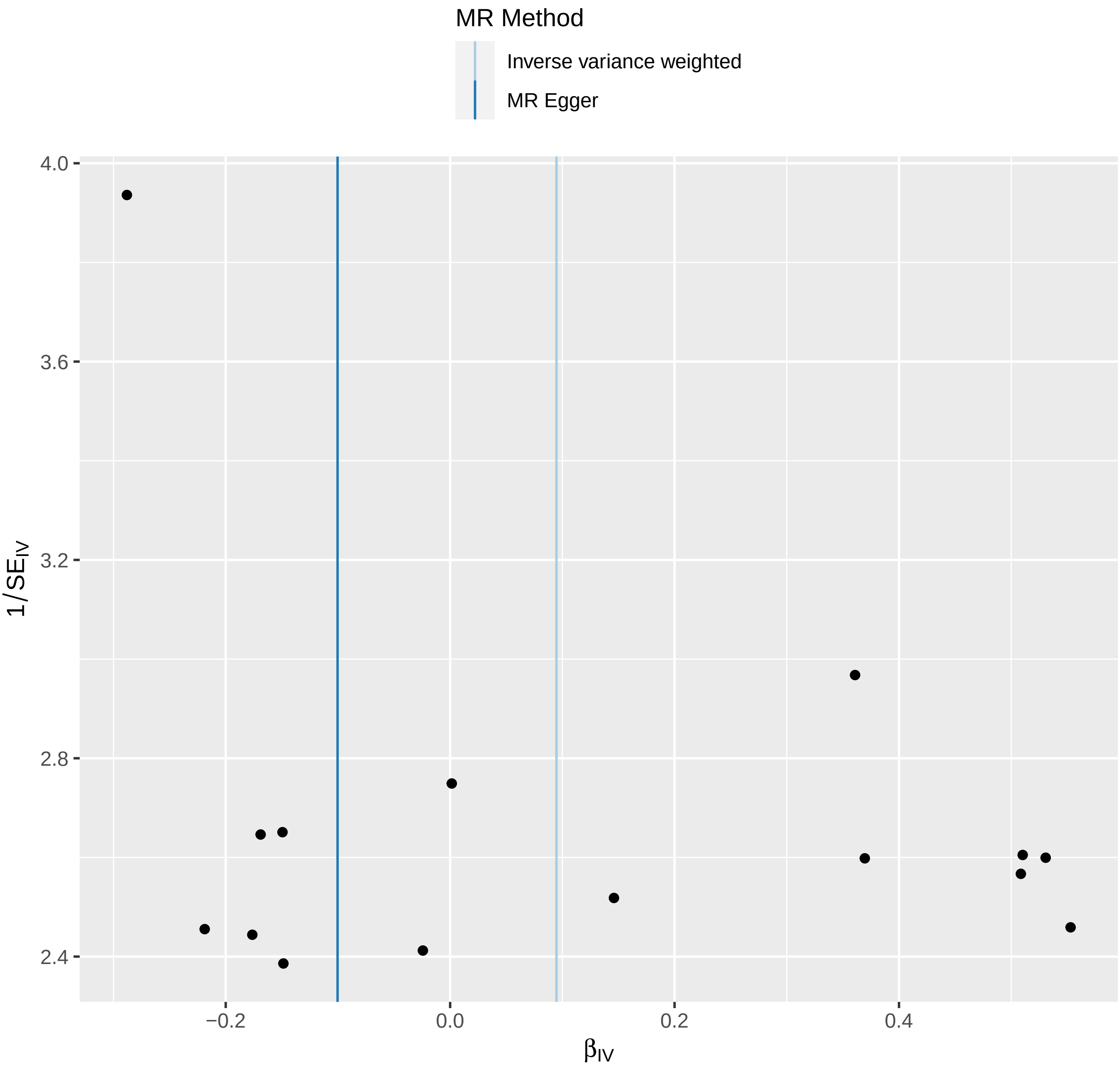

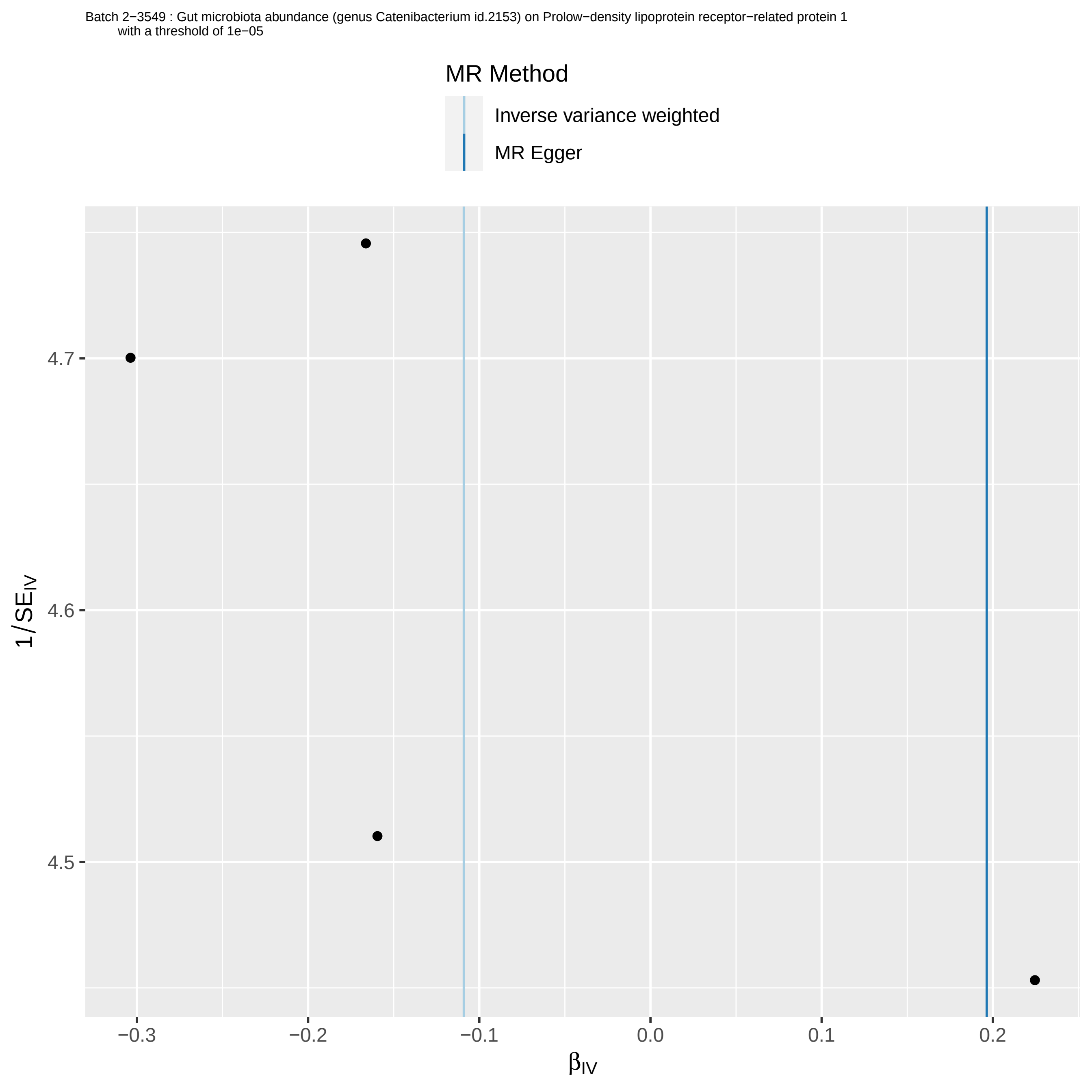
Batch 2−3549 : Gut microbiota abundance (genus Catenibacterium id.2153) on Prolow−density lipoprotein receptor−related protein 1
with a threshold of 1e−05
MR Method
Inverse variance weighted
MR Egger
4.7
4.6
4.5
1/SE_IV
−0.3
−0.2
−0.1
0.0
0.1
0.2
β_IV

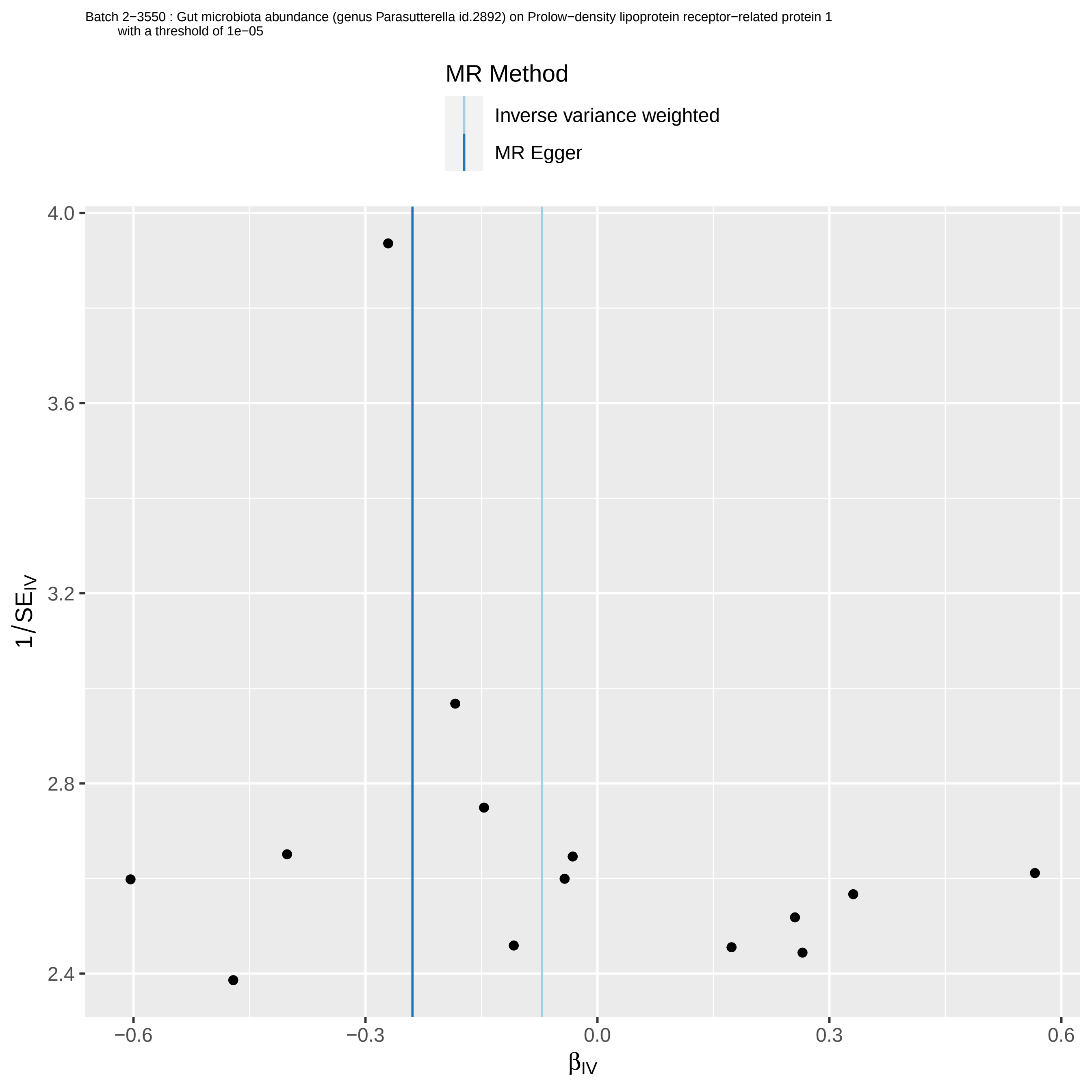

Batch 2−3550 : Gut microbiota abundance (genus Parasutterella id.2892) on Prolow−density lipoprotein receptor−related protein 1
with a threshold of 1e−05
MR Method
Inverse variance weighted
MR Egger
4.0
3.6
3.2
2.8
2.4
1/SE_IV
−0.6
−0.3
0.0
0.3
0.6
β_IV

Batch 2−3551 : Gut microbiota abundance (genus Catenibacterium id.2153) on Low−density lipoprotein receptor−related protein 1, soluble with a threshold of 1e−05

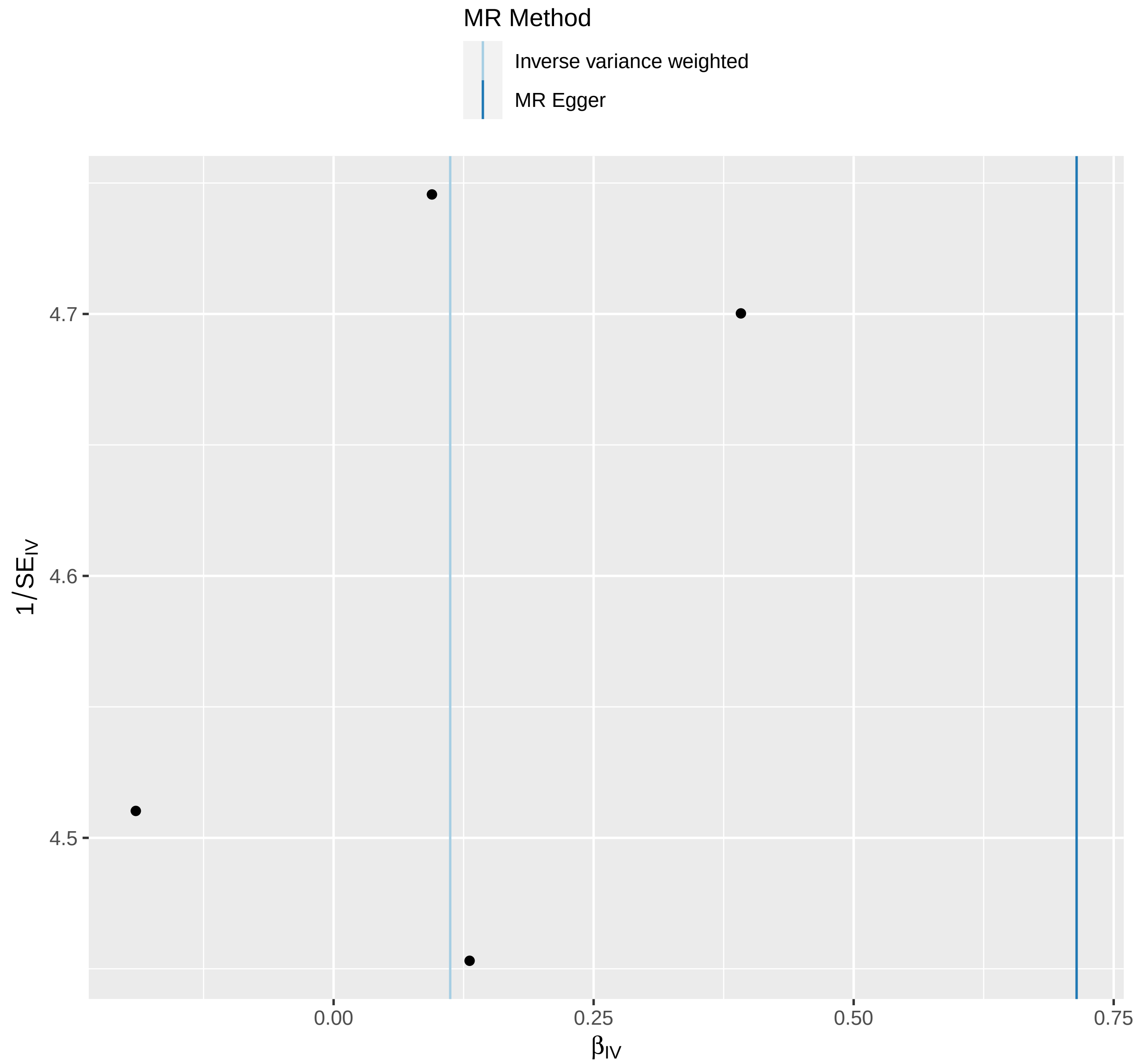

Batch 2−3552 : Gut microbiota abundance (genus Parasutterella id.2892) on Low−density lipoprotein receptor−related protein 1, soluble
with a threshold of 1e−05

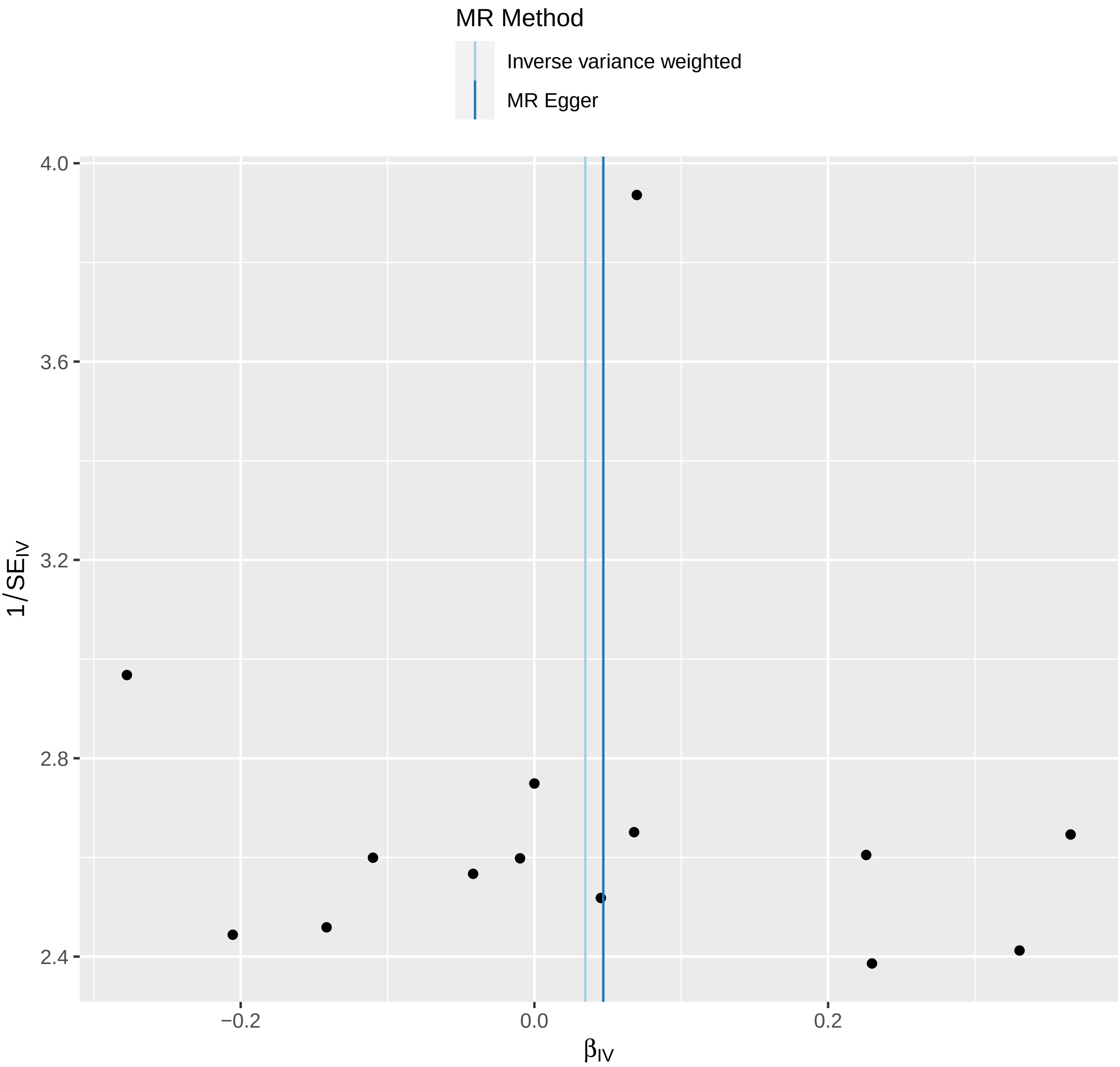

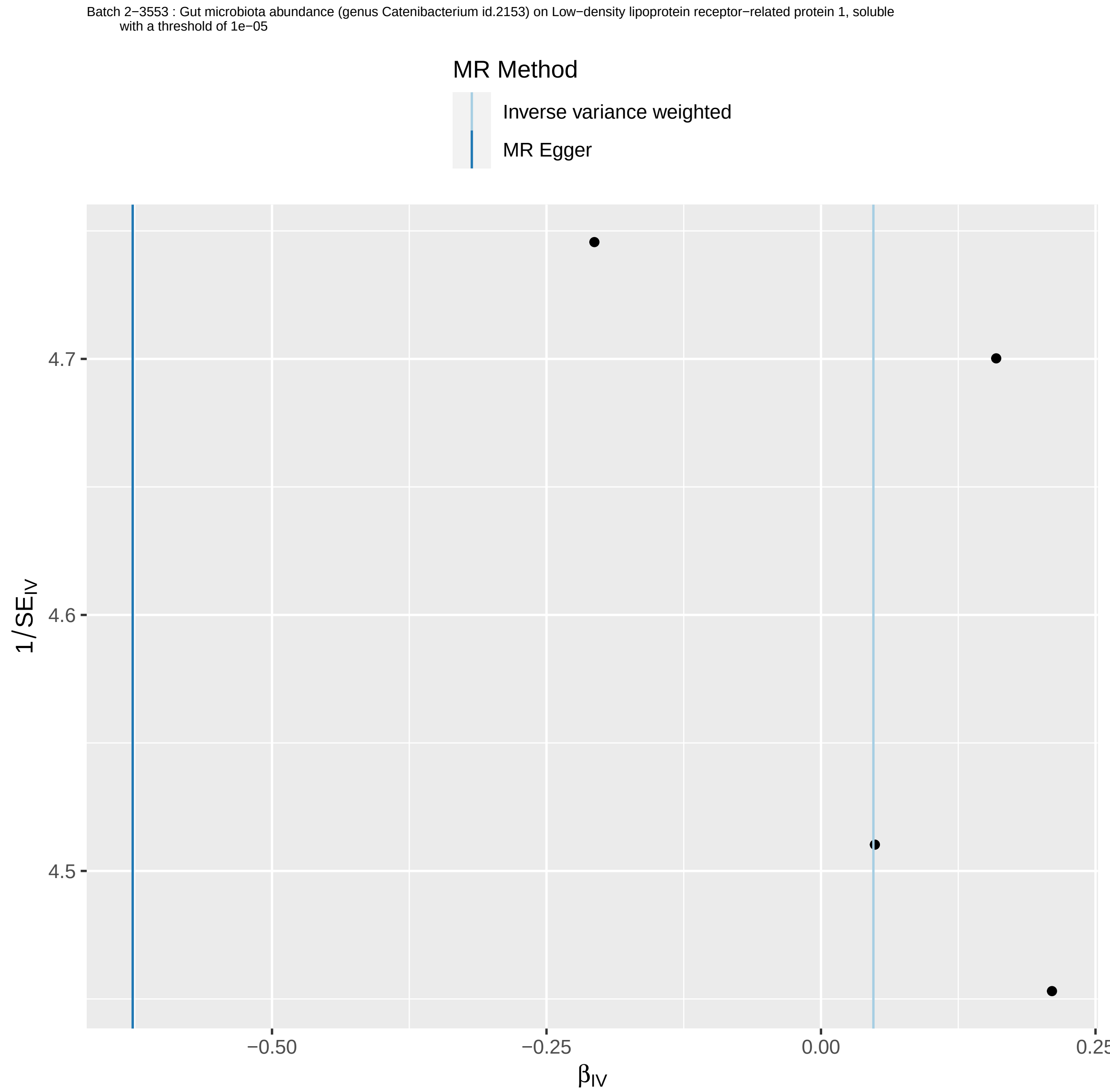
Batch 2−3553 : Gut microbiota abundance (genus Catenibacterium id.2153) on Low−density lipoprotein receptor−related protein 1, soluble
with a threshold of 1e−05
MR Method
Inverse variance weighted
MR Egger
4.7
4.6
4.5
1/SE_IV
−0.50
−0.25
0.00
0.25
β_IV

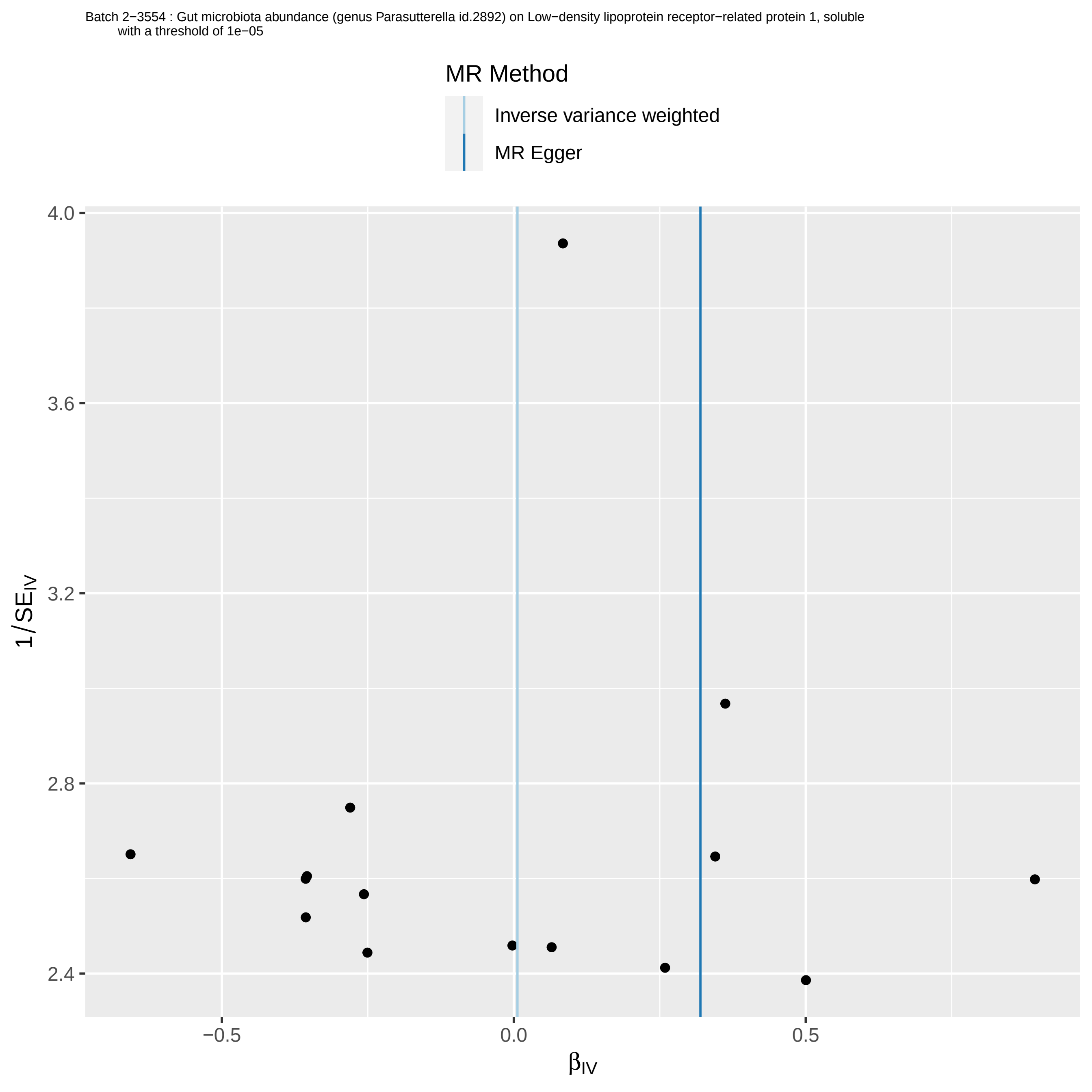

Batch 2−3554 : Gut microbiota abundance (genus Parasutterella id.2892) on Low−density lipoprotein receptor−related protein 1, soluble
with a threshold of 1e−05
MR Method
Inverse variance weighted
MR Egger
4.0
3.6
3.2
2.8
2.4
1/SE_IV
−0.5
0.0
0.5
β_IV

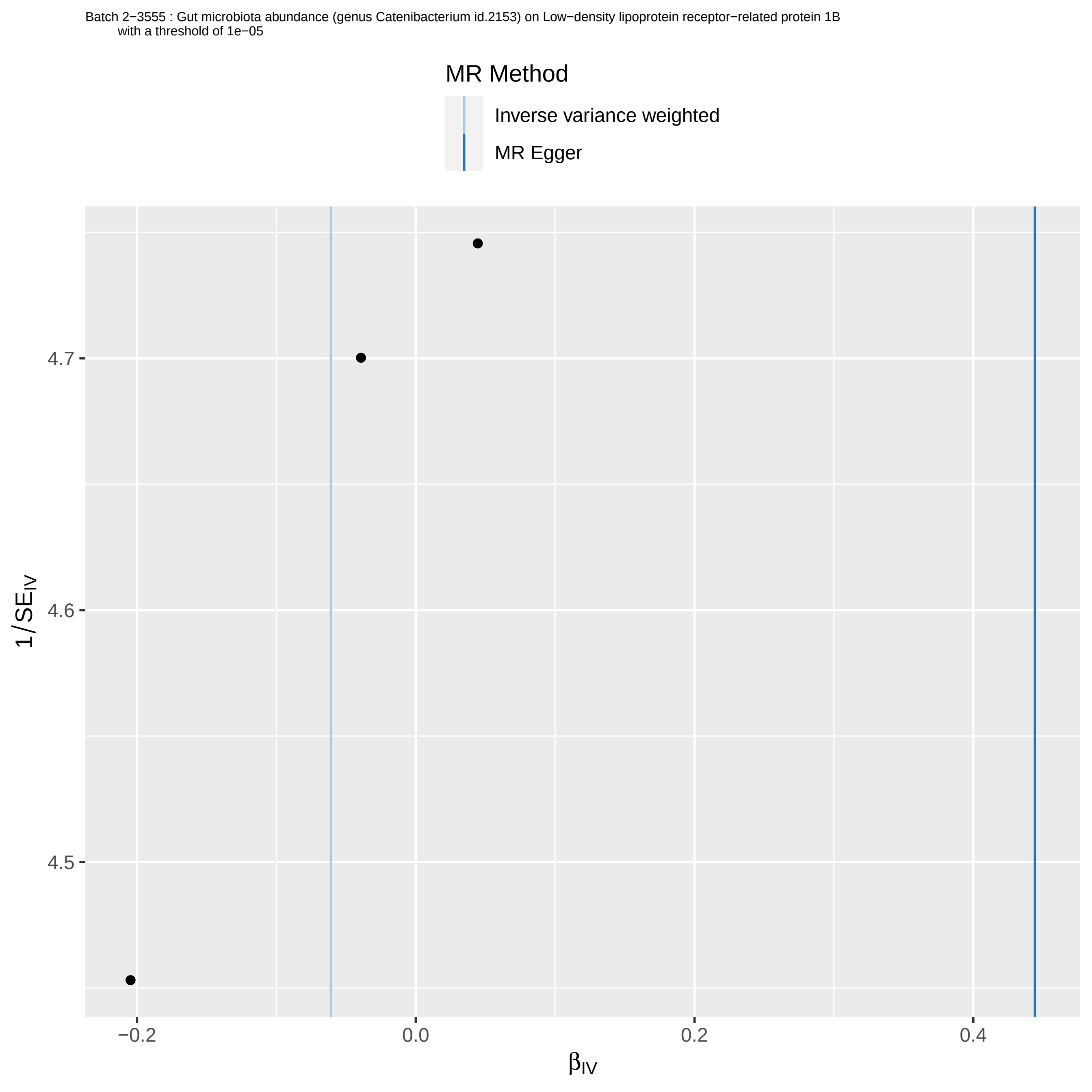
Batch 2−3555 : Gut microbiota abundance (genus Catenibacterium id.2153) on Low−density lipoprotein receptor−related protein 1B
with a threshold of 1e−05
MR Method
Inverse variance weighted
MR Egger
4.7
4.6
4.5
1/SE_IV
−0.2
0.0
0.2
0.4
β_IV

Batch 2−3556 : Gut microbiota abundance (genus Parasutterella id.2892) on Low−density lipoprotein receptor−related protein 1B
with a threshold of 1e−05

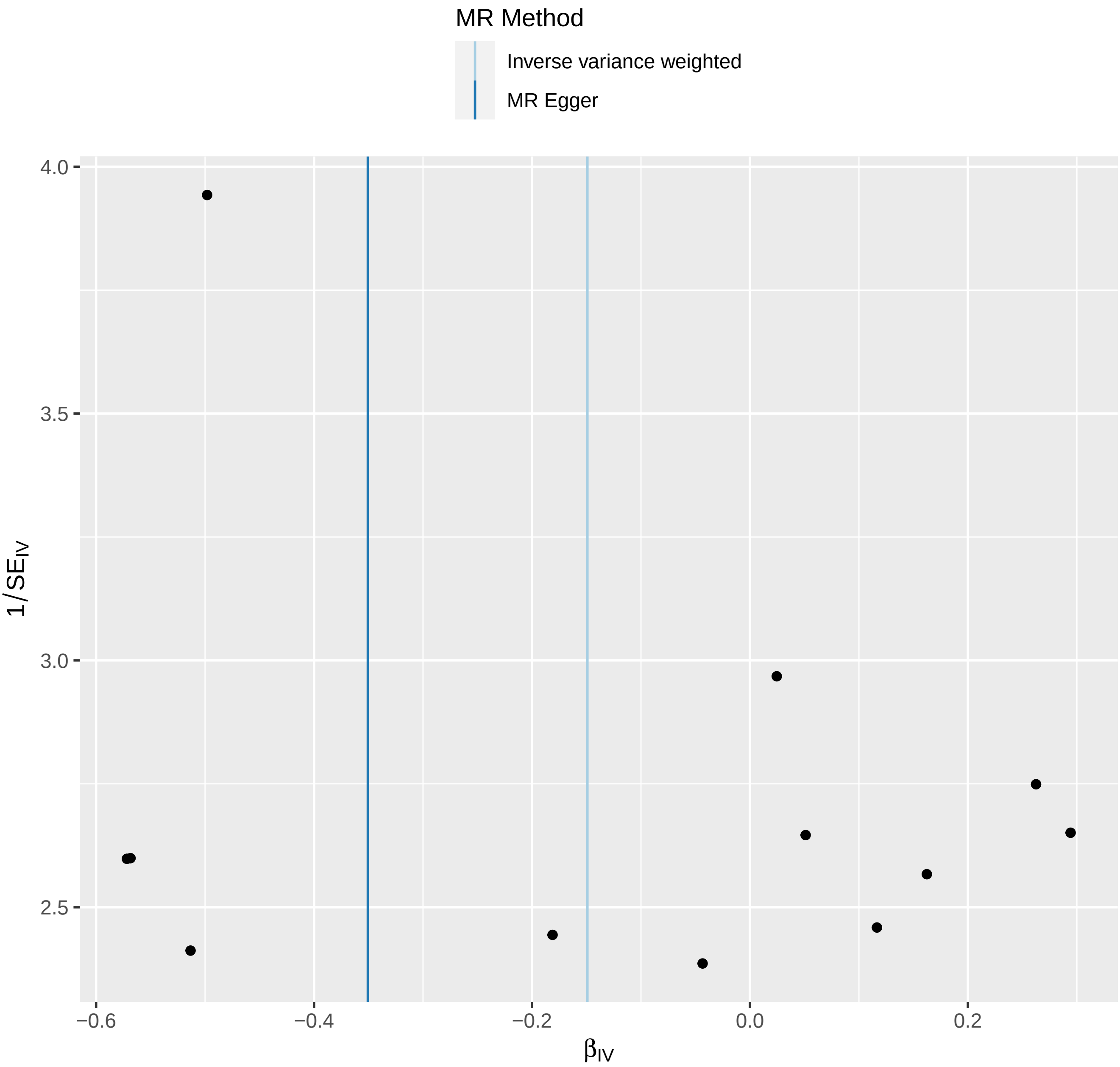

Batch 2−3557 : Gut microbiota abundance (genus Catenibacterium id.2153) on Low−density lipoprotein receptor−related protein 1B
with a threshold of 1e−05

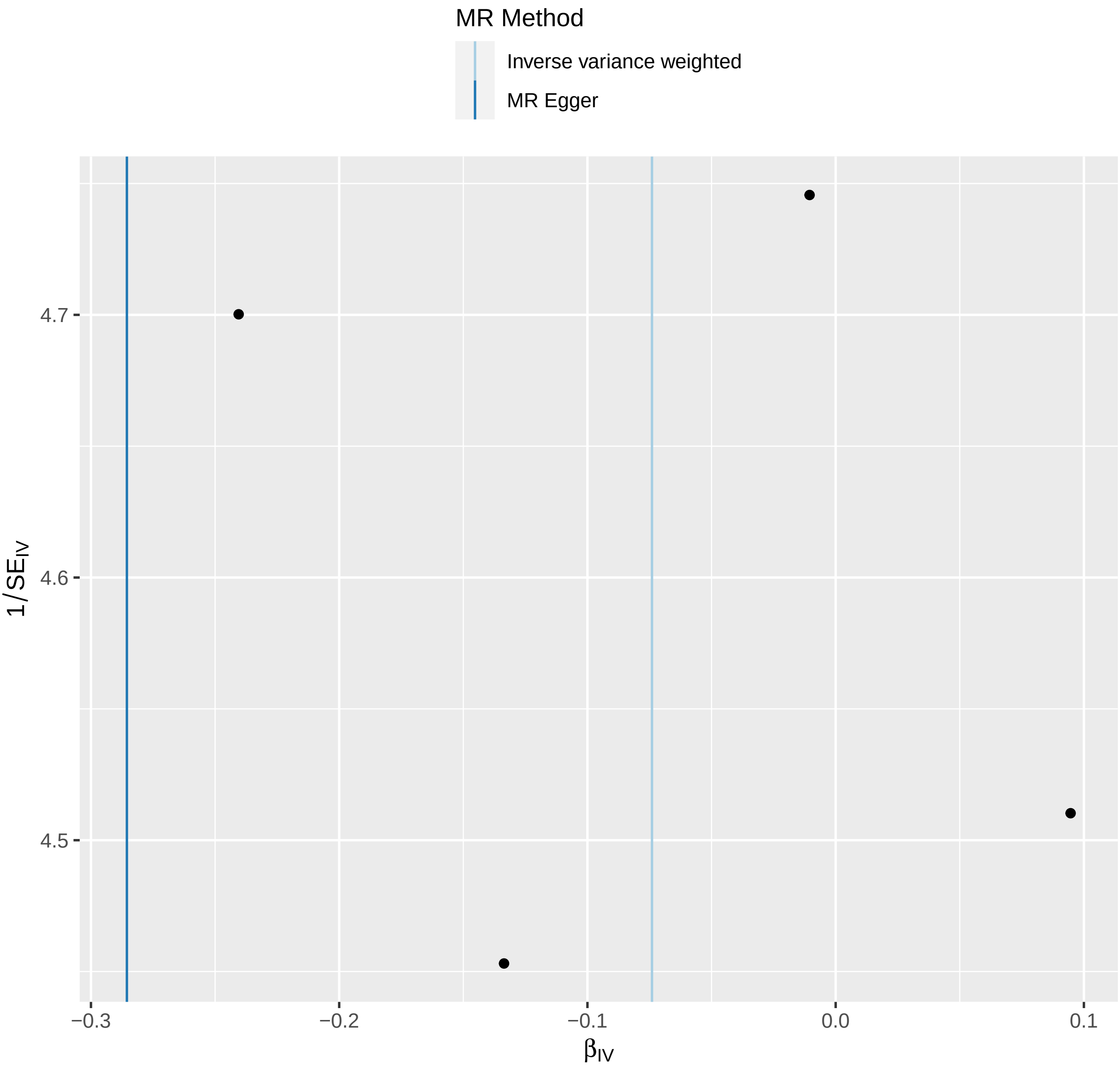

Batch 2−3558 : Gut microbiota abundance (genus Parasutterella id.2892) on Low−density lipoprotein receptor−related protein 1B
with a threshold of 1e−05

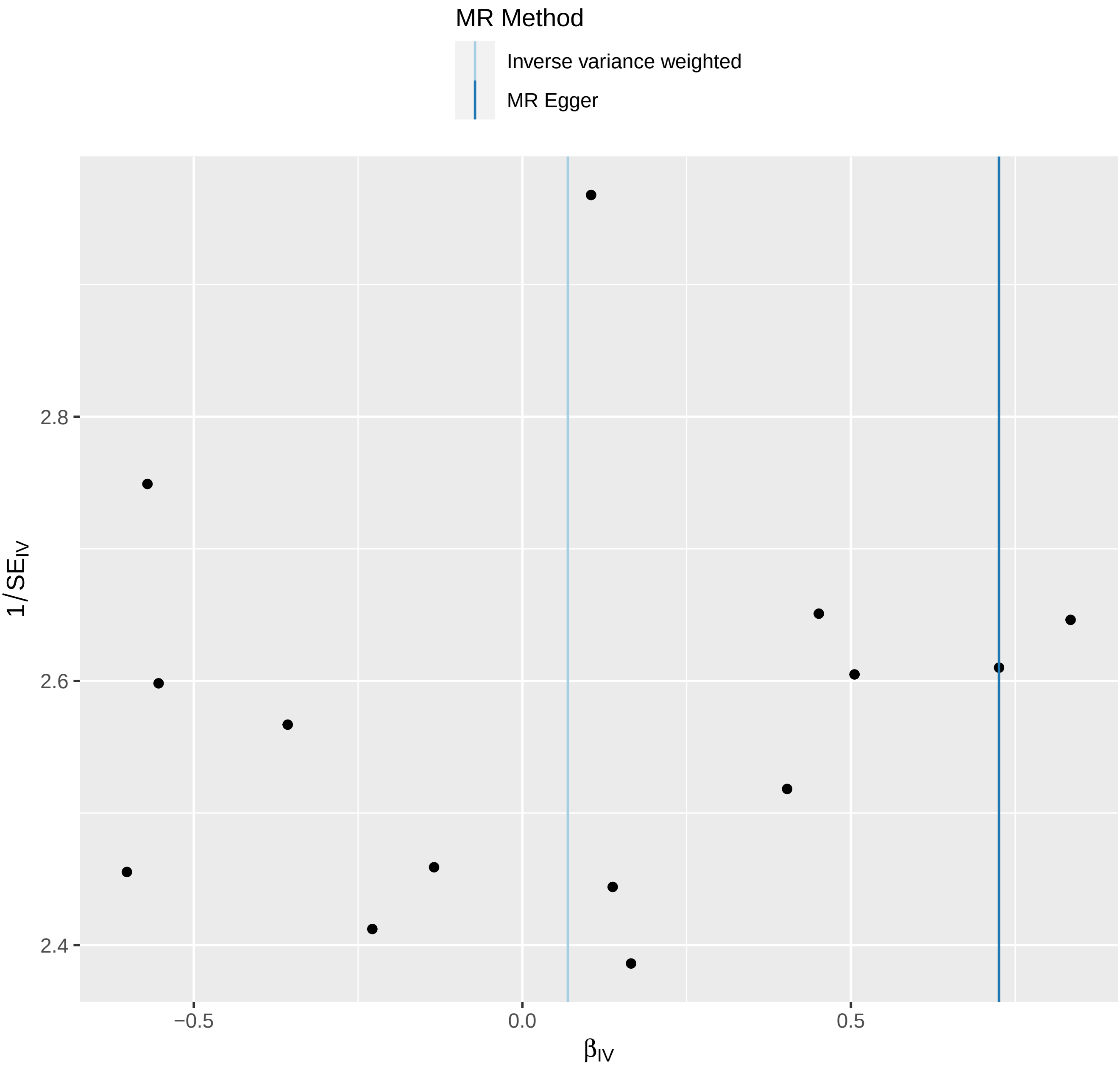

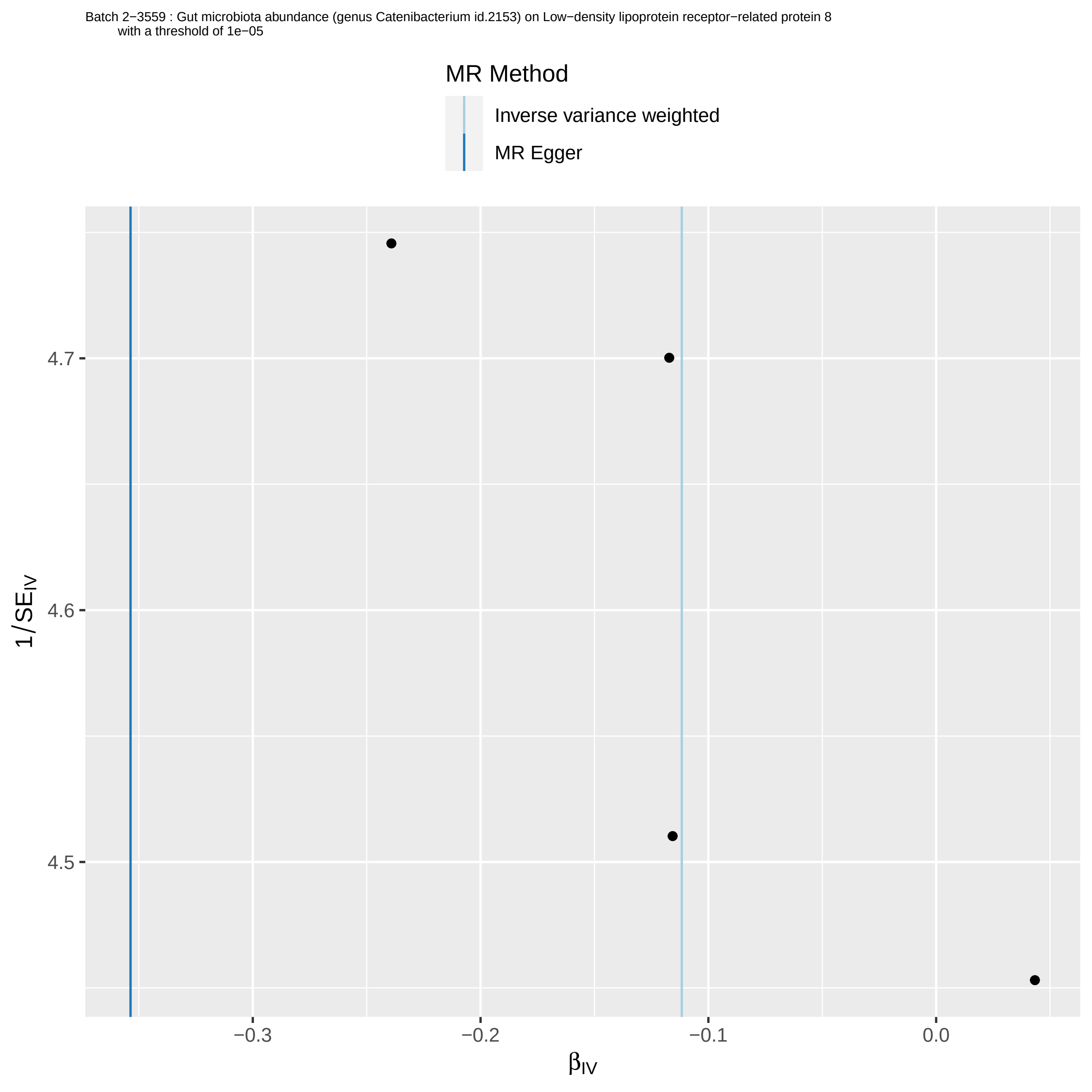

Batch 2−3559 : Gut microbiota abundance (genus Catenibacterium id.2153) on Low−density lipoprotein receptor−related protein 8
with a threshold of 1e−05
MR Method
Inverse variance weighted
MR Egger
4.7
4.6
4.5
1/SE$_{IV}$
−0.3
−0.2
−0.1
0.0
β_{IV}

Batch 2−3560 : Gut microbiota abundance (genus Parasutterella id.2892) on Low−density lipoprotein receptor−related protein 8 with a threshold of 1e−05

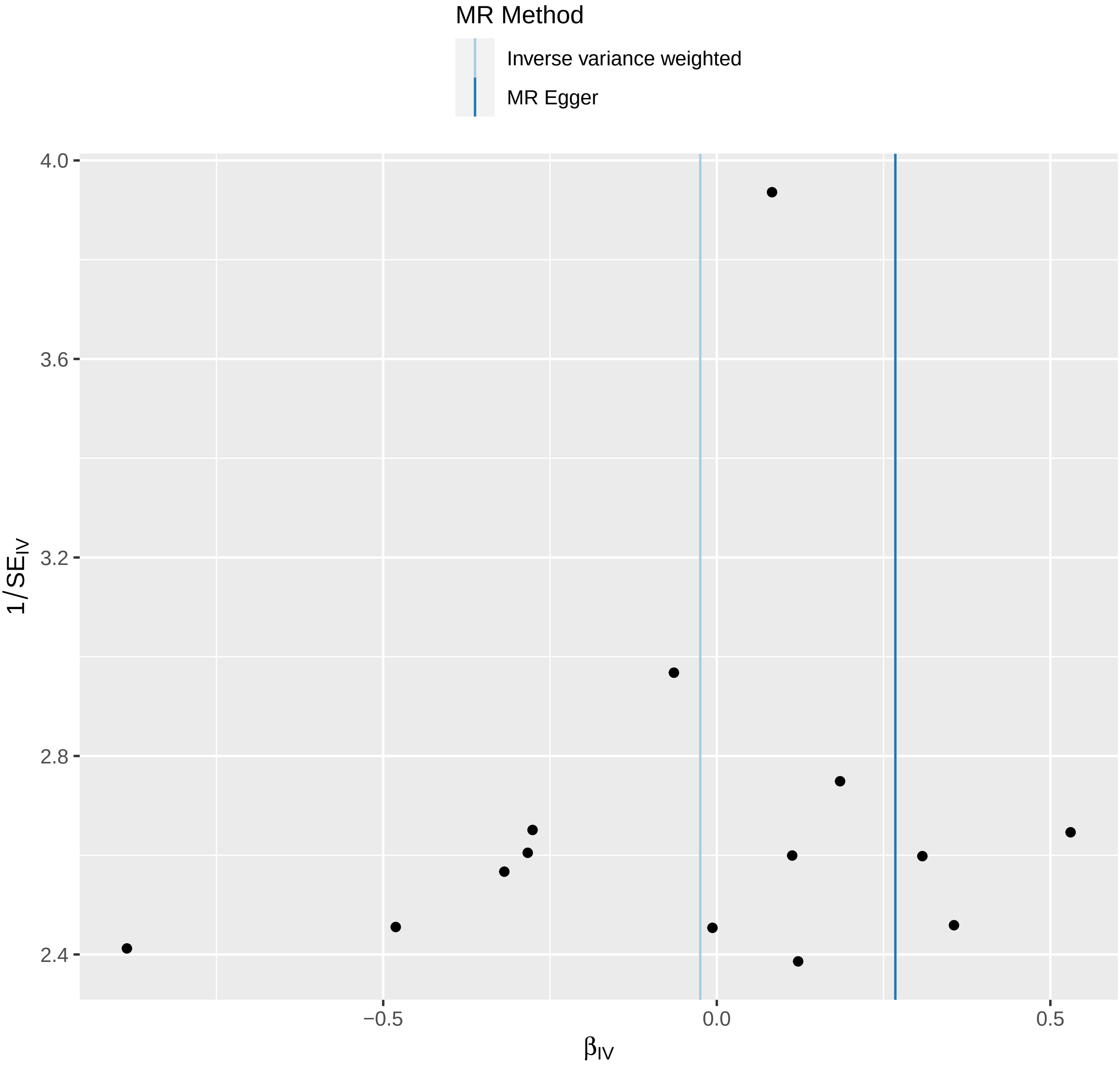

Batch 2−3561 : Gut microbiota abundance (genus Catenibacterium id.2153) on alpha−2−macroglobulin receptor−associated protein with a threshold of 1e−05

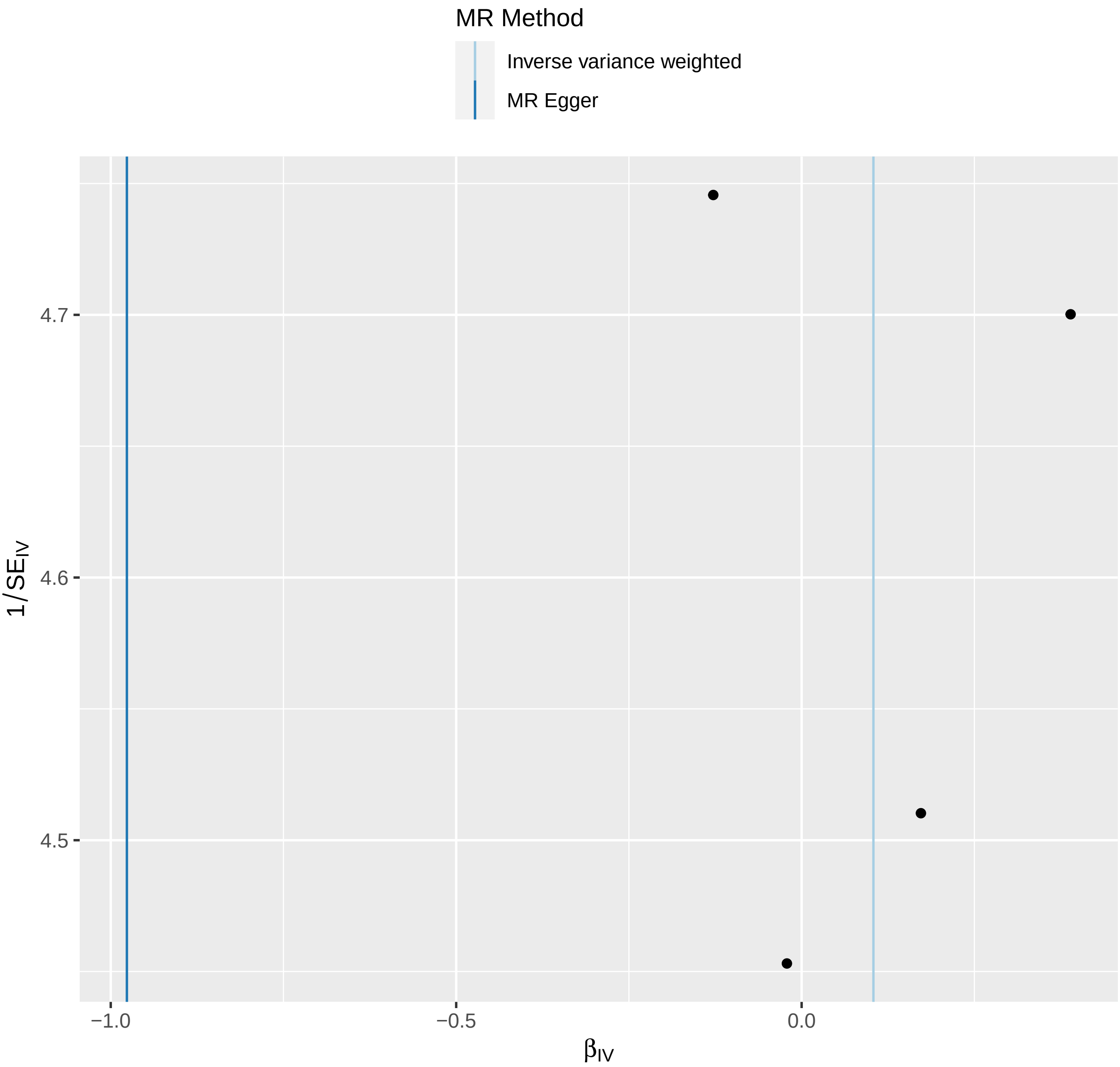

Batch 2−3562 : Gut microbiota abundance (genus Parasutterella id.2892) on alpha−2−macroglobulin receptor−associated protein
with a threshold of 1e−05

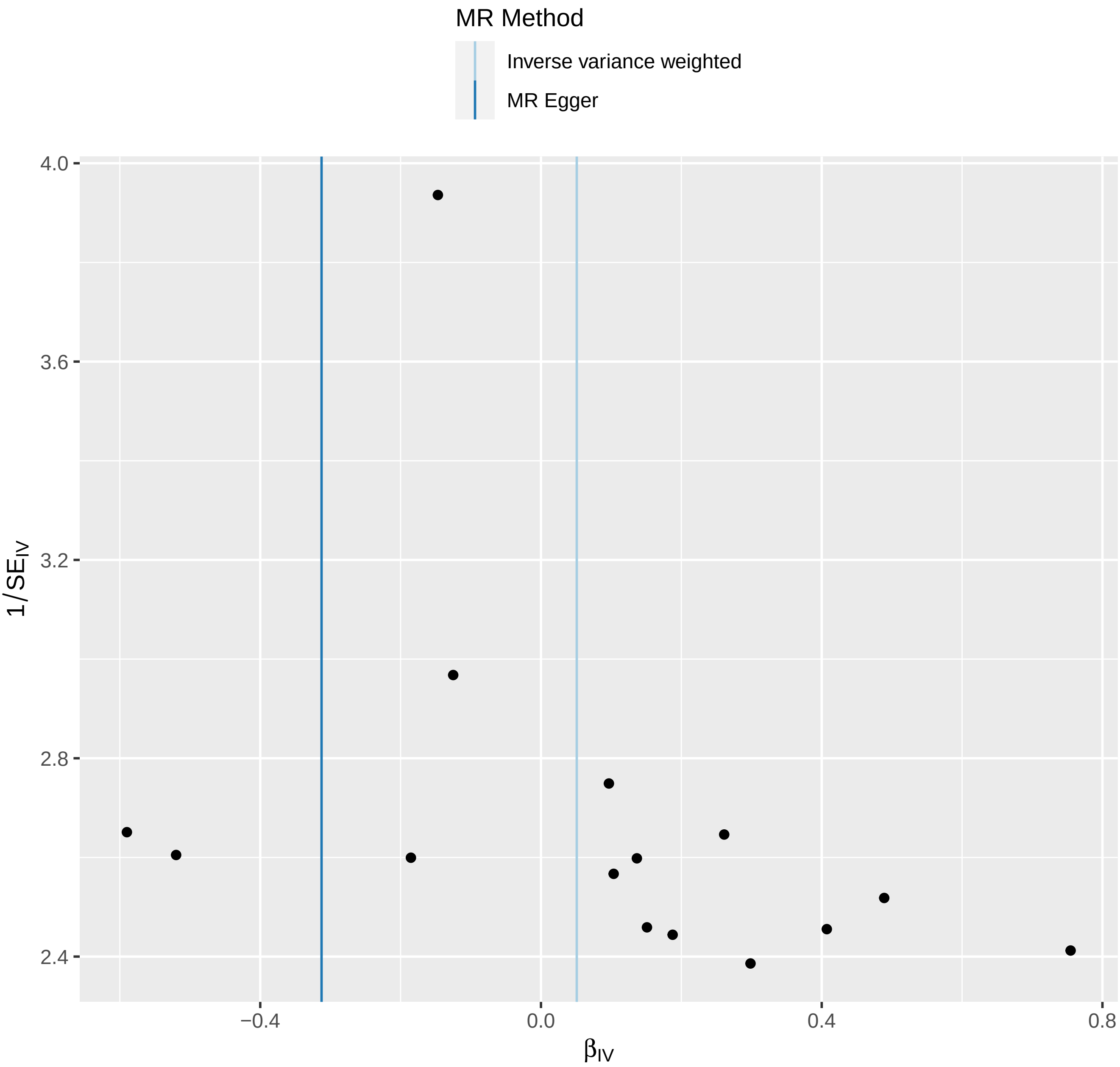

Batch 2−3563 : Gut microbiota abundance (genus Catenibacterium id.2153) on alpha−2−macroglobulin receptor−associated protein with a threshold of 1e−05

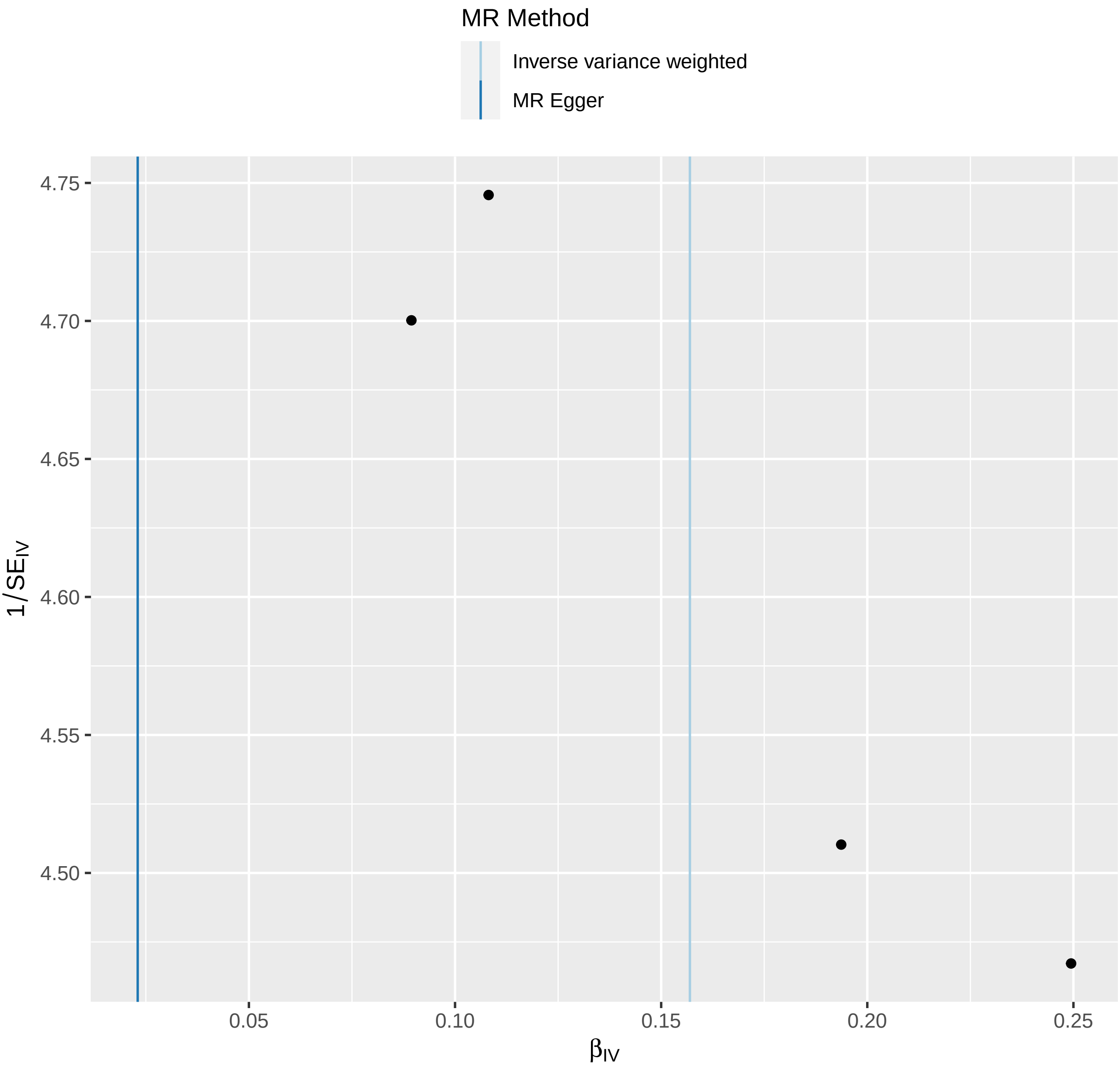

Batch 2−3564 : Gut microbiota abundance (genus Parasutterella id.2892) on alpha−2−macroglobulin receptor−associated protein with a threshold of 1e−05

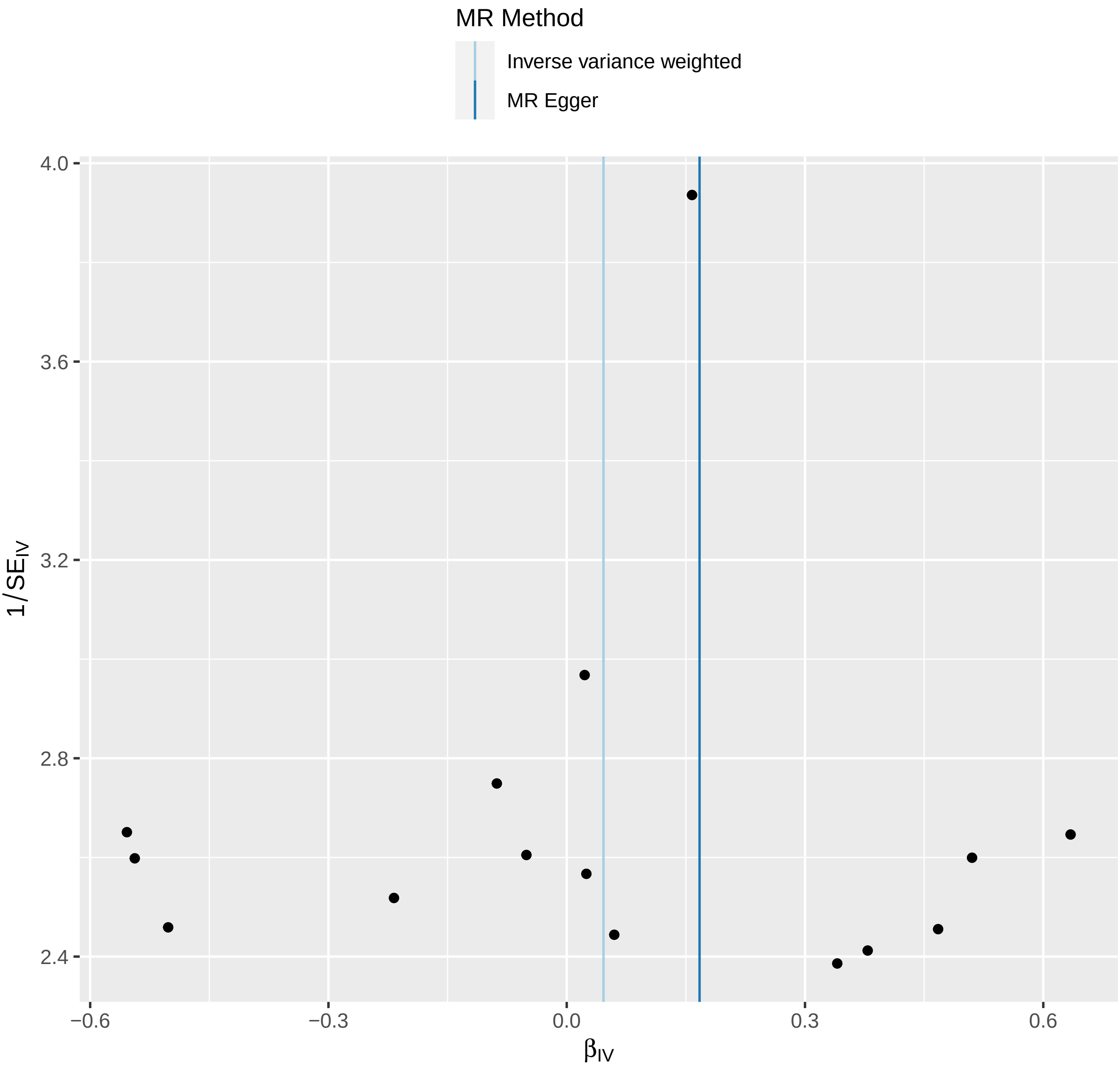

Batch 2−3565 : Gut microbiota abundance (genus Catenibacterium id.2153) on Leucine−rich PPR motif−containing protein, mitochondrial
with a threshold of 1e−05

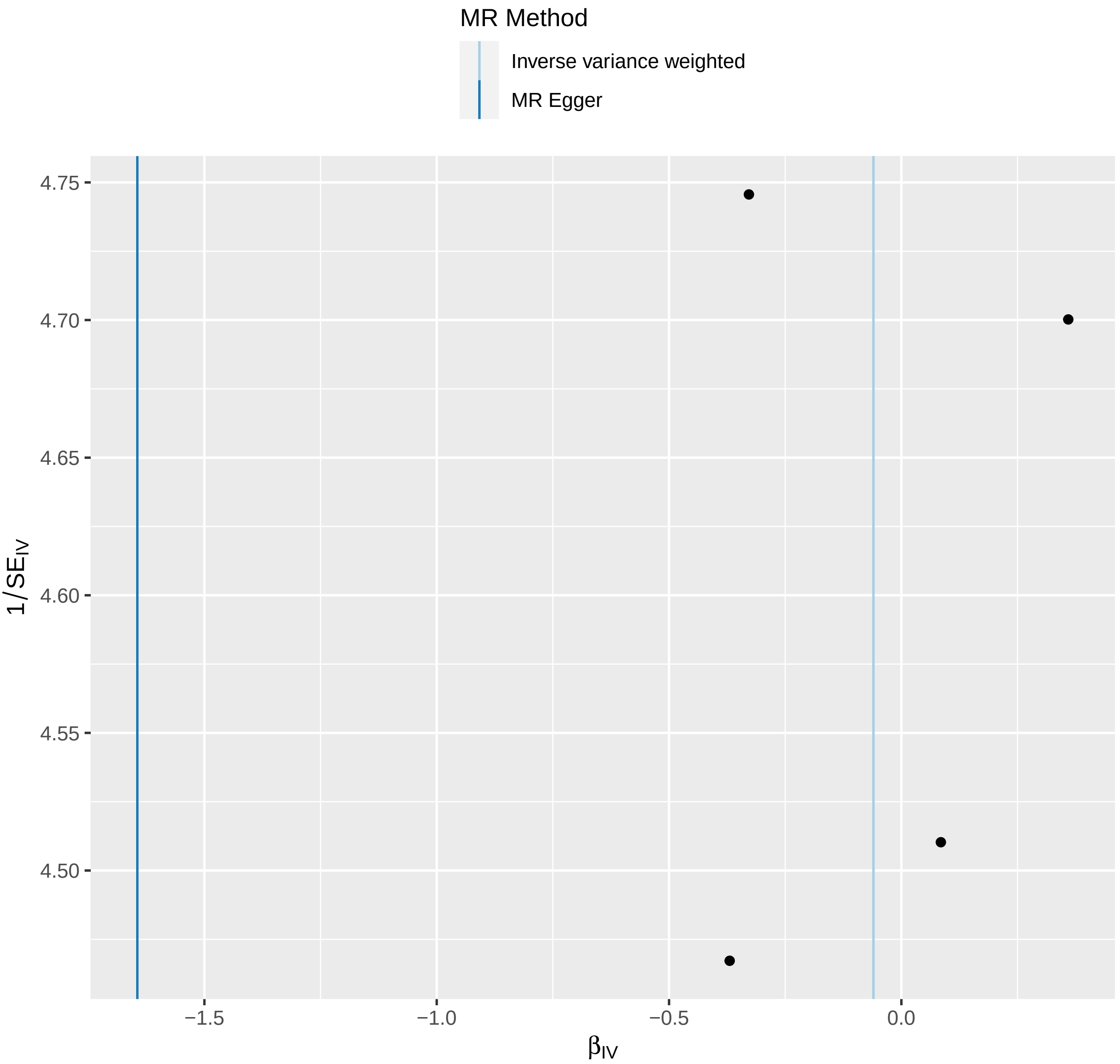

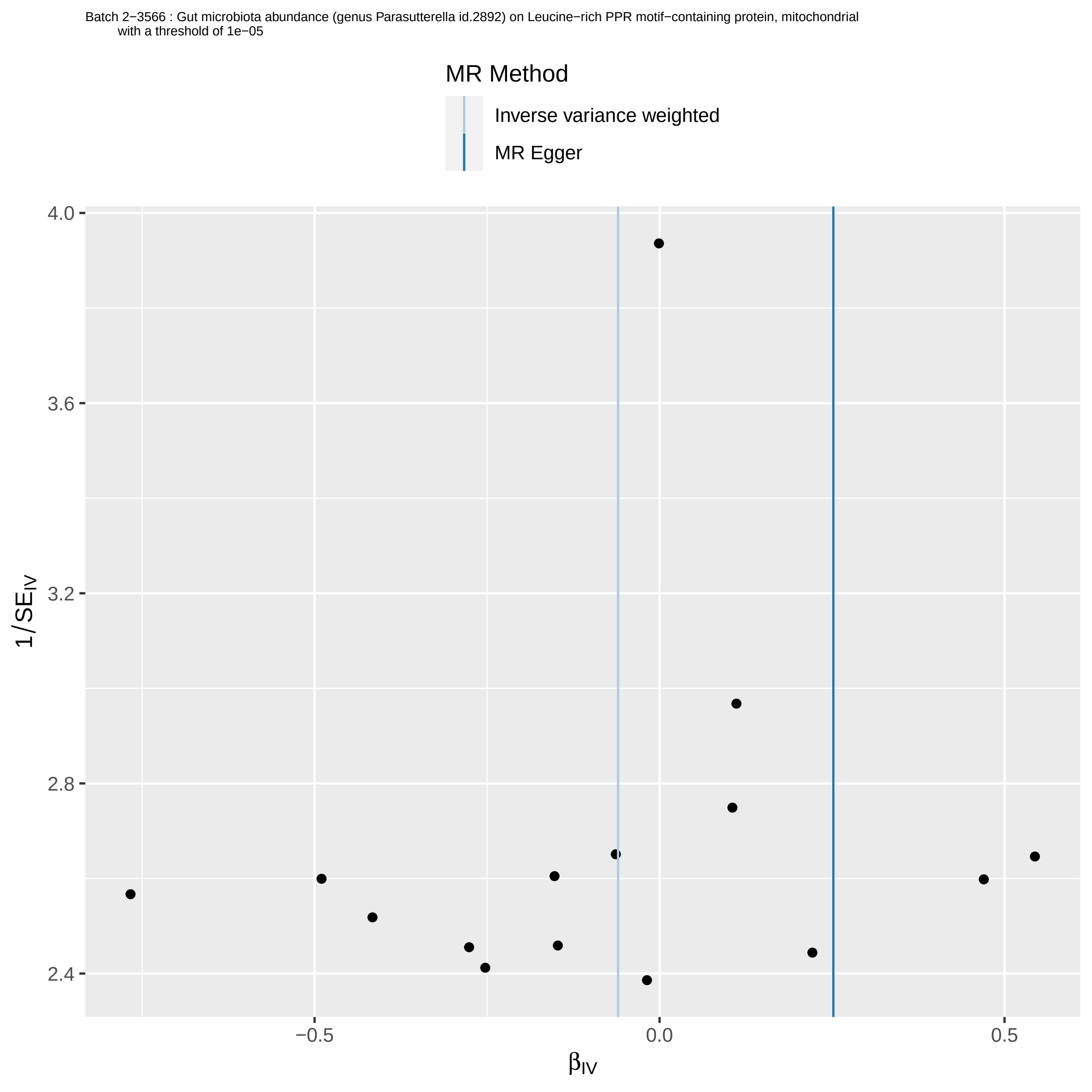
Batch 2−3566 : Gut microbiota abundance (genus Parasutterella id.2892) on Leucine−rich PPR motif−containing protein, mitochondrial
with a threshold of 1e−05
MR Method
Inverse variance weighted
MR Egger
4.0
3.6
3.2
2.8
2.4
1/SE_IV
−0.5
0.0
0.5
β_IV

Batch 2−3567 : Gut microbiota abundance (genus Catenibacterium id.2153) on Leucine−rich repeat−containing protein 15
with a threshold of 1e−05

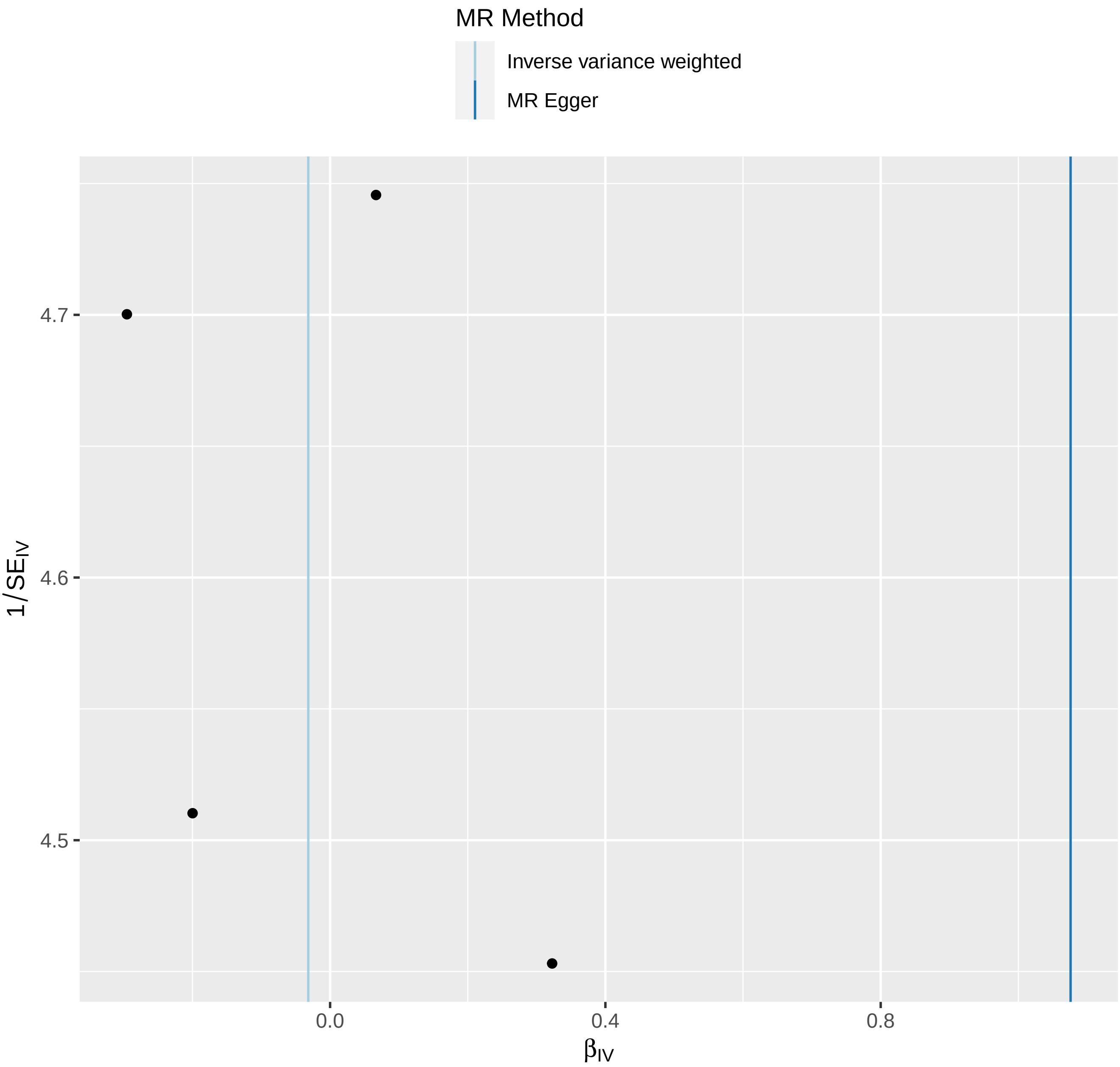

Batch 2−3568 : Gut microbiota abundance (genus Parasutterella id.2892) on Leucine−rich repeat−containing protein 15
with a threshold of 1e−05

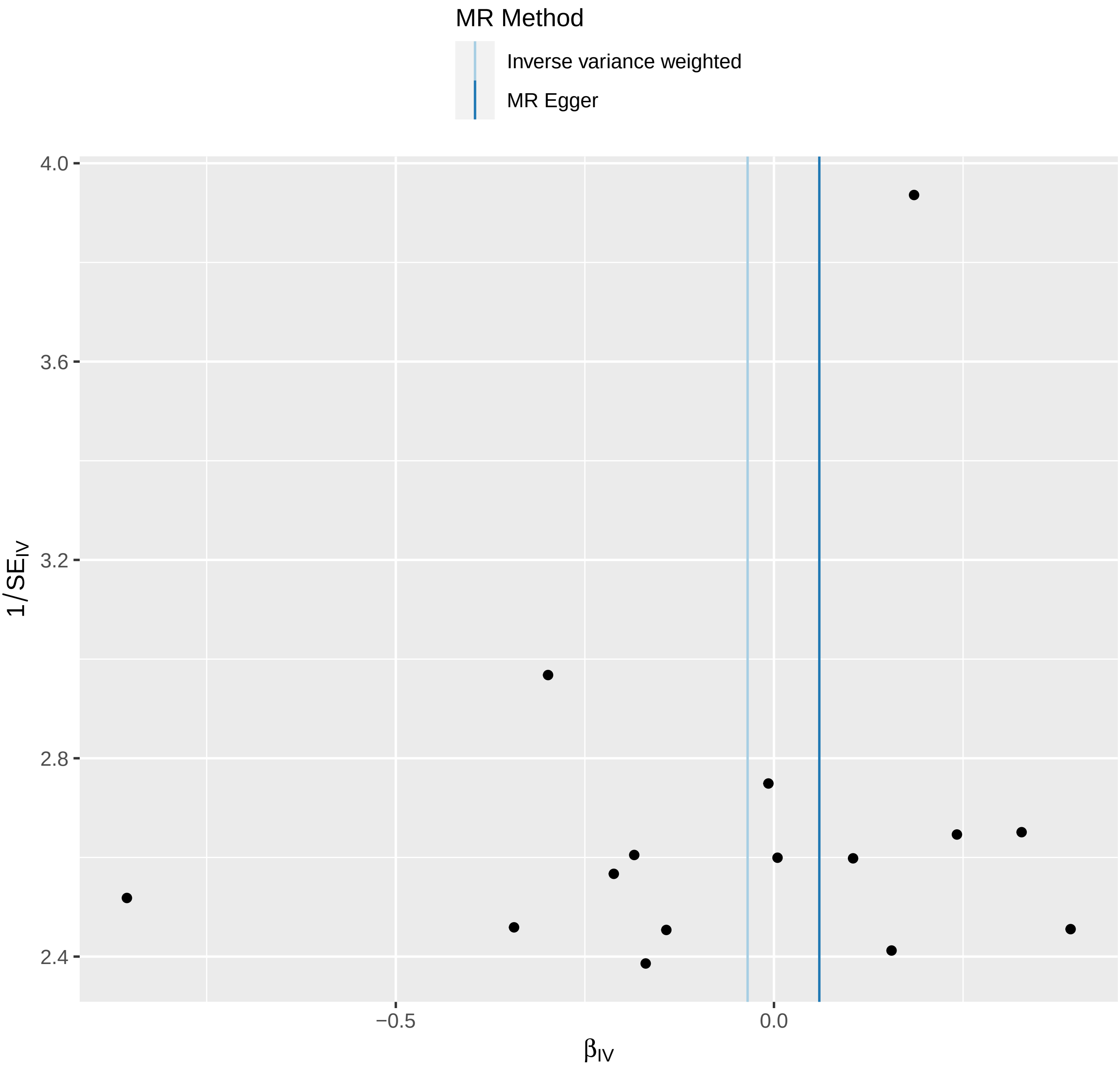

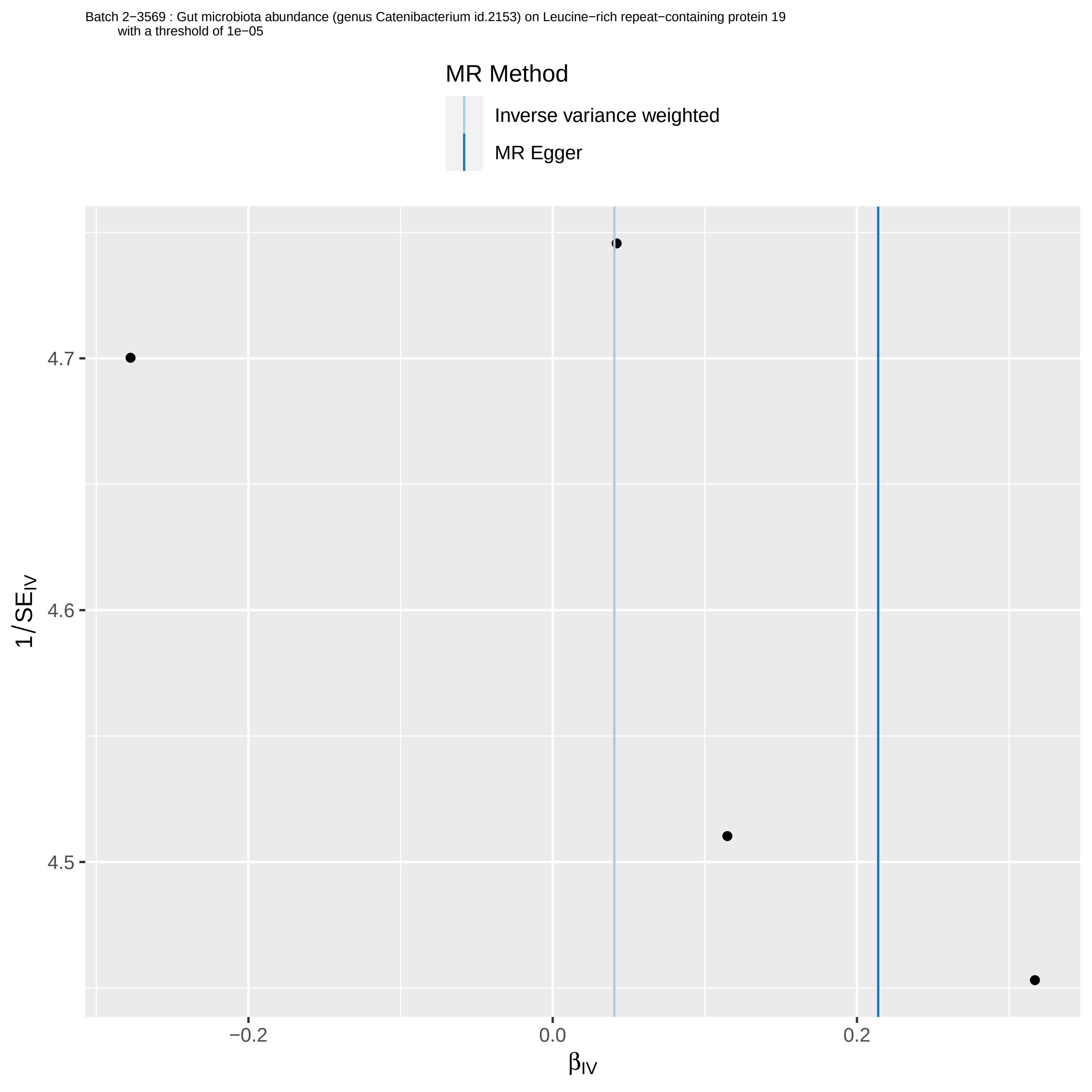

Batch 2−3569 : Gut microbiota abundance (genus Catenibacterium id.2153) on Leucine−rich repeat−containing protein 19
with a threshold of 1e−05
MR Method
Inverse variance weighted
MR Egger
4.7
4.6
4.5
1/SE_IV
−0.2
0.0
0.2
β_IV

Batch 2−3570 : Gut microbiota abundance (genus Parasutterella id.2892) on Leucine−rich repeat−containing protein 19
with a threshold of 1e−05

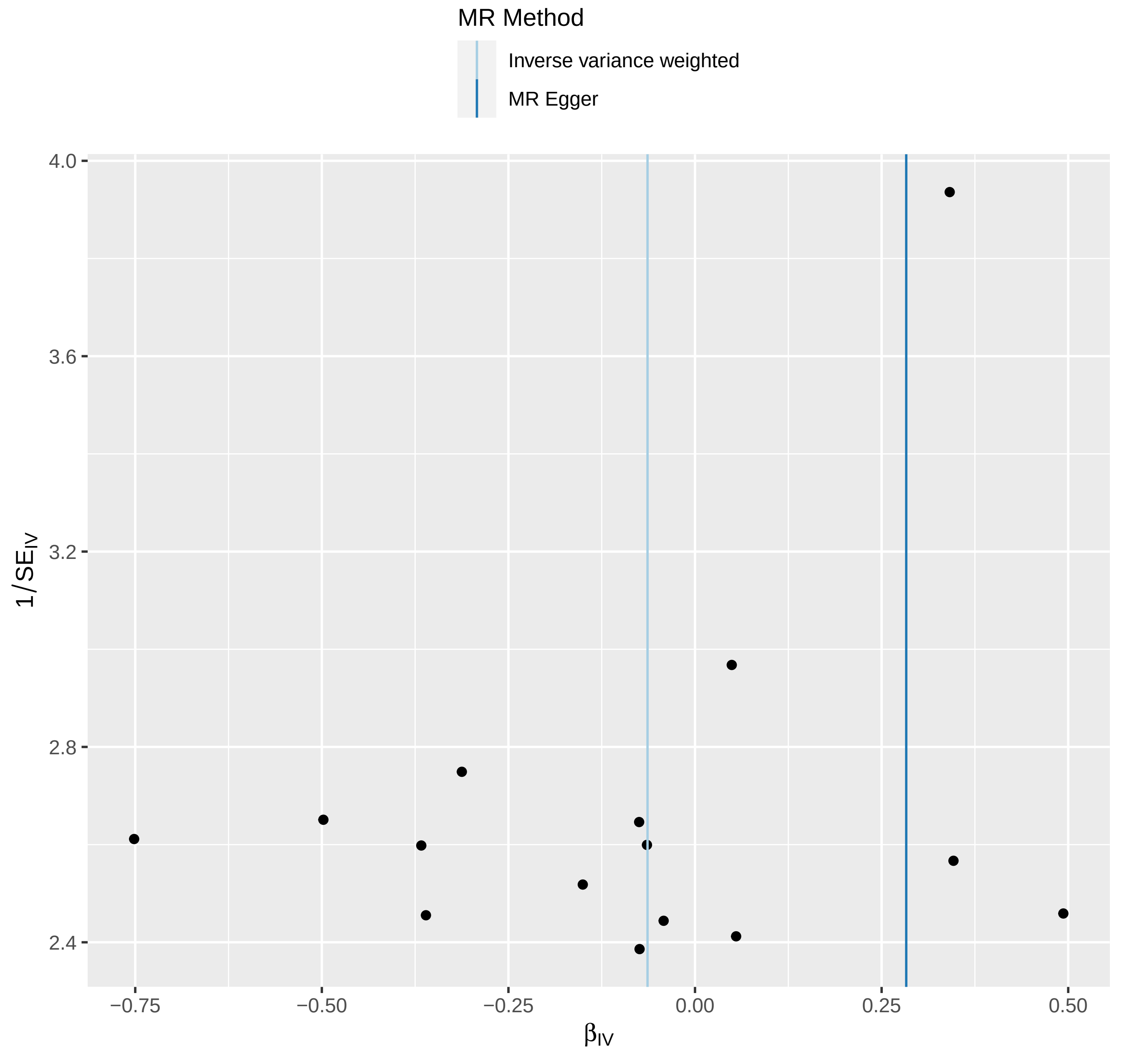

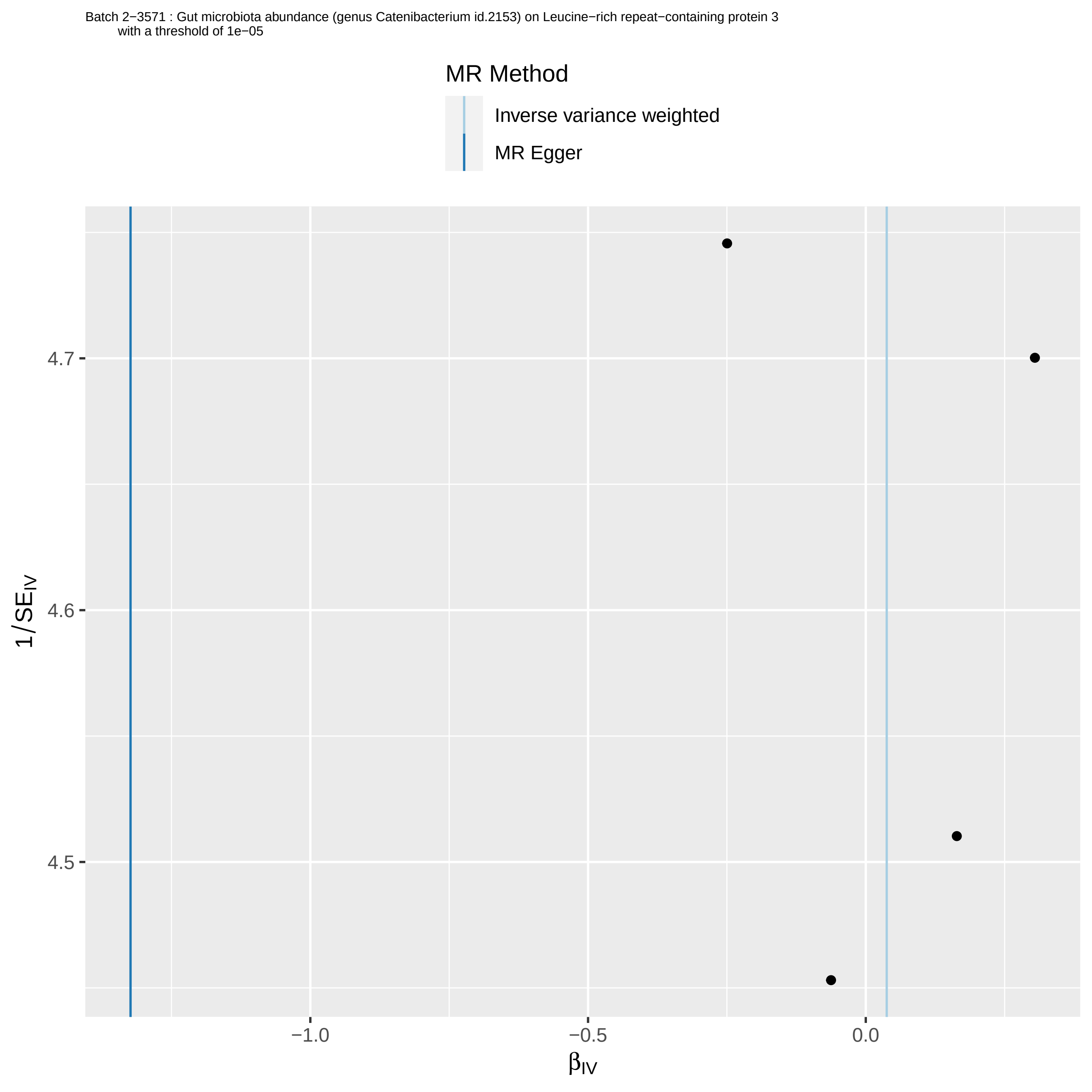
Batch 2−3571 : Gut microbiota abundance (genus Catenibacterium id.2153) on Leucine−rich repeat−containing protein 3
with a threshold of 1e−05
MR Method
Inverse variance weighted
MR Egger
4.7
4.6
4.5
1/SE_IV
−1.0
−0.5
0.0
β_IV

Batch 2−3572 : Gut microbiota abundance (genus Parasutterella id.2892) on Leucine−rich repeat−containing protein 3
with a threshold of 1e−05

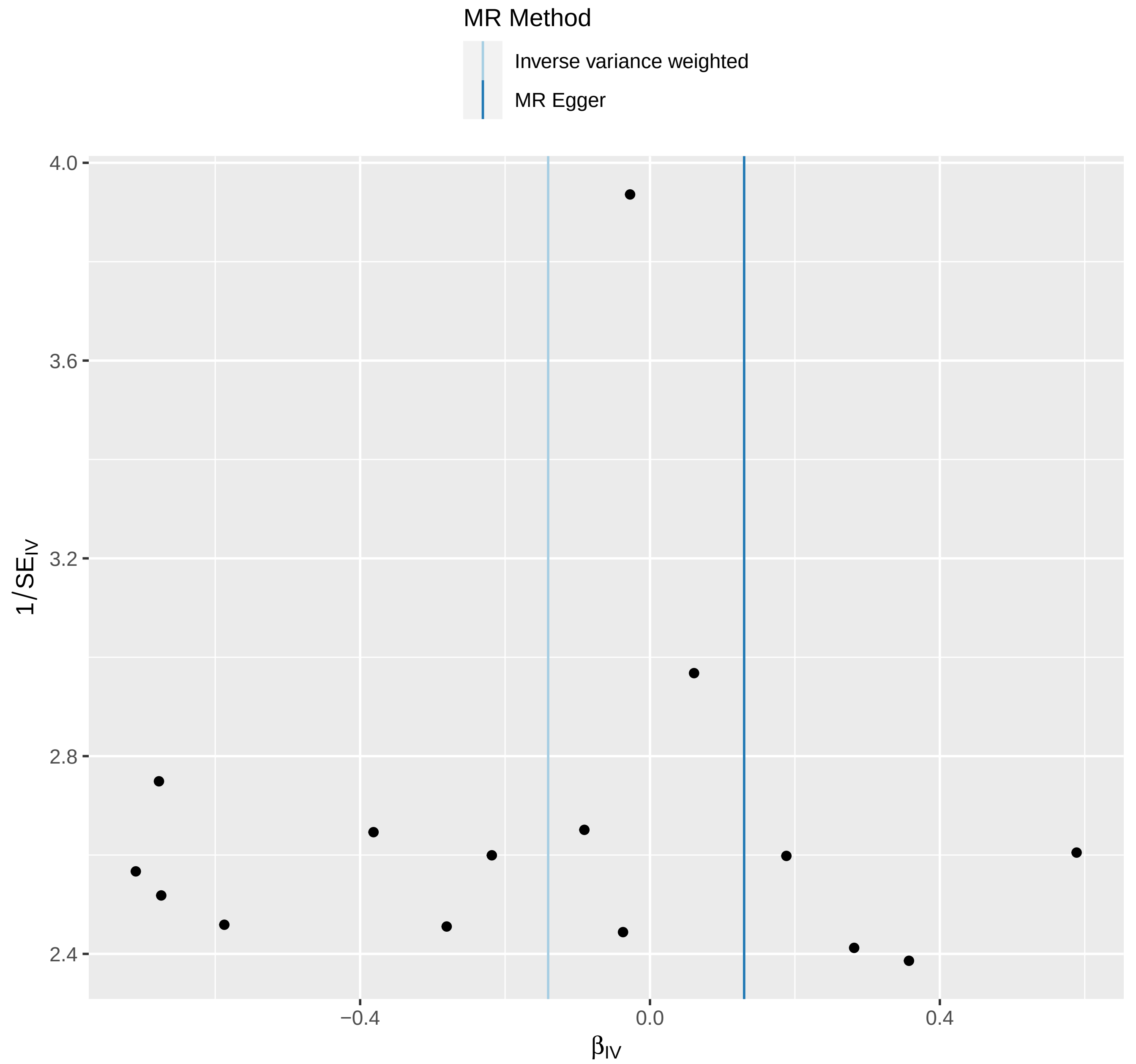

Batch 2−3573 : Gut microbiota abundance (genus Catenibacterium id.2153) on Leucine−rich repeat−containing protein 37A2
with a threshold of 1e−05

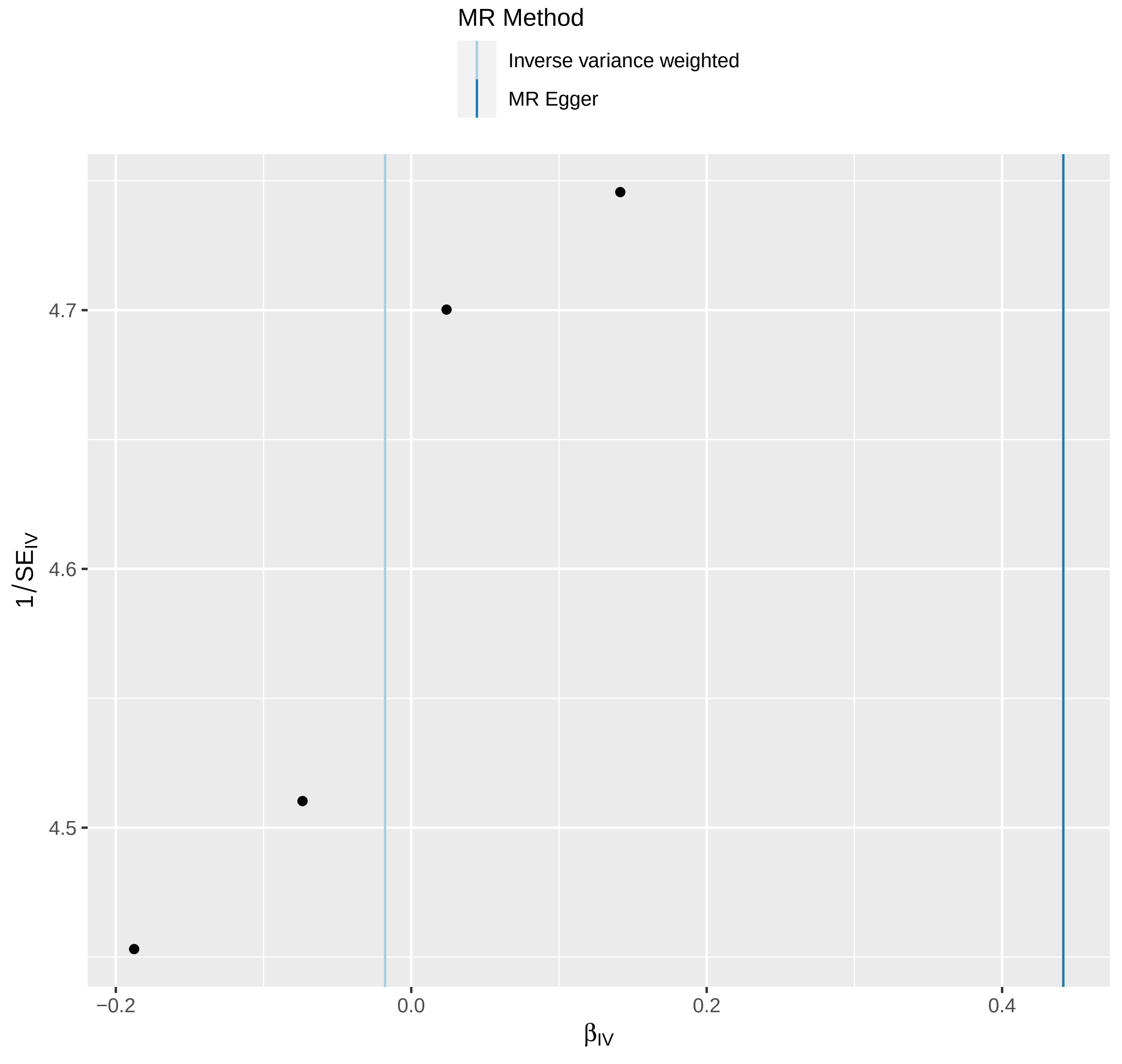

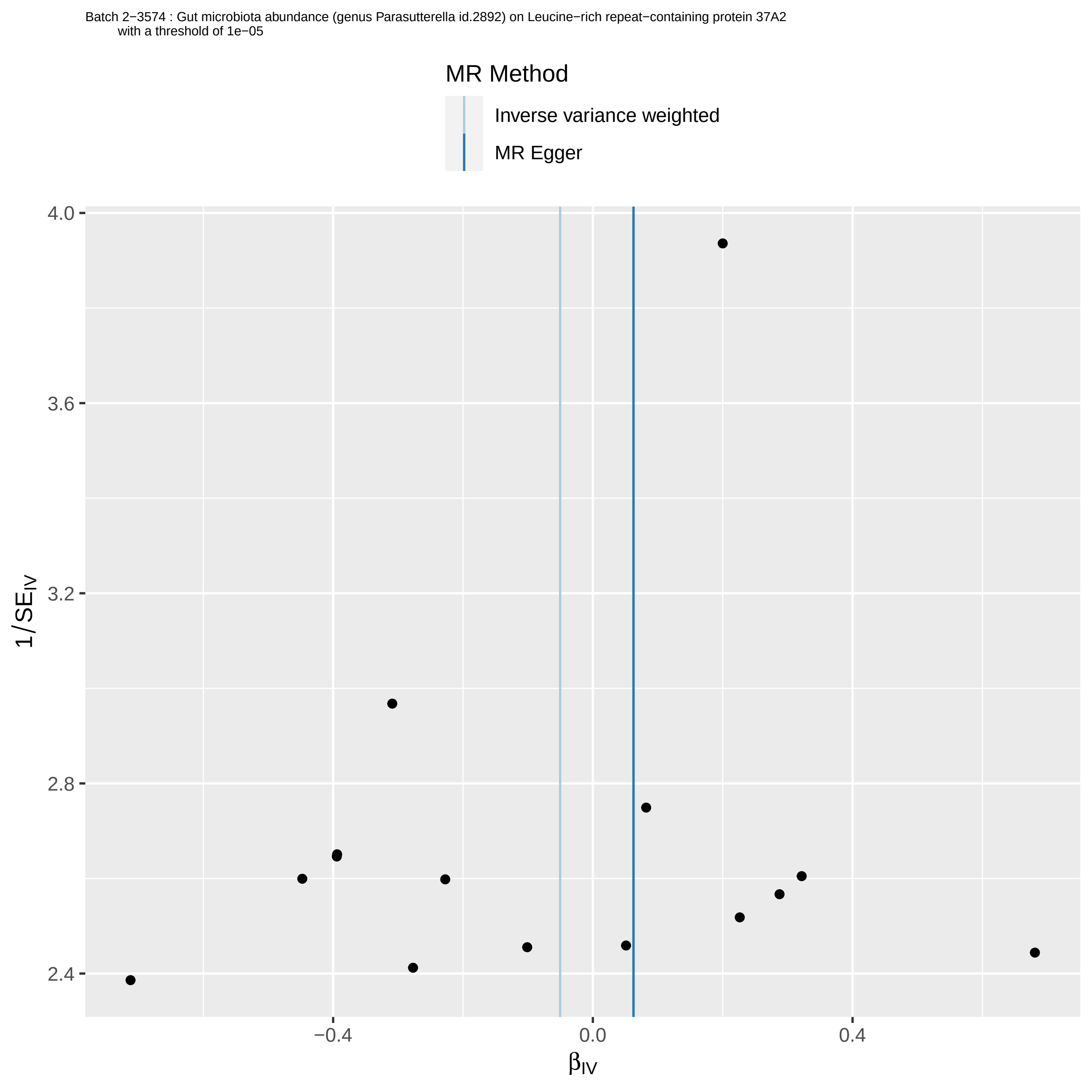

Batch 2−3574 : Gut microbiota abundance (genus Parasutterella id.2892) on Leucine−rich repeat−containing protein 37A2
with a threshold of 1e−05
MR Method
Inverse variance weighted
MR Egger
4.0
3.6
3.2
2.8
2.4
1/SE_IV
−0.4
0.0
0.4
β_IV

Batch 2−3575 : Gut microbiota abundance (genus Catenibacterium id.2153) on Leucine−rich repeat−containing protein 4B with a threshold of 1e−05

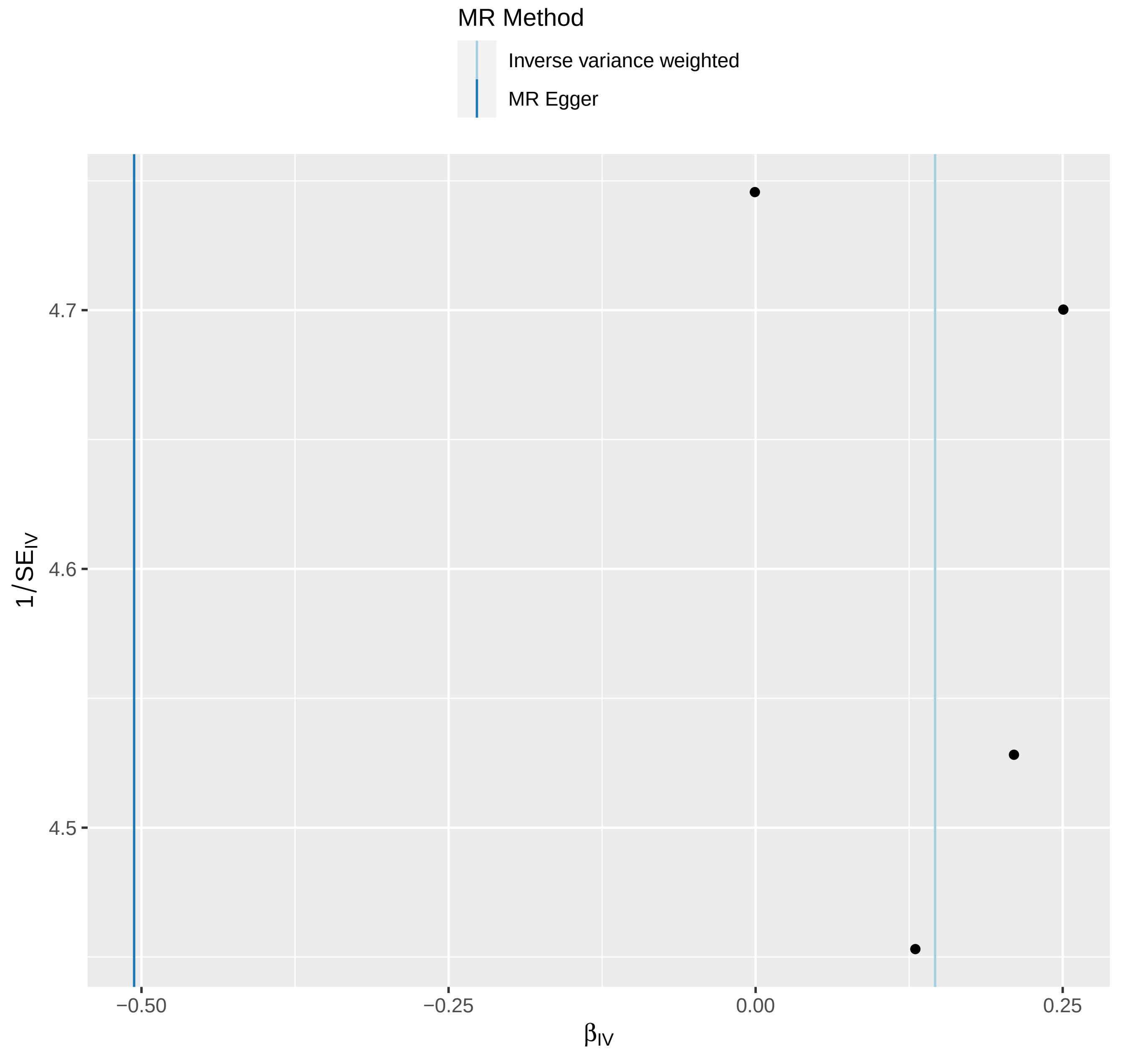

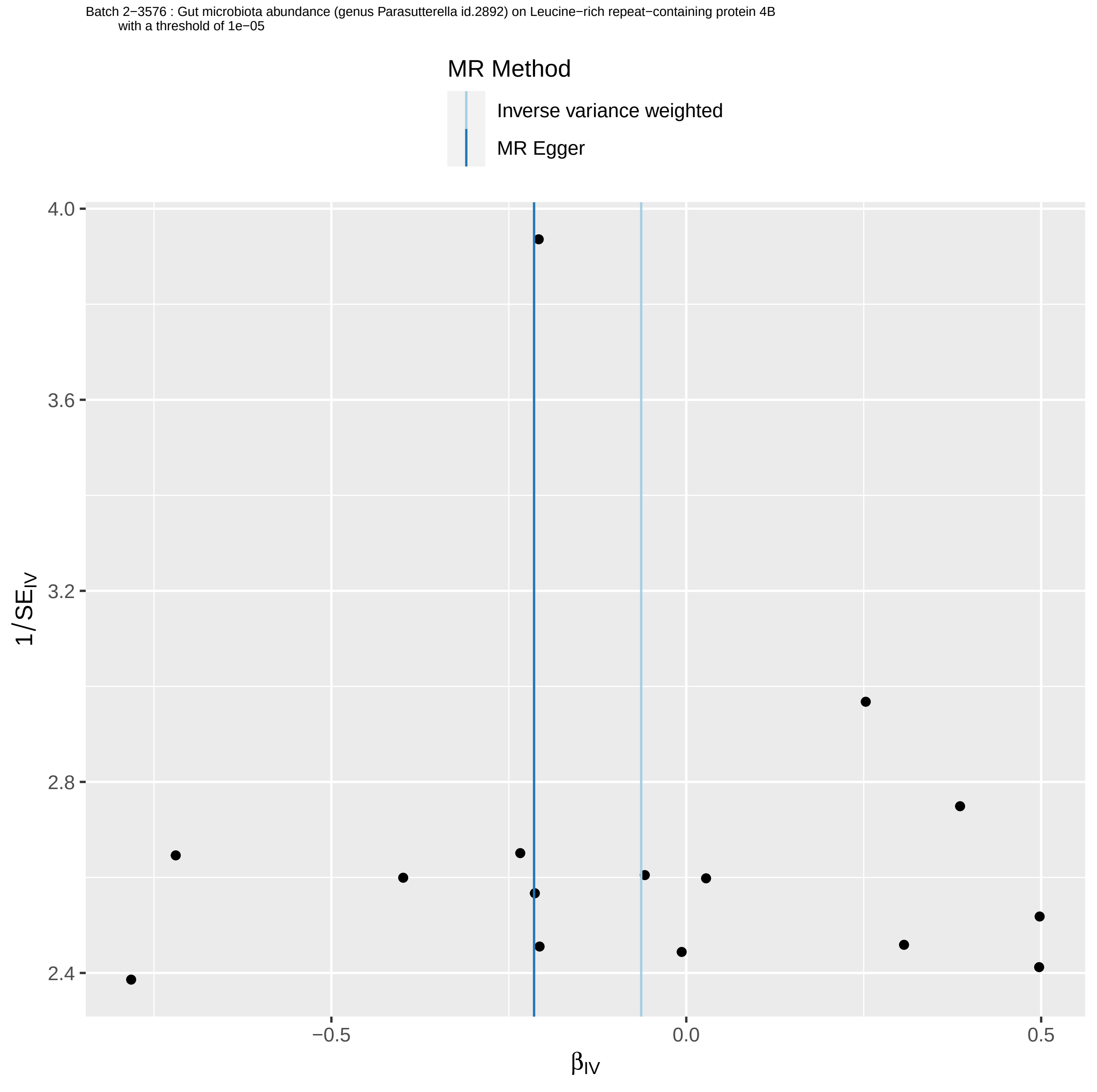
Batch 2−3576 : Gut microbiota abundance (genus Parasutterella id.2892) on Leucine−rich repeat−containing protein 4B
with a threshold of 1e−05
MR Method
Inverse variance weighted
MR Egger
4.0
3.6
3.2
2.8
2.4
1/SE_IV
−0.5
0.0
0.5
β_IV

Batch 2−3577 : Gut microbiota abundance (genus Catenibacterium id.2153) on Leucine−rich repeat−containing protein 4C with a threshold of 1e−05

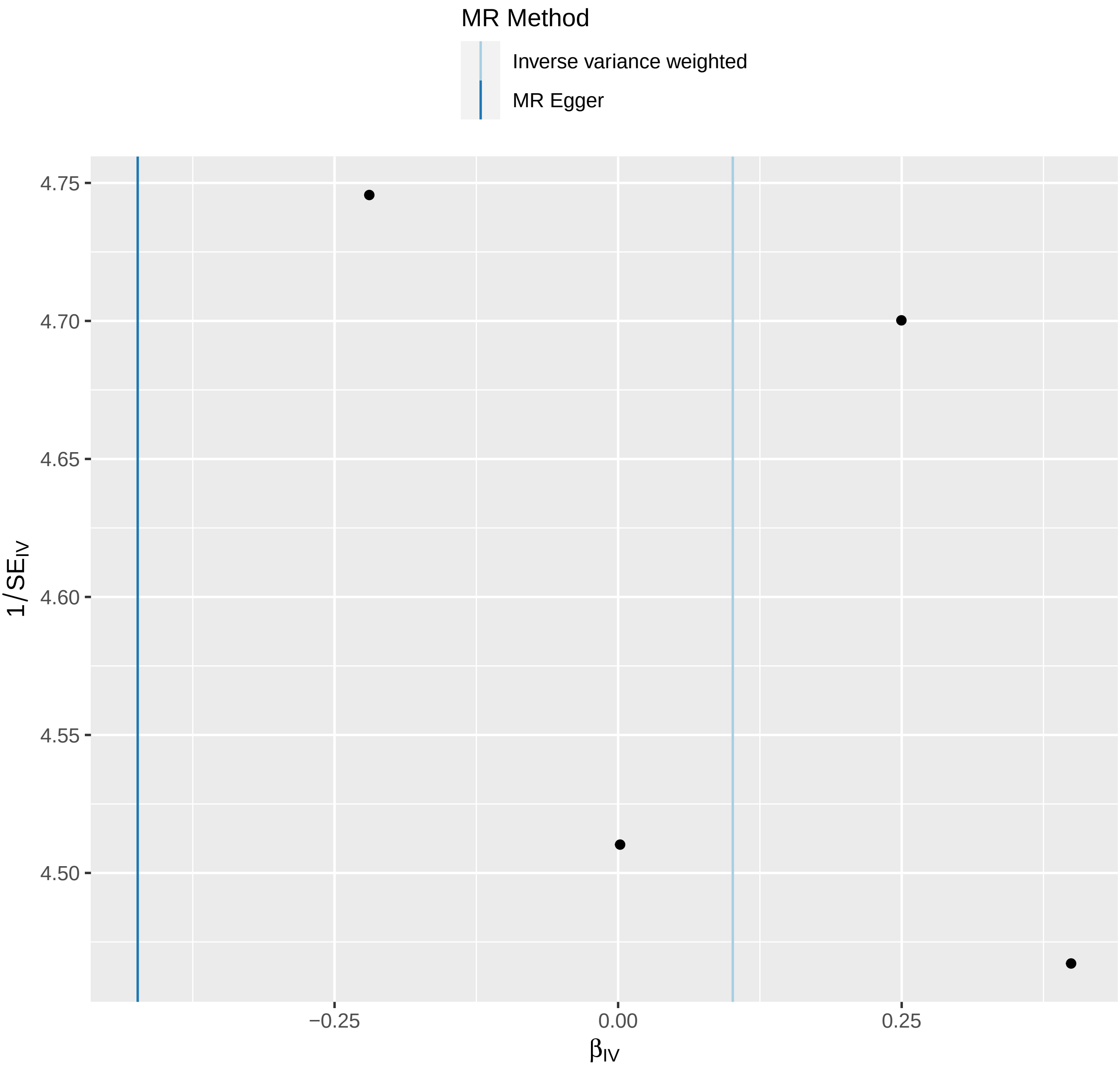

Batch 2−3578 : Gut microbiota abundance (genus Parasutterella id.2892) on Leucine−rich repeat−containing protein 4C
with a threshold of 1e−05

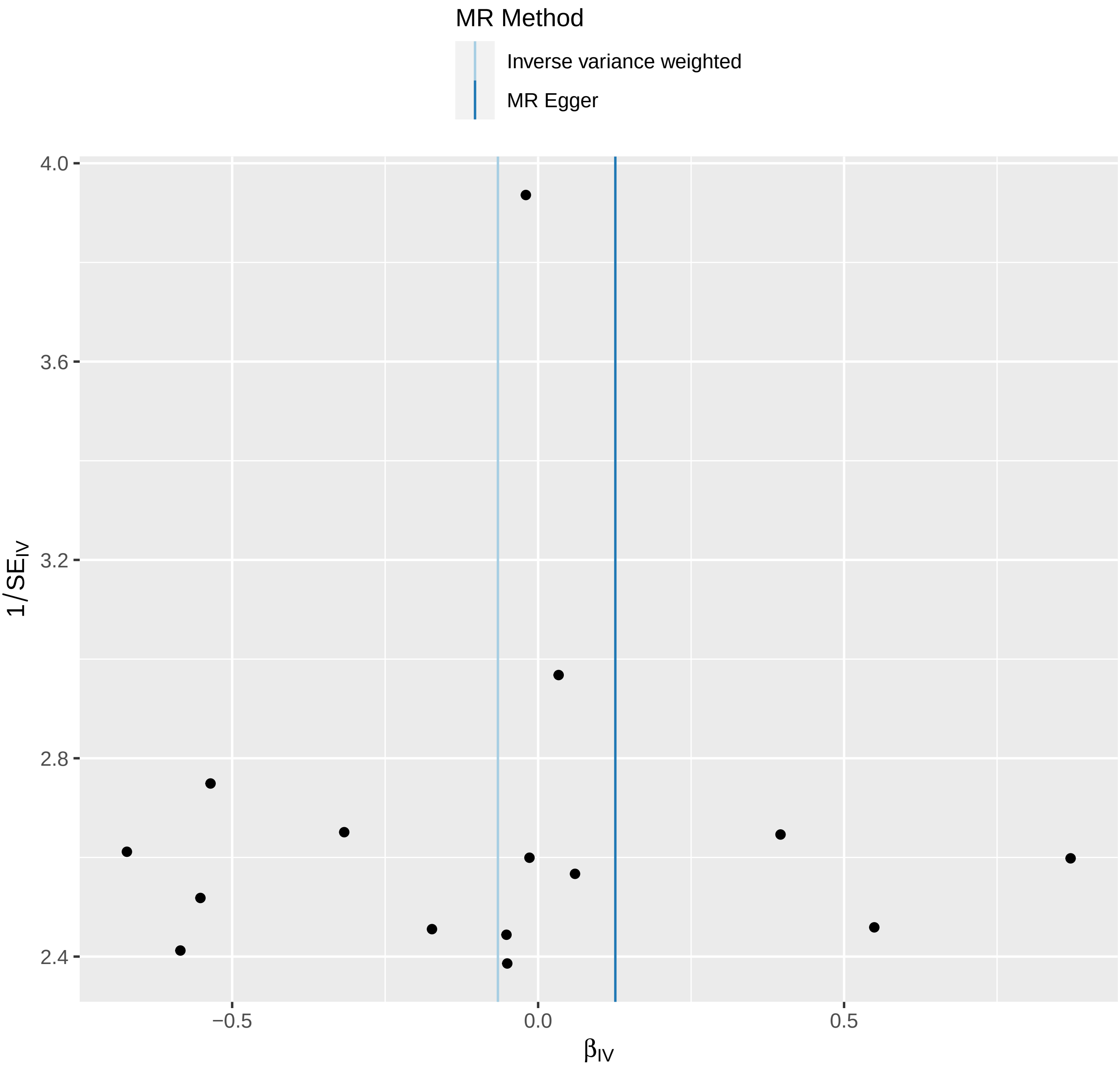

Batch 2−3579 : Gut microbiota abundance (genus Catenibacterium id.2153) on Leucine−rich repeat−containing protein 74A
with a threshold of 1e−05

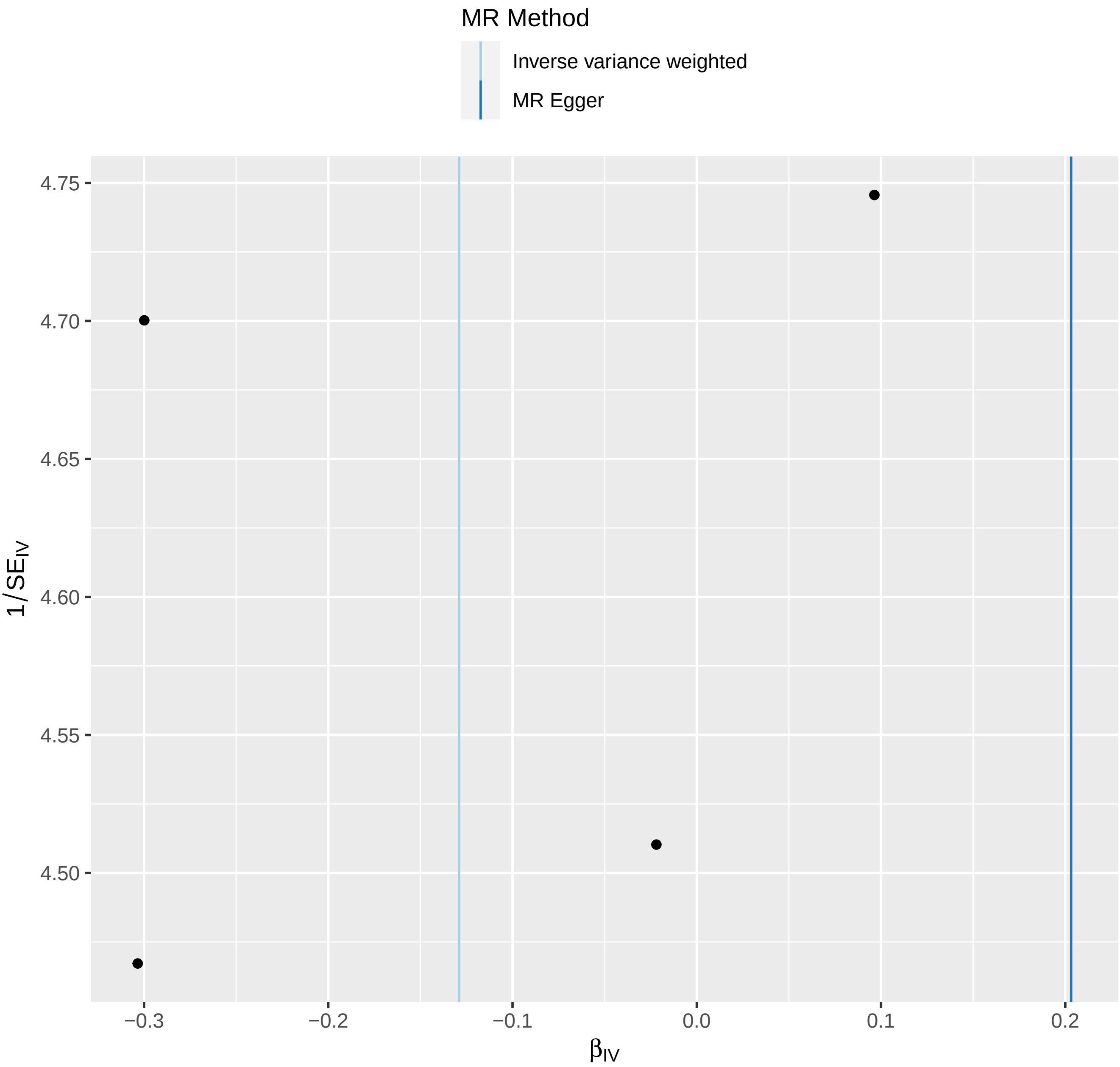

Batch 2−3580 : Gut microbiota abundance (genus Parasutterella id.2892) on Leucine−rich repeat−containing protein 74A with a threshold of 1e−05

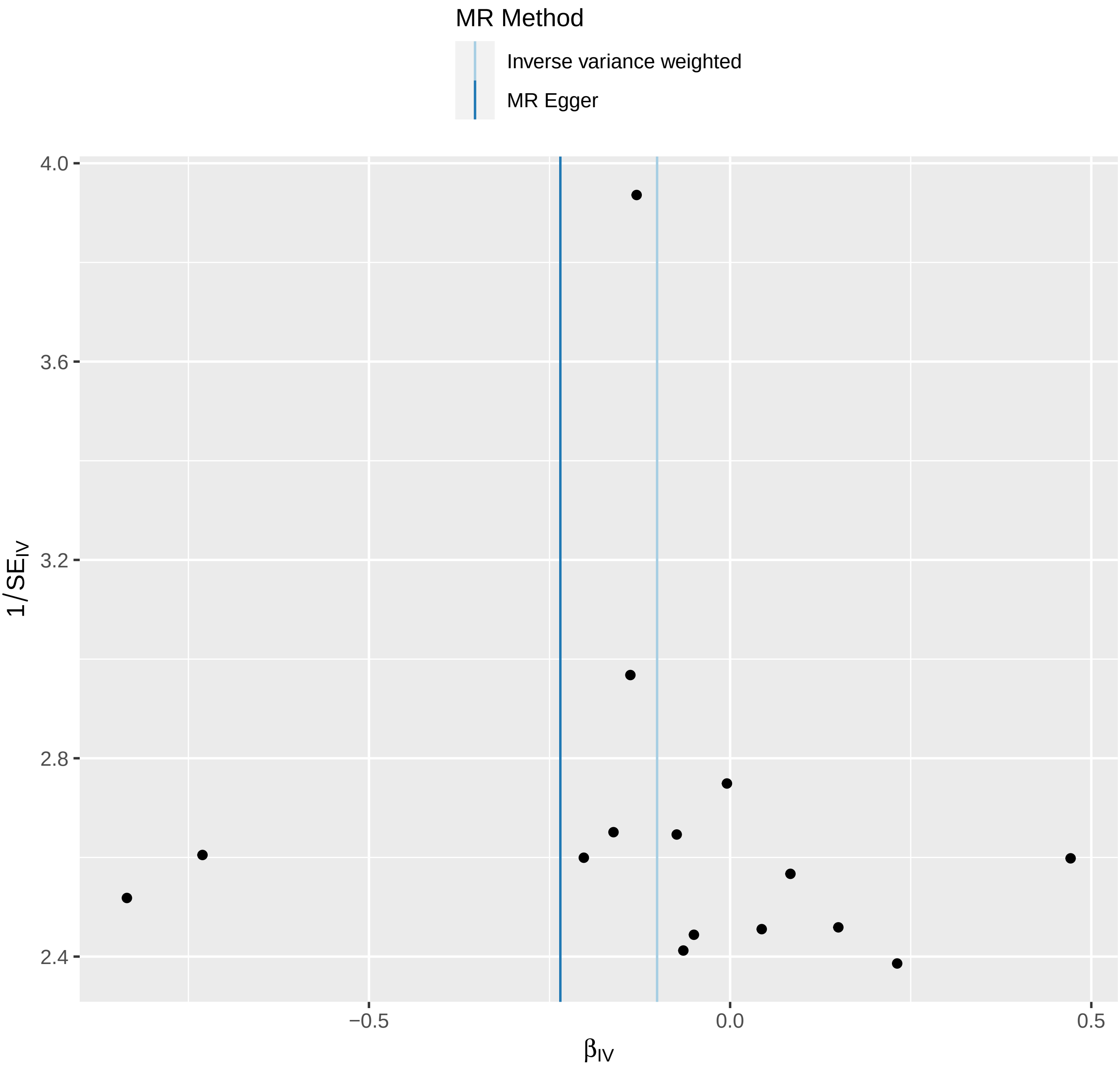

Batch 2−3581 : Gut microbiota abundance (genus Catenibacterium id.2153) on Leucine−rich repeat serine/threonine−protein kinase 2 with a threshold of 1e−05

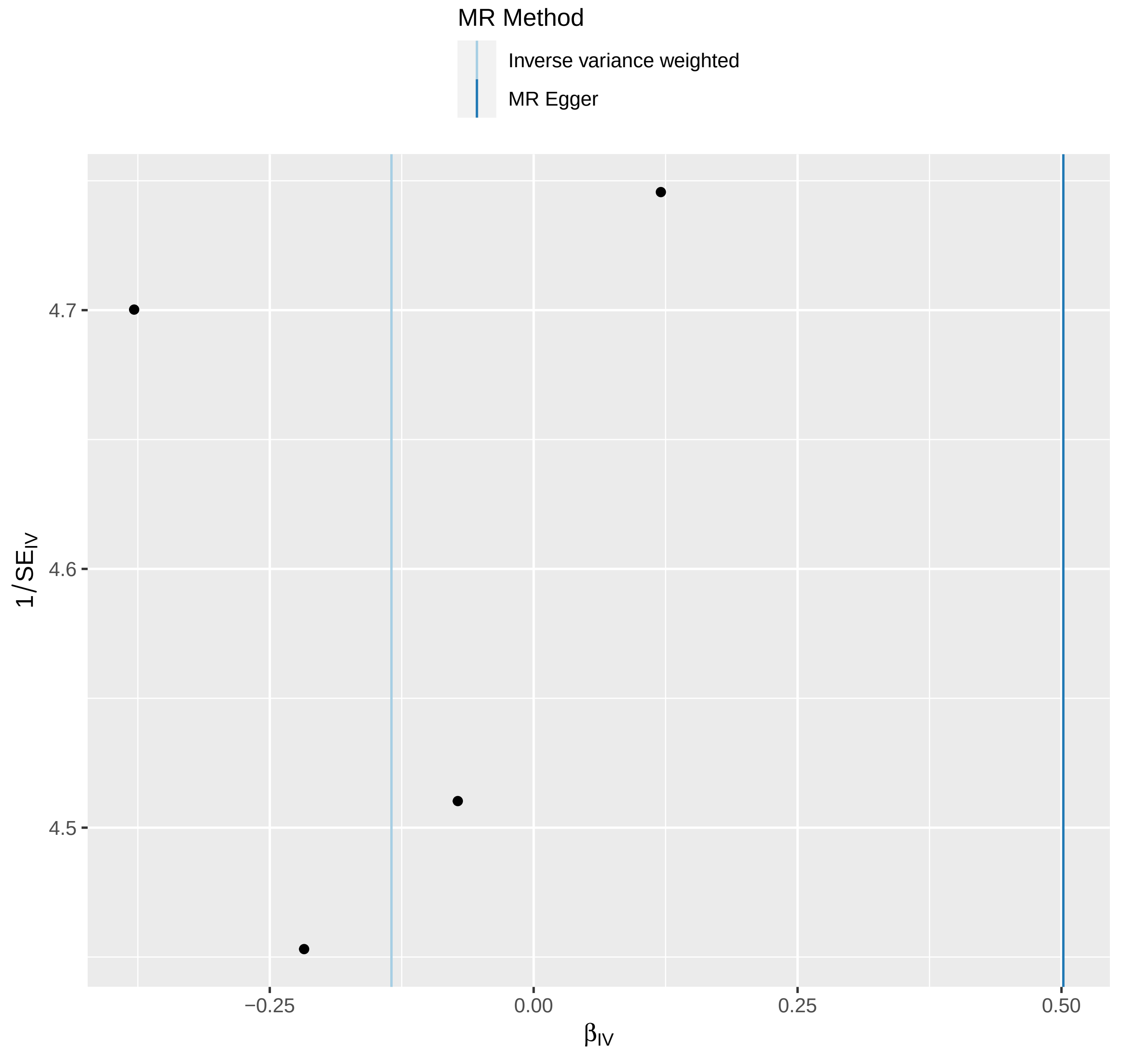

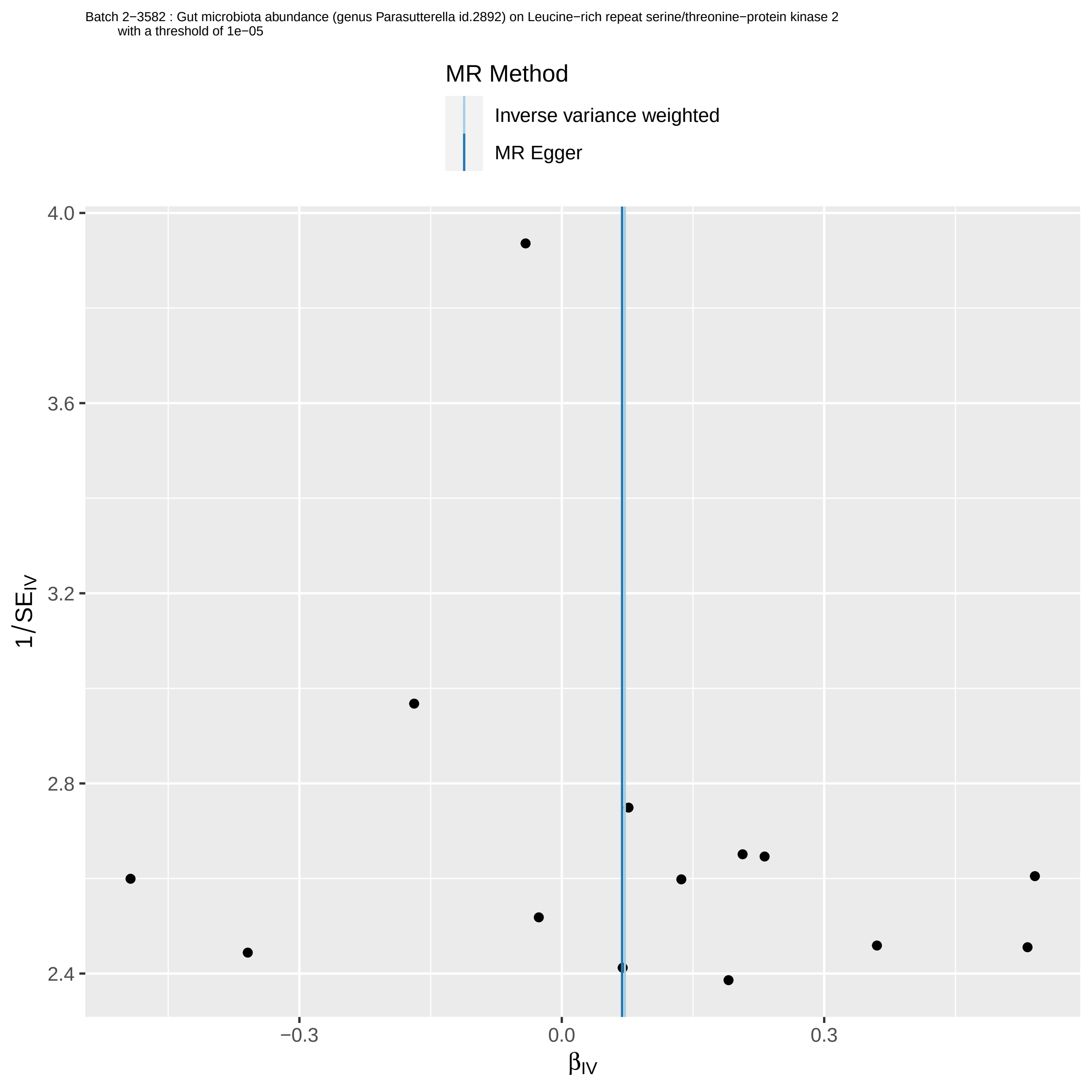
Batch 2−3582 : Gut microbiota abundance (genus Parasutterella id.2892) on Leucine−rich repeat serine/threonine−protein kinase 2
with a threshold of 1e−05
MR Method
Inverse variance weighted
MR Egger
4.0
3.6
3.2
2.8
2.4
1/SE_IV
−0.3
0.0
0.3
β_IV

Batch 2−3583 : Gut microbiota abundance (genus Catenibacterium id.2153) on Leucine−rich repeat neuronal protein 1
with a threshold of 1e−05

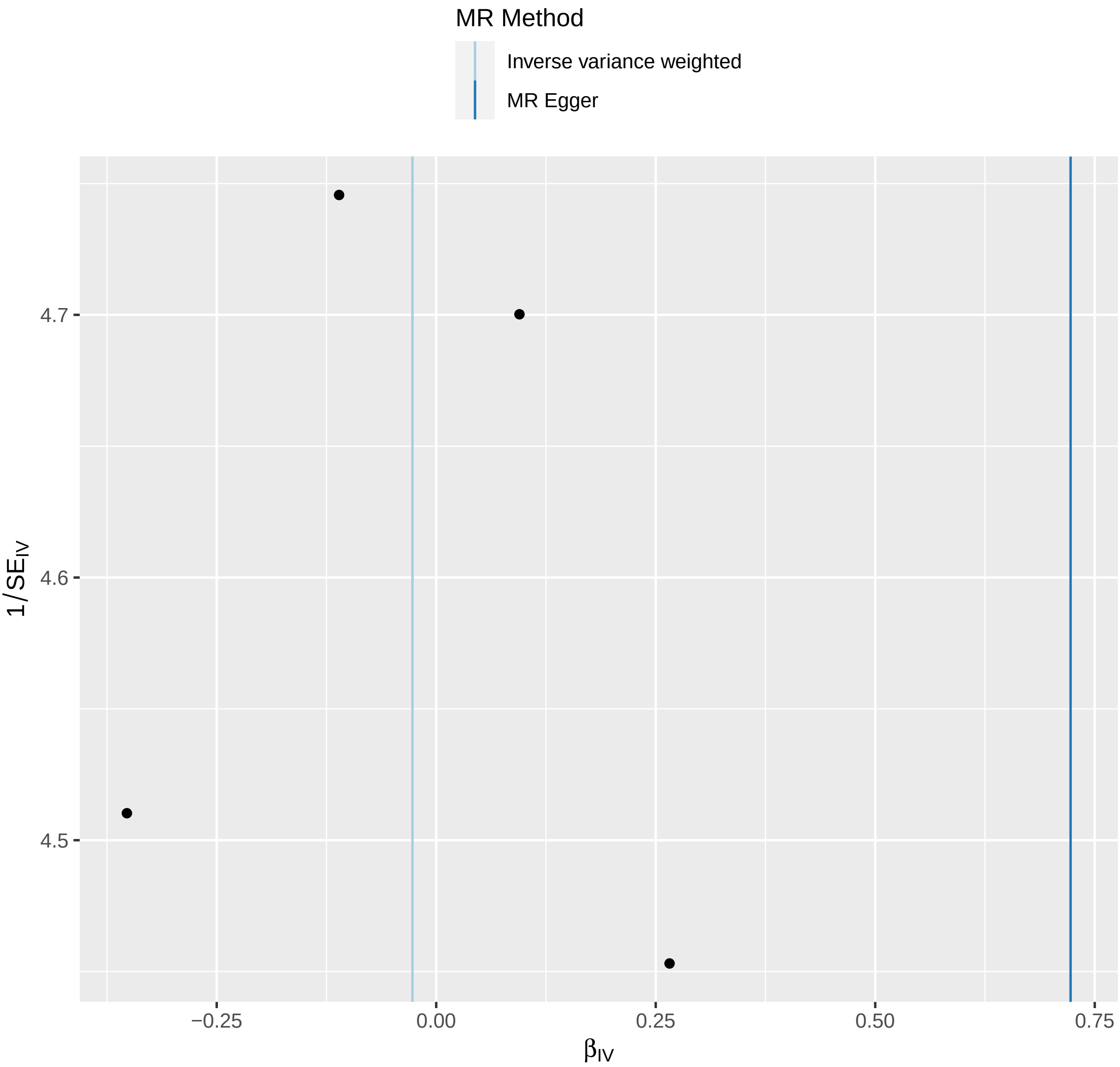

Batch 2−3584 : Gut microbiota abundance (genus Parasutterella id.2892) on Leucine−rich repeat neuronal protein 1
with a threshold of 1e−05

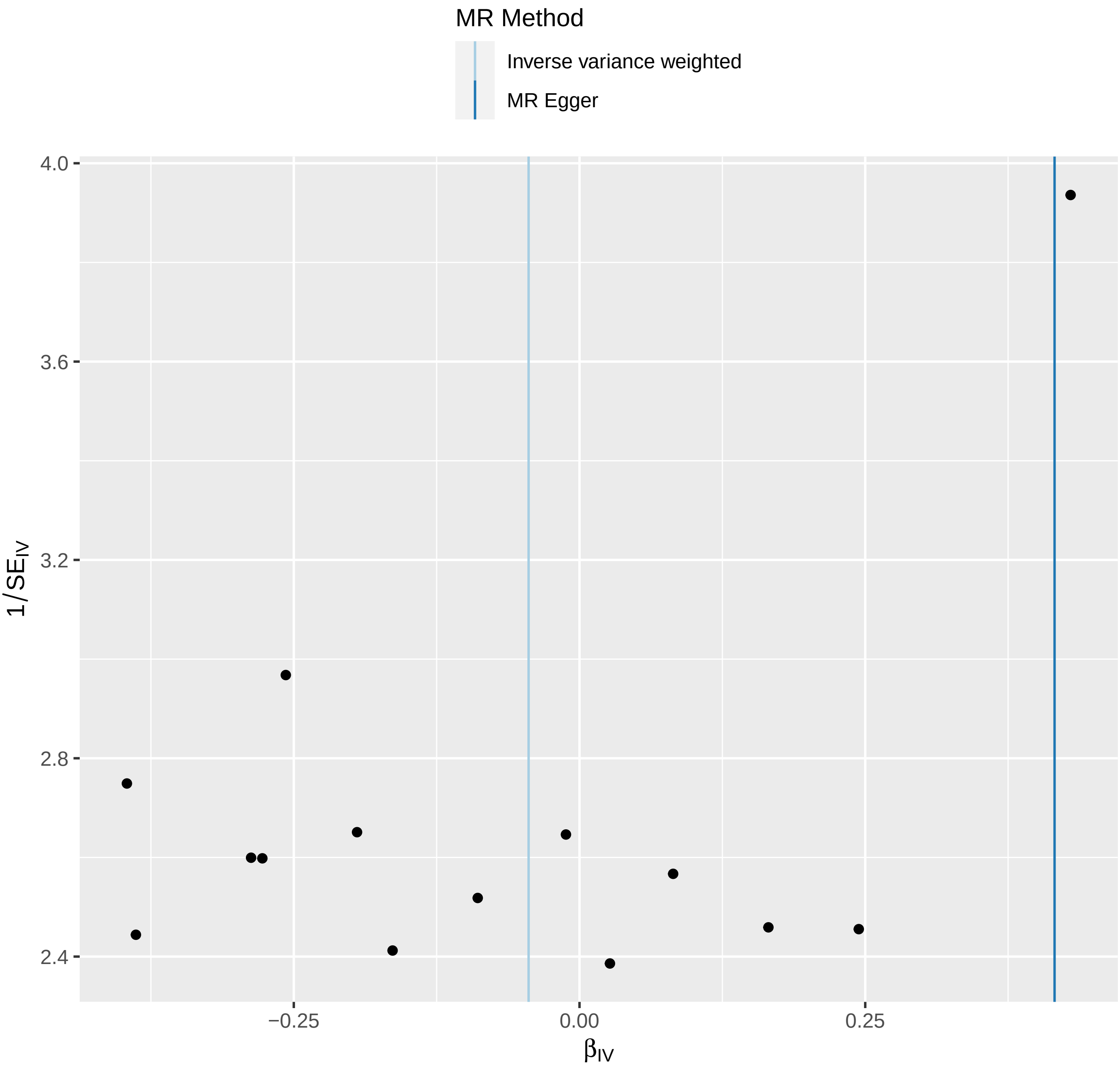

Batch 2−3585 : Gut microbiota abundance (genus Catenibacterium id.2153) on Leucine−rich repeat neuronal protein 1
with a threshold of 1e−05

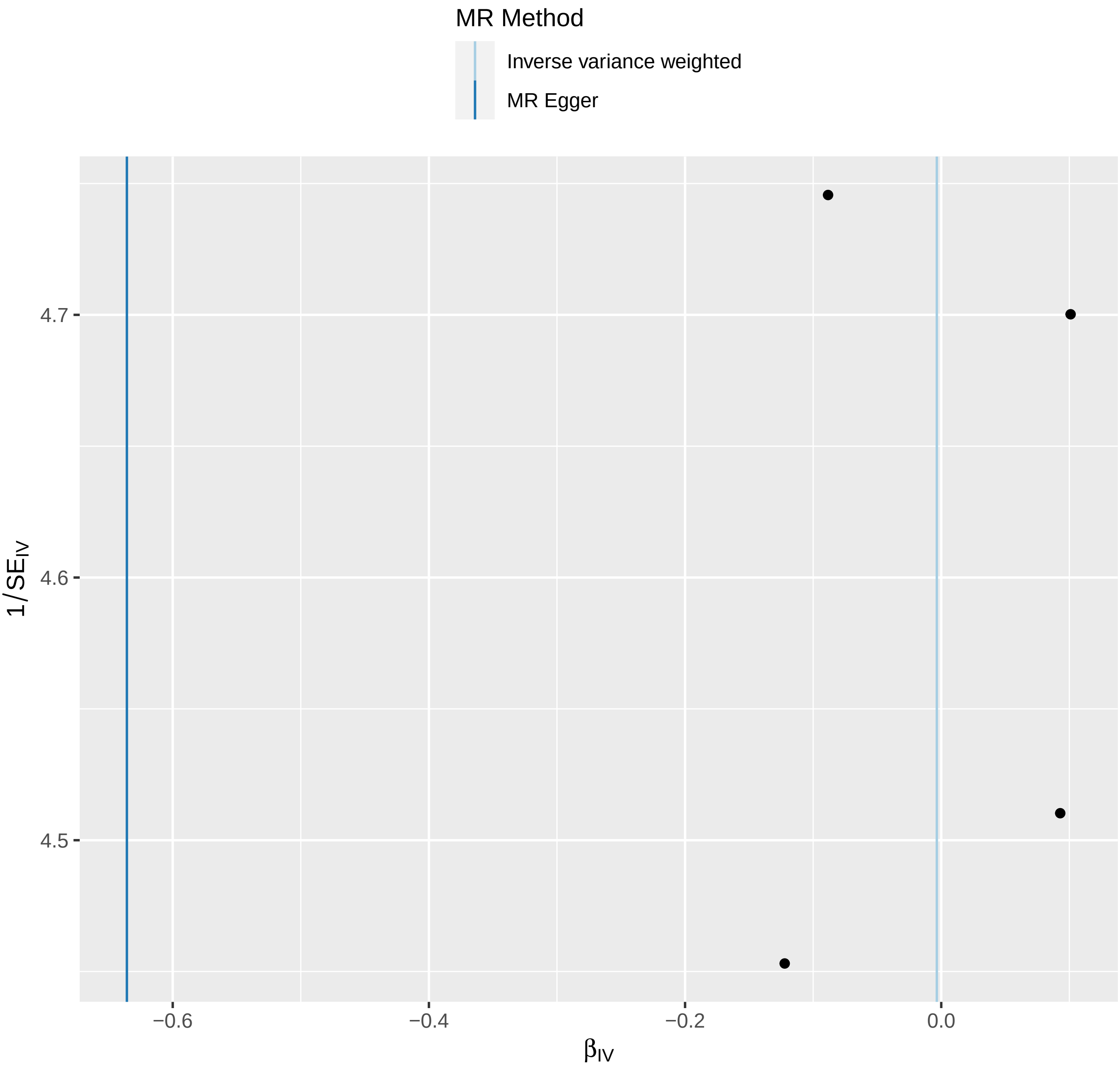

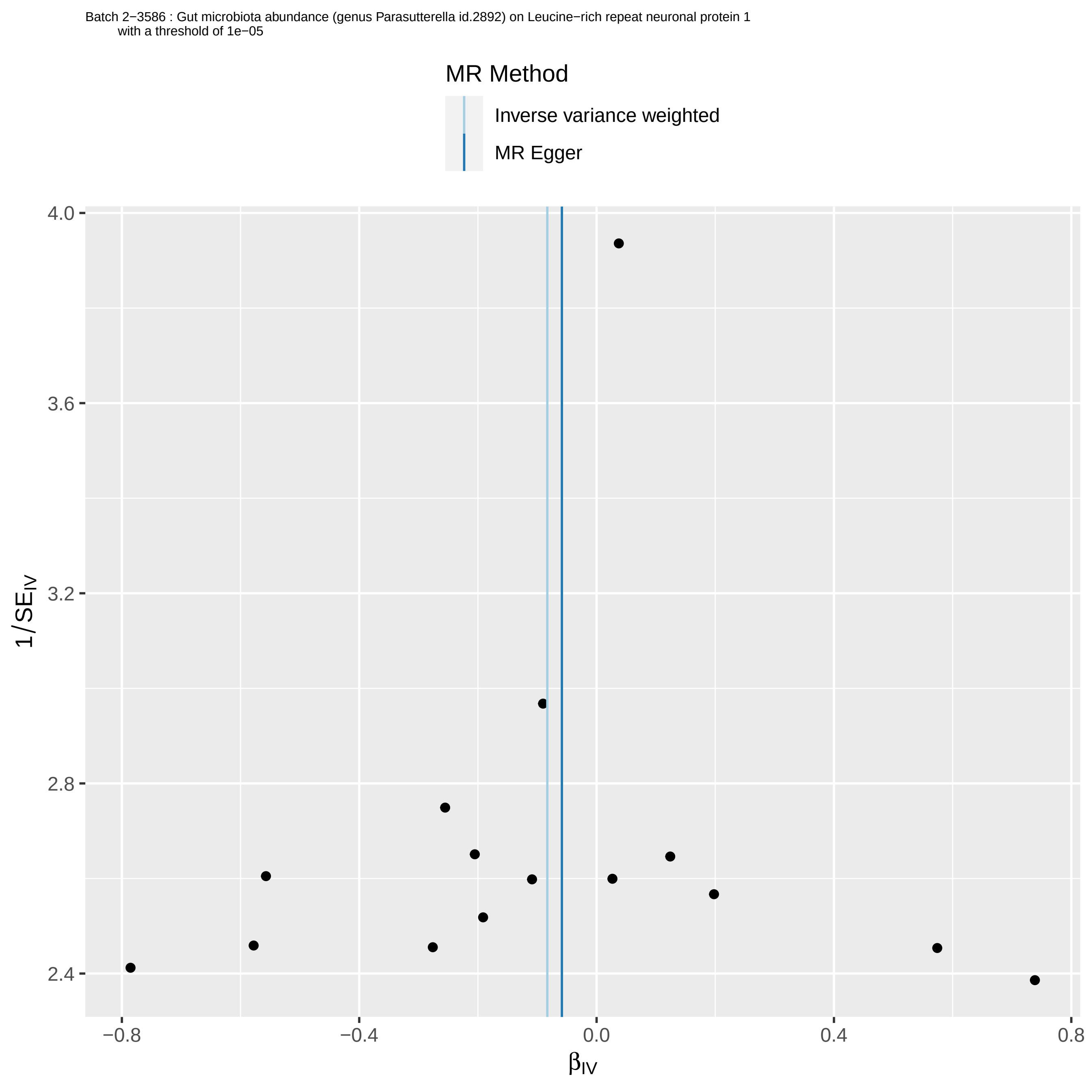
Batch 2−3586 : Gut microbiota abundance (genus Parasutterella id.2892) on Leucine−rich repeat neuronal protein 1
with a threshold of 1e−05
MR Method
Inverse variance weighted
MR Egger
4.0
3.6
3.2
2.8
2.4
1/SE_IV
−0.8
−0.4
0.0
0.4
0.8
β_IV

Batch 2−3587 : Gut microbiota abundance (genus Catenibacterium id.2153) on Leucine−rich repeat neuronal protein 3
with a threshold of 1e−05

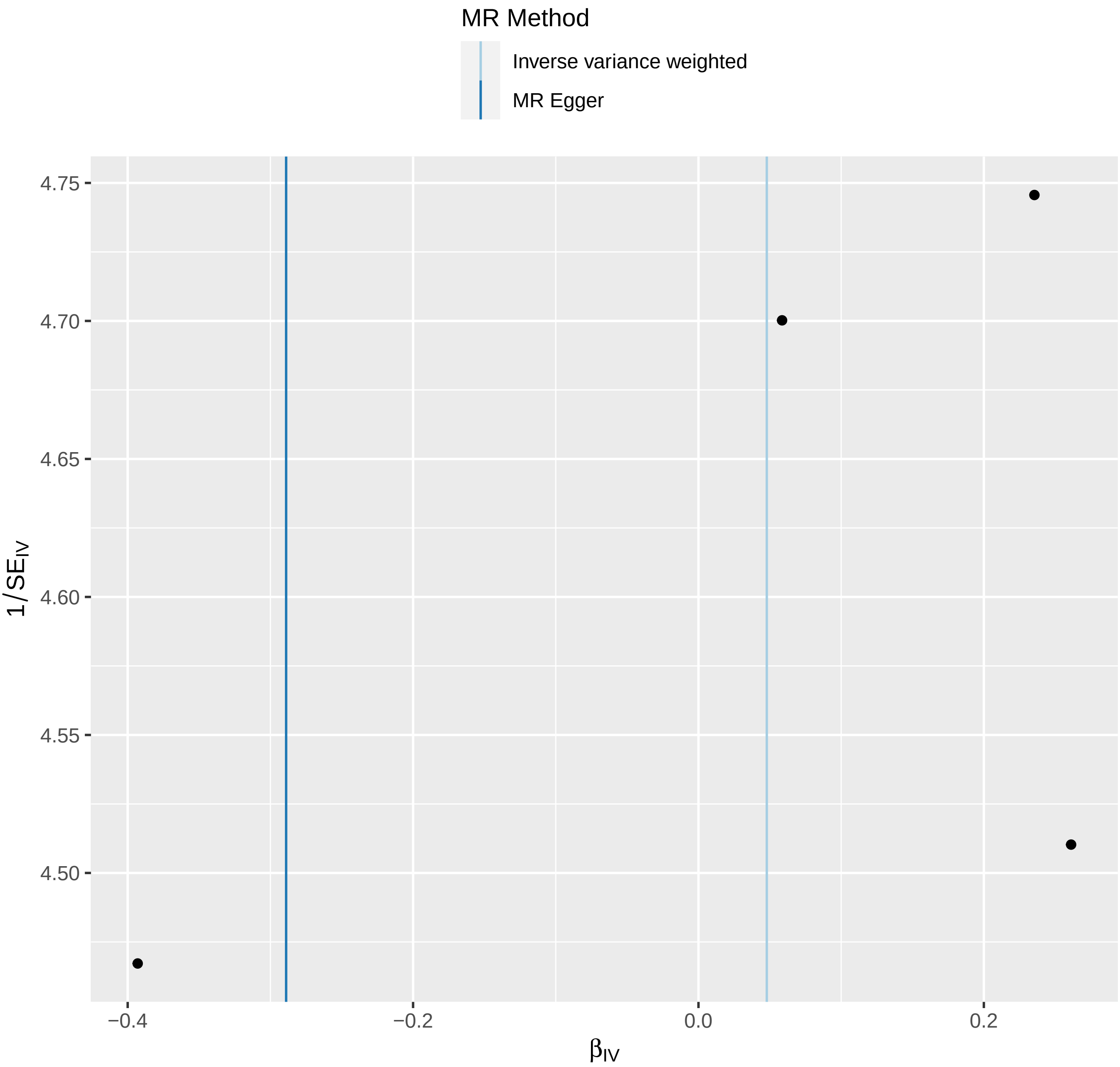

Batch 2−3588 : Gut microbiota abundance (genus Parasutterella id.2892) on Leucine−rich repeat neuronal protein 3
with a threshold of 1e−05

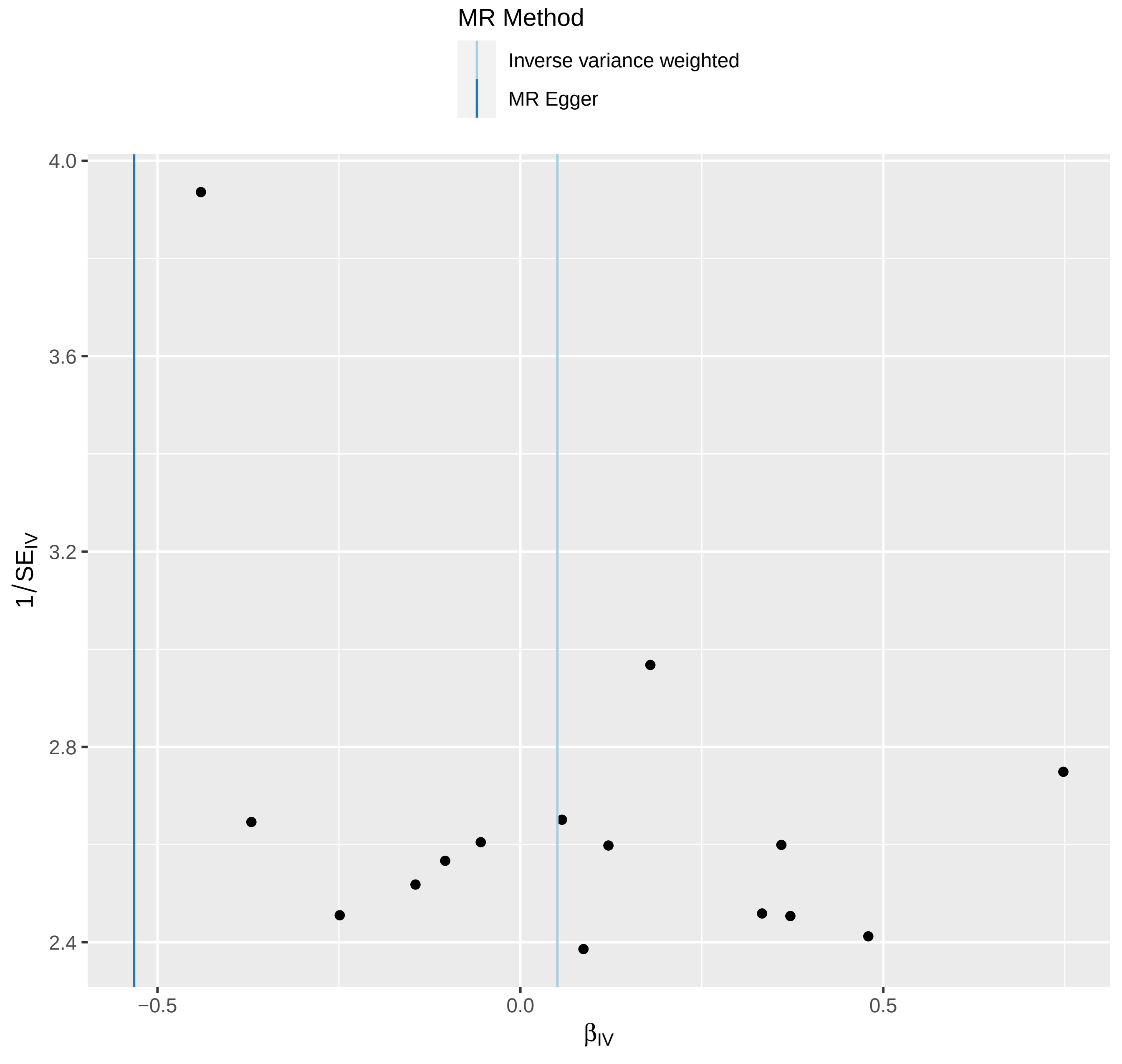

Batch 2−3589 : Gut microbiota abundance (genus Catenibacterium id.2153) on Leucine−rich repeat transmembrane neuronal protein 1 with a threshold of 1e−05

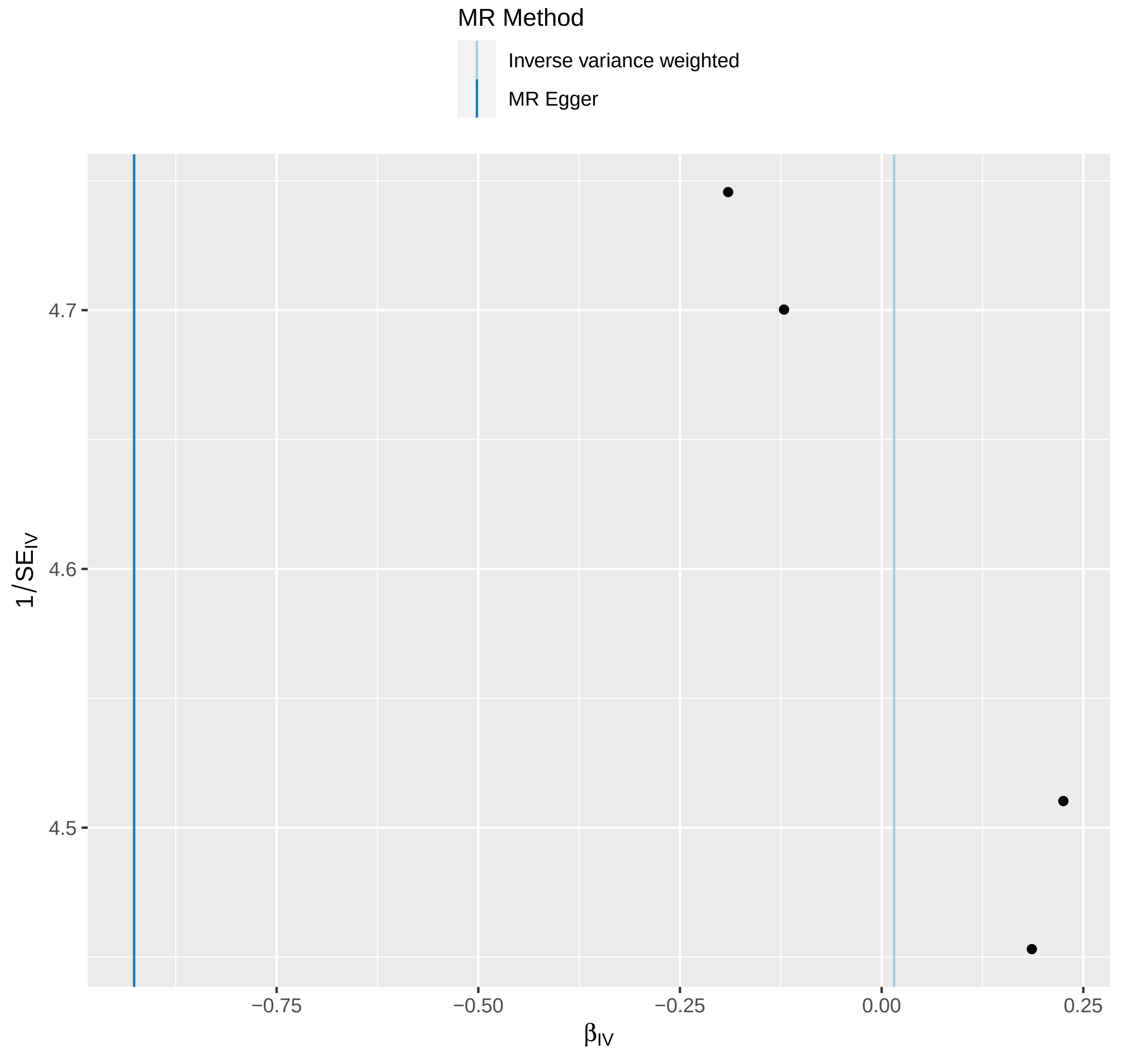

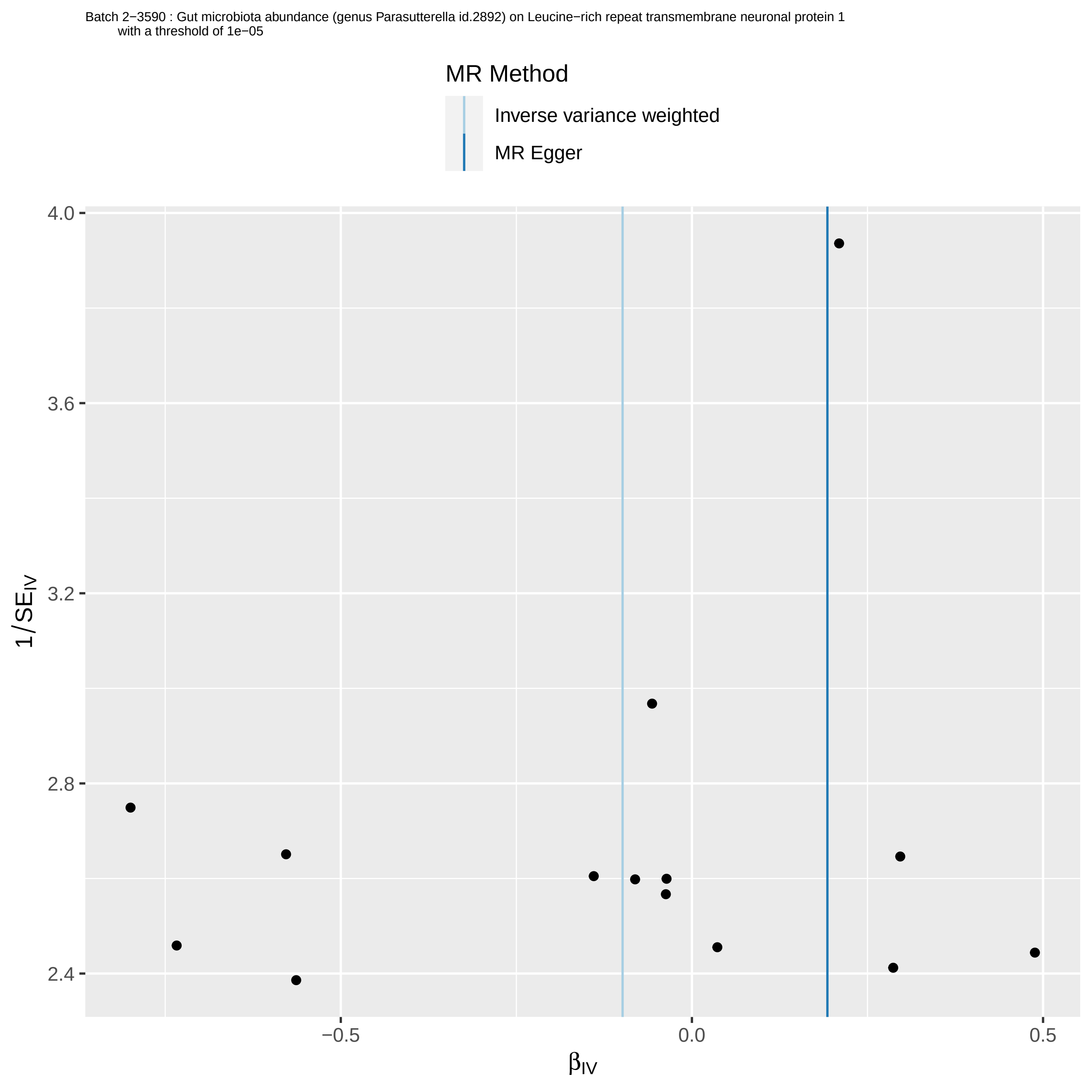

Batch 2−3590 : Gut microbiota abundance (genus Parasutterella id.2892) on Leucine−rich repeat transmembrane neuronal protein 1
with a threshold of 1e−05
MR Method
Inverse variance weighted
MR Egger
4.0
3.6
3.2
2.8
2.4
1/SE_IV
−0.5
0.0
0.5
β_IV

Batch 2−3591 : Gut microbiota abundance (genus Catenibacterium id.2153) on Leucine−rich repeat transmembrane neuronal protein 2
with a threshold of 1e−05

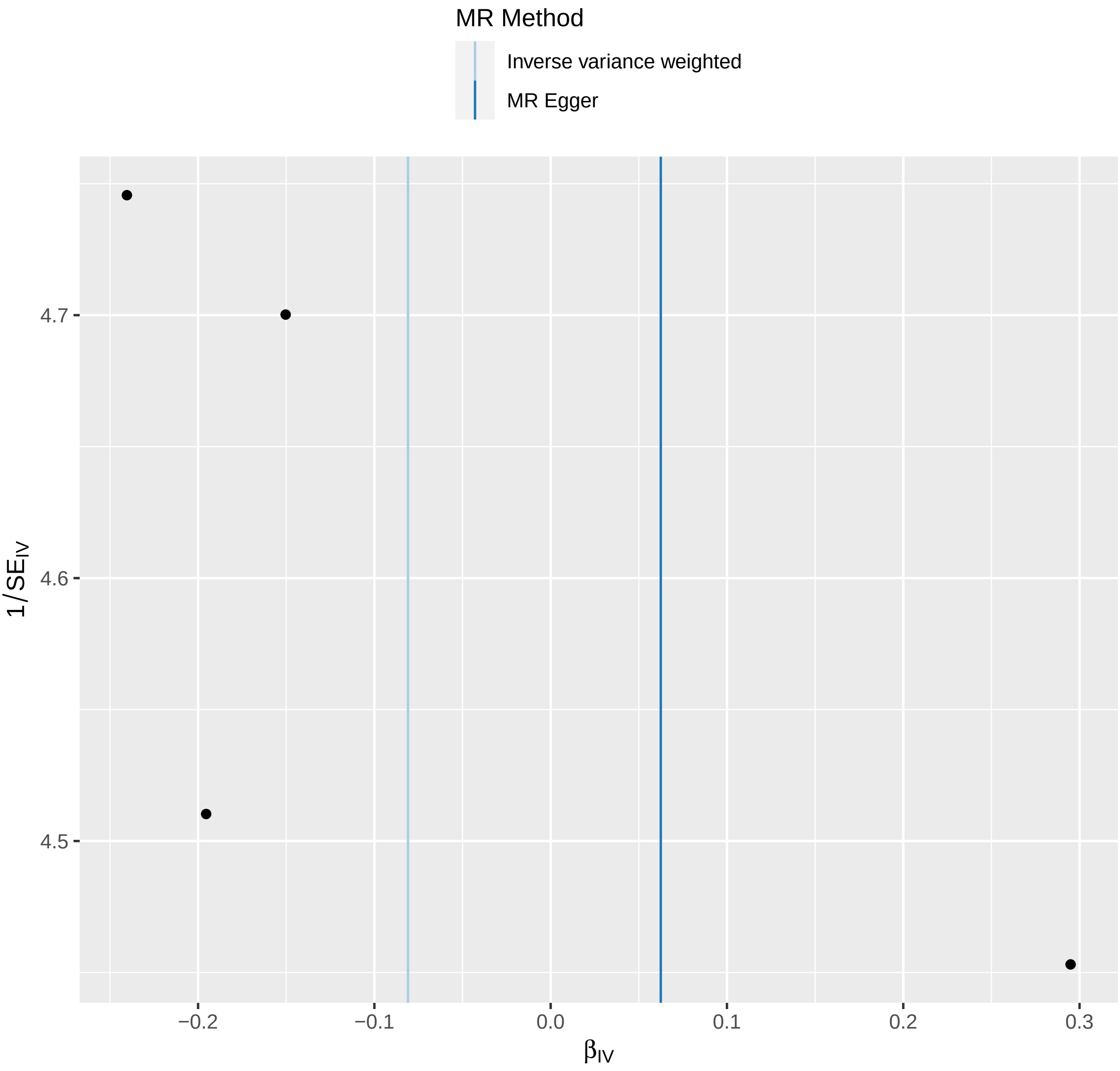

Batch 2−3592 : Gut microbiota abundance (genus Parasutterella id.2892) on Leucine−rich repeat transmembrane neuronal protein 2 with a threshold of 1e−05

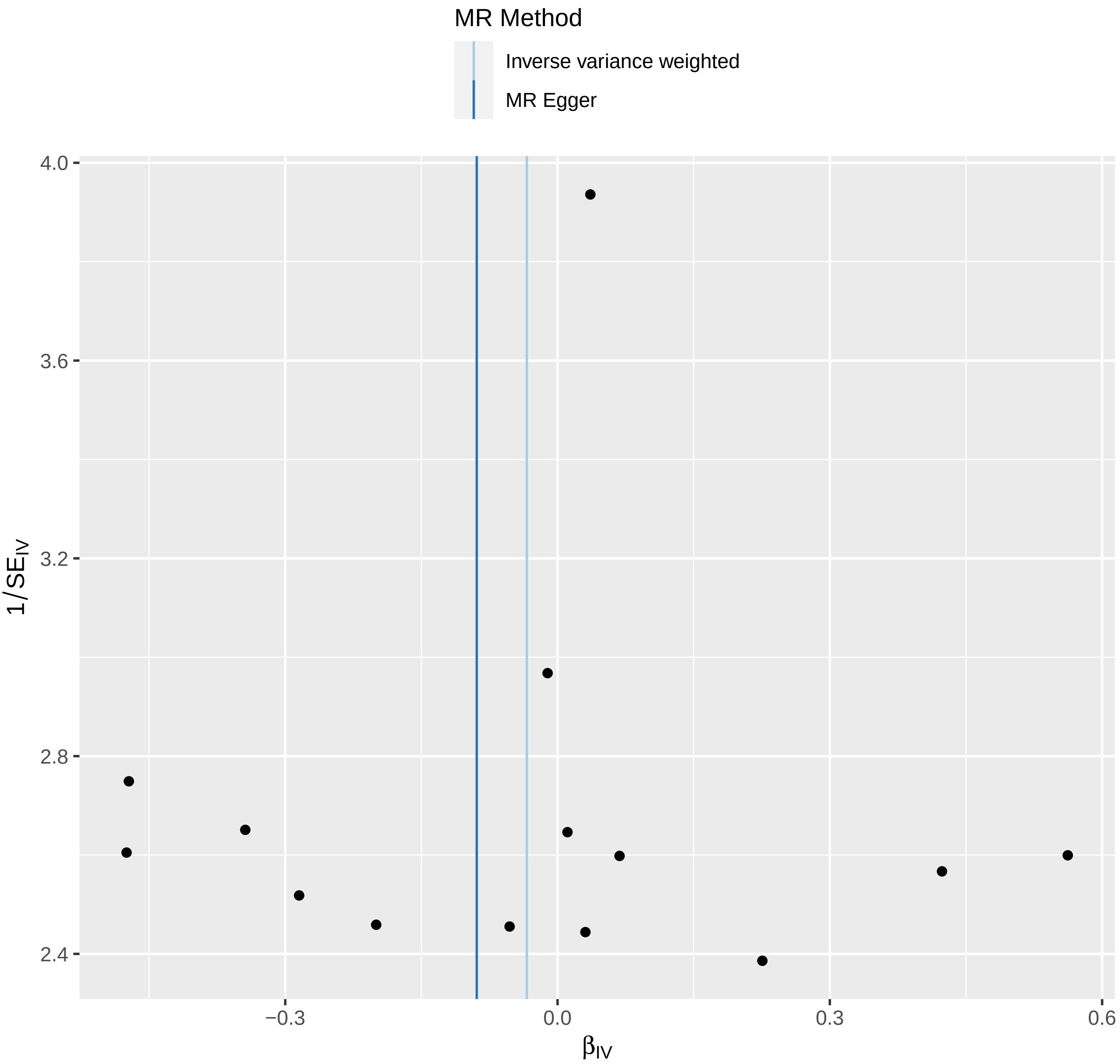

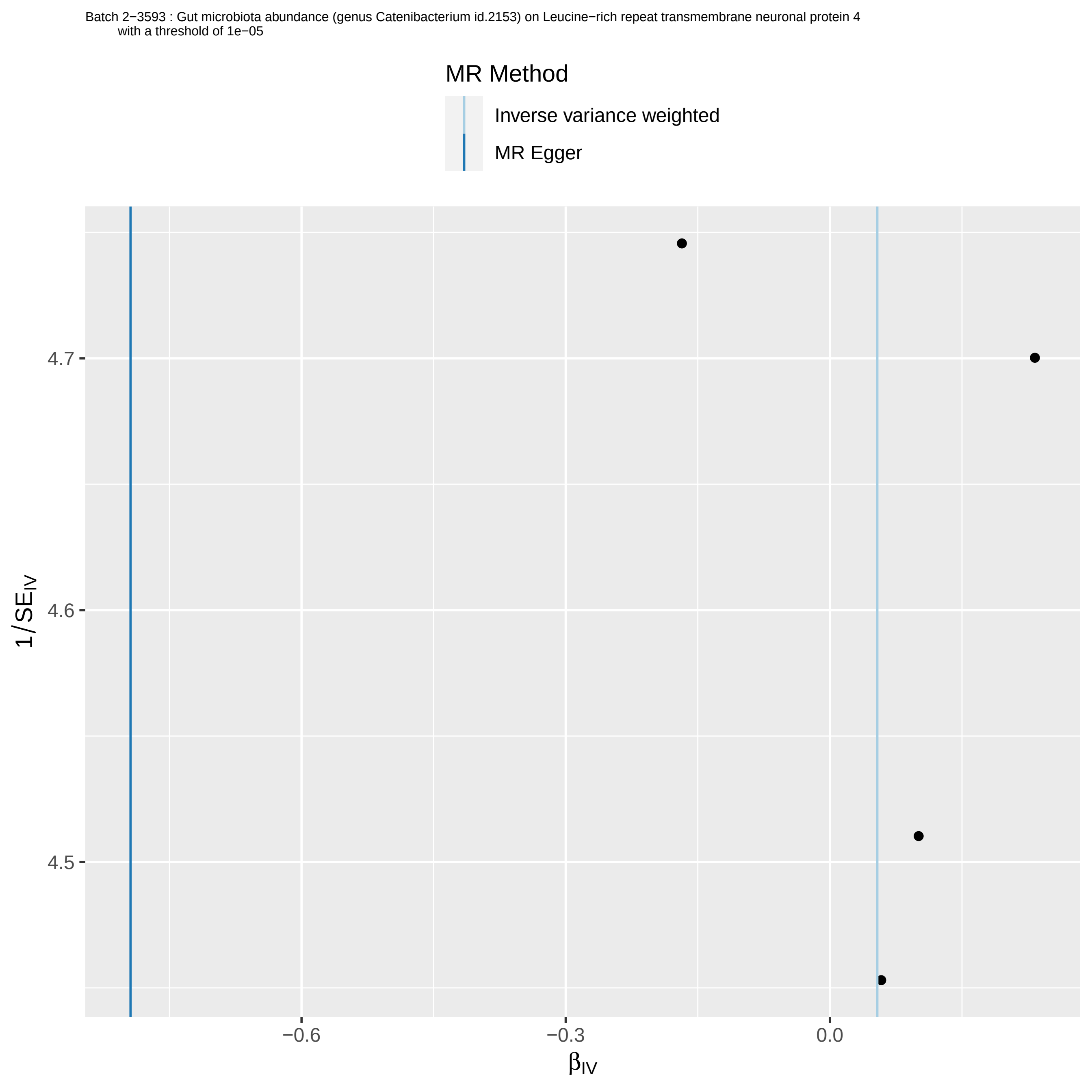
Batch 2−3593 : Gut microbiota abundance (genus Catenibacterium id.2153) on Leucine−rich repeat transmembrane neuronal protein 4
with a threshold of 1e−05
MR Method
Inverse variance weighted
MR Egger
4.7
4.6
4.5
1/SE_IV
−0.6
−0.3
0.0
β_IV

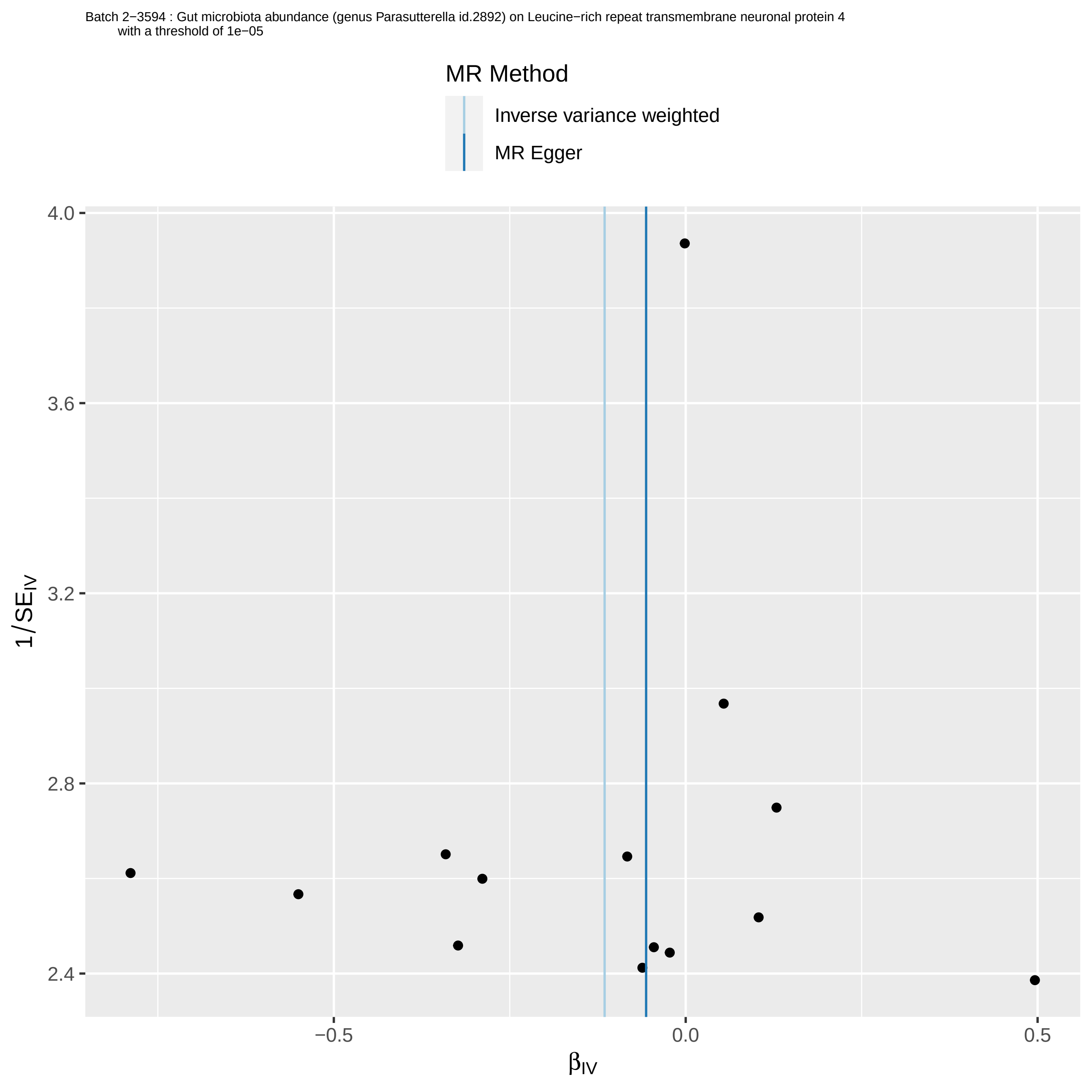

Batch 2−3594 : Gut microbiota abundance (genus Parasutterella id.2892) on Leucine−rich repeat transmembrane neuronal protein 4
with a threshold of 1e−05
MR Method
Inverse variance weighted
MR Egger
4.0
3.6
3.2
2.8
2.4
1/SE_IV
−0.5
0.0
0.5
β_IV

Batch 2−3595 : Gut microbiota abundance (genus Catenibacterium id.2153) on Leucine−rich repeat transmembrane neuronal protein 4 with a threshold of 1e−05

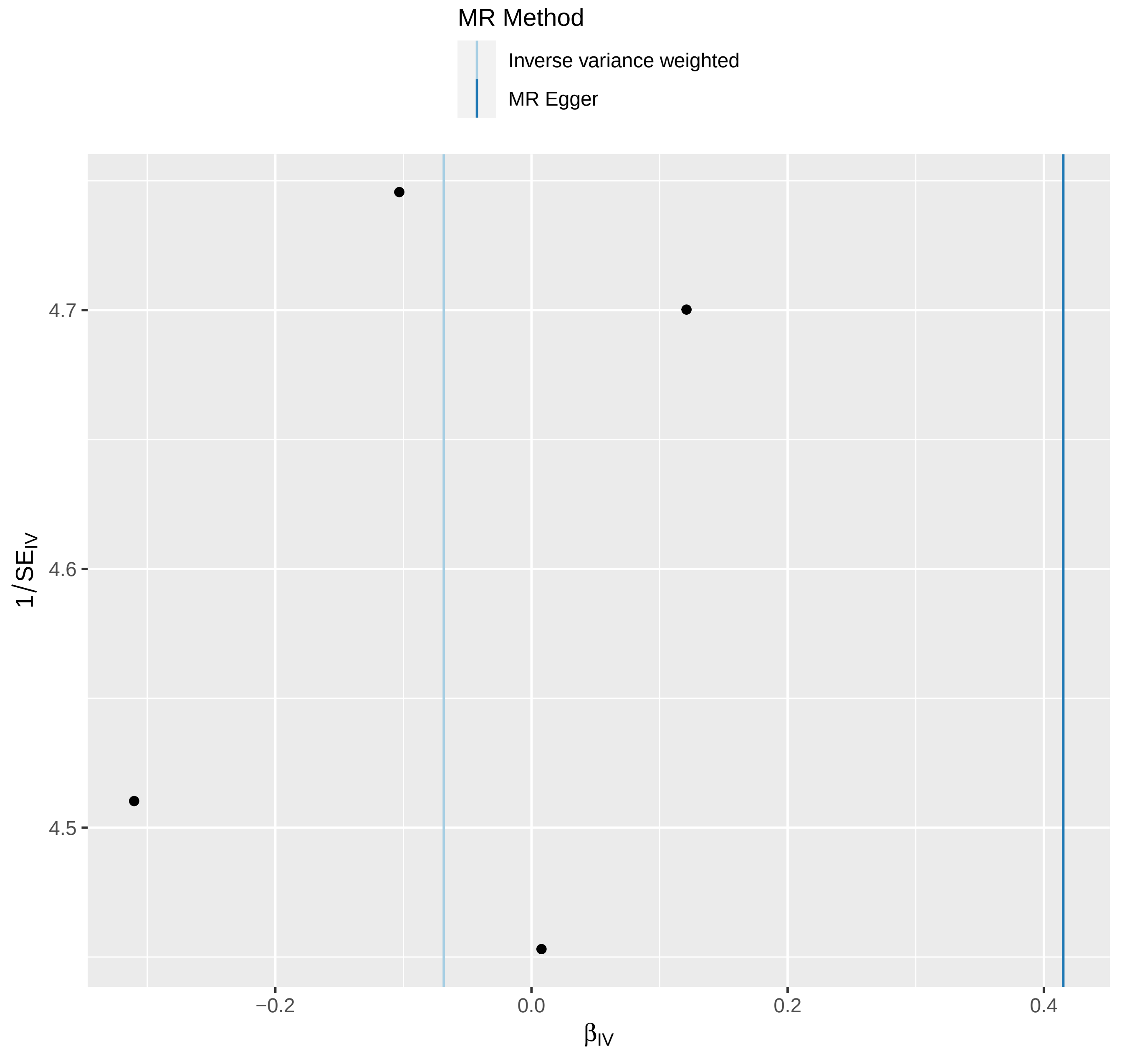

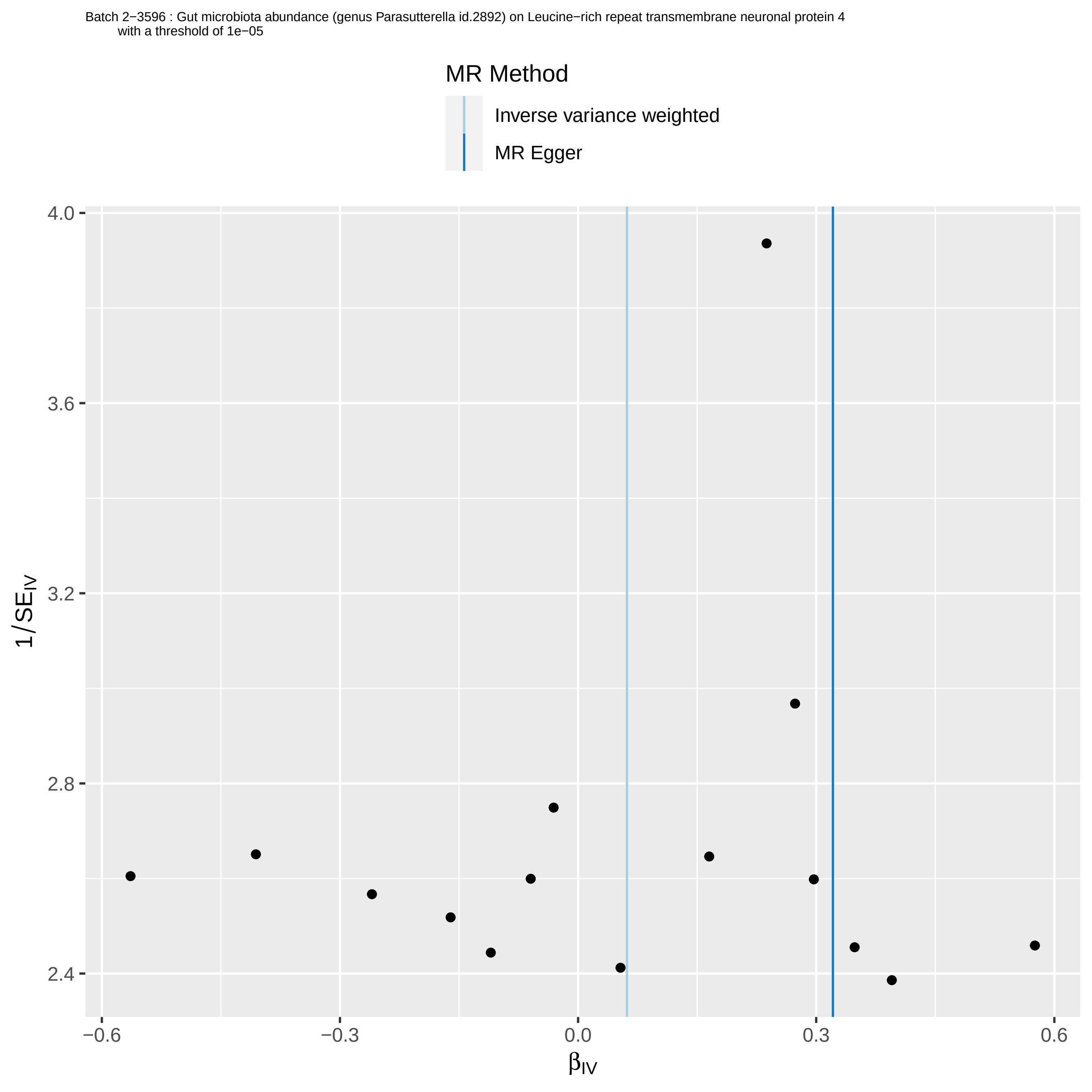
Batch 2−3596 : Gut microbiota abundance (genus Parasutterella id.2892) on Leucine−rich repeat transmembrane neuronal protein 4
with a threshold of 1e−05
MR Method
Inverse variance weighted
MR Egger
4.0
3.6
3.2
2.8
2.4
$1/SE_{IV}$
−0.6
−0.3
0.0
0.3
0.6
β_{IV}

Batch 2−3597 : Gut microbiota abundance (genus Catenibacterium id.2153) on Leucine−rich repeat and transmembrane domain−containing protein 1 with a threshold of 1e−05

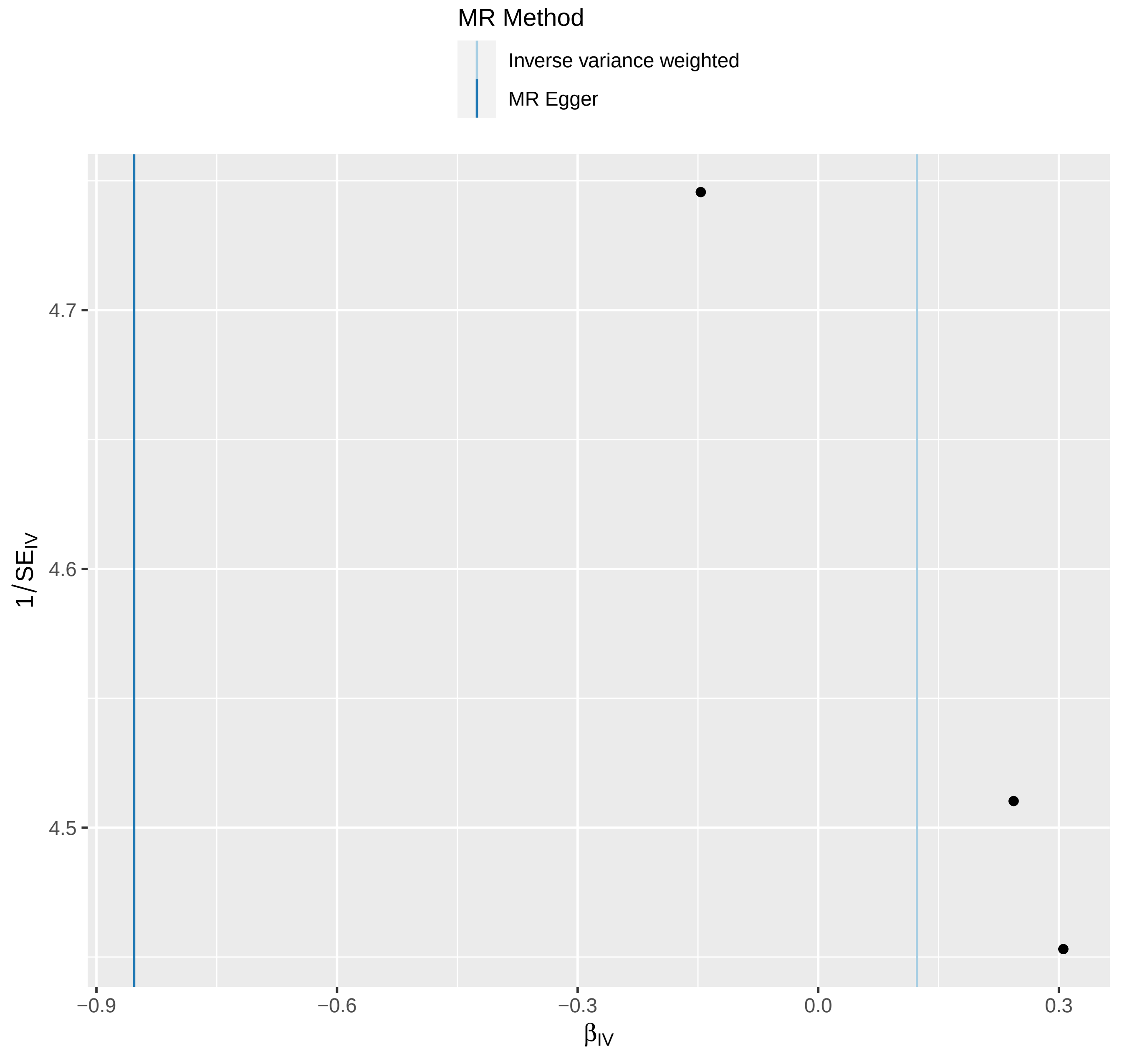

Batch 2−3598 : Gut microbiota abundance (genus Parasutterella id.2892) on Leucine−rich repeat and transmembrane domain−containing protein 1 with a threshold of 1e−05

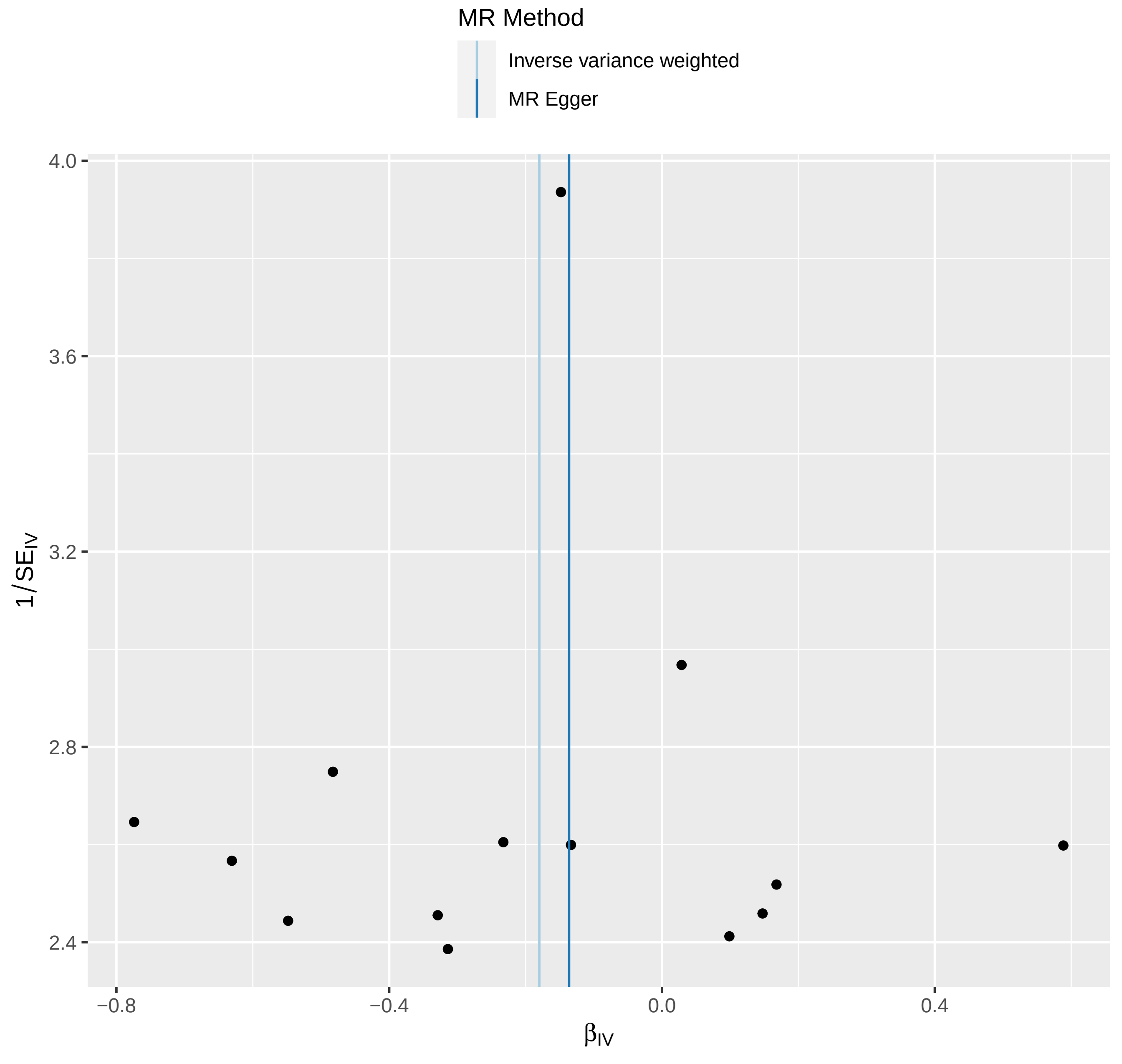

Batch 2−3599 : Gut microbiota abundance (genus Catenibacterium id.2153) on Leucine−rich repeat and transmembrane domain−containing protein 2 with a threshold of 1e−05

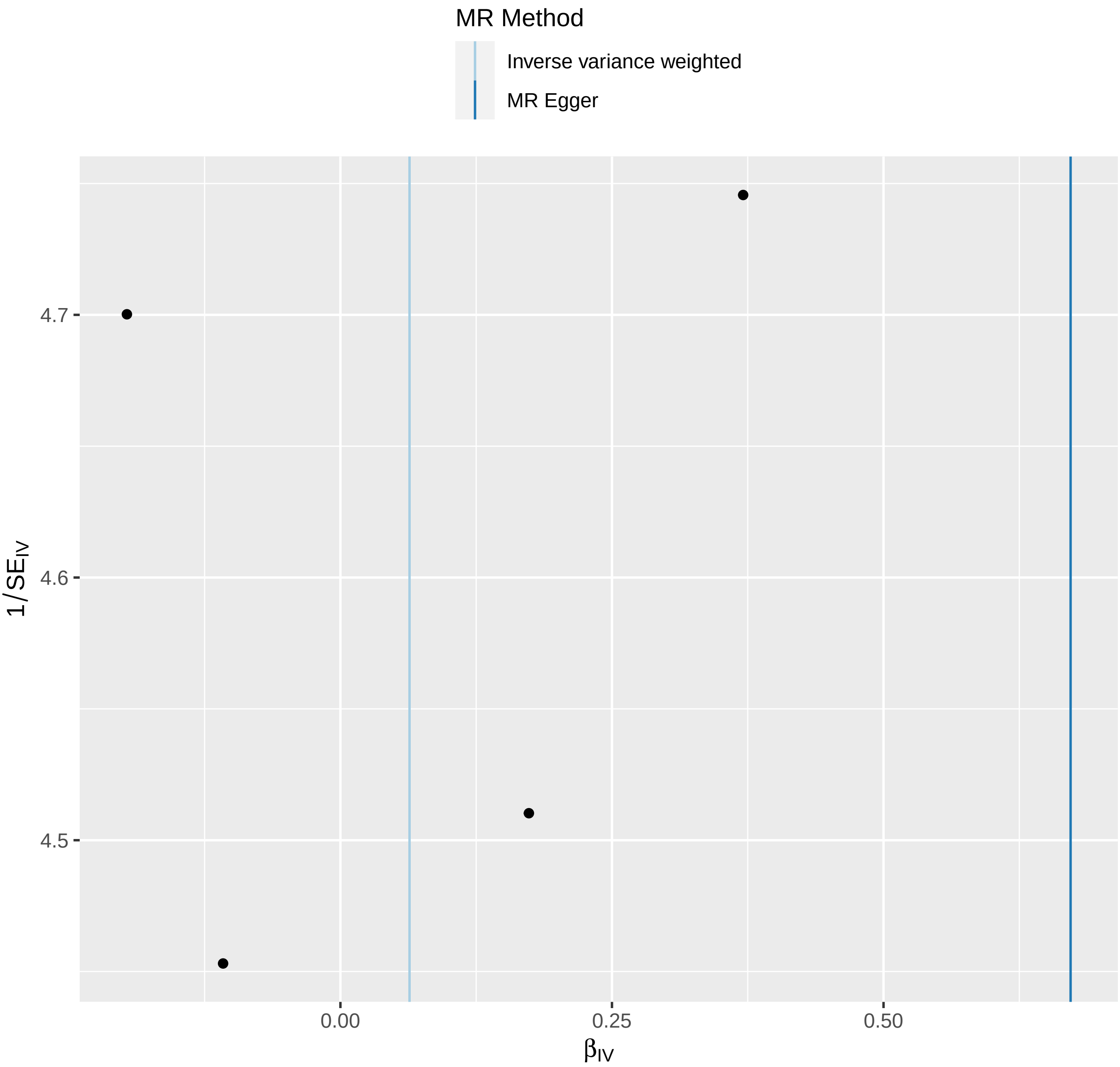

Batch 2−3600 : Gut microbiota abundance (genus Parasutterella id.2892) on Leucine−rich repeat and transmembrane domain−containing protein 2
with a threshold of 1e−05

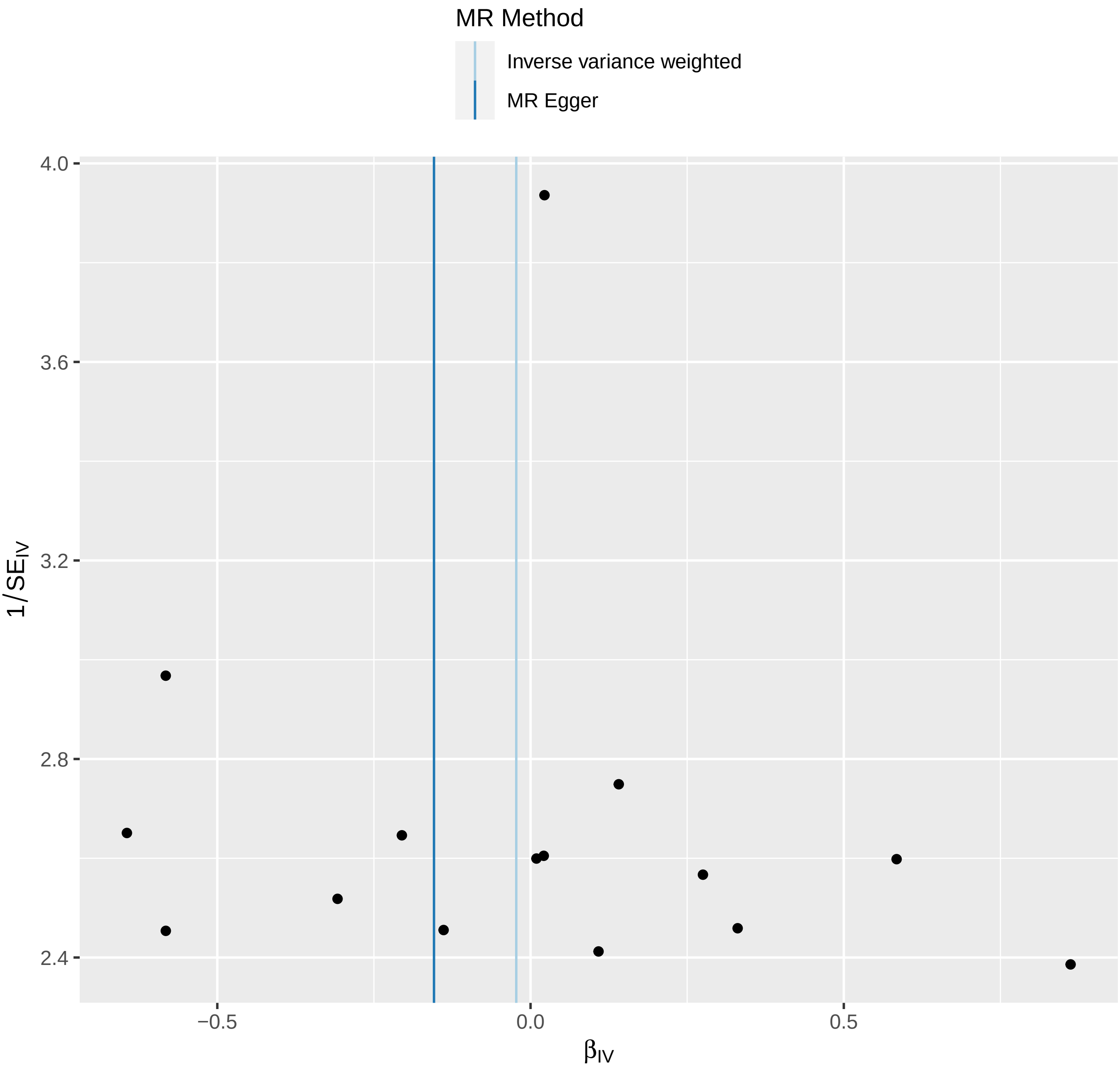

Batch 2−3601 : Gut microbiota abundance (genus Catenibacterium id.2153) on Limbic system−associated membrane protein
with a threshold of 1e−05

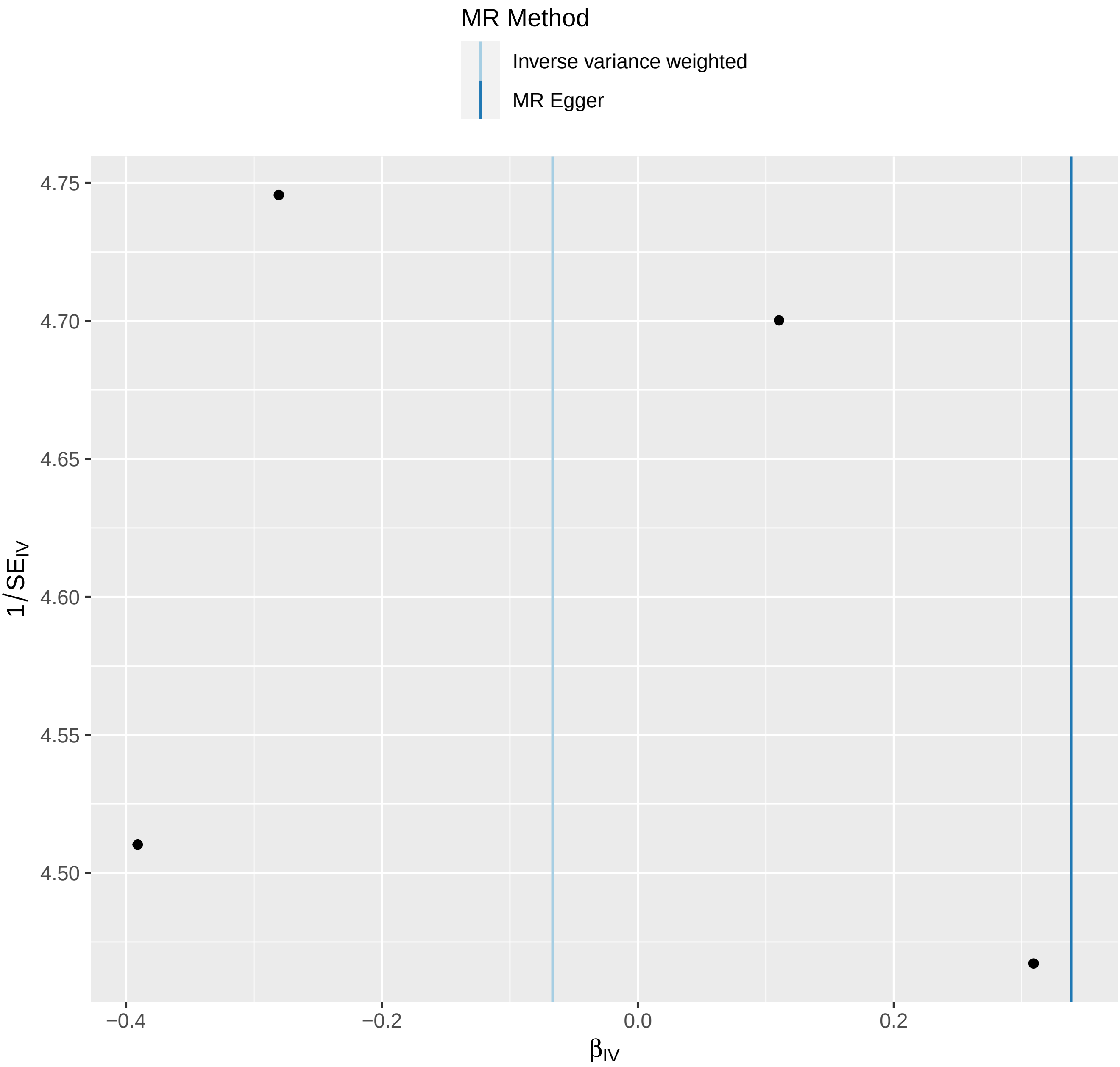

Batch 2−3602 : Gut microbiota abundance (genus Parasutterella id.2892) on Limbic system−associated membrane protein
with a threshold of 1e−05

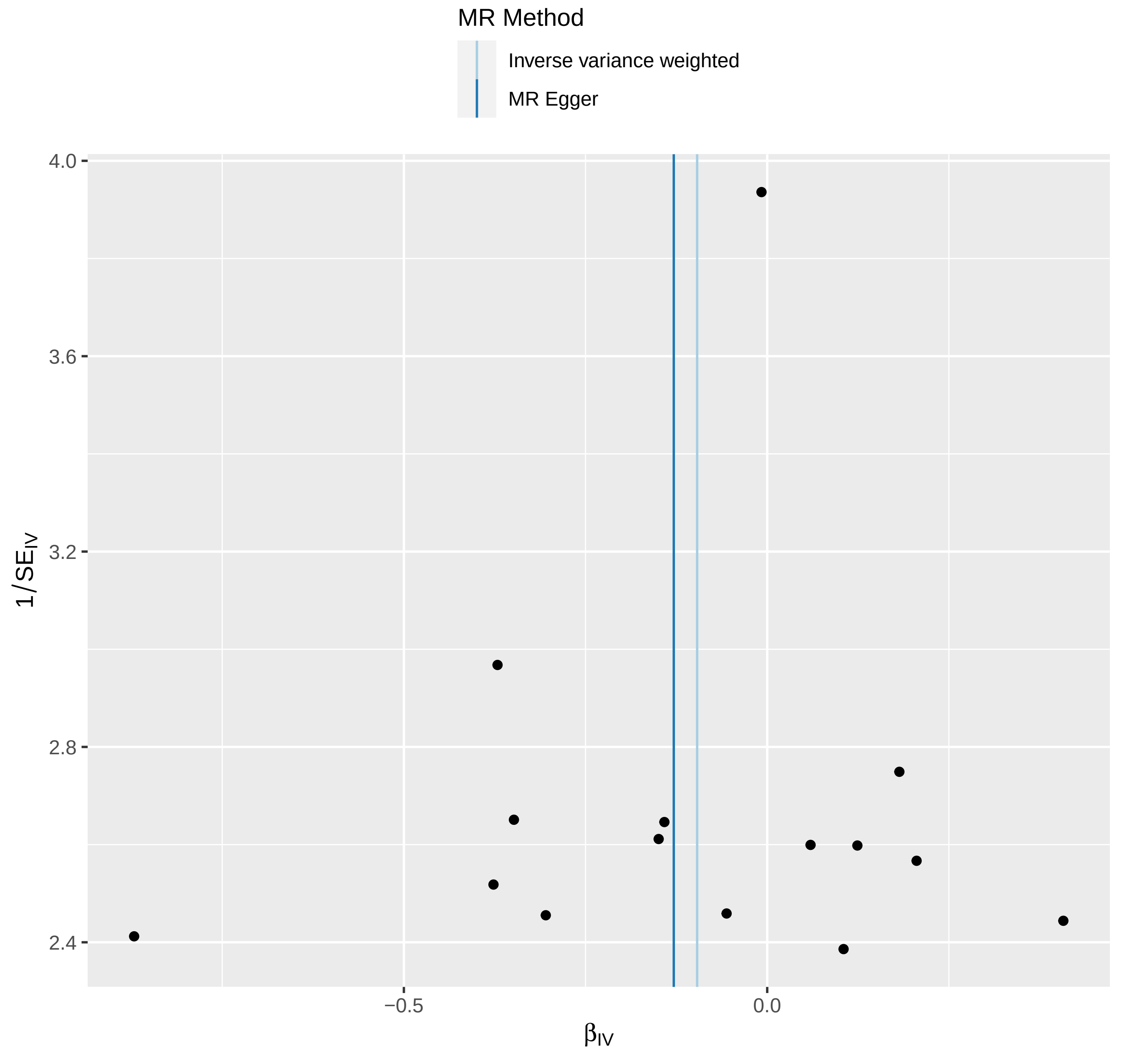

Batch 2−3603 : Gut microbiota abundance (genus Catenibacterium id.2153) on Leukocyte−specific transcript 1 protein with a threshold of 1e−05

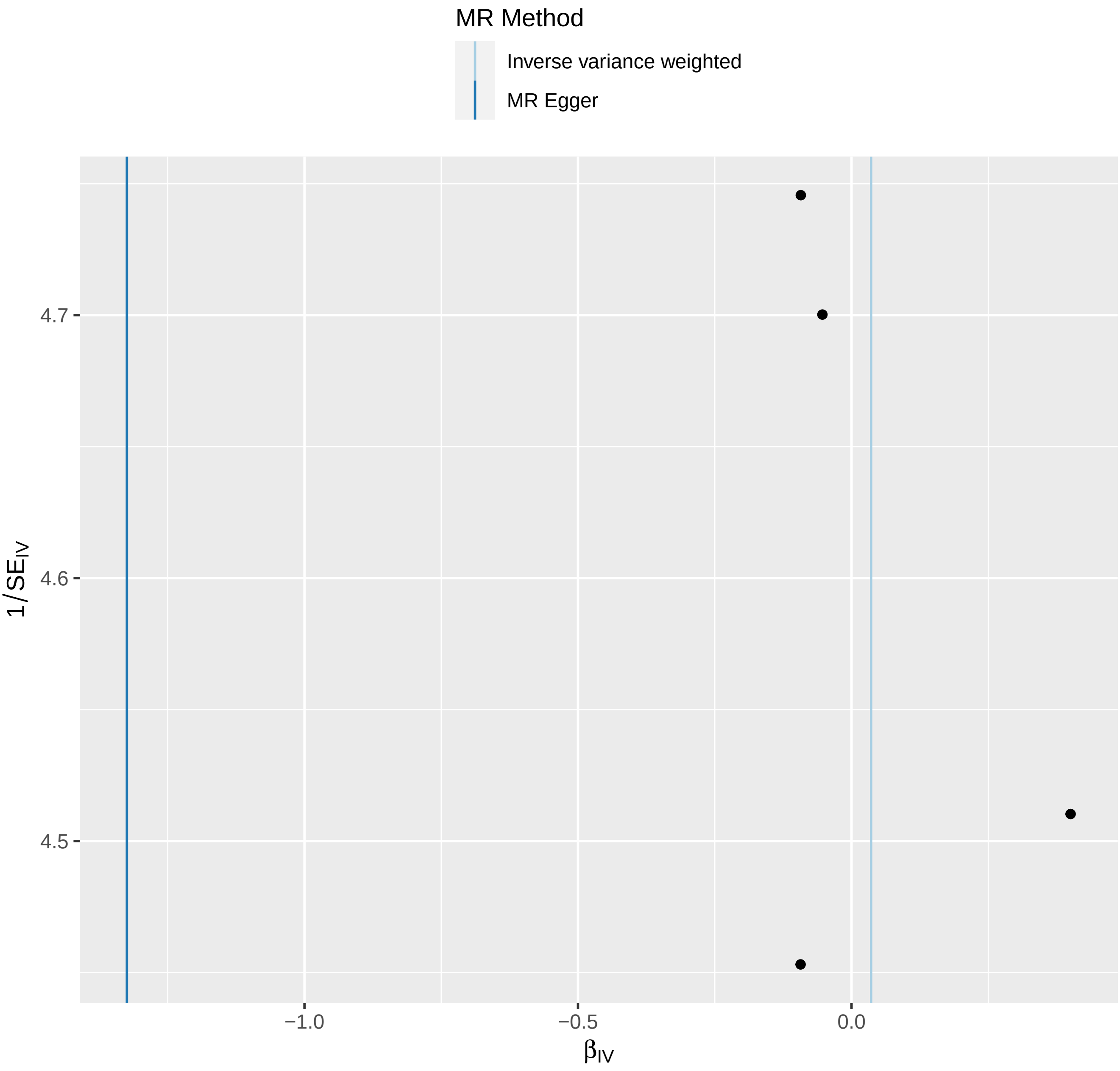

Batch 2−3604 : Gut microbiota abundance (genus Parasutterella id.2892) on Leukocyte−specific transcript 1 protein
with a threshold of 1e−05

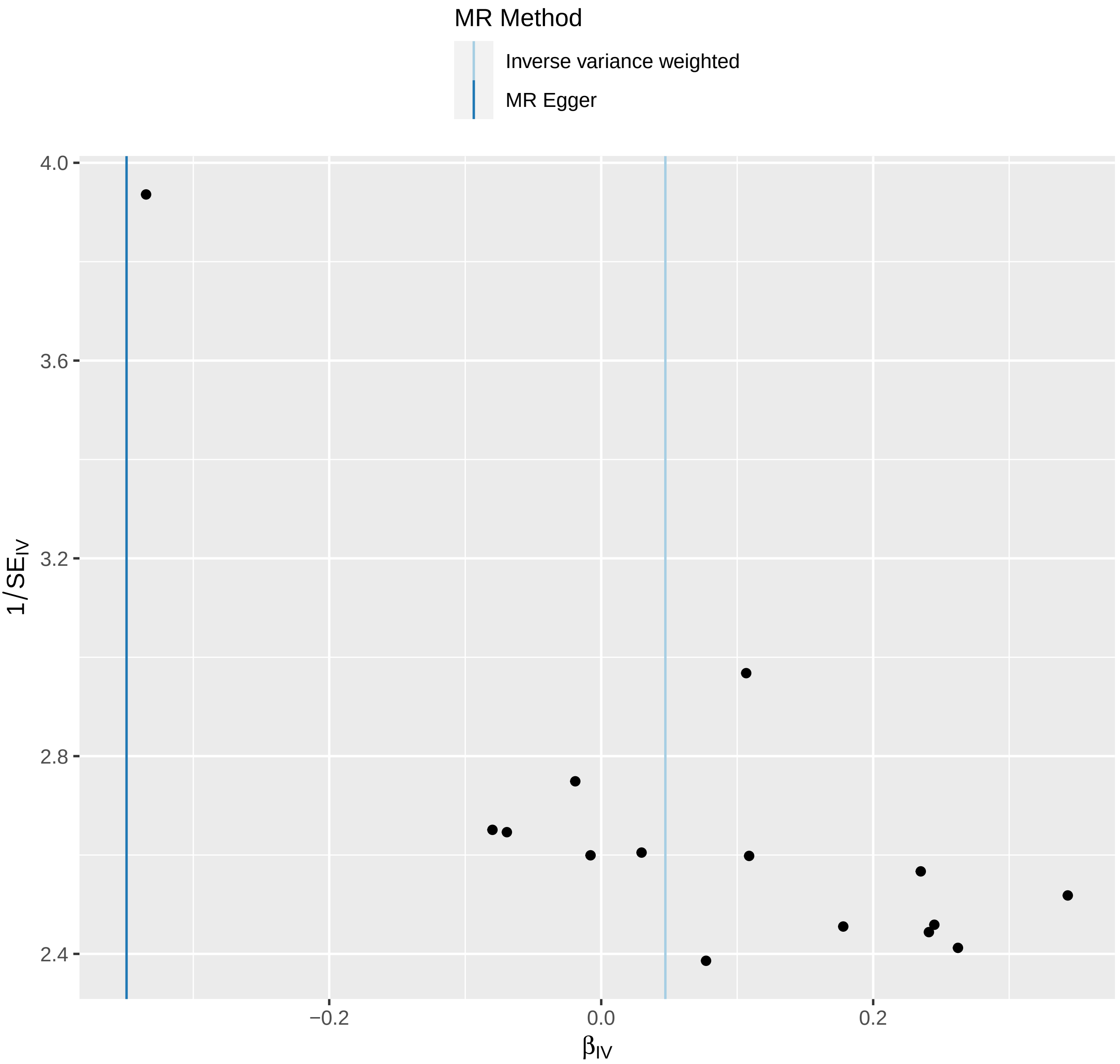

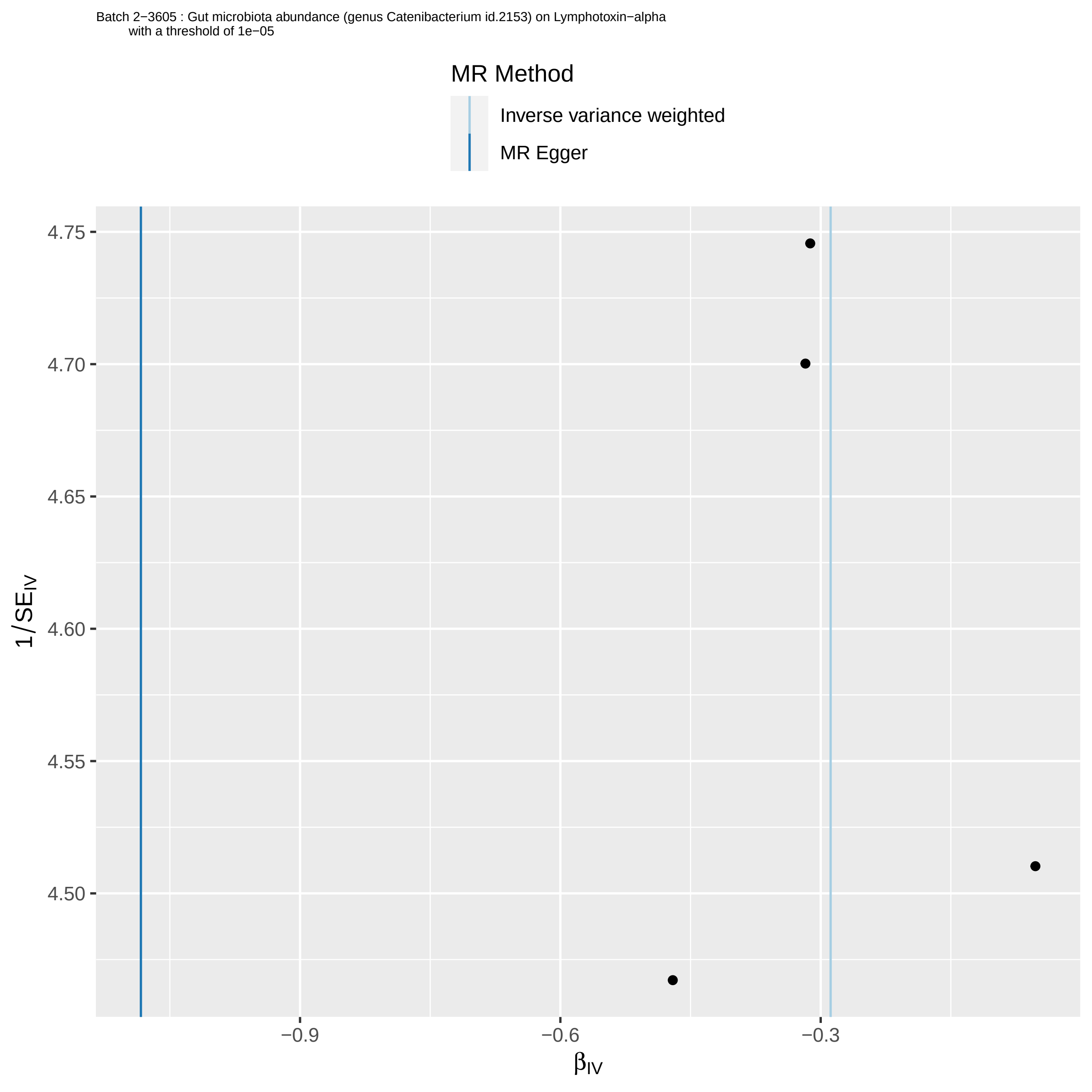

Batch 2−3605 : Gut microbiota abundance (genus Catenibacterium id.2153) on Lymphotoxin−alpha
with a threshold of 1e−05
MR Method
Inverse variance weighted
MR Egger
4.75
4.70
4.65
4.60
4.55
4.50
1/SE_IV
−0.9
−0.6
−0.3
β_IV

Batch 2−3606 : Gut microbiota abundance (genus Parasutterella id.2892) on Lymphotoxin−alpha
with a threshold of 1e−05

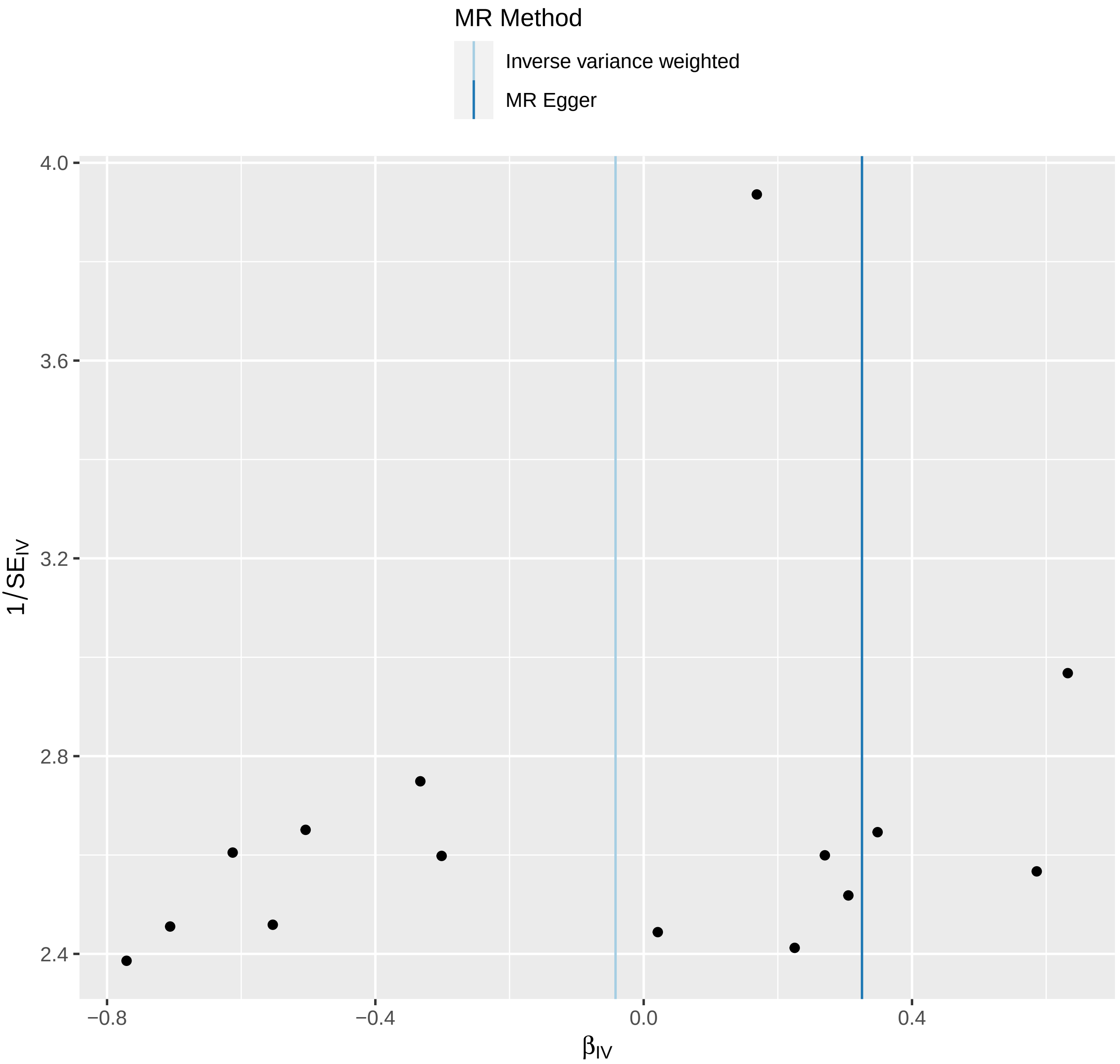

Batch 2−3607 : Gut microbiota abundance (genus Catenibacterium id.2153) on Leukotriene A−4 hydrolase
with a threshold of 1e−05

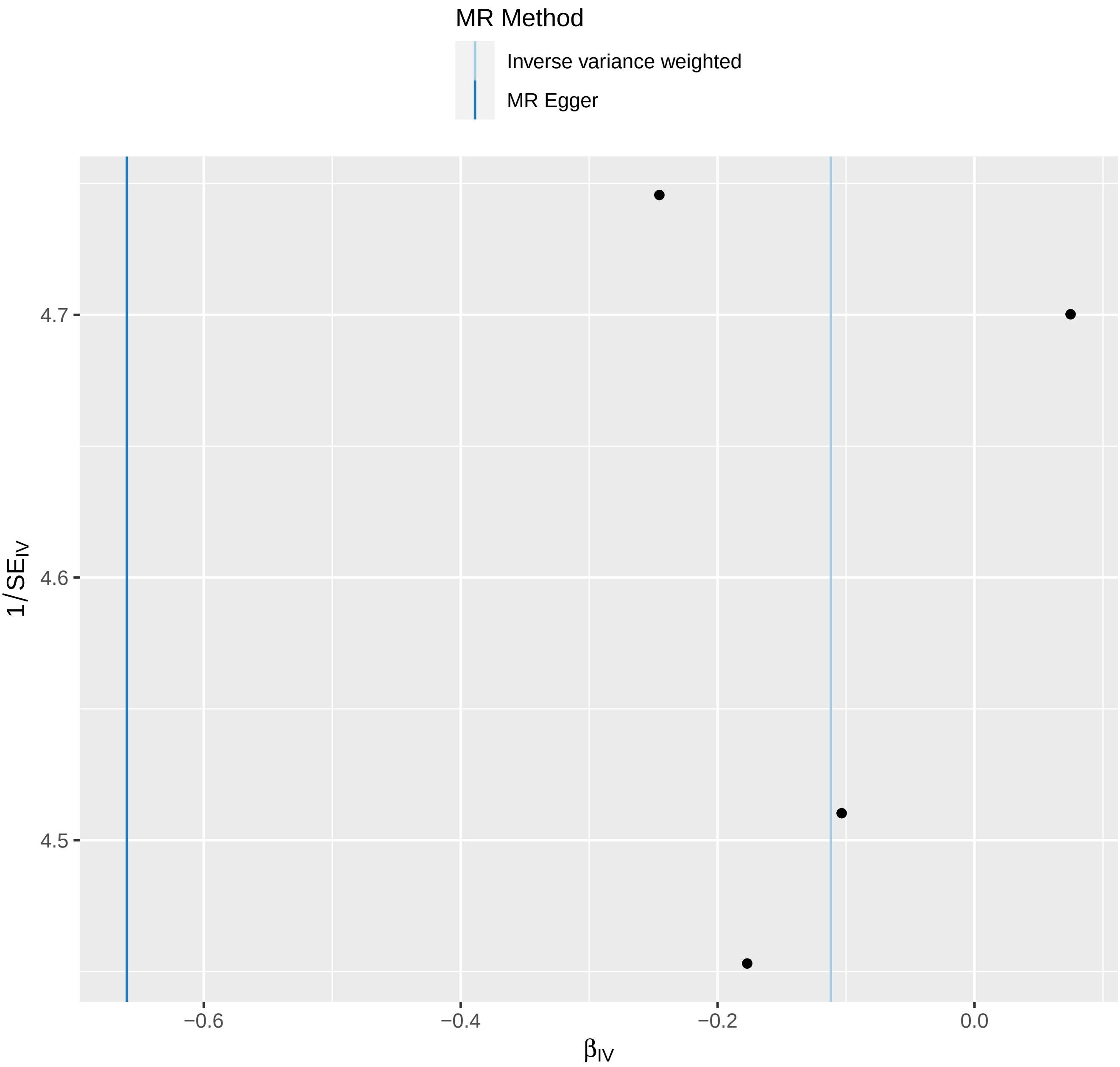

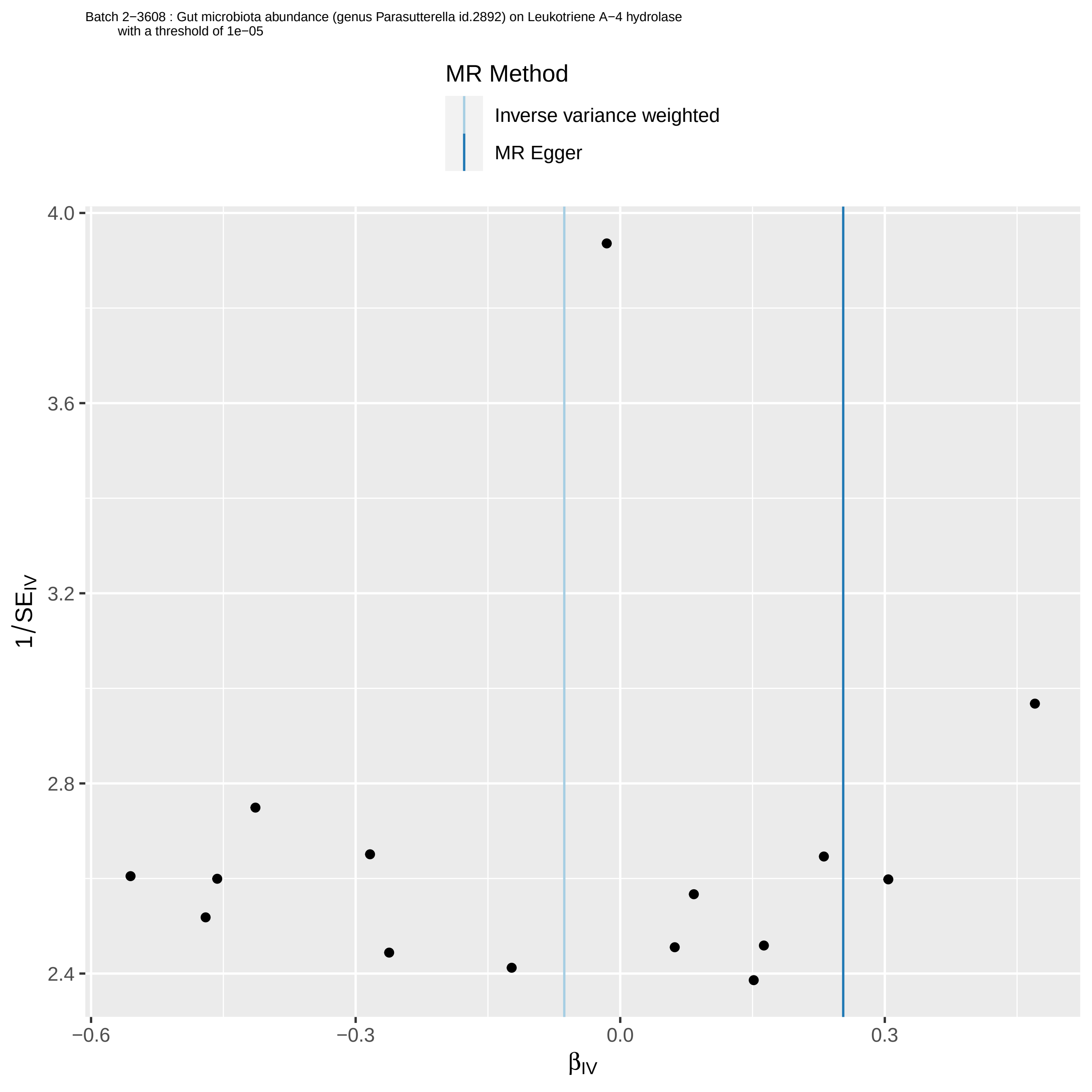

Batch 2−3608 : Gut microbiota abundance (genus Parasutterella id.2892) on Leukotriene A−4 hydrolase
with a threshold of 1e−05
MR Method
Inverse variance weighted
MR Egger
4.0
3.6
3.2
2.8
2.4
1/SE_IV
−0.6
−0.3
0.0
0.3
β_IV

Batch 2−3609 : Gut microbiota abundance (genus Catenibacterium id.2153) on Lymphotoxin alpha2:beta1
with a threshold of 1e−05

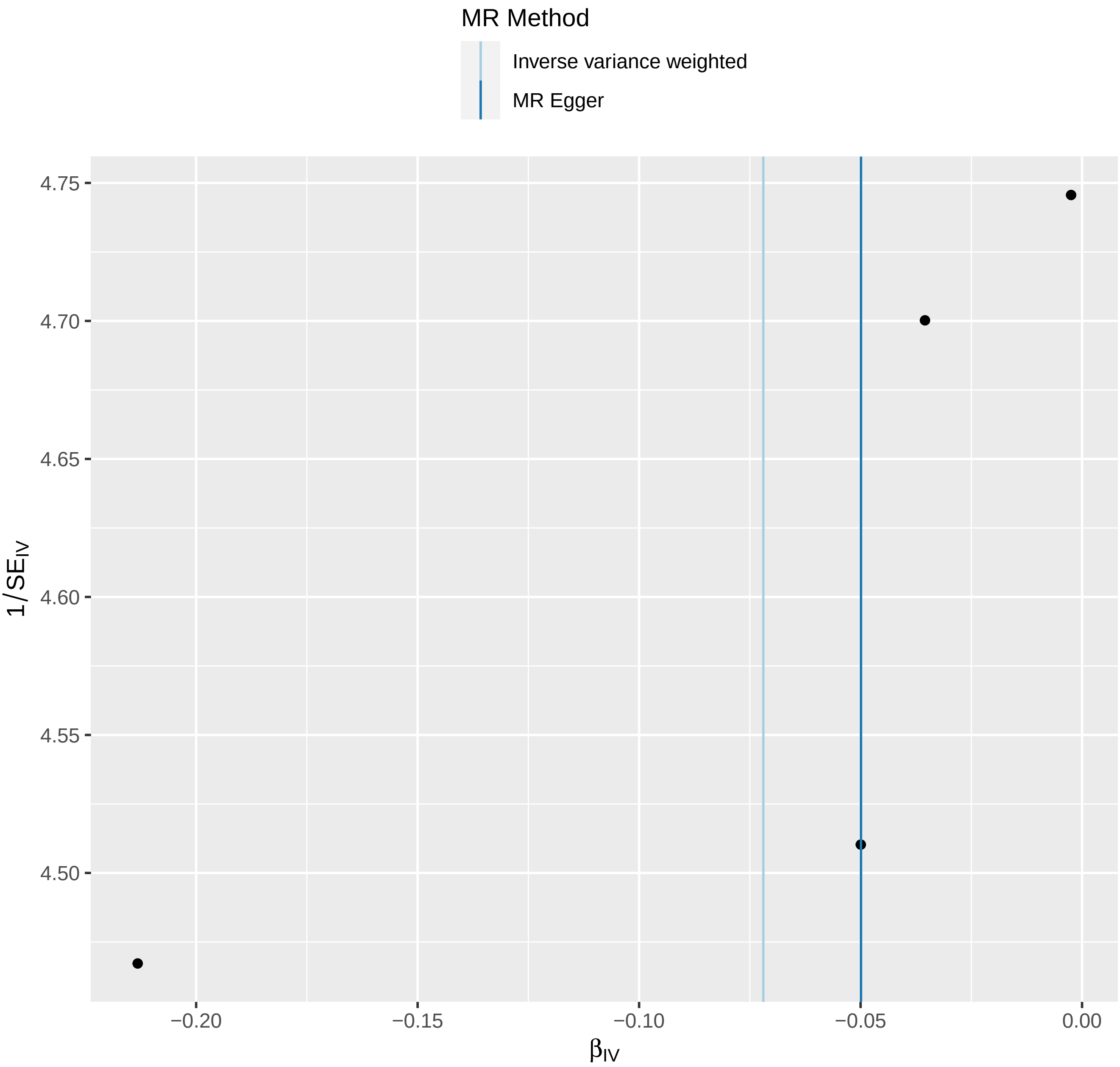

Batch 2−3610 : Gut microbiota abundance (genus Parasutterella id.2892) on Lymphotoxin alpha2:beta1
with a threshold of 1e−05

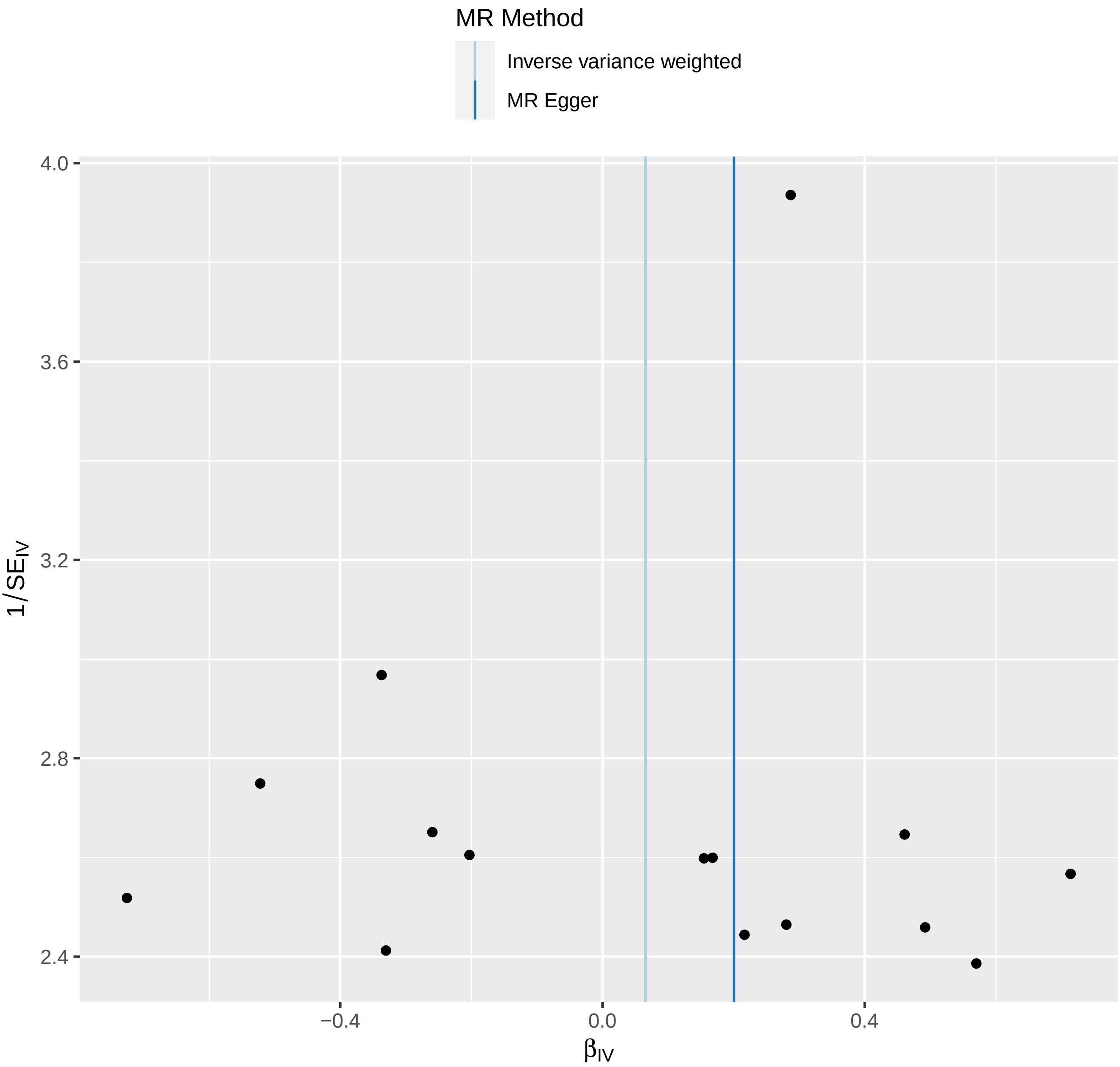

Batch 2−3611 : Gut microbiota abundance (genus Catenibacterium id.2153) on Latent−transforming growth factor beta−binding protein 4 with a threshold of 1e−05

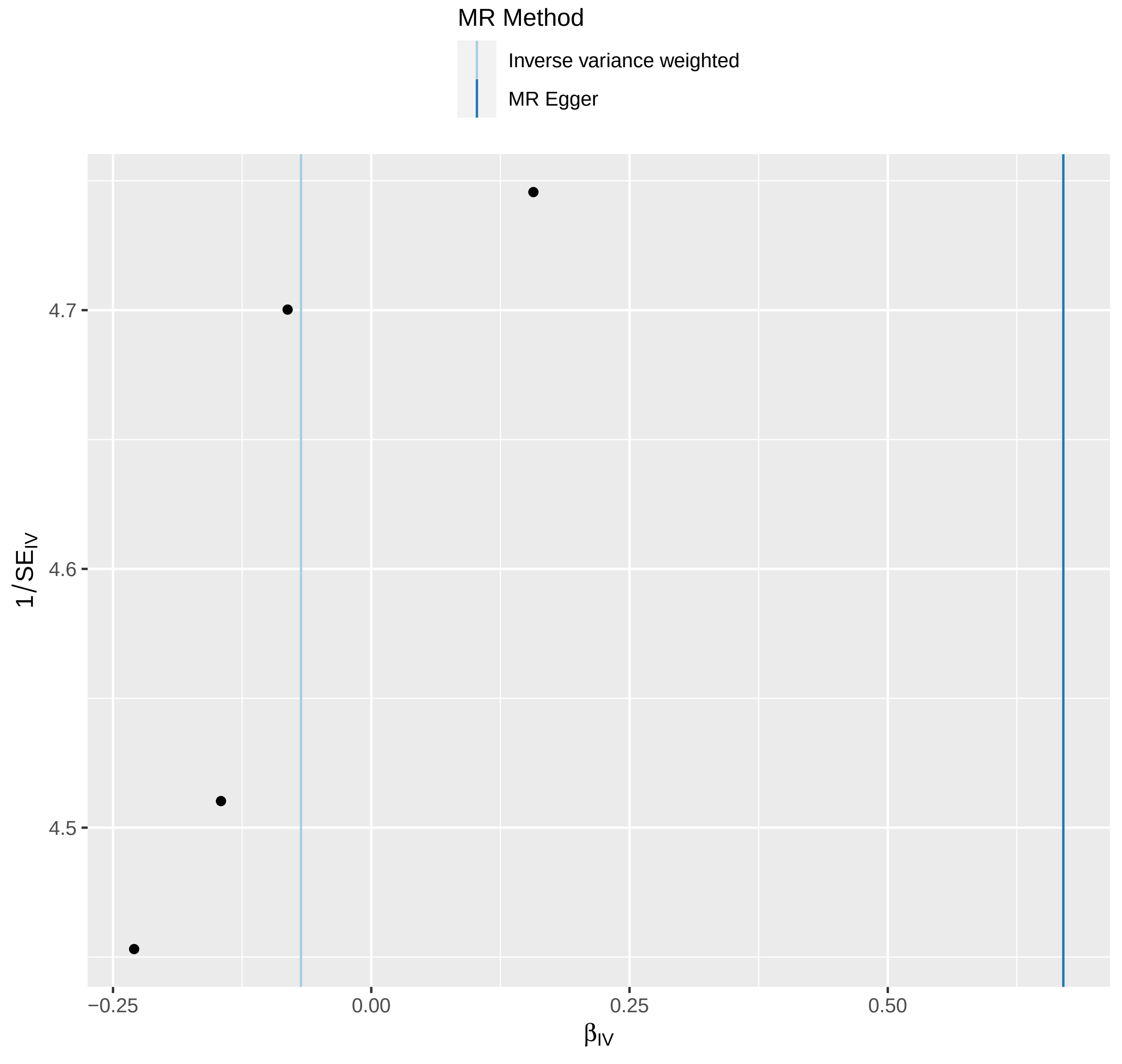

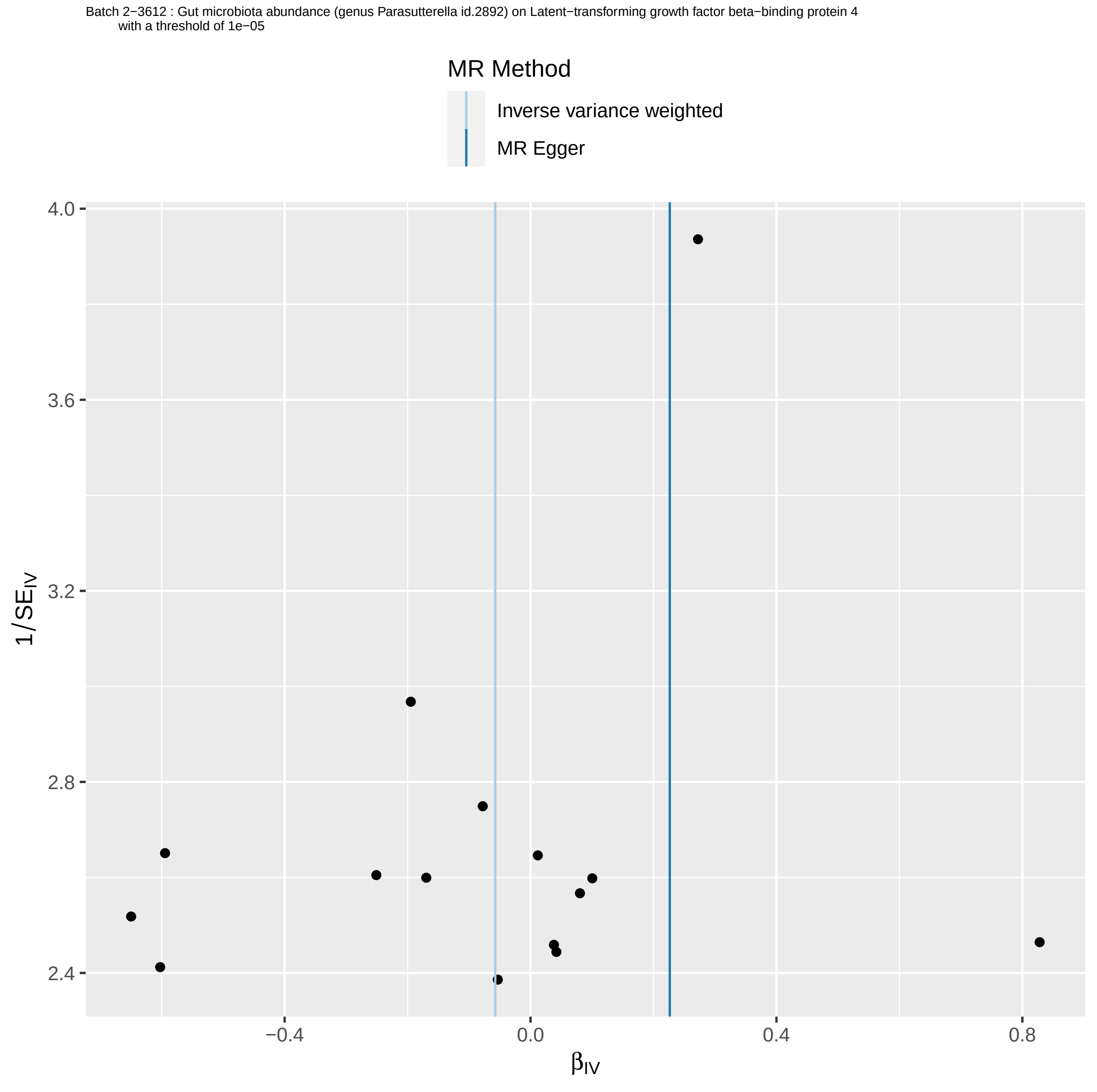
Batch 2−3612 : Gut microbiota abundance (genus Parasutterella id.2892) on Latent−transforming growth factor beta−binding protein 4
with a threshold of 1e−05
MR Method
Inverse variance weighted
MR Egger
4.0
3.6
3.2
2.8
2.4
1/SE_IV
−0.4
0.0
0.4
0.8
β_IV

Batch 2−3613 : Gut microbiota abundance (genus Catenibacterium id.2153) on Tumor necrosis factor receptor superfamily member 3 with a threshold of 1e−05

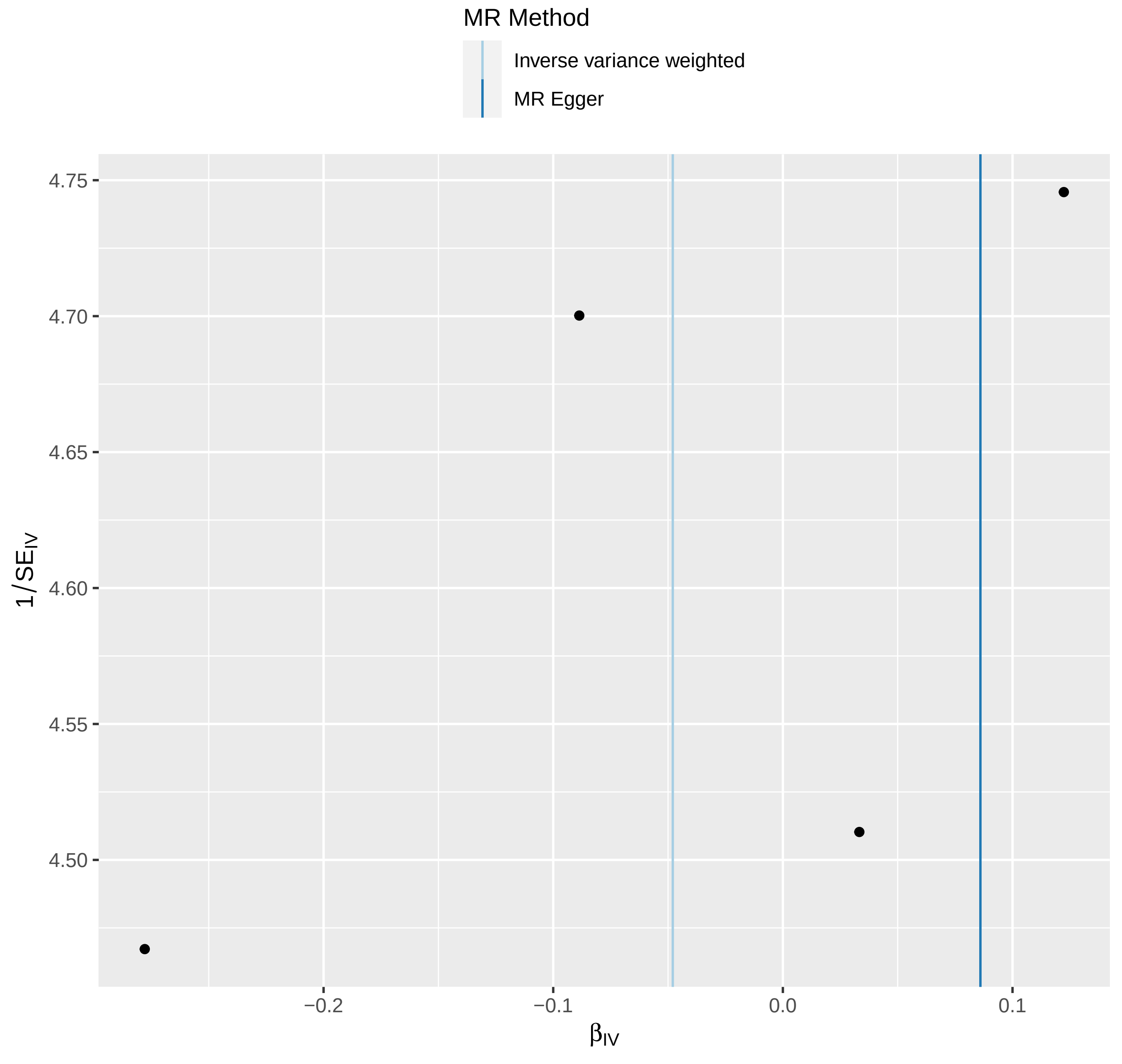

Batch 2−3614 : Gut microbiota abundance (genus Parasutterella id.2892) on Tumor necrosis factor receptor superfamily member 3
with a threshold of 1e−05

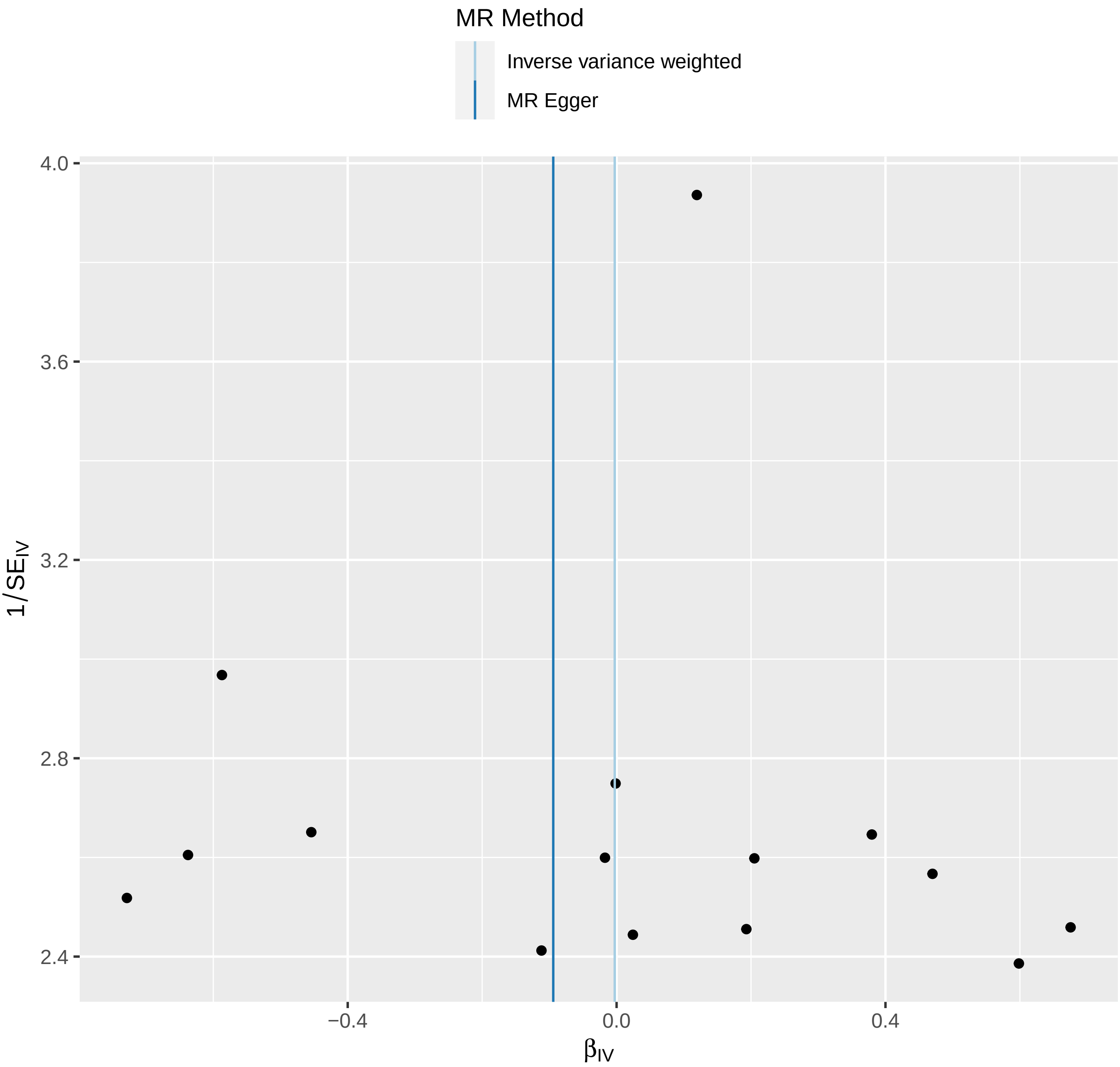

Batch 2−3615 : Gut microbiota abundance (genus Catenibacterium id.2153) on Lactotransferrin
with a threshold of 1e−05

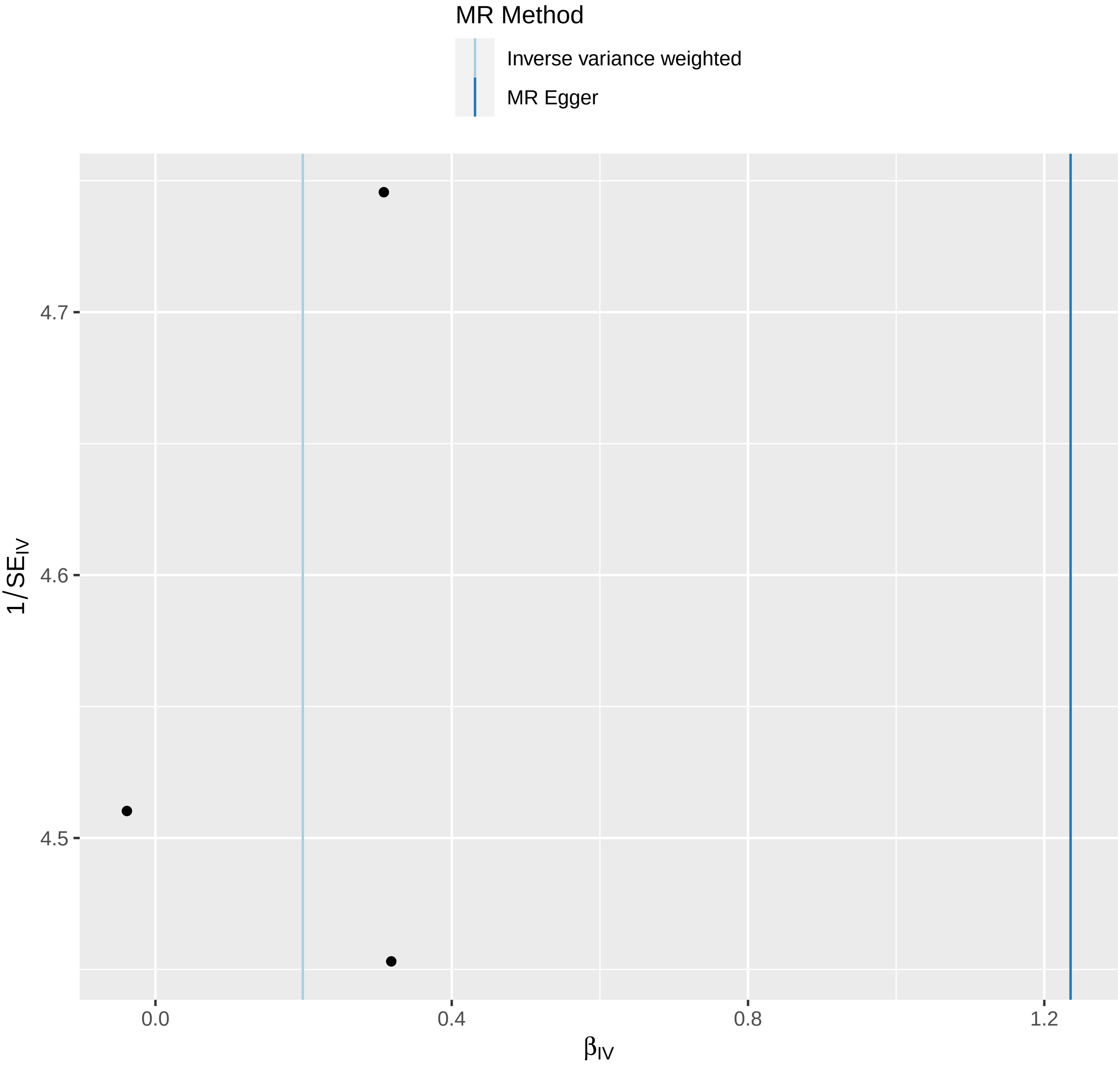

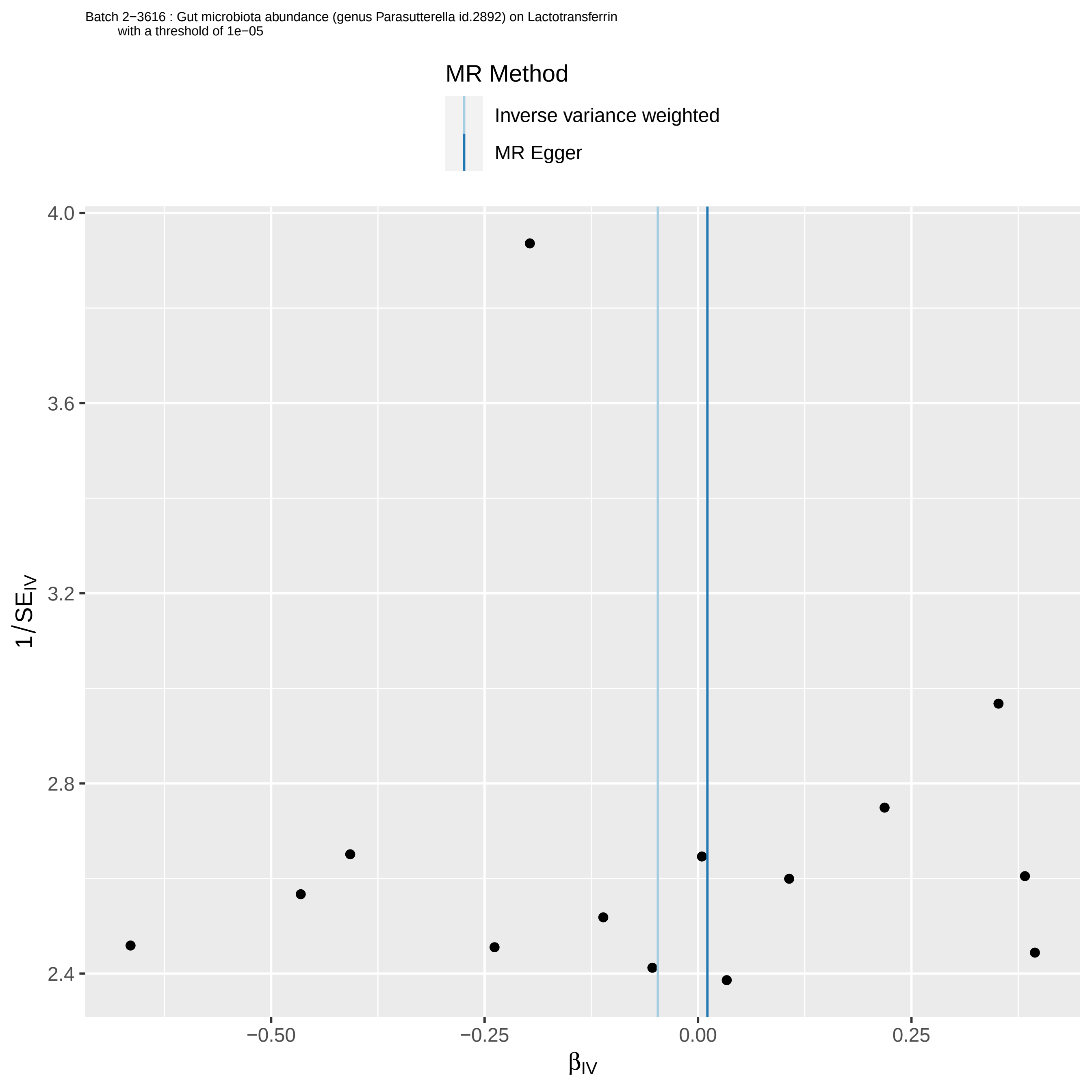
Batch 2−3616 : Gut microbiota abundance (genus Parasutterella id.2892) on Lactotransferrin
with a threshold of 1e−05
MR Method
Inverse variance weighted
MR Egger
1/SE_IV
4.0
3.6
3.2
2.8
2.4
−0.50
−0.25
0.00
0.25
β_IV

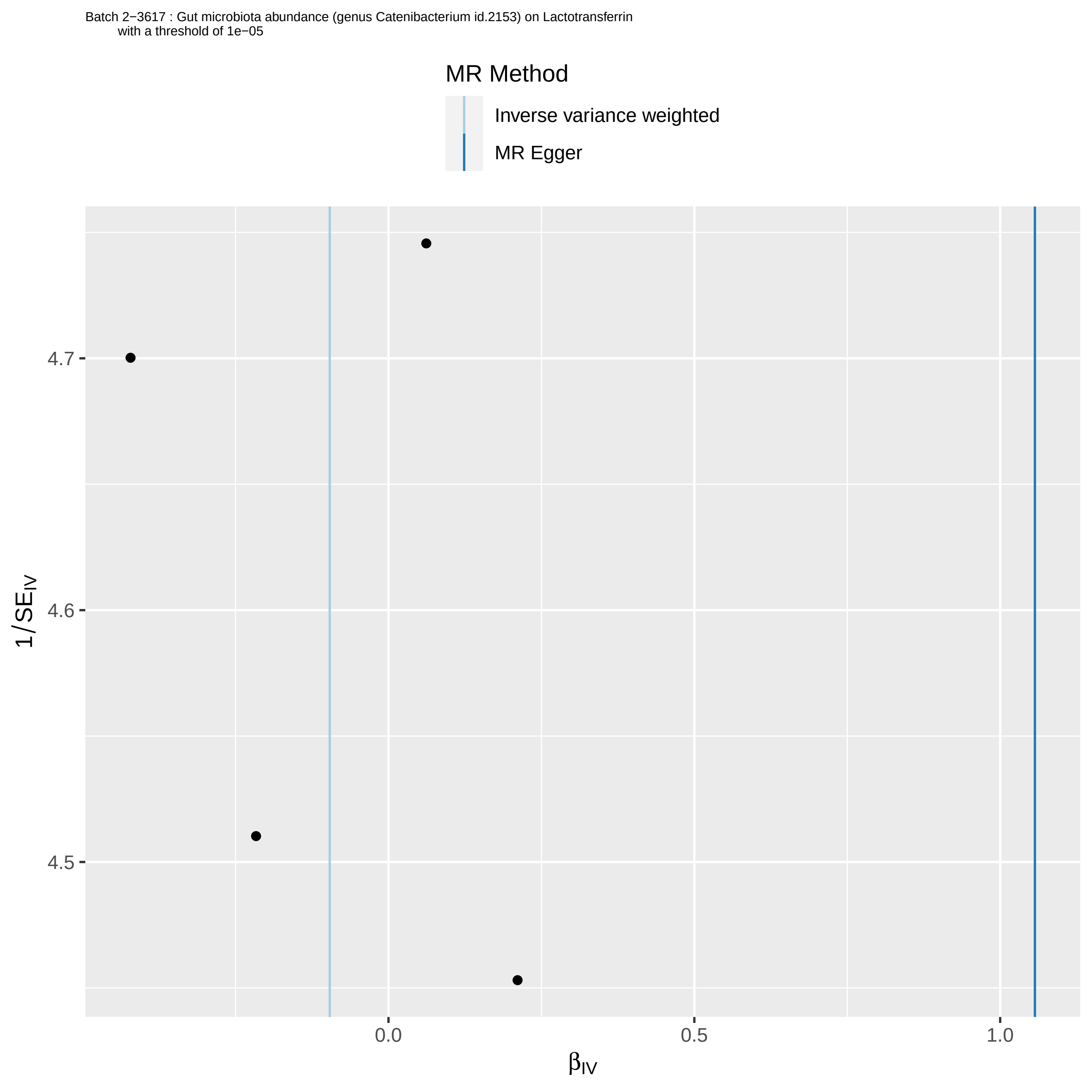
Batch 2−3617 : Gut microbiota abundance (genus Catenibacterium id.2153) on Lactotransferrin
with a threshold of 1e−05
MR Method
Inverse variance weighted
MR Egger
4.7
4.6
4.5
1/SE_IV
0.0
0.5
1.0
β_IV

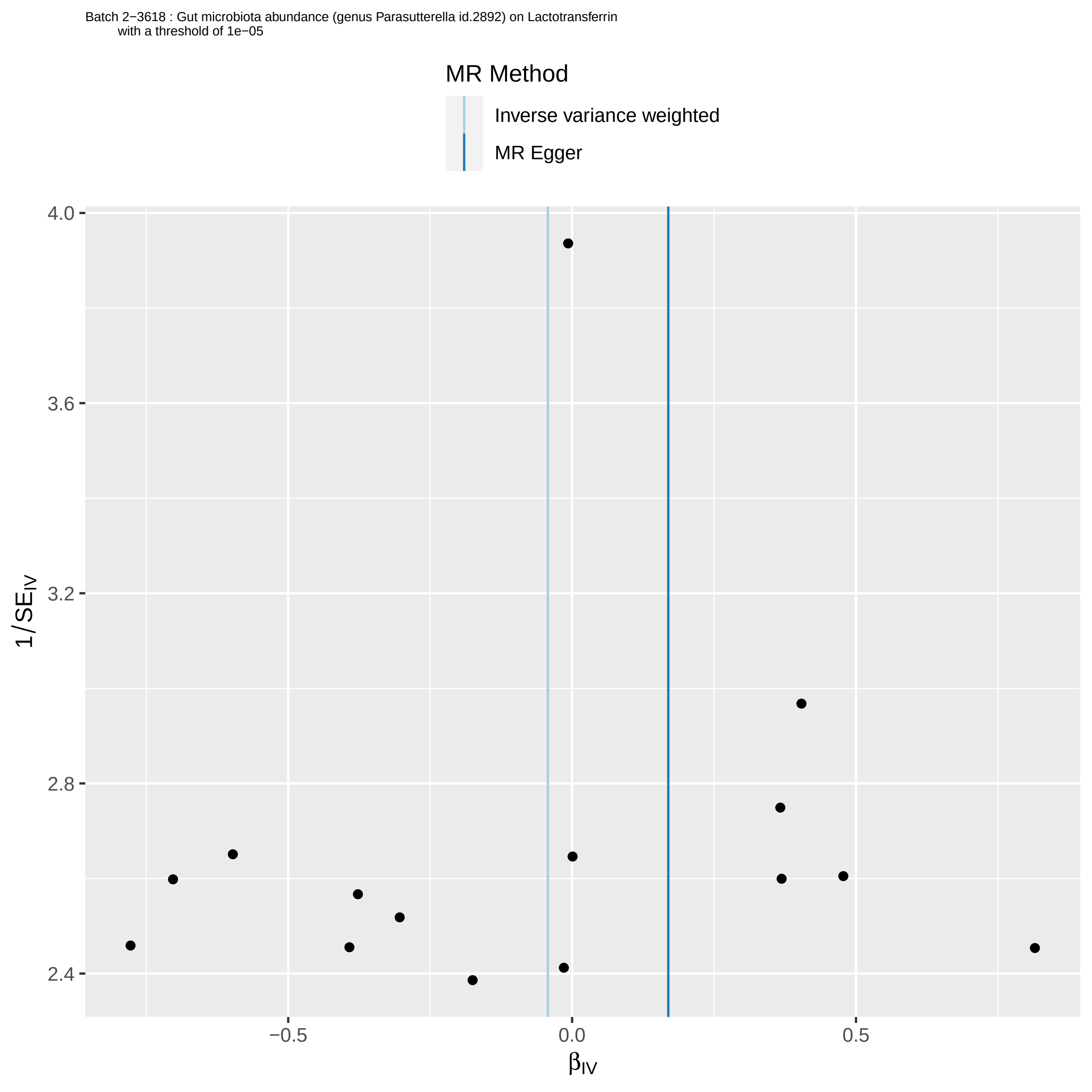
Batch 2−3618 : Gut microbiota abundance (genus Parasutterella id.2892) on Lactotransferrin
with a threshold of 1e−05
MR Method
Inverse variance weighted
MR Egger
4.0
3.6
3.2
2.8
2.4
1/SE$_{IV}$
−0.5
0.0
0.5
β$_{IV}$

Batch 2−3619 : Gut microbiota abundance (genus Catenibacterium id.2153) on Lymphocyte antigen 6 complex locus protein G6c
with a threshold of 1e−05

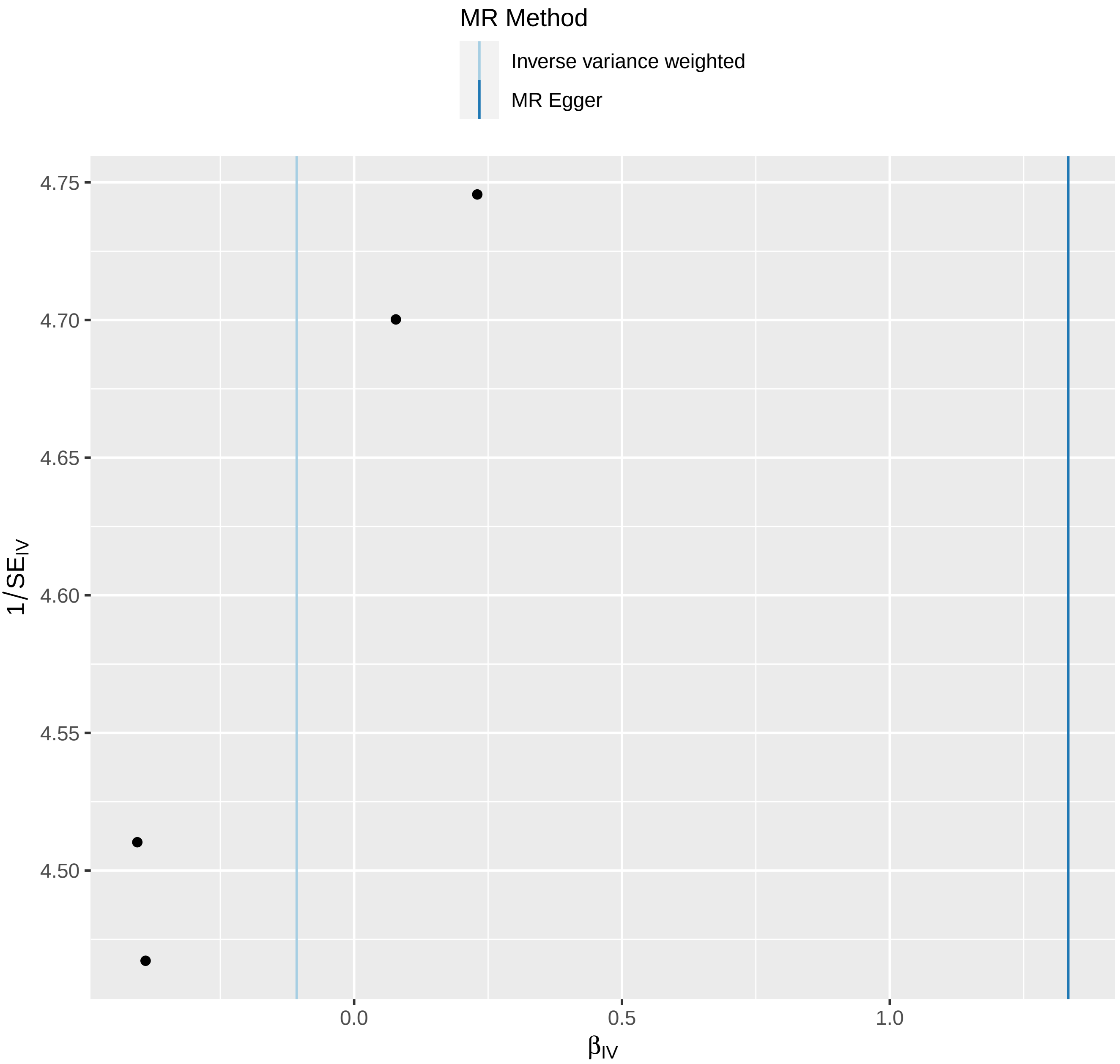

Batch 2−3620 : Gut microbiota abundance (genus Parasutterella id.2892) on Lymphocyte antigen 6 complex locus protein G6c
with a threshold of 1e−05

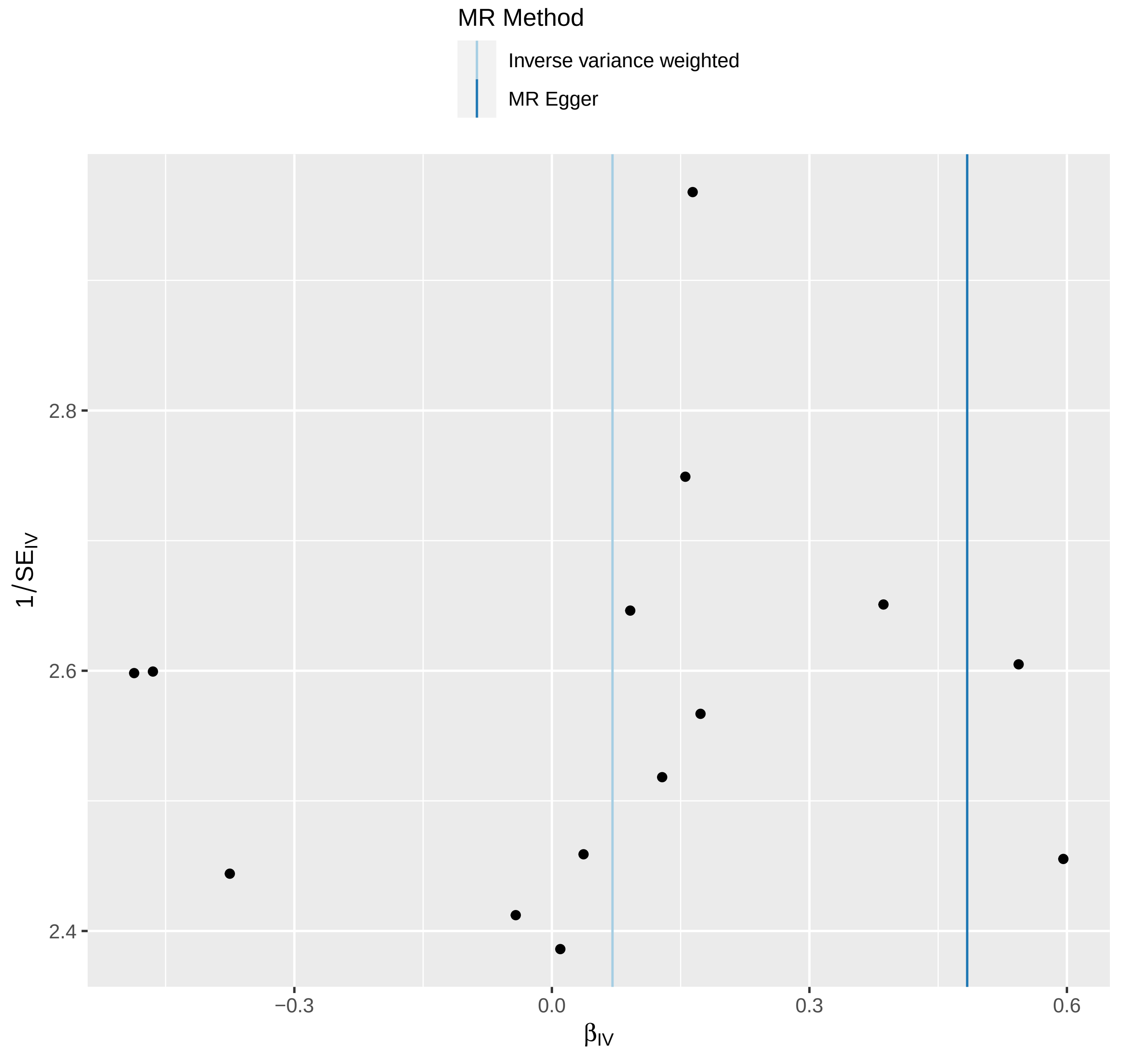

Batch 2−3621 : Gut microbiota abundance (genus Catenibacterium id.2153) on Lymphocyte antigen 6 complex locus protein G6d
with a threshold of 1e−05

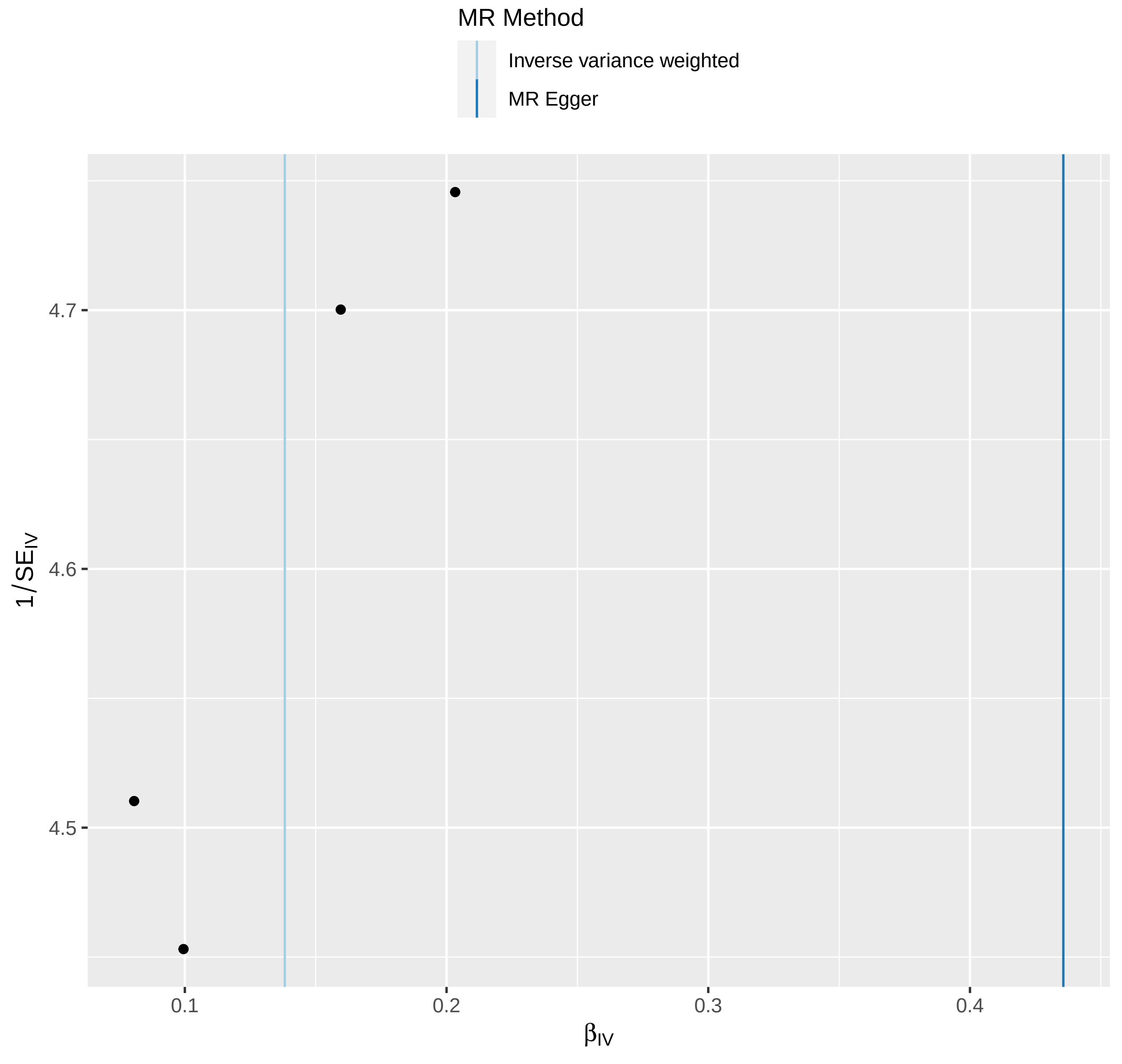

Batch 2−3622 : Gut microbiota abundance (genus Parasutterella id.2892) on Lymphocyte antigen 6 complex locus protein G6d
with a threshold of 1e−05

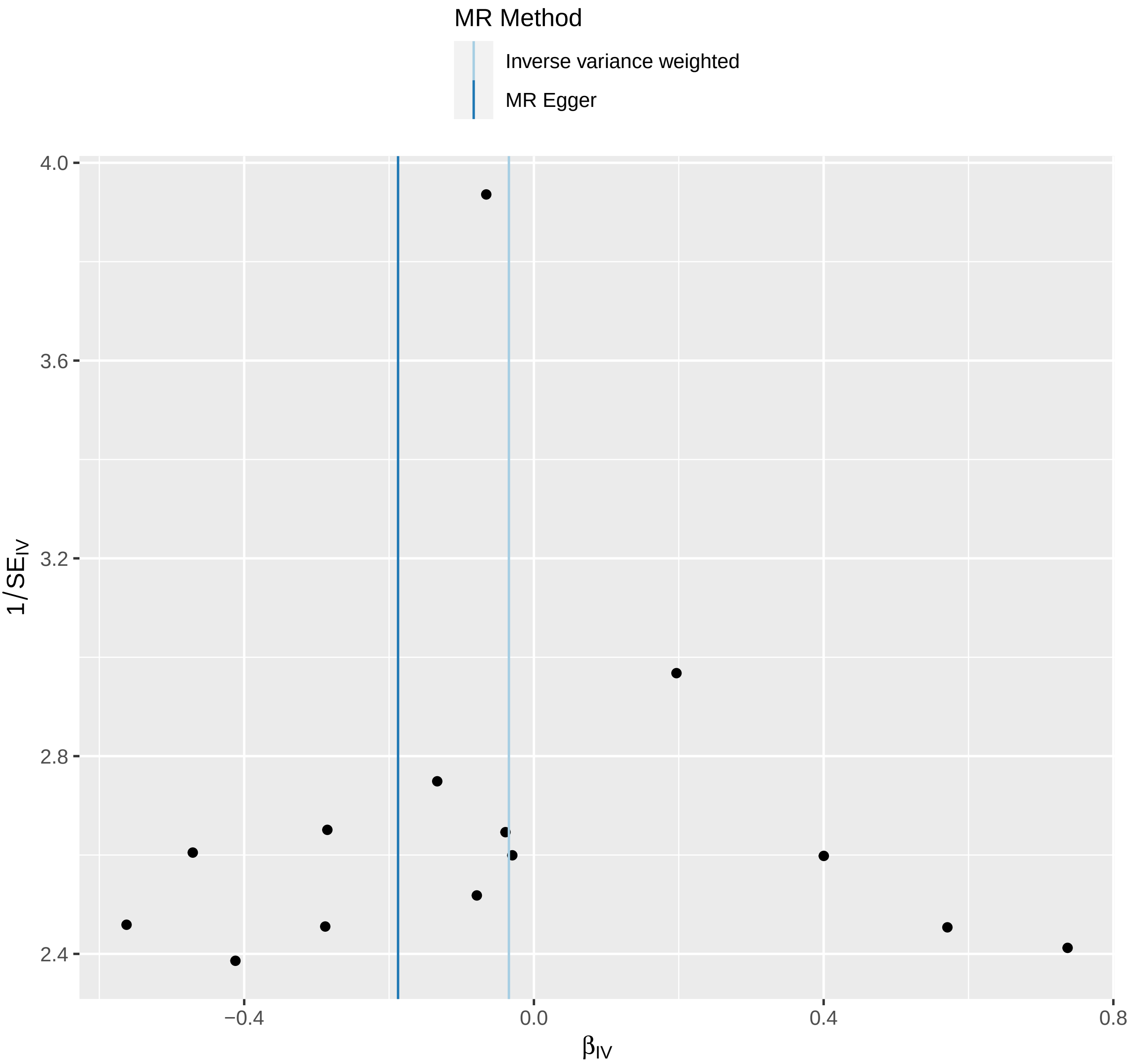

Batch 2−3623 : Gut microbiota abundance (genus Catenibacterium id.2153) on Lymphocyte antigen 86
with a threshold of 1e−05

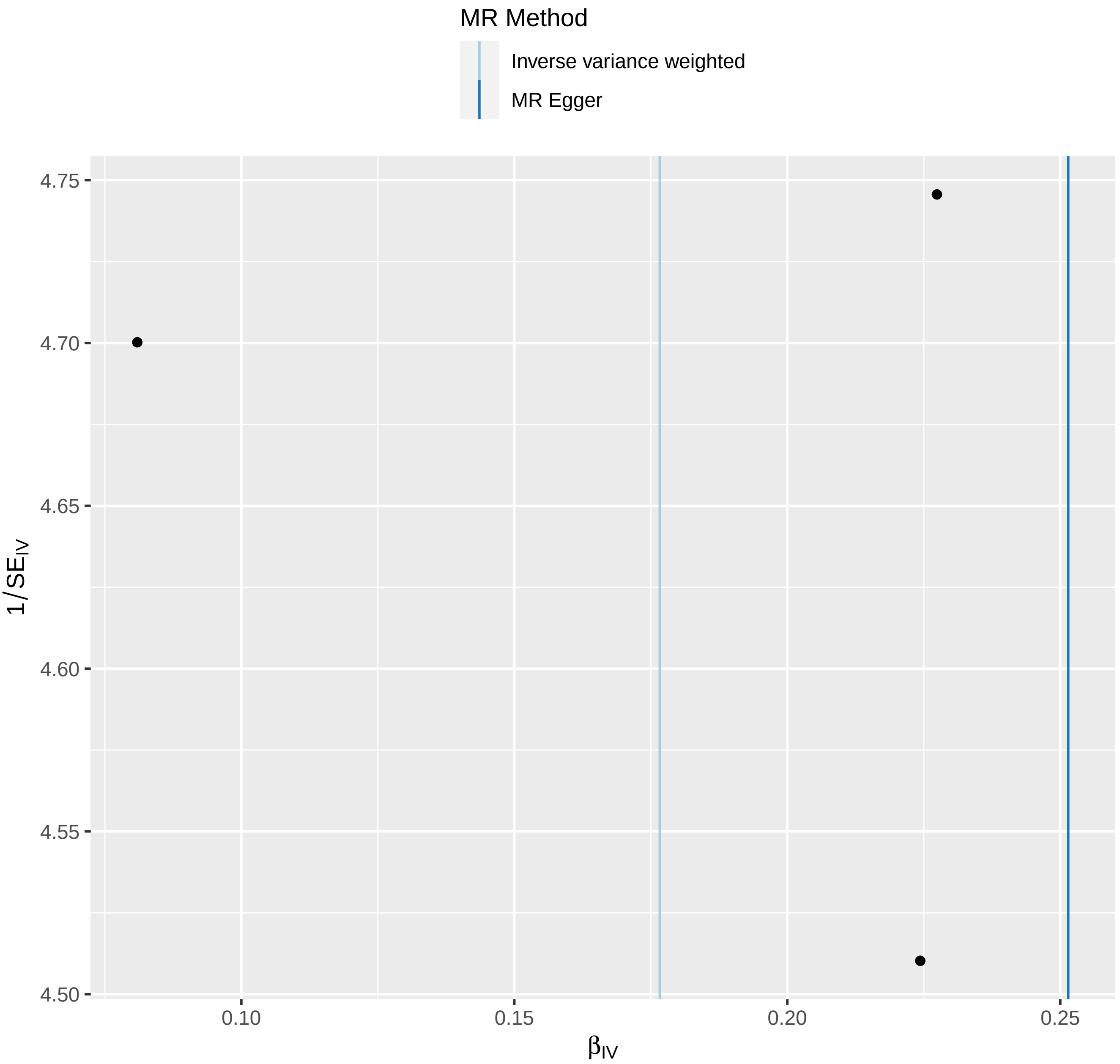

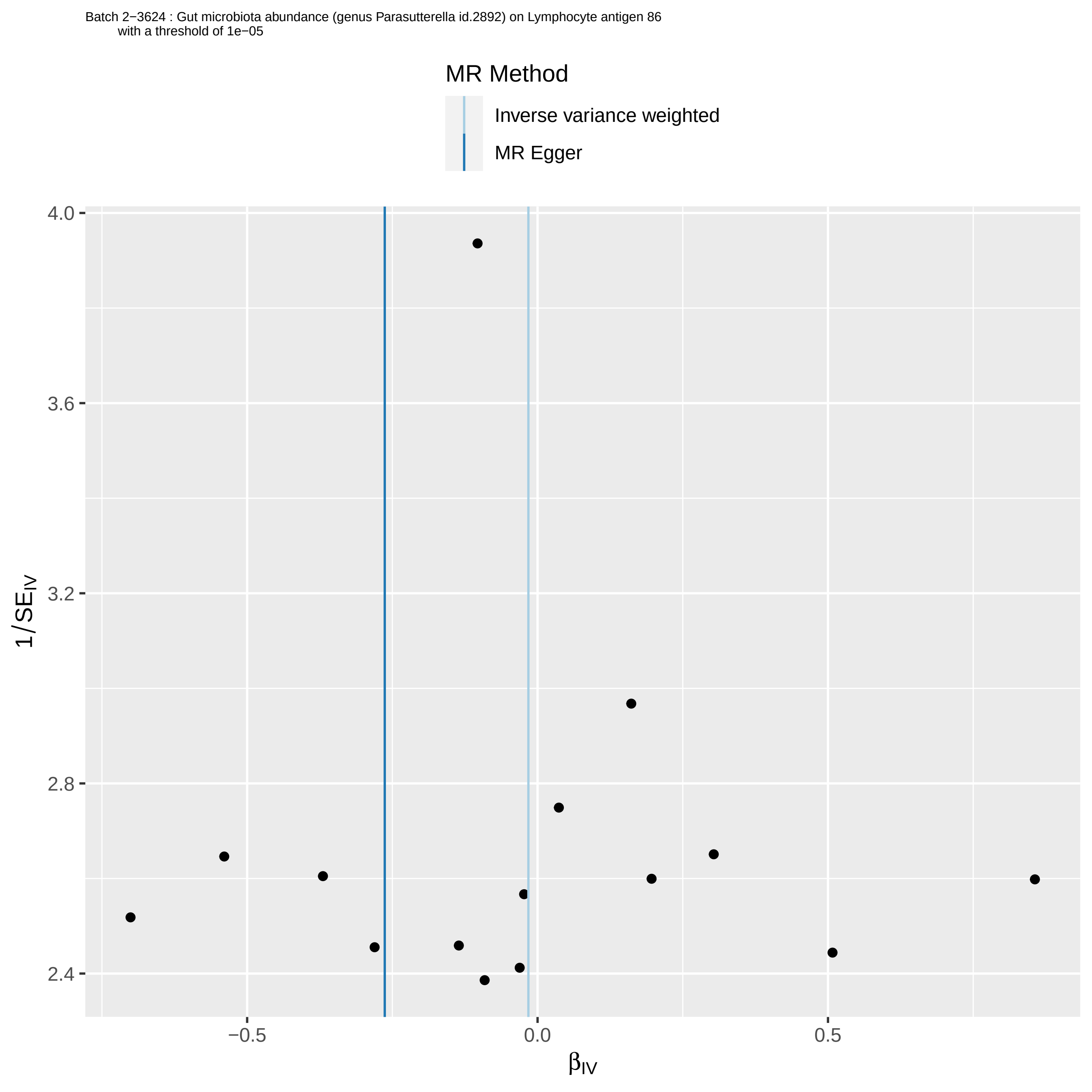

Batch 2−3624 : Gut microbiota abundance (genus Parasutterella id.2892) on Lymphocyte antigen 86
with a threshold of 1e−05
MR Method
Inverse variance weighted
MR Egger
4.0
3.6
3.2
2.8
2.4
1/SE$_{IV}$
−0.5
0.0
0.5
β$_{IV}$

Batch 2−3625 : Gut microbiota abundance (genus Catenibacterium id.2153) on T−lymphocyte surface antigen Ly−9
with a threshold of 1e−05

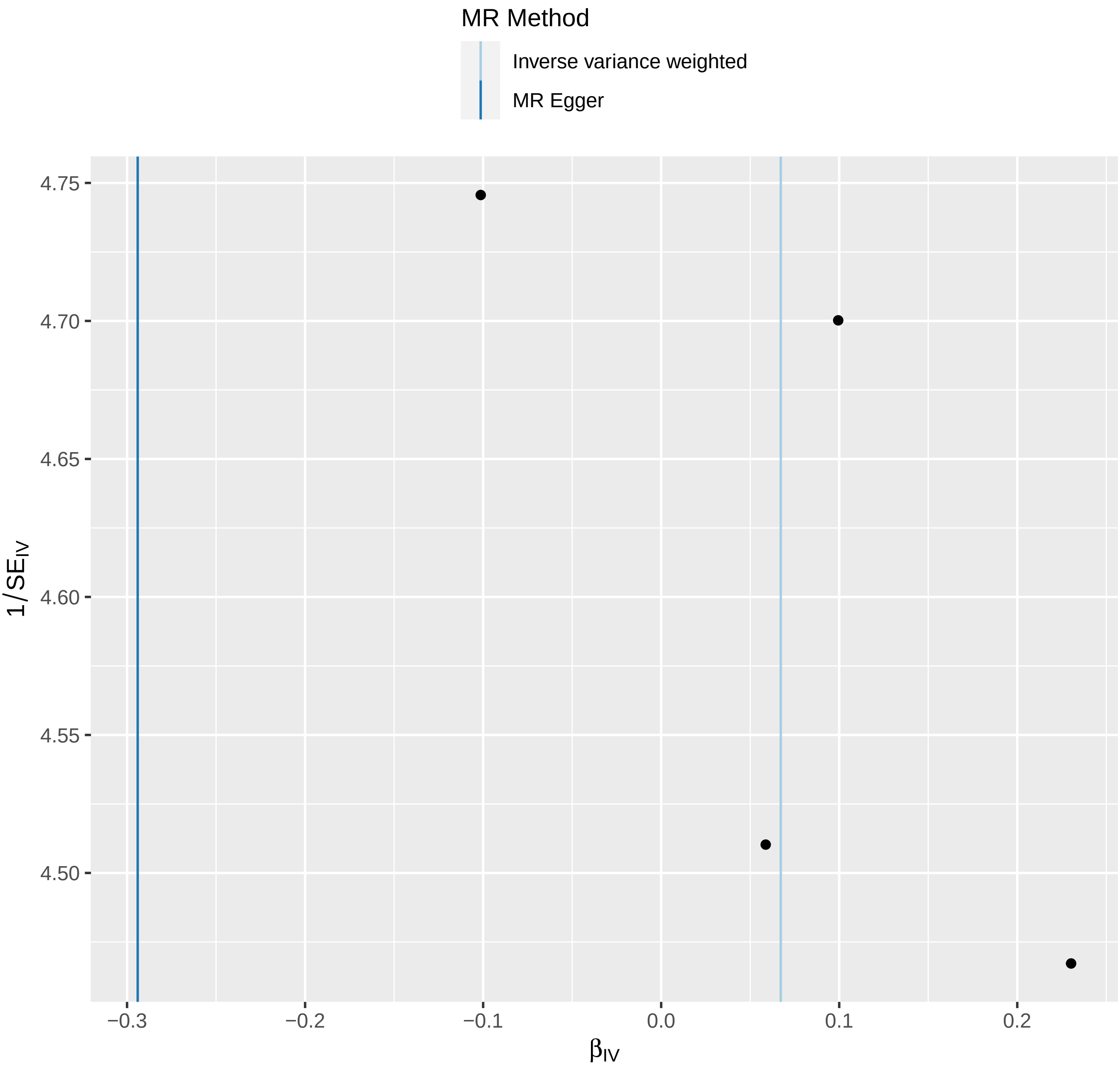

Batch 2−3626 : Gut microbiota abundance (genus Parasutterella id.2892) on T−lymphocyte surface antigen Ly−9
with a threshold of 1e−05

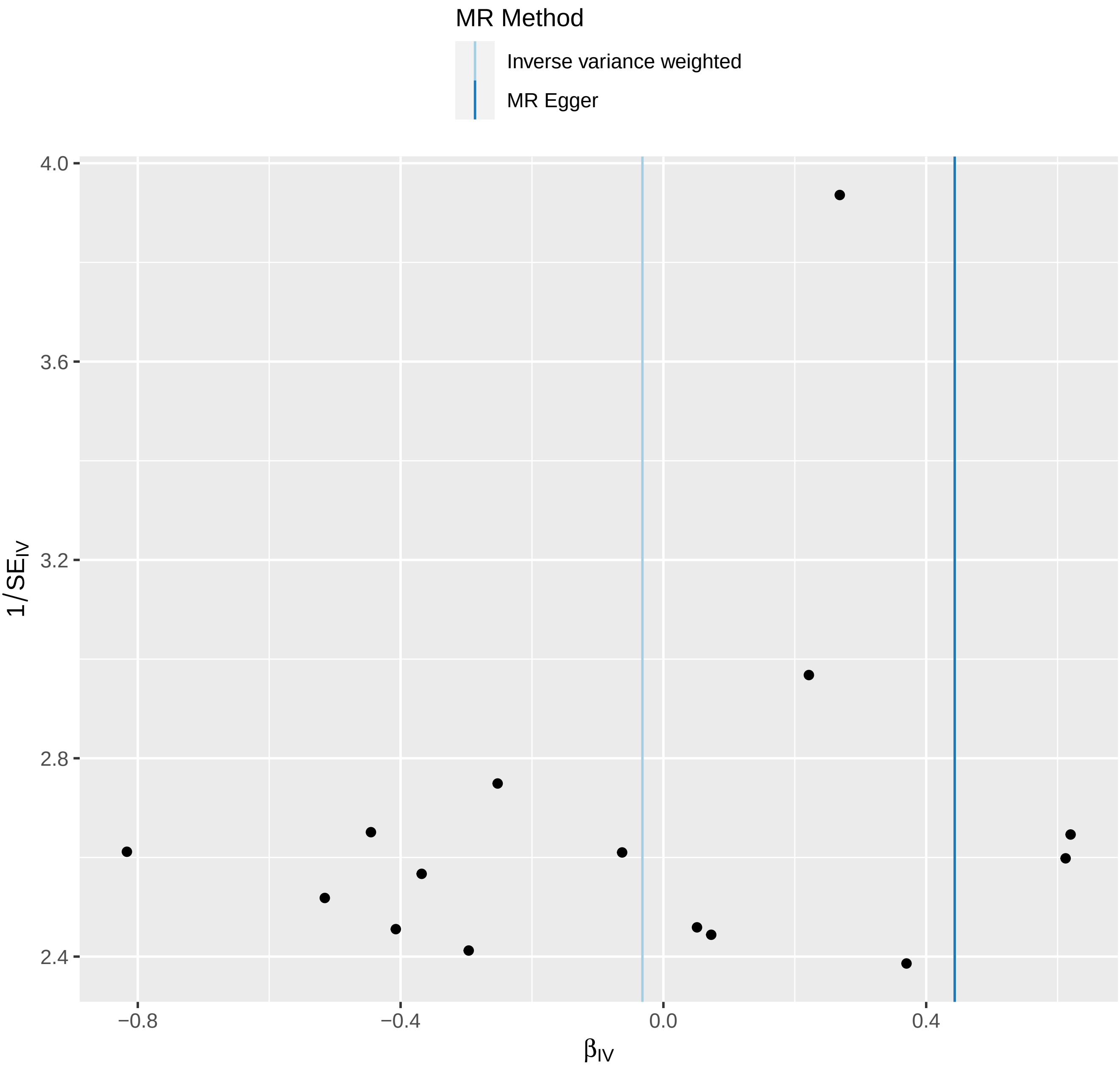

Batch 2−3627 : Gut microbiota abundance (genus Catenibacterium id.2153) on Lysozyme g−like protein 1
with a threshold of 1e−05

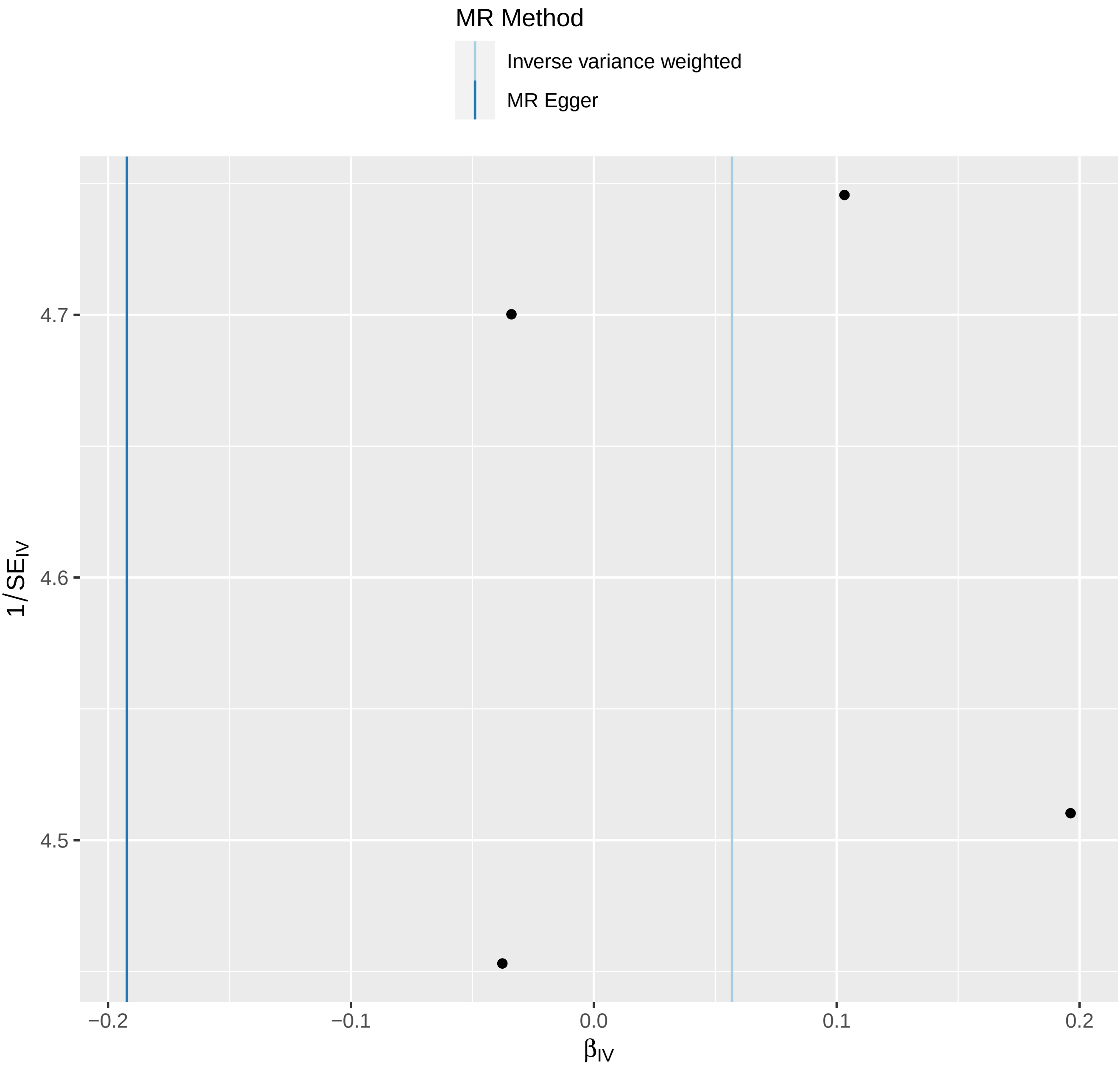

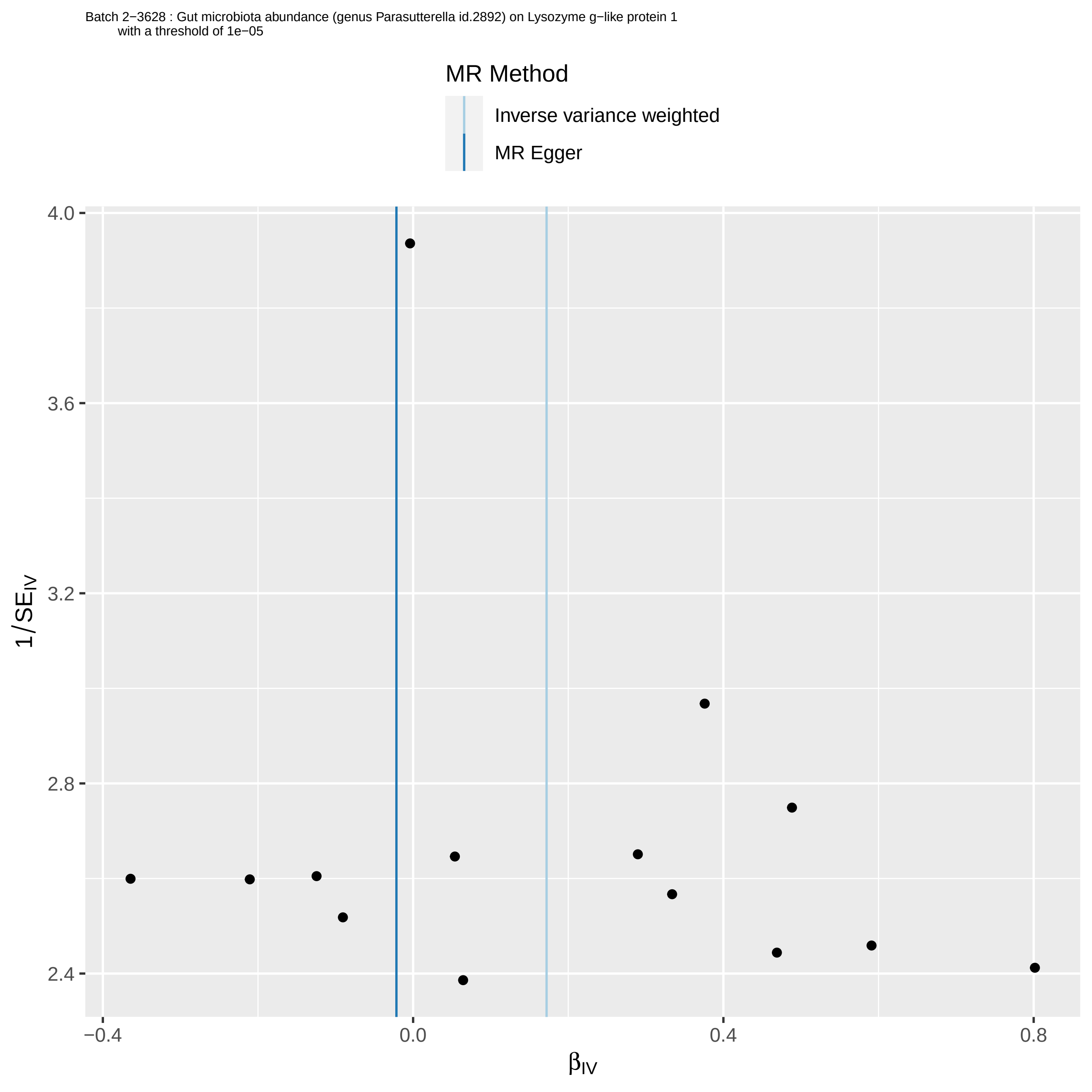

Batch 2−3628 : Gut microbiota abundance (genus Parasutterella id.2892) on Lysozyme g−like protein 1
with a threshold of 1e−05
MR Method
Inverse variance weighted
MR Egger
4.0
3.6
3.2
2.8
2.4
1/SE_IV
−0.4
0.0
0.4
0.8
β_IV

Batch 2−3629 : Gut microbiota abundance (genus Catenibacterium id.2153) on Tyrosine−protein kinase Lyn, isoform B
with a threshold of 1e−05

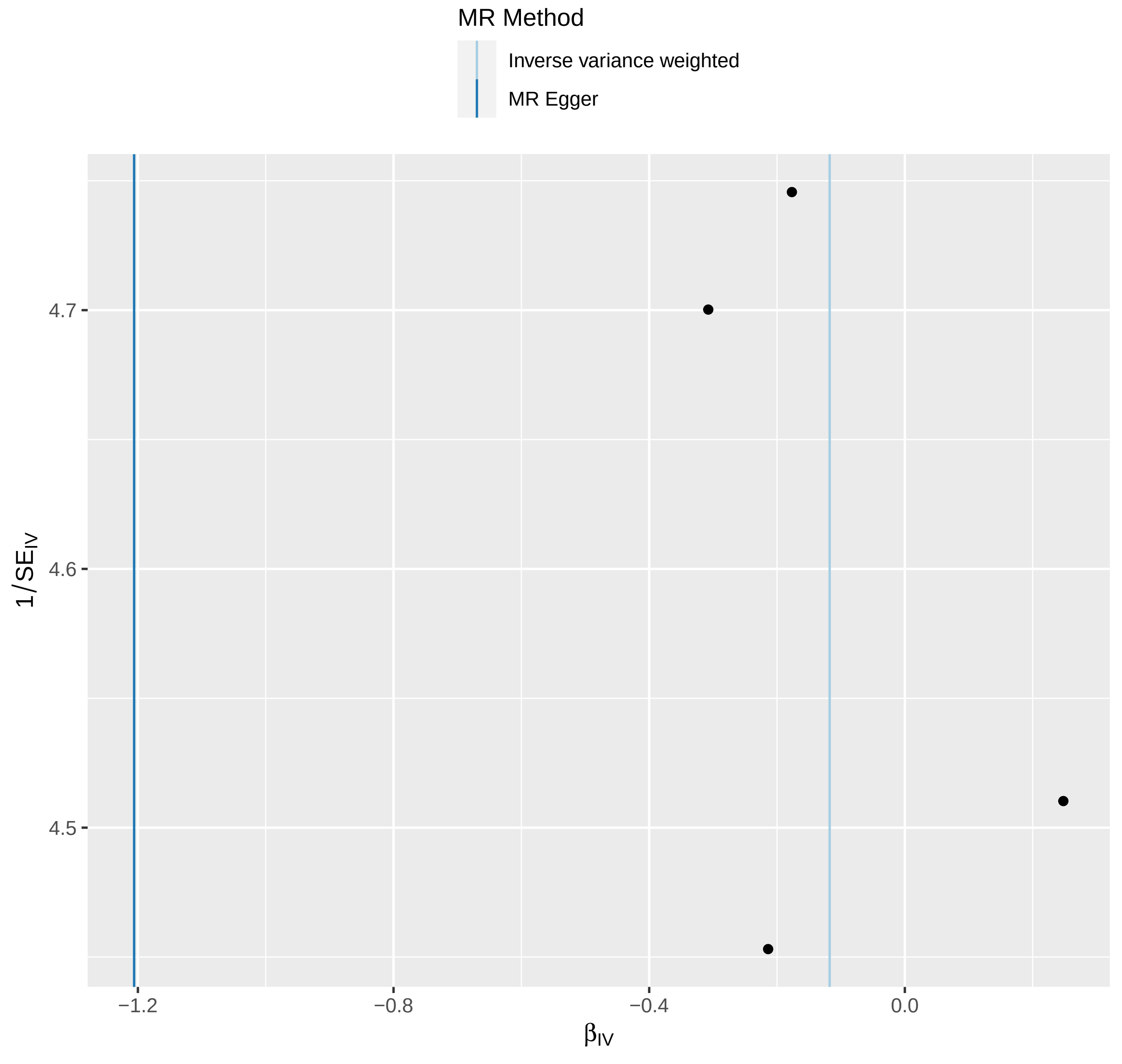

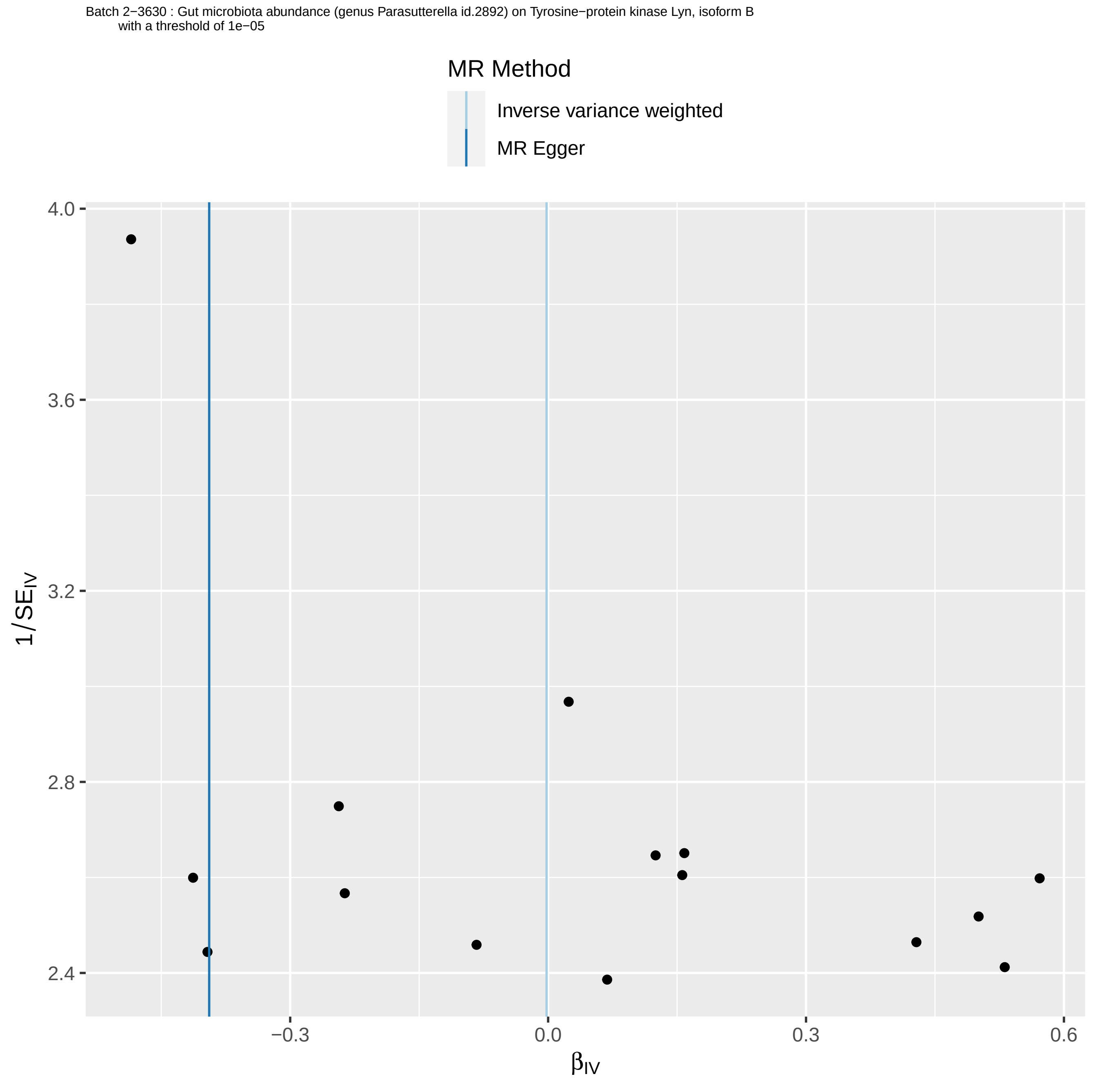
Batch 2−3630 : Gut microbiota abundance (genus Parasutterella id.2892) on Tyrosine−protein kinase Lyn, isoform B
with a threshold of 1e−05
MR Method
Inverse variance weighted
MR Egger
4.0
3.6
3.2
2.8
2.4
1/SE_IV
−0.3
0.0
0.3
0.6
β_IV

Batch 2−3631 : Gut microbiota abundance (genus Catenibacterium id.2153) on Tyrosine−protein kinase Lyn
with a threshold of 1e−05

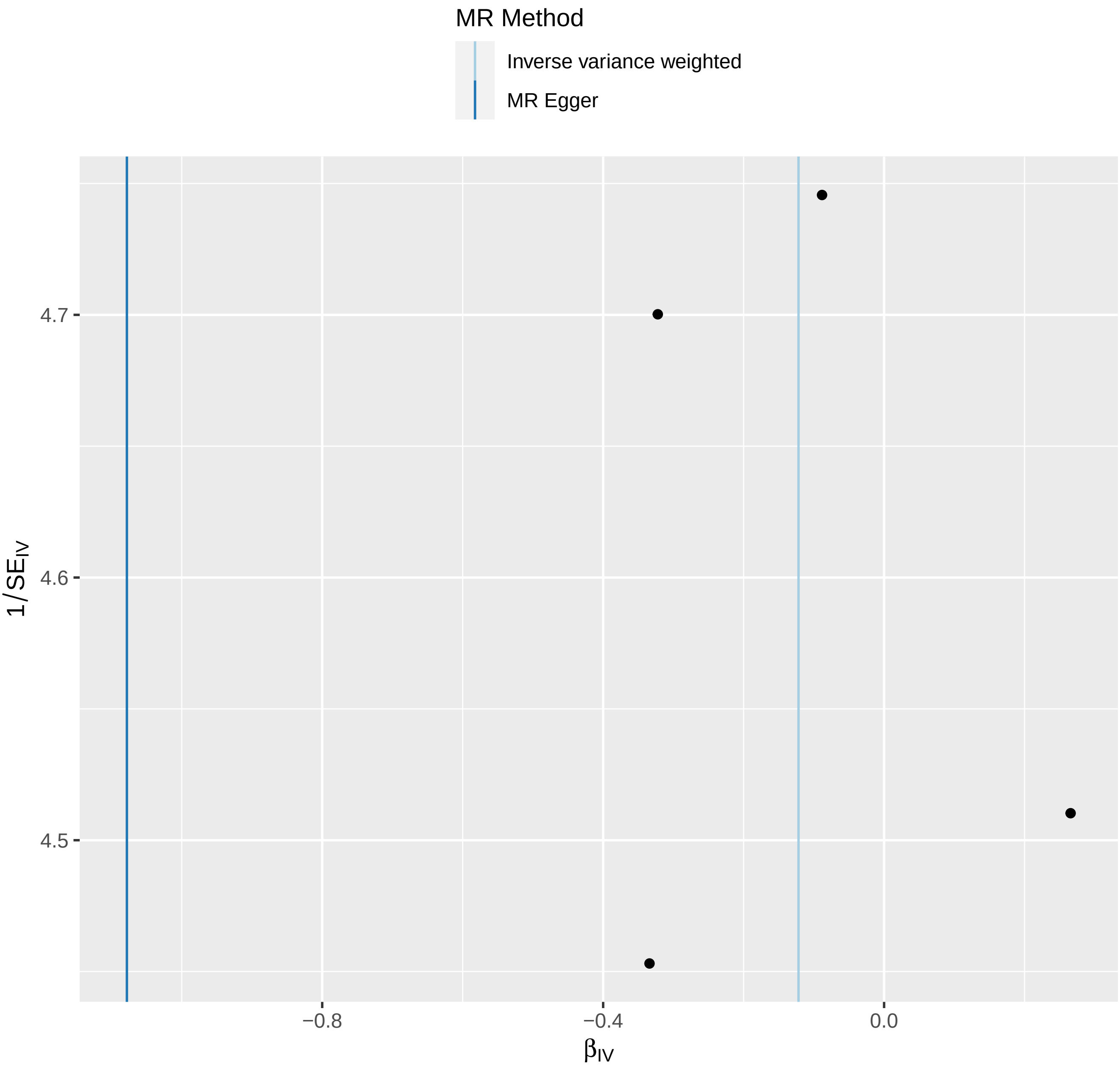

Batch 2−3632 : Gut microbiota abundance (genus Parasutterella id.2892) on Tyrosine−protein kinase Lyn
with a threshold of 1e−05

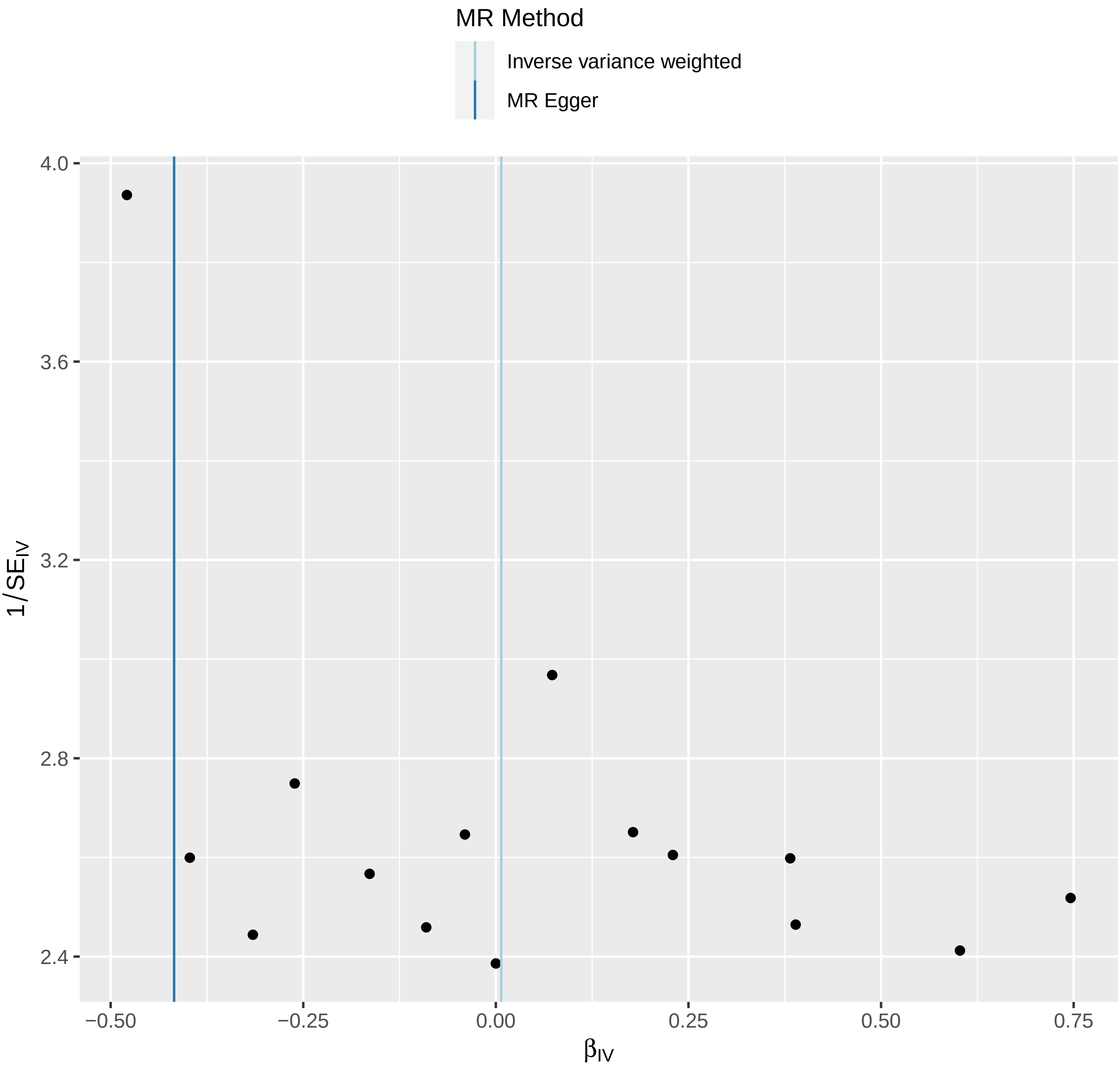

Batch 2−3633 : Gut microbiota abundance (genus Catenibacterium id.2153) on Ly6/PLAUR domain−containing protein 3
with a threshold of 1e−05

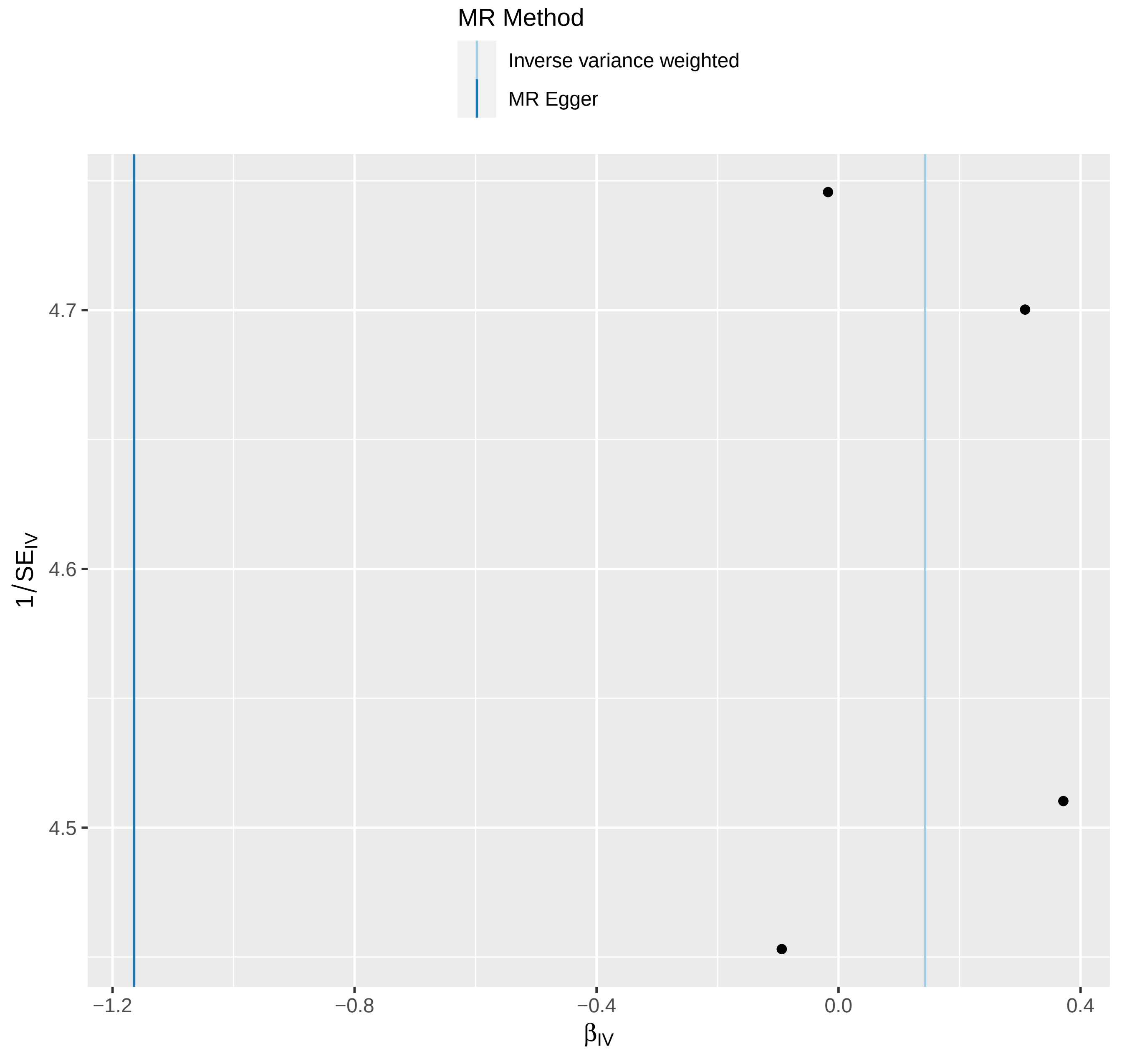

Batch 2−3634 : Gut microbiota abundance (genus Parasutterella id.2892) on Ly6/PLAUR domain−containing protein 3
with a threshold of 1e−05

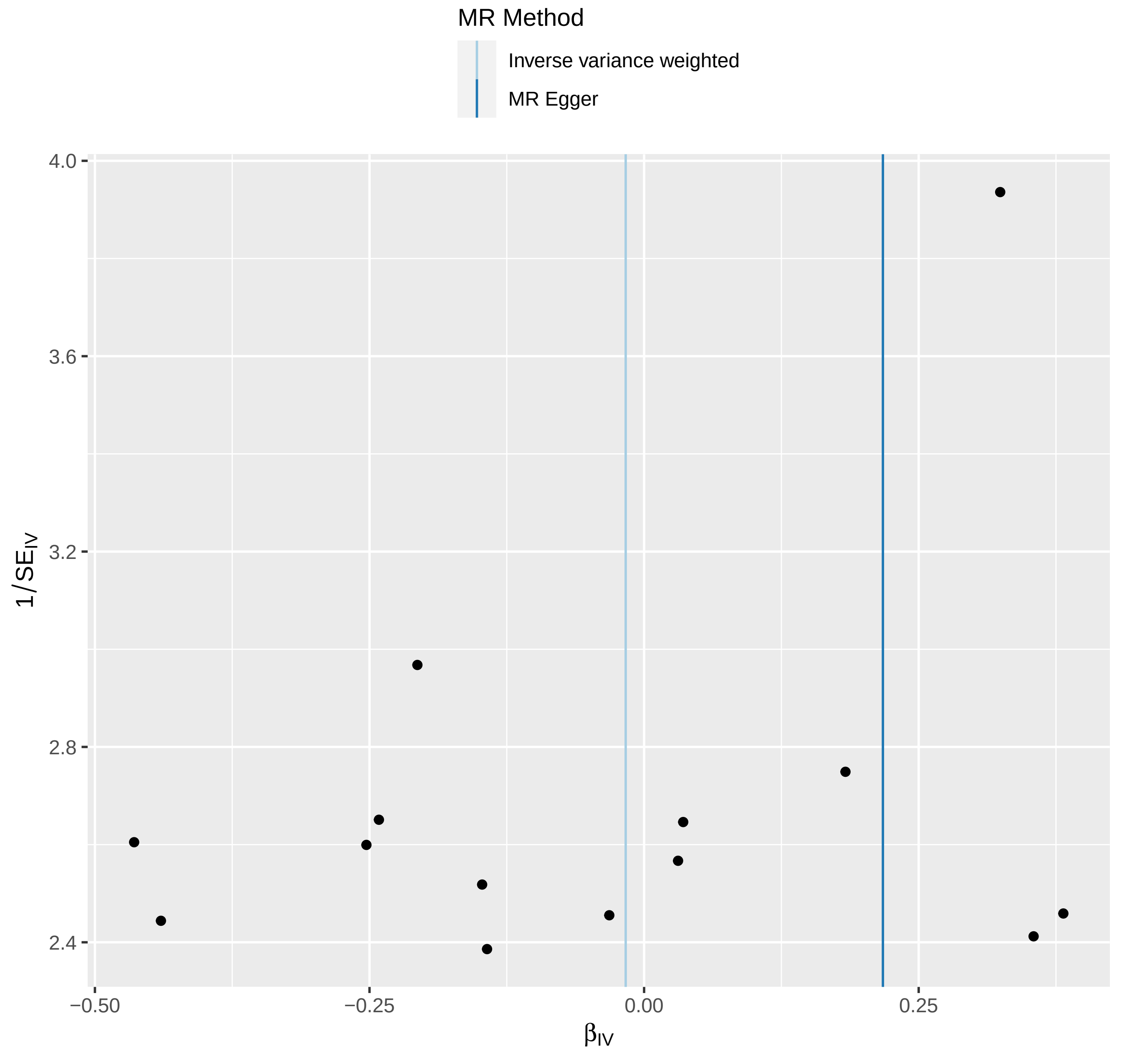

Batch 2−3635 : Gut microbiota abundance (genus Catenibacterium id.2153) on Lysophospholipase−like protein 1
with a threshold of 1e−05

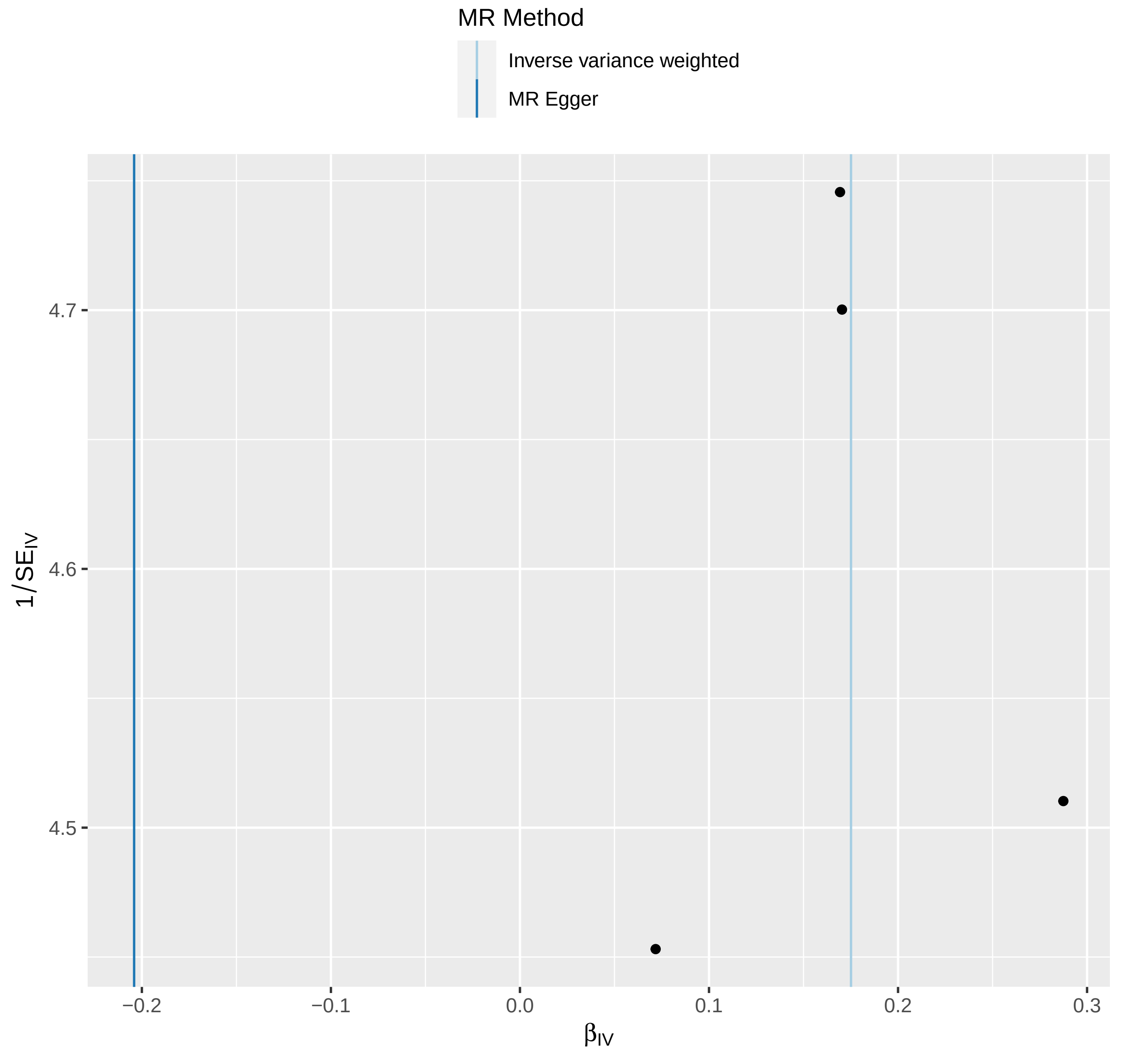

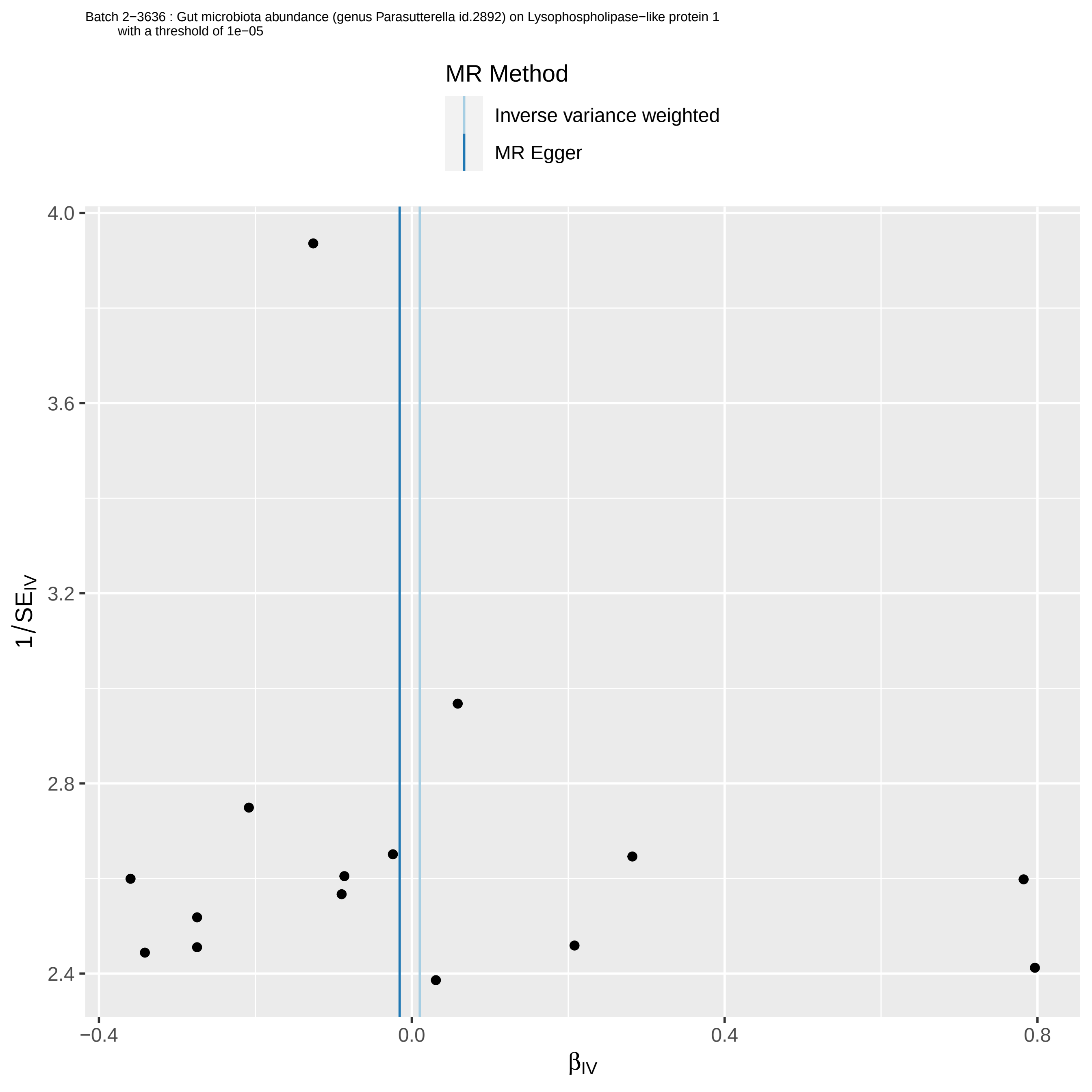
Batch 2−3636 : Gut microbiota abundance (genus Parasutterella id.2892) on Lysophospholipase−like protein 1
with a threshold of 1e−05
MR Method
Inverse variance weighted
MR Egger
4.0
3.6
3.2
2.8
2.4
1/SE_IV
−0.4
0.0
0.4
0.8
β_IV

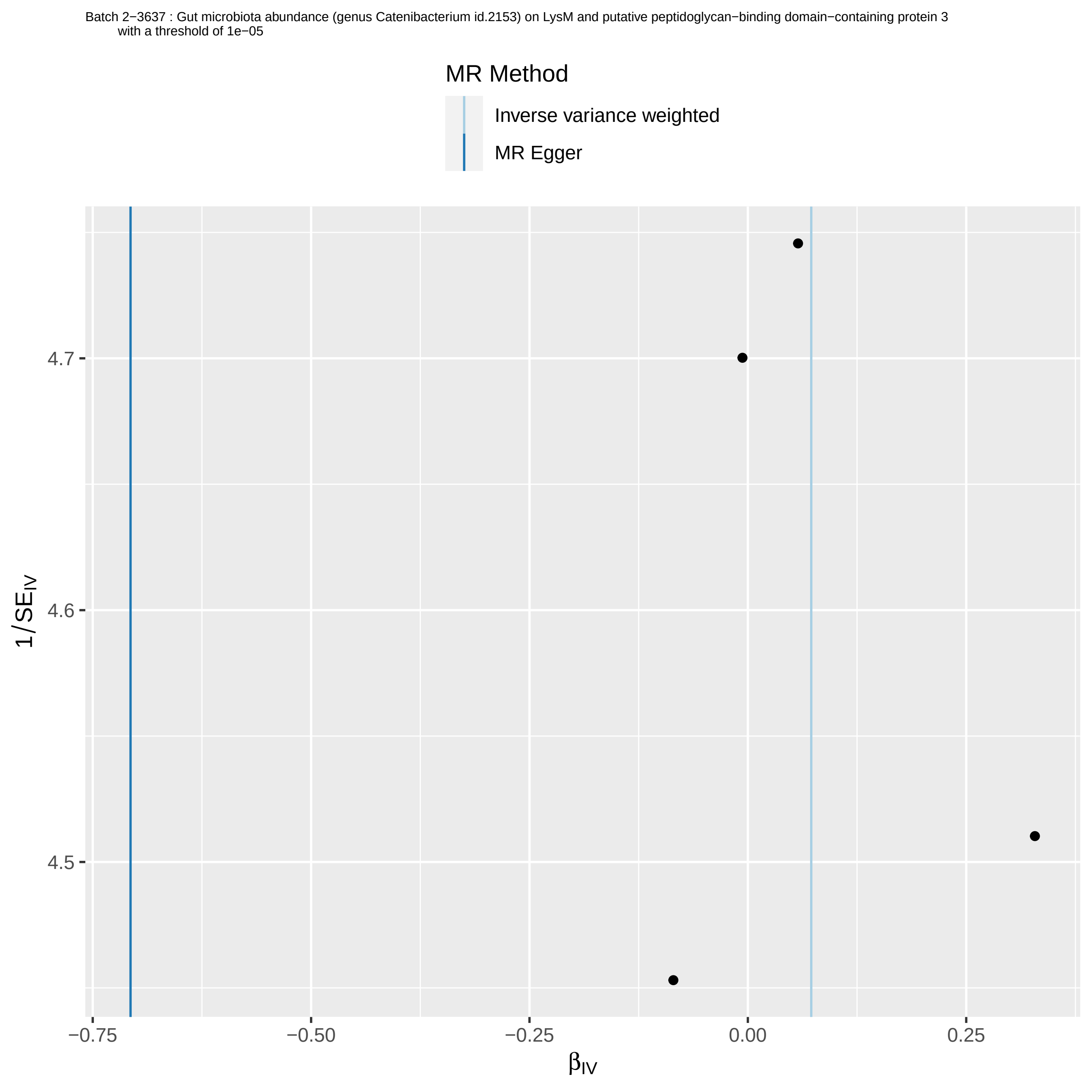

Batch 2−3637 : Gut microbiota abundance (genus Catenibacterium id.2153) on LysM and putative peptidoglycan−binding domain−containing protein 3
with a threshold of 1e−05
MR Method
Inverse variance weighted
MR Egger
4.7
4.6
4.5
1/SE_IV
−0.75
−0.50
−0.25
0.00
0.25
β_IV

Batch 2−3638 : Gut microbiota abundance (genus Parasutterella id.2892) on LysM and putative peptidoglycan−binding domain−containing protein 3 with a threshold of 1e−05

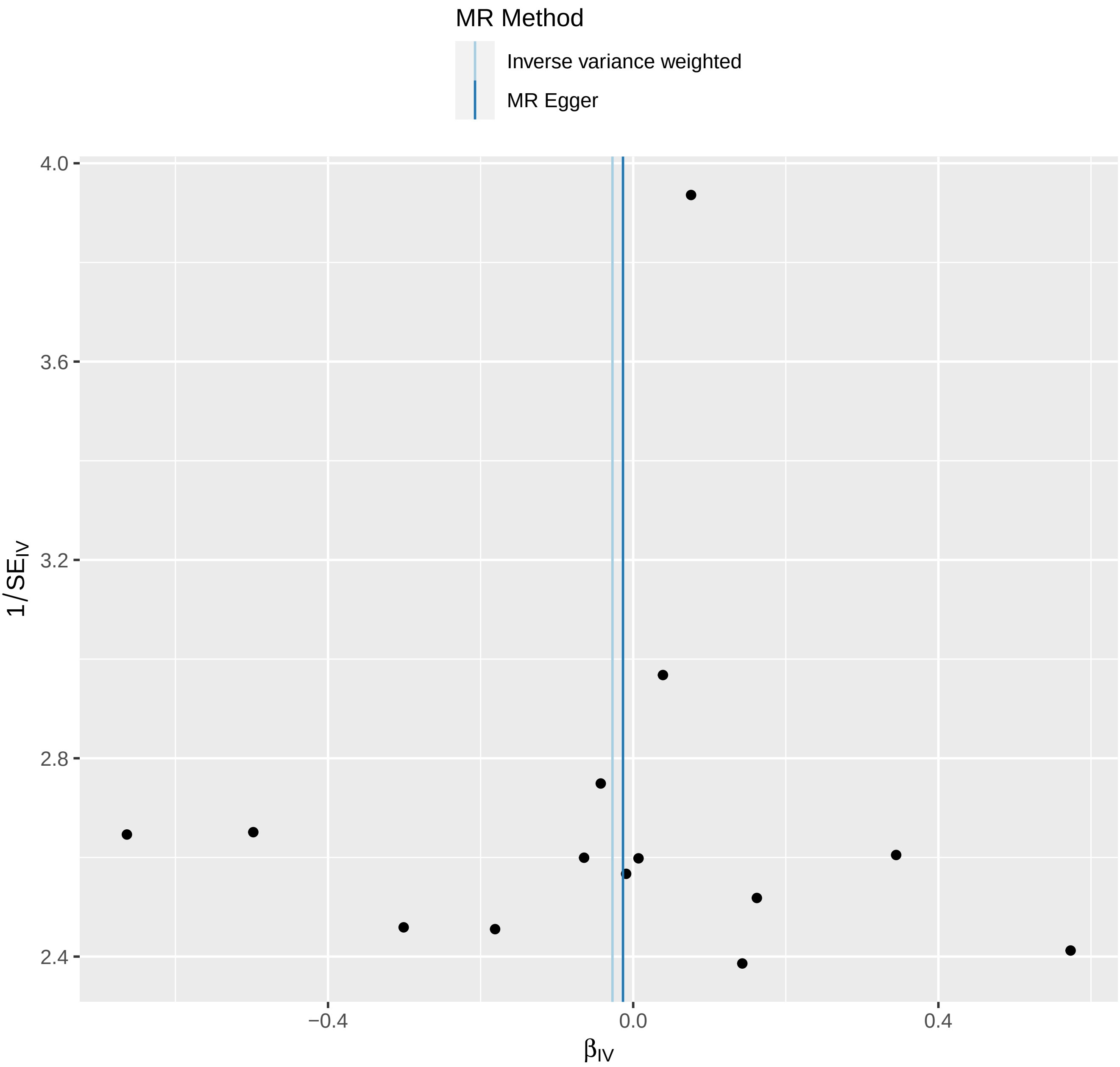

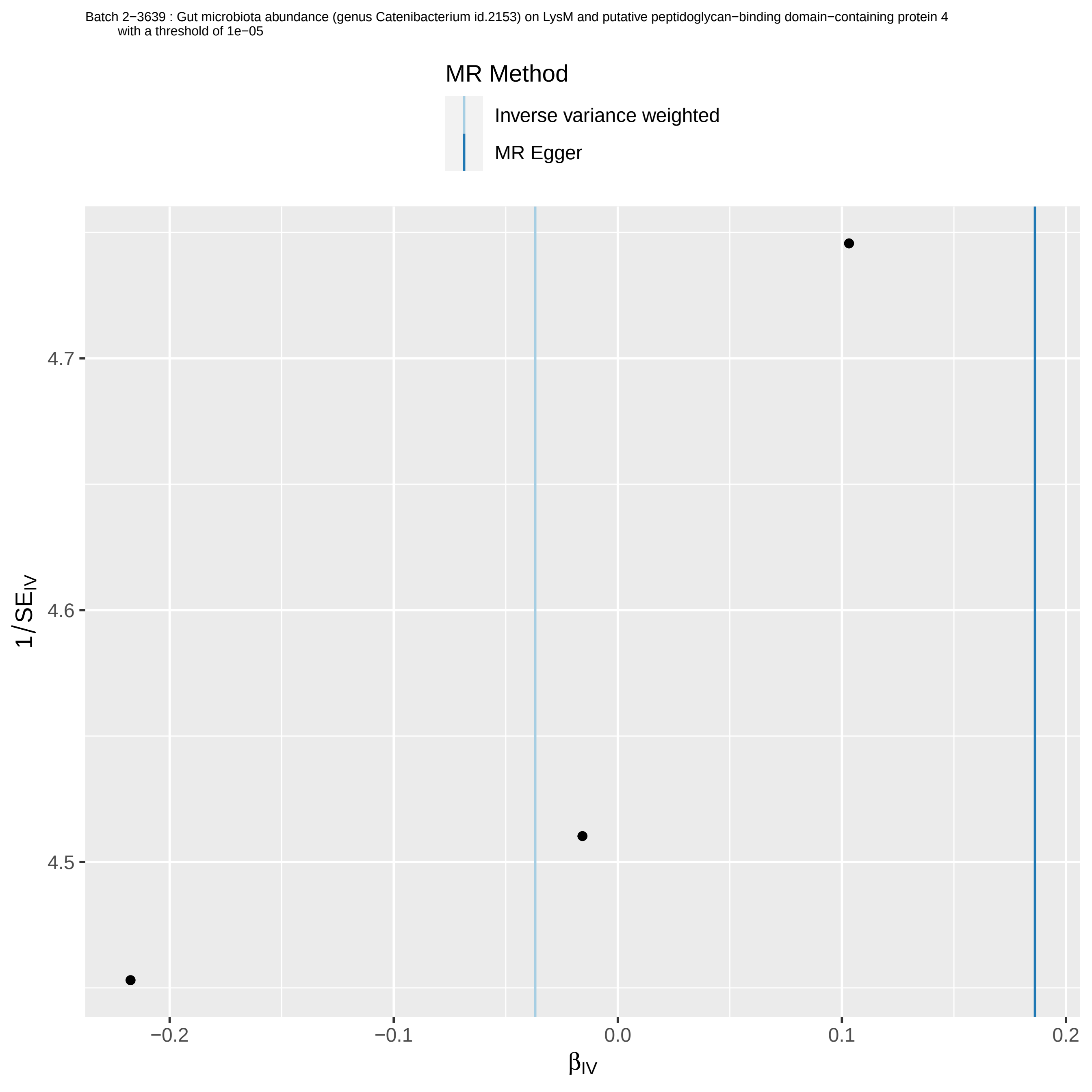

Batch 2−3639 : Gut microbiota abundance (genus Catenibacterium id.2153) on LysM and putative peptidoglycan−binding domain−containing protein 4
with a threshold of 1e−05
MR Method
Inverse variance weighted
MR Egger
4.7
4.6
4.5
1/SE_IV
−0.2
−0.1
0.0
0.1
0.2
β_IV

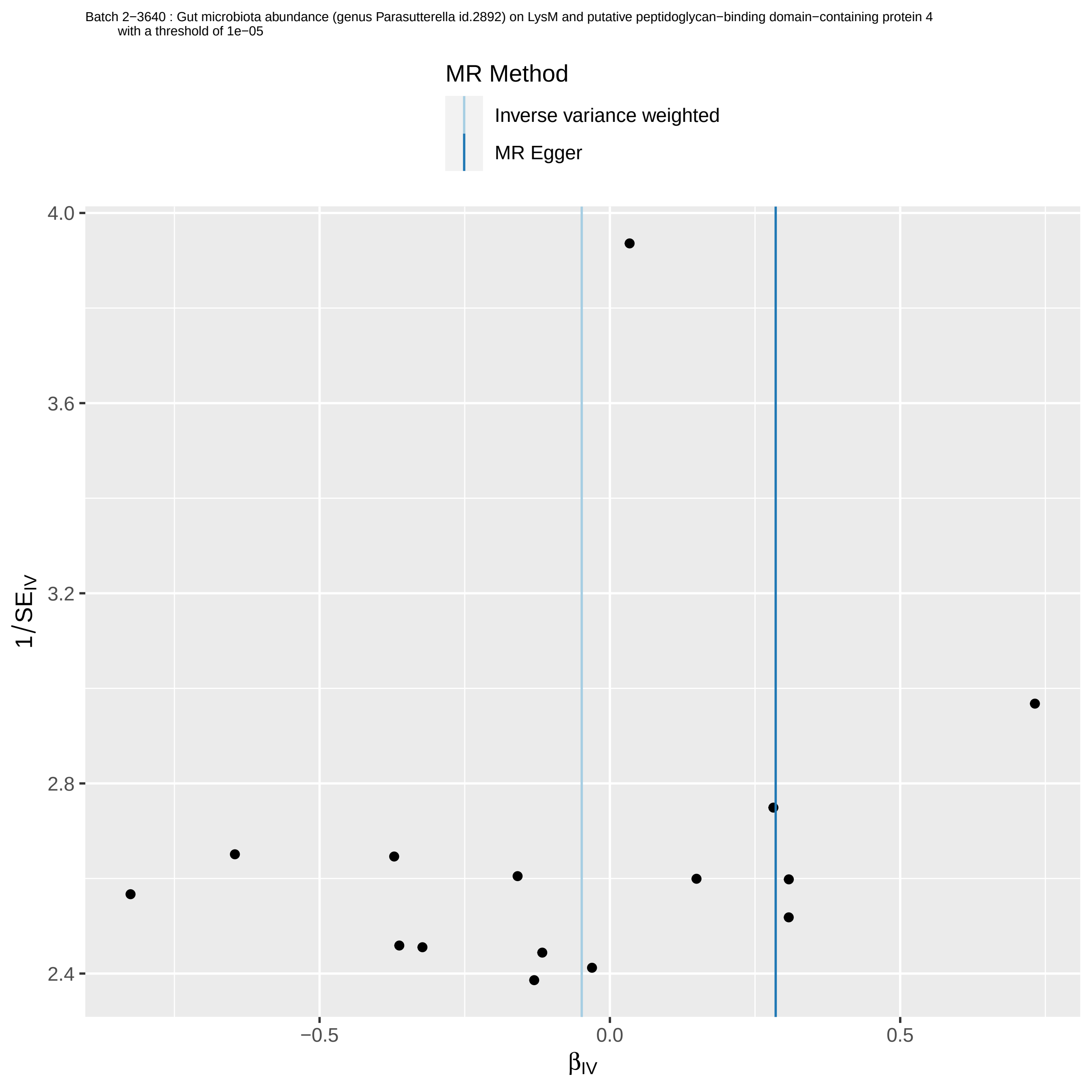

Batch 2−3640 : Gut microbiota abundance (genus Parasutterella id.2892) on LysM and putative peptidoglycan−binding domain−containing protein 4 with a threshold of 1e−05
MR Method
Inverse variance weighted
MR Egger
4.0
3.6
3.2
2.8
2.4
1/SE_IV
−0.5
0.0
0.5
β_IV

Batch 2−3641 : Gut microbiota abundance (genus Catenibacterium id.2153) on Lysozyme C
with a threshold of 1e−05

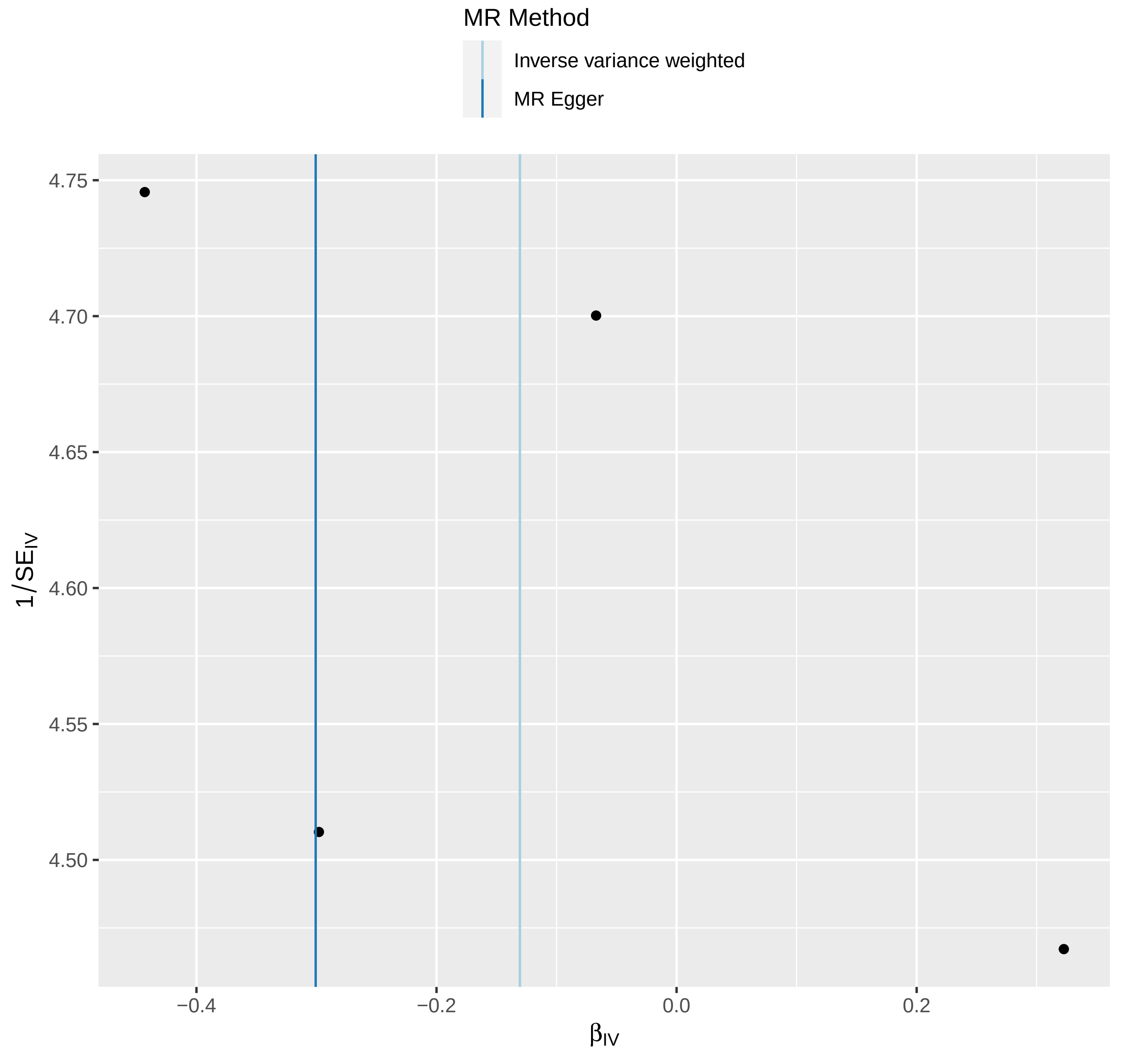

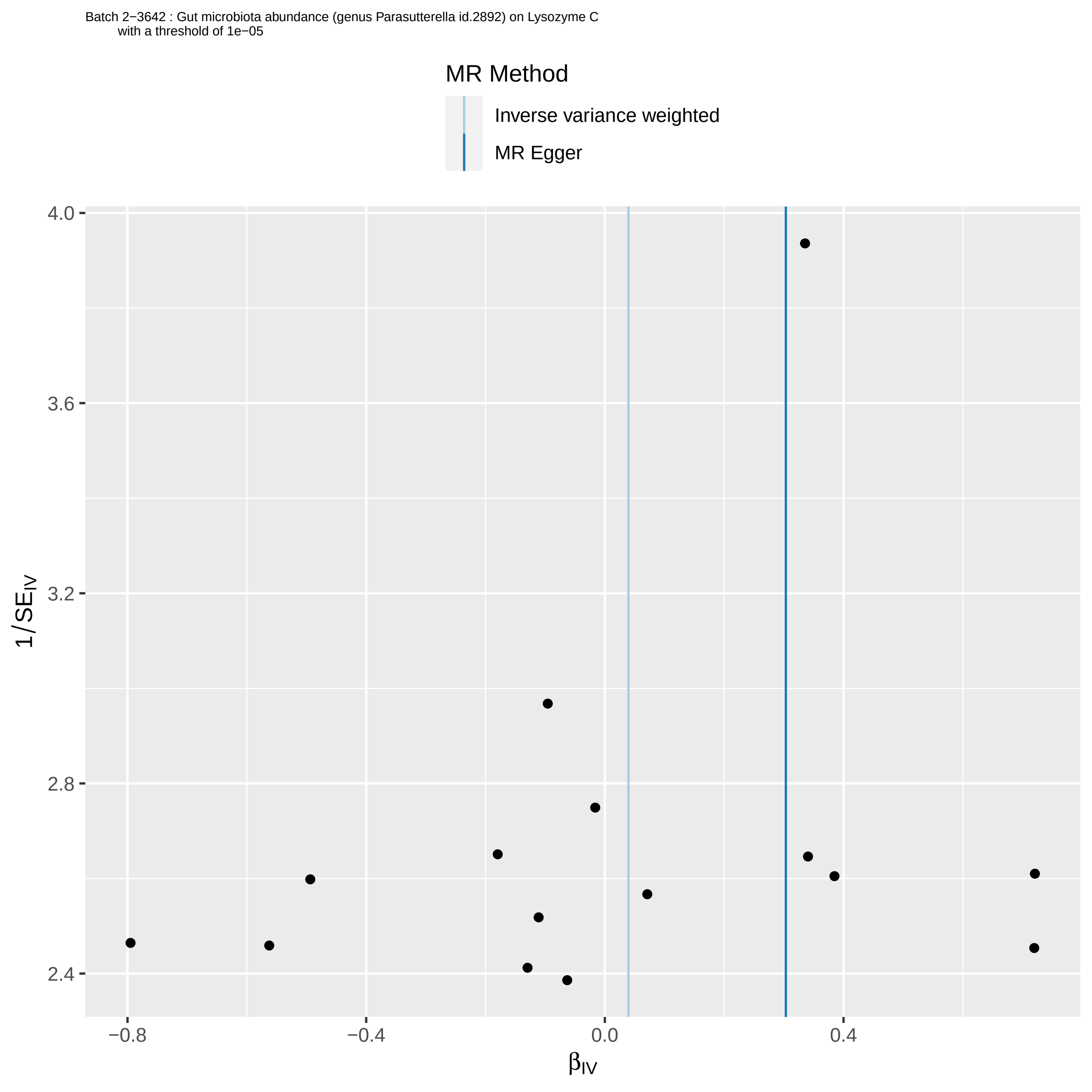
Batch 2−3642 : Gut microbiota abundance (genus Parasutterella id.2892) on Lysozyme C
with a threshold of 1e−05
MR Method
Inverse variance weighted
MR Egger
4.0
3.6
3.2
2.8
2.4
1/SE_IV
−0.8
−0.4
0.0
0.4
β_IV

Batch 2−3643 : Gut microbiota abundance (genus Catenibacterium id.2153) on Lysozyme−like protein 2
with a threshold of 1e−05

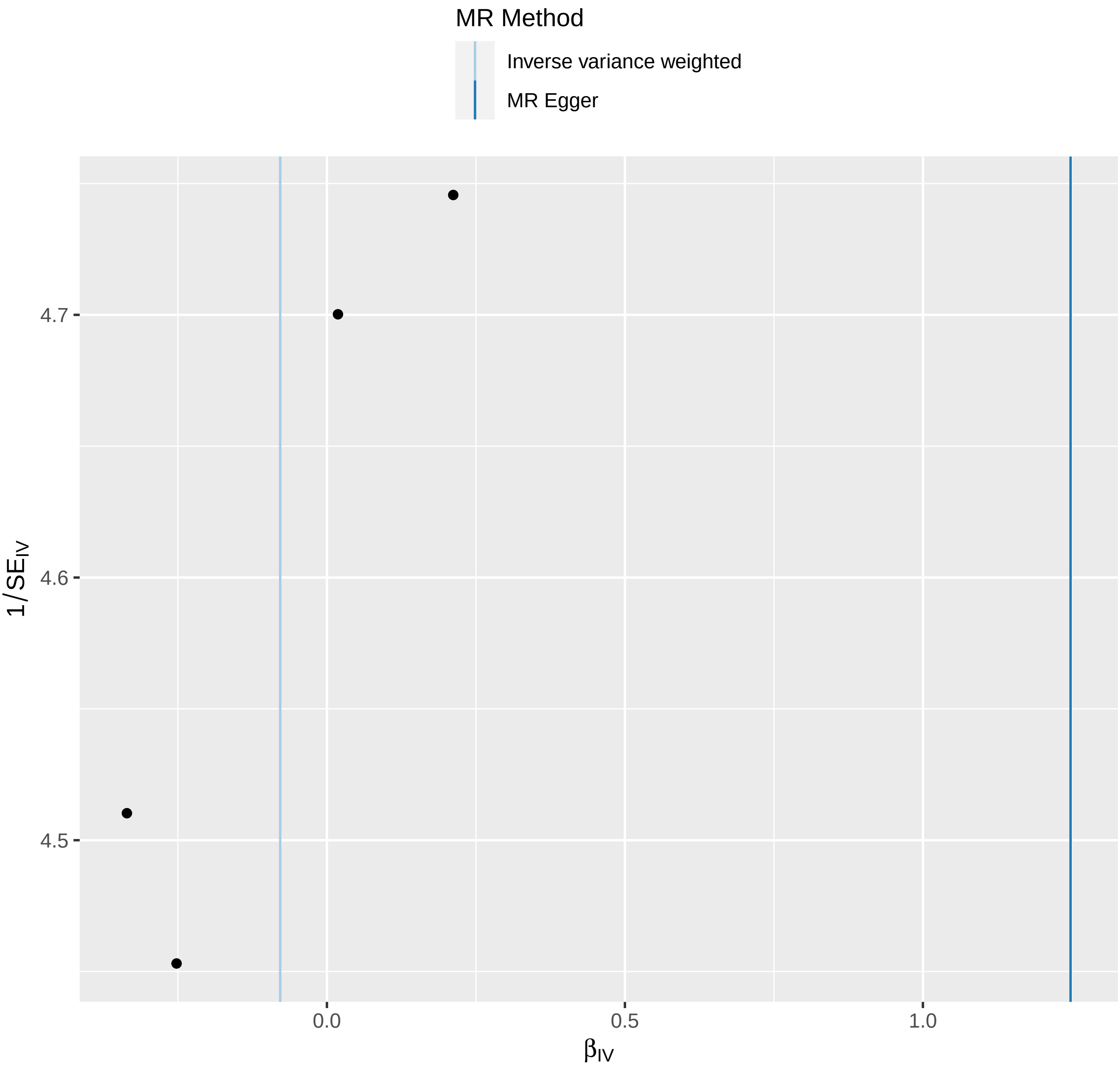

Batch 2−3644 : Gut microbiota abundance (genus Parasutterella id.2892) on Lysozyme−like protein 2
with a threshold of 1e−05

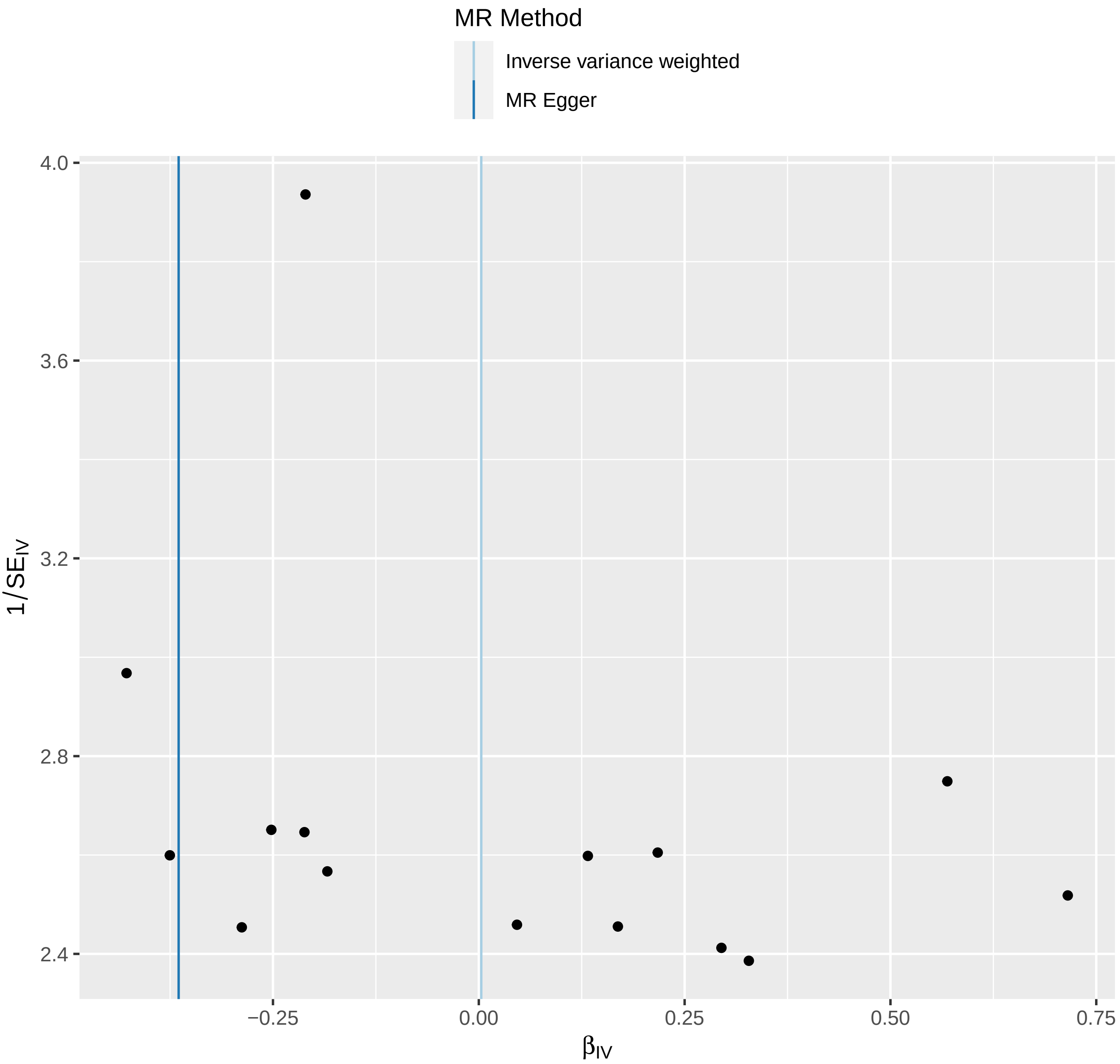

Batch 2−3645 : Gut microbiota abundance (genus Catenibacterium id.2153) on Cation−dependent mannose−6−phosphate receptor
with a threshold of 1e−05

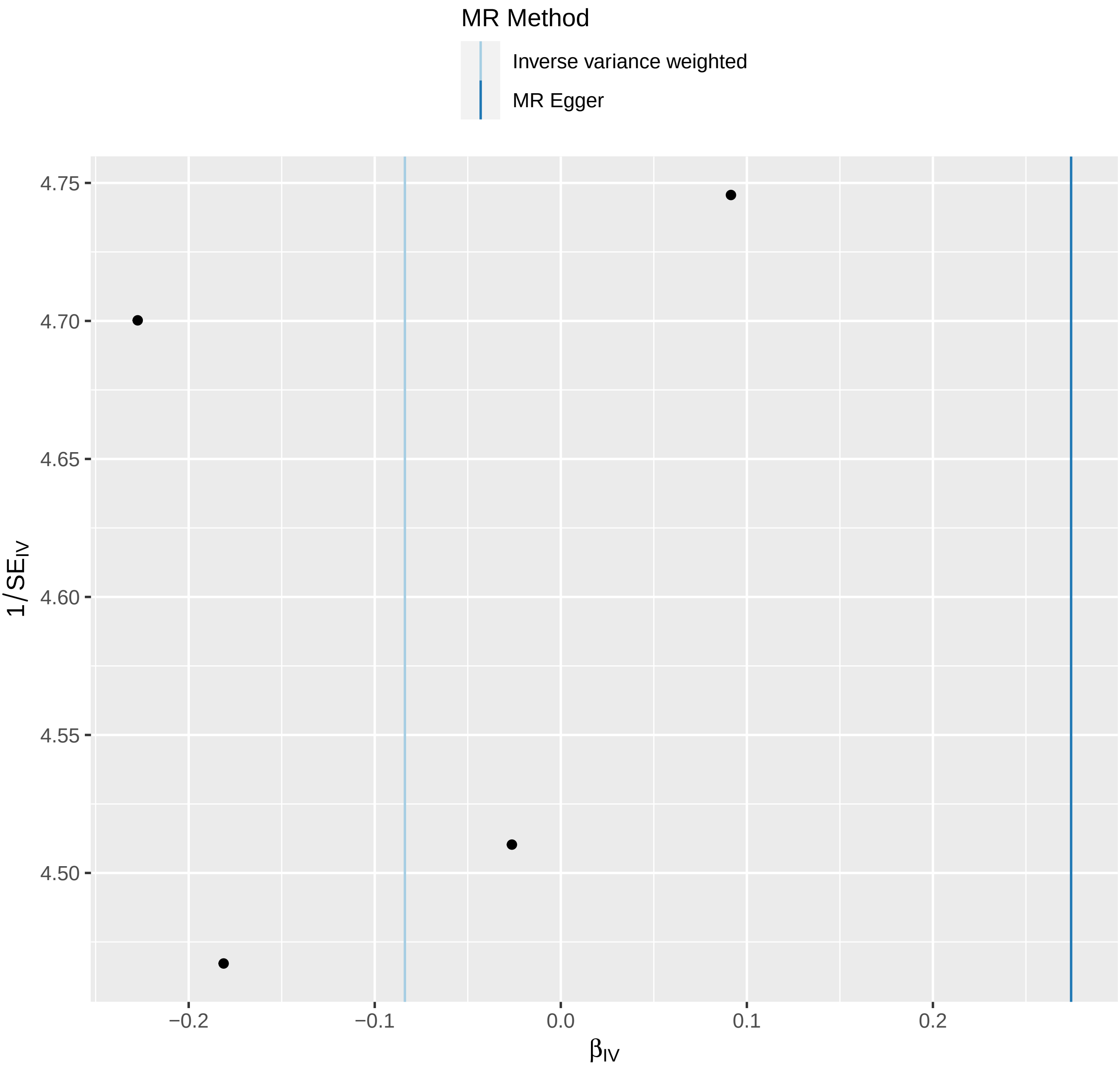

Batch 2−3646 : Gut microbiota abundance (genus Parasutterella id.2892) on Cation−dependent mannose−6−phosphate receptor with a threshold of 1e−05

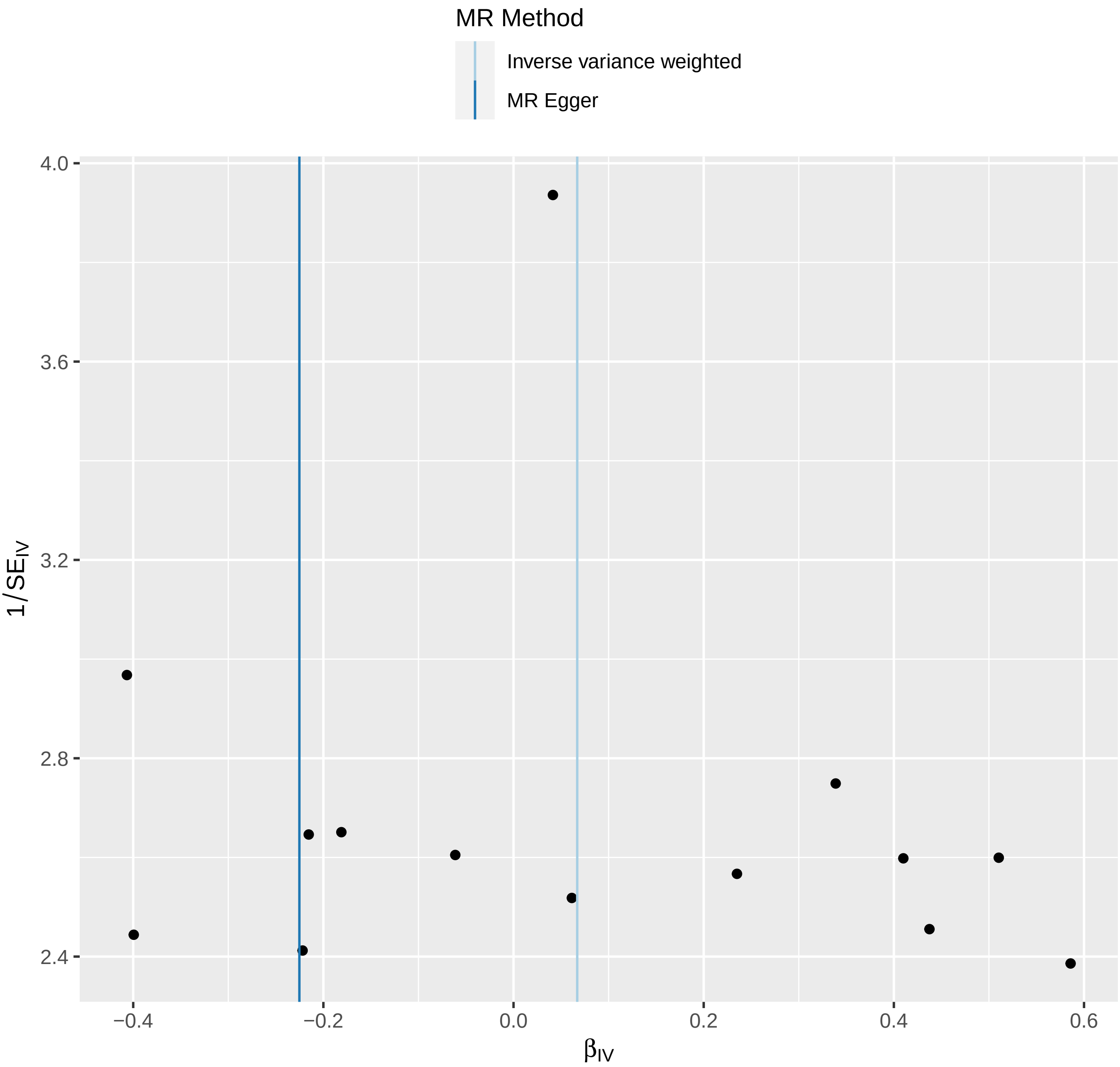

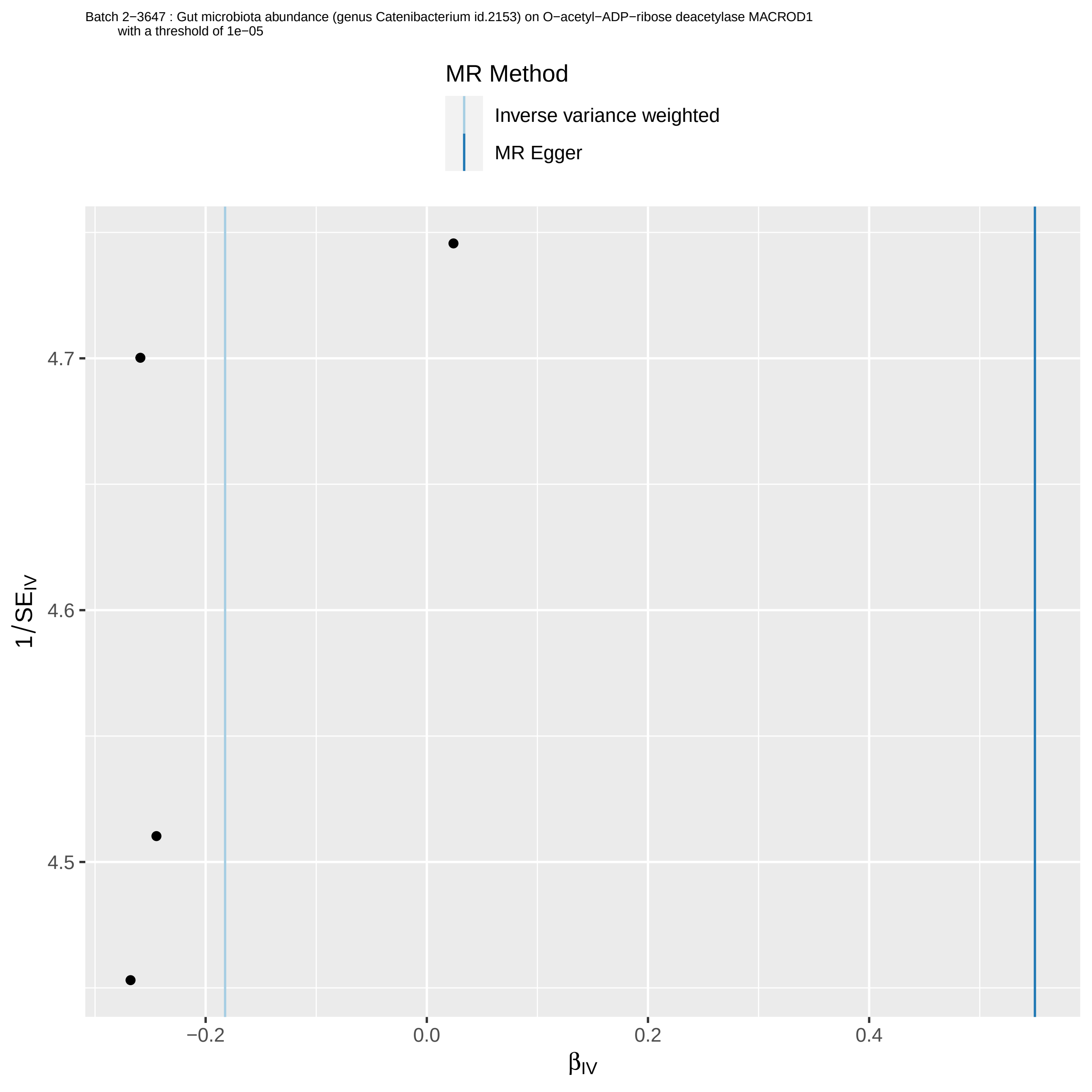
Batch 2−3647 : Gut microbiota abundance (genus Catenibacterium id.2153) on O−acetyl−ADP−ribose deacetylase MACROD1
with a threshold of 1e−05
MR Method
Inverse variance weighted
MR Egger
4.7
4.6
4.5
1/SE_IV
−0.2
0.0
0.2
0.4
β_IV

Batch 2−3648 : Gut microbiota abundance (genus Parasutterella id.2892) on O−acetyl−ADP−ribose deacetylase MACROD1 with a threshold of 1e−05

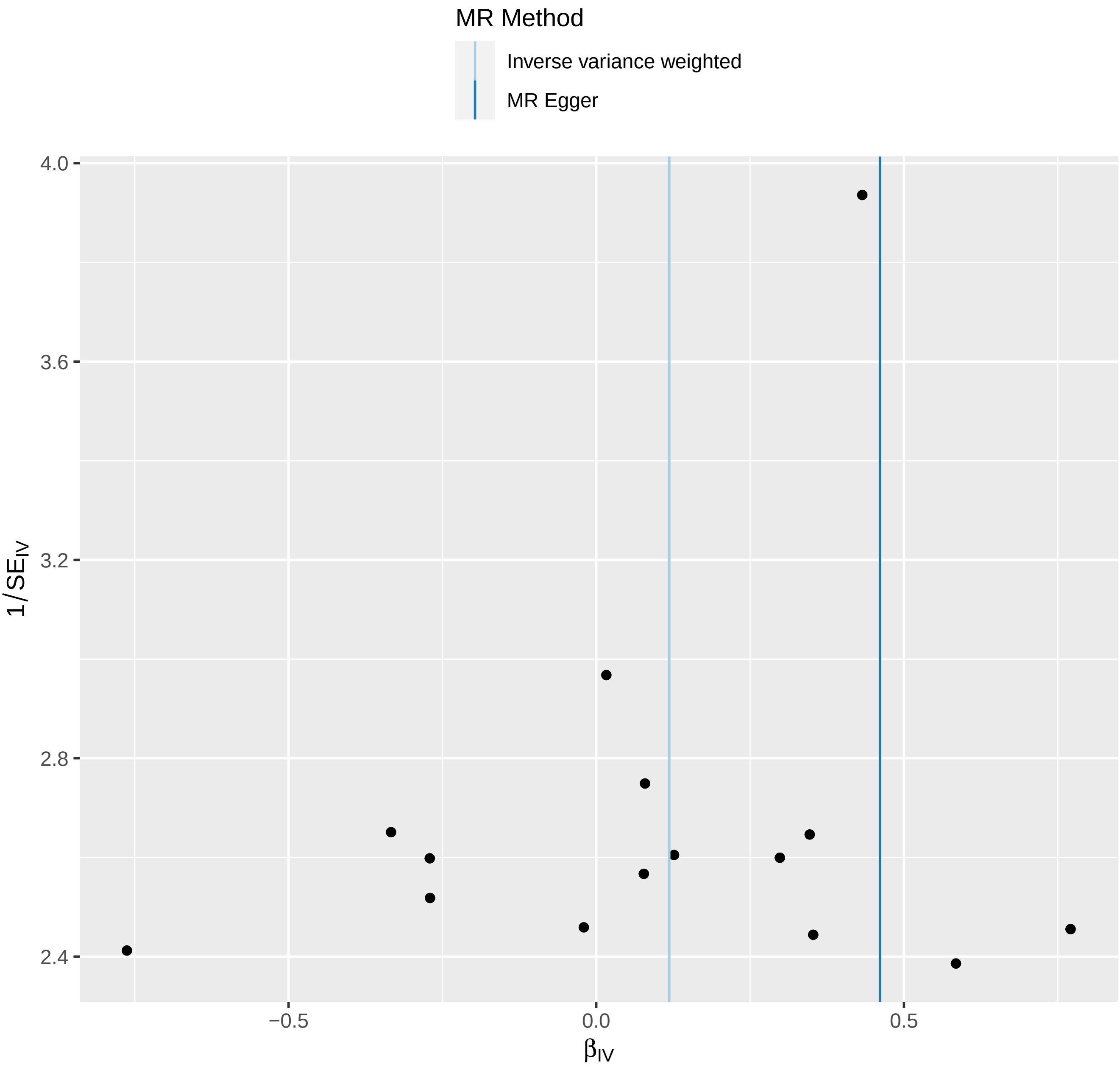

Batch 2−3649 : Gut microbiota abundance (genus Catenibacterium id.2153) on Mitotic spindle assembly checkpoint protein MAD1
with a threshold of 1e−05

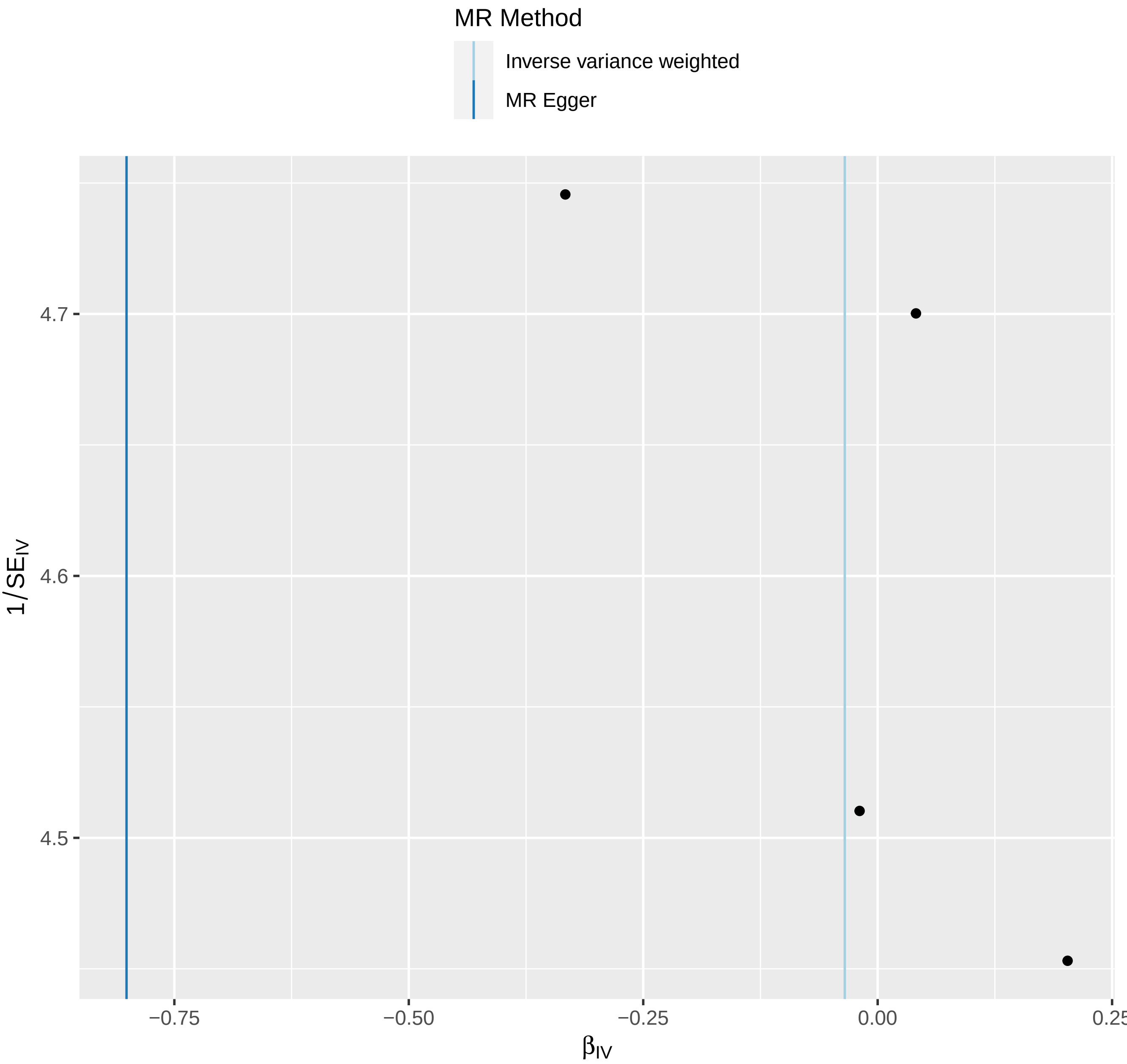

Batch 2−3650 : Gut microbiota abundance (genus Parasutterella id.2892) on Mitotic spindle assembly checkpoint protein MAD1
with a threshold of 1e−05

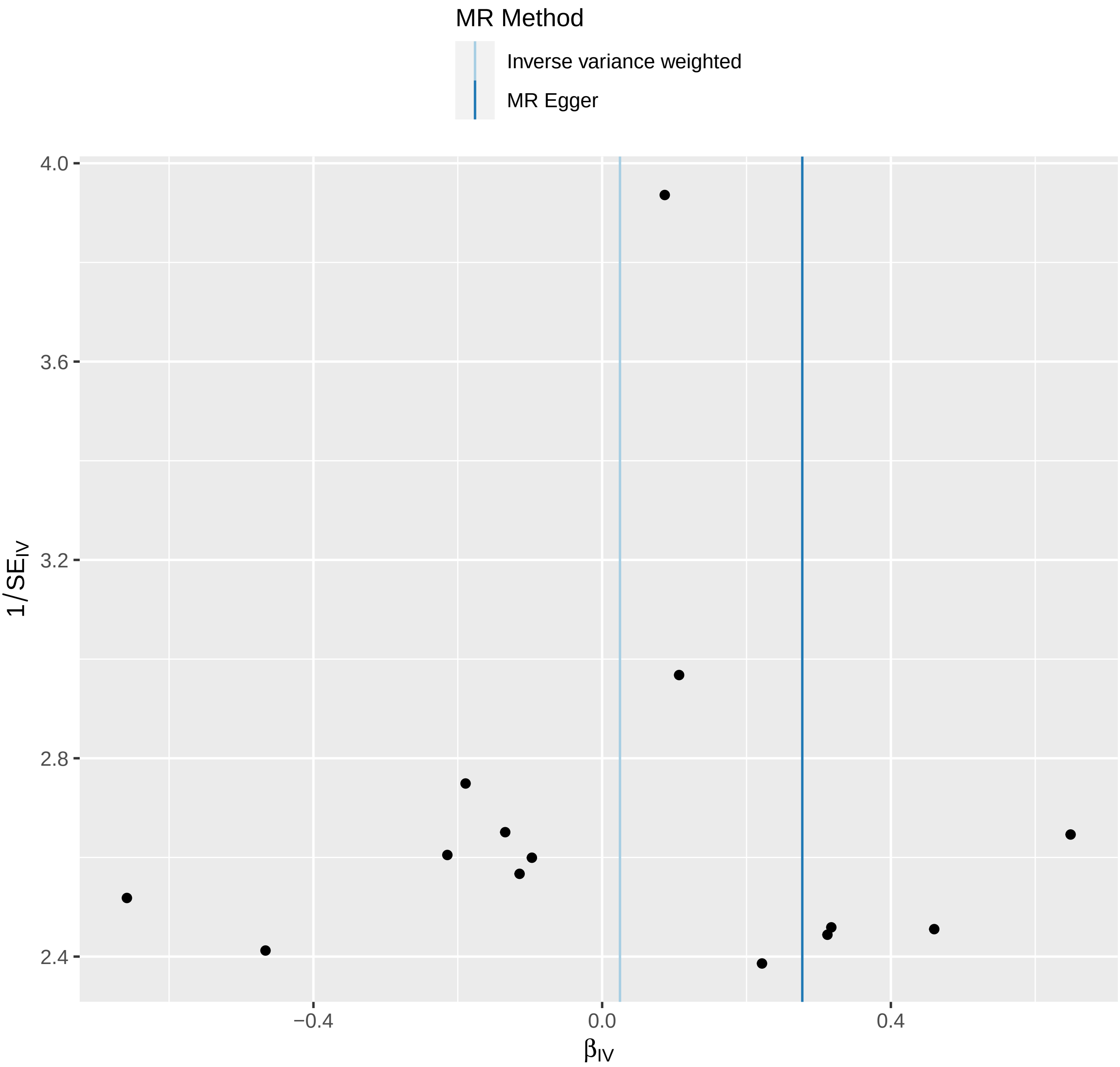

Batch 2−3651 : Gut microbiota abundance (genus Catenibacterium id.2153) on Mucosal addressin cell adhesion molecule 1
with a threshold of 1e−05

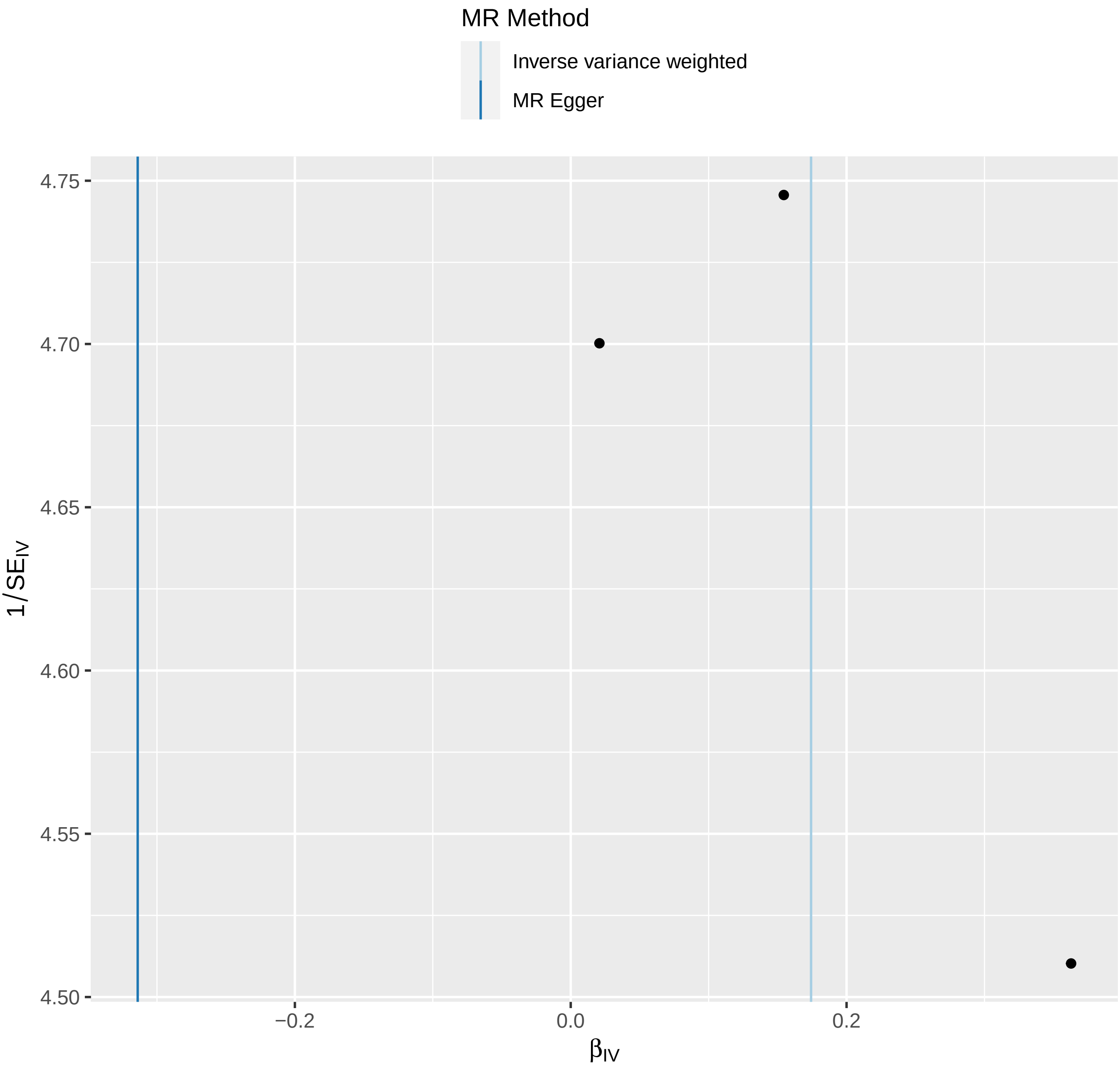

Batch 2−3652 : Gut microbiota abundance (genus Parasutterella id.2892) on Mucosal addressin cell adhesion molecule 1 with a threshold of 1e−05

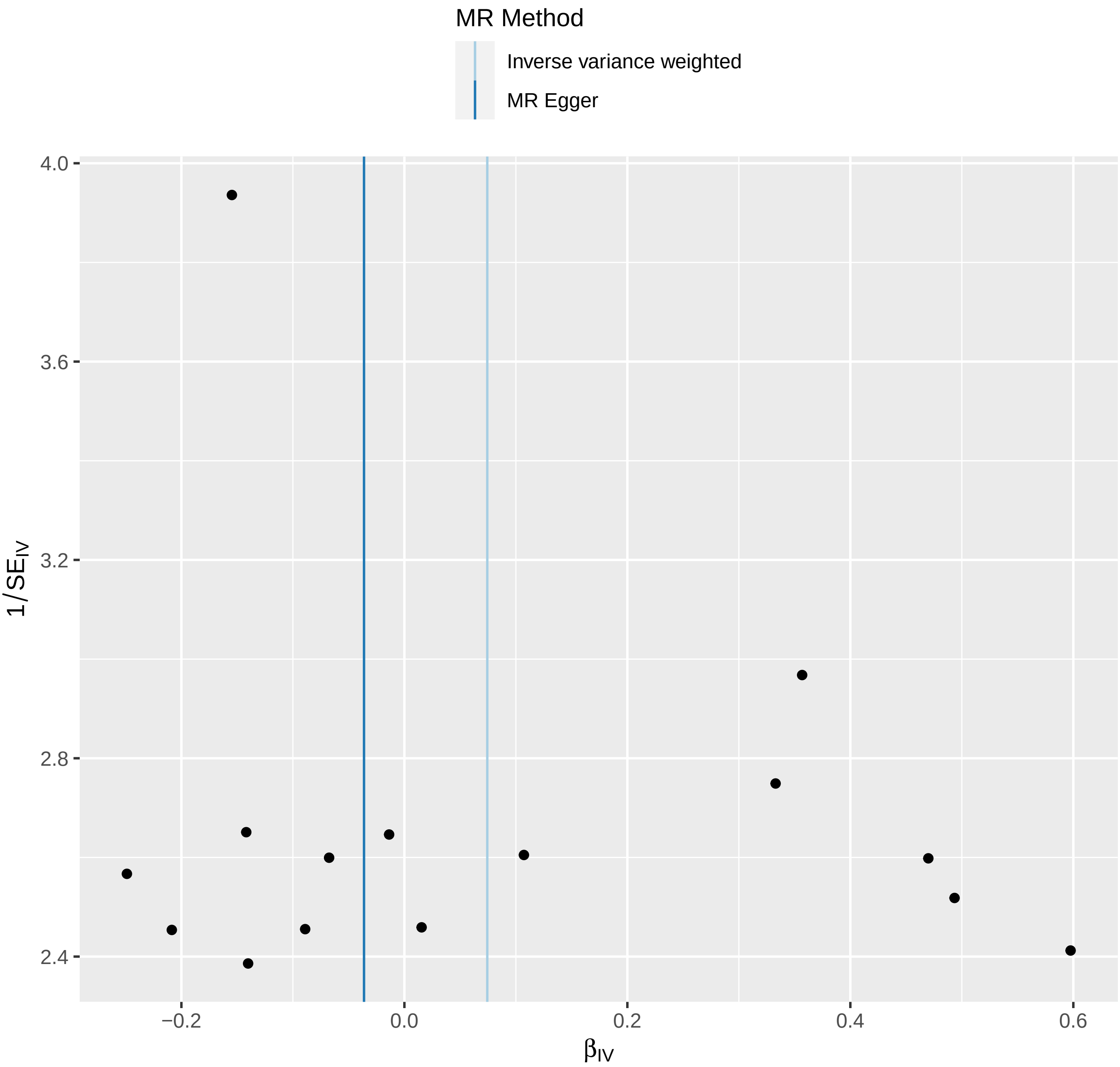

Batch 2−3653 : Gut microbiota abundance (genus Catenibacterium id.2153) on Melanoma−associated antigen 10
with a threshold of 1e−05

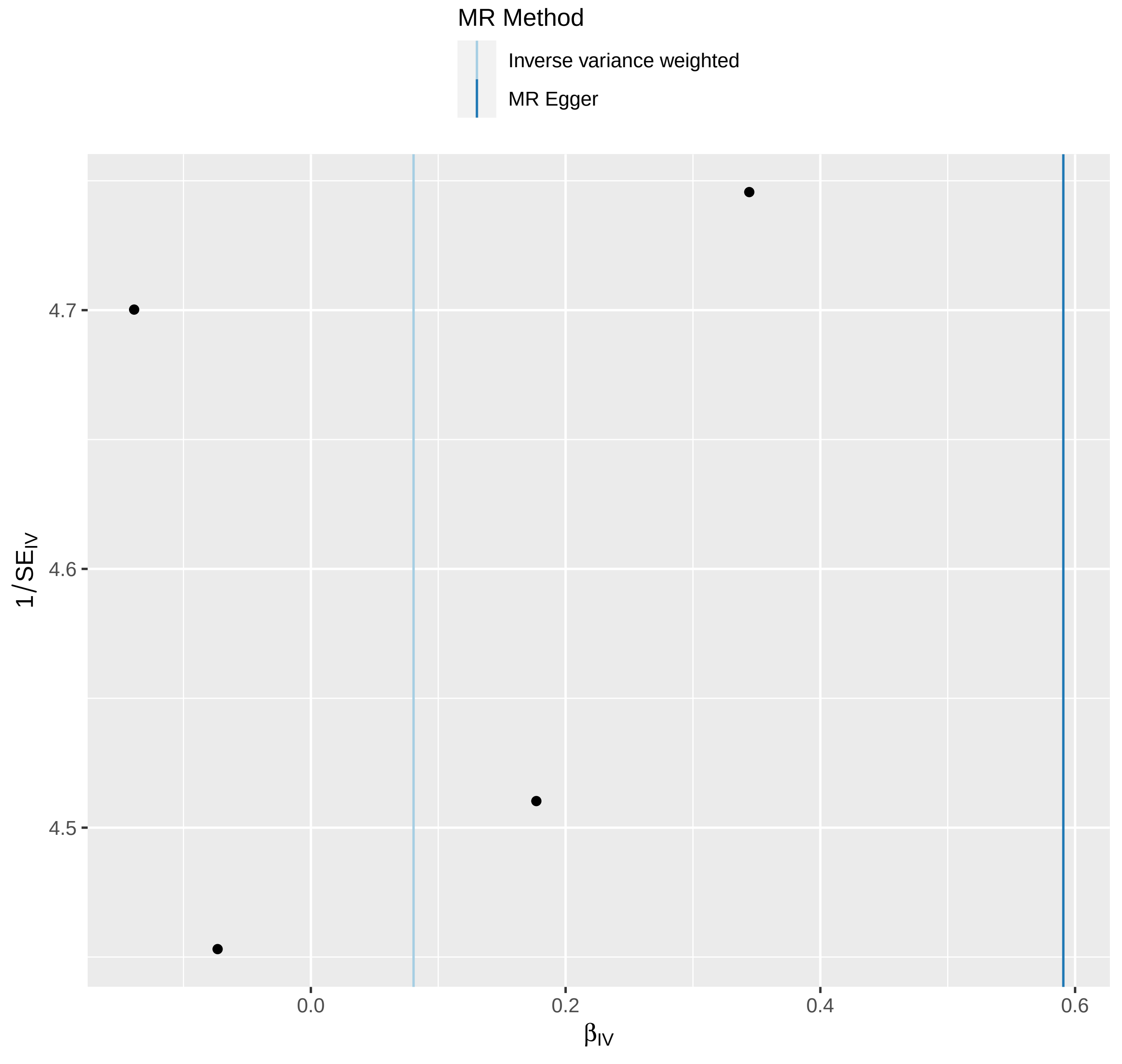

Batch 2−3654 : Gut microbiota abundance (genus Parasutterella id.2892) on Melanoma−associated antigen 10
with a threshold of 1e−05

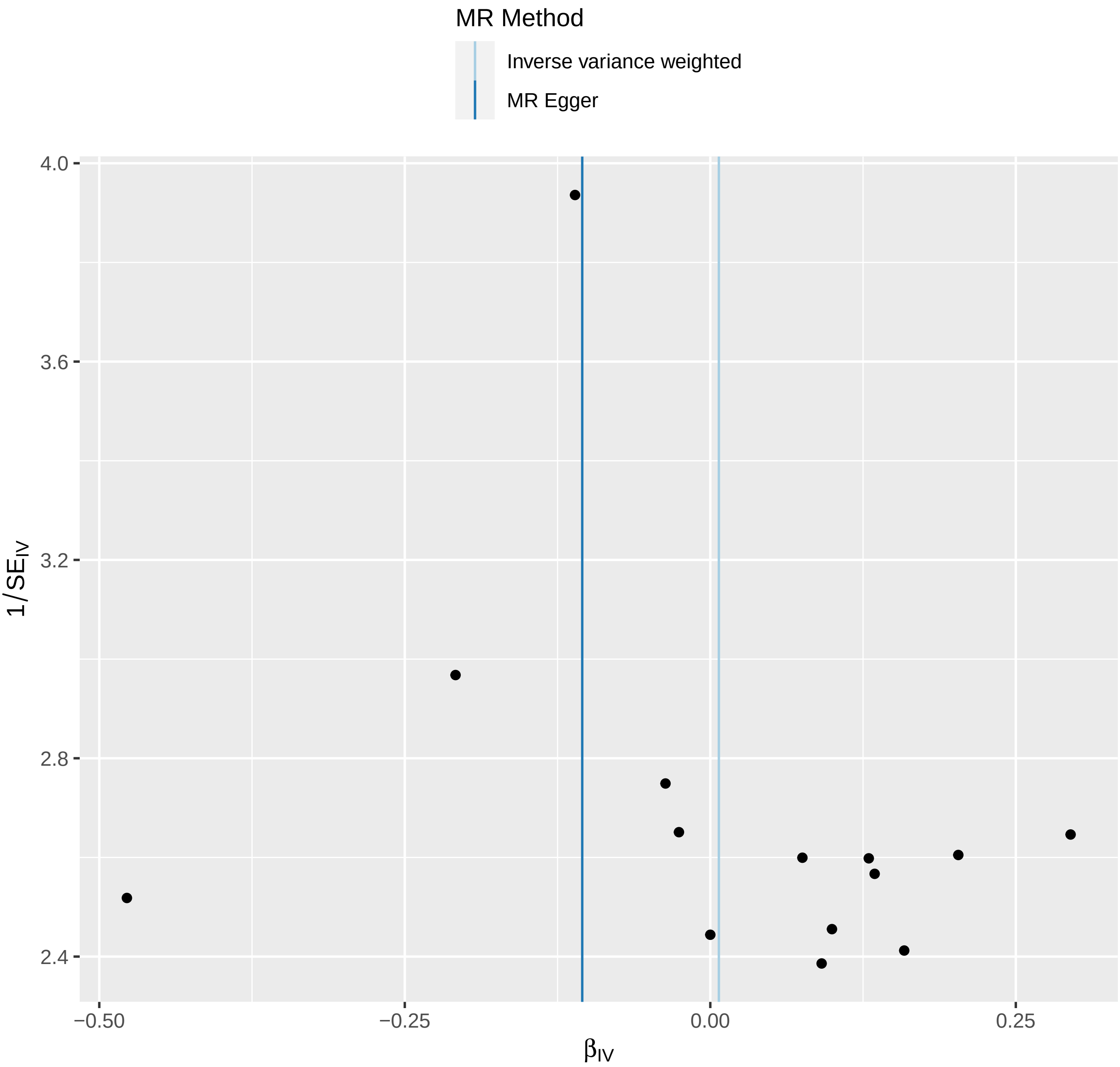

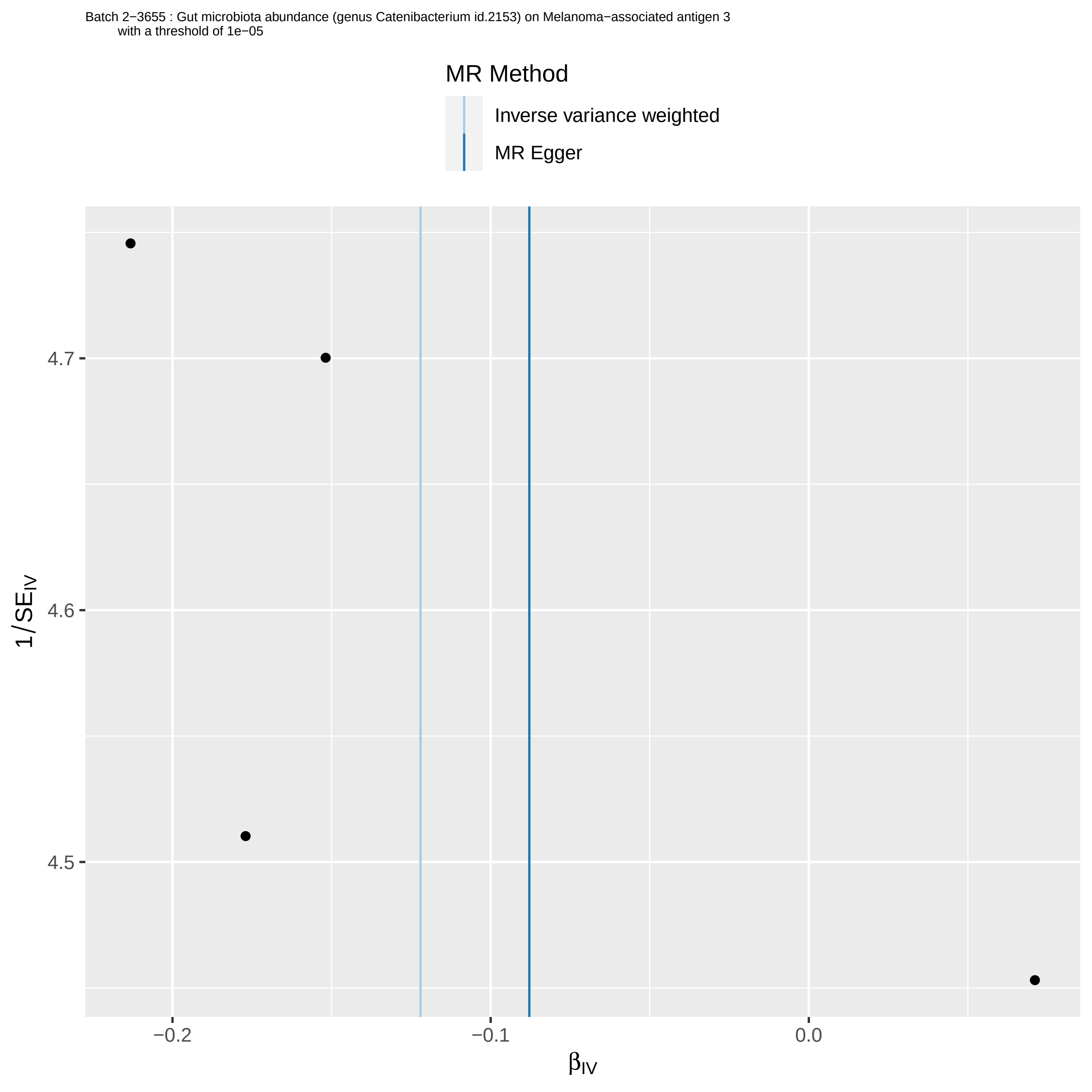
Batch 2−3655 : Gut microbiota abundance (genus Catenibacterium id.2153) on Melanoma−associated antigen 3
with a threshold of 1e−05
MR Method
Inverse variance weighted
MR Egger
4.7
4.6
4.5
1/SE_IV
−0.2
−0.1
0.0
β_IV

Batch 2−3656 : Gut microbiota abundance (genus Parasutterella id.2892) on Melanoma−associated antigen 3
with a threshold of 1e−05

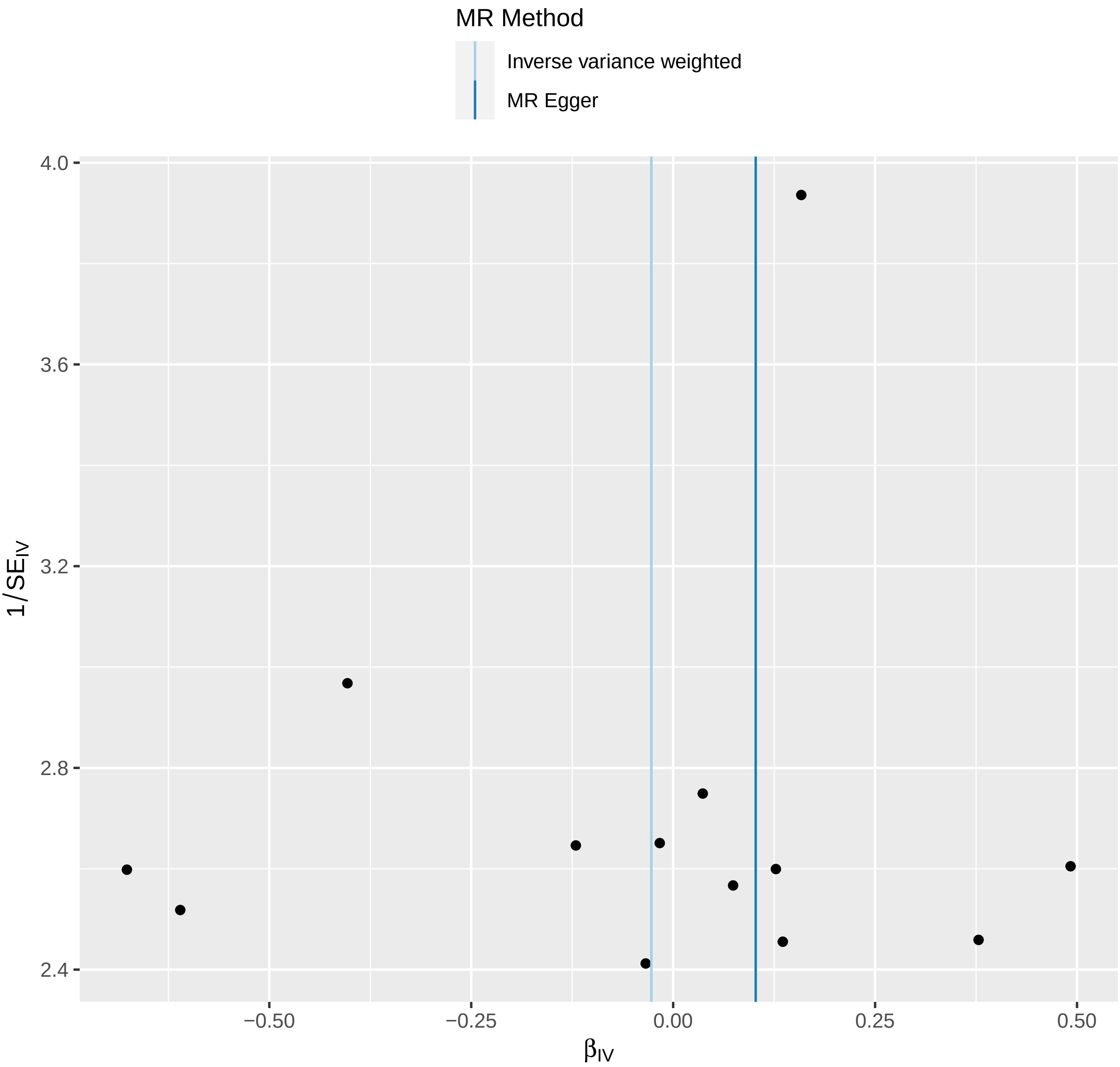

Batch 2−3657 : Gut microbiota abundance (genus Catenibacterium id.2153) on Melanoma−associated antigen B10
with a threshold of 1e−05

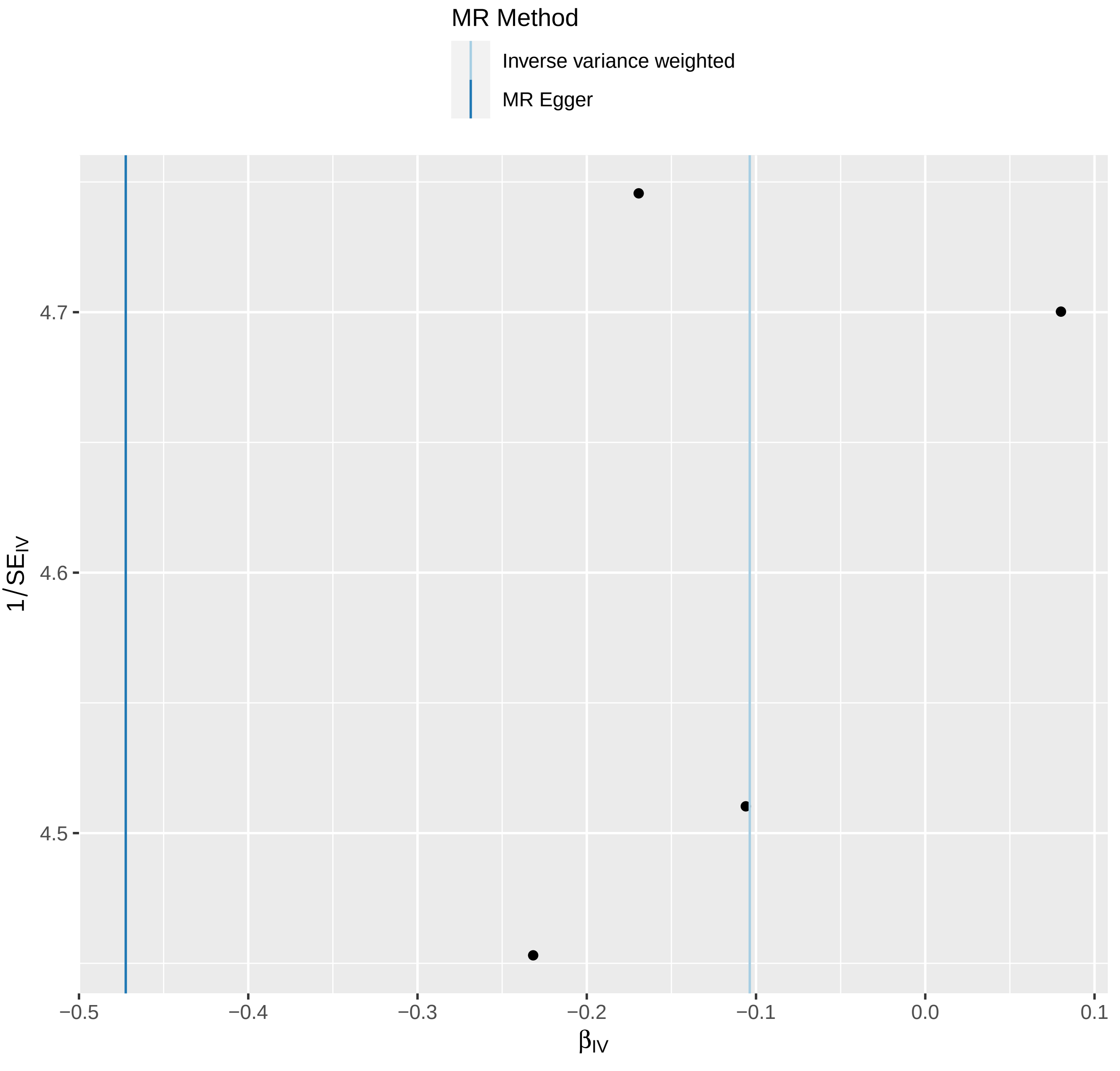

Batch 2−3658 : Gut microbiota abundance (genus Parasutterella id.2892) on Melanoma−associated antigen B10
with a threshold of 1e−05

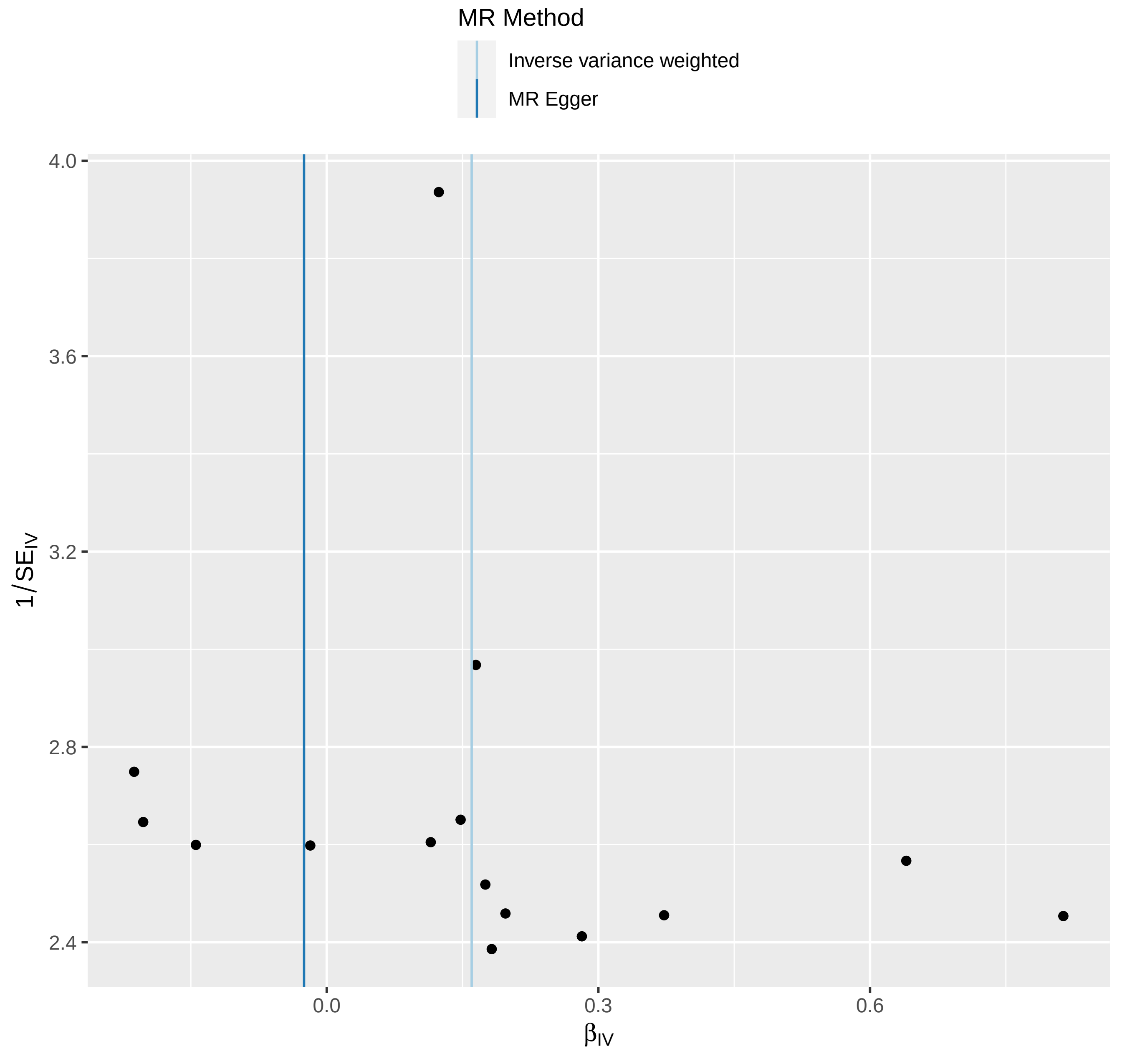

Batch 2−3659 : Gut microbiota abundance (genus Catenibacterium id.2153) on Membrane−associated guanylate kinase, WW and PDZ domain−containing protein 2 with a threshold of 1e−05

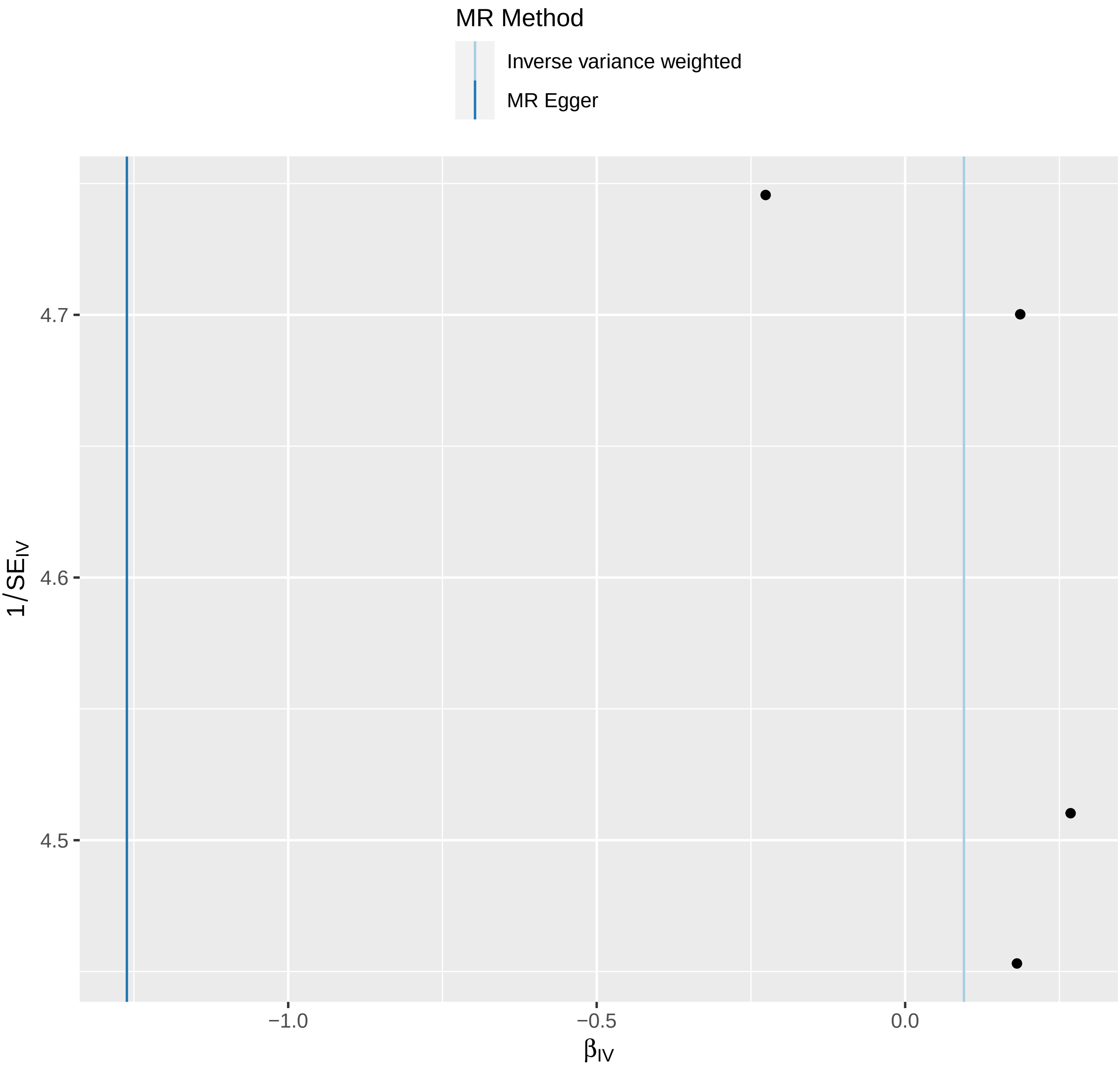

Batch 2−3660 : Gut microbiota abundance (genus Parasutterella id.2892) on Membrane−associated guanylate kinase, WW and PDZ domain−containing protein 2 with a threshold of 1e−05

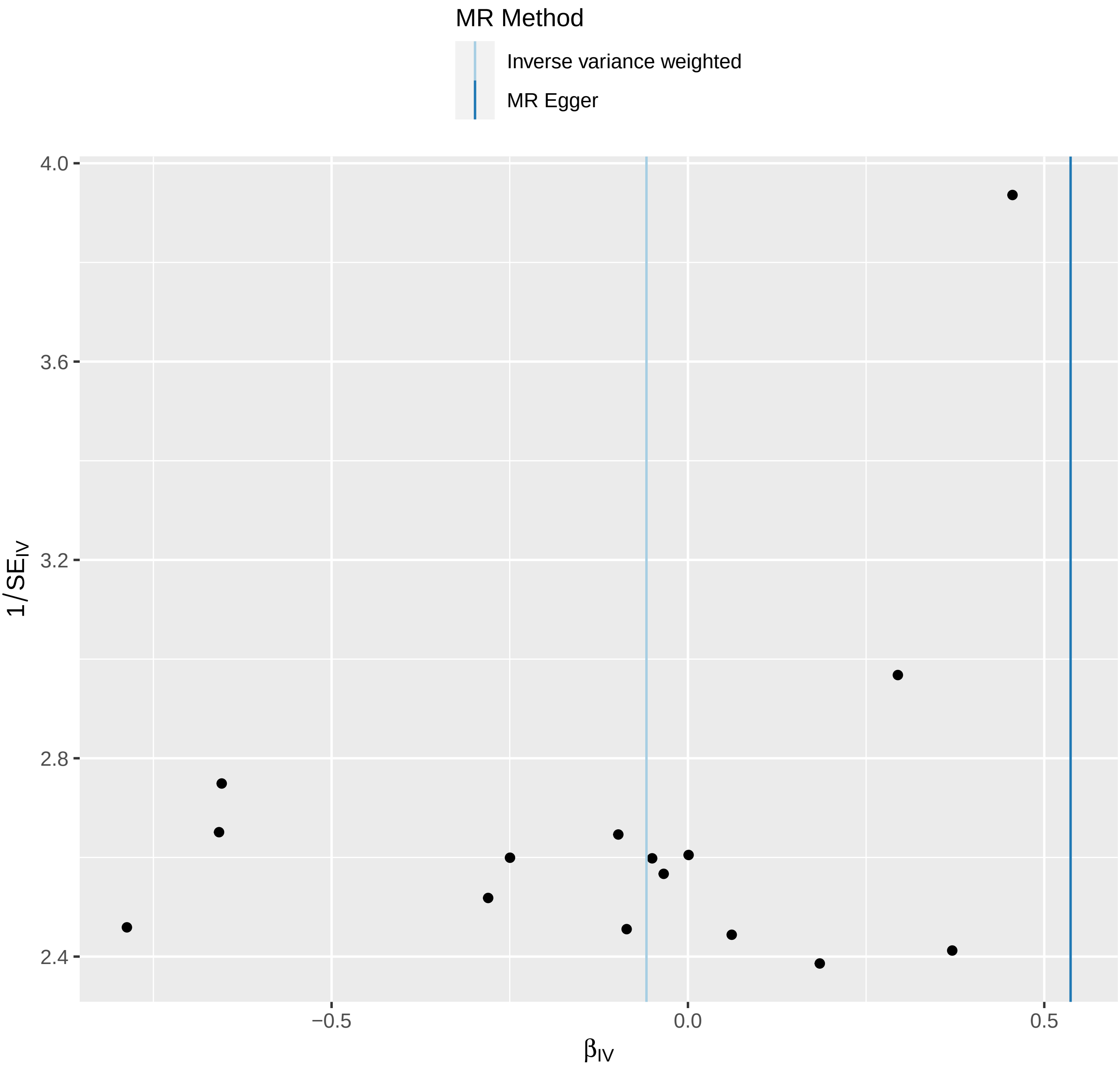

Batch 2−3661 : Gut microbiota abundance (genus Catenibacterium id.2153) on MAM domain−containing protein 2
with a threshold of 1e−05

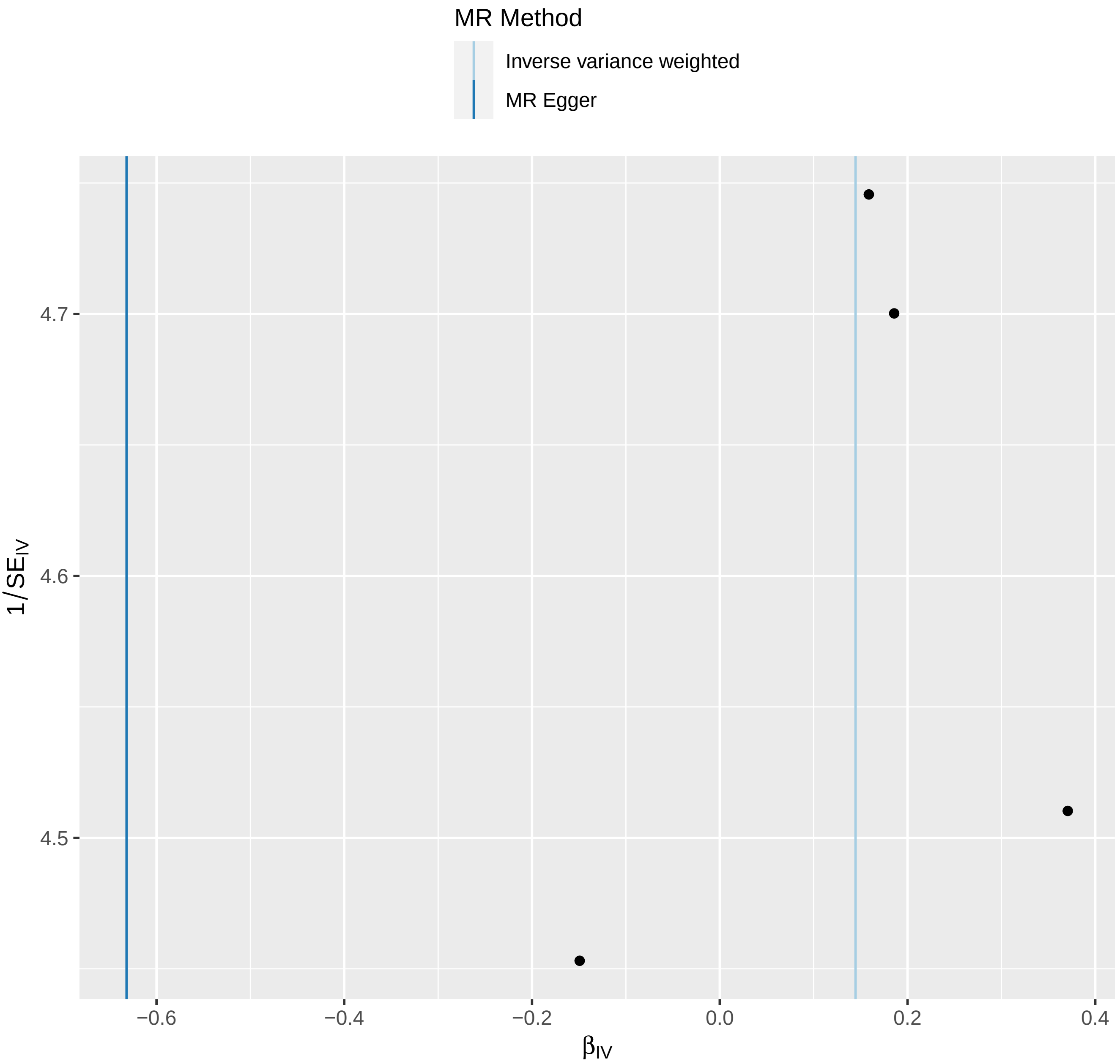

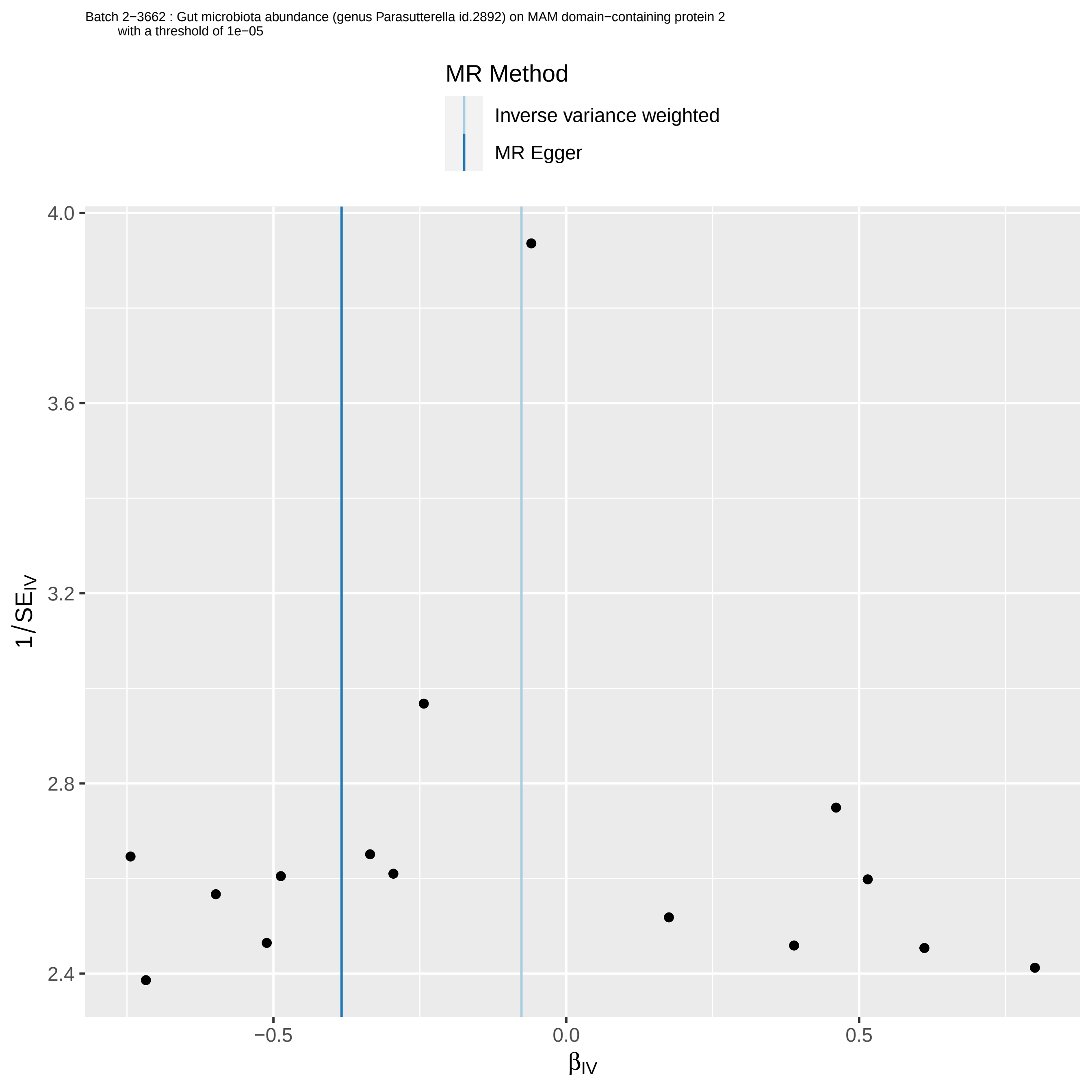

Batch 2−3662 : Gut microbiota abundance (genus Parasutterella id.2892) on MAM domain−containing protein 2
with a threshold of 1e−05
MR Method
Inverse variance weighted
MR Egger
4.0
3.6
3.2
2.8
2.4
1/SE_IV
−0.5
0.0
0.5
β_IV

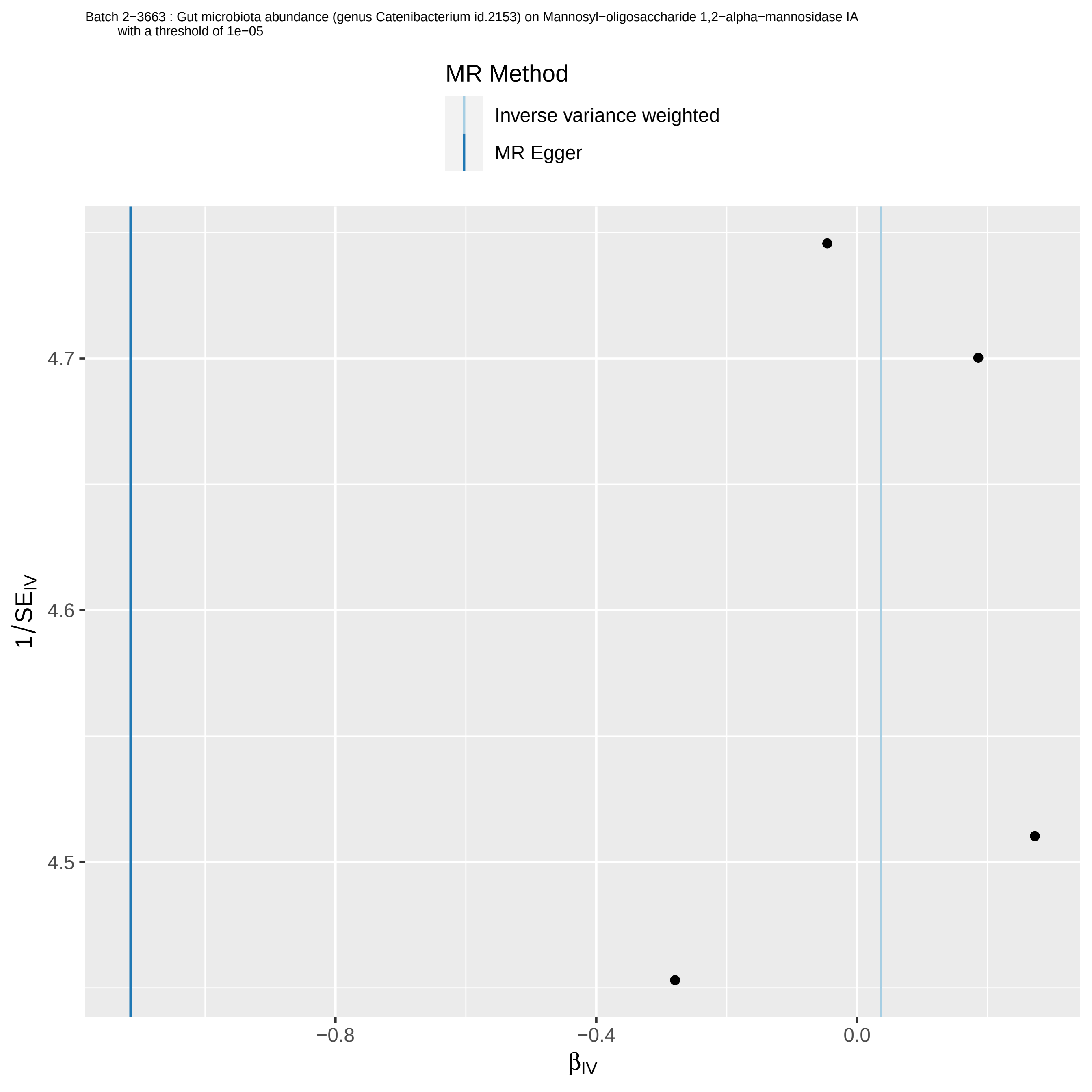

Batch 2−3663 : Gut microbiota abundance (genus Catenibacterium id.2153) on Mannosyl−oligosaccharide 1,2−alpha−mannosidase IA
with a threshold of 1e−05
MR Method
Inverse variance weighted
MR Egger
4.7
4.6
4.5
1/SE_IV
−0.8
−0.4
0.0
β_IV

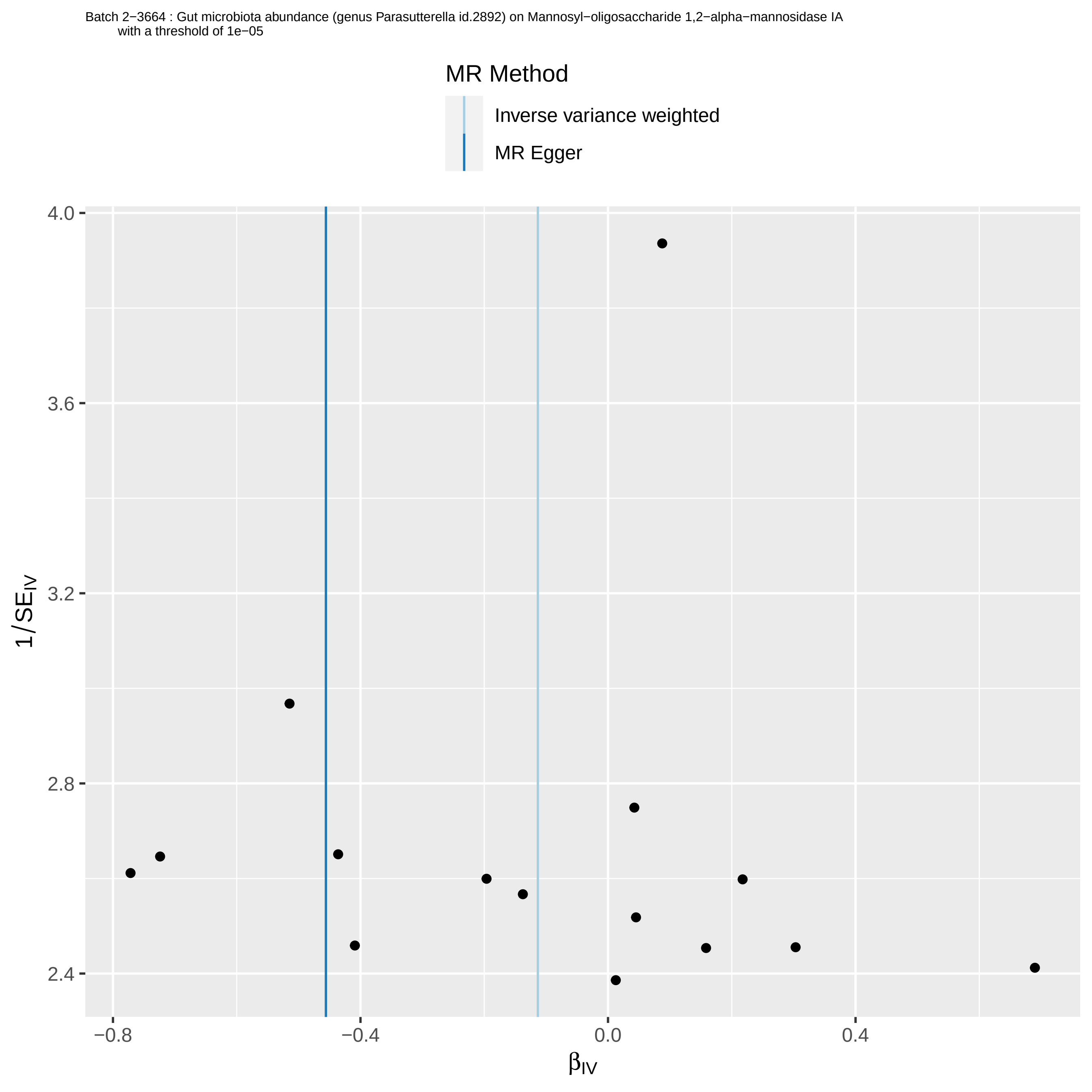
Batch 2−3664 : Gut microbiota abundance (genus Parasutterella id.2892) on Mannosyl−oligosaccharide 1,2−alpha−mannosidase IA with a threshold of 1e−05
MR Method
Inverse variance weighted
MR Egger
4.0
3.6
3.2
2.8
2.4
1/SE$_{IV}$
−0.8
−0.4
0.0
0.4
β$_{IV}$

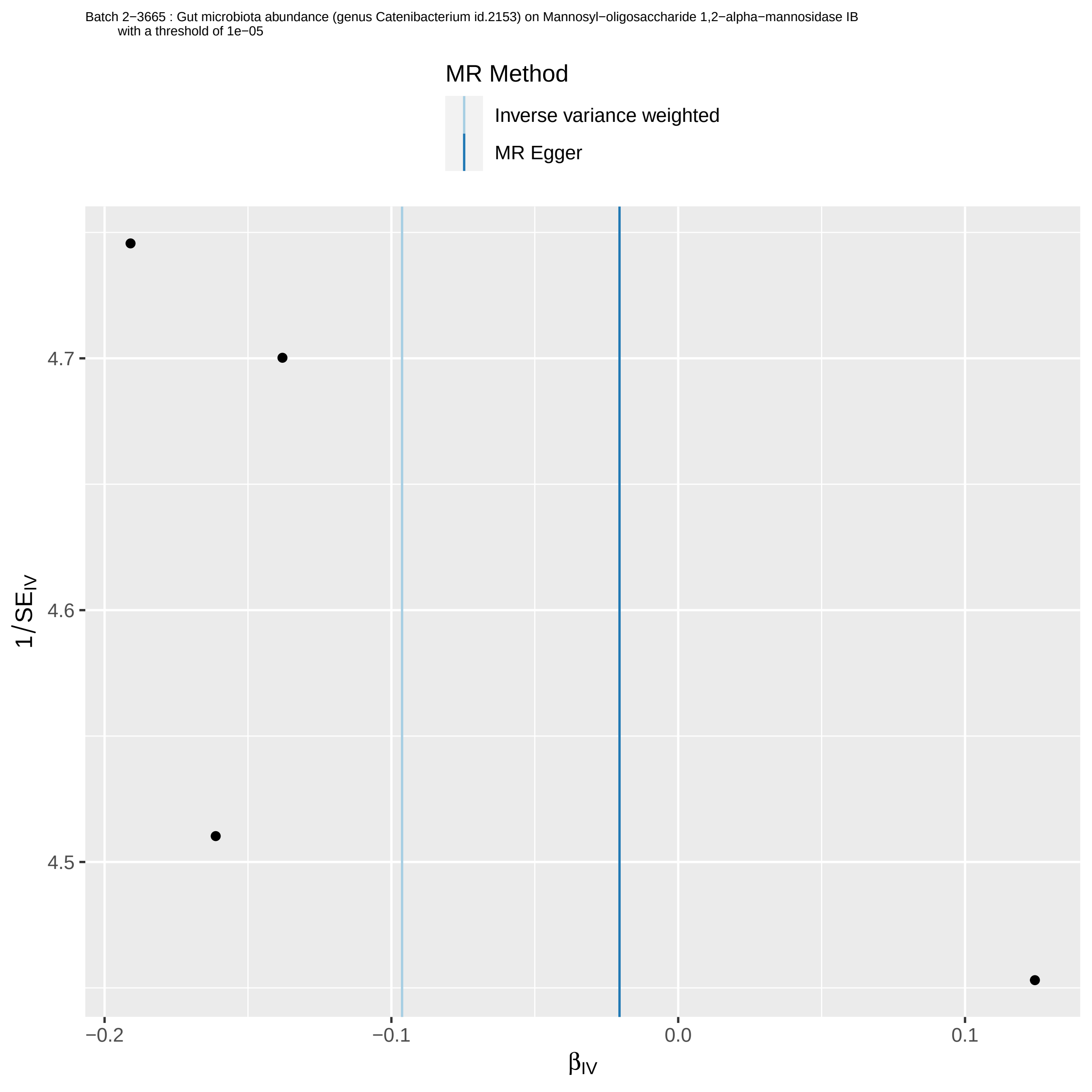

Batch 2−3665 : Gut microbiota abundance (genus Catenibacterium id.2153) on Mannosyl−oligosaccharide 1,2−alpha−mannosidase IB
with a threshold of 1e−05
MR Method
Inverse variance weighted
MR Egger
4.7
4.6
4.5
1/SE_IV
−0.2
−0.1
0.0
0.1
β_IV

Batch 2−3666 : Gut microbiota abundance (genus Parasutterella id.2892) on Mannosyl−oligosaccharide 1,2−alpha−mannosidase IB with a threshold of 1e−05

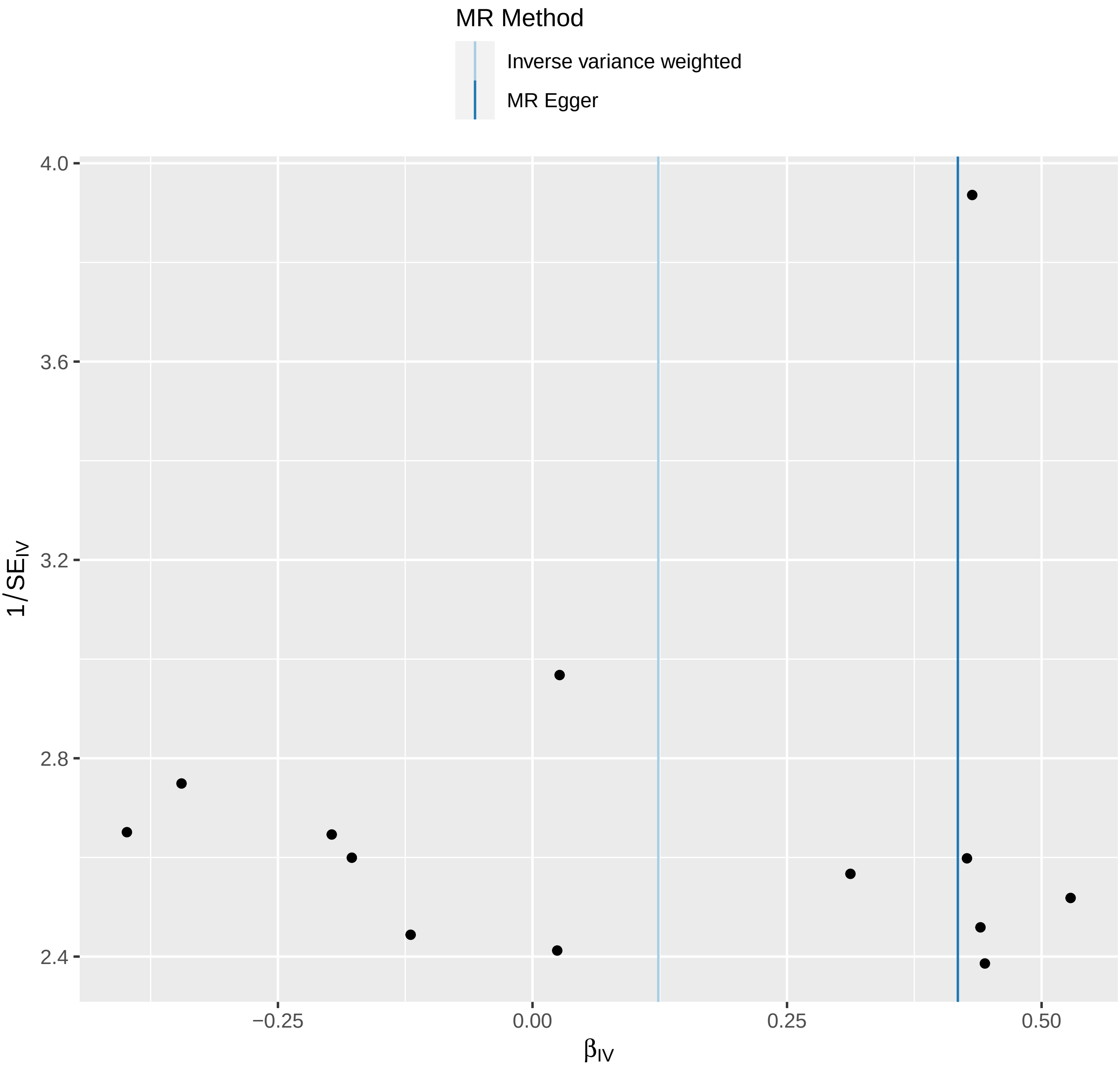

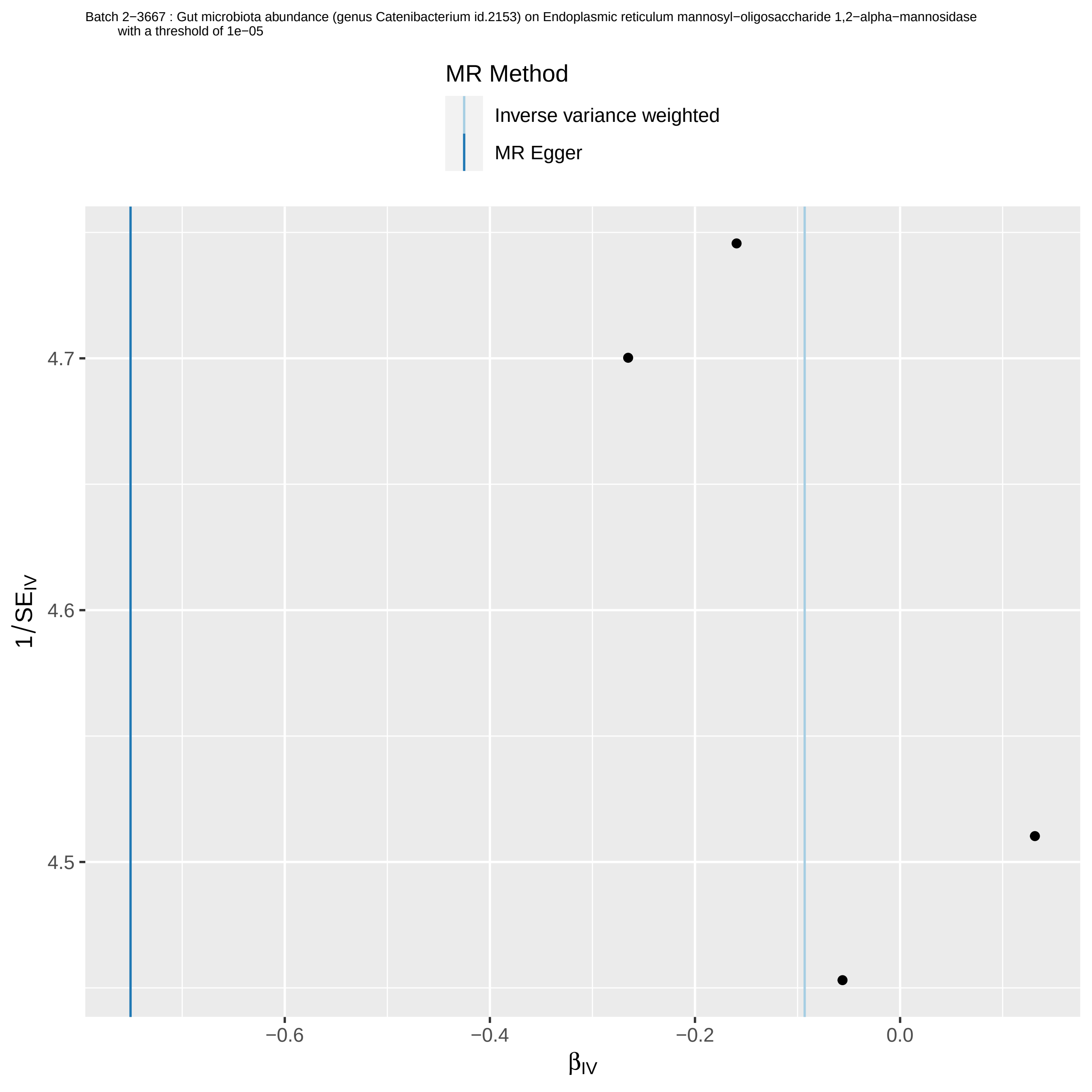
Batch 2−3667 : Gut microbiota abundance (genus Catenibacterium id.2153) on Endoplasmic reticulum mannosyl−oligosaccharide 1,2−alpha−mannosidase
with a threshold of 1e−05
MR Method
Inverse variance weighted
MR Egger
4.7
4.6
4.5
1/SE_IV
−0.6
−0.4
−0.2
0.0
β_IV

Batch 2−3668 : Gut microbiota abundance (genus Parasutterella id.2892) on Endoplasmic reticulum mannosyl−oligosaccharide 1,2−alpha−mannosidase with a threshold of 1e−05

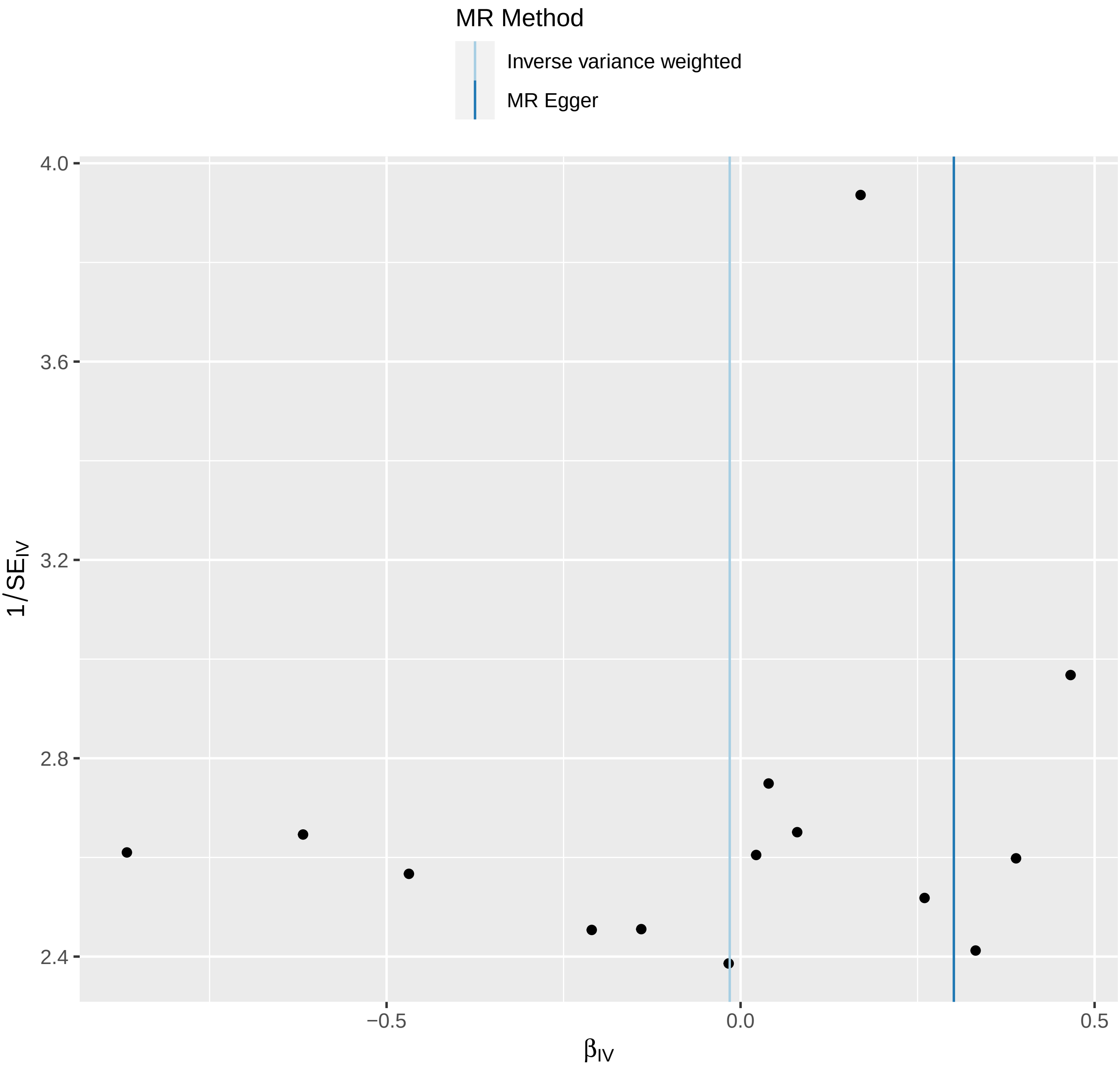

Batch 2−3669 : Gut microbiota abundance (genus Catenibacterium id.2153) on Epididymis−specific alpha−mannosidase
with a threshold of 1e−05

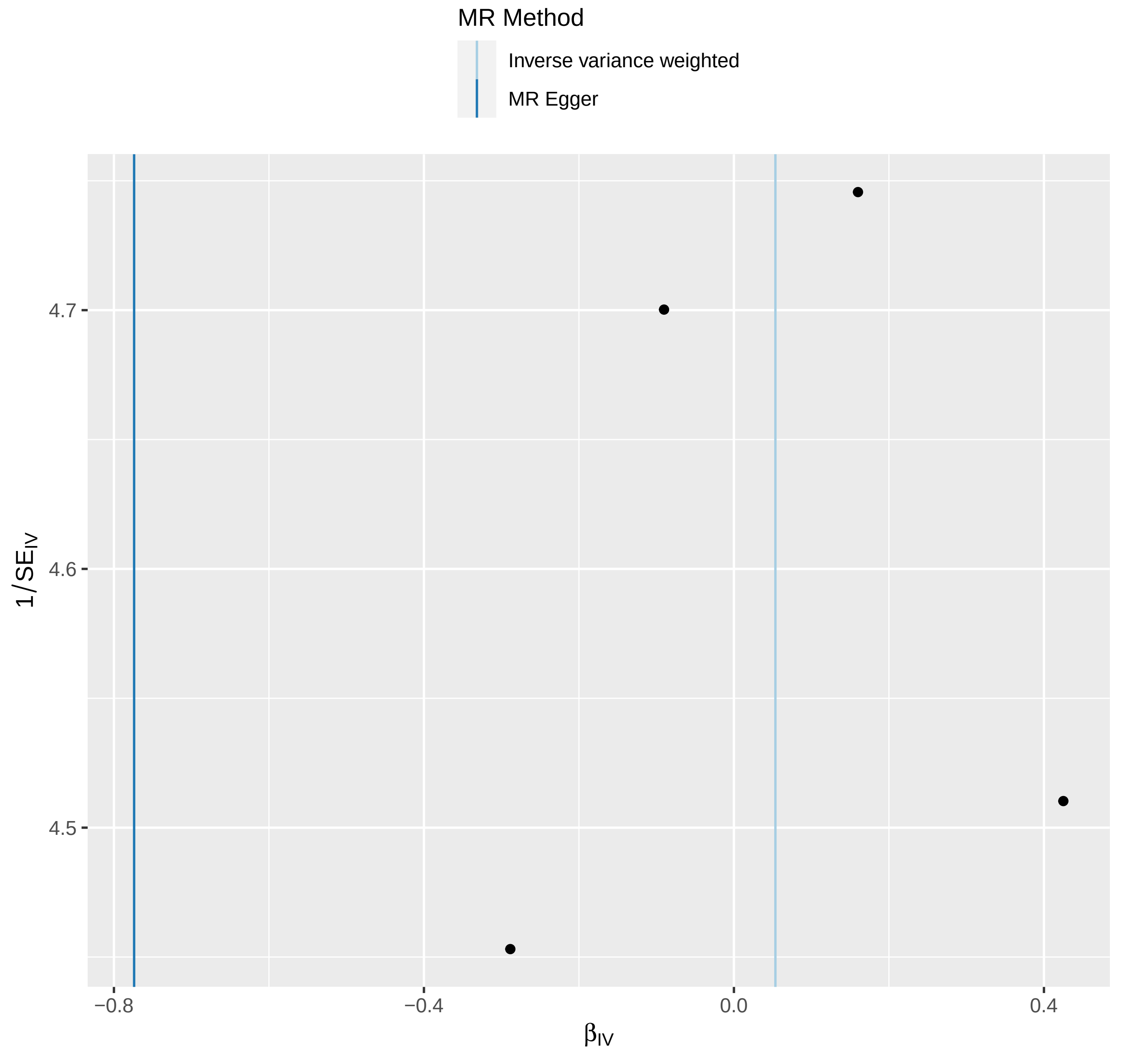

Batch 2−3670 : Gut microbiota abundance (genus Parasutterella id.2892) on Epididymis−specific alpha−mannosidase
with a threshold of 1e−05

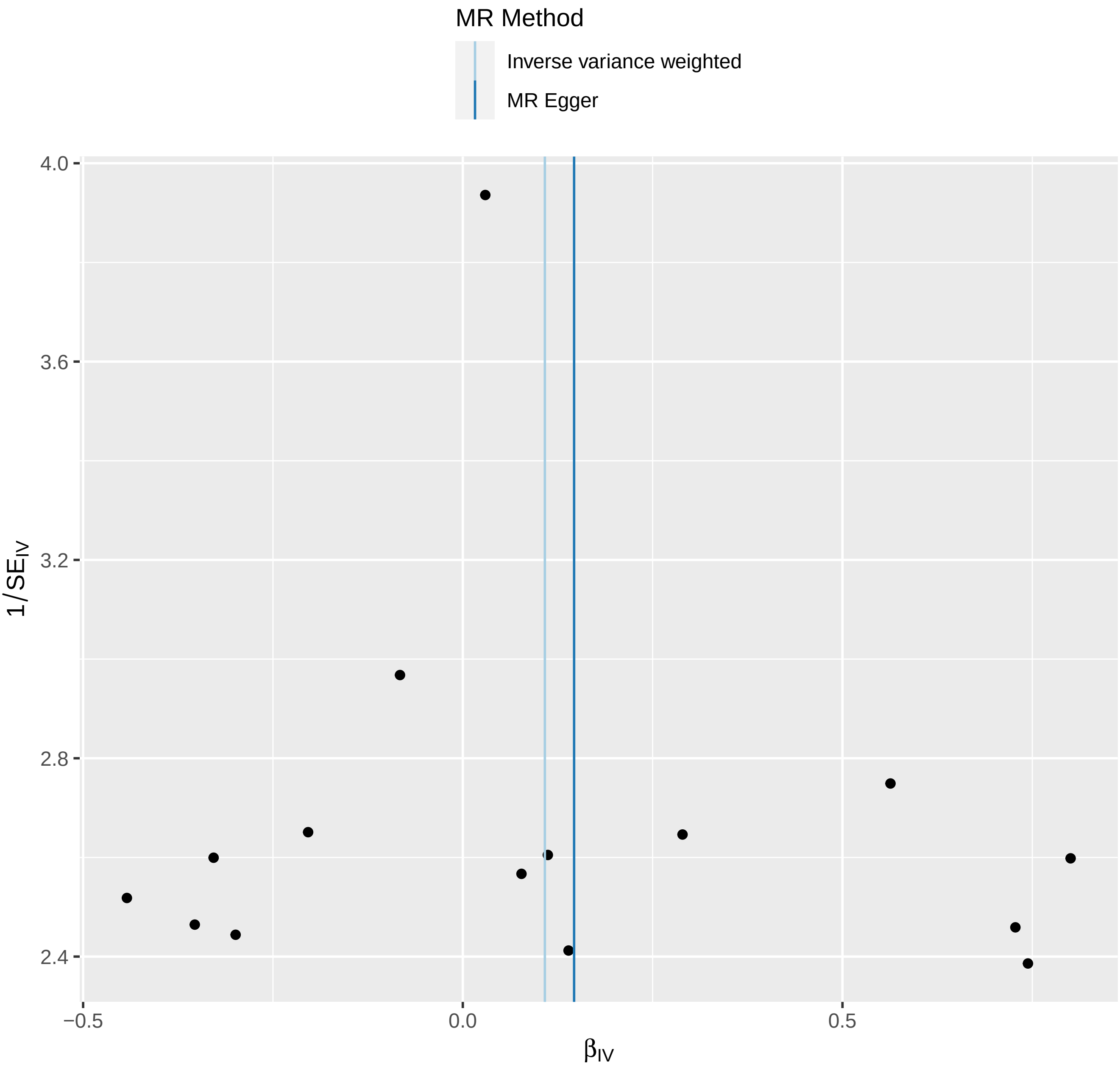

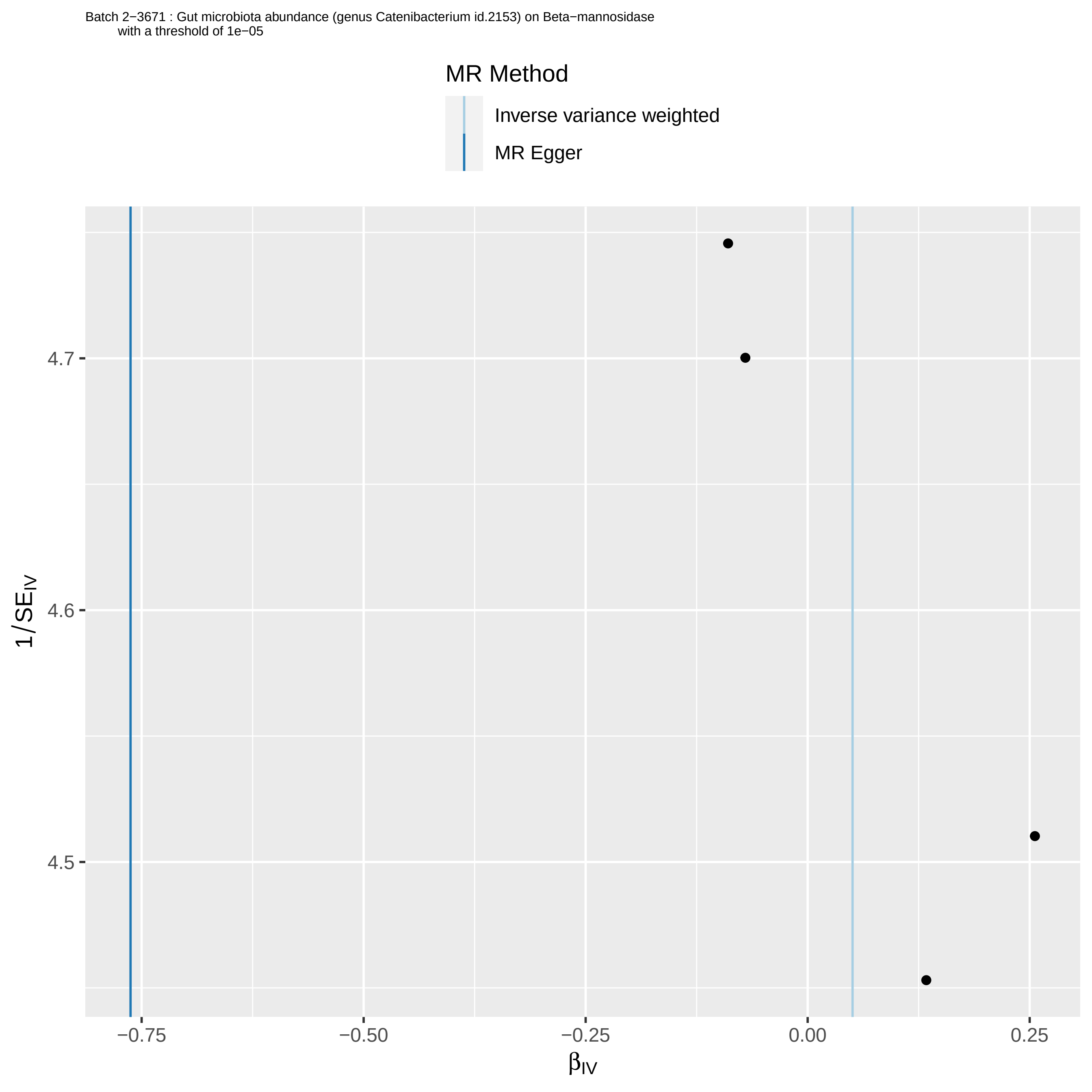
Batch 2−3671 : Gut microbiota abundance (genus Catenibacterium id.2153) on Beta−mannosidase
with a threshold of 1e−05
MR Method
Inverse variance weighted
MR Egger
4.7
4.6
4.5
1/SE$_{IV}$
−0.75
−0.50
−0.25
0.00
0.25
β_{IV}

Batch 2−3672 : Gut microbiota abundance (genus Parasutterella id.2892) on Beta−mannosidase
with a threshold of 1e−05

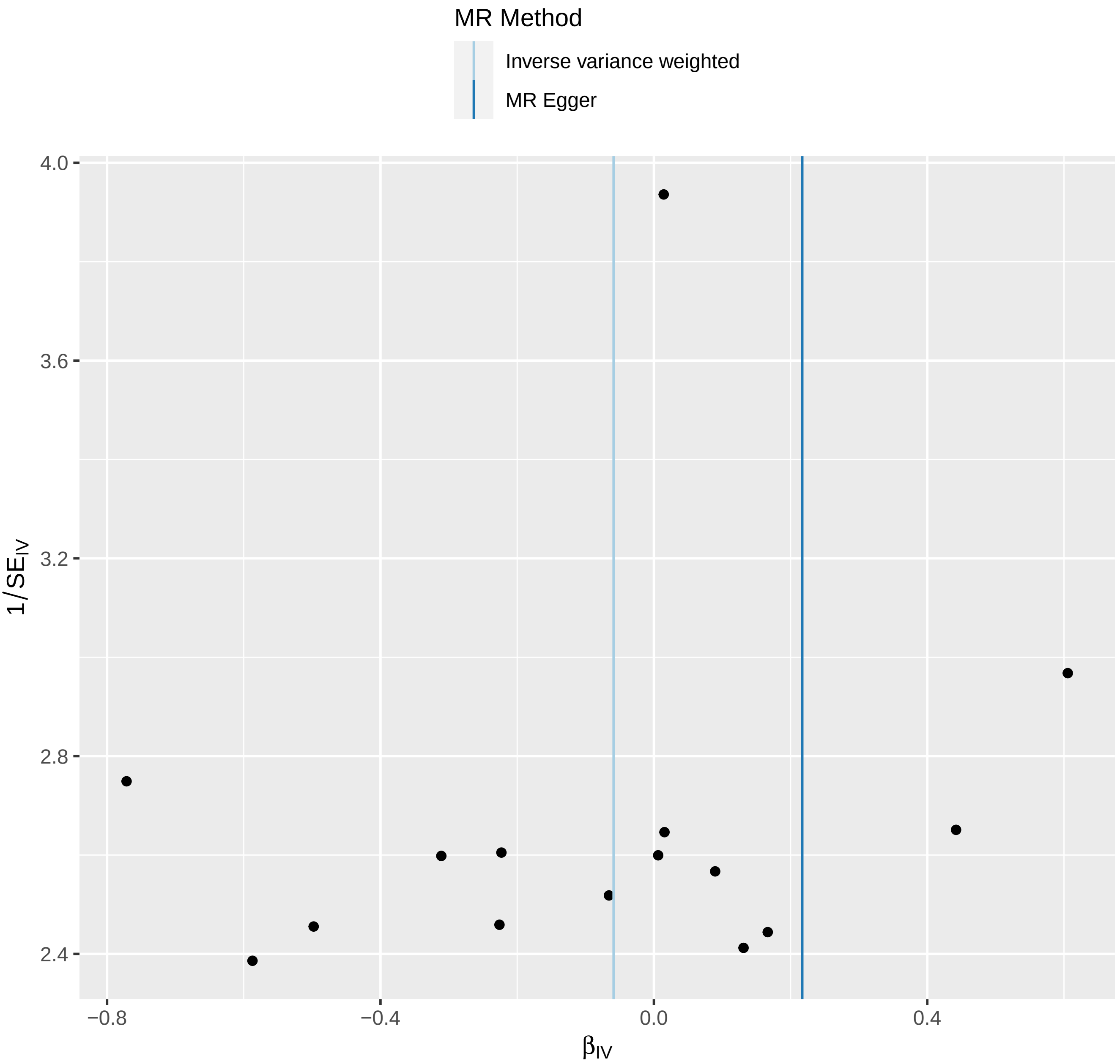

Batch 2−3673 : Gut microbiota abundance (genus Catenibacterium id.2153) on Glycoprotein endo−alpha−1,2−mannosidase with a threshold of 1e−05

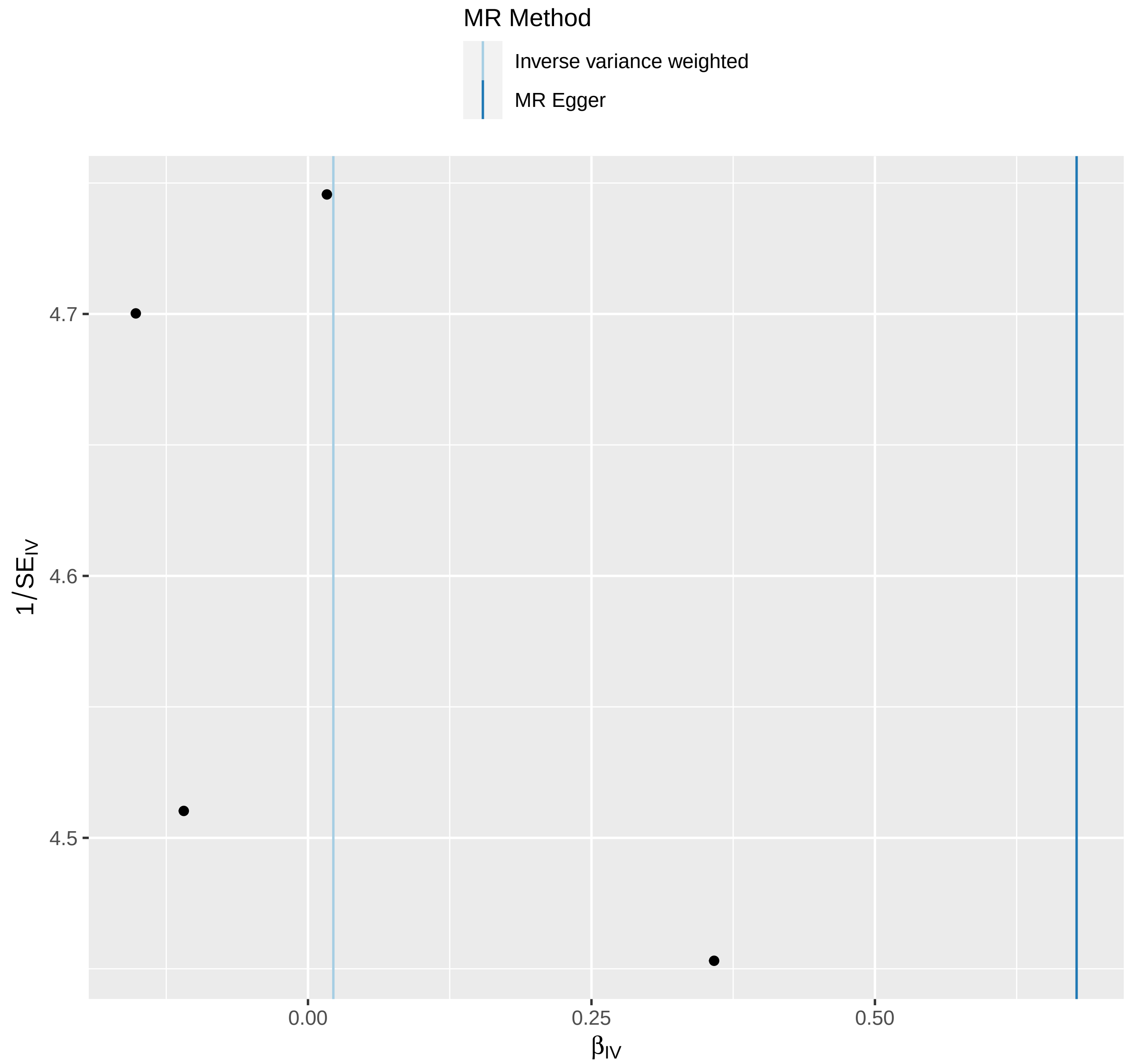

Batch 2−3674 : Gut microbiota abundance (genus Parasutterella id.2892) on Glycoprotein endo−alpha−1,2−mannosidase
with a threshold of 1e−05

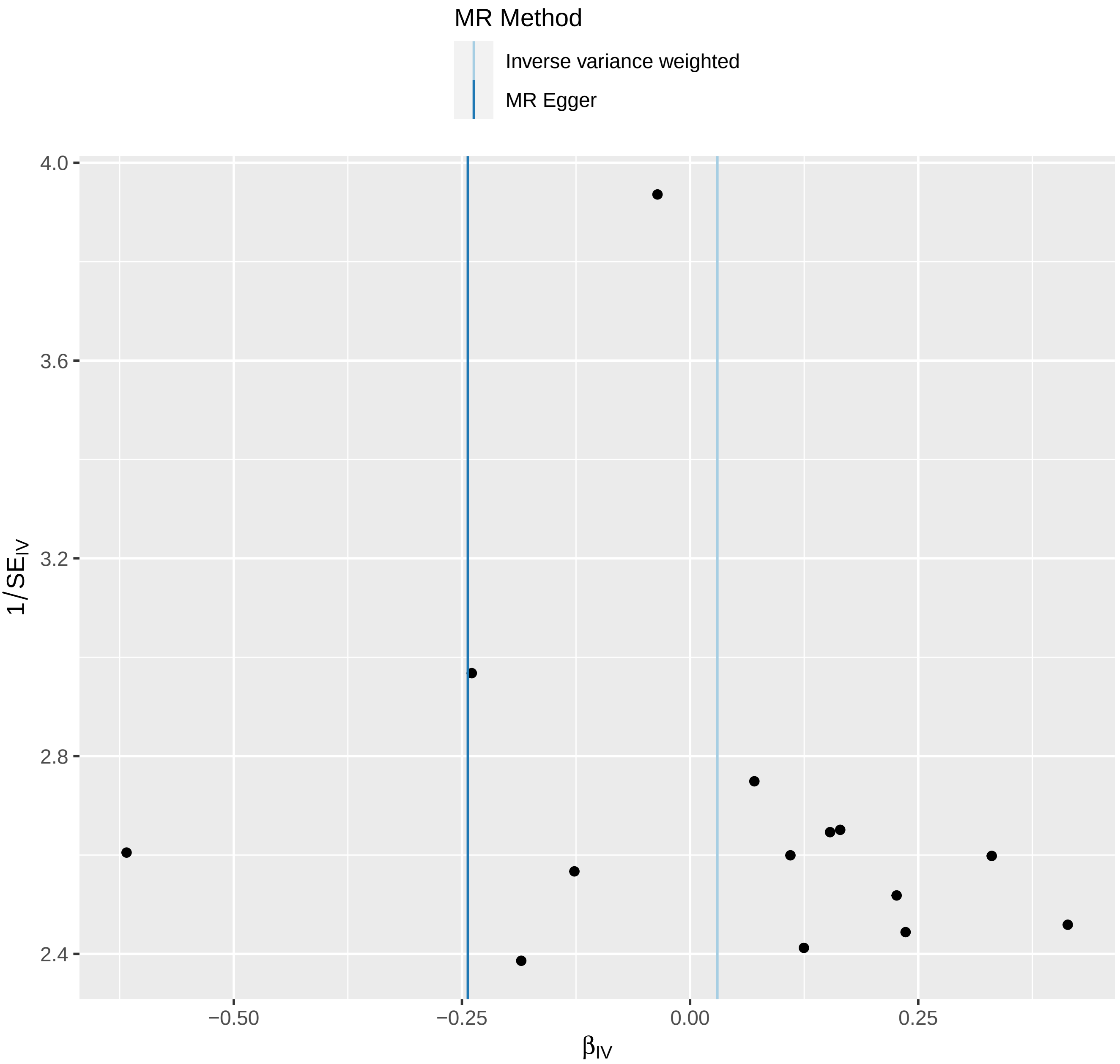

Batch 2−3675 : Gut microbiota abundance (genus Catenibacterium id.2153) on MANSC domain−containing protein 1
with a threshold of 1e−05

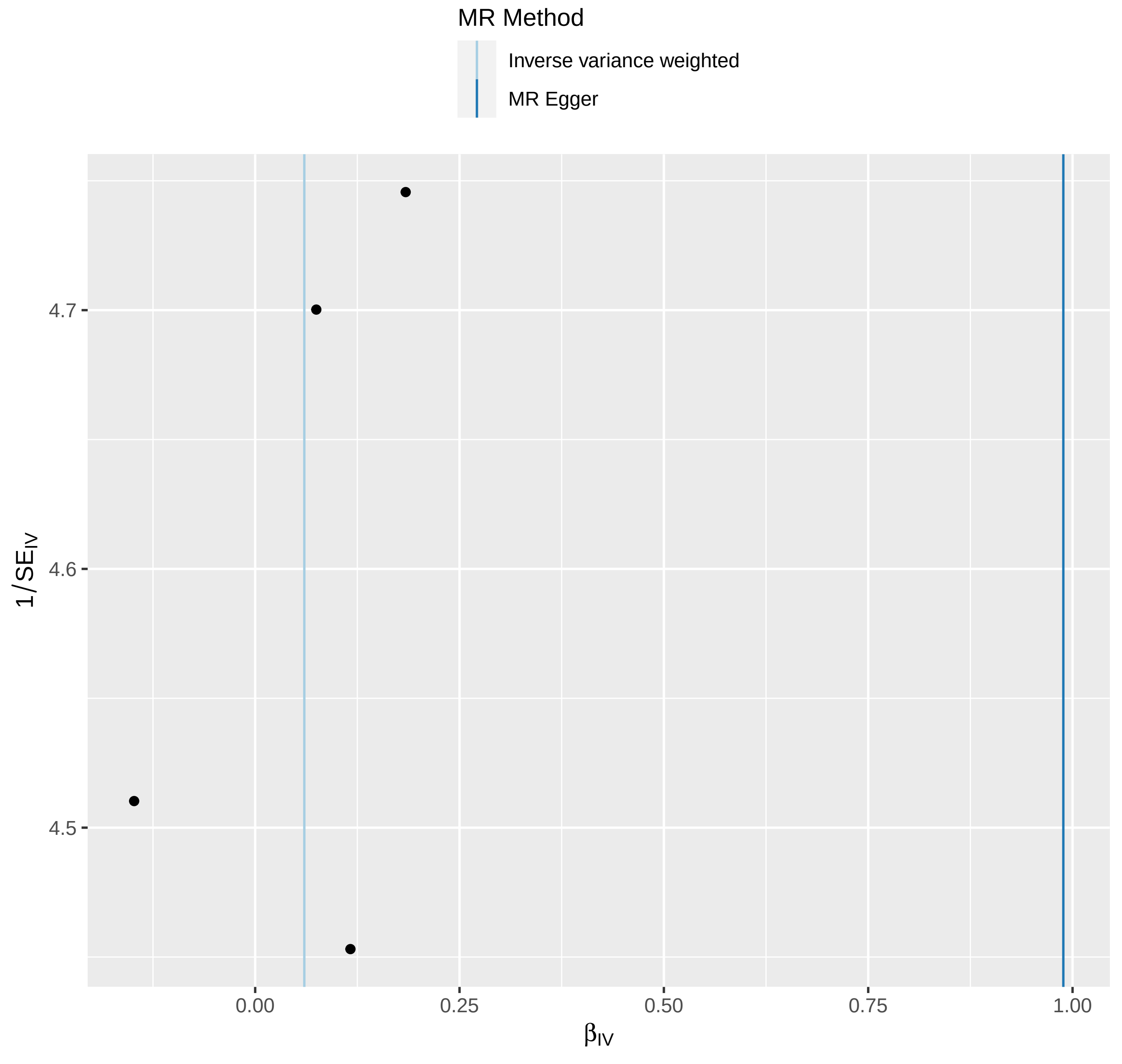

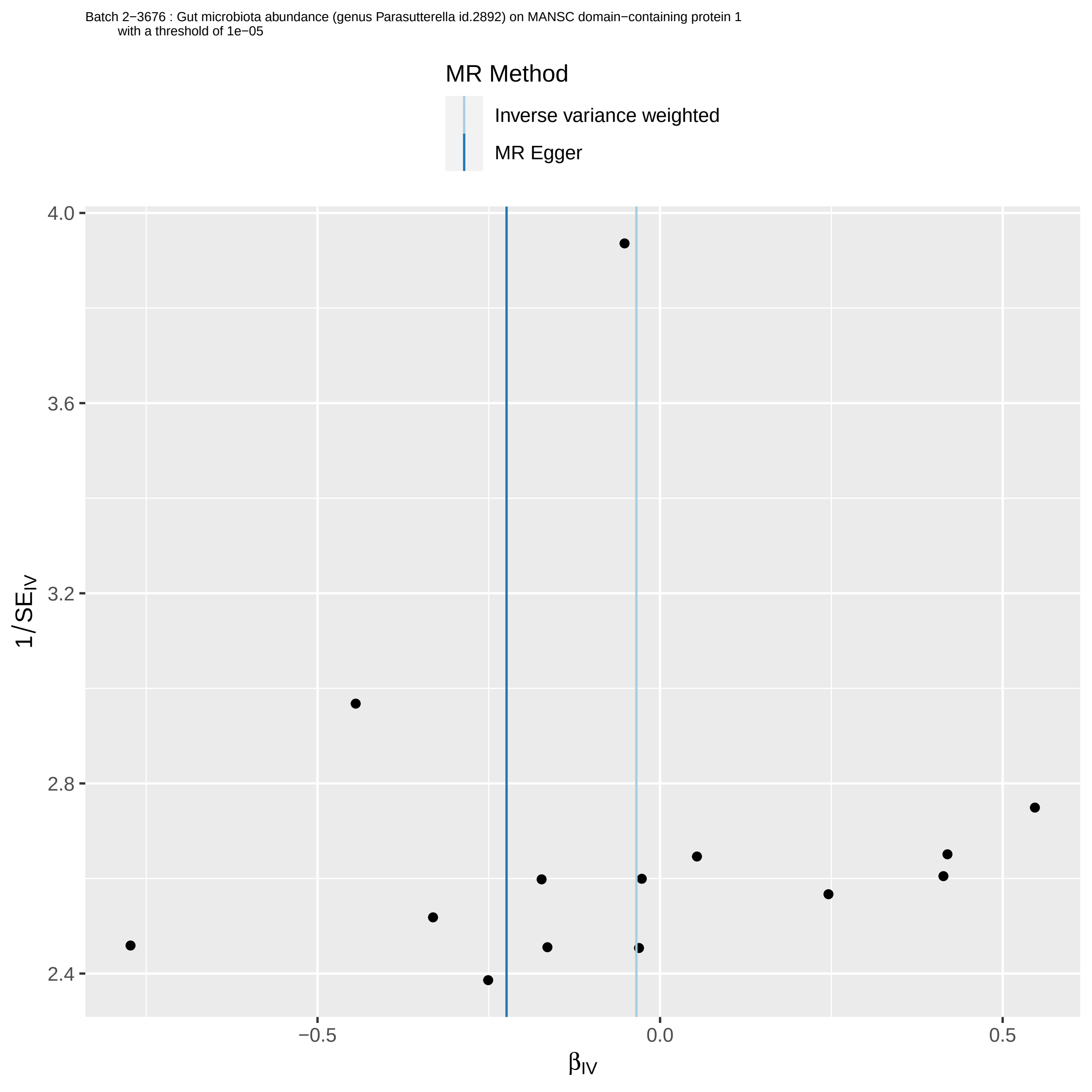

Batch 2−3676 : Gut microbiota abundance (genus Parasutterella id.2892) on MANSC domain−containing protein 1
with a threshold of 1e−05
MR Method
Inverse variance weighted
MR Egger
4.0
3.6
3.2
2.8
2.4
1/SE_IV
−0.5
0.0
0.5
β_IV

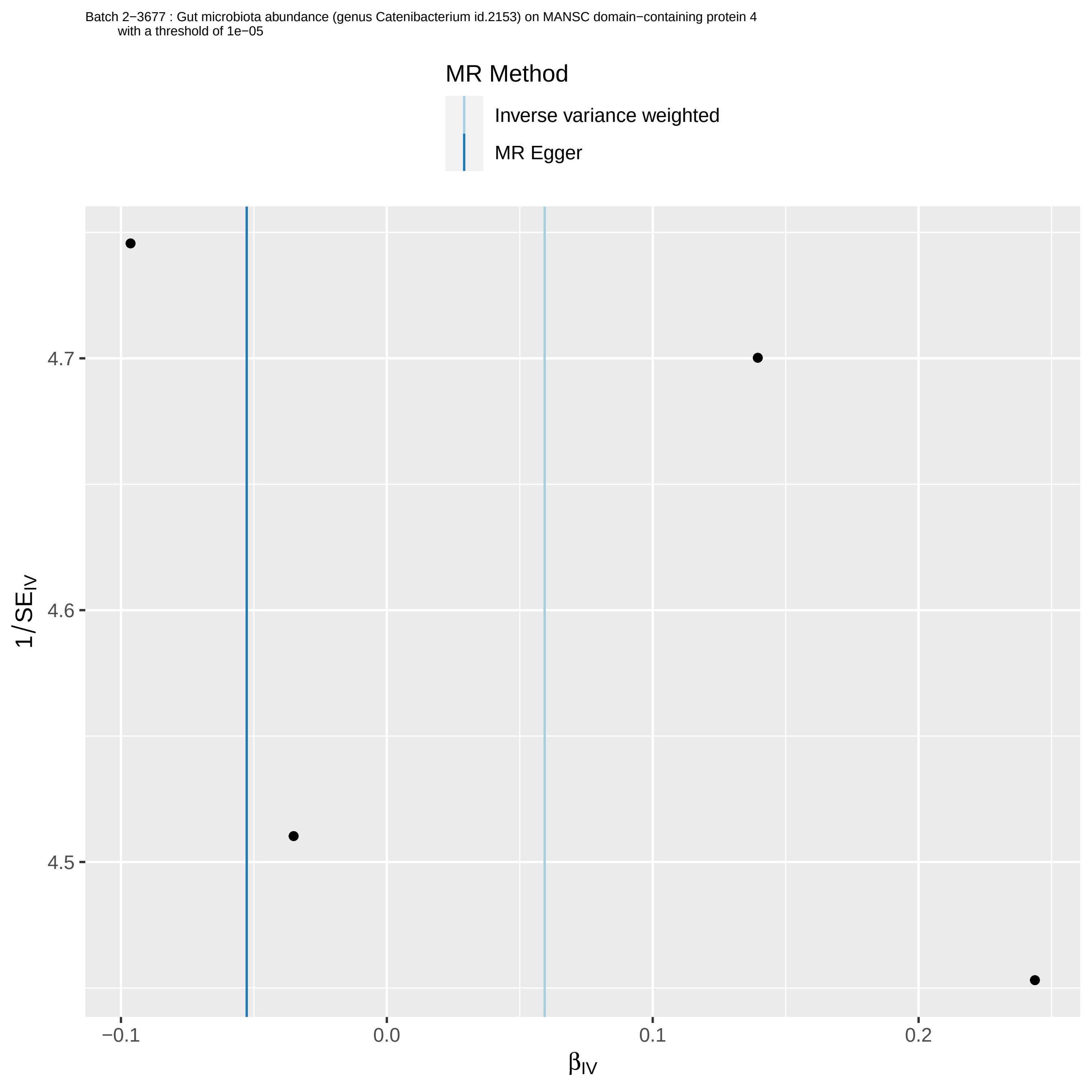
Batch 2−3677 : Gut microbiota abundance (genus Catenibacterium id.2153) on MANSC domain−containing protein 4
with a threshold of 1e−05
MR Method
Inverse variance weighted
MR Egger
1/SE_IV
4.7
4.6
4.5
−0.1
0.0
0.1
0.2
β_IV

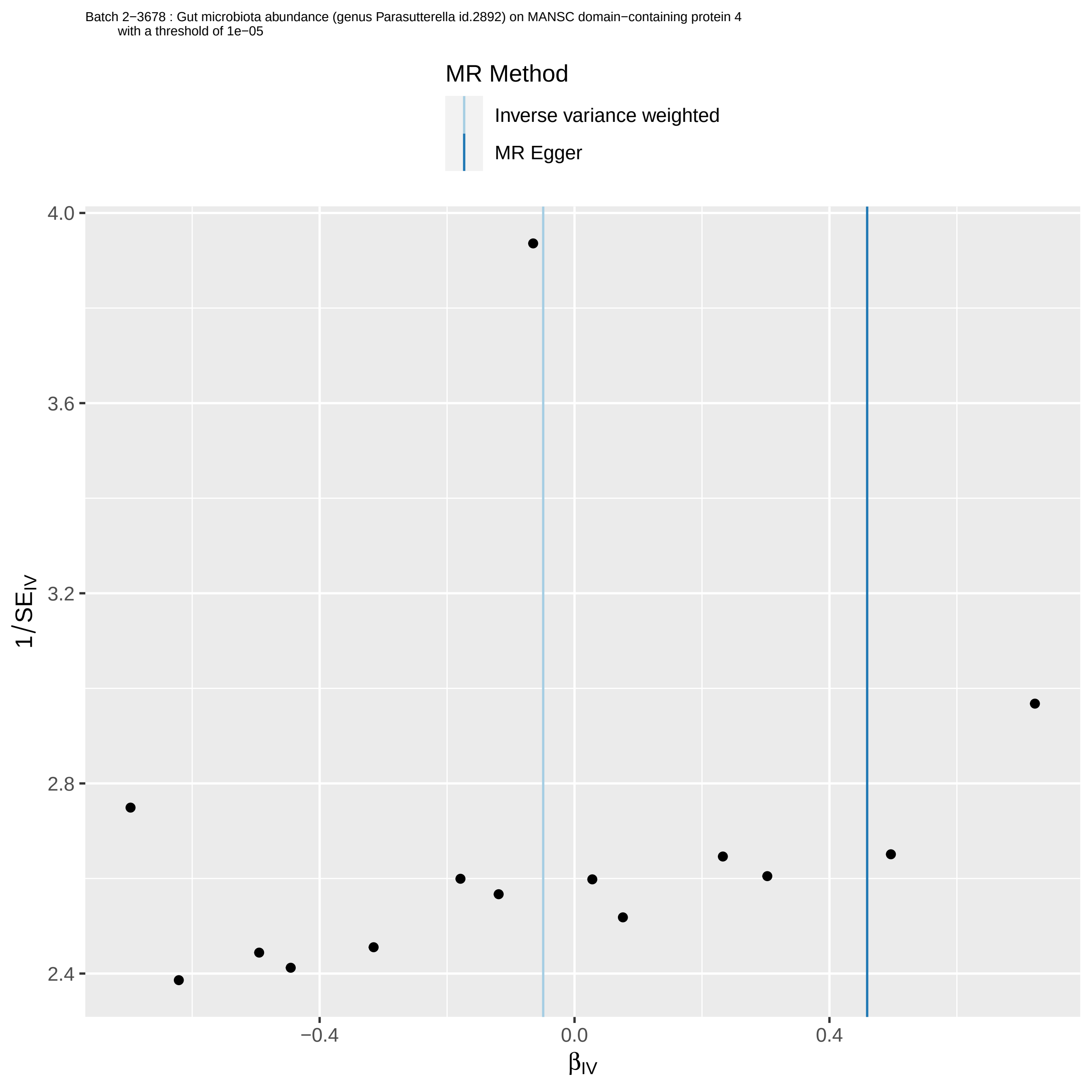
Batch 2−3678 : Gut microbiota abundance (genus Parasutterella id.2892) on MANSC domain−containing protein 4
with a threshold of 1e−05
MR Method
Inverse variance weighted
MR Egger
4.0
3.6
3.2
2.8
2.4
1/SE$_{IV}$
−0.4
0.0
0.4
β_{IV}

Batch 2−3679 : Gut microbiota abundance (genus Catenibacterium id.2153) on Dual specificity mitogen−activated protein kinase kinase 1 with a threshold of 1e−05

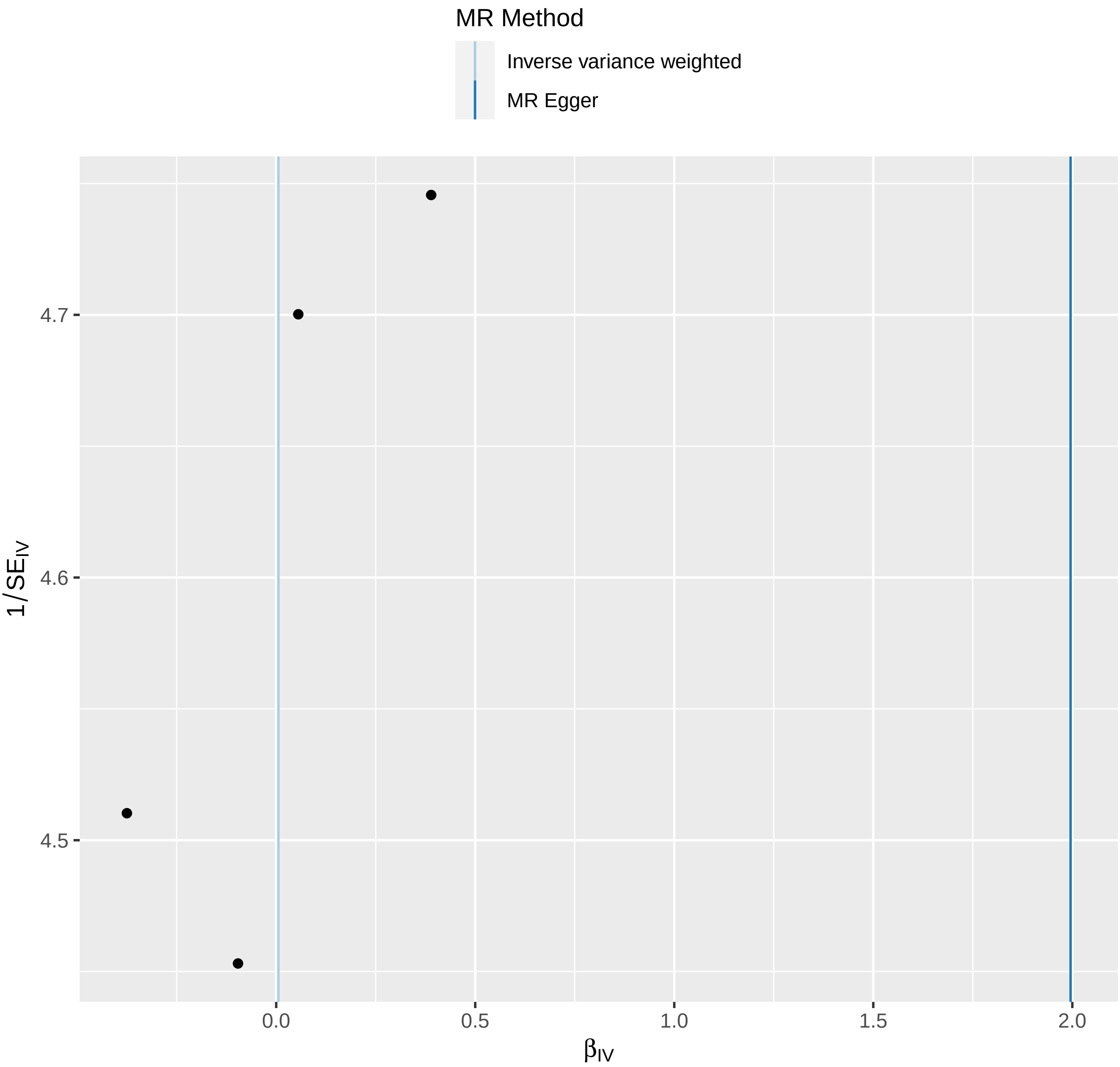

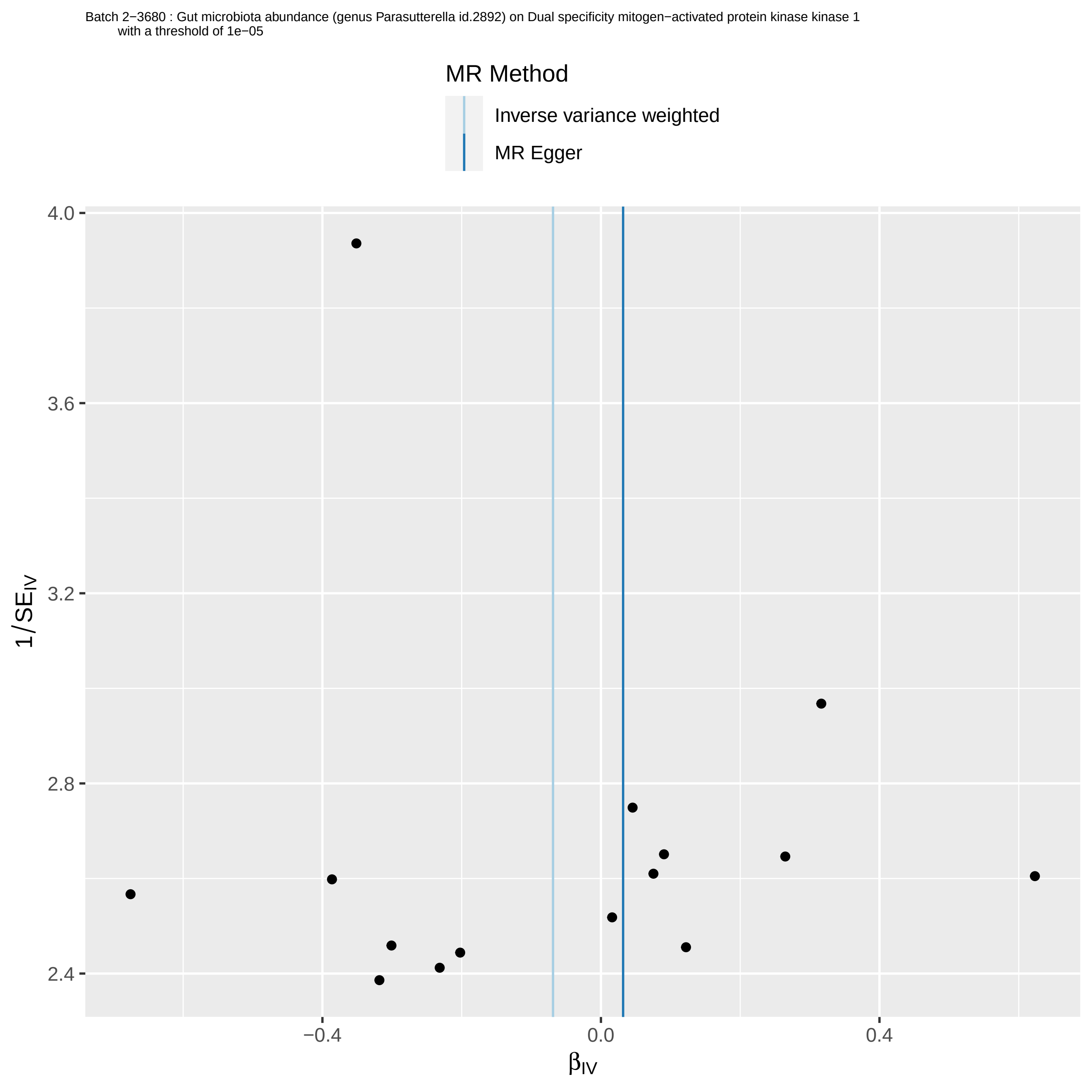

Batch 2−3680 : Gut microbiota abundance (genus Parasutterella id.2892) on Dual specificity mitogen−activated protein kinase kinase 1
with a threshold of 1e−05
MR Method
Inverse variance weighted
MR Egger
4.0
3.6
3.2
2.8
2.4
1/SE$_{IV}$
−0.4
0.0
0.4
β_{IV}

Batch 2−3681 : Gut microbiota abundance (genus Catenibacterium id.2153) on Dual specificity mitogen−activated protein kinase kinase 3 with a threshold of 1e−05

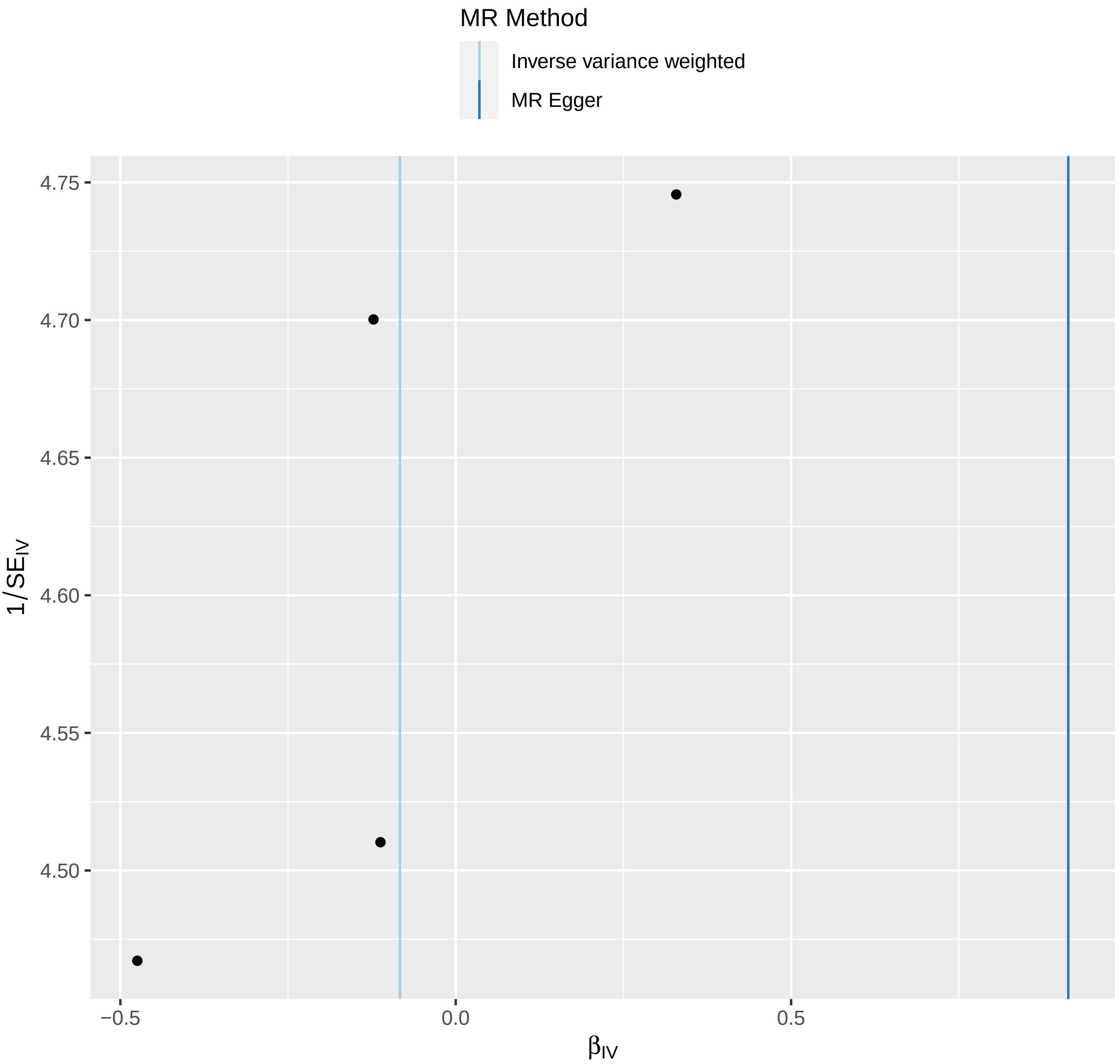

Batch 2−3682 : Gut microbiota abundance (genus Parasutterella id.2892) on Dual specificity mitogen−activated protein kinase kinase 3
with a threshold of 1e−05

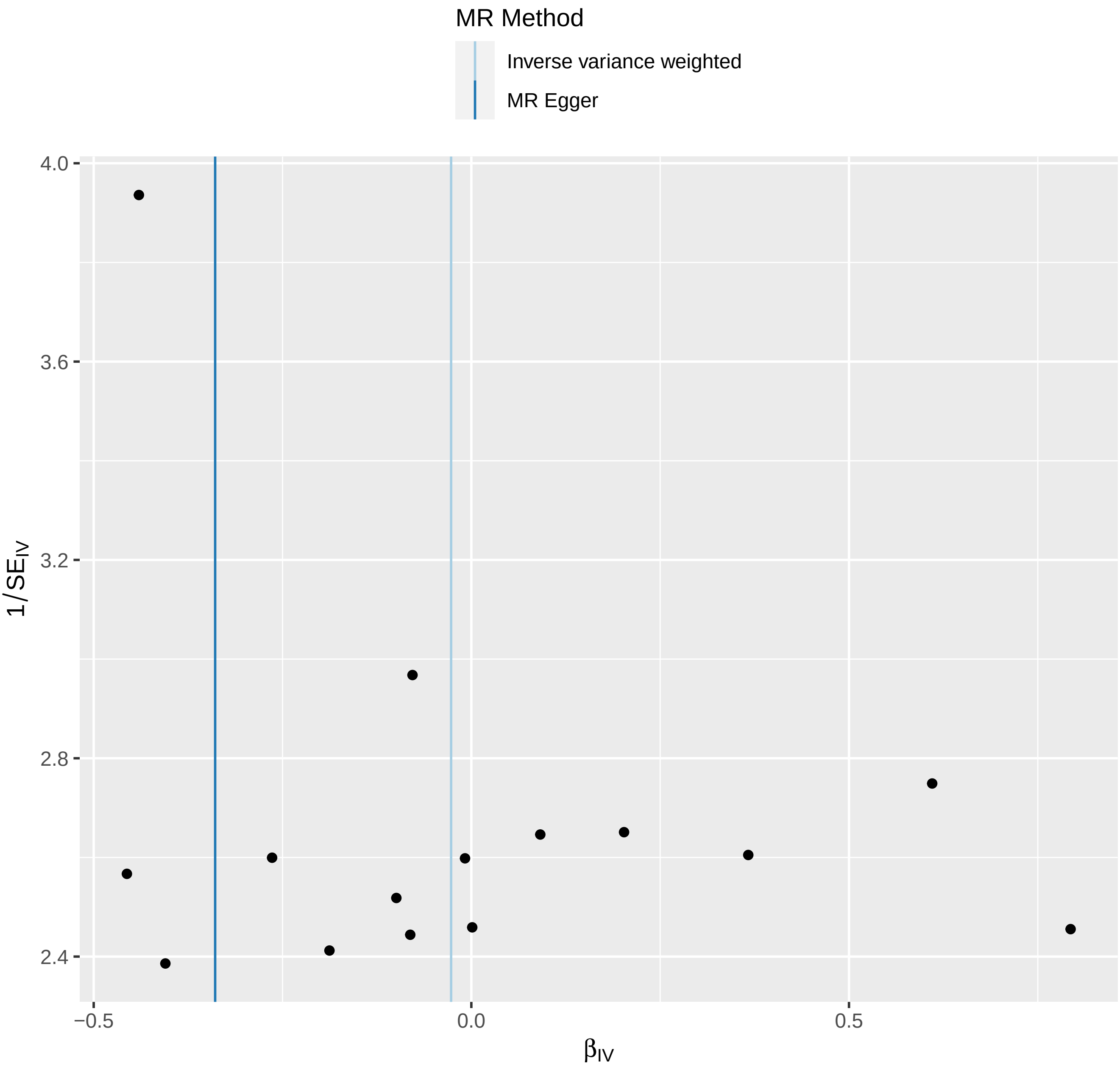

Batch 2−3683 : Gut microbiota abundance (genus Catenibacterium id.2153) on Dual specificity mitogen−activated protein kinase kinase 4 with a threshold of 1e−05

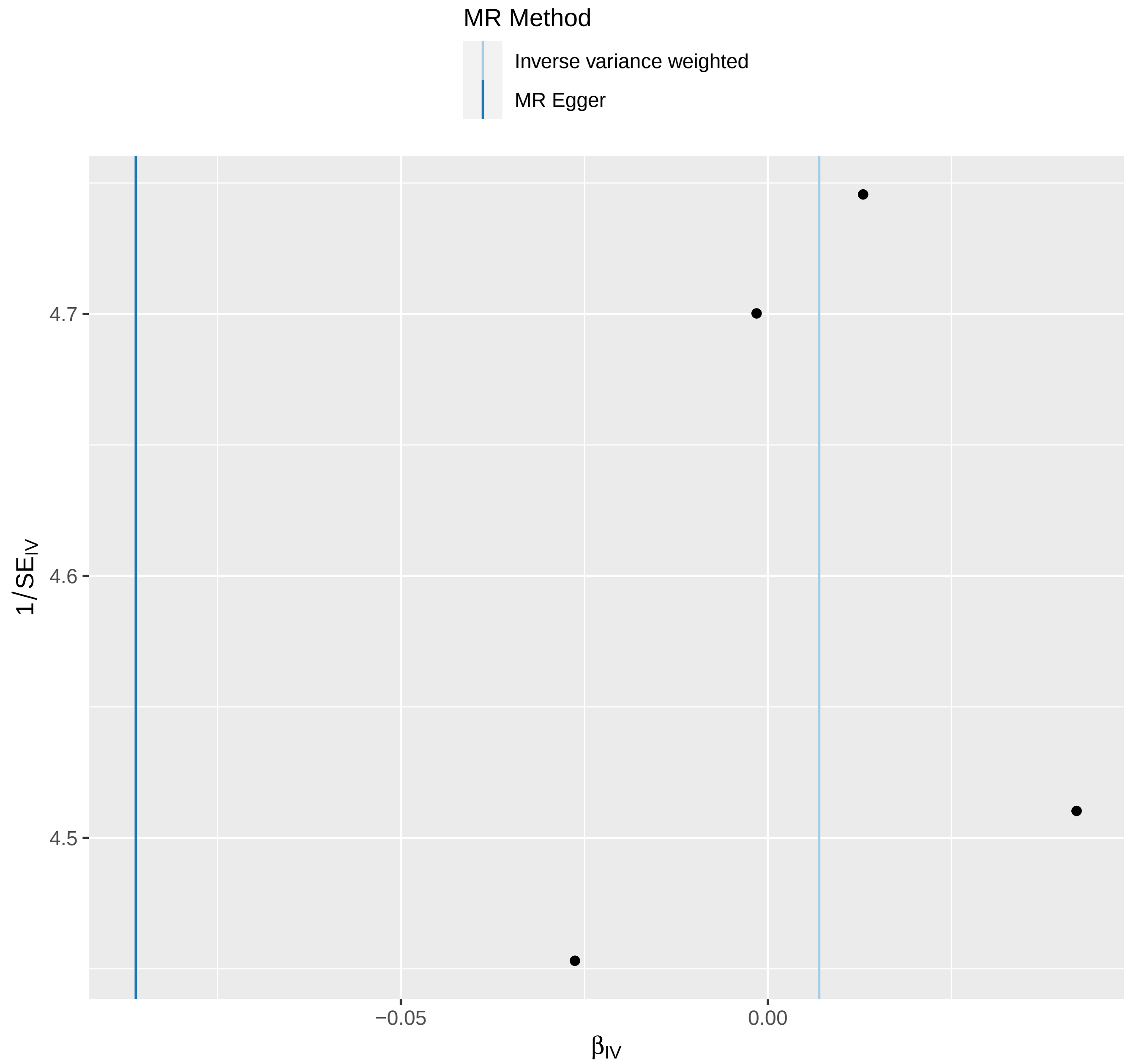

Batch 2−3684 : Gut microbiota abundance (genus Parasutterella id.2892) on Dual specificity mitogen−activated protein kinase kinase 4
with a threshold of 1e−05

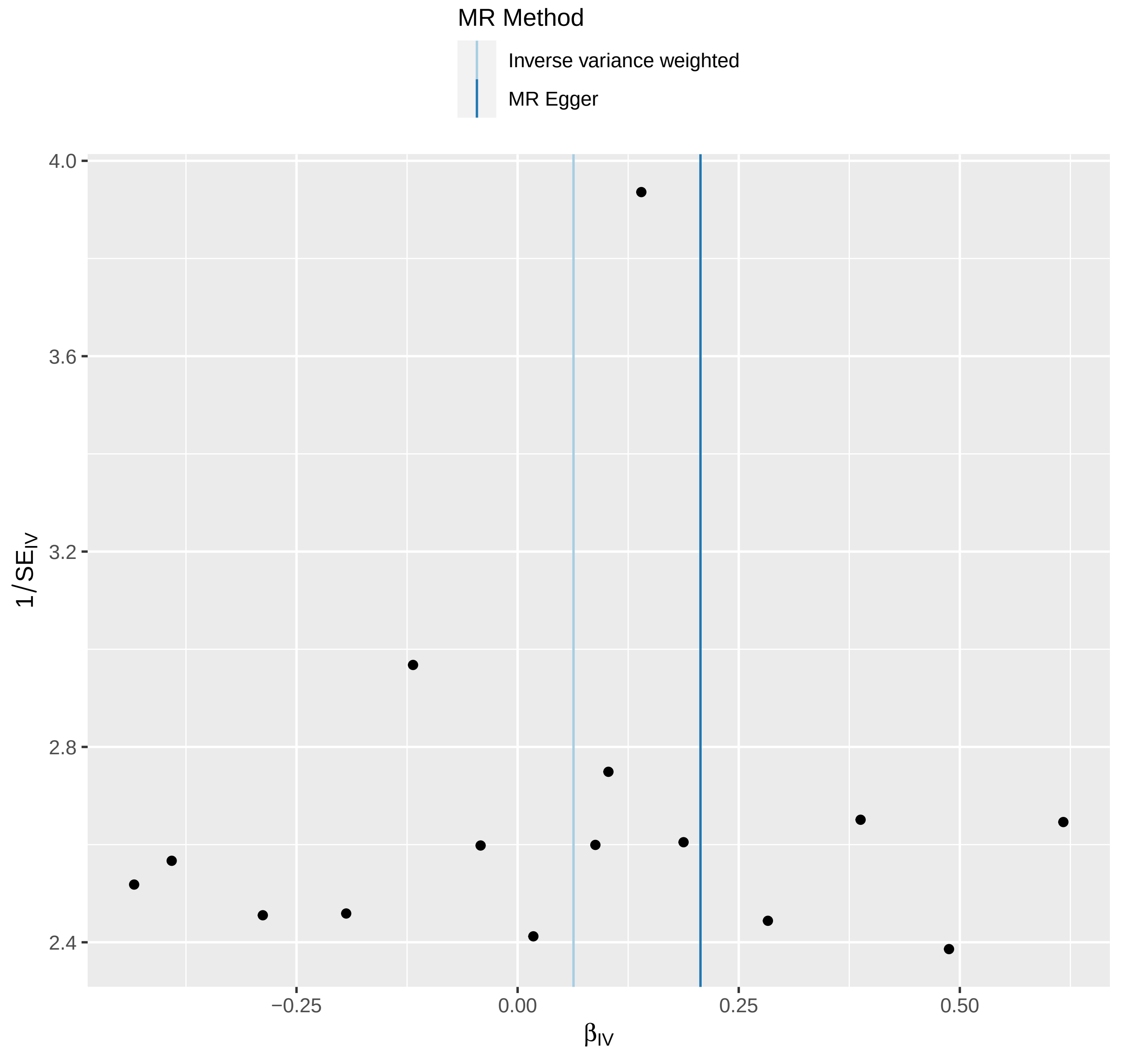

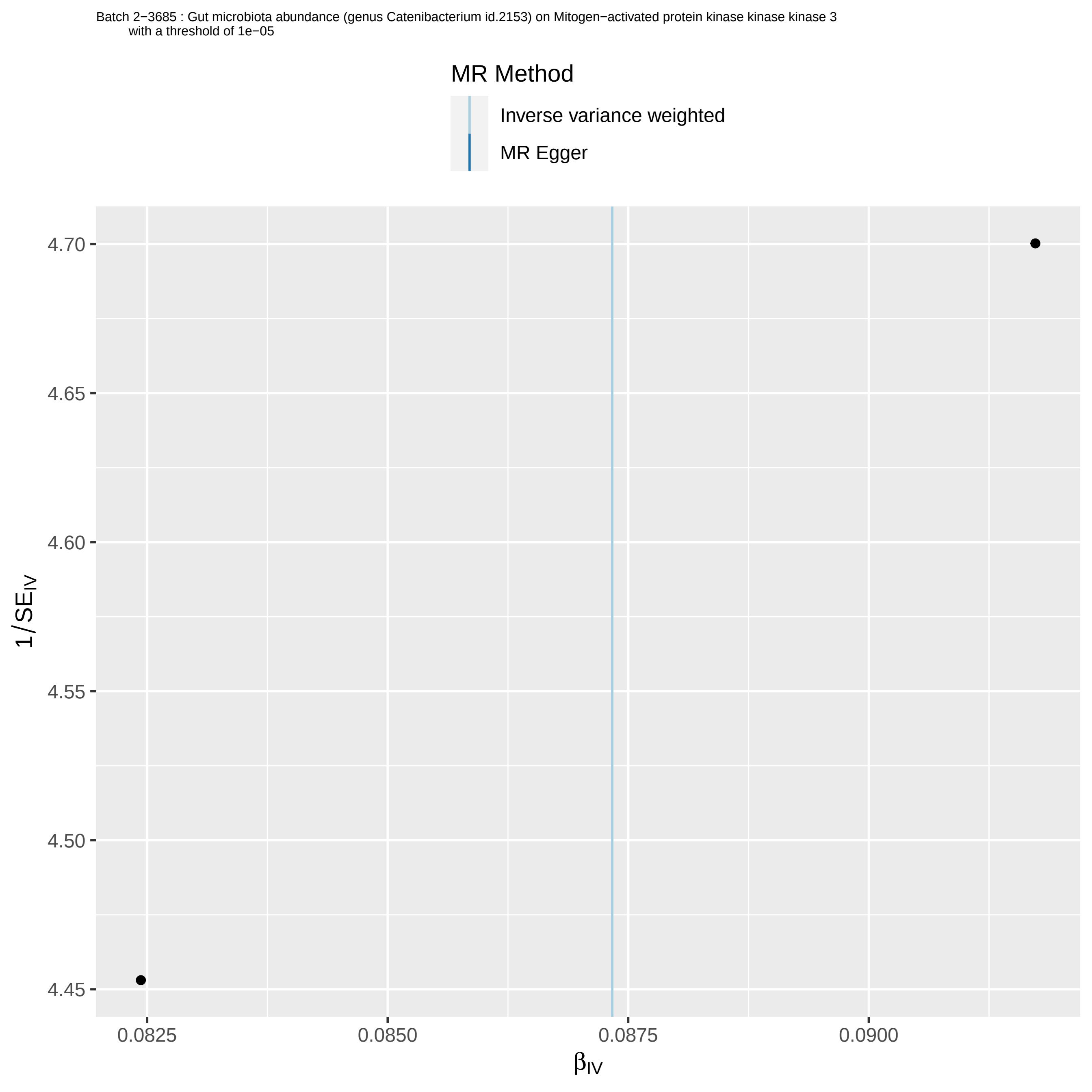

Batch 2−3685 : Gut microbiota abundance (genus Catenibacterium id.2153) on Mitogen−activated protein kinase kinase kinase 3
with a threshold of 1e−05
MR Method
Inverse variance weighted
MR Egger
4.70
4.65
4.60
4.55
4.50
4.45
1/SE$_{IV}$
0.0825
0.0850
0.0875
0.0900
β$_{IV}$

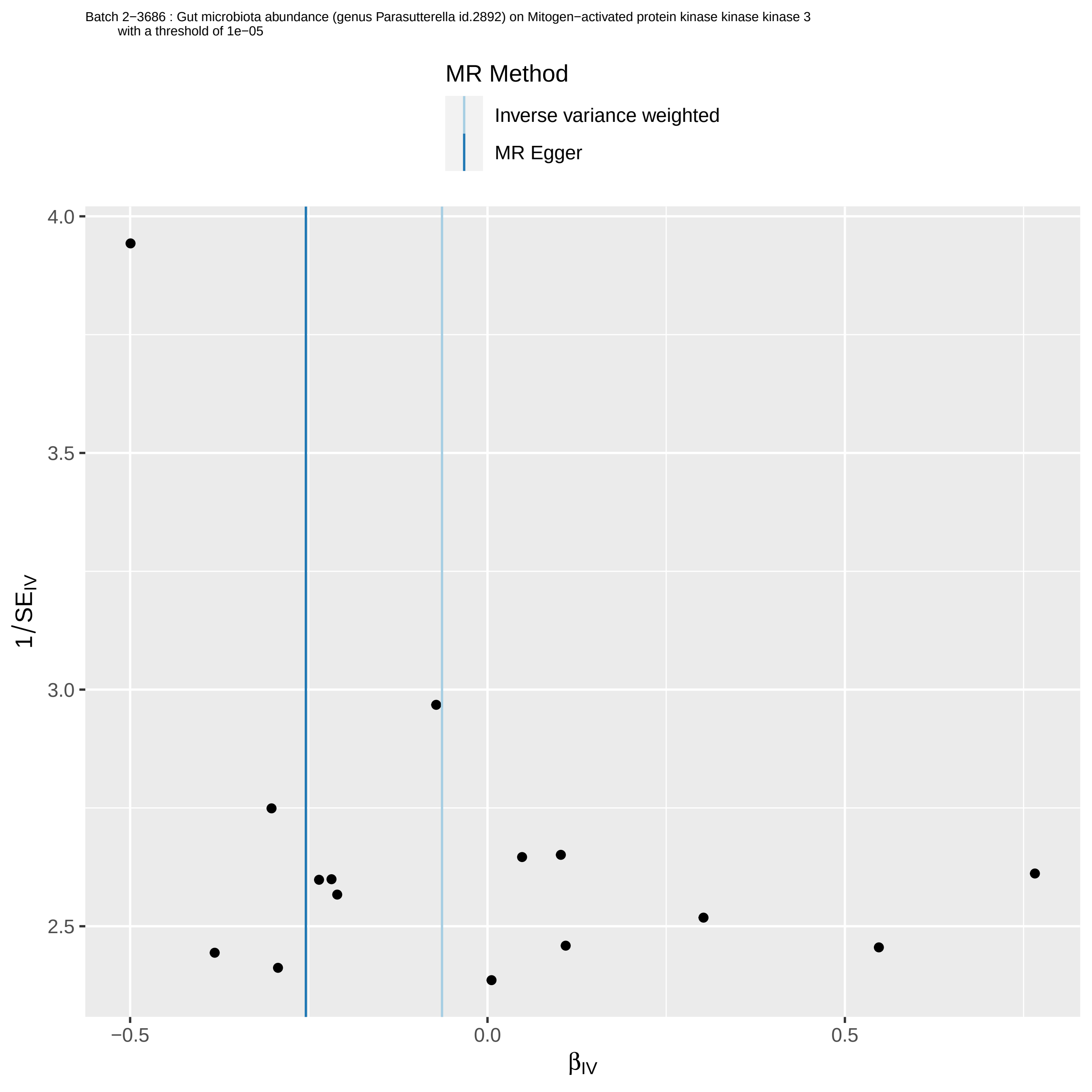
Batch 2−3686 : Gut microbiota abundance (genus Parasutterella id.2892) on Mitogen−activated protein kinase kinase kinase 3
with a threshold of 1e−05
MR Method
Inverse variance weighted
MR Egger
4.0
3.5
3.0
2.5
1/SE_IV
−0.5
0.0
0.5
β_IV

Batch 2−3687 : Gut microbiota abundance (genus Catenibacterium id.2153) on Mitogen−activated protein kinase kinase kinase 7:TGF−beta−activated kinase 1 and MAP3K7−b
with a threshold of 1e−05

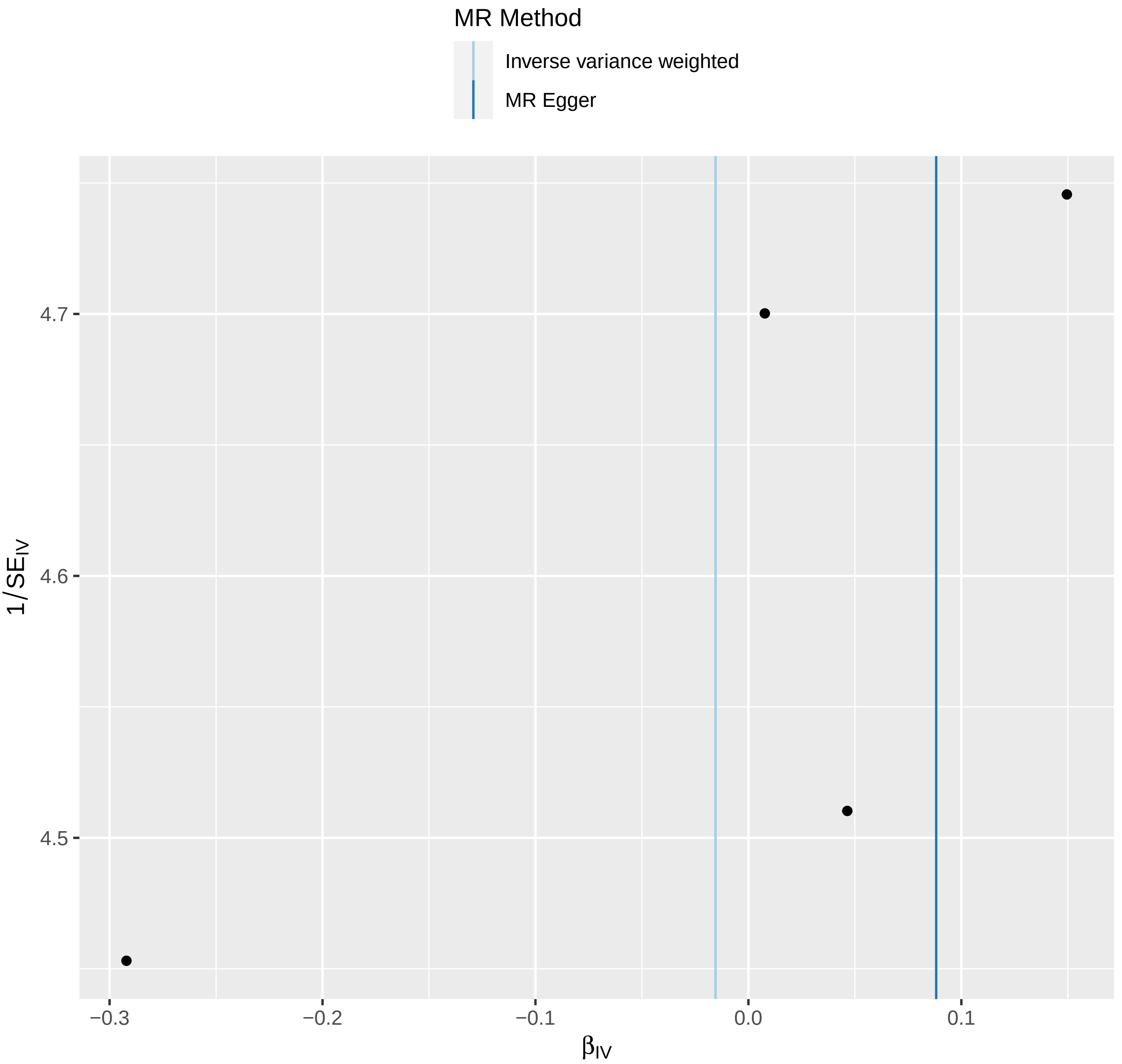

Batch 2−3688 : Gut microbiota abundance (genus Parasutterella id.2892) on Mitogen−activated protein kinase kinase kinase 7:TGF−beta−activated kinase 1 and MAP3K7−binc
with a threshold of 1e−05

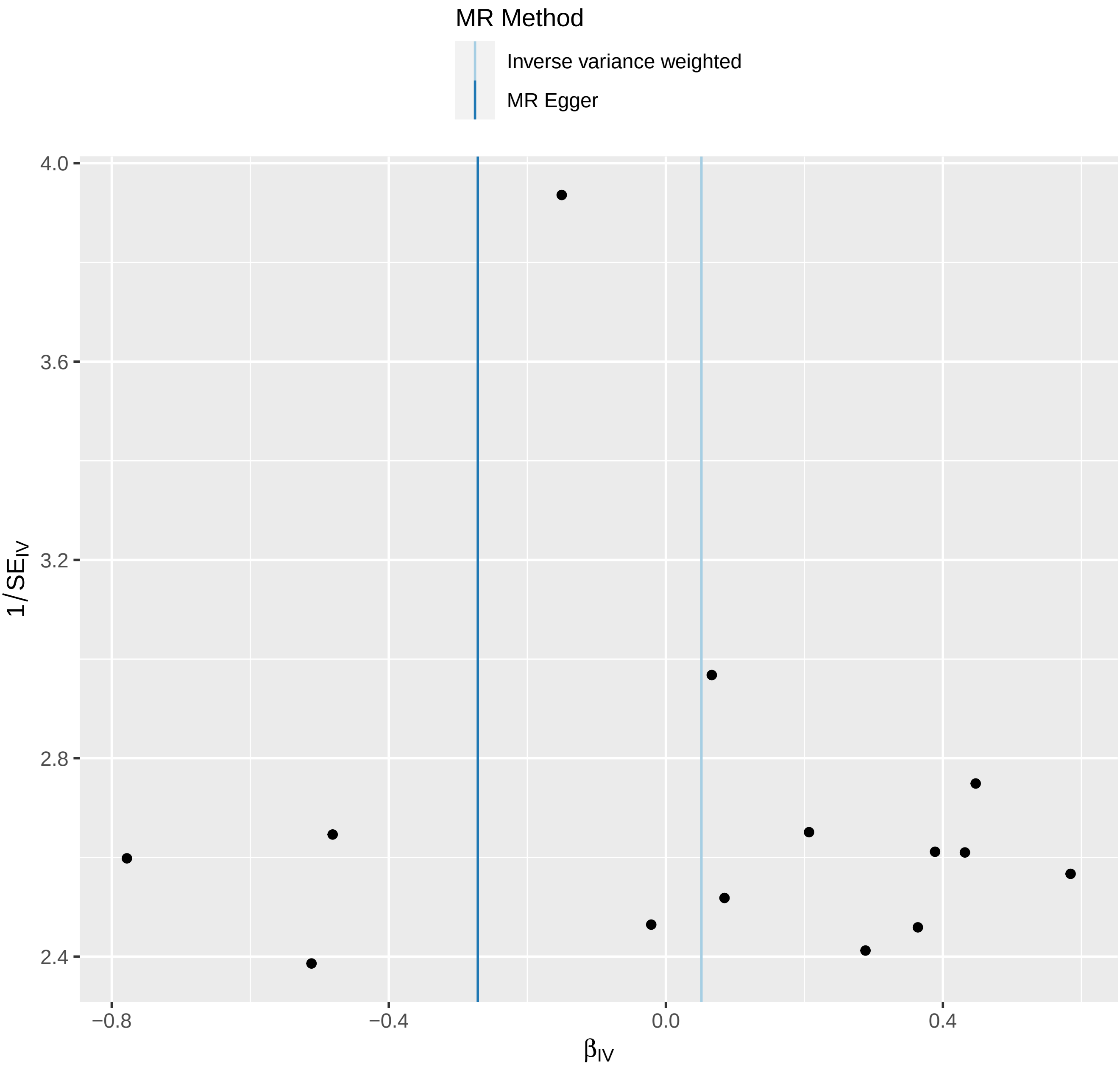

Batch 2−3689 : Gut microbiota abundance (genus Catenibacterium id.2153) on Mitogen−activated protein kinase 1
with a threshold of 1e−05

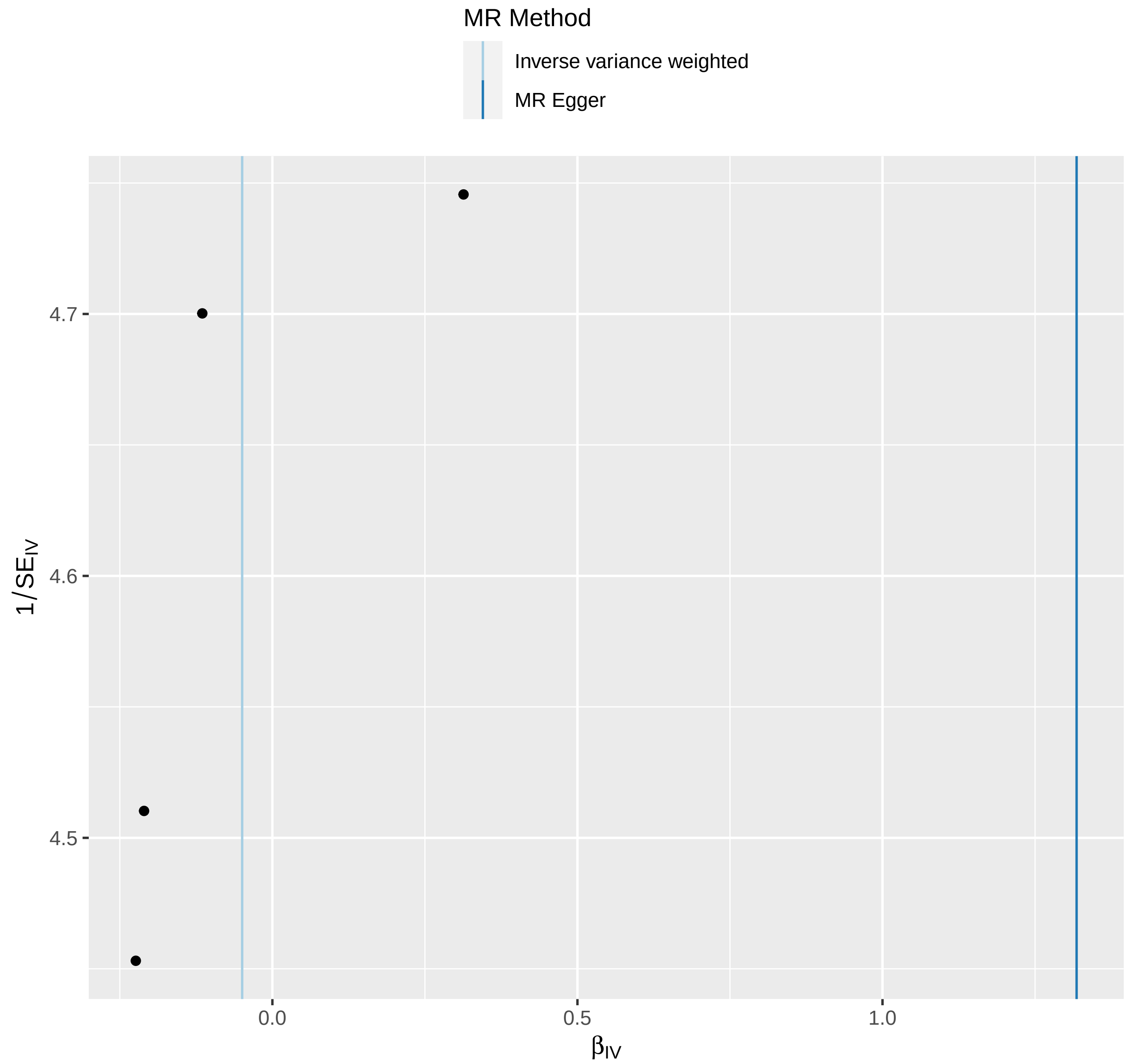

Batch 2−3690 : Gut microbiota abundance (genus Parasutterella id.2892) on Mitogen−activated protein kinase 1
with a threshold of 1e−05

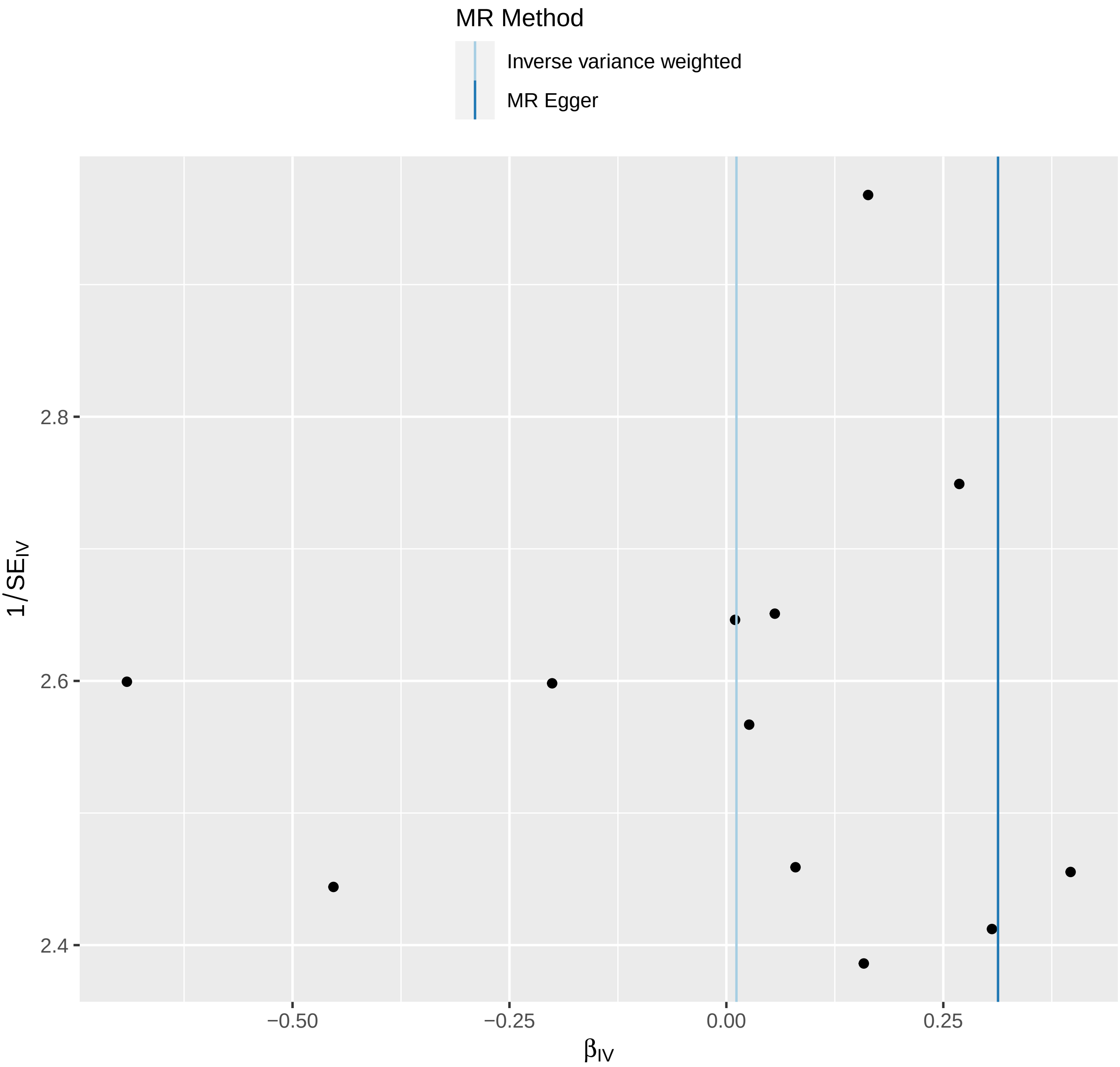

Batch 2−3691 : Gut microbiota abundance (genus Catenibacterium id.2153) on Mitogen−activated protein kinase 13
with a threshold of 1e−05

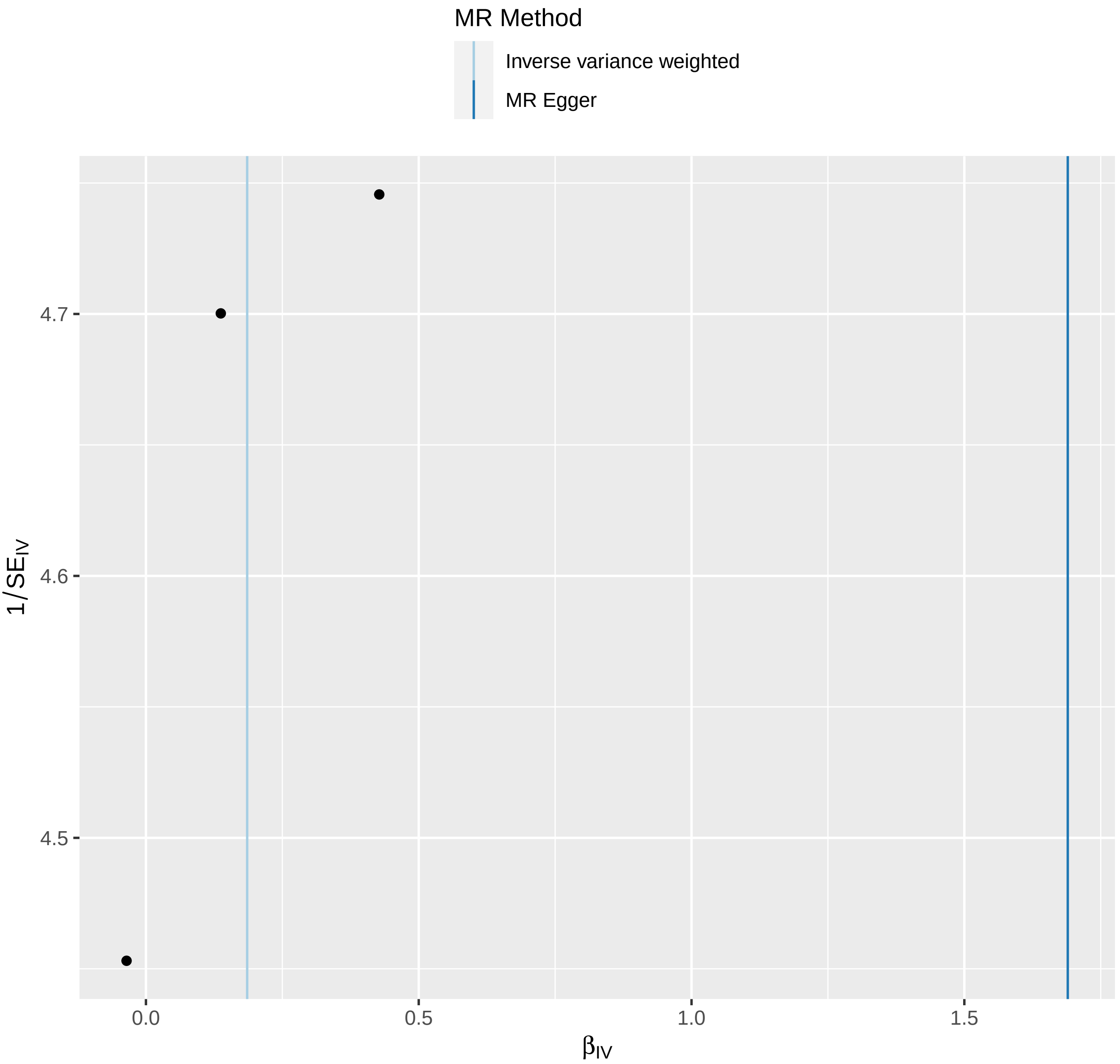

Batch 2−3692 : Gut microbiota abundance (genus Parasutterella id.2892) on Mitogen−activated protein kinase 13 with a threshold of 1e−05

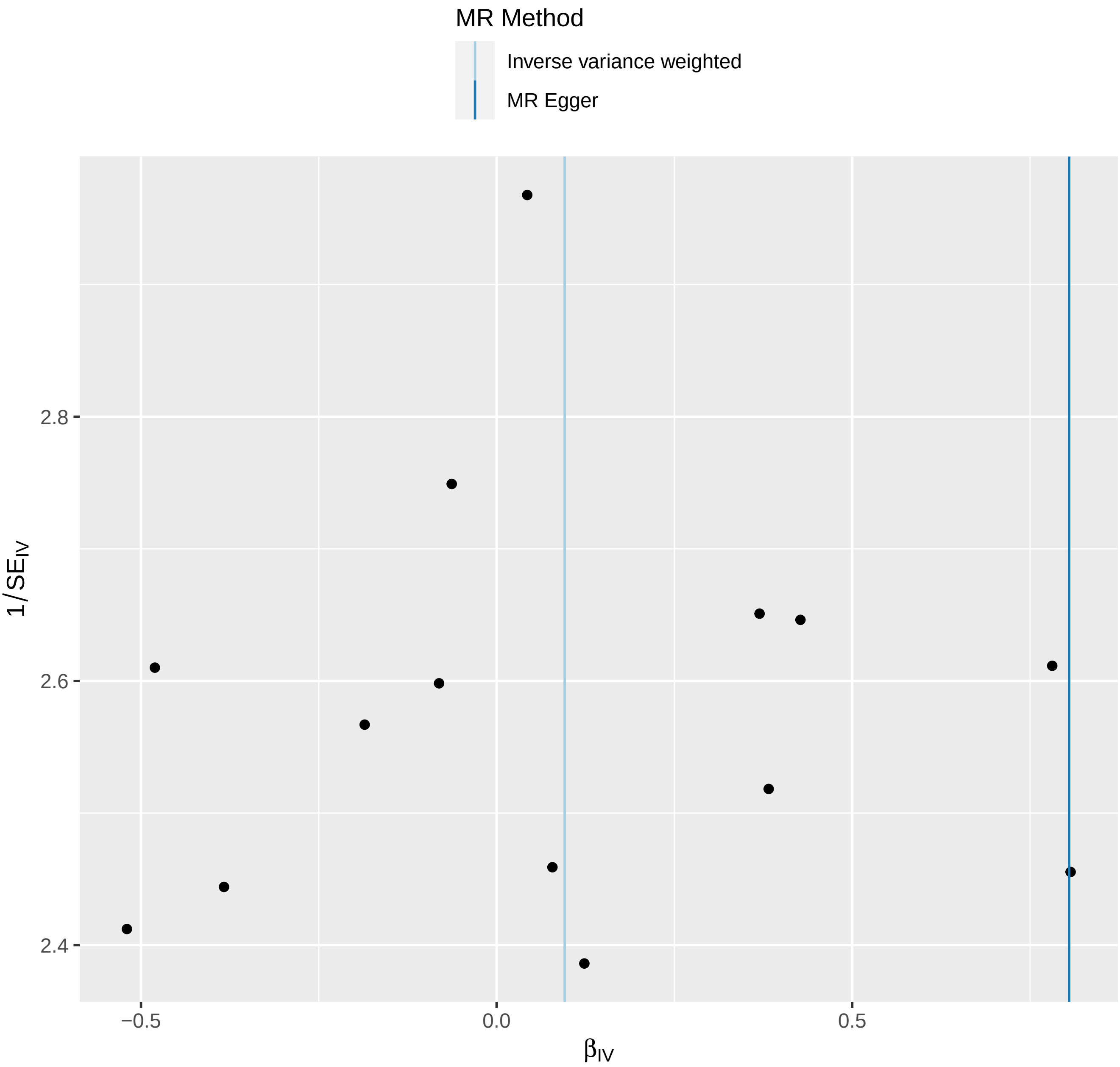

Batch 2−3693 : Gut microbiota abundance (genus Catenibacterium id.2153) on Mitogen−activated protein kinase 14
with a threshold of 1e−05

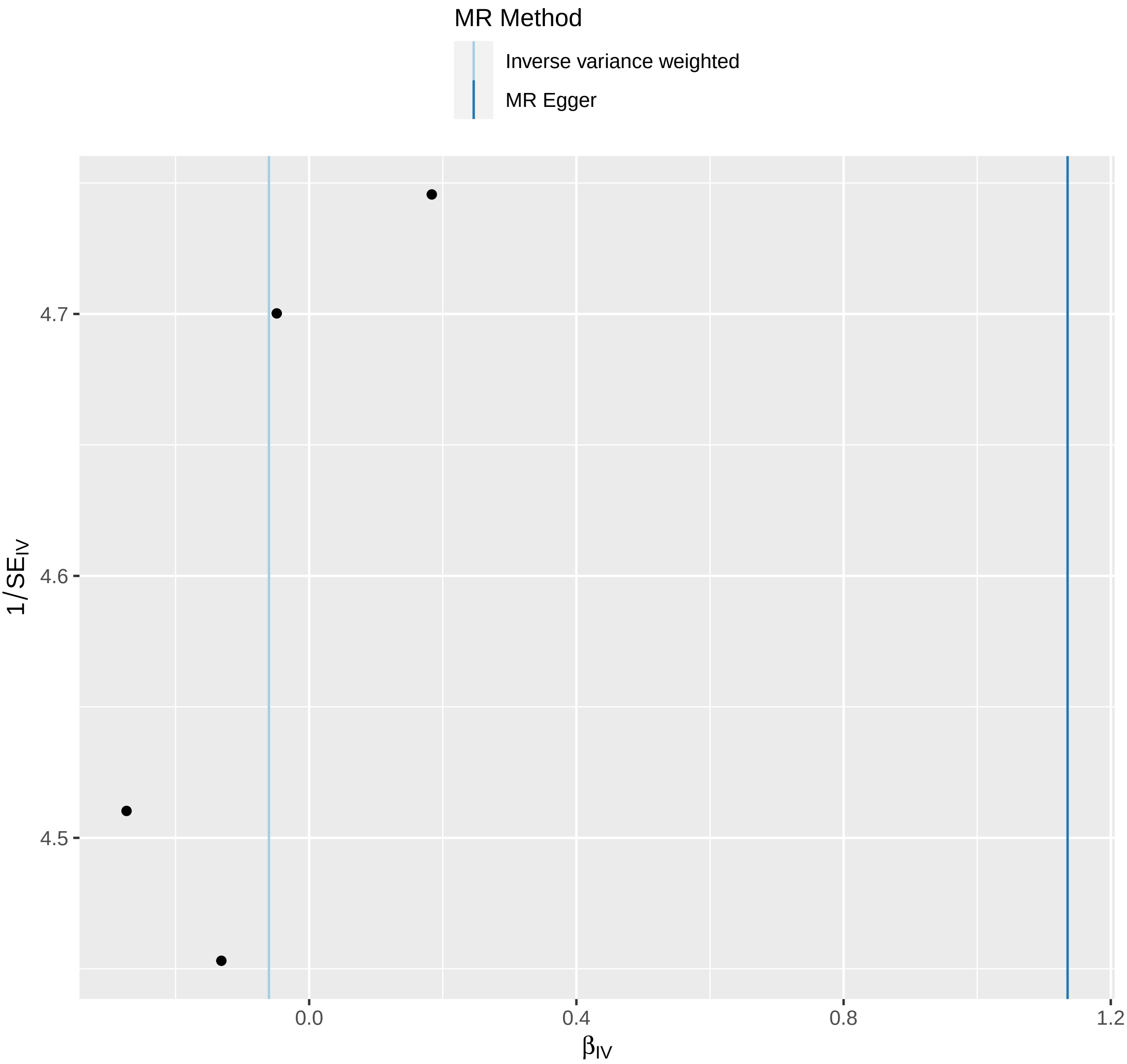

Batch 2−3694 : Gut microbiota abundance (genus Parasutterella id.2892) on Mitogen−activated protein kinase 14
with a threshold of 1e−05

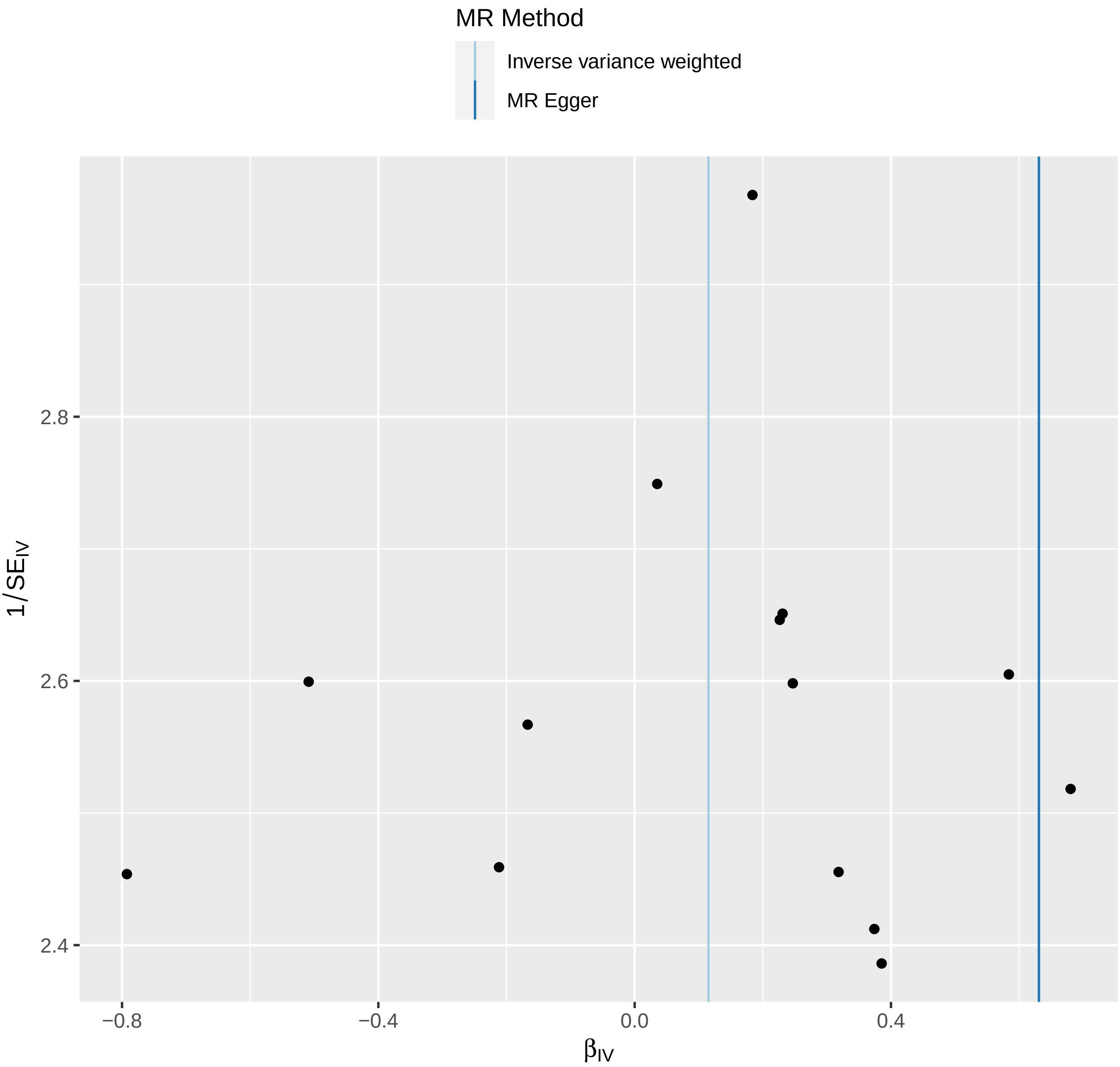

Batch 2−3695 : Gut microbiota abundance (genus Catenibacterium id.2153) on Mitogen−activated protein kinase 3
with a threshold of 1e−05

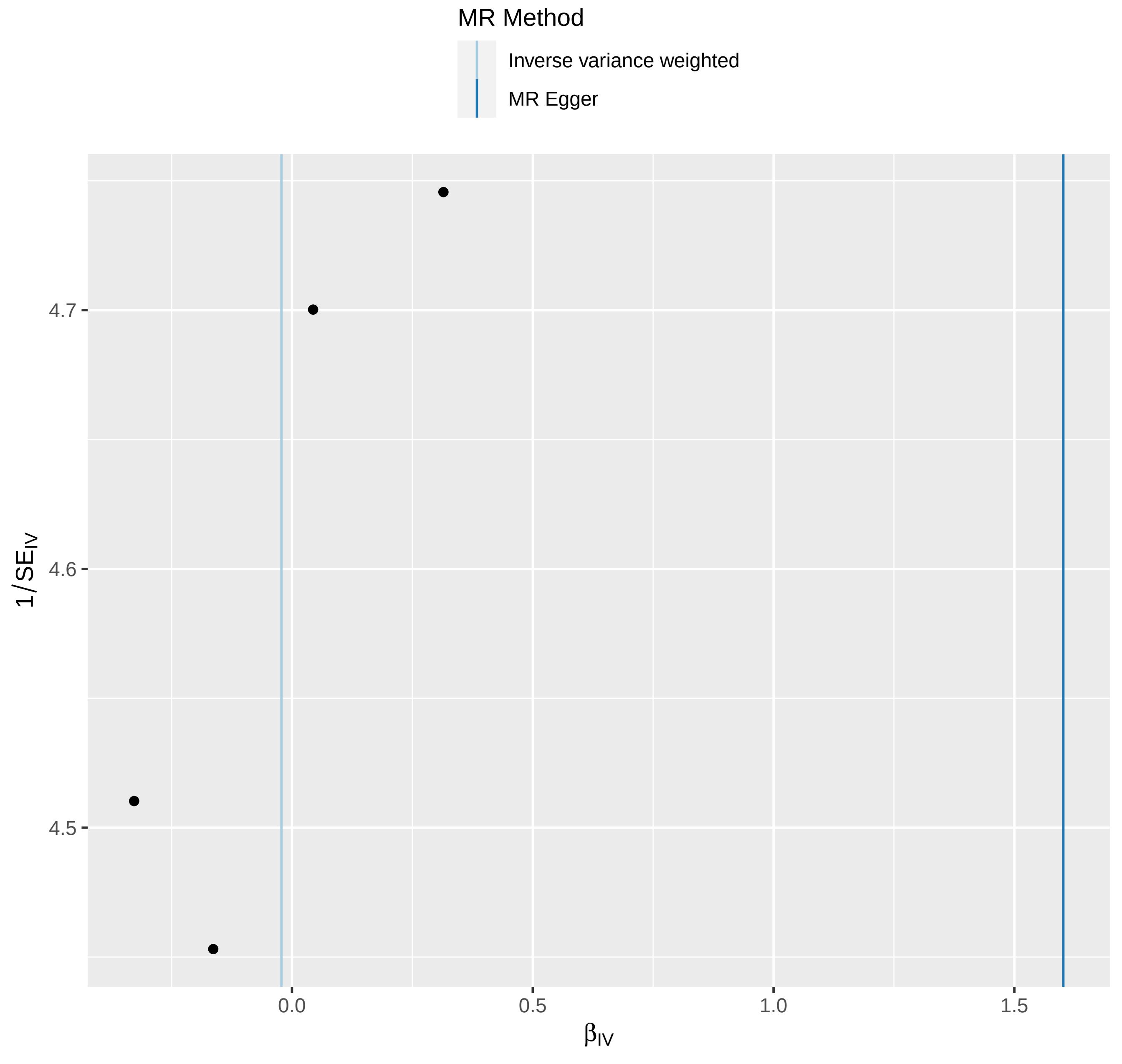

Batch 2−3696 : Gut microbiota abundance (genus Parasutterella id.2892) on Mitogen−activated protein kinase 3
with a threshold of 1e−05

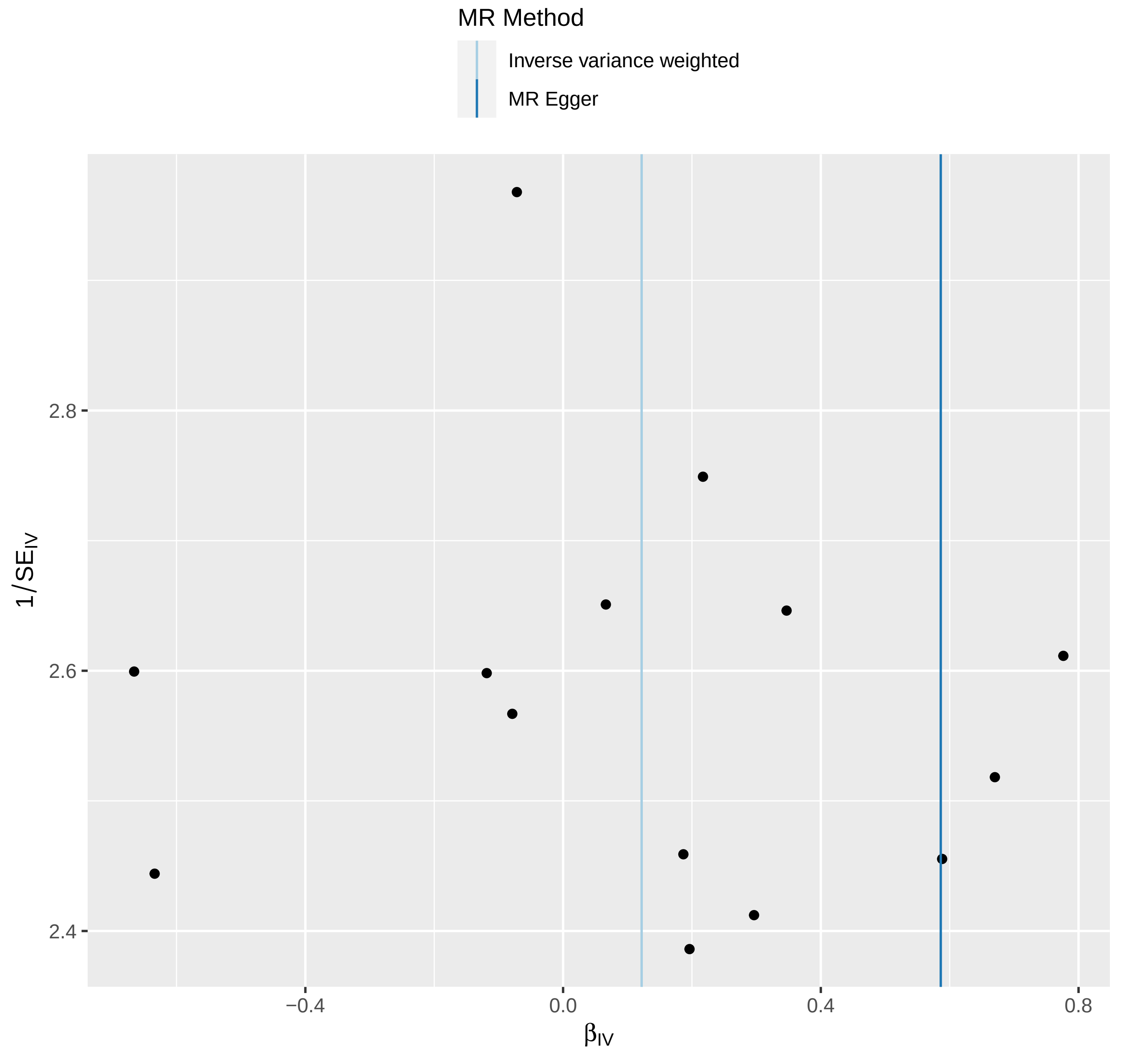

Batch 2−3697 : Gut microbiota abundance (genus Catenibacterium id.2153) on Mitogen−activated protein kinase 8
with a threshold of 1e−05

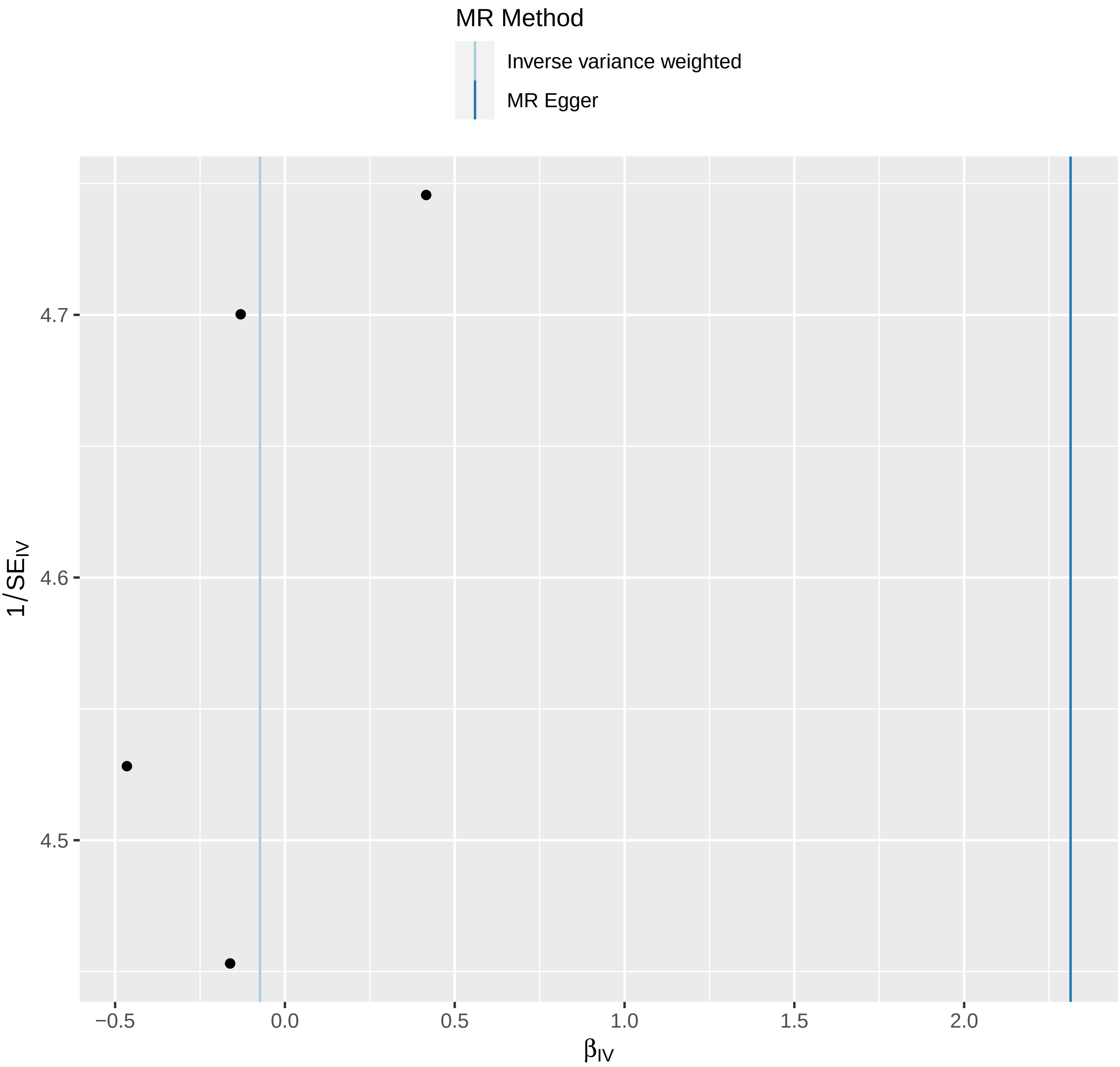

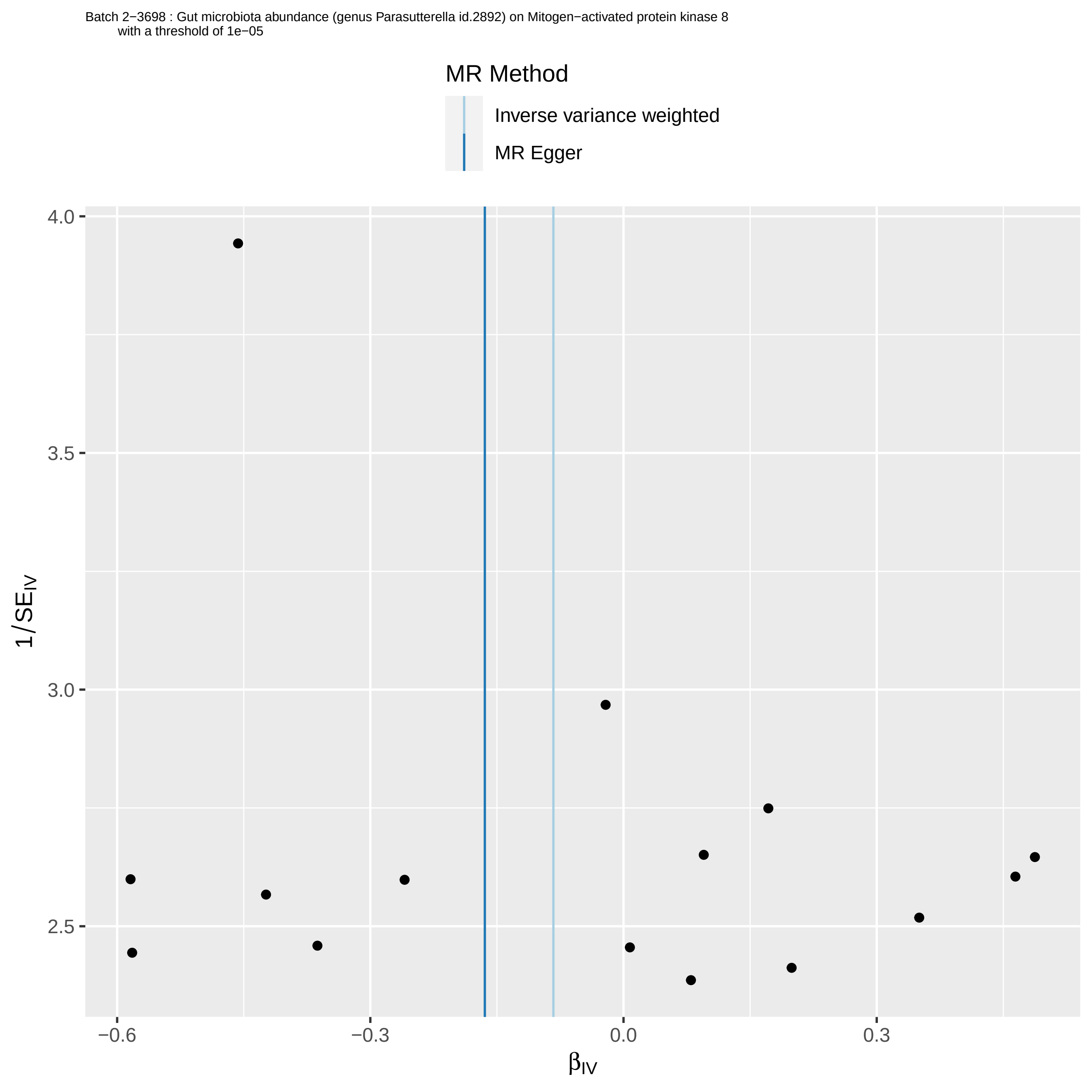
Batch 2−3698 : Gut microbiota abundance (genus Parasutterella id.2892) on Mitogen−activated protein kinase 8
with a threshold of 1e−05
MR Method
Inverse variance weighted
MR Egger
4.0
3.5
3.0
2.5
1/SE_IV
−0.6
−0.3
0.0
0.3
β_IV

Batch 2−3699 : Gut microbiota abundance (genus Catenibacterium id.2153) on Mitogen−activated protein kinase 9
with a threshold of 1e−05

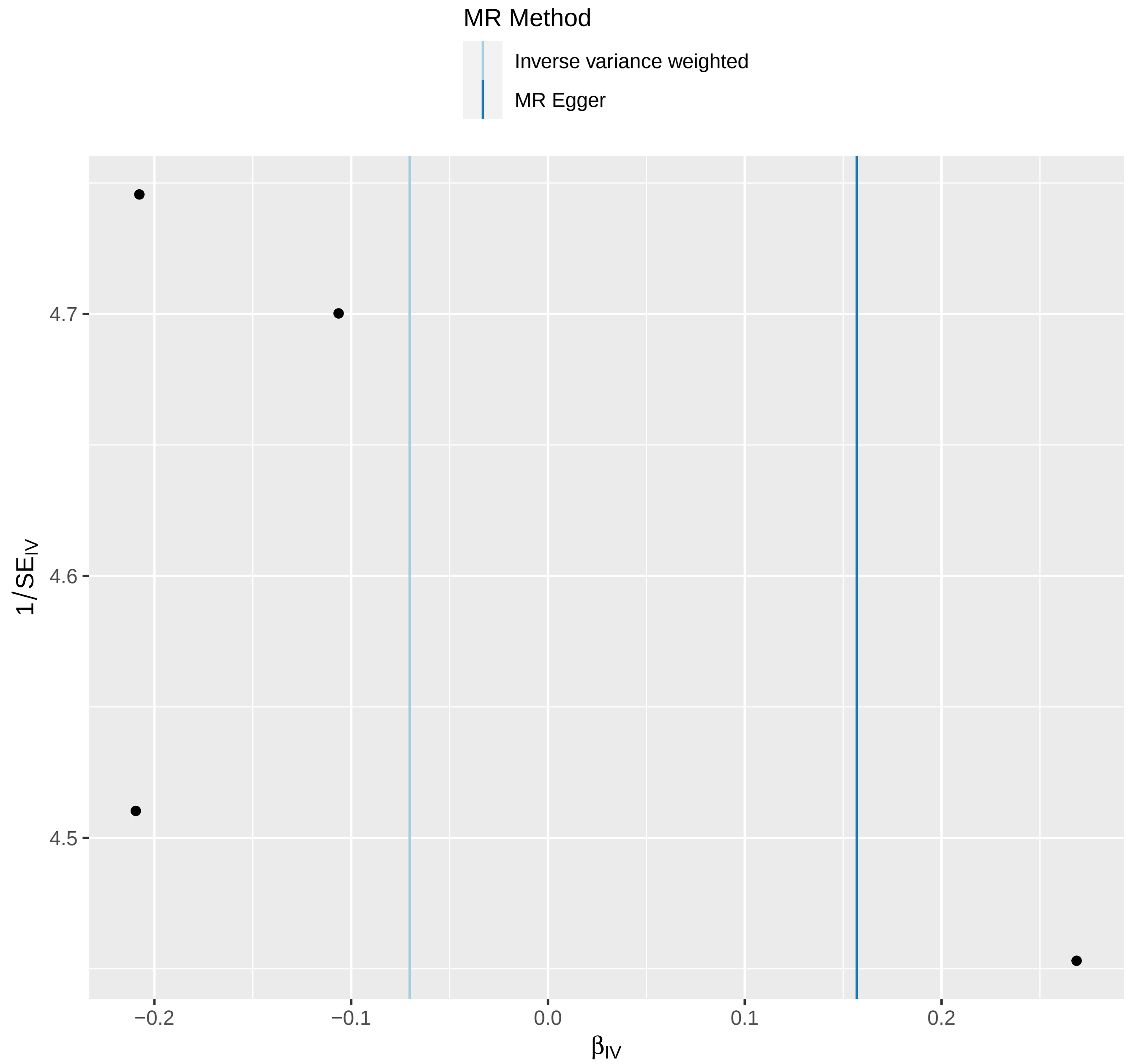

Batch 2−3700 : Gut microbiota abundance (genus Parasutterella id.2892) on Mitogen−activated protein kinase 9
with a threshold of 1e−05

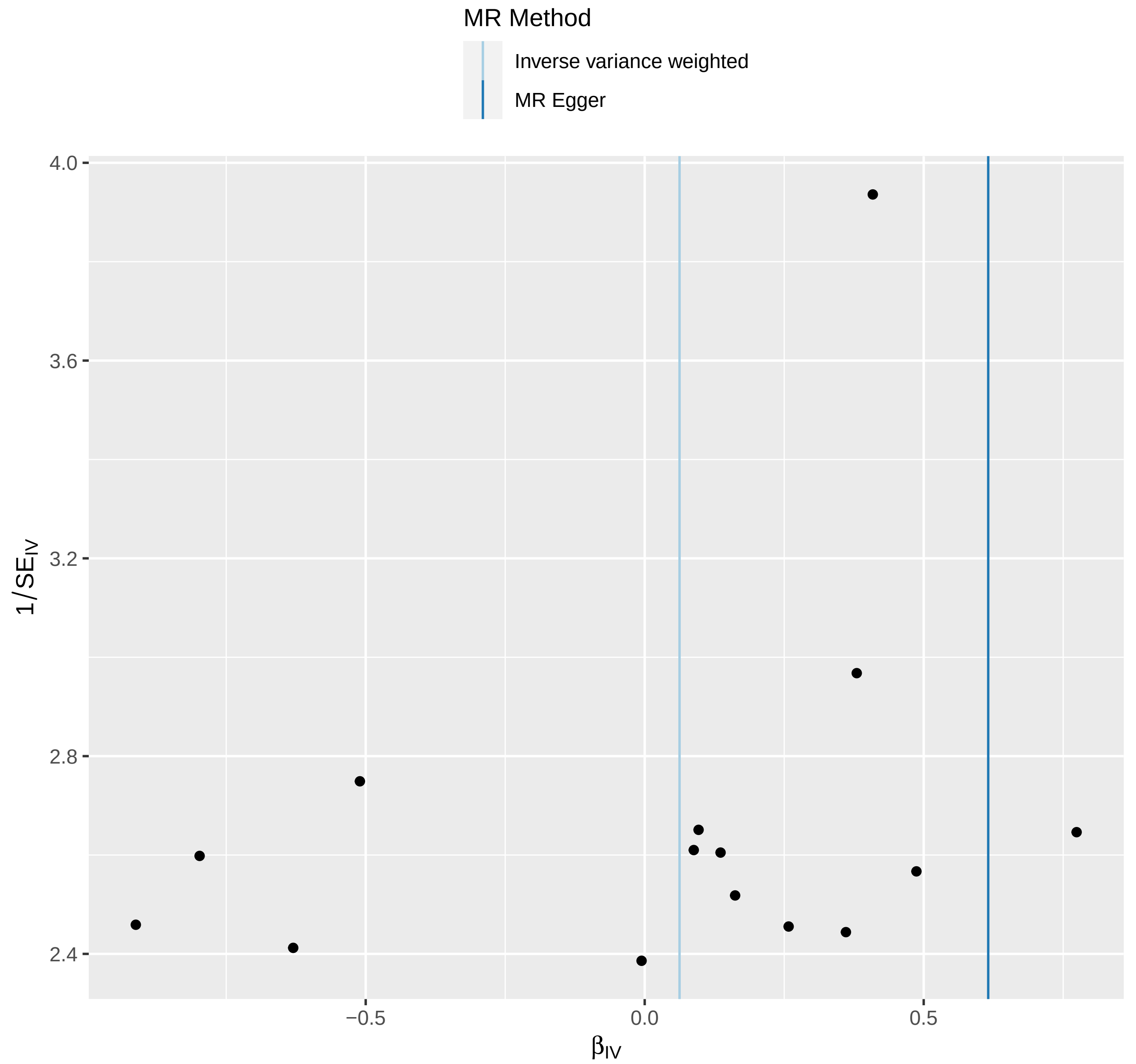

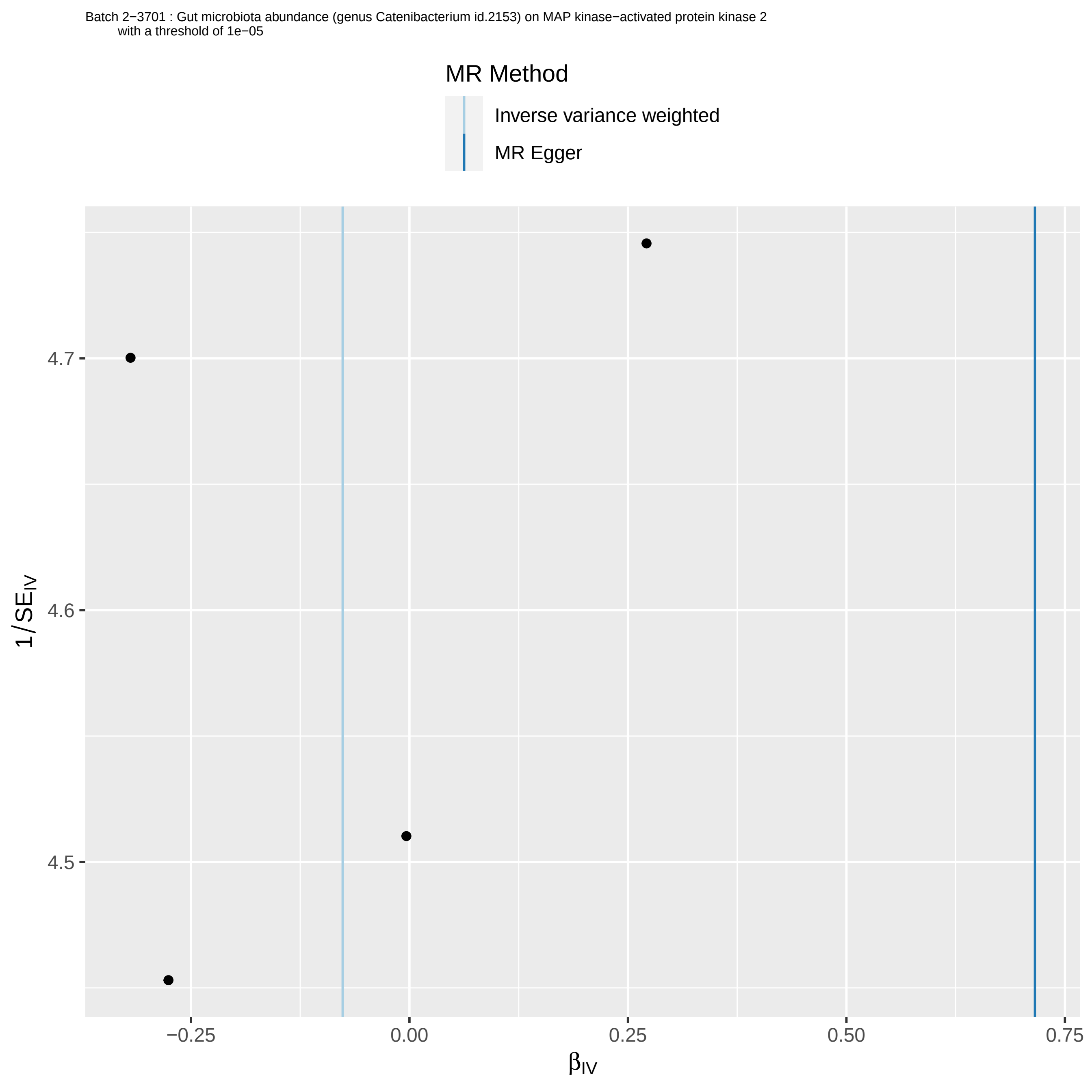
Batch 2−3701 : Gut microbiota abundance (genus Catenibacterium id.2153) on MAP kinase−activated protein kinase 2
with a threshold of 1e−05
MR Method
Inverse variance weighted
MR Egger
4.7
4.6
4.5
1/SE$_{IV}$
−0.25
0.00
0.25
0.50
0.75
β$_{IV}$

Batch 2−3702 : Gut microbiota abundance (genus Parasutterella id.2892) on MAP kinase−activated protein kinase 2 with a threshold of 1e−05

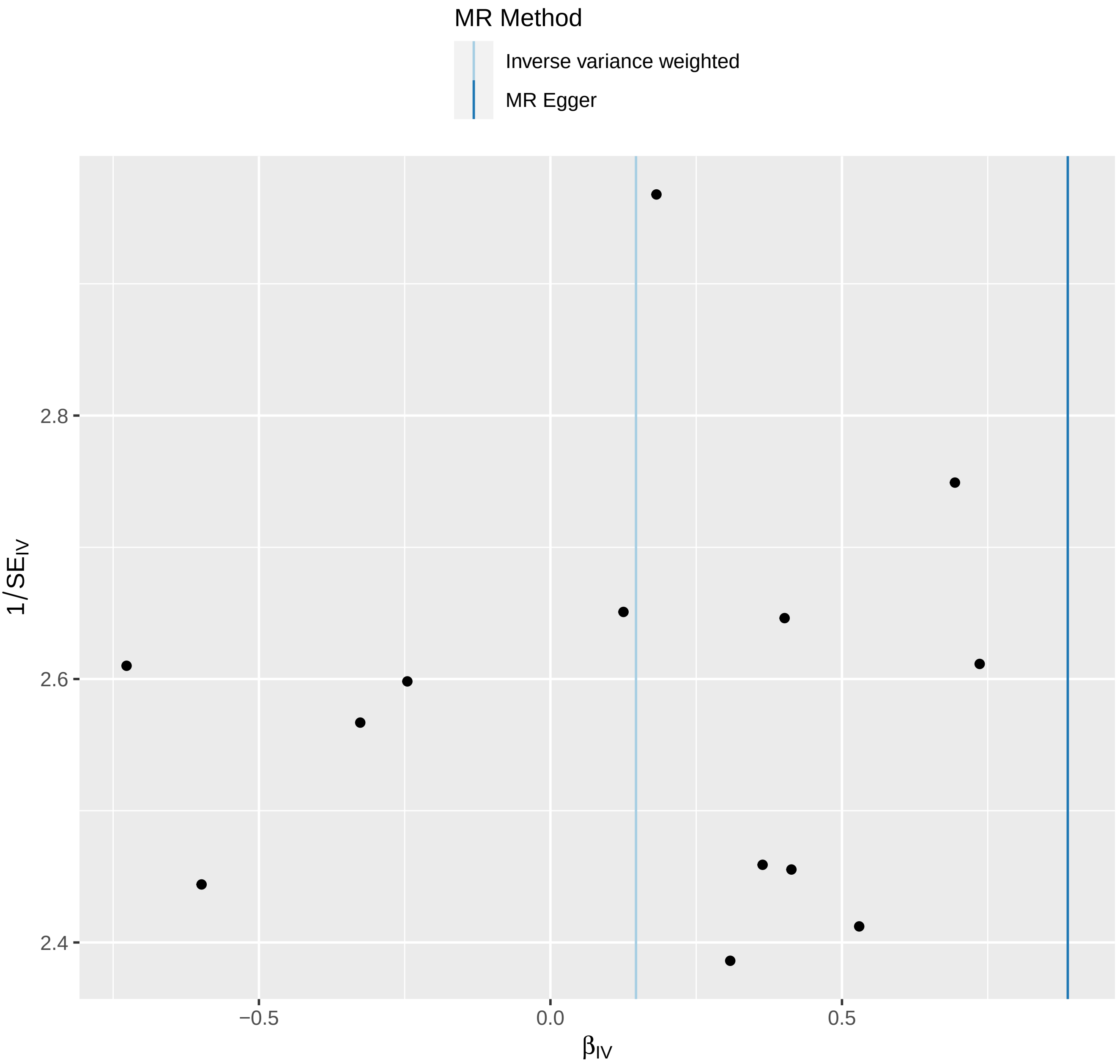

Batch 2−3703 : Gut microbiota abundance (genus Catenibacterium id.2153) on MAP kinase−activated protein kinase 3
with a threshold of 1e−05

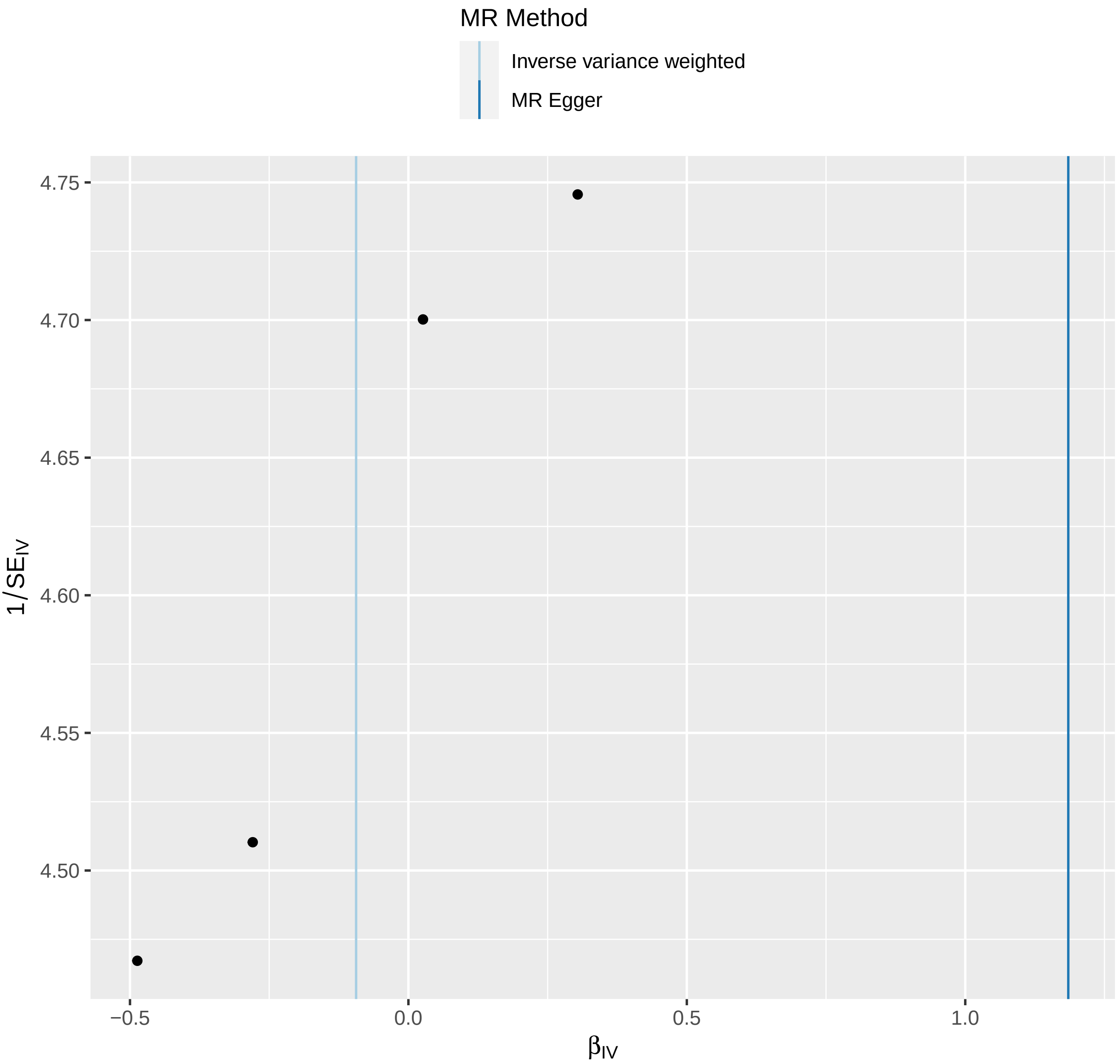

Batch 2−3704 : Gut microbiota abundance (genus Parasutterella id.2892) on MAP kinase−activated protein kinase 3
with a threshold of 1e−05

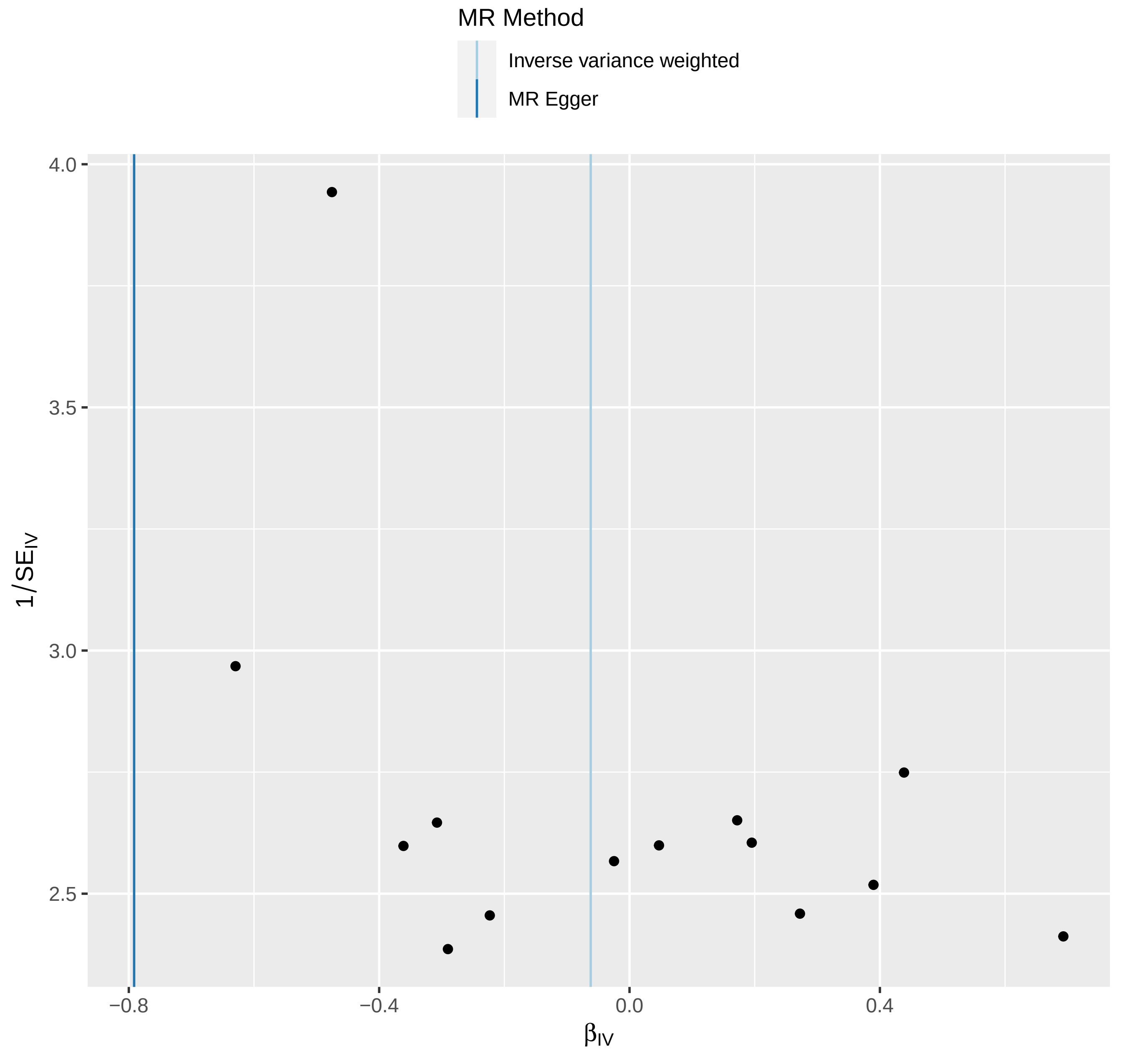

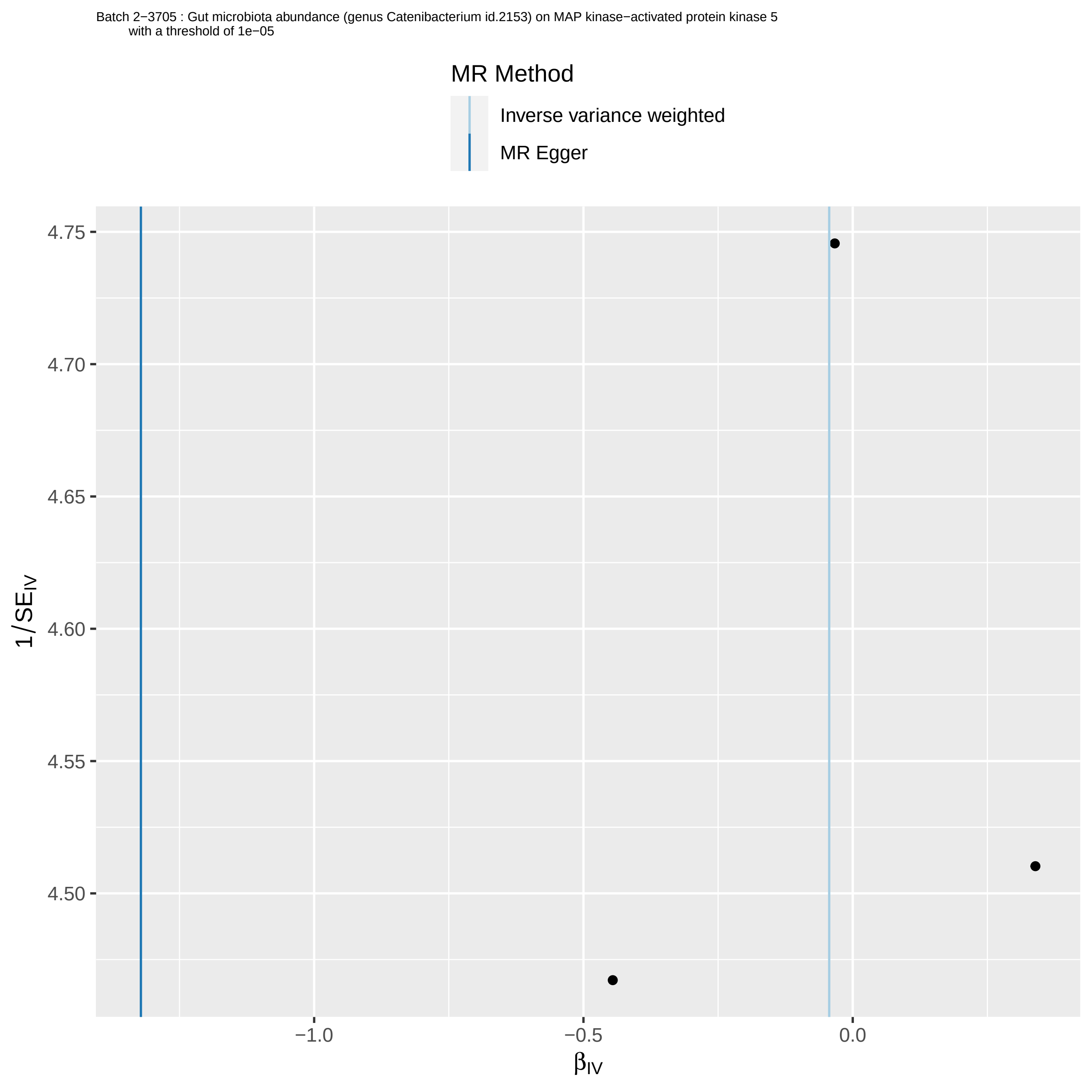
Batch 2−3705 : Gut microbiota abundance (genus Catenibacterium id.2153) on MAP kinase−activated protein kinase 5
with a threshold of 1e−05
MR Method
Inverse variance weighted
MR Egger
4.75
4.70
4.65
4.60
4.55
4.50
1/SE_IV
−1.0
−0.5
0.0
β_IV

Batch 2−3706 : Gut microbiota abundance (genus Parasutterella id.2892) on MAP kinase−activated protein kinase 5
with a threshold of 1e−05

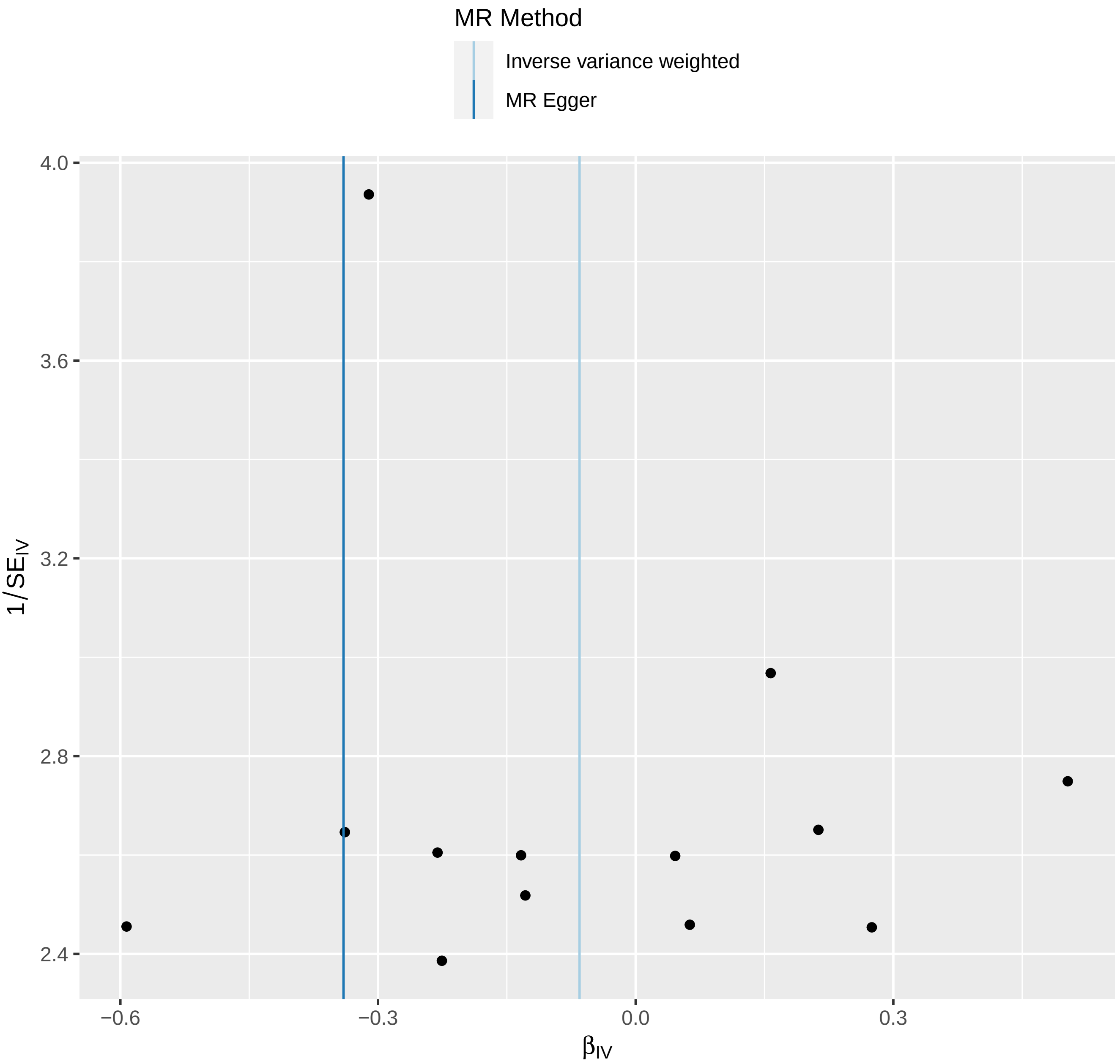

Batch 2−3707 : Gut microbiota abundance (genus Catenibacterium id.2153) on MAP kinase−activated protein kinase 5
with a threshold of 1e−05

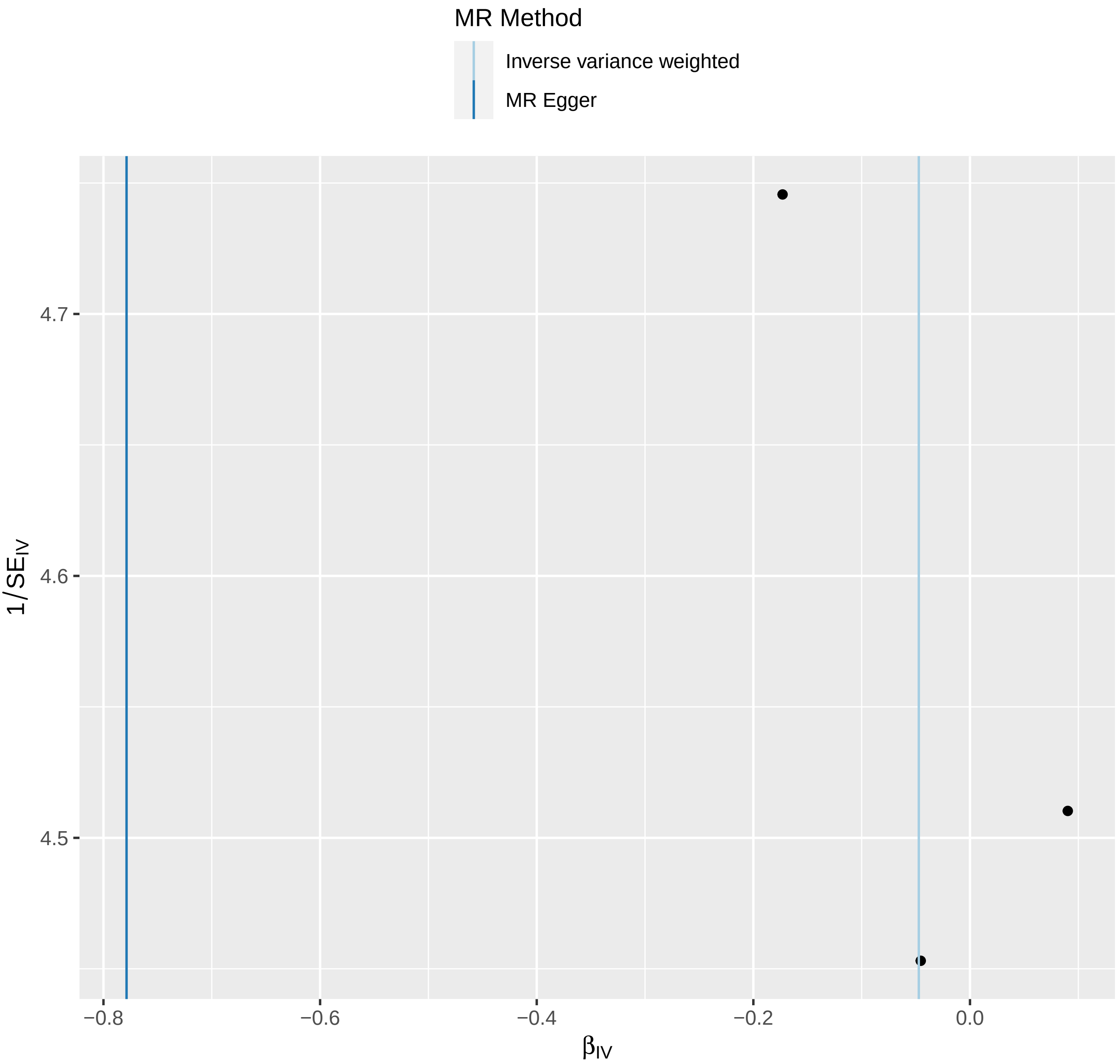

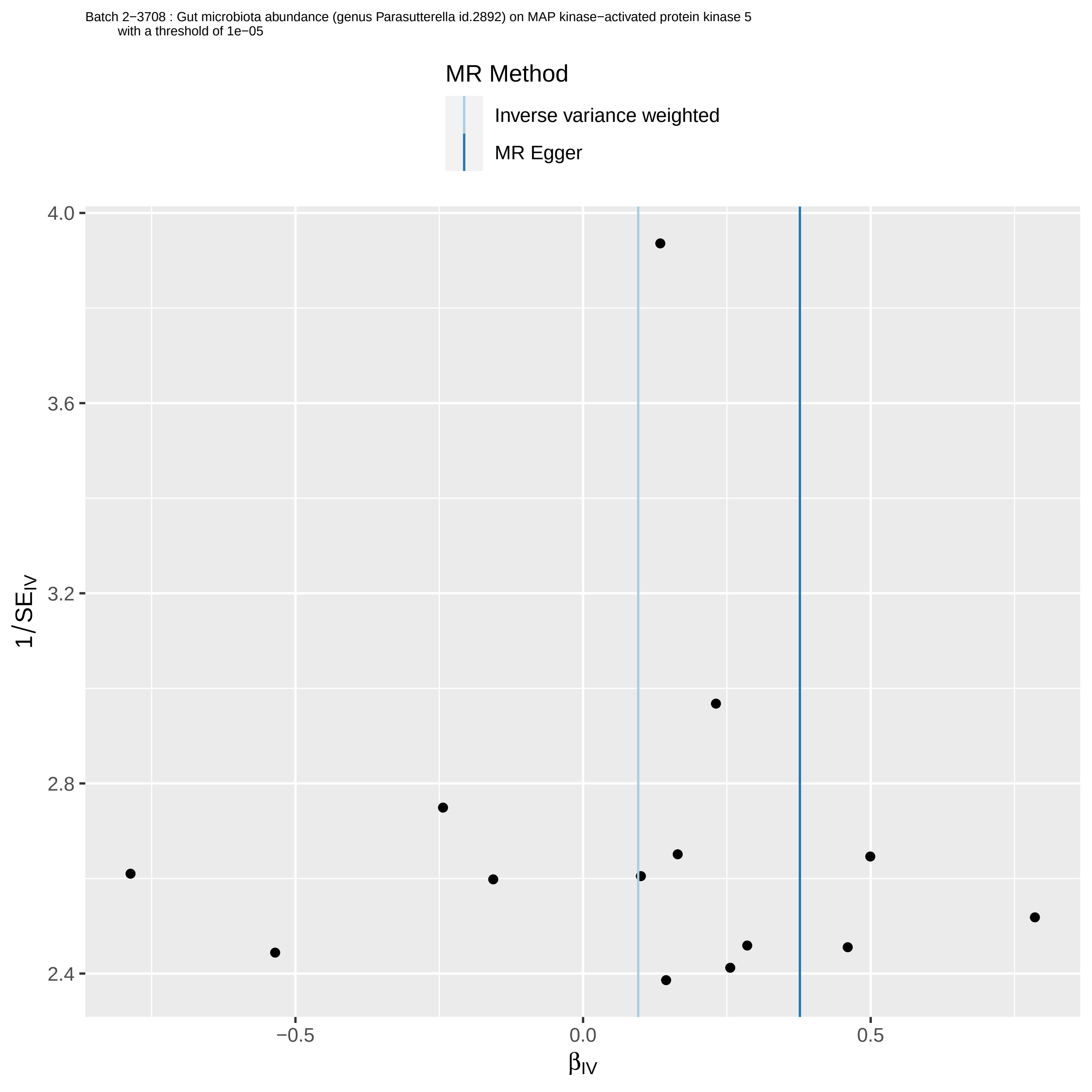

Batch 2−3708 : Gut microbiota abundance (genus Parasutterella id.2892) on MAP kinase−activated protein kinase 5
with a threshold of 1e−05
MR Method
Inverse variance weighted
MR Egger
4.0
3.6
3.2
2.8
2.4
1/SE$_{IV}$
−0.5
0.0
0.5
β_{IV}

Batch 2−3709 : Gut microbiota abundance (genus Catenibacterium id.2153) on Mannan−binding lectin serine protease 1
with a threshold of 1e−05

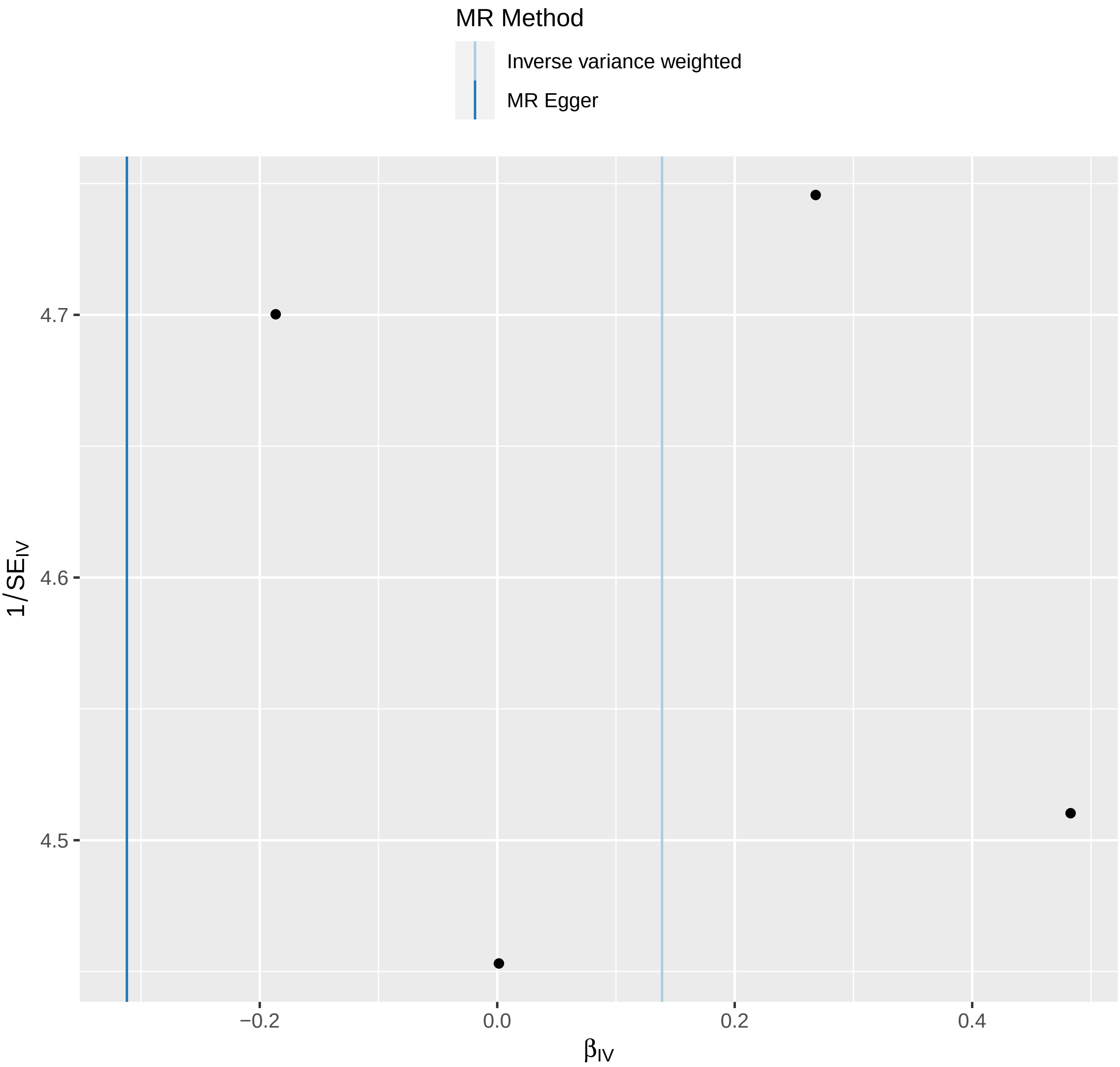

Batch 2−3710 : Gut microbiota abundance (genus Parasutterella id.2892) on Mannan−binding lectin serine protease 1
with a threshold of 1e−05

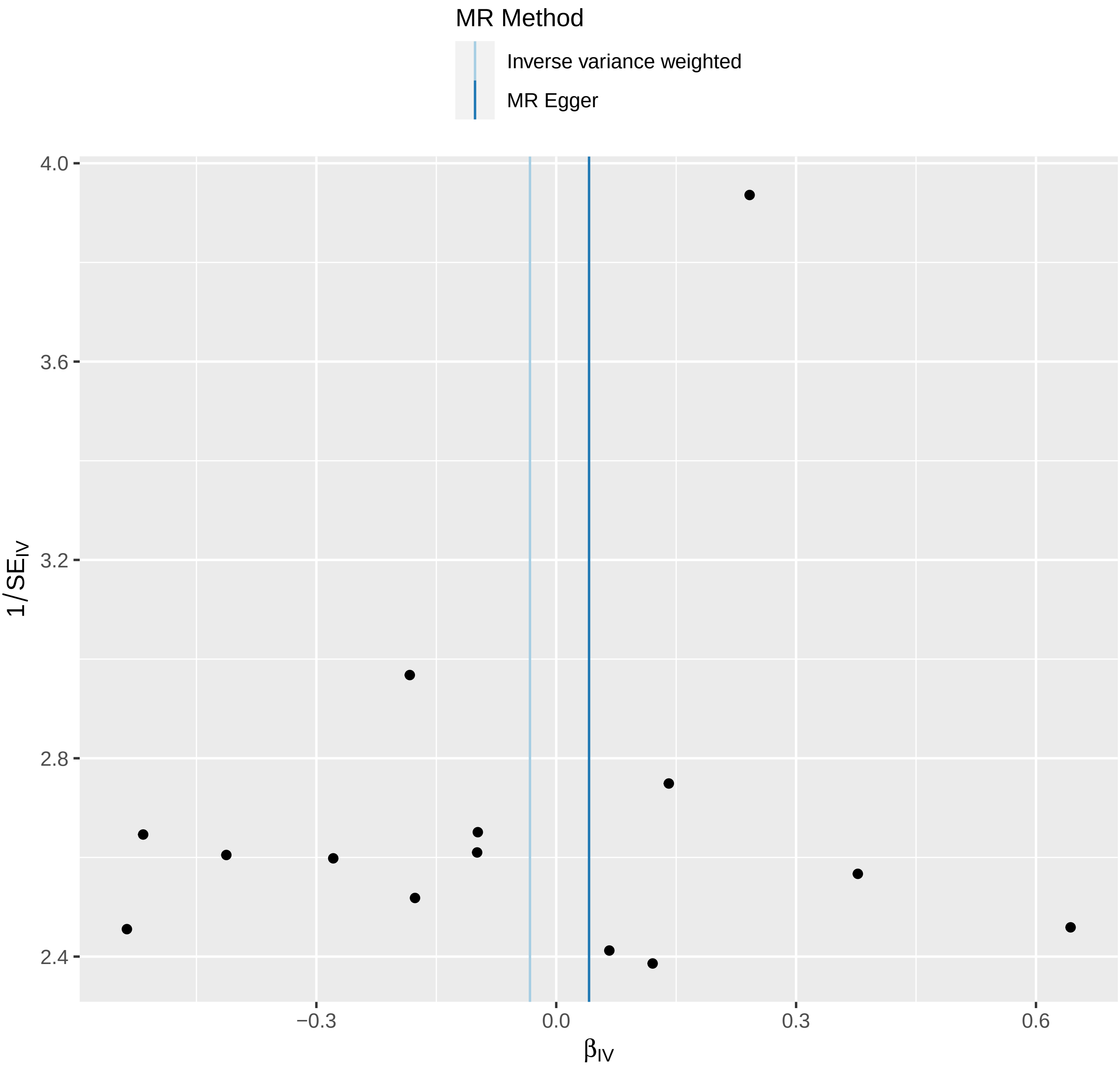

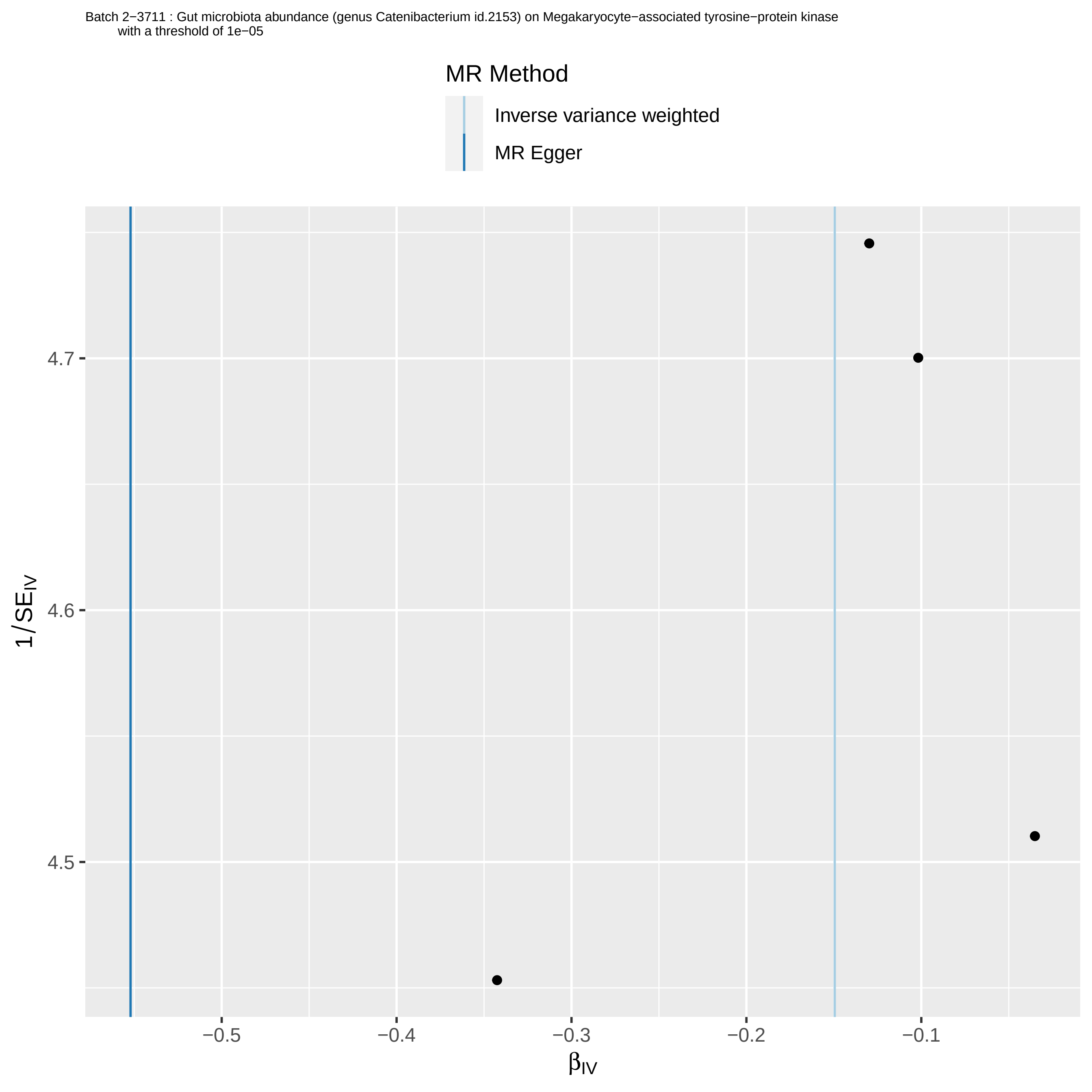

Batch 2−3711 : Gut microbiota abundance (genus Catenibacterium id.2153) on Megakaryocyte−associated tyrosine−protein kinase with a threshold of 1e−05
MR Method
Inverse variance weighted
MR Egger
4.7
4.6
4.5
1/SE_IV
−0.5
−0.4
−0.3
−0.2
−0.1
β_IV

Batch 2−3712 : Gut microbiota abundance (genus Parasutterella id.2892) on Megakaryocyte−associated tyrosine−protein kinase with a threshold of 1e−05

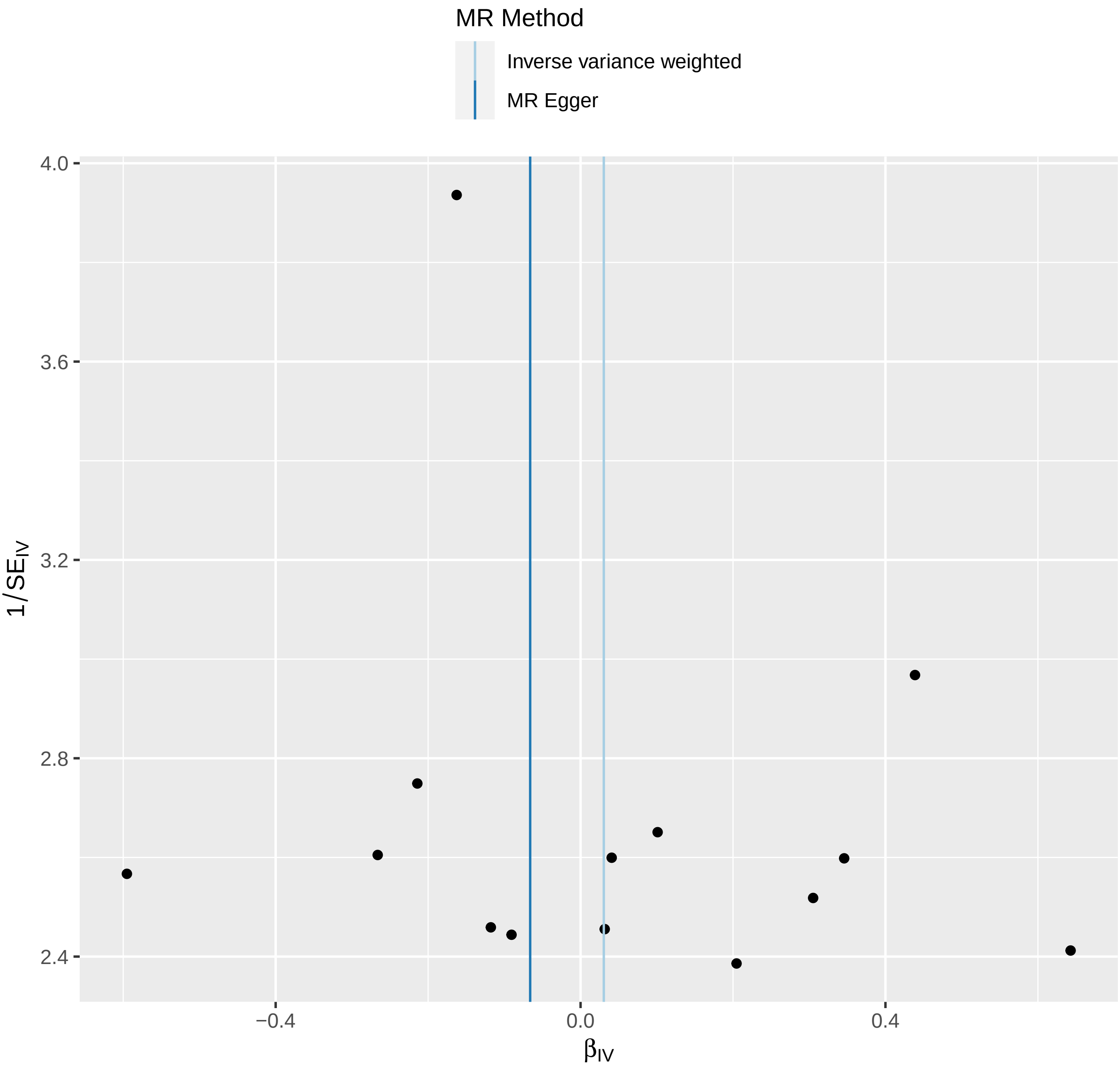

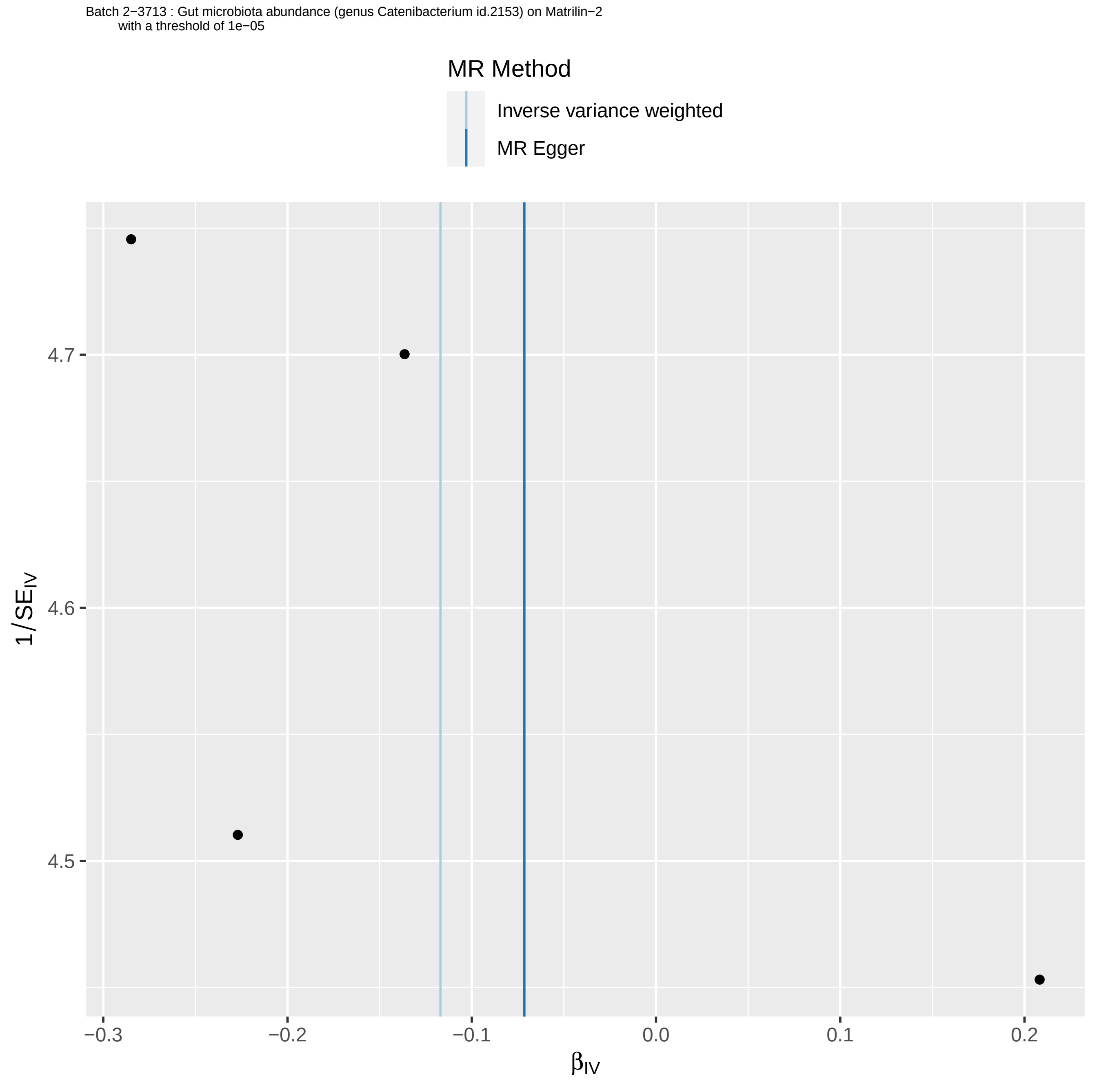

Batch 2−3713 : Gut microbiota abundance (genus Catenibacterium id.2153) on Matrilin−2
with a threshold of 1e−05
MR Method
Inverse variance weighted
MR Egger
4.7
4.6
4.5
1/SE$_{IV}$
−0.3
−0.2
−0.1
0.0
0.1
0.2
β$_{IV}$

Batch 2−3714 : Gut microbiota abundance (genus Parasutterella id.2892) on Matrilin−2
with a threshold of 1e−05

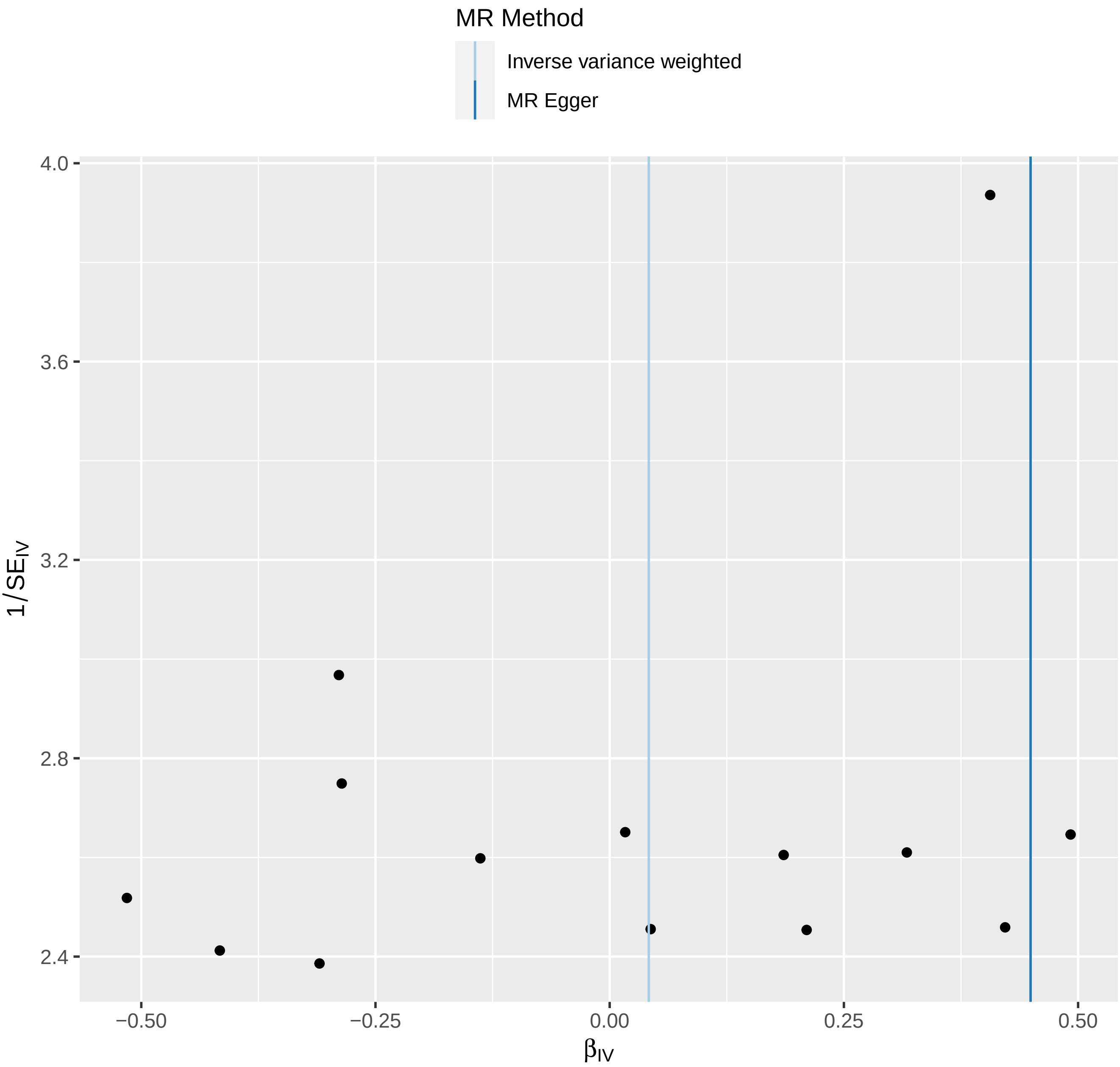

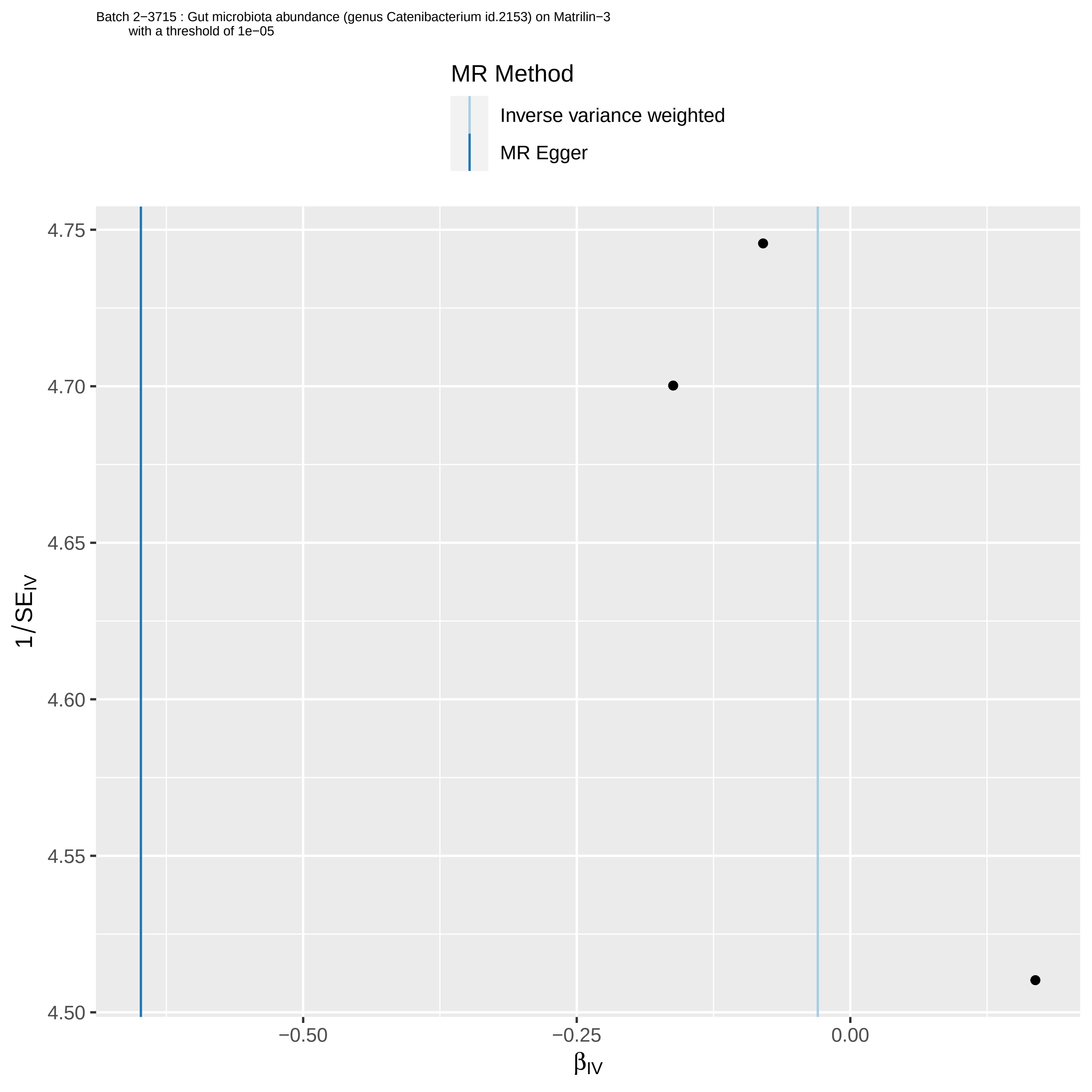

Batch 2−3715 : Gut microbiota abundance (genus Catenibacterium id.2153) on Matrilin−3
with a threshold of 1e−05
MR Method
Inverse variance weighted
MR Egger
4.75
4.70
4.65
4.60
4.55
4.50
1/SE_IV
−0.50
−0.25
0.00
β_IV

Batch 2−3716 : Gut microbiota abundance (genus Parasutterella id.2892) on Matrilin−3
with a threshold of 1e−05

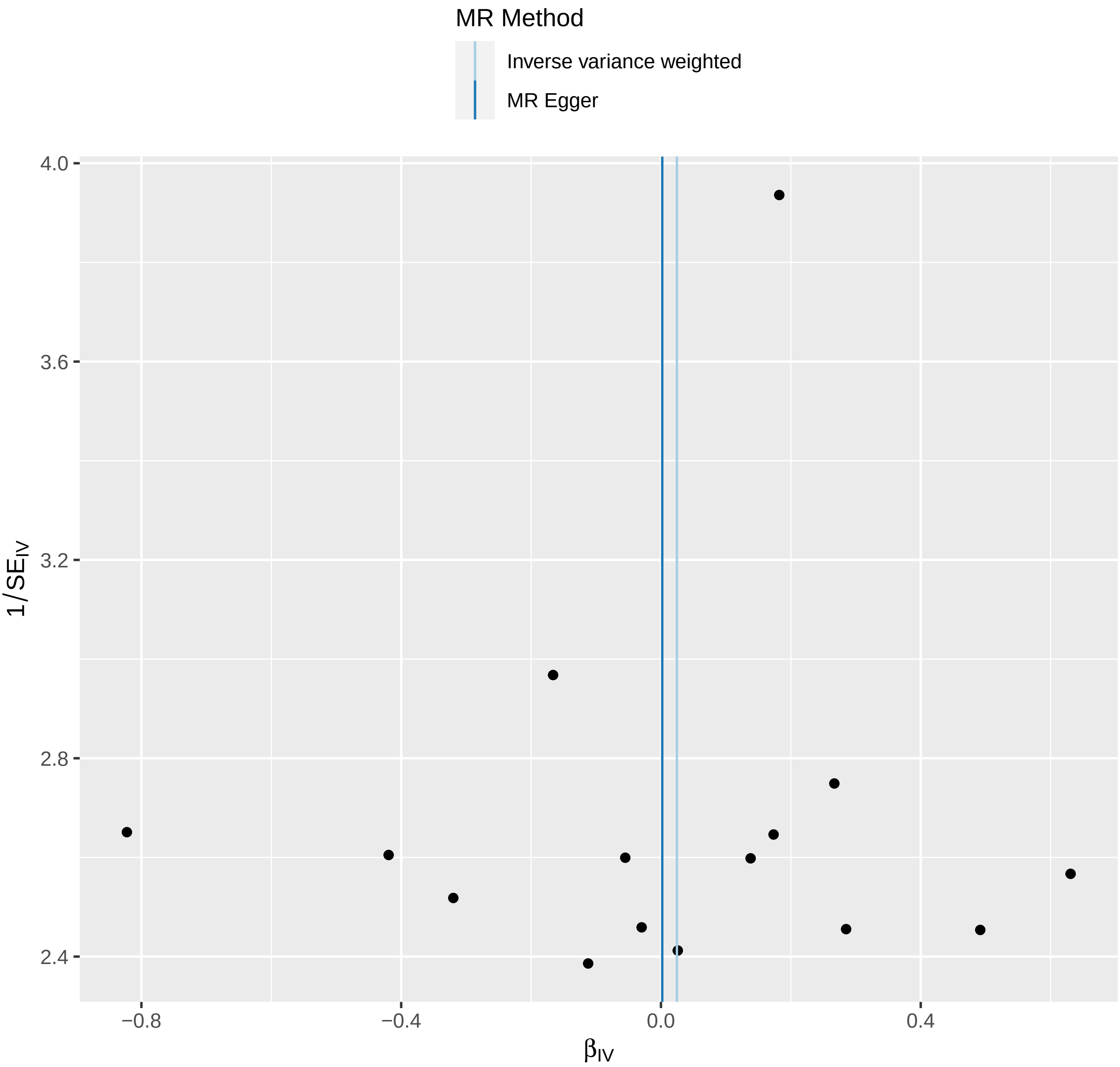

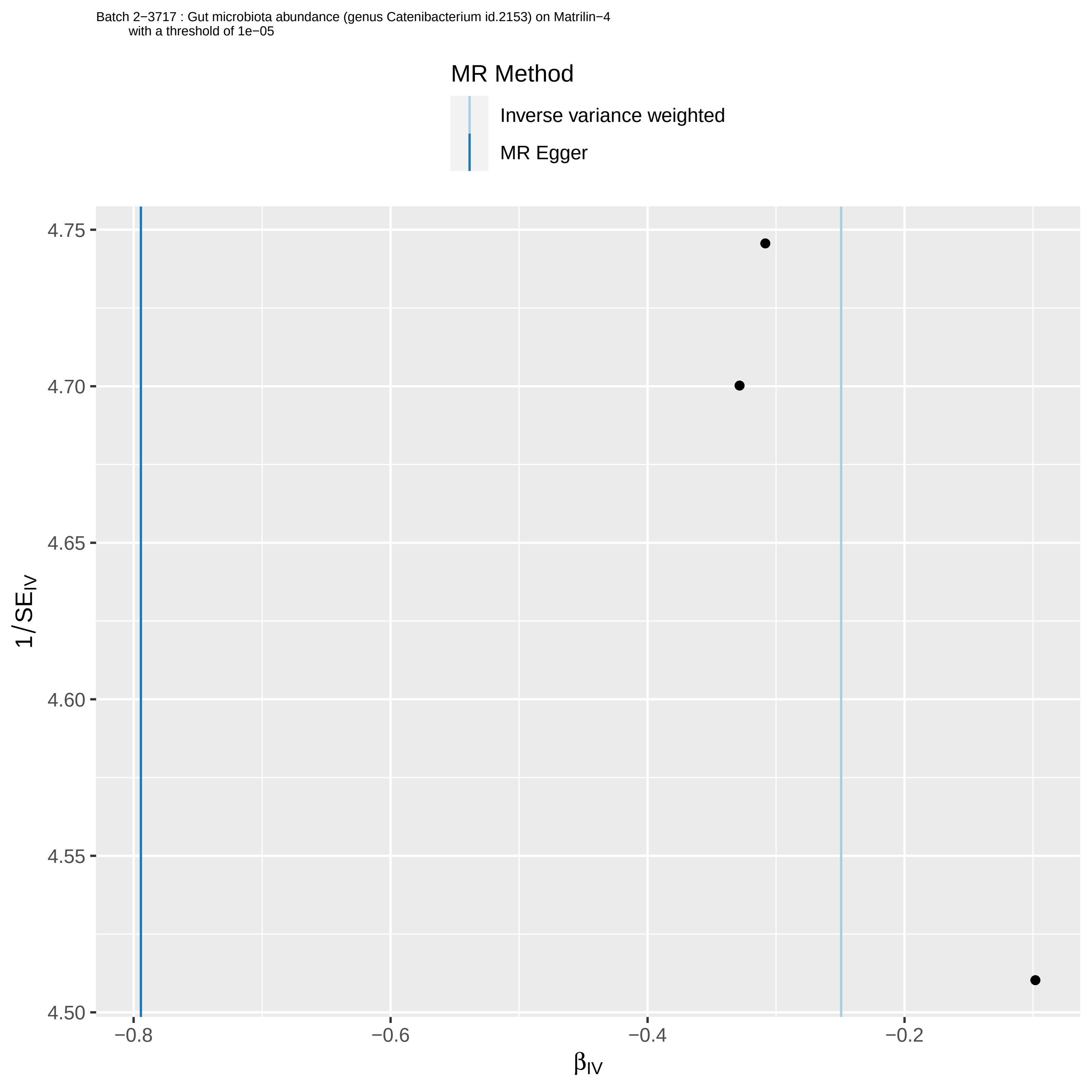

Batch 2−3717 : Gut microbiota abundance (genus Catenibacterium id.2153) on Matrilin−4
with a threshold of 1e−05
MR Method
Inverse variance weighted
MR Egger
4.75
4.70
4.65
4.60
4.55
4.50
1/SE_IV
−0.8
−0.6
−0.4
−0.2
β_IV

Batch 2−3718 : Gut microbiota abundance (genus Parasutterella id.2892) on Matrilin−4
with a threshold of 1e−05

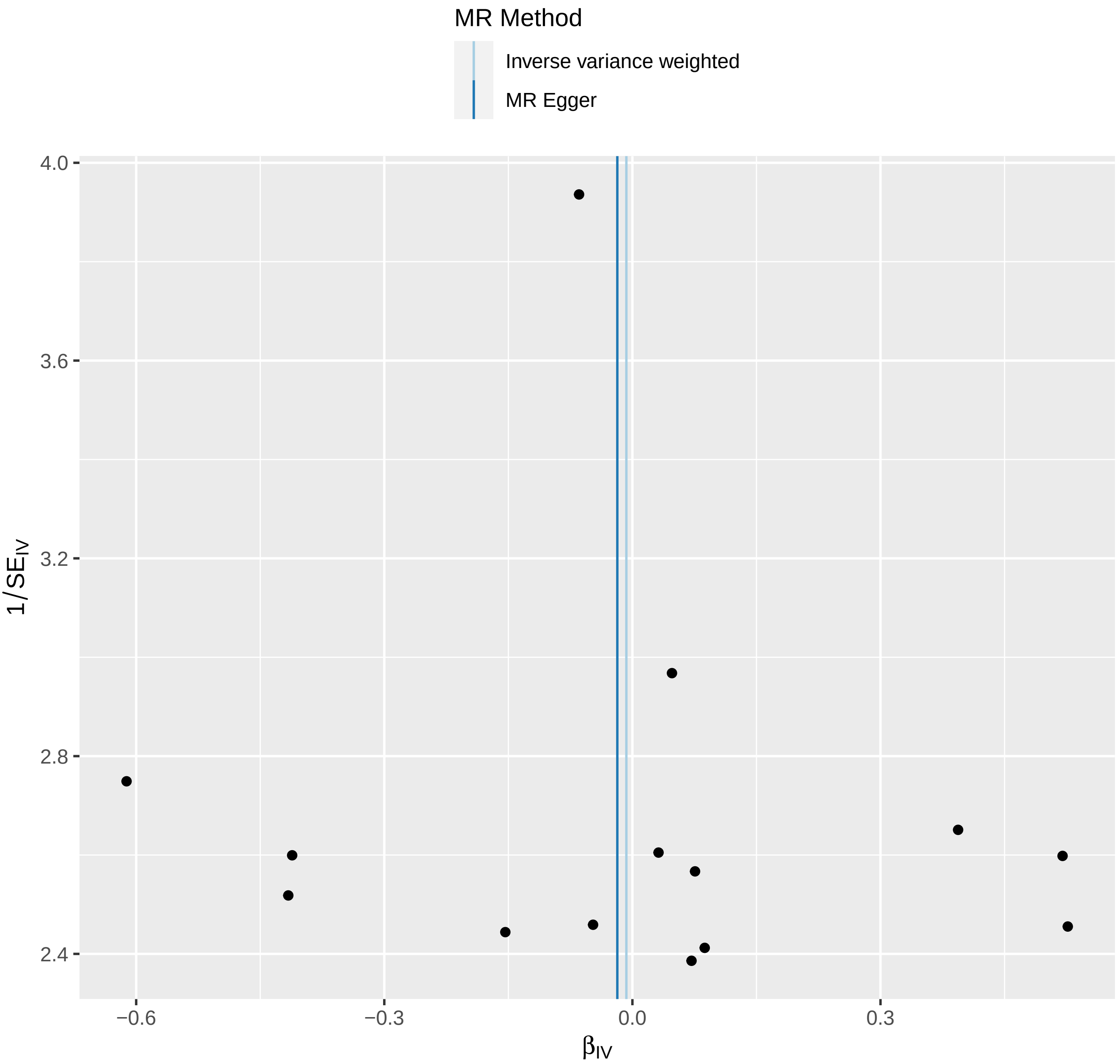

Batch 2−3719 : Gut microbiota abundance (genus Catenibacterium id.2153) on Myc−associated zinc finger protein
with a threshold of 1e−05

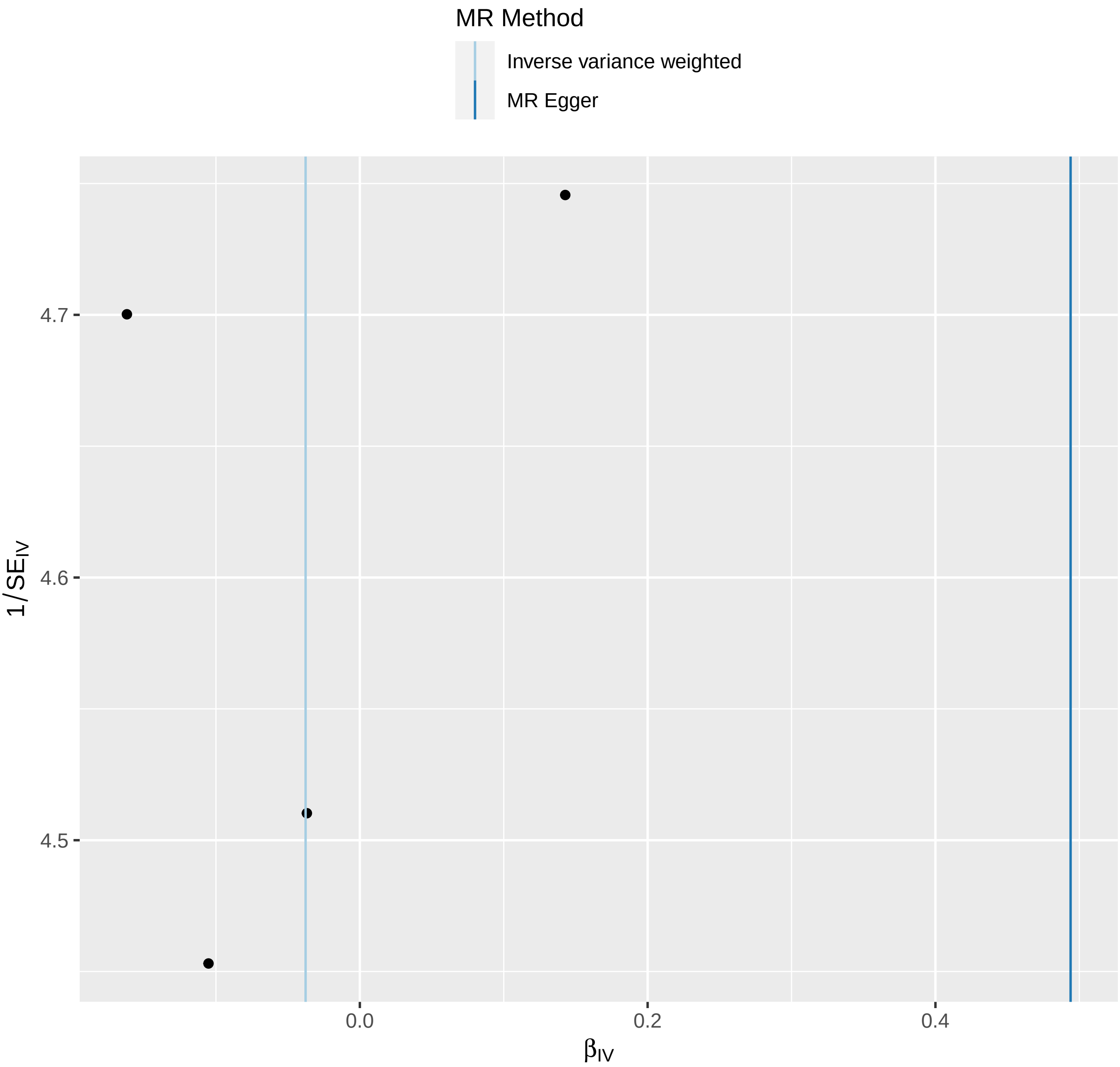

Batch 2−3720 : Gut microbiota abundance (genus Parasutterella id.2892) on Myc−associated zinc finger protein
with a threshold of 1e−05

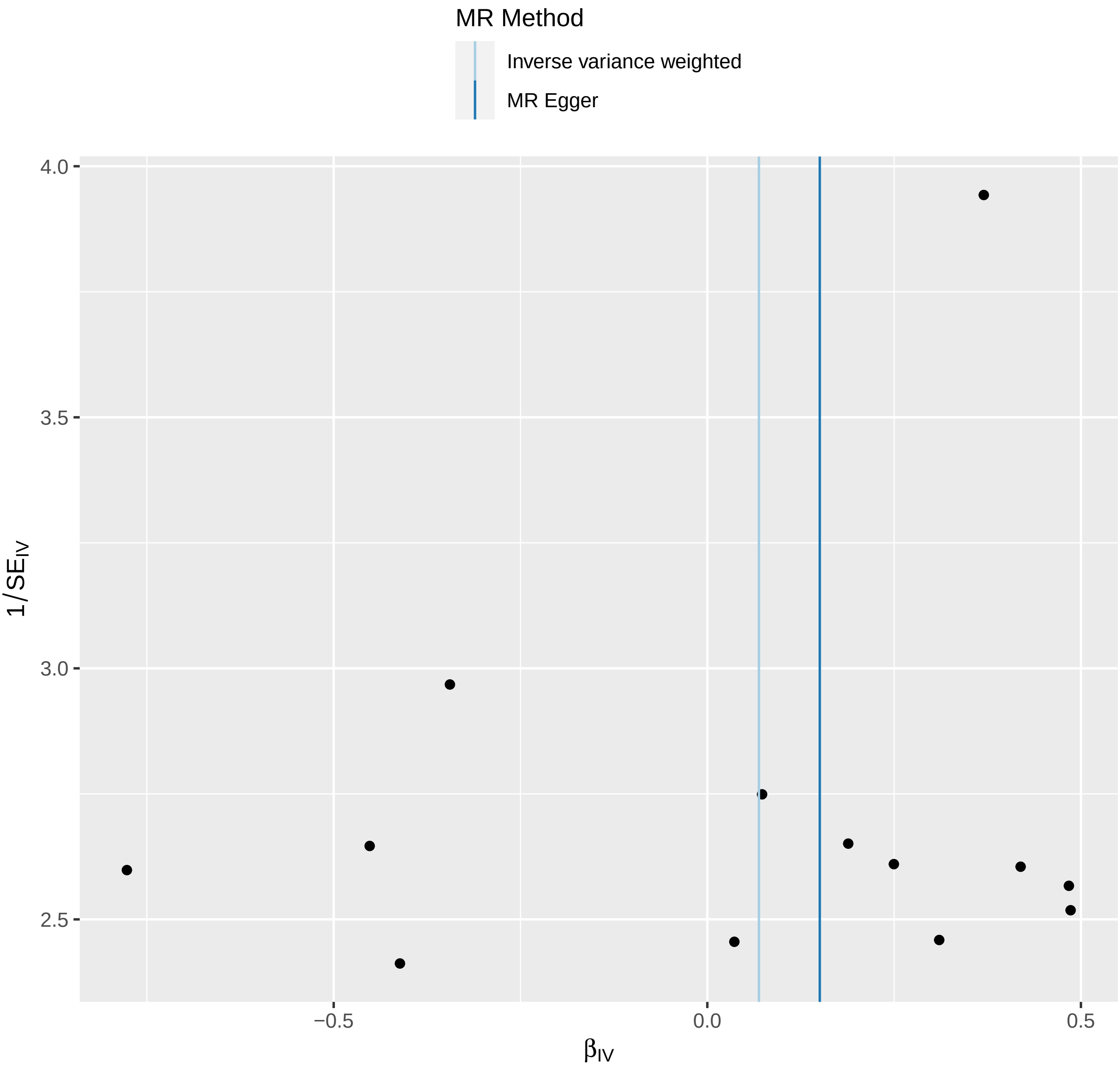

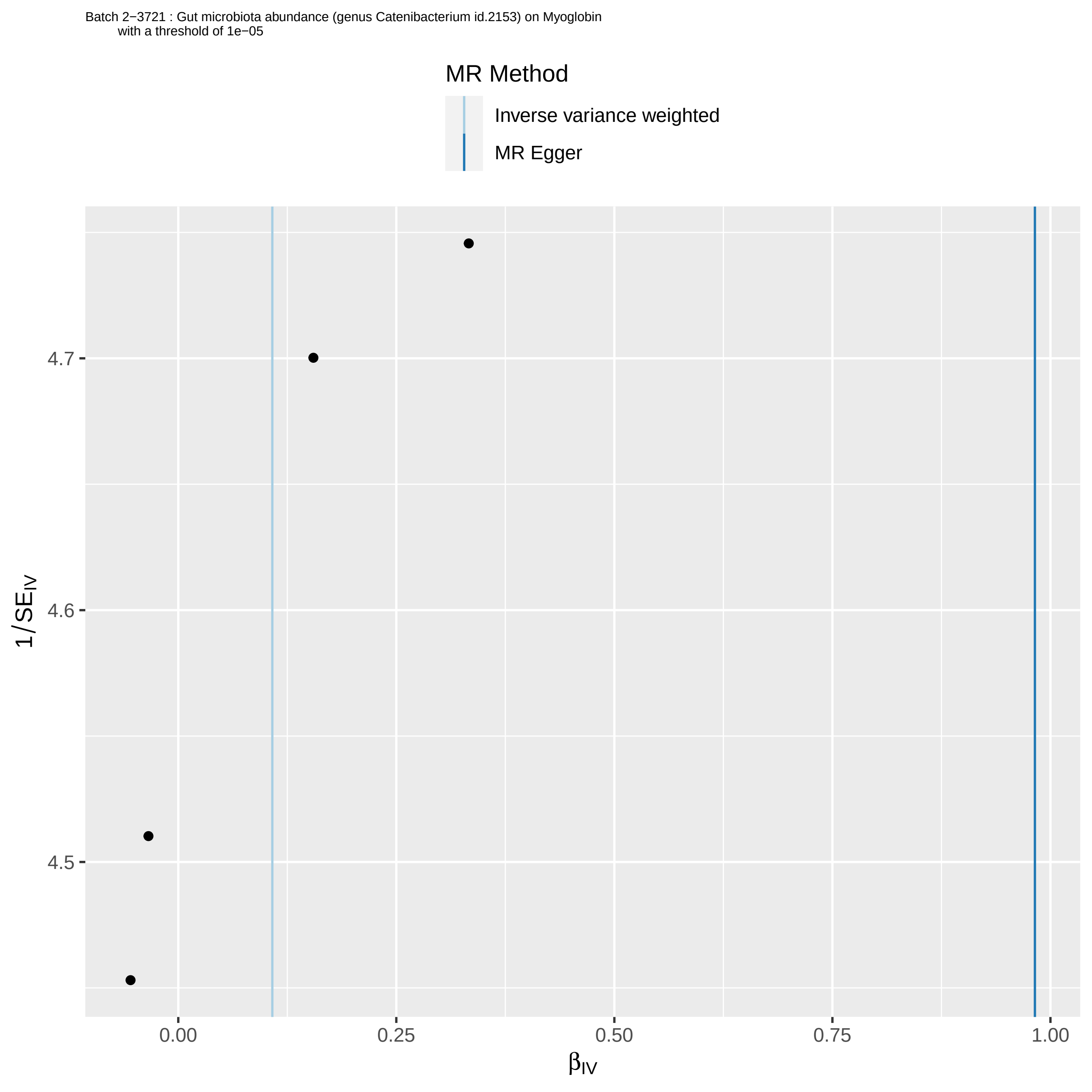
Batch 2−3721 : Gut microbiota abundance (genus Catenibacterium id.2153) on Myoglobin
with a threshold of 1e−05
MR Method
Inverse variance weighted
MR Egger
4.7
4.6
4.5
1/SE_IV
0.00
0.25
0.50
0.75
1.00
β_IV

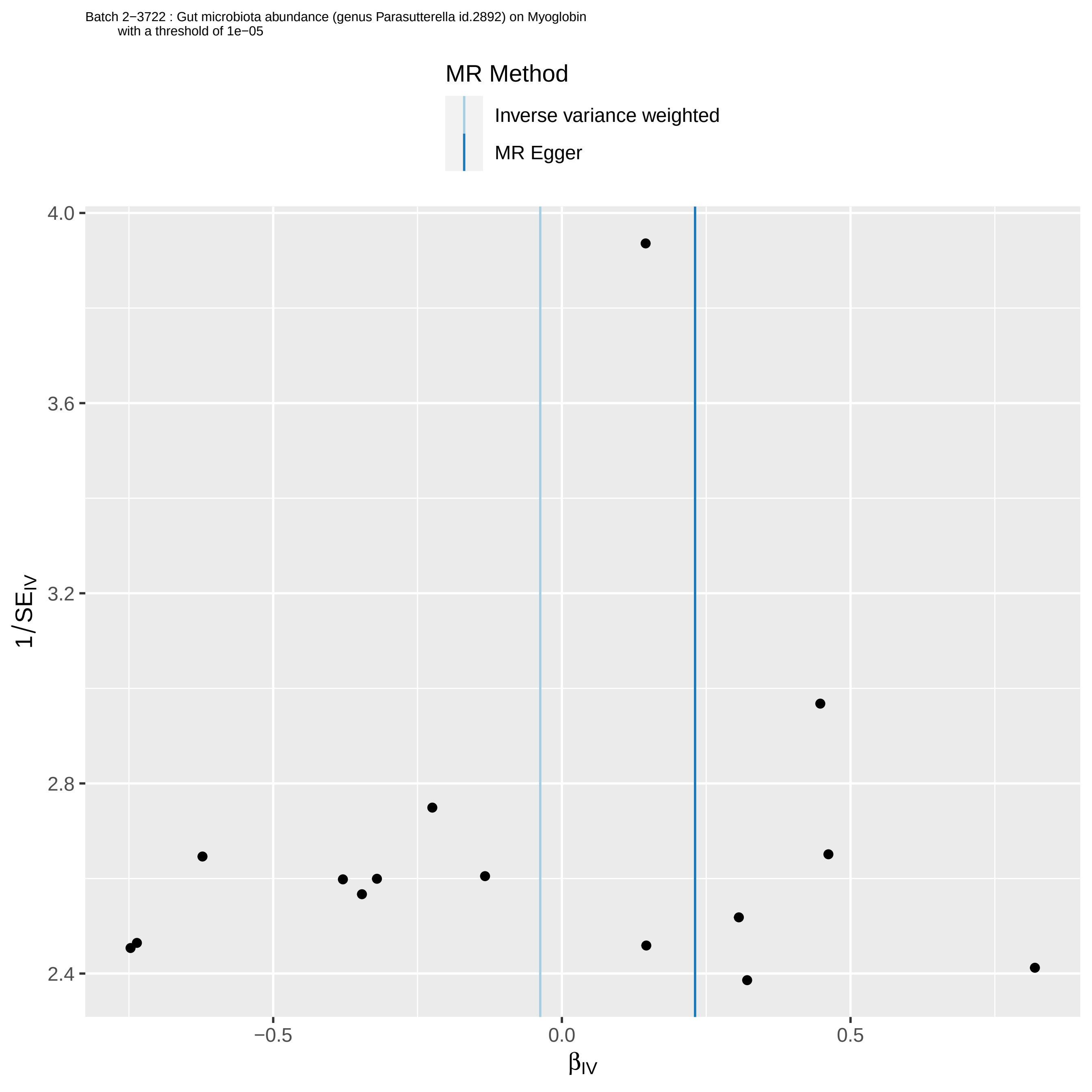

Batch 2−3722 : Gut microbiota abundance (genus Parasutterella id.2892) on Myoglobin
with a threshold of 1e−05
MR Method
Inverse variance weighted
MR Egger
4.0
3.6
3.2
2.8
2.4
1/SE_IV
−0.5
0.0
0.5
β_IV

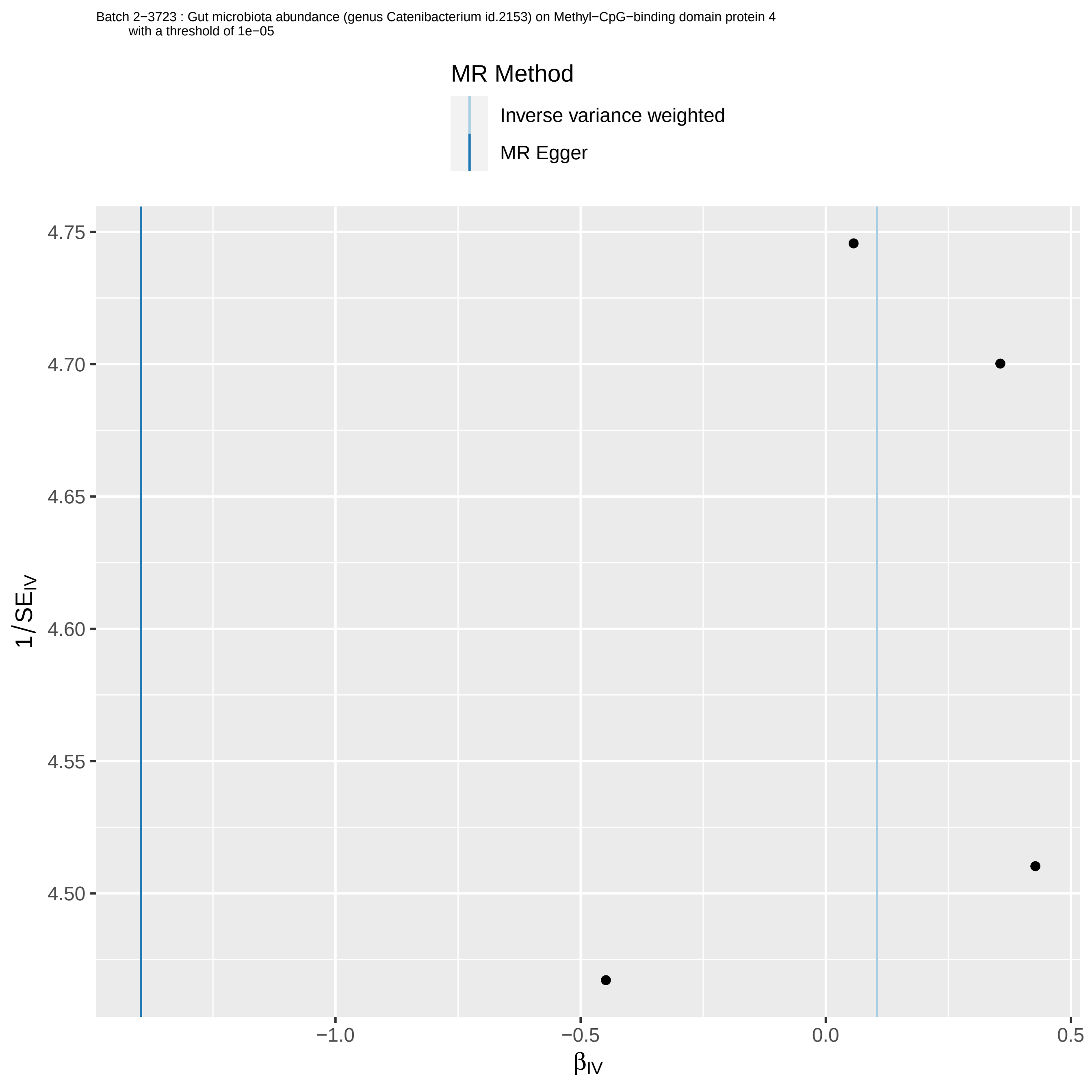

Batch 2−3723 : Gut microbiota abundance (genus Catenibacterium id.2153) on Methyl−CpG−binding domain protein 4
with a threshold of 1e−05
MR Method
Inverse variance weighted
MR Egger
4.75
4.70
4.65
4.60
4.55
4.50
1/SE_IV
−1.0
−0.5
0.0
0.5
β_IV

Batch 2−3724 : Gut microbiota abundance (genus Parasutterella id.2892) on Methyl−CpG−binding domain protein 4
with a threshold of 1e−05

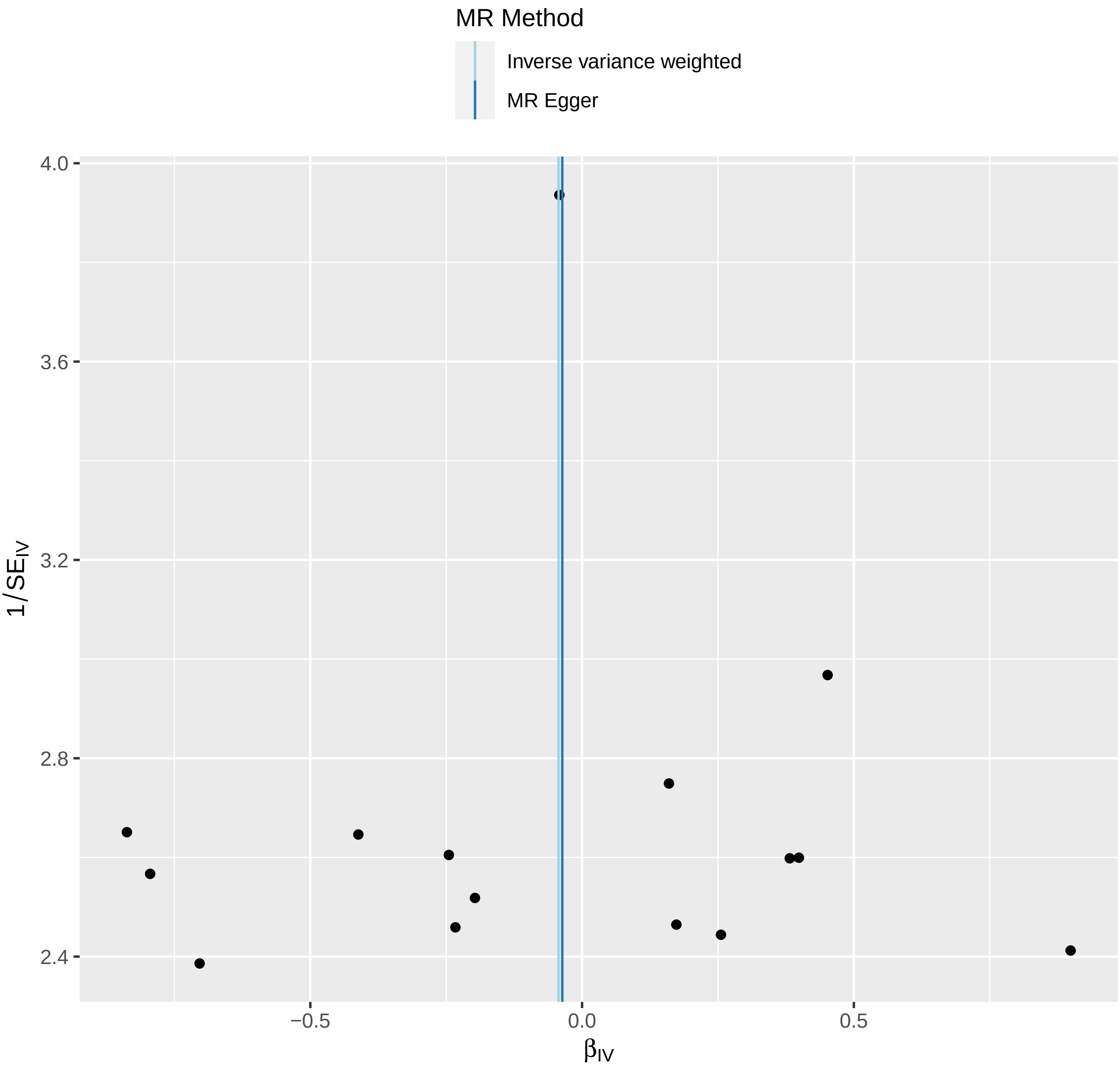

Batch 2−3725 : Gut microbiota abundance (genus Catenibacterium id.2153) on Mannose−binding protein C
with a threshold of 1e−05

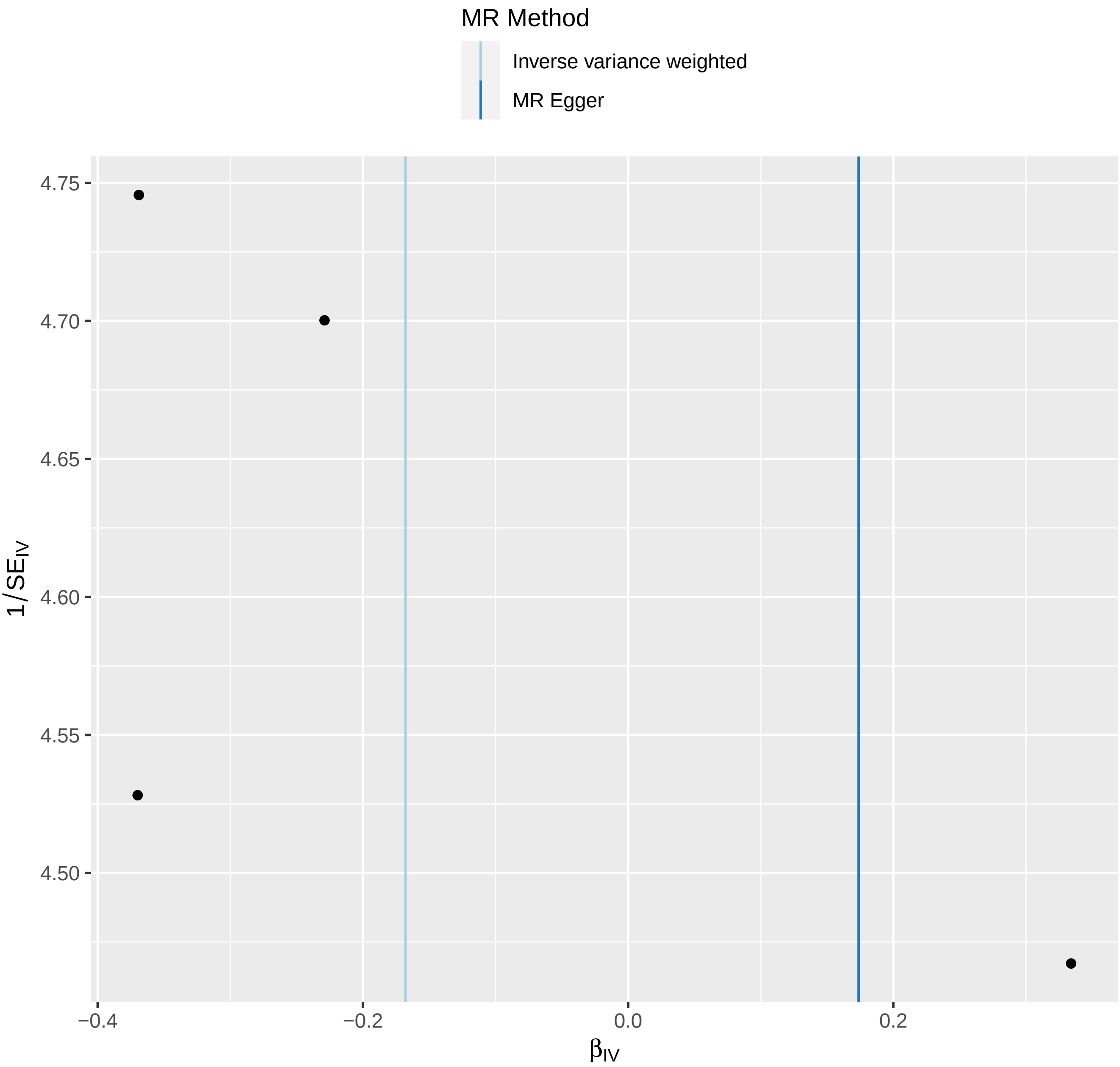

Batch 2−3726 : Gut microbiota abundance (genus Parasutterella id.2892) on Mannose−binding protein C
with a threshold of 1e−05

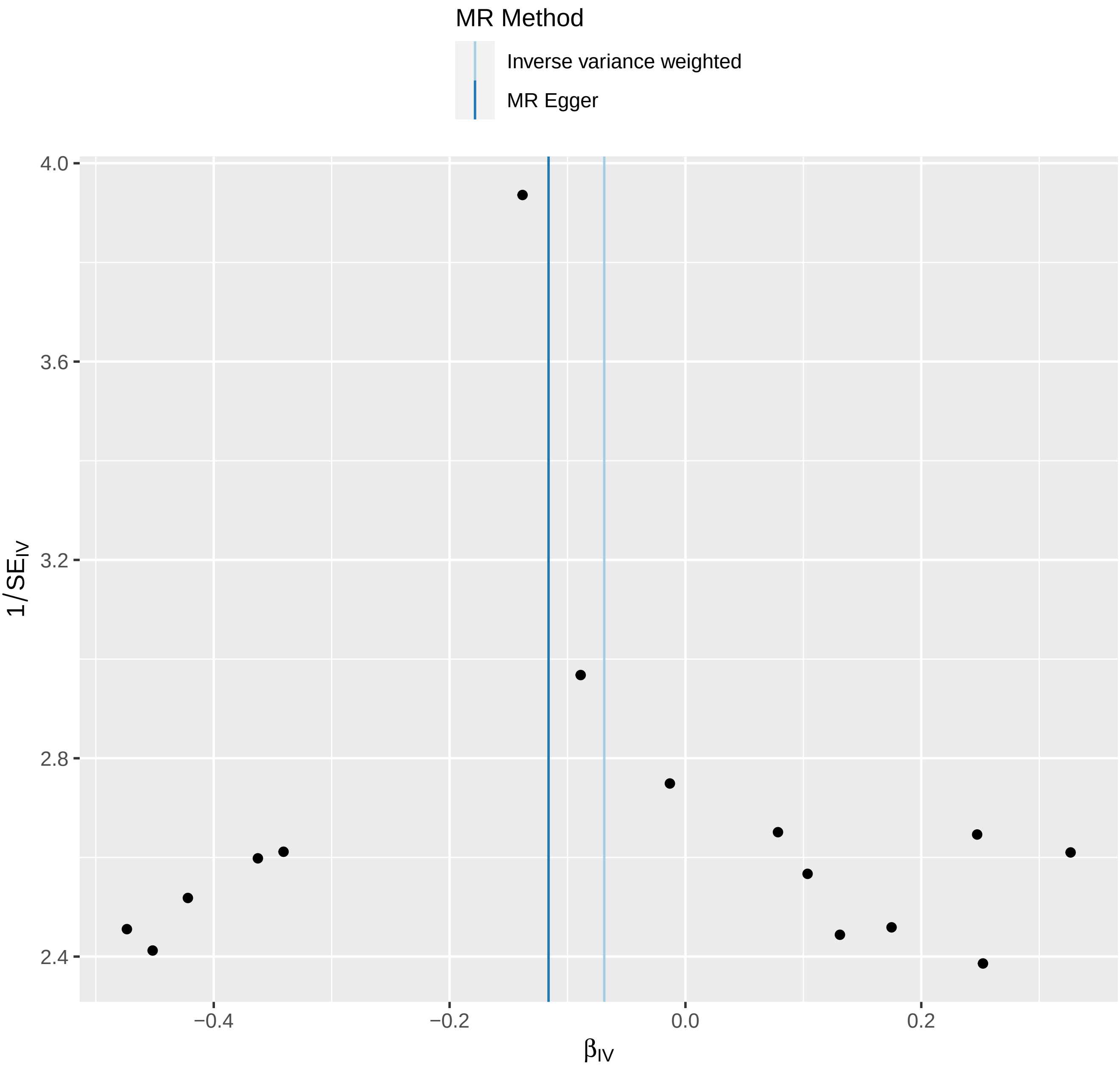

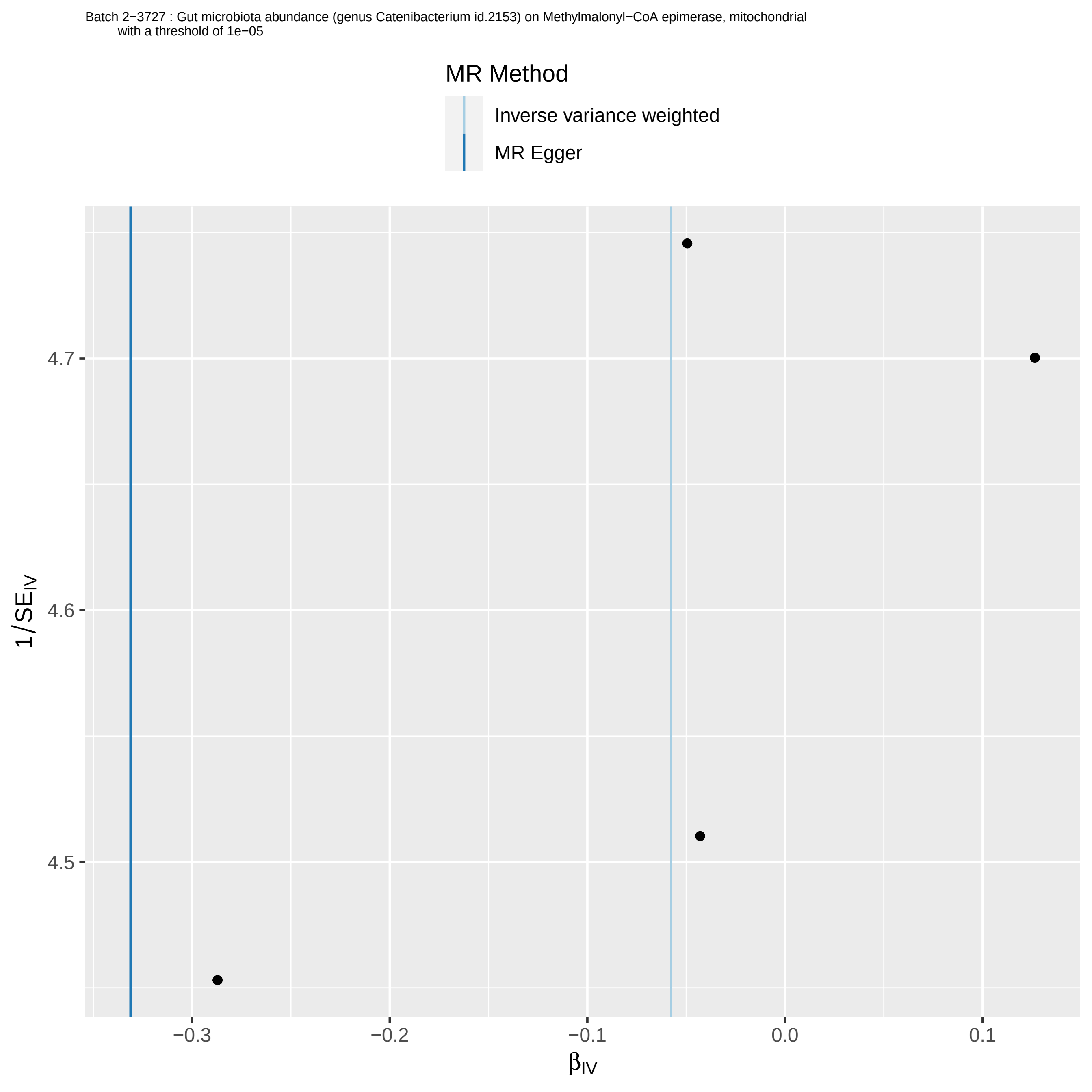
Batch 2−3727 : Gut microbiota abundance (genus Catenibacterium id.2153) on Methylmalonyl−CoA epimerase, mitochondrial
with a threshold of 1e−05
MR Method
Inverse variance weighted
MR Egger
4.7
4.6
4.5
1/SE_IV
−0.3
−0.2
−0.1
0.0
0.1
β_IV

Batch 2−3728 : Gut microbiota abundance (genus Parasutterella id.2892) on Methylmalonyl−CoA epimerase, mitochondrial
with a threshold of 1e−05

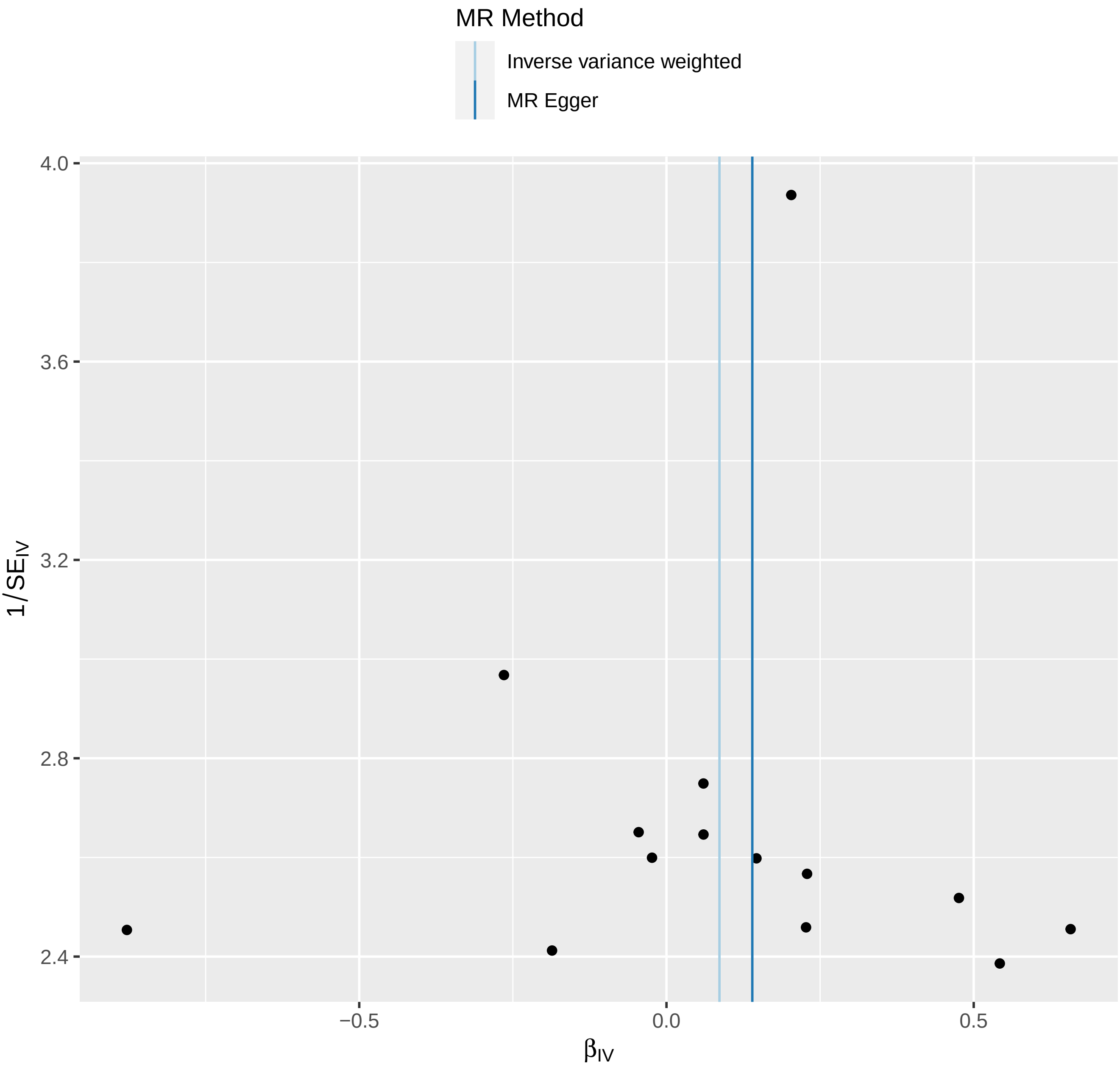

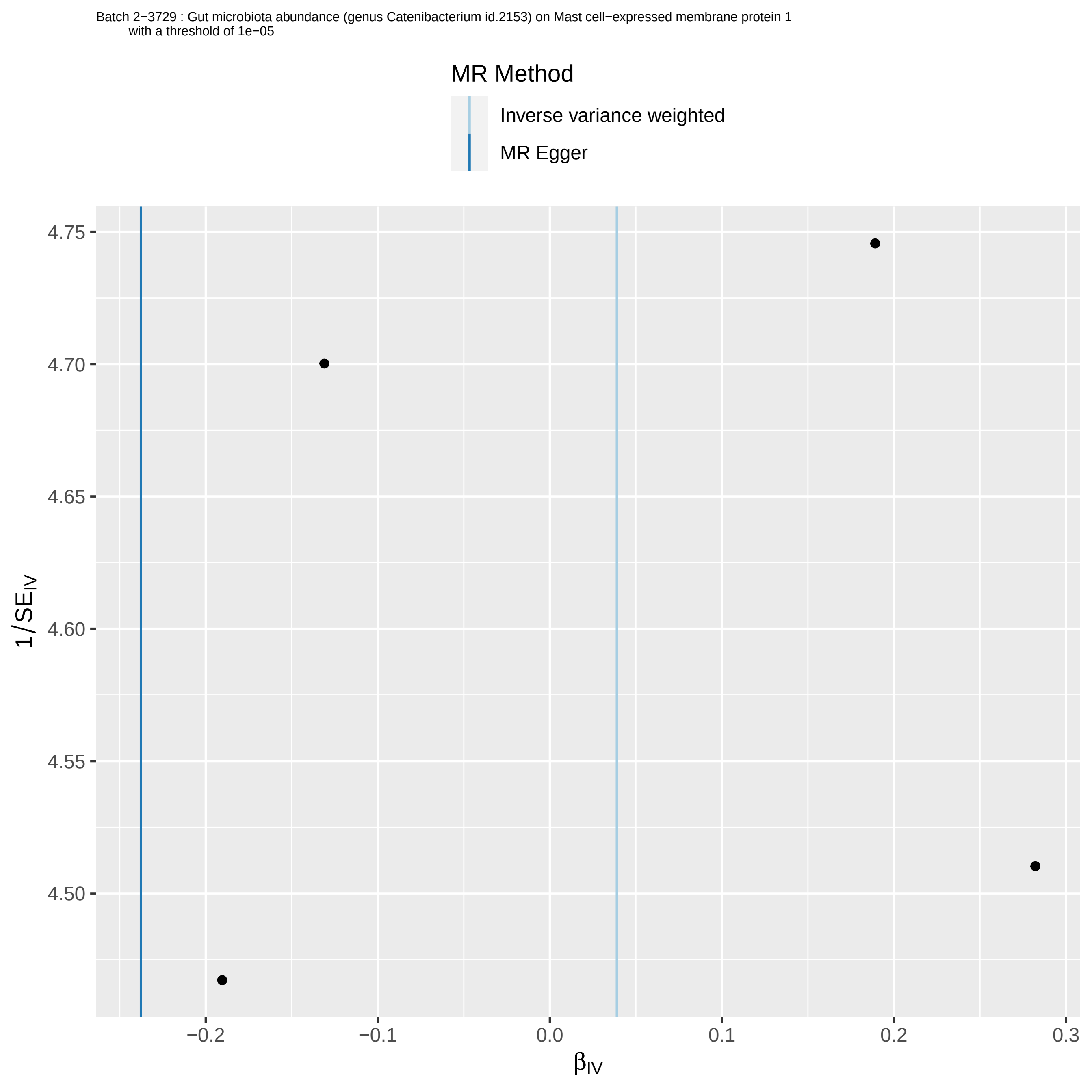
Batch 2−3729 : Gut microbiota abundance (genus Catenibacterium id.2153) on Mast cell−expressed membrane protein 1
with a threshold of 1e−05
MR Method
Inverse variance weighted
MR Egger
4.75
4.70
4.65
4.60
4.55
4.50
1/SEIV
−0.2
−0.1
0.0
0.1
0.2
0.3
βIV

Batch 2−3730 : Gut microbiota abundance (genus Parasutterella id.2892) on Mast cell−expressed membrane protein 1
with a threshold of 1e−05

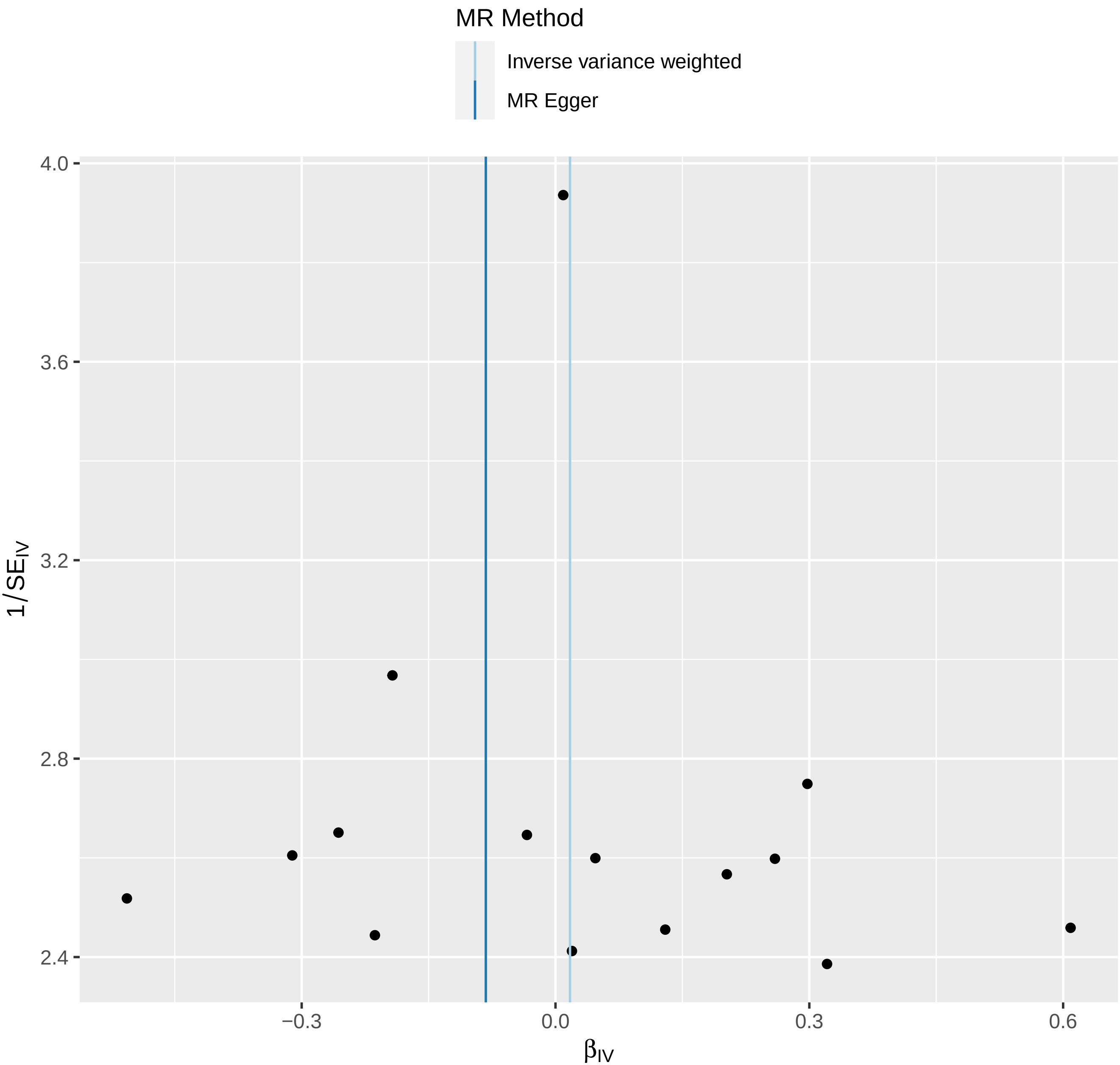

Batch 2−3731 : Gut microbiota abundance (genus Catenibacterium id.2153) on Guanine nucleotide exchange factor DBS
with a threshold of 1e−05

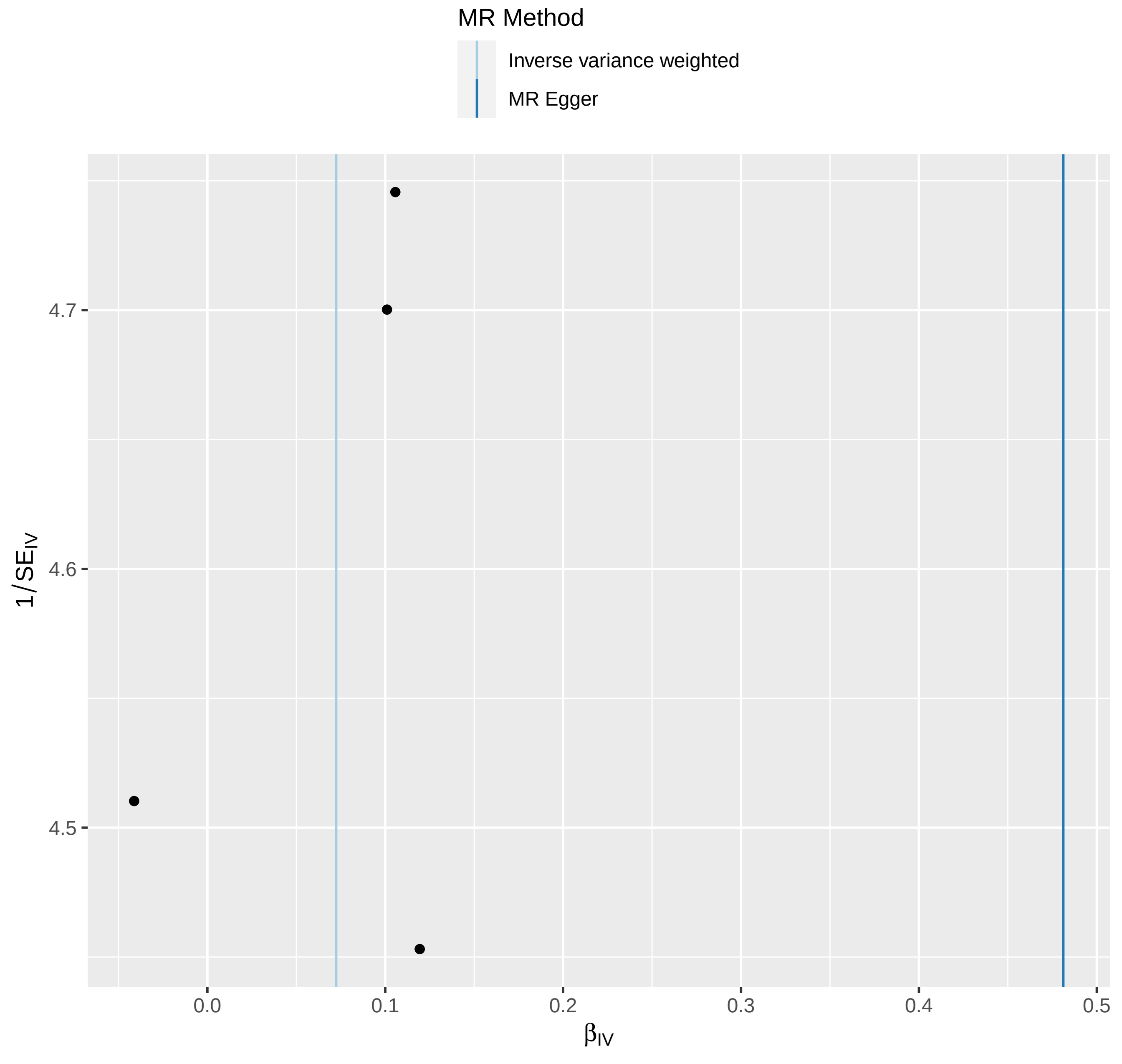

Batch 2−3732 : Gut microbiota abundance (genus Parasutterella id.2892) on Guanine nucleotide exchange factor DBS
with a threshold of 1e−05

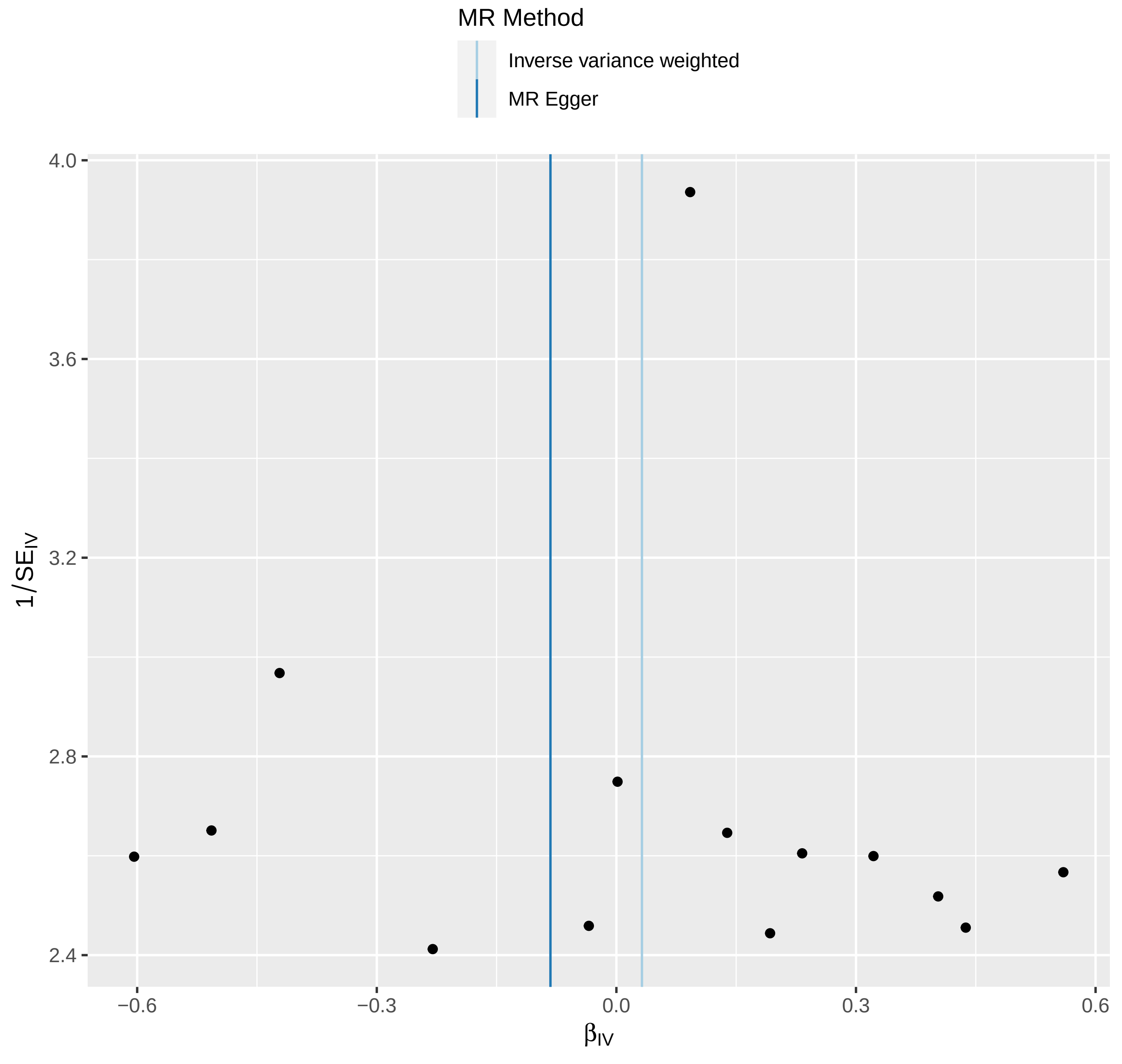

Batch 2−3733 : Gut microbiota abundance (genus Catenibacterium id.2153) on Multiple coagulation factor deficiency protein 2
with a threshold of 1e−05

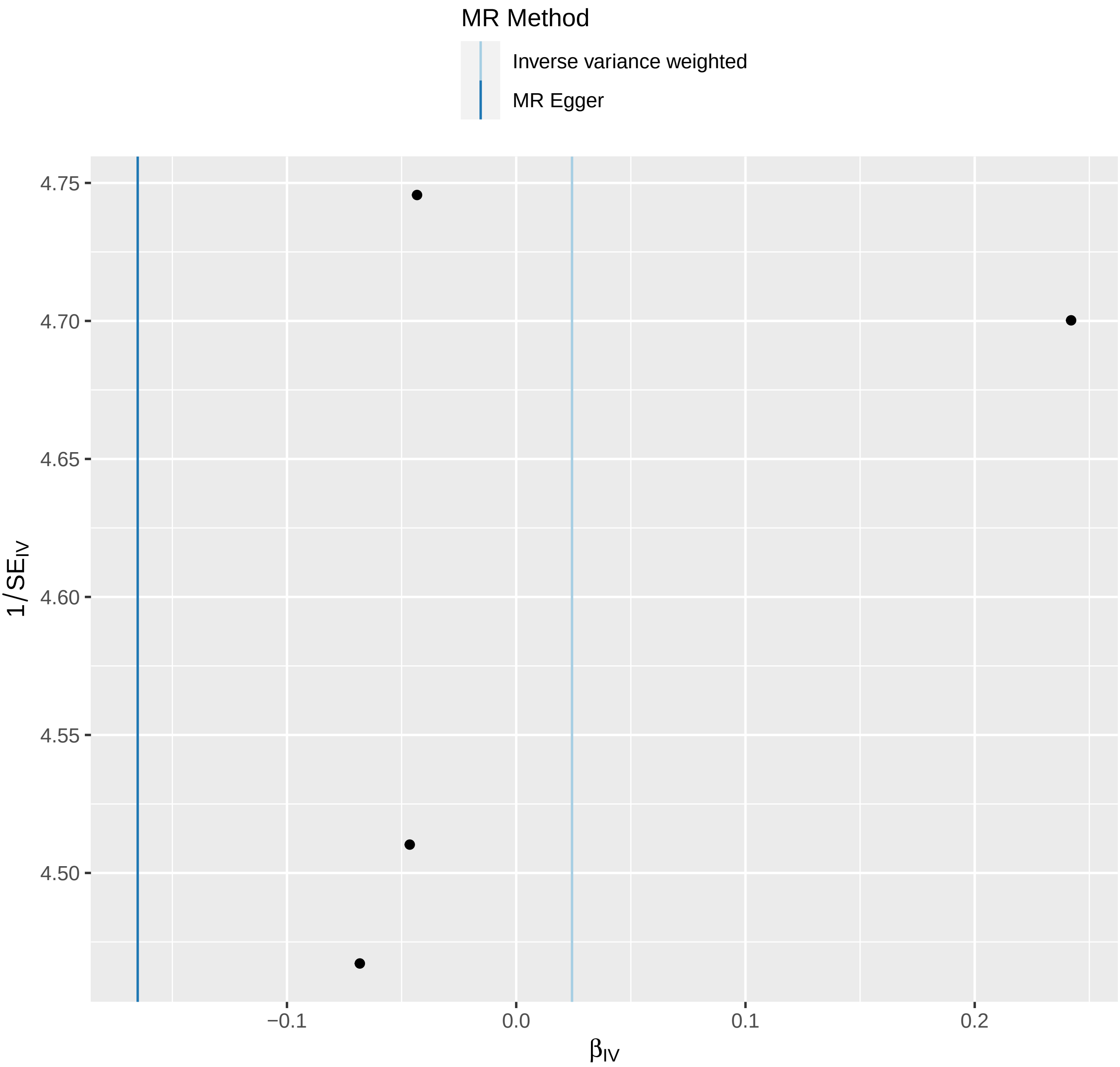

Batch 2−3734 : Gut microbiota abundance (genus Parasutterella id.2892) on Multiple coagulation factor deficiency protein 2
with a threshold of 1e−05

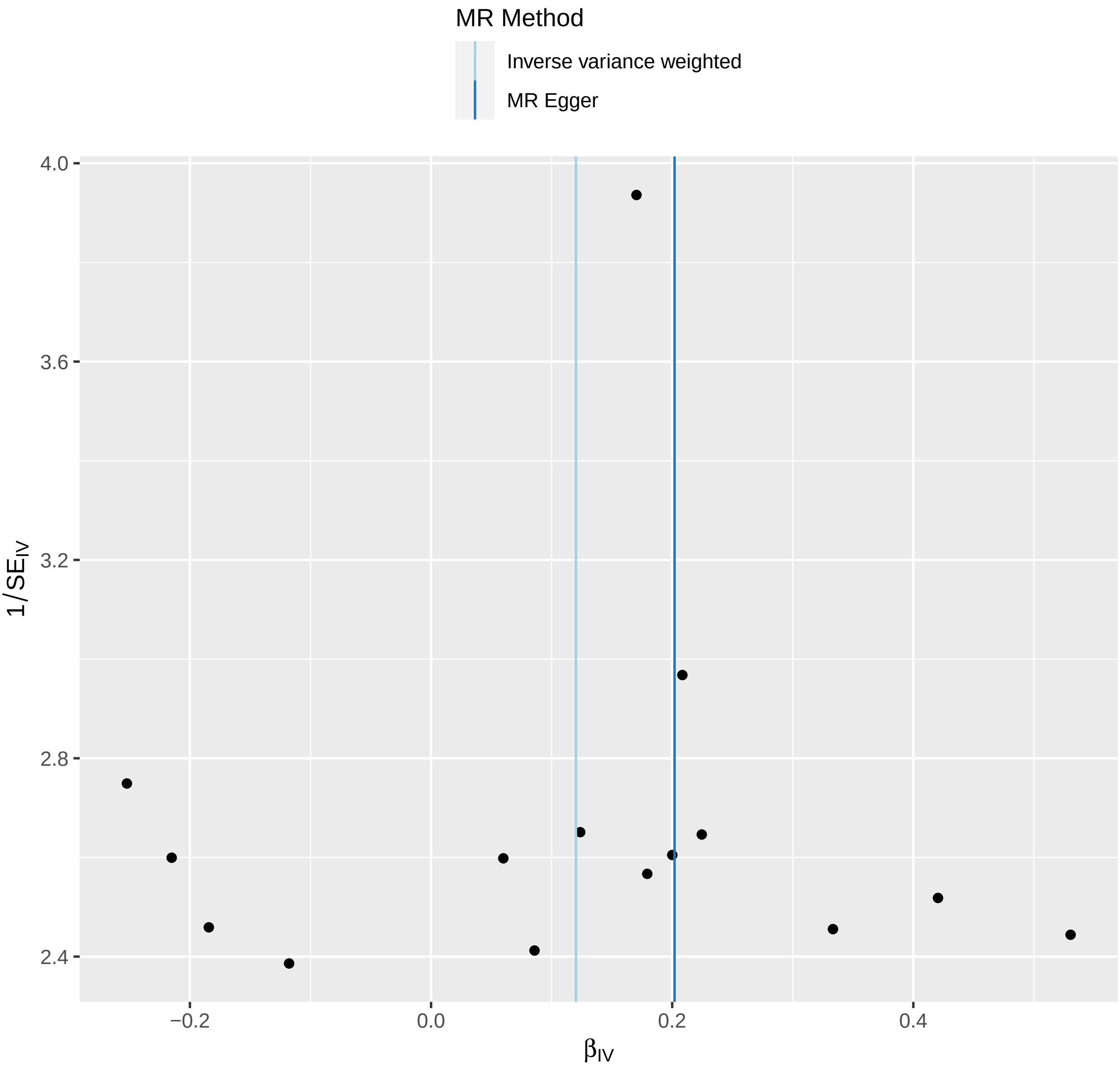

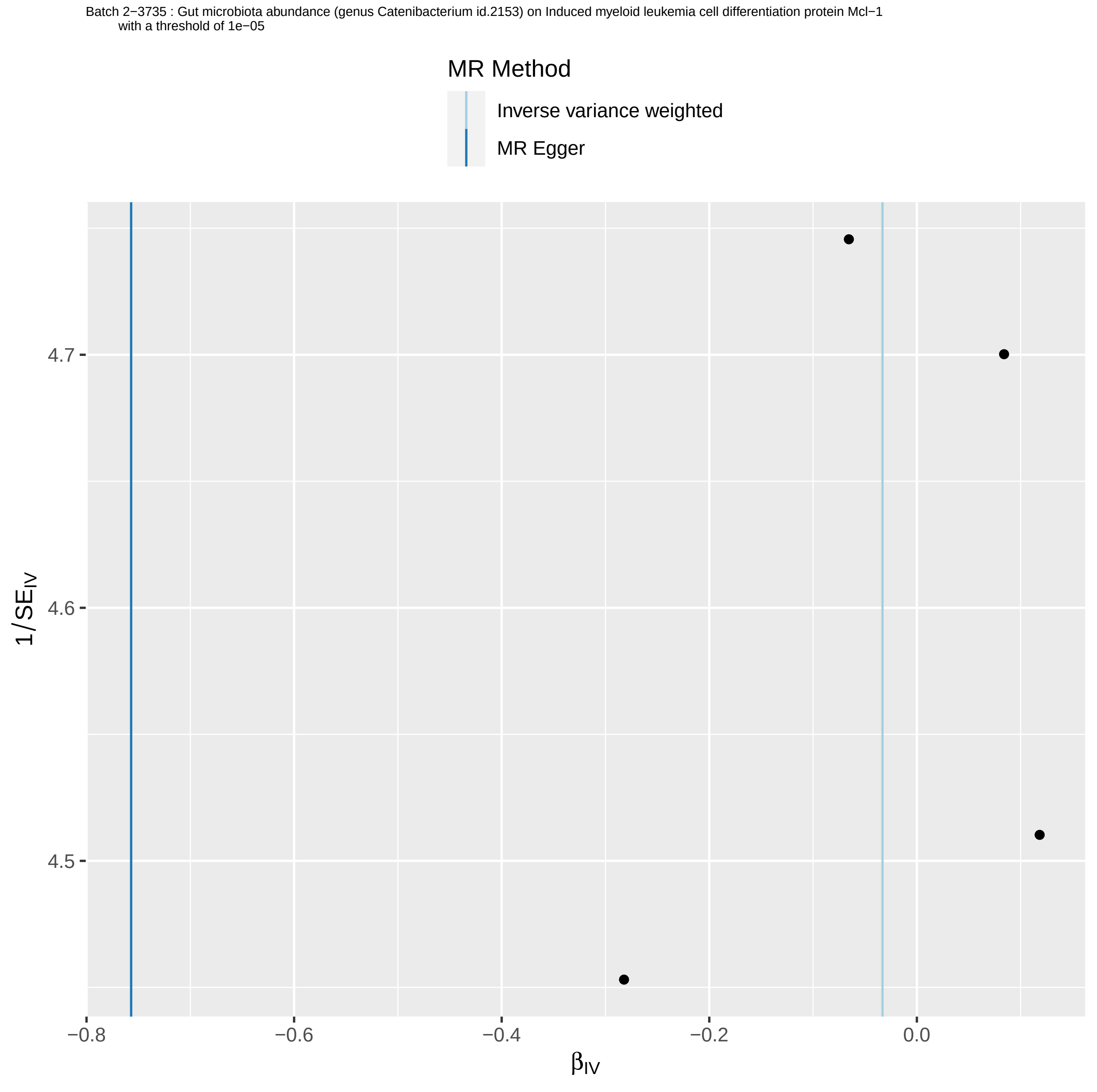

Batch 2−3735 : Gut microbiota abundance (genus Catenibacterium id.2153) on Induced myeloid leukemia cell differentiation protein Mcl−1
with a threshold of 1e−05
MR Method
Inverse variance weighted
MR Egger
4.7
4.6
4.5
1/SE$_{IV}$
−0.8
−0.6
−0.4
−0.2
0.0
β$_{IV}$

Batch 2−3736 : Gut microbiota abundance (genus Parasutterella id.2892) on Induced myeloid leukemia cell differentiation protein Mcl−1
with a threshold of 1e−05

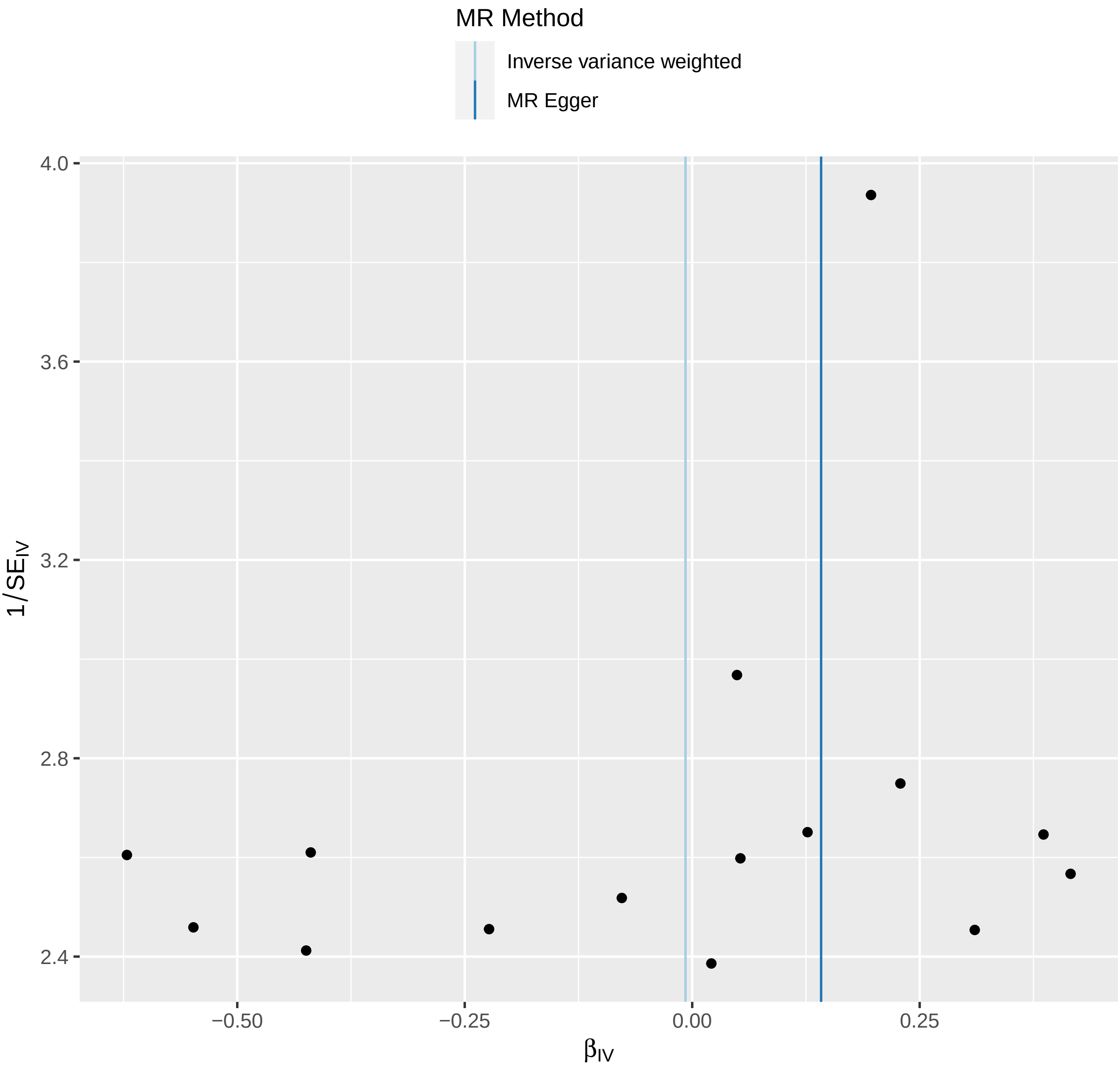

Batch 2−3737 : Gut microbiota abundance (genus Catenibacterium id.2153) on Malignant T−cell−amplified sequence 1
with a threshold of 1e−05

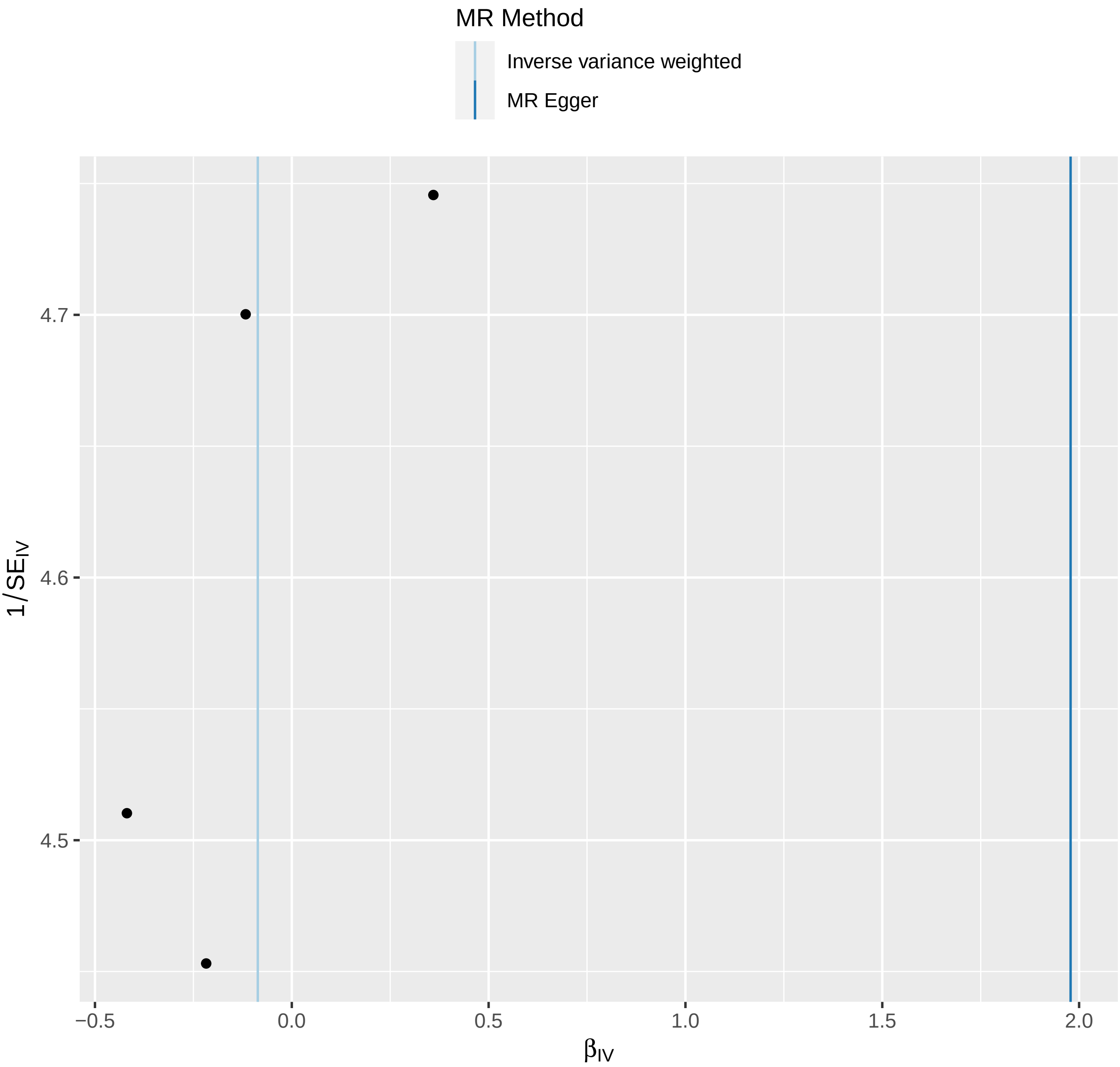

Batch 2−3738 : Gut microbiota abundance (genus Parasutterella id.2892) on Malignant T−cell−amplified sequence 1 with a threshold of 1e−05

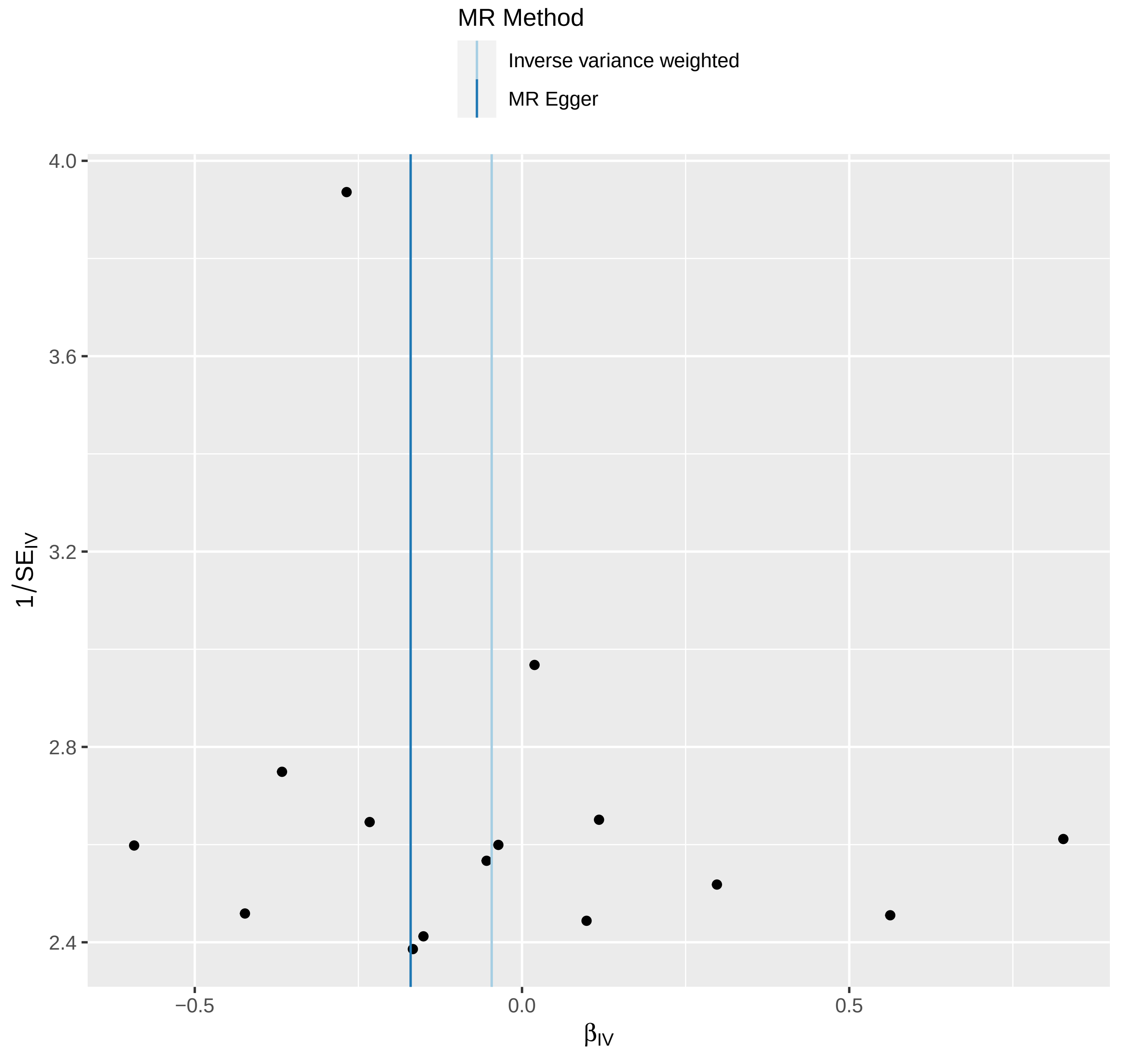

Batch 2−3739 : Gut microbiota abundance (genus Catenibacterium id.2153) on Malate dehydrogenase, cytoplasmic
with a threshold of 1e−05

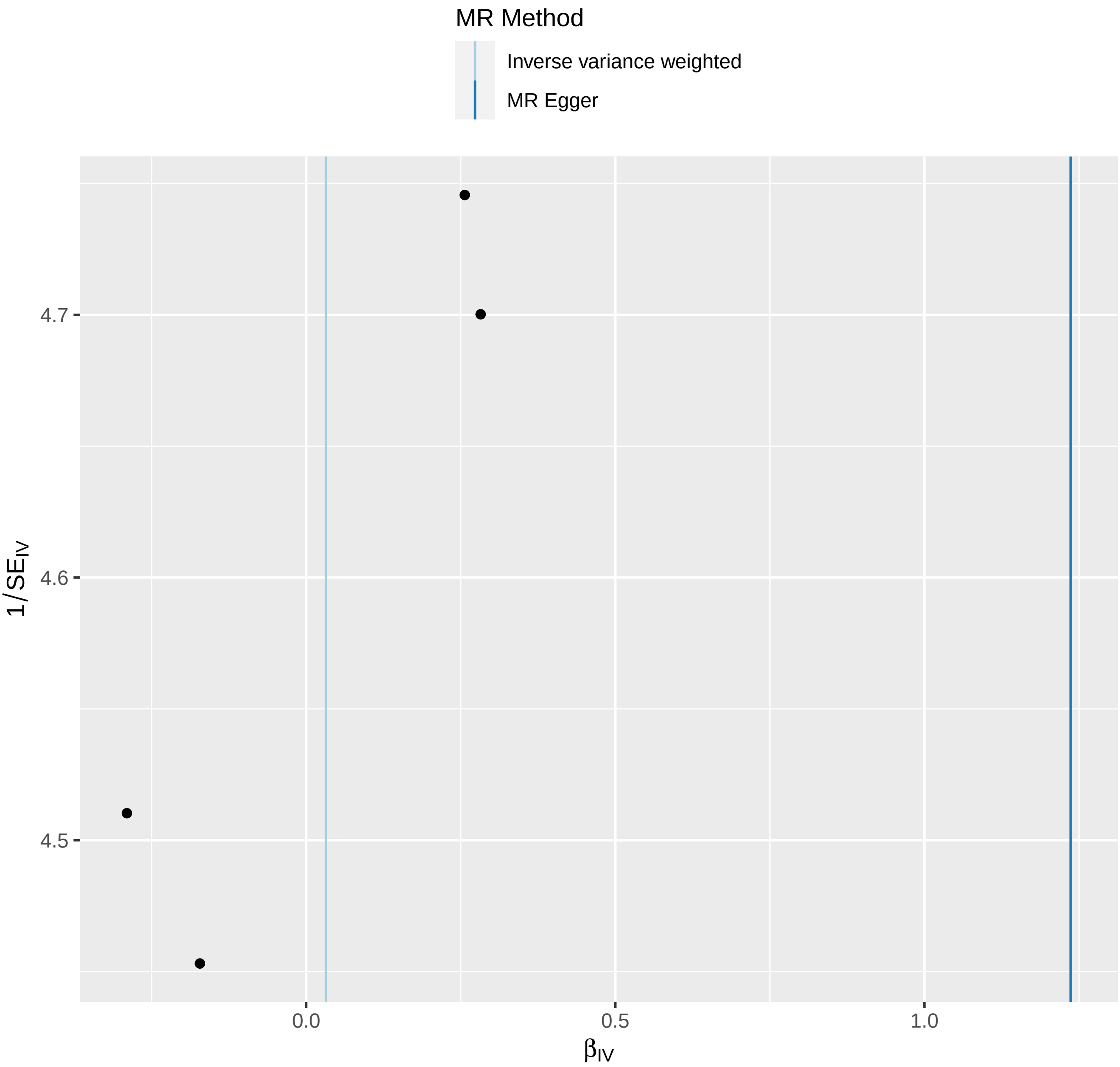

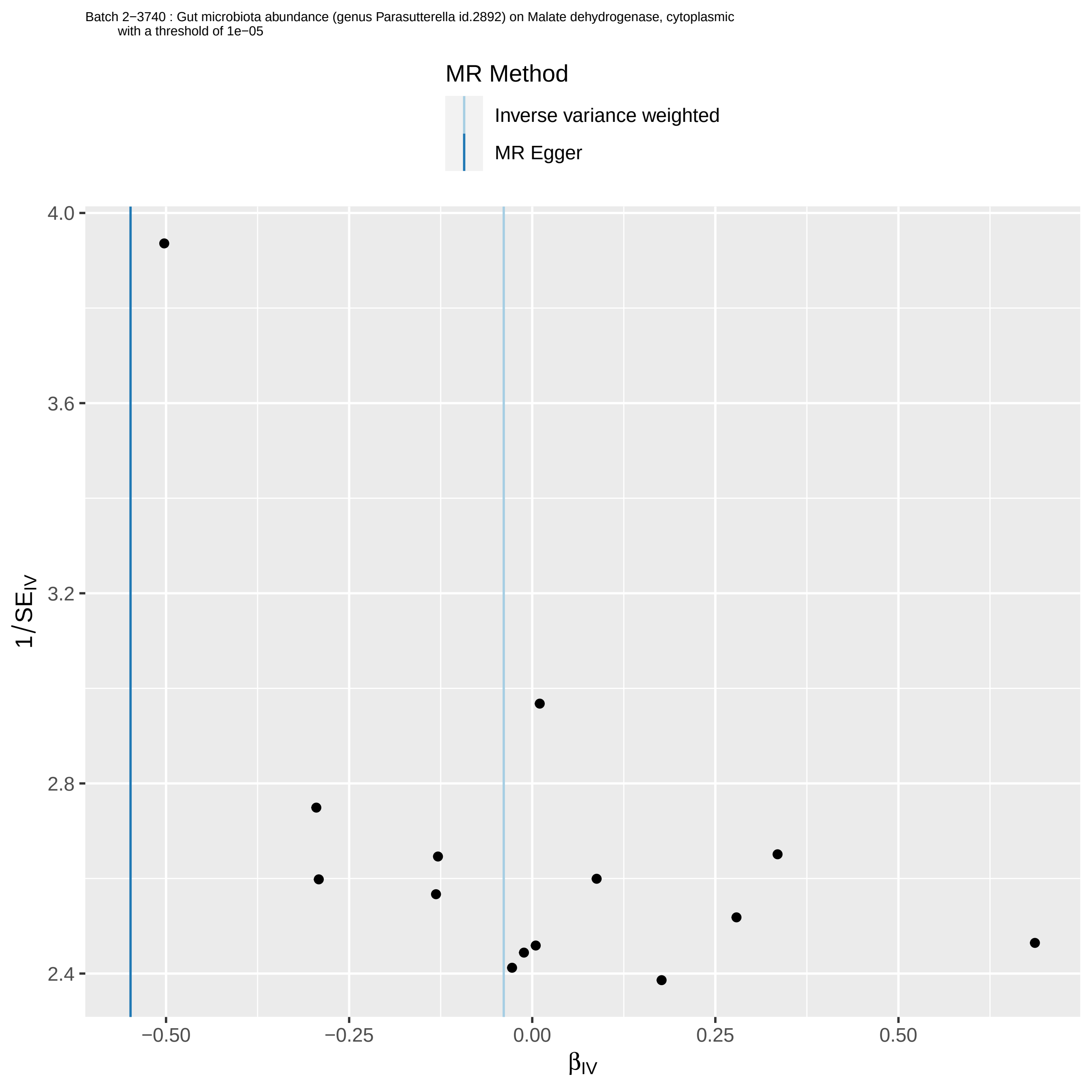

Batch 2−3740 : Gut microbiota abundance (genus Parasutterella id.2892) on Malate dehydrogenase, cytoplasmic
with a threshold of 1e−05
MR Method
Inverse variance weighted
MR Egger
4.0
3.6
3.2
2.8
2.4
1/SE_IV
−0.50
−0.25
0.00
0.25
0.50
β_IV

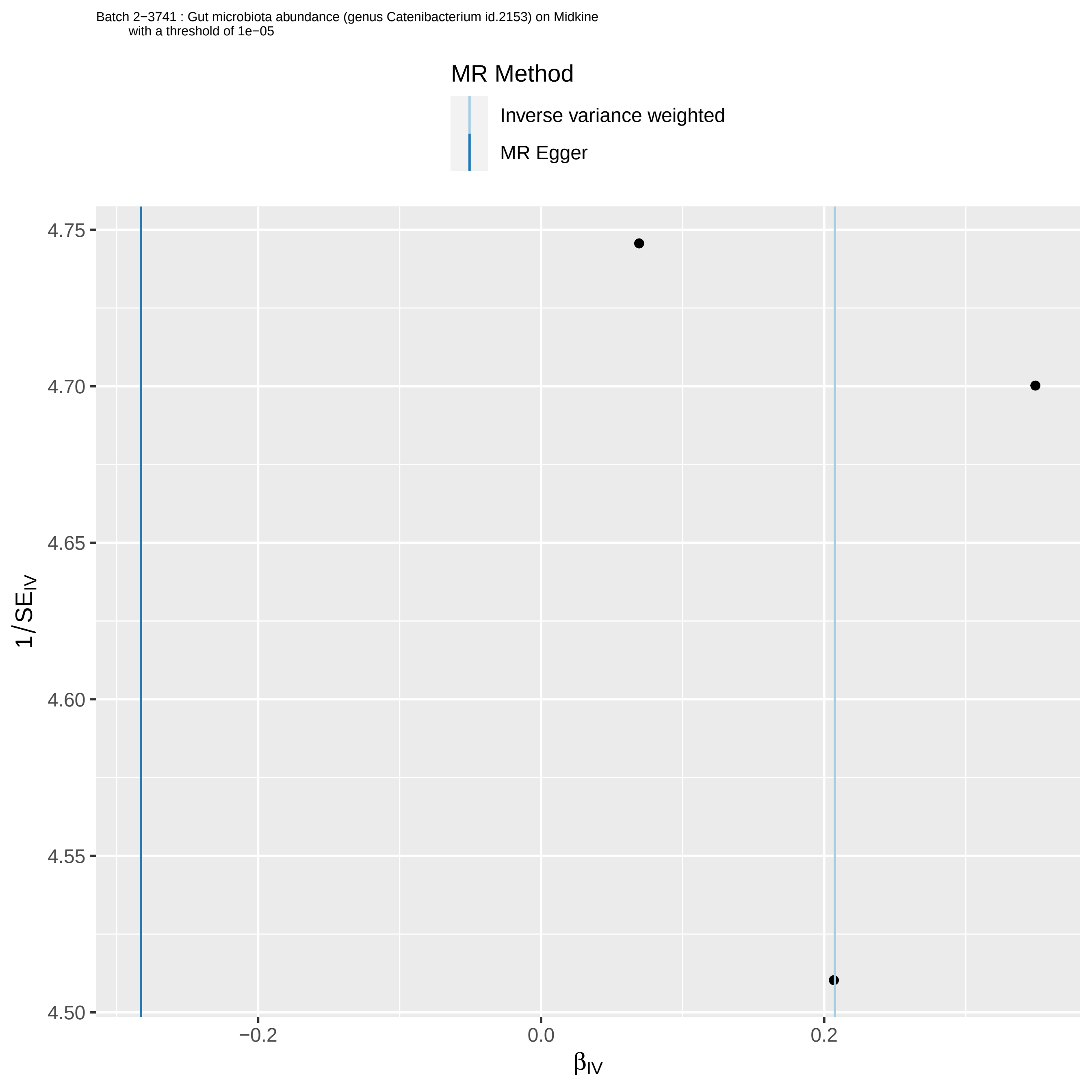

Batch 2−3741 : Gut microbiota abundance (genus Catenibacterium id.2153) on Midkine
with a threshold of 1e−05
MR Method
Inverse variance weighted
MR Egger
4.75
4.70
4.65
4.60
4.55
4.50
1/SE_IV
−0.2
0.0
0.2
β_IV

Batch 2−3742 : Gut microbiota abundance (genus Parasutterella id.2892) on Midkine
with a threshold of 1e−05

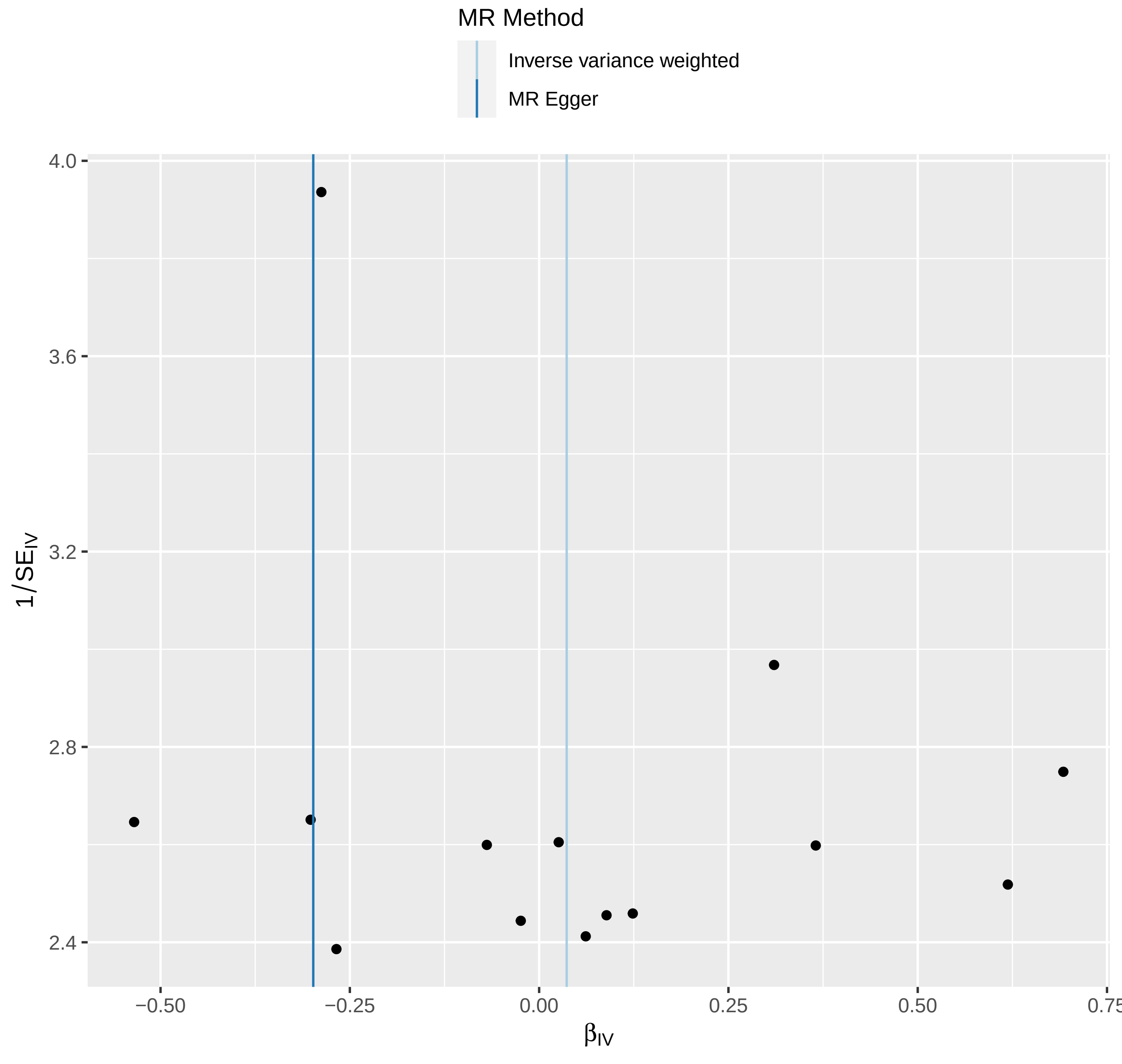

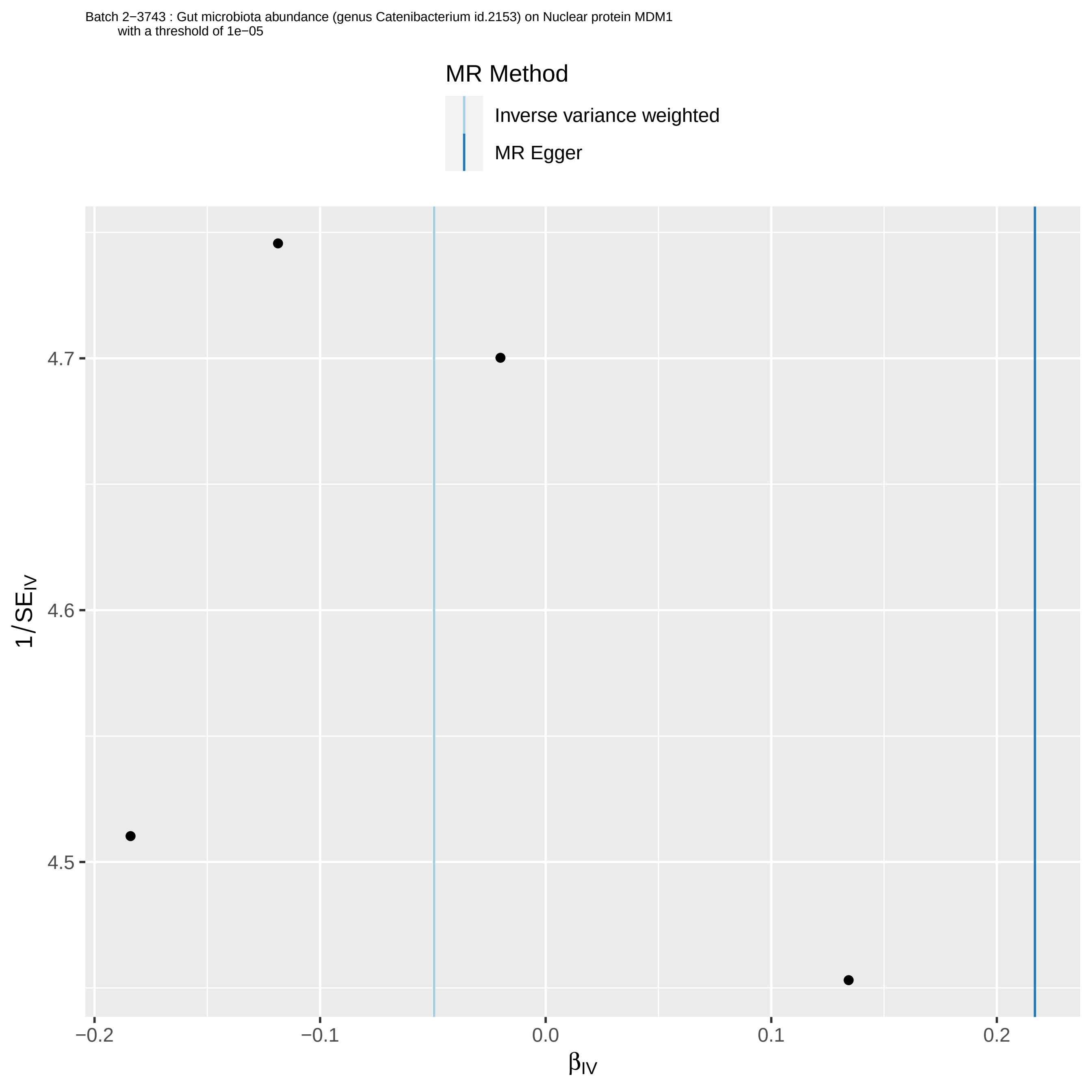

Batch 2−3743 : Gut microbiota abundance (genus Catenibacterium id.2153) on Nuclear protein MDM1
with a threshold of 1e−05
MR Method
Inverse variance weighted
MR Egger
4.7
4.6
4.5
1/SE_IV
−0.2
−0.1
0.0
0.1
0.2
β_IV

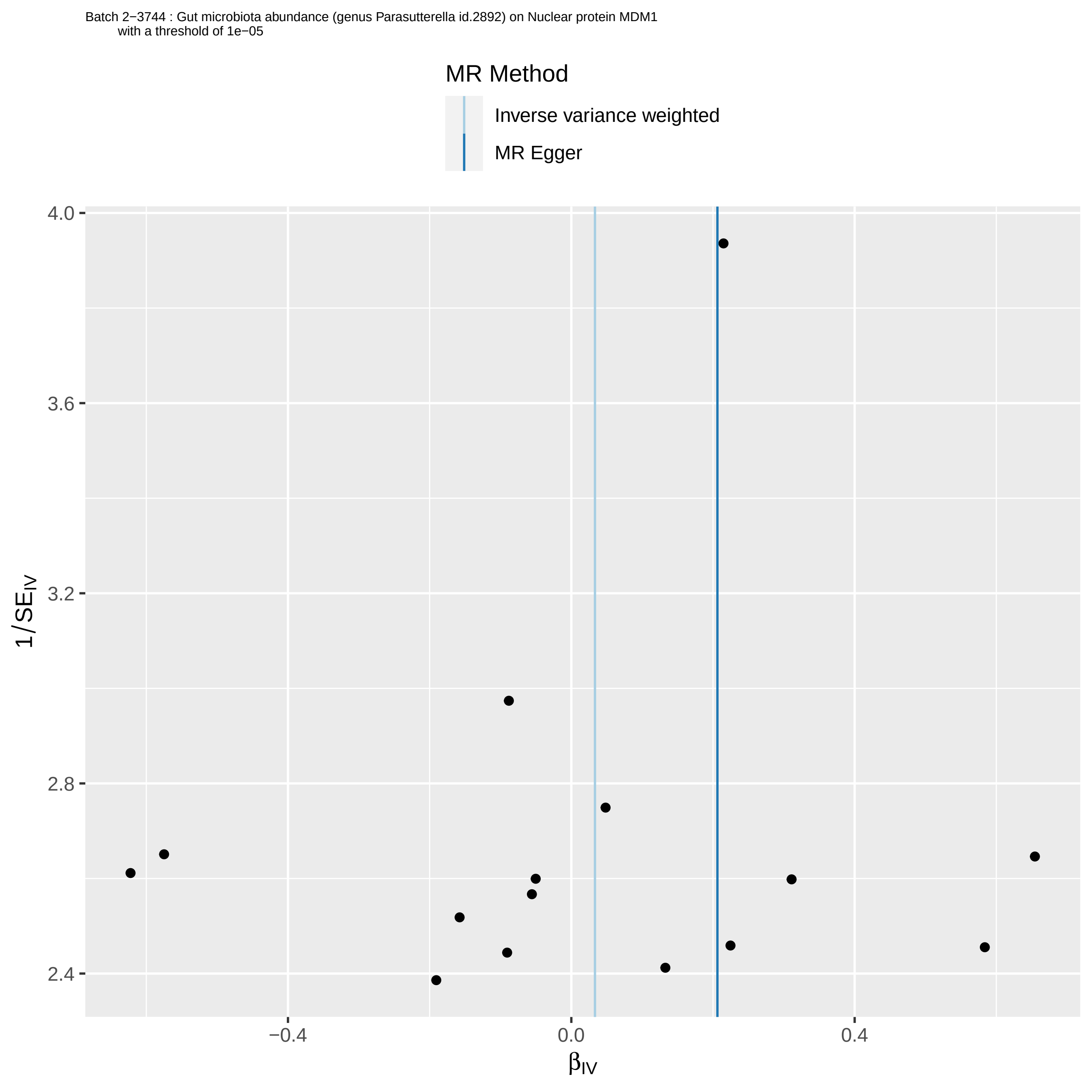

Batch 2−3744 : Gut microbiota abundance (genus Parasutterella id.2892) on Nuclear protein MDM1
with a threshold of 1e−05
MR Method
Inverse variance weighted
MR Egger
4.0
3.6
3.2
2.8
2.4
1/SE_IV
−0.4
0.0
0.4
β_IV

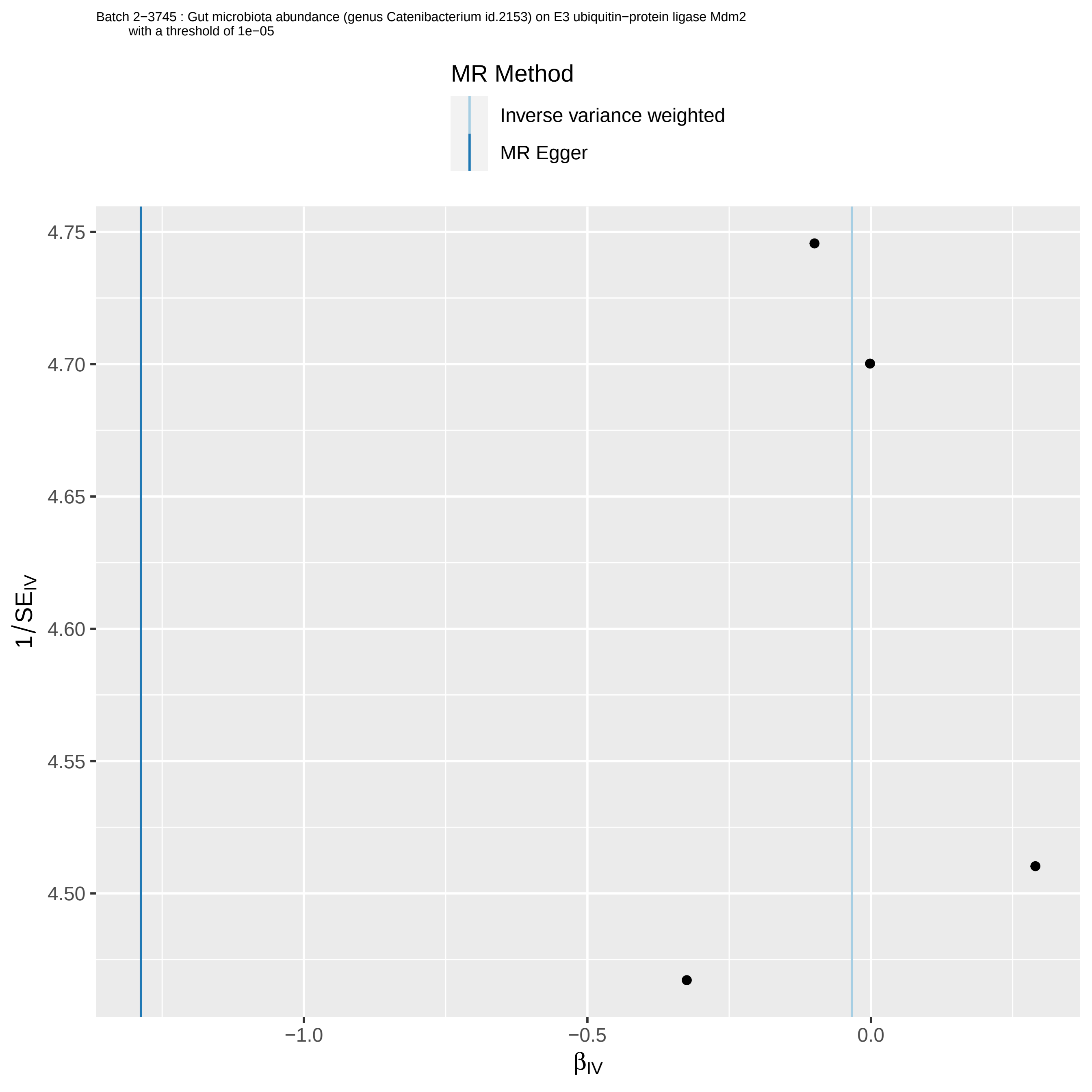
Batch 2−3745 : Gut microbiota abundance (genus Catenibacterium id.2153) on E3 ubiquitin−protein ligase Mdm2
with a threshold of 1e−05
MR Method
Inverse variance weighted
MR Egger
4.75
4.70
4.65
4.60
4.55
4.50
1/SE$_{IV}$
−1.0
−0.5
0.0
β$_{IV}$

Batch 2−3746 : Gut microbiota abundance (genus Parasutterella id.2892) on E3 ubiquitin−protein ligase Mdm2 with a threshold of 1e−05

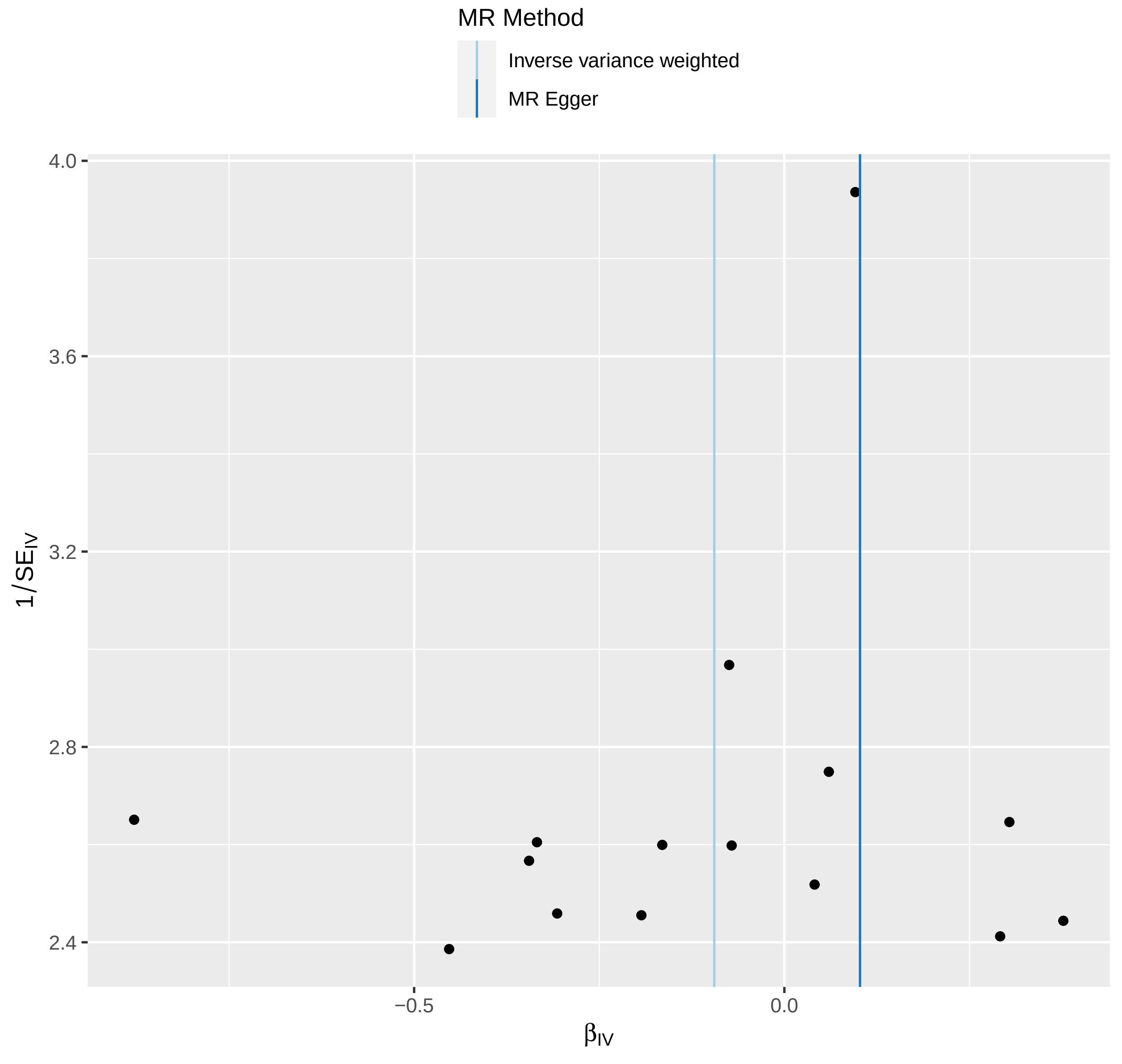

Batch 2−3747 : Gut microbiota abundance (genus Catenibacterium id.2153) on Mediator of RNA polymerase II transcription subunit 1 with a threshold of 1e−05

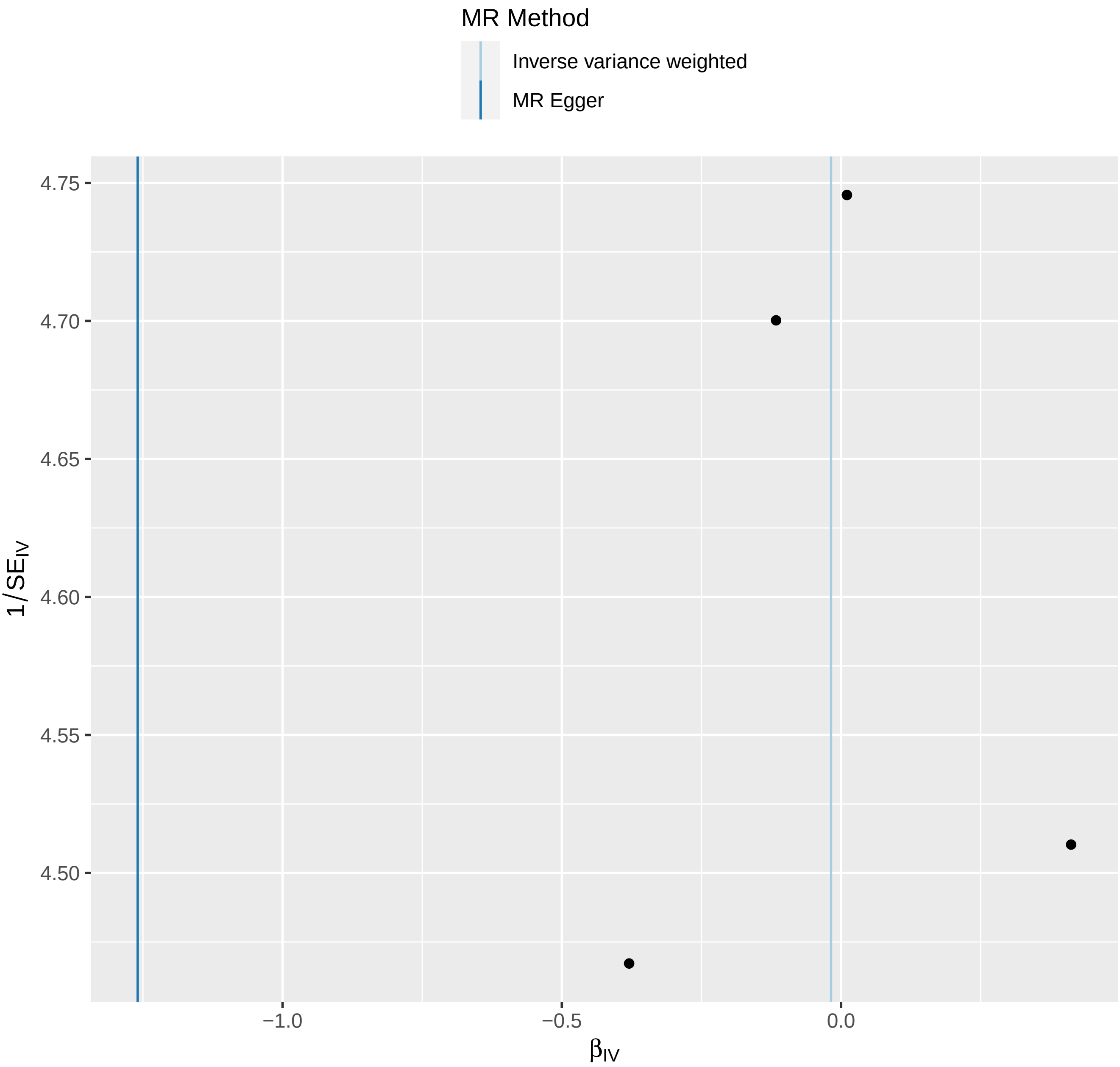

Batch 2−3748 : Gut microbiota abundance (genus Parasutterella id.2892) on Mediator of RNA polymerase II transcription subunit 1 with a threshold of 1e−05

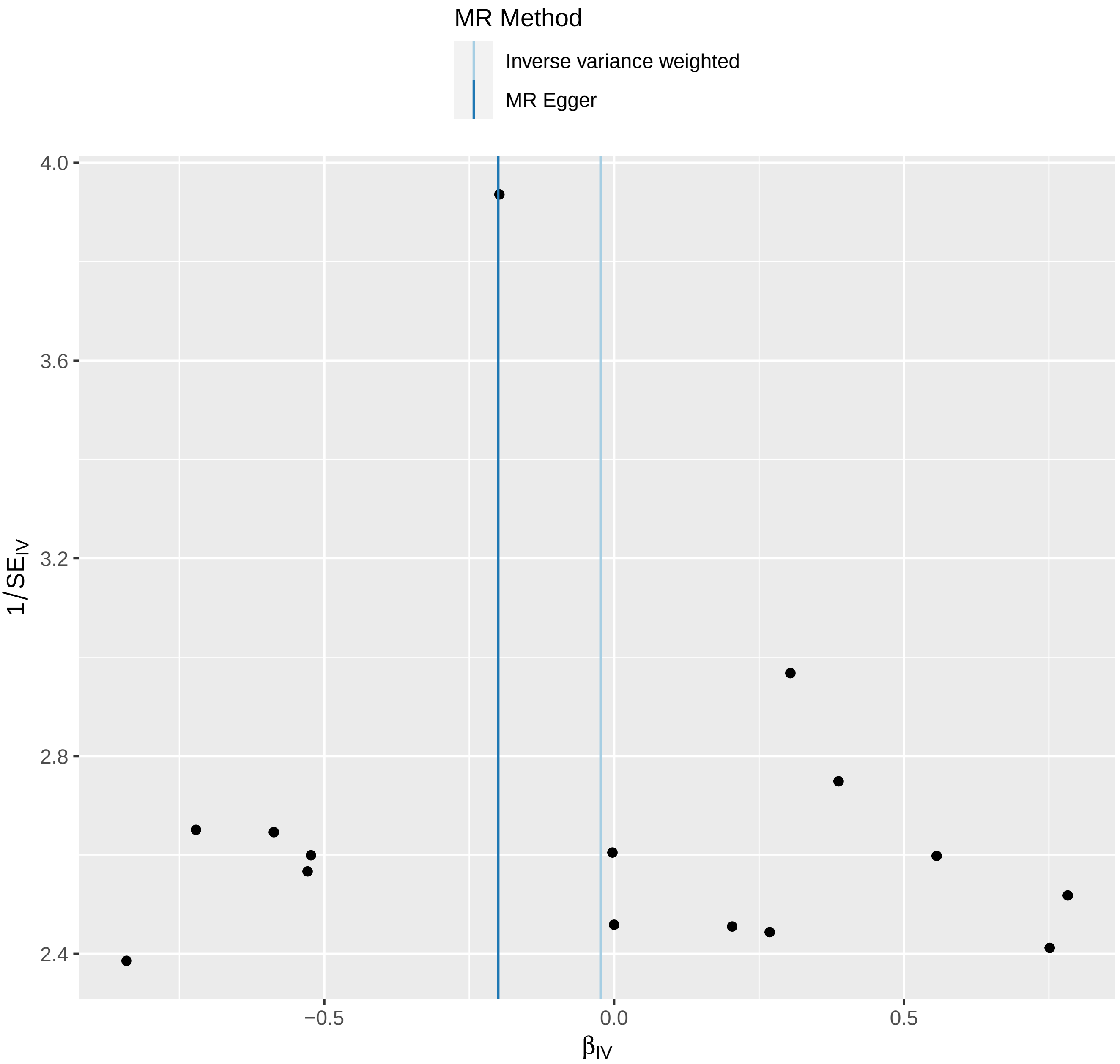

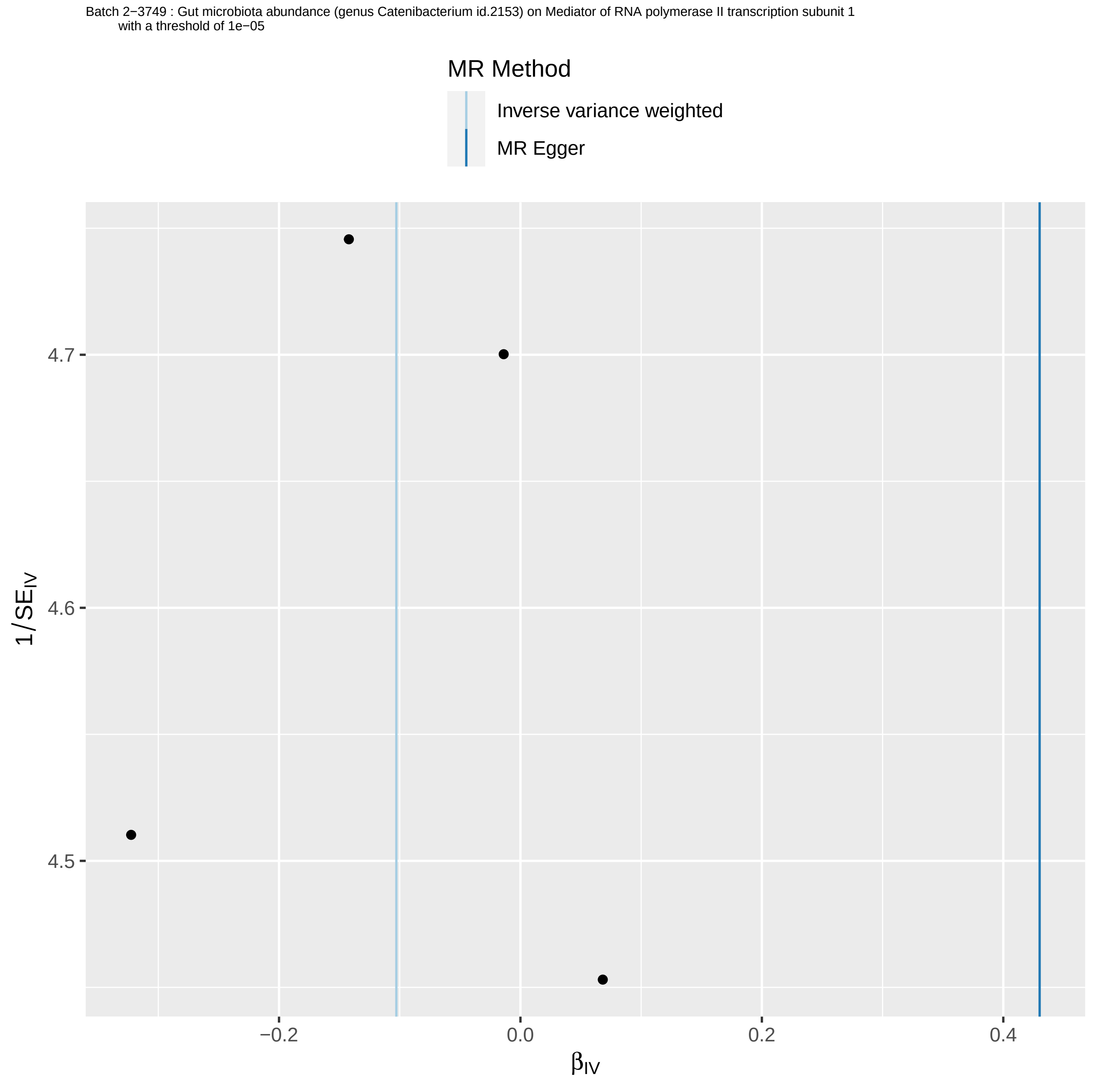

Batch 2−3749 : Gut microbiota abundance (genus Catenibacterium id.2153) on Mediator of RNA polymerase II transcription subunit 1
with a threshold of 1e−05
MR Method
Inverse variance weighted
MR Egger
4.7
4.6
4.5
1/SE_IV
−0.2
0.0
0.2
0.4
β_IV

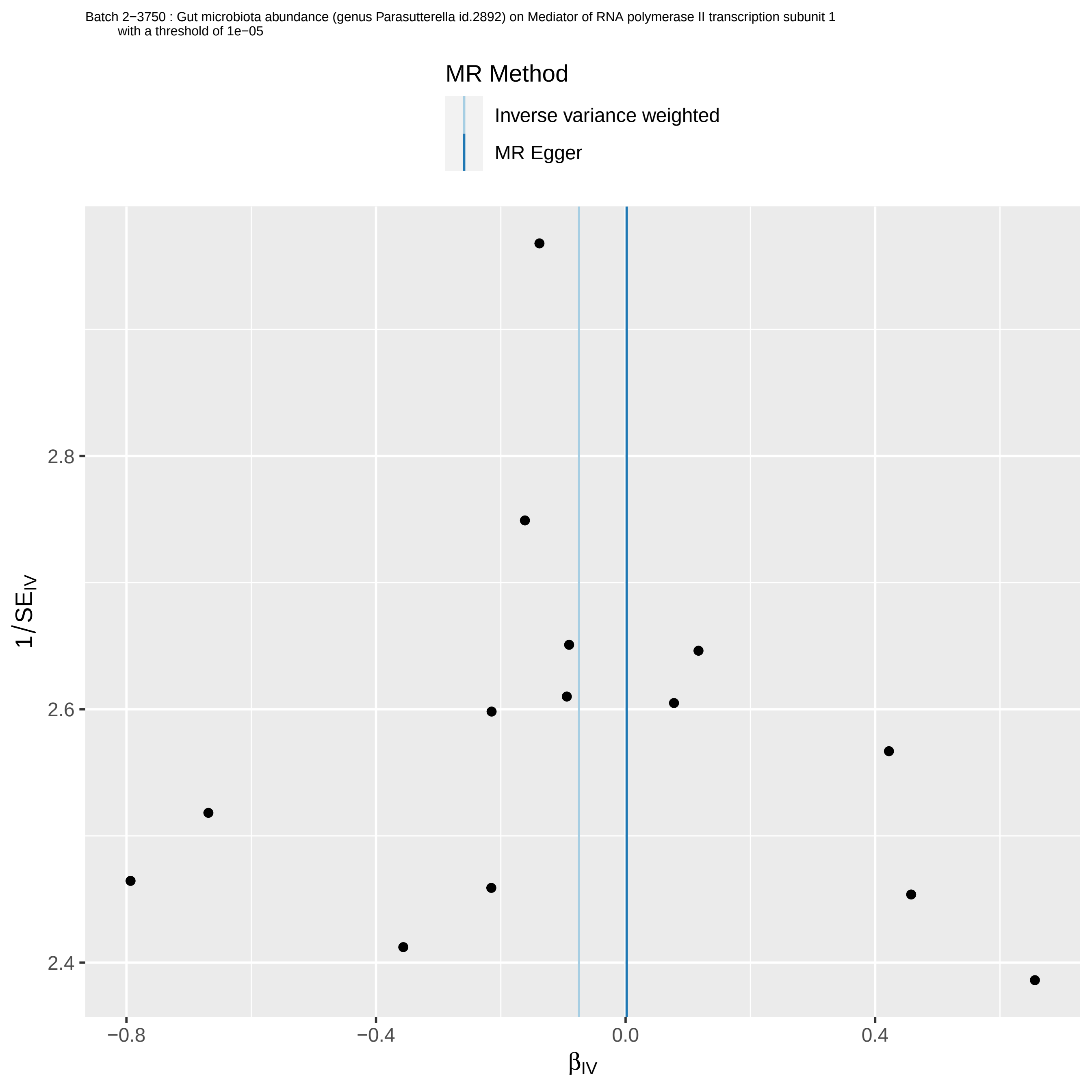

Batch 2−3750 : Gut microbiota abundance (genus Parasutterella id.2892) on Mediator of RNA polymerase II transcription subunit 1
with a threshold of 1e−05
MR Method
Inverse variance weighted
MR Egger
2.8
2.6
2.4
1/SE_IV
−0.8
−0.4
0.0
0.4
β_IV

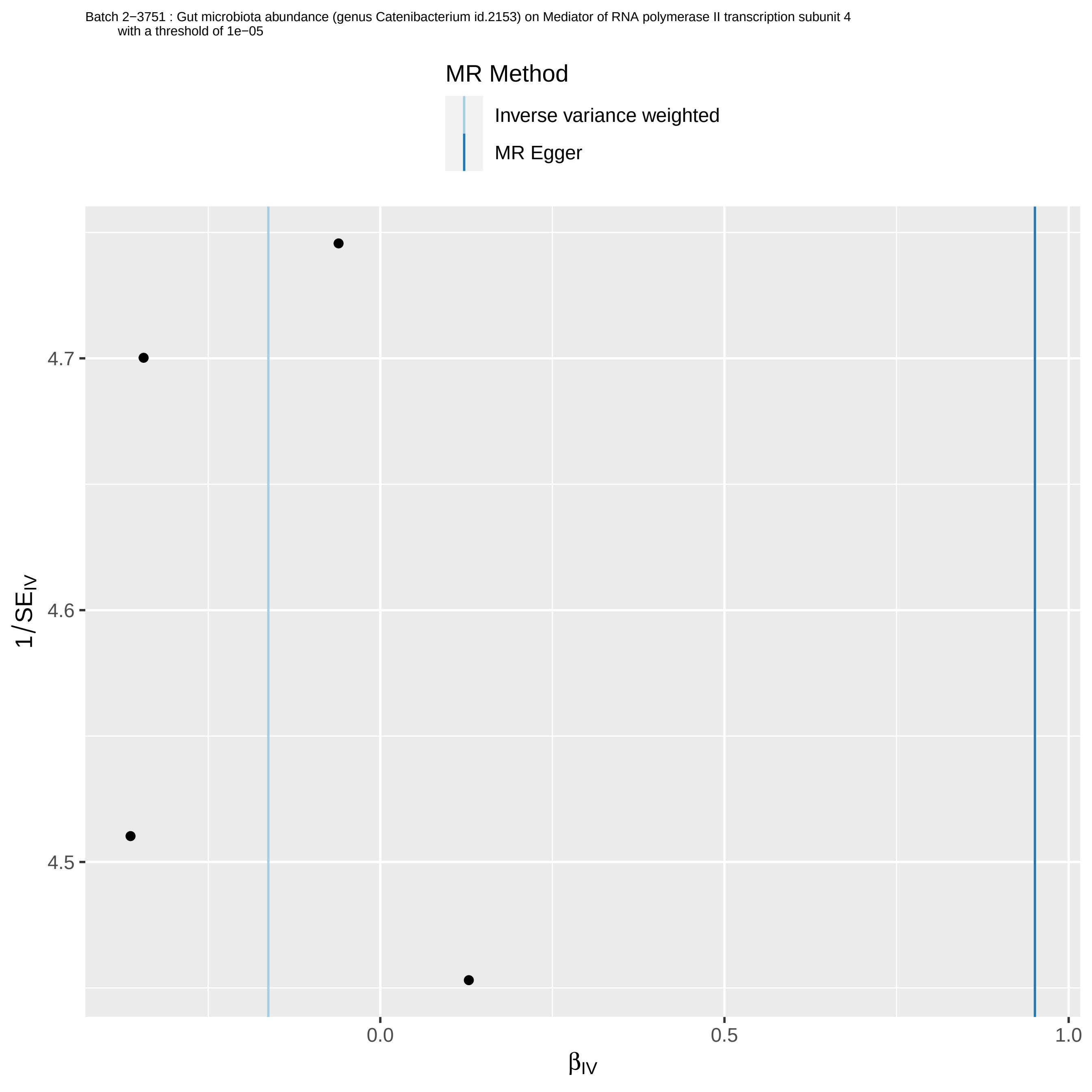
Batch 2−3751 : Gut microbiota abundance (genus Catenibacterium id.2153) on Mediator of RNA polymerase II transcription subunit 4
with a threshold of 1e−05
MR Method
Inverse variance weighted
MR Egger
4.7
4.6
4.5
1/SE_IV
0.0
0.5
1.0
β_IV

Batch 2−3752 : Gut microbiota abundance (genus Parasutterella id.2892) on Mediator of RNA polymerase II transcription subunit 4 with a threshold of 1e−05

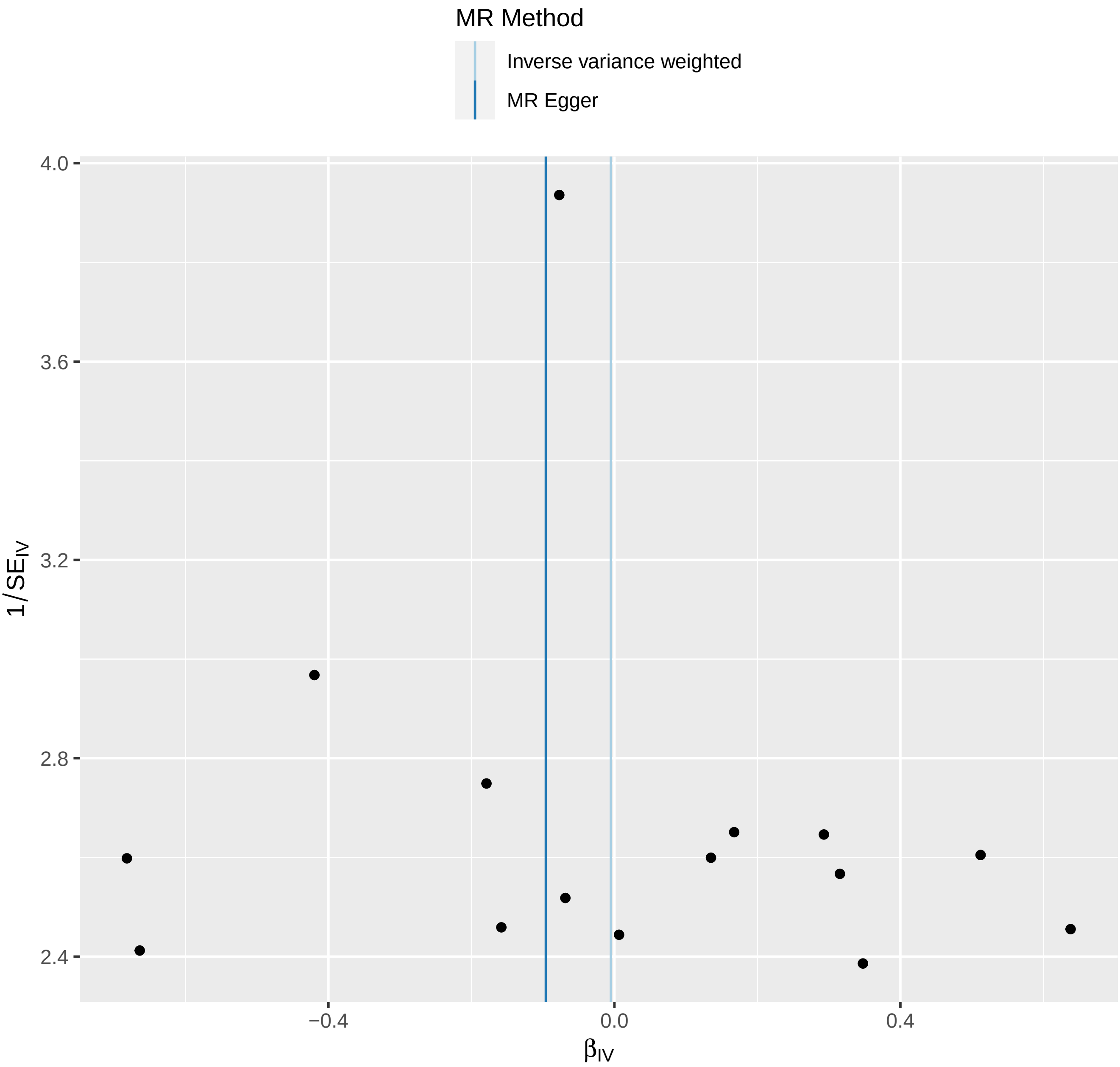

Batch 2−3753 : Gut microbiota abundance (genus Catenibacterium id.2153) on Myocyte−specific enhancer factor 2C
with a threshold of 1e−05

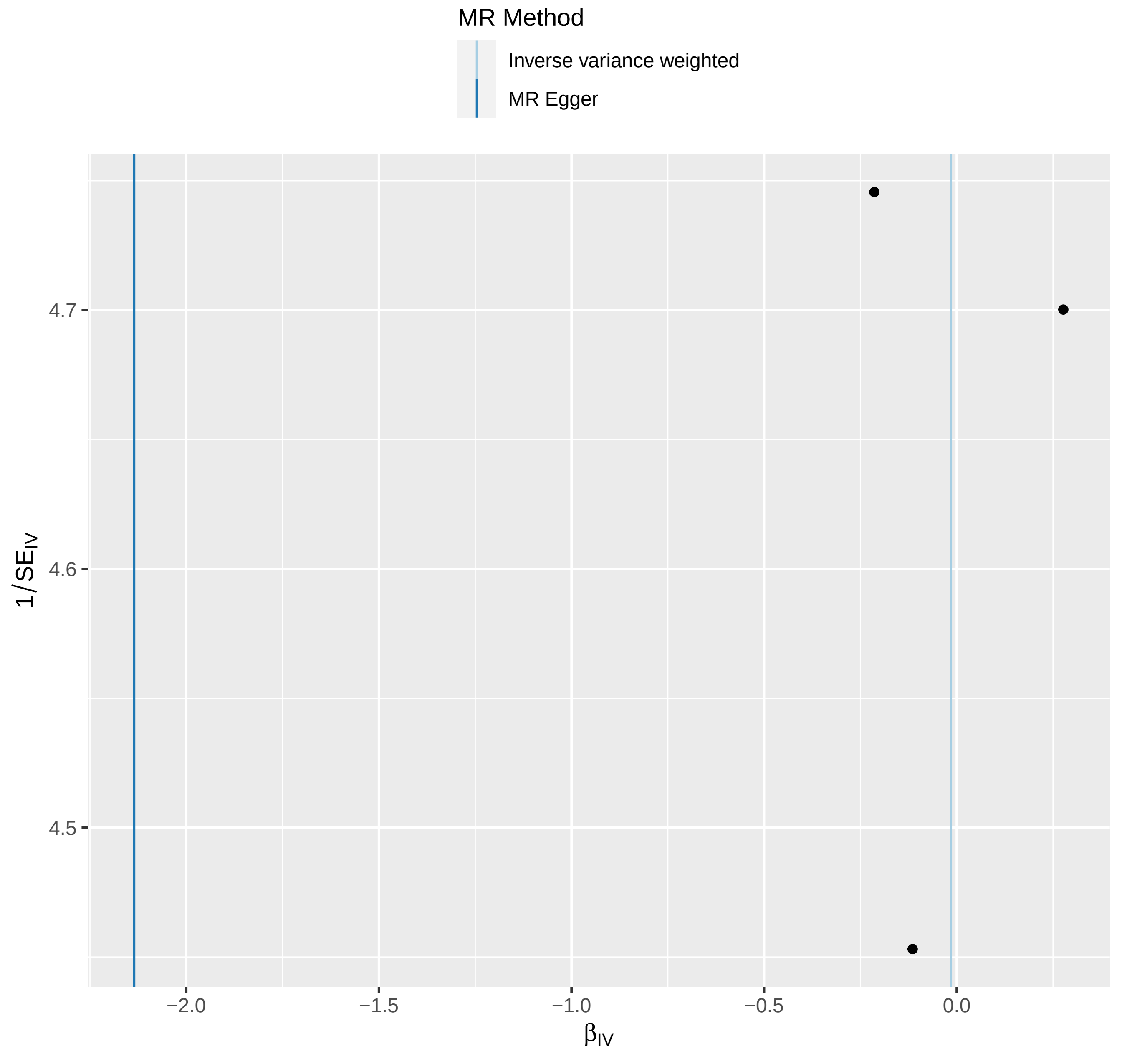

Batch 2−3754 : Gut microbiota abundance (genus Parasutterella id.2892) on Myocyte−specific enhancer factor 2C
with a threshold of 1e−05

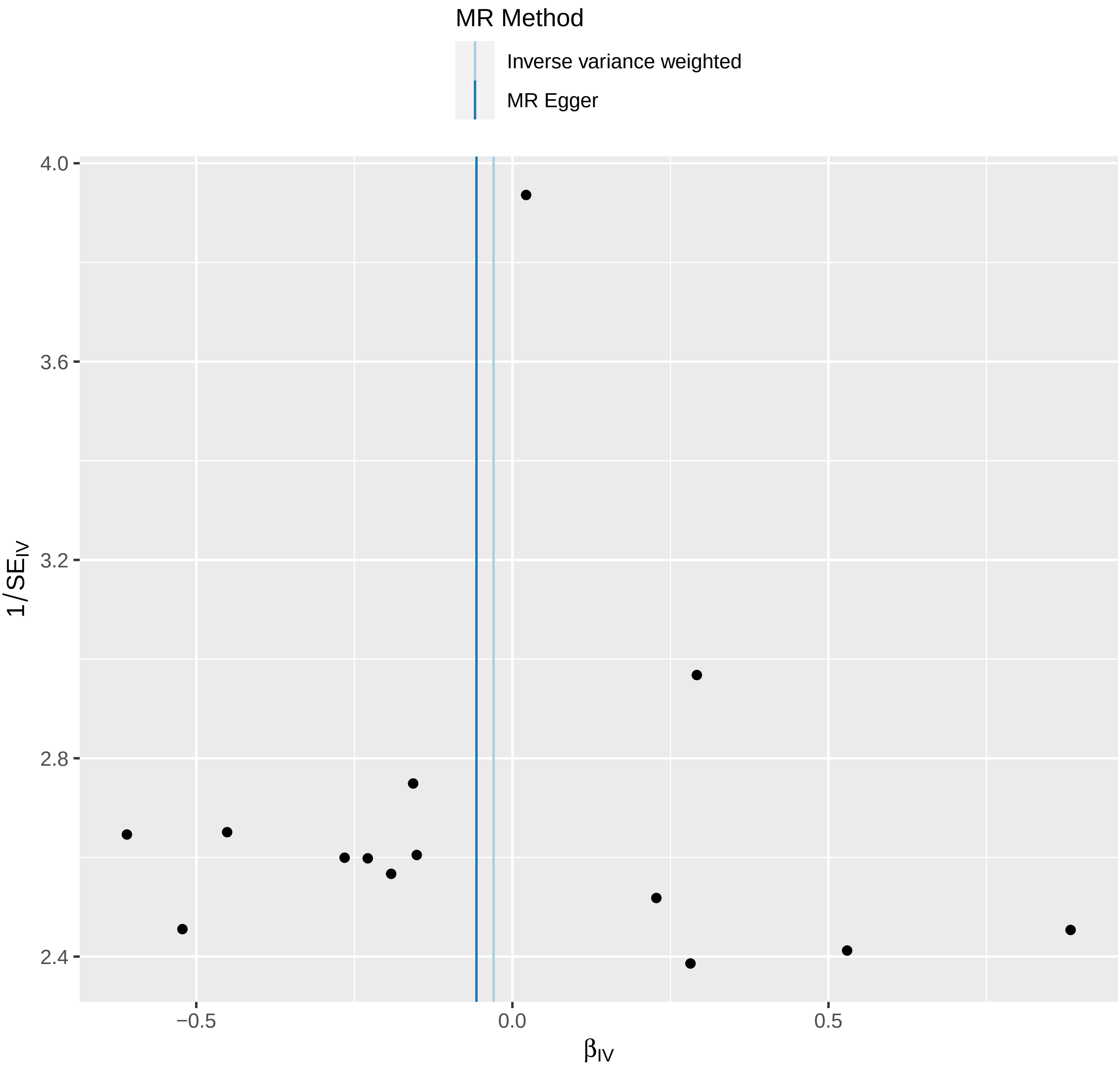

Batch 2−3755 : Gut microbiota abundance (genus Catenibacterium id.2153) on Multiple epidermal growth factor−like domains protein 10 with a threshold of 1e−05

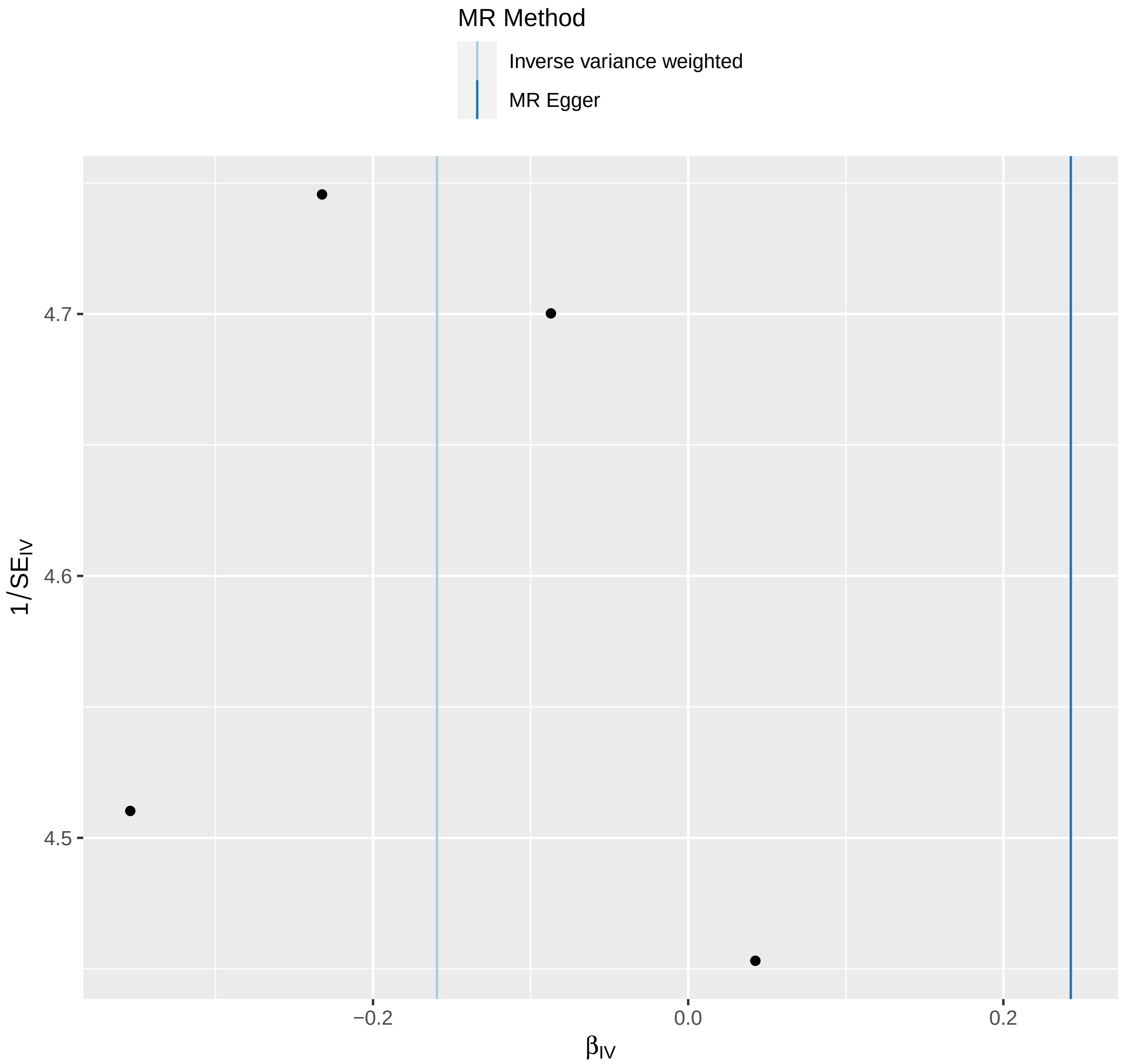

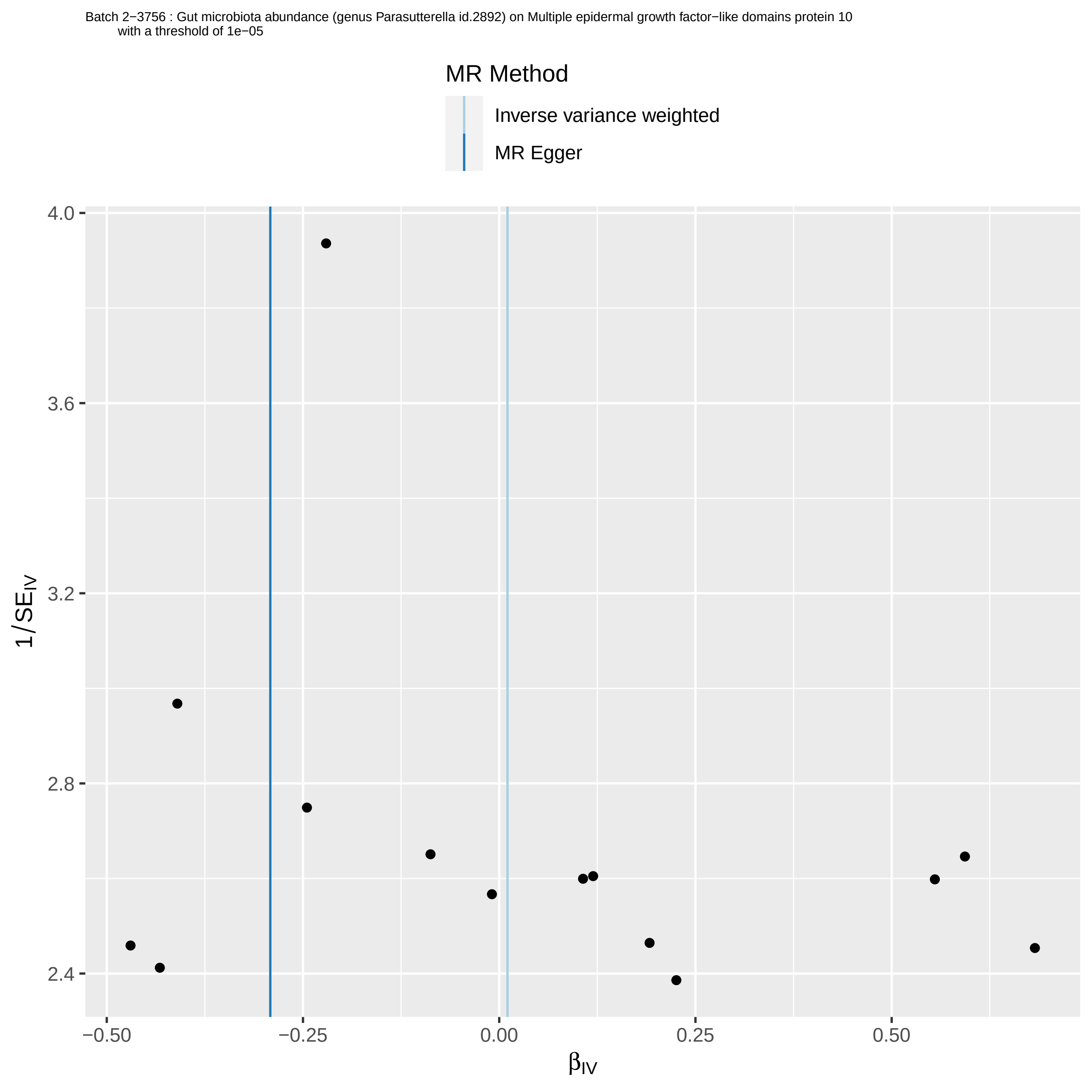
Batch 2−3756 : Gut microbiota abundance (genus Parasutterella id.2892) on Multiple epidermal growth factor−like domains protein 10
with a threshold of 1e−05
MR Method
Inverse variance weighted
MR Egger
4.0
3.6
3.2
2.8
2.4
1/SE_IV
−0.50
−0.25
0.00
0.25
0.50
β_IV

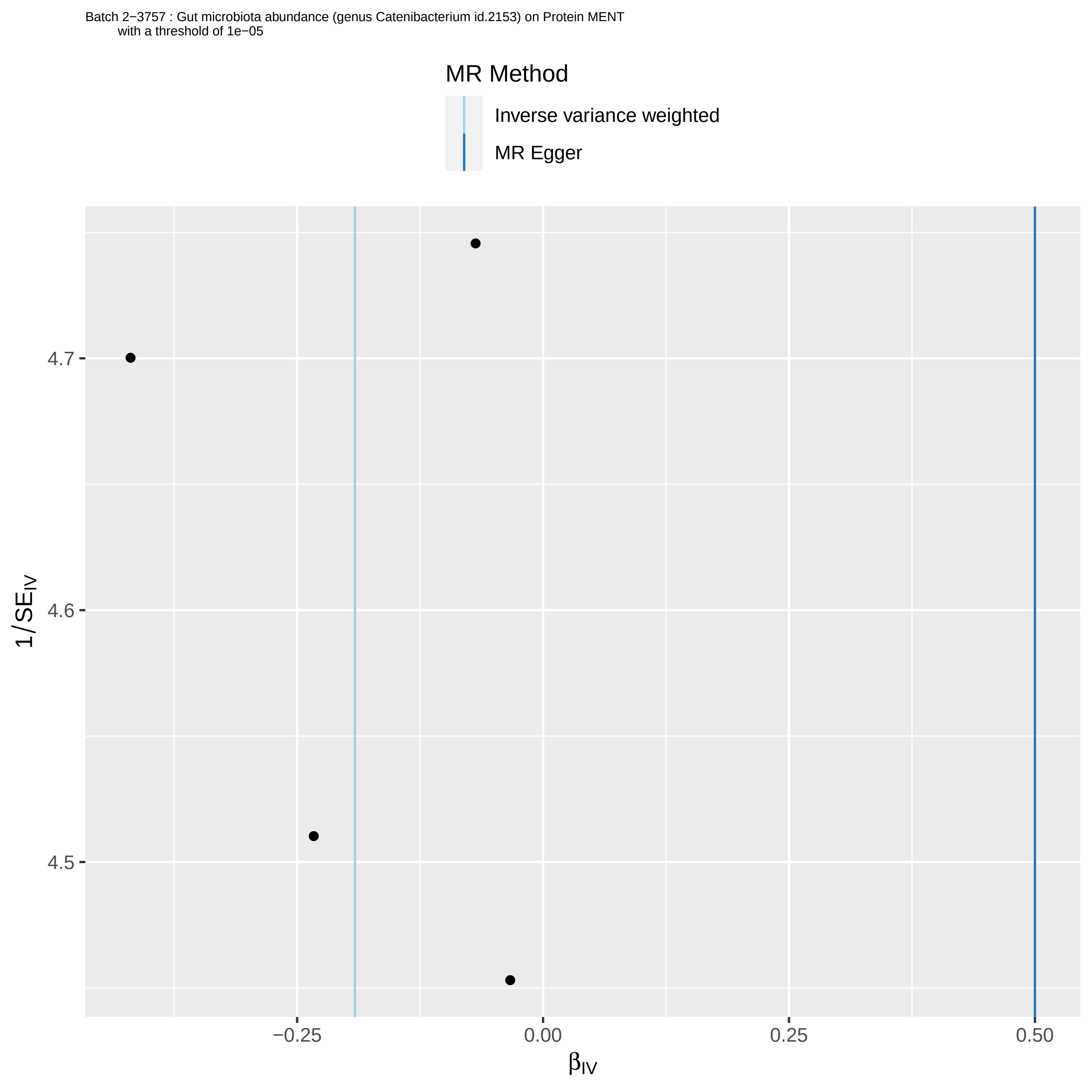
Batch 2−3757 : Gut microbiota abundance (genus Catenibacterium id.2153) on Protein MENT
with a threshold of 1e−05
MR Method
Inverse variance weighted
MR Egger
4.7
4.6
4.5
1/SE_IV
−0.25
0.00
0.25
0.50
β_IV

Batch 2−3758 : Gut microbiota abundance (genus Parasutterella id.2892) on Protein MENT
with a threshold of 1e−05

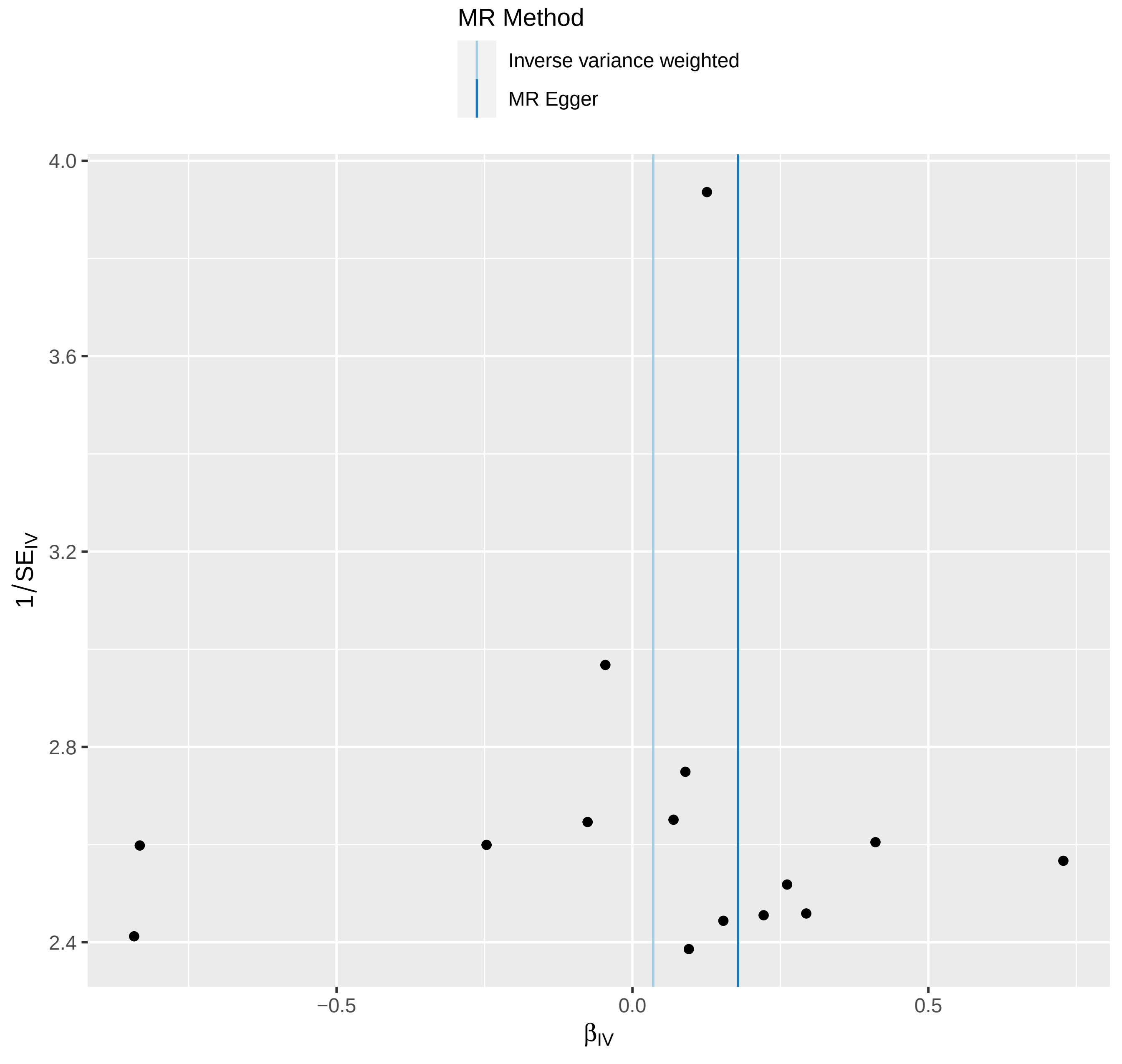

Batch 2−3759 : Gut microbiota abundance (genus Catenibacterium id.2153) on Matrix extracellular phosphoglycoprotein
with a threshold of 1e−05

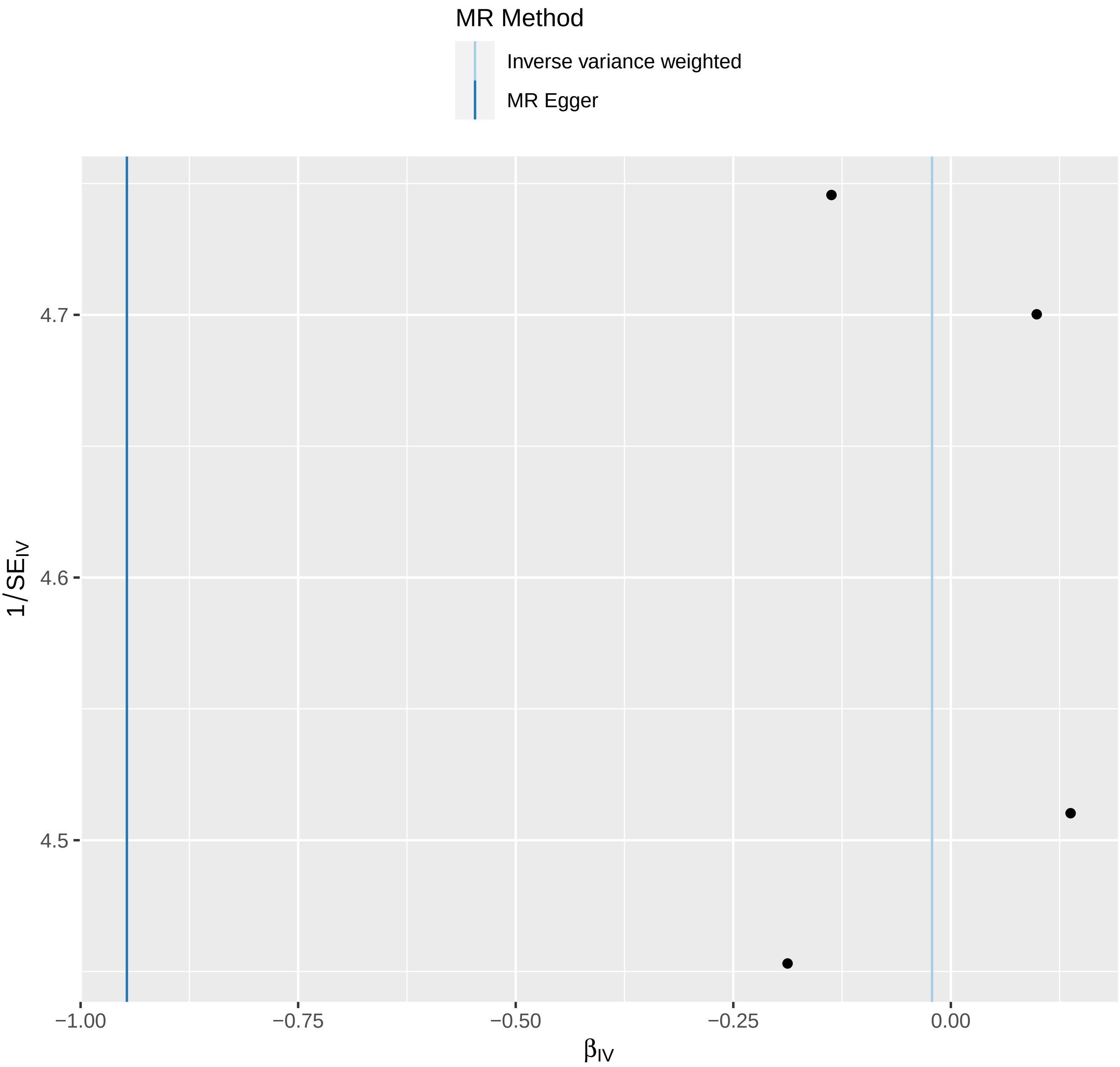

Batch 2−3760 : Gut microbiota abundance (genus Parasutterella id.2892) on Matrix extracellular phosphoglycoprotein
with a threshold of 1e−05

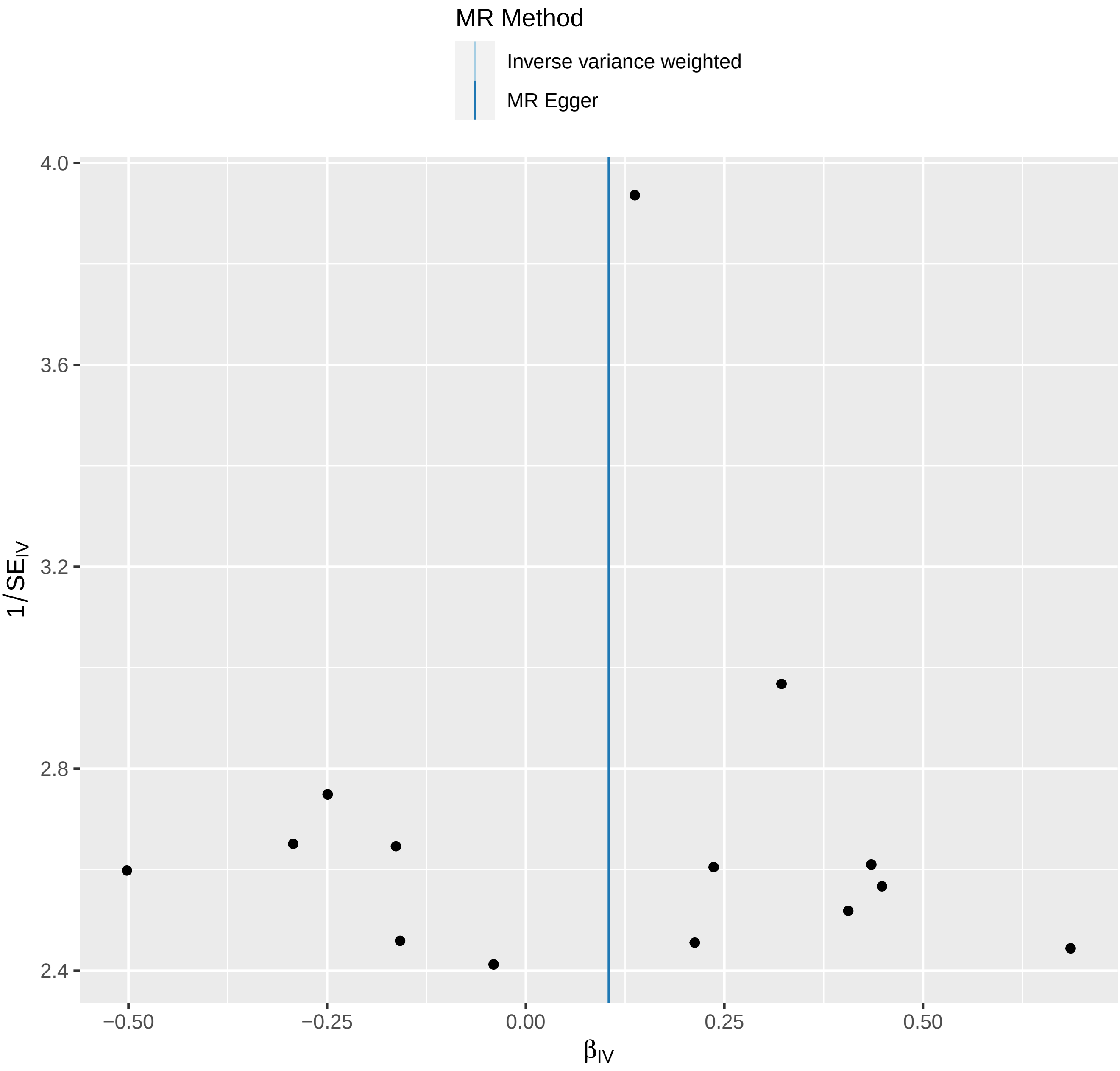

Batch 2−3761 : Gut microbiota abundance (genus Catenibacterium id.2153) on LDLR chaperone MESD
with a threshold of 1e−05

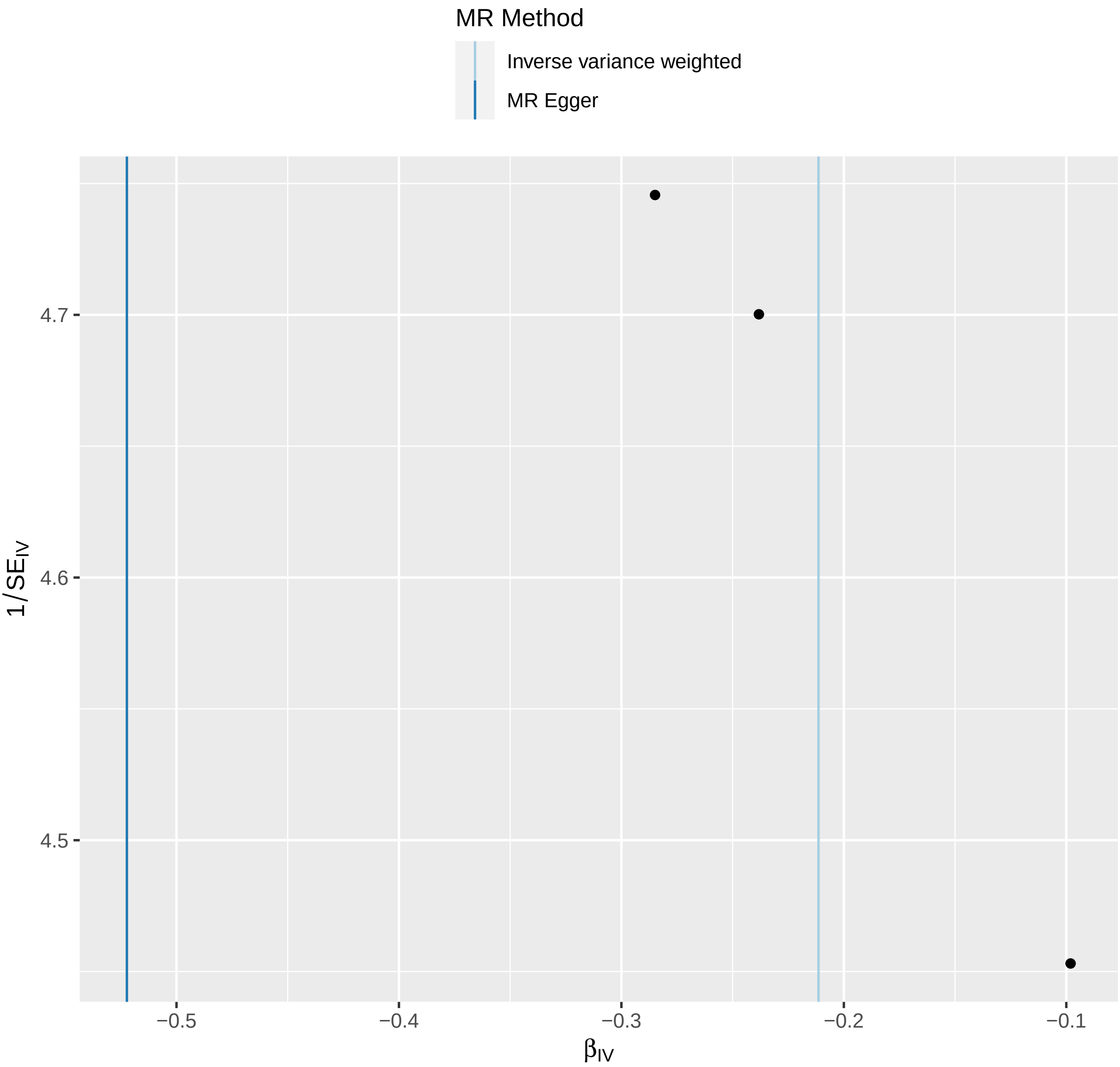

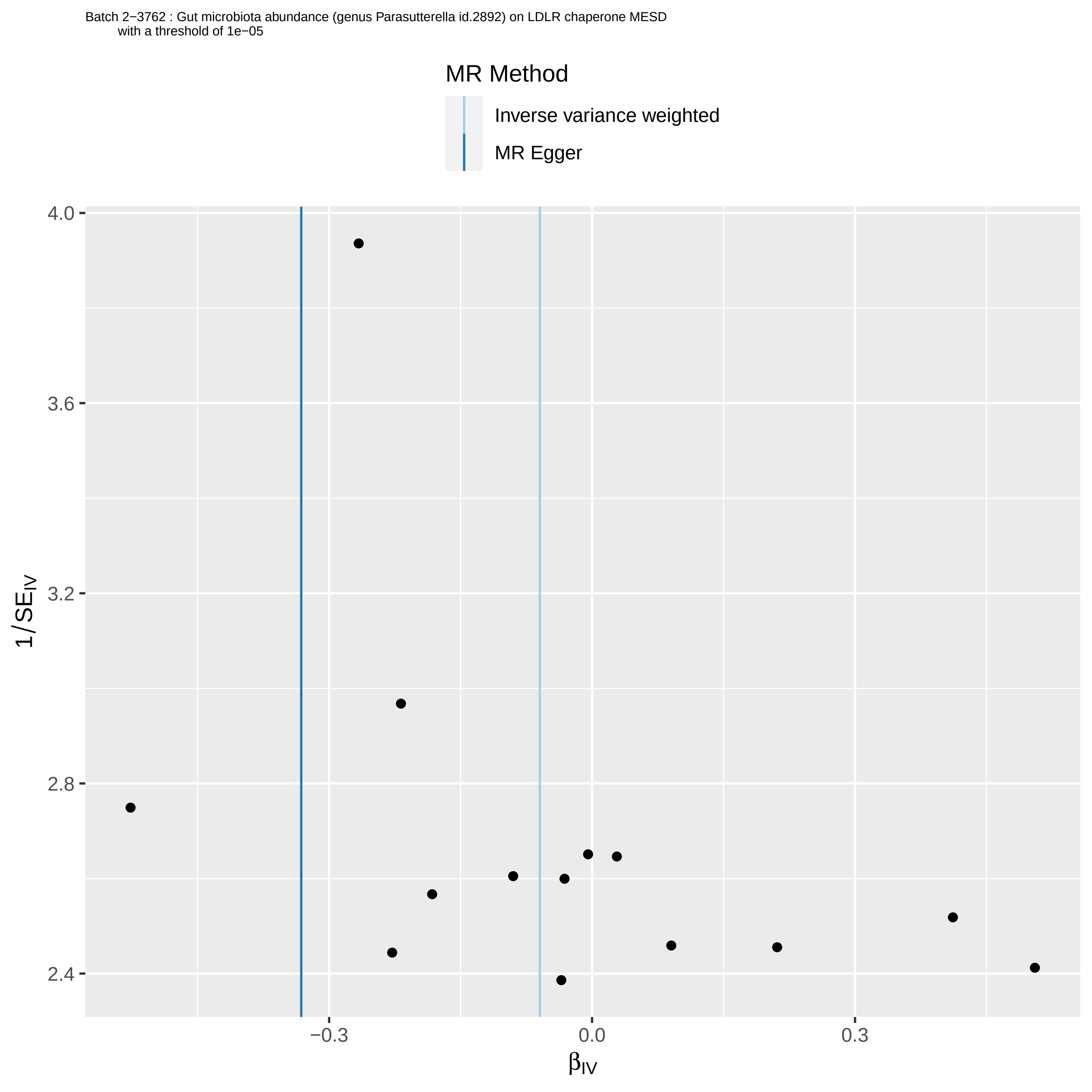
Batch 2−3762 : Gut microbiota abundance (genus Parasutterella id.2892) on LDLR chaperone MESD
with a threshold of 1e−05
MR Method
Inverse variance weighted
MR Egger
4.0
3.6
3.2
2.8
2.4
1/SE_IV
−0.3
0.0
0.3
β_IV

Batch 2−3763 : Gut microbiota abundance (genus Catenibacterium id.2153) on Hepatocyte growth factor receptor
with a threshold of 1e−05

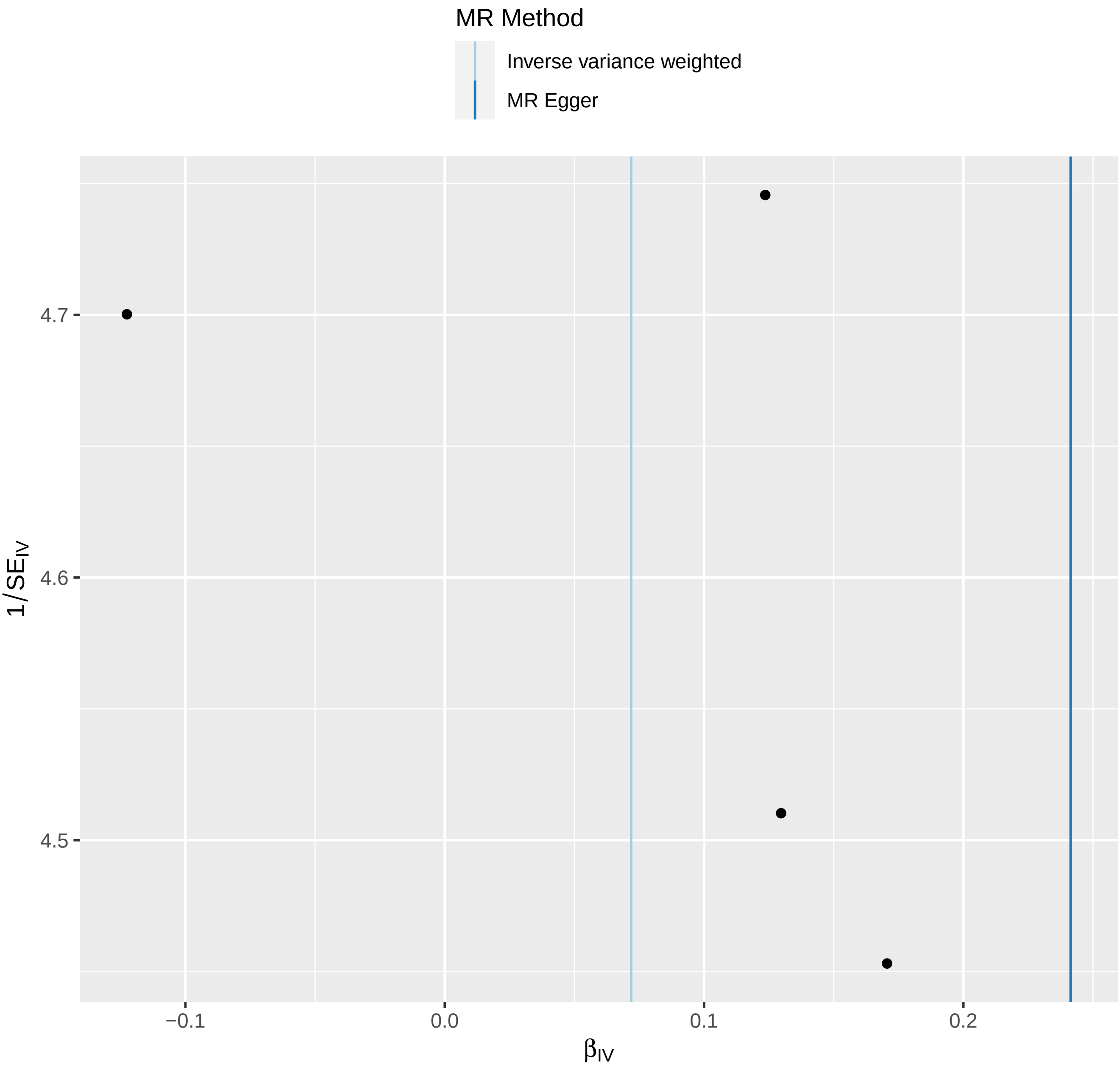

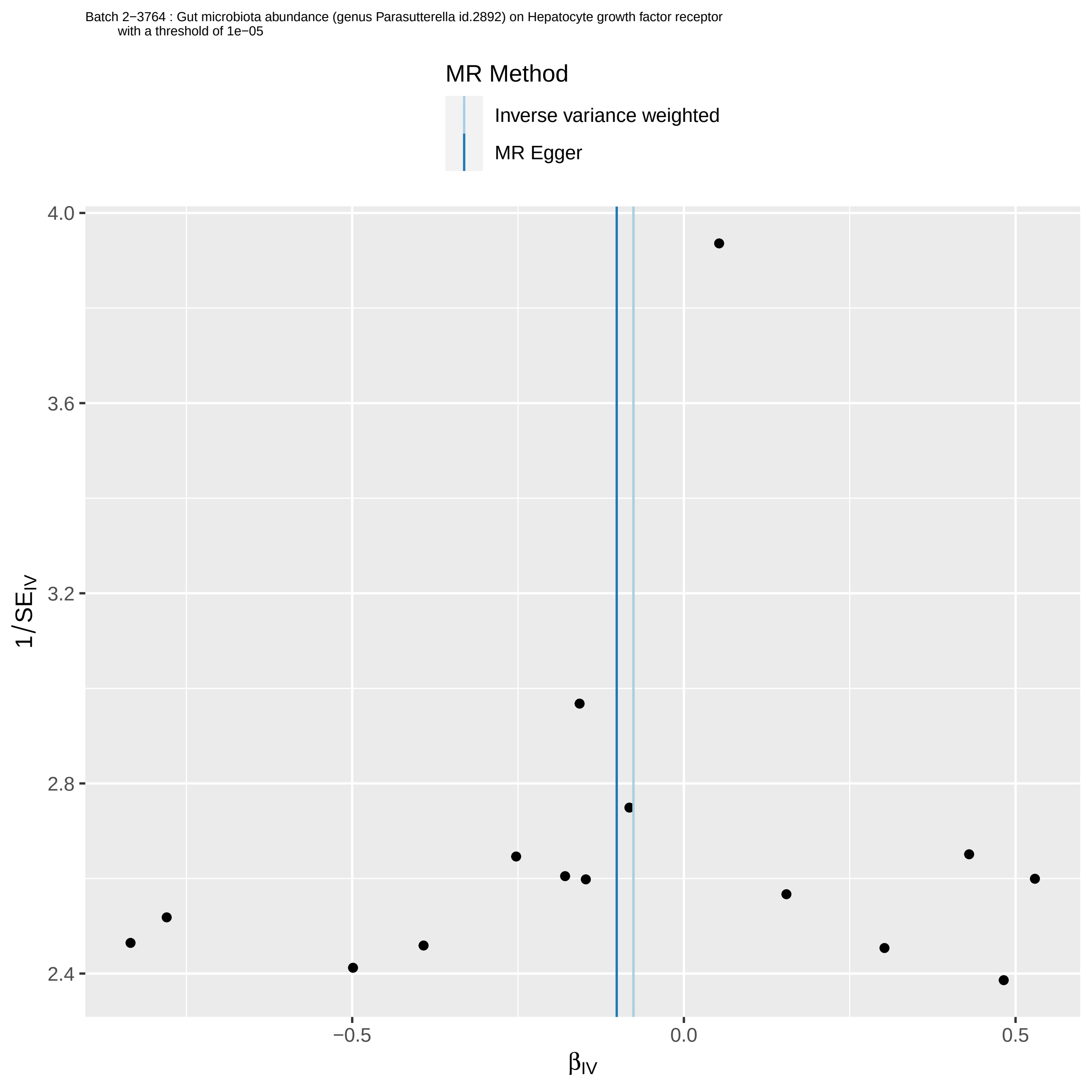
Batch 2−3764 : Gut microbiota abundance (genus Parasutterella id.2892) on Hepatocyte growth factor receptor
with a threshold of 1e−05
MR Method
Inverse variance weighted
MR Egger
4.0
3.6
3.2
2.8
2.4
1/SE_IV
−0.5
0.0
0.5
β_IV

Batch 2−3765 : Gut microbiota abundance (genus Catenibacterium id.2153) on tRNA (guanine−N(7)−)−methyltransferase
with a threshold of 1e−05

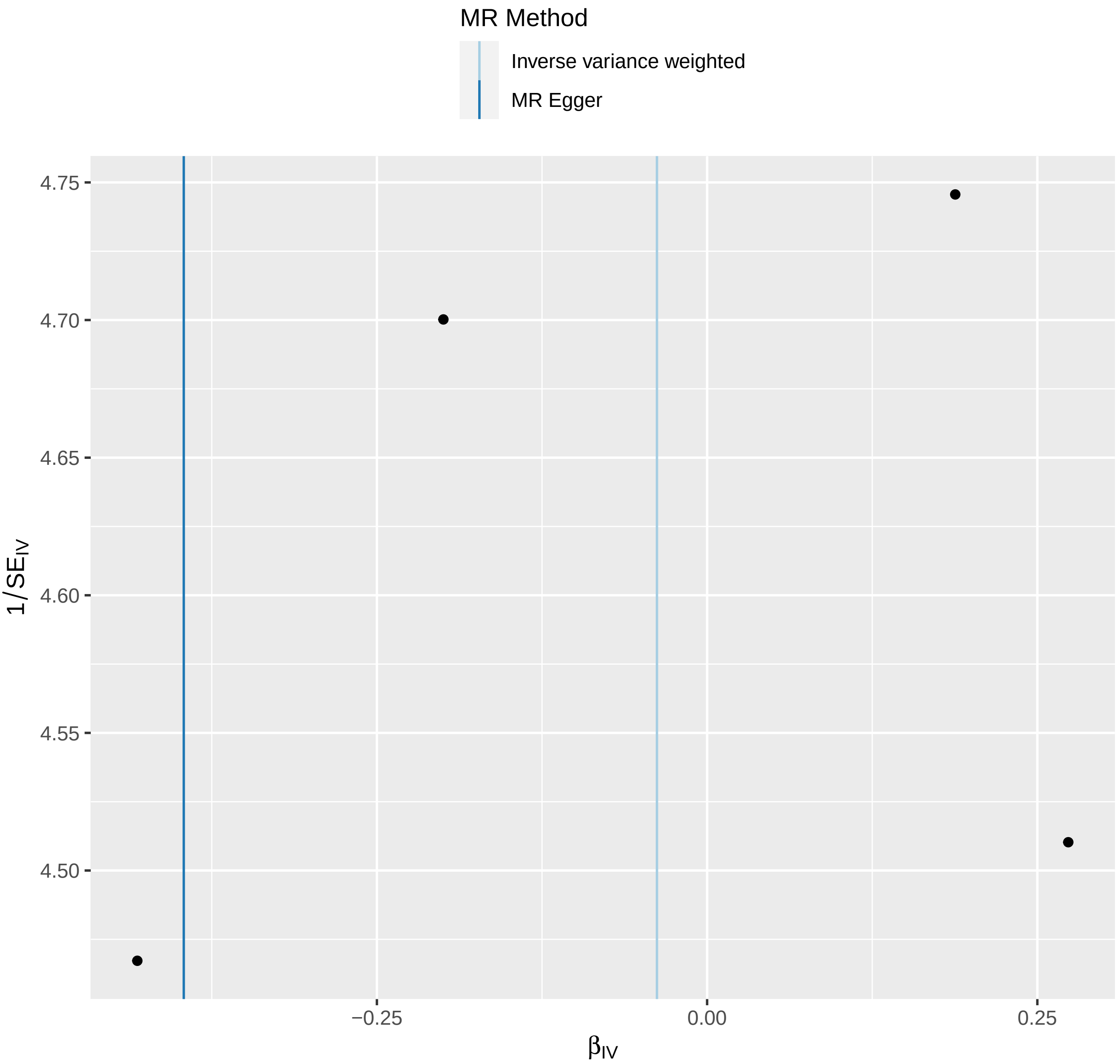

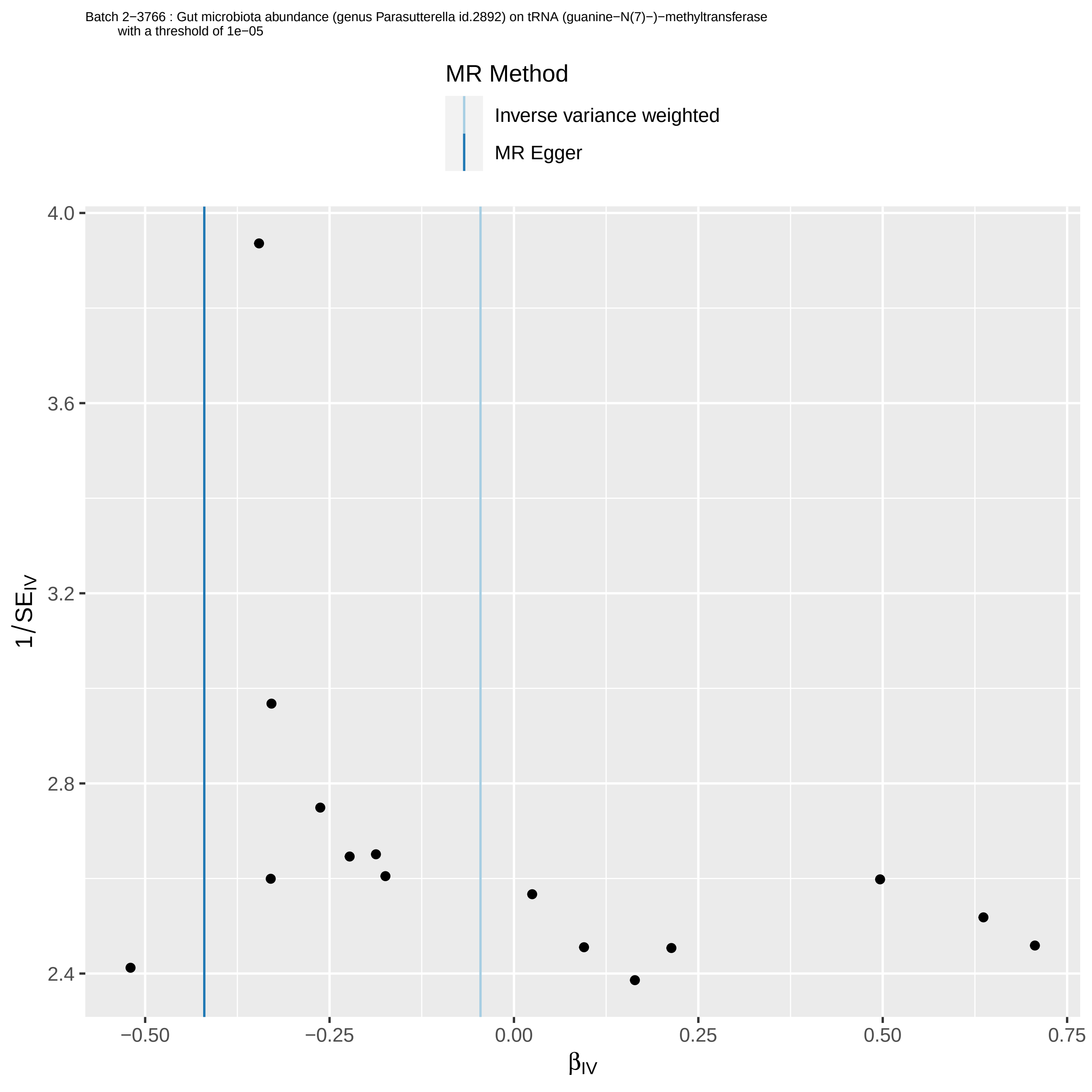

Batch 2−3766 : Gut microbiota abundance (genus Parasutterella id.2892) on tRNA (guanine−N(7)−)−methyltransferase
with a threshold of 1e−05
MR Method
Inverse variance weighted
MR Egger
4.0
3.6
3.2
2.8
2.4
$1/SE_{IV}$
−0.50
−0.25
0.00
0.25
0.50
0.75
β_{IV}

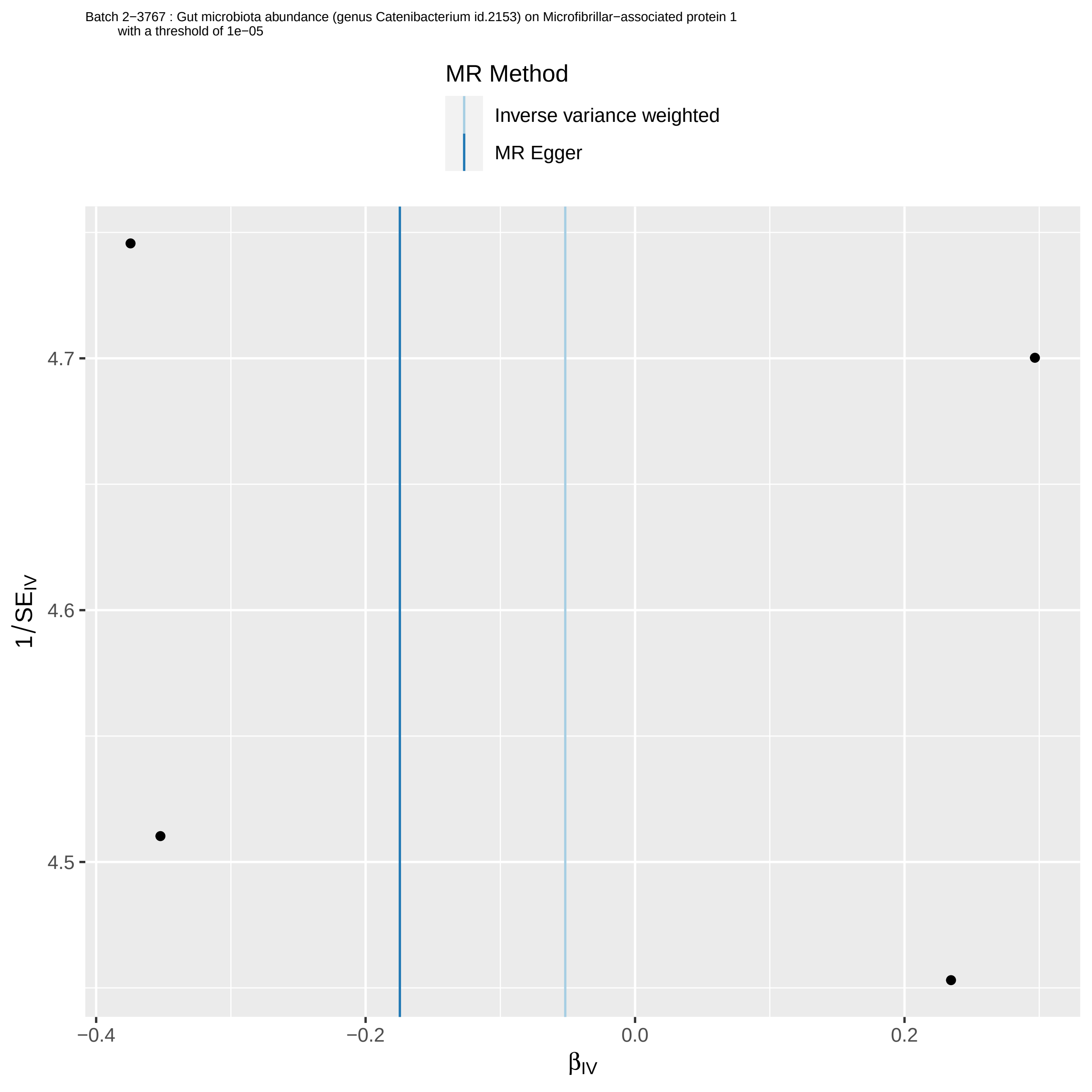
Batch 2−3767 : Gut microbiota abundance (genus Catenibacterium id.2153) on Microfibrillar−associated protein 1
with a threshold of 1e−05
MR Method
Inverse variance weighted
MR Egger
4.7
4.6
4.5
1/SE$_{IV}$
−0.4
−0.2
0.0
0.2
β$_{IV}$

Batch 2−3768 : Gut microbiota abundance (genus Parasutterella id.2892) on Microfibrillar−associated protein 1 with a threshold of 1e−05

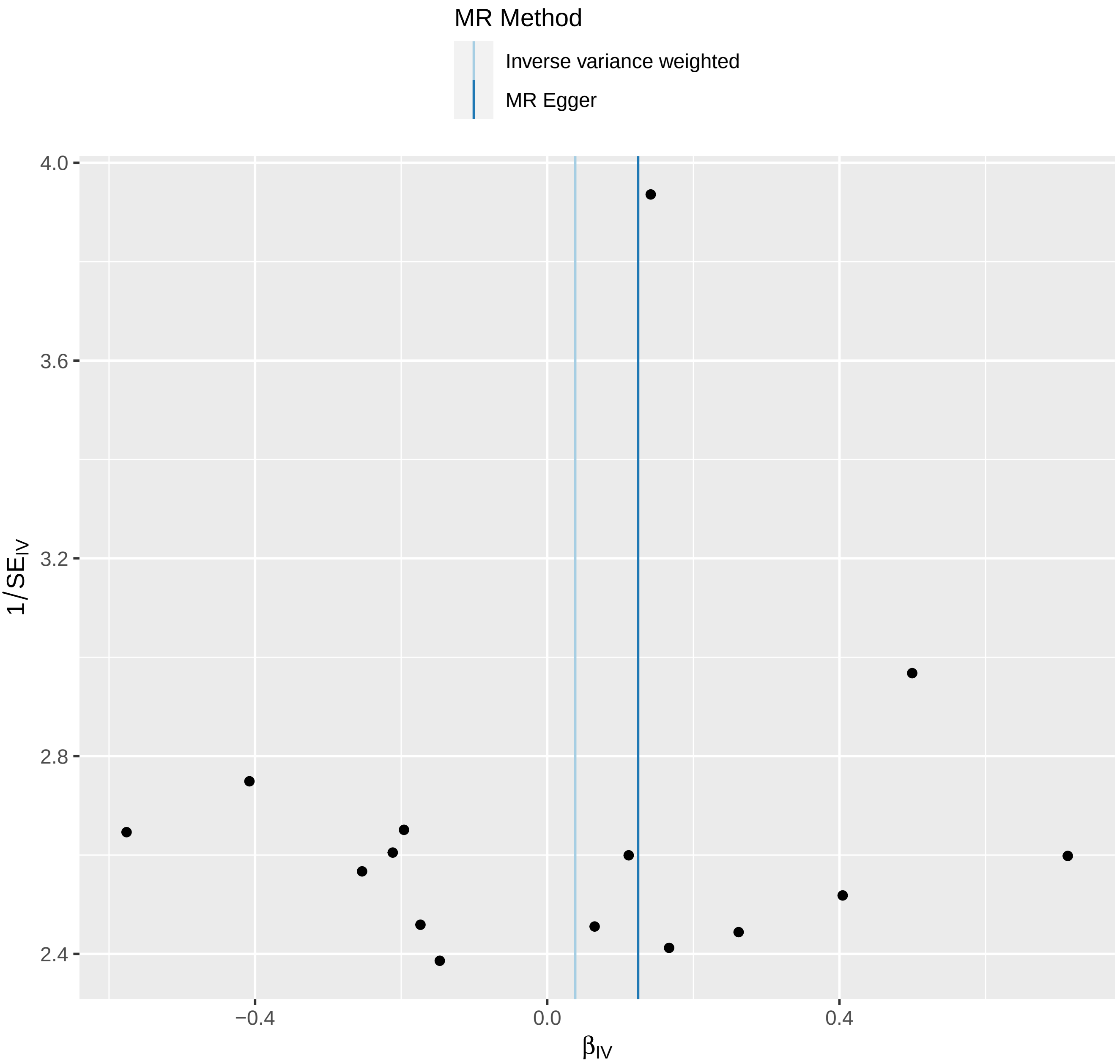

Batch 2−3769 : Gut microbiota abundance (genus Catenibacterium id.2153) on Microfibrillar−associated protein 2
with a threshold of 1e−05

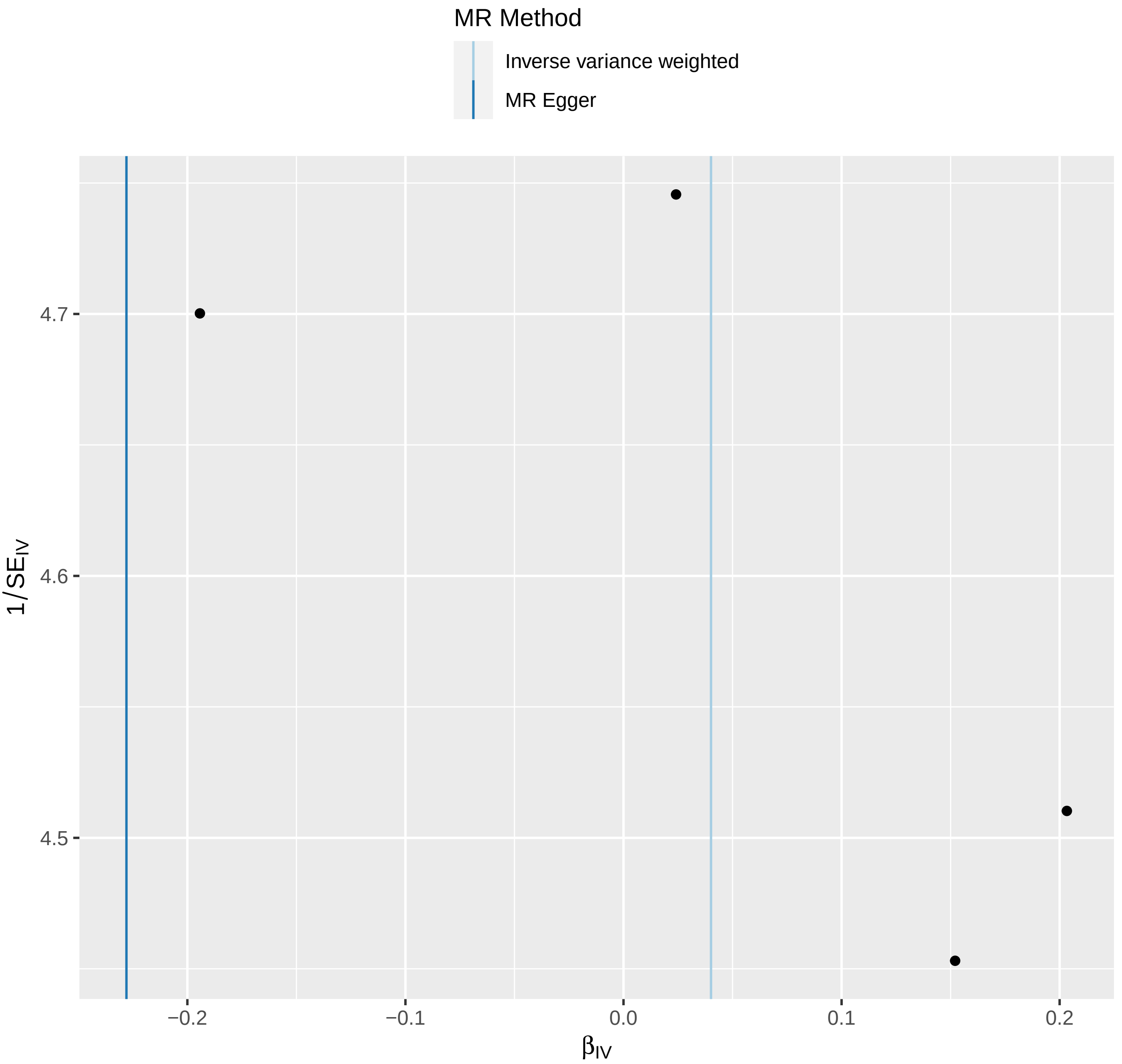

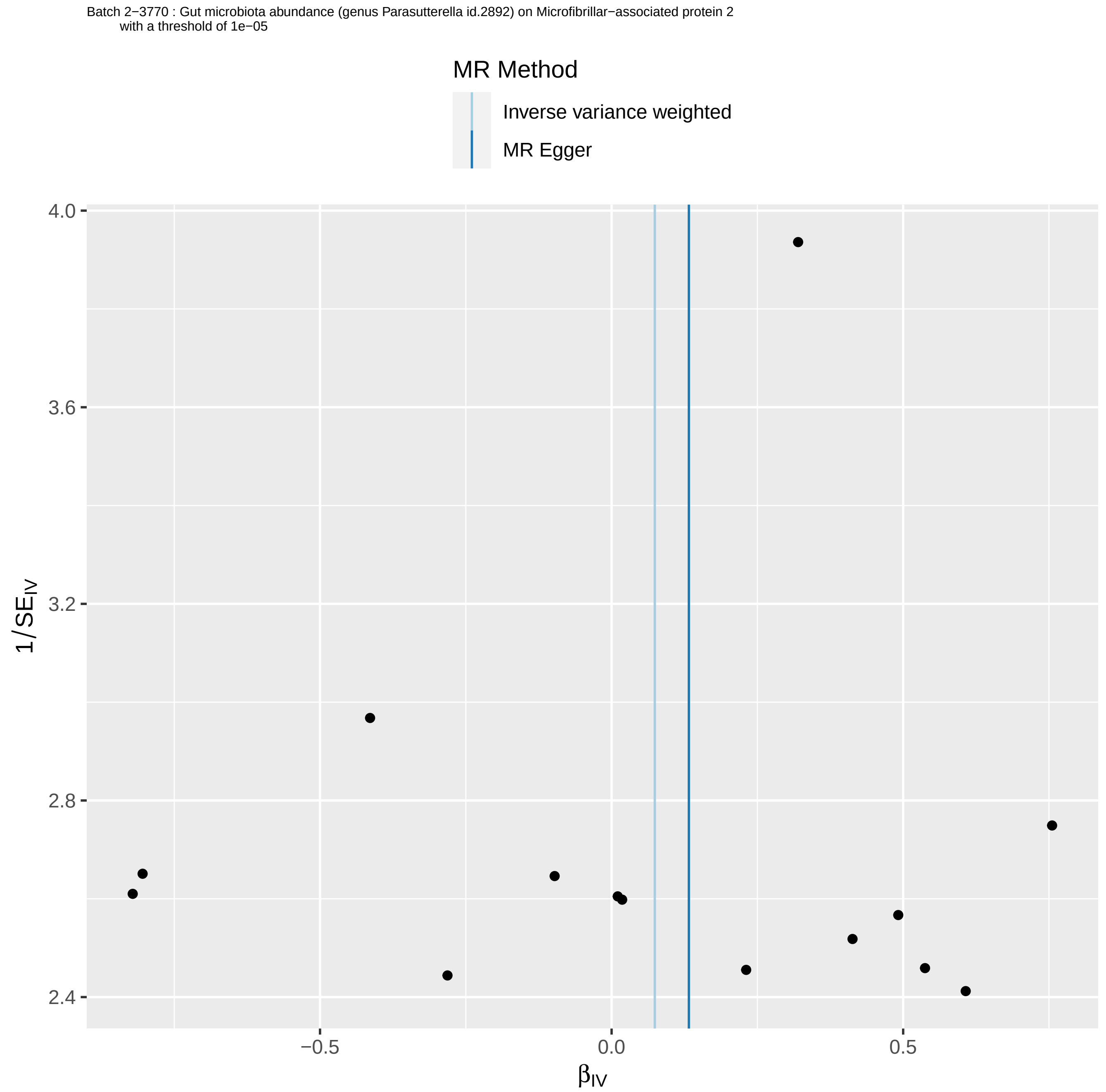

Batch 2−3770 : Gut microbiota abundance (genus Parasutterella id.2892) on Microfibrillar−associated protein 2
with a threshold of 1e−05
MR Method
Inverse variance weighted
MR Egger
4.0
3.6
3.2
2.8
2.4
1/SE_IV
−0.5
0.0
0.5
β_IV

Batch 2−3771 : Gut microbiota abundance (genus Catenibacterium id.2153) on Microfibril−associated glycoprotein 4
with a threshold of 1e−05

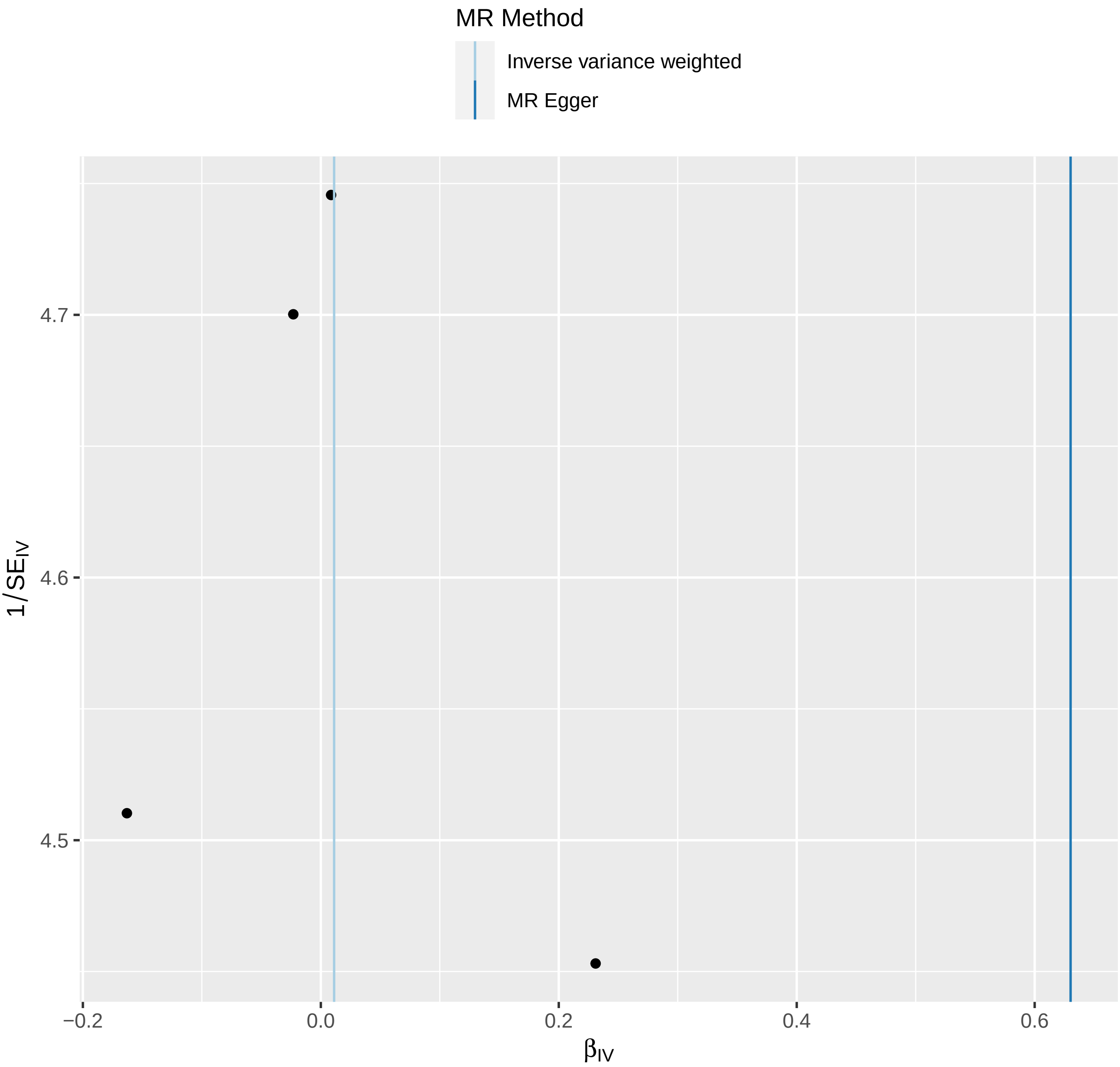

Batch 2−3772 : Gut microbiota abundance (genus Parasutterella id.2892) on Microfibril−associated glycoprotein 4
with a threshold of 1e−05

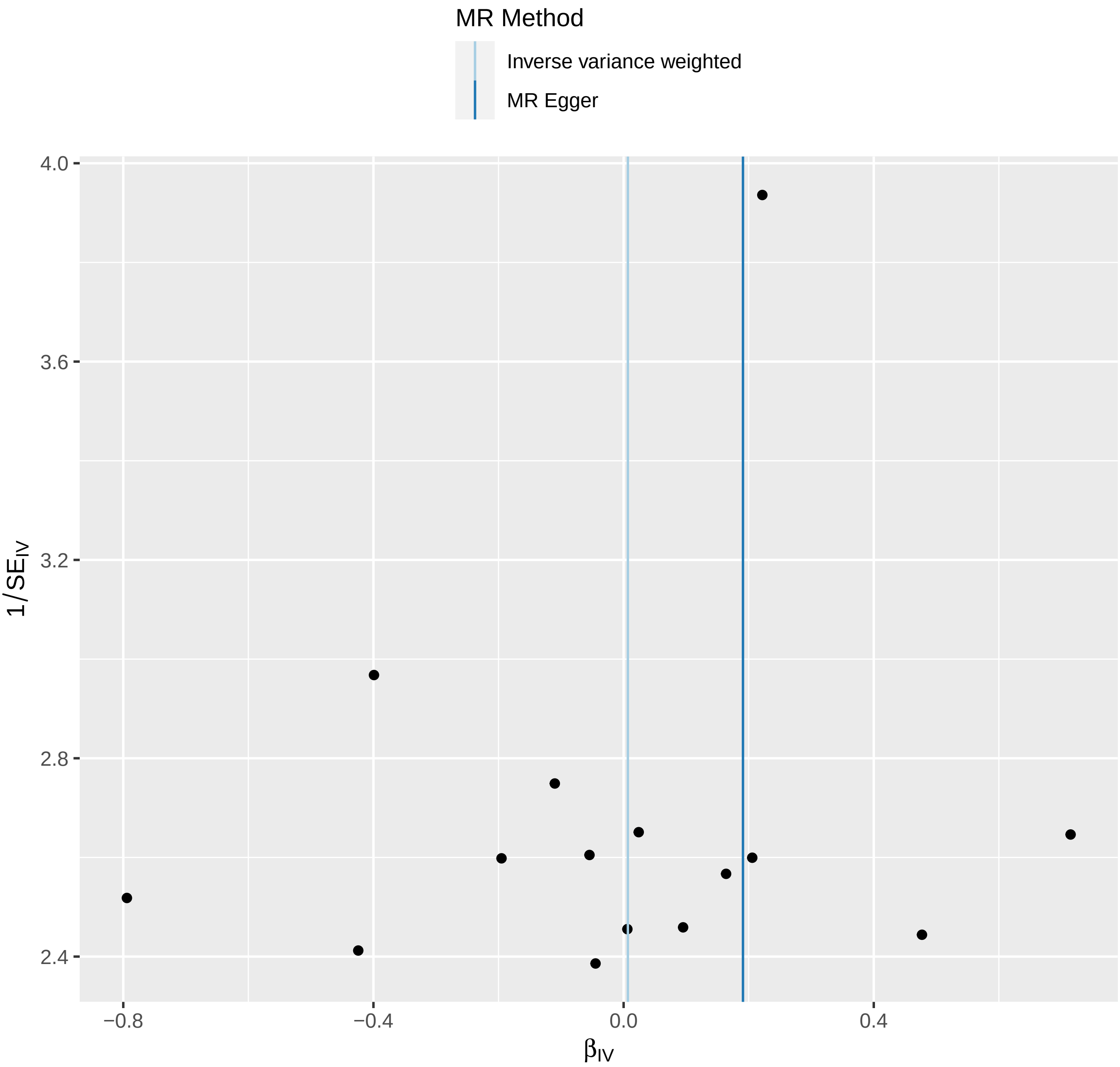

Batch 2−3773 : Gut microbiota abundance (genus Catenibacterium id.2153) on Microfibrillar−associated protein 5
with a threshold of 1e−05

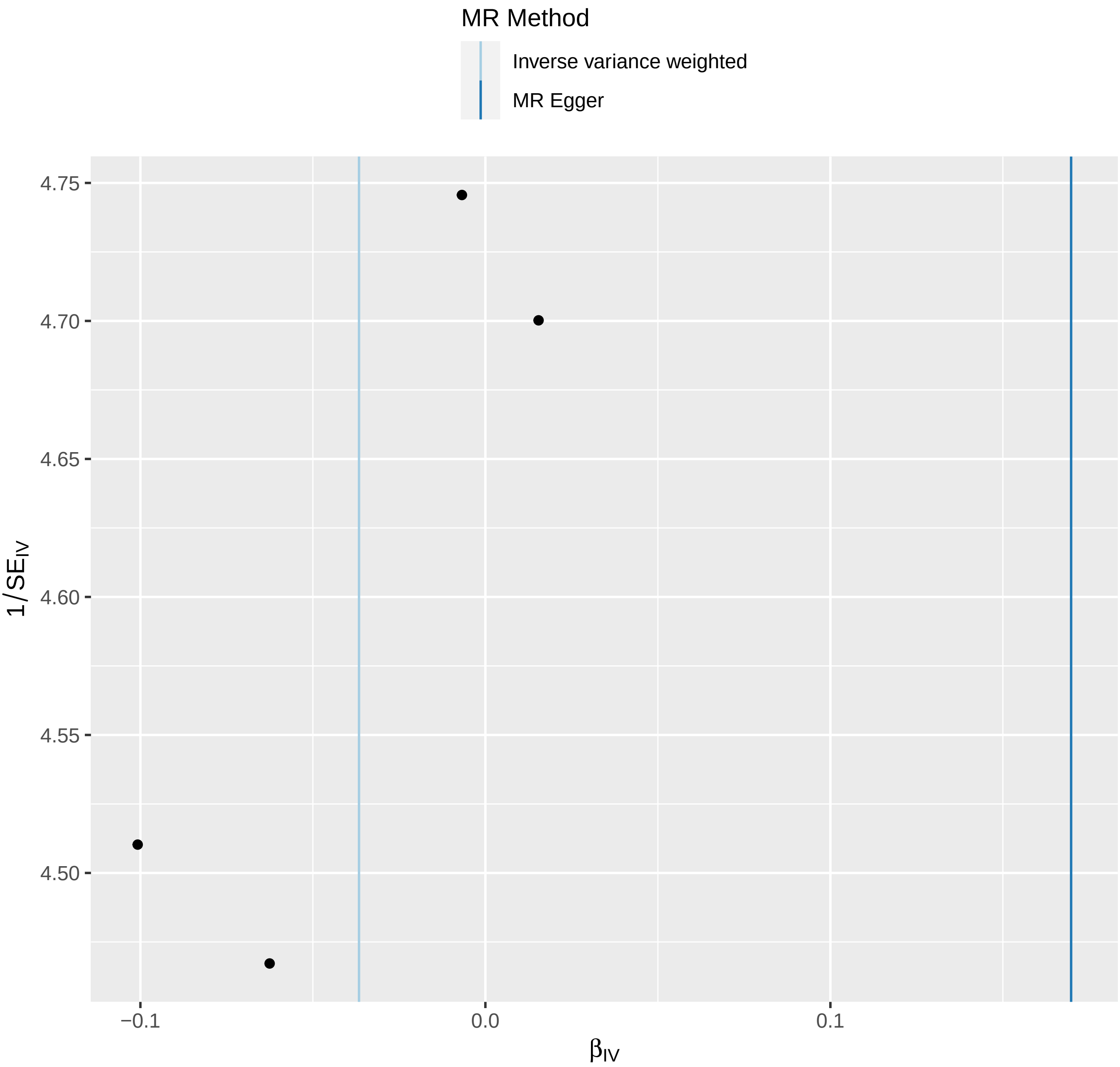

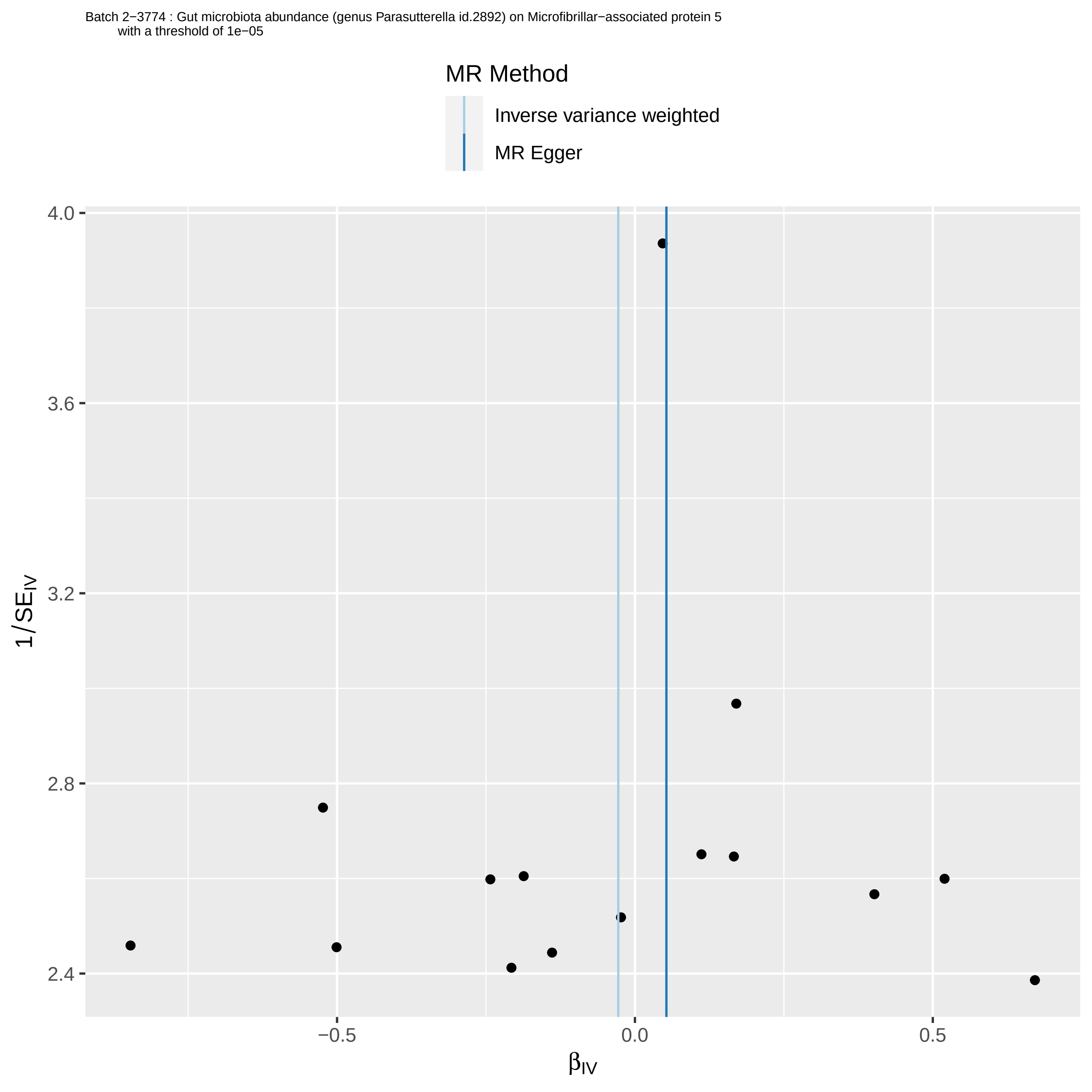

Batch 2−3774 : Gut microbiota abundance (genus Parasutterella id.2892) on Microfibrillar−associated protein 5
with a threshold of 1e−05
MR Method
Inverse variance weighted
MR Egger
4.0
3.6
3.2
2.8
2.4
1/SE_IV
−0.5
0.0
0.5
β_IV

Batch 2−3775 : Gut microbiota abundance (genus Catenibacterium id.2153) on Lactadherin
with a threshold of 1e−05

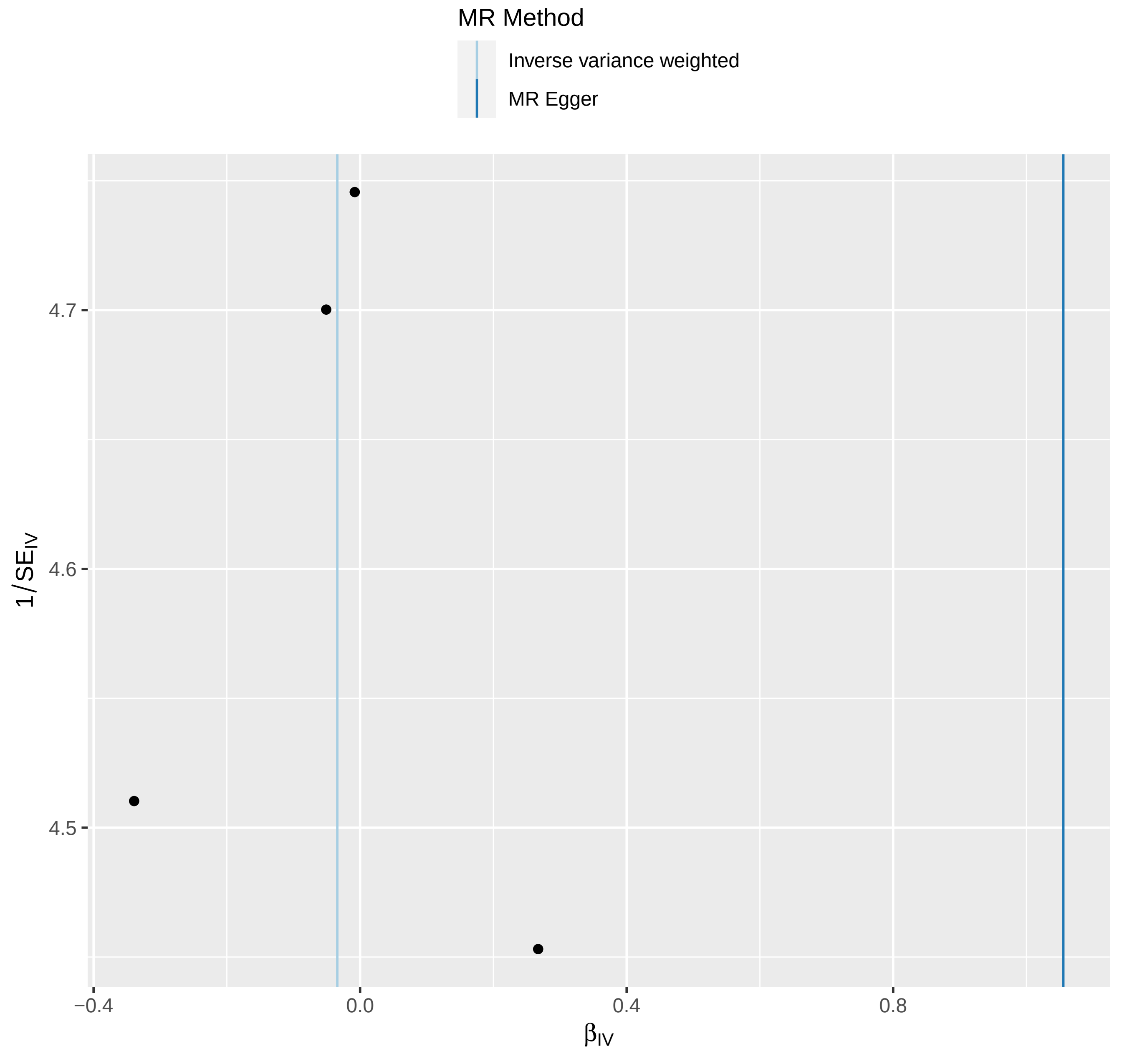

Batch 2−3776 : Gut microbiota abundance (genus Parasutterella id.2892) on Lactadherin
with a threshold of 1e−05

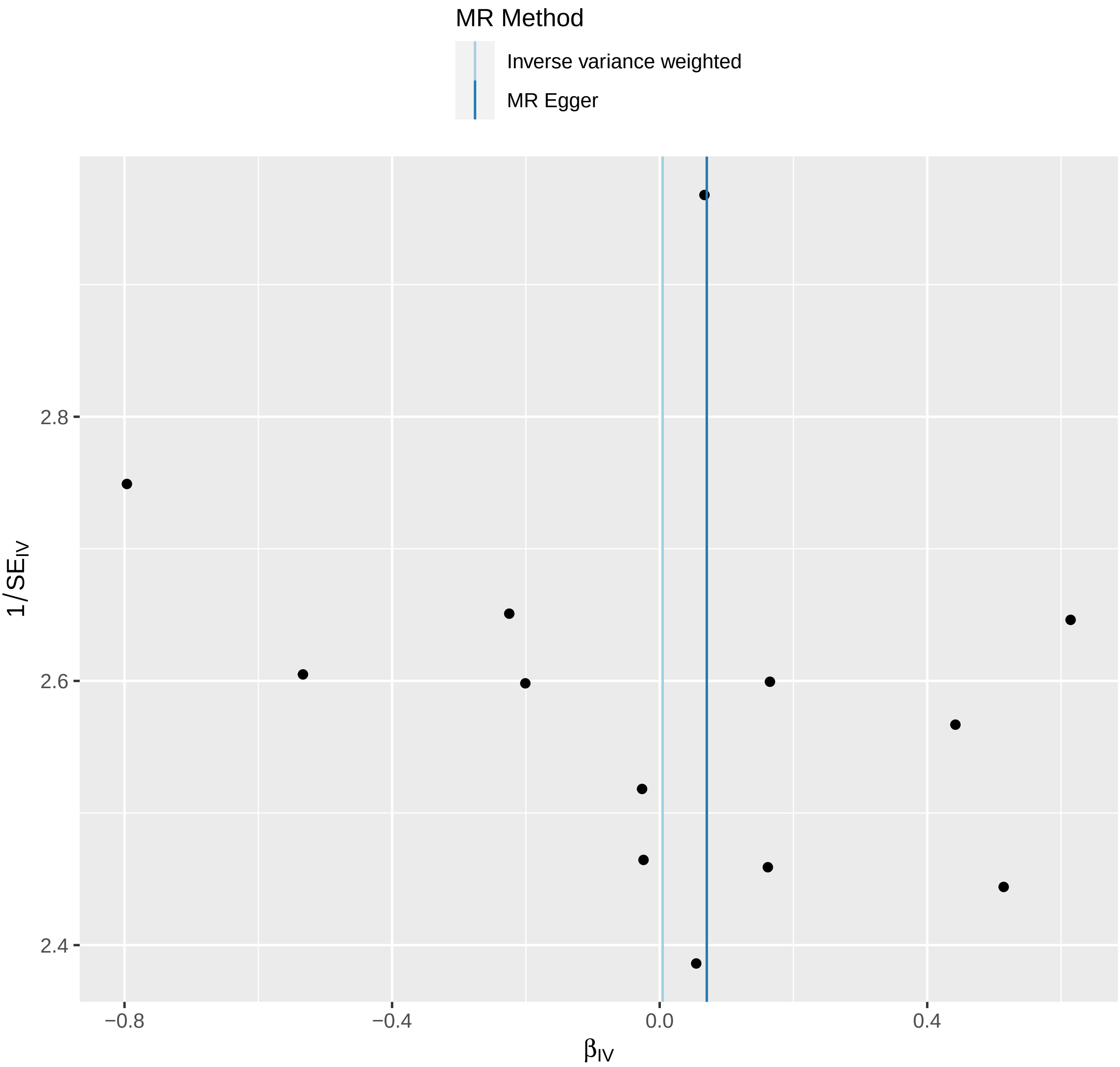

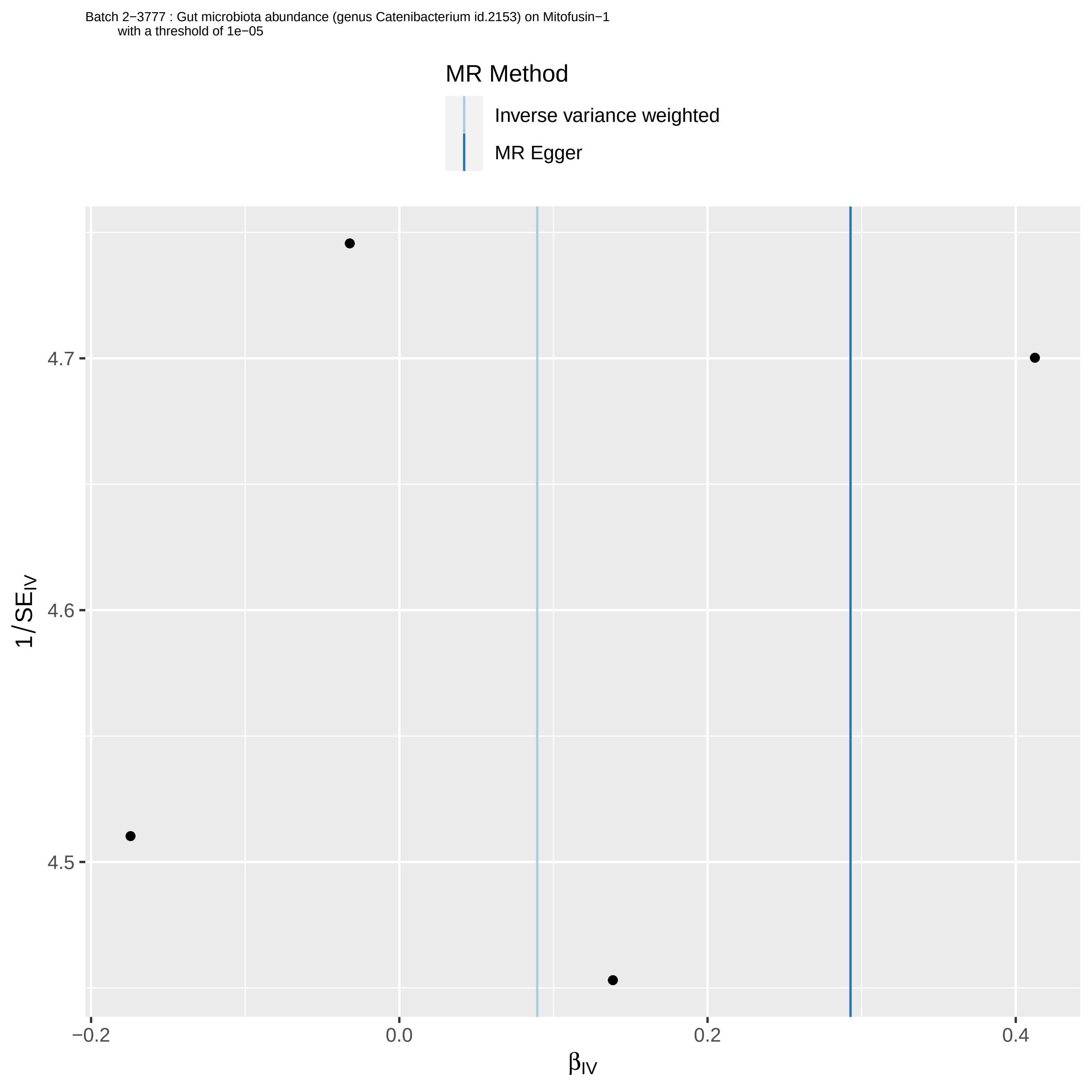

Batch 2−3777 : Gut microbiota abundance (genus Catenibacterium id.2153) on Mitofusin−1
with a threshold of 1e−05
MR Method
Inverse variance weighted
MR Egger
4.7
4.6
4.5
1/SE_IV
−0.2
0.0
0.2
0.4
β_IV

Batch 2−3778 : Gut microbiota abundance (genus Parasutterella id.2892) on Mitofusin−1
with a threshold of 1e−05

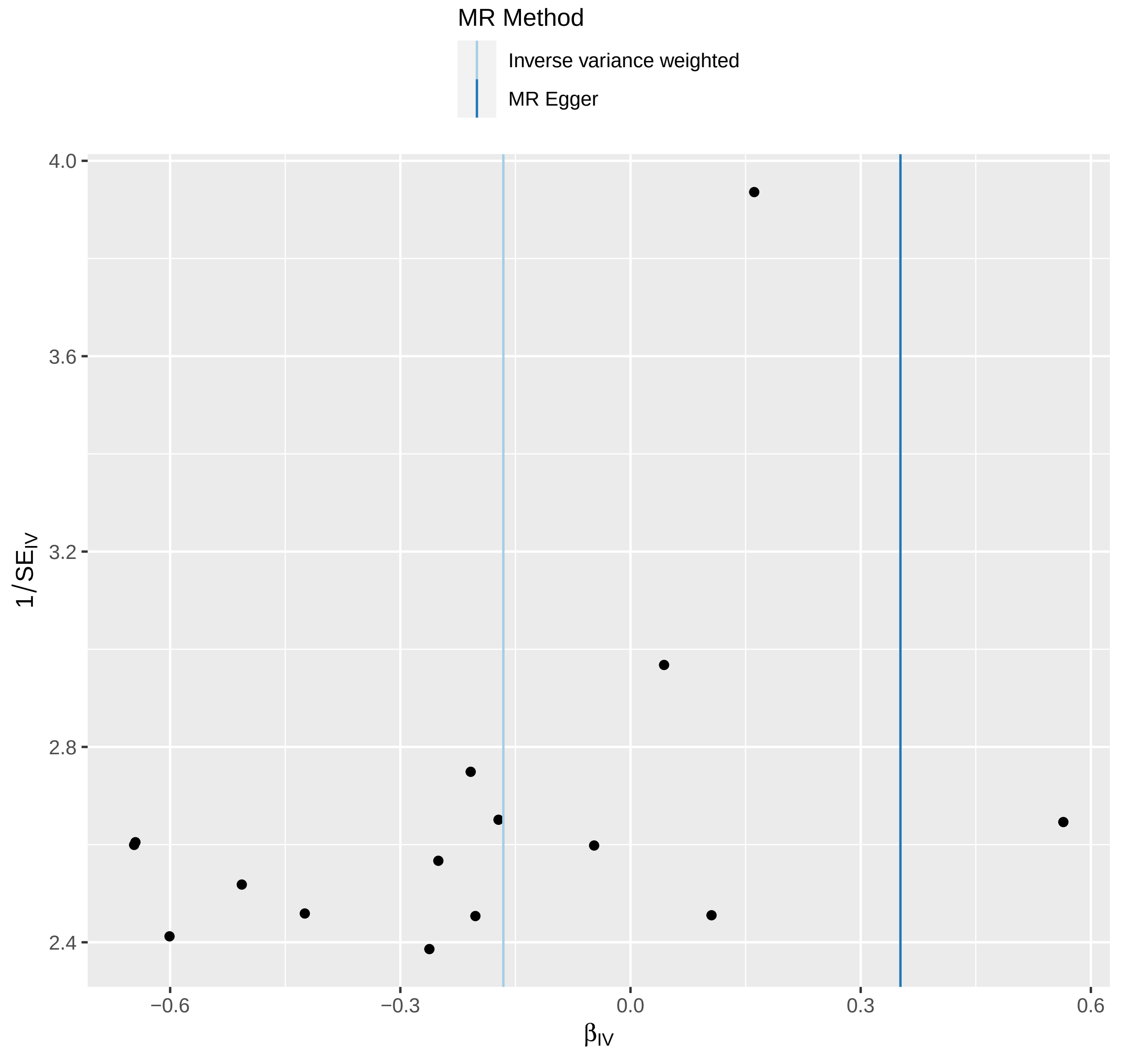

Batch 2−3779 : Gut microbiota abundance (genus Catenibacterium id.2153) on Beta−1,3−N−acetylglucosaminyltransferase manic fringe with a threshold of 1e−05

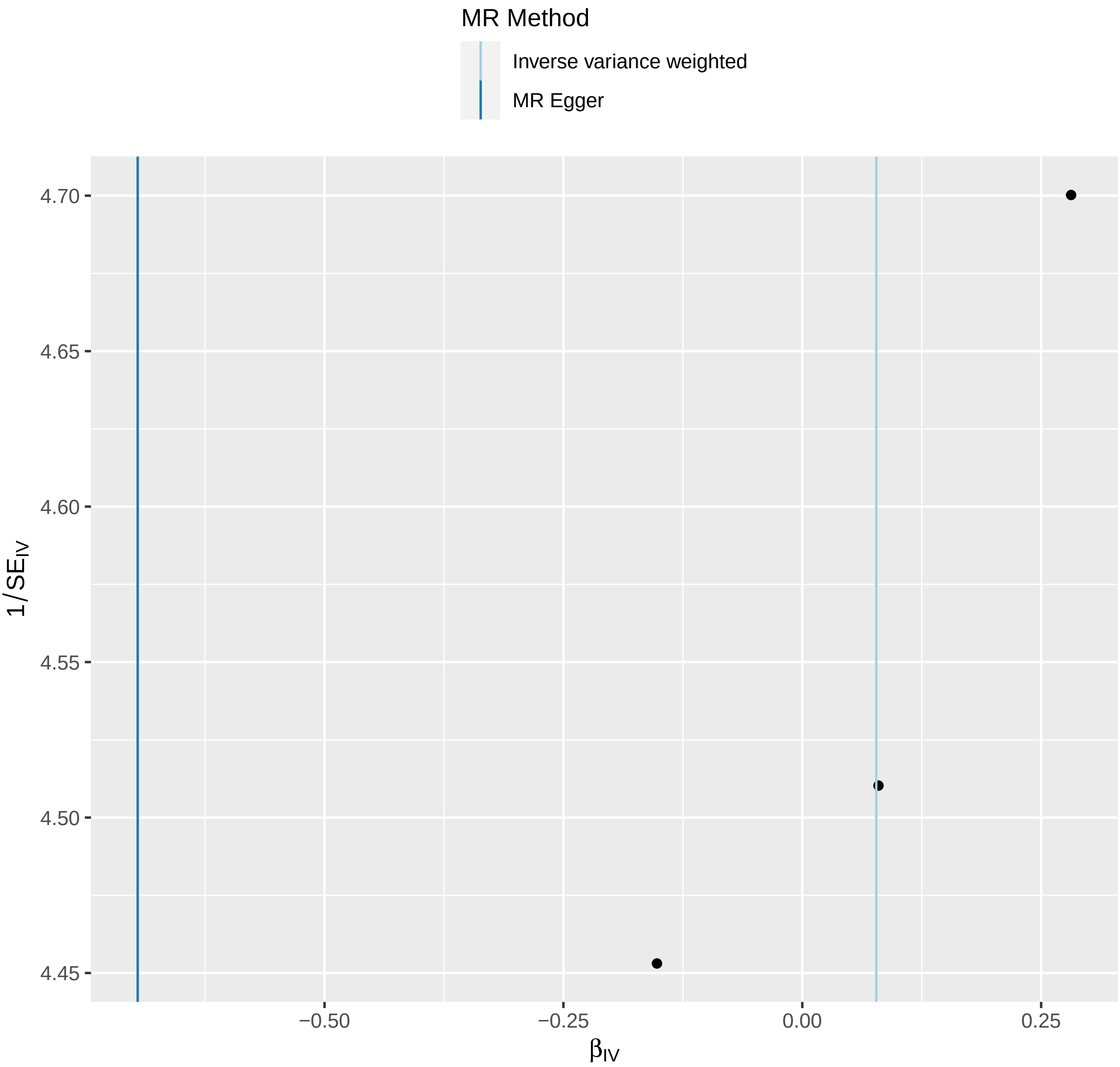

Batch 2−3780 : Gut microbiota abundance (genus Parasutterella id.2892) on Beta−1,3−N−acetylglucosaminyltransferase manic fringe with a threshold of 1e−05

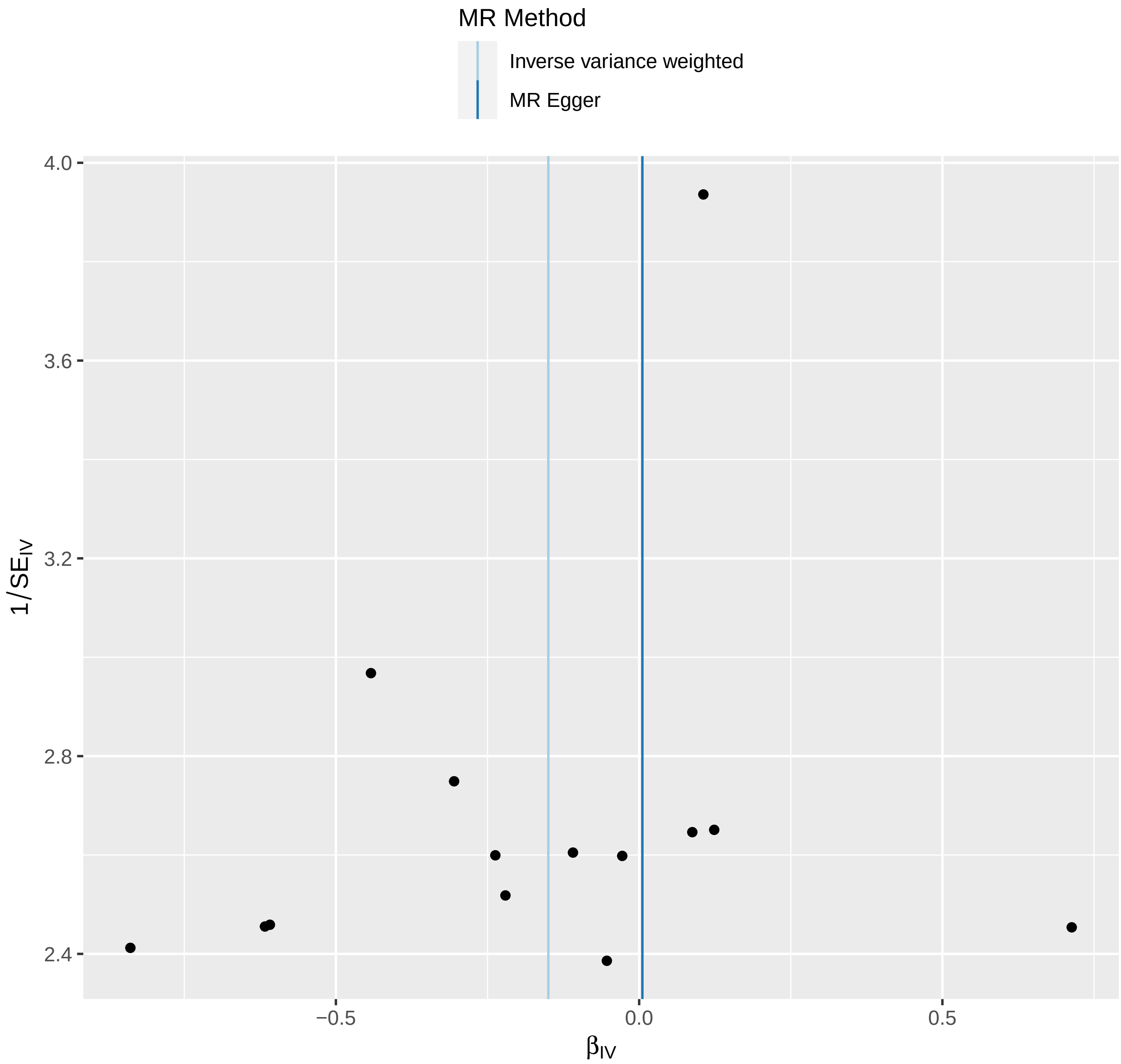

Batch 2−3781 : Gut microbiota abundance (genus Catenibacterium id.2153) on MAX gene−associated protein
with a threshold of 1e−05

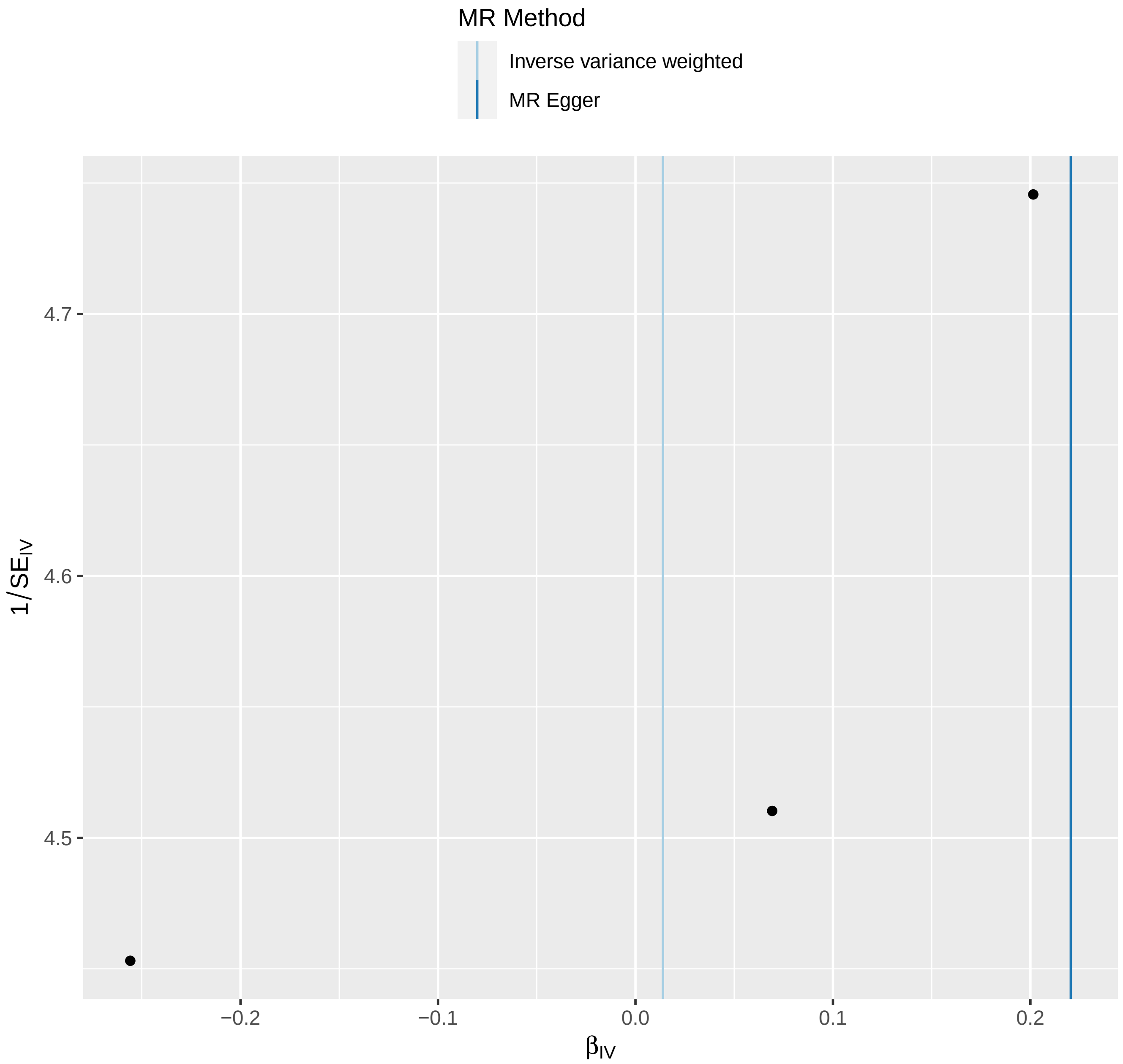

Batch 2−3782 : Gut microbiota abundance (genus Parasutterella id.2892) on MAX gene−associated protein with a threshold of 1e−05

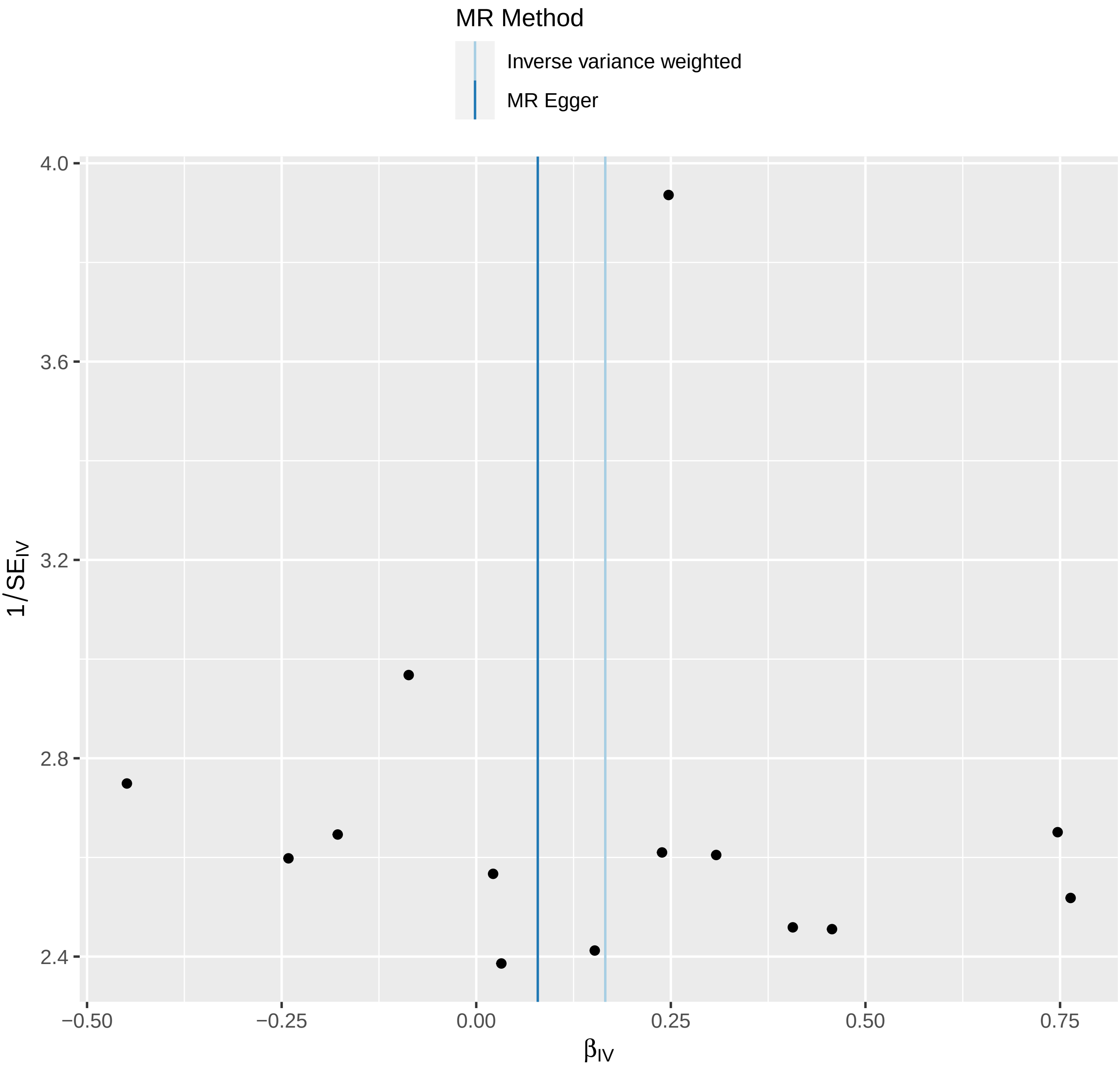

Batch 2−3783 : Gut microbiota abundance (genus Catenibacterium id.2153) on Alpha−1,3−mannosyl−glycoprotein 2−beta−N−acetylglucosaminyltransferase with a threshold of 1e−05

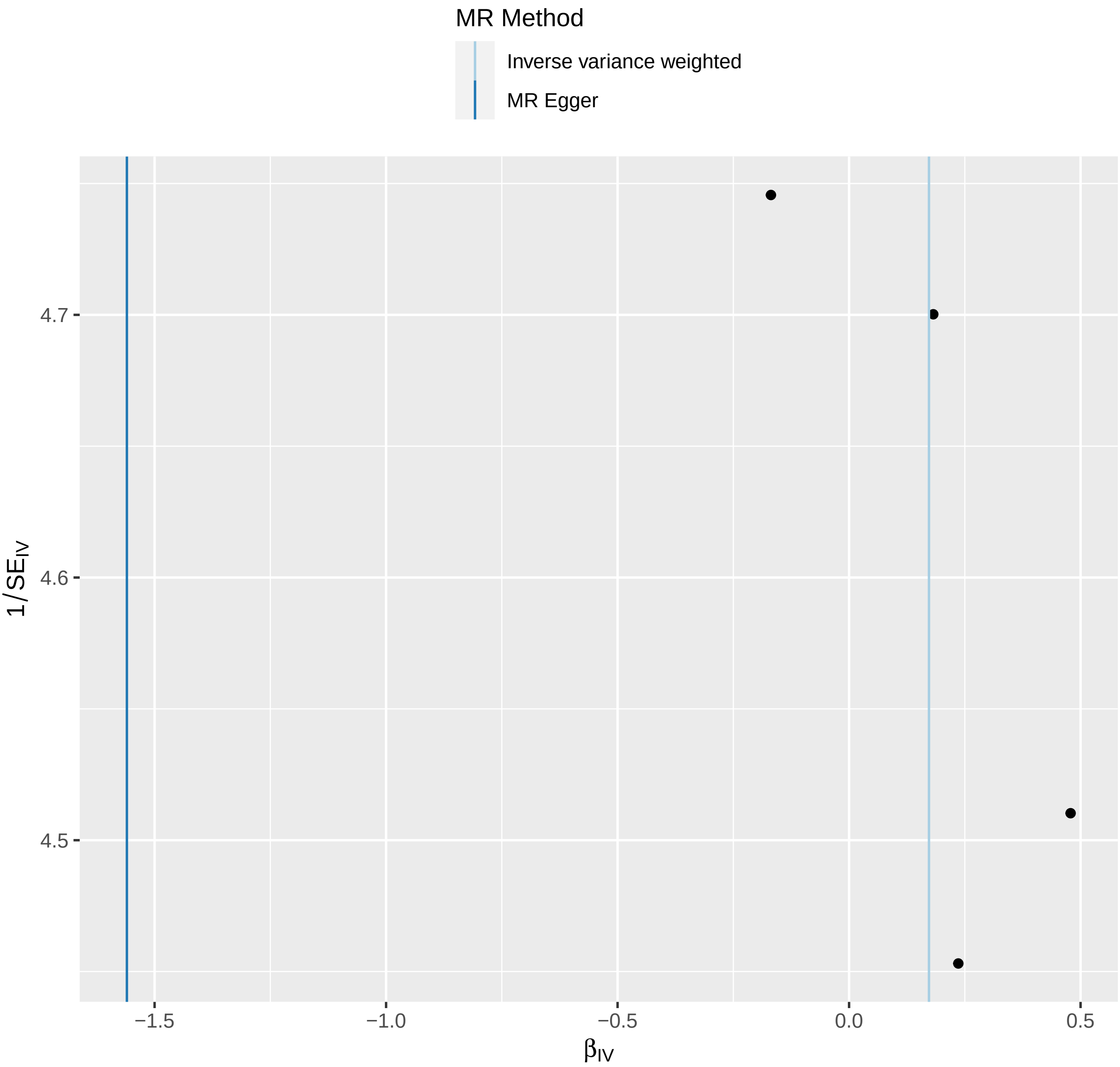

Batch 2−3784 : Gut microbiota abundance (genus Parasutterella id.2892) on Alpha−1,3−mannosyl−glycoprotein 2−beta−N−acetylglucosaminyltransferase with a threshold of 1e−05

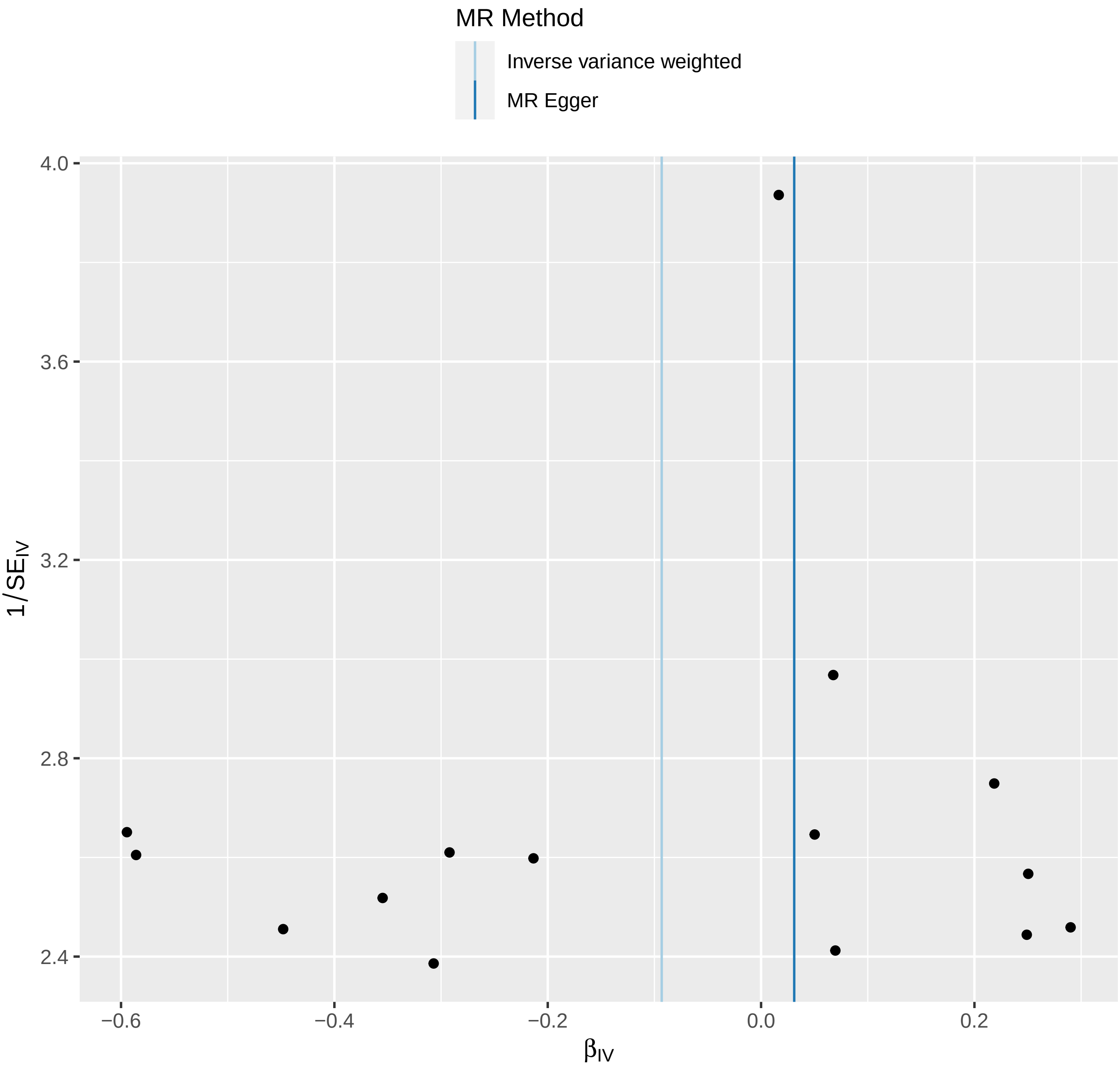

Batch 2−3785 : Gut microbiota abundance (genus Catenibacterium id.2153) on Alpha−1,6−mannosyl−glycoprotein 2−beta−N−acetylglucosaminyltransferase with a threshold of 1e−05

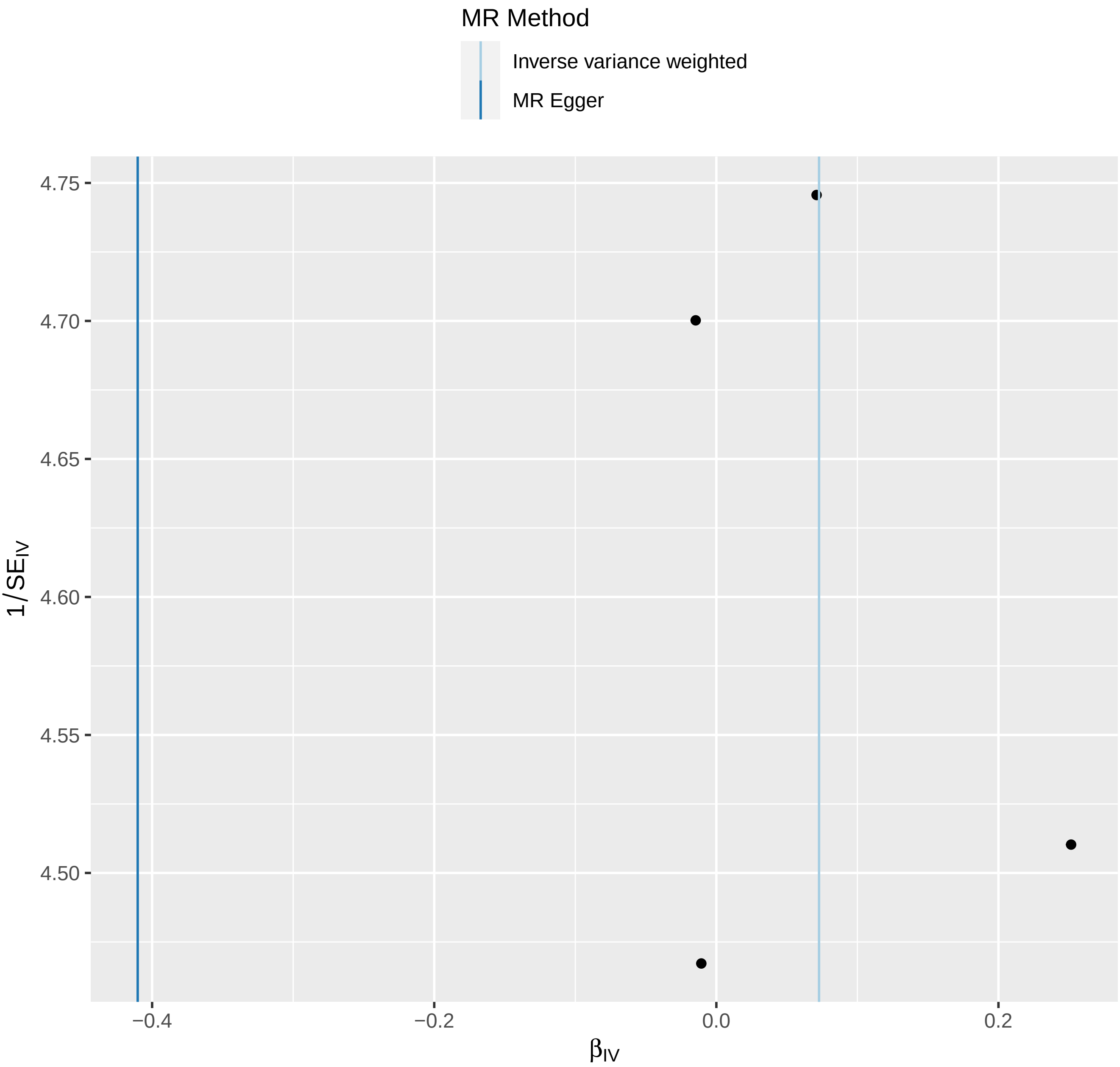

Batch 2−3786 : Gut microbiota abundance (genus Parasutterella id.2892) on Alpha−1,6−mannosyl−glycoprotein 2−beta−N−acetylglucosaminyltransferase
with a threshold of 1e−05

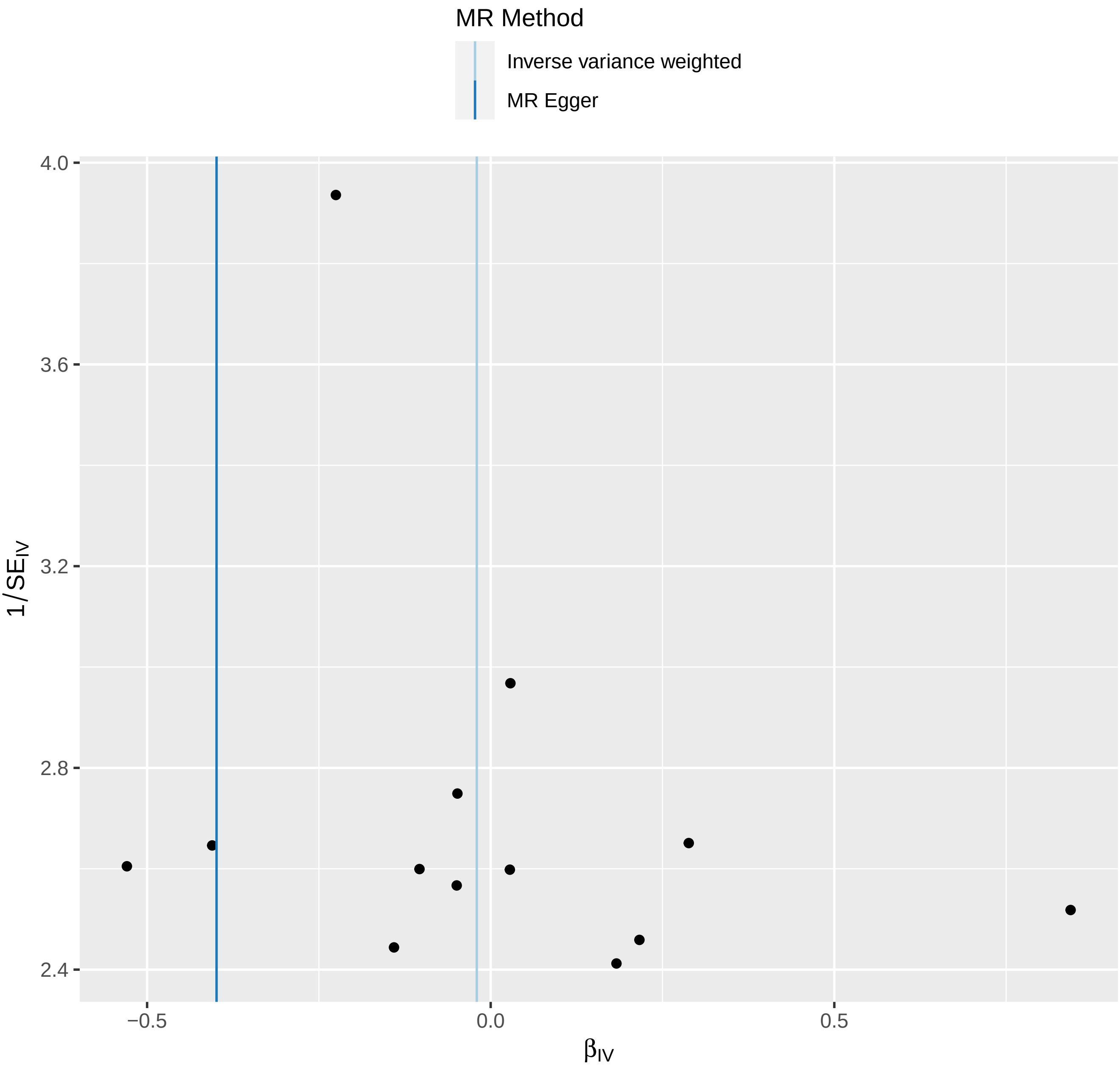

Batch 2−3787 : Gut microbiota abundance (genus Catenibacterium id.2153) on Alpha−1,3−mannosyl−glycoprotein 4−beta−N−acetylglucosaminyltransferase B with a threshold of 1e−05

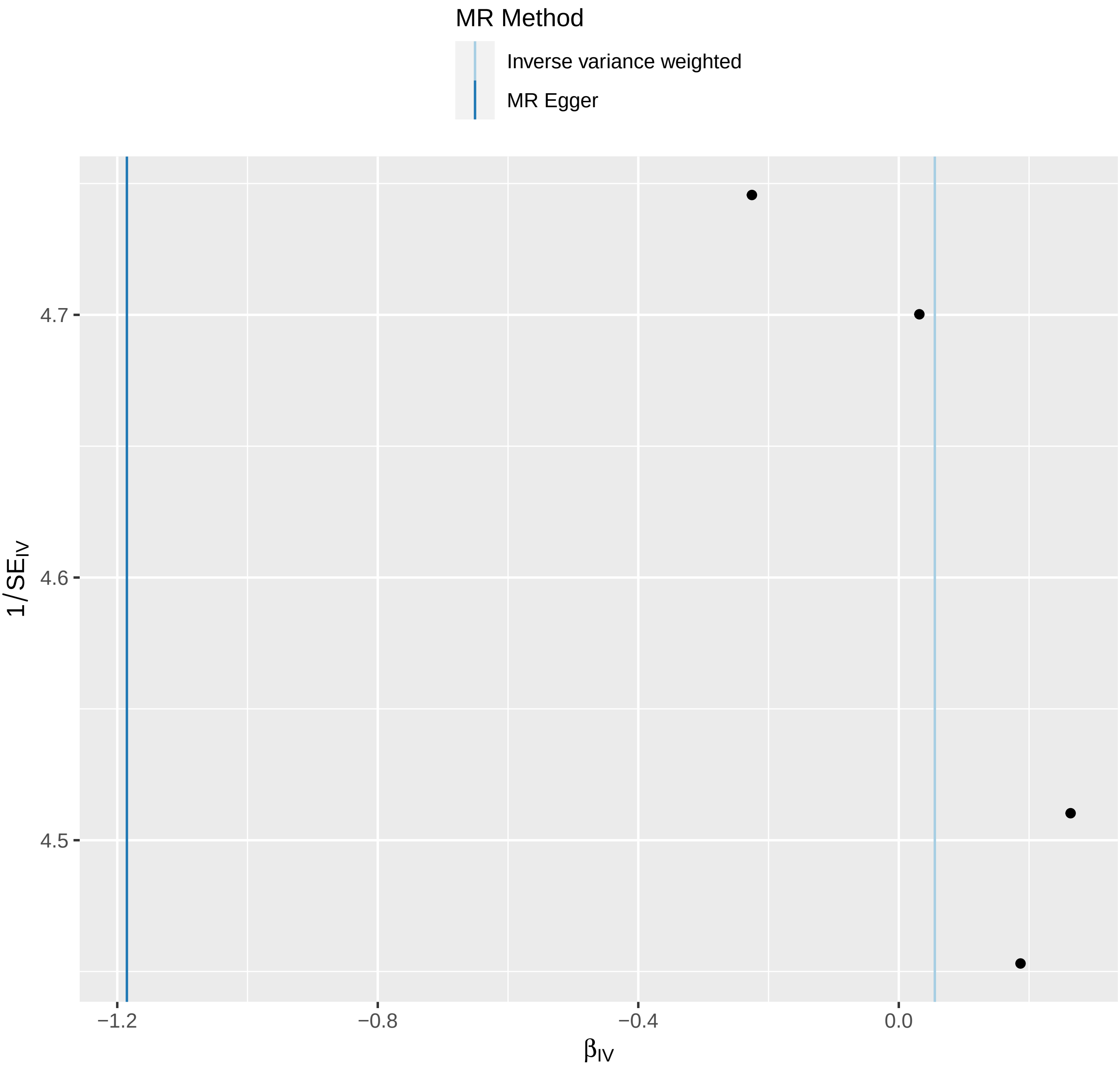

Batch 2−3788 : Gut microbiota abundance (genus Parasutterella id.2892) on Alpha−1,3−mannosyl−glycoprotein 4−beta−N−acetylglucosaminyltransferase B with a threshold of 1e−05

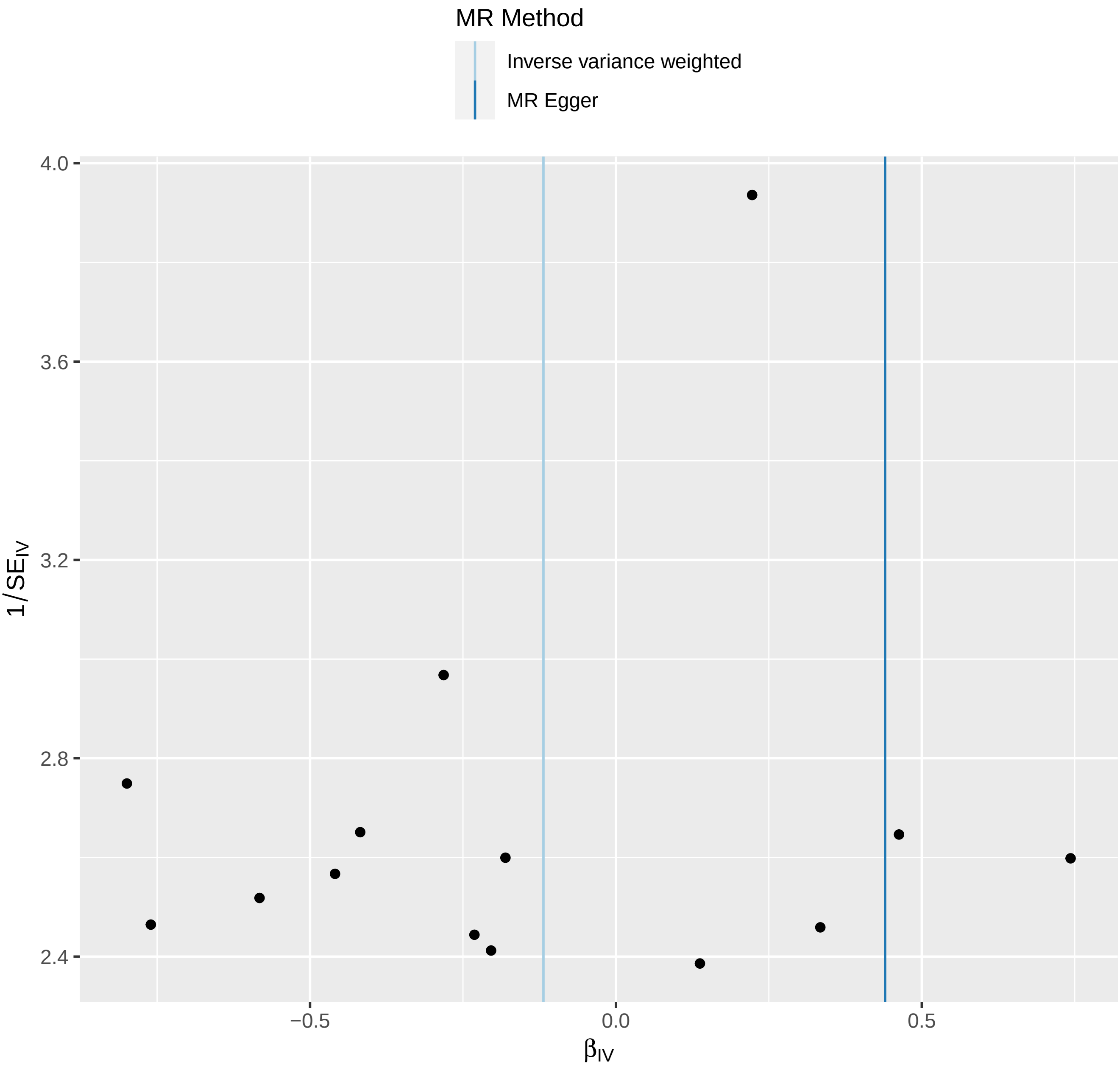

Batch 2−3789 : Gut microbiota abundance (genus Catenibacterium id.2153) on Alpha−1,3−mannosyl−glycoprotein 4−beta−N−acetylglucosaminyltransferase C with a threshold of 1e−05

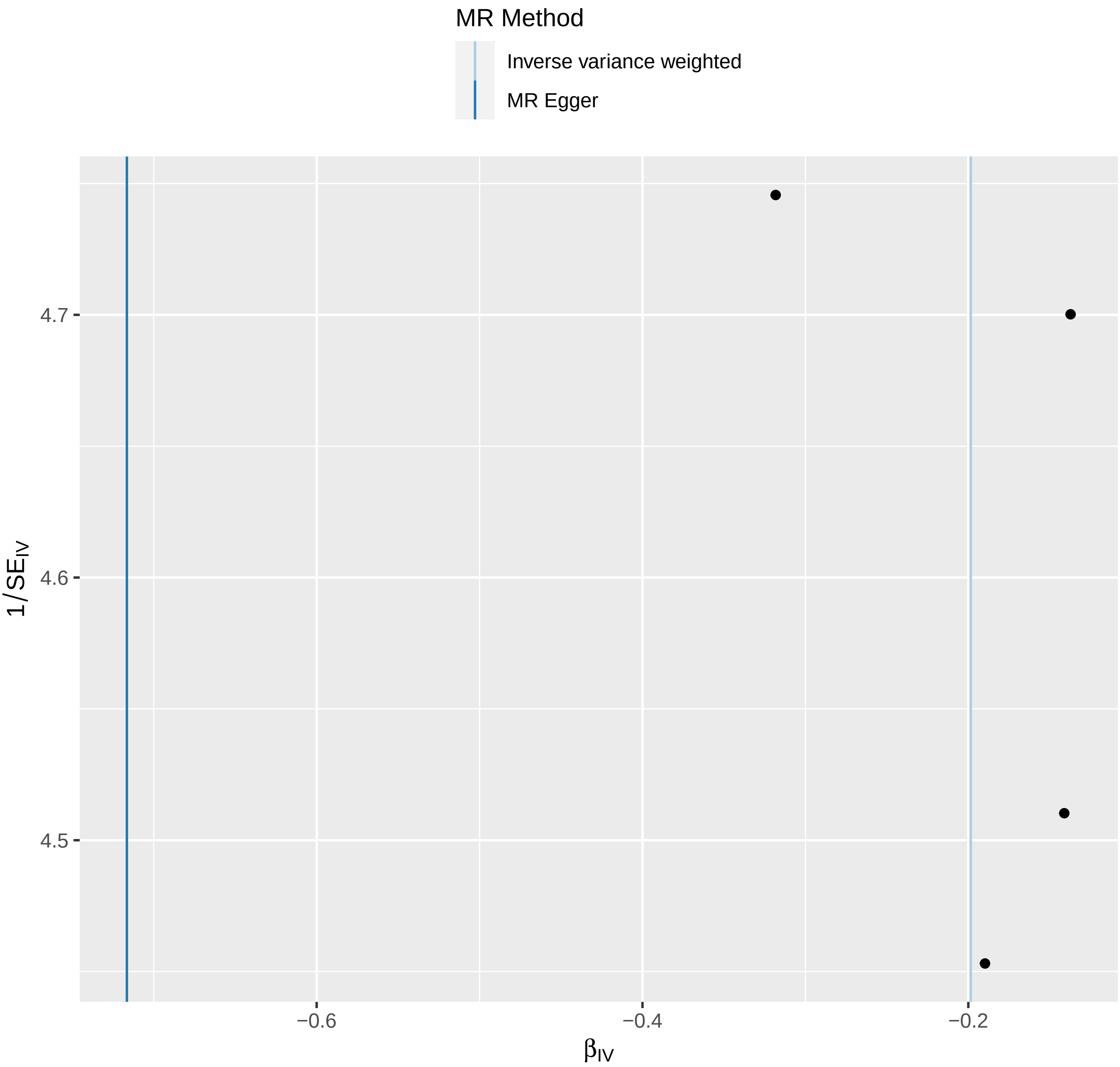

Batch 2−3790 : Gut microbiota abundance (genus Parasutterella id.2892) on Alpha−1,3−mannosyl−glycoprotein 4−beta−N−acetylglucosaminyltransferase C
with a threshold of 1e−05

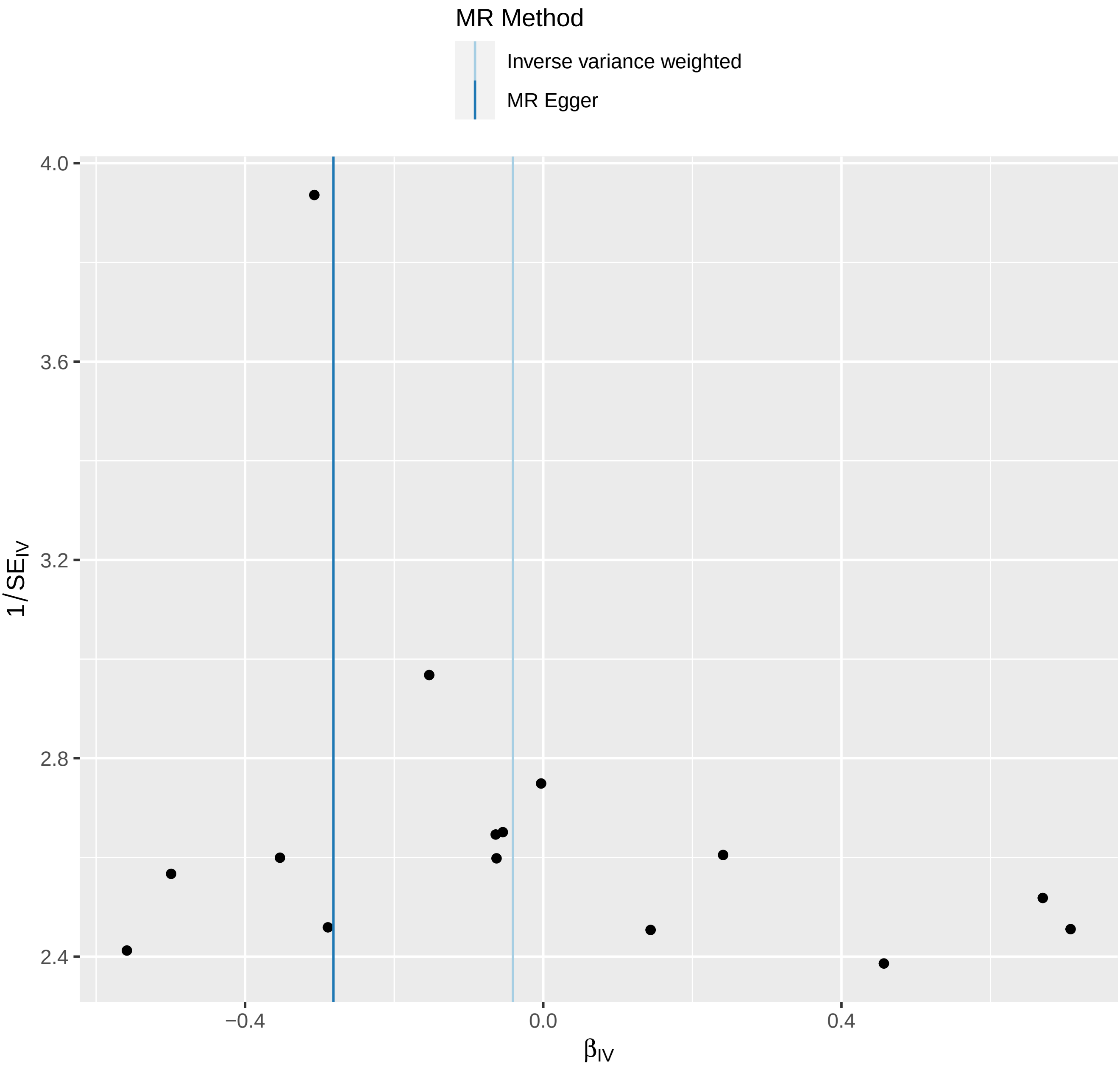

Batch 2−3791 : Gut microbiota abundance (genus Catenibacterium id.2153) on Melanoma−derived growth regulatory protein with a threshold of 1e−05

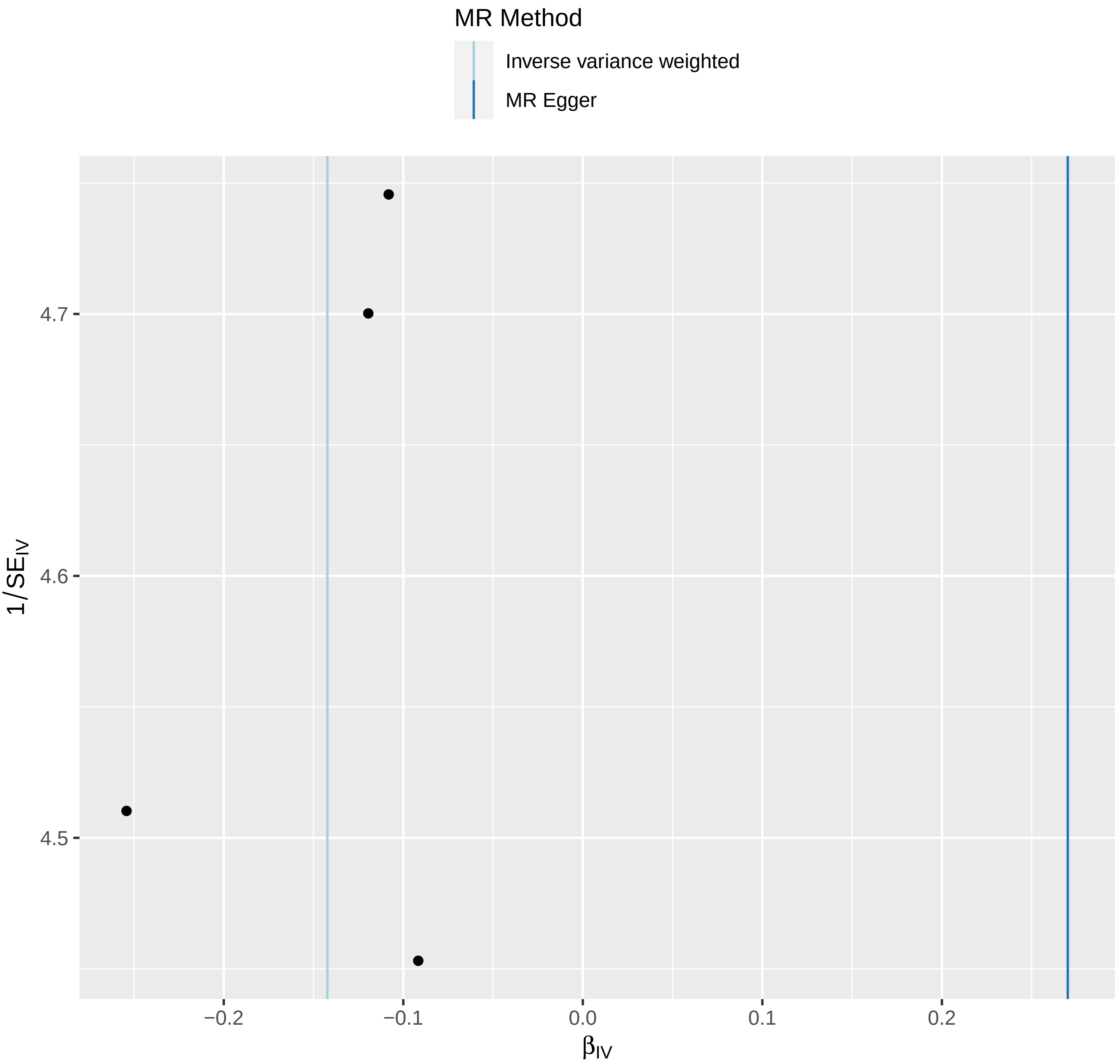

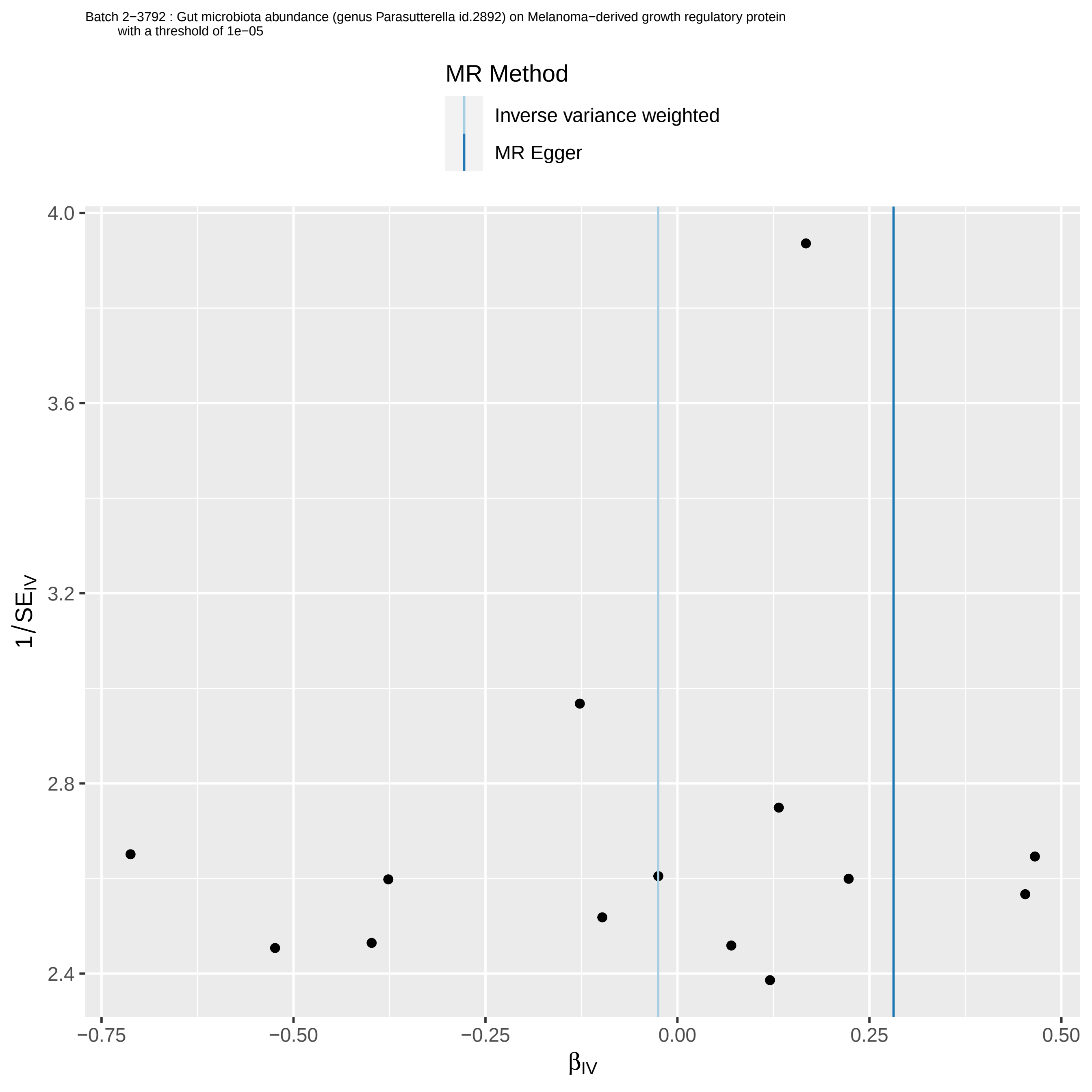

Batch 2−3792 : Gut microbiota abundance (genus Parasutterella id.2892) on Melanoma−derived growth regulatory protein
with a threshold of 1e−05
MR Method
Inverse variance weighted
MR Egger
4.0
3.6
3.2
2.8
2.4
1/SE_IV
−0.75
−0.50
−0.25
0.00
0.25
0.50
β_IV

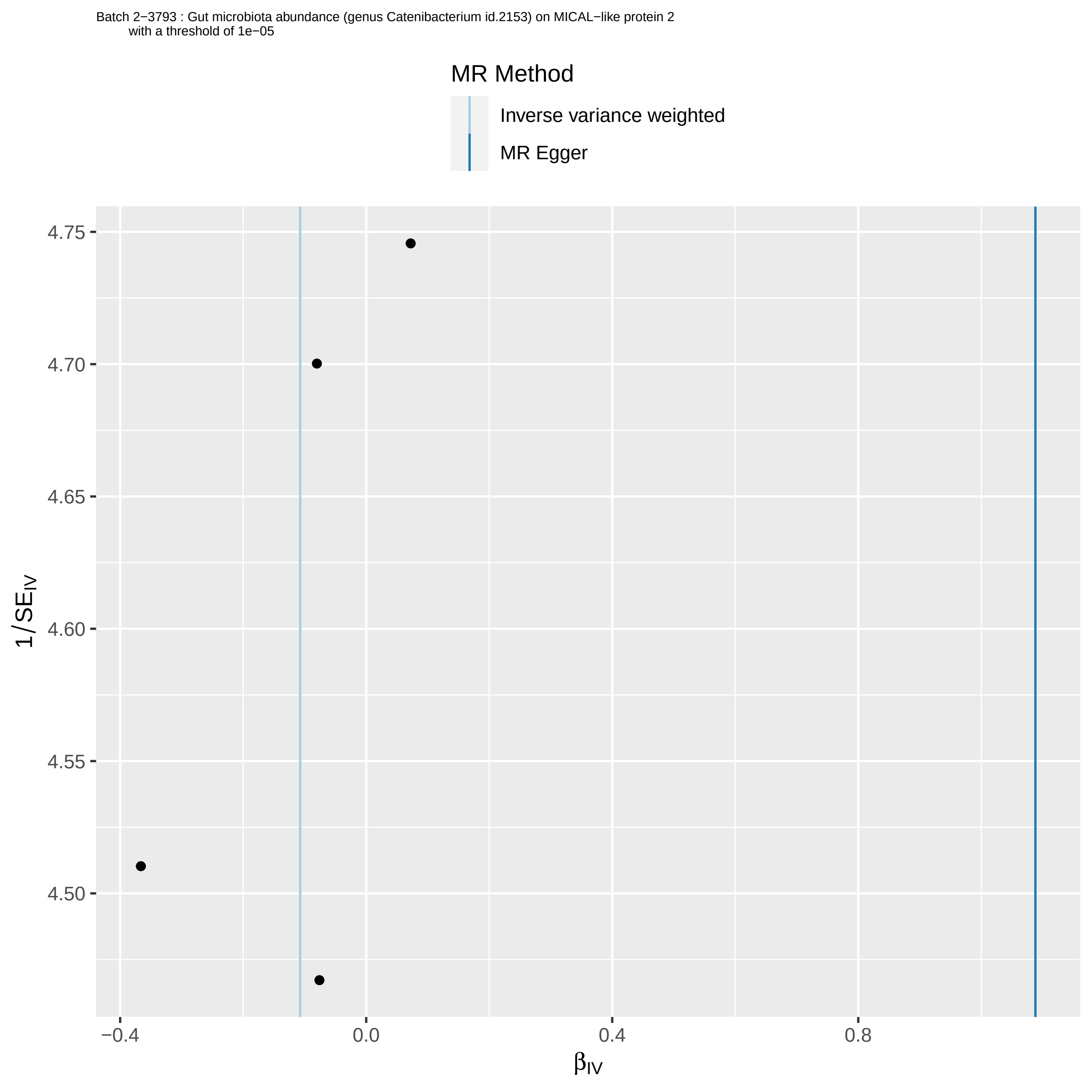
Batch 2−3793 : Gut microbiota abundance (genus Catenibacterium id.2153) on MICAL−like protein 2
with a threshold of 1e−05
MR Method
Inverse variance weighted
MR Egger
4.75
4.70
4.65
4.60
4.55
4.50
1/SE_IV
−0.4
0.0
0.4
0.8
β_IV

Batch 2−3794 : Gut microbiota abundance (genus Parasutterella id.2892) on MICAL−like protein 2
with a threshold of 1e−05

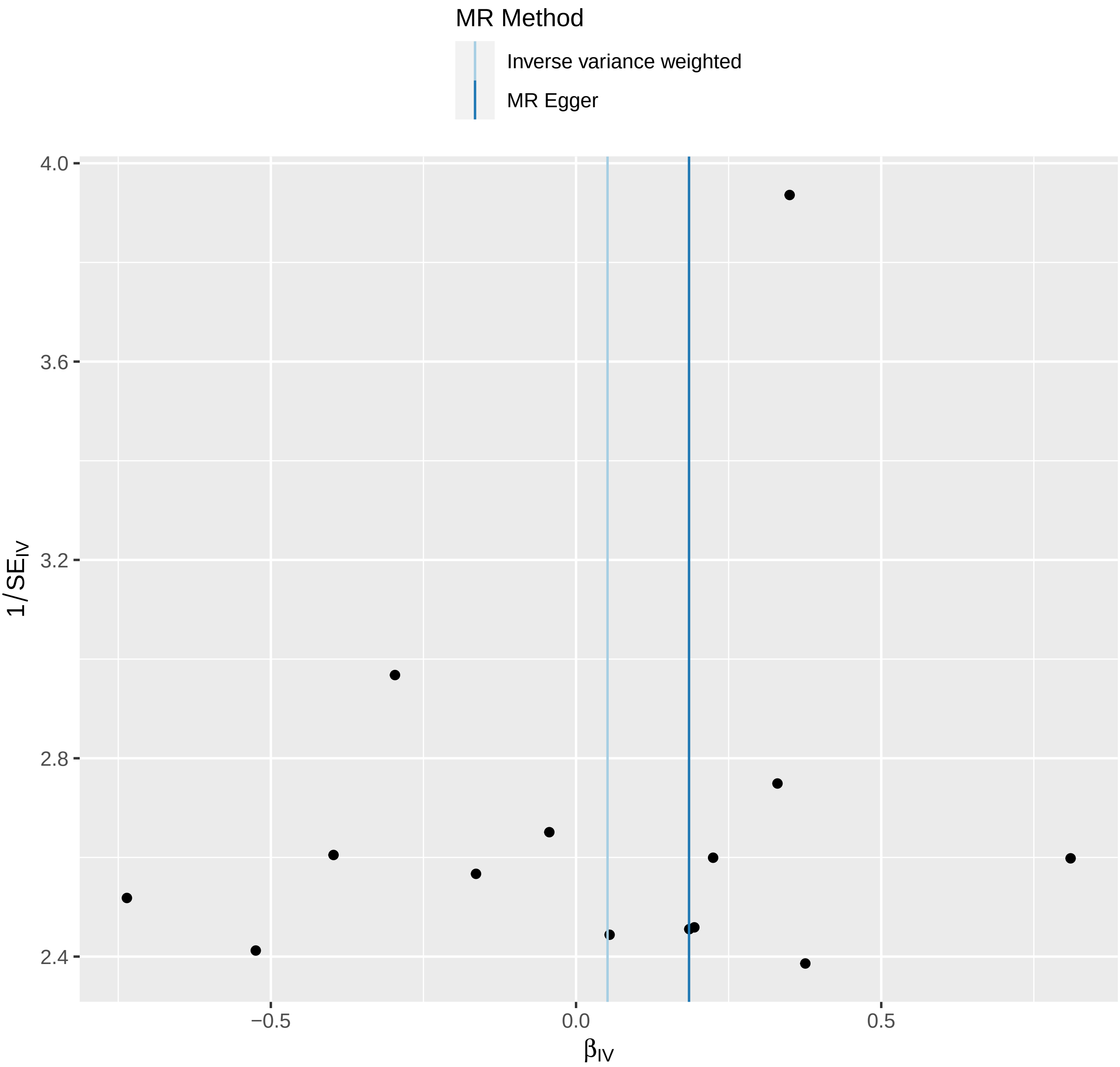

Batch 2−3795 : Gut microbiota abundance (genus Catenibacterium id.2153) on MHC class I polypeptide−related sequence B
with a threshold of 1e−05

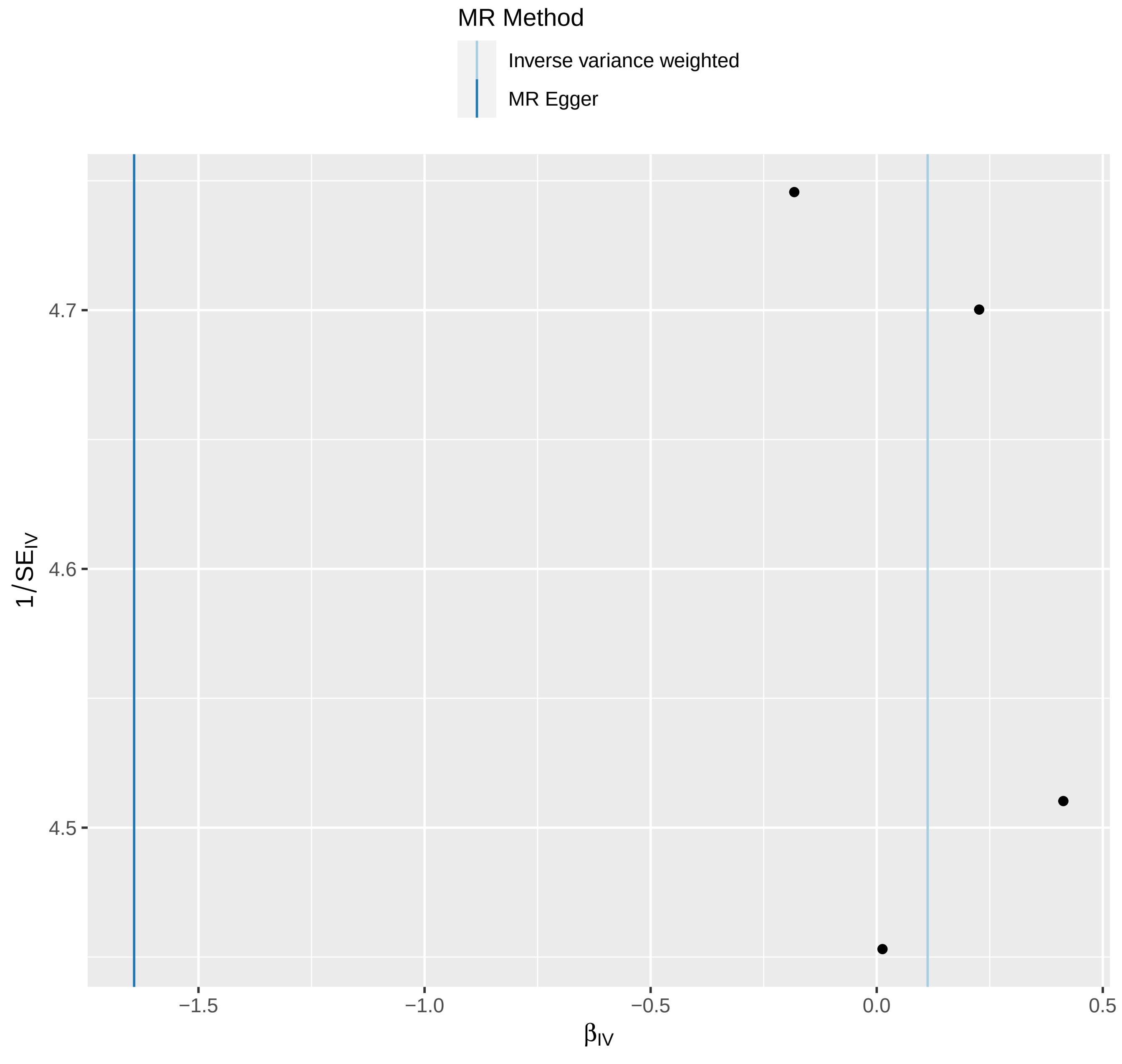

Batch 2−3796 : Gut microbiota abundance (genus Parasutterella id.2892) on MHC class I polypeptide−related sequence B
with a threshold of 1e−05

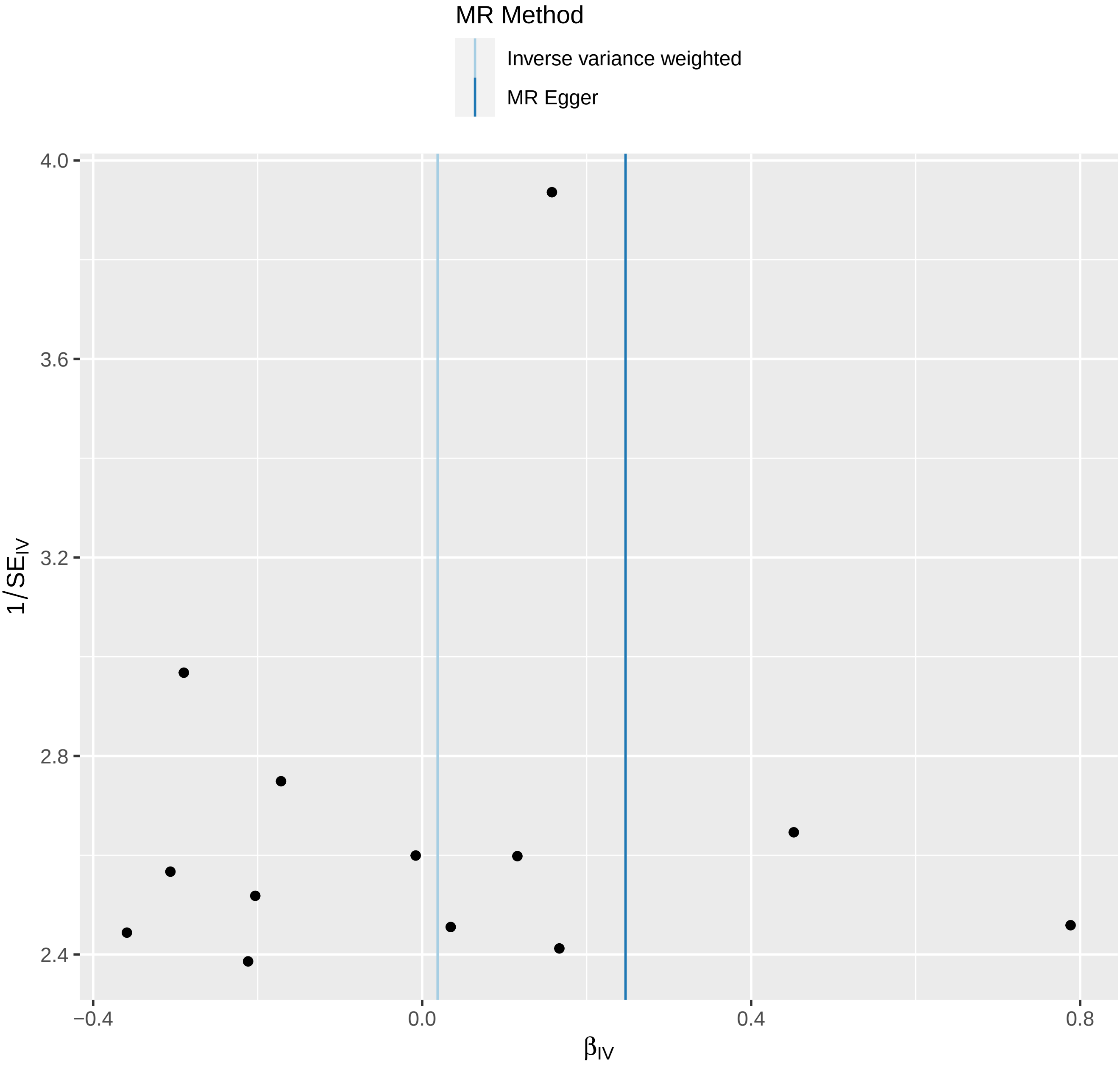

Batch 2−3797 : Gut microbiota abundance (genus Catenibacterium id.2153) on Probable E3 ubiquitin−protein ligase MID2
with a threshold of 1e−05

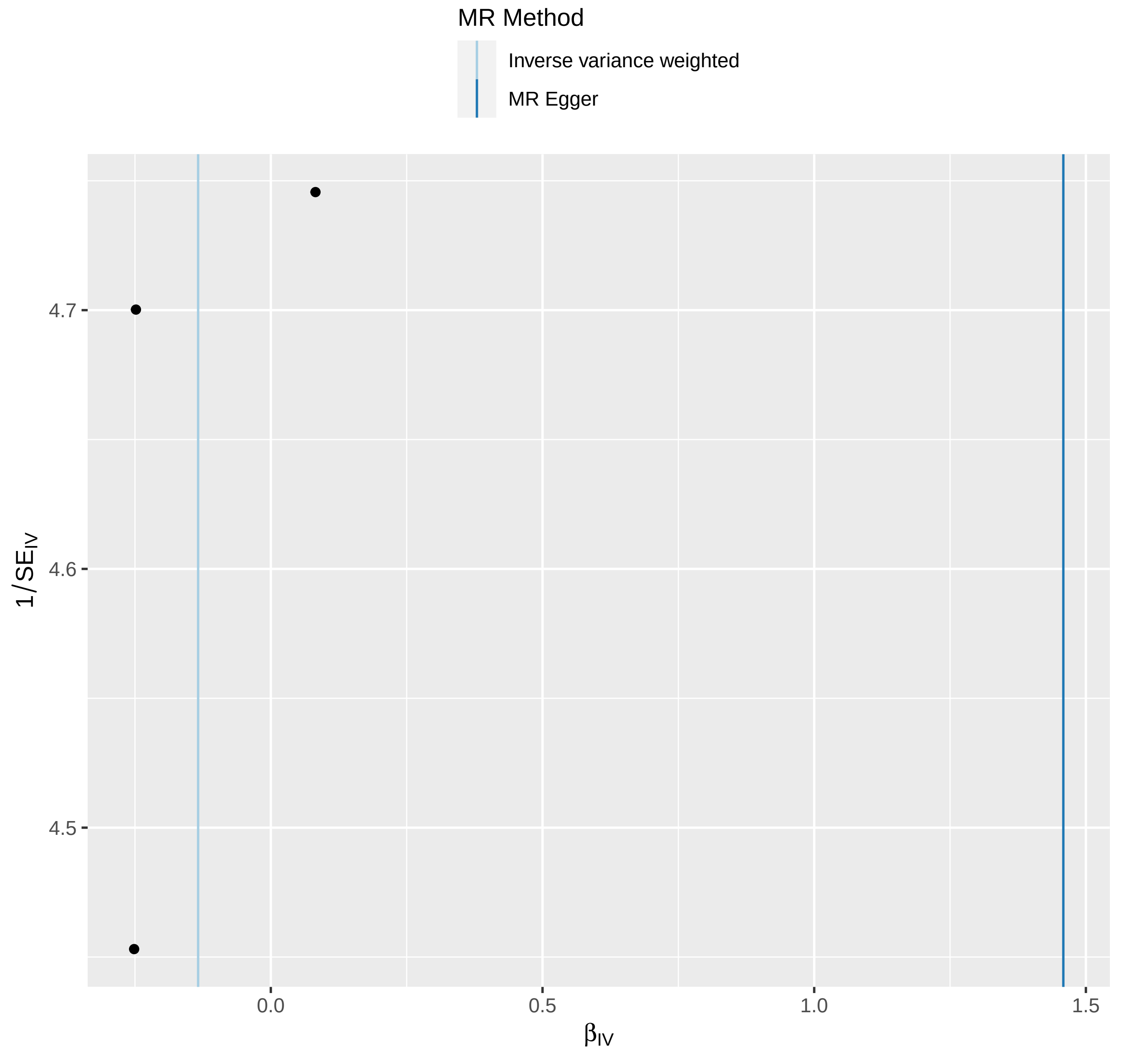

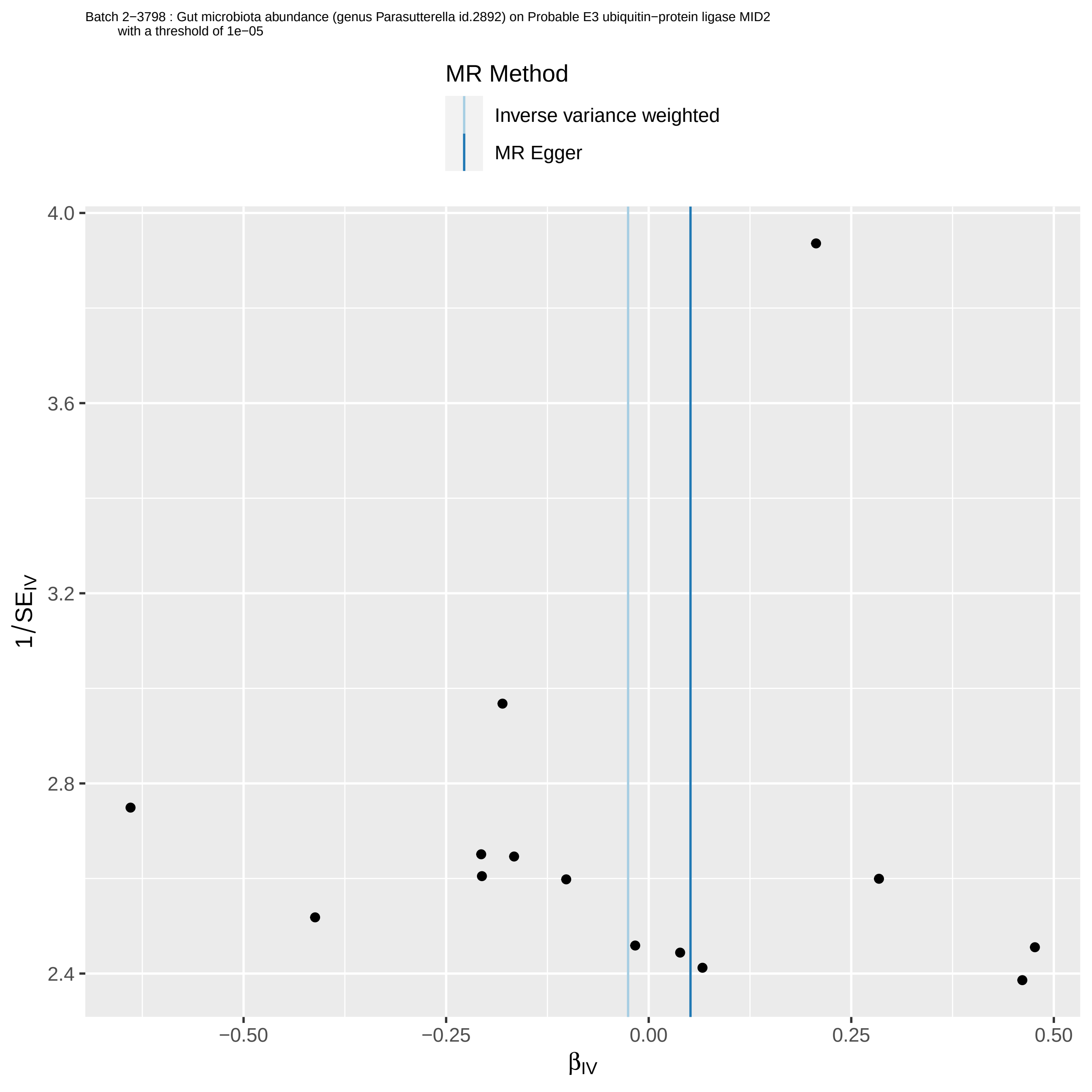

Batch 2−3798 : Gut microbiota abundance (genus Parasutterella id.2892) on Probable E3 ubiquitin−protein ligase MID2
with a threshold of 1e−05
MR Method
Inverse variance weighted
MR Egger
4.0
3.6
3.2
2.8
2.4
1/SE_IV
−0.50
−0.25
0.00
0.25
0.50
β_IV

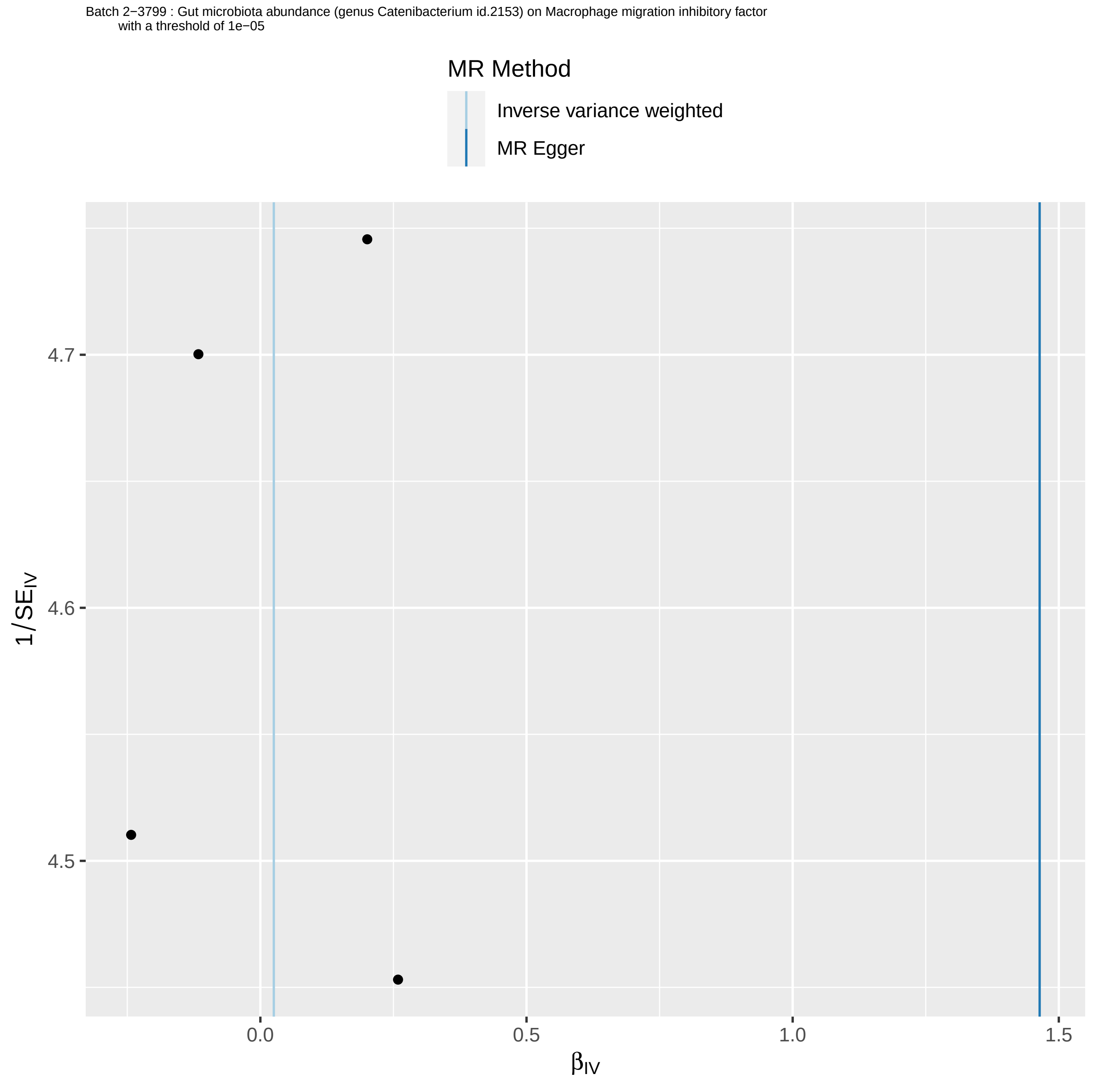

Batch 2−3799 : Gut microbiota abundance (genus Catenibacterium id.2153) on Macrophage migration inhibitory factor
with a threshold of 1e−05
MR Method
Inverse variance weighted
MR Egger
4.7
4.6
4.5
1/SE$_{IV}$
0.0
0.5
1.0
1.5
β_{IV}

Batch 2−3800 : Gut microbiota abundance (genus Parasutterella id.2892) on Macrophage migration inhibitory factor
with a threshold of 1e−05

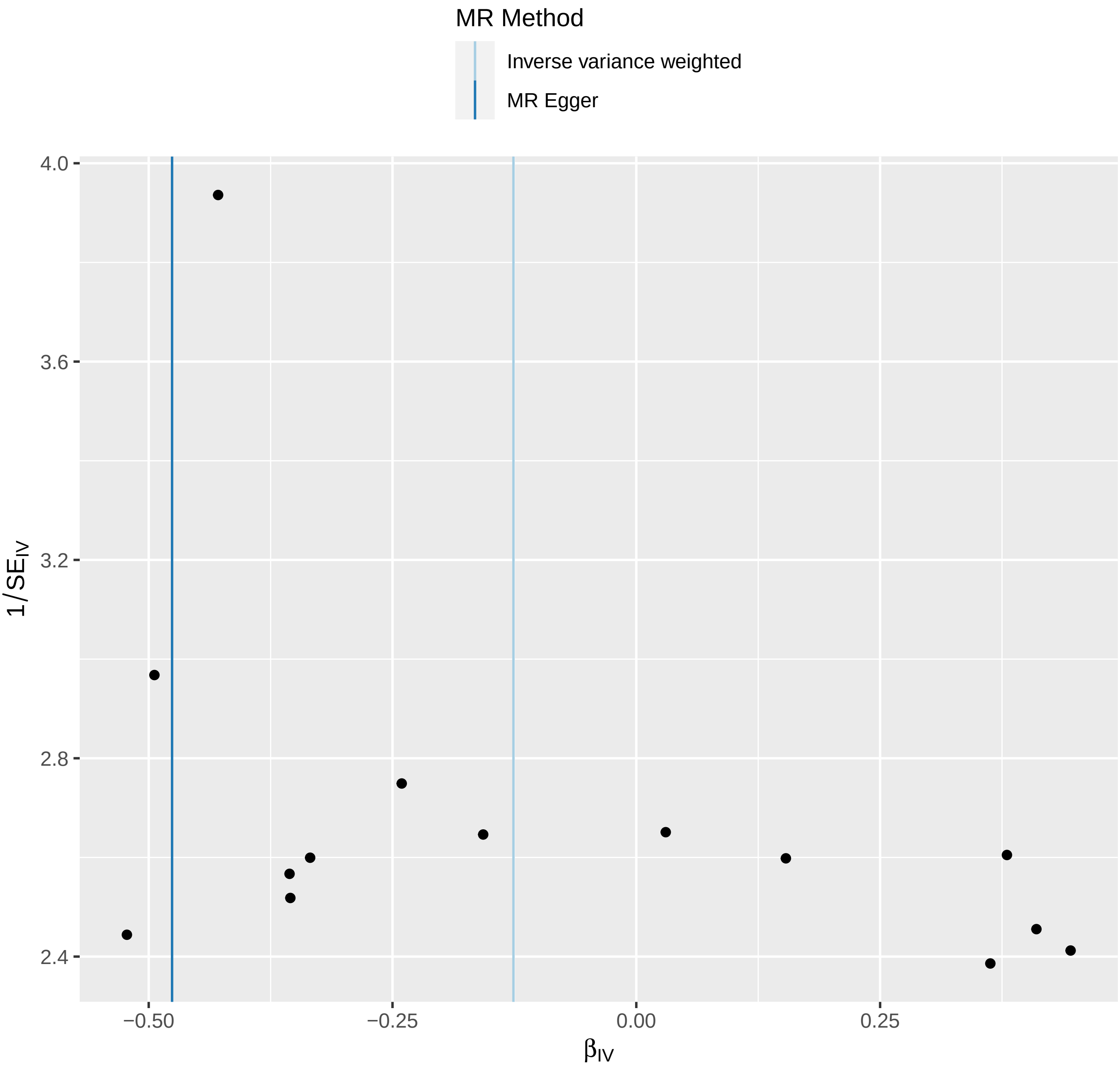

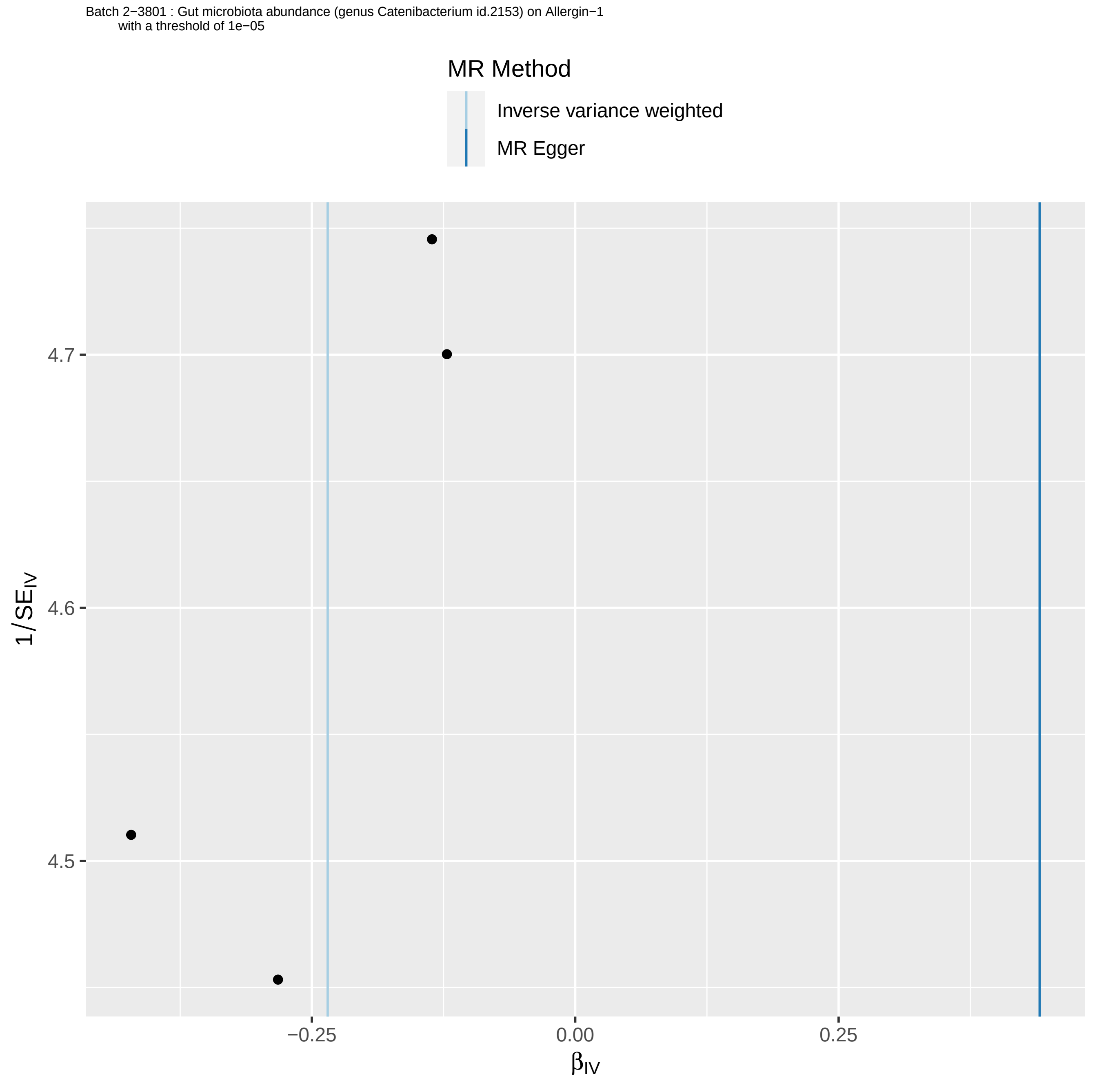

Batch 2−3801 : Gut microbiota abundance (genus Catenibacterium id.2153) on Allergin−1
with a threshold of 1e−05
MR Method
Inverse variance weighted
MR Egger
4.7
4.6
4.5
1/SE_IV
−0.25
0.00
0.25
β_IV

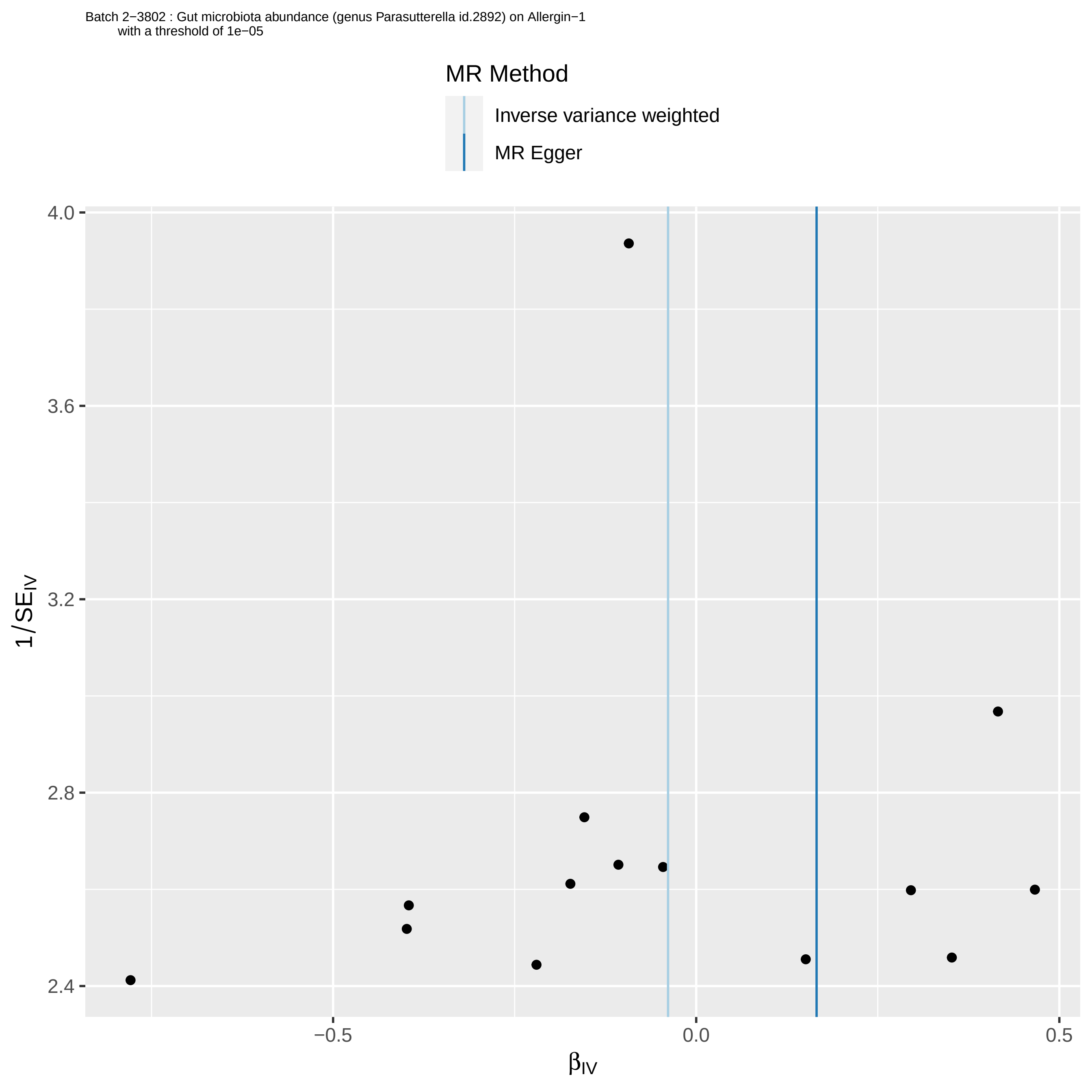
Batch 2−3802 : Gut microbiota abundance (genus Parasutterella id.2892) on Allergin−1
with a threshold of 1e−05
MR Method
Inverse variance weighted
MR Egger
4.0
3.6
3.2
2.8
2.4
1/SE_IV
−0.5
0.0
0.5
β_IV

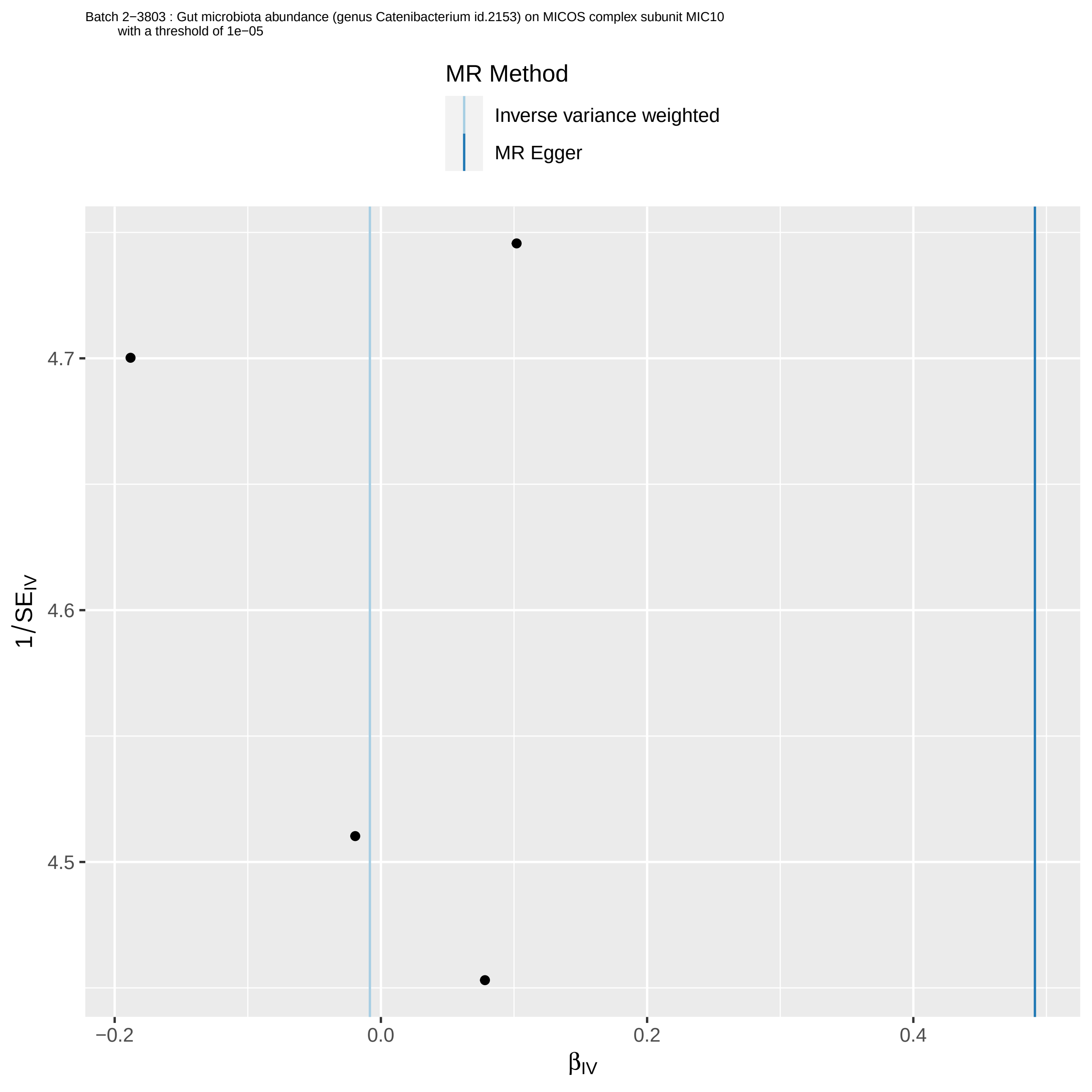

Batch 2−3803 : Gut microbiota abundance (genus Catenibacterium id.2153) on MICOS complex subunit MIC10
with a threshold of 1e−05
MR Method
Inverse variance weighted
MR Egger
4.7
4.6
4.5
1/SE$_{IV}$
−0.2
0.0
0.2
0.4
β_{IV}

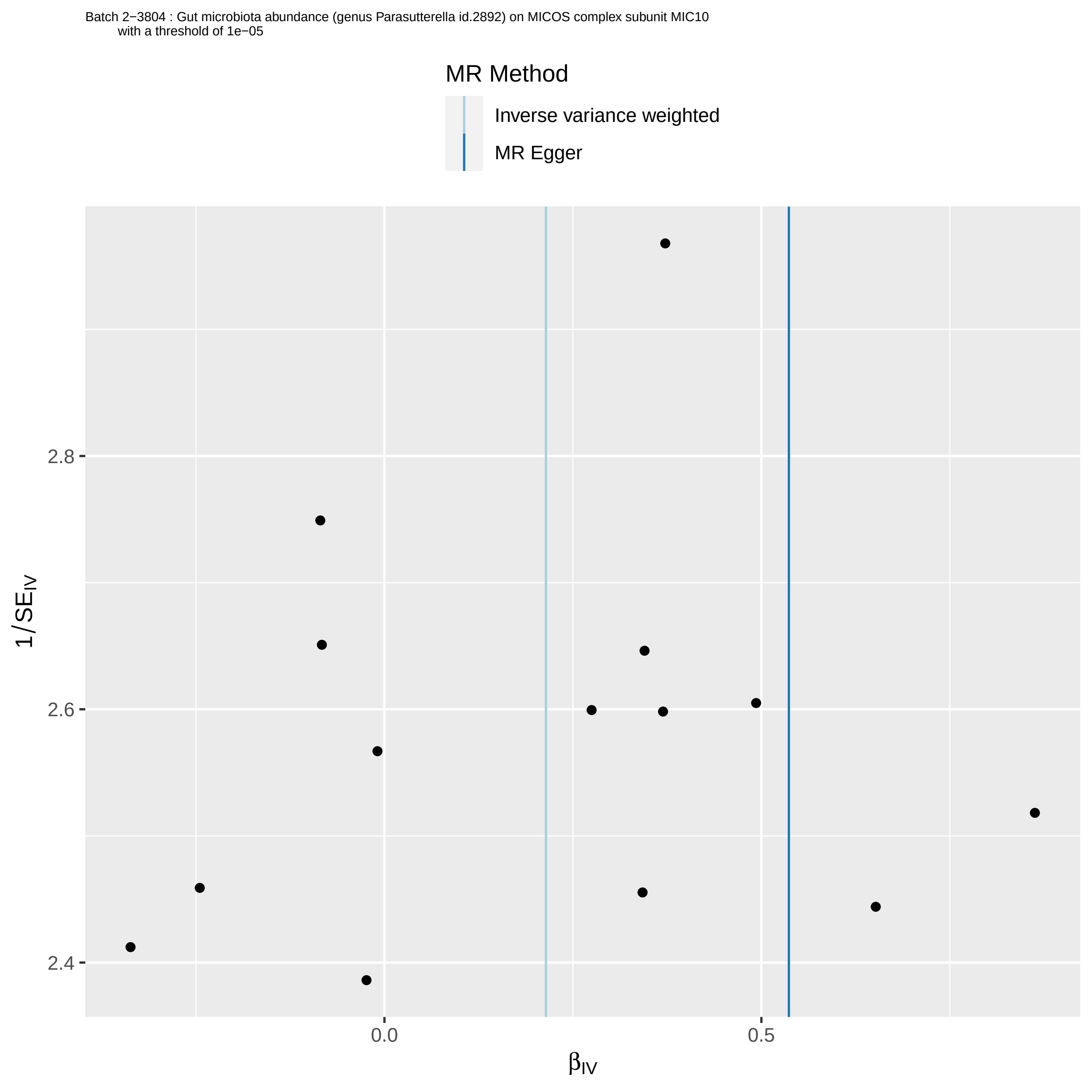
Batch 2−3804 : Gut microbiota abundance (genus Parasutterella id.2892) on MICOS complex subunit MIC10
with a threshold of 1e−05
MR Method
Inverse variance weighted
MR Egger
2.8
2.6
2.4
1/SE_IV
0.0
0.5
β_IV

Batch 2−3805 : Gut microbiota abundance (genus Catenibacterium id.2153) on Multiple inositol polyphosphate phosphatase 1
with a threshold of 1e−05

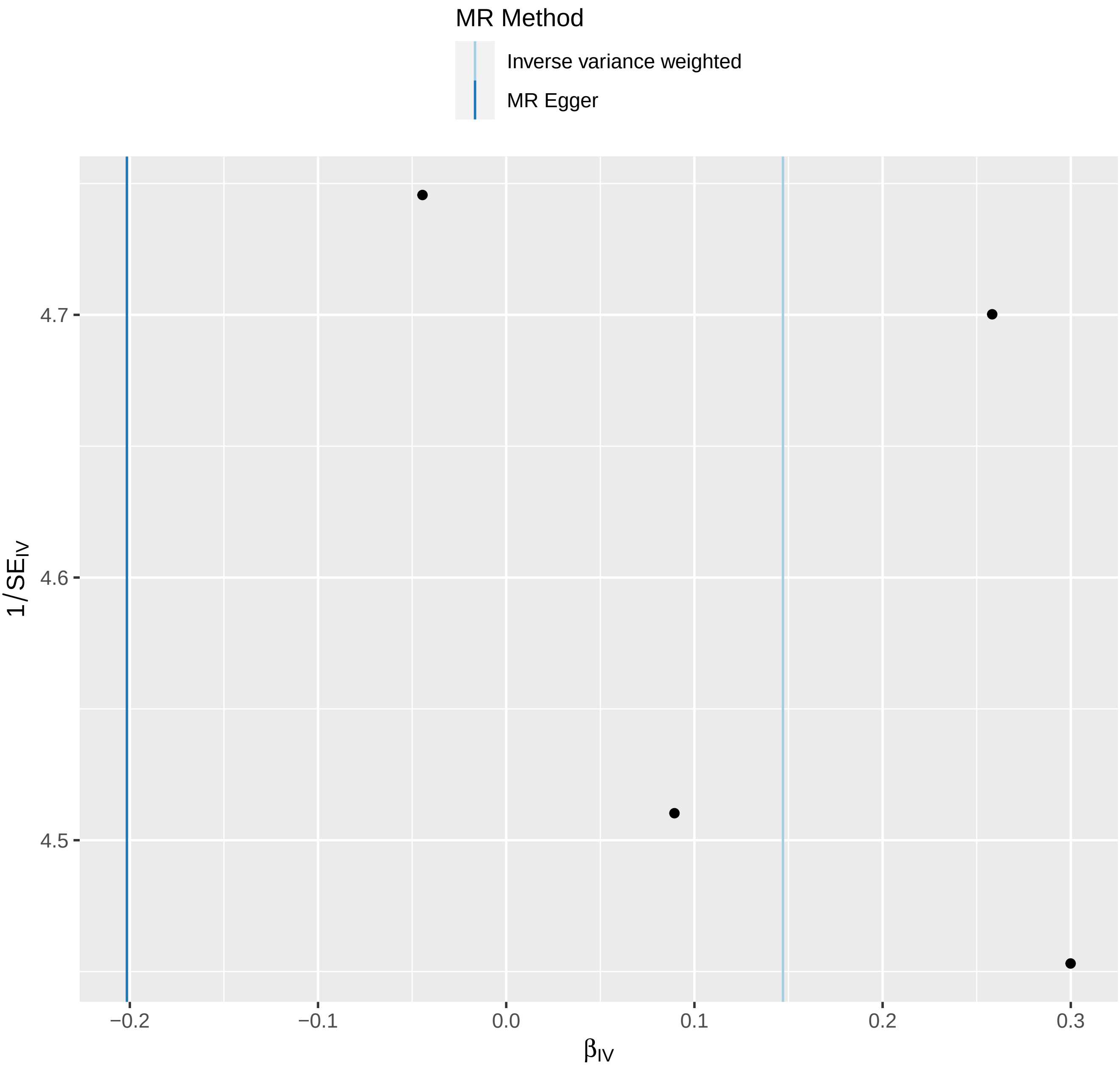

Batch 2−3806 : Gut microbiota abundance (genus Parasutterella id.2892) on Multiple inositol polyphosphate phosphatase 1
with a threshold of 1e−05

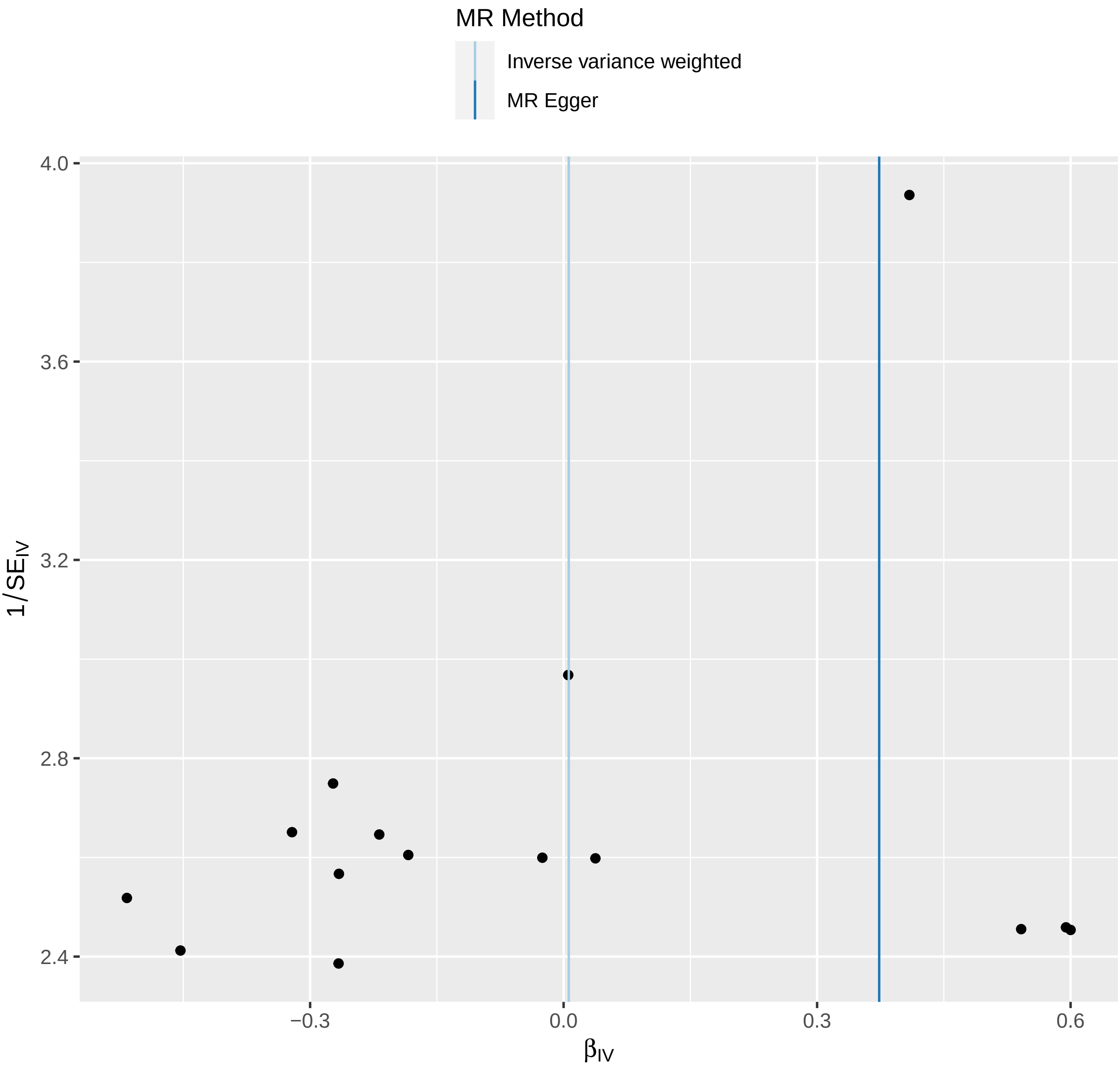

Batch 2−3807 : Gut microbiota abundance (genus Catenibacterium id.2153) on Malectin
with a threshold of 1e−05

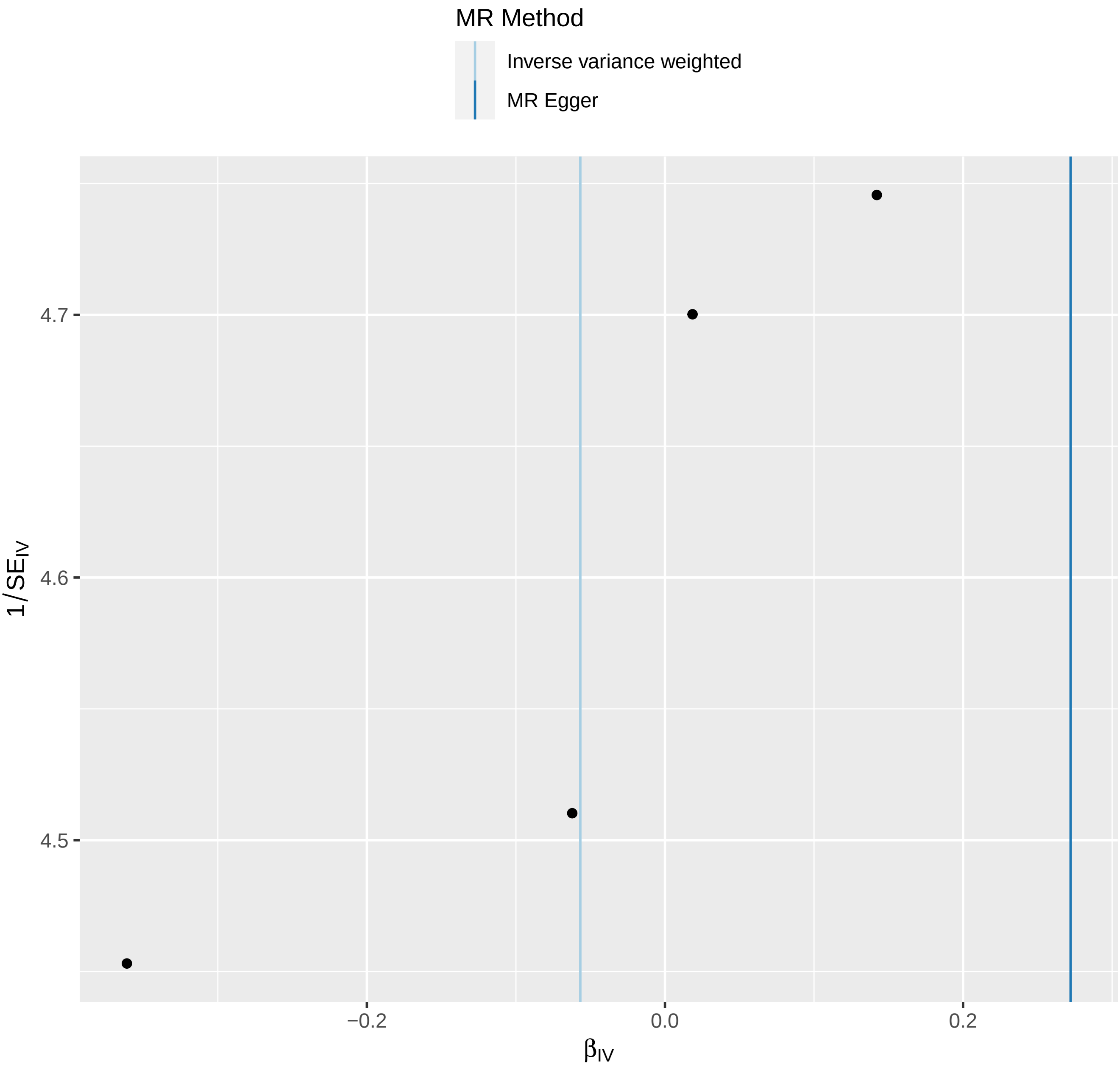

Batch 2−3808 : Gut microbiota abundance (genus Parasutterella id.2892) on Malectin
with a threshold of 1e−05

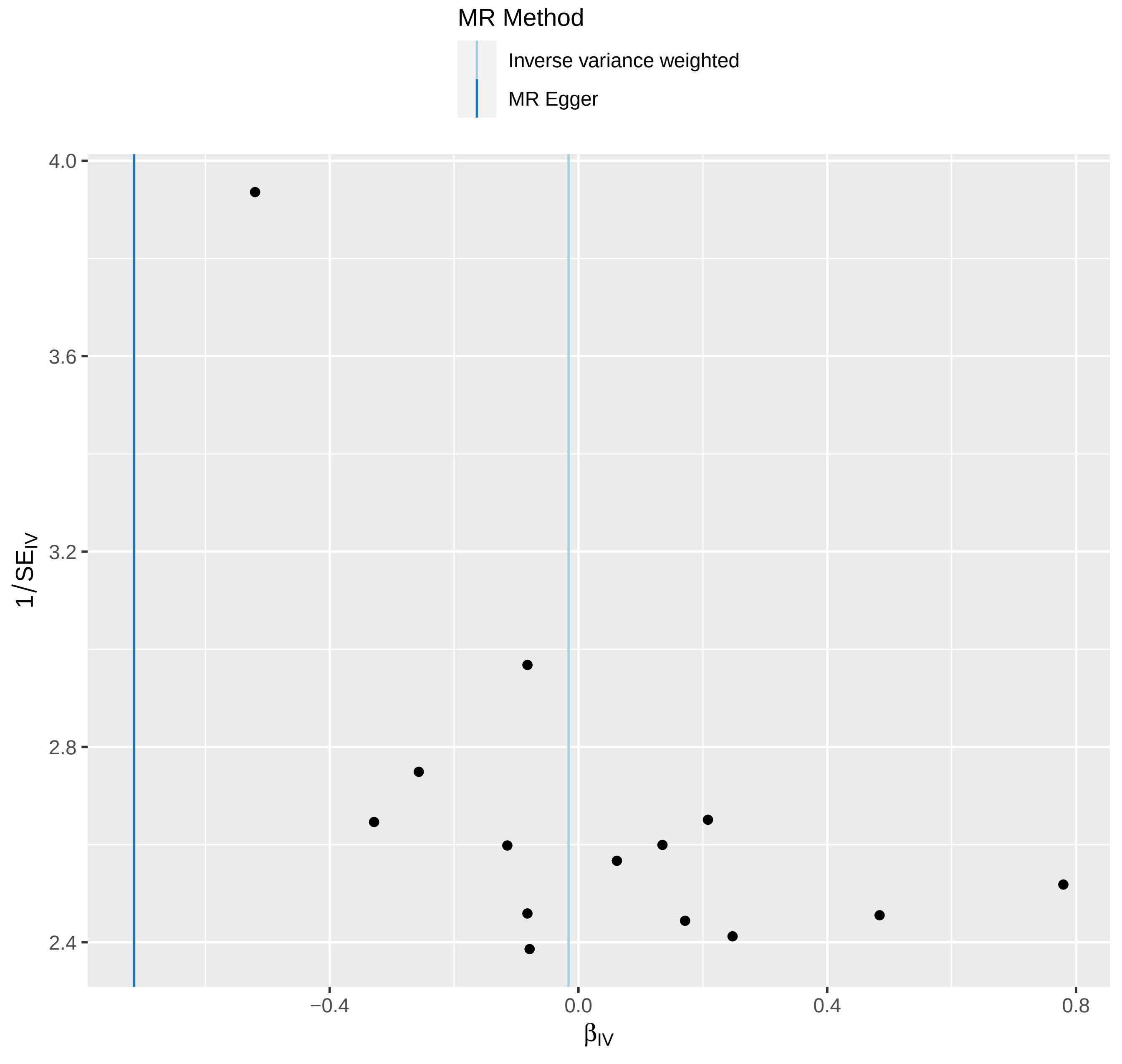

Batch 2−3809 : Gut microbiota abundance (genus Catenibacterium id.2153) on Histone−lysine N−methyltransferase 2D
with a threshold of 1e−05

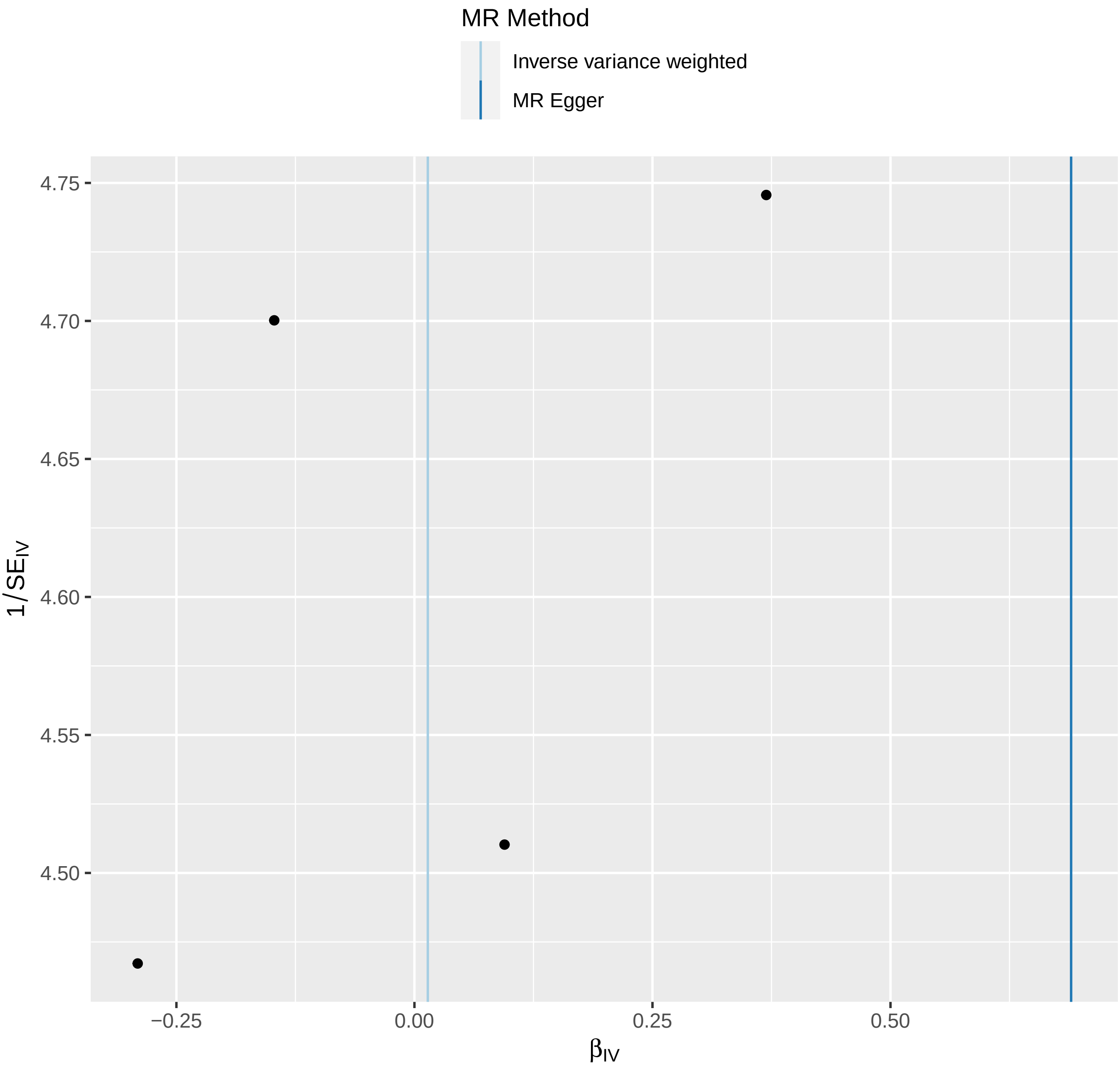

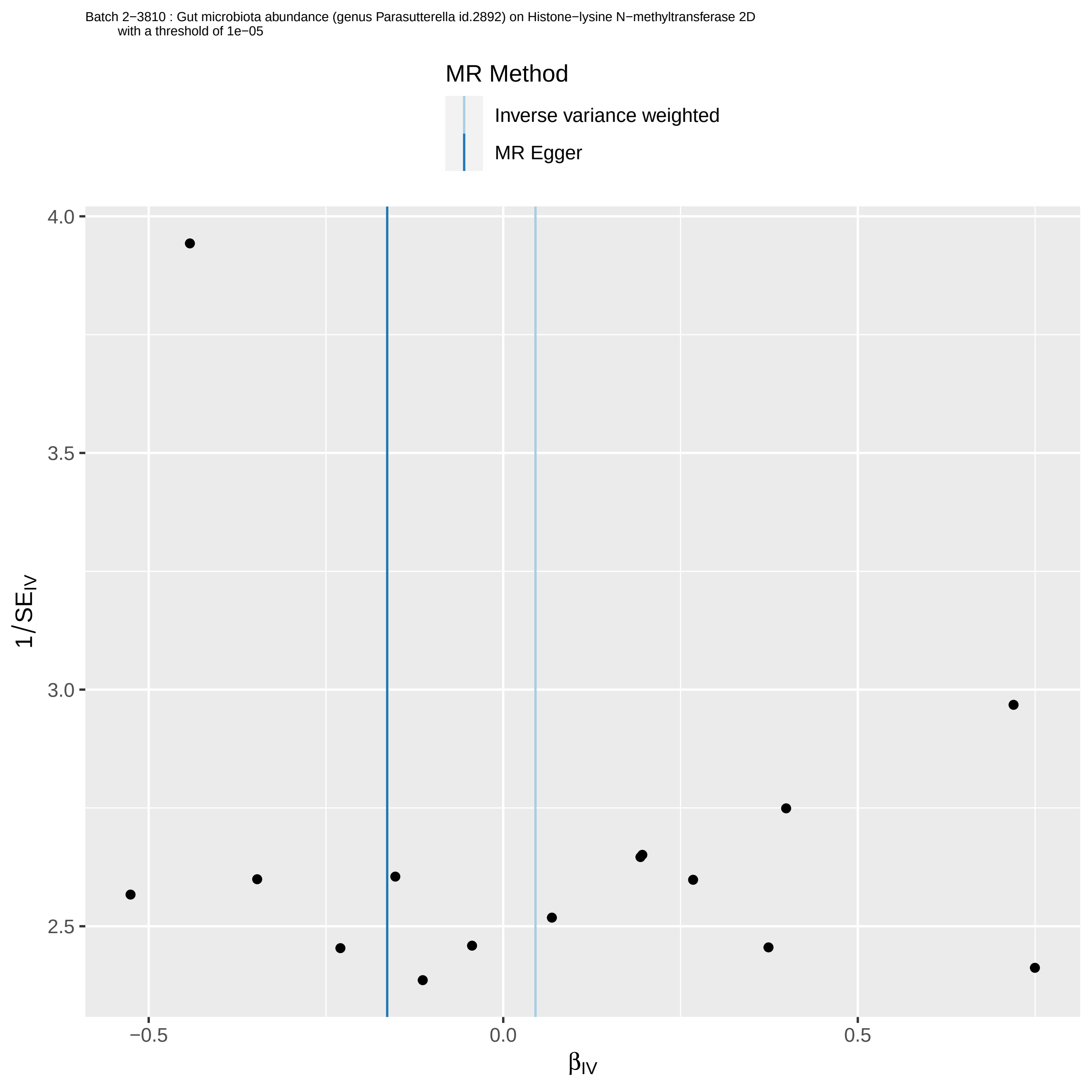
Batch 2−3810 : Gut microbiota abundance (genus Parasutterella id.2892) on Histone−lysine N−methyltransferase 2D
with a threshold of 1e−05
MR Method
Inverse variance weighted
MR Egger
4.0
3.5
3.0
2.5
1/SE$_{IV}$
−0.5
0.0
0.5
β_{IV}

Batch 2−3811 : Gut microbiota abundance (genus Catenibacterium id.2153) on Promotilin
with a threshold of 1e−05

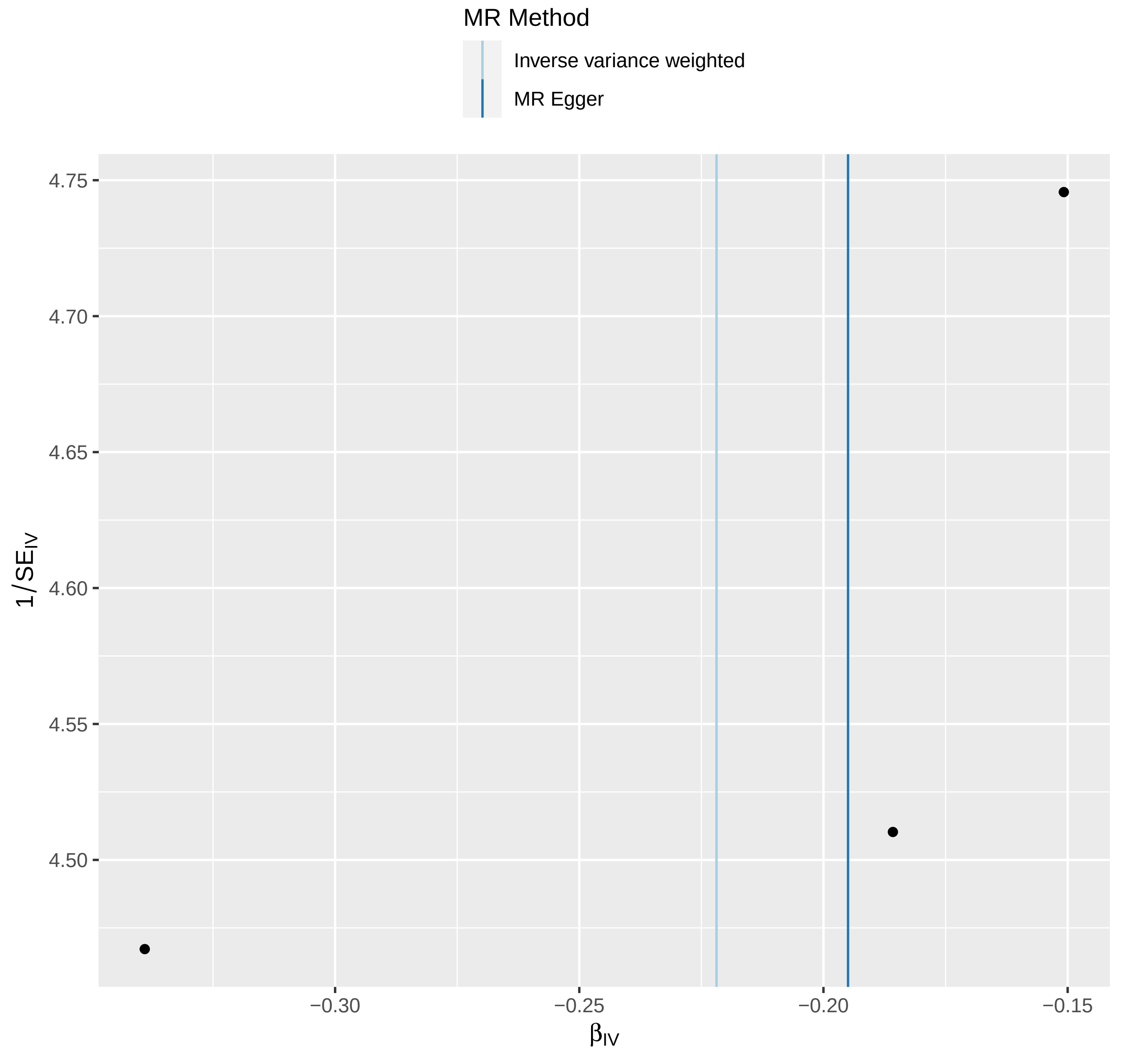

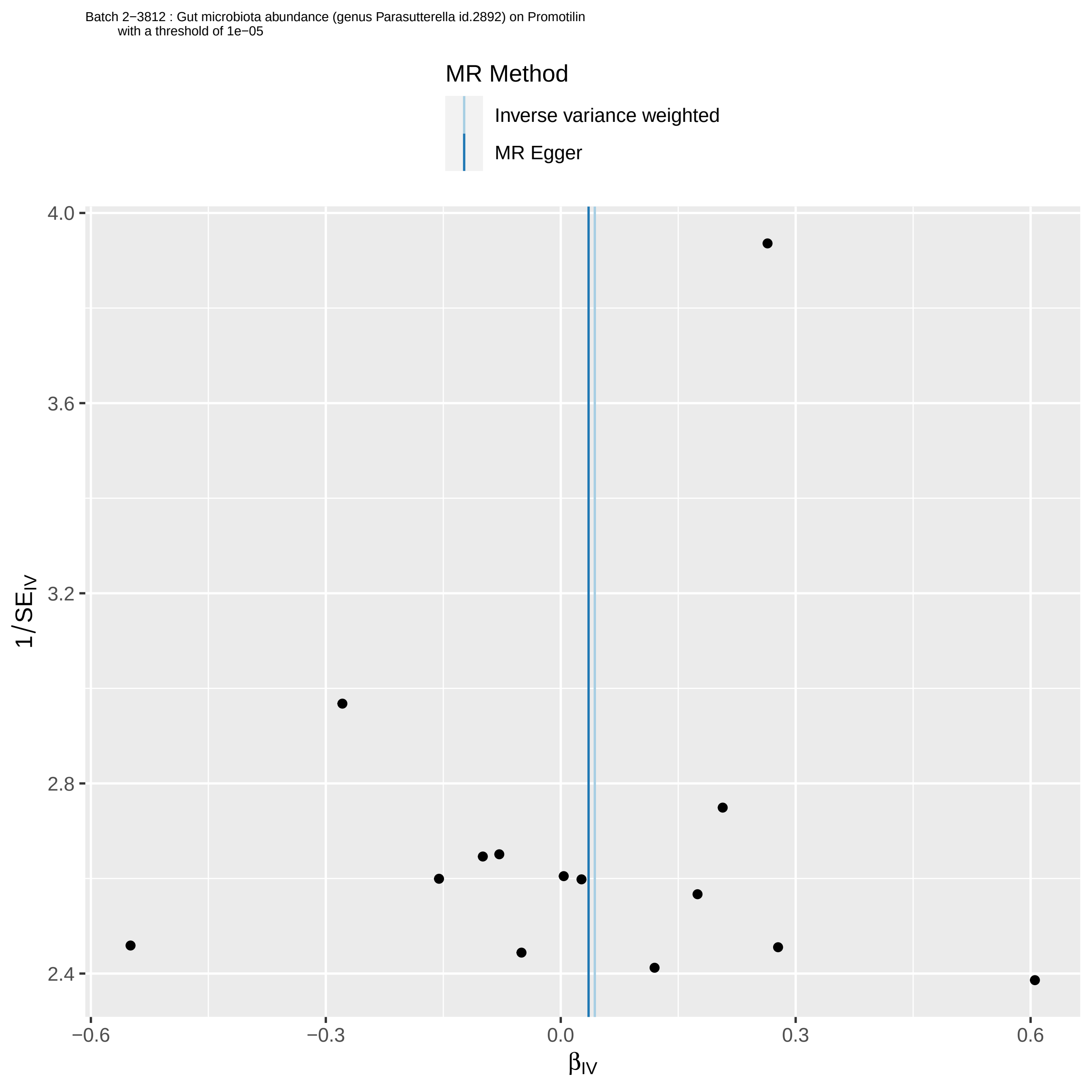
Batch 2−3812 : Gut microbiota abundance (genus Parasutterella id.2892) on Promotilin
with a threshold of 1e−05
MR Method
Inverse variance weighted
MR Egger
4.0
3.6
3.2
2.8
2.4
1/SE$_{IV}$
−0.6
−0.3
0.0
0.3
0.6
β_{IV}

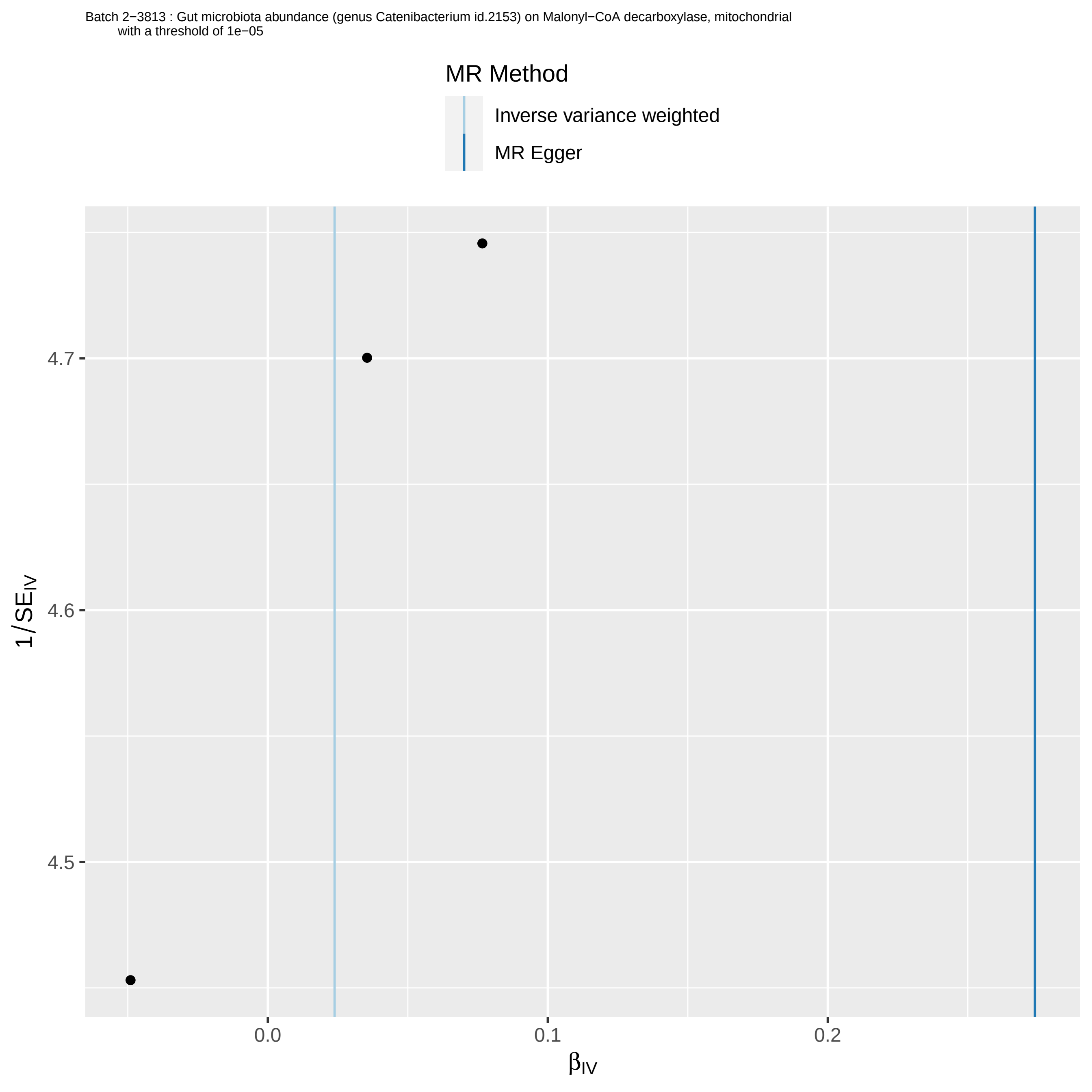
Batch 2−3813 : Gut microbiota abundance (genus Catenibacterium id.2153) on Malonyl−CoA decarboxylase, mitochondrial
with a threshold of 1e−05
MR Method
Inverse variance weighted
MR Egger
4.7
4.6
4.5
1/SE_IV
0.0
0.1
0.2
β_IV

Batch 2−3814 : Gut microbiota abundance (genus Parasutterella id.2892) on Malonyl−CoA decarboxylase, mitochondrial
with a threshold of 1e−05

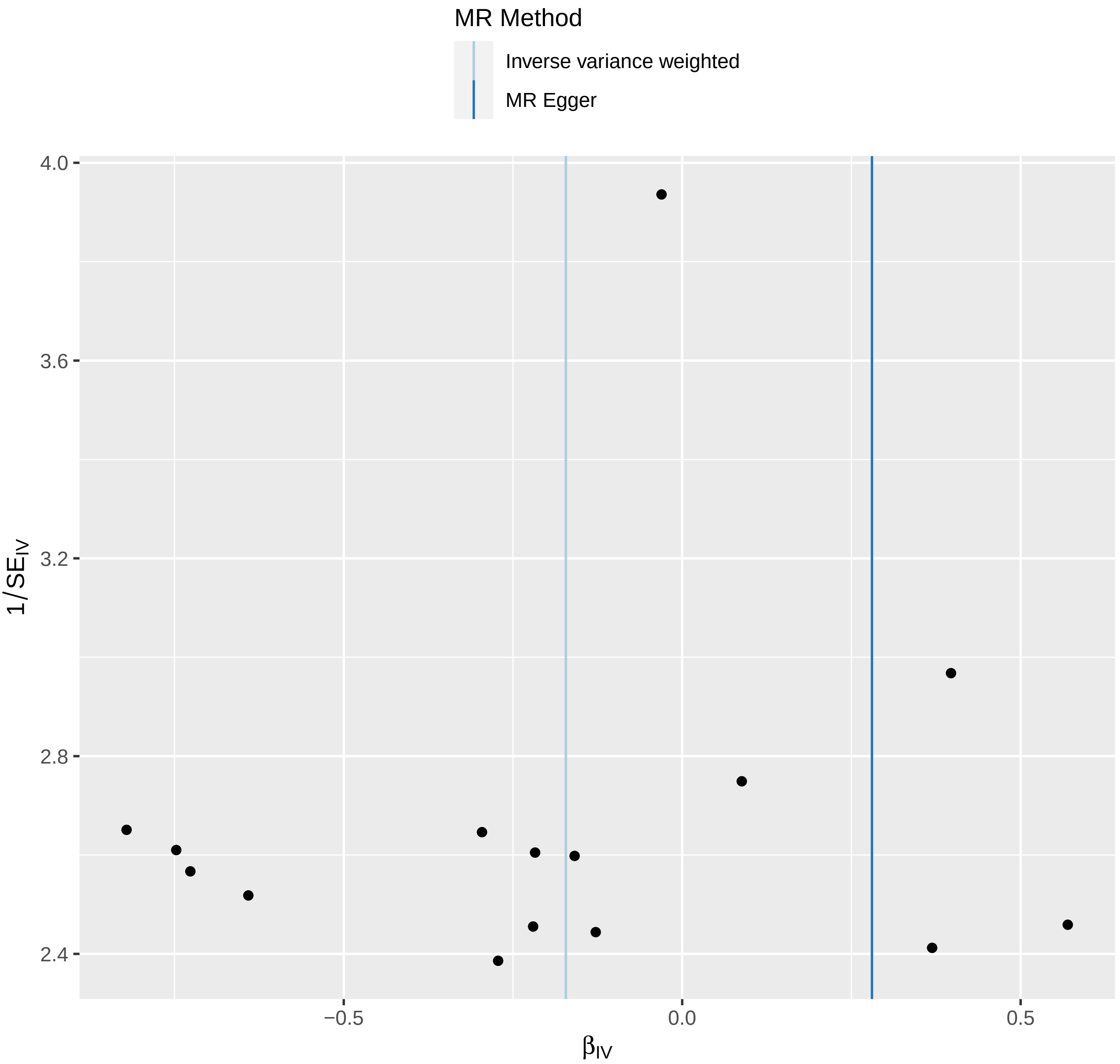

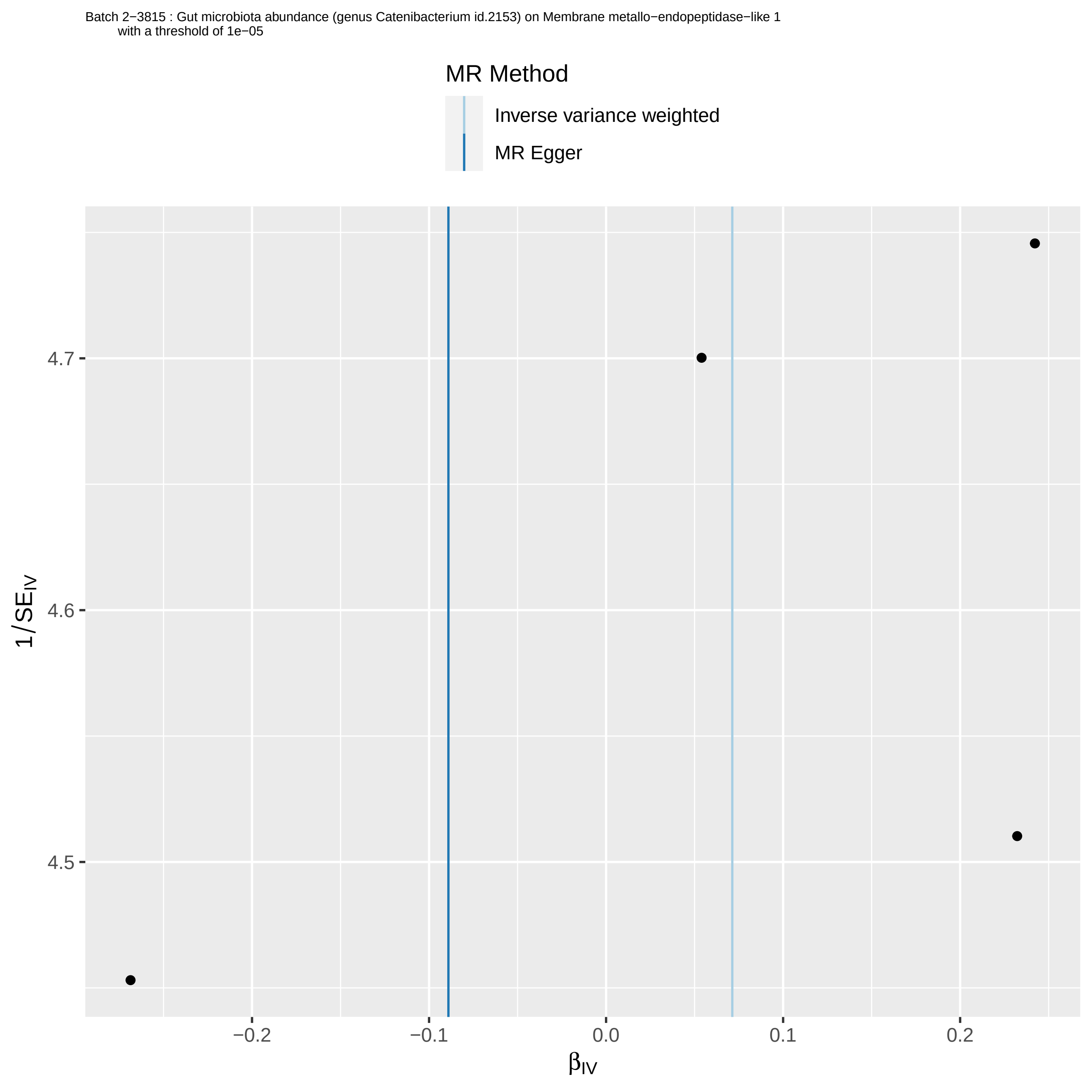
Batch 2−3815 : Gut microbiota abundance (genus Catenibacterium id.2153) on Membrane metallo−endopeptidase−like 1
with a threshold of 1e−05
MR Method
Inverse variance weighted
MR Egger
4.7
4.6
4.5
1/SE_IV
−0.2
−0.1
0.0
0.1
0.2
β_IV

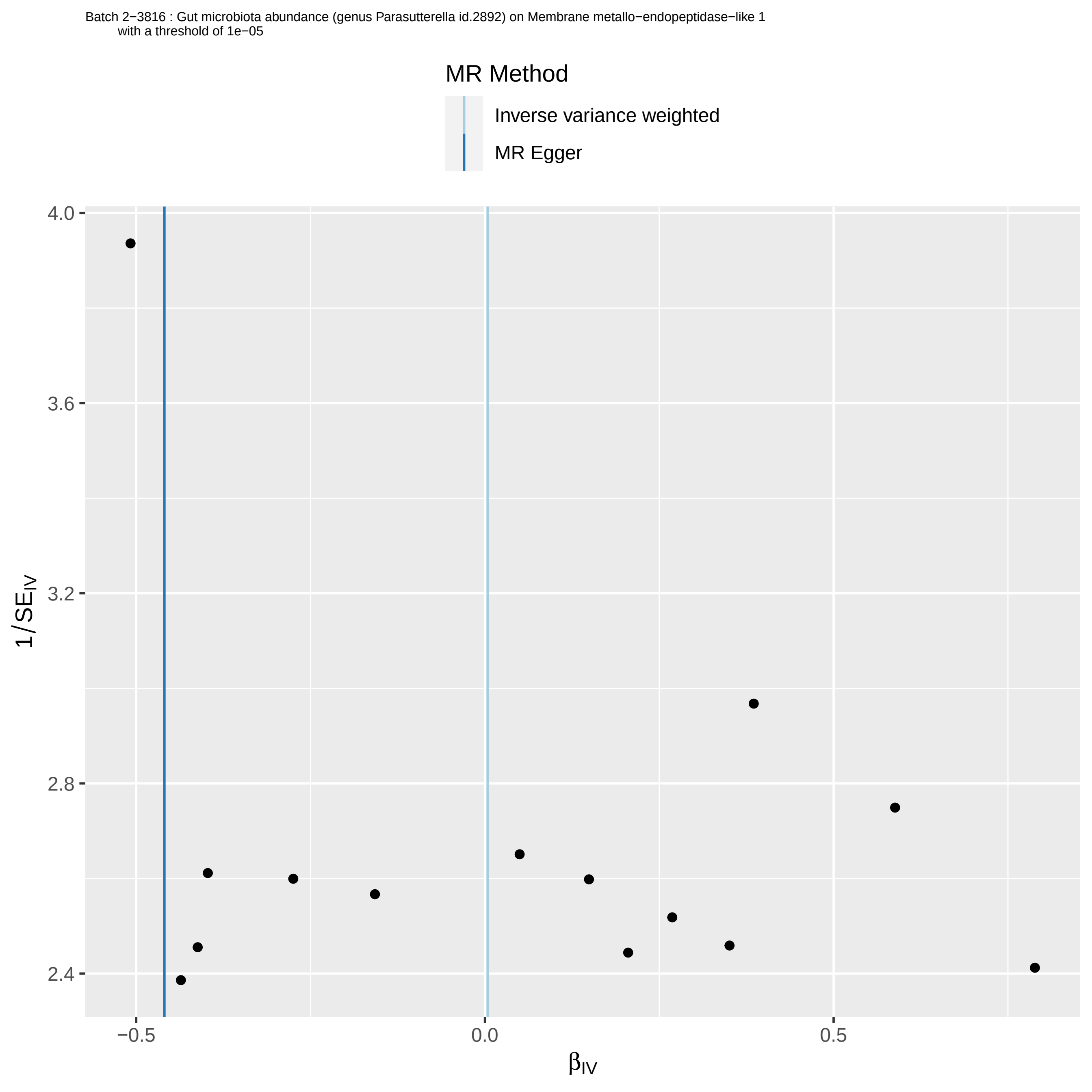
Batch 2−3816 : Gut microbiota abundance (genus Parasutterella id.2892) on Membrane metallo−endopeptidase−like 1
with a threshold of 1e−05
MR Method
Inverse variance weighted
MR Egger
4.0
3.6
3.2
2.8
2.4
1/SE_IV
−0.5
0.0
0.5
β_IV

Batch 2−3817 : Gut microbiota abundance (genus Catenibacterium id.2153) on Membrane magnesium transporter 1
with a threshold of 1e−05

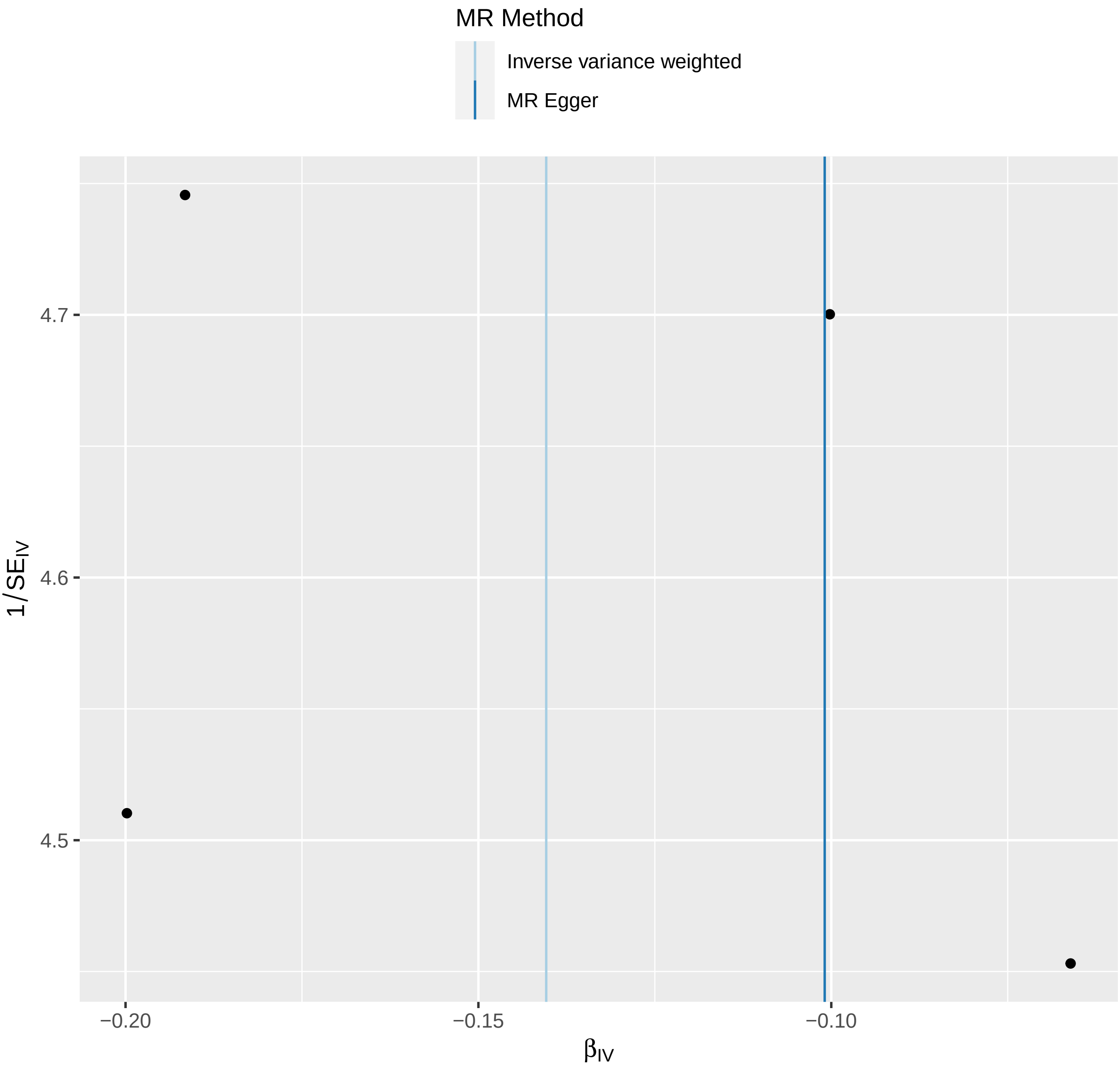

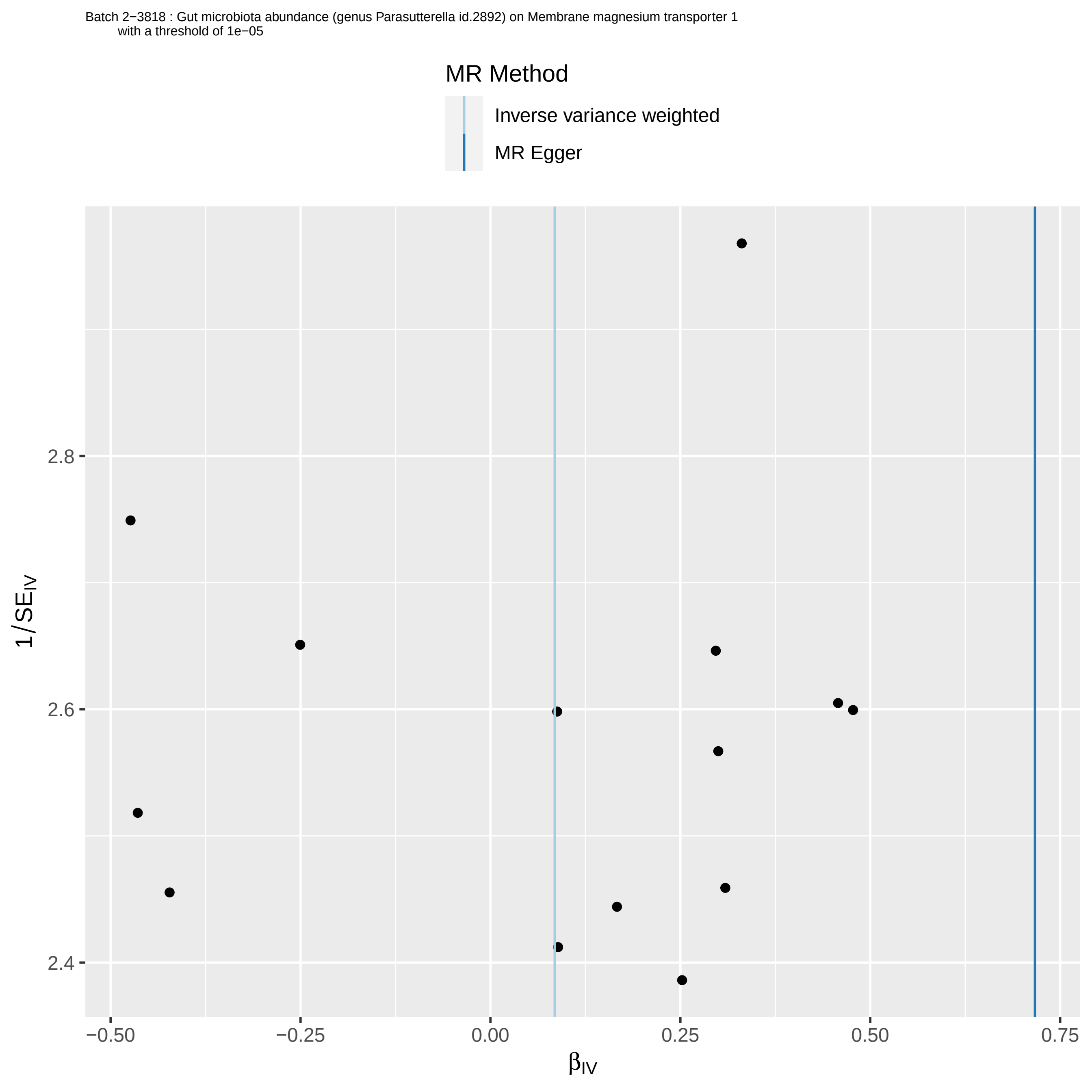

Batch 2−3818 : Gut microbiota abundance (genus Parasutterella id.2892) on Membrane magnesium transporter 1
with a threshold of 1e−05
MR Method
Inverse variance weighted
MR Egger
2.8
2.6
2.4
1/SE_IV
−0.50
−0.25
0.00
0.25
0.50
0.75
β_IV

Batch 2−3819 : Gut microbiota abundance (genus Catenibacterium id.2153) on Stromelysin−2
with a threshold of 1e−05

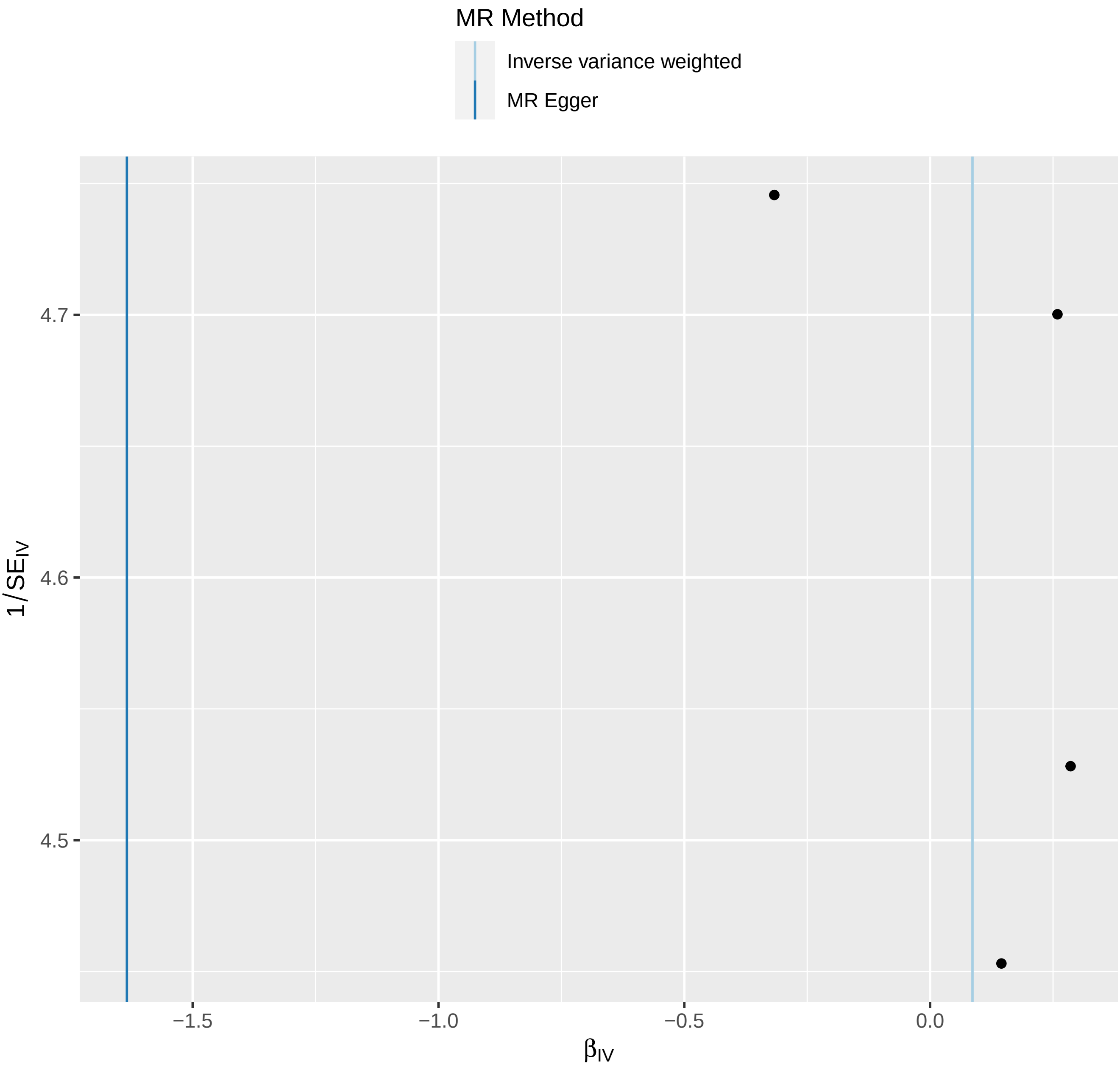

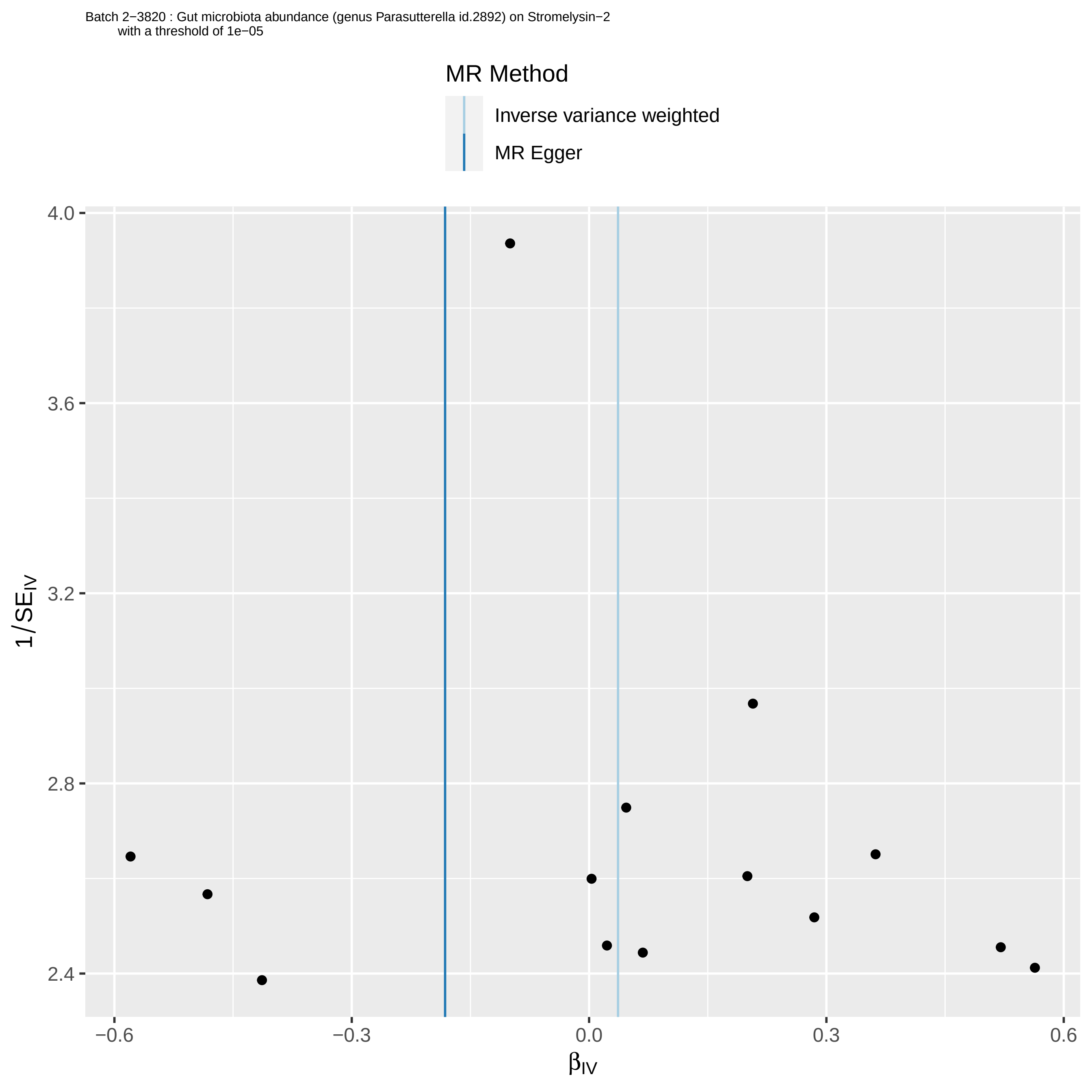

Batch 2−3820 : Gut microbiota abundance (genus Parasutterella id.2892) on Stromelysin−2
with a threshold of 1e−05
MR Method
Inverse variance weighted
MR Egger
4.0
3.6
3.2
2.8
2.4
1/SE_IV
−0.6
−0.3
0.0
0.3
0.6
β_IV

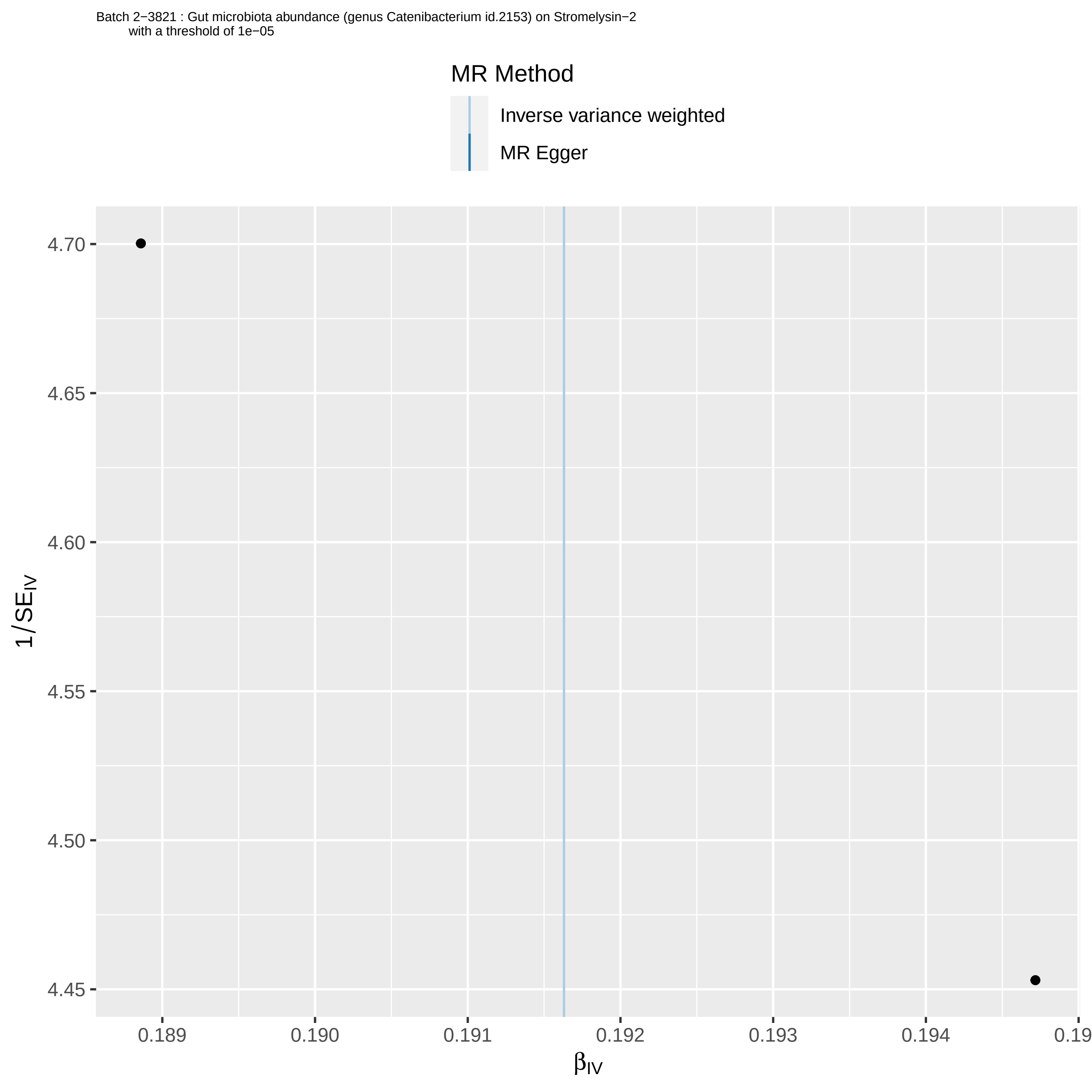

Batch 2−3821 : Gut microbiota abundance (genus Catenibacterium id.2153) on Stromelysin−2
with a threshold of 1e−05
MR Method
Inverse variance weighted
MR Egger
4.70
4.65
4.60
4.55
4.50
4.45
1/SE$_{IV}$
0.189
0.190
0.191
0.192
0.193
0.194
0.19
β$_{IV}$

Batch 2−3822 : Gut microbiota abundance (genus Parasutterella id.2892) on Stromelysin−2
with a threshold of 1e−05

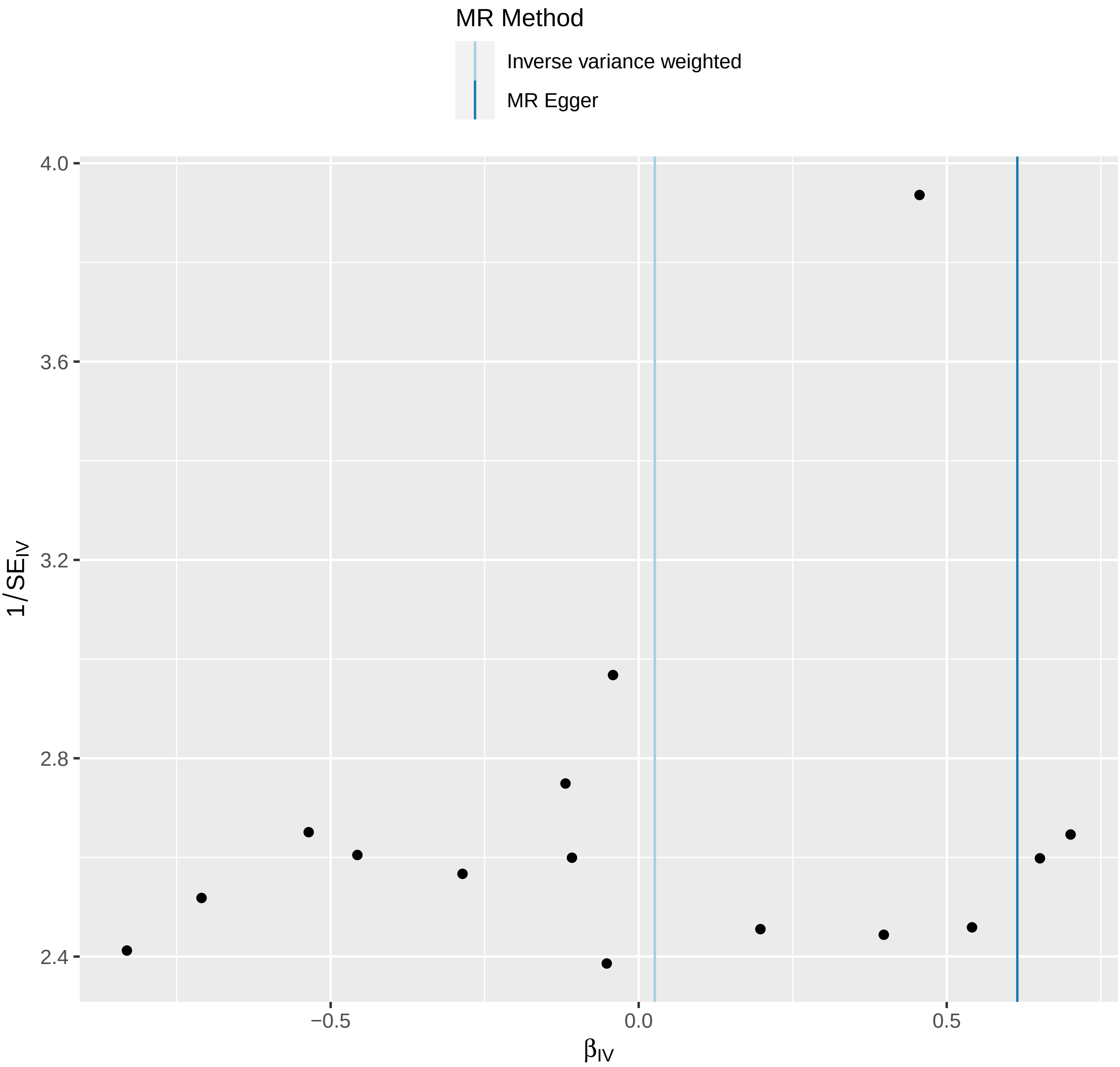

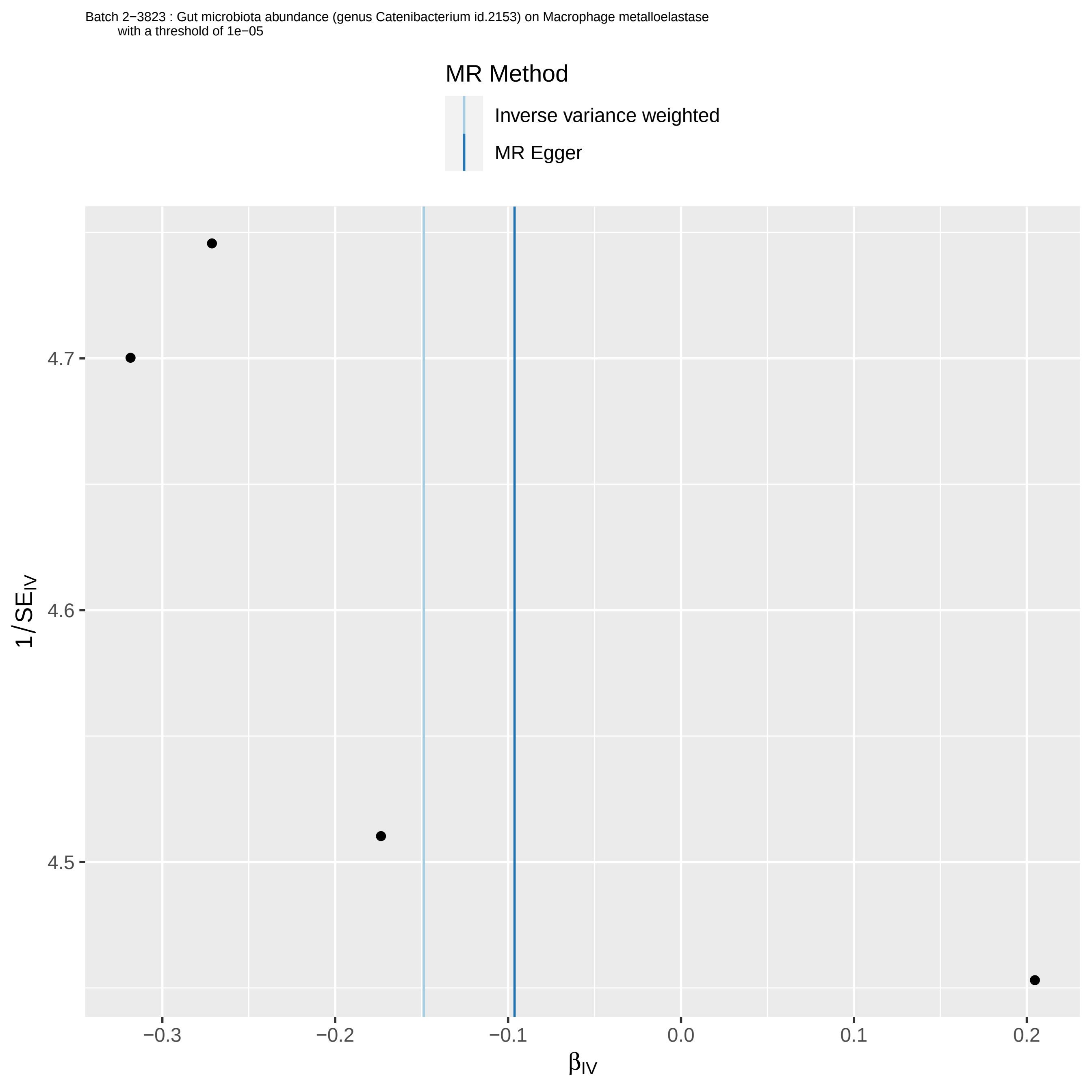

Batch 2−3823 : Gut microbiota abundance (genus Catenibacterium id.2153) on Macrophage metalloelastase
with a threshold of 1e−05
MR Method
Inverse variance weighted
MR Egger
4.7
4.6
4.5
1/SE_IV
−0.3
−0.2
−0.1
0.0
0.1
0.2
β_IV

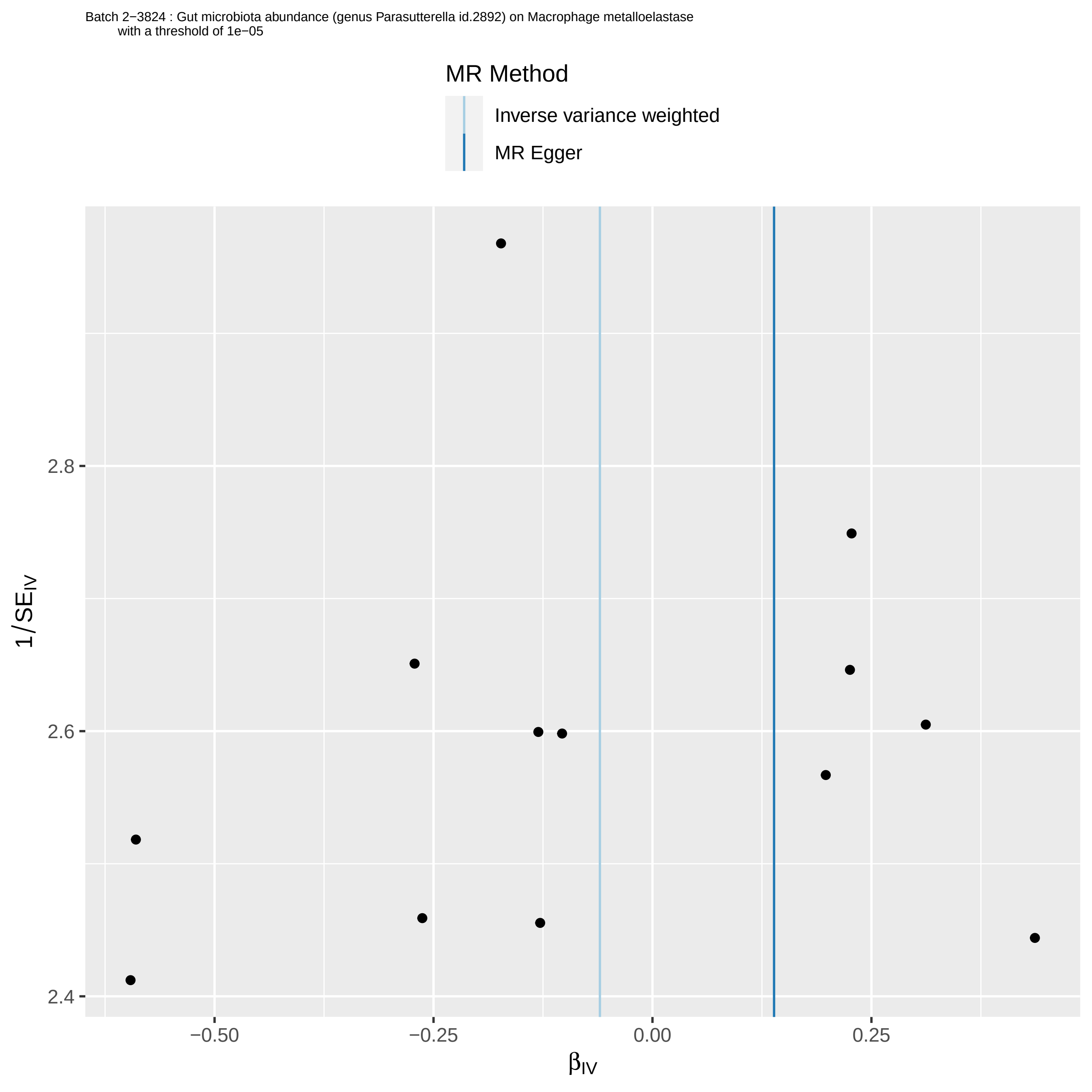

Batch 2−3824 : Gut microbiota abundance (genus Parasutterella id.2892) on Macrophage metalloelastase
with a threshold of 1e−05
MR Method
Inverse variance weighted
MR Egger
1/SE_IV
2.8
2.6
2.4
−0.50
−0.25
0.00
0.25
β_IV

Batch 2−3825 : Gut microbiota abundance (genus Catenibacterium id.2153) on Collagenase 3
with a threshold of 1e−05

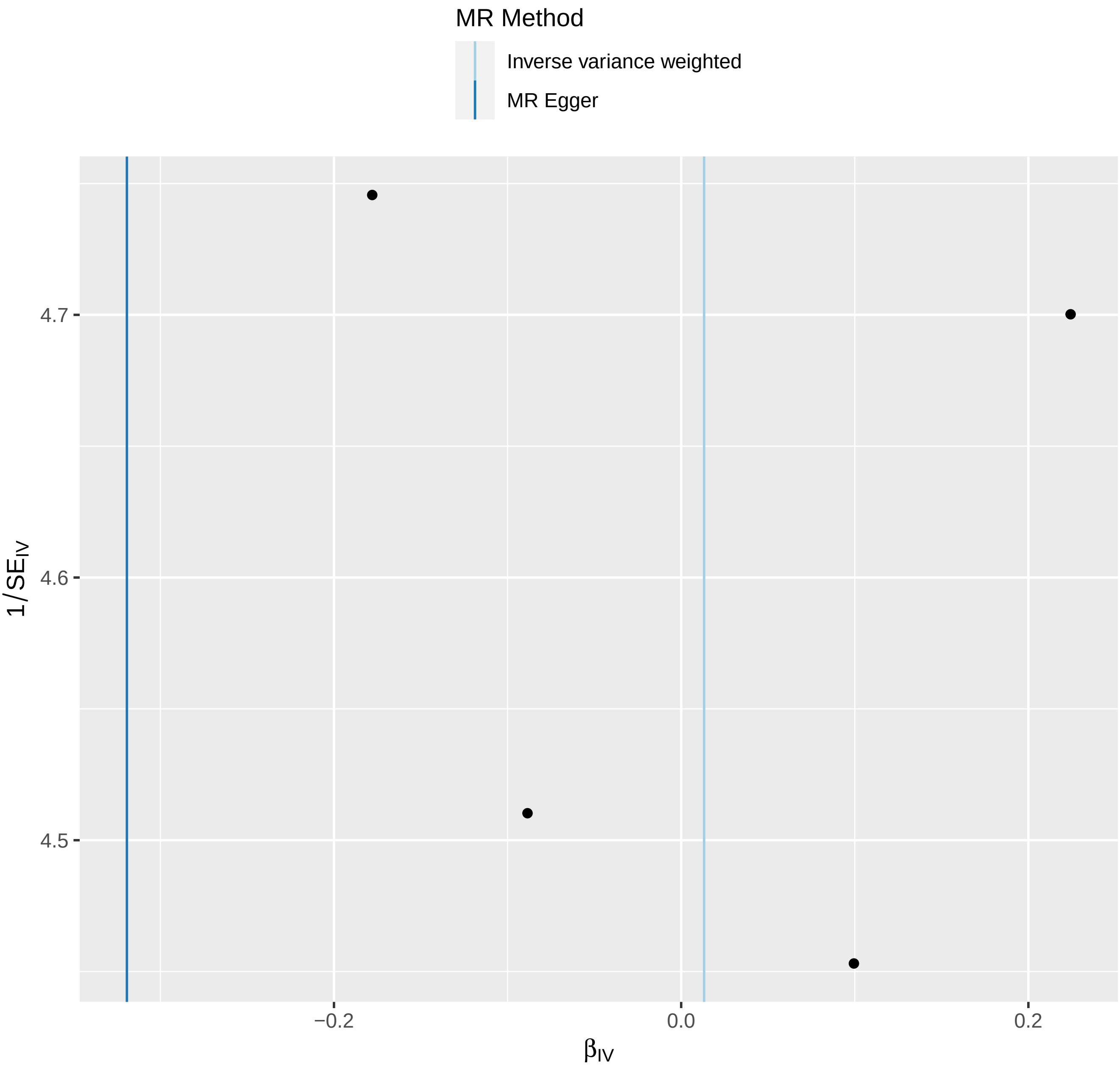

Batch 2−3826 : Gut microbiota abundance (genus Parasutterella id.2892) on Collagenase 3
with a threshold of 1e−05

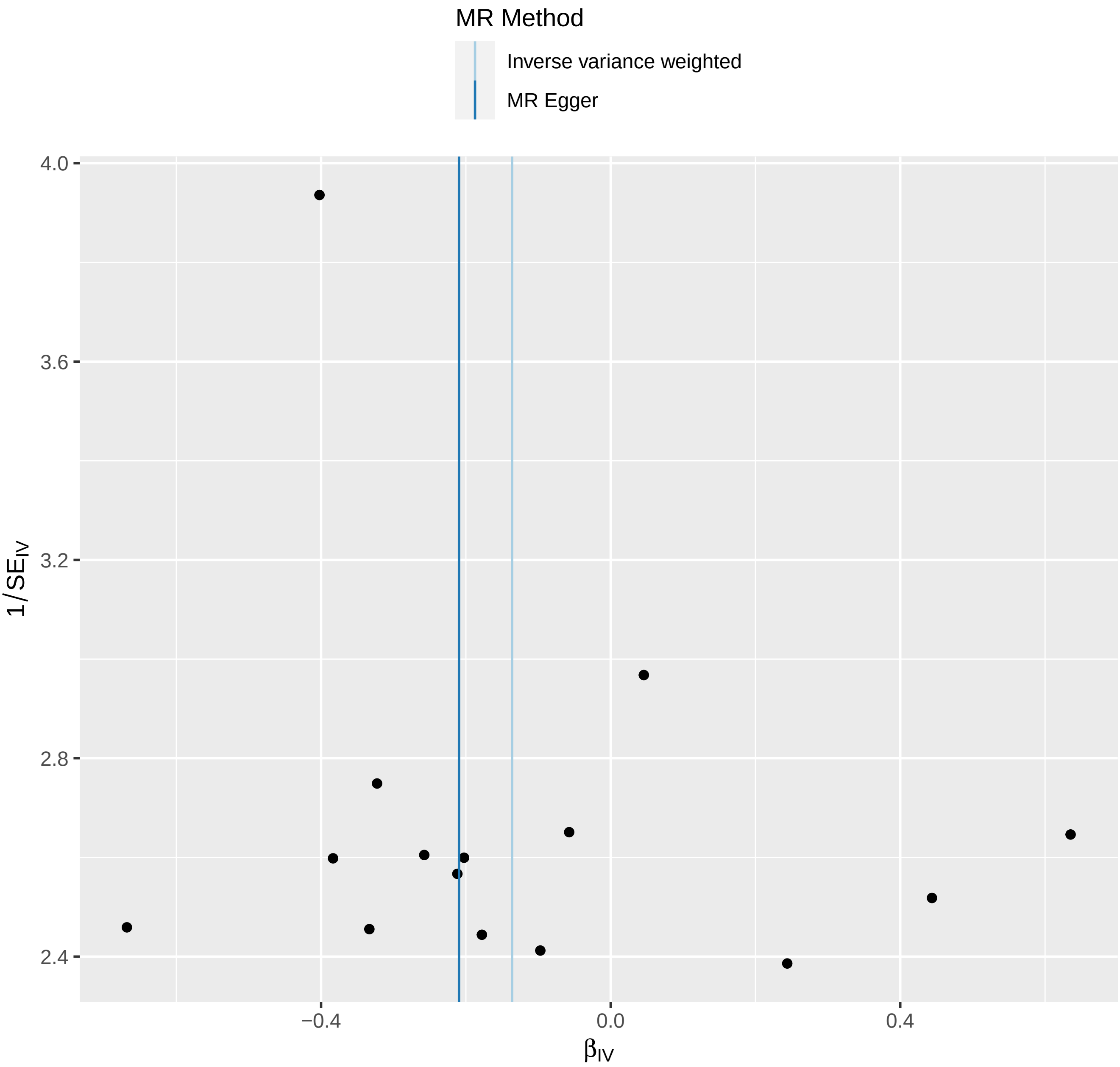

Batch 2−3827 : Gut microbiota abundance (genus Catenibacterium id.2153) on Matrix metalloproteinase−14
with a threshold of 1e−05

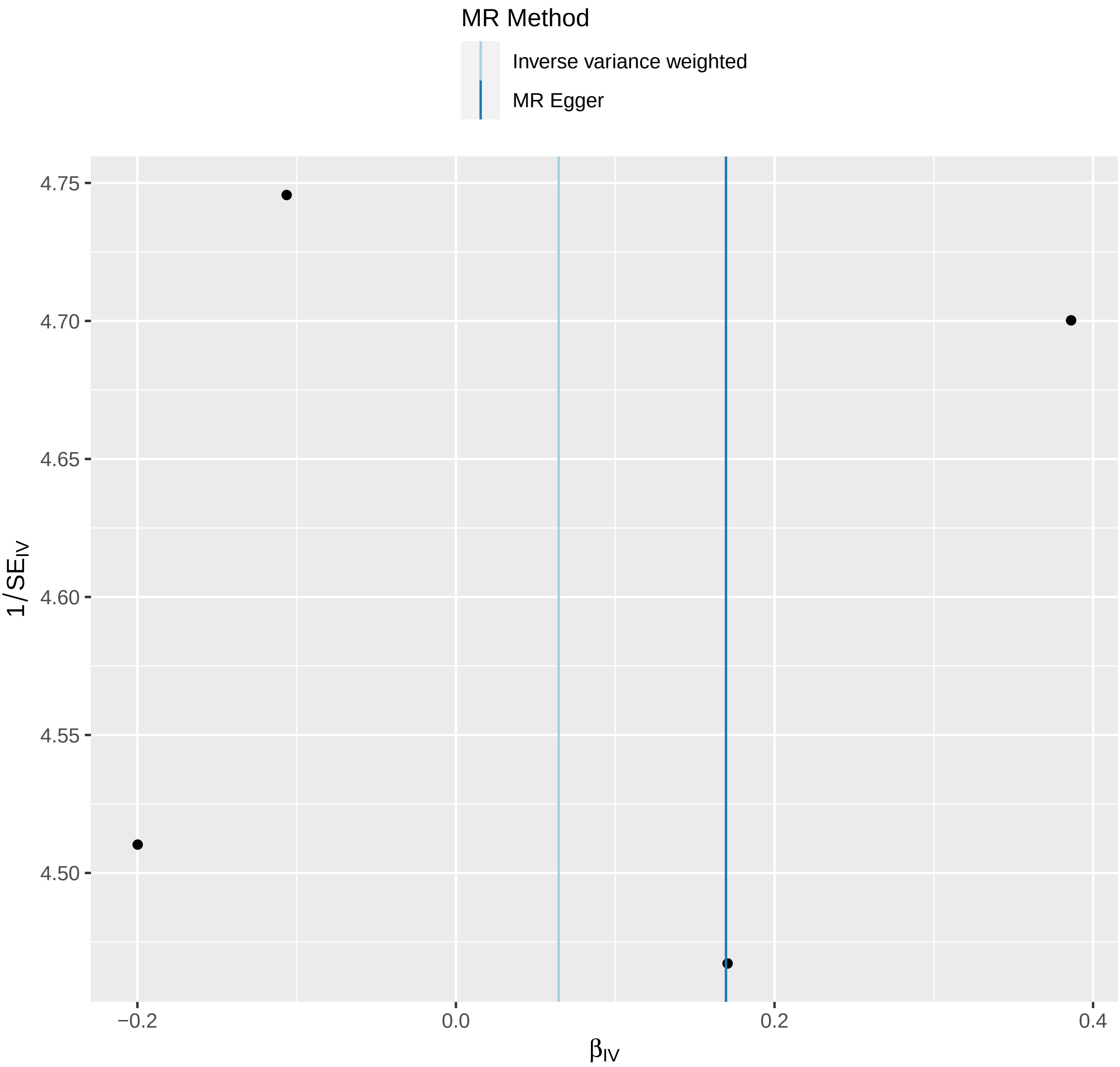

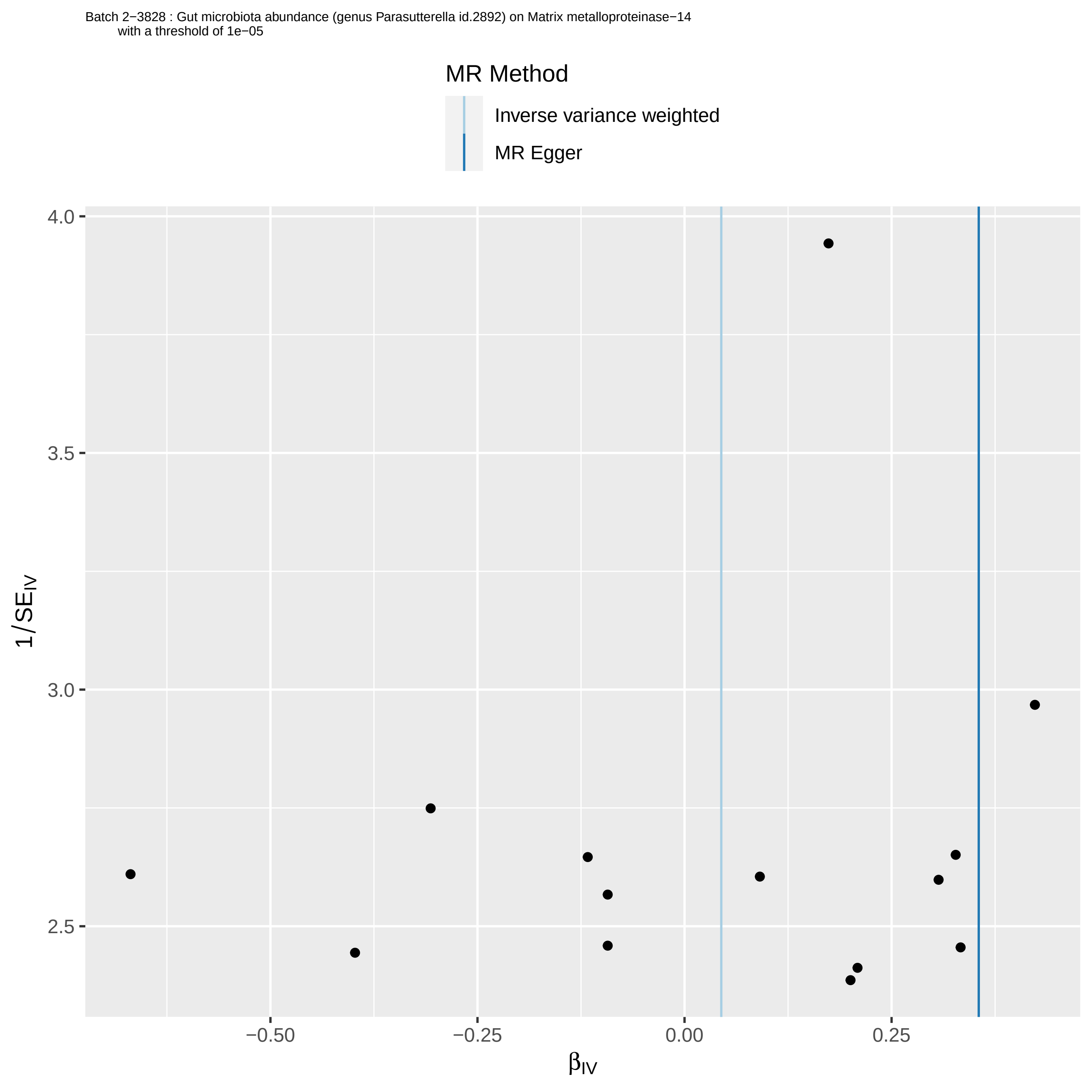

Batch 2−3828 : Gut microbiota abundance (genus Parasutterella id.2892) on Matrix metalloproteinase−14
with a threshold of 1e−05
MR Method
Inverse variance weighted
MR Egger
4.0
3.5
3.0
2.5
1/SE_IV
−0.50
−0.25
0.00
0.25
β_IV

Batch 2−3829 : Gut microbiota abundance (genus Catenibacterium id.2153) on Matrix metalloproteinase−16
with a threshold of 1e−05

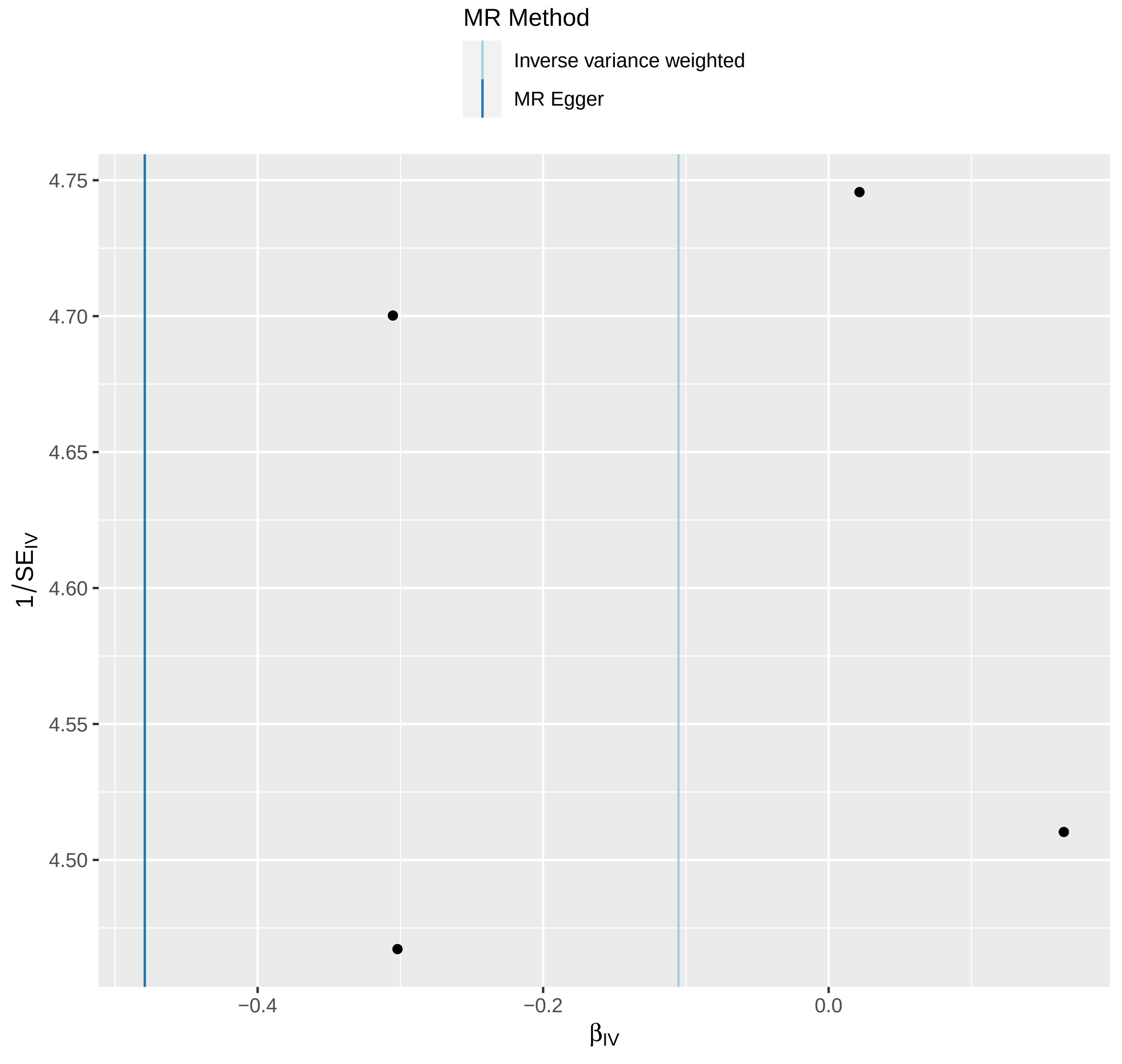

Batch 2−3830 : Gut microbiota abundance (genus Parasutterella id.2892) on Matrix metalloproteinase−16
with a threshold of 1e−05

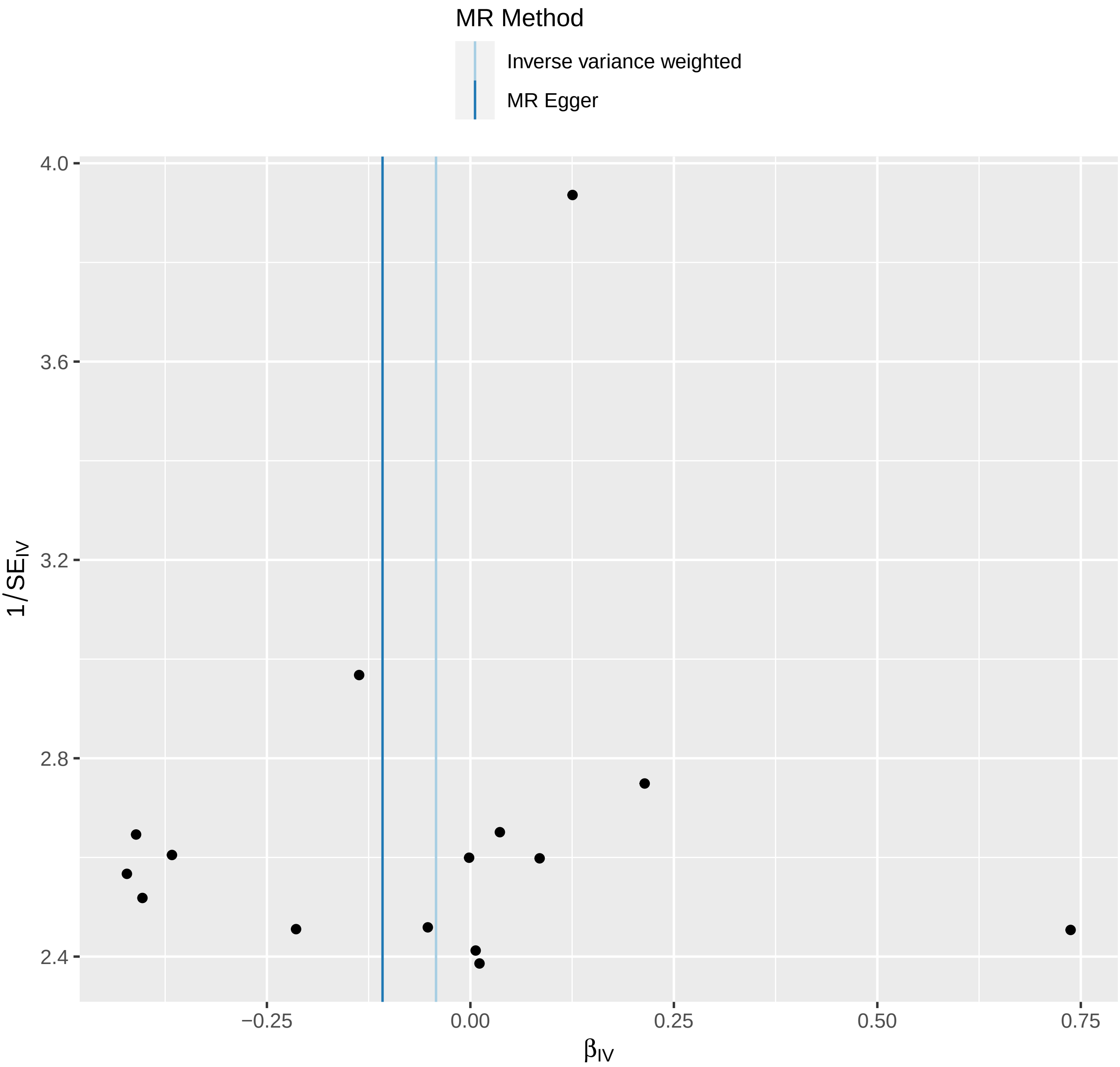

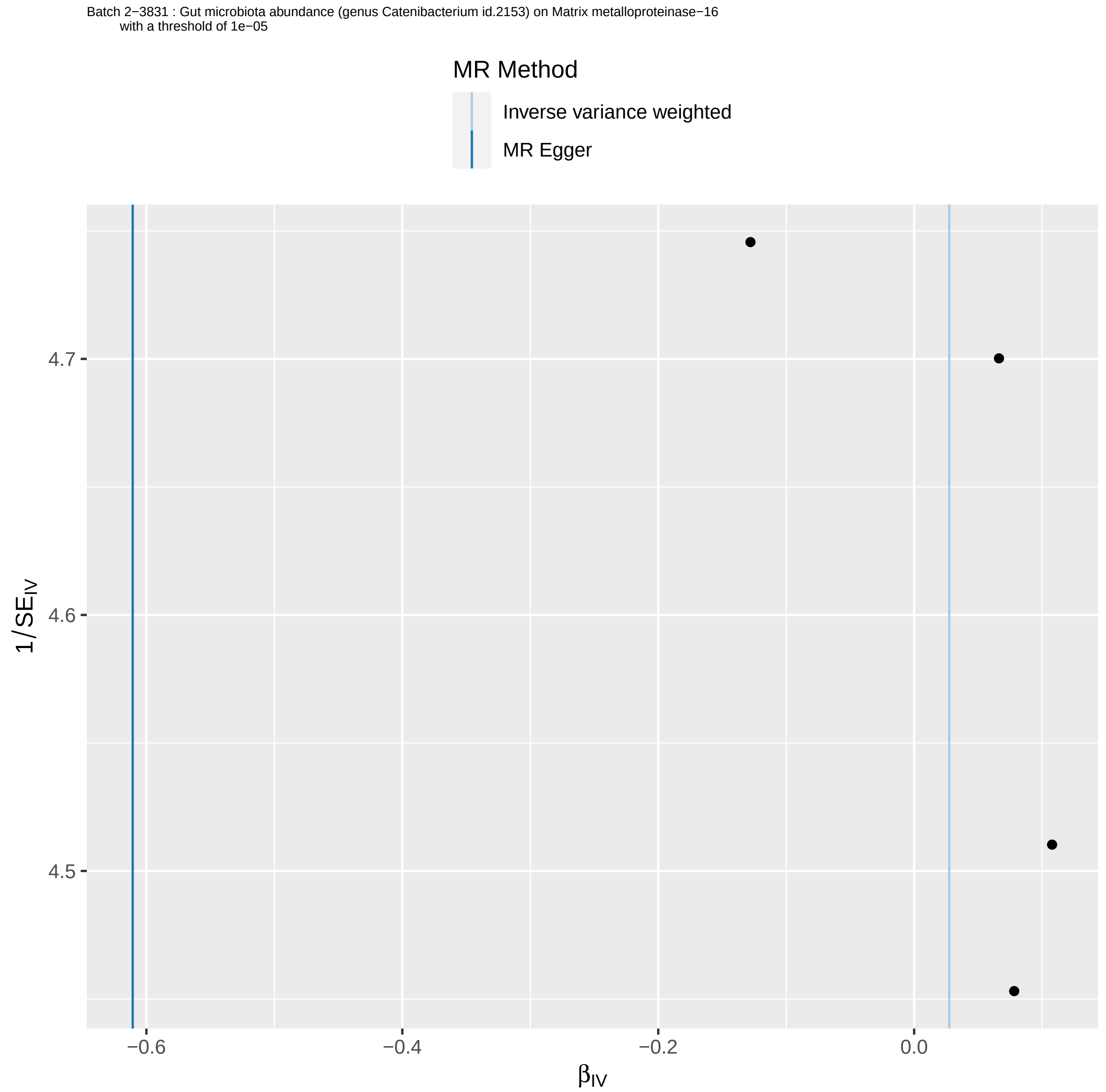

Batch 2−3831 : Gut microbiota abundance (genus Catenibacterium id.2153) on Matrix metalloproteinase−16
with a threshold of 1e−05
MR Method
Inverse variance weighted
MR Egger
4.7
4.6
4.5
1/SE_IV
−0.6
−0.4
−0.2
0.0
β_IV

Batch 2−3832 : Gut microbiota abundance (genus Parasutterella id.2892) on Matrix metalloproteinase−16
with a threshold of 1e−05

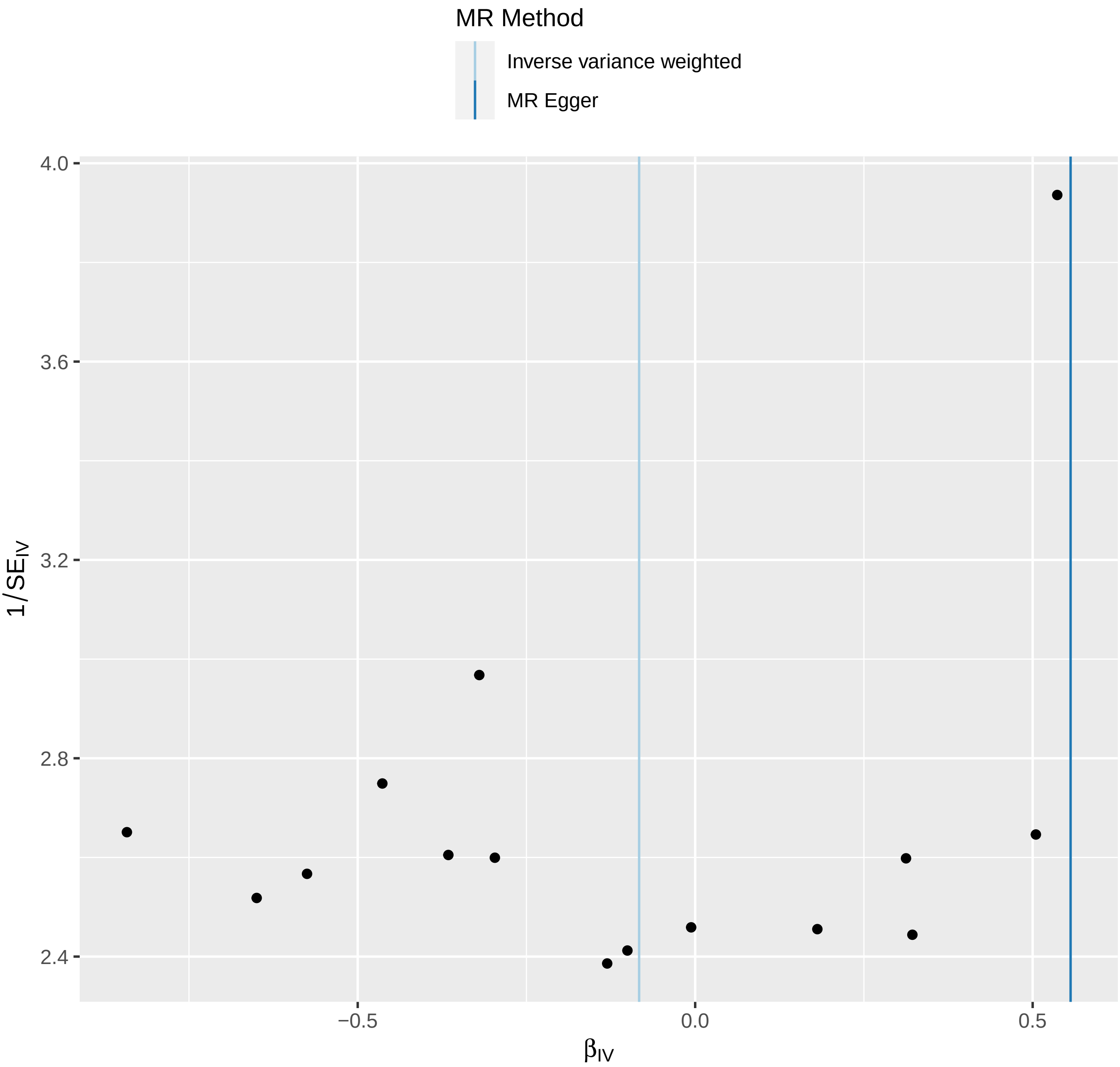

Batch 2−3833 : Gut microbiota abundance (genus Catenibacterium id.2153) on Matrix metalloproteinase−17
with a threshold of 1e−05

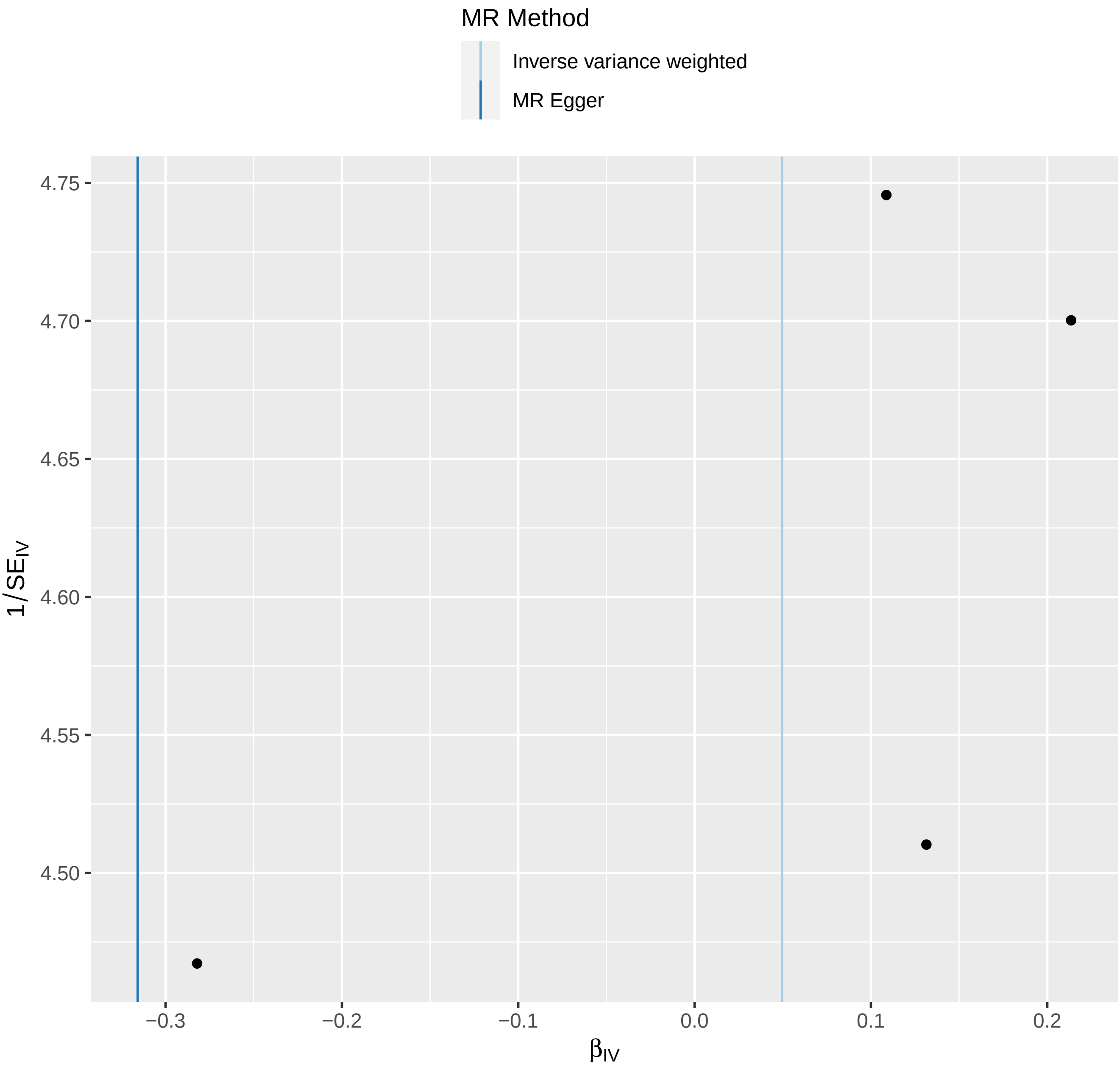

Batch 2−3834 : Gut microbiota abundance (genus Parasutterella id.2892) on Matrix metalloproteinase−17
with a threshold of 1e−05

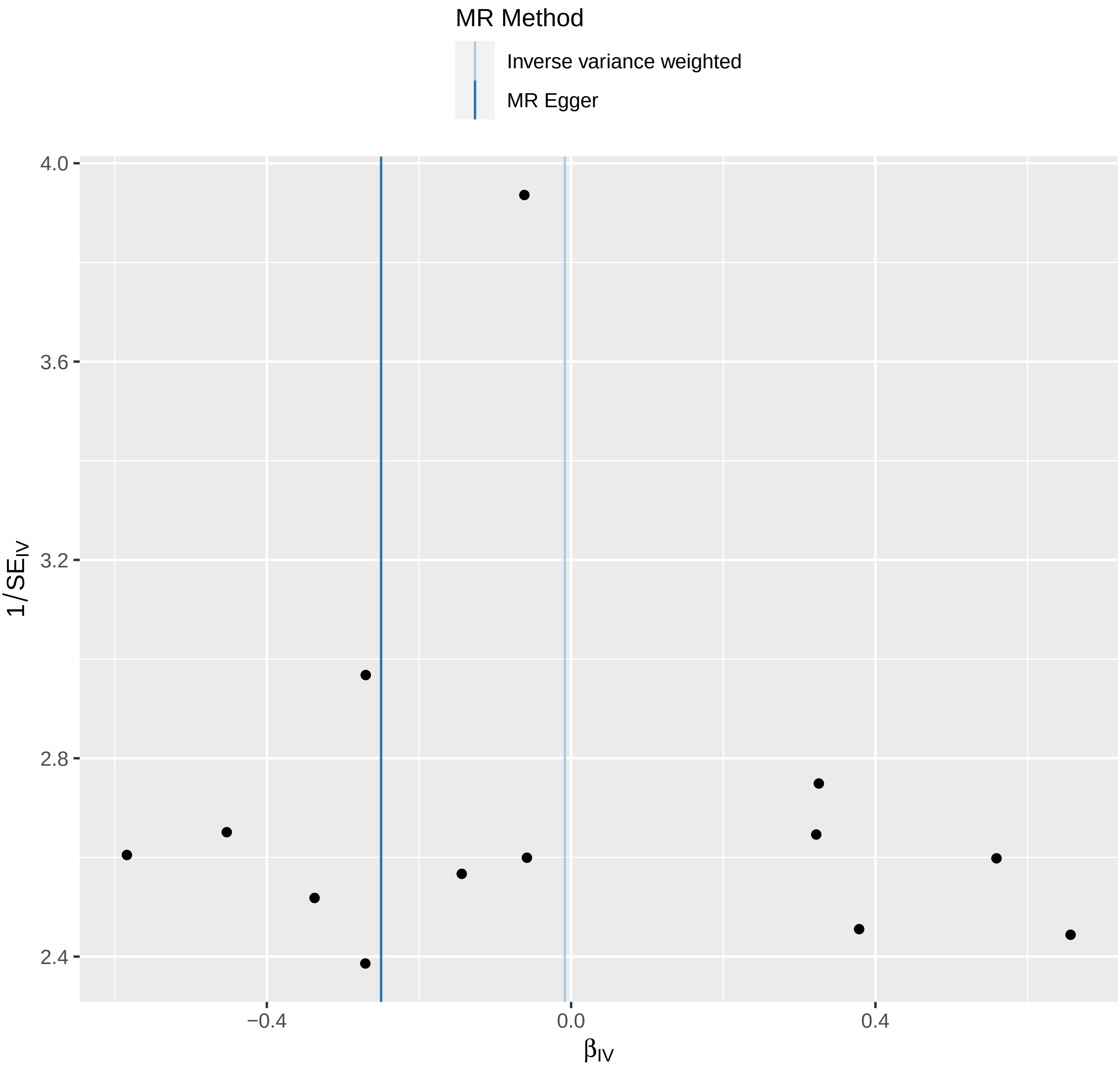

Batch 2−3835 : Gut microbiota abundance (genus Catenibacterium id.2153) on 72 kDa type IV collagenase
with a threshold of 1e−05

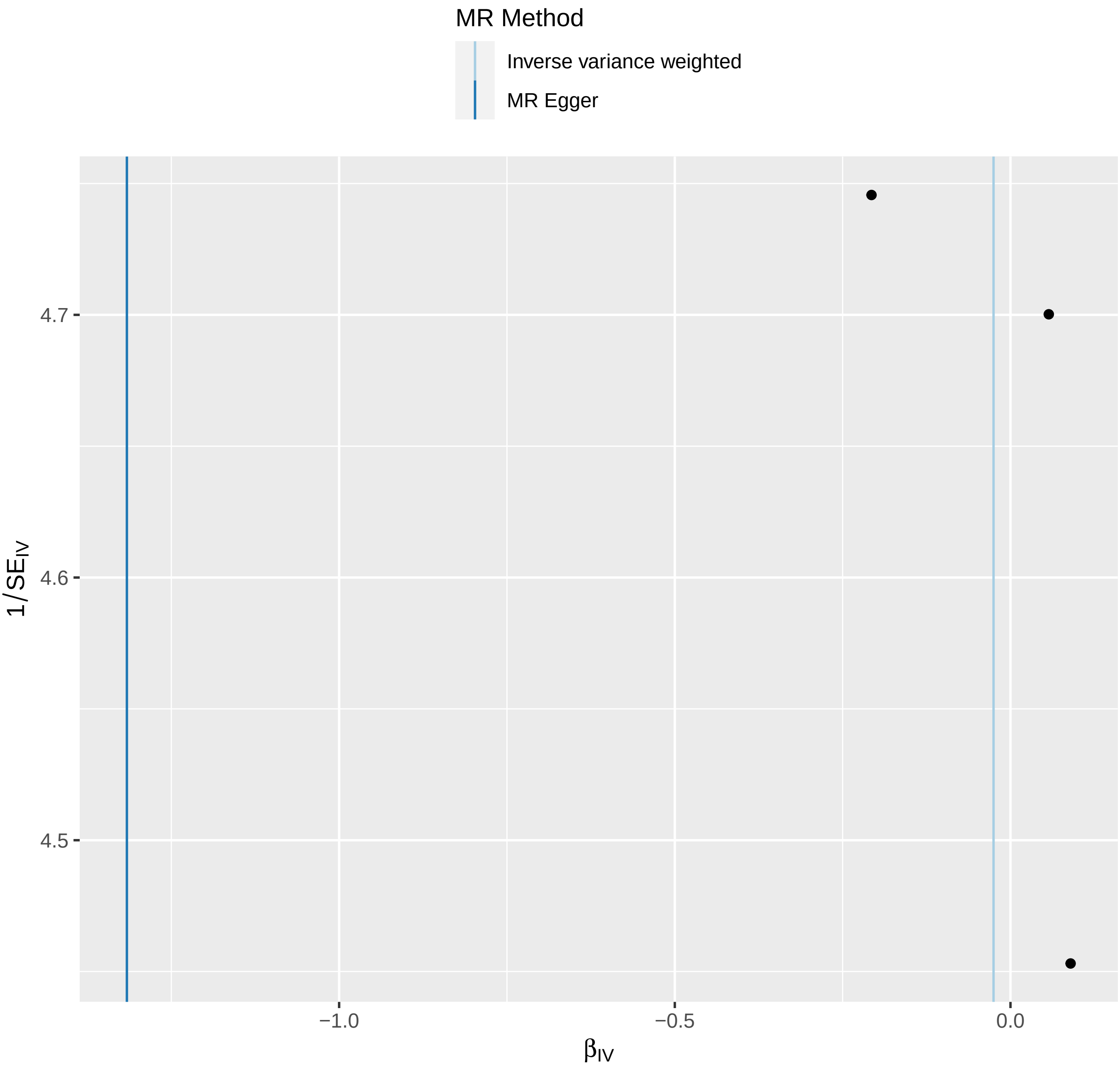

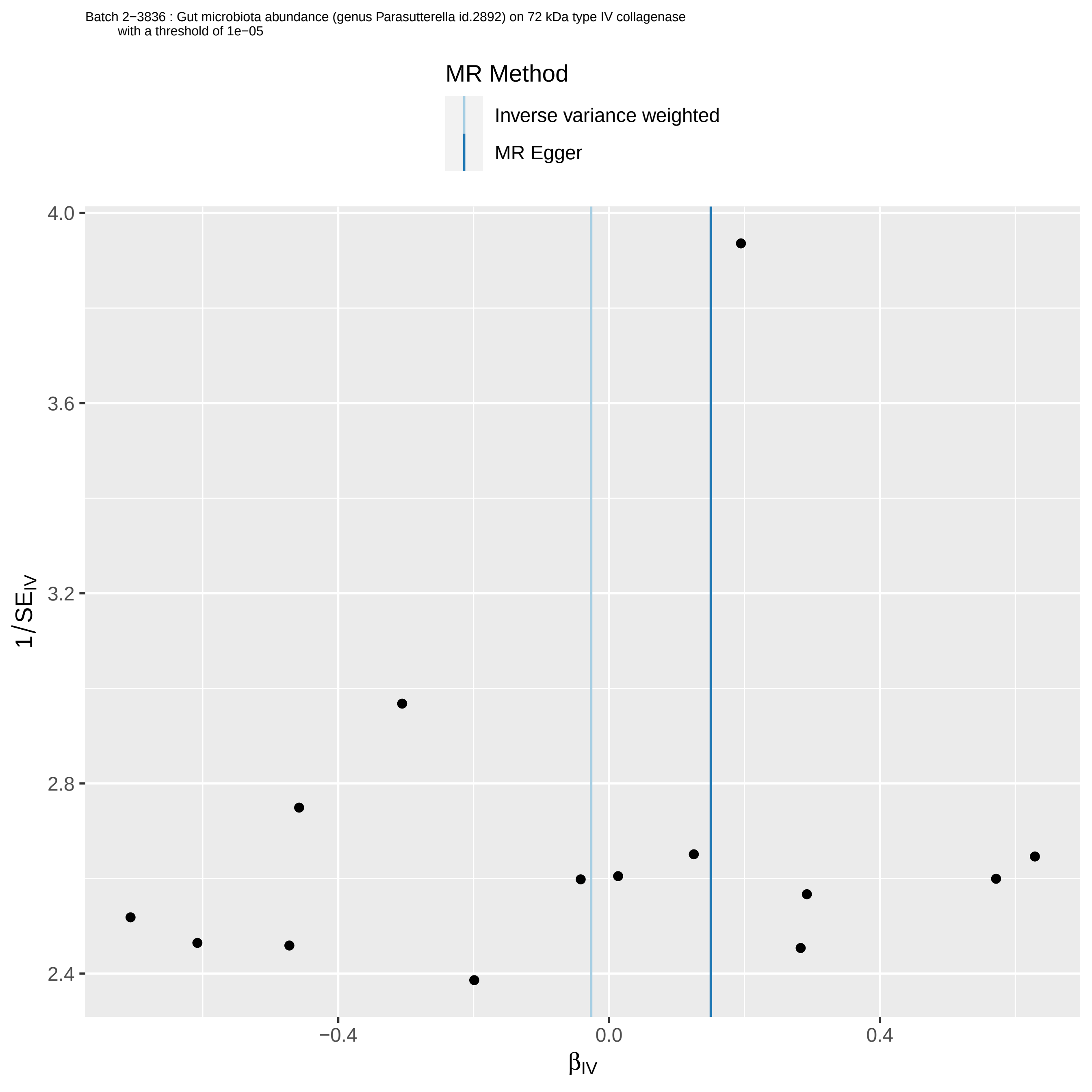

Batch 2−3836 : Gut microbiota abundance (genus Parasutterella id.2892) on 72 kDa type IV collagenase
with a threshold of 1e−05
MR Method
Inverse variance weighted
MR Egger
4.0
3.6
3.2
2.8
2.4
1/SE$_{IV}$
−0.4
0.0
0.4
β_{IV}

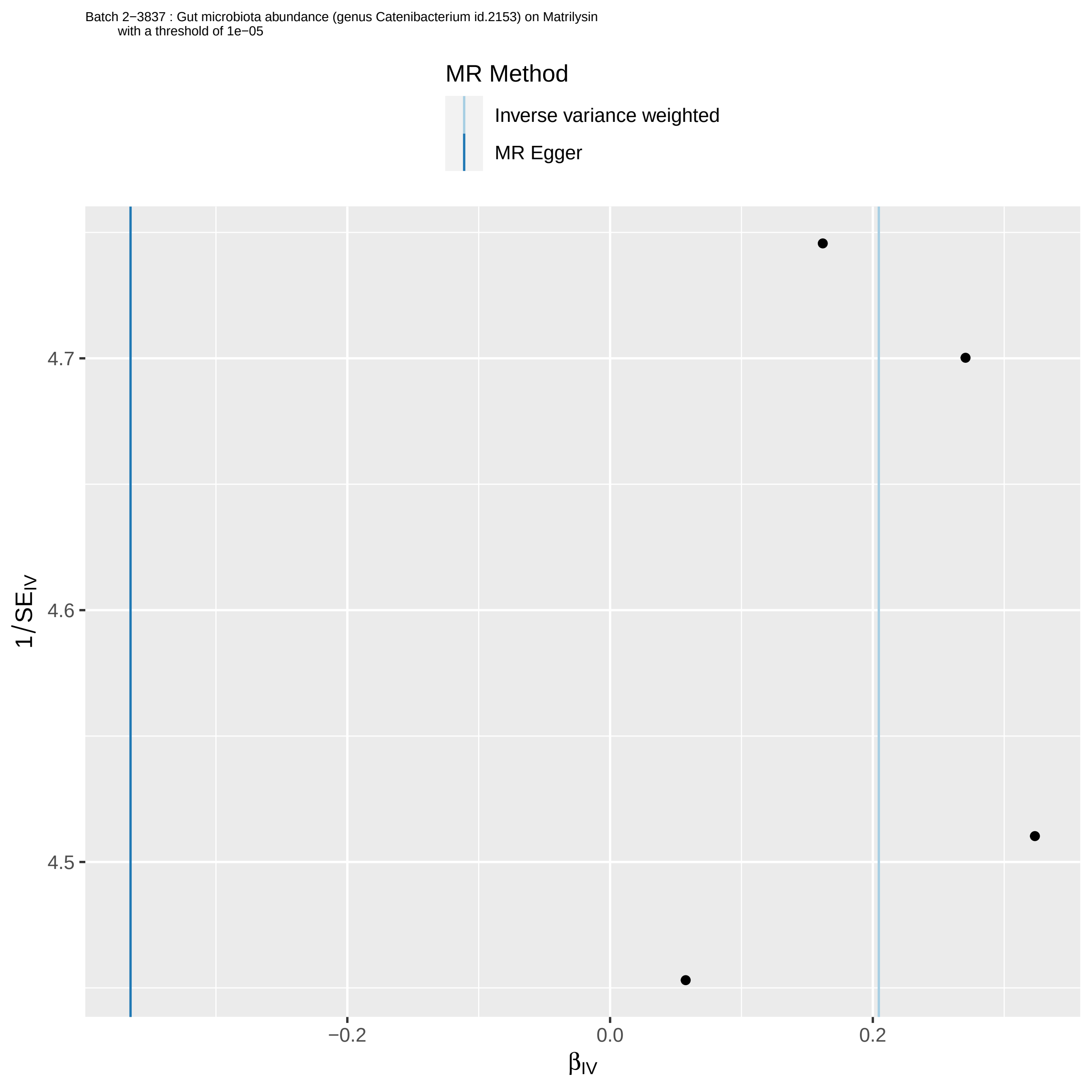
Batch 2−3837 : Gut microbiota abundance (genus Catenibacterium id.2153) on Matrilysin
with a threshold of 1e−05
MR Method
Inverse variance weighted
MR Egger
4.7
4.6
4.5
1/SE_IV
−0.2
0.0
0.2
β_IV

Batch 2−3838 : Gut microbiota abundance (genus Parasutterella id.2892) on Matrilysin
with a threshold of 1e−05

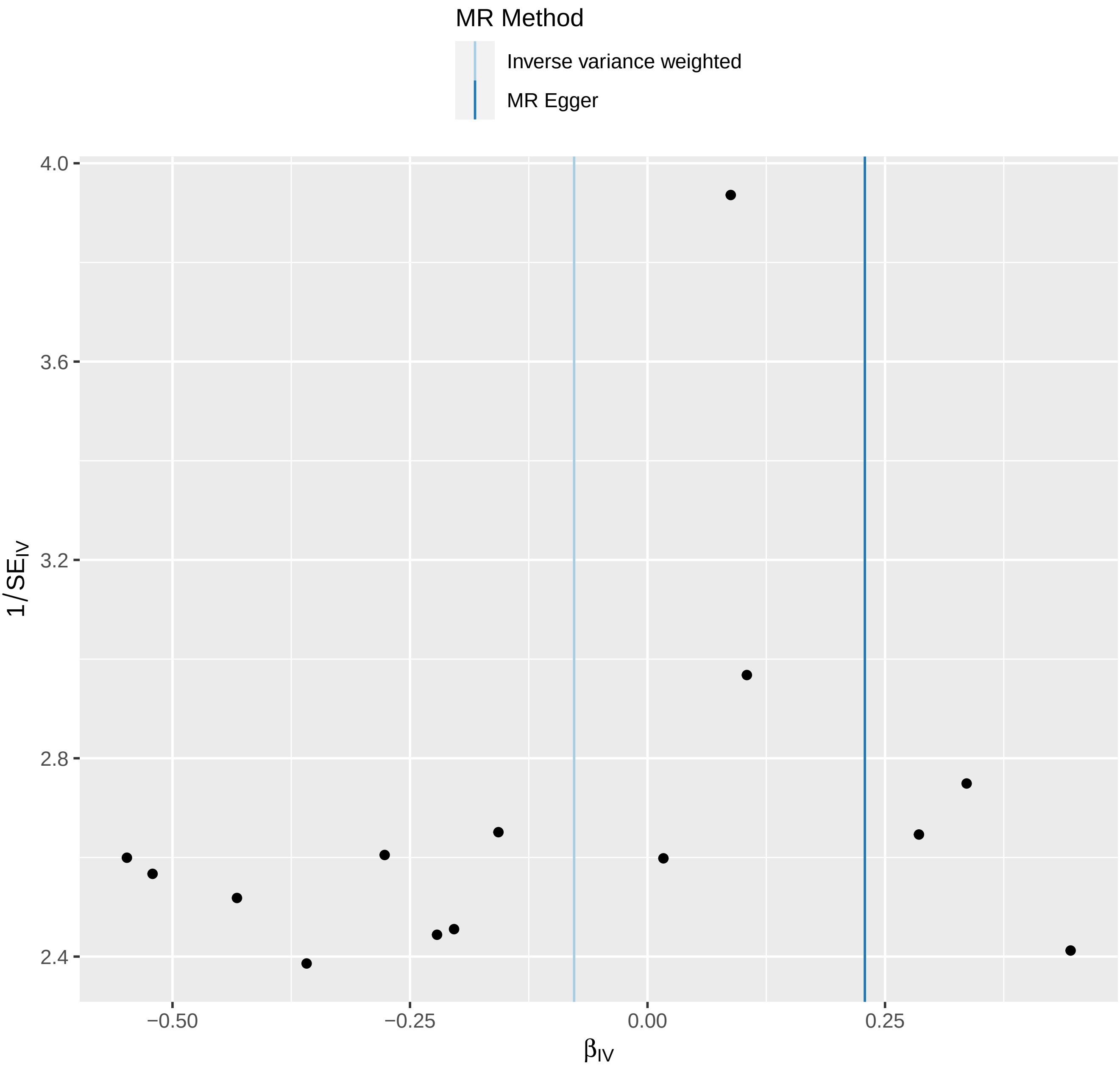

Batch 2−3839 : Gut microbiota abundance (genus Catenibacterium id.2153) on Neutrophil collagenase with a threshold of 1e−05

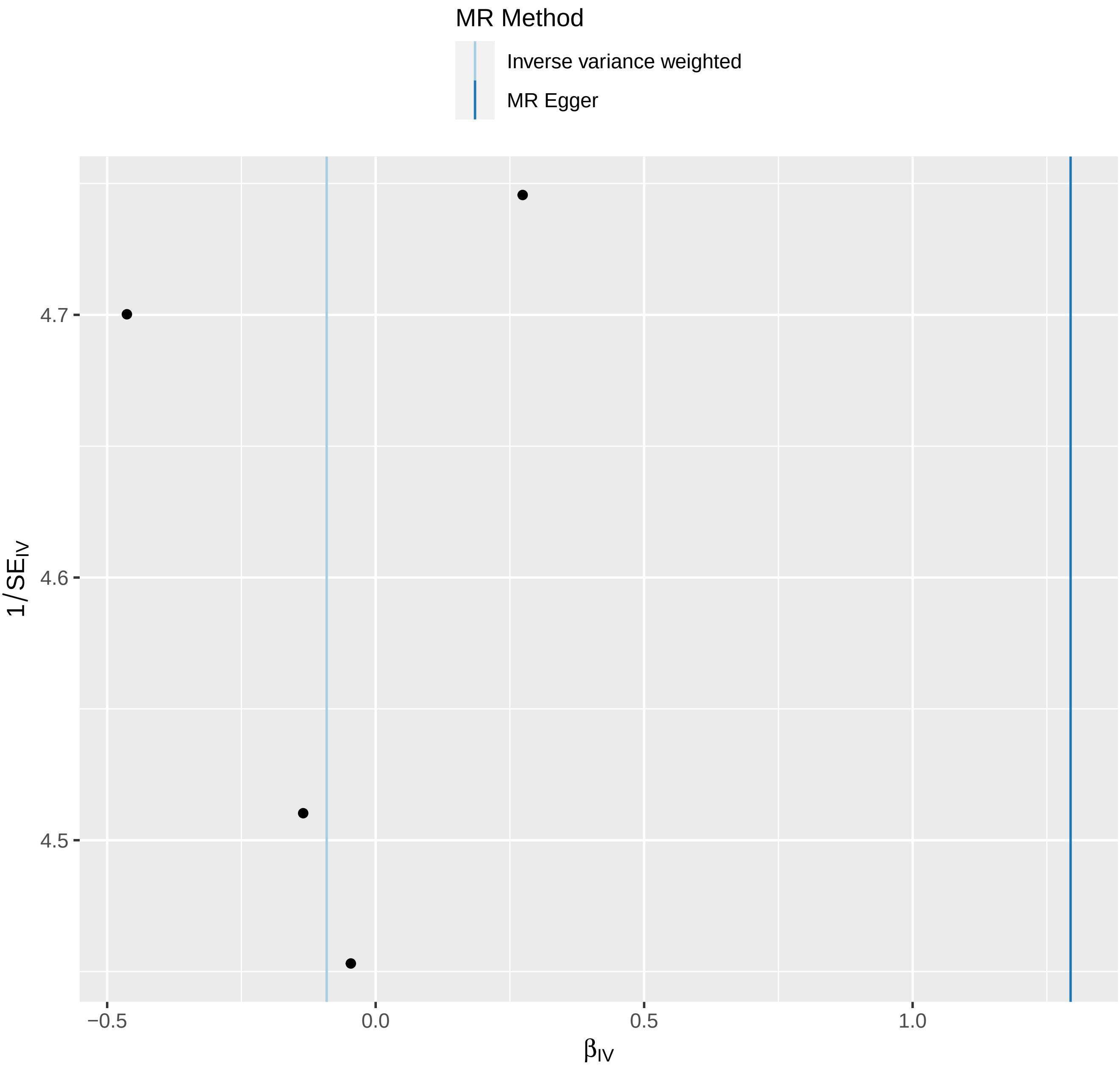

Batch 2−3840 : Gut microbiota abundance (genus Parasutterella id.2892) on Neutrophil collagenase
with a threshold of 1e−05

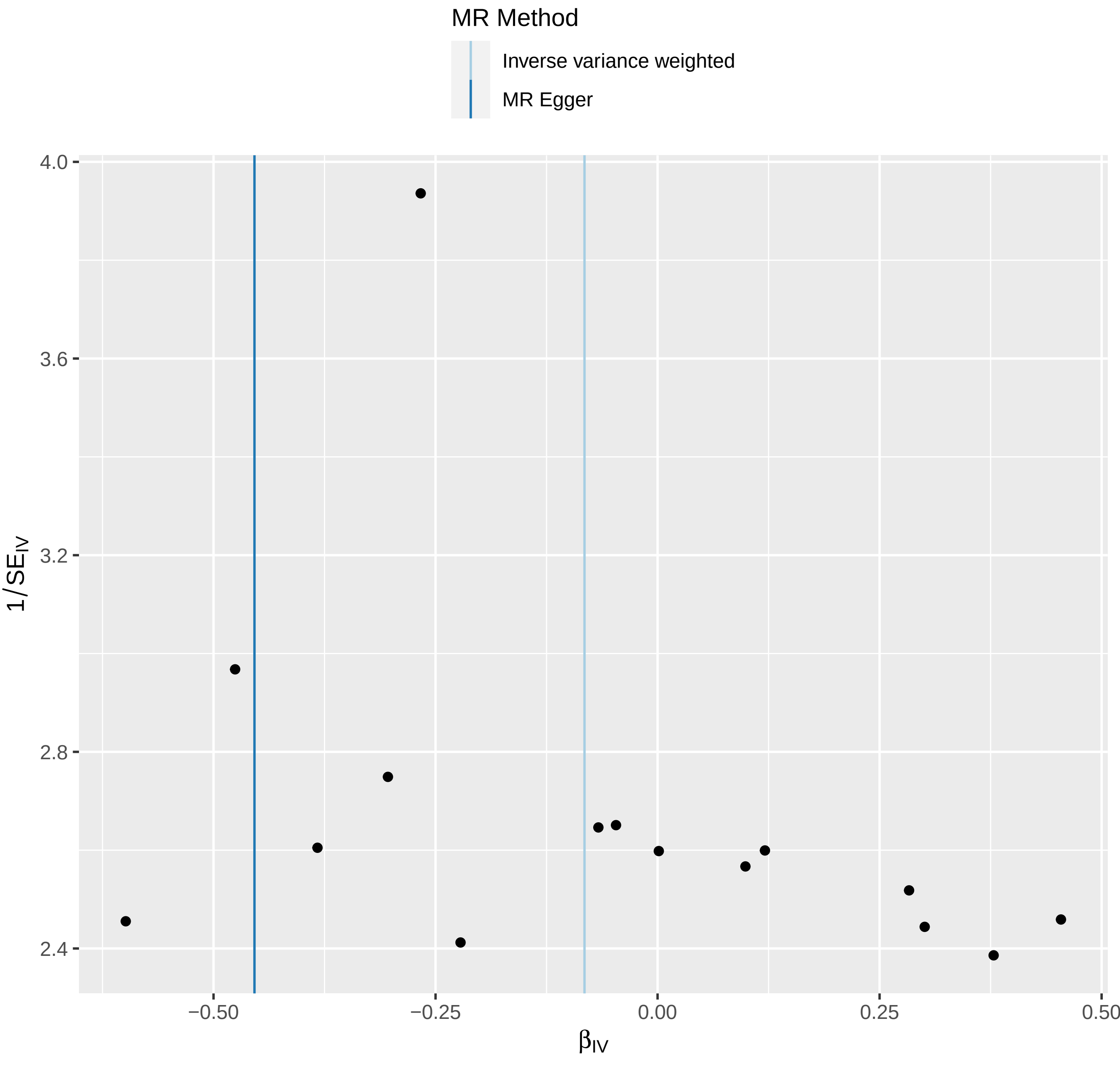

Batch 2−3841 : Gut microbiota abundance (genus Catenibacterium id.2153) on Matrix metalloproteinase−9
with a threshold of 1e−05

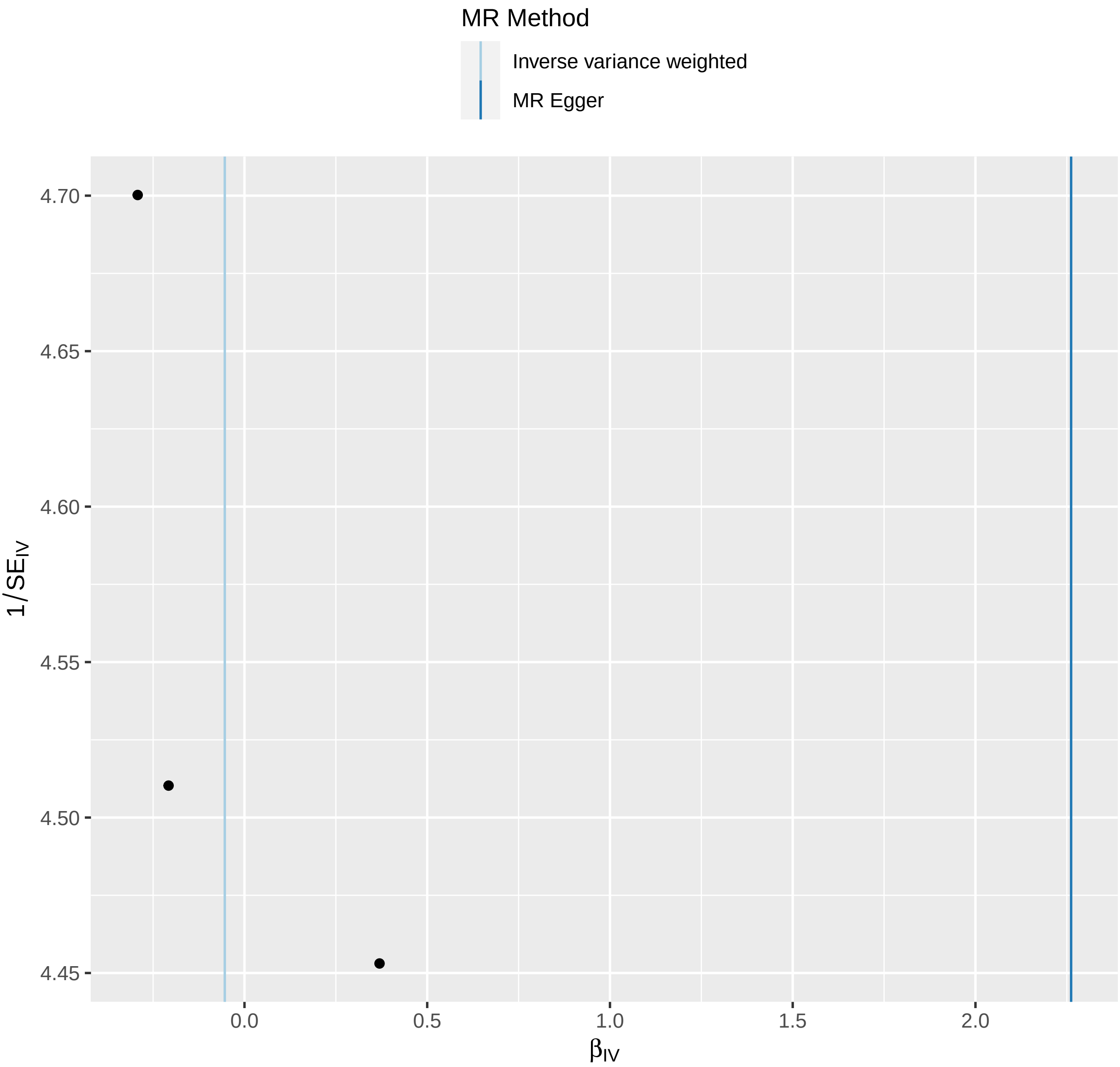

Batch 2−3842 : Gut microbiota abundance (genus Parasutterella id.2892) on Matrix metalloproteinase−9
with a threshold of 1e−05

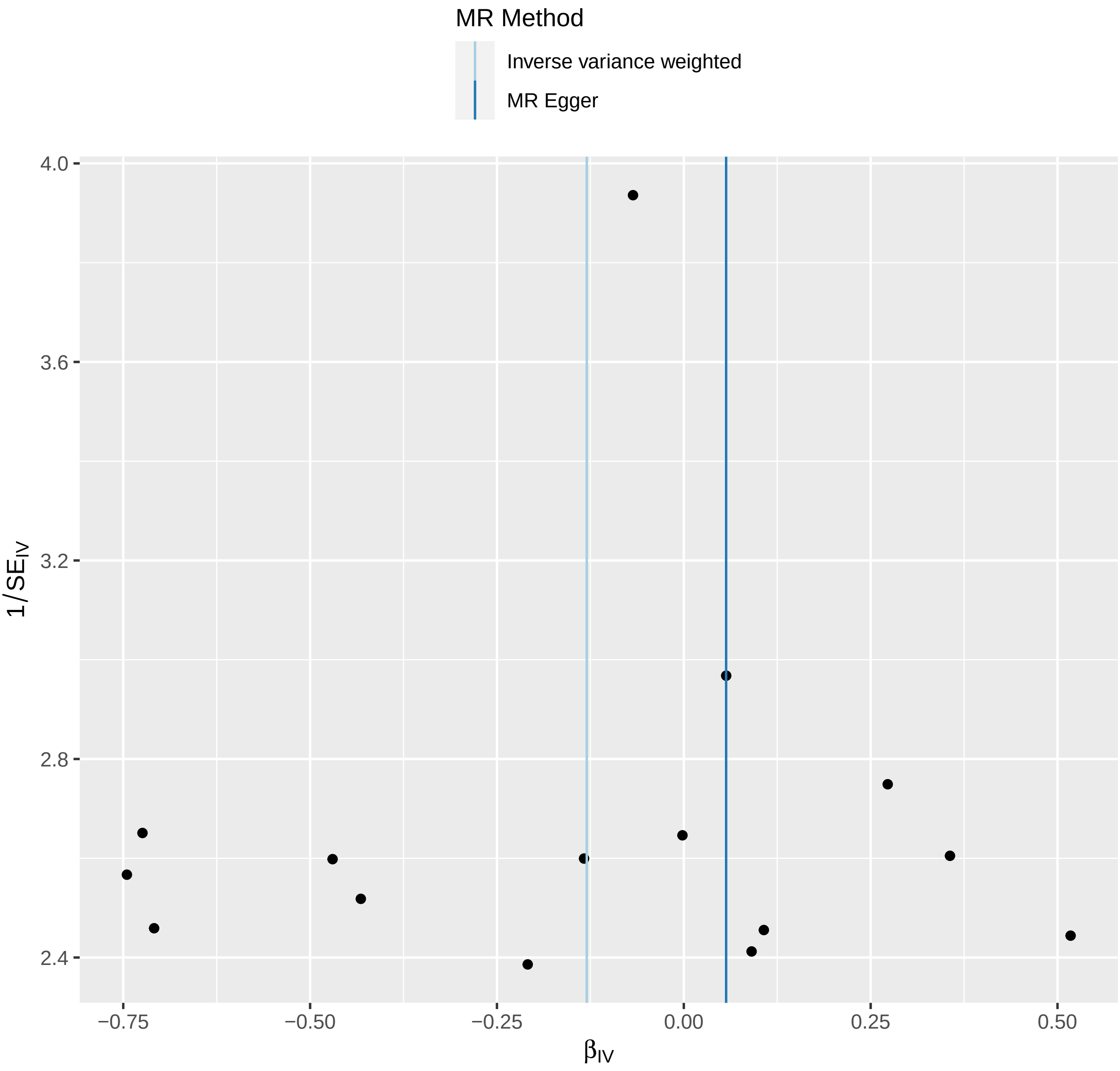

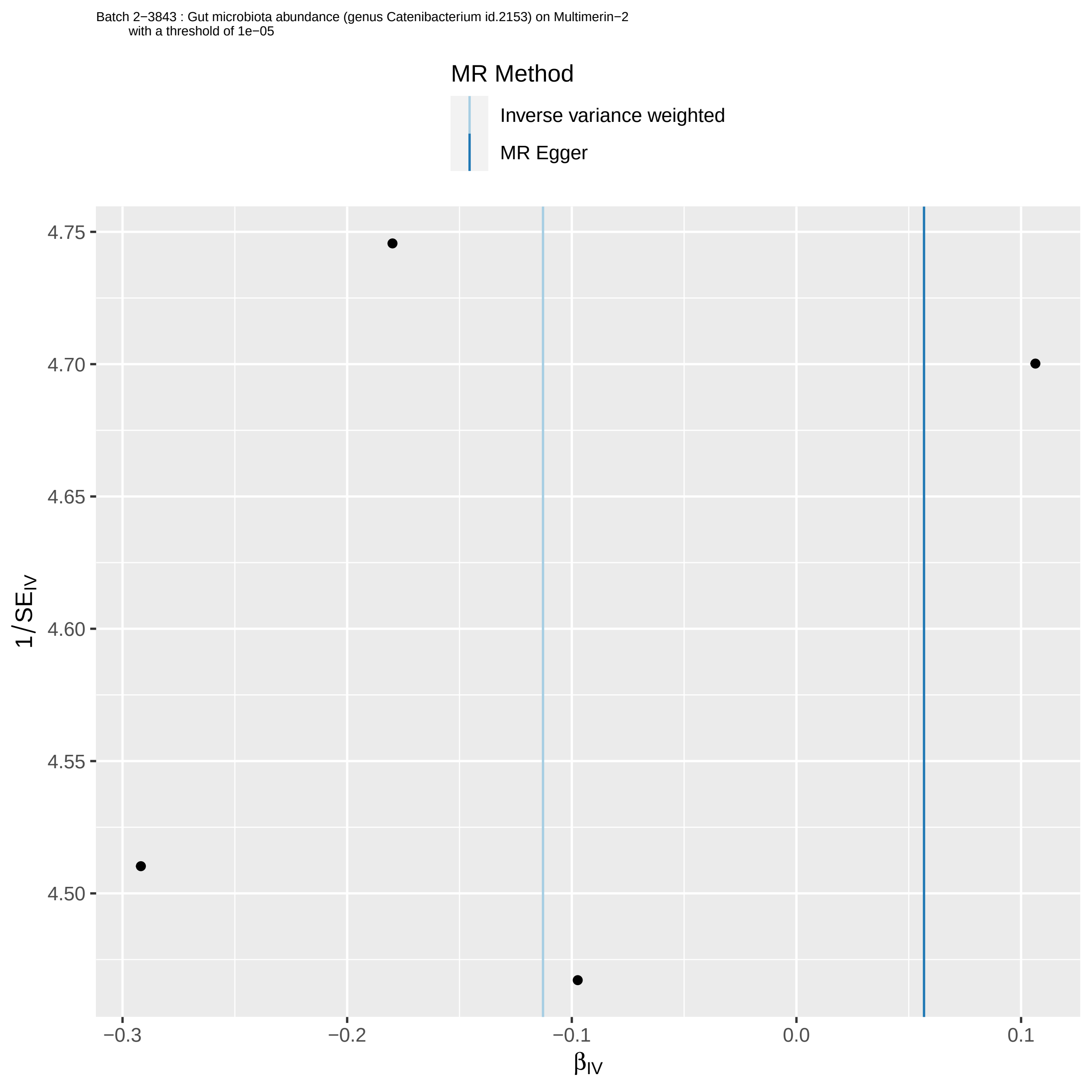

Batch 2−3843 : Gut microbiota abundance (genus Catenibacterium id.2153) on Multimerin−2
with a threshold of 1e−05
MR Method
Inverse variance weighted
MR Egger
4.75
4.70
4.65
4.60
4.55
4.50
1/SE_IV
−0.3
−0.2
−0.1
0.0
0.1
β_IV

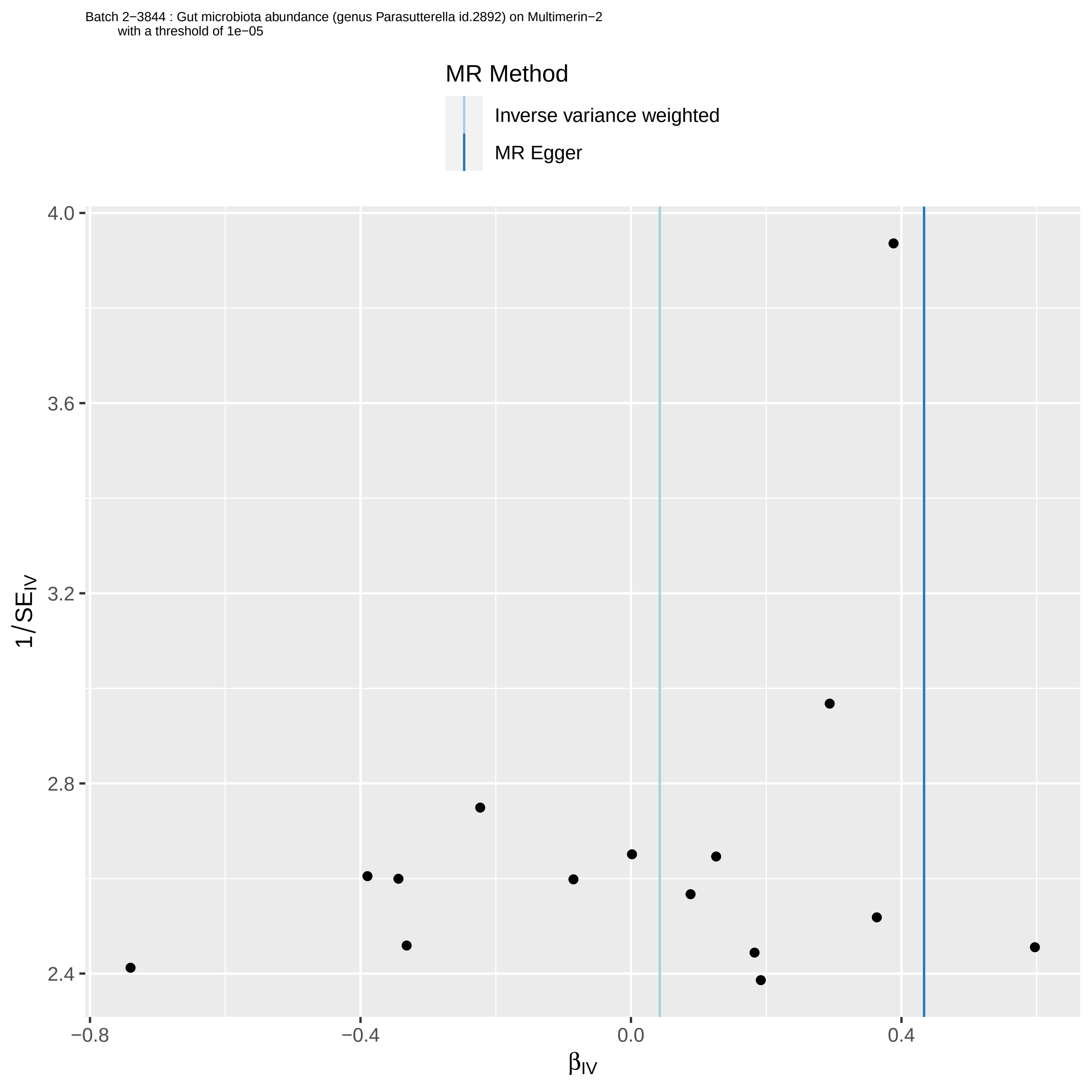
Batch 2−3844 : Gut microbiota abundance (genus Parasutterella id.2892) on Multimerin−2
with a threshold of 1e−05
MR Method
Inverse variance weighted
MR Egger
4.0
3.6
3.2
2.8
2.4
1/SE$_{IV}$
−0.8
−0.4
0.0
0.4
β_{IV}

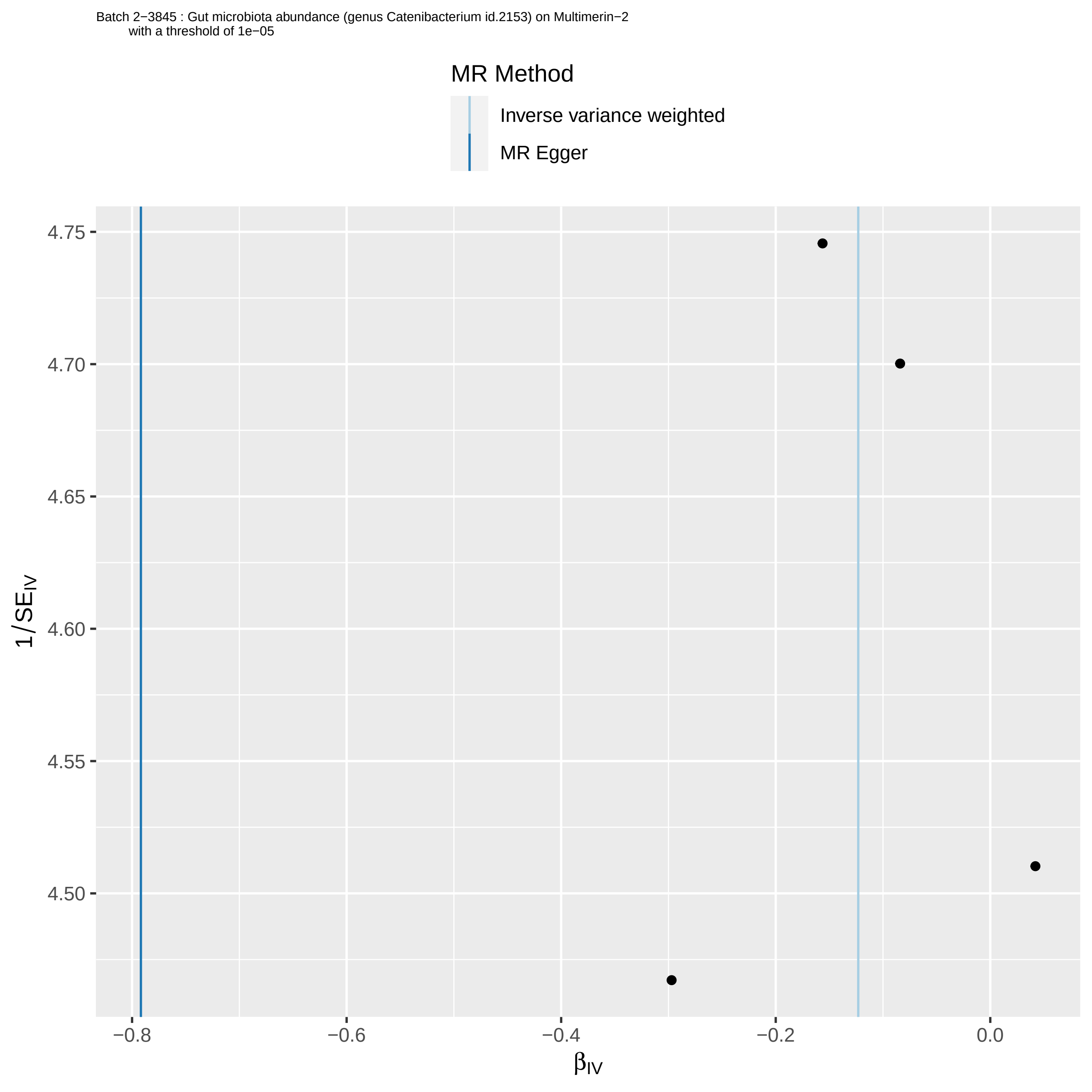
Batch 2−3845 : Gut microbiota abundance (genus Catenibacterium id.2153) on Multimerin−2
with a threshold of 1e−05
MR Method
Inverse variance weighted
MR Egger
4.75
4.70
4.65
4.60
4.55
4.50
1/SE_IV
−0.8
−0.6
−0.4
−0.2
0.0
β_IV

Batch 2−3846 : Gut microbiota abundance (genus Parasutterella id.2892) on Multimerin−2
with a threshold of 1e−05

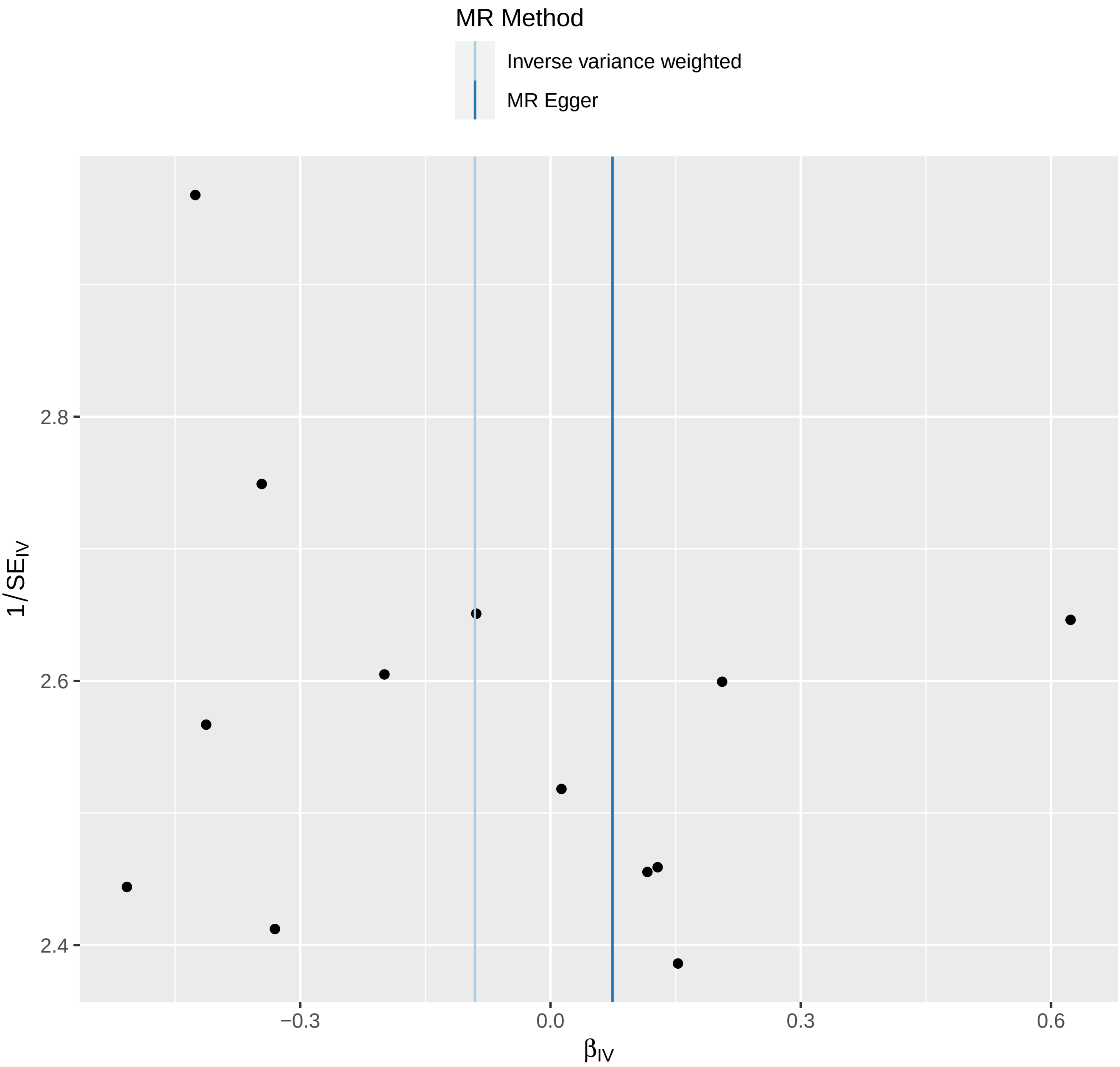

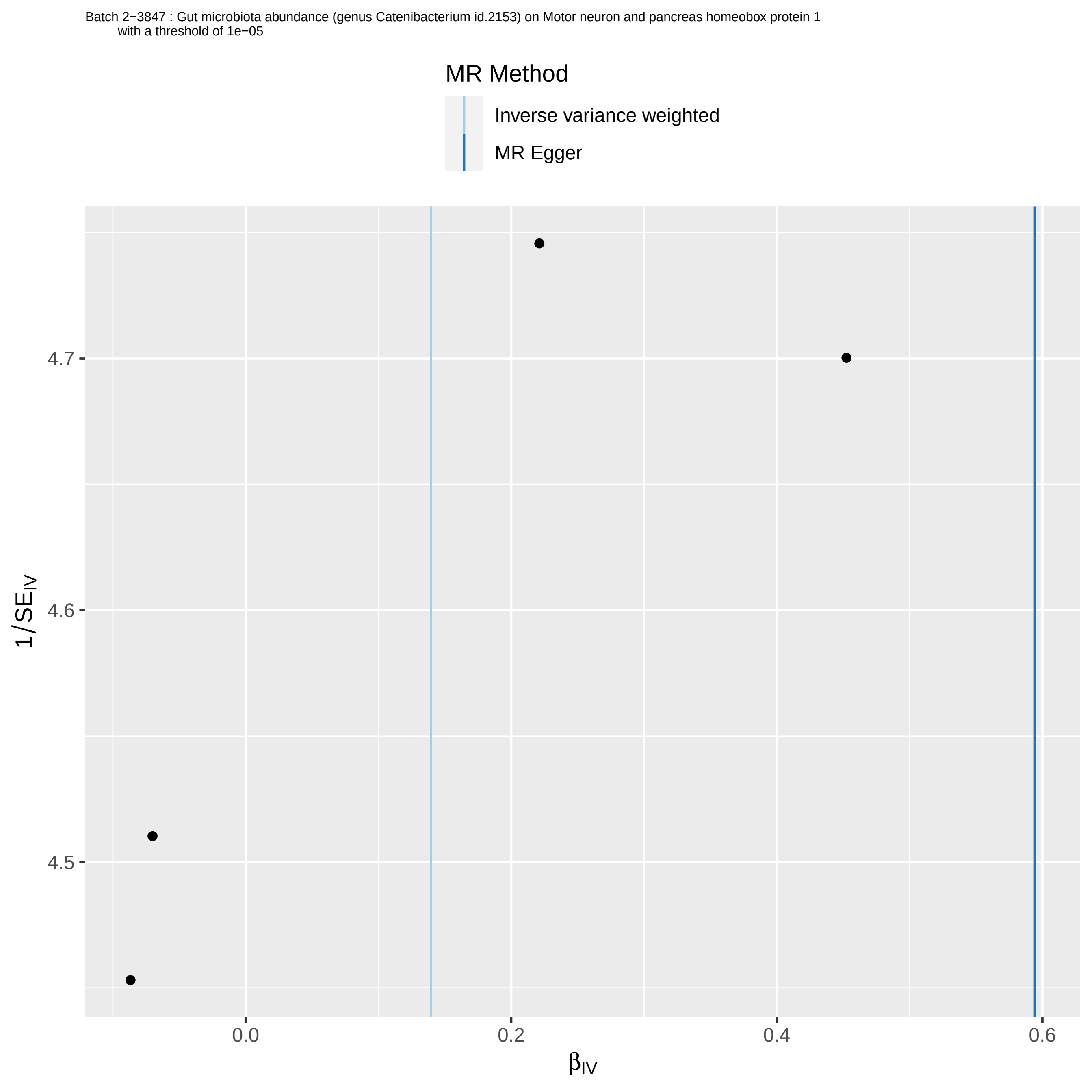
Batch 2−3847 : Gut microbiota abundance (genus Catenibacterium id.2153) on Motor neuron and pancreas homeobox protein 1
with a threshold of 1e−05
MR Method
Inverse variance weighted
MR Egger
4.7
4.6
4.5
1/SE_IV
0.0
0.2
0.4
0.6
β_IV

Batch 2−3848 : Gut microbiota abundance (genus Parasutterella id.2892) on Motor neuron and pancreas homeobox protein 1 with a threshold of 1e−05

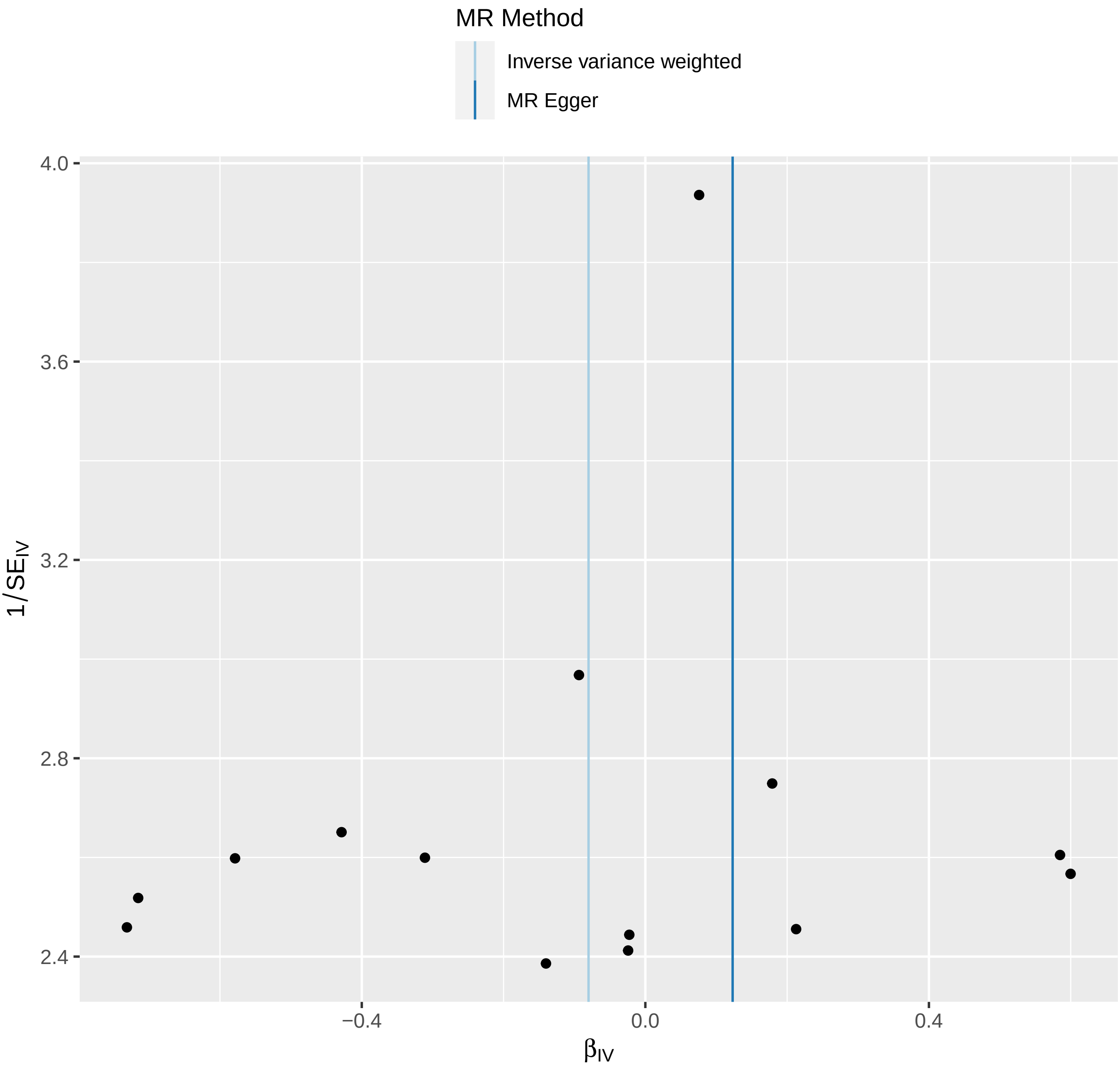

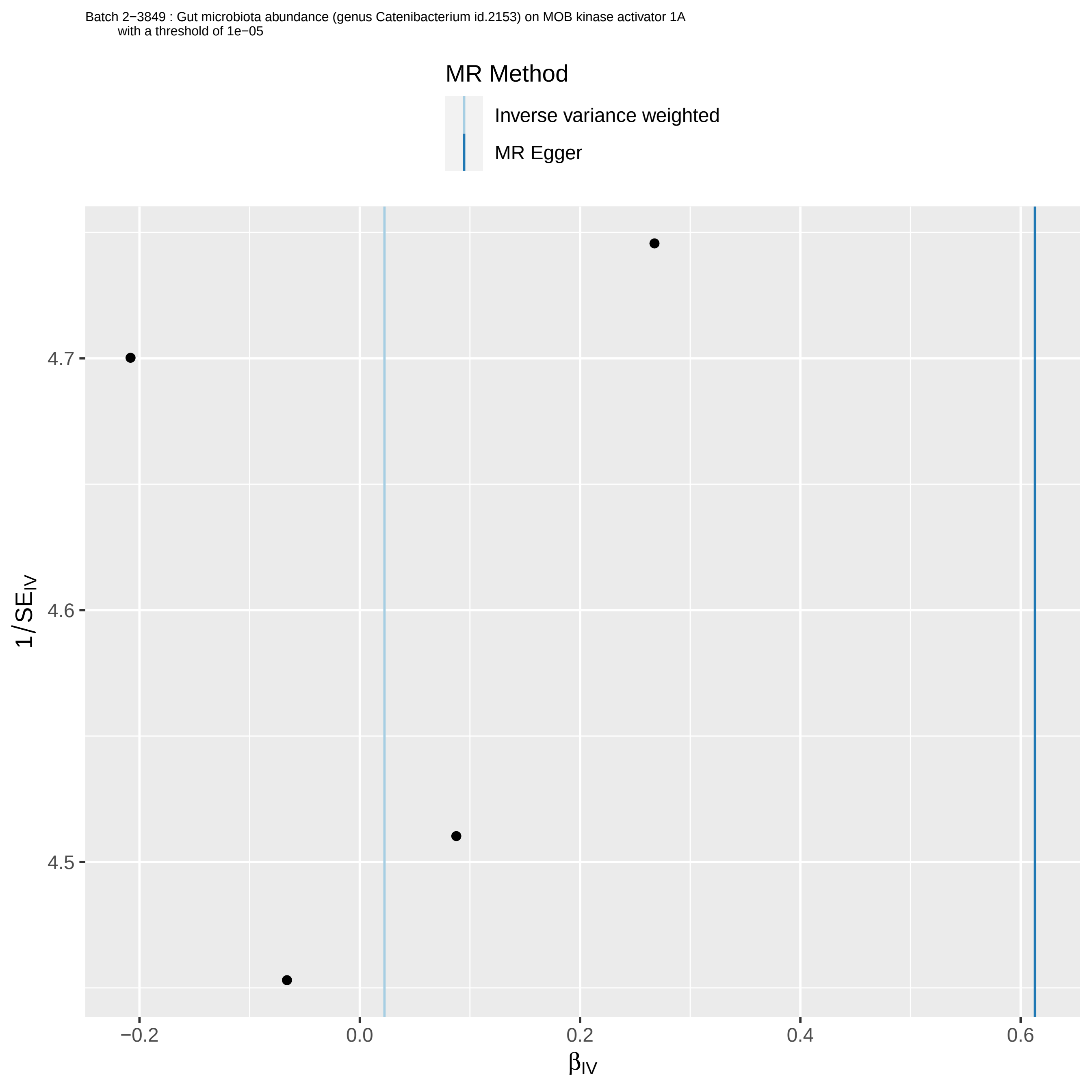
Batch 2−3849 : Gut microbiota abundance (genus Catenibacterium id.2153) on MOB kinase activator 1A
with a threshold of 1e−05
MR Method
Inverse variance weighted
MR Egger
4.7
4.6
4.5
1/SE_IV
−0.2
0.0
0.2
0.4
0.6
β_IV

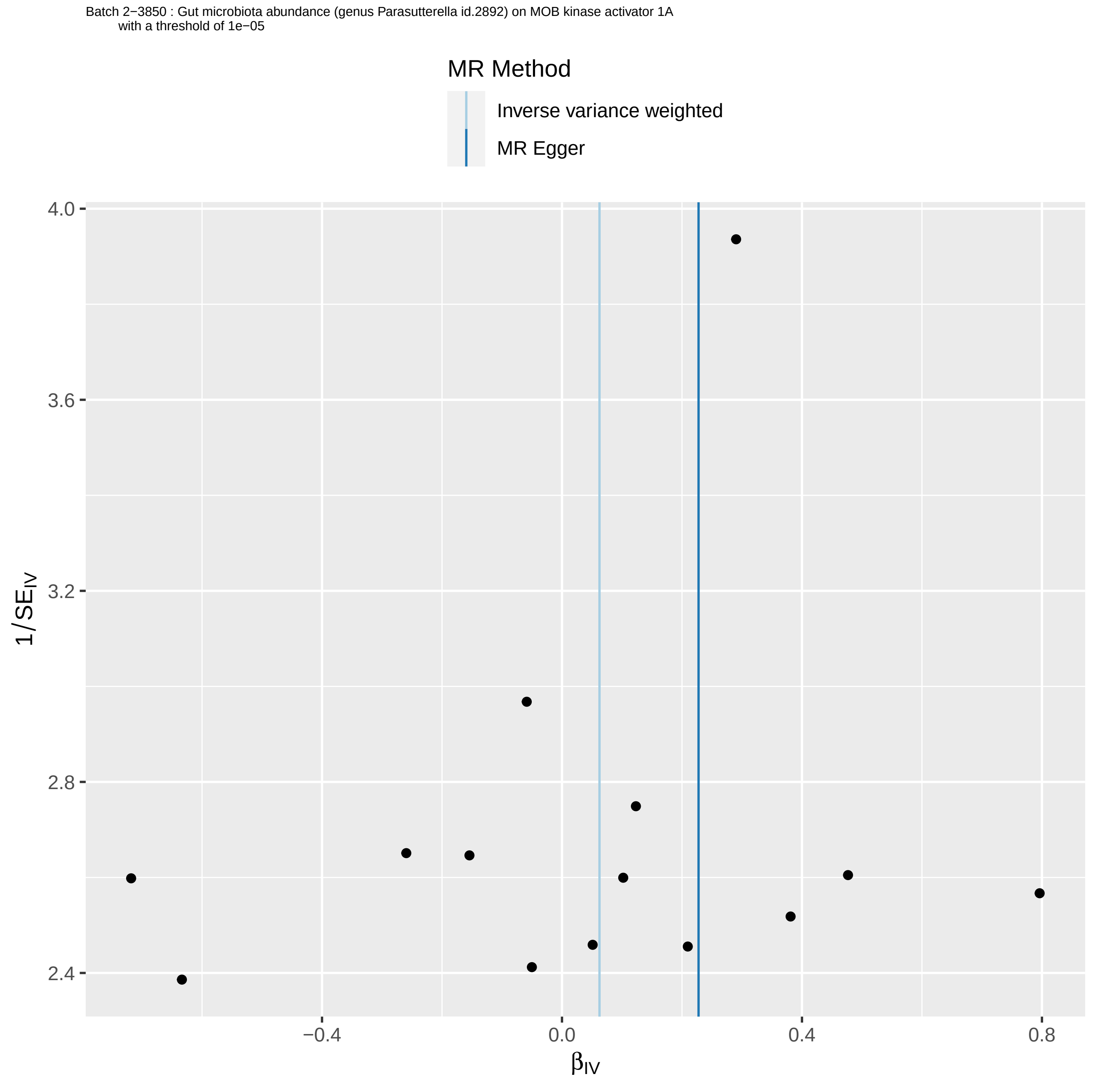
Batch 2−3850 : Gut microbiota abundance (genus Parasutterella id.2892) on MOB kinase activator 1A
with a threshold of 1e−05
MR Method
Inverse variance weighted
MR Egger
4.0
3.6
3.2
2.8
2.4
1/SE_IV
−0.4
0.0
0.4
0.8
β_IV

Batch 2−3851 : Gut microbiota abundance (genus Catenibacterium id.2153) on Adenylyltransferase and sulfurtransferase MOCS3
with a threshold of 1e−05

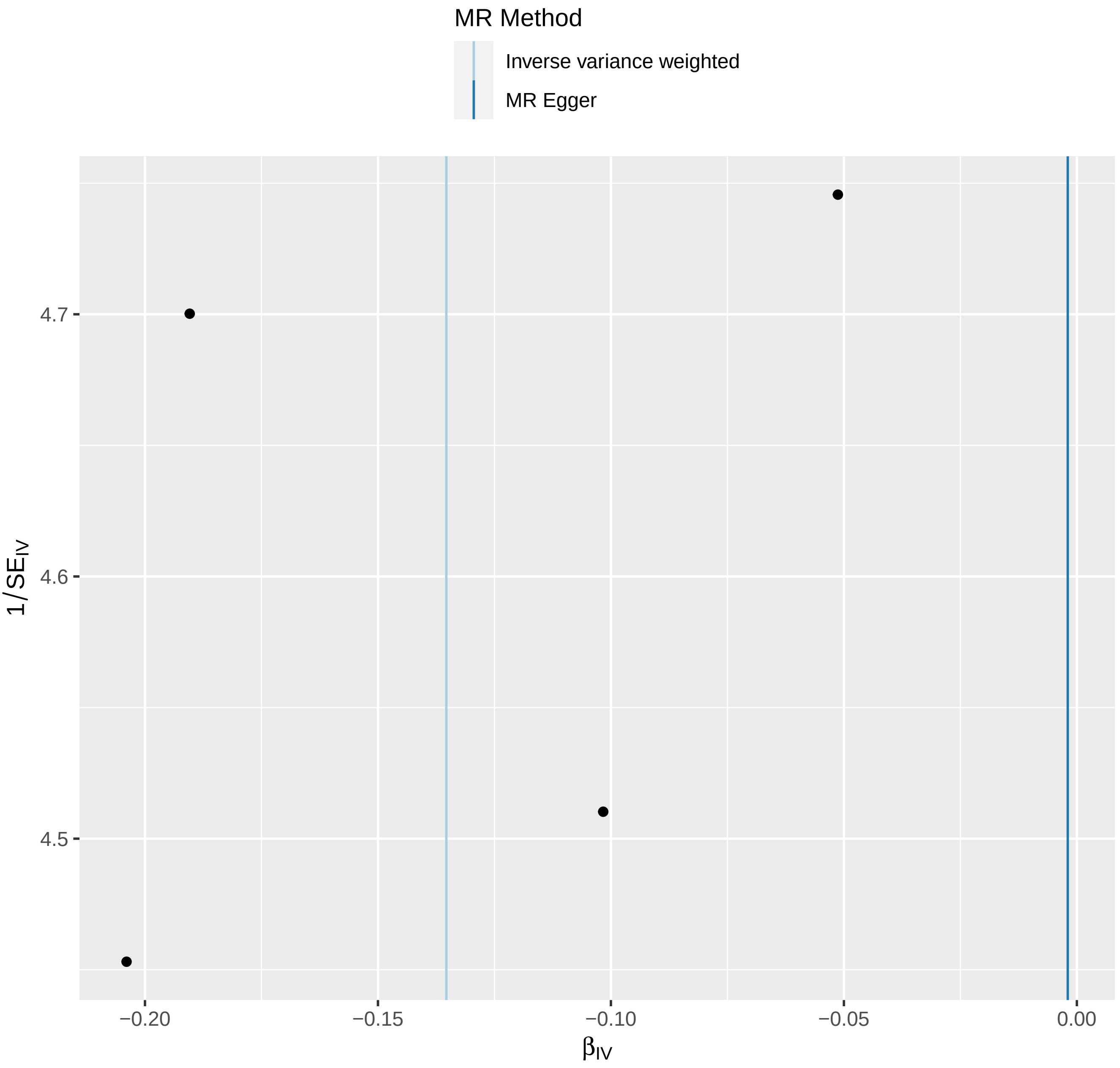

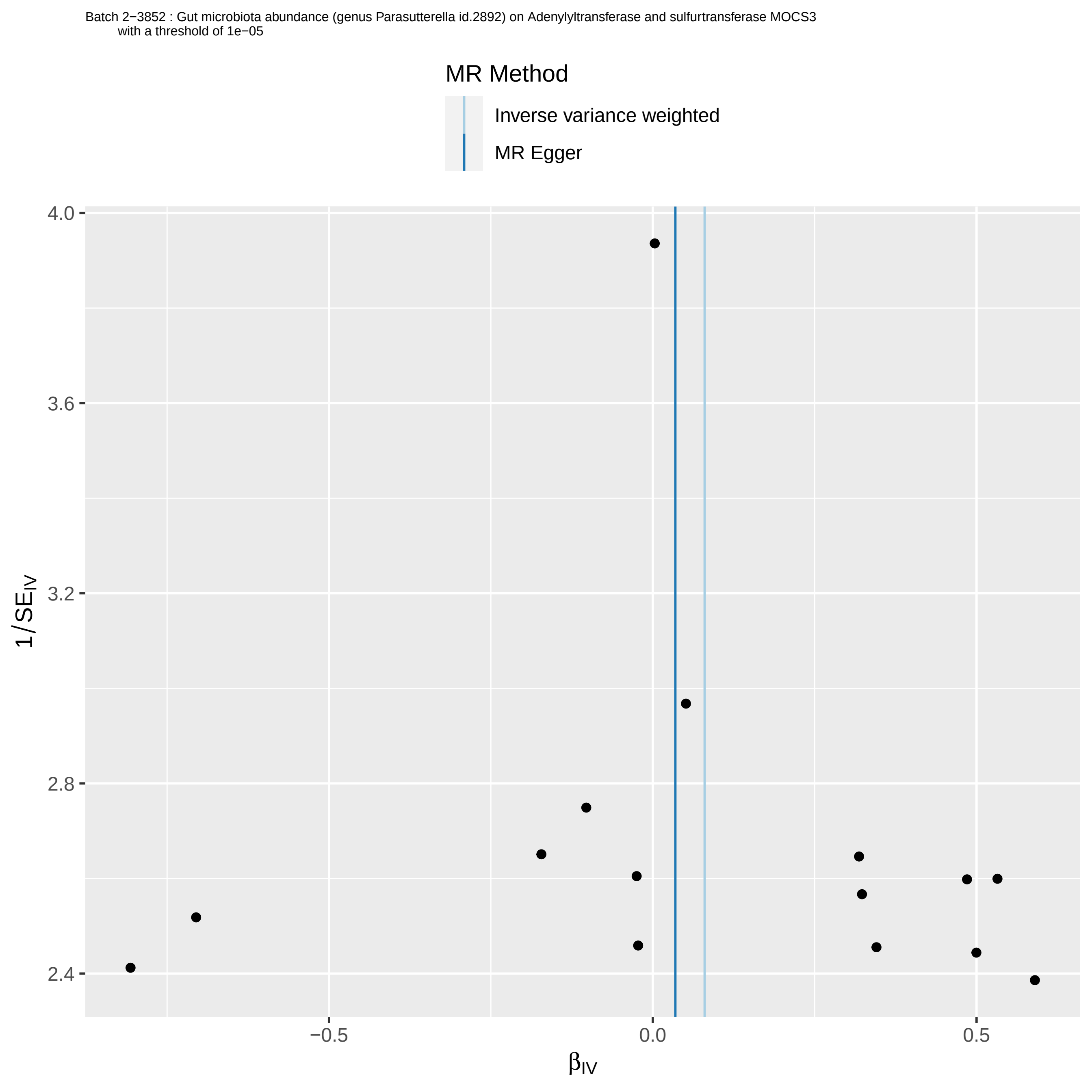

Batch 2−3852 : Gut microbiota abundance (genus Parasutterella id.2892) on Adenylyltransferase and sulfurtransferase MOCS3
with a threshold of 1e−05
MR Method
Inverse variance weighted
MR Egger
4.0
3.6
3.2
2.8
2.4
1/SE_IV
−0.5
0.0
0.5
β_IV

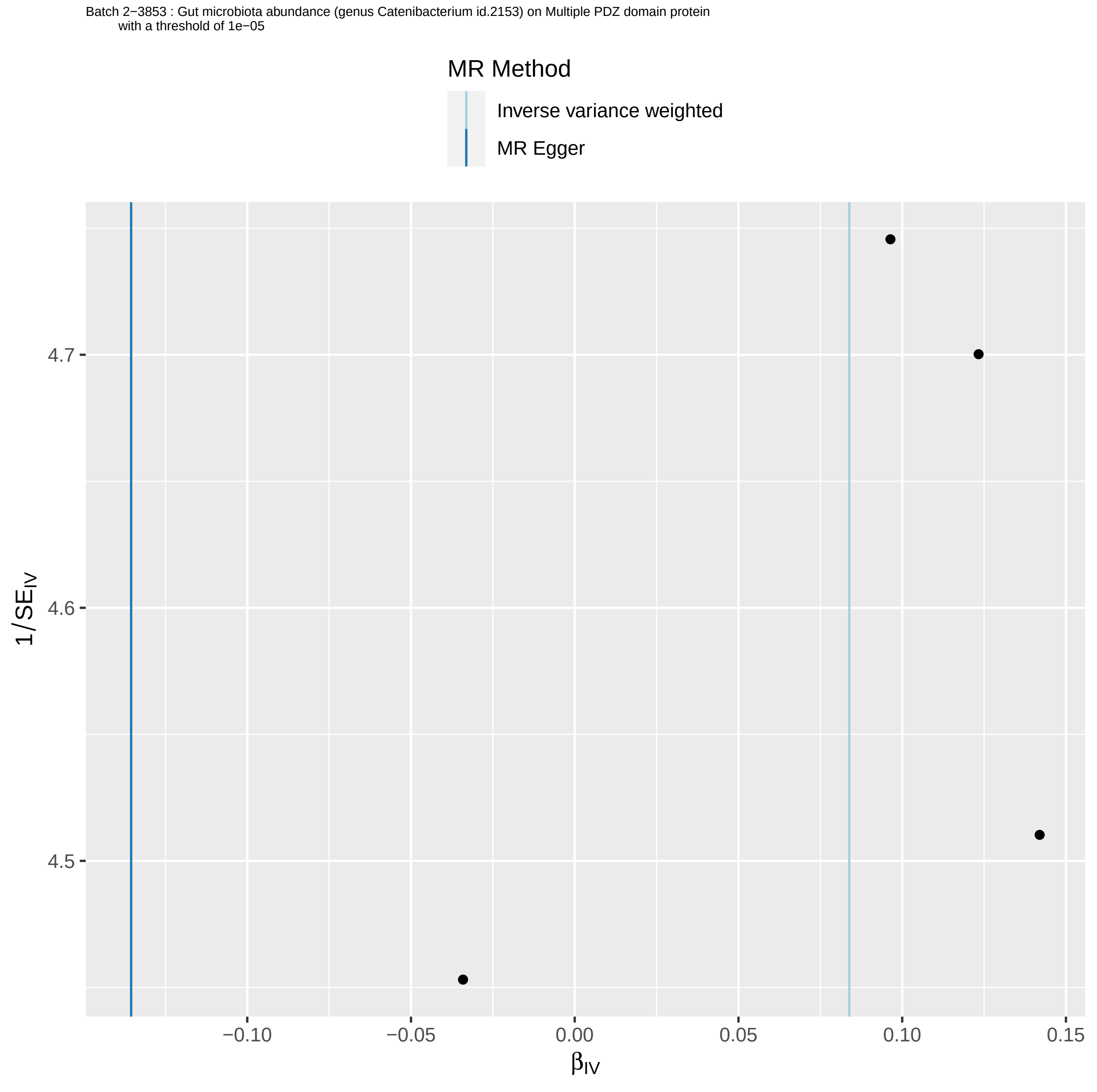
Batch 2−3853 : Gut microbiota abundance (genus Catenibacterium id.2153) on Multiple PDZ domain protein
with a threshold of 1e−05
MR Method
Inverse variance weighted
MR Egger
4.7
4.6
4.5
$1/SE_{IV}$
−0.10
−0.05
0.00
0.05
0.10
0.15
β_{IV}

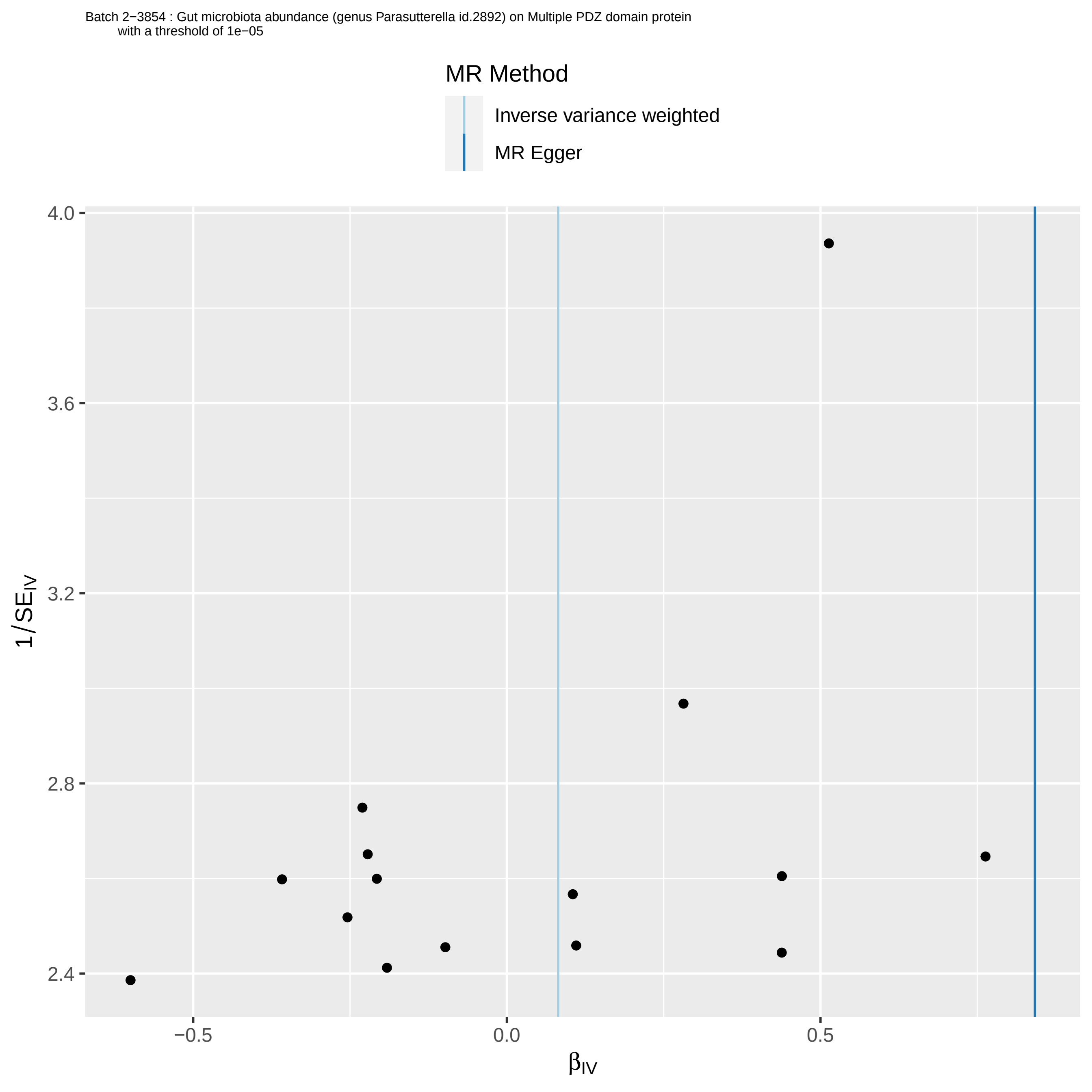
Batch 2−3854 : Gut microbiota abundance (genus Parasutterella id.2892) on Multiple PDZ domain protein
with a threshold of 1e−05
MR Method
Inverse variance weighted
MR Egger
4.0
3.6
3.2
2.8
2.4
1/SE_IV
−0.5
0.0
0.5
β_IV

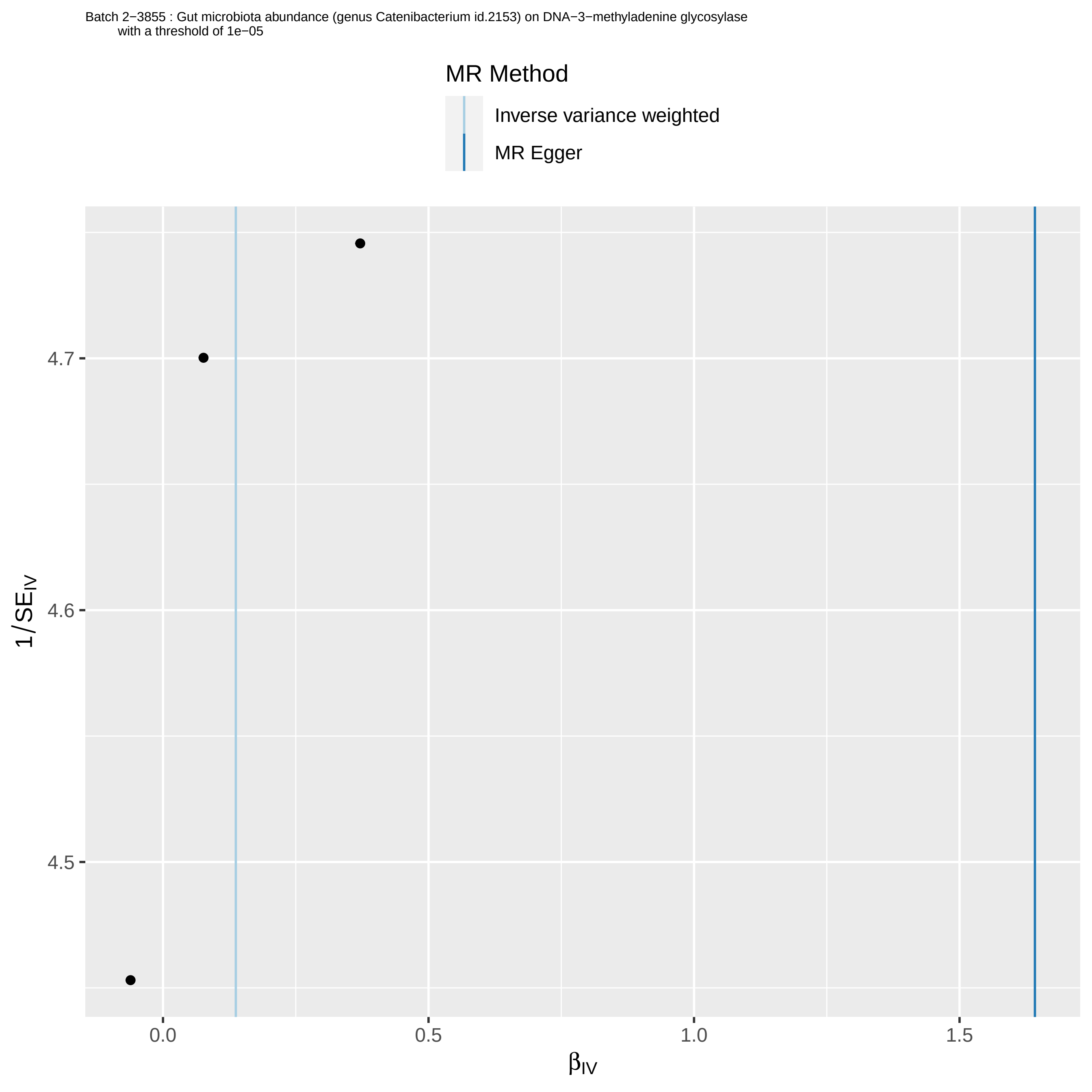

Batch 2−3855 : Gut microbiota abundance (genus Catenibacterium id.2153) on DNA−3−methyladenine glycosylase
with a threshold of 1e−05
MR Method
Inverse variance weighted
MR Egger
4.7
4.6
4.5
1/SE_IV
0.0
0.5
1.0
1.5
β_IV

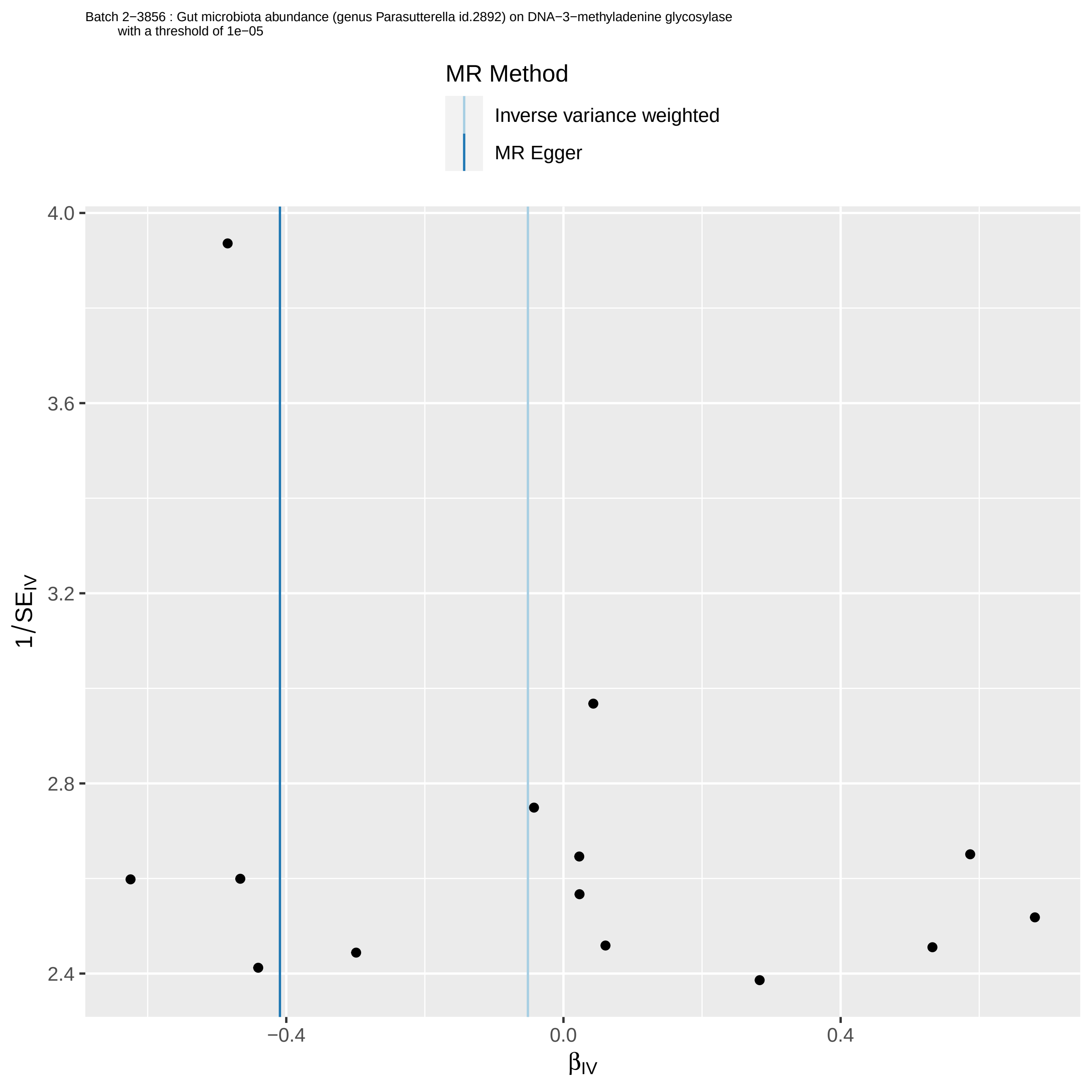
Batch 2−3856 : Gut microbiota abundance (genus Parasutterella id.2892) on DNA−3−methyladenine glycosylase
with a threshold of 1e−05
MR Method
Inverse variance weighted
MR Egger
4.0
3.6
3.2
2.8
2.4
1/SE_IV
−0.4
0.0
0.4
β_IV

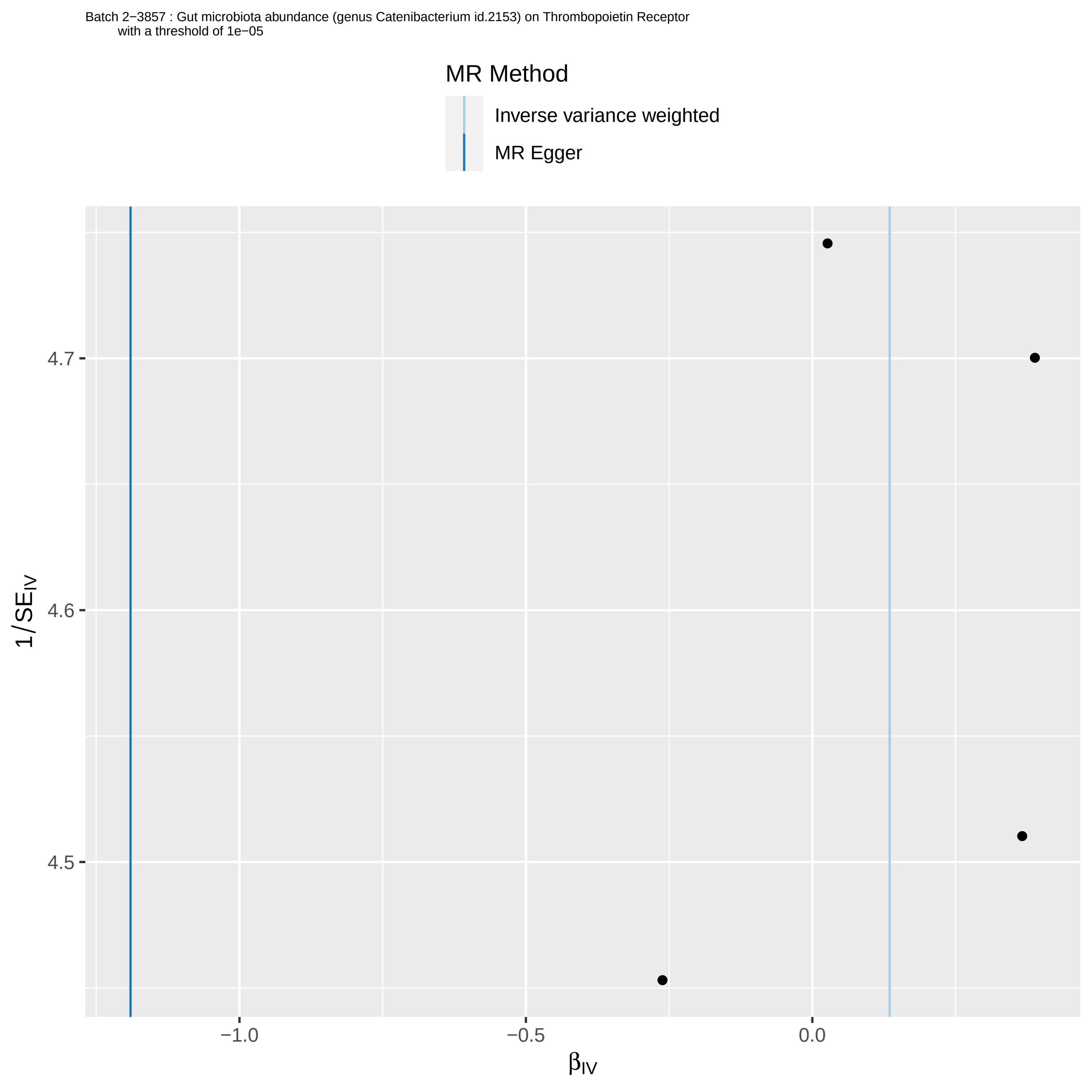

Batch 2−3857 : Gut microbiota abundance (genus Catenibacterium id.2153) on Thrombopoietin Receptor
with a threshold of 1e−05
MR Method
Inverse variance weighted
MR Egger
4.7
4.6
4.5
1/SE_IV
−1.0
−0.5
0.0
β_IV

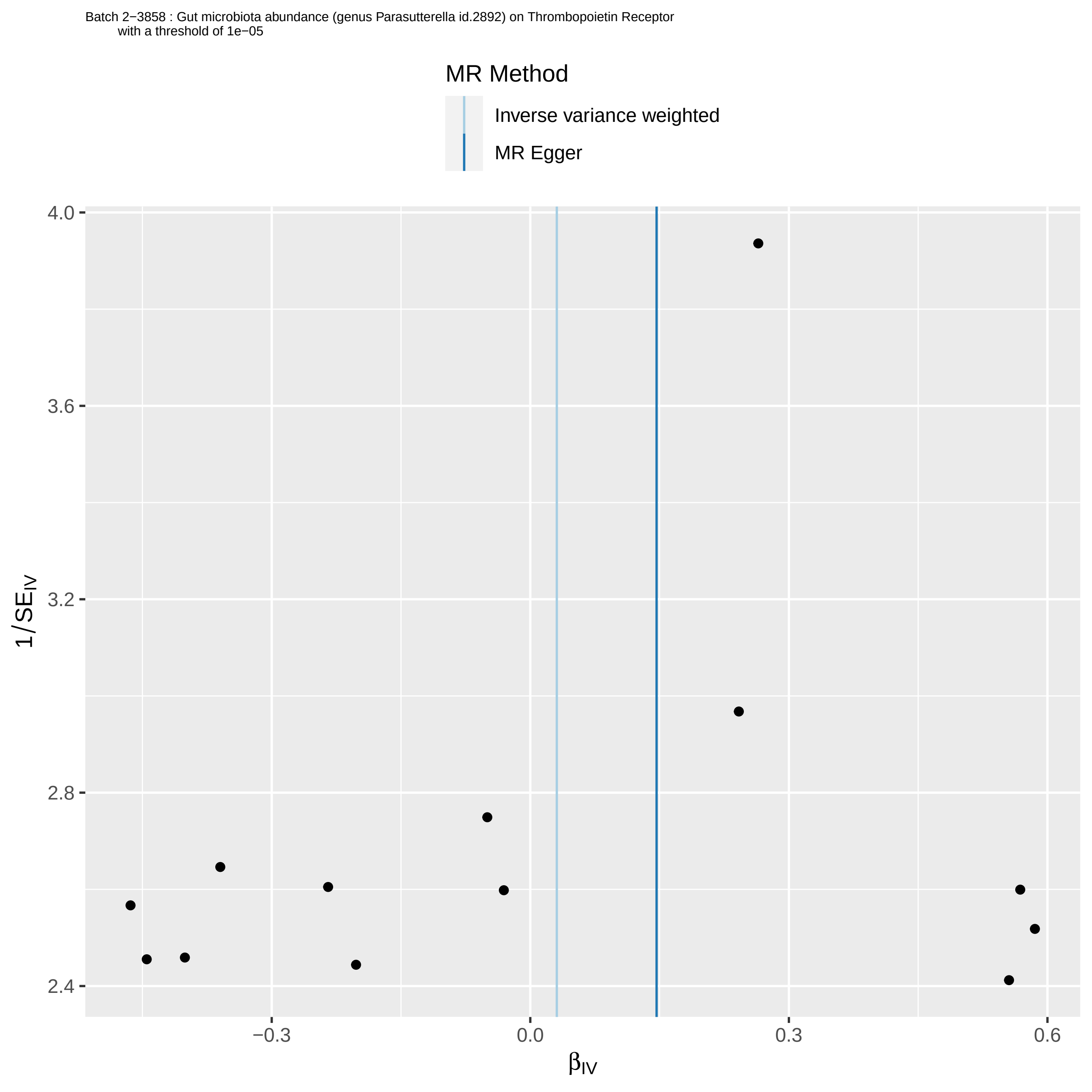

Batch 2−3858 : Gut microbiota abundance (genus Parasutterella id.2892) on Thrombopoietin Receptor
with a threshold of 1e−05
MR Method
Inverse variance weighted
MR Egger
4.0
3.6
3.2
2.8
2.4
1/SE_IV
−0.3
0.0
0.3
0.6
β_IV

Batch 2−3859 : Gut microbiota abundance (genus Catenibacterium id.2153) on Myeloperoxidase
with a threshold of 1e−05

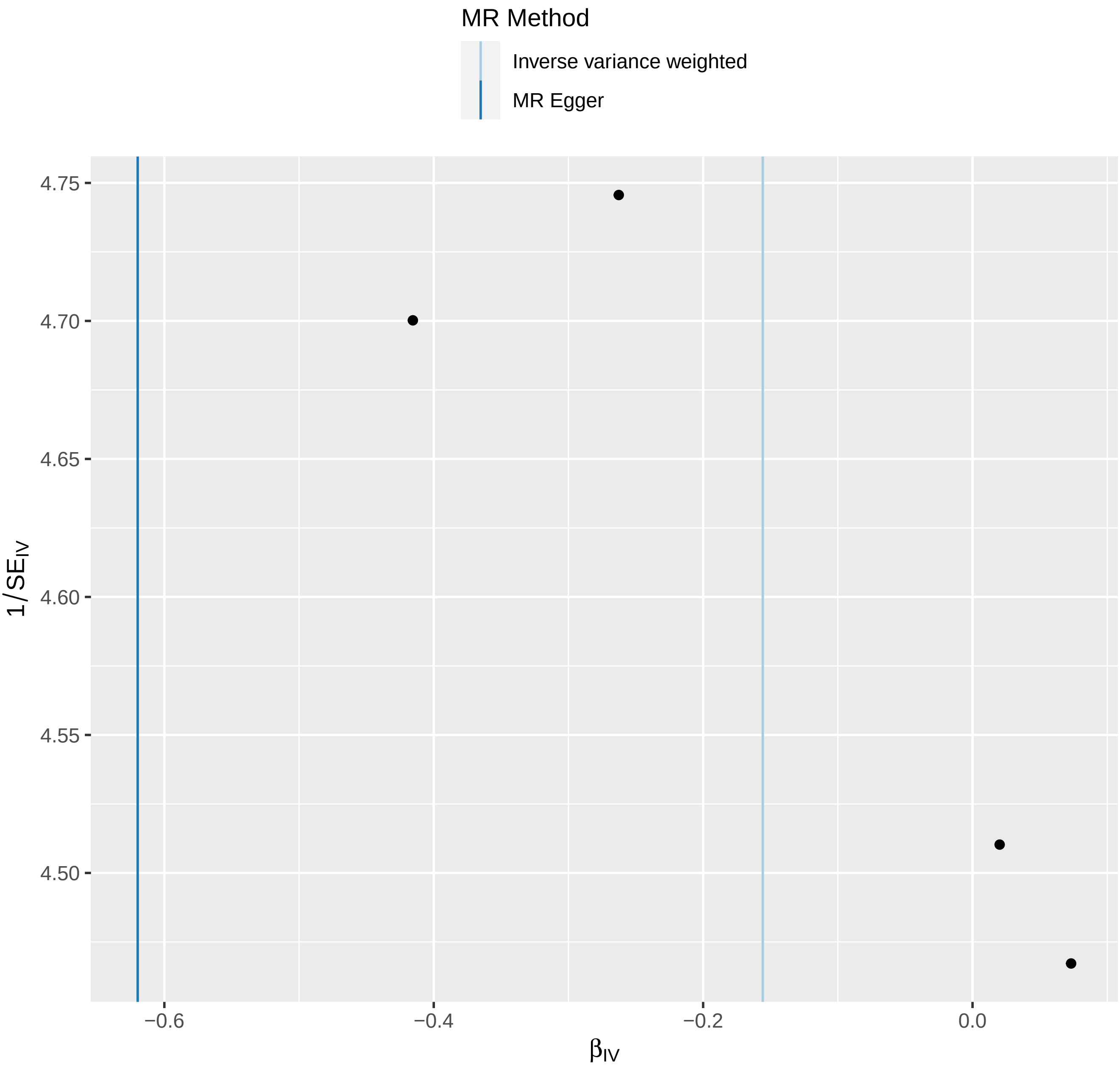

Batch 2−3860 : Gut microbiota abundance (genus Parasutterella id.2892) on Myeloperoxidase
with a threshold of 1e−05

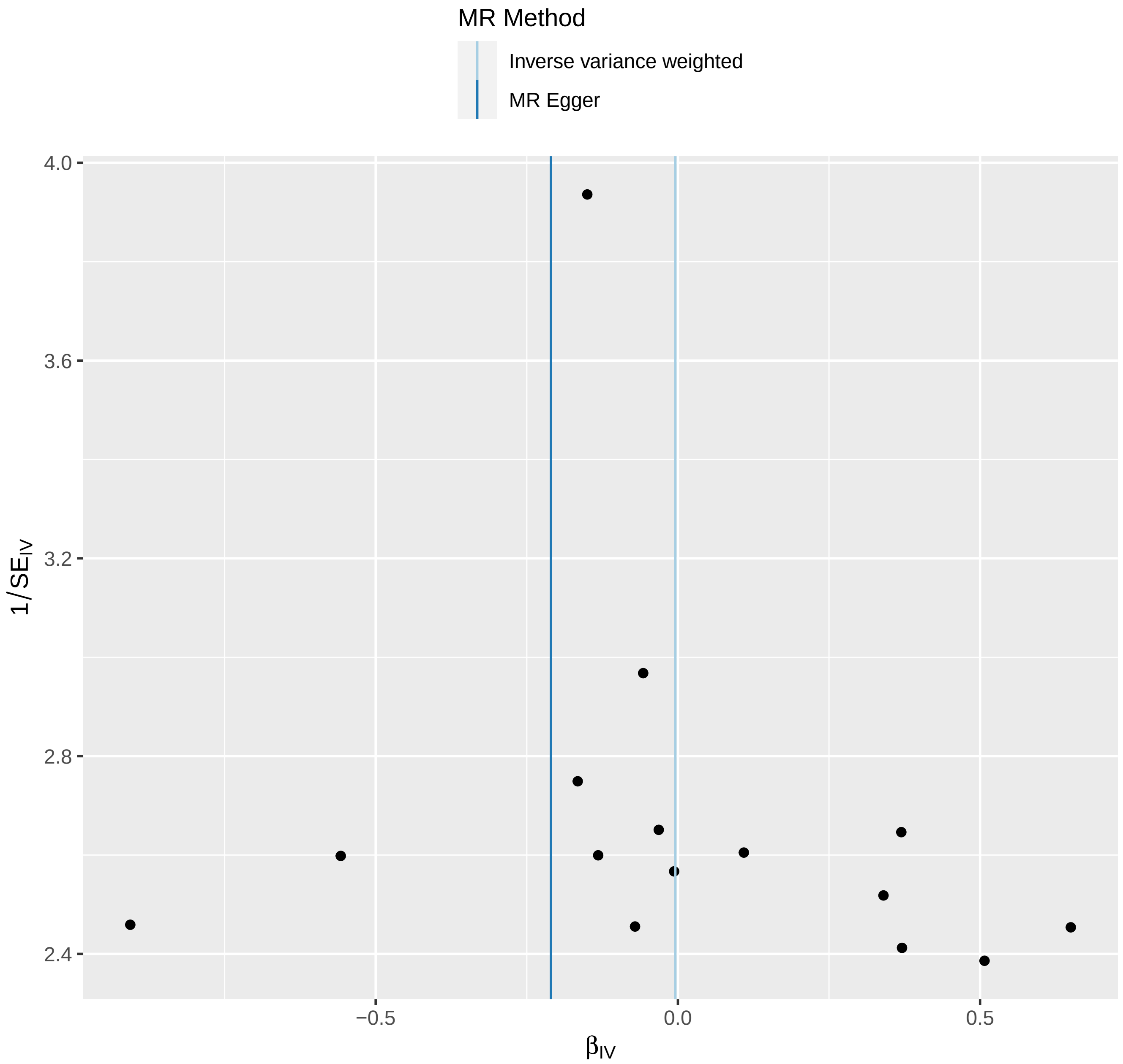

Batch 2−3861 : Gut microbiota abundance (genus Catenibacterium id.2153) on MAGUK p55 subfamily member 6 with a threshold of 1e−05

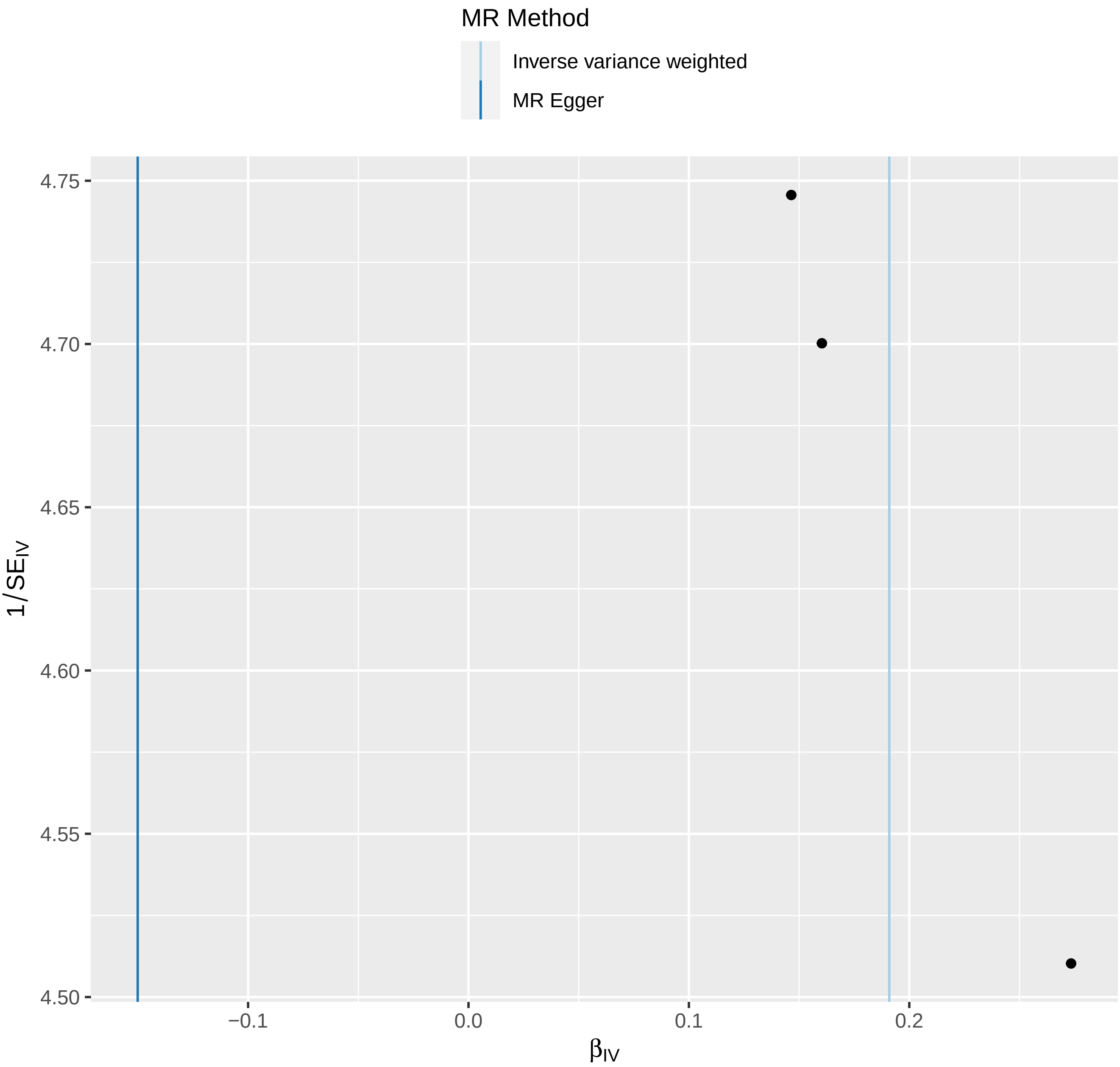

Batch 2−3862 : Gut microbiota abundance (genus Parasutterella id.2892) on MAGUK p55 subfamily member 6
with a threshold of 1e−05

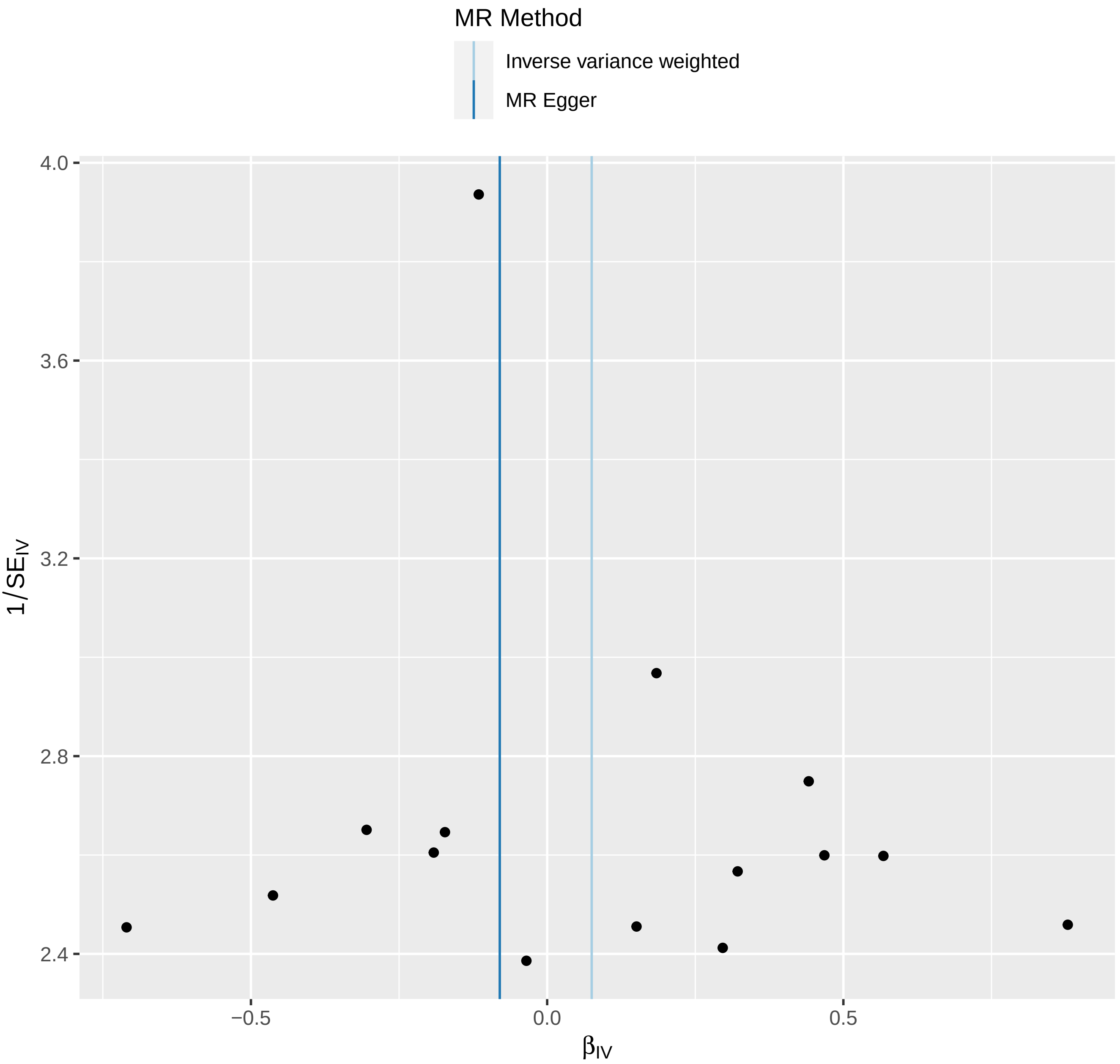

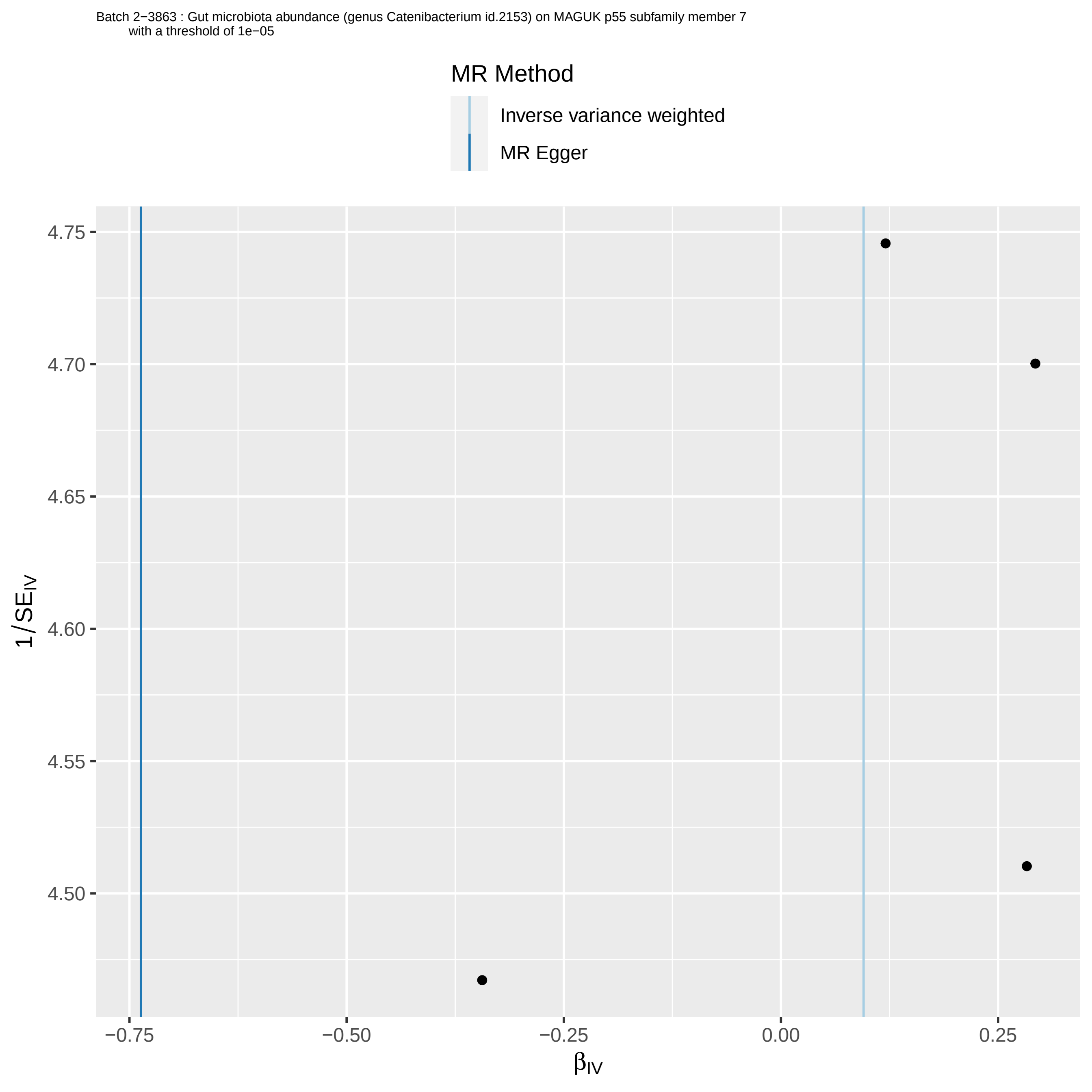
Batch 2−3863 : Gut microbiota abundance (genus Catenibacterium id.2153) on MAGUK p55 subfamily member 7
with a threshold of 1e−05
MR Method
Inverse variance weighted
MR Egger
4.75
4.70
4.65
4.60
4.55
4.50
1/SE$_{IV}$
−0.75
−0.50
−0.25
0.00
0.25
β_{IV}

Batch 2−3864 : Gut microbiota abundance (genus Parasutterella id.2892) on MAGUK p55 subfamily member 7
with a threshold of 1e−05

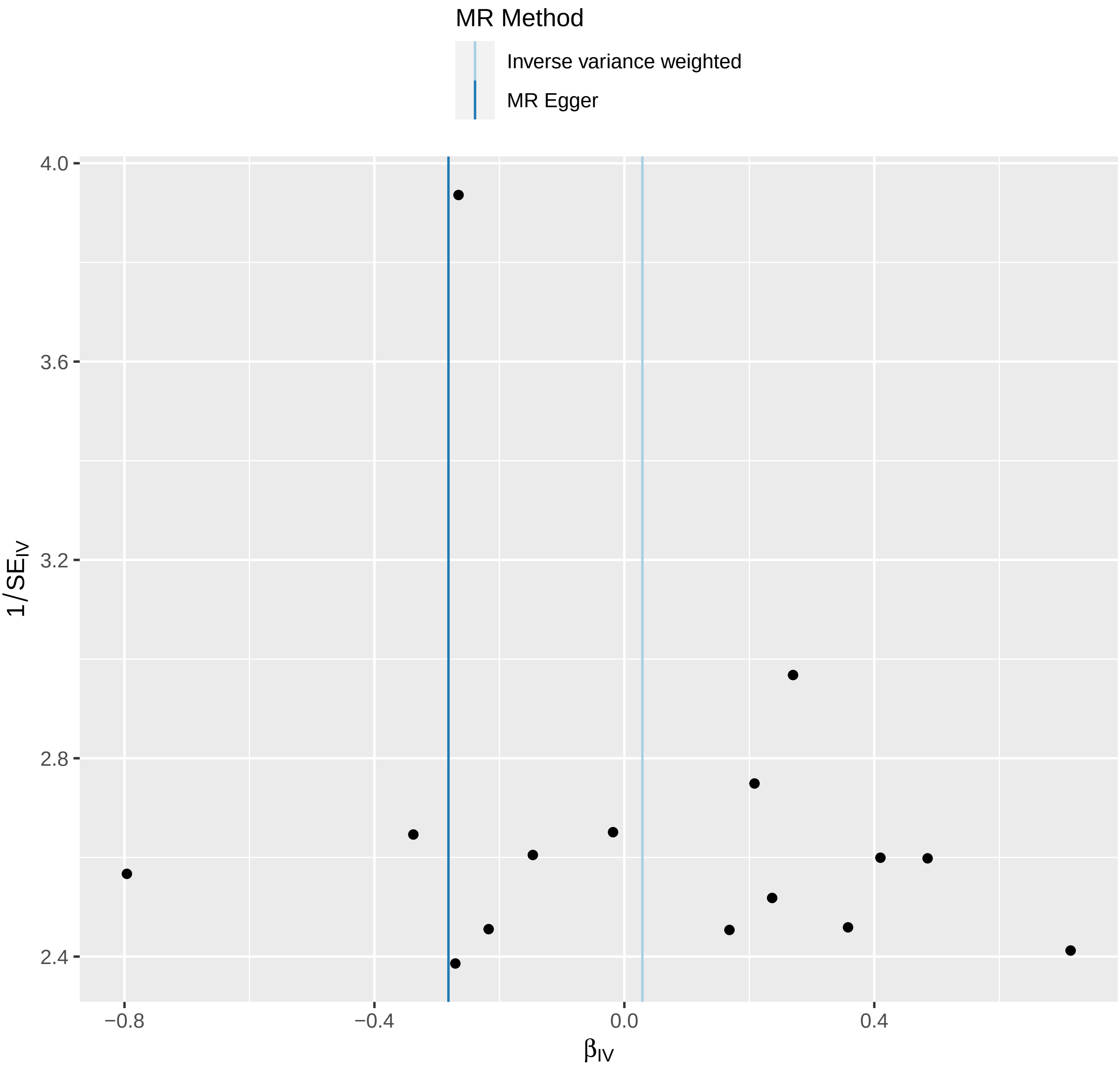

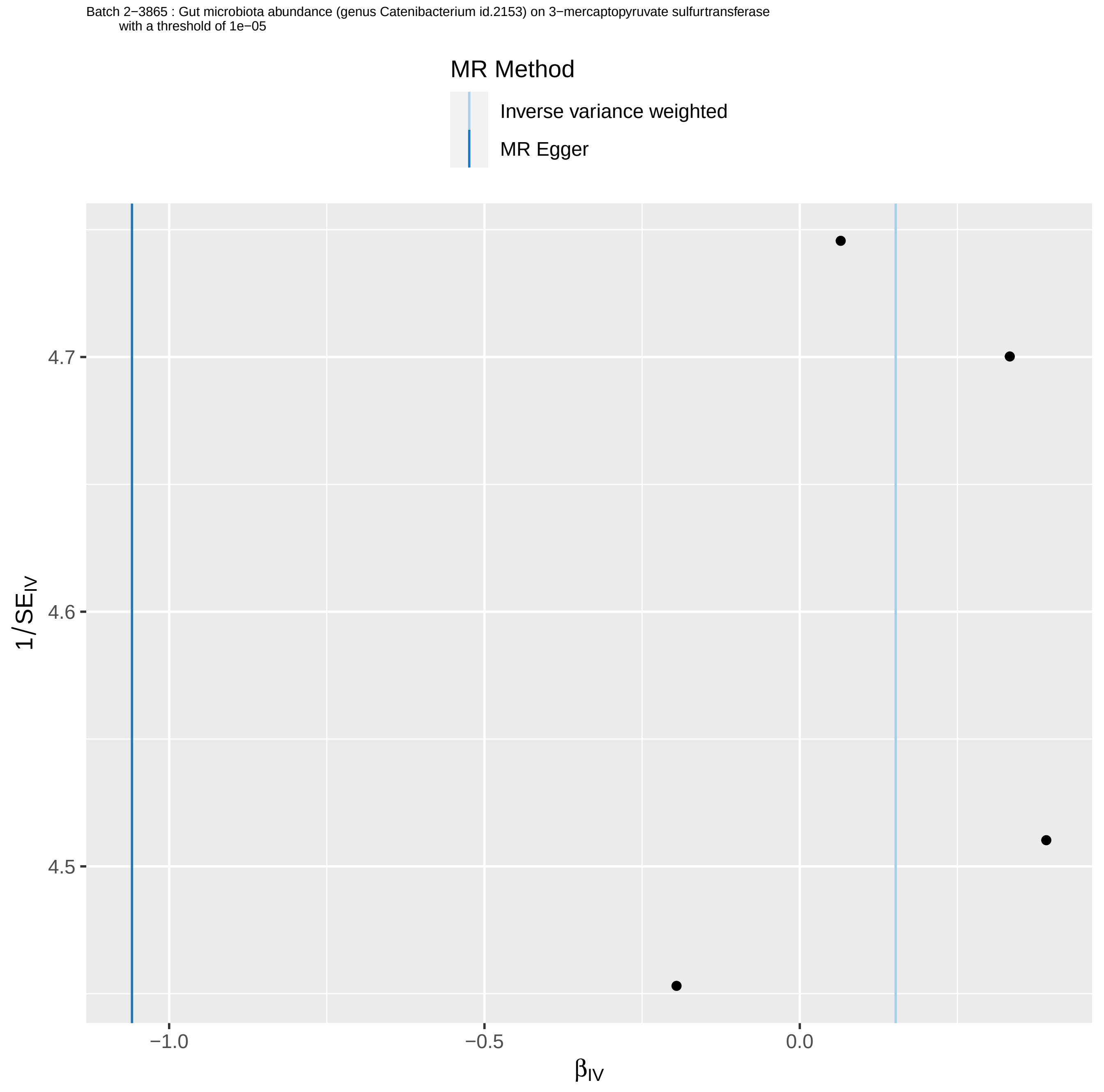
Batch 2−3865 : Gut microbiota abundance (genus Catenibacterium id.2153) on 3−mercaptopyruvate sulfurtransferase
with a threshold of 1e−05
MR Method
Inverse variance weighted
MR Egger
4.7
4.6
4.5
1/SE_IV
−1.0
−0.5
0.0
β_IV

Batch 2−3866 : Gut microbiota abundance (genus Parasutterella id.2892) on 3−mercaptopyruvate sulfurtransferase with a threshold of 1e−05

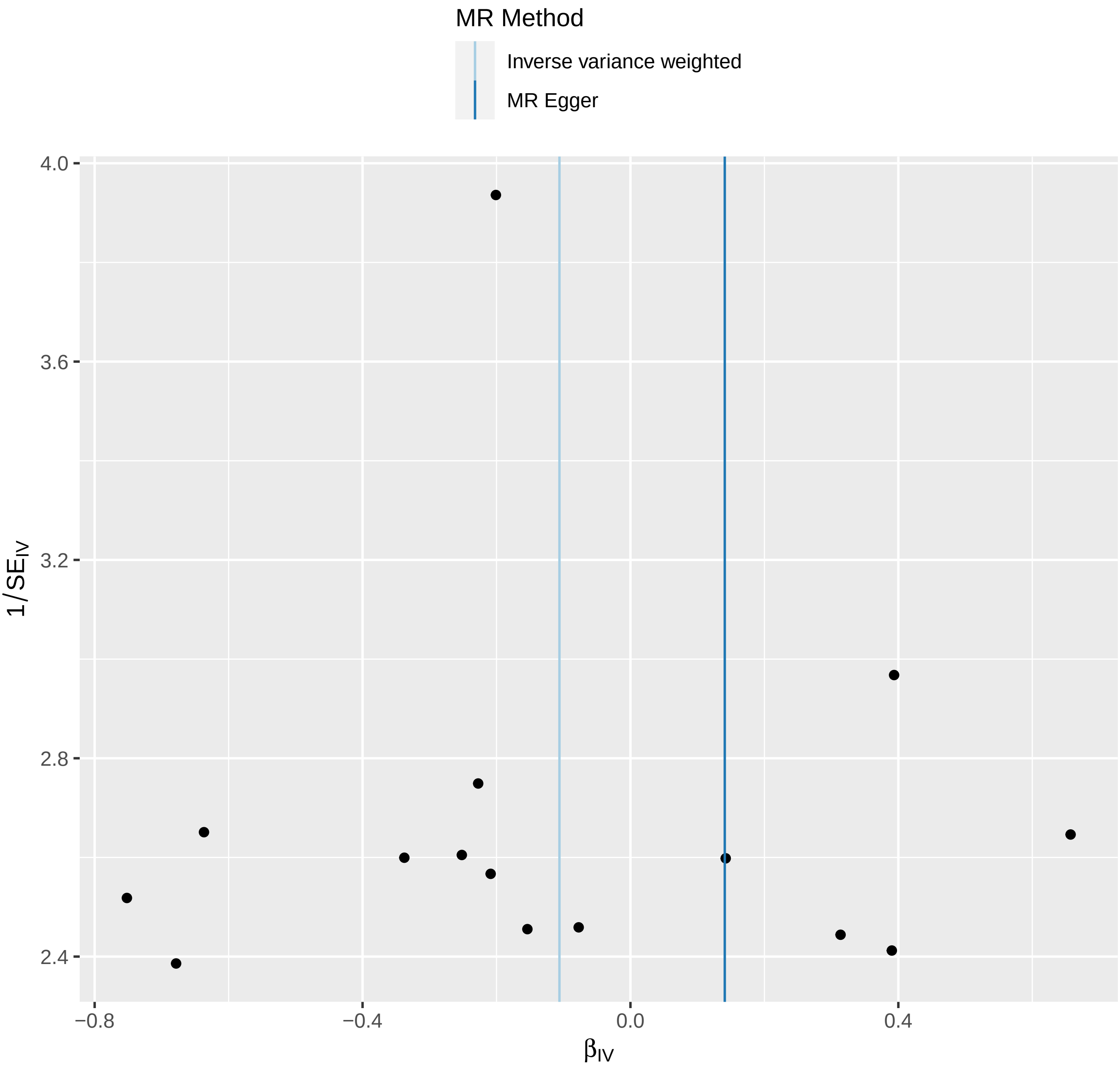

Batch 2−3867 : Gut microbiota abundance (genus Catenibacterium id.2153) on Myelin protein P0
with a threshold of 1e−05

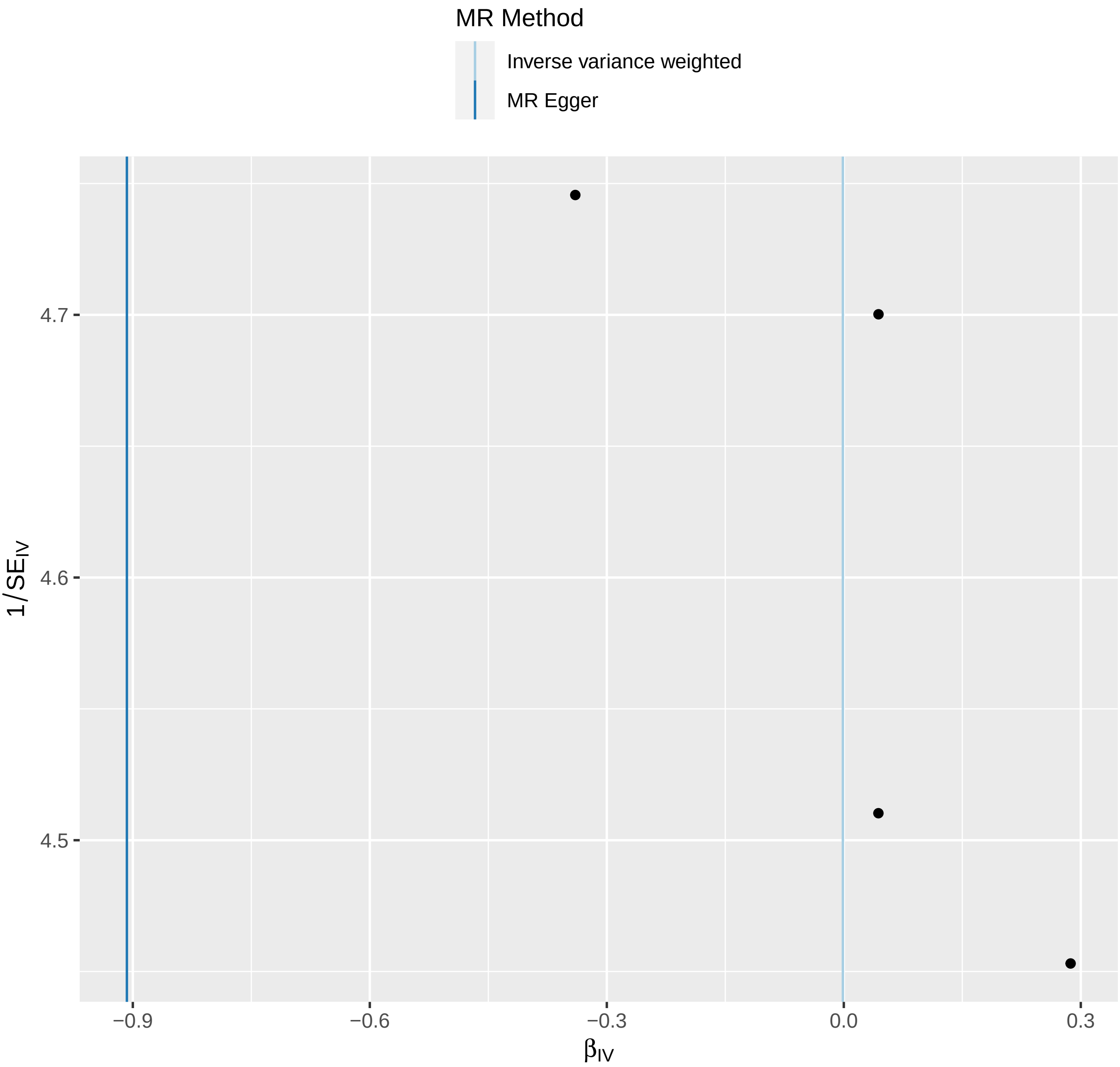

Batch 2−3868 : Gut microbiota abundance (genus Parasutterella id.2892) on Myelin protein P0
with a threshold of 1e−05

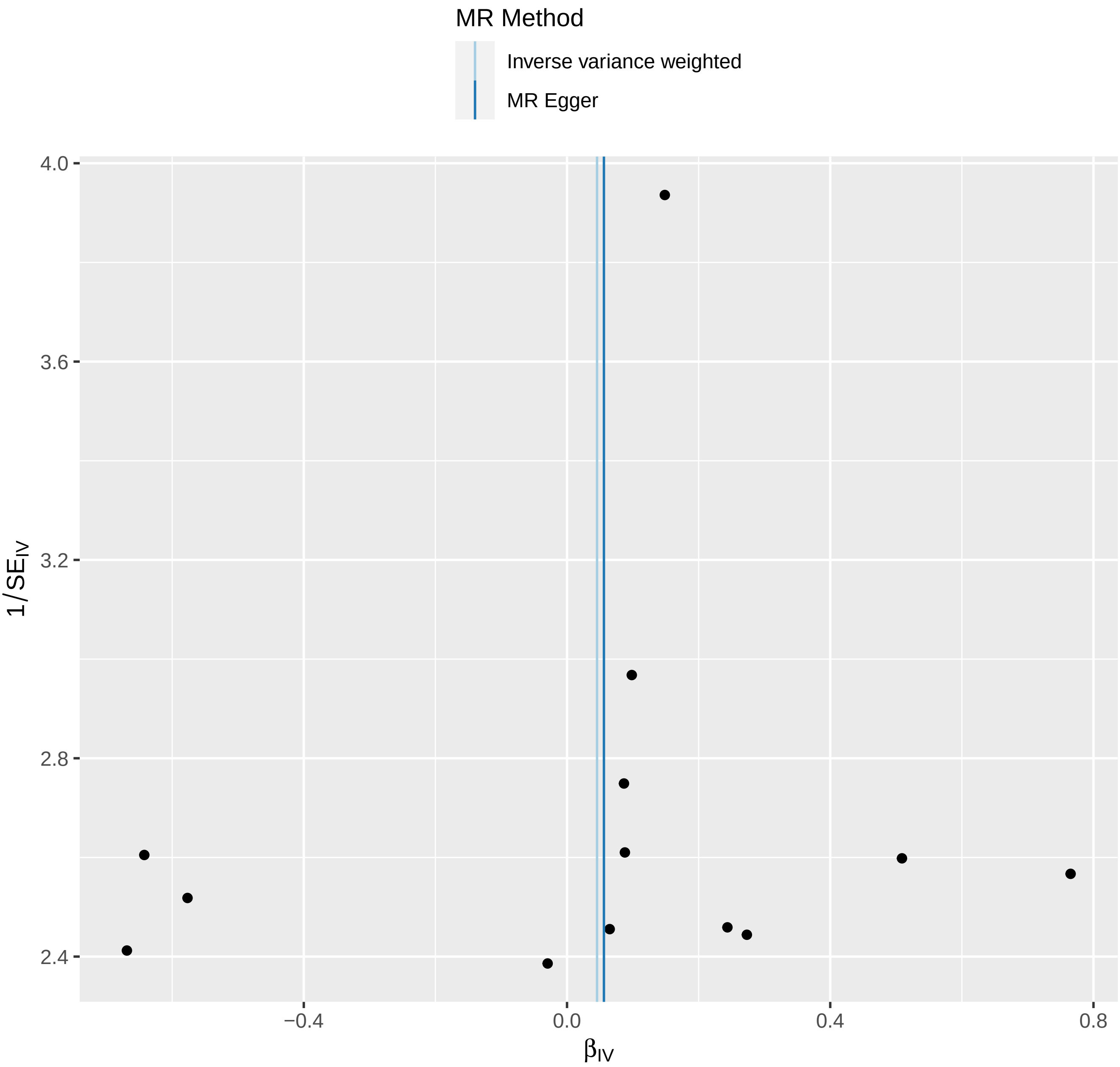

Batch 2−3869 : Gut microbiota abundance (genus Catenibacterium id.2153) on Melanocortin−2 receptor accessory protein 2 with a threshold of 1e−05

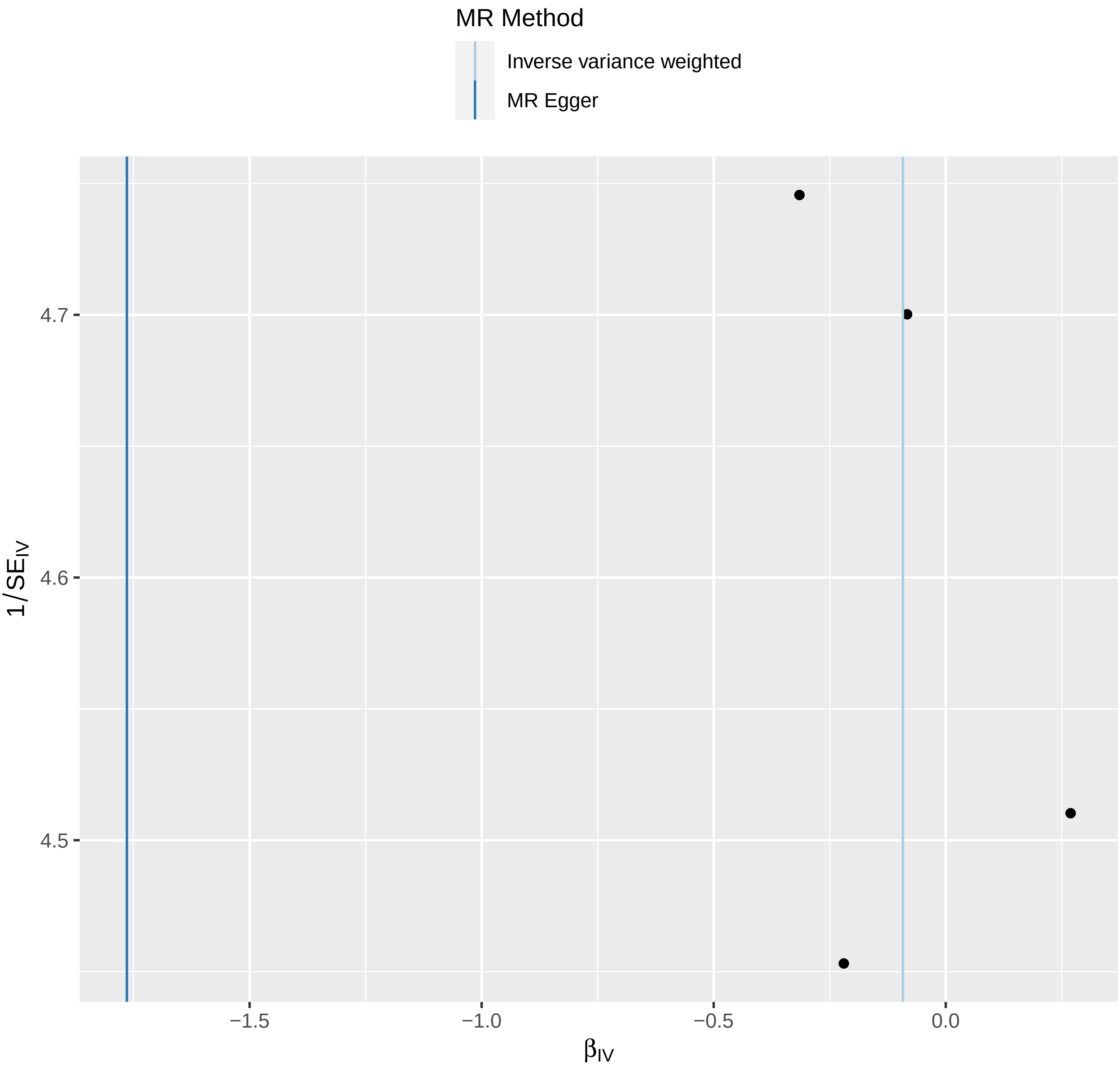

Batch 2−3870 : Gut microbiota abundance (genus Parasutterella id.2892) on Melanocortin−2 receptor accessory protein 2 with a threshold of 1e−05

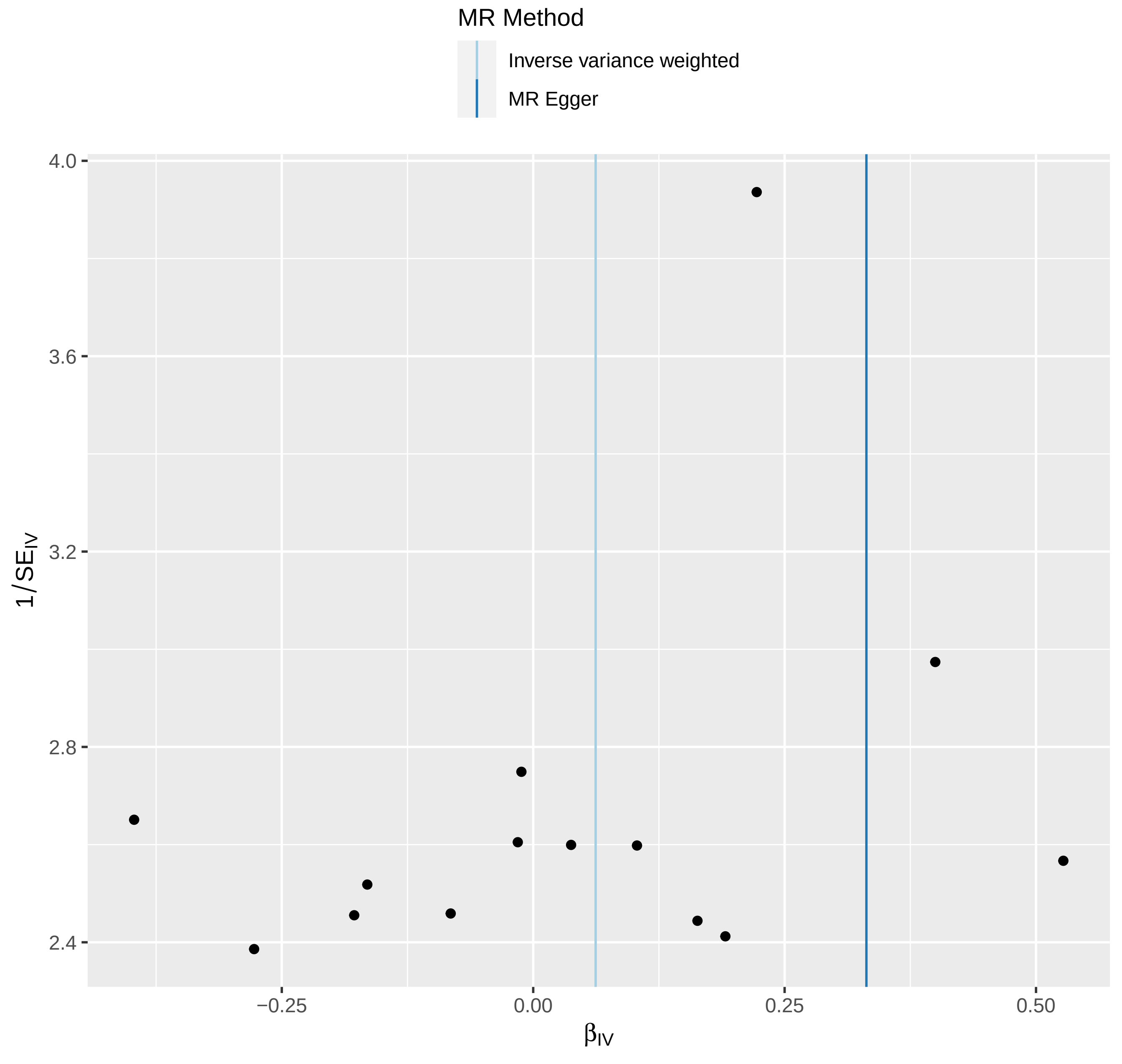

Batch 2−3871 : Gut microbiota abundance (genus Catenibacterium id.2153) on Melanocortin−2 receptor accessory protein
with a threshold of 1e−05

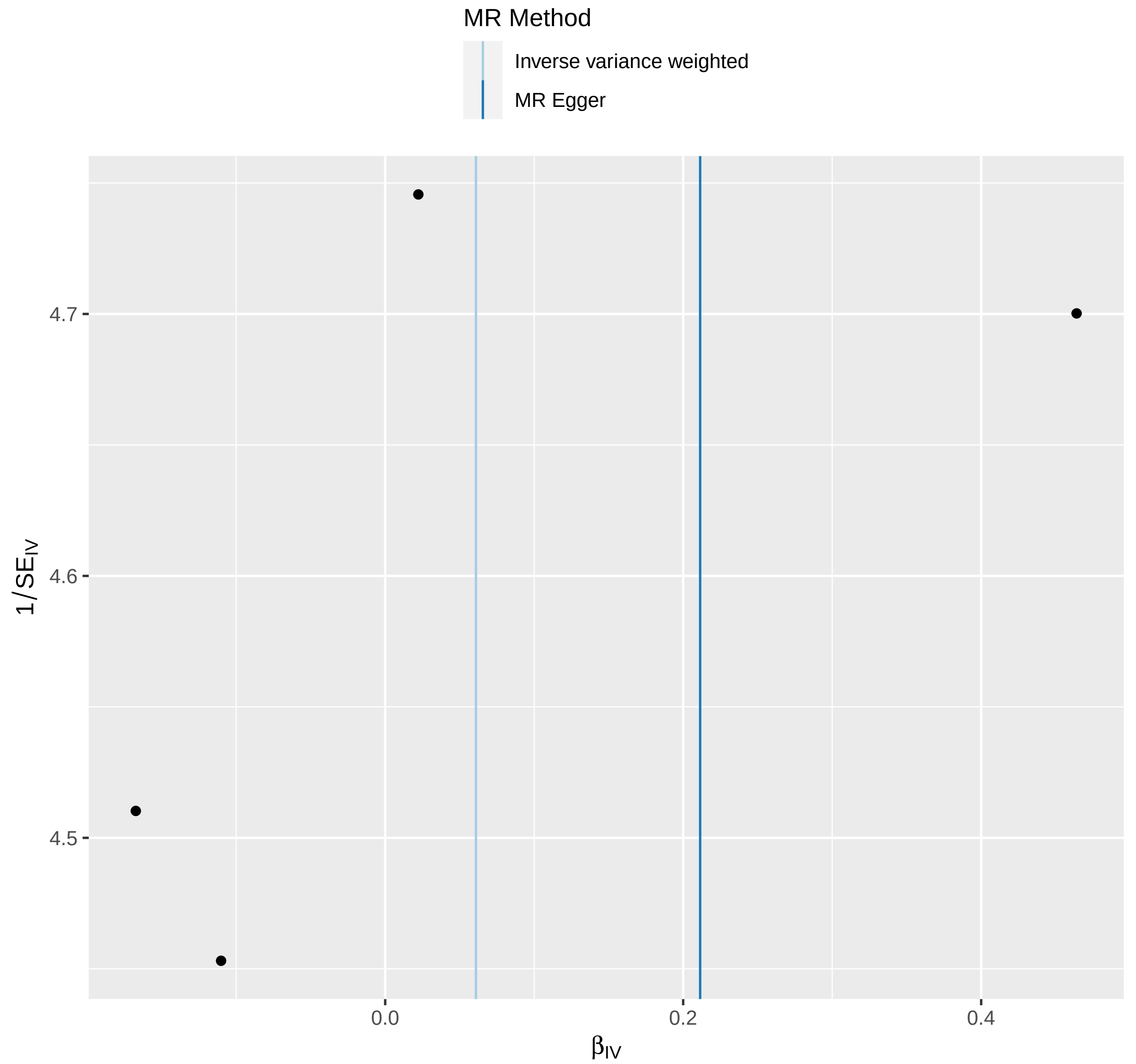

Batch 2−3872 : Gut microbiota abundance (genus Parasutterella id.2892) on Melanocortin−2 receptor accessory protein
with a threshold of 1e−05

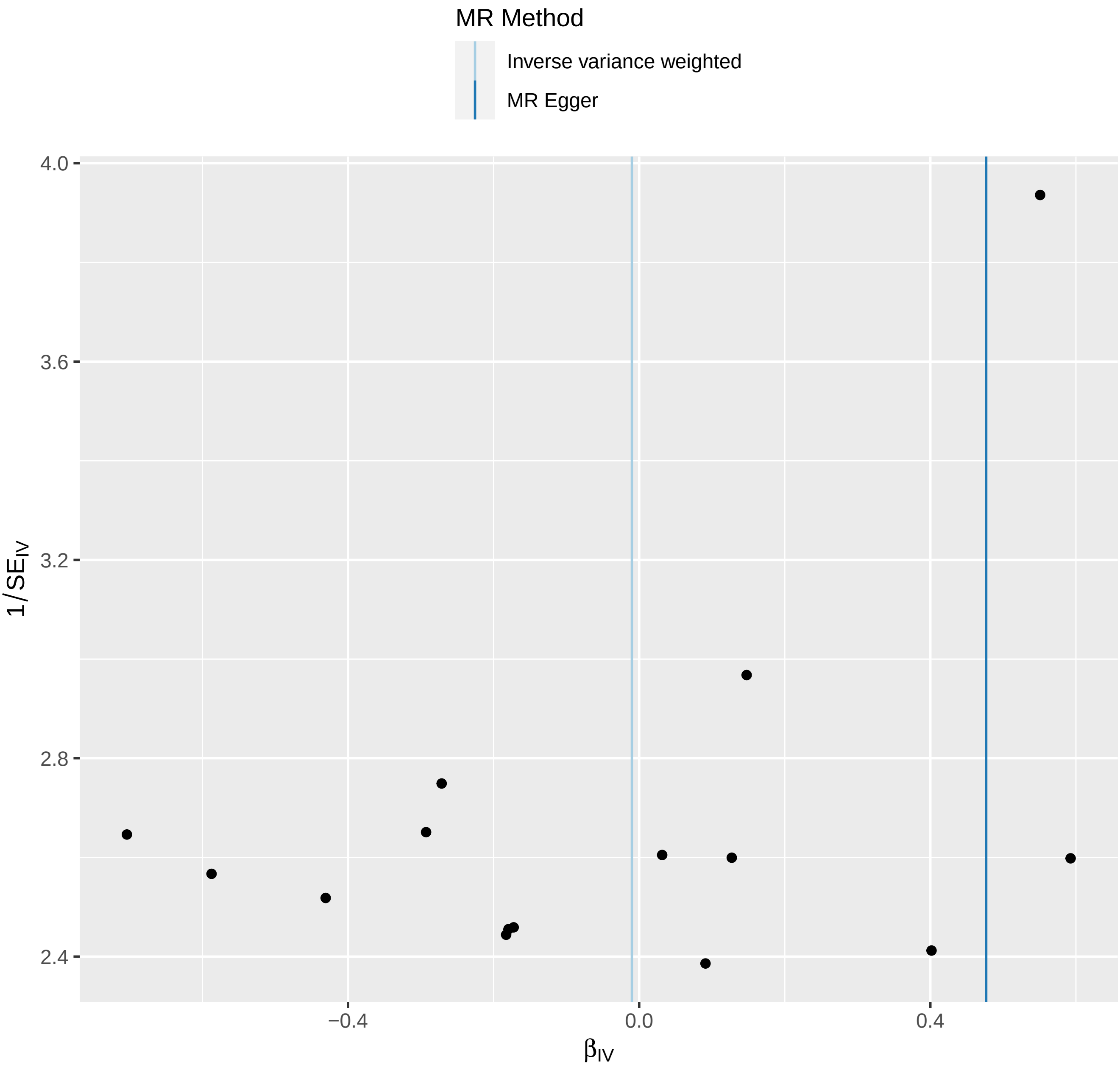

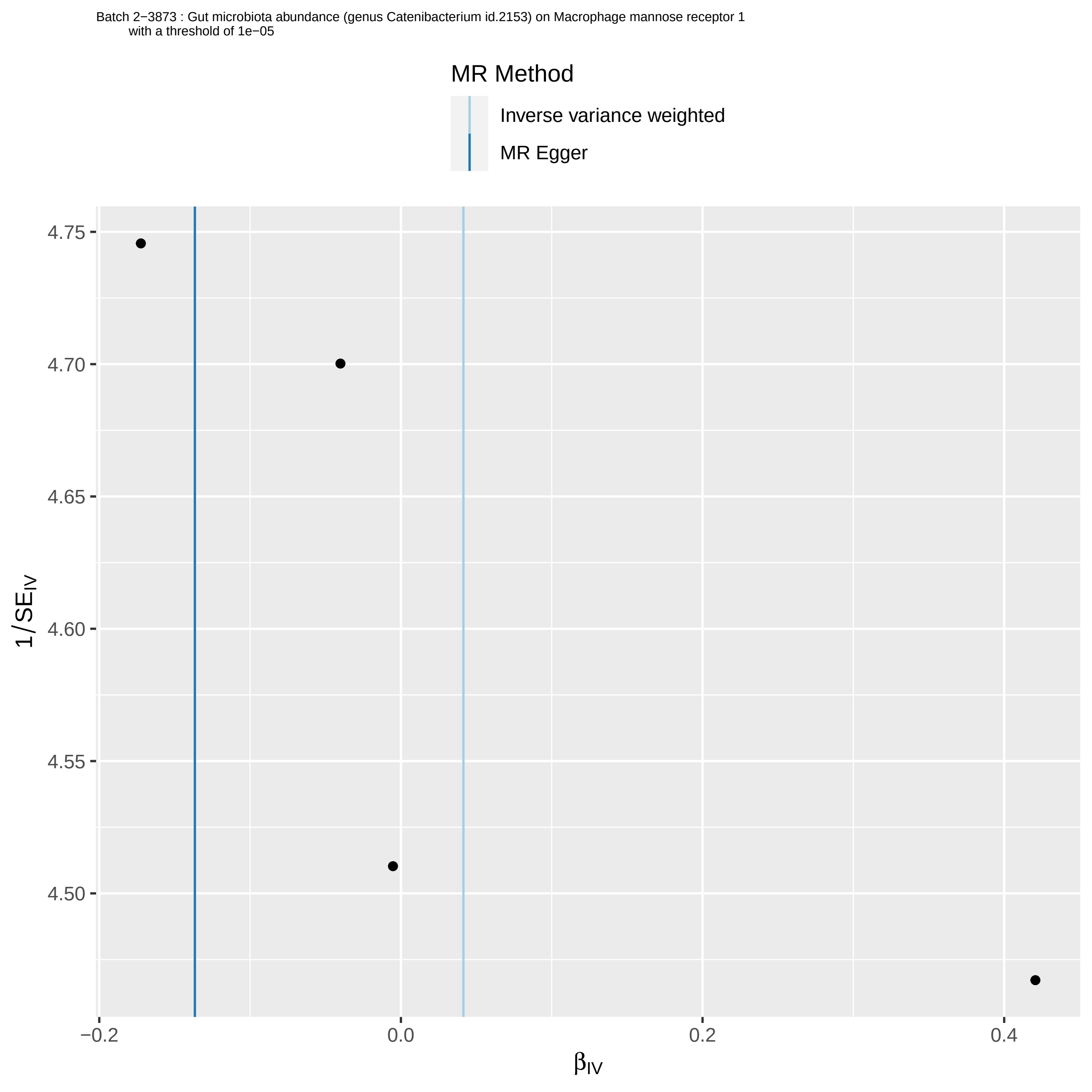

Batch 2−3873 : Gut microbiota abundance (genus Catenibacterium id.2153) on Macrophage mannose receptor 1
with a threshold of 1e−05
MR Method
Inverse variance weighted
MR Egger
4.75
4.70
4.65
4.60
4.55
4.50
1/SE_IV
−0.2
0.0
0.2
0.4
β_IV

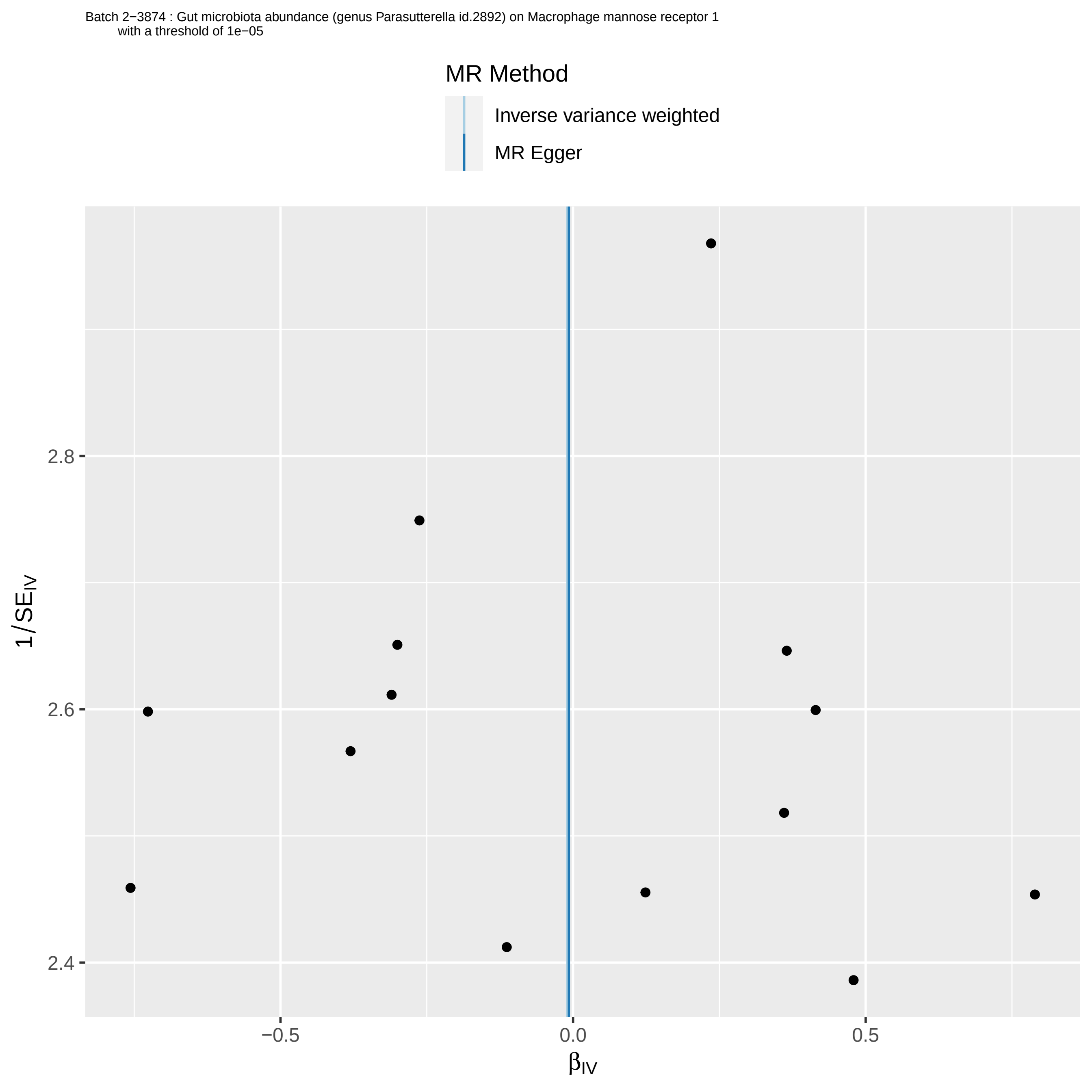
Batch 2−3874 : Gut microbiota abundance (genus Parasutterella id.2892) on Macrophage mannose receptor 1
with a threshold of 1e−05
MR Method
Inverse variance weighted
MR Egger
2.8
2.6
2.4
1/SE$_{IV}$
−0.5
0.0
0.5
β_{IV}

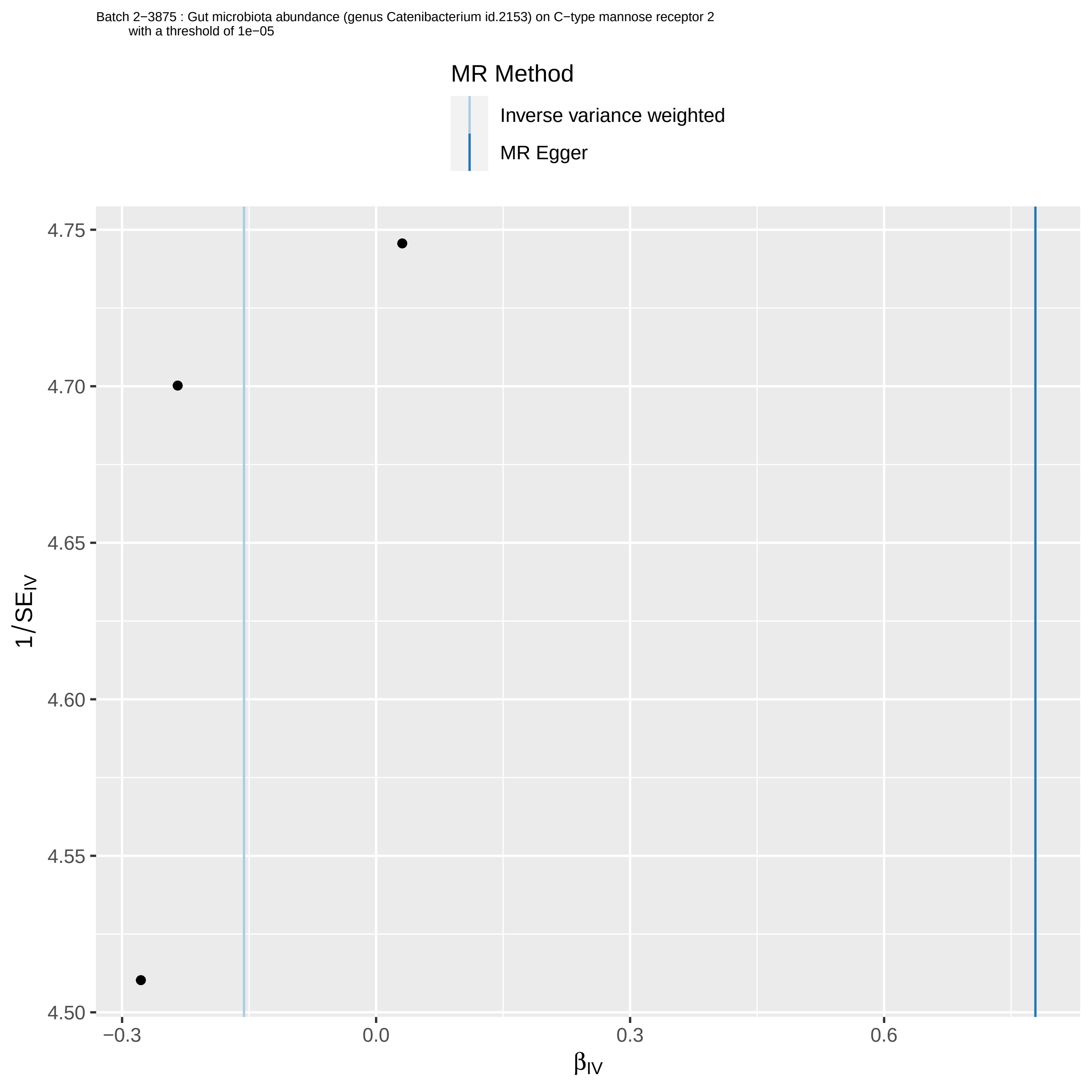
Batch 2−3875 : Gut microbiota abundance (genus Catenibacterium id.2153) on C−type mannose receptor 2
with a threshold of 1e−05
MR Method
Inverse variance weighted
MR Egger
4.75
4.70
4.65
4.60
4.55
4.50
1/SE_IV
−0.3
0.0
0.3
0.6
β_IV

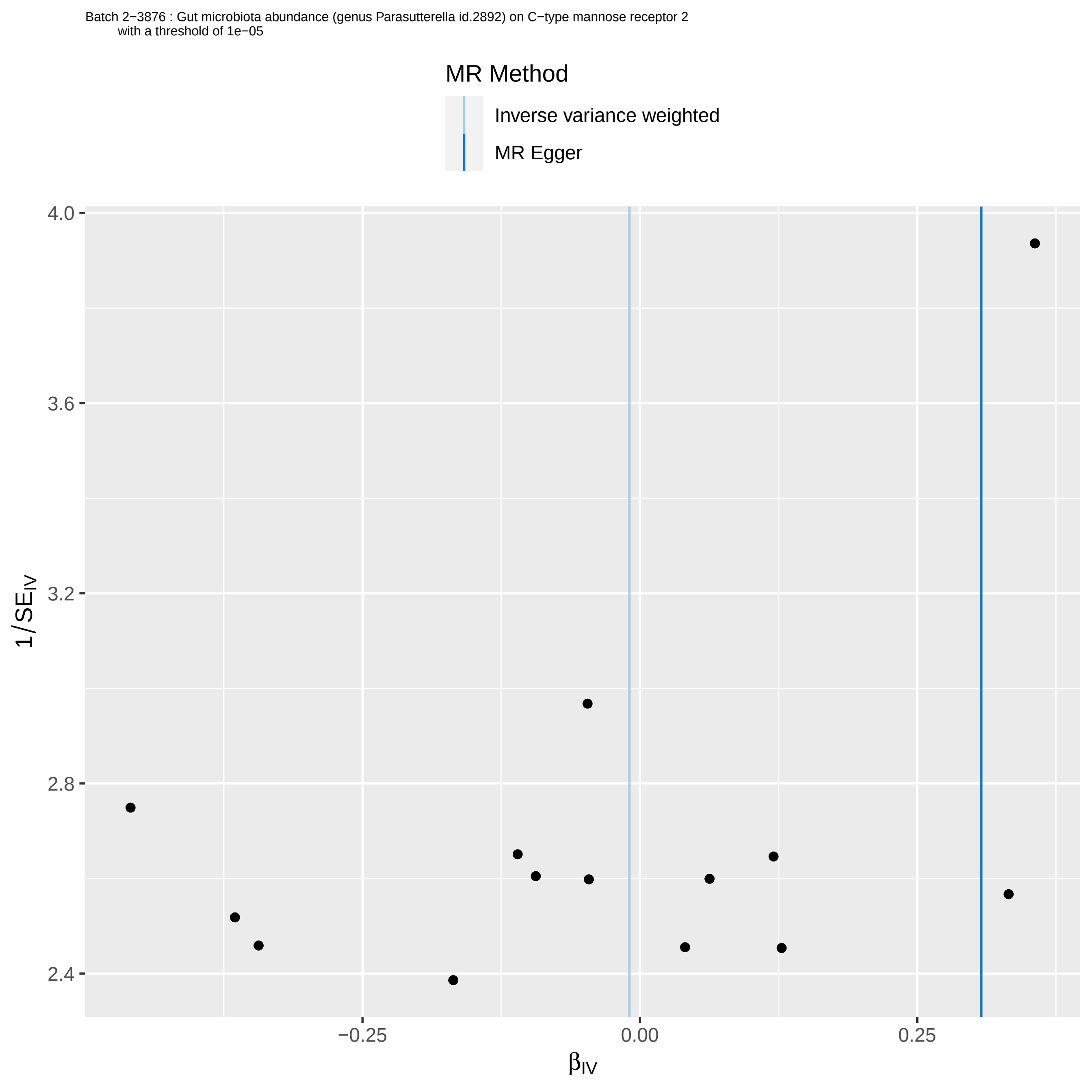

Batch 2−3876 : Gut microbiota abundance (genus Parasutterella id.2892) on C−type mannose receptor 2
with a threshold of 1e−05
MR Method
Inverse variance weighted
MR Egger
4.0
3.6
3.2
2.8
2.4
1/SE_IV
−0.25
0.00
0.25
β_IV

Batch 2−3877 : Gut microbiota abundance (genus Catenibacterium id.2153) on Double−strand break repair protein MRE11A
with a threshold of 1e−05

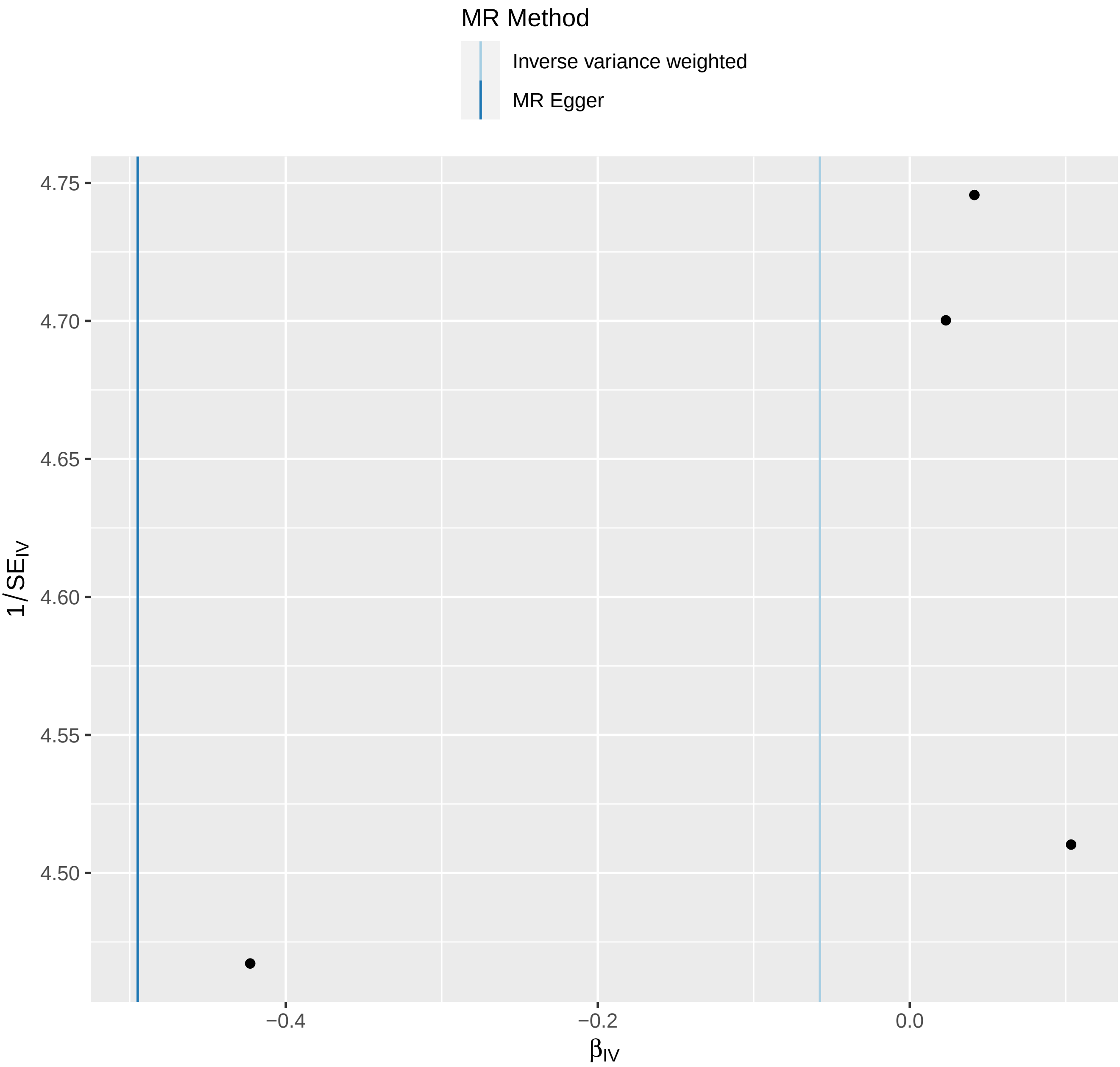

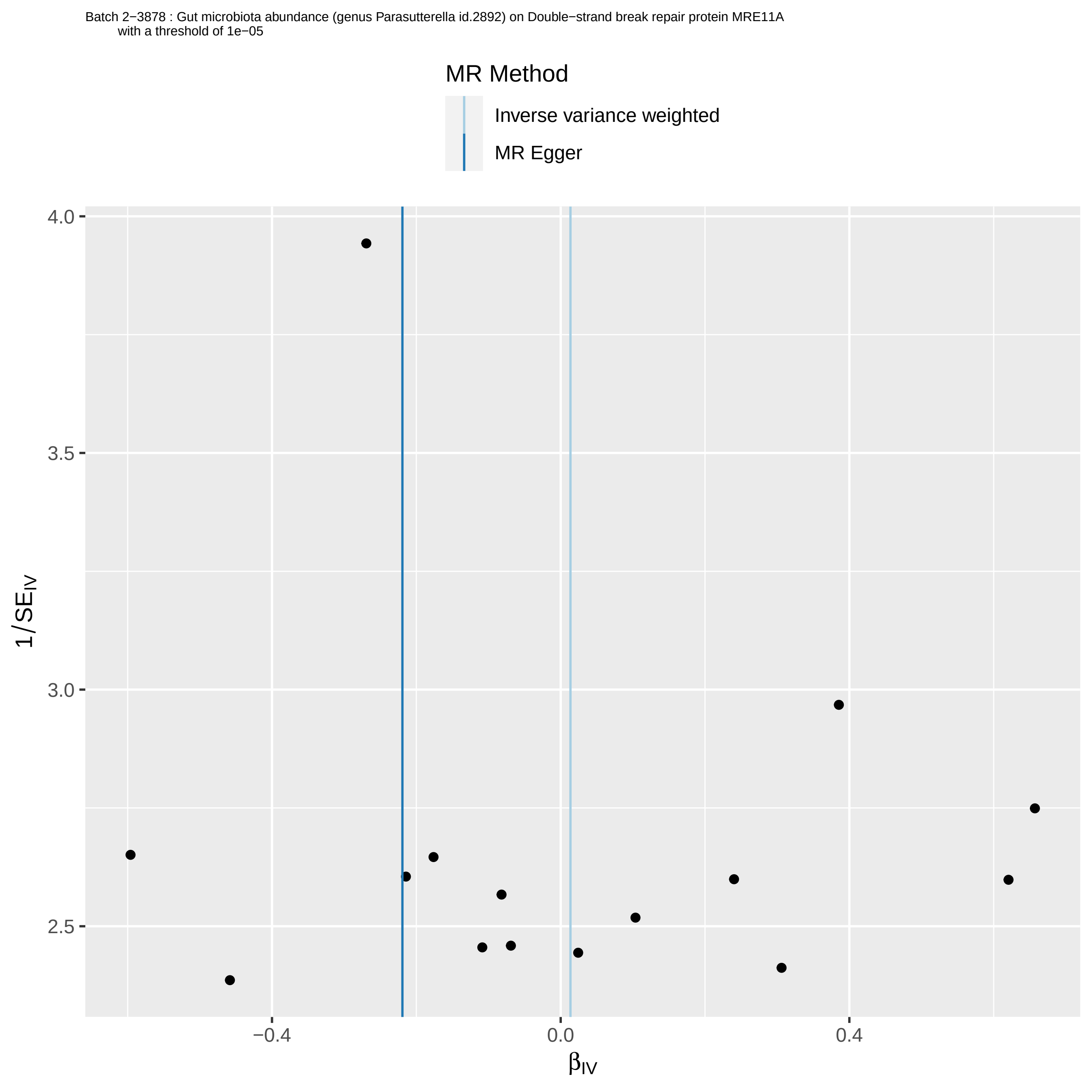
Batch 2−3878 : Gut microbiota abundance (genus Parasutterella id.2892) on Double−strand break repair protein MRE11A
with a threshold of 1e−05
MR Method
Inverse variance weighted
MR Egger
4.0
3.5
3.0
2.5
1/SEIV
−0.4
0.0
0.4
βIV

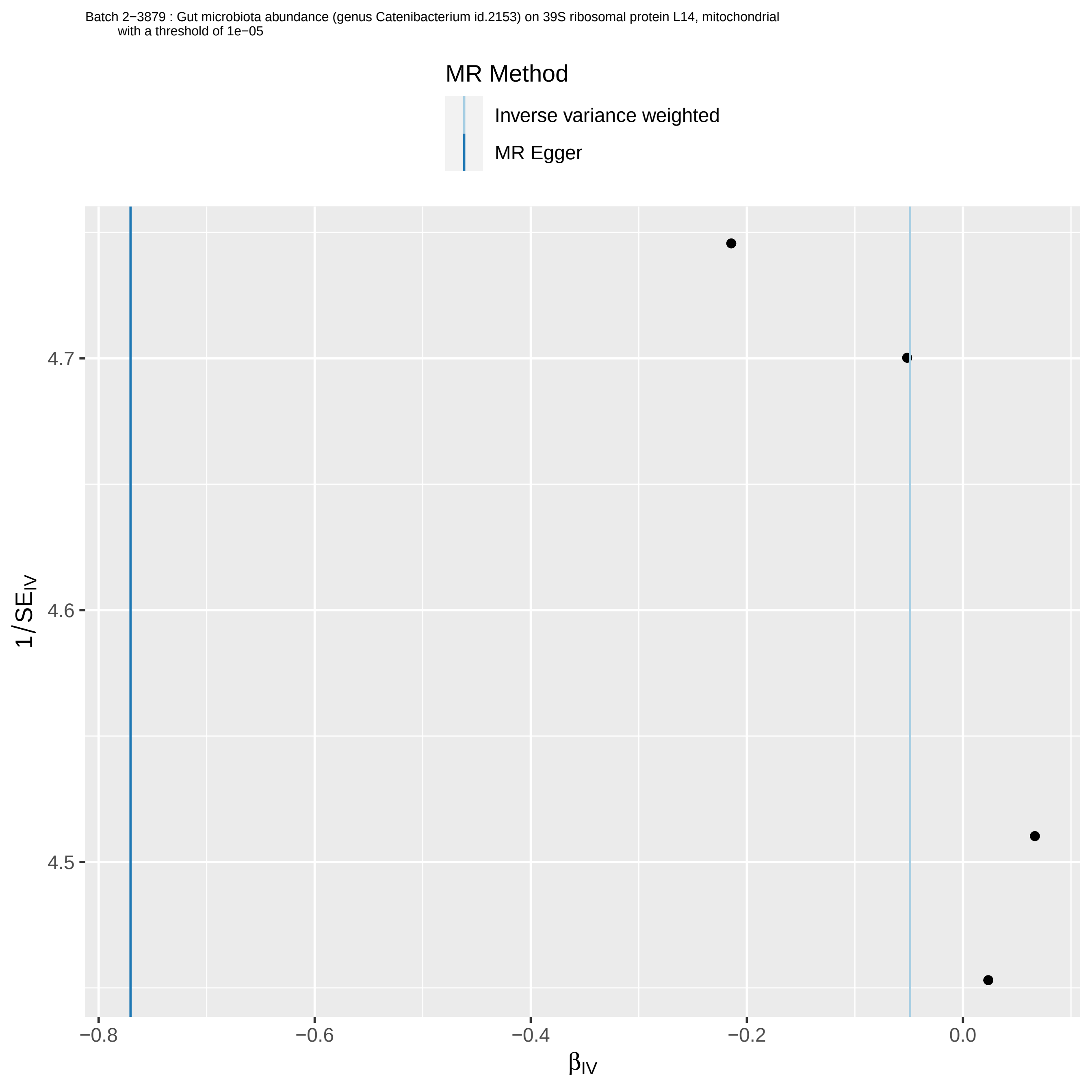
Batch 2−3879 : Gut microbiota abundance (genus Catenibacterium id.2153) on 39S ribosomal protein L14, mitochondrial
with a threshold of 1e−05
MR Method
Inverse variance weighted
MR Egger
4.7
4.6
4.5
1/SE_IV
−0.8
−0.6
−0.4
−0.2
0.0
β_IV

Batch 2−3880 : Gut microbiota abundance (genus Parasutterella id.2892) on 39S ribosomal protein L14, mitochondrial
with a threshold of 1e−05

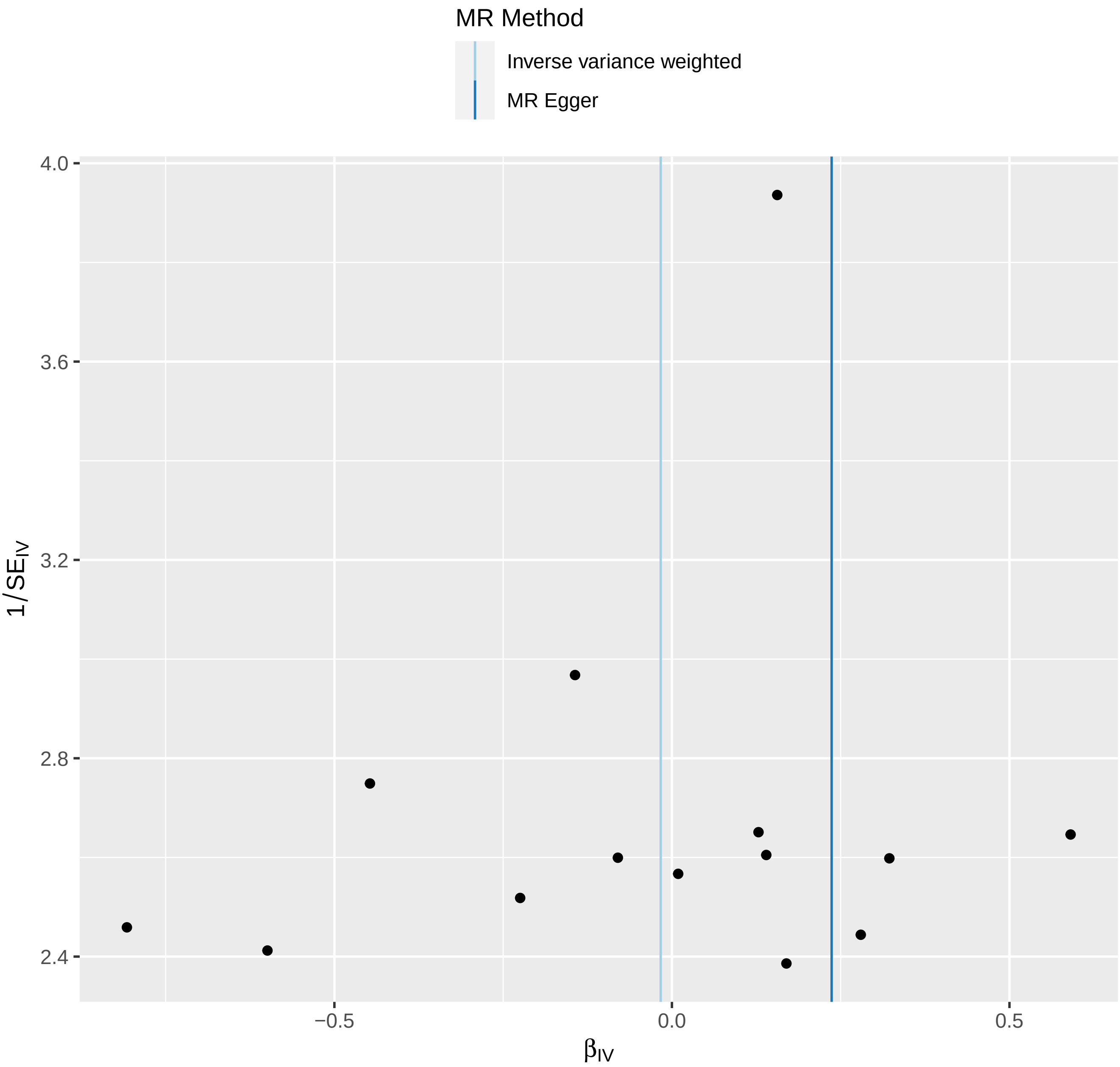

Batch 2−3881 : Gut microbiota abundance (genus Catenibacterium id.2153) on 39S ribosomal protein L32, mitochondrial with a threshold of 1e−05

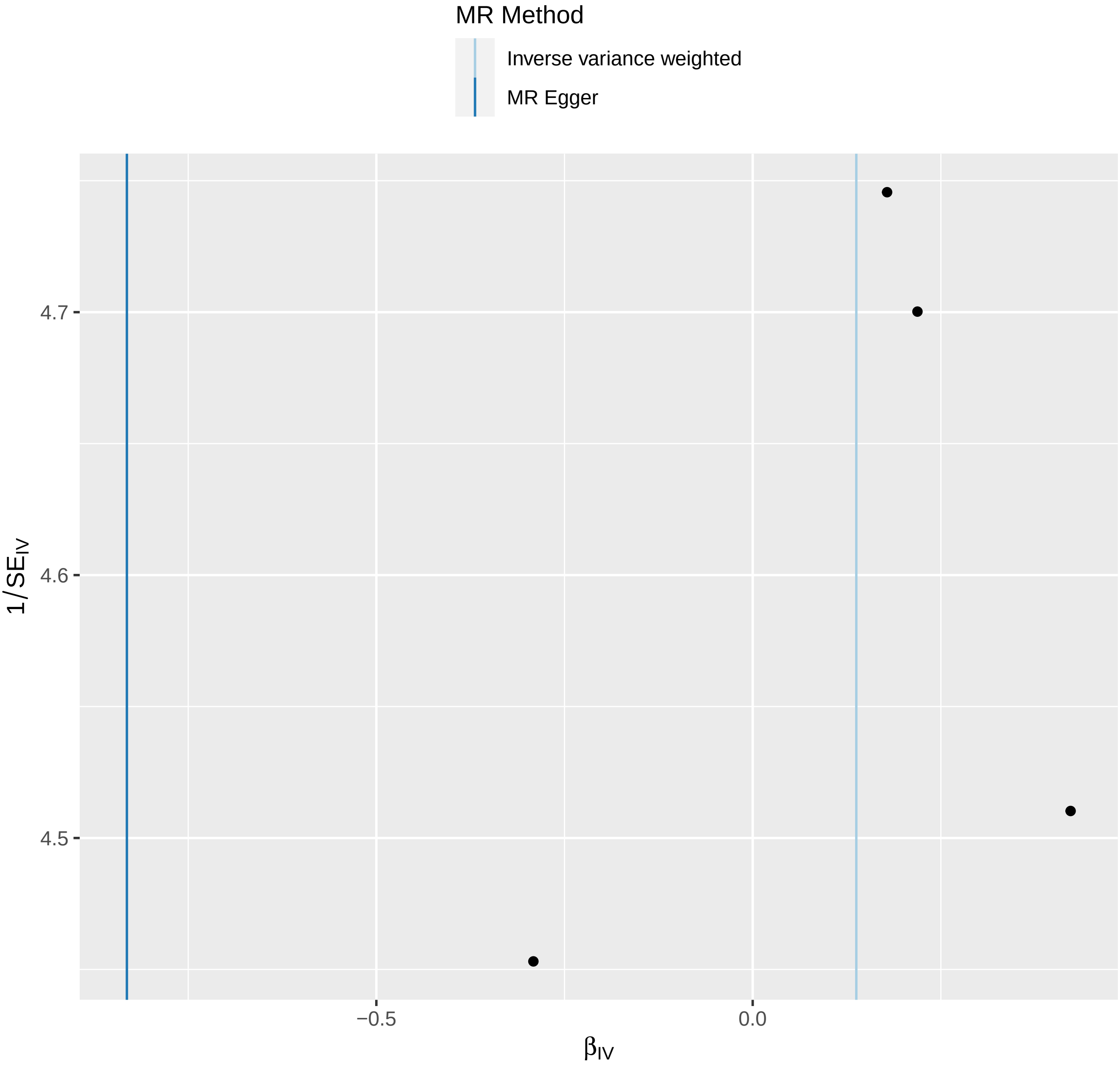

Batch 2−3882 : Gut microbiota abundance (genus Parasutterella id.2892) on 39S ribosomal protein L32, mitochondrial
with a threshold of 1e−05

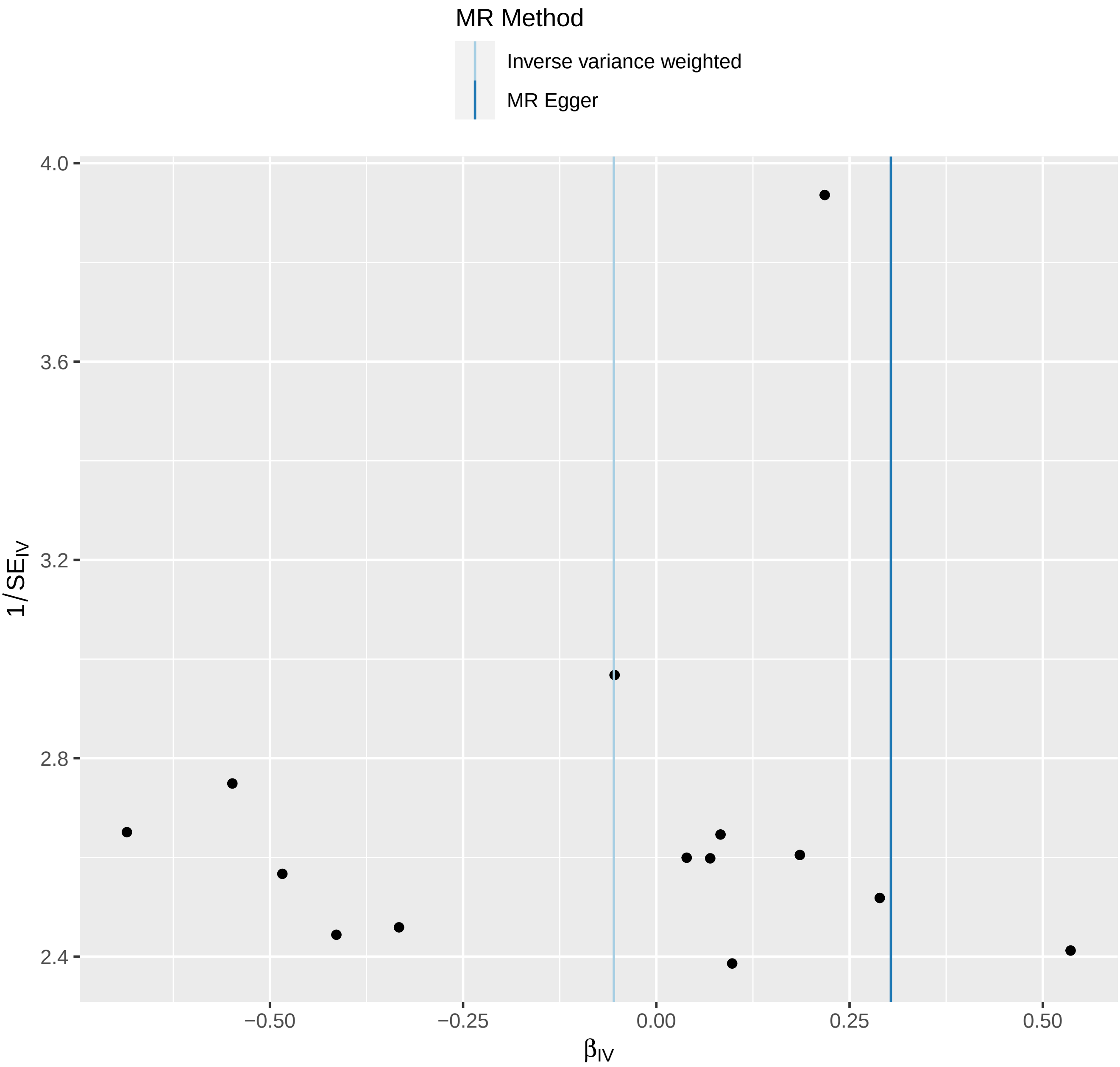

Batch 2−3883 : Gut microbiota abundance (genus Catenibacterium id.2153) on 39S ribosomal protein L33, mitochondrial with a threshold of 1e−05

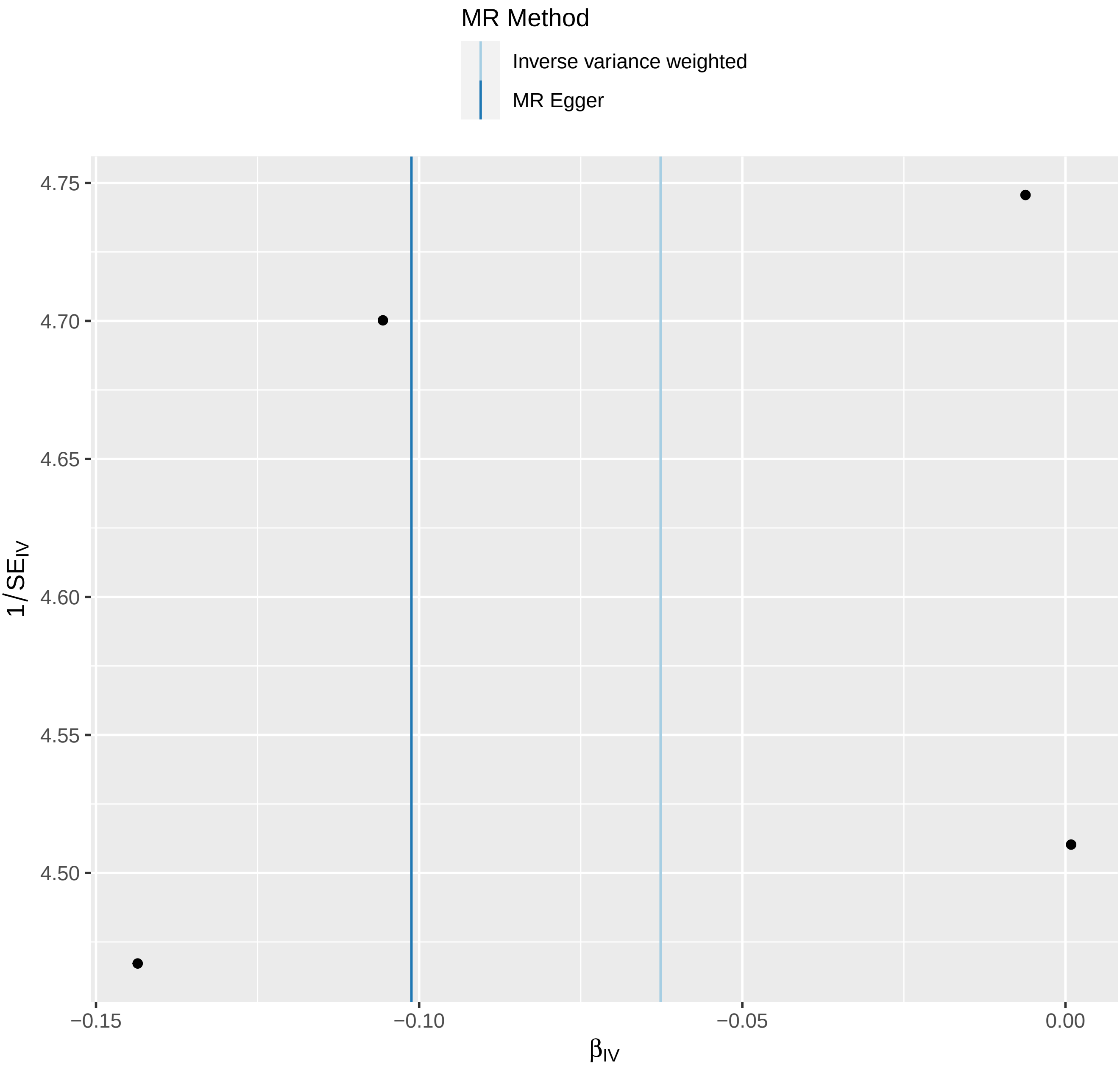

Batch 2−3884 : Gut microbiota abundance (genus Parasutterella id.2892) on 39S ribosomal protein L33, mitochondrial
with a threshold of 1e−05

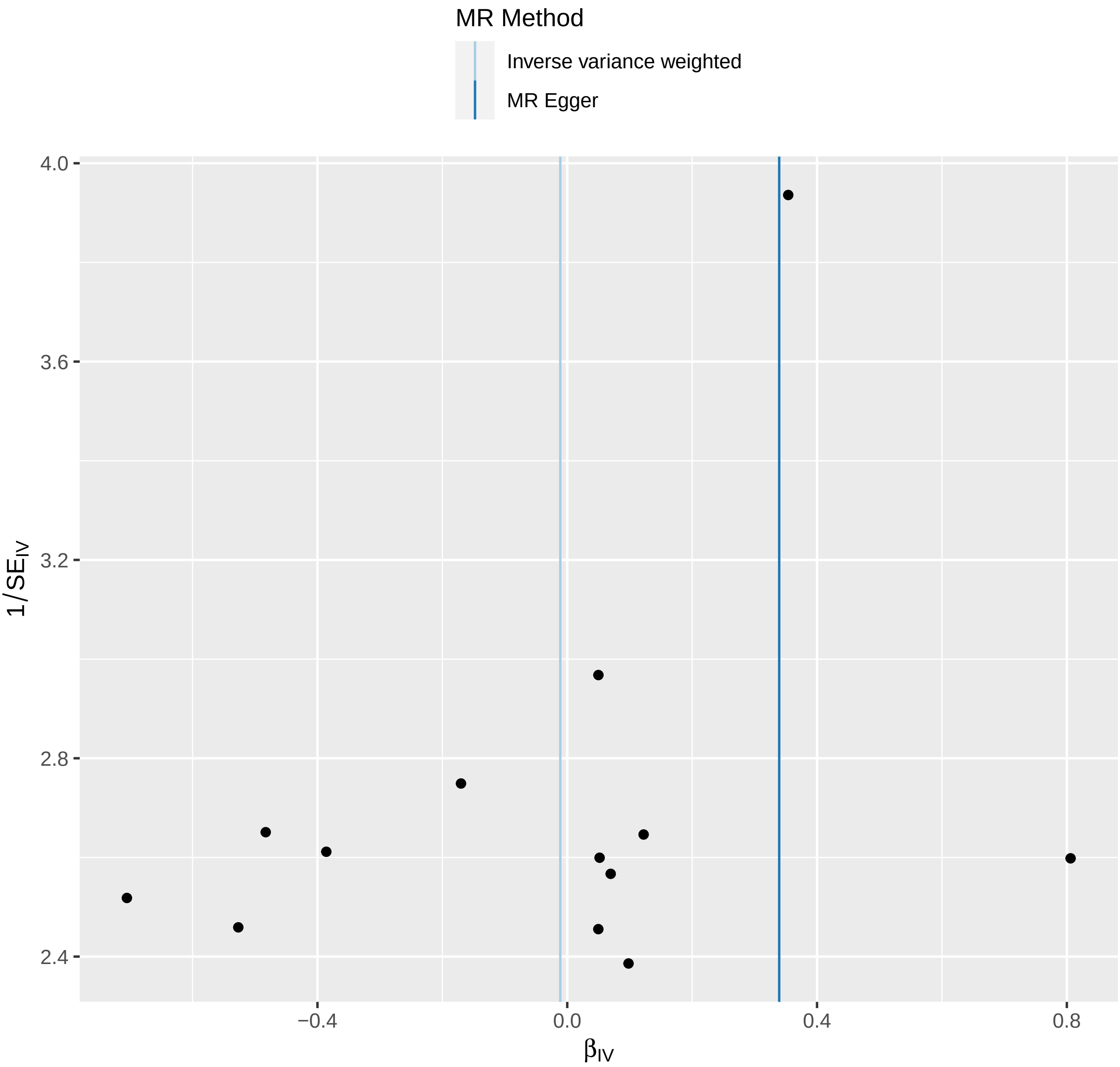

Batch 2−3885 : Gut microbiota abundance (genus Catenibacterium id.2153) on 39S ribosomal protein L34, mitochondrial
with a threshold of 1e−05

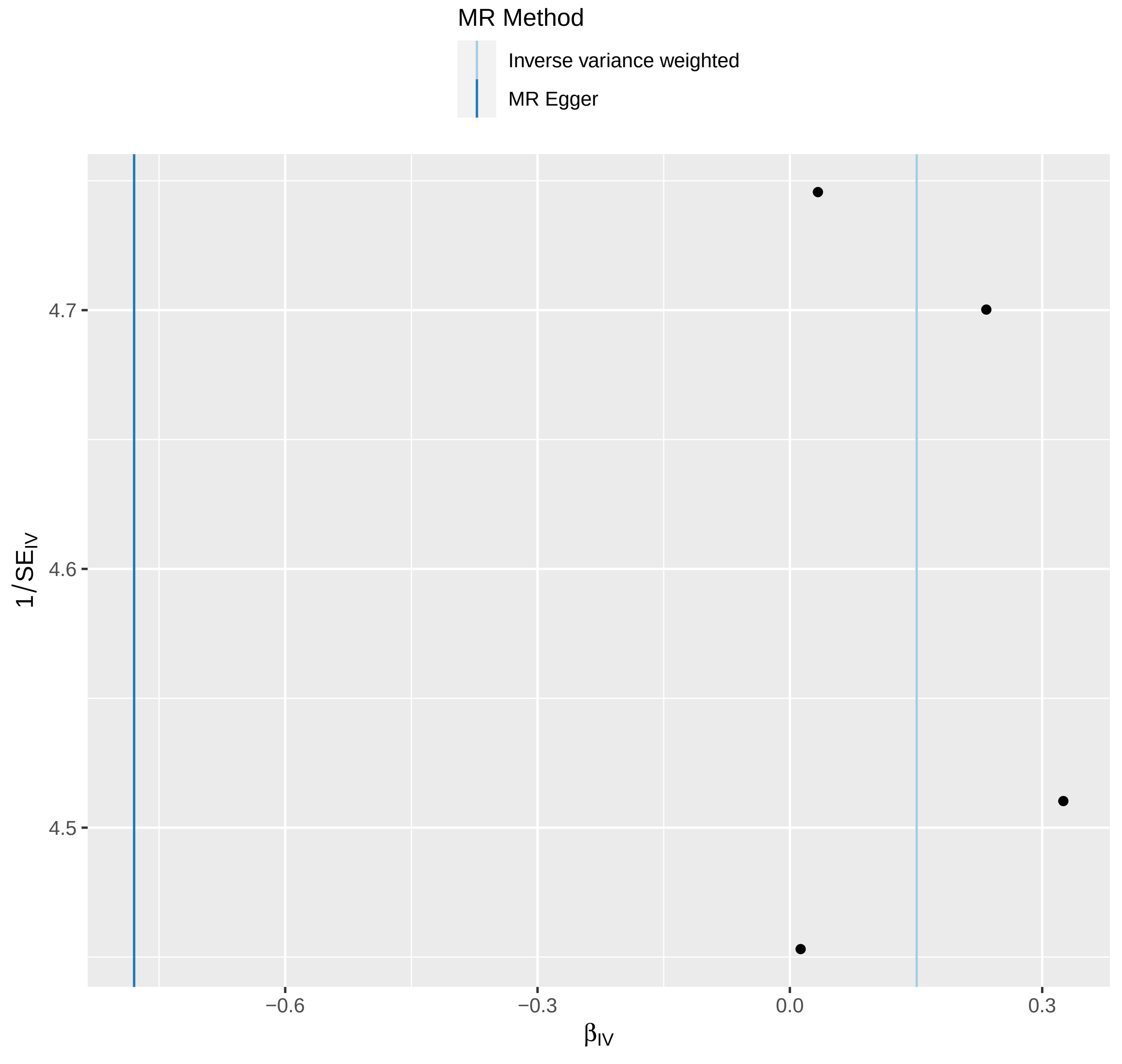

Batch 2−3886 : Gut microbiota abundance (genus Parasutterella id.2892) on 39S ribosomal protein L34, mitochondrial
with a threshold of 1e−05

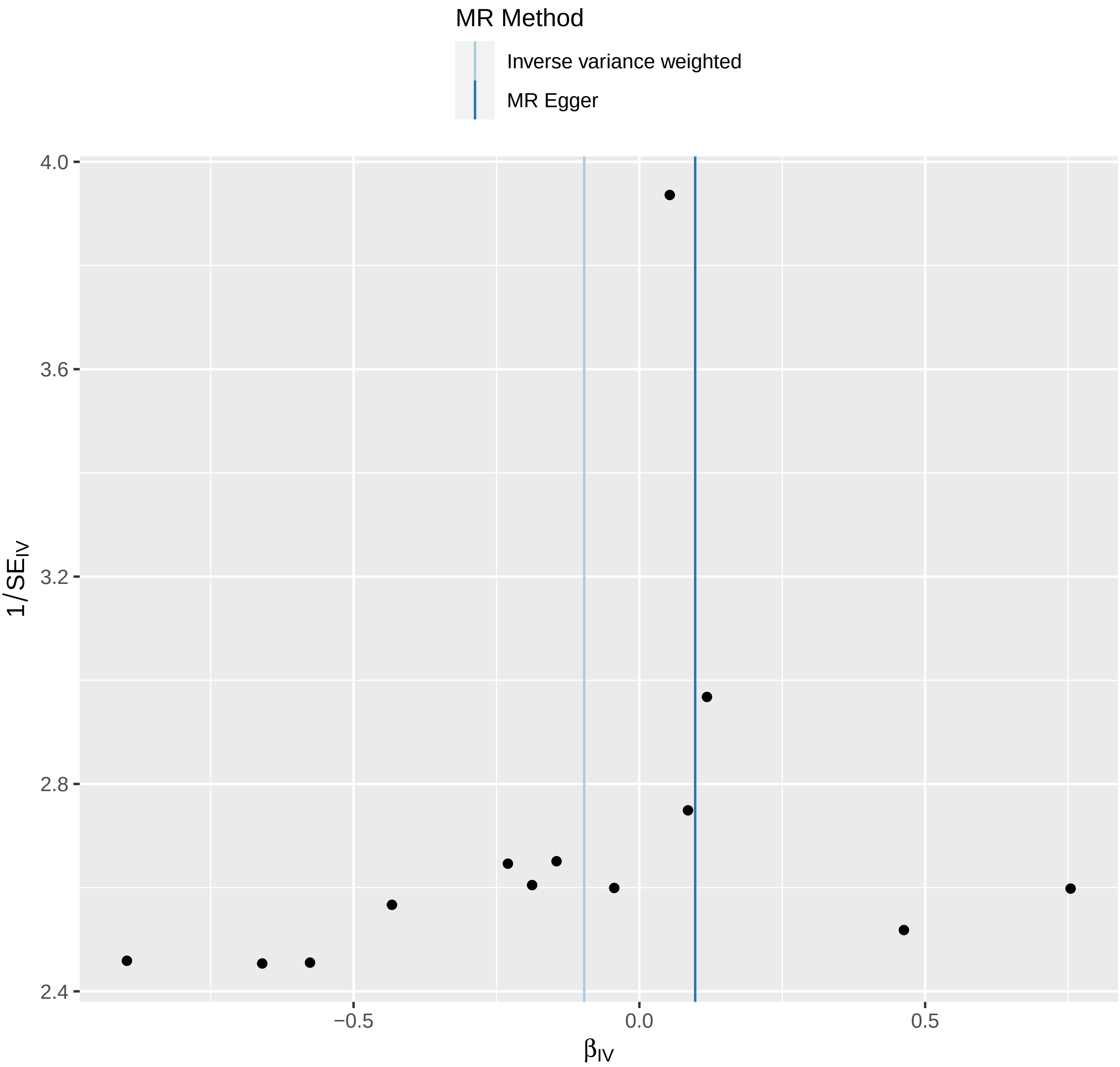

Batch 2−3887 : Gut microbiota abundance (genus Catenibacterium id.2153) on 39S ribosomal protein L52, mitochondrial
with a threshold of 1e−05

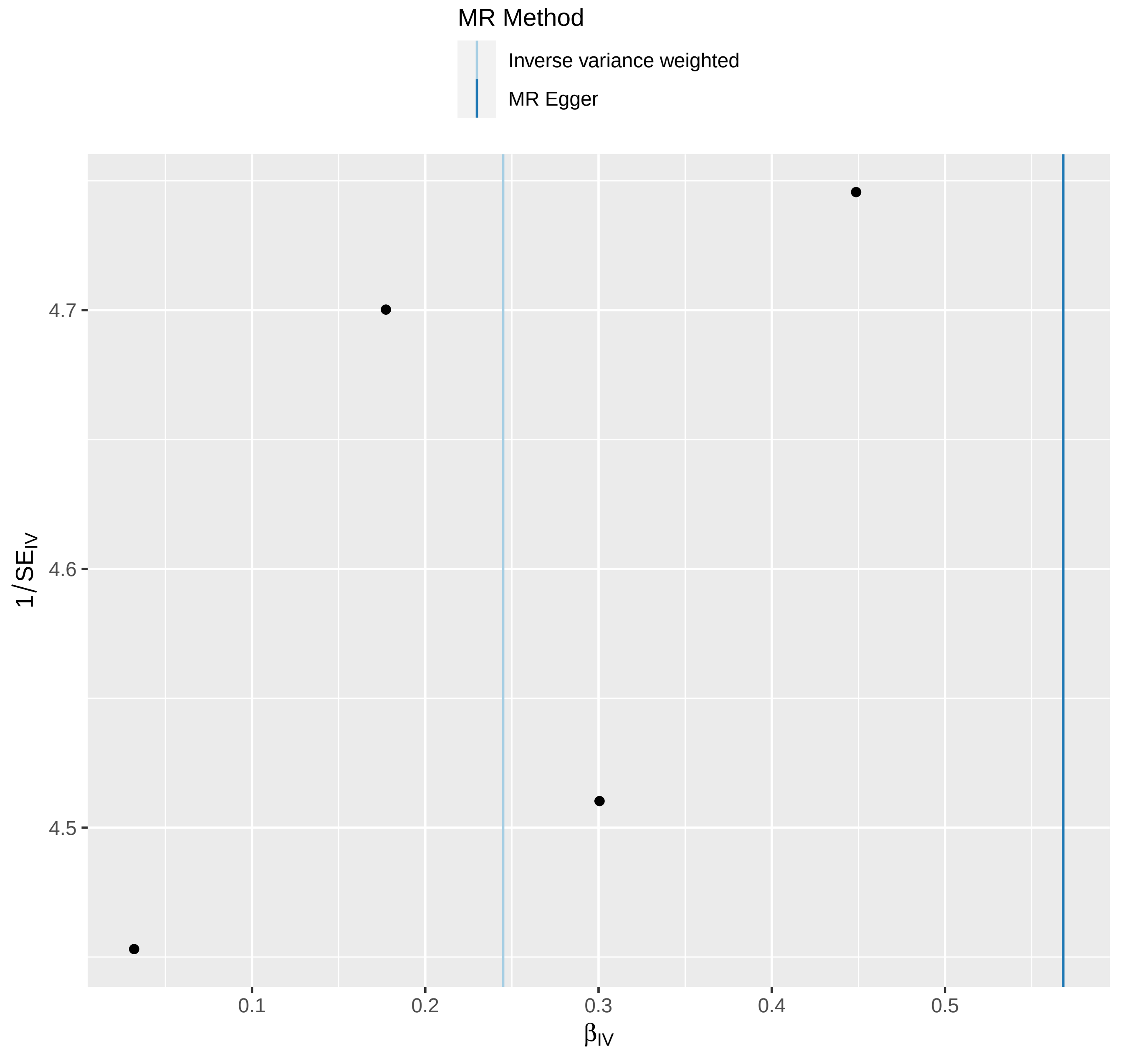

Batch 2−3888 : Gut microbiota abundance (genus Parasutterella id.2892) on 39S ribosomal protein L52, mitochondrial
with a threshold of 1e−05

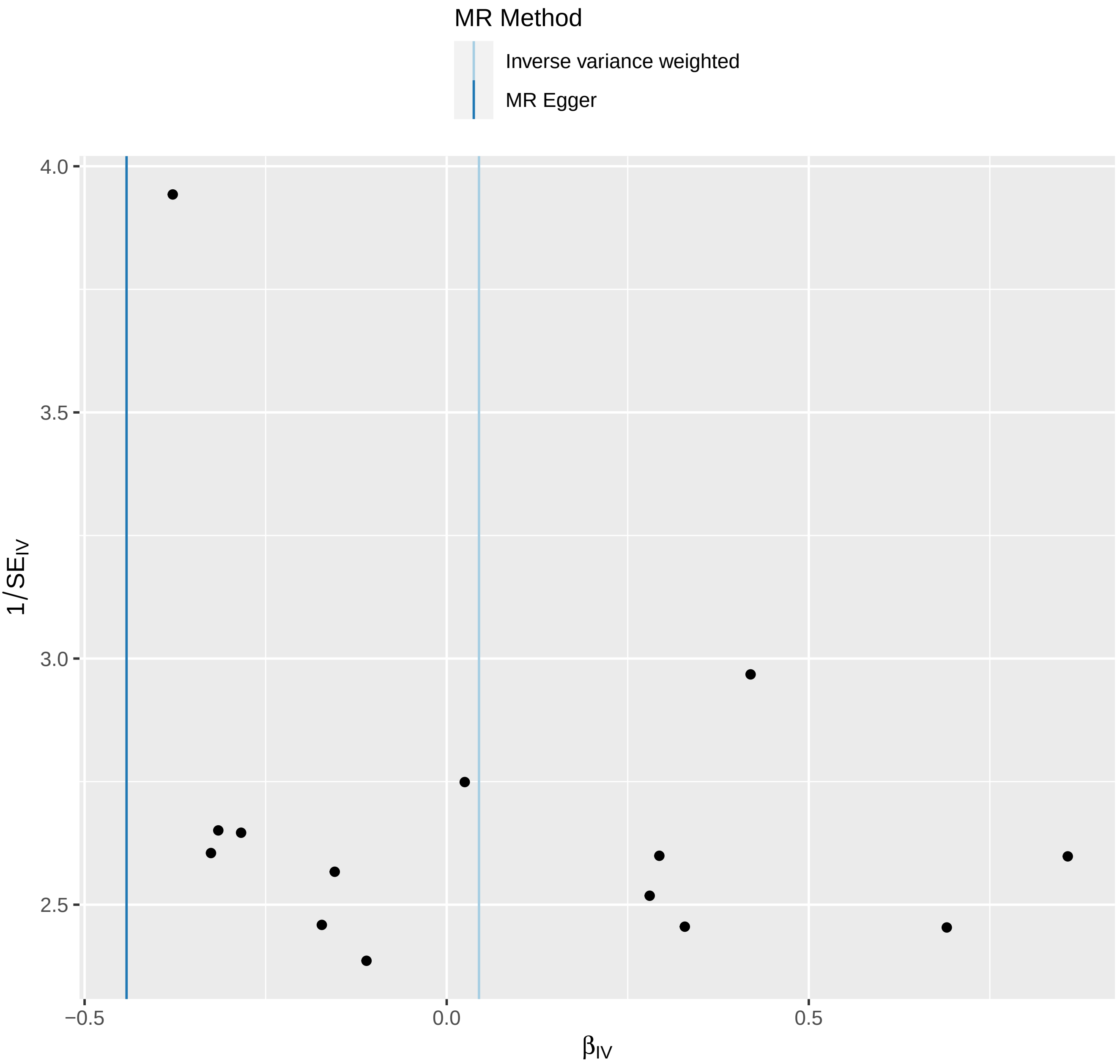

Batch 2−3889 : Gut microbiota abundance (genus Catenibacterium id.2153) on Ribosome−recycling factor, mitochondrial
with a threshold of 1e−05

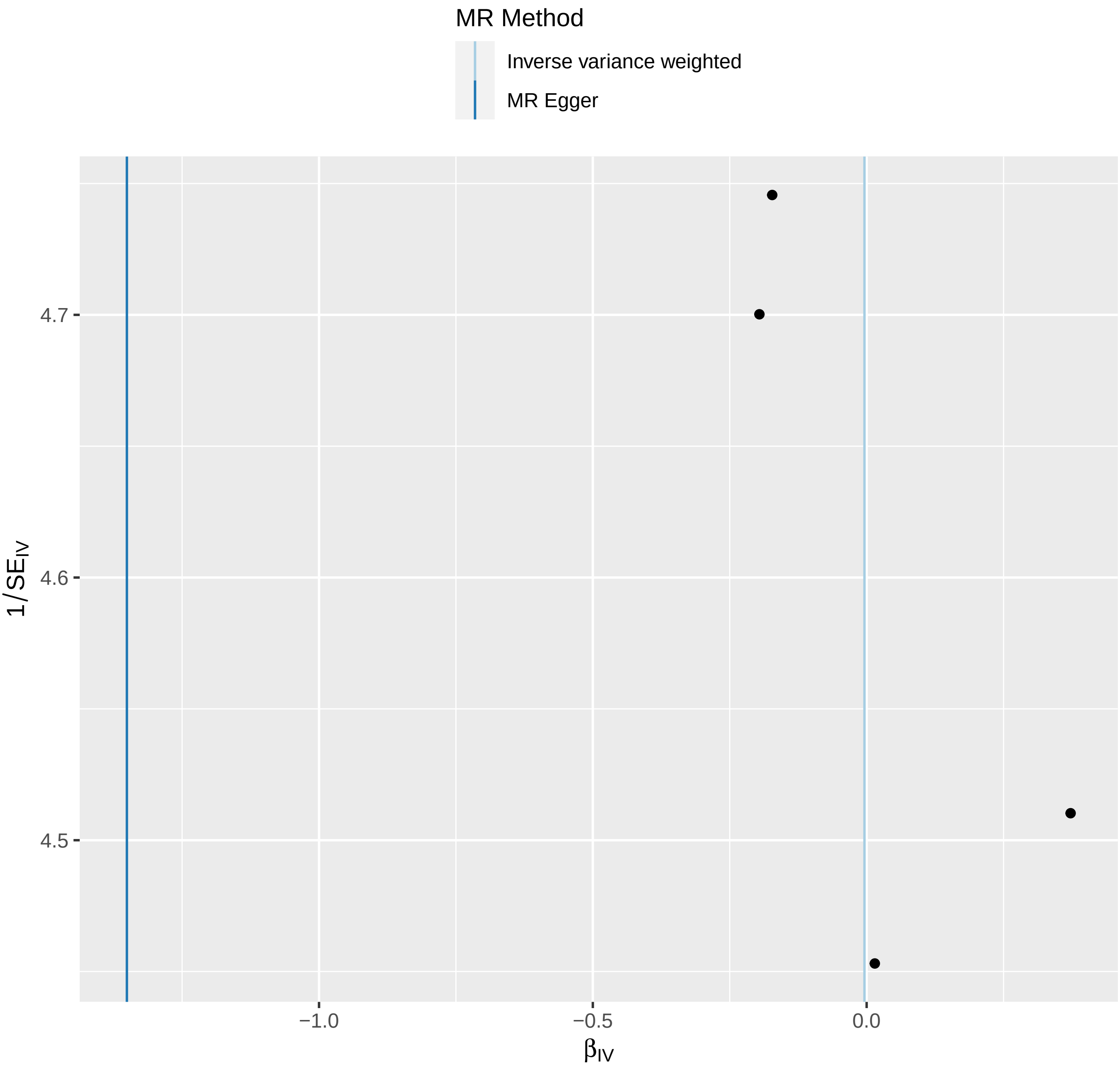

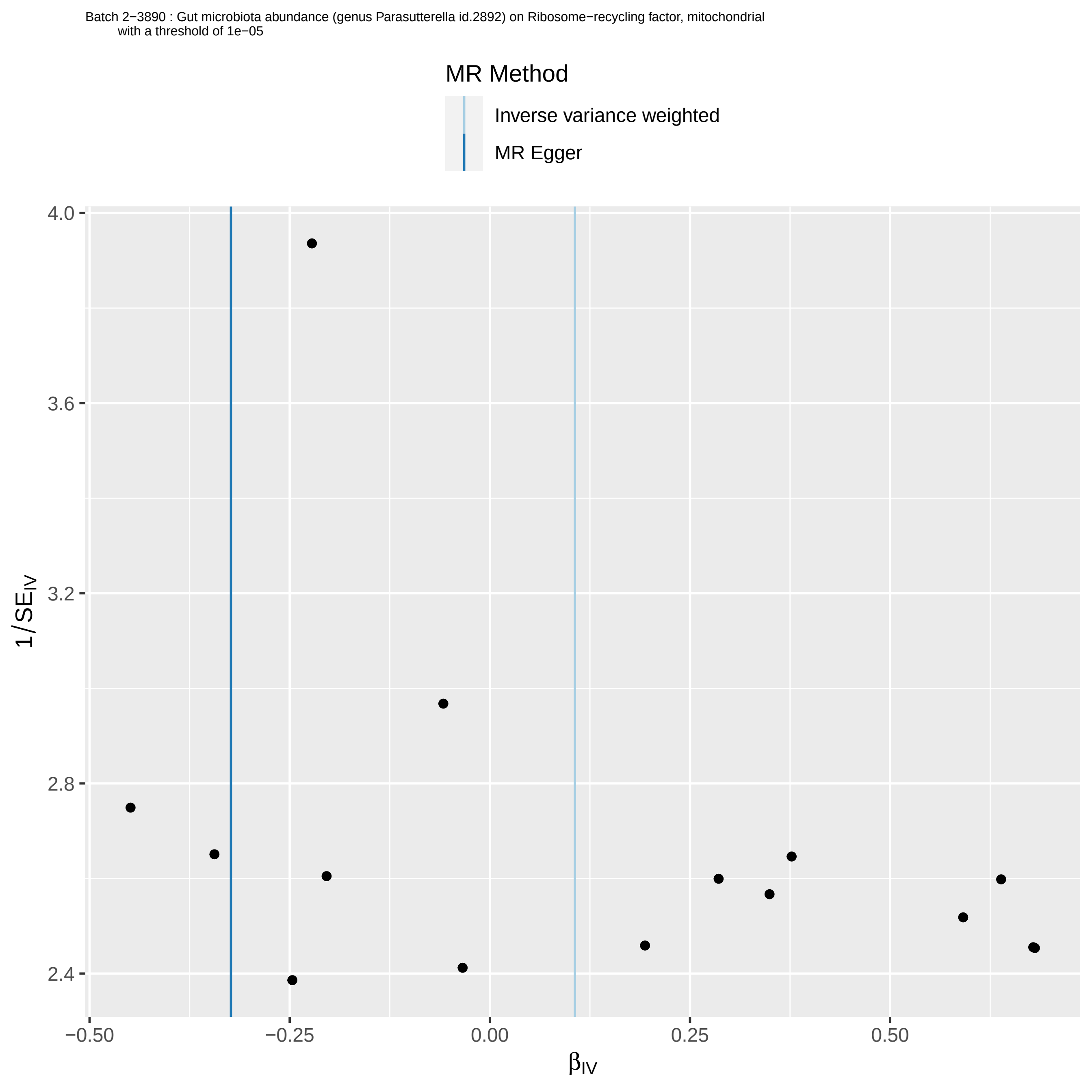
Batch 2−3890 : Gut microbiota abundance (genus Parasutterella id.2892) on Ribosome−recycling factor, mitochondrial
with a threshold of 1e−05
MR Method
Inverse variance weighted
MR Egger
4.0
3.6
3.2
2.8
2.4
1/SE_IV
−0.50
−0.25
0.00
0.25
0.50
β_IV

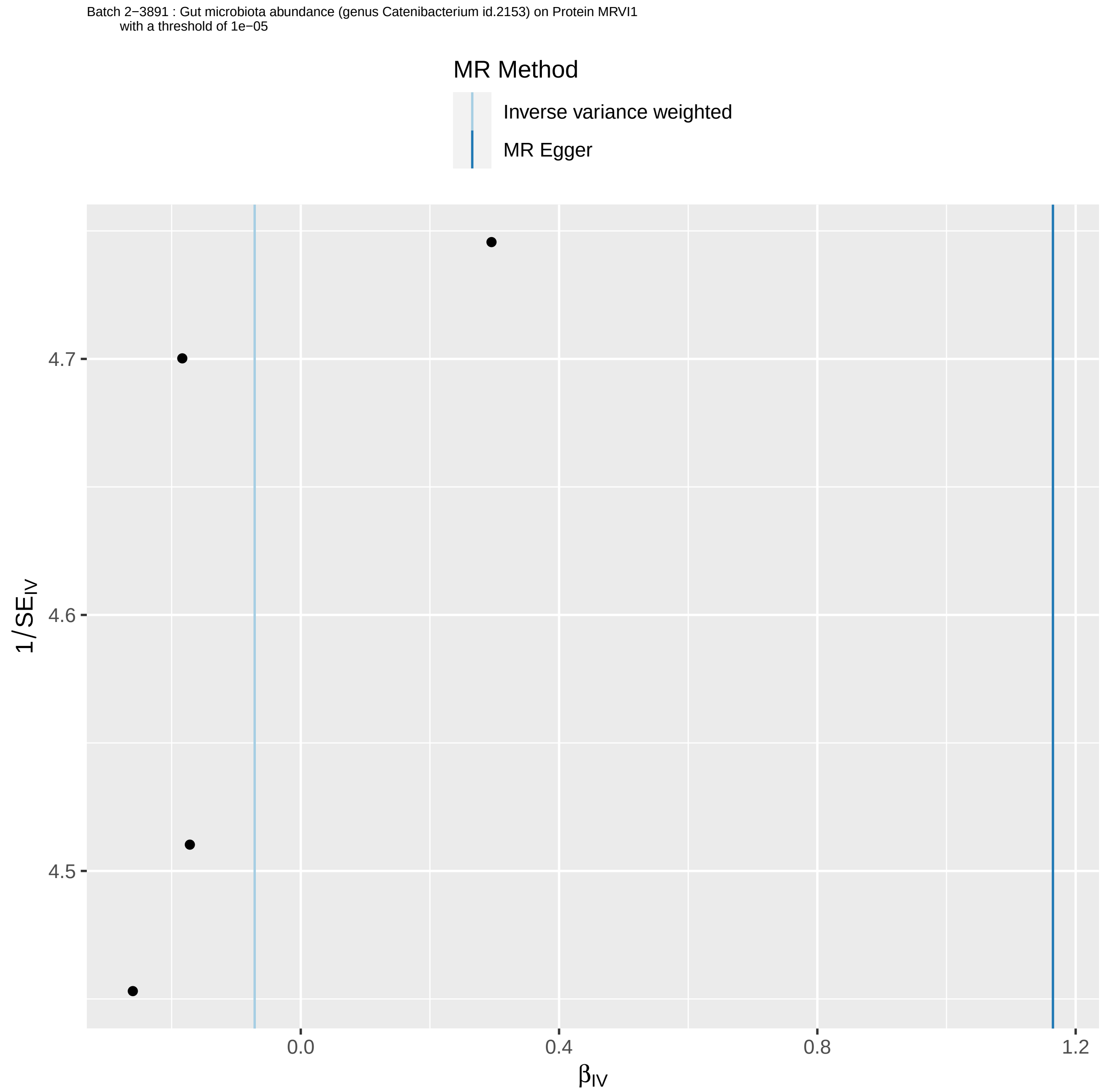
Batch 2−3891 : Gut microbiota abundance (genus Catenibacterium id.2153) on Protein MRVI1
with a threshold of 1e−05
MR Method
Inverse variance weighted
MR Egger
4.7
4.6
4.5
1/SE_IV
0.0
0.4
0.8
1.2
β_IV

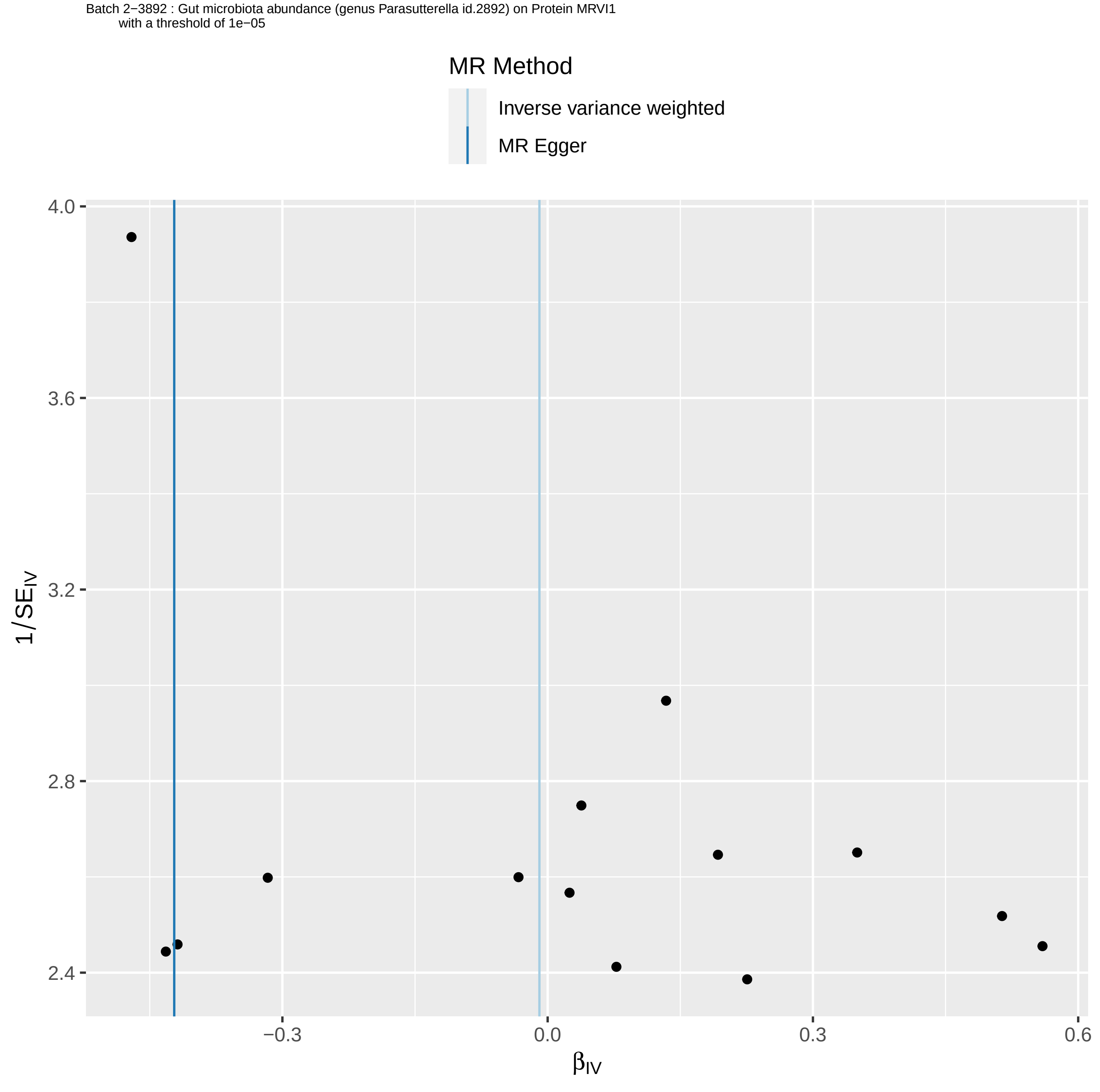

Batch 2−3892 : Gut microbiota abundance (genus Parasutterella id.2892) on Protein MRVI1
with a threshold of 1e−05
MR Method
Inverse variance weighted
MR Egger
4.0
3.6
3.2
2.8
2.4
1/SE_IV
−0.3
0.0
0.3
0.6
β_IV

Batch 2−3893 : Gut microbiota abundance (genus Catenibacterium id.2153) on Mesothelin
with a threshold of 1e−05

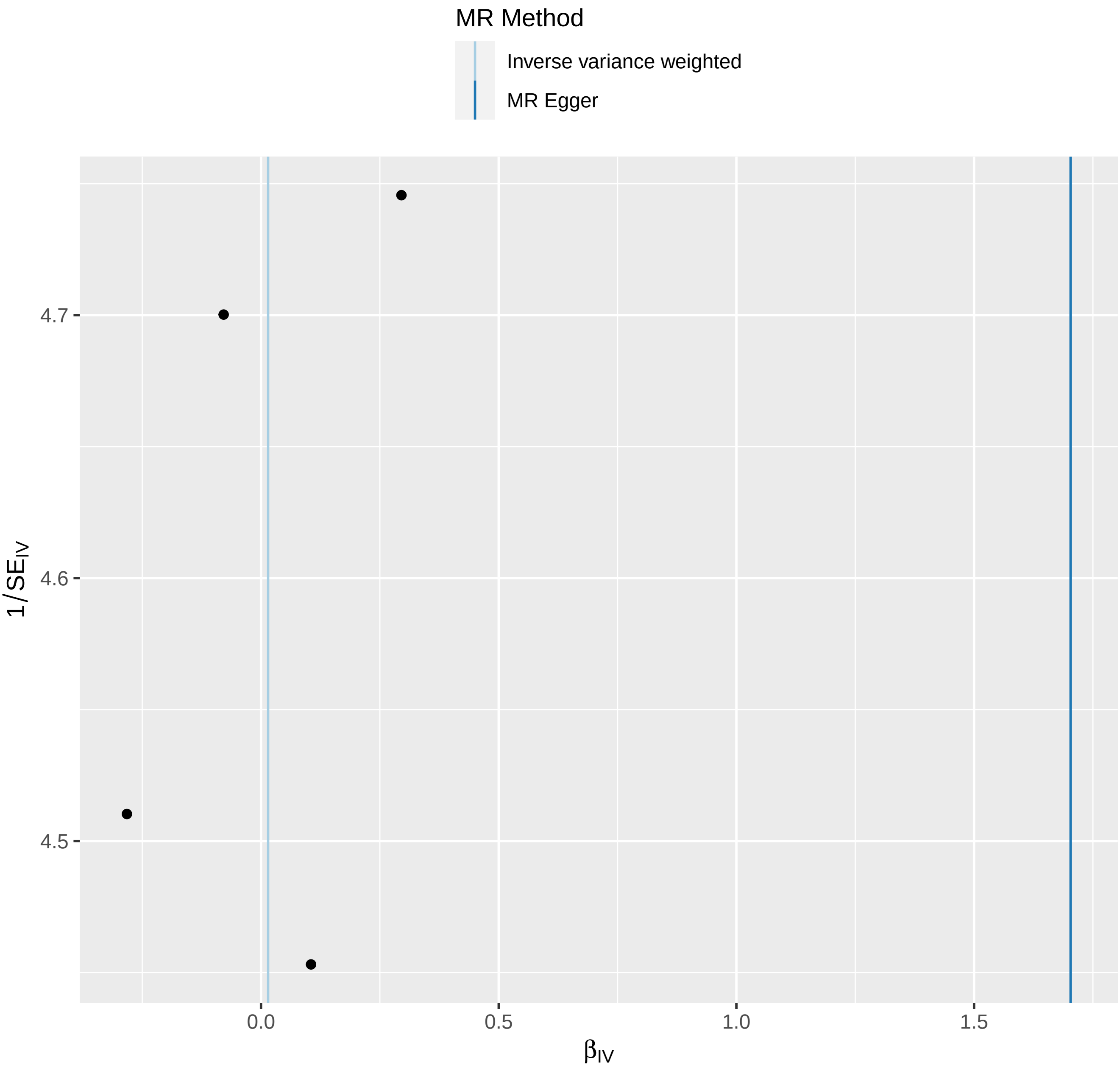

Batch 2−3894 : Gut microbiota abundance (genus Parasutterella id.2892) on Mesothelin
with a threshold of 1e−05

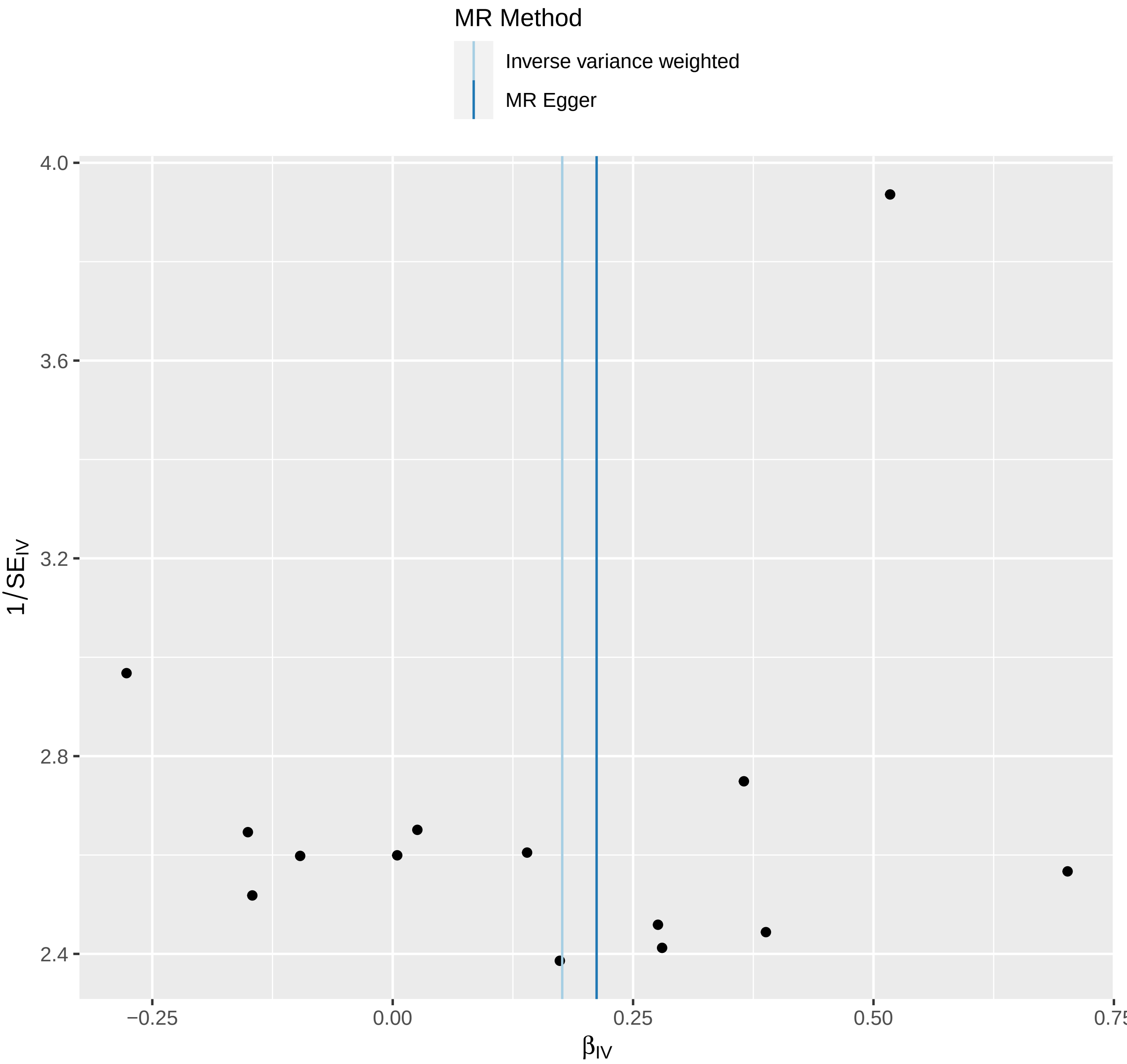

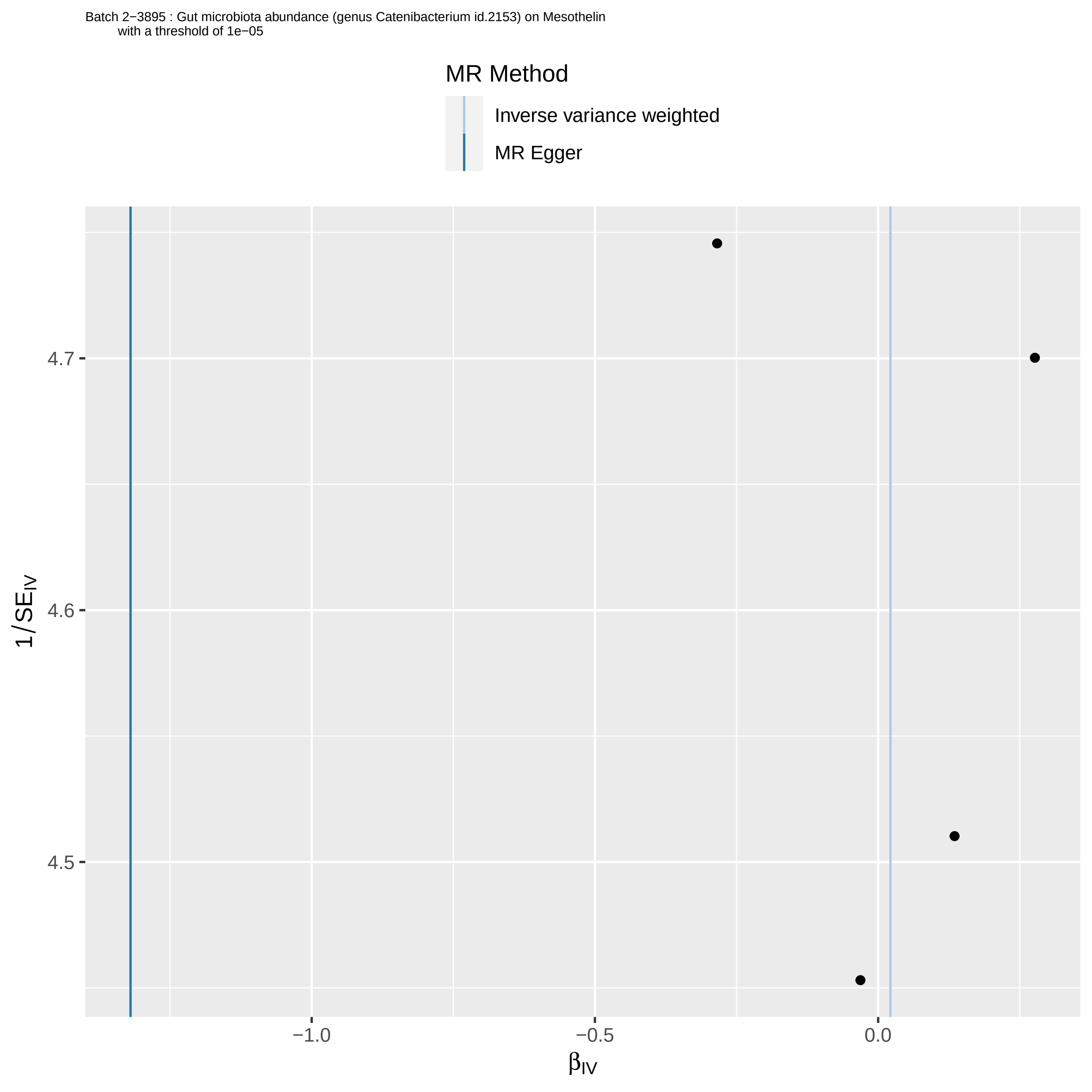

Batch 2−3895 : Gut microbiota abundance (genus Catenibacterium id.2153) on Mesothelin
with a threshold of 1e−05
MR Method
Inverse variance weighted
MR Egger
4.7
4.6
4.5
1/SE_IV
−1.0
−0.5
0.0
β_IV

Batch 2−3896 : Gut microbiota abundance (genus Parasutterella id.2892) on Mesothelin
with a threshold of 1e−05

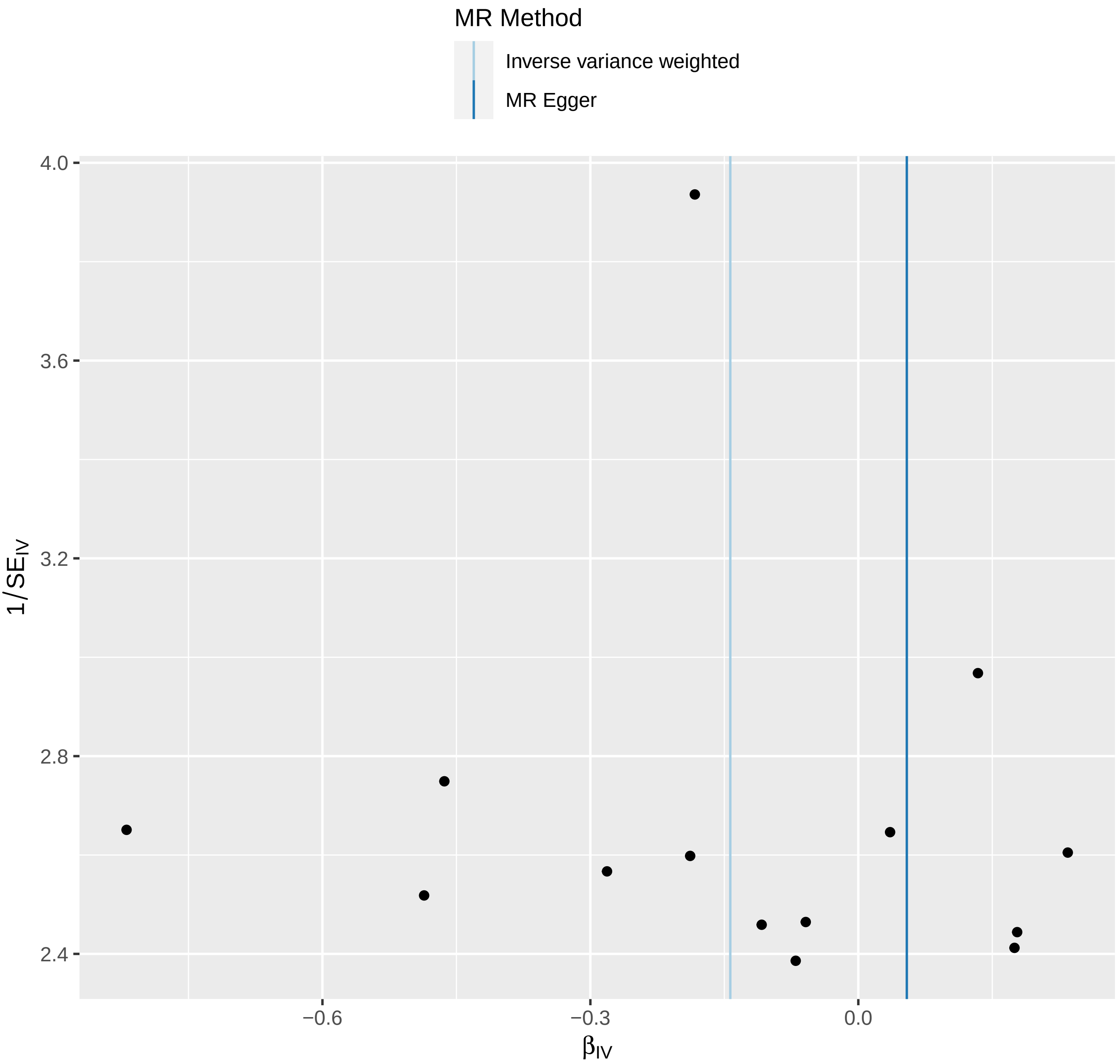

Batch 2−3897 : Gut microbiota abundance (genus Catenibacterium id.2153) on Beta−microseminoprotein
with a threshold of 1e−05

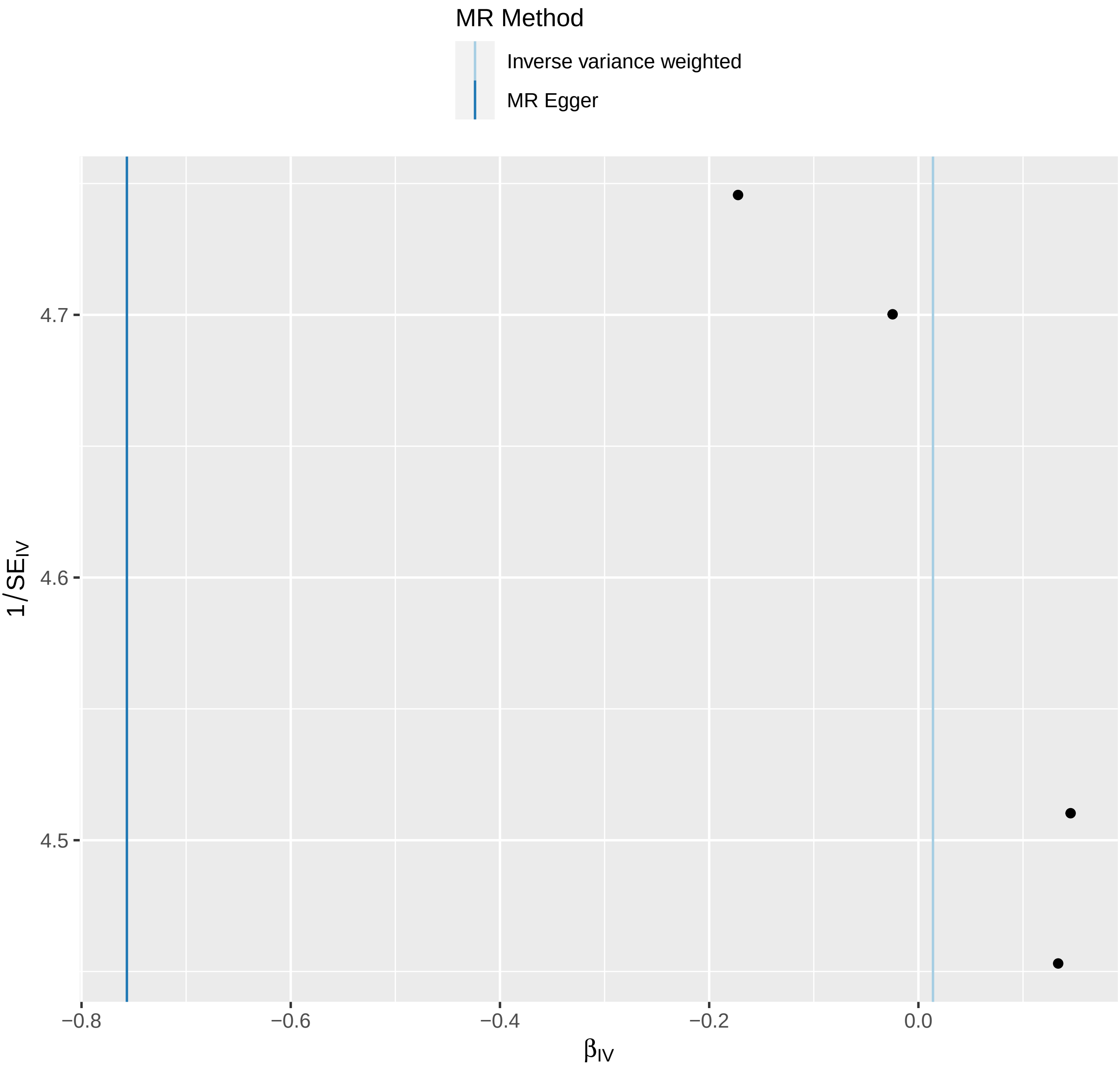

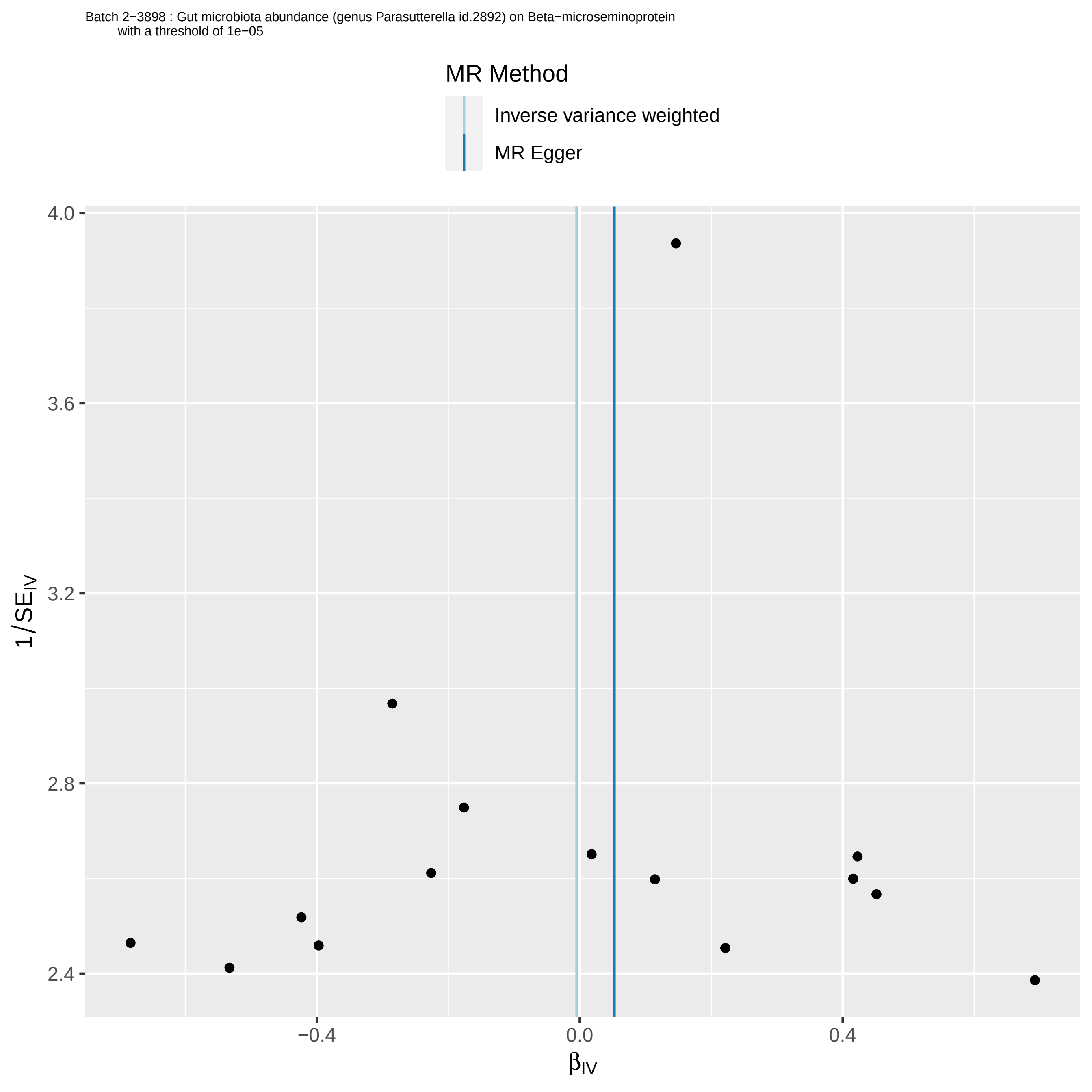

Batch 2−3898 : Gut microbiota abundance (genus Parasutterella id.2892) on Beta−microseminoprotein
with a threshold of 1e−05
MR Method
Inverse variance weighted
MR Egger
4.0
3.6
3.2
2.8
2.4
1/SE_IV
−0.4
0.0
0.4
β_IV

Batch 2−3899 : Gut microbiota abundance (genus Catenibacterium id.2153) on Prostate−associated microseminoprotein
with a threshold of 1e−05

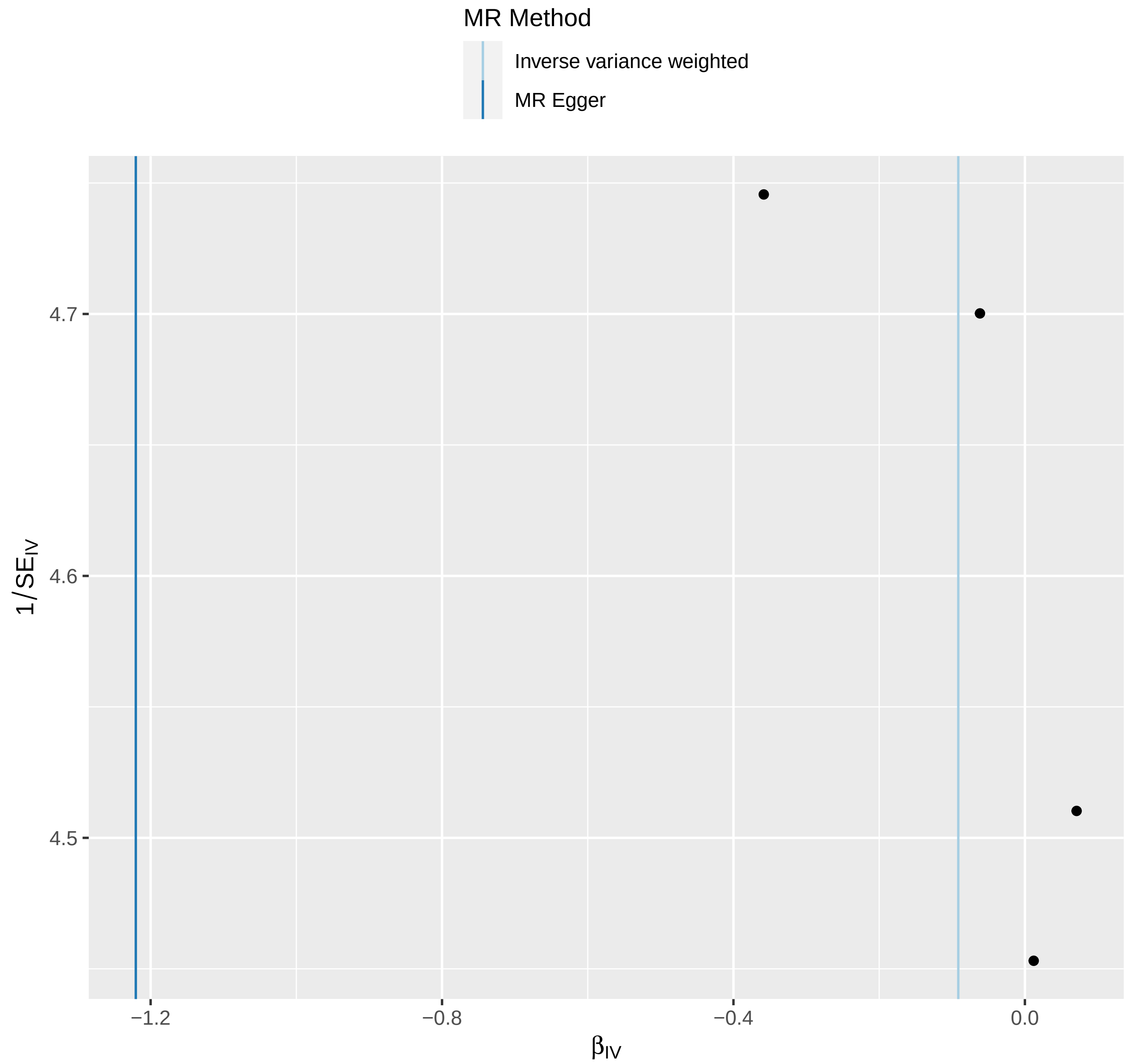

Batch 2−3900 : Gut microbiota abundance (genus Parasutterella id.2892) on Prostate−associated microseminoprotein
with a threshold of 1e−05

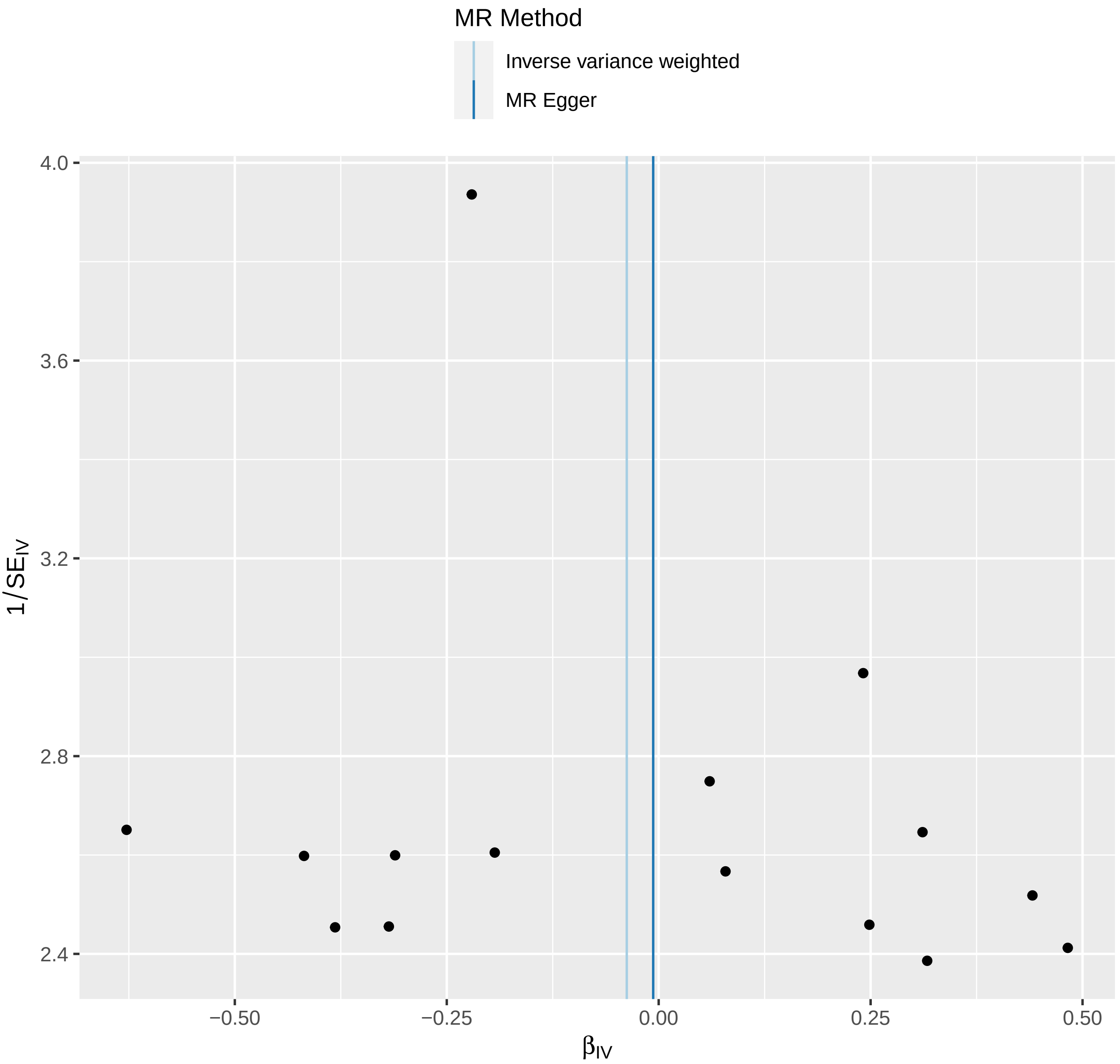

Batch 2−3901 : Gut microbiota abundance (genus Catenibacterium id.2153) on Macrophage scavenger receptor types I and II
with a threshold of 1e−05

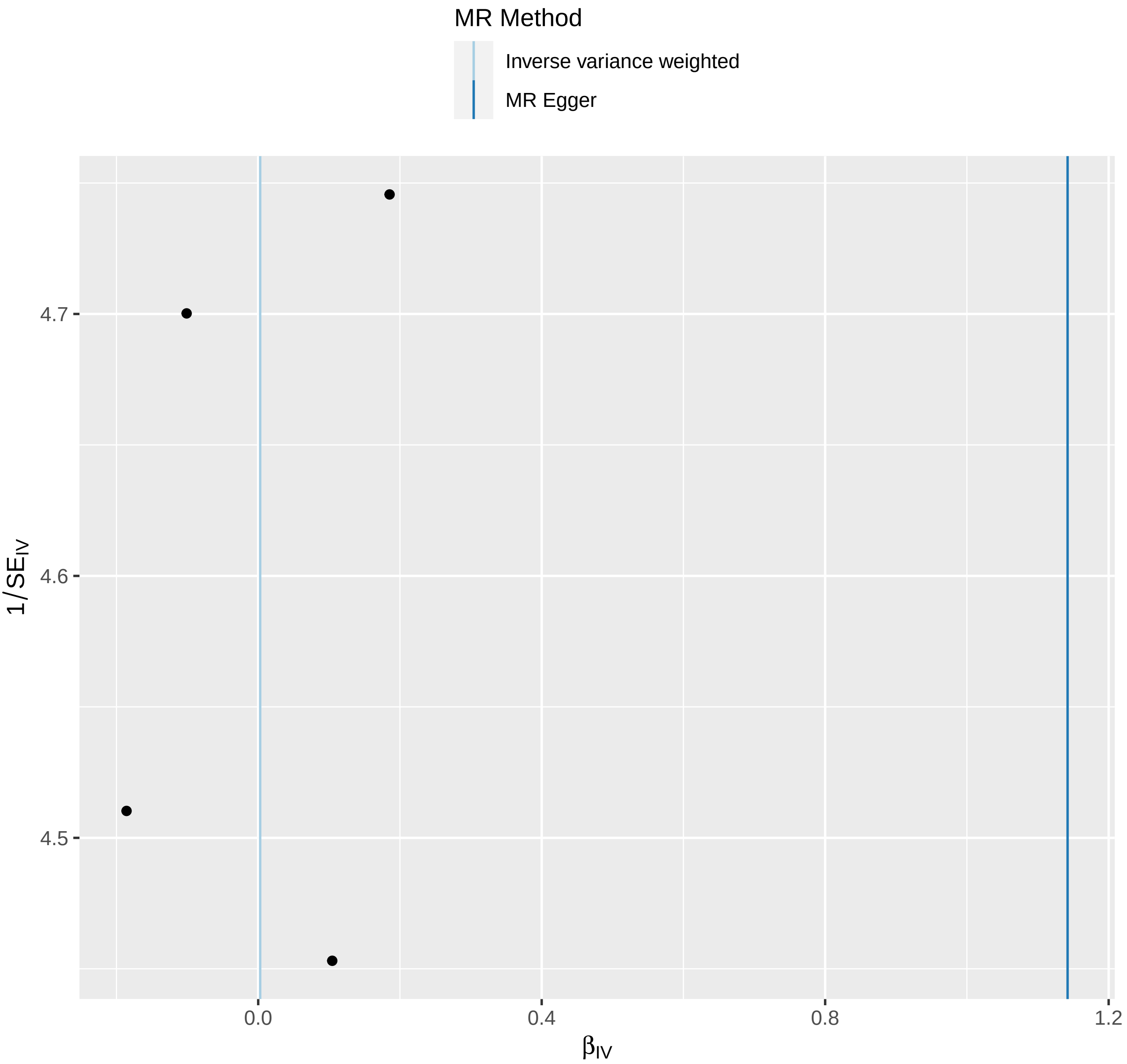

Batch 2−3902 : Gut microbiota abundance (genus Parasutterella id.2892) on Macrophage scavenger receptor types I and II with a threshold of 1e−05

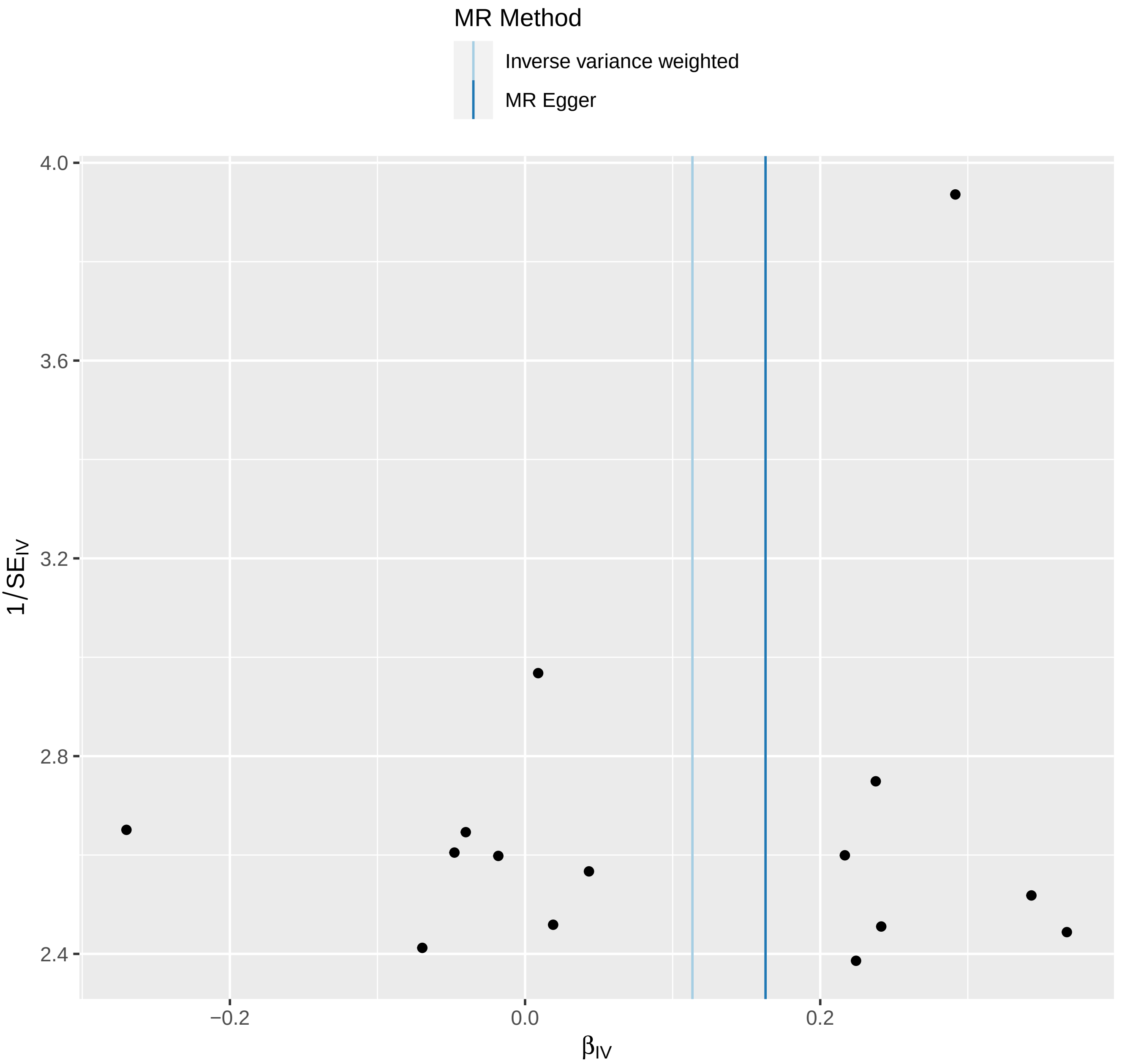

Batch 2−3903 : Gut microbiota abundance (genus Catenibacterium id.2153) on Macrophage scavenger receptor types I and II
with a threshold of 1e−05

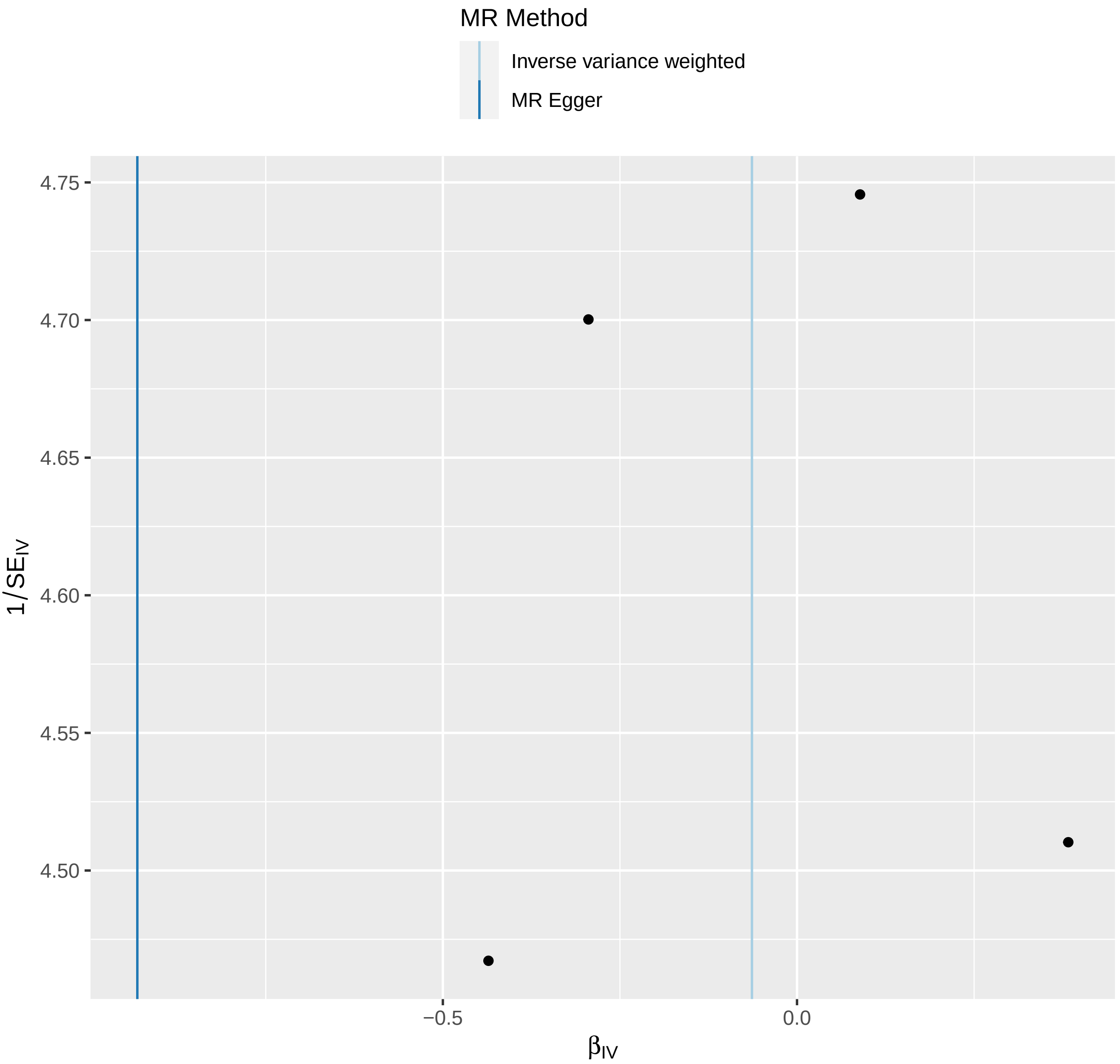

Batch 2−3904 : Gut microbiota abundance (genus Parasutterella id.2892) on Macrophage scavenger receptor types I and II with a threshold of 1e−05

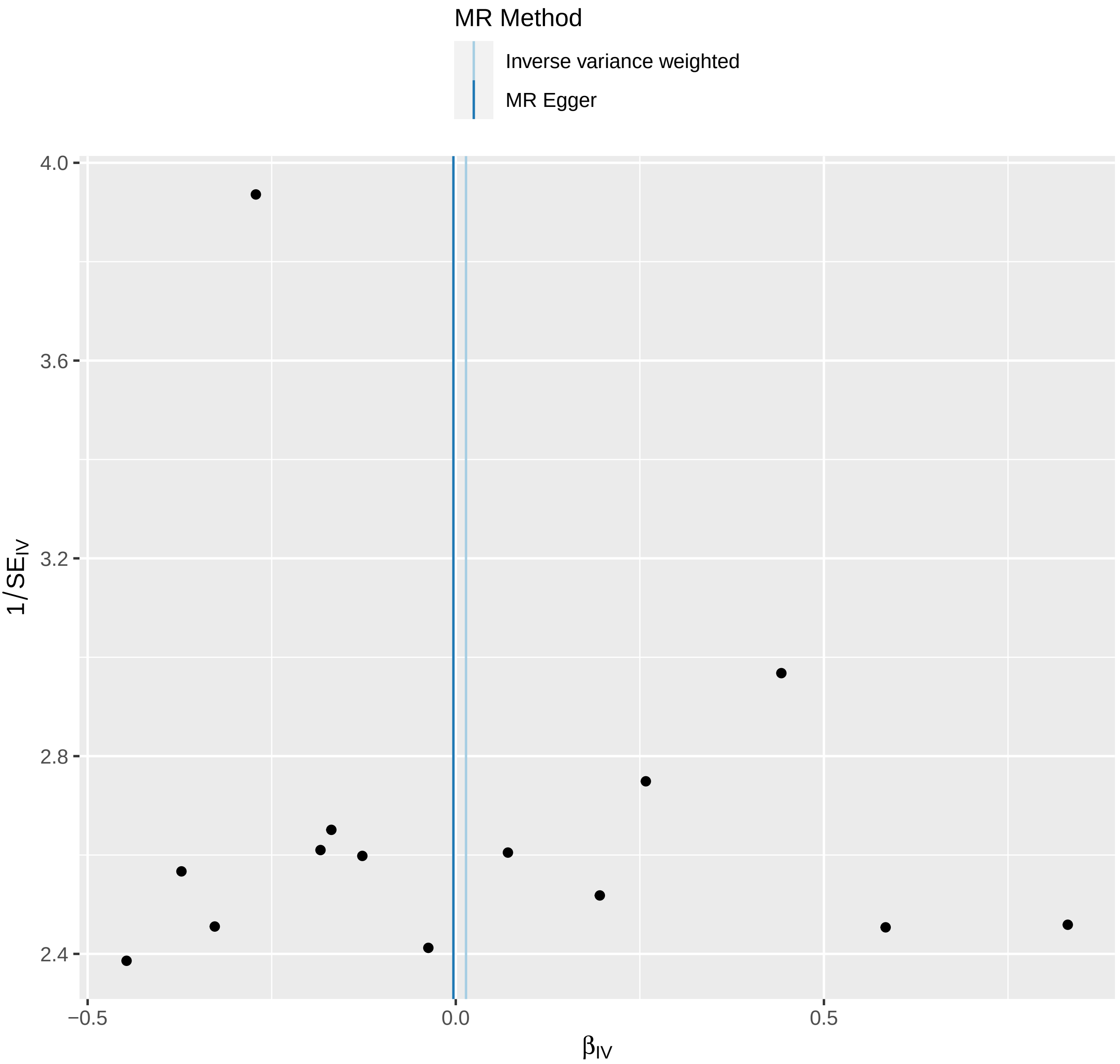

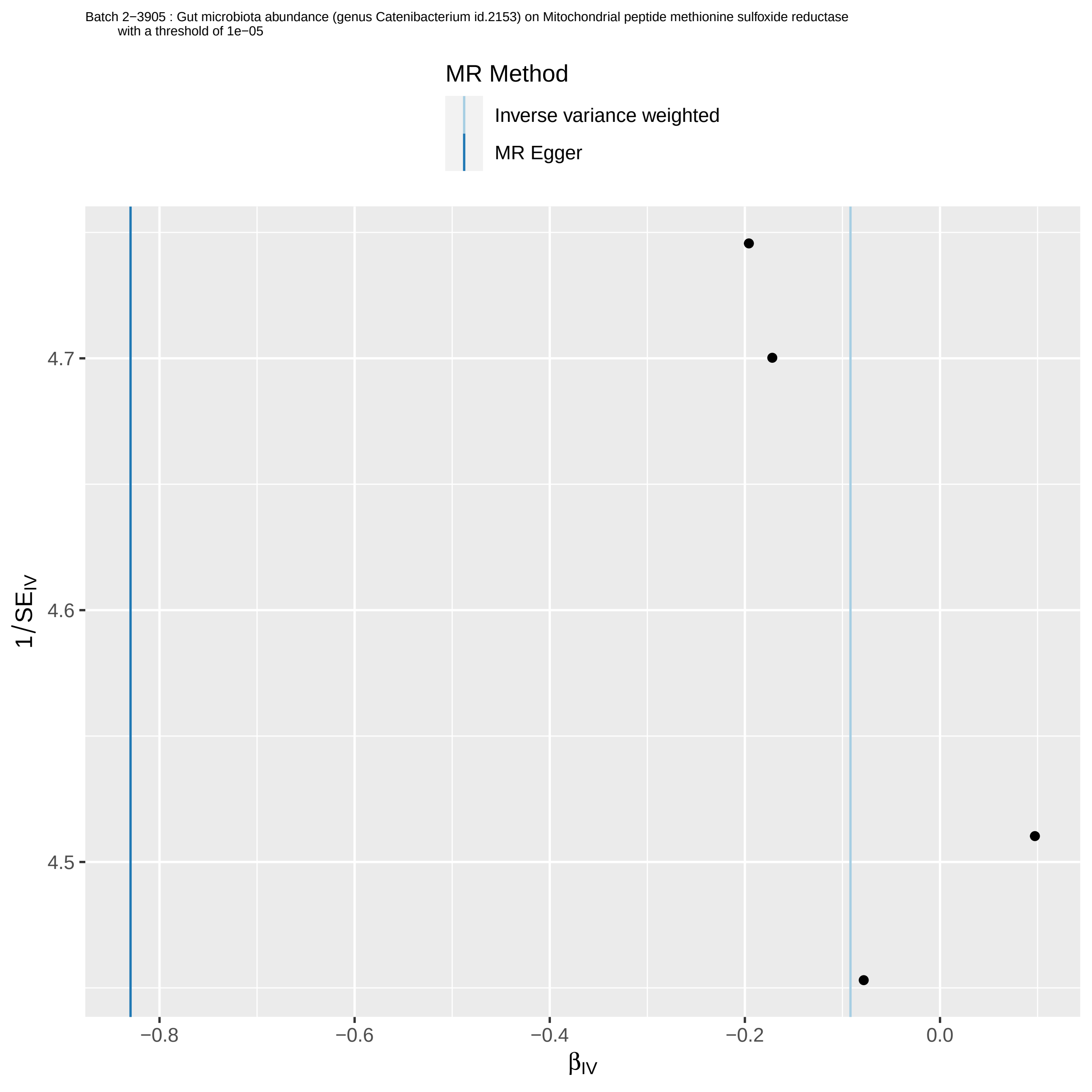
Batch 2−3905 : Gut microbiota abundance (genus Catenibacterium id.2153) on Mitochondrial peptide methionine sulfoxide reductase
with a threshold of 1e−05
MR Method
Inverse variance weighted
MR Egger
4.7
4.6
4.5
1/SE_IV
−0.8
−0.6
−0.4
−0.2
0.0
β_IV

Batch 2−3906 : Gut microbiota abundance (genus Parasutterella id.2892) on Mitochondrial peptide methionine sulfoxide reductase with a threshold of 1e−05

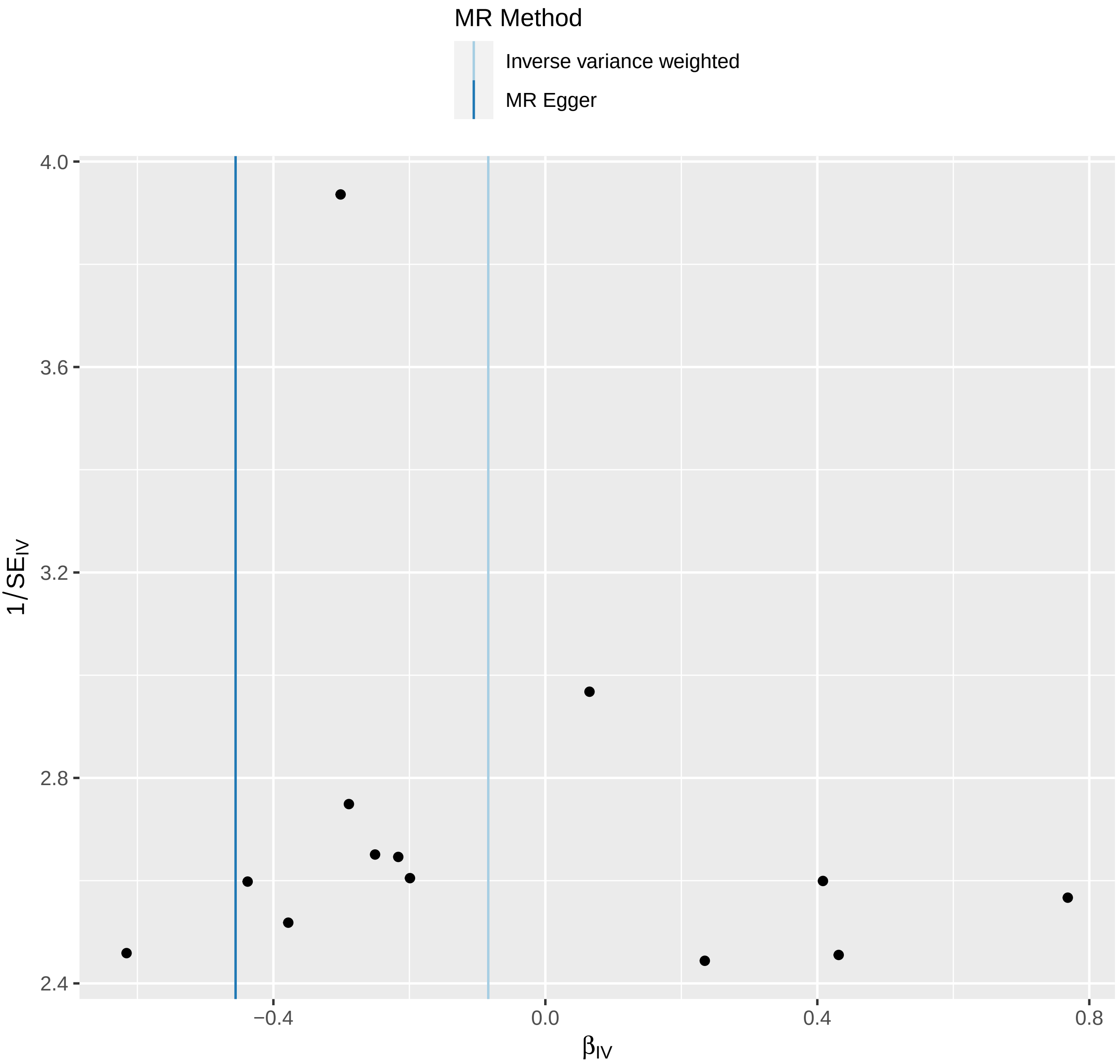

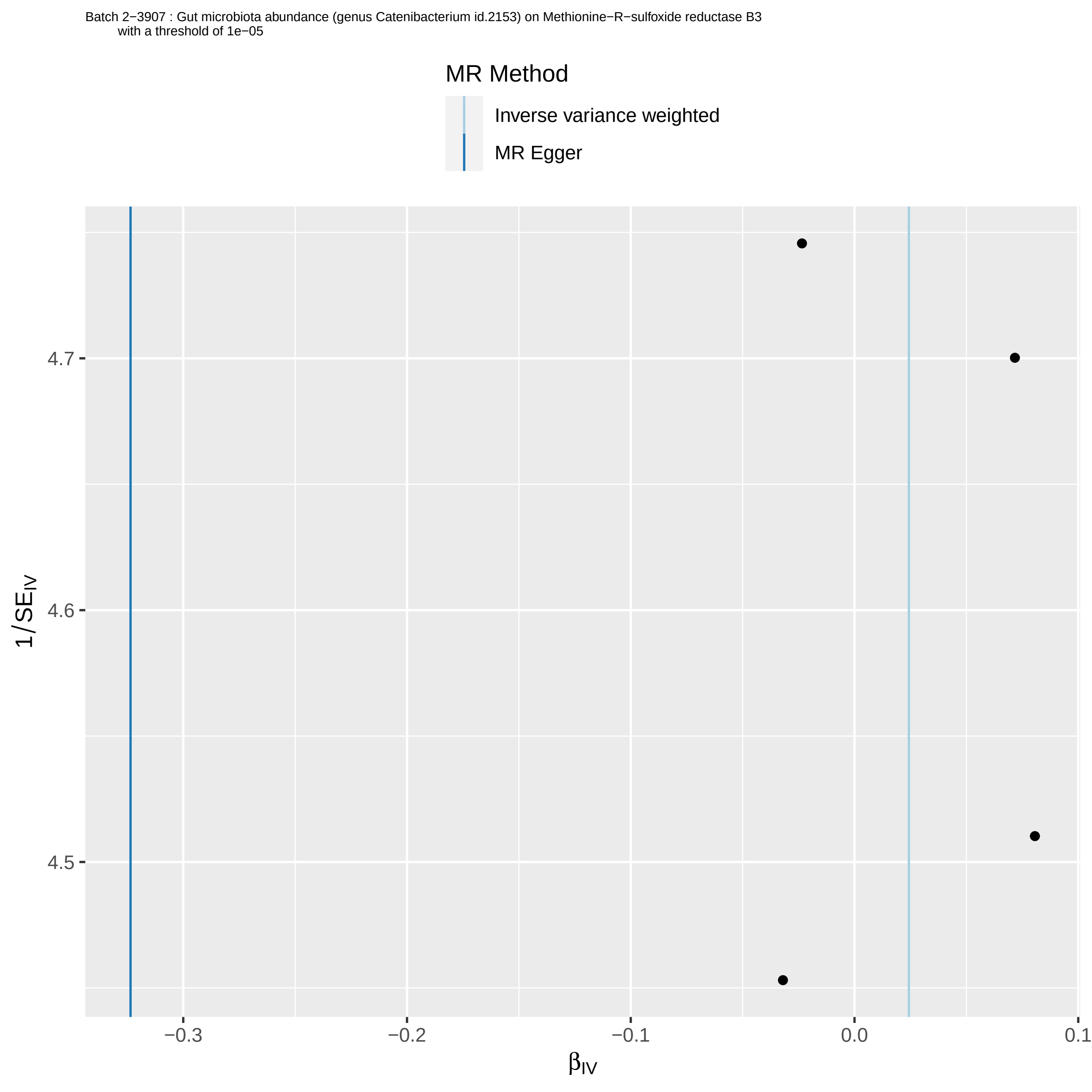

Batch 2−3907 : Gut microbiota abundance (genus Catenibacterium id.2153) on Methionine−R−sulfoxide reductase B3
with a threshold of 1e−05
MR Method
Inverse variance weighted
MR Egger
4.7
4.6
4.5
1/SEIV
−0.3
−0.2
−0.1
0.0
0.1
βIV

Batch 2−3908 : Gut microbiota abundance (genus Parasutterella id.2892) on Methionine−R−sulfoxide reductase B3
with a threshold of 1e−05

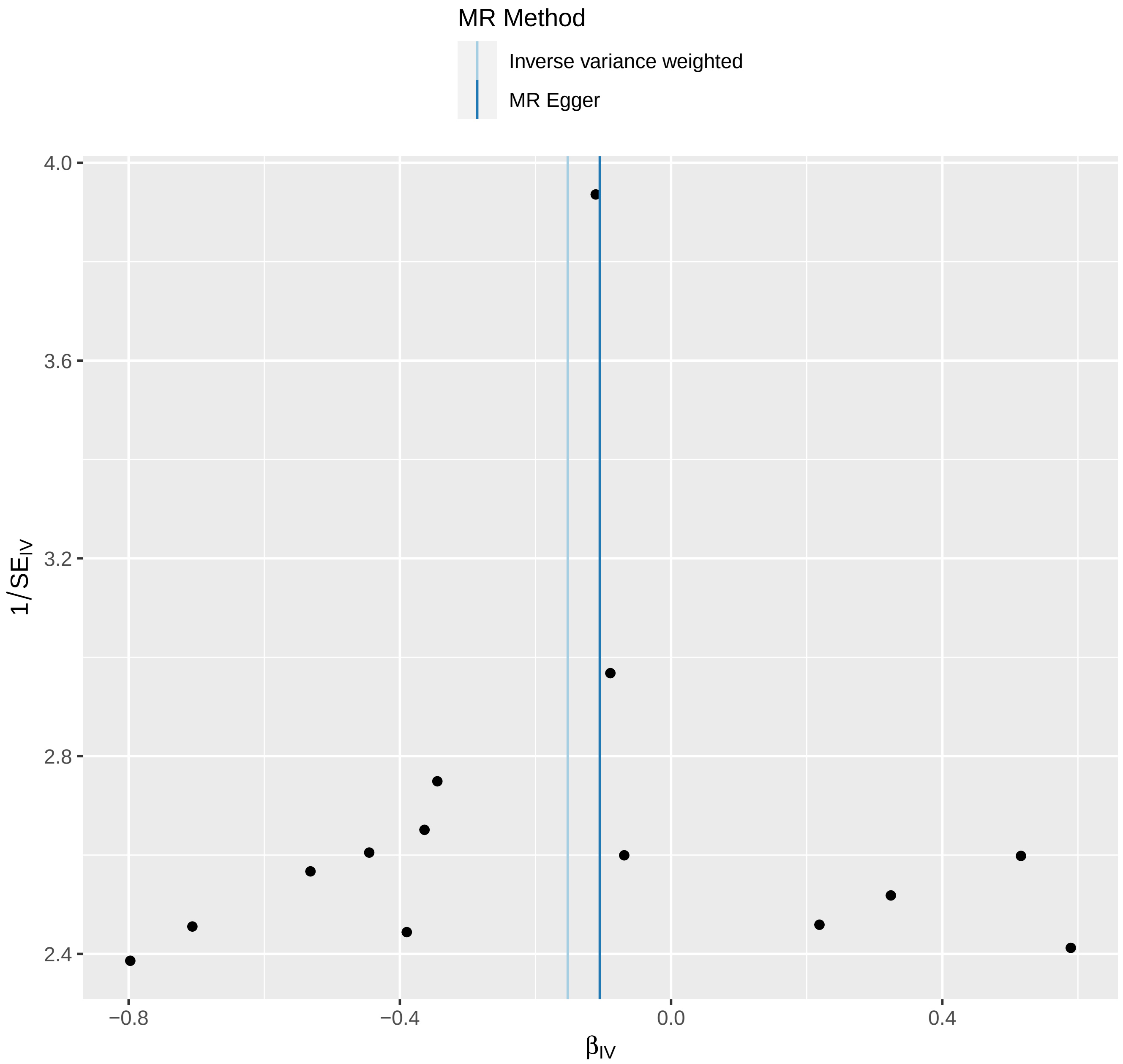

Batch 2−3909 : Gut microbiota abundance (genus Catenibacterium id.2153) on Macrophage−stimulating protein receptor
with a threshold of 1e−05

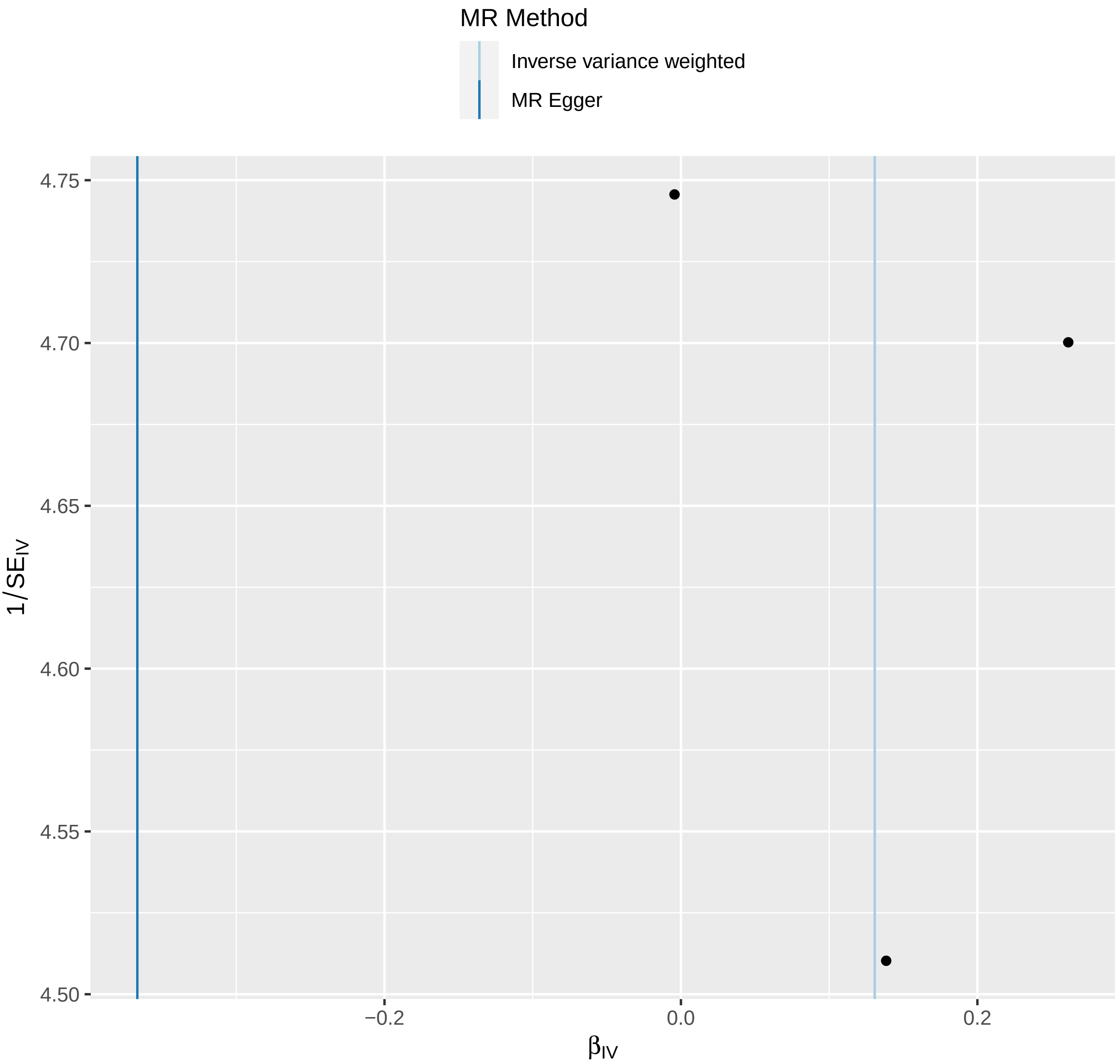

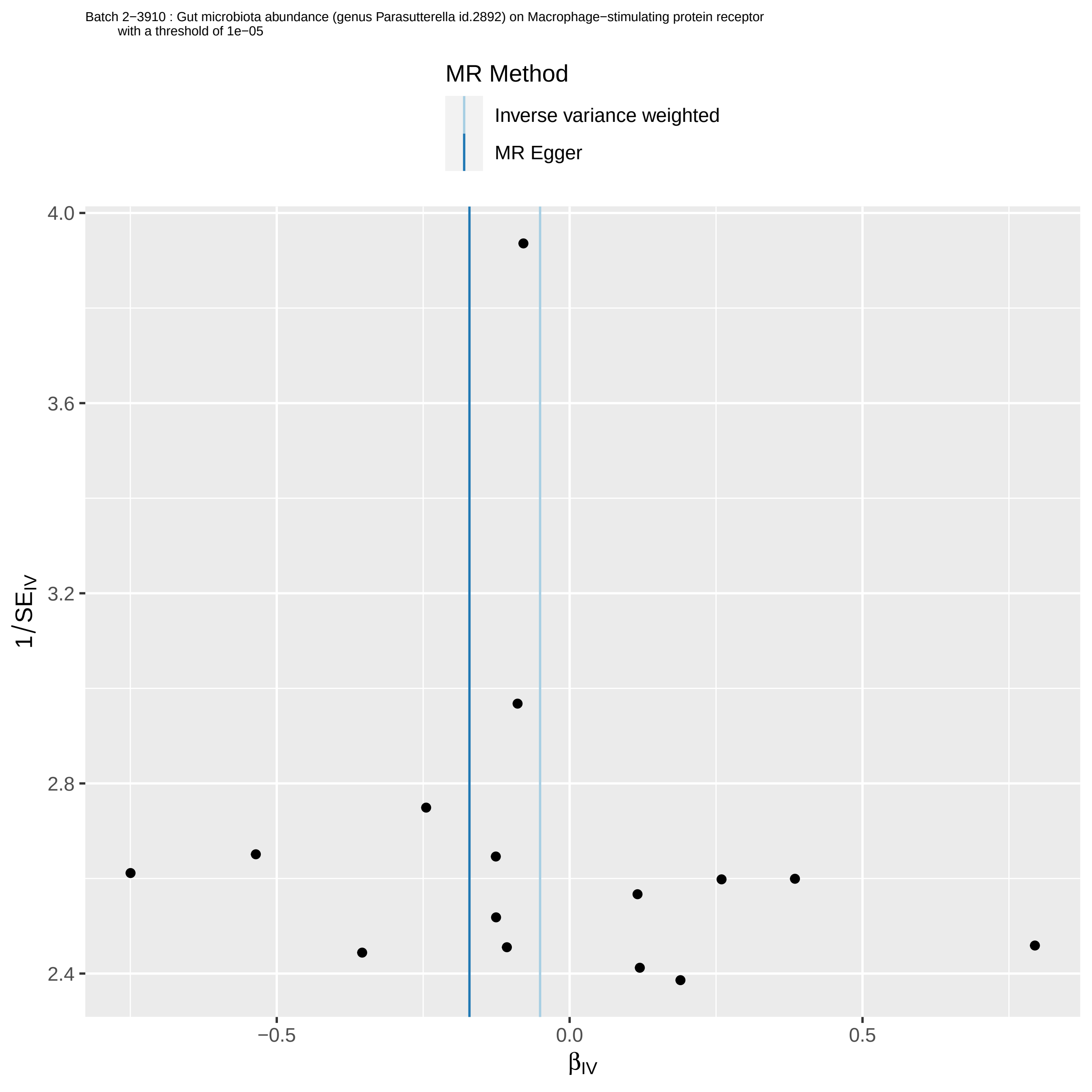

Batch 2−3910 : Gut microbiota abundance (genus Parasutterella id.2892) on Macrophage−stimulating protein receptor
with a threshold of 1e−05
MR Method
Inverse variance weighted
MR Egger
4.0
3.6
3.2
2.8
2.4
1/SE_IV
−0.5
0.0
0.5
β_IV

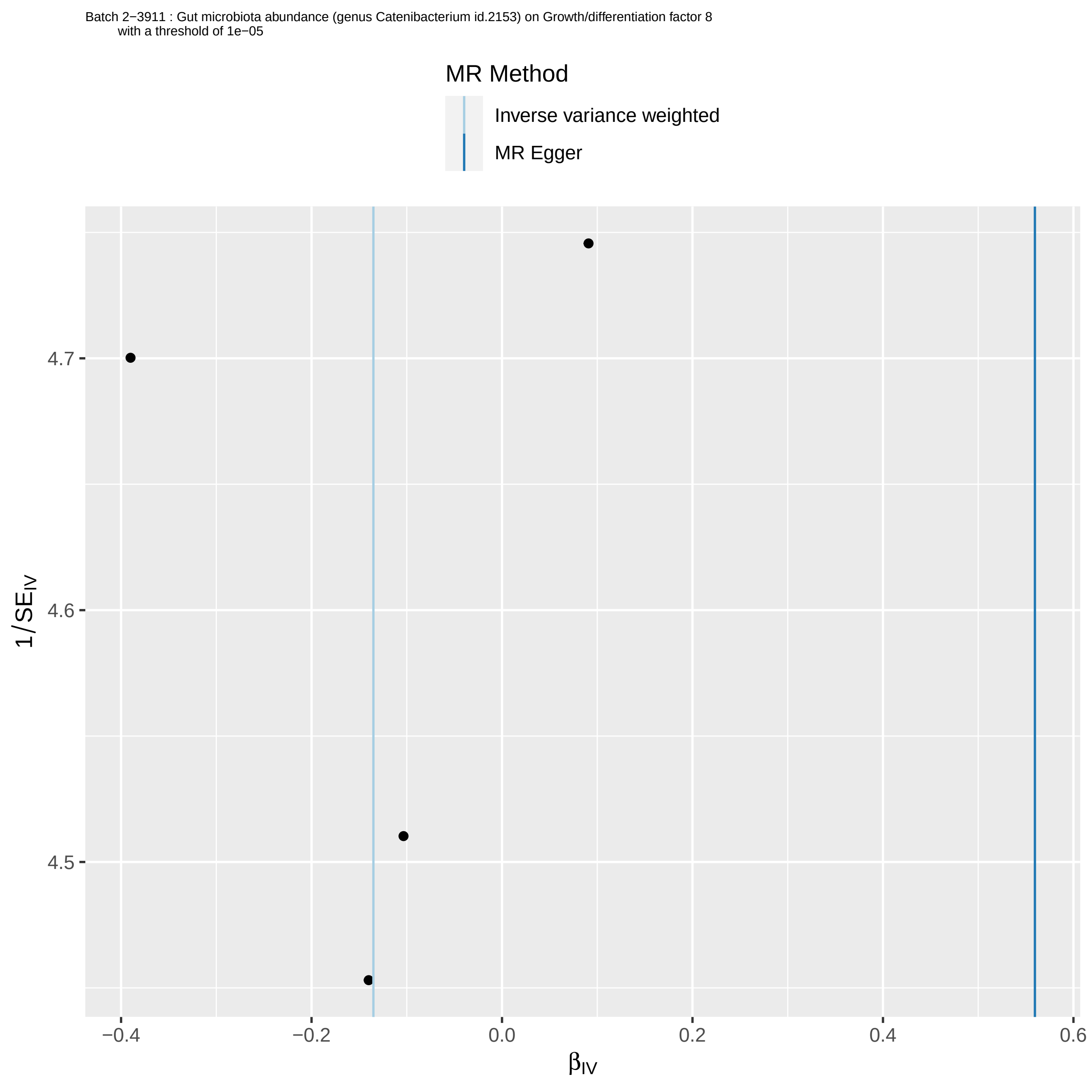

Batch 2−3911 : Gut microbiota abundance (genus Catenibacterium id.2153) on Growth/differentiation factor 8
with a threshold of 1e−05
MR Method
Inverse variance weighted
MR Egger
4.7
4.6
4.5
1/SE_IV
−0.4
−0.2
0.0
0.2
0.4
0.6
β_IV

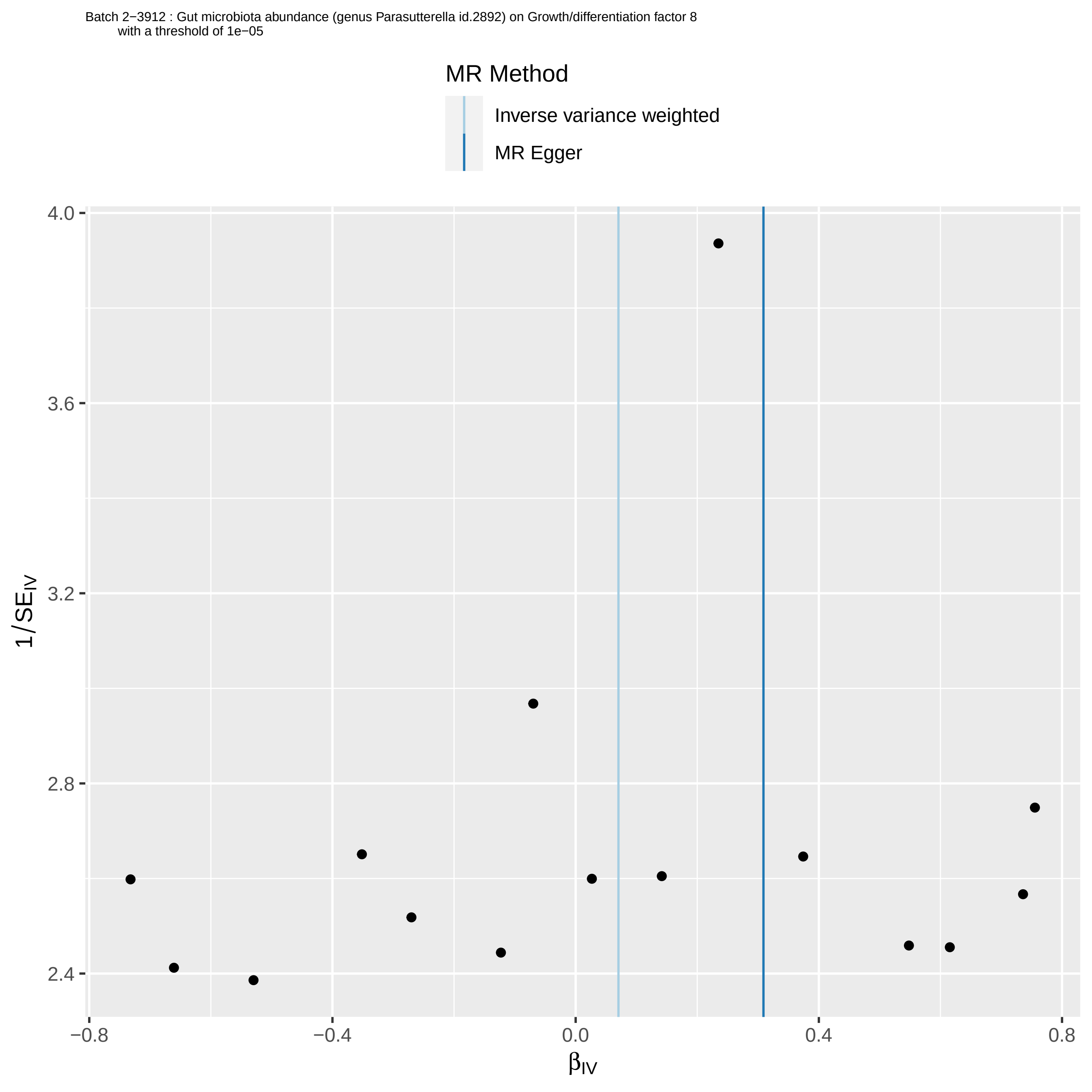

Batch 2−3912 : Gut microbiota abundance (genus Parasutterella id.2892) on Growth/differentiation factor 8
with a threshold of 1e−05
MR Method
Inverse variance weighted
MR Egger
4.0
3.6
3.2
2.8
2.4
1/SE$_{IV}$
−0.8
−0.4
0.0
0.4
0.8
β$_{IV}$

Batch 2−3913 : Gut microbiota abundance (genus Catenibacterium id.2153) on Growth/differentiation factor 8
with a threshold of 1e−05

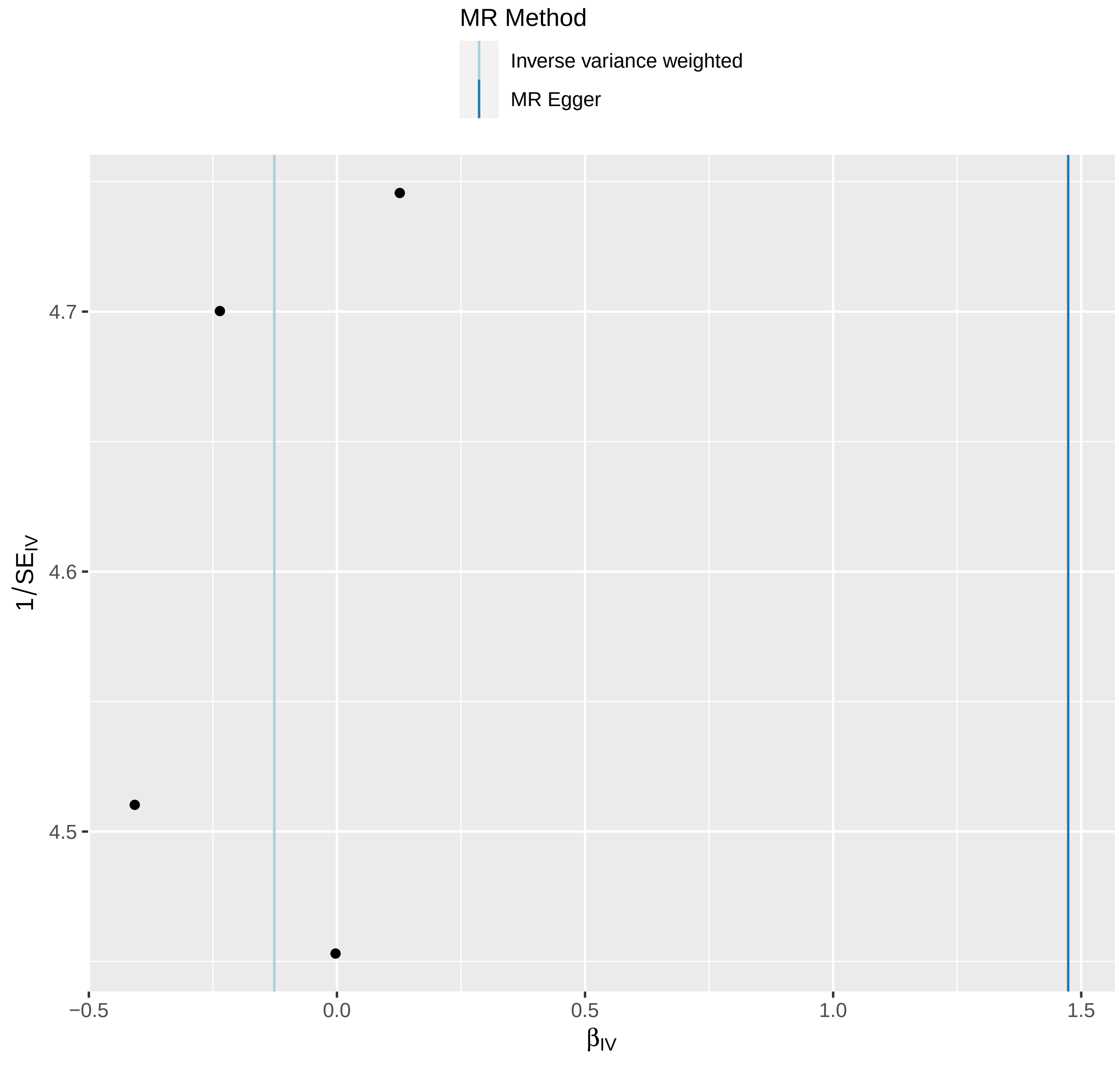

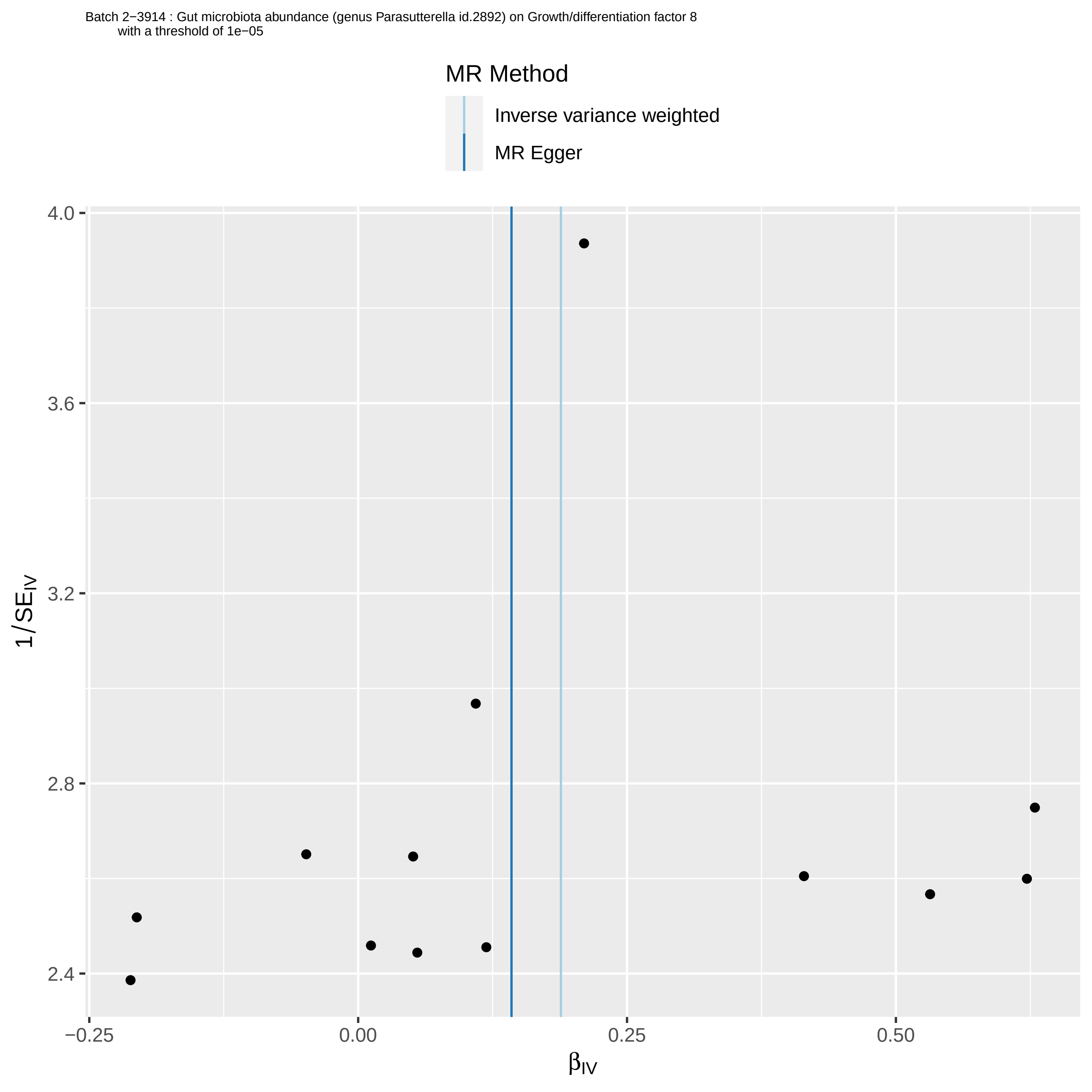

Batch 2−3914 : Gut microbiota abundance (genus Parasutterella id.2892) on Growth/differentiation factor 8
with a threshold of 1e−05
MR Method
Inverse variance weighted
MR Egger
4.0
3.6
3.2
2.8
2.4
1/SE_IV
−0.25
0.00
0.25
0.50
β_IV

Batch 2−3915 : Gut microbiota abundance (genus Catenibacterium id.2153) on Metallothionein−1F
with a threshold of 1e−05

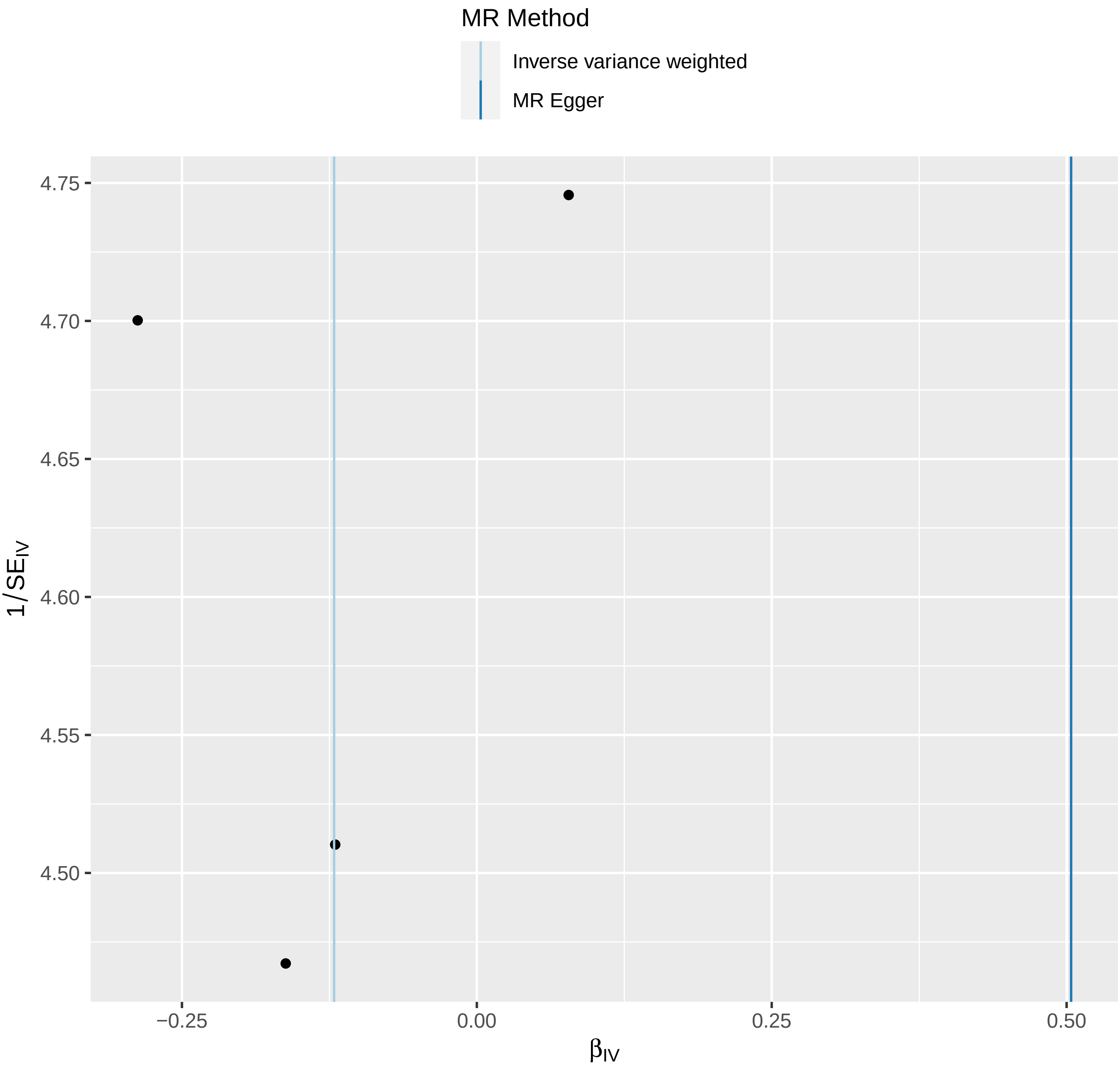

Batch 2−3916 : Gut microbiota abundance (genus Parasutterella id.2892) on Metallothionein−1F
with a threshold of 1e−05

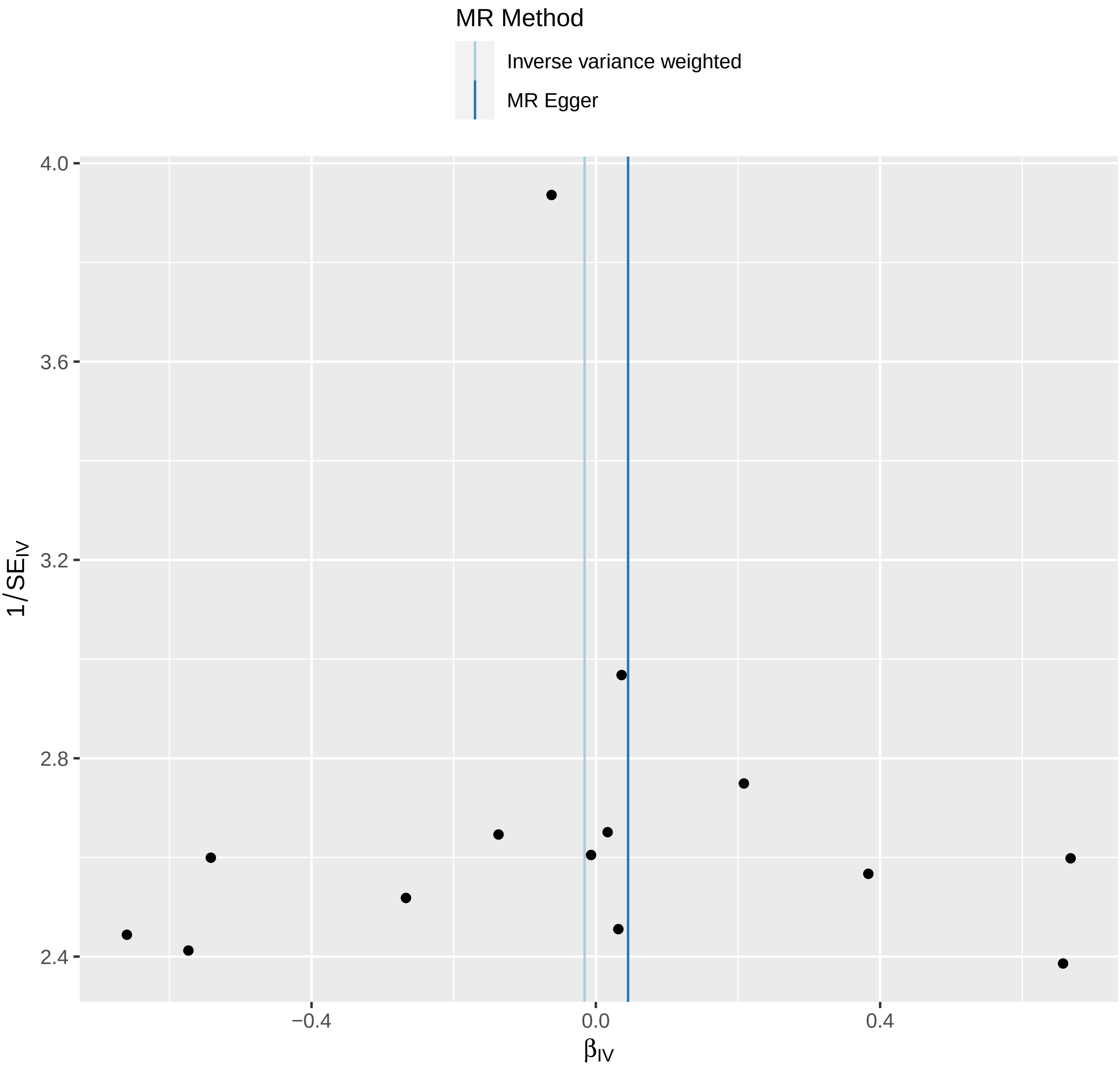

Batch 2−3917 : Gut microbiota abundance (genus Catenibacterium id.2153) on S−methyl−5'−thioadenosine phosphorylase
with a threshold of 1e−05

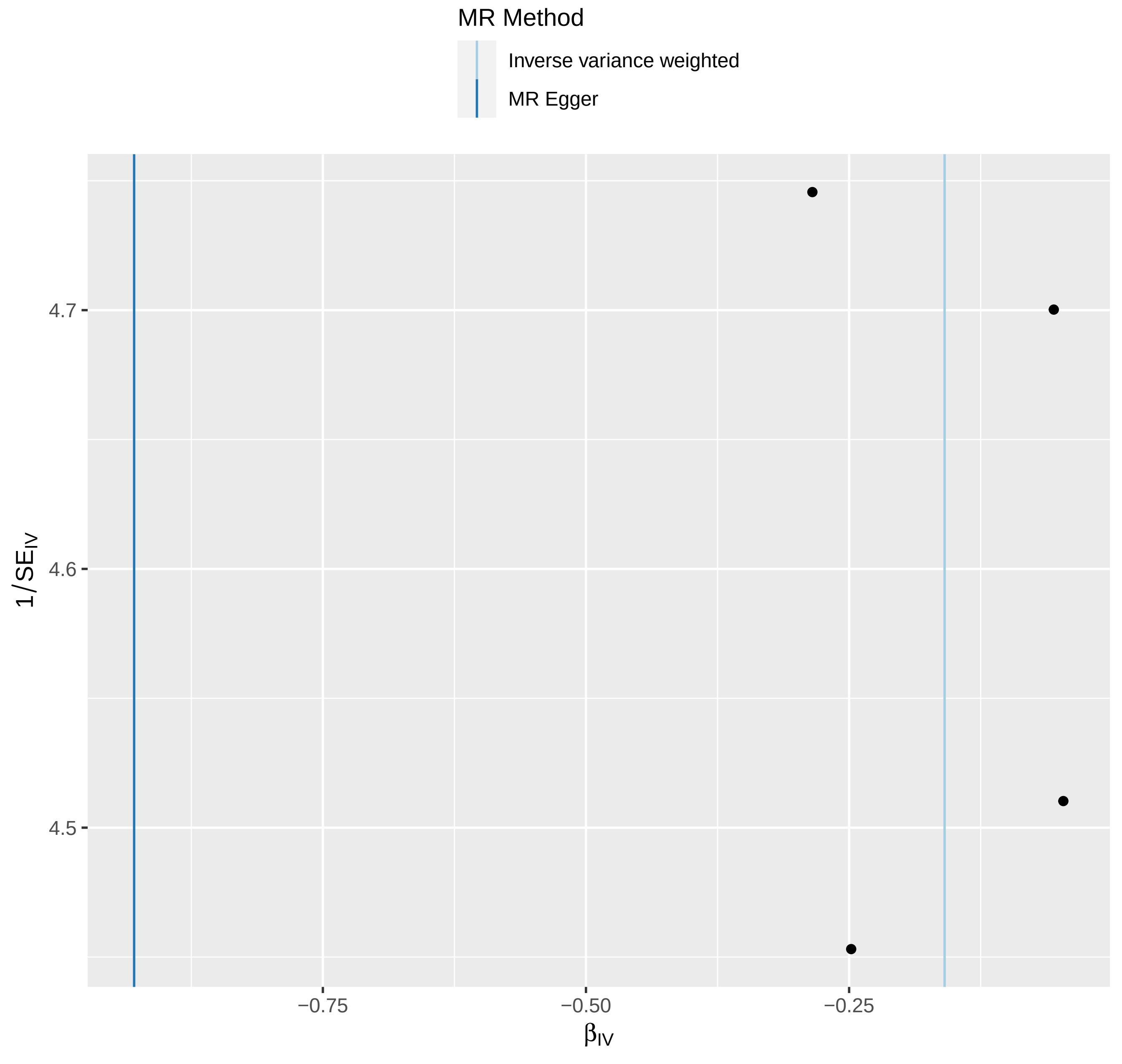

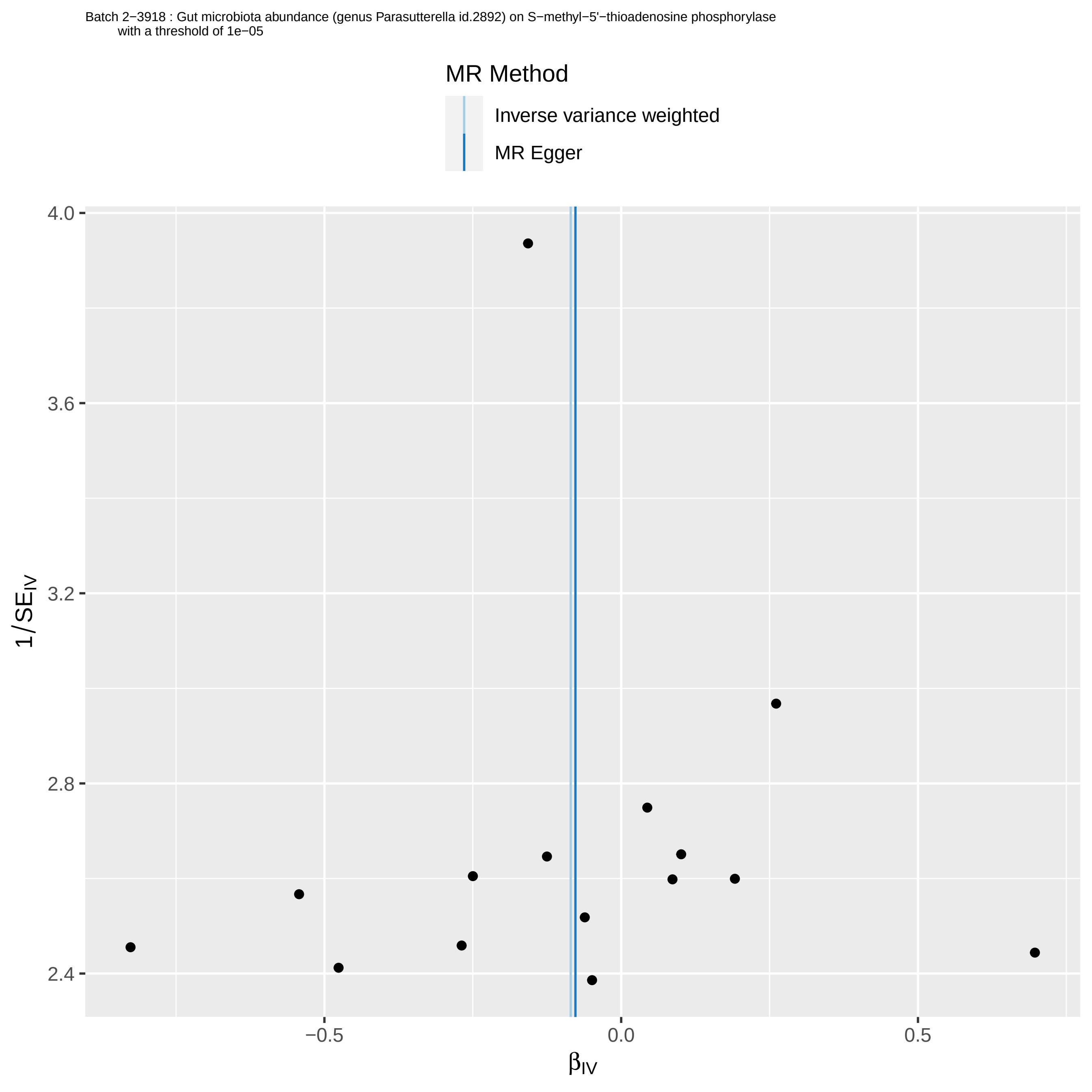
Batch 2−3918 : Gut microbiota abundance (genus Parasutterella id.2892) on S−methyl−5'−thioadenosine phosphorylase
with a threshold of 1e−05
MR Method
Inverse variance weighted
MR Egger
4.0
3.6
3.2
2.8
2.4
1/SE$_{IV}$
−0.5
0.0
0.5
β_{IV}

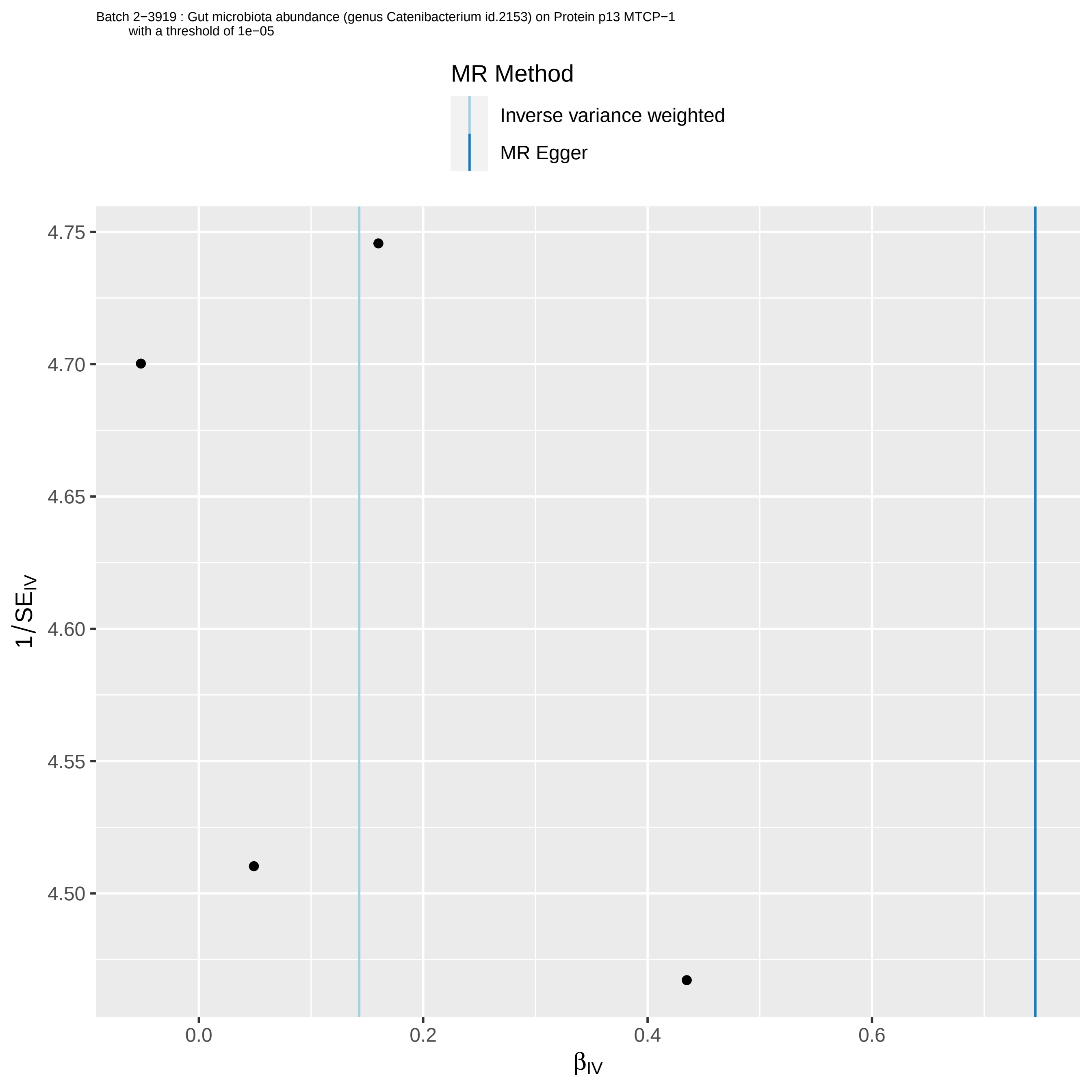

Batch 2−3919 : Gut microbiota abundance (genus Catenibacterium id.2153) on Protein p13 MTCP−1
with a threshold of 1e−05
MR Method
Inverse variance weighted
MR Egger
4.75
4.70
4.65
4.60
4.55
4.50
1/SE_IV
0.0
0.2
0.4
0.6
β_IV

Batch 2−3920 : Gut microbiota abundance (genus Parasutterella id.2892) on Protein p13 MTCP−1
with a threshold of 1e−05

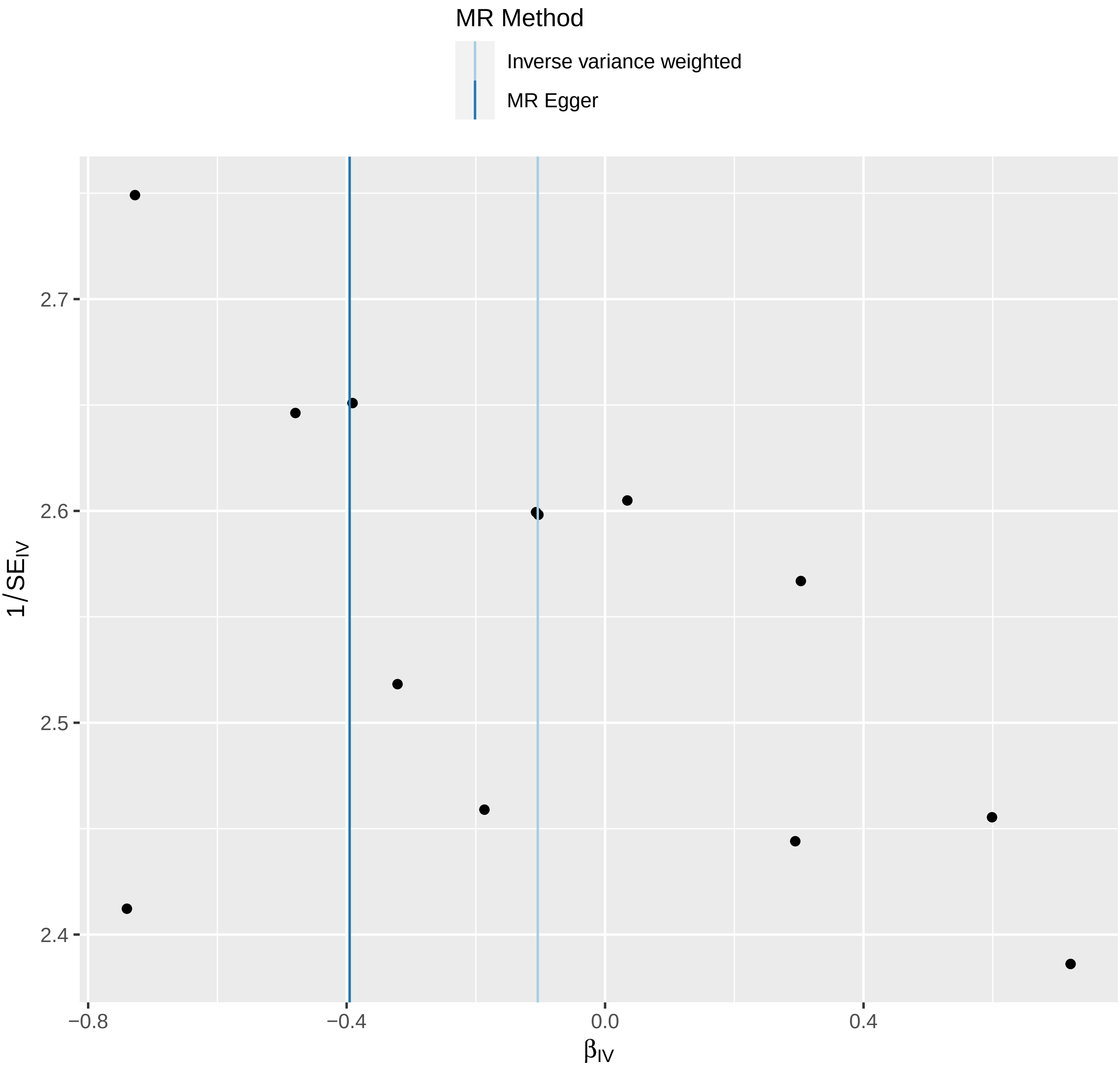

Batch 2−3921 : Gut microbiota abundance (genus Catenibacterium id.2153) on Mitochondrial fission regulator 1
with a threshold of 1e−05

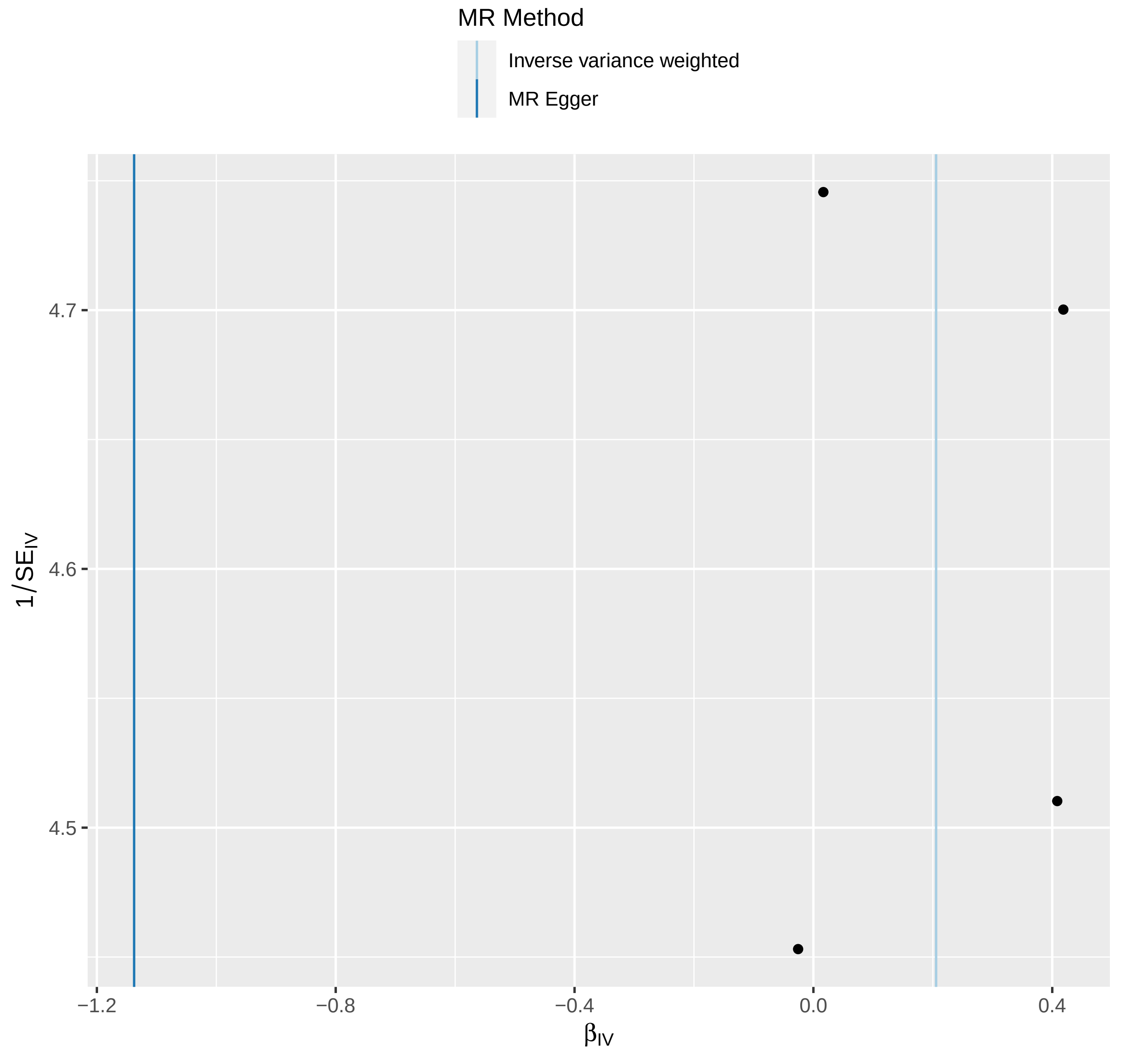

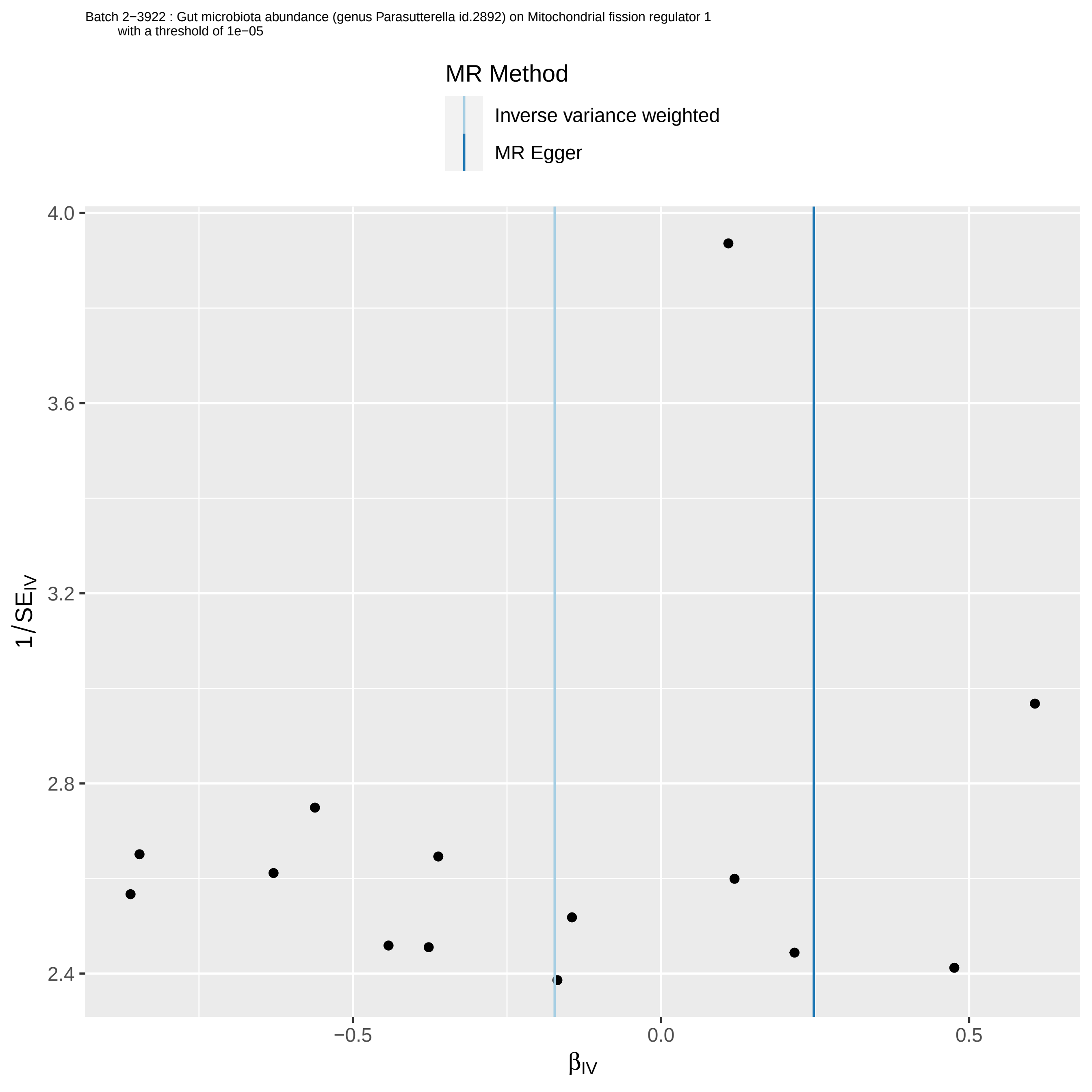
Batch 2−3922 : Gut microbiota abundance (genus Parasutterella id.2892) on Mitochondrial fission regulator 1
with a threshold of 1e−05
MR Method
Inverse variance weighted
MR Egger
4.0
3.6
3.2
2.8
2.4
−0.5
0.0
0.5
1/SE_IV
β_IV

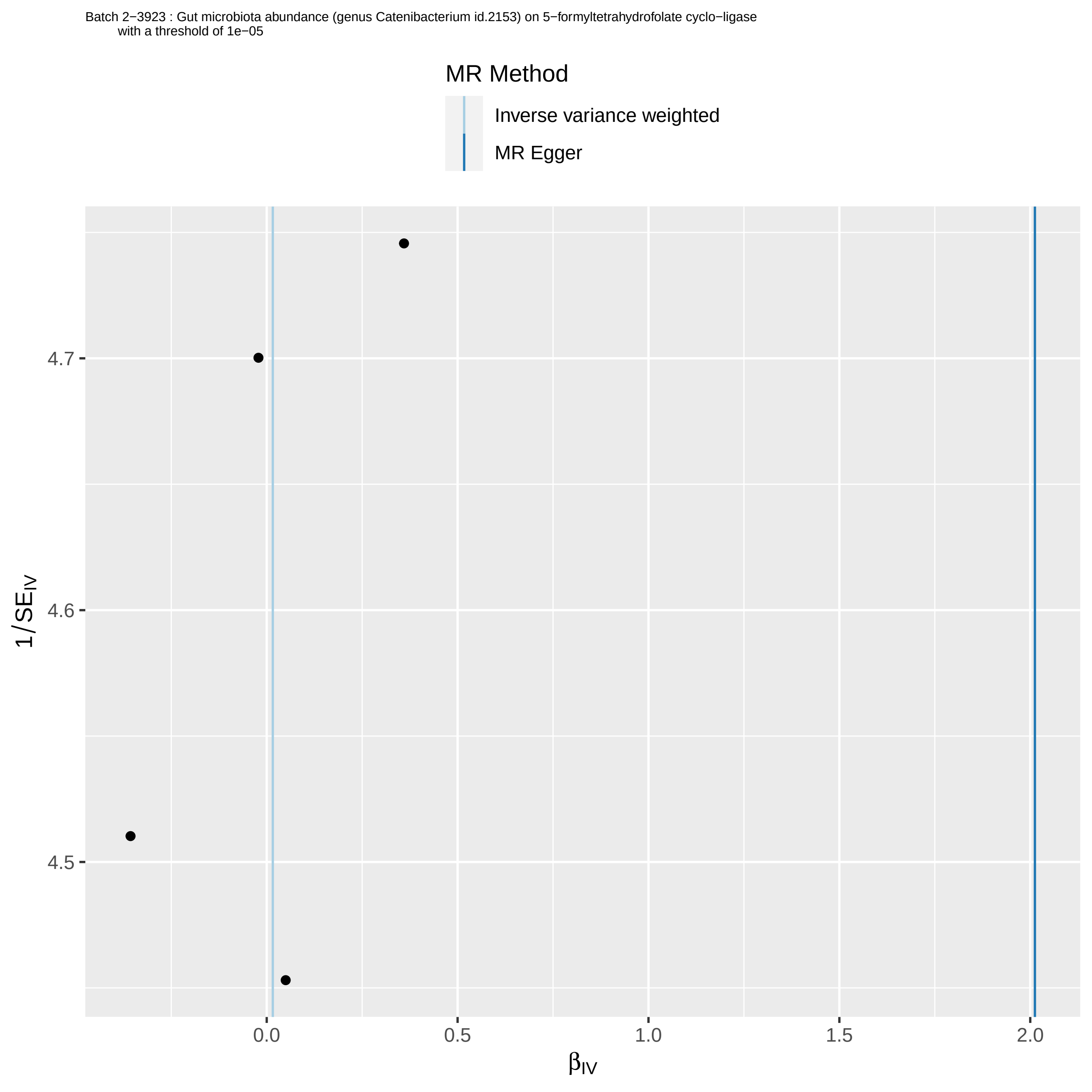
Batch 2−3923 : Gut microbiota abundance (genus Catenibacterium id.2153) on 5−formyltetrahydrofolate cyclo−ligase
with a threshold of 1e−05
MR Method
Inverse variance weighted
MR Egger
4.7
4.6
4.5
1/SE_IV
0.0
0.5
1.0
1.5
2.0
β_IV

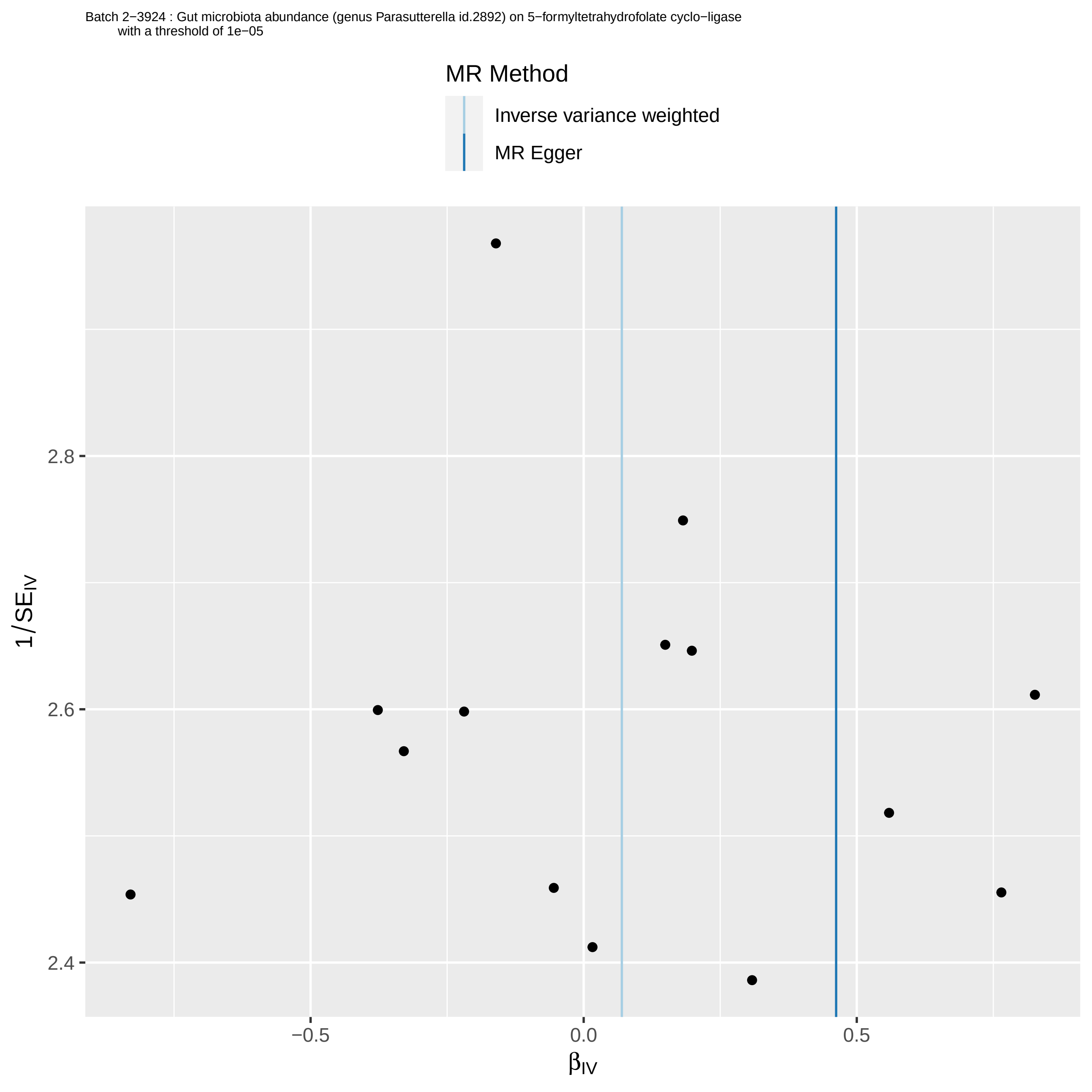

Batch 2−3924 : Gut microbiota abundance (genus Parasutterella id.2892) on 5−formyltetrahydrofolate cyclo−ligase
with a threshold of 1e−05
MR Method
Inverse variance weighted
MR Egger
2.8
2.6
2.4
1/SE_IV
−0.5
0.0
0.5
β_IV

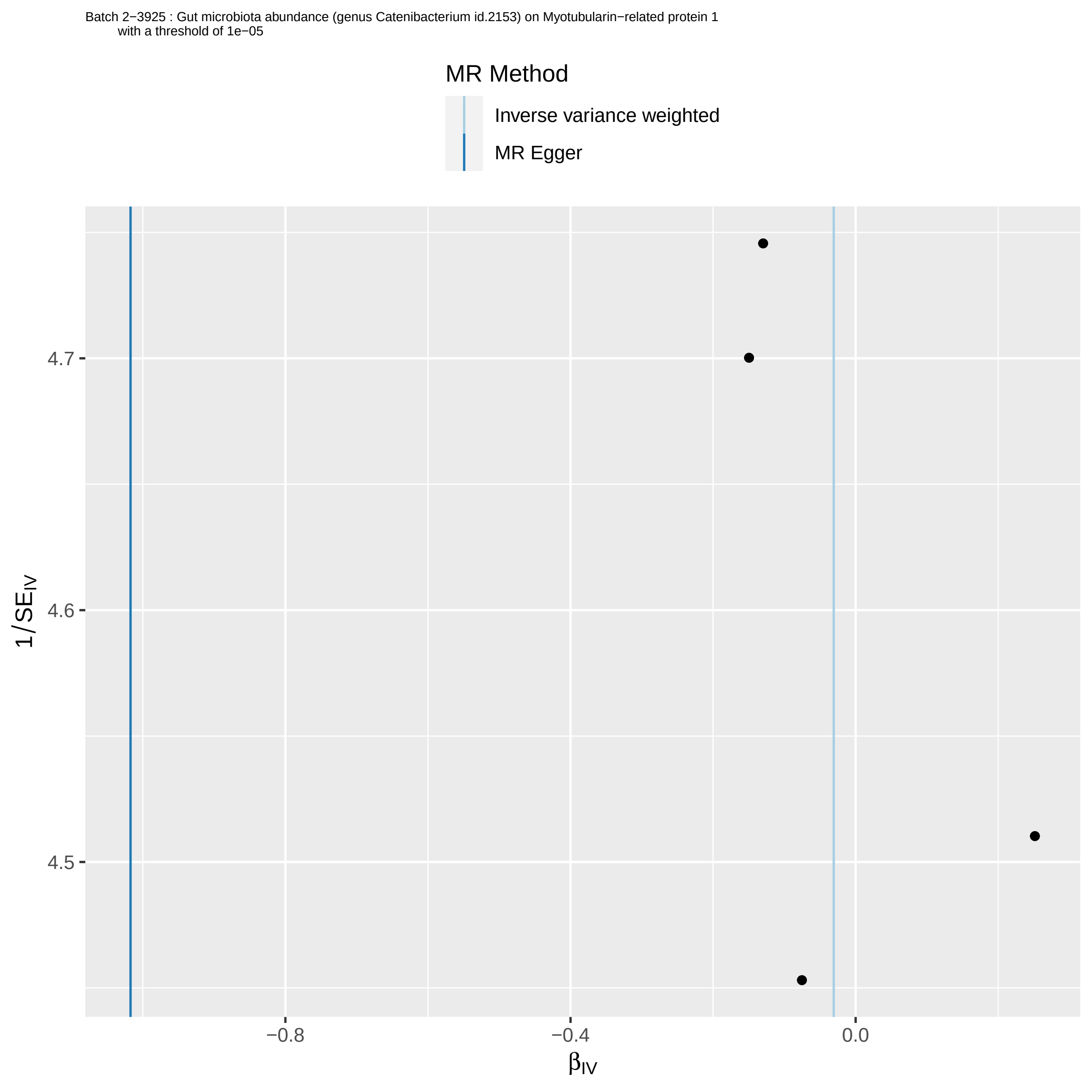

Batch 2−3925 : Gut microbiota abundance (genus Catenibacterium id.2153) on Myotubularin−related protein 1
with a threshold of 1e−05
MR Method
Inverse variance weighted
MR Egger
4.7
4.6
4.5
1/SE_IV
−0.8
−0.4
0.0
β_IV

Batch 2−3926 : Gut microbiota abundance (genus Parasutterella id.2892) on Myotubularin−related protein 1
with a threshold of 1e−05

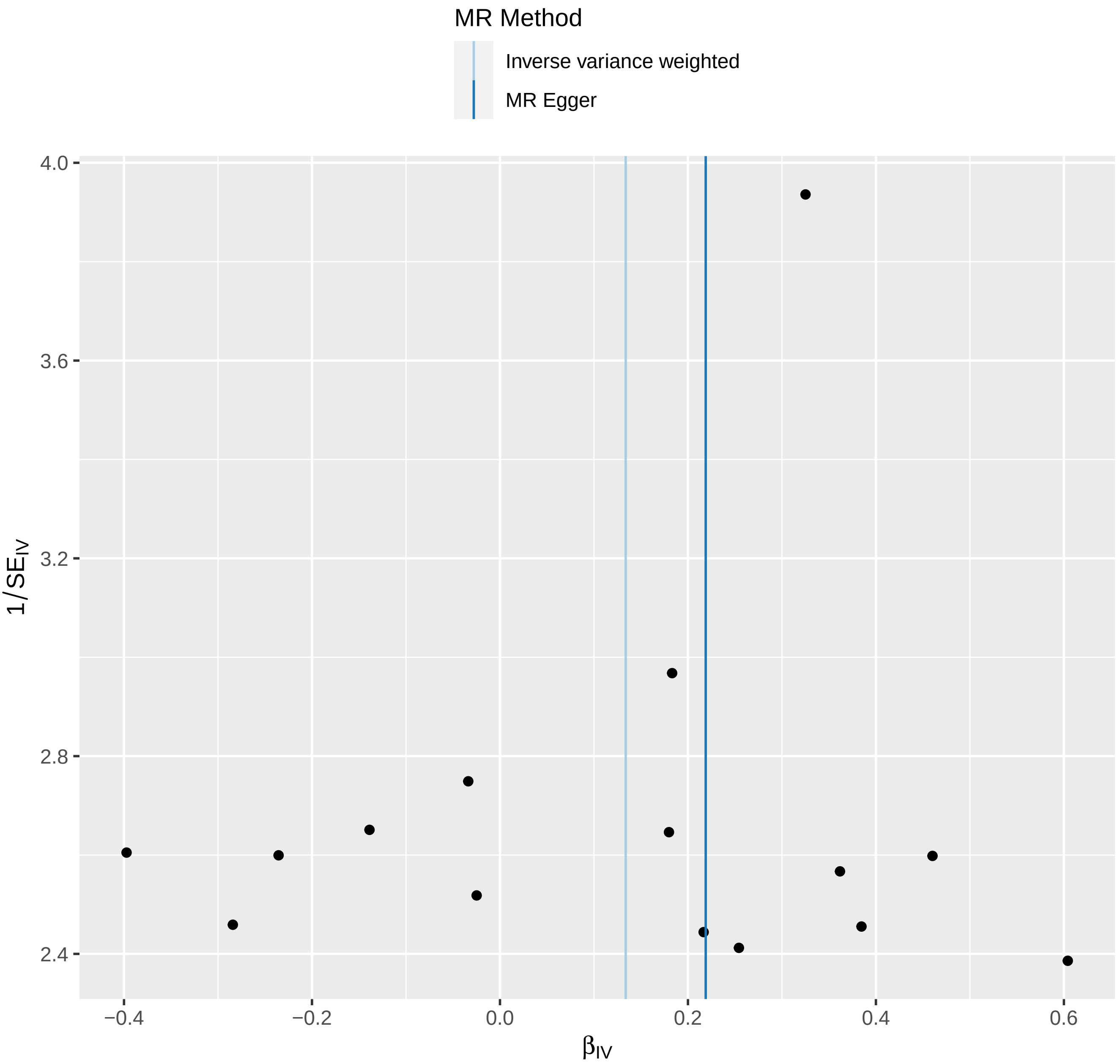

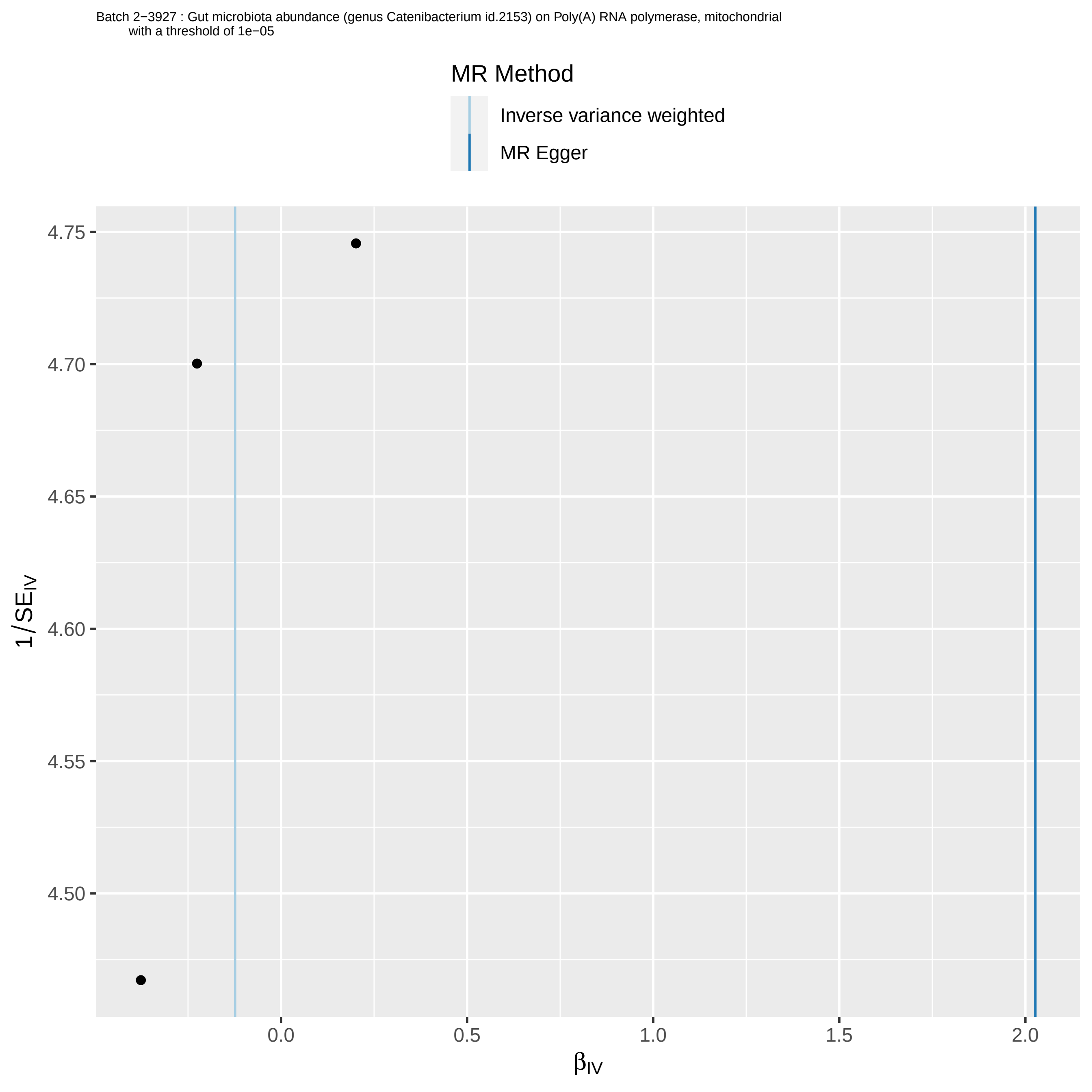

Batch 2−3927 : Gut microbiota abundance (genus Catenibacterium id.2153) on Poly(A) RNA polymerase, mitochondrial
with a threshold of 1e−05
MR Method
Inverse variance weighted
MR Egger
4.75
4.70
4.65
4.60
4.55
4.50
1/SE$_{IV}$
0.0
0.5
1.0
1.5
2.0
β$_{IV}$

Batch 2−3928 : Gut microbiota abundance (genus Parasutterella id.2892) on Poly(A) RNA polymerase, mitochondrial
with a threshold of 1e−05

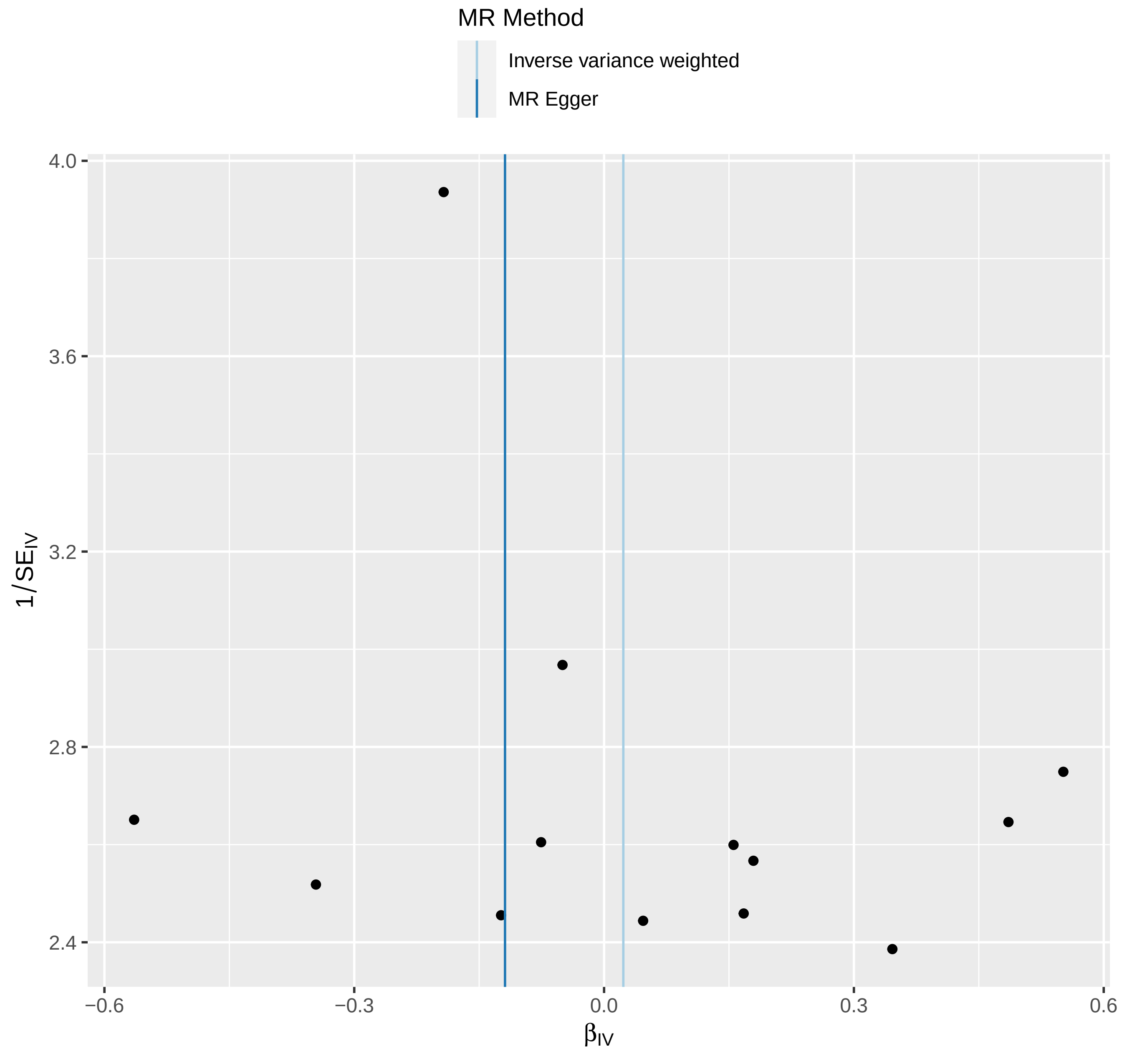

Batch 2−3929 : Gut microbiota abundance (genus Catenibacterium id.2153) on Peptide chain release factor 1−like, mitochondrial
with a threshold of 1e−05

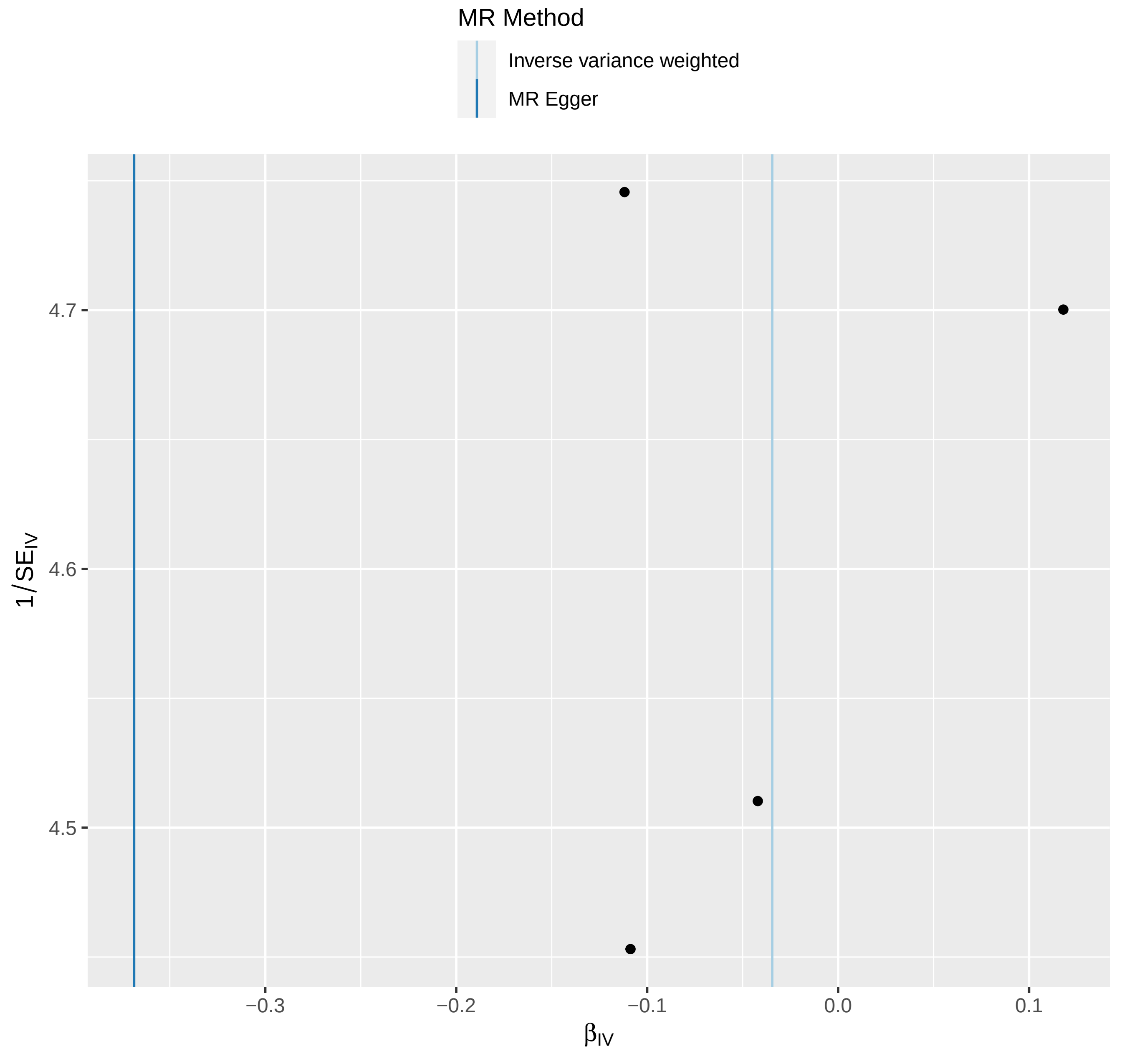

Batch 2−3930 : Gut microbiota abundance (genus Parasutterella id.2892) on Peptide chain release factor 1−like, mitochondrial
with a threshold of 1e−05

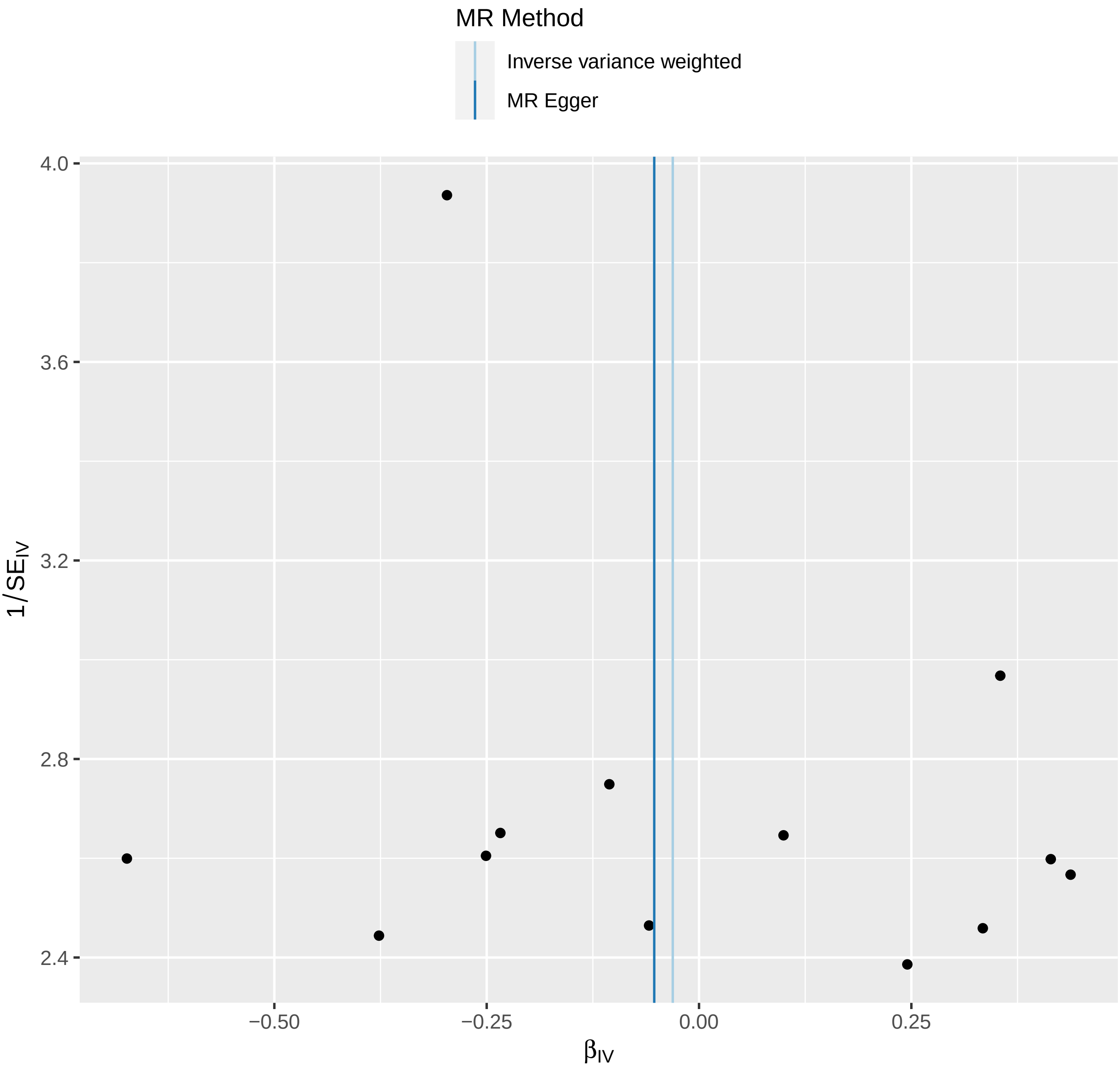

Batch 2−3931 : Gut microbiota abundance (genus Catenibacterium id.2153) on Mucin−1
with a threshold of 1e−05

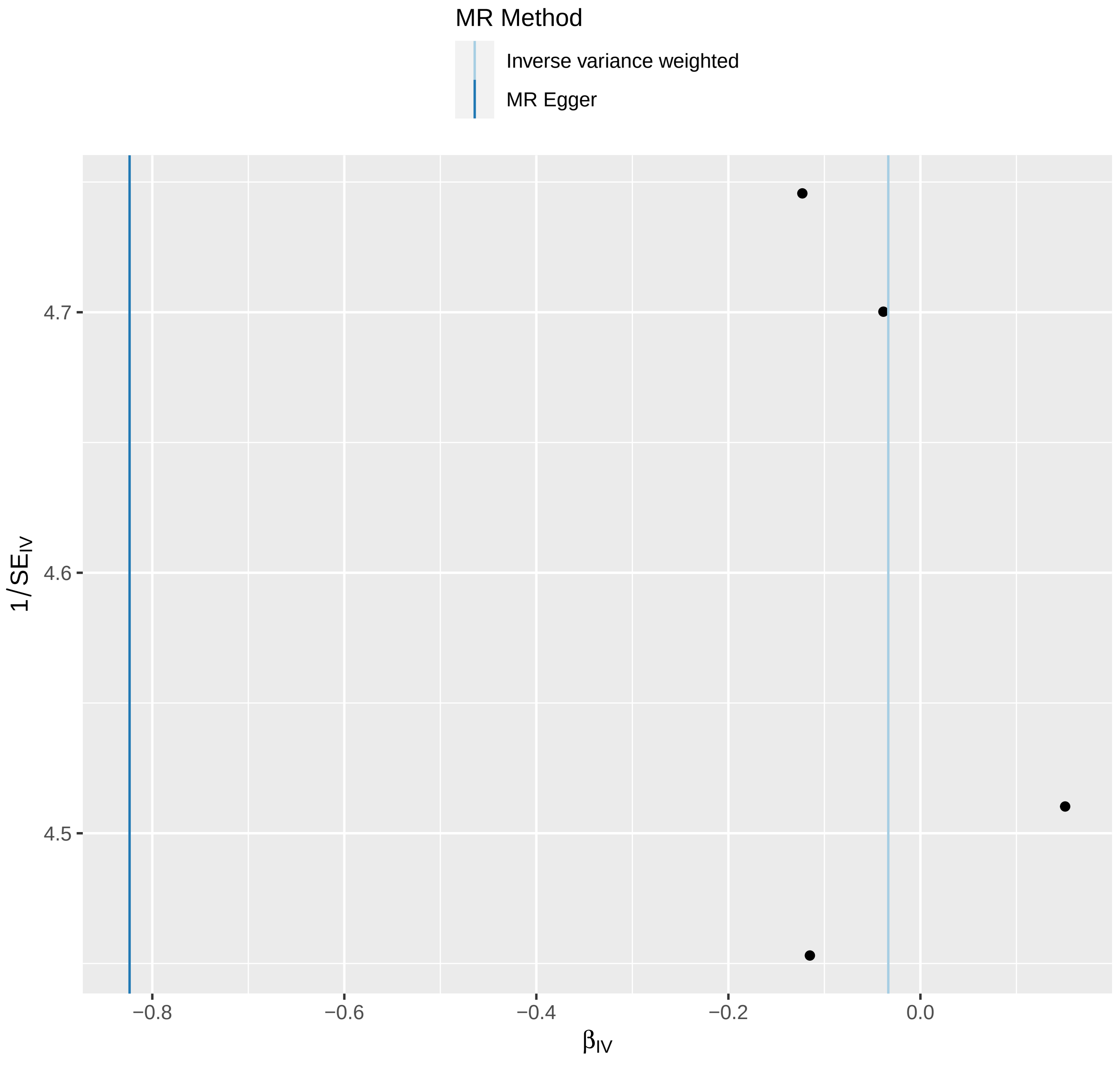

Batch 2−3932 : Gut microbiota abundance (genus Parasutterella id.2892) on Mucin−1
with a threshold of 1e−05

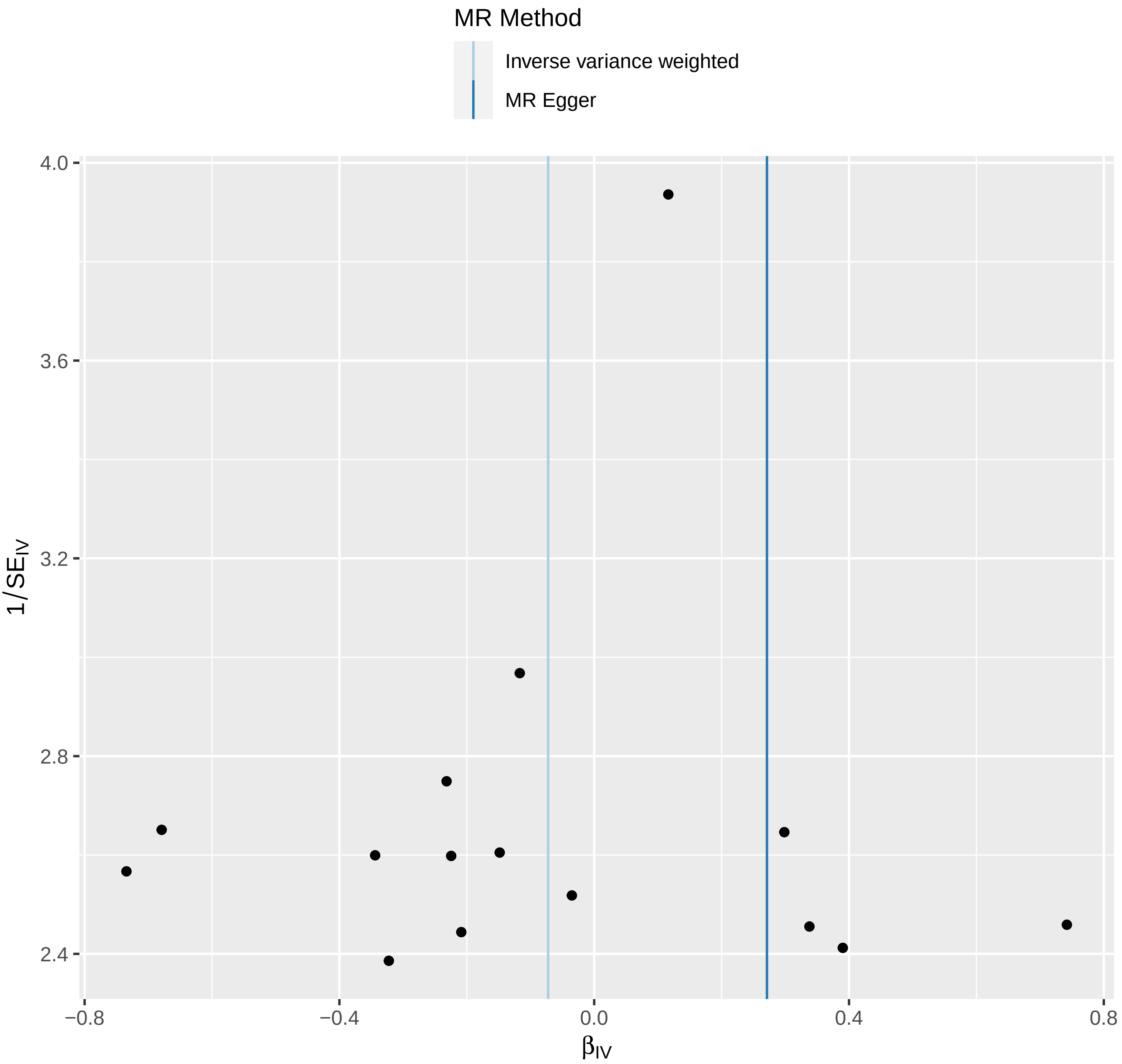

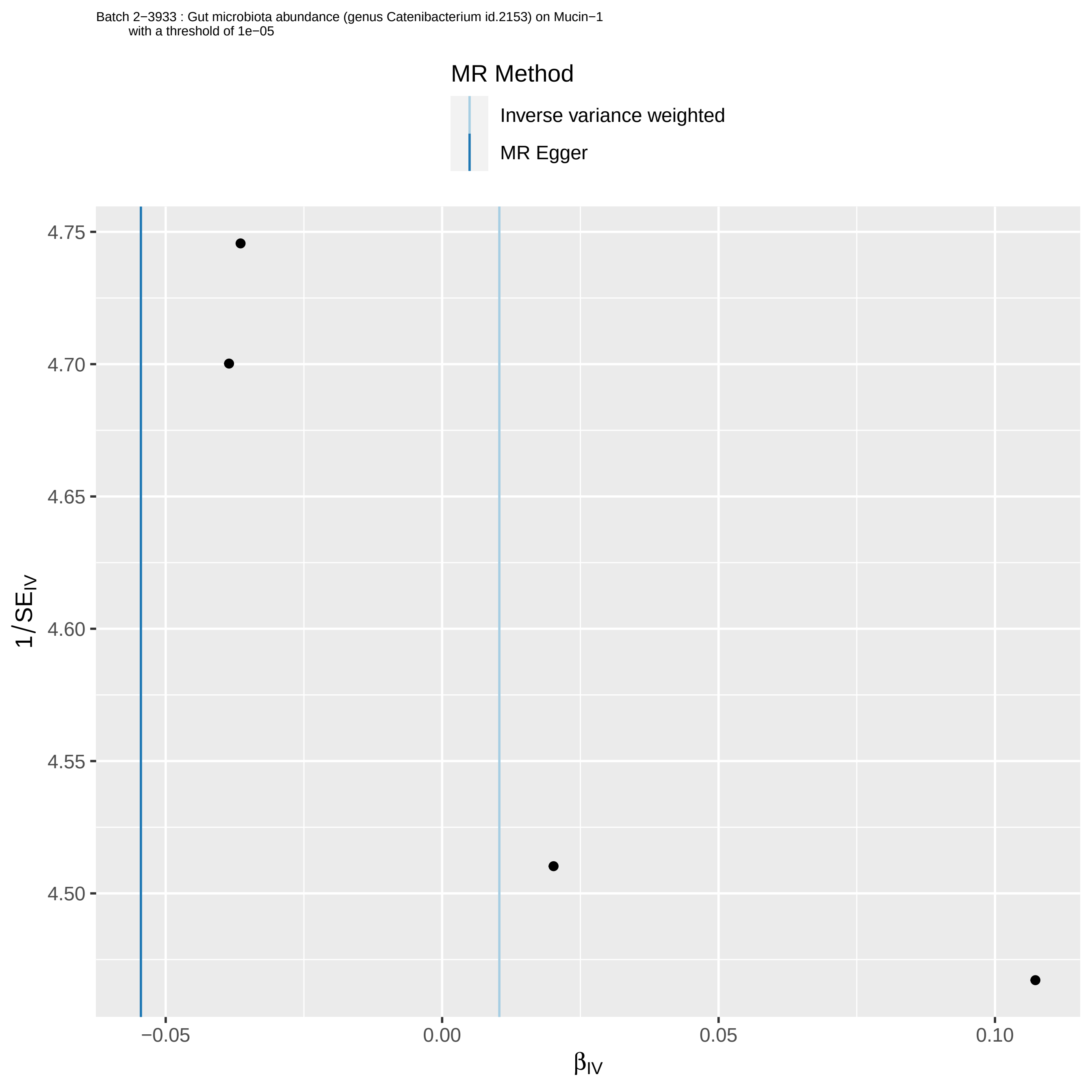
Batch 2−3933 : Gut microbiota abundance (genus Catenibacterium id.2153) on Mucin−1
with a threshold of 1e−05
MR Method
Inverse variance weighted
MR Egger
4.75
4.70
4.65
4.60
4.55
4.50
1/SE_IV
−0.05
0.00
0.05
0.10
β_IV

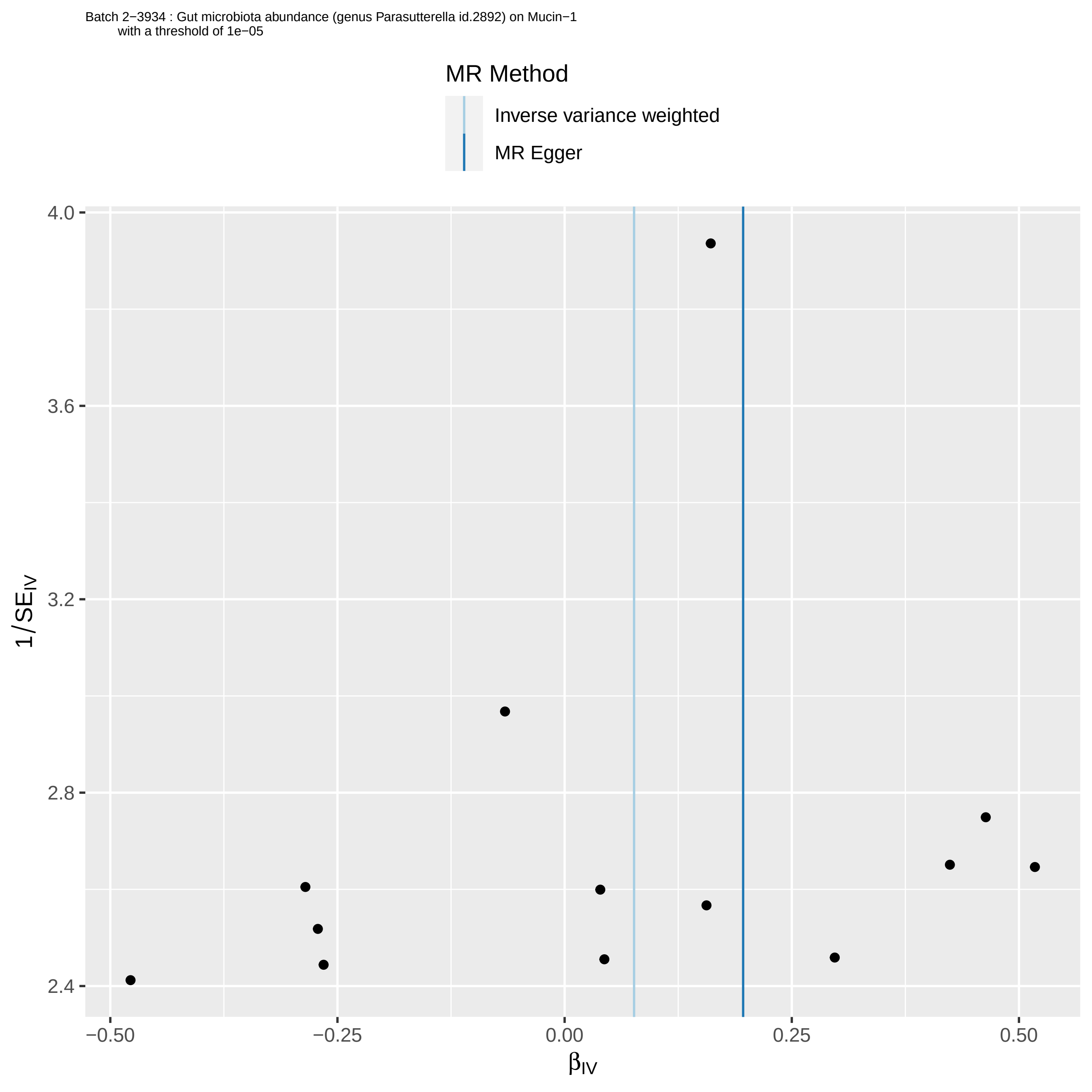

Batch 2−3934 : Gut microbiota abundance (genus Parasutterella id.2892) on Mucin−1
with a threshold of 1e−05
MR Method
Inverse variance weighted
MR Egger
4.0
3.6
3.2
2.8
2.4
1/SE$_{IV}$
−0.50
−0.25
0.00
0.25
0.50
β_{IV}

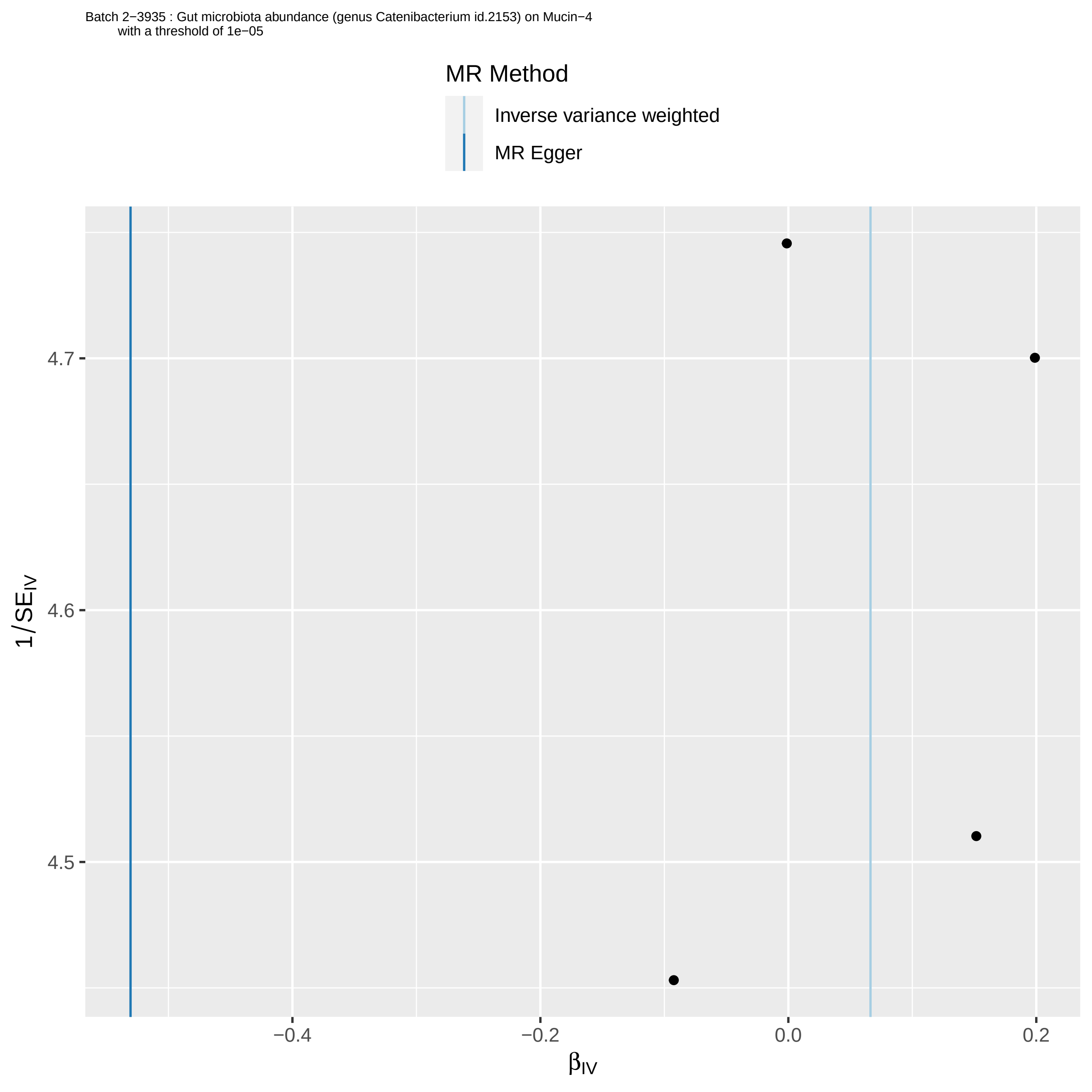
Batch 2−3935 : Gut microbiota abundance (genus Catenibacterium id.2153) on Mucin−4
with a threshold of 1e−05
MR Method
Inverse variance weighted
MR Egger
4.7
4.6
4.5
1/SE_IV
−0.4
−0.2
0.0
0.2
β_IV

Batch 2−3936 : Gut microbiota abundance (genus Parasutterella id.2892) on Mucin−4
with a threshold of 1e−05

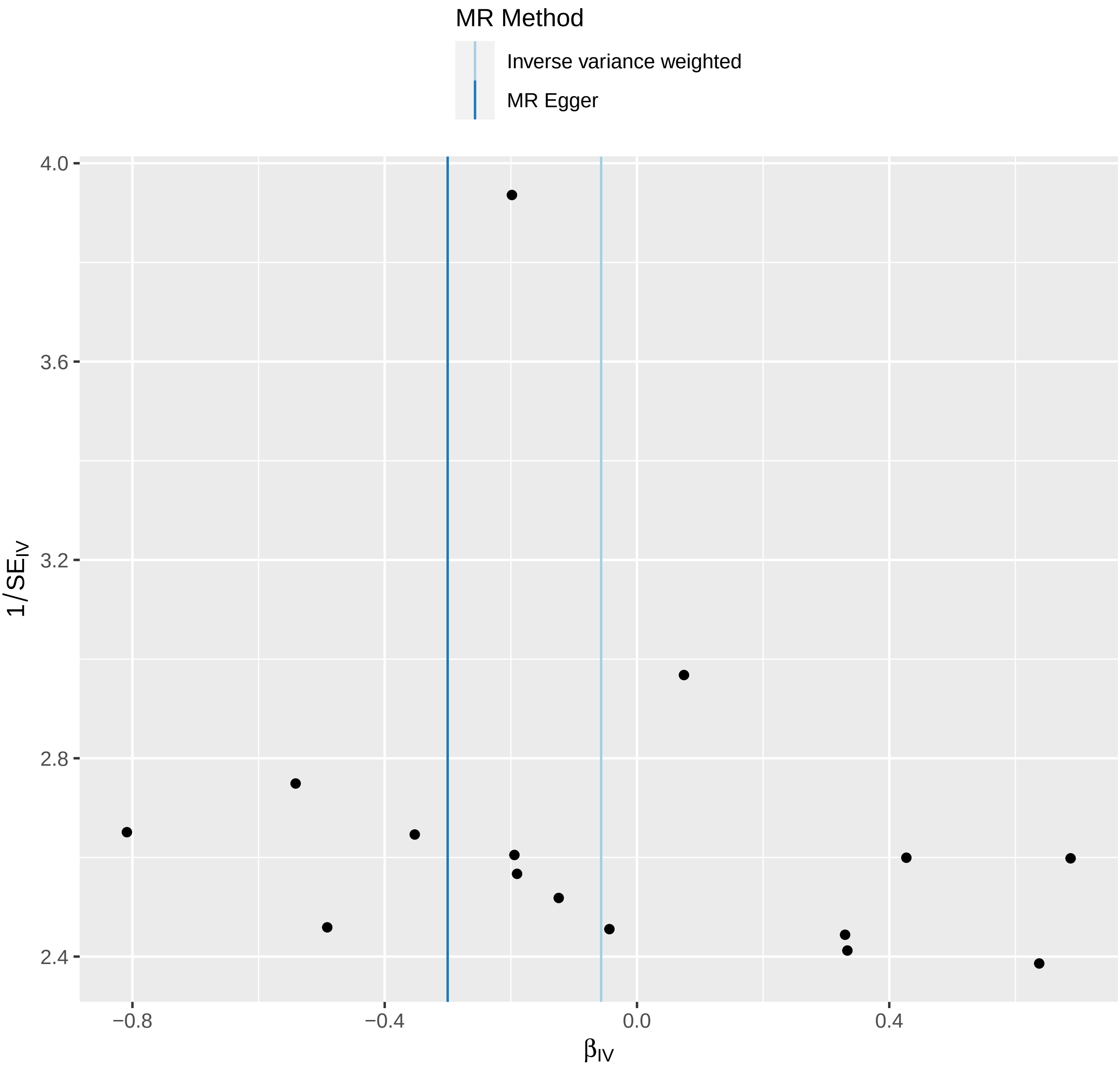

Batch 2−3937 : Gut microbiota abundance (genus Catenibacterium id.2153) on Mitochondrial ubiquitin ligase activator of NFKB 1
with a threshold of 1e−05

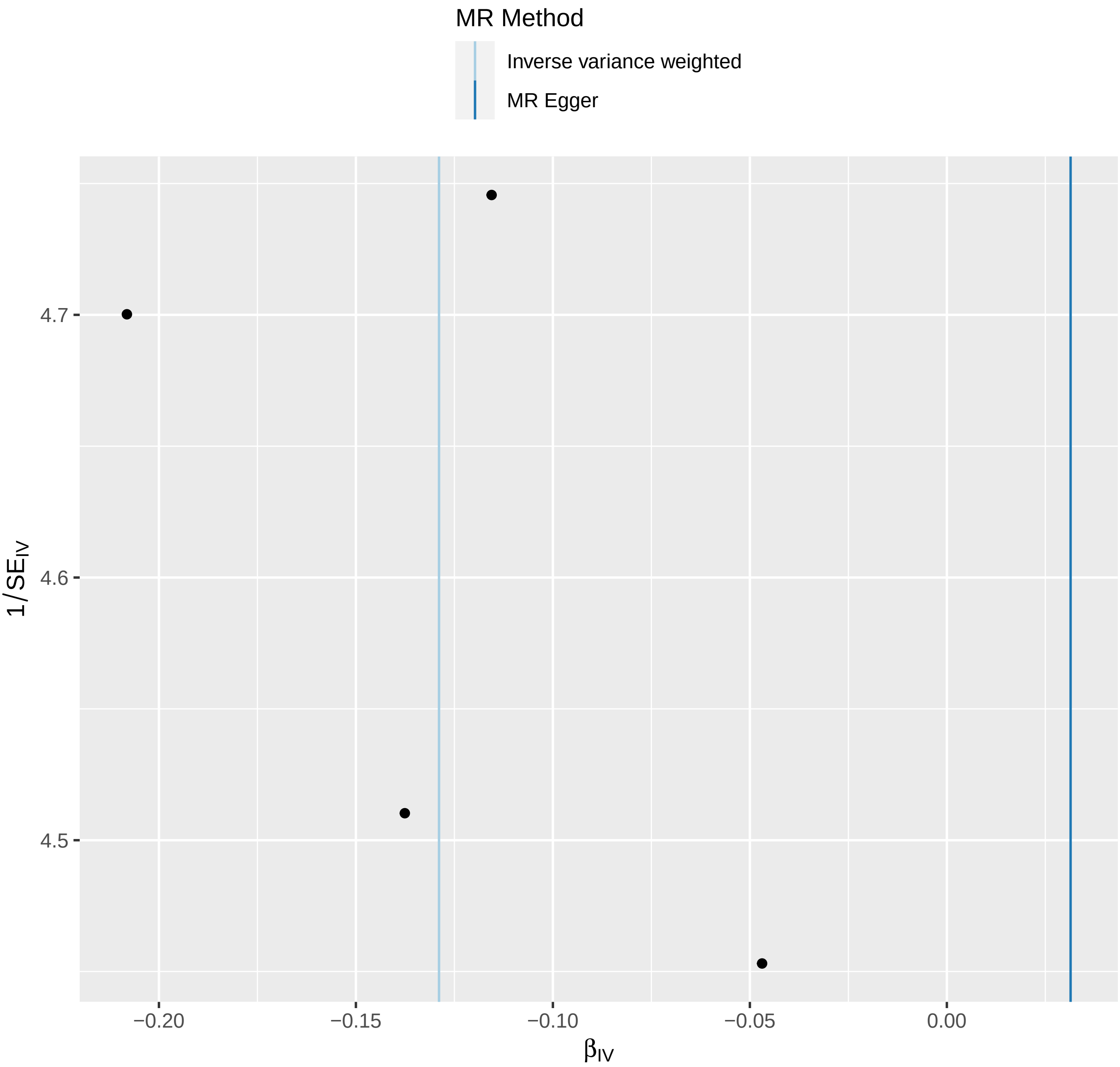

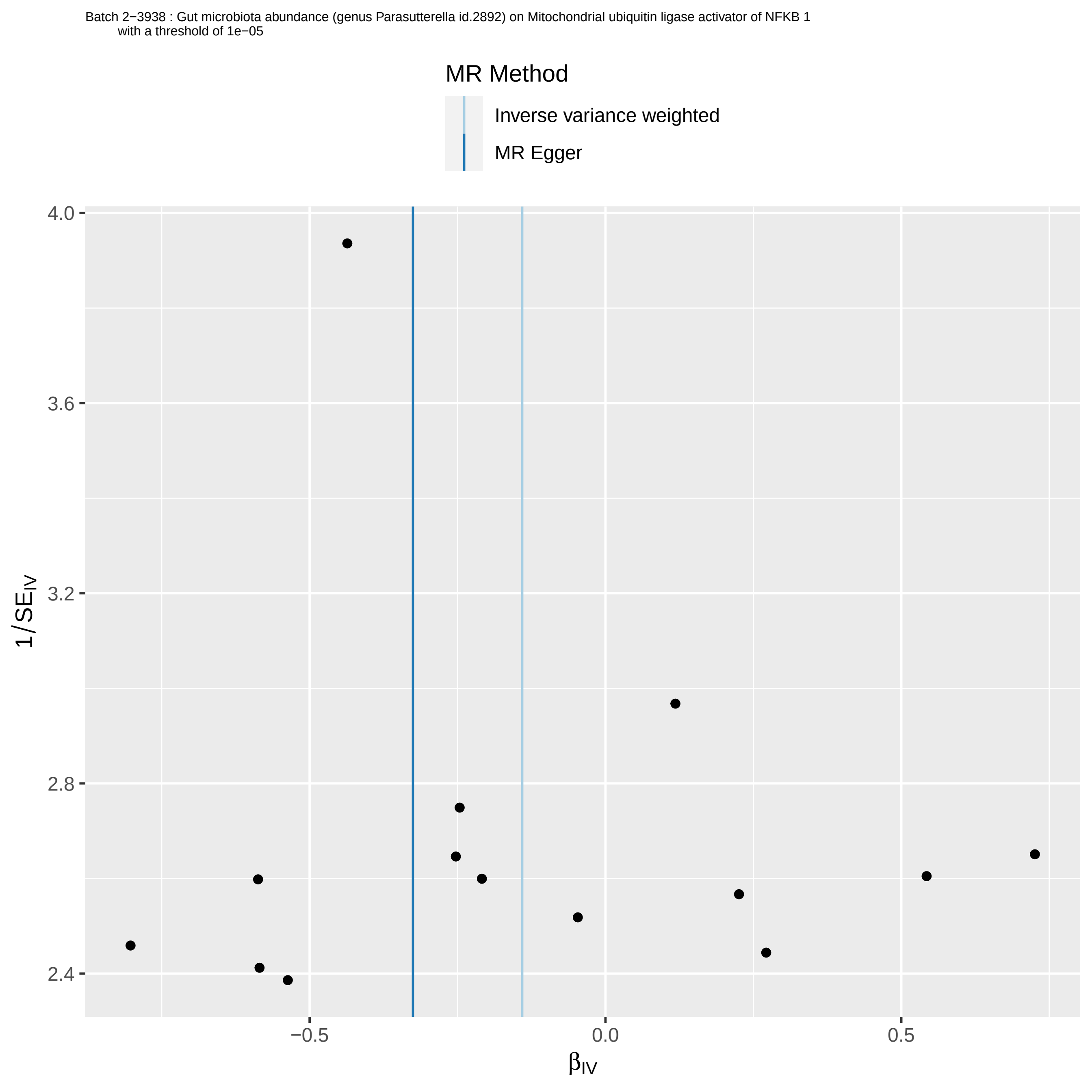
Batch 2−3938 : Gut microbiota abundance (genus Parasutterella id.2892) on Mitochondrial ubiquitin ligase activator of NFKB 1
with a threshold of 1e−05
MR Method
Inverse variance weighted
MR Egger
4.0
3.6
3.2
2.8
2.4
1/SE_IV
−0.5
0.0
0.5
β_IV

Batch 2−3939 : Gut microbiota abundance (genus Catenibacterium id.2153) on Mitochondrial ubiquitin ligase activator of NFKB 1
with a threshold of 1e−05

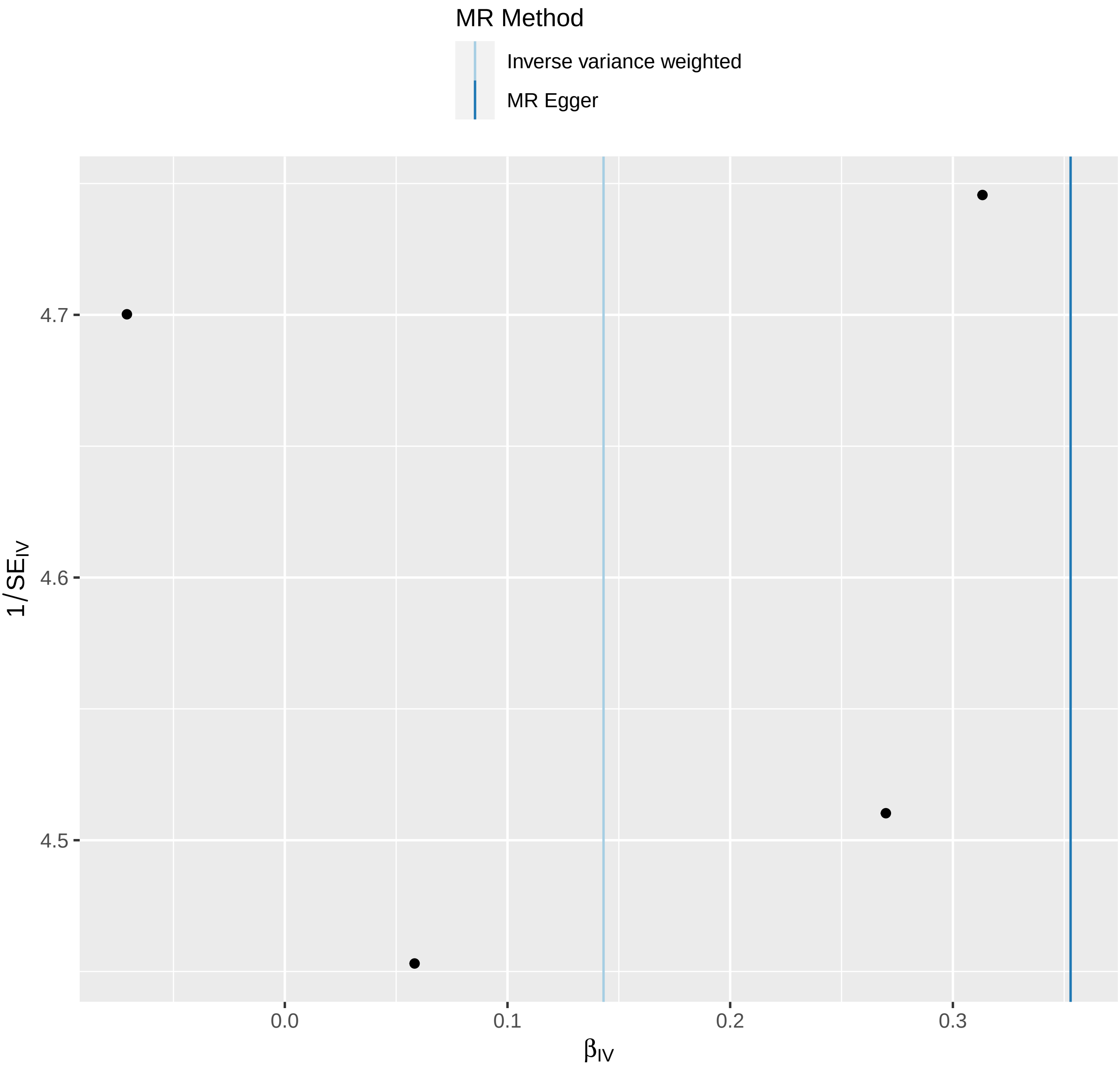

Batch 2−3940 : Gut microbiota abundance (genus Parasutterella id.2892) on Mitochondrial ubiquitin ligase activator of NFKB 1
with a threshold of 1e−05

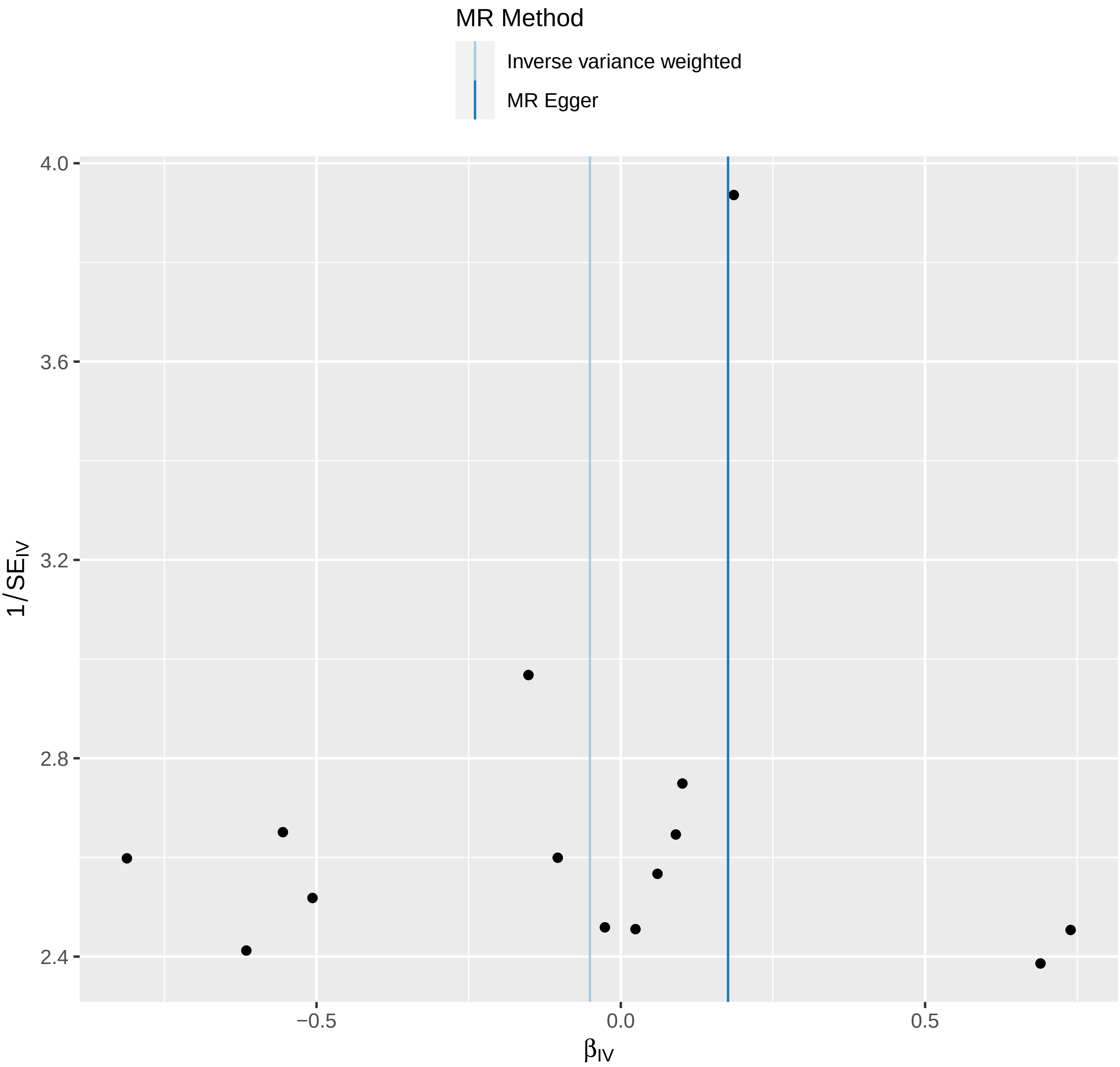

Batch 2−3941 : Gut microbiota abundance (genus Catenibacterium id.2153) on Muscle, skeletal receptor tyrosine−protein kinase
with a threshold of 1e−05

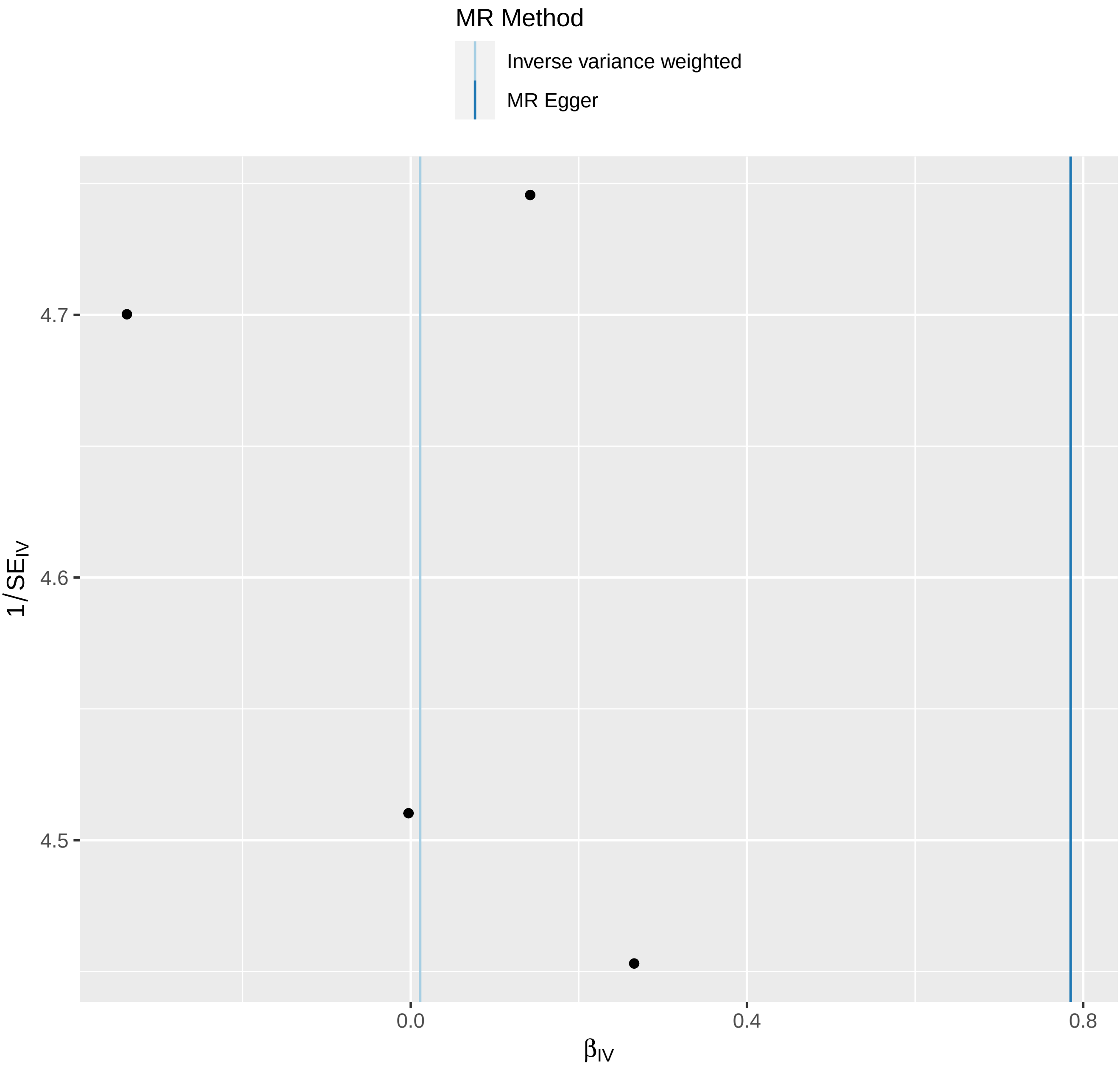

Batch 2−3942 : Gut microbiota abundance (genus Parasutterella id.2892) on Muscle, skeletal receptor tyrosine−protein kinase
with a threshold of 1e−05

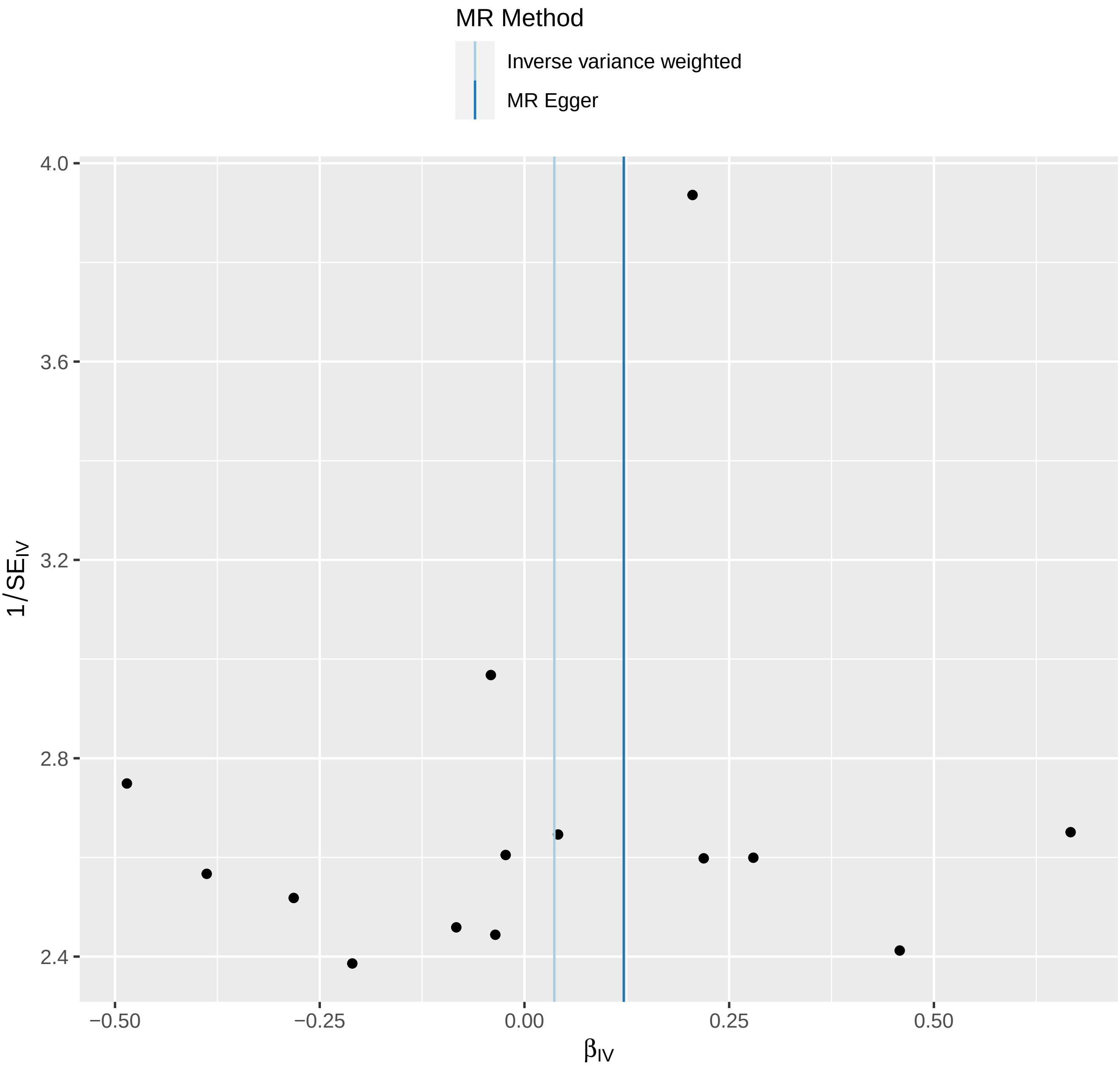

Batch 2−3943 : Gut microbiota abundance (genus Catenibacterium id.2153) on A/G−specific adenine DNA glycosylase with a threshold of 1e−05

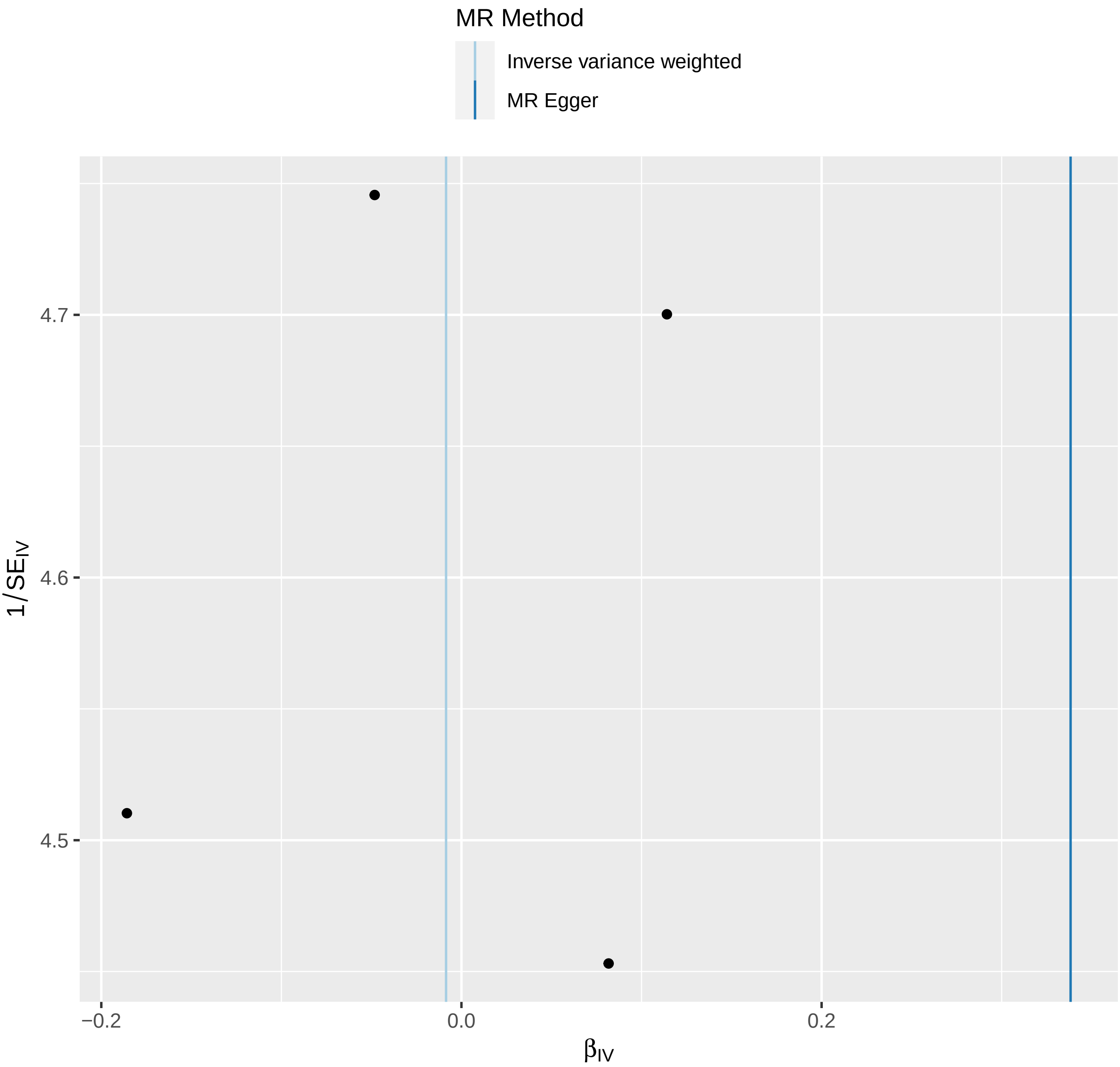

Batch 2−3944 : Gut microbiota abundance (genus Parasutterella id.2892) on A/G−specific adenine DNA glycosylase with a threshold of 1e−05

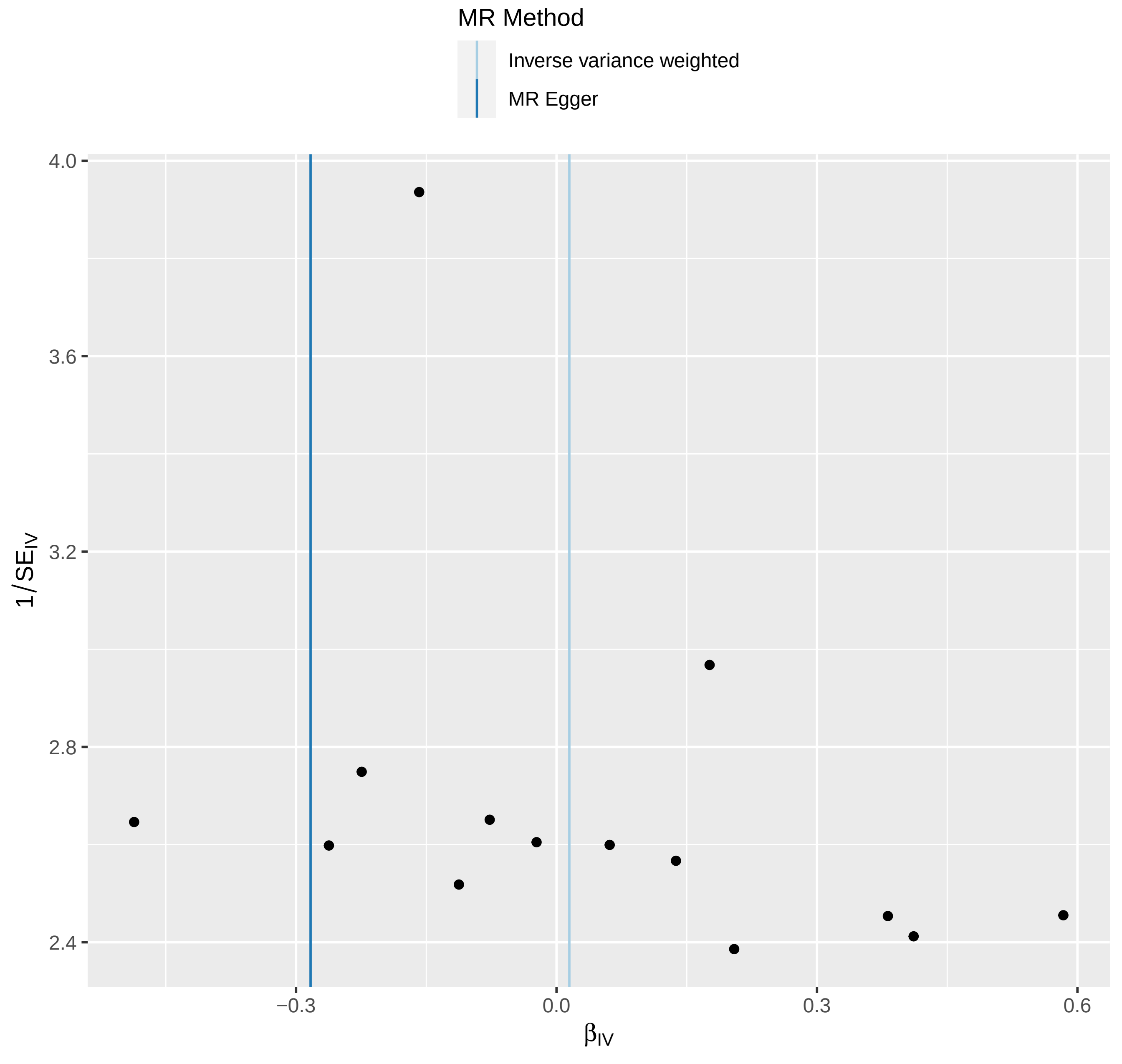

Batch 2−3945 : Gut microbiota abundance (genus Catenibacterium id.2153) on MAX−interacting protein 1
with a threshold of 1e−05

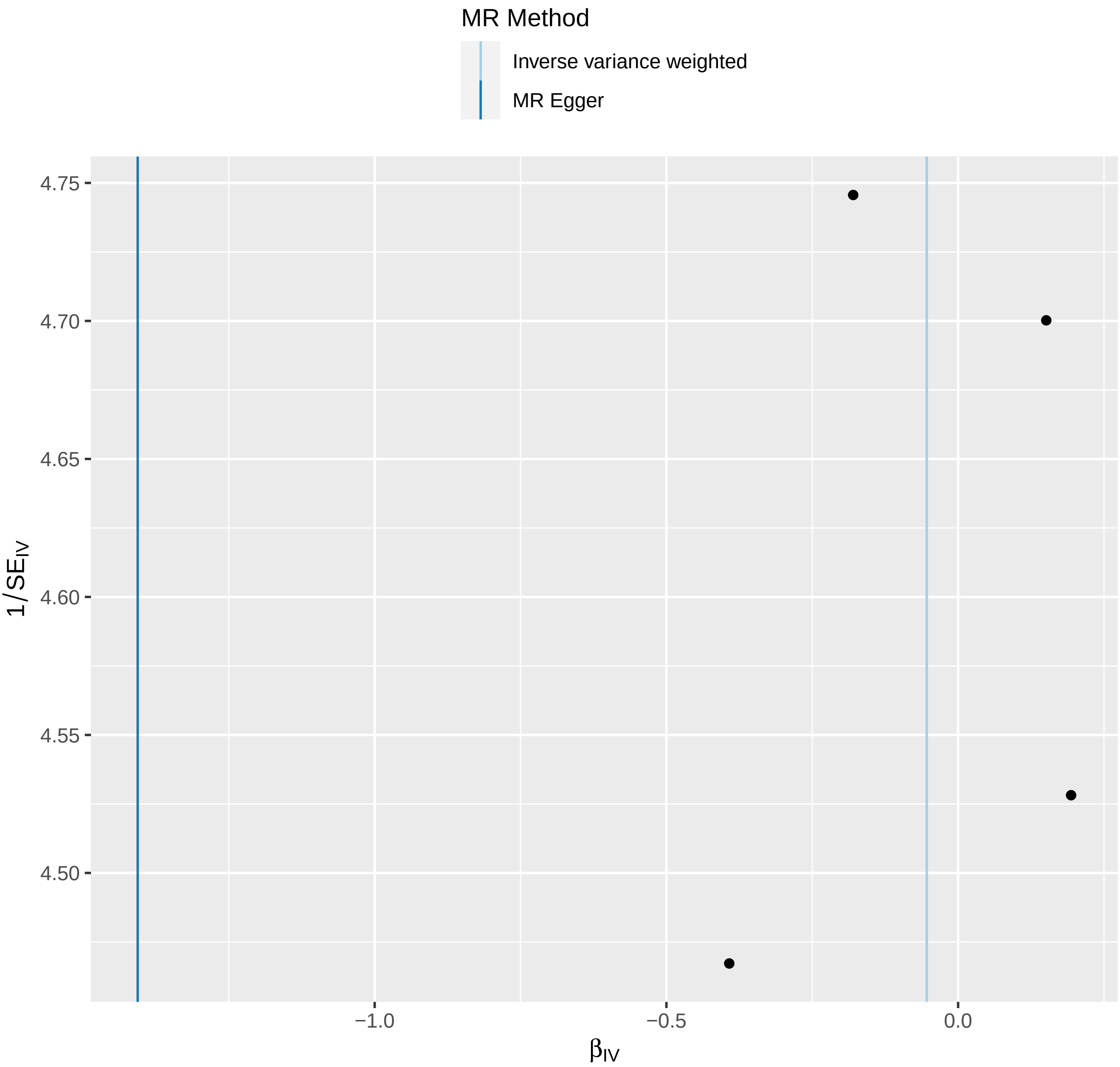

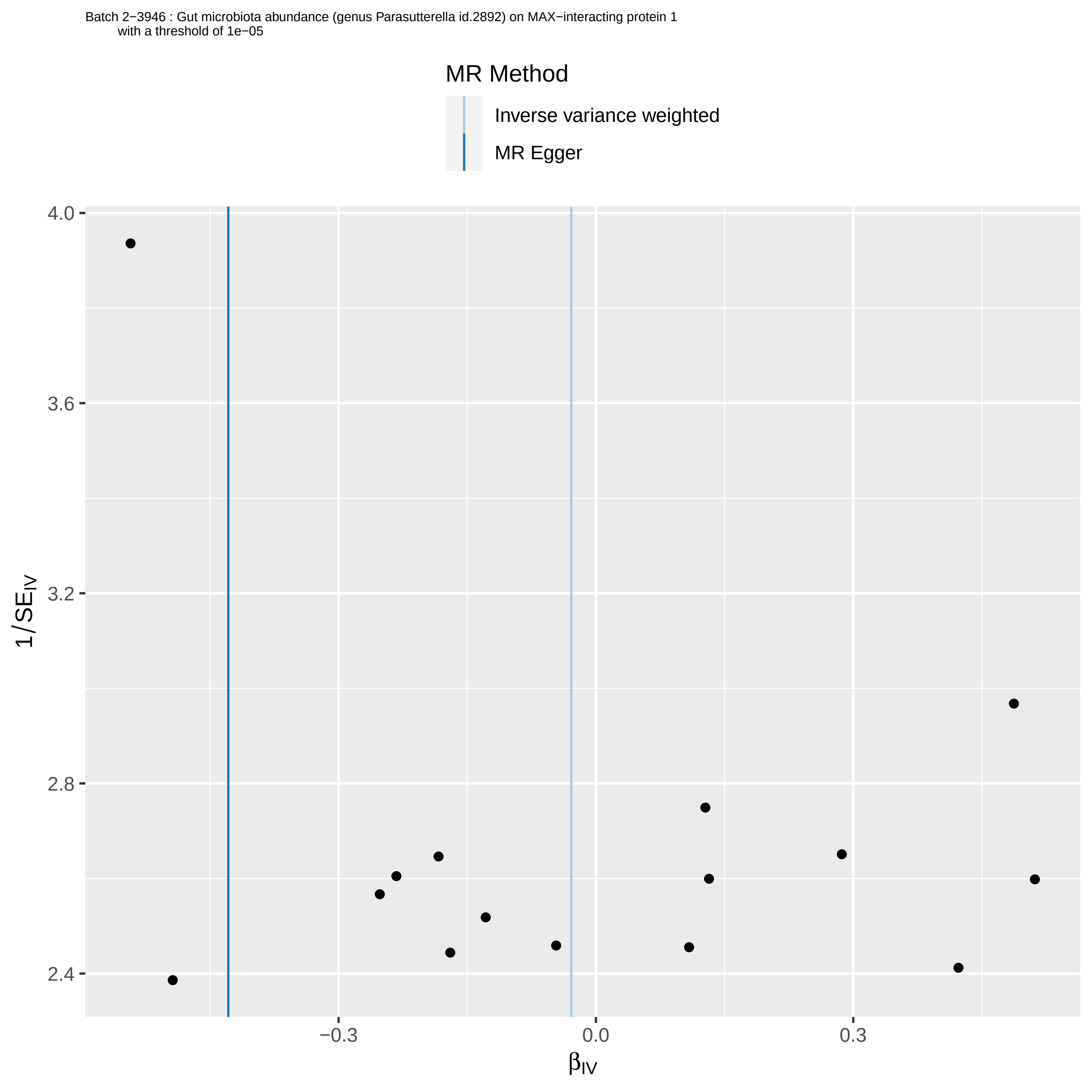
Batch 2−3946 : Gut microbiota abundance (genus Parasutterella id.2892) on MAX−interacting protein 1
with a threshold of 1e−05
MR Method
Inverse variance weighted
MR Egger
4.0
3.6
3.2
2.8
2.4
1/SE_IV
−0.3
0.0
0.3
β_IV

Batch 2−3947 : Gut microbiota abundance (genus Catenibacterium id.2153) on Matrix−remodeling−associated protein 7 with a threshold of 1e−05

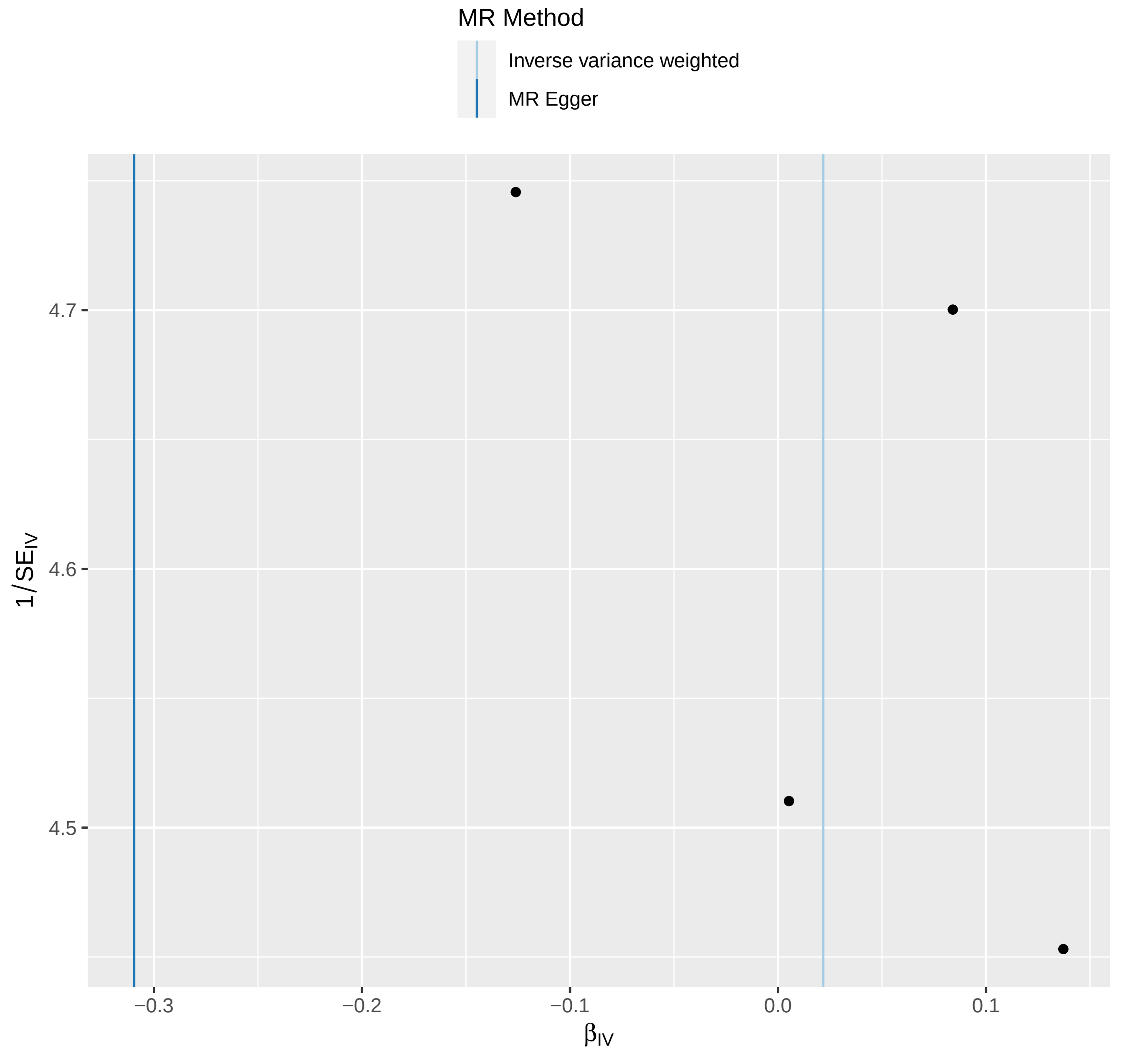

Batch 2−3948 : Gut microbiota abundance (genus Parasutterella id.2892) on Matrix−remodeling−associated protein 7 with a threshold of 1e−05

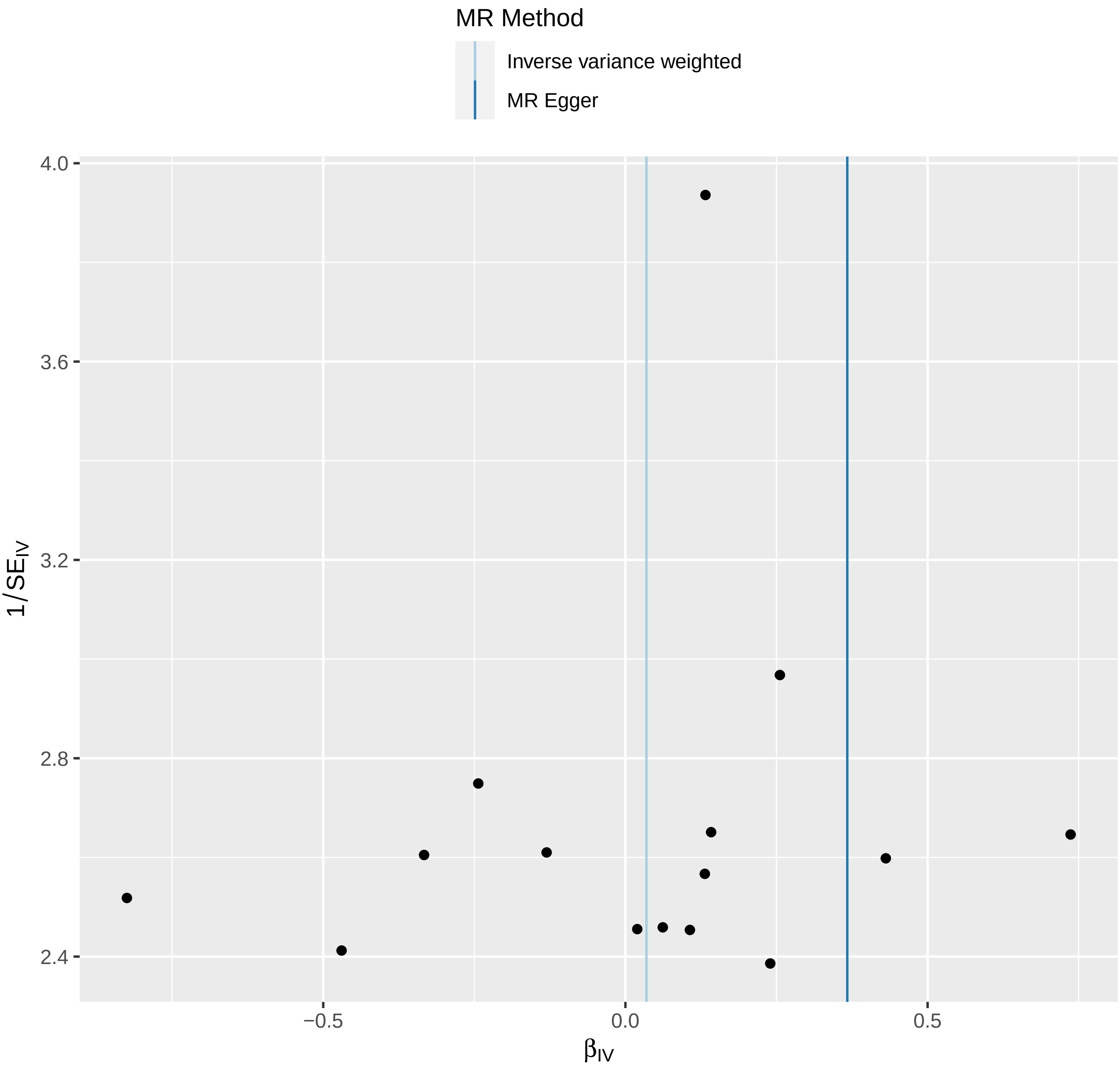

Batch 2−3949 : Gut microbiota abundance (genus Catenibacterium id.2153) on Matrix−remodeling−associated protein 8
with a threshold of 1e−05

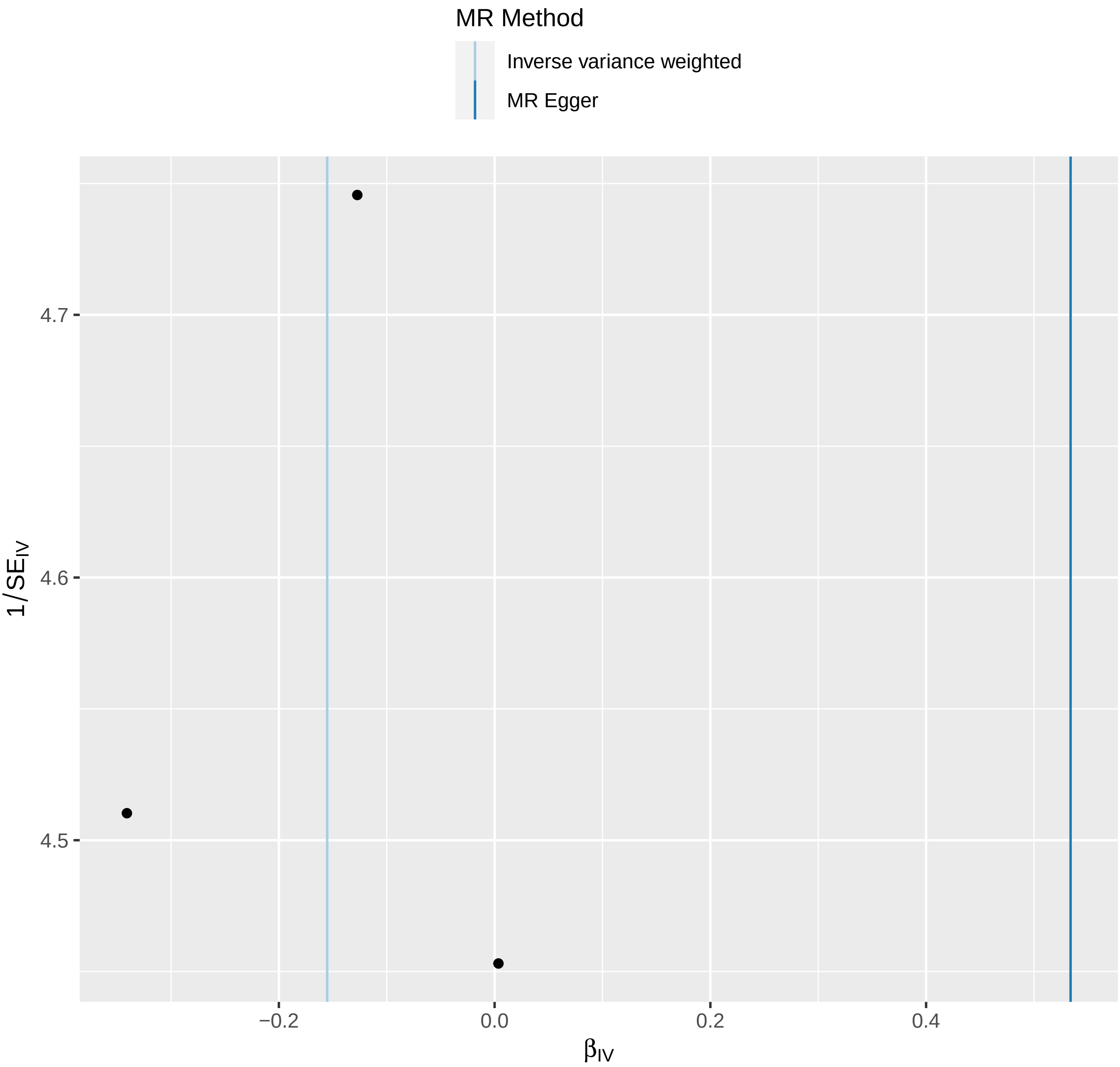

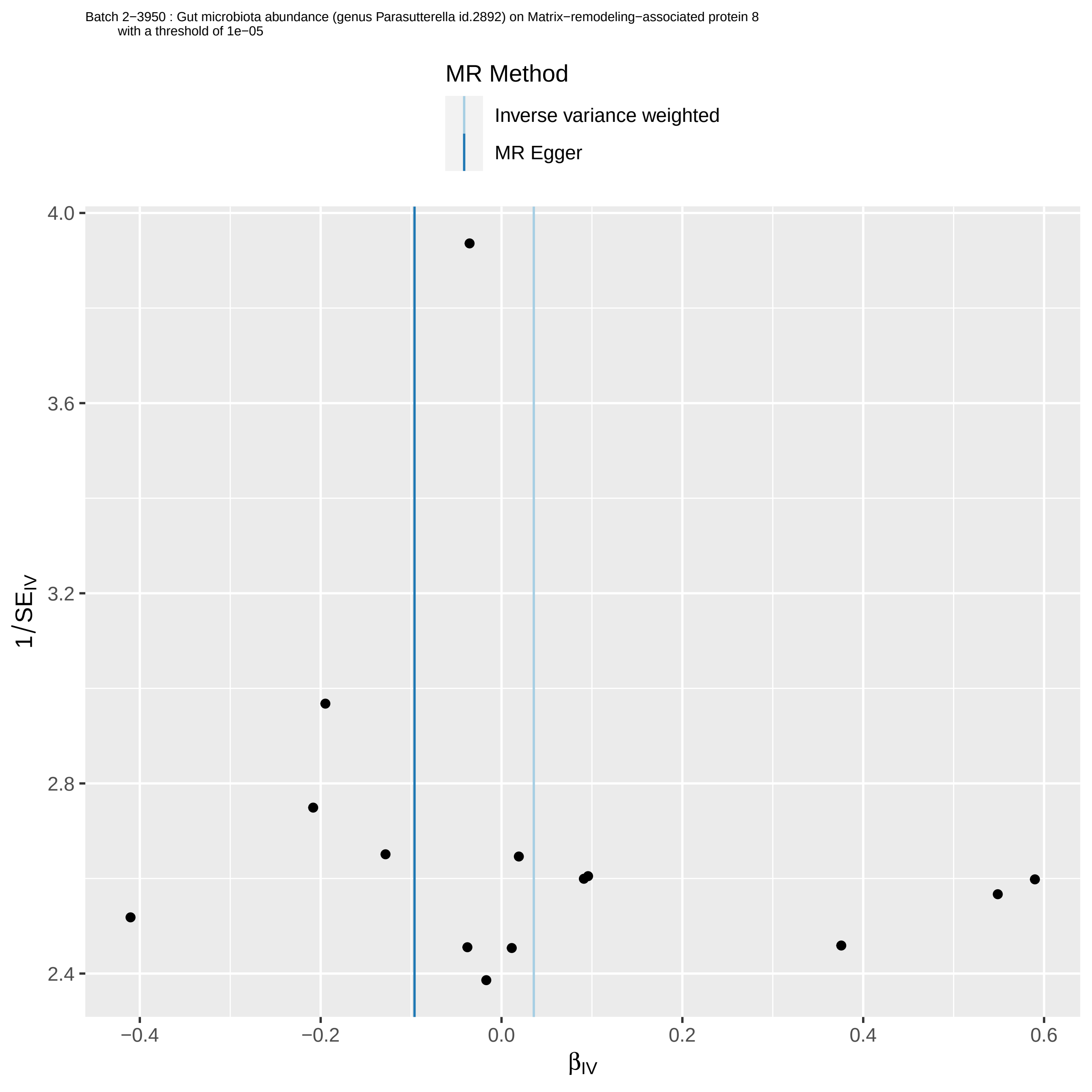
Batch 2−3950 : Gut microbiota abundance (genus Parasutterella id.2892) on Matrix−remodeling−associated protein 8
with a threshold of 1e−05
MR Method
Inverse variance weighted
MR Egger
4.0
3.6
3.2
2.8
2.4
1/SE_IV
−0.4
−0.2
0.0
0.2
0.4
0.6
β_IV

Batch 2−3951 : Gut microbiota abundance (genus Catenibacterium id.2153) on Matrix−remodeling−associated protein 8
with a threshold of 1e−05

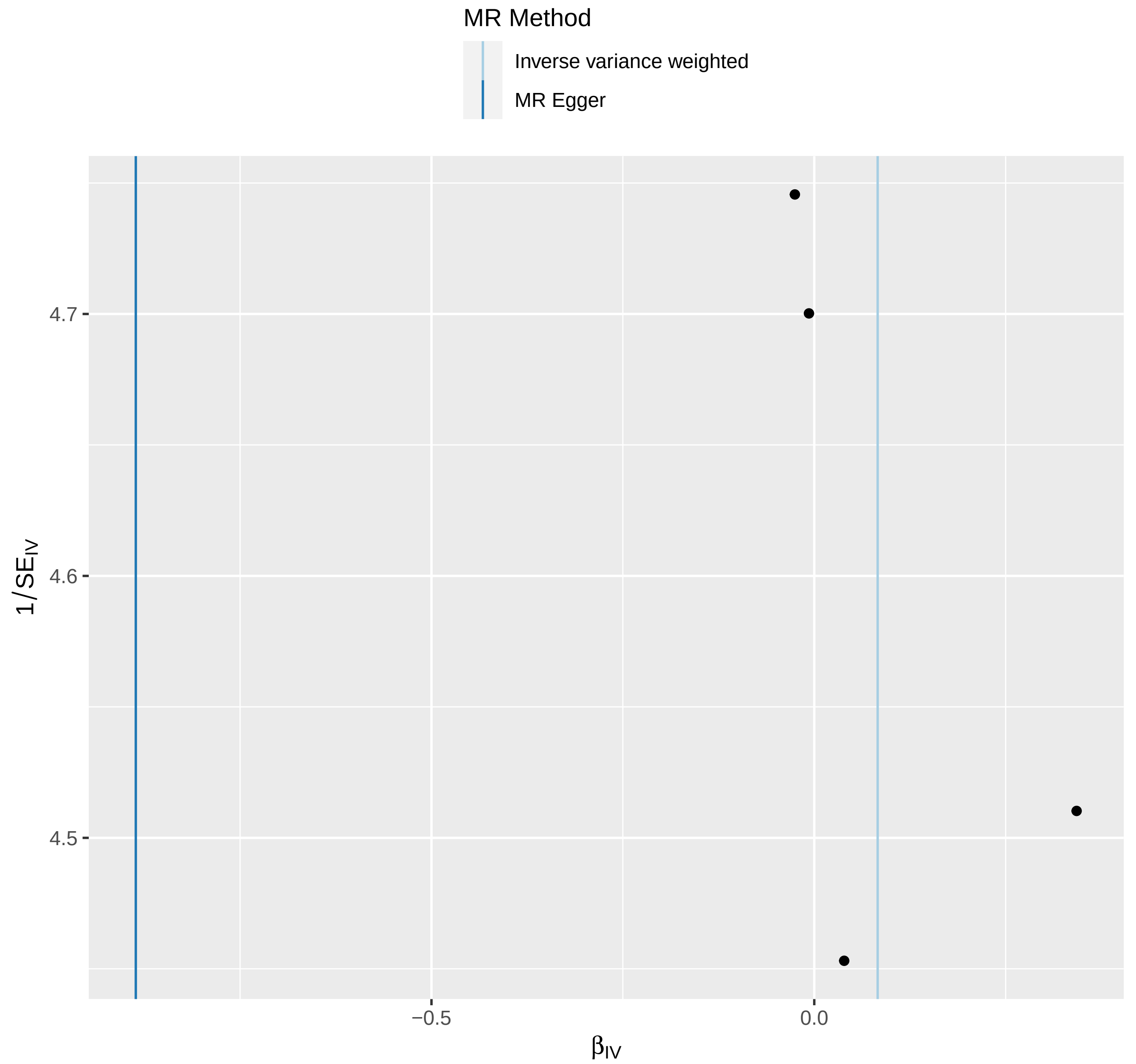

Batch 2−3952 : Gut microbiota abundance (genus Parasutterella id.2892) on Matrix−remodeling−associated protein 8
with a threshold of 1e−05

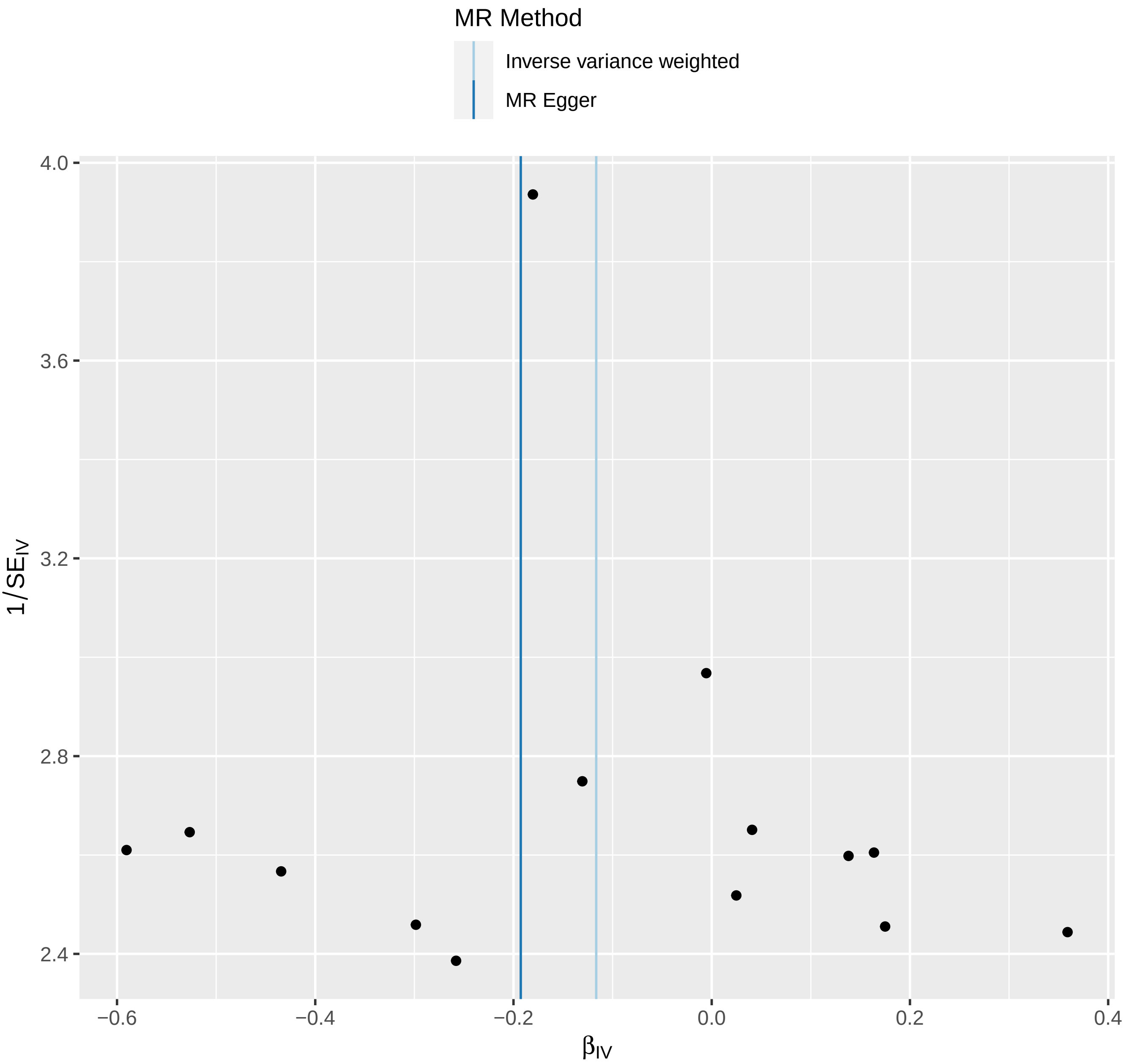

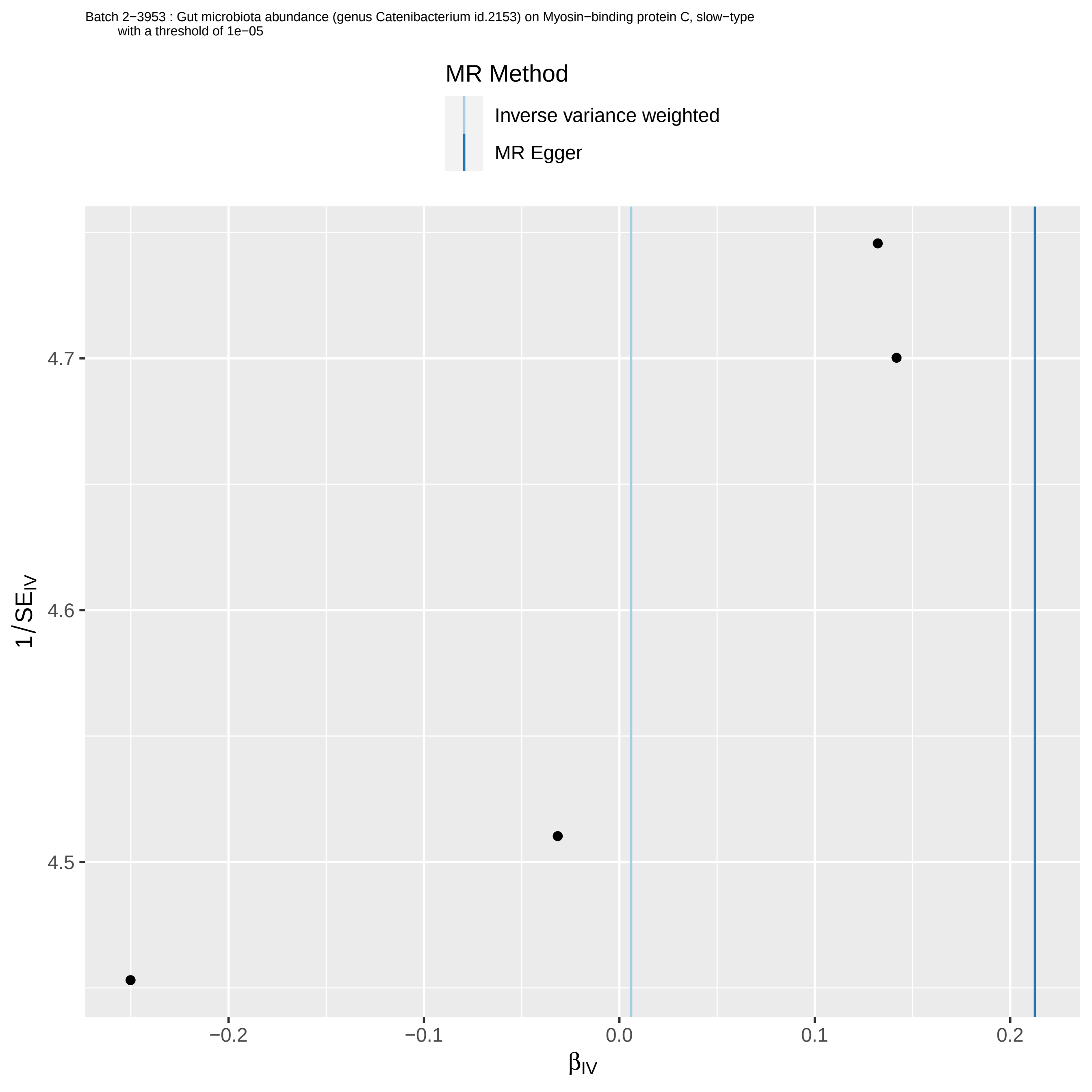

Batch 2−3953 : Gut microbiota abundance (genus Catenibacterium id.2153) on Myosin−binding protein C, slow−type
with a threshold of 1e−05
MR Method
Inverse variance weighted
MR Egger
4.7
4.6
4.5
1/SE_IV
−0.2
−0.1
0.0
0.1
0.2
β_IV

Batch 2−3954 : Gut microbiota abundance (genus Parasutterella id.2892) on Myosin−binding protein C, slow−type
with a threshold of 1e−05

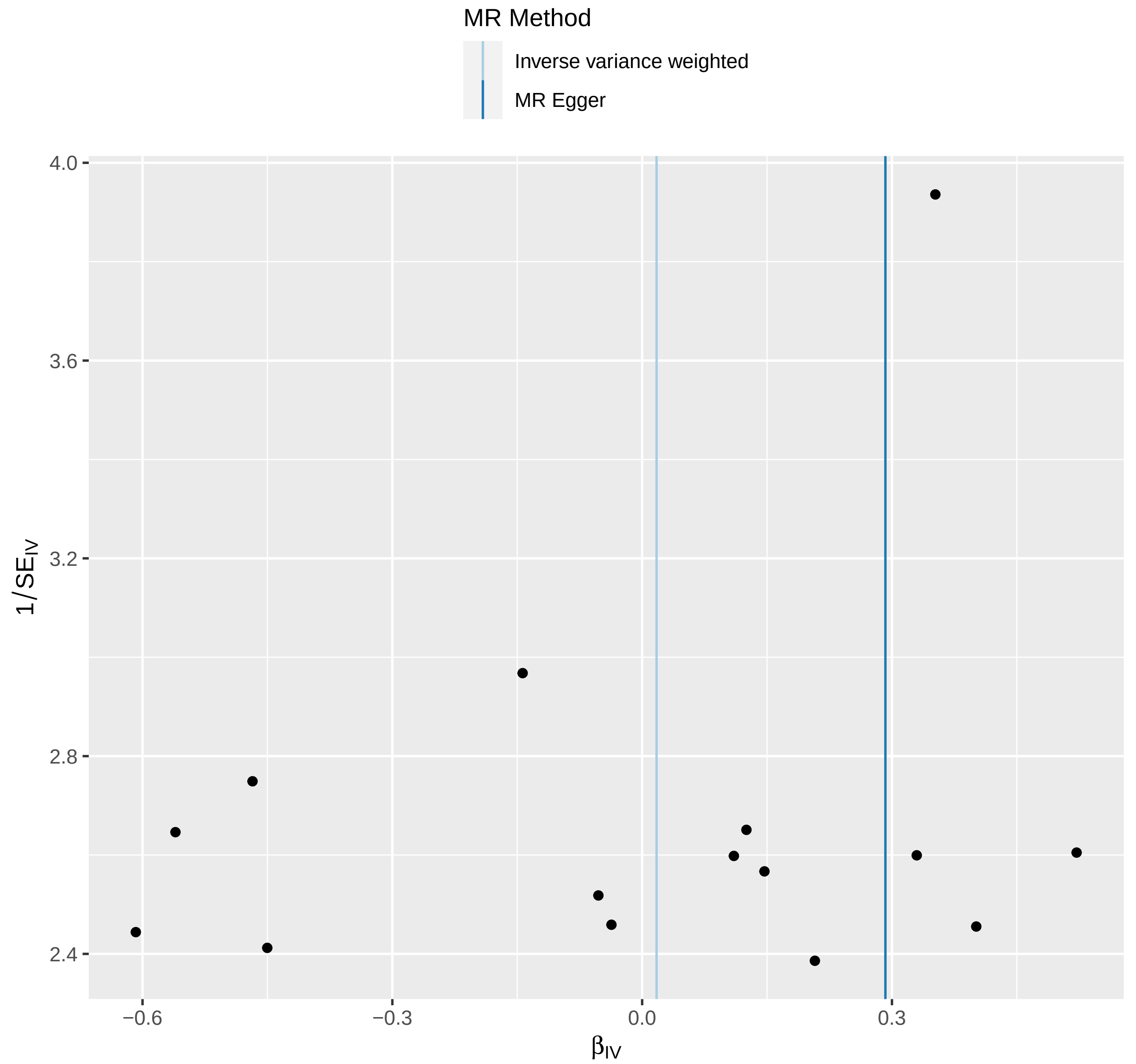

Batch 2−3955 : Gut microbiota abundance (genus Catenibacterium id.2153) on Myc proto−oncogene protein
with a threshold of 1e−05

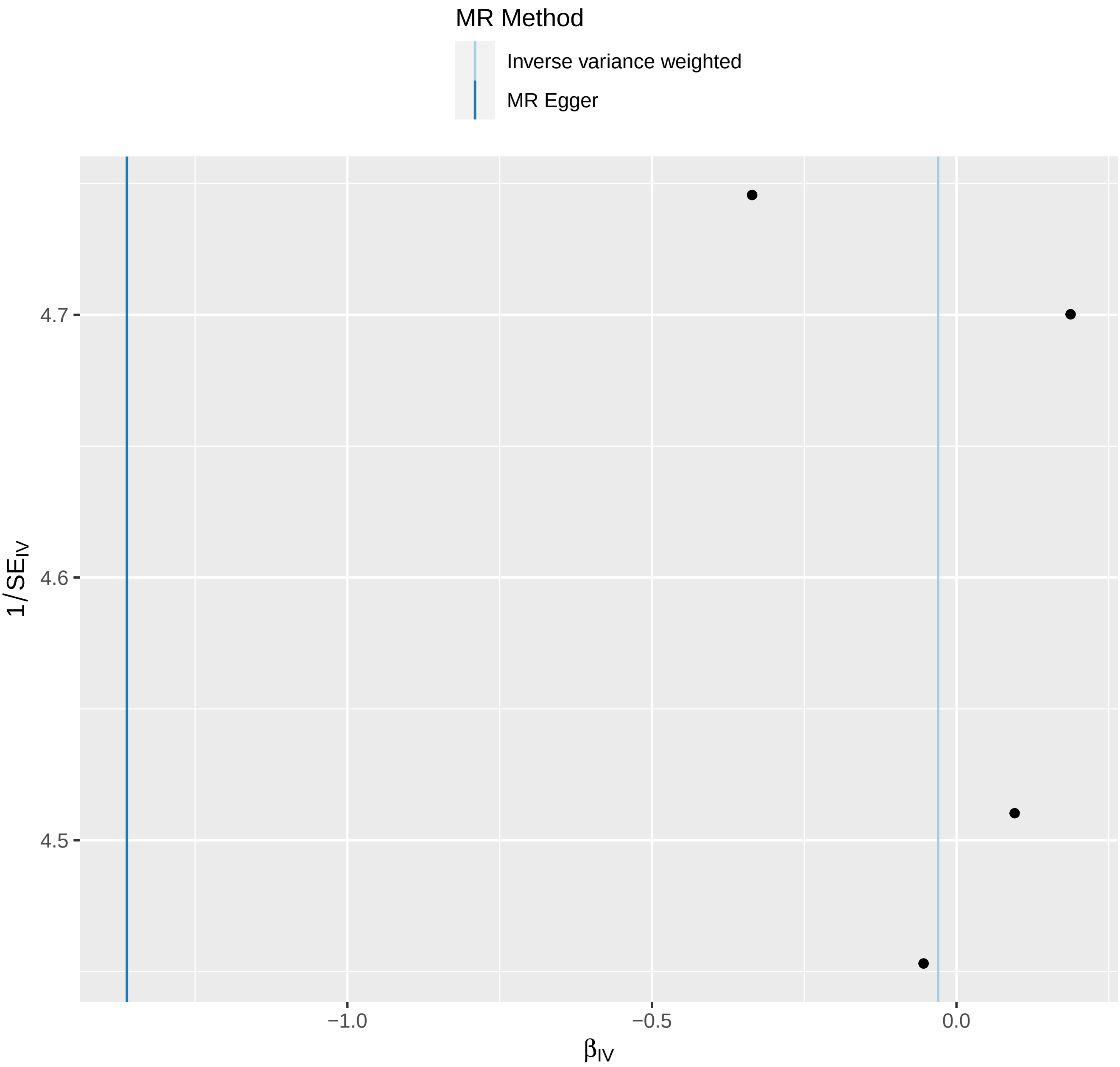

Batch 2−3956 : Gut microbiota abundance (genus Parasutterella id.2892) on Myc proto−oncogene protein
with a threshold of 1e−05

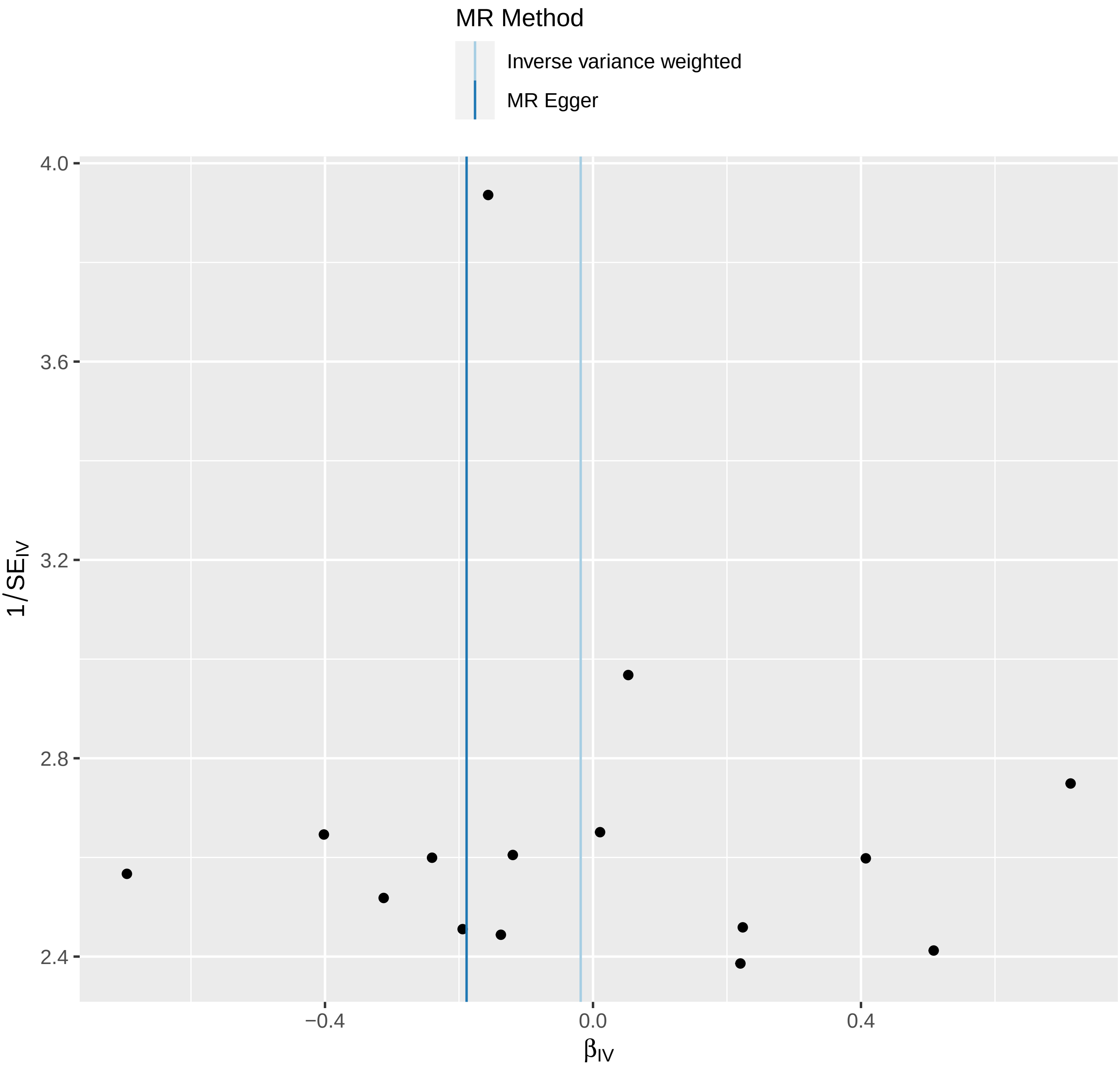

Batch 2−3957 : Gut microbiota abundance (genus Catenibacterium id.2153) on Myc target protein 1
with a threshold of 1e−05

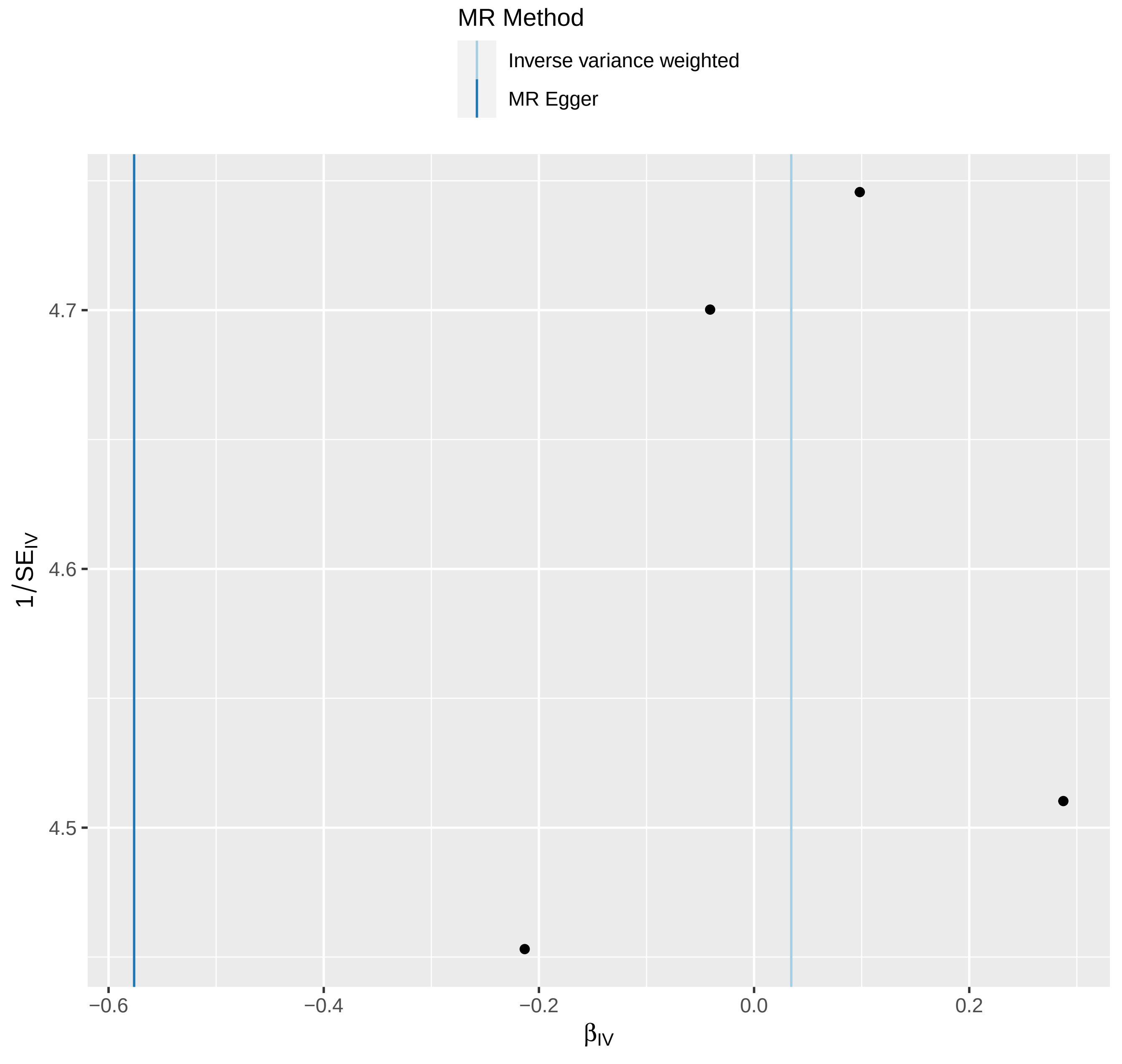

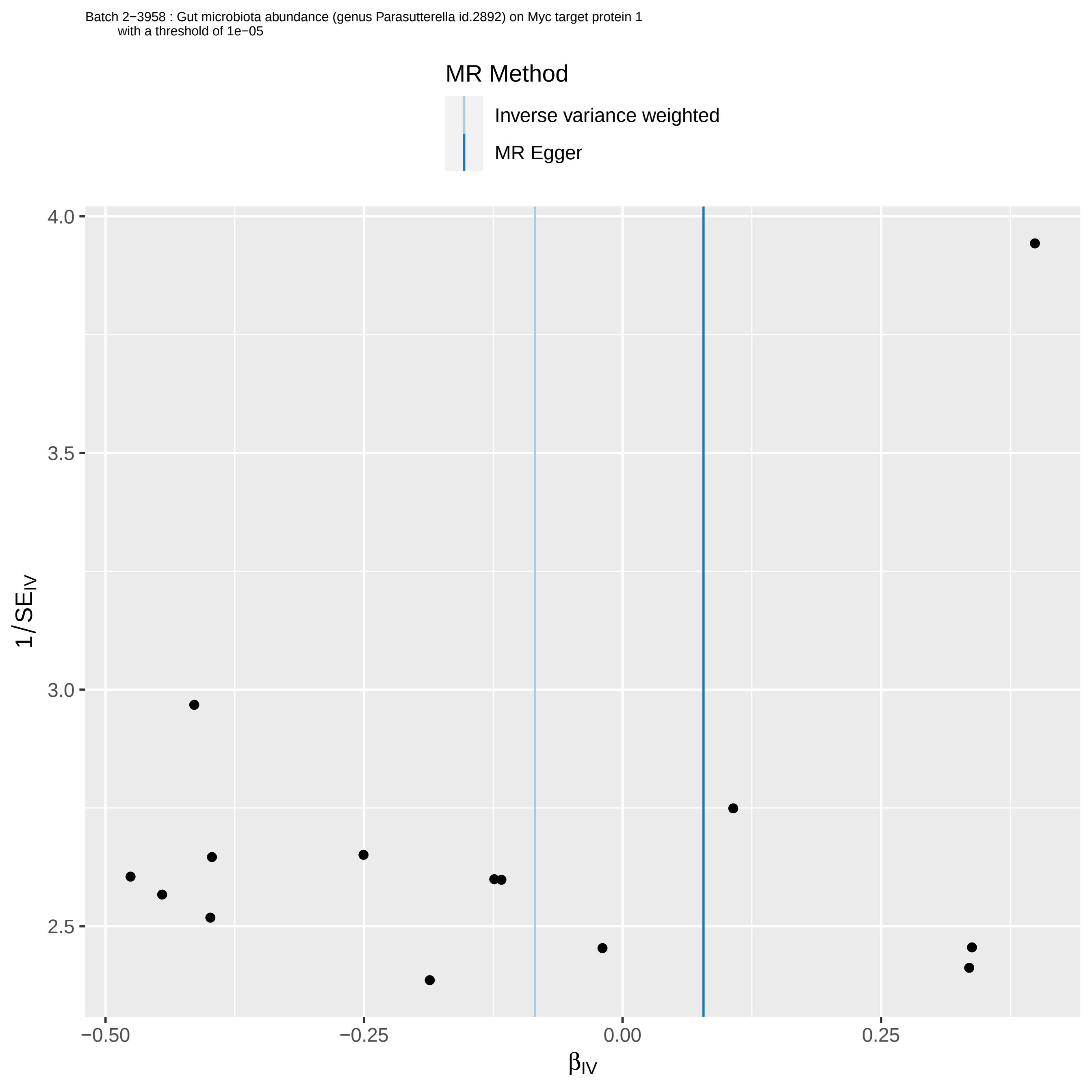

Batch 2−3958 : Gut microbiota abundance (genus Parasutterella id.2892) on Myc target protein 1
with a threshold of 1e−05
MR Method
Inverse variance weighted
MR Egger
4.0
3.5
3.0
2.5
1/SE_IV
−0.50
−0.25
0.00
0.25
β_IV

Batch 2−3959 : Gut microbiota abundance (genus Catenibacterium id.2153) on Unconventional myosin−VI
with a threshold of 1e−05

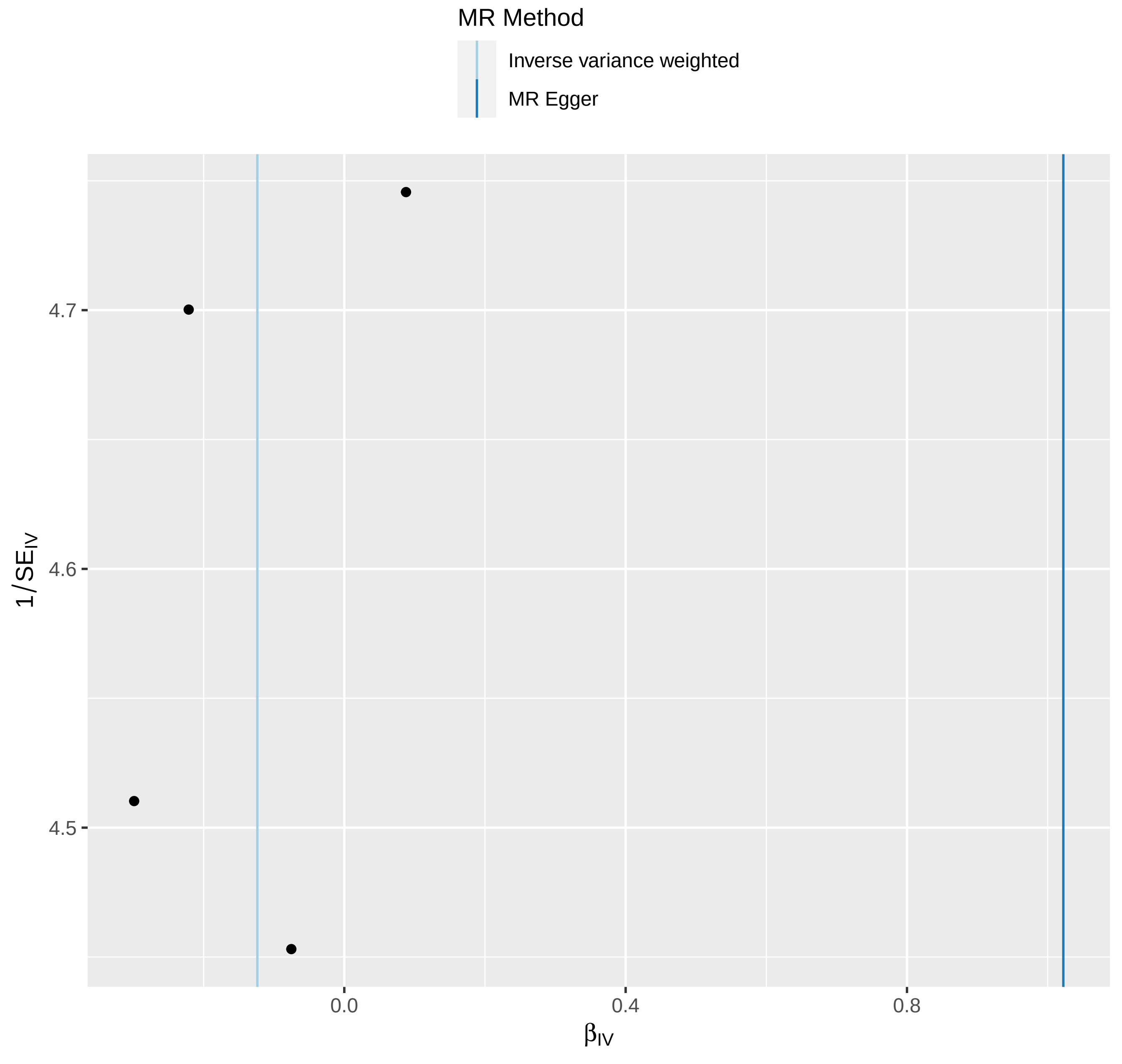

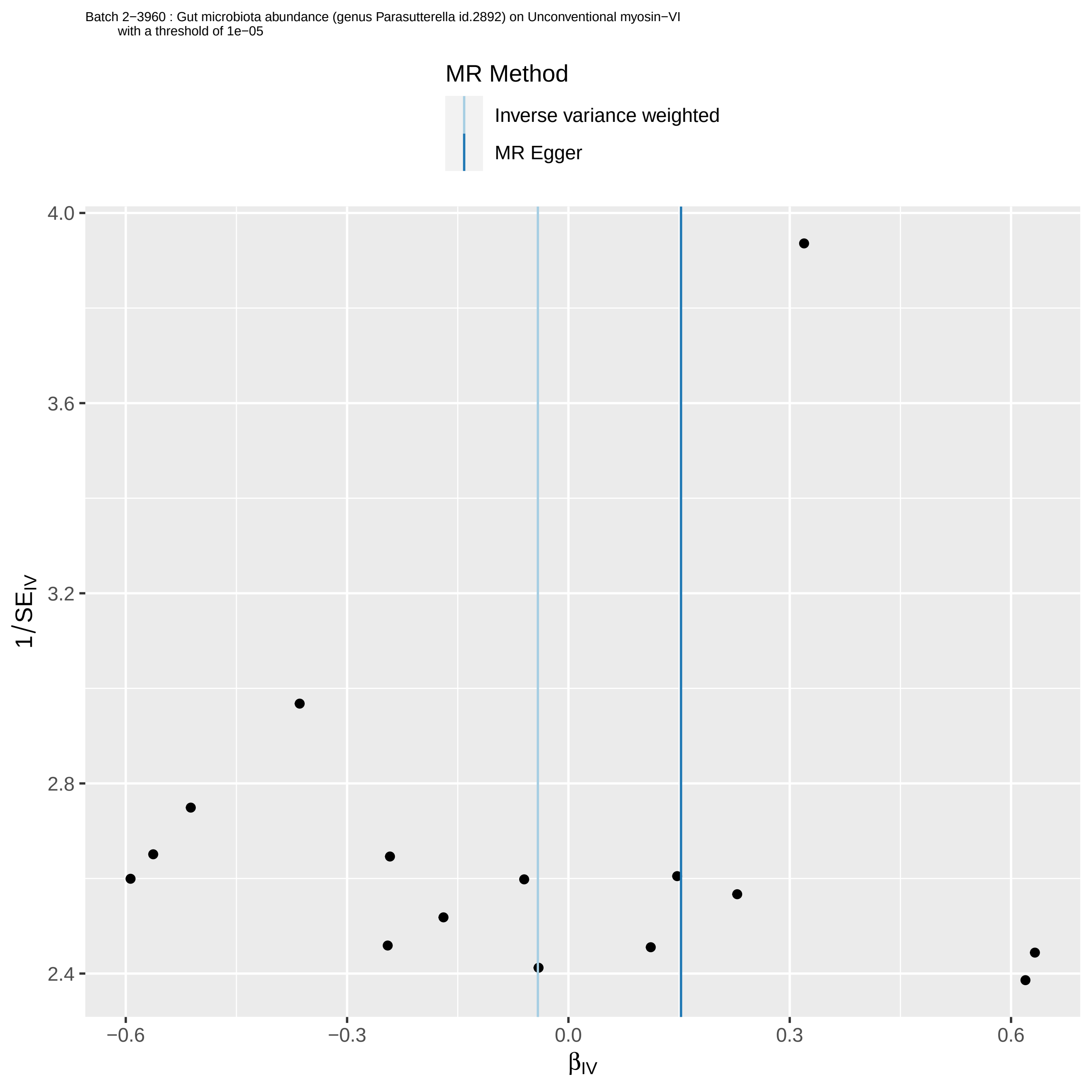

Batch 2−3960 : Gut microbiota abundance (genus Parasutterella id.2892) on Unconventional myosin−VI
with a threshold of 1e−05
MR Method
Inverse variance weighted
MR Egger
4.0
3.6
3.2
2.8
2.4
1/SE_IV
−0.6
−0.3
0.0
0.3
0.6
β_IV

Batch 2−3961 : Gut microbiota abundance (genus Catenibacterium id.2153) on Myomesin−2
with a threshold of 1e−05

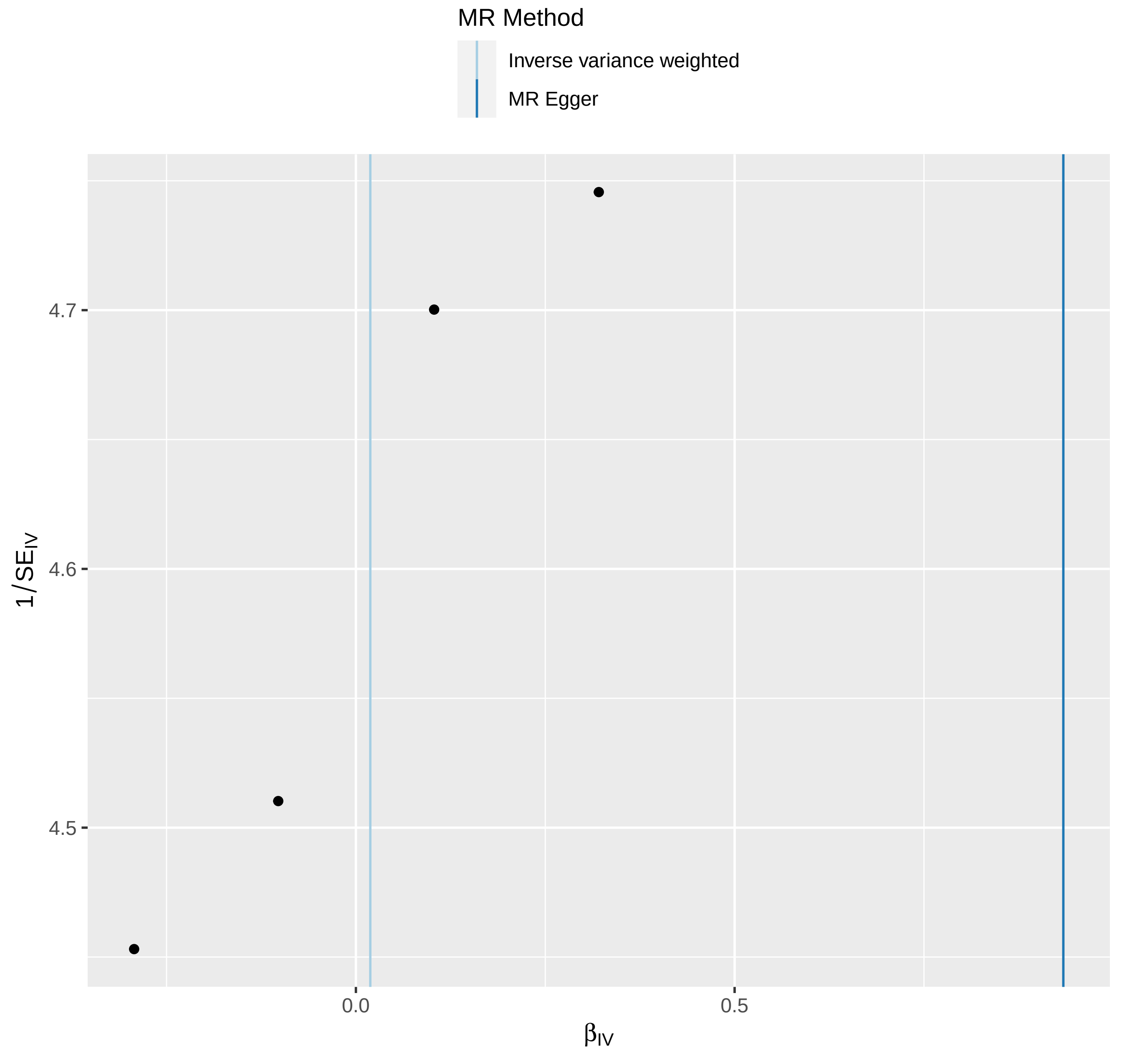

Batch 2−3962 : Gut microbiota abundance (genus Parasutterella id.2892) on Myomesin−2
with a threshold of 1e−05

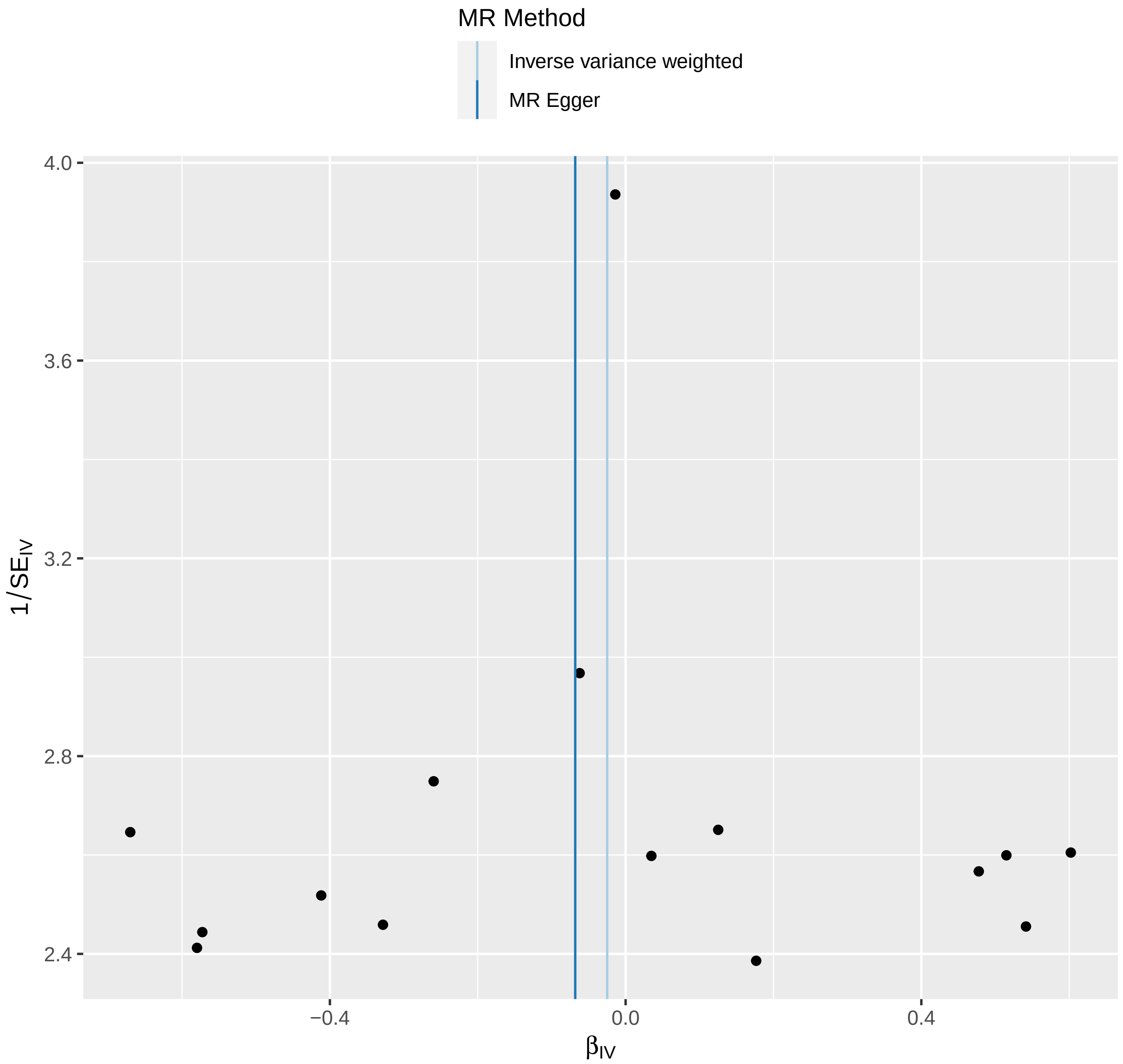

Batch 2−3963 : Gut microbiota abundance (genus Catenibacterium id.2153) on Myelin regulatory factor
with a threshold of 1e−05

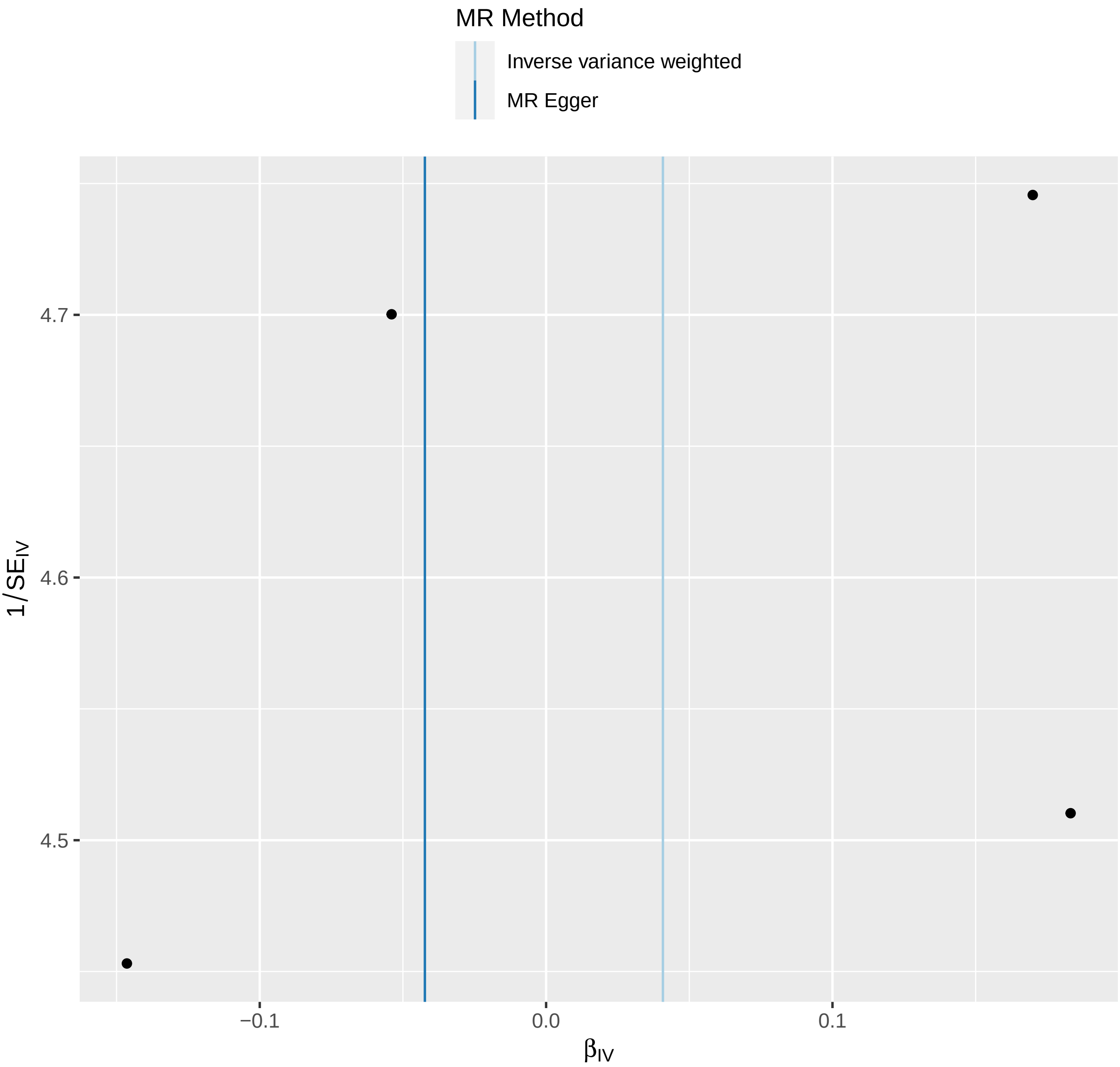

Batch 2−3964 : Gut microbiota abundance (genus Parasutterella id.2892) on Myelin regulatory factor
with a threshold of 1e−05

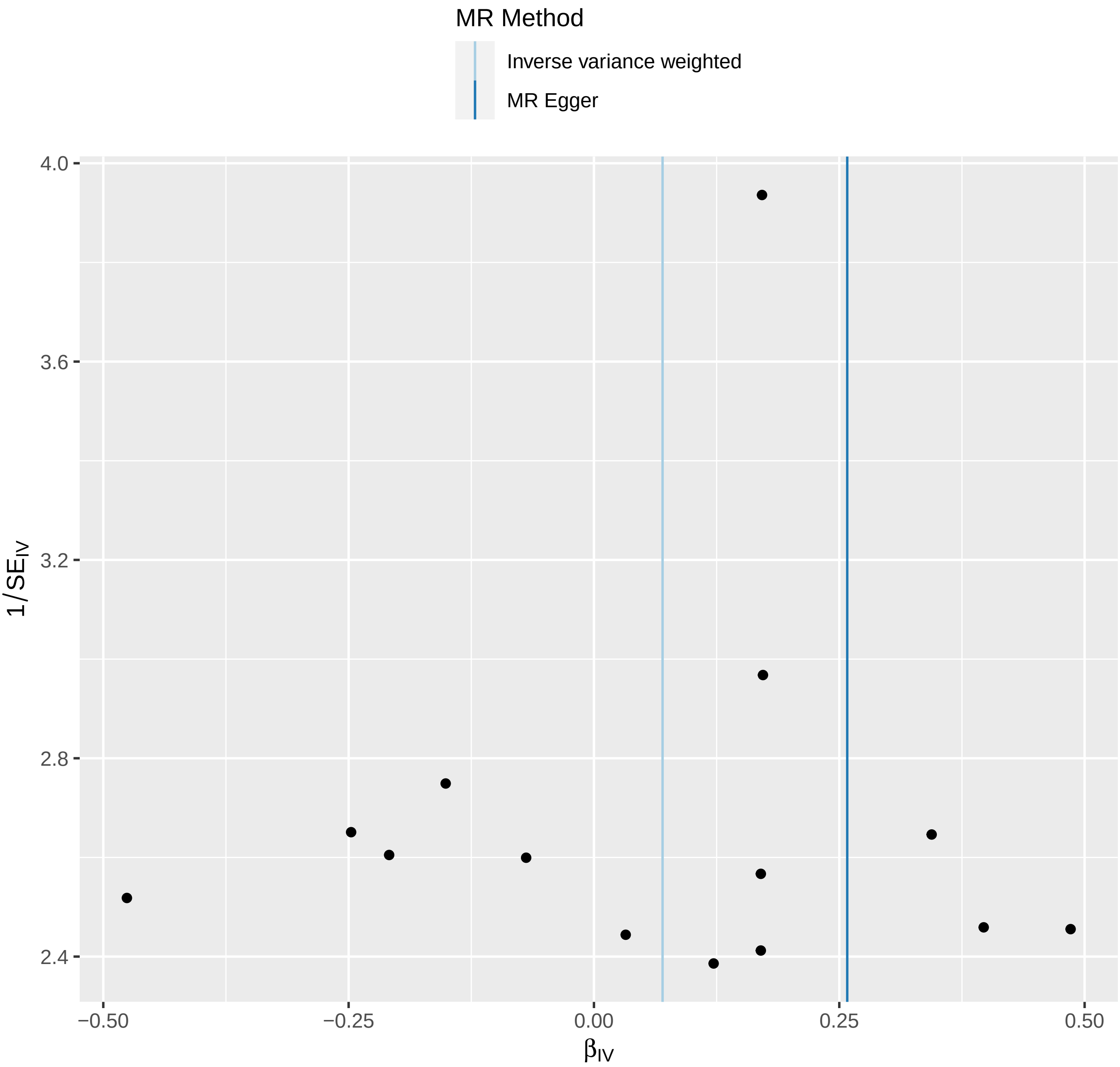

Batch 2−3965 : Gut microbiota abundance (genus Catenibacterium id.2153) on Histone H2A deubiquitinase MYSM1
with a threshold of 1e−05

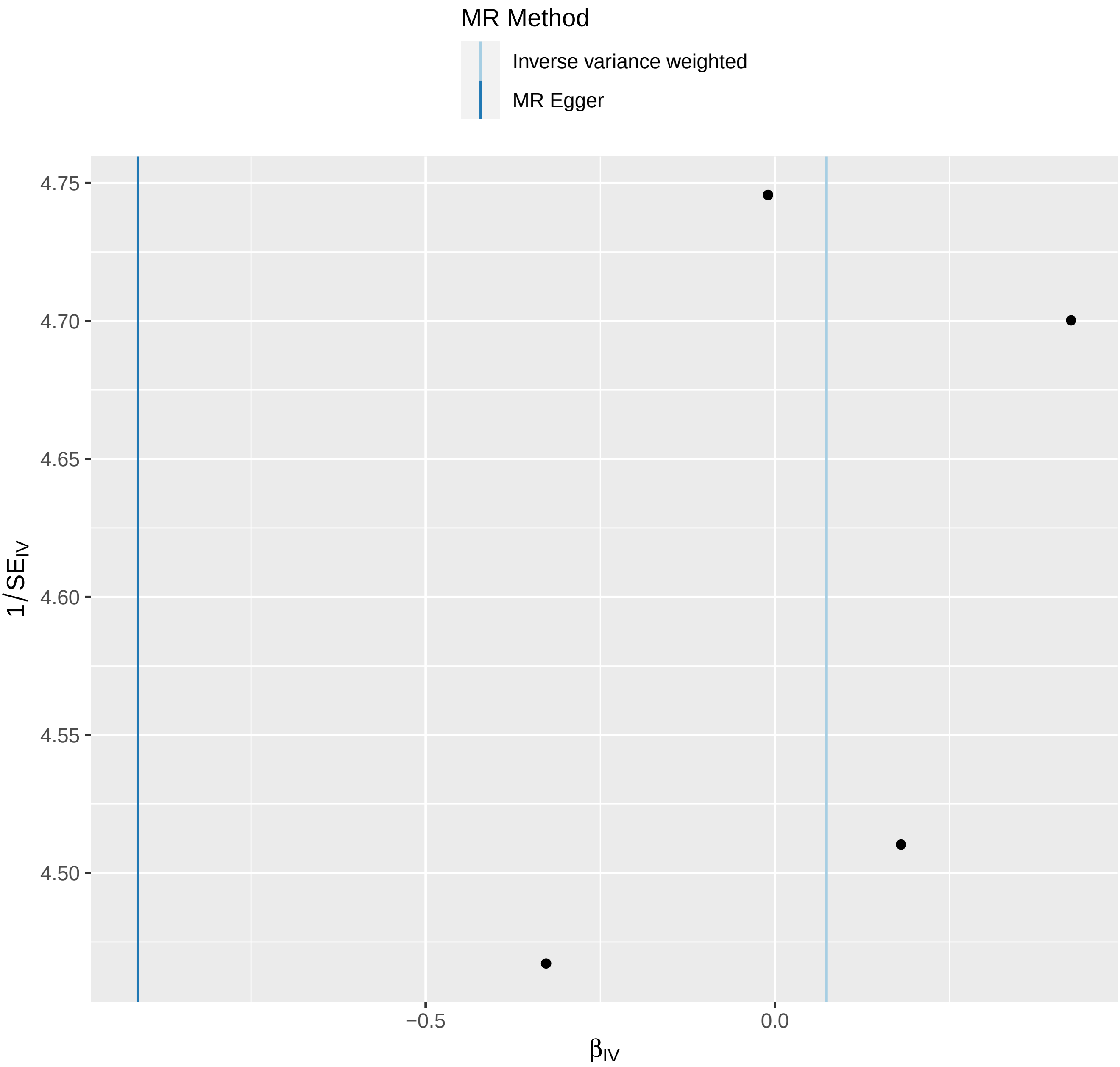

Batch 2−3966 : Gut microbiota abundance (genus Parasutterella id.2892) on Histone H2A deubiquitinase MYSM1
with a threshold of 1e−05

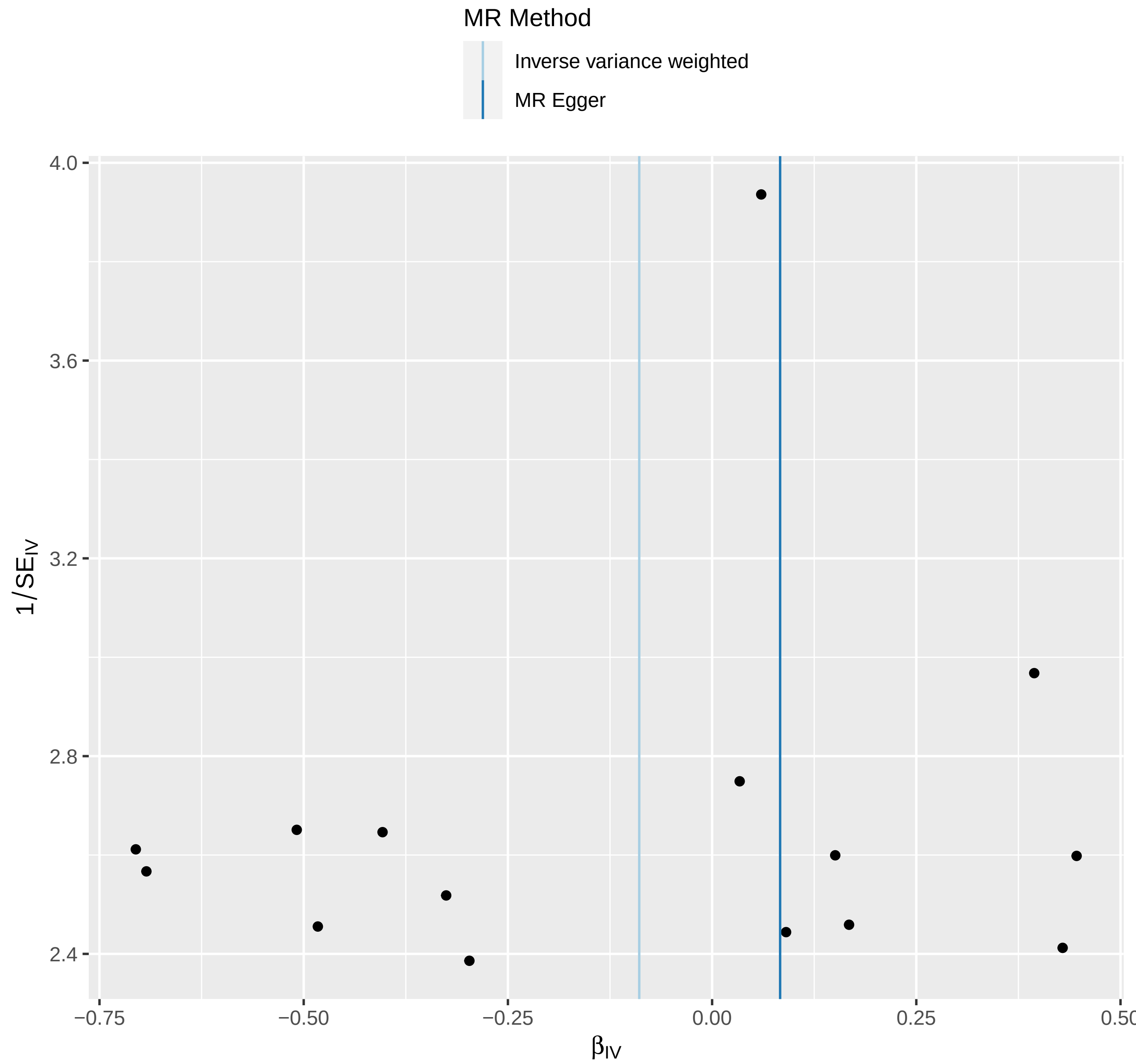

Batch 2−3967 : Gut microbiota abundance (genus Catenibacterium id.2153) on Myocardial zonula adherens protein with a threshold of 1e−05

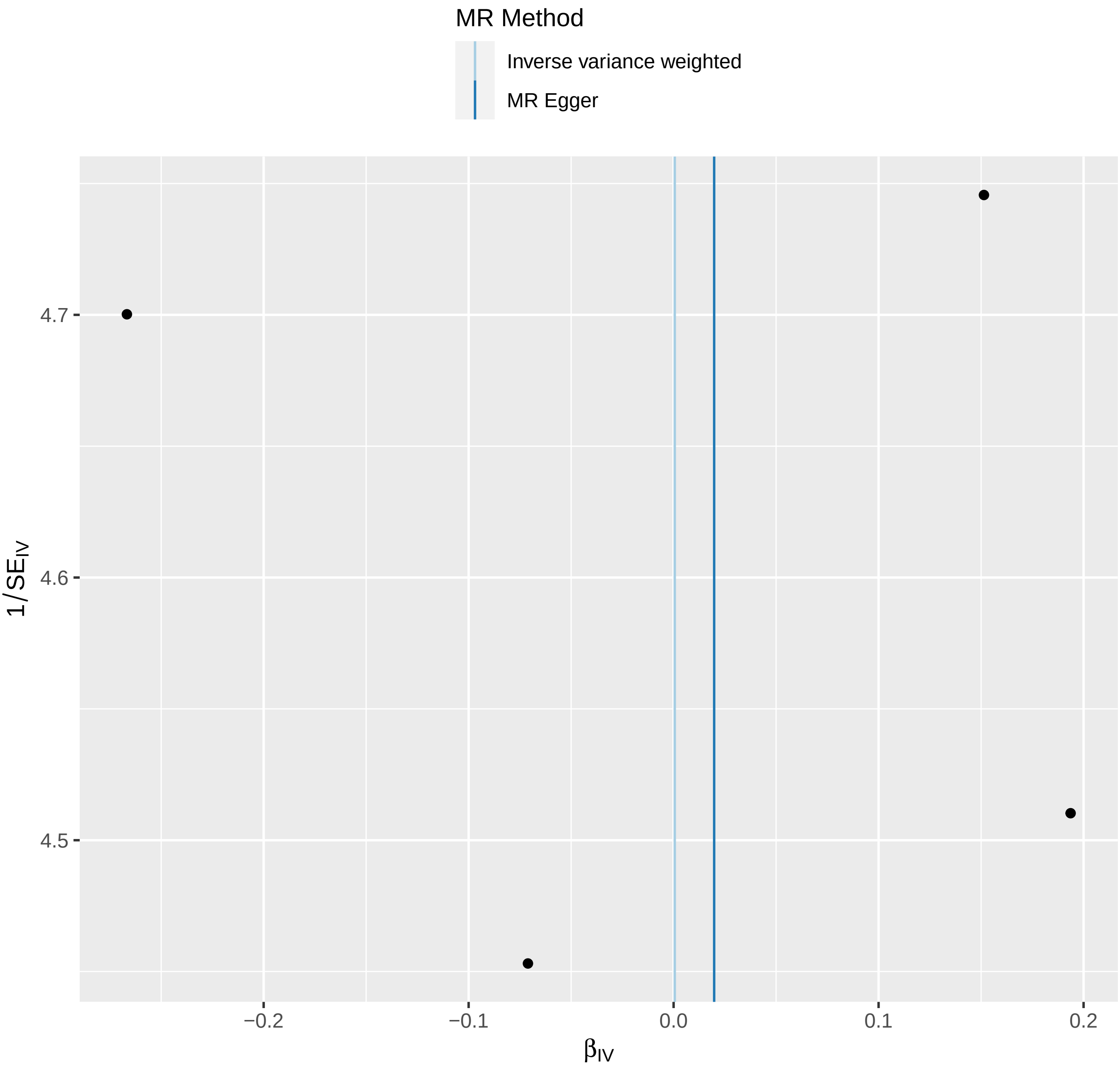

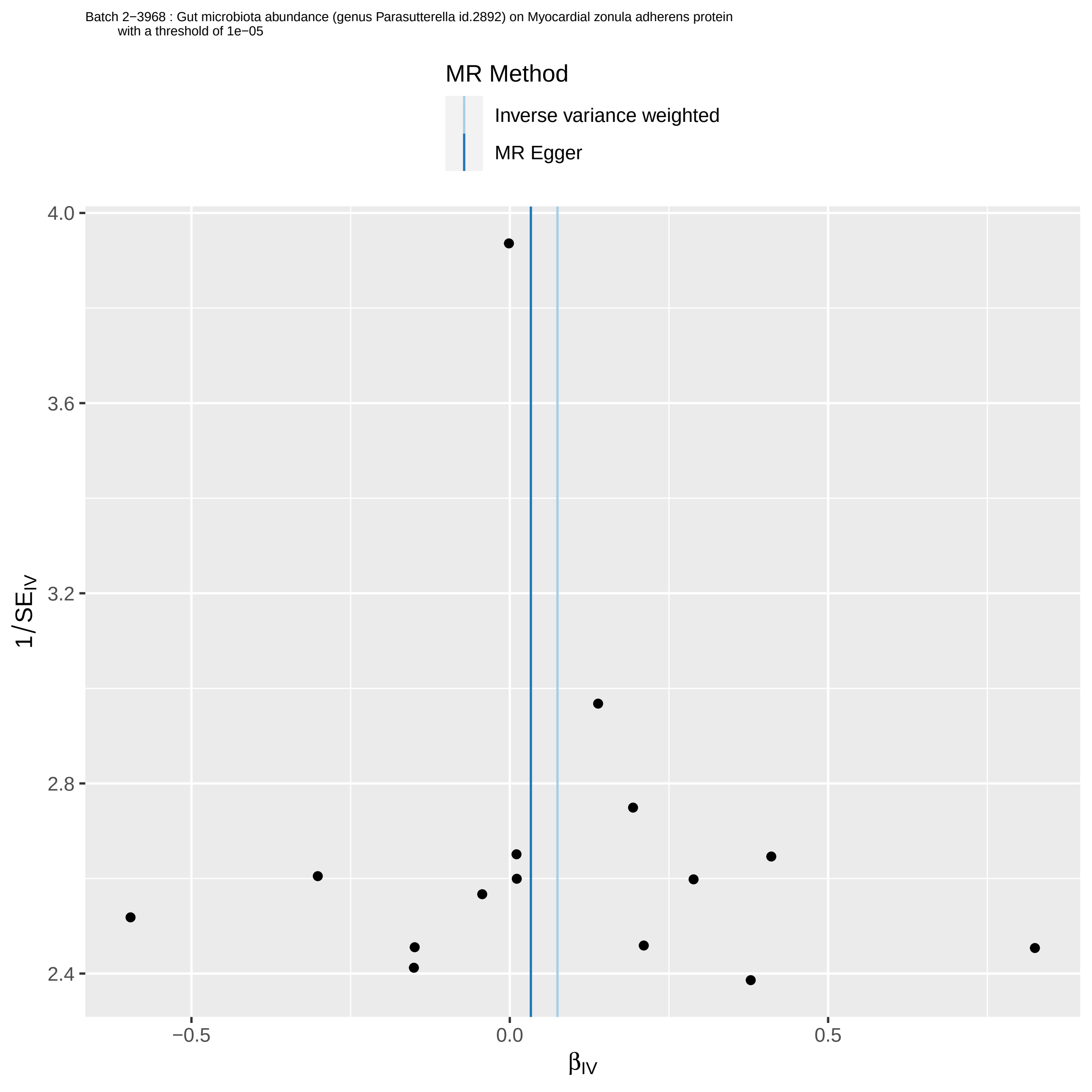

Batch 2−3968 : Gut microbiota abundance (genus Parasutterella id.2892) on Myocardial zonula adherens protein
with a threshold of 1e−05
MR Method
Inverse variance weighted
MR Egger
4.0
3.6
3.2
2.8
2.4
1/SE_IV
−0.5
0.0
0.5
β_IV

Batch 2−3969 : Gut microbiota abundance (genus Catenibacterium id.2153) on Myocardial zonula adherens protein
with a threshold of 1e−05

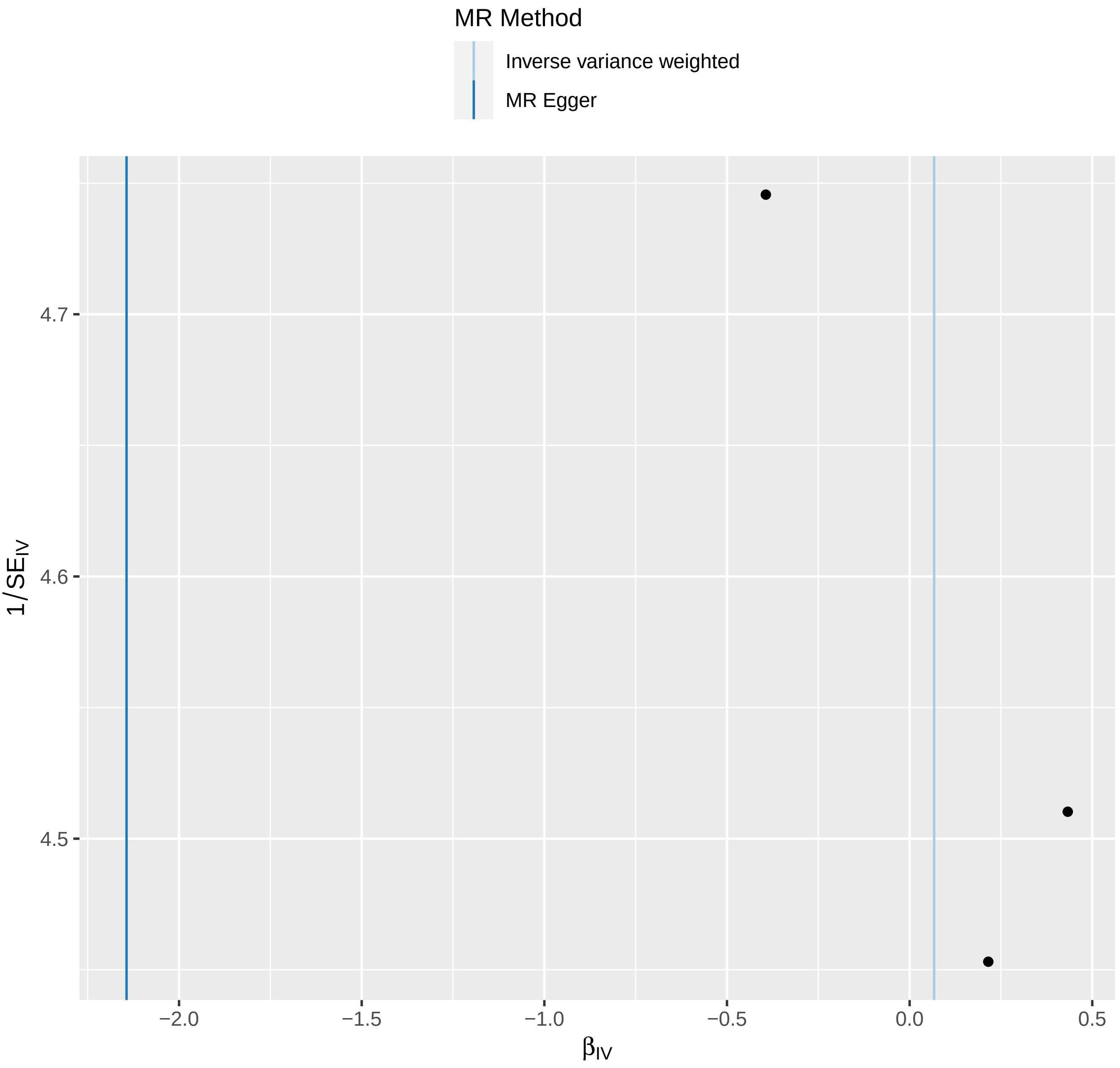

Batch 2−3970 : Gut microbiota abundance (genus Parasutterella id.2892) on Myocardial zonula adherens protein
with a threshold of 1e−05

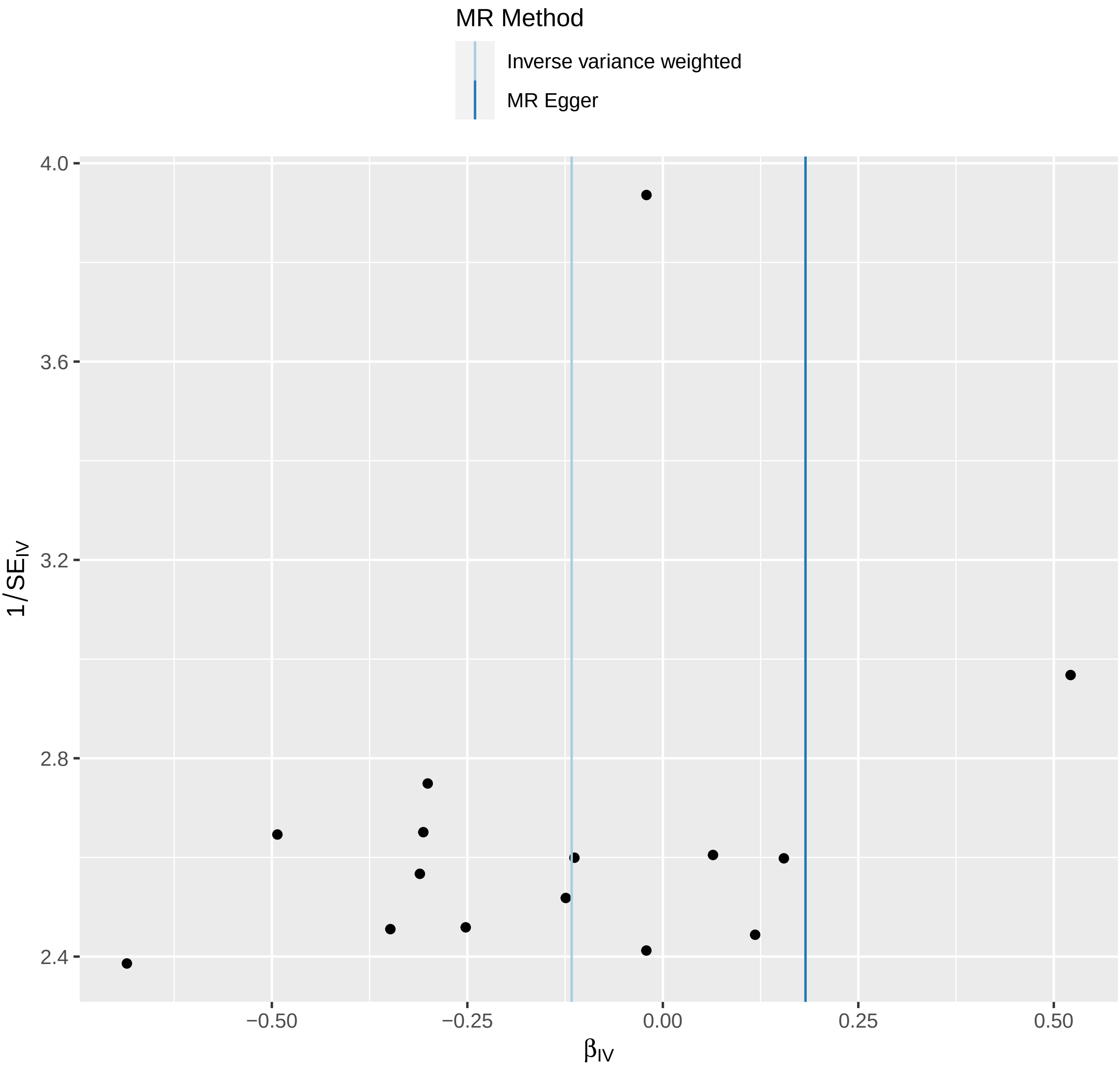

Batch 2−3971 : Gut microbiota abundance (genus Catenibacterium id.2153) on Myocardial zonula adherens protein
with a threshold of 1e−05

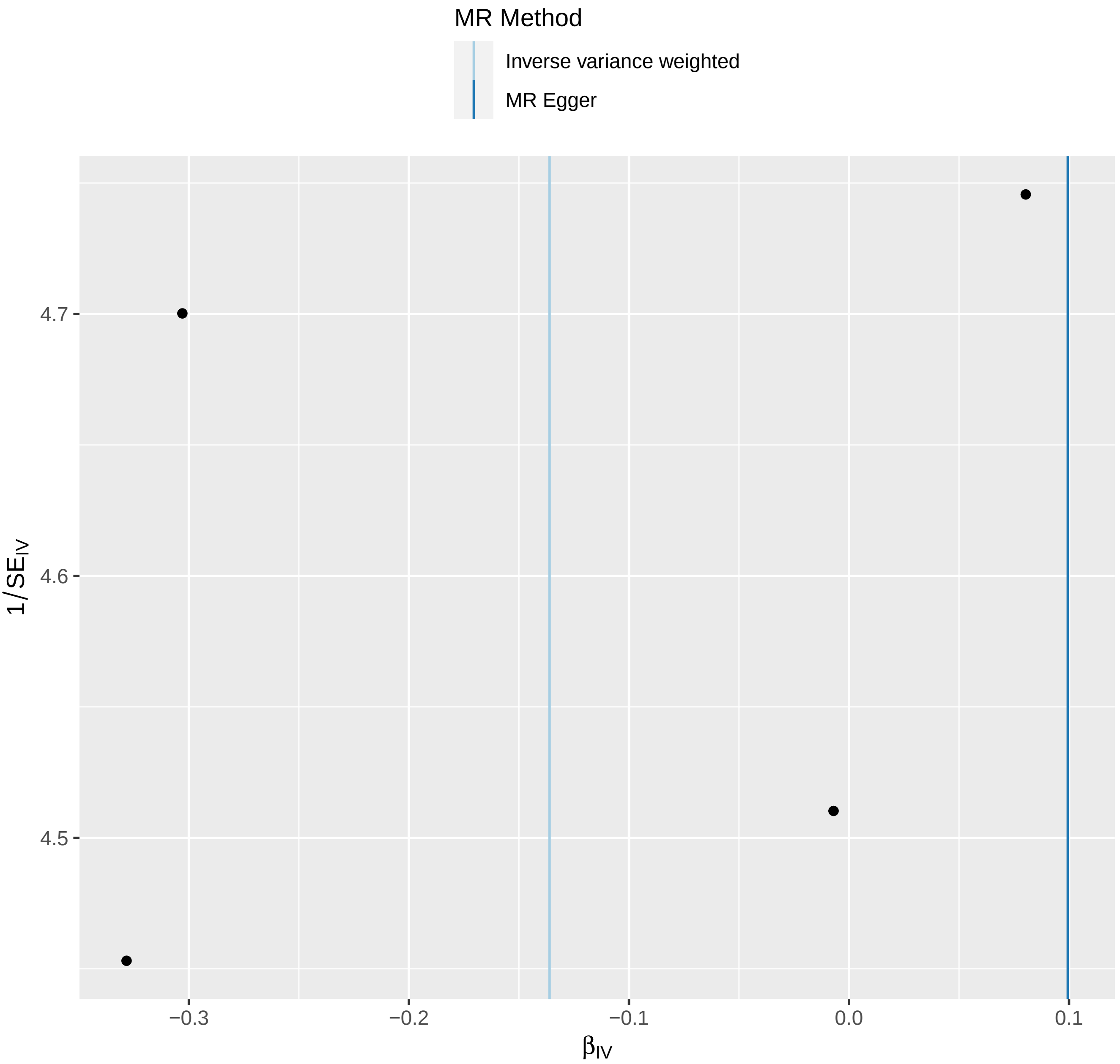

Batch 2−3972 : Gut microbiota abundance (genus Parasutterella id.2892) on Myocardial zonula adherens protein
with a threshold of 1e−05

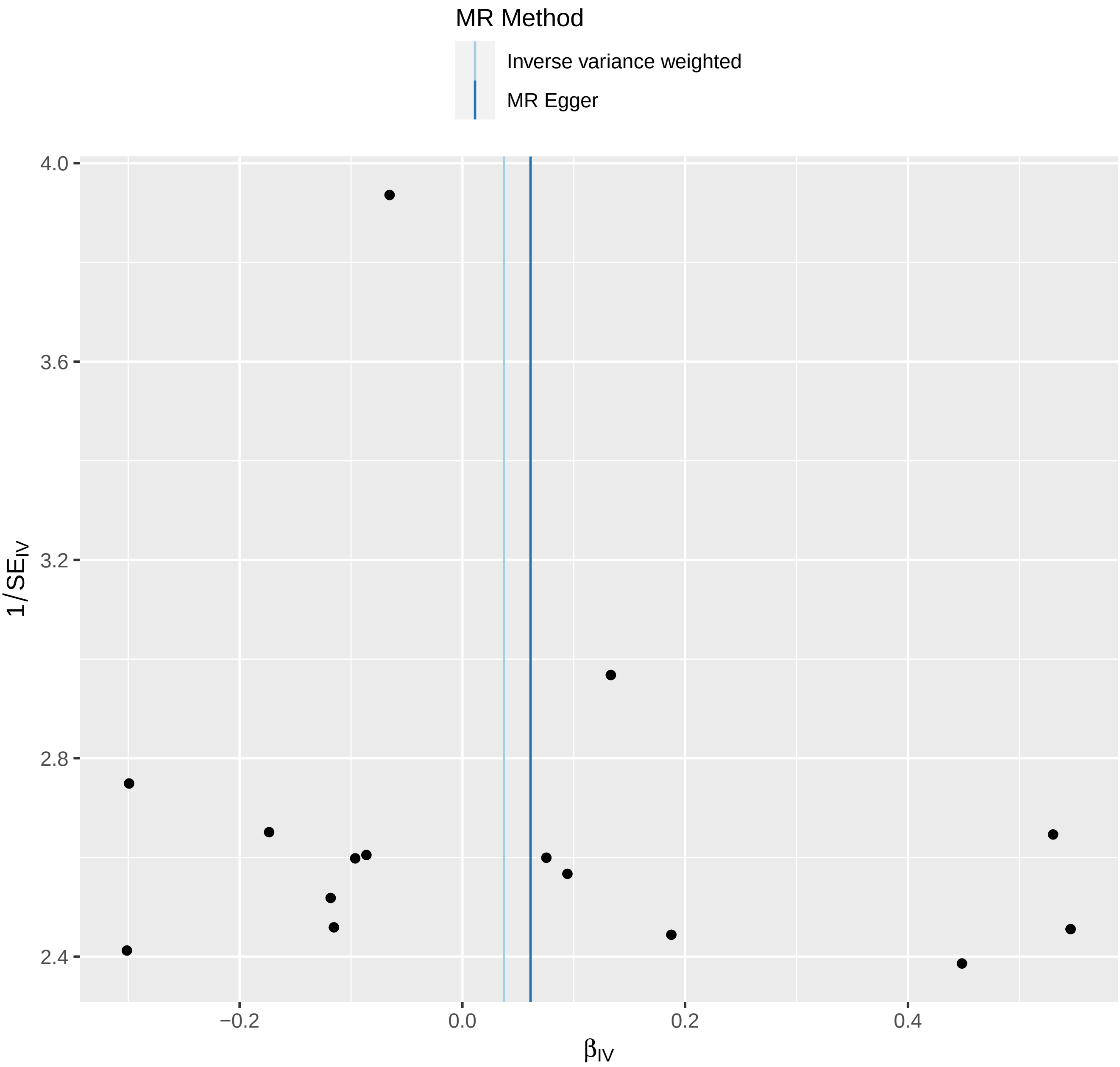

Batch 2−3973 : Gut microbiota abundance (genus Catenibacterium id.2153) on Myeloid zinc finger 1
with a threshold of 1e−05

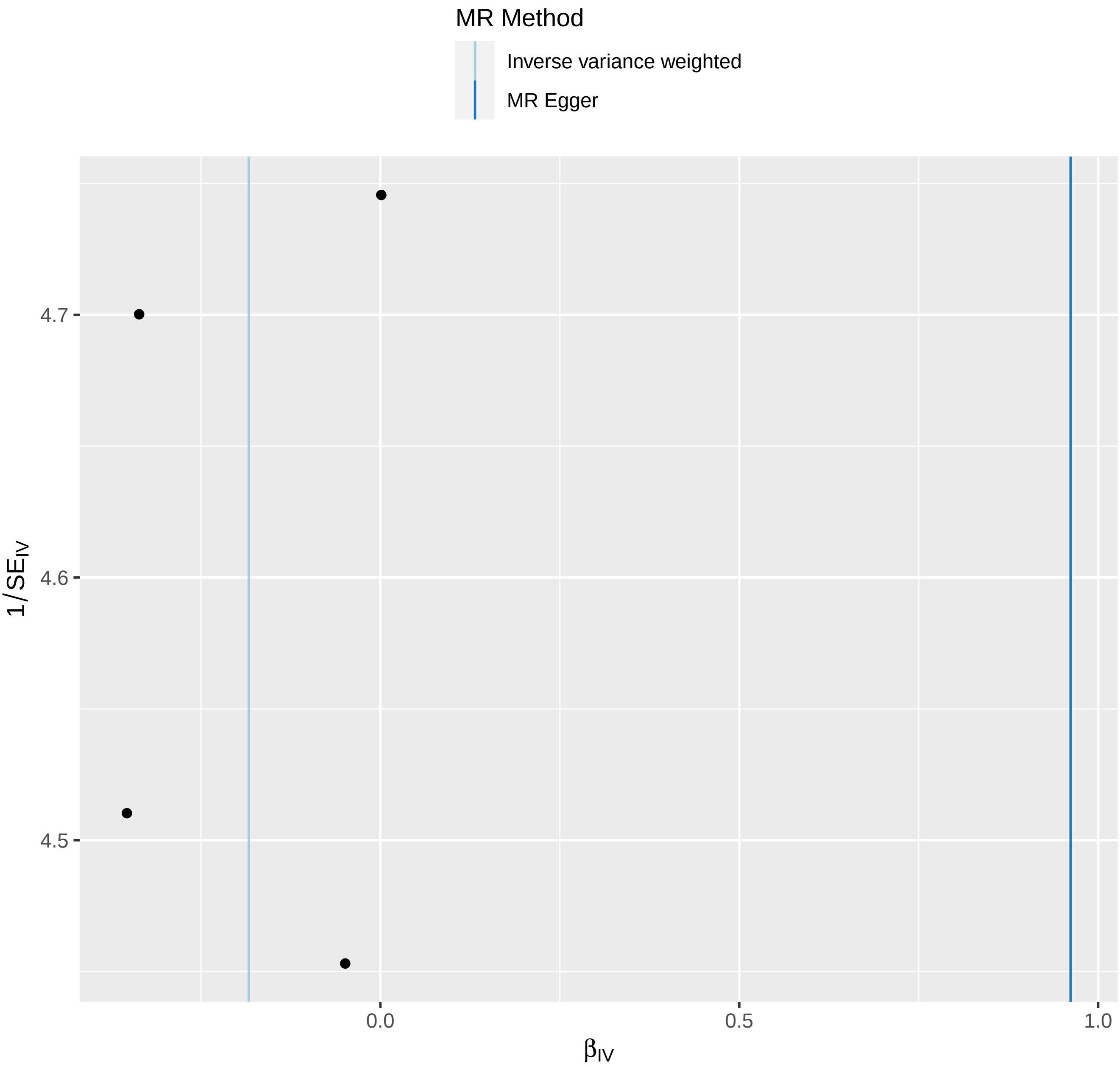

Batch 2−3974 : Gut microbiota abundance (genus Parasutterella id.2892) on Myeloid zinc finger 1
with a threshold of 1e−05

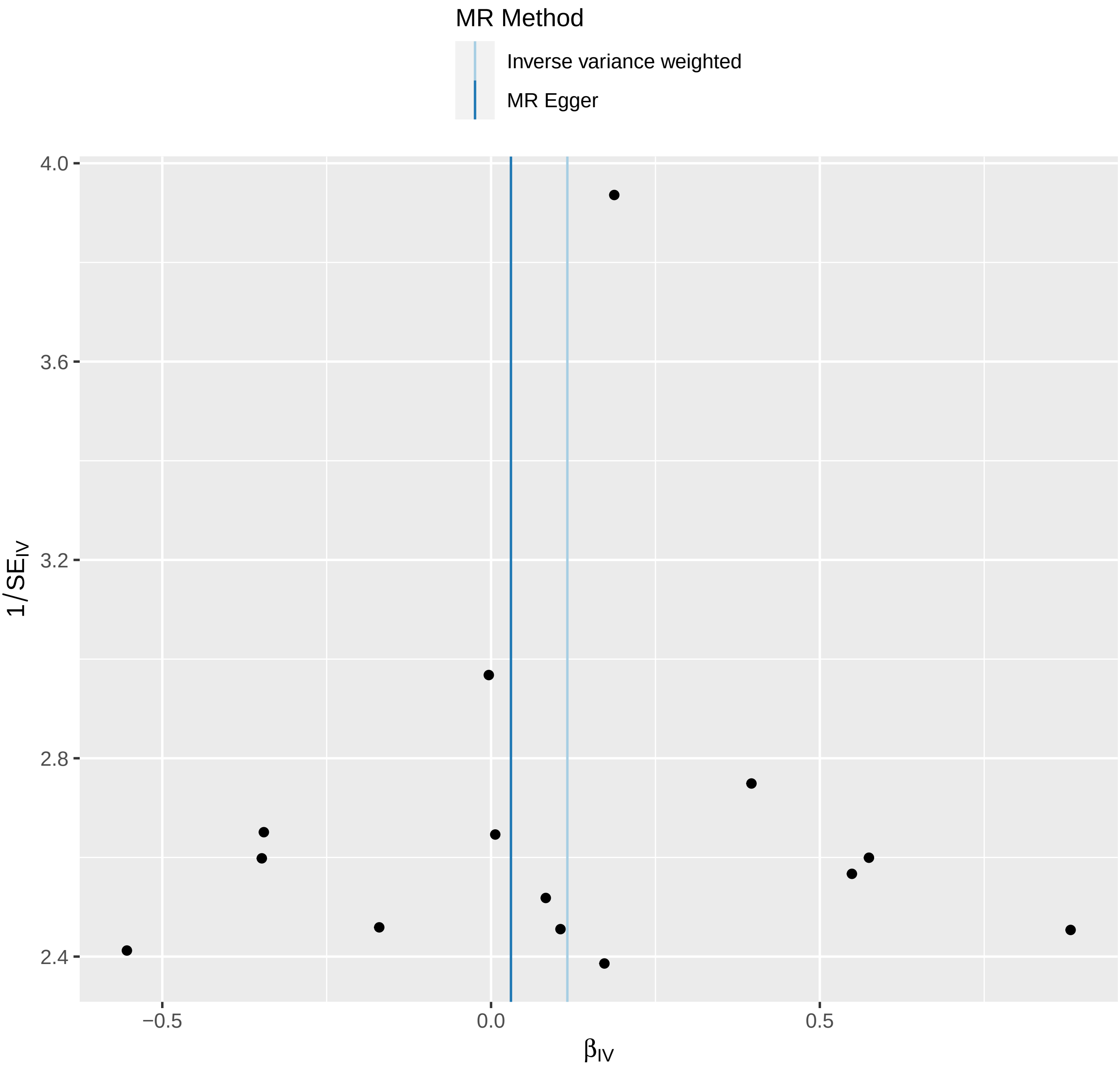

Batch 2−3975 : Gut microbiota abundance (genus Catenibacterium id.2153) on Mitotic−spindle organizing protein 1 with a threshold of 1e−05

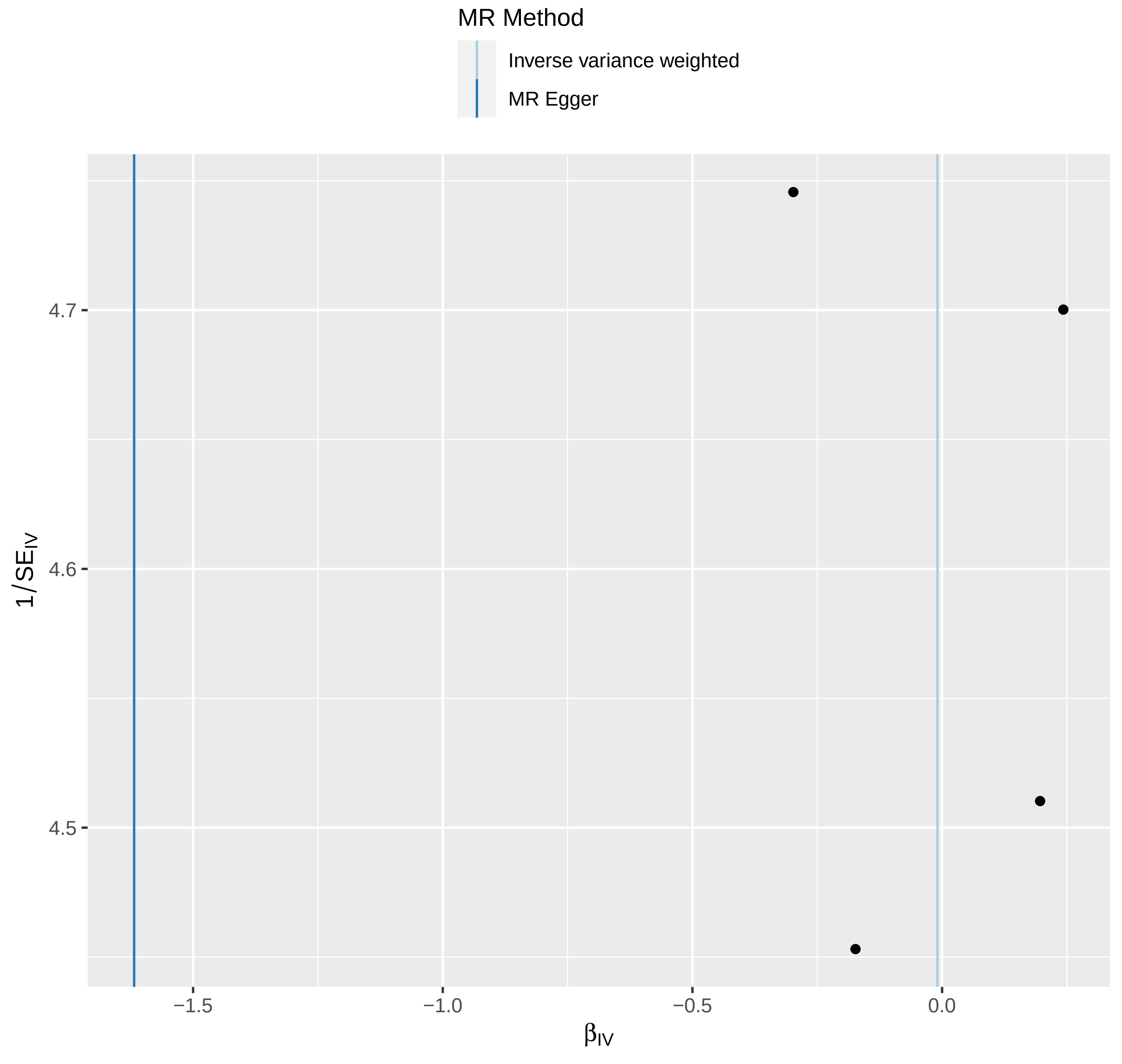

Batch 2−3976 : Gut microbiota abundance (genus Parasutterella id.2892) on Mitotic−spindle organizing protein 1
with a threshold of 1e−05

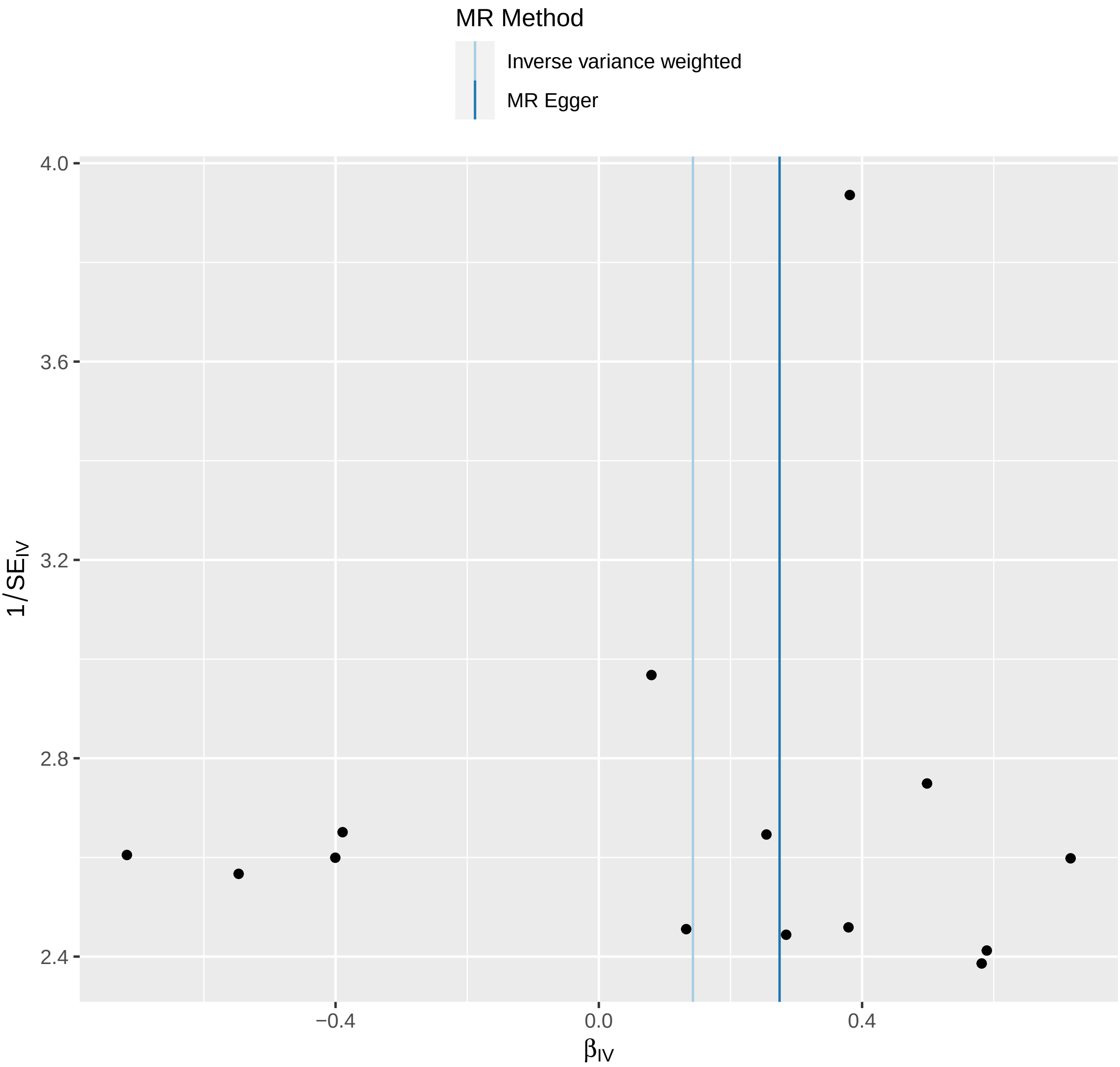

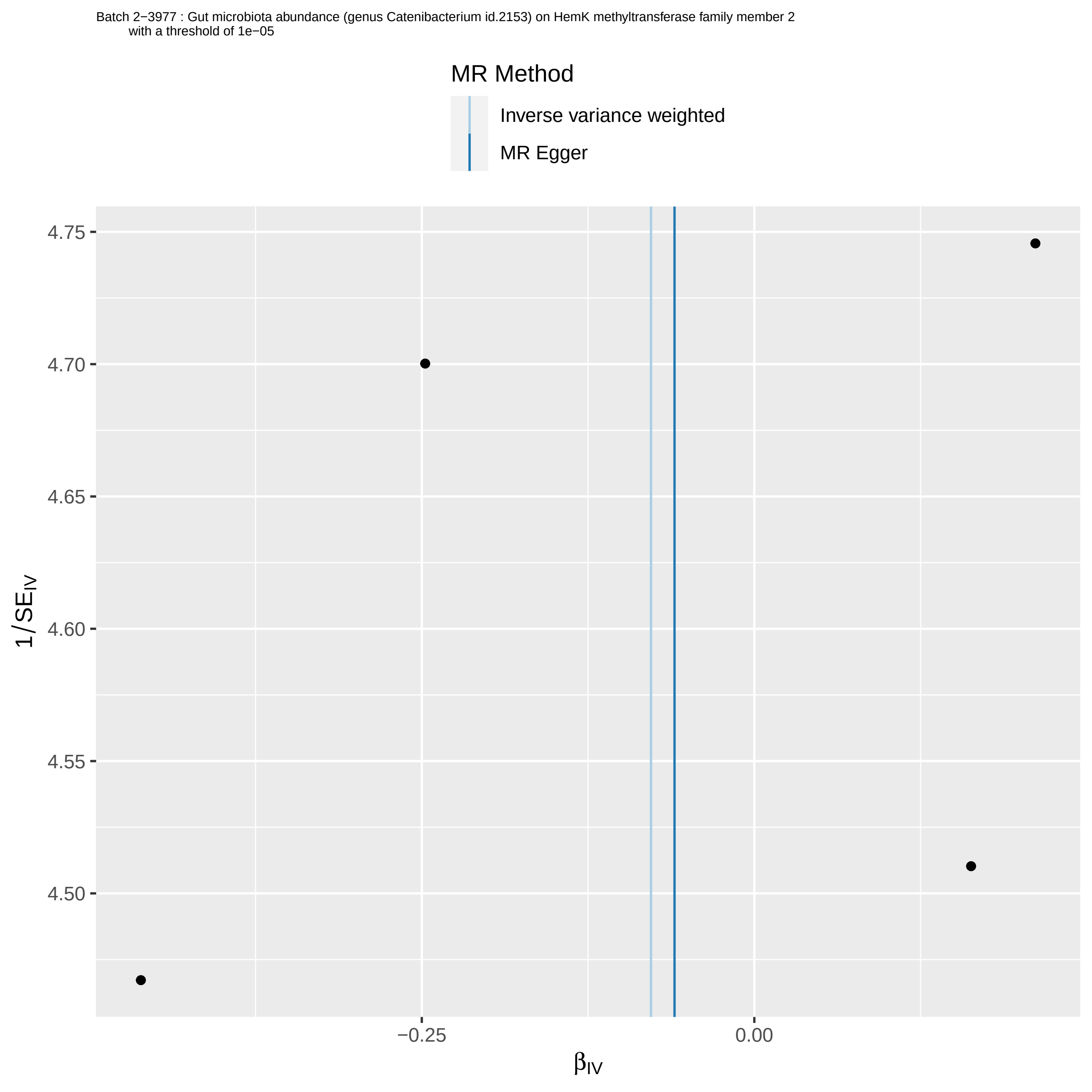

Batch 2−3977 : Gut microbiota abundance (genus Catenibacterium id.2153) on HemK methyltransferase family member 2
with a threshold of 1e−05
MR Method
Inverse variance weighted
MR Egger
4.75
4.70
4.65
4.60
4.55
4.50
1/SE_IV
−0.25
0.00
β_IV

Batch 2−3978 : Gut microbiota abundance (genus Parasutterella id.2892) on HemK methyltransferase family member 2
with a threshold of 1e−05

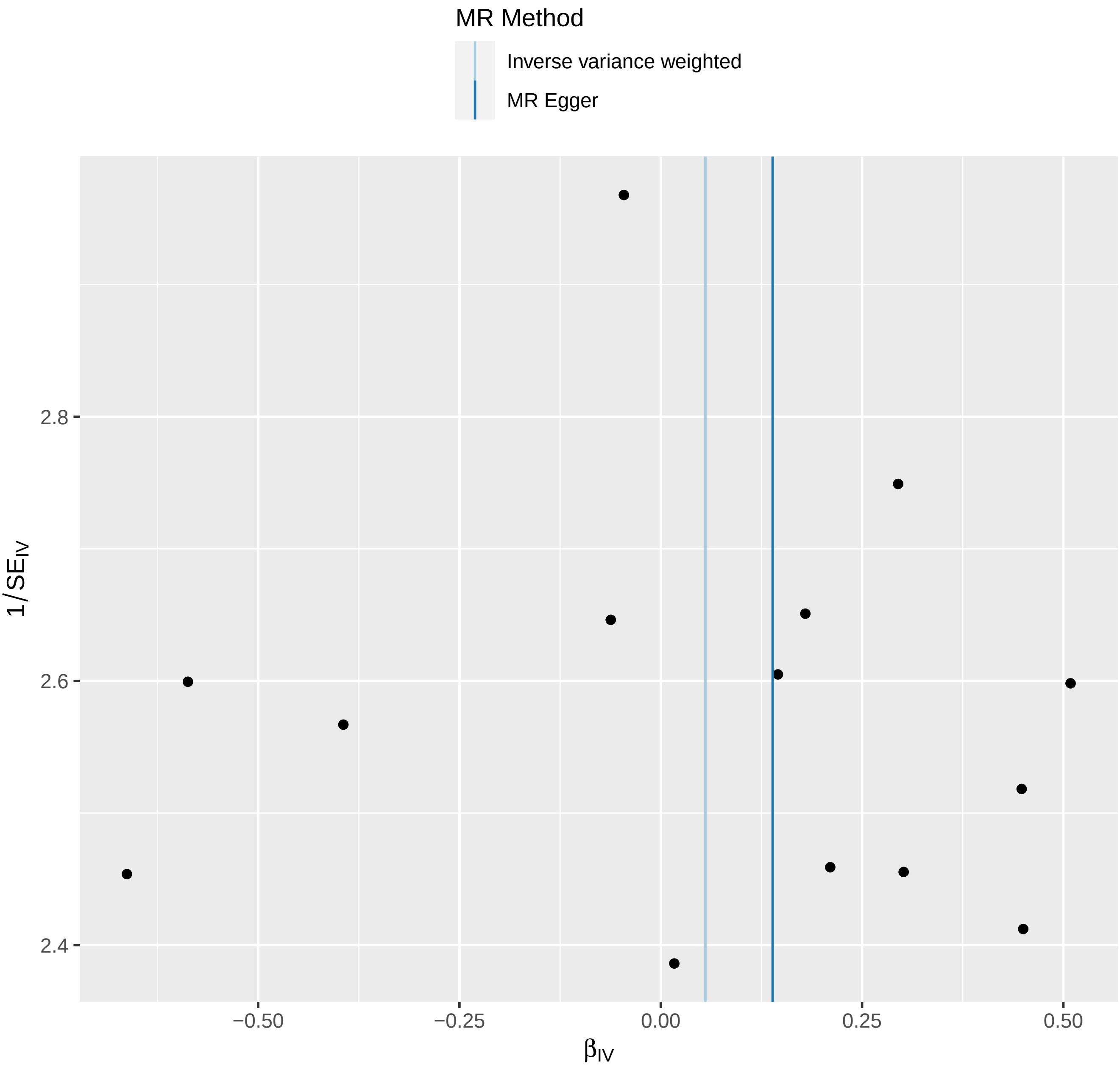

Batch 2−3979 : Gut microbiota abundance (genus Catenibacterium id.2153) on N−acylethanolamine−hydrolyzing acid amidase
with a threshold of 1e−05

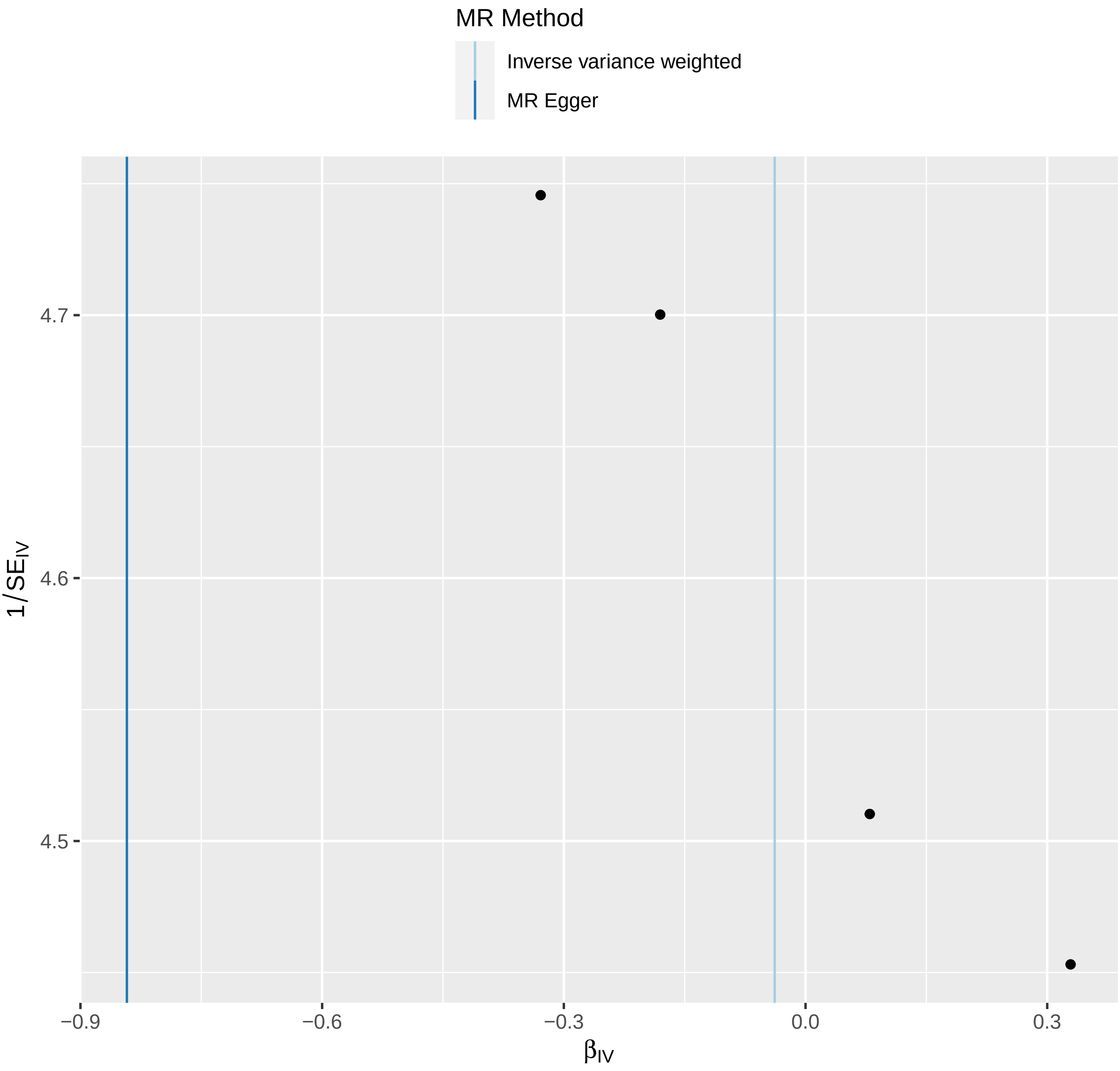

Batch 2−3980 : Gut microbiota abundance (genus Parasutterella id.2892) on N−acylethanolamine−hydrolyzing acid amidase with a threshold of 1e−05

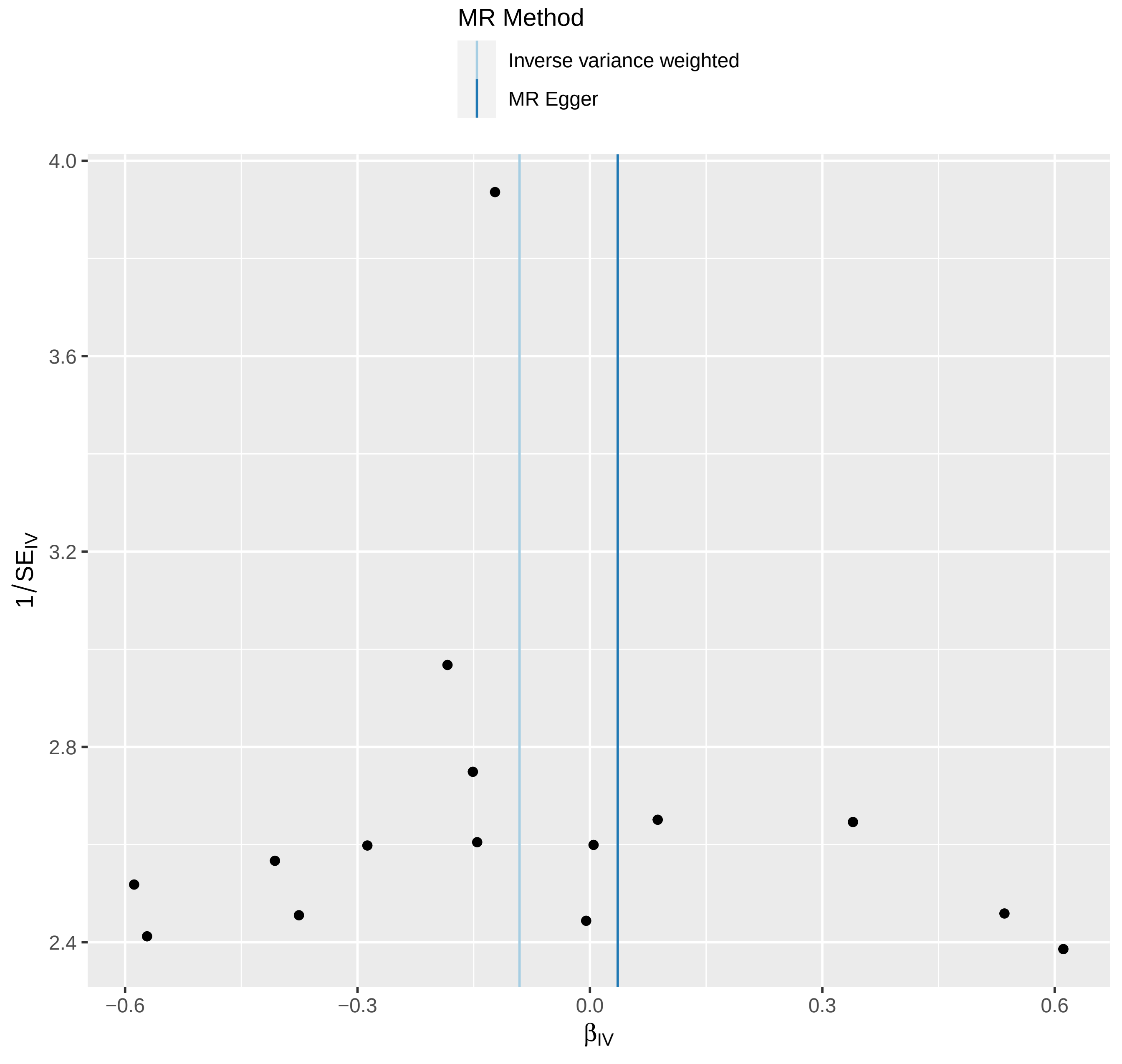

Batch 2−3981 : Gut microbiota abundance (genus Catenibacterium id.2153) on N−acetylated−alpha−linked acidic dipeptidase 2
with a threshold of 1e−05

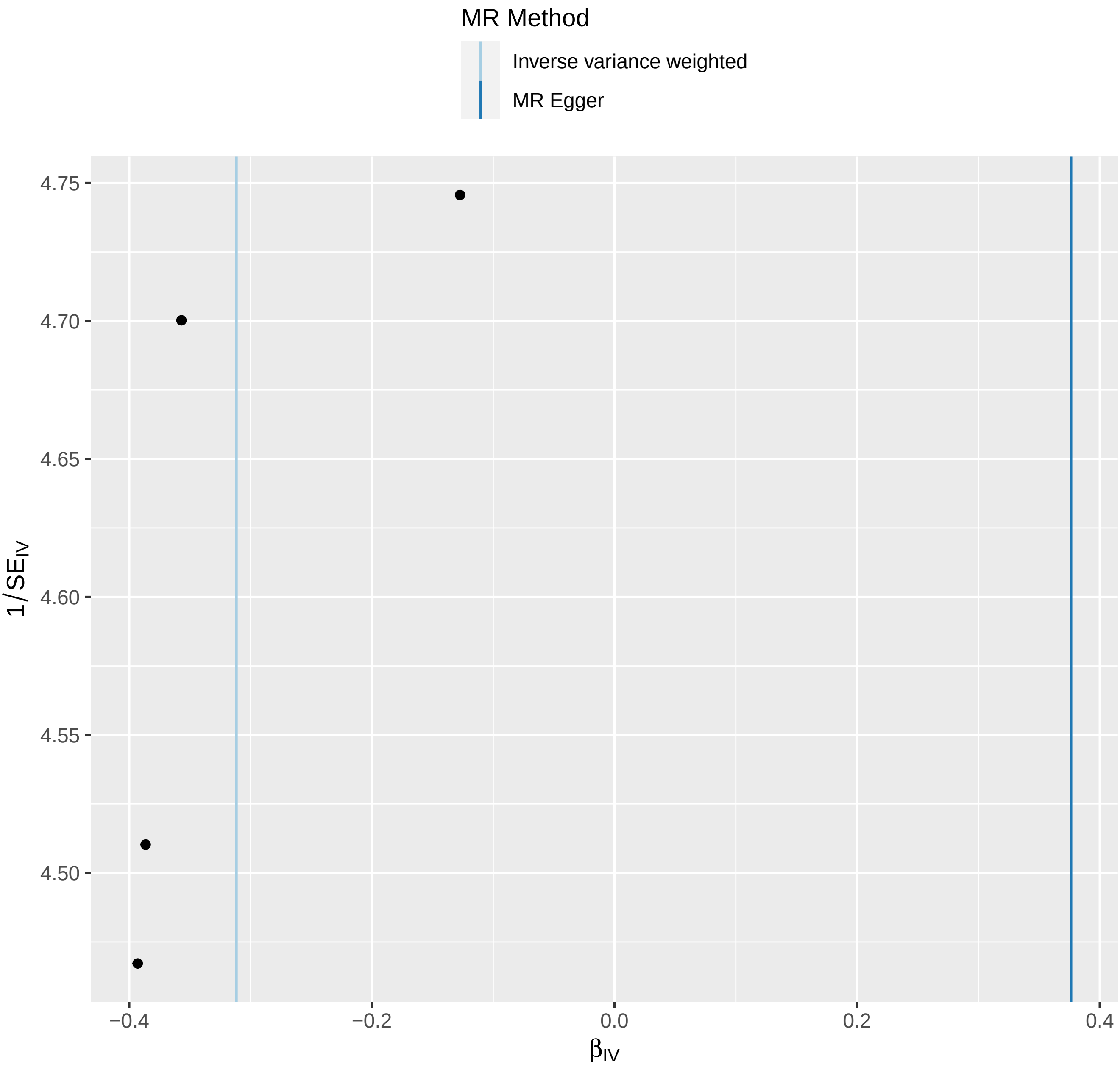

Batch 2−3982 : Gut microbiota abundance (genus Parasutterella id.2892) on N−acetylated−alpha−linked acidic dipeptidase 2 with a threshold of 1e−05

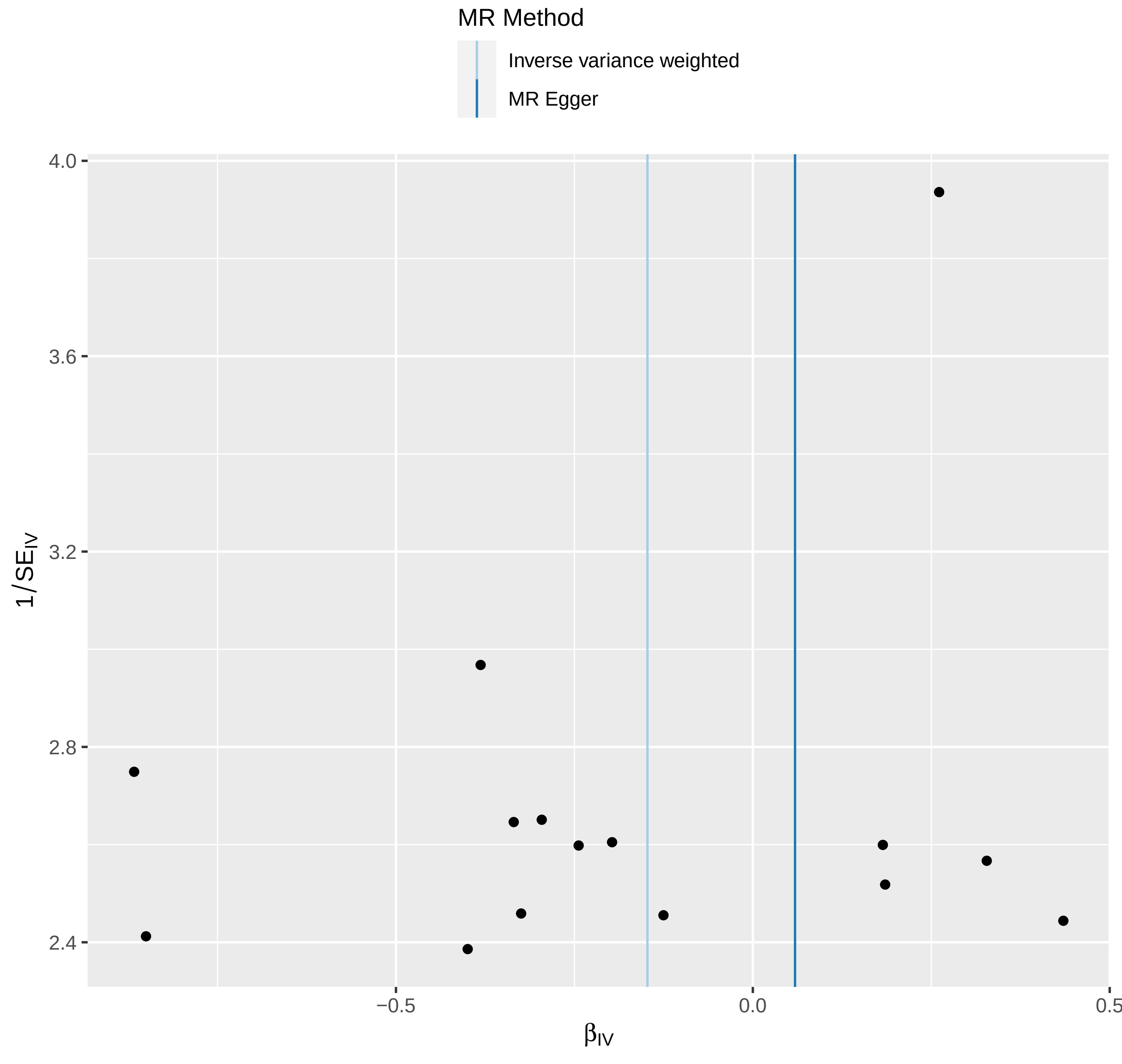

Batch 2−3983 : Gut microbiota abundance (genus Catenibacterium id.2153) on NGFI−A−binding protein 1
with a threshold of 1e−05

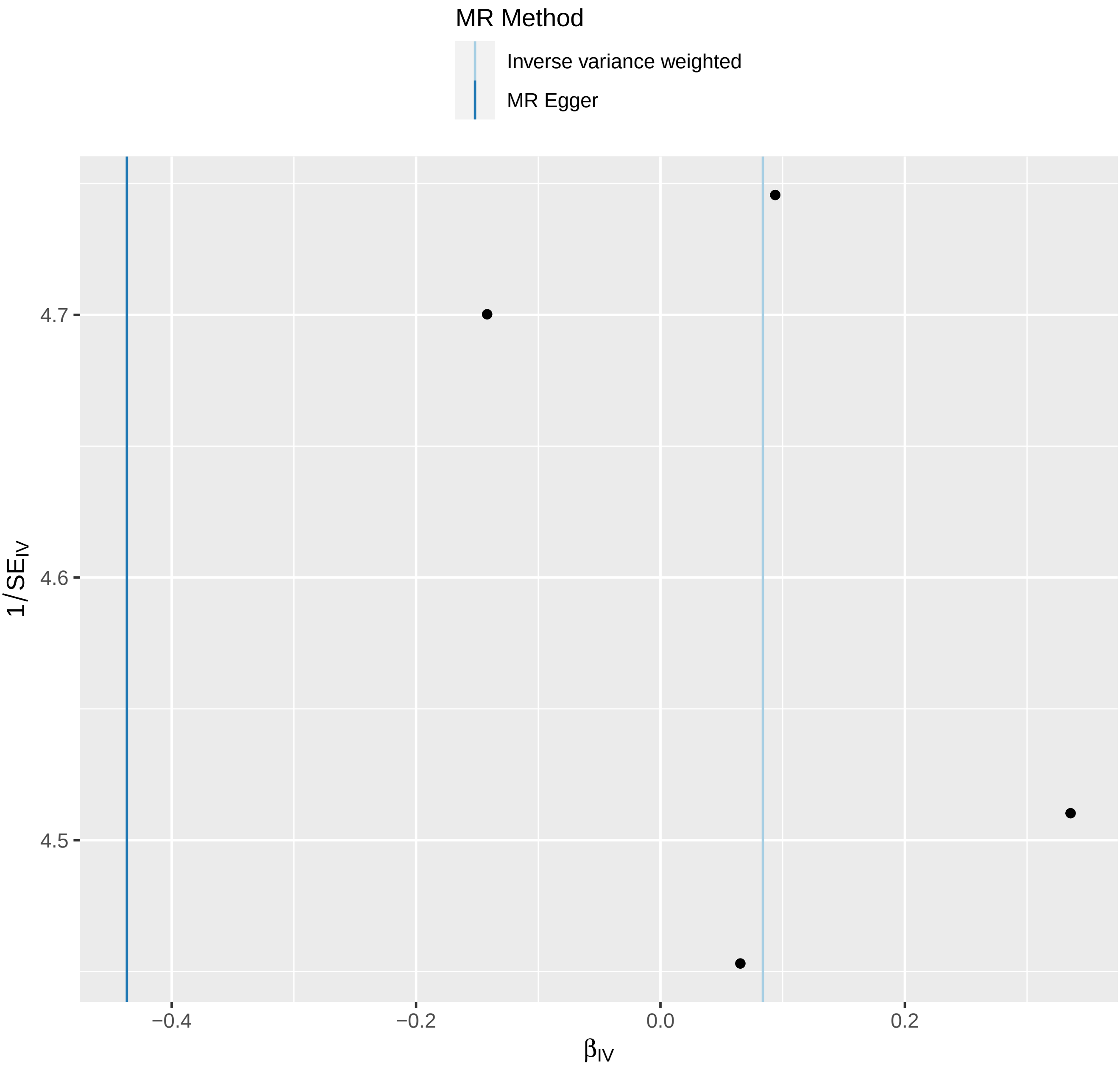

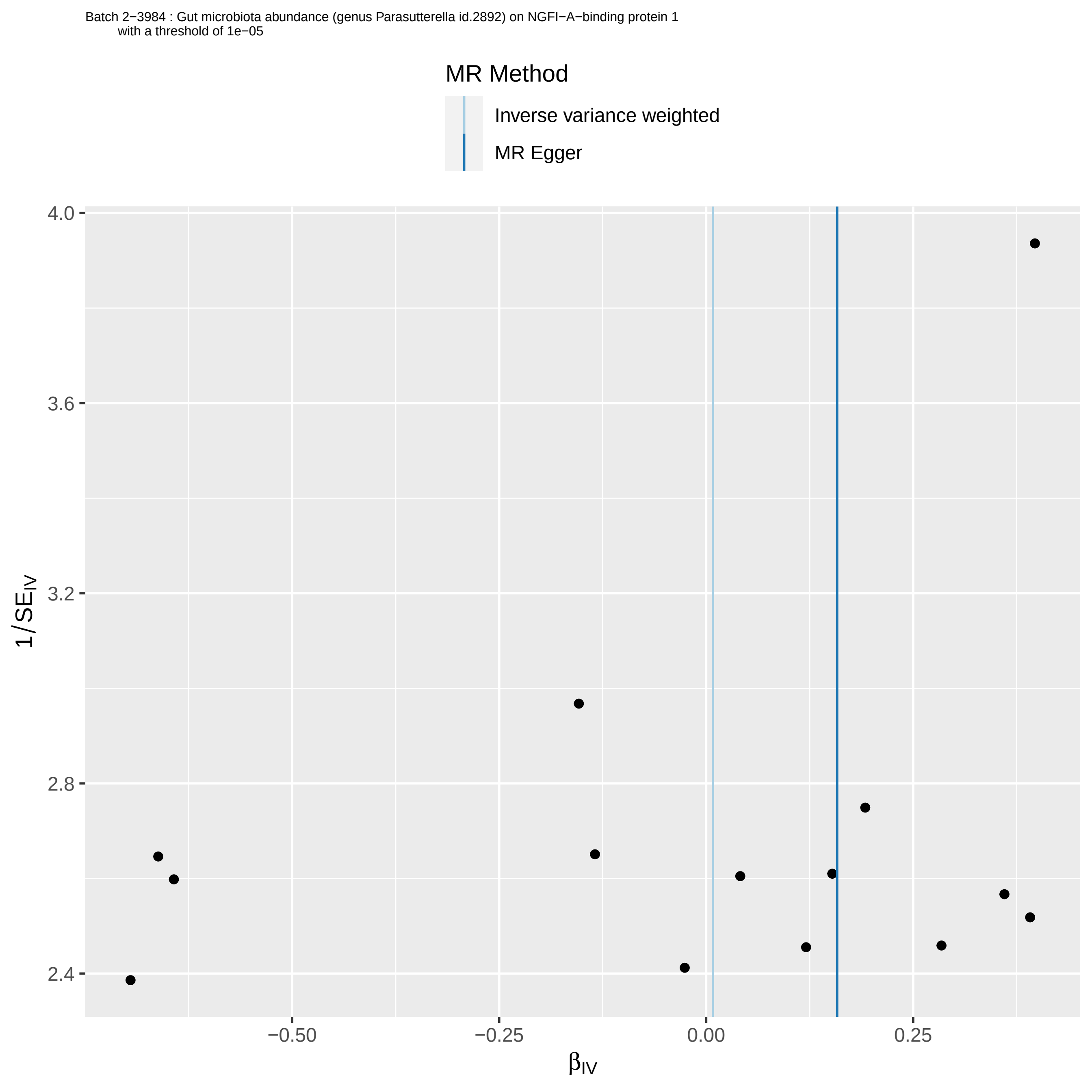

Batch 2−3984 : Gut microbiota abundance (genus Parasutterella id.2892) on NGFI−A−binding protein 1
with a threshold of 1e−05
MR Method
Inverse variance weighted
MR Egger
4.0
3.6
3.2
2.8
2.4
1/SE_IV
−0.50
−0.25
0.00
0.25
β_IV

Batch 2−3985 : Gut microbiota abundance (genus Catenibacterium id.2153) on NGFI−A−binding protein 2
with a threshold of 1e−05

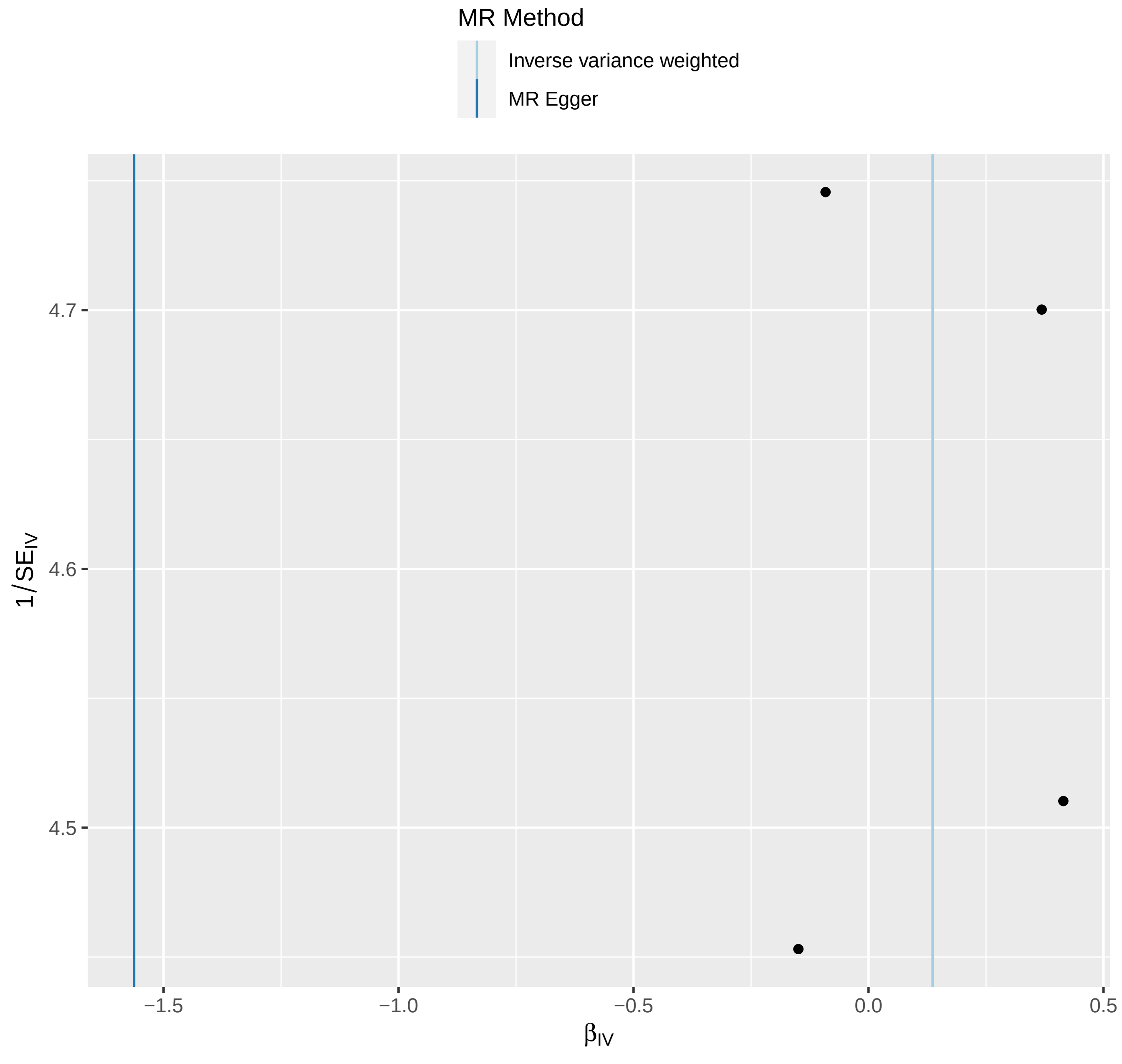

Batch 2−3986 : Gut microbiota abundance (genus Parasutterella id.2892) on NGFI−A−binding protein 2
with a threshold of 1e−05

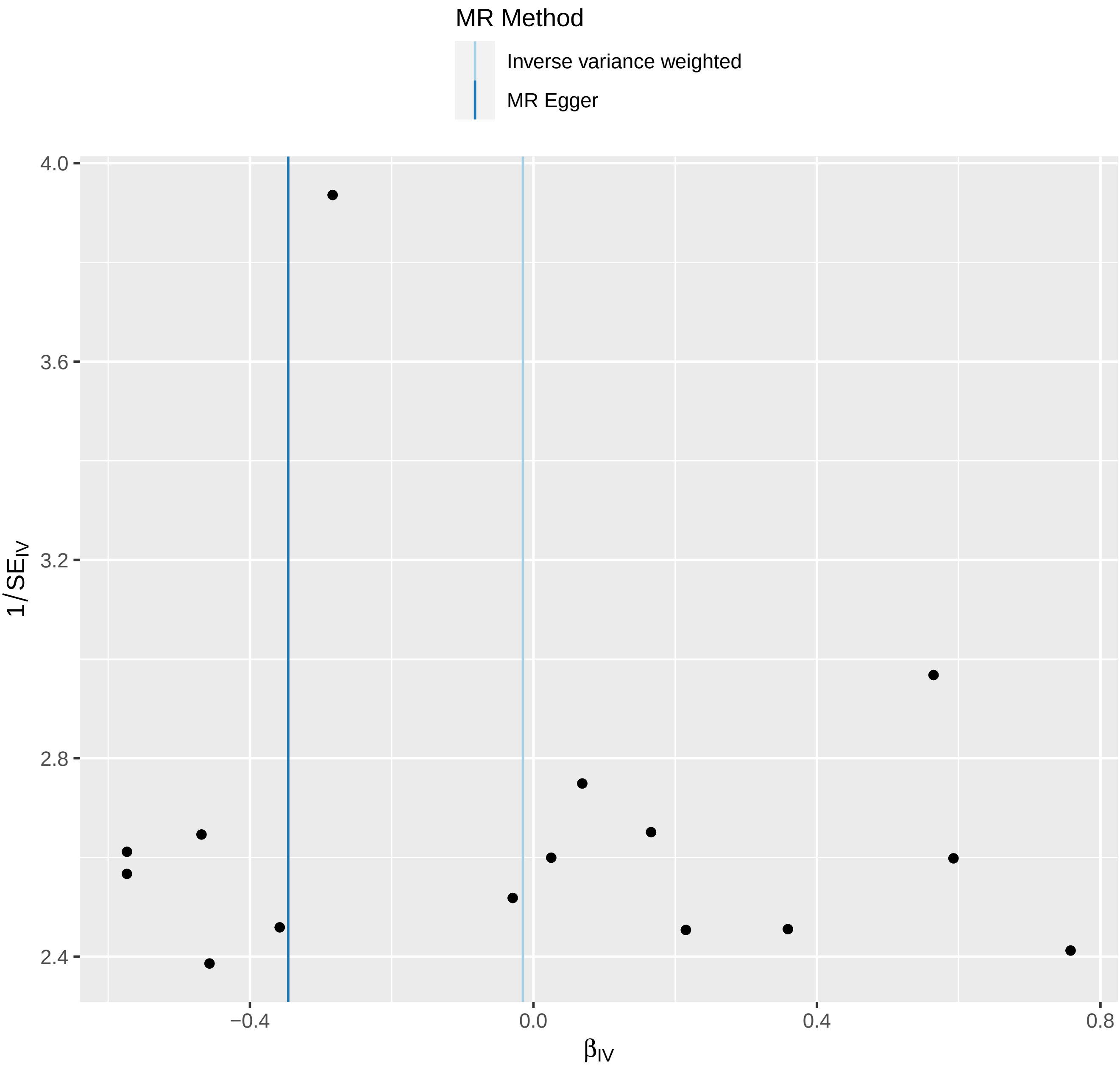

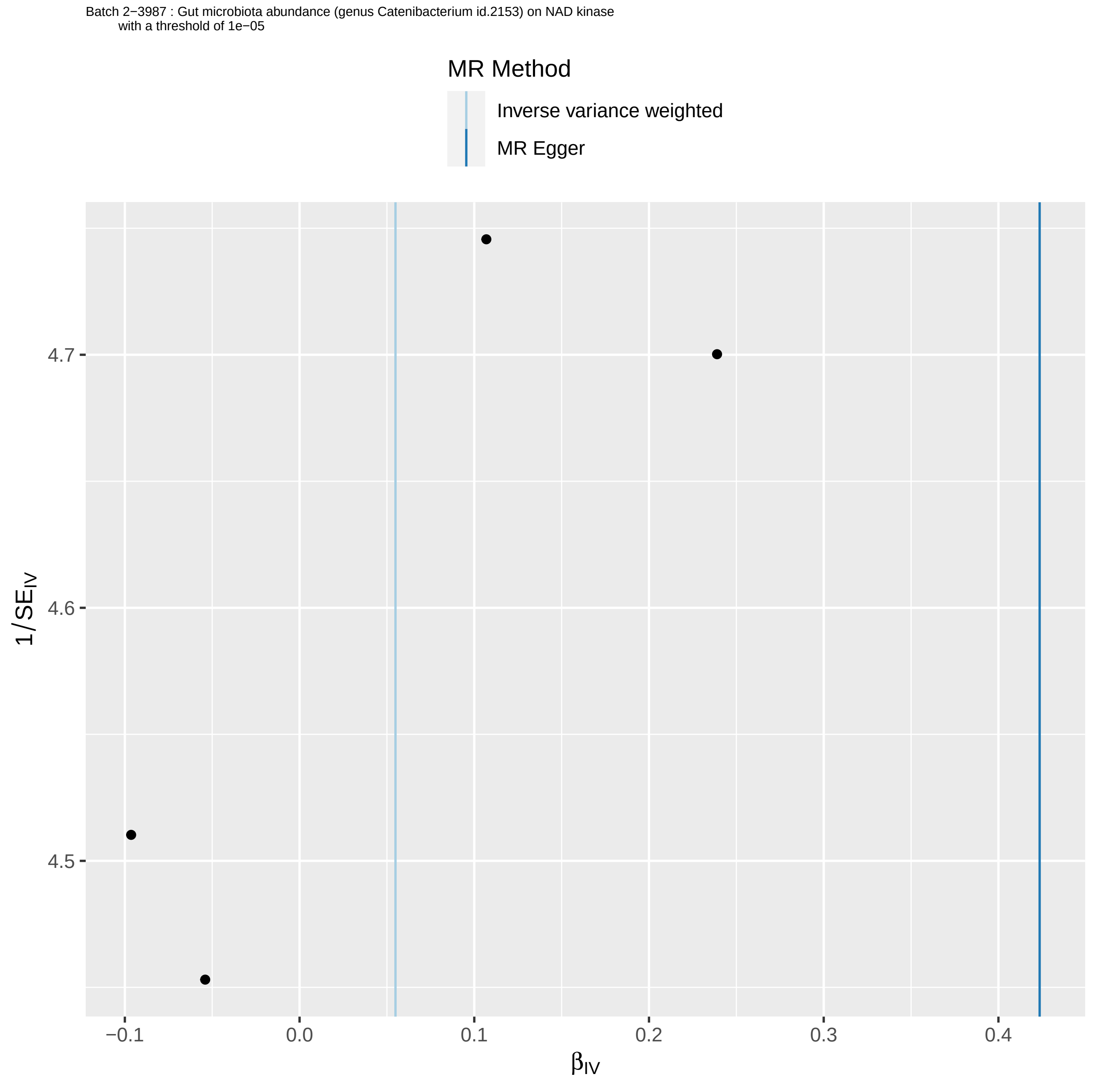
Batch 2−3987 : Gut microbiota abundance (genus Catenibacterium id.2153) on NAD kinase
with a threshold of 1e−05
MR Method
Inverse variance weighted
MR Egger
4.7
4.6
4.5
1/SE$_{IV}$
−0.1
0.0
0.1
0.2
0.3
0.4
β$_{IV}$

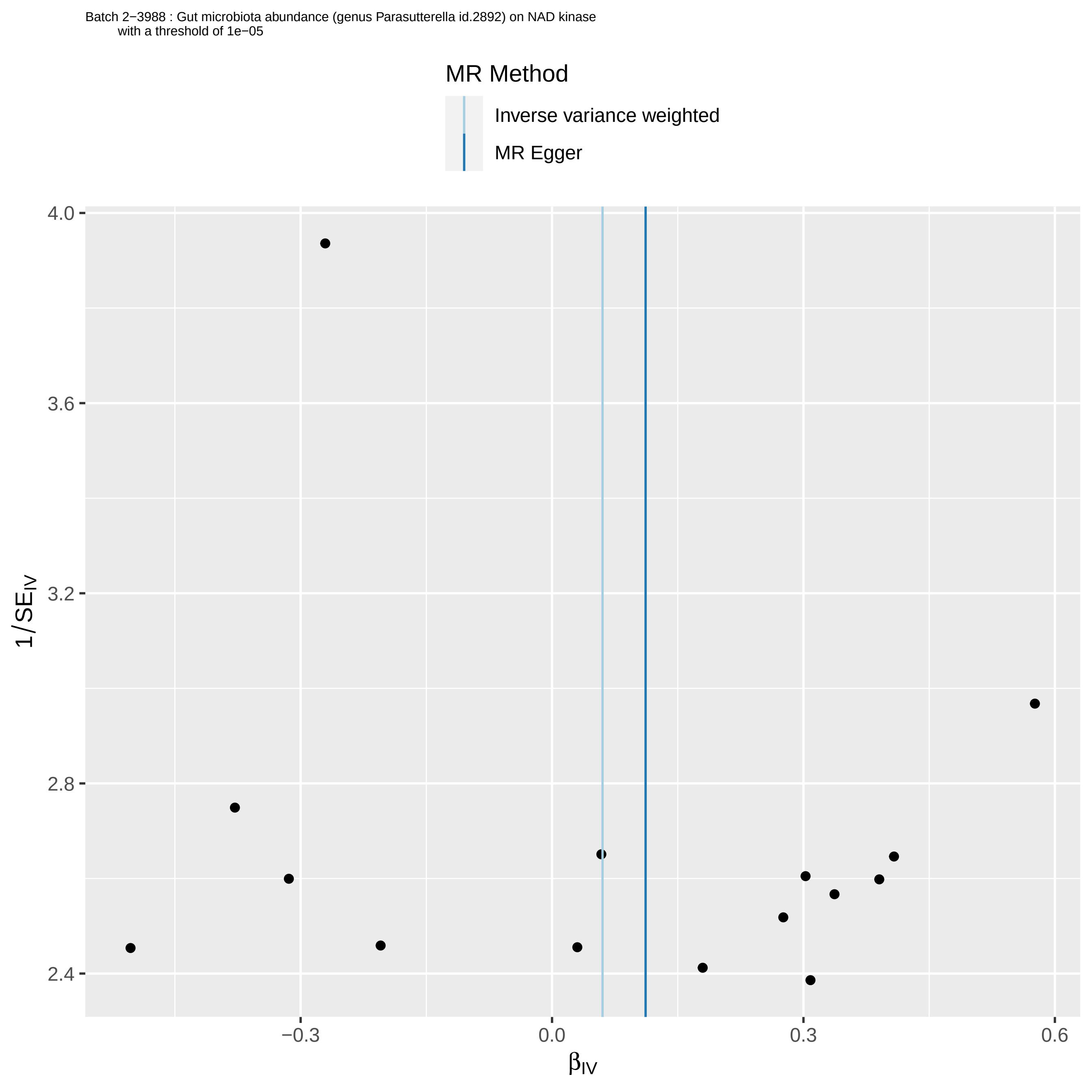

Batch 2−3988 : Gut microbiota abundance (genus Parasutterella id.2892) on NAD kinase
with a threshold of 1e−05
MR Method
Inverse variance weighted
MR Egger
4.0
3.6
3.2
2.8
2.4
1/SE$_{IV}$
−0.3
0.0
0.3
0.6
β_{IV}

Batch 2−3989 : Gut microbiota abundance (genus Catenibacterium id.2153) on N−acetyl−D−glucosamine kinase
with a threshold of 1e−05

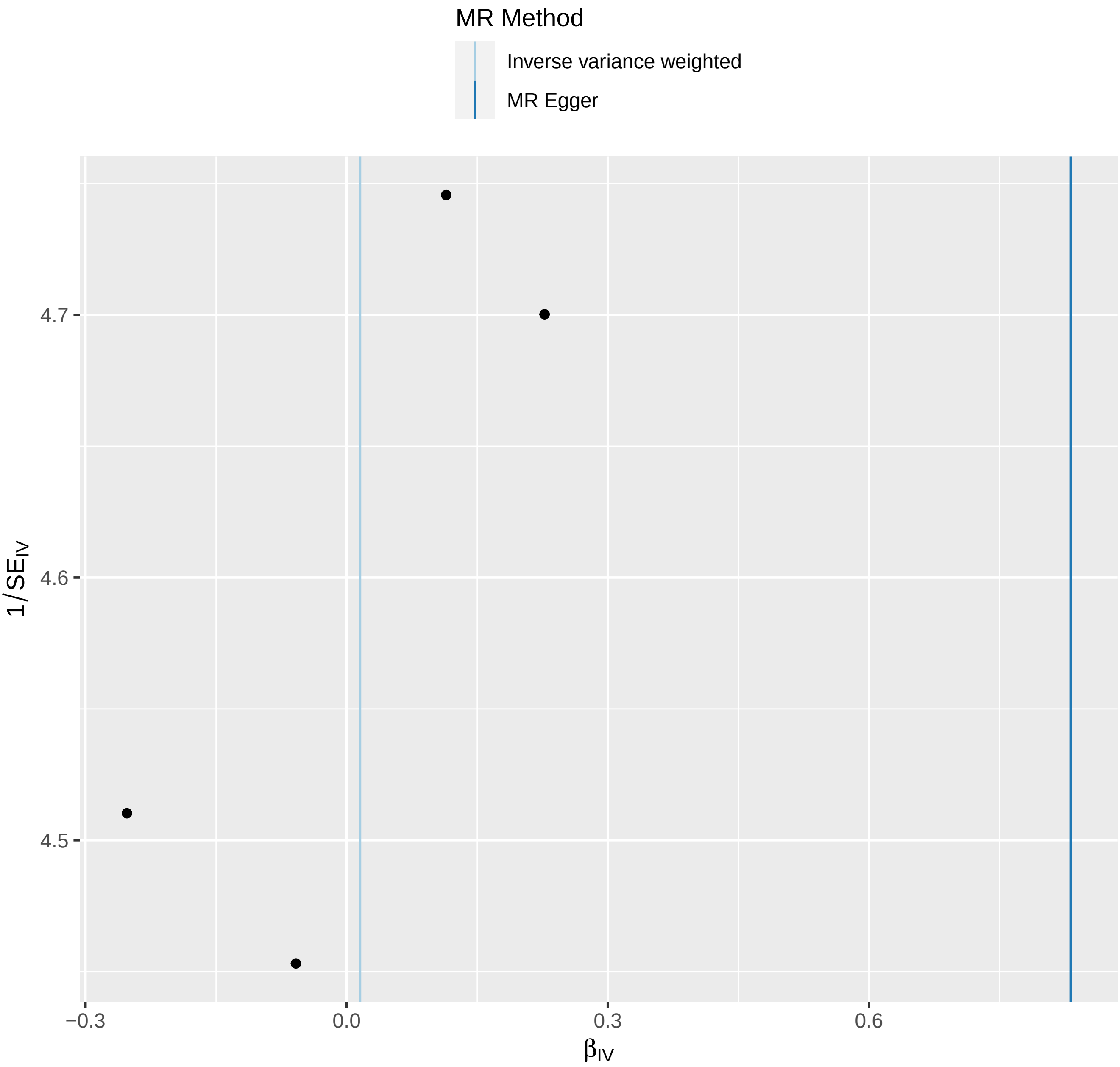

Batch 2−3990 : Gut microbiota abundance (genus Parasutterella id.2892) on N−acetyl−D−glucosamine kinase
with a threshold of 1e−05

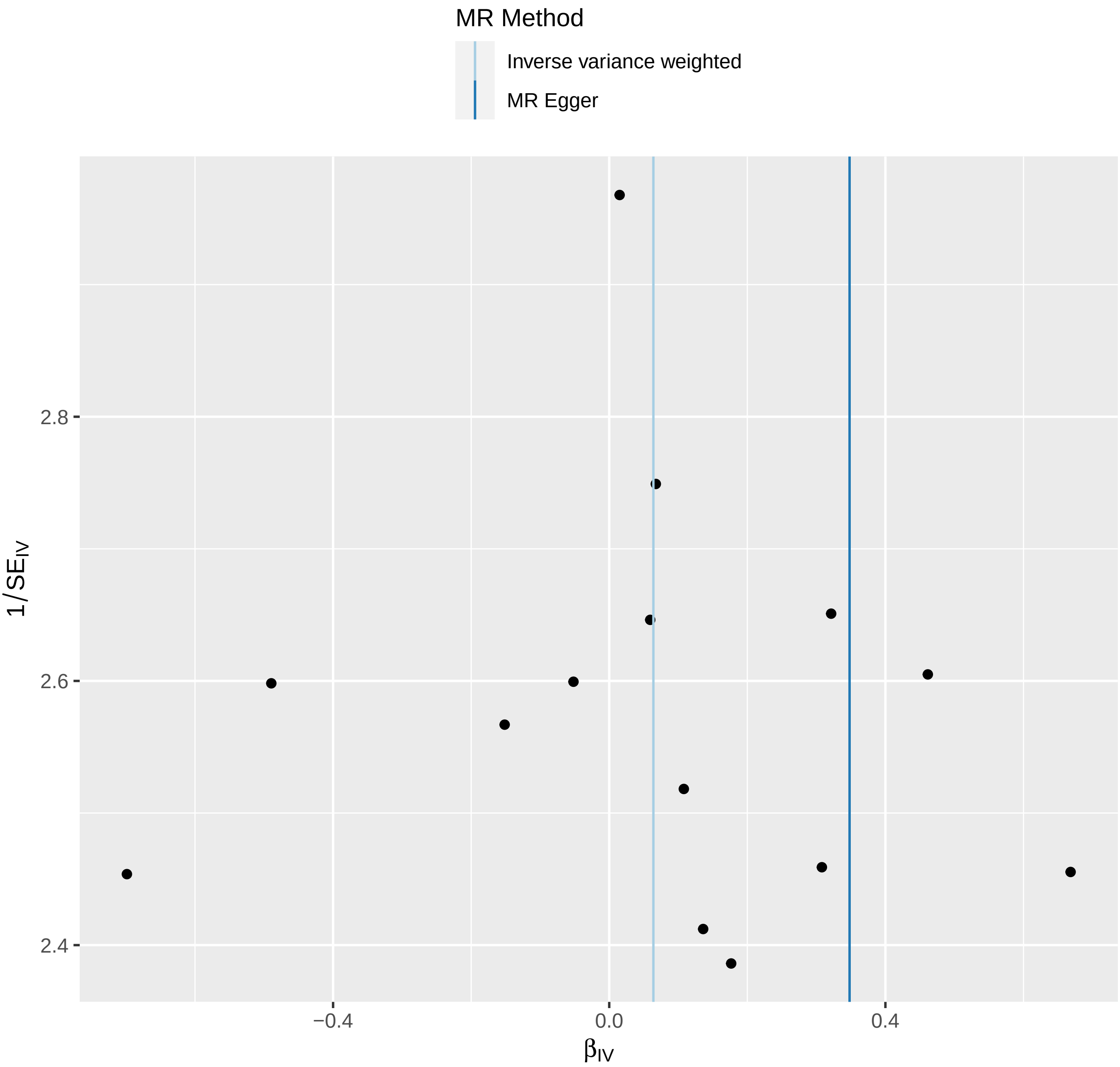

Batch 2−3991 : Gut microbiota abundance (genus Catenibacterium id.2153) on N−acetylglucosamine−1−phosphodiester alpha−N−acetylglucosaminidase with a threshold of 1e−05

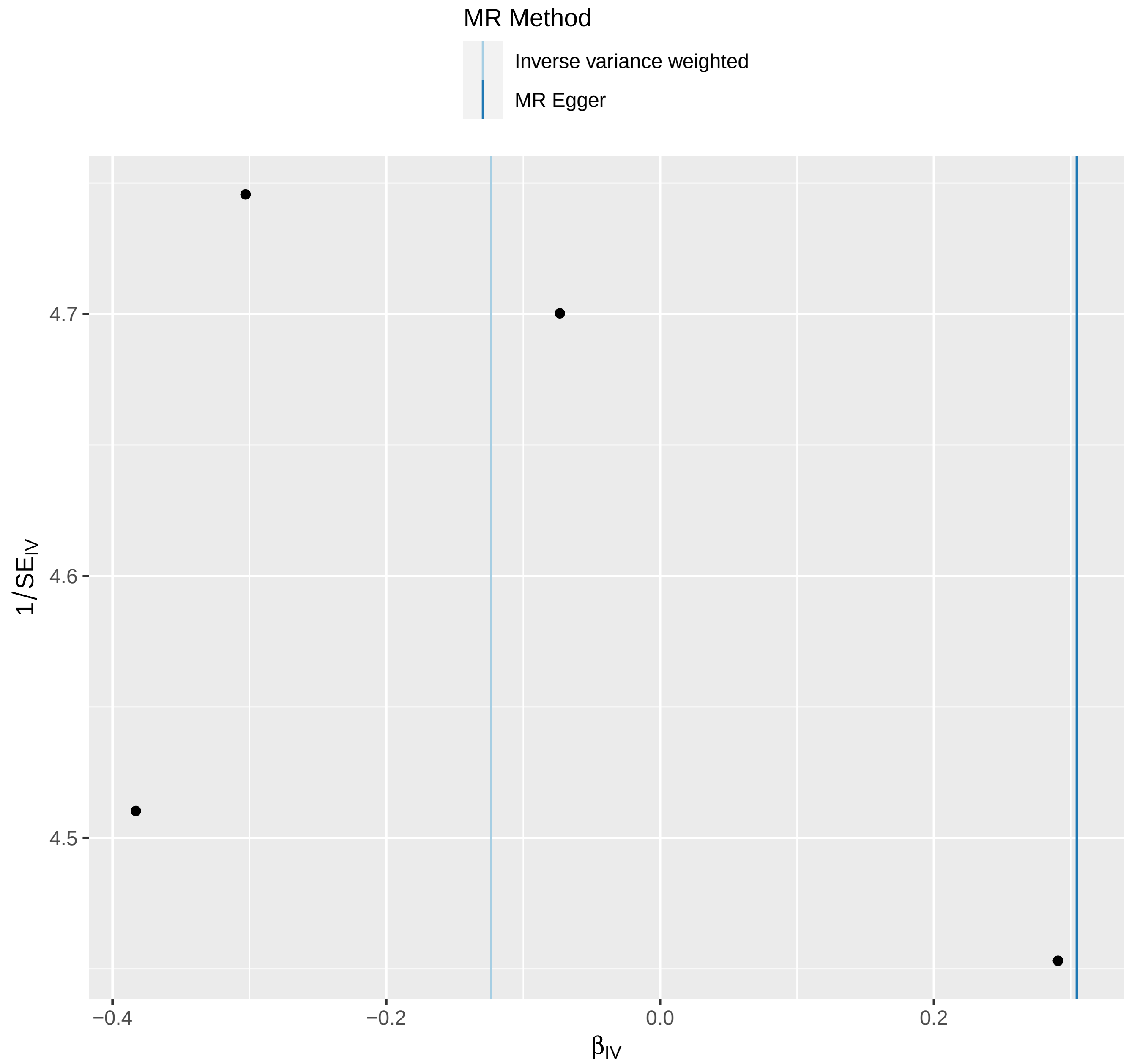

Batch 2−3992 : Gut microbiota abundance (genus Parasutterella id.2892) on N−acetylglucosamine−1−phosphodiester alpha−N−acetylglucosaminidase with a threshold of 1e−05

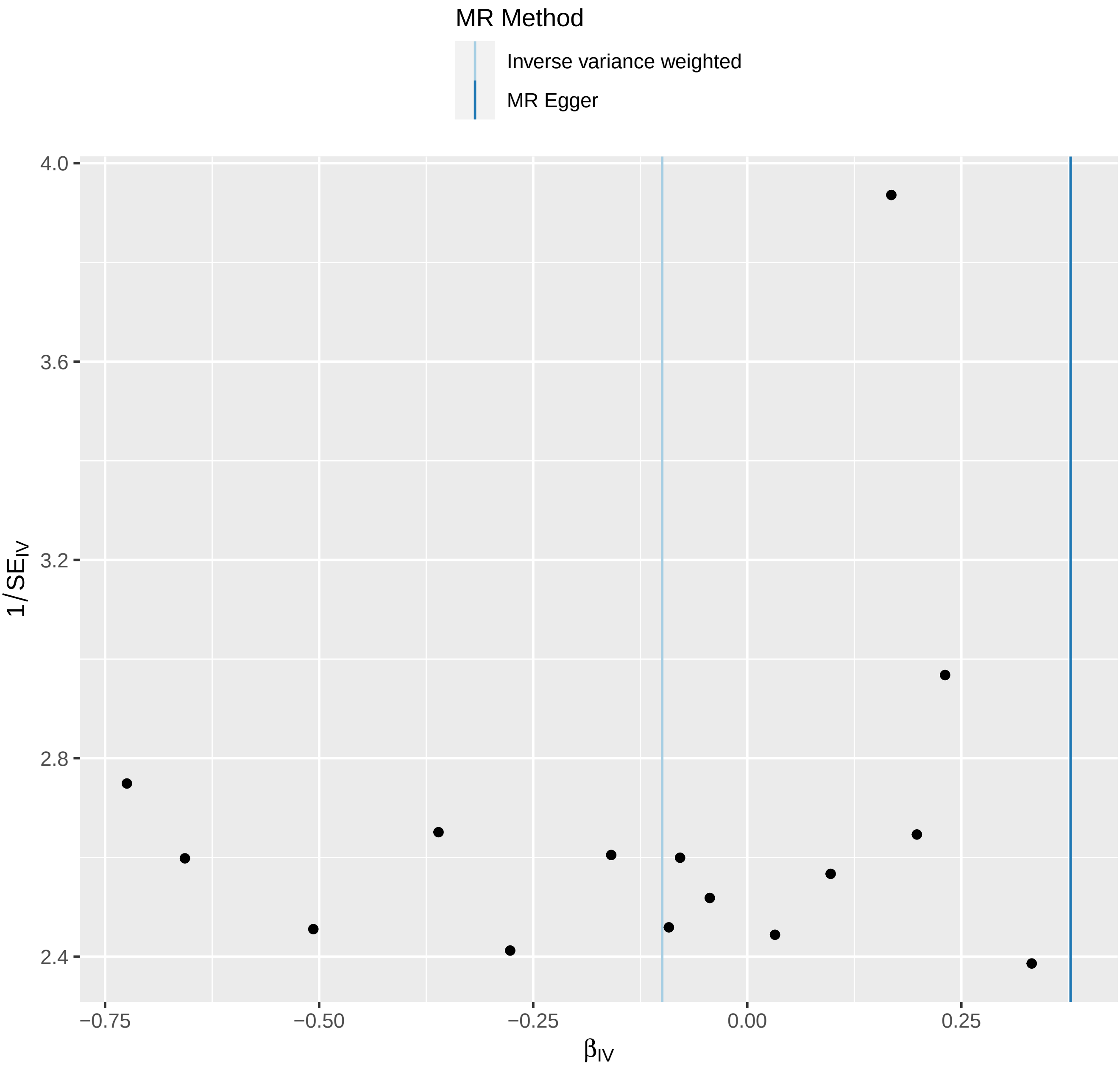

Batch 2−3993 : Gut microbiota abundance (genus Catenibacterium id.2153) on N−acetylglutamate synthase, mitochondrial
with a threshold of 1e−05

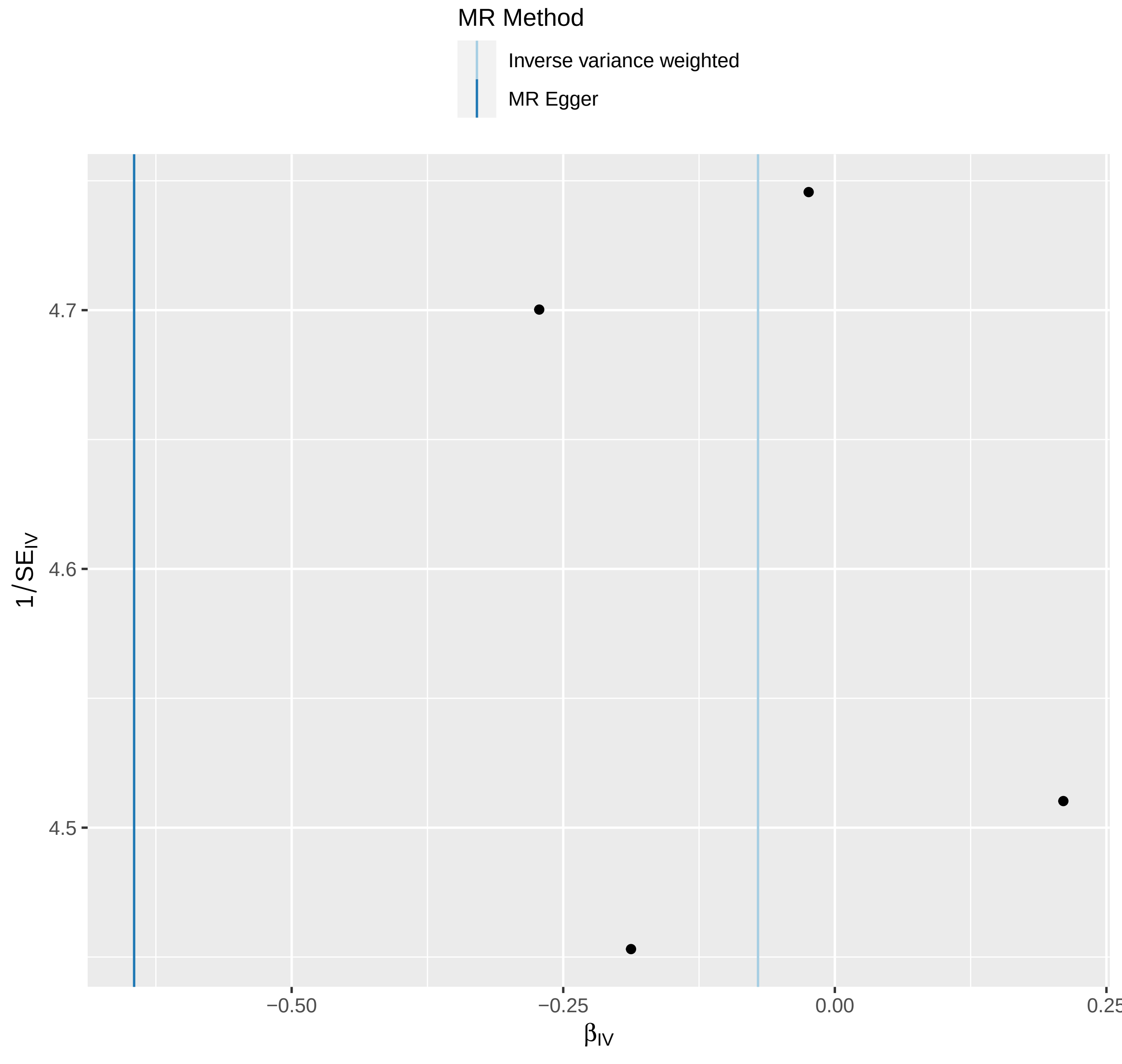

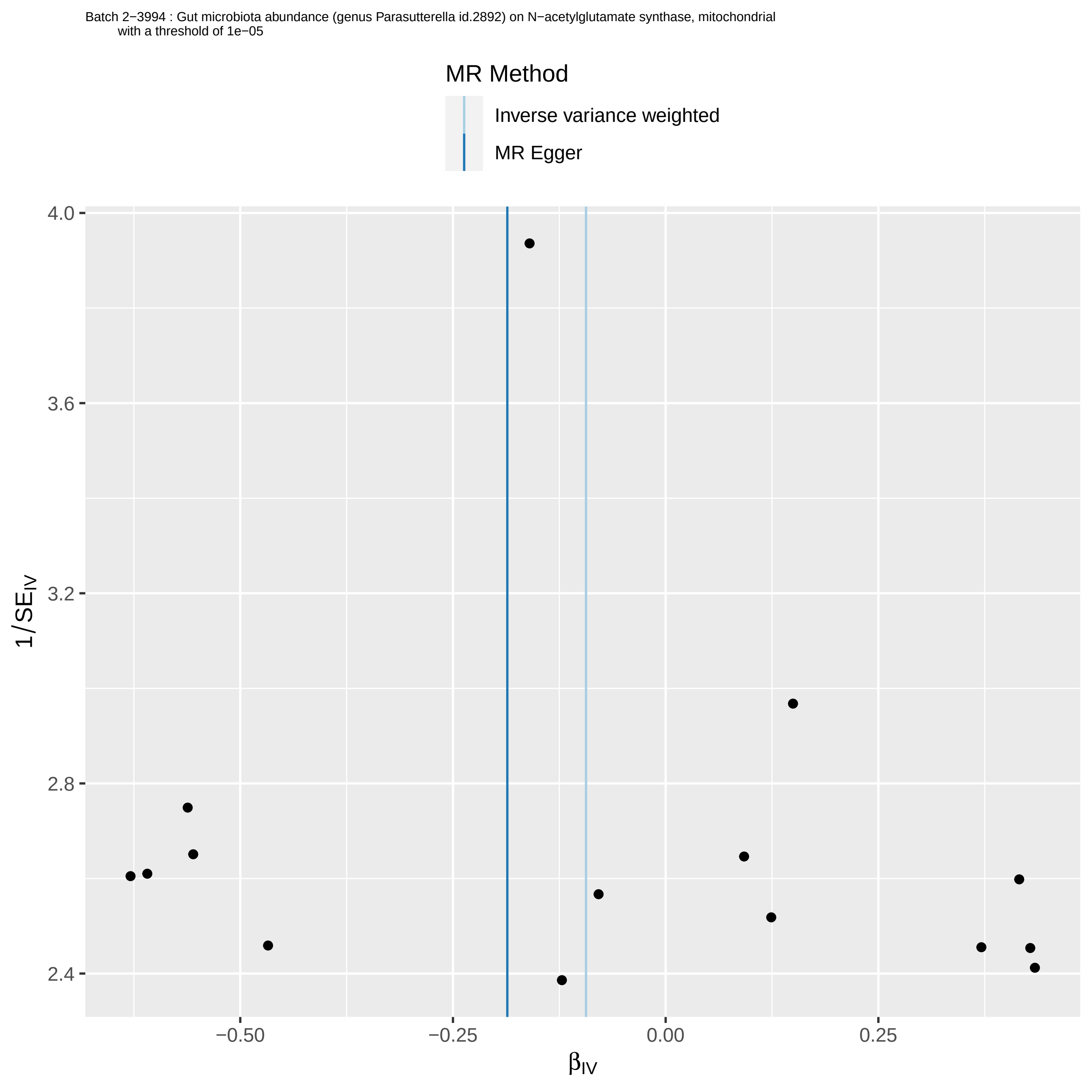
Batch 2−3994 : Gut microbiota abundance (genus Parasutterella id.2892) on N−acetylglutamate synthase, mitochondrial
with a threshold of 1e−05
MR Method
Inverse variance weighted
MR Egger
4.0
3.6
3.2
2.8
2.4
1/SE_IV
−0.50
−0.25
0.00
0.25
β_IV

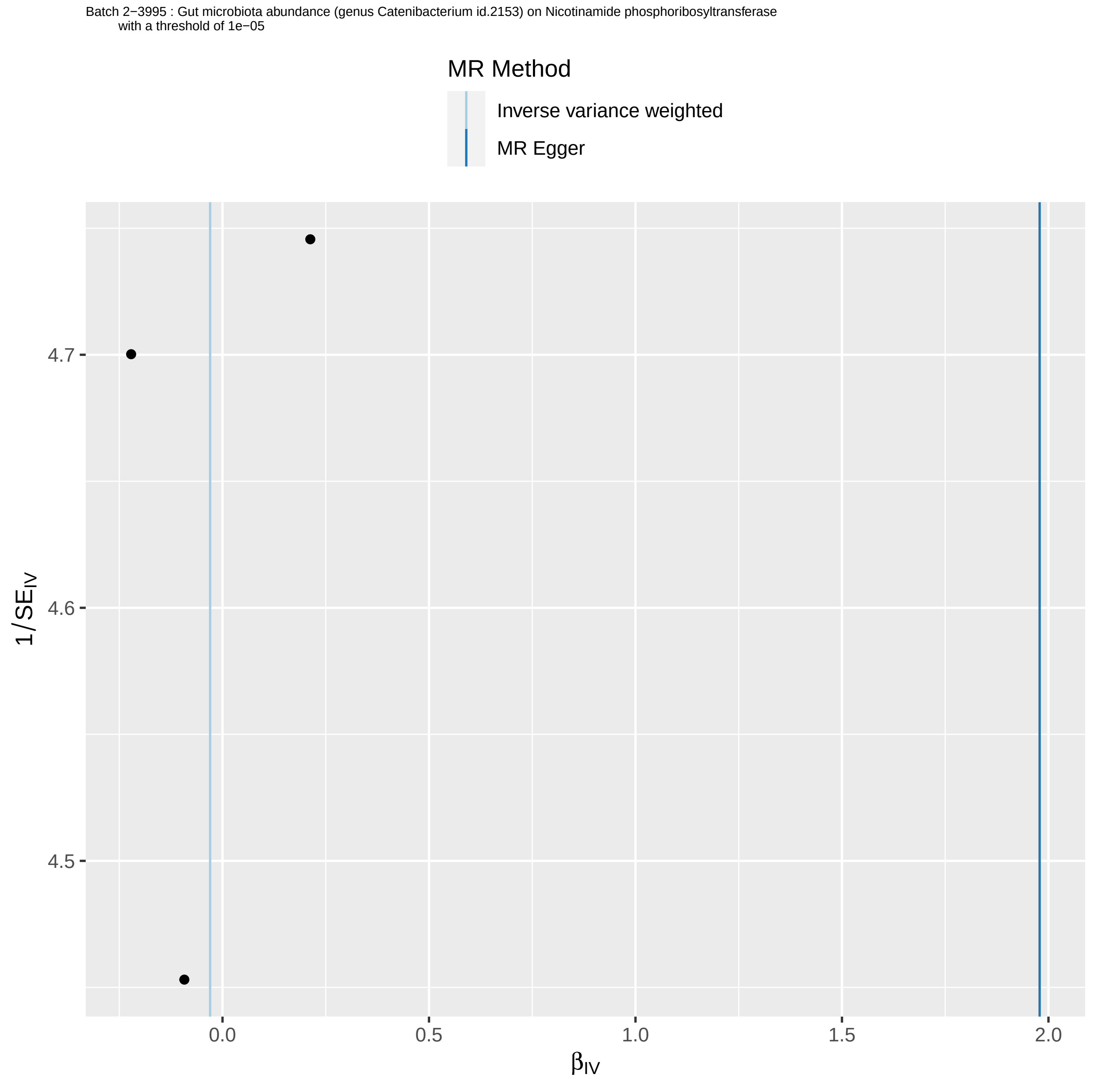

Batch 2−3995 : Gut microbiota abundance (genus Catenibacterium id.2153) on Nicotinamide phosphoribosyltransferase
with a threshold of 1e−05
MR Method
Inverse variance weighted
MR Egger
4.7
4.6
4.5
1/SE_IV
0.0
0.5
1.0
1.5
2.0
β_IV

Batch 2−3996 : Gut microbiota abundance (genus Parasutterella id.2892) on Nicotinamide phosphoribosyltransferase
with a threshold of 1e−05

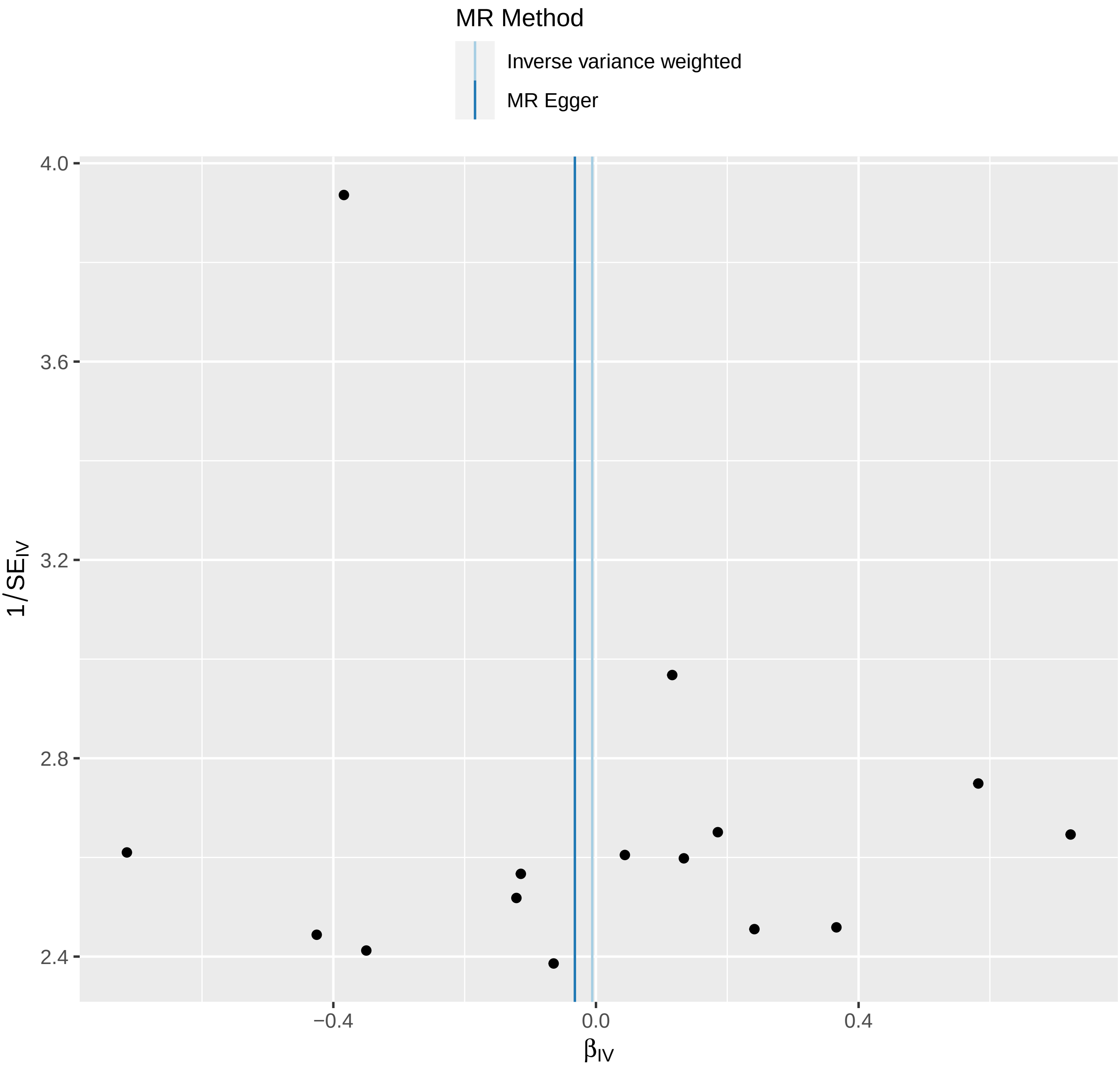

Batch 2−3997 : Gut microbiota abundance (genus Catenibacterium id.2153) on Nucleosome assembly protein 1−like 2
with a threshold of 1e−05

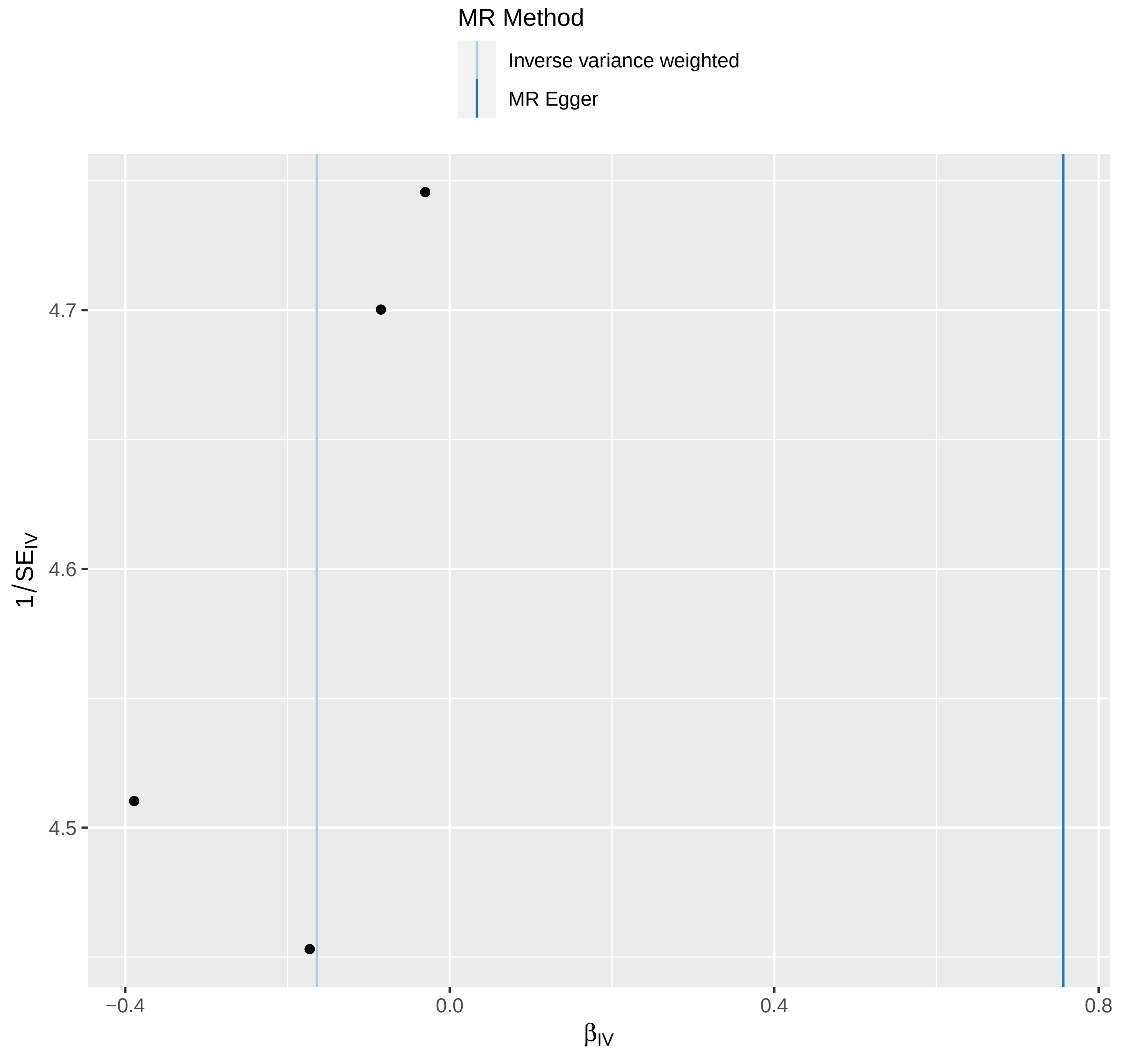

Batch 2−3998 : Gut microbiota abundance (genus Parasutterella id.2892) on Nucleosome assembly protein 1−like 2
with a threshold of 1e−05

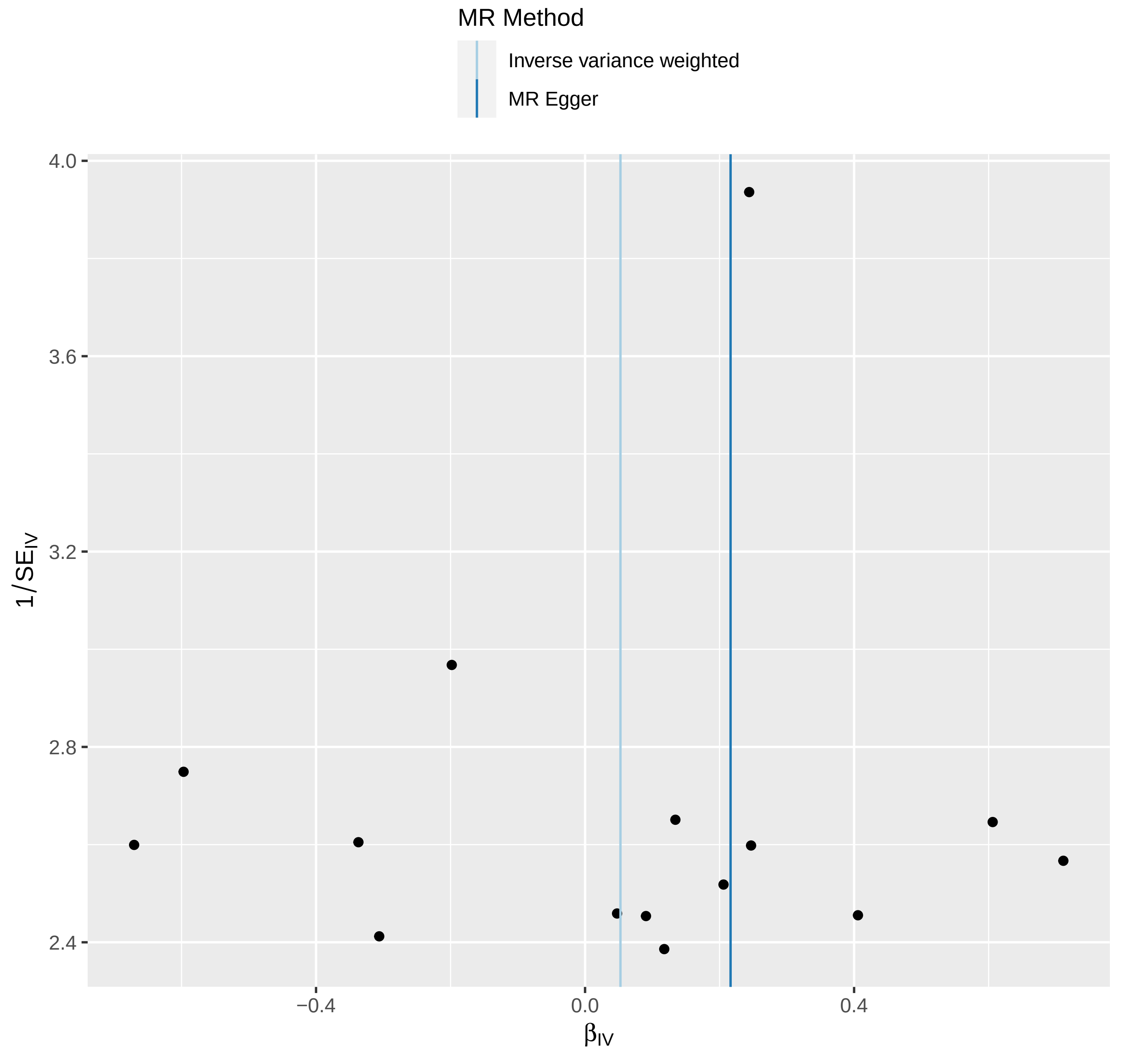

Batch 2−3999 : Gut microbiota abundance (genus Catenibacterium id.2153) on Beta−soluble NSF attachment protein with a threshold of 1e−05

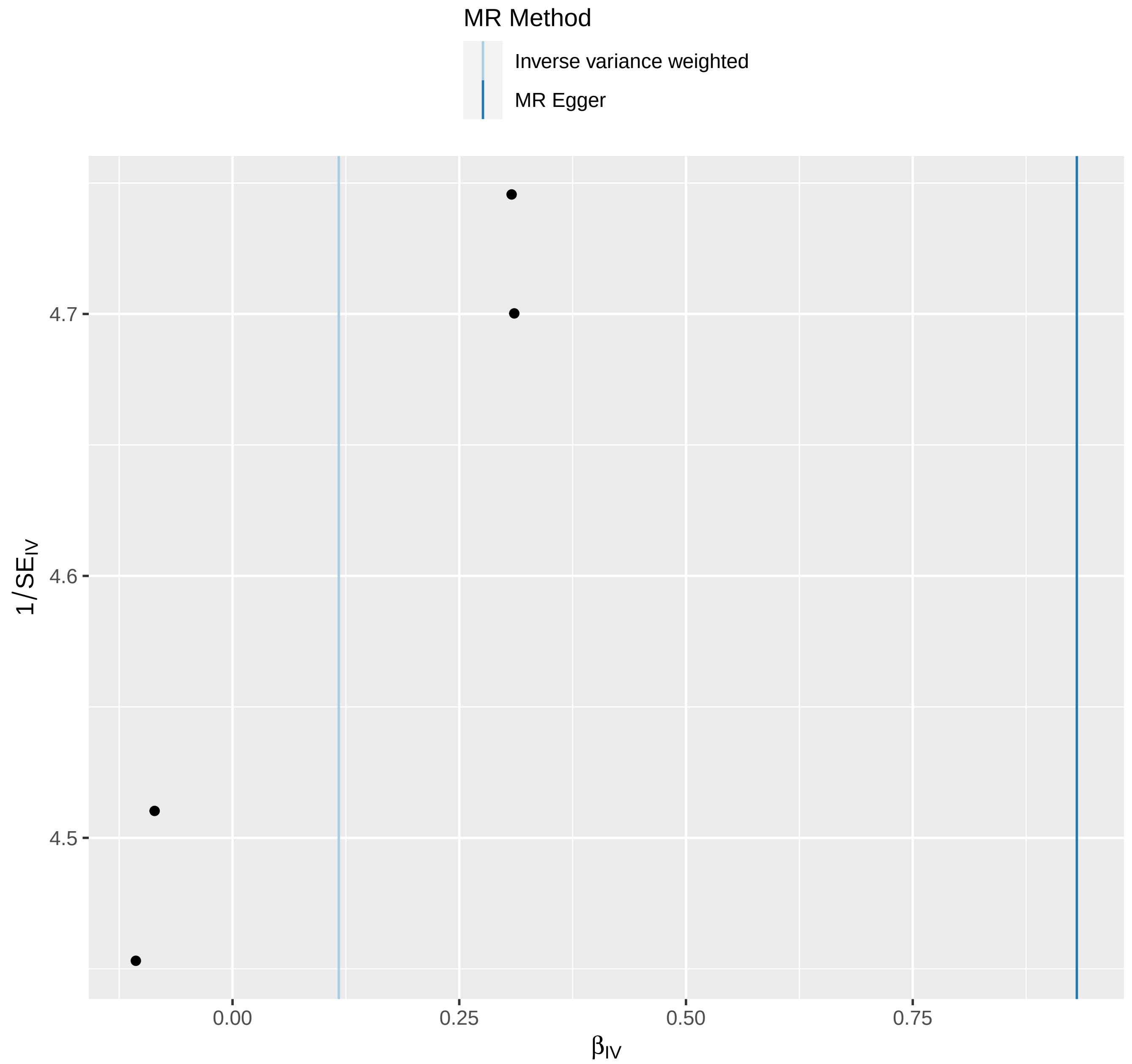

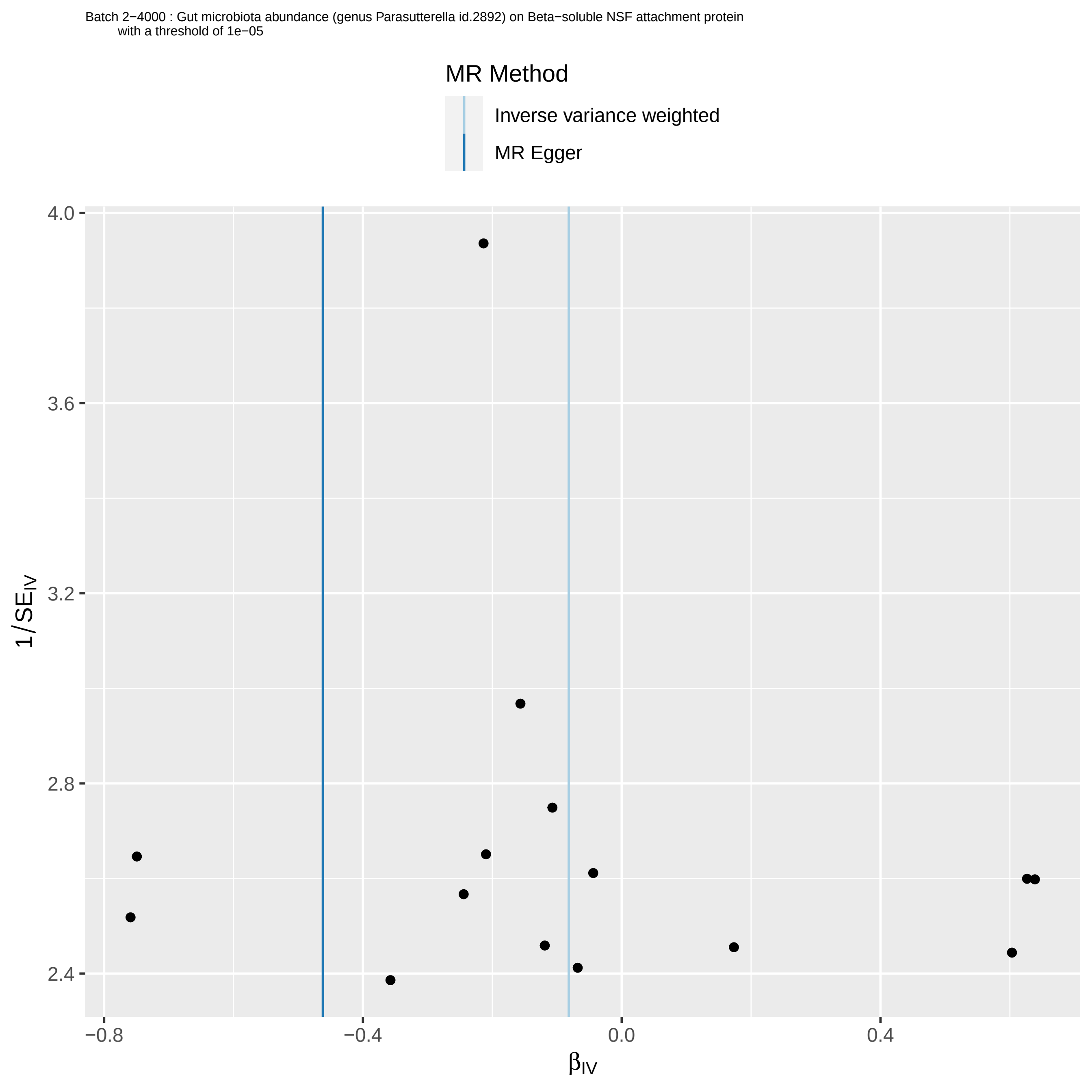

Batch 2−4000 : Gut microbiota abundance (genus Parasutterella id.2892) on Beta−soluble NSF attachment protein
with a threshold of 1e−05
MR Method
Inverse variance weighted
MR Egger
4.0
3.6
3.2
2.8
2.4
1/SE_IV
−0.8
−0.4
0.0
0.4
β_IV

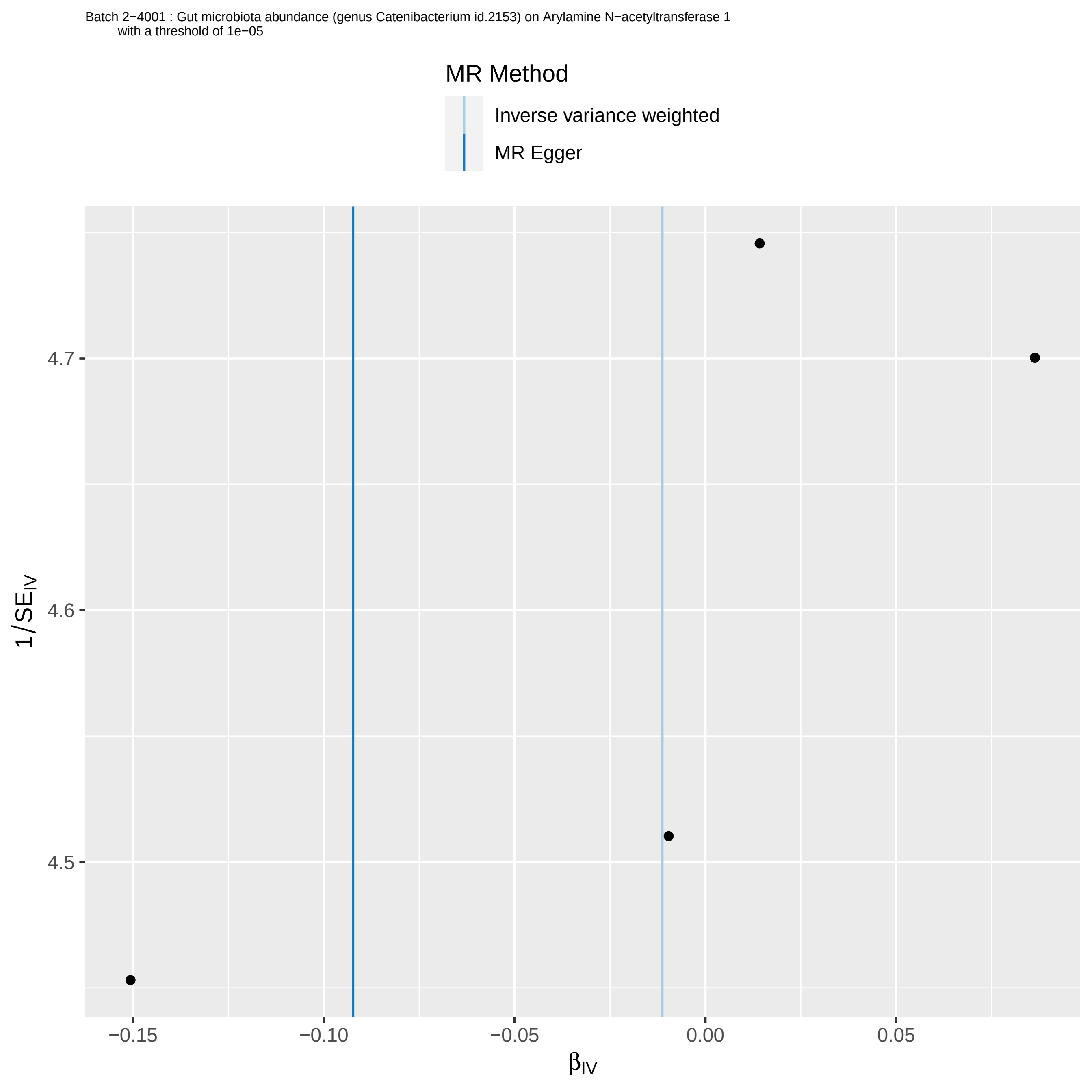

Batch 2−4001 : Gut microbiota abundance (genus Catenibacterium id.2153) on Arylamine N−acetyltransferase 1
with a threshold of 1e−05
MR Method
Inverse variance weighted
MR Egger
4.7
4.6
4.5
1/SE_IV
−0.15
−0.10
−0.05
0.00
0.05
β_IV

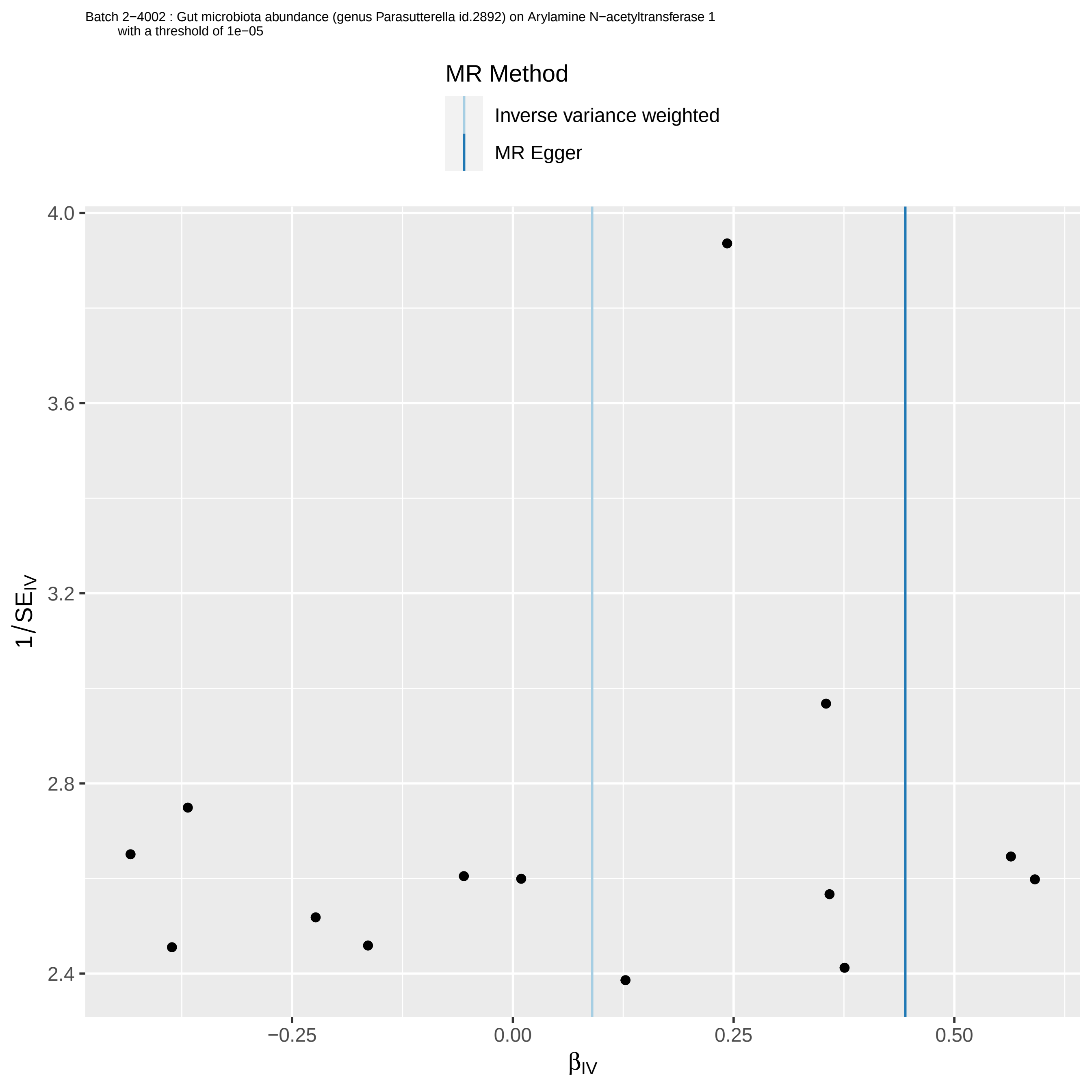

Batch 2−4002 : Gut microbiota abundance (genus Parasutterella id.2892) on Arylamine N−acetyltransferase 1
with a threshold of 1e−05
MR Method
Inverse variance weighted
MR Egger
4.0
3.6
3.2
2.8
2.4
1/SE_IV
−0.25
0.00
0.25
0.50
β_IV

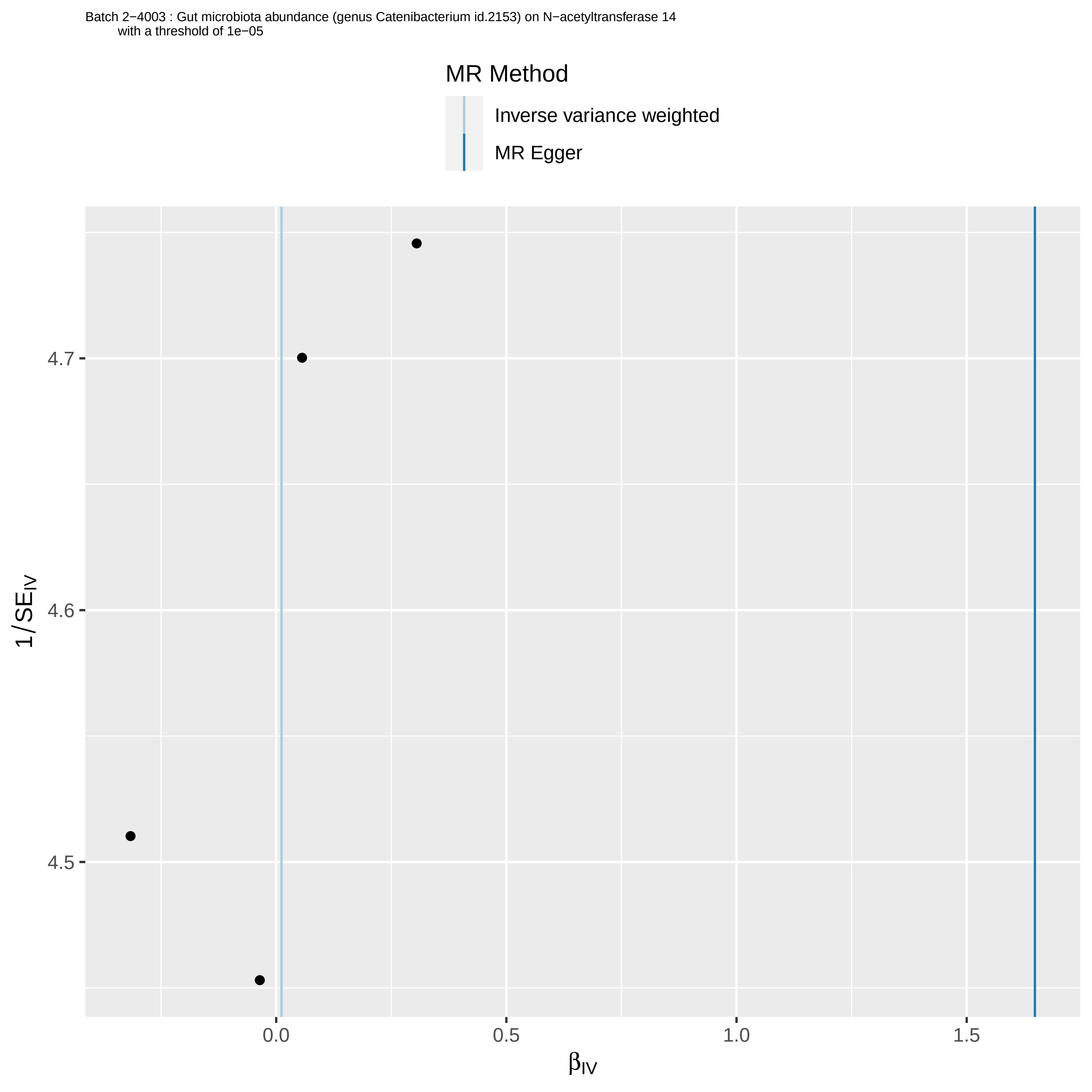
Batch 2−4003 : Gut microbiota abundance (genus Catenibacterium id.2153) on N−acetyltransferase 14
with a threshold of 1e−05
MR Method
Inverse variance weighted
MR Egger
4.7
4.6
4.5
$1/SE_{IV}$
0.0
0.5
1.0
1.5
β_{IV}

Batch 2−4004 : Gut microbiota abundance (genus Parasutterella id.2892) on N−acetyltransferase 14
with a threshold of 1e−05

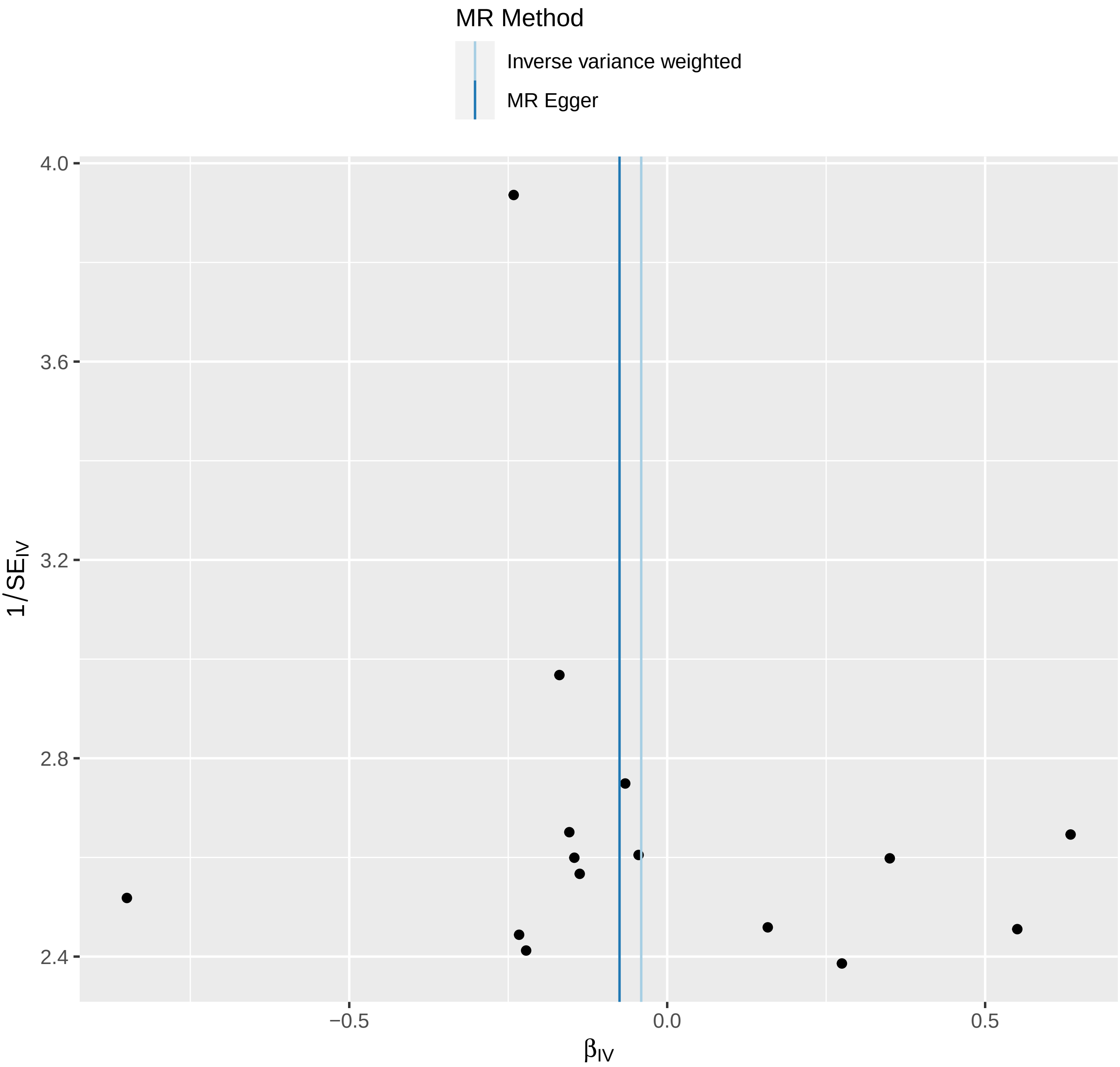

Batch 2−4005 : Gut microbiota abundance (genus Catenibacterium id.2153) on Neuroblastoma suppressor of tumorigenicity 1
with a threshold of 1e−05

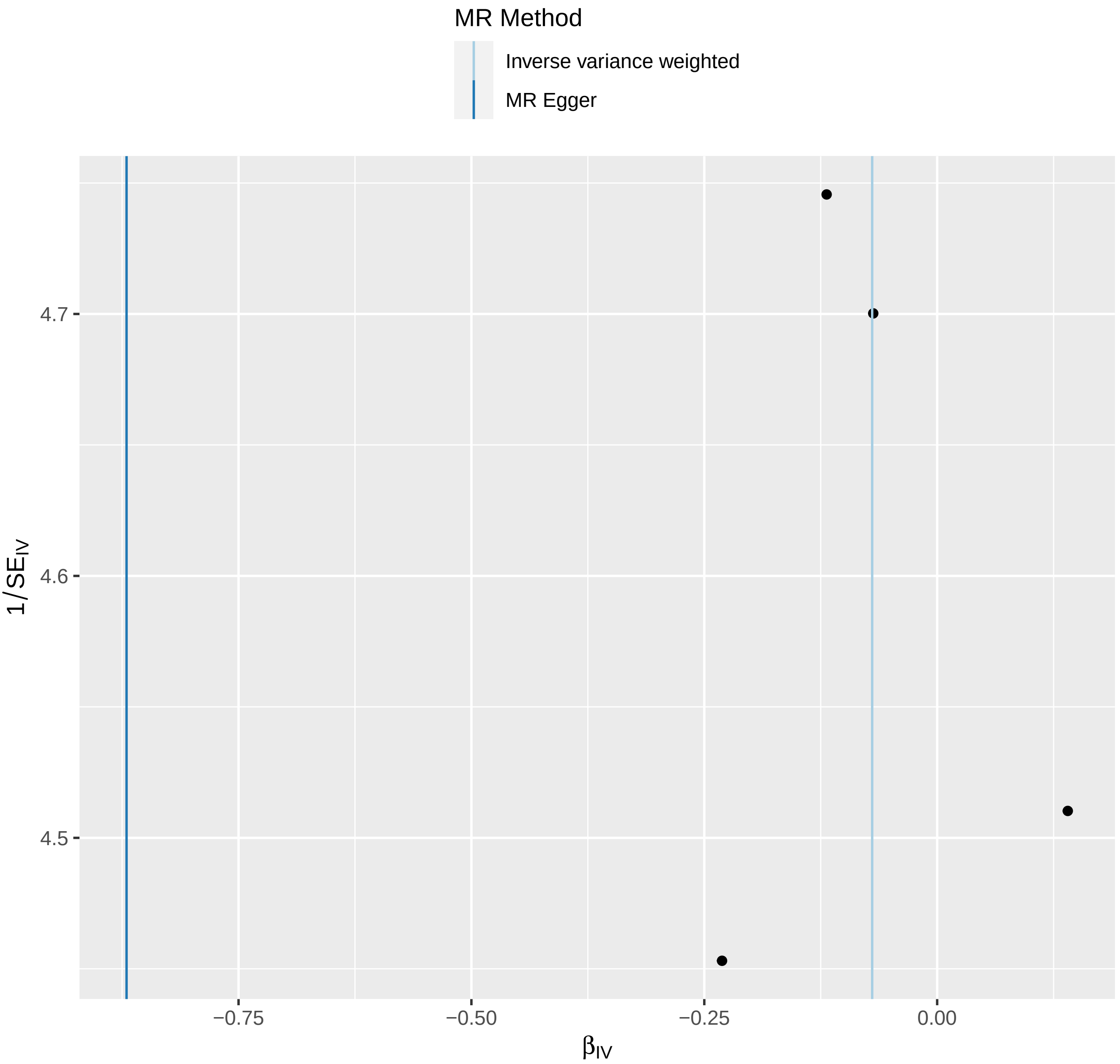

Batch 2−4006 : Gut microbiota abundance (genus Parasutterella id.2892) on Neuroblastoma suppressor of tumorigenicity 1
with a threshold of 1e−05

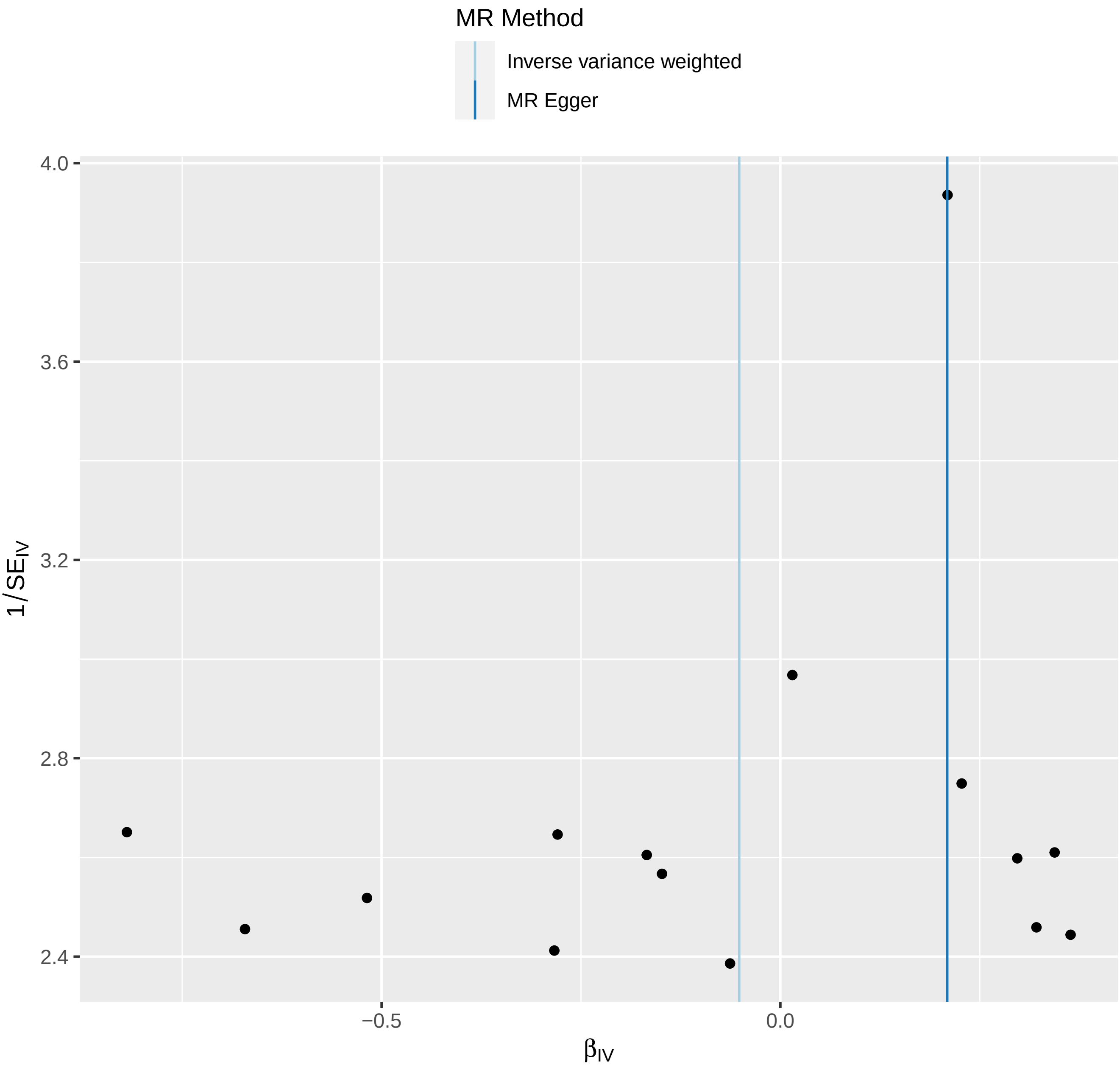

Batch 2−4007 : Gut microbiota abundance (genus Catenibacterium id.2153) on Next to BRCA1 gene 1 protein with a threshold of 1e−05

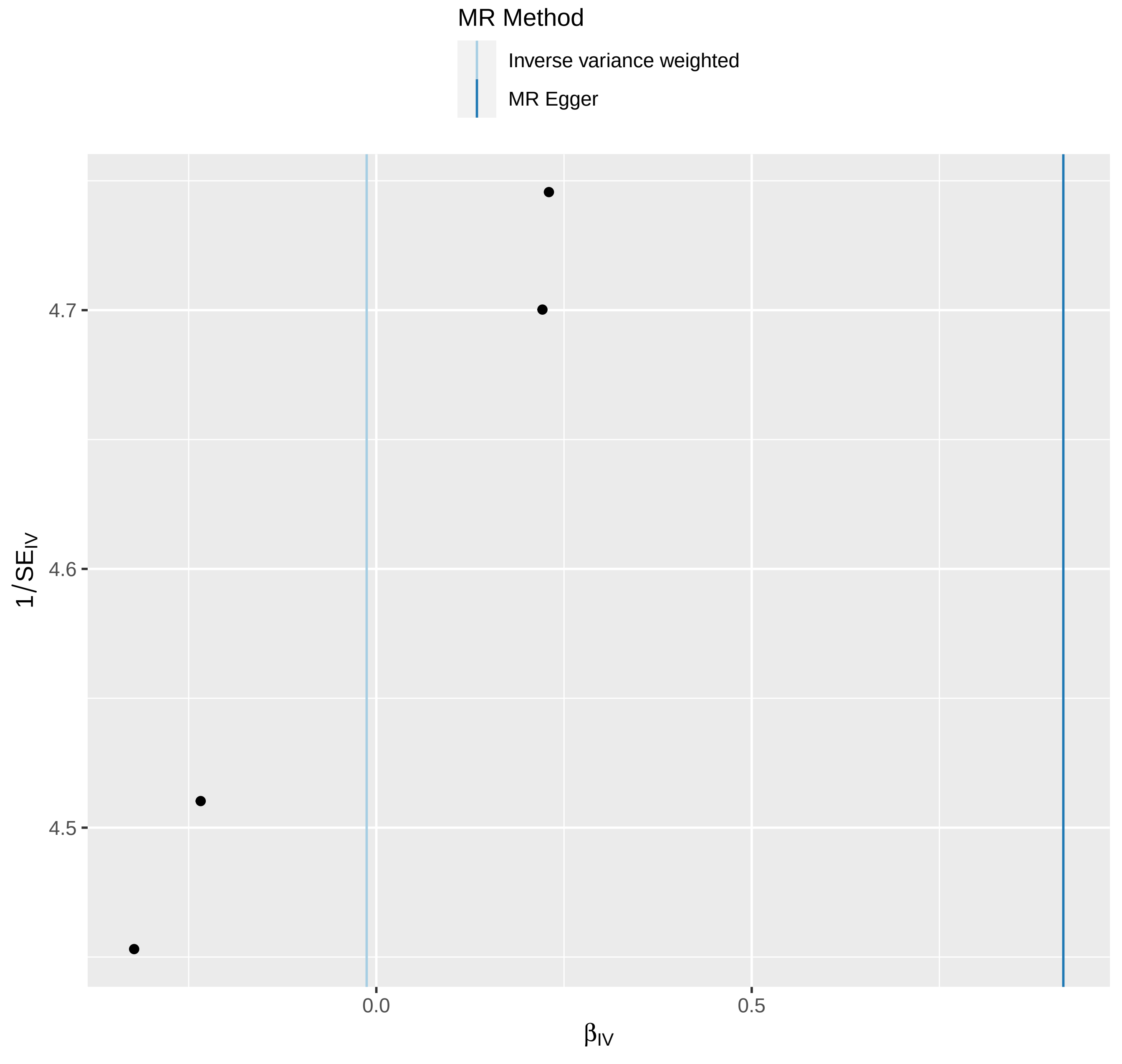

Batch 2−4008 : Gut microbiota abundance (genus Parasutterella id.2892) on Next to BRCA1 gene 1 protein
with a threshold of 1e−05

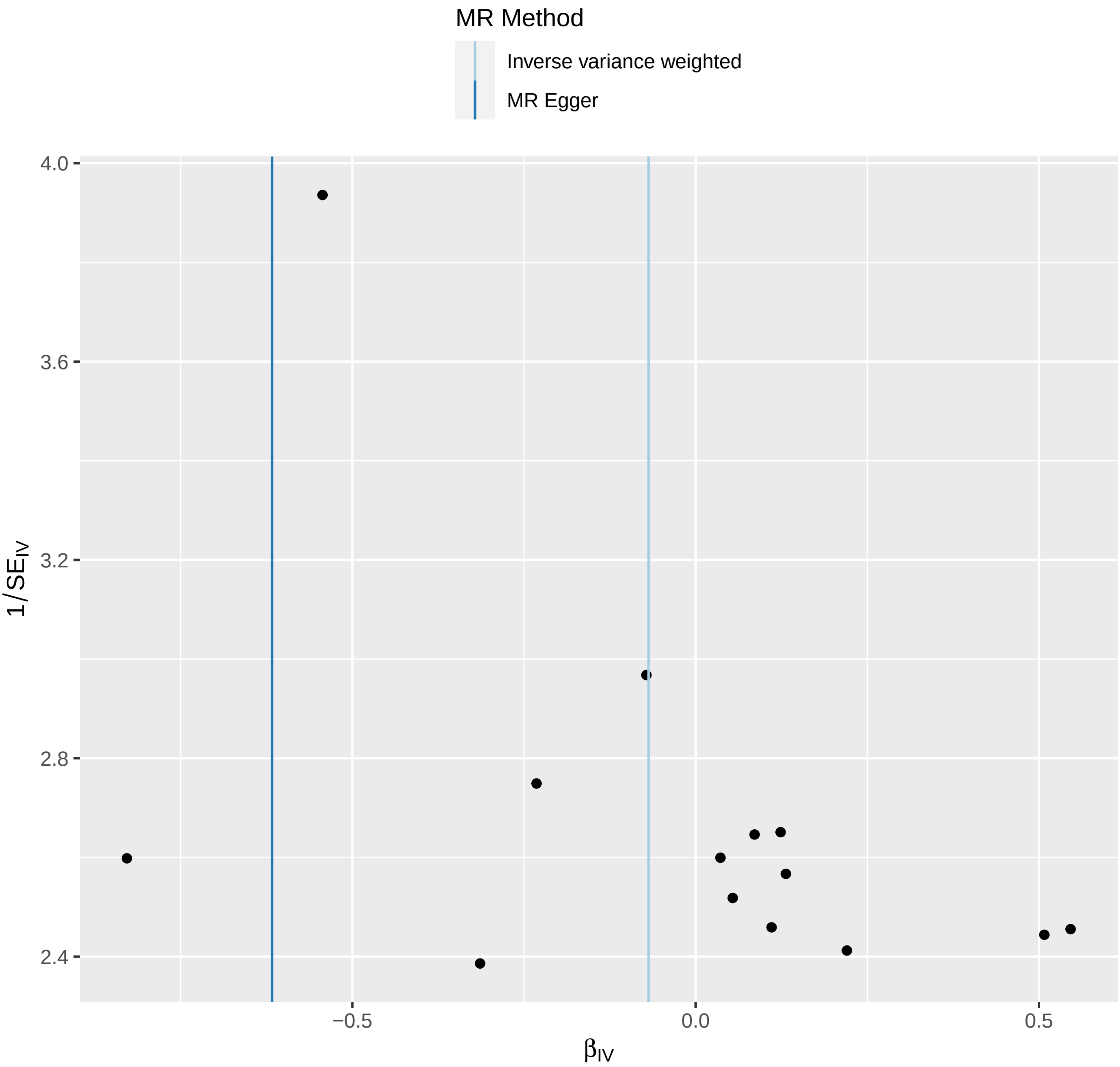

Batch 2−4009 : Gut microbiota abundance (genus Catenibacterium id.2153) on Next to BRCA1 gene 1 protein
with a threshold of 1e−05

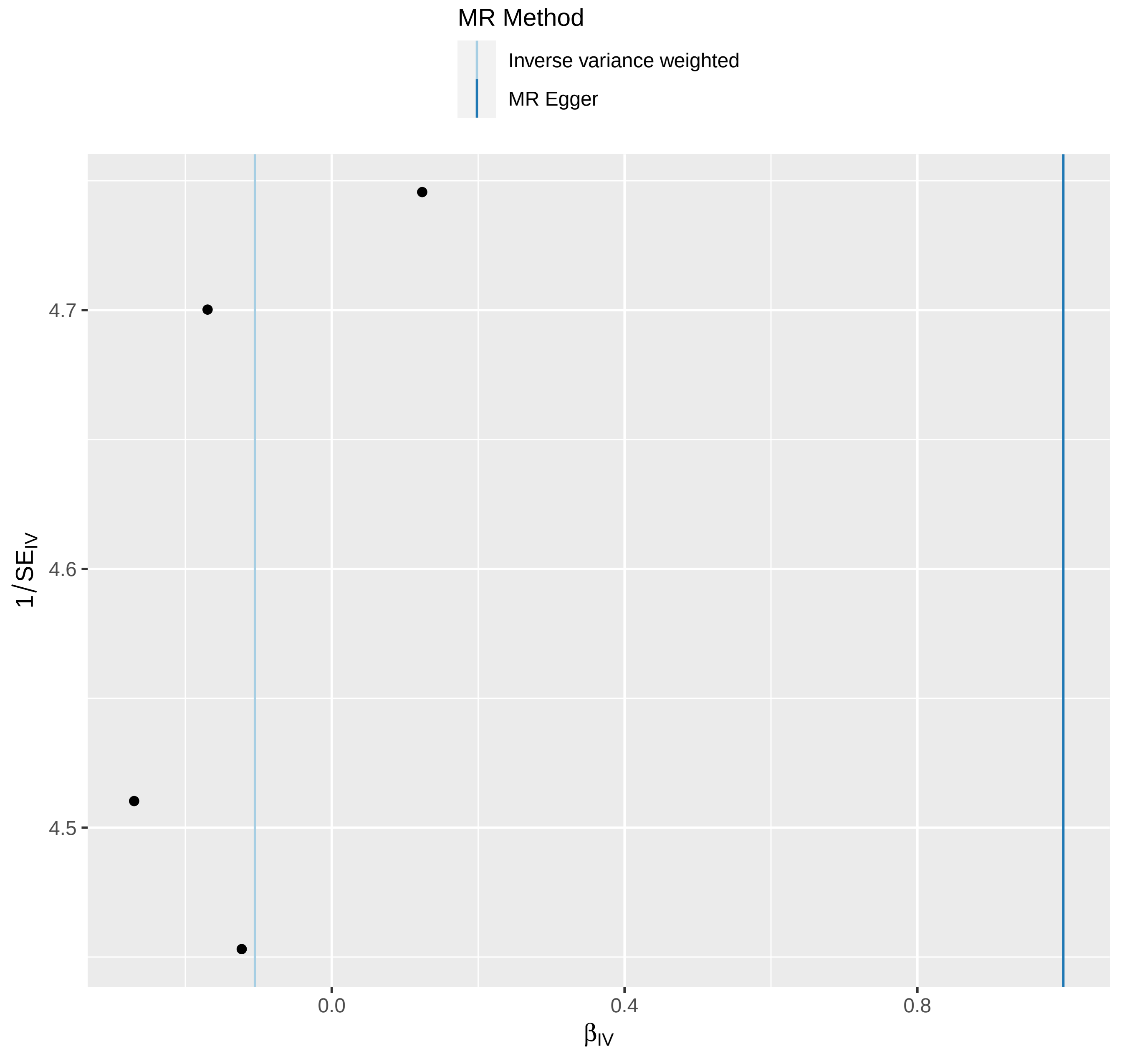

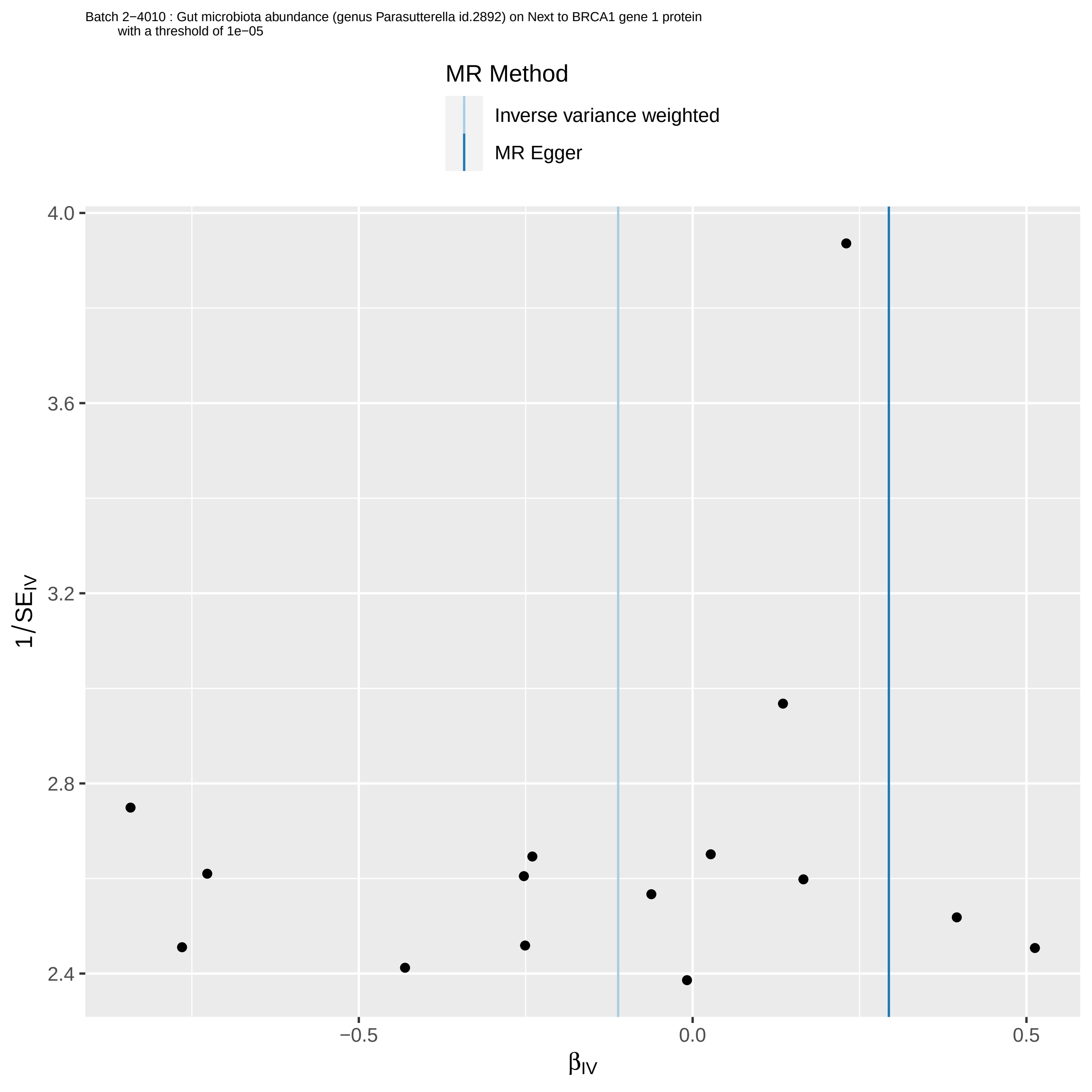
Batch 2−4010 : Gut microbiota abundance (genus Parasutterella id.2892) on Next to BRCA1 gene 1 protein
with a threshold of 1e−05
MR Method
Inverse variance weighted
MR Egger
4.0
3.6
3.2
2.8
2.4
1/SE_IV
−0.5
0.0
0.5
β_IV

Batch 2−4011 : Gut microbiota abundance (genus Catenibacterium id.2153) on Neural cell adhesion molecule 1, 120 kDa isoform
with a threshold of 1e−05

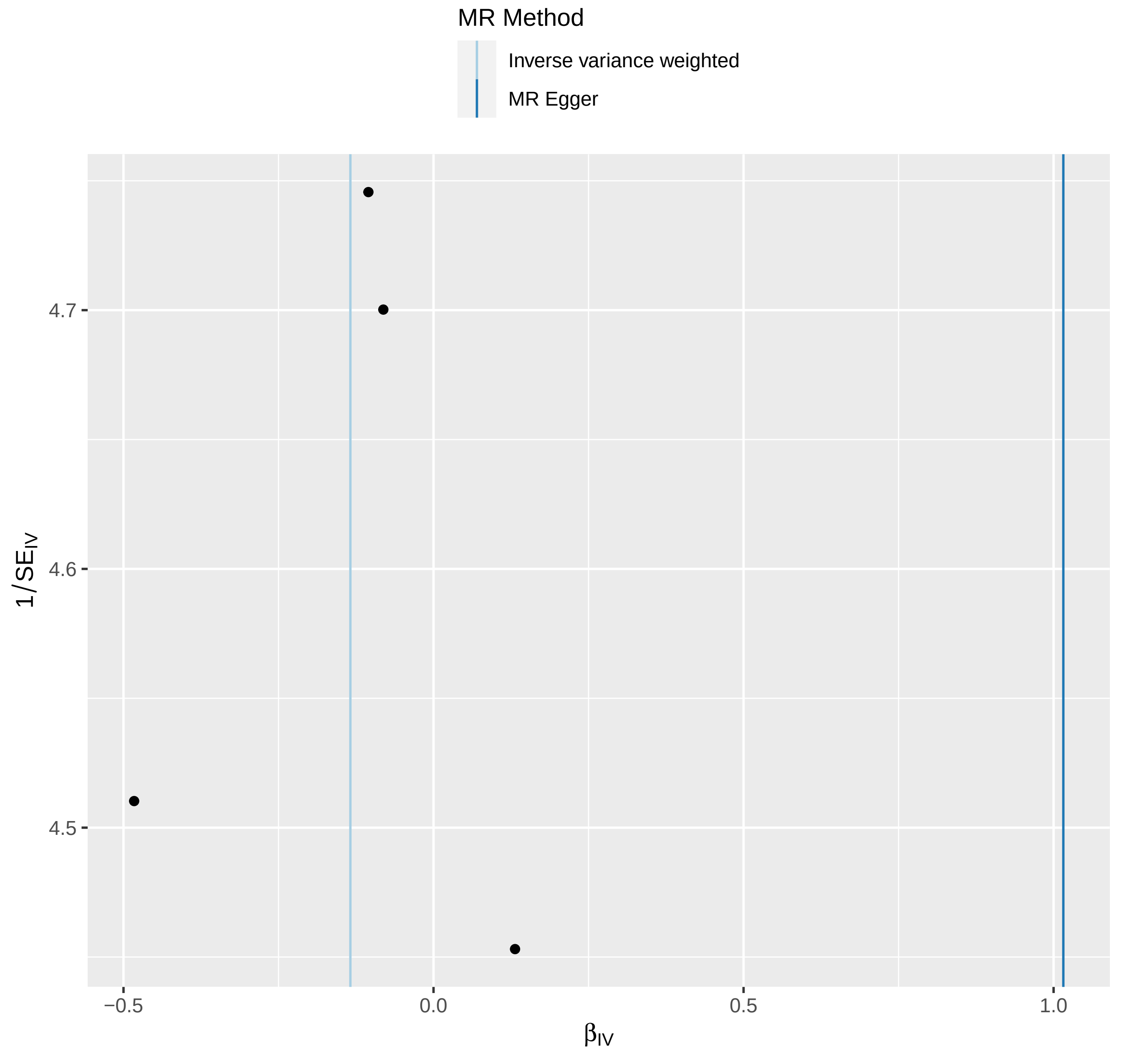

Batch 2−4012 : Gut microbiota abundance (genus Parasutterella id.2892) on Neural cell adhesion molecule 1, 120 kDa isoform with a threshold of 1e−05

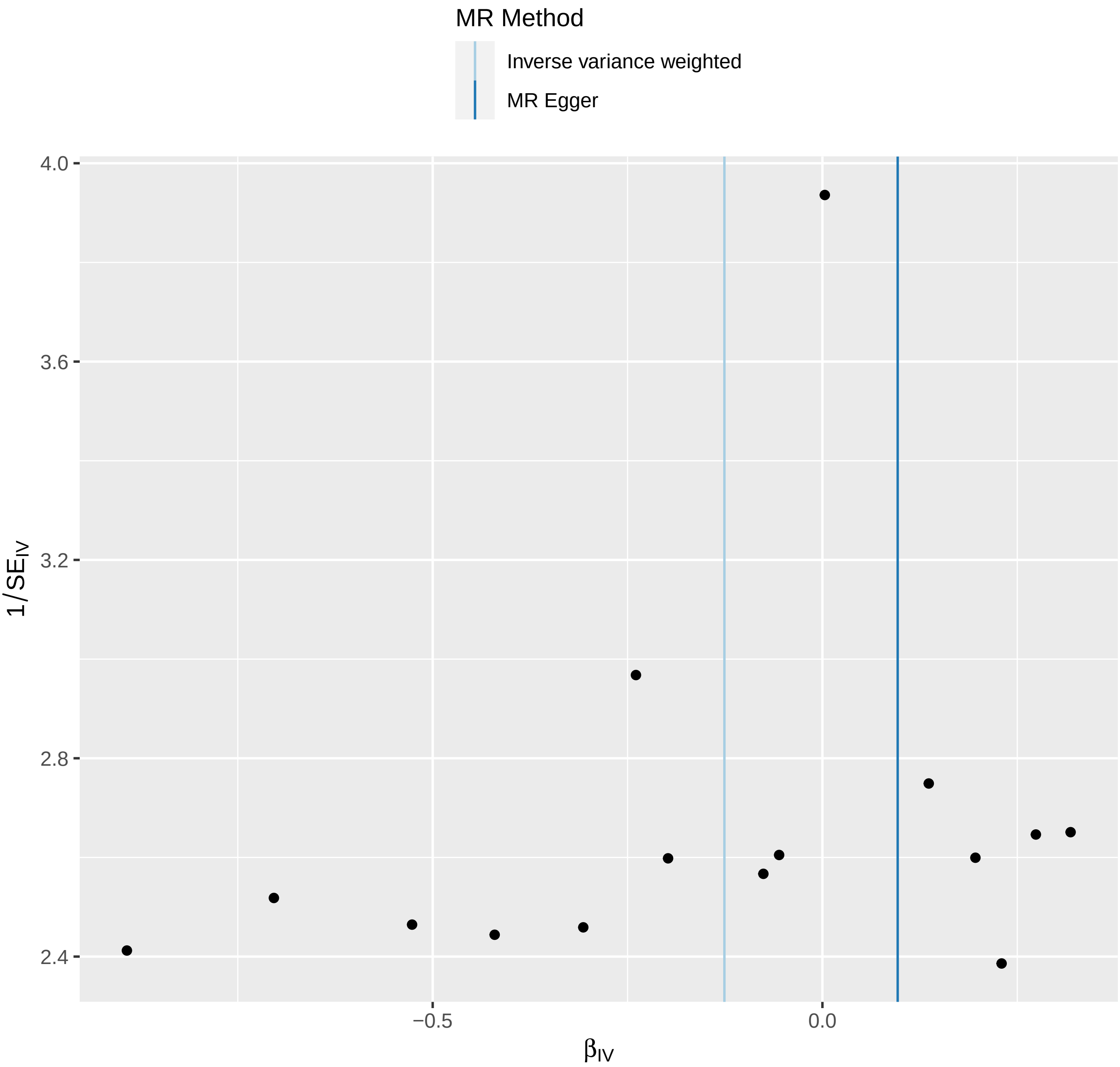

Batch 2−4013 : Gut microbiota abundance (genus Catenibacterium id.2153) on Neural cell adhesion molecule 1
with a threshold of 1e−05

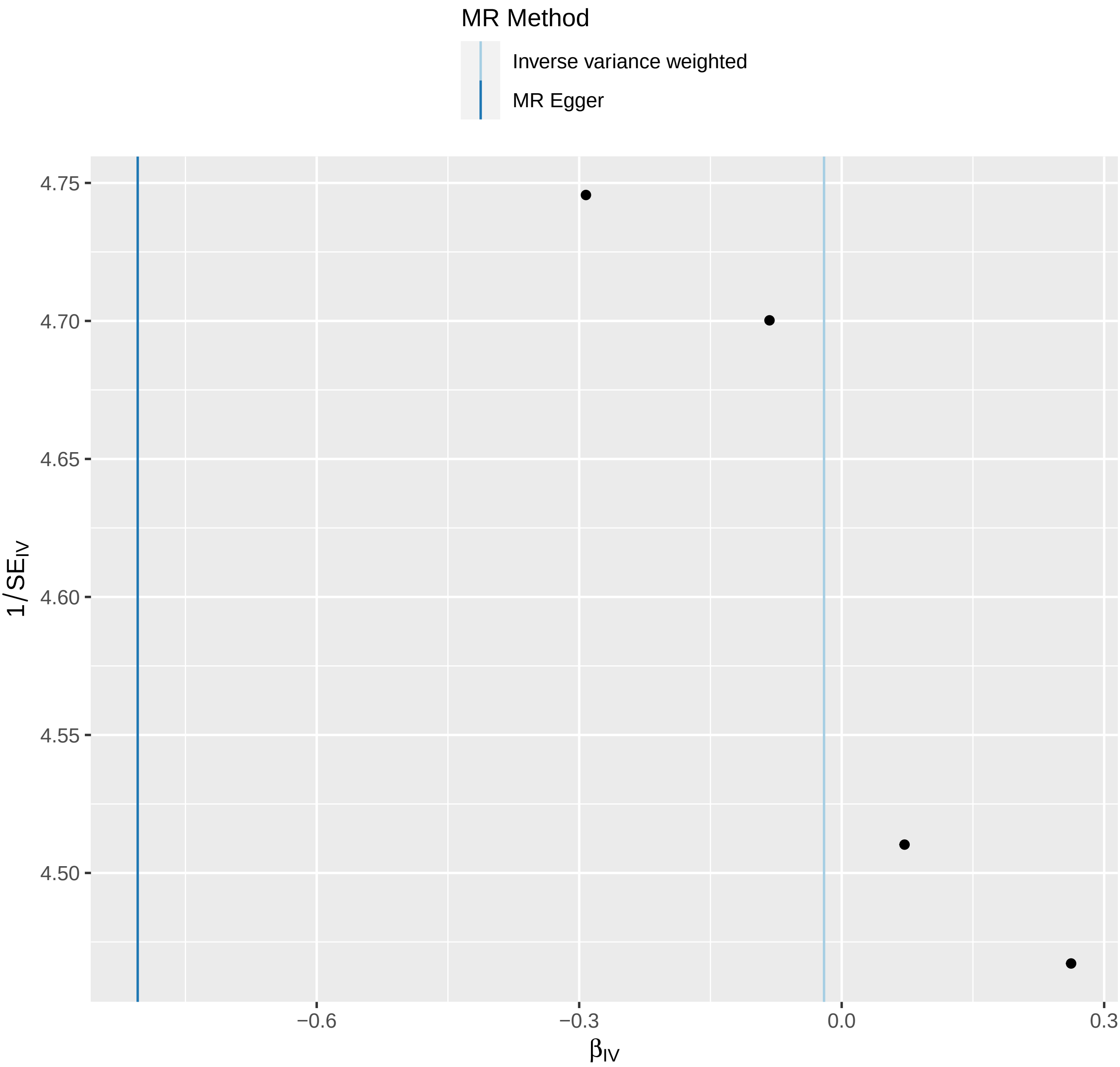

Batch 2−4014 : Gut microbiota abundance (genus Parasutterella id.2892) on Neural cell adhesion molecule 1
with a threshold of 1e−05

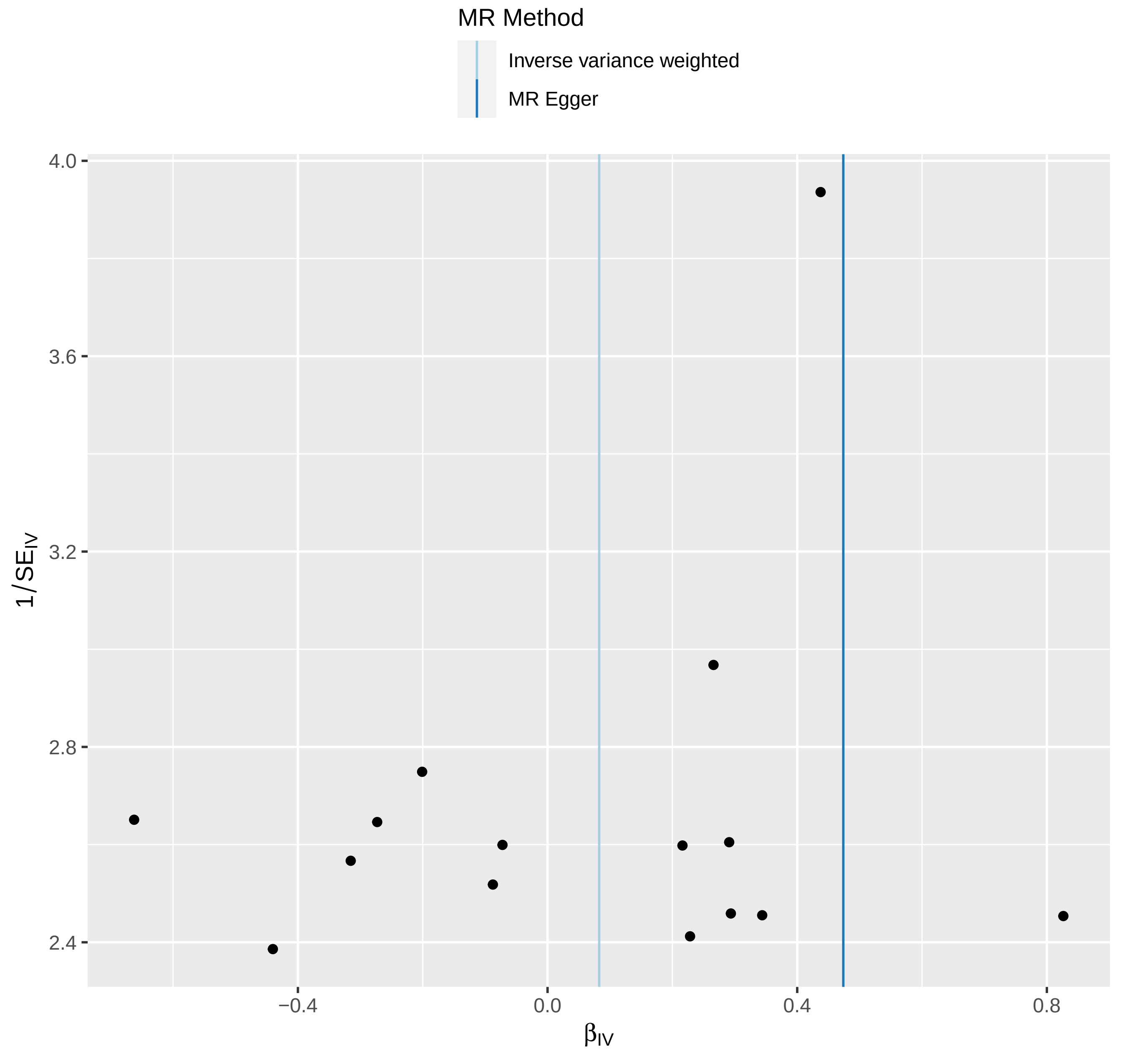

Batch 2−4015 : Gut microbiota abundance (genus Catenibacterium id.2153) on Neural cell adhesion molecule 2
with a threshold of 1e−05

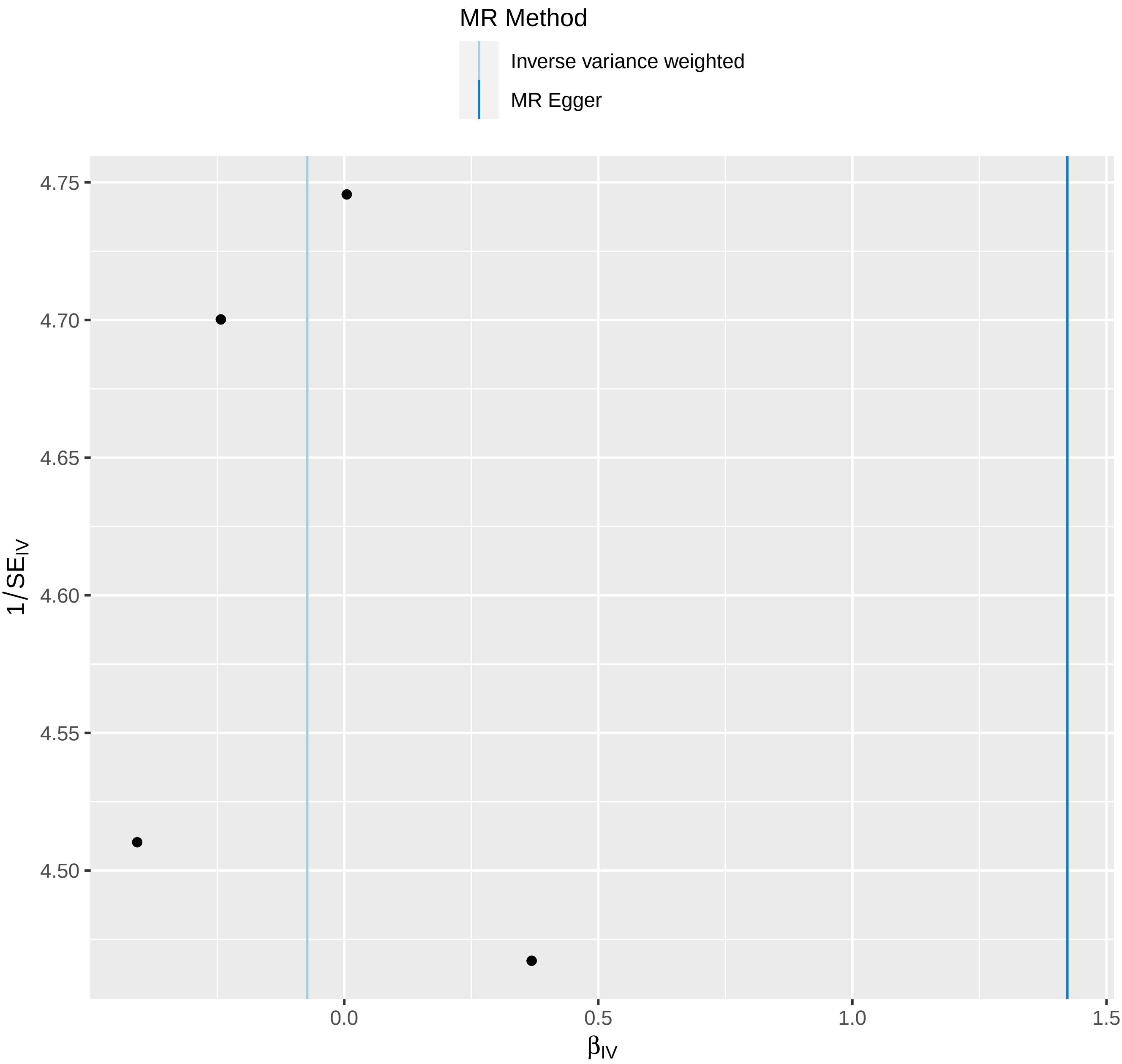

Batch 2−4016 : Gut microbiota abundance (genus Parasutterella id.2892) on Neural cell adhesion molecule 2
with a threshold of 1e−05

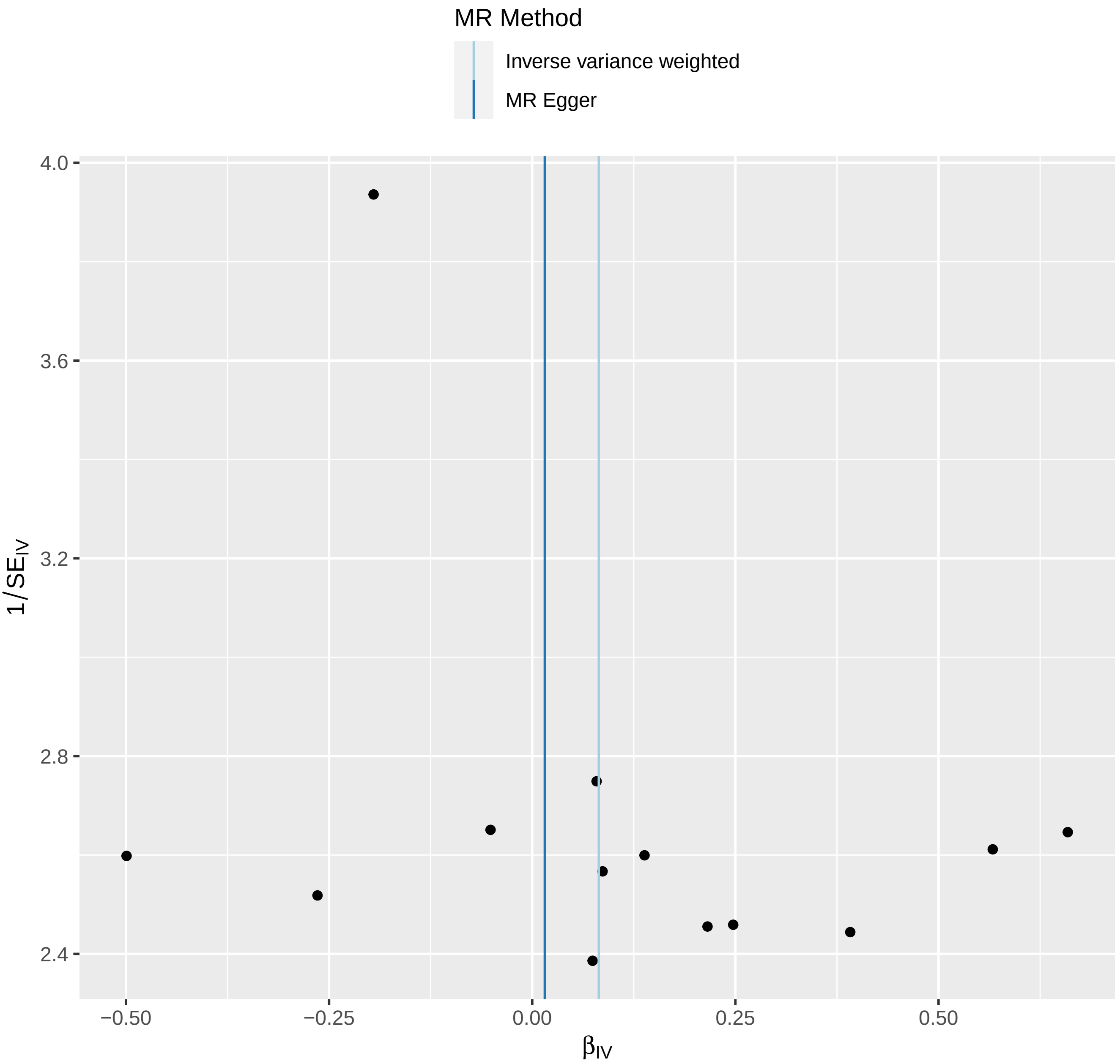

Batch 2−4017 : Gut microbiota abundance (genus Catenibacterium id.2153) on Neutrophil cytosol factor 2
with a threshold of 1e−05

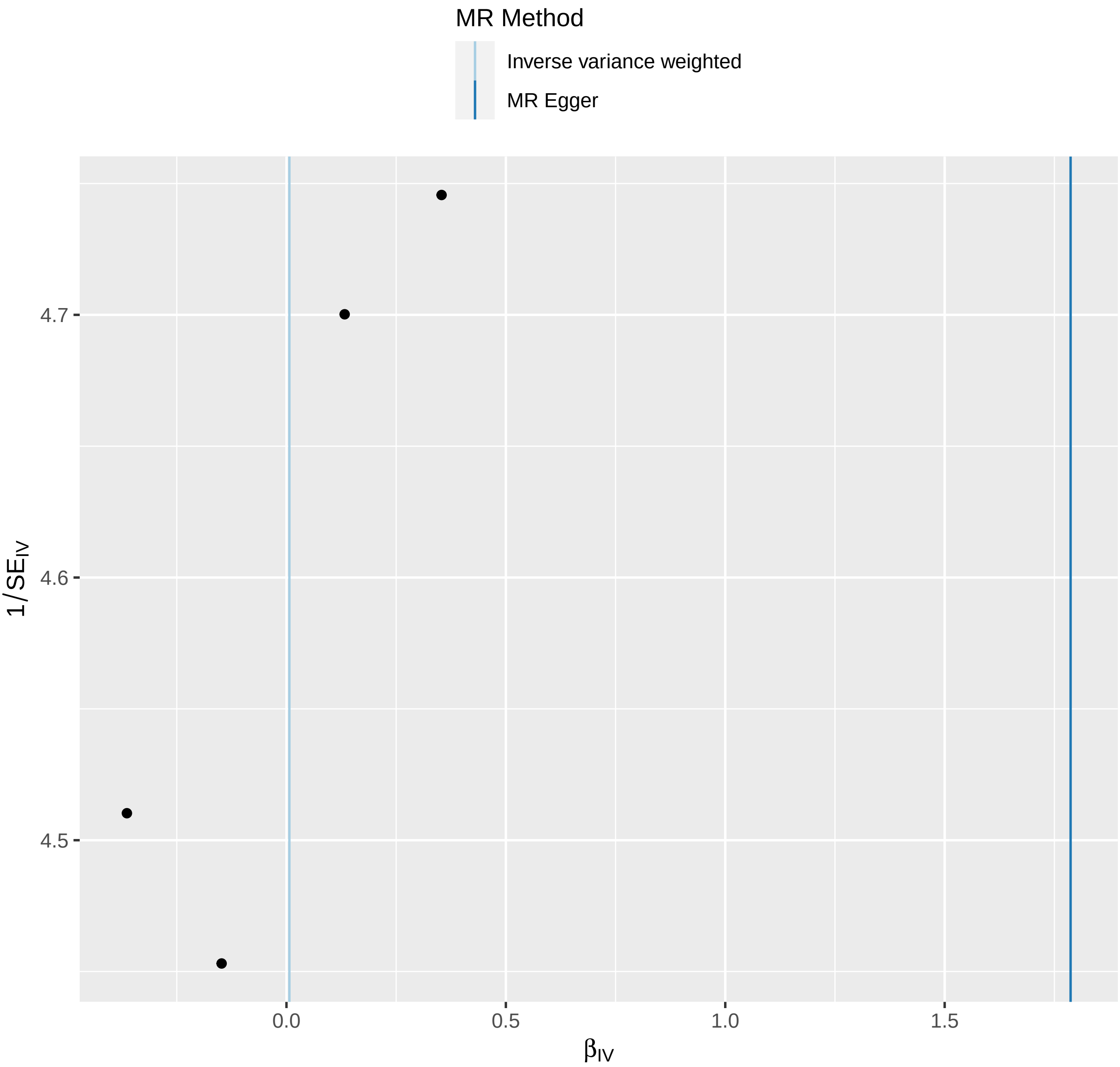

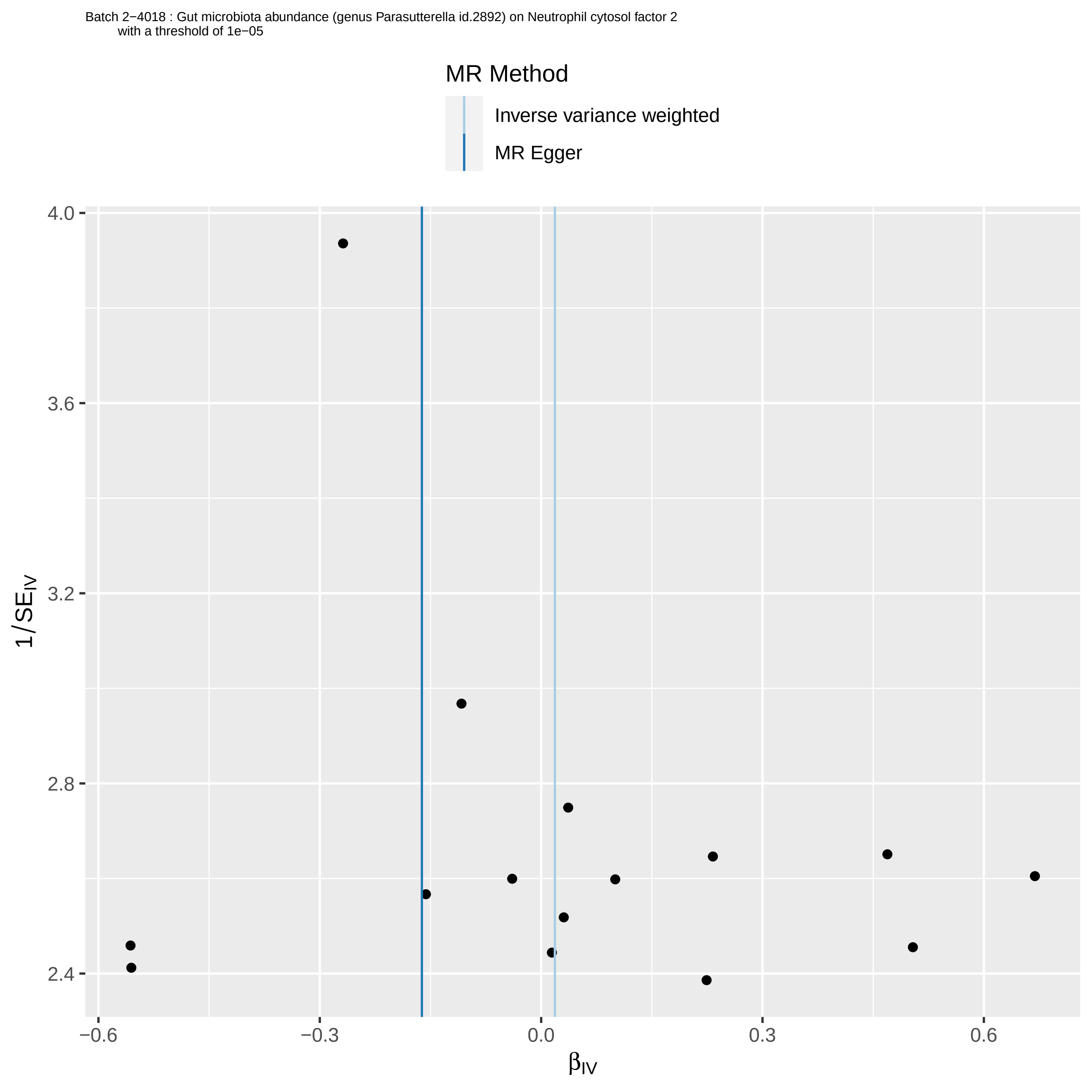

Batch 2−4018 : Gut microbiota abundance (genus Parasutterella id.2892) on Neutrophil cytosol factor 2
with a threshold of 1e−05
MR Method
Inverse variance weighted
MR Egger
4.0
3.6
3.2
2.8
2.4
1/SE_IV
−0.6
−0.3
0.0
0.3
0.6
β_IV

Batch 2−4019 : Gut microbiota abundance (genus Catenibacterium id.2153) on Nucleolin
with a threshold of 1e−05

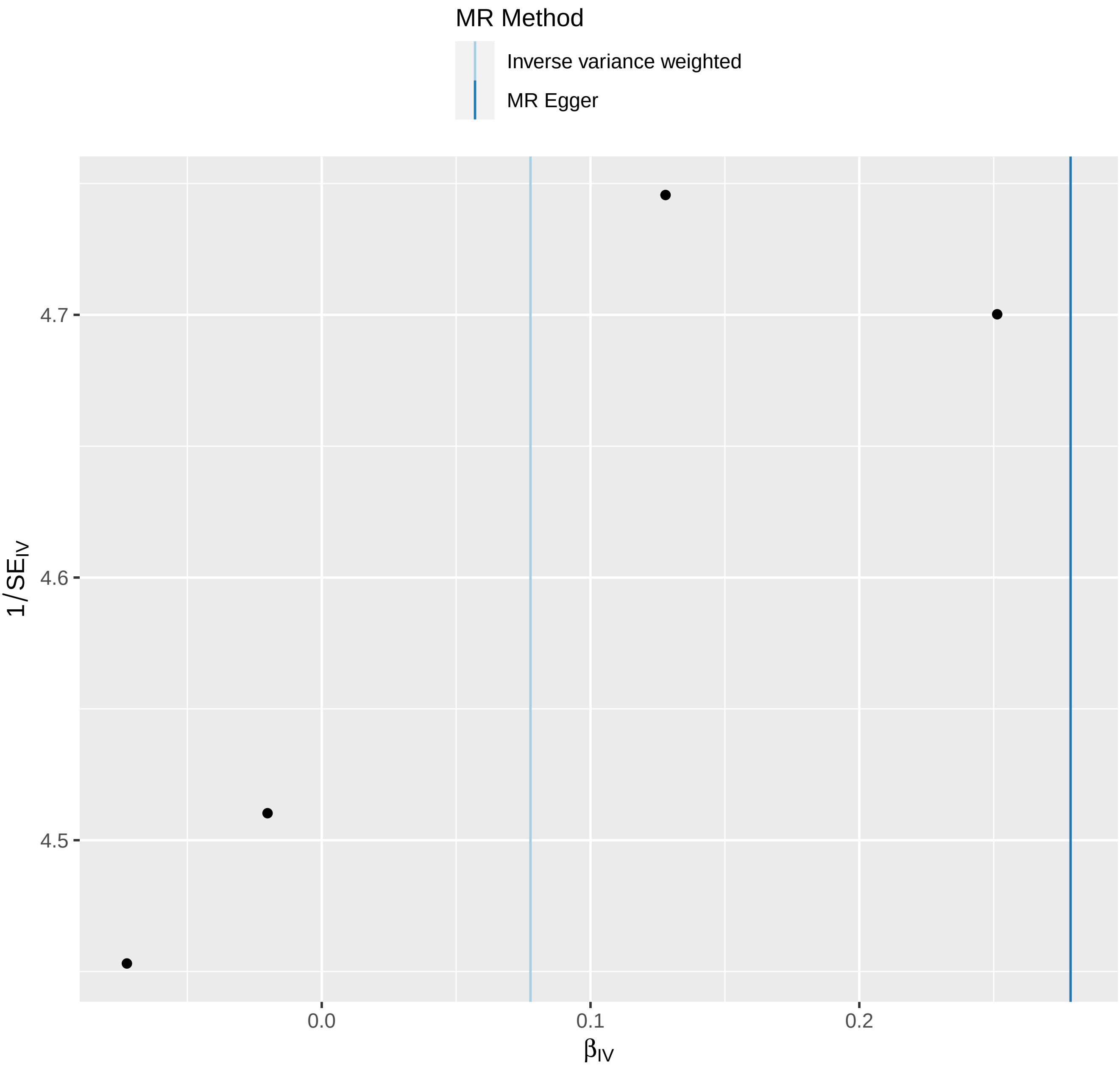

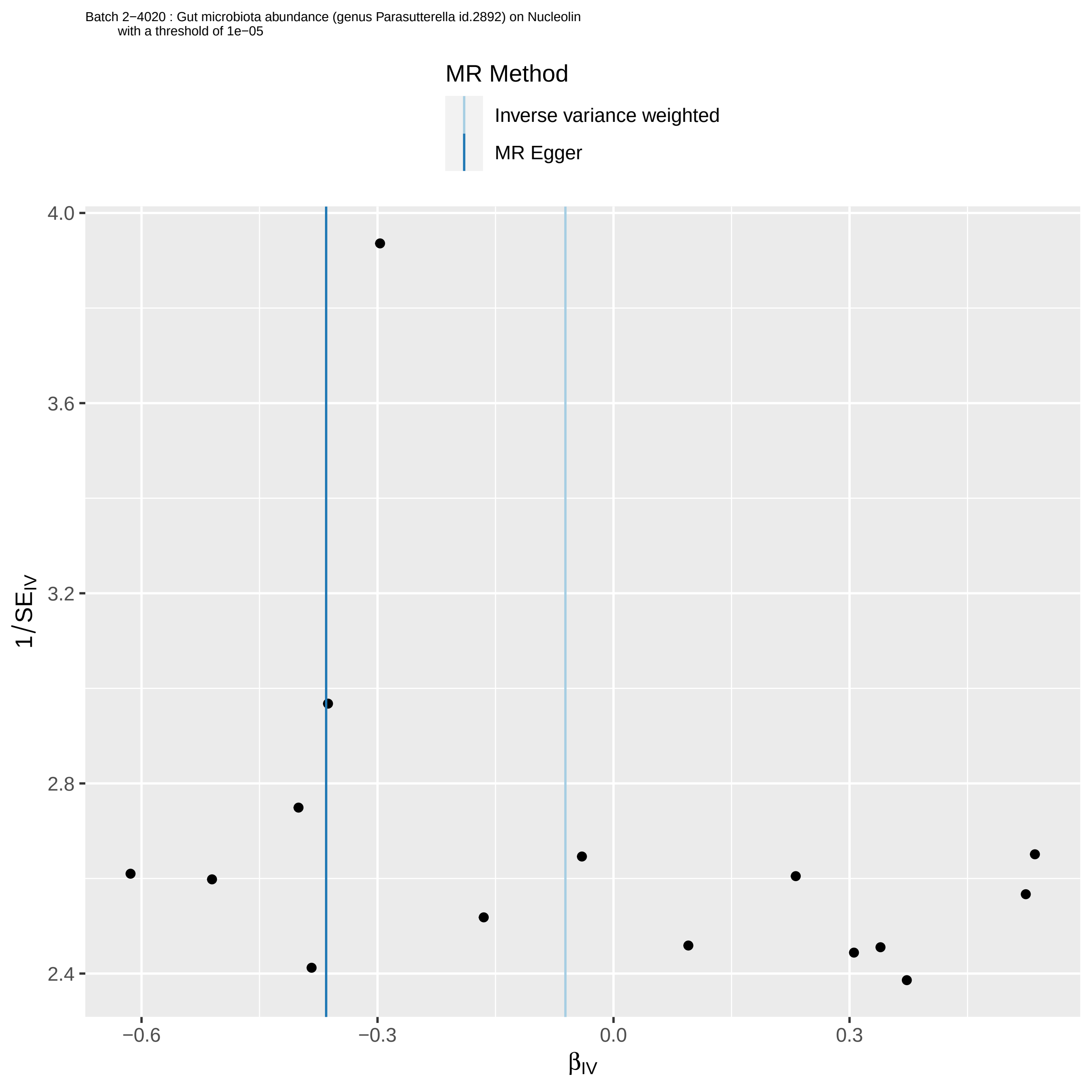

Batch 2−4020 : Gut microbiota abundance (genus Parasutterella id.2892) on Nucleolin
with a threshold of 1e−05
MR Method
Inverse variance weighted
MR Egger
4.0
3.6
3.2
2.8
2.4
1/SE_IV
−0.6
−0.3
0.0
0.3
β_IV

Batch 2−4021 : Gut microbiota abundance (genus Catenibacterium id.2153) on Noncompact myelin−associated protein with a threshold of 1e−05

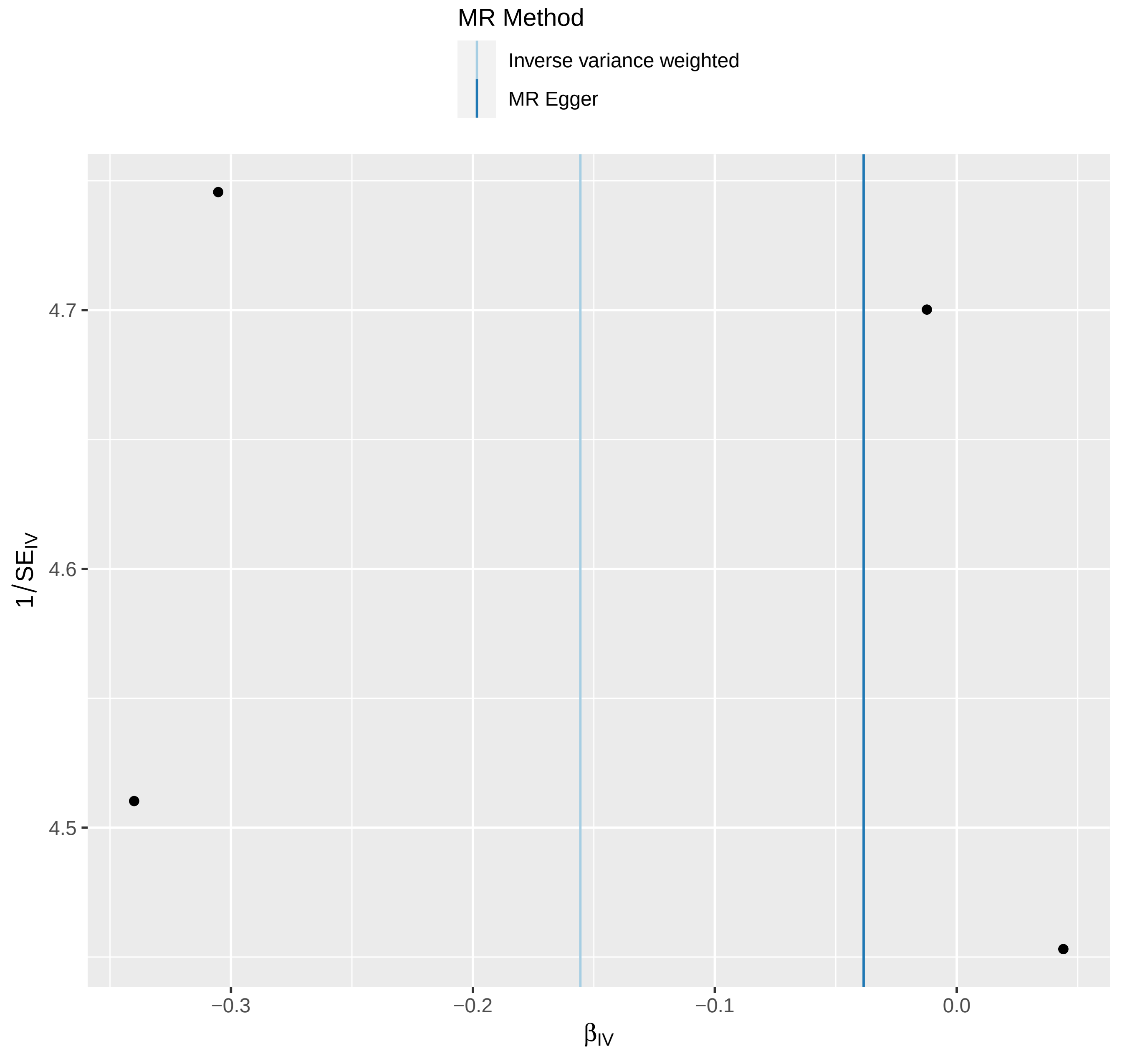

Batch 2−4022 : Gut microbiota abundance (genus Parasutterella id.2892) on Noncompact myelin−associated protein
with a threshold of 1e−05

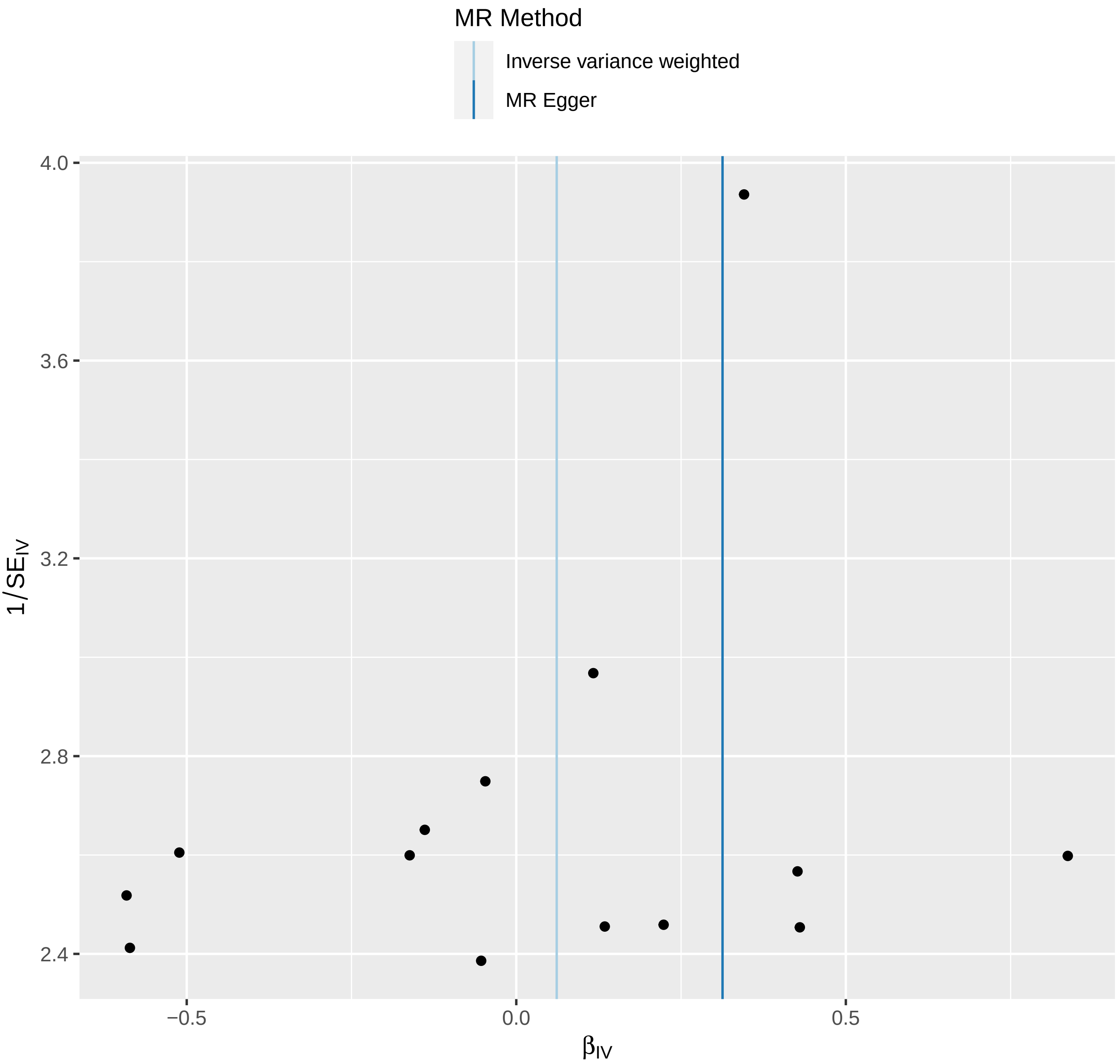

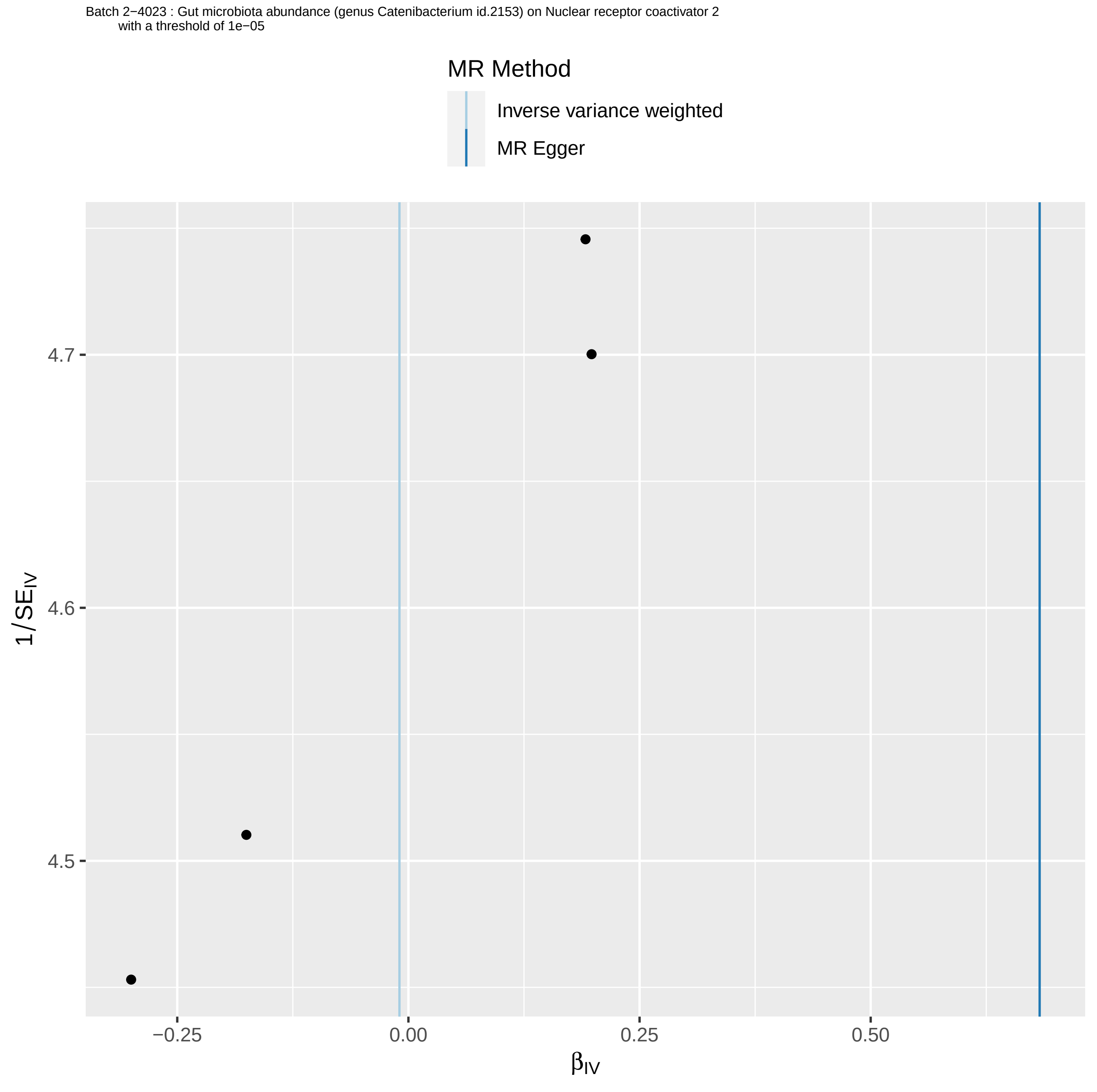
Batch 2−4023 : Gut microbiota abundance (genus Catenibacterium id.2153) on Nuclear receptor coactivator 2
with a threshold of 1e−05
MR Method
Inverse variance weighted
MR Egger
4.7
4.6
4.5
1/SE_IV
−0.25
0.00
0.25
0.50
β_IV

Batch 2−4024 : Gut microbiota abundance (genus Parasutterella id.2892) on Nuclear receptor coactivator 2
with a threshold of 1e−05

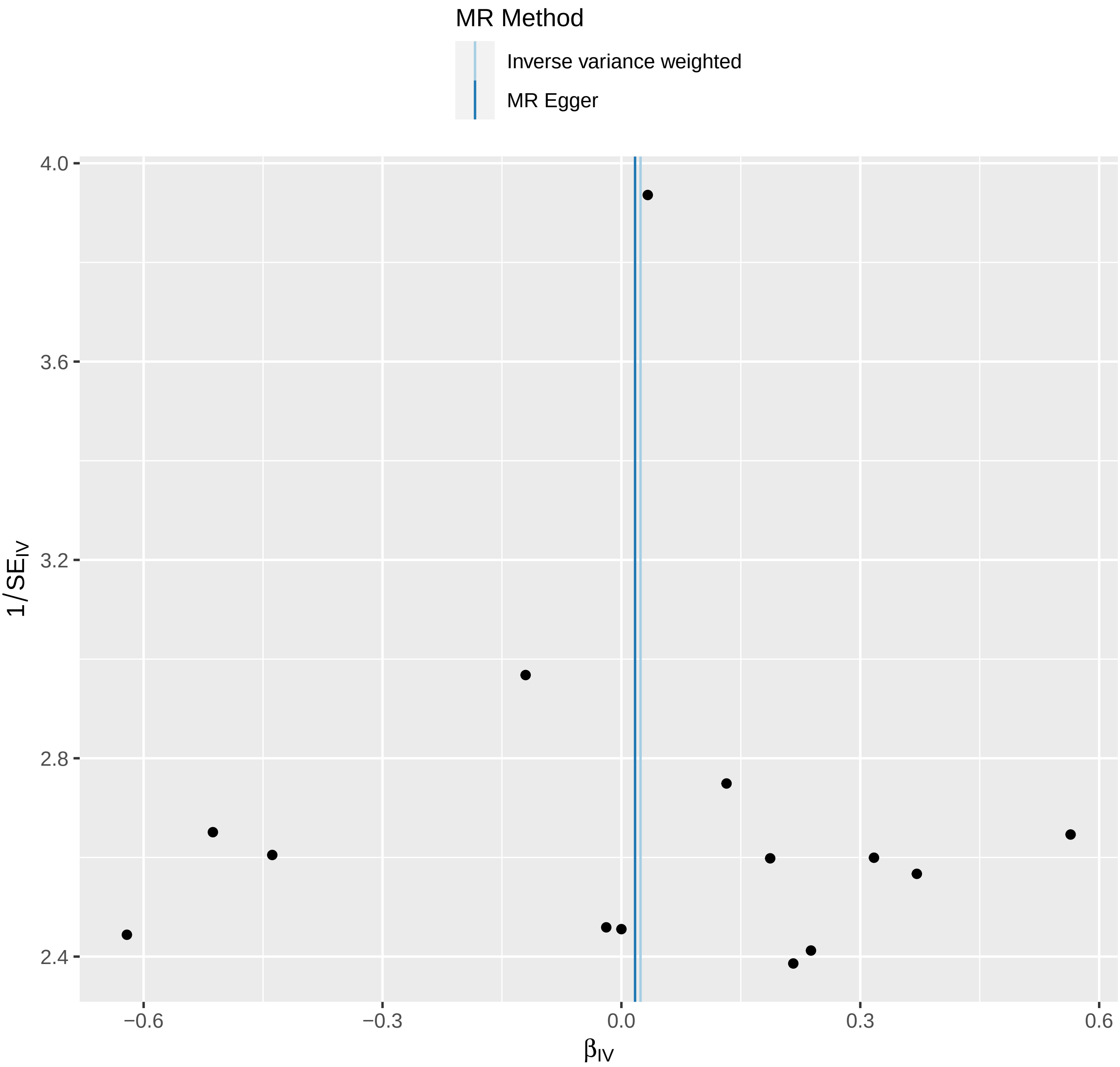

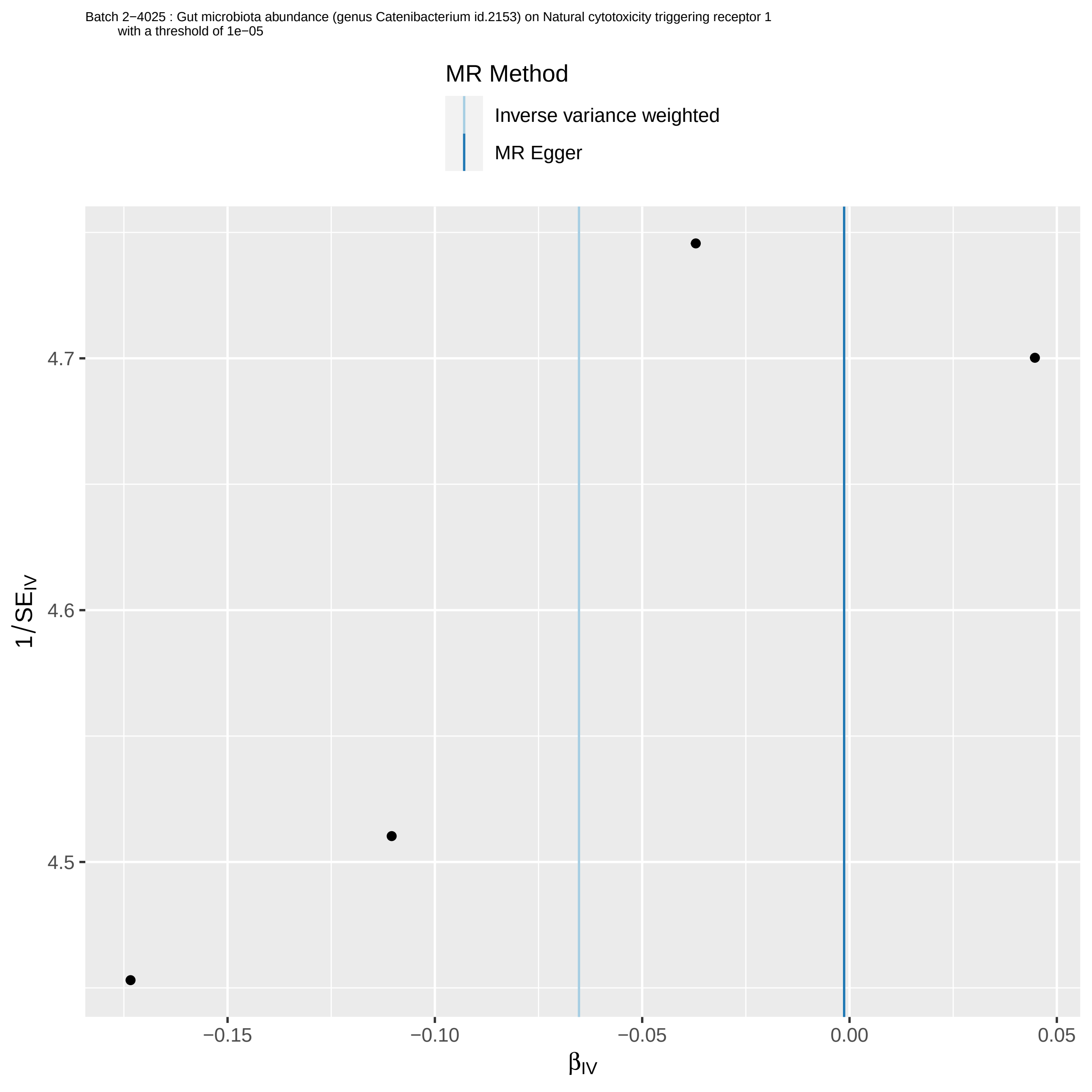

Batch 2−4025 : Gut microbiota abundance (genus Catenibacterium id.2153) on Natural cytotoxicity triggering receptor 1
with a threshold of 1e−05
MR Method
Inverse variance weighted
MR Egger
4.7
4.6
4.5
1/SE_IV
−0.15
−0.10
−0.05
0.00
0.05
β_IV

Batch 2−4026 : Gut microbiota abundance (genus Parasutterella id.2892) on Natural cytotoxicity triggering receptor 1
with a threshold of 1e−05

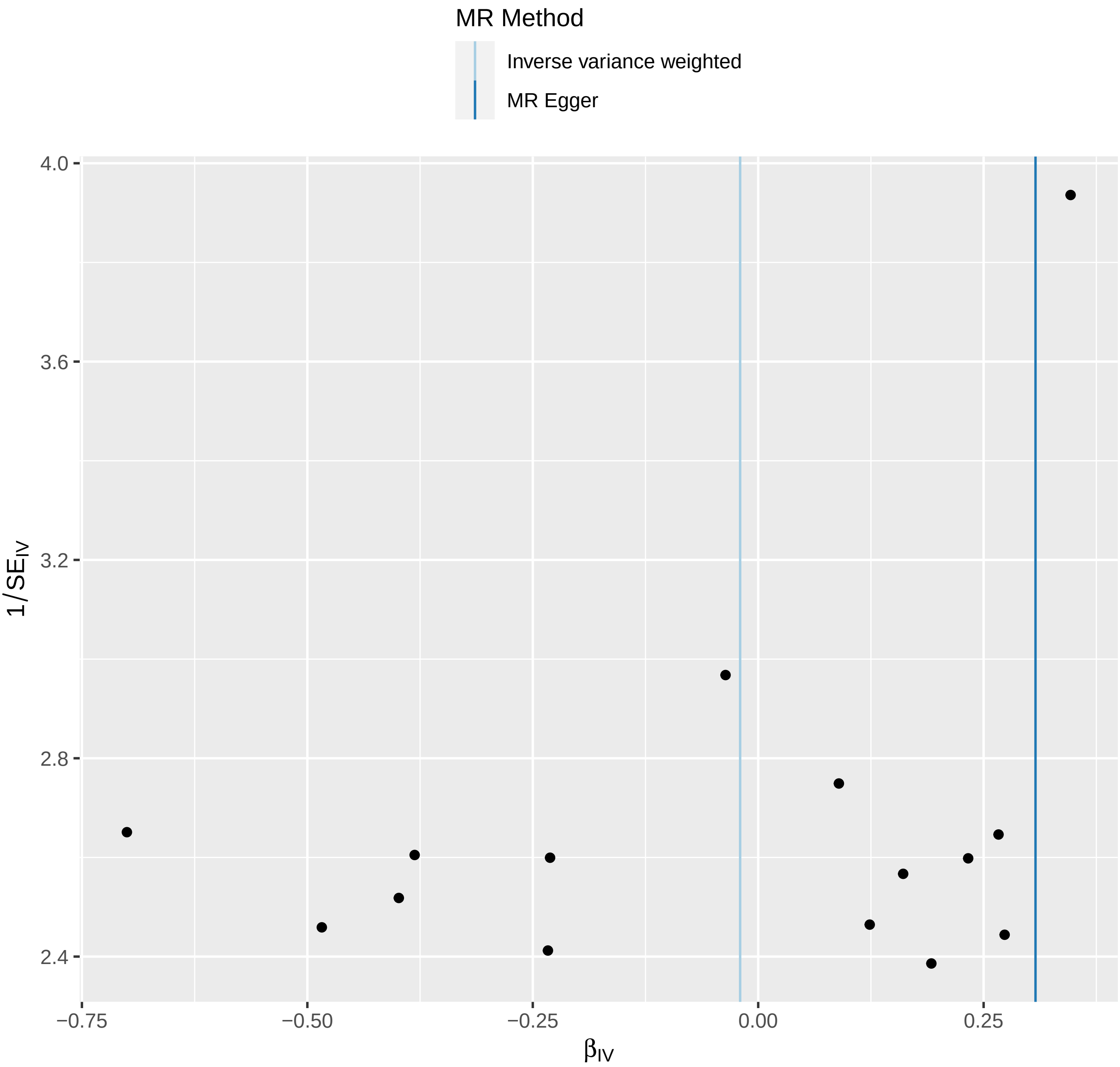

Batch 2−4027 : Gut microbiota abundance (genus Catenibacterium id.2153) on Natural cytotoxicity triggering receptor 1
with a threshold of 1e−05

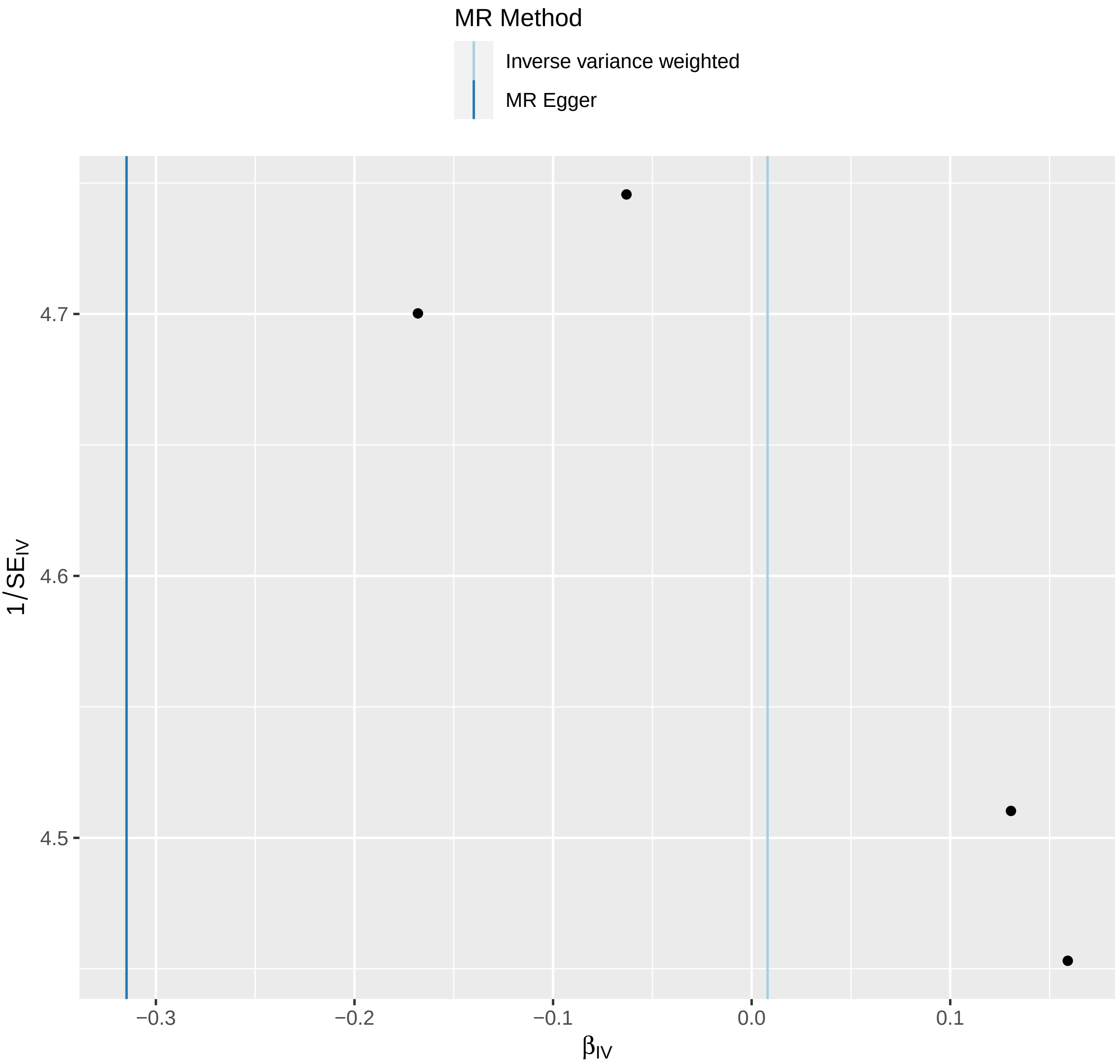

Batch 2−4028 : Gut microbiota abundance (genus Parasutterella id.2892) on Natural cytotoxicity triggering receptor 1
with a threshold of 1e−05

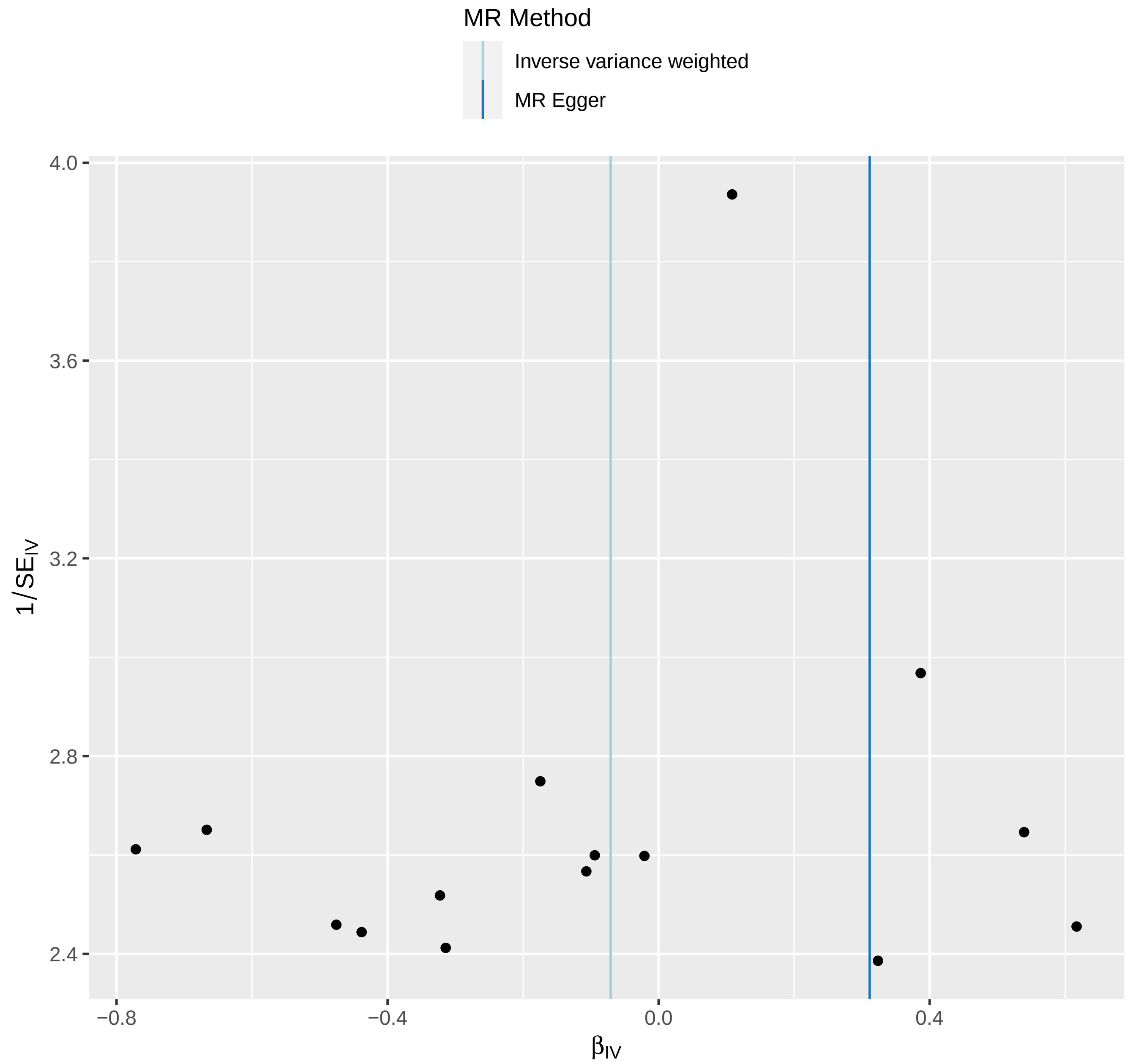

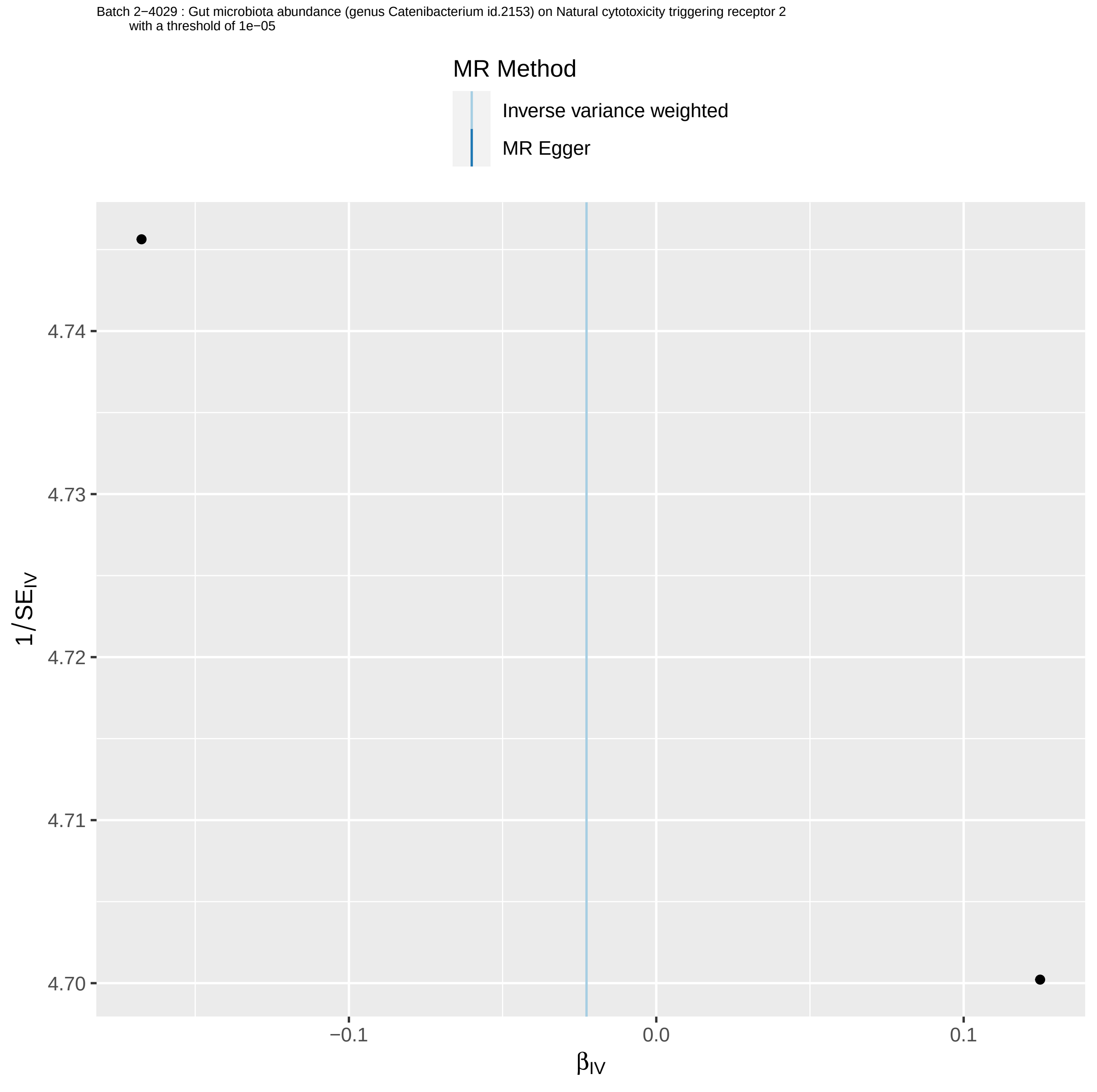
Batch 2−4029 : Gut microbiota abundance (genus Catenibacterium id.2153) on Natural cytotoxicity triggering receptor 2
with a threshold of 1e−05
MR Method
Inverse variance weighted
MR Egger
4.74
4.73
4.72
4.71
4.70
1/SE_IV
−0.1
0.0
0.1
β_IV

Batch 2−4030 : Gut microbiota abundance (genus Parasutterella id.2892) on Natural cytotoxicity triggering receptor 2
with a threshold of 1e−05

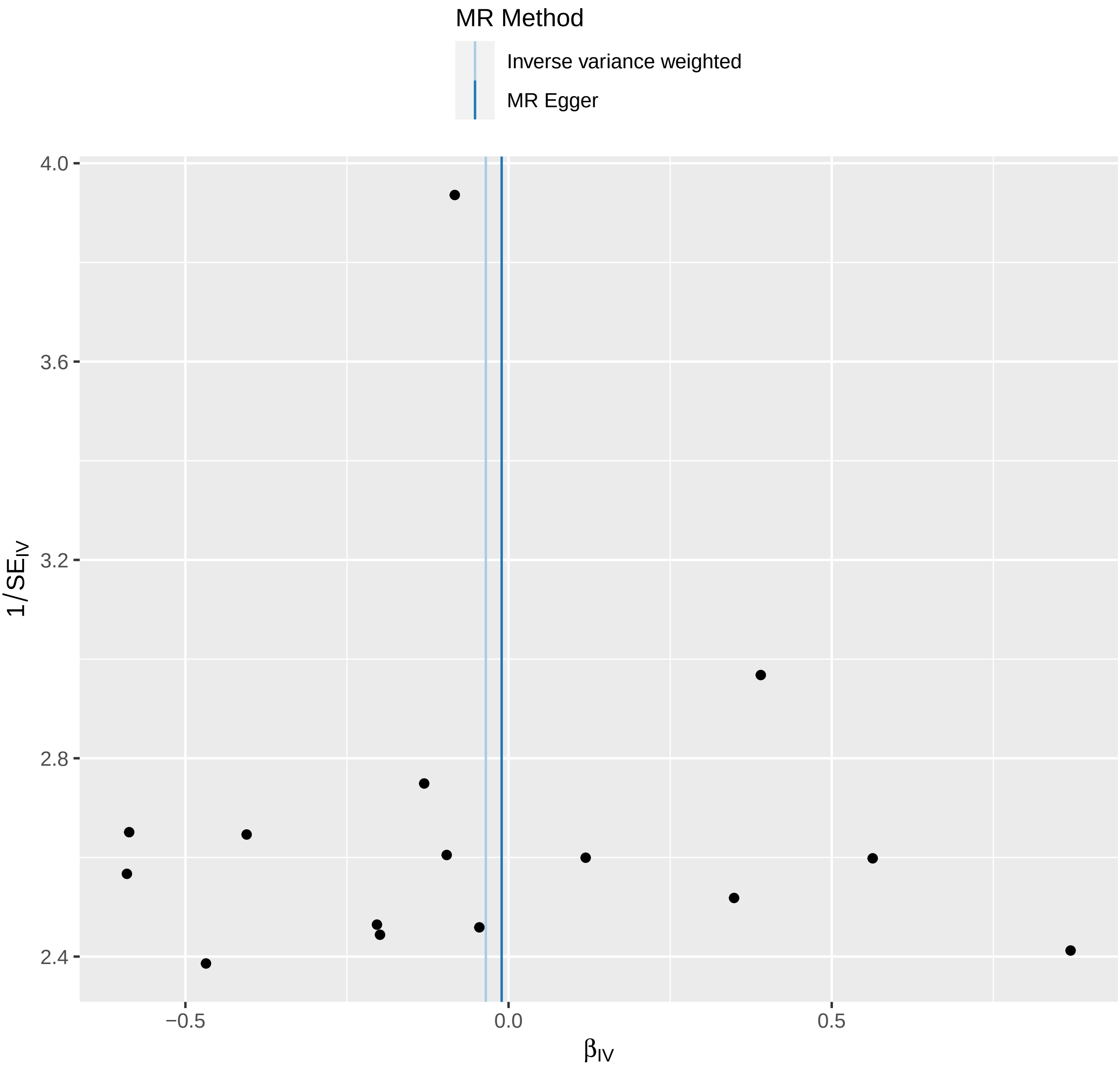

Batch 2−4031 : Gut microbiota abundance (genus Catenibacterium id.2153) on Natural cytotoxicity triggering receptor 3
with a threshold of 1e−05

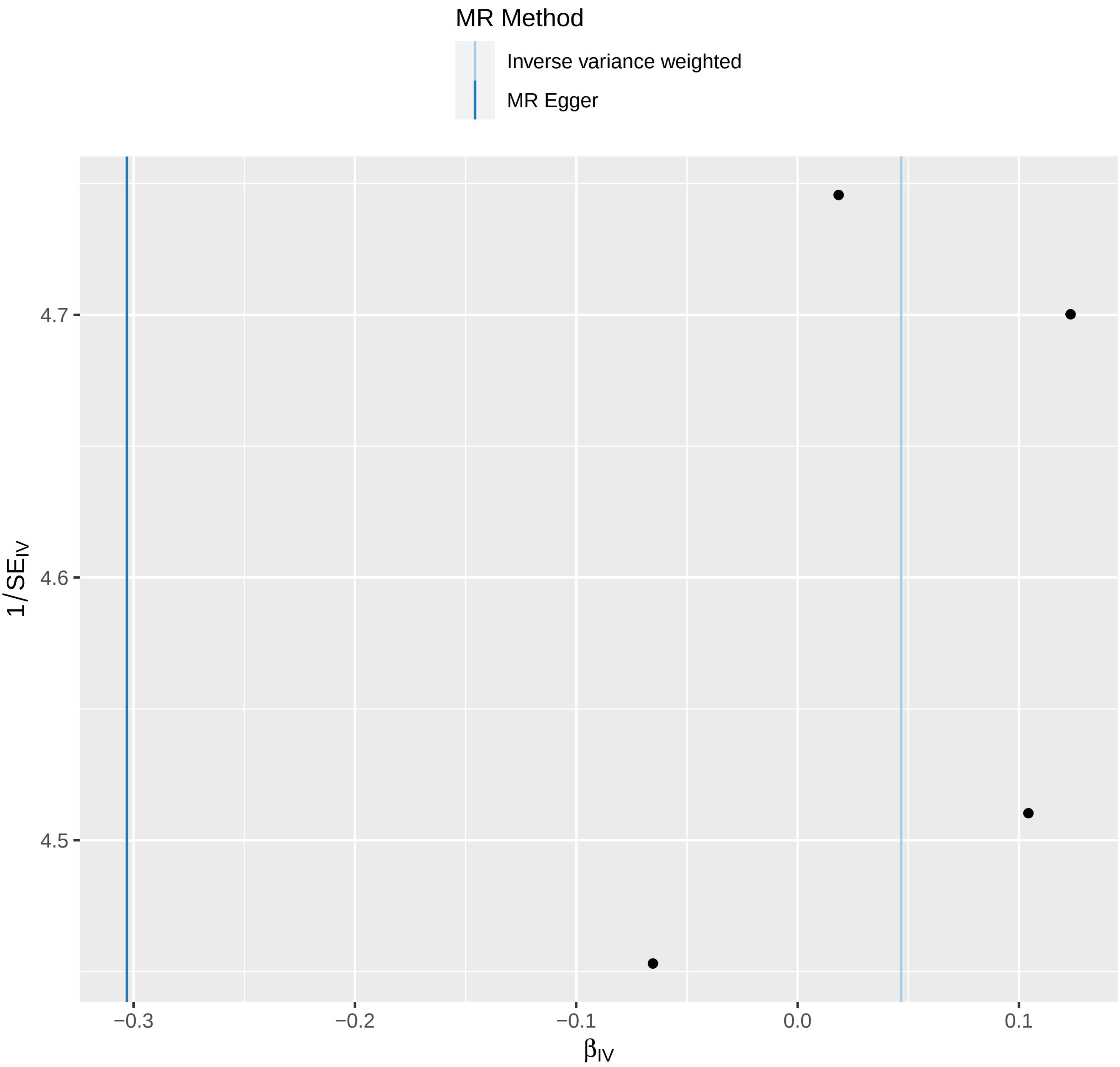

Batch 2−4032 : Gut microbiota abundance (genus Parasutterella id.2892) on Natural cytotoxicity triggering receptor 3 with a threshold of 1e−05

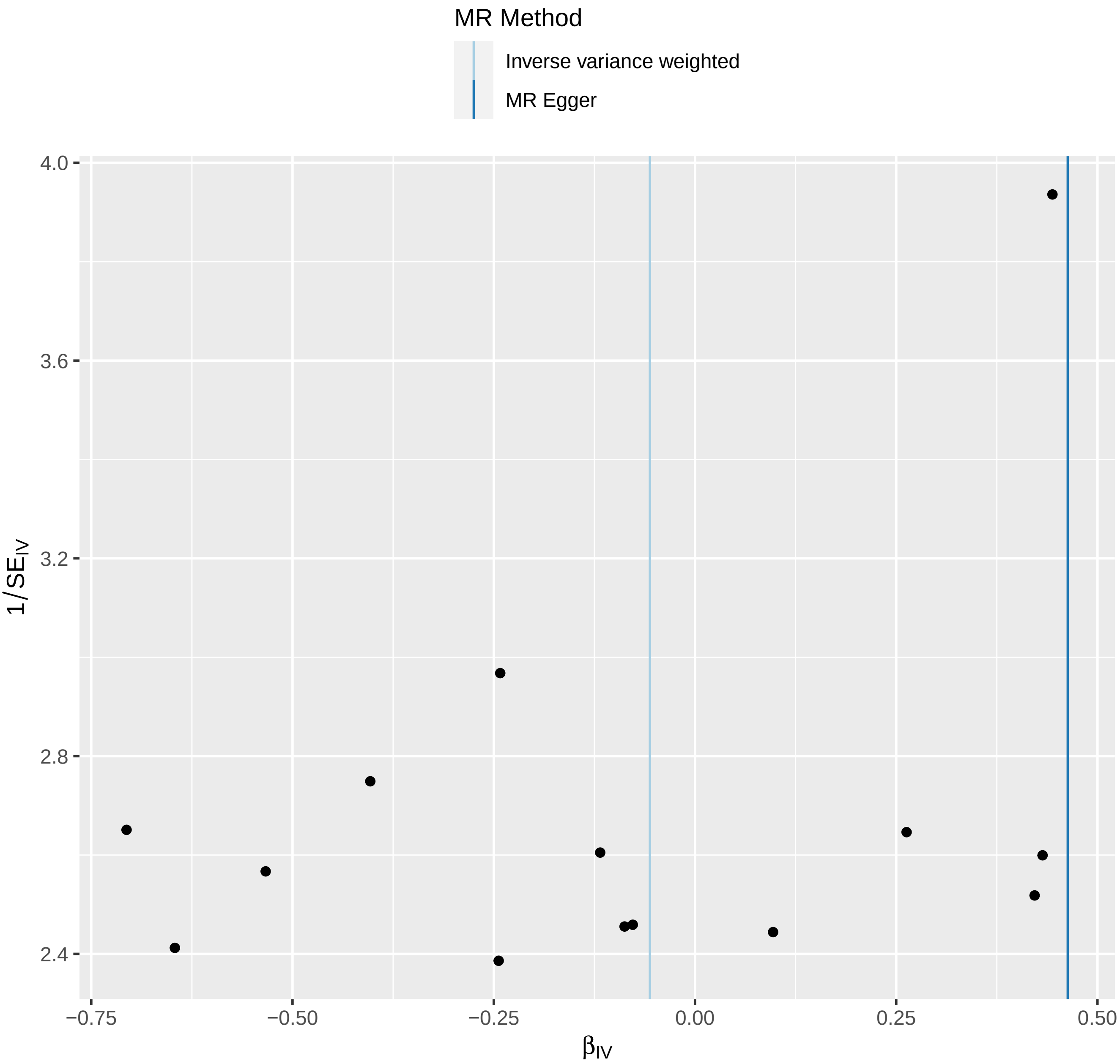

Batch 2−4033 : Gut microbiota abundance (genus Catenibacterium id.2153) on Natural cytotoxicity triggering receptor 3 ligand 1 with a threshold of 1e−05

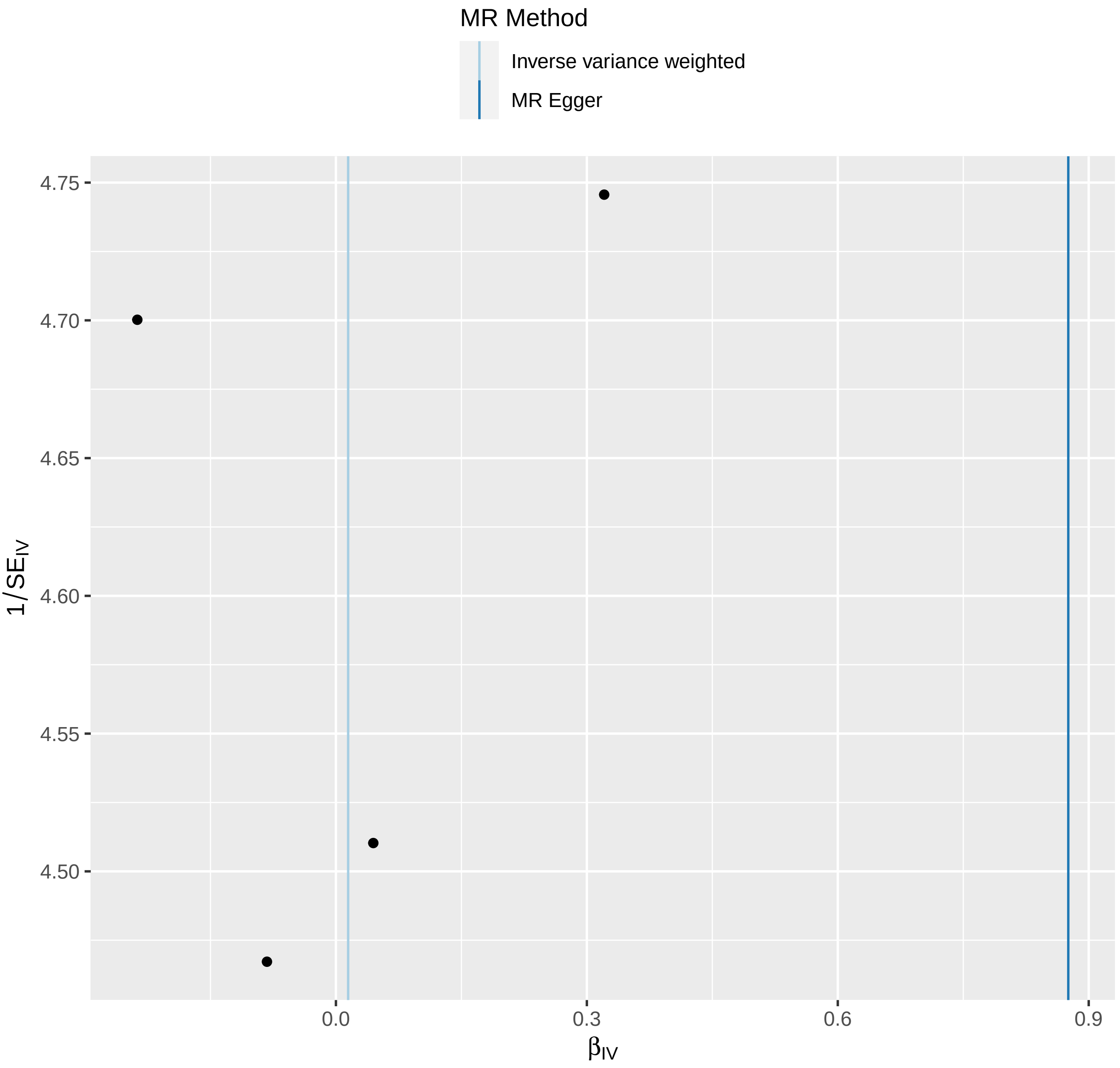

Batch 2−4034 : Gut microbiota abundance (genus Parasutterella id.2892) on Natural cytotoxicity triggering receptor 3 ligand 1
with a threshold of 1e−05

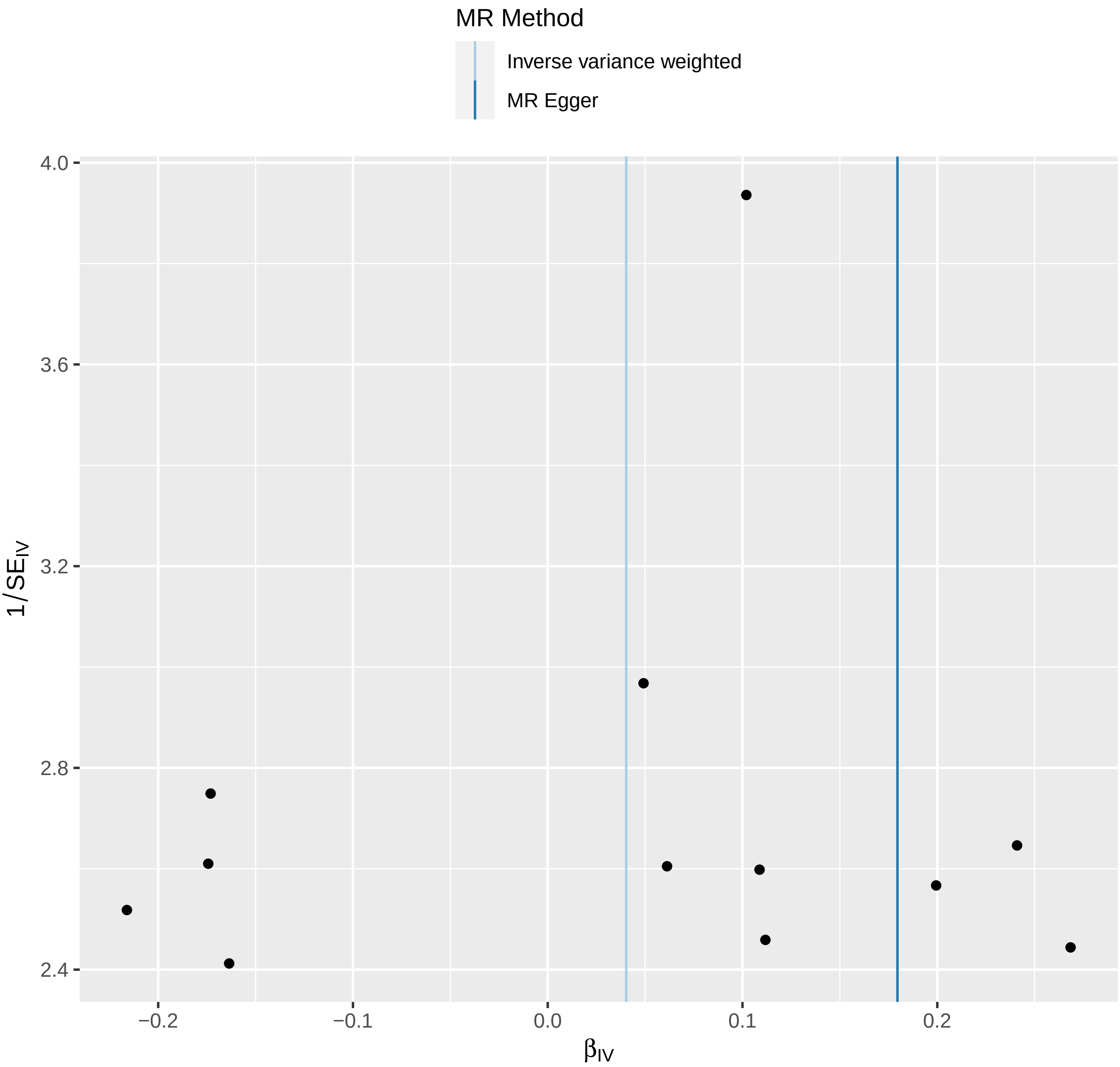

Batch 2−4035 : Gut microbiota abundance (genus Catenibacterium id.2153) on Kinetochore protein NDC80 homolog
with a threshold of 1e−05

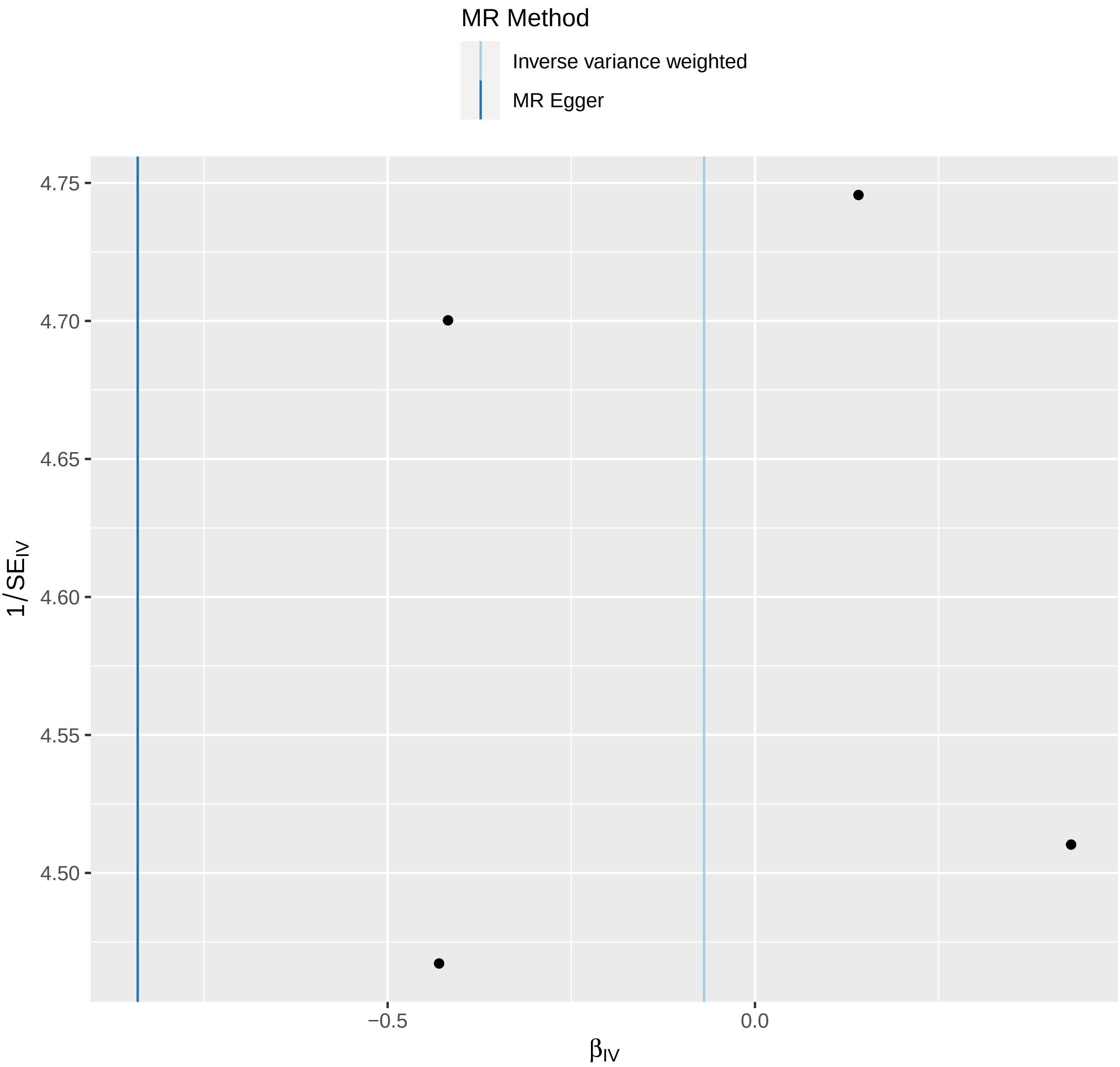

Batch 2−4036 : Gut microbiota abundance (genus Parasutterella id.2892) on Kinetochore protein NDC80 homolog
with a threshold of 1e−05

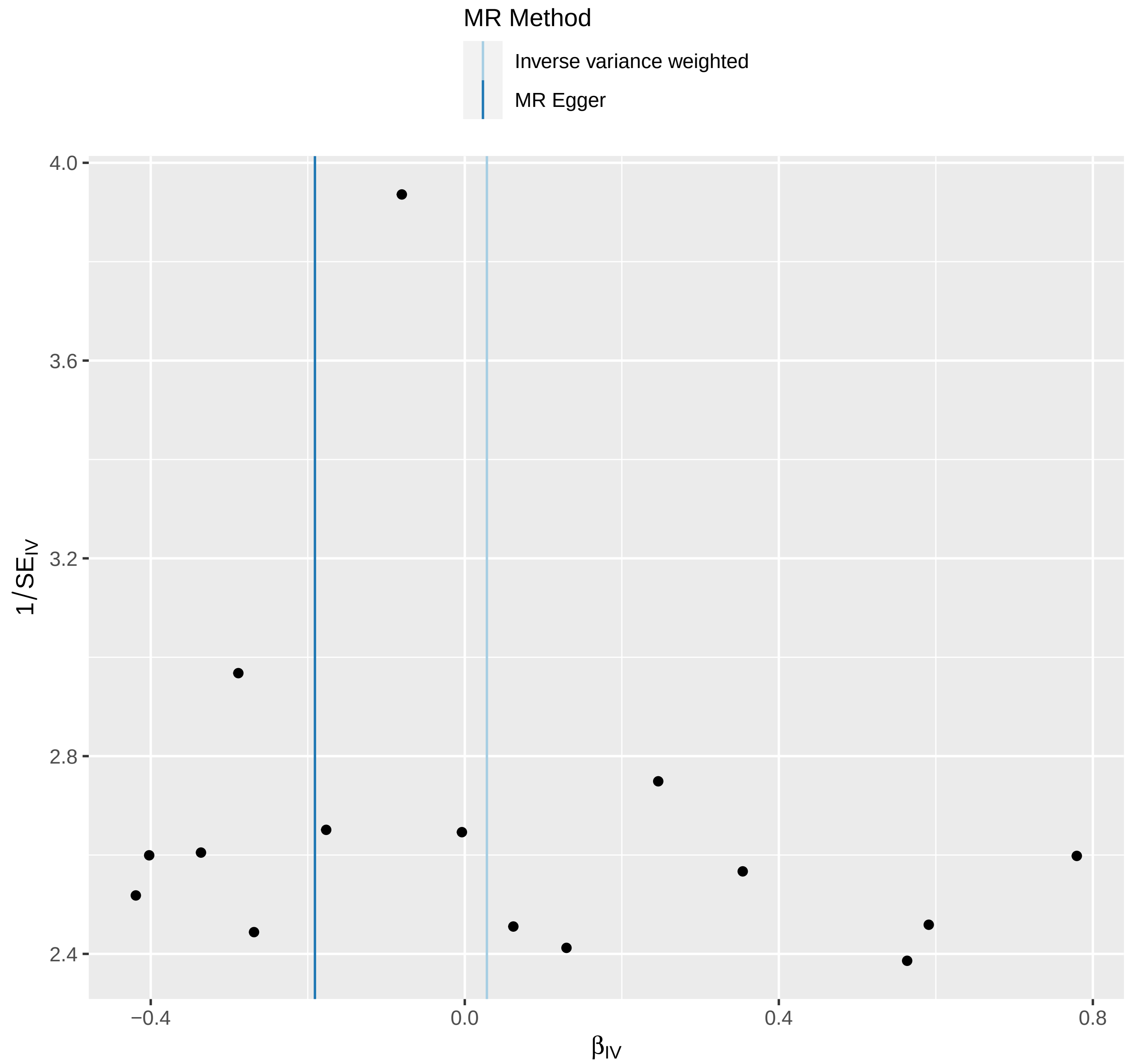

Batch 2−4037 : Gut microbiota abundance (genus Catenibacterium id.2153) on Protein NDNF
with a threshold of 1e−05

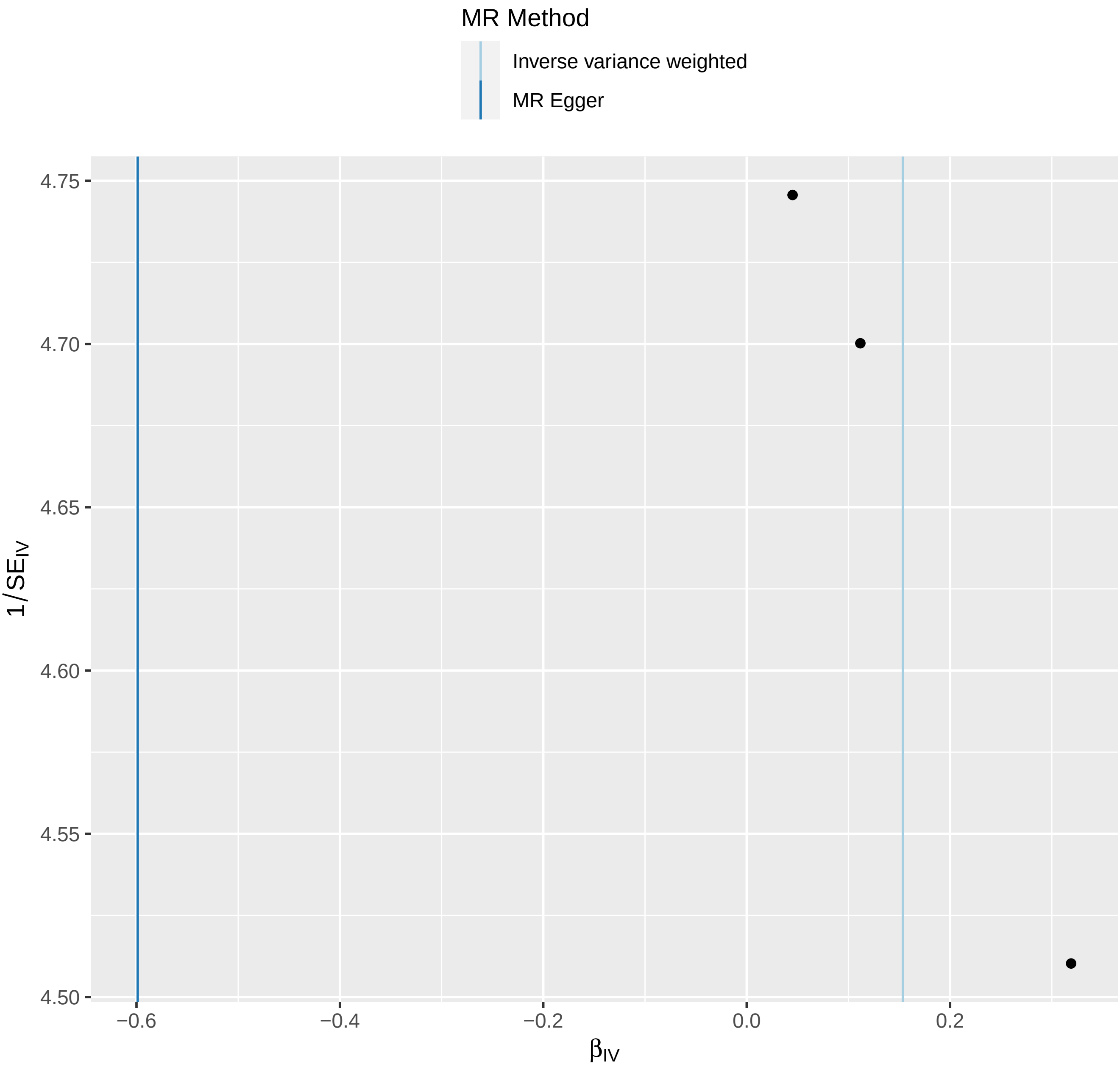

Batch 2−4038 : Gut microbiota abundance (genus Parasutterella id.2892) on Protein NDNF
with a threshold of 1e−05

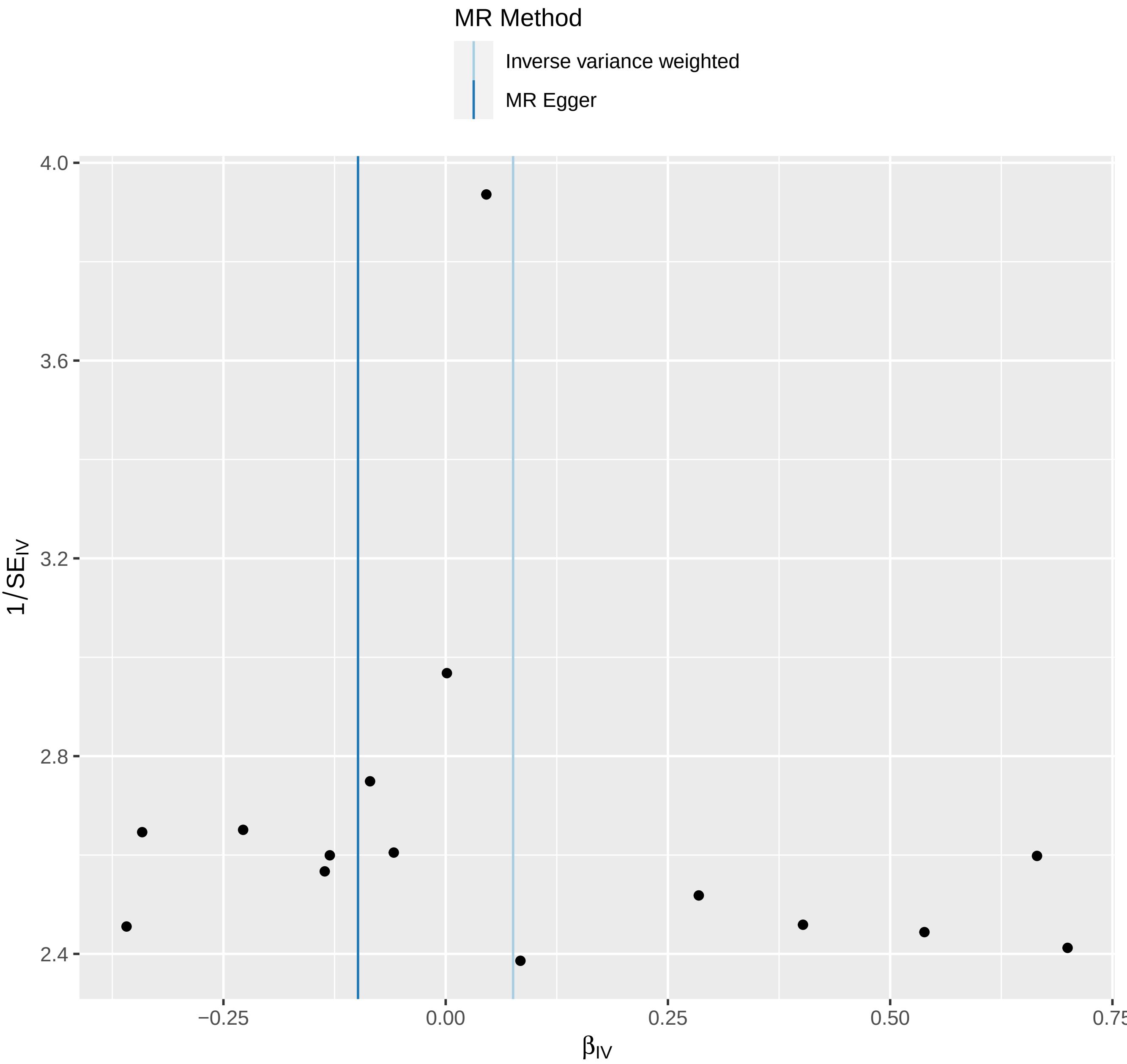

Batch 2−4039 : Gut microbiota abundance (genus Catenibacterium id.2153) on Protein NDRG4
with a threshold of 1e−05

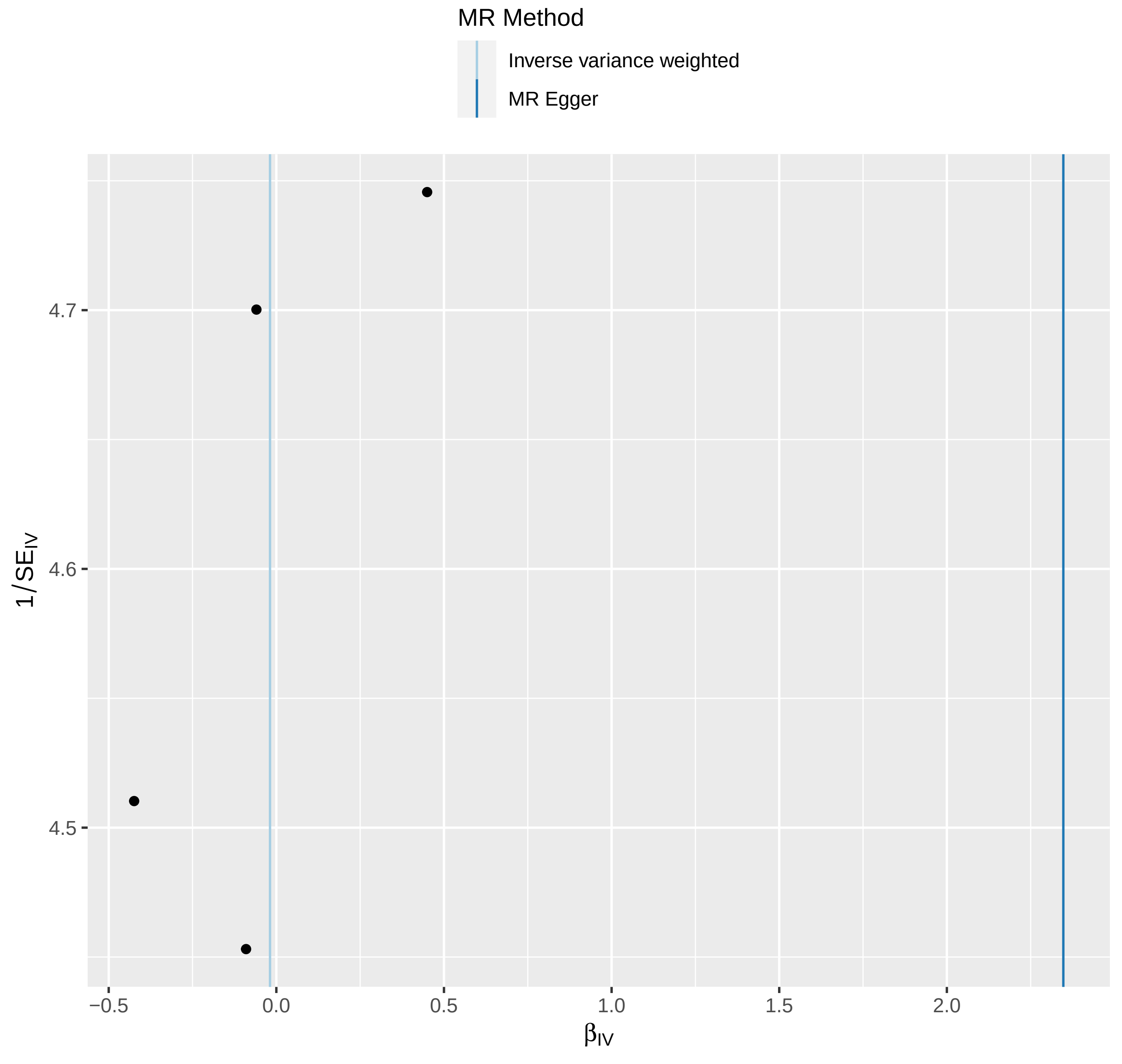

Batch 2−4040 : Gut microbiota abundance (genus Parasutterella id.2892) on Protein NDRG4
with a threshold of 1e−05

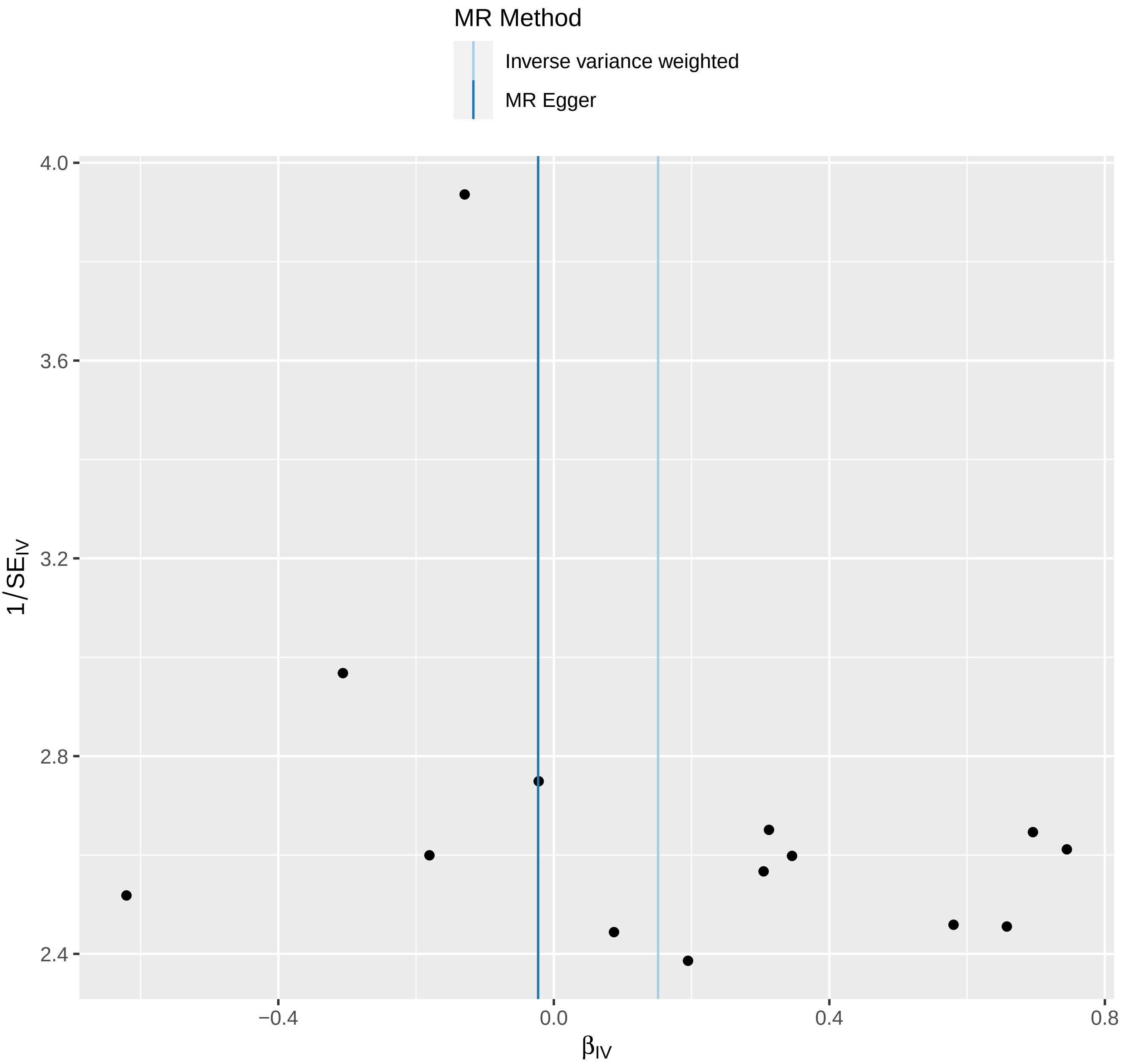

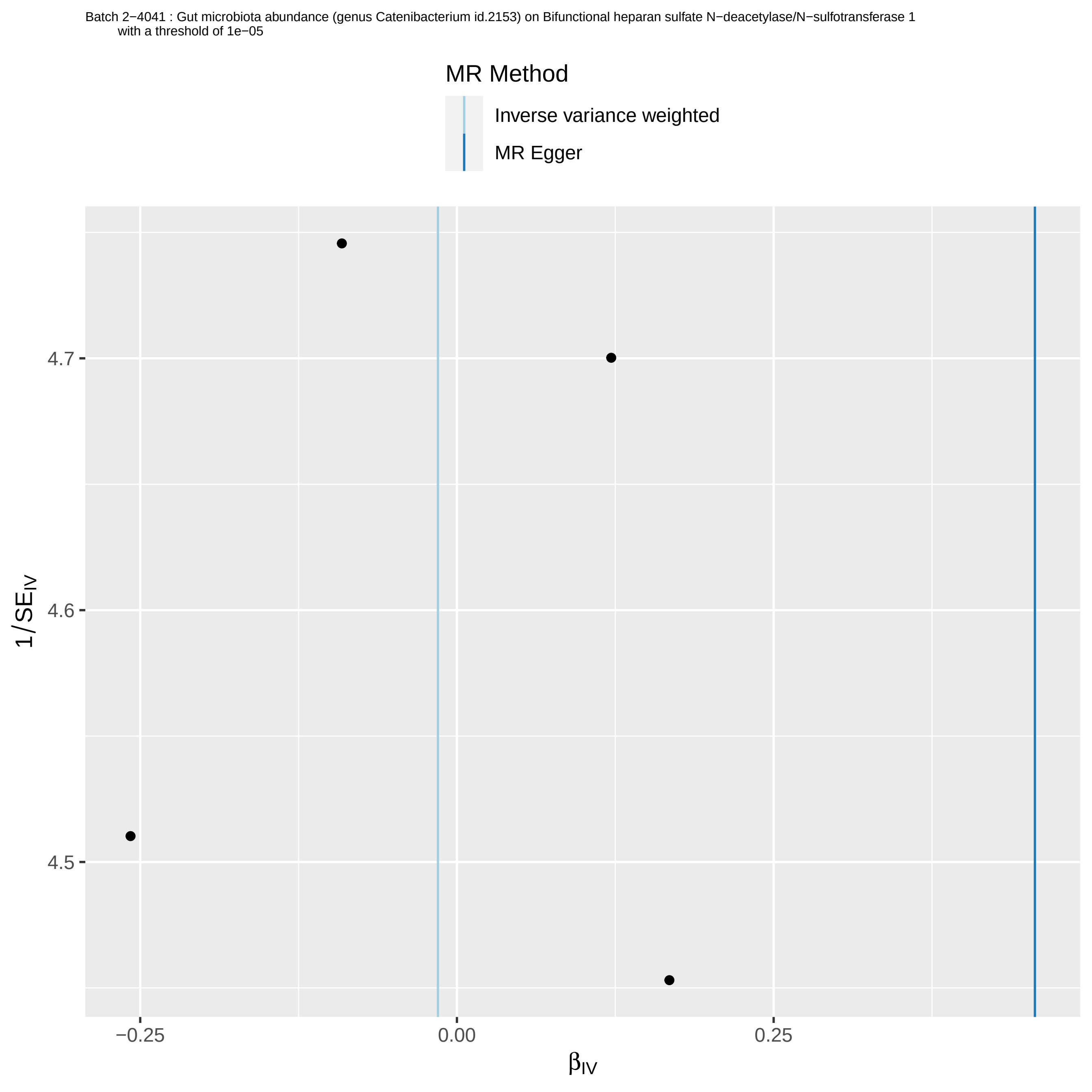

Batch 2−4041 : Gut microbiota abundance (genus Catenibacterium id.2153) on Bifunctional heparan sulfate N−deacetylase/N−sulfotransferase 1
with a threshold of 1e−05
MR Method
Inverse variance weighted
MR Egger
4.7
4.6
4.5
1/SE_IV
−0.25
0.00
0.25
β_IV

Batch 2−4042 : Gut microbiota abundance (genus Parasutterella id.2892) on Bifunctional heparan sulfate N−deacetylase/N−sulfotransferase 1 with a threshold of 1e−05

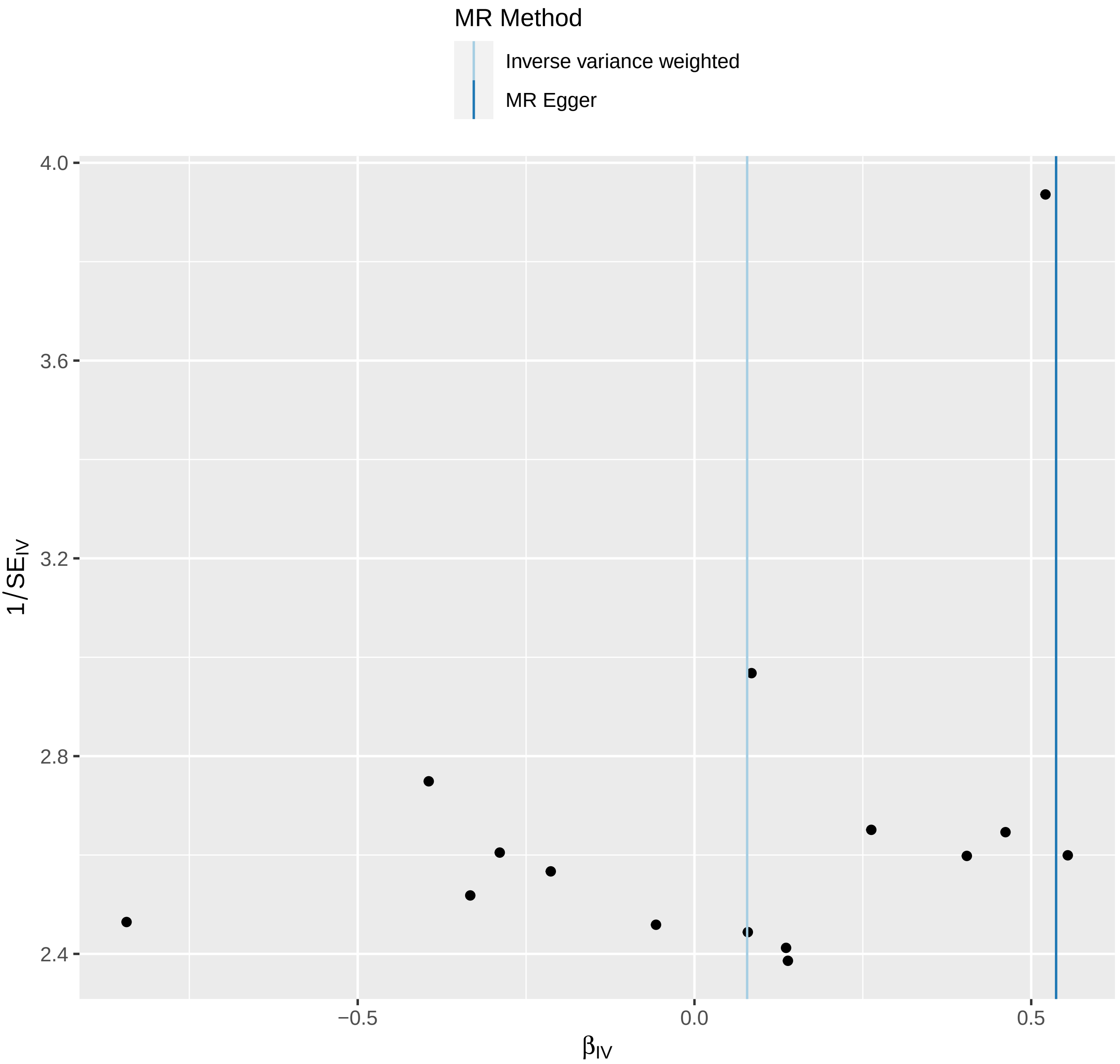

Batch 2−4043 : Gut microbiota abundance (genus Catenibacterium id.2153) on NADH dehydrogenase [ubiquinone] 1 beta subcomplex subunit 11, mitochondrial with a threshold of 1e−05

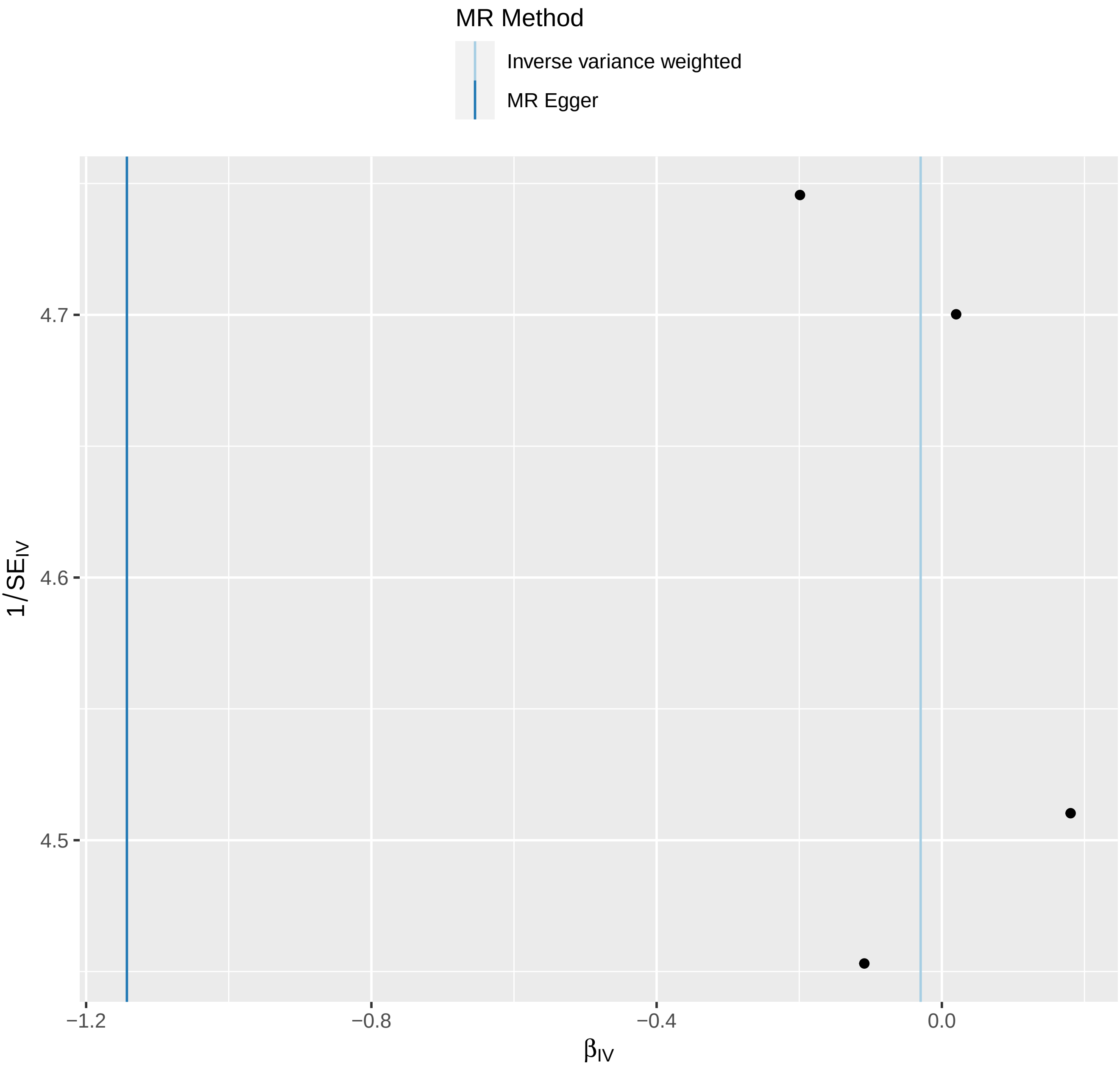

Batch 2−4044 : Gut microbiota abundance (genus Parasutterella id.2892) on NADH dehydrogenase [ubiquinone] 1 beta subcomplex subunit 11, mitochondrial with a threshold of 1e−05

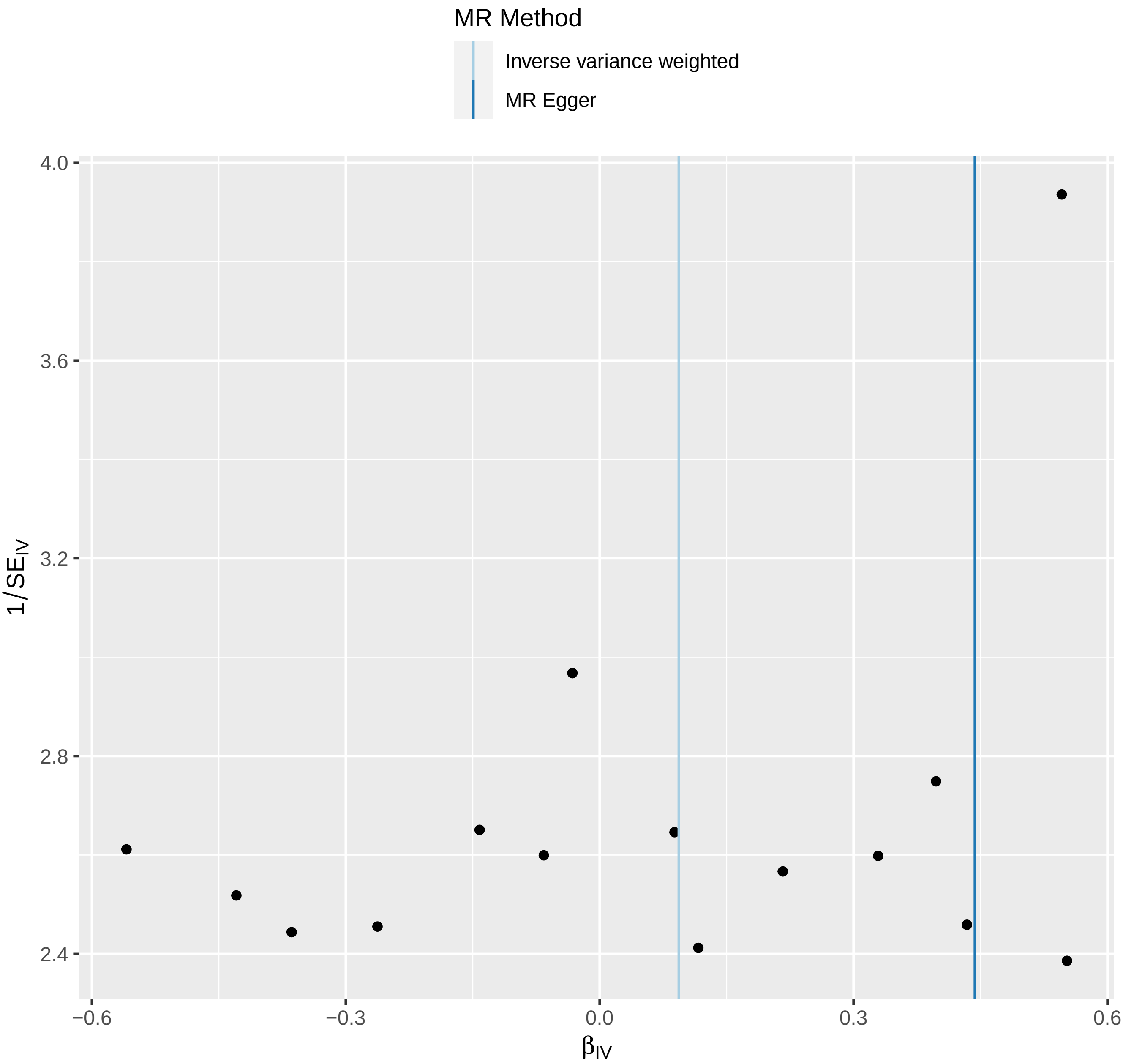

Batch 2−4045 : Gut microbiota abundance (genus Catenibacterium id.2153) on NADH dehydrogenase [ubiquinone] 1 beta subcomplex subunit 4 with a threshold of 1e−05

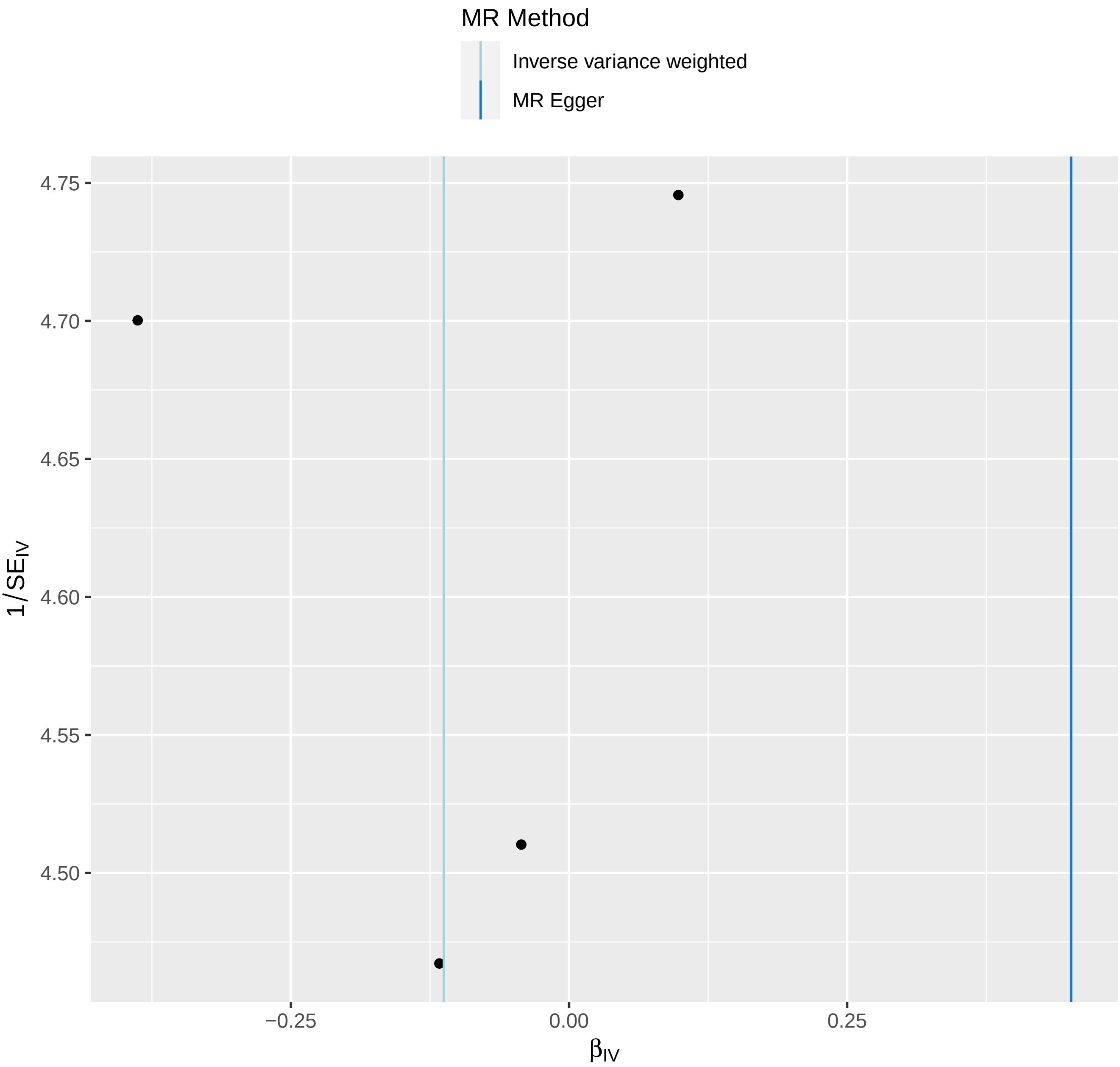

Batch 2−4046 : Gut microbiota abundance (genus Parasutterella id.2892) on NADH dehydrogenase [ubiquinone] 1 beta subcomplex subunit 4 with a threshold of 1e−05

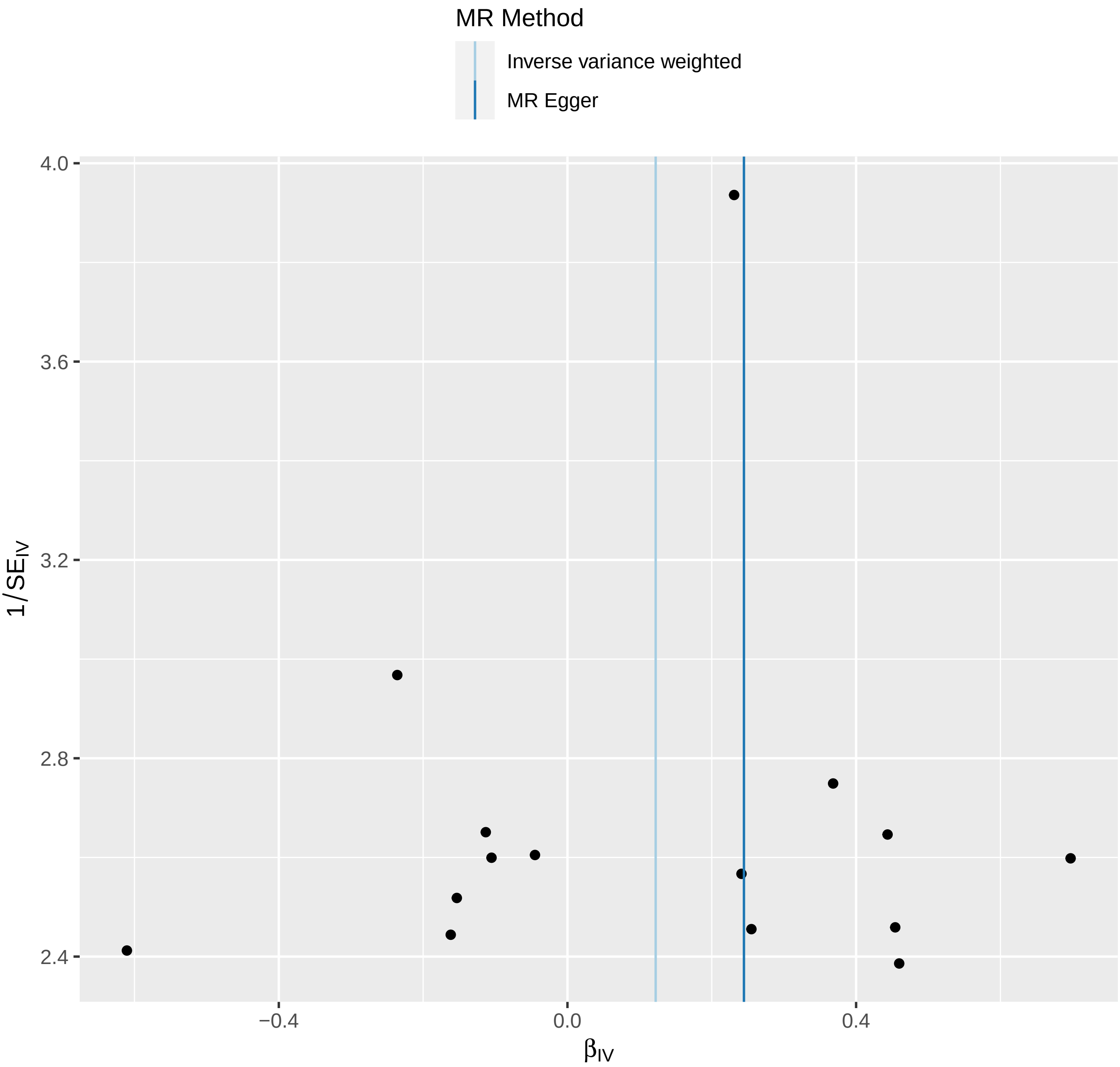

Batch 2−4047 : Gut microbiota abundance (genus Catenibacterium id.2153) on NADH dehydrogenase [ubiquinone] 1 beta subcomplex subunit 8, mitochondrial with a threshold of 1e−05

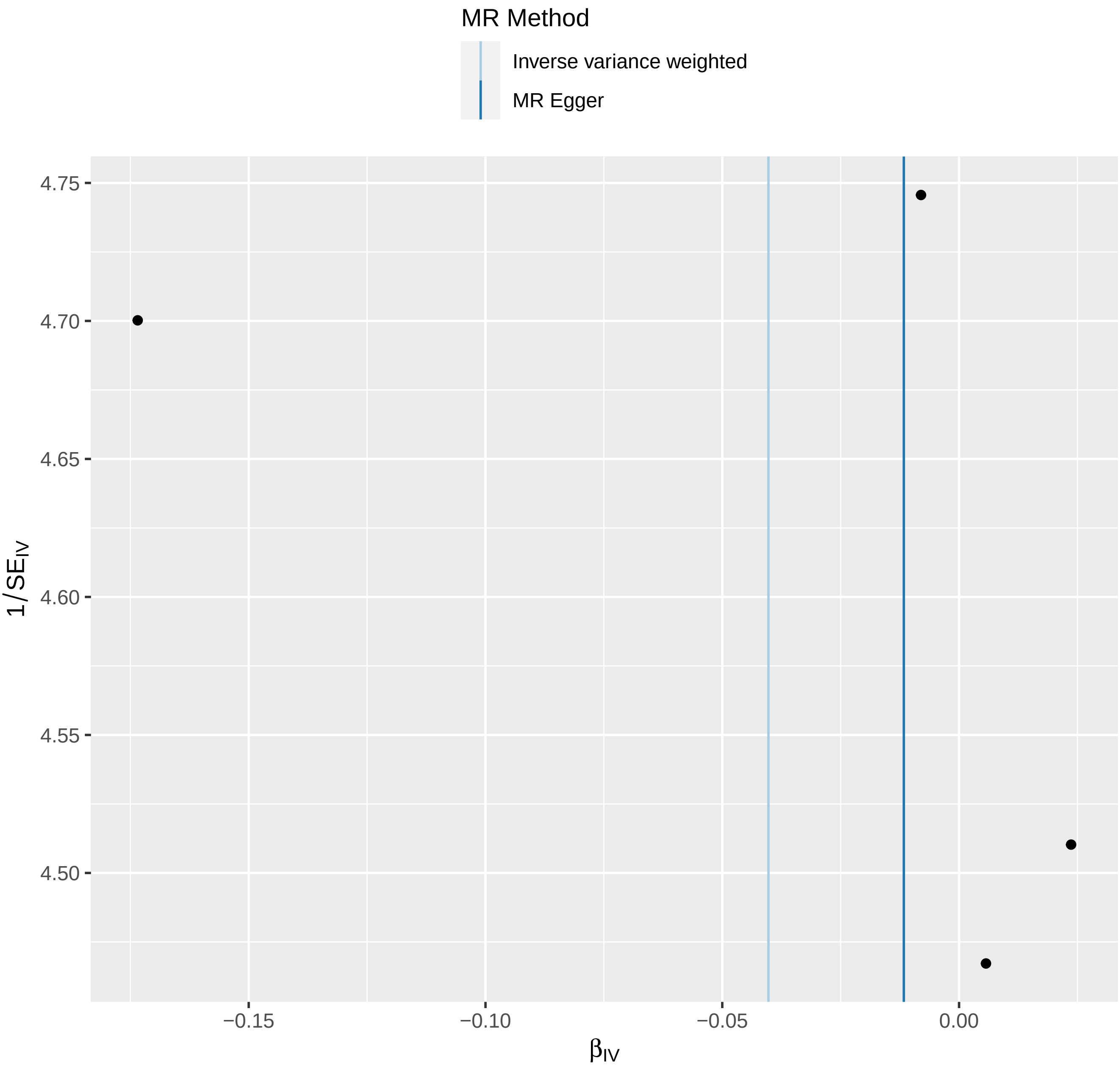

Batch 2−4048 : Gut microbiota abundance (genus Parasutterella id.2892) on NADH dehydrogenase [ubiquinone] 1 beta subcomplex subunit 8, mitochondrial with a threshold of 1e−05

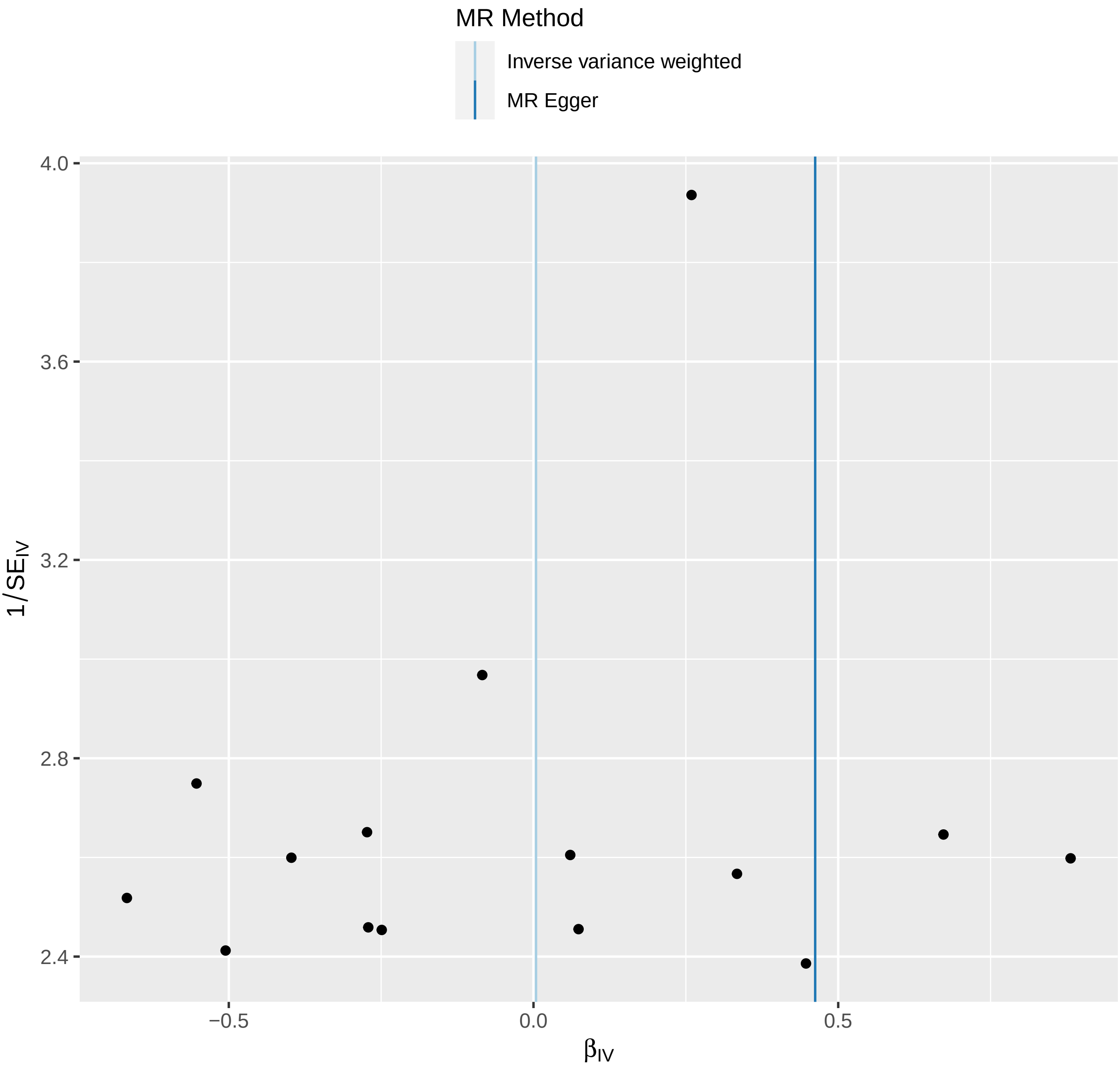

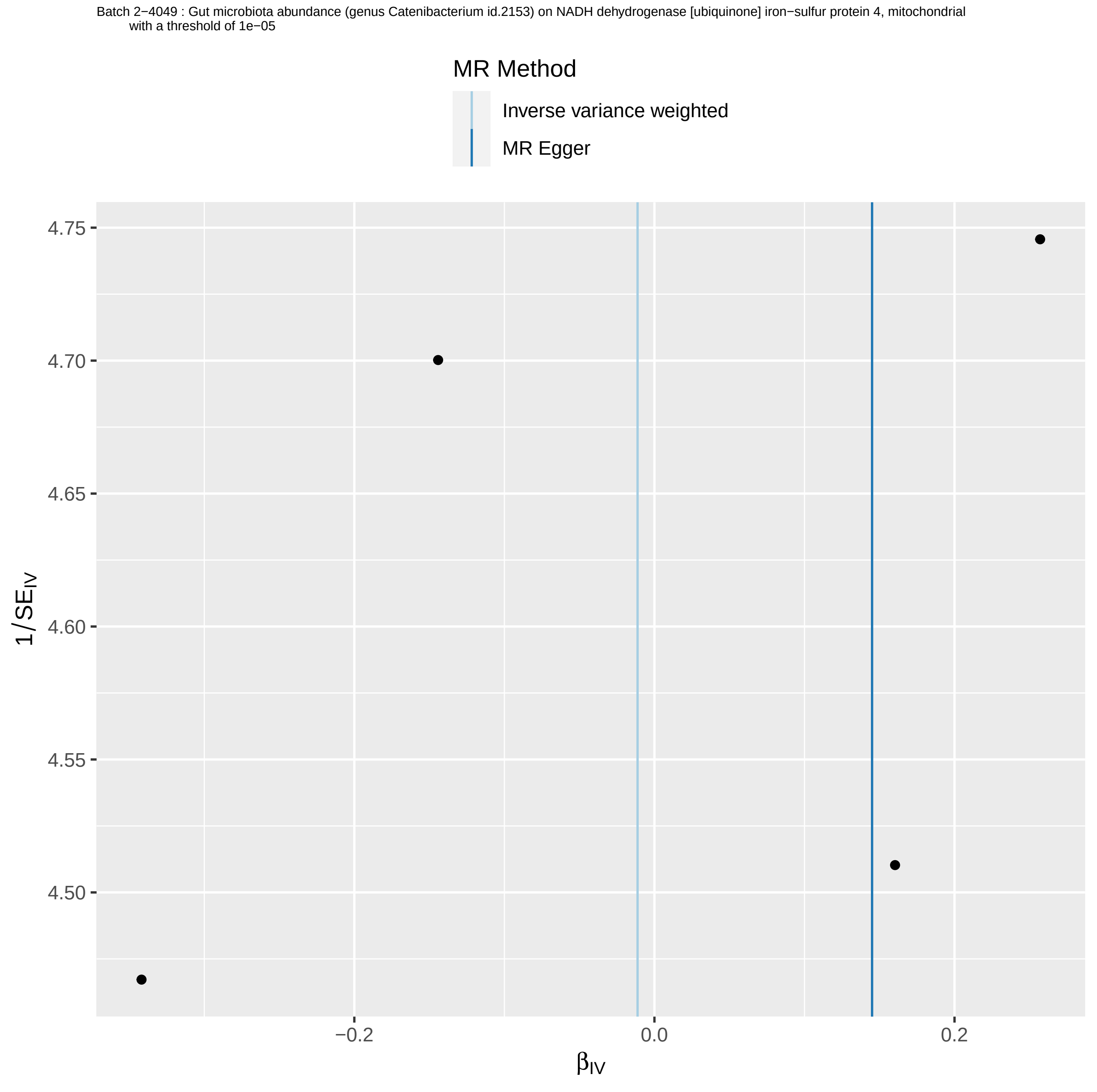
Batch 2−4049 : Gut microbiota abundance (genus Catenibacterium id.2153) on NADH dehydrogenase [ubiquinone] iron−sulfur protein 4, mitochondrial
with a threshold of 1e−05
MR Method
Inverse variance weighted
MR Egger
4.75
4.70
4.65
4.60
4.55
4.50
1/SE_IV
−0.2
0.0
0.2
β_IV

Batch 2−4050 : Gut microbiota abundance (genus Parasutterella id.2892) on NADH dehydrogenase [ubiquinone] iron−sulfur protein 4, mitochondrial with a threshold of 1e−05

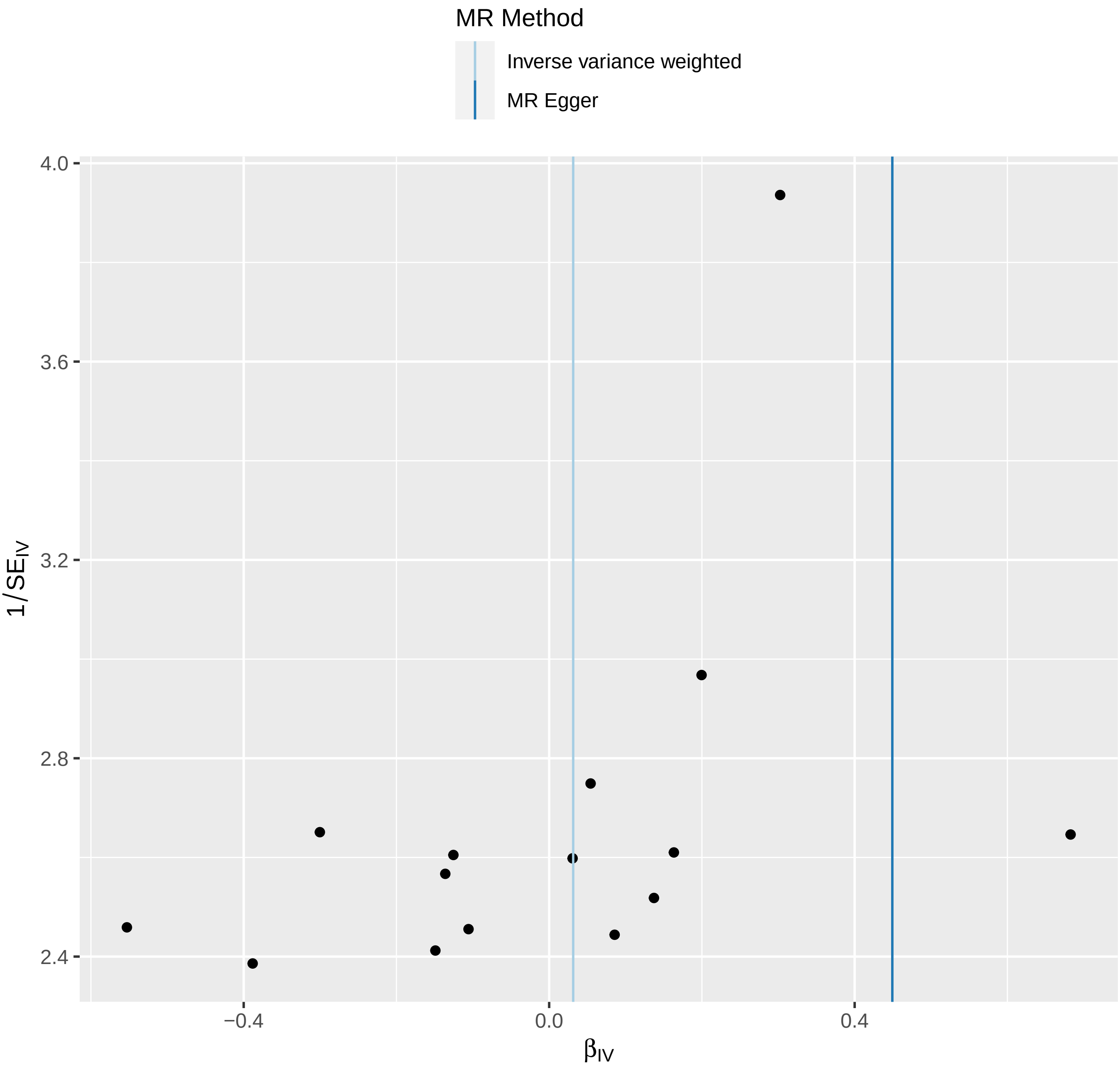

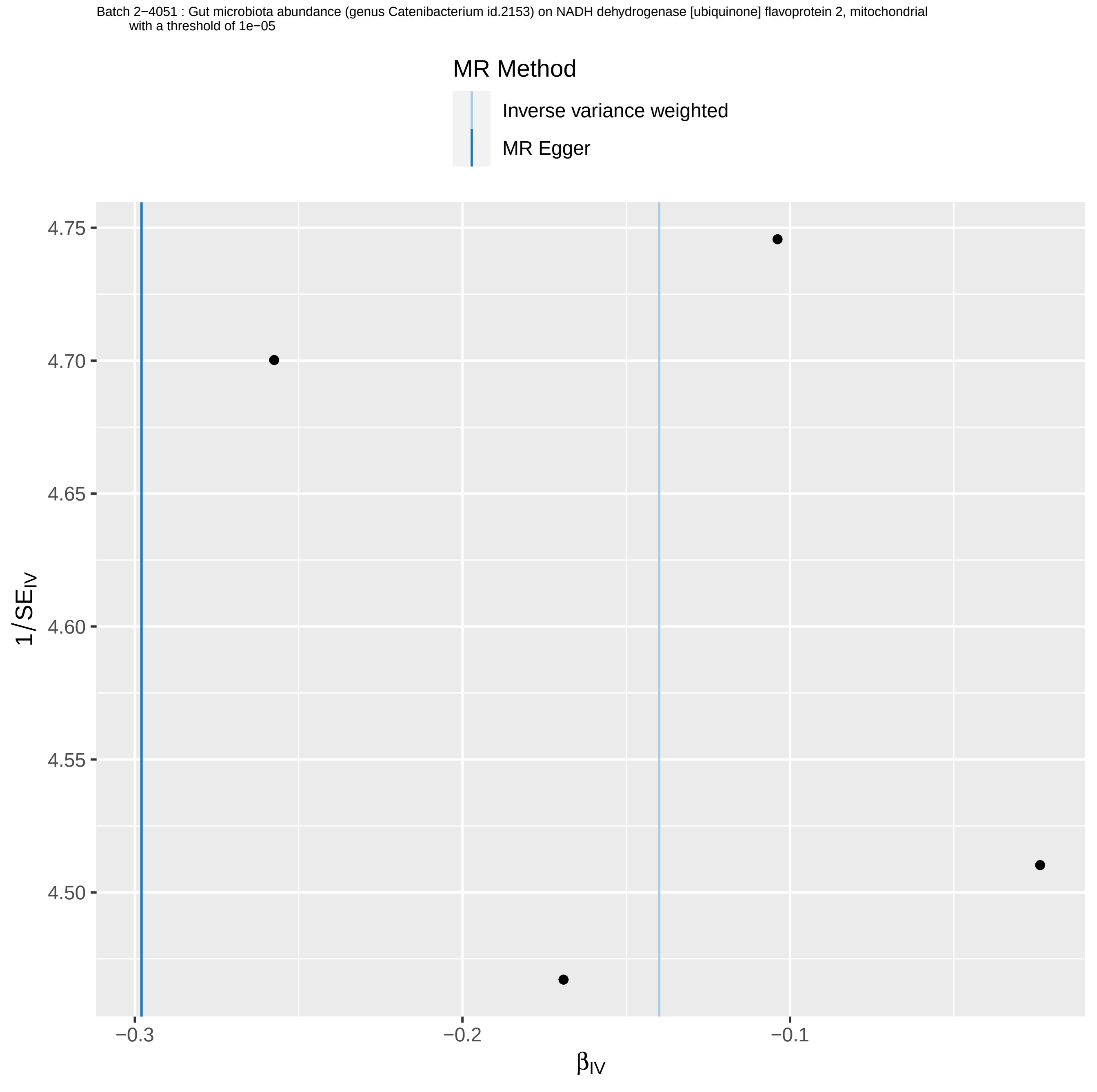

Batch 2−4051 : Gut microbiota abundance (genus Catenibacterium id.2153) on NADH dehydrogenase [ubiquinone] flavoprotein 2, mitochondrial with a threshold of 1e−05
MR Method
Inverse variance weighted
MR Egger
4.75
4.70
4.65
4.60
4.55
4.50
1/SE_IV
−0.3
−0.2
−0.1
β_IV

Batch 2−4052 : Gut microbiota abundance (genus Parasutterella id.2892) on NADH dehydrogenase [ubiquinone] flavoprotein 2, mitochondrial
with a threshold of 1e−05

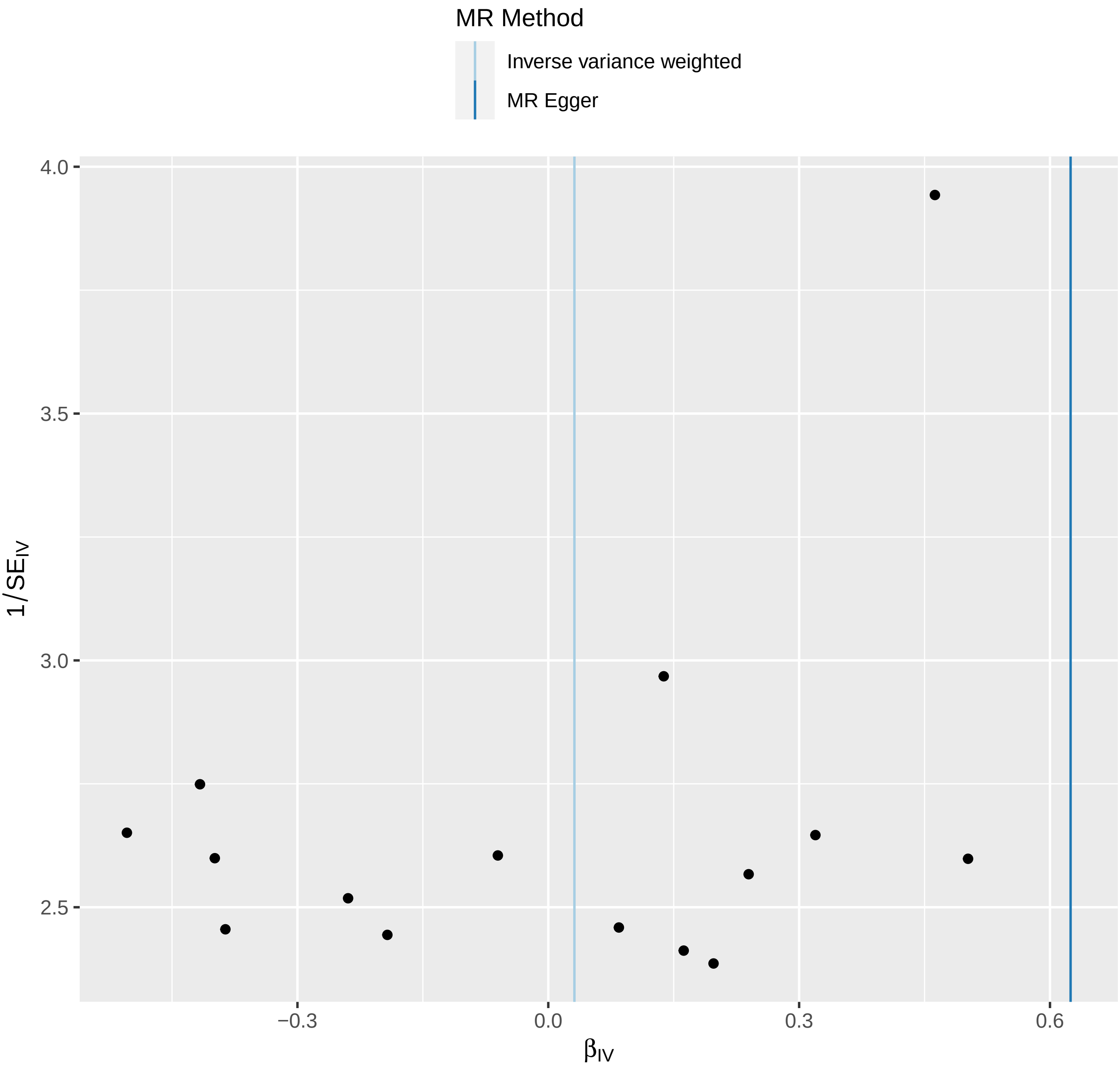

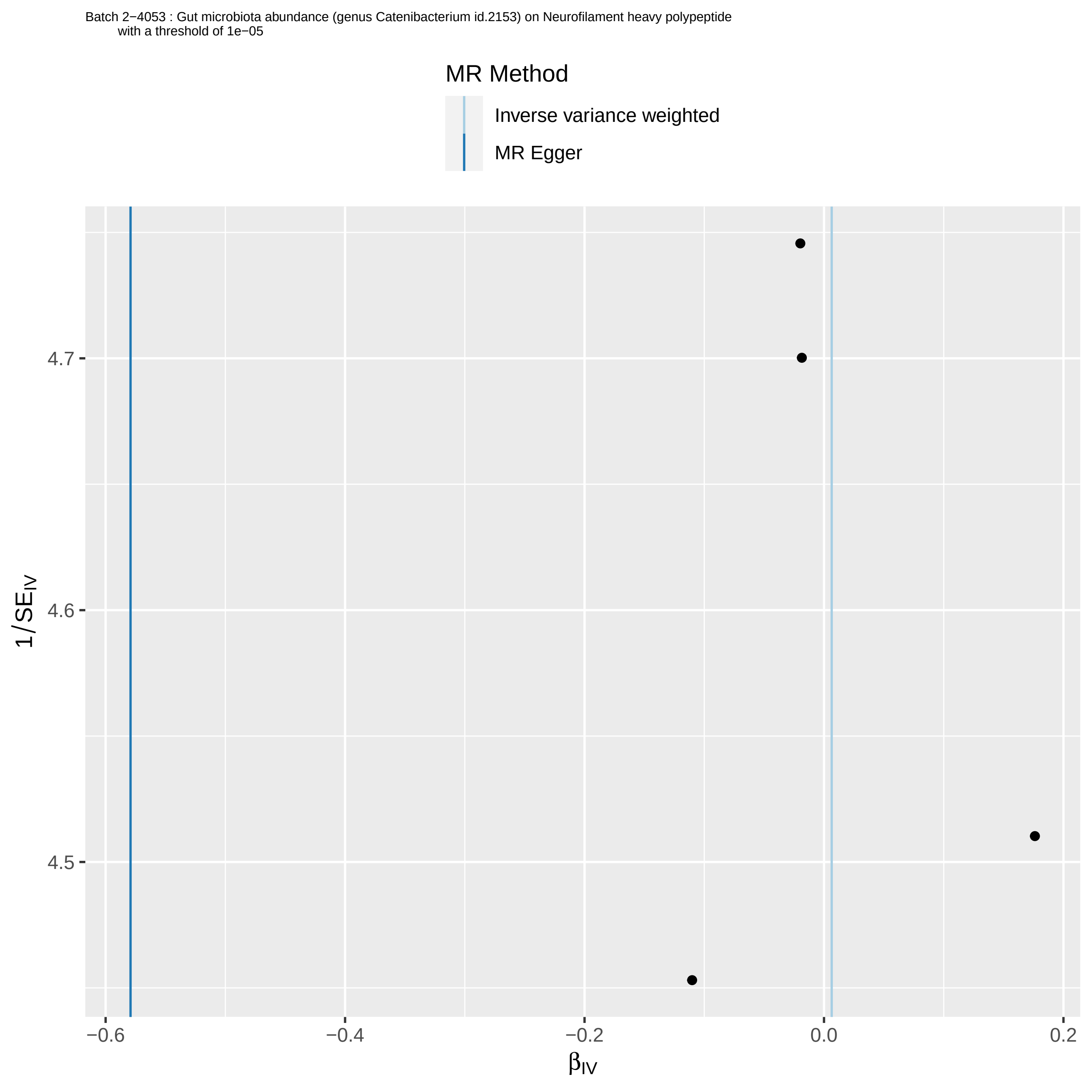
Batch 2−4053 : Gut microbiota abundance (genus Catenibacterium id.2153) on Neurofilament heavy polypeptide
with a threshold of 1e−05
MR Method
Inverse variance weighted
MR Egger
4.7
4.6
4.5
1/SE$_{IV}$
−0.6
−0.4
−0.2
0.0
0.2
β$_{IV}$

Batch 2−4054 : Gut microbiota abundance (genus Parasutterella id.2892) on Neurofilament heavy polypeptide
with a threshold of 1e−05

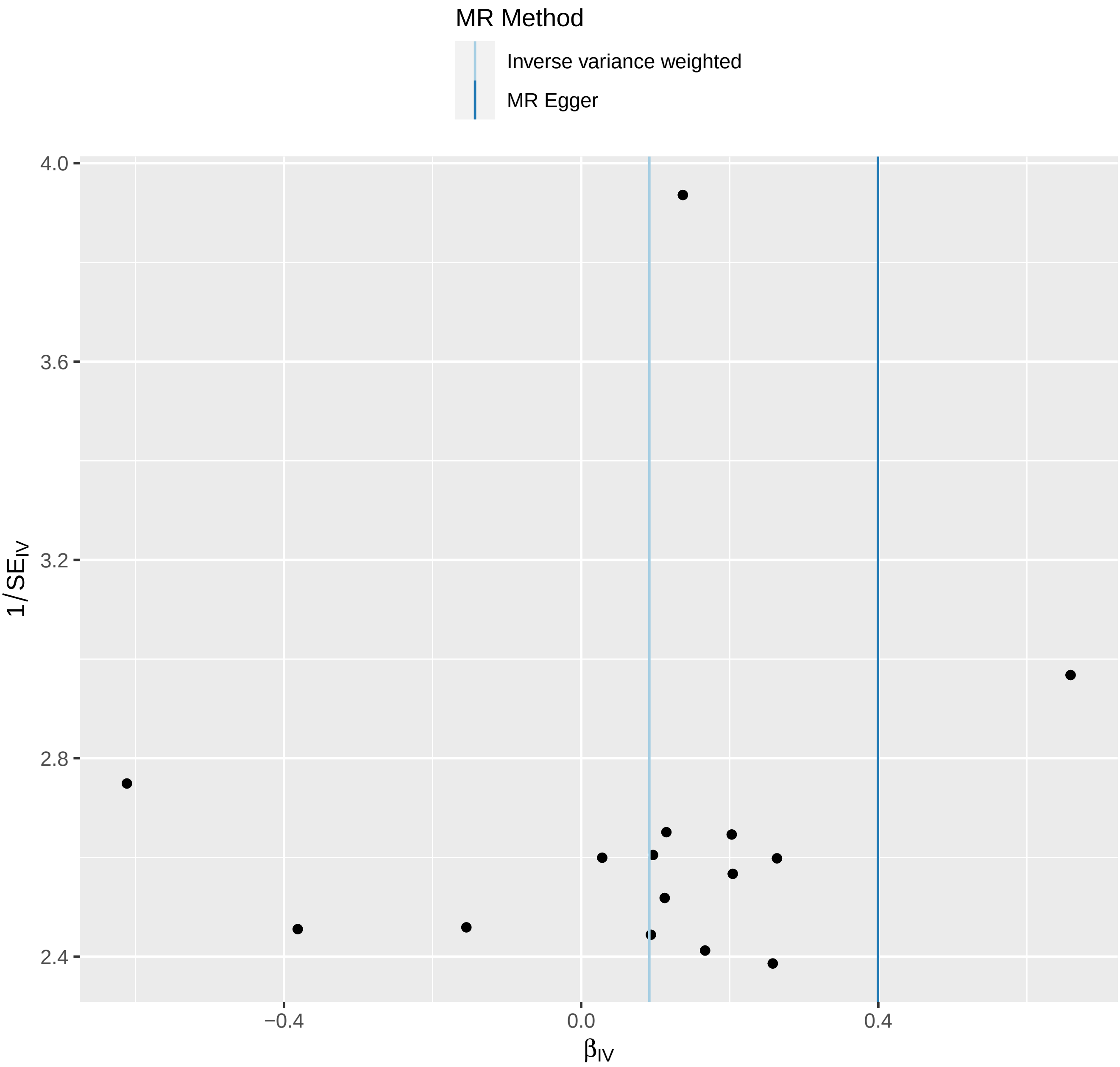

Batch 2−4055 : Gut microbiota abundance (genus Catenibacterium id.2153) on Neuronal growth regulator 1
with a threshold of 1e−05

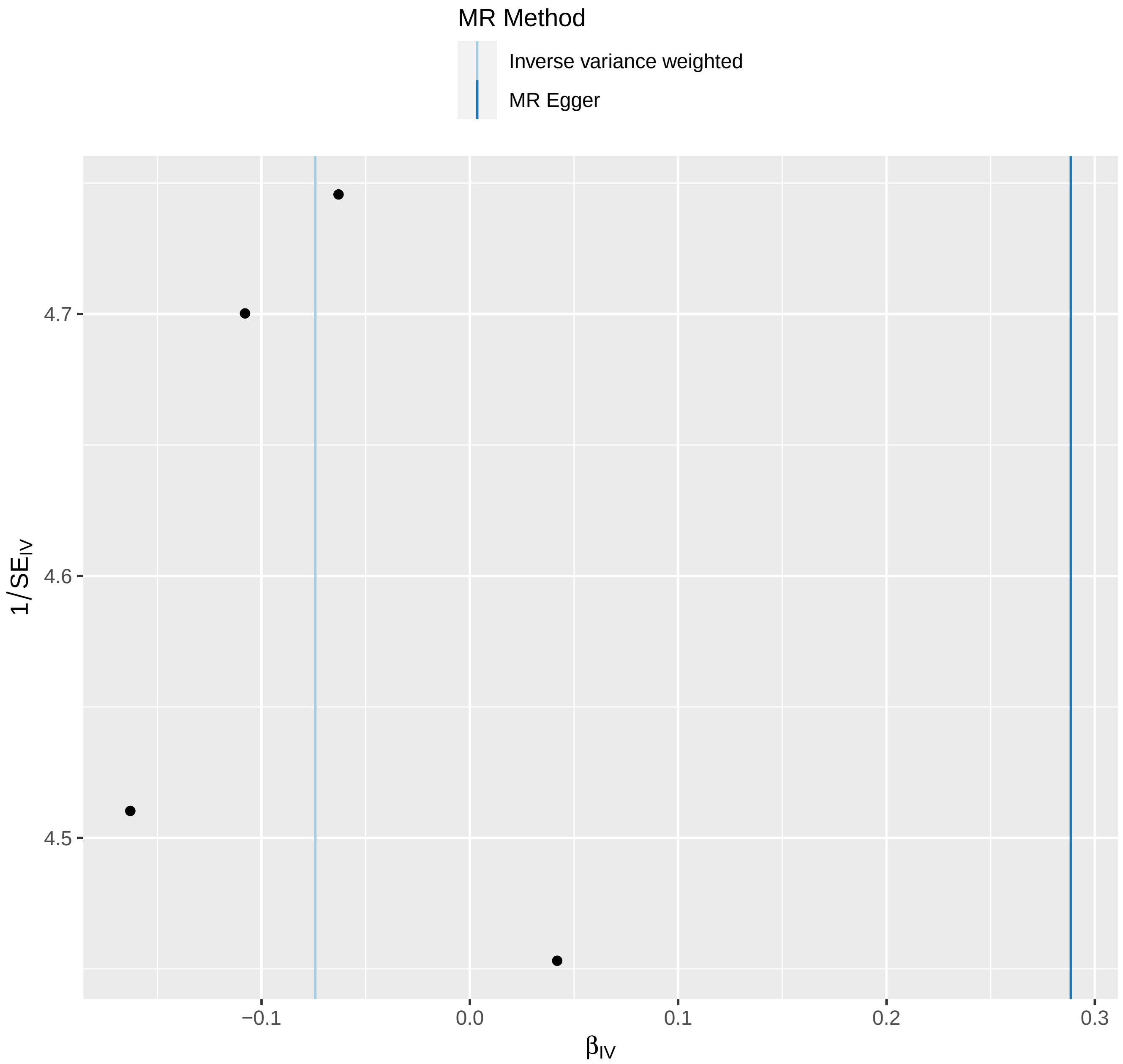

Batch 2−4056 : Gut microbiota abundance (genus Parasutterella id.2892) on Neuronal growth regulator 1
with a threshold of 1e−05

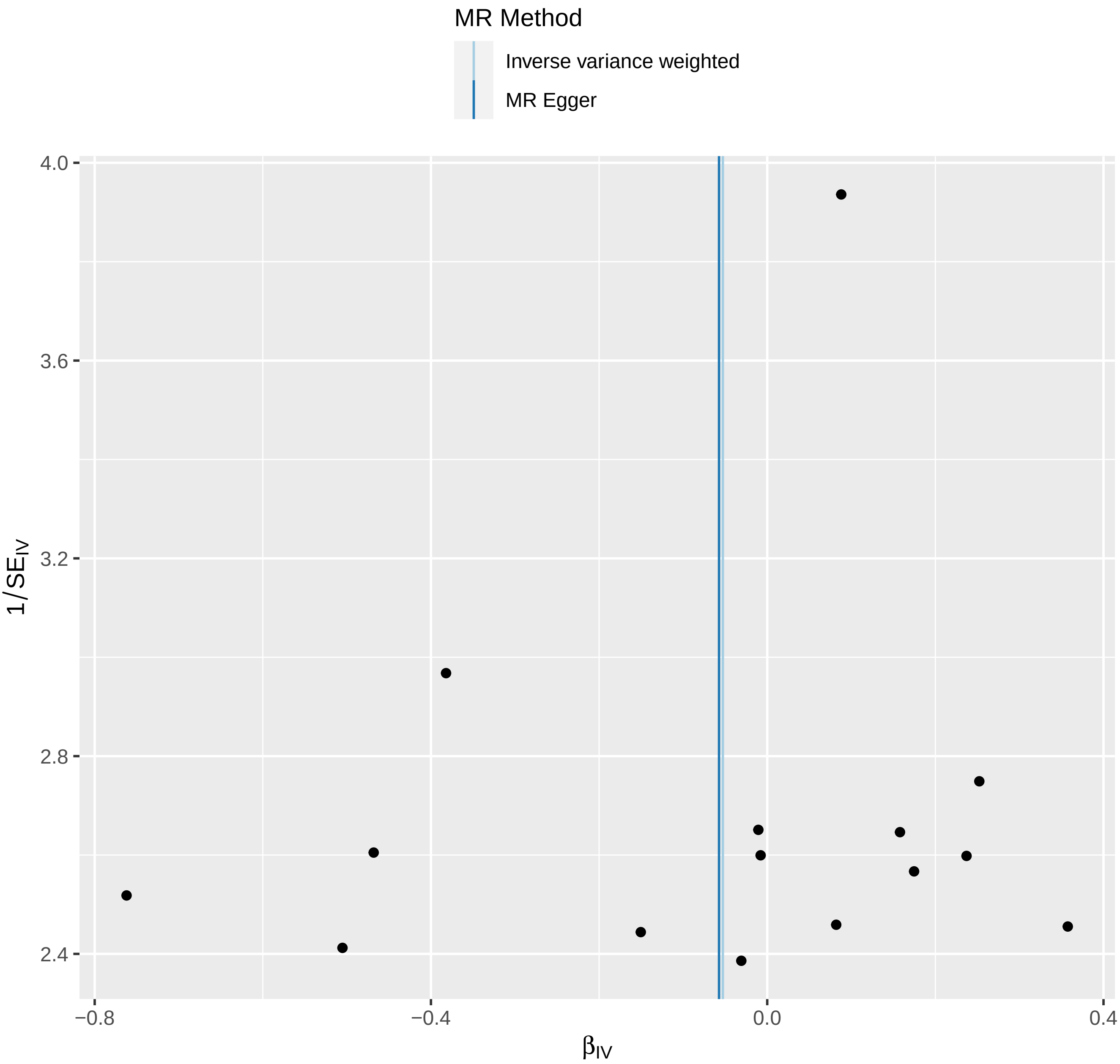

Batch 2−4057 : Gut microbiota abundance (genus Catenibacterium id.2153) on Neuronal growth regulator 1
with a threshold of 1e−05

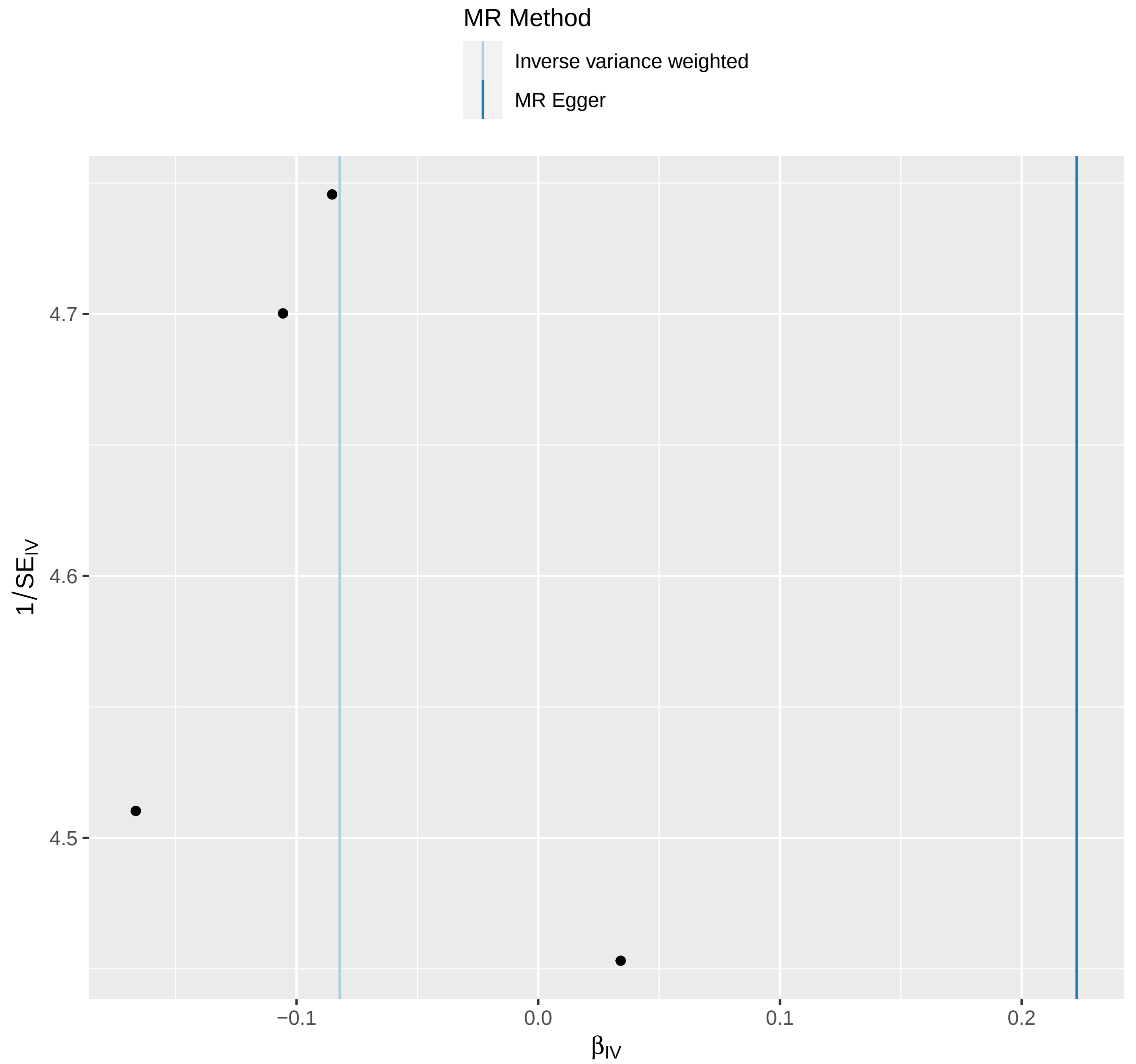

Batch 2−4058 : Gut microbiota abundance (genus Parasutterella id.2892) on Neuronal growth regulator 1
with a threshold of 1e−05

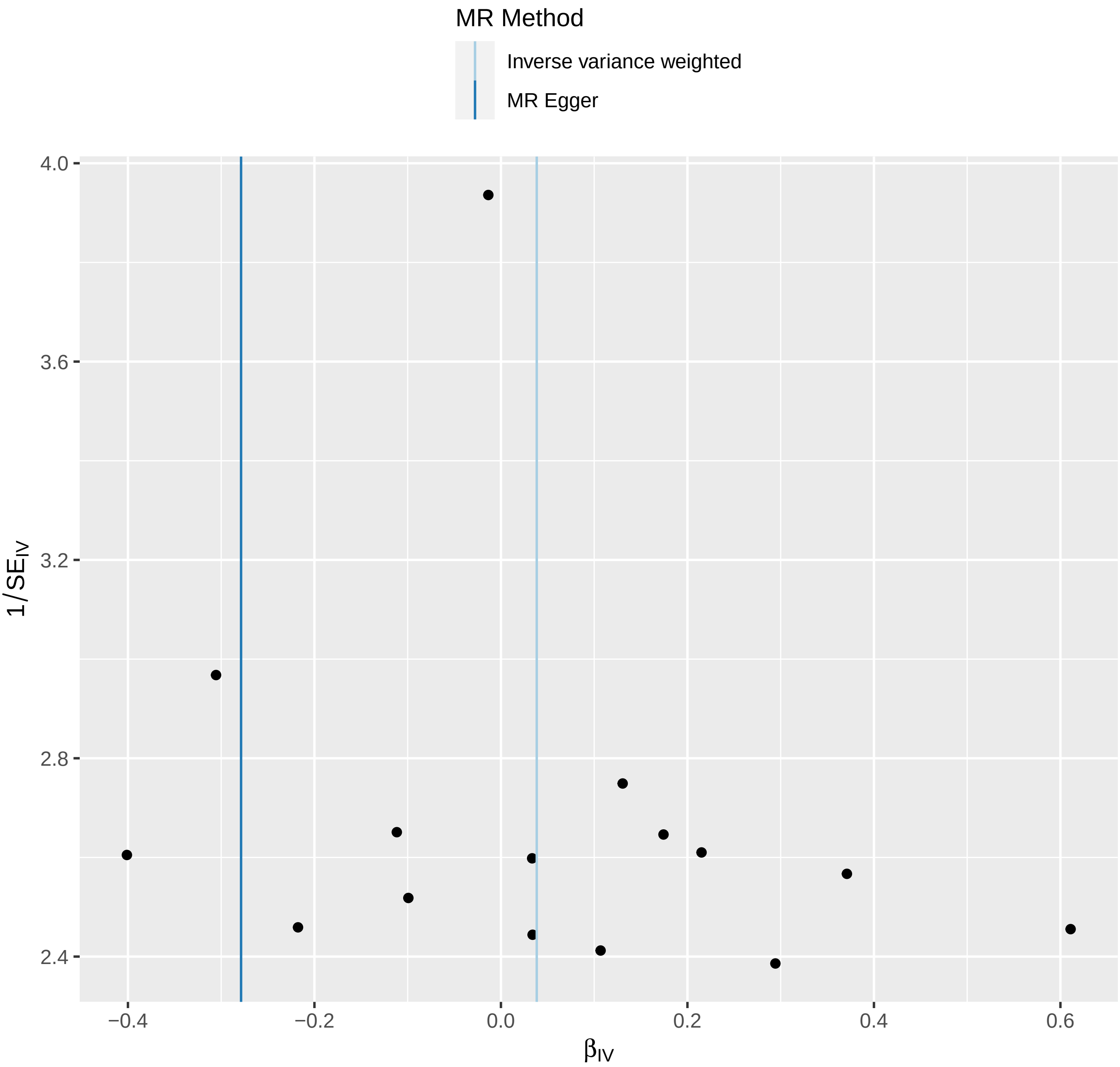

Batch 2−4059 : Gut microbiota abundance (genus Catenibacterium id.2153) on Protein kinase C−binding protein NELL1
with a threshold of 1e−05

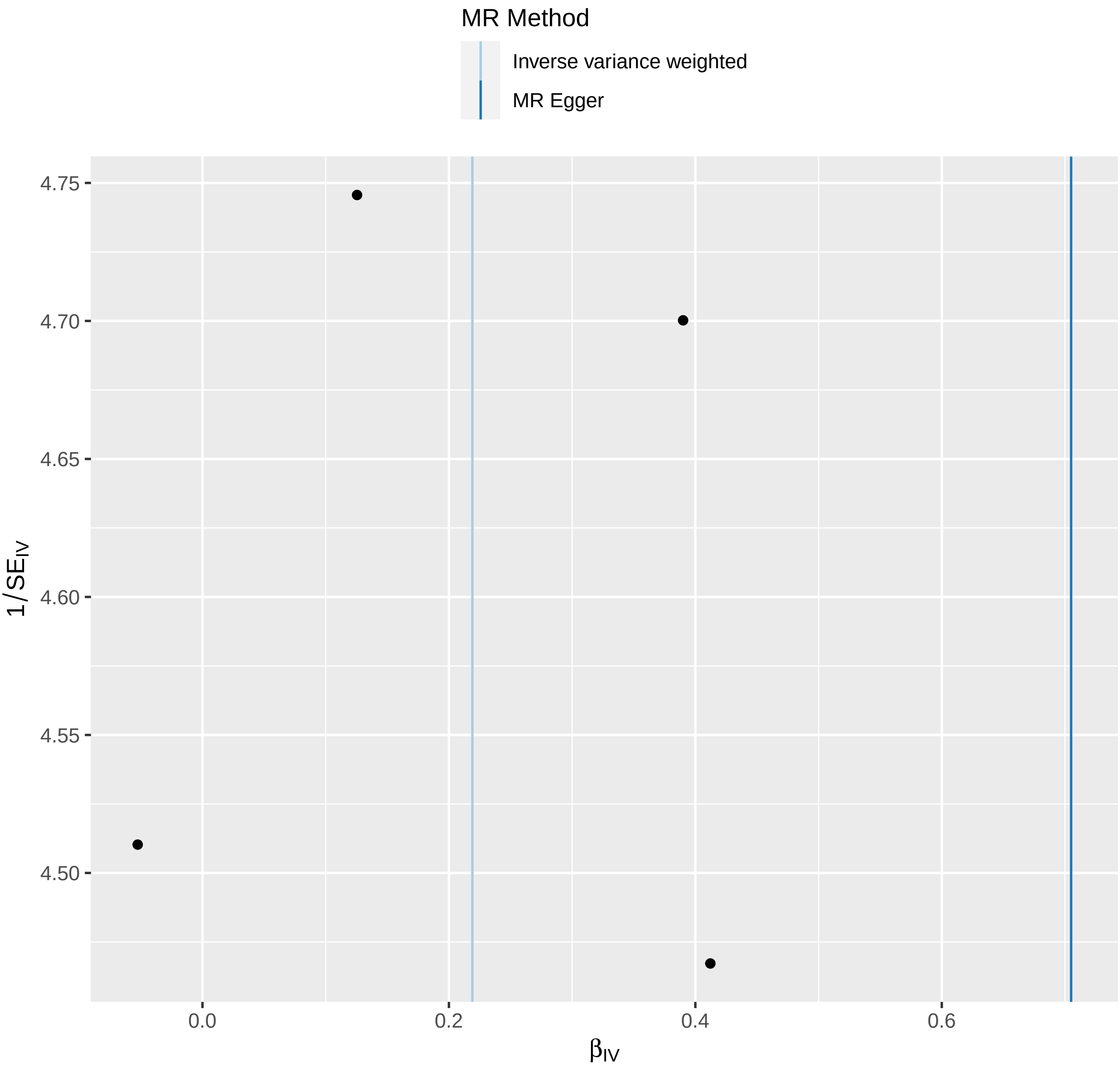

Batch 2−4060 : Gut microbiota abundance (genus Parasutterella id.2892) on Protein kinase C−binding protein NELL1 with a threshold of 1e−05

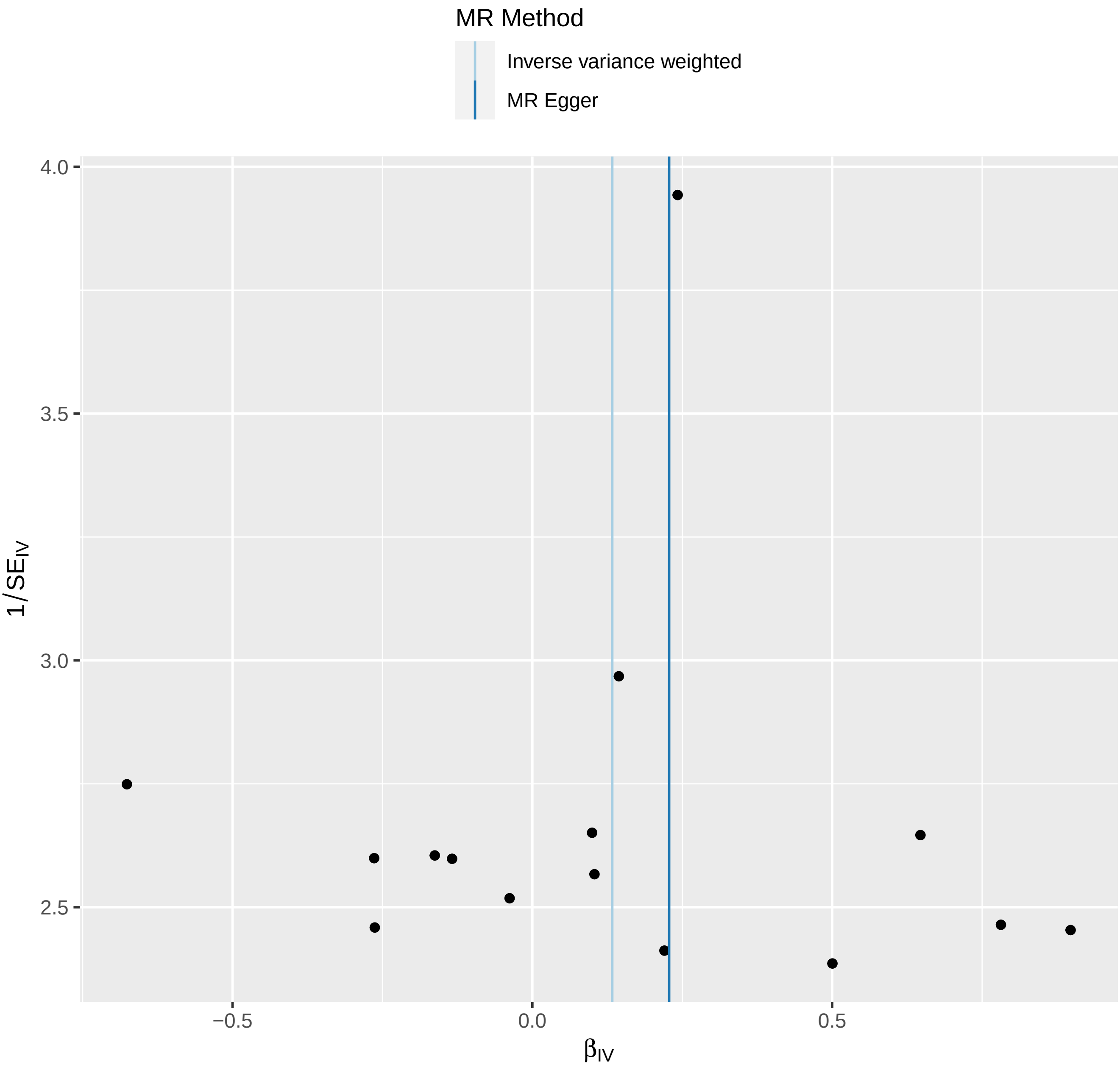

Batch 2−4061 : Gut microbiota abundance (genus Catenibacterium id.2153) on Protein kinase C−binding protein NELL2 with a threshold of 1e−05

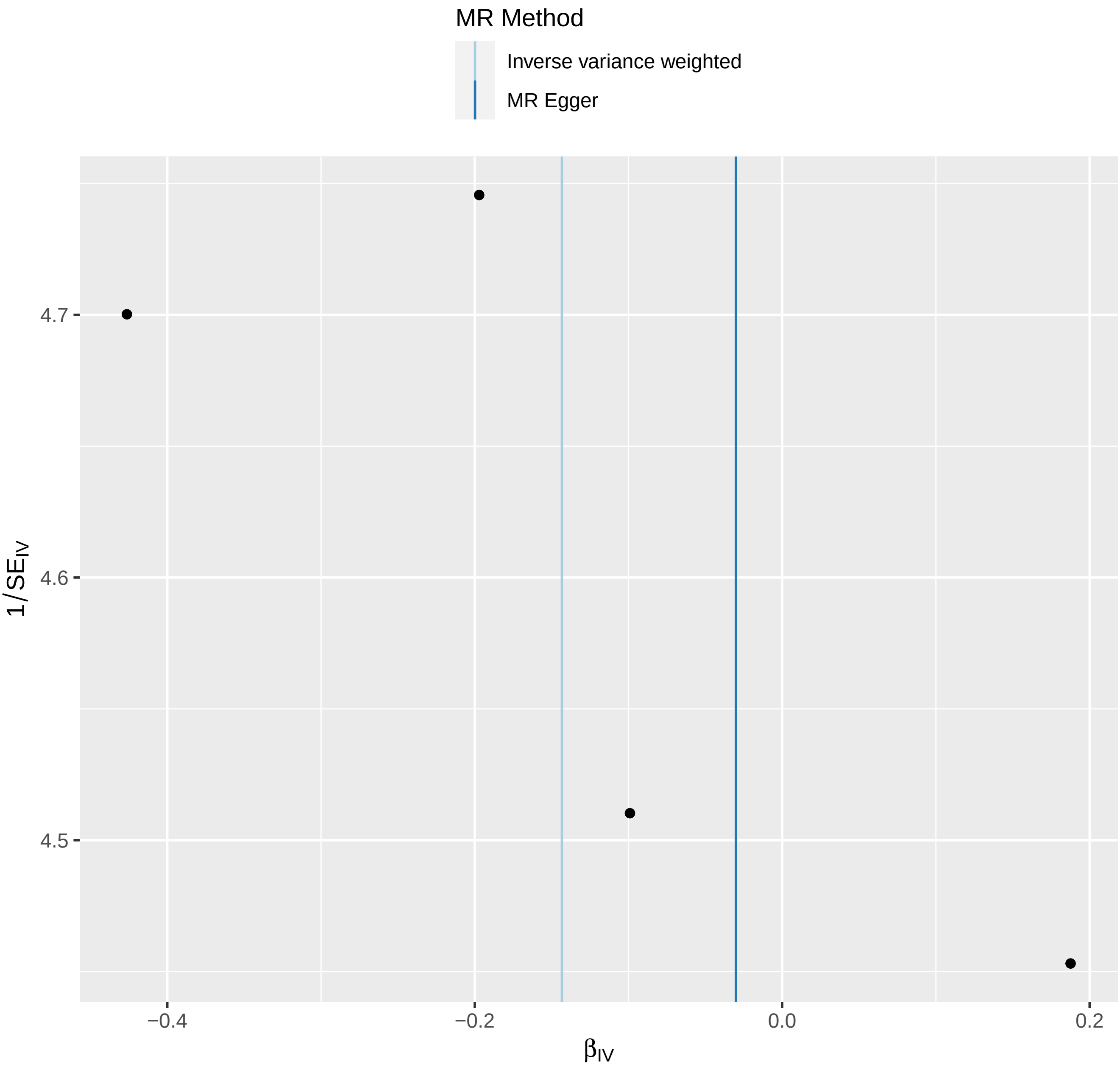

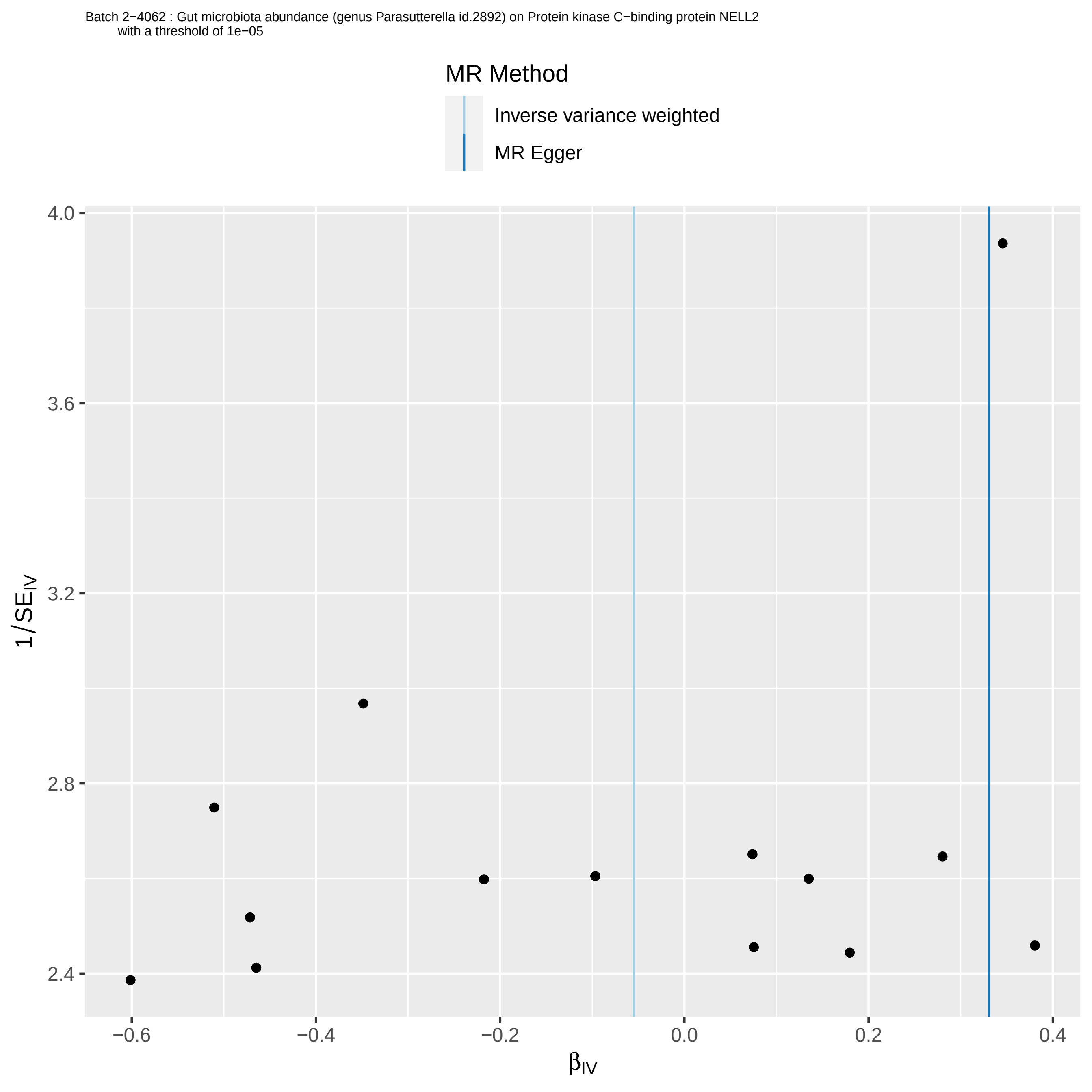
Batch 2−4062 : Gut microbiota abundance (genus Parasutterella id.2892) on Protein kinase C−binding protein NELL2
with a threshold of 1e−05
MR Method
Inverse variance weighted
MR Egger
4.0
3.6
3.2
2.8
2.4
1/SE_IV
−0.6
−0.4
−0.2
0.0
0.2
0.4
β_IV

Batch 2−4063 : Gut microbiota abundance (genus Catenibacterium id.2153) on Neudesin
with a threshold of 1e−05

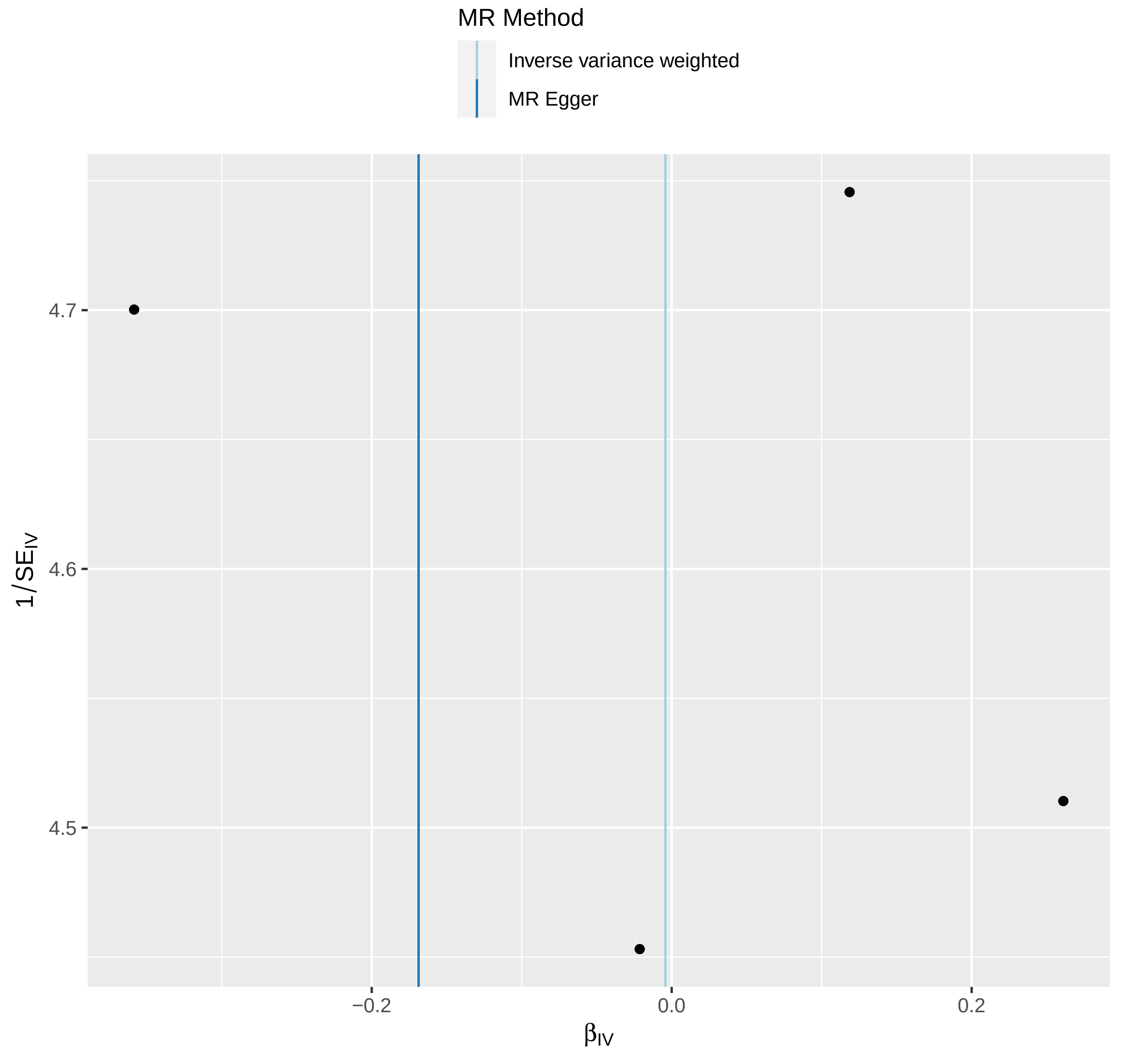

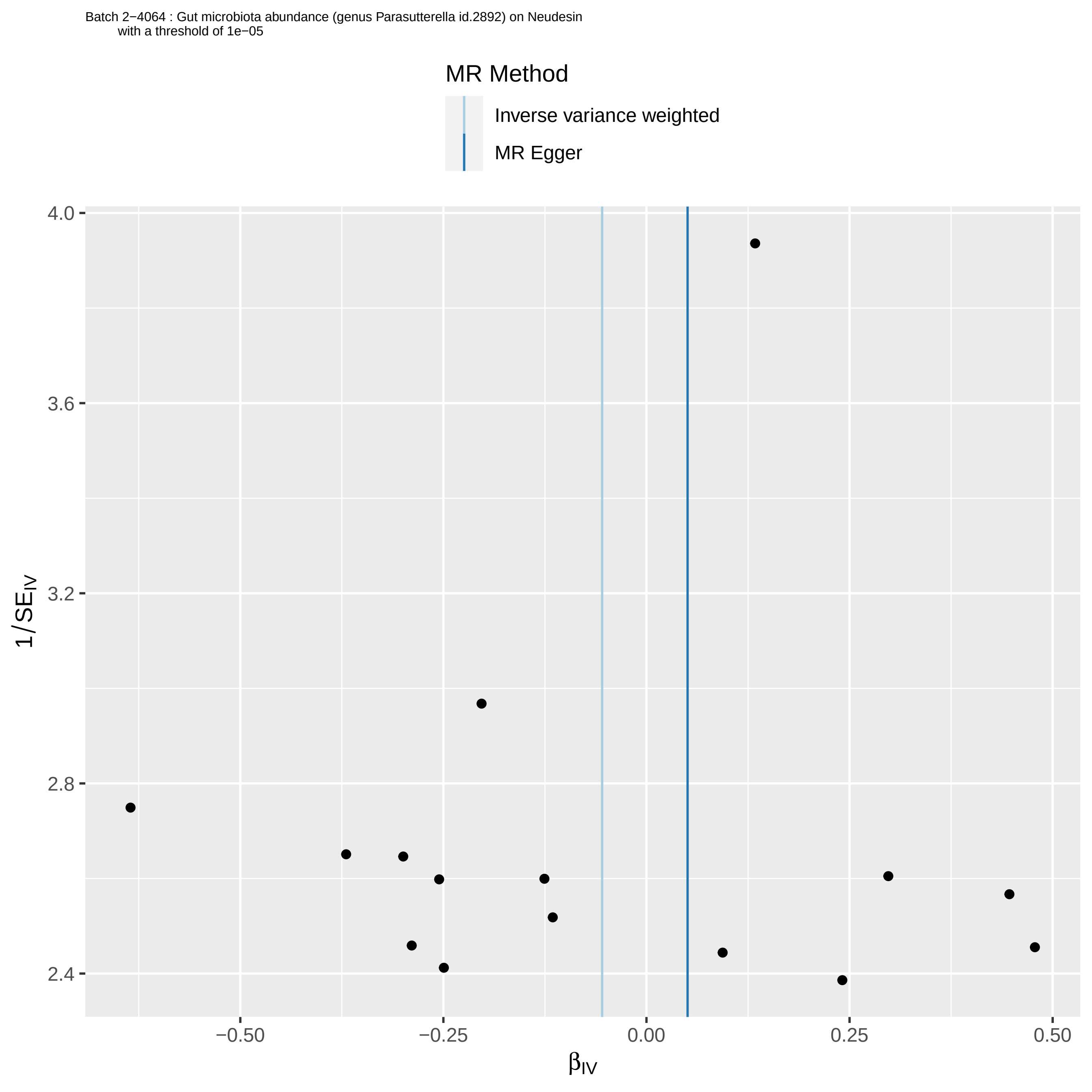
Batch 2−4064 : Gut microbiota abundance (genus Parasutterella id.2892) on Neudesin
with a threshold of 1e−05
MR Method
Inverse variance weighted
MR Egger
4.0
3.6
3.2
2.8
2.4
1/SE_IV
−0.50
−0.25
0.00
0.25
0.50
β_IV

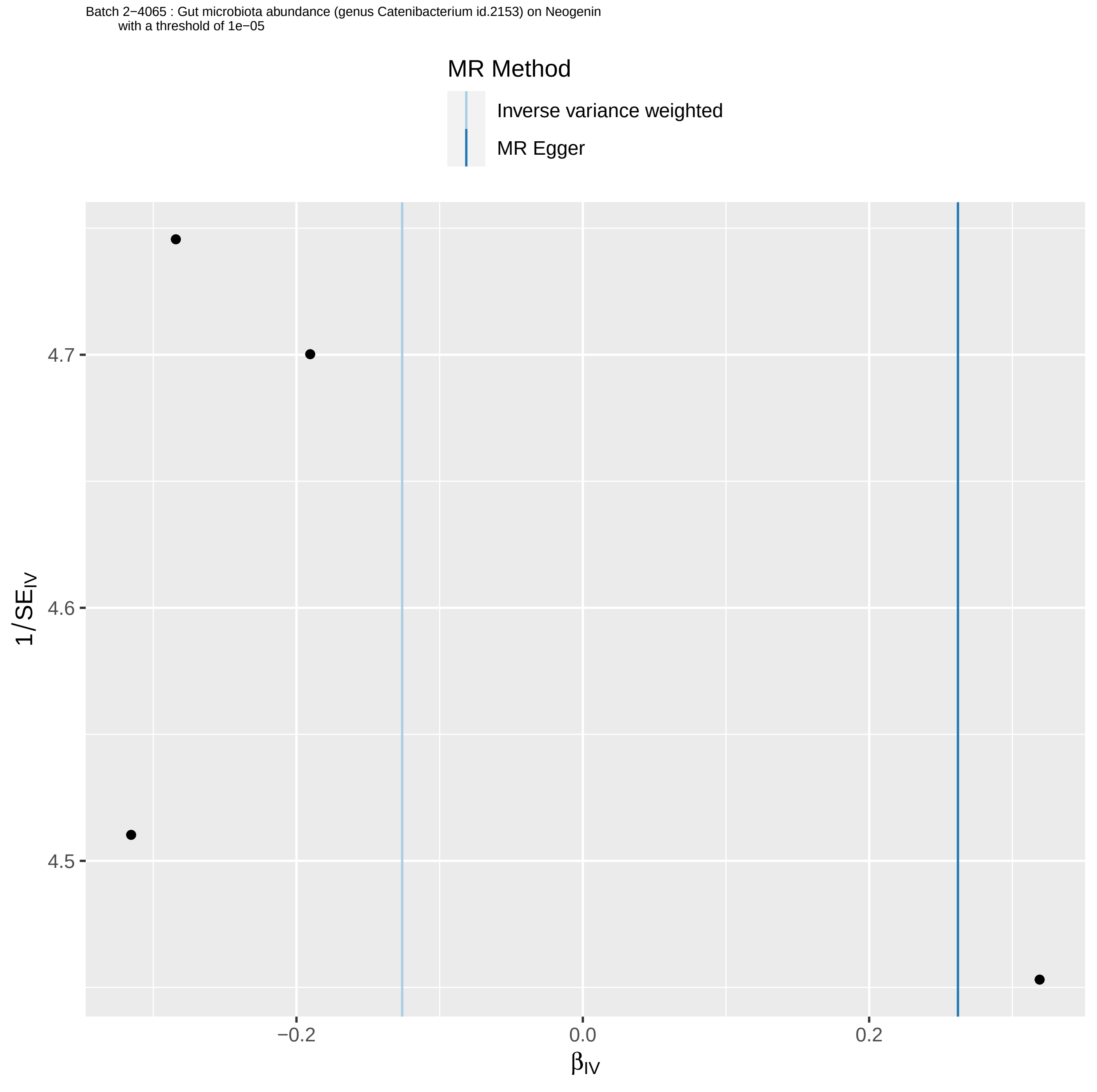
Batch 2−4065 : Gut microbiota abundance (genus Catenibacterium id.2153) on Neogenin
with a threshold of 1e−05
MR Method
Inverse variance weighted
MR Egger
4.7
4.6
4.5
1/SE$_{IV}$
−0.2
0.0
0.2
β$_{IV}$

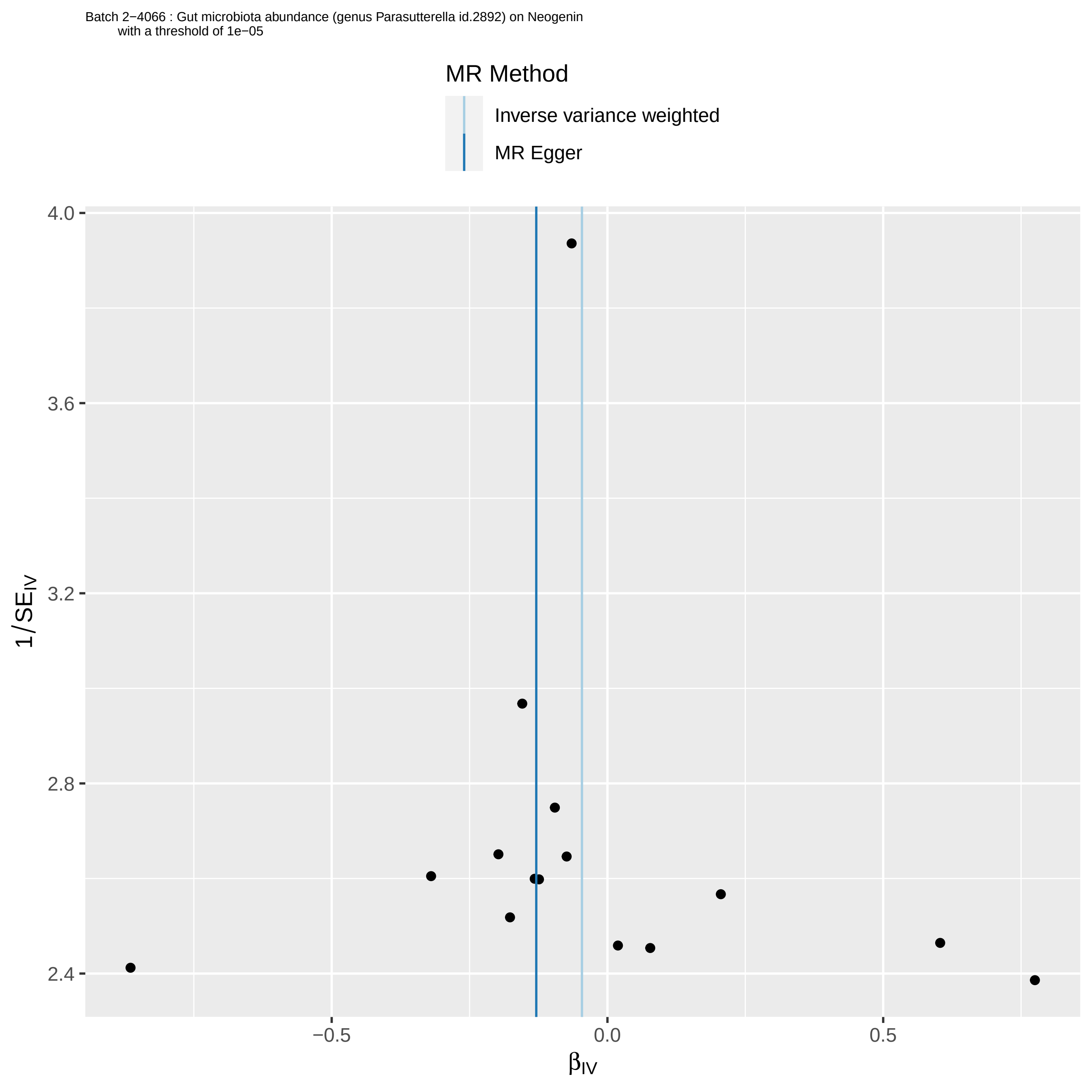

Batch 2−4066 : Gut microbiota abundance (genus Parasutterella id.2892) on Neogenin
with a threshold of 1e−05
MR Method
Inverse variance weighted
MR Egger
4.0
3.6
3.2
2.8
2.4
1/SE_IV
−0.5
0.0
0.5
β_IV

Batch 2−4067 : Gut microbiota abundance (genus Catenibacterium id.2153) on Neuroepithelial cell−transforming gene 1 protein with a threshold of 1e−05

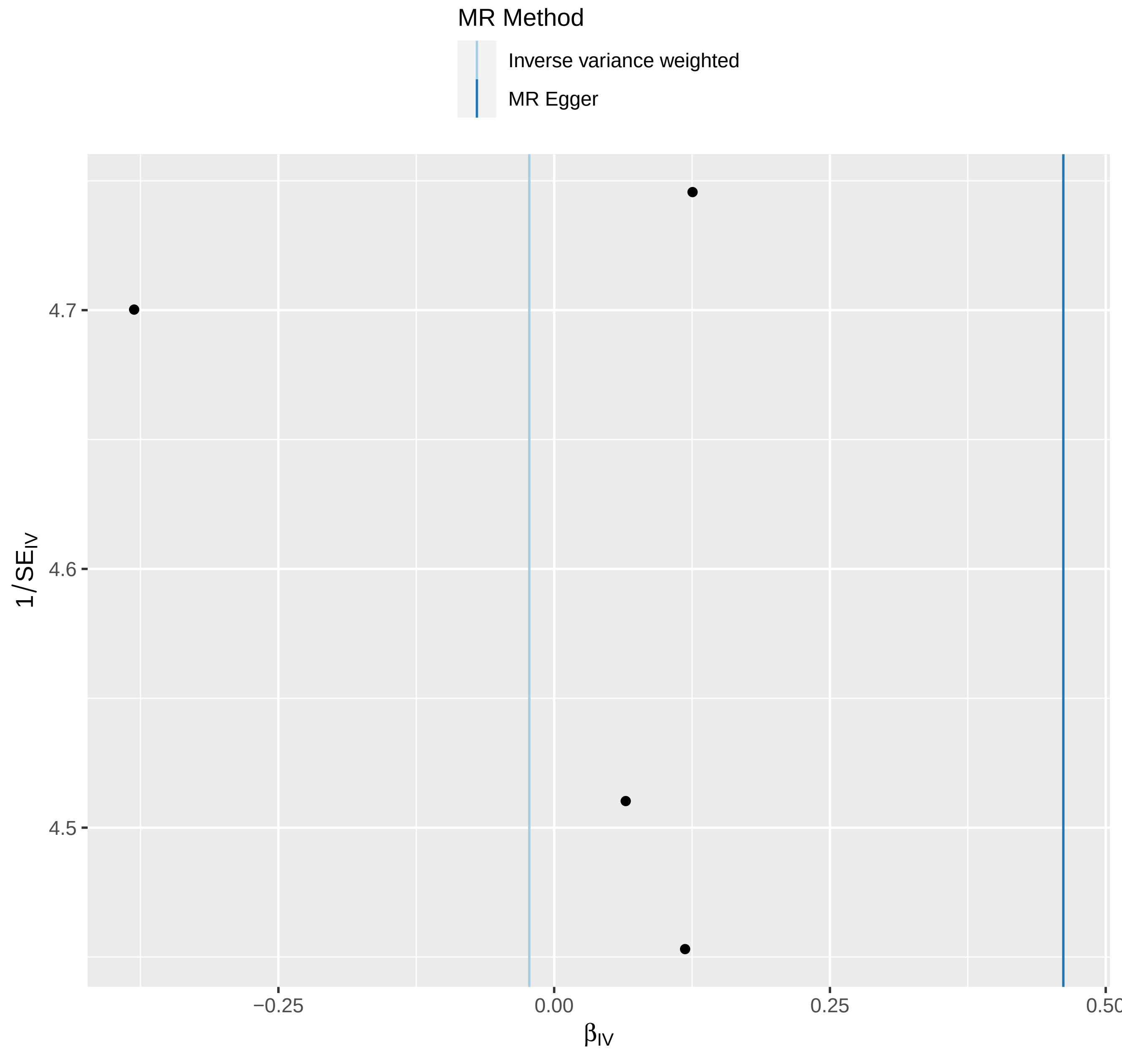

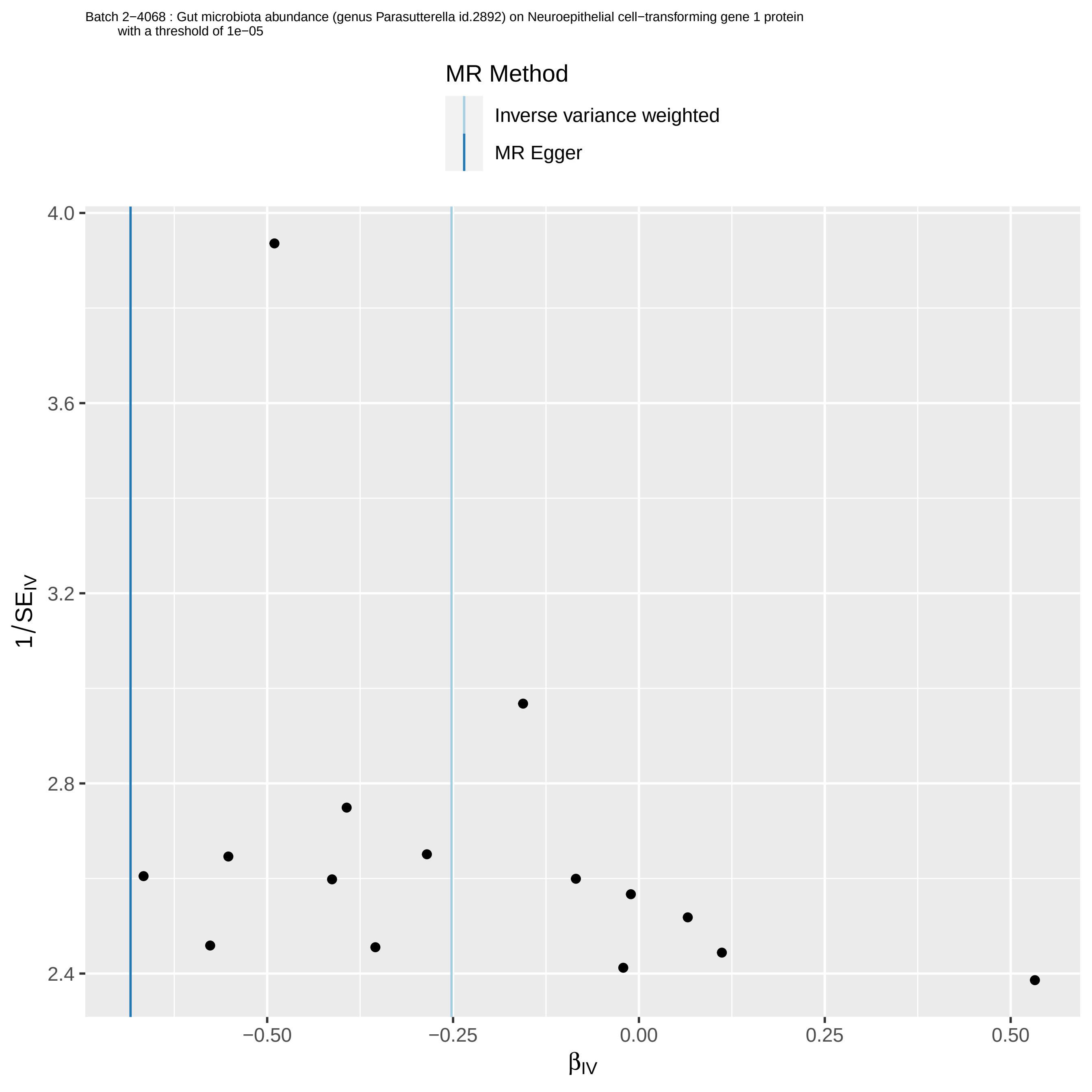
Batch 2−4068 : Gut microbiota abundance (genus Parasutterella id.2892) on Neuroepithelial cell−transforming gene 1 protein
with a threshold of 1e−05
MR Method
Inverse variance weighted
MR Egger
4.0
3.6
3.2
2.8
2.4
1/SE_IV
−0.50
−0.25
0.00
0.25
0.50
β_IV

Batch 2−4069 : Gut microbiota abundance (genus Catenibacterium id.2153) on Neuropilin and tolloid−like protein 1
with a threshold of 1e−05

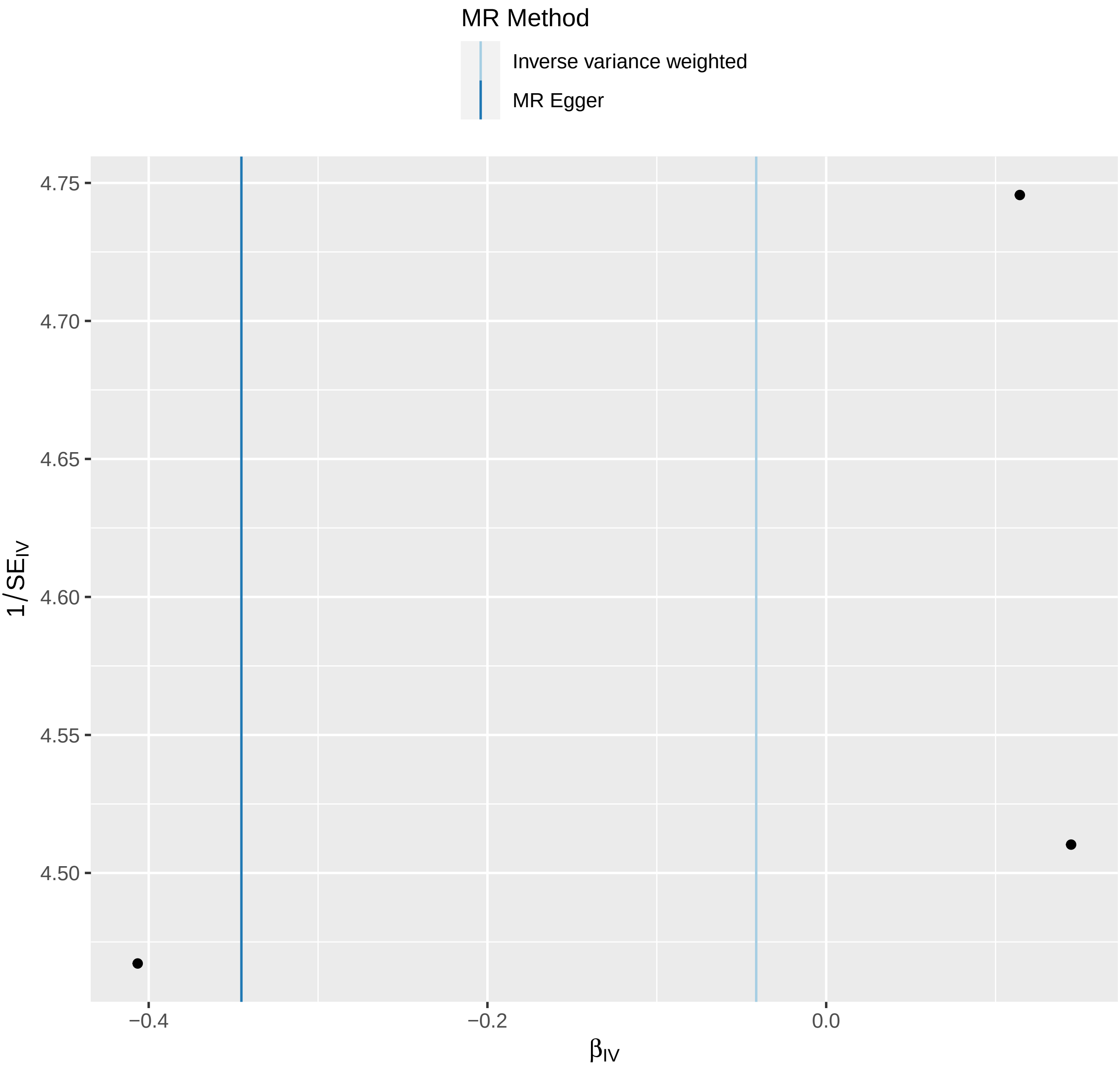

Batch 2−4070 : Gut microbiota abundance (genus Parasutterella id.2892) on Neuropilin and tolloid−like protein 1
with a threshold of 1e−05

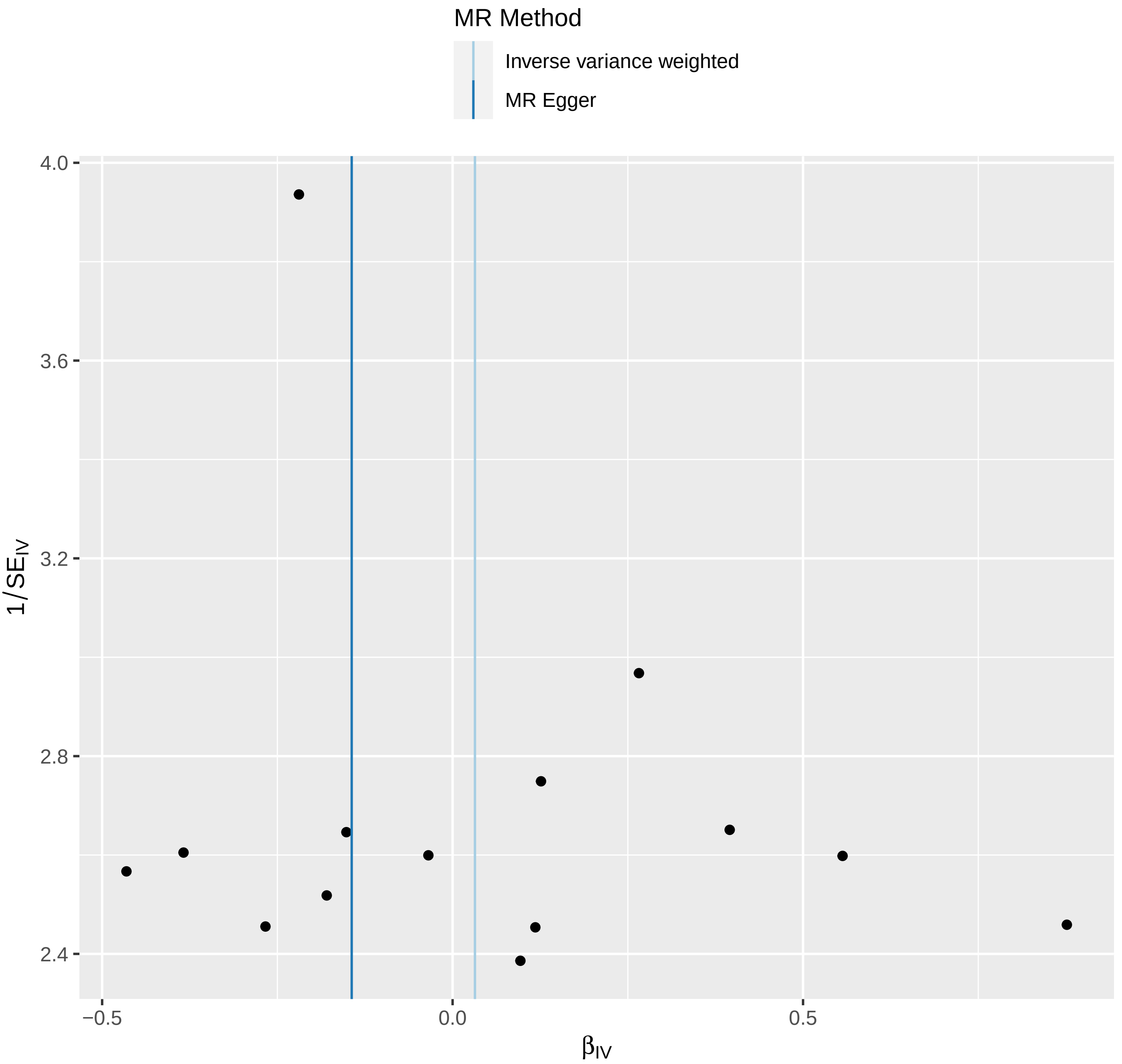

Batch 2−4071 : Gut microbiota abundance (genus Catenibacterium id.2153) on Neuropilin and tolloid−like protein 2
with a threshold of 1e−05

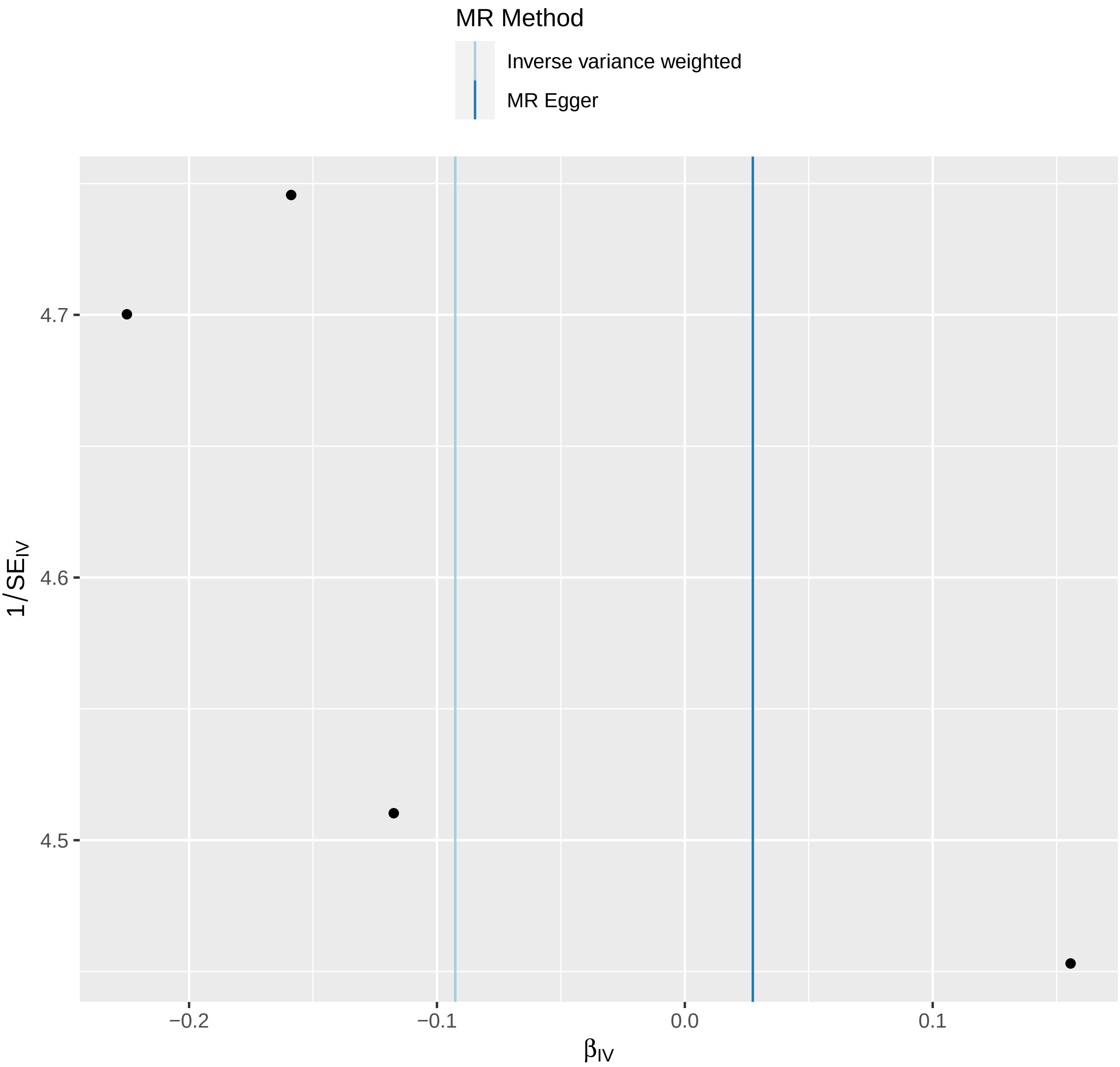

Batch 2−4072 : Gut microbiota abundance (genus Parasutterella id.2892) on Neuropilin and tolloid−like protein 2 with a threshold of 1e−05

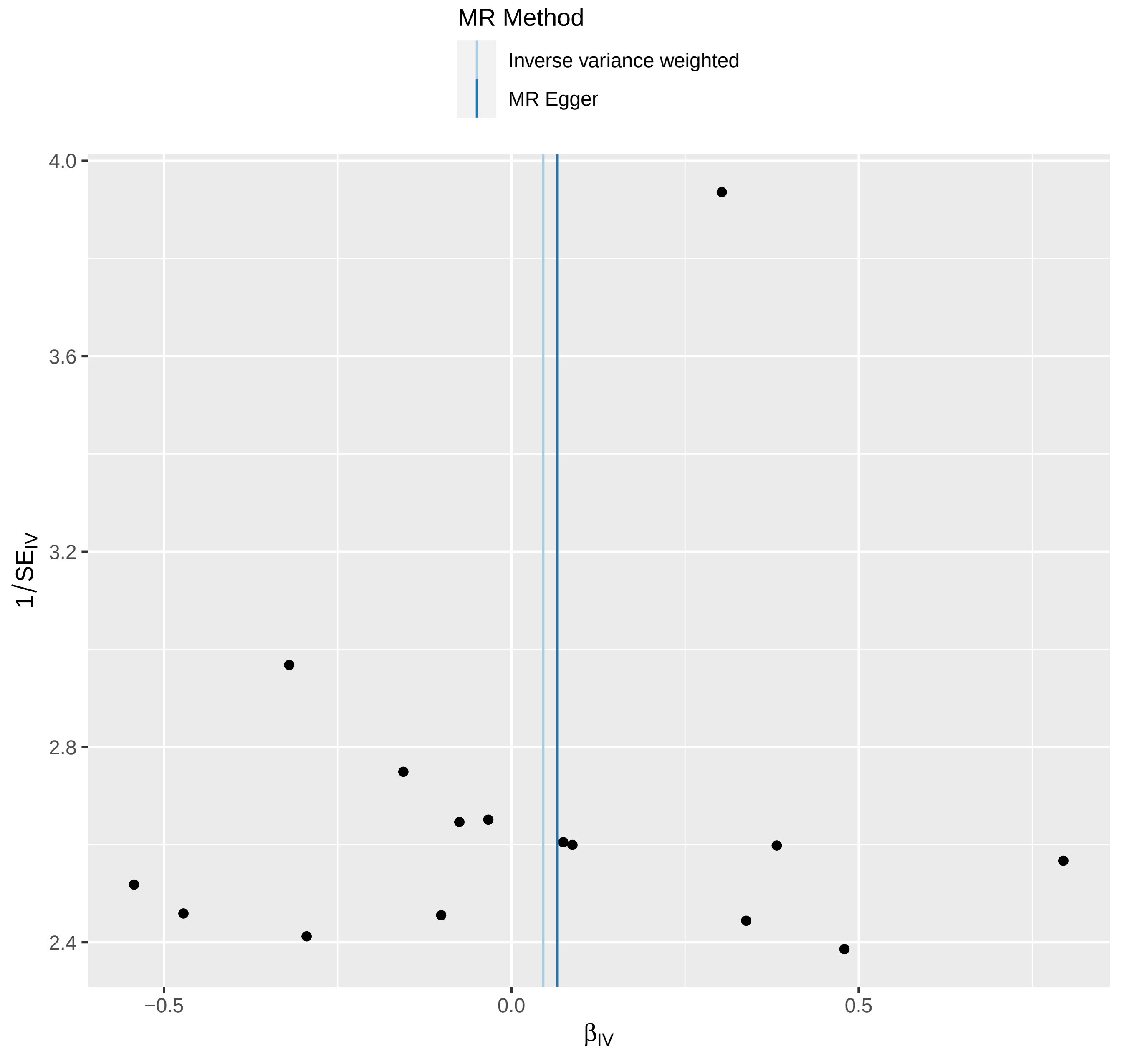

Batch 2−4073 : Gut microbiota abundance (genus Catenibacterium id.2153) on E3 ubiquitin−protein ligase NEURL1
with a threshold of 1e−05

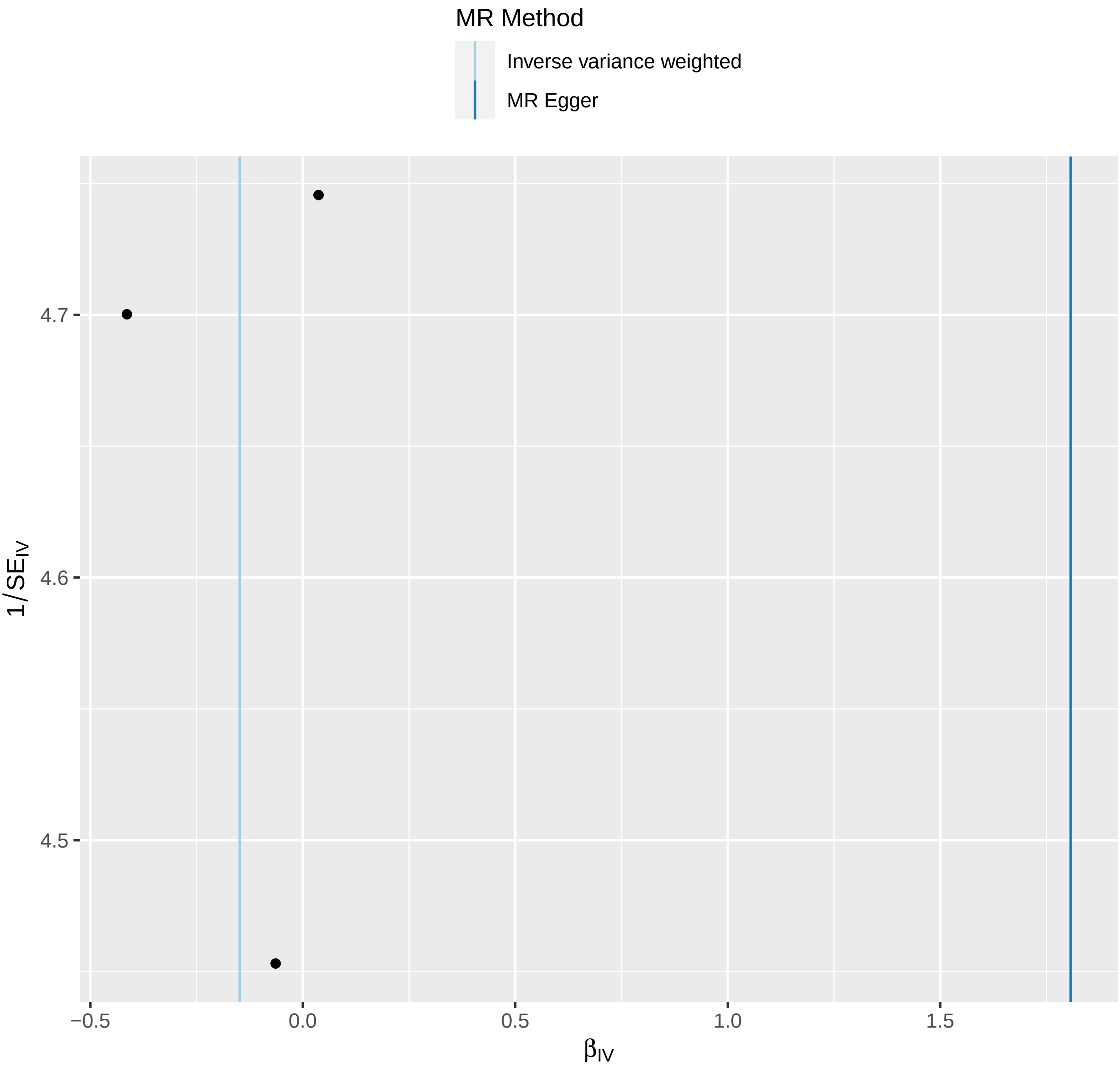

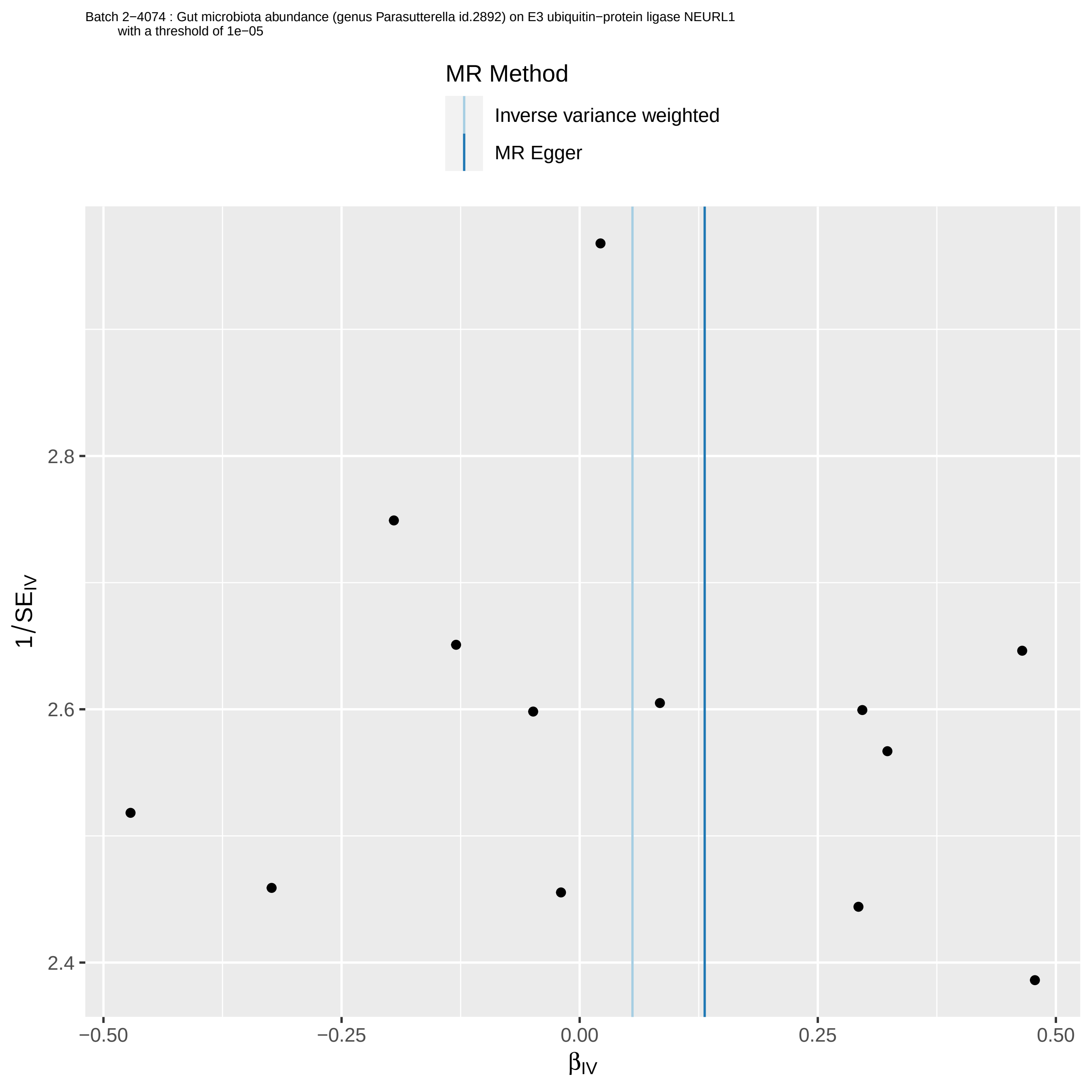

Batch 2−4074 : Gut microbiota abundance (genus Parasutterella id.2892) on E3 ubiquitin−protein ligase NEURL1
with a threshold of 1e−05
MR Method
Inverse variance weighted
MR Egger
2.8
2.6
2.4
1/SE_IV
−0.50
−0.25
0.00
0.25
0.50
β_IV

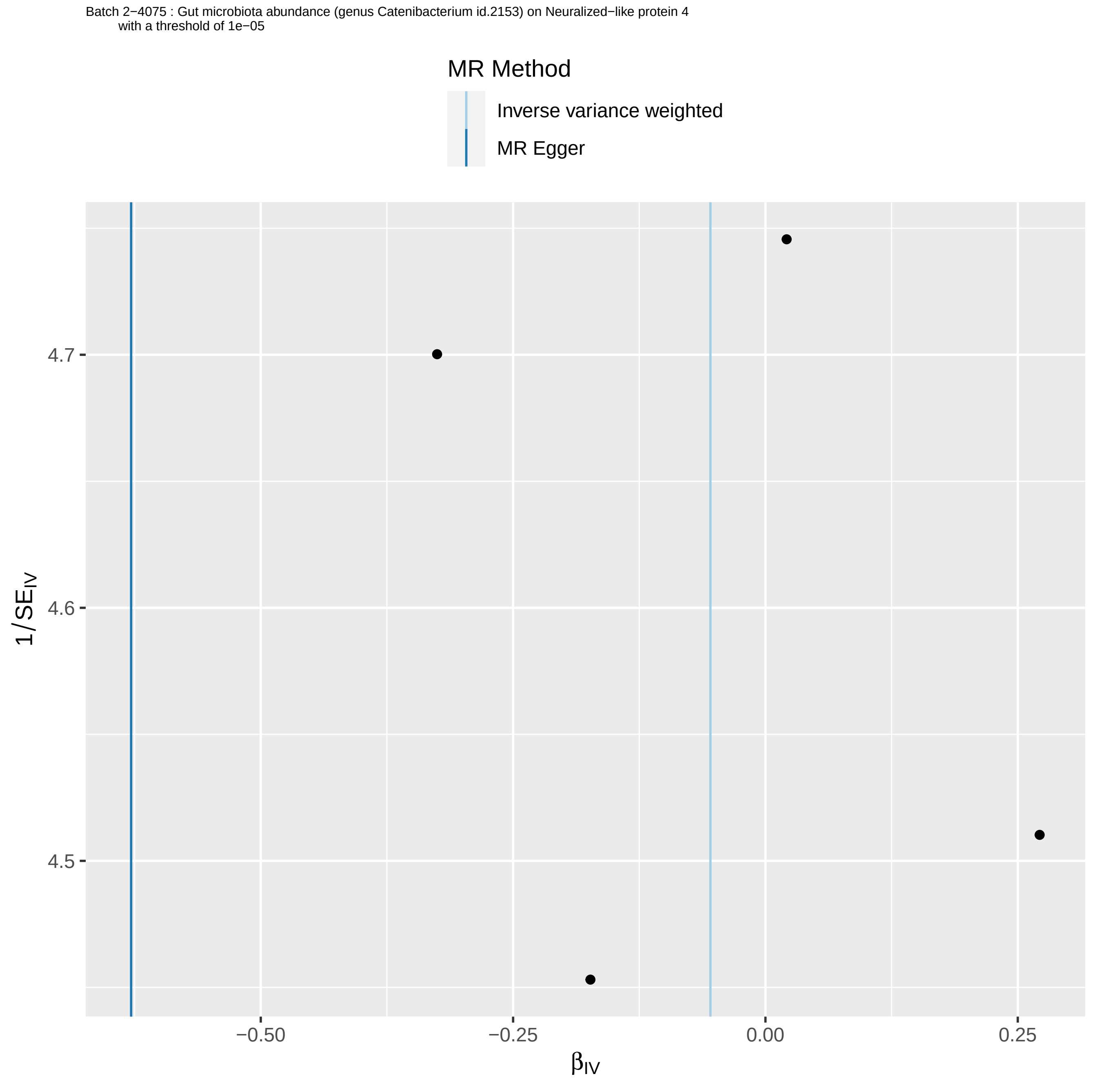

Batch 2−4075 : Gut microbiota abundance (genus Catenibacterium id.2153) on Neuralized−like protein 4
with a threshold of 1e−05
MR Method
Inverse variance weighted
MR Egger
4.7
4.6
4.5
1/SE_IV
−0.50
−0.25
0.00
0.25
β_IV

Batch 2−4076 : Gut microbiota abundance (genus Parasutterella id.2892) on Neuralized−like protein 4
with a threshold of 1e−05

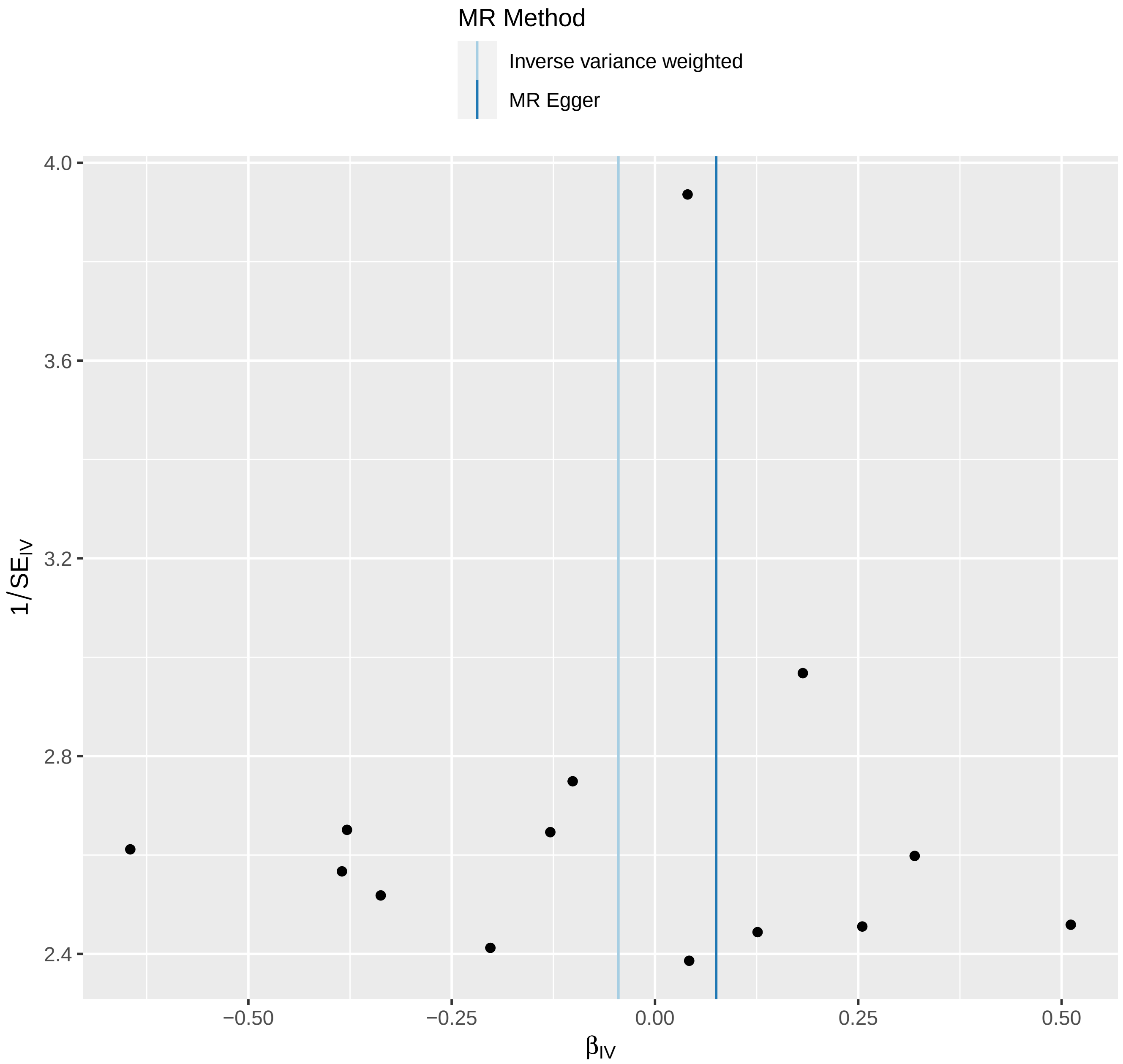

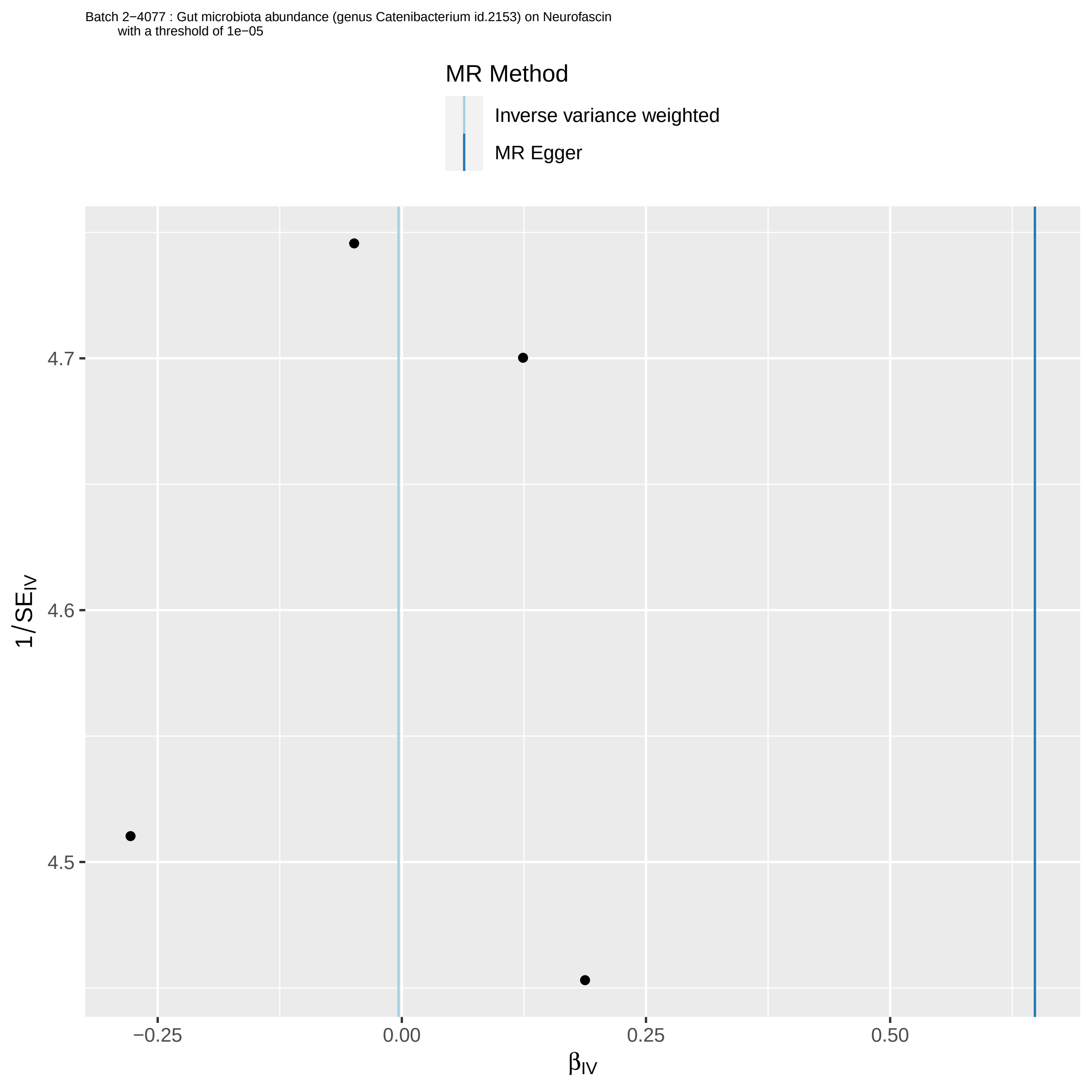
Batch 2−4077 : Gut microbiota abundance (genus Catenibacterium id.2153) on Neurofascin
with a threshold of 1e−05
MR Method
Inverse variance weighted
MR Egger
4.7
4.6
4.5
1/SE_IV
−0.25
0.00
0.25
0.50
β_IV

Batch 2−4078 : Gut microbiota abundance (genus Parasutterella id.2892) on Neurofascin
with a threshold of 1e−05

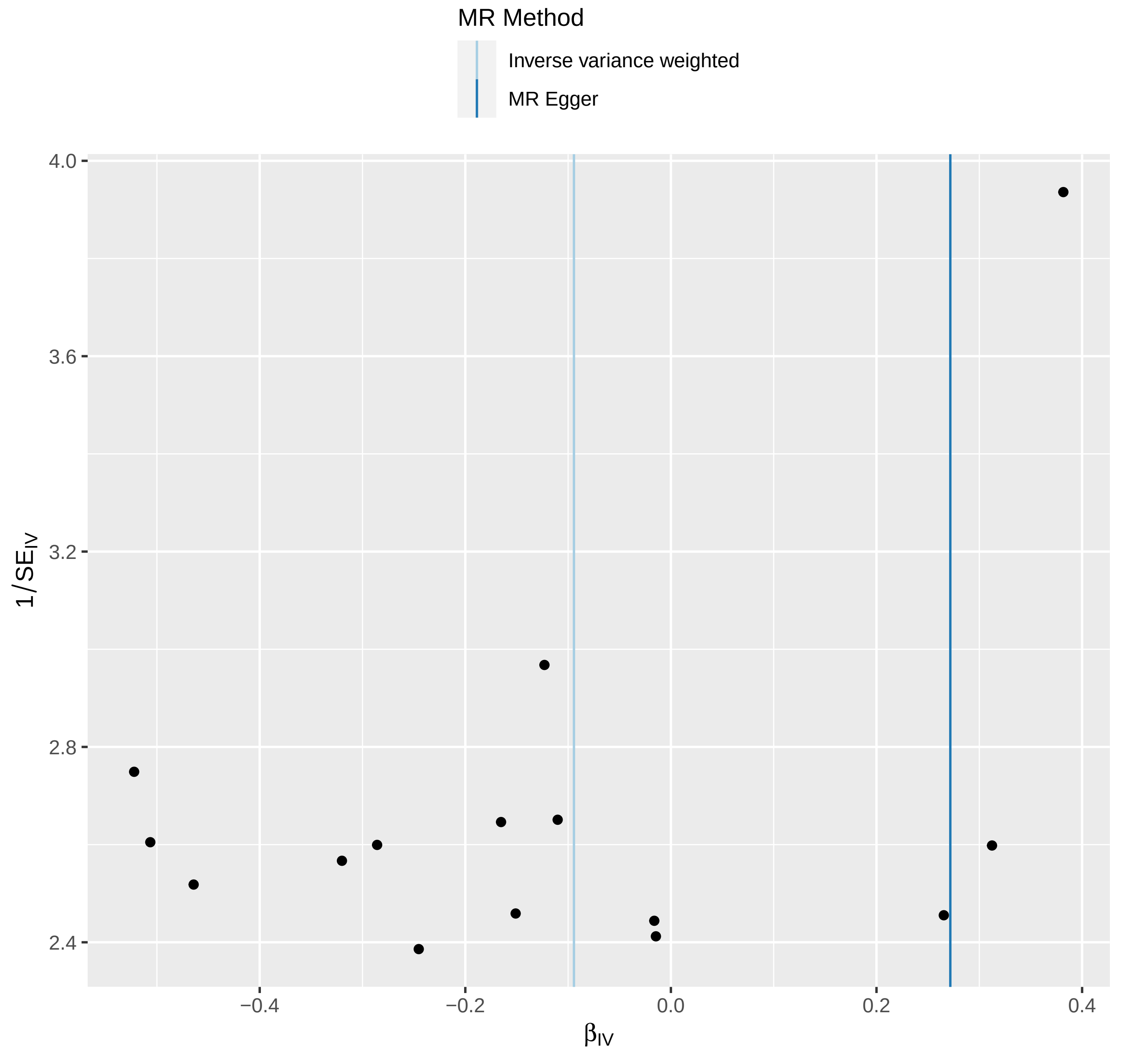

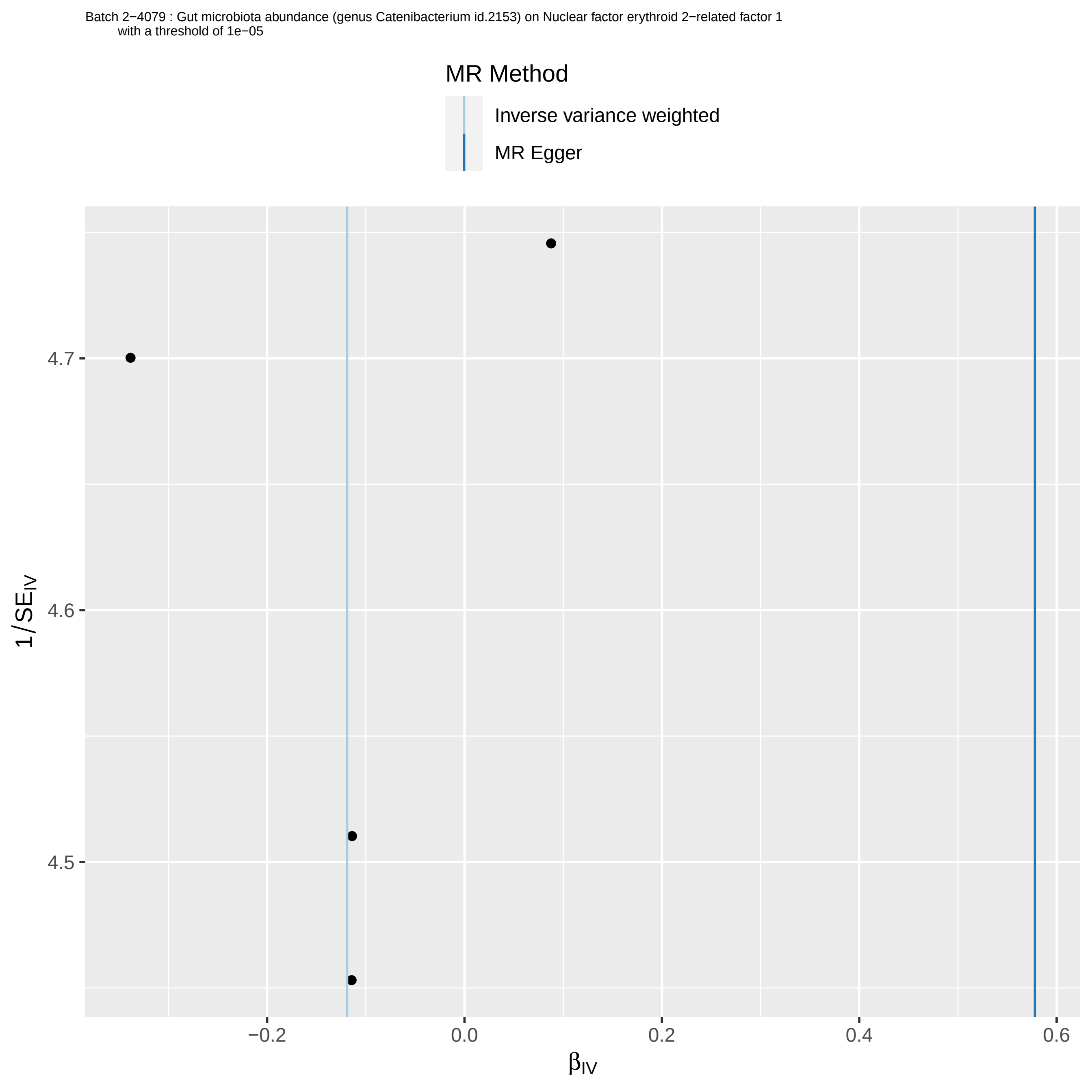
Batch 2−4079 : Gut microbiota abundance (genus Catenibacterium id.2153) on Nuclear factor erythroid 2−related factor 1
with a threshold of 1e−05
MR Method
Inverse variance weighted
MR Egger
4.7
4.6
4.5
1/SE_IV
−0.2
0.0
0.2
0.4
0.6
β_IV

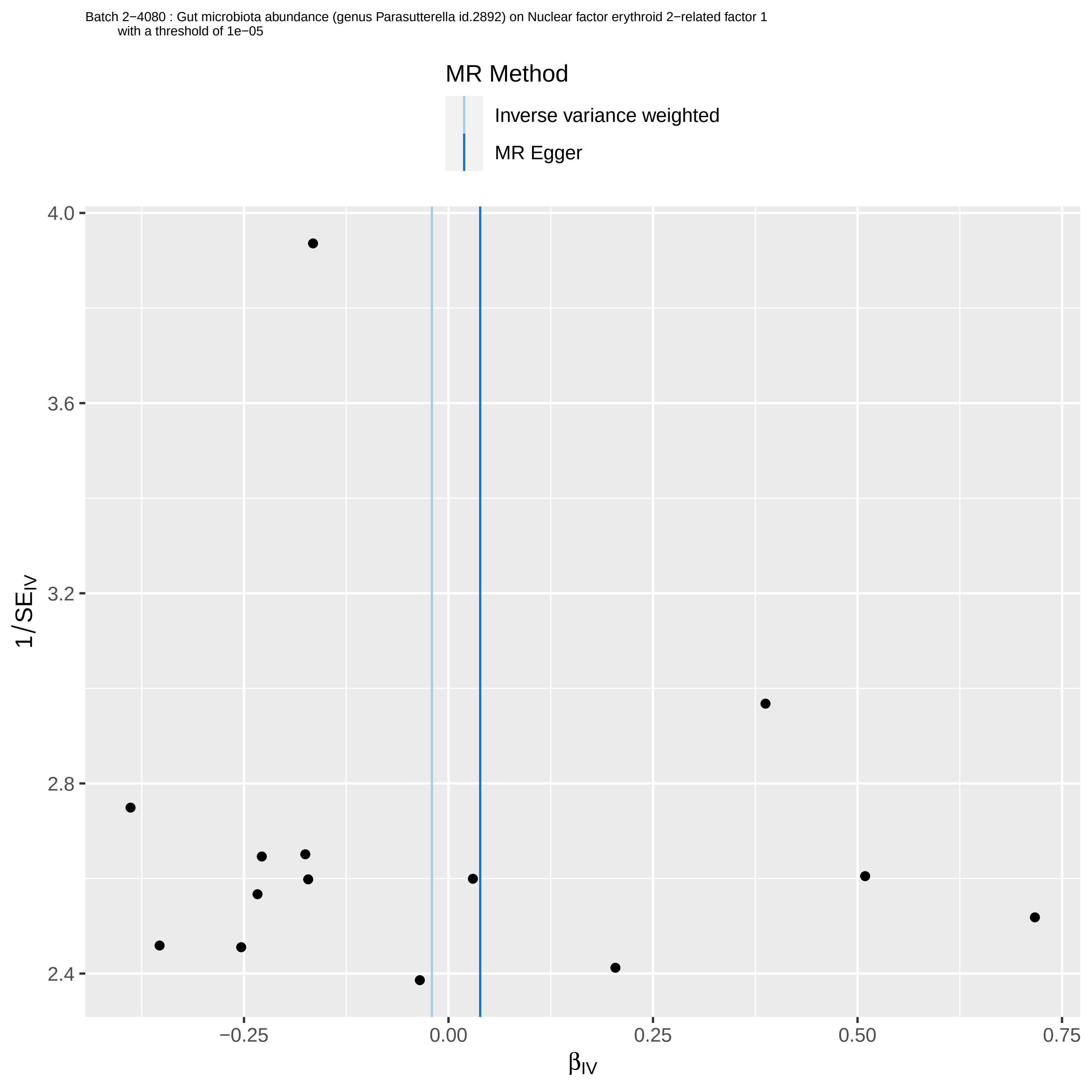
Batch 2−4080 : Gut microbiota abundance (genus Parasutterella id.2892) on Nuclear factor erythroid 2−related factor 1
with a threshold of 1e−05
MR Method
Inverse variance weighted
MR Egger
4.0
3.6
3.2
2.8
2.4
1/SE_IV
−0.25
0.00
0.25
0.50
0.75
β_IV

Batch 2−4081 : Gut microbiota abundance (genus Catenibacterium id.2153) on NFU1 iron−sulfur cluster scaffold homolog, mitochondrial
with a threshold of 1e−05

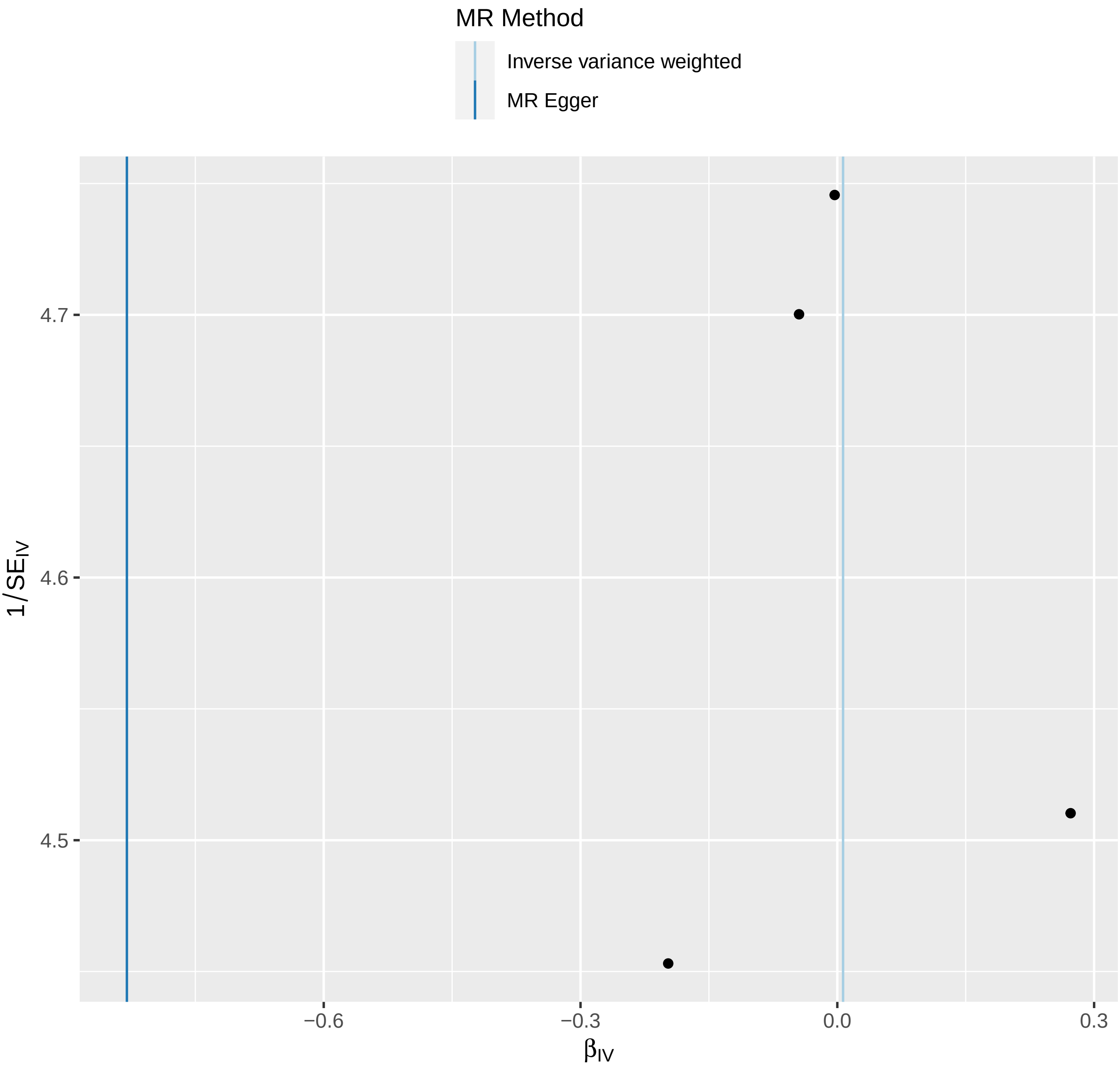

Batch 2−4082 : Gut microbiota abundance (genus Parasutterella id.2892) on NFU1 iron−sulfur cluster scaffold homolog, mitochondrial
with a threshold of 1e−05

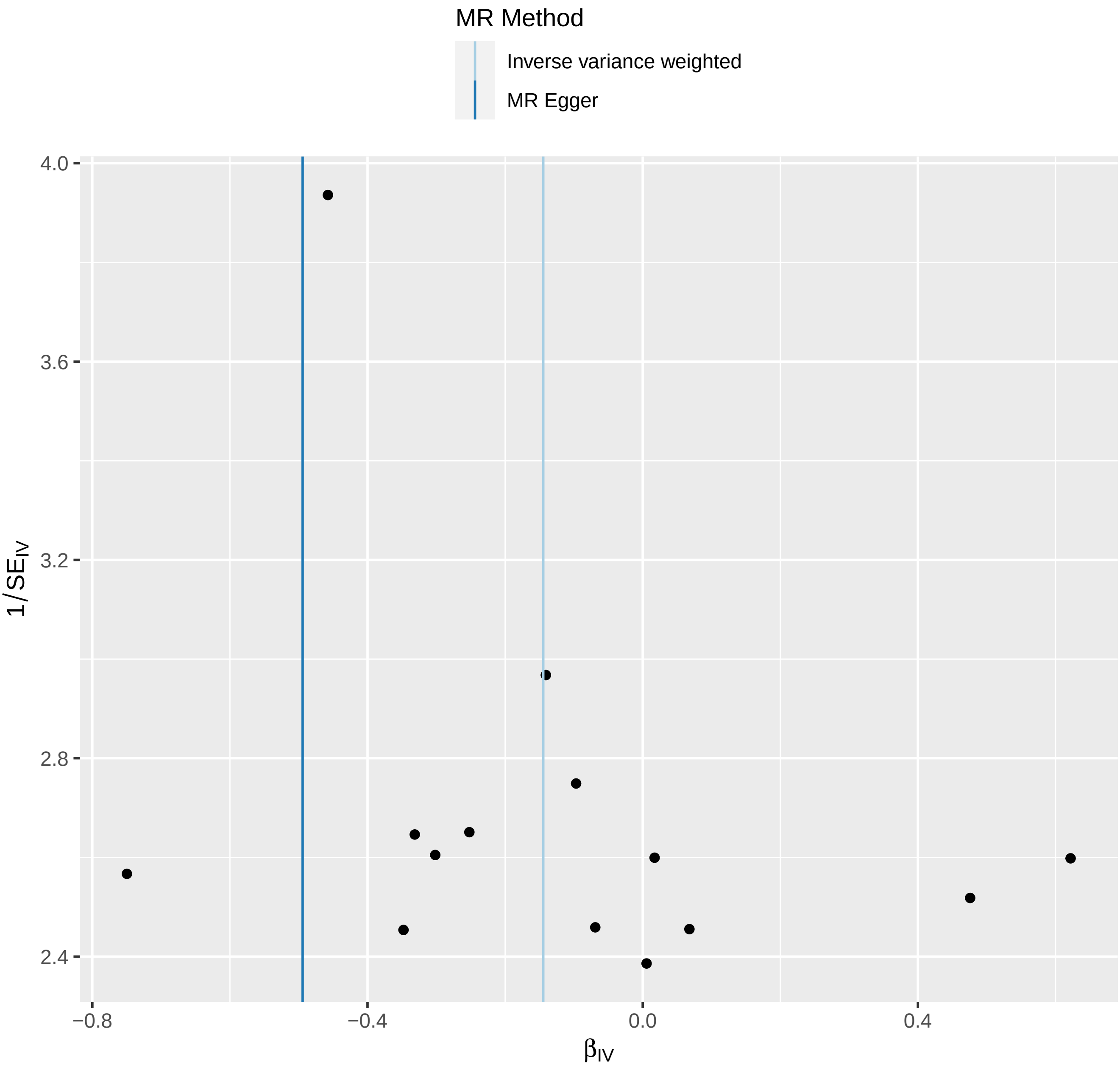

Batch 2−4083 : Gut microbiota abundance (genus Catenibacterium id.2153) on beta−nerve growth factor
with a threshold of 1e−05

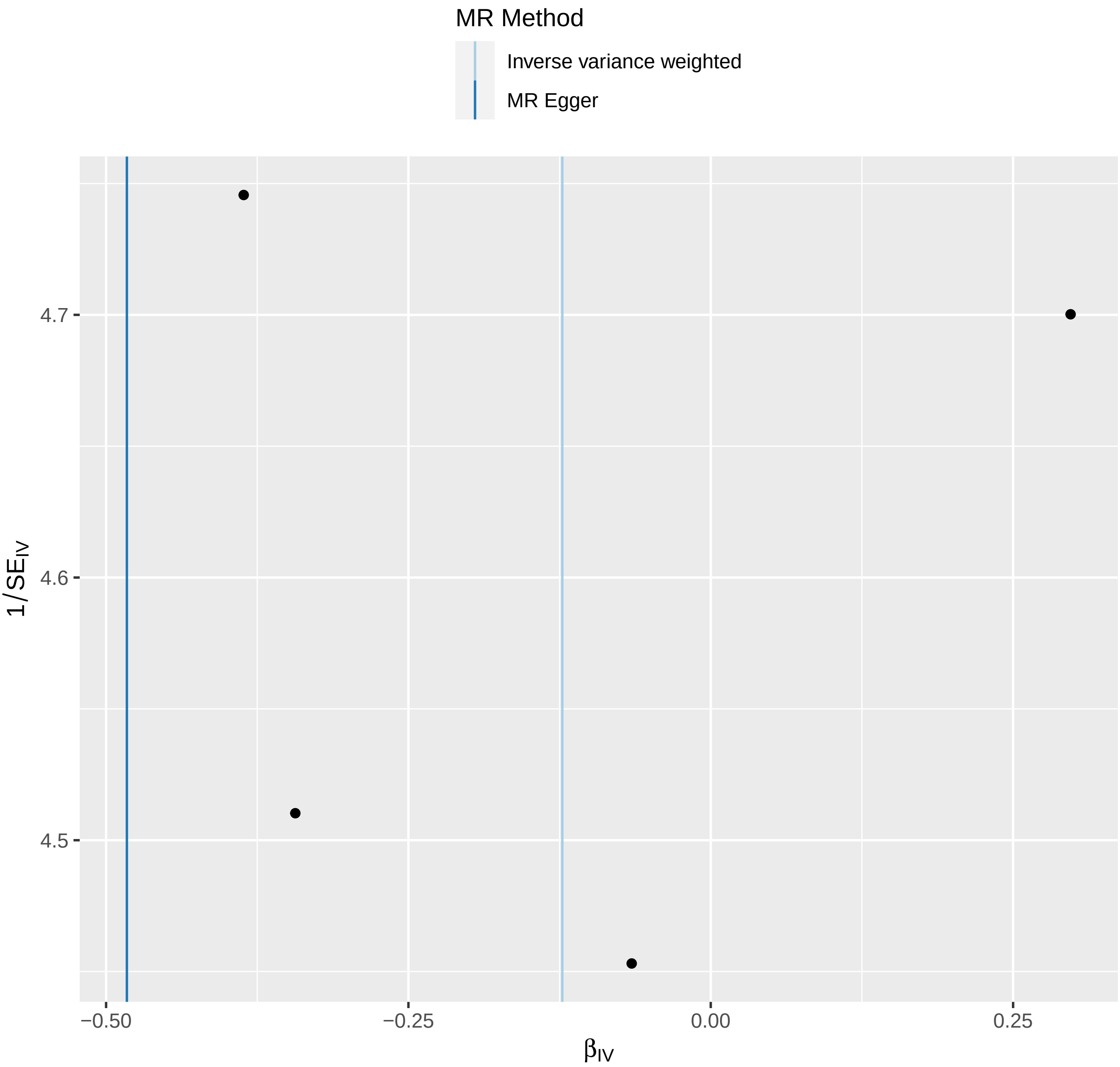

Batch 2−4084 : Gut microbiota abundance (genus Parasutterella id.2892) on beta−nerve growth factor with a threshold of 1e−05

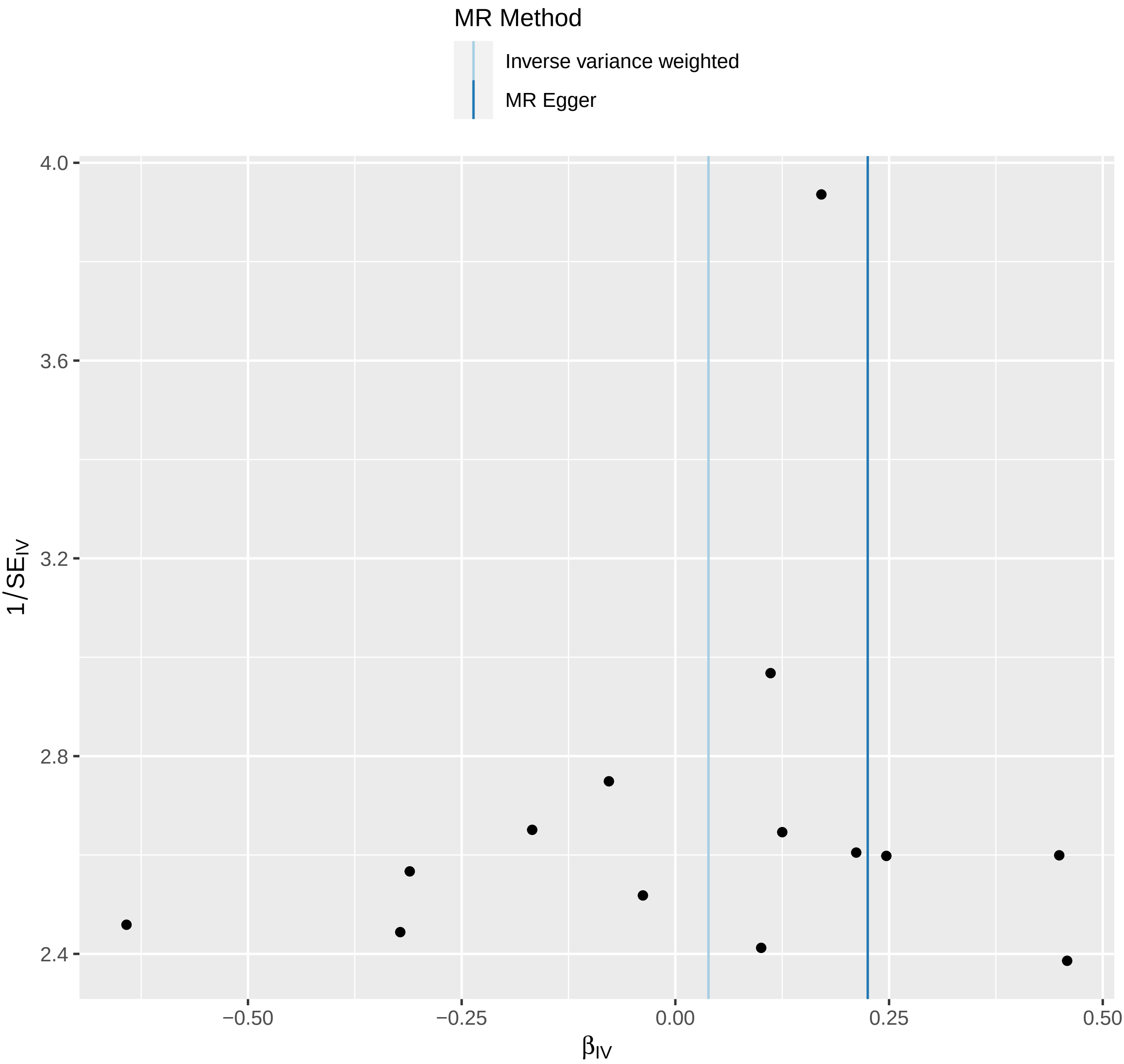

Batch 2−4085 : Gut microbiota abundance (genus Catenibacterium id.2153) on Tumor necrosis factor receptor superfamily member 16
with a threshold of 1e−05

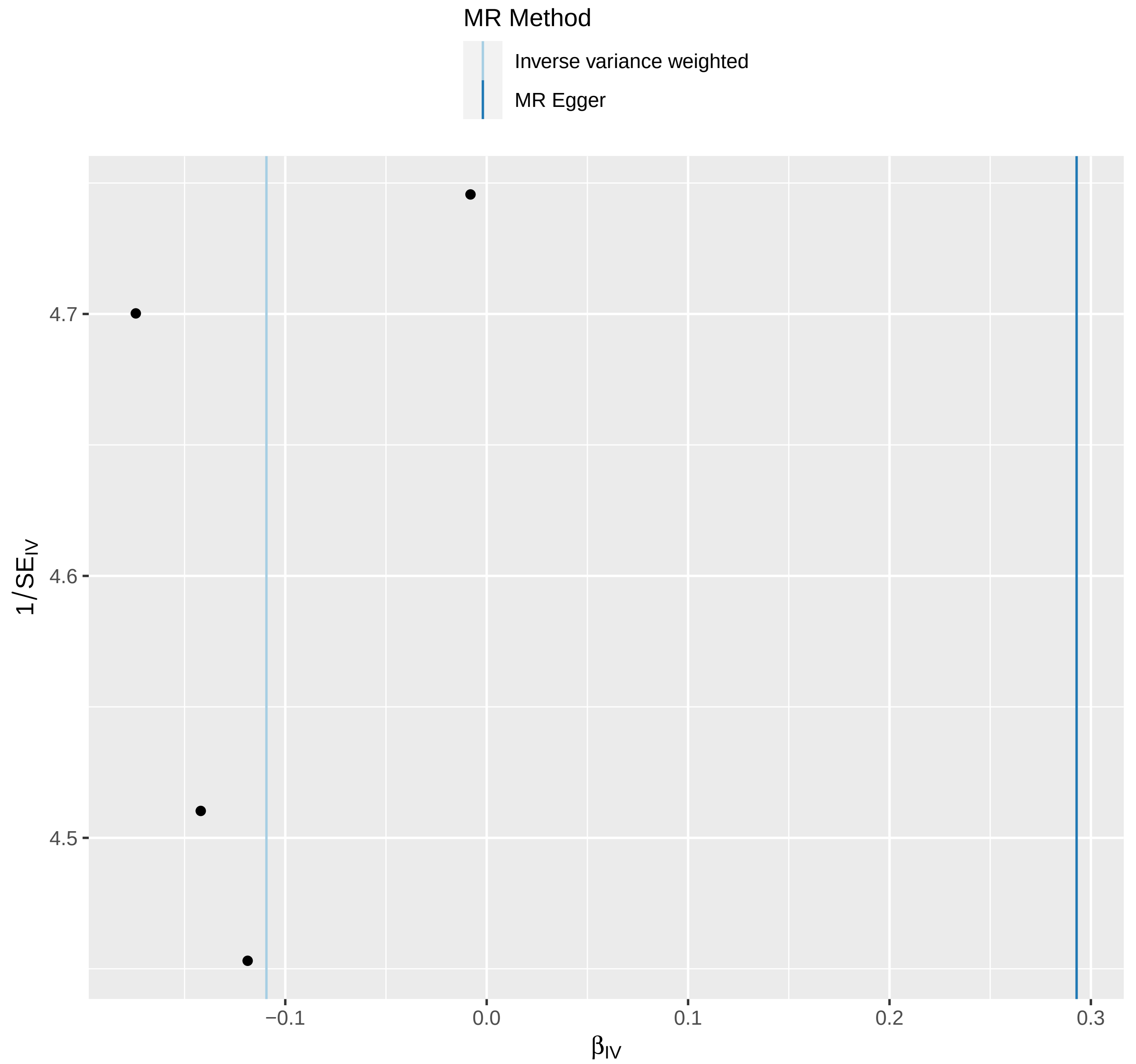

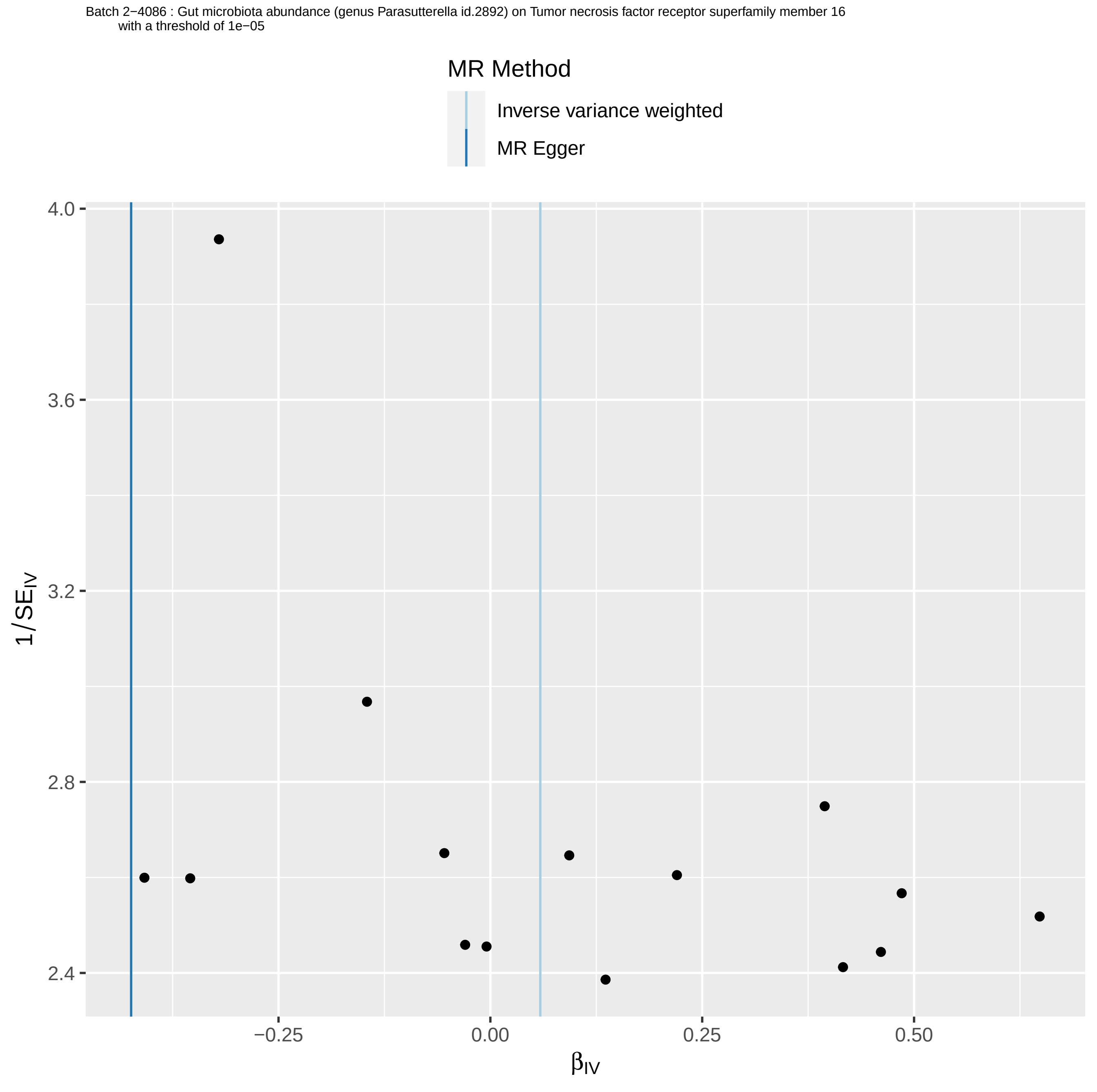
Batch 2−4086 : Gut microbiota abundance (genus Parasutterella id.2892) on Tumor necrosis factor receptor superfamily member 16
with a threshold of 1e−05
MR Method
Inverse variance weighted
MR Egger
4.0
3.6
3.2
2.8
2.4
1/SE$_{IV}$
−0.25
0.00
0.25
0.50
β$_{IV}$

Batch 2−4087 : Gut microbiota abundance (genus Catenibacterium id.2153) on Tumor necrosis factor receptor superfamily member 16
with a threshold of 1e−05

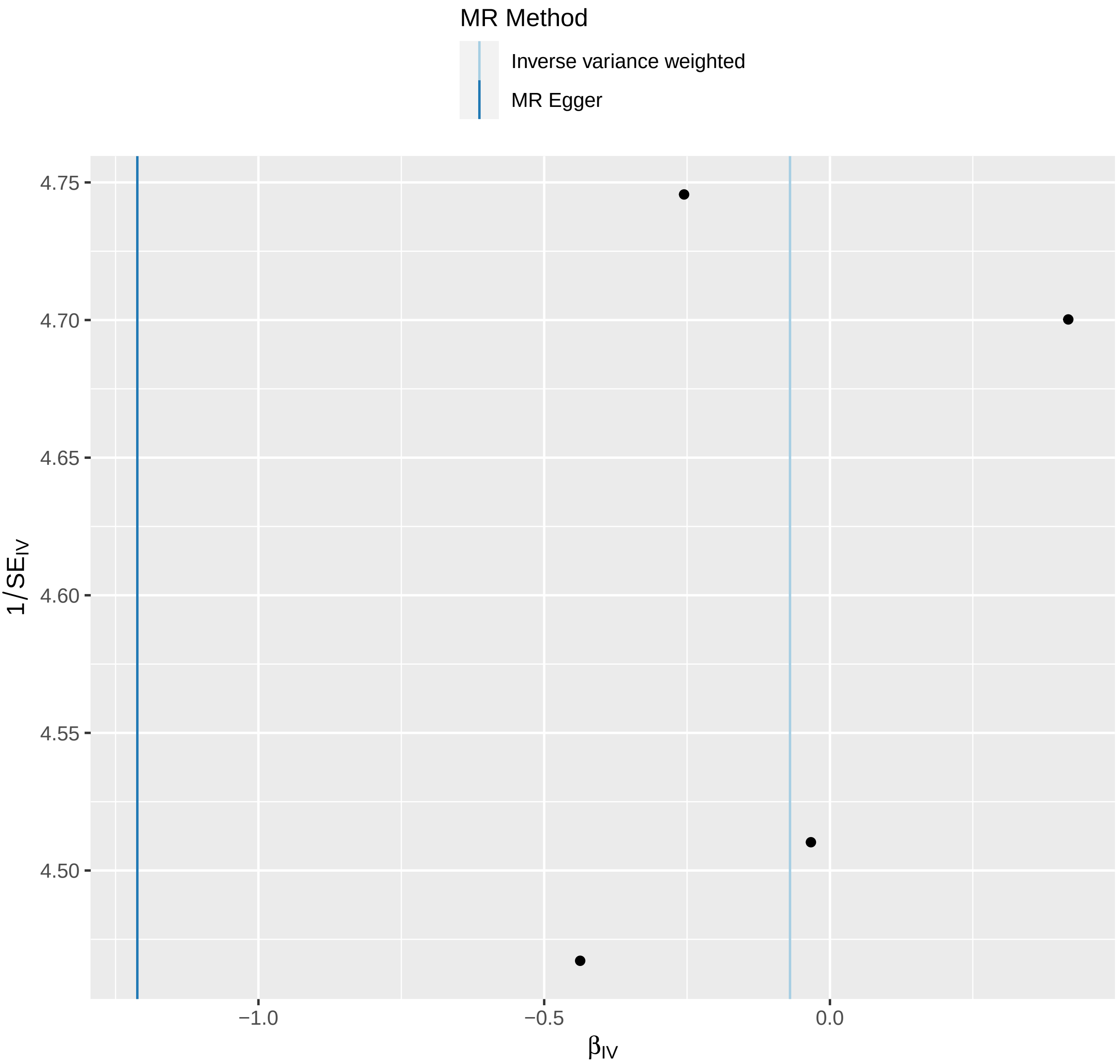

Batch 2−4088 : Gut microbiota abundance (genus Parasutterella id.2892) on Tumor necrosis factor receptor superfamily member 16
with a threshold of 1e−05

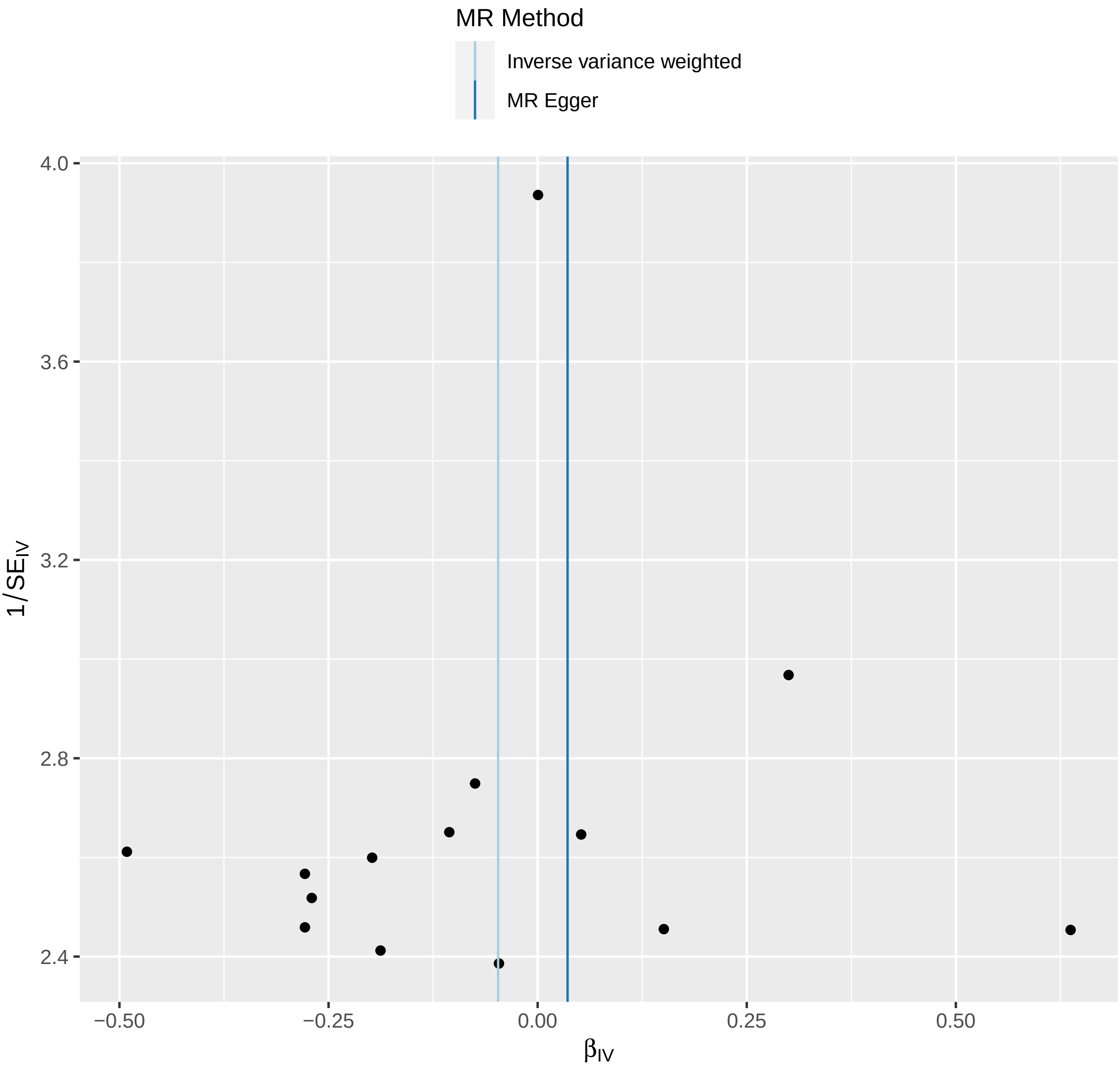

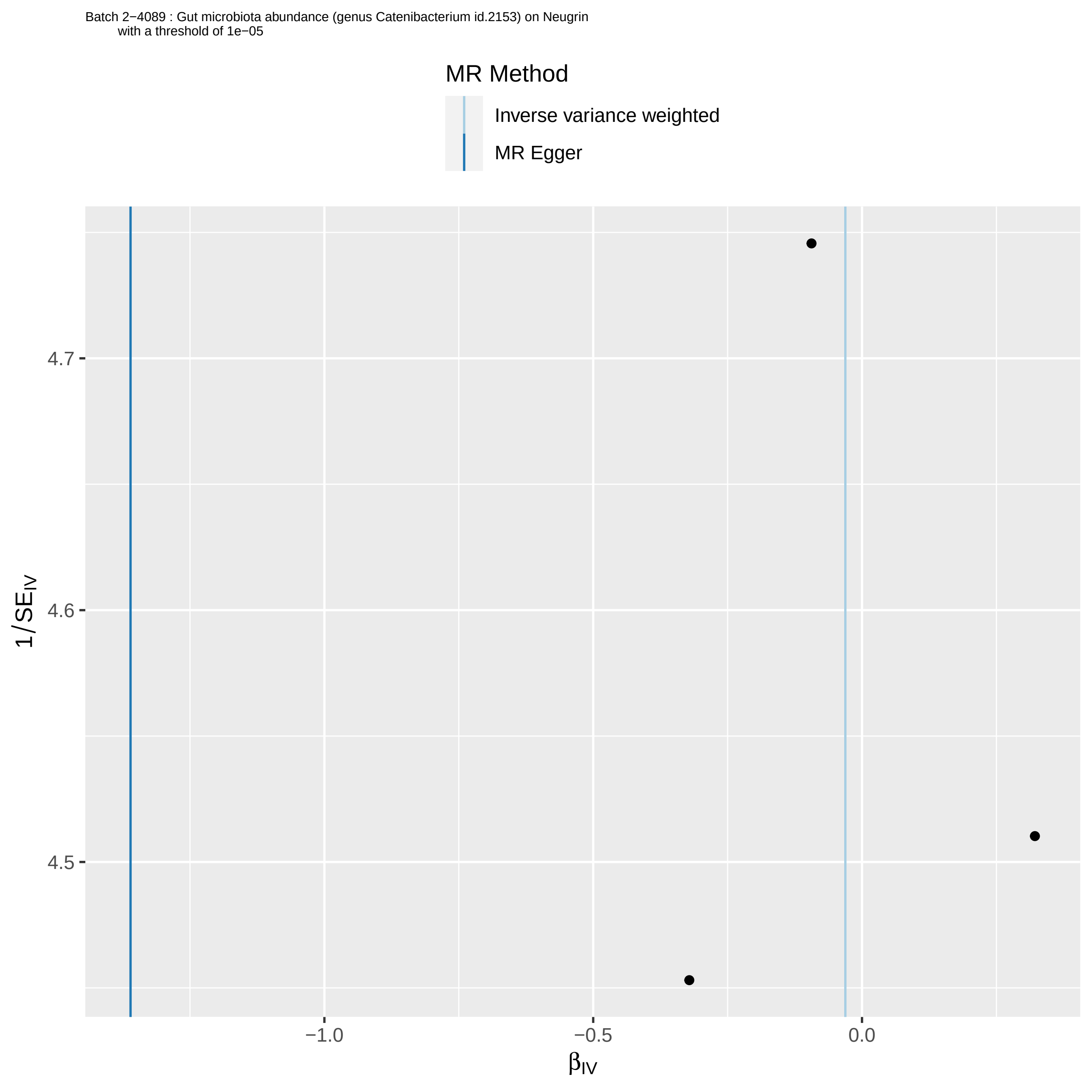
Batch 2−4089 : Gut microbiota abundance (genus Catenibacterium id.2153) on Neugrin
with a threshold of 1e−05
MR Method
Inverse variance weighted
MR Egger
4.7
4.6
4.5
1/SE$_{IV}$
−1.0
−0.5
0.0
β$_{IV}$

Batch 2−4090 : Gut microbiota abundance (genus Parasutterella id.2892) on Neugrin
with a threshold of 1e−05

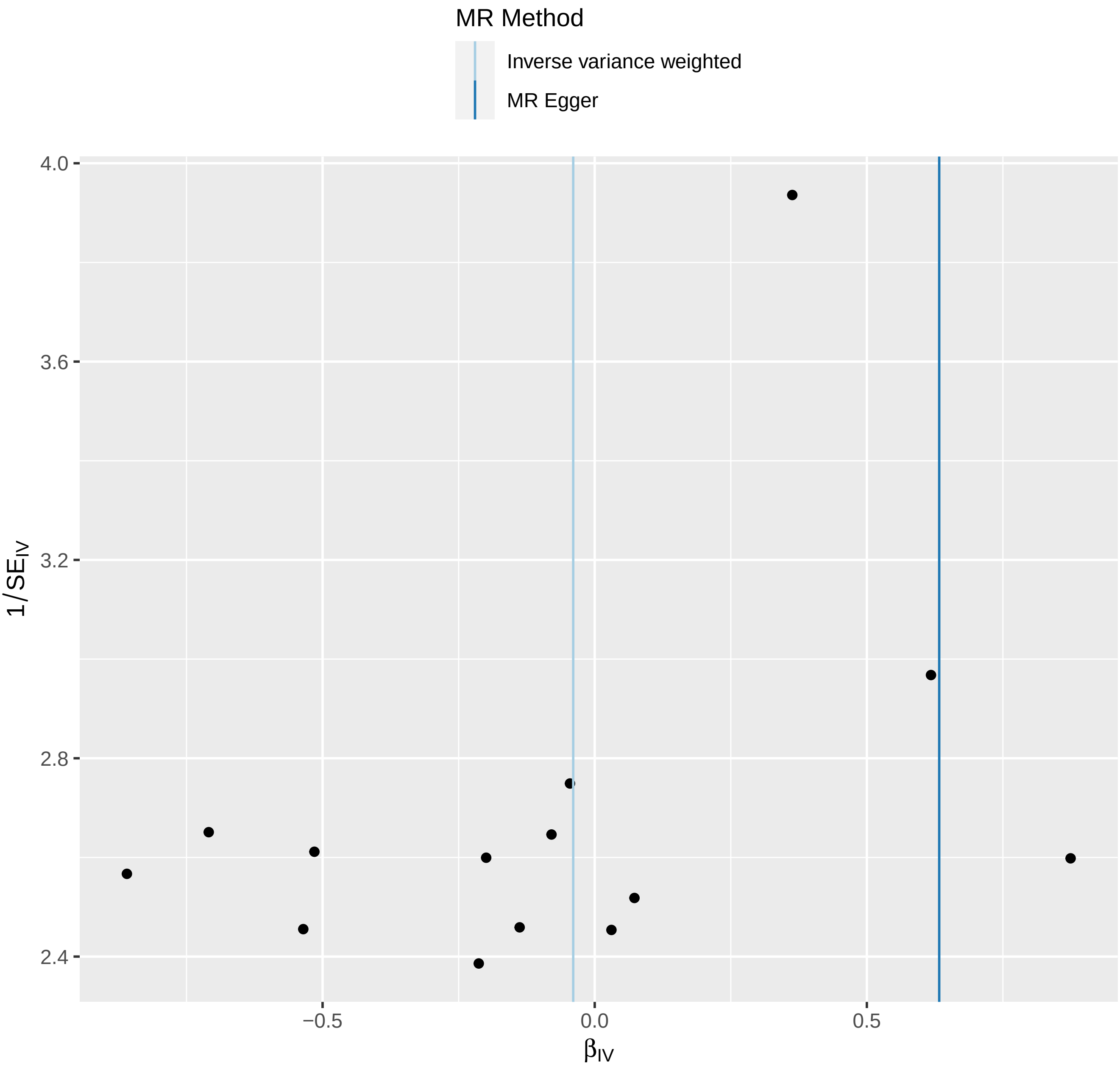

Batch 2−4091 : Gut microbiota abundance (genus Catenibacterium id.2153) on Non−homologous end−joining factor 1
with a threshold of 1e−05

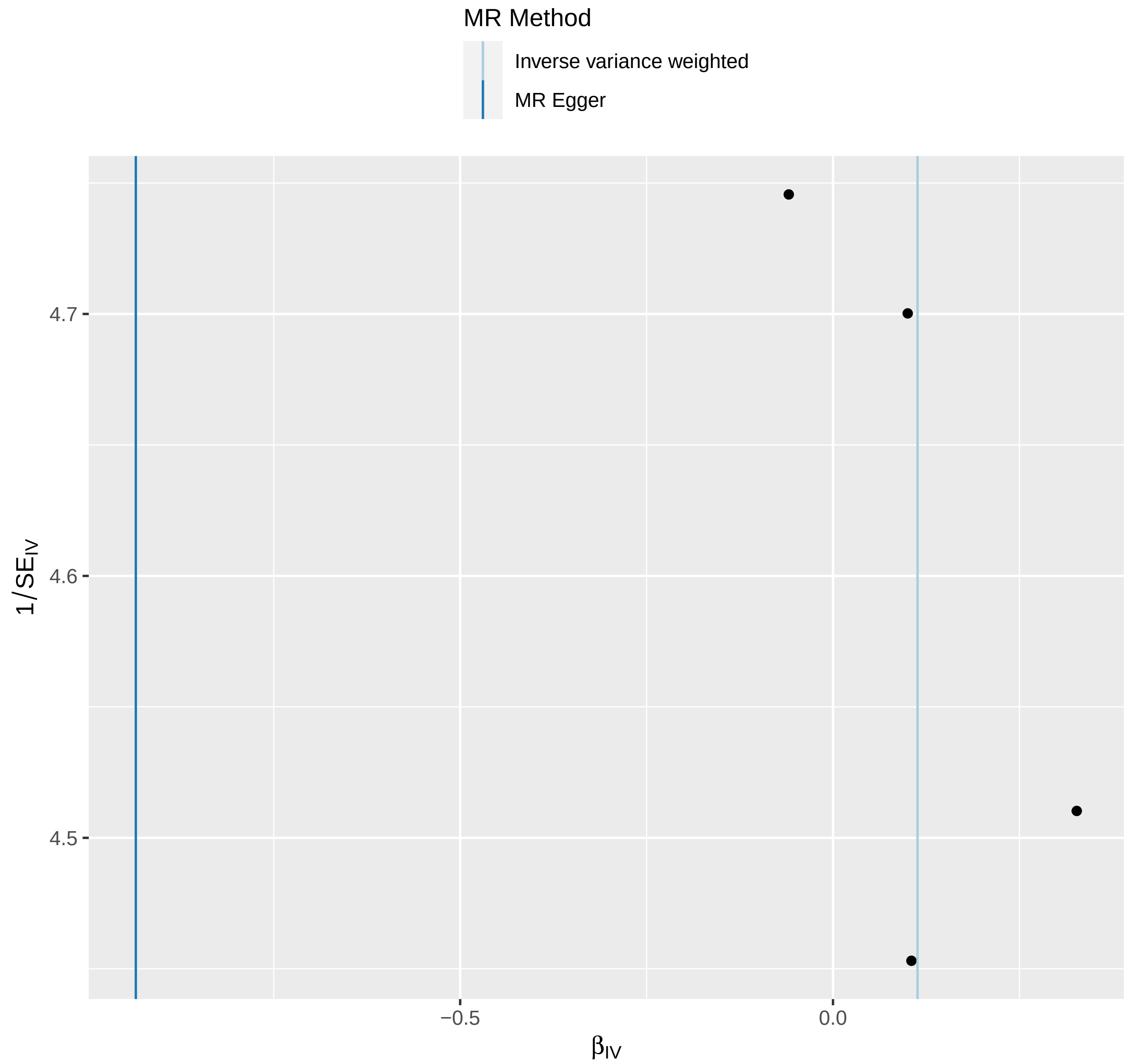

Batch 2−4092 : Gut microbiota abundance (genus Parasutterella id.2892) on Non−homologous end−joining factor 1
with a threshold of 1e−05

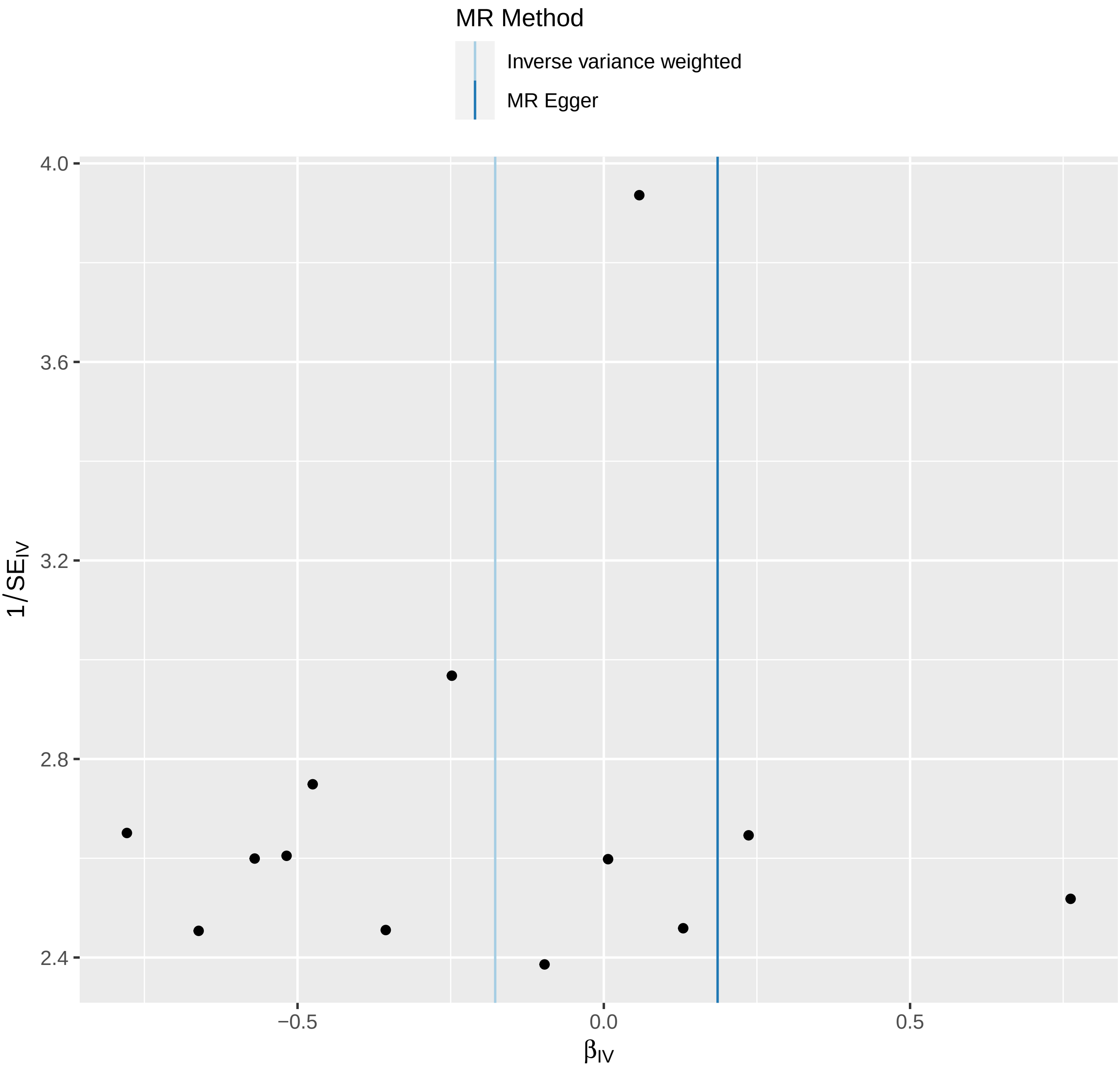

Batch 2−4093 : Gut microbiota abundance (genus Catenibacterium id.2153) on NHL repeat−containing protein 3
with a threshold of 1e−05

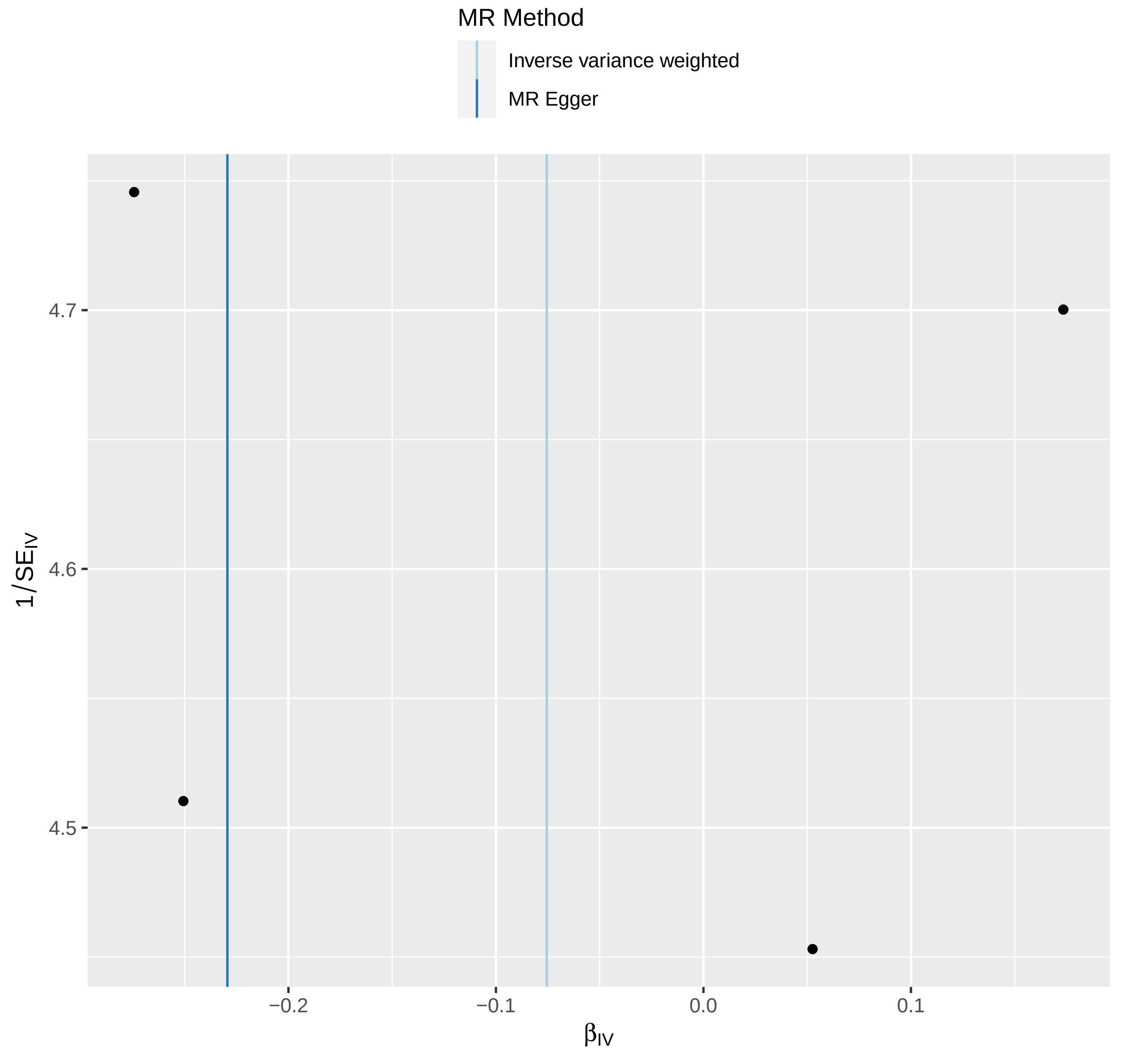

Batch 2−4094 : Gut microbiota abundance (genus Parasutterella id.2892) on NHL repeat−containing protein 3
with a threshold of 1e−05

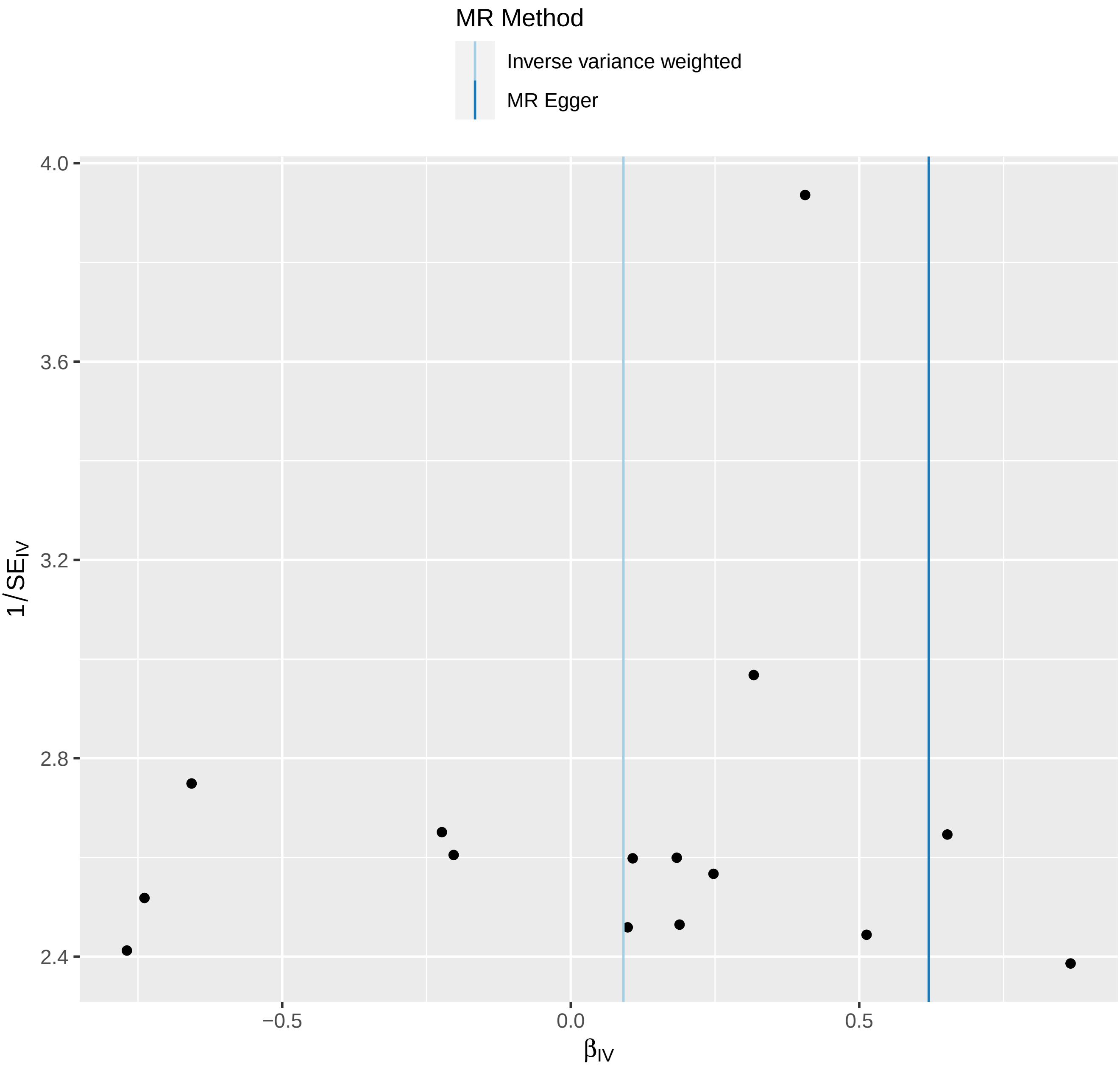

Batch 2−4095 : Gut microbiota abundance (genus Catenibacterium id.2153) on NHP2−like protein 1
with a threshold of 1e−05

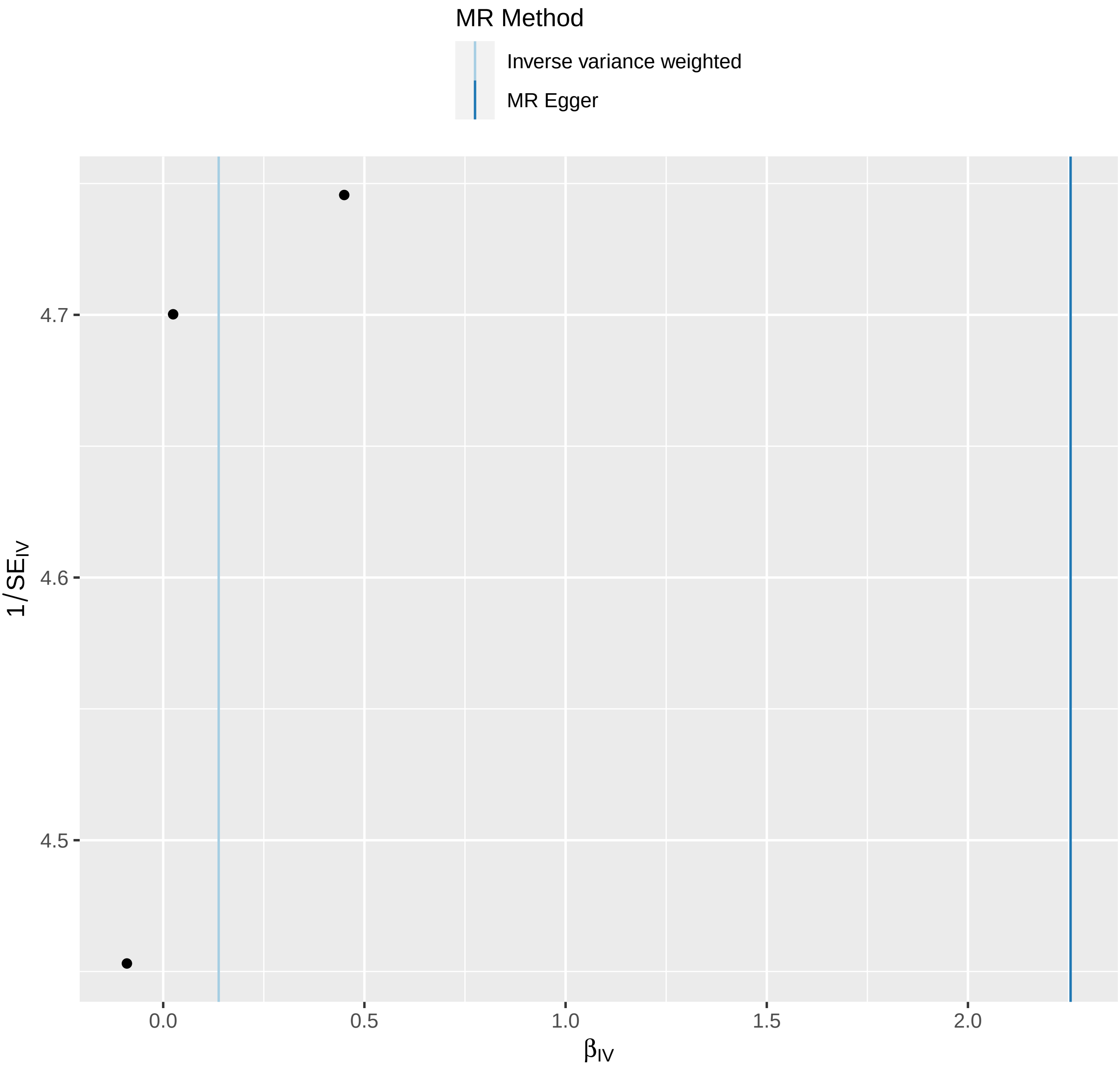

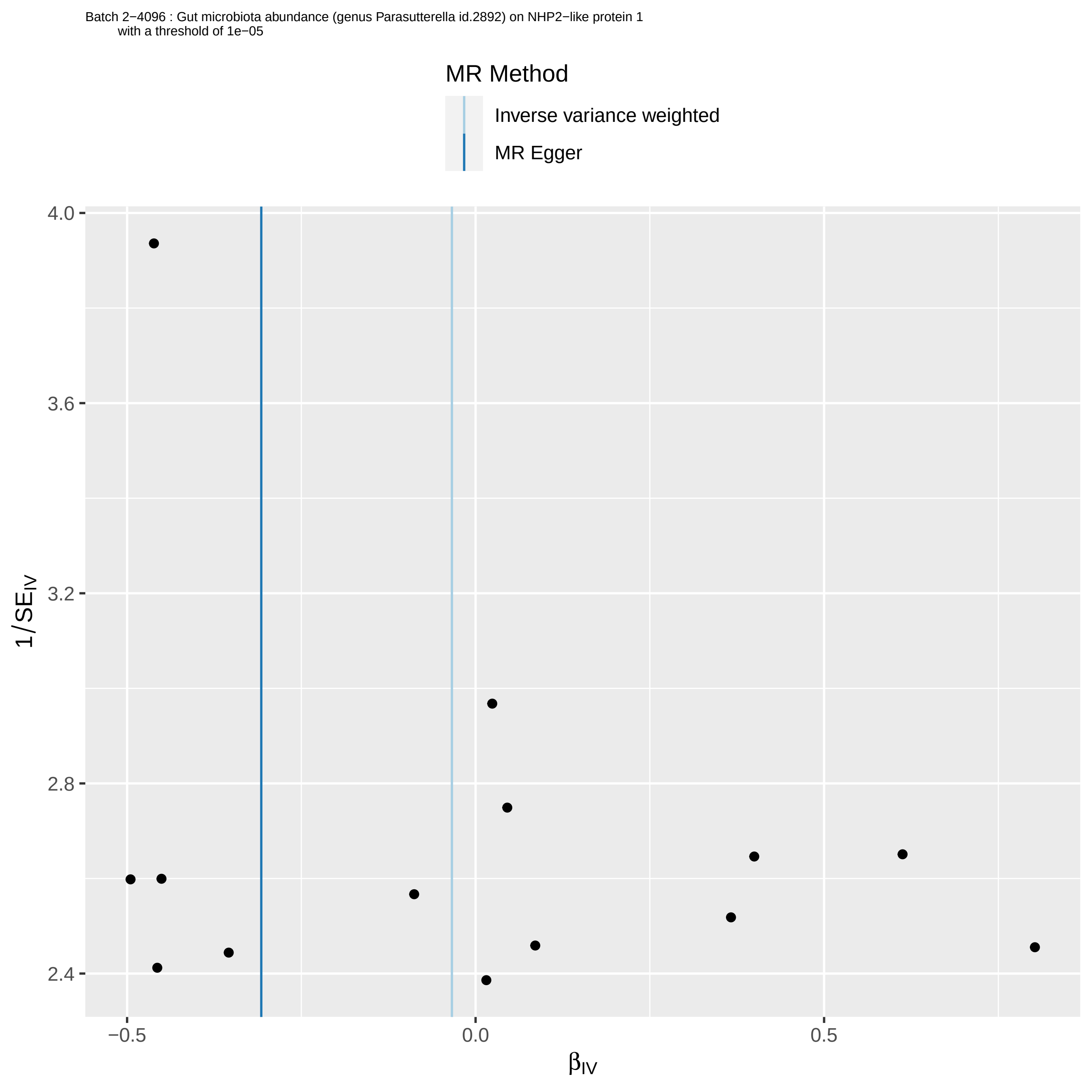

Batch 2−4096 : Gut microbiota abundance (genus Parasutterella id.2892) on NHP2−like protein 1
with a threshold of 1e−05
MR Method
Inverse variance weighted
MR Egger
4.0
3.6
3.2
2.8
2.4
1/SE_IV
−0.5
0.0
0.5
β_IV

Batch 2−4097 : Gut microbiota abundance (genus Catenibacterium id.2153) on Nidogen−1
with a threshold of 1e−05

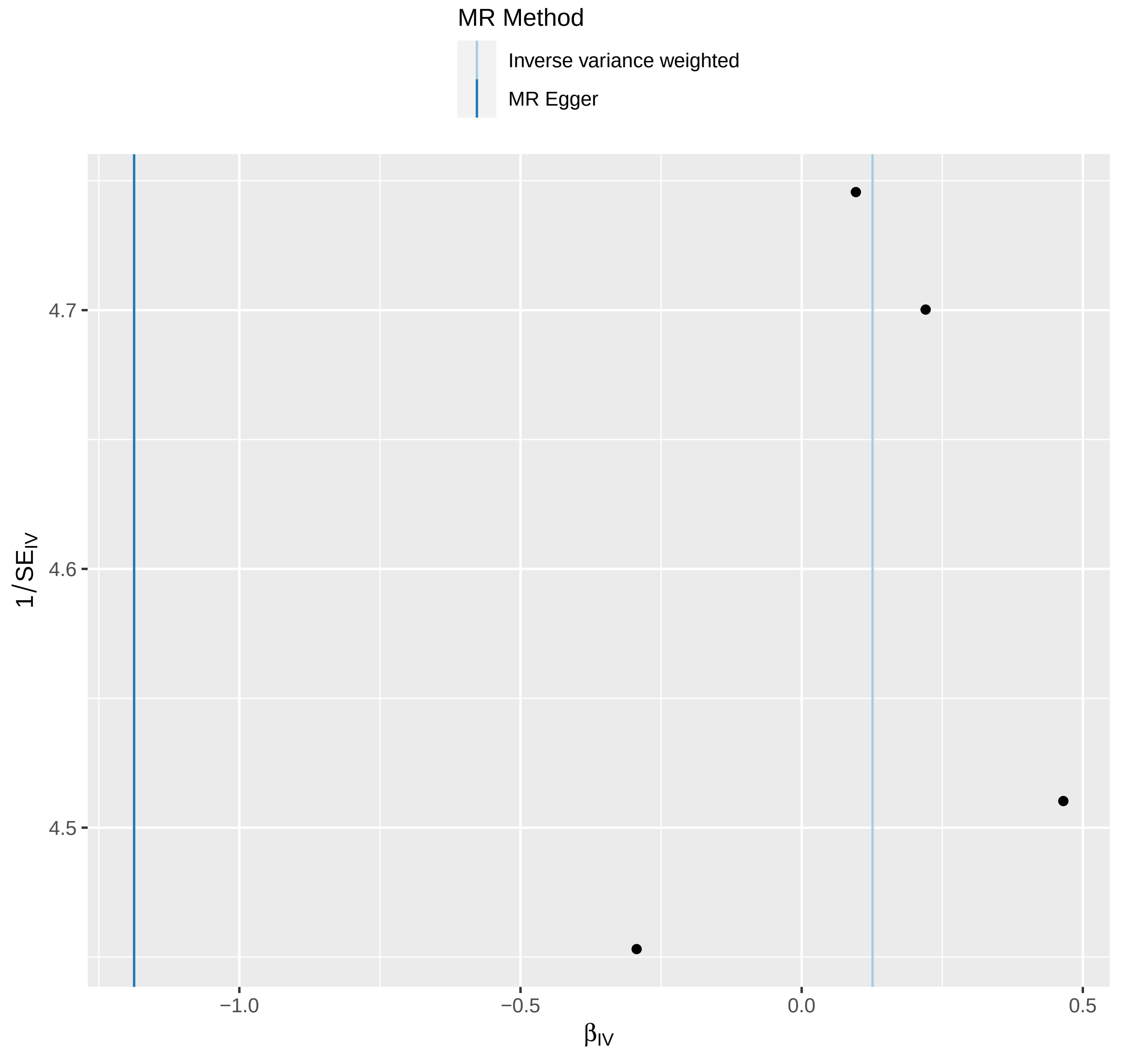

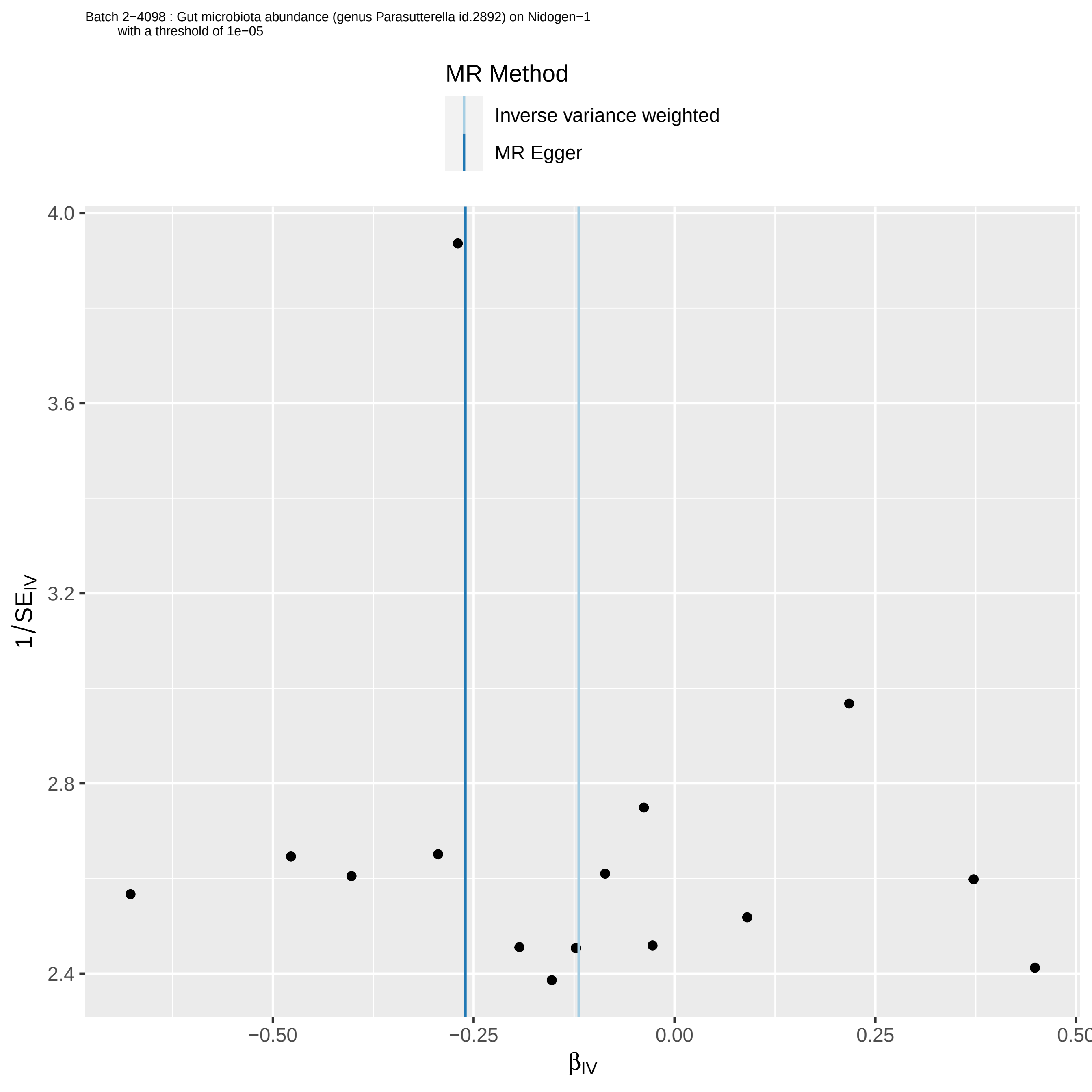

Batch 2−4098 : Gut microbiota abundance (genus Parasutterella id.2892) on Nidogen−1
with a threshold of 1e−05
MR Method
Inverse variance weighted
MR Egger
4.0
3.6
3.2
2.8
2.4
1/SE_IV
−0.50
−0.25
0.00
0.25
0.50
β_IV

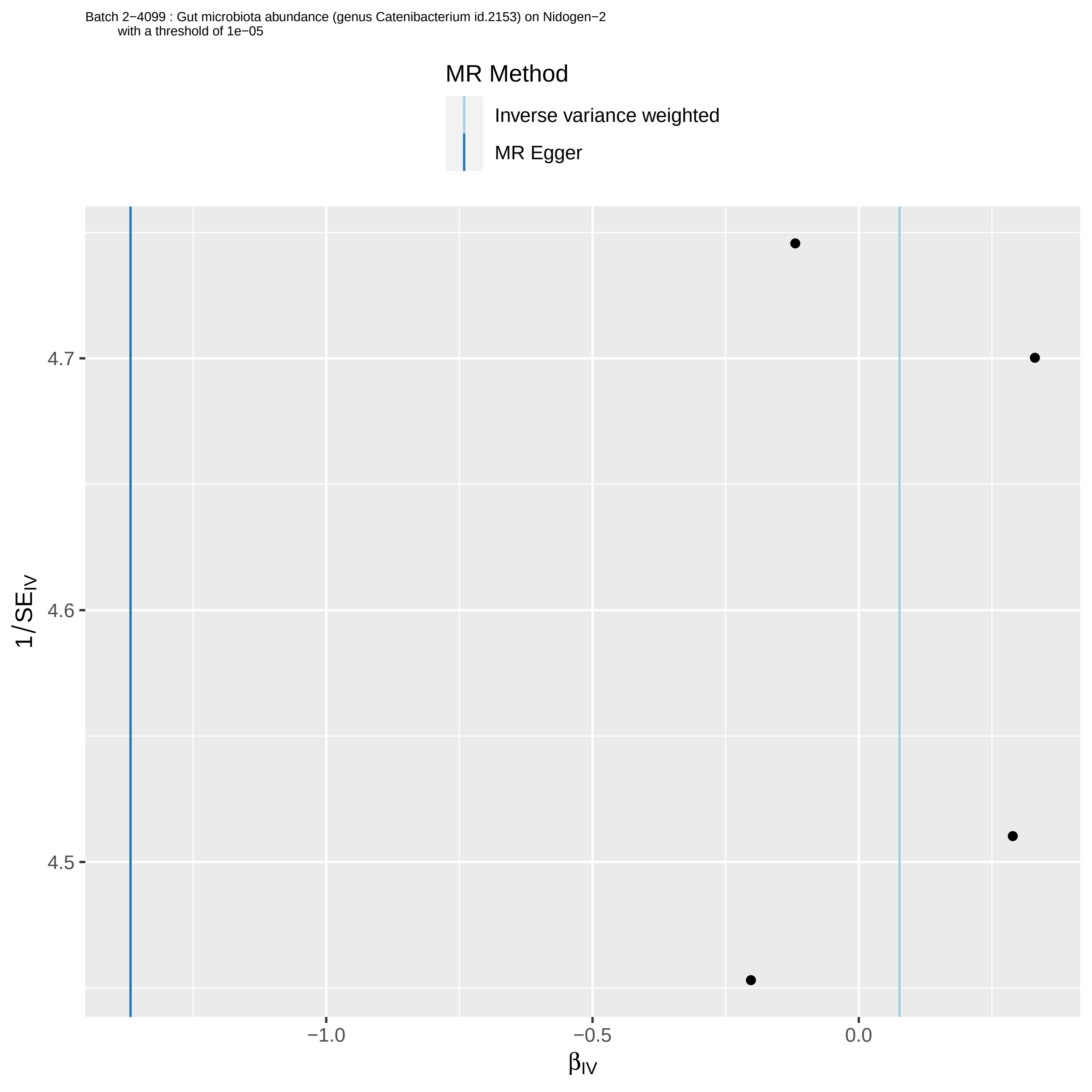
Batch 2−4099 : Gut microbiota abundance (genus Catenibacterium id.2153) on Nidogen−2
with a threshold of 1e−05
MR Method
Inverse variance weighted
MR Egger
4.7
4.6
4.5
1/SE_IV
−1.0
−0.5
0.0
β_IV

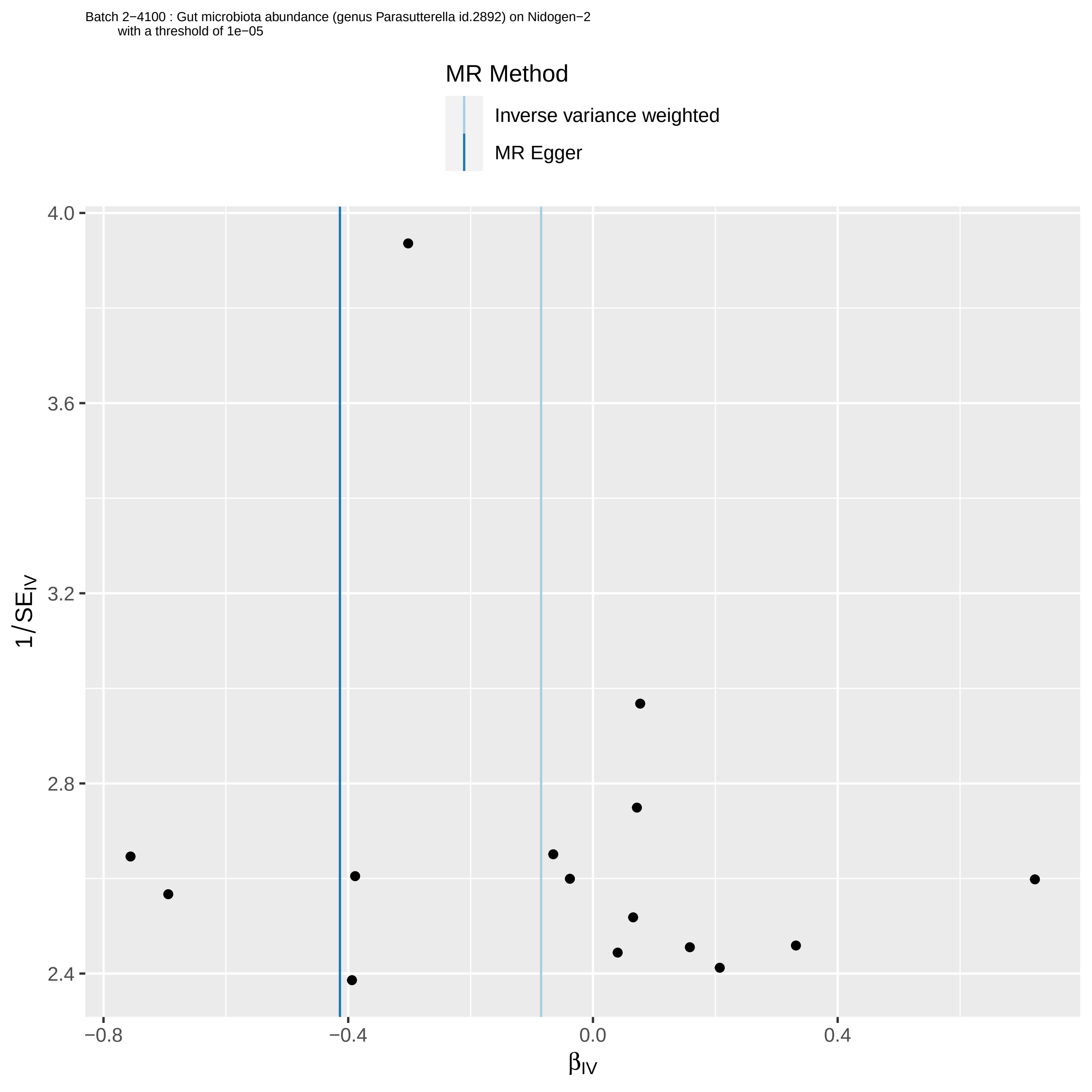

Batch 2−4100 : Gut microbiota abundance (genus Parasutterella id.2892) on Nidogen−2
with a threshold of 1e−05
MR Method
Inverse variance weighted
MR Egger
4.0
3.6
3.2
2.8
2.4
1/SE_IV
−0.8
−0.4
0.0
0.4
β_IV

Batch 2−4101 : Gut microbiota abundance (genus Catenibacterium id.2153) on Magnesium transporter NIPA4
with a threshold of 1e−05

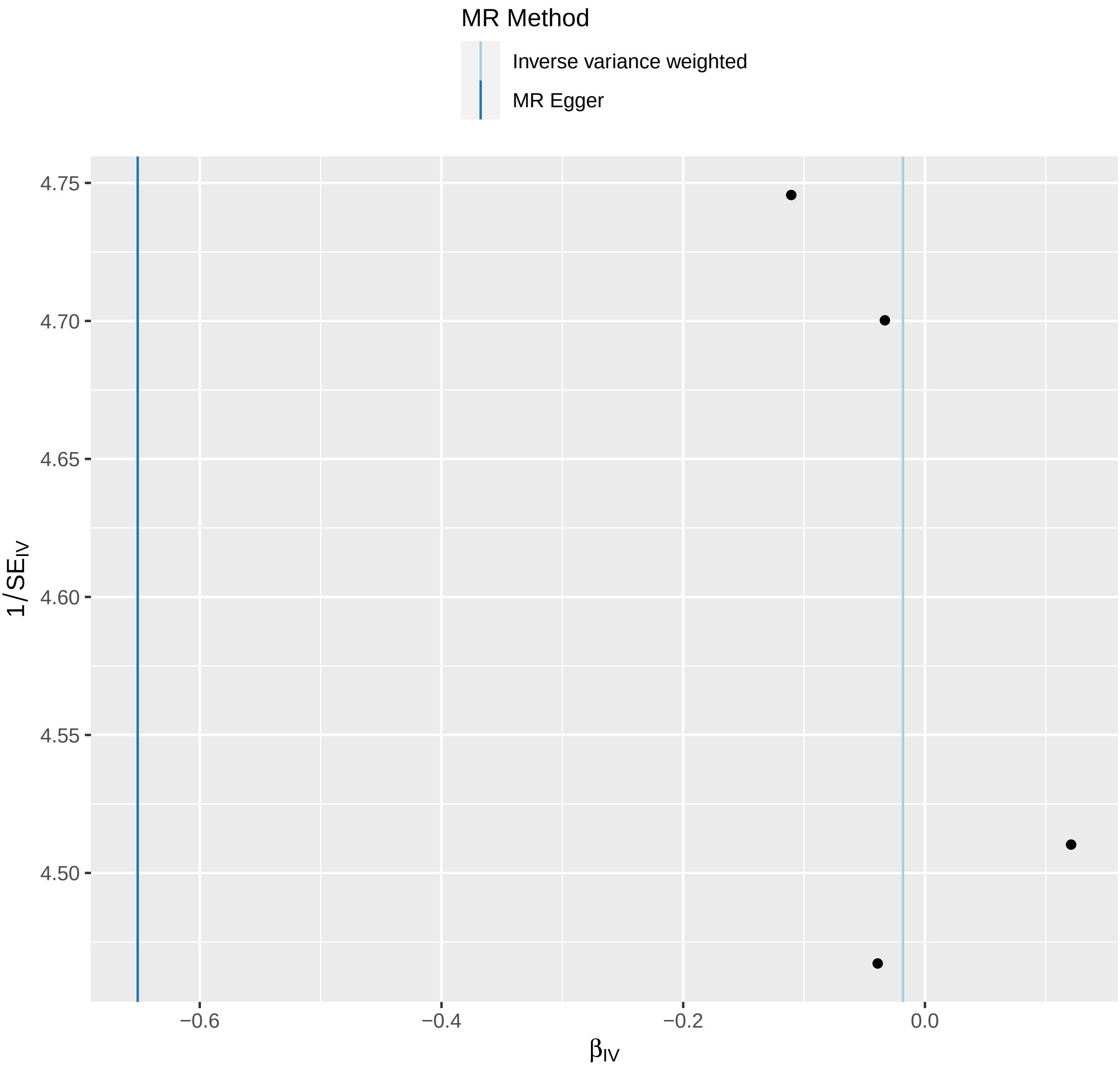

Batch 2−4102 : Gut microbiota abundance (genus Parasutterella id.2892) on Magnesium transporter NIPA4
with a threshold of 1e−05

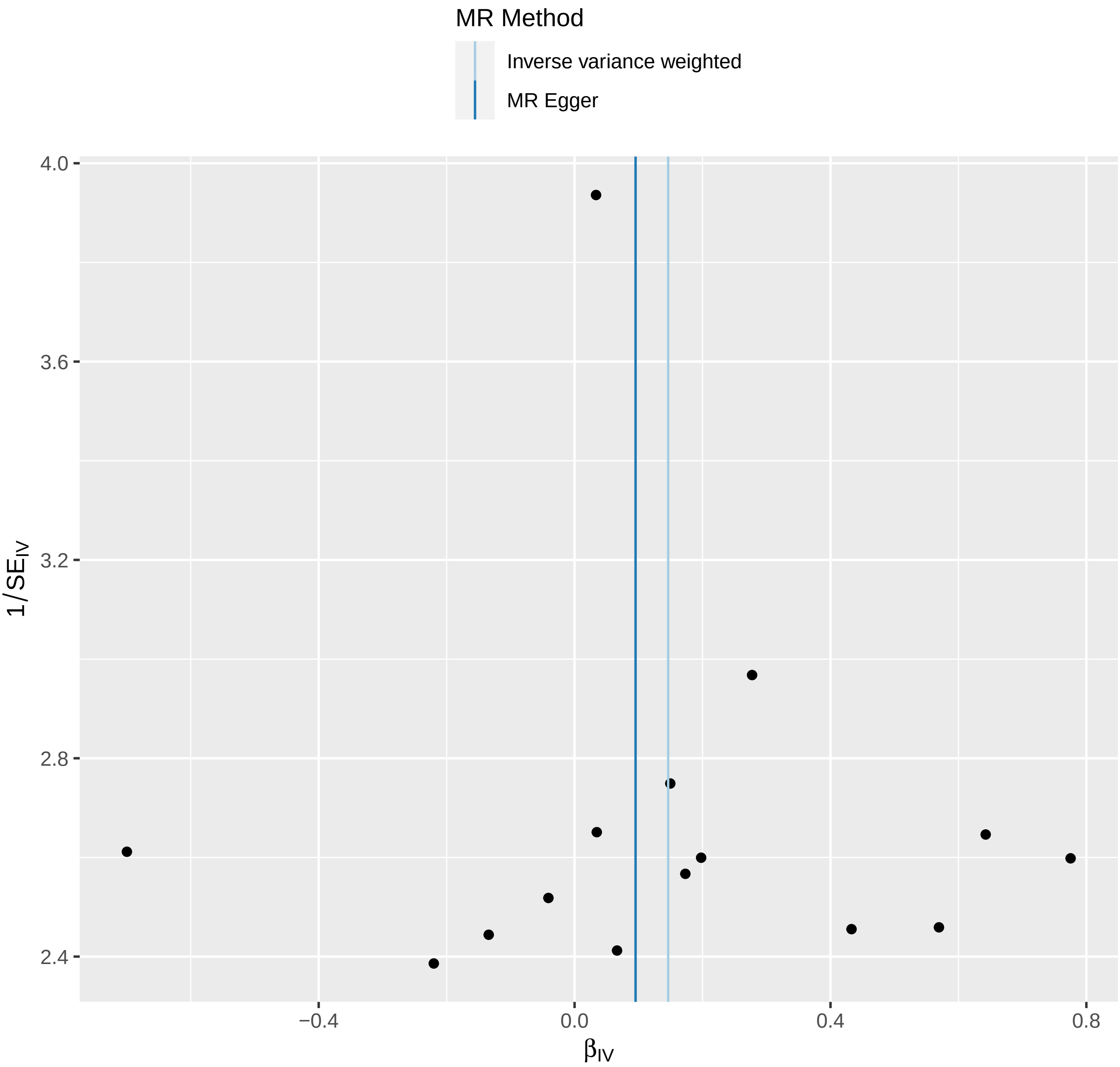

Batch 2−4103 : Gut microbiota abundance (genus Catenibacterium id.2153) on Nischarin
with a threshold of 1e−05

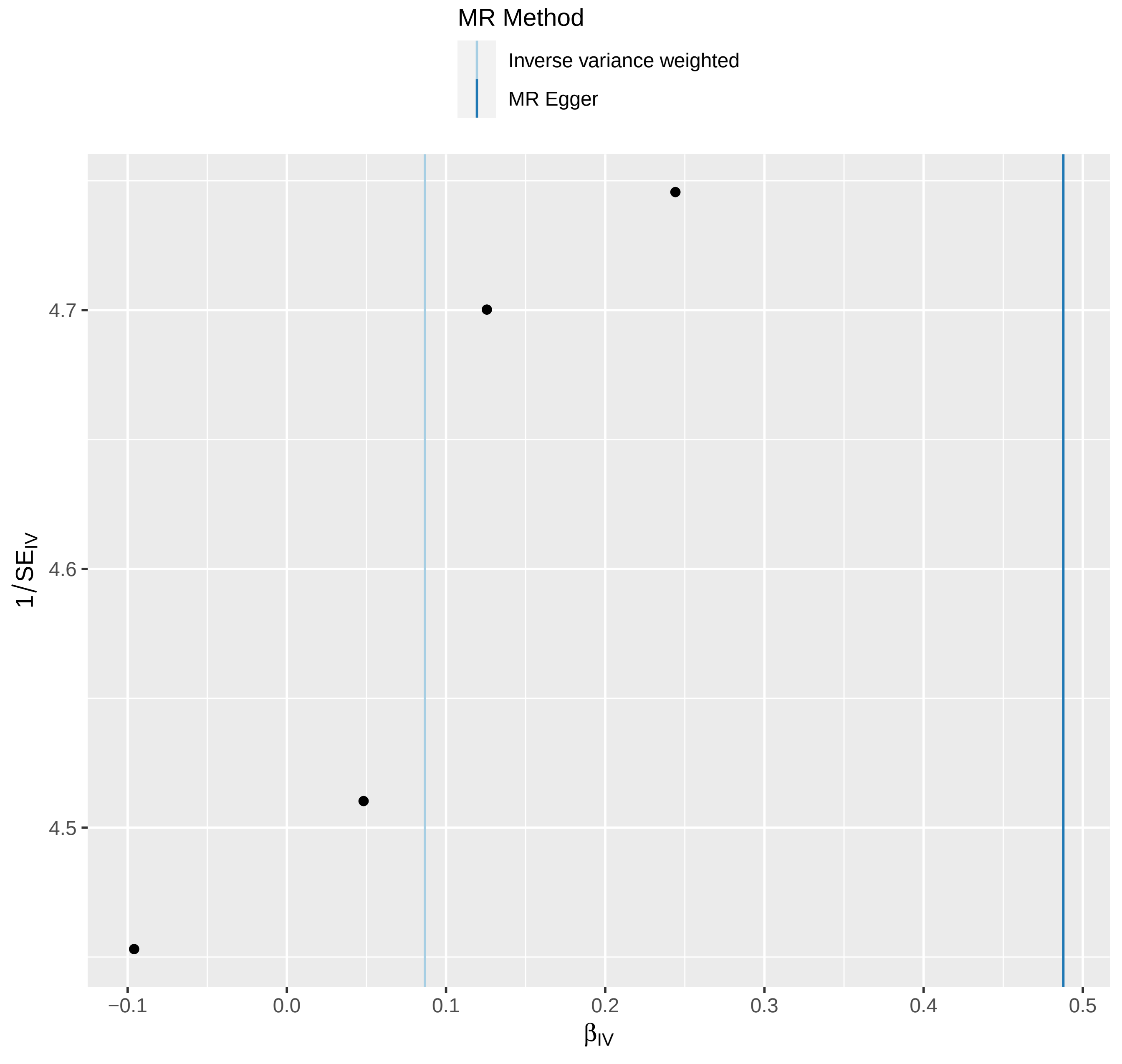

Batch 2−4104 : Gut microbiota abundance (genus Parasutterella id.2892) on Nischarin
with a threshold of 1e−05

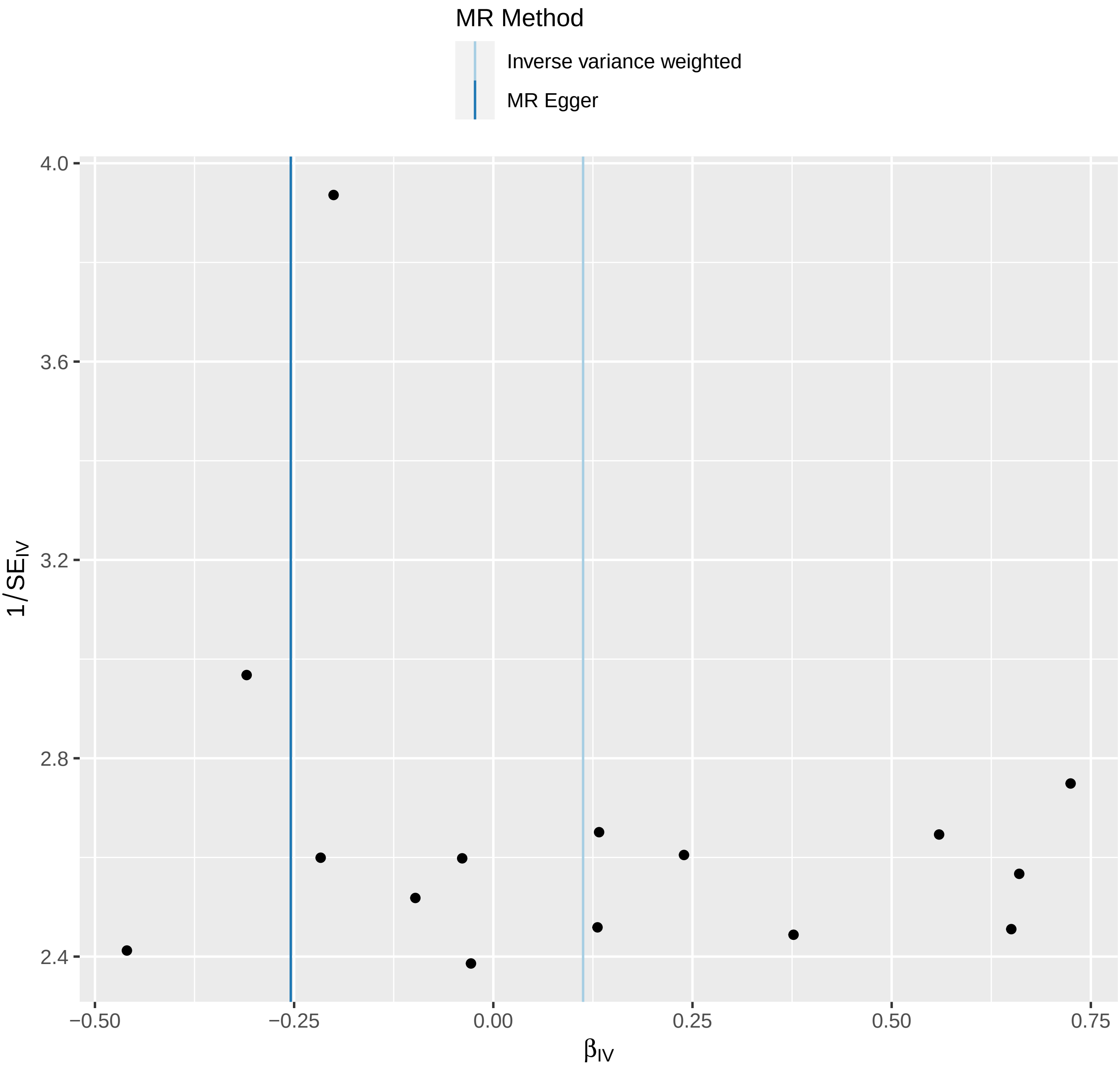

Batch 2−4105 : Gut microbiota abundance (genus Catenibacterium id.2153) on Neuroligin−1
with a threshold of 1e−05

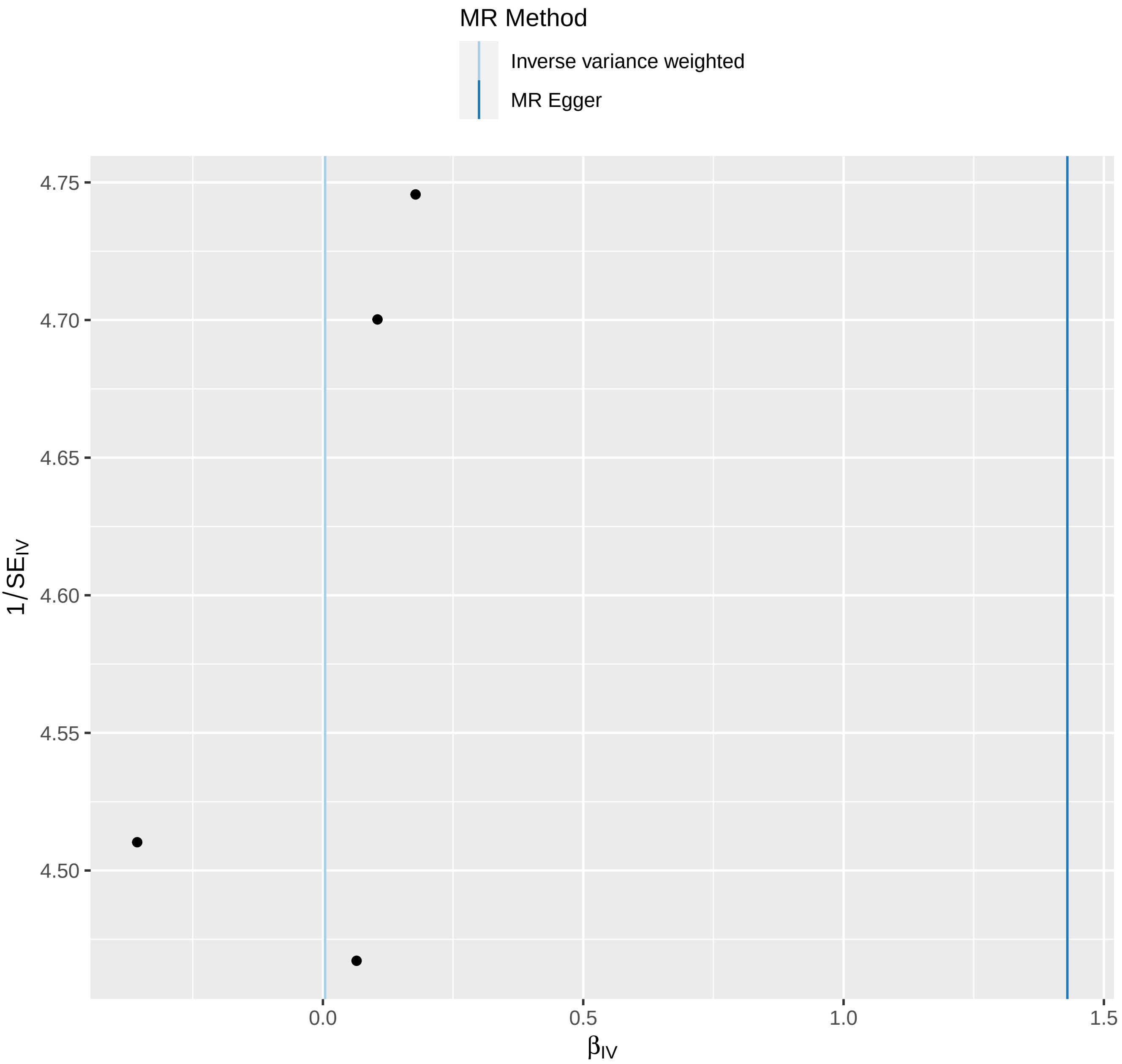

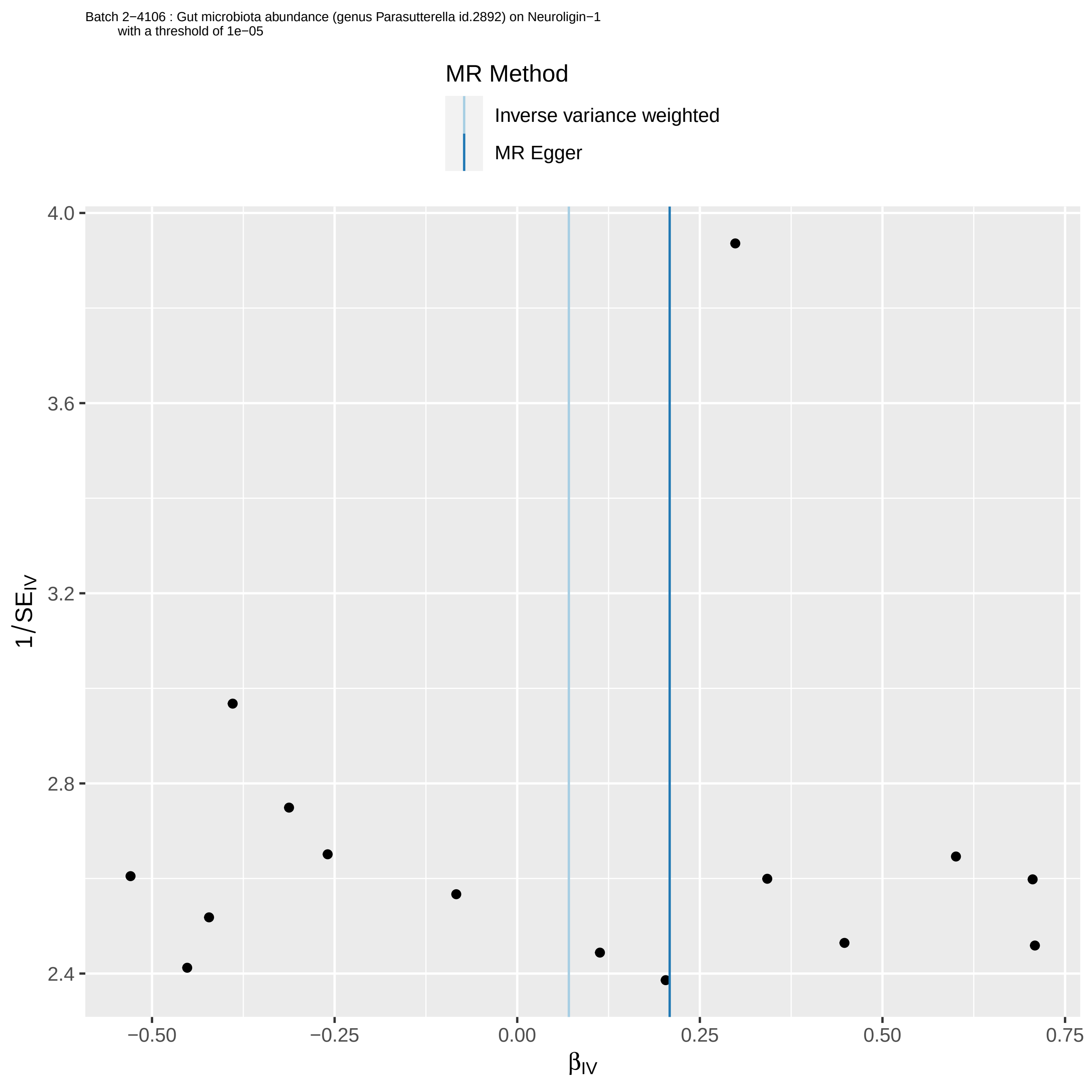

Batch 2−4106 : Gut microbiota abundance (genus Parasutterella id.2892) on Neuroligin−1
with a threshold of 1e−05
MR Method
Inverse variance weighted
MR Egger
4.0
3.6
3.2
2.8
2.4
1/SE_IV
−0.50
−0.25
0.00
0.25
0.50
0.75
β_IV

Batch 2−4107 : Gut microbiota abundance (genus Catenibacterium id.2153) on Neuroligin−4, X−linked
with a threshold of 1e−05

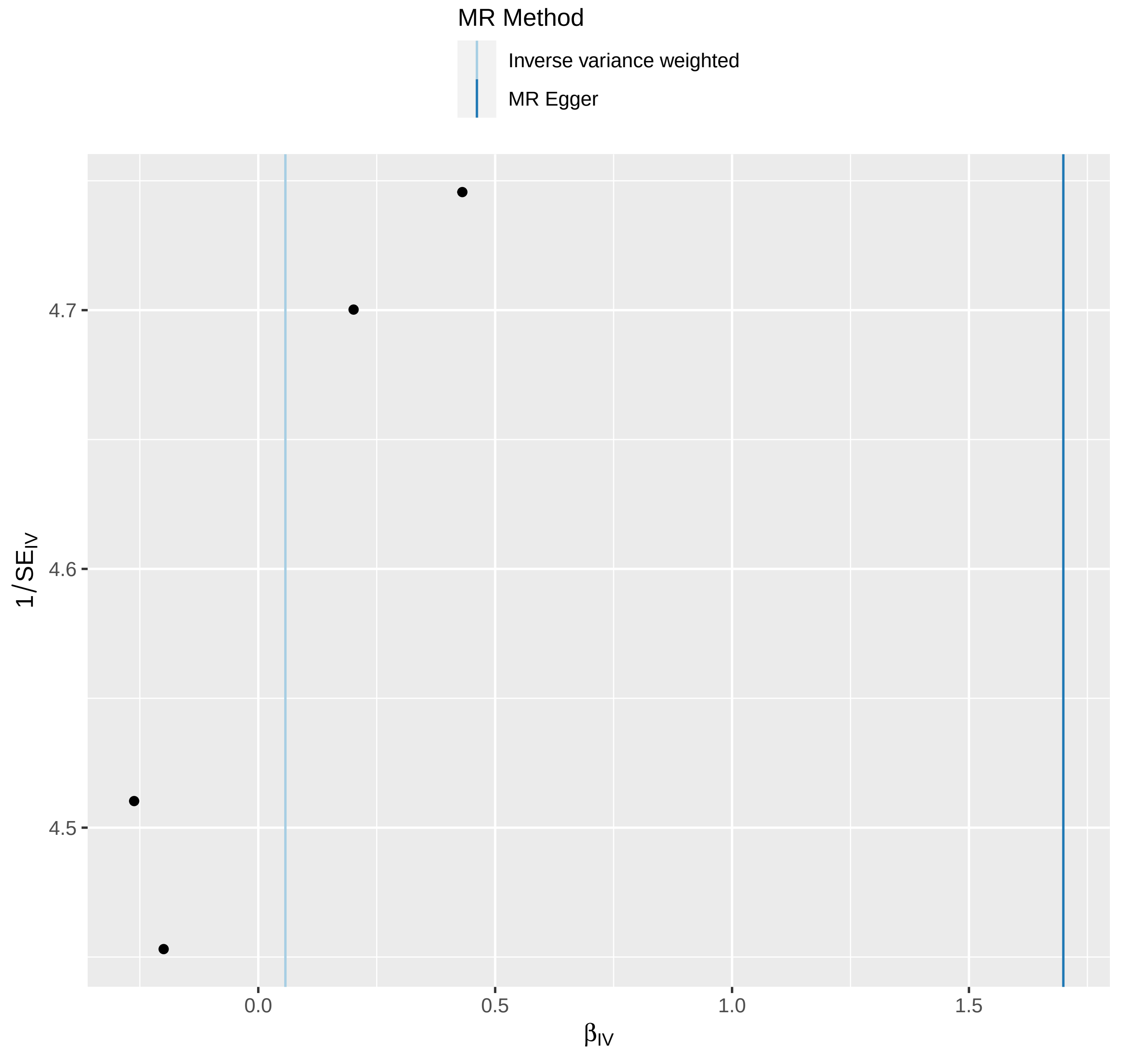

Batch 2−4108 : Gut microbiota abundance (genus Parasutterella id.2892) on Neuroligin−4, X−linked
with a threshold of 1e−05

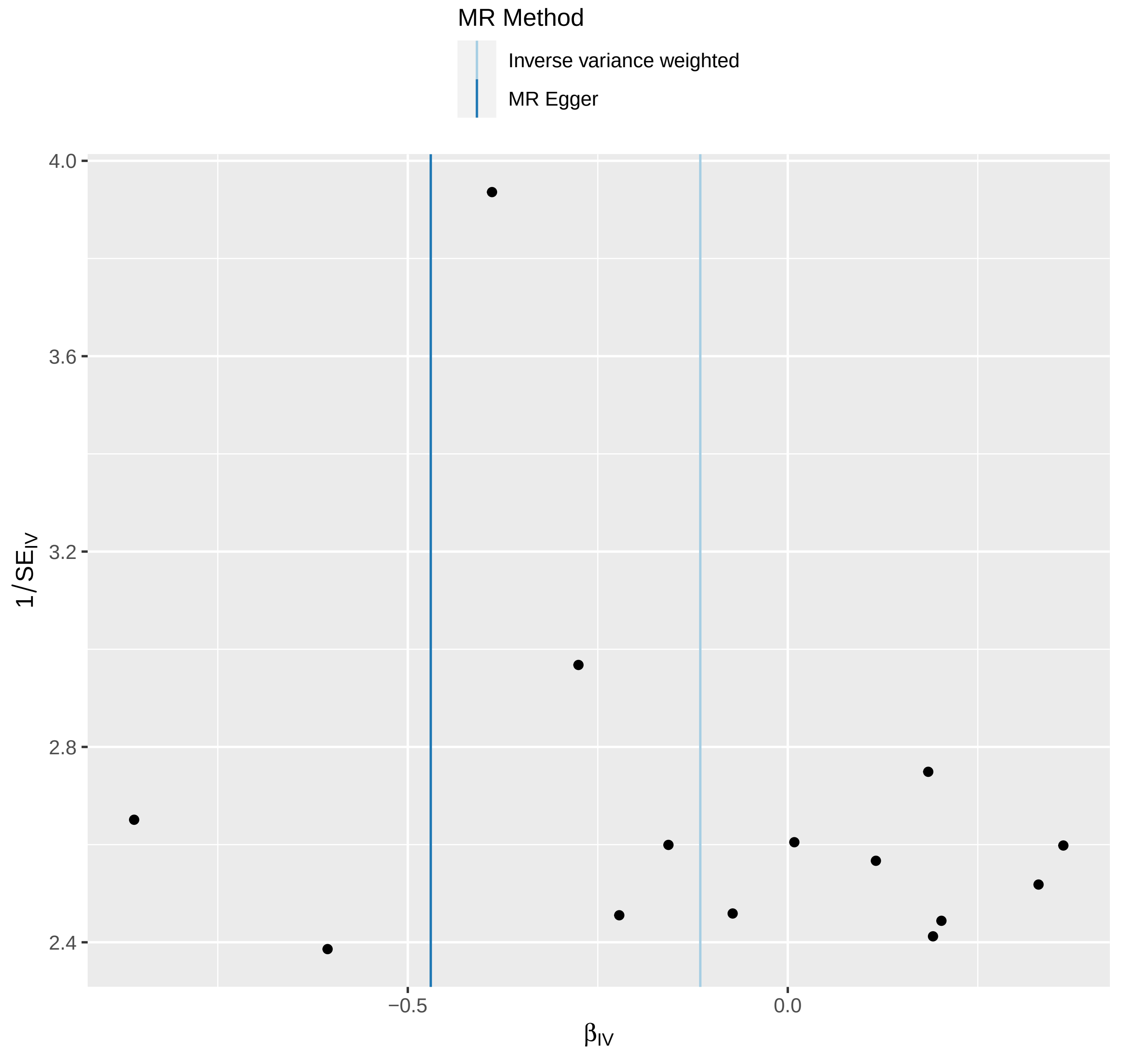

Batch 2−4109 : Gut microbiota abundance (genus Catenibacterium id.2153) on NACHT, LRR and PYD domains−containing protein 10
with a threshold of 1e−05

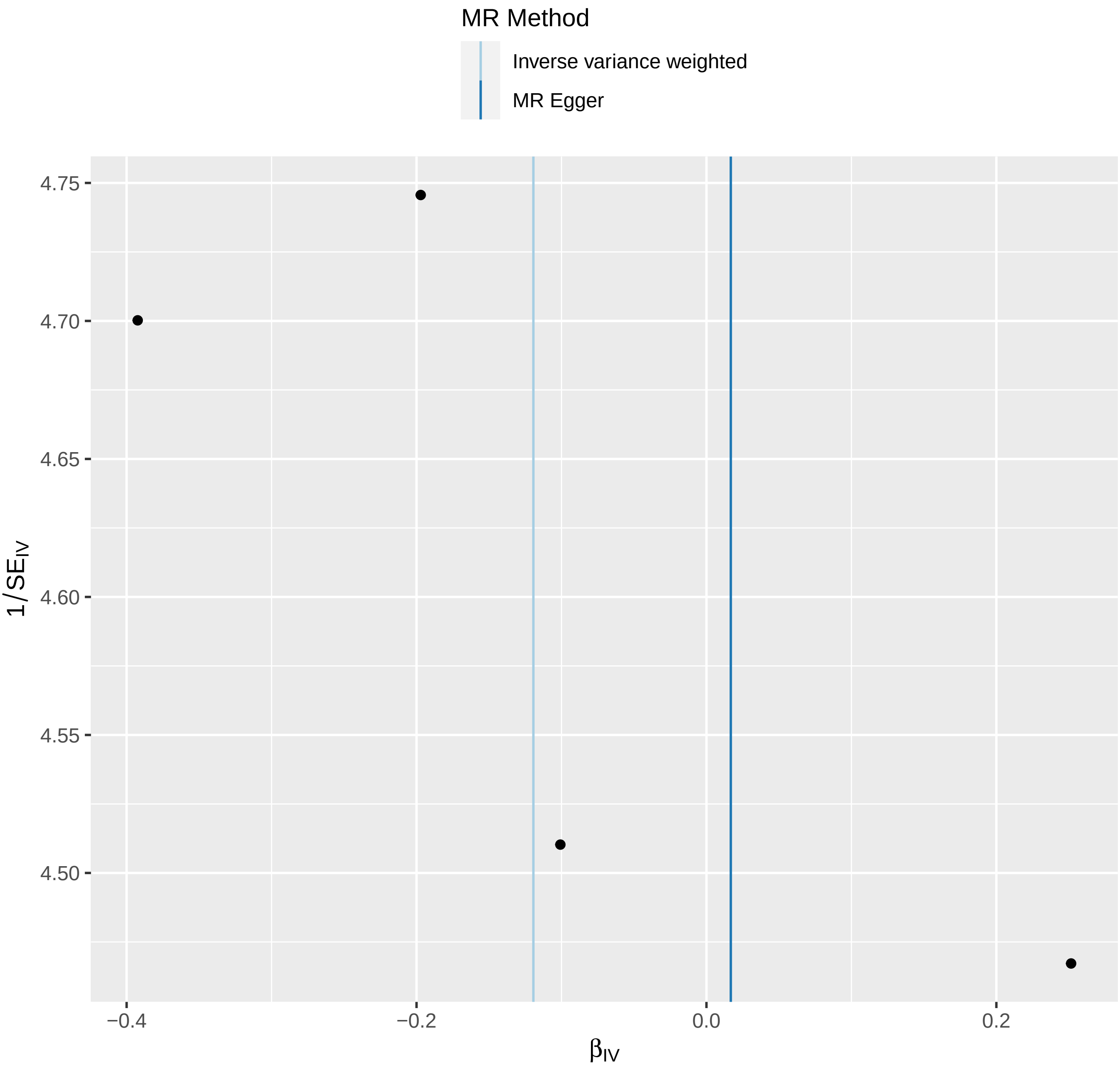

Batch 2−4110 : Gut microbiota abundance (genus Parasutterella id.2892) on NACHT, LRR and PYD domains−containing protein 10 with a threshold of 1e−05

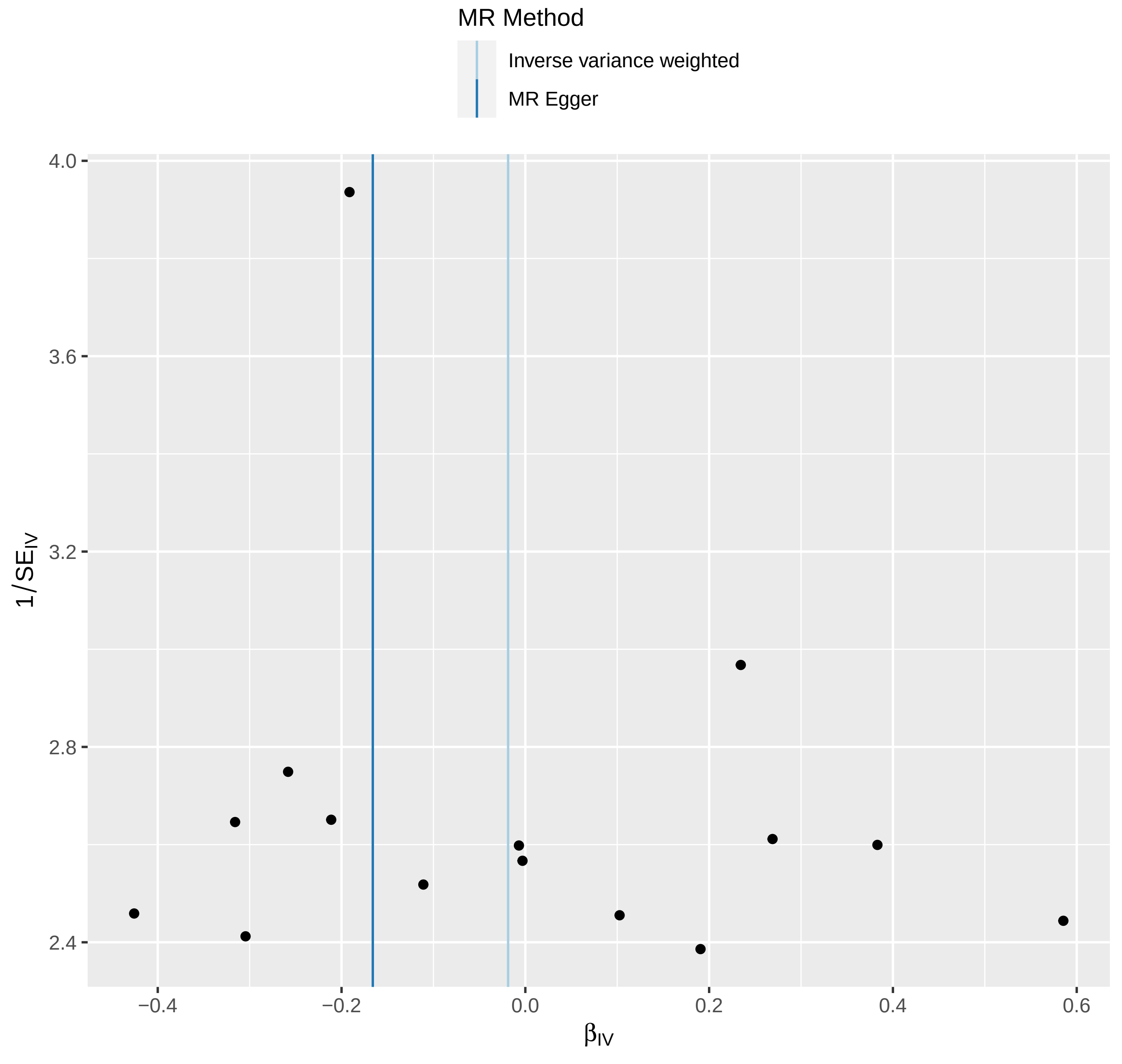

Batch 2−4111 : Gut microbiota abundance (genus Catenibacterium id.2153) on NACHT, LRR and PYD domains−containing protein 1 with a threshold of 1e−05

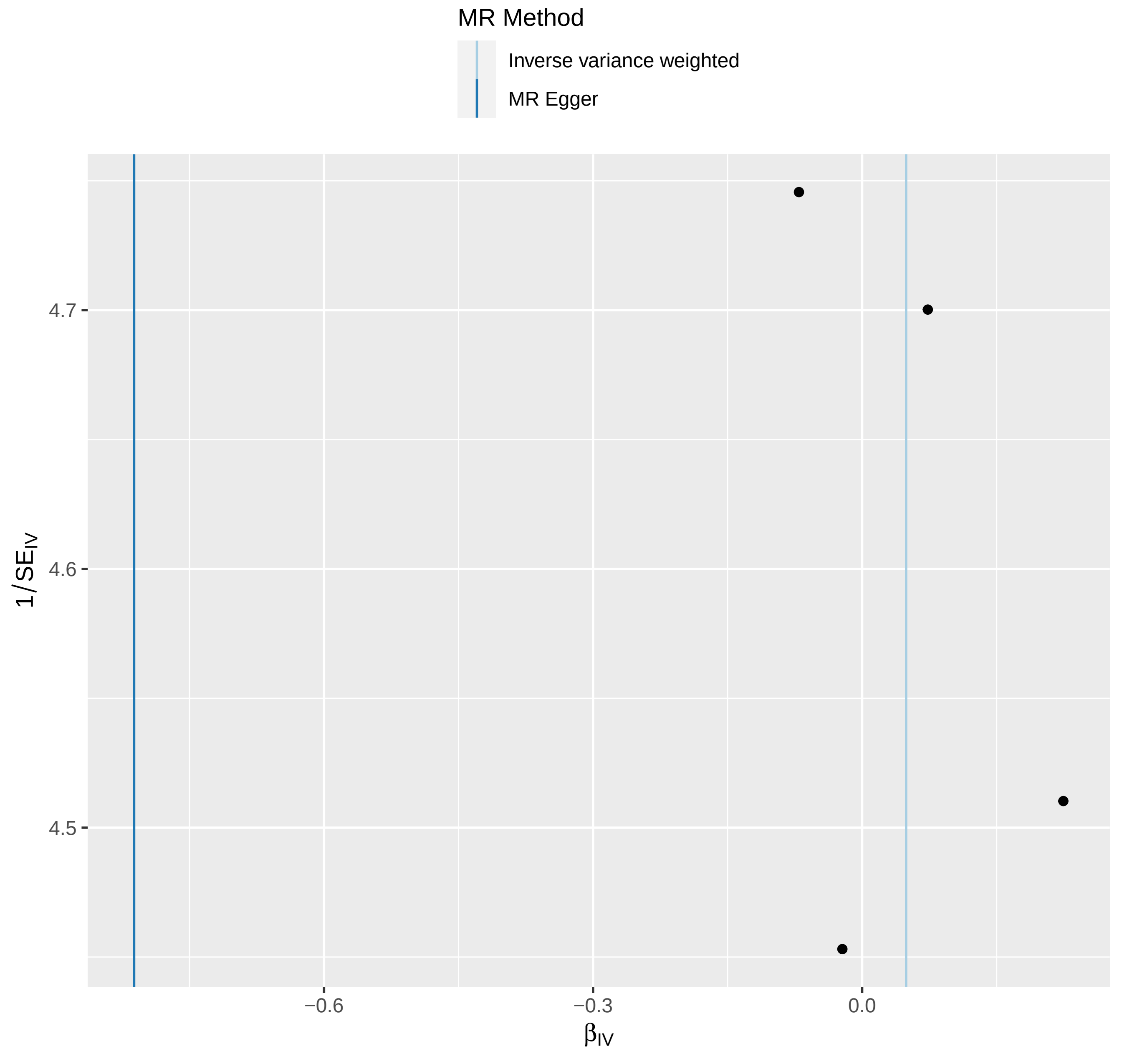

Batch 2−4112 : Gut microbiota abundance (genus Parasutterella id.2892) on NACHT, LRR and PYD domains−containing protein 1
with a threshold of 1e−05

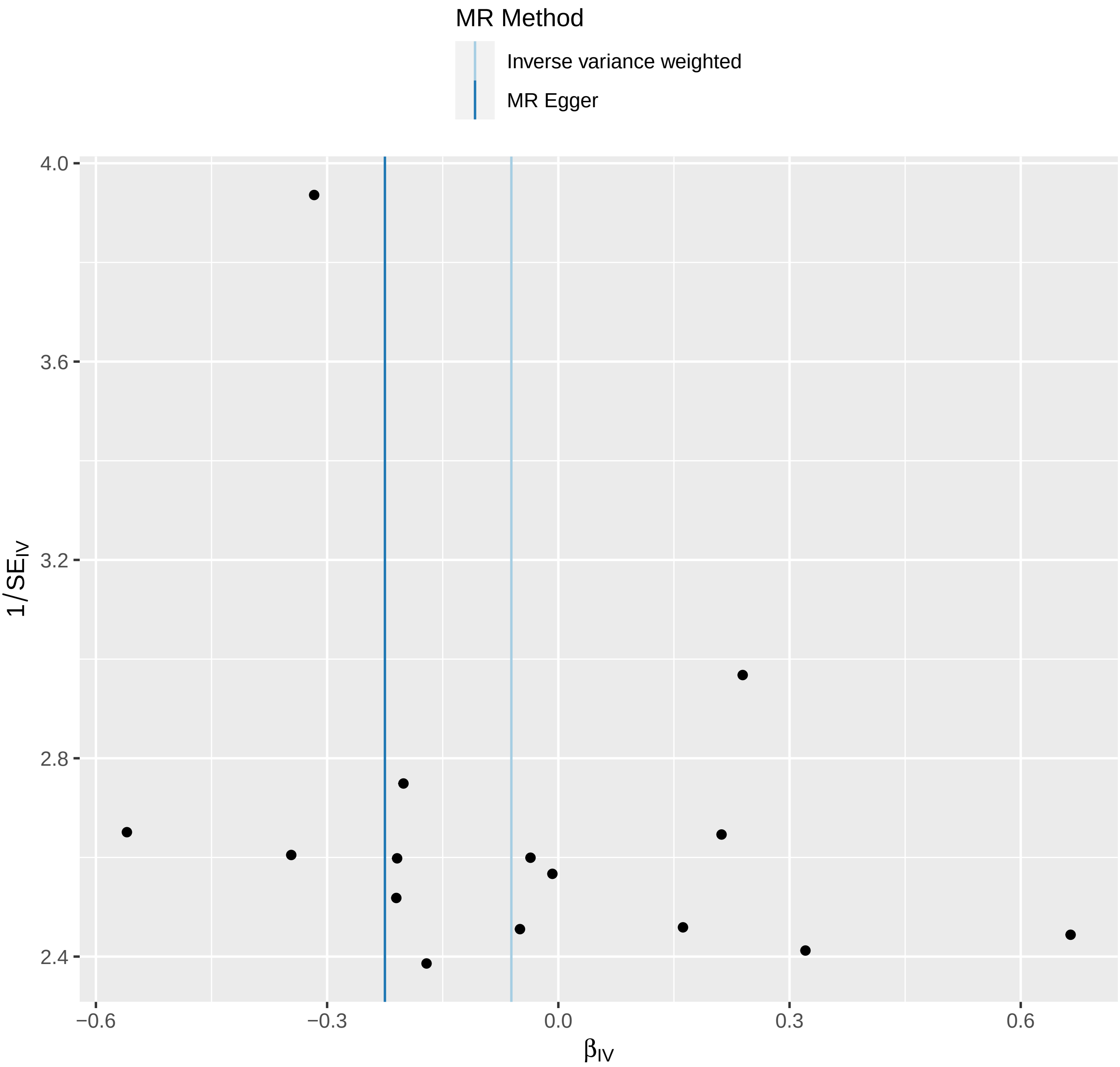

Batch 2−4113 : Gut microbiota abundance (genus Catenibacterium id.2153) on NACHT, LRR and PYD domains−containing protein 4 with a threshold of 1e−05

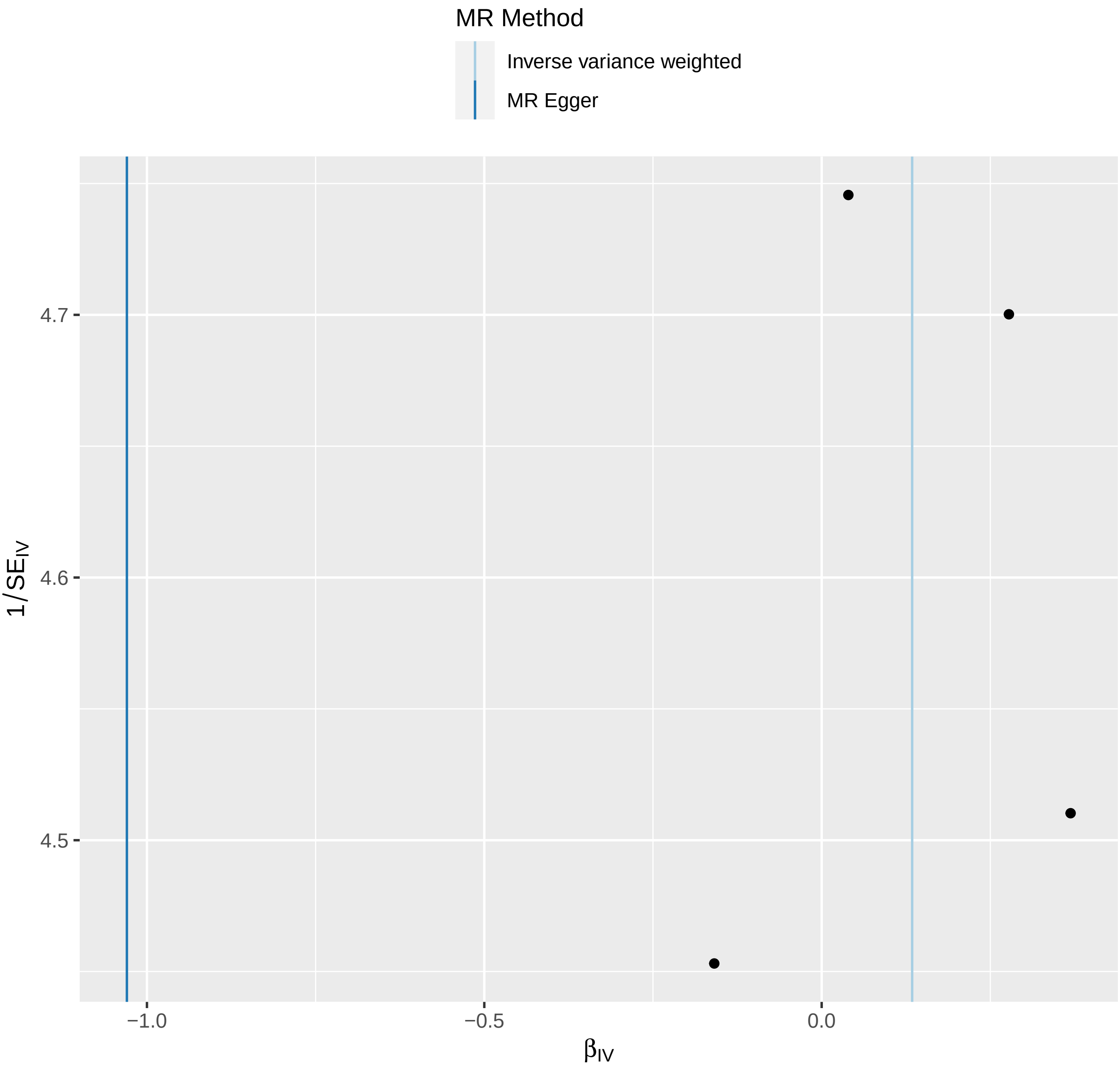

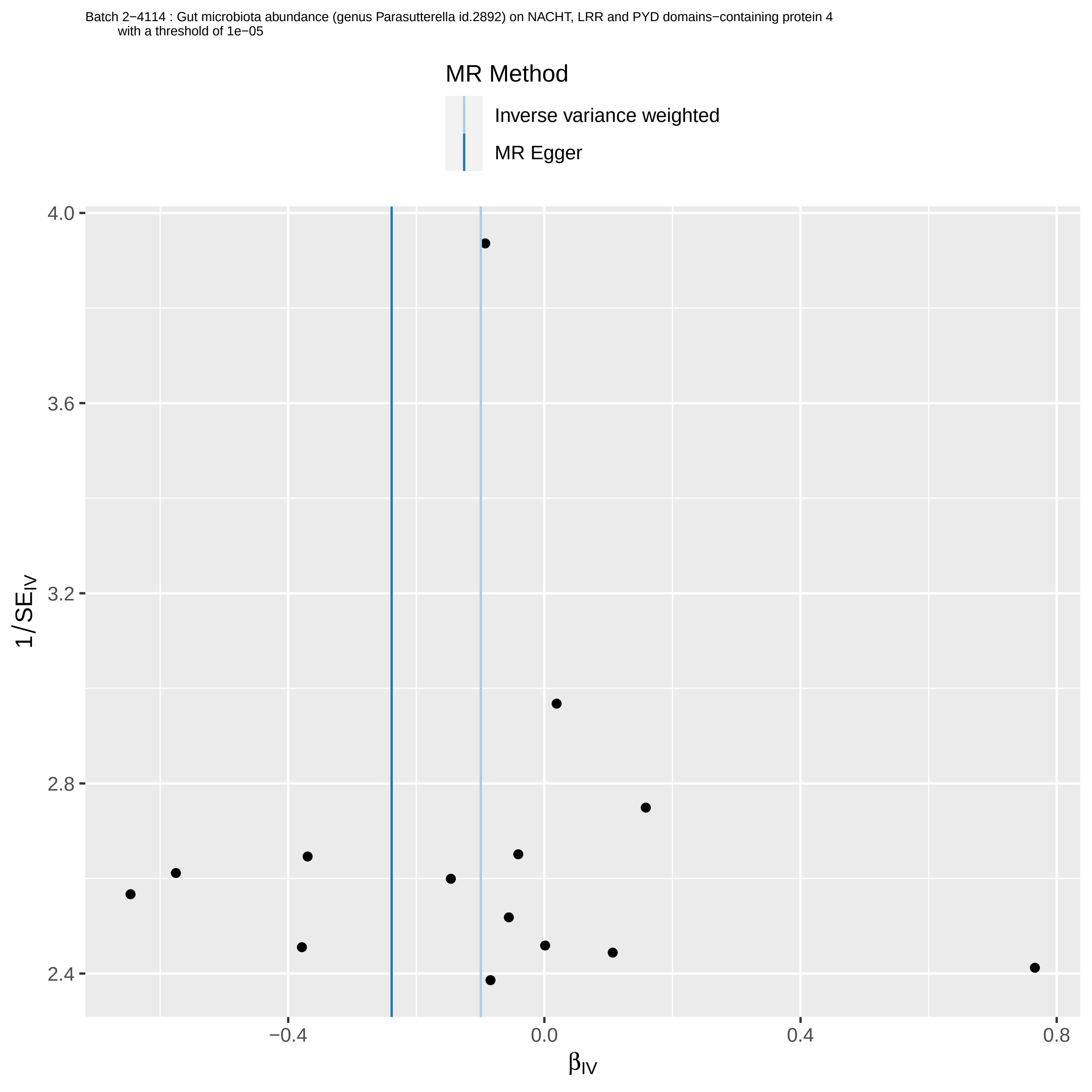
Batch 2−4114 : Gut microbiota abundance (genus Parasutterella id.2892) on NACHT, LRR and PYD domains−containing protein 4
with a threshold of 1e−05
MR Method
Inverse variance weighted
MR Egger
4.0
3.6
3.2
2.8
2.4
1/SE_IV
−0.4
0.0
0.4
0.8
β_IV

Batch 2−4115 : Gut microbiota abundance (genus Catenibacterium id.2153) on Neuromedin−B
with a threshold of 1e−05

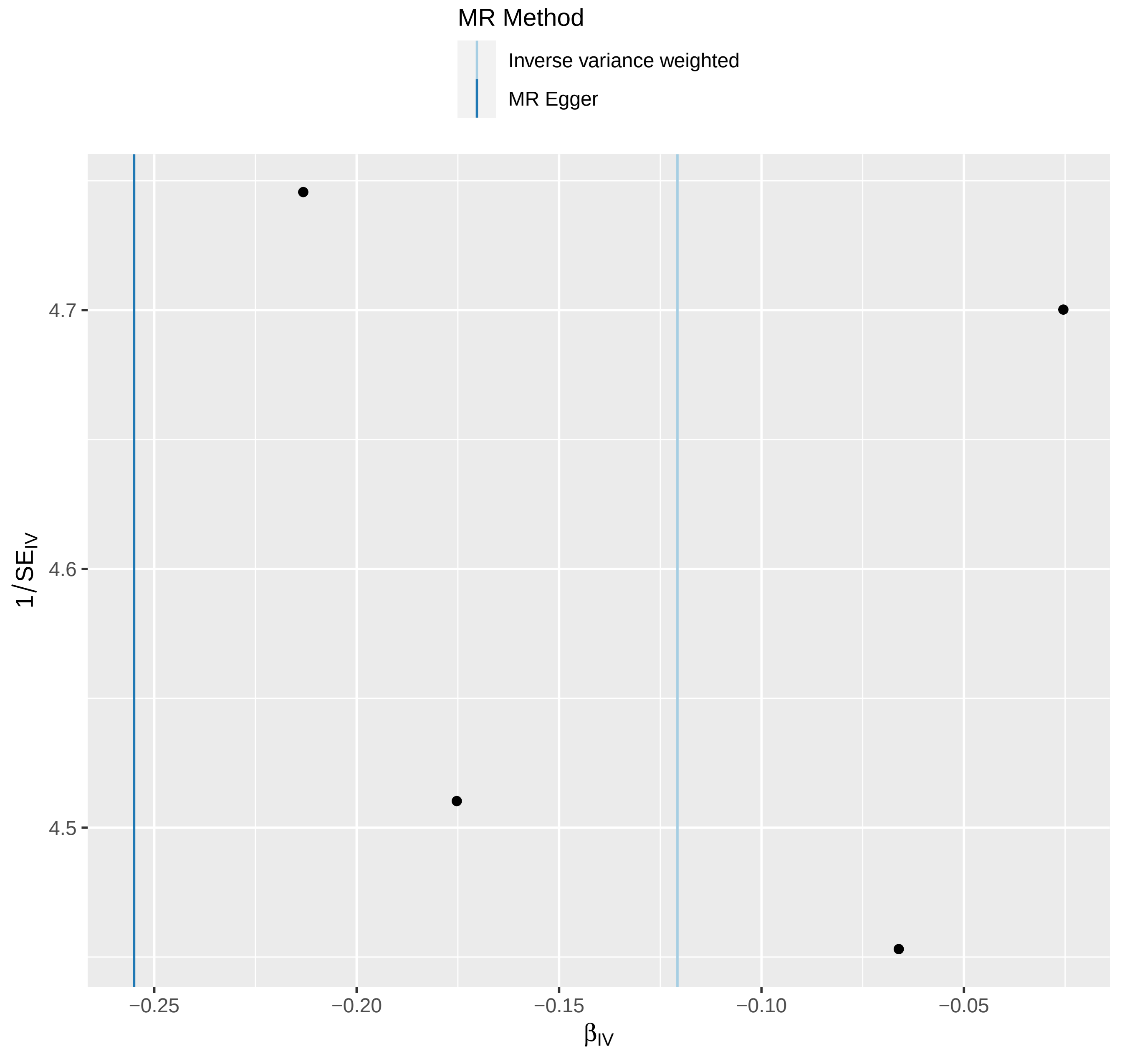

Batch 2−4116 : Gut microbiota abundance (genus Parasutterella id.2892) on Neuromedin−B
with a threshold of 1e−05

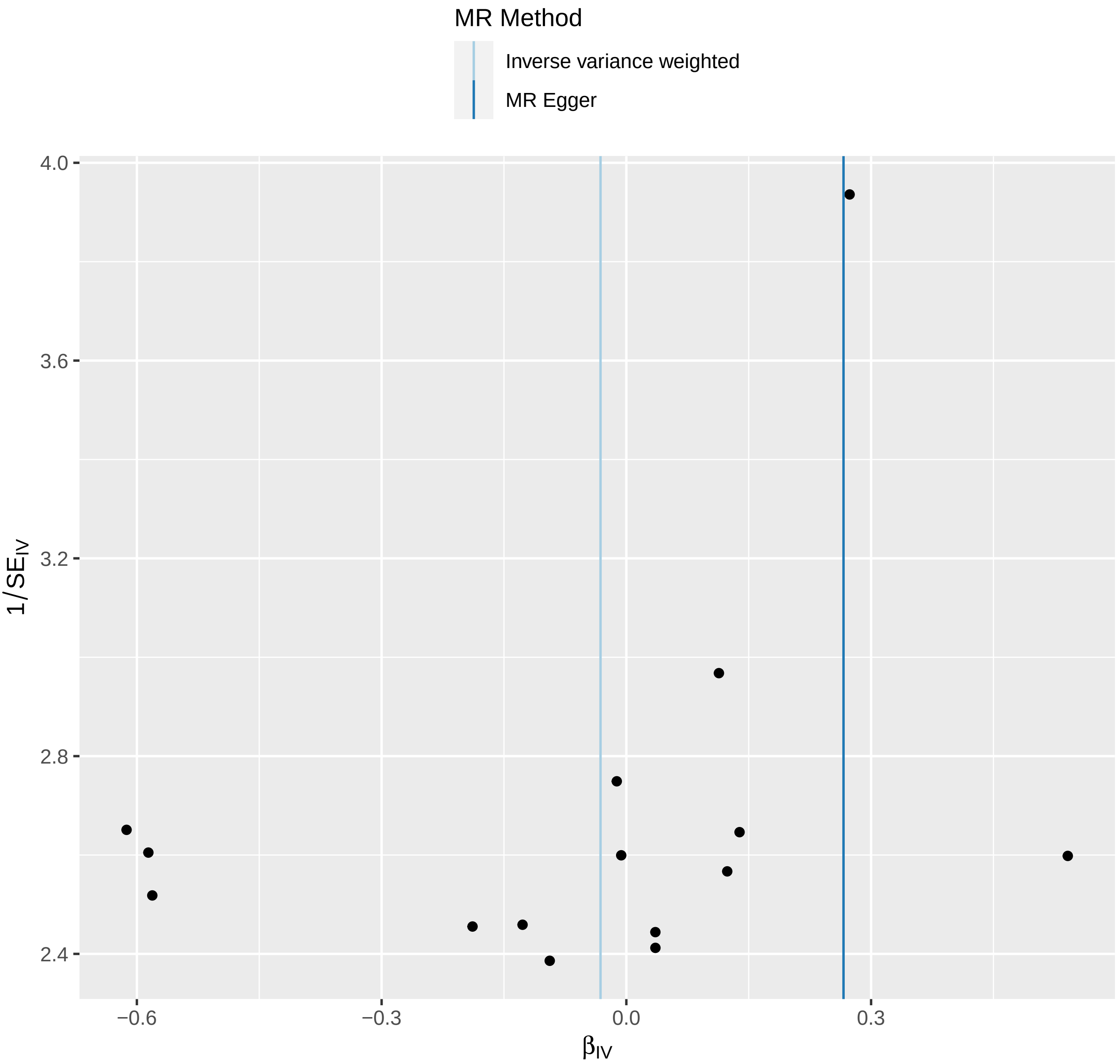

Batch 2−4117 : Gut microbiota abundance (genus Catenibacterium id.2153) on Nucleoside diphosphate kinase A
with a threshold of 1e−05

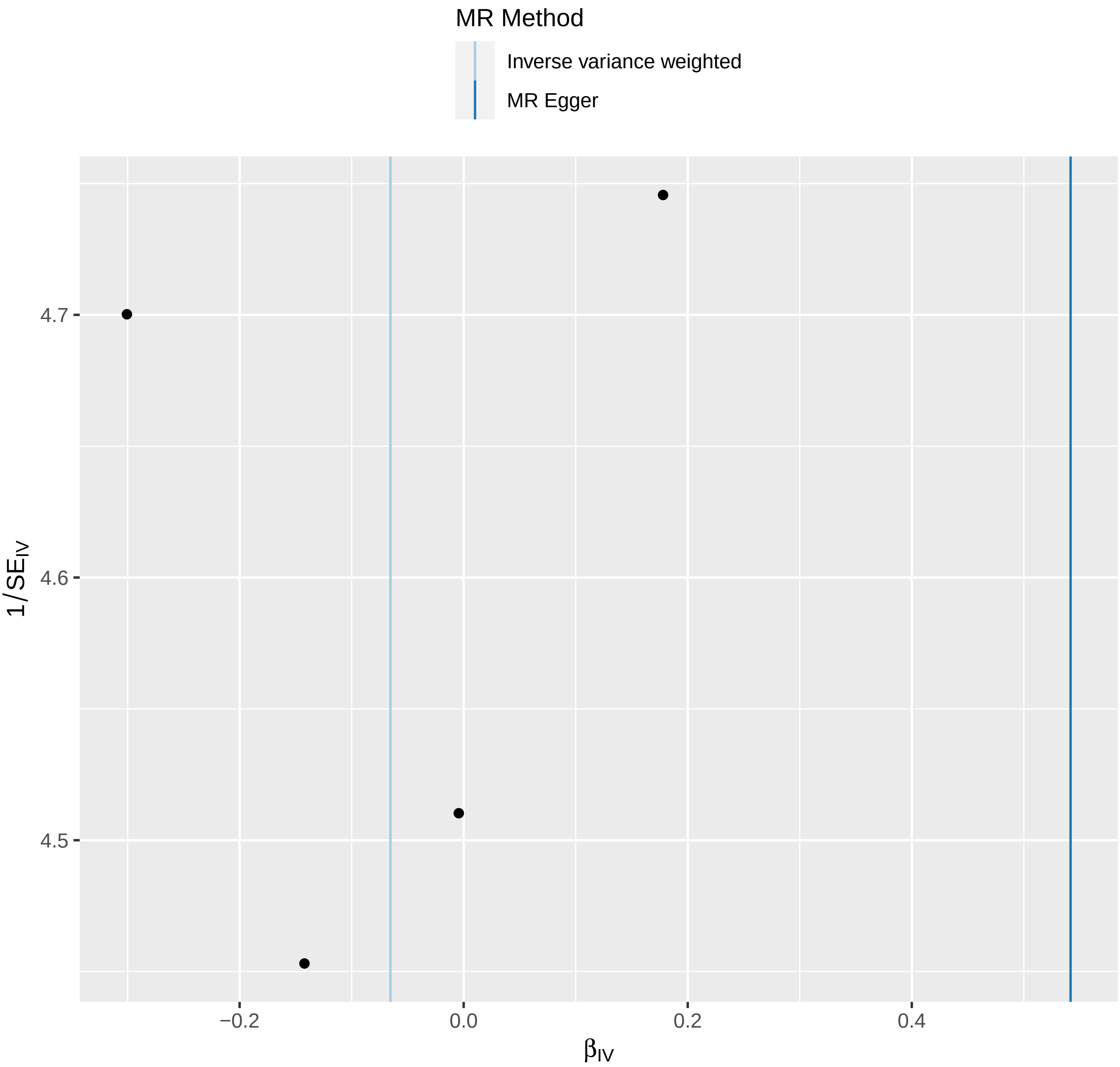

Batch 2−4118 : Gut microbiota abundance (genus Parasutterella id.2892) on Nucleoside diphosphate kinase A
with a threshold of 1e−05

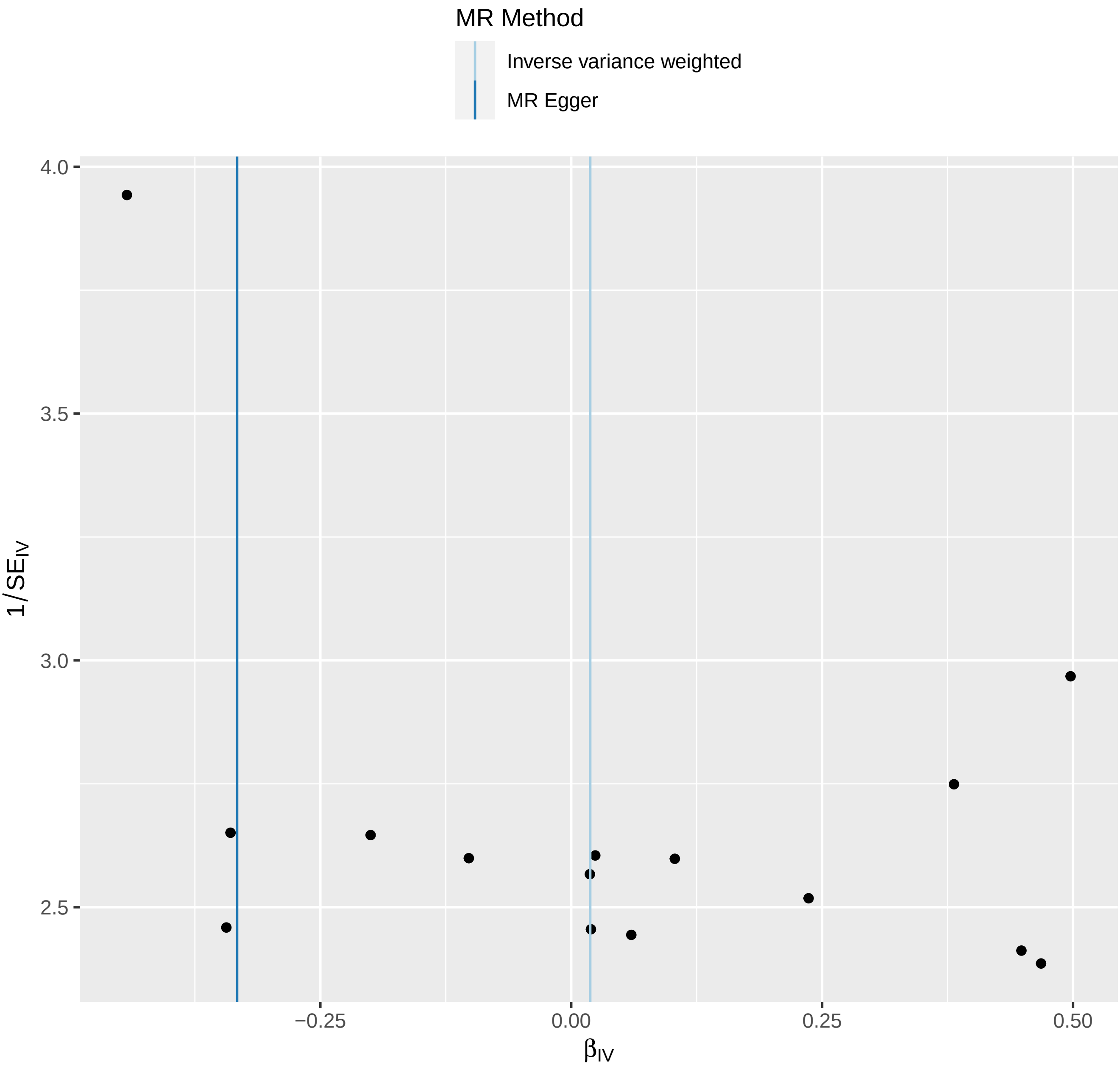

Batch 2−4119 : Gut microbiota abundance (genus Catenibacterium id.2153) on Nucleoside diphosphate kinase B
with a threshold of 1e−05

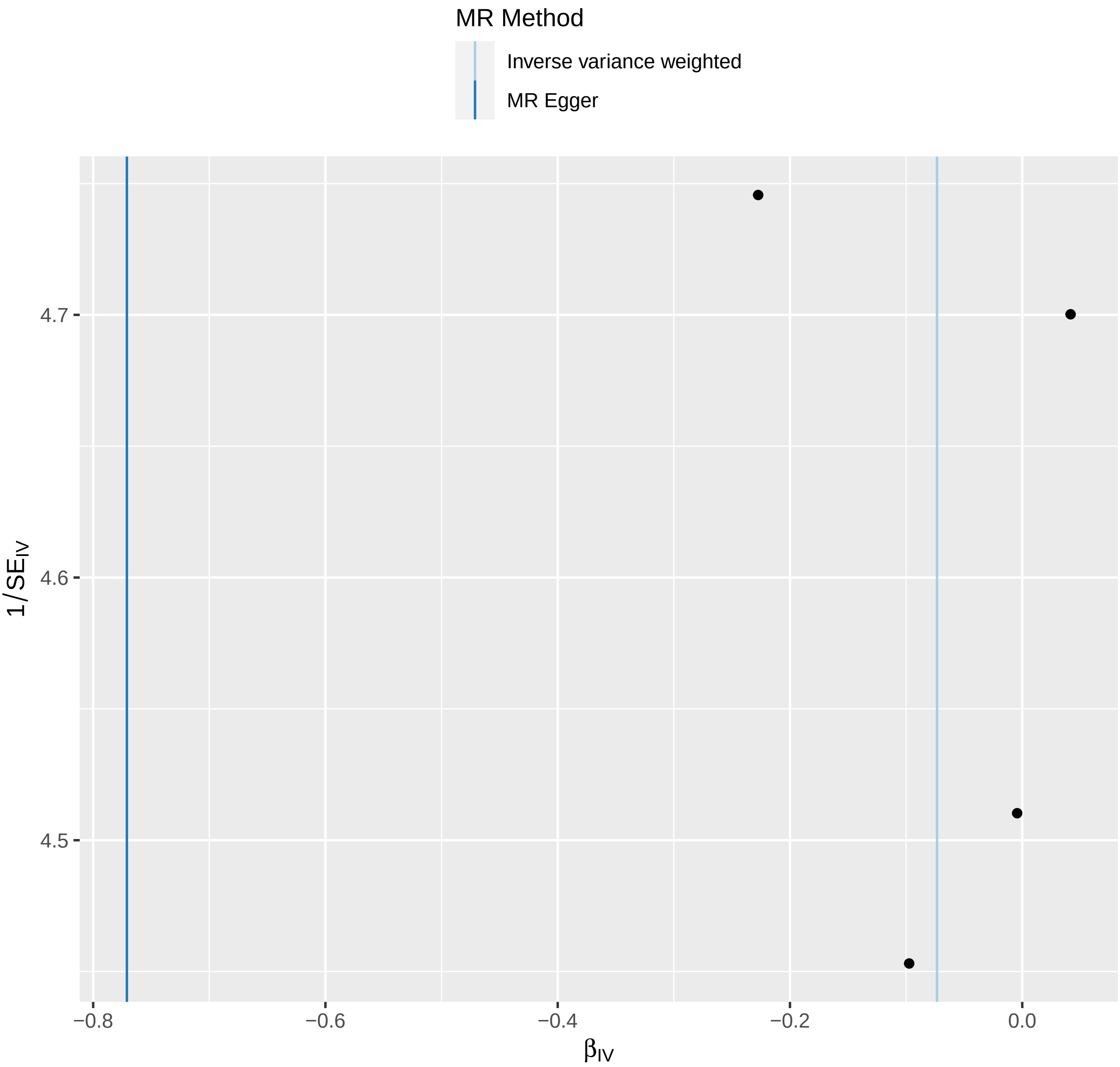

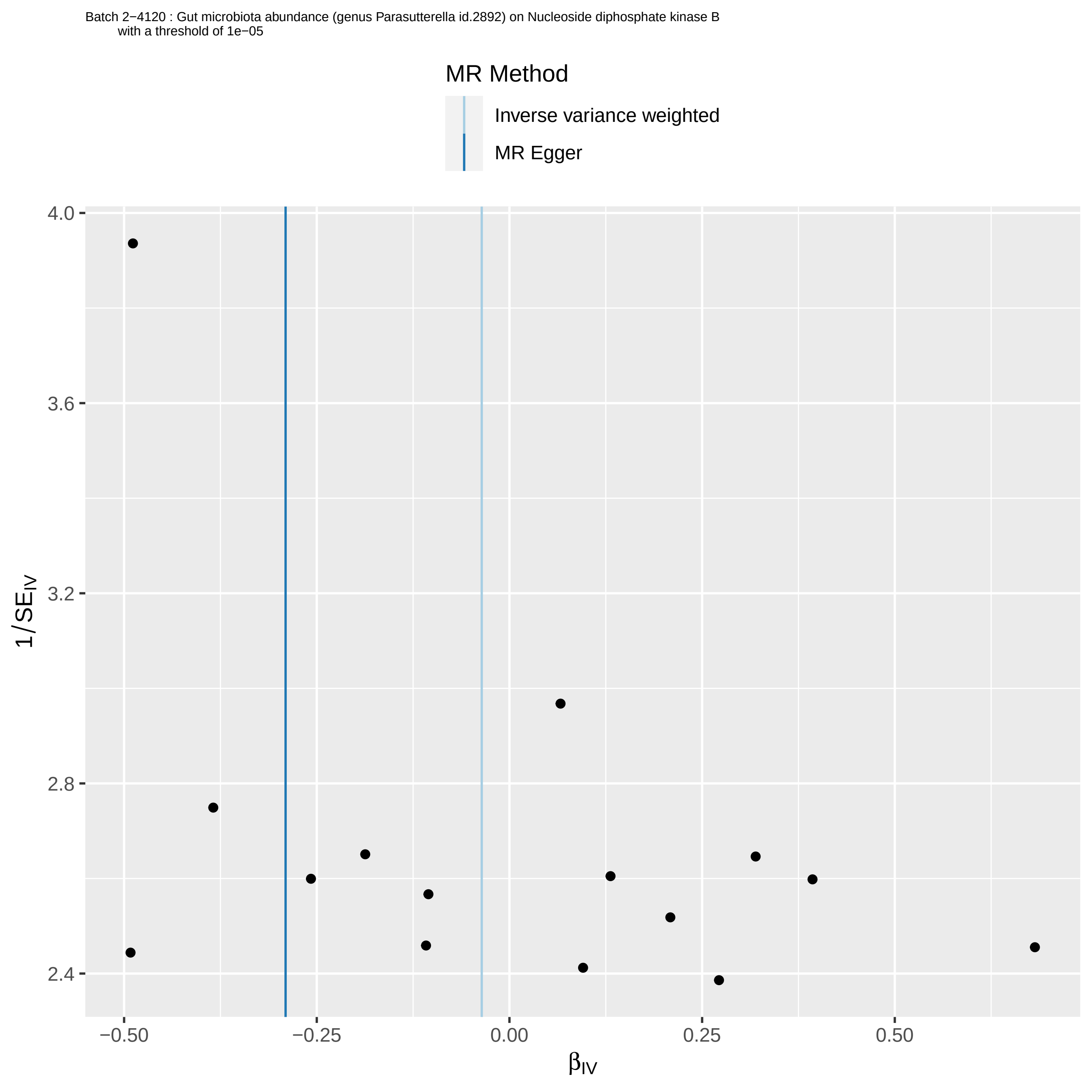
Batch 2−4120 : Gut microbiota abundance (genus Parasutterella id.2892) on Nucleoside diphosphate kinase B
with a threshold of 1e−05
MR Method
Inverse variance weighted
MR Egger
4.0
3.6
3.2
2.8
2.4
1/SE_IV
−0.50
−0.25
0.00
0.25
0.50
β_IV

Batch 2−4121 : Gut microbiota abundance (genus Catenibacterium id.2153) on Normal mucosa of esophagus−specific gene 1 protein with a threshold of 1e−05

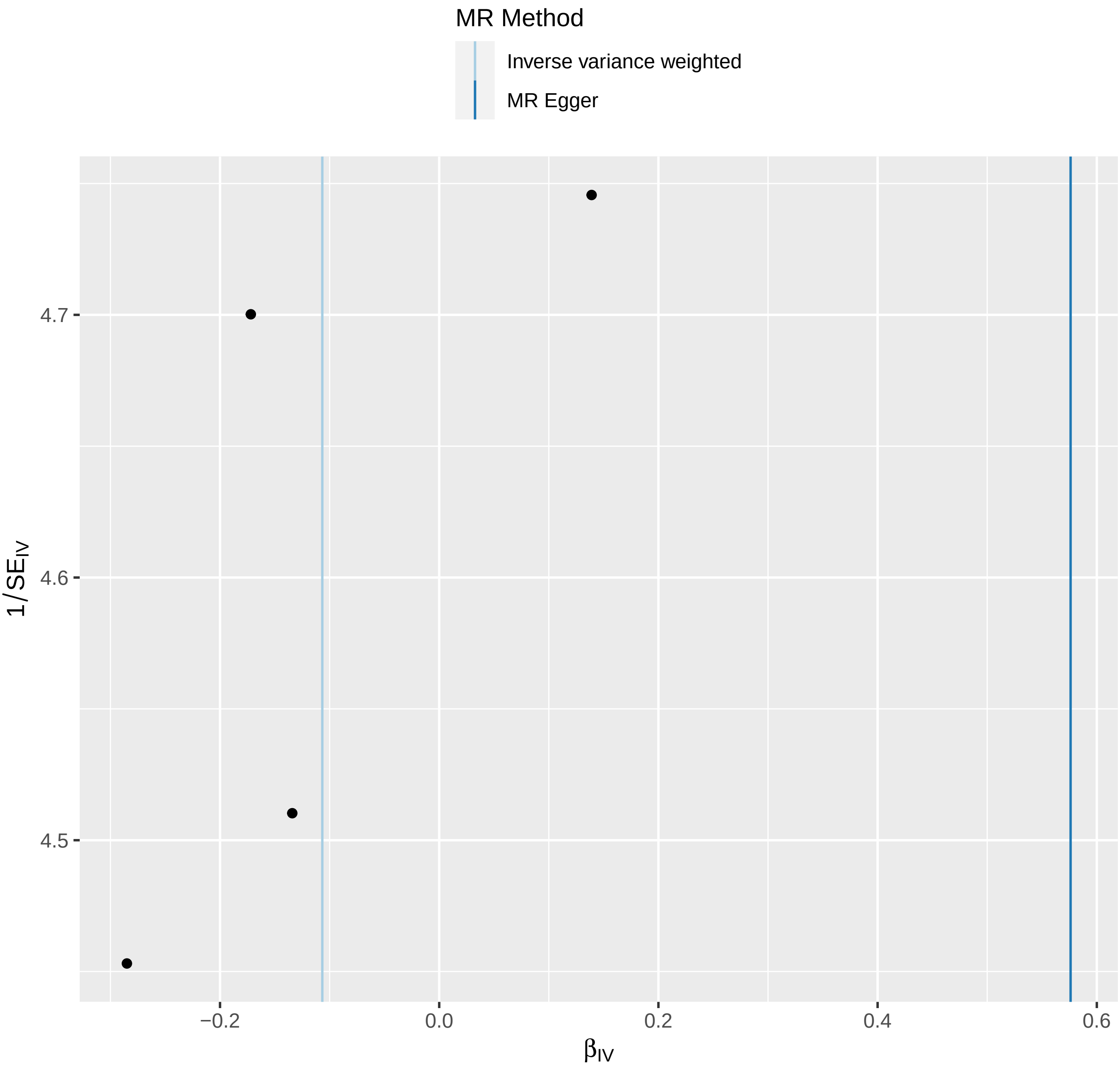

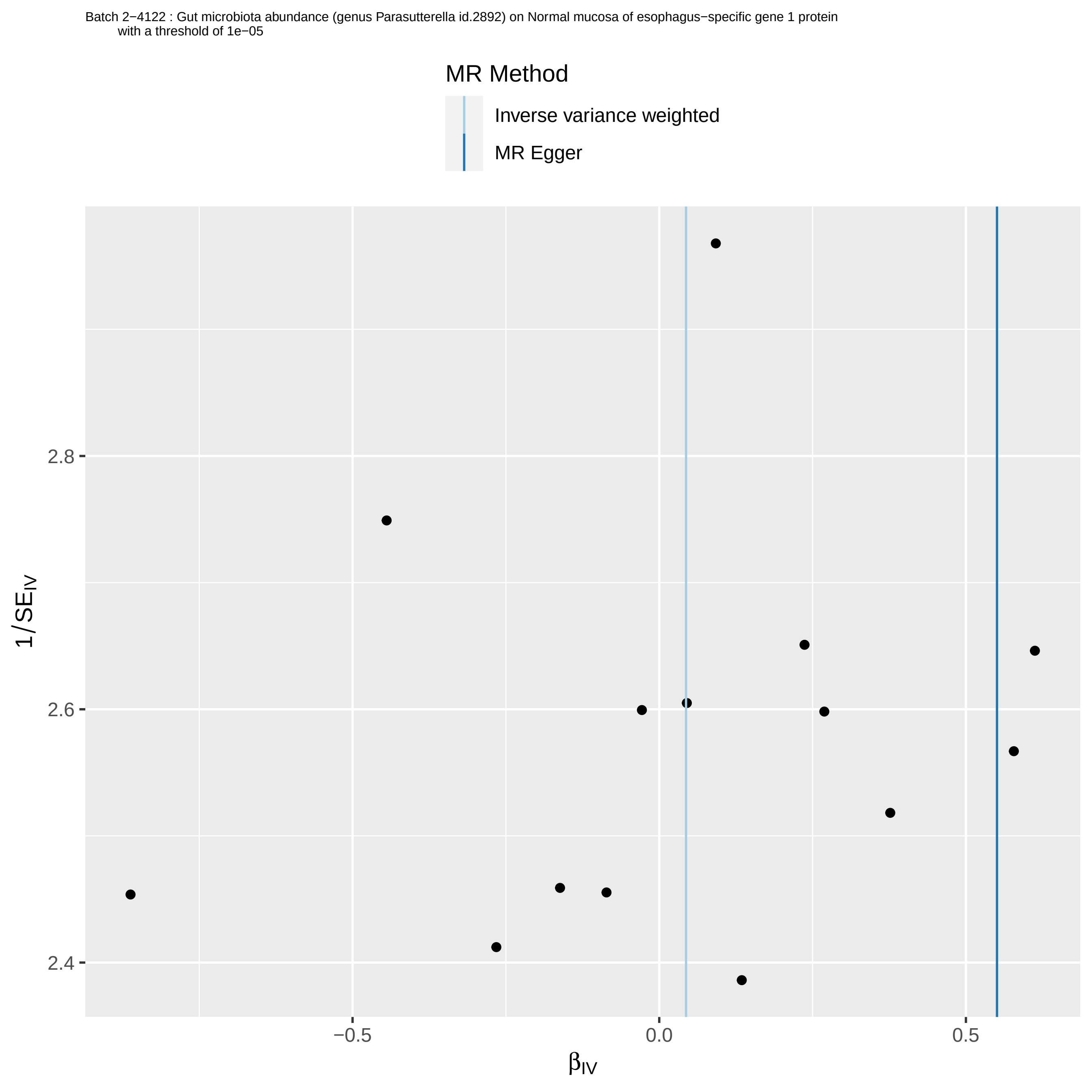
Batch 2−4122 : Gut microbiota abundance (genus Parasutterella id.2892) on Normal mucosa of esophagus−specific gene 1 protein
with a threshold of 1e−05
MR Method
Inverse variance weighted
MR Egger
2.8
2.6
2.4
1/SE_IV
−0.5
0.0
0.5
β_IV

Batch 2−4123 : Gut microbiota abundance (genus Catenibacterium id.2153) on NmrA−like family domain−containing protein 1 with a threshold of 1e−05

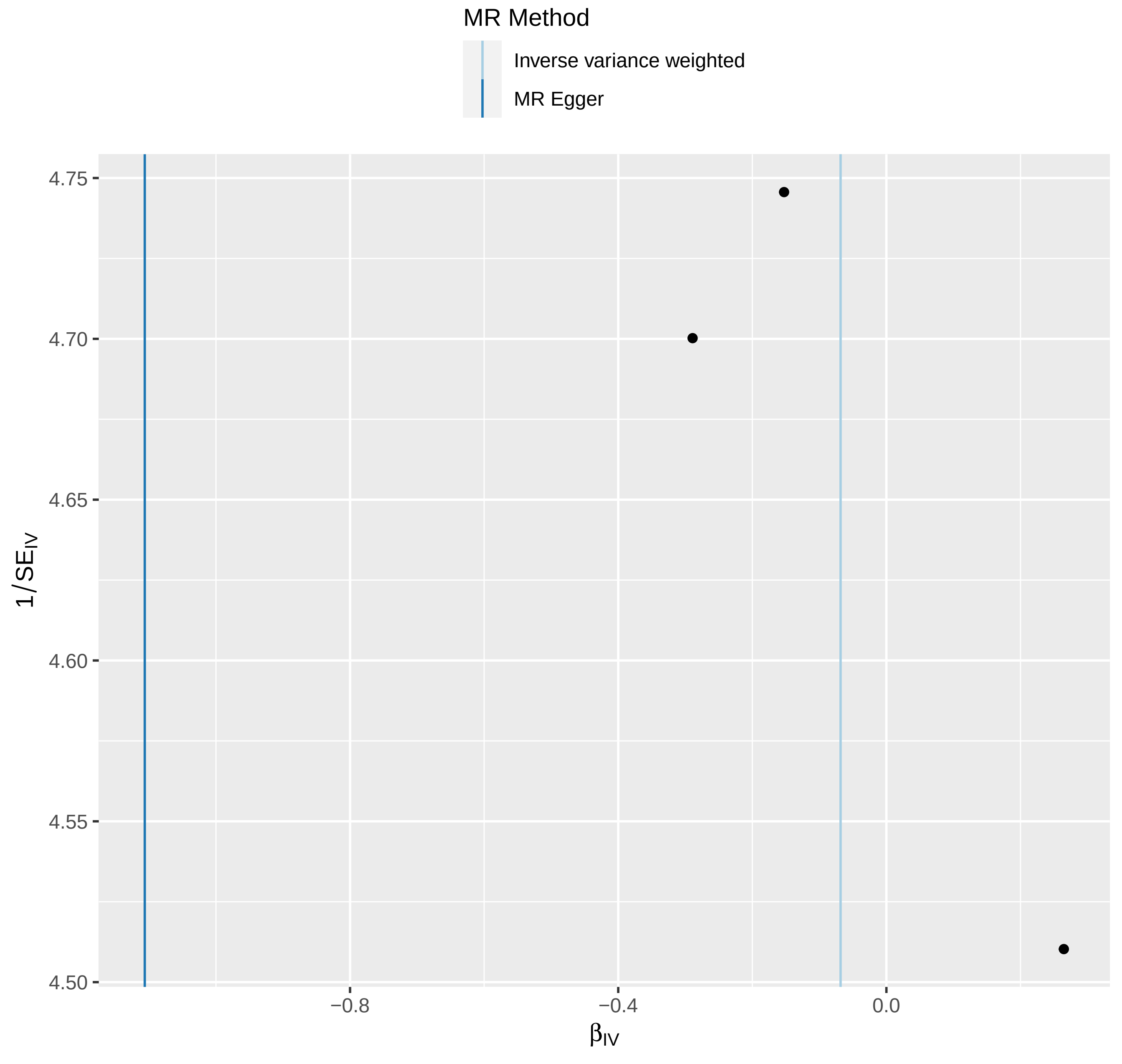

Batch 2−4124 : Gut microbiota abundance (genus Parasutterella id.2892) on NmrA−like family domain−containing protein 1 with a threshold of 1e−05

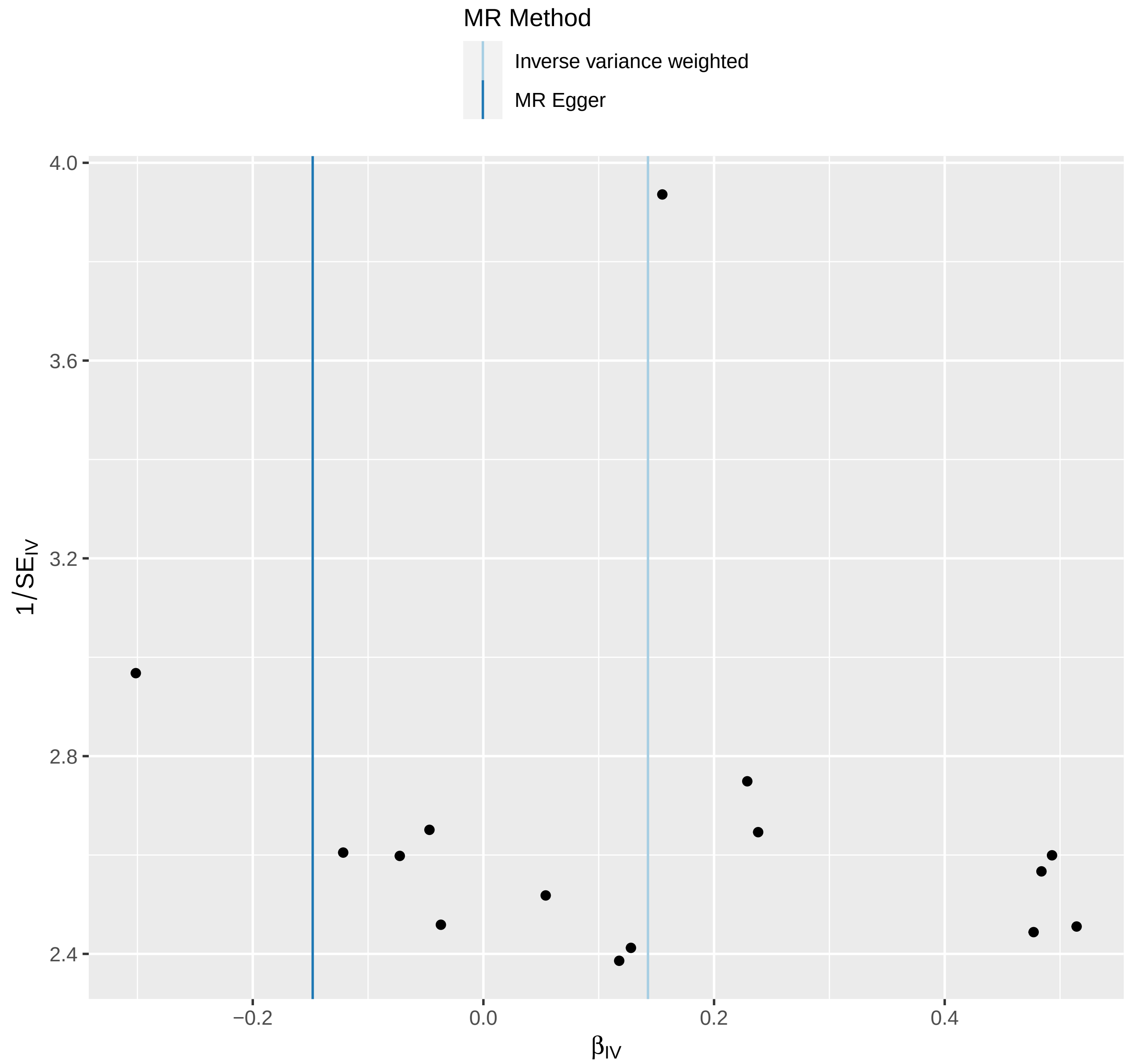

Batch 2−4125 : Gut microbiota abundance (genus Catenibacterium id.2153) on Glycylpeptide N−tetradecanoyltransferase 1
with a threshold of 1e−05

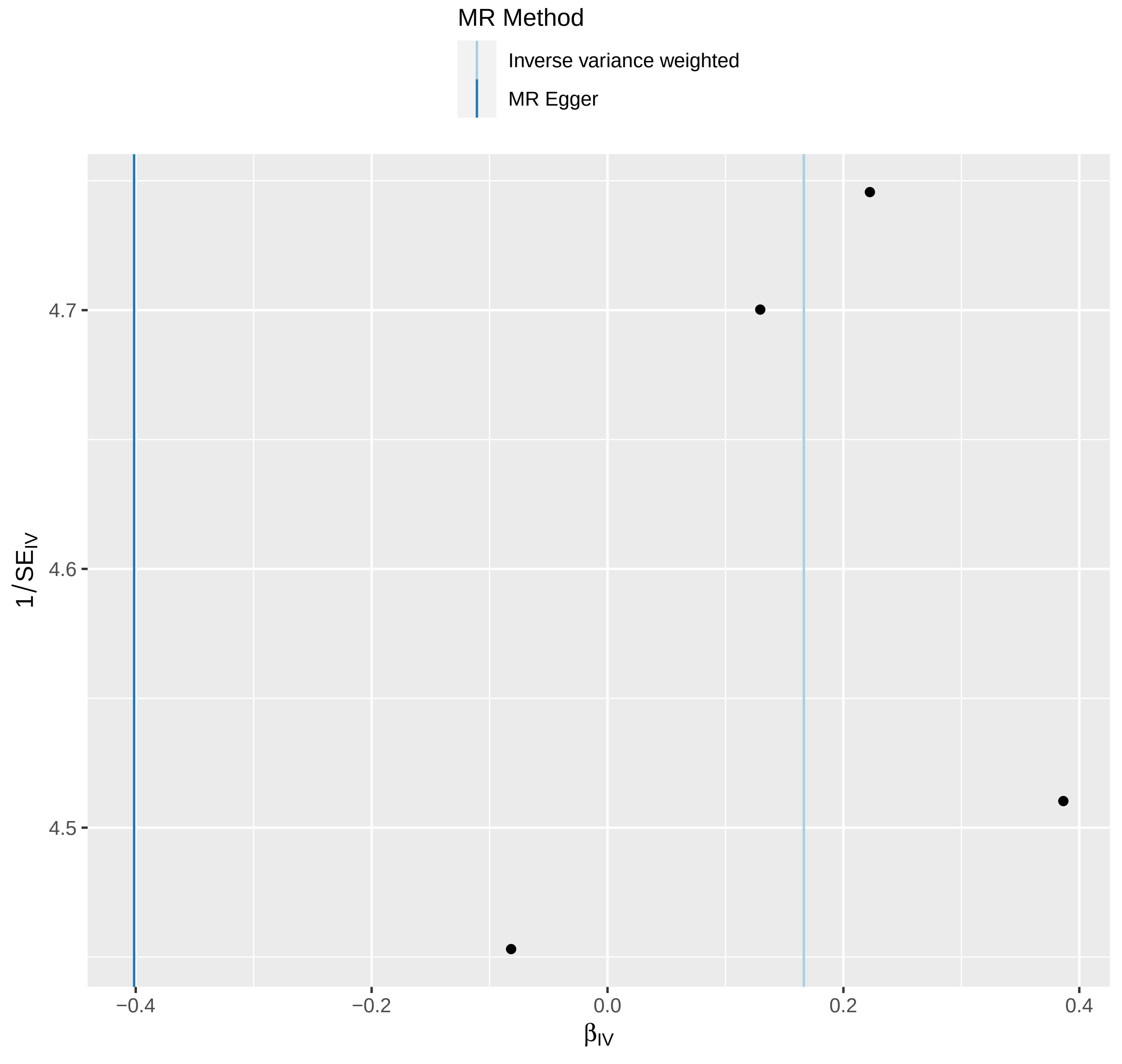

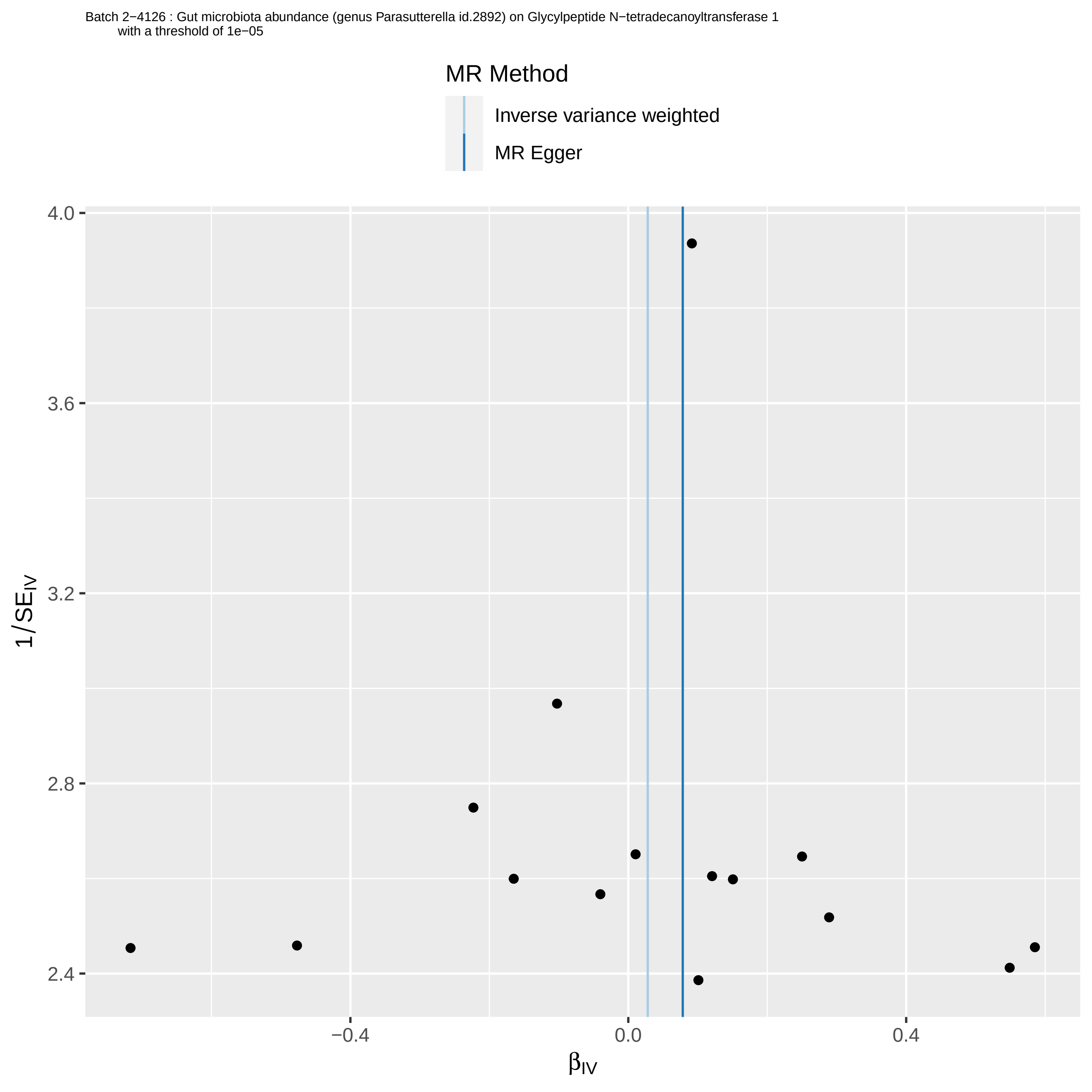
Batch 2−4126 : Gut microbiota abundance (genus Parasutterella id.2892) on Glycylpeptide N−tetradecanoyltransferase 1
with a threshold of 1e−05
MR Method
Inverse variance weighted
MR Egger
4.0
3.6
3.2
2.8
2.4
1/SE$_{IV}$
−0.4
0.0
0.4
β$_{IV}$

Batch 2−4127 : Gut microbiota abundance (genus Catenibacterium id.2153) on Noggin
with a threshold of 1e−05

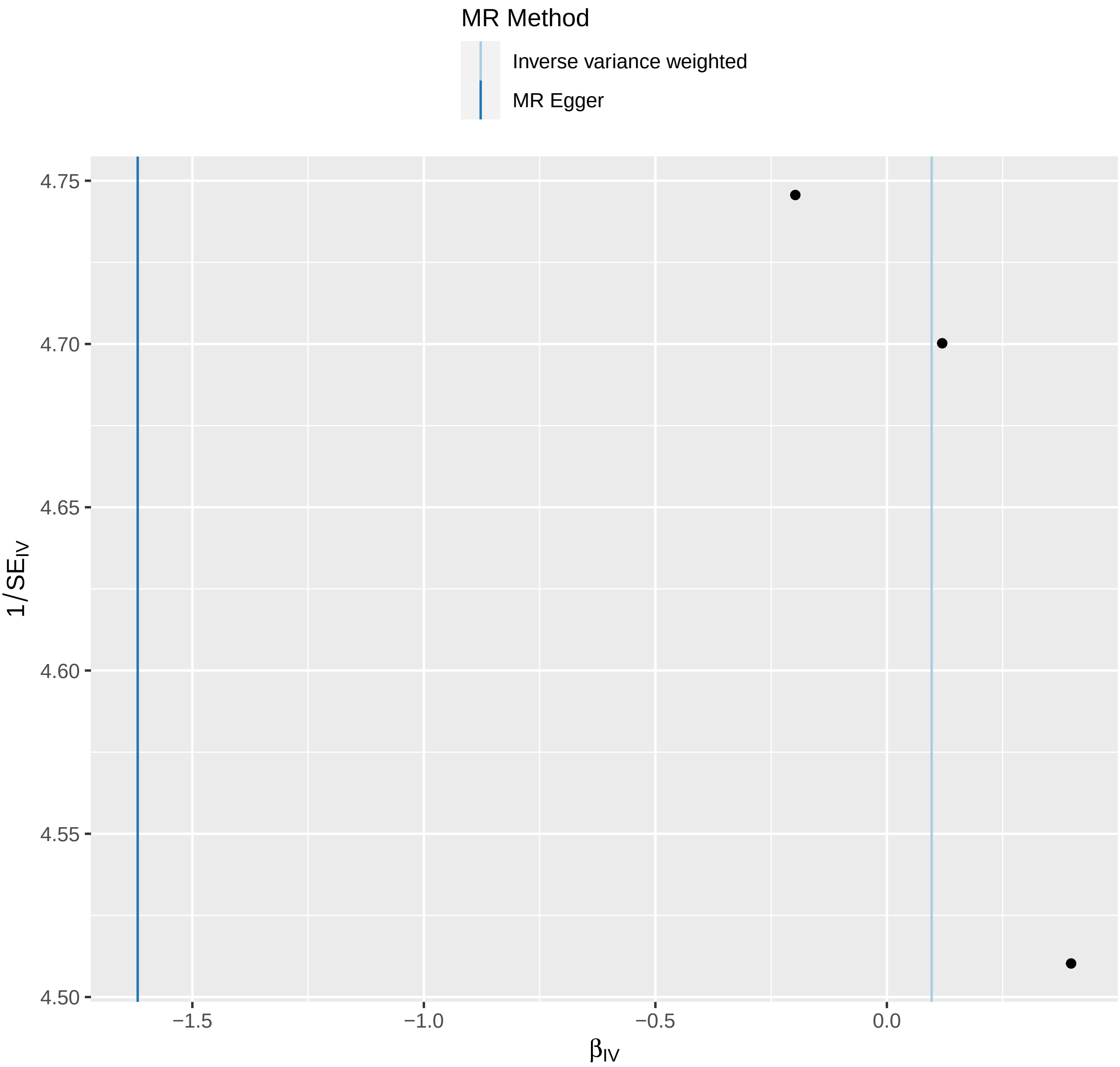

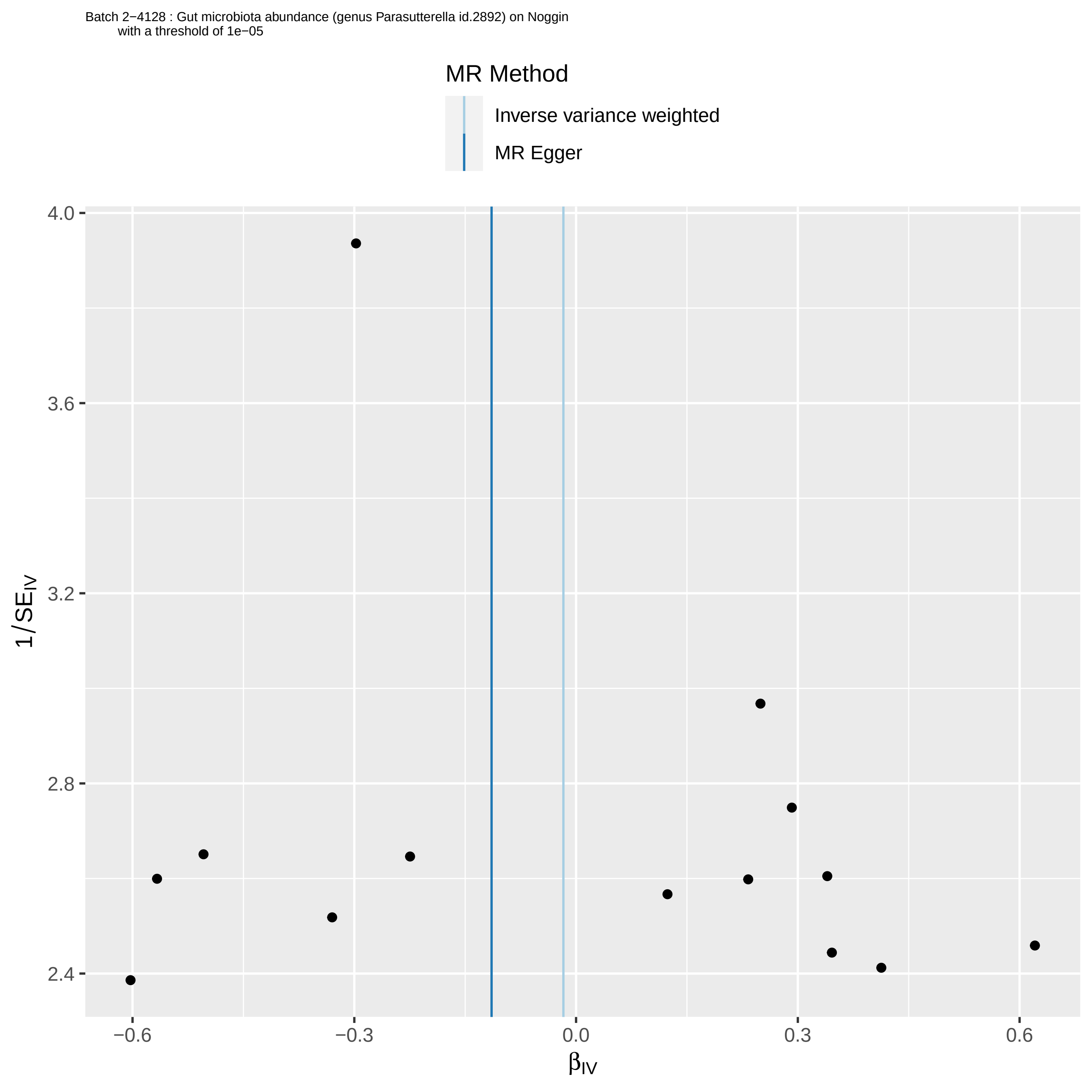
Batch 2−4128 : Gut microbiota abundance (genus Parasutterella id.2892) on Noggin
with a threshold of 1e−05
MR Method
Inverse variance weighted
MR Egger
4.0
3.6
3.2
2.8
2.4
1/SE_IV
−0.6
−0.3
0.0
0.3
0.6
β_IV

Batch 2−4129 : Gut microbiota abundance (genus Catenibacterium id.2153) on NoneX
with a threshold of 1e−05

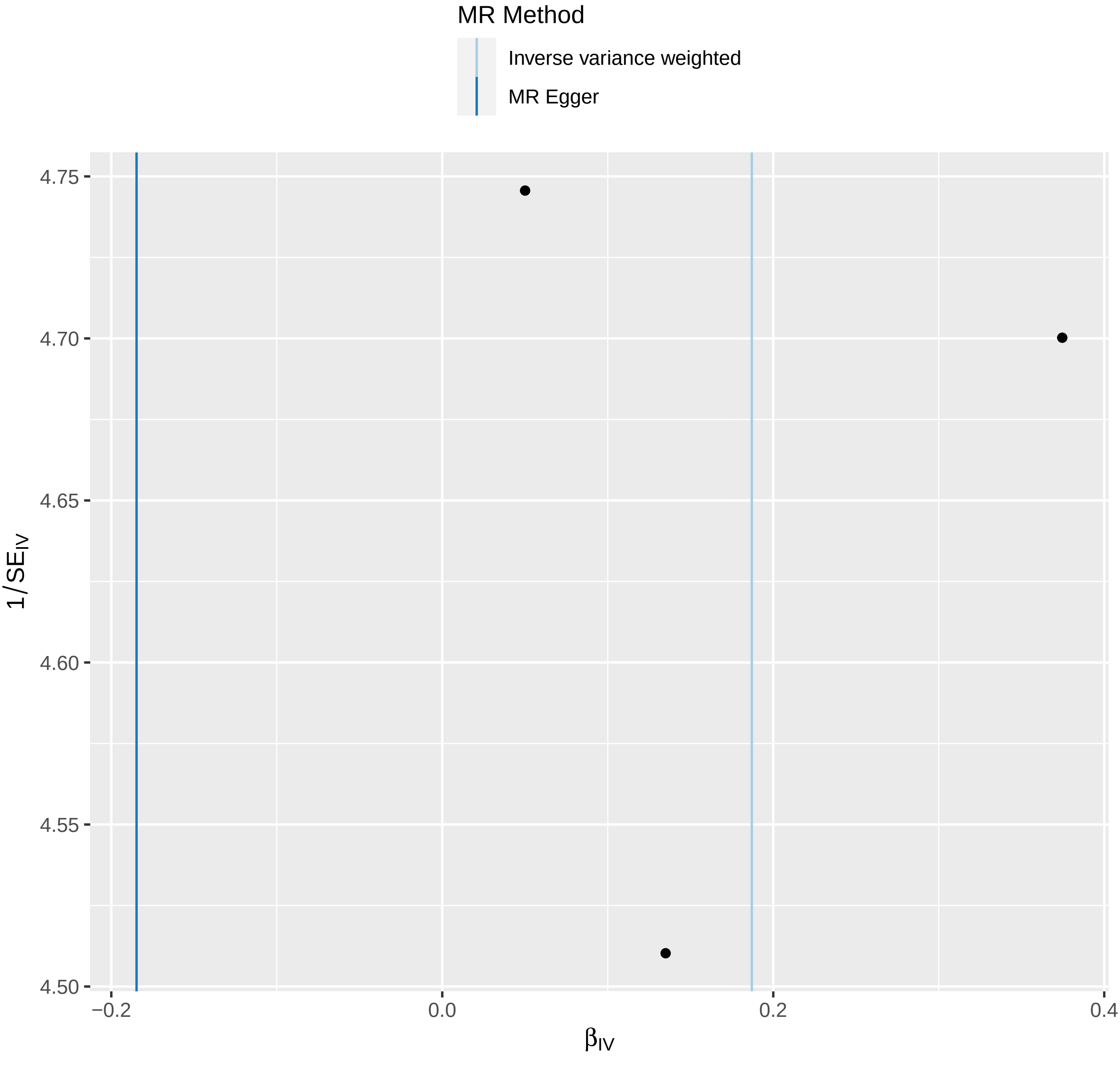

Batch 2−4130 : Gut microbiota abundance (genus Parasutterella id.2892) on NoneX
with a threshold of 1e−05

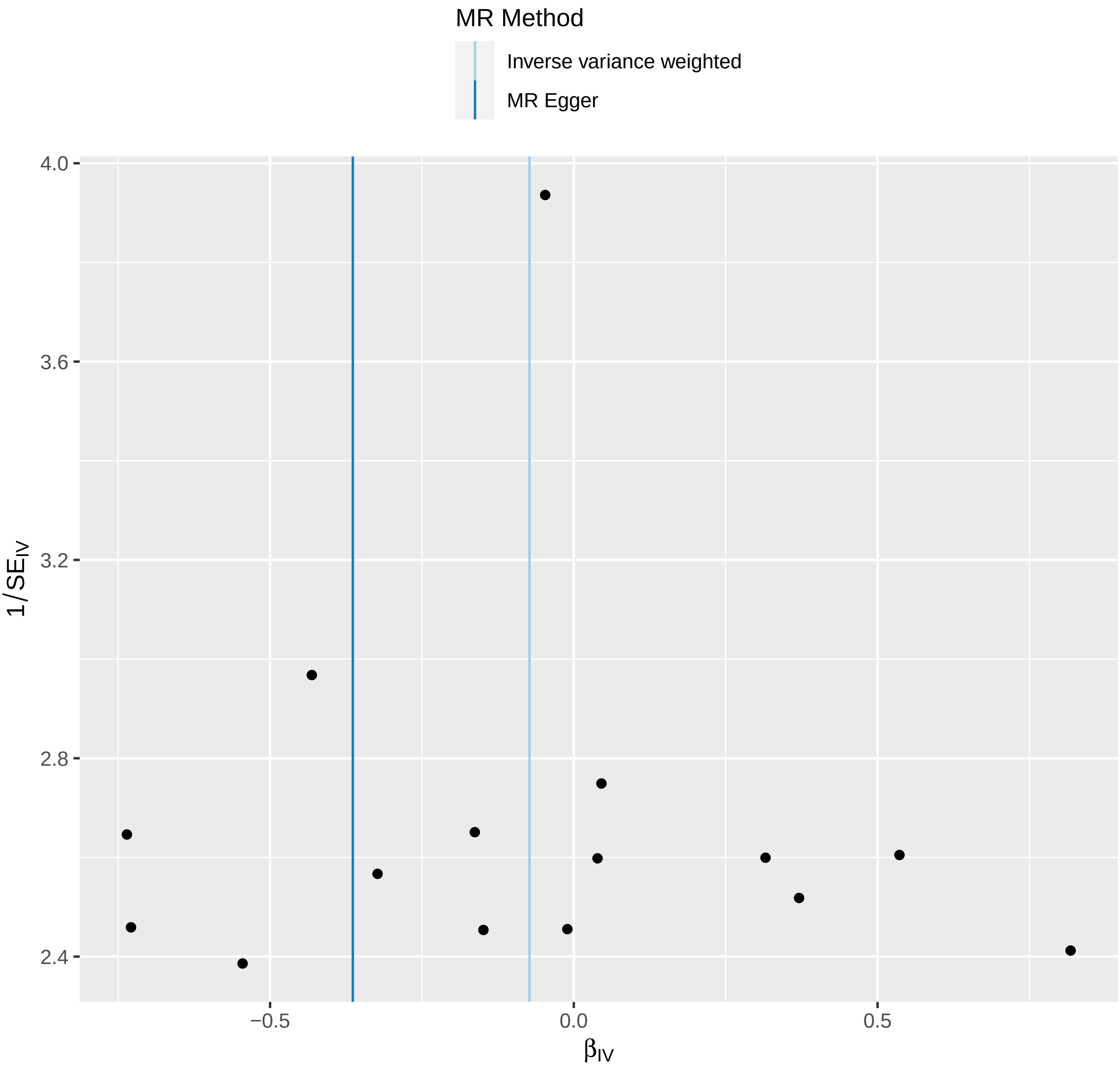

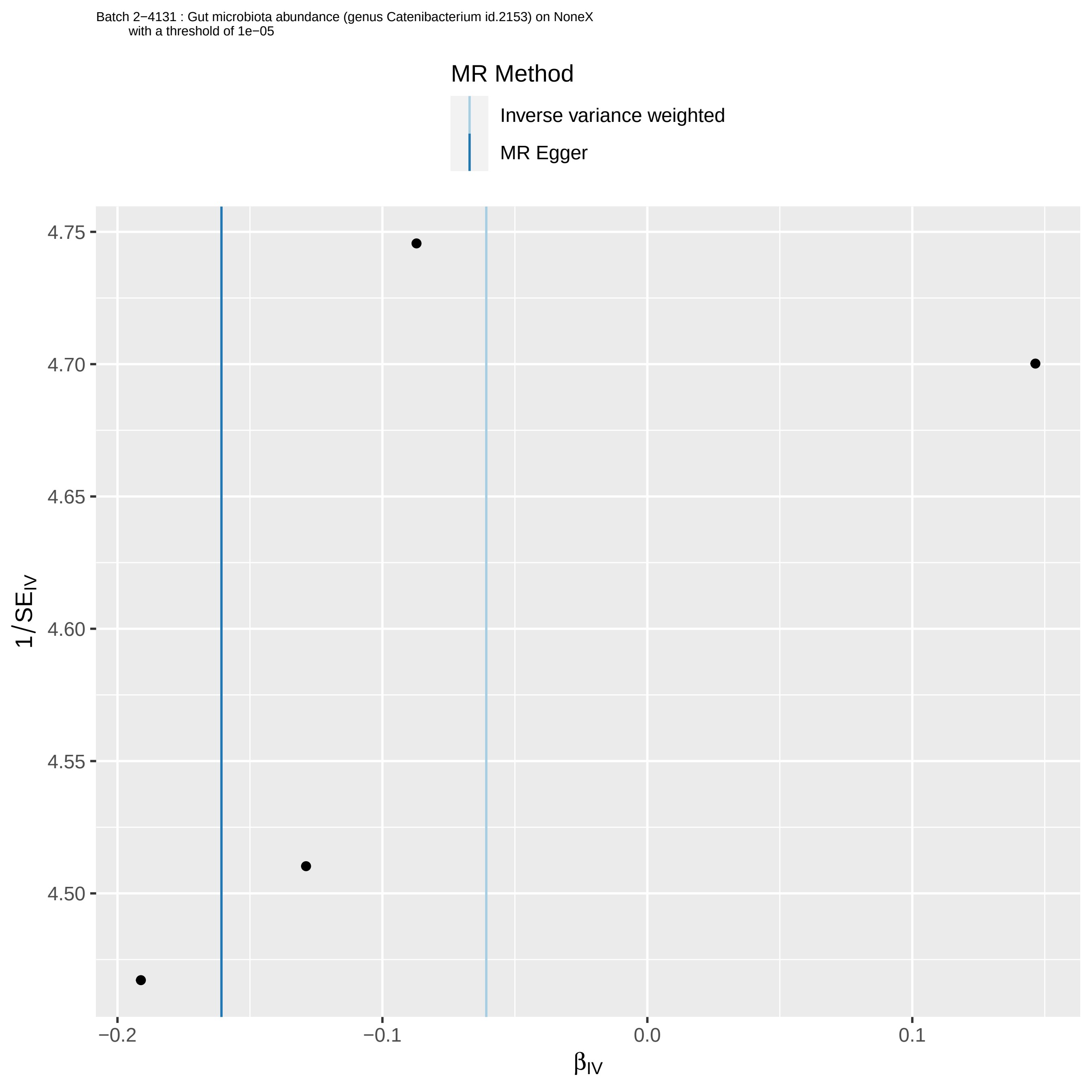

Batch 2−4131 : Gut microbiota abundance (genus Catenibacterium id.2153) on NoneX
with a threshold of 1e−05
MR Method
Inverse variance weighted
MR Egger
4.75
4.70
4.65
4.60
4.55
4.50
1/SE_IV
−0.2
−0.1
0.0
0.1
β_IV

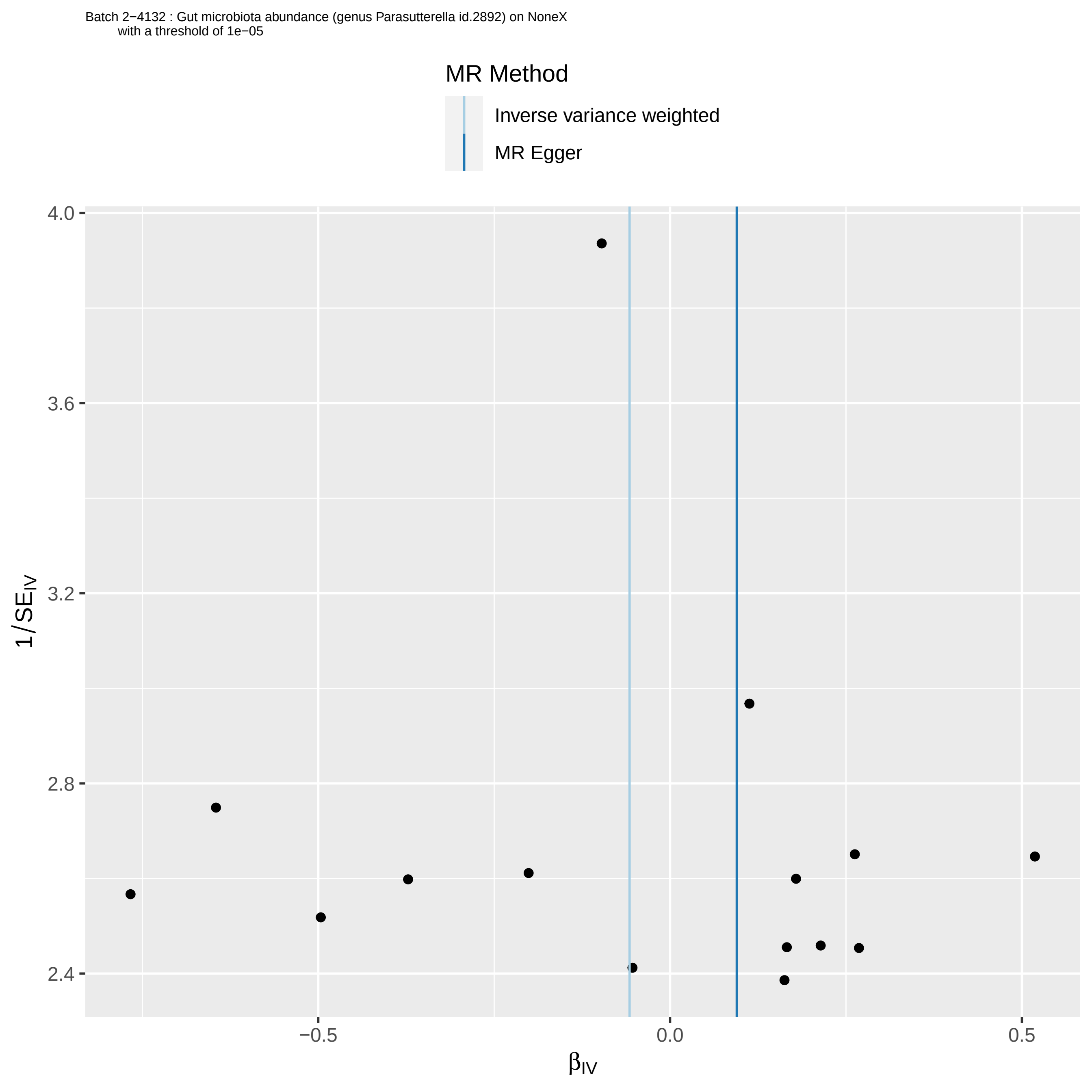

Batch 2−4132 : Gut microbiota abundance (genus Parasutterella id.2892) on NoneX
with a threshold of 1e−05
MR Method
Inverse variance weighted
MR Egger
4.0
3.6
3.2
2.8
2.4
1/SE_IV
−0.5
0.0
0.5
β_IV

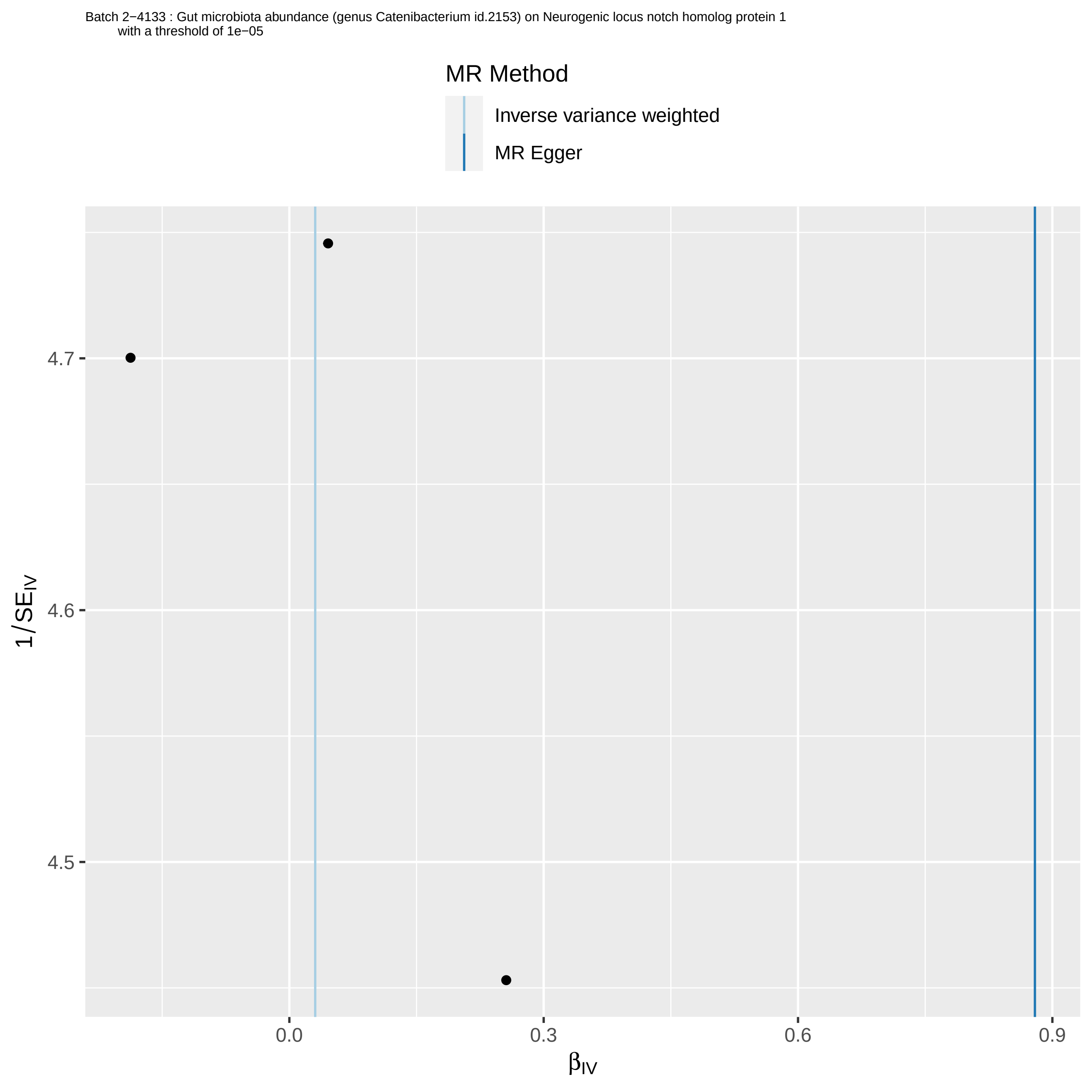

Batch 2−4133 : Gut microbiota abundance (genus Catenibacterium id.2153) on Neurogenic locus notch homolog protein 1
with a threshold of 1e−05
MR Method
Inverse variance weighted
MR Egger
4.7
4.6
4.5
1/SE$_{IV}$
0.0
0.3
0.6
0.9
β$_{IV}$

Batch 2−4134 : Gut microbiota abundance (genus Parasutterella id.2892) on Neurogenic locus notch homolog protein 1
with a threshold of 1e−05

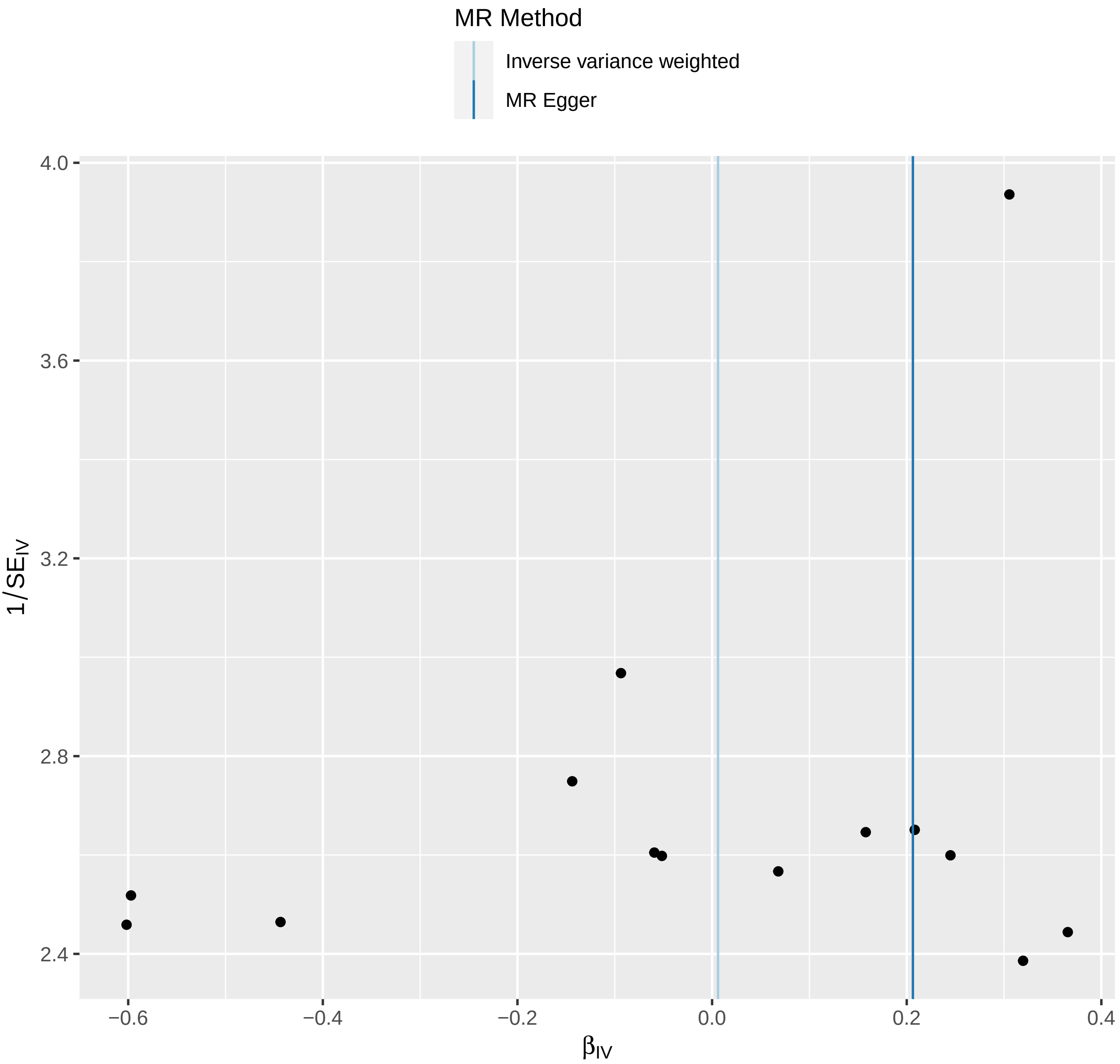

Batch 2−4135 : Gut microbiota abundance (genus Catenibacterium id.2153) on Palmitoleoyl−protein carboxylesterase NOTUM
with a threshold of 1e−05

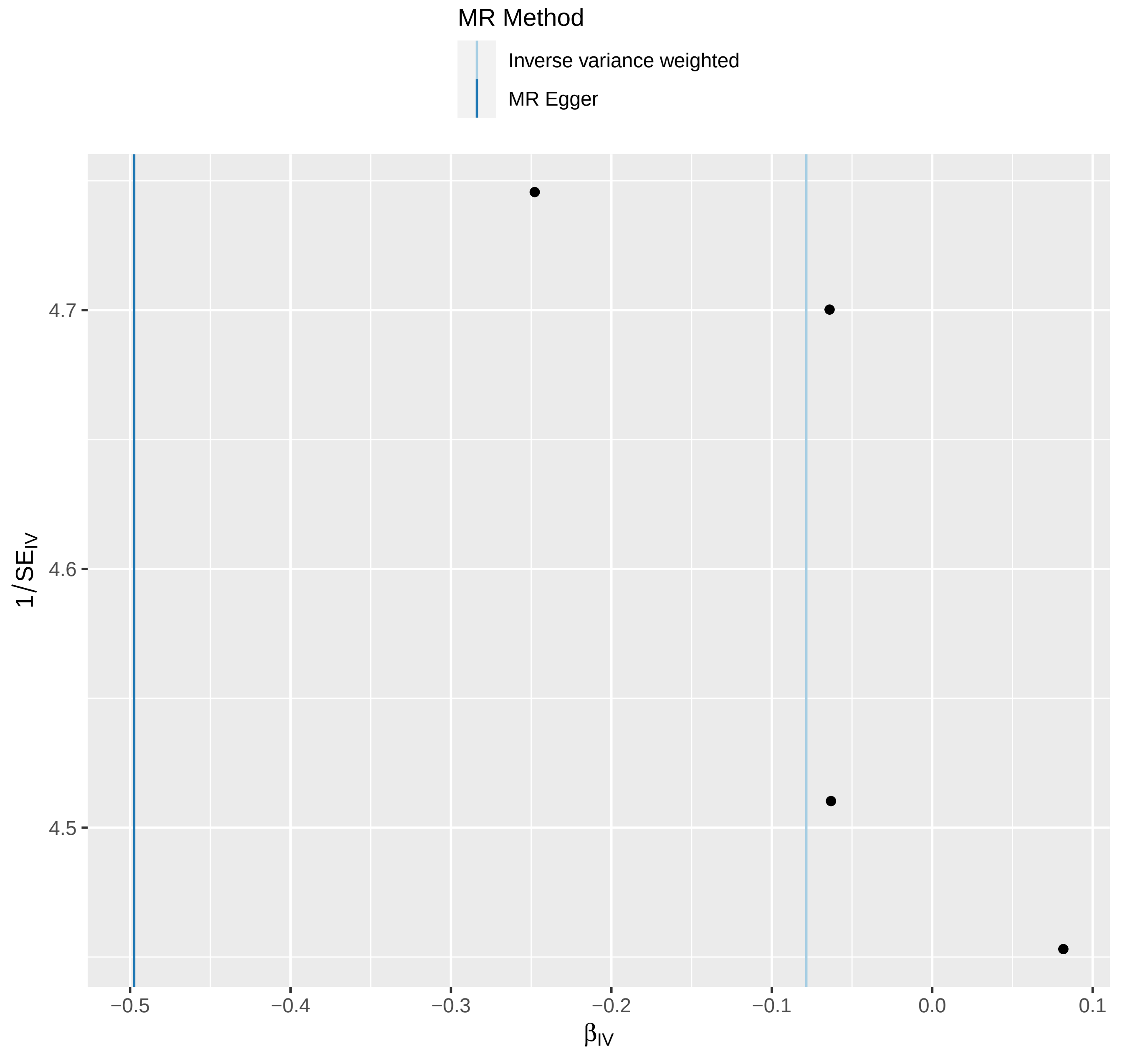

Batch 2−4136 : Gut microbiota abundance (genus Parasutterella id.2892) on Palmitoleoyl−protein carboxylesterase NOTUM
with a threshold of 1e−05

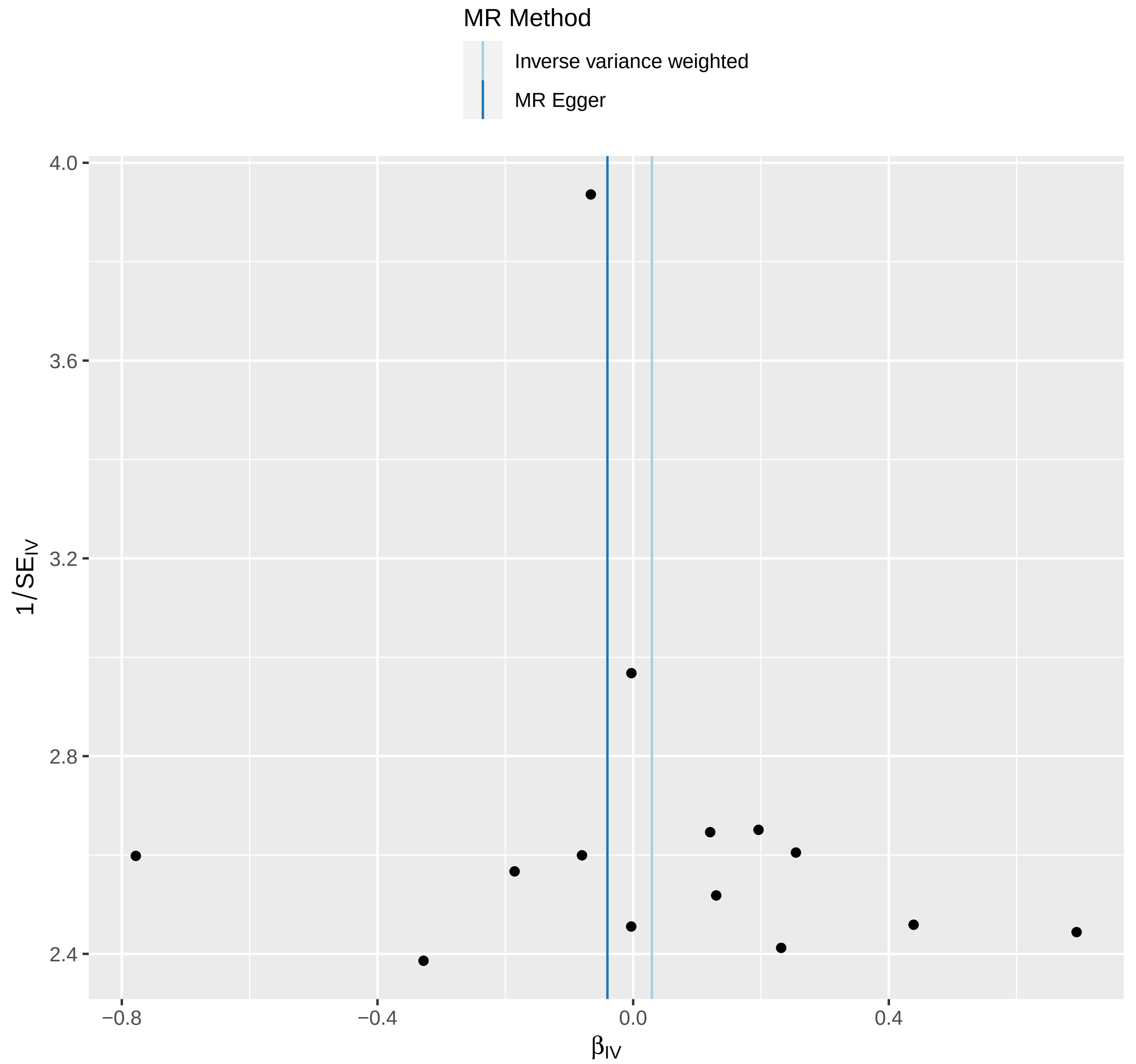

Batch 2−4137 : Gut microbiota abundance (genus Catenibacterium id.2153) on Protein NOV homolog
with a threshold of 1e−05

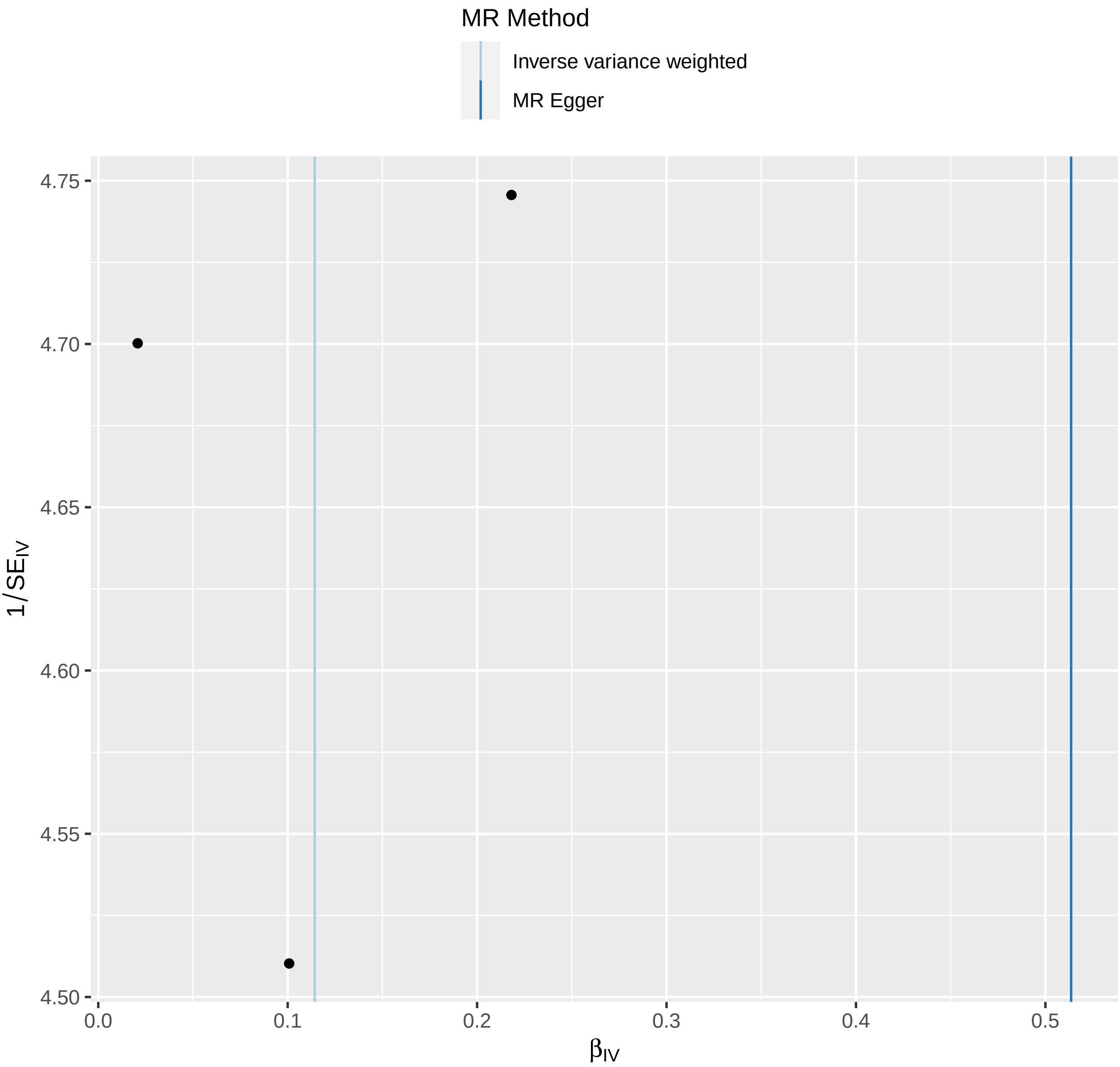

Batch 2−4138 : Gut microbiota abundance (genus Parasutterella id.2892) on Protein NOV homolog
with a threshold of 1e−05

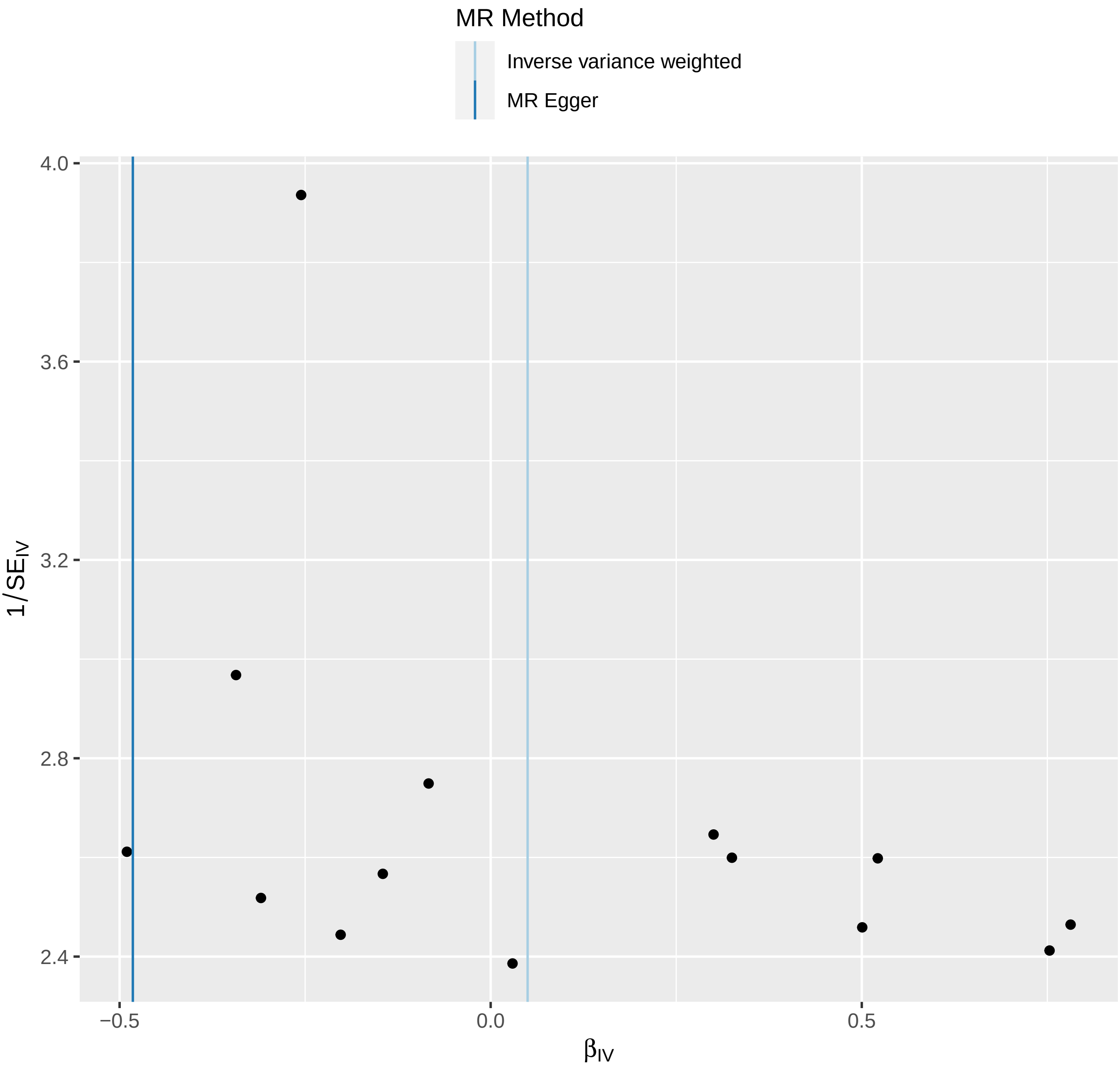

Batch 2−4139 : Gut microbiota abundance (genus Catenibacterium id.2153) on RNA−binding protein Nova−1
with a threshold of 1e−05

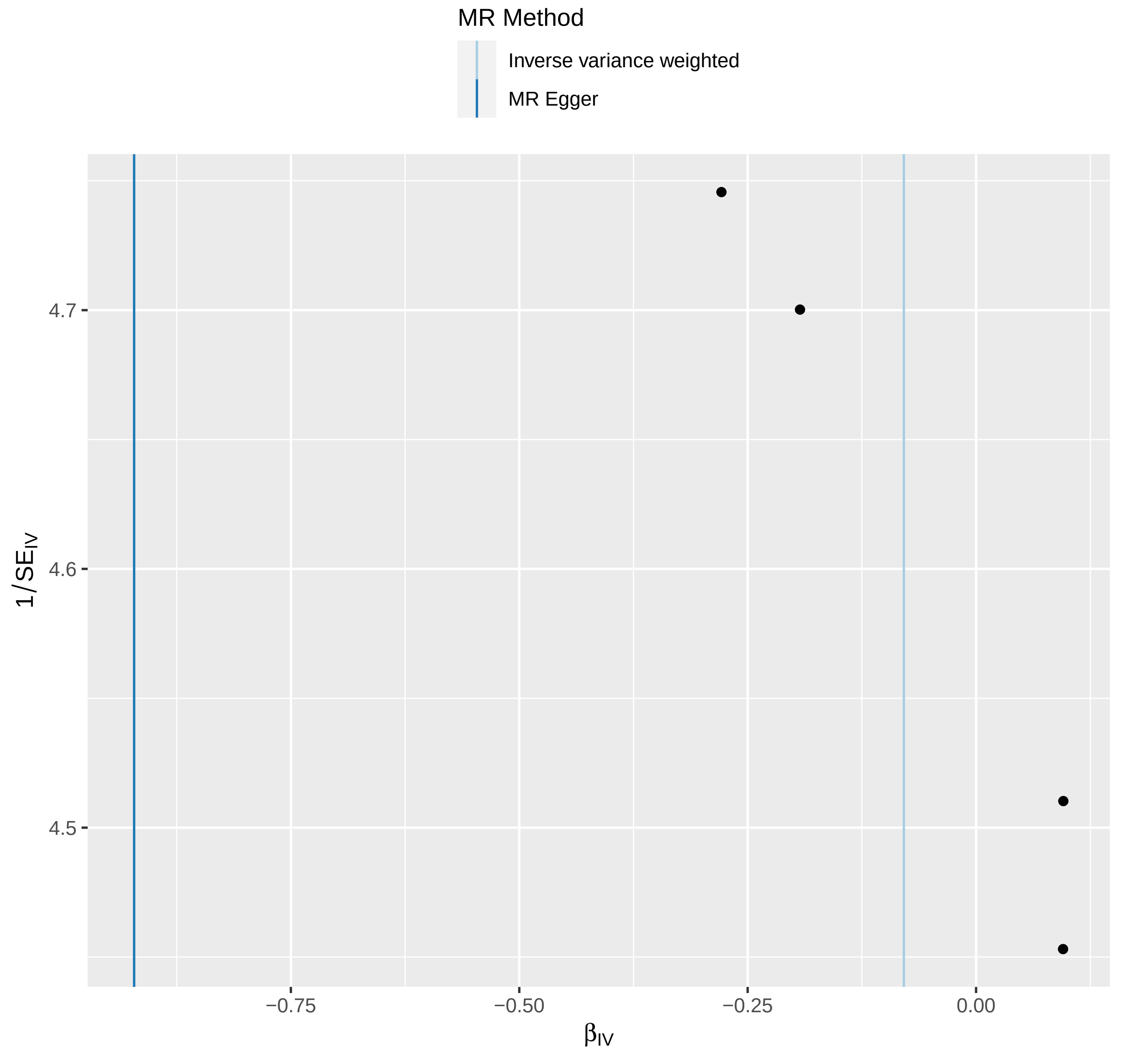

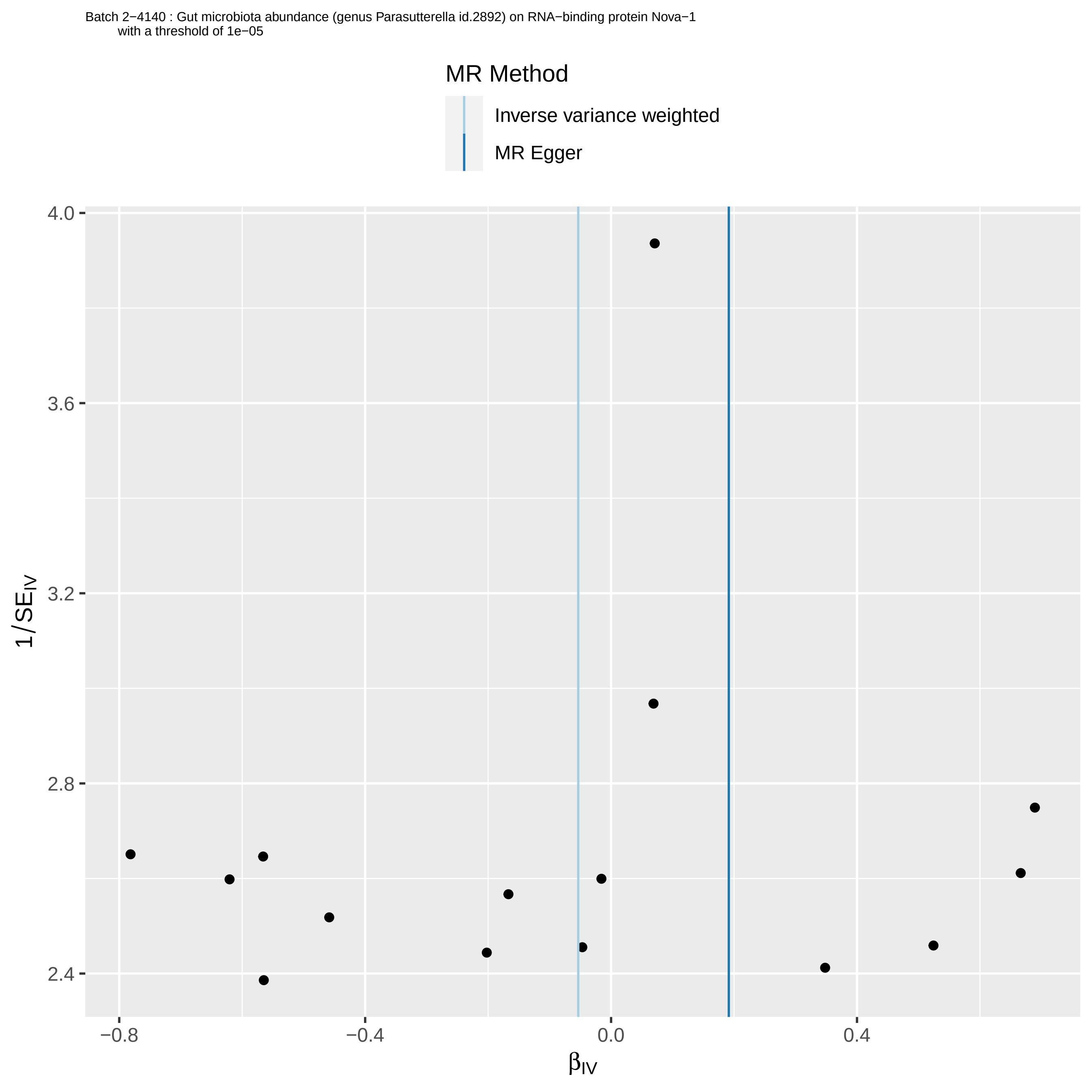
Batch 2−4140 : Gut microbiota abundance (genus Parasutterella id.2892) on RNA−binding protein Nova−1
with a threshold of 1e−05
MR Method
Inverse variance weighted
MR Egger
4.0
3.6
3.2
2.8
2.4
1/SE_IV
−0.8
−0.4
0.0
0.4
β_IV

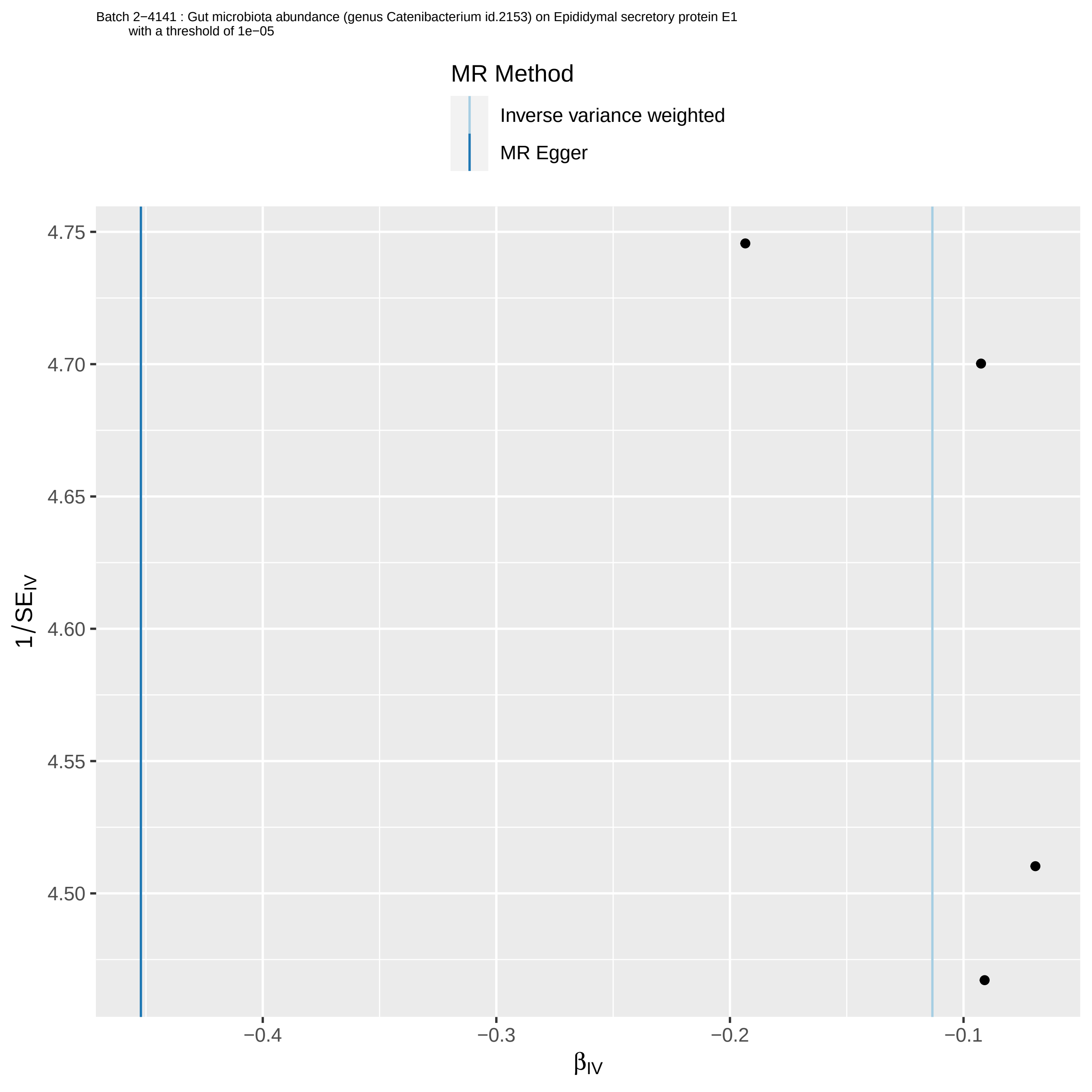
Batch 2−4141 : Gut microbiota abundance (genus Catenibacterium id.2153) on Epididymal secretory protein E1
with a threshold of 1e−05
MR Method
Inverse variance weighted
MR Egger
4.75
4.70
4.65
4.60
4.55
4.50
1/SE_IV
−0.4
−0.3
−0.2
−0.1
β_IV

Batch 2−4142 : Gut microbiota abundance (genus Parasutterella id.2892) on Epididymal secretory protein E1
with a threshold of 1e−05

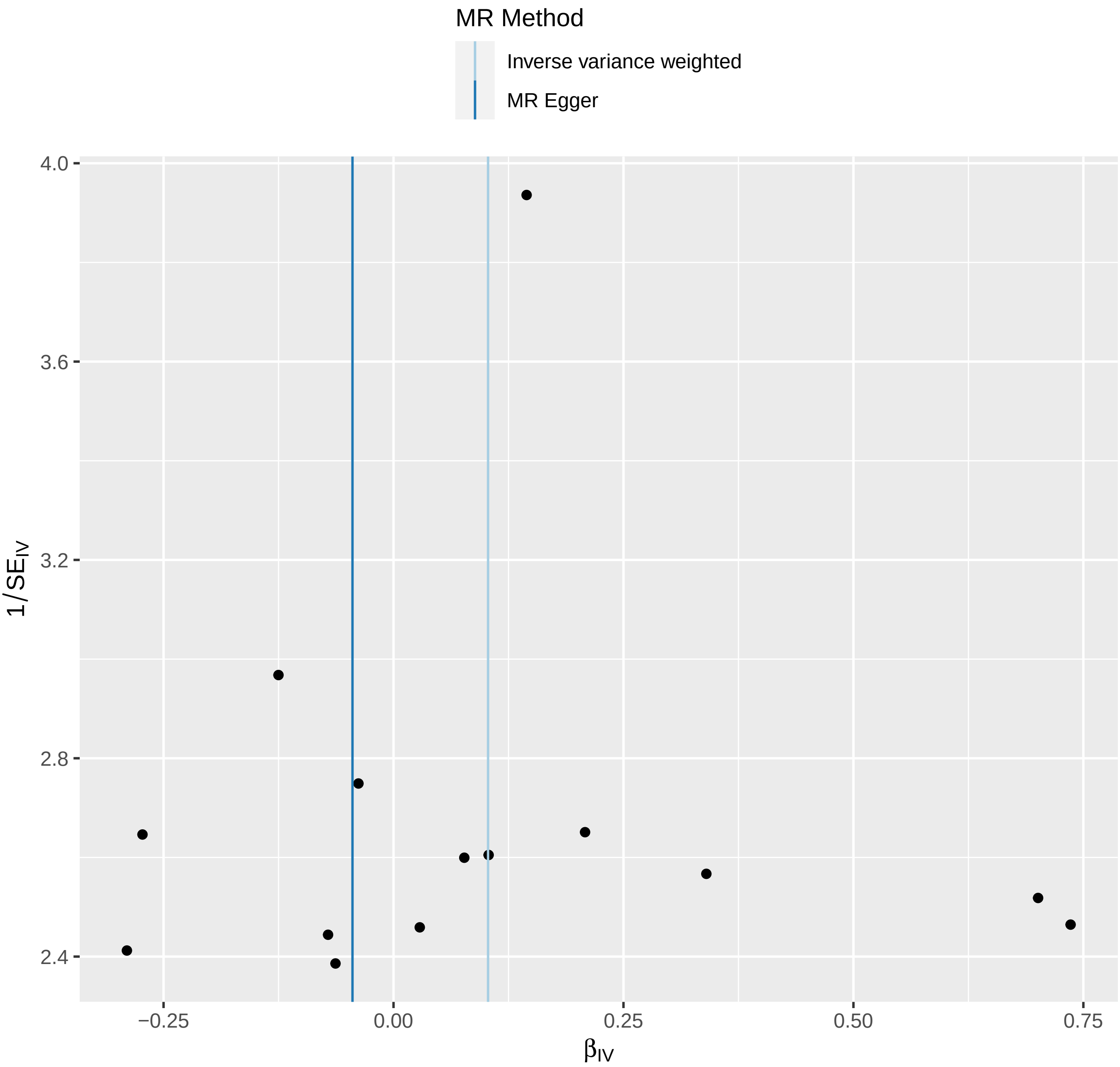

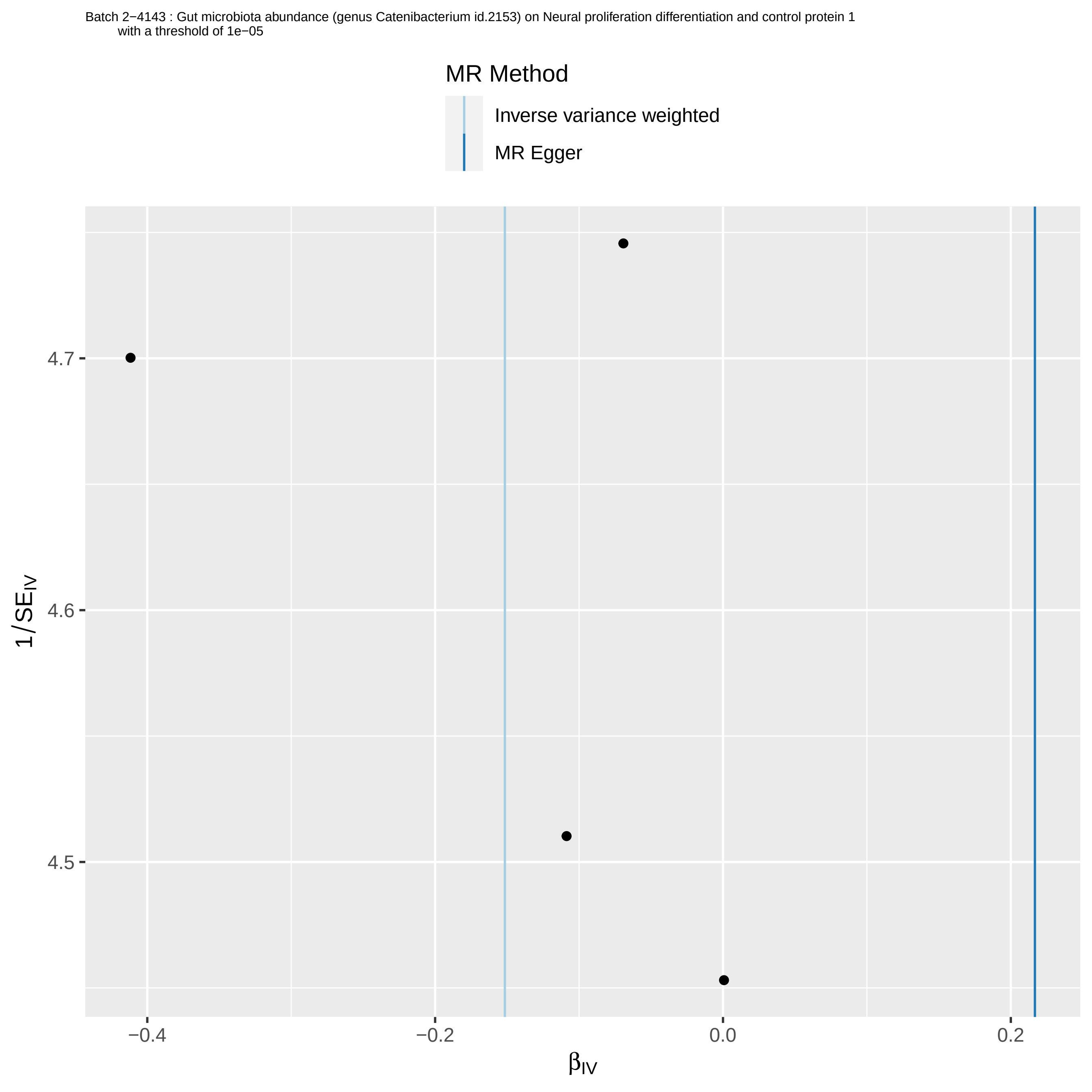

Batch 2−4143 : Gut microbiota abundance (genus Catenibacterium id.2153) on Neural proliferation differentiation and control protein 1
with a threshold of 1e−05
MR Method
Inverse variance weighted
MR Egger
4.7
4.6
4.5
1/SE_IV
−0.4
−0.2
0.0
0.2
β_IV

Batch 2−4144 : Gut microbiota abundance (genus Parasutterella id.2892) on Neural proliferation differentiation and control protein 1 with a threshold of 1e−05

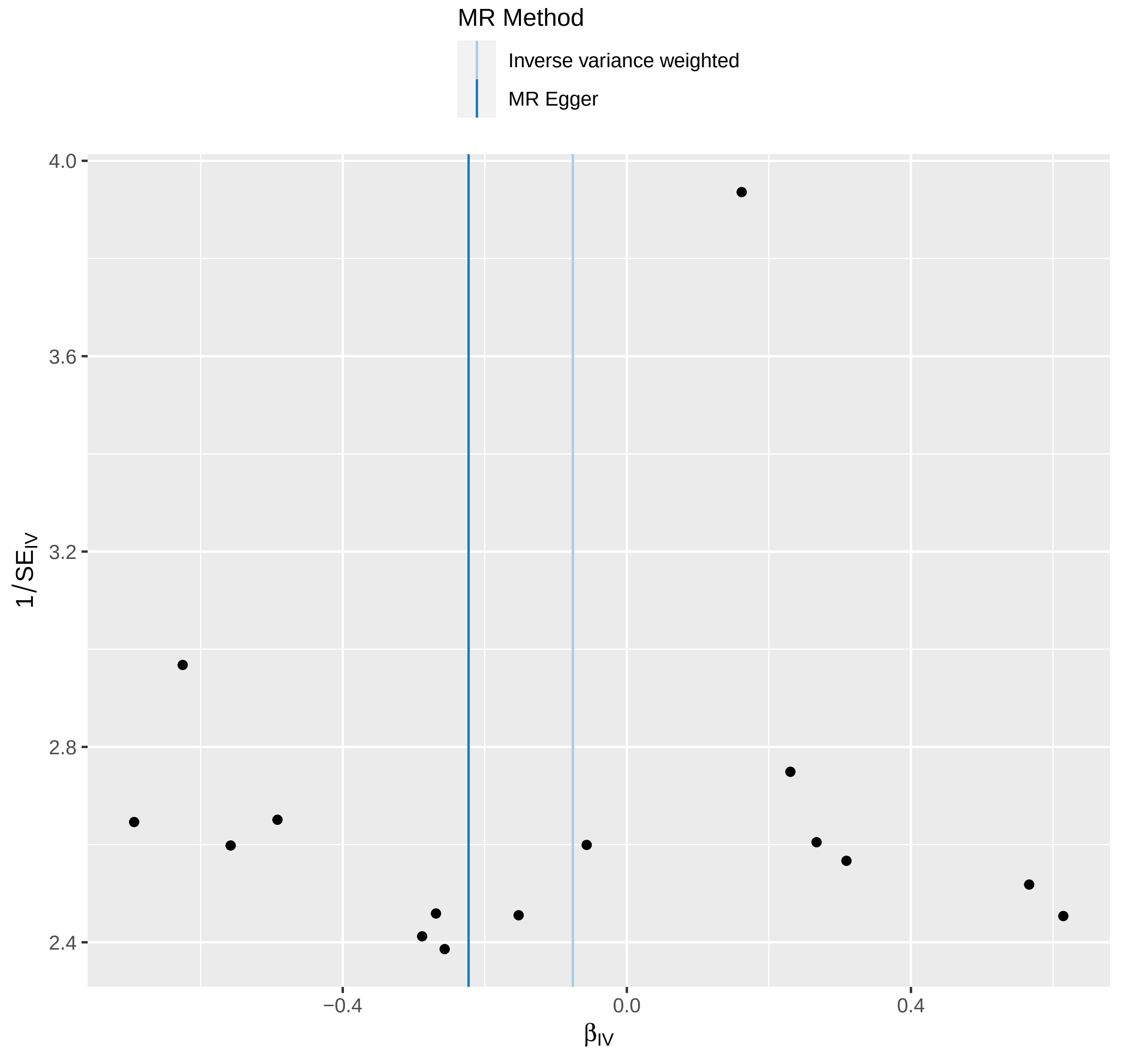

Batch 2−4145 : Gut microbiota abundance (genus Catenibacterium id.2153) on Pro−FMRFamide−related neuropeptide FF
with a threshold of 1e−05

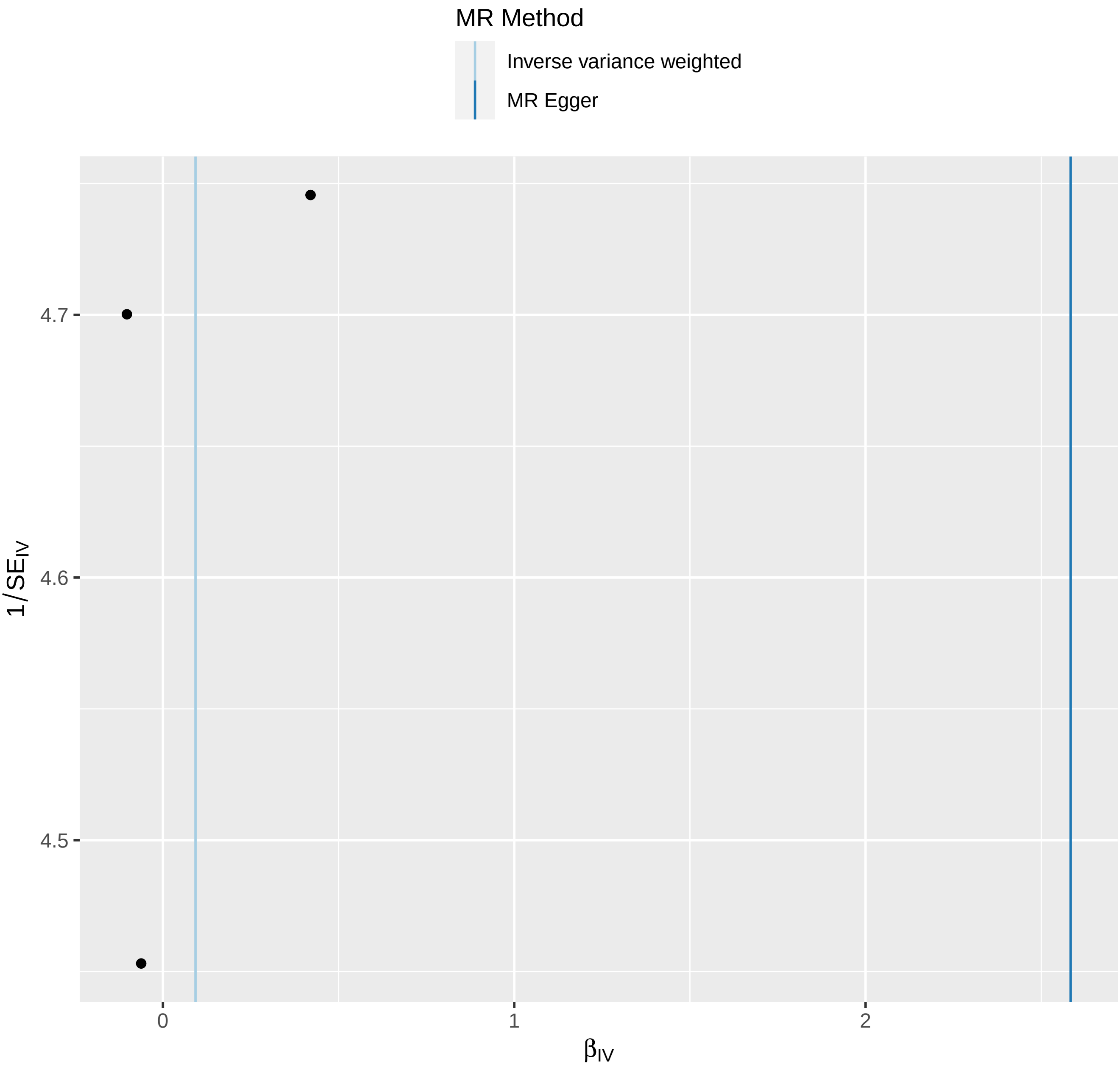

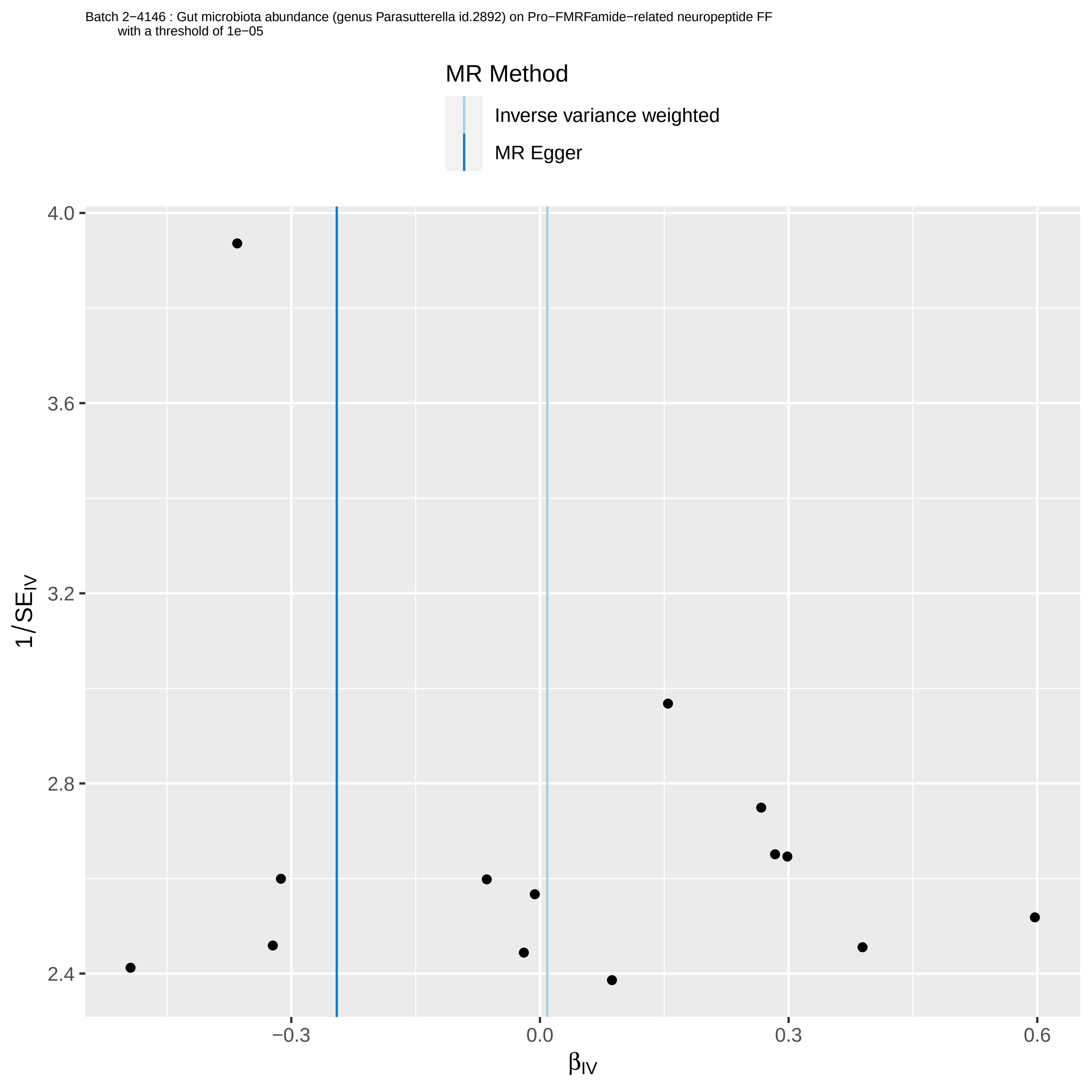

Batch 2−4146 : Gut microbiota abundance (genus Parasutterella id.2892) on Pro−FMRFamide−related neuropeptide FF
with a threshold of 1e−05
MR Method
Inverse variance weighted
MR Egger
4.0
3.6
3.2
2.8
2.4
1/SE$_{IV}$
−0.3
0.0
0.3
0.6
β_{IV}

Batch 2−4147 : Gut microbiota abundance (genus Catenibacterium id.2153) on Nuclear pore complex−interacting protein family member B3
with a threshold of 1e−05

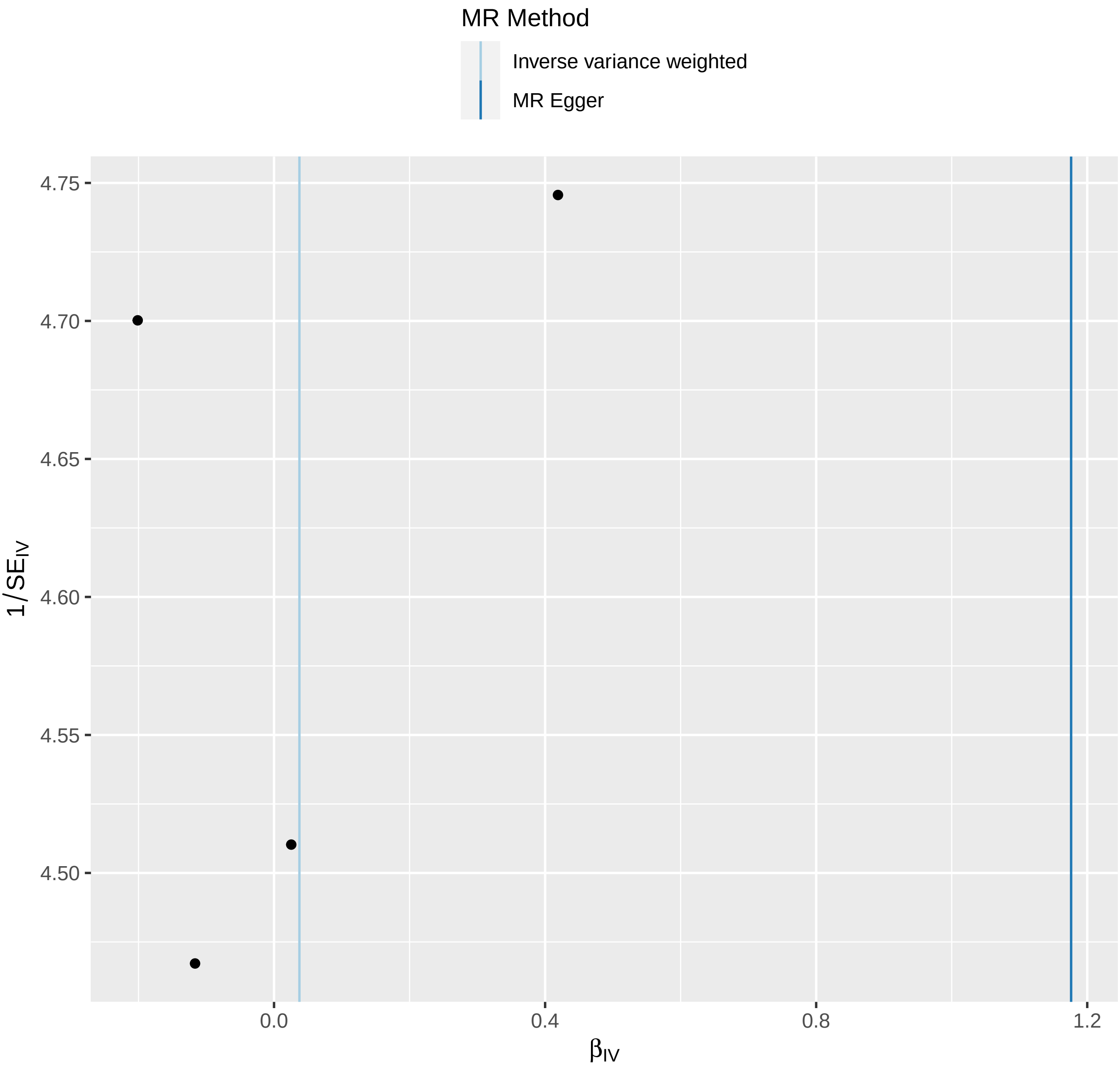

Batch 2−4148 : Gut microbiota abundance (genus Parasutterella id.2892) on Nuclear pore complex−interacting protein family member B3 with a threshold of 1e−05

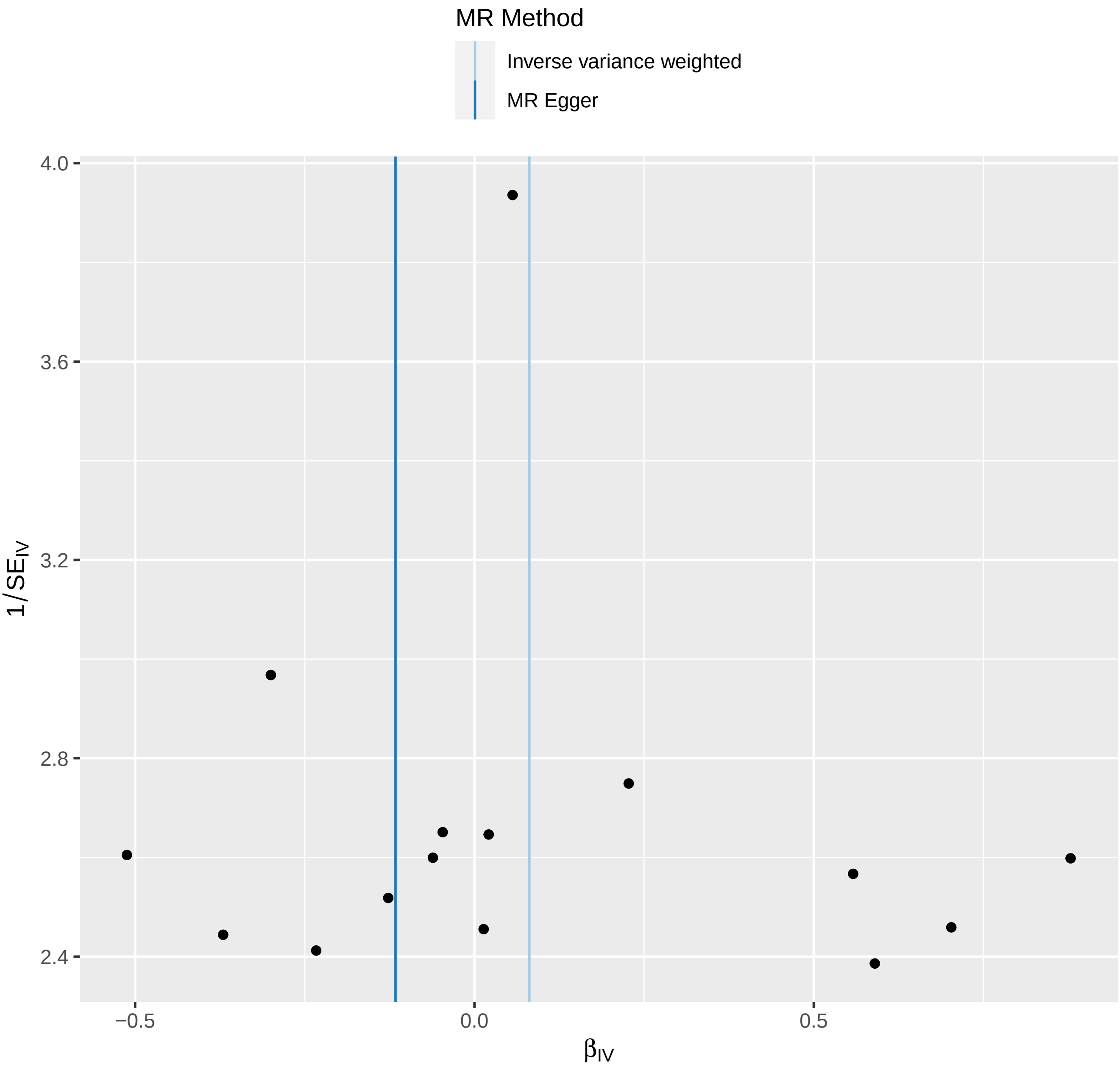

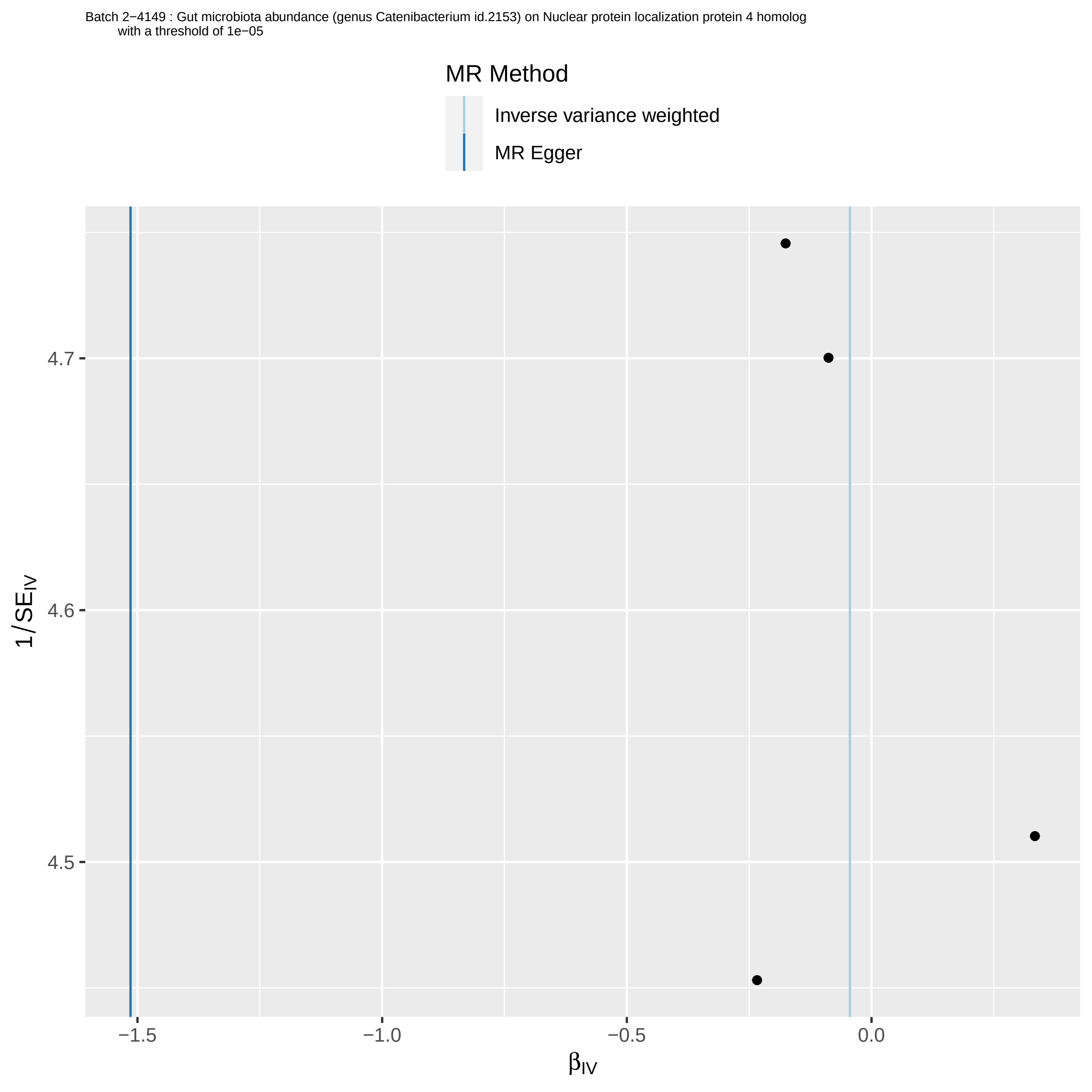
Batch 2−4149 : Gut microbiota abundance (genus Catenibacterium id.2153) on Nuclear protein localization protein 4 homolog
with a threshold of 1e−05
MR Method
Inverse variance weighted
MR Egger
4.7
4.6
4.5
1/SE_IV
−1.5
−1.0
−0.5
0.0
β_IV

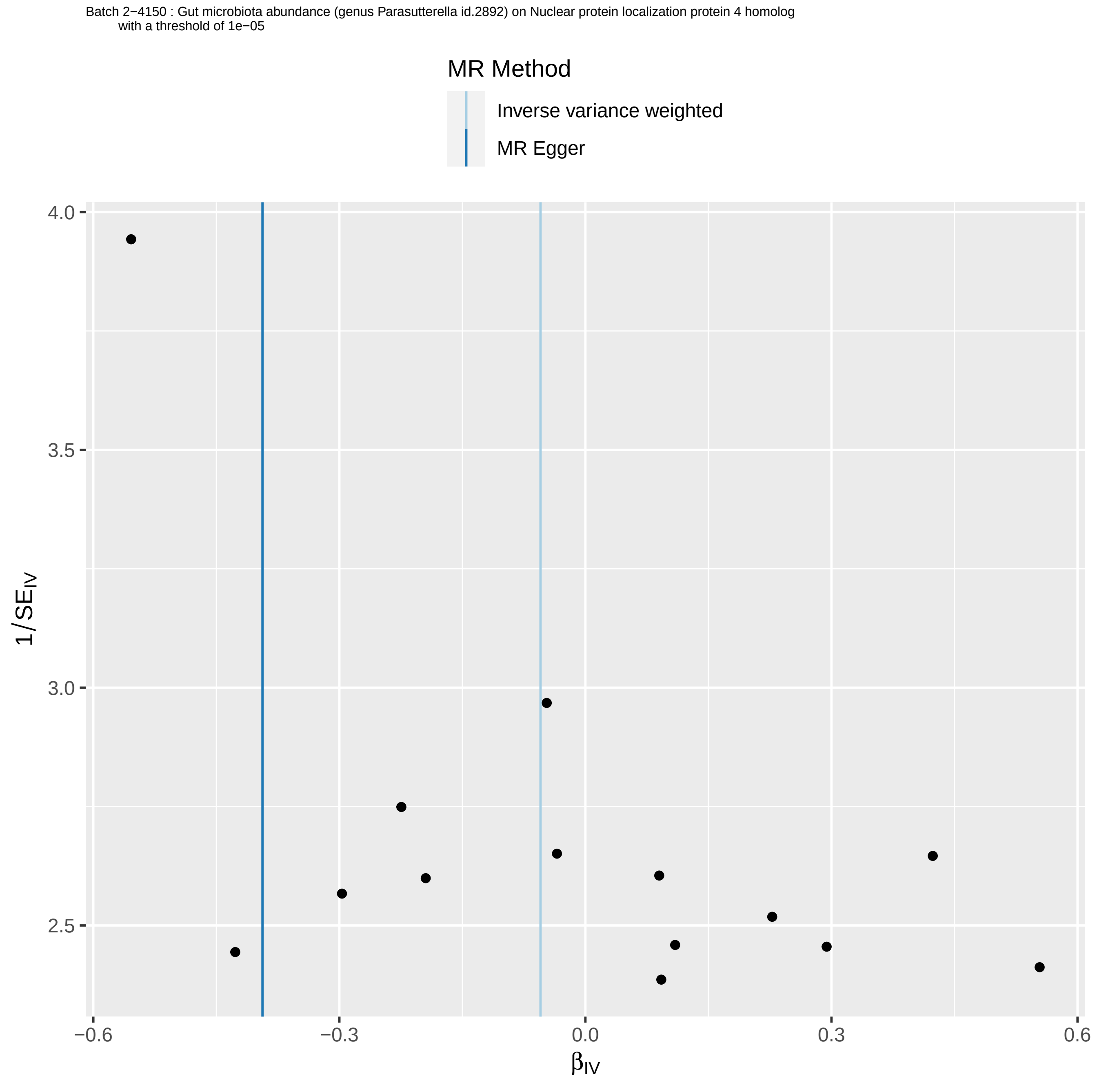
Batch 2−4150 : Gut microbiota abundance (genus Parasutterella id.2892) on Nuclear protein localization protein 4 homolog
with a threshold of 1e−05
MR Method
Inverse variance weighted
MR Egger
1/SE_IV
4.0
3.5
3.0
2.5
−0.6
−0.3
0.0
0.3
0.6
β_IV

Batch 2−4151 : Gut microbiota abundance (genus Catenibacterium id.2153) on Atrial natriuretic factor
with a threshold of 1e−05

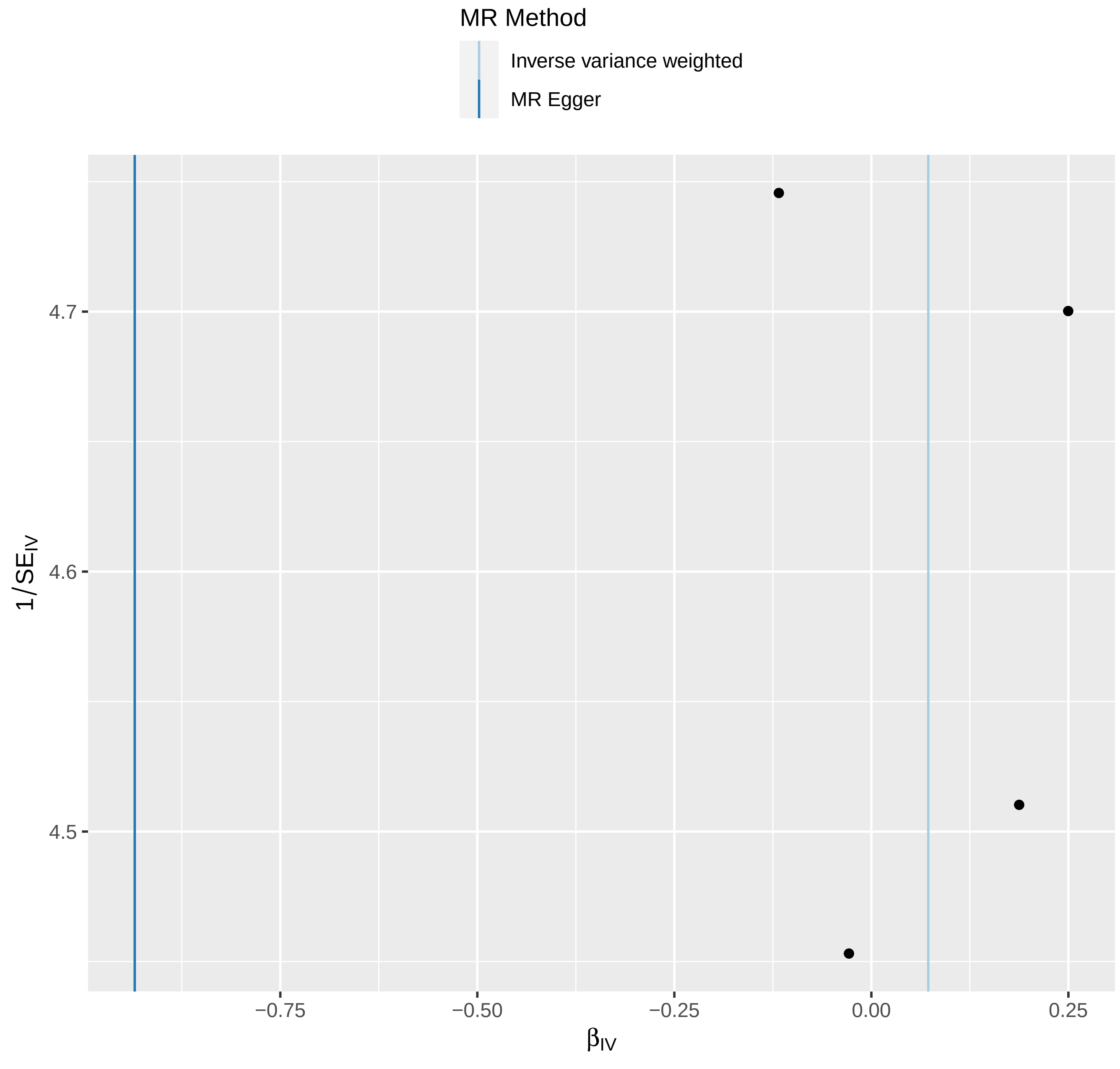

Batch 2−4152 : Gut microbiota abundance (genus Parasutterella id.2892) on Atrial natriuretic factor
with a threshold of 1e−05

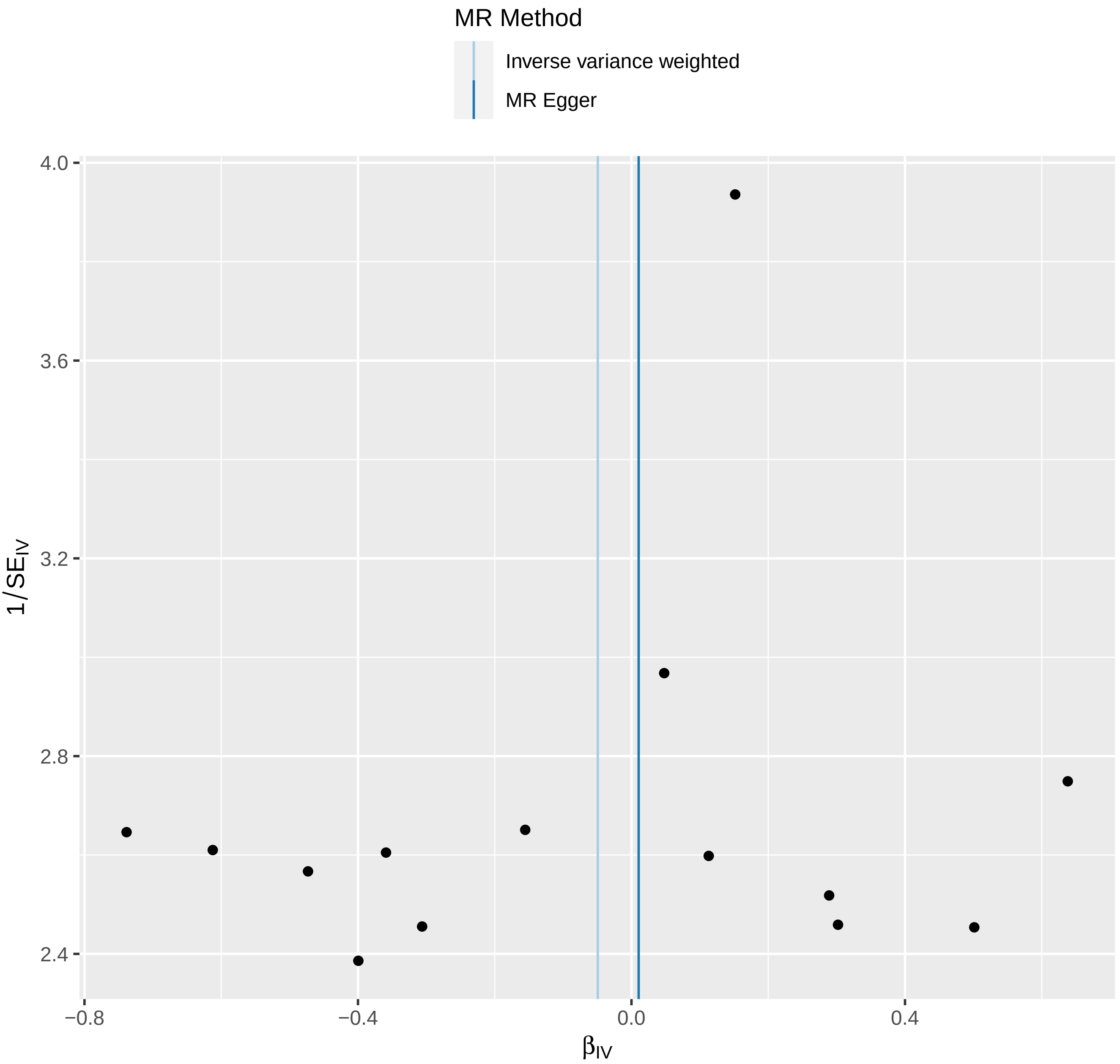

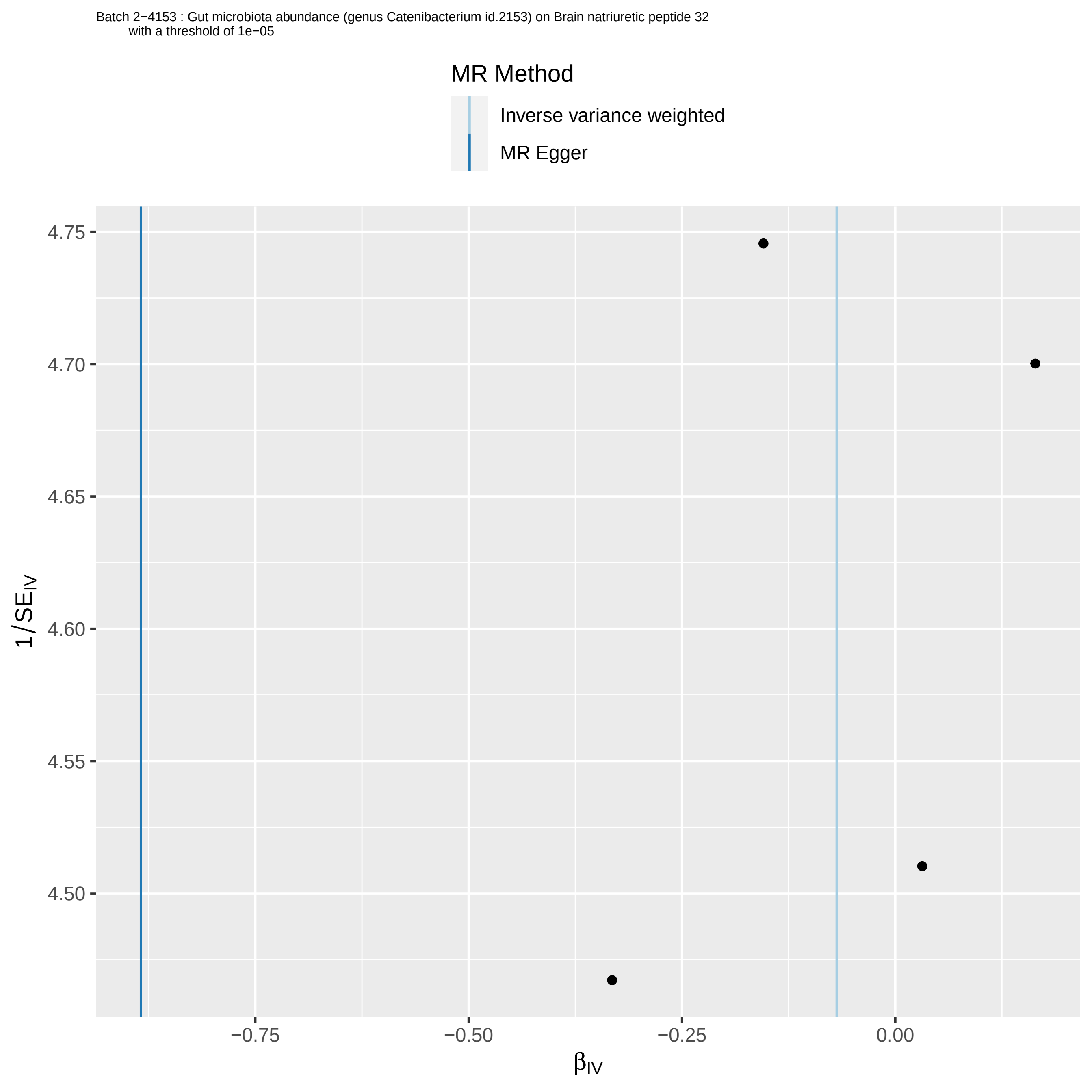
Batch 2−4153 : Gut microbiota abundance (genus Catenibacterium id.2153) on Brain natriuretic peptide 32
with a threshold of 1e−05
MR Method
Inverse variance weighted
MR Egger
4.75
4.70
4.65
4.60
4.55
4.50
1/SE_IV
−0.75
−0.50
−0.25
0.00
β_IV

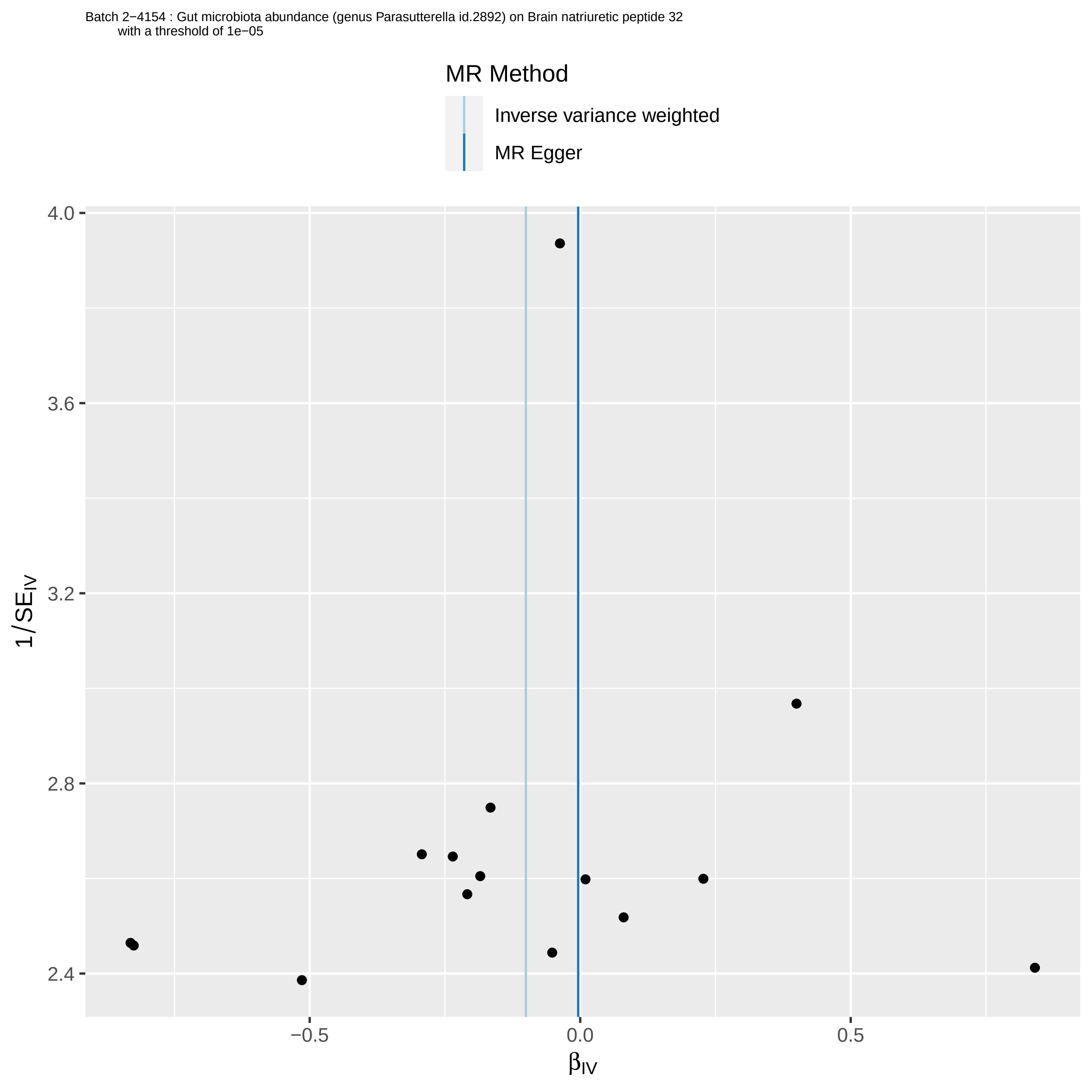
Batch 2−4154 : Gut microbiota abundance (genus Parasutterella id.2892) on Brain natriuretic peptide 32
with a threshold of 1e−05
MR Method
Inverse variance weighted
MR Egger
4.0
3.6
3.2
2.8
2.4
1/SE_IV
−0.5
0.0
0.5
β_IV

Batch 2−4155 : Gut microbiota abundance (genus Catenibacterium id.2153) on N−terminal pro−BNP
with a threshold of 1e−05

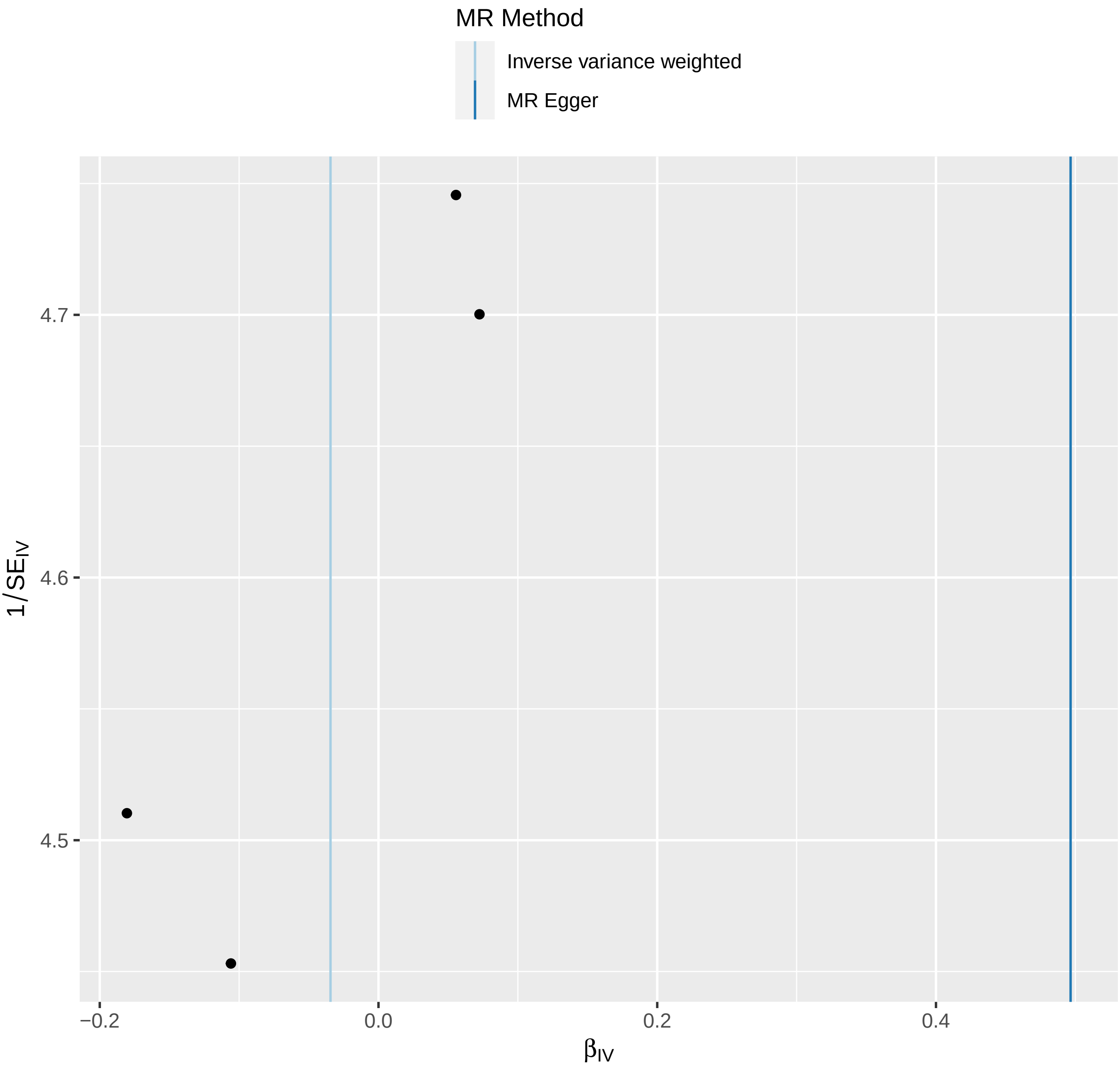

Batch 2−4156 : Gut microbiota abundance (genus Parasutterella id.2892) on N−terminal pro−BNP
with a threshold of 1e−05

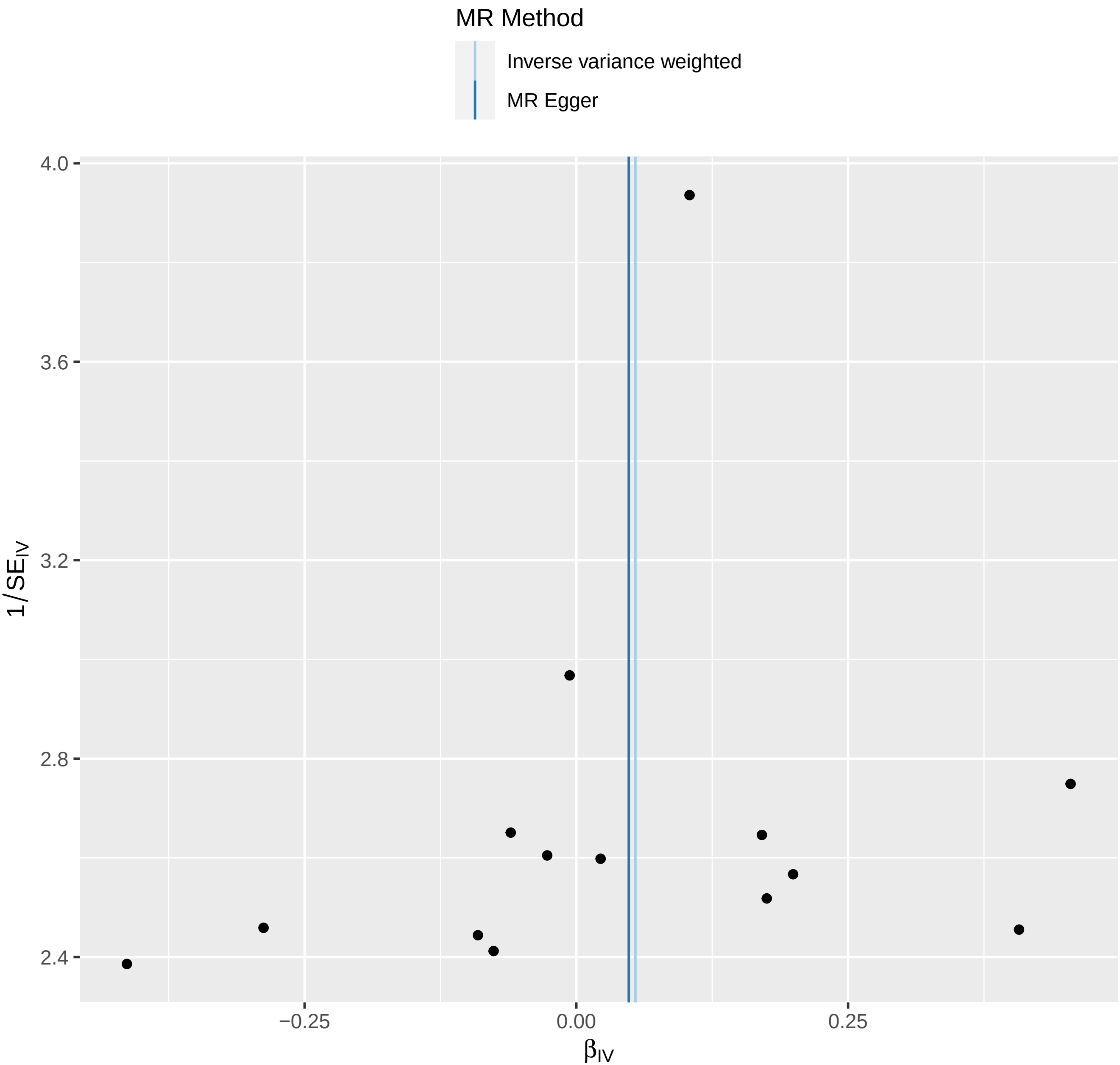

Batch 2−4157 : Gut microbiota abundance (genus Catenibacterium id.2153) on Neuropeptide S
with a threshold of 1e−05

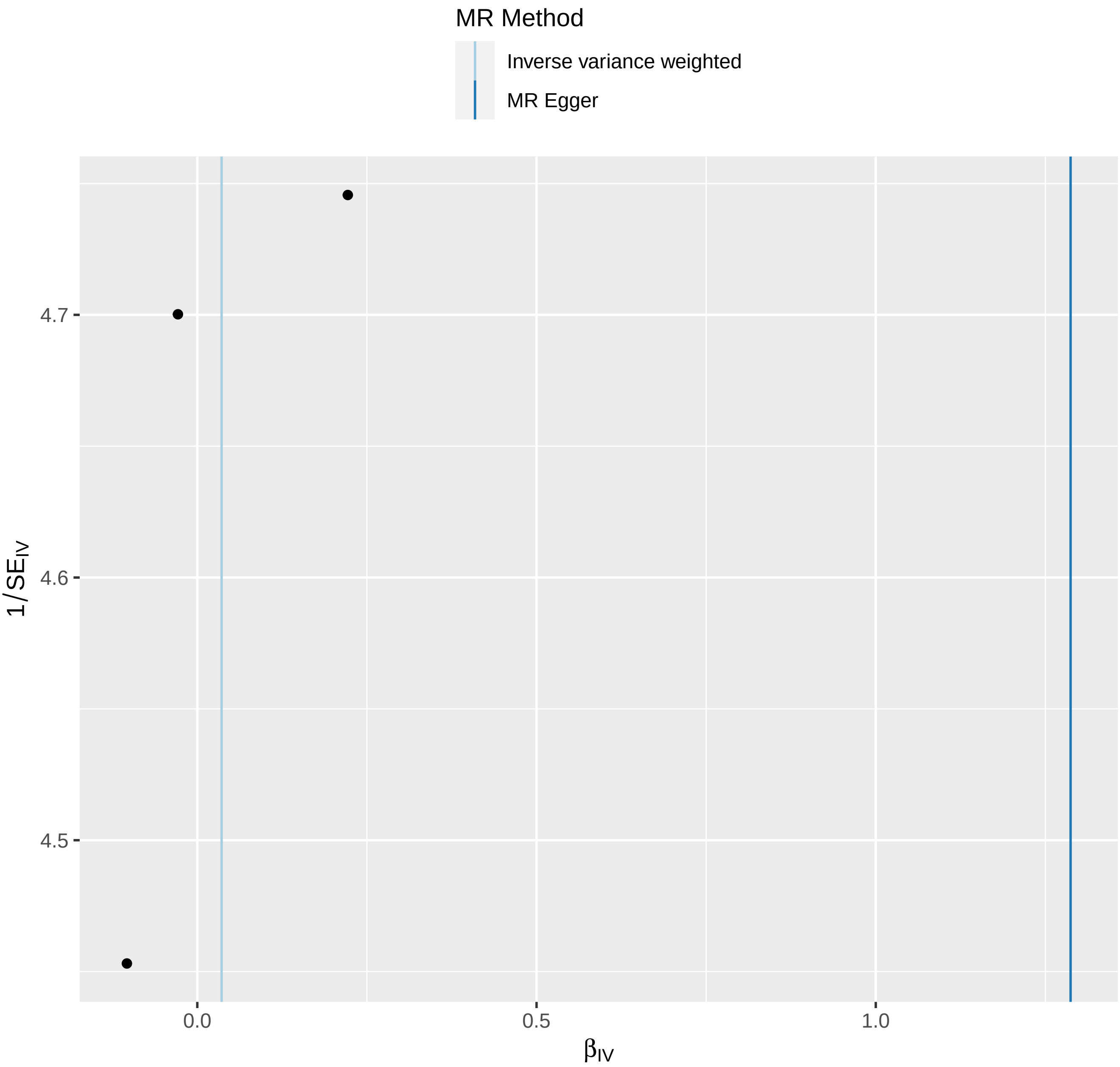

Batch 2−4158 : Gut microbiota abundance (genus Parasutterella id.2892) on Neuropeptide S
with a threshold of 1e−05

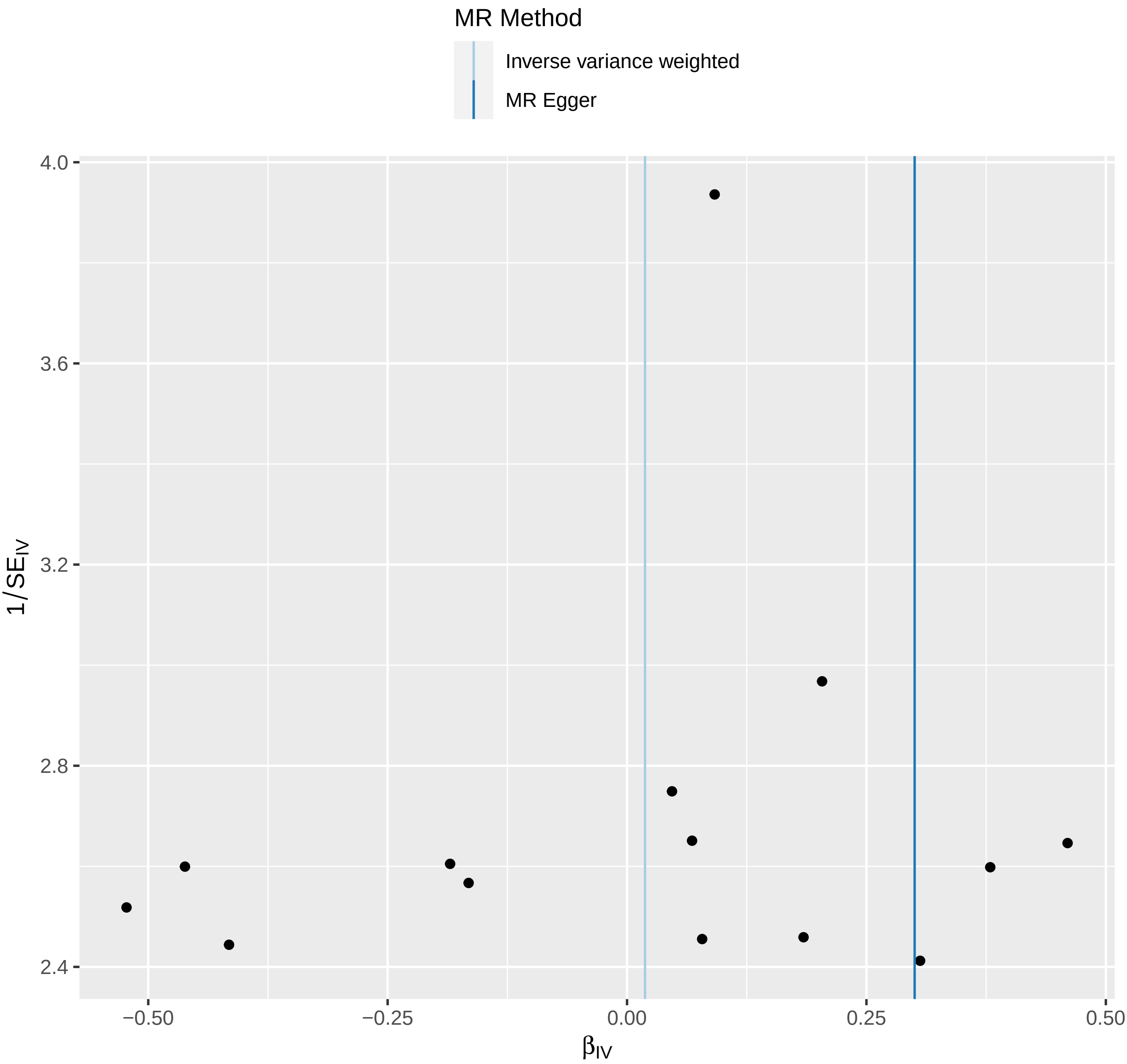

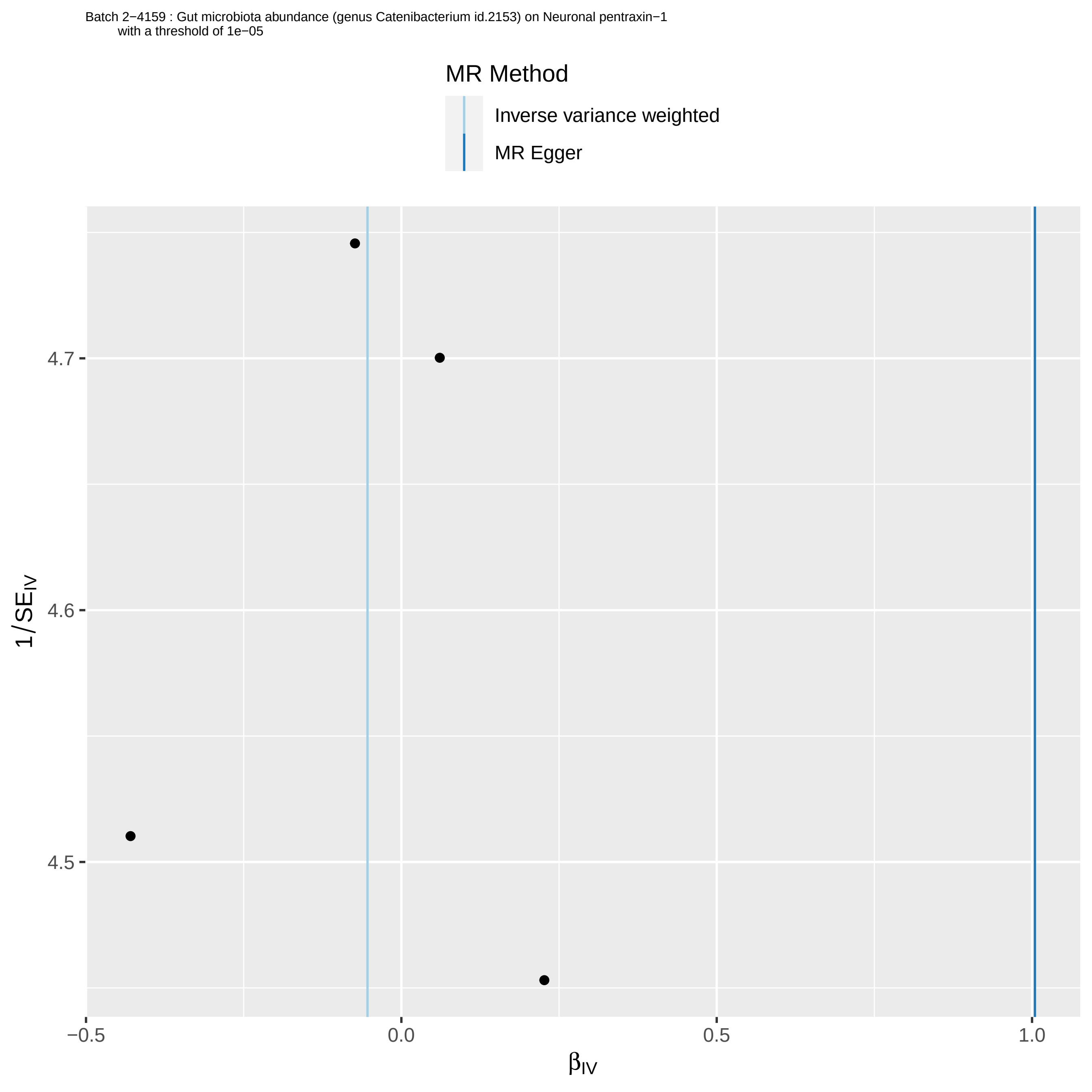
Batch 2−4159 : Gut microbiota abundance (genus Catenibacterium id.2153) on Neuronal pentraxin−1
with a threshold of 1e−05
MR Method
Inverse variance weighted
MR Egger
4.7
4.6
4.5
1/SE_IV
−0.5
0.0
0.5
1.0
β_IV

Batch 2−4160 : Gut microbiota abundance (genus Parasutterella id.2892) on Neuronal pentraxin−1
with a threshold of 1e−05

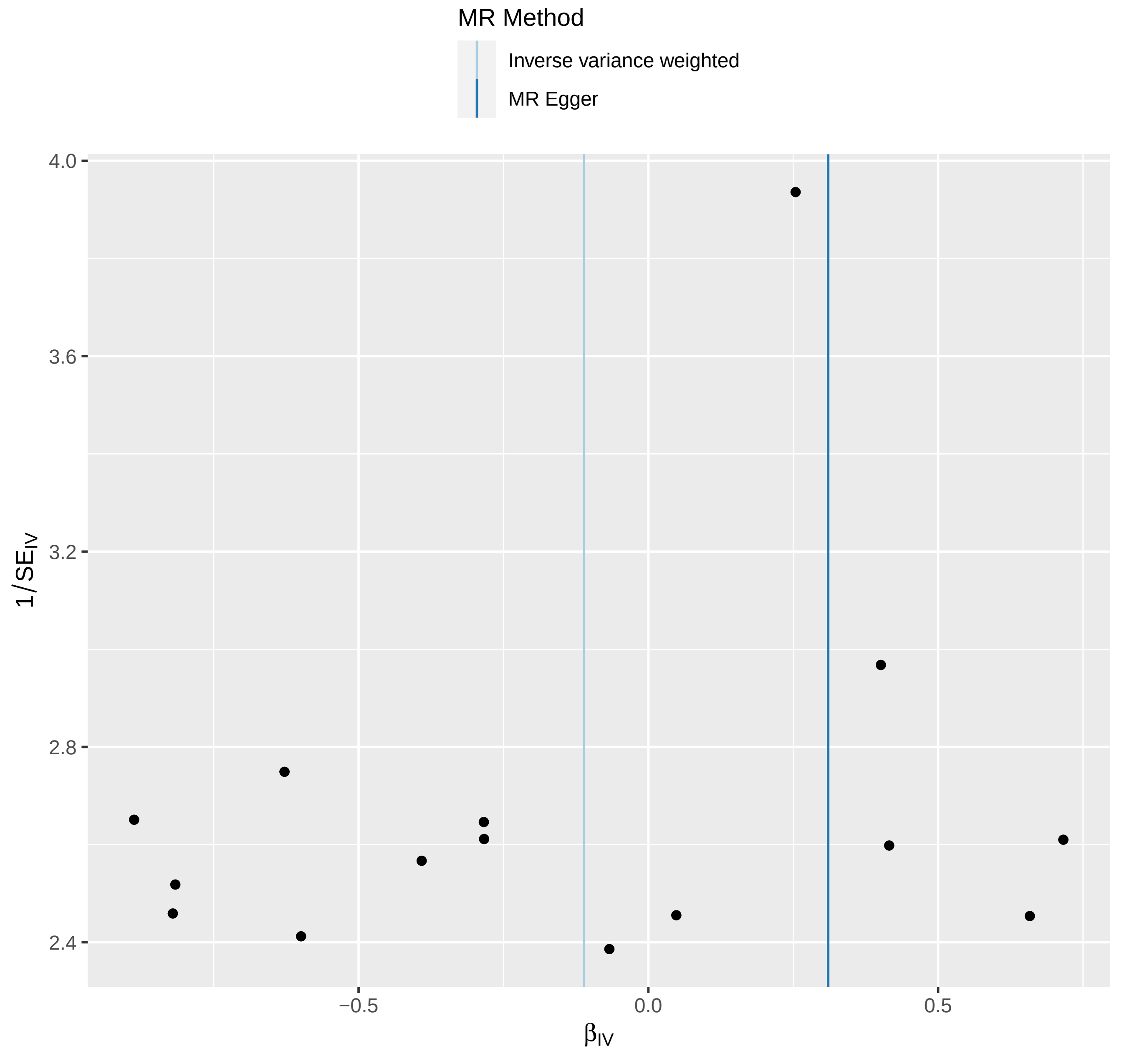

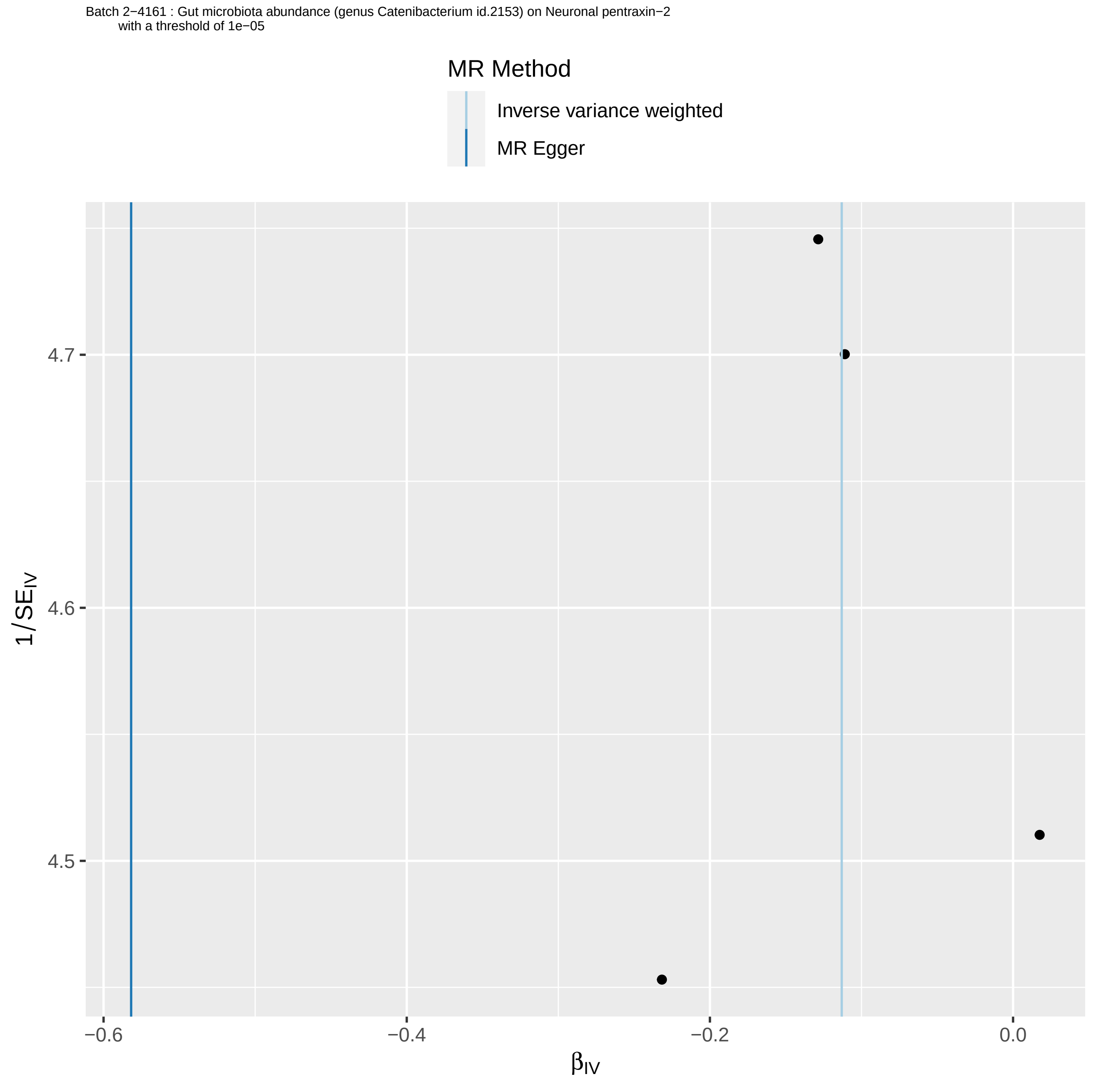
Batch 2−4161 : Gut microbiota abundance (genus Catenibacterium id.2153) on Neuronal pentraxin−2
with a threshold of 1e−05
MR Method
Inverse variance weighted
MR Egger
4.7
4.6
4.5
1/SE_IV
−0.6
−0.4
−0.2
0.0
β_IV

Batch 2−4162 : Gut microbiota abundance (genus Parasutterella id.2892) on Neuronal pentraxin−2
with a threshold of 1e−05

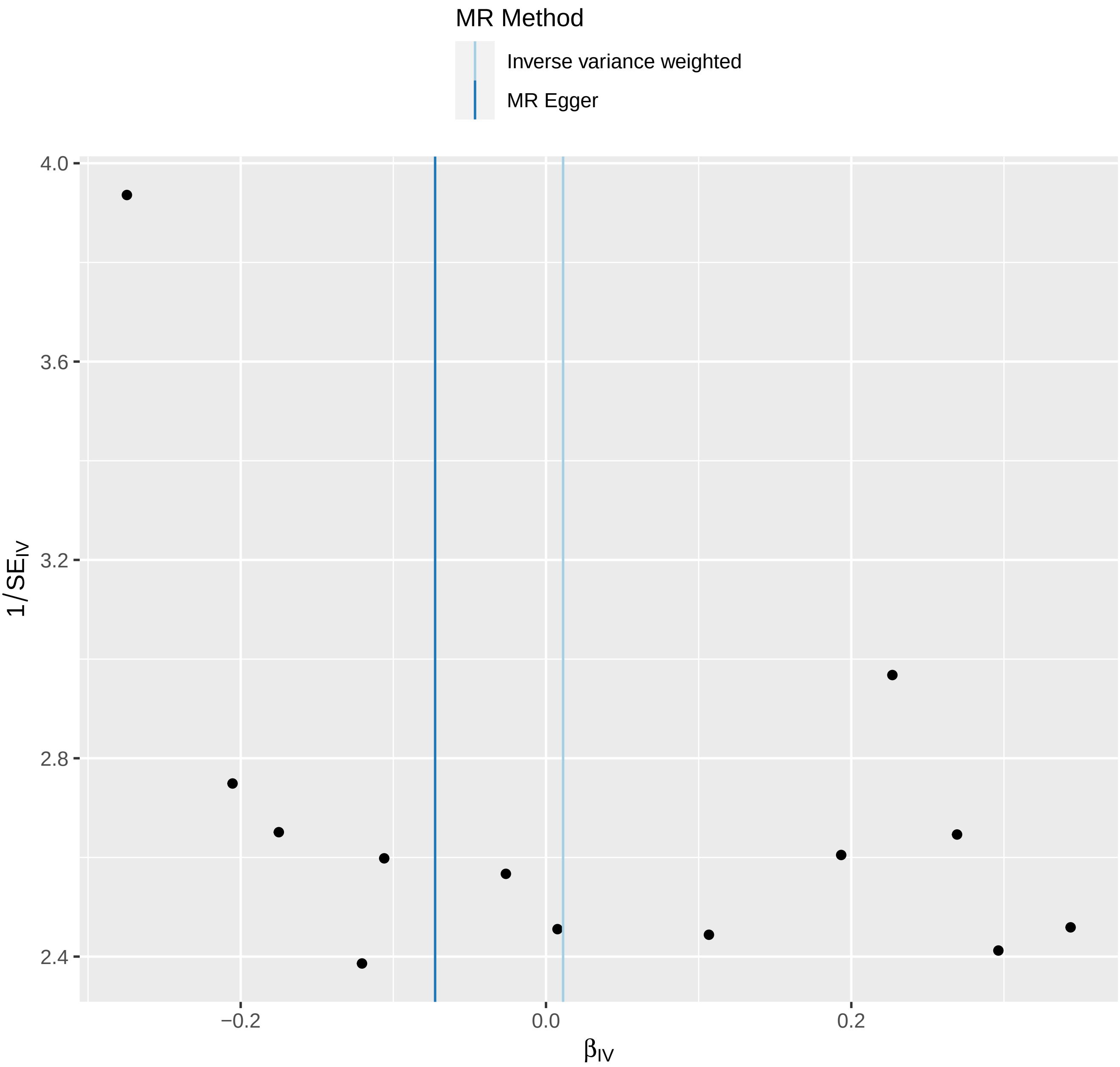

Batch 2−4163 : Gut microbiota abundance (genus Catenibacterium id.2153) on Neuropeptide W
with a threshold of 1e−05

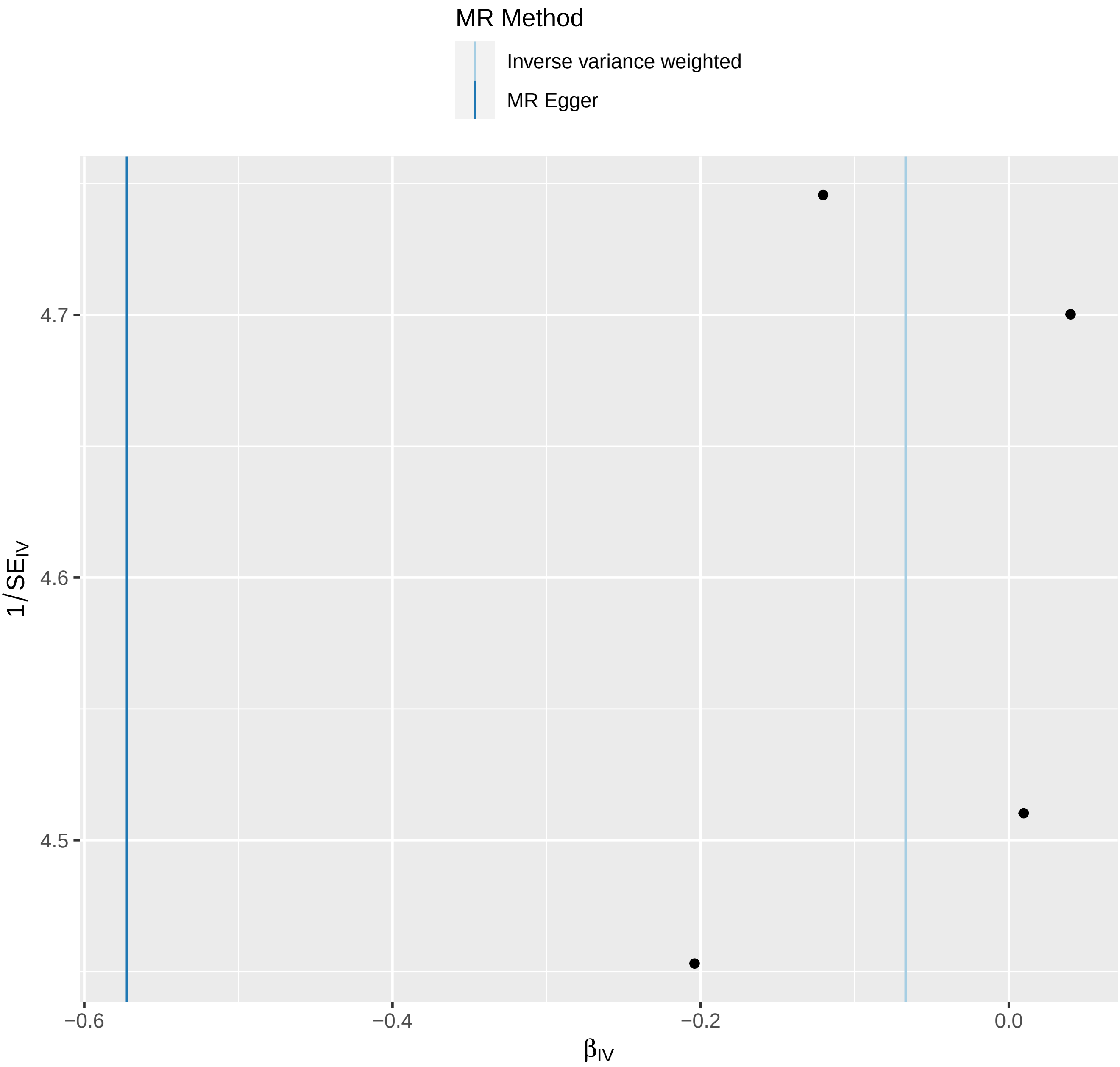

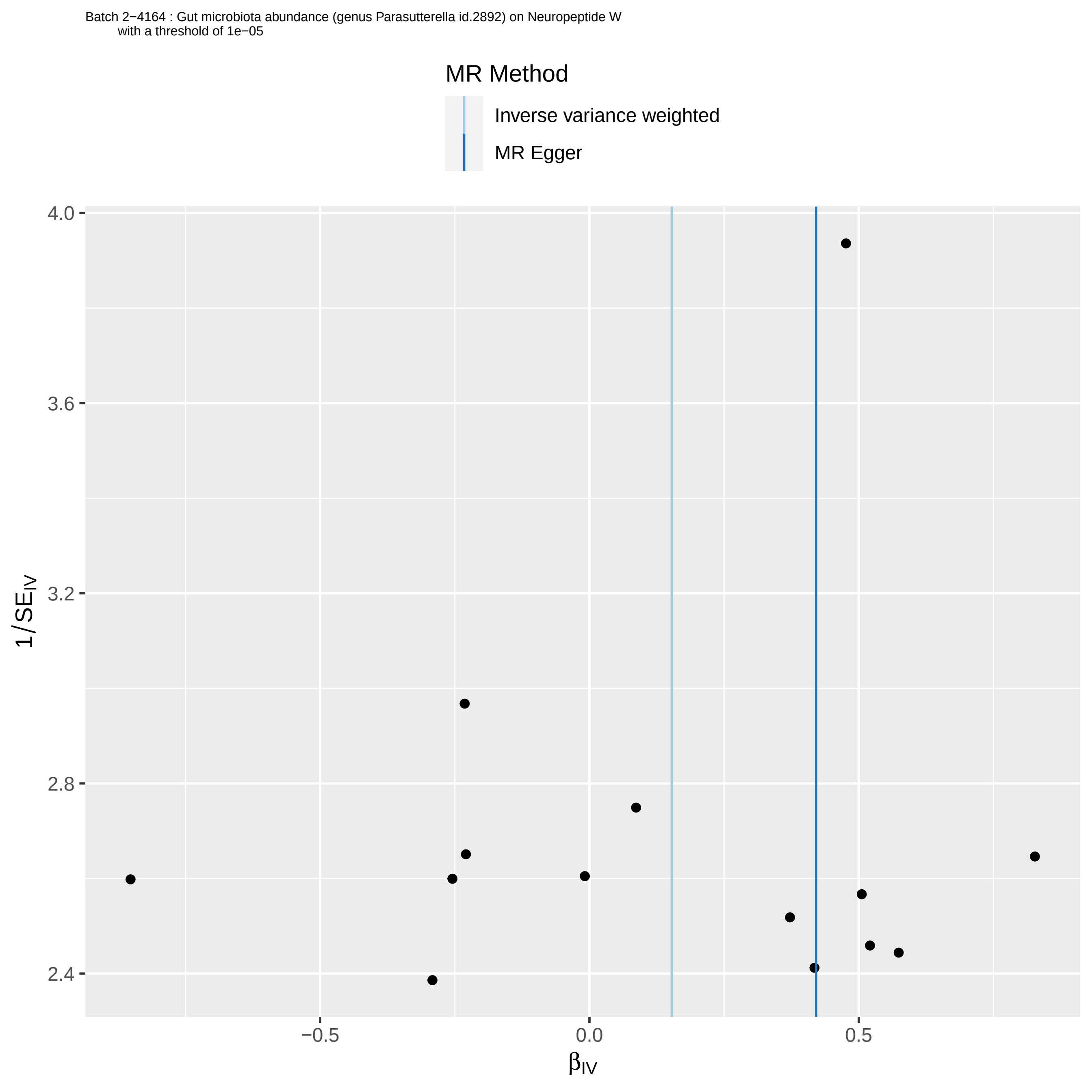
Batch 2−4164 : Gut microbiota abundance (genus Parasutterella id.2892) on Neuropeptide W
with a threshold of 1e−05
MR Method
Inverse variance weighted
MR Egger
1/SE_IV
4.0
3.6
3.2
2.8
2.4
−0.5
0.0
0.5
β_IV

Batch 2−4165 : Gut microbiota abundance (genus Catenibacterium id.2153) on NAD(P)H dehydrogenase [quinone] 1
with a threshold of 1e−05

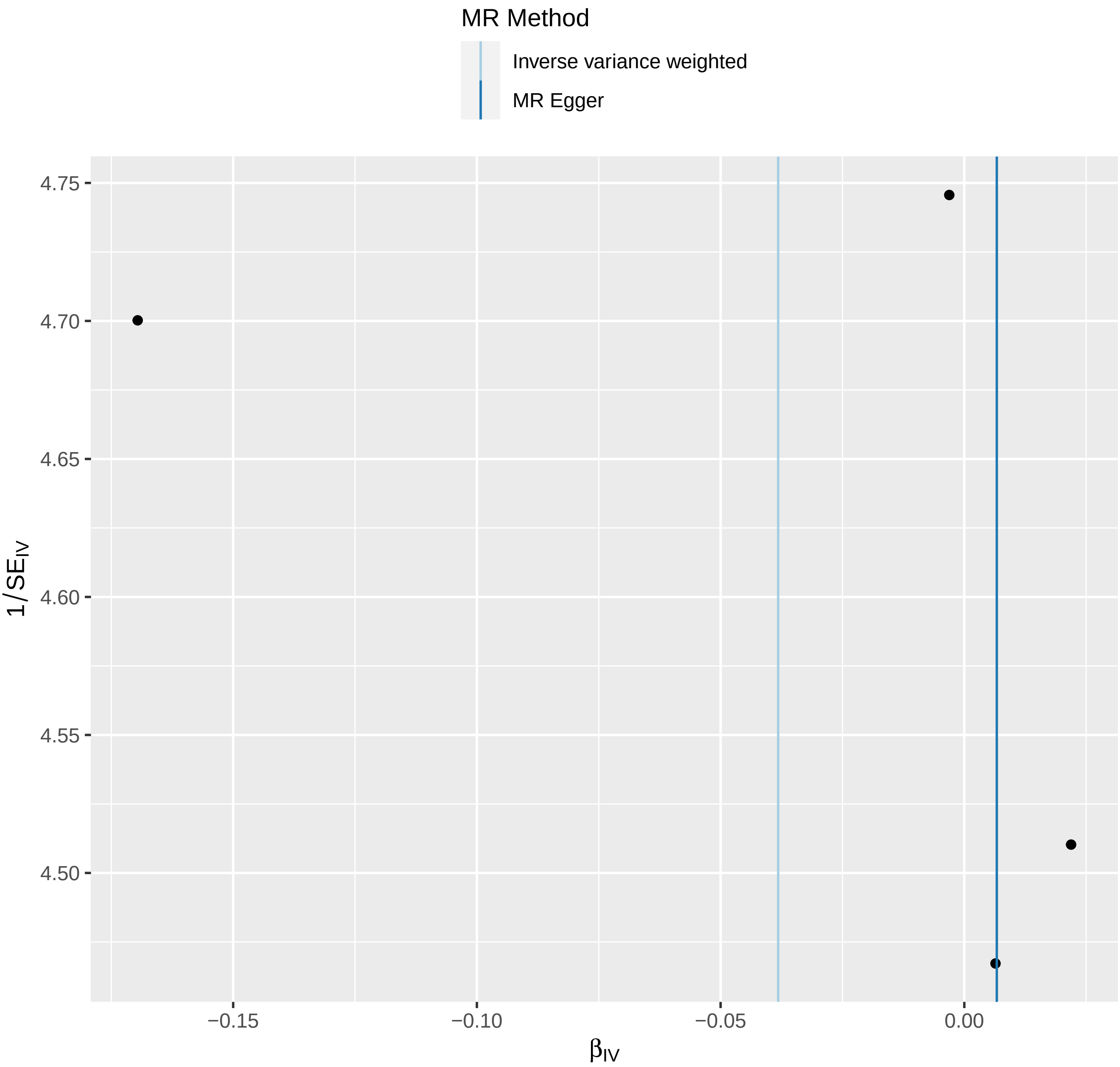

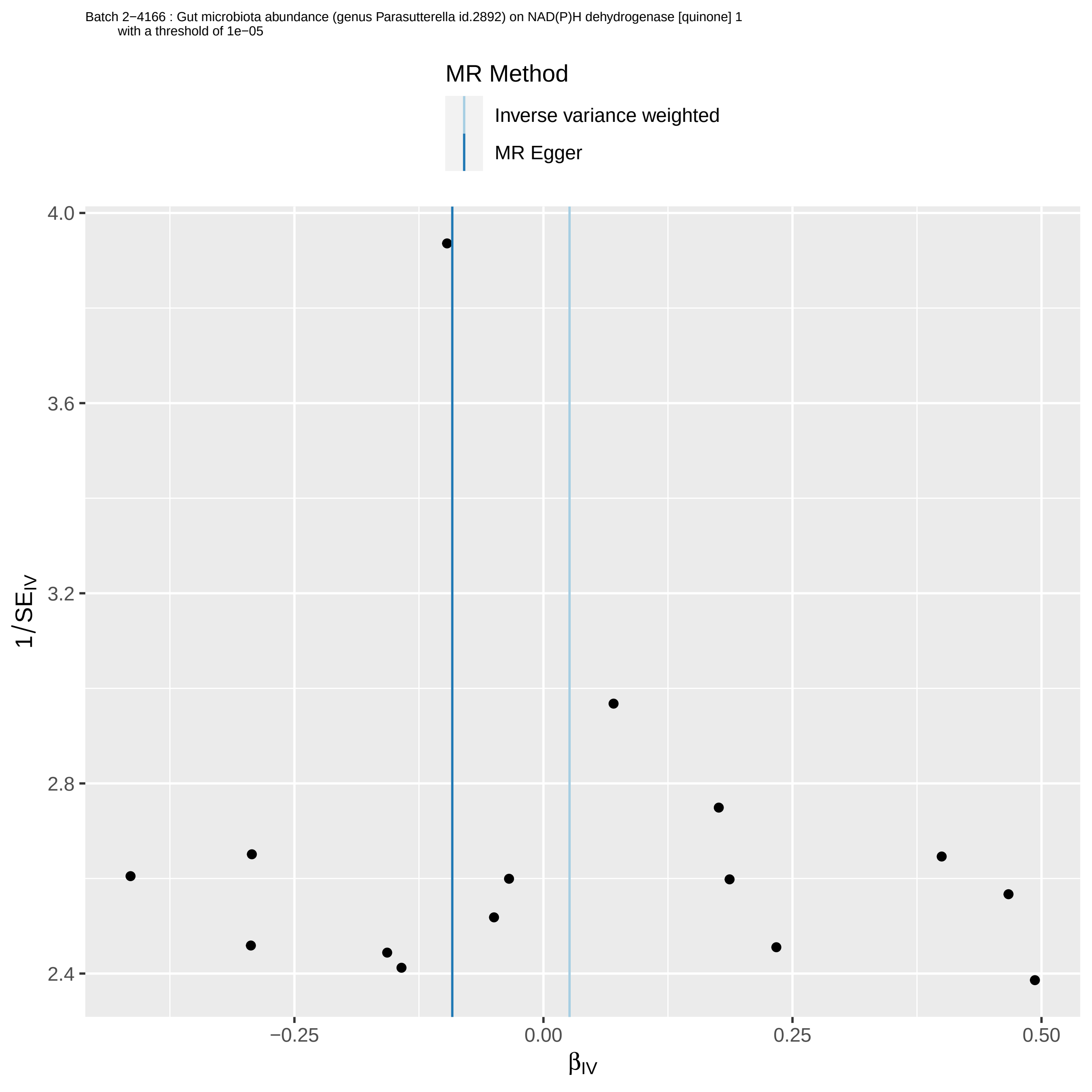
Batch 2−4166 : Gut microbiota abundance (genus Parasutterella id.2892) on NAD(P)H dehydrogenase [quinone] 1
with a threshold of 1e−05
MR Method
Inverse variance weighted
MR Egger
4.0
3.6
3.2
2.8
2.4
1/SE$_{IV}$
−0.25
0.00
0.25
0.50
β$_{IV}$

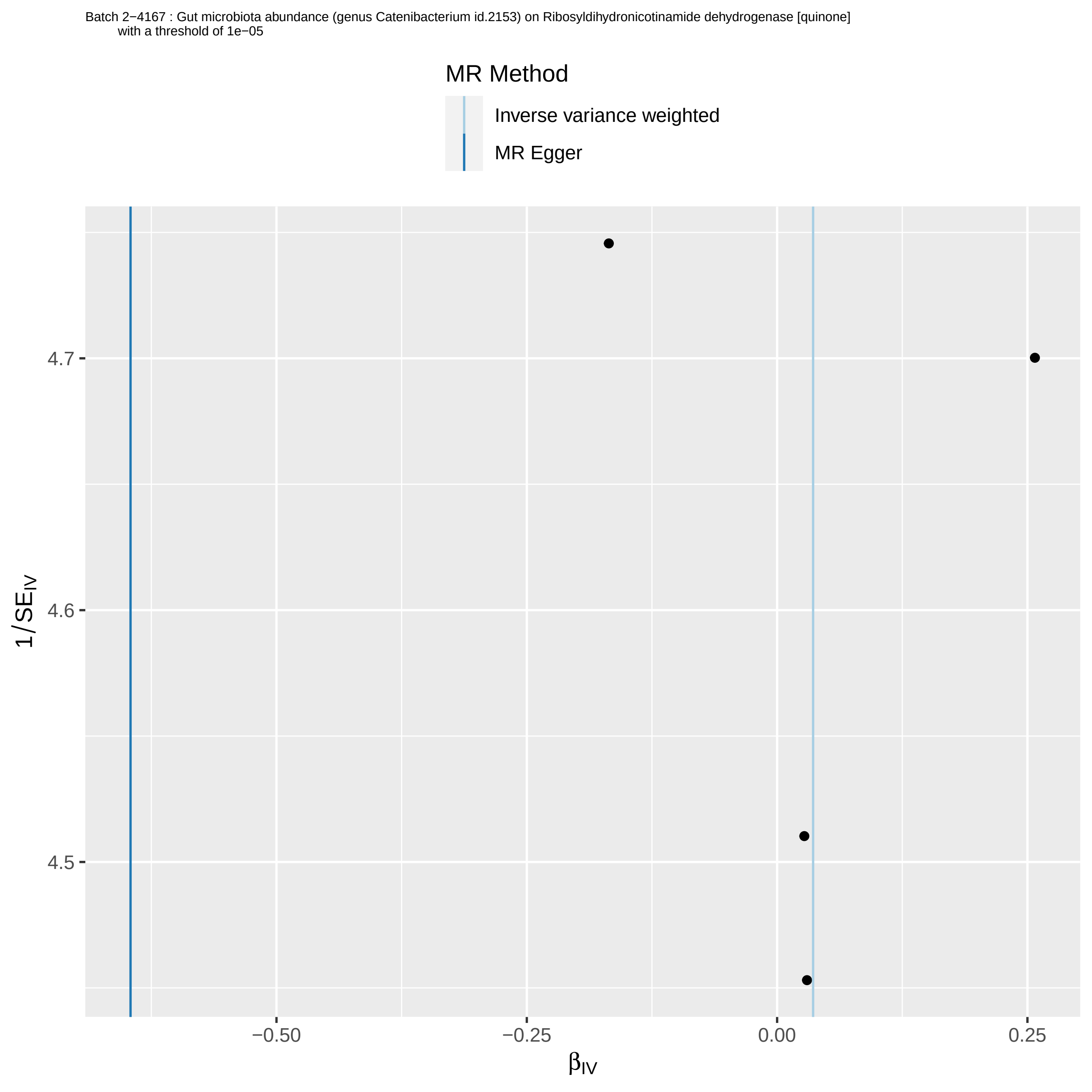

Batch 2−4167 : Gut microbiota abundance (genus Catenibacterium id.2153) on Ribosyldihydronicotinamide dehydrogenase [quinone] with a threshold of 1e−05
MR Method
Inverse variance weighted
MR Egger
4.7
4.6
4.5
1/SE_IV
−0.50
−0.25
0.00
0.25
β_IV

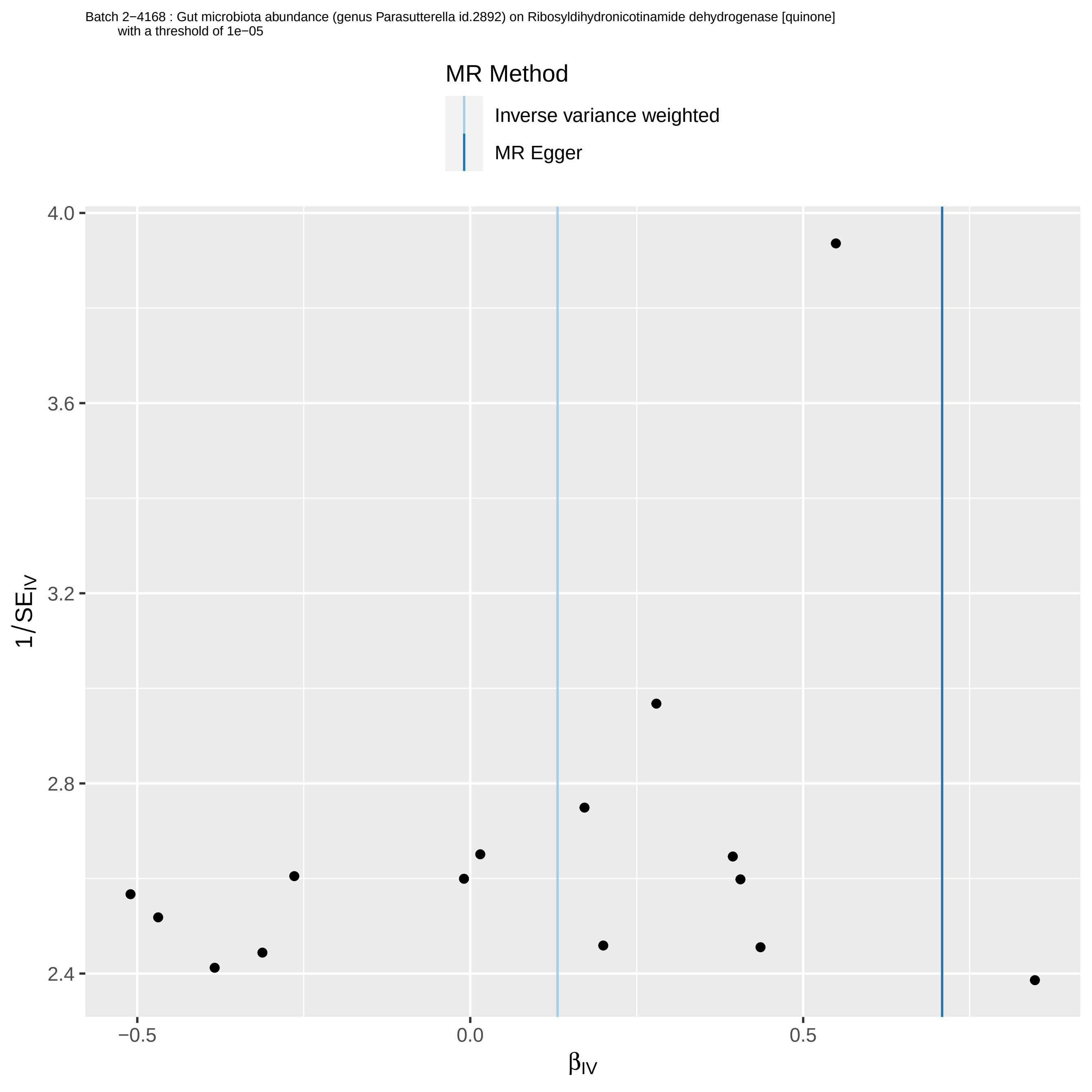

Batch 2−4168 : Gut microbiota abundance (genus Parasutterella id.2892) on Ribosyldihydronicotinamide dehydrogenase [quinone]
with a threshold of 1e−05
MR Method
Inverse variance weighted
MR Egger
4.0
3.6
3.2
2.8
2.4
1/SE_IV
−0.5
0.0
0.5
β_IV

Batch 2−4169 : Gut microbiota abundance (genus Catenibacterium id.2153) on Nuclear receptor subfamily 1 group D member 1
with a threshold of 1e−05

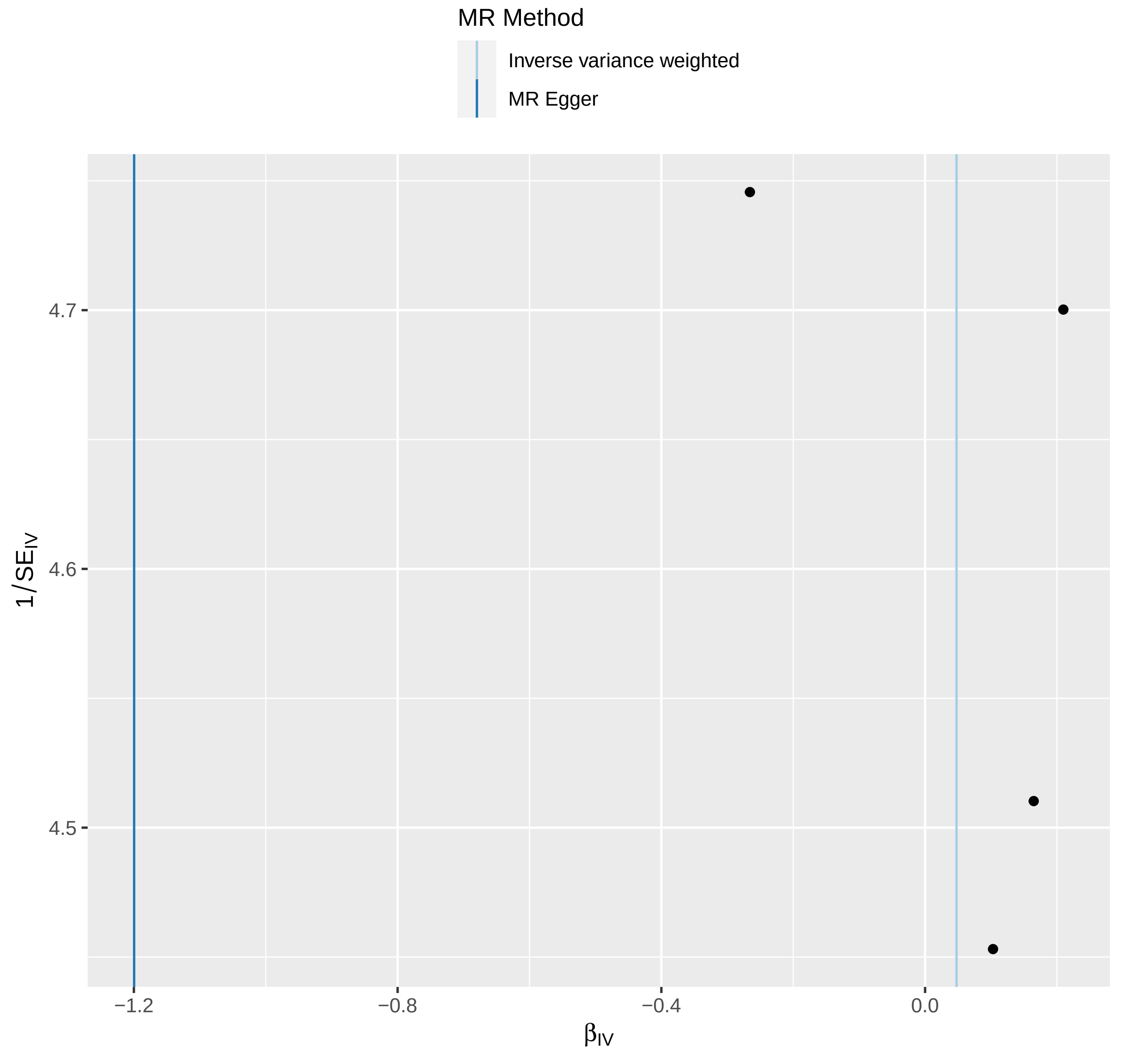

Batch 2−4170 : Gut microbiota abundance (genus Parasutterella id.2892) on Nuclear receptor subfamily 1 group D member 1
with a threshold of 1e−05

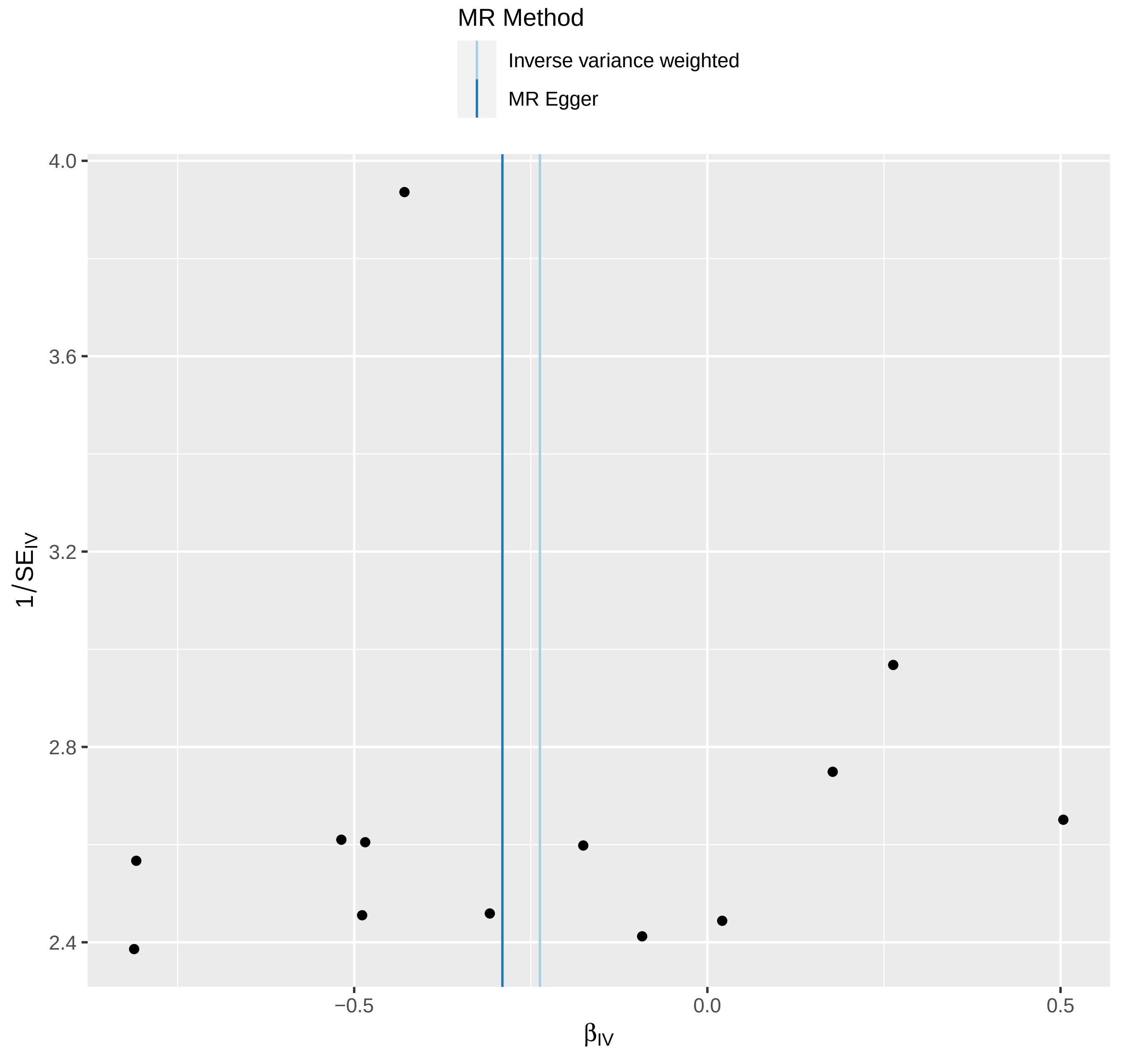

Batch 2−4171 : Gut microbiota abundance (genus Catenibacterium id.2153) on Nuclear receptor subfamily 1 group D member 2
with a threshold of 1e−05

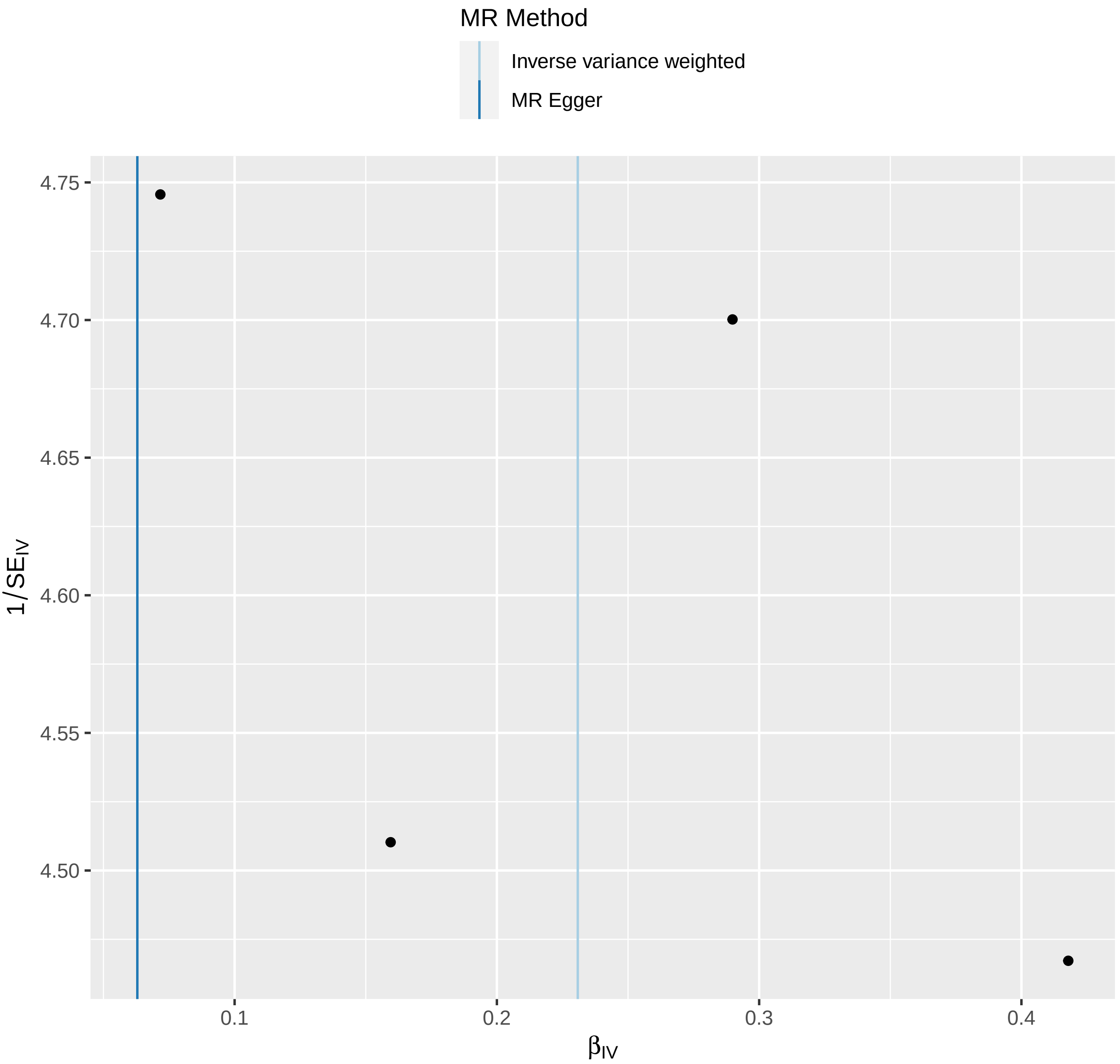

Batch 2−4172 : Gut microbiota abundance (genus Parasutterella id.2892) on Nuclear receptor subfamily 1 group D member 2
with a threshold of 1e−05

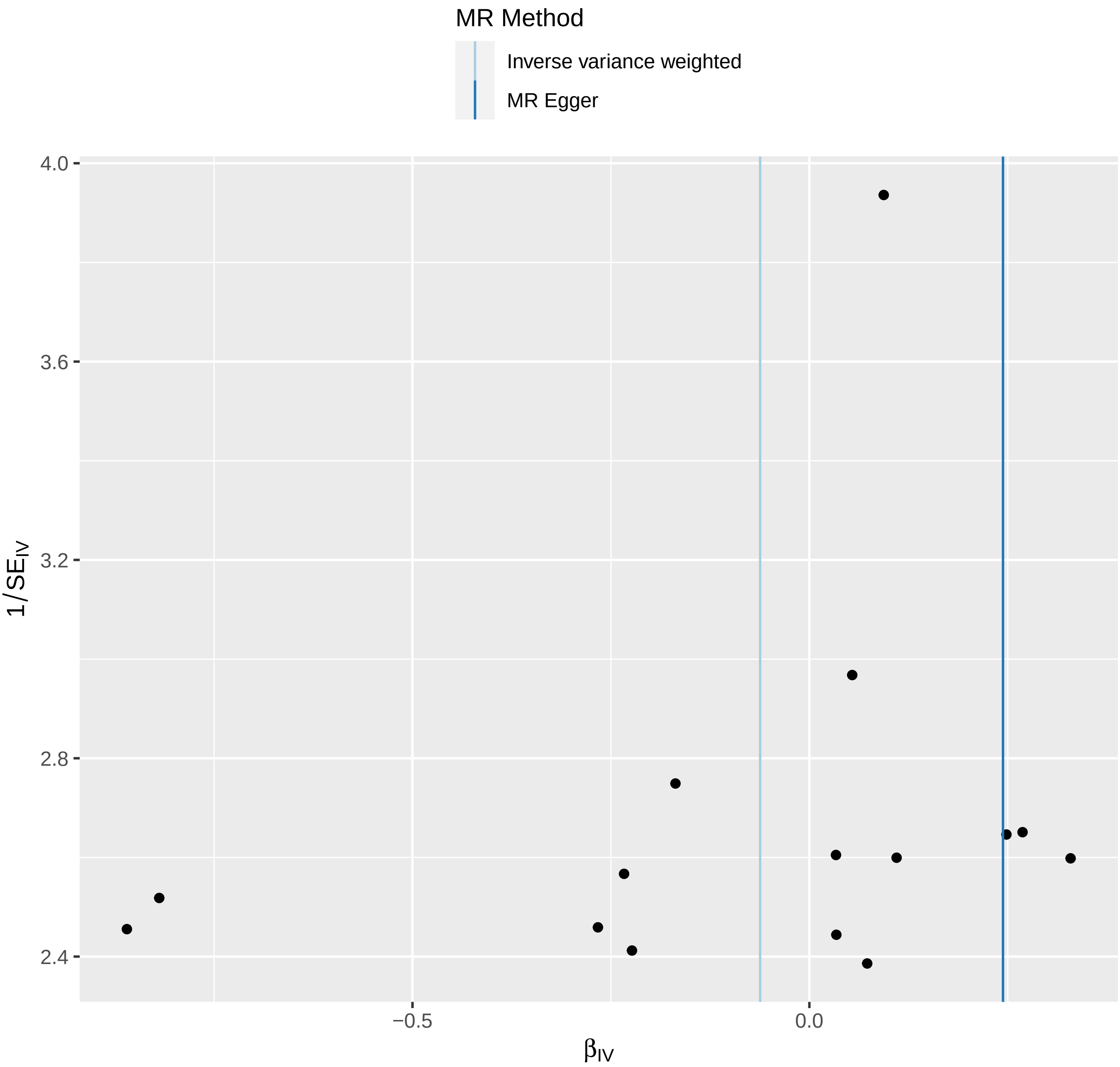

Batch 2−4173 : Gut microbiota abundance (genus Catenibacterium id.2153) on Oxysterols receptor LXR−beta
with a threshold of 1e−05

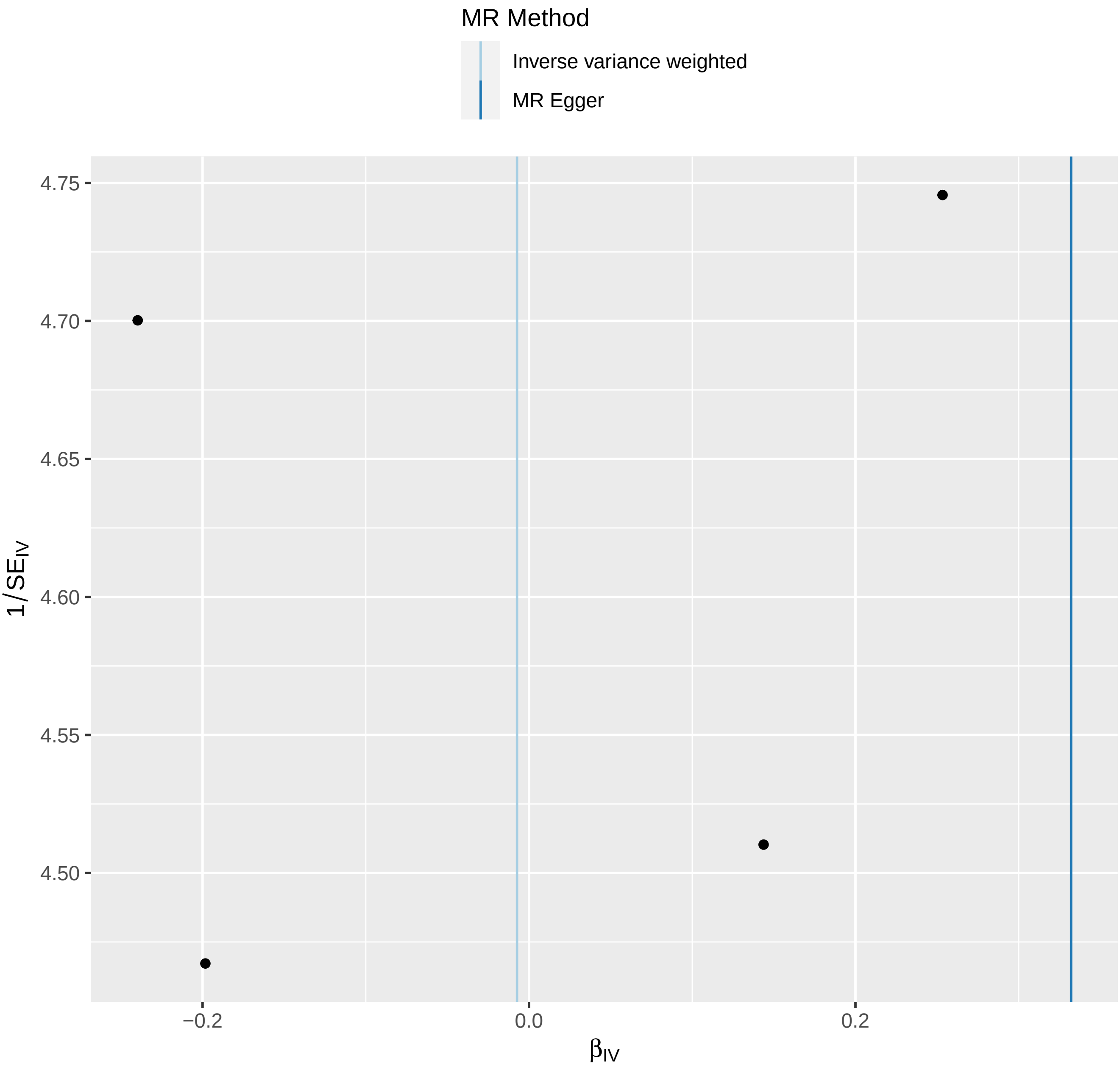

Batch 2−4174 : Gut microbiota abundance (genus Parasutterella id.2892) on Oxysterols receptor LXR−beta
with a threshold of 1e−05

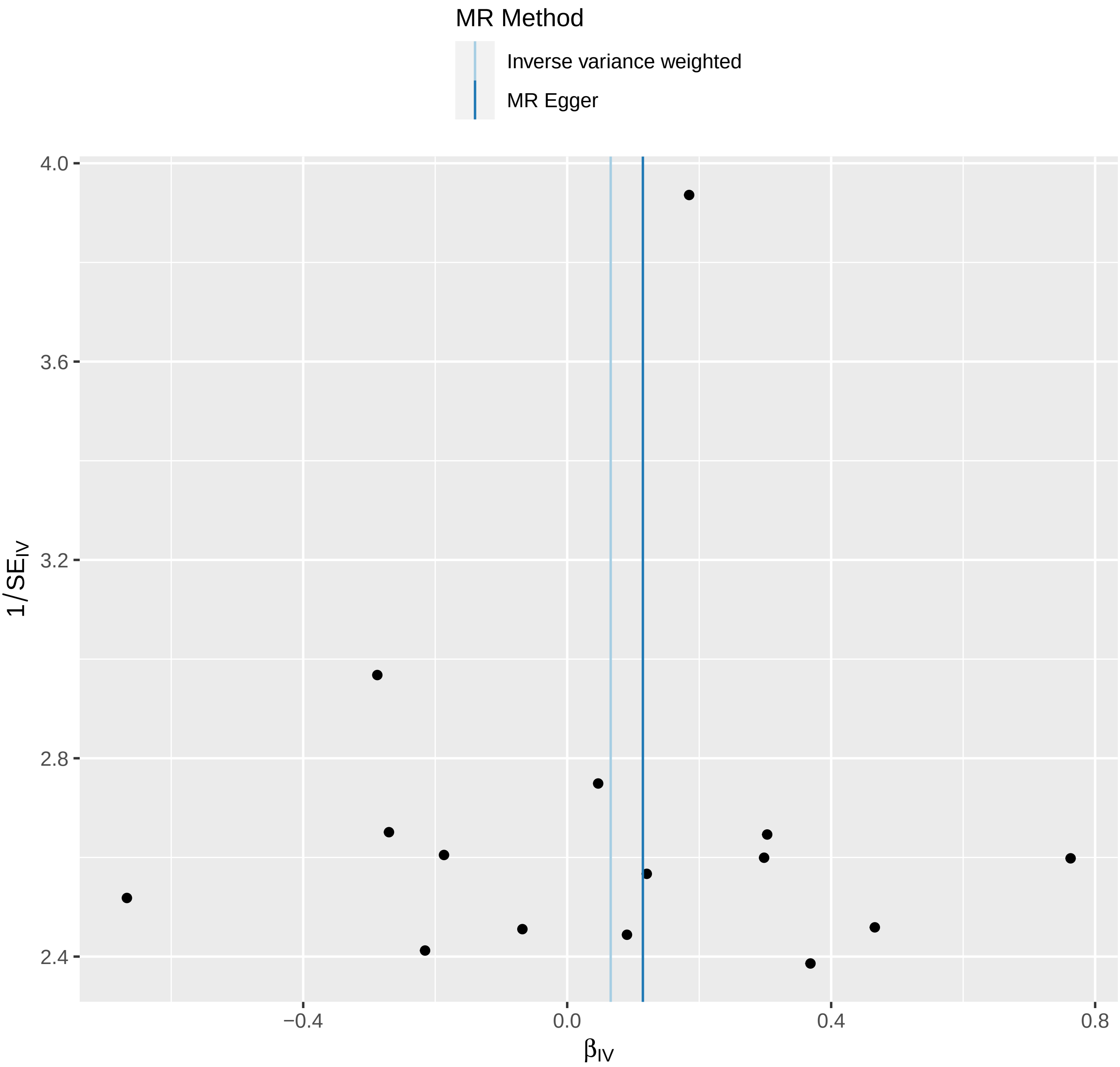

Batch 2−4175 : Gut microbiota abundance (genus Catenibacterium id.2153) on Glucocorticoid receptor
with a threshold of 1e−05

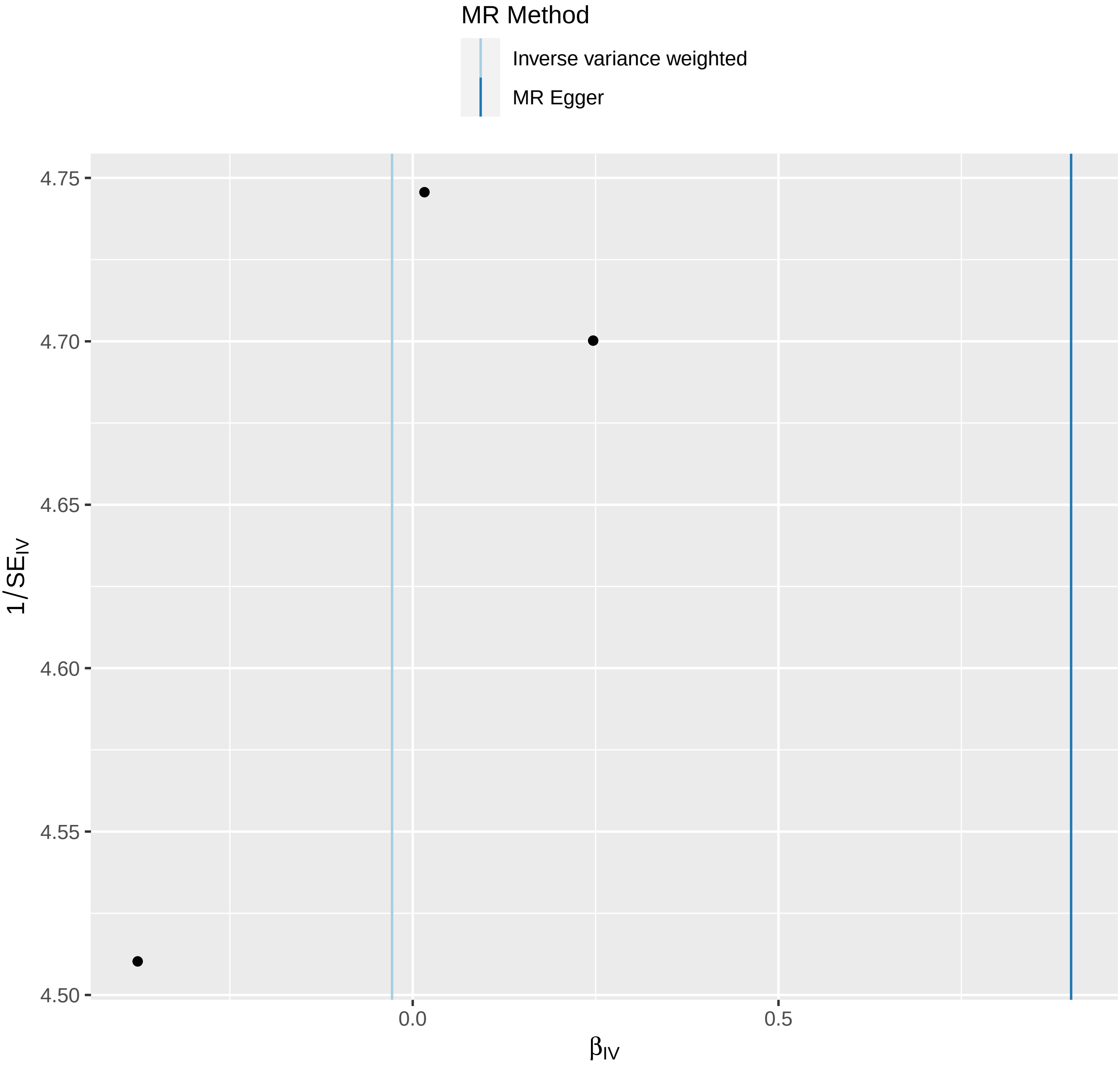

Batch 2−4176 : Gut microbiota abundance (genus Parasutterella id.2892) on Glucocorticoid receptor
with a threshold of 1e−05

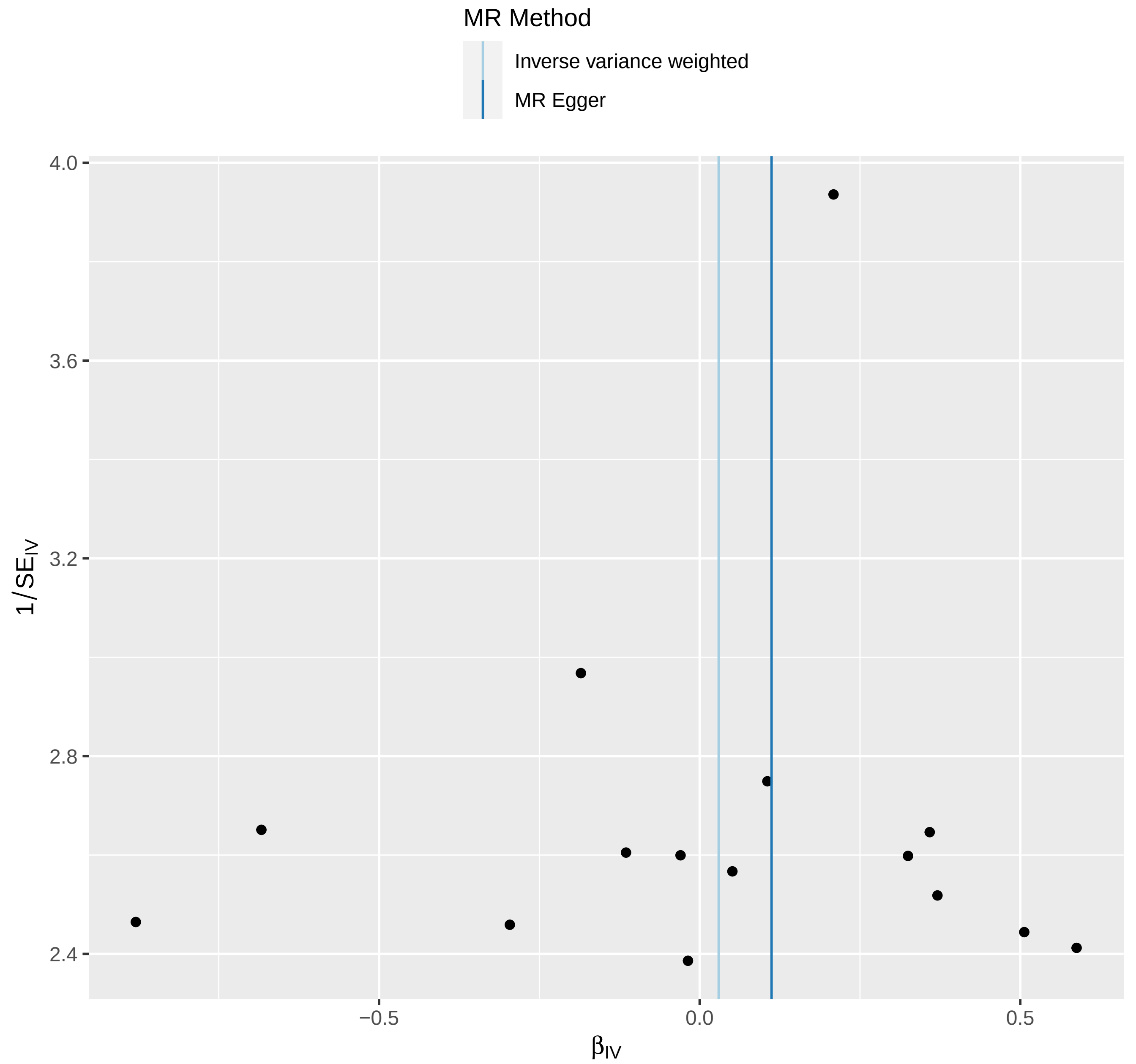

Batch 2−4177 : Gut microbiota abundance (genus Catenibacterium id.2153) on Mineralocorticoid receptor
with a threshold of 1e−05

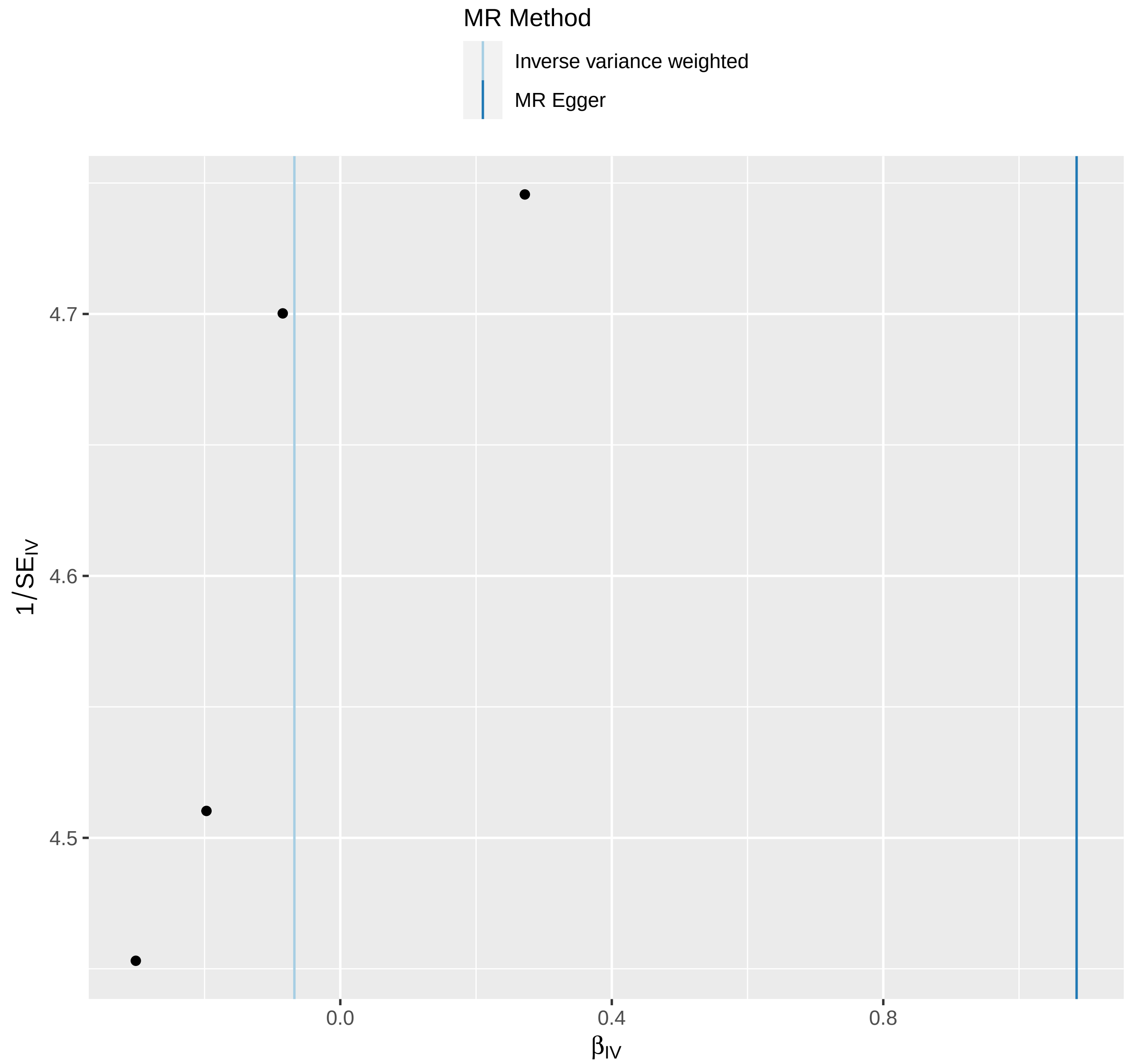

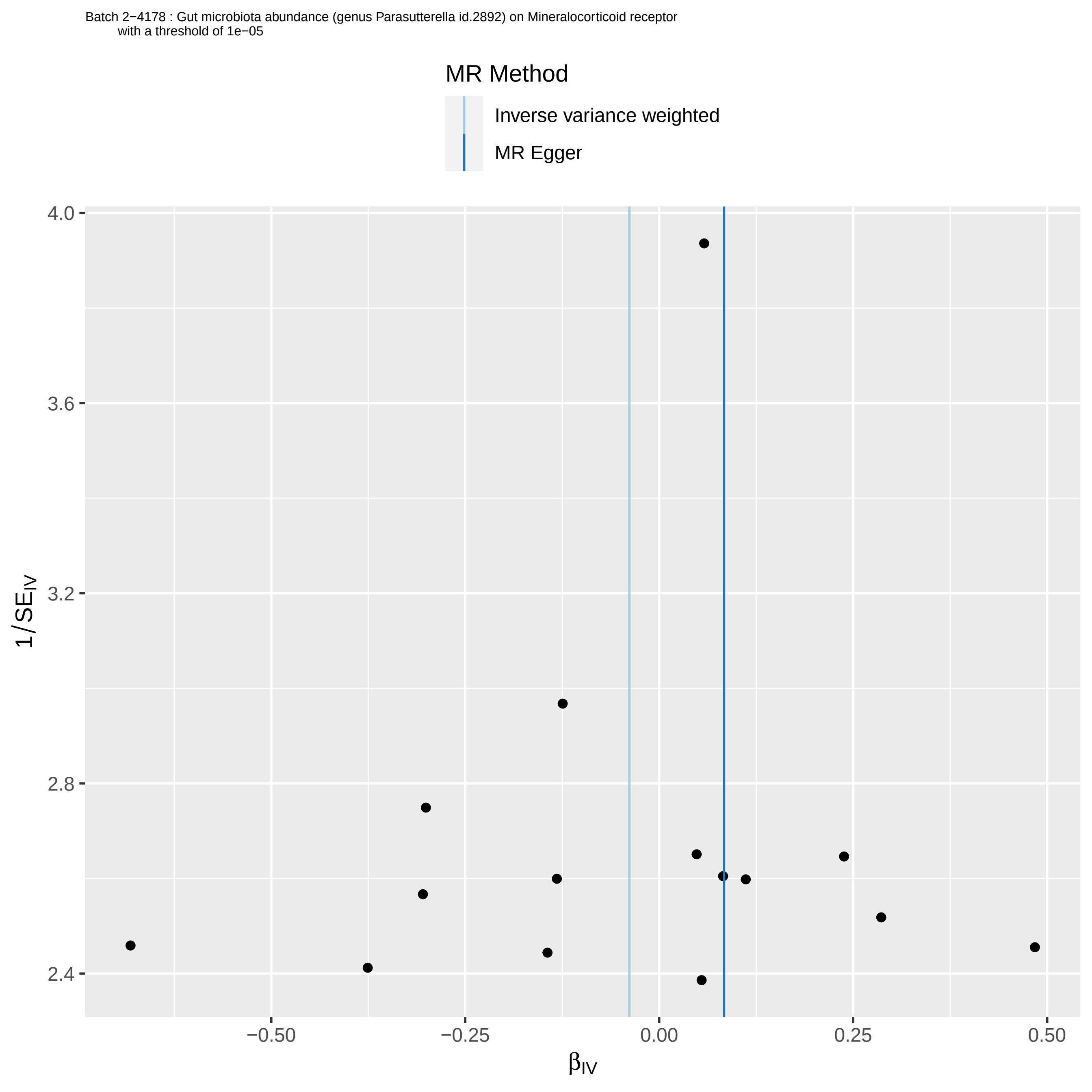

Batch 2−4178 : Gut microbiota abundance (genus Parasutterella id.2892) on Mineralocorticoid receptor
with a threshold of 1e−05
MR Method
Inverse variance weighted
MR Egger
4.0
3.6
3.2
2.8
2.4
1/SE_IV
−0.50
−0.25
0.00
0.25
0.50
β_IV

Batch 2−4179 : Gut microbiota abundance (genus Catenibacterium id.2153) on Nuclear receptor subfamily 4 group A member 1
with a threshold of 1e−05

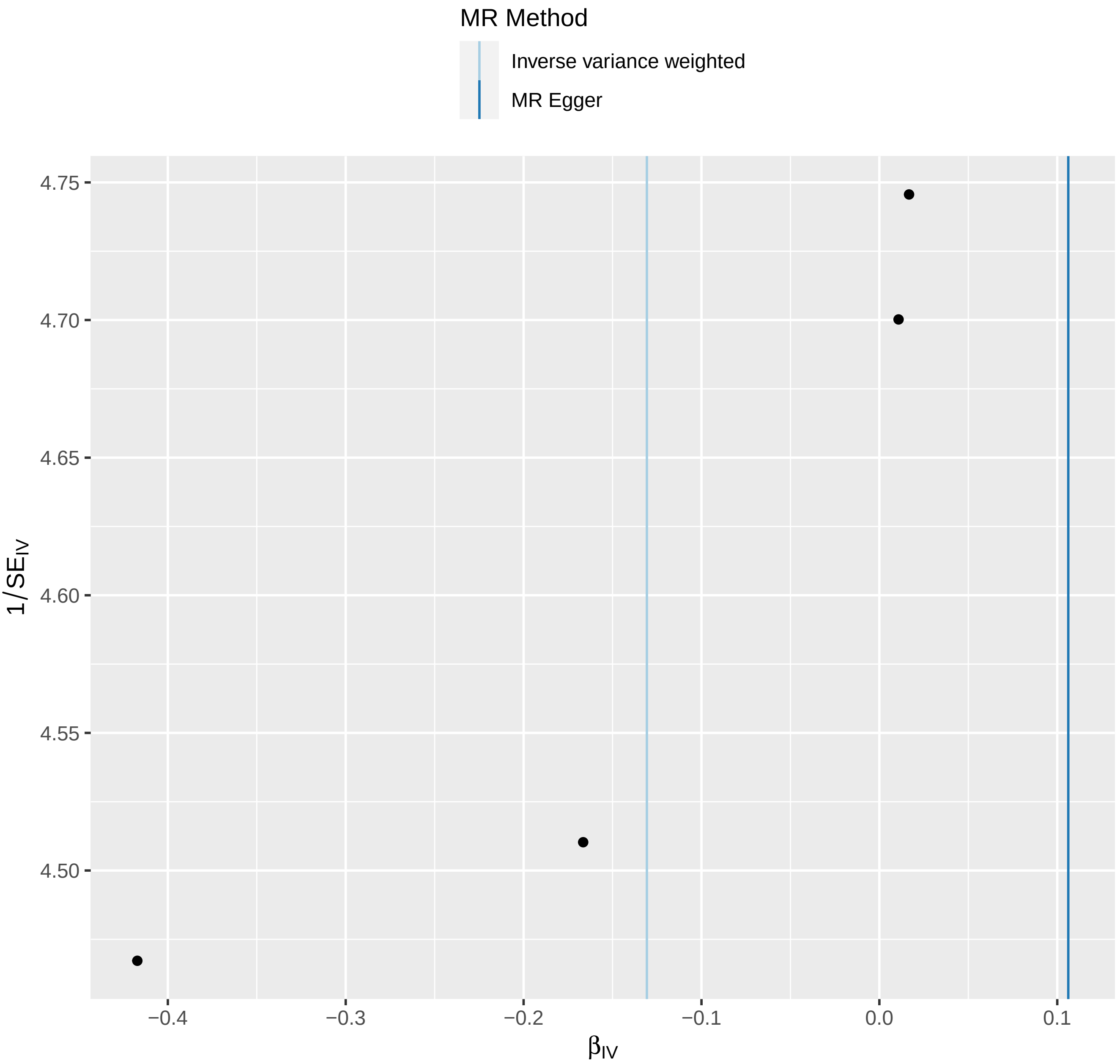

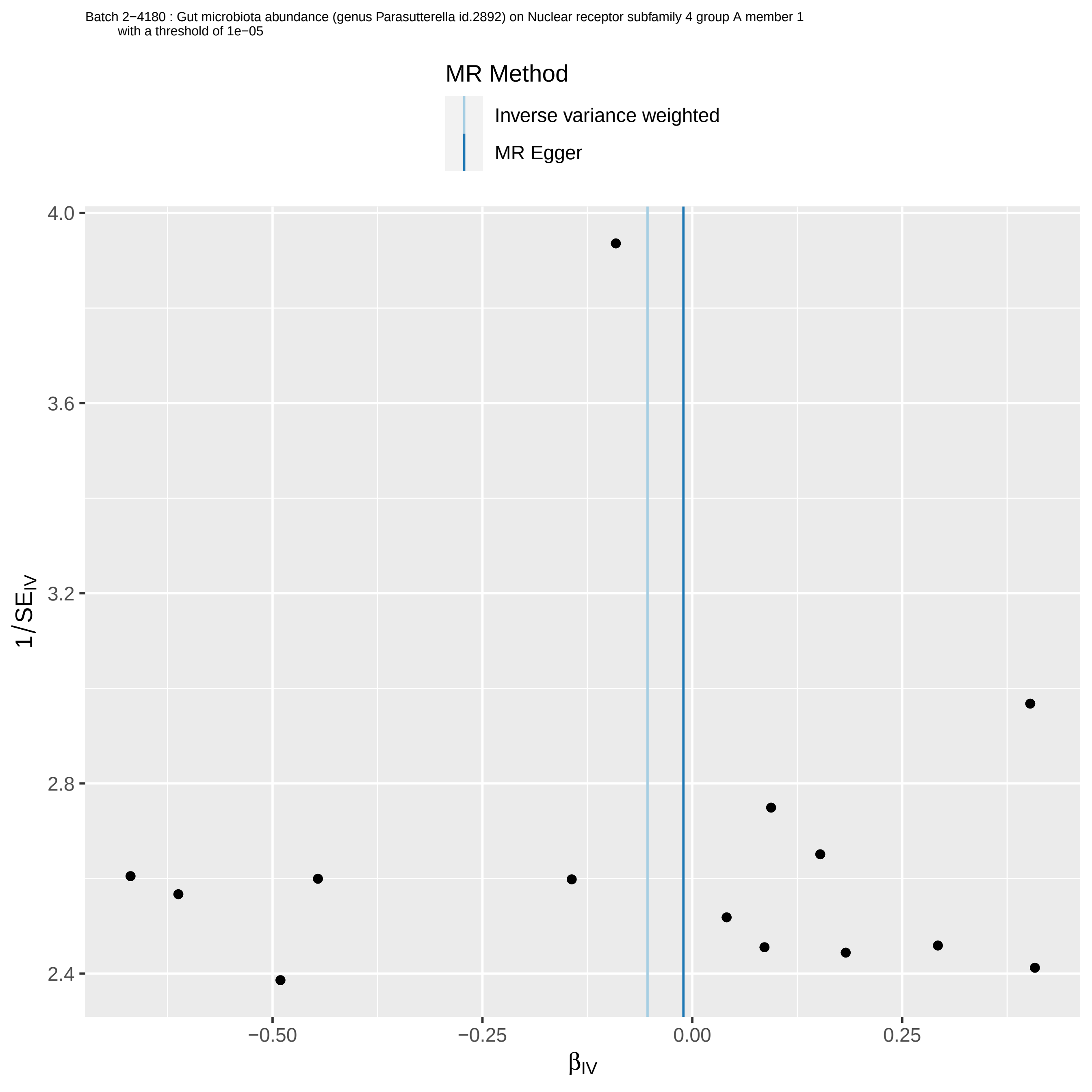

Batch 2−4180 : Gut microbiota abundance (genus Parasutterella id.2892) on Nuclear receptor subfamily 4 group A member 1
with a threshold of 1e−05
MR Method
Inverse variance weighted
MR Egger
4.0
3.6
3.2
2.8
2.4
$1/SE_{IV}$
−0.50
−0.25
0.00
0.25
β_{IV}

Batch 2−4181 : Gut microbiota abundance (genus Catenibacterium id.2153) on Nuclear receptor subfamily 5 group A member 2
with a threshold of 1e−05

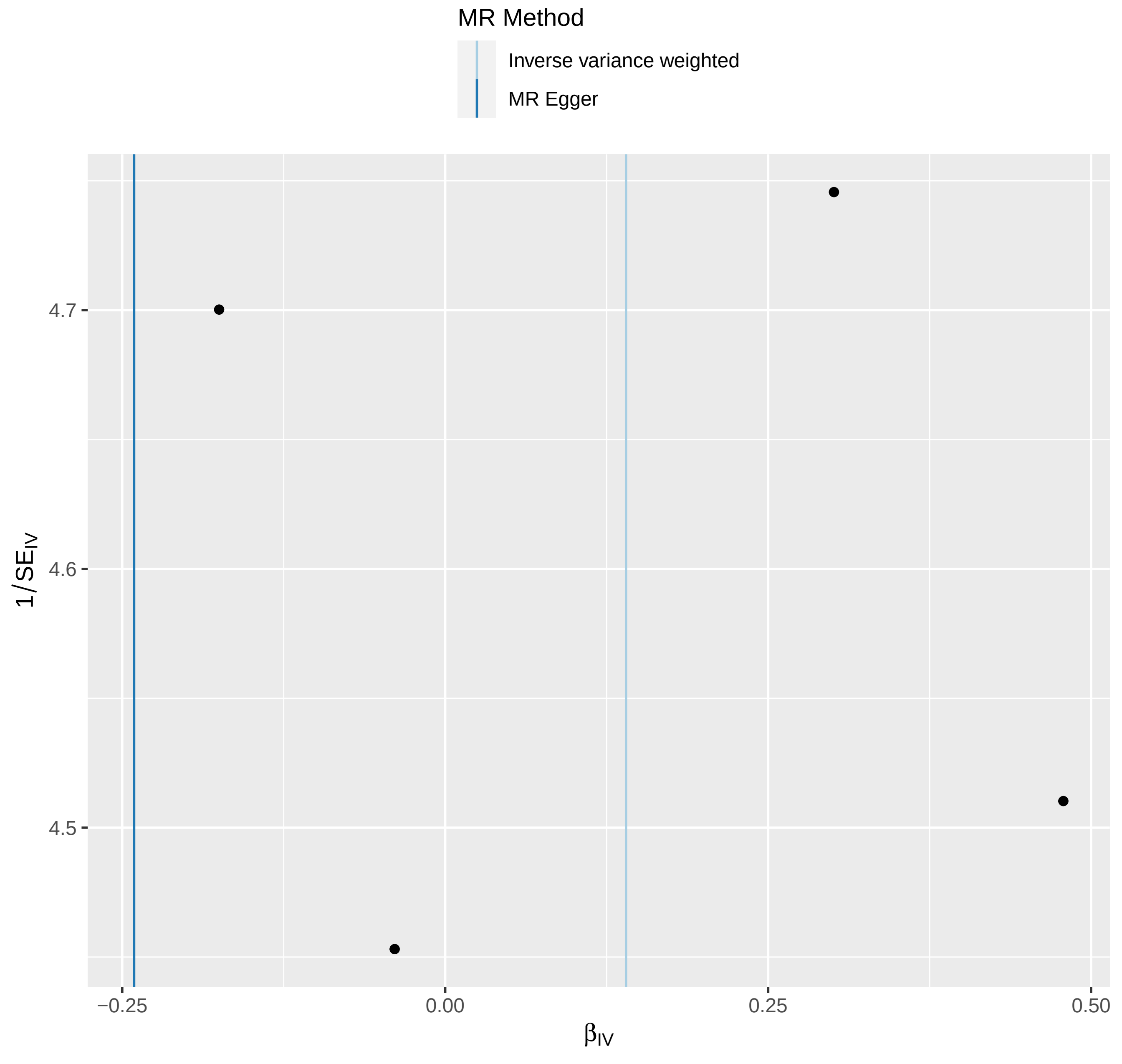

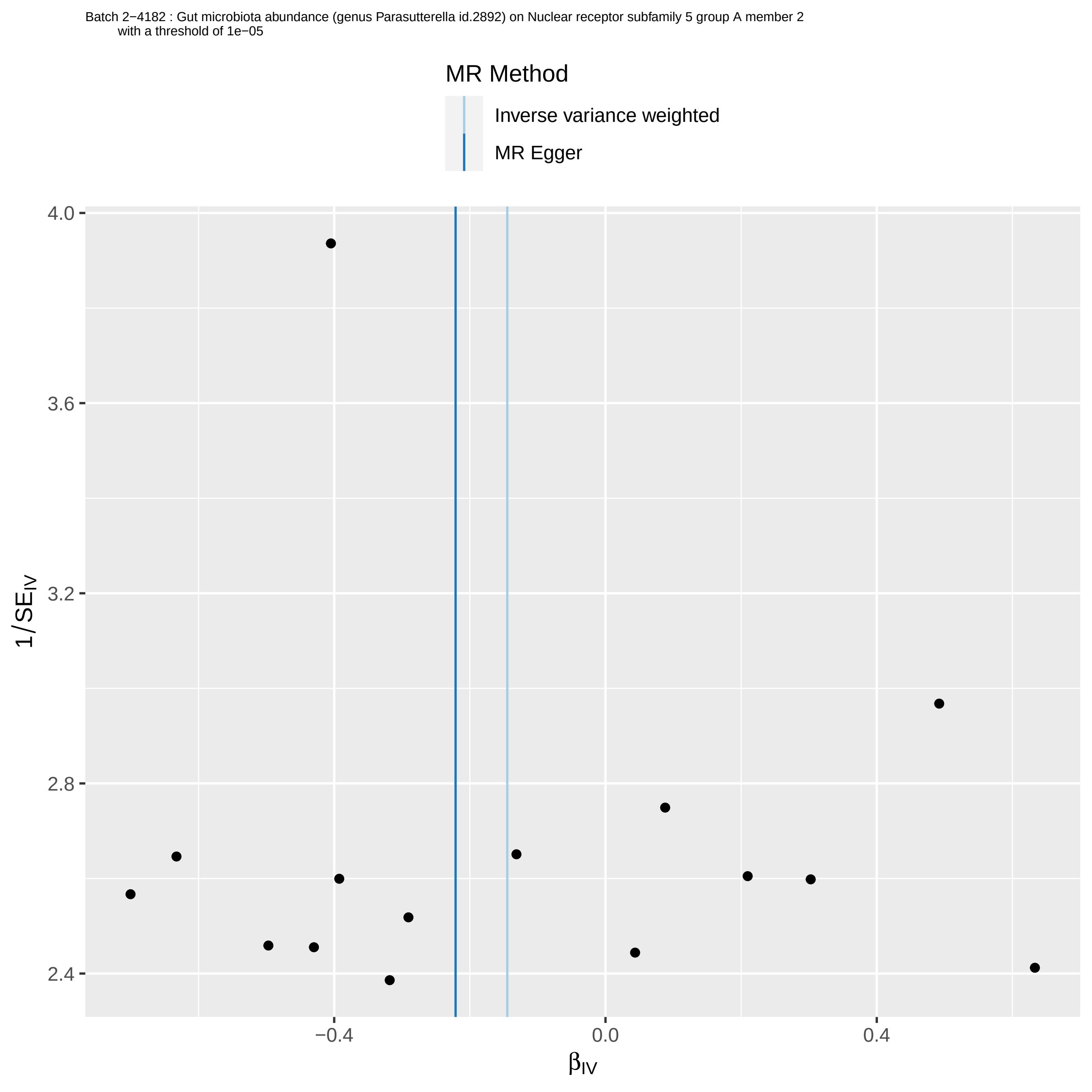

Batch 2−4182 : Gut microbiota abundance (genus Parasutterella id.2892) on Nuclear receptor subfamily 5 group A member 2
with a threshold of 1e−05
MR Method
Inverse variance weighted
MR Egger
4.0
3.6
3.2
2.8
2.4
1/SE_IV
−0.4
0.0
0.4
β_IV

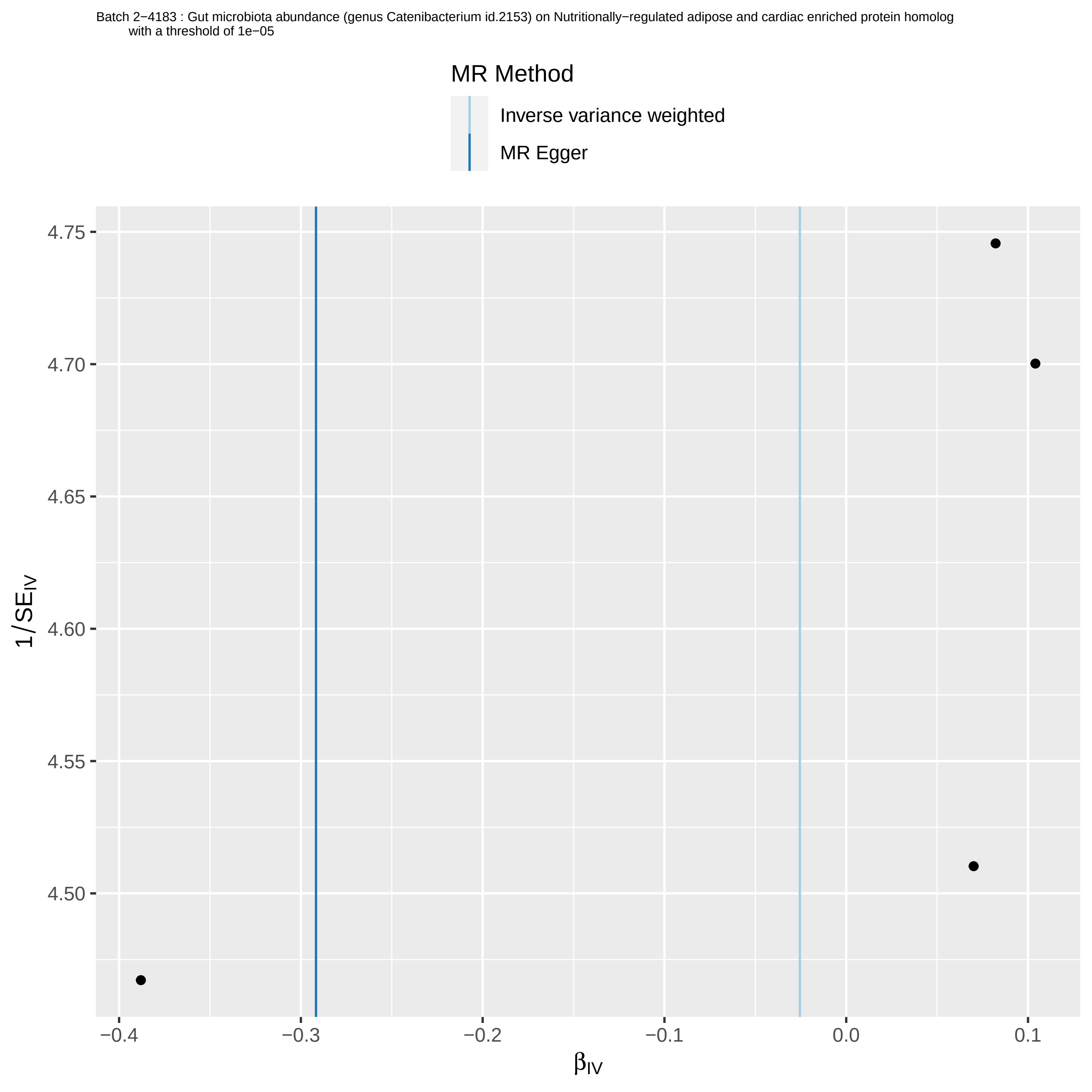

Batch 2−4183 : Gut microbiota abundance (genus Catenibacterium id.2153) on Nutritionally−regulated adipose and cardiac enriched protein homolog with a threshold of 1e−05
MR Method
Inverse variance weighted
MR Egger
4.75
4.70
4.65
4.60
4.55
4.50
1/SE_IV
−0.4
−0.3
−0.2
−0.1
0.0
0.1
β_IV

Batch 2−4184 : Gut microbiota abundance (genus Parasutterella id.2892) on Nutritionally−regulated adipose and cardiac enriched protein homolog
with a threshold of 1e−05

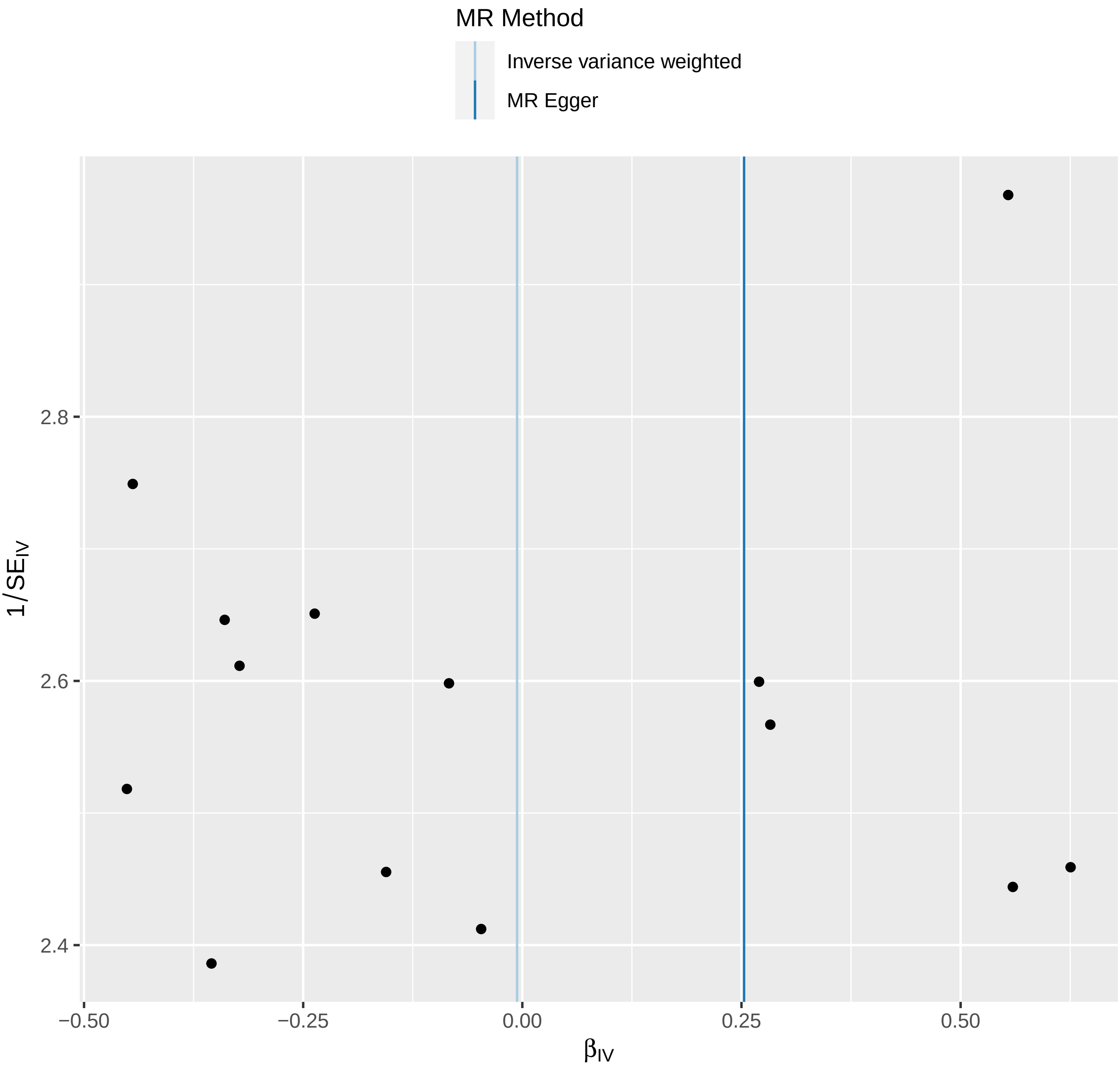

Batch 2−4185 : Gut microbiota abundance (genus Catenibacterium id.2153) on Nuclear receptor−binding protein
with a threshold of 1e−05

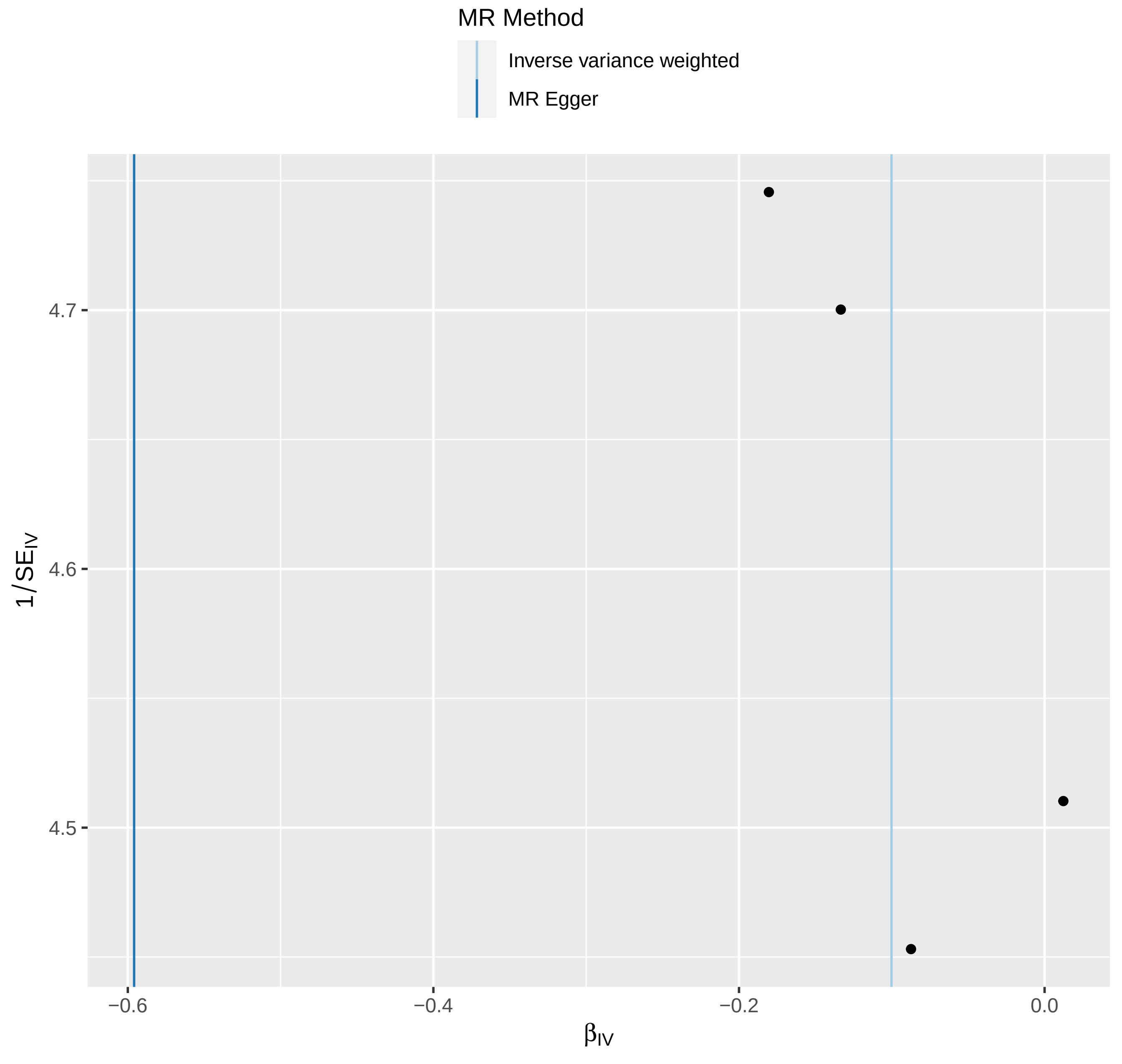

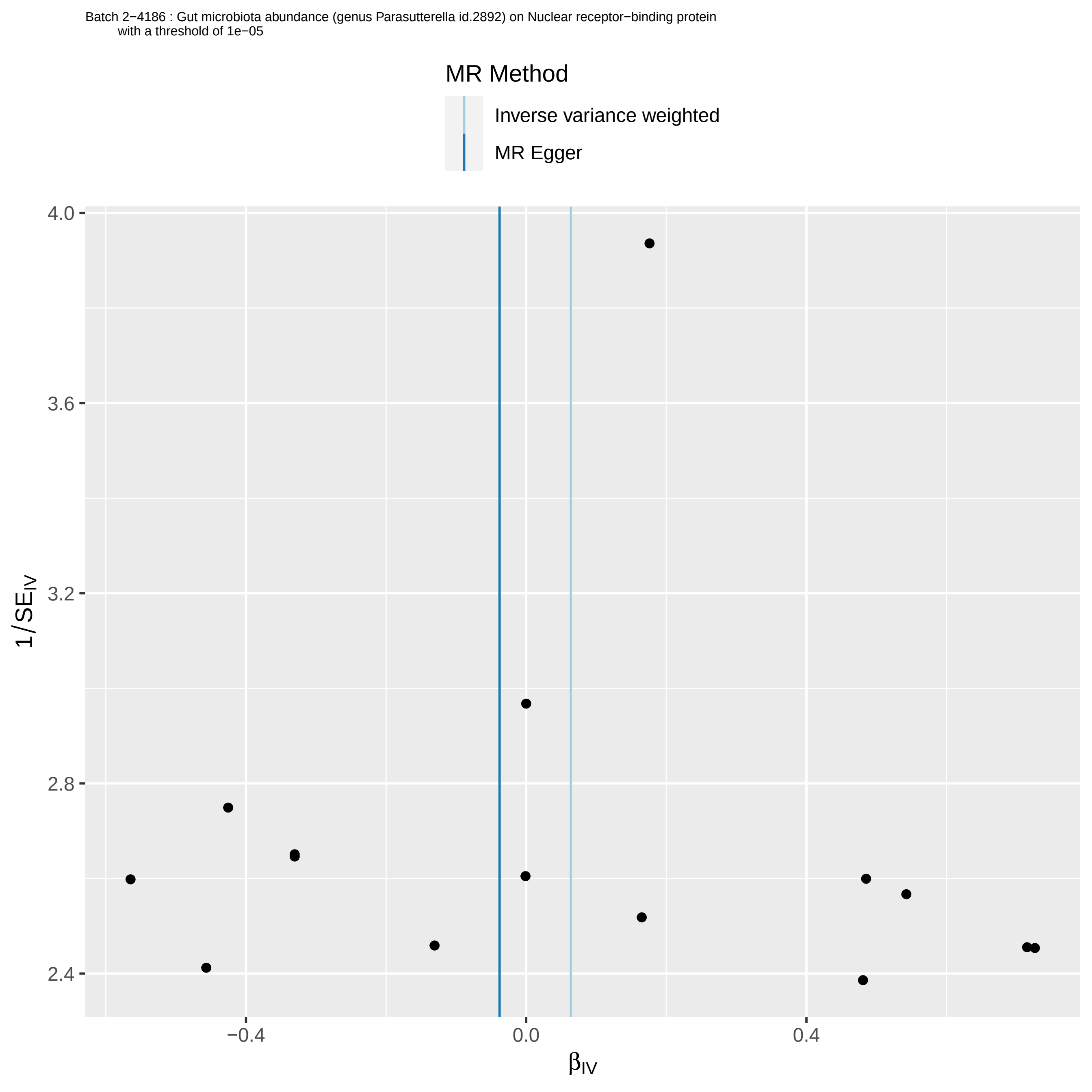
Batch 2−4186 : Gut microbiota abundance (genus Parasutterella id.2892) on Nuclear receptor−binding protein
with a threshold of 1e−05
MR Method
Inverse variance weighted
MR Egger
4.0
3.6
3.2
2.8
2.4
1/SE$_{IV}$
−0.4
0.0
0.4
β$_{IV}$

Batch 2−4187 : Gut microbiota abundance (genus Catenibacterium id.2153) on Neuronal cell adhesion molecule
with a threshold of 1e−05

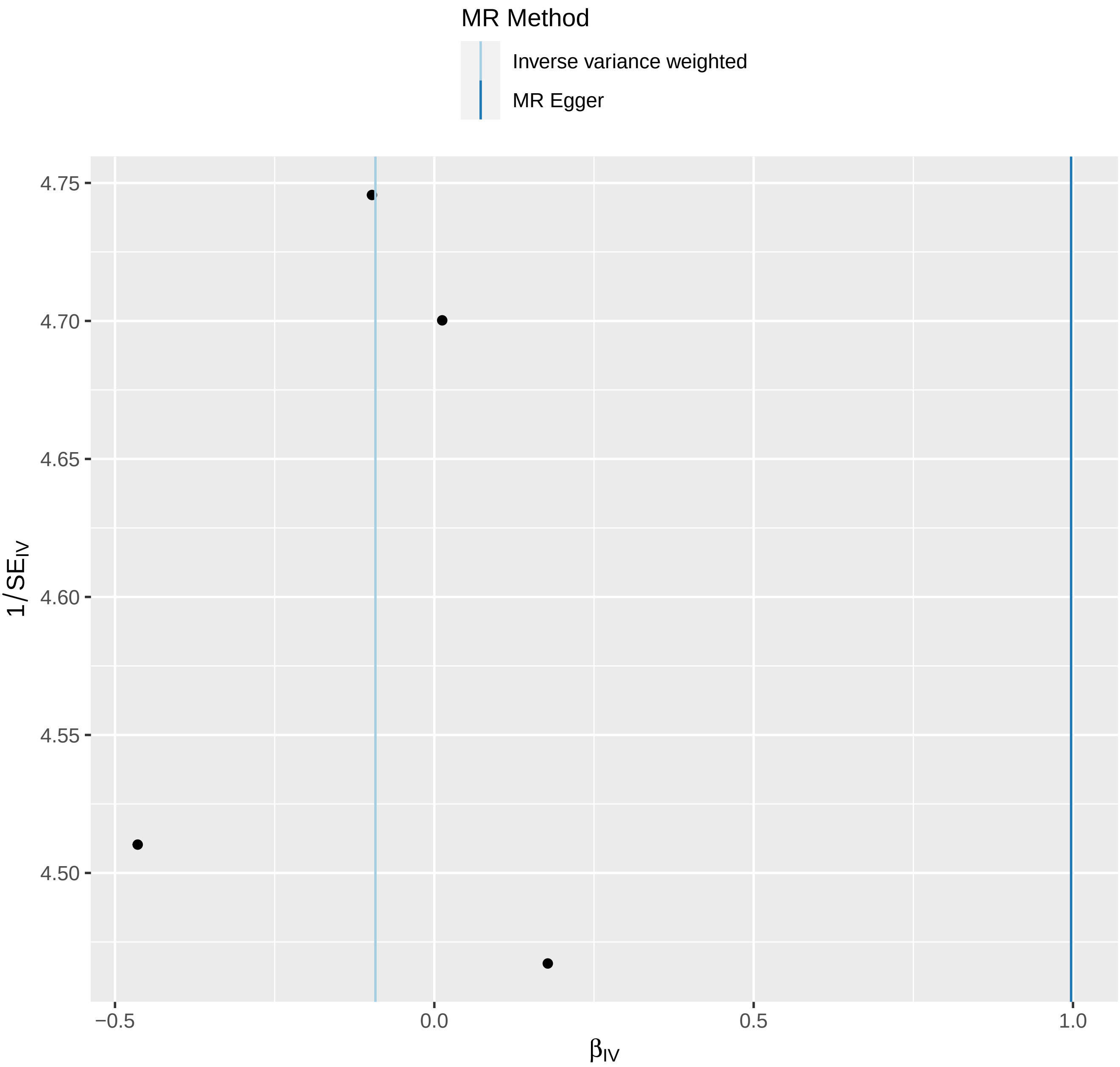

Batch 2−4188 : Gut microbiota abundance (genus Parasutterella id.2892) on Neuronal cell adhesion molecule
with a threshold of 1e−05

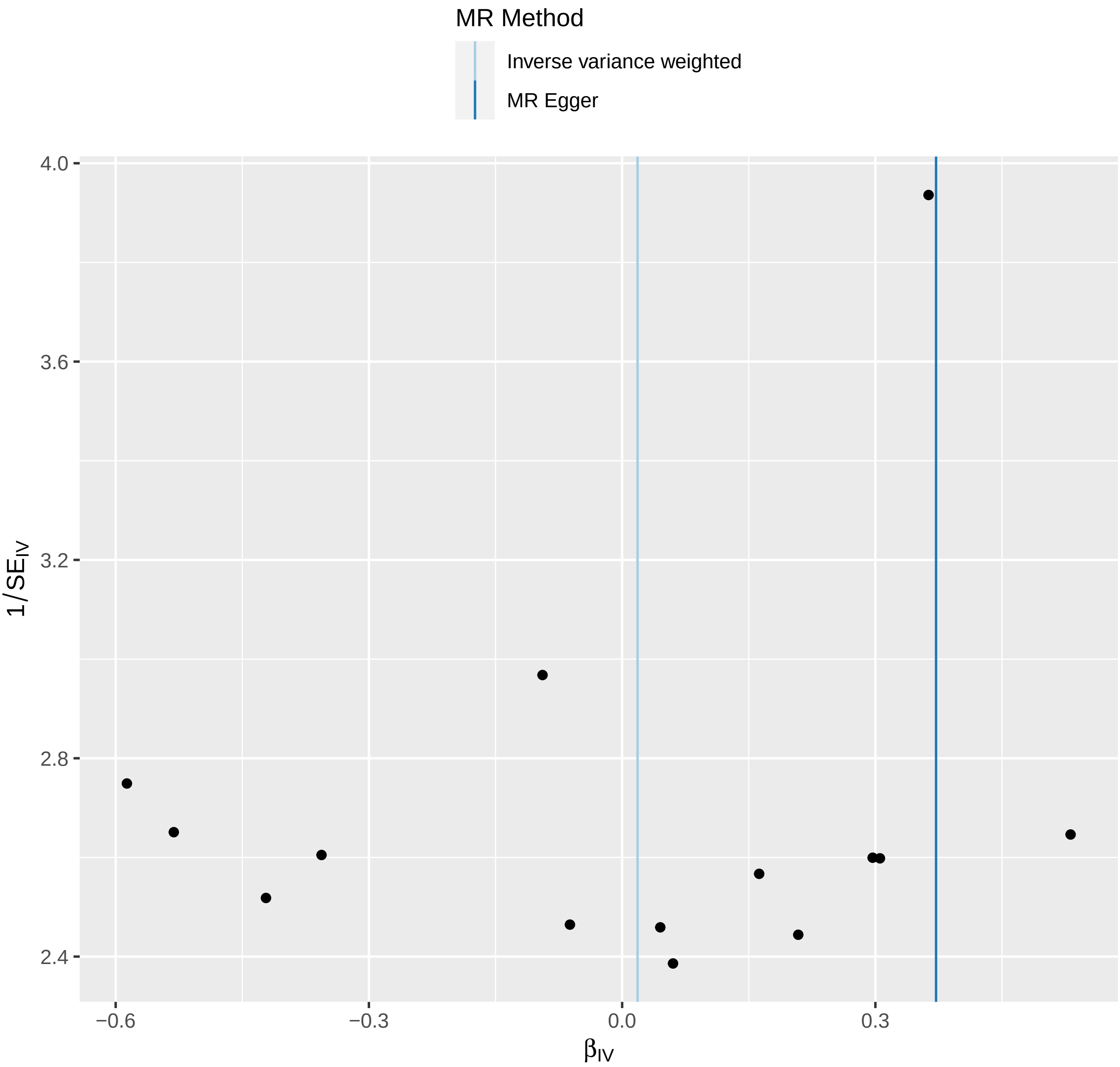

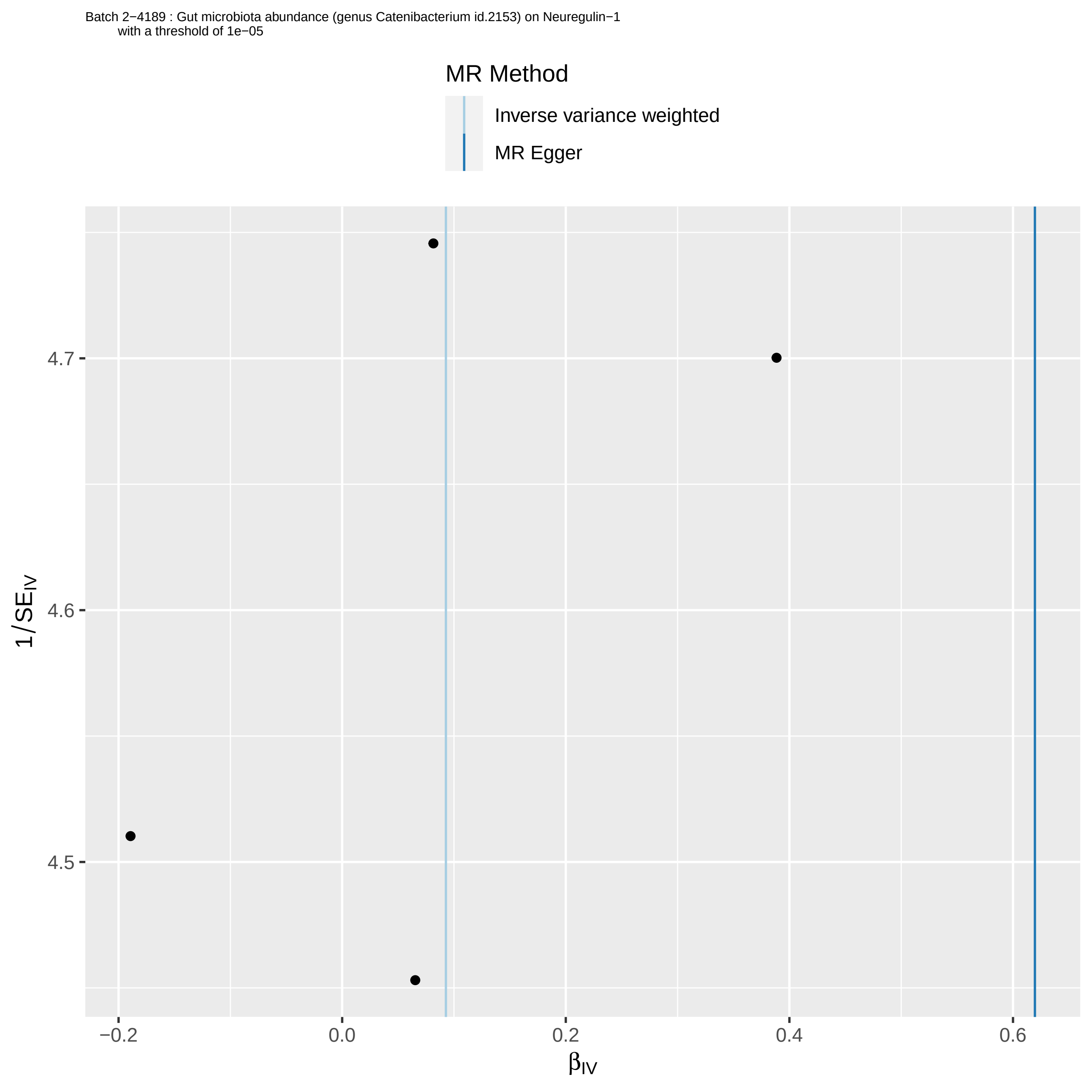

Batch 2−4189 : Gut microbiota abundance (genus Catenibacterium id.2153) on Neuregulin−1
with a threshold of 1e−05
MR Method
Inverse variance weighted
MR Egger
4.7
4.6
4.5
1/SE$_{IV}$
−0.2
0.0
0.2
0.4
0.6
β$_{IV}$

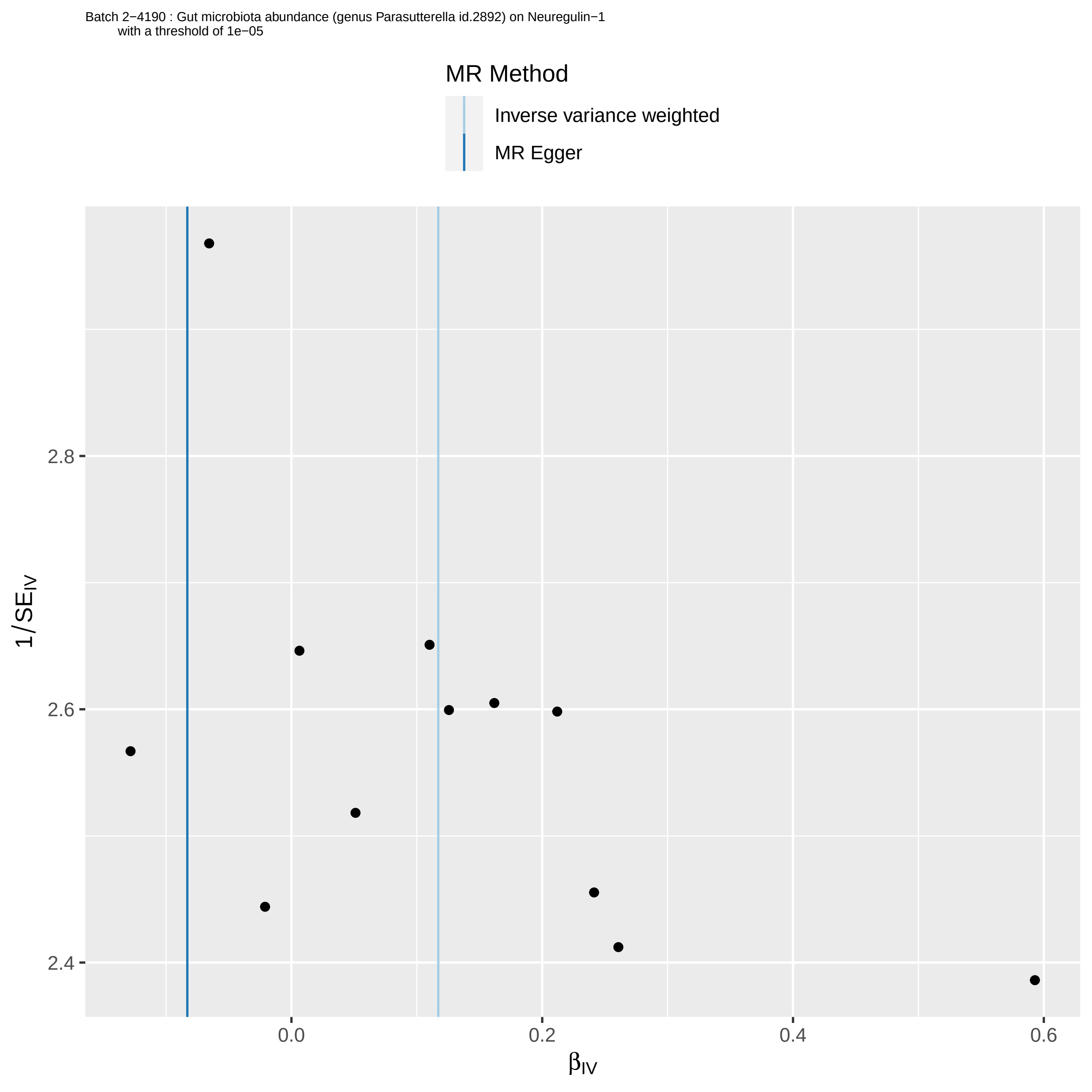

Batch 2−4190 : Gut microbiota abundance (genus Parasutterella id.2892) on Neuregulin−1
with a threshold of 1e−05
MR Method
Inverse variance weighted
MR Egger
2.8
2.6
2.4
1/SE$_{IV}$
0.0
0.2
0.4
0.6
β_{IV}

Batch 2−4191 : Gut microbiota abundance (genus Catenibacterium id.2153) on Pro−neuregulin−2, membrane−bound isoform
with a threshold of 1e−05

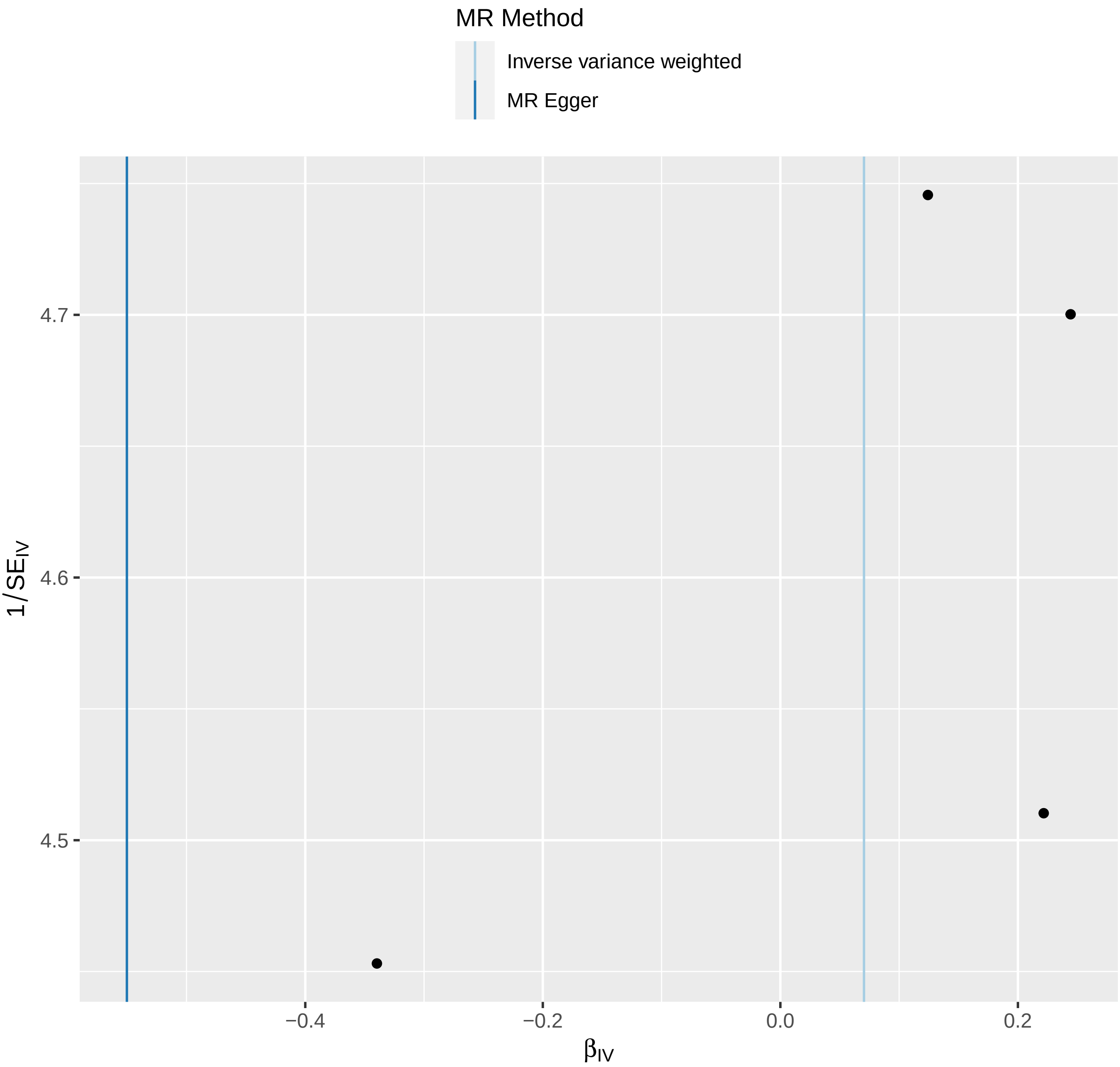

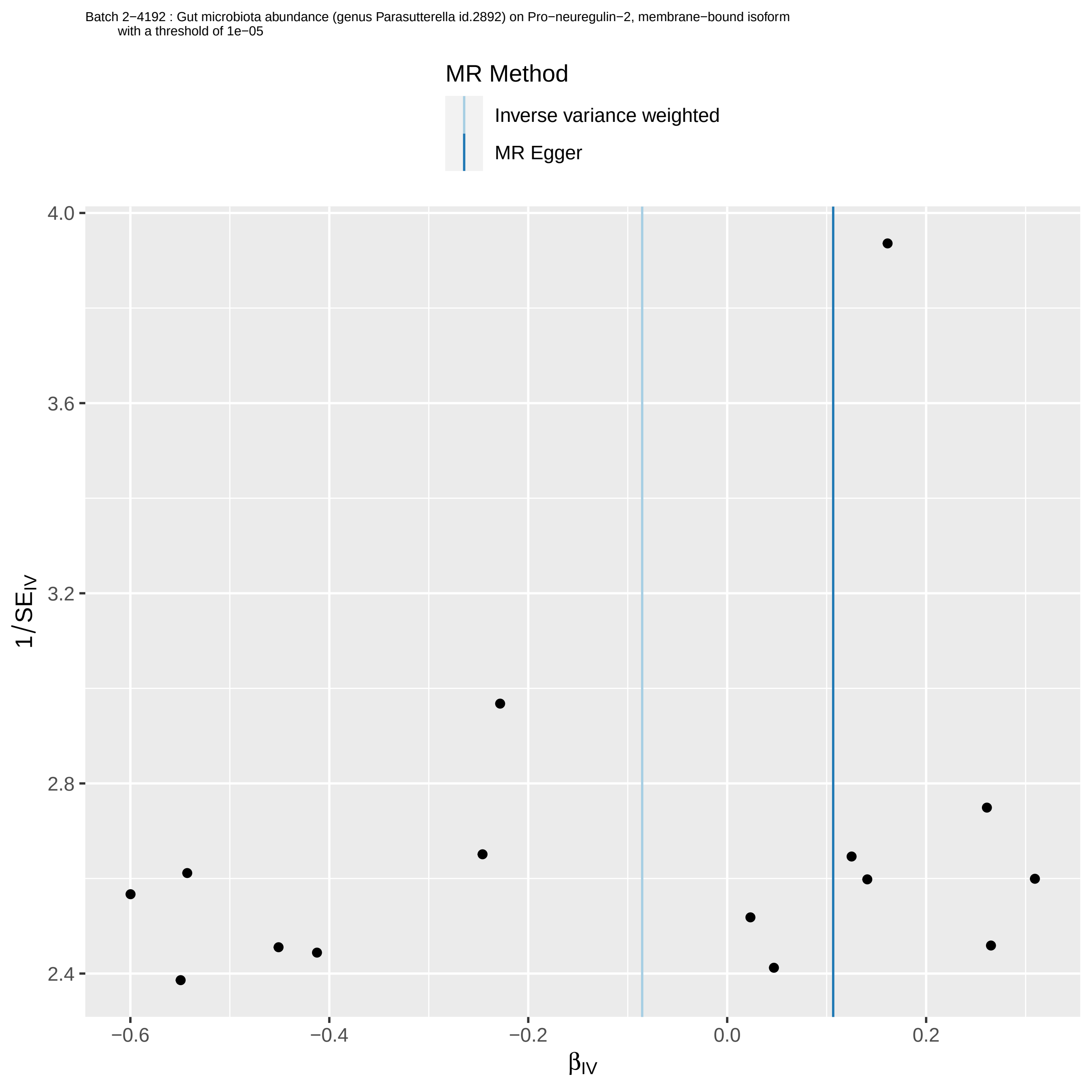
Batch 2−4192 : Gut microbiota abundance (genus Parasutterella id.2892) on Pro−neuregulin−2, membrane−bound isoform
with a threshold of 1e−05
MR Method
Inverse variance weighted
MR Egger
4.0
3.6
3.2
2.8
2.4
1/SE_IV
−0.6
−0.4
−0.2
0.0
0.2
β_IV

Batch 2−4193 : Gut microbiota abundance (genus Catenibacterium id.2153) on Neuregulin−4
with a threshold of 1e−05

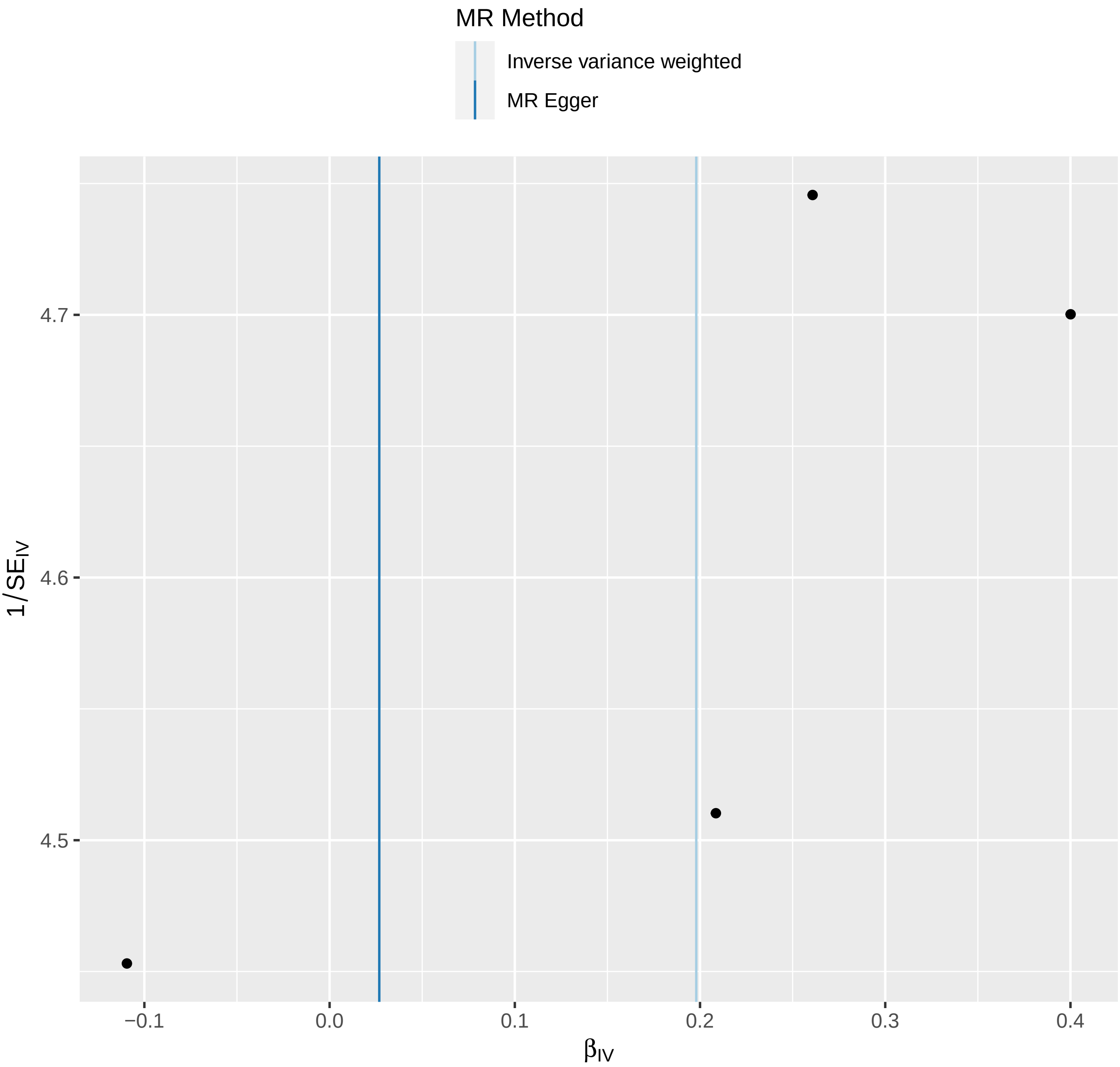

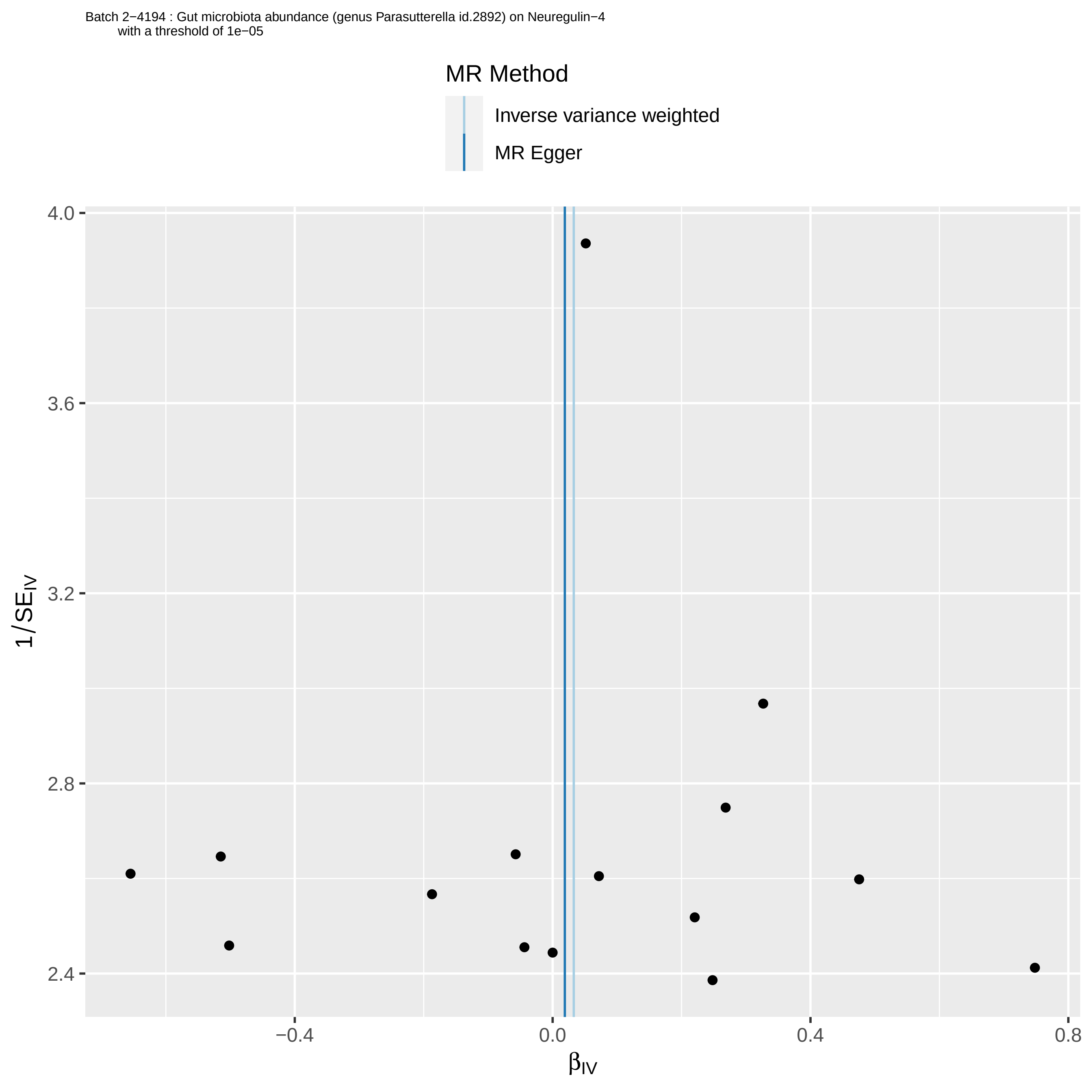
Batch 2−4194 : Gut microbiota abundance (genus Parasutterella id.2892) on Neuregulin−4
with a threshold of 1e−05
MR Method
Inverse variance weighted
MR Egger
4.0
3.6
3.2
2.8
2.4
1/SE_IV
−0.4
0.0
0.4
0.8
β_IV

Batch 2−4195 : Gut microbiota abundance (genus Catenibacterium id.2153) on Neuritin
with a threshold of 1e−05

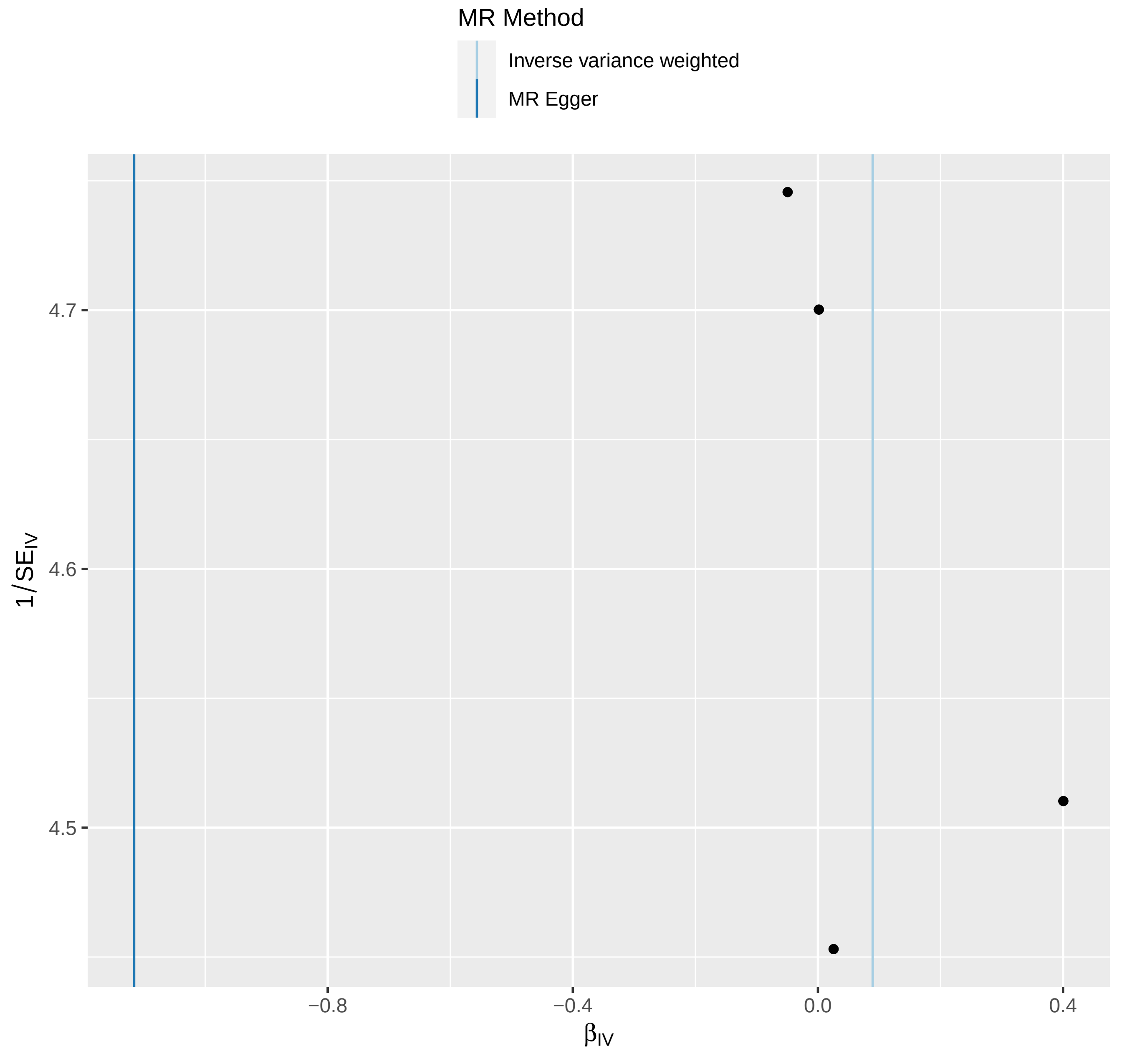

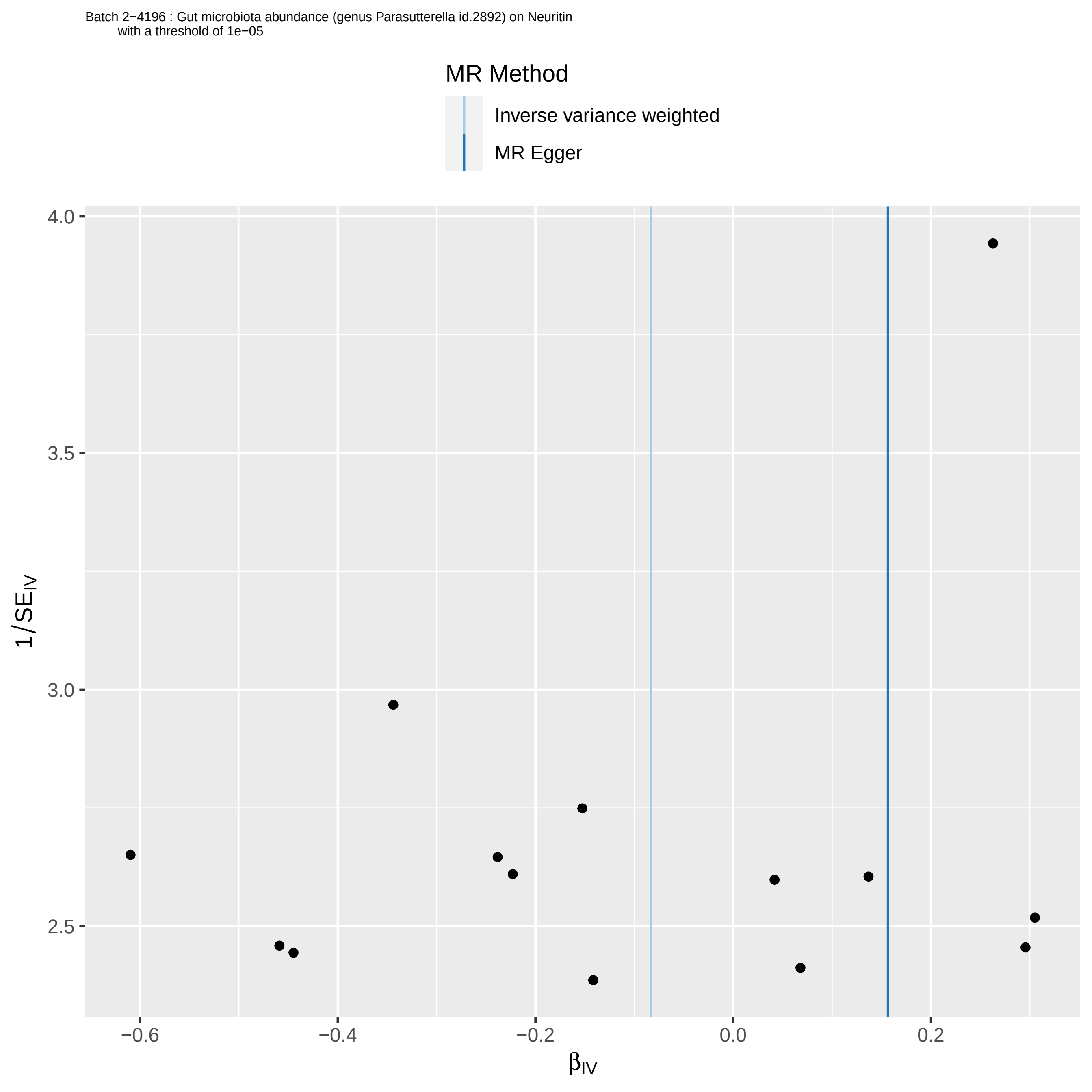
Batch 2−4196 : Gut microbiota abundance (genus Parasutterella id.2892) on Neuritin
with a threshold of 1e−05
MR Method
Inverse variance weighted
MR Egger
4.0
3.5
3.0
2.5
1/SE_IV
−0.6
−0.4
−0.2
0.0
0.2
β_IV

Batch 2−4197 : Gut microbiota abundance (genus Catenibacterium id.2153) on Neuropilin−1
with a threshold of 1e−05

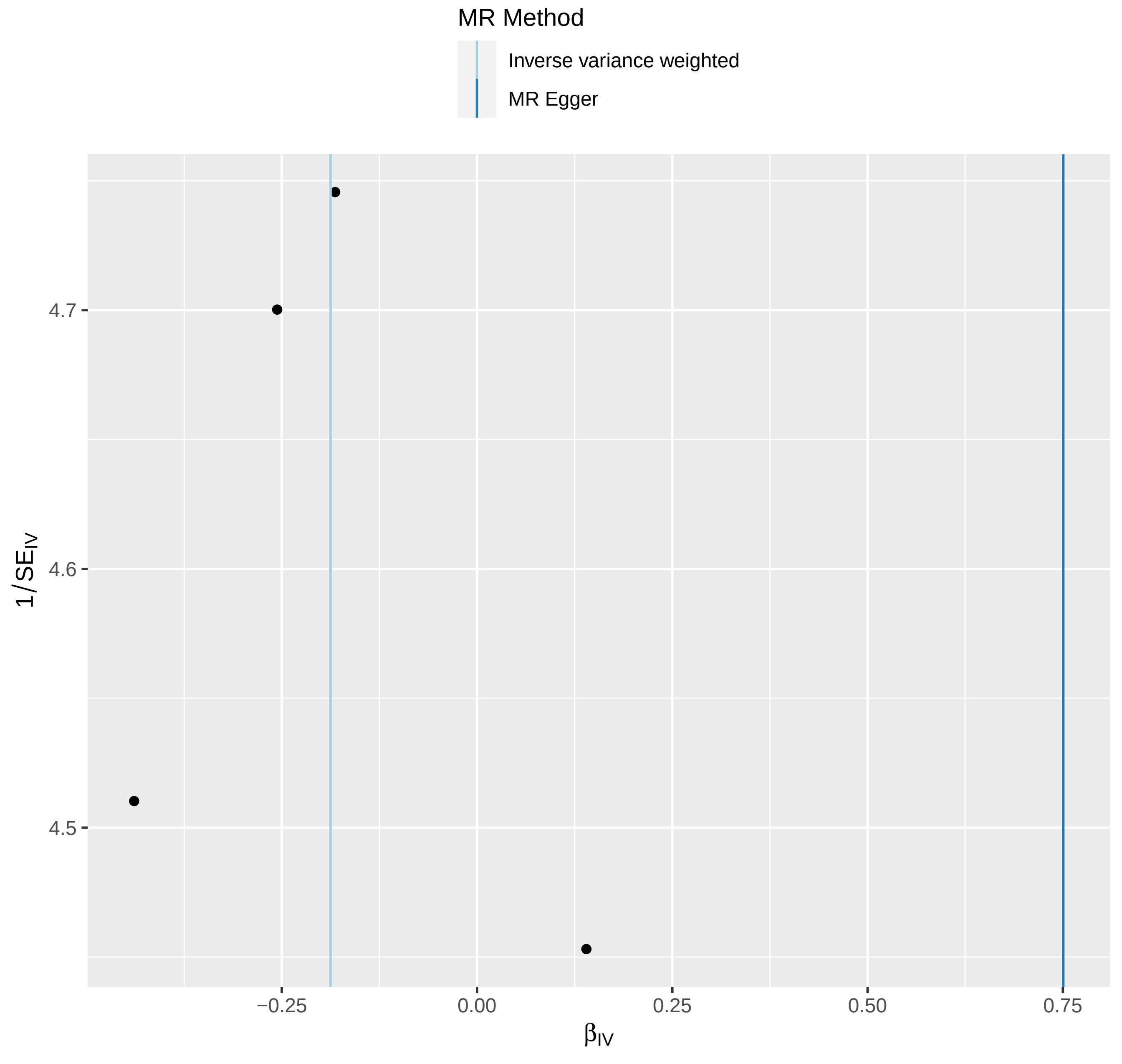

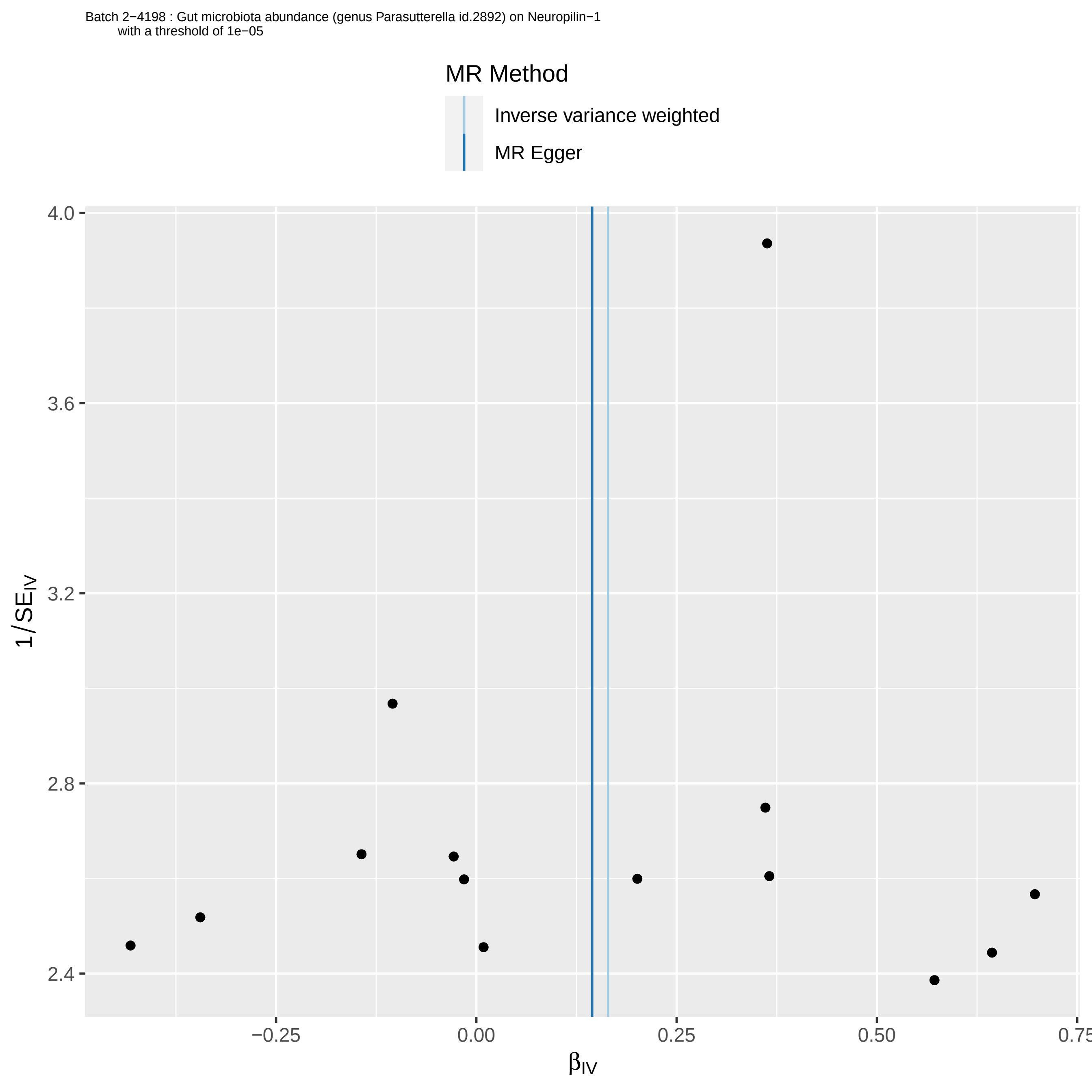
Batch 2−4198 : Gut microbiota abundance (genus Parasutterella id.2892) on Neuropilin−1
with a threshold of 1e−05
MR Method
Inverse variance weighted
MR Egger
4.0
3.6
3.2
2.8
2.4
1/SE$_{IV}$
−0.25
0.00
0.25
0.50
0.75
β$_{IV}$

Batch 2−4199 : Gut microbiota abundance (genus Catenibacterium id.2153) on Neuropilin−2
with a threshold of 1e−05

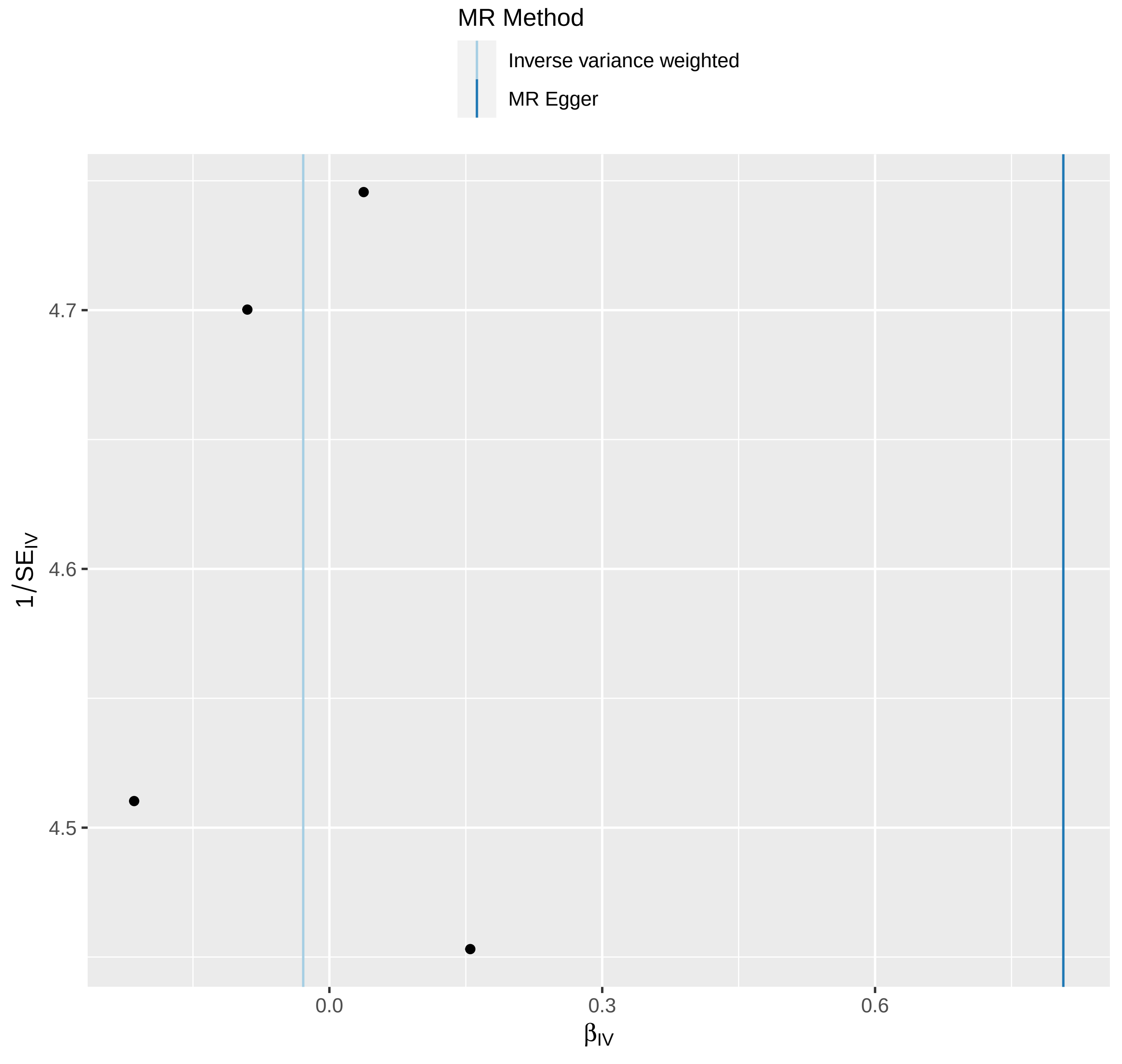

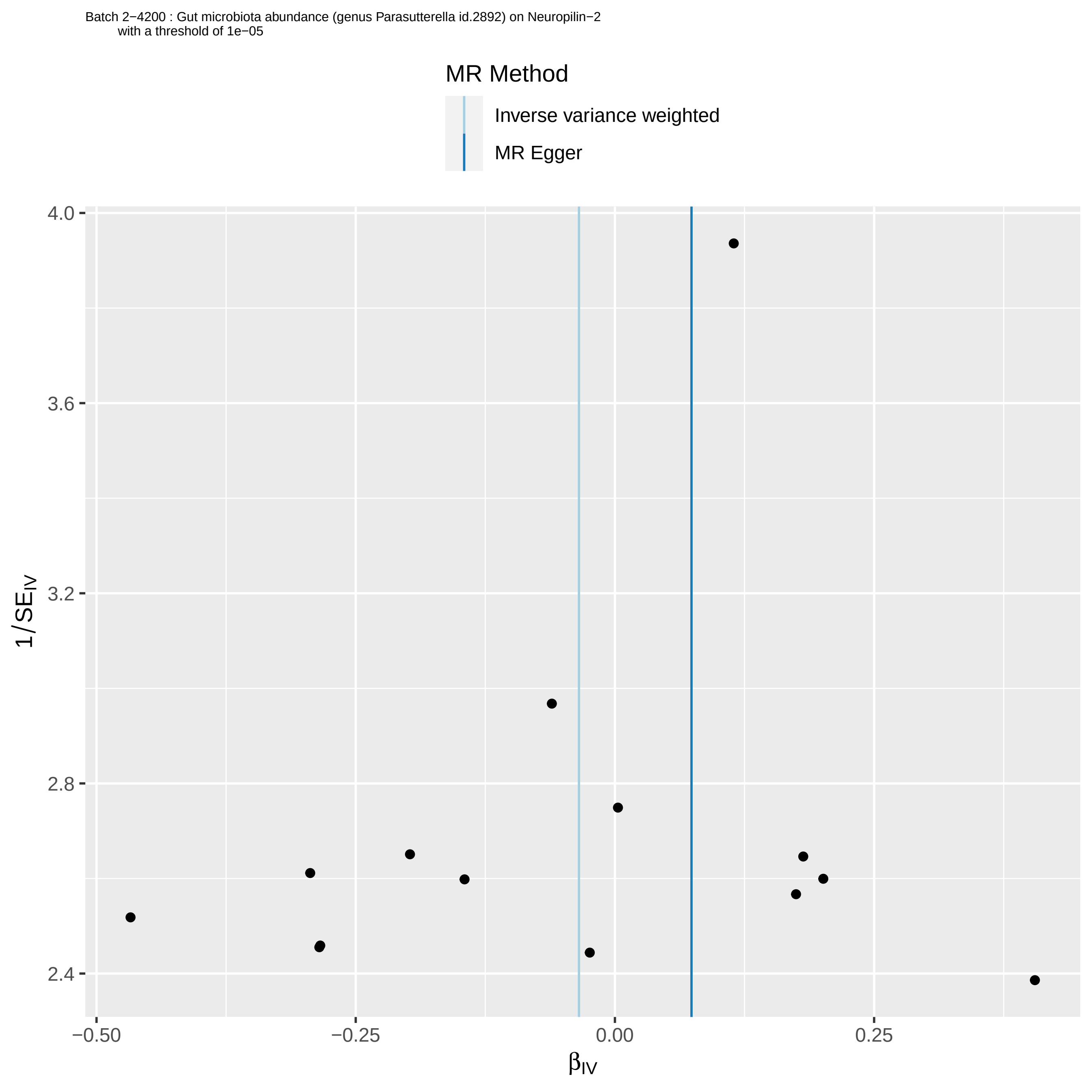

Batch 2−4200 : Gut microbiota abundance (genus Parasutterella id.2892) on Neuropilin−2
with a threshold of 1e−05
MR Method
Inverse variance weighted
MR Egger
4.0
3.6
3.2
2.8
2.4
1/SE_IV
−0.50
−0.25
0.00
0.25
β_IV

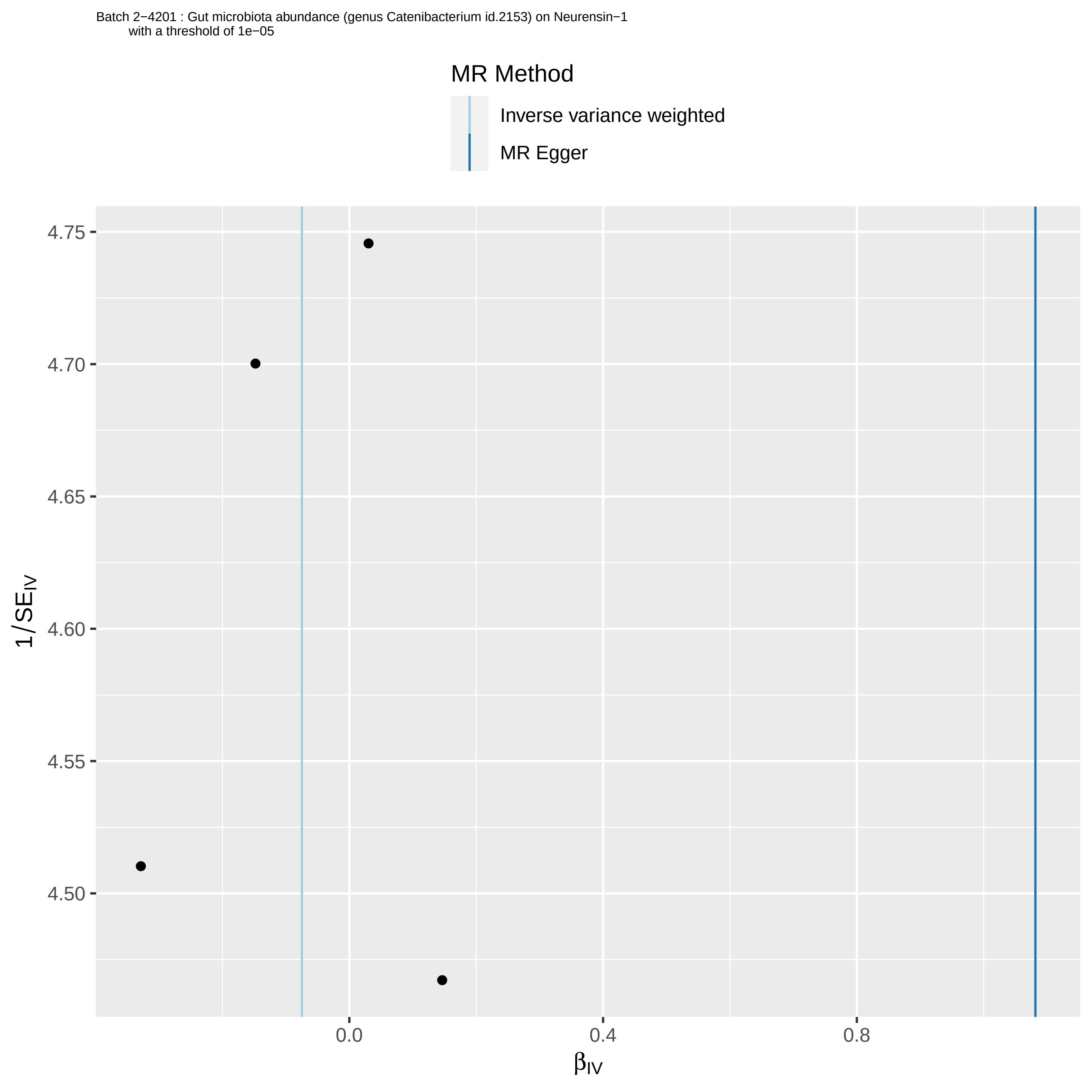
Batch 2−4201 : Gut microbiota abundance (genus Catenibacterium id.2153) on Neurensin−1
with a threshold of 1e−05
MR Method
Inverse variance weighted
MR Egger
4.75
4.70
4.65
4.60
4.55
4.50
1/SE_IV
0.0
0.4
0.8
β_IV

Batch 2−4202 : Gut microbiota abundance (genus Parasutterella id.2892) on Neurensin−1
with a threshold of 1e−05

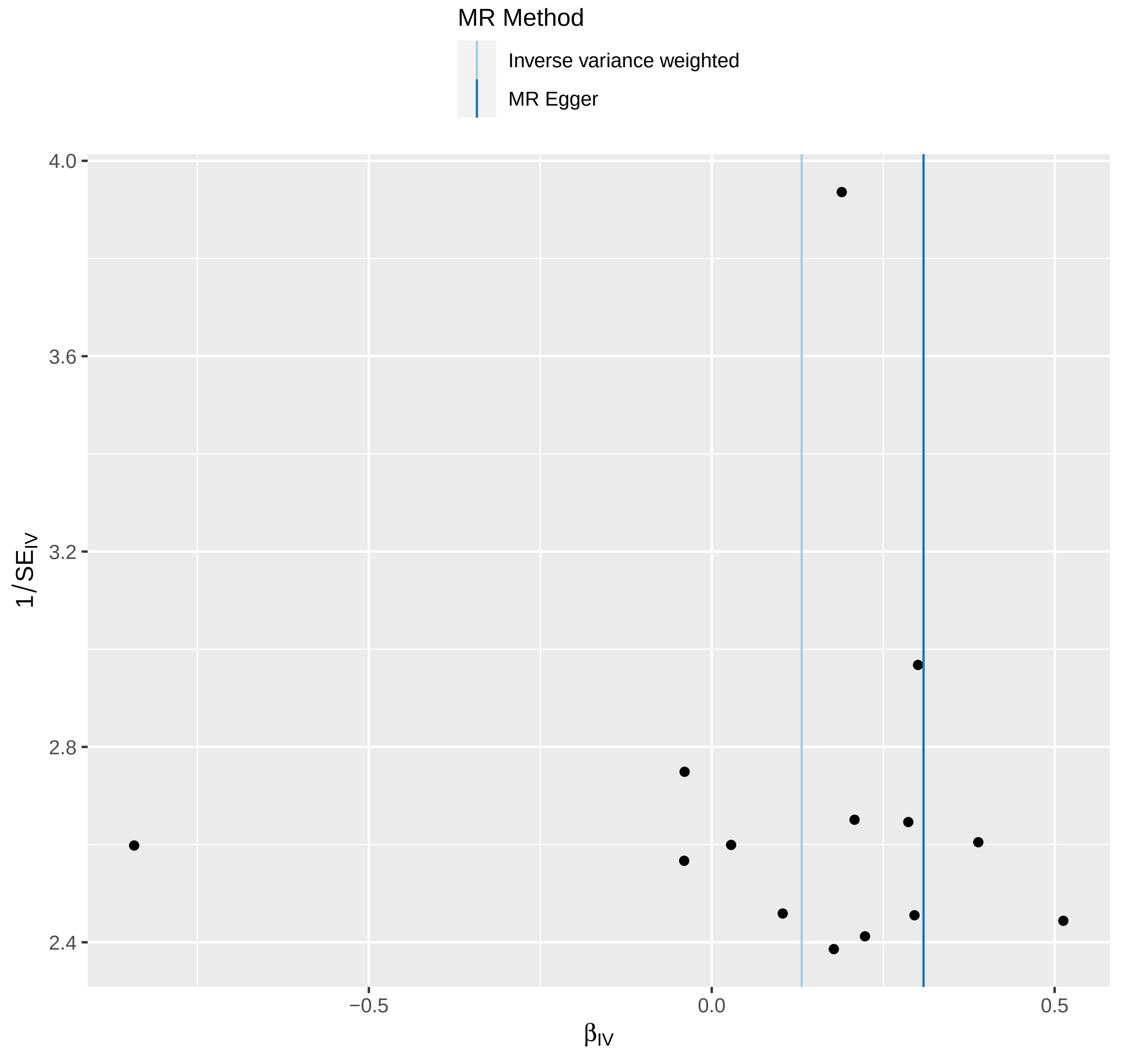

Batch 2−4203 : Gut microbiota abundance (genus Catenibacterium id.2153) on Neurexin−1−beta
with a threshold of 1e−05

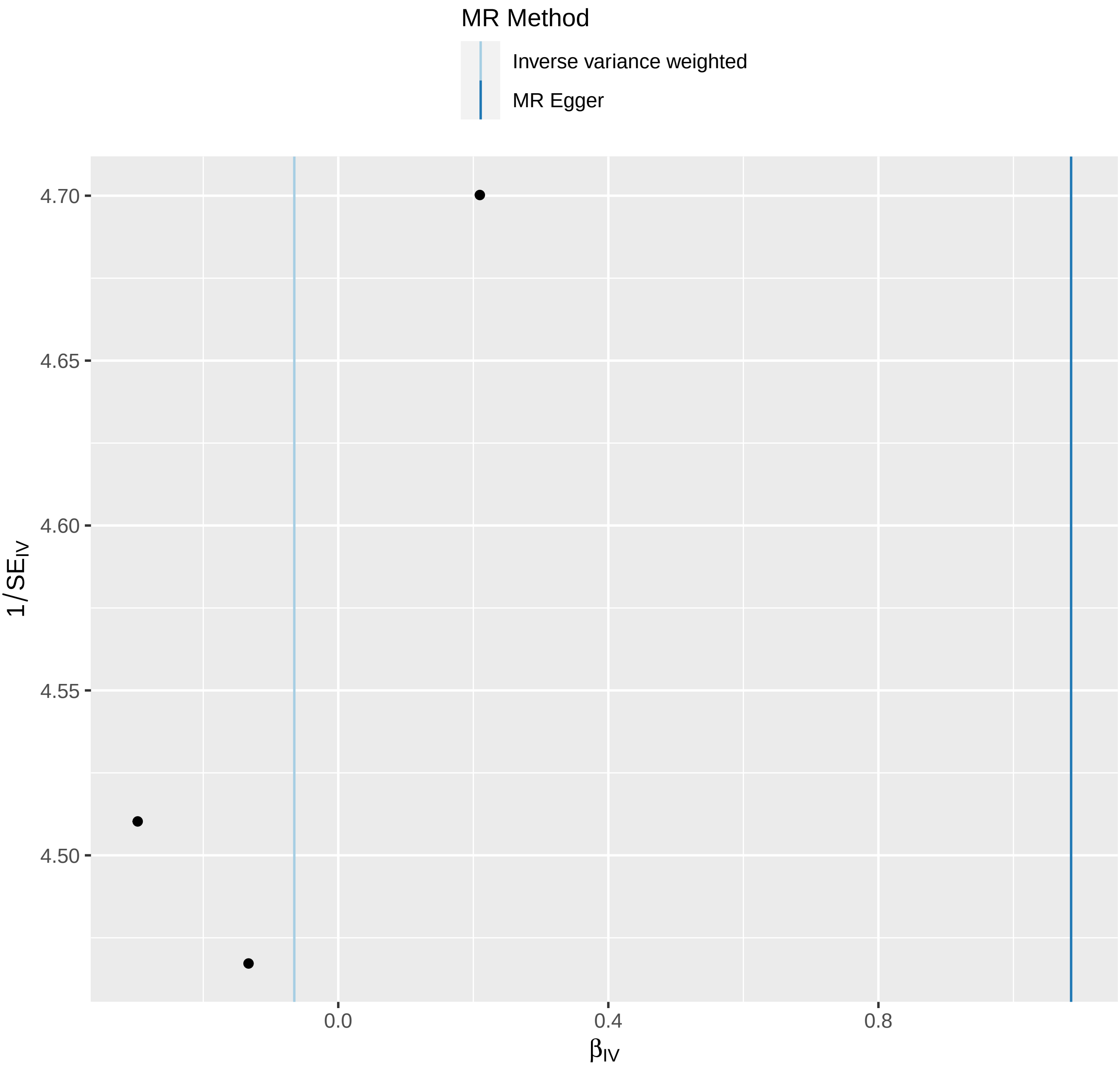

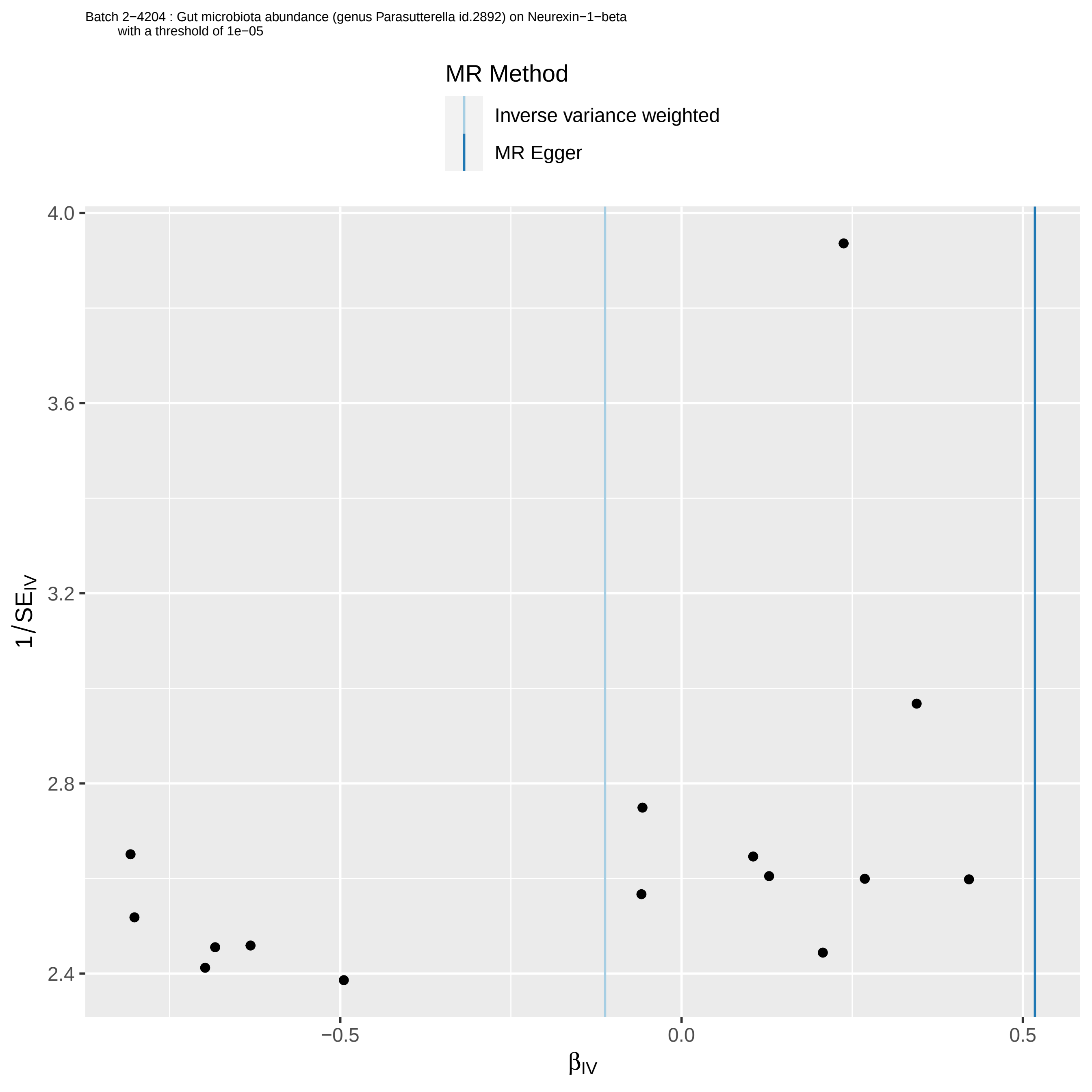

Batch 2−4204 : Gut microbiota abundance (genus Parasutterella id.2892) on Neurexin−1−beta
with a threshold of 1e−05
MR Method
Inverse variance weighted
MR Egger
4.0
3.6
3.2
2.8
2.4
1/SE_IV
−0.5
0.0
0.5
β_IV

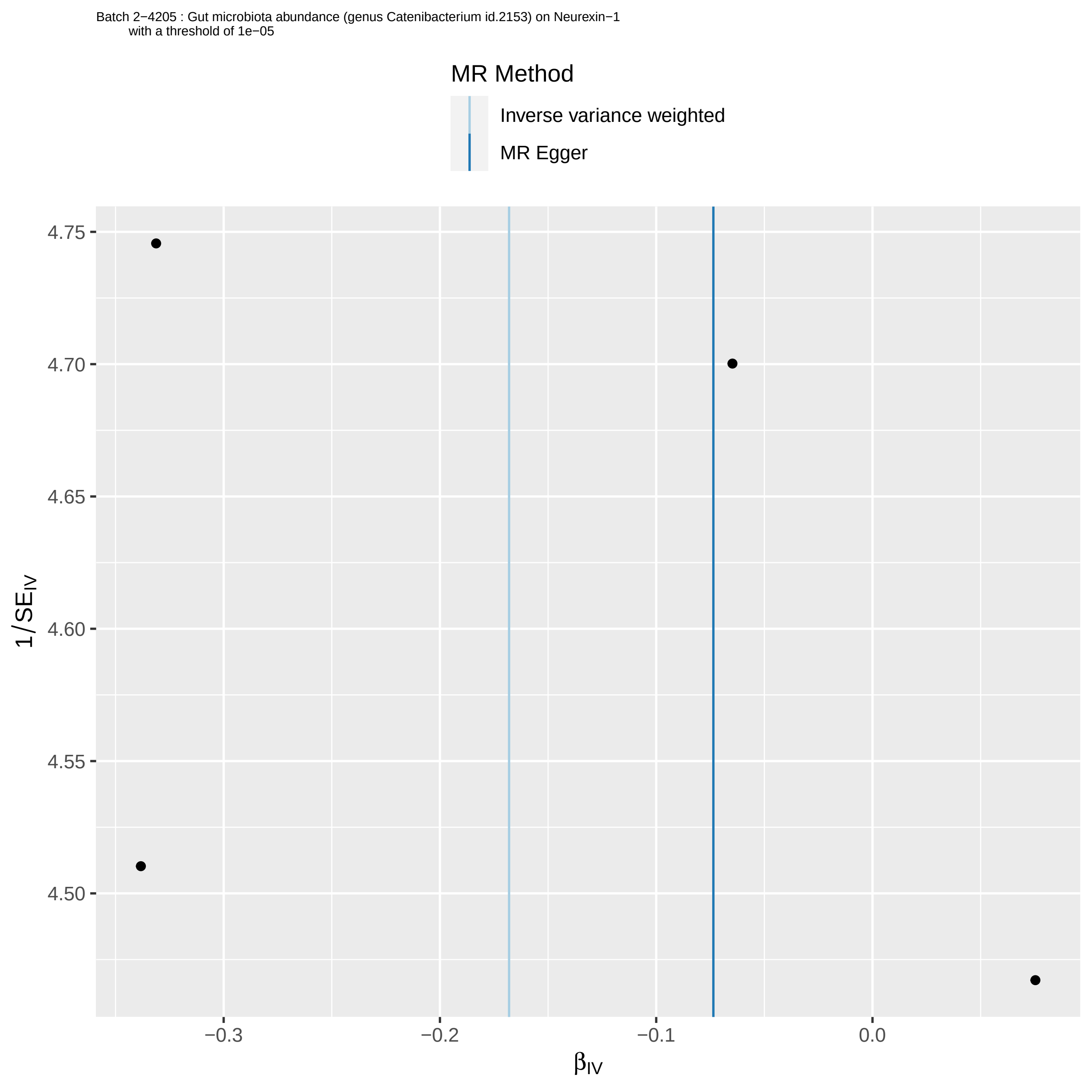
Batch 2−4205 : Gut microbiota abundance (genus Catenibacterium id.2153) on Neurexin−1
with a threshold of 1e−05
MR Method
Inverse variance weighted
MR Egger
1/SE_IV
4.75
4.70
4.65
4.60
4.55
4.50
−0.3
−0.2
−0.1
0.0
β_IV

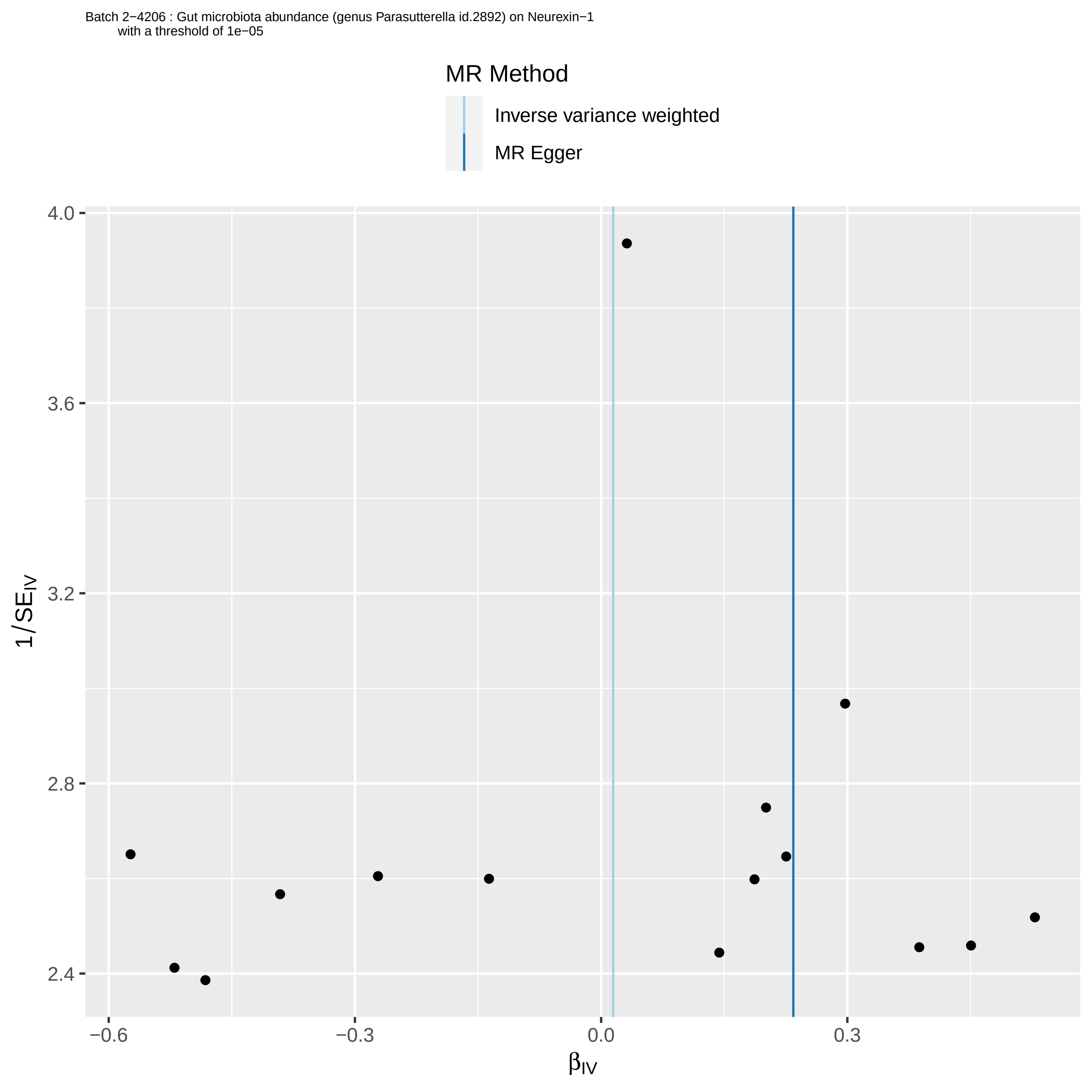
Batch 2−4206 : Gut microbiota abundance (genus Parasutterella id.2892) on Neurexin−1
with a threshold of 1e−05
MR Method
Inverse variance weighted
MR Egger
4.0
3.6
3.2
2.8
2.4
$1/SE_{IV}$
−0.6
−0.3
0.0
0.3
β_{IV}

Batch 2−4207 : Gut microbiota abundance (genus Catenibacterium id.2153) on Neurexin−2−beta
with a threshold of 1e−05

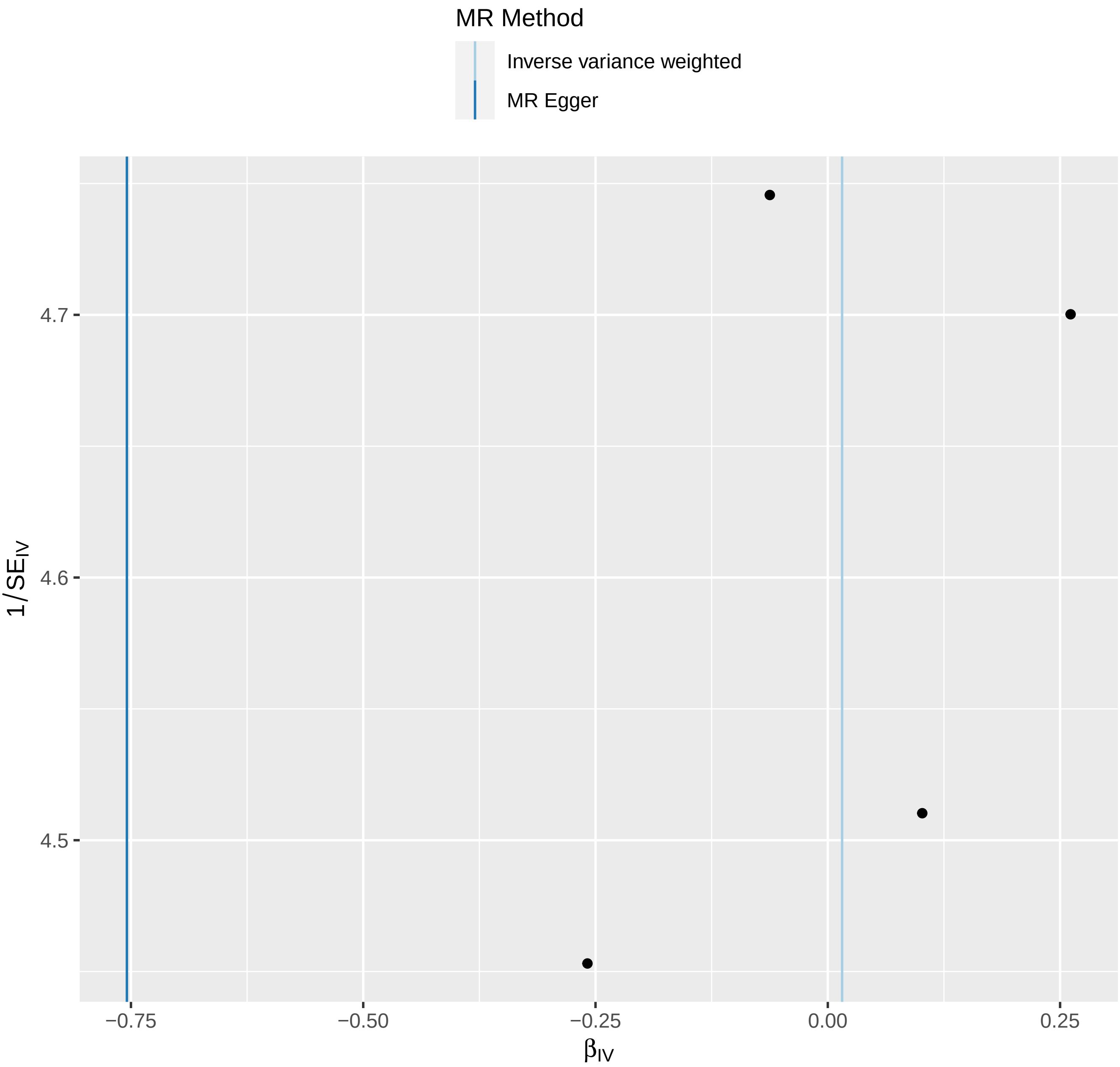

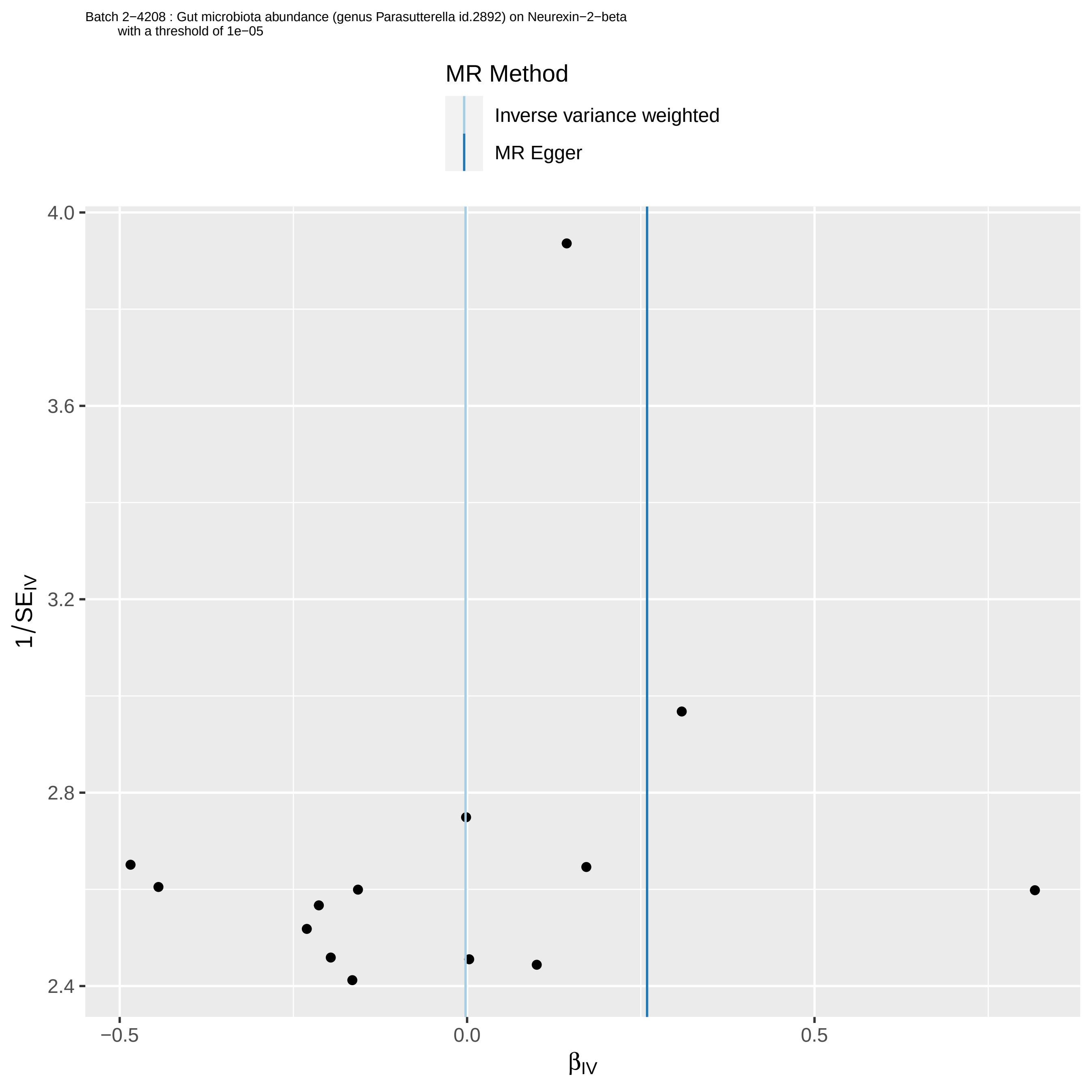

Batch 2−4208 : Gut microbiota abundance (genus Parasutterella id.2892) on Neurexin−2−beta
with a threshold of 1e−05
MR Method
Inverse variance weighted
MR Egger
4.0
3.6
3.2
2.8
2.4
1/SE_IV
−0.5
0.0
0.5
β_IV

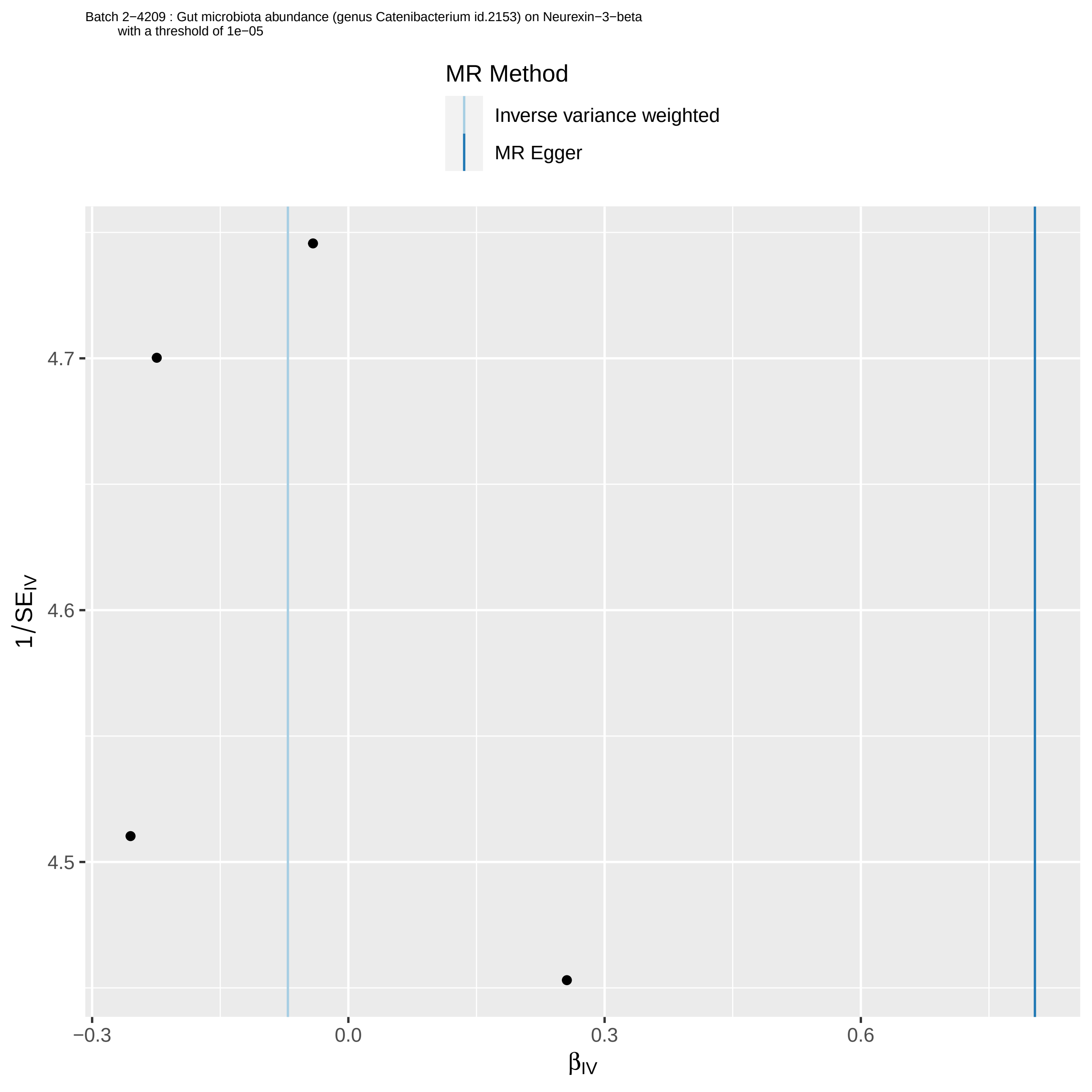
Batch 2−4209 : Gut microbiota abundance (genus Catenibacterium id.2153) on Neurexin−3−beta
with a threshold of 1e−05
MR Method
Inverse variance weighted
MR Egger
4.7
4.6
4.5
1/SE_IV
−0.3
0.0
0.3
0.6
β_IV

Batch 2−4210 : Gut microbiota abundance (genus Parasutterella id.2892) on Neurexin−3−beta
with a threshold of 1e−05

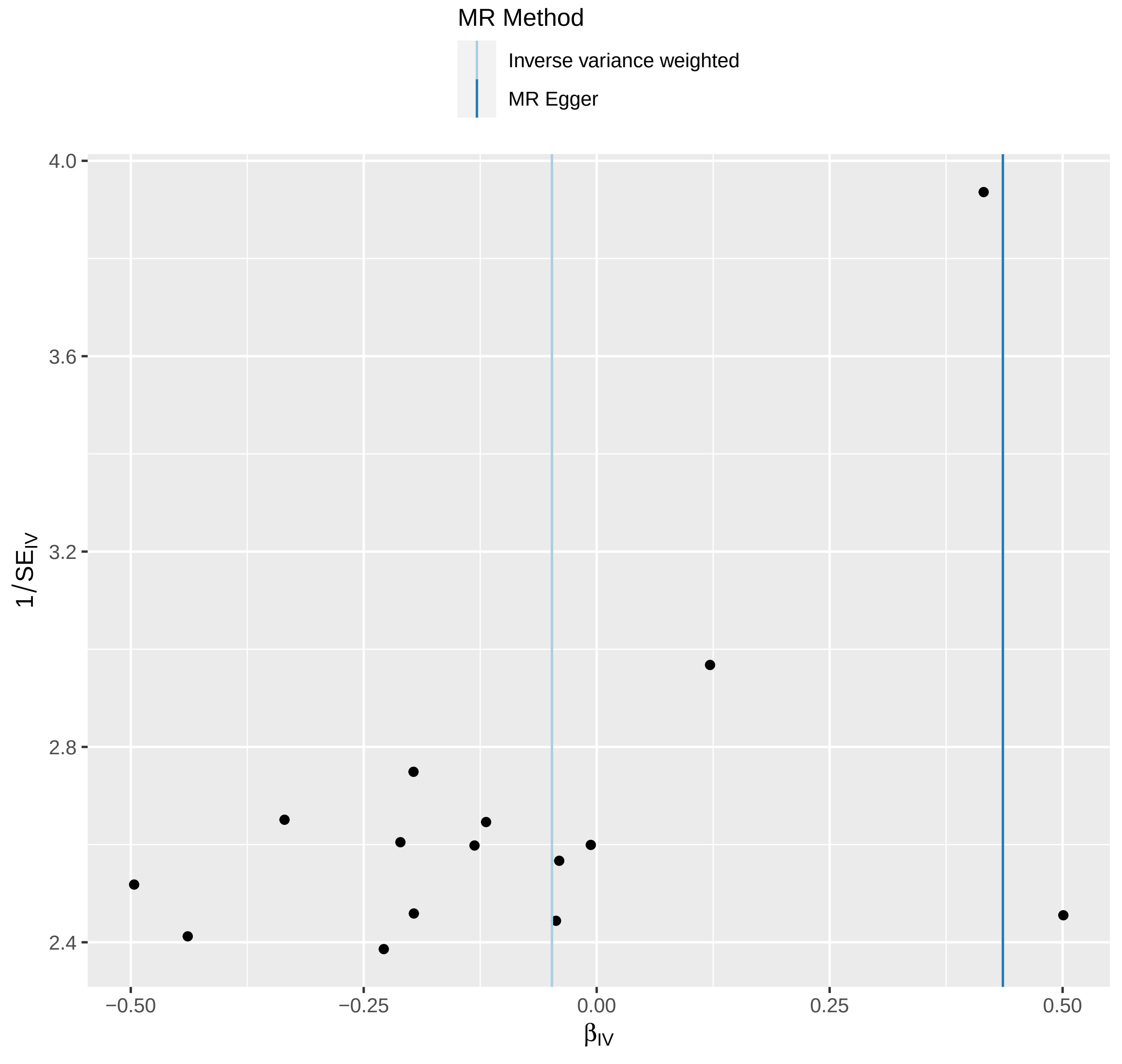

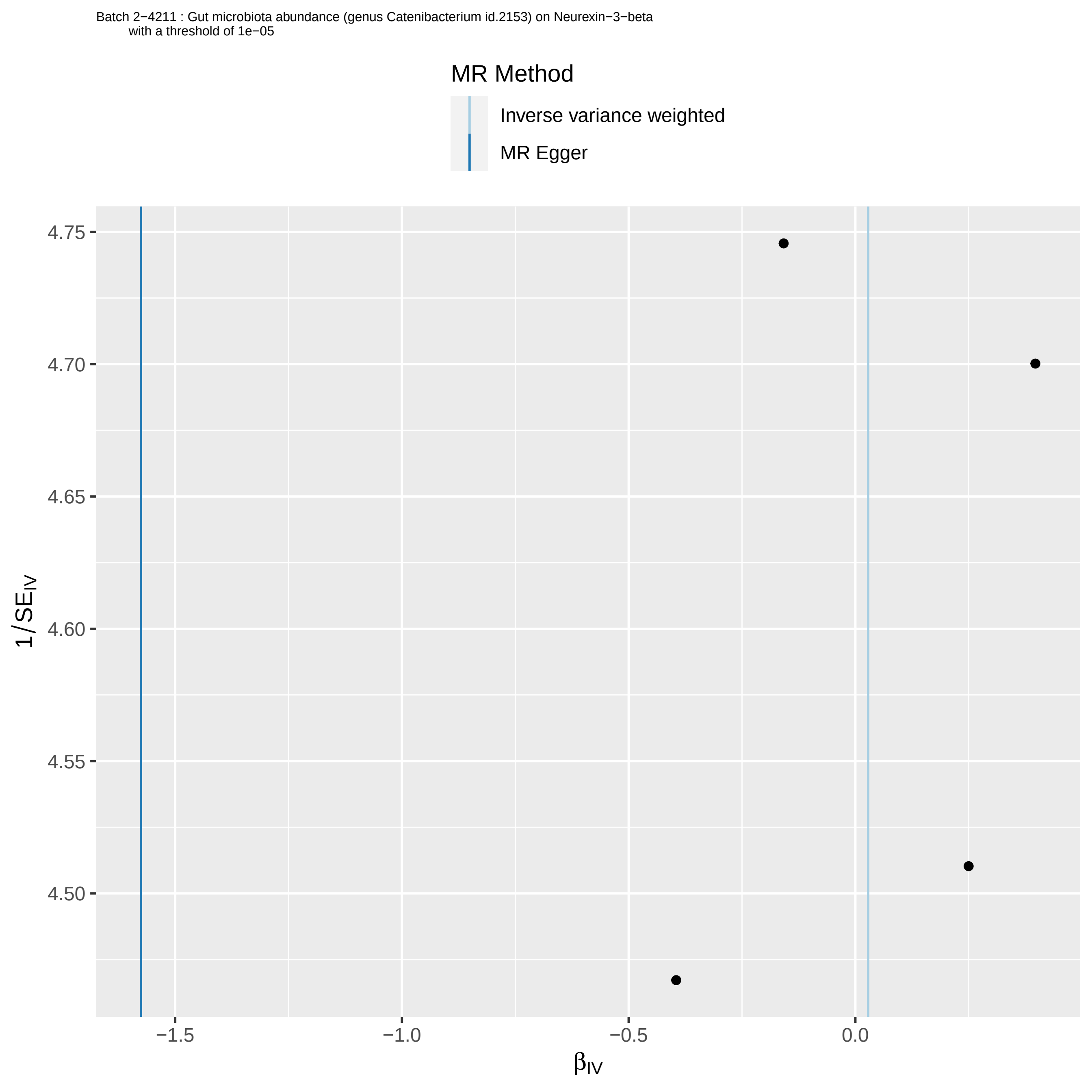
Batch 2−4211 : Gut microbiota abundance (genus Catenibacterium id.2153) on Neurexin−3−beta
with a threshold of 1e−05
MR Method
Inverse variance weighted
MR Egger
4.75
4.70
4.65
4.60
4.55
4.50
$1/SE_{IV}$
−1.5
−1.0
−0.5
0.0
β_{IV}

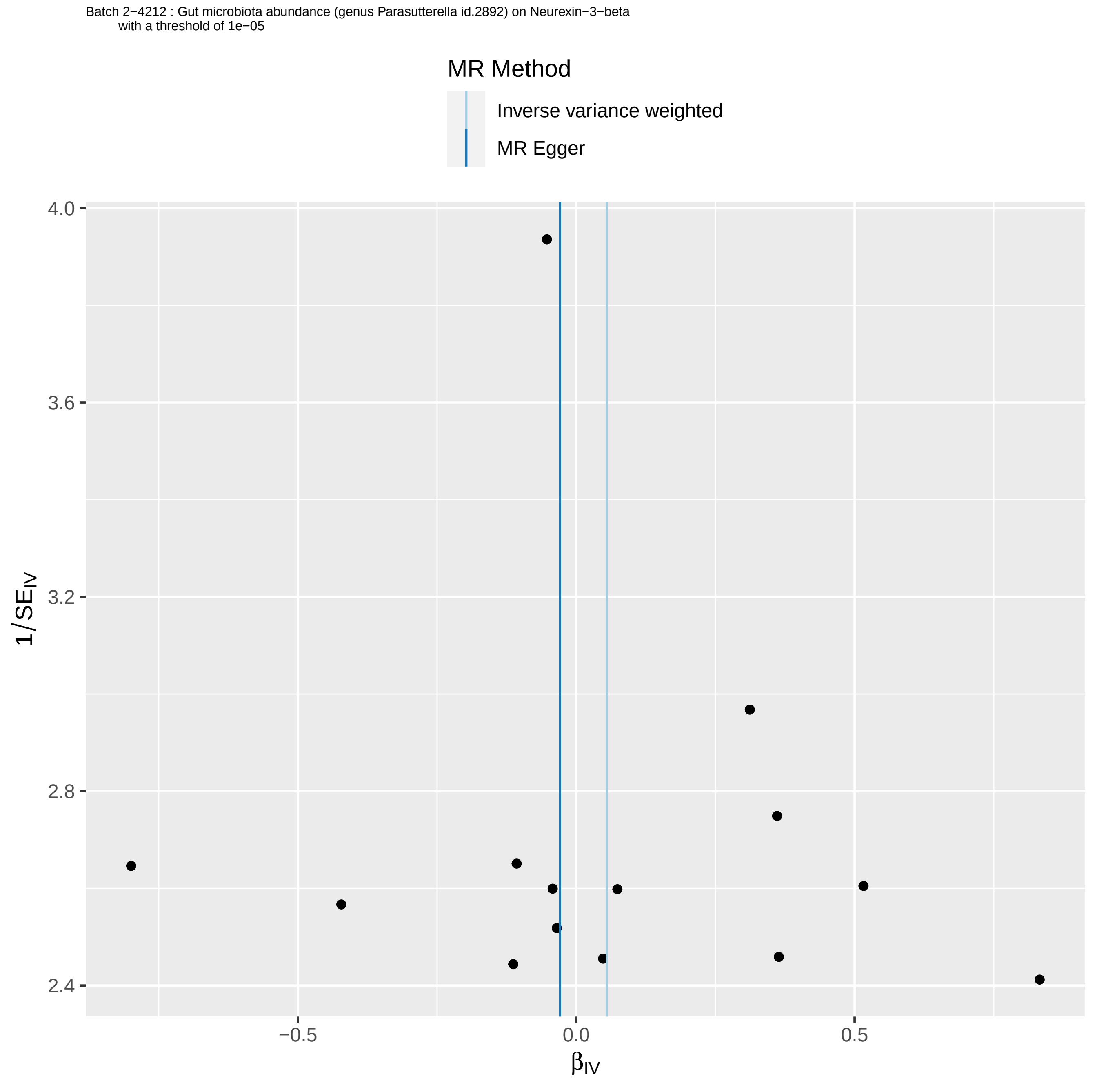
Batch 2−4212 : Gut microbiota abundance (genus Parasutterella id.2892) on Neurexin−3−beta
with a threshold of 1e−05
MR Method
Inverse variance weighted
MR Egger
4.0
3.6
3.2
2.8
2.4
1/SE_IV
−0.5
0.0
0.5
β_IV

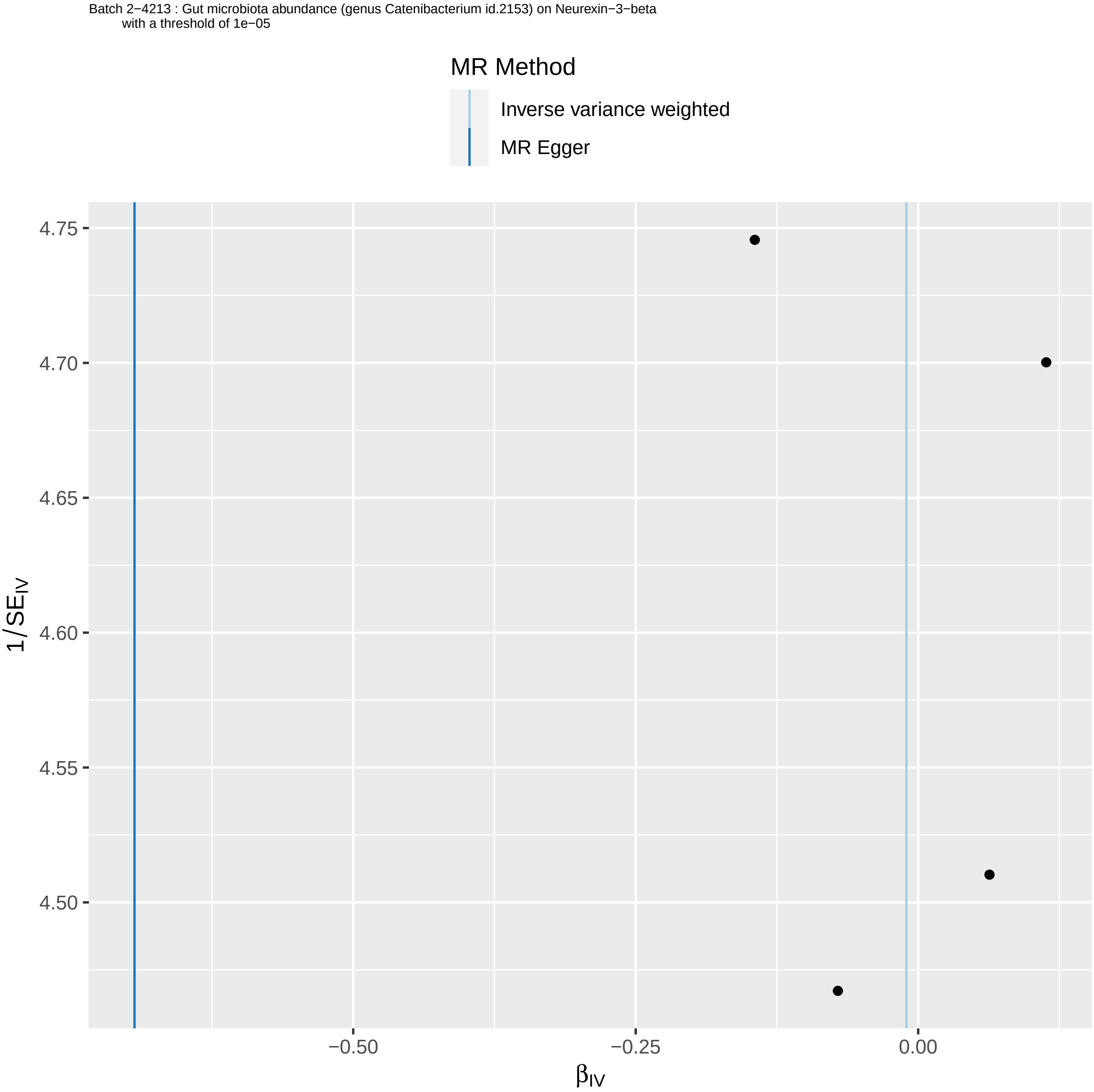
Batch 2−4213 : Gut microbiota abundance (genus Catenibacterium id.2153) on Neurexin−3−beta
with a threshold of 1e−05
MR Method
Inverse variance weighted
MR Egger
4.75
4.70
4.65
4.60
4.55
4.50
1/SE_IV
−0.50
−0.25
0.00
β_IV

Batch 2−4214 : Gut microbiota abundance (genus Parasutterella id.2892) on Neurexin−3−beta
with a threshold of 1e−05

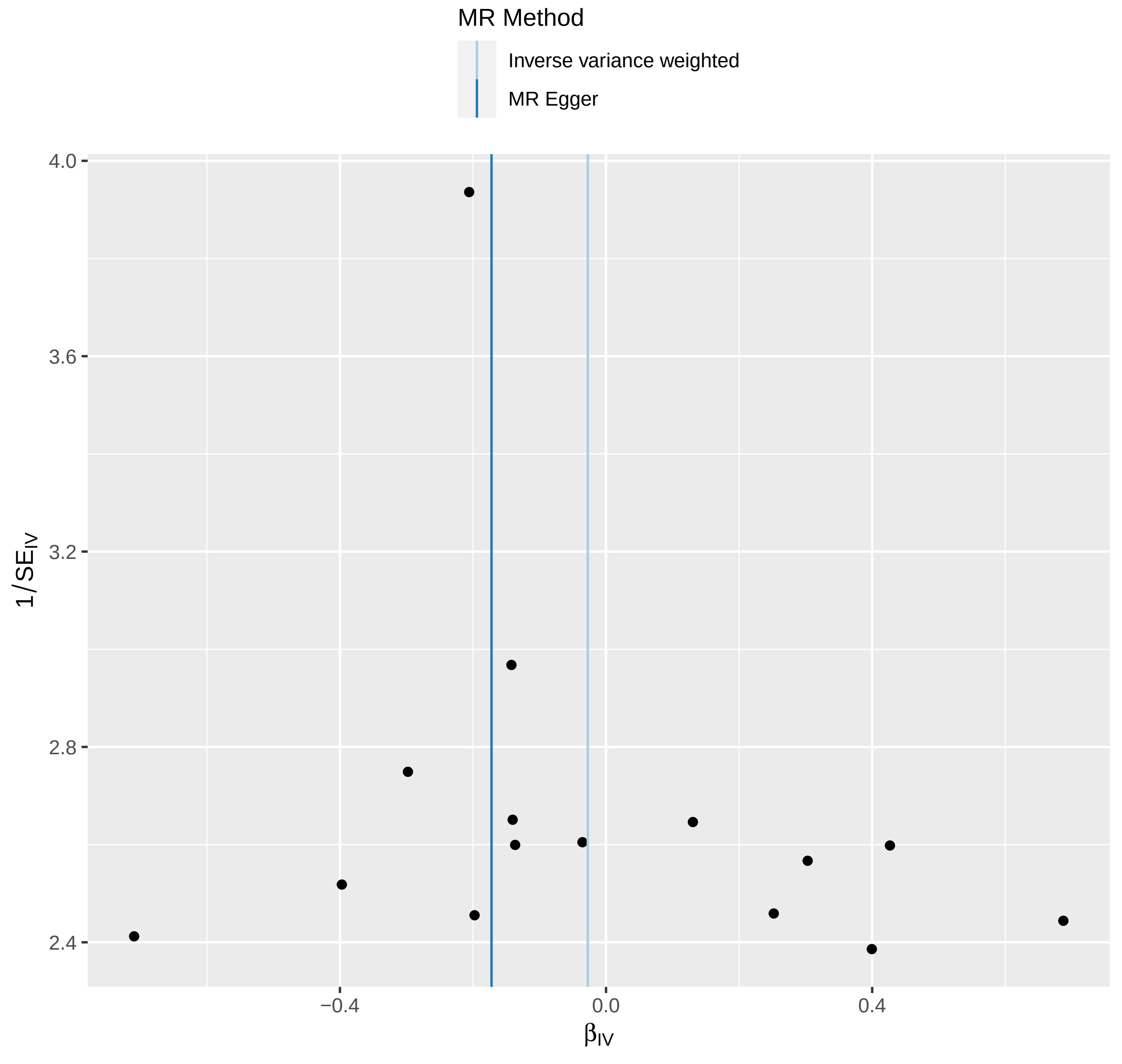

Batch 2−4215 : Gut microbiota abundance (genus Catenibacterium id.2153) on Sterol−4−alpha−carboxylate 3−dehydrogenase, decarboxylating with a threshold of 1e−05

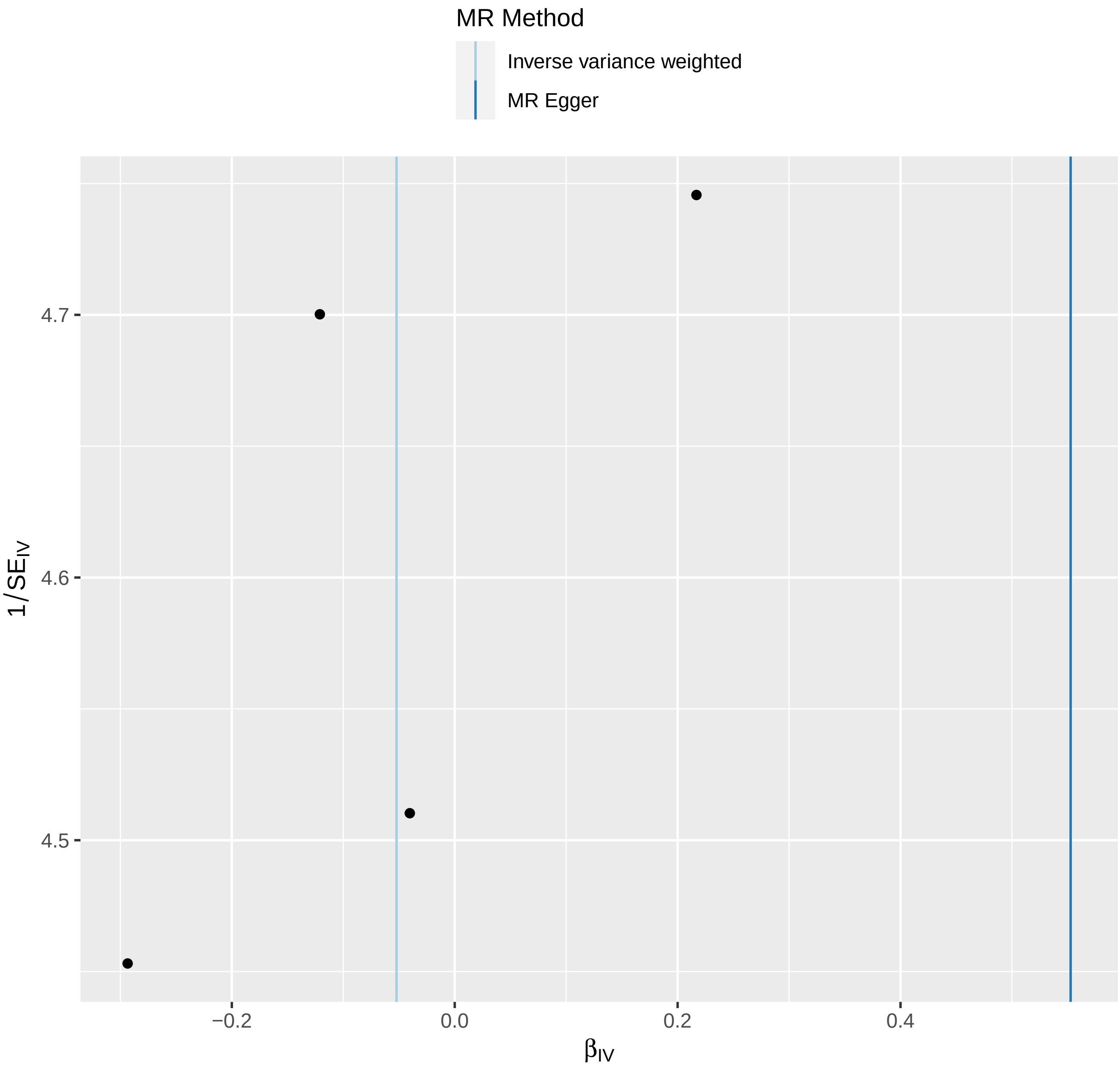

Batch 2−4216 : Gut microbiota abundance (genus Parasutterella id.2892) on Sterol−4−alpha−carboxylate 3−dehydrogenase, decarboxylating
with a threshold of 1e−05

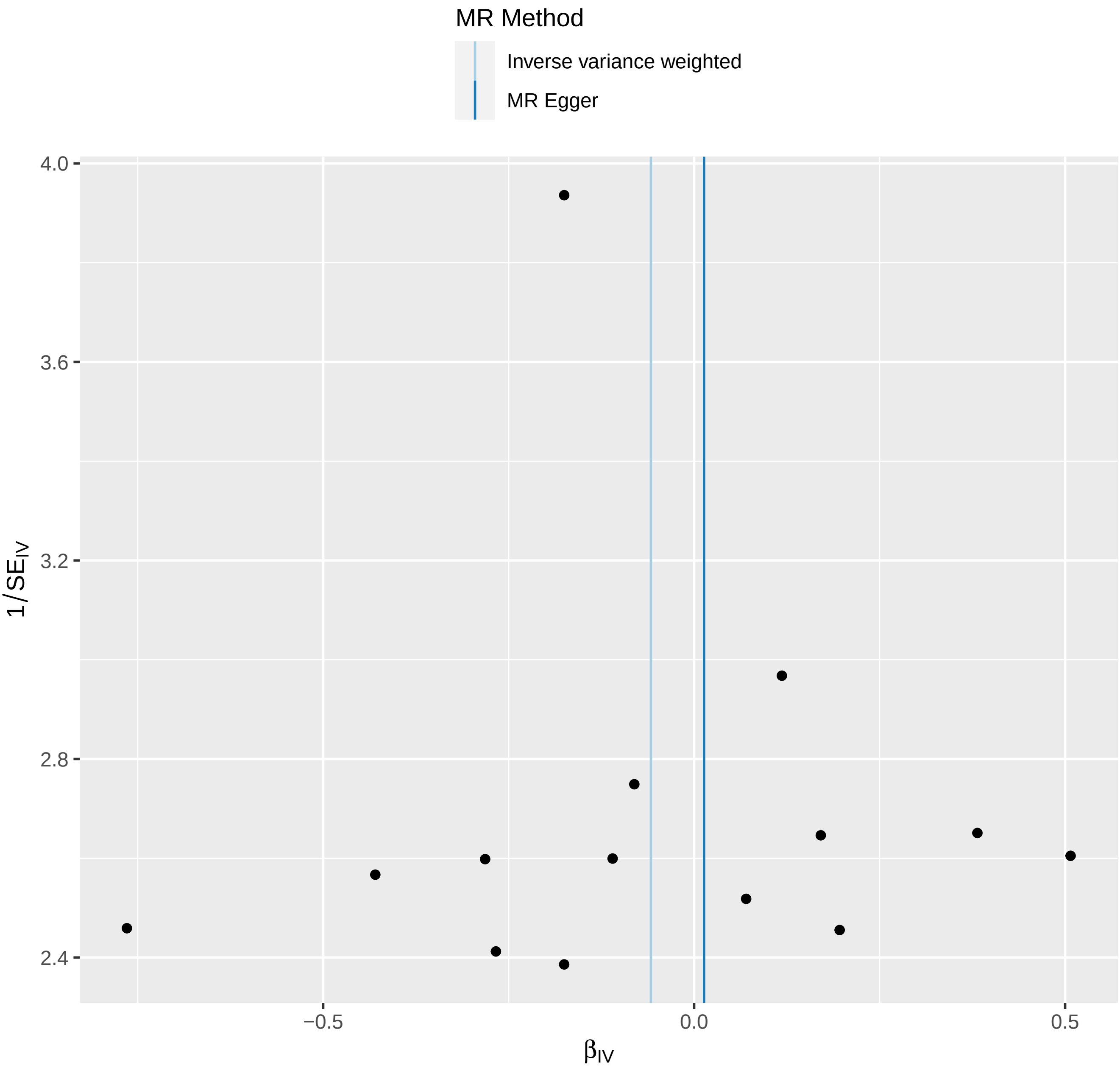

Batch 2−4217 : Gut microbiota abundance (genus Catenibacterium id.2153) on Vesicle−fusing ATPase
with a threshold of 1e−05

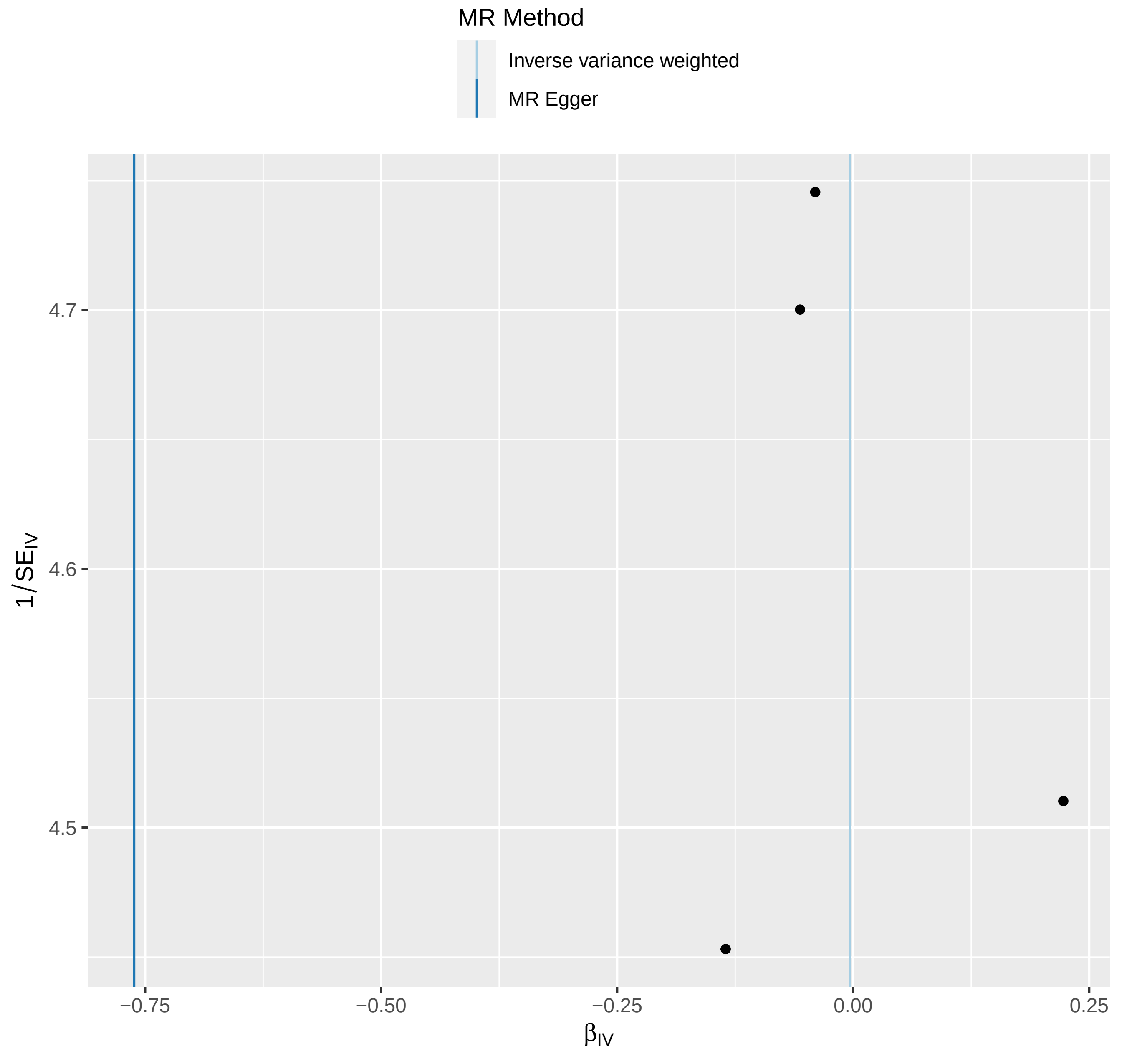

Batch 2−4218 : Gut microbiota abundance (genus Parasutterella id.2892) on Vesicle−fusing ATPase
with a threshold of 1e−05

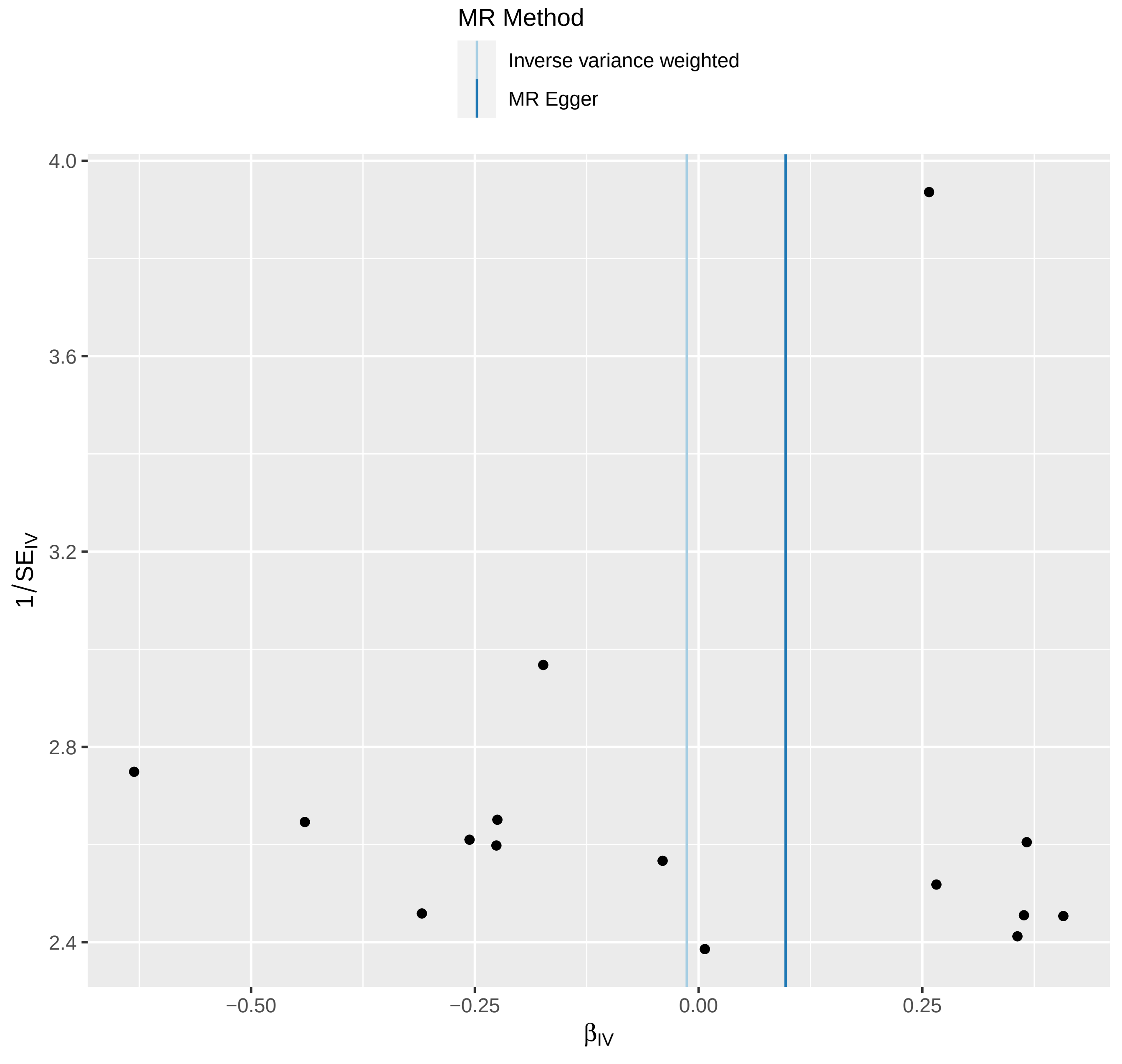

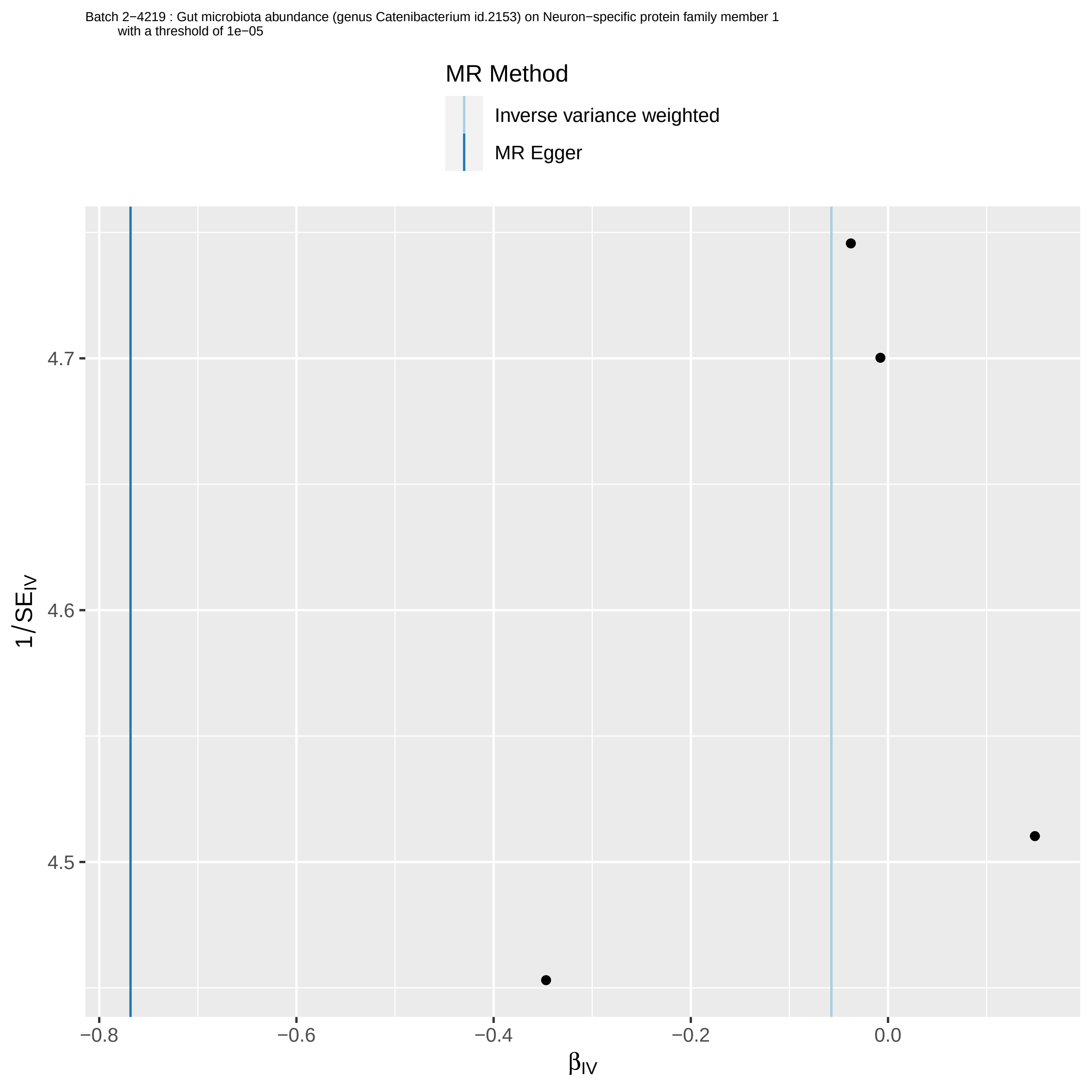

Batch 2−4219 : Gut microbiota abundance (genus Catenibacterium id.2153) on Neuron−specific protein family member 1
with a threshold of 1e−05
MR Method
Inverse variance weighted
MR Egger
4.7
4.6
4.5
1/SE_IV
−0.8
−0.6
−0.4
−0.2
0.0
β_IV

Batch 2−4220 : Gut microbiota abundance (genus Parasutterella id.2892) on Neuron−specific protein family member 1
with a threshold of 1e−05

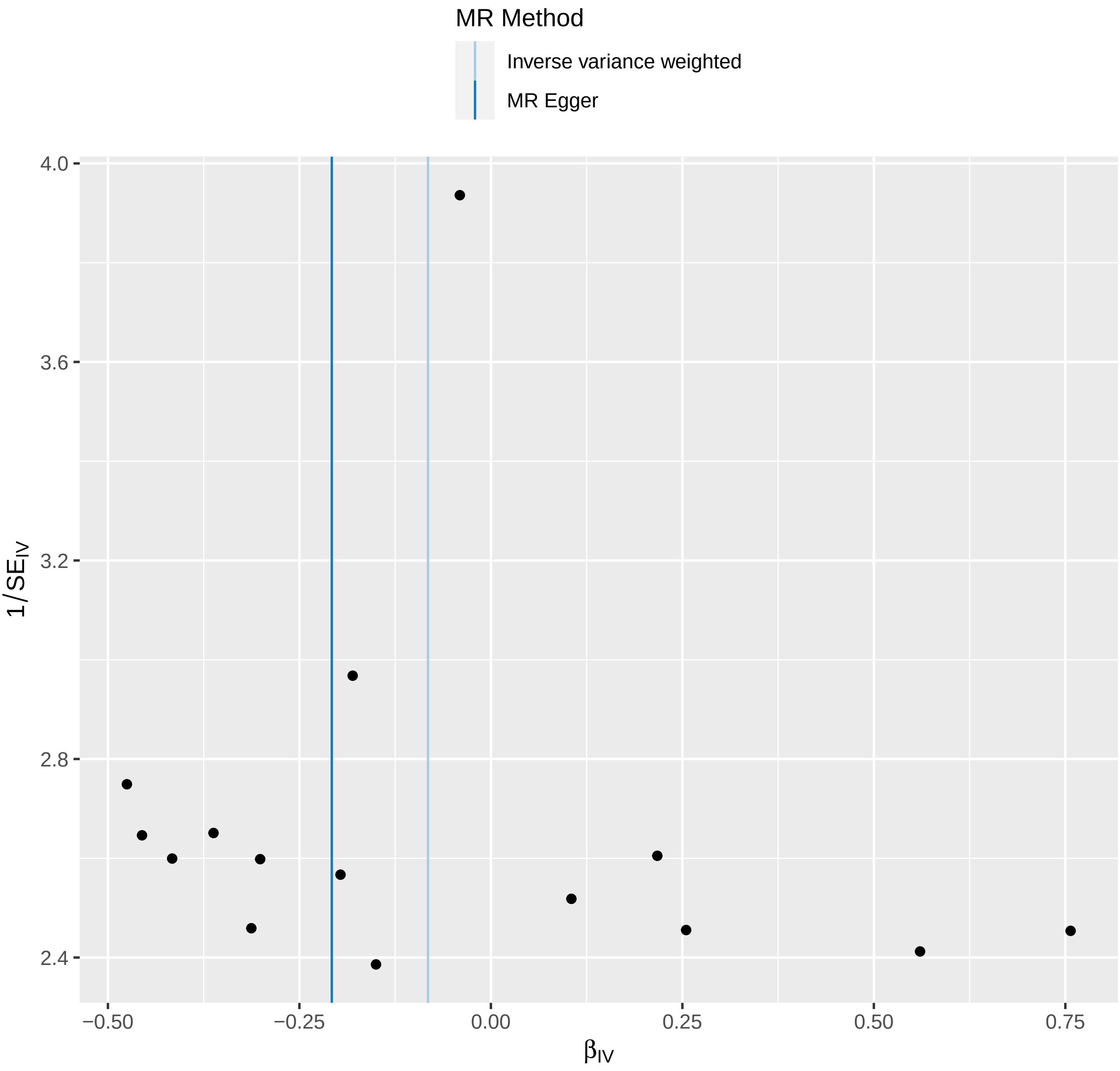

Batch 2−4221 : Gut microbiota abundance (genus Catenibacterium id.2153) on Neuron−specific protein family member 2
with a threshold of 1e−05

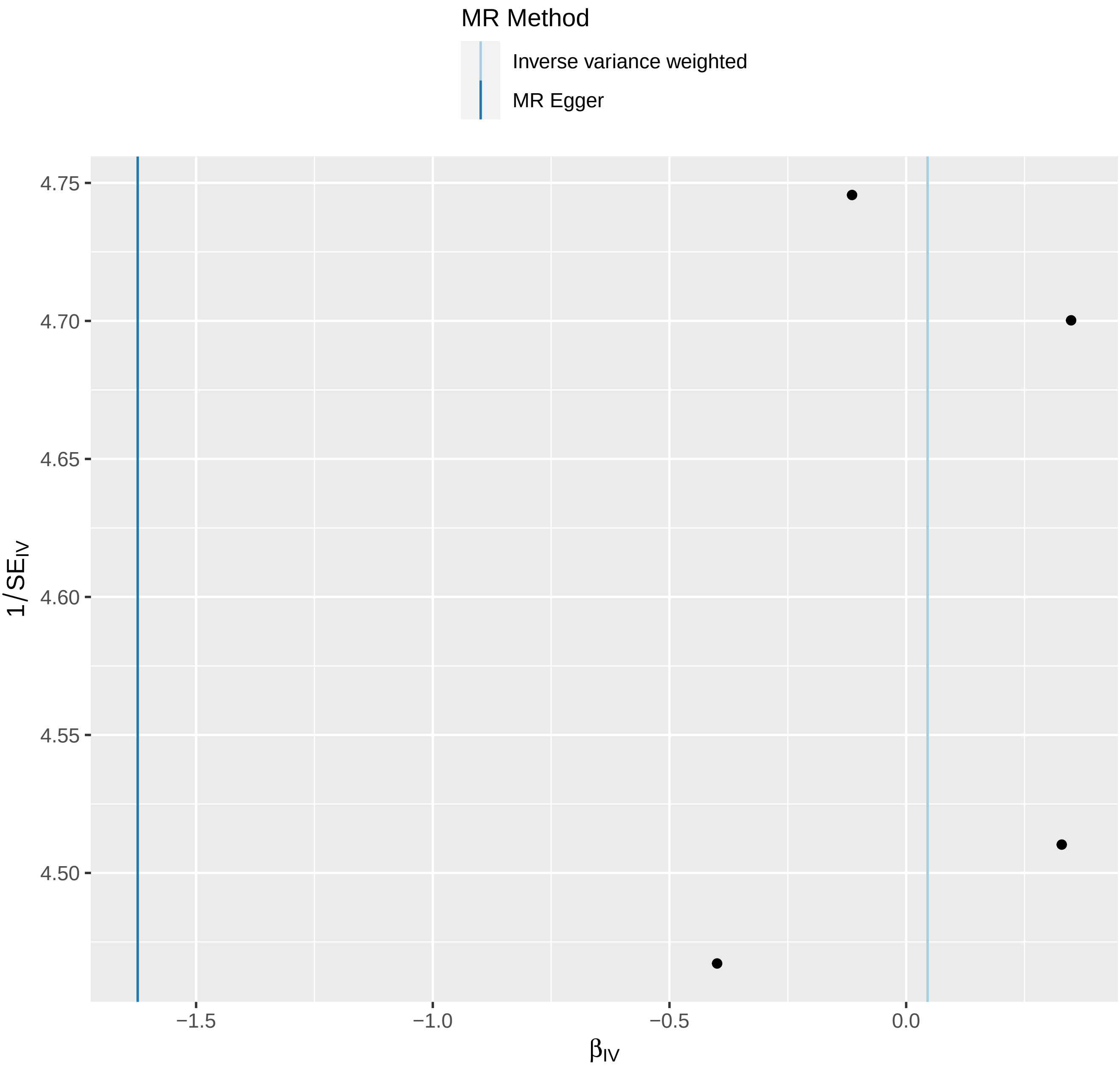

Batch 2−4222 : Gut microbiota abundance (genus Parasutterella id.2892) on Neuron−specific protein family member 2
with a threshold of 1e−05

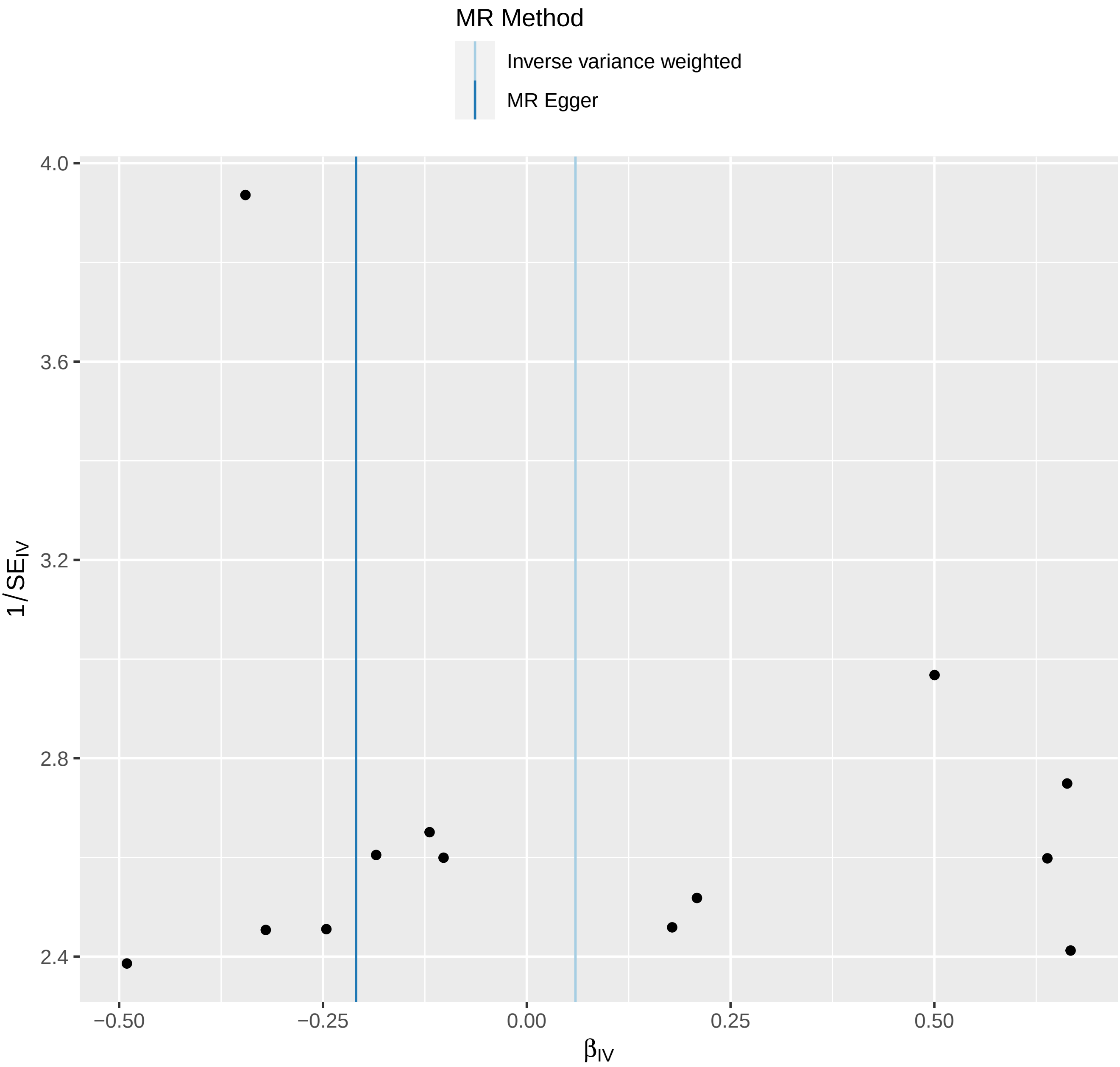

Batch 2−4223 : Gut microbiota abundance (genus Catenibacterium id.2153) on 5'(3')−deoxyribonucleotidase, cytosolic type with a threshold of 1e−05

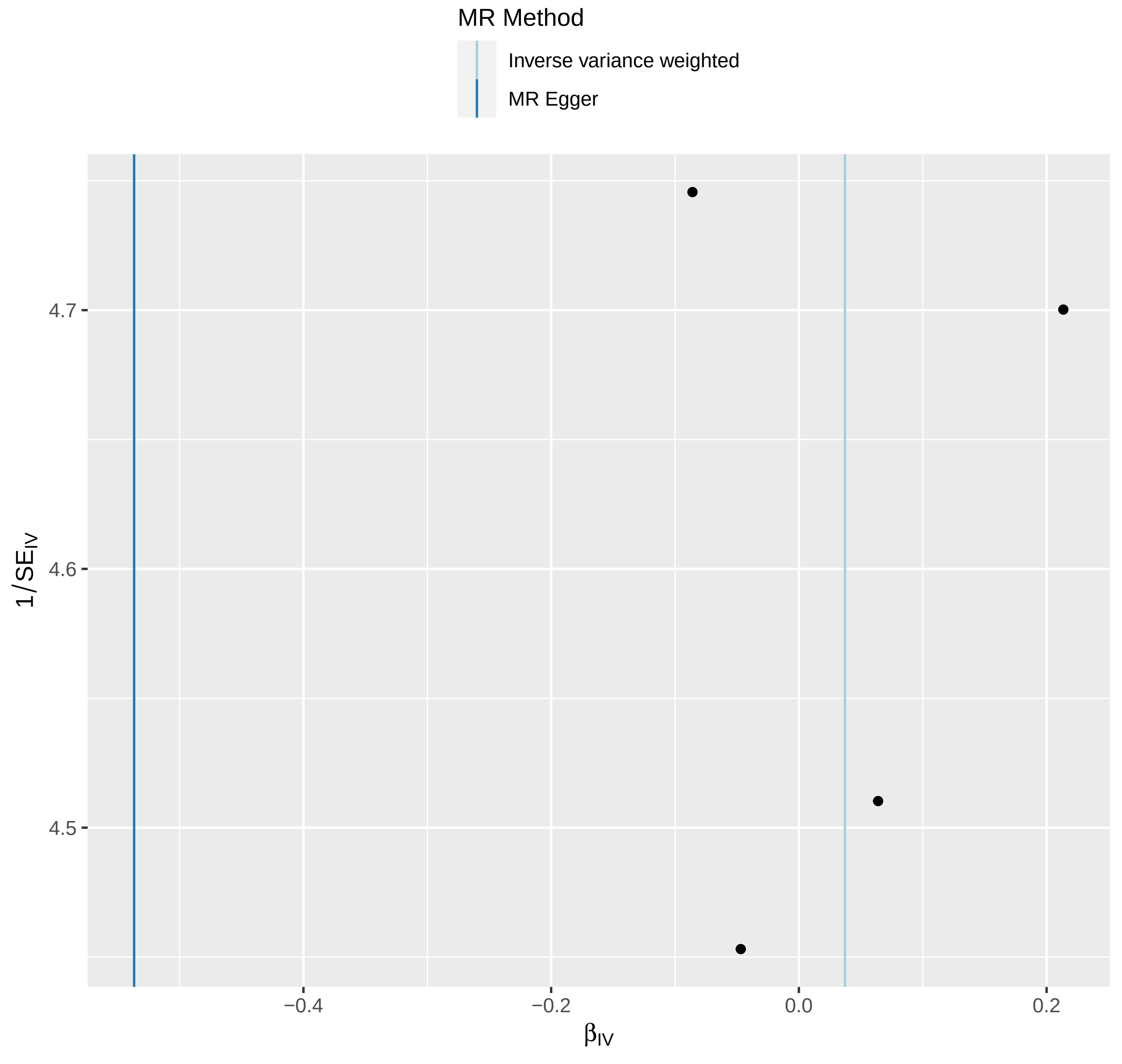

Batch 2−4224 : Gut microbiota abundance (genus Parasutterella id.2892) on 5'(3')−deoxyribonucleotidase, cytosolic type
with a threshold of 1e−05

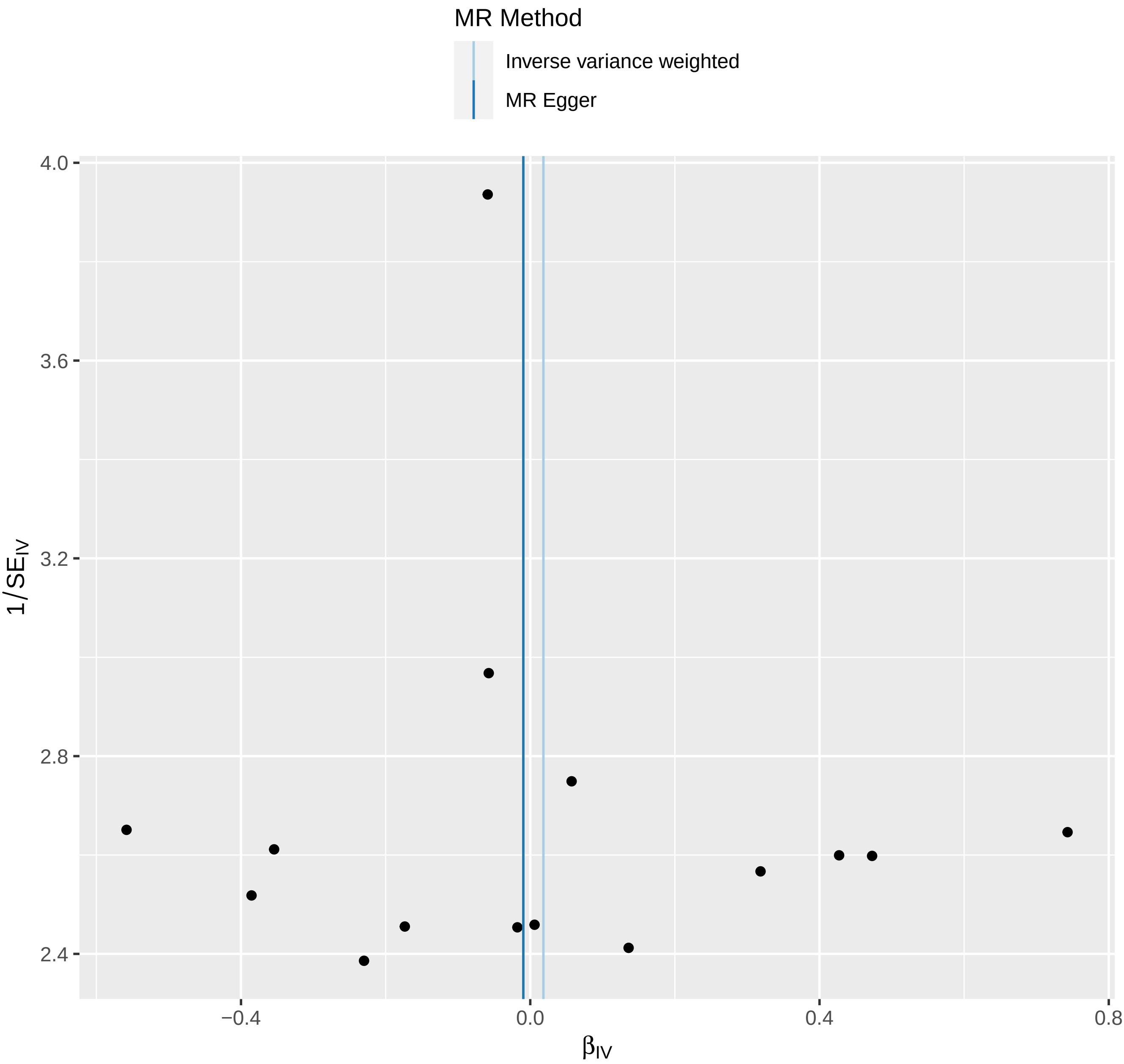

Batch 2−4225 : Gut microbiota abundance (genus Catenibacterium id.2153) on 5'−Nucleotidase
with a threshold of 1e−05

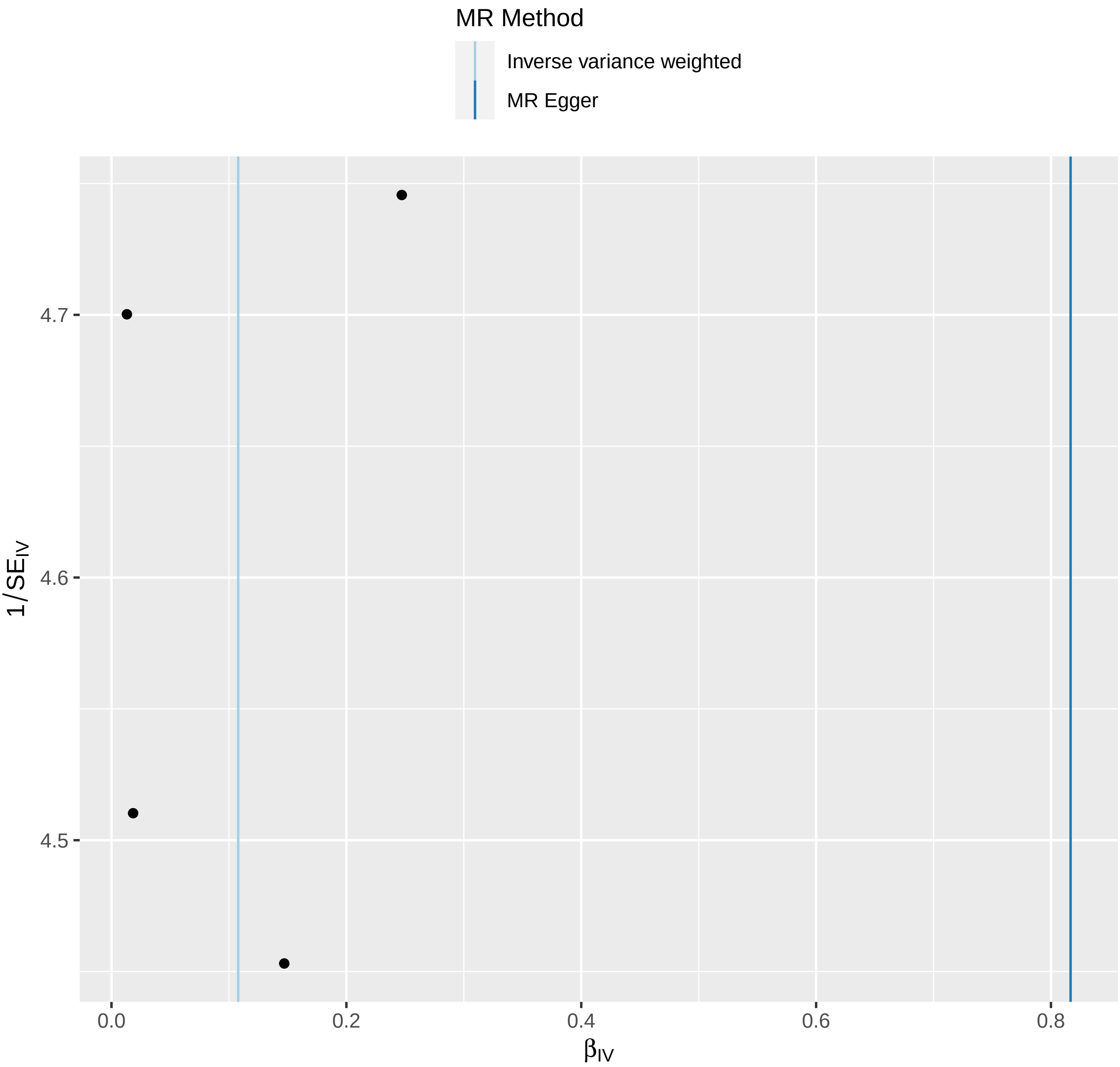

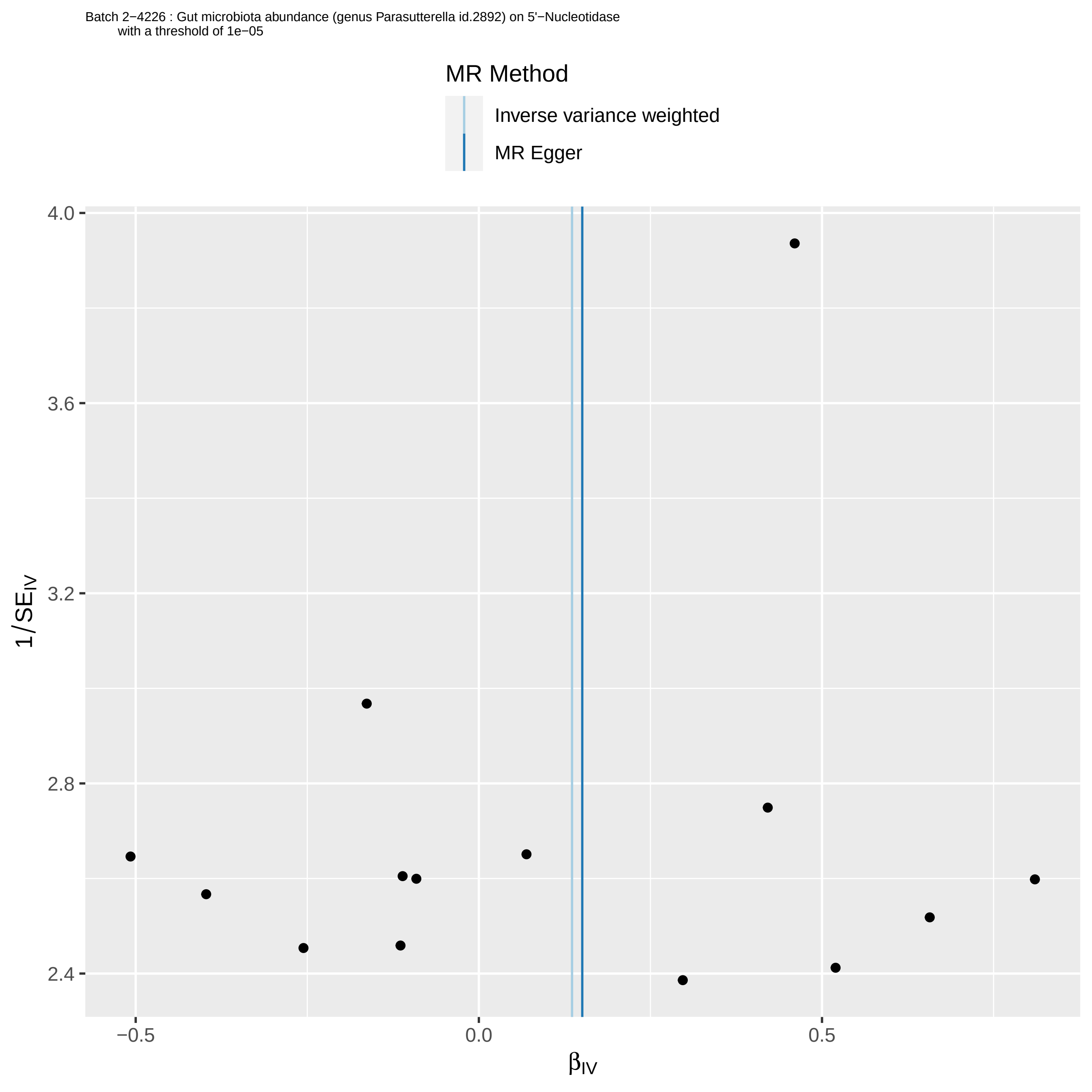
Batch 2−4226 : Gut microbiota abundance (genus Parasutterella id.2892) on 5'−Nucleotidase
with a threshold of 1e−05
MR Method
Inverse variance weighted
MR Egger
4.0
3.6
3.2
2.8
2.4
1/SE_IV
−0.5
0.0
0.5
β_IV

Batch 2−4227 : Gut microbiota abundance (genus Catenibacterium id.2153) on Neurotrophin−3
with a threshold of 1e−05

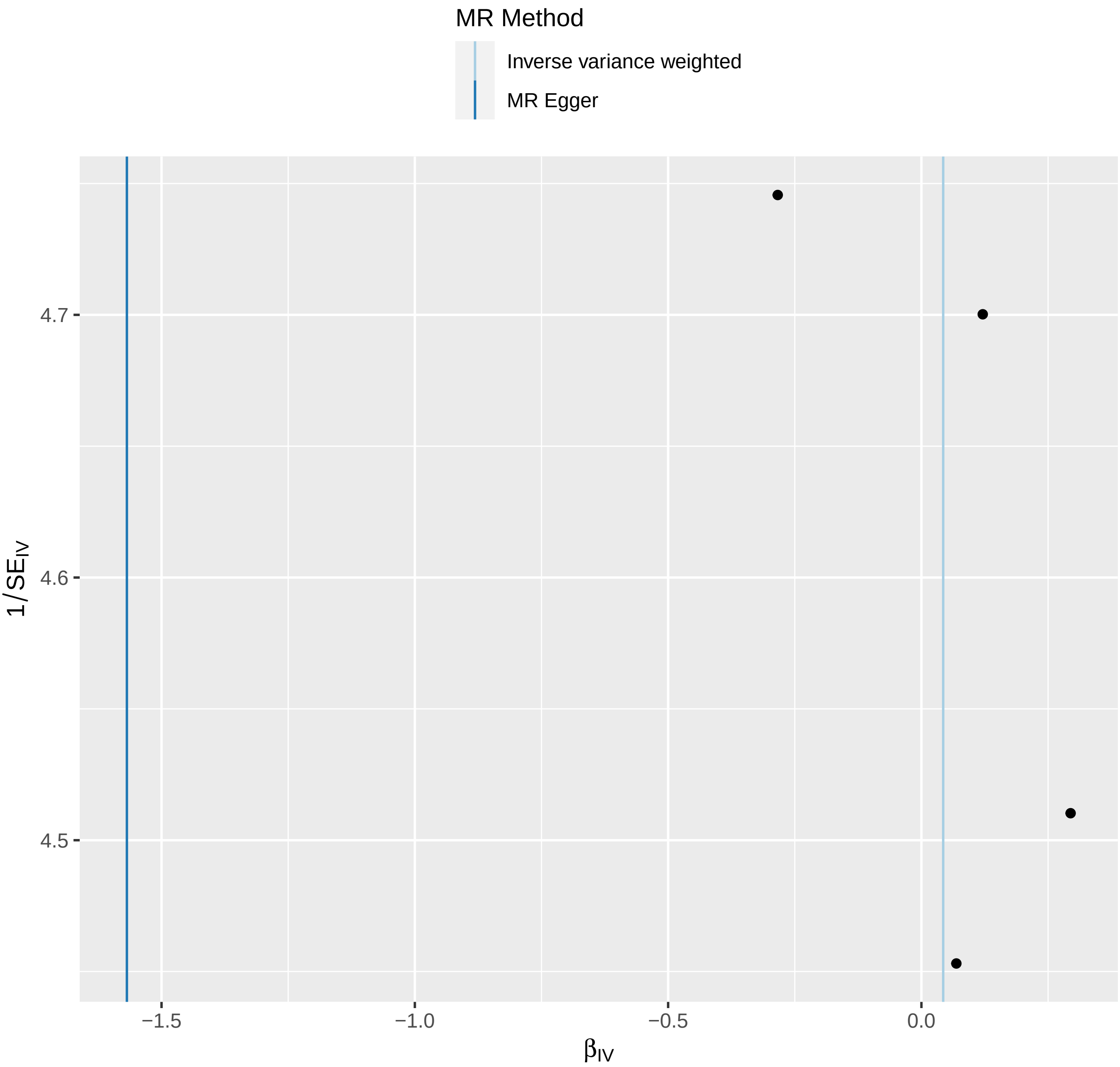

Batch 2−4228 : Gut microbiota abundance (genus Parasutterella id.2892) on Neurotrophin−3
with a threshold of 1e−05

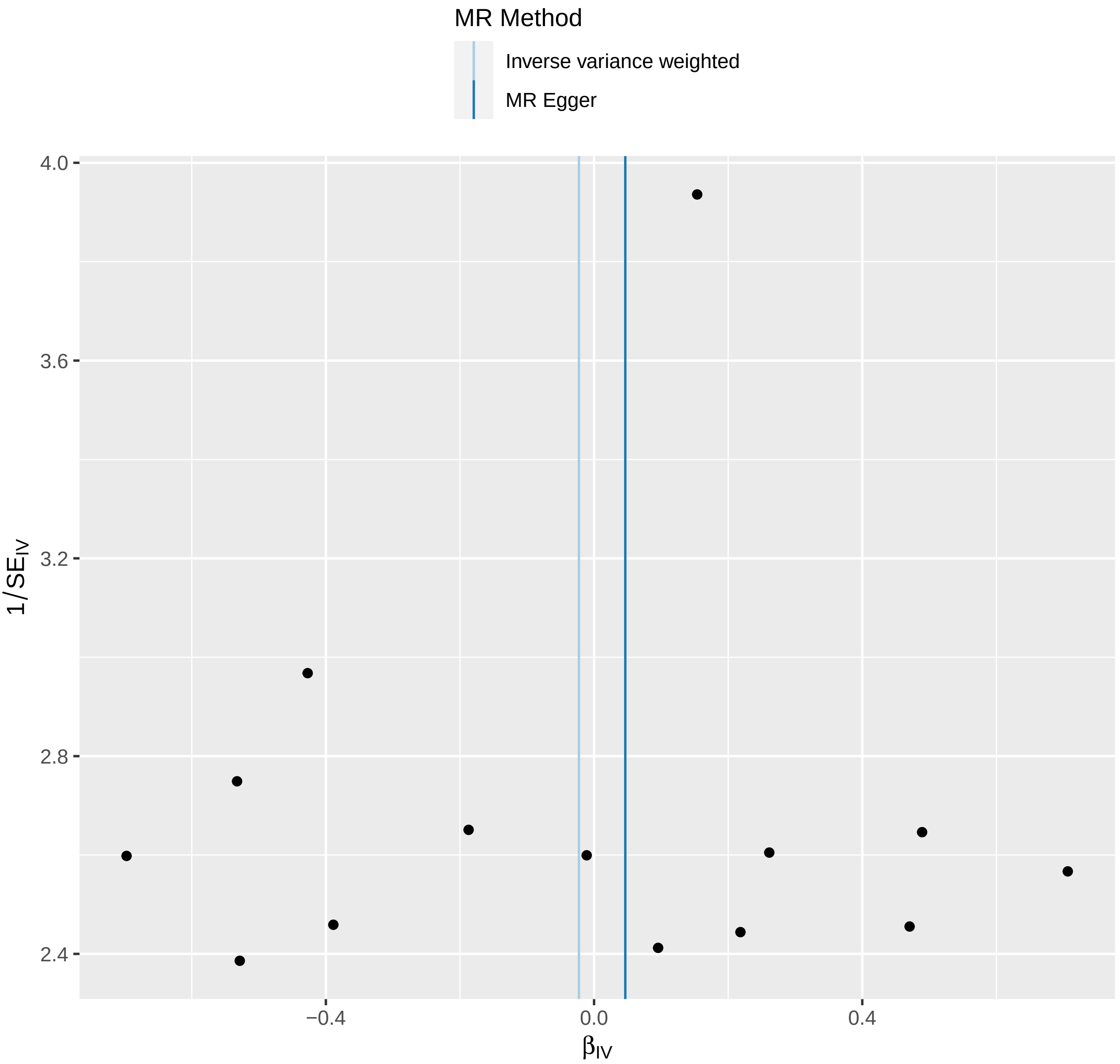

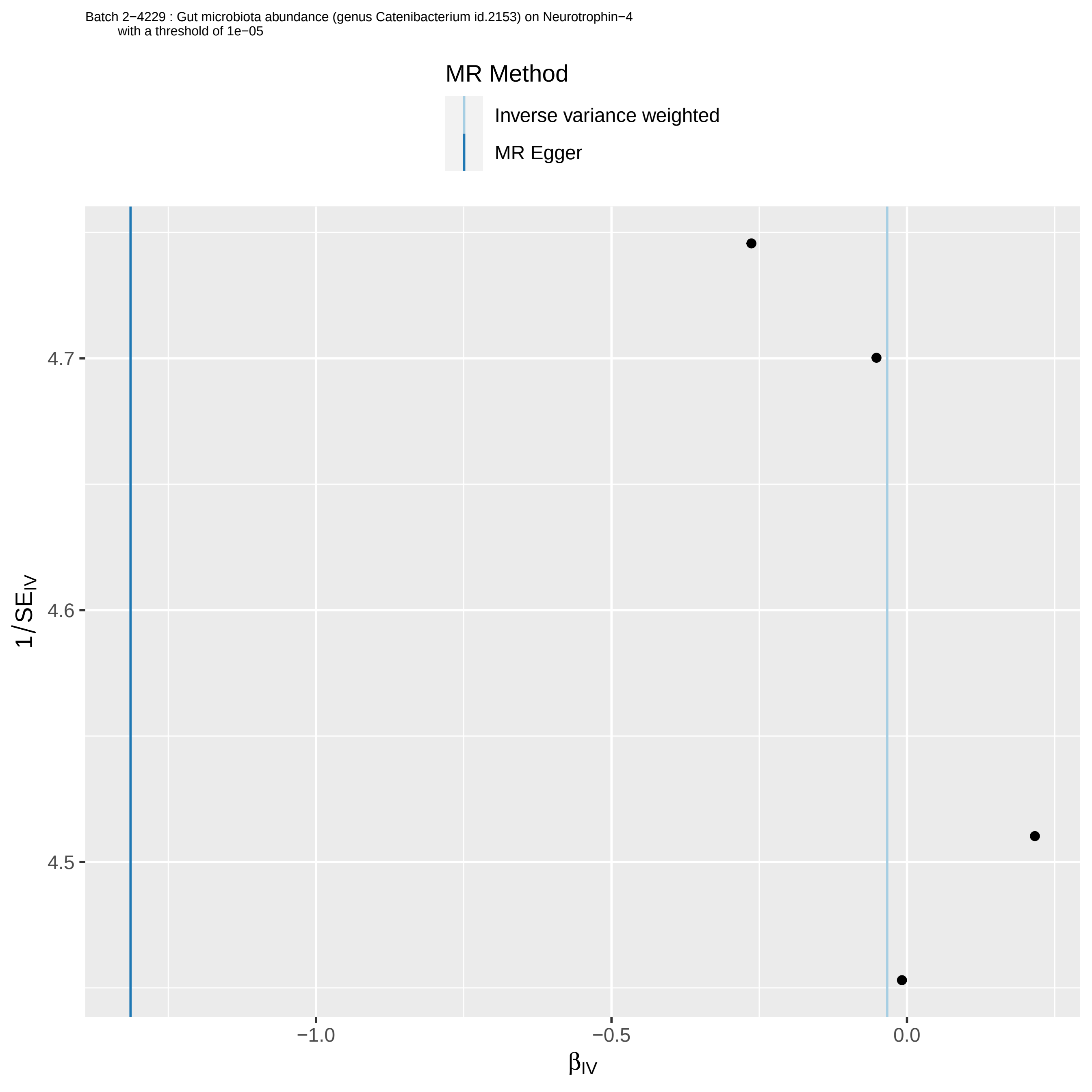
Batch 2−4229 : Gut microbiota abundance (genus Catenibacterium id.2153) on Neurotrophin−4
with a threshold of 1e−05
MR Method
Inverse variance weighted
MR Egger
4.7
4.6
4.5
1/SE_IV
−1.0
−0.5
0.0
β_IV

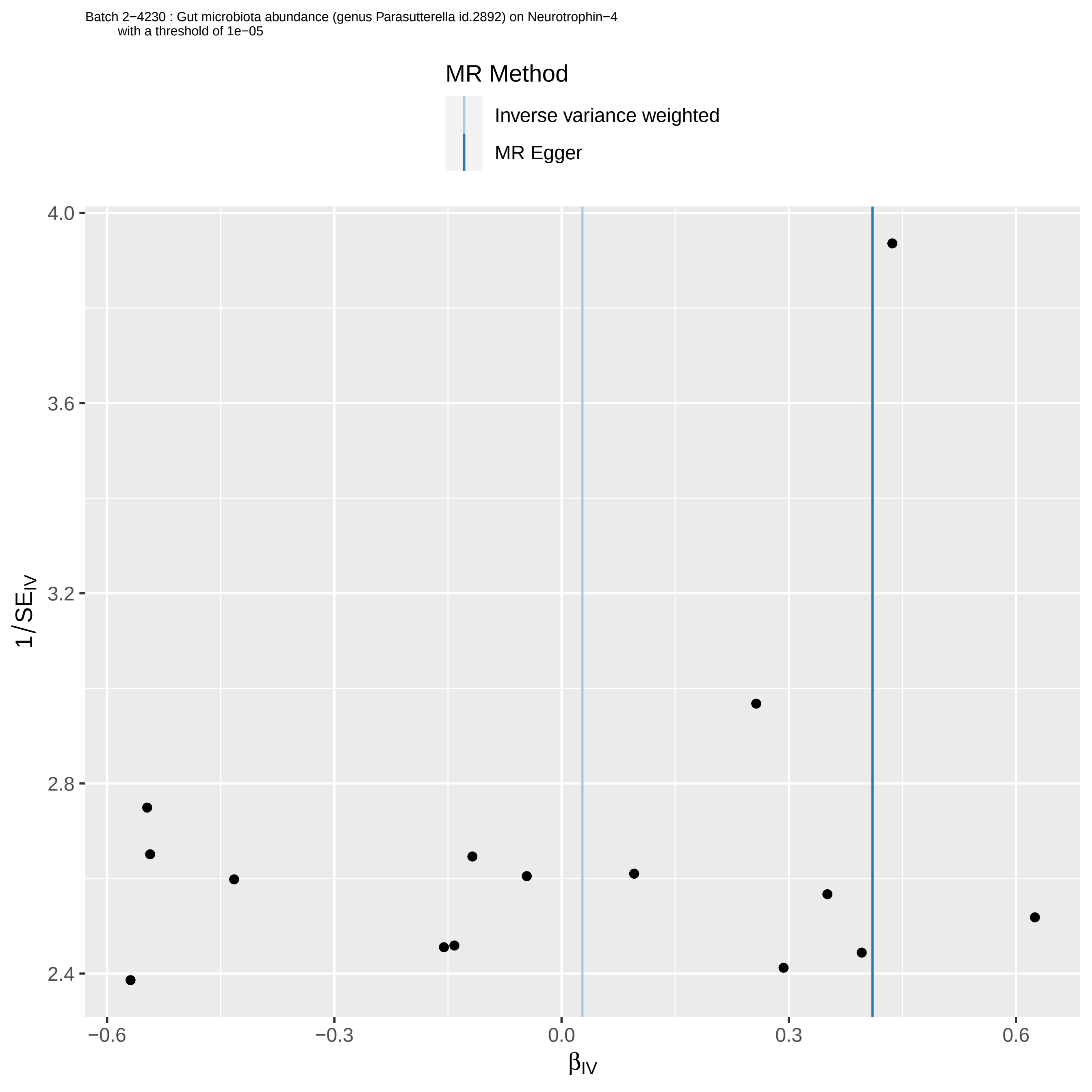

Batch 2−4230 : Gut microbiota abundance (genus Parasutterella id.2892) on Neurotrophin−4
with a threshold of 1e−05
MR Method
Inverse variance weighted
MR Egger
4.0
3.6
3.2
2.8
2.4
1/SE_IV
−0.6
−0.3
0.0
0.3
0.6
β_IV

Batch 2−4231 : Gut microbiota abundance (genus Catenibacterium id.2153) on N−terminal Xaa−Pro−Lys N−methyltransferase 1
with a threshold of 1e−05

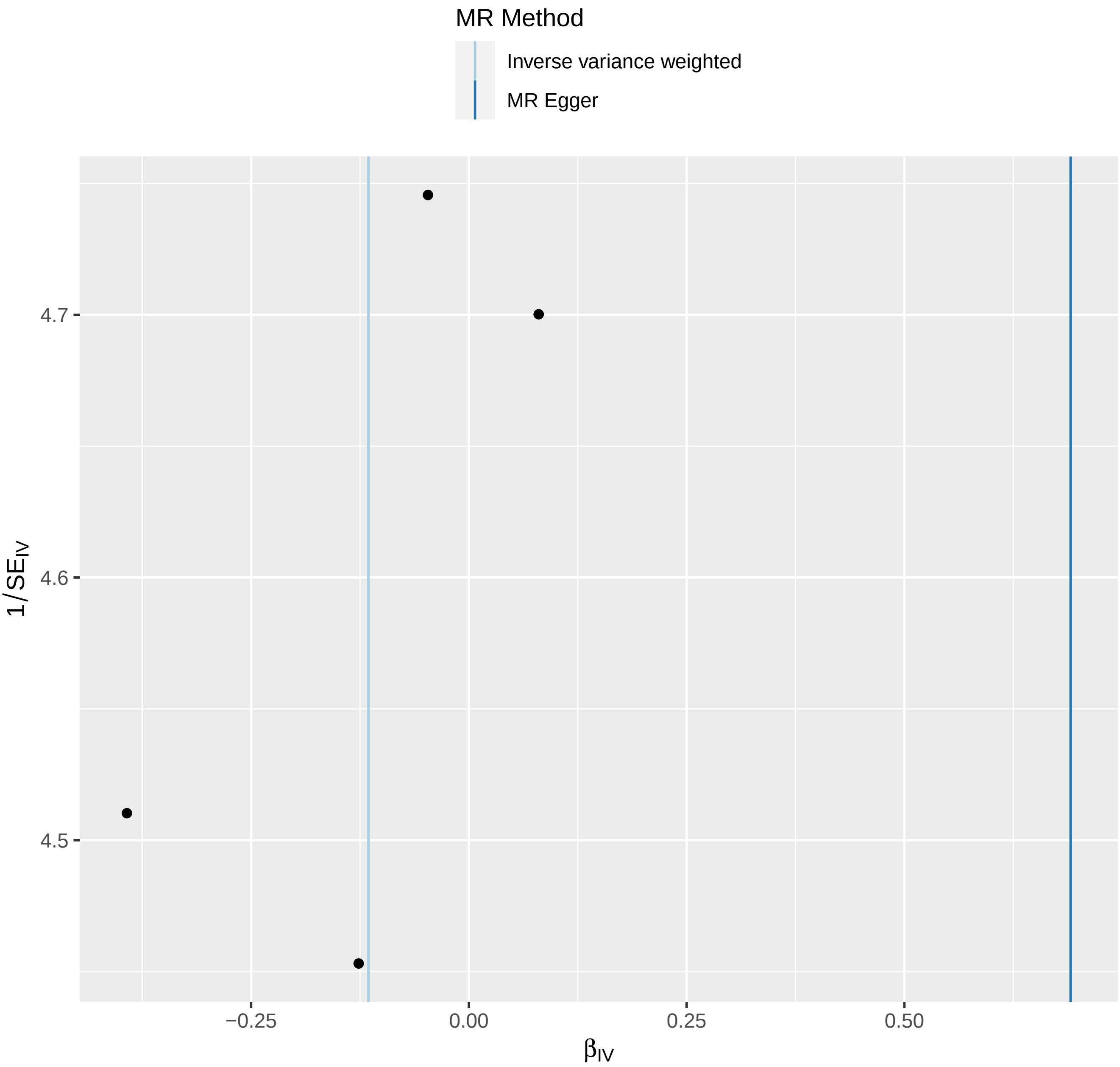

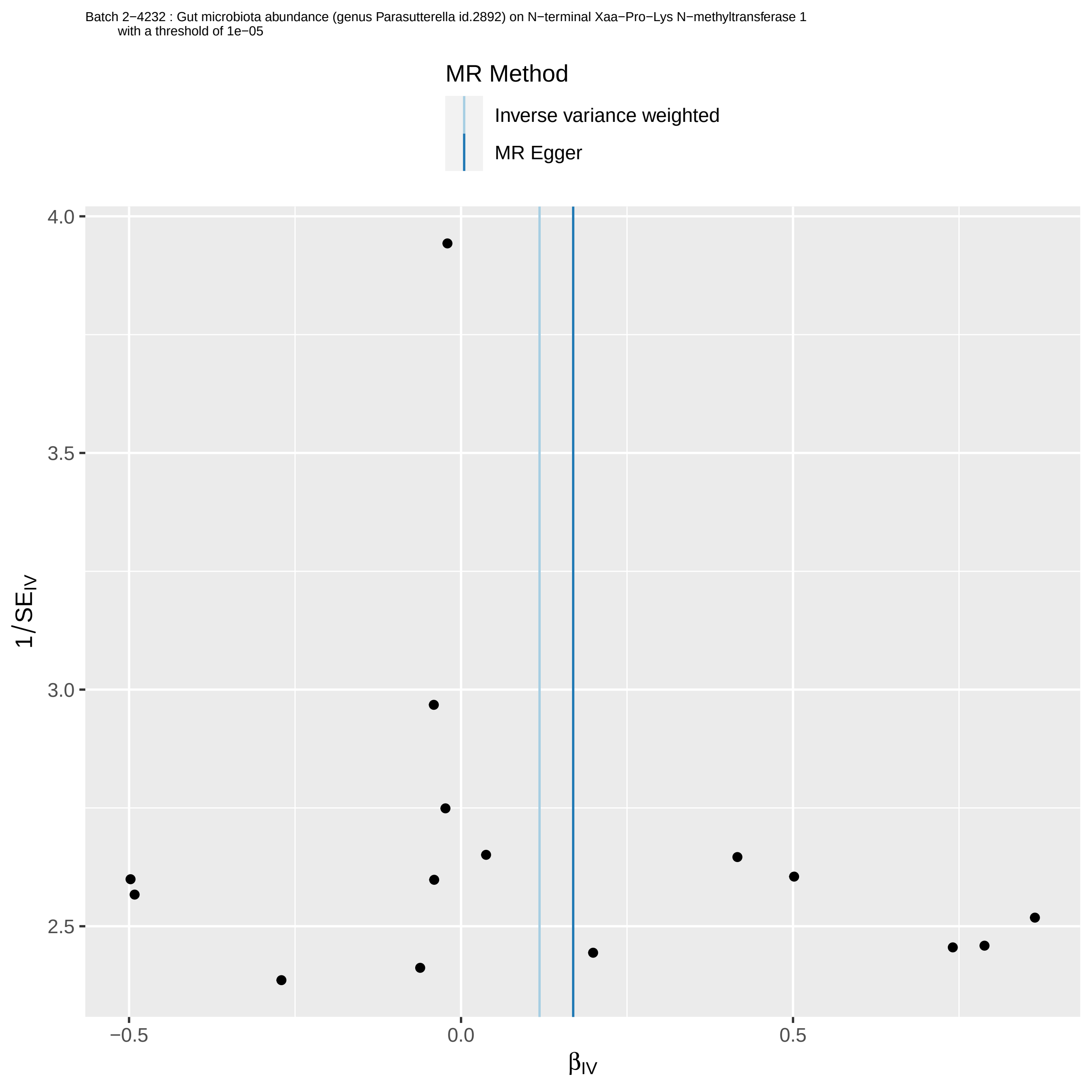

Batch 2−4232 : Gut microbiota abundance (genus Parasutterella id.2892) on N−terminal Xaa−Pro−Lys N−methyltransferase 1
with a threshold of 1e−05
MR Method
Inverse variance weighted
MR Egger
4.0
3.5
3.0
2.5
1/SE_IV
−0.5
0.0
0.5
β_IV

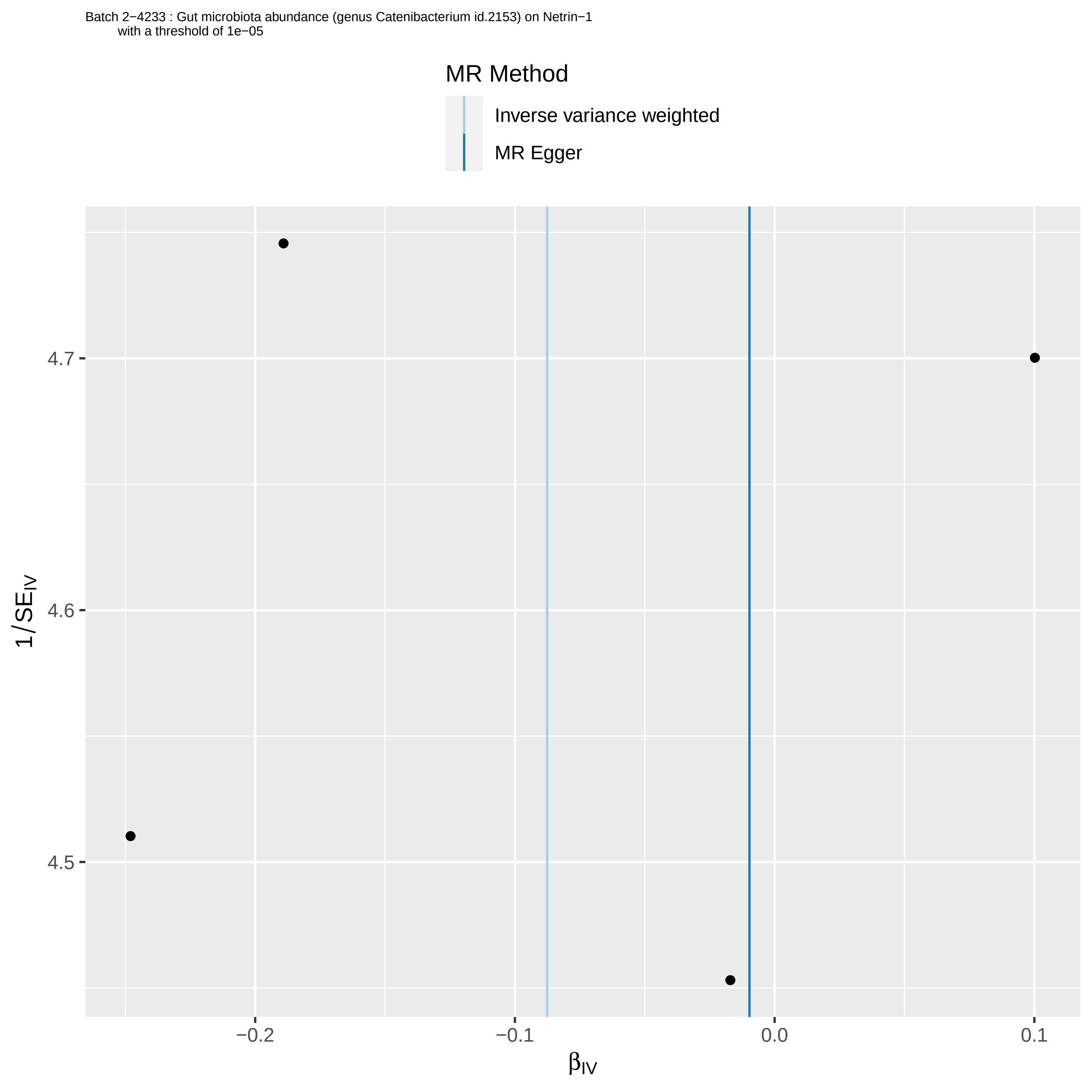
Batch 2−4233 : Gut microbiota abundance (genus Catenibacterium id.2153) on Netrin−1
with a threshold of 1e−05
MR Method
Inverse variance weighted
MR Egger
4.7
4.6
4.5
1/SE_IV
−0.2
−0.1
0.0
0.1
β_IV

Batch 2−4234 : Gut microbiota abundance (genus Parasutterella id.2892) on Netrin−1
with a threshold of 1e−05

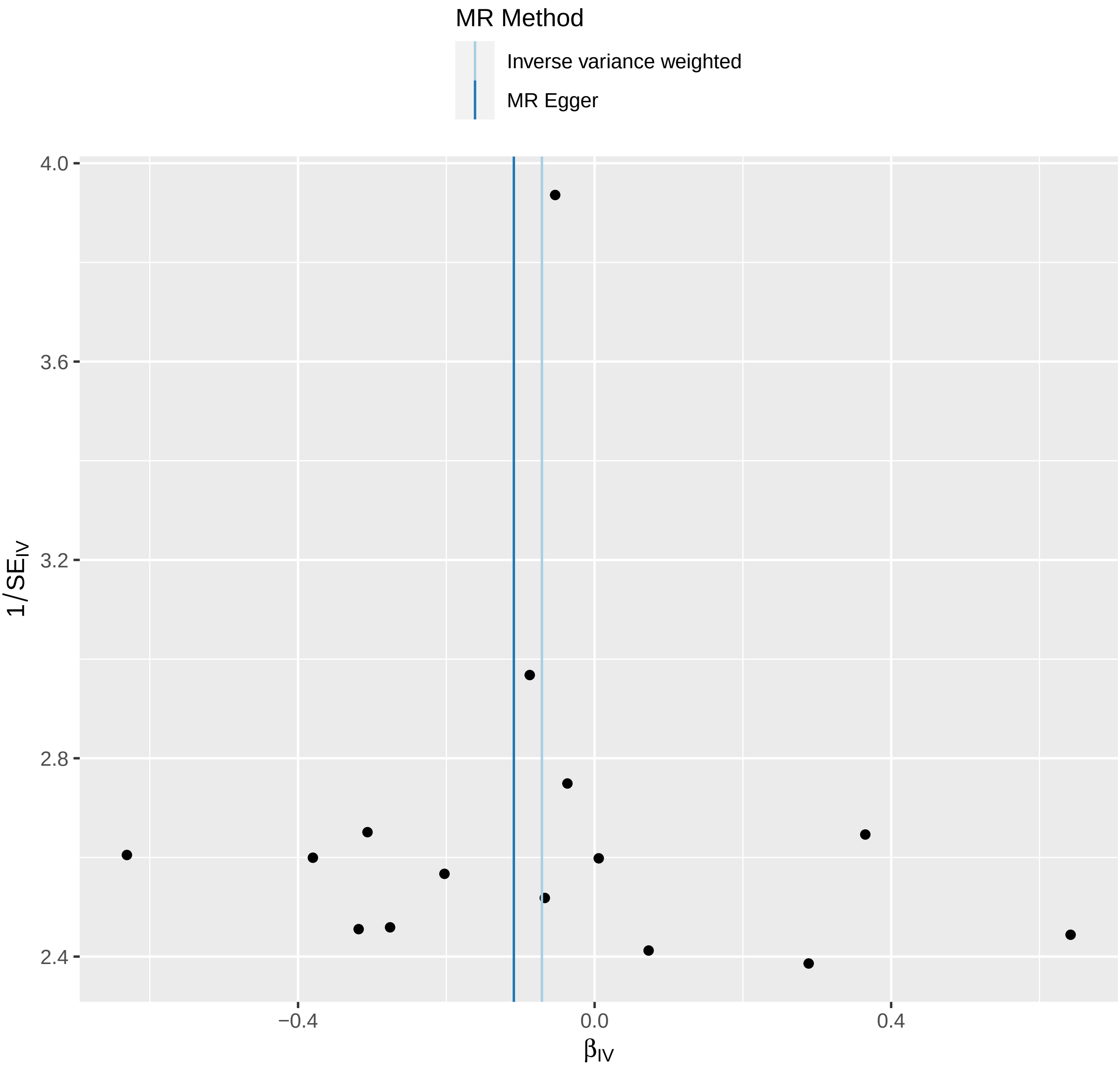

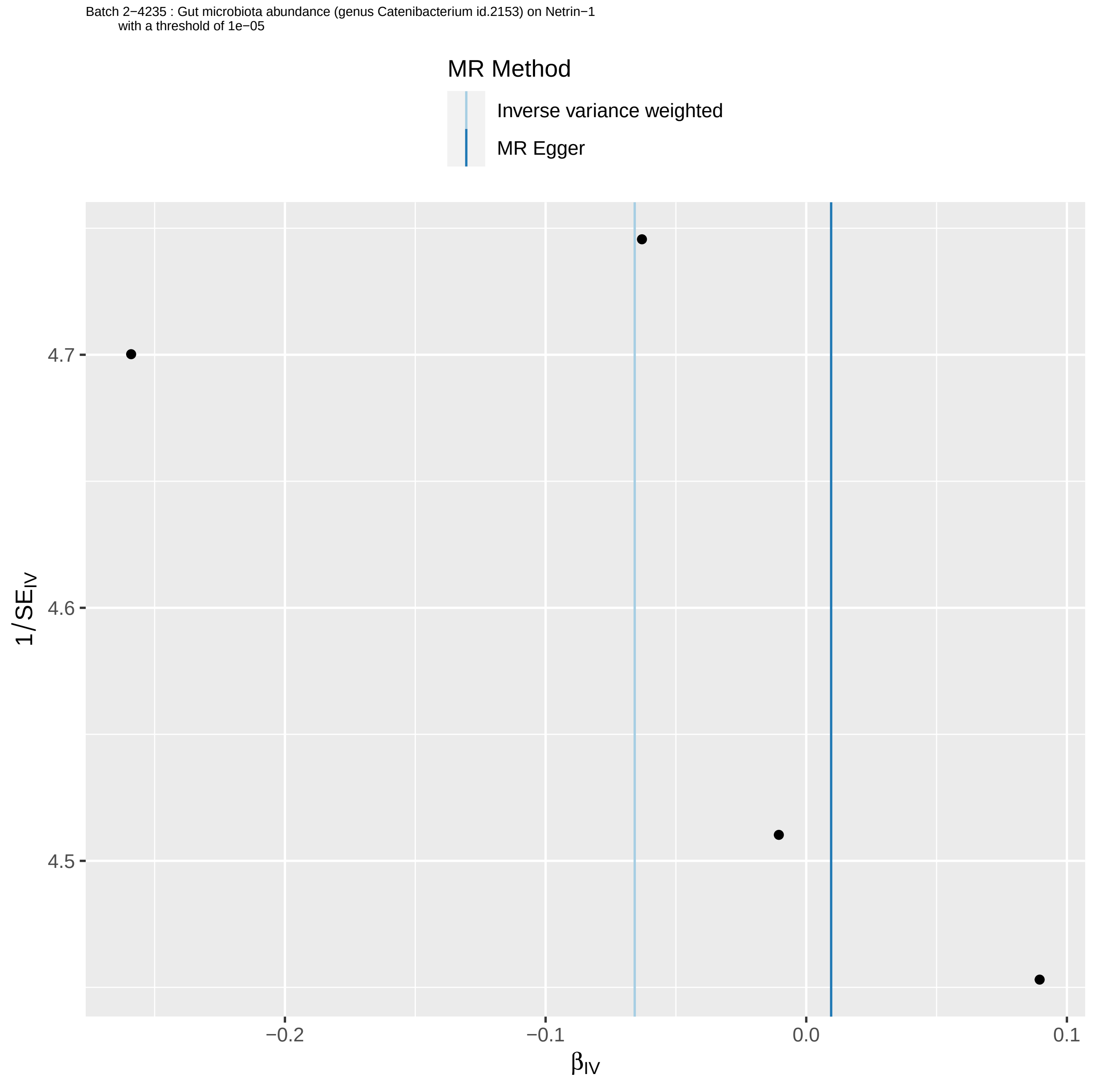

Batch 2−4235 : Gut microbiota abundance (genus Catenibacterium id.2153) on Netrin−1
with a threshold of 1e−05
MR Method
Inverse variance weighted
MR Egger
4.7
4.6
4.5
1/SE_IV
−0.2
−0.1
0.0
0.1
β_IV

Batch 2−4236 : Gut microbiota abundance (genus Parasutterella id.2892) on Netrin−1
with a threshold of 1e−05

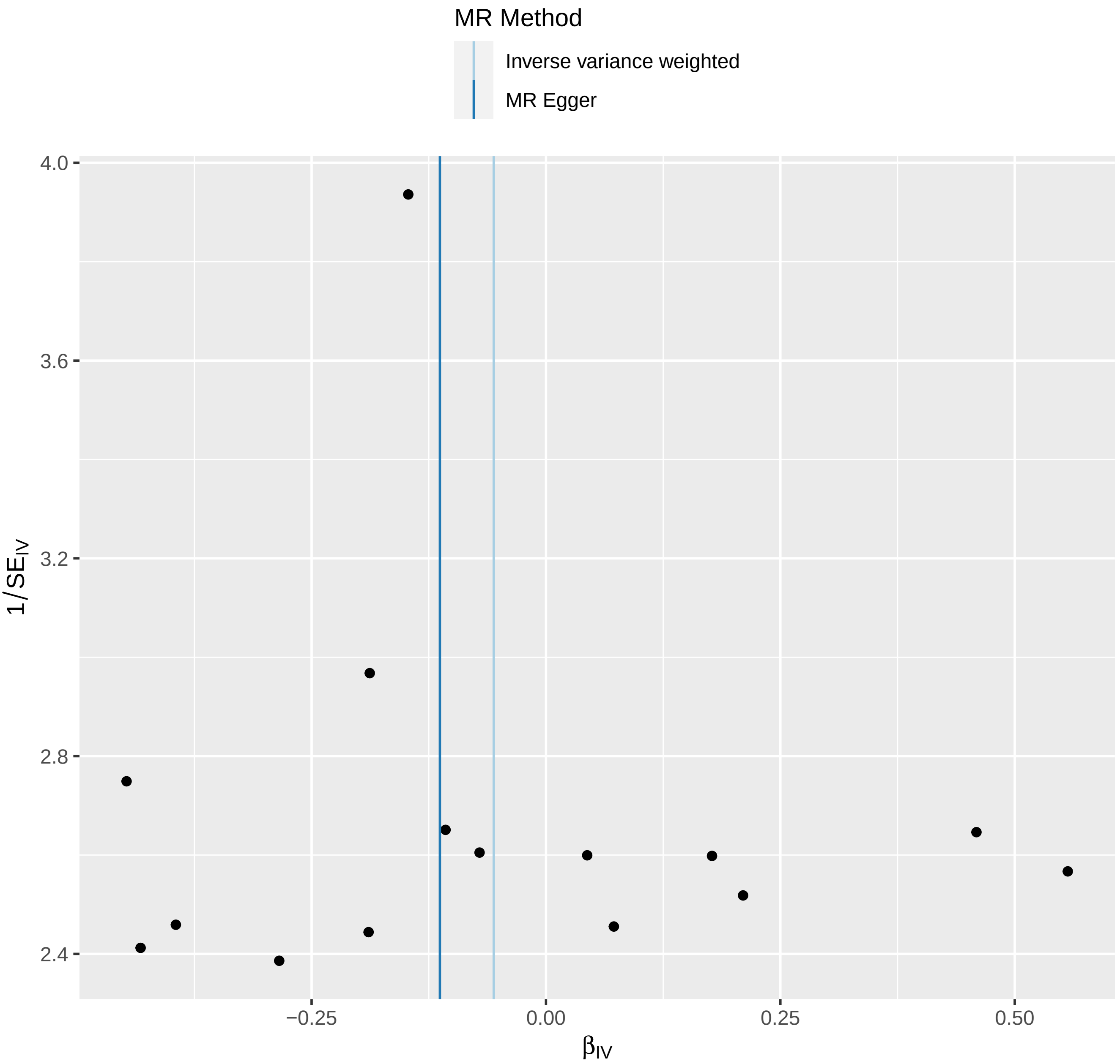

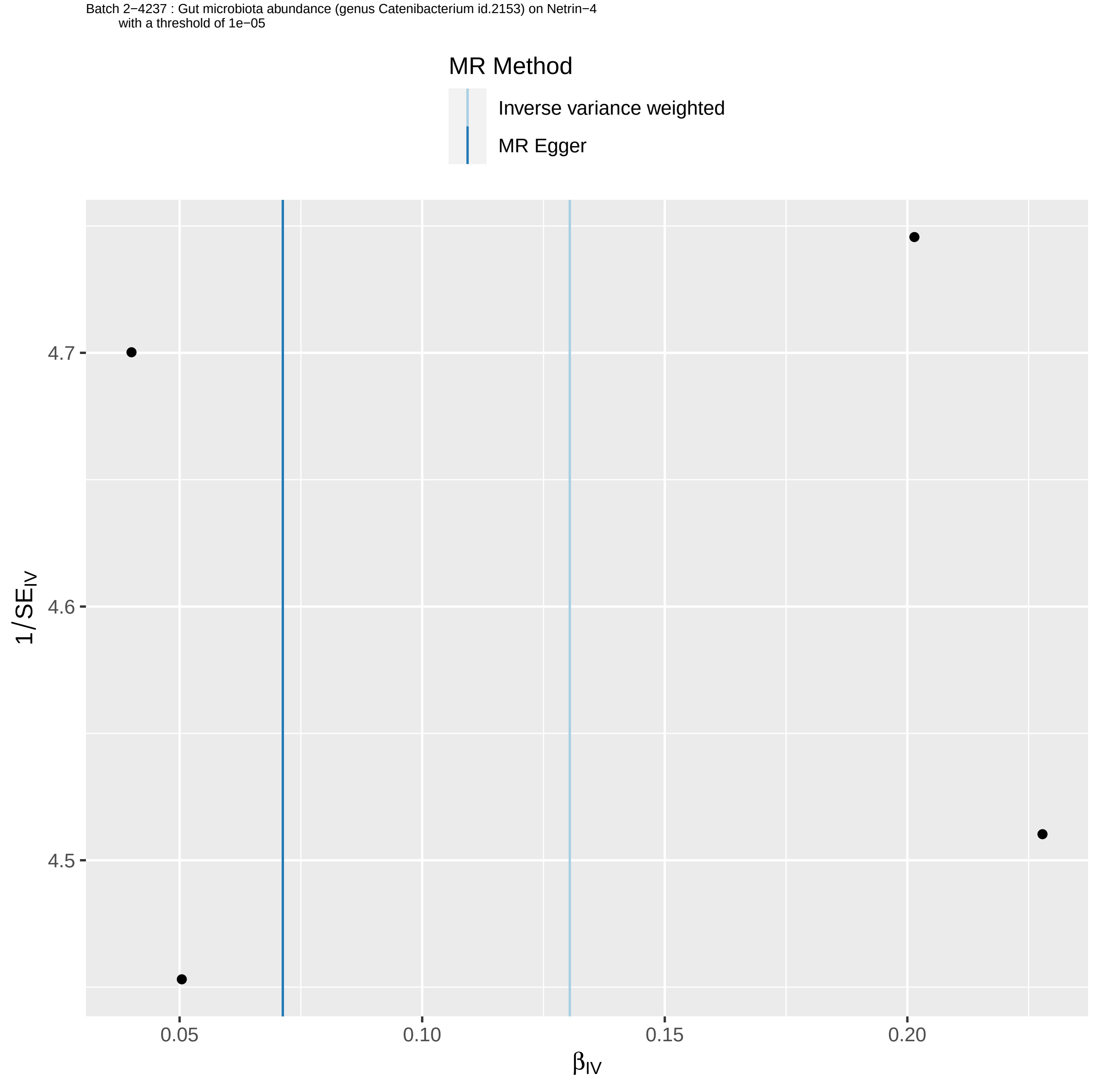
Batch 2−4237 : Gut microbiota abundance (genus Catenibacterium id.2153) on Netrin−4
with a threshold of 1e−05
MR Method
Inverse variance weighted
MR Egger
4.7
4.6
4.5
1/SE_IV
0.05
0.10
0.15
0.20
β_IV

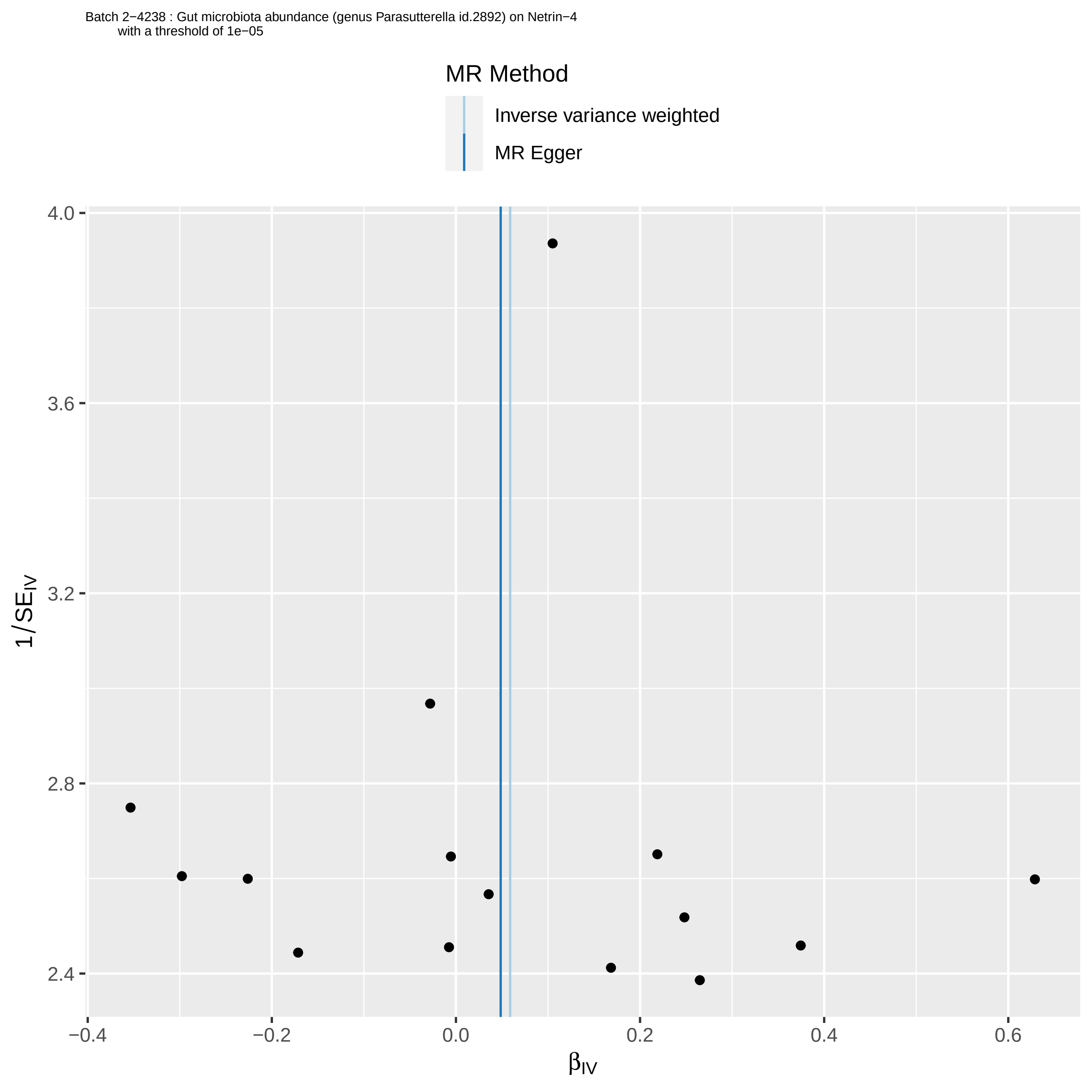

Batch 2−4238 : Gut microbiota abundance (genus Parasutterella id.2892) on Netrin−4
with a threshold of 1e−05
MR Method
Inverse variance weighted
MR Egger
4.0
3.6
3.2
2.8
2.4
$1/SE_{IV}$
−0.4
−0.2
0.0
0.2
0.4
0.6
β_{IV}

Batch 2−4239 : Gut microbiota abundance (genus Catenibacterium id.2153) on Netrin−G1
with a threshold of 1e−05

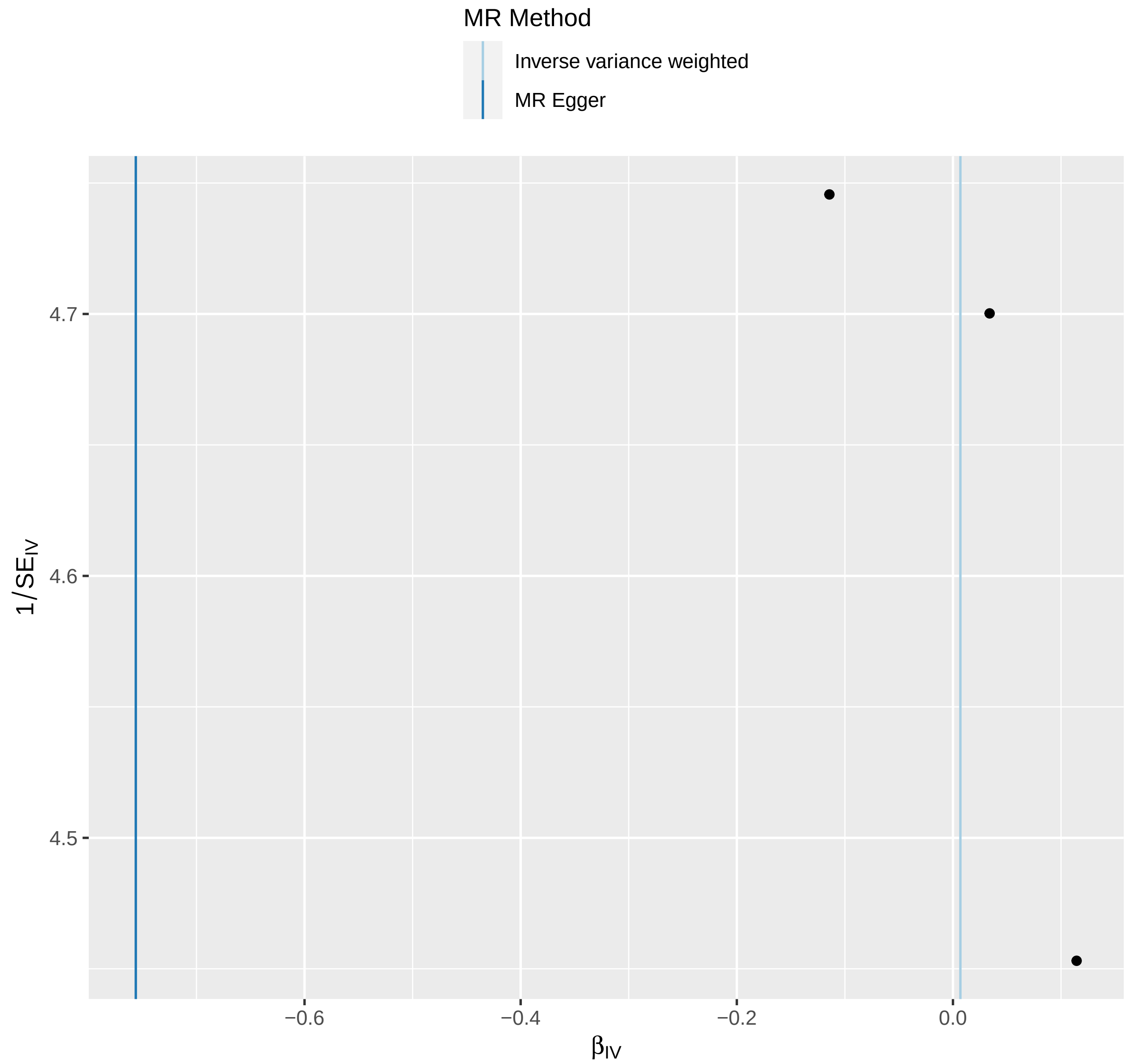

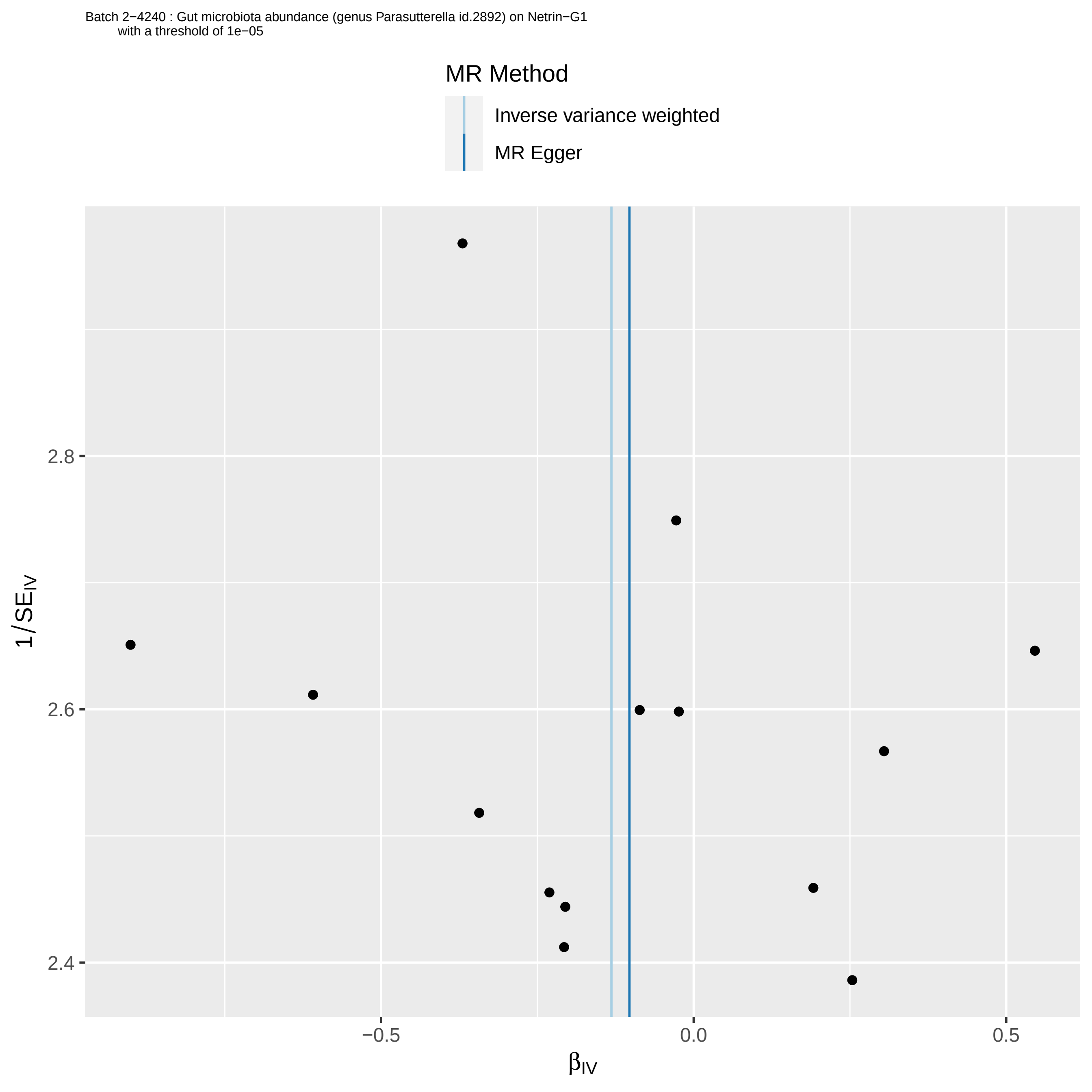

Batch 2−4240 : Gut microbiota abundance (genus Parasutterella id.2892) on Netrin−G1
with a threshold of 1e−05
MR Method
Inverse variance weighted
MR Egger
2.8
2.6
2.4
1/SE_IV
−0.5
0.0
0.5
β_IV

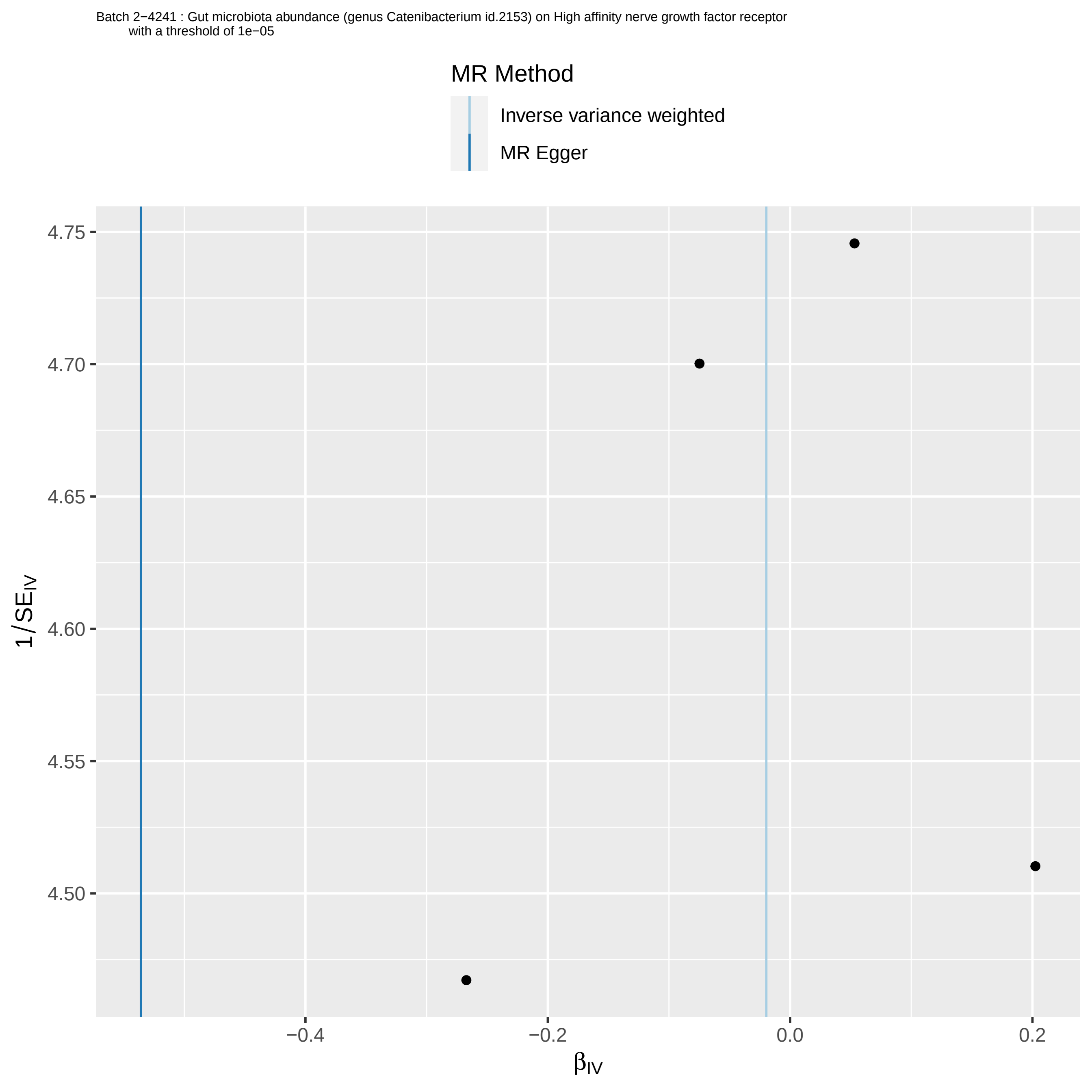
Batch 2−4241 : Gut microbiota abundance (genus Catenibacterium id.2153) on High affinity nerve growth factor receptor
with a threshold of 1e−05
MR Method
Inverse variance weighted
MR Egger
4.75
4.70
4.65
4.60
4.55
4.50
1/SE_IV
−0.4
−0.2
0.0
0.2
β_IV

Batch 2−4242 : Gut microbiota abundance (genus Parasutterella id.2892) on High affinity nerve growth factor receptor with a threshold of 1e−05

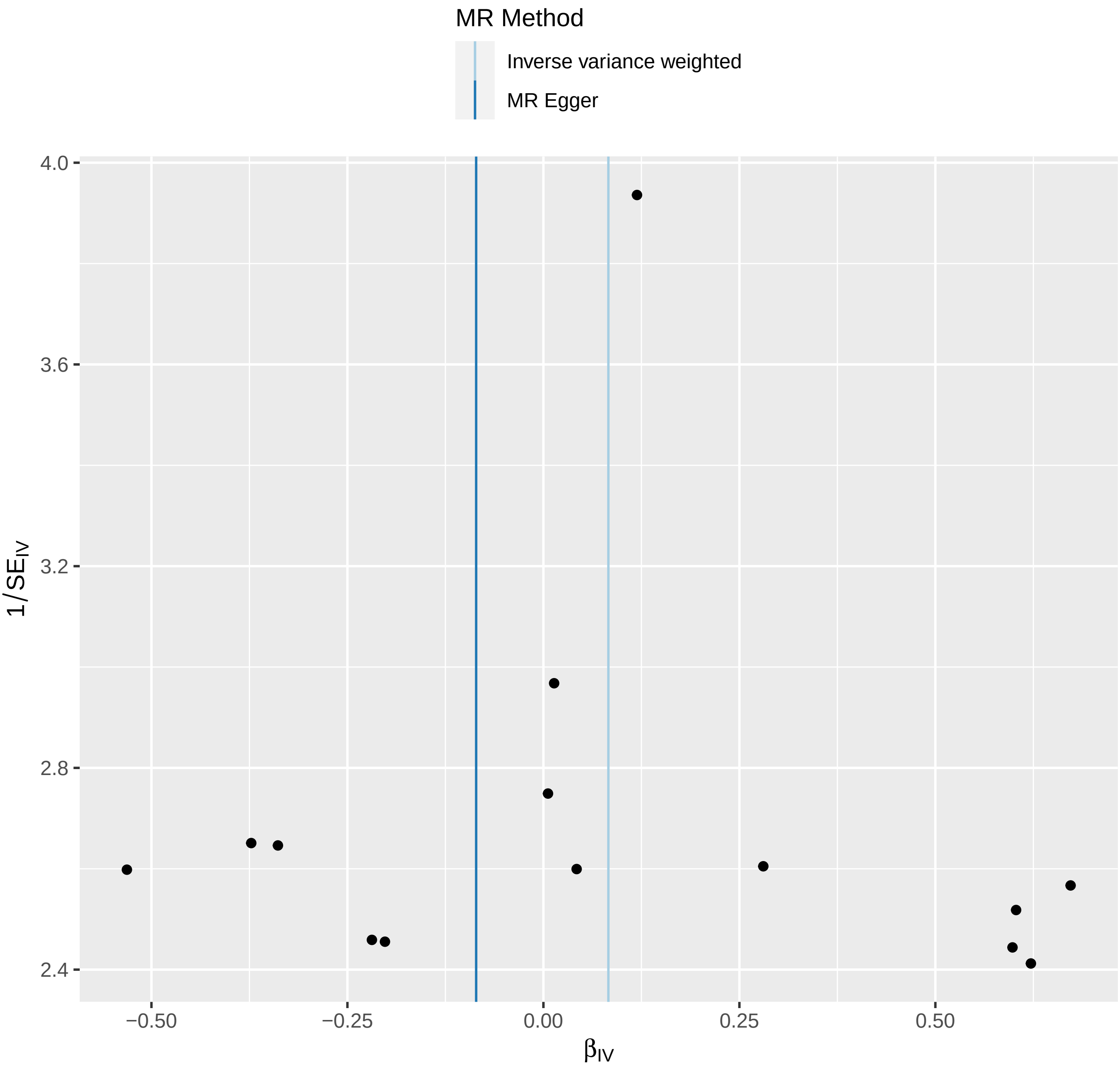

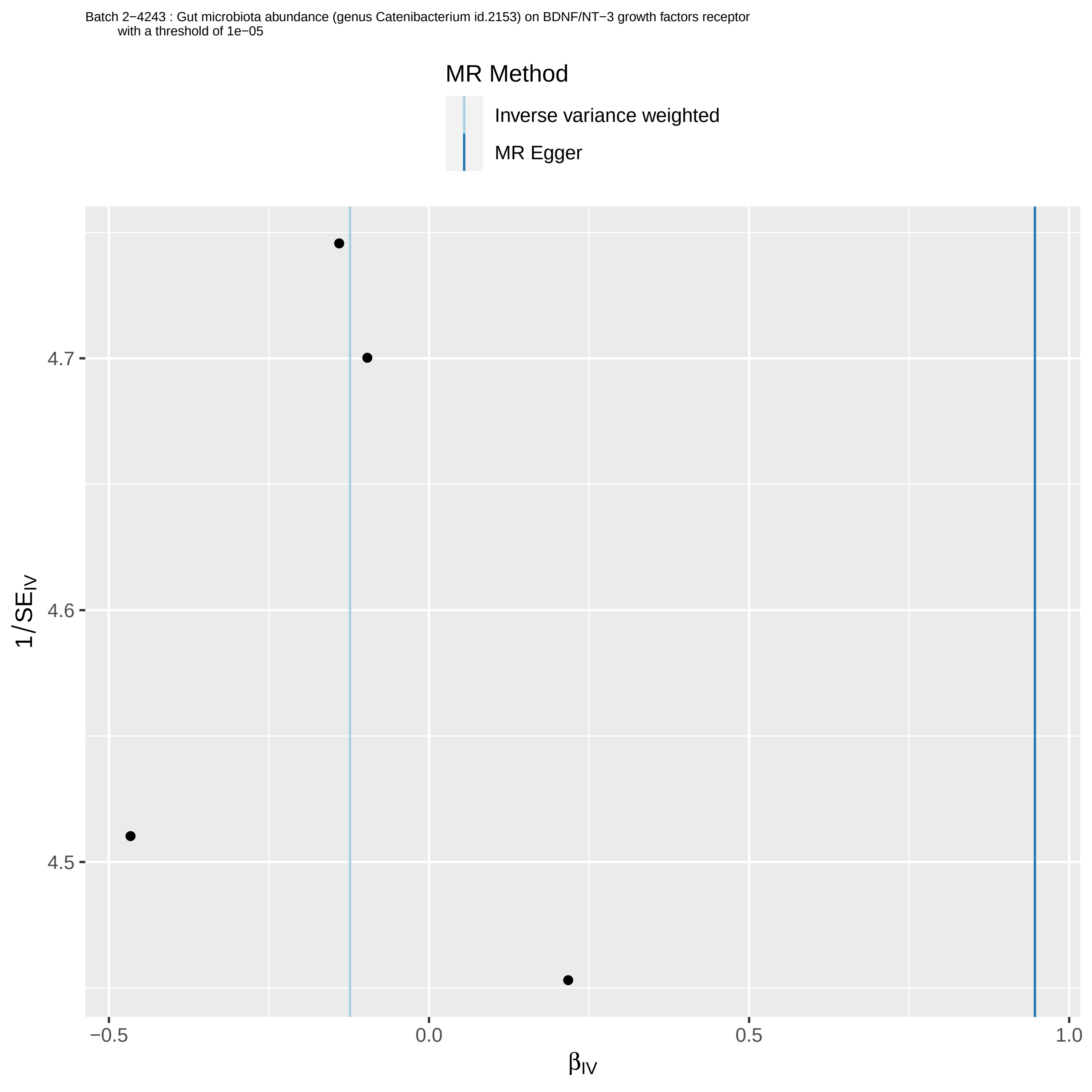
Batch 2−4243 : Gut microbiota abundance (genus Catenibacterium id.2153) on BDNF/NT−3 growth factors receptor
with a threshold of 1e−05
MR Method
Inverse variance weighted
MR Egger
4.7
4.6
4.5
1/SE_IV
−0.5
0.0
0.5
1.0
β_IV

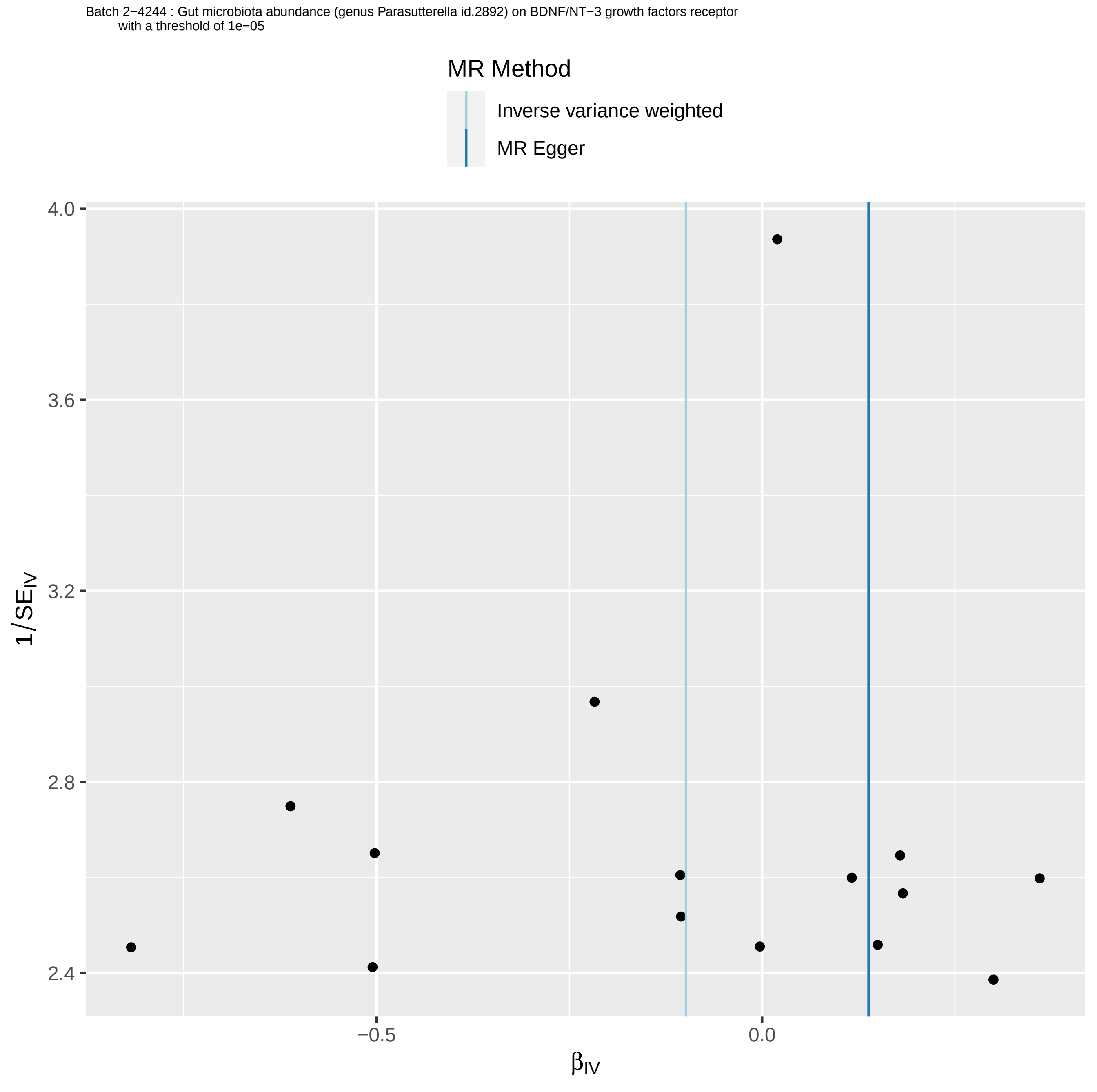

Batch 2−4244 : Gut microbiota abundance (genus Parasutterella id.2892) on BDNF/NT−3 growth factors receptor
with a threshold of 1e−05
MR Method
Inverse variance weighted
MR Egger
4.0
3.6
3.2
2.8
2.4
1/SE_IV
−0.5
0.0
β_IV

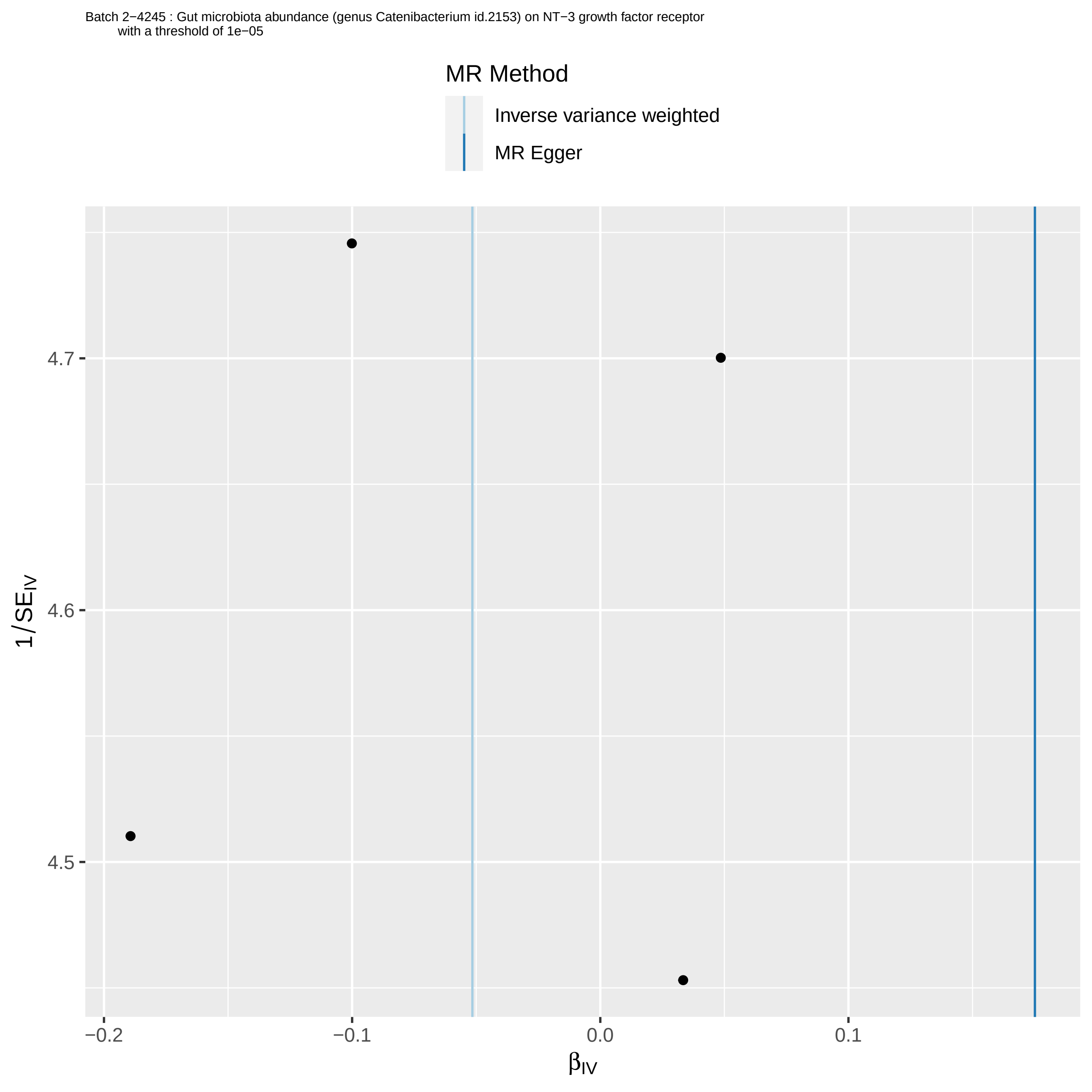
Batch 2−4245 : Gut microbiota abundance (genus Catenibacterium id.2153) on NT−3 growth factor receptor
with a threshold of 1e−05
MR Method
Inverse variance weighted
MR Egger
4.7
4.6
4.5
1/SE_IV
−0.2
−0.1
0.0
0.1
β_IV

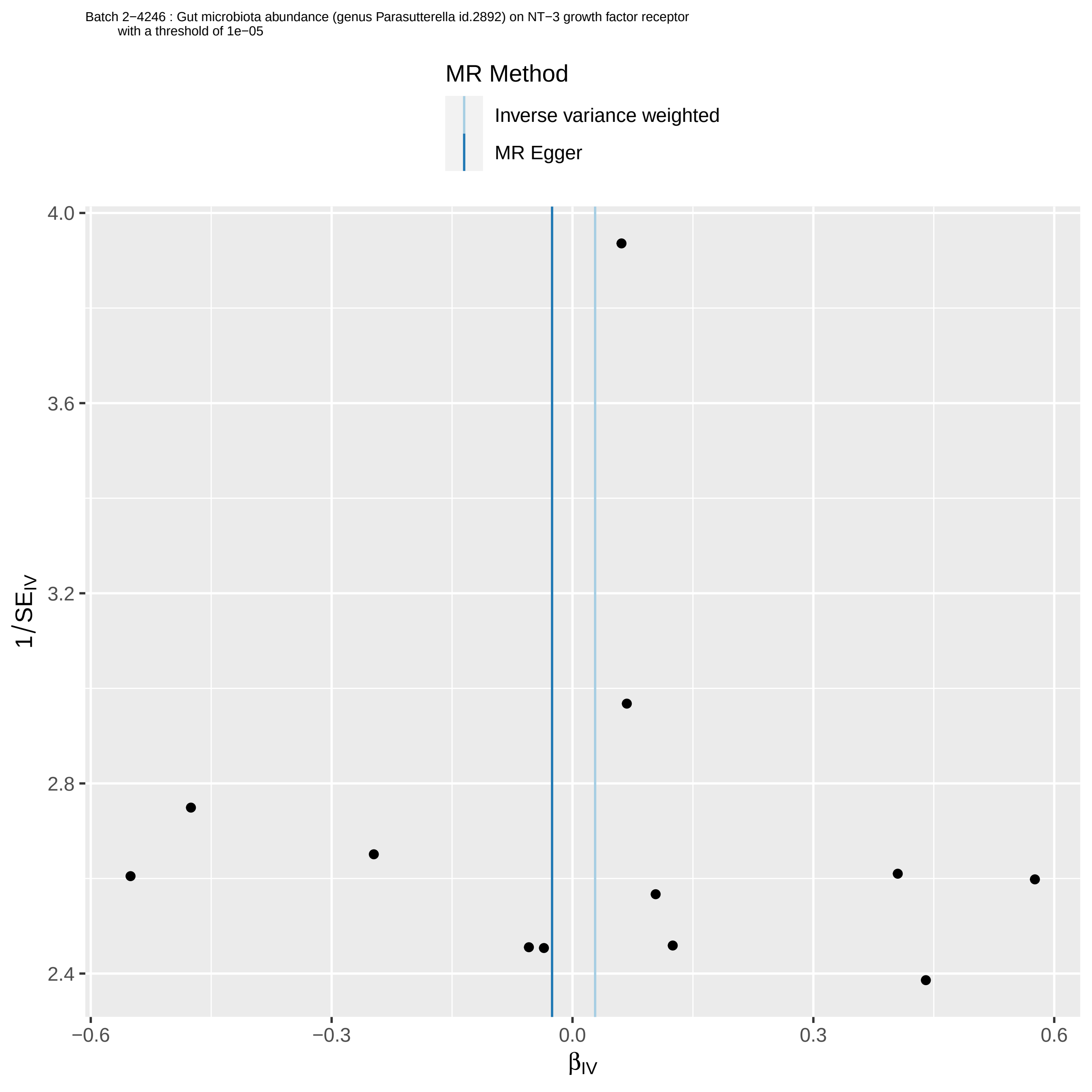
Batch 2−4246 : Gut microbiota abundance (genus Parasutterella id.2892) on NT−3 growth factor receptor
with a threshold of 1e−05
MR Method
Inverse variance weighted
MR Egger
4.0
3.6
3.2
2.8
2.4
1/SE_IV
−0.6
−0.3
0.0
0.3
0.6
β_IV

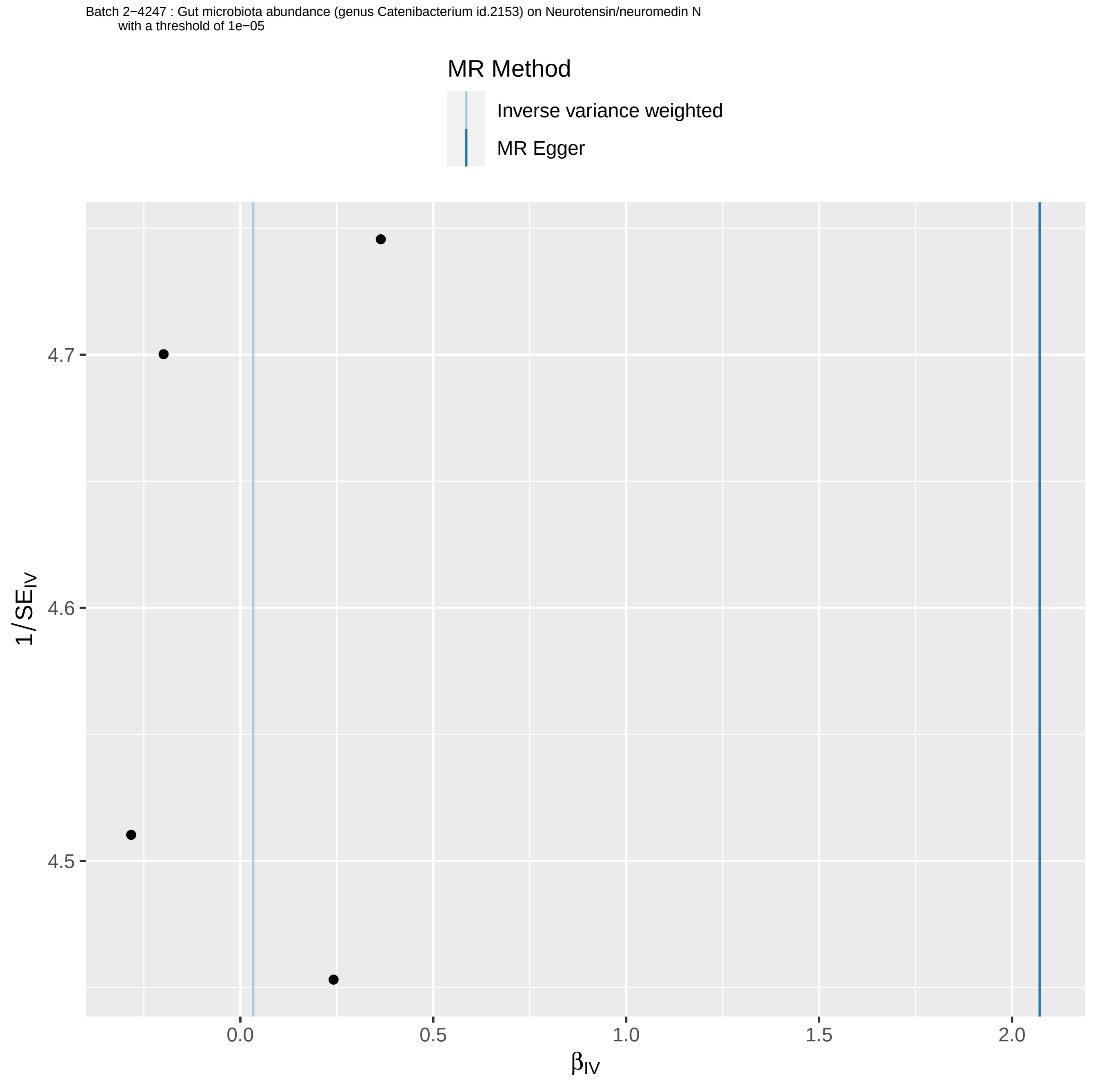
Batch 2−4247 : Gut microbiota abundance (genus Catenibacterium id.2153) on Neurotensin/neuromedin N
with a threshold of 1e−05
MR Method
Inverse variance weighted
MR Egger
4.7
4.6
4.5
1/SE_IV
0.0
0.5
1.0
1.5
2.0
β_IV

Batch 2−4248 : Gut microbiota abundance (genus Parasutterella id.2892) on Neurotensin/neuromedin N
with a threshold of 1e−05

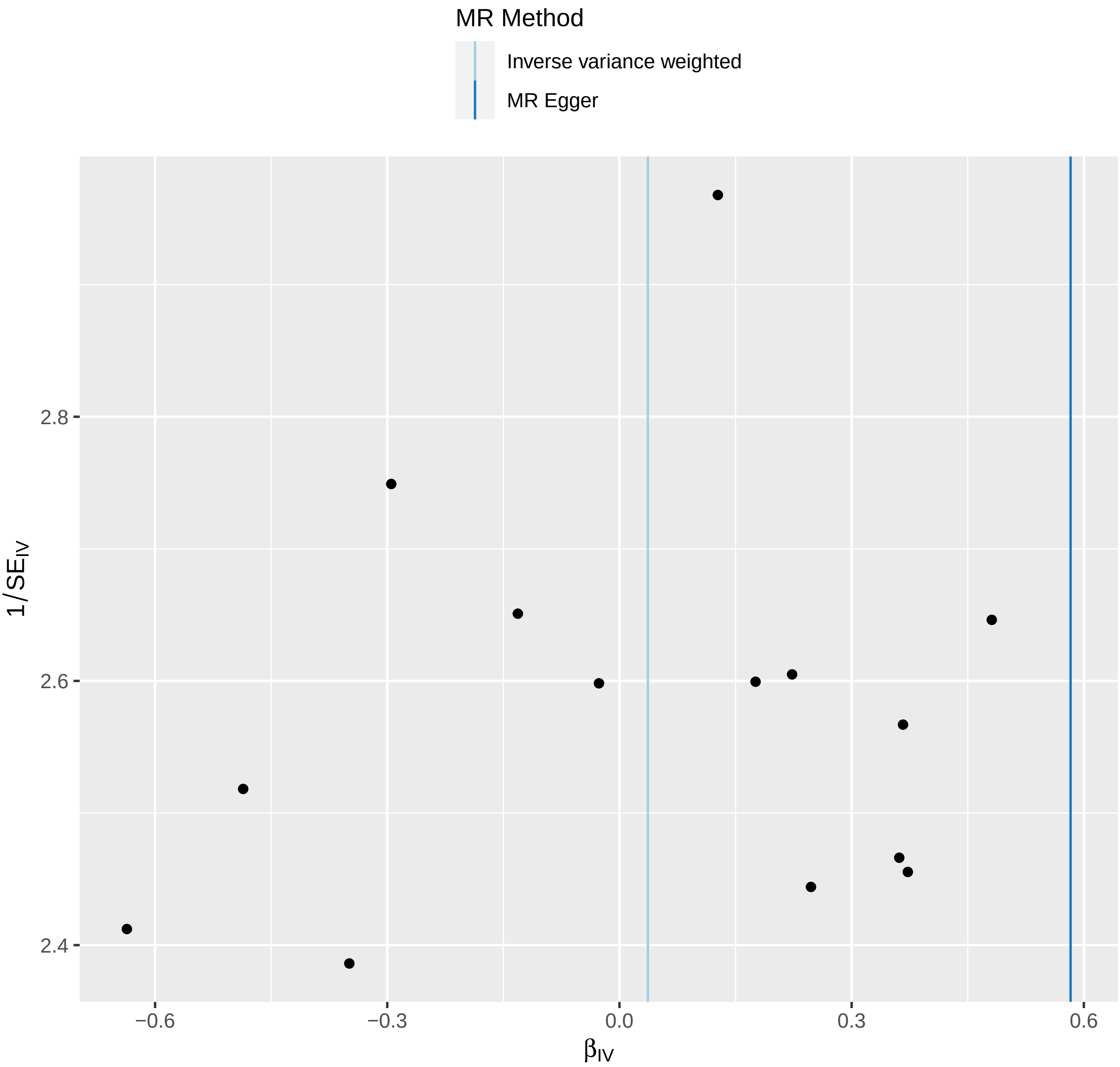

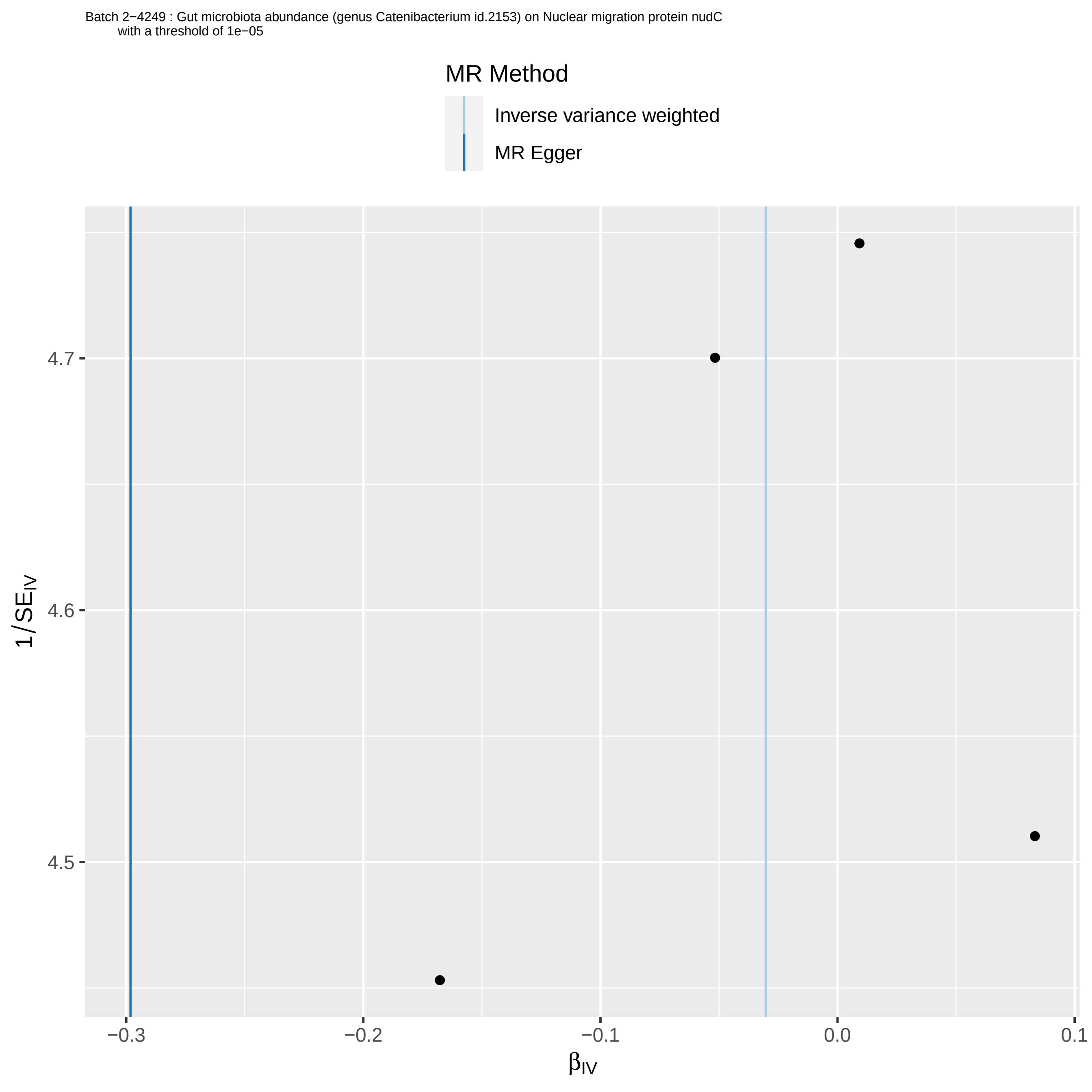

Batch 2−4249 : Gut microbiota abundance (genus Catenibacterium id.2153) on Nuclear migration protein nudC
with a threshold of 1e−05
MR Method
Inverse variance weighted
MR Egger
4.7
4.6
4.5
1/SE_IV
−0.3
−0.2
−0.1
0.0
0.1
β_IV

Batch 2−4250 : Gut microbiota abundance (genus Parasutterella id.2892) on Nuclear migration protein nudC
with a threshold of 1e−05

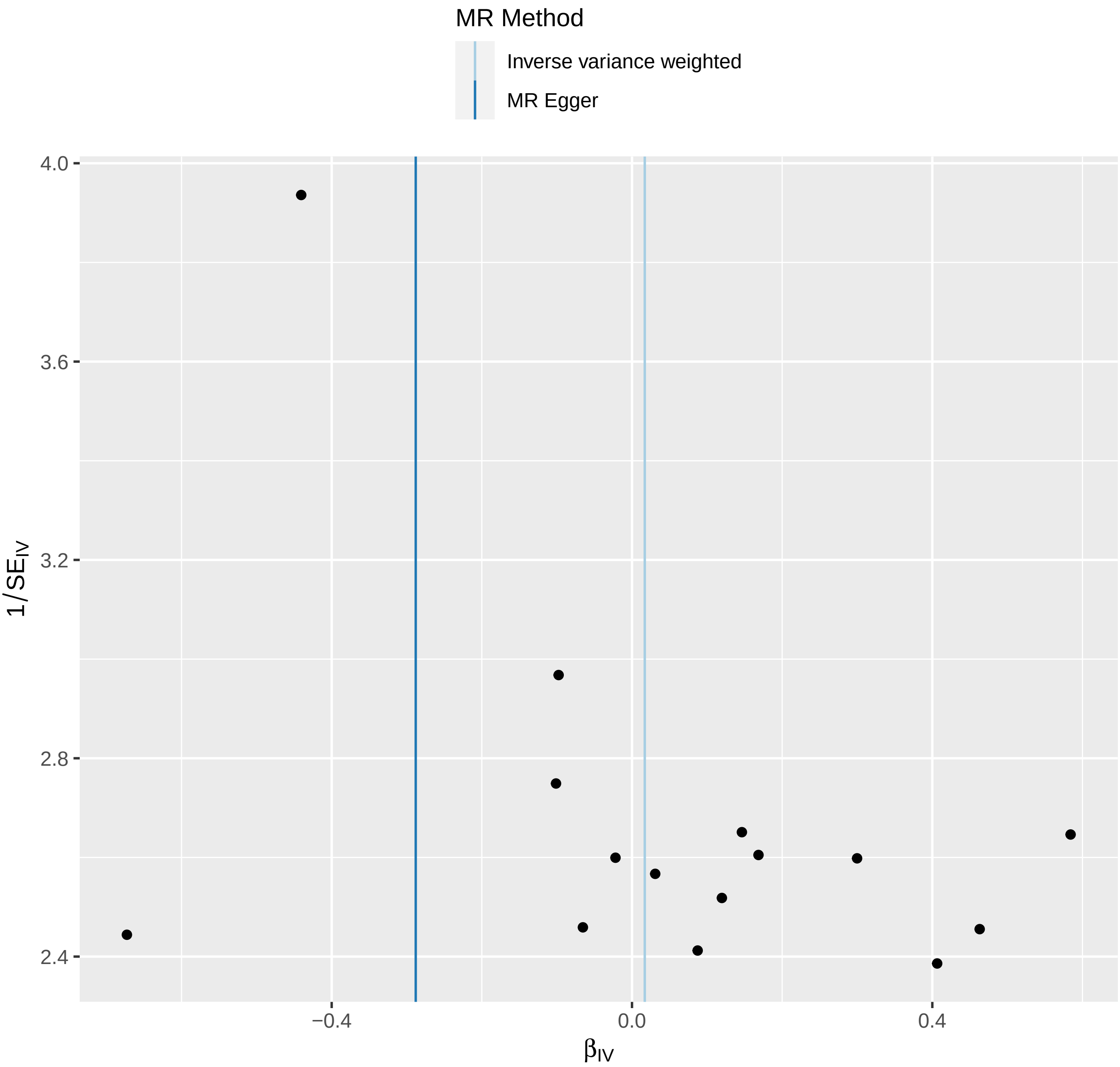

Batch 2−4251 : Gut microbiota abundance (genus Catenibacterium id.2153) on Peroxisomal NADH pyrophosphatase NUDT12 with a threshold of 1e−05

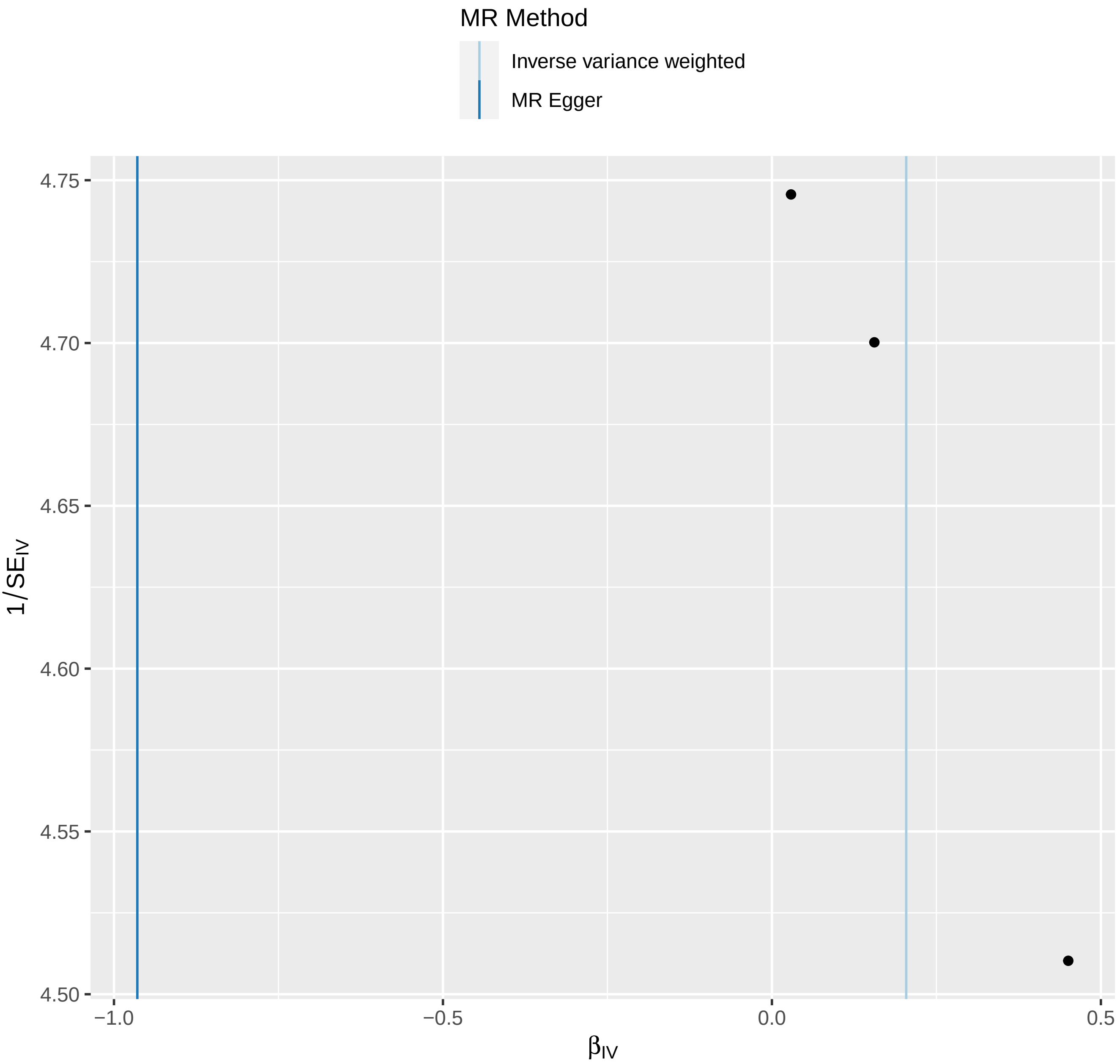

Batch 2−4252 : Gut microbiota abundance (genus Parasutterella id.2892) on Peroxisomal NADH pyrophosphatase NUDT12
with a threshold of 1e−05

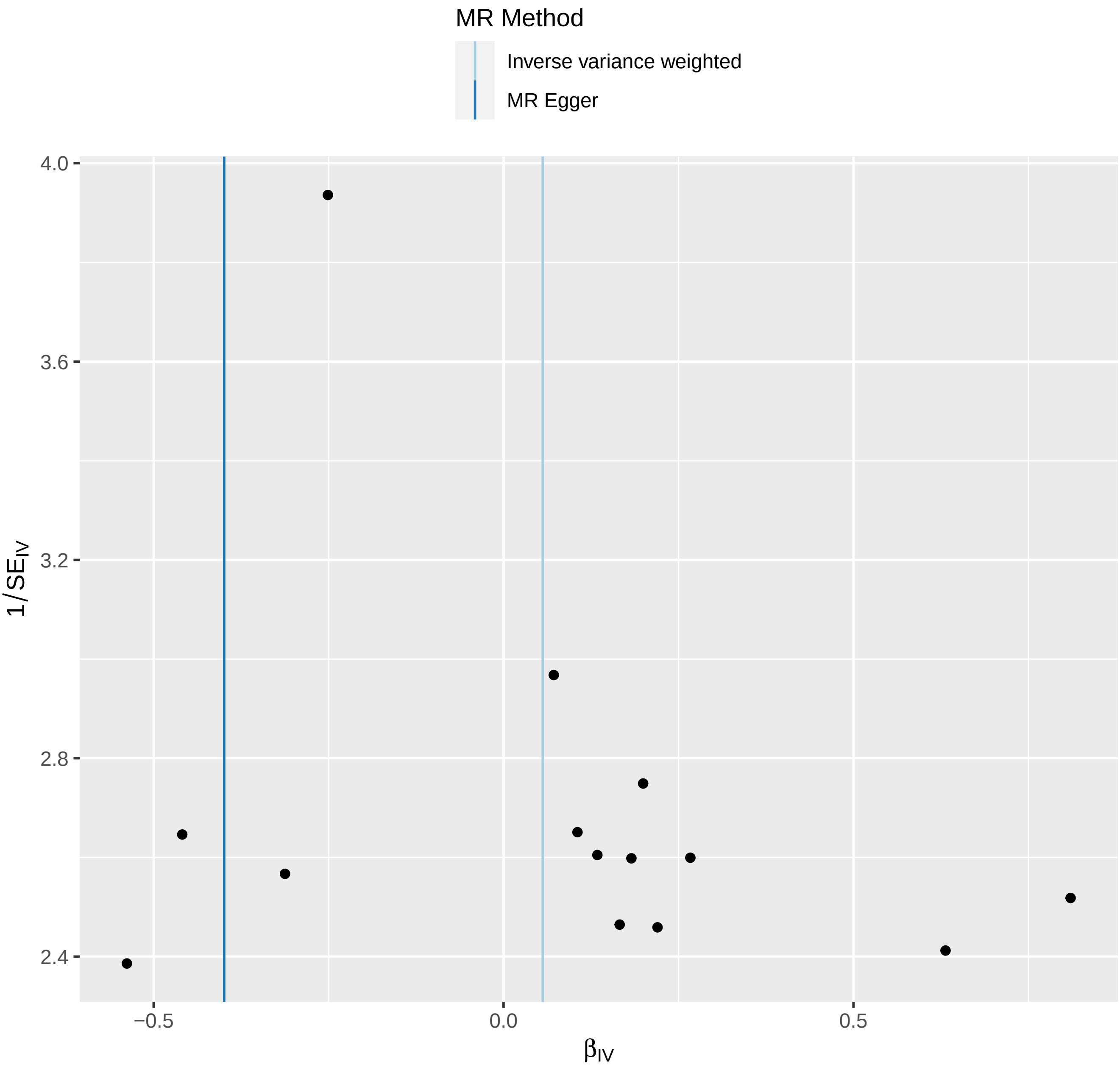

Batch 2−4253 : Gut microbiota abundance (genus Catenibacterium id.2153) on Protein syndesmos
with a threshold of 1e−05

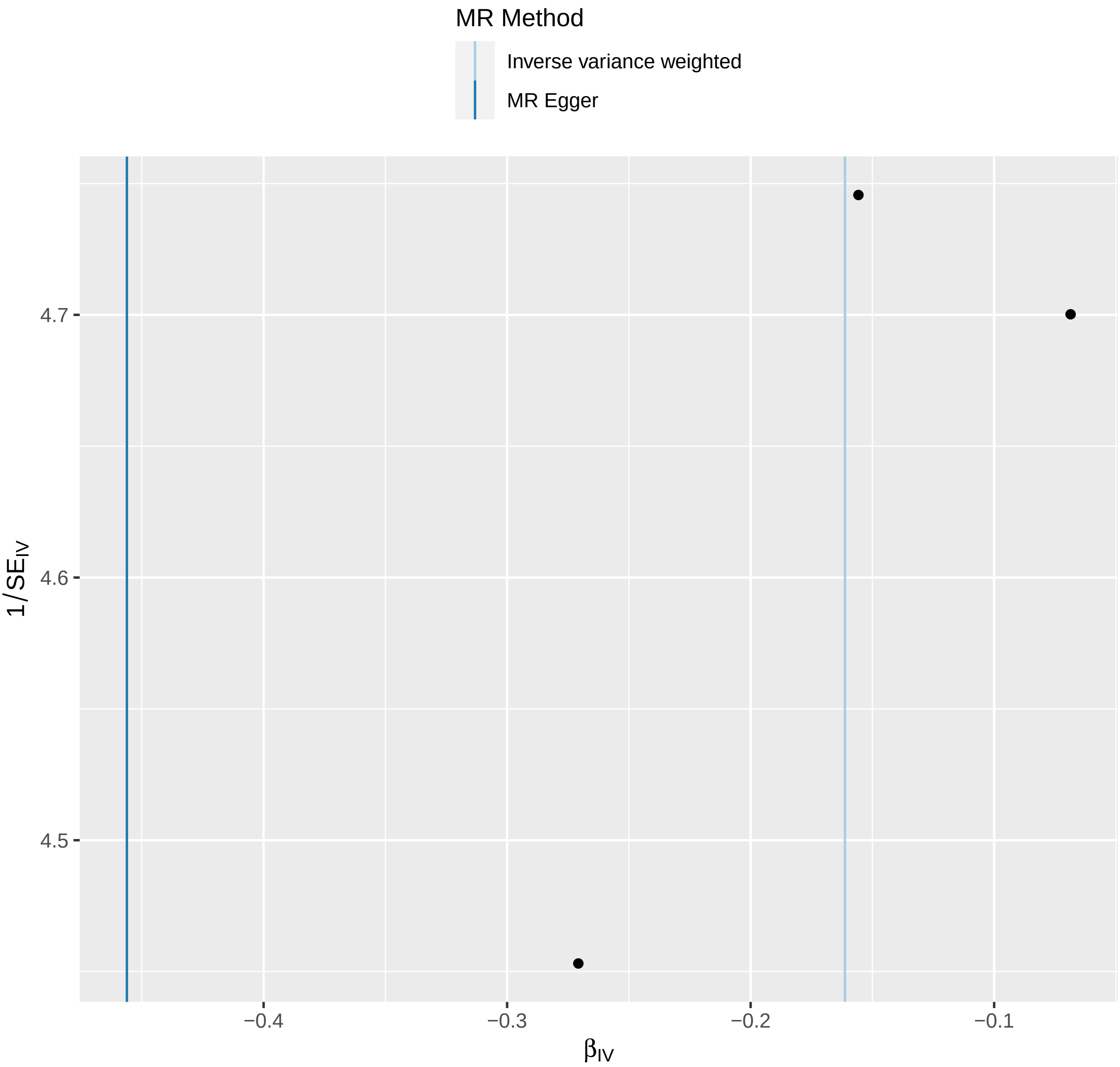

Batch 2−4254 : Gut microbiota abundance (genus Parasutterella id.2892) on Protein syndesmos
with a threshold of 1e−05

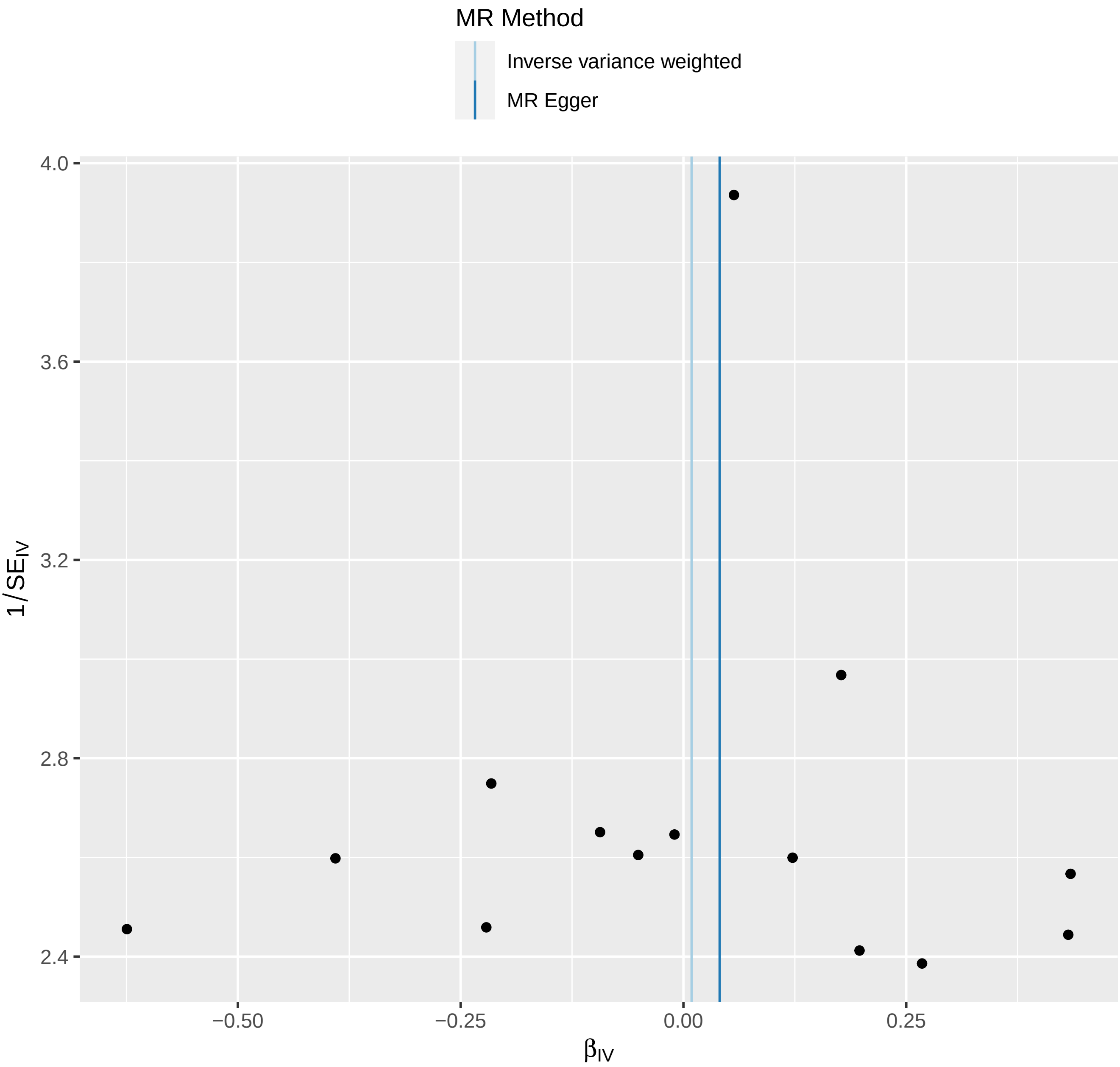

Batch 2−4255 : Gut microbiota abundance (genus Catenibacterium id.2153) on Nucleoside diphosphate−linked moiety X motif 8, mitochondrial
with a threshold of 1e−05

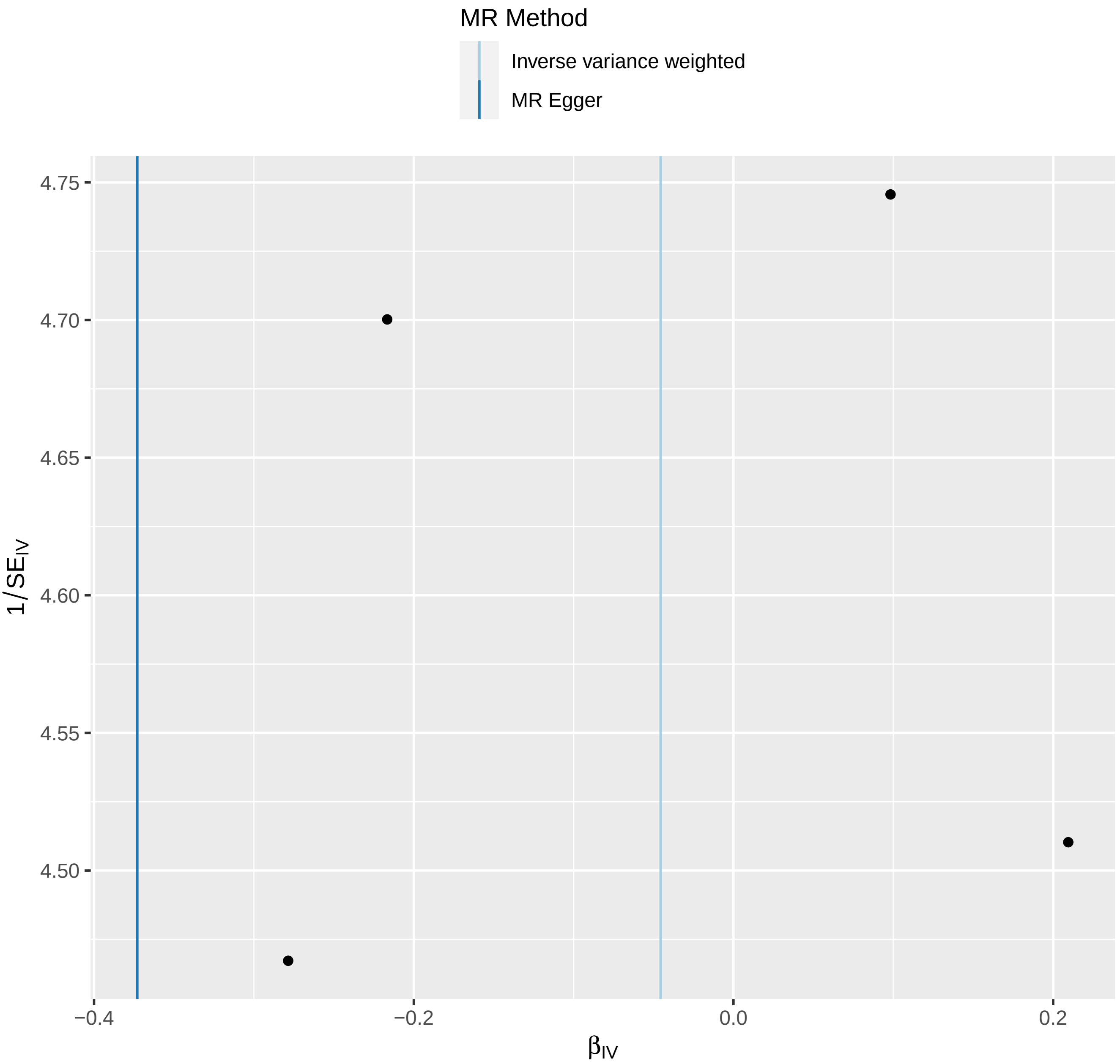

Batch 2−4256 : Gut microbiota abundance (genus Parasutterella id.2892) on Nucleoside diphosphate−linked moiety X motif 8, mitochondrial
with a threshold of 1e−05

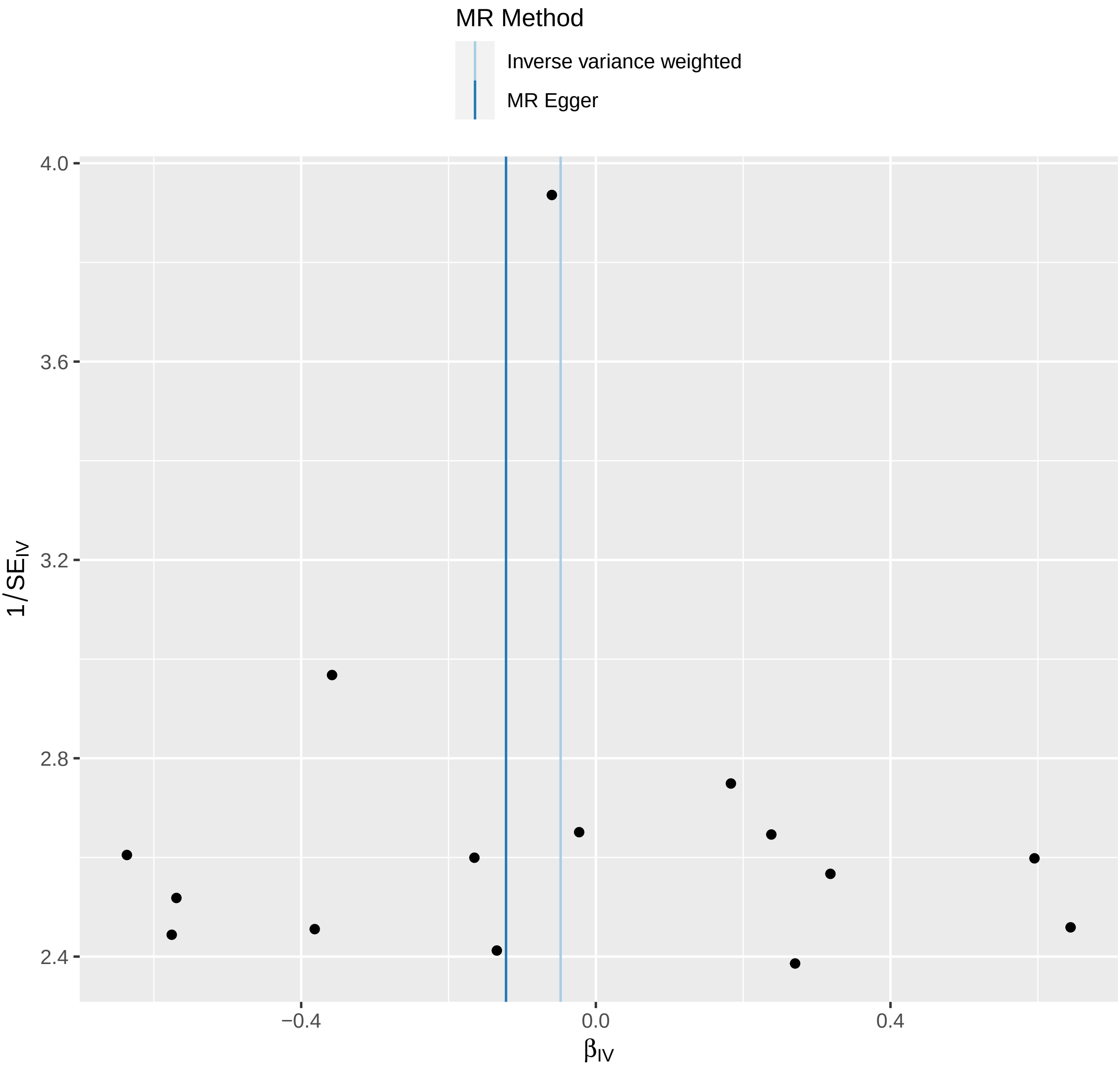

Batch 2−4257 : Gut microbiota abundance (genus Catenibacterium id.2153) on ADP−ribose pyrophosphatase, mitochondrial
with a threshold of 1e−05

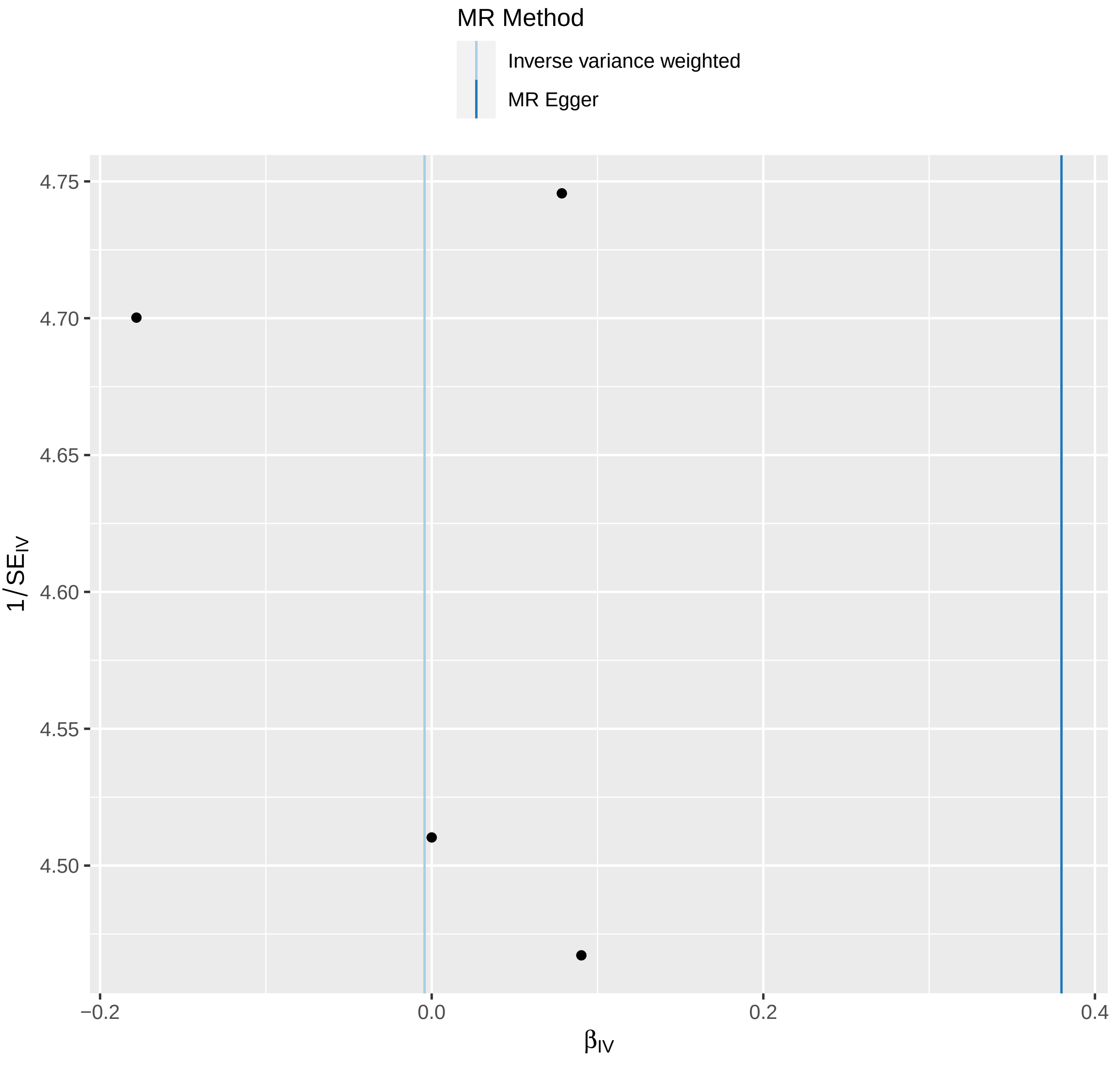

Batch 2−4258 : Gut microbiota abundance (genus Parasutterella id.2892) on ADP−ribose pyrophosphatase, mitochondrial
with a threshold of 1e−05

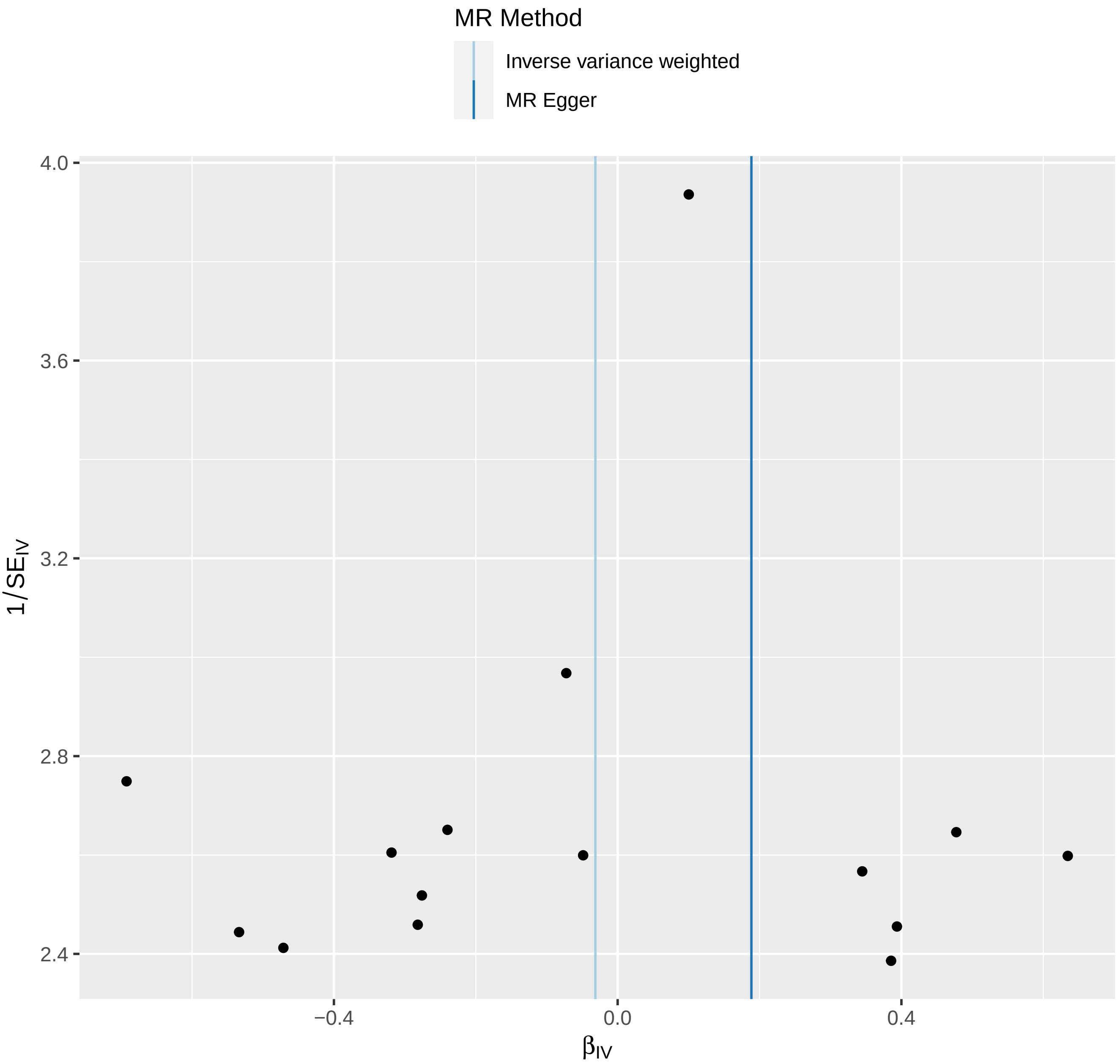

Batch 2−4259 : Gut microbiota abundance (genus Catenibacterium id.2153) on Nuclear pore membrane glycoprotein 210−like
with a threshold of 1e−05

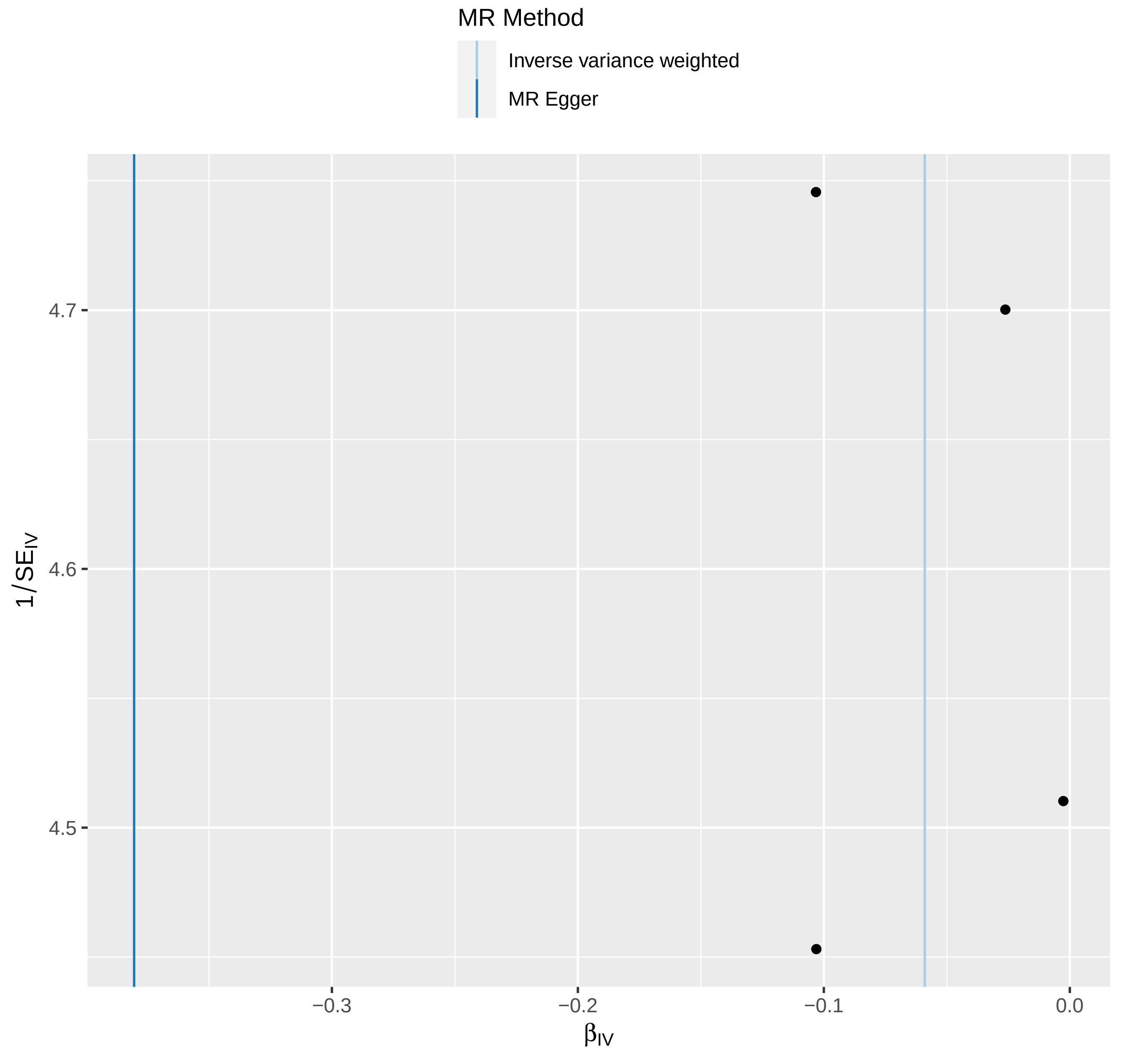

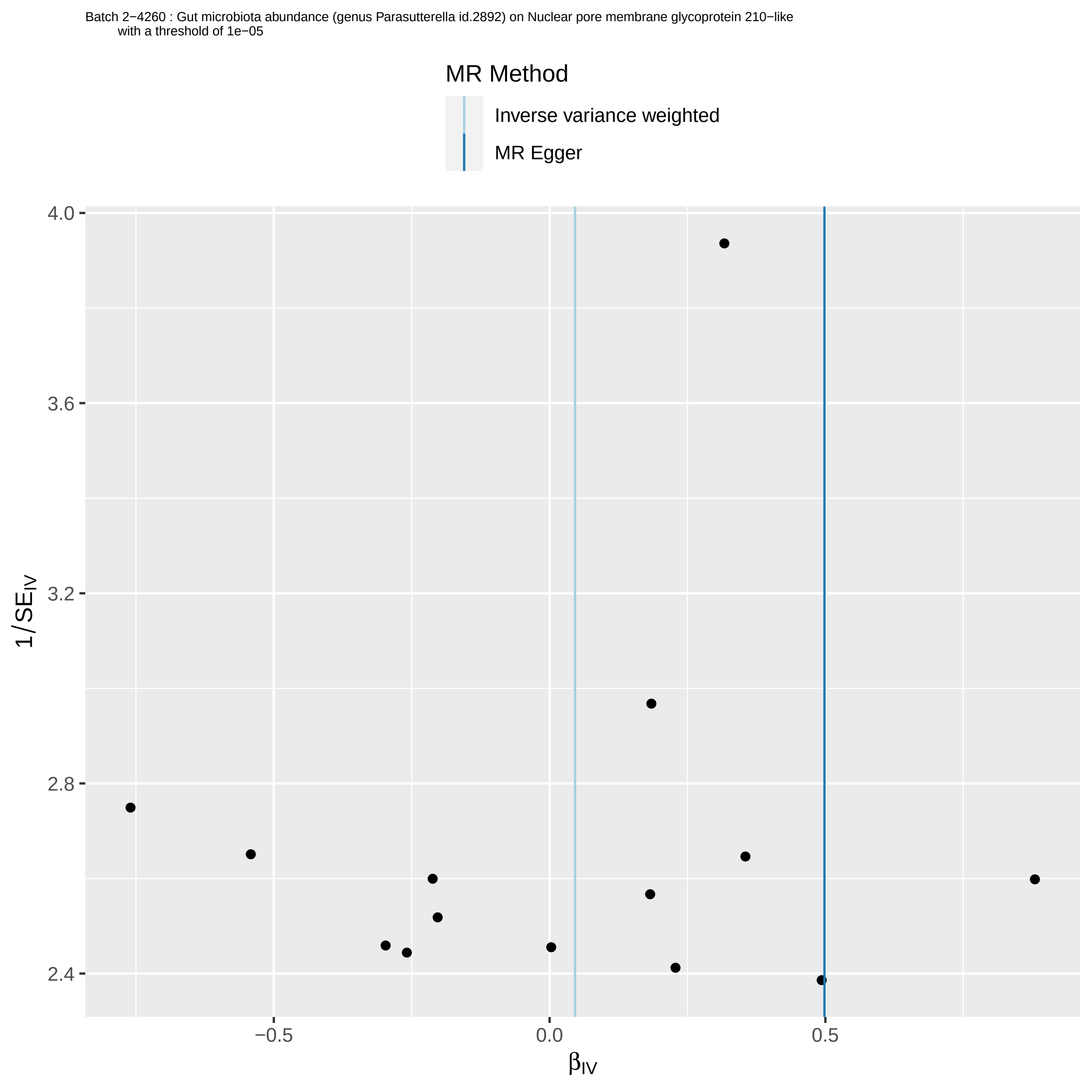

Batch 2−4260 : Gut microbiota abundance (genus Parasutterella id.2892) on Nuclear pore membrane glycoprotein 210−like
with a threshold of 1e−05
MR Method
Inverse variance weighted
MR Egger
4.0
3.6
3.2
2.8
2.4
1/SE_IV
−0.5
0.0
0.5
β_IV

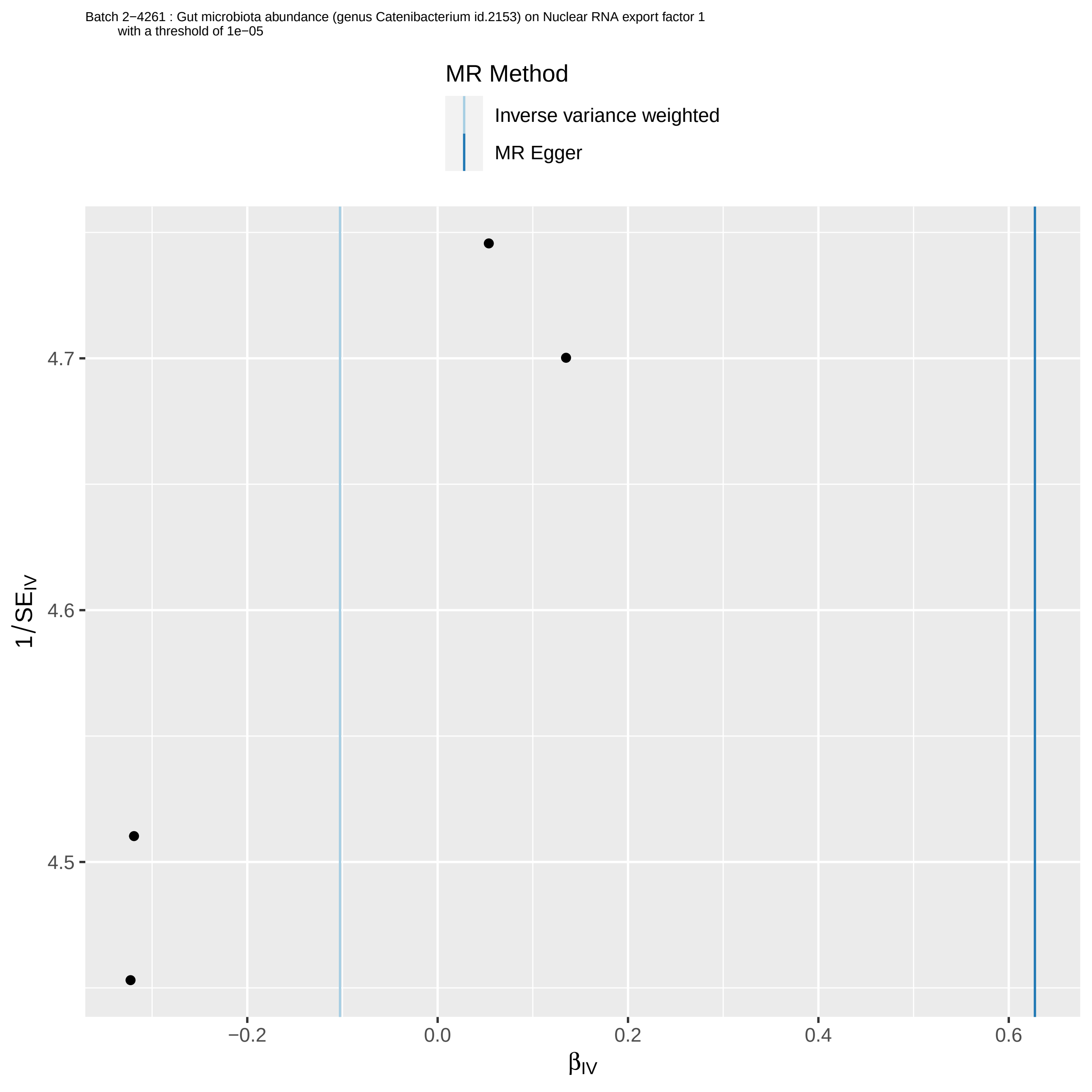

Batch 2−4261 : Gut microbiota abundance (genus Catenibacterium id.2153) on Nuclear RNA export factor 1
with a threshold of 1e−05
MR Method
Inverse variance weighted
MR Egger
4.7
4.6
4.5
1/SE$_{IV}$
−0.2
0.0
0.2
0.4
0.6
β$_{IV}$

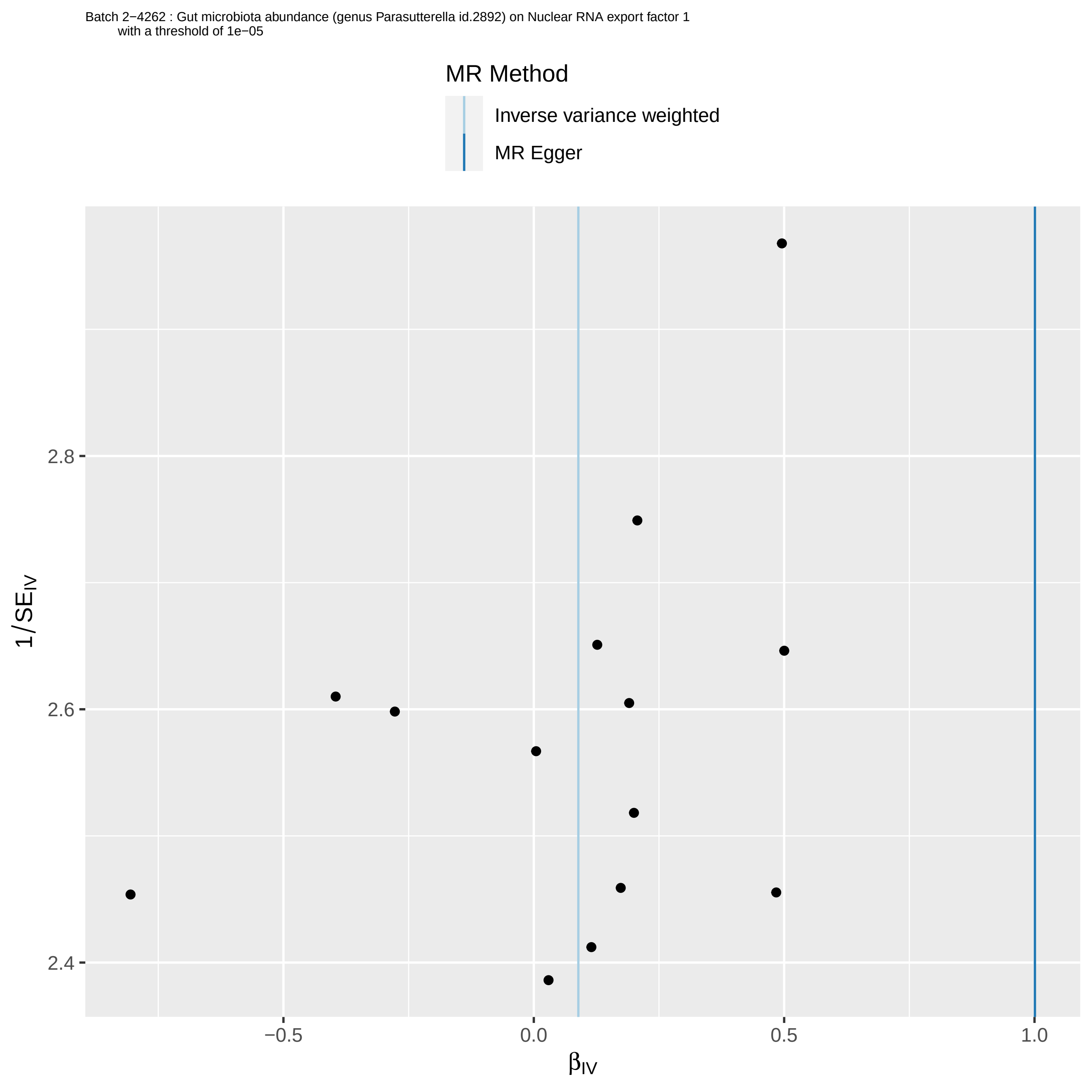

Batch 2−4262 : Gut microbiota abundance (genus Parasutterella id.2892) on Nuclear RNA export factor 1
with a threshold of 1e−05
MR Method
Inverse variance weighted
MR Egger
1/SE_IV
2.4
2.6
2.8
−0.5
0.0
0.5
1.0
β_IV

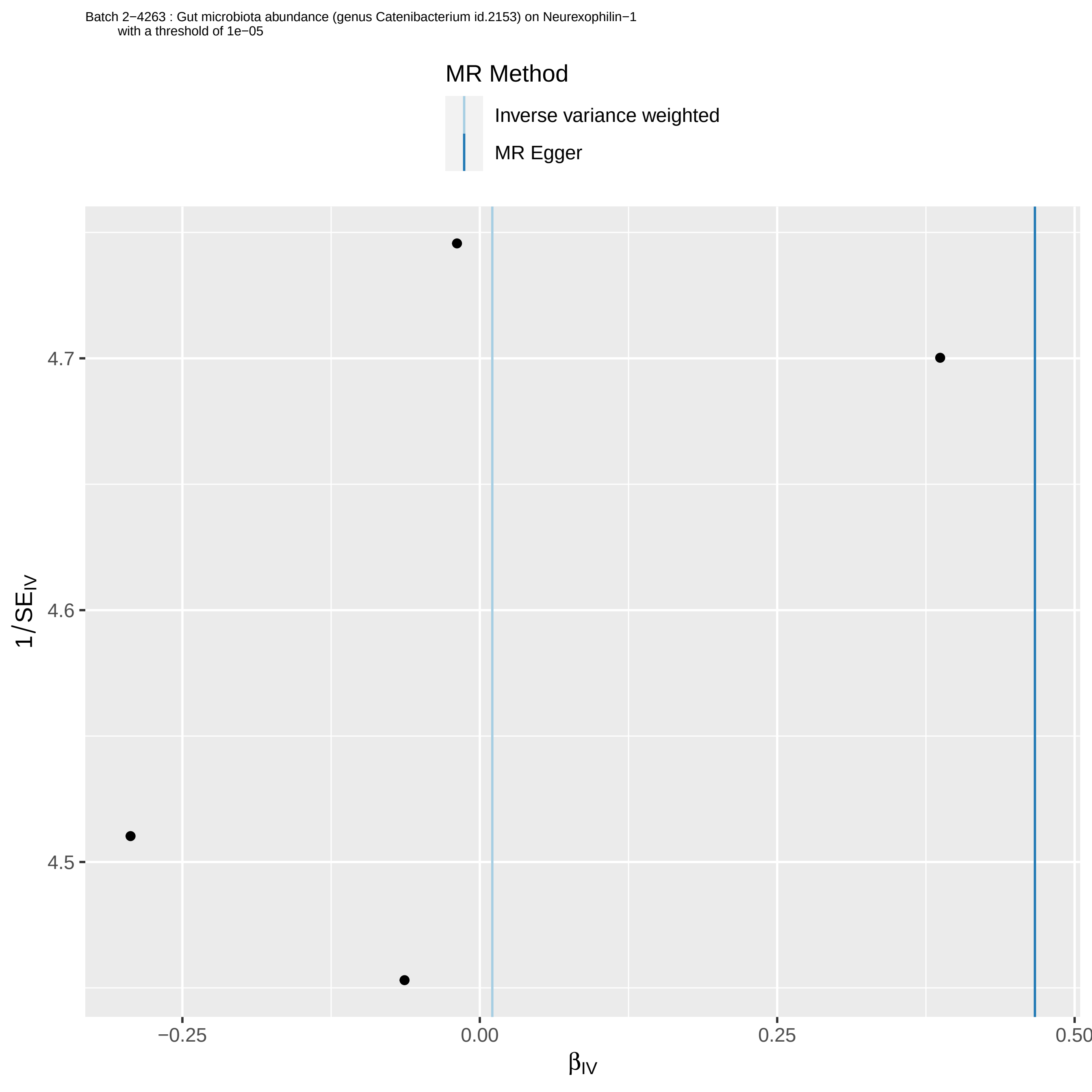

Batch 2−4263 : Gut microbiota abundance (genus Catenibacterium id.2153) on Neurexophilin−1
with a threshold of 1e−05
MR Method
Inverse variance weighted
MR Egger
4.7
4.6
4.5
1/SE_IV
−0.25
0.00
0.25
0.50
β_IV

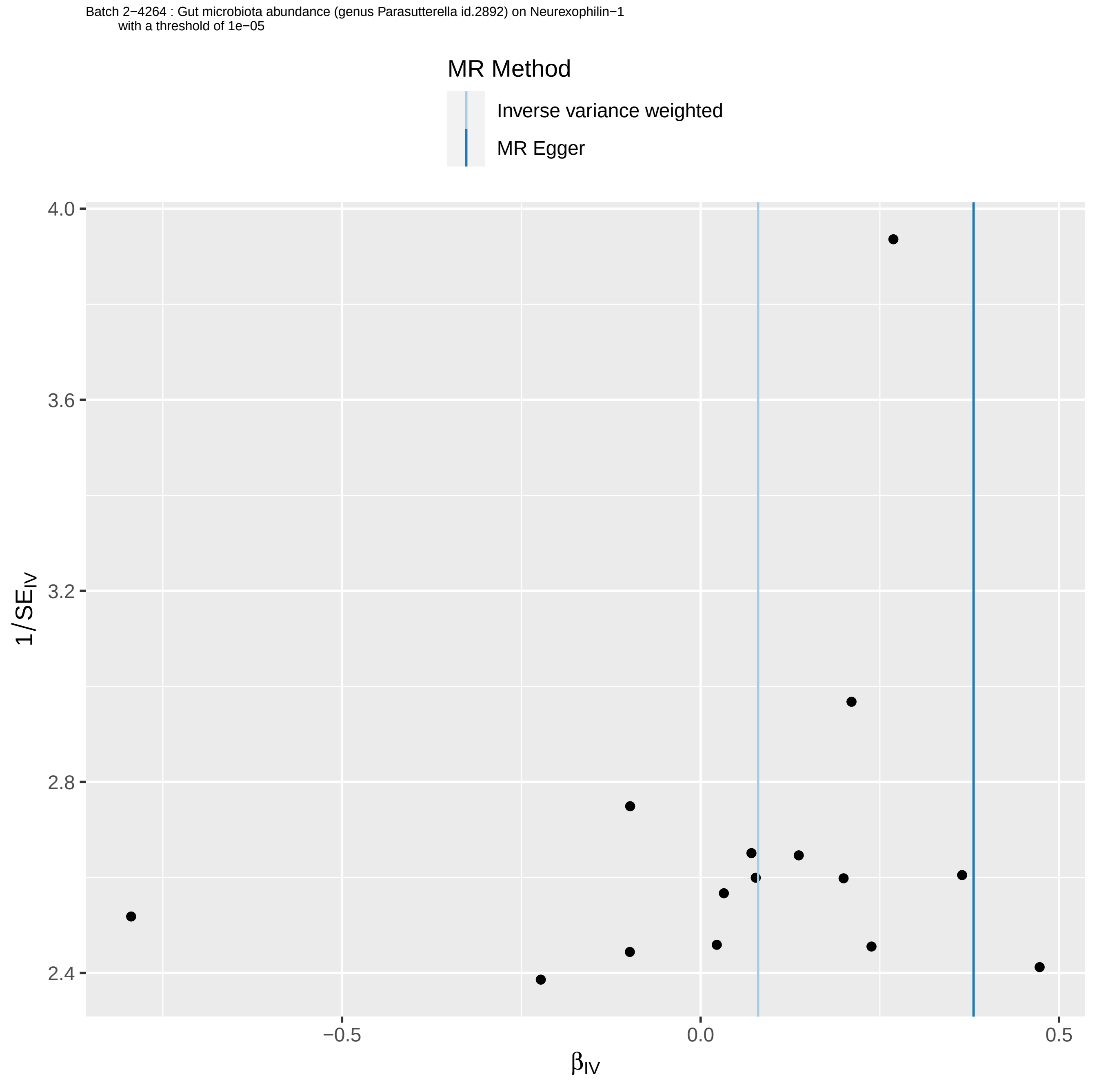

Batch 2−4264 : Gut microbiota abundance (genus Parasutterella id.2892) on Neurexophilin−1
with a threshold of 1e−05
MR Method
Inverse variance weighted
MR Egger
4.0
3.6
3.2
2.8
2.4
1/SE_IV
−0.5
0.0
0.5
β_IV

Batch 2−4265 : Gut microbiota abundance (genus Catenibacterium id.2153) on Neurexophilin−3
with a threshold of 1e−05

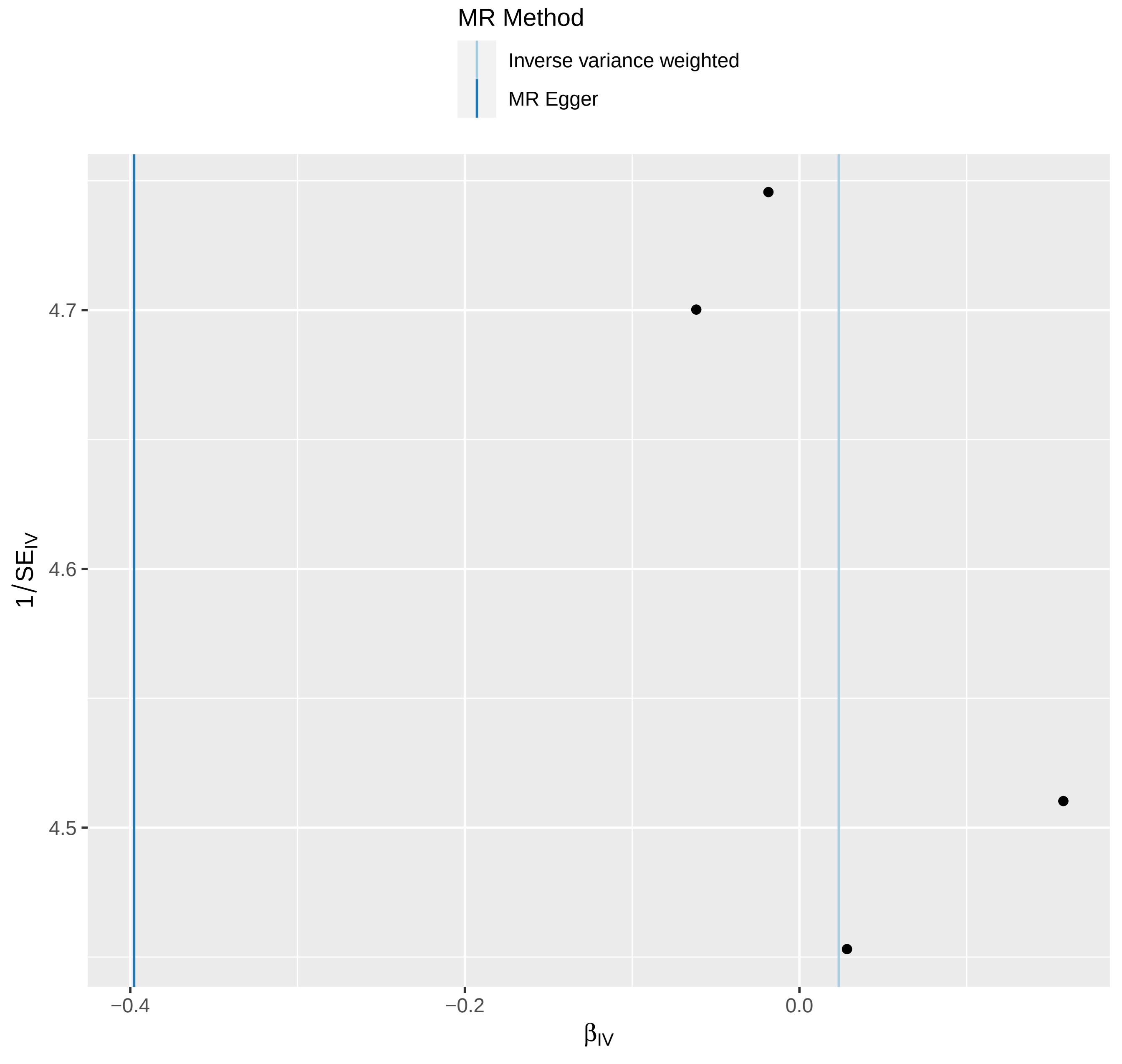

Batch 2−4266 : Gut microbiota abundance (genus Parasutterella id.2892) on Neurexophilin−3
with a threshold of 1e−05

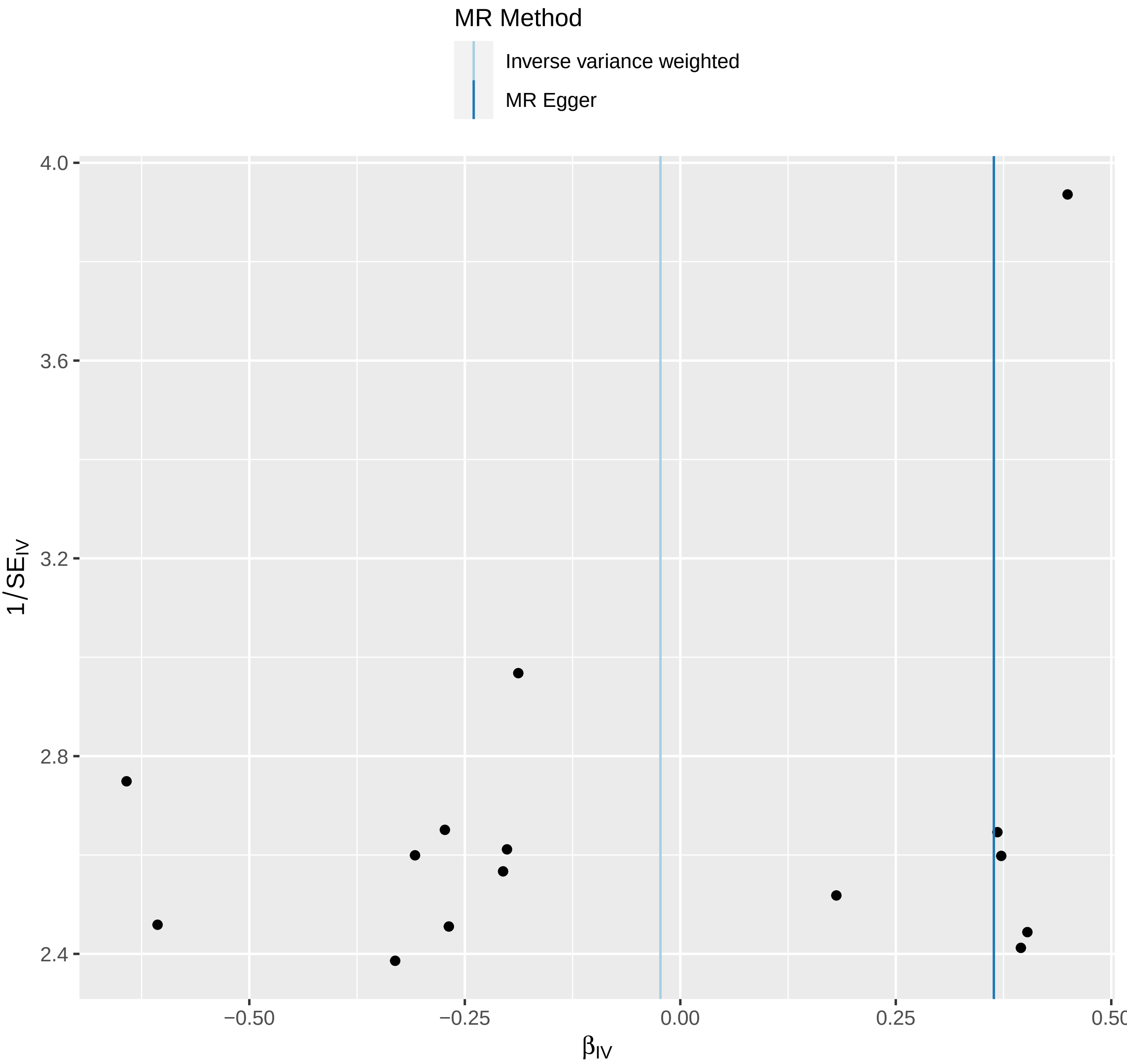

Batch 2−4267 : Gut microbiota abundance (genus Catenibacterium id.2153) on NTF2−related export protein 1
with a threshold of 1e−05

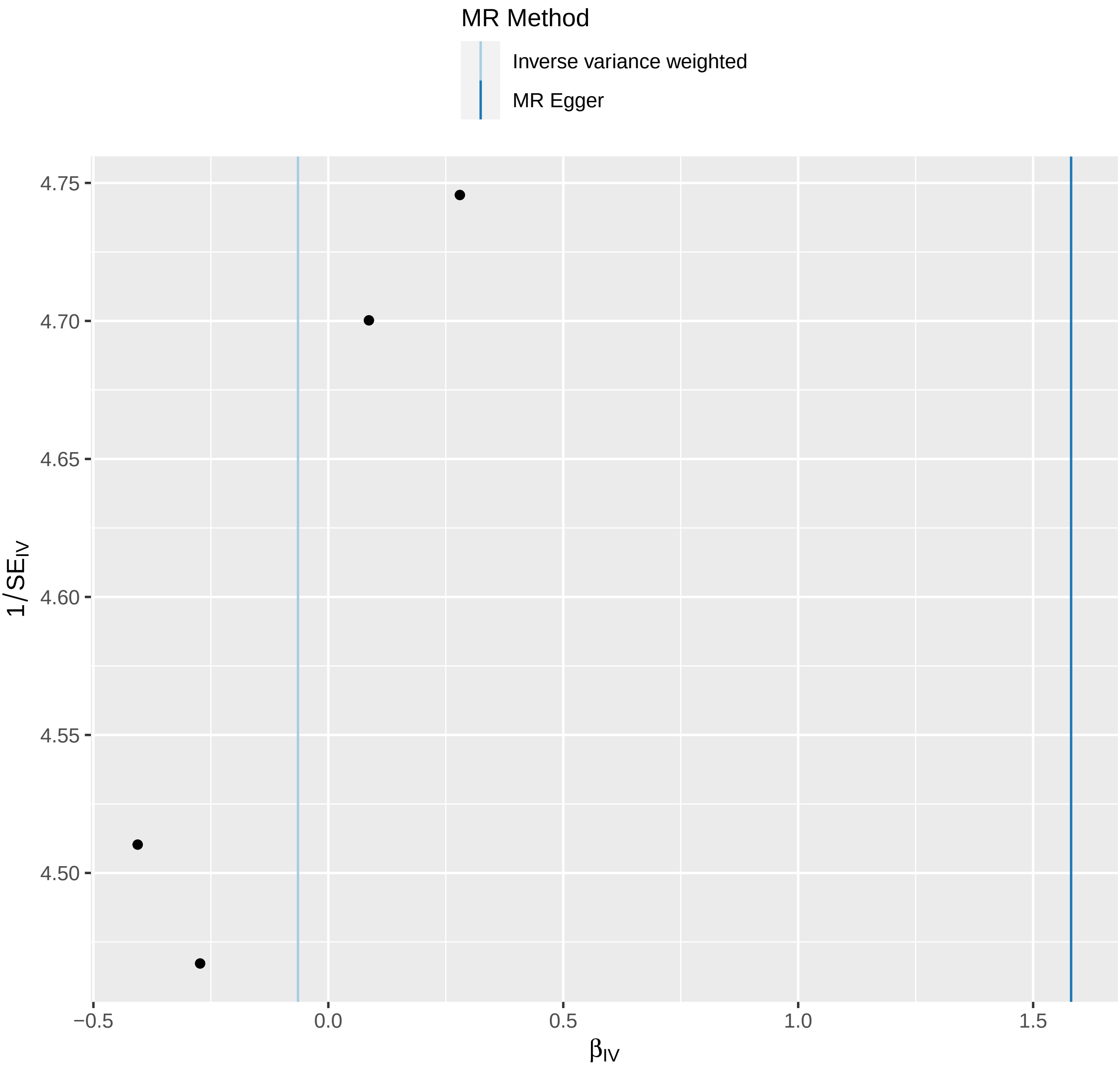

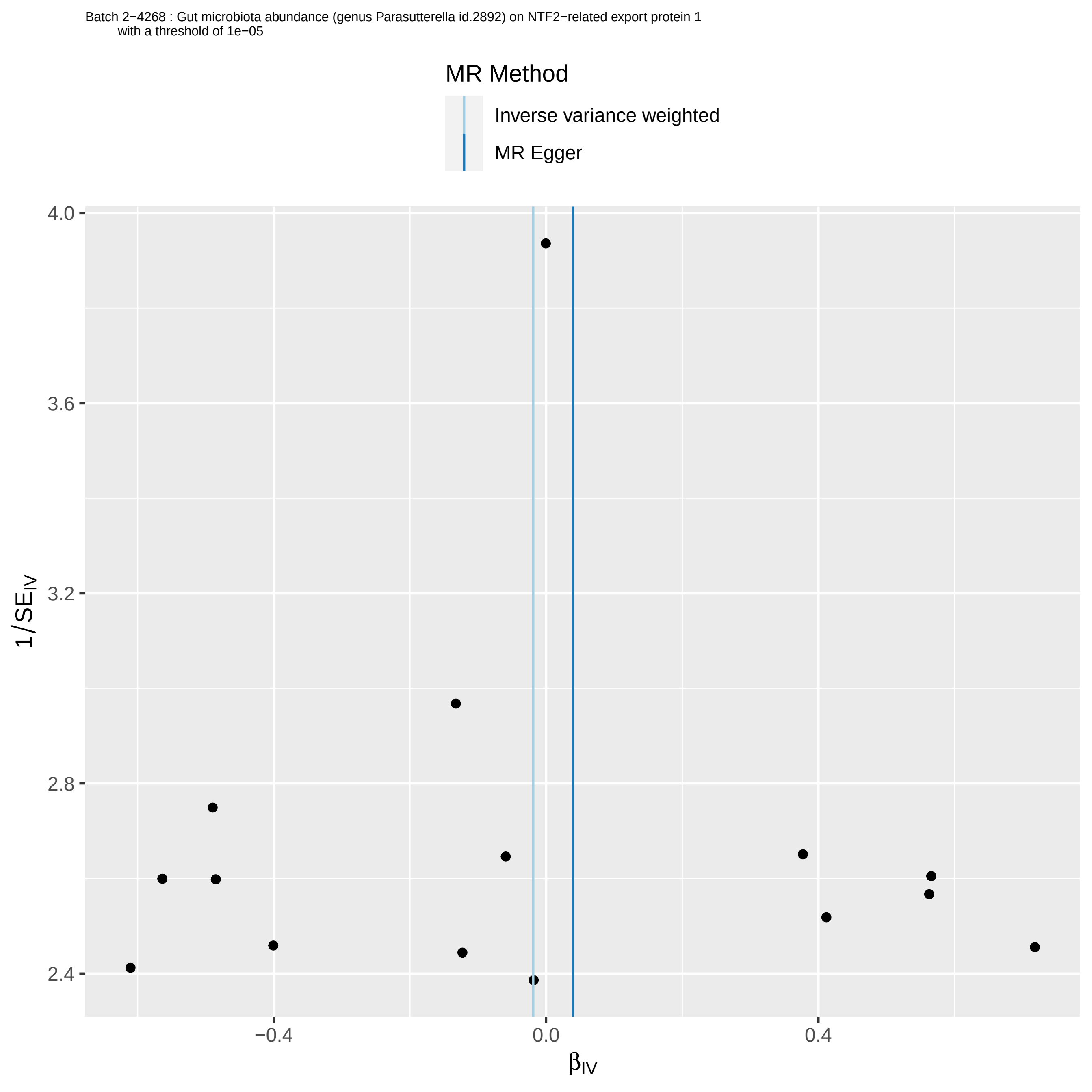
Batch 2−4268 : Gut microbiota abundance (genus Parasutterella id.2892) on NTF2−related export protein 1
with a threshold of 1e−05
MR Method
Inverse variance weighted
MR Egger
1/SE_IV
4.0
3.6
3.2
2.8
2.4
−0.4
0.0
0.4
β_IV

Batch 2−4269 : Gut microbiota abundance (genus Catenibacterium id.2153) on Out at first protein homolog
with a threshold of 1e−05

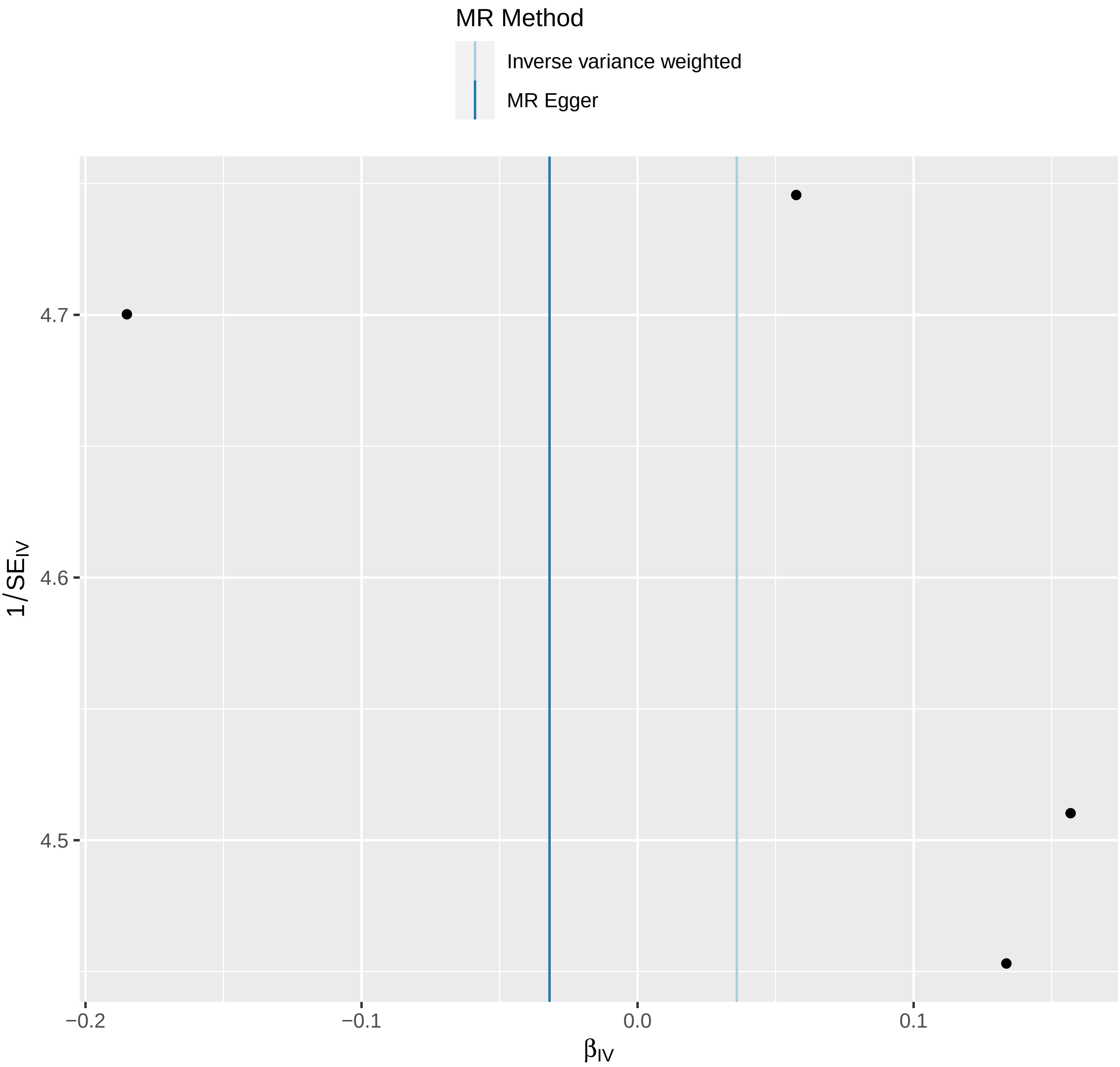

Batch 2−4270 : Gut microbiota abundance (genus Parasutterella id.2892) on Out at first protein homolog
with a threshold of 1e−05

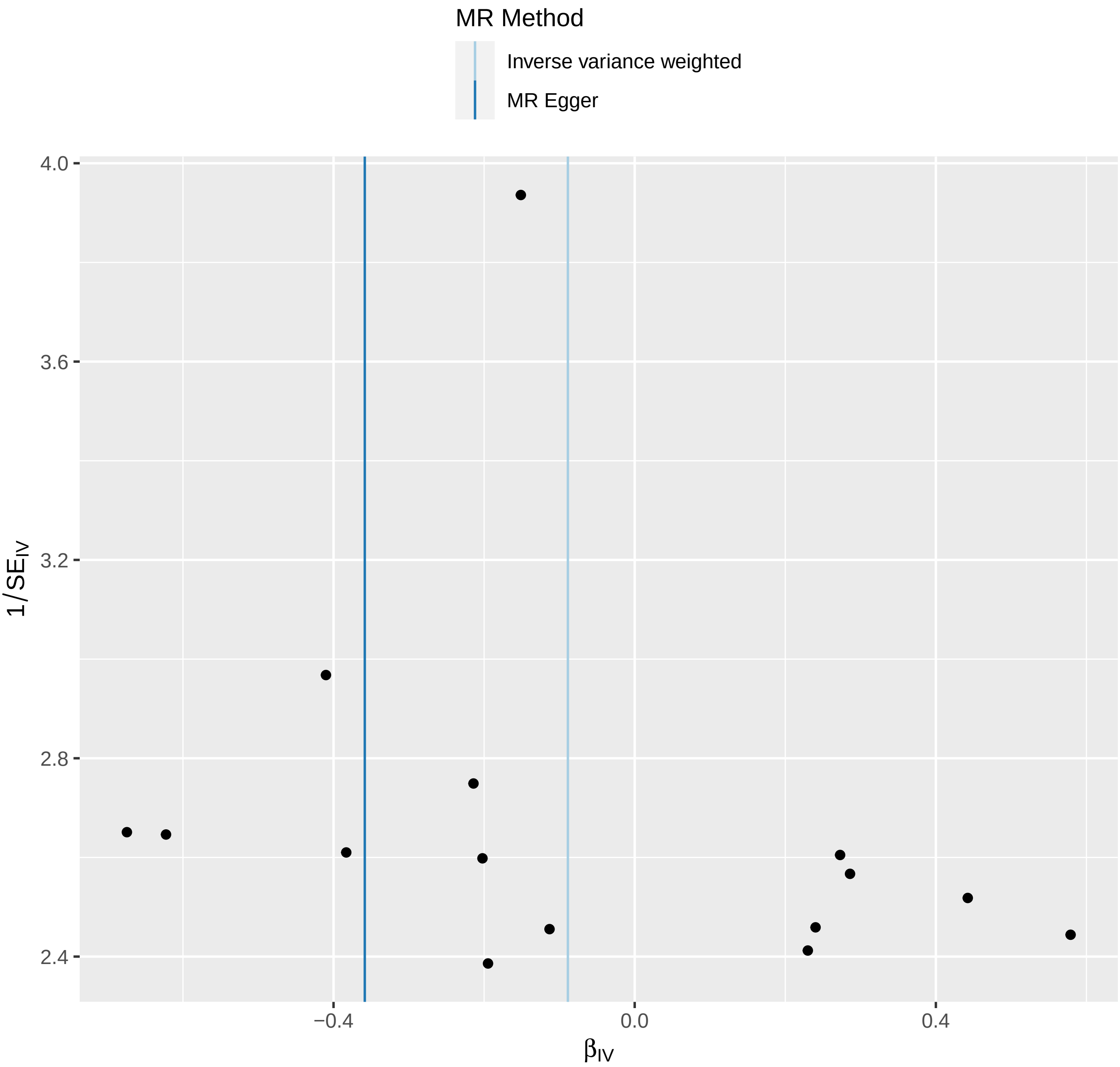

Batch 2−4271 : Gut microbiota abundance (genus Catenibacterium id.2153) on 2'−5'−oligoadenylate synthase 1 with a threshold of 1e−05

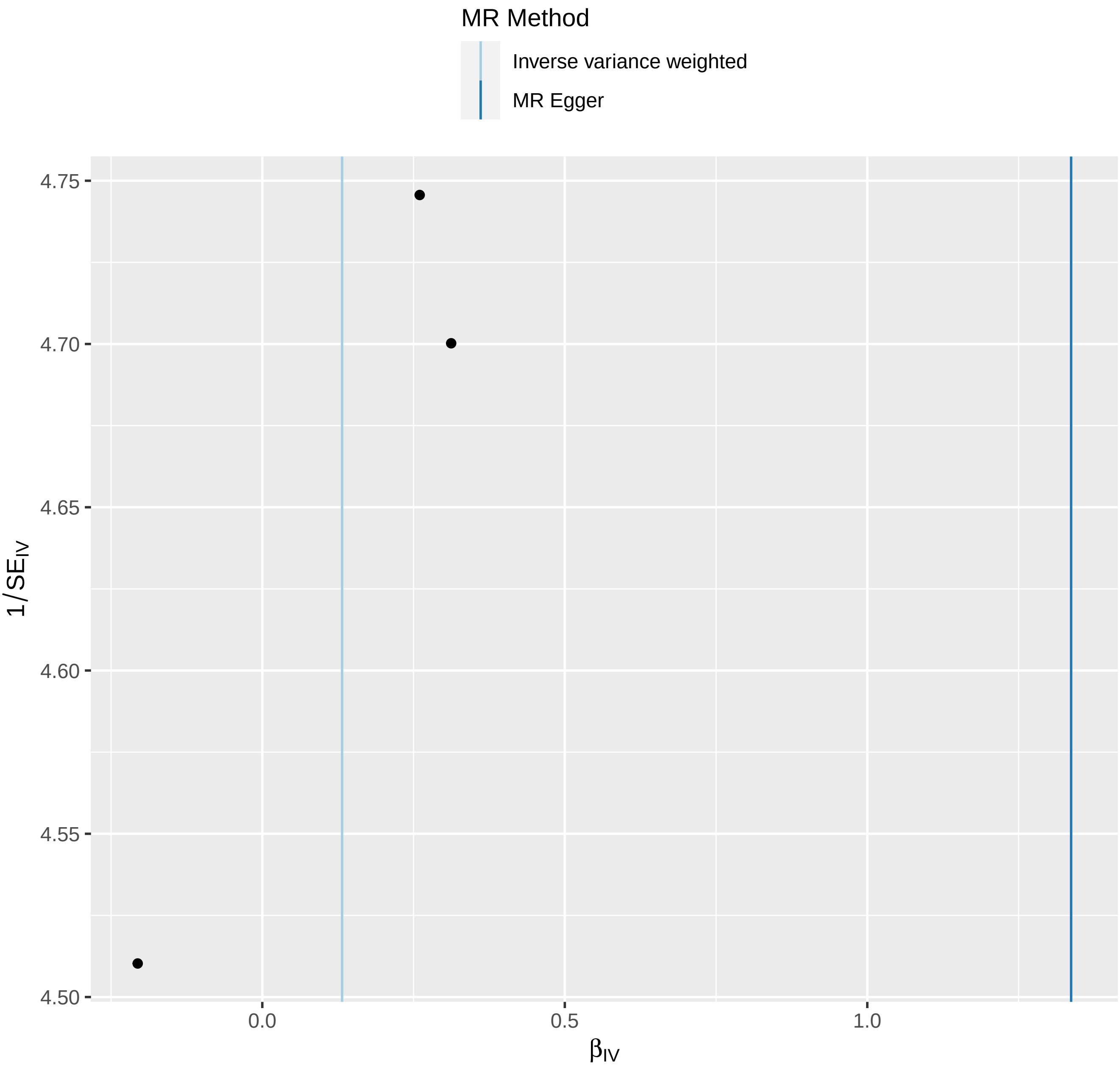

Batch 2−4272 : Gut microbiota abundance (genus Parasutterella id.2892) on 2'−5'−oligoadenylate synthase 1
with a threshold of 1e−05

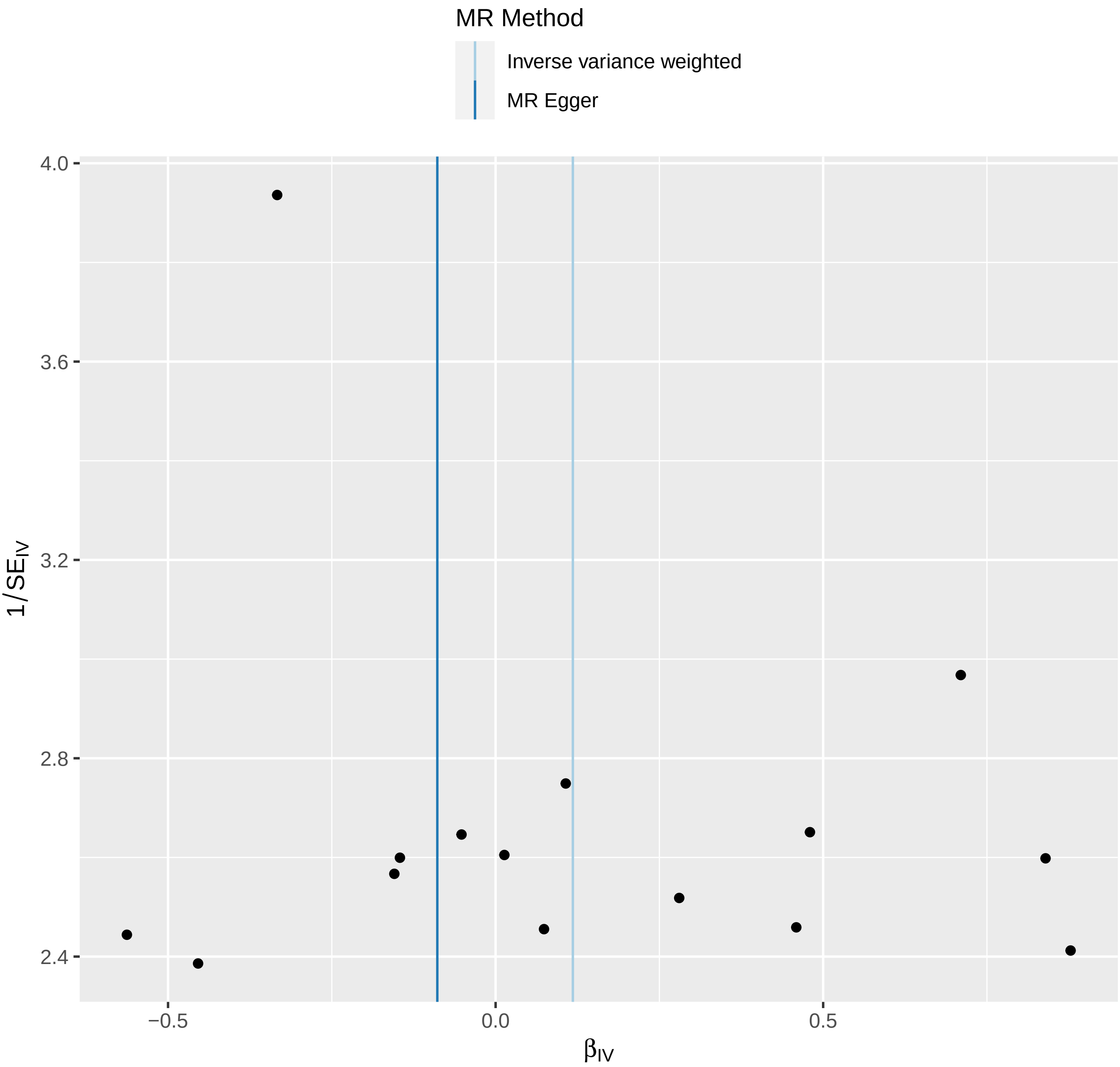

Batch 2−4273 : Gut microbiota abundance (genus Catenibacterium id.2153) on Ornithine decarboxylase antizyme 1
with a threshold of 1e−05

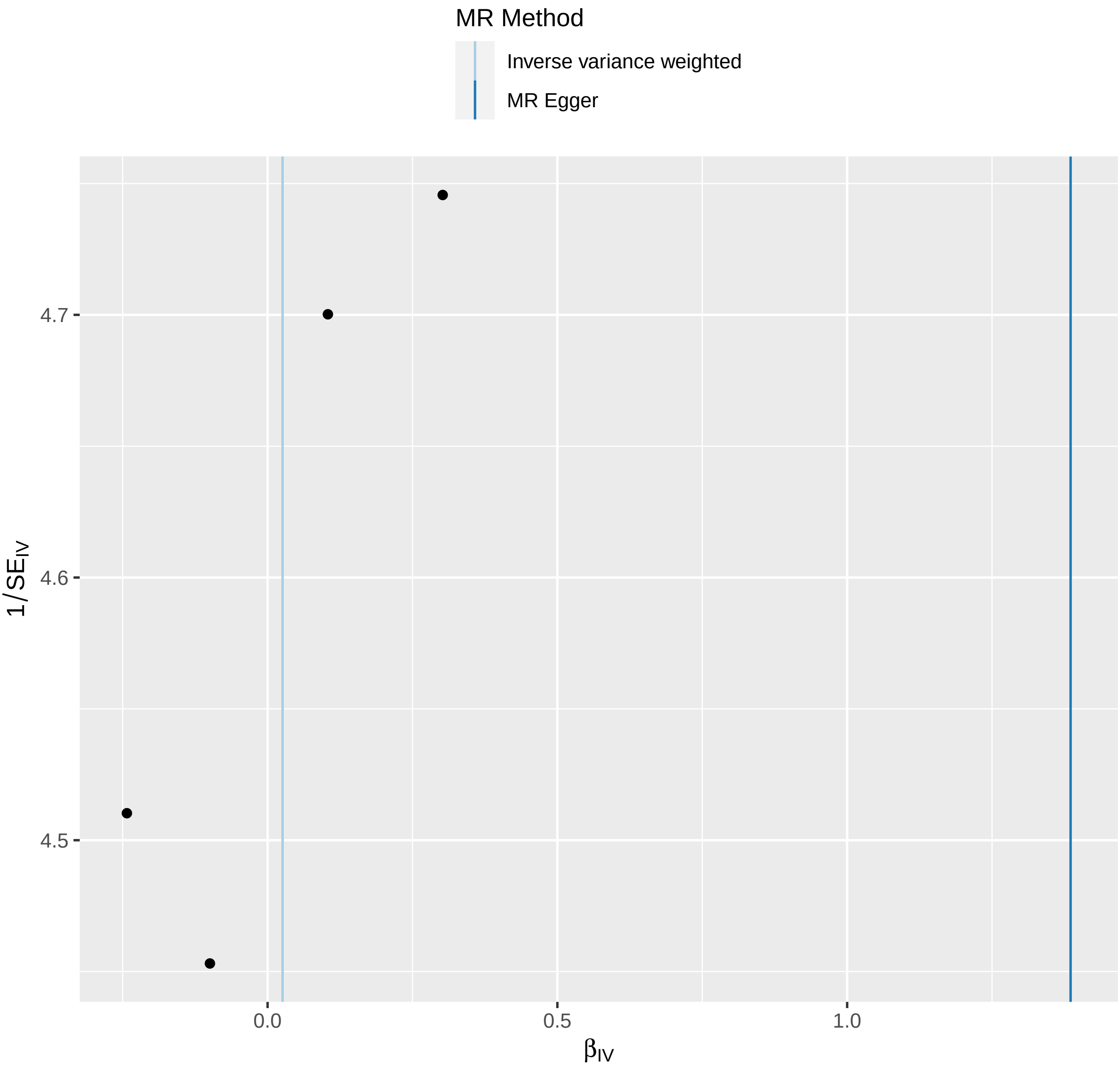

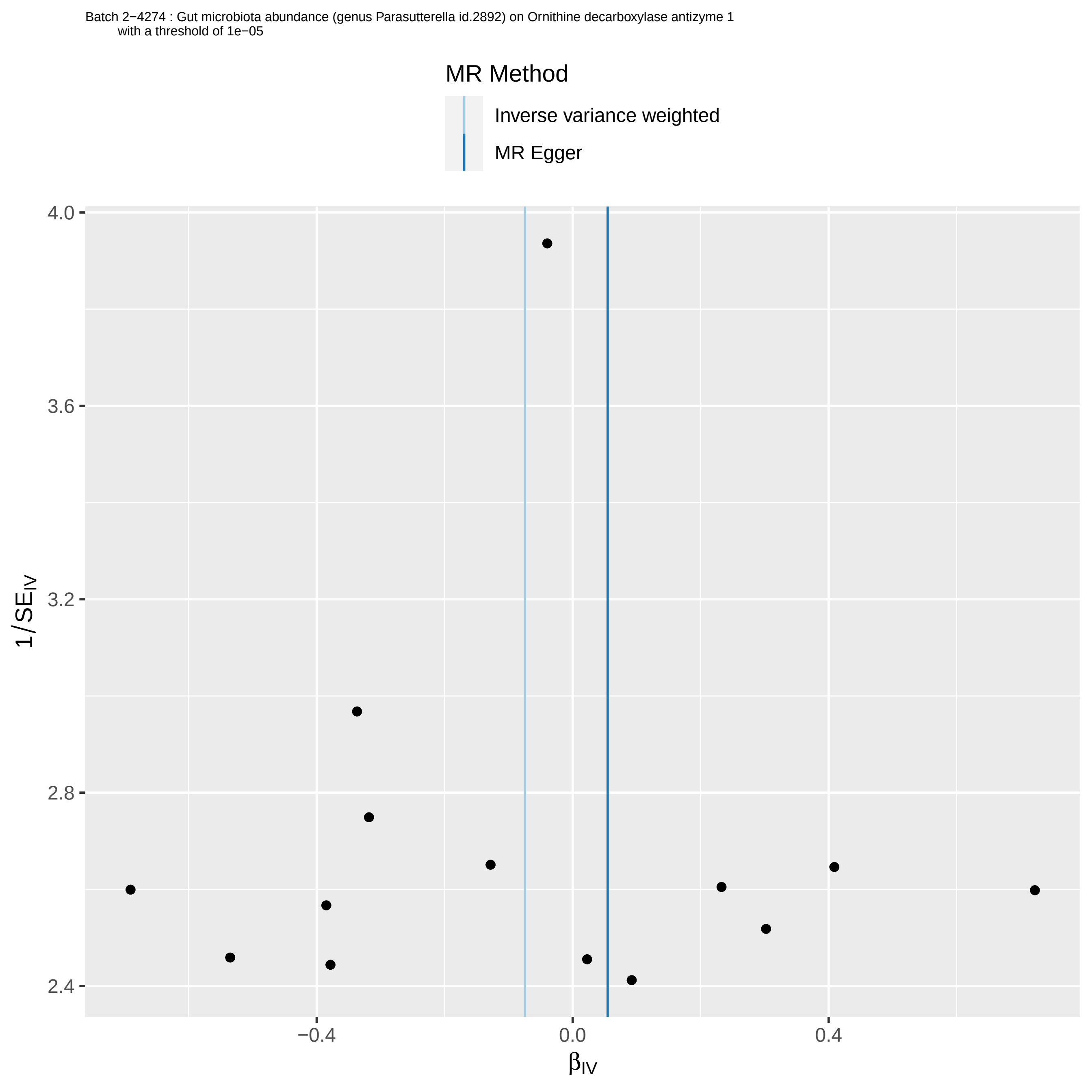

Batch 2−4274 : Gut microbiota abundance (genus Parasutterella id.2892) on Ornithine decarboxylase antizyme 1
with a threshold of 1e−05
MR Method
Inverse variance weighted
MR Egger
4.0
3.6
3.2
2.8
2.4
1/SE$_{IV}$
−0.4
0.0
0.4
β_{IV}

Batch 2−4275 : Gut microbiota abundance (genus Catenibacterium id.2153) on Odorant−binding protein 2a
with a threshold of 1e−05

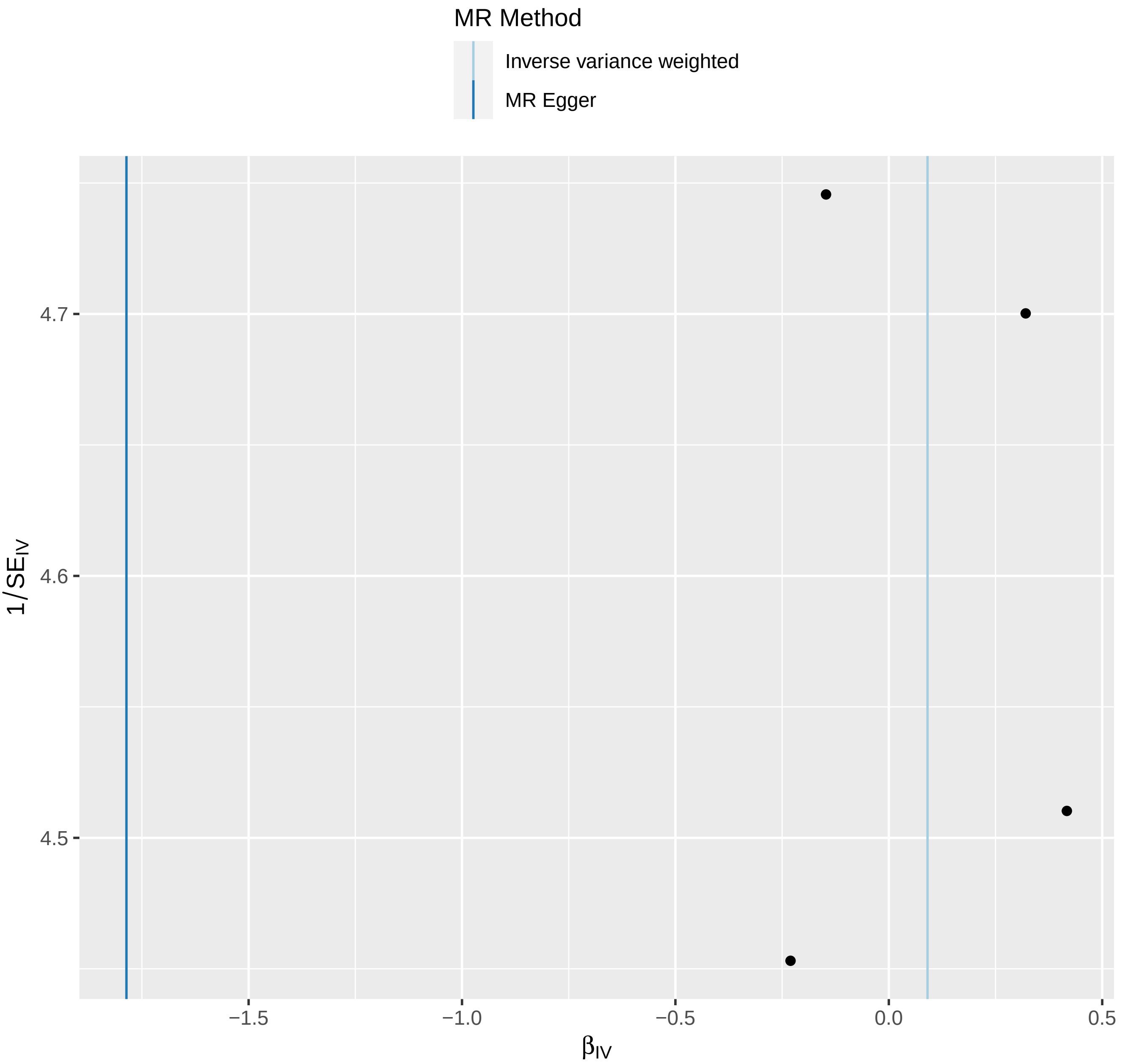

Batch 2−4276 : Gut microbiota abundance (genus Parasutterella id.2892) on Odorant−binding protein 2a
with a threshold of 1e−05

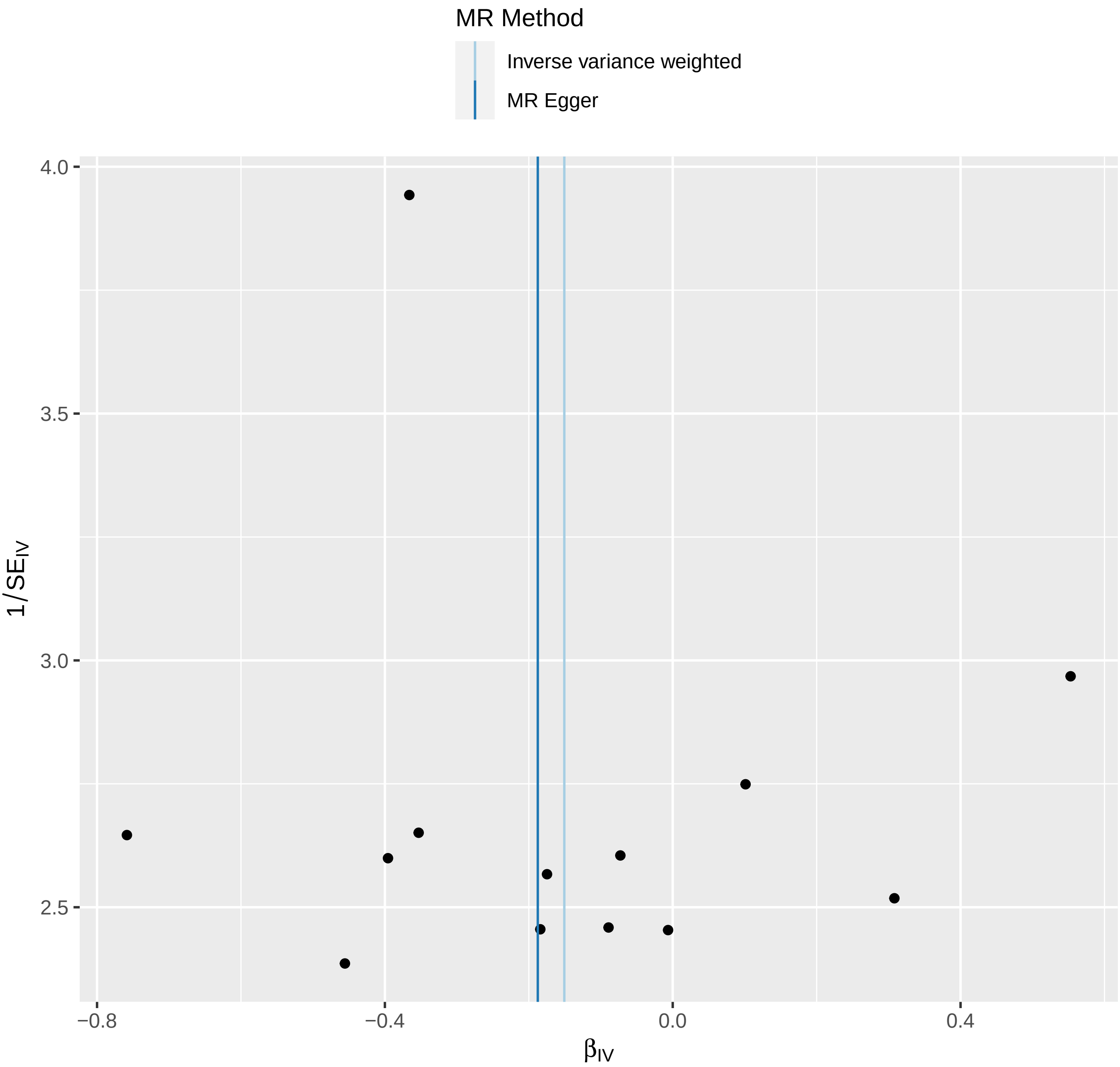

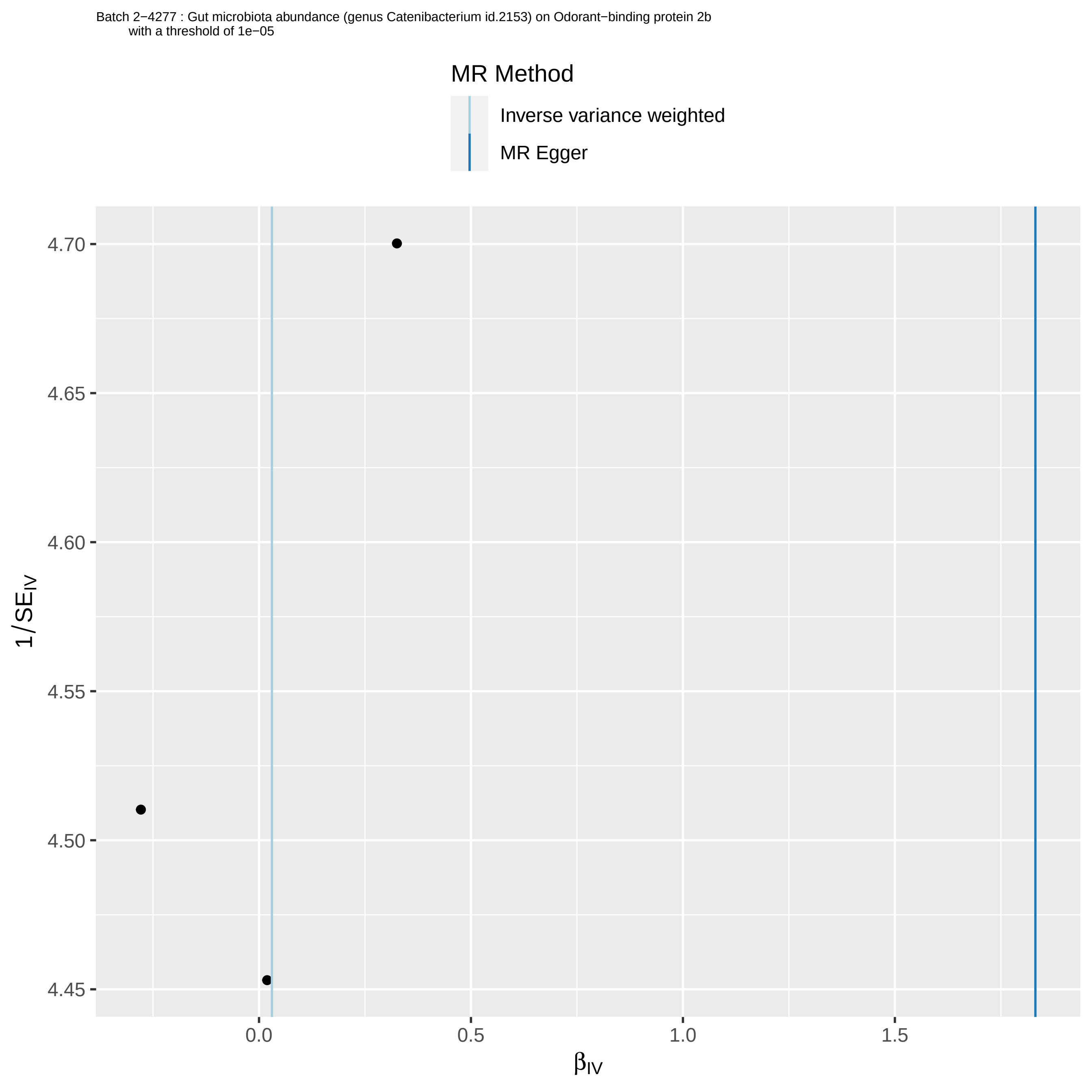

Batch 2−4277 : Gut microbiota abundance (genus Catenibacterium id.2153) on Odorant−binding protein 2b
with a threshold of 1e−05
MR Method
Inverse variance weighted
MR Egger
4.70
4.65
4.60
4.55
4.50
4.45
1/SE$_{IV}$
0.0
0.5
1.0
1.5
β$_{IV}$

Batch 2−4278 : Gut microbiota abundance (genus Parasutterella id.2892) on Odorant−binding protein 2b
with a threshold of 1e−05

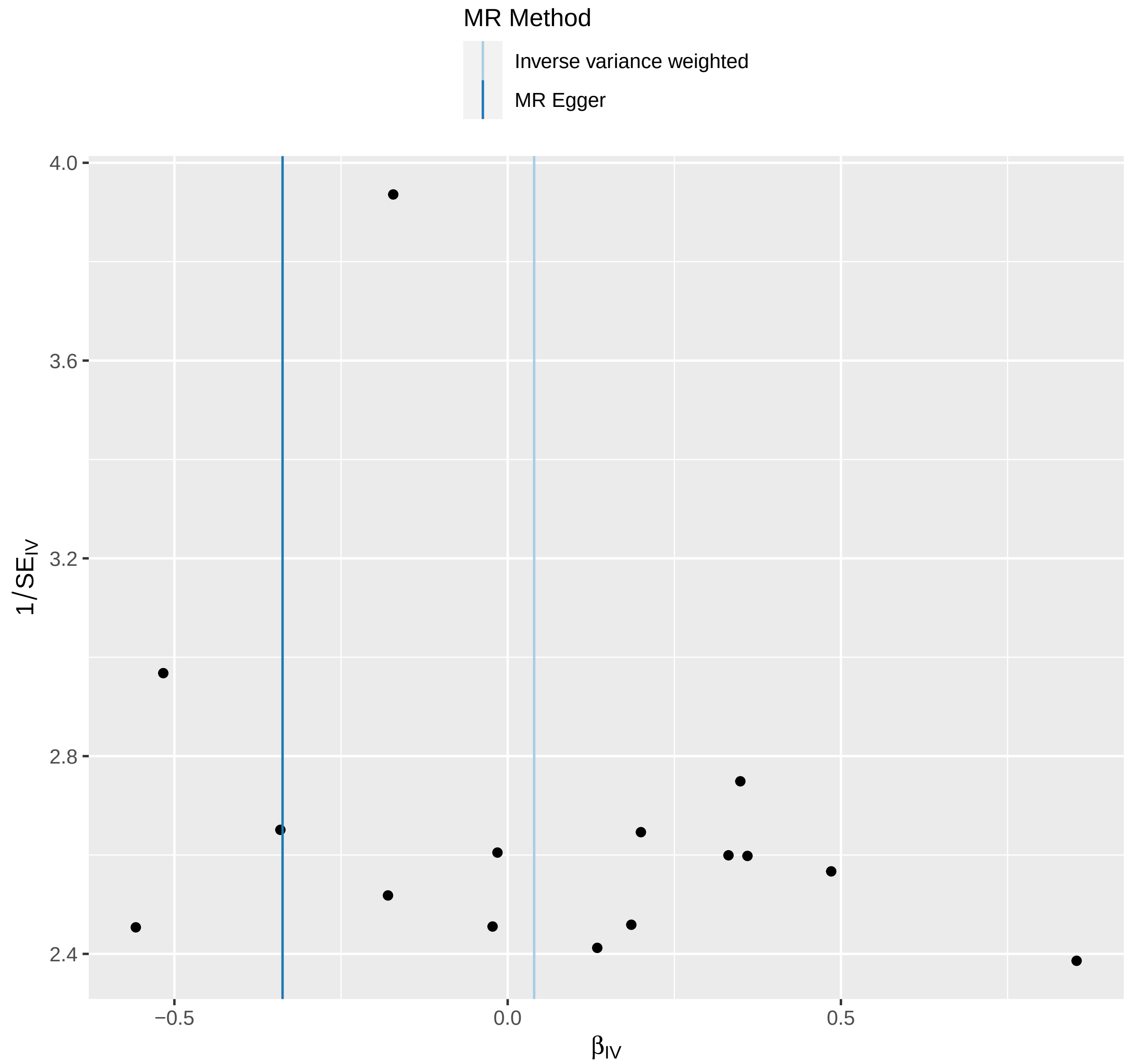

Batch 2−4279 : Gut microbiota abundance (genus Catenibacterium id.2153) on OCIA domain−containing protein 1
with a threshold of 1e−05

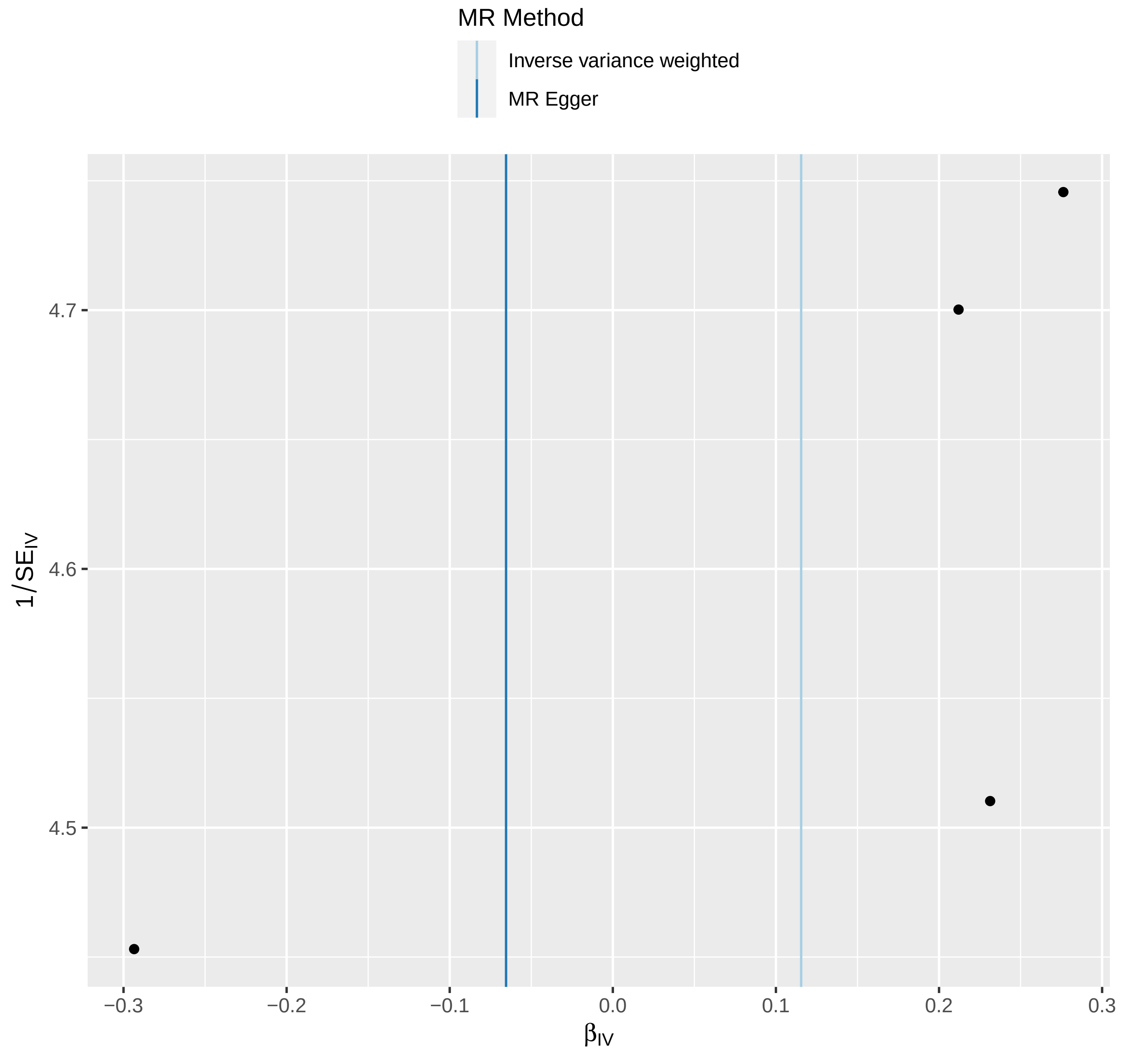

Batch 2−4280 : Gut microbiota abundance (genus Parasutterella id.2892) on OCIA domain−containing protein 1 with a threshold of 1e−05

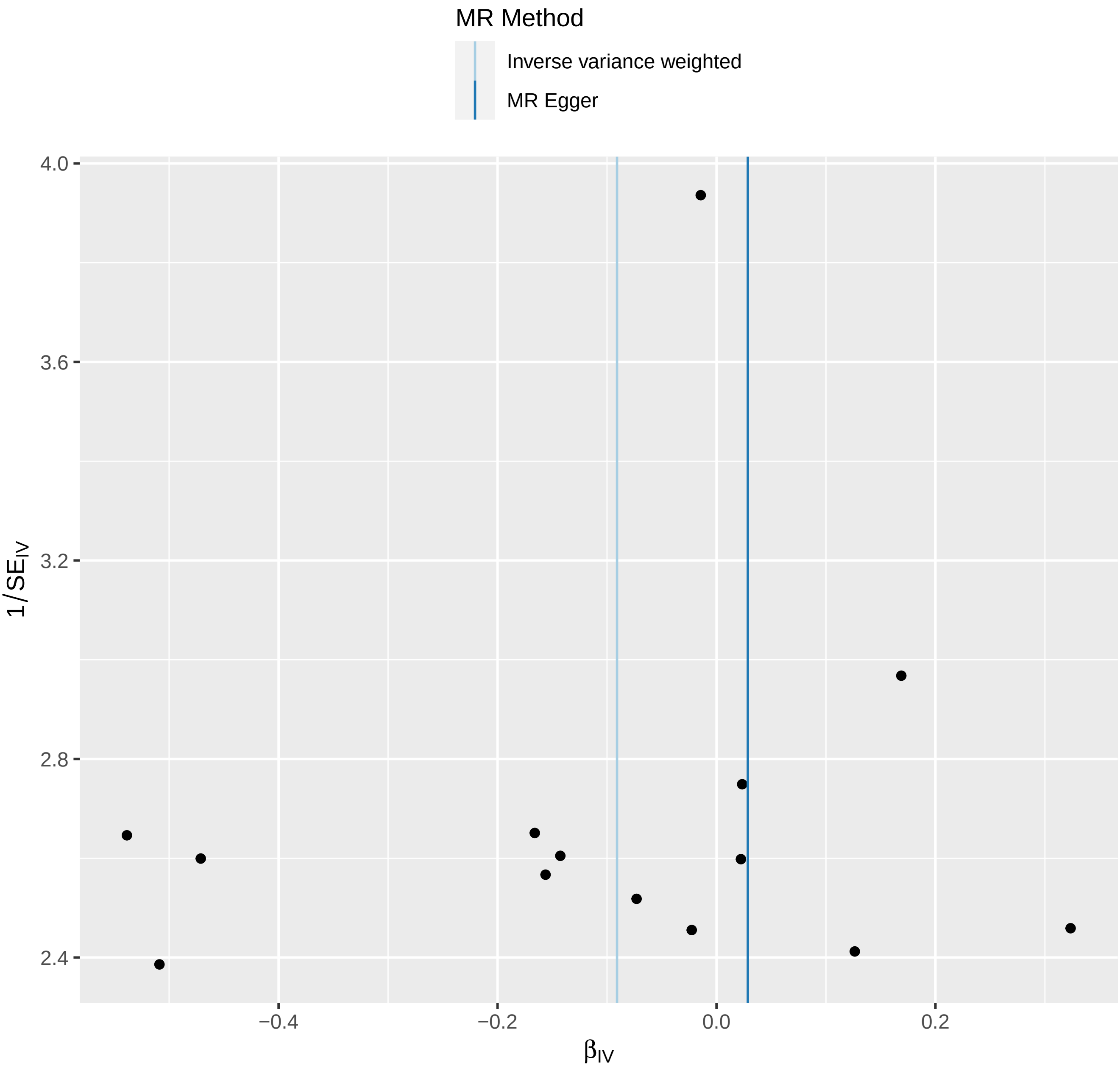

Batch 2−4281 : Gut microbiota abundance (genus Catenibacterium id.2153) on Inositol polyphosphate 5−phosphatase OCRL−1
with a threshold of 1e−05

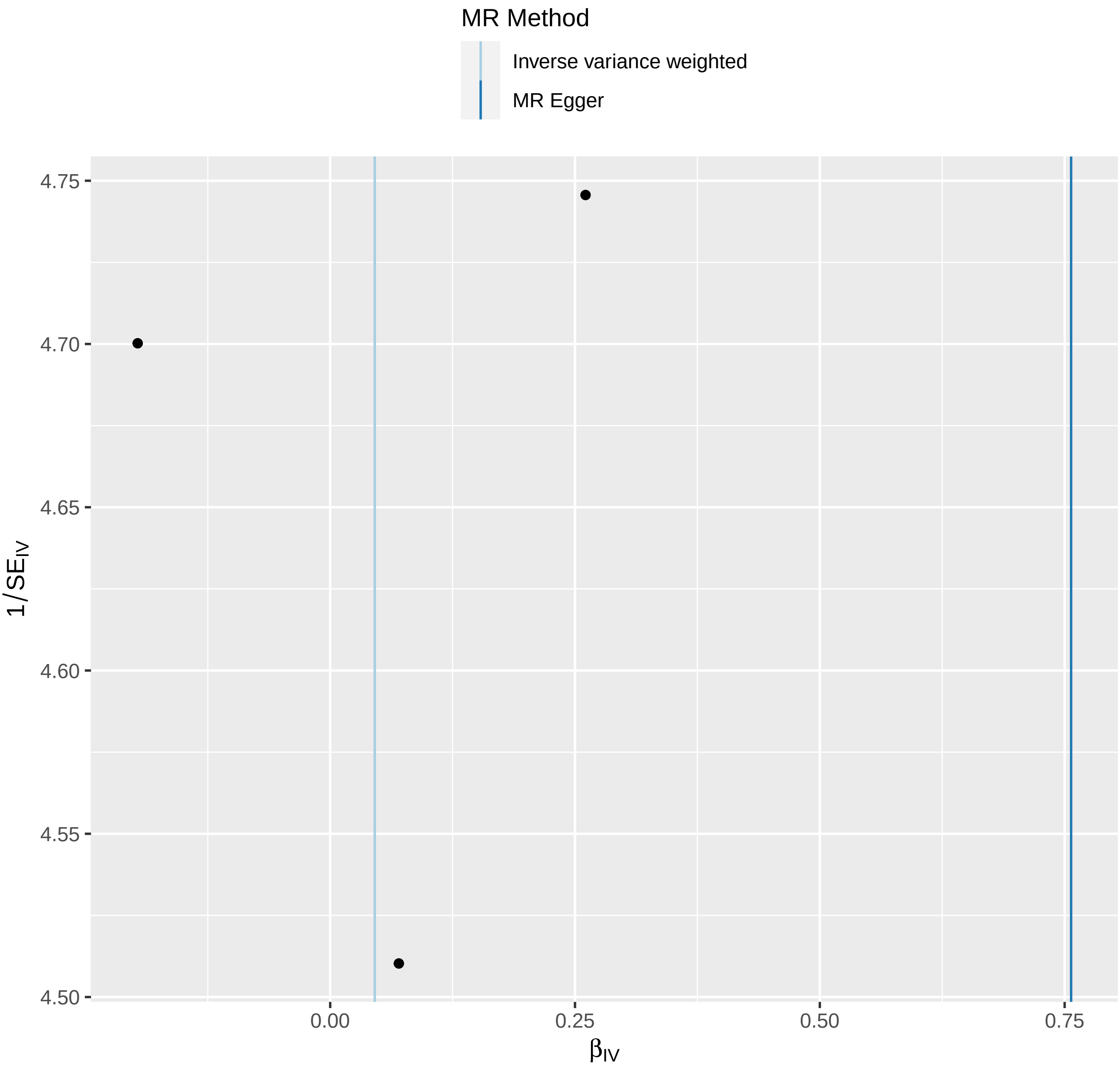

Batch 2−4282 : Gut microbiota abundance (genus Parasutterella id.2892) on Inositol polyphosphate 5−phosphatase OCRL−1
with a threshold of 1e−05

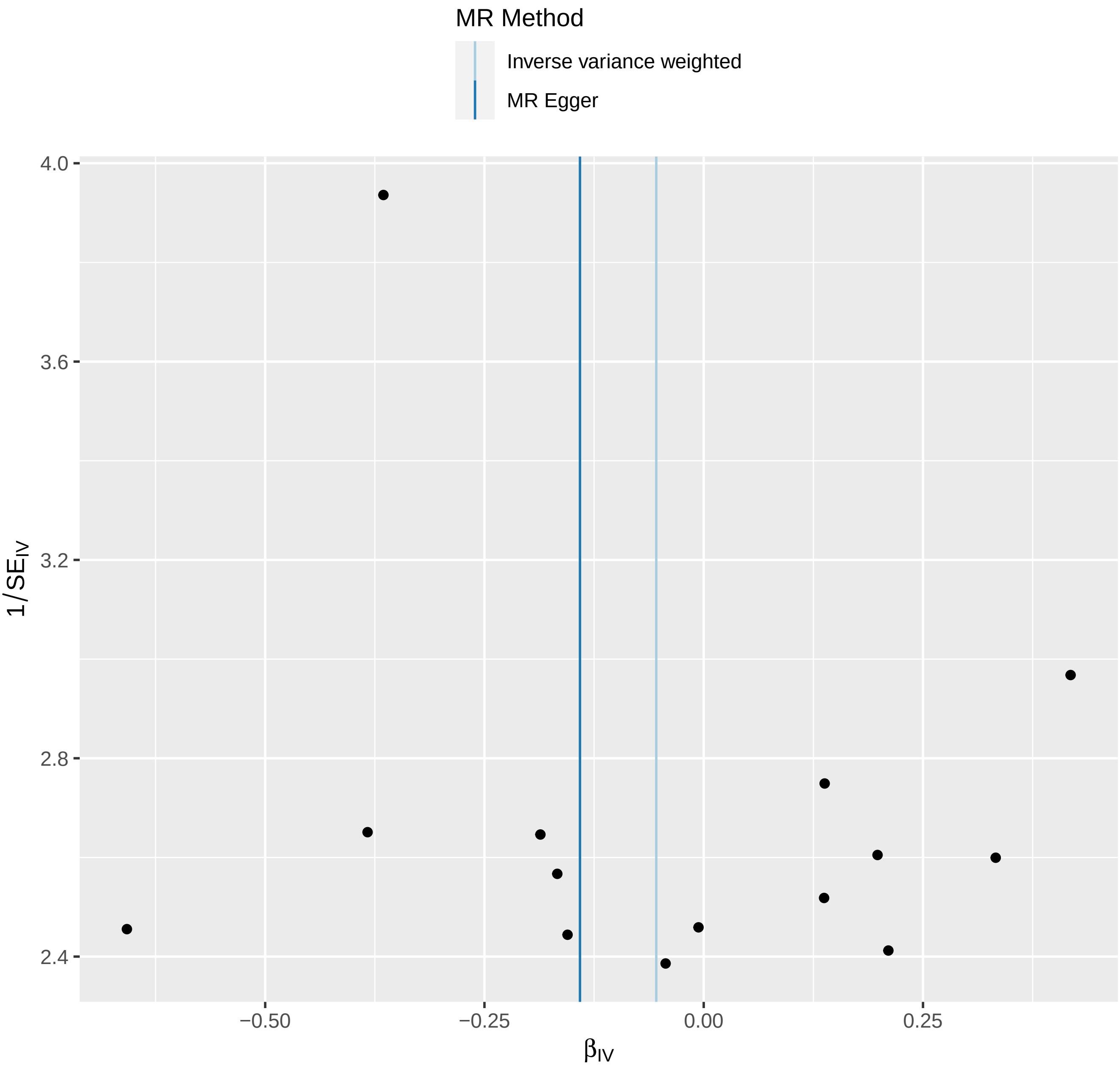

Batch 2−4283 : Gut microbiota abundance (genus Catenibacterium id.2153) on Ornithine decarboxylase
with a threshold of 1e−05

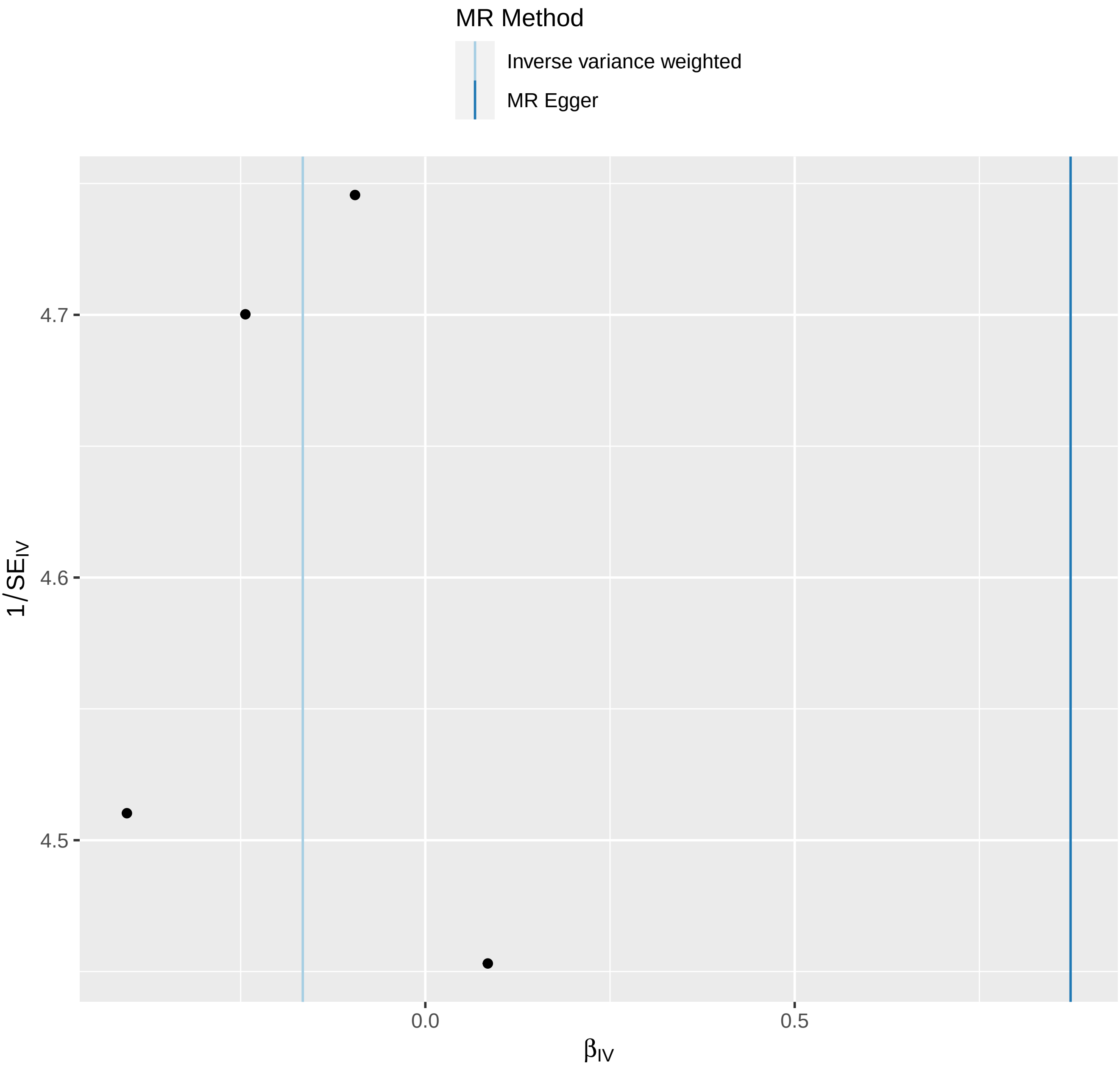

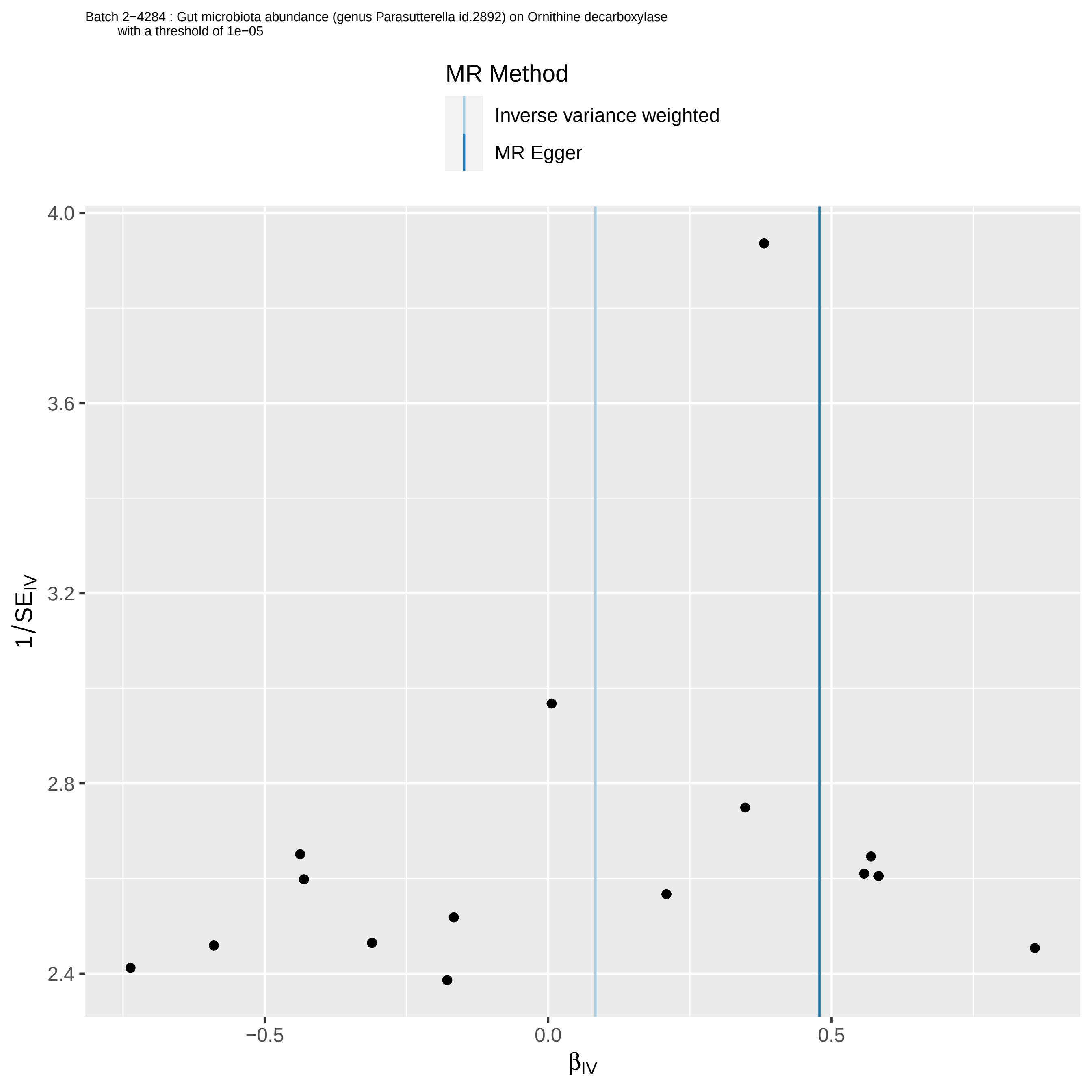

Batch 2−4284 : Gut microbiota abundance (genus Parasutterella id.2892) on Ornithine decarboxylase
with a threshold of 1e−05
MR Method
Inverse variance weighted
MR Egger
4.0
3.6
3.2
2.8
2.4
1/SE_IV
−0.5
0.0
0.5
β_IV

Batch 2−4285 : Gut microbiota abundance (genus Catenibacterium id.2153) on Obg−like ATPase 1
with a threshold of 1e−05

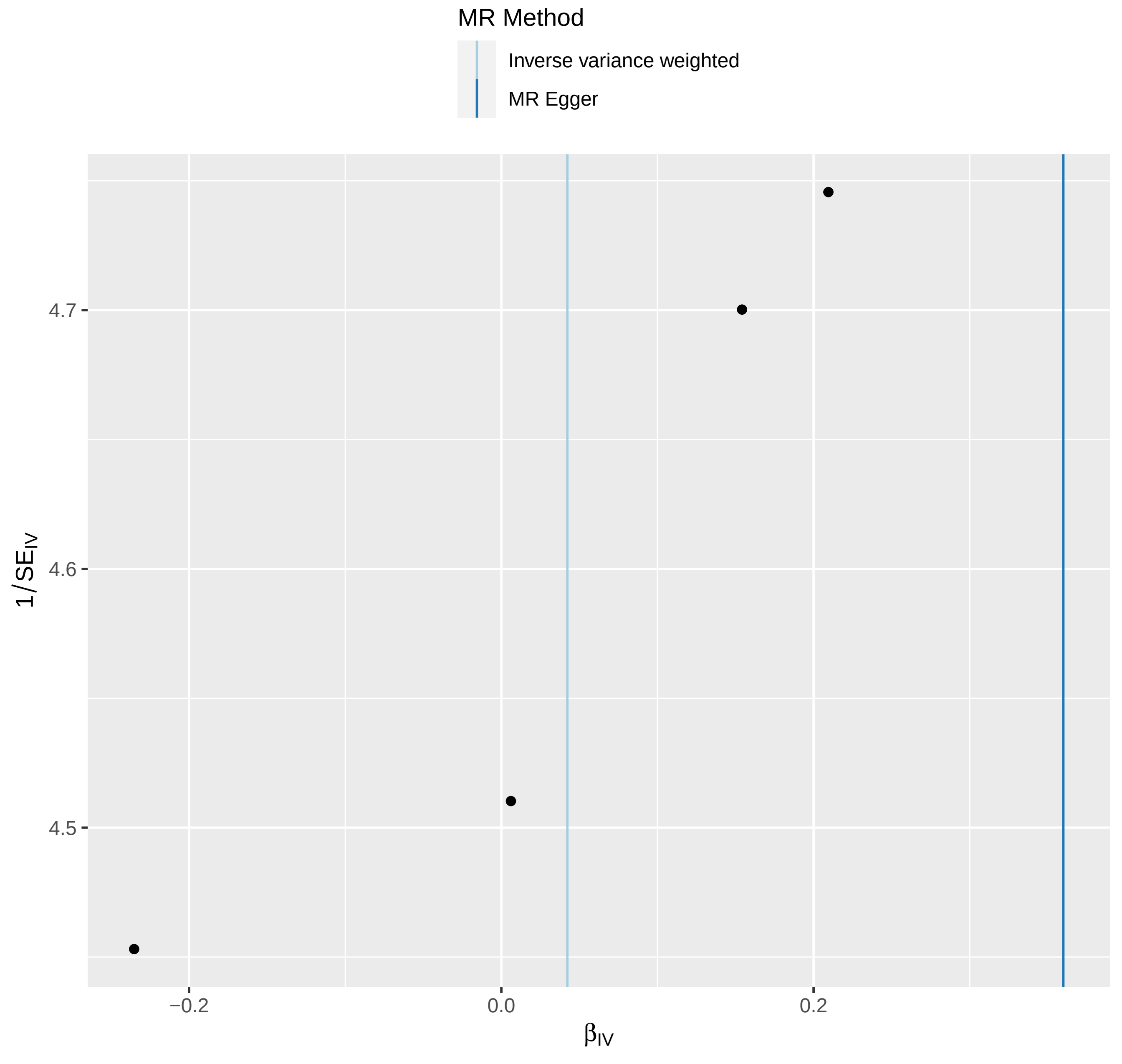

Batch 2−4286 : Gut microbiota abundance (genus Parasutterella id.2892) on Obg−like ATPase 1
with a threshold of 1e−05

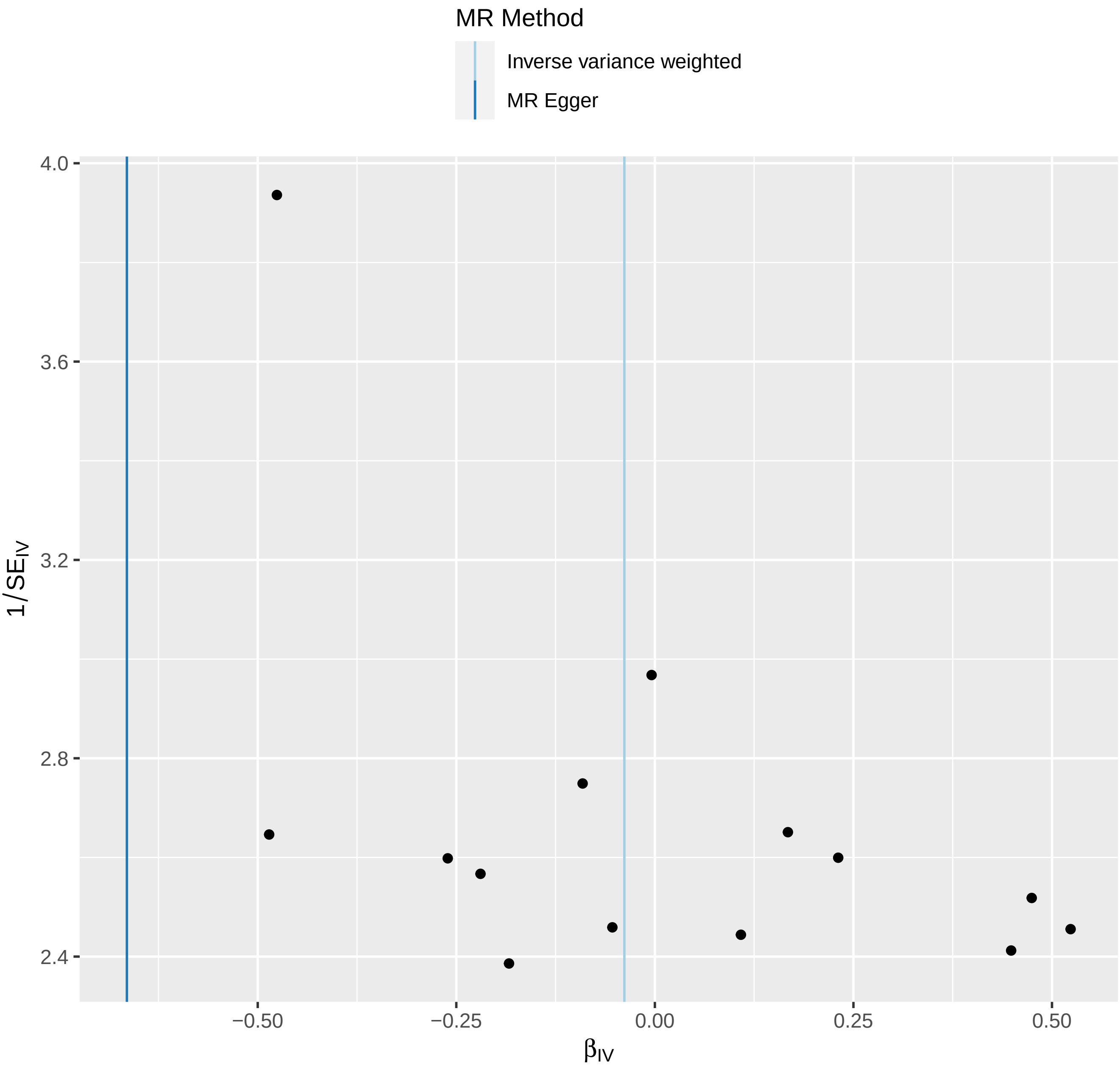

Batch 2−4287 : Gut microbiota abundance (genus Catenibacterium id.2153) on Olfactomedin−like protein 3
with a threshold of 1e−05

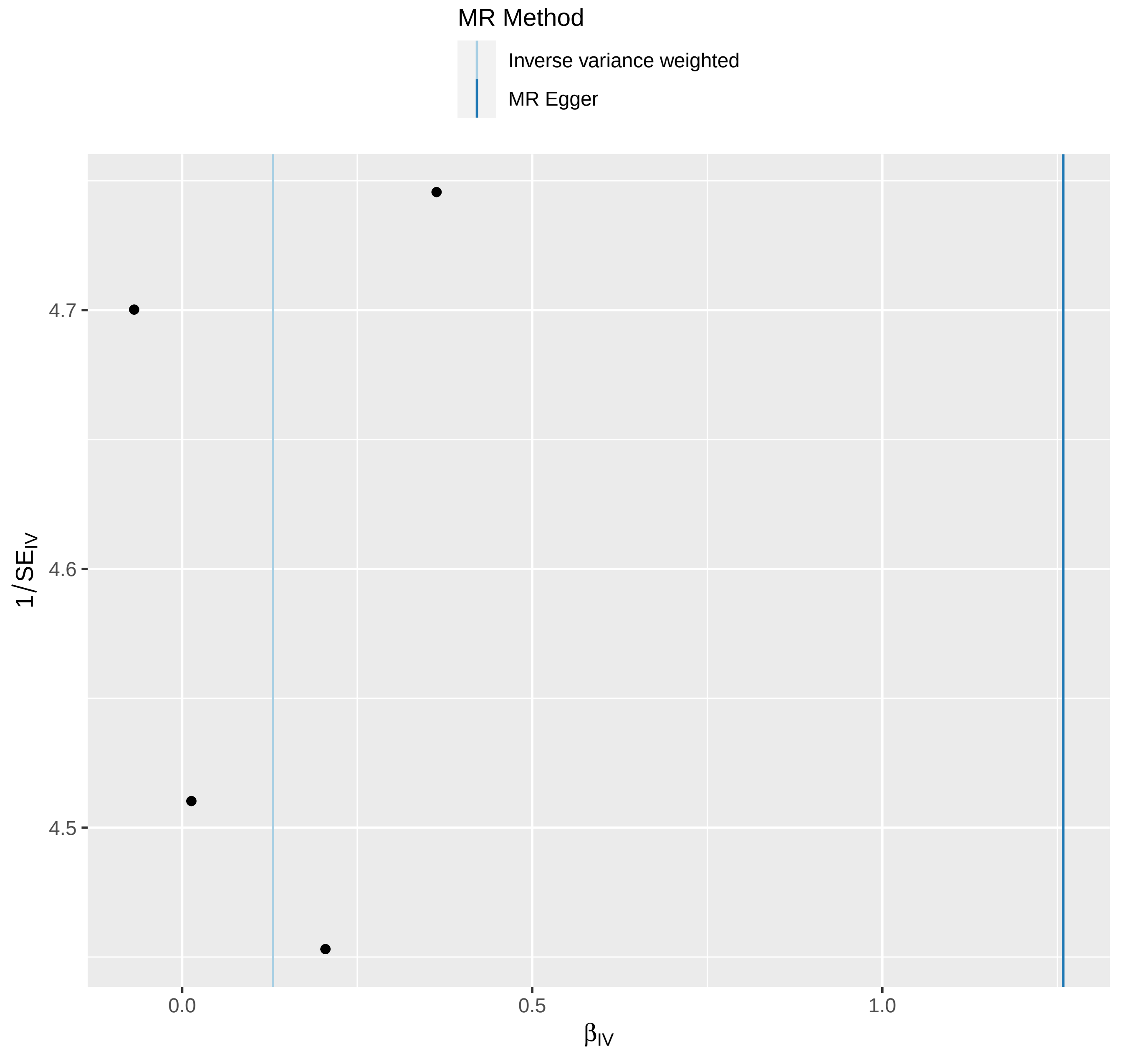

Batch 2−4288 : Gut microbiota abundance (genus Parasutterella id.2892) on Olfactomedin−like protein 3
with a threshold of 1e−05

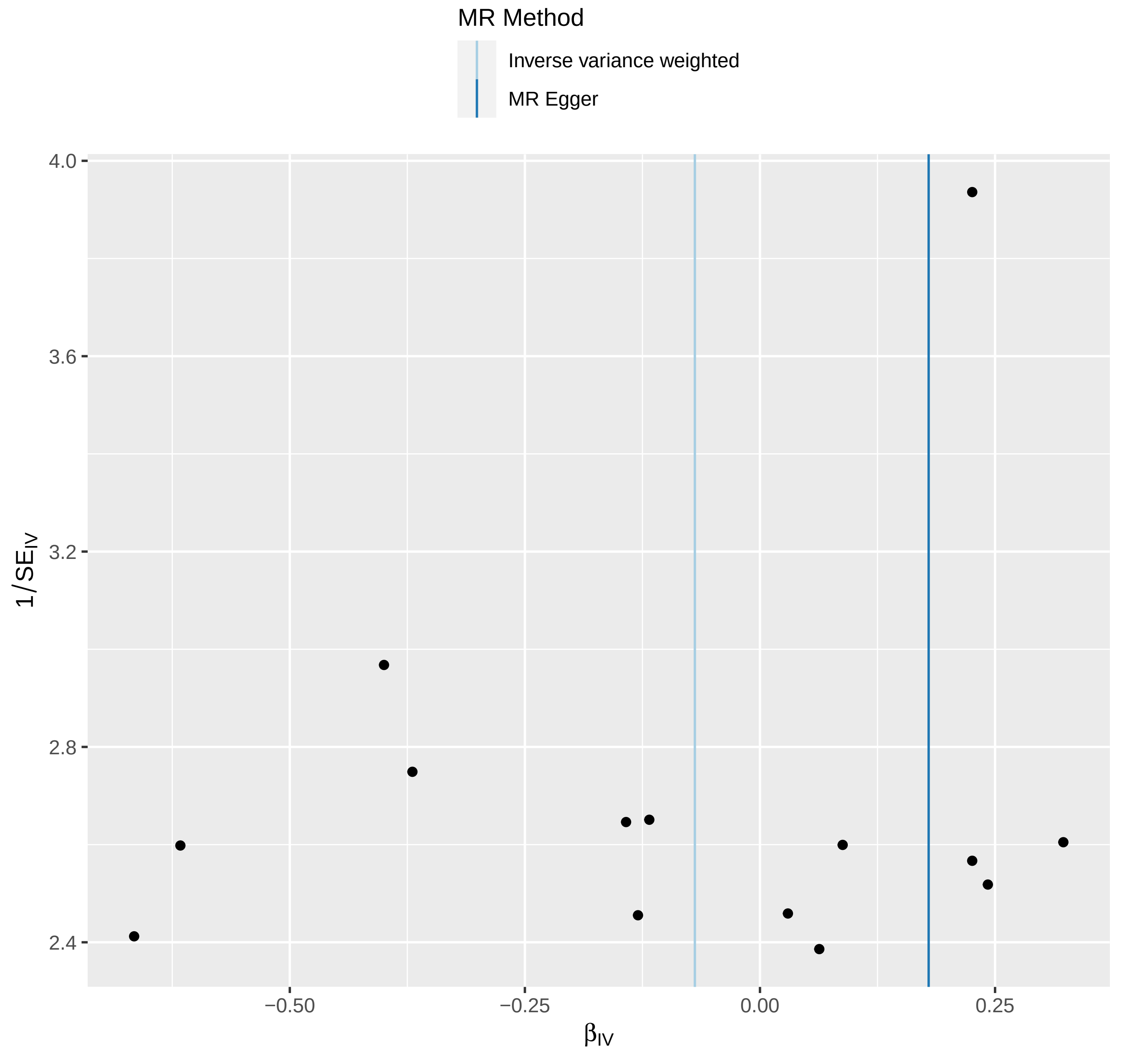

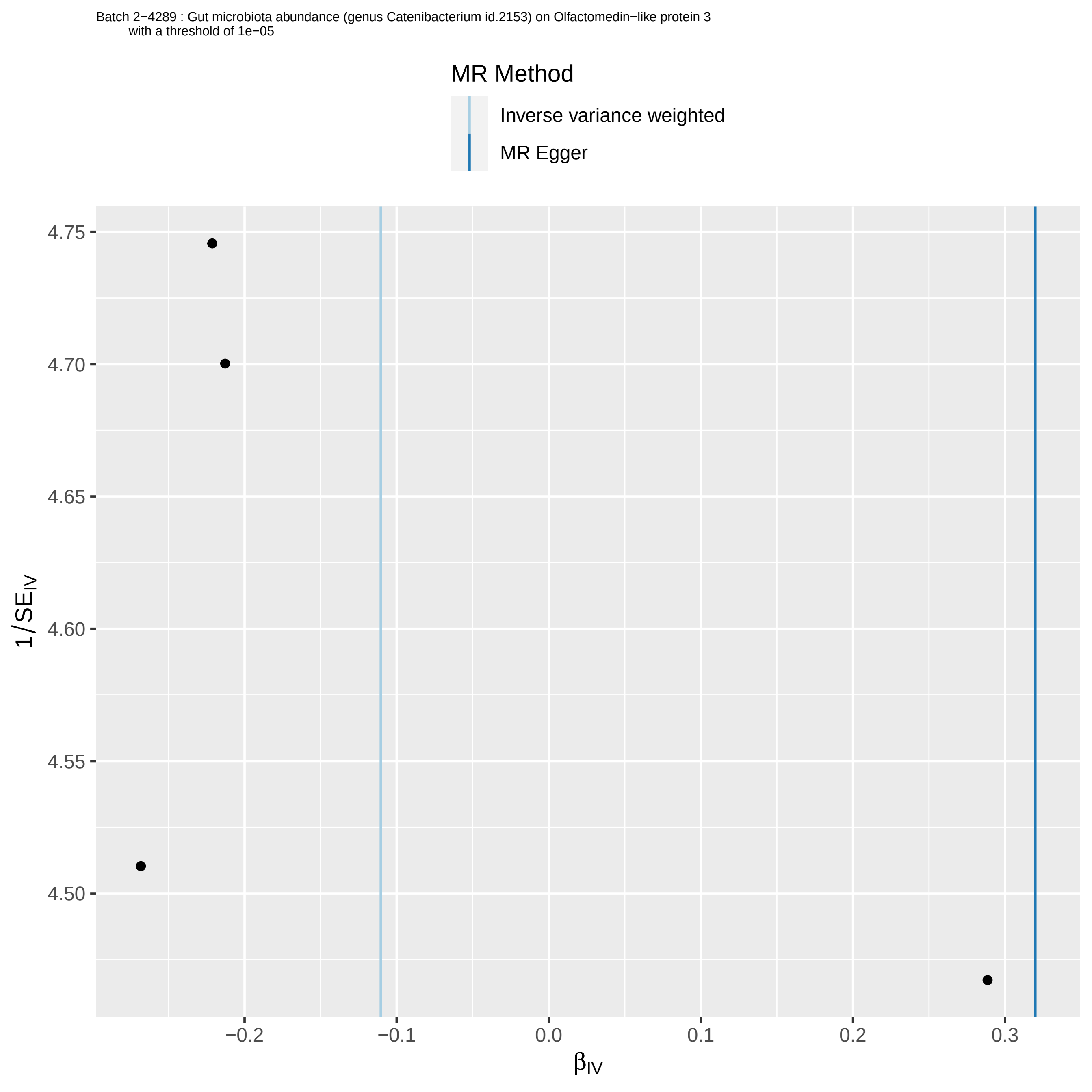

Batch 2−4289 : Gut microbiota abundance (genus Catenibacterium id.2153) on Olfactomedin−like protein 3
with a threshold of 1e−05
MR Method
Inverse variance weighted
MR Egger
4.75
4.70
4.65
4.60
4.55
4.50
1/SE_IV
−0.2
−0.1
0.0
0.1
0.2
0.3
β_IV

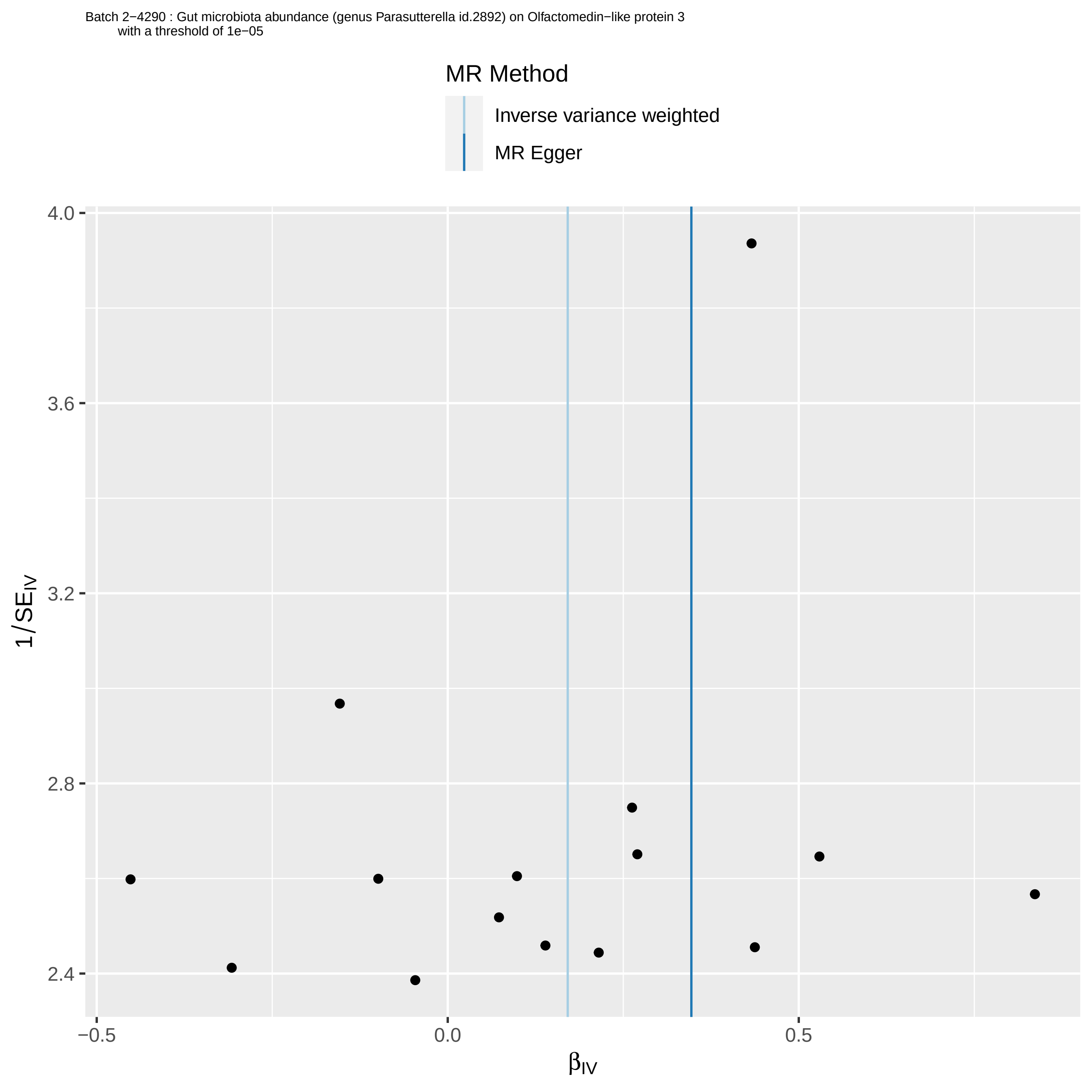

Batch 2−4290 : Gut microbiota abundance (genus Parasutterella id.2892) on Olfactomedin−like protein 3
with a threshold of 1e−05
MR Method
Inverse variance weighted
MR Egger
4.0
3.6
3.2
2.8
2.4
$1/SE_{IV}$
−0.5
0.0
0.5
β_{IV}

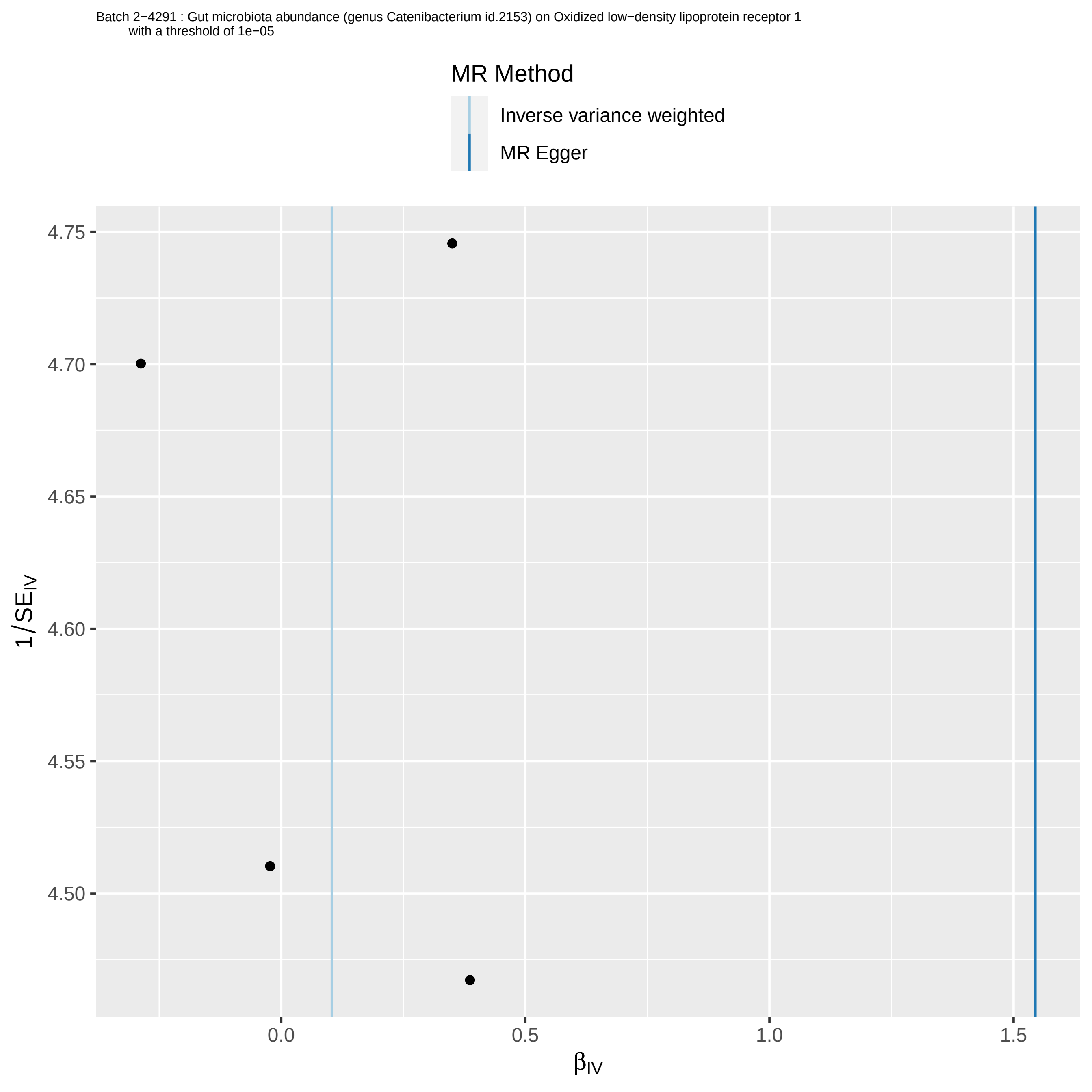

Batch 2−4291 : Gut microbiota abundance (genus Catenibacterium id.2153) on Oxidized low−density lipoprotein receptor 1
with a threshold of 1e−05
MR Method
Inverse variance weighted
MR Egger
4.75
4.70
4.65
4.60
4.55
4.50
1/SE_IV
0.0
0.5
1.0
1.5
β_IV

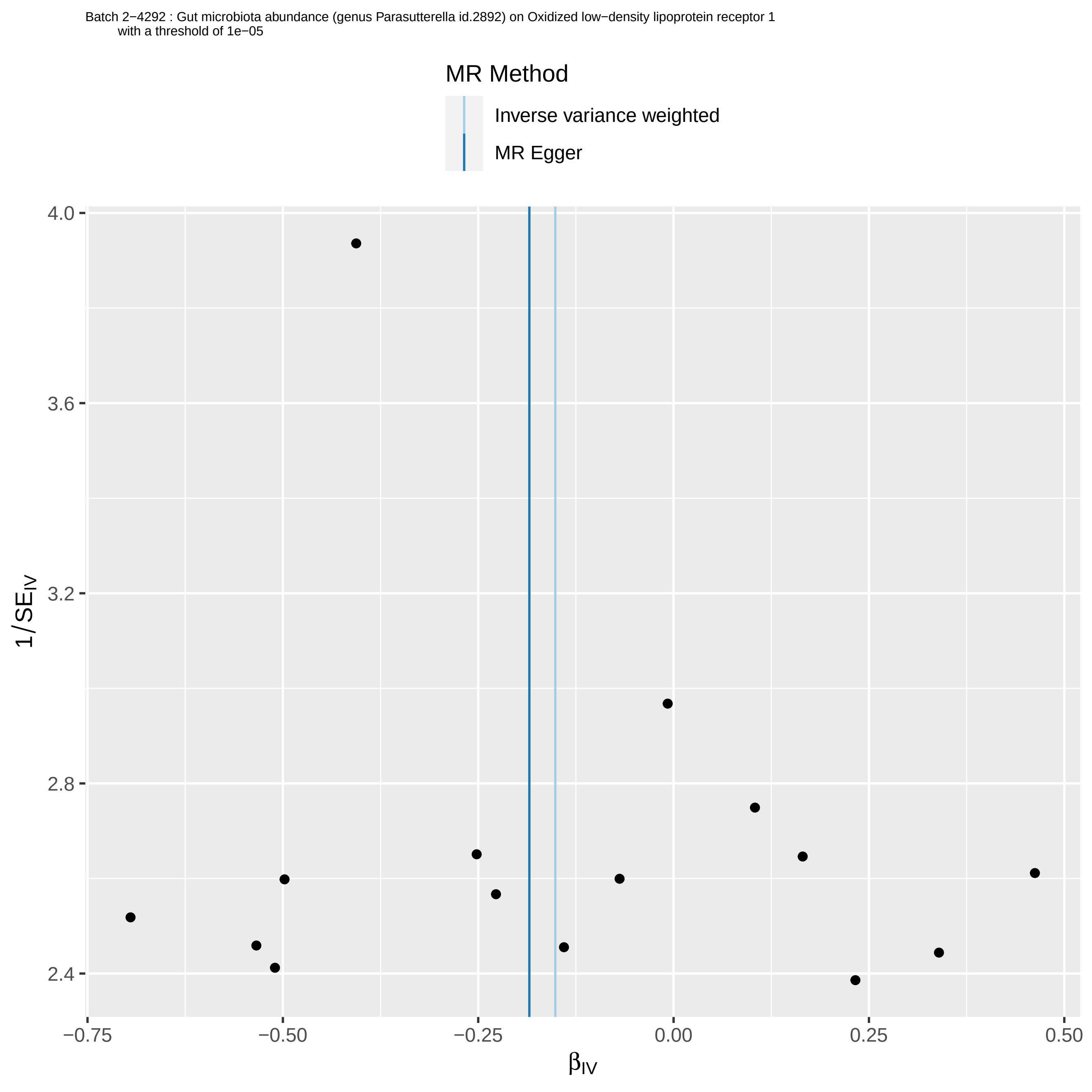

Batch 2−4292 : Gut microbiota abundance (genus Parasutterella id.2892) on Oxidized low−density lipoprotein receptor 1
with a threshold of 1e−05
MR Method
Inverse variance weighted
MR Egger
4.0
3.6
3.2
2.8
2.4
1/SE_IV
−0.75
−0.50
−0.25
0.00
0.25
0.50
β_IV

Batch 2−4293 : Gut microbiota abundance (genus Catenibacterium id.2153) on Osteomodulin
with a threshold of 1e−05

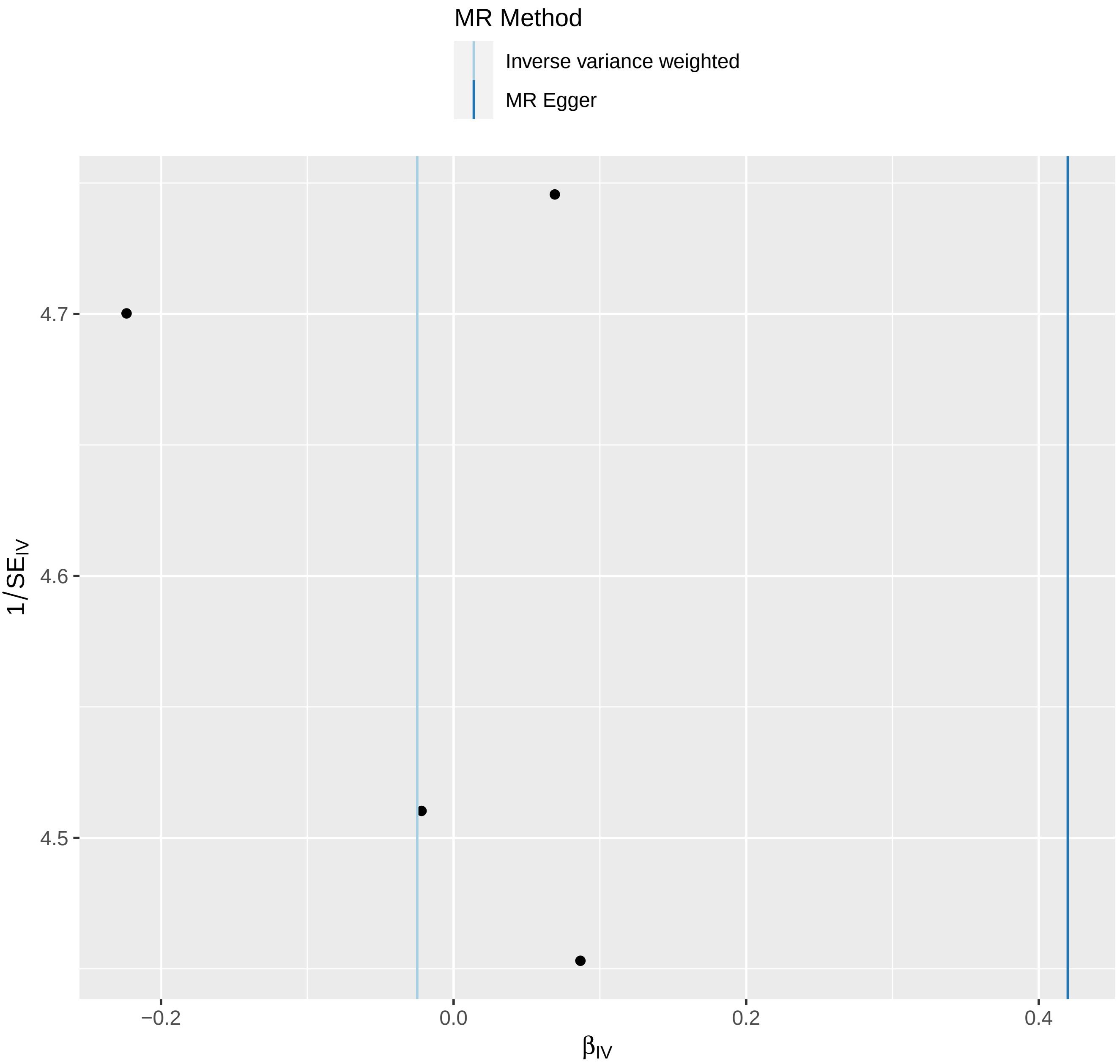

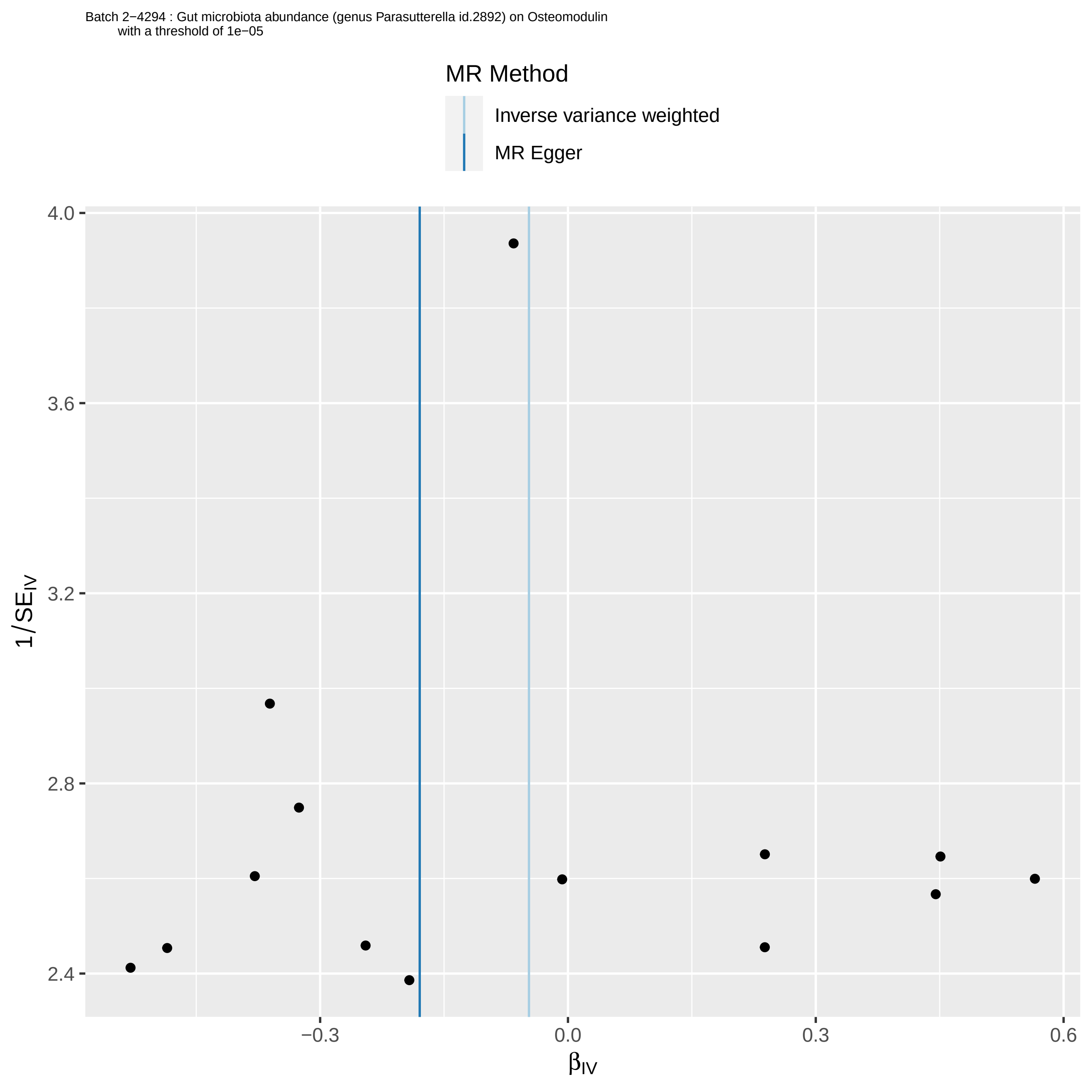
Batch 2−4294 : Gut microbiota abundance (genus Parasutterella id.2892) on Osteomodulin
with a threshold of 1e−05
MR Method
Inverse variance weighted
MR Egger
4.0
3.6
3.2
2.8
2.4
1/SE$_{IV}$
−0.3
0.0
0.3
0.6
β$_{IV}$

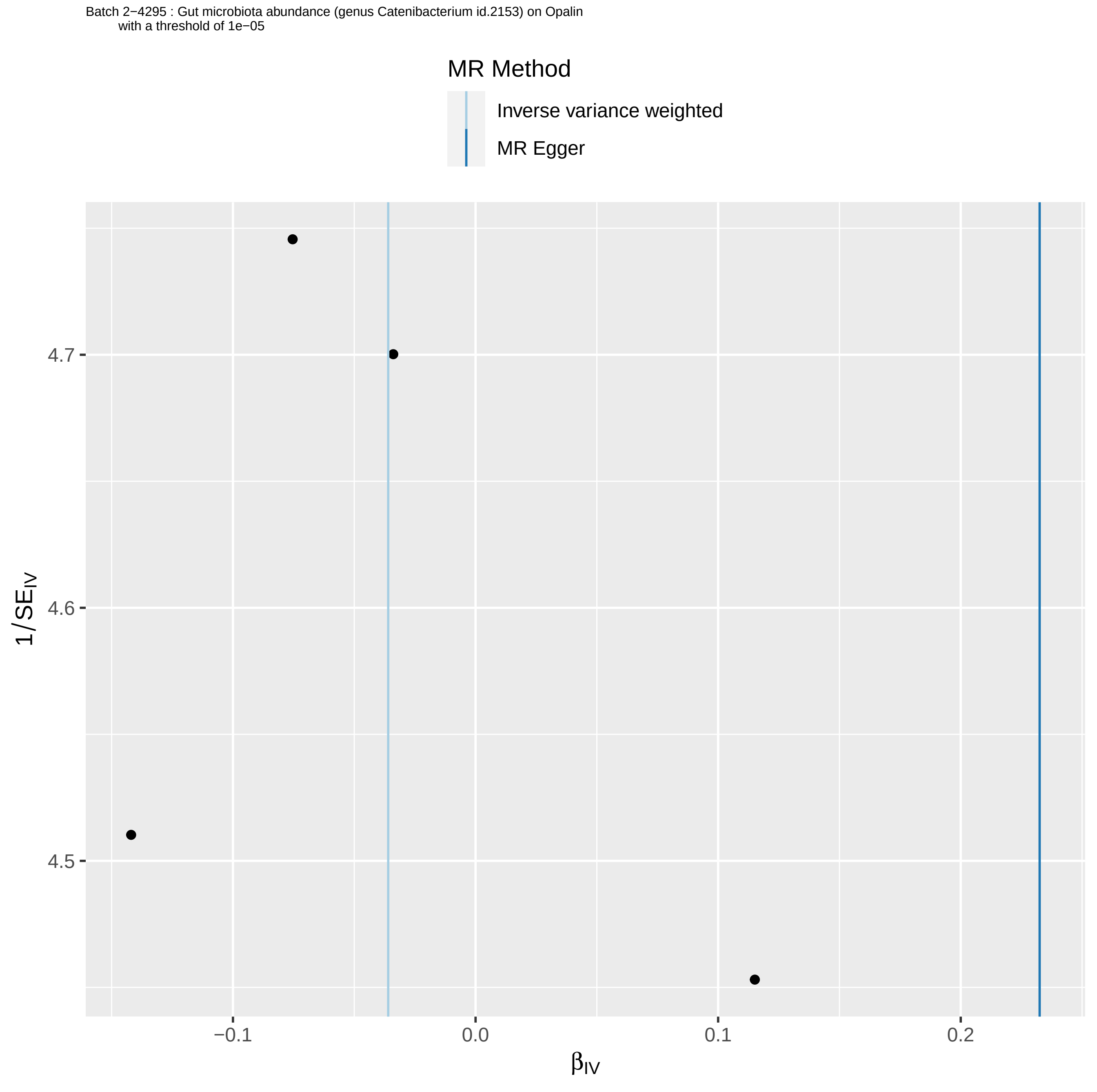

Batch 2−4295 : Gut microbiota abundance (genus Catenibacterium id.2153) on Opalin
with a threshold of 1e−05
MR Method
Inverse variance weighted
MR Egger
4.7
4.6
4.5
1/SE_IV
−0.1
0.0
0.1
0.2
β_IV

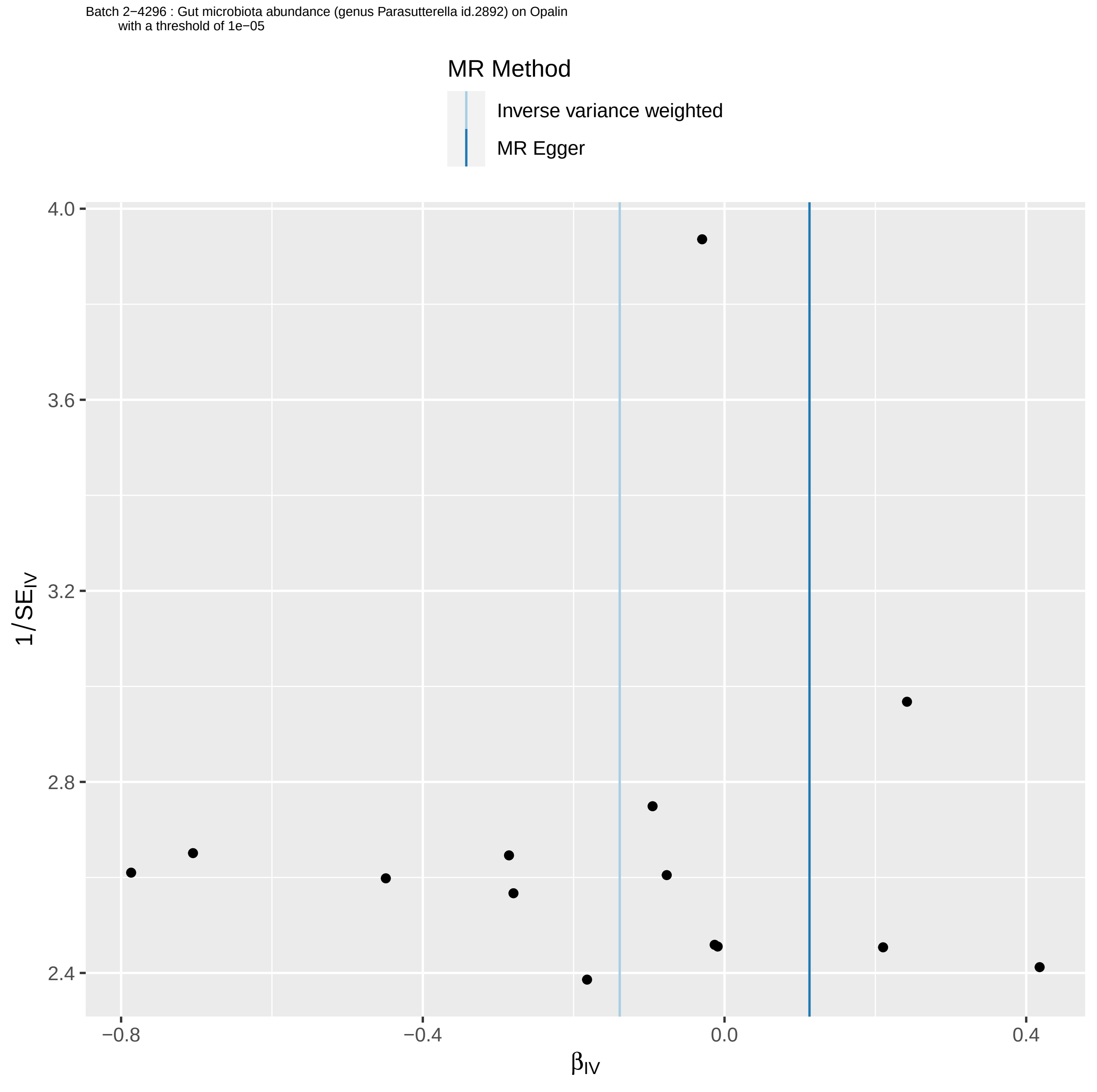
Batch 2−4296 : Gut microbiota abundance (genus Parasutterella id.2892) on Opalin
with a threshold of 1e−05
MR Method
Inverse variance weighted
MR Egger
1/SE$_{IV}$
4.0
3.6
3.2
2.8
2.4
−0.8
−0.4
0.0
0.4
β$_{IV}$

Batch 2−4297 : Gut microbiota abundance (genus Catenibacterium id.2153) on Opioid−binding protein/cell adhesion molecule with a threshold of 1e−05

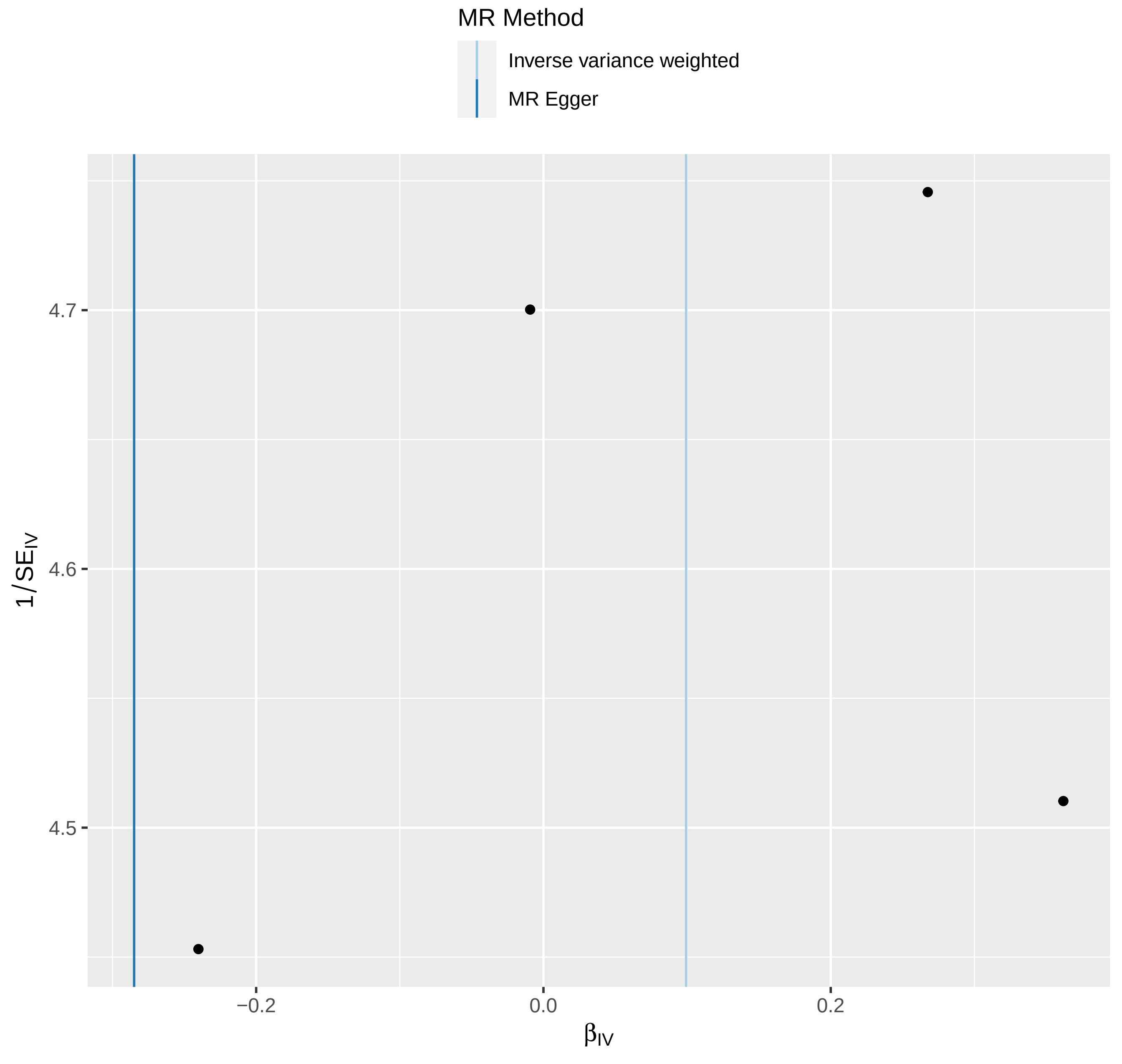

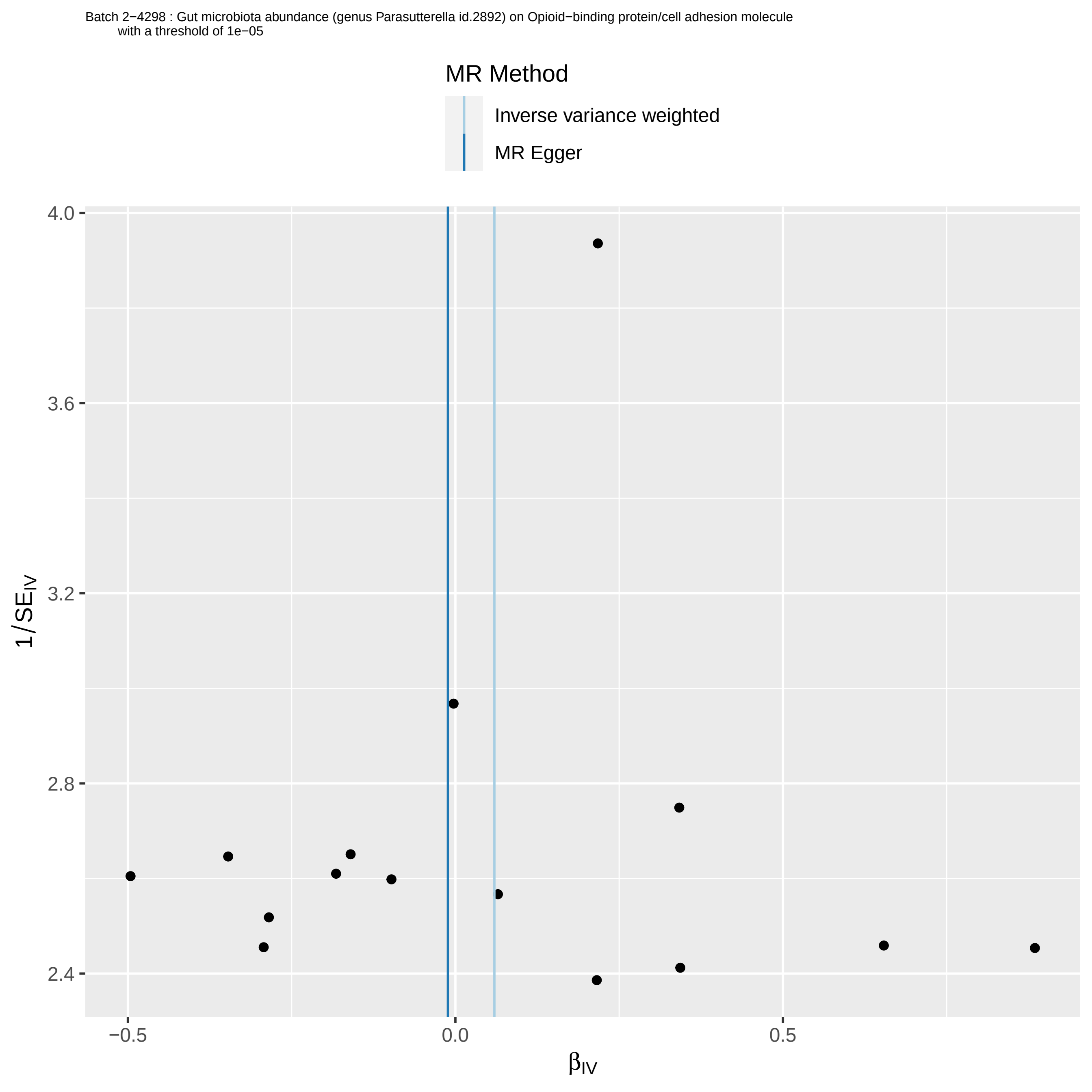
Batch 2−4298 : Gut microbiota abundance (genus Parasutterella id.2892) on Opioid−binding protein/cell adhesion molecule
with a threshold of 1e−05
MR Method
Inverse variance weighted
MR Egger
4.0
3.6
3.2
2.8
2.4
1/SE_IV
−0.5
0.0
0.5
β_IV

Batch 2−4299 : Gut microbiota abundance (genus Catenibacterium id.2153) on Oligophrenin−1
with a threshold of 1e−05

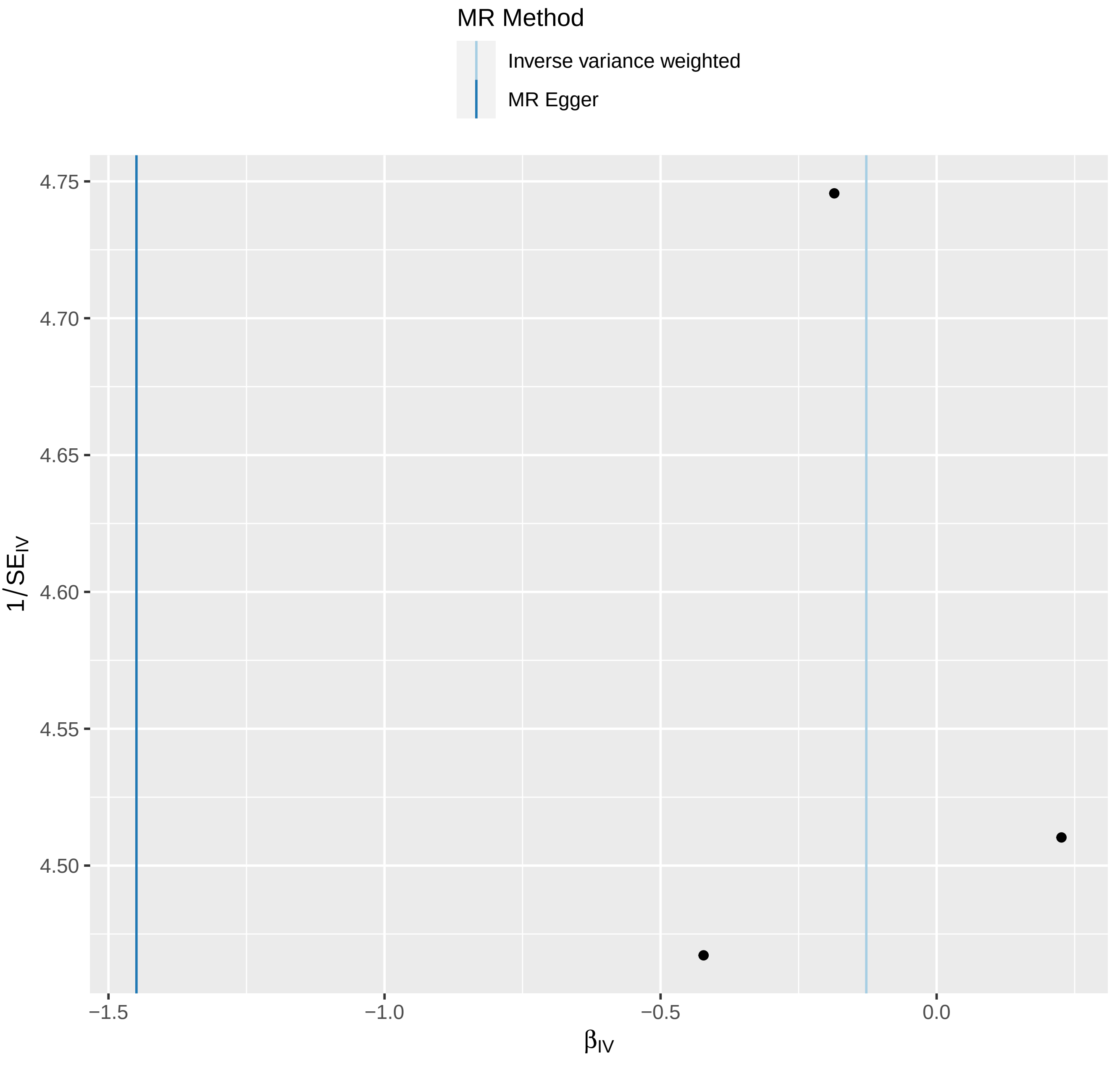

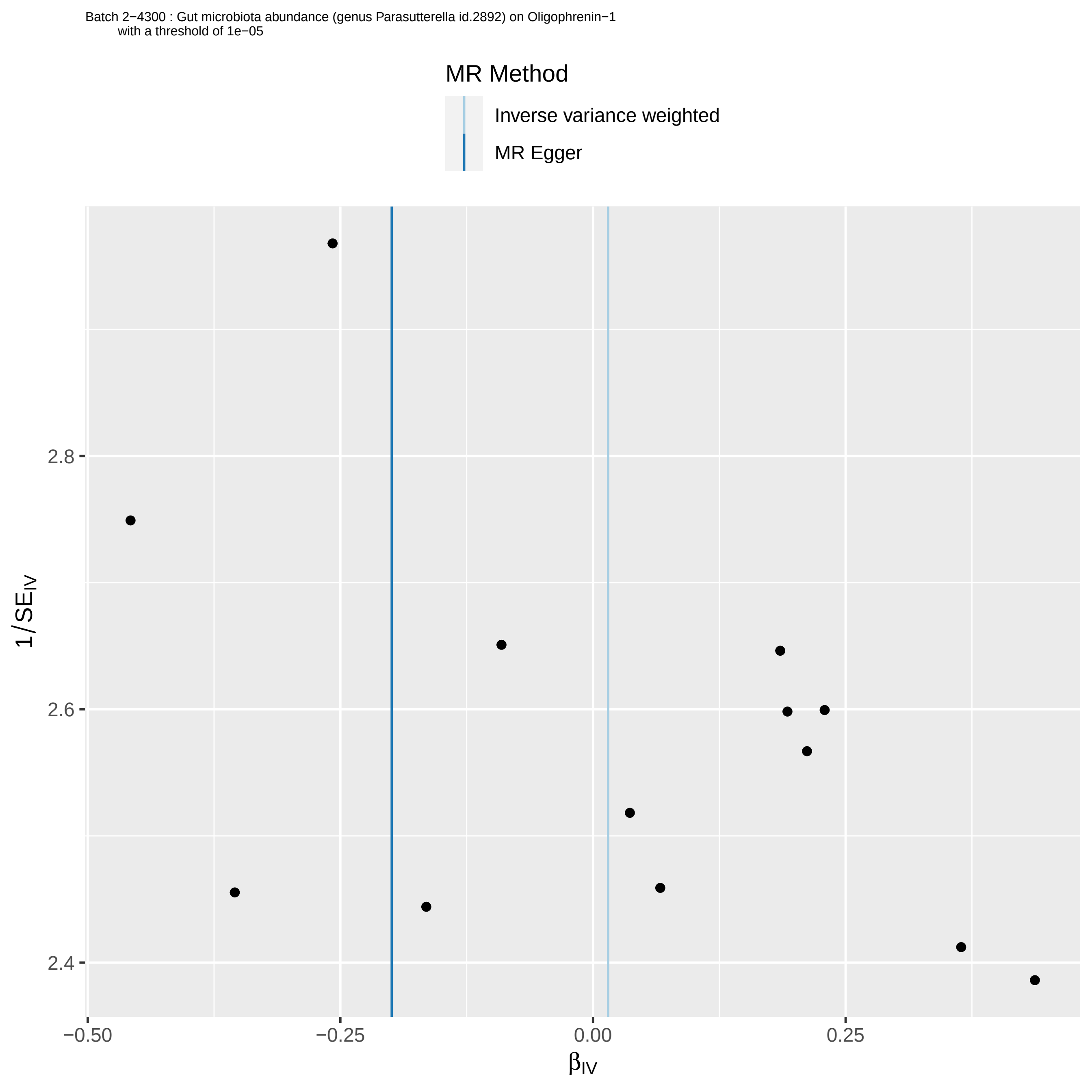
Batch 2−4300 : Gut microbiota abundance (genus Parasutterella id.2892) on Oligophrenin−1
with a threshold of 1e−05
MR Method
Inverse variance weighted
MR Egger
2.8
2.6
2.4
1/SE$_{IV}$
−0.50
−0.25
0.00
0.25
β$_{IV}$

Batch 2−4301 : Gut microbiota abundance (genus Catenibacterium id.2153) on Origin recognition complex subunit 6
with a threshold of 1e−05

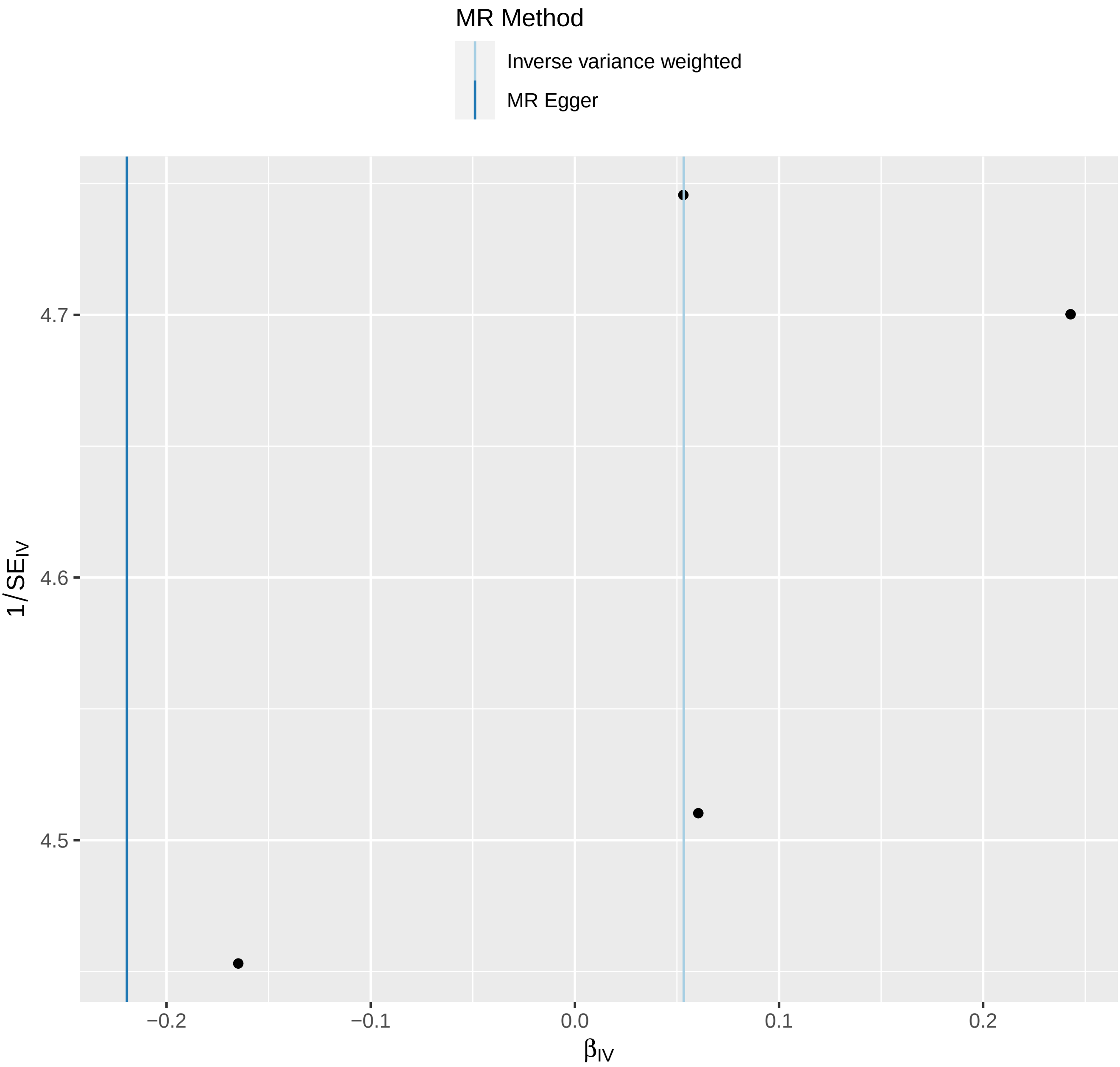

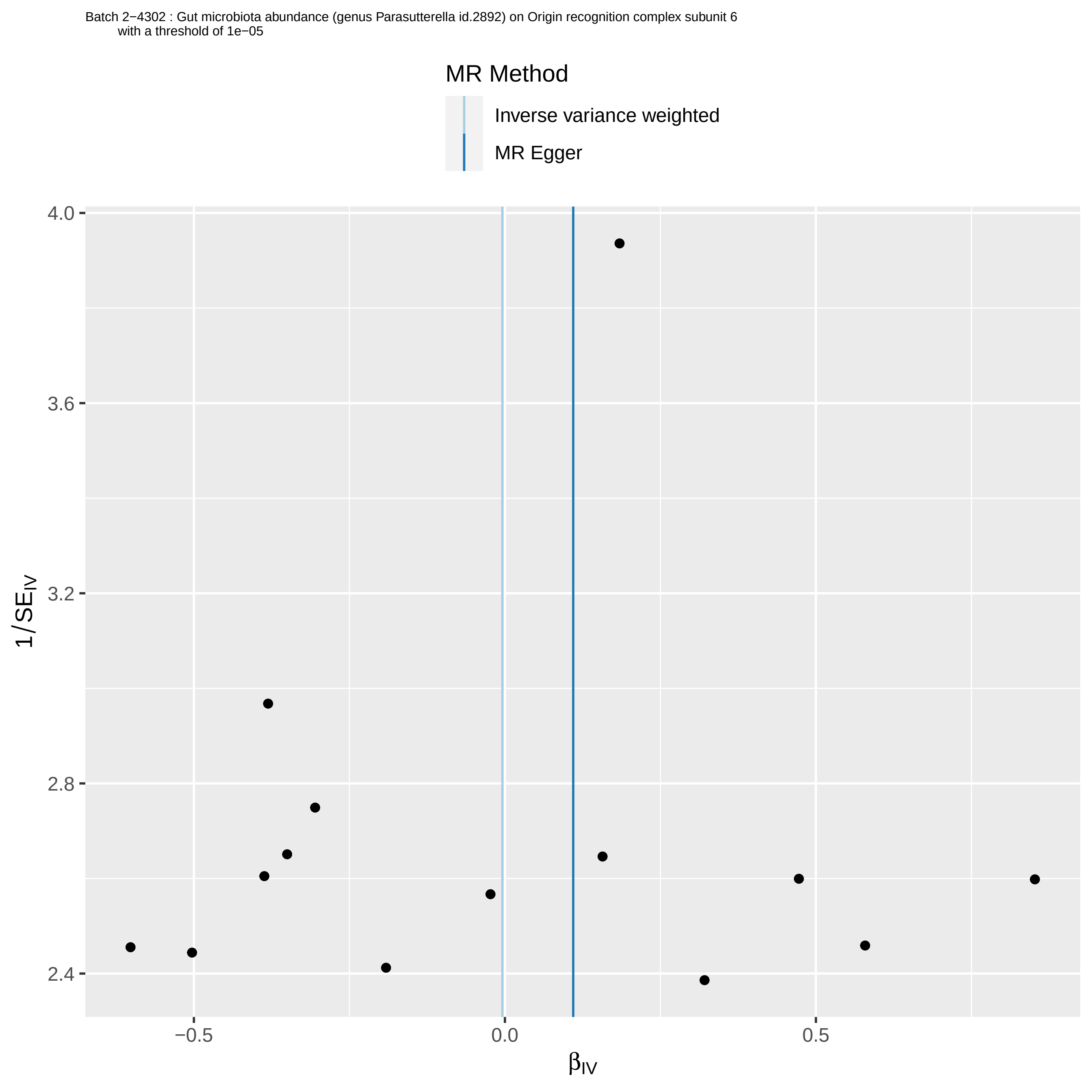

Batch 2−4302 : Gut microbiota abundance (genus Parasutterella id.2892) on Origin recognition complex subunit 6
with a threshold of 1e−05
MR Method
Inverse variance weighted
MR Egger
4.0
3.6
3.2
2.8
2.4
1/SE_IV
−0.5
0.0
0.5
β_IV

Batch 2−4303 : Gut microbiota abundance (genus Catenibacterium id.2153) on Oxysterol−binding protein−related protein 11
with a threshold of 1e−05

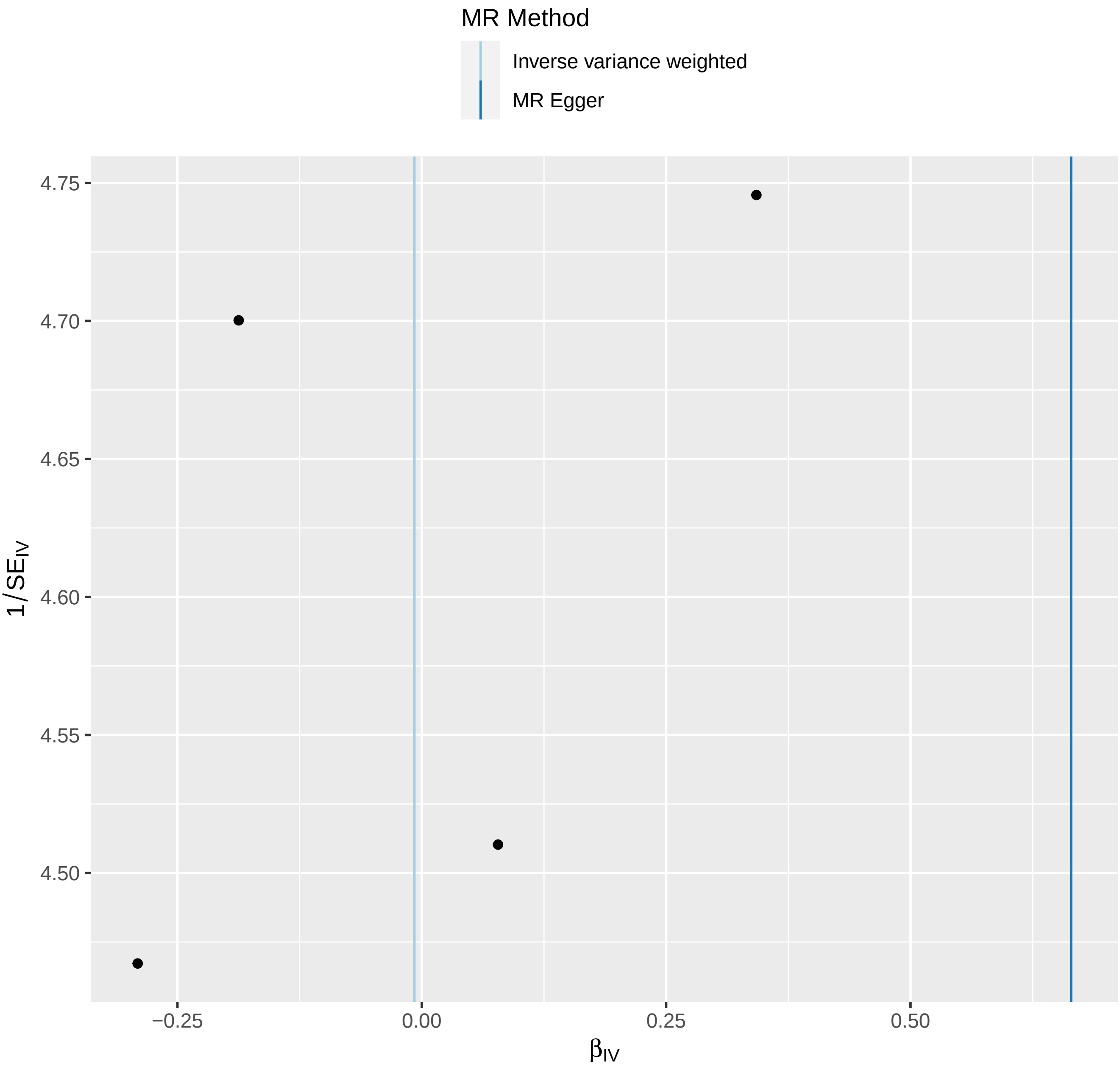

Batch 2−4304 : Gut microbiota abundance (genus Parasutterella id.2892) on Oxysterol−binding protein−related protein 11
with a threshold of 1e−05

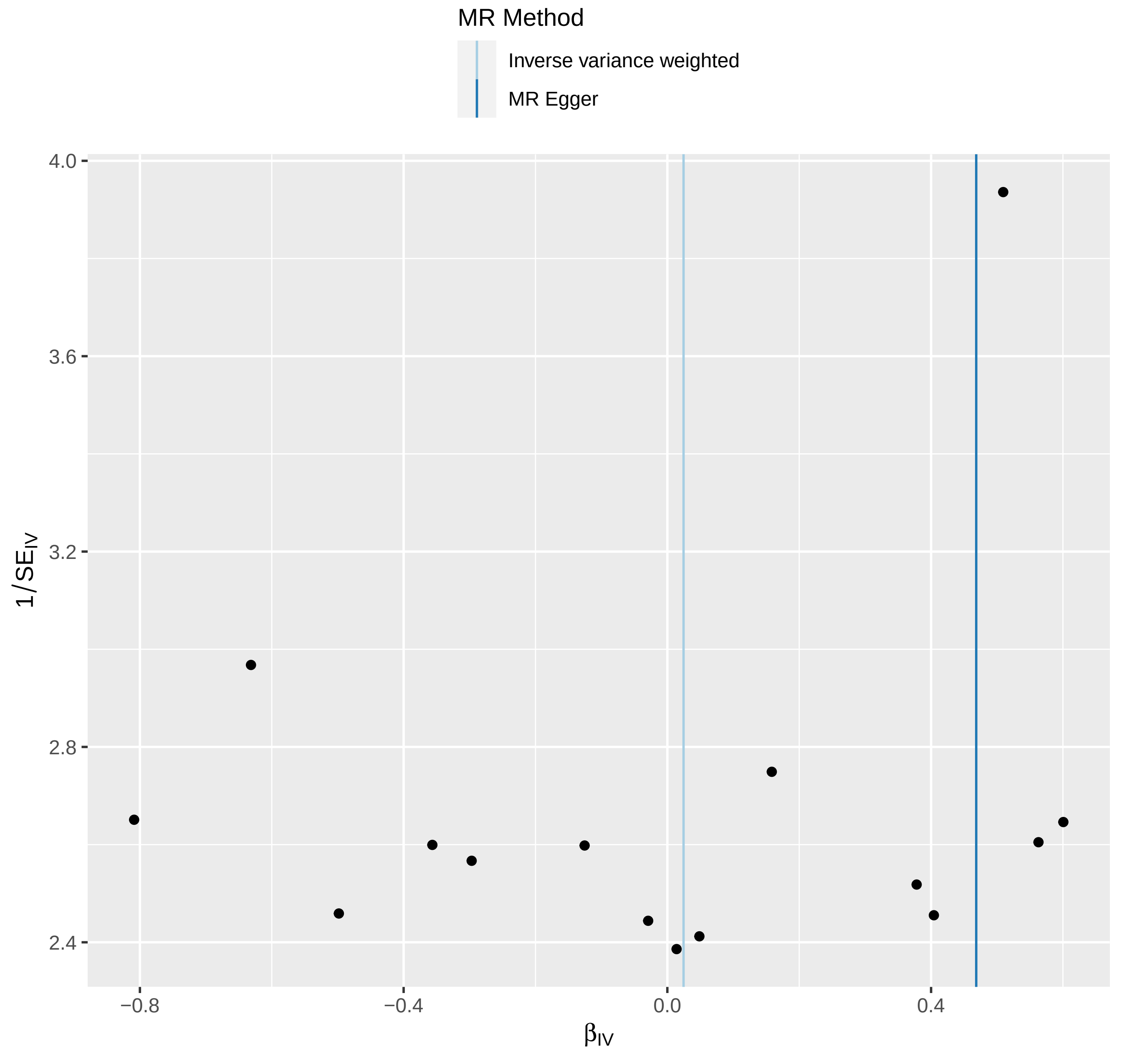

Batch 2−4305 : Gut microbiota abundance (genus Catenibacterium id.2153) on Osteoclast−associated immunoglobulin−like receptor
with a threshold of 1e−05

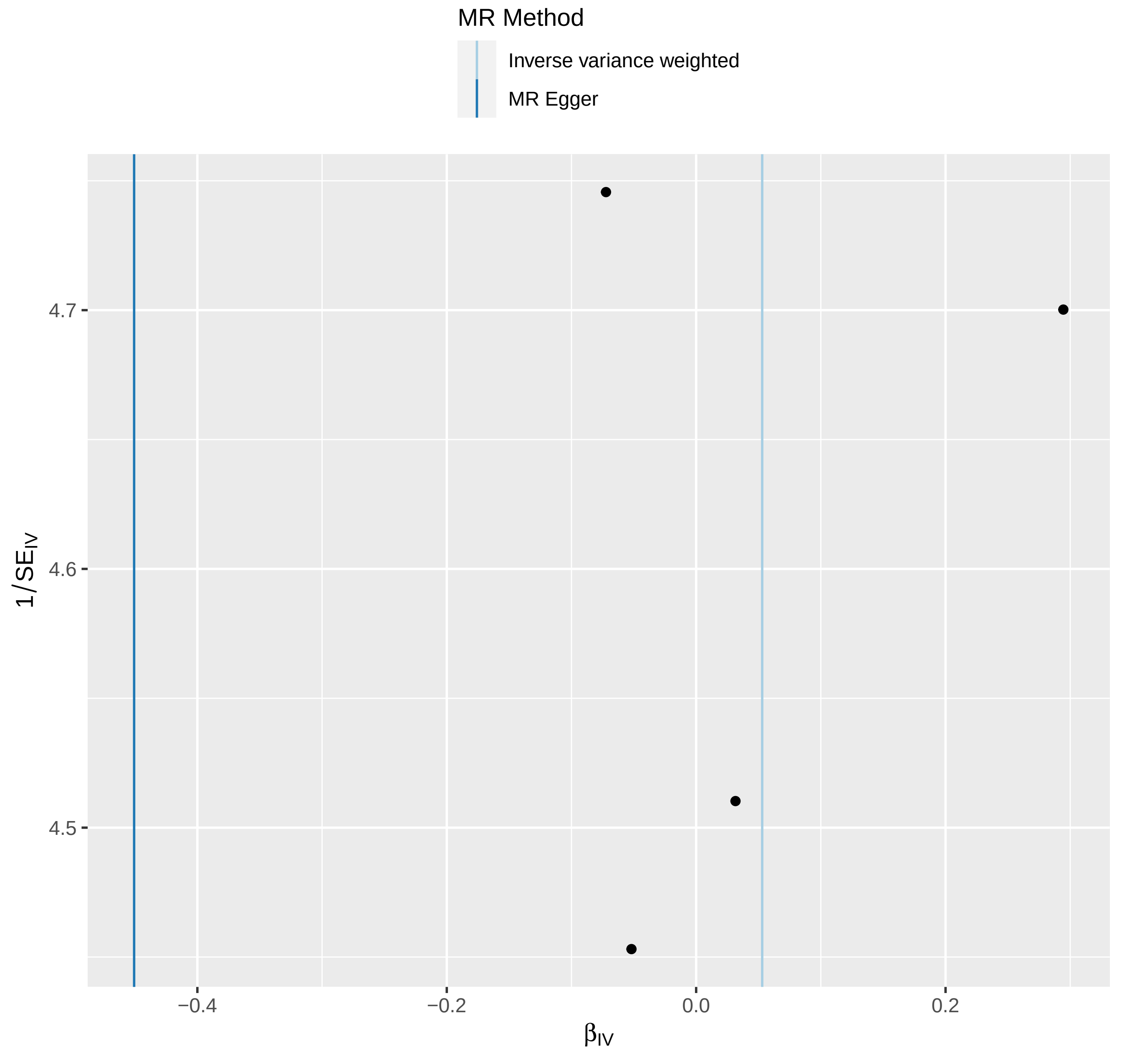

Batch 2−4306 : Gut microbiota abundance (genus Parasutterella id.2892) on Osteoclast−associated immunoglobulin−like receptor with a threshold of 1e−05

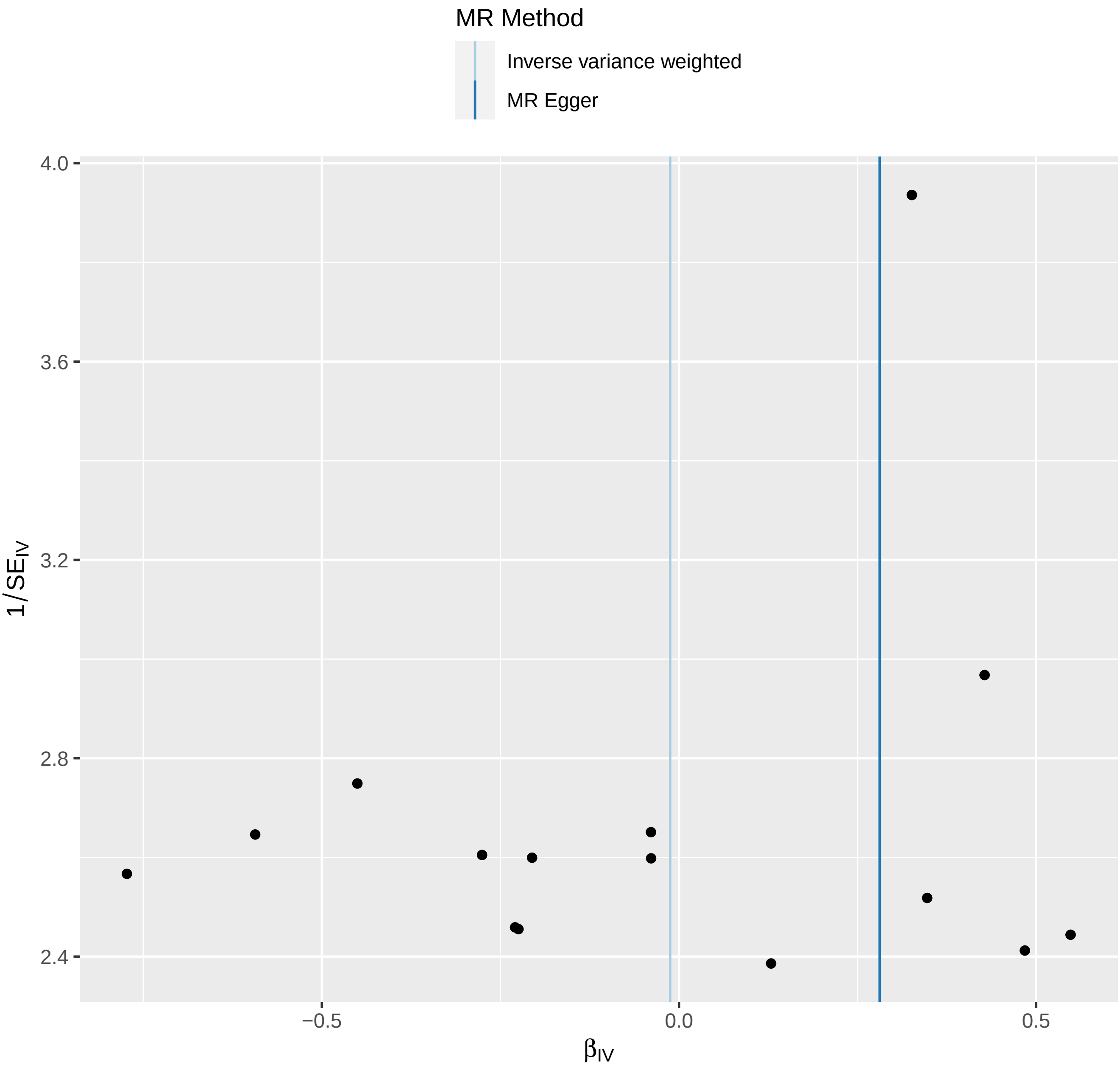

Batch 2−4307 : Gut microbiota abundance (genus Catenibacterium id.2153) on Oncostatin−M
with a threshold of 1e−05

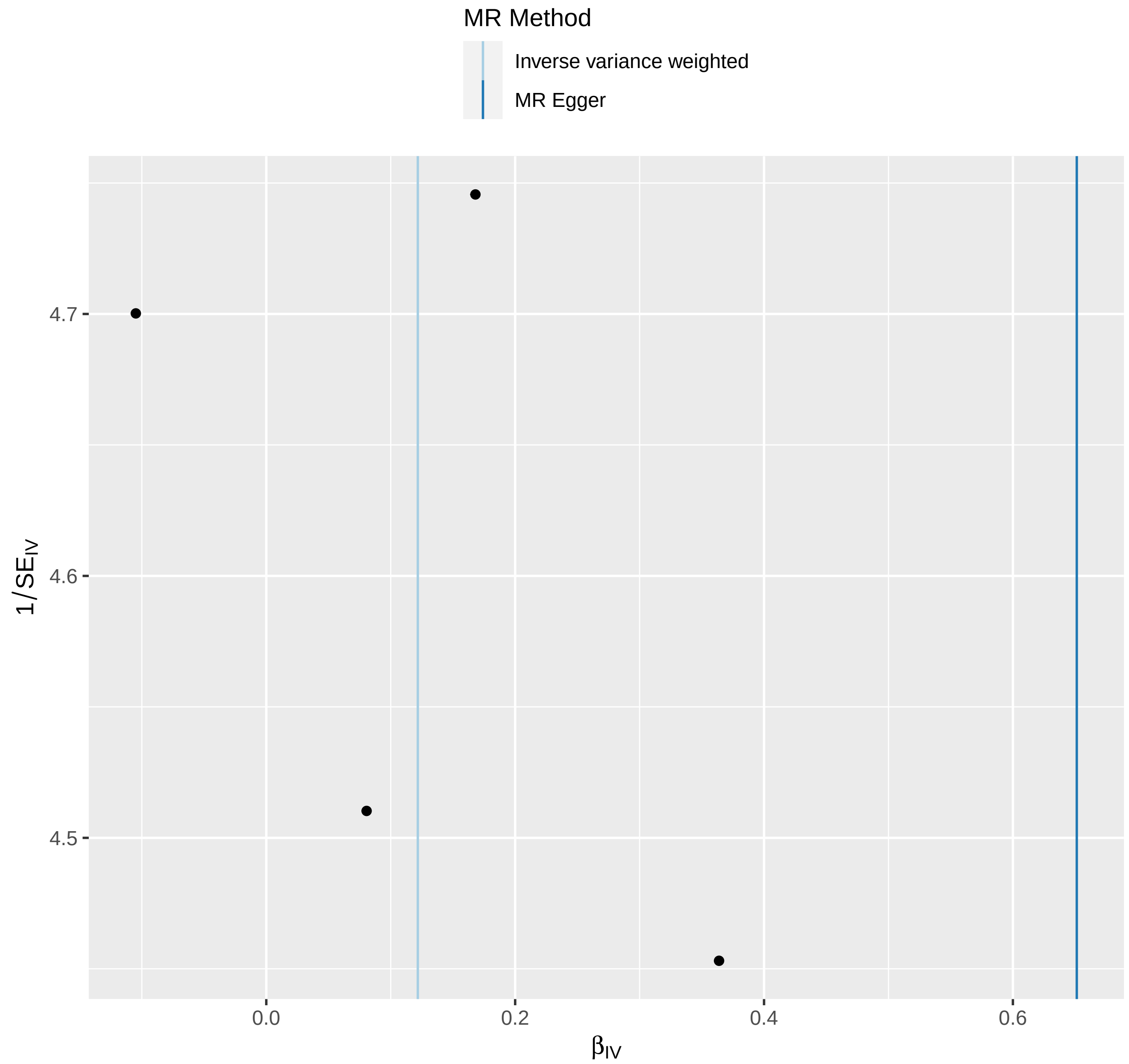

Batch 2−4308 : Gut microbiota abundance (genus Parasutterella id.2892) on Oncostatin−M
with a threshold of 1e−05

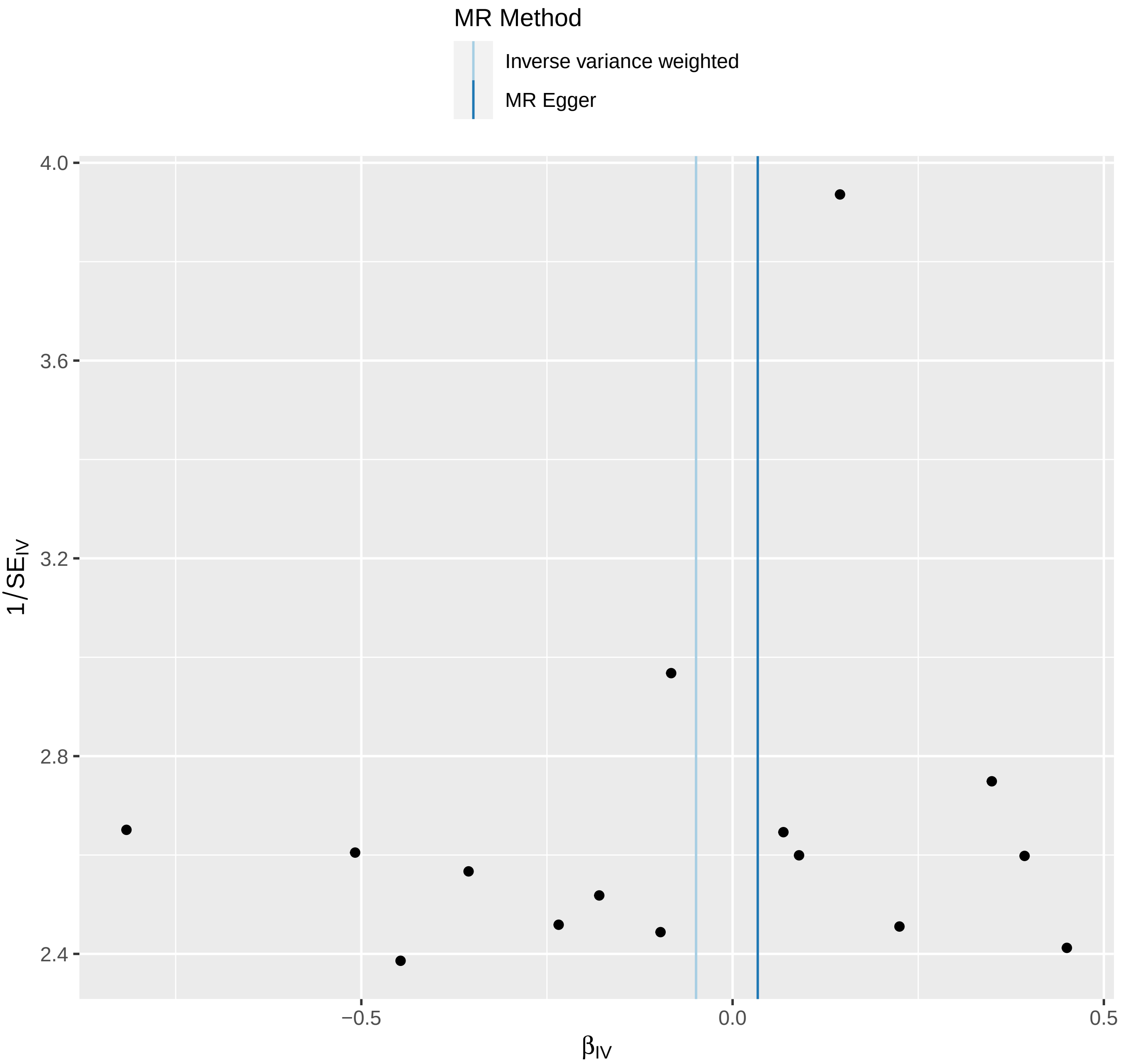

Batch 2−4309 : Gut microbiota abundance (genus Catenibacterium id.2153) on Oncostatin−M
with a threshold of 1e−05

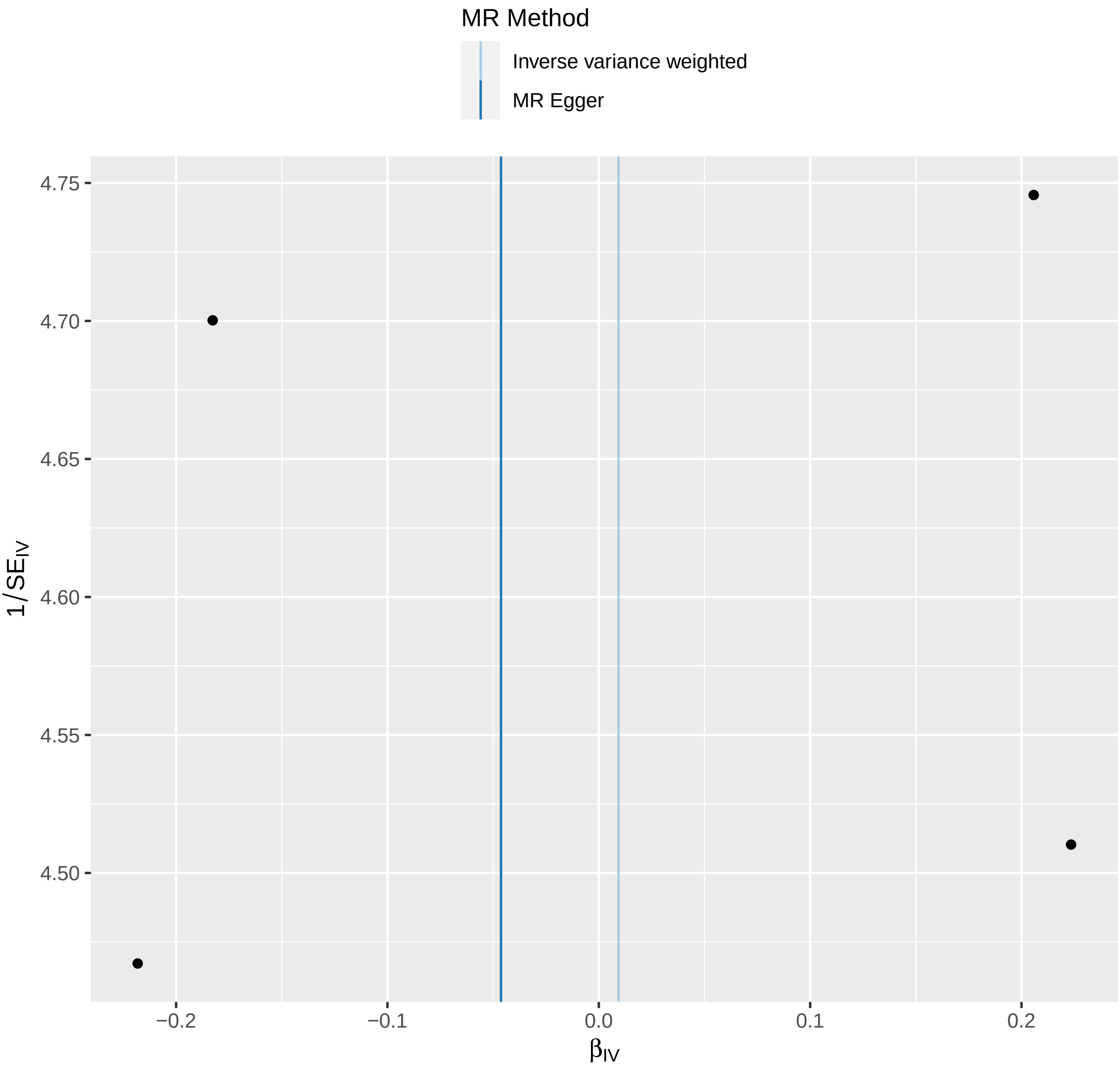

Batch 2−4310 : Gut microbiota abundance (genus Parasutterella id.2892) on Oncostatin−M
with a threshold of 1e−05

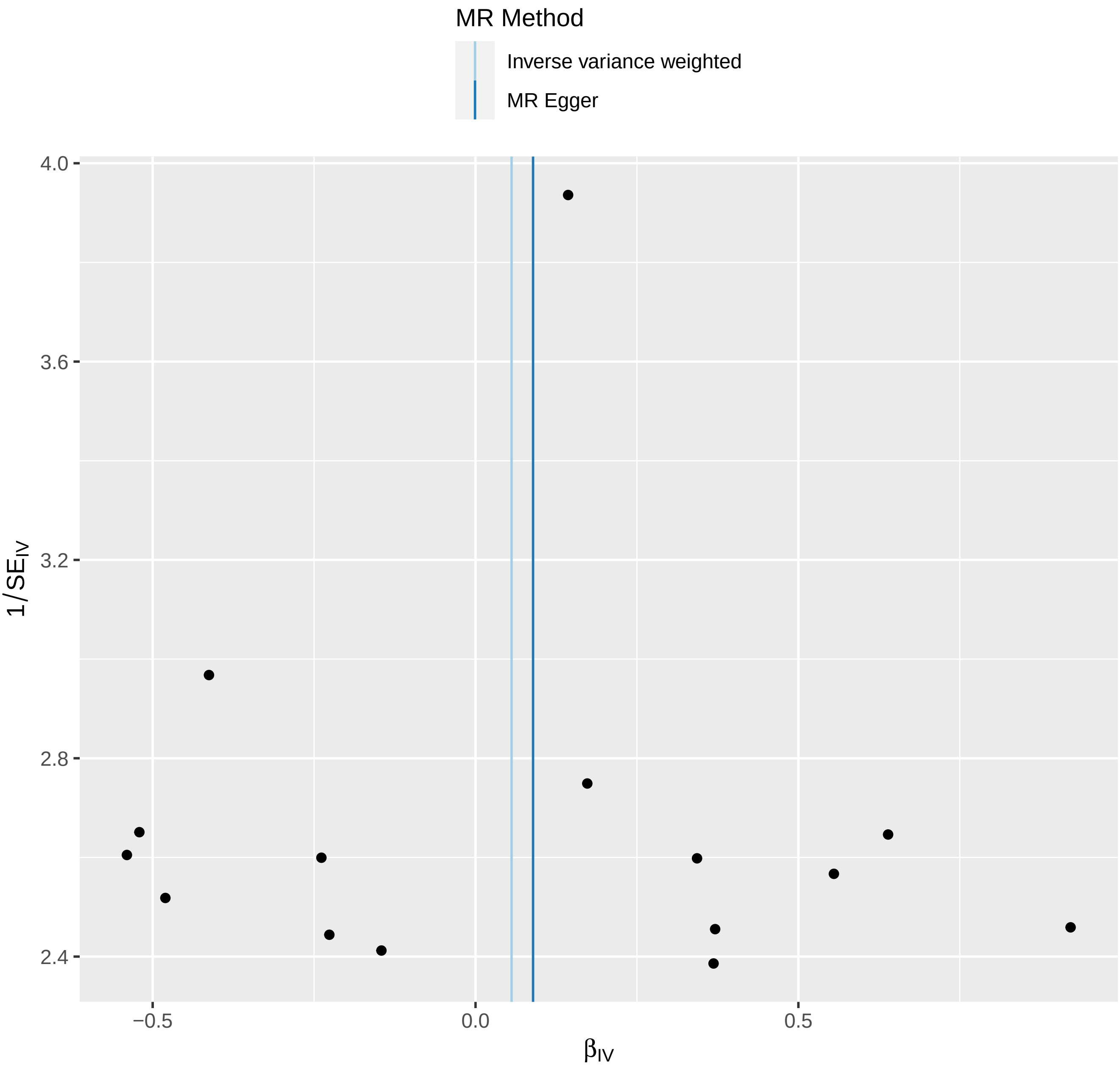

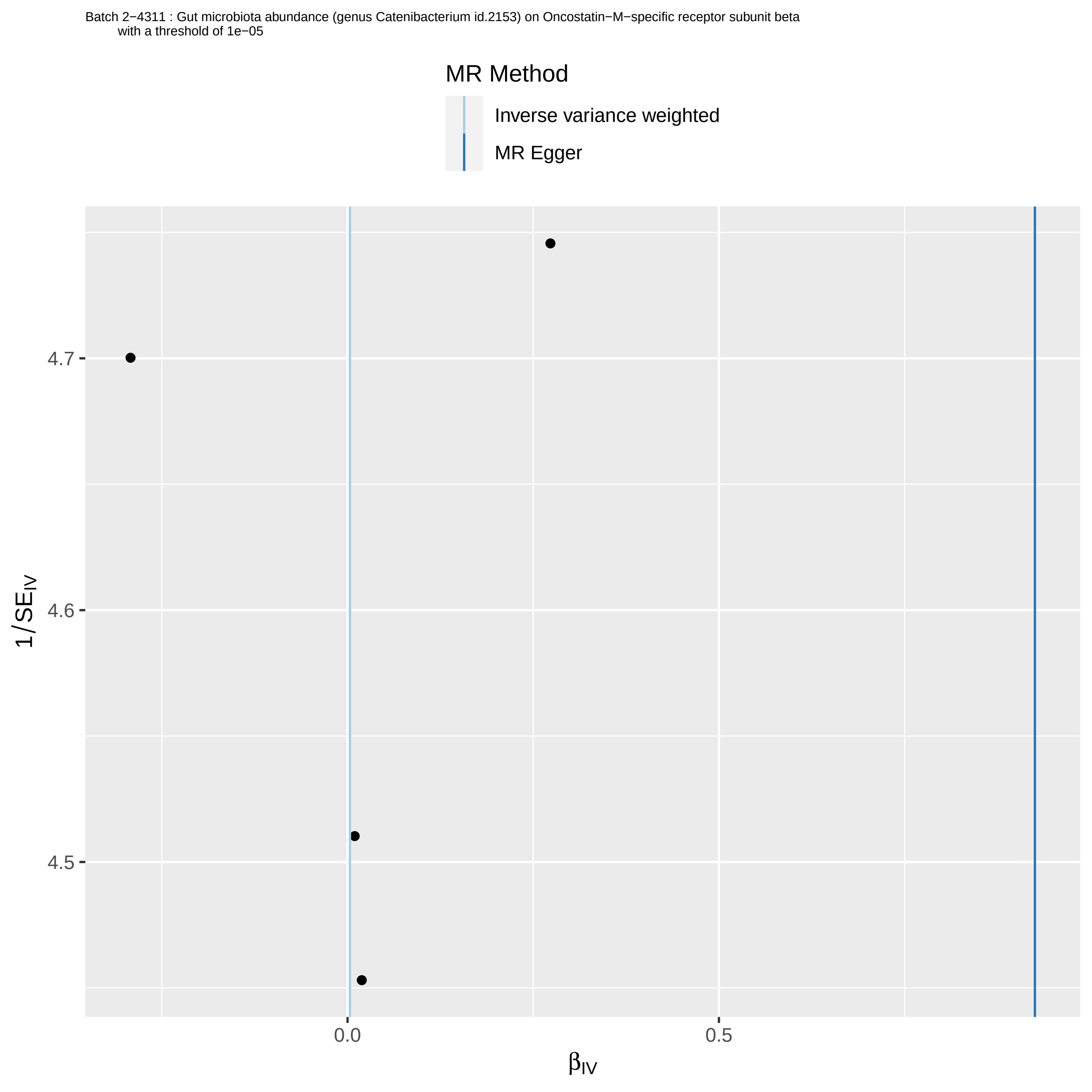

Batch 2−4311 : Gut microbiota abundance (genus Catenibacterium id.2153) on Oncostatin−M−specific receptor subunit beta
with a threshold of 1e−05
MR Method
Inverse variance weighted
MR Egger
4.7
4.6
4.5
1/SE_IV
0.0
0.5
β_IV

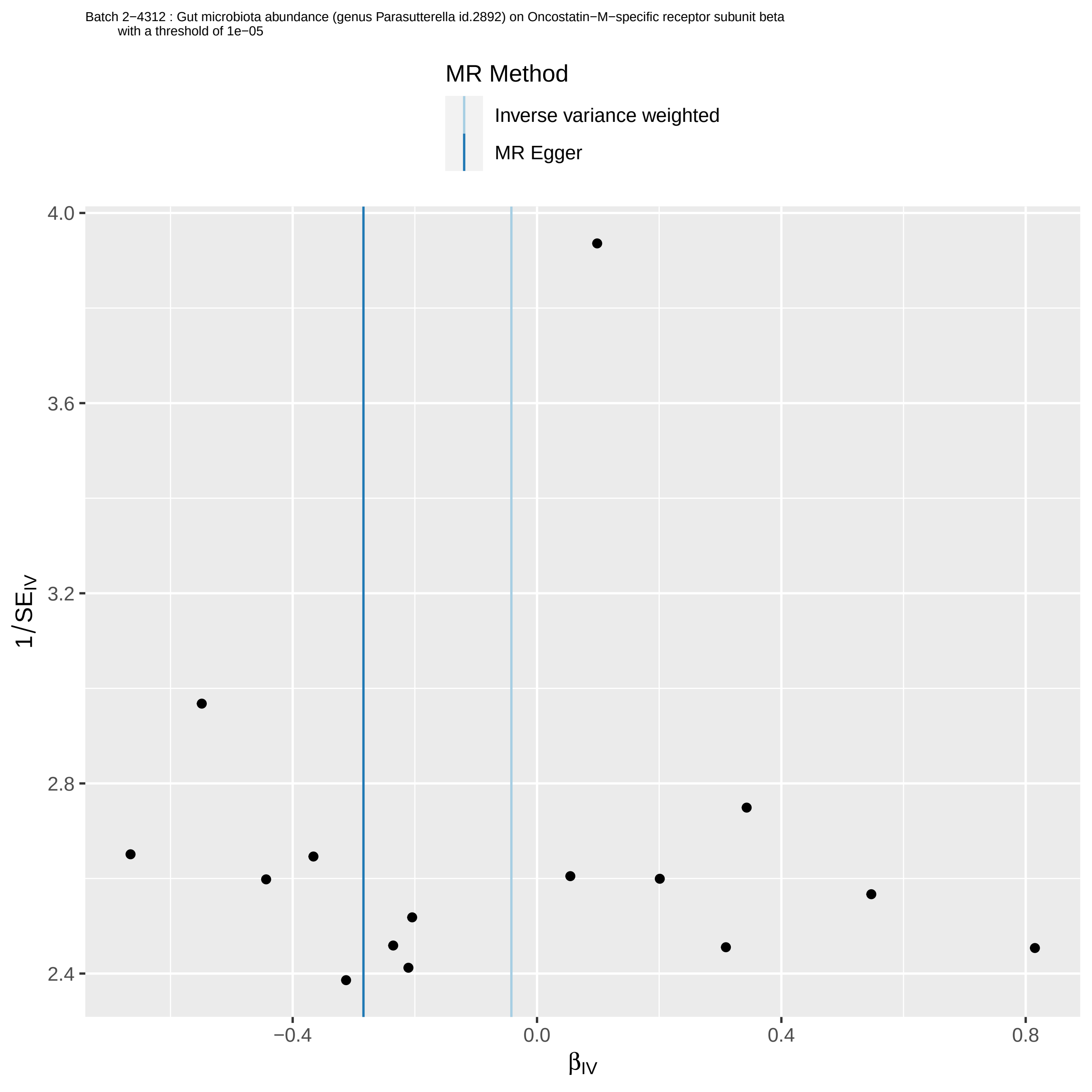
Batch 2−4312 : Gut microbiota abundance (genus Parasutterella id.2892) on Oncostatin−M−specific receptor subunit beta
with a threshold of 1e−05
MR Method
Inverse variance weighted
MR Egger
1/SE_IV
β_IV
4.0
3.6
3.2
2.8
2.4
−0.4
0.0
0.4
0.8

Batch 2−4313 : Gut microbiota abundance (genus Catenibacterium id.2153) on Osteocrin
with a threshold of 1e−05

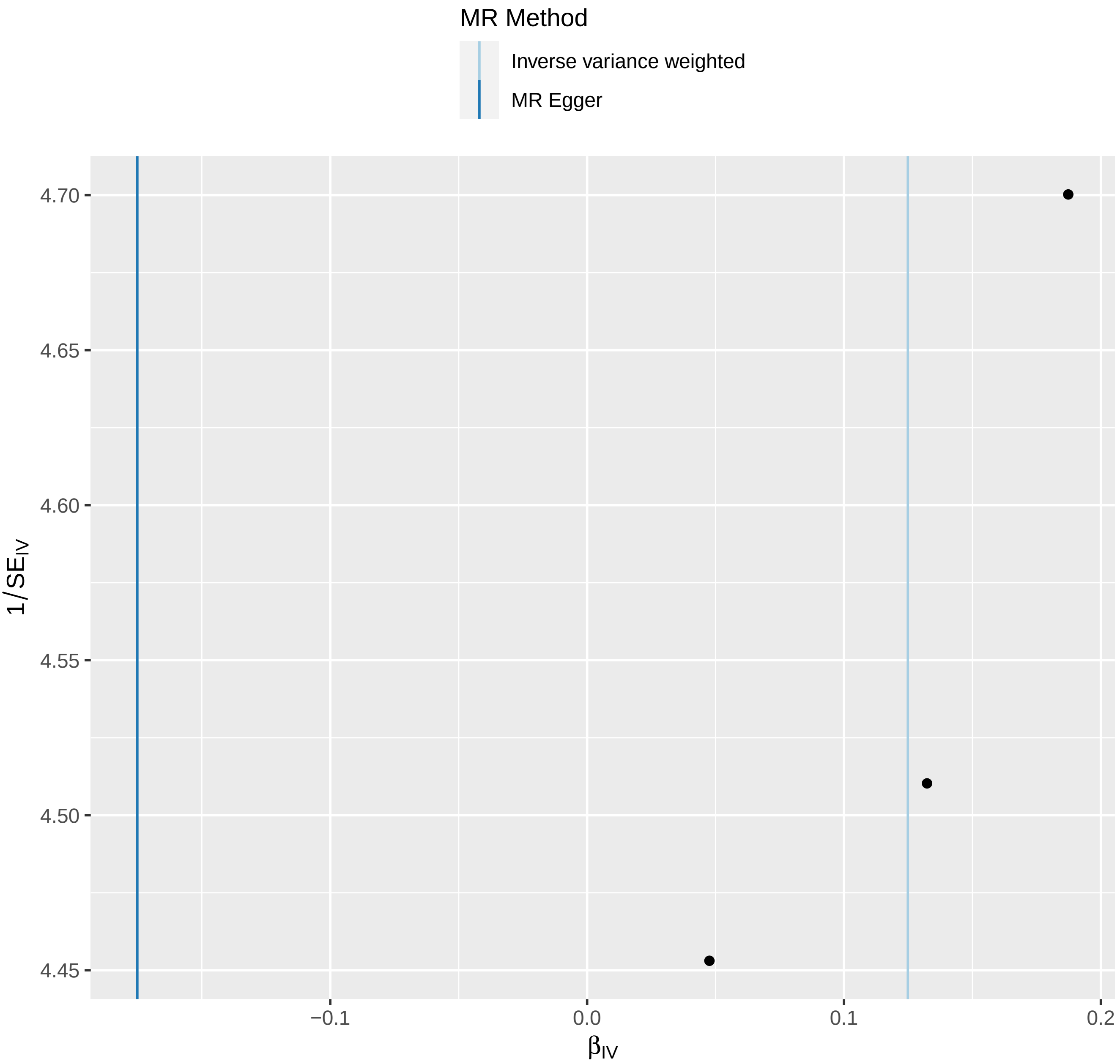

Batch 2−4314 : Gut microbiota abundance (genus Parasutterella id.2892) on Osteocrin
with a threshold of 1e−05

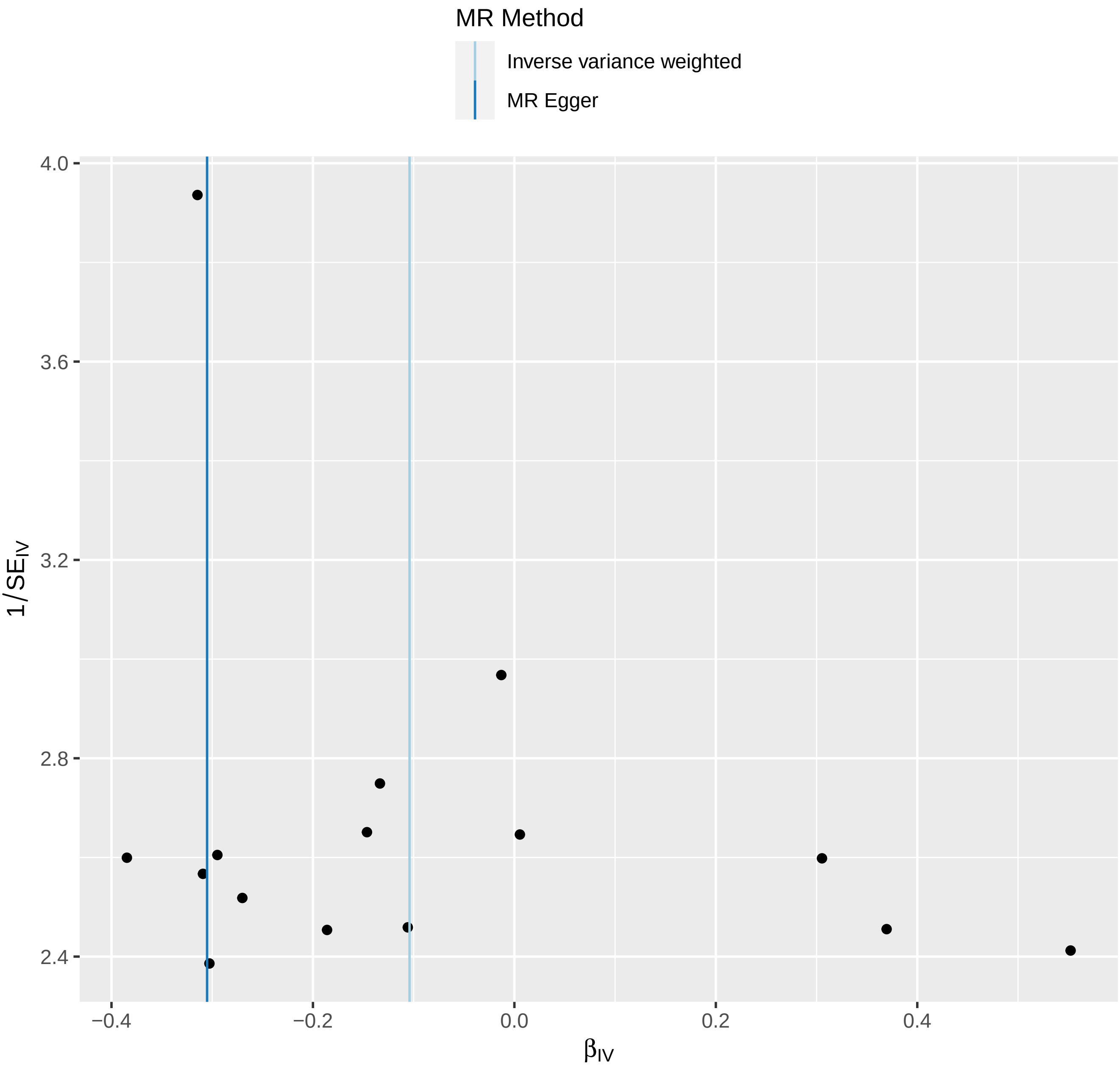

Batch 2−4315 : Gut microbiota abundance (genus Catenibacterium id.2153) on Ubiquitin thioesterase OTUB2
with a threshold of 1e−05

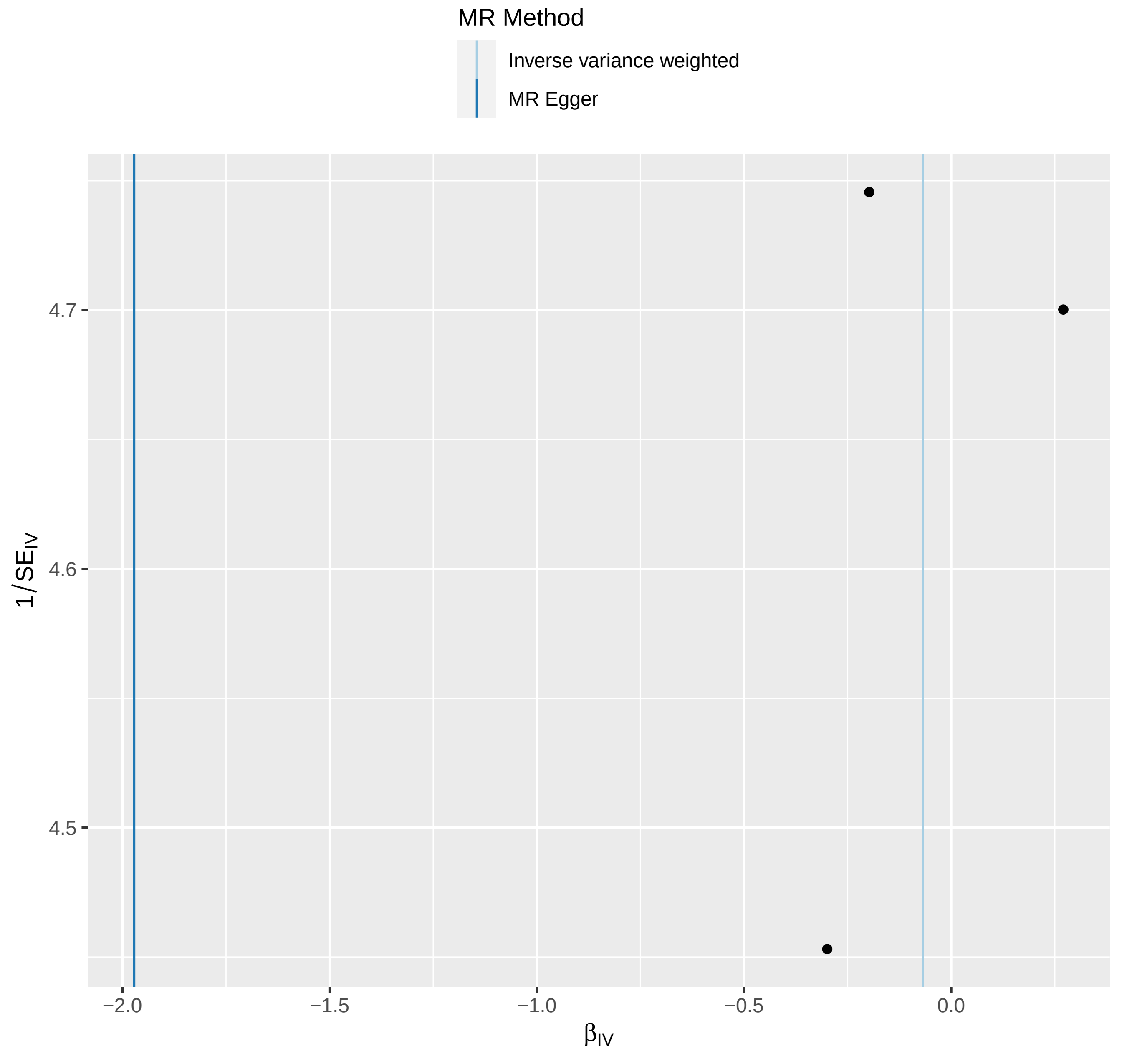

Batch 2−4316 : Gut microbiota abundance (genus Parasutterella id.2892) on Ubiquitin thioesterase OTUB2
with a threshold of 1e−05

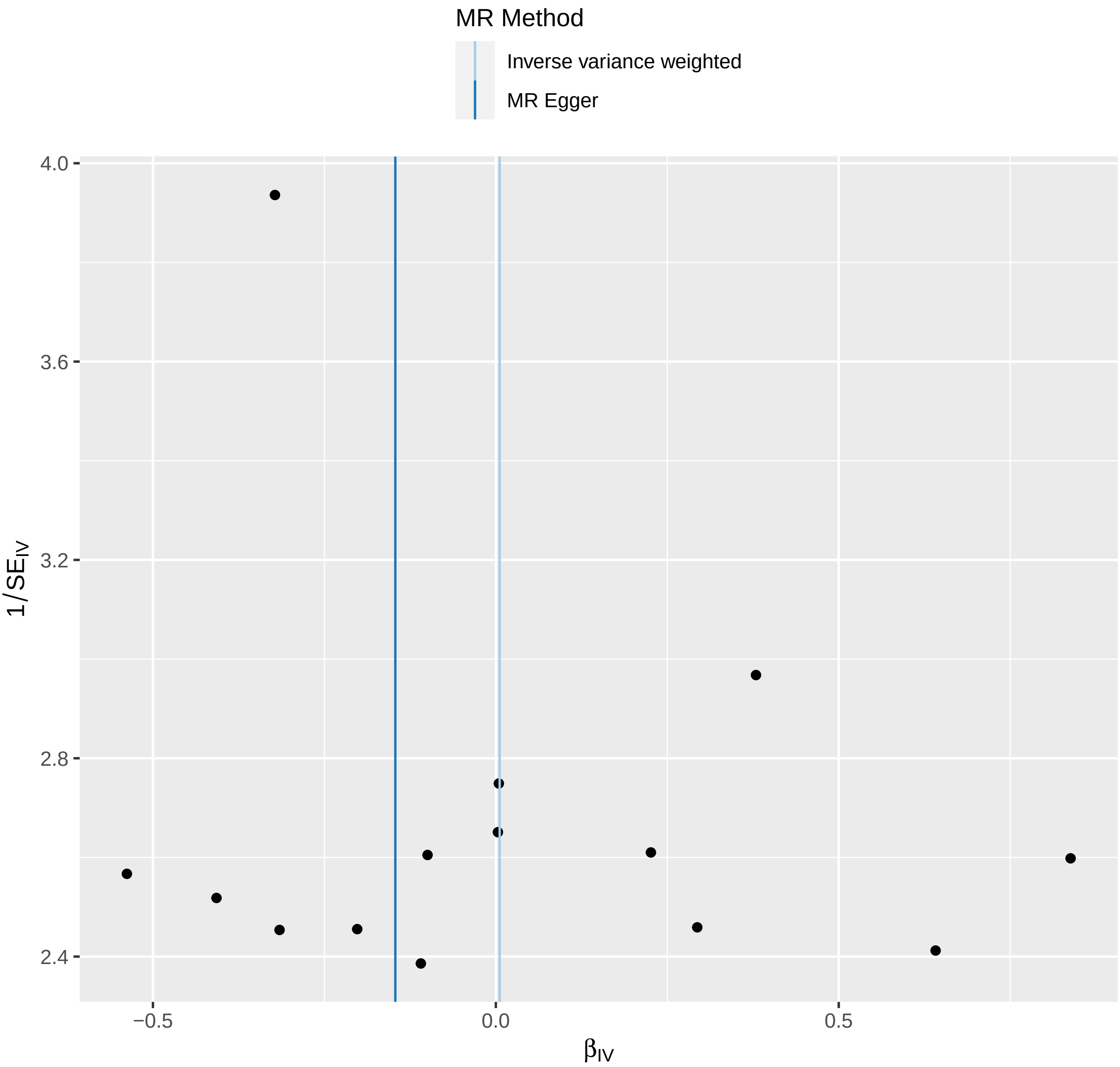

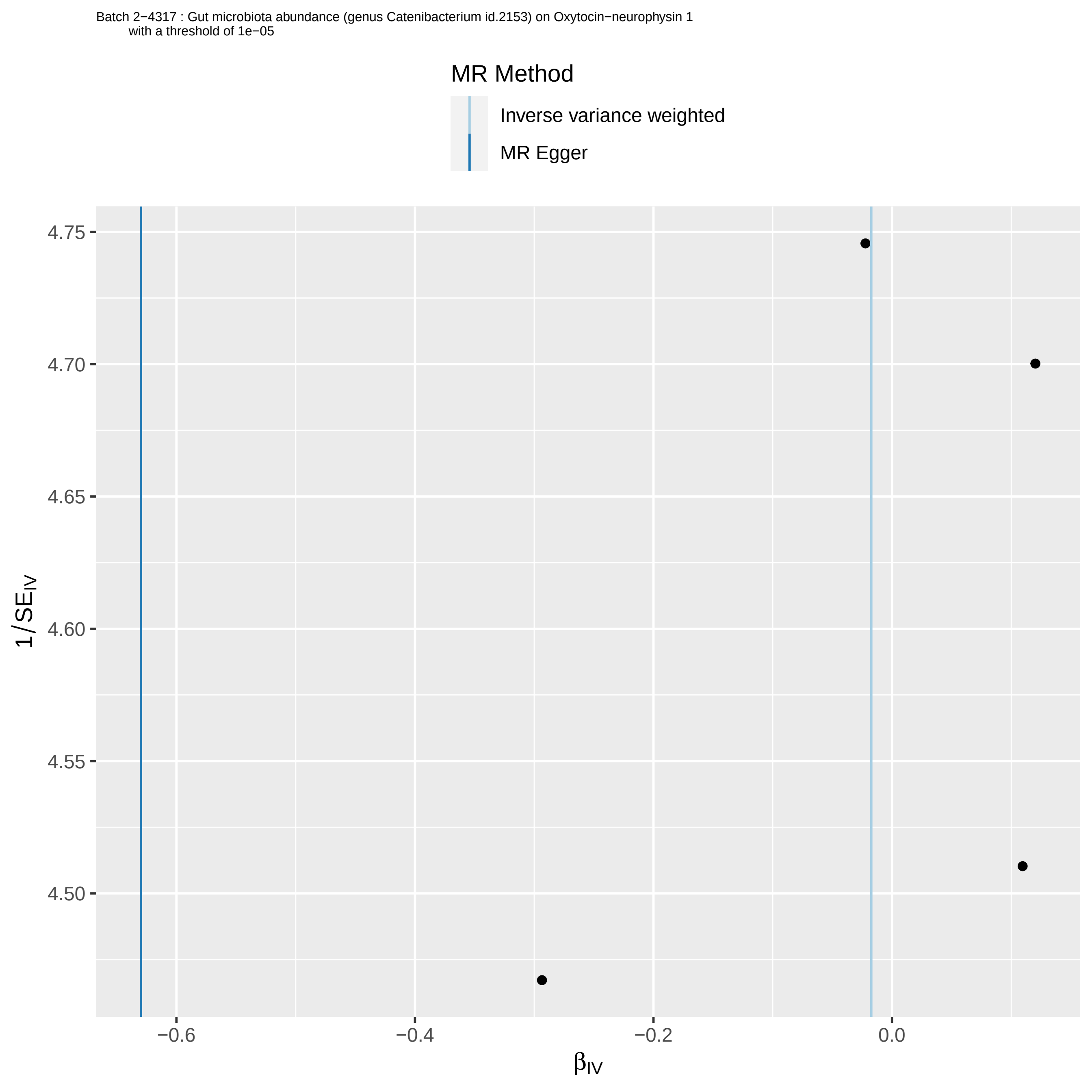
Batch 2−4317 : Gut microbiota abundance (genus Catenibacterium id.2153) on Oxytocin−neurophysin 1
with a threshold of 1e−05
MR Method
Inverse variance weighted
MR Egger
4.75
4.70
4.65
4.60
4.55
4.50
1/SE$_{IV}$
−0.6
−0.4
−0.2
0.0
β$_{IV}$

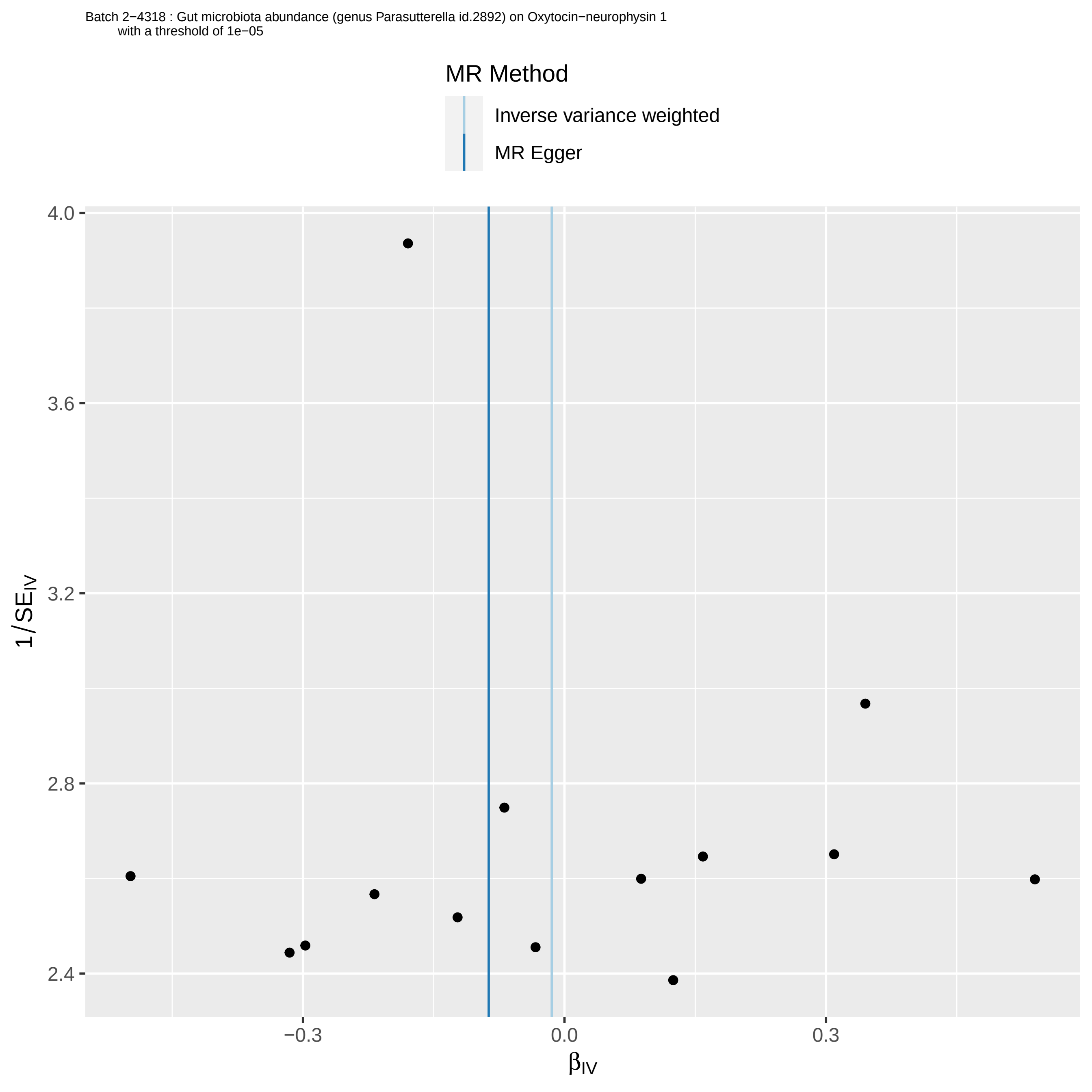
Batch 2−4318 : Gut microbiota abundance (genus Parasutterella id.2892) on Oxytocin−neurophysin 1
with a threshold of 1e−05
MR Method
Inverse variance weighted
MR Egger
4.0
3.6
3.2
2.8
2.4
1/SE_IV
−0.3
0.0
0.3
β_IV

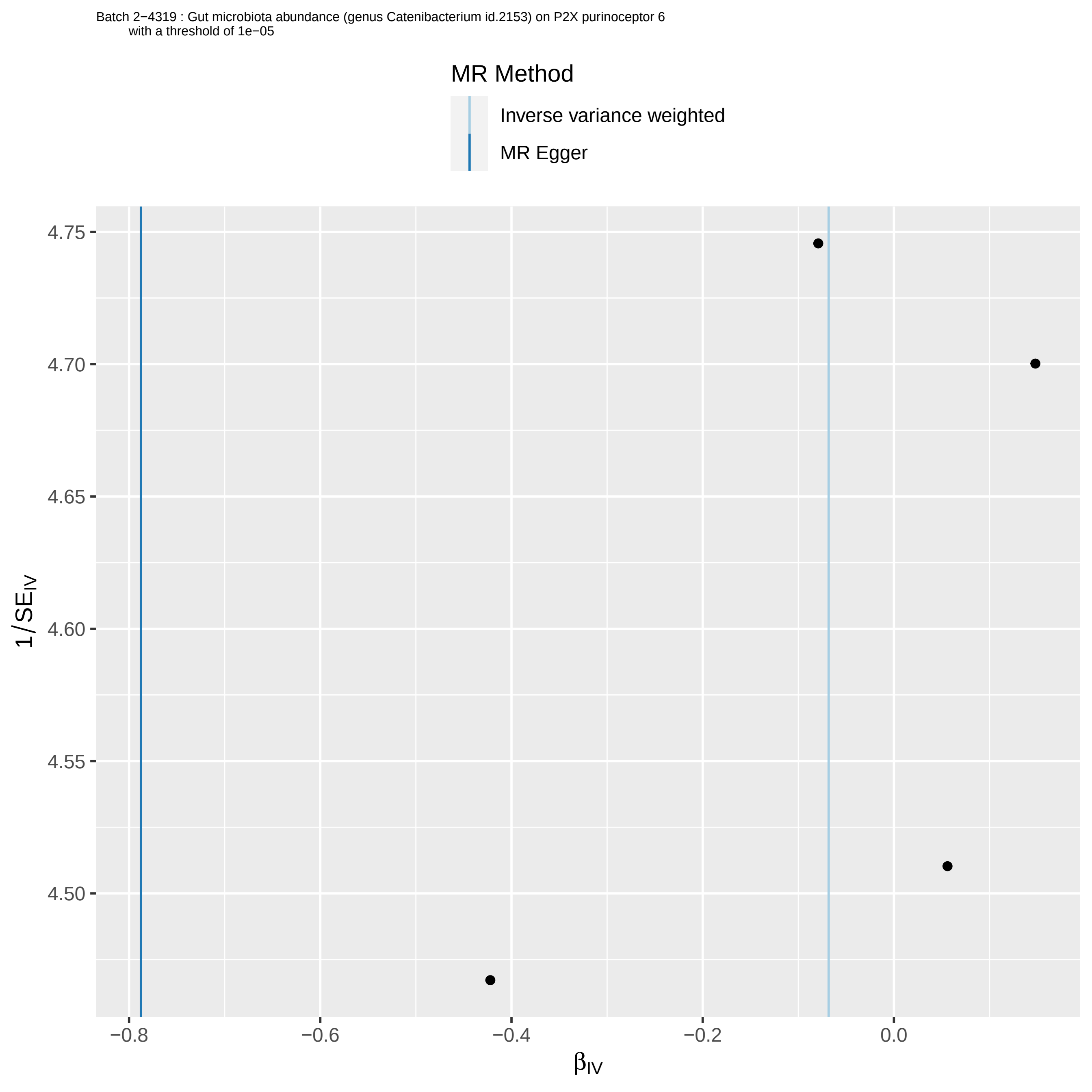
Batch 2−4319 : Gut microbiota abundance (genus Catenibacterium id.2153) on P2X purinoceptor 6
with a threshold of 1e−05
MR Method
Inverse variance weighted
MR Egger
4.75
4.70
4.65
4.60
4.55
4.50
1/SE_IV
−0.8
−0.6
−0.4
−0.2
0.0
β_IV

Batch 2−4320 : Gut microbiota abundance (genus Parasutterella id.2892) on P2X purinoceptor 6
with a threshold of 1e−05

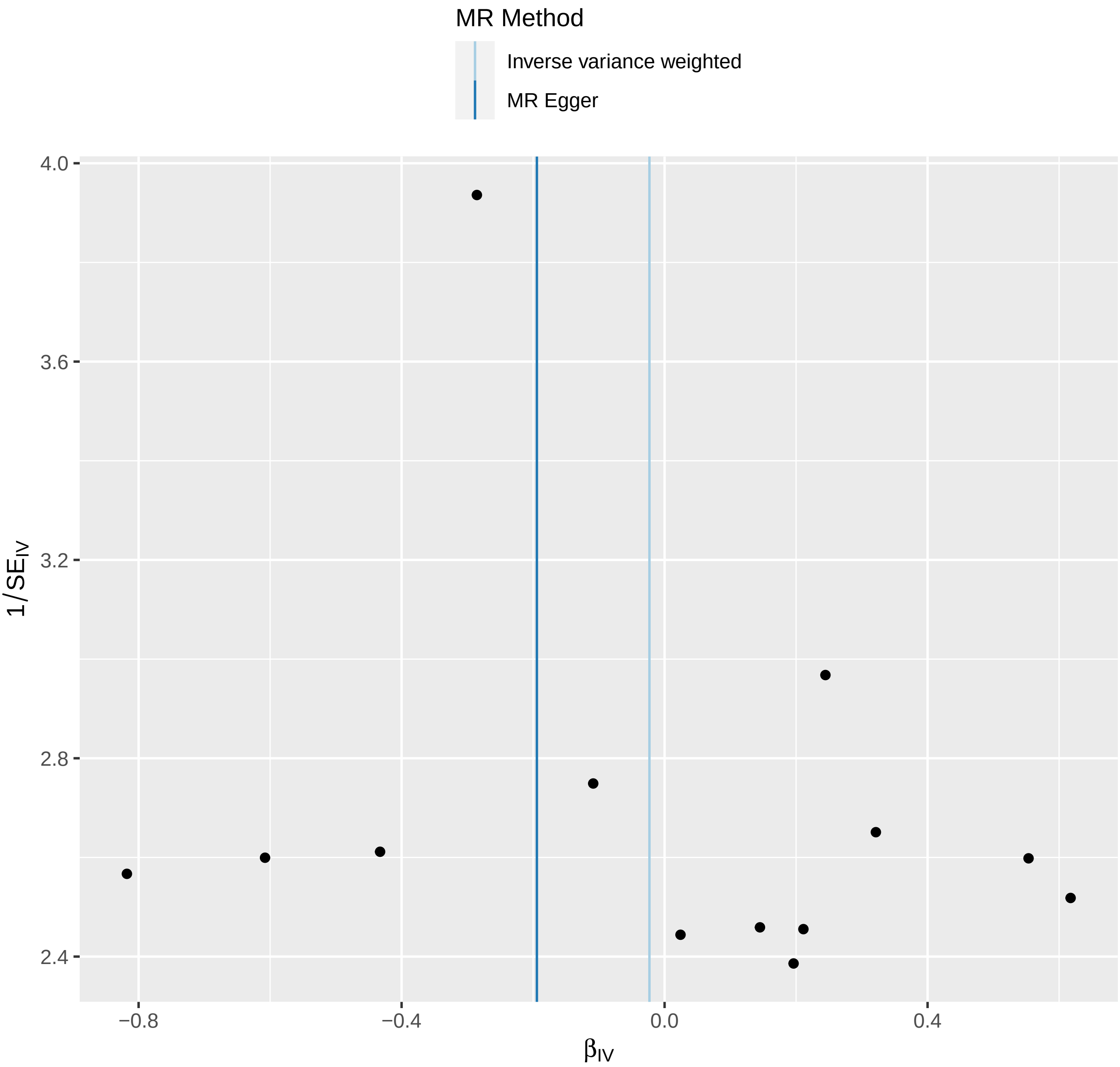

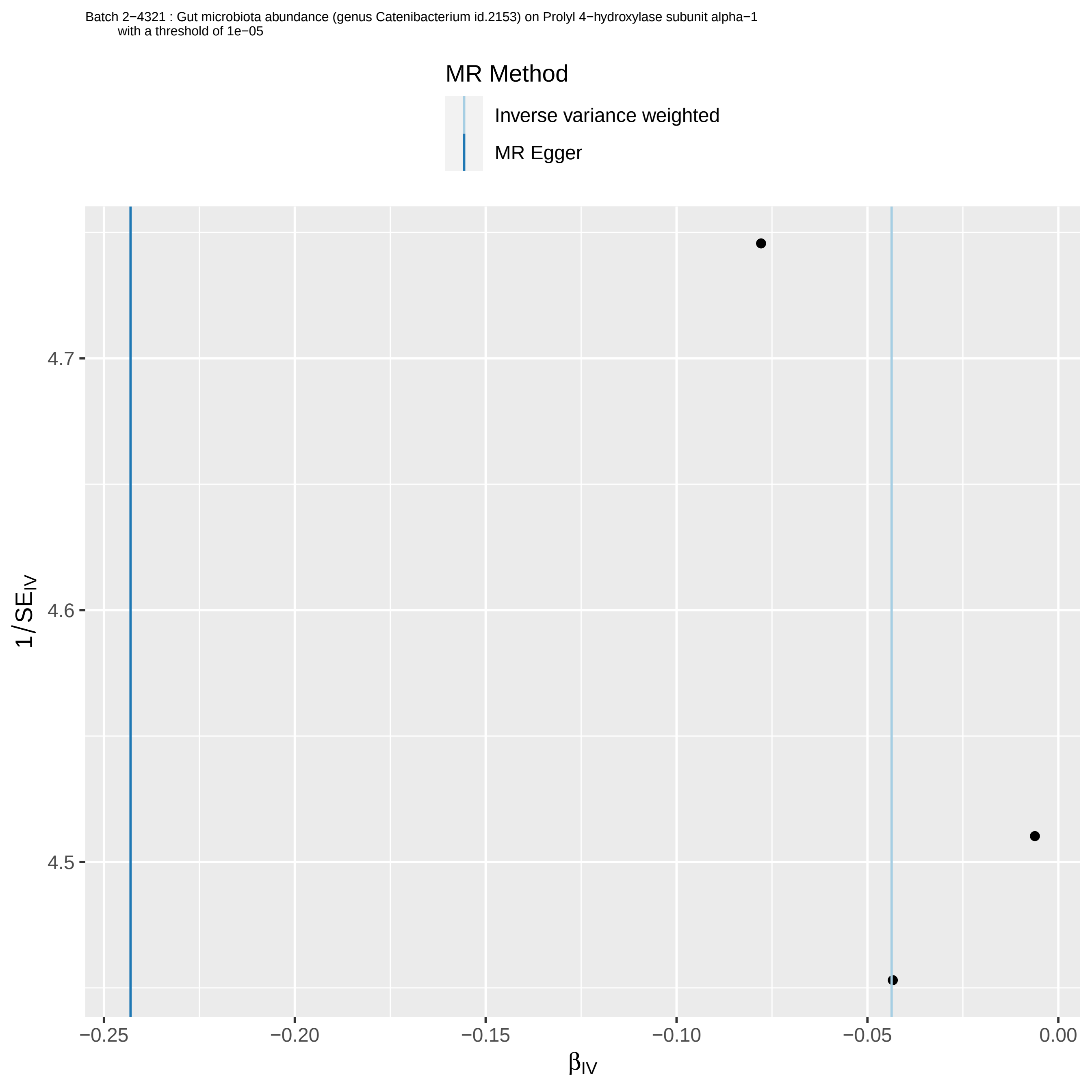

Batch 2−4321 : Gut microbiota abundance (genus Catenibacterium id.2153) on Prolyl 4−hydroxylase subunit alpha−1
with a threshold of 1e−05
MR Method
Inverse variance weighted
MR Egger
4.7
4.6
4.5
1/SE_IV
−0.25
−0.20
−0.15
−0.10
−0.05
0.00
β_IV

Batch 2−4322 : Gut microbiota abundance (genus Parasutterella id.2892) on Prolyl 4−hydroxylase subunit alpha−1
with a threshold of 1e−05

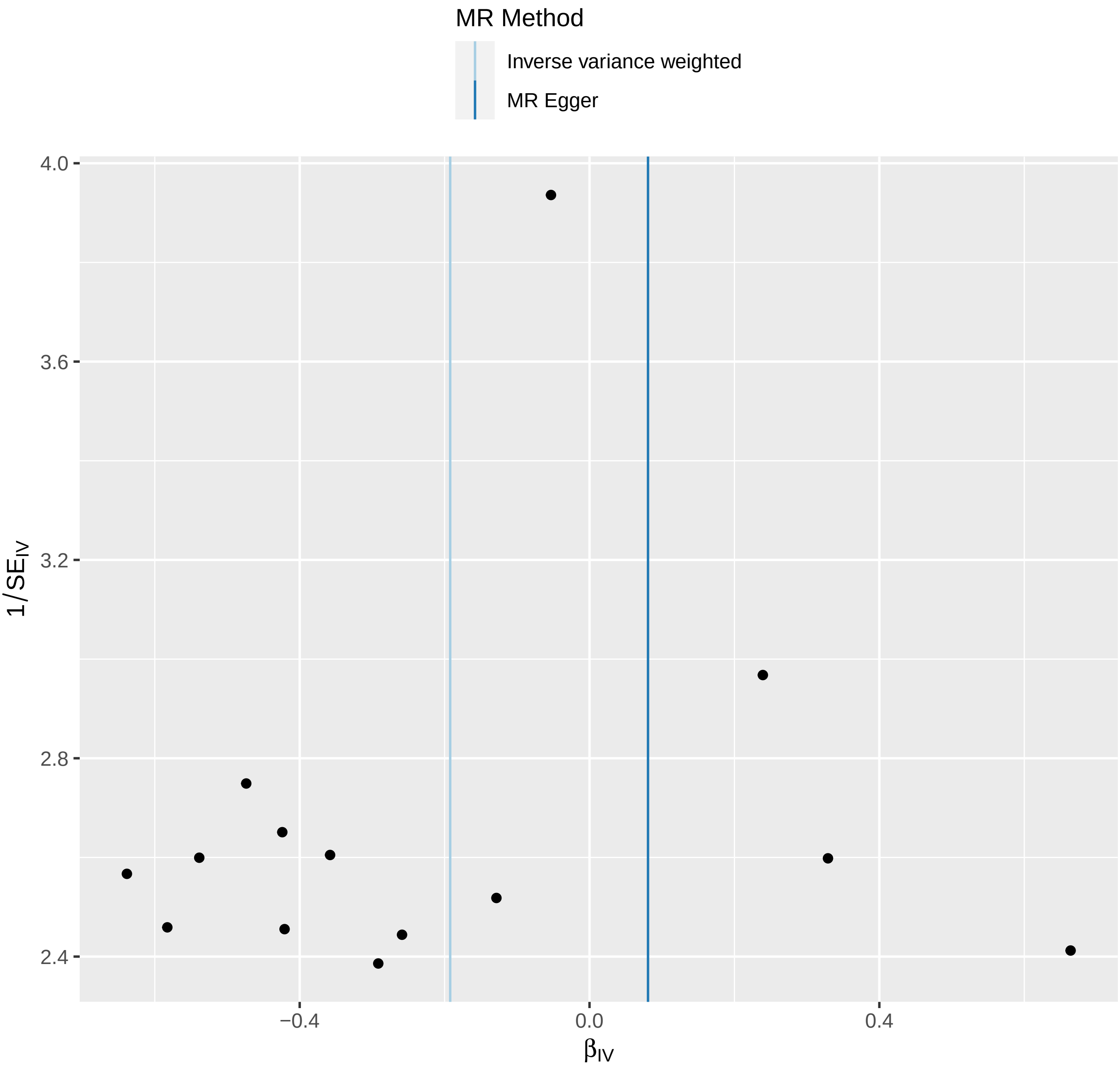

Batch 2−4323 : Gut microbiota abundance (genus Catenibacterium id.2153) on Prolyl 4−hydroxylase subunit alpha−2
with a threshold of 1e−05

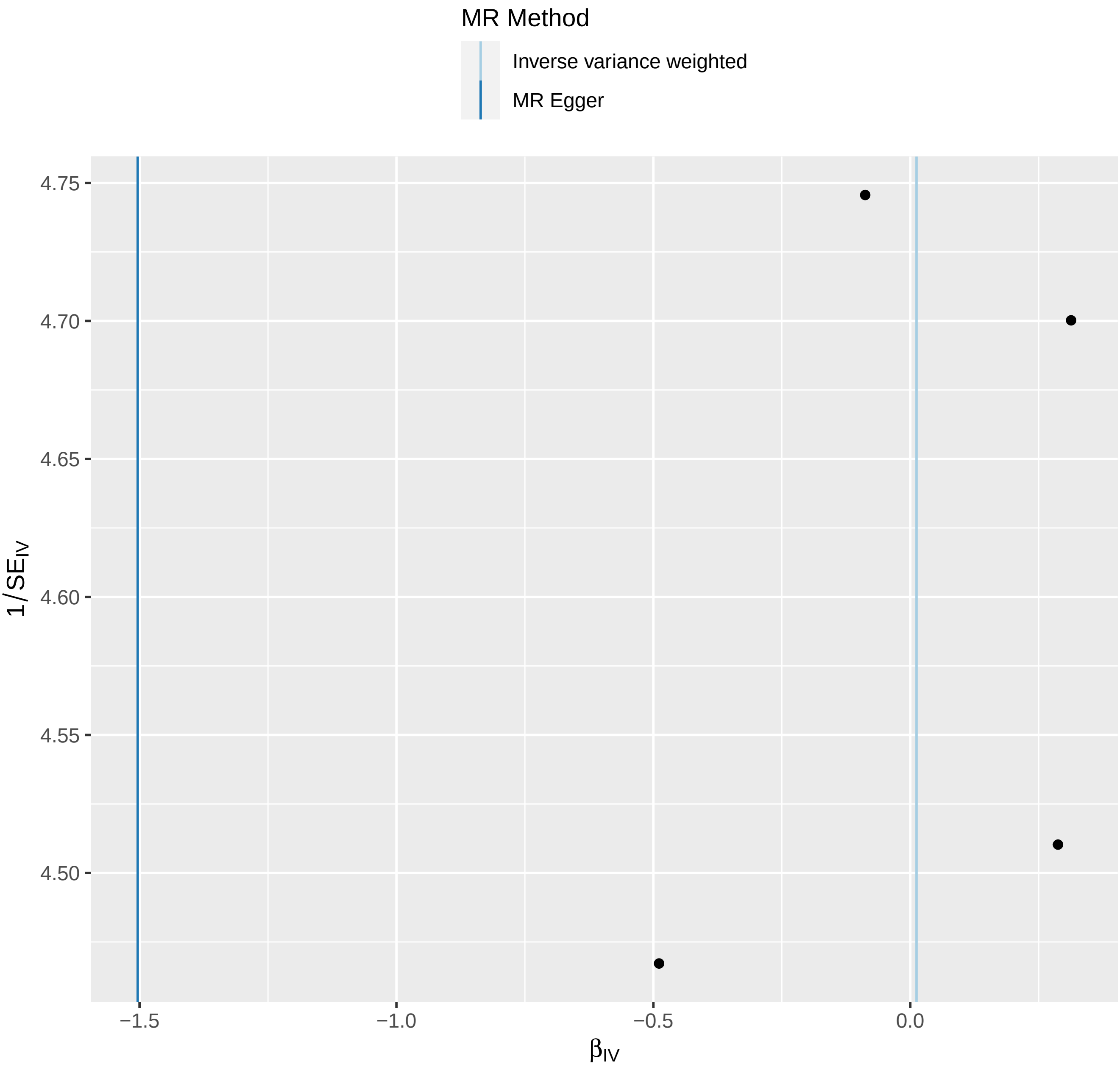

Batch 2−4324 : Gut microbiota abundance (genus Parasutterella id.2892) on Prolyl 4−hydroxylase subunit alpha−2 with a threshold of 1e−05

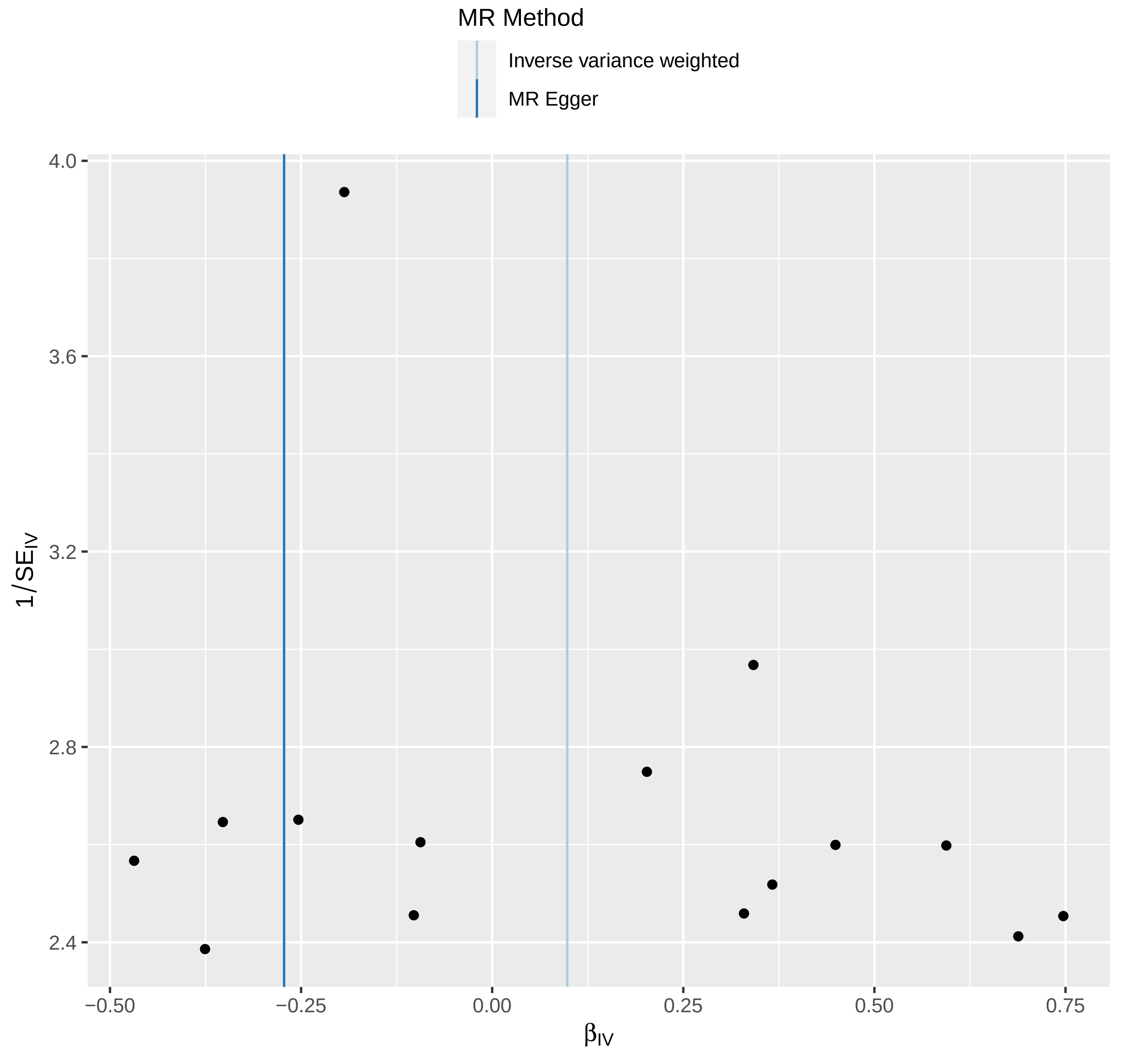

Batch 2−4325 : Gut microbiota abundance (genus Catenibacterium id.2153) on Polyadenylate−binding protein 3
with a threshold of 1e−05

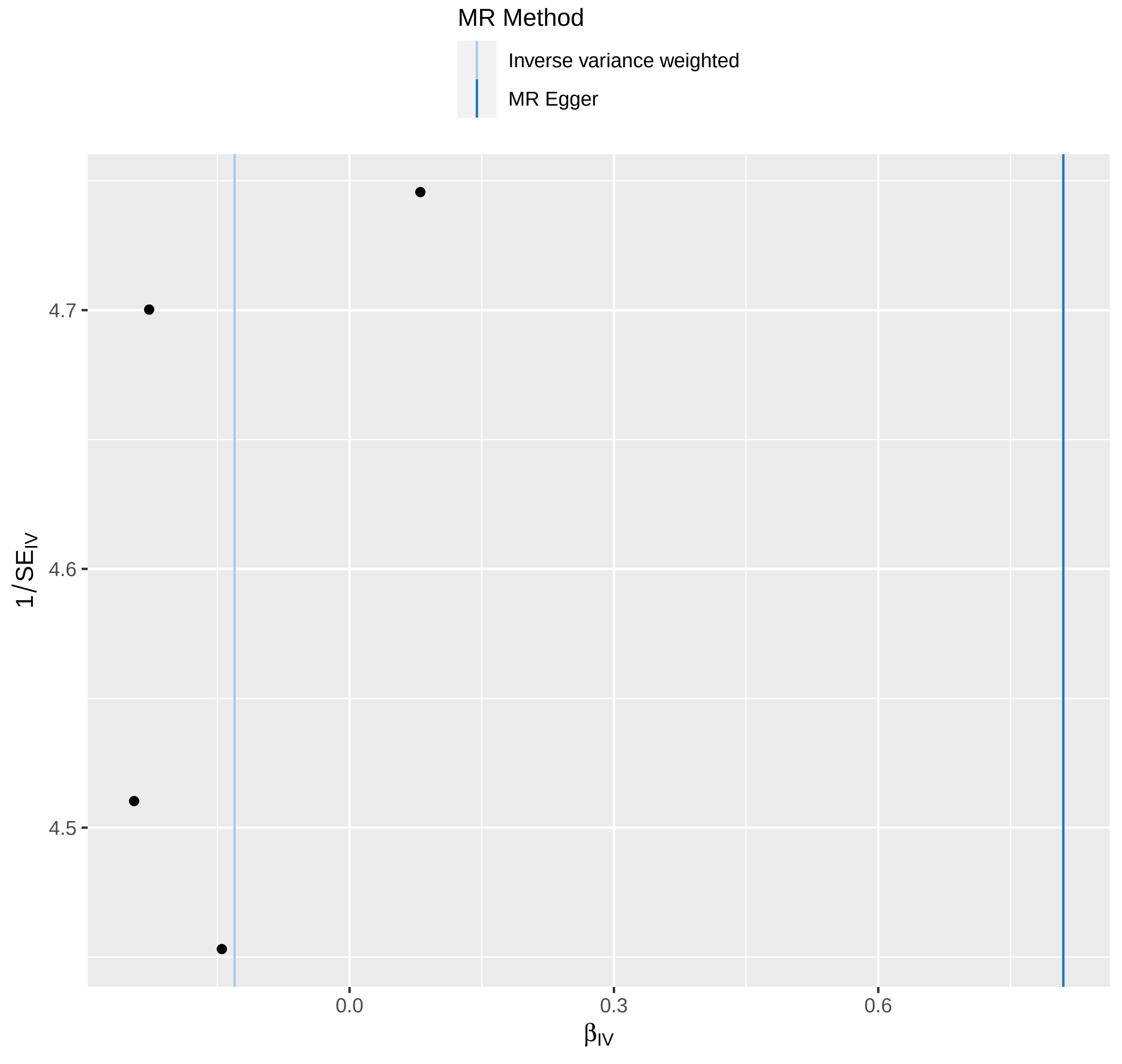

Batch 2−4326 : Gut microbiota abundance (genus Parasutterella id.2892) on Polyadenylate−binding protein 3
with a threshold of 1e−05

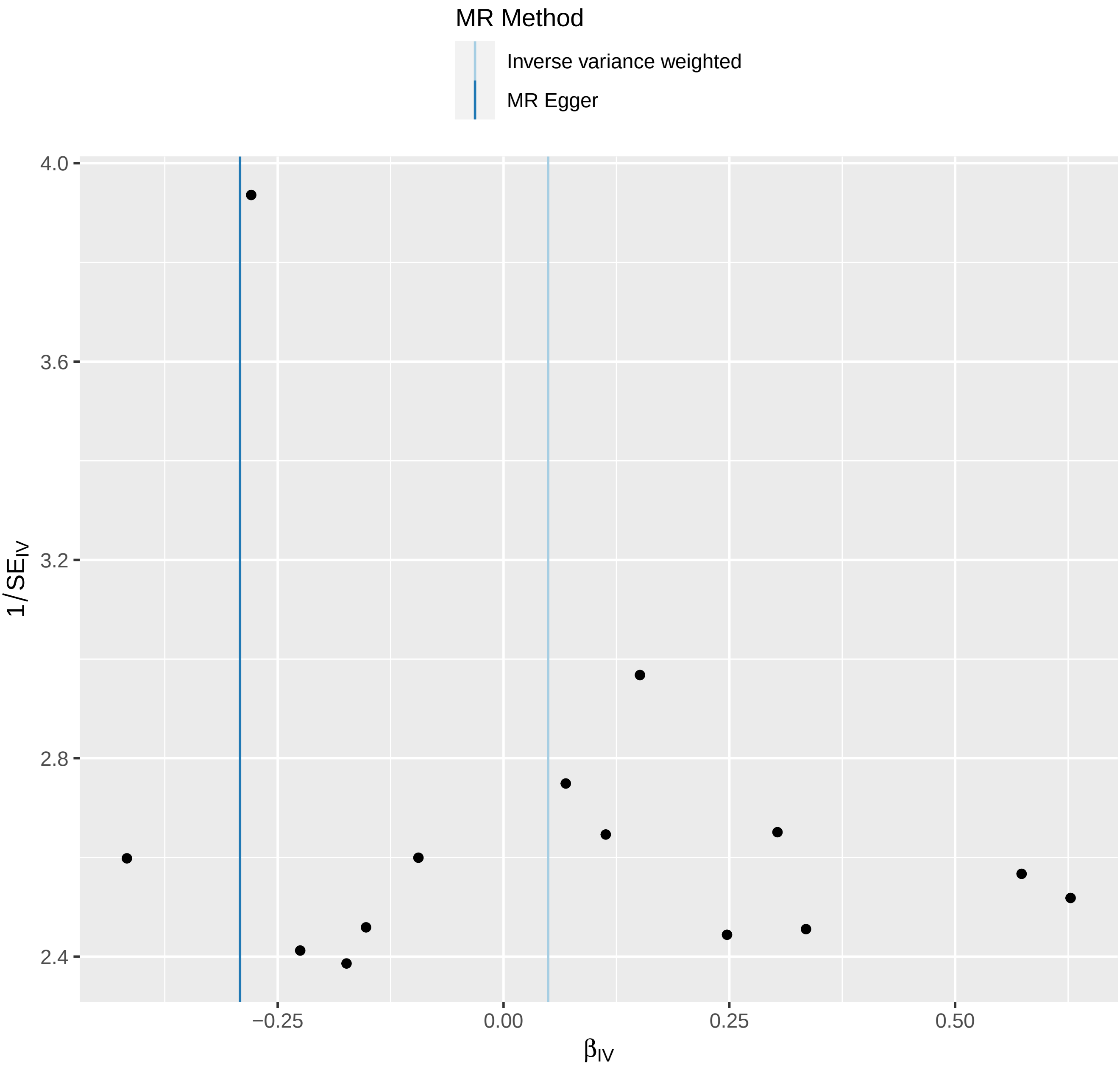

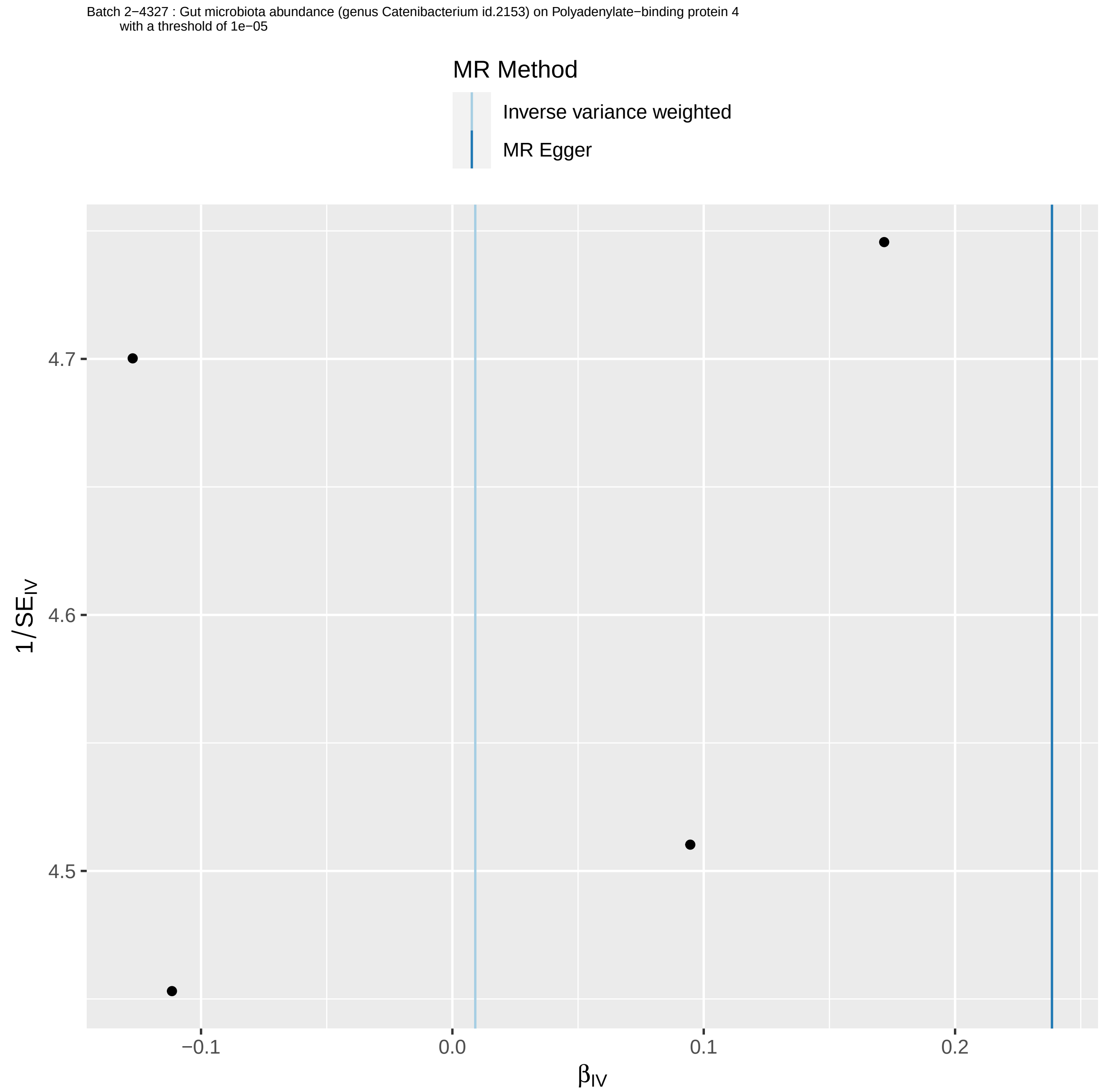

Batch 2−4327 : Gut microbiota abundance (genus Catenibacterium id.2153) on Polyadenylate−binding protein 4
with a threshold of 1e−05
MR Method
Inverse variance weighted
MR Egger
4.7
4.6
4.5
1/SE$_{IV}$
−0.1
0.0
0.1
0.2
β_{IV}

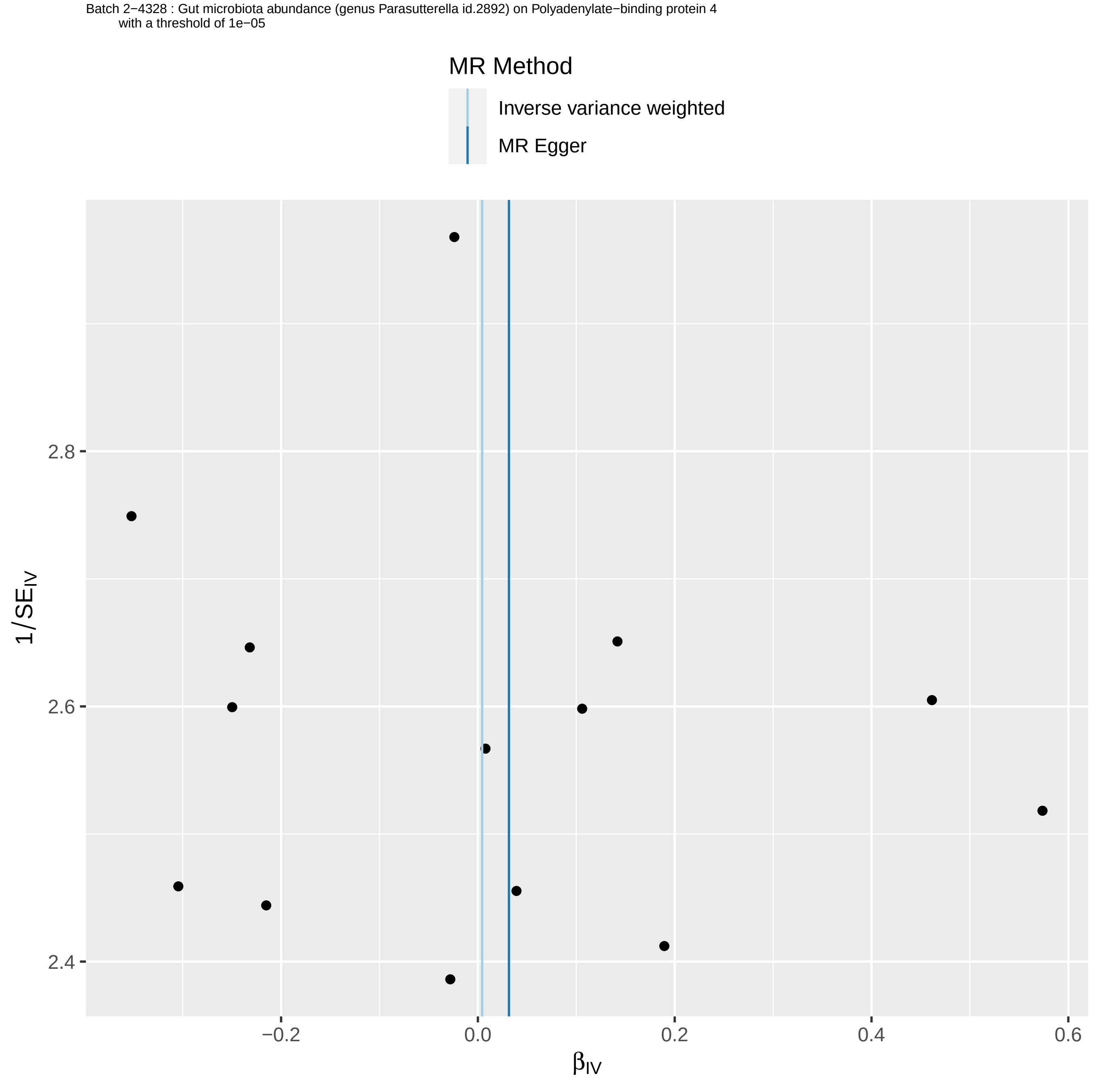
Batch 2−4328 : Gut microbiota abundance (genus Parasutterella id.2892) on Polyadenylate−binding protein 4
with a threshold of 1e−05
MR Method
Inverse variance weighted
MR Egger
2.8
2.6
2.4
$1/SE_{IV}$
−0.2
0.0
0.2
0.4
0.6
β_{IV}

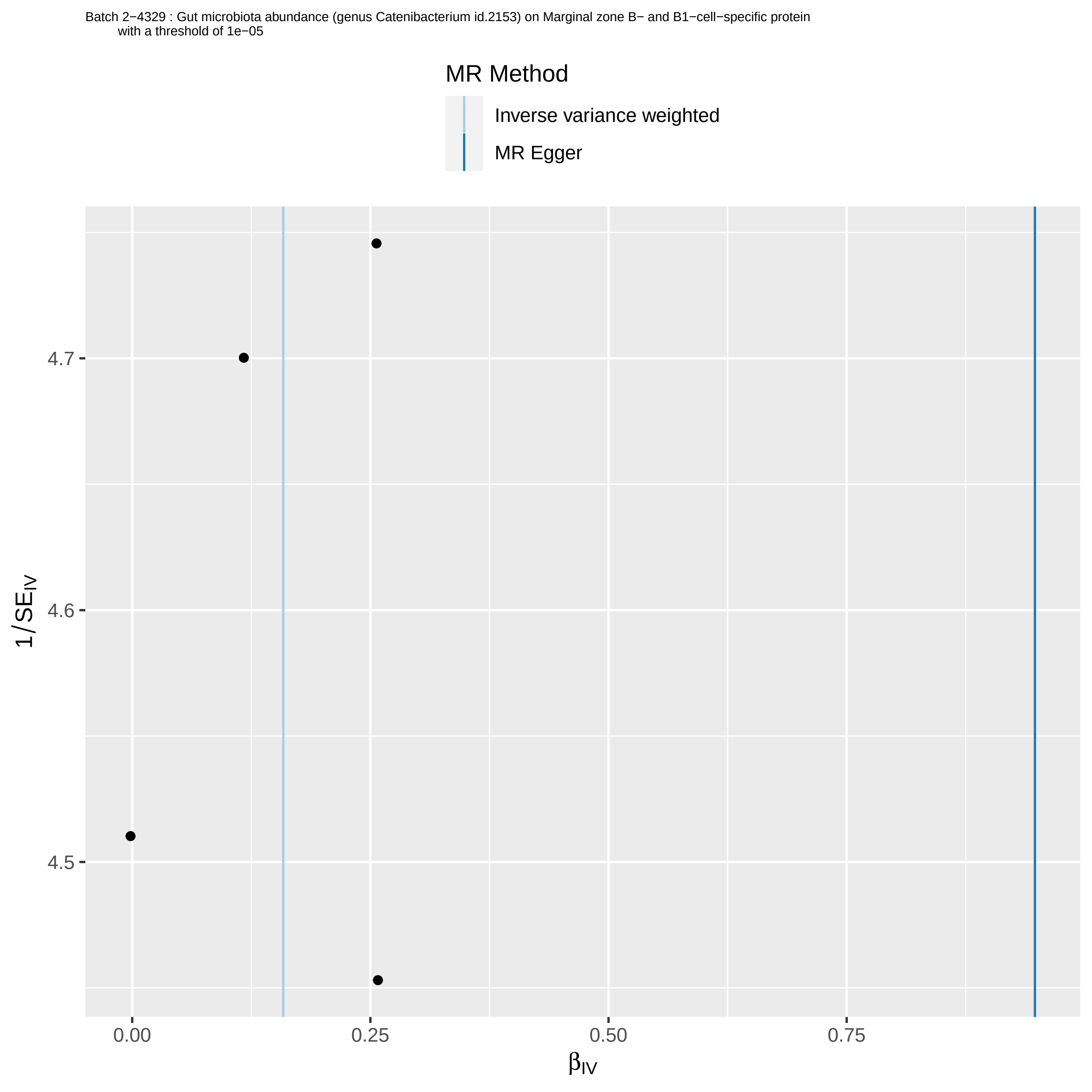

Batch 2−4329 : Gut microbiota abundance (genus Catenibacterium id.2153) on Marginal zone B− and B1−cell−specific protein
with a threshold of 1e−05
MR Method
Inverse variance weighted
MR Egger
4.7
4.6
4.5
1/SE_IV
0.00
0.25
0.50
0.75
β_IV

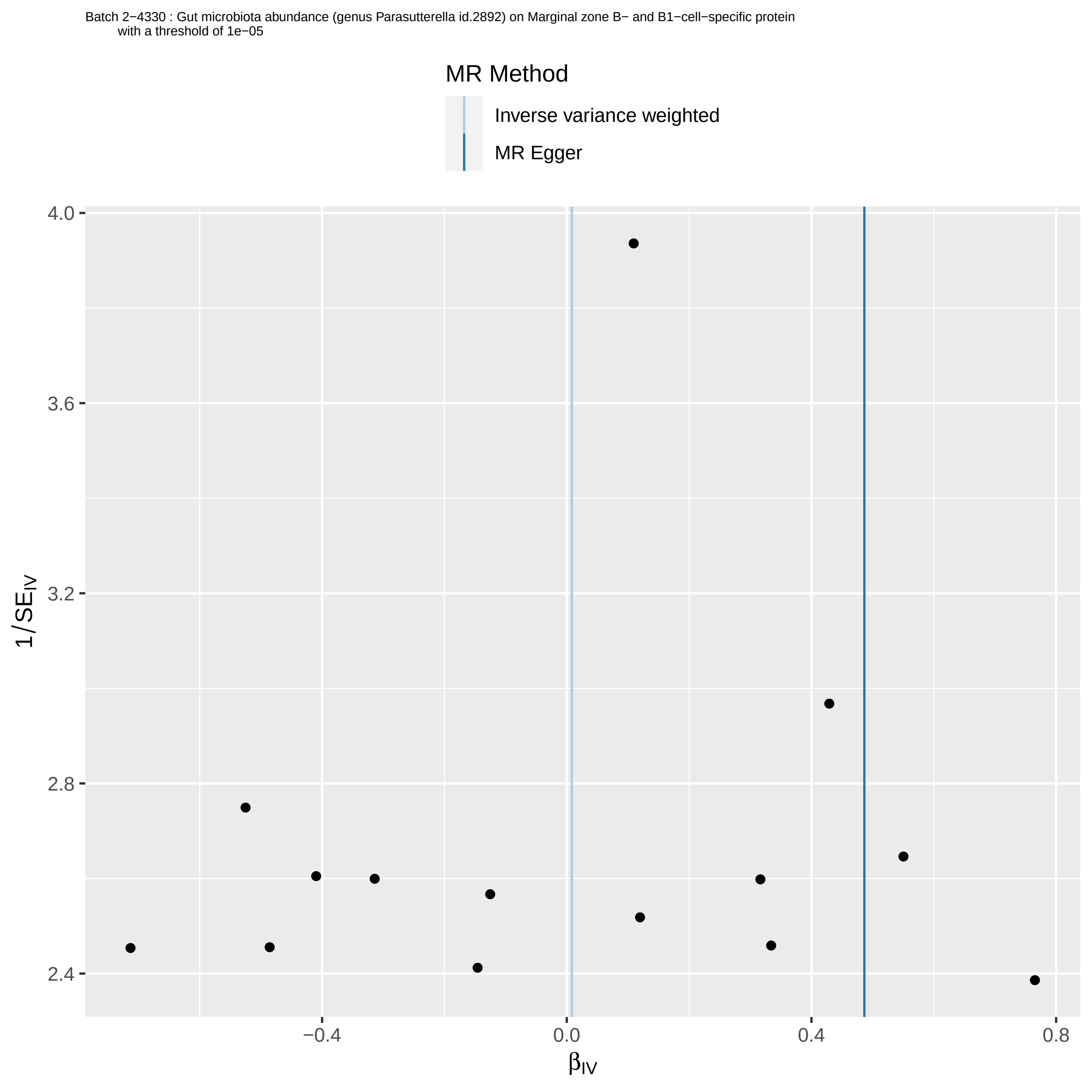
Batch 2−4330 : Gut microbiota abundance (genus Parasutterella id.2892) on Marginal zone B− and B1−cell−specific protein
with a threshold of 1e−05
MR Method
Inverse variance weighted
MR Egger
4.0
3.6
3.2
2.8
2.4
1/SE_IV
−0.4
0.0
0.4
0.8
β_IV

Batch 2−4331 : Gut microbiota abundance (genus Catenibacterium id.2153) on Platelet−activating factor acetylhydrolase IB subunit beta
with a threshold of 1e−05

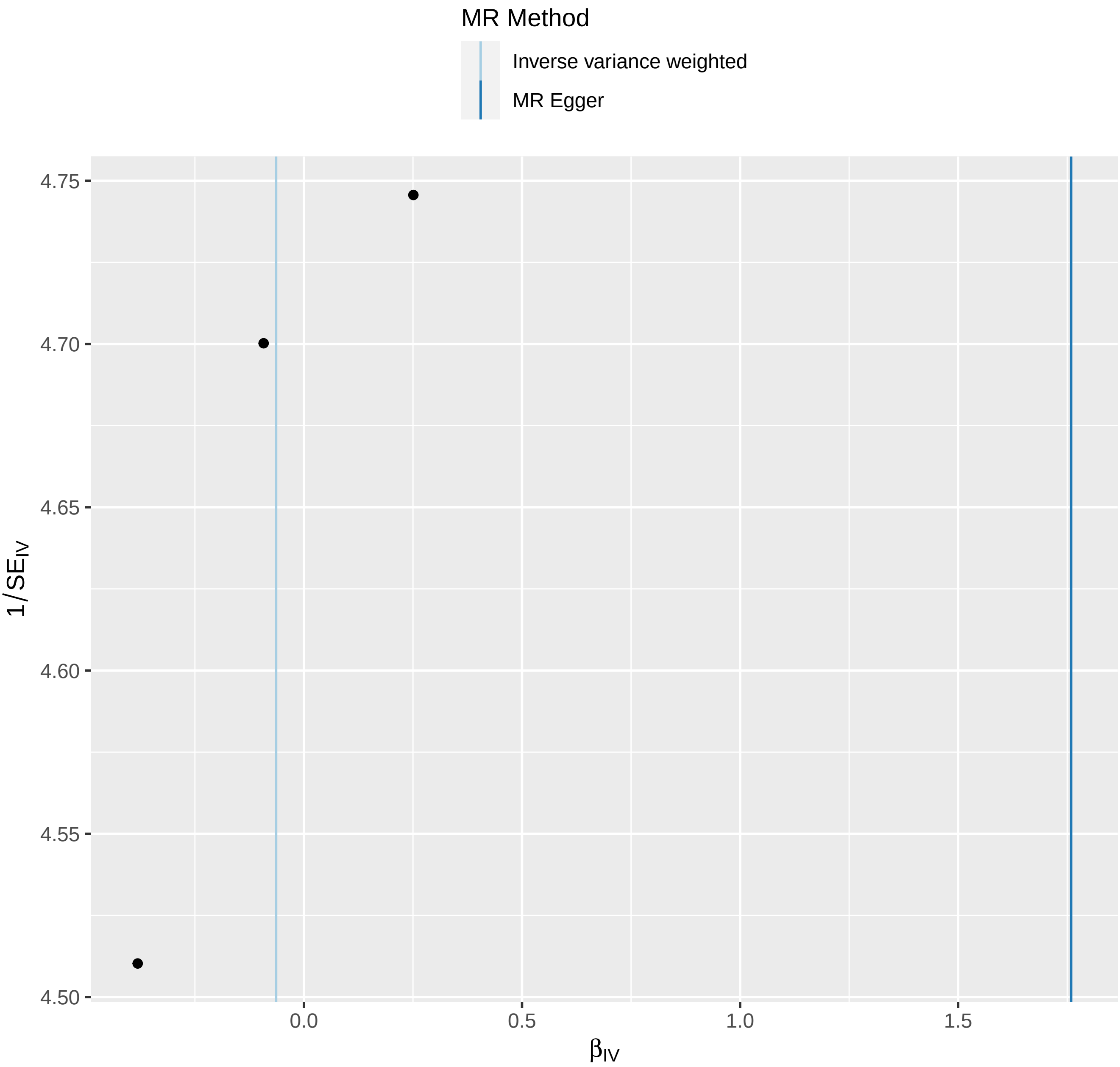

Batch 2−4332 : Gut microbiota abundance (genus Parasutterella id.2892) on Platelet−activating factor acetylhydrolase IB subunit beta
with a threshold of 1e−05

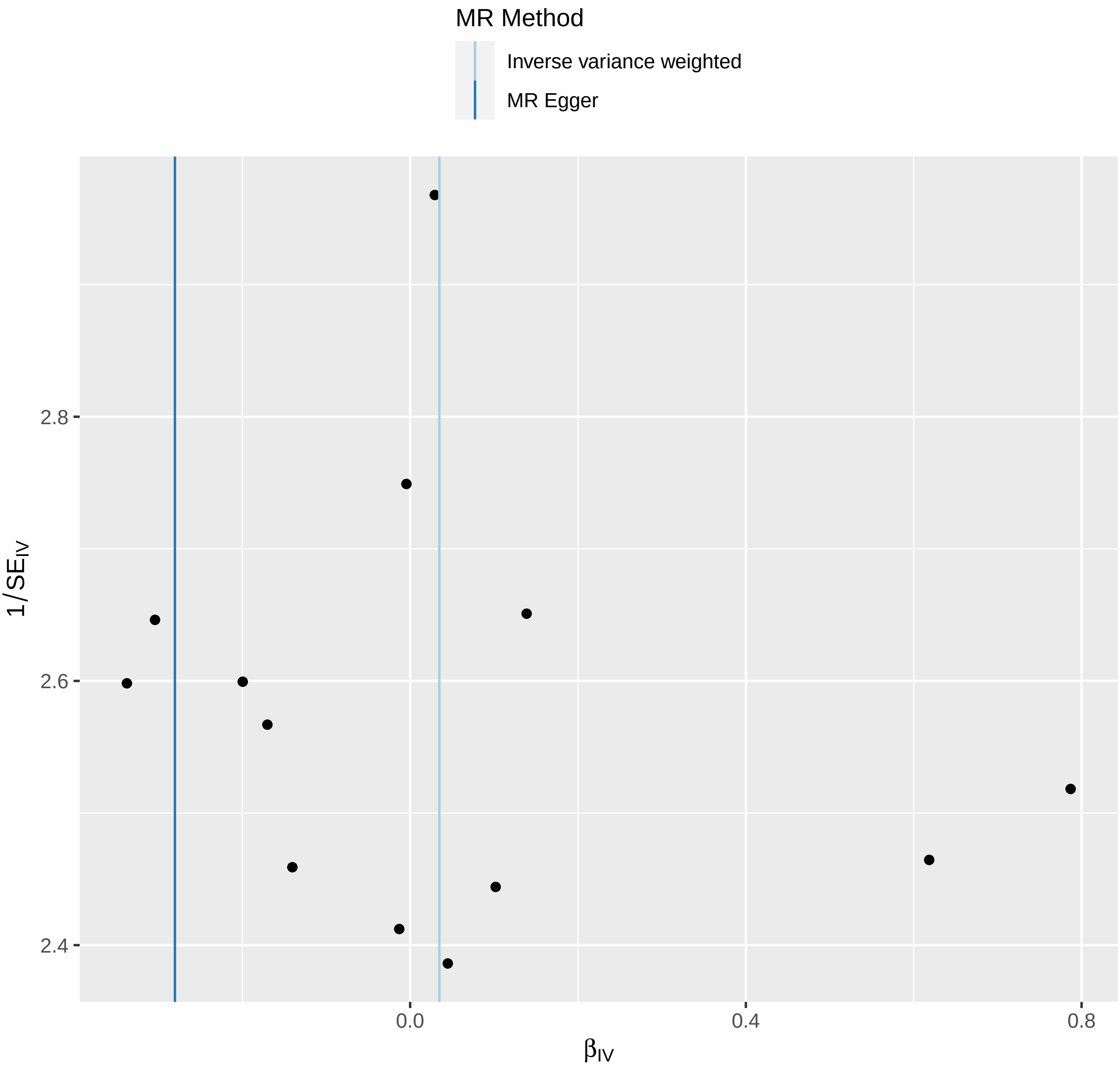

Batch 2−4333 : Gut microbiota abundance (genus Catenibacterium id.2153) on Multifunctional protein ADE2
with a threshold of 1e−05

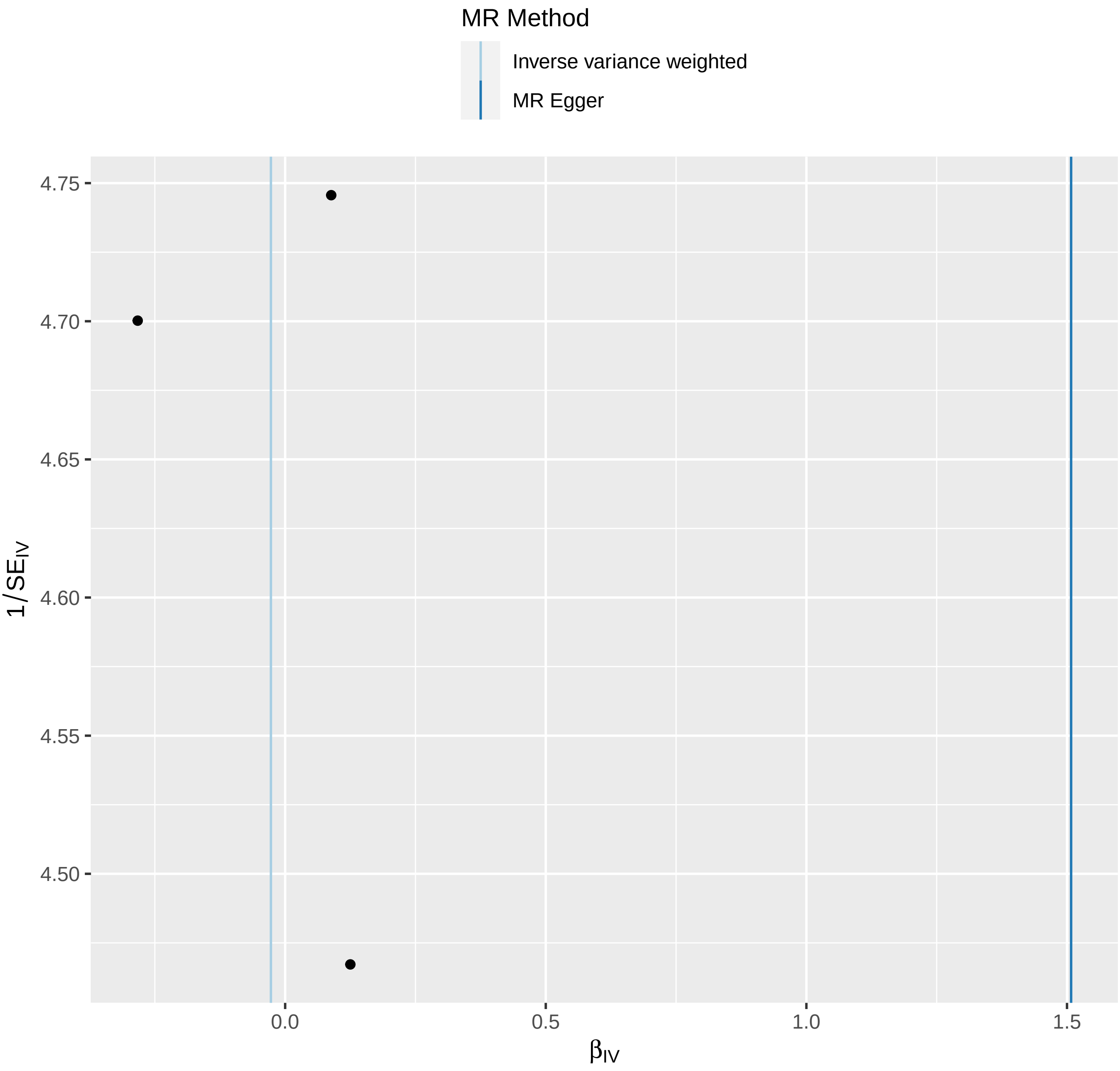

Batch 2−4334 : Gut microbiota abundance (genus Parasutterella id.2892) on Multifunctional protein ADE2
with a threshold of 1e−05

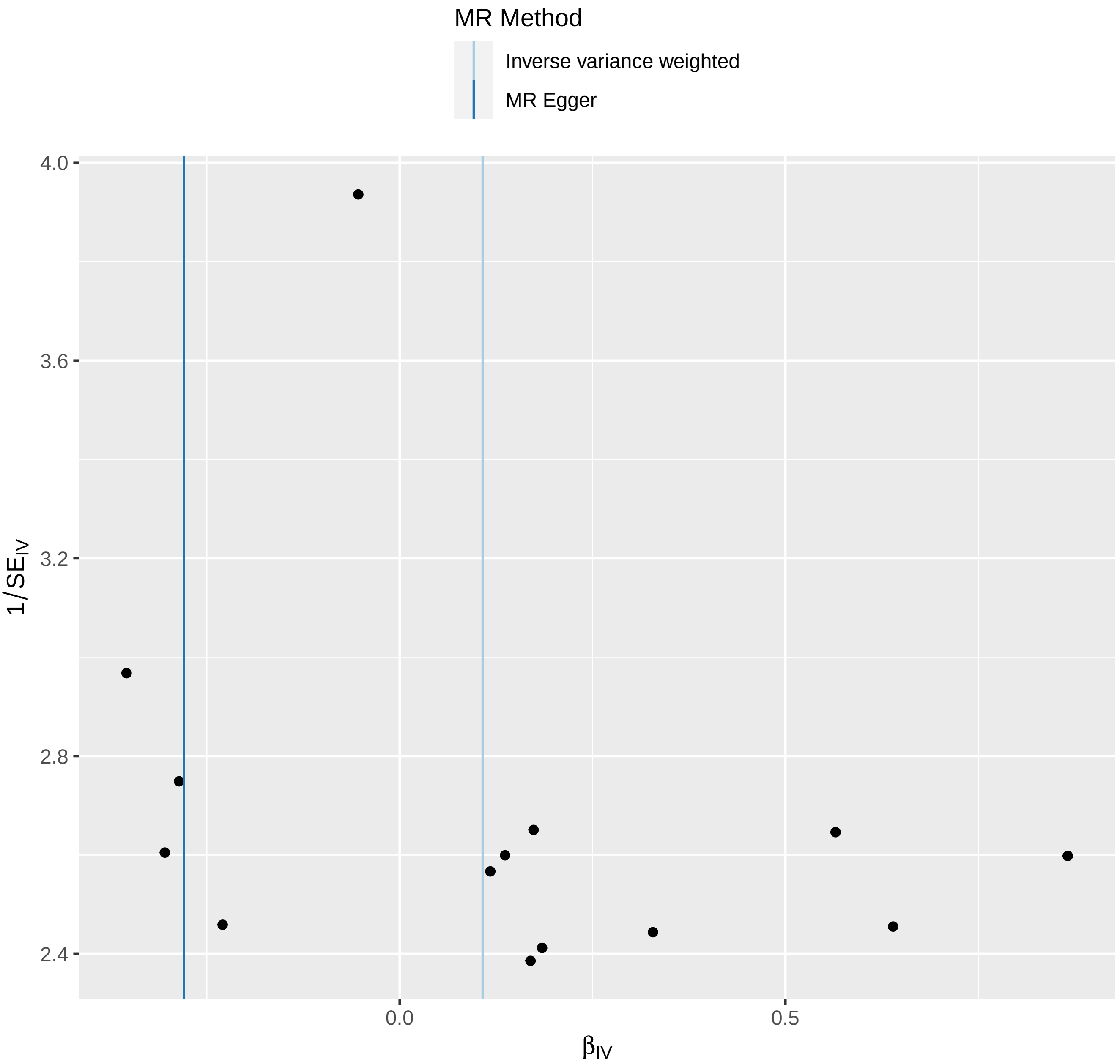

Batch 2−4335 : Gut microbiota abundance (genus Catenibacterium id.2153) on Polyadenylate−binding protein−interacting protein 1 with a threshold of 1e−05

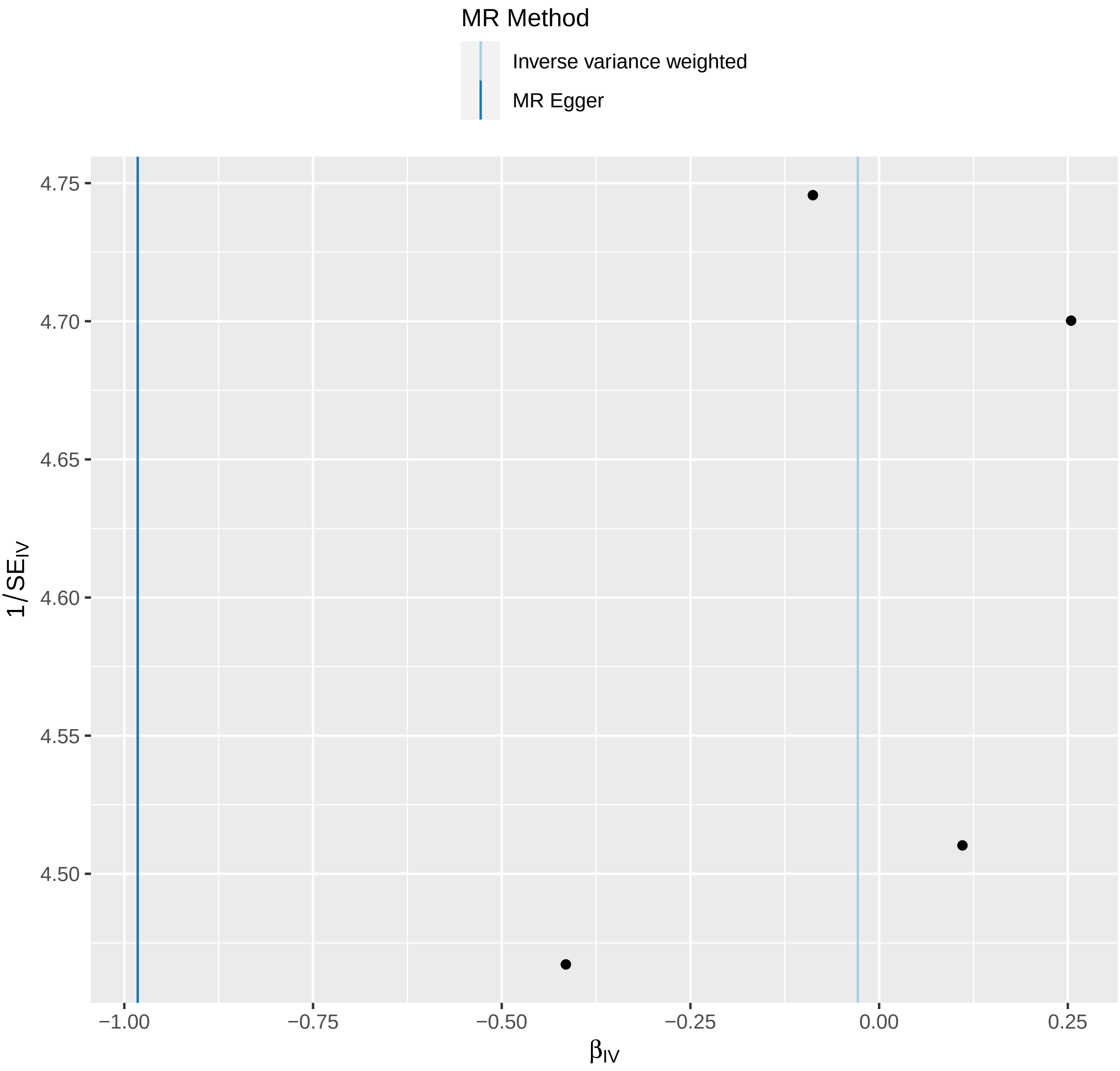

Batch 2−4336 : Gut microbiota abundance (genus Parasutterella id.2892) on Polyadenylate−binding protein−interacting protein 1
with a threshold of 1e−05

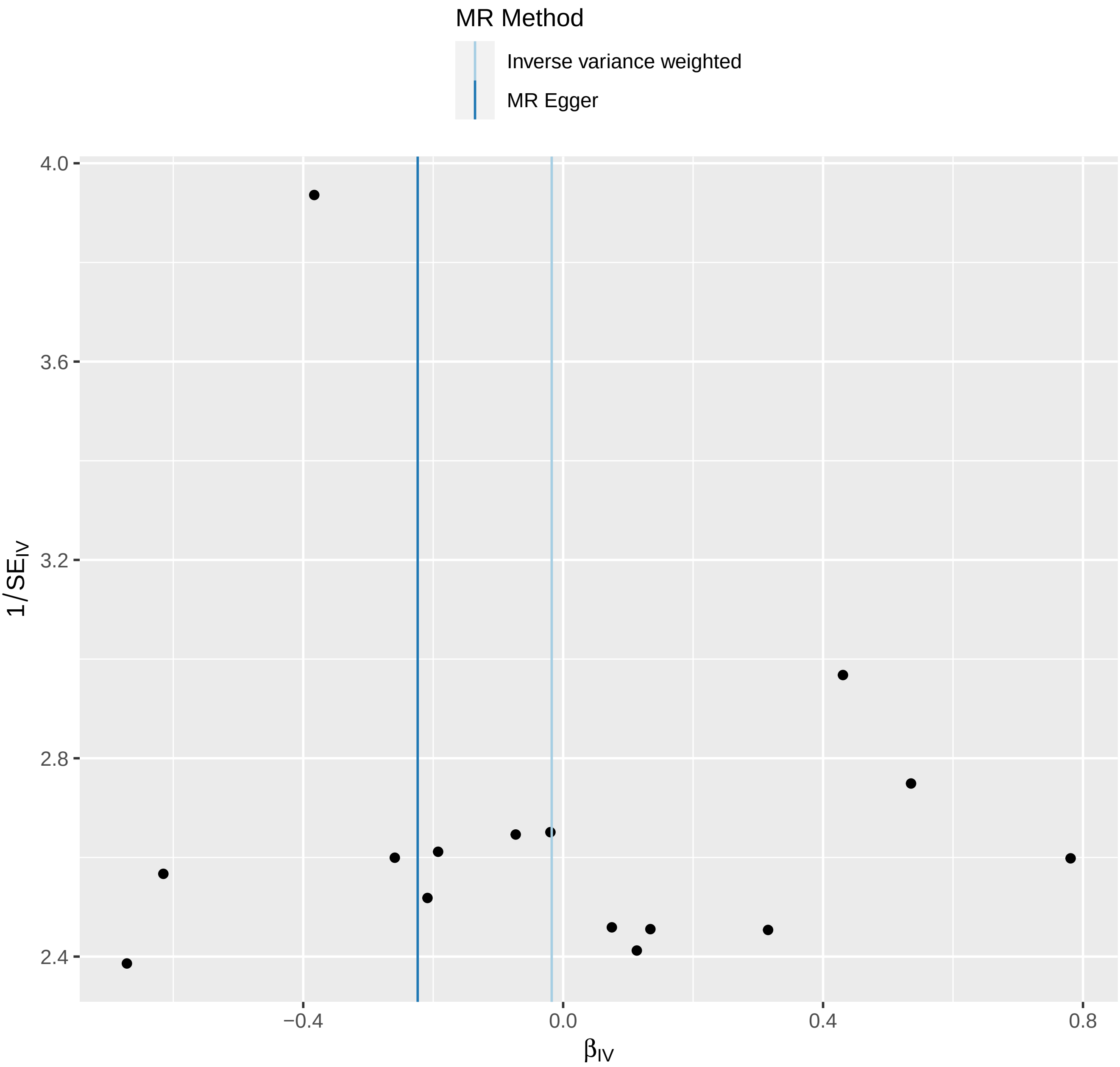

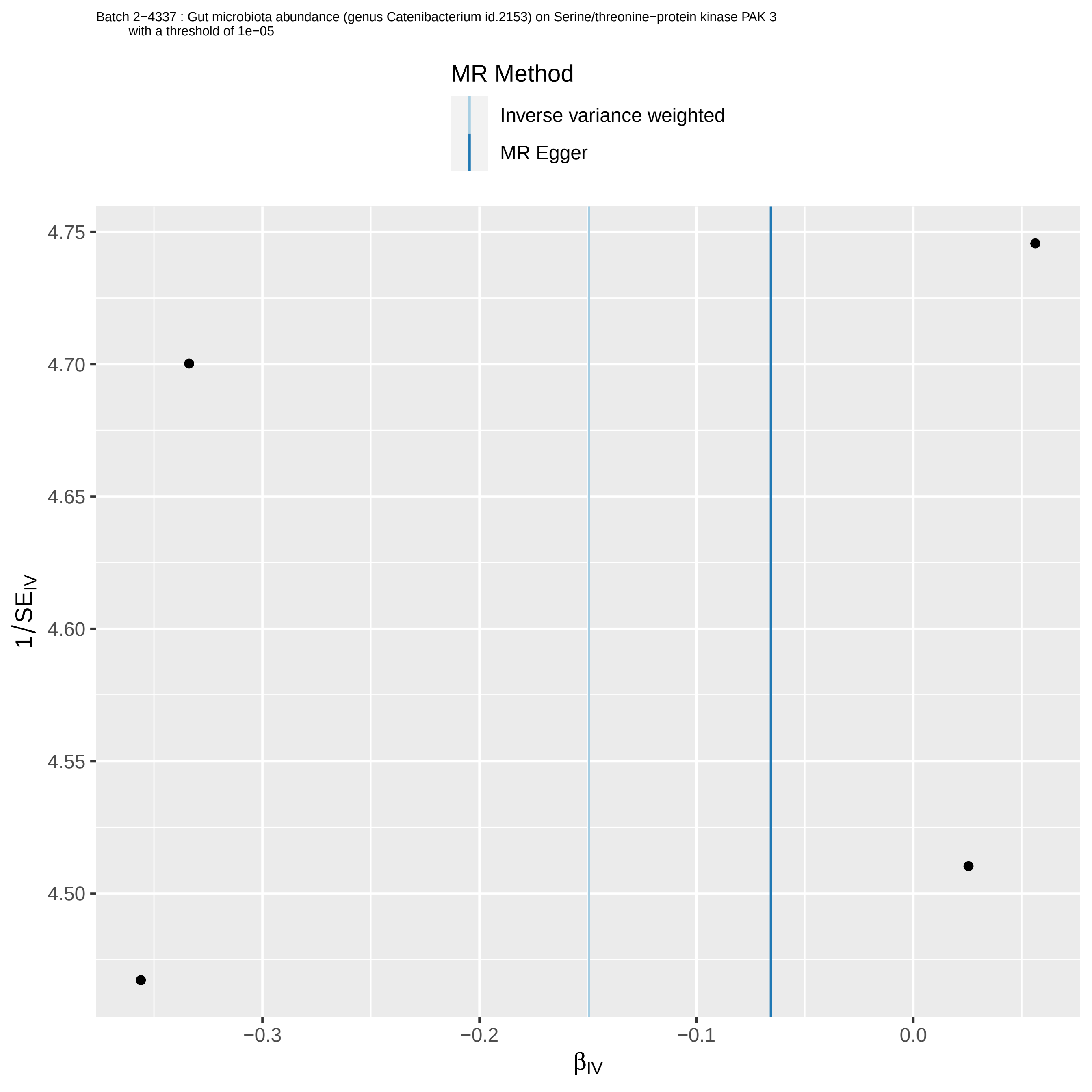
Batch 2−4337 : Gut microbiota abundance (genus Catenibacterium id.2153) on Serine/threonine−protein kinase PAK 3
with a threshold of 1e−05
MR Method
Inverse variance weighted
MR Egger
4.75
4.70
4.65
4.60
4.55
4.50
1/SE_IV
−0.3
−0.2
−0.1
0.0
β_IV

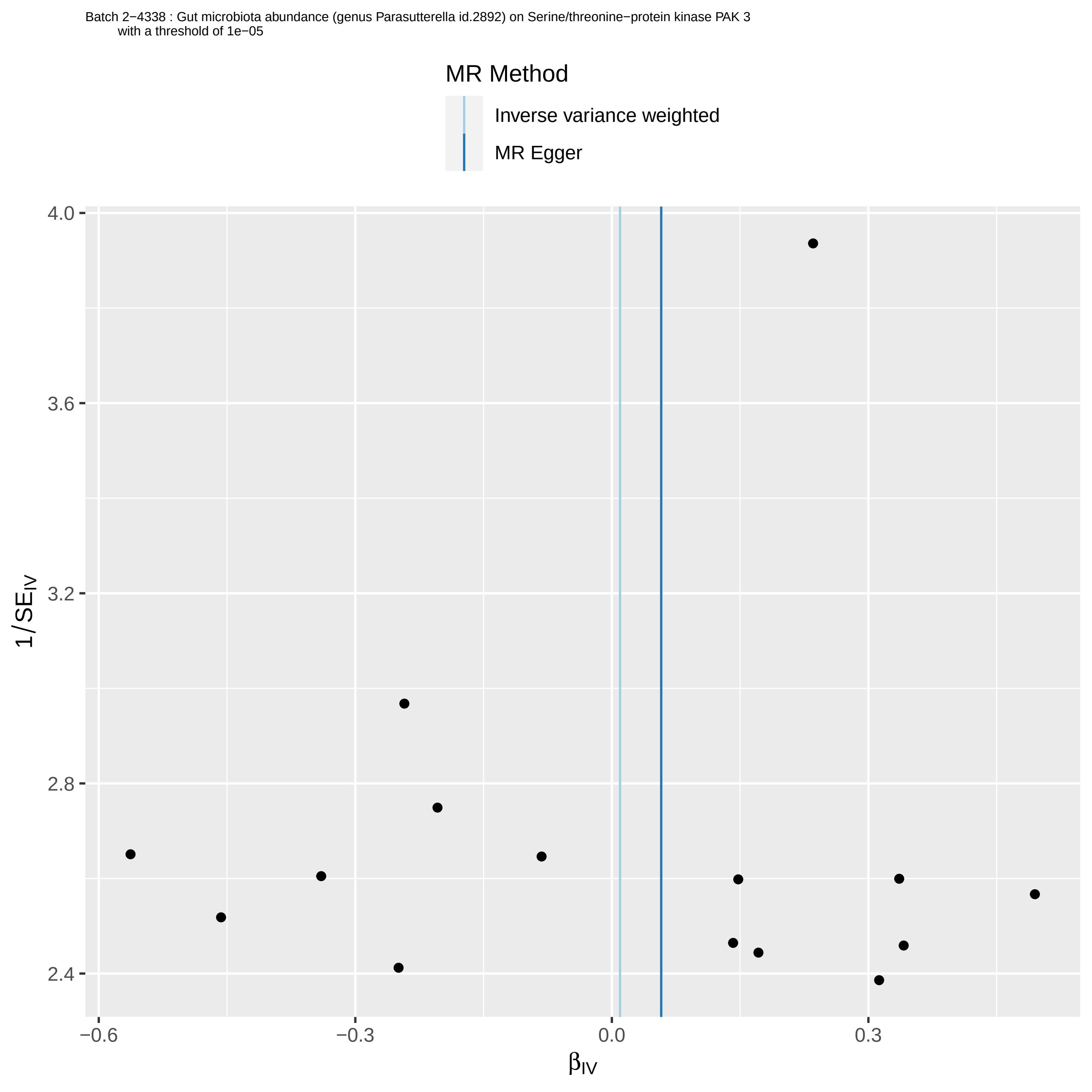

Batch 2−4338 : Gut microbiota abundance (genus Parasutterella id.2892) on Serine/threonine−protein kinase PAK 3
with a threshold of 1e−05
MR Method
Inverse variance weighted
MR Egger
4.0
3.6
3.2
2.8
2.4
1/SE_IV
−0.6
−0.3
0.0
0.3
β_IV

Batch 2−4339 : Gut microbiota abundance (genus Catenibacterium id.2153) on Serine/threonine−protein kinase PAK 4
with a threshold of 1e−05

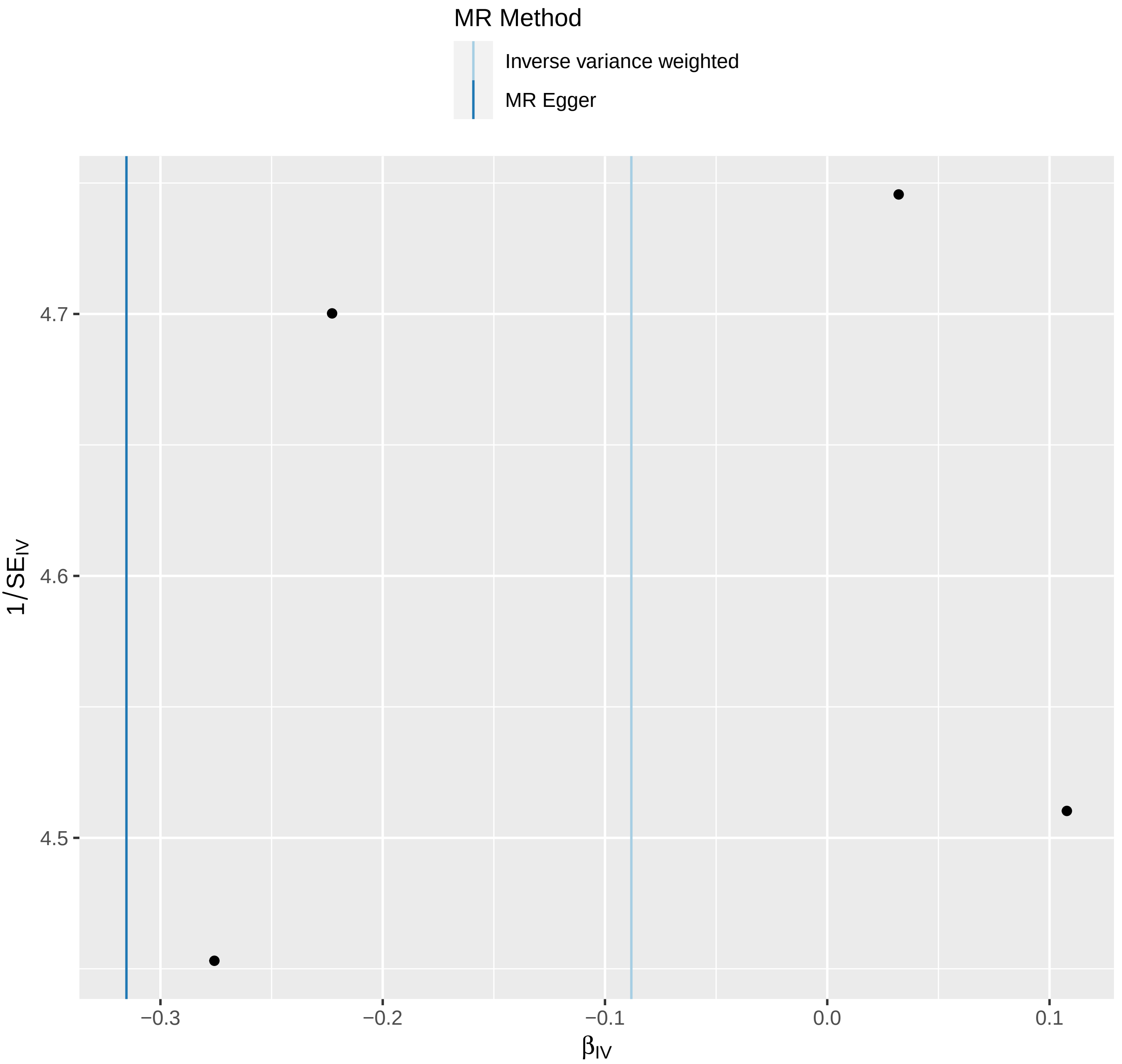

Batch 2−4340 : Gut microbiota abundance (genus Parasutterella id.2892) on Serine/threonine−protein kinase PAK 4
with a threshold of 1e−05

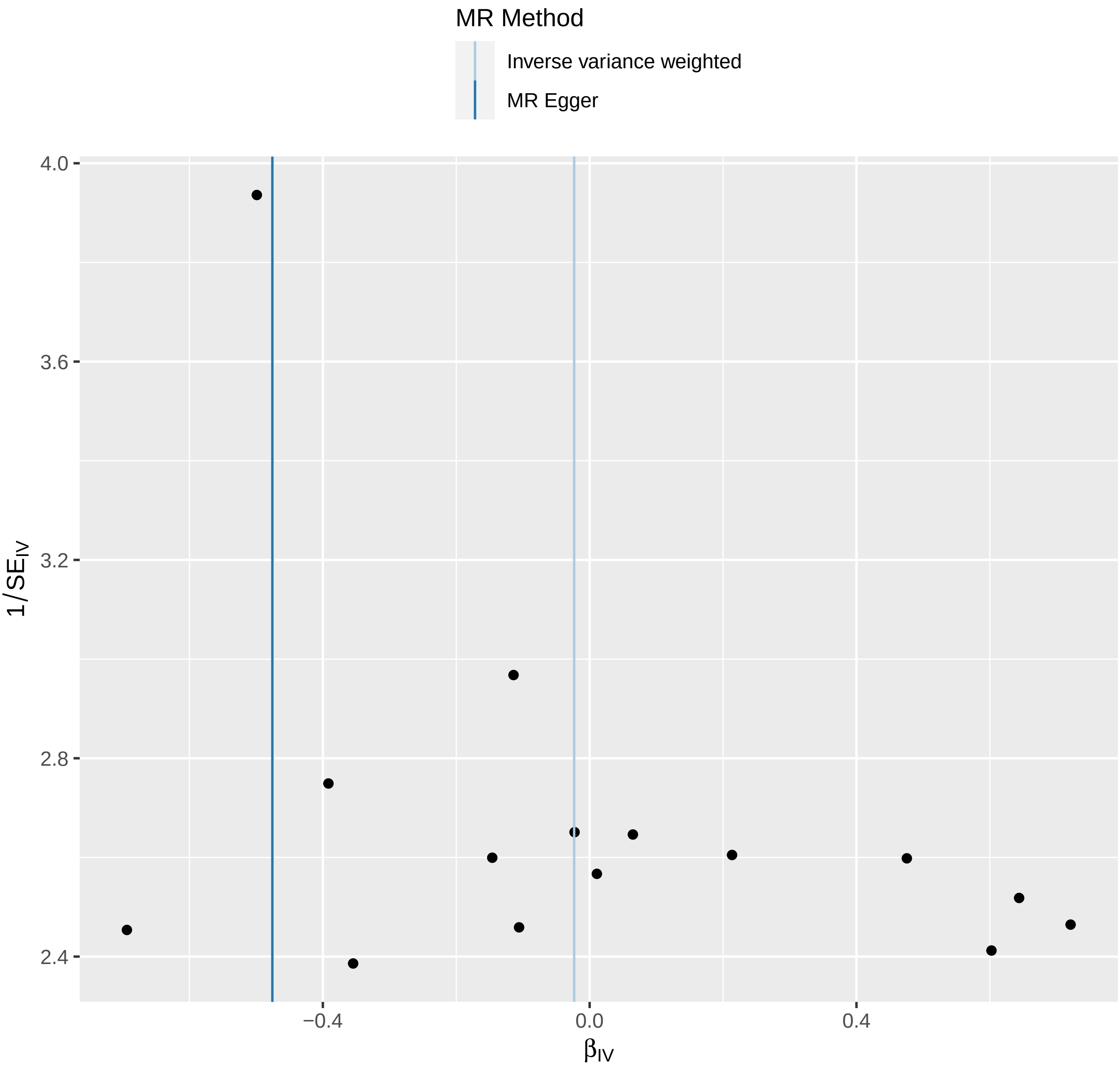

Batch 2−4341 : Gut microbiota abundance (genus Catenibacterium id.2153) on Serine/threonine−protein kinase PAK 7
with a threshold of 1e−05

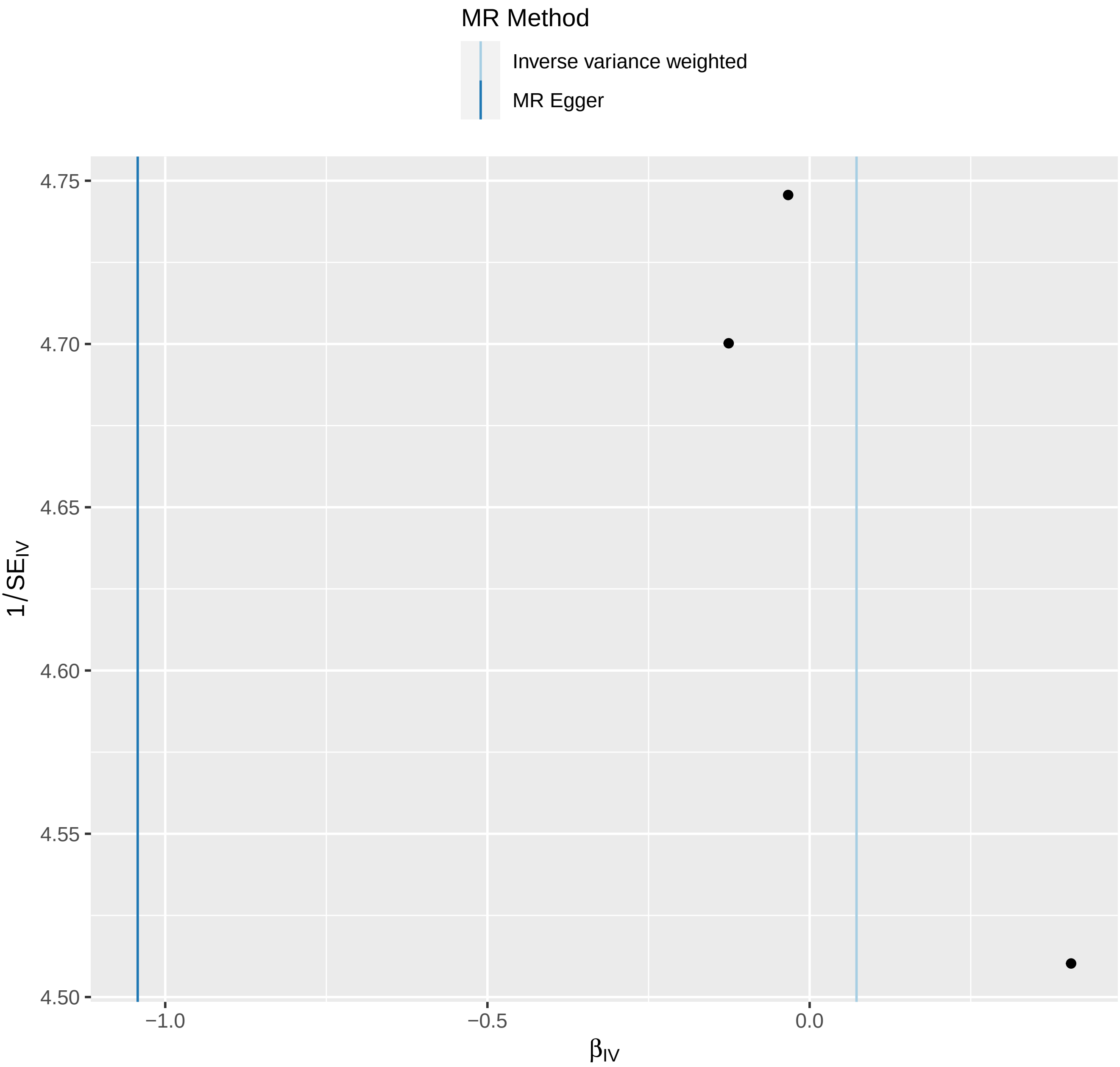

Batch 2−4342 : Gut microbiota abundance (genus Parasutterella id.2892) on Serine/threonine−protein kinase PAK 7 with a threshold of 1e−05

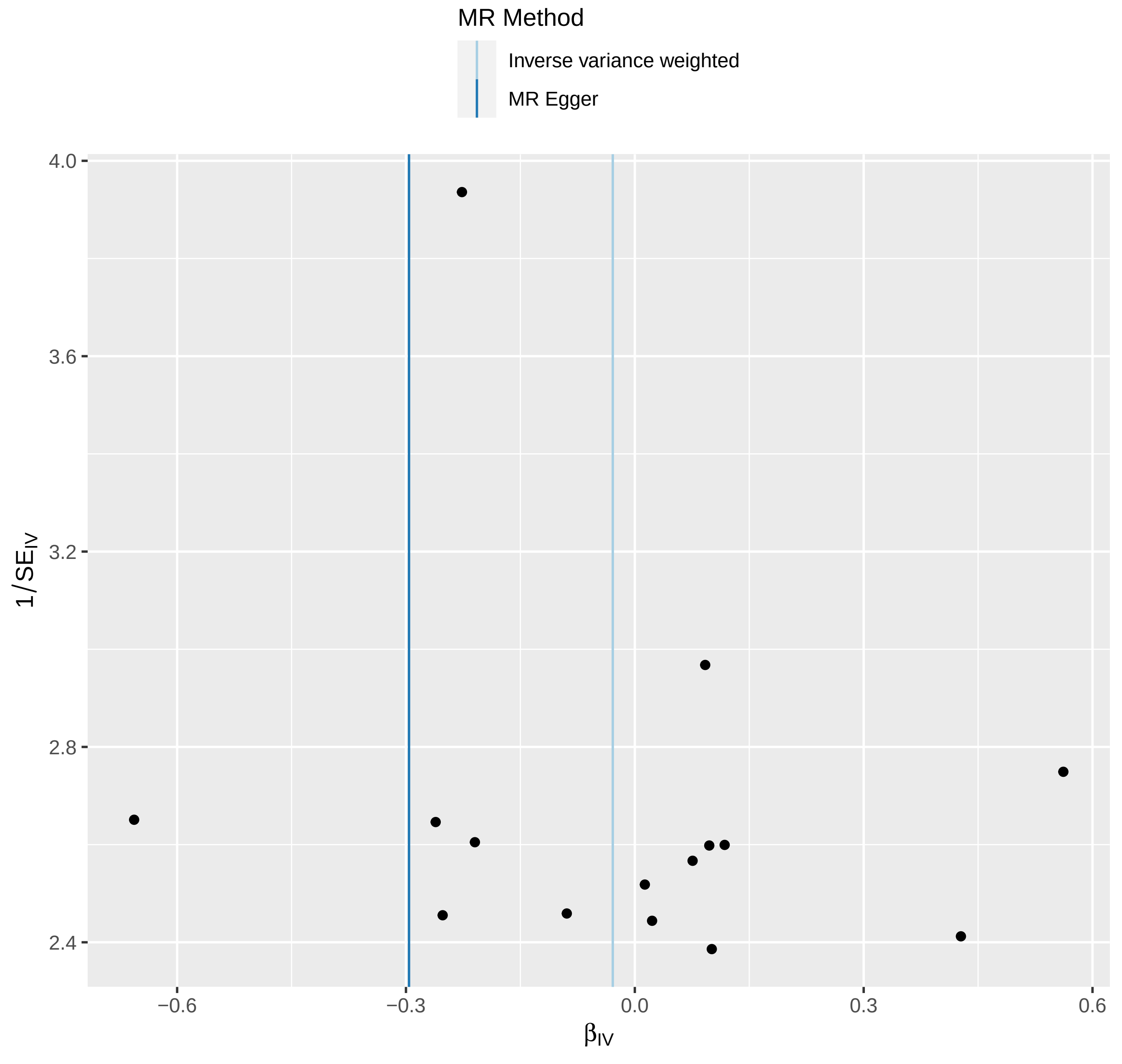

Batch 2−4343 : Gut microbiota abundance (genus Catenibacterium id.2153) on Peptidyl−glycine alpha−amidating monooxygenase with a threshold of 1e−05

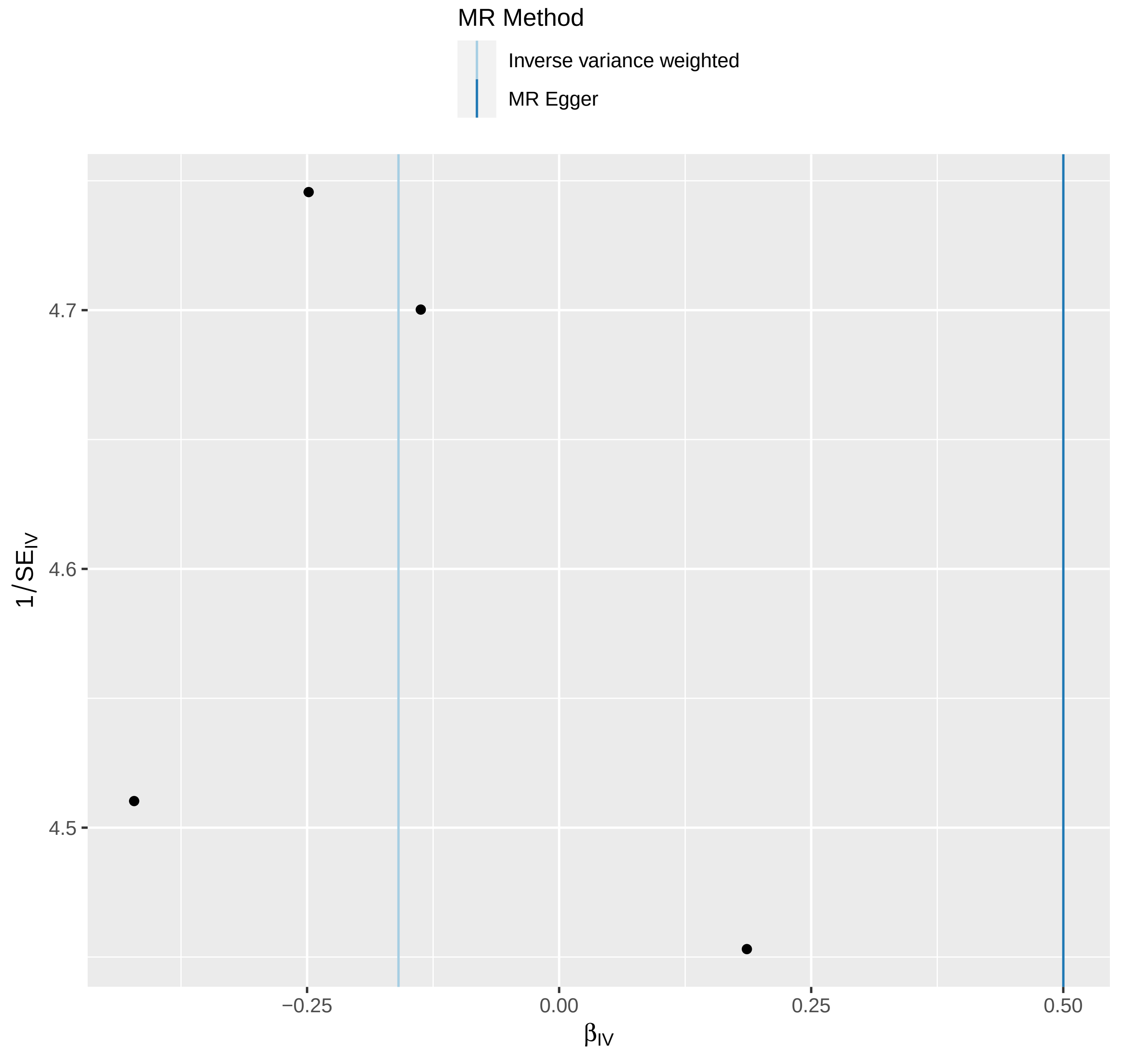

Batch 2−4344 : Gut microbiota abundance (genus Parasutterella id.2892) on Peptidyl−glycine alpha−amidating monooxygenase with a threshold of 1e−05

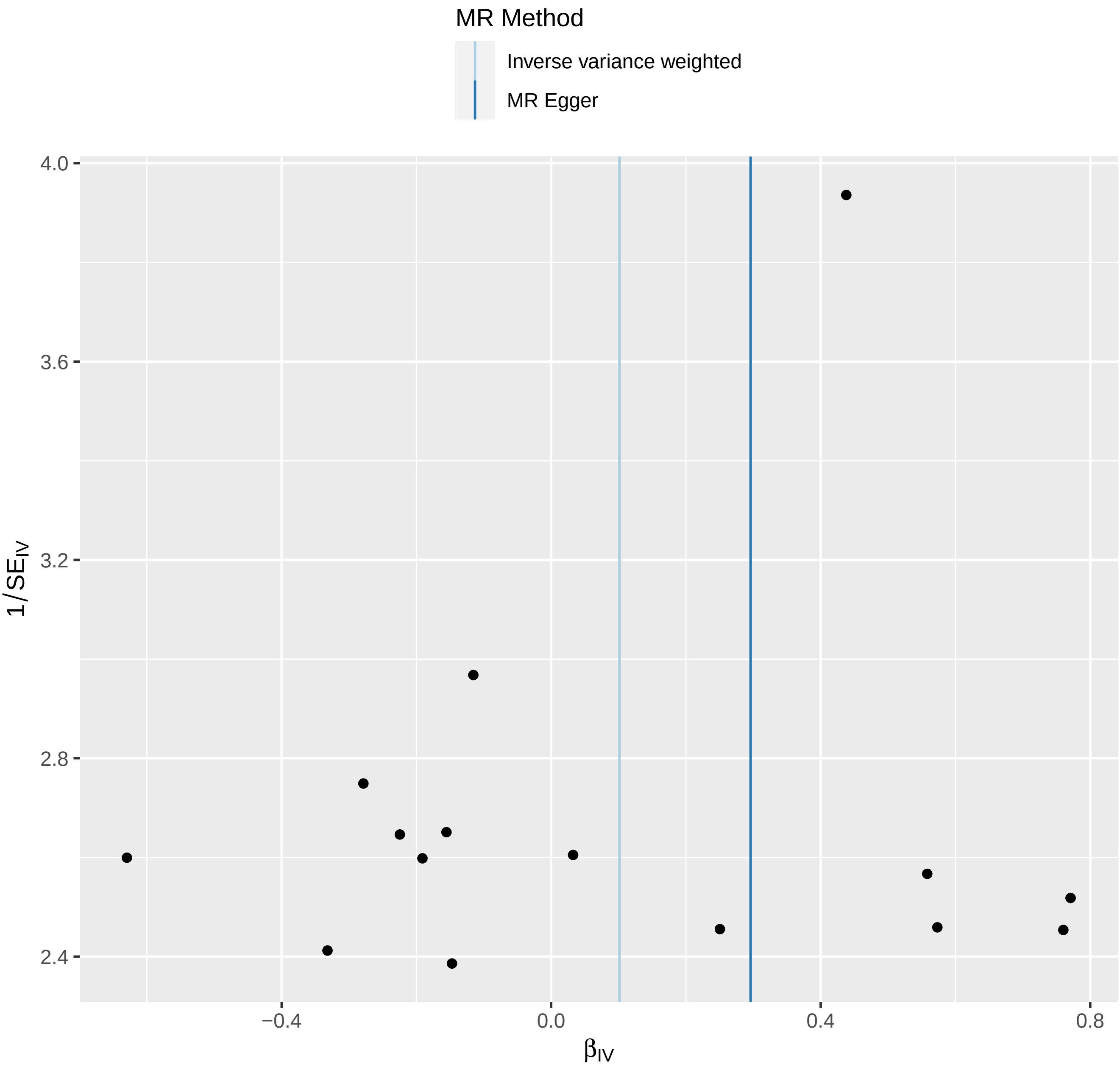

Batch 2−4345 : Gut microbiota abundance (genus Catenibacterium id.2153) on Pantothenate kinase 3
with a threshold of 1e−05

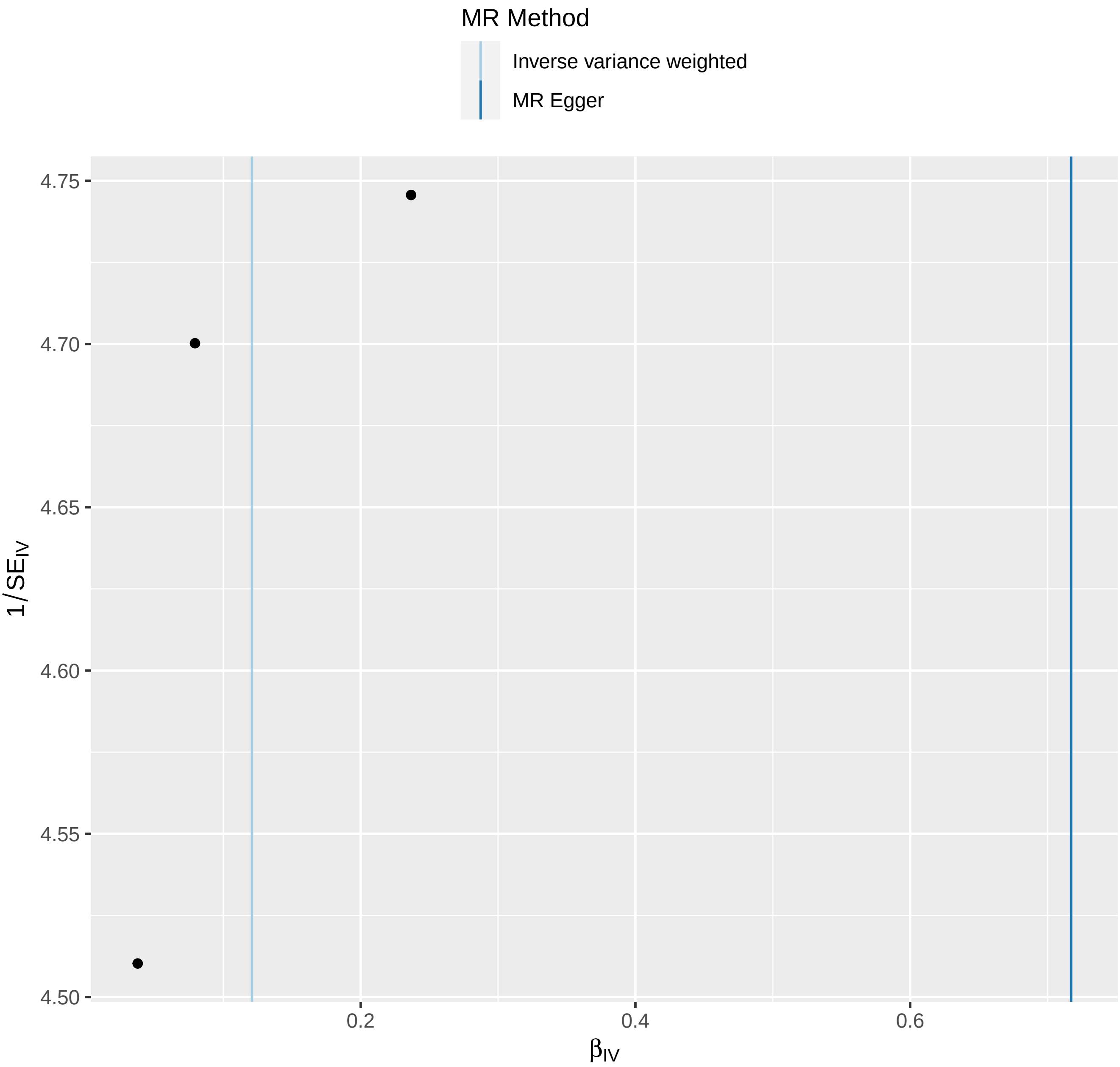

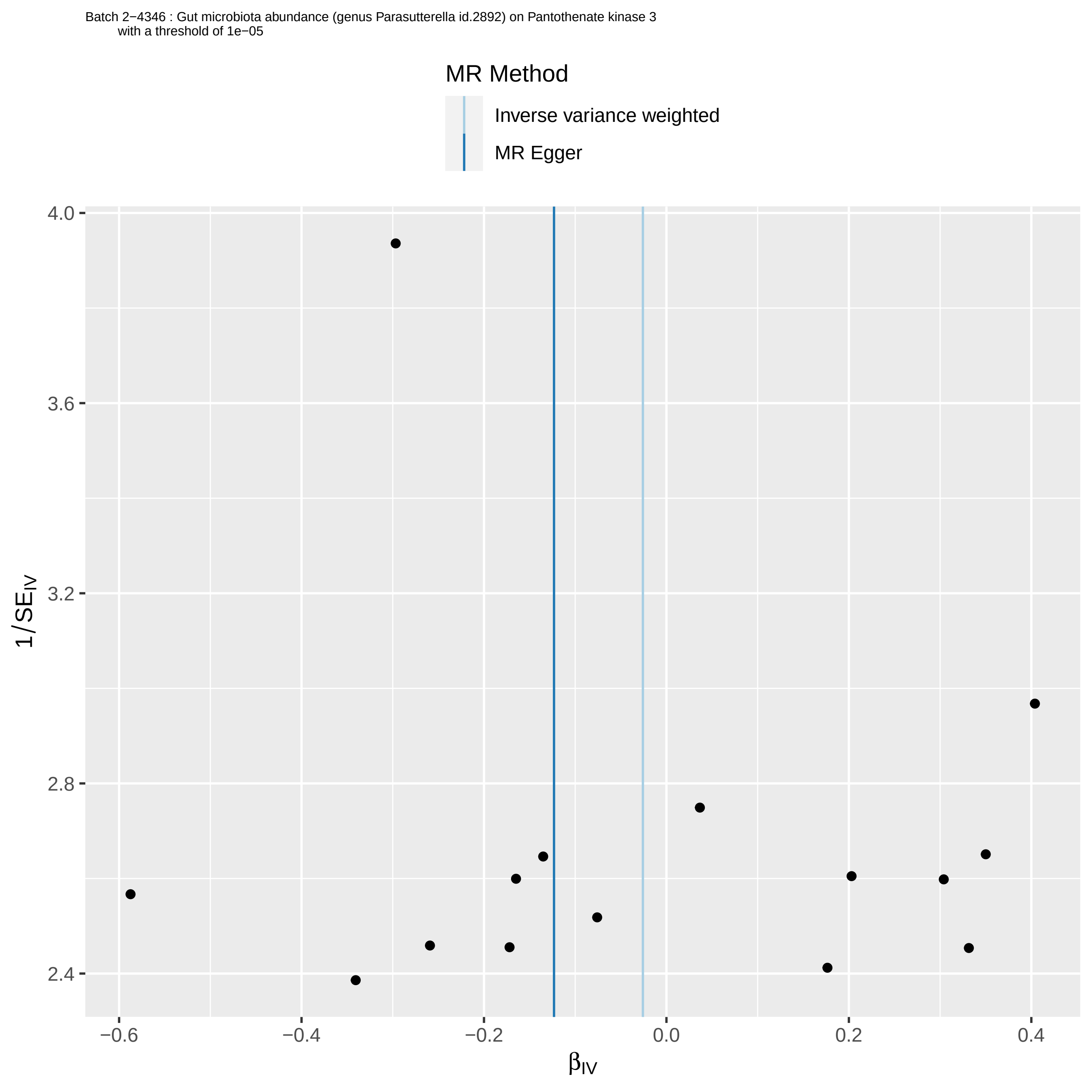

Batch 2−4346 : Gut microbiota abundance (genus Parasutterella id.2892) on Pantothenate kinase 3
with a threshold of 1e−05
MR Method
Inverse variance weighted
MR Egger
4.0
3.6
3.2
2.8
2.4
1/SE_IV
−0.6
−0.4
−0.2
0.0
0.2
0.4
β_IV

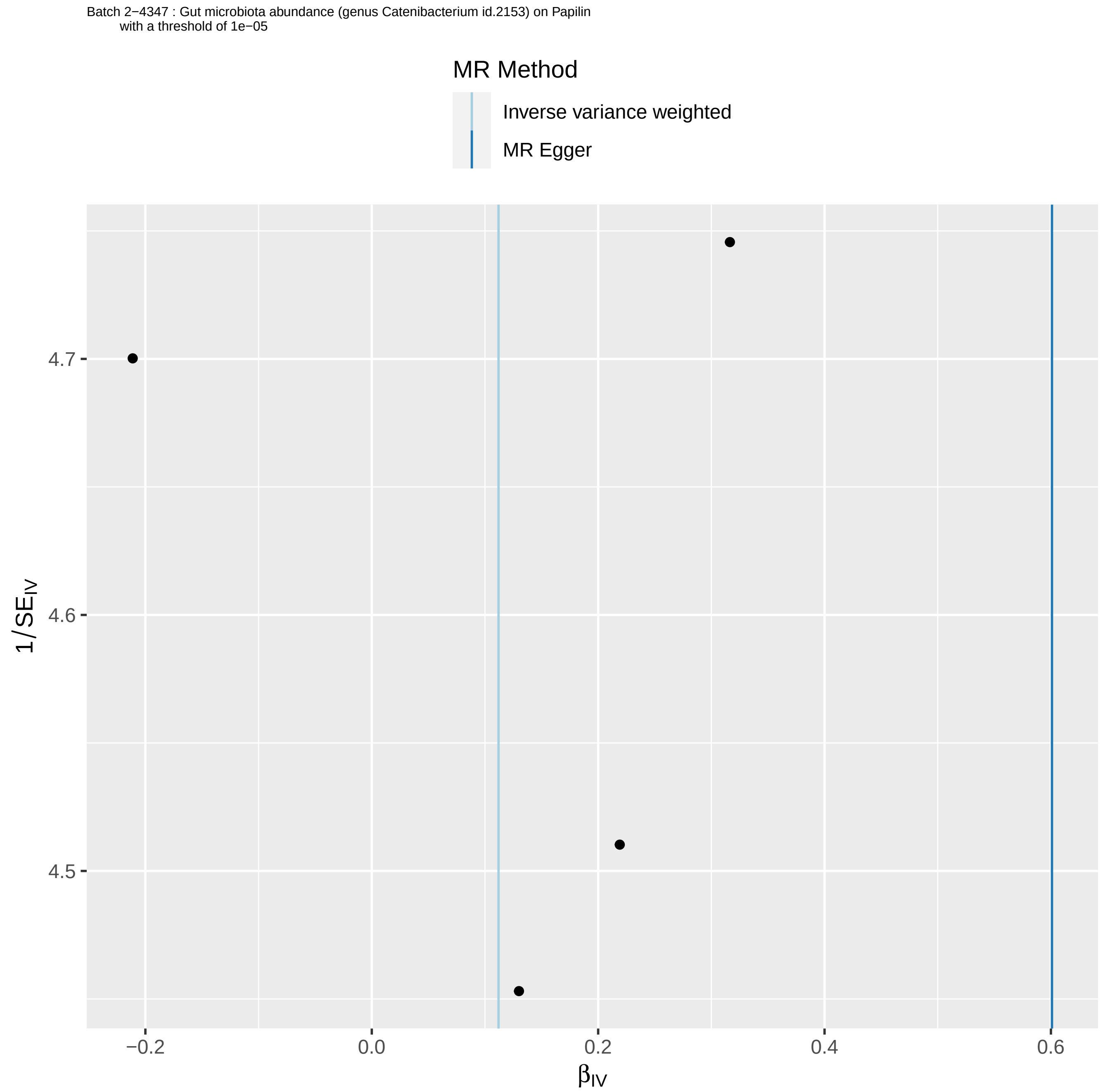

Batch 2−4347 : Gut microbiota abundance (genus Catenibacterium id.2153) on Papilin
with a threshold of 1e−05
MR Method
Inverse variance weighted
MR Egger
4.7
4.6
4.5
1/SE$_{IV}$
−0.2
0.0
0.2
0.4
0.6
β_{IV}

Batch 2−4348 : Gut microbiota abundance (genus Parasutterella id.2892) on Papilin
with a threshold of 1e−05

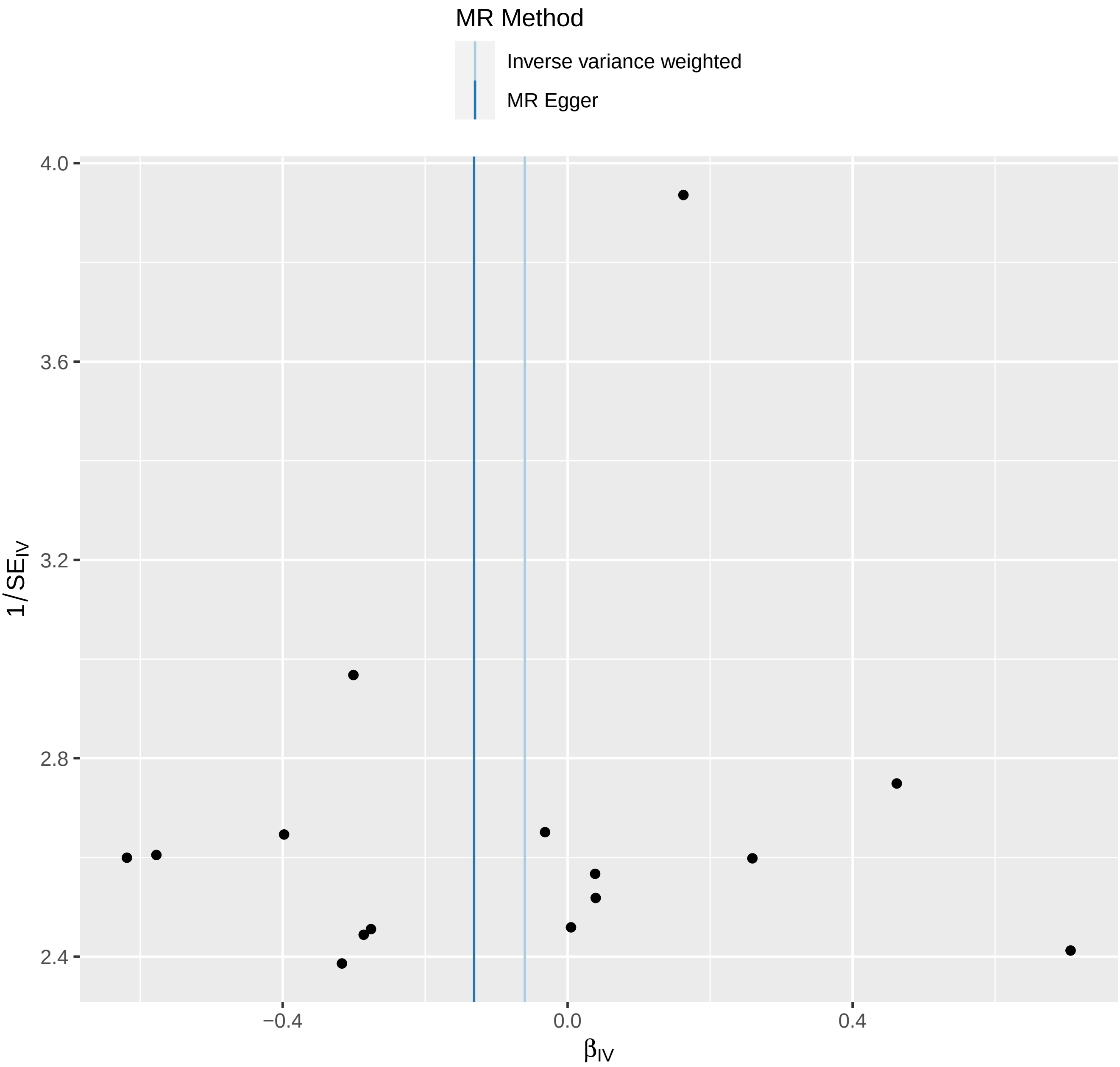

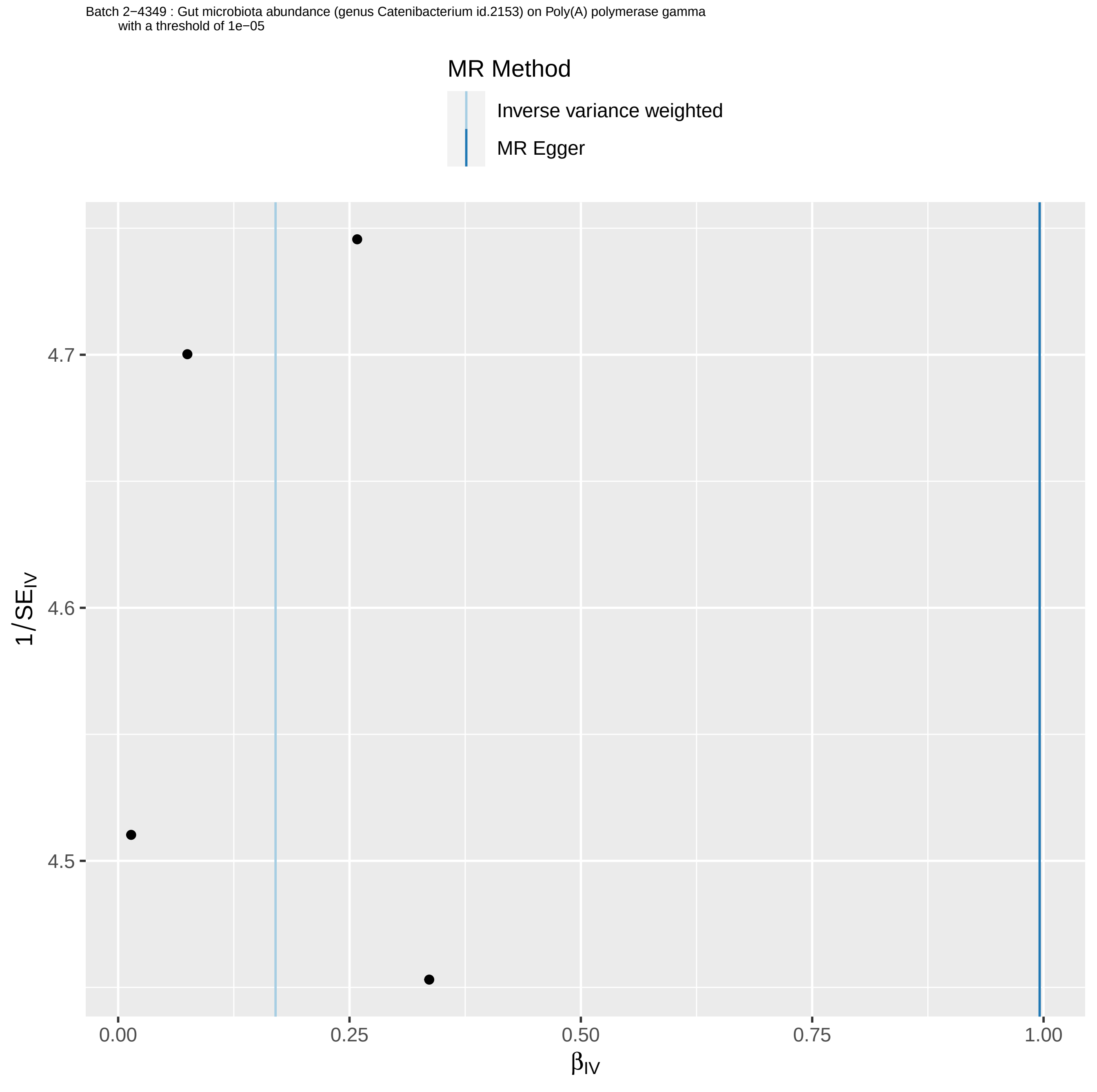
Batch 2−4349 : Gut microbiota abundance (genus Catenibacterium id.2153) on Poly(A) polymerase gamma
with a threshold of 1e−05
MR Method
Inverse variance weighted
MR Egger
4.7
4.6
4.5
1/SE$_{IV}$
0.00
0.25
0.50
0.75
1.00
β_{IV}

Batch 2−4350 : Gut microbiota abundance (genus Parasutterella id.2892) on Poly(A) polymerase gamma
with a threshold of 1e−05

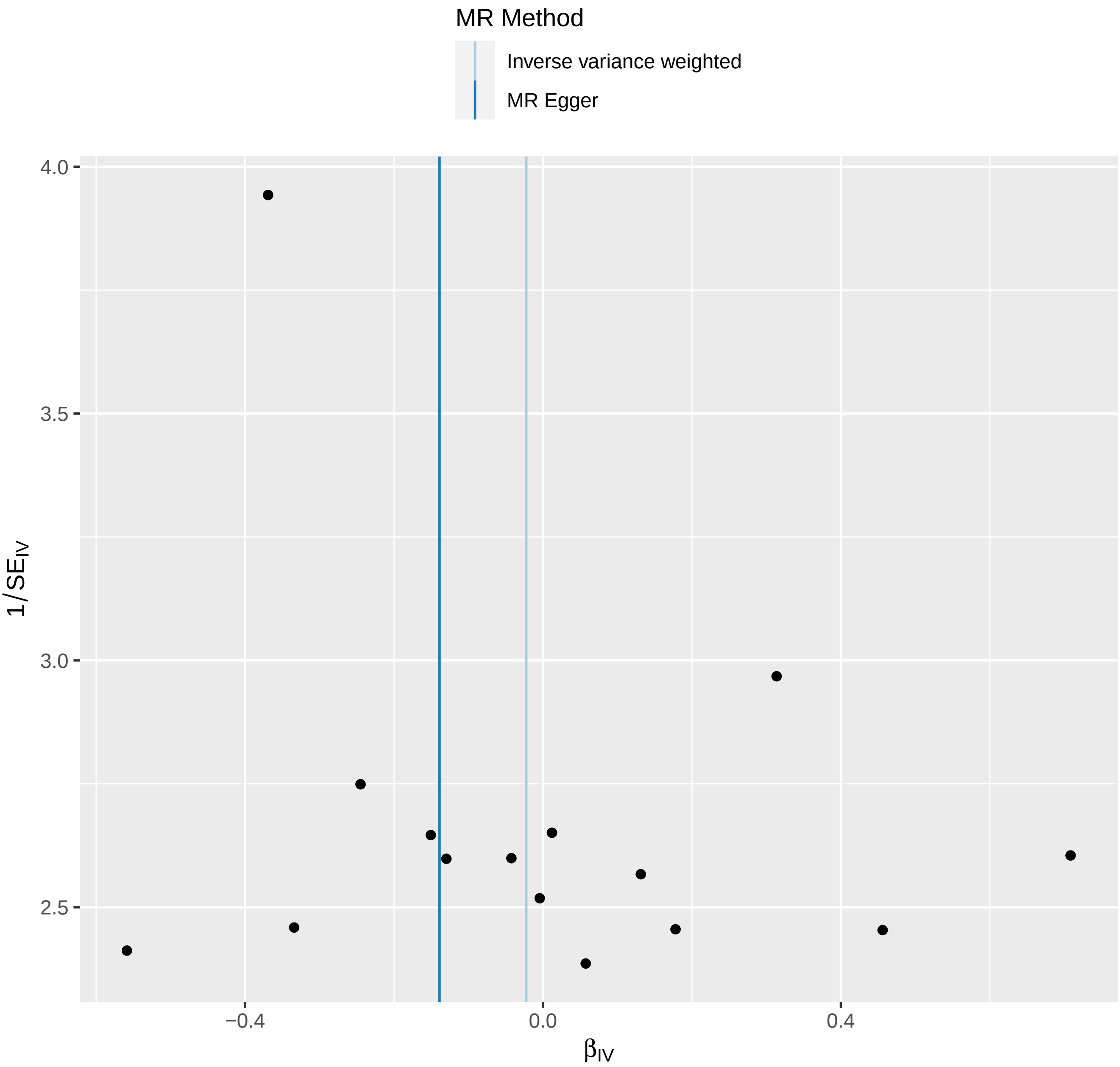

Batch 2−4351 : Gut microbiota abundance (genus Catenibacterium id.2153) on Pappalysin−2
with a threshold of 1e−05

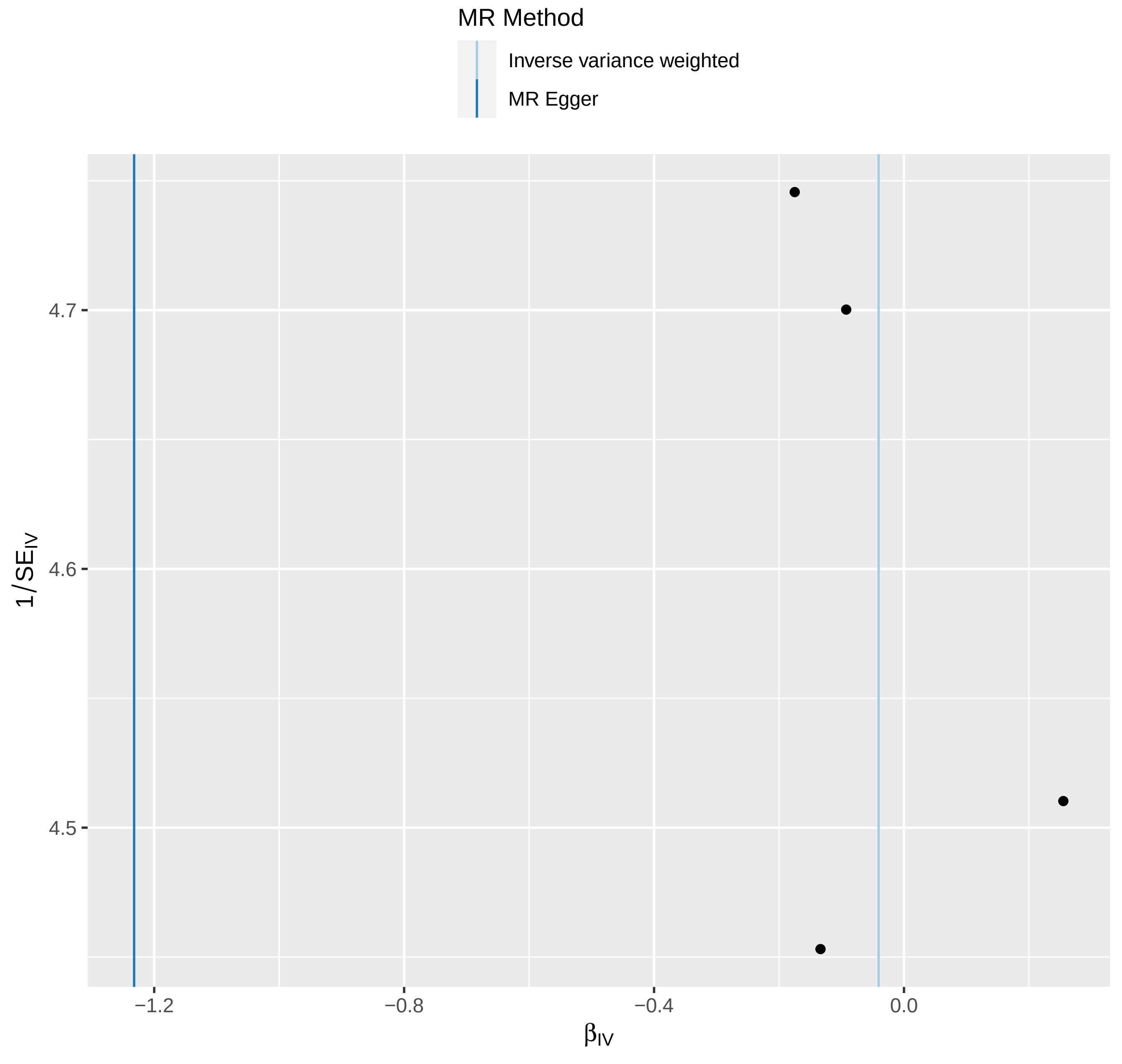

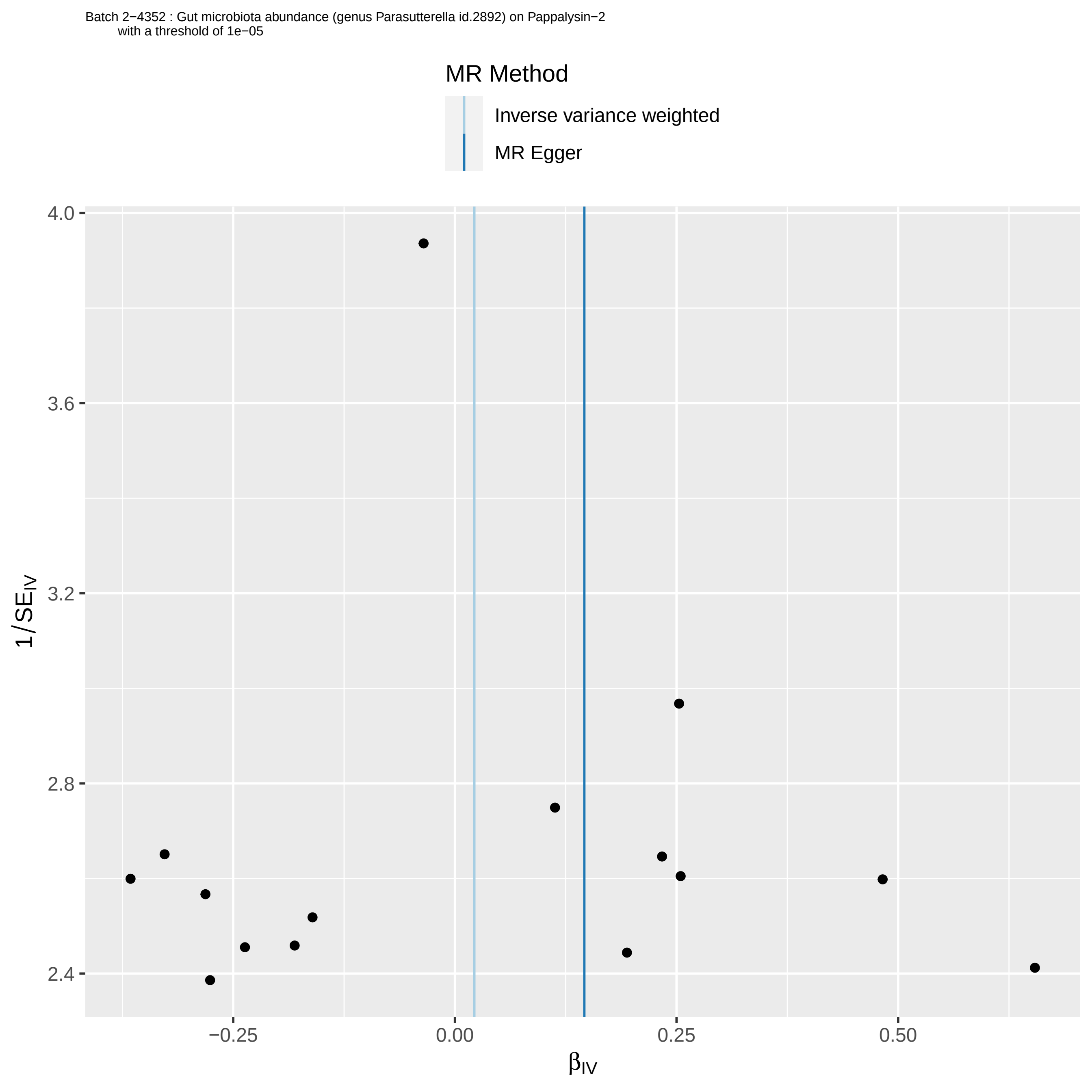

Batch 2−4352 : Gut microbiota abundance (genus Parasutterella id.2892) on Pappalysin−2
with a threshold of 1e−05
MR Method
Inverse variance weighted
MR Egger
4.0
3.6
3.2
2.8
2.4
1/SE_IV
−0.25
0.00
0.25
0.50
β_IV

Batch 2−4353 : Gut microbiota abundance (genus Catenibacterium id.2153) on Pappalysin−1
with a threshold of 1e−05

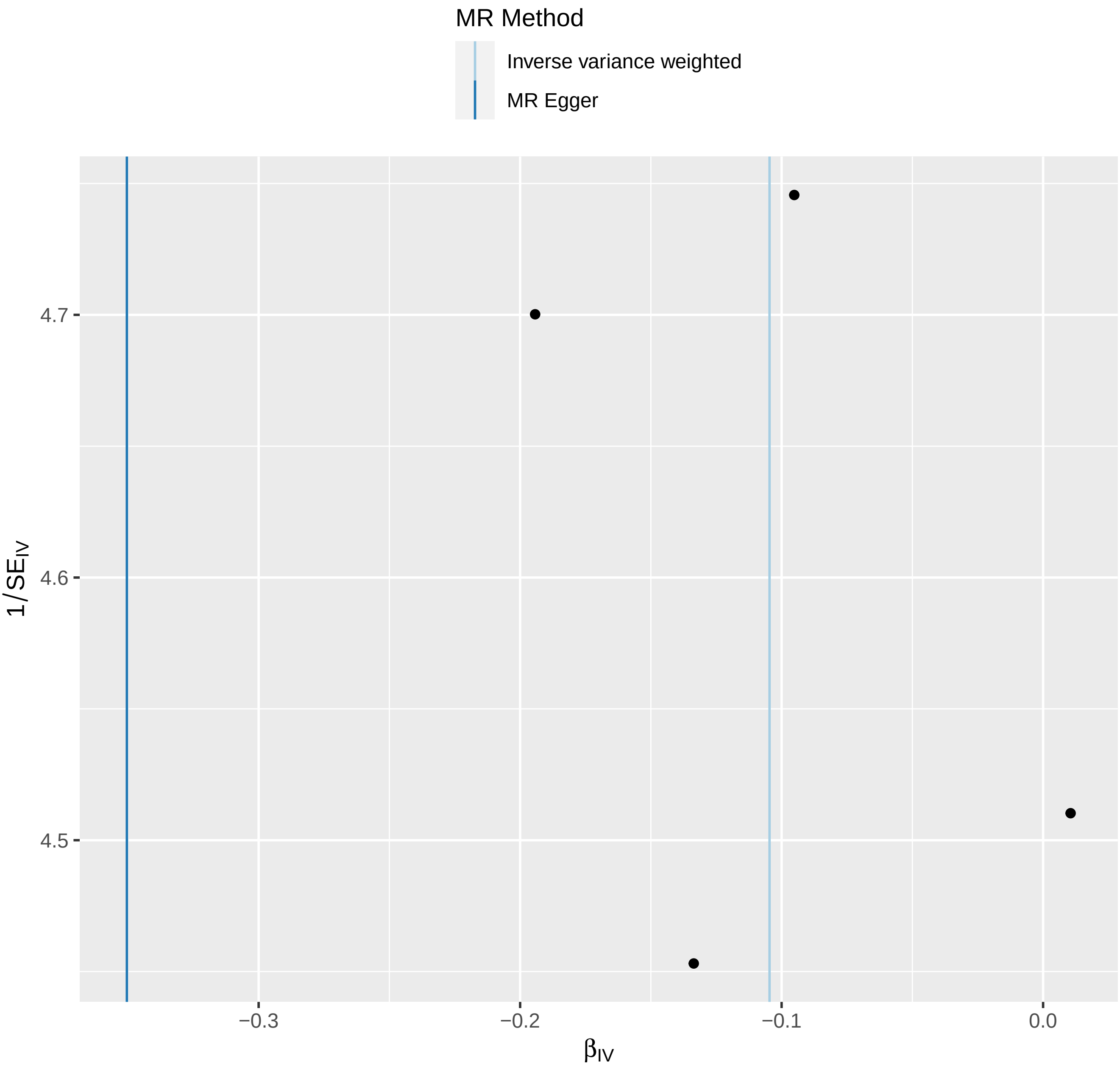

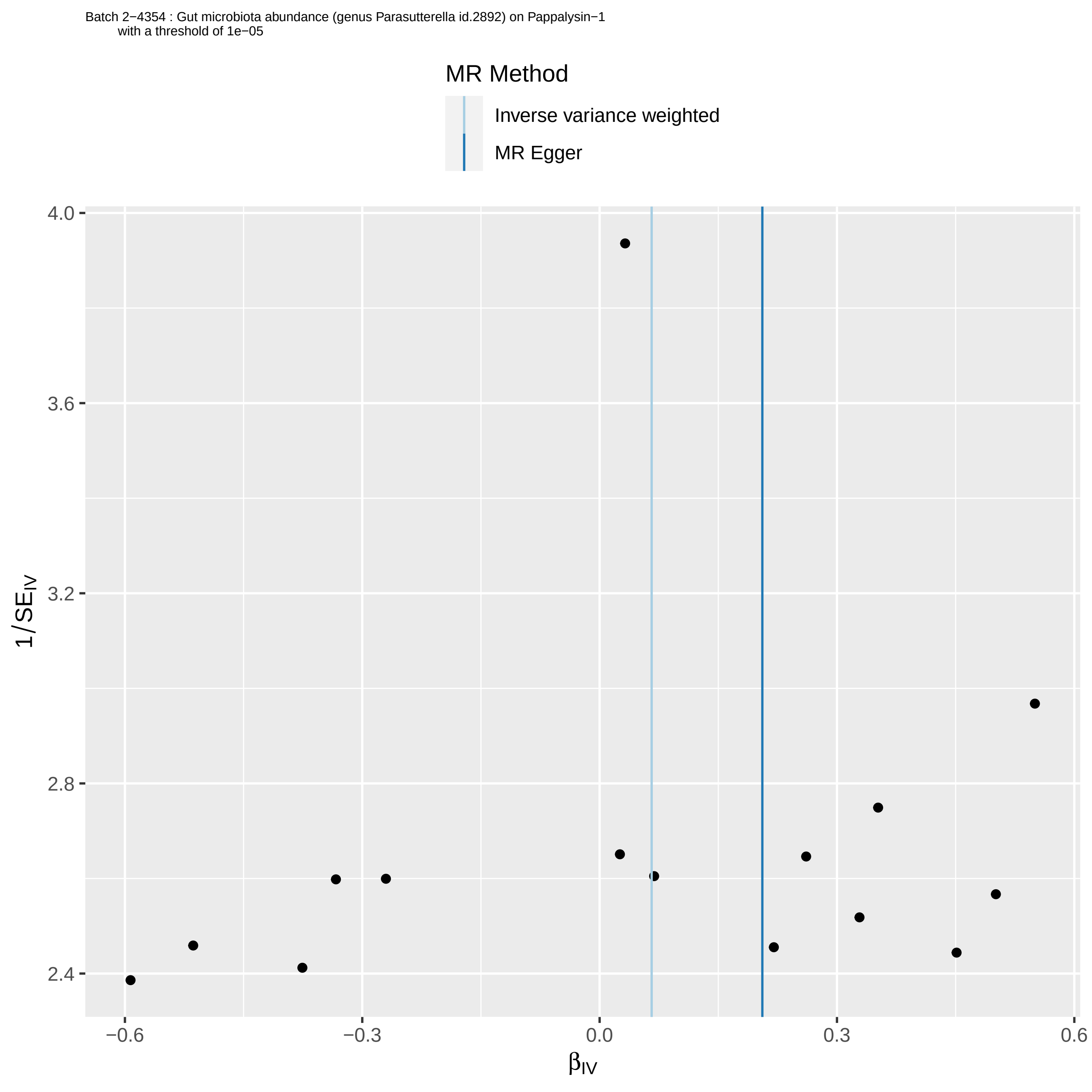
Batch 2−4354 : Gut microbiota abundance (genus Parasutterella id.2892) on Pappalysin−1
with a threshold of 1e−05
MR Method
Inverse variance weighted
MR Egger
4.0
3.6
3.2
2.8
2.4
1/SE_IV
−0.6
−0.3
0.0
0.3
0.6
β_IV

Batch 2−4355 : Gut microbiota abundance (genus Catenibacterium id.2153) on Bifunctional 3'−phosphoadenosine 5'−phosphosulfate synthase 1 with a threshold of 1e−05

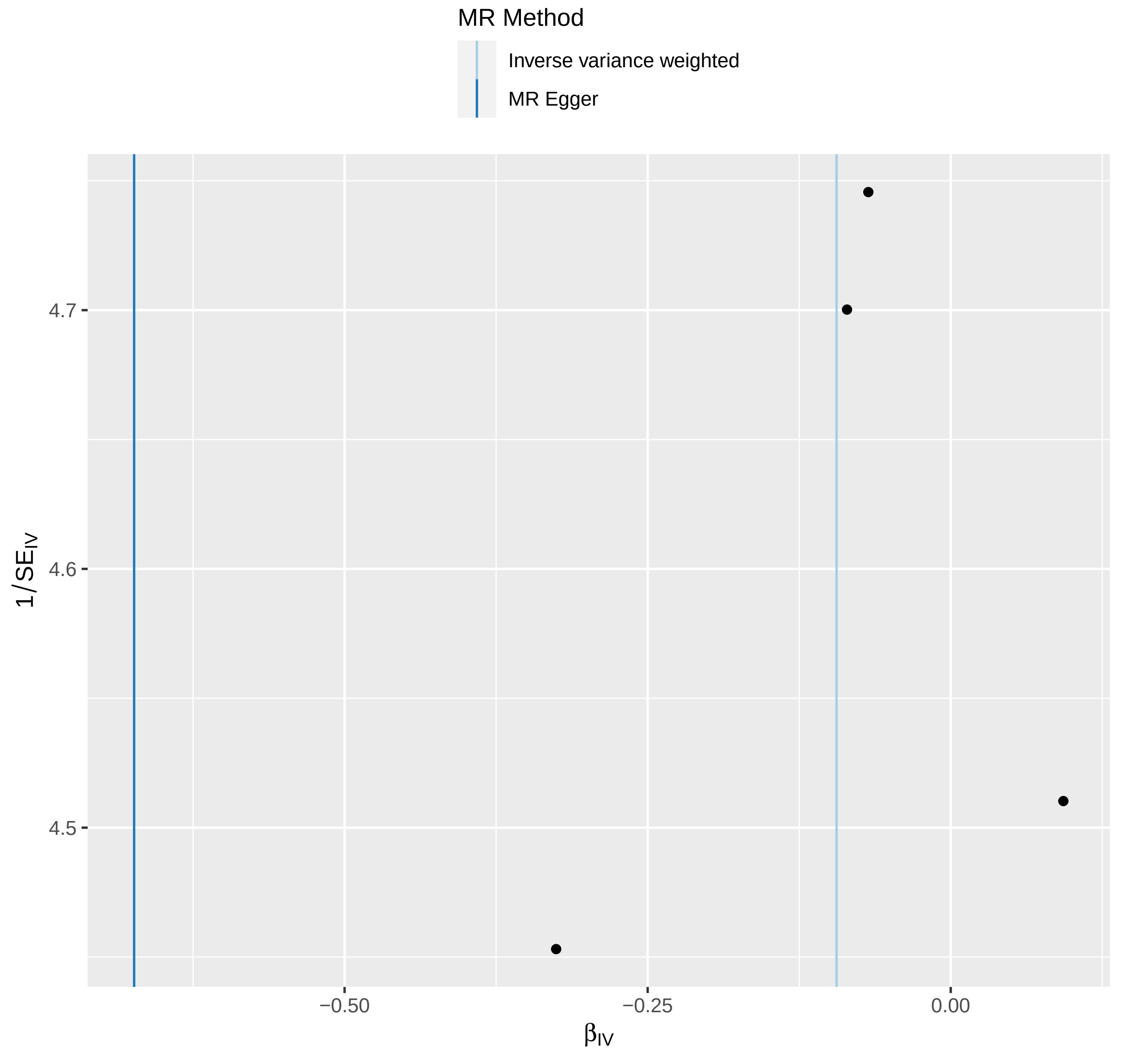

Batch 2−4356 : Gut microbiota abundance (genus Parasutterella id.2892) on Bifunctional 3'−phosphoadenosine 5'−phosphosulfate synthase 1 with a threshold of 1e−05

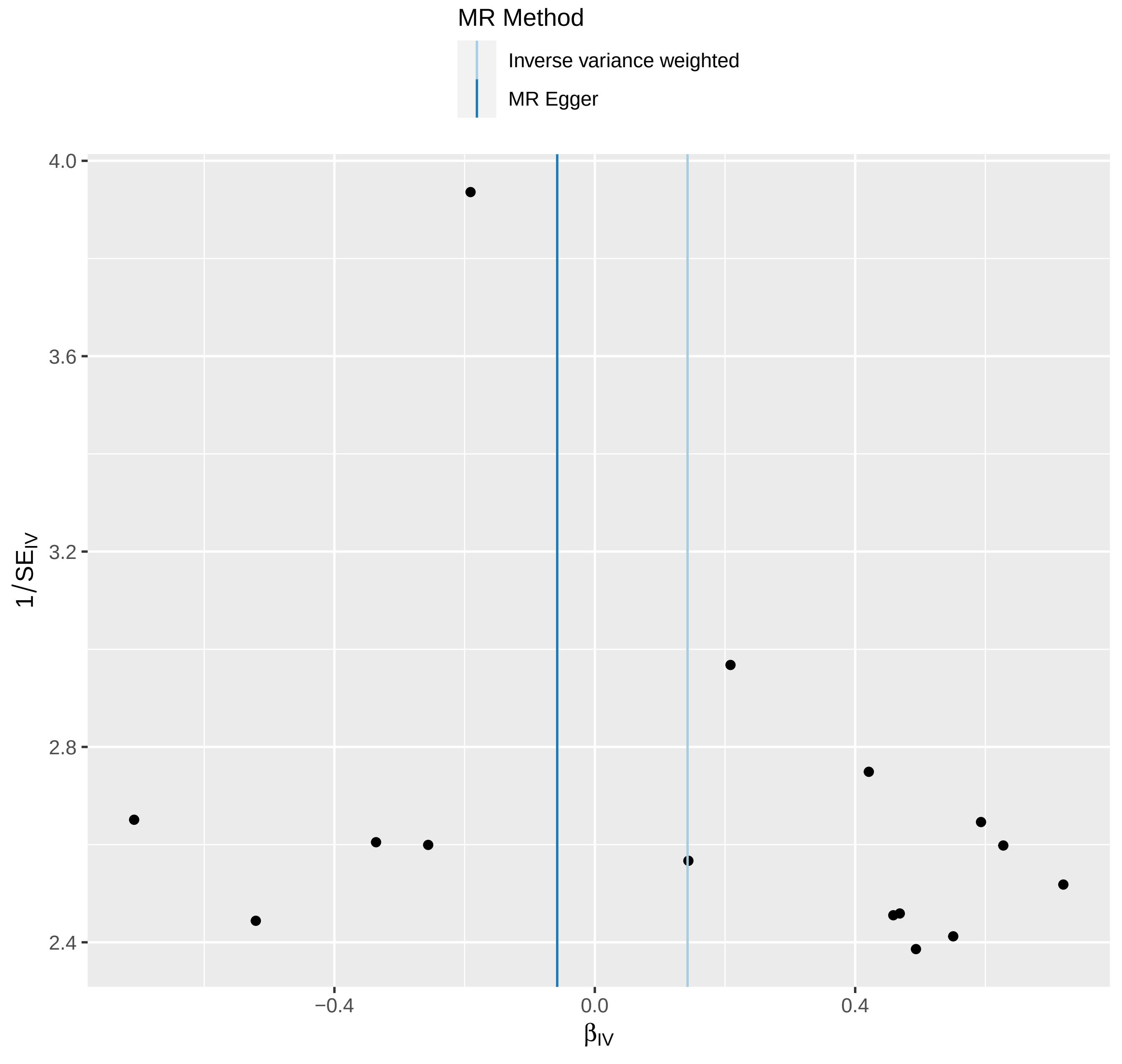

Batch 2−4357 : Gut microbiota abundance (genus Catenibacterium id.2153) on E3 ubiquitin−protein ligase parkin
with a threshold of 1e−05

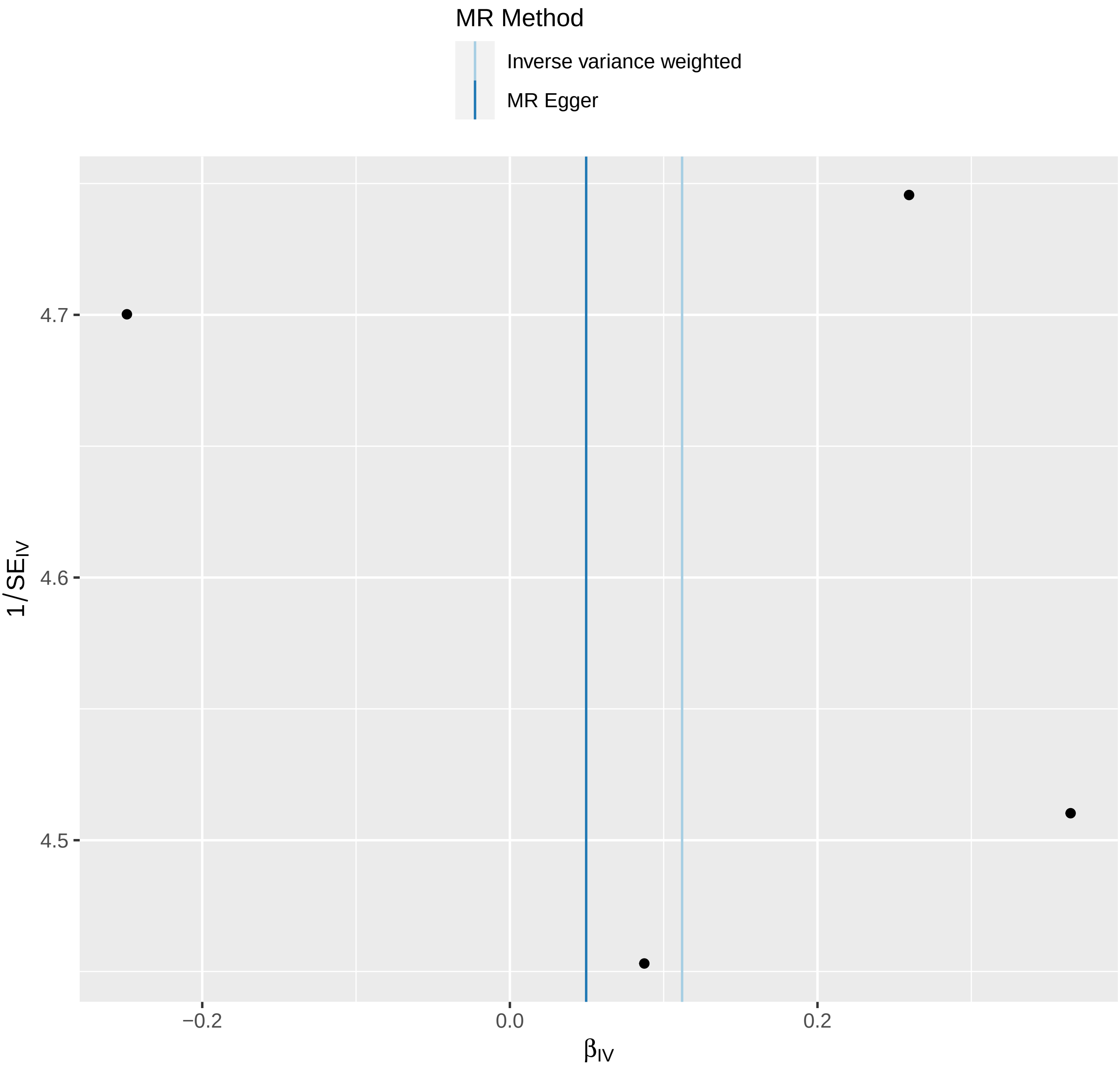

Batch 2−4358 : Gut microbiota abundance (genus Parasutterella id.2892) on E3 ubiquitin−protein ligase parkin
with a threshold of 1e−05

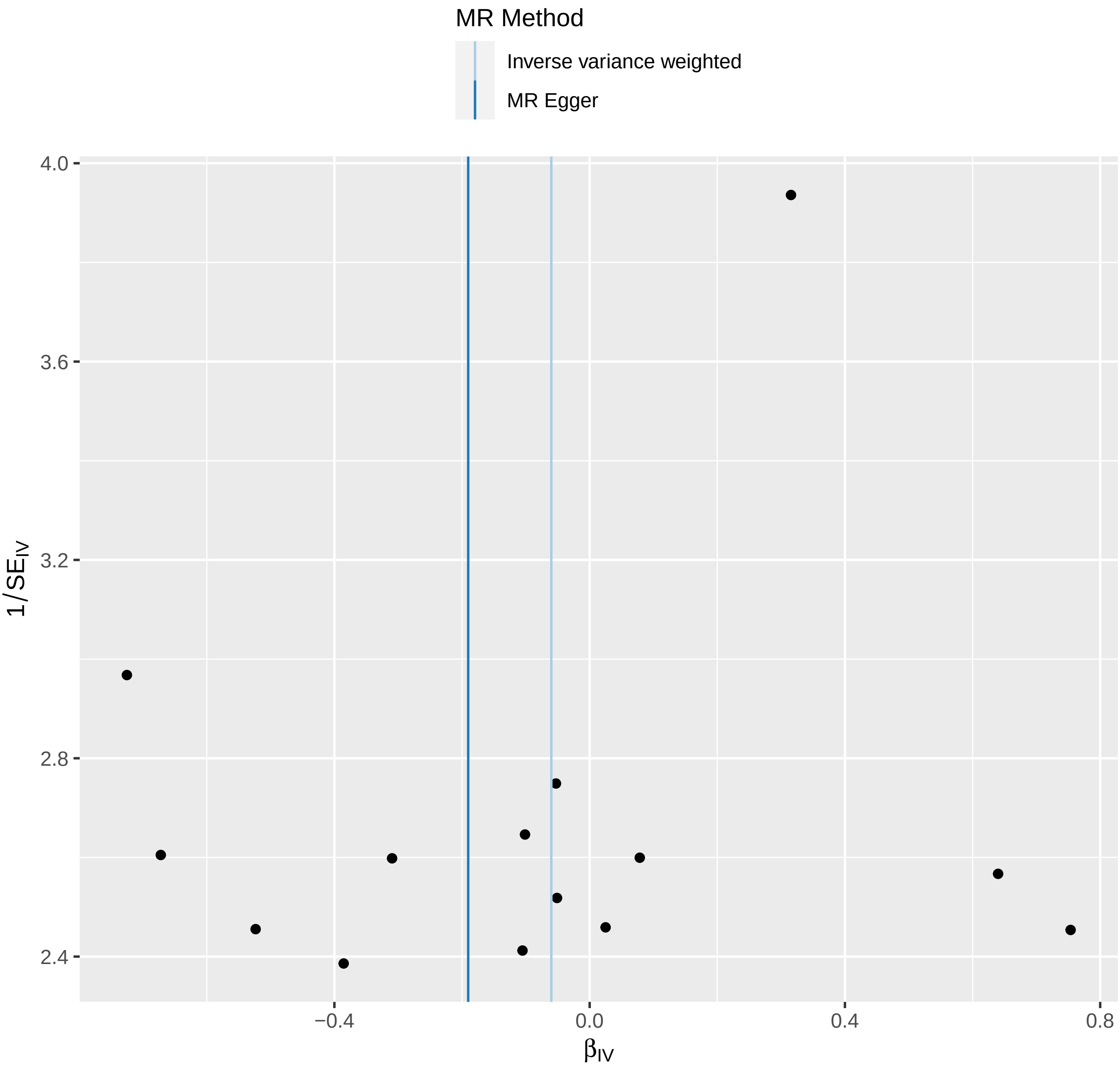

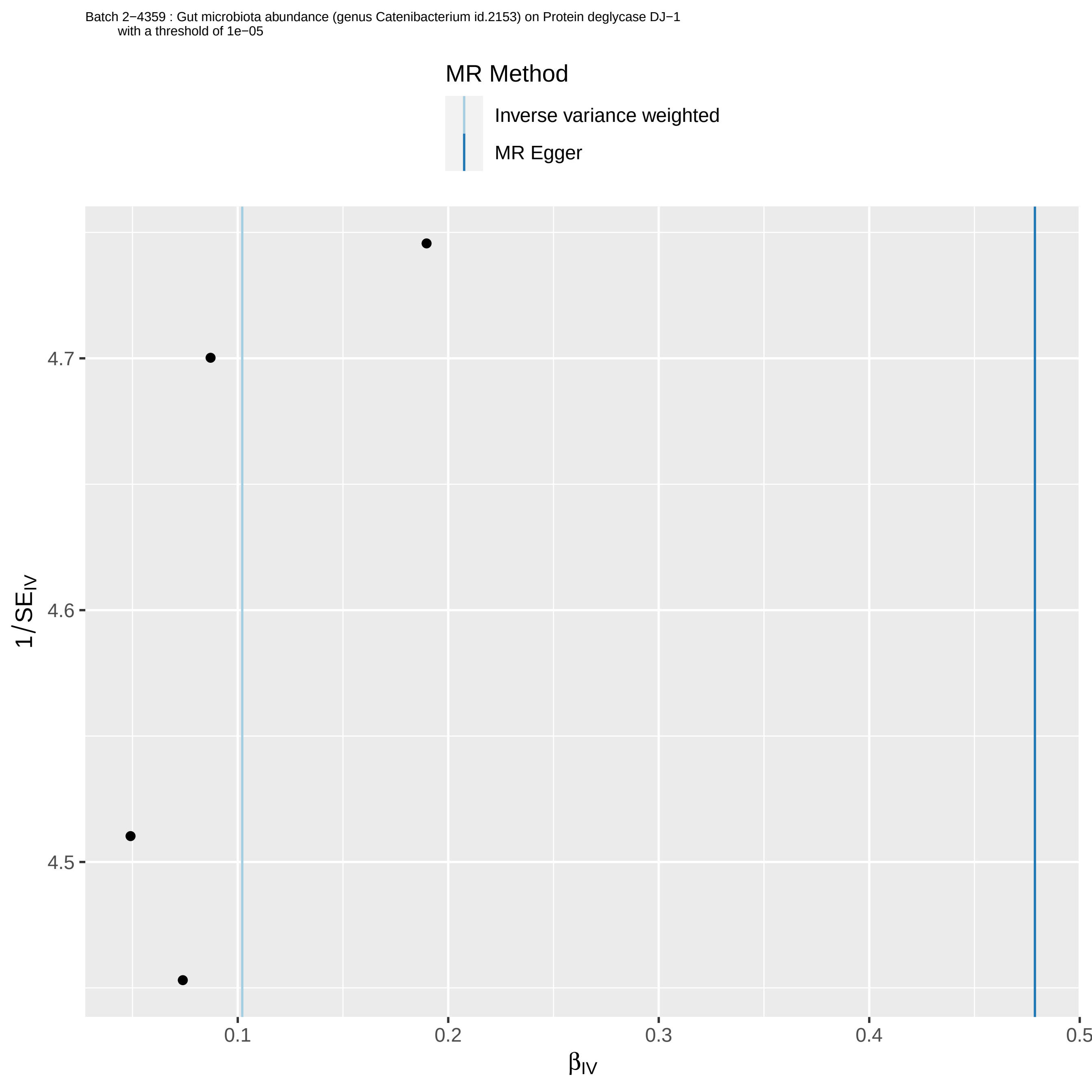
Batch 2−4359 : Gut microbiota abundance (genus Catenibacterium id.2153) on Protein deglycase DJ−1
with a threshold of 1e−05
MR Method
Inverse variance weighted
MR Egger
4.7
4.6
4.5
1/SE_IV
0.1
0.2
0.3
0.4
0.5
β_IV

Batch 2−4360 : Gut microbiota abundance (genus Parasutterella id.2892) on Protein deglycase DJ−1
with a threshold of 1e−05

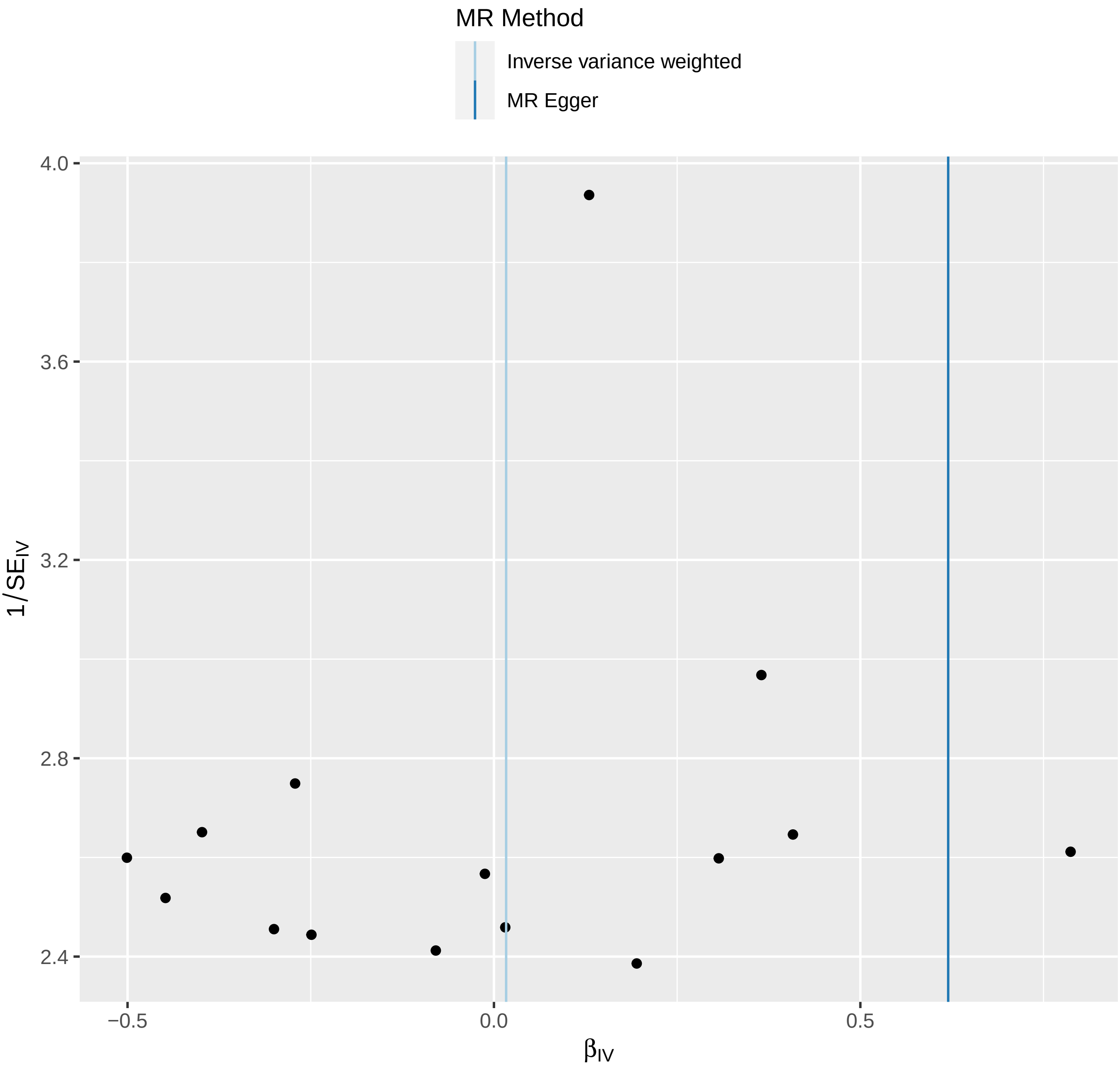

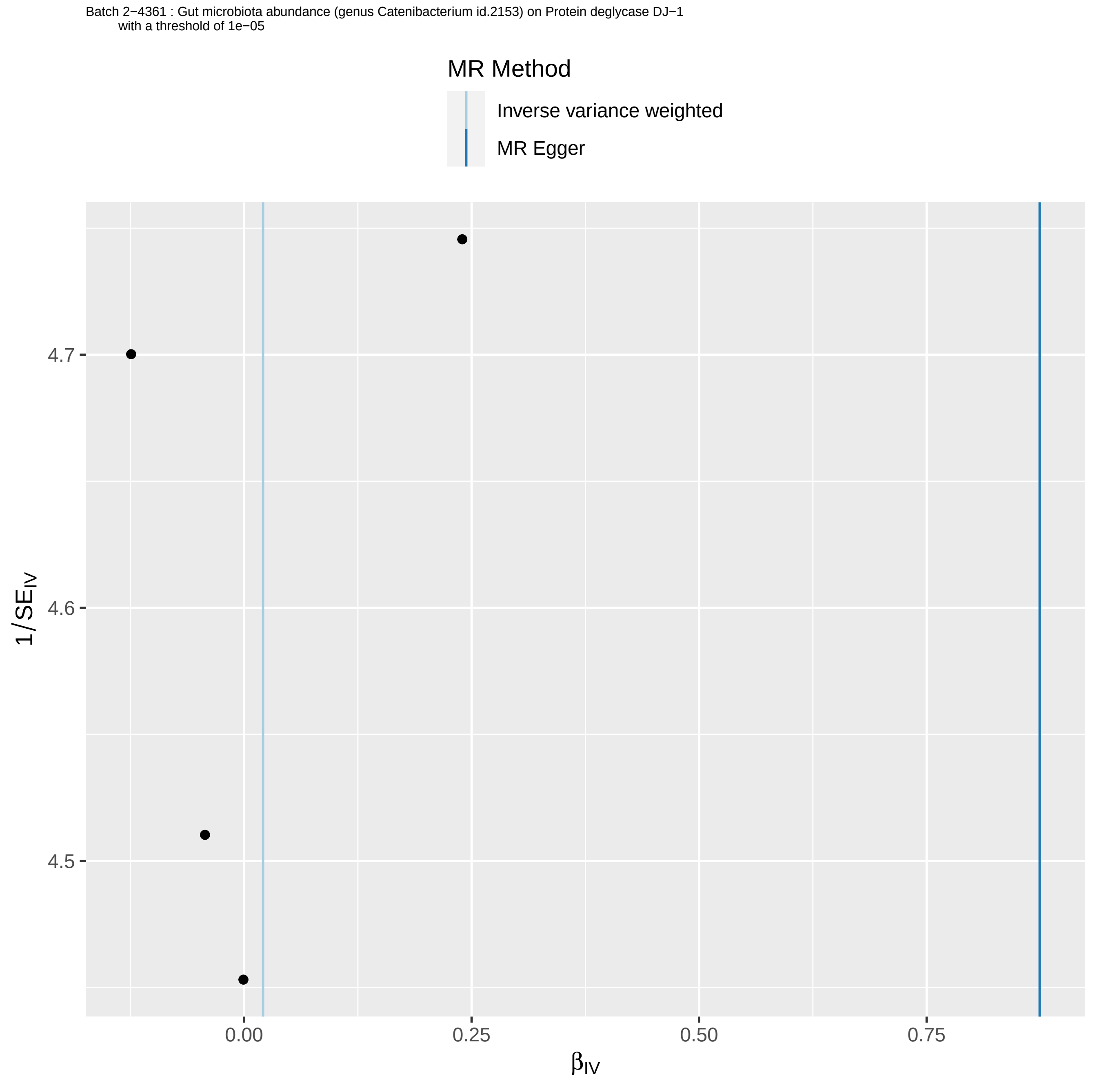
Batch 2−4361 : Gut microbiota abundance (genus Catenibacterium id.2153) on Protein deglycase DJ−1
with a threshold of 1e−05
MR Method
Inverse variance weighted
MR Egger
4.7
4.6
4.5
1/SE_IV
0.00
0.25
0.50
0.75
β_IV

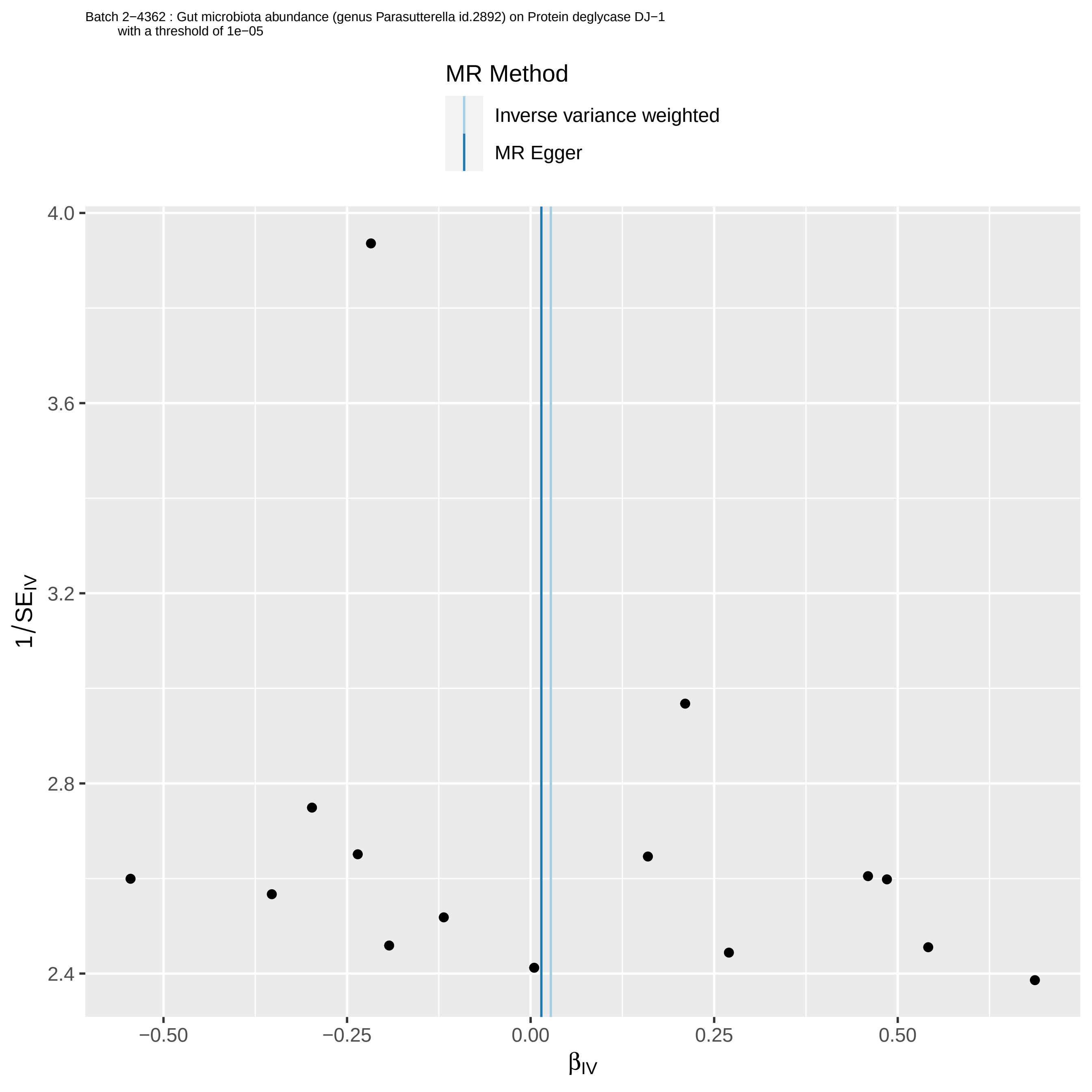
Batch 2−4362 : Gut microbiota abundance (genus Parasutterella id.2892) on Protein deglycase DJ−1
with a threshold of 1e−05
MR Method
Inverse variance weighted
MR Egger
4.0
3.6
3.2
2.8
2.4
1/SE$_{IV}$
−0.50
−0.25
0.00
0.25
0.50
β$_{IV}$

Batch 2−4363 : Gut microbiota abundance (genus Catenibacterium id.2153) on Poly [ADP−ribose] polymerase 1
with a threshold of 1e−05

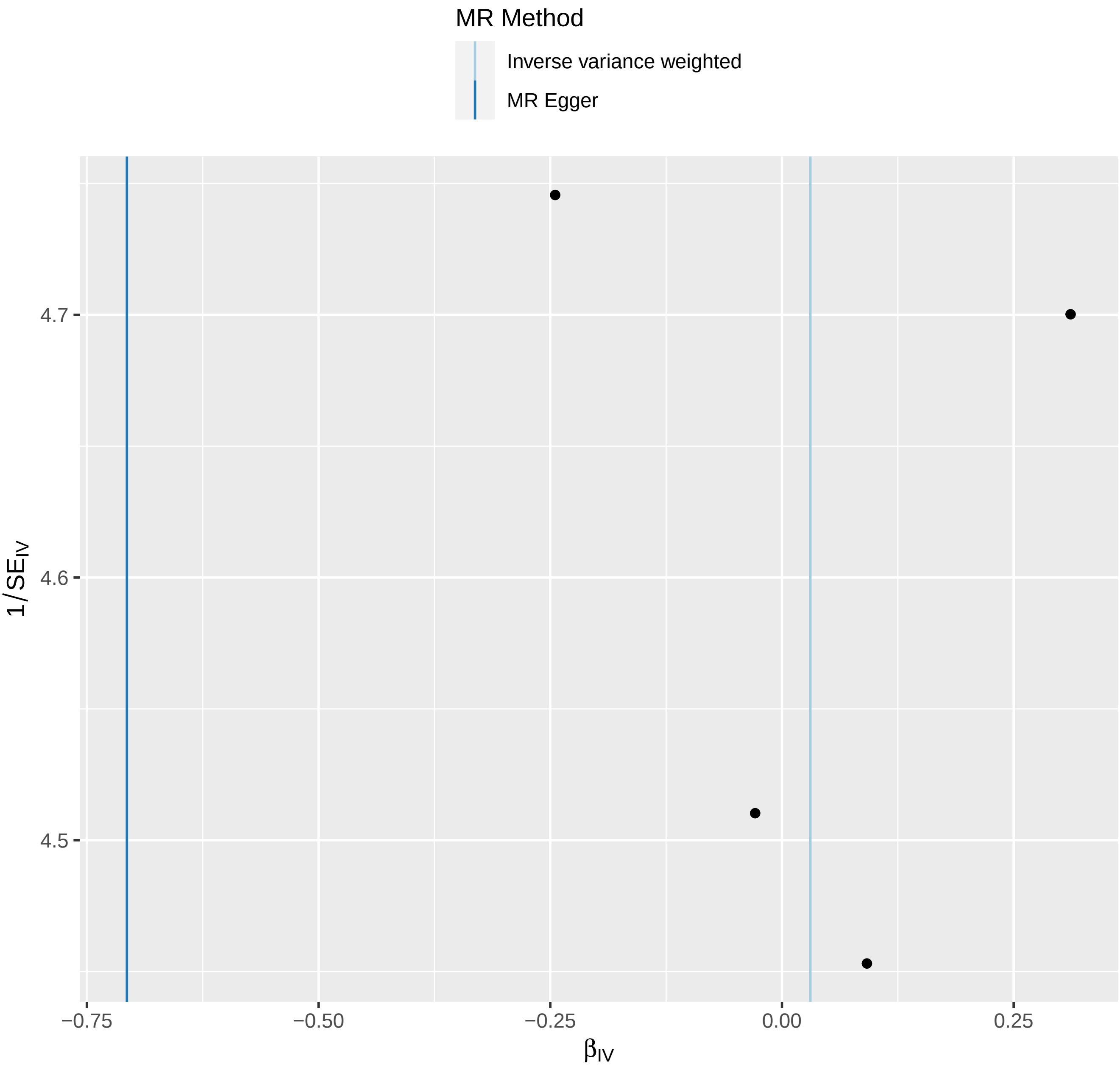

Batch 2−4364 : Gut microbiota abundance (genus Parasutterella id.2892) on Poly [ADP−ribose] polymerase 1
with a threshold of 1e−05

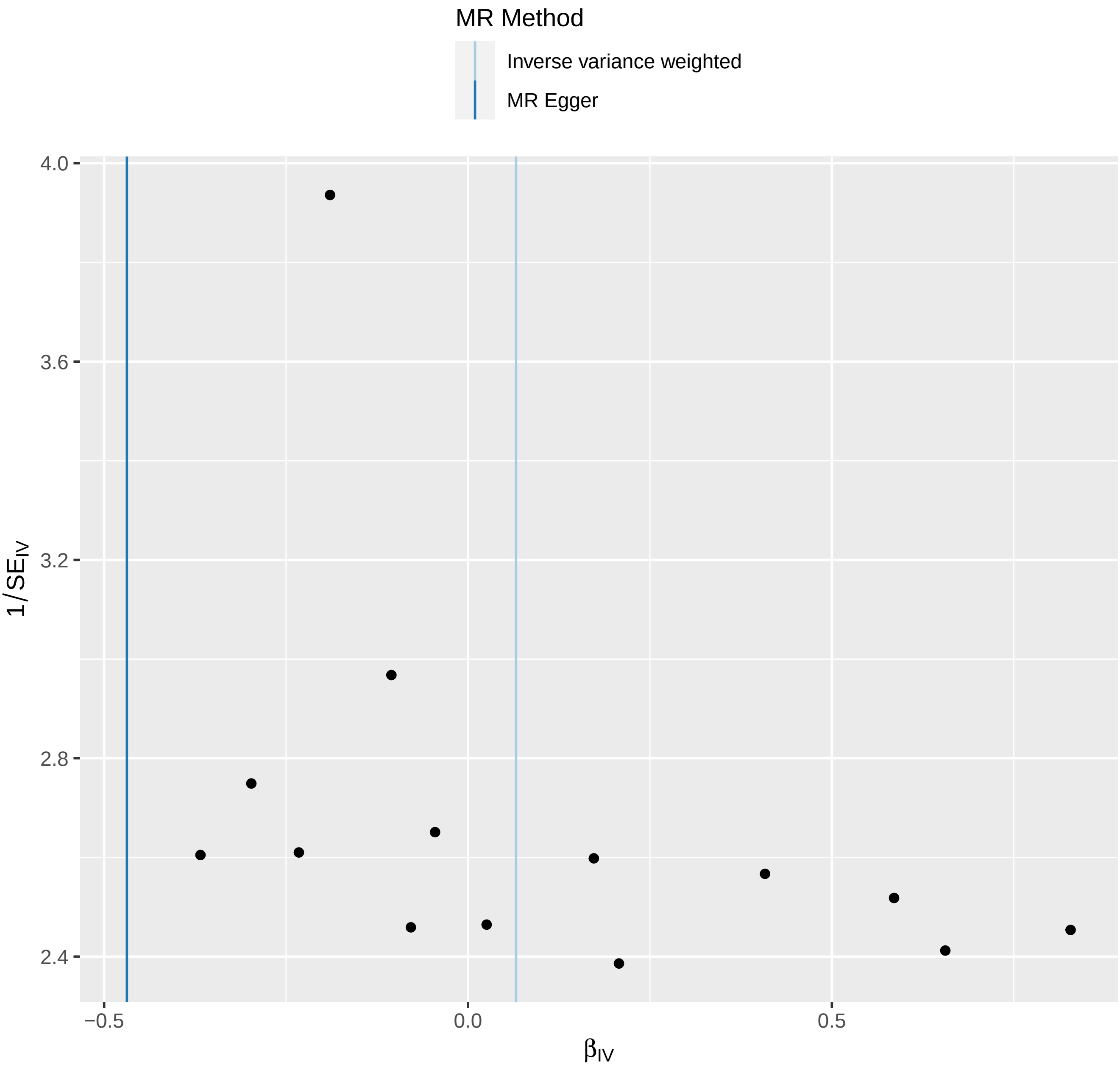

Batch 2−4365 : Gut microbiota abundance (genus Catenibacterium id.2153) on Poly [ADP−ribose] polymerase 11
with a threshold of 1e−05

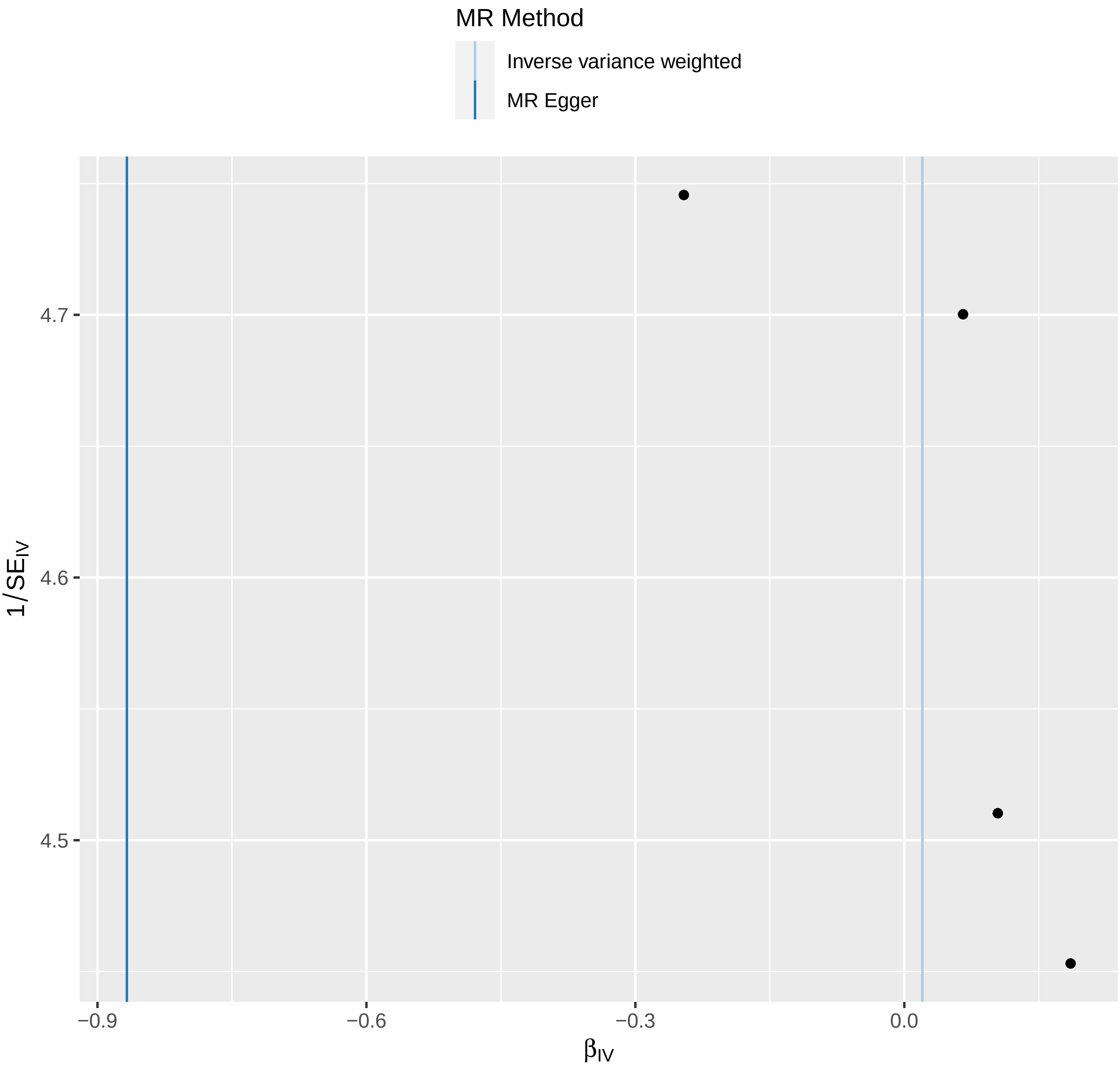

Batch 2−4366 : Gut microbiota abundance (genus Parasutterella id.2892) on Poly [ADP−ribose] polymerase 11 with a threshold of 1e−05

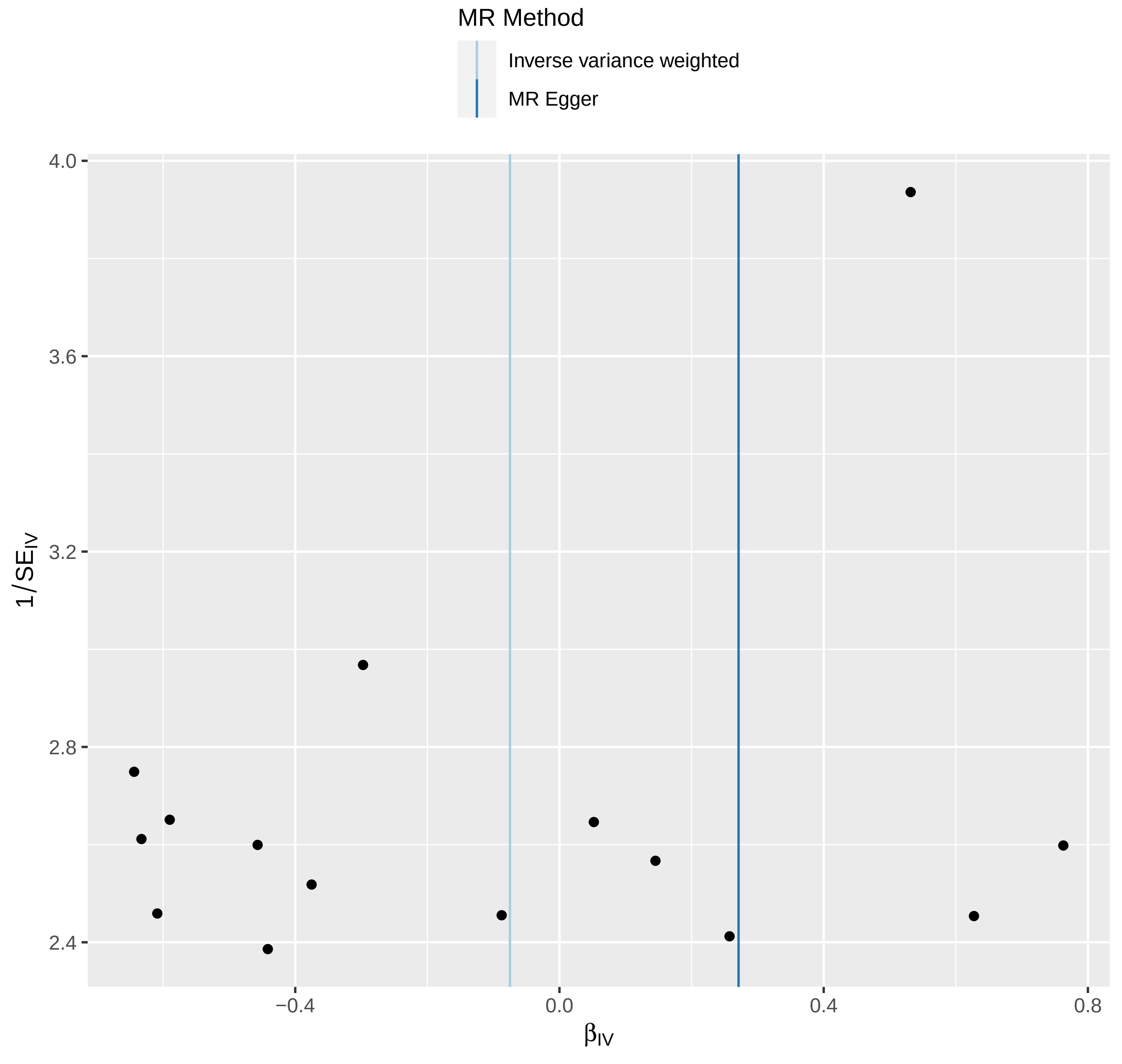

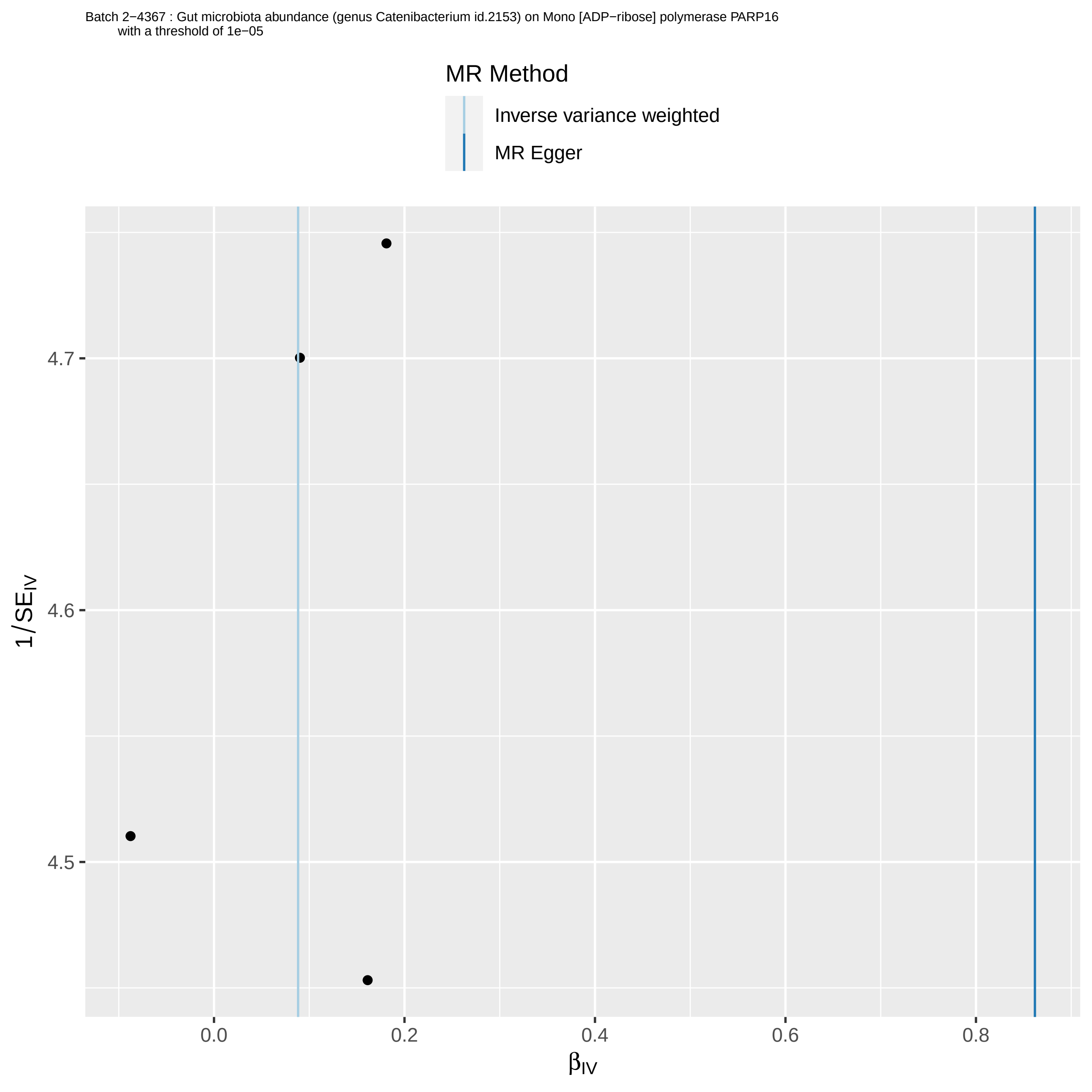

Batch 2−4367 : Gut microbiota abundance (genus Catenibacterium id.2153) on Mono [ADP−ribose] polymerase PARP16
with a threshold of 1e−05
MR Method
Inverse variance weighted
MR Egger
4.7
4.6
4.5
1/SE_IV
0.0
0.2
0.4
0.6
0.8
β_IV

Batch 2−4368 : Gut microbiota abundance (genus Parasutterella id.2892) on Mono [ADP−ribose] polymerase PARP16 with a threshold of 1e−05

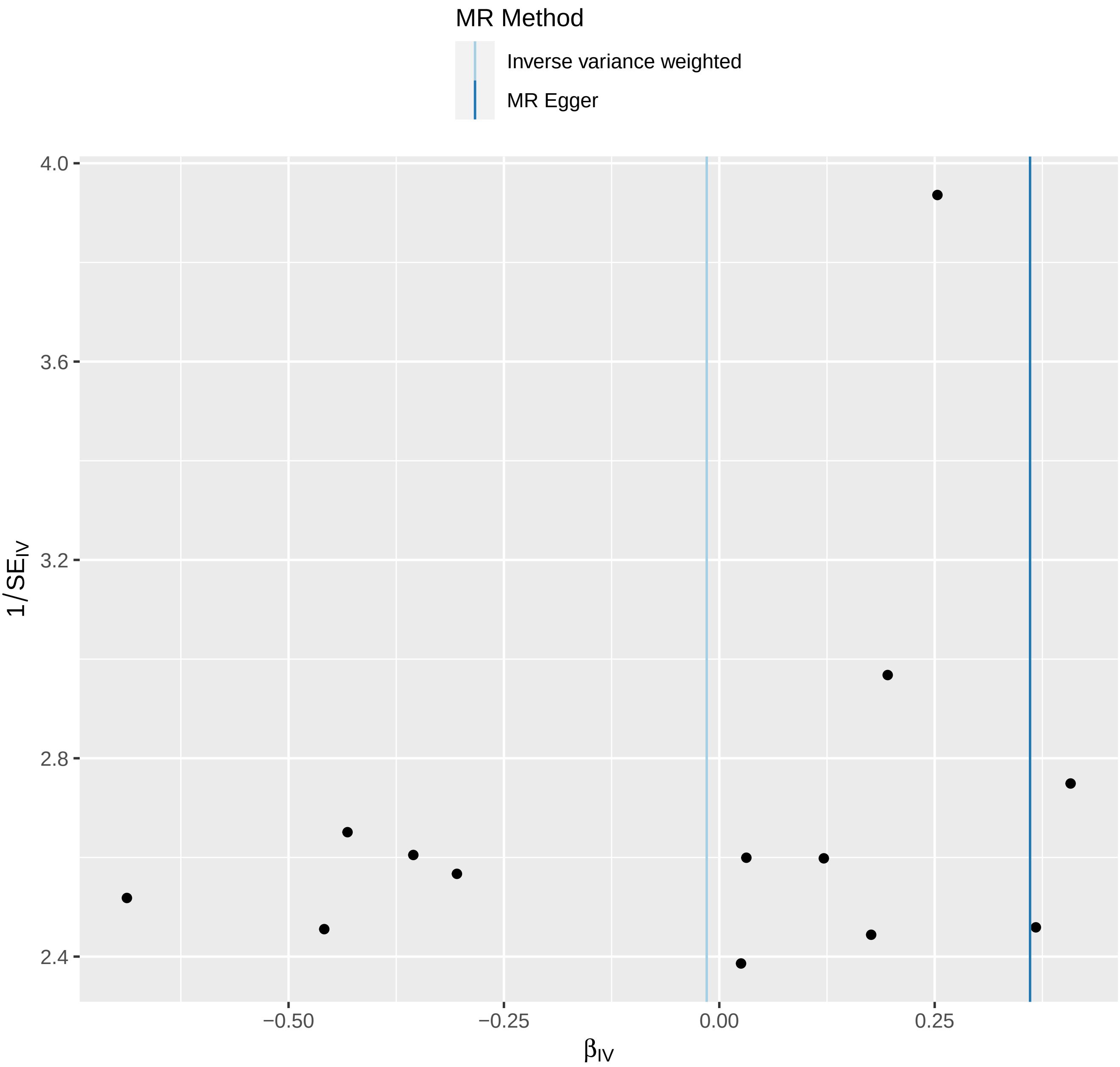

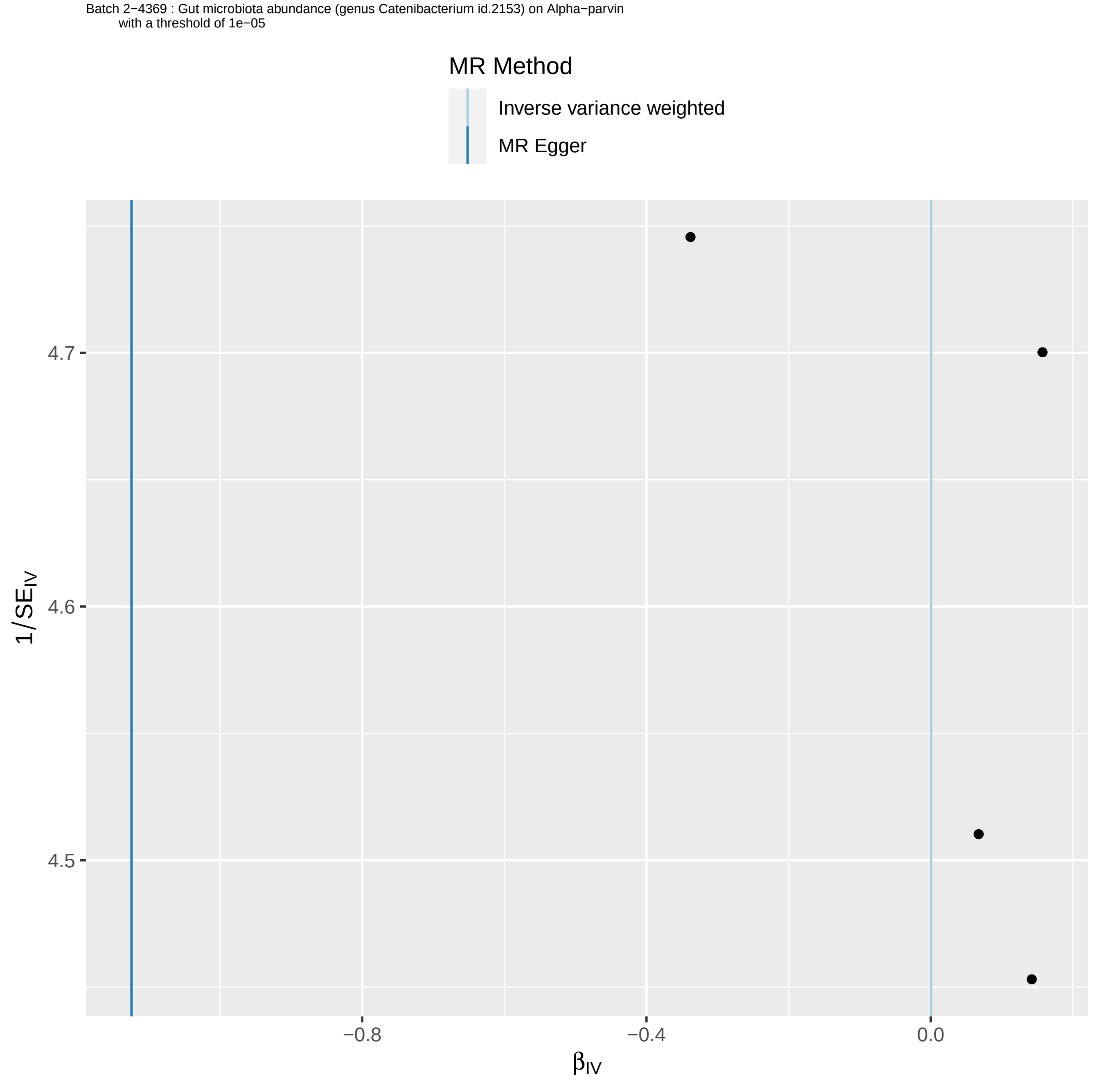

Batch 2−4369 : Gut microbiota abundance (genus Catenibacterium id.2153) on Alpha−parvin
with a threshold of 1e−05
MR Method
Inverse variance weighted
MR Egger
4.7
4.6
4.5
1/SE_IV
−0.8
−0.4
0.0
β_IV

Batch 2−4370 : Gut microbiota abundance (genus Parasutterella id.2892) on Alpha−parvin
with a threshold of 1e−05

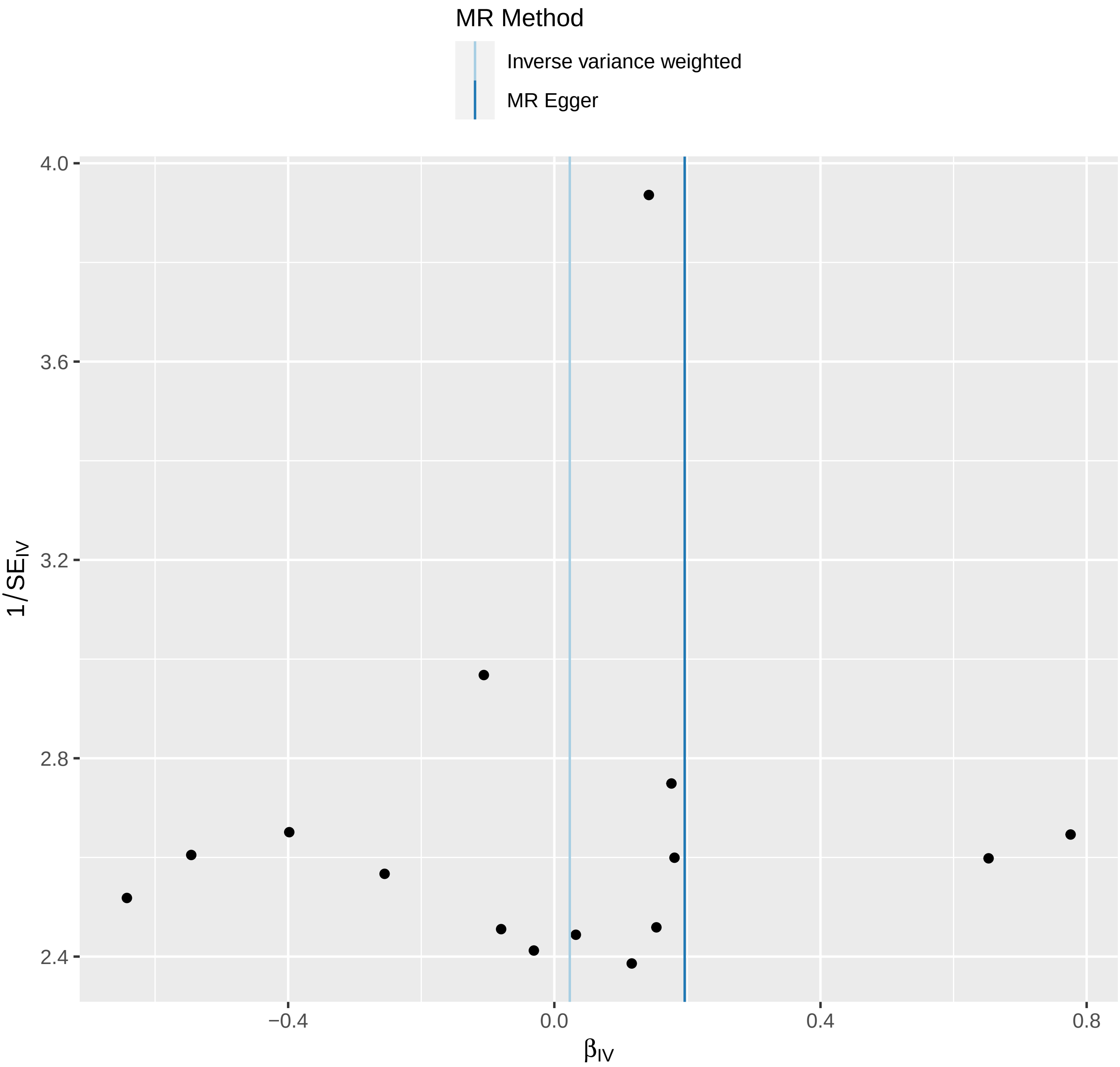

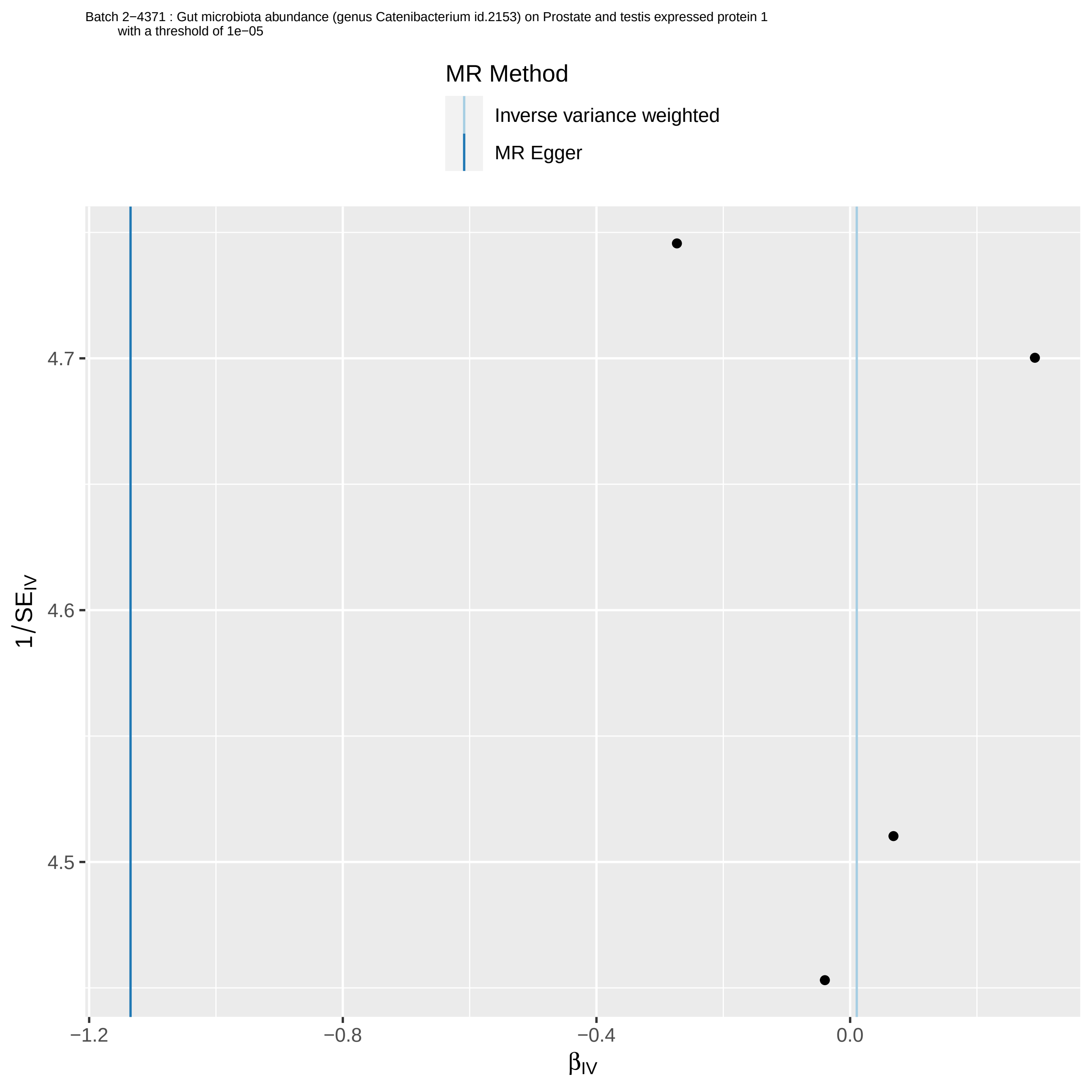
Batch 2−4371 : Gut microbiota abundance (genus Catenibacterium id.2153) on Prostate and testis expressed protein 1
with a threshold of 1e−05
MR Method
Inverse variance weighted
MR Egger
4.7
4.6
4.5
1/SE$_{IV}$
−1.2
−0.8
−0.4
0.0
β$_{IV}$

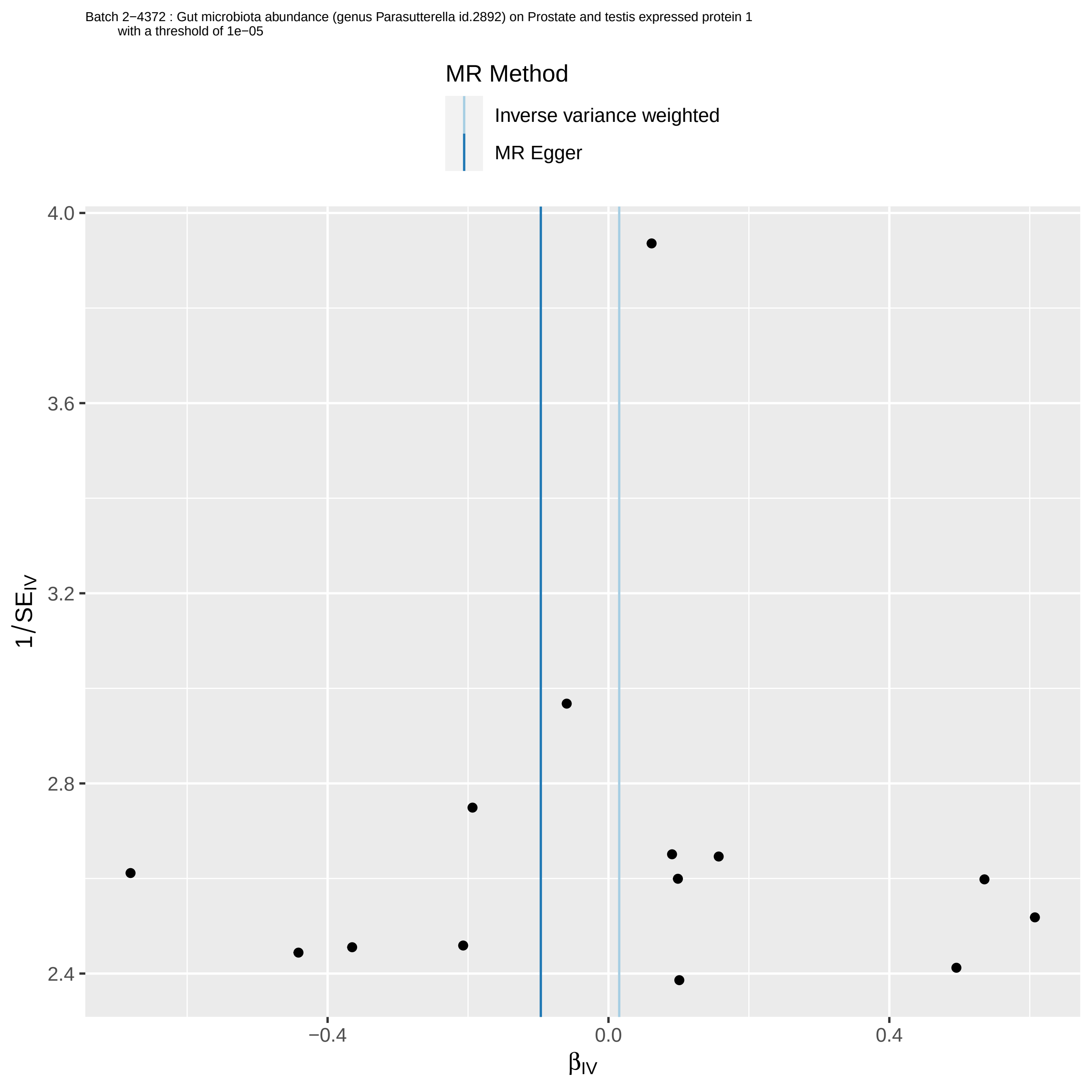
Batch 2−4372 : Gut microbiota abundance (genus Parasutterella id.2892) on Prostate and testis expressed protein 1
with a threshold of 1e−05
MR Method
Inverse variance weighted
MR Egger
4.0
3.6
3.2
2.8
2.4
1/SE$_{IV}$
−0.4
0.0
0.4
β$_{IV}$

Batch 2−4373 : Gut microbiota abundance (genus Catenibacterium id.2153) on Prostate and testis expressed protein 4
with a threshold of 1e−05

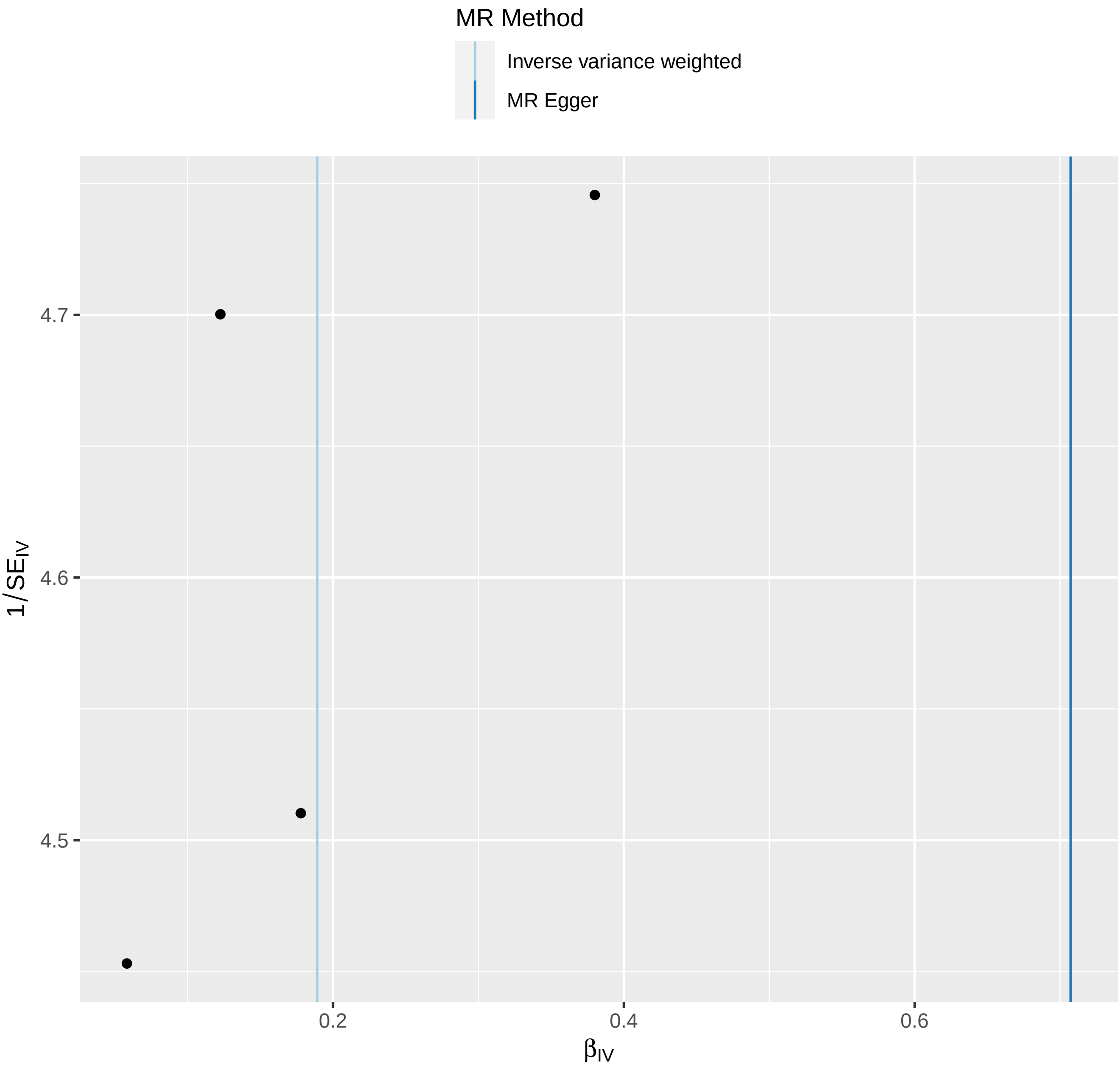

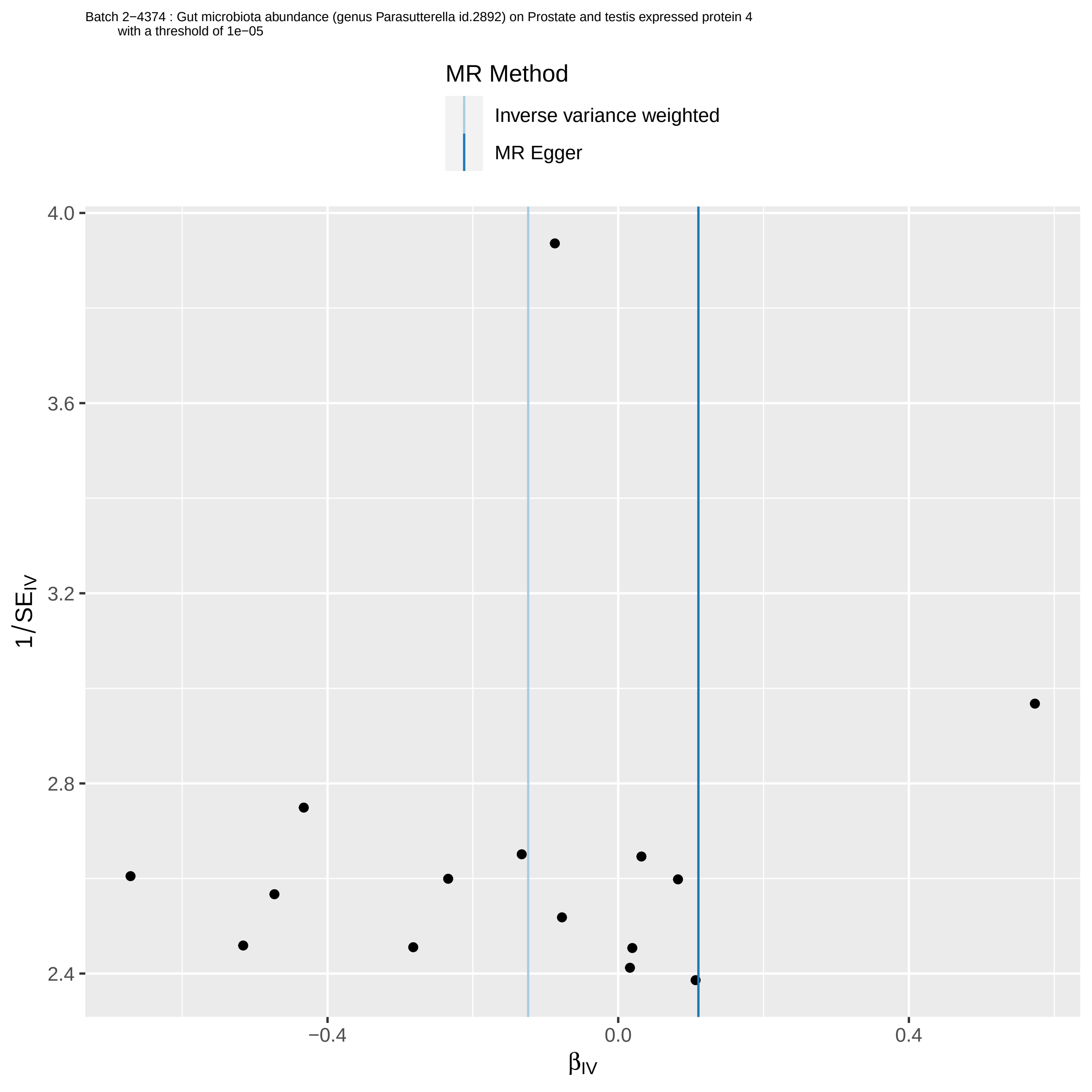

Batch 2−4374 : Gut microbiota abundance (genus Parasutterella id.2892) on Prostate and testis expressed protein 4
with a threshold of 1e−05
MR Method
Inverse variance weighted
MR Egger
4.0
3.6
3.2
2.8
2.4
1/SE_IV
−0.4
0.0
0.4
β_IV

Batch 2−4375 : Gut microbiota abundance (genus Catenibacterium id.2153) on Paired box protein Pax−4
with a threshold of 1e−05

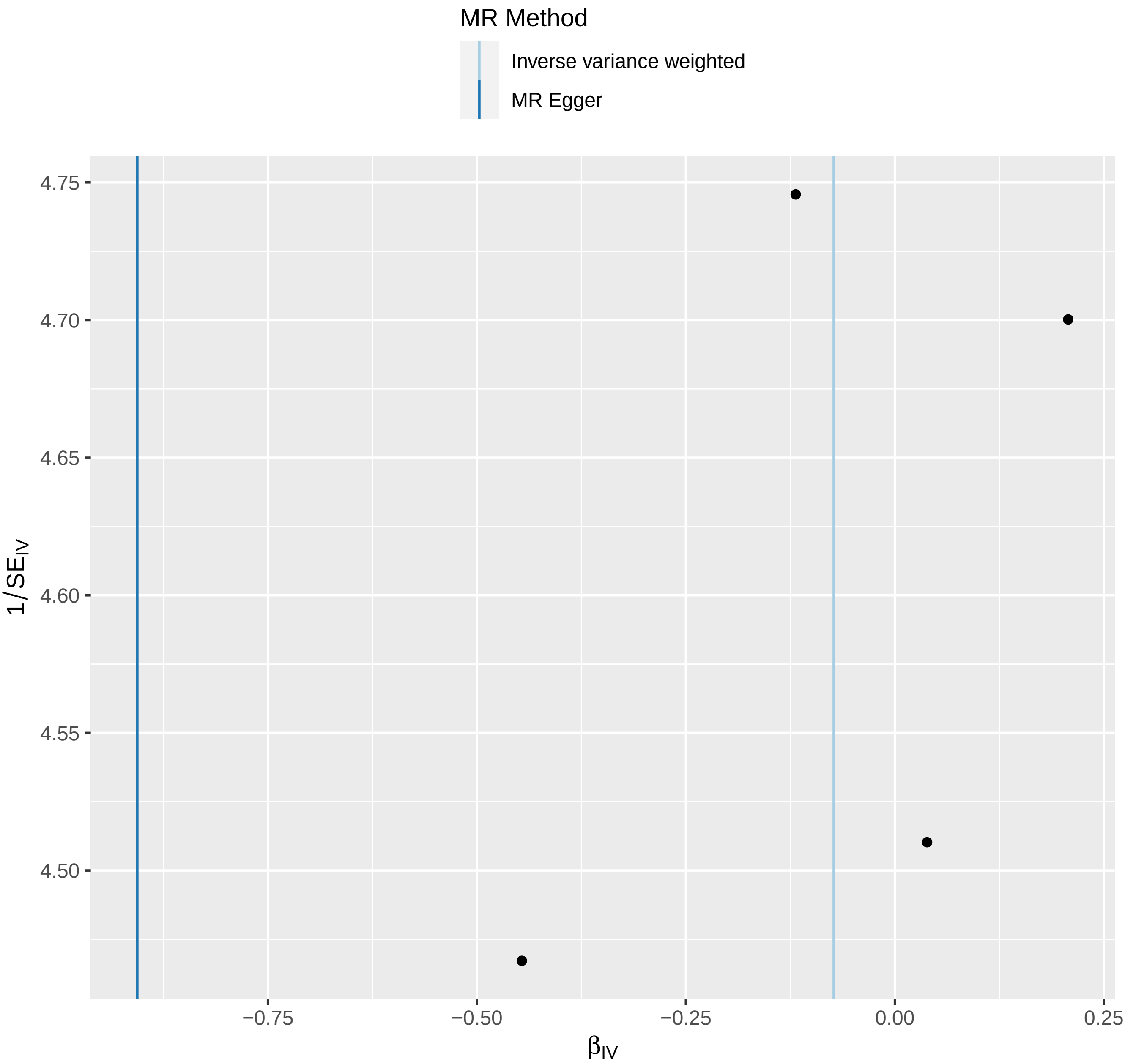

Batch 2−4376 : Gut microbiota abundance (genus Parasutterella id.2892) on Paired box protein Pax−4
with a threshold of 1e−05

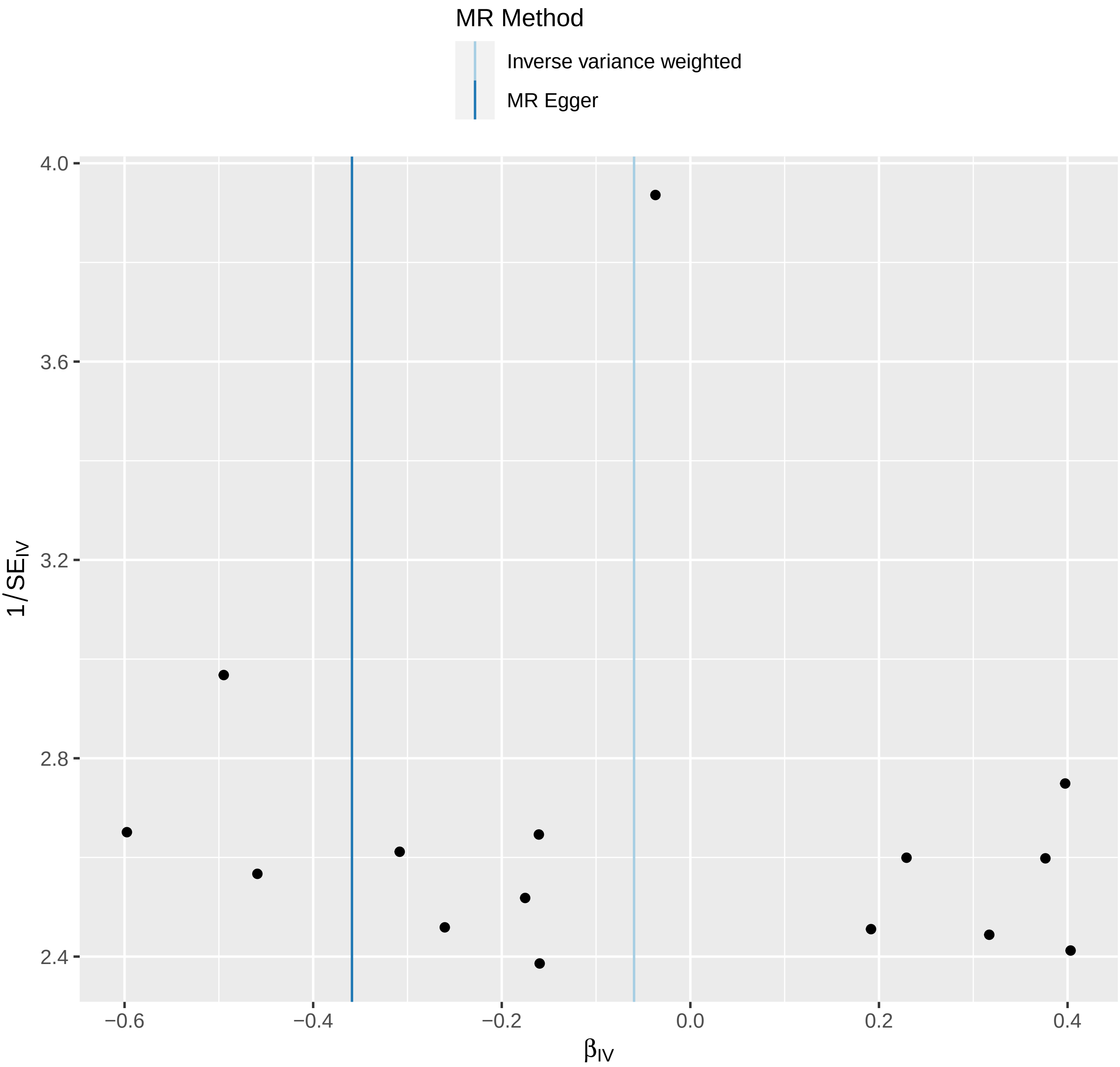

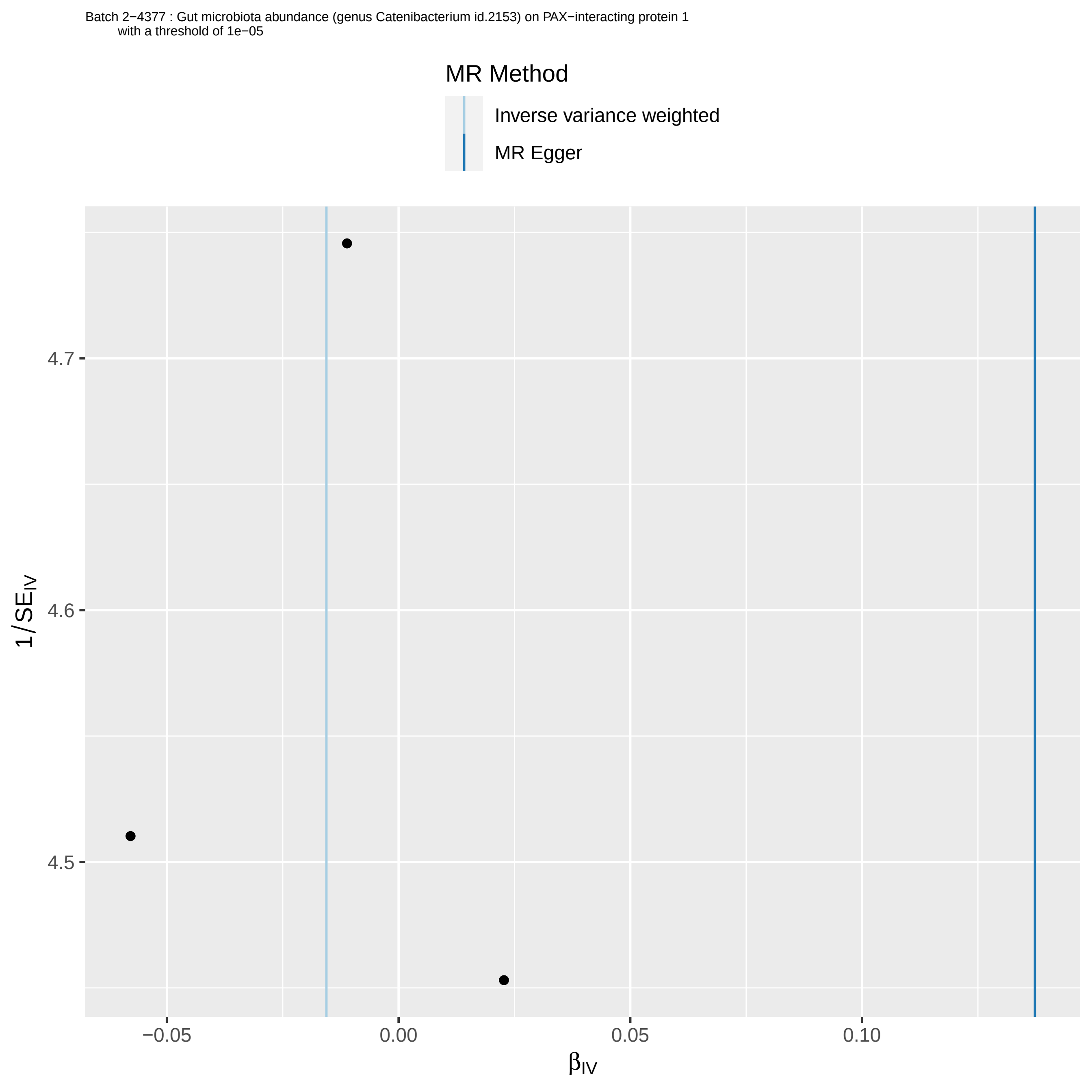
Batch 2−4377 : Gut microbiota abundance (genus Catenibacterium id.2153) on PAX−interacting protein 1
with a threshold of 1e−05
MR Method
Inverse variance weighted
MR Egger
4.7
4.6
4.5
1/SE_IV
−0.05
0.00
0.05
0.10
β_IV

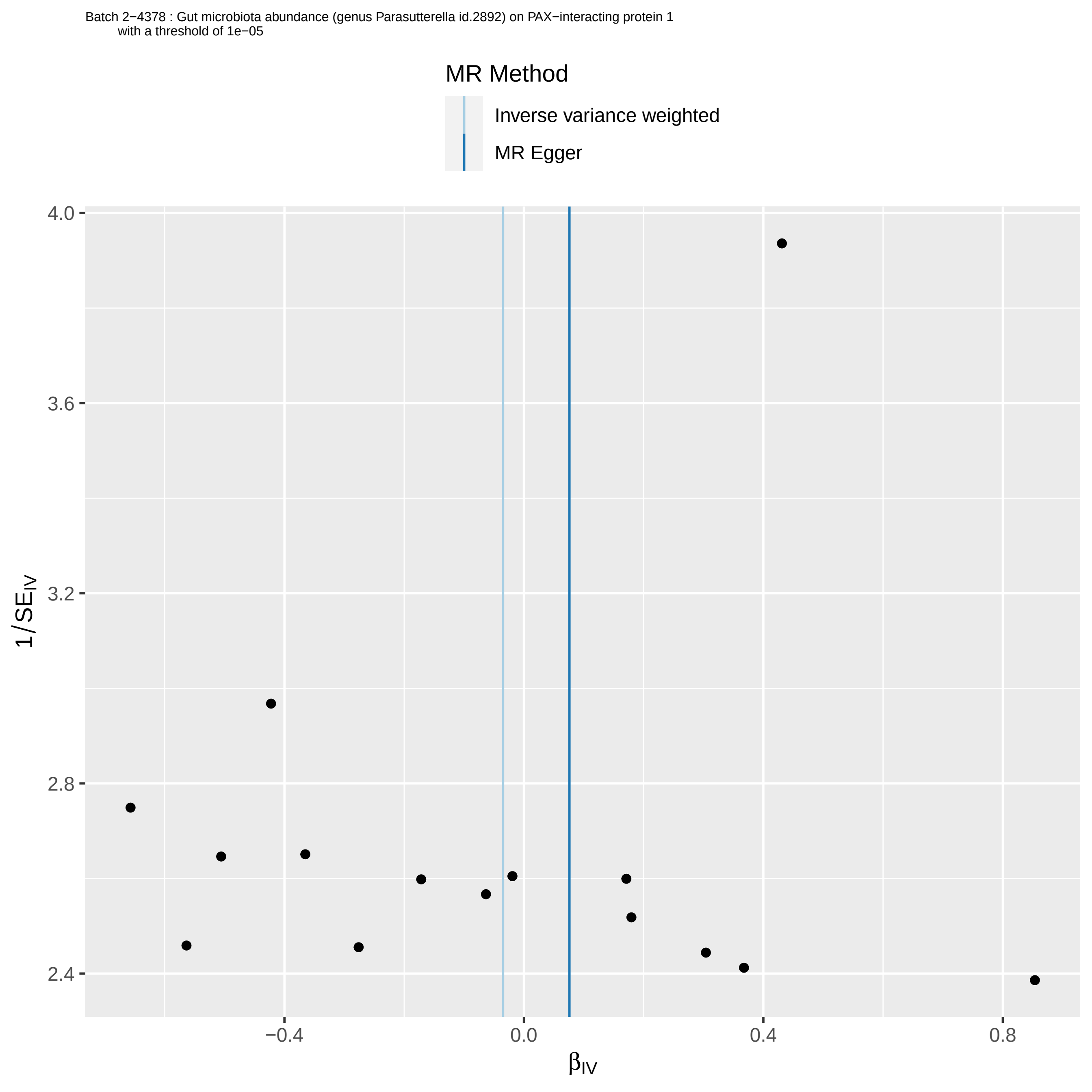
Batch 2−4378 : Gut microbiota abundance (genus Parasutterella id.2892) on PAX−interacting protein 1
with a threshold of 1e−05
MR Method
Inverse variance weighted
MR Egger
4.0
3.6
3.2
2.8
2.4
1/SE$_{IV}$
−0.4
0.0
0.4
0.8
β_{IV}

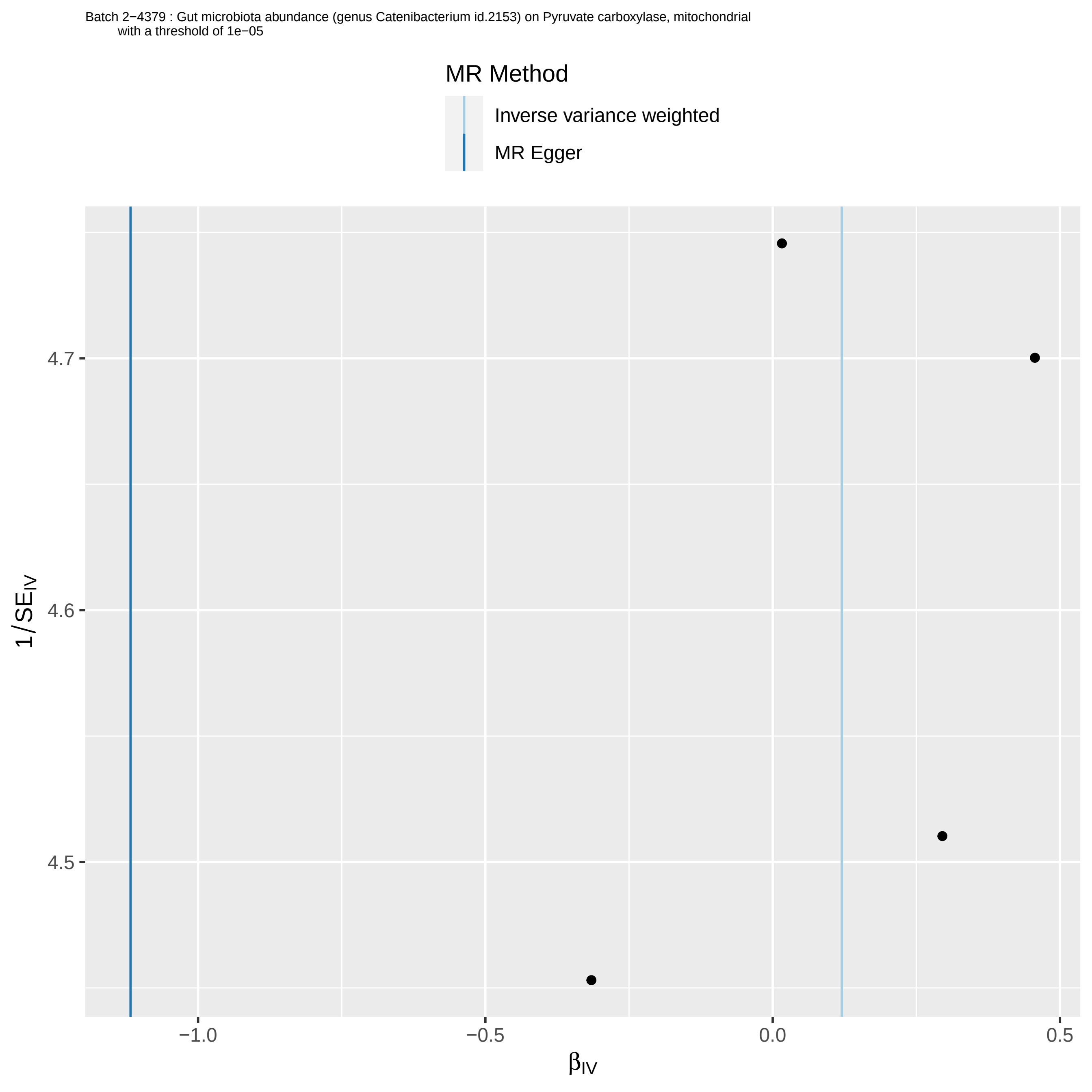
Batch 2−4379 : Gut microbiota abundance (genus Catenibacterium id.2153) on Pyruvate carboxylase, mitochondrial
with a threshold of 1e−05
MR Method
Inverse variance weighted
MR Egger
4.7
4.6
4.5
1/SE_IV
−1.0
−0.5
0.0
0.5
β_IV

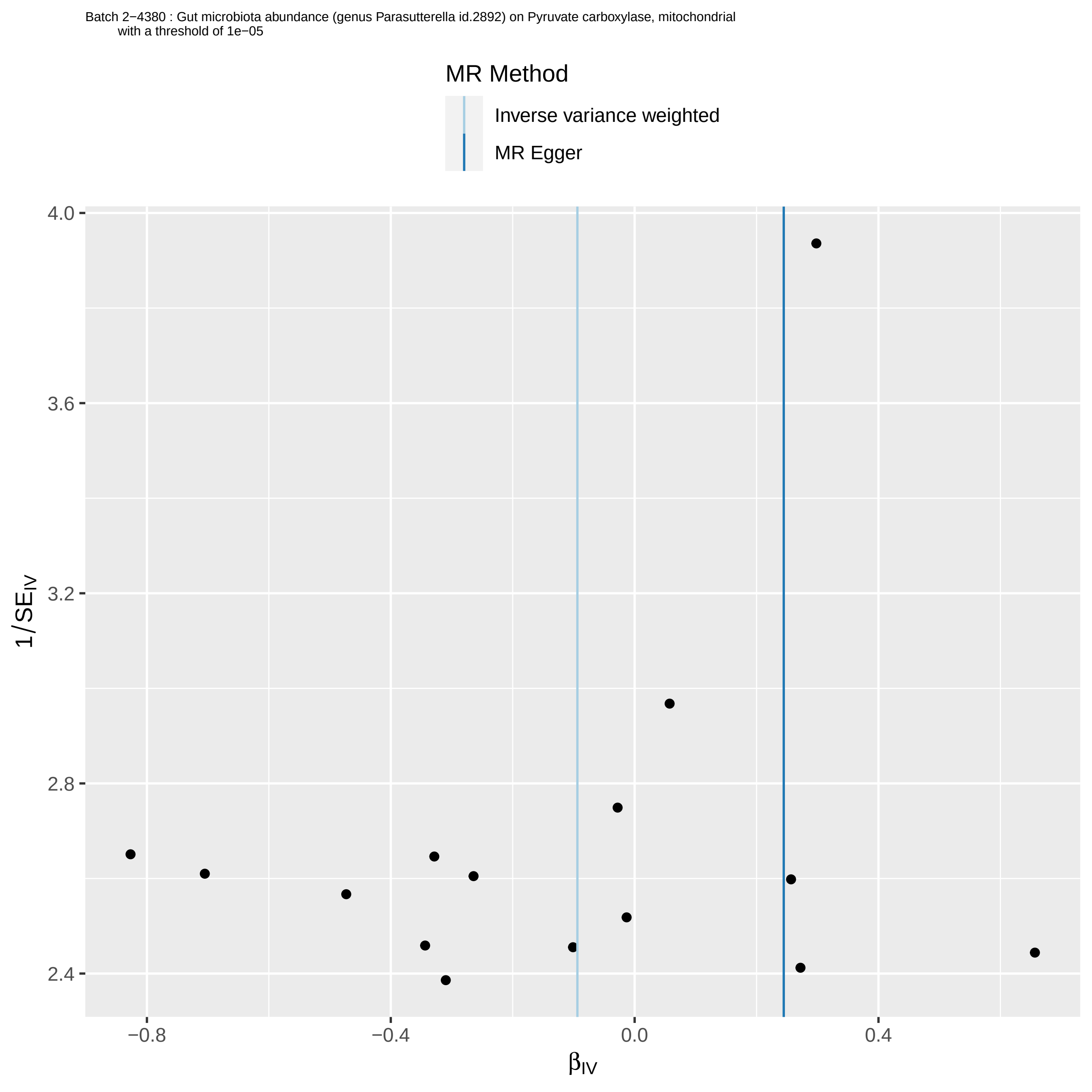

Batch 2−4380 : Gut microbiota abundance (genus Parasutterella id.2892) on Pyruvate carboxylase, mitochondrial
with a threshold of 1e−05
MR Method
Inverse variance weighted
MR Egger
4.0
3.6
3.2
2.8
2.4
1/SE$_{IV}$
−0.8
−0.4
0.0
0.4
β$_{IV}$

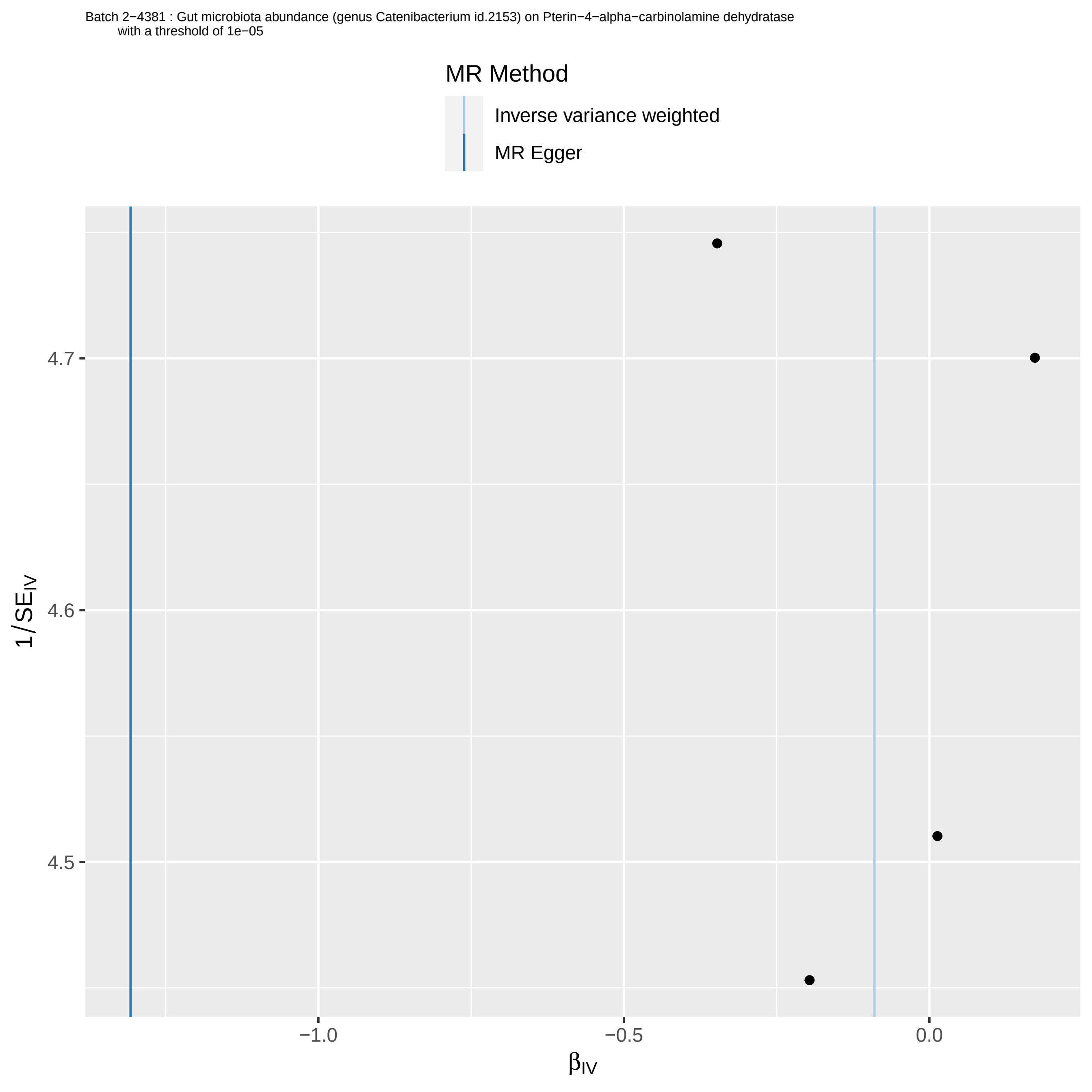
Batch 2−4381 : Gut microbiota abundance (genus Catenibacterium id.2153) on Pterin−4−alpha−carbinolamine dehydratase
with a threshold of 1e−05
MR Method
Inverse variance weighted
MR Egger
4.7
4.6
4.5
$1/SE_{IV}$
−1.0
−0.5
0.0
β_{IV}

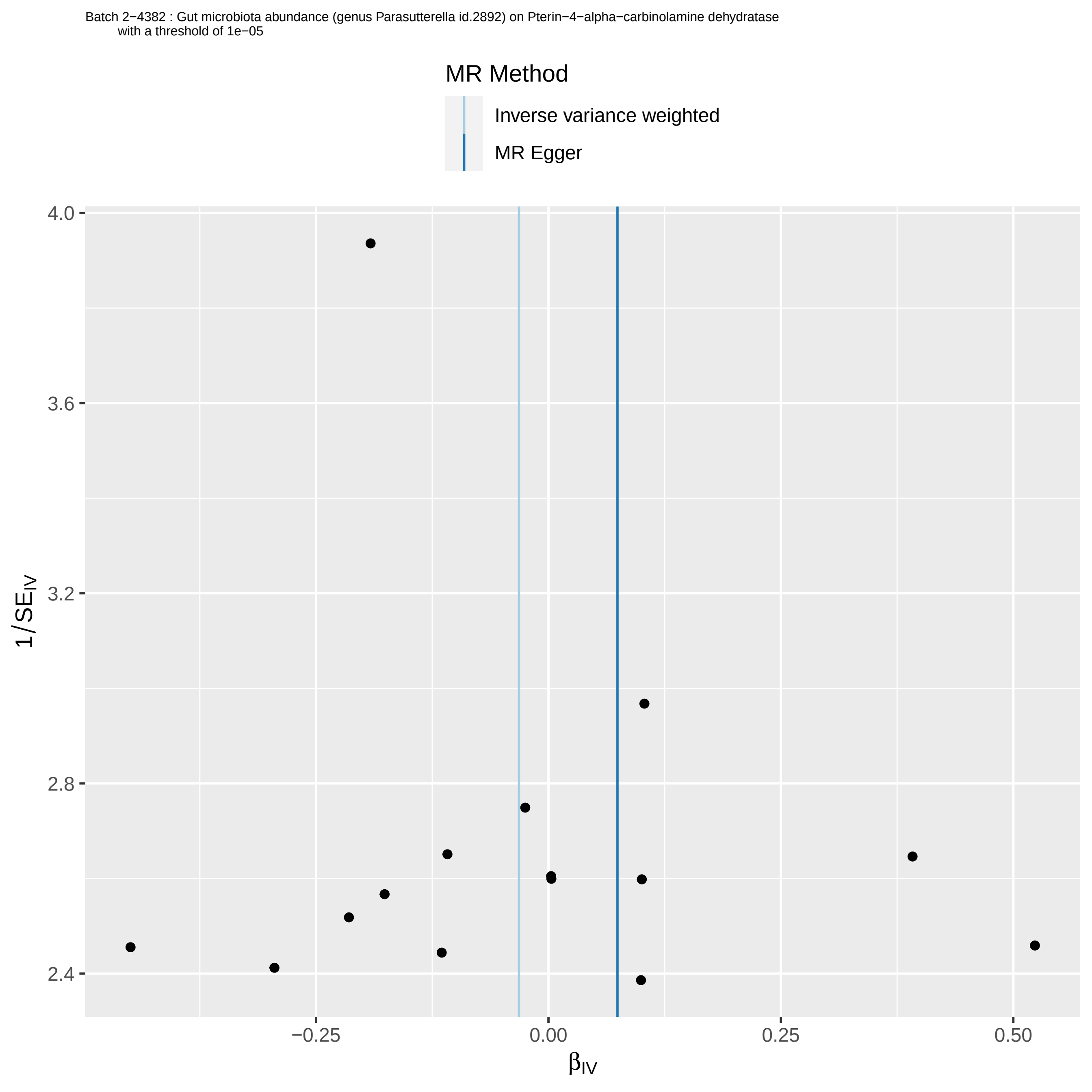

Batch 2−4382 : Gut microbiota abundance (genus Parasutterella id.2892) on Pterin−4−alpha−carbinolamine dehydratase
with a threshold of 1e−05
MR Method
Inverse variance weighted
MR Egger
4.0
3.6
3.2
2.8
2.4
1/SE_IV
−0.25
0.00
0.25
0.50
β_IV

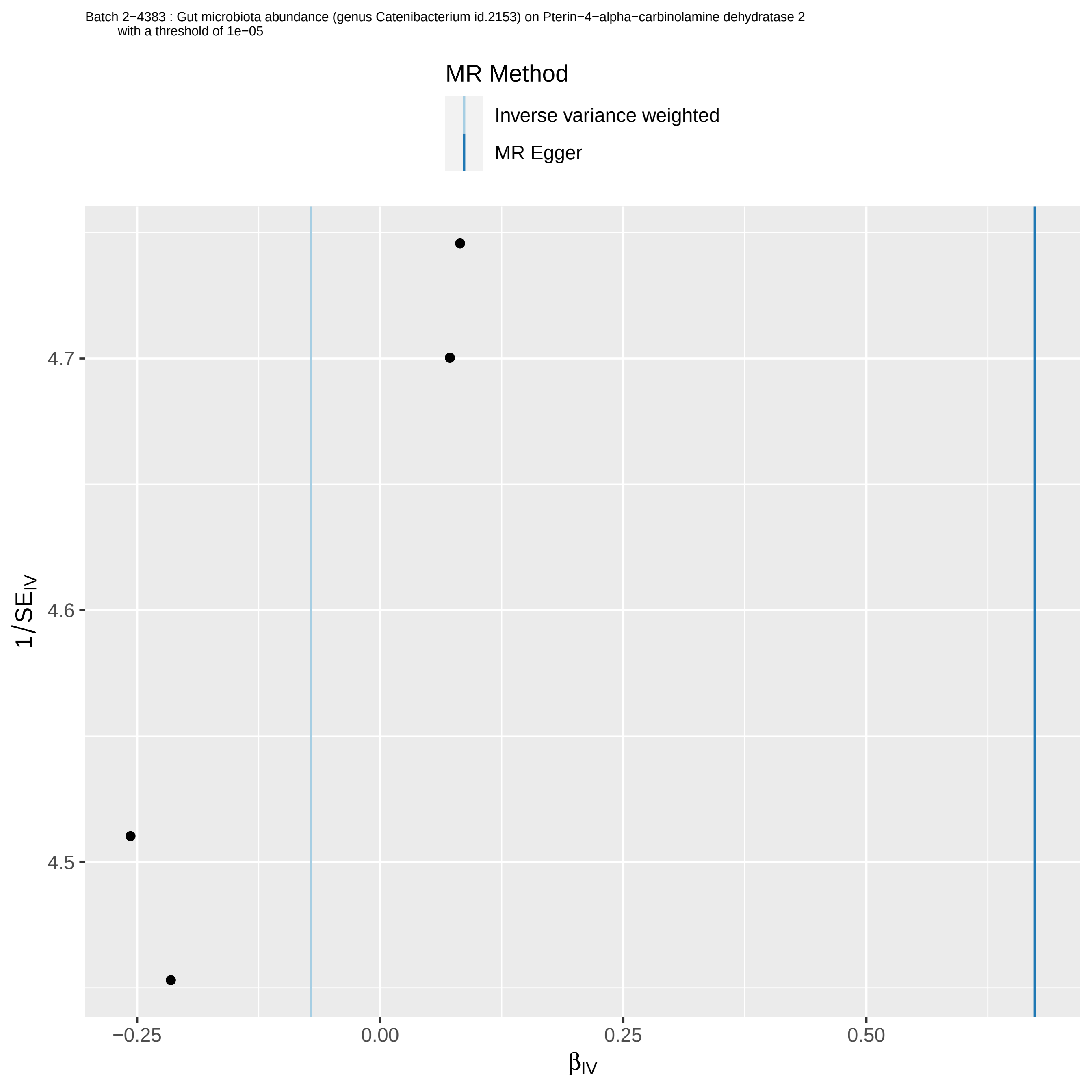
Batch 2−4383 : Gut microbiota abundance (genus Catenibacterium id.2153) on Pterin−4−alpha−carbinolamine dehydratase 2
with a threshold of 1e−05
MR Method
Inverse variance weighted
MR Egger
4.7
4.6
4.5
1/SE_IV
−0.25
0.00
0.25
0.50
β_IV

Batch 2−4384 : Gut microbiota abundance (genus Parasutterella id.2892) on Pterin−4−alpha−carbinolamine dehydratase 2 with a threshold of 1e−05

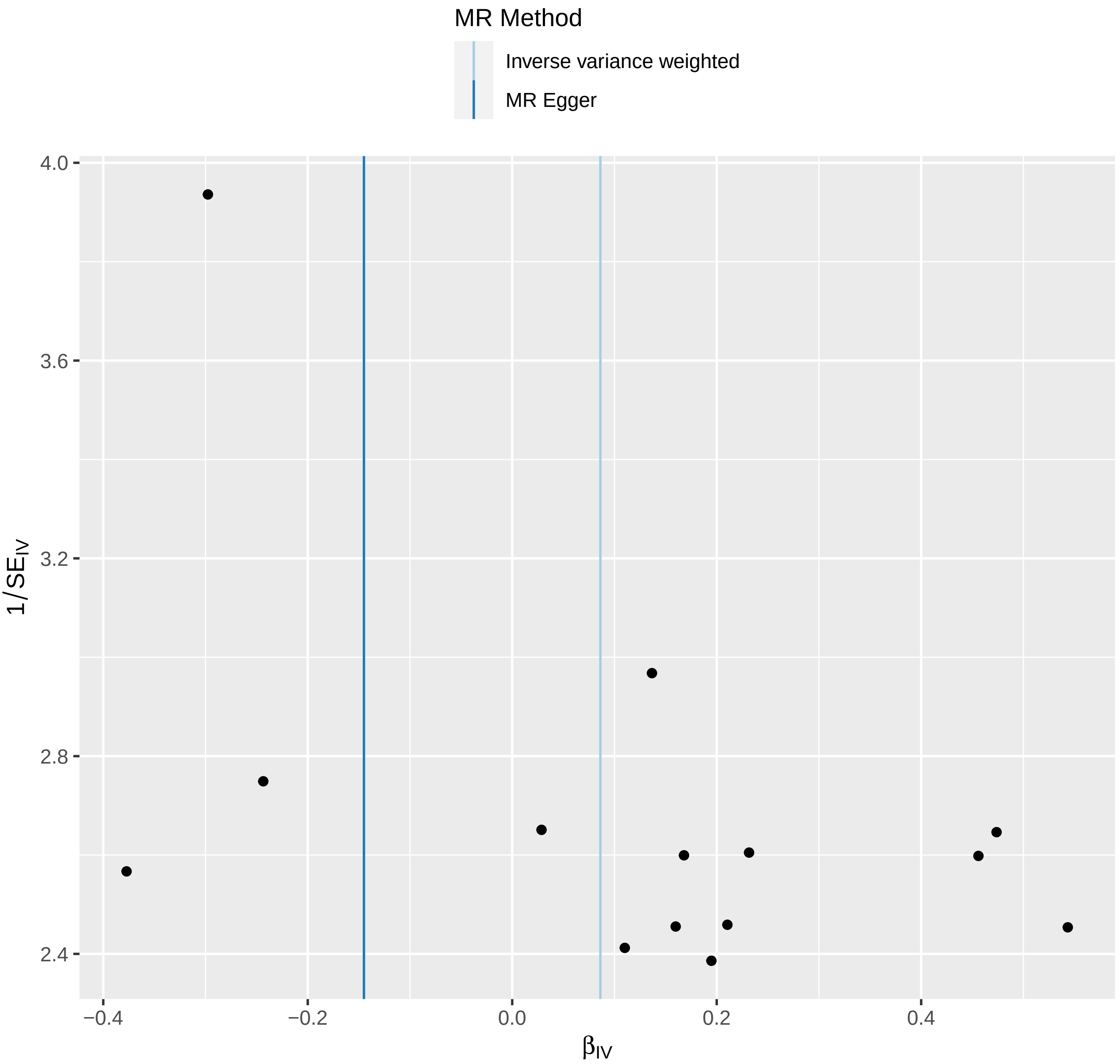

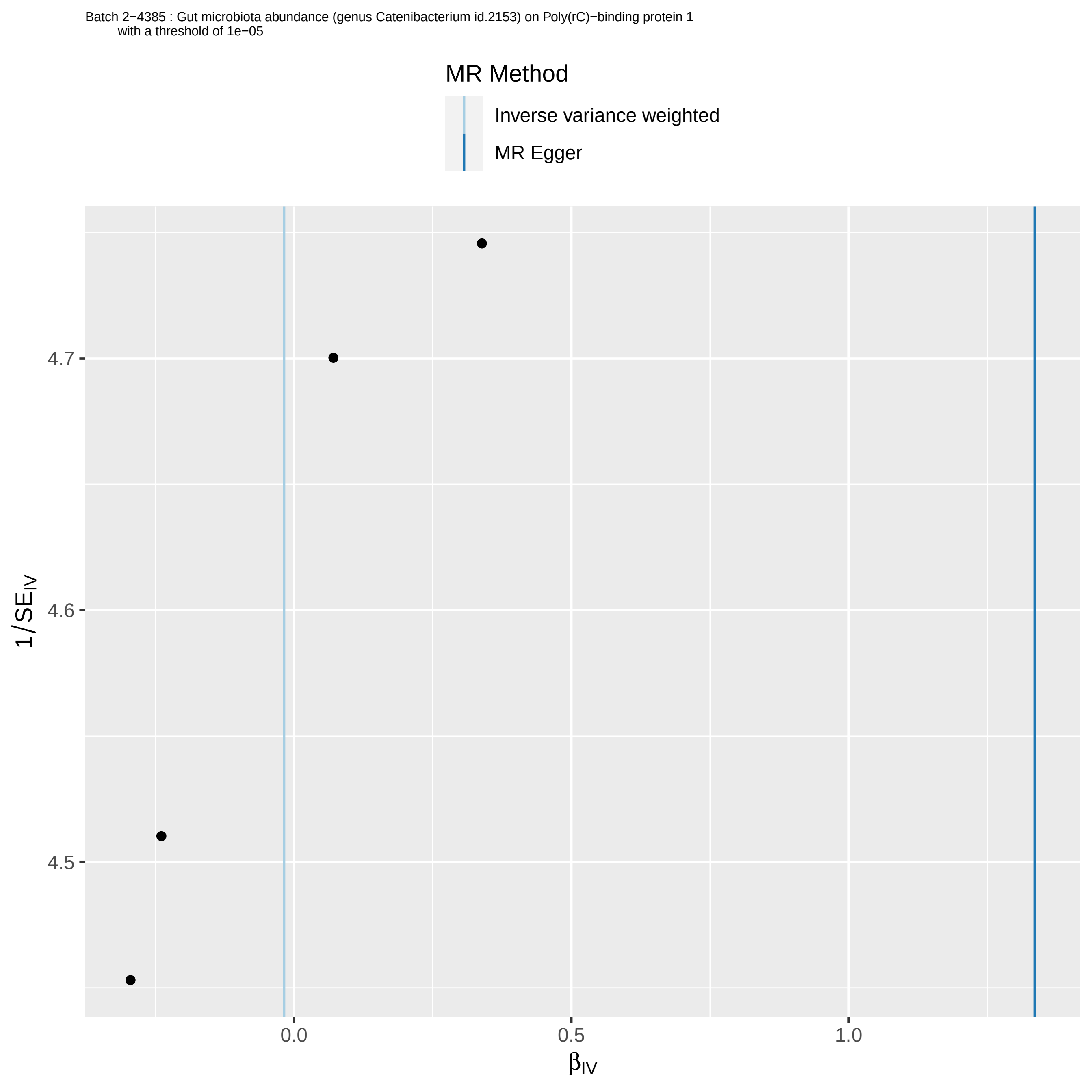
Batch 2−4385 : Gut microbiota abundance (genus Catenibacterium id.2153) on Poly(rC)−binding protein 1
with a threshold of 1e−05
MR Method
Inverse variance weighted
MR Egger
4.7
4.6
4.5
1/SE_IV
0.0
0.5
1.0
β_IV

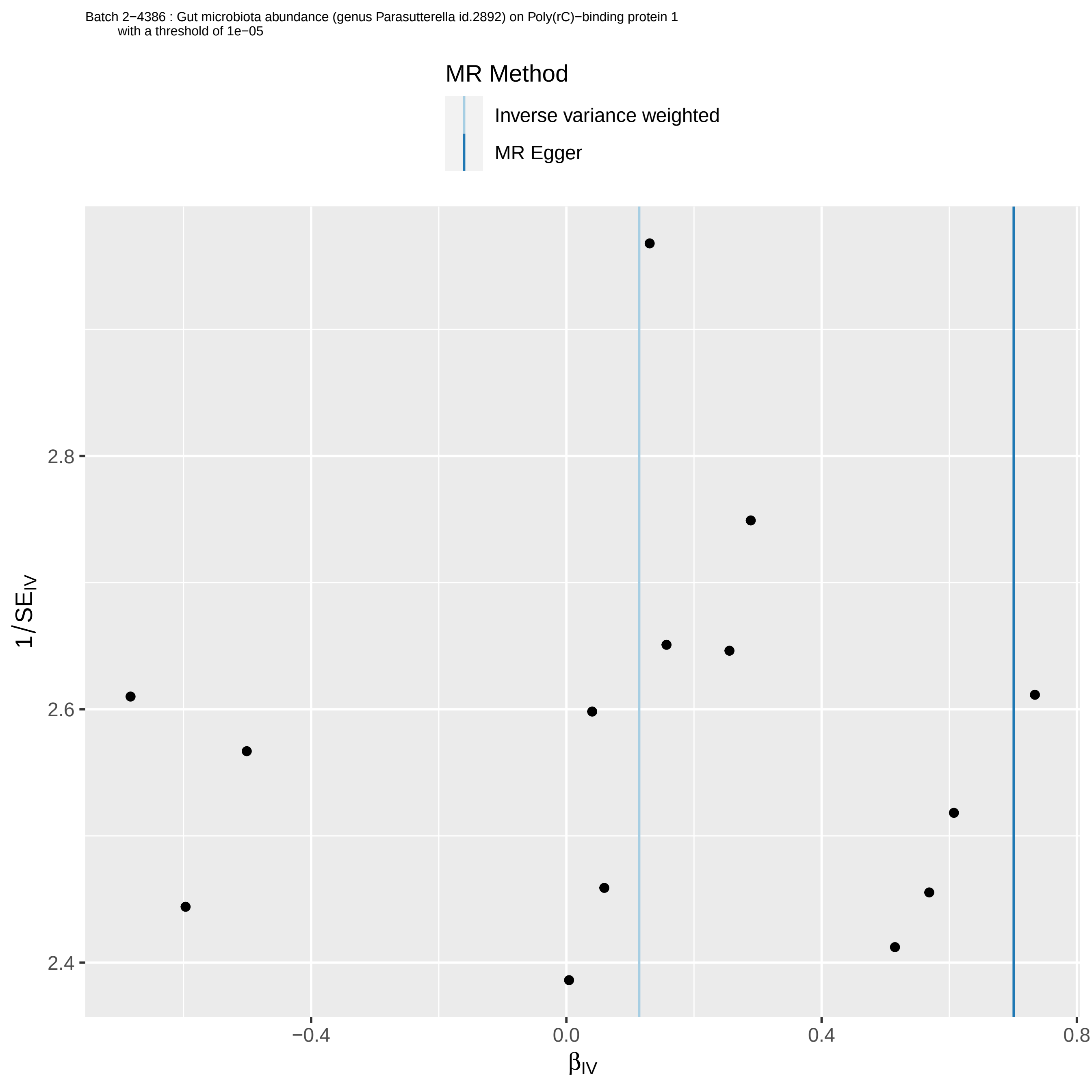
Batch 2−4386 : Gut microbiota abundance (genus Parasutterella id.2892) on Poly(rC)−binding protein 1
with a threshold of 1e−05
MR Method
Inverse variance weighted
MR Egger
2.8
2.6
2.4
1/SE_IV
−0.4
0.0
0.4
0.8
β_IV

Batch 2−4387 : Gut microbiota abundance (genus Catenibacterium id.2153) on Protocadherin−10
with a threshold of 1e−05

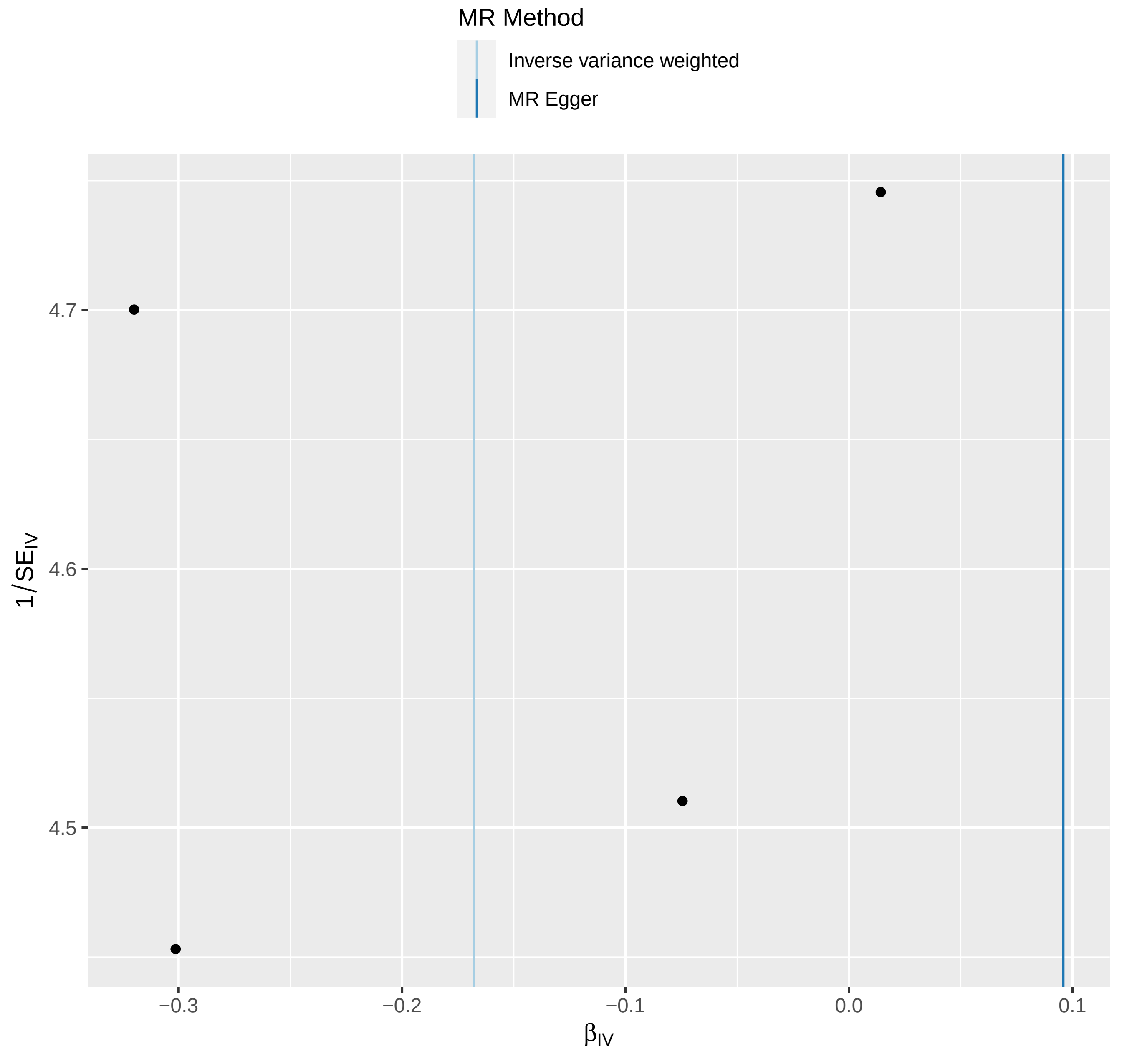

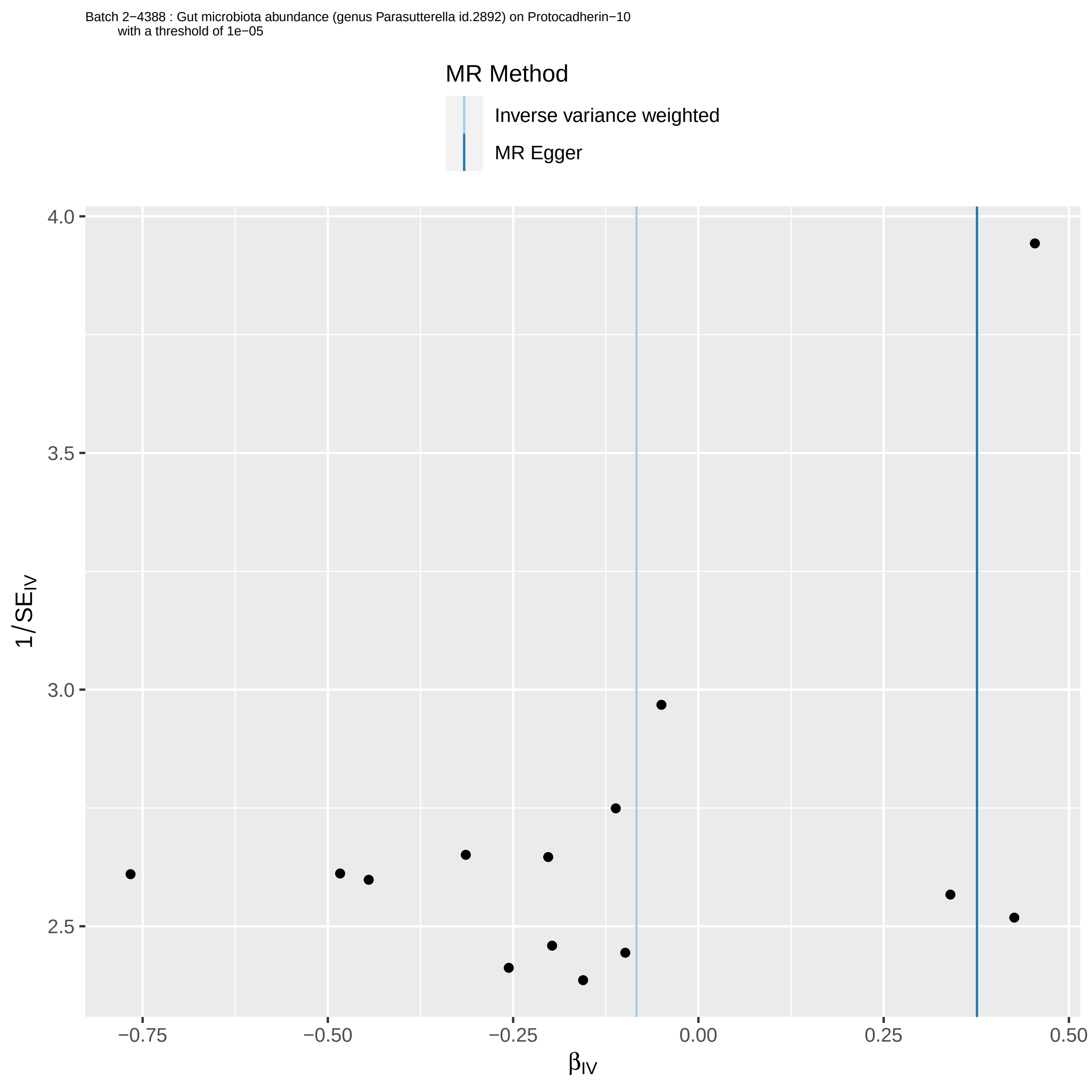
Batch 2−4388 : Gut microbiota abundance (genus Parasutterella id.2892) on Protocadherin−10
with a threshold of 1e−05
MR Method
Inverse variance weighted
MR Egger
4.0
3.5
3.0
2.5
1/SE_IV
−0.75
−0.50
−0.25
0.00
0.25
0.50
β_IV

Batch 2−4389 : Gut microbiota abundance (genus Catenibacterium id.2153) on Protocadherin−10
with a threshold of 1e−05

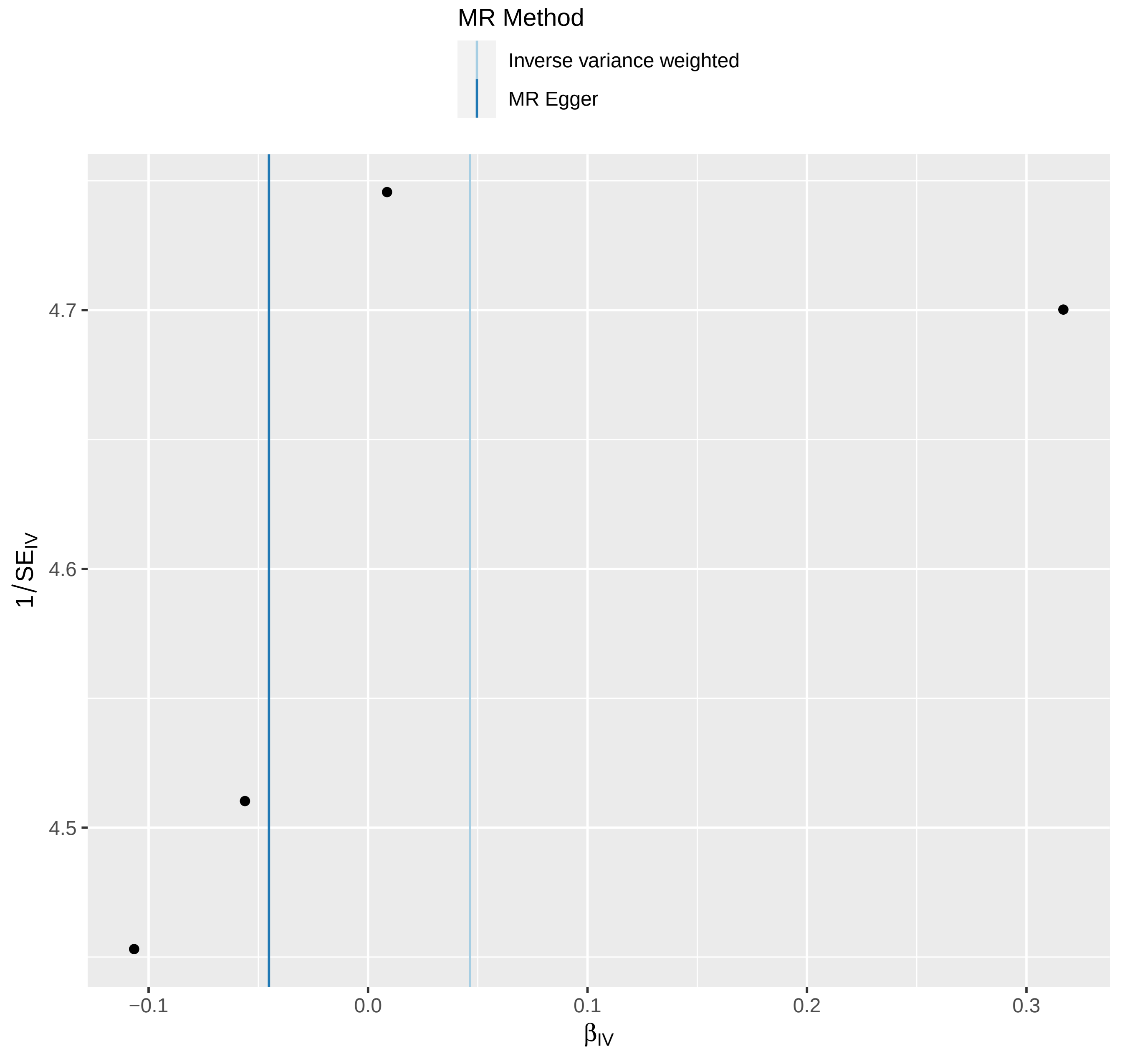

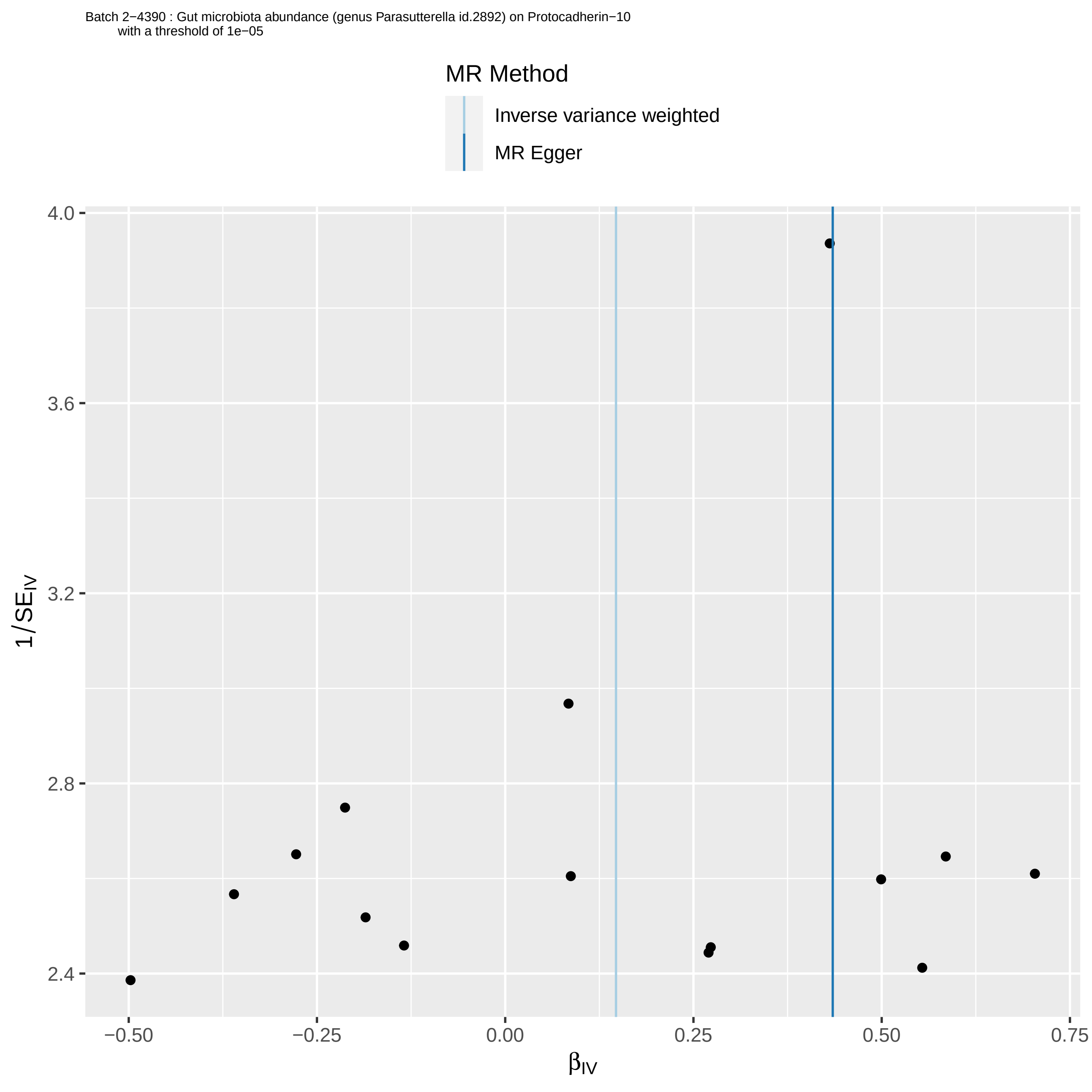

Batch 2−4390 : Gut microbiota abundance (genus Parasutterella id.2892) on Protocadherin−10
with a threshold of 1e−05
MR Method
Inverse variance weighted
MR Egger
4.0
3.6
3.2
2.8
2.4
1/SE_IV
−0.50
−0.25
0.00
0.25
0.50
0.75
β_IV

Batch 2−4391 : Gut microbiota abundance (genus Catenibacterium id.2153) on Protocadherin−15
with a threshold of 1e−05

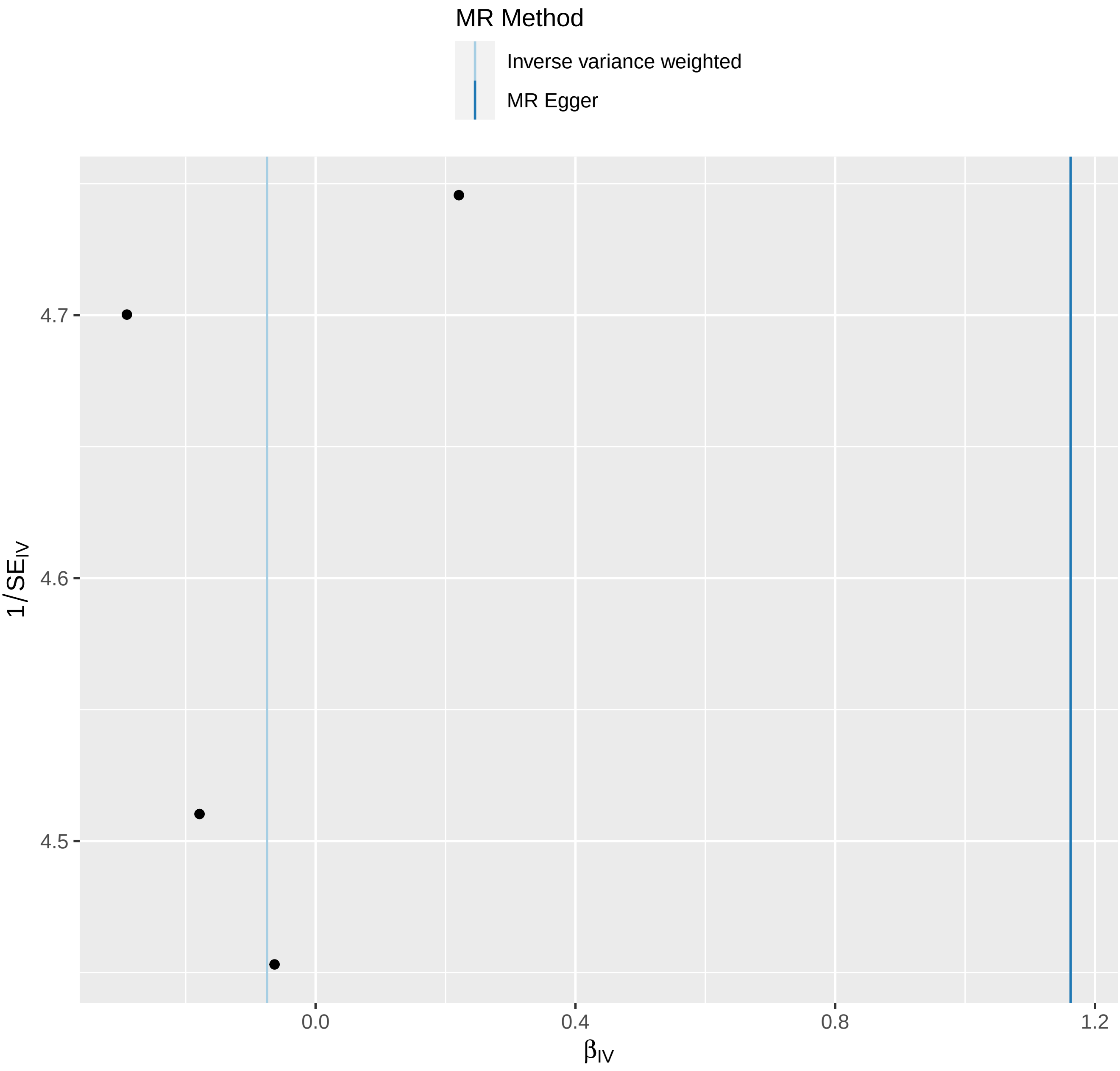

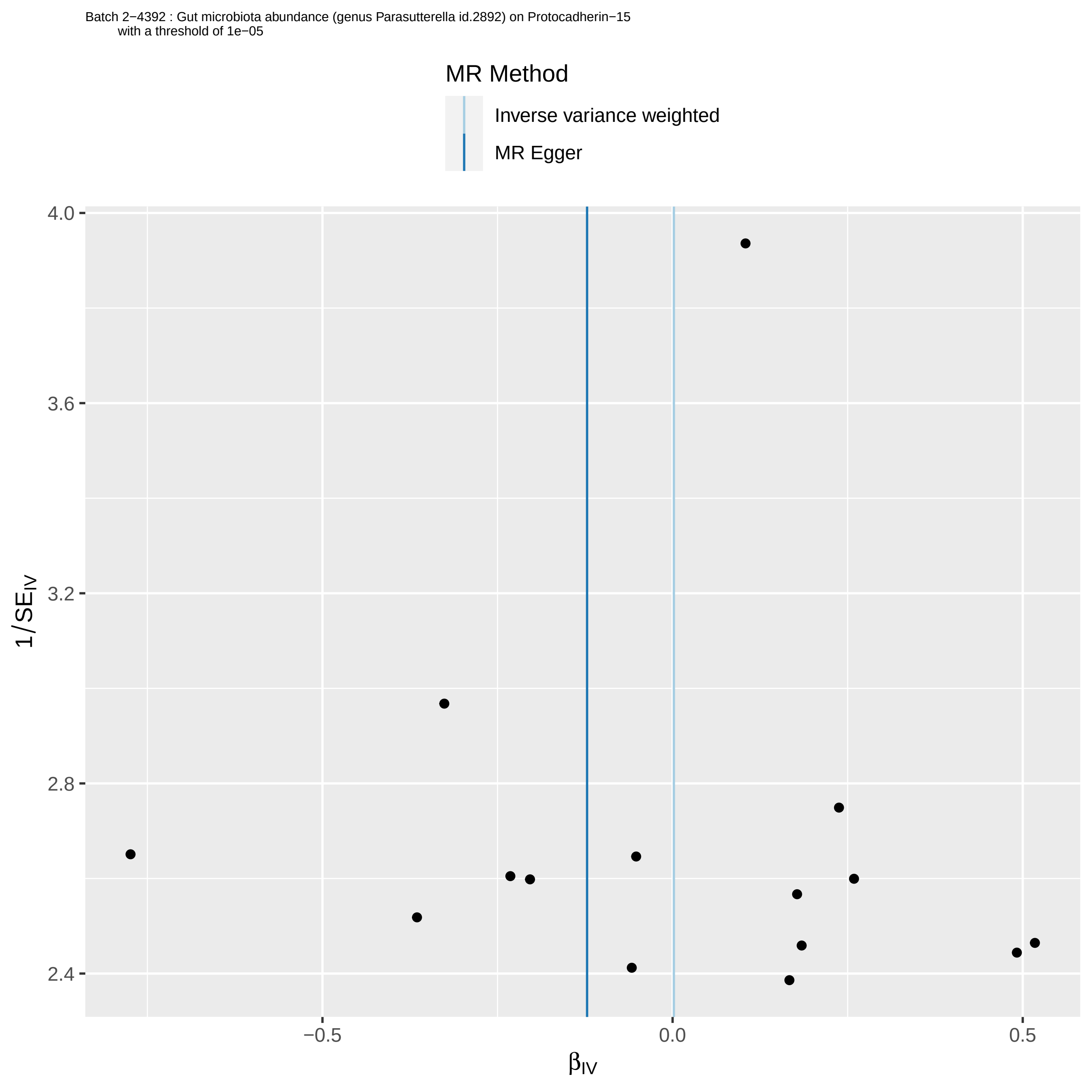
Batch 2−4392 : Gut microbiota abundance (genus Parasutterella id.2892) on Protocadherin−15
with a threshold of 1e−05
MR Method
Inverse variance weighted
MR Egger
4.0
3.6
3.2
2.8
2.4
1/SE$_{IV}$
−0.5
0.0
0.5
β$_{IV}$

Batch 2−4393 : Gut microbiota abundance (genus Catenibacterium id.2153) on Protocadherin−9
with a threshold of 1e−05

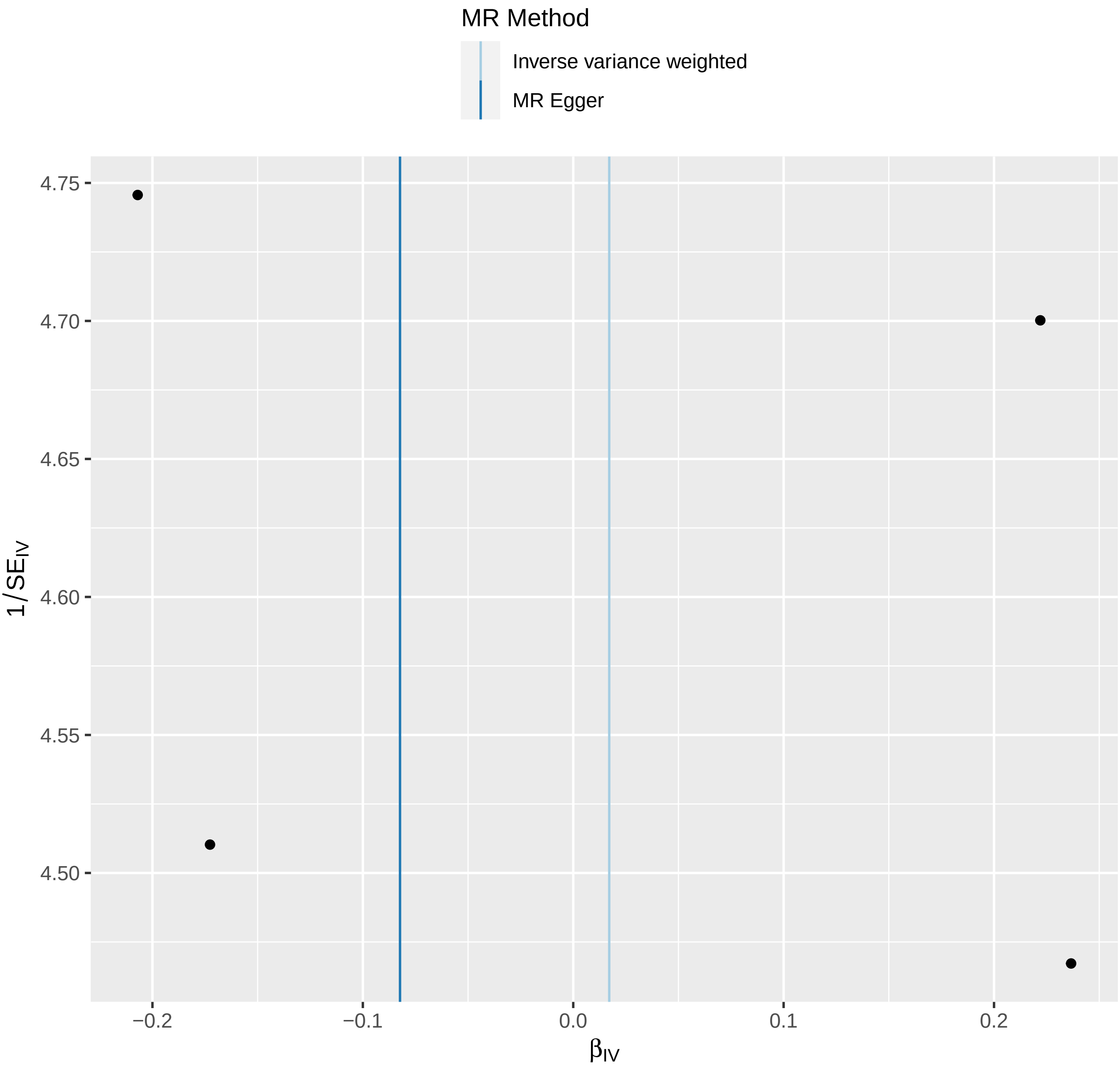

Batch 2−4394 : Gut microbiota abundance (genus Parasutterella id.2892) on Protocadherin−9
with a threshold of 1e−05

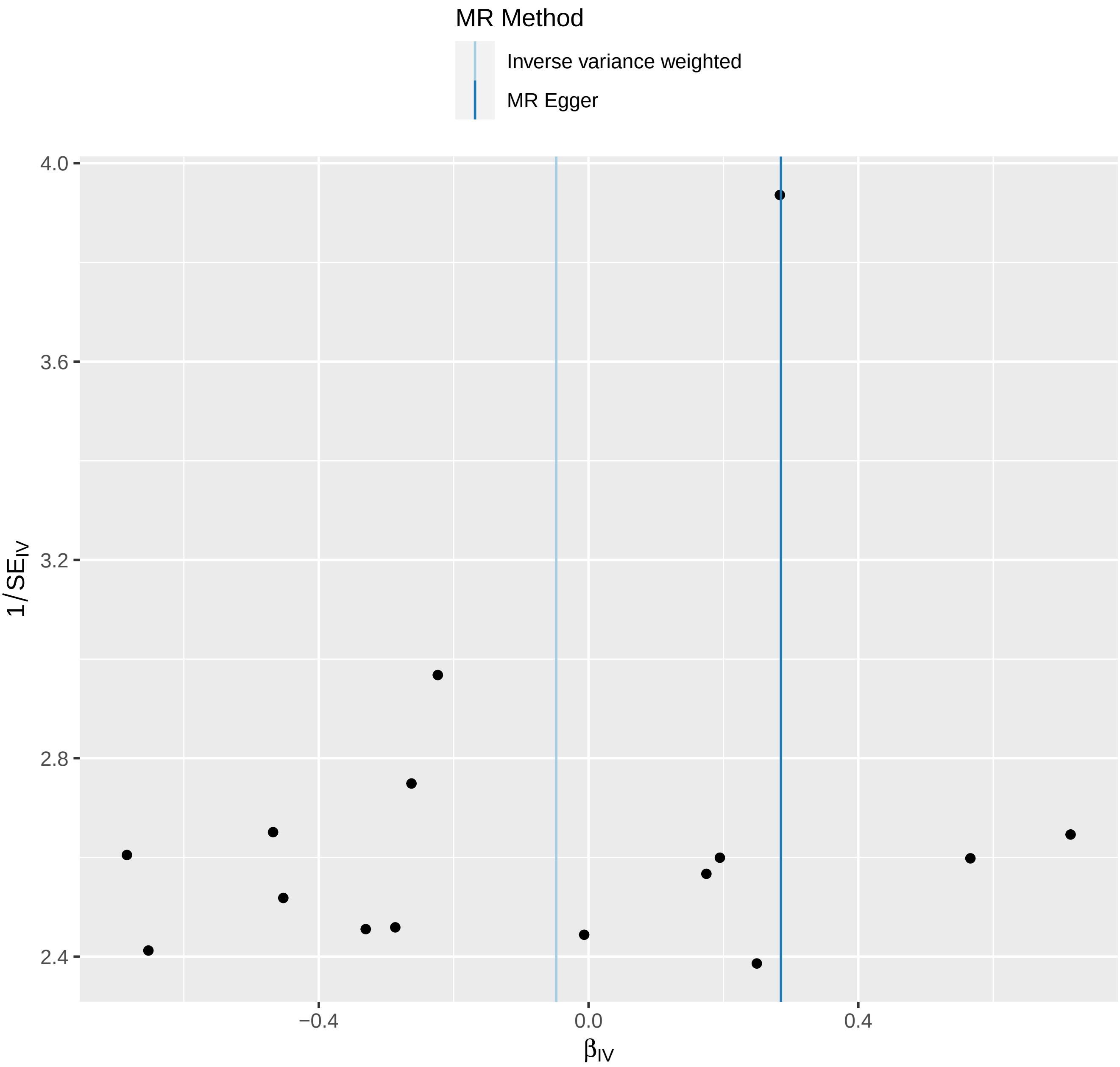

Batch 2−4395 : Gut microbiota abundance (genus Catenibacterium id.2153) on Protocadherin alpha−4
with a threshold of 1e−05

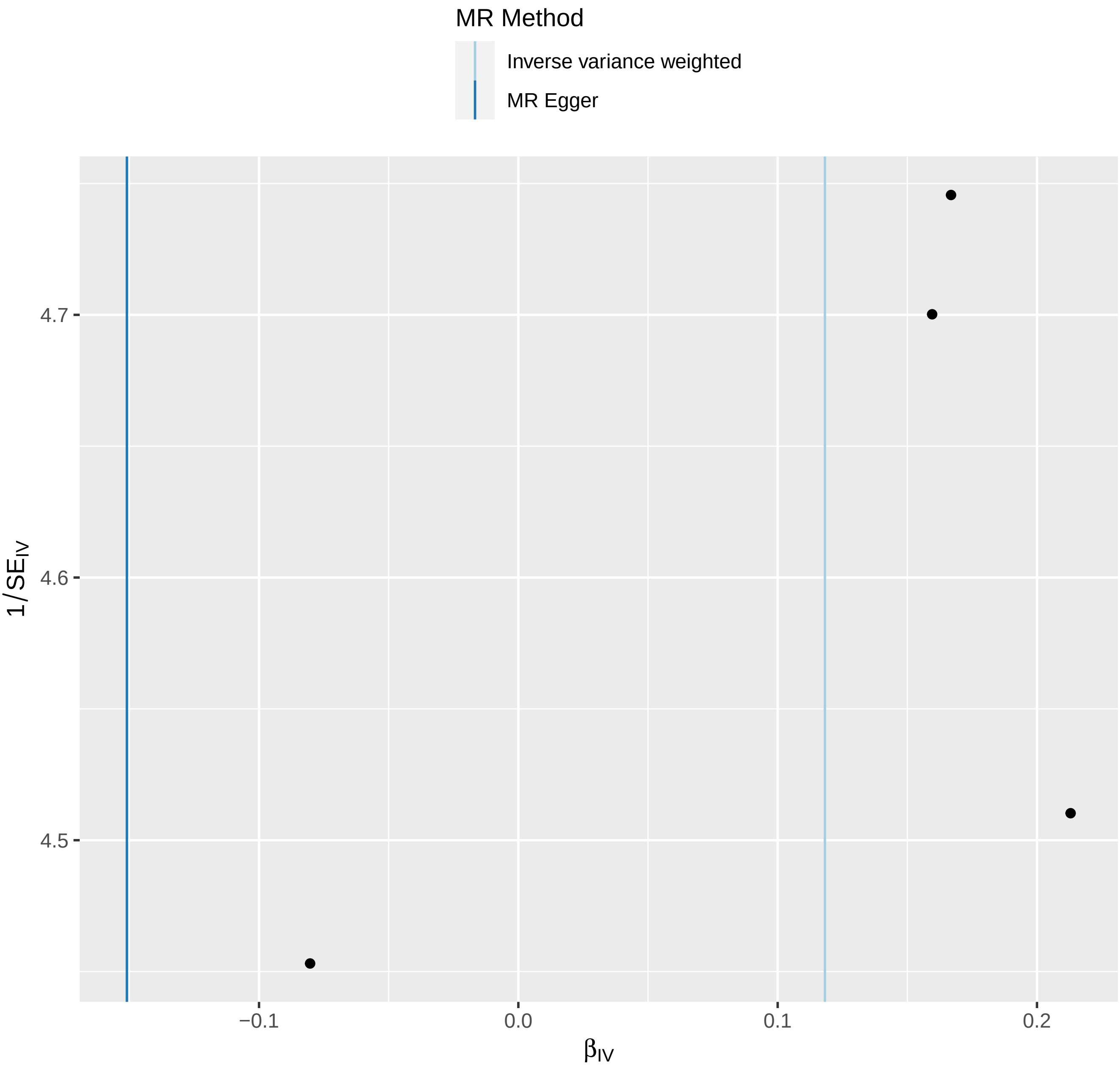

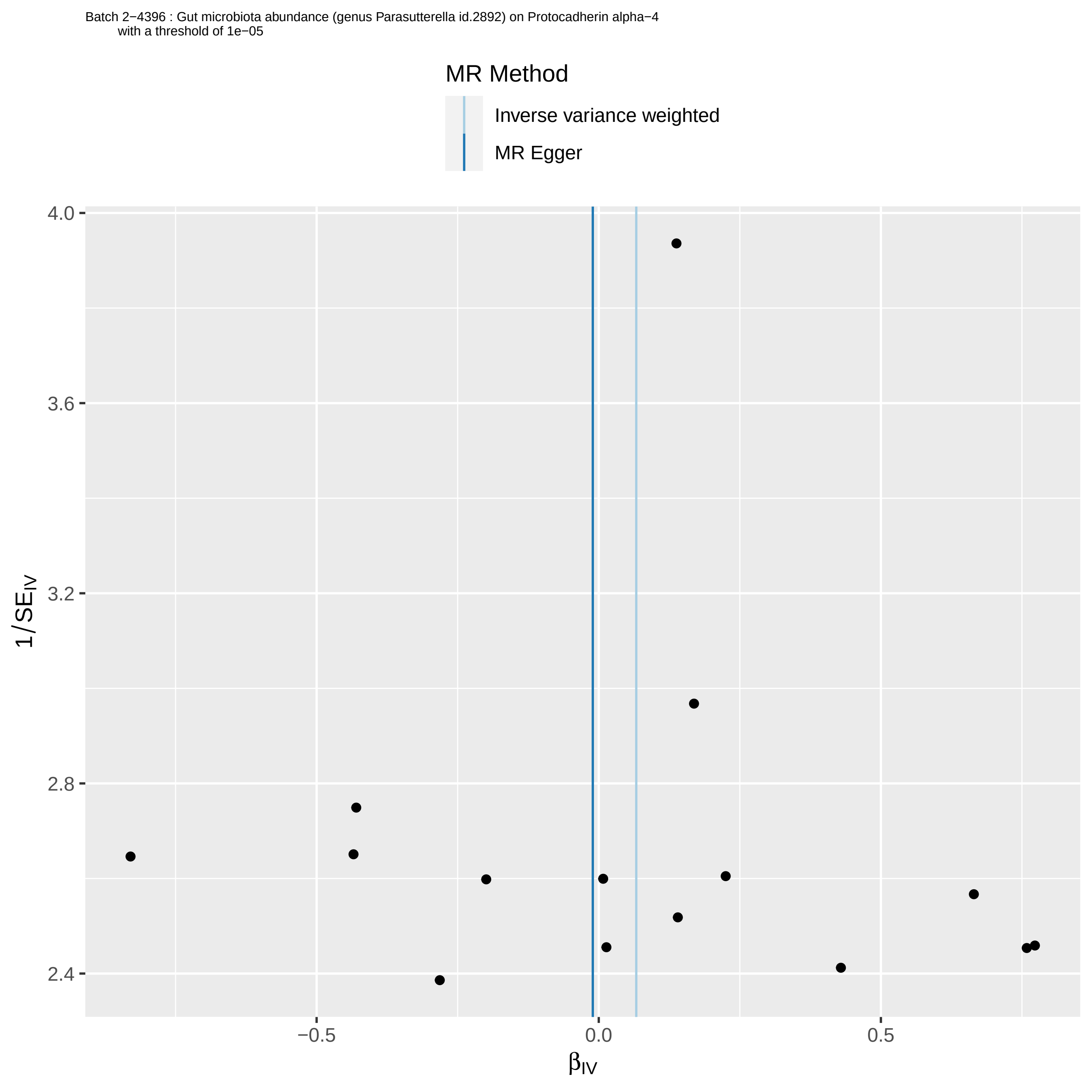

Batch 2−4396 : Gut microbiota abundance (genus Parasutterella id.2892) on Protocadherin alpha−4
with a threshold of 1e−05
MR Method
Inverse variance weighted
MR Egger
4.0
3.6
3.2
2.8
2.4
1/SE_IV
−0.5
0.0
0.5
β_IV

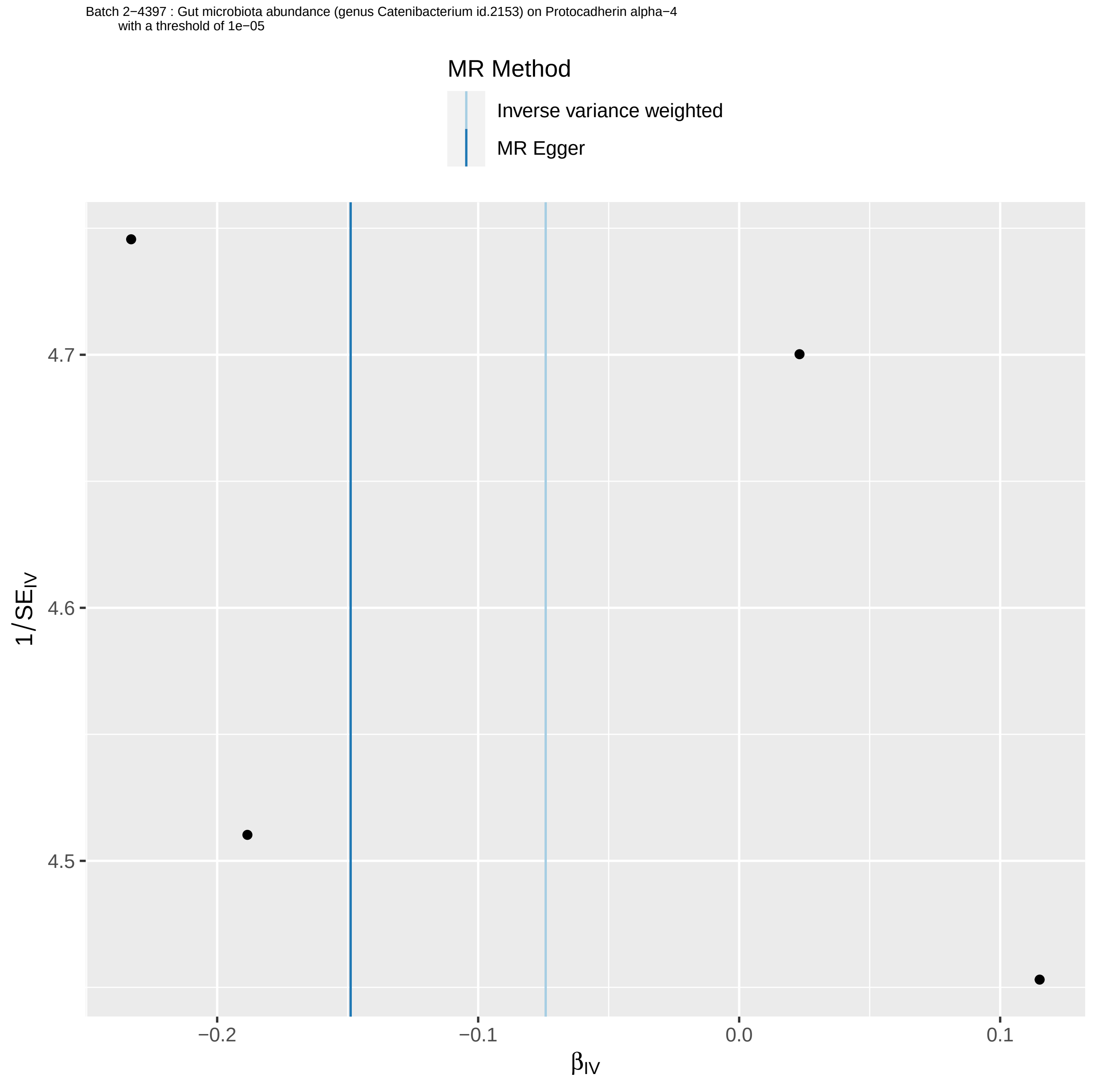
Batch 2−4397 : Gut microbiota abundance (genus Catenibacterium id.2153) on Protocadherin alpha−4
with a threshold of 1e−05
MR Method
Inverse variance weighted
MR Egger
4.7
4.6
4.5
1/SE_IV
−0.2
−0.1
0.0
0.1
β_IV

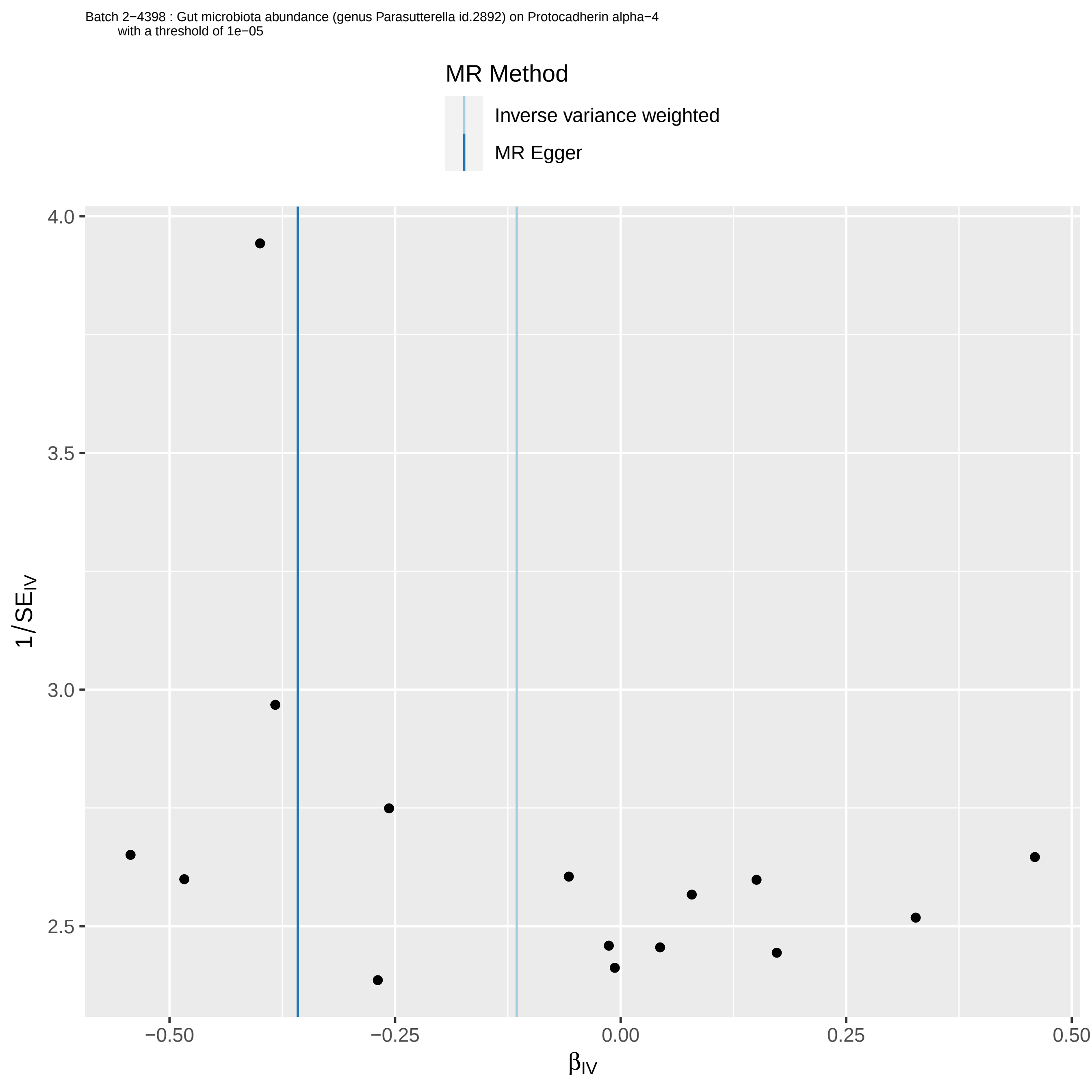
Batch 2−4398 : Gut microbiota abundance (genus Parasutterella id.2892) on Protocadherin alpha−4
with a threshold of 1e−05
MR Method
Inverse variance weighted
MR Egger
4.0
3.5
3.0
2.5
1/SE_IV
−0.50
−0.25
0.00
0.25
0.50
β_IV

Batch 2−4399 : Gut microbiota abundance (genus Catenibacterium id.2153) on Protocadherin alpha−7
with a threshold of 1e−05

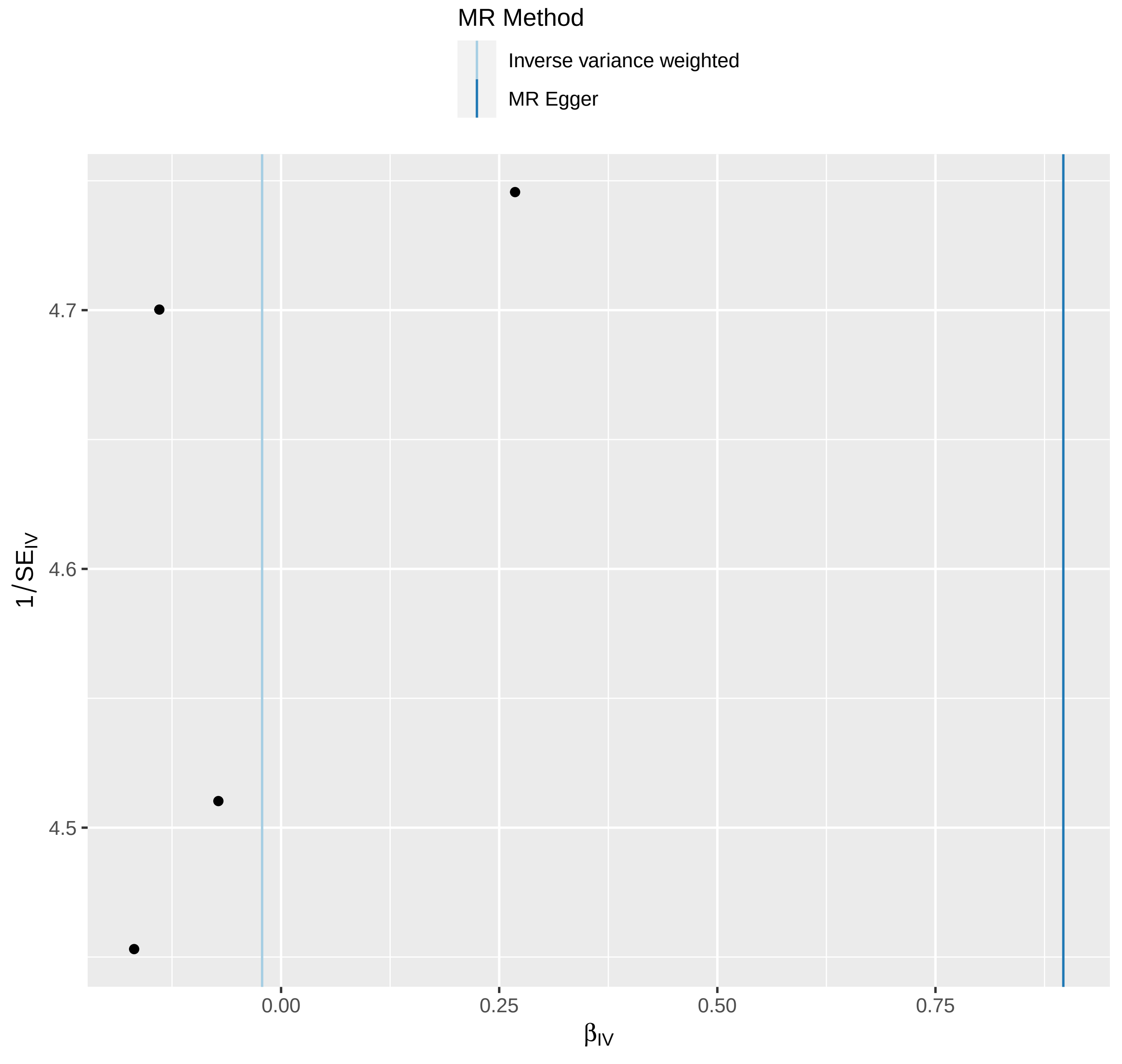

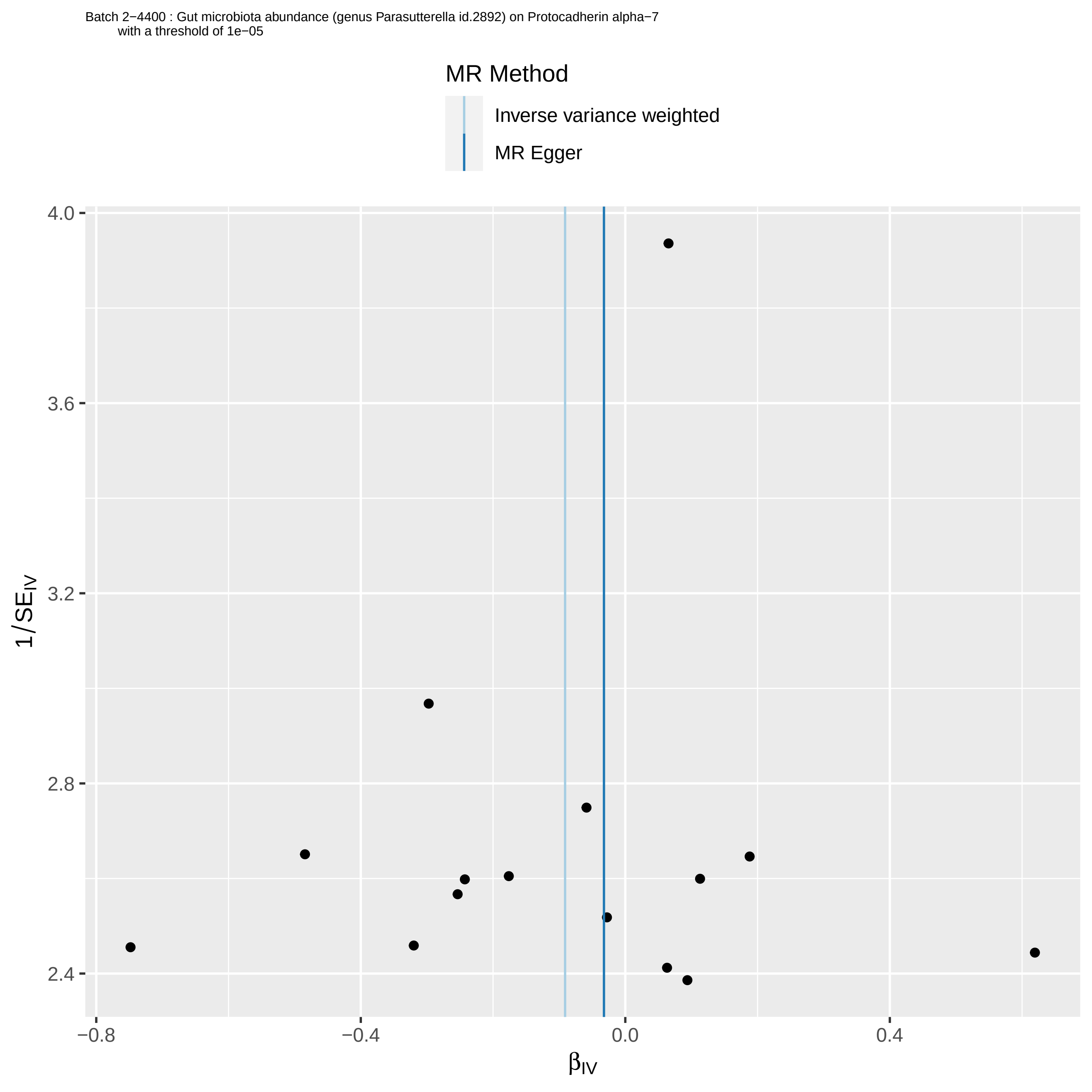
Batch 2−4400 : Gut microbiota abundance (genus Parasutterella id.2892) on Protocadherin alpha−7
with a threshold of 1e−05
MR Method
Inverse variance weighted
MR Egger
4.0
3.6
3.2
2.8
2.4
1/SE_IV
−0.8
−0.4
0.0
0.4
β_IV

Batch 2−4401 : Gut microbiota abundance (genus Catenibacterium id.2153) on Protocadherin alpha−C2
with a threshold of 1e−05

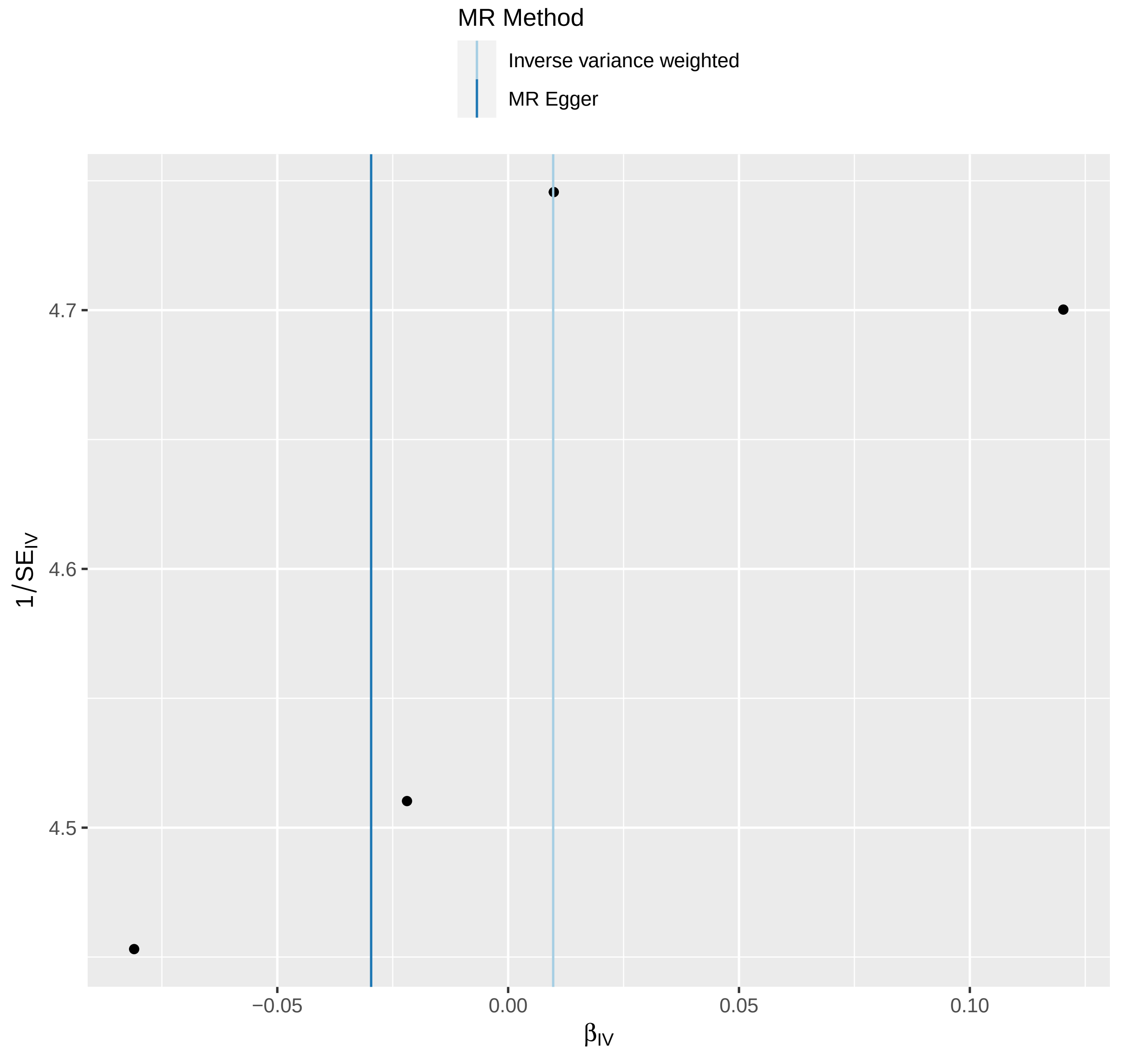

Batch 2−4402 : Gut microbiota abundance (genus Parasutterella id.2892) on Protocadherin alpha−C2
with a threshold of 1e−05

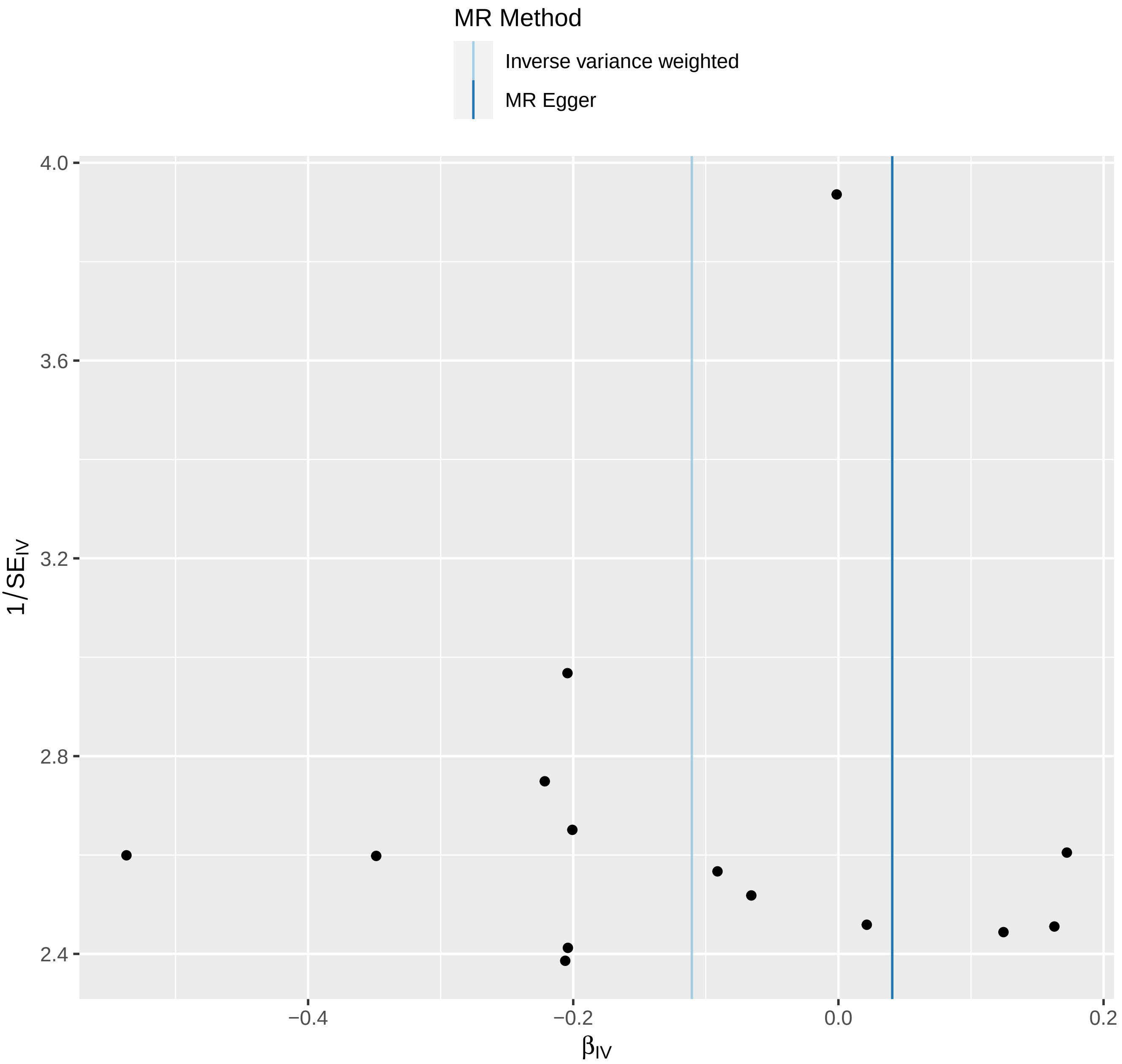

Batch 2−4403 : Gut microbiota abundance (genus Catenibacterium id.2153) on Protocadherin beta−10
with a threshold of 1e−05

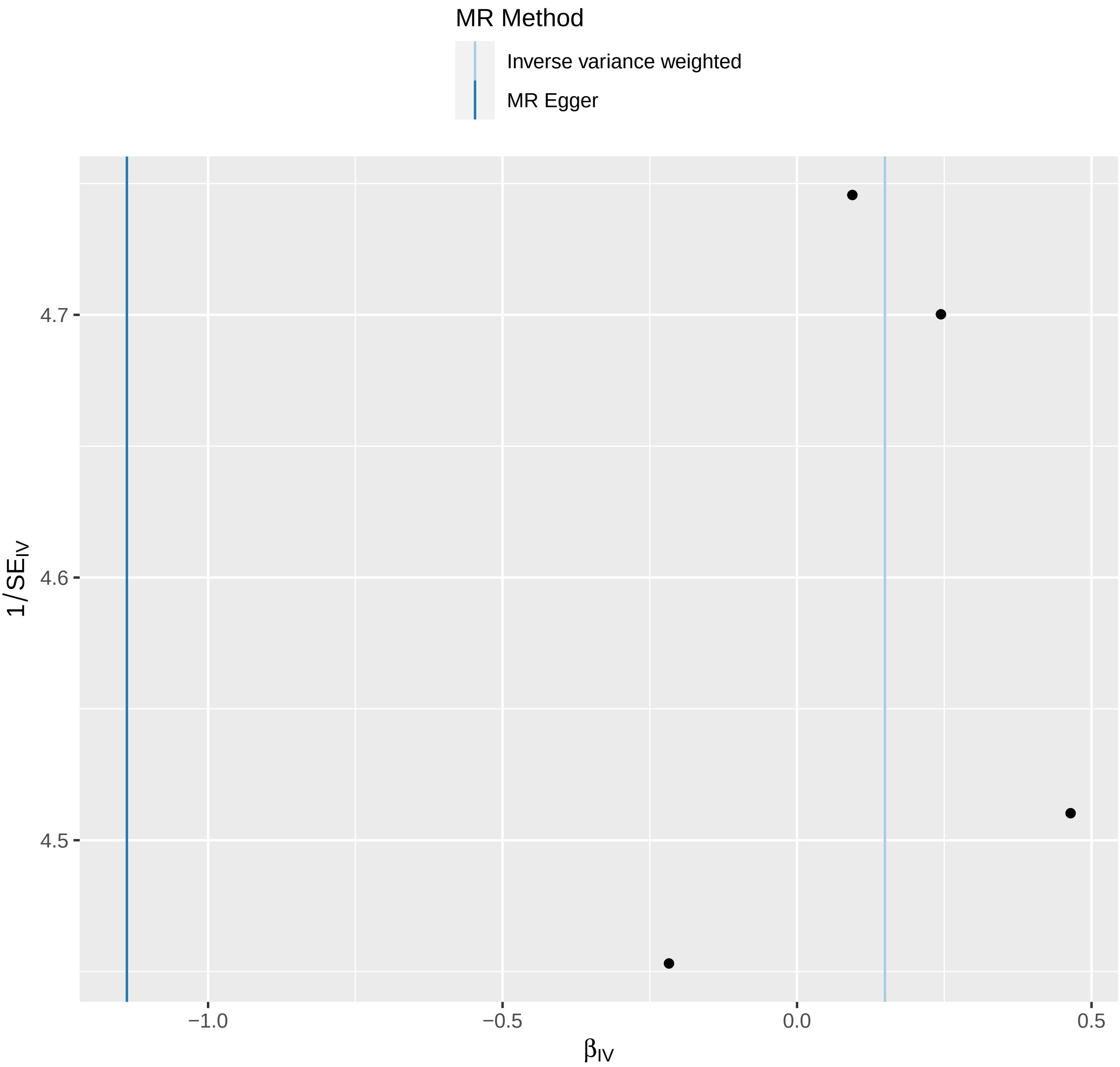

Batch 2−4404 : Gut microbiota abundance (genus Parasutterella id.2892) on Protocadherin beta−10
with a threshold of 1e−05

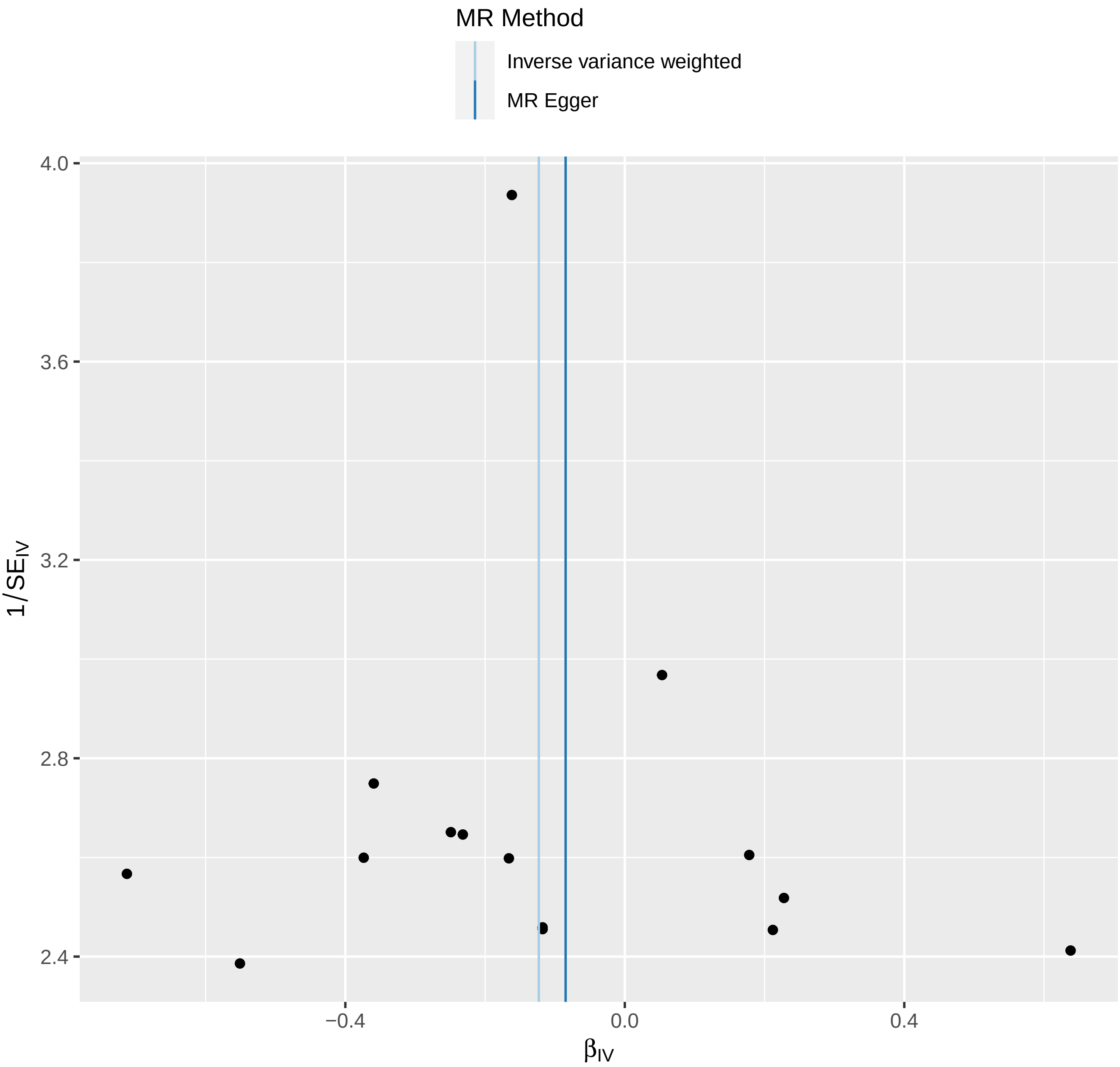

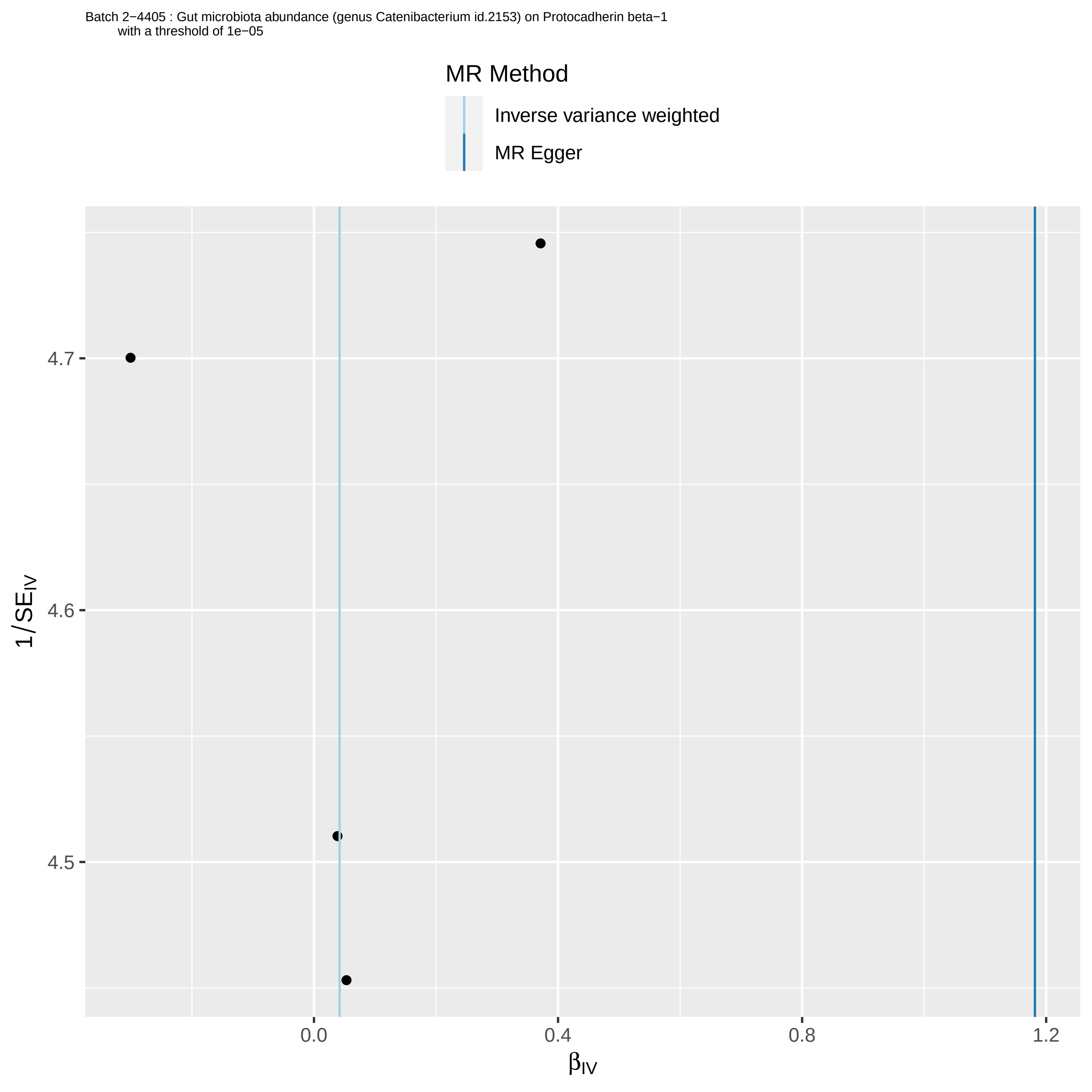
Batch 2−4405 : Gut microbiota abundance (genus Catenibacterium id.2153) on Protocadherin beta−1
with a threshold of 1e−05
MR Method
Inverse variance weighted
MR Egger
4.7
4.6
4.5
1/SE_IV
0.0
0.4
0.8
1.2
β_IV

Batch 2−4406 : Gut microbiota abundance (genus Parasutterella id.2892) on Protocadherin beta−1
with a threshold of 1e−05

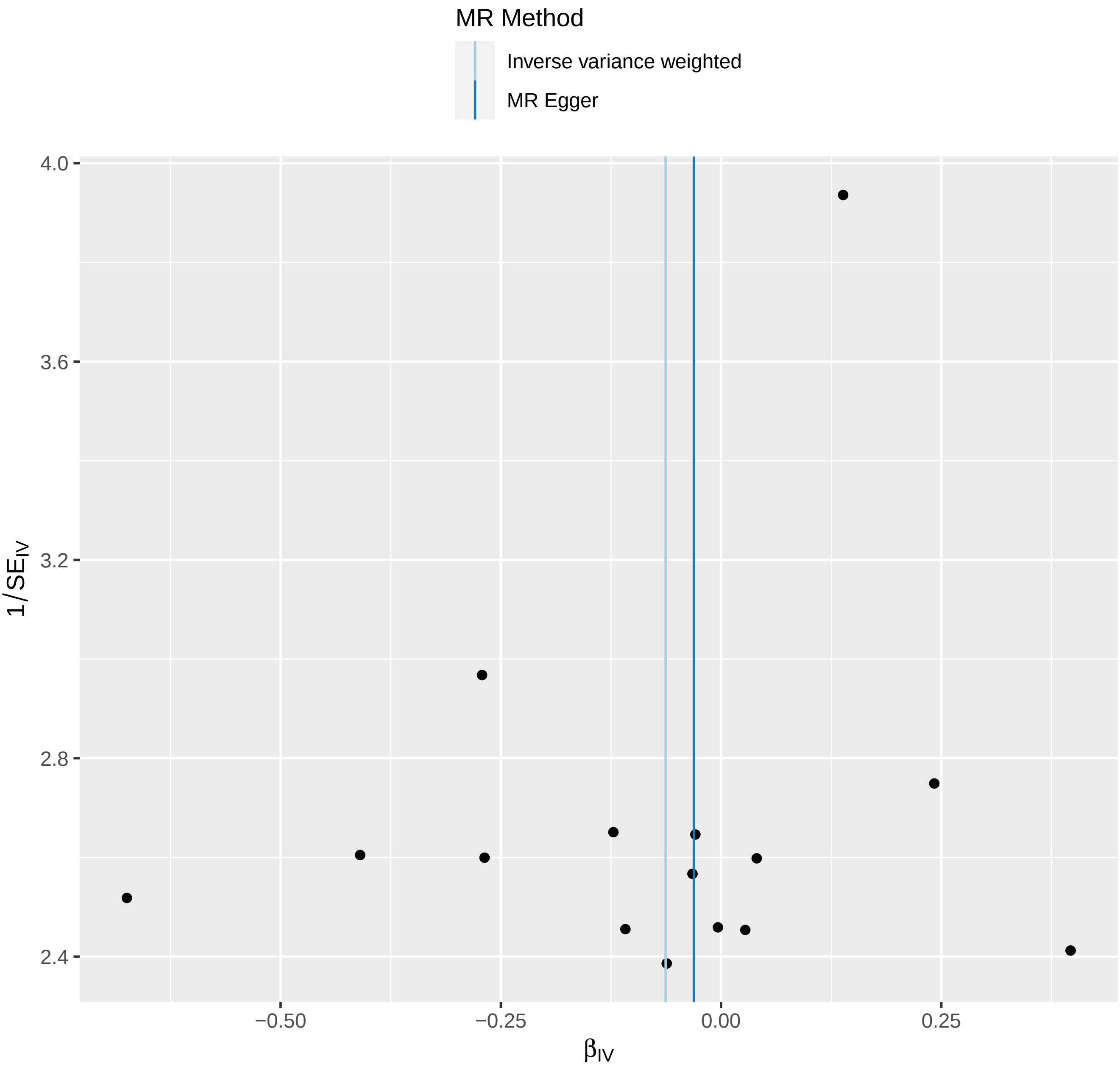

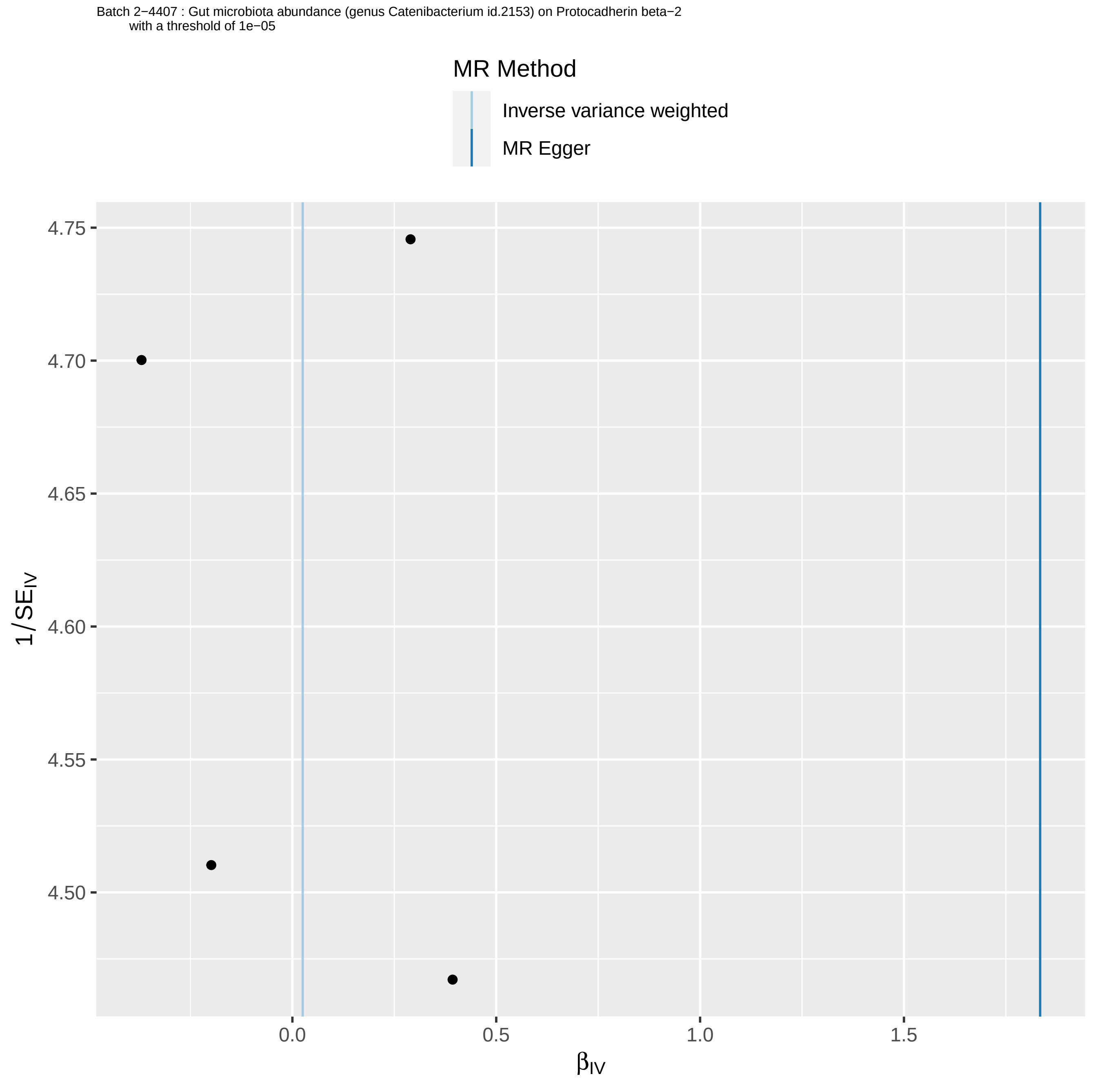

Batch 2−4407 : Gut microbiota abundance (genus Catenibacterium id.2153) on Protocadherin beta−2
with a threshold of 1e−05
MR Method
Inverse variance weighted
MR Egger
4.75
4.70
4.65
4.60
4.55
4.50
1/SE_IV
0.0
0.5
1.0
1.5
β_IV

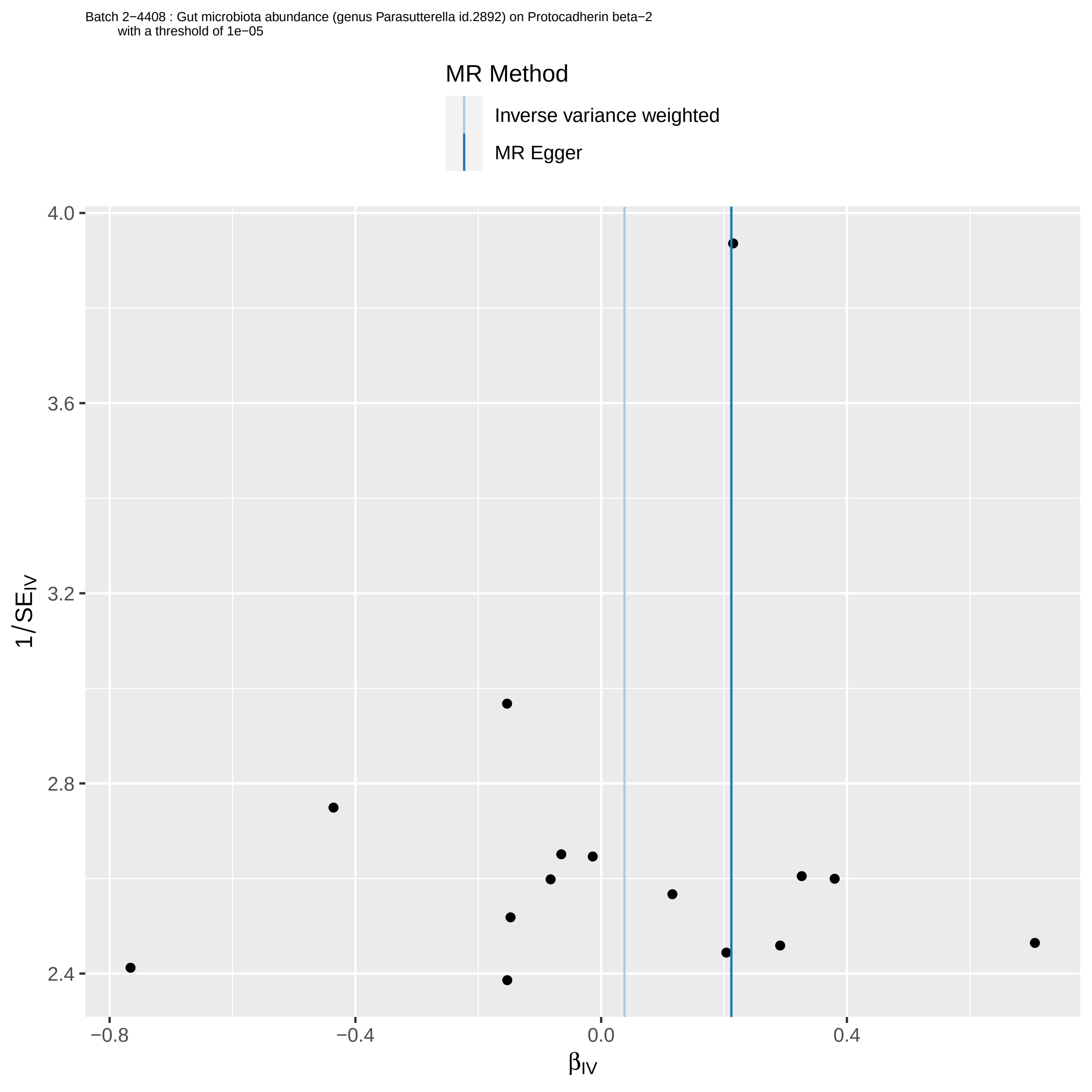

Batch 2−4408 : Gut microbiota abundance (genus Parasutterella id.2892) on Protocadherin beta−2
with a threshold of 1e−05
MR Method
Inverse variance weighted
MR Egger
4.0
3.6
3.2
2.8
2.4
1/SE_IV
−0.8
−0.4
0.0
0.4
β_IV

Batch 2−4409 : Gut microbiota abundance (genus Catenibacterium id.2153) on Protocadherin beta−4
with a threshold of 1e−05

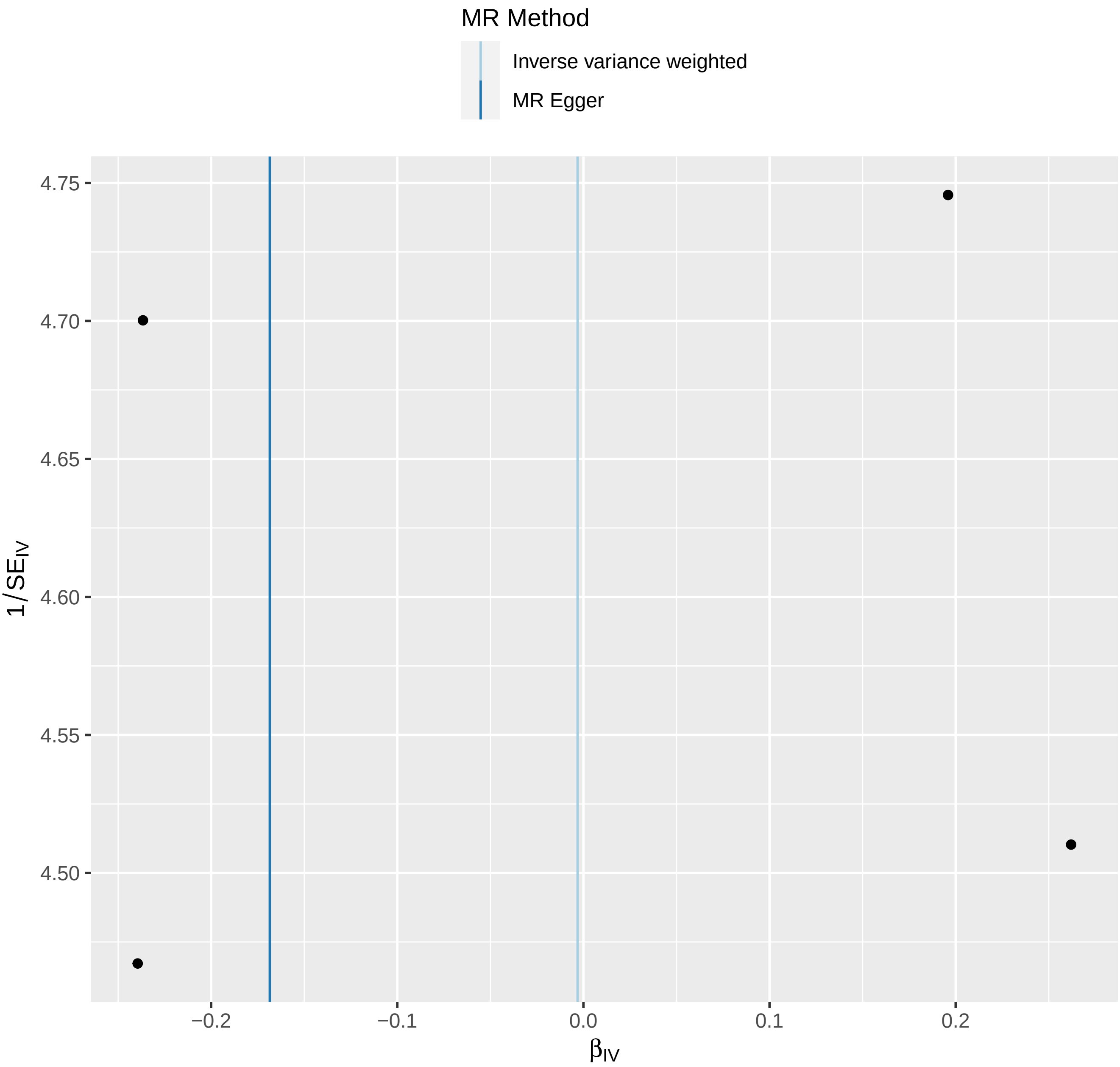

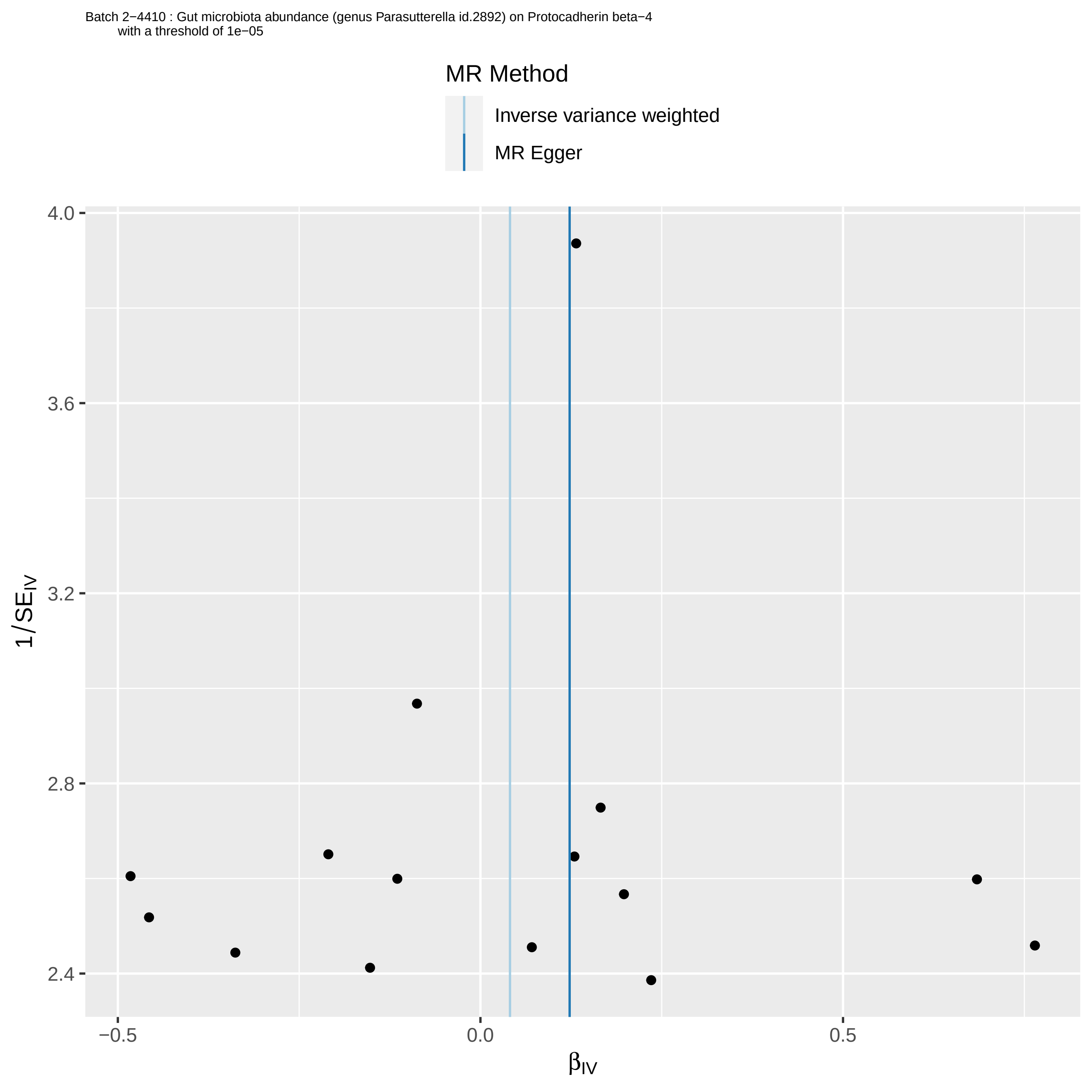
Batch 2−4410 : Gut microbiota abundance (genus Parasutterella id.2892) on Protocadherin beta−4
with a threshold of 1e−05
MR Method
Inverse variance weighted
MR Egger
4.0
3.6
3.2
2.8
2.4
1/SE_IV
−0.5
0.0
0.5
β_IV

Batch 2−4411 : Gut microbiota abundance (genus Catenibacterium id.2153) on Protocadherin gamma−A8
with a threshold of 1e−05

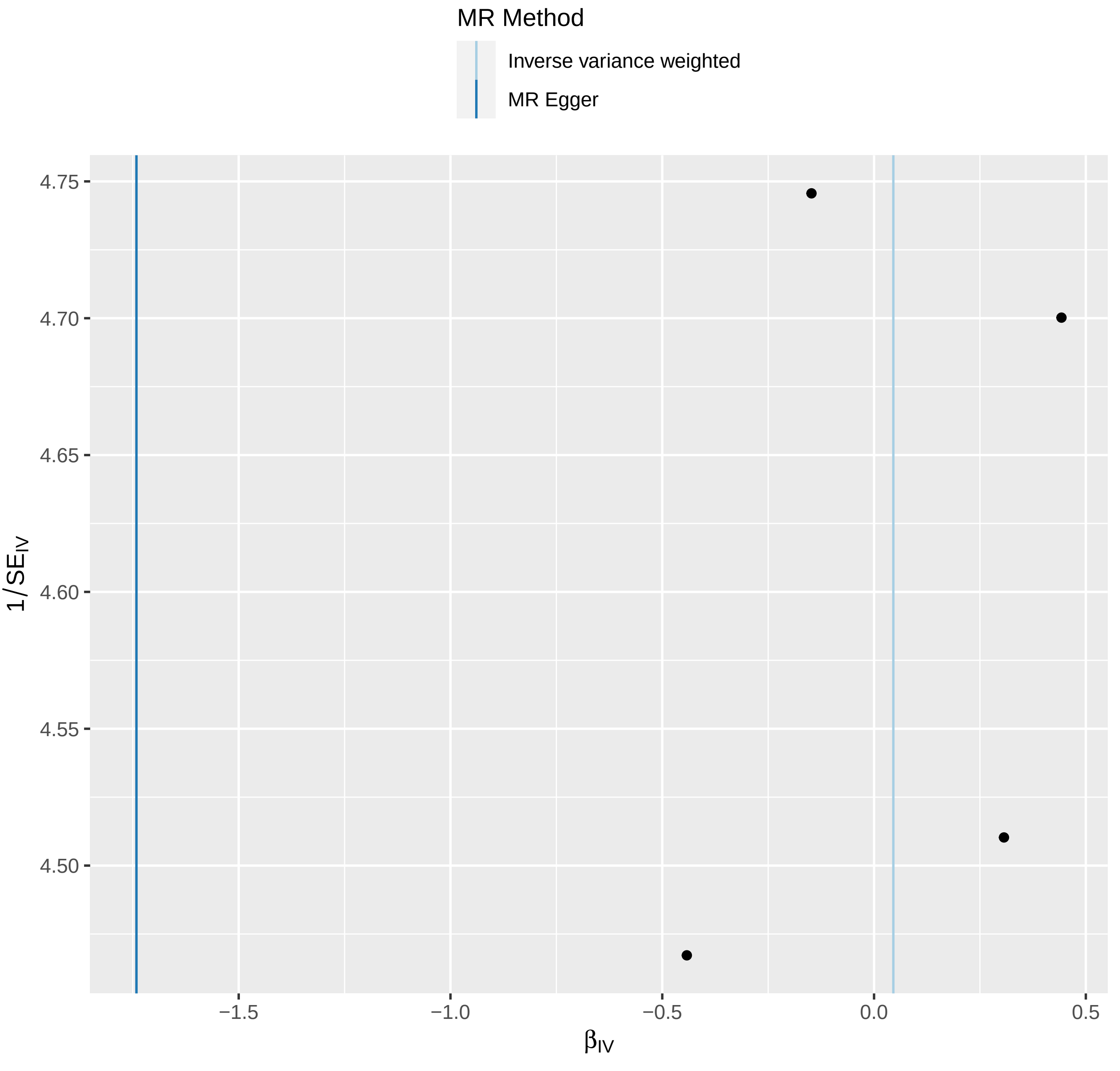

Batch 2−4412 : Gut microbiota abundance (genus Parasutterella id.2892) on Protocadherin gamma−A8
with a threshold of 1e−05

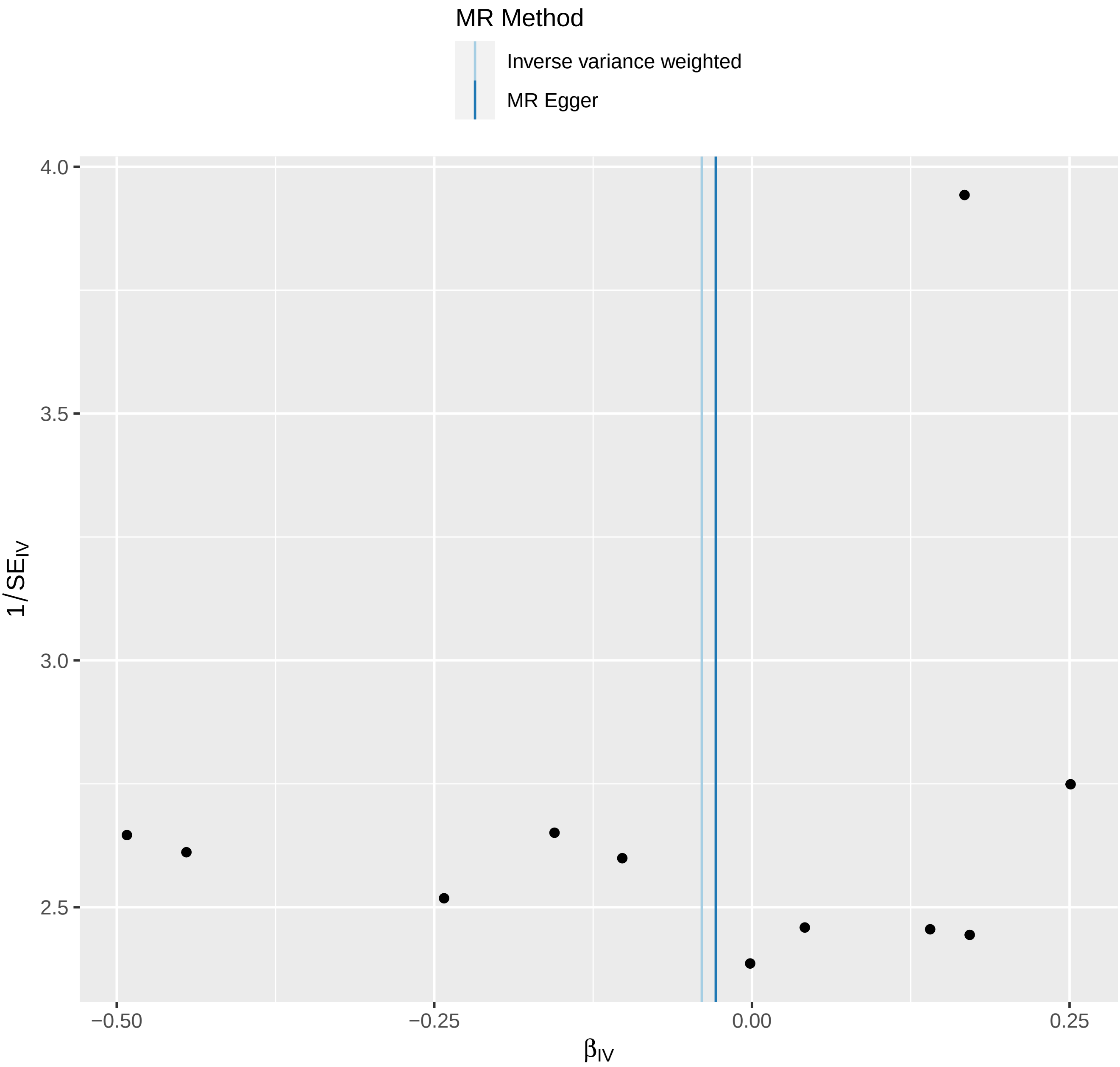

Batch 2−4413 : Gut microbiota abundance (genus Catenibacterium id.2153) on Protocadherin gamma−C5
with a threshold of 1e−05

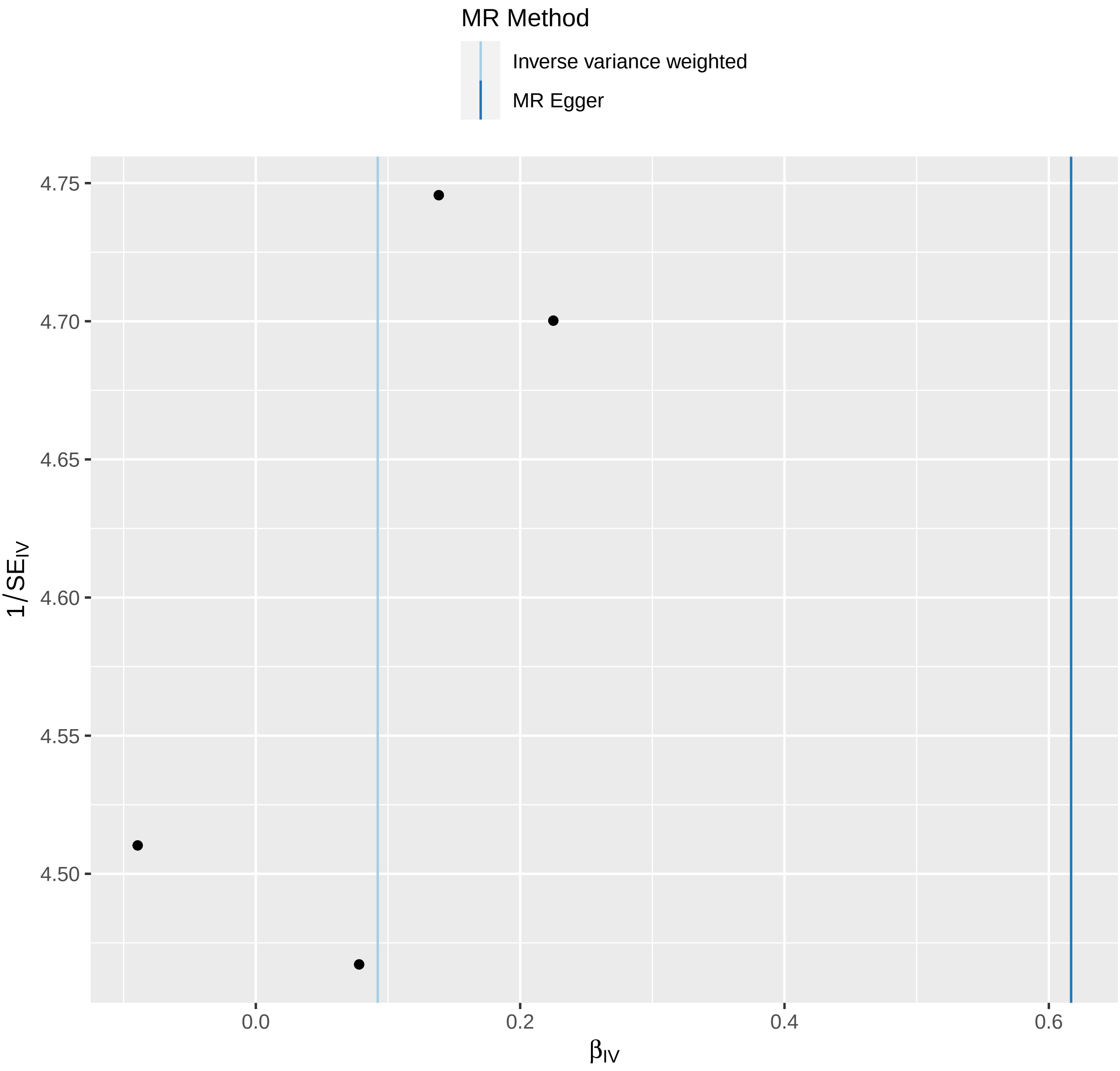

Batch 2−4414 : Gut microbiota abundance (genus Parasutterella id.2892) on Protocadherin gamma−C5
with a threshold of 1e−05

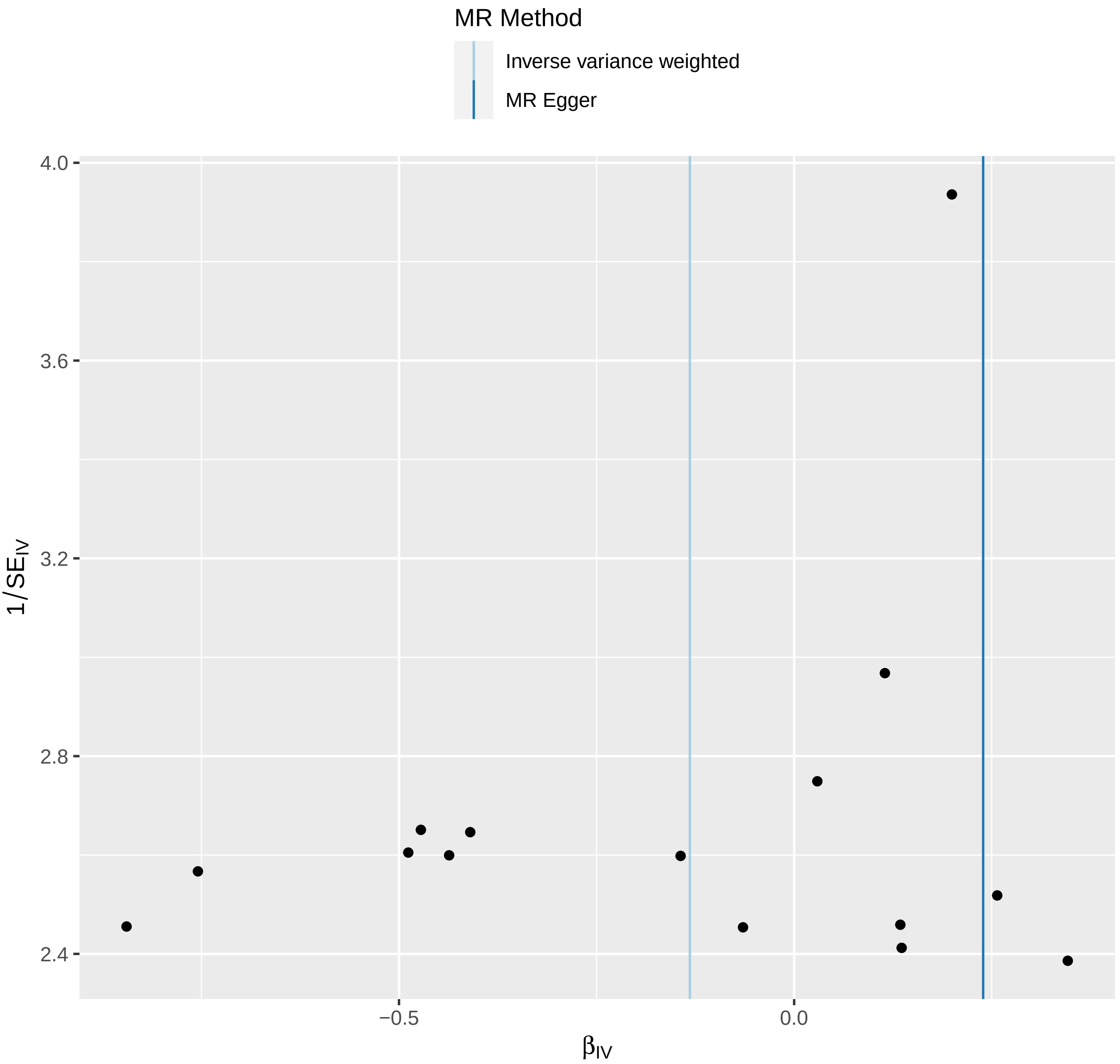

Batch 2−4415 : Gut microbiota abundance (genus Catenibacterium id.2153) on Proliferating cell nuclear antigen
with a threshold of 1e−05

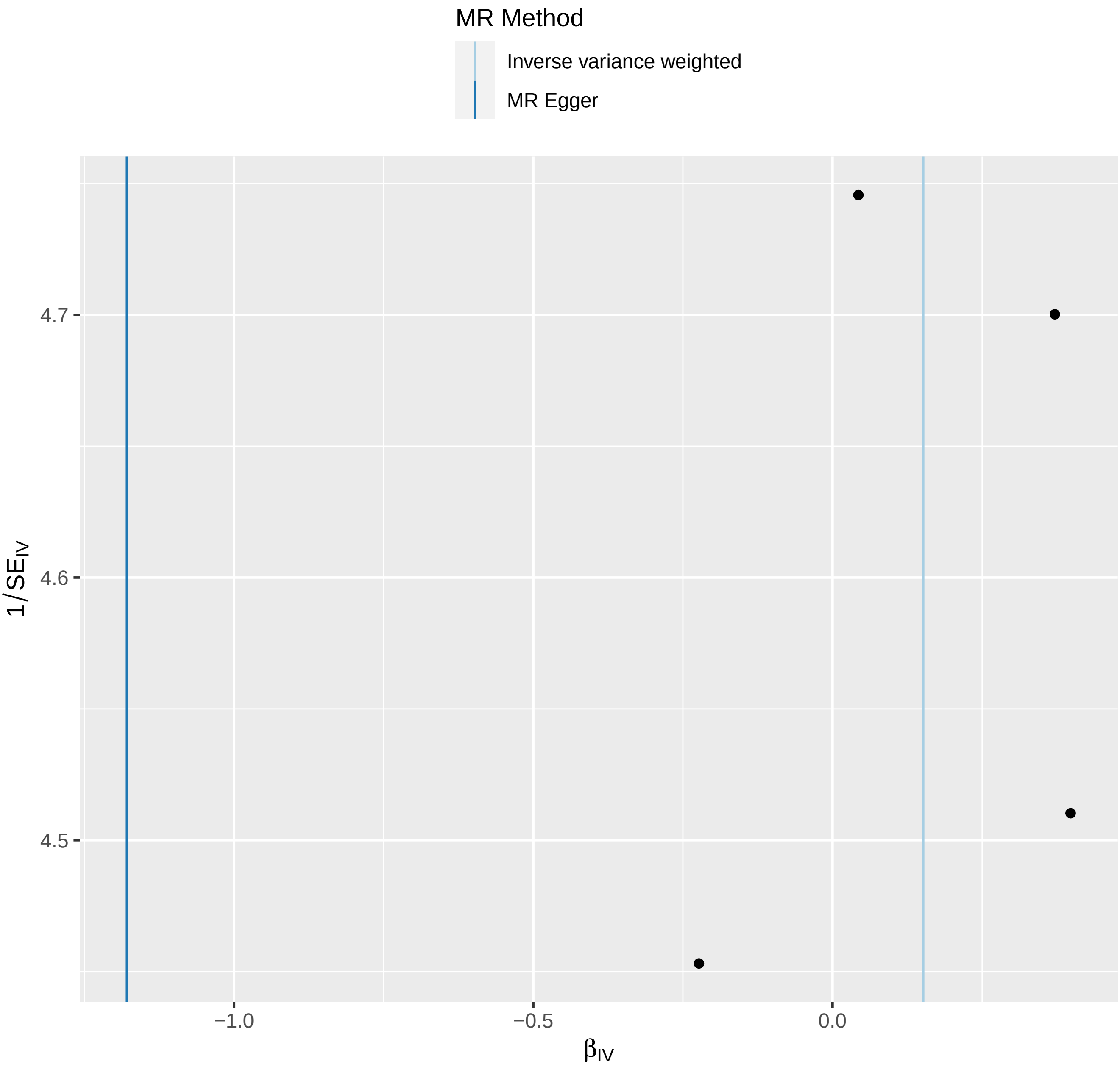

Batch 2−4416 : Gut microbiota abundance (genus Parasutterella id.2892) on Proliferating cell nuclear antigen
with a threshold of 1e−05

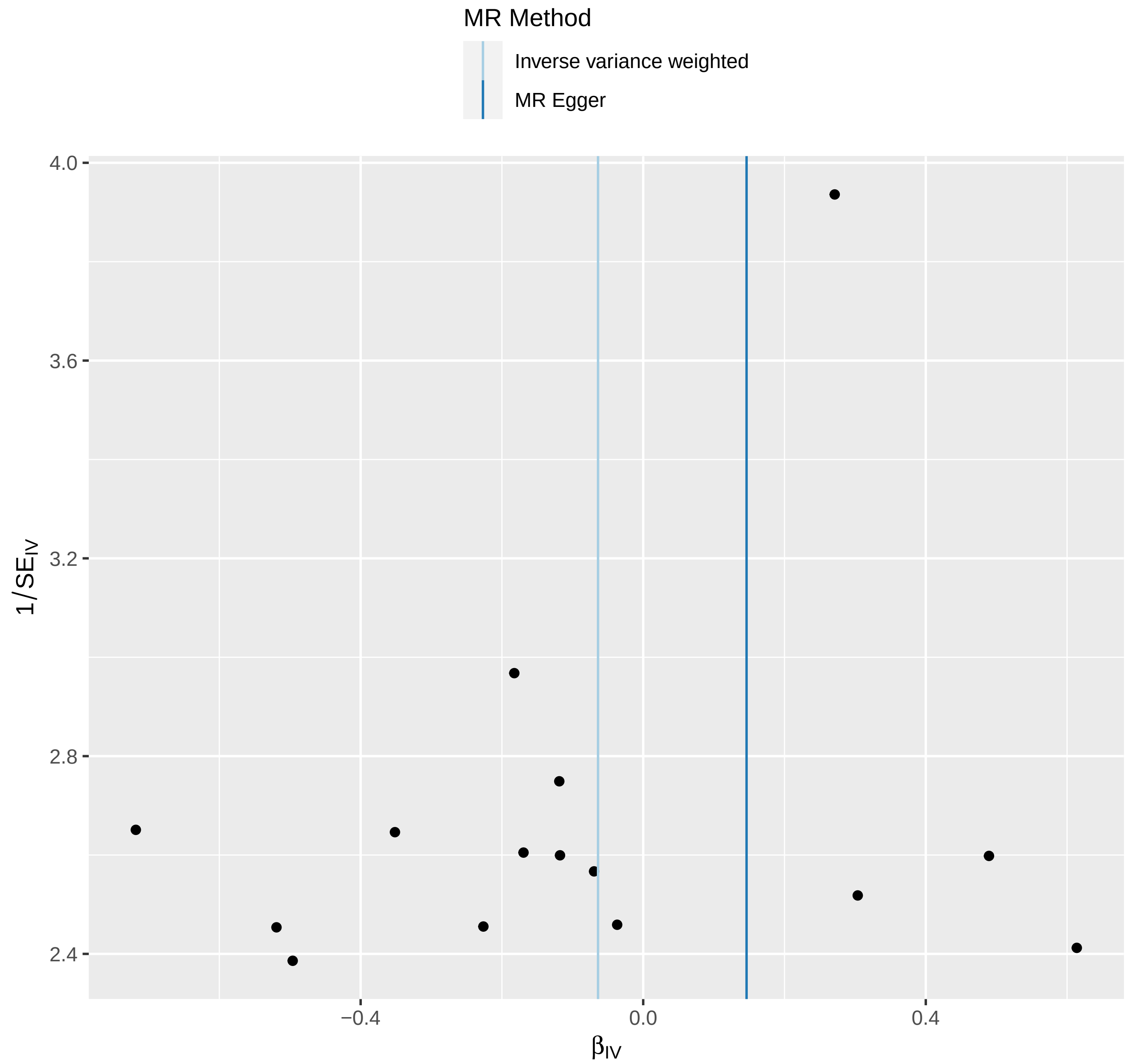

Batch 2−4417 : Gut microbiota abundance (genus Catenibacterium id.2153) on Procollagen C−endopeptidase enhancer 1
with a threshold of 1e−05

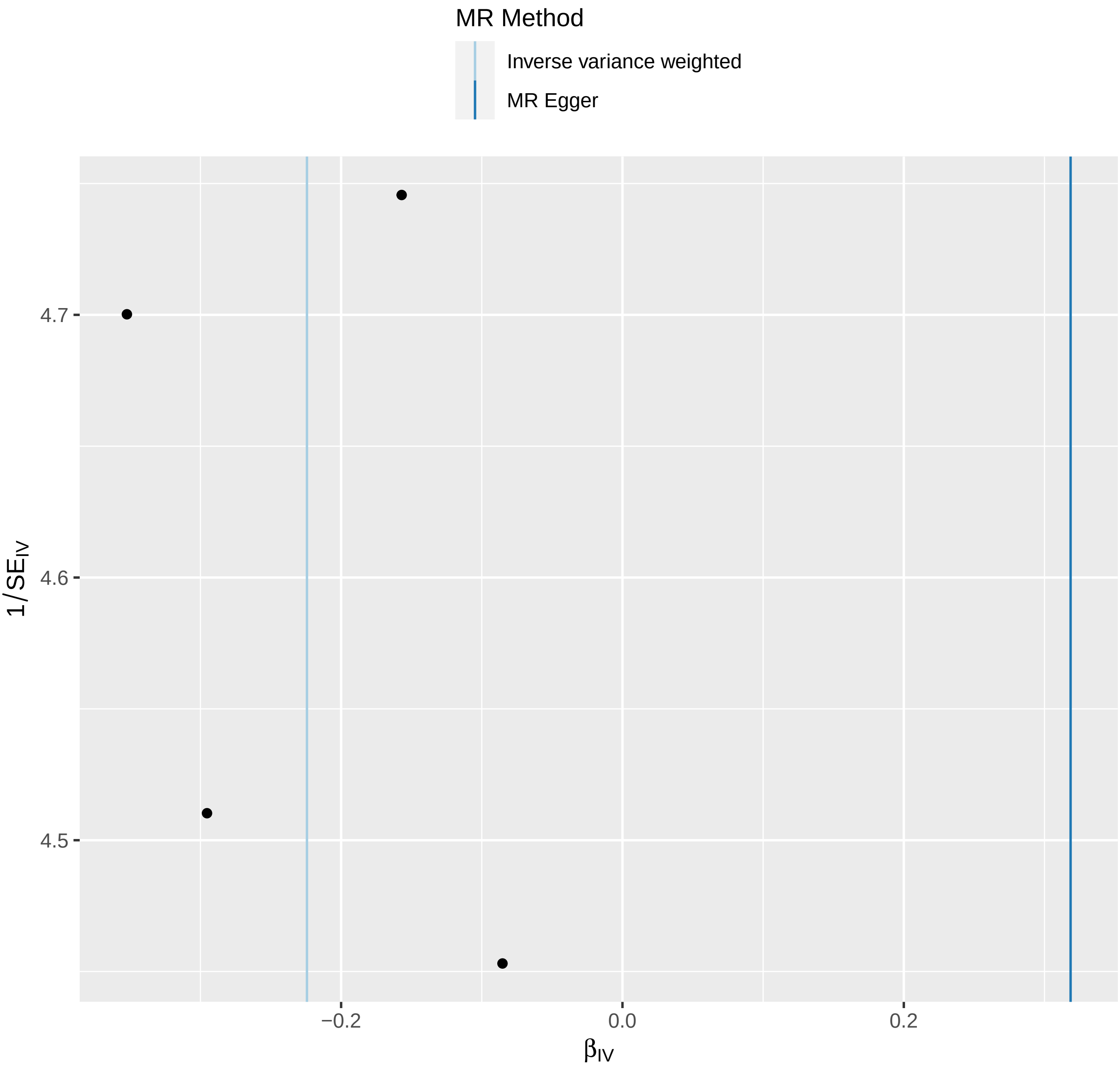

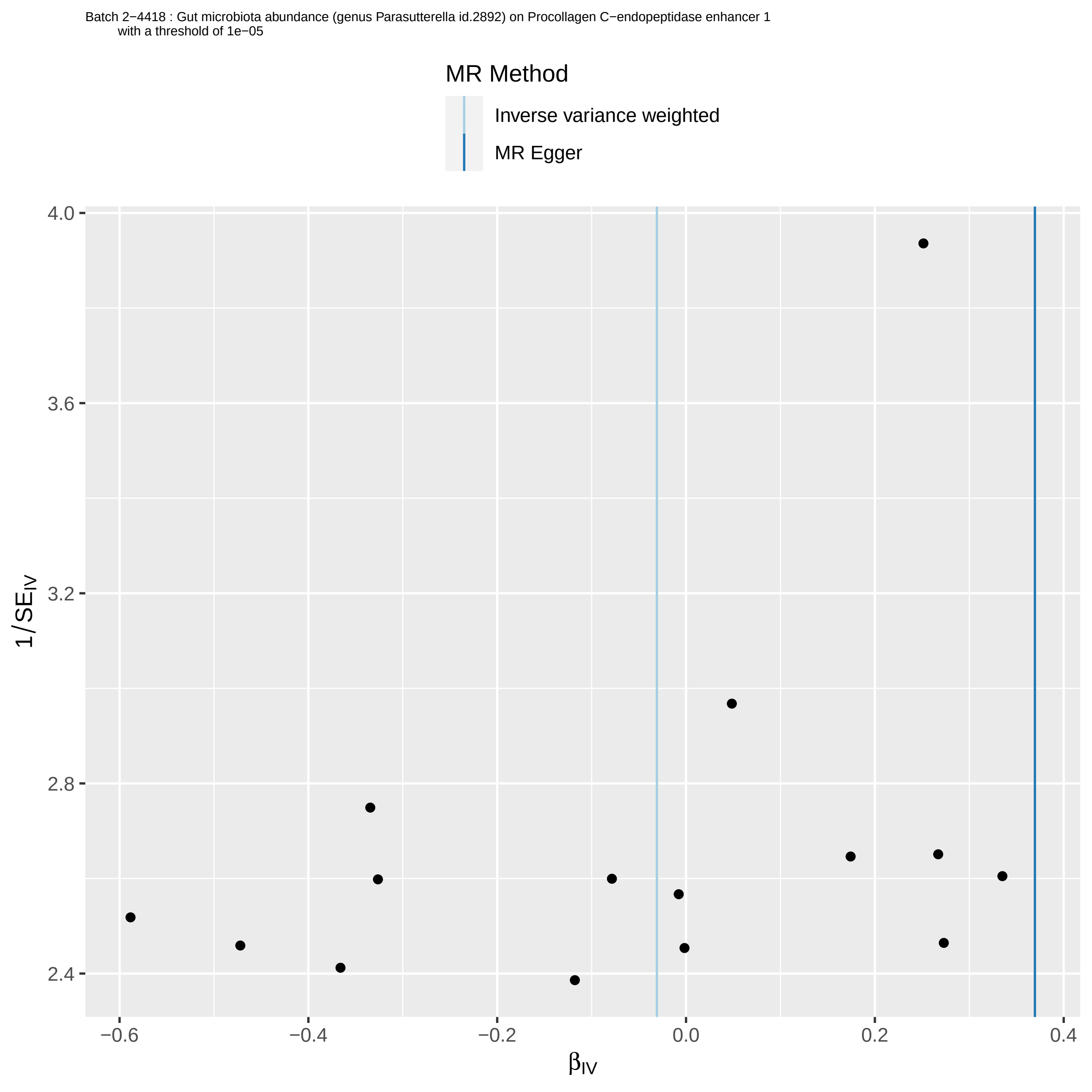
Batch 2−4418 : Gut microbiota abundance (genus Parasutterella id.2892) on Procollagen C−endopeptidase enhancer 1
with a threshold of 1e−05
MR Method
Inverse variance weighted
MR Egger
4.0
3.6
3.2
2.8
2.4
1/SE_IV
−0.6
−0.4
−0.2
0.0
0.2
0.4
β_IV

Batch 2−4419 : Gut microbiota abundance (genus Catenibacterium id.2153) on Procollagen C−endopeptidase enhancer 2
with a threshold of 1e−05

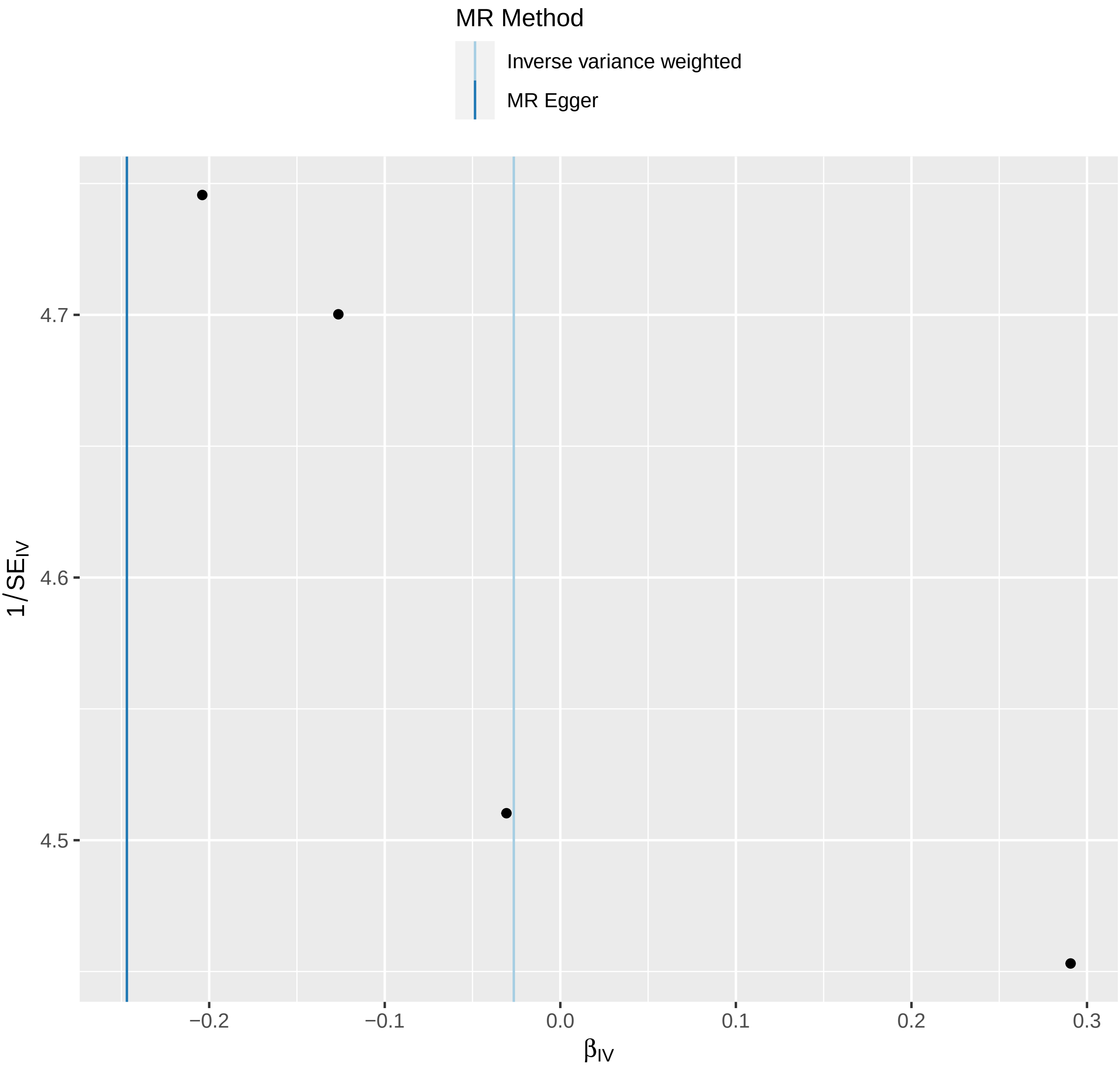

Batch 2−4420 : Gut microbiota abundance (genus Parasutterella id.2892) on Procollagen C−endopeptidase enhancer 2
with a threshold of 1e−05

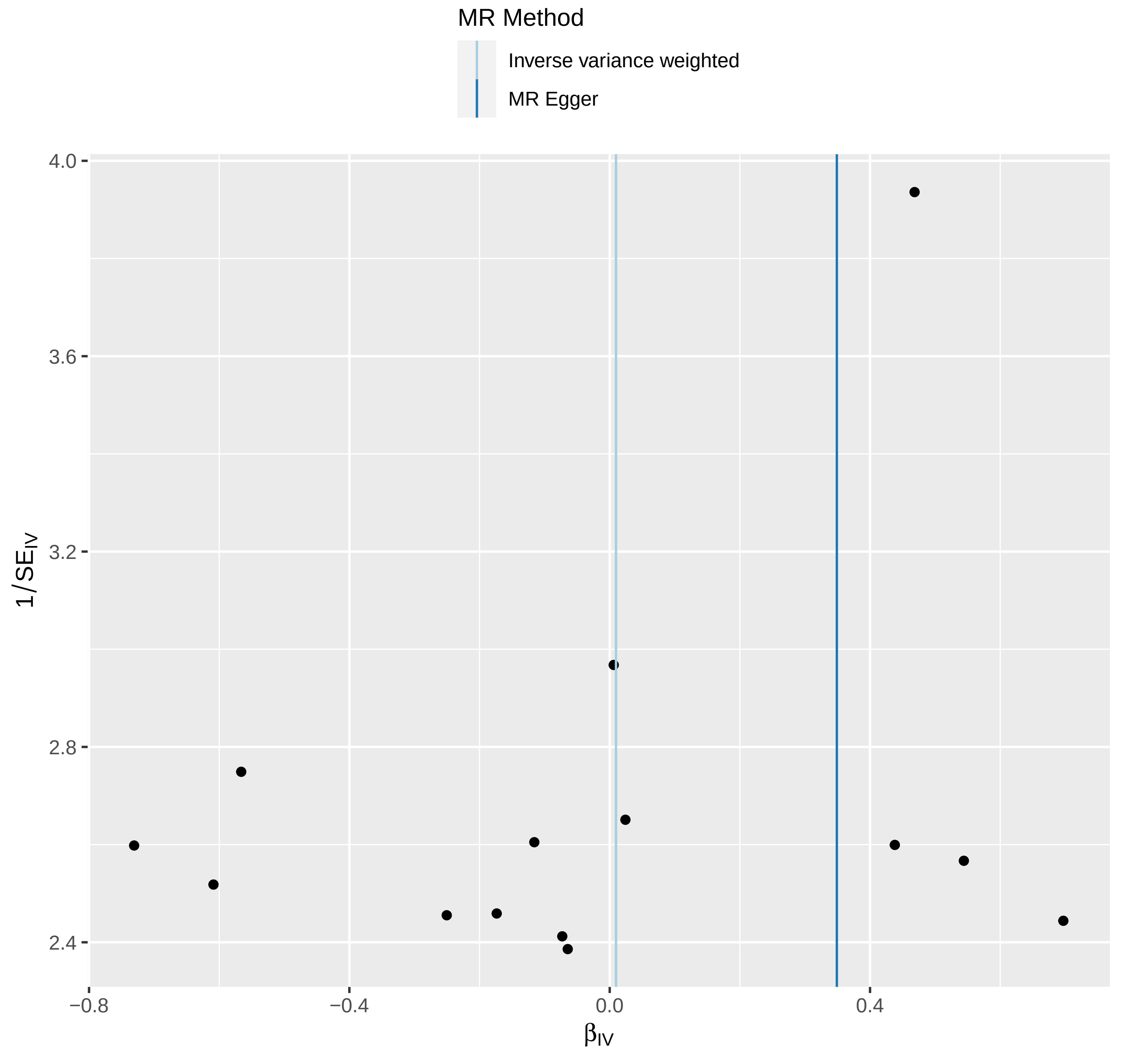

Batch 2−4421 : Gut microbiota abundance (genus Catenibacterium id.2153) on Neuroendocrine convertase 1 with a threshold of 1e−05

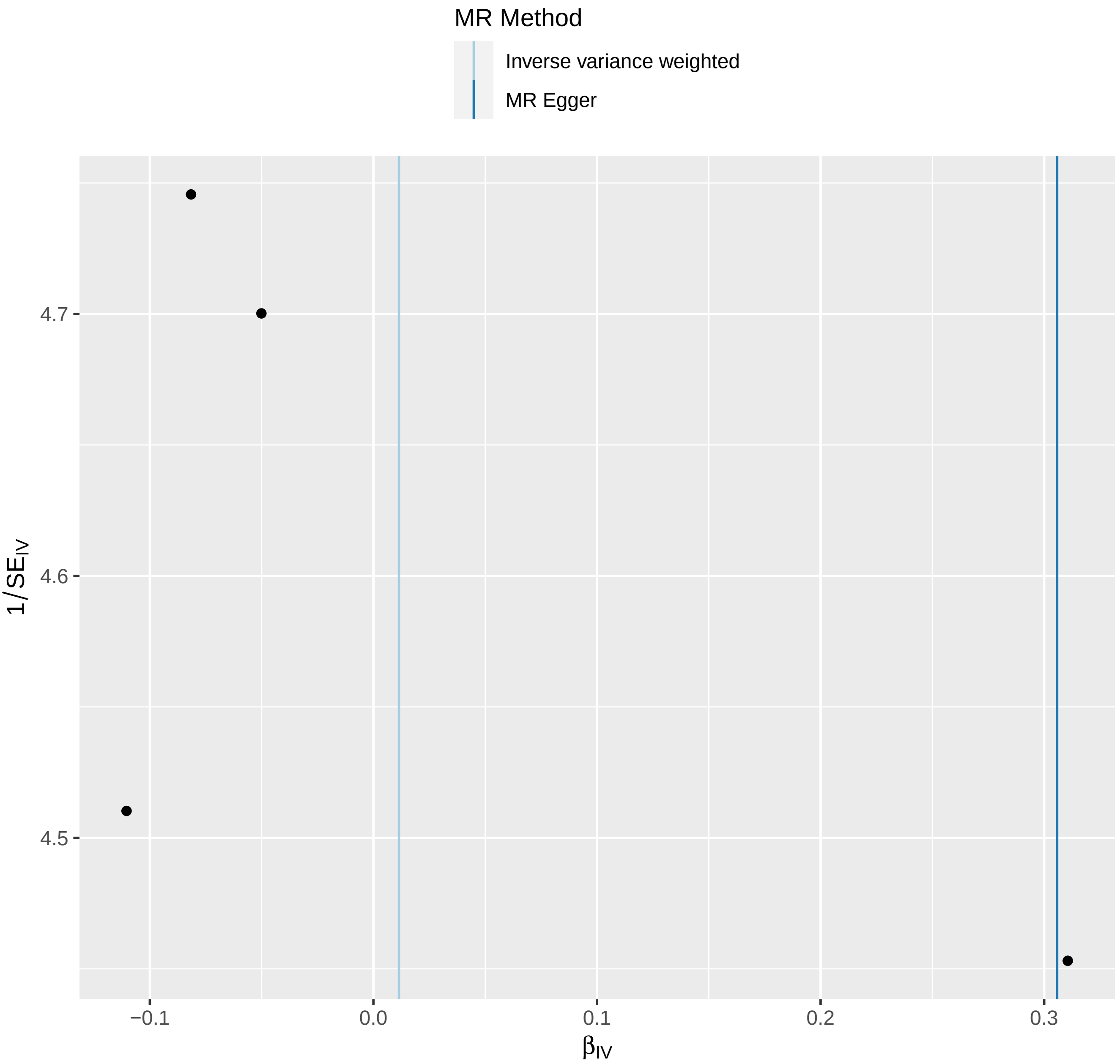

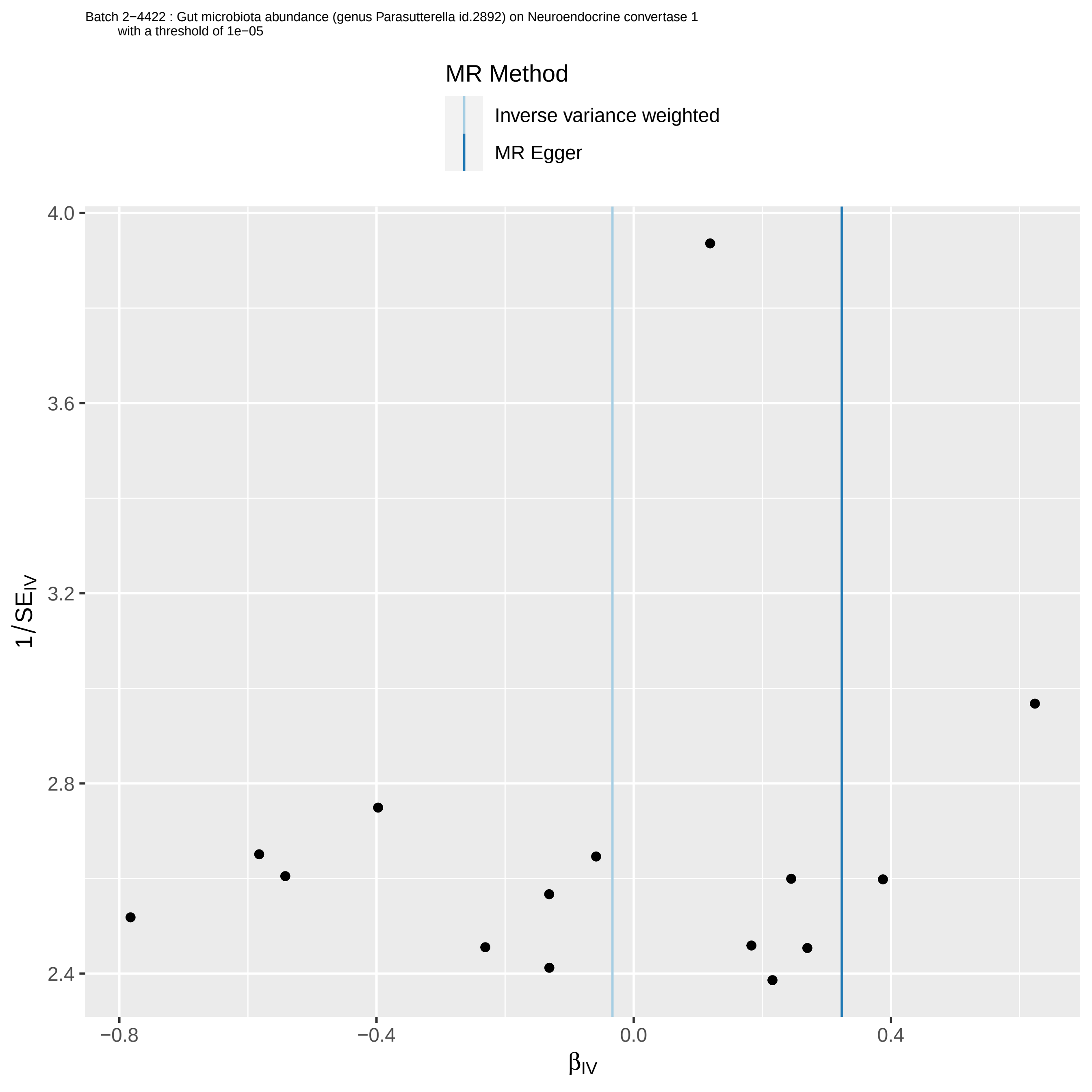
Batch 2−4422 : Gut microbiota abundance (genus Parasutterella id.2892) on Neuroendocrine convertase 1
with a threshold of 1e−05
MR Method
Inverse variance weighted
MR Egger
4.0
3.6
3.2
2.8
2.4
1/SE_IV
−0.8
−0.4
0.0
0.4
β_IV

Batch 2−4423 : Gut microbiota abundance (genus Catenibacterium id.2153) on Proprotein convertase subtilisin/kexin type 7
with a threshold of 1e−05

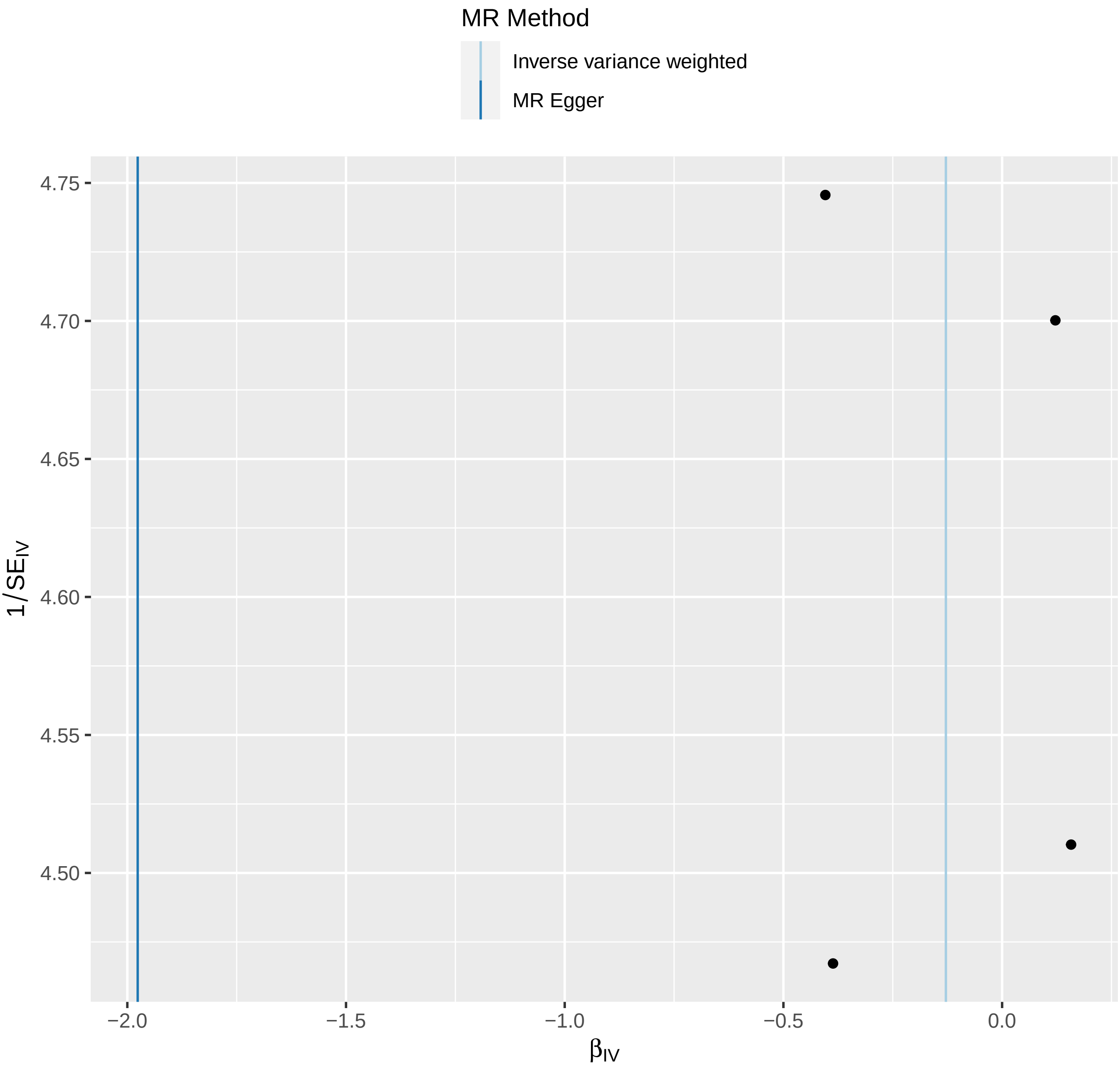

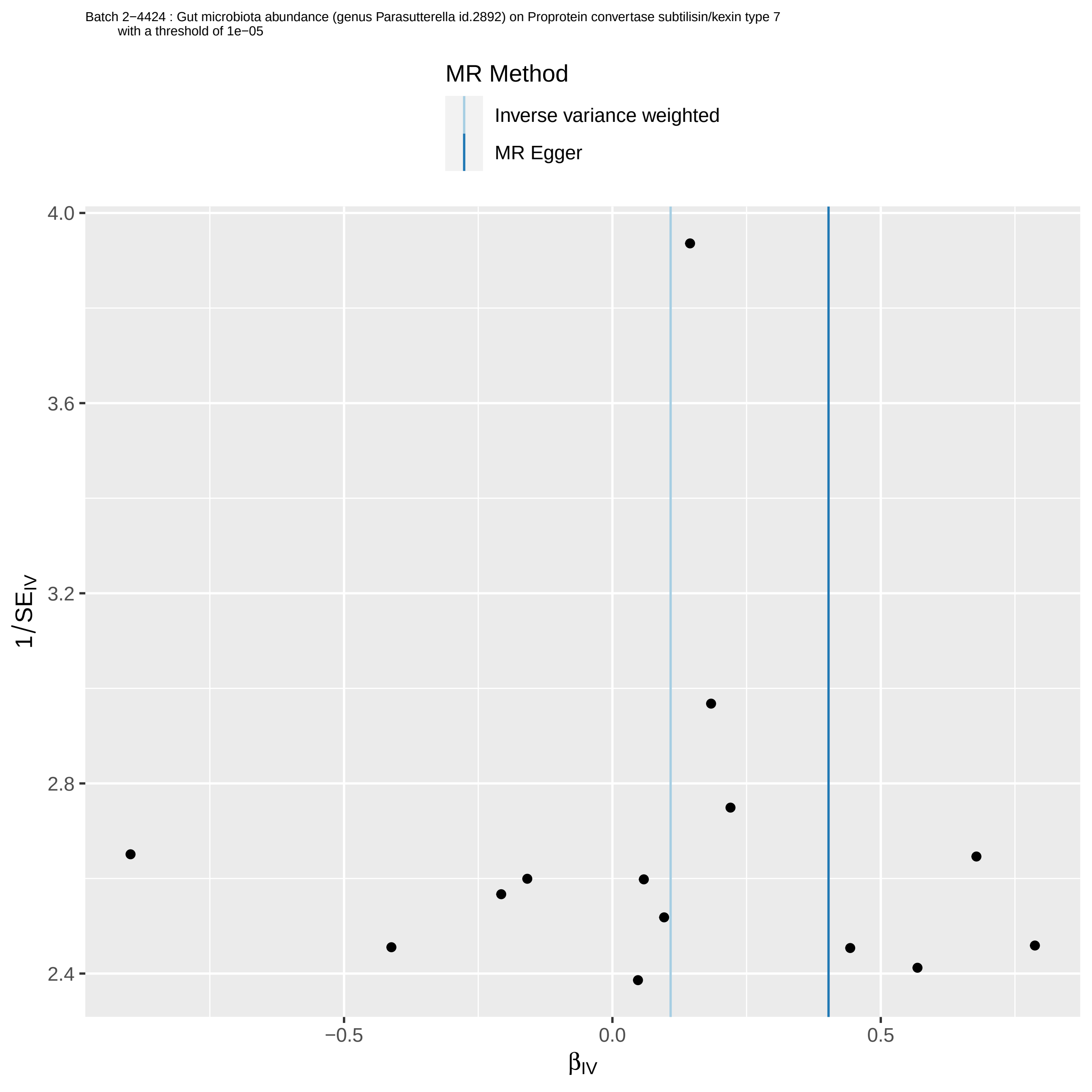

Batch 2−4424 : Gut microbiota abundance (genus Parasutterella id.2892) on Proprotein convertase subtilisin/kexin type 7
with a threshold of 1e−05
MR Method
Inverse variance weighted
MR Egger
4.0
3.6
3.2
2.8
2.4
1/SE$_{IV}$
−0.5
0.0
0.5
β$_{IV}$

Batch 2−4425 : Gut microbiota abundance (genus Catenibacterium id.2153) on Prenylcysteine oxidase−like
with a threshold of 1e−05

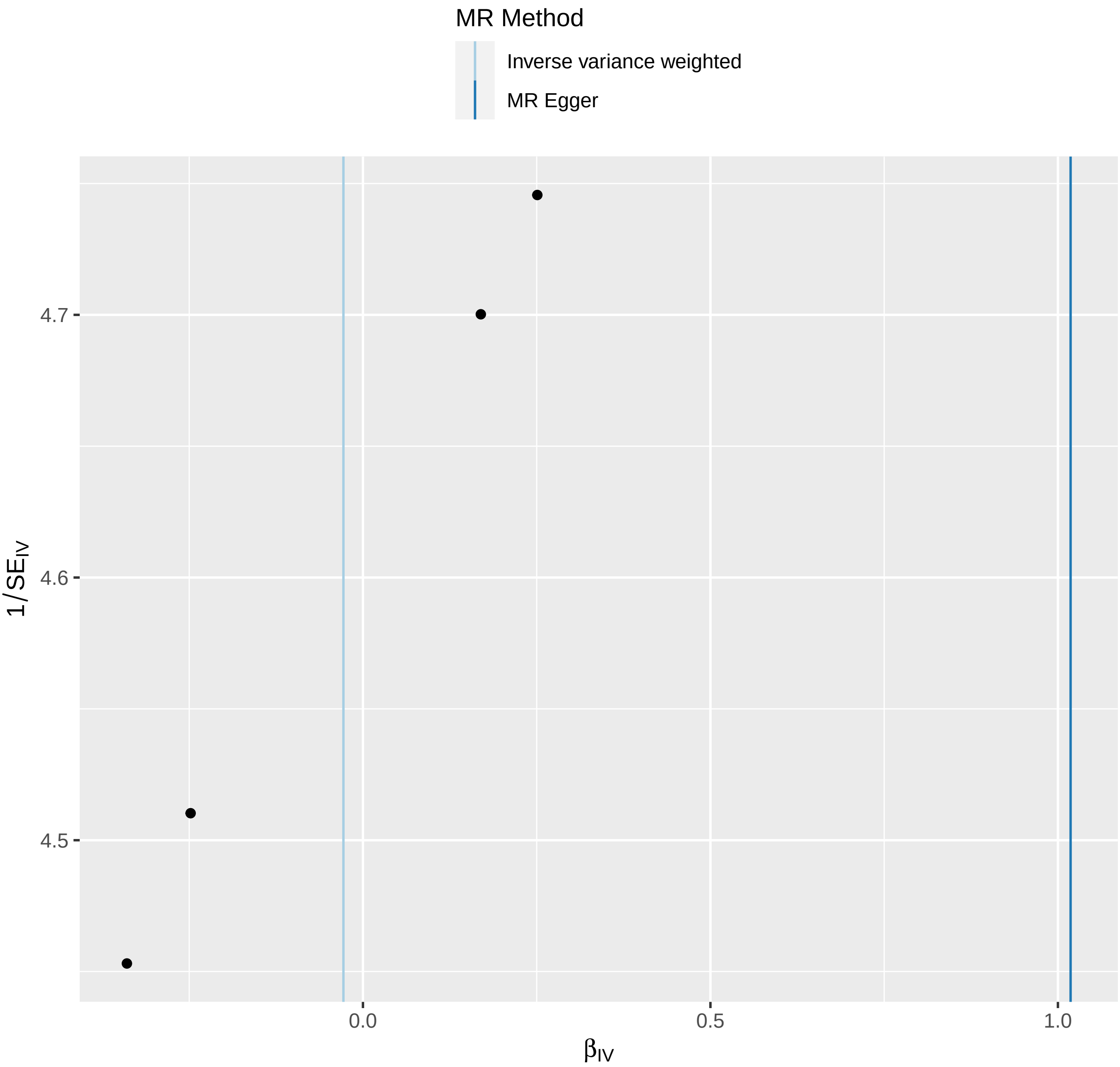

Batch 2−4426 : Gut microbiota abundance (genus Parasutterella id.2892) on Prenylcysteine oxidase−like
with a threshold of 1e−05

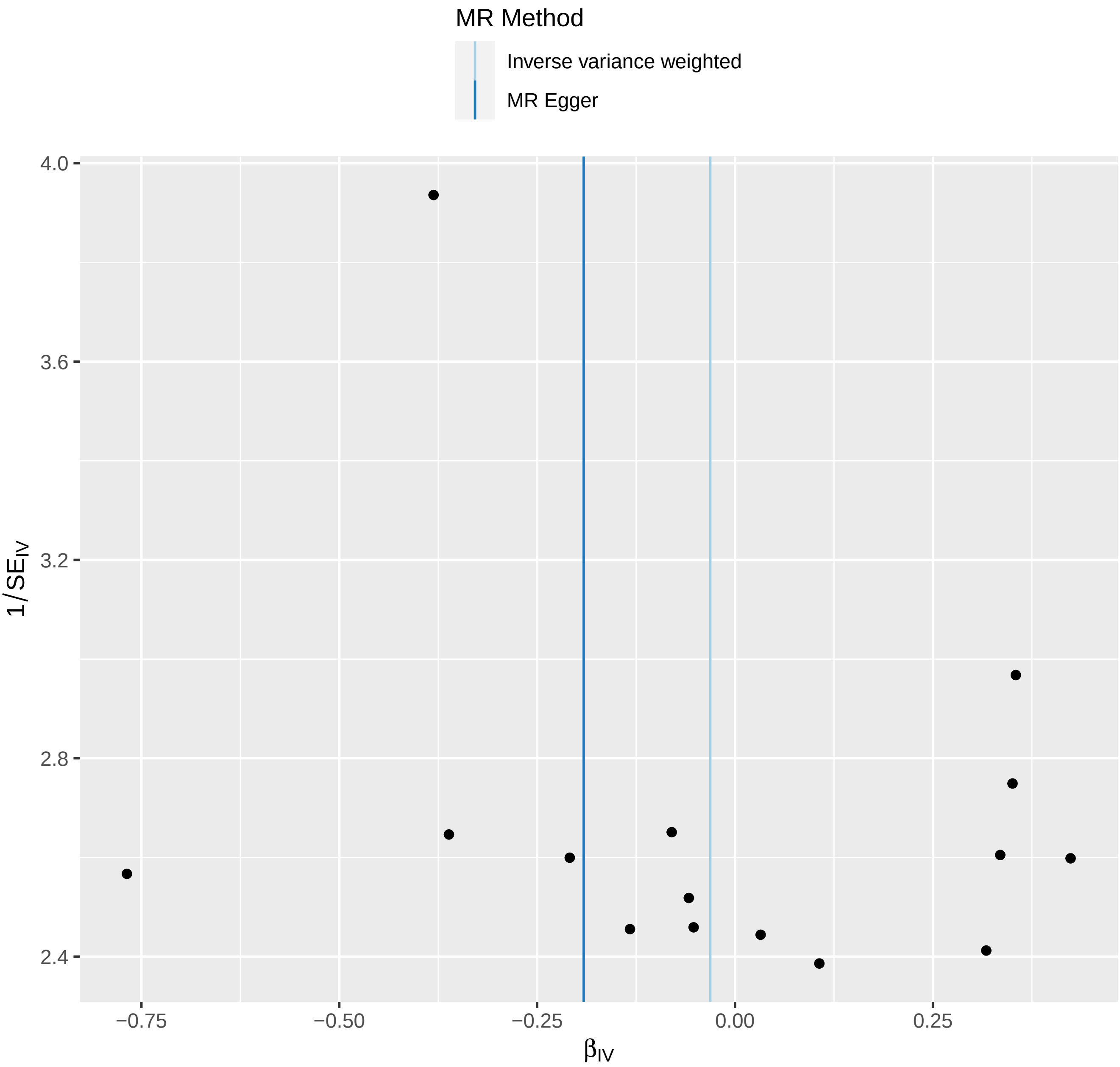

Batch 2−4427 : Gut microbiota abundance (genus Catenibacterium id.2153) on Programmed cell death protein 1
with a threshold of 1e−05

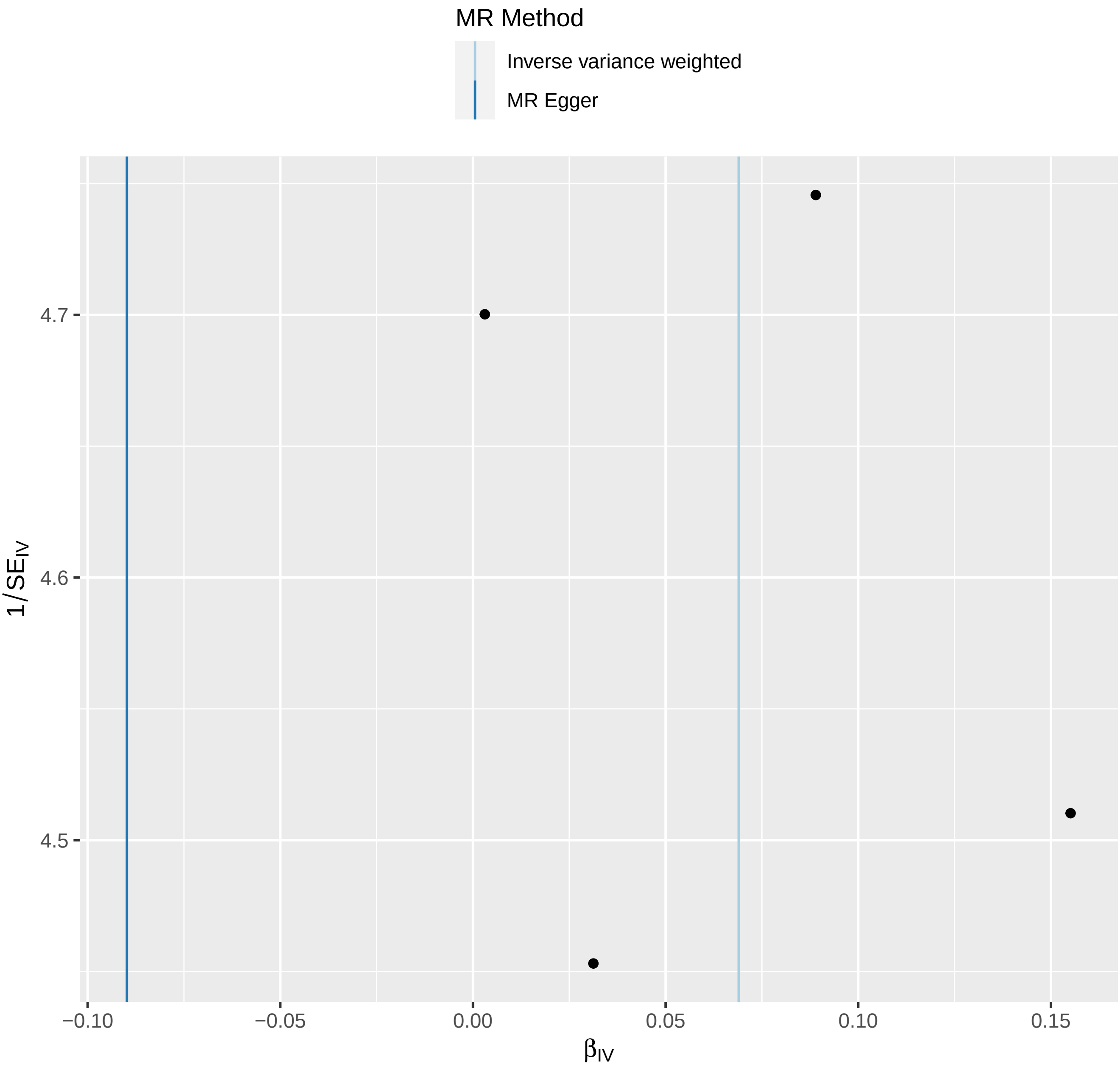

Batch 2−4428 : Gut microbiota abundance (genus Parasutterella id.2892) on Programmed cell death protein 1
with a threshold of 1e−05

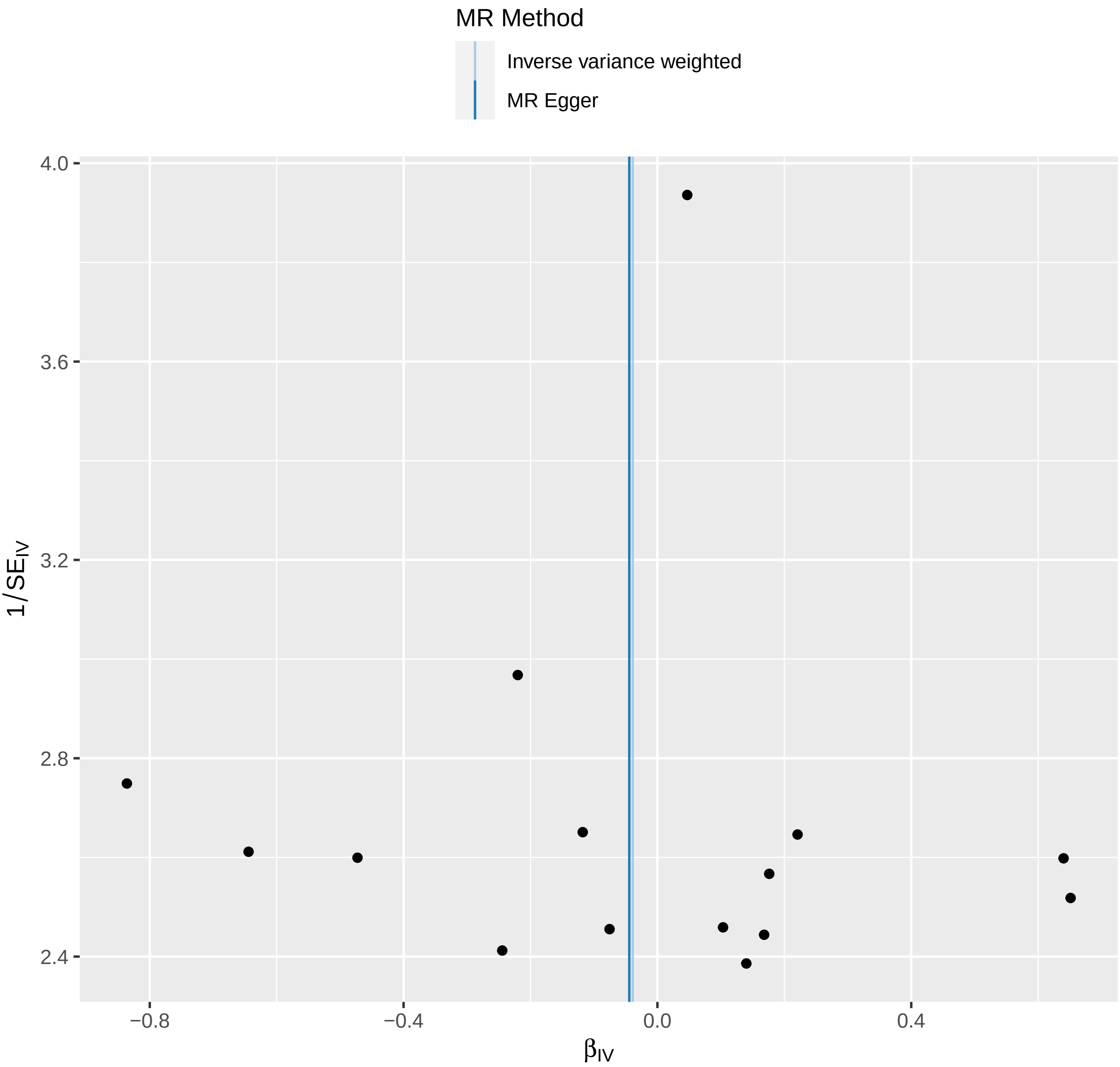

Batch 2−4429 : Gut microbiota abundance (genus Catenibacterium id.2153) on Programmed cell death 1 ligand 2 with a threshold of 1e−05

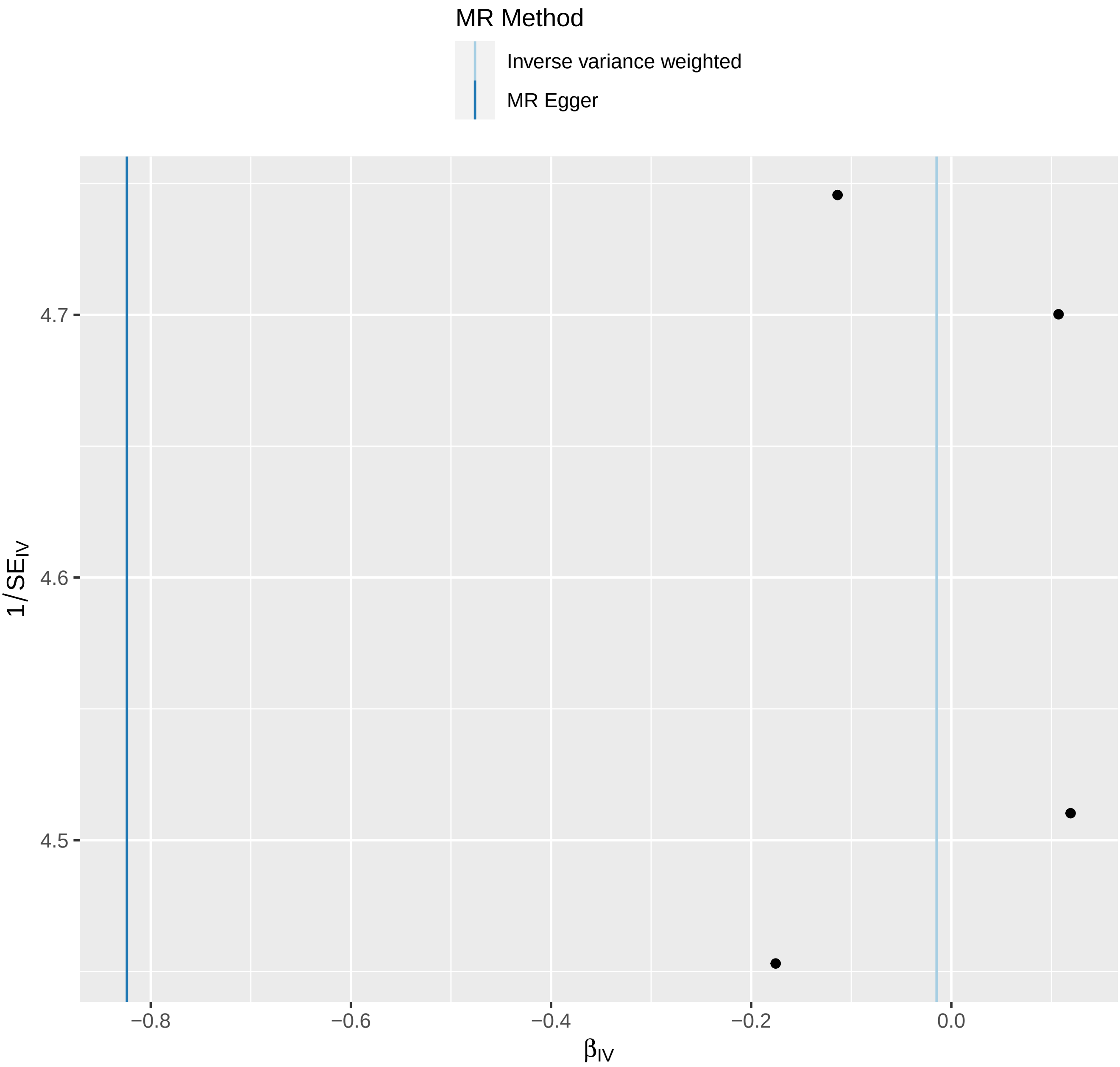

Batch 2−4430 : Gut microbiota abundance (genus Parasutterella id.2892) on Programmed cell death 1 ligand 2
with a threshold of 1e−05

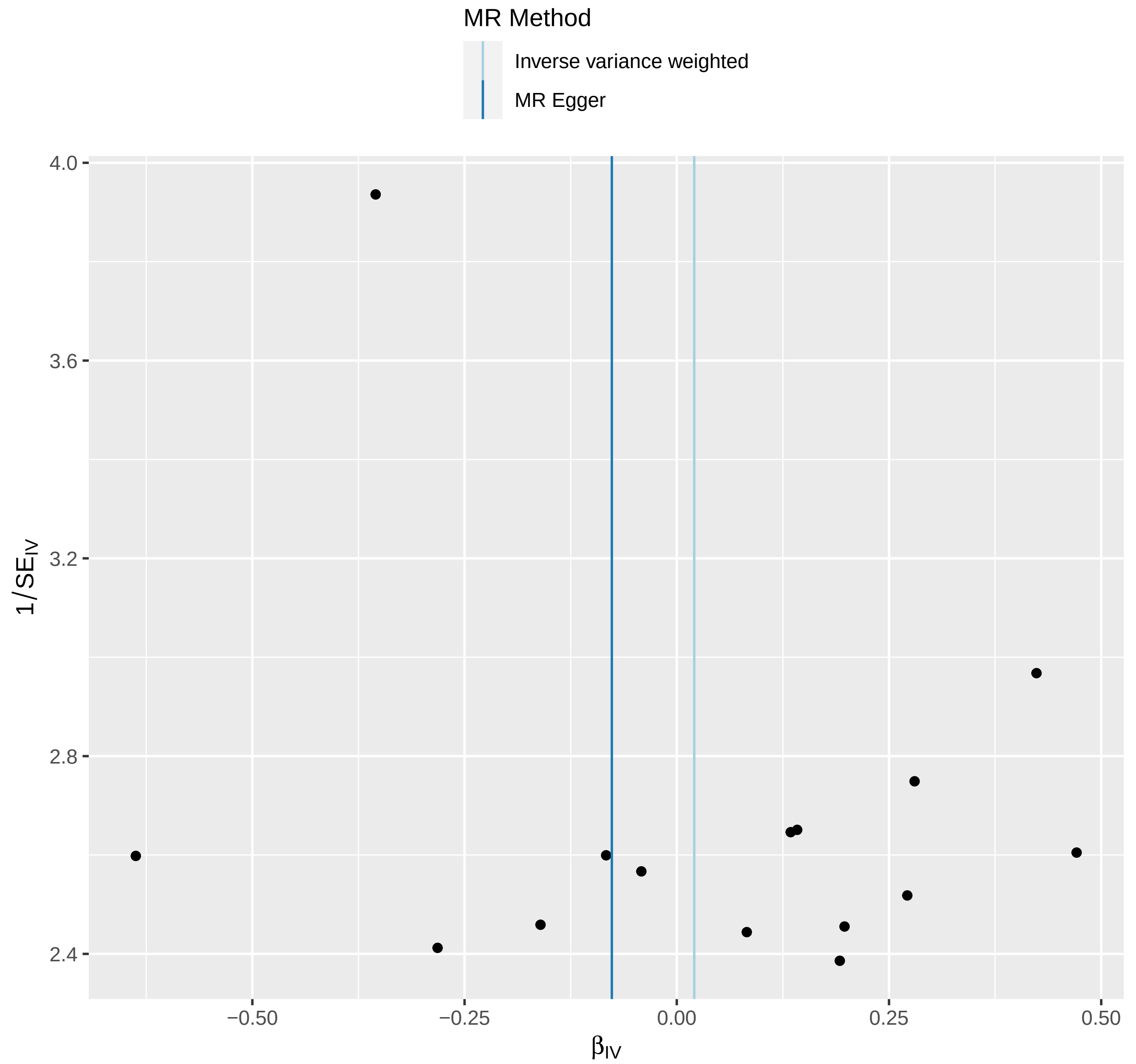

Batch 2−4431 : Gut microbiota abundance (genus Catenibacterium id.2153) on Programmed cell death protein 5
with a threshold of 1e−05

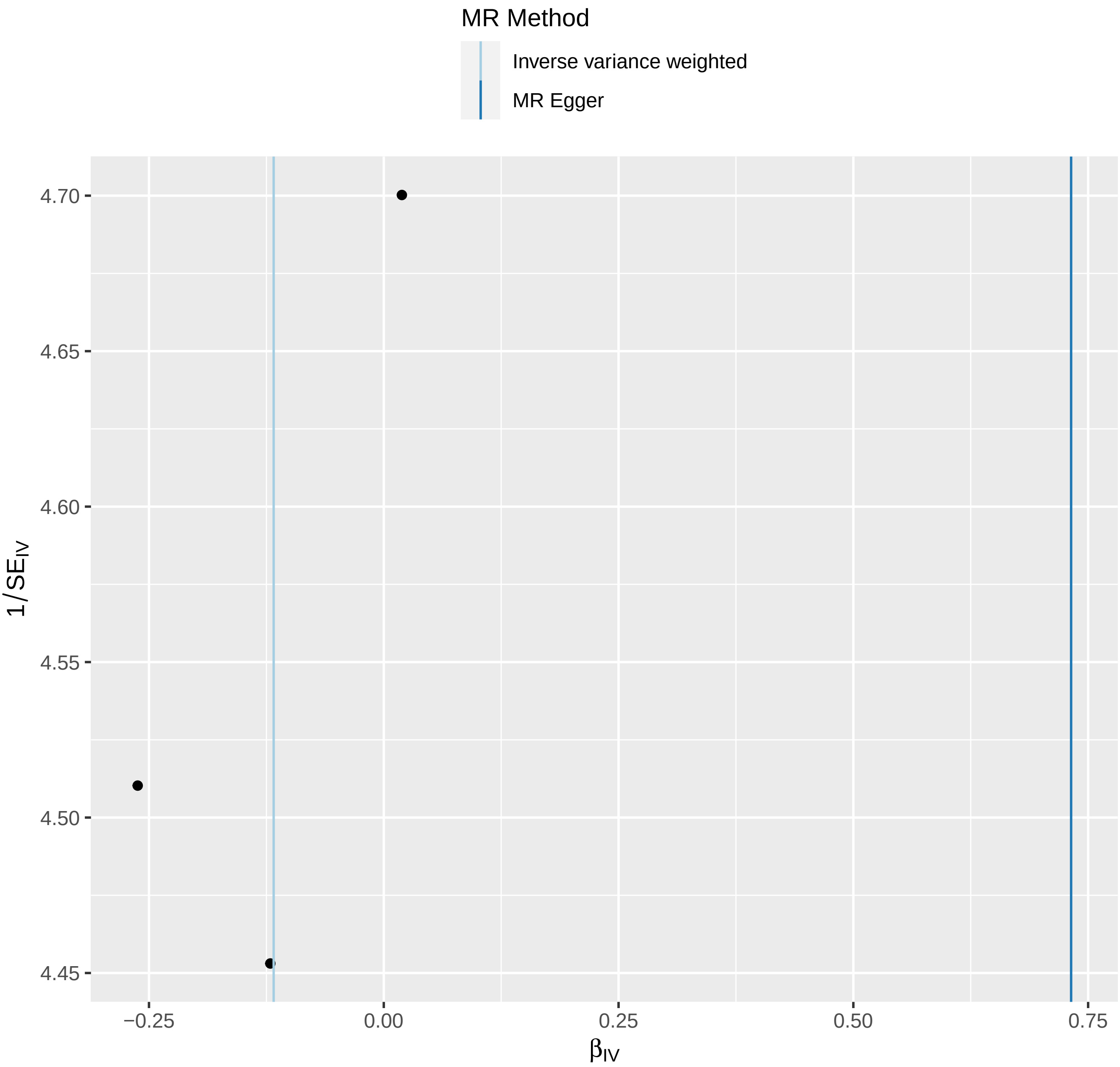

Batch 2−4432 : Gut microbiota abundance (genus Parasutterella id.2892) on Programmed cell death protein 5
with a threshold of 1e−05

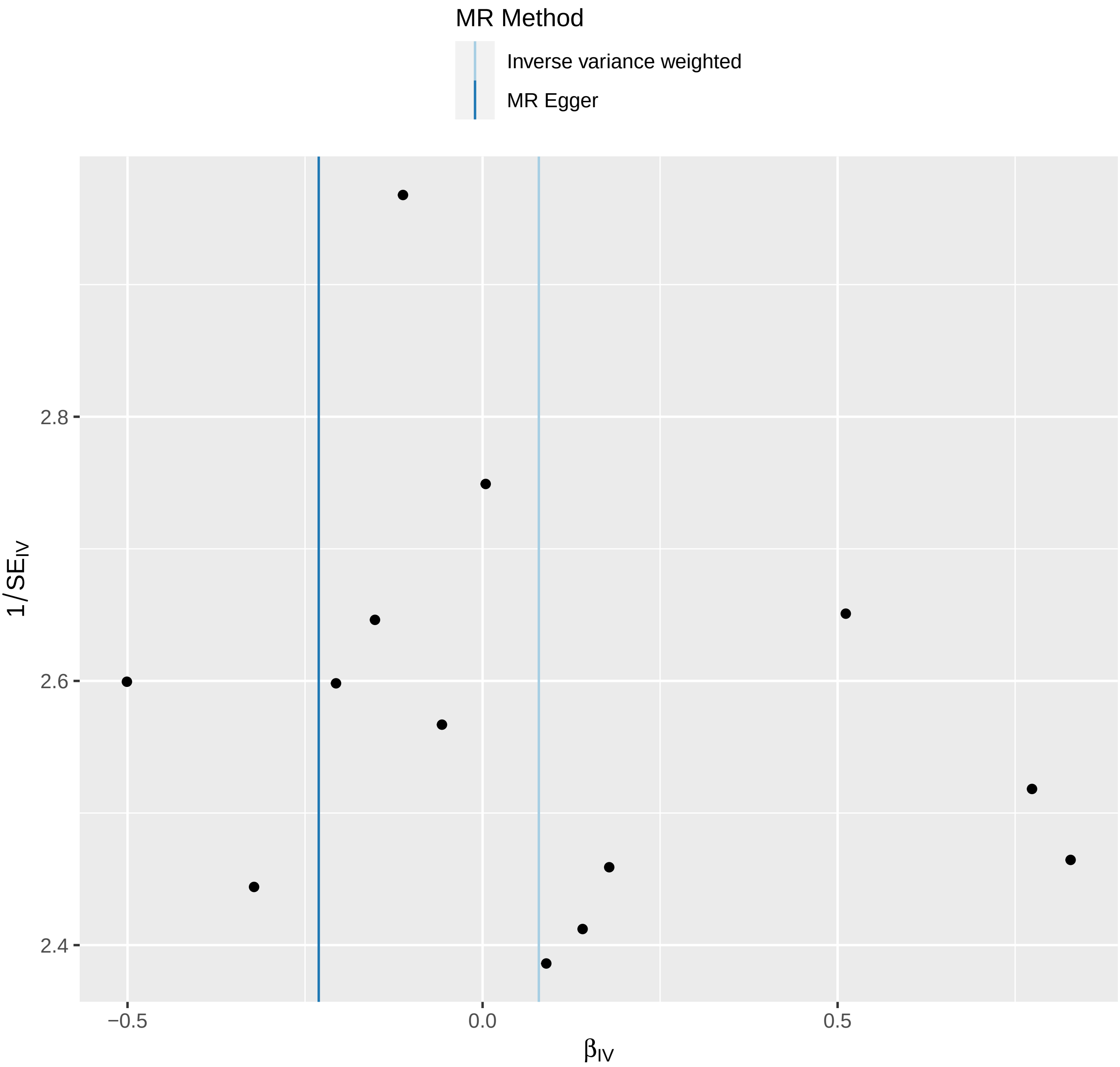

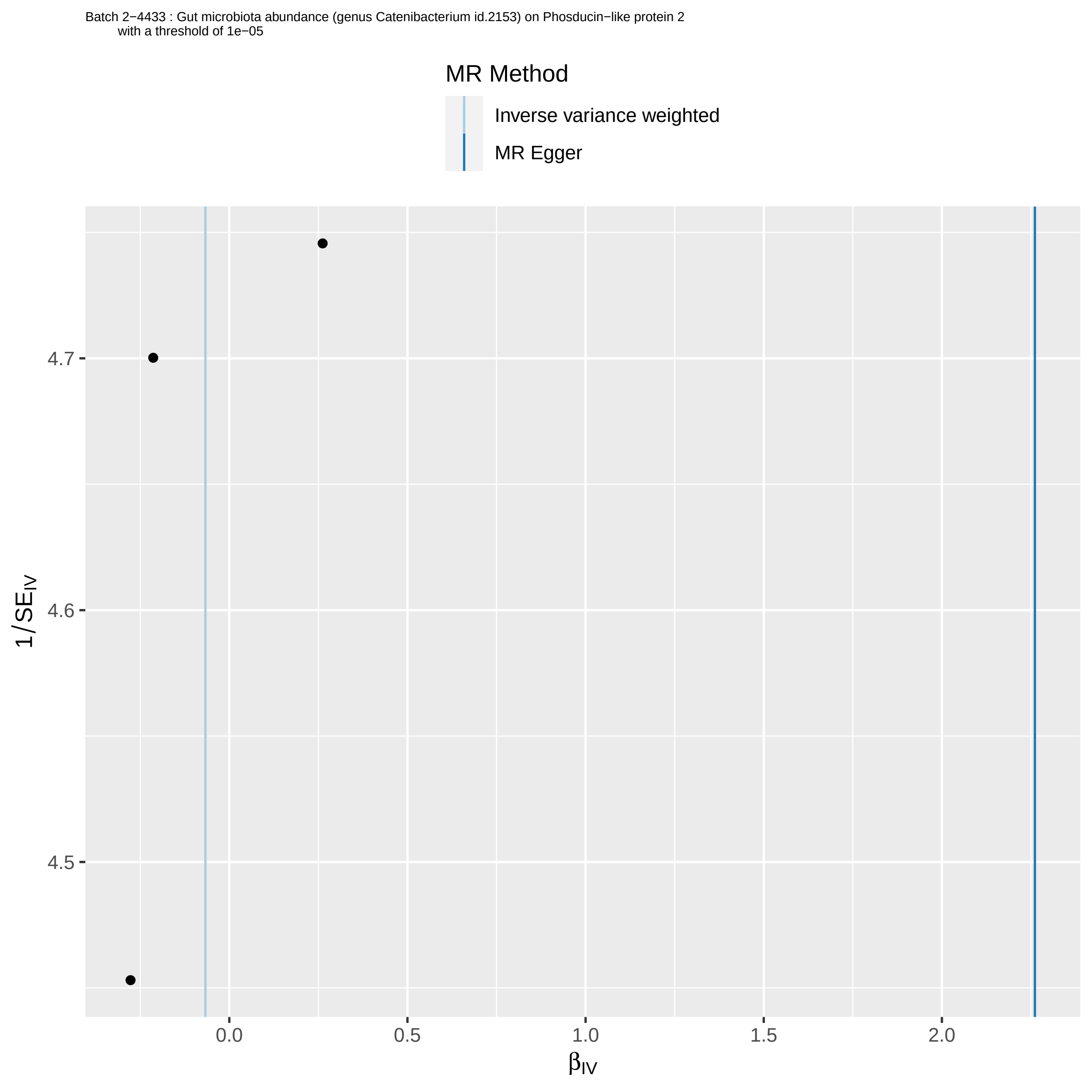

Batch 2−4433 : Gut microbiota abundance (genus Catenibacterium id.2153) on Phosducin−like protein 2
with a threshold of 1e−05
MR Method
Inverse variance weighted
MR Egger
4.7
4.6
4.5
1/SE$_{IV}$
0.0
0.5
1.0
1.5
2.0
β$_{IV}$

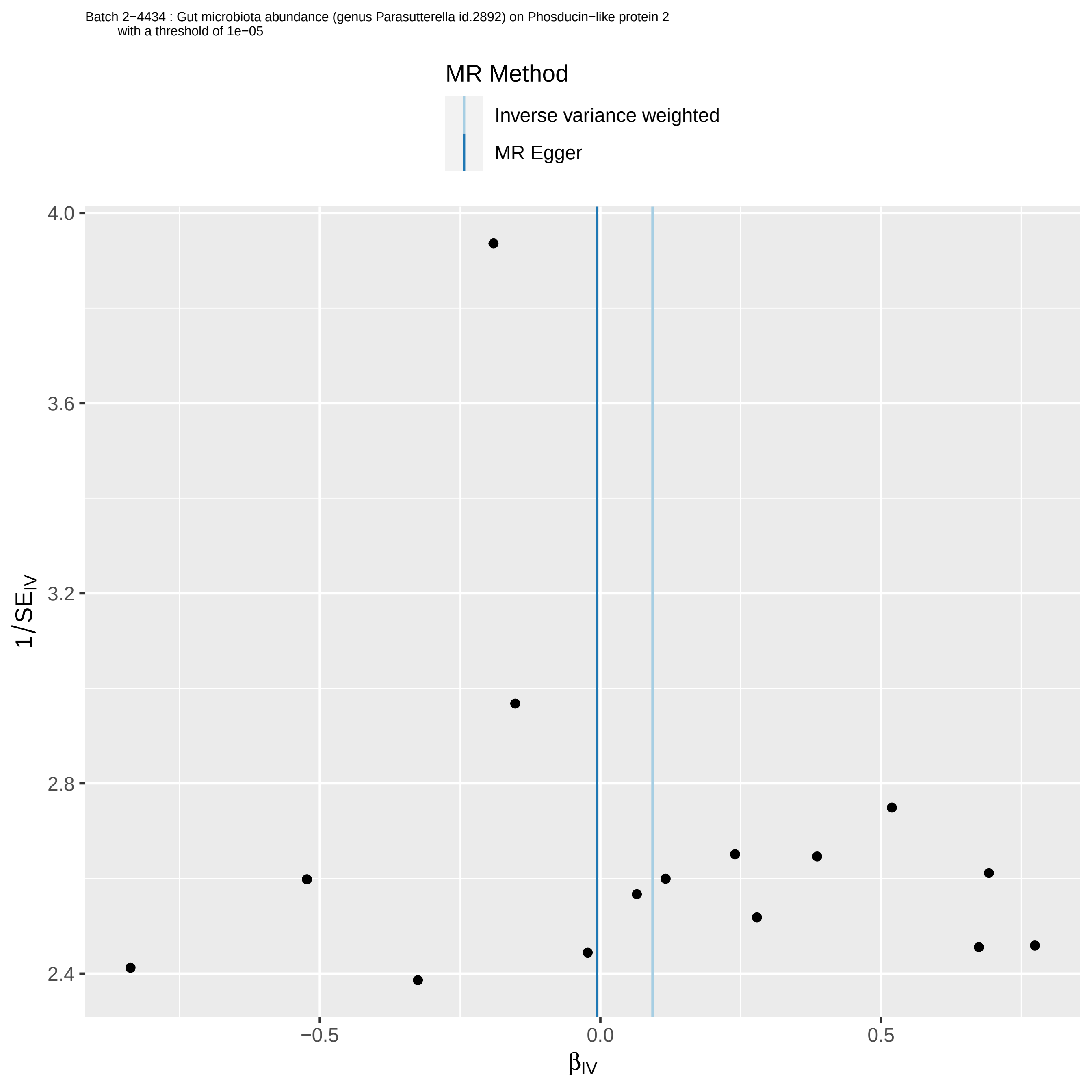

Batch 2−4434 : Gut microbiota abundance (genus Parasutterella id.2892) on Phosducin−like protein 2
with a threshold of 1e−05
MR Method
Inverse variance weighted
MR Egger
4.0
3.6
3.2
2.8
2.4
1/SE_IV
−0.5
0.0
0.5
β_IV

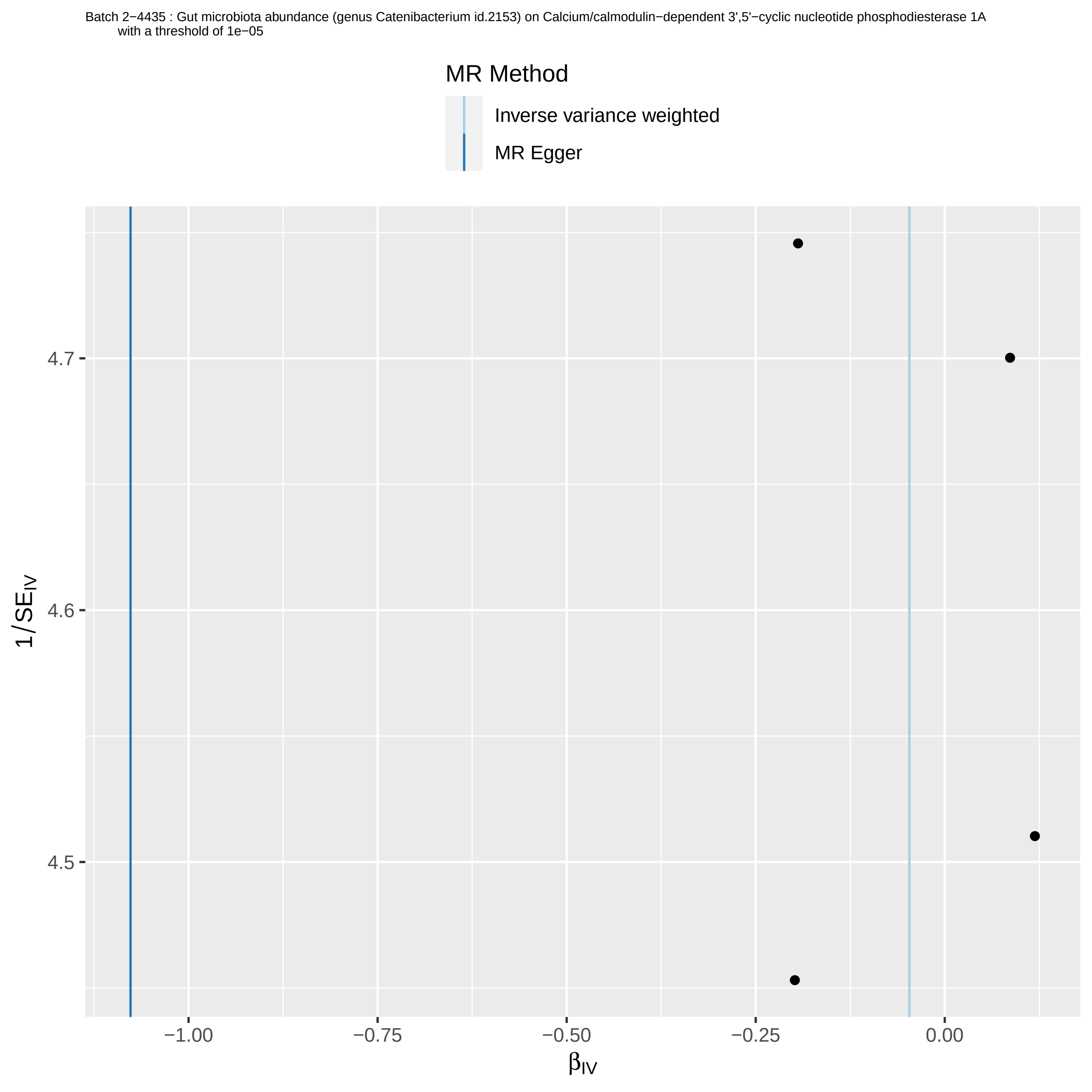

Batch 2−4435 : Gut microbiota abundance (genus Catenibacterium id.2153) on Calcium/calmodulin−dependent 3',5'−cyclic nucleotide phosphodiesterase 1A
with a threshold of 1e−05
MR Method
Inverse variance weighted
MR Egger
4.7
4.6
4.5
1/SE_IV
−1.00
−0.75
−0.50
−0.25
0.00
β_IV

Batch 2−4436 : Gut microbiota abundance (genus Parasutterella id.2892) on Calcium/calmodulin−dependent 3',5'−cyclic nucleotide phosphodiesterase 1A with a threshold of 1e−05

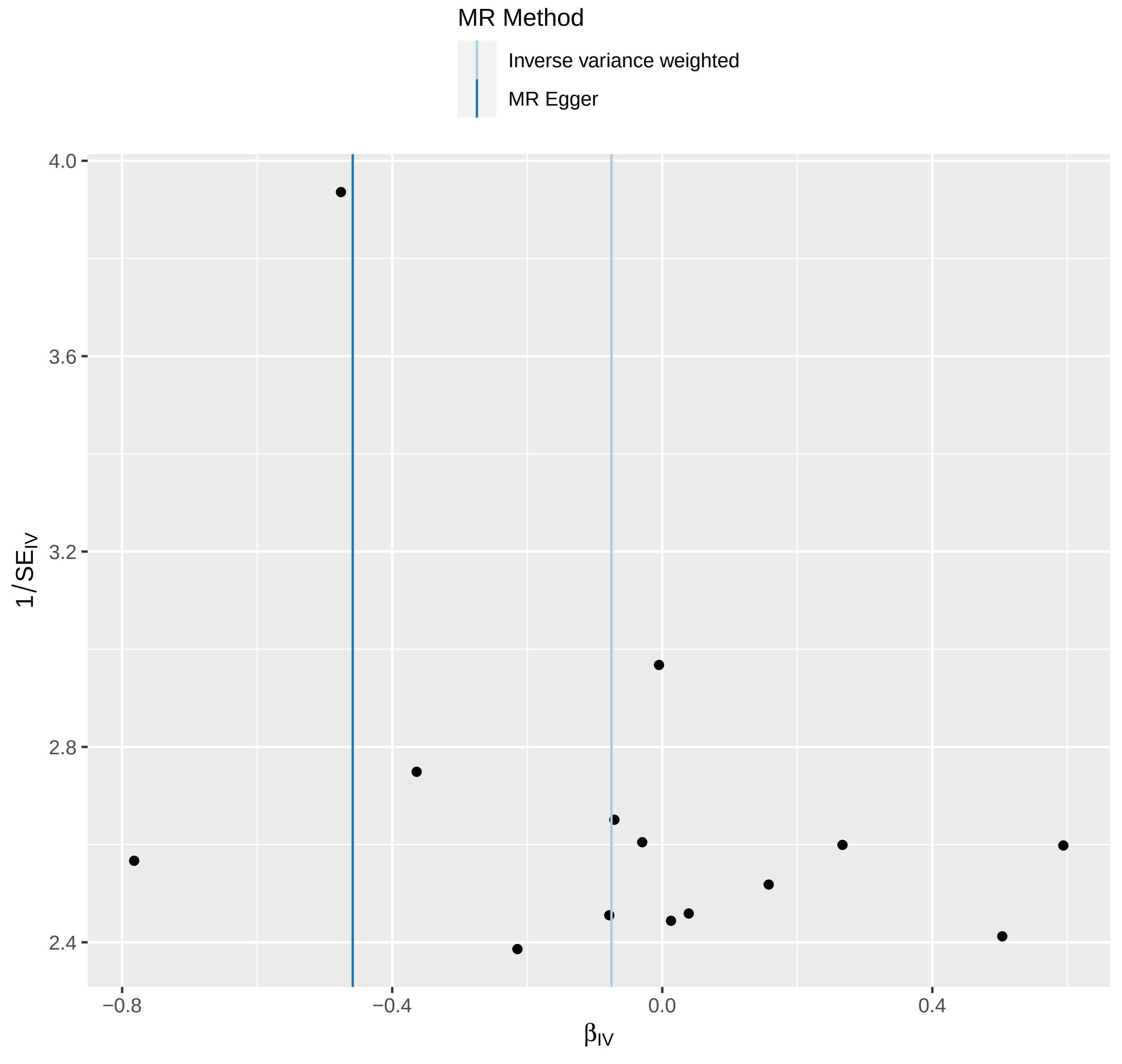

Batch 2−4437 : Gut microbiota abundance (genus Catenibacterium id.2153) on cGMP−dependent 3',5'−cyclic phosphodiesterase with a threshold of 1e−05

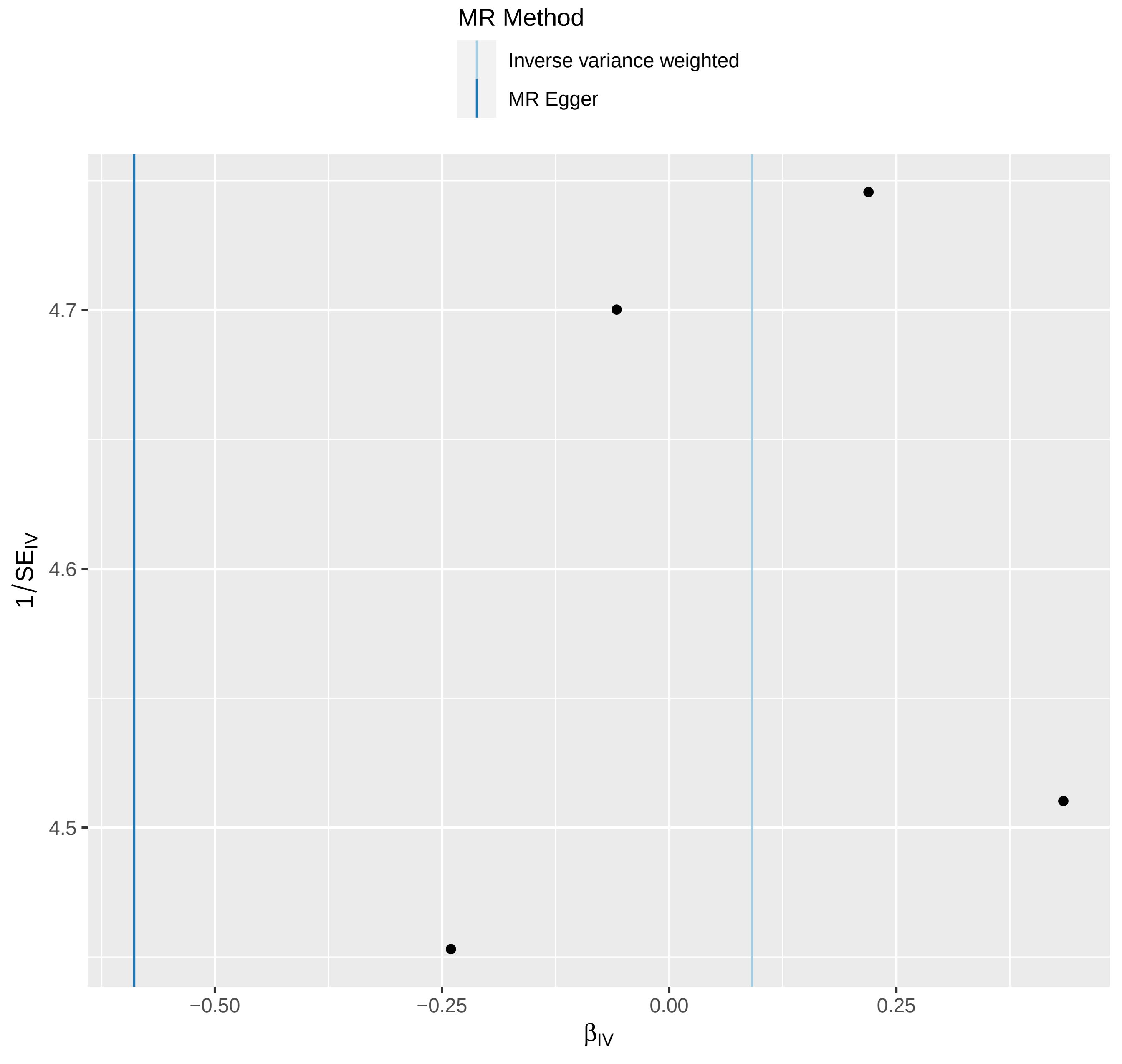

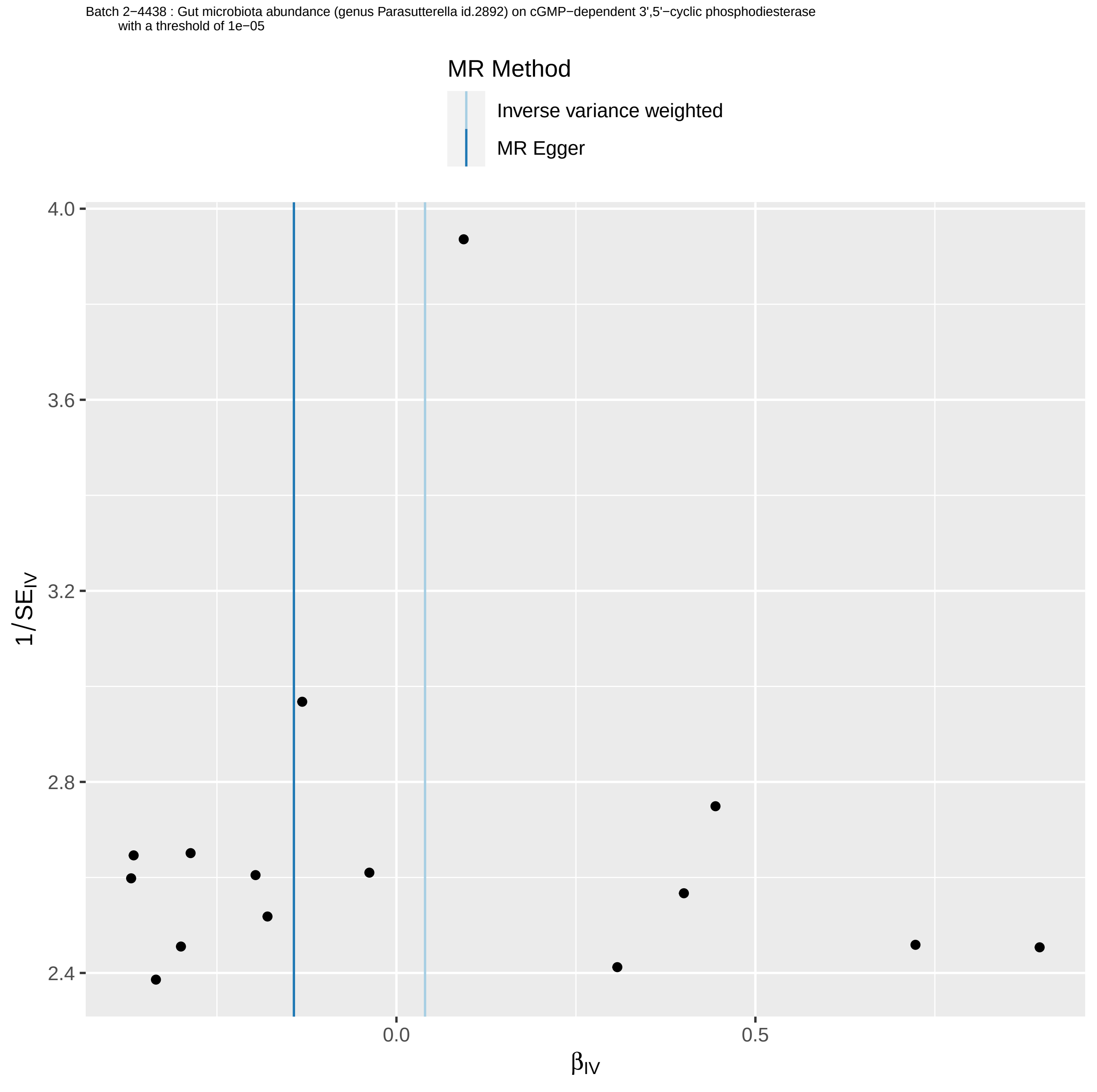
Batch 2−4438 : Gut microbiota abundance (genus Parasutterella id.2892) on cGMP−dependent 3',5'−cyclic phosphodiesterase
with a threshold of 1e−05
MR Method
Inverse variance weighted
MR Egger
4.0
3.6
3.2
2.8
2.4
1/SE_IV
0.0
0.5
β_IV

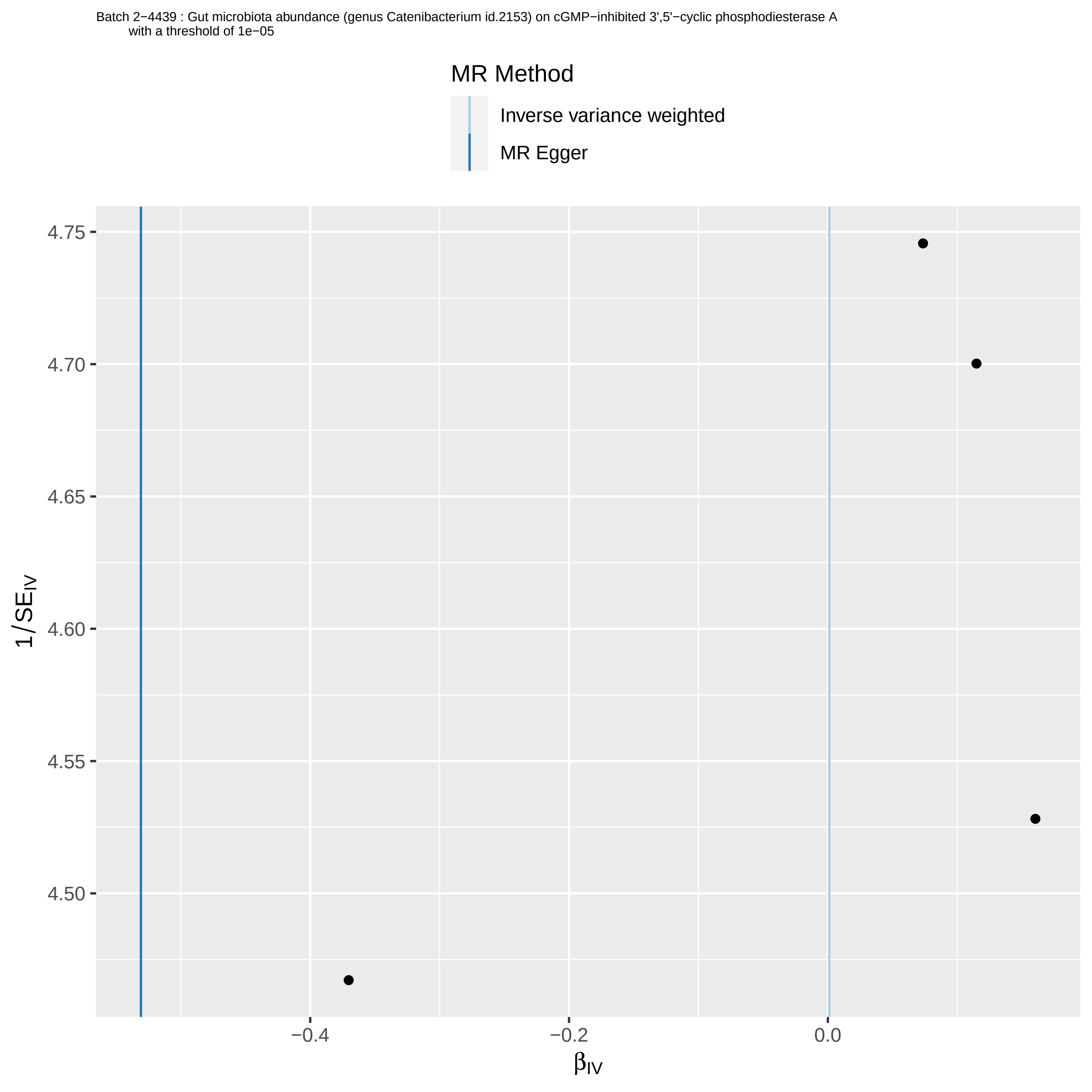
Batch 2−4439 : Gut microbiota abundance (genus Catenibacterium id.2153) on cGMP−inhibited 3',5'−cyclic phosphodiesterase A
with a threshold of 1e−05
MR Method
Inverse variance weighted
MR Egger
4.75
4.70
4.65
4.60
4.55
4.50
1/SE_IV
−0.4
−0.2
0.0
β_IV

Batch 2−4440 : Gut microbiota abundance (genus Parasutterella id.2892) on cGMP−inhibited 3',5'−cyclic phosphodiesterase A
with a threshold of 1e−05

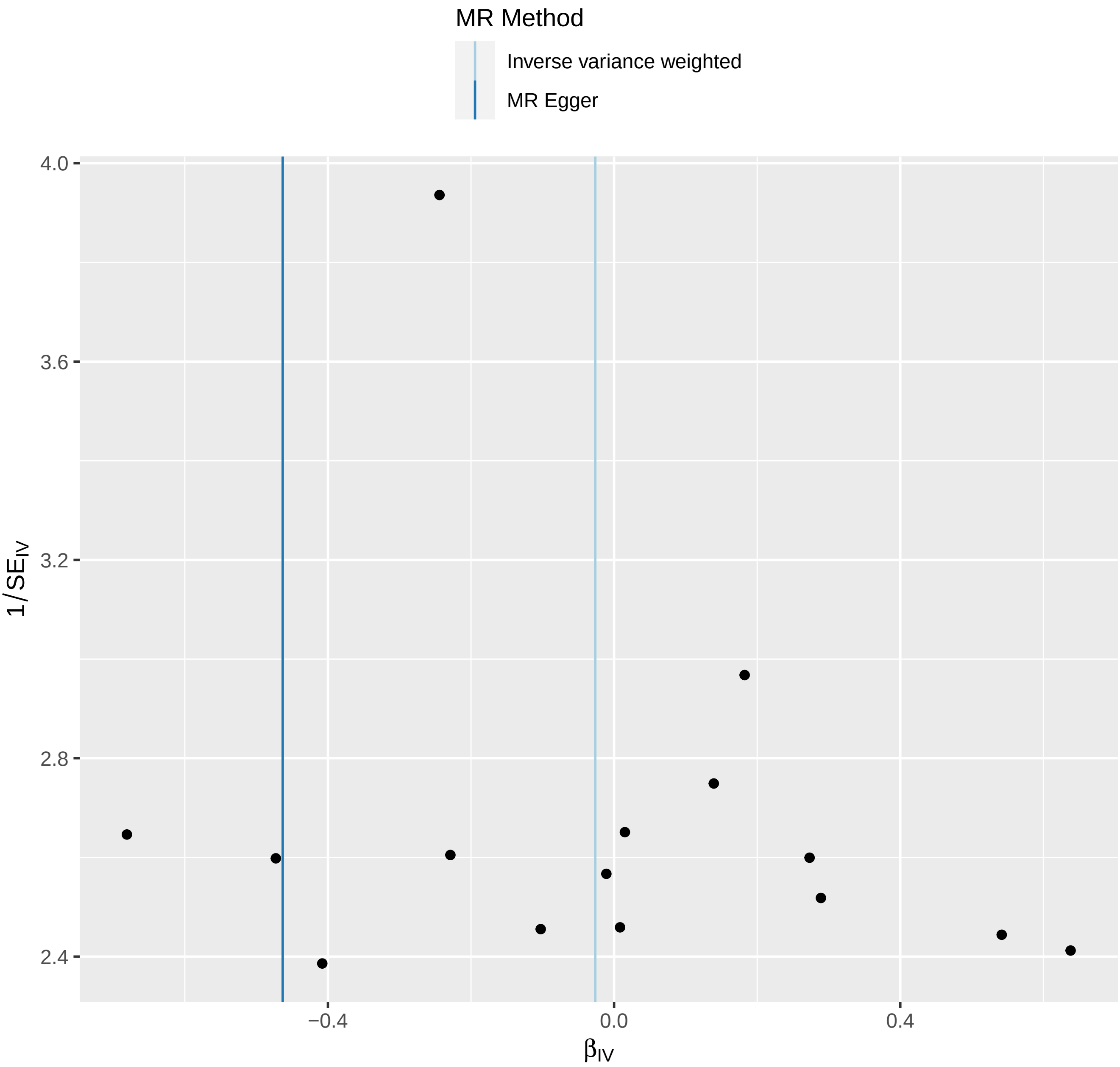

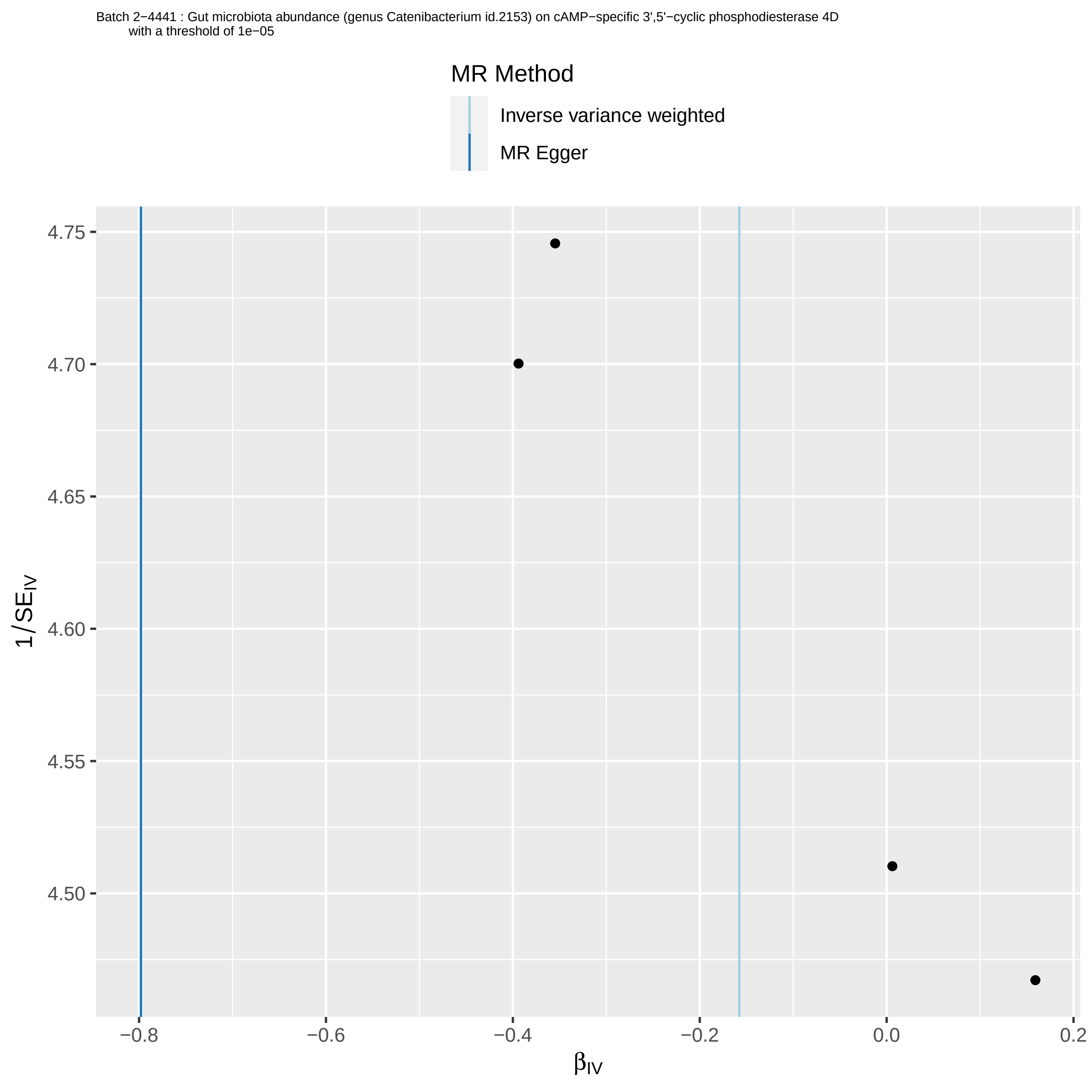

Batch 2−4441 : Gut microbiota abundance (genus Catenibacterium id.2153) on cAMP−specific 3',5'−cyclic phosphodiesterase 4D
with a threshold of 1e−05
MR Method
Inverse variance weighted
MR Egger
4.75
4.70
4.65
4.60
4.55
4.50
1/SE_IV
−0.8
−0.6
−0.4
−0.2
0.0
0.2
β_IV

Batch 2−4442 : Gut microbiota abundance (genus Parasutterella id.2892) on cAMP−specific 3',5'−cyclic phosphodiesterase 4D with a threshold of 1e−05

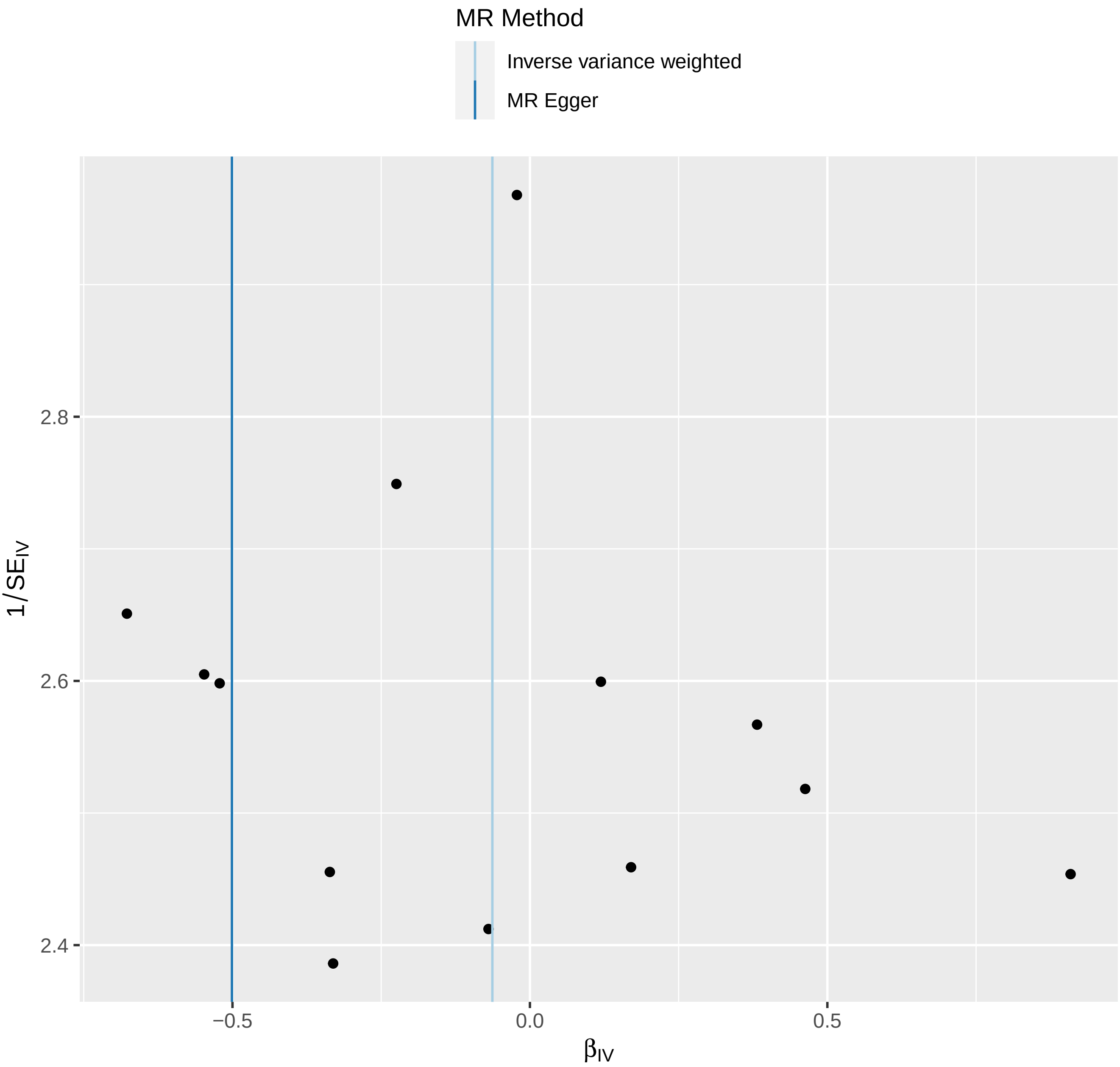

Batch 2−4443 : Gut microbiota abundance (genus Catenibacterium id.2153) on Retinal rod rhodopsin−sensitive cGMP 3',5'−cyclic phosphodiesterase subunit delta with a threshold of 1e−05

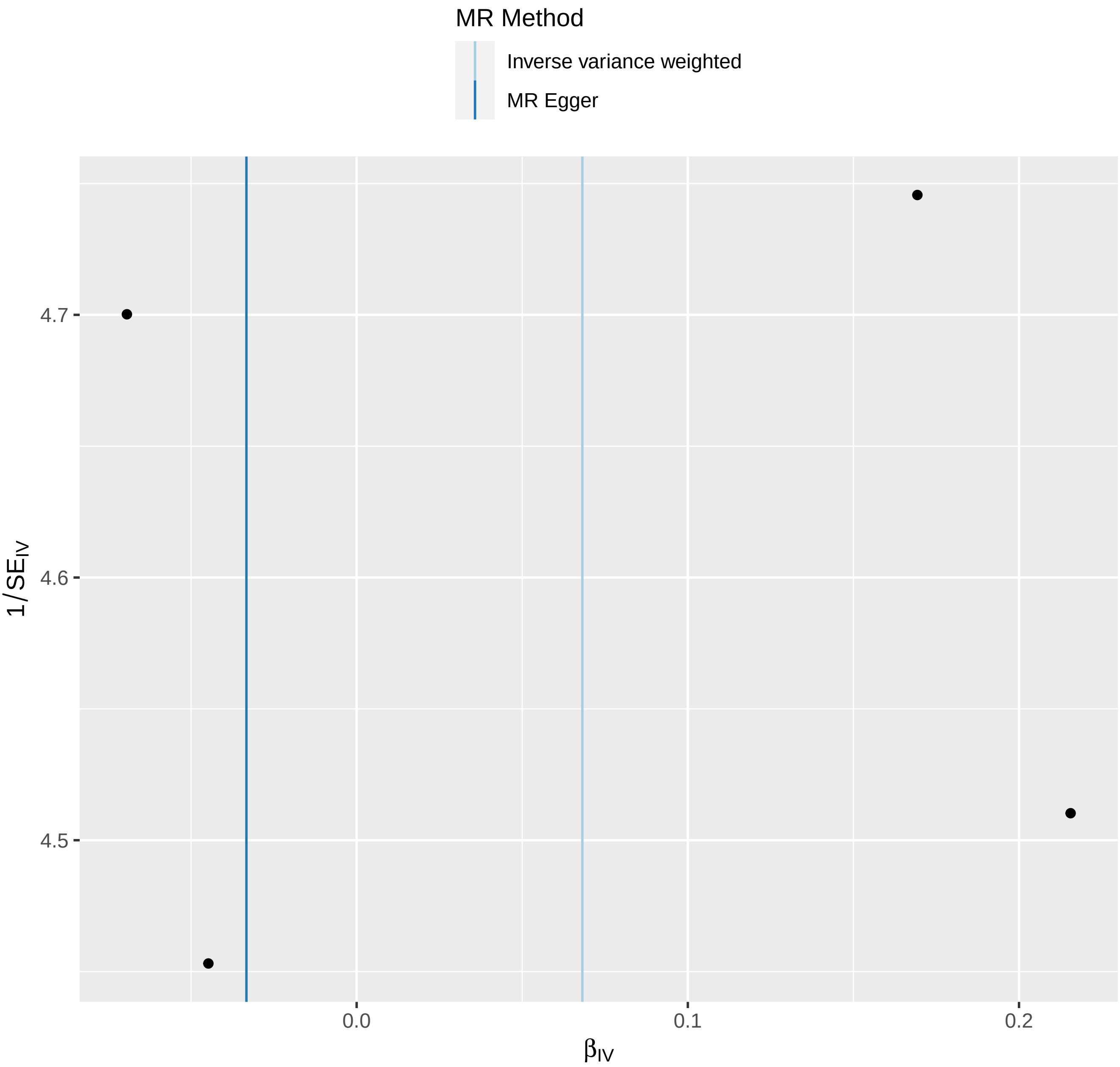

Batch 2−4444 : Gut microbiota abundance (genus Parasutterella id.2892) on Retinal rod rhodopsin−sensitive cGMP 3',5'−cyclic phosphodiesterase subunit delta with a threshold of 1e−05

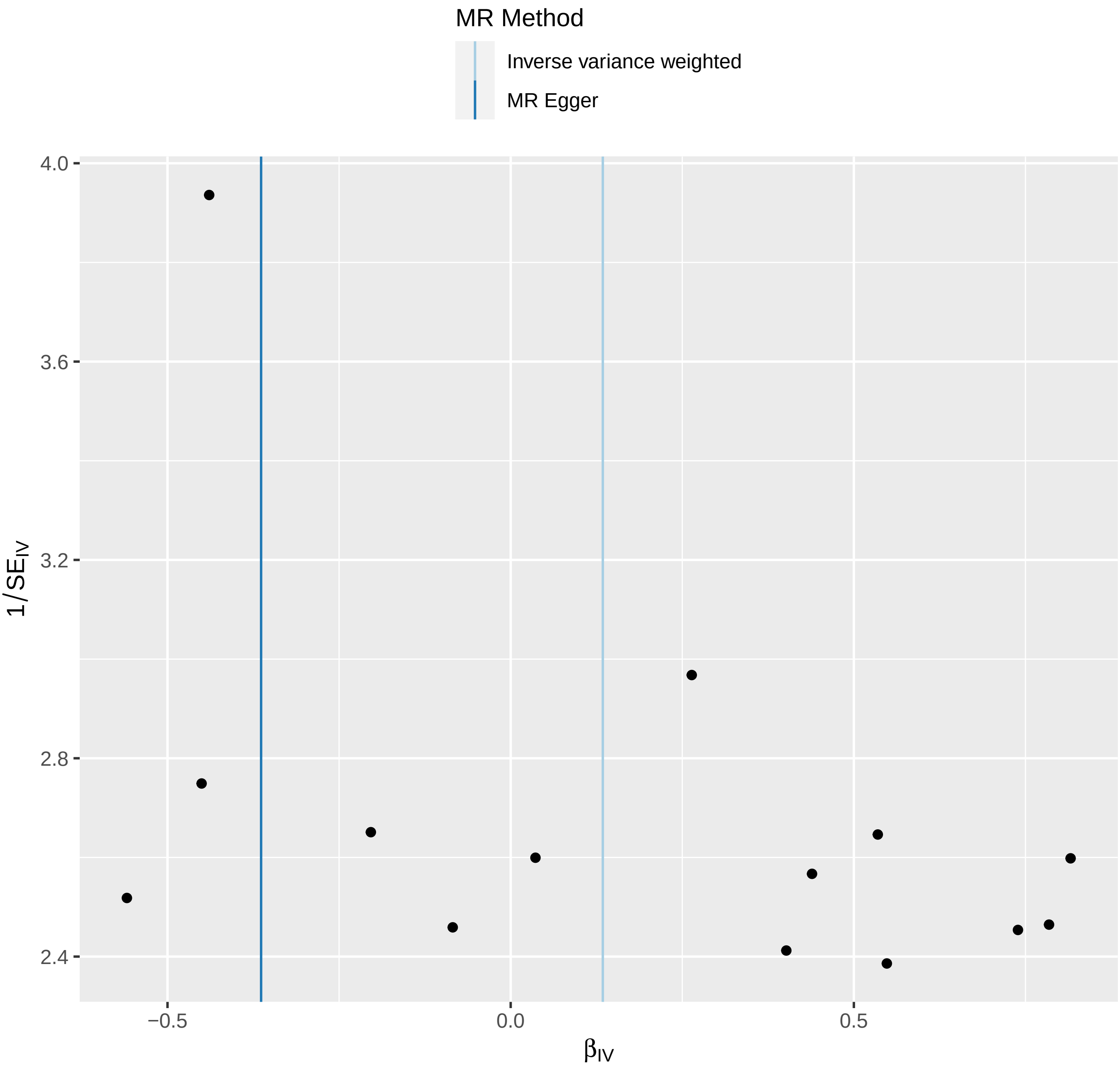

Batch 2−4445 : Gut microbiota abundance (genus Catenibacterium id.2153) on High affinity cAMP−specific 3',5'−cyclic phosphodiesterase 7A with a threshold of 1e−05

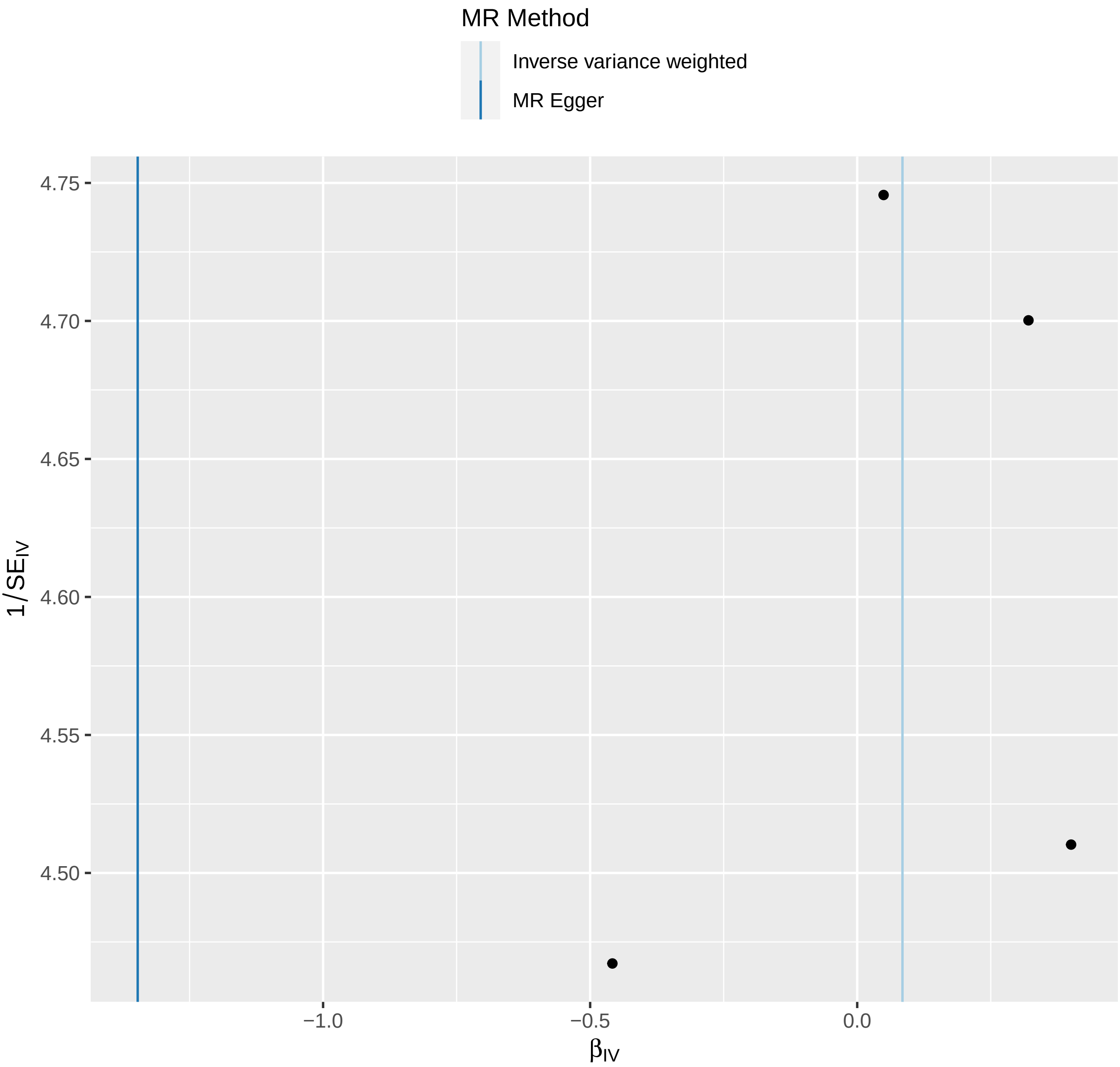

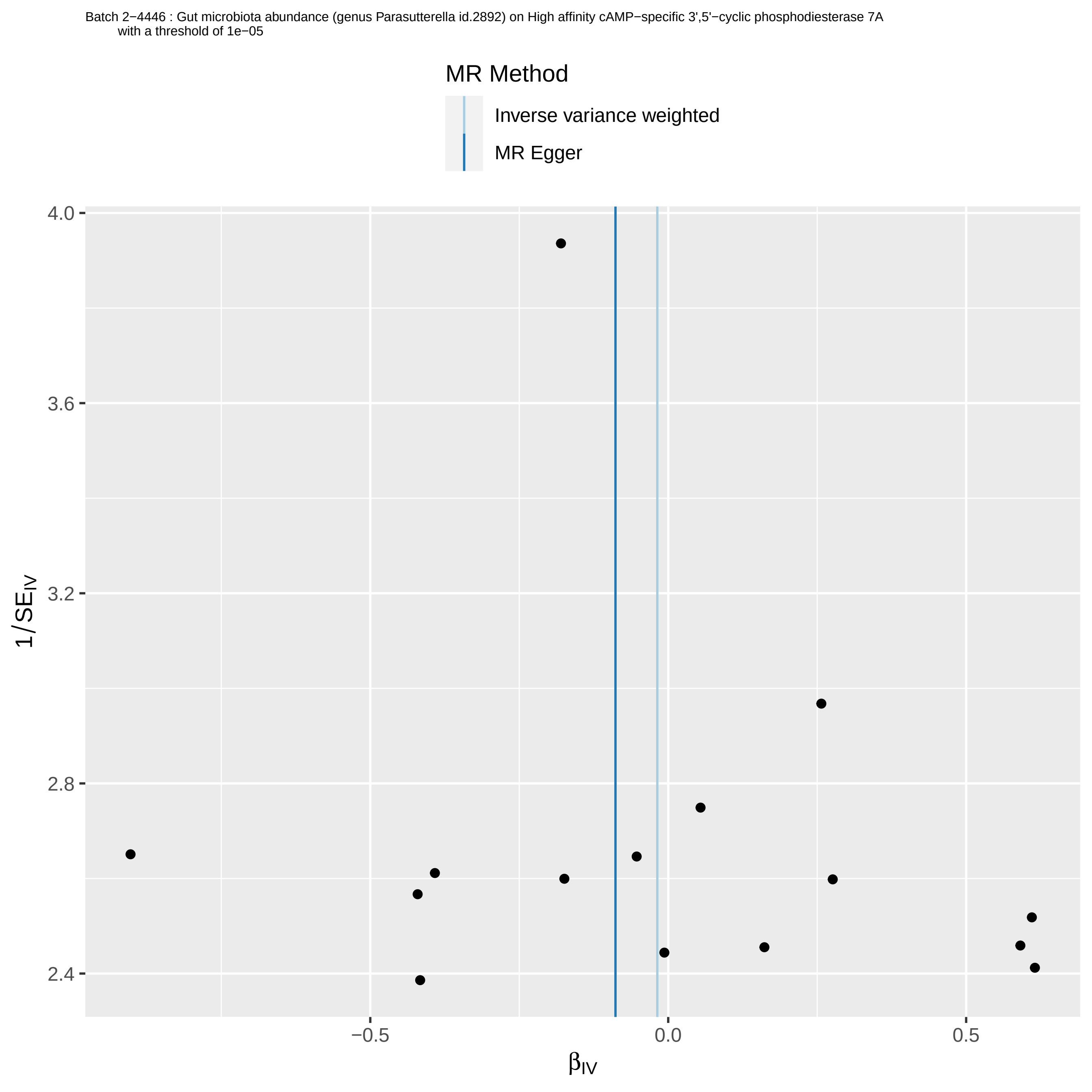
Batch 2−4446 : Gut microbiota abundance (genus Parasutterella id.2892) on High affinity cAMP−specific 3',5'−cyclic phosphodiesterase 7A
with a threshold of 1e−05
MR Method
Inverse variance weighted
MR Egger
4.0
3.6
3.2
2.8
2.4
1/SE_IV
−0.5
0.0
0.5
β_IV

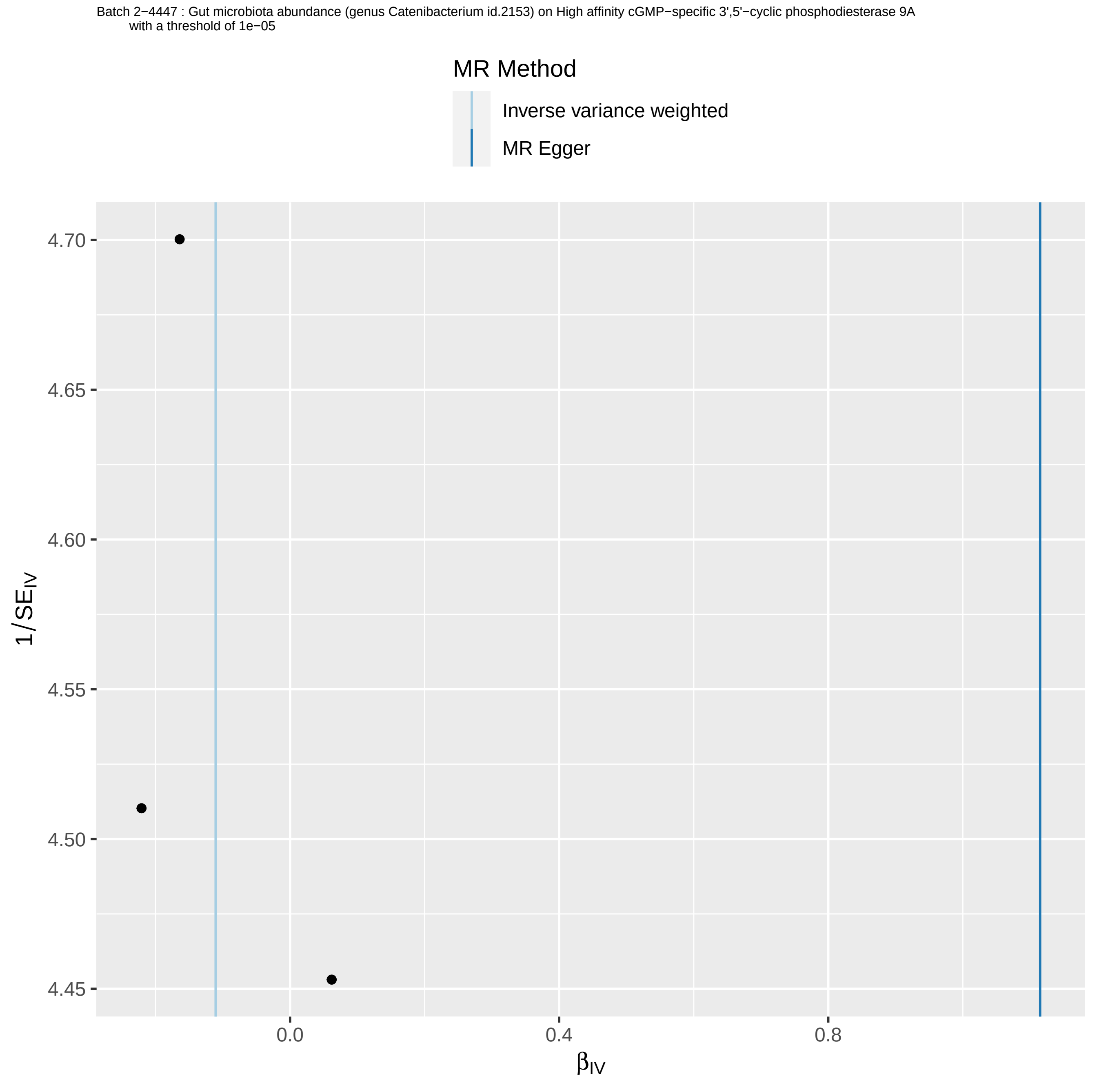
Batch 2−4447 : Gut microbiota abundance (genus Catenibacterium id.2153) on High affinity cGMP−specific 3',5'−cyclic phosphodiesterase 9A
with a threshold of 1e−05
MR Method
Inverse variance weighted
MR Egger
4.70
4.65
4.60
4.55
4.50
4.45
1/SE_IV
0.0
0.4
0.8
β_IV

Batch 2−4448 : Gut microbiota abundance (genus Parasutterella id.2892) on High affinity cGMP−specific 3',5'−cyclic phosphodiesterase 9A
with a threshold of 1e−05

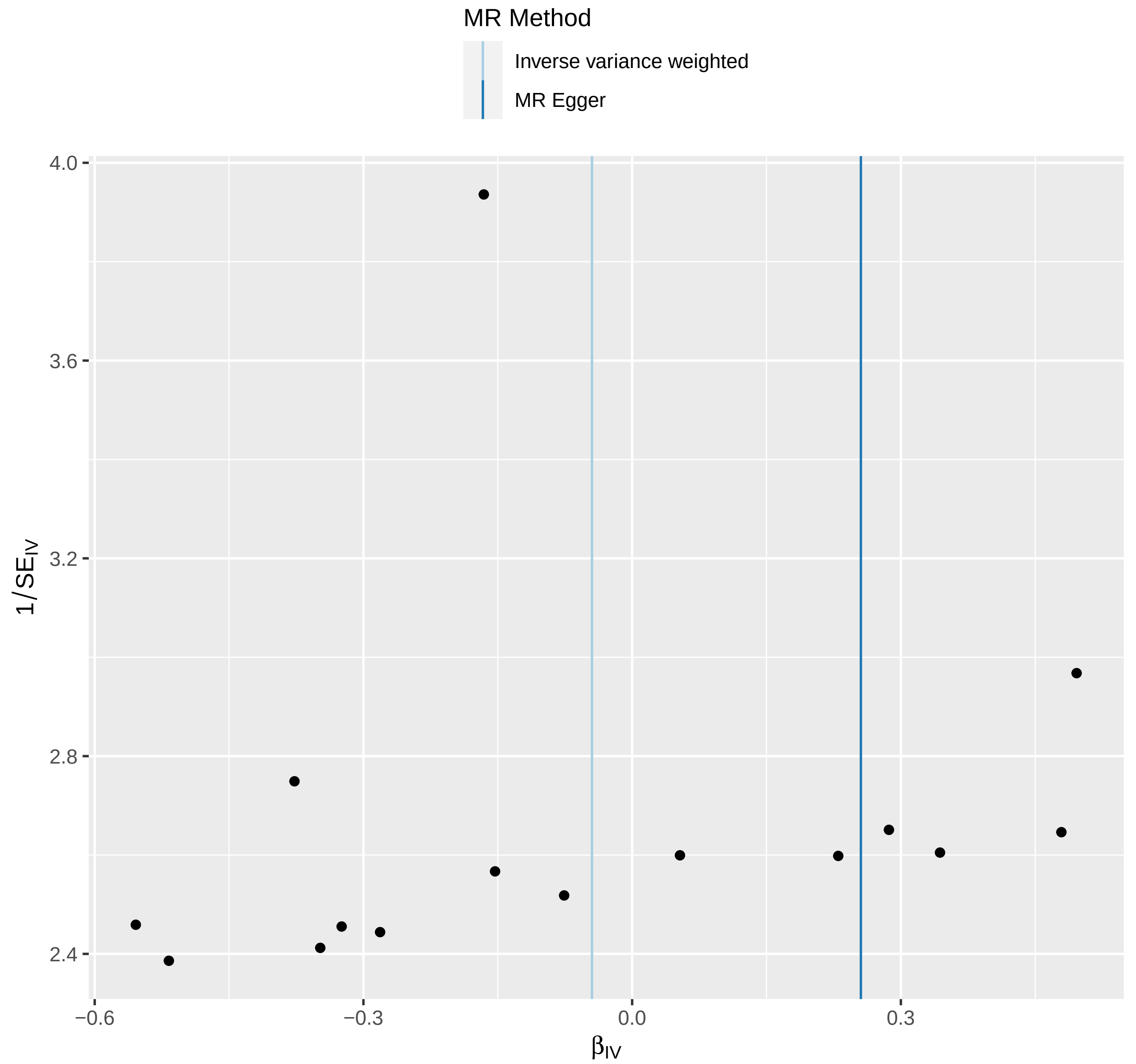

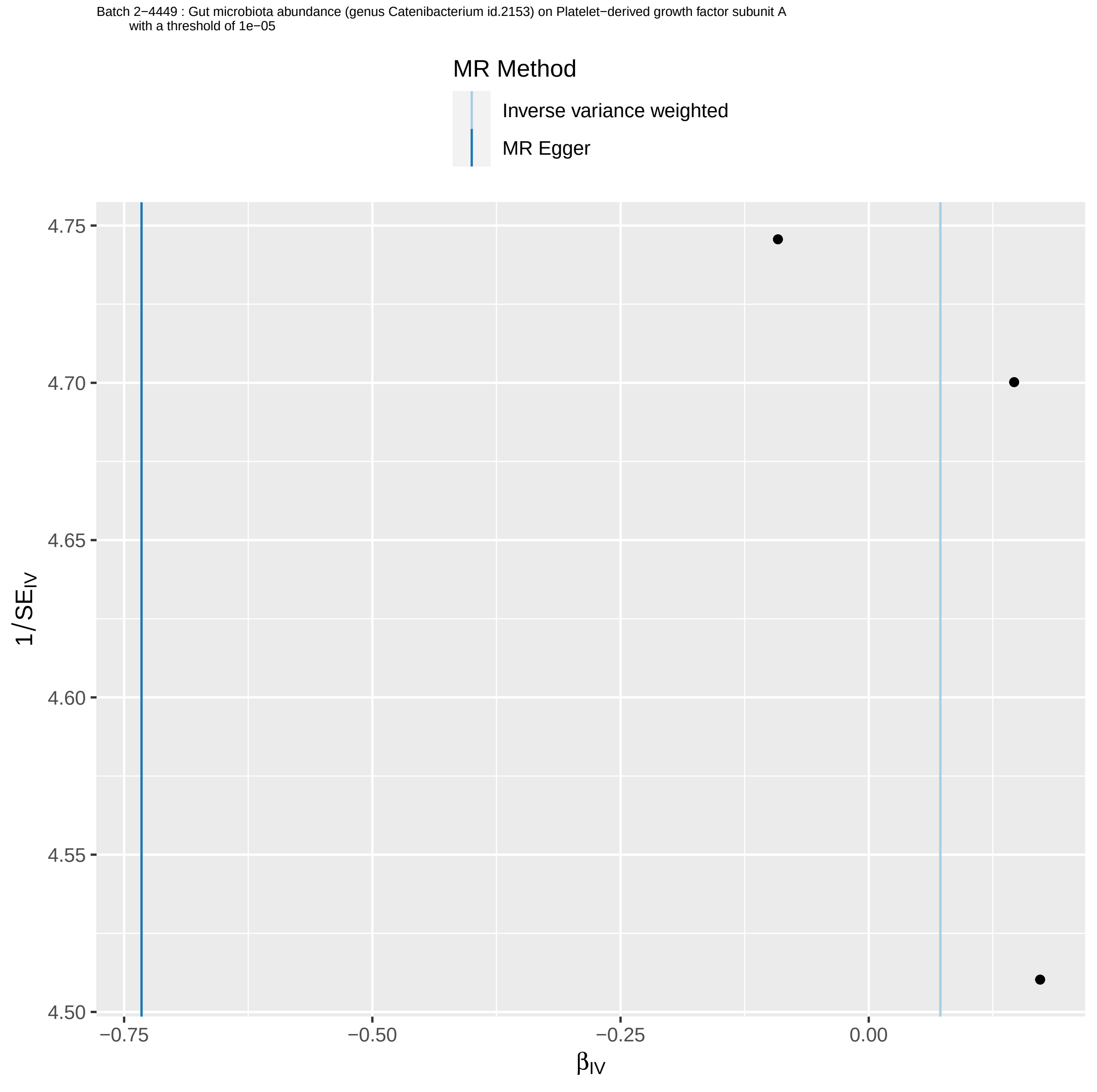
Batch 2−4449 : Gut microbiota abundance (genus Catenibacterium id.2153) on Platelet−derived growth factor subunit A
with a threshold of 1e−05
MR Method
Inverse variance weighted
MR Egger
4.75
4.70
4.65
4.60
4.55
4.50
1/SE_IV
−0.75
−0.50
−0.25
0.00
β_IV

Batch 2−4450 : Gut microbiota abundance (genus Parasutterella id.2892) on Platelet−derived growth factor subunit A with a threshold of 1e−05

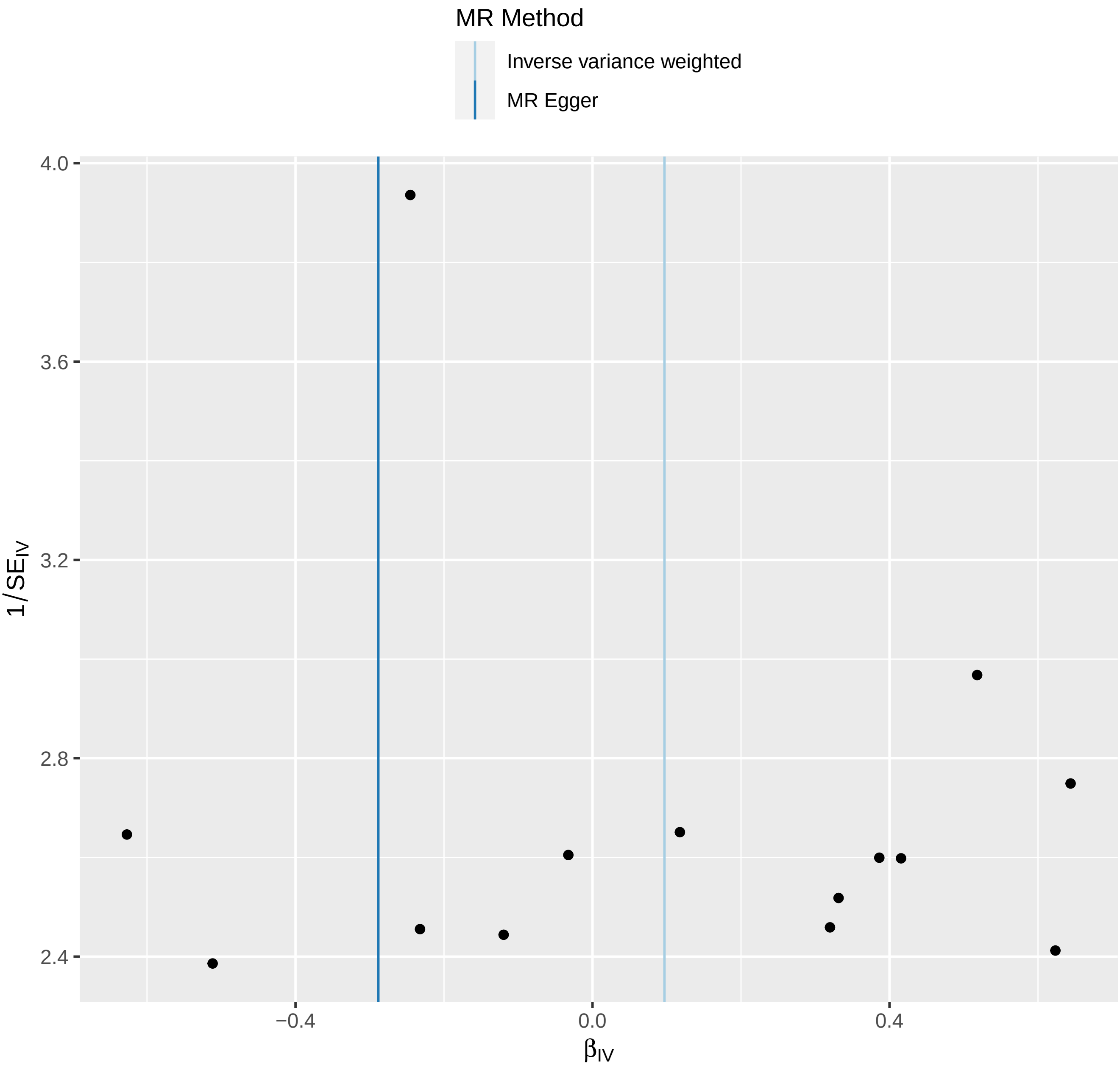

Batch 2−4451 : Gut microbiota abundance (genus Catenibacterium id.2153) on Platelet−derived growth factor subunit B
with a threshold of 1e−05

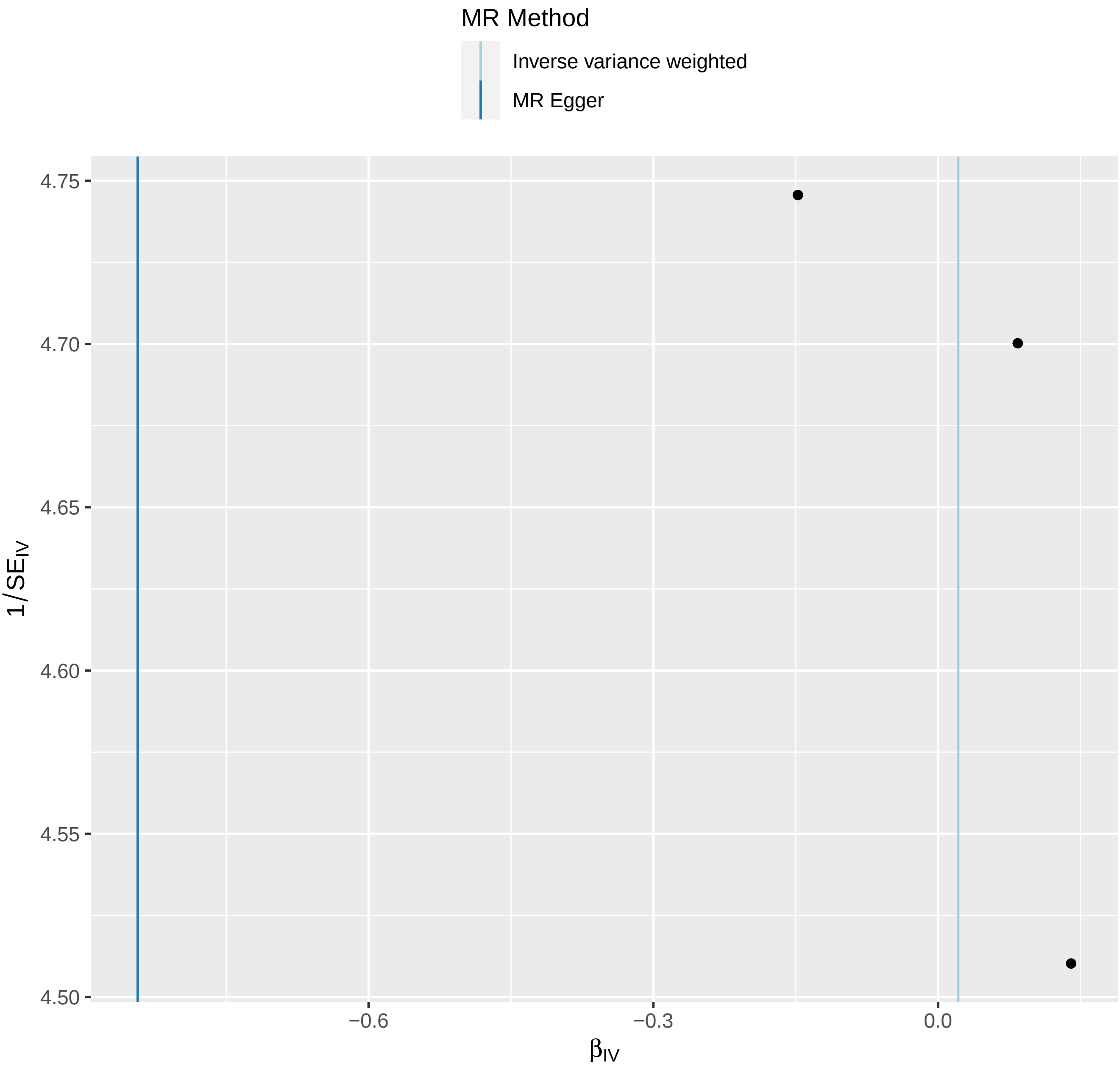

Batch 2−4452 : Gut microbiota abundance (genus Parasutterella id.2892) on Platelet−derived growth factor subunit B with a threshold of 1e−05

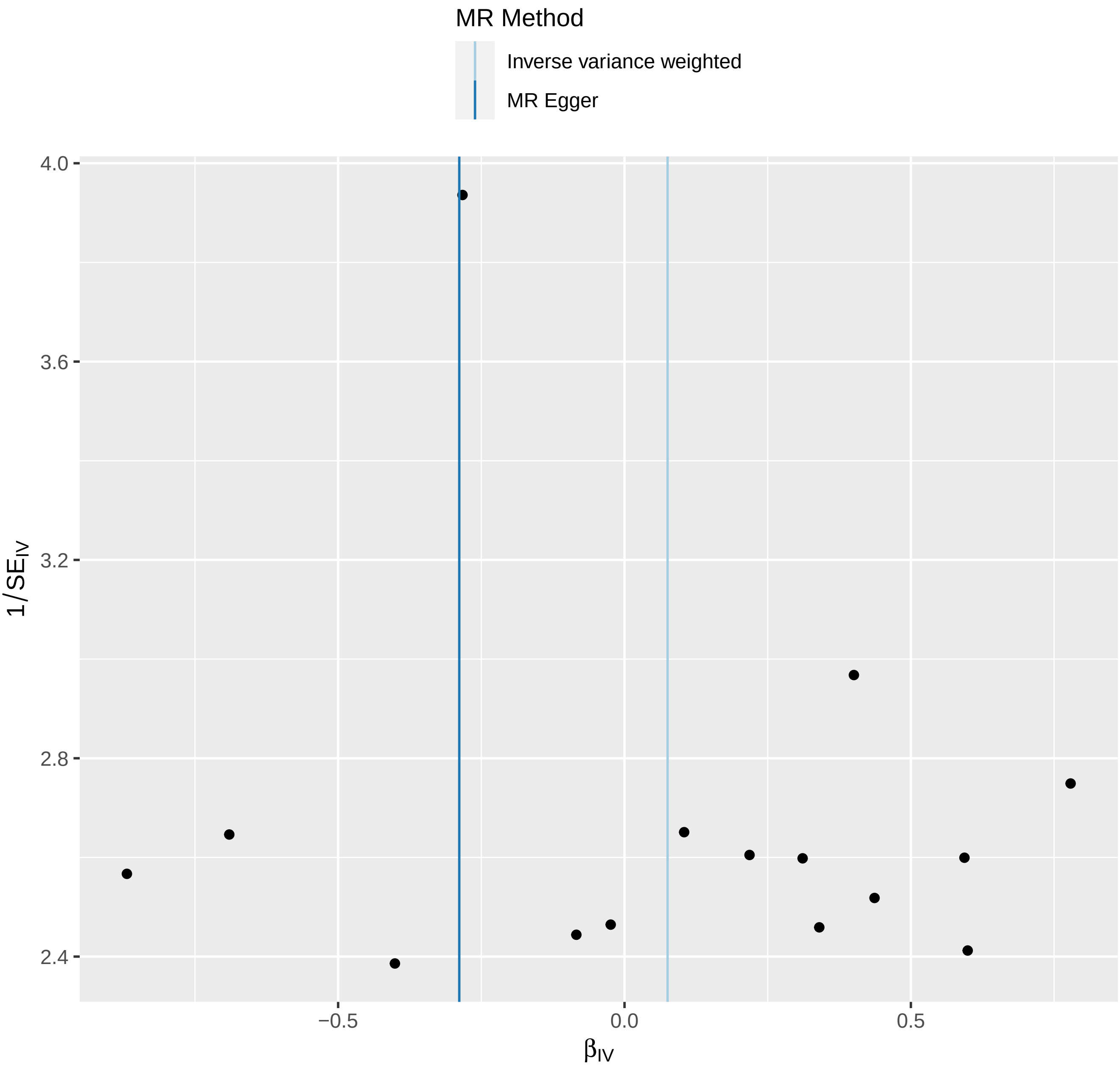

Batch 2−4453 : Gut microbiota abundance (genus Catenibacterium id.2153) on Platelet−derived growth factor C
with a threshold of 1e−05

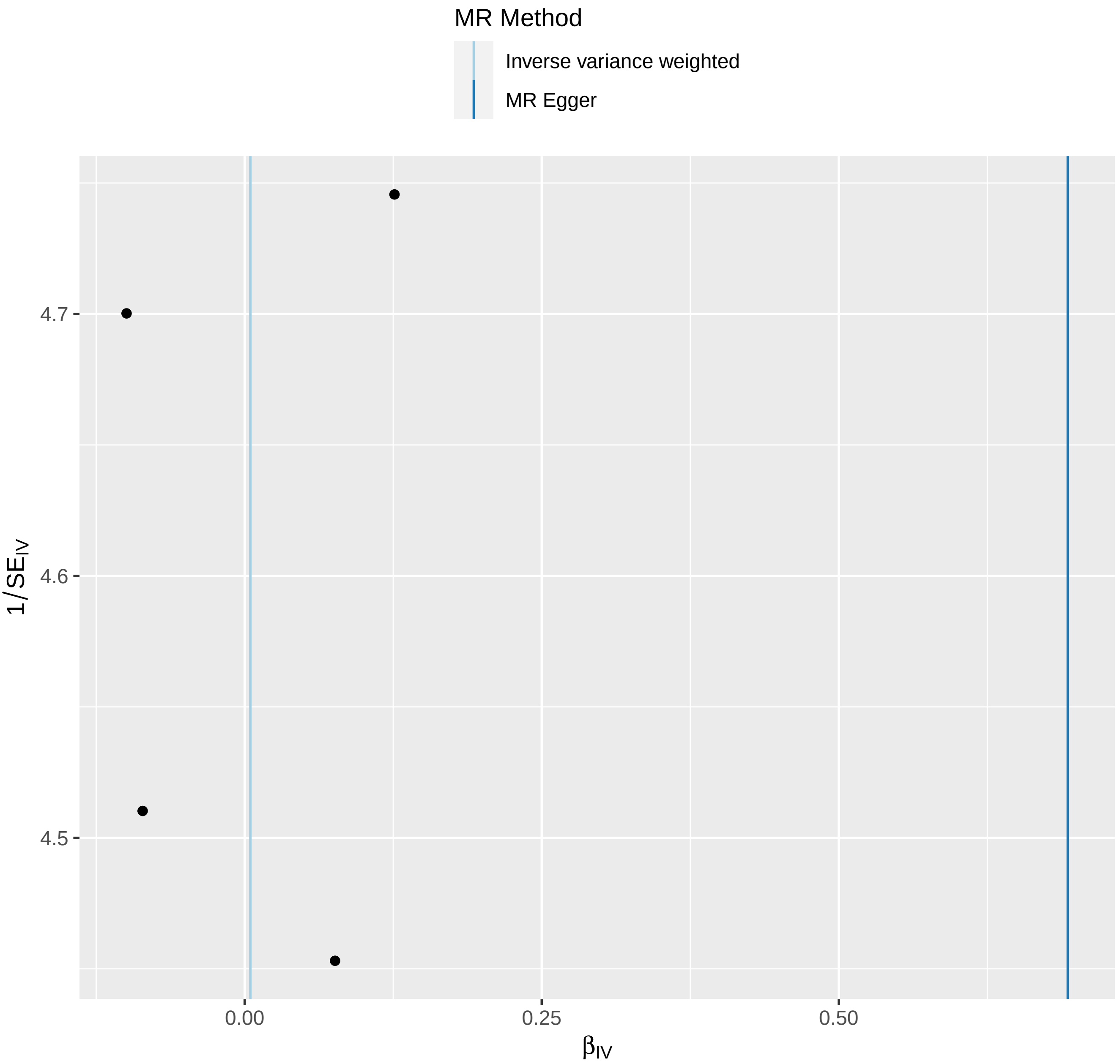

Batch 2−4454 : Gut microbiota abundance (genus Parasutterella id.2892) on Platelet−derived growth factor C
with a threshold of 1e−05

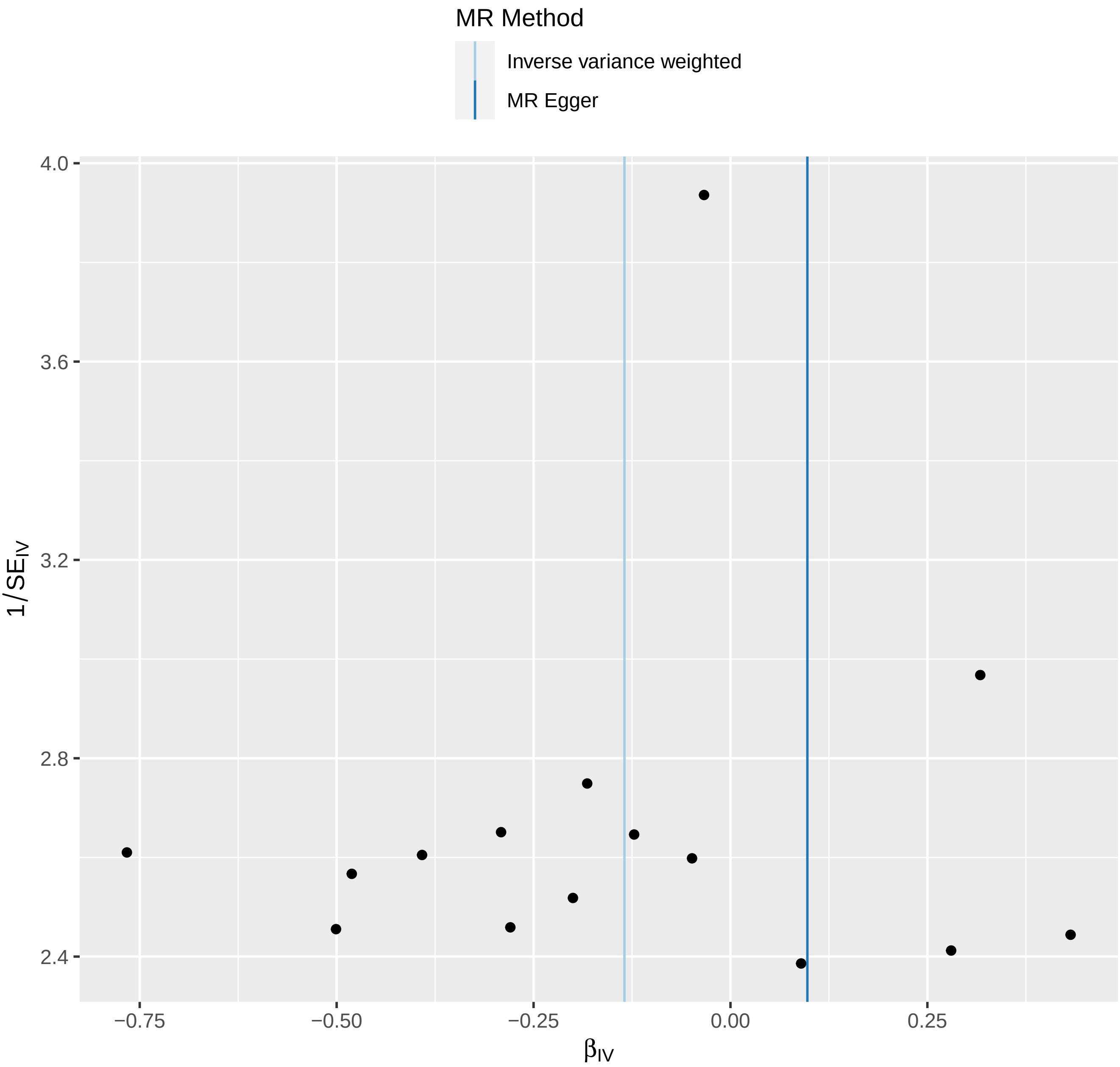

Batch 2−4455 : Gut microbiota abundance (genus Catenibacterium id.2153) on Platelet−derived growth factor D with a threshold of 1e−05

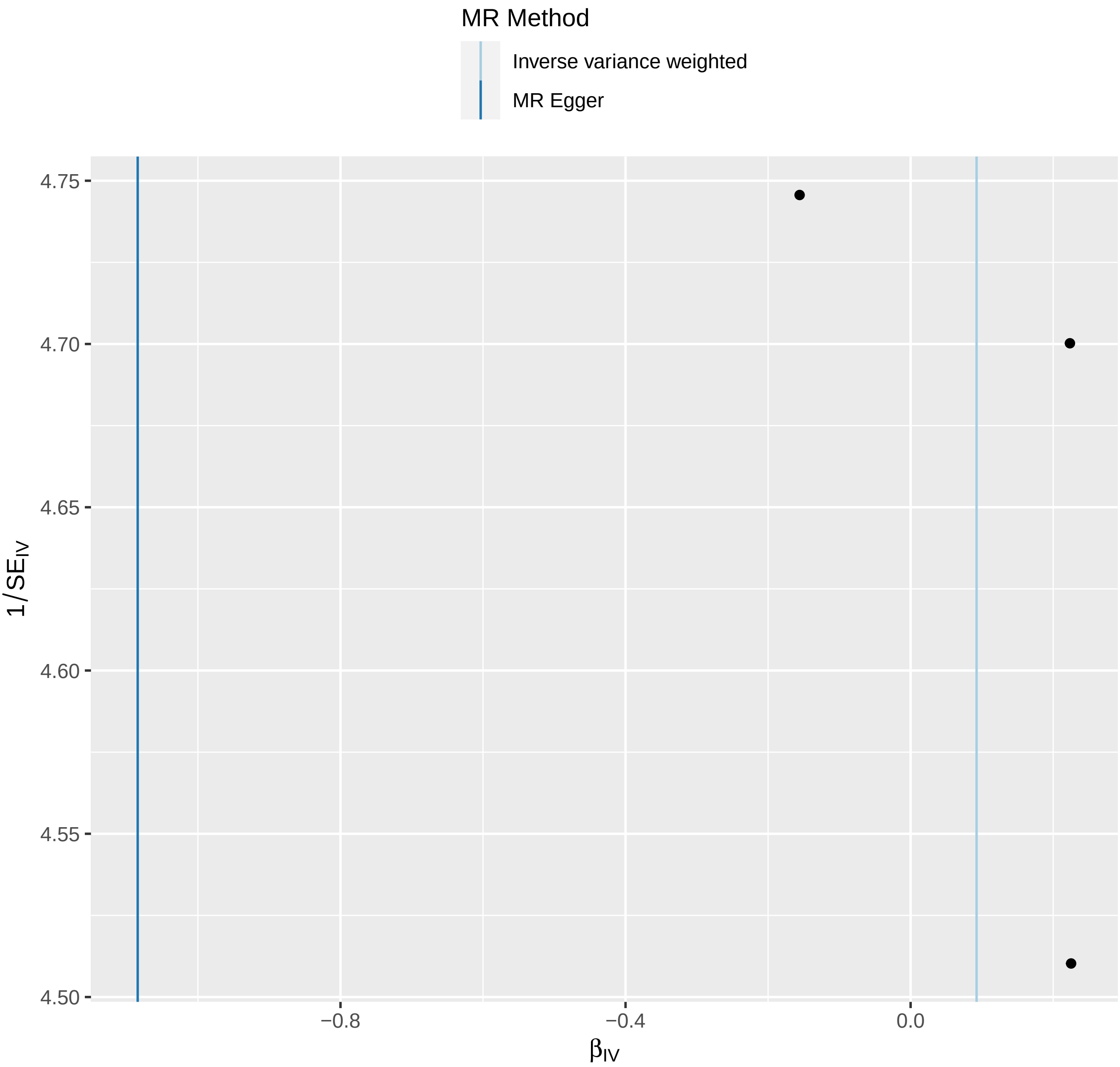

Batch 2−4456 : Gut microbiota abundance (genus Parasutterella id.2892) on Platelet−derived growth factor D
with a threshold of 1e−05

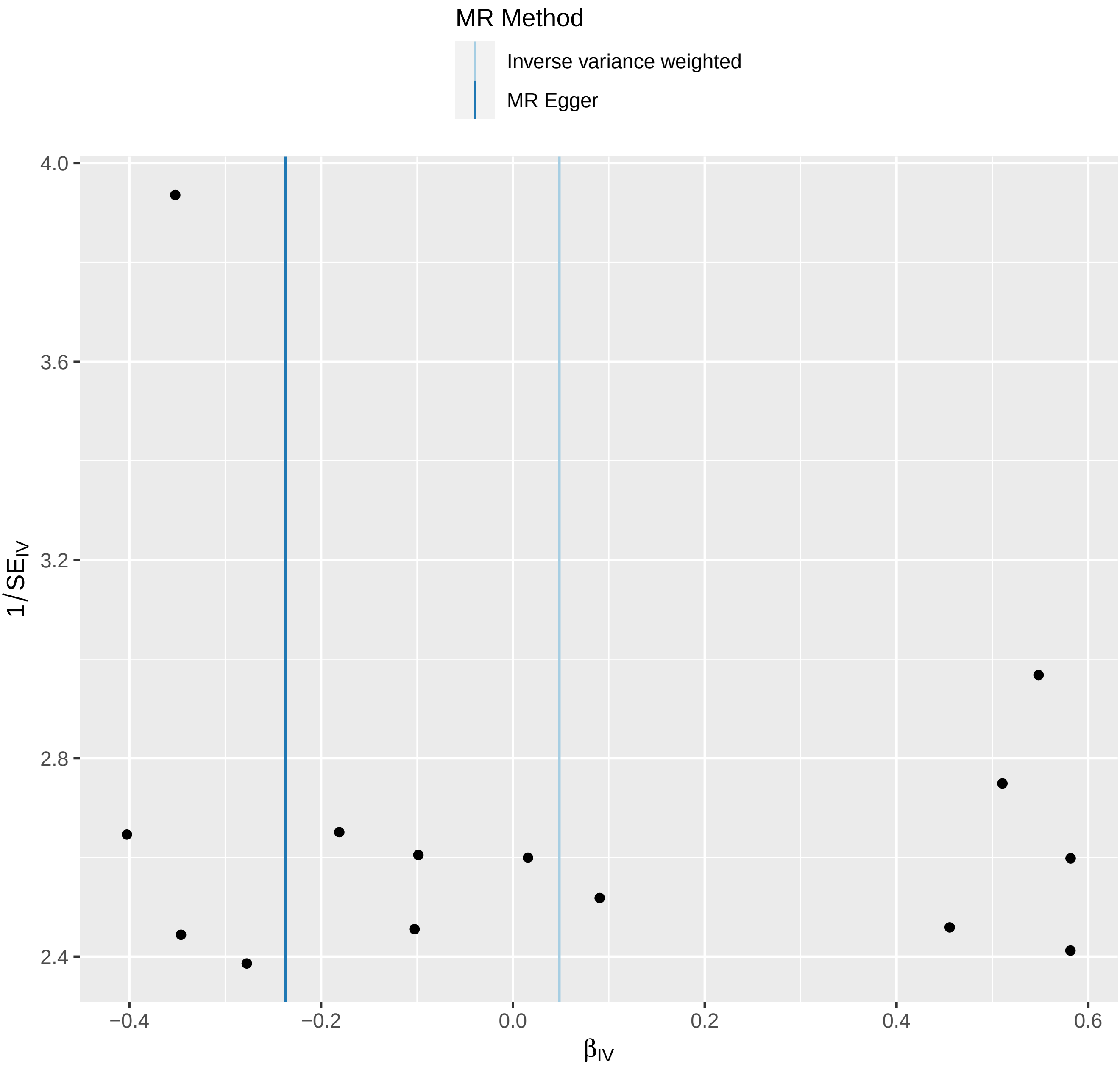

Batch 2−4457 : Gut microbiota abundance (genus Catenibacterium id.2153) on Platelet−derived growth factor receptor alpha with a threshold of 1e−05

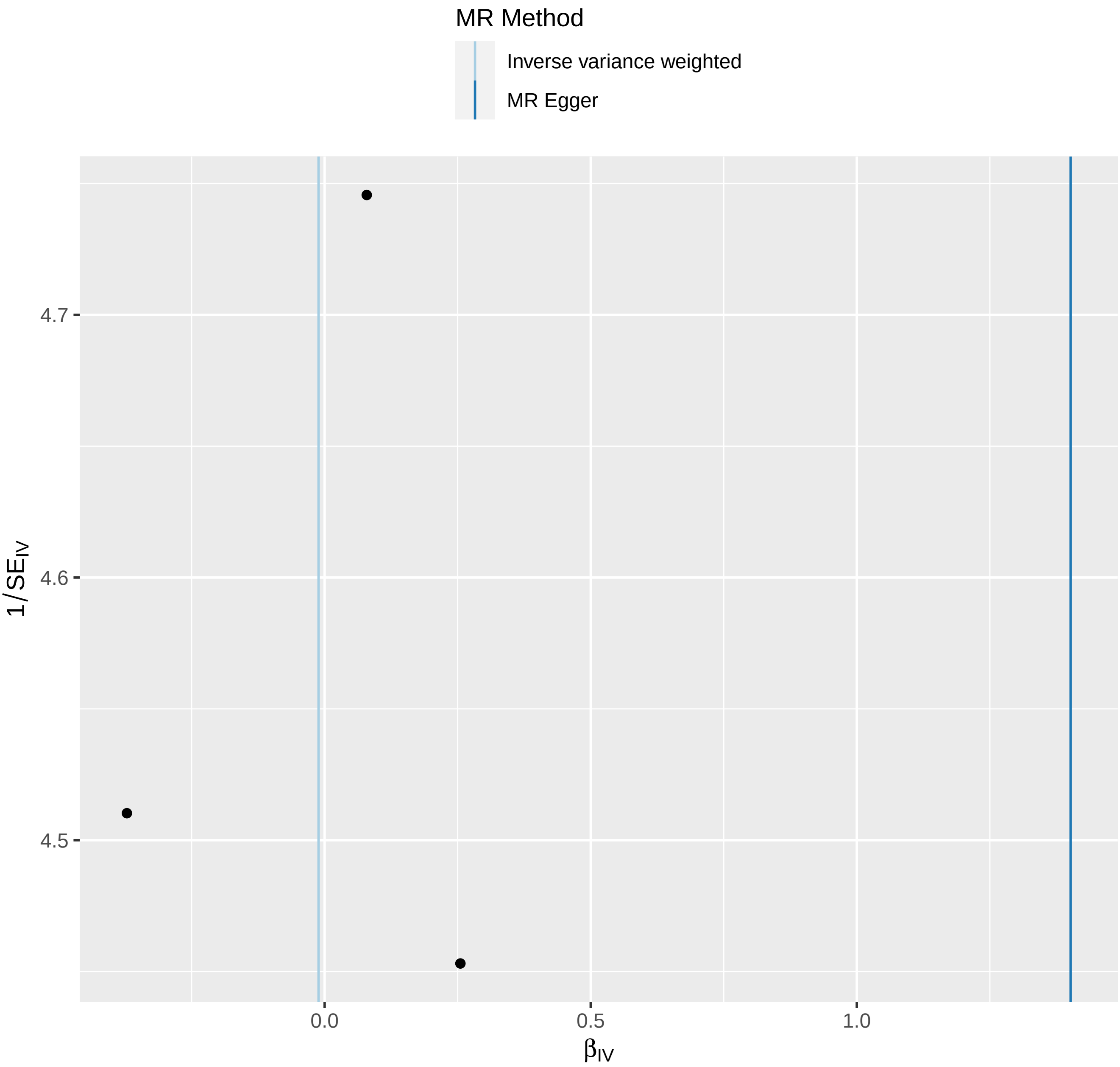

Batch 2−4458 : Gut microbiota abundance (genus Parasutterella id.2892) on Platelet−derived growth factor receptor alpha
with a threshold of 1e−05

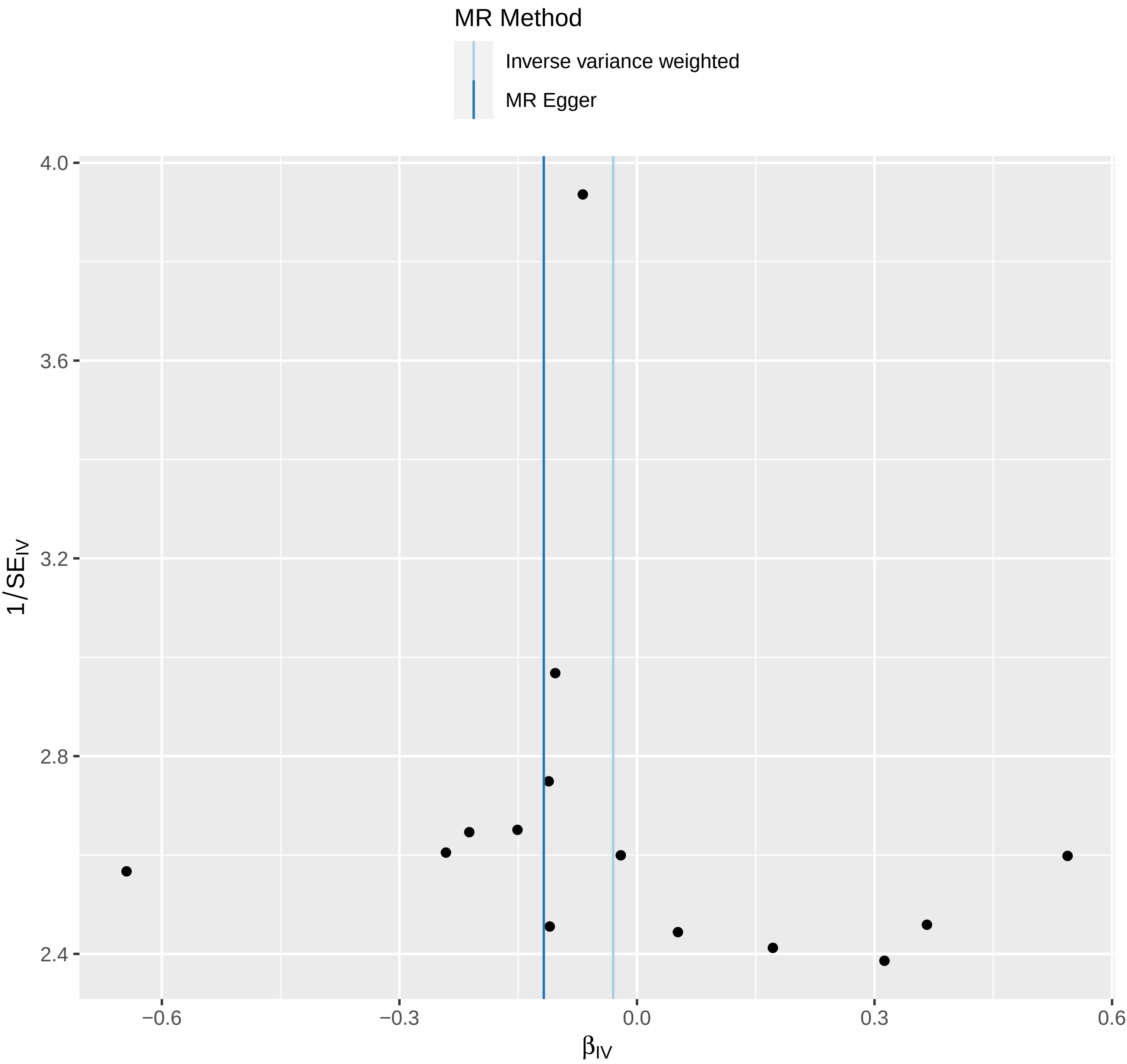

Batch 2−4459 : Gut microbiota abundance (genus Catenibacterium id.2153) on Platelet−derived growth factor receptor beta
with a threshold of 1e−05

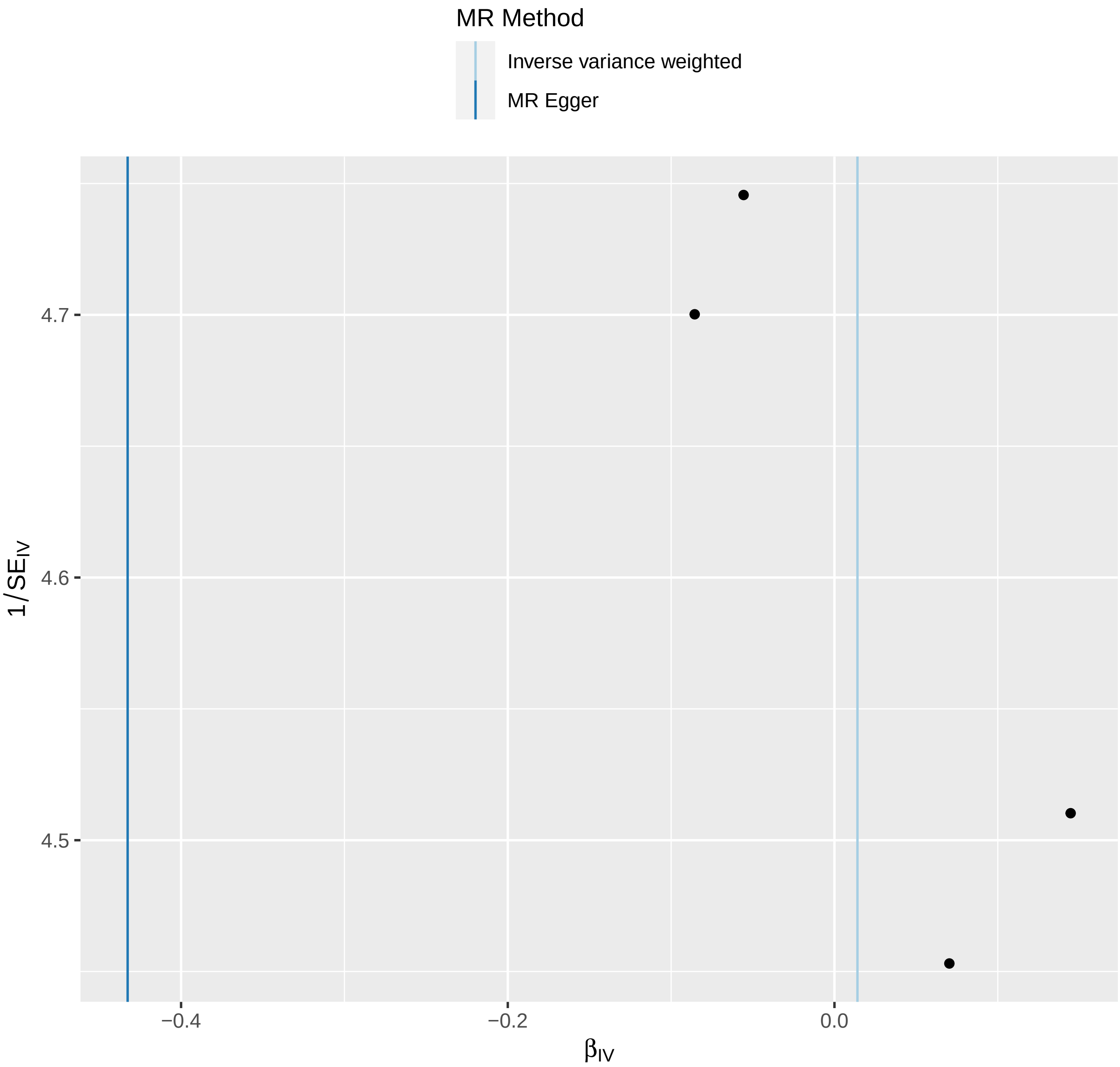

Batch 2−4460 : Gut microbiota abundance (genus Parasutterella id.2892) on Platelet−derived growth factor receptor beta
with a threshold of 1e−05

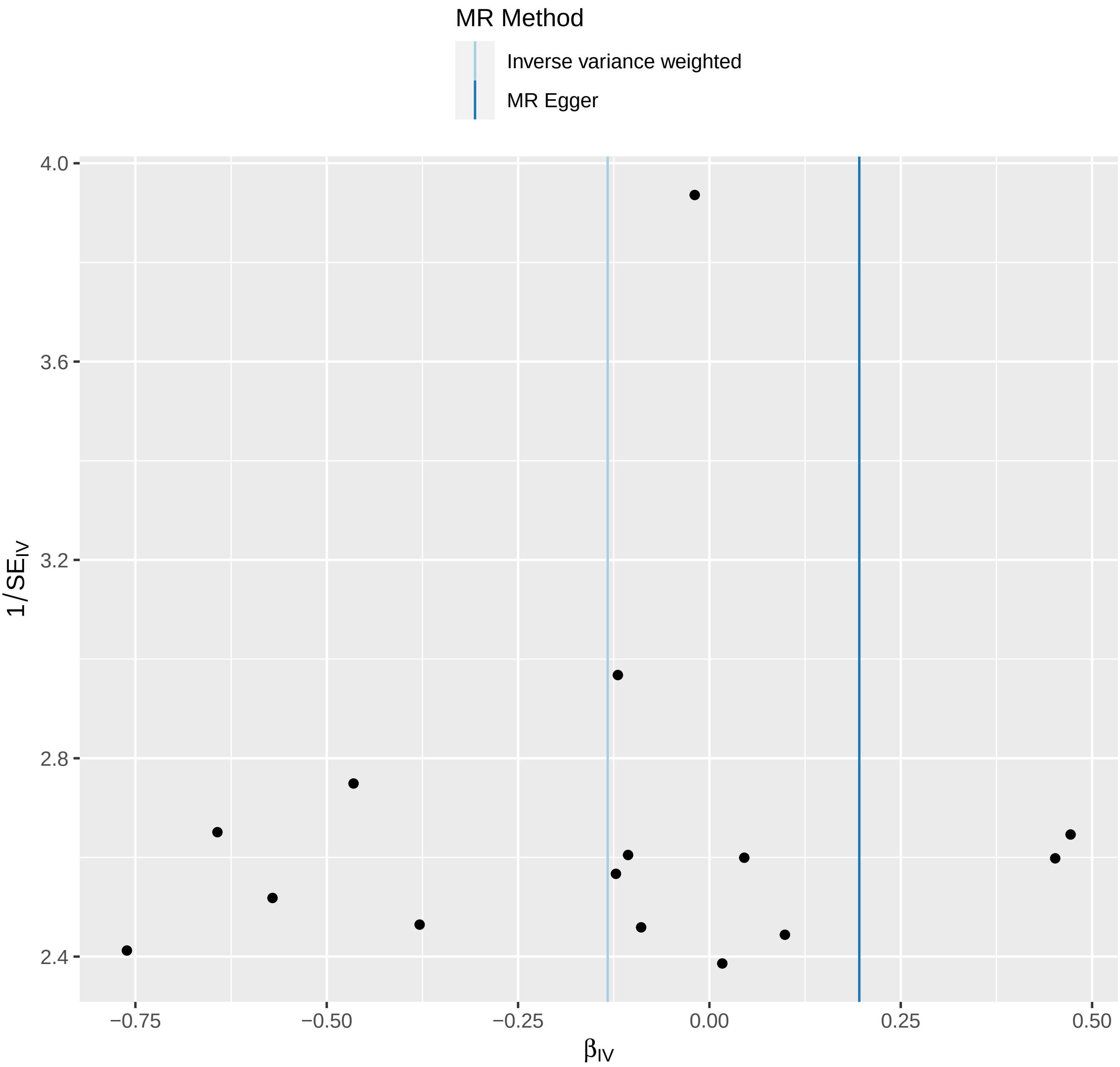

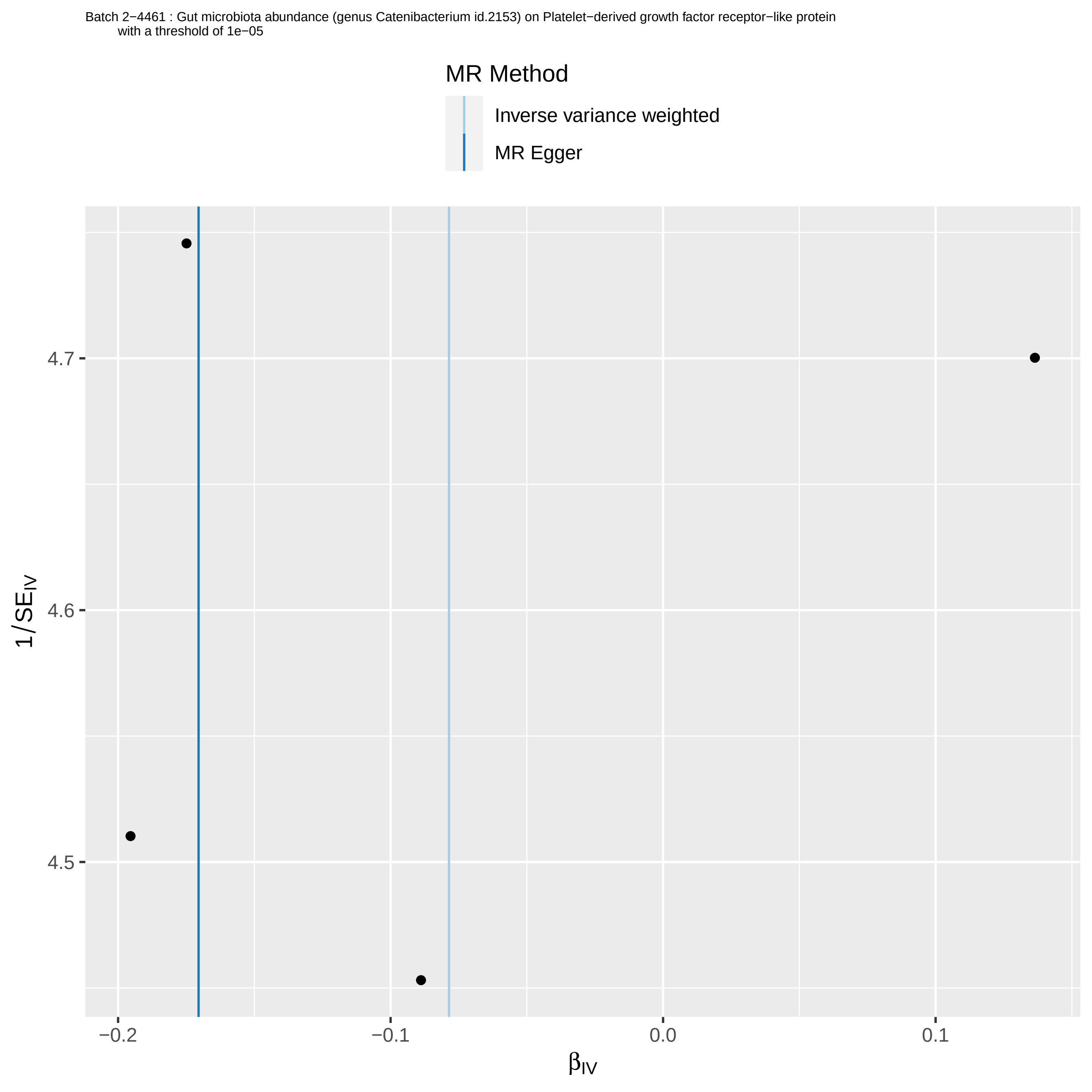
Batch 2−4461 : Gut microbiota abundance (genus Catenibacterium id.2153) on Platelet−derived growth factor receptor−like protein
with a threshold of 1e−05
MR Method
Inverse variance weighted
MR Egger
4.7
4.6
4.5
1/SE_IV
−0.2
−0.1
0.0
0.1
β_IV

Batch 2−4462 : Gut microbiota abundance (genus Parasutterella id.2892) on Platelet−derived growth factor receptor−like protein with a threshold of 1e−05

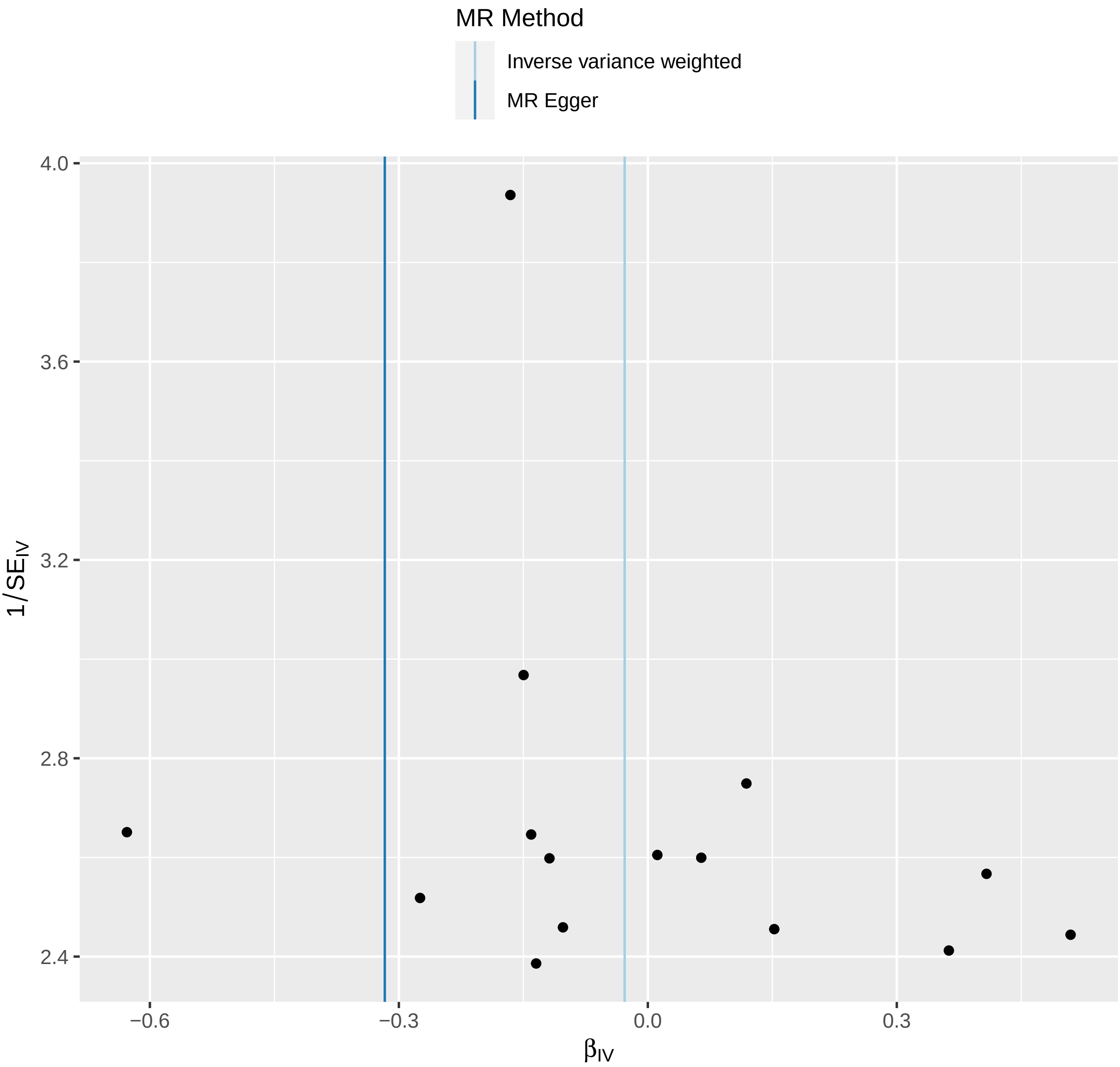

Batch 2−4463 : Gut microbiota abundance (genus Catenibacterium id.2153) on Protein disulfide−isomerase A3
with a threshold of 1e−05

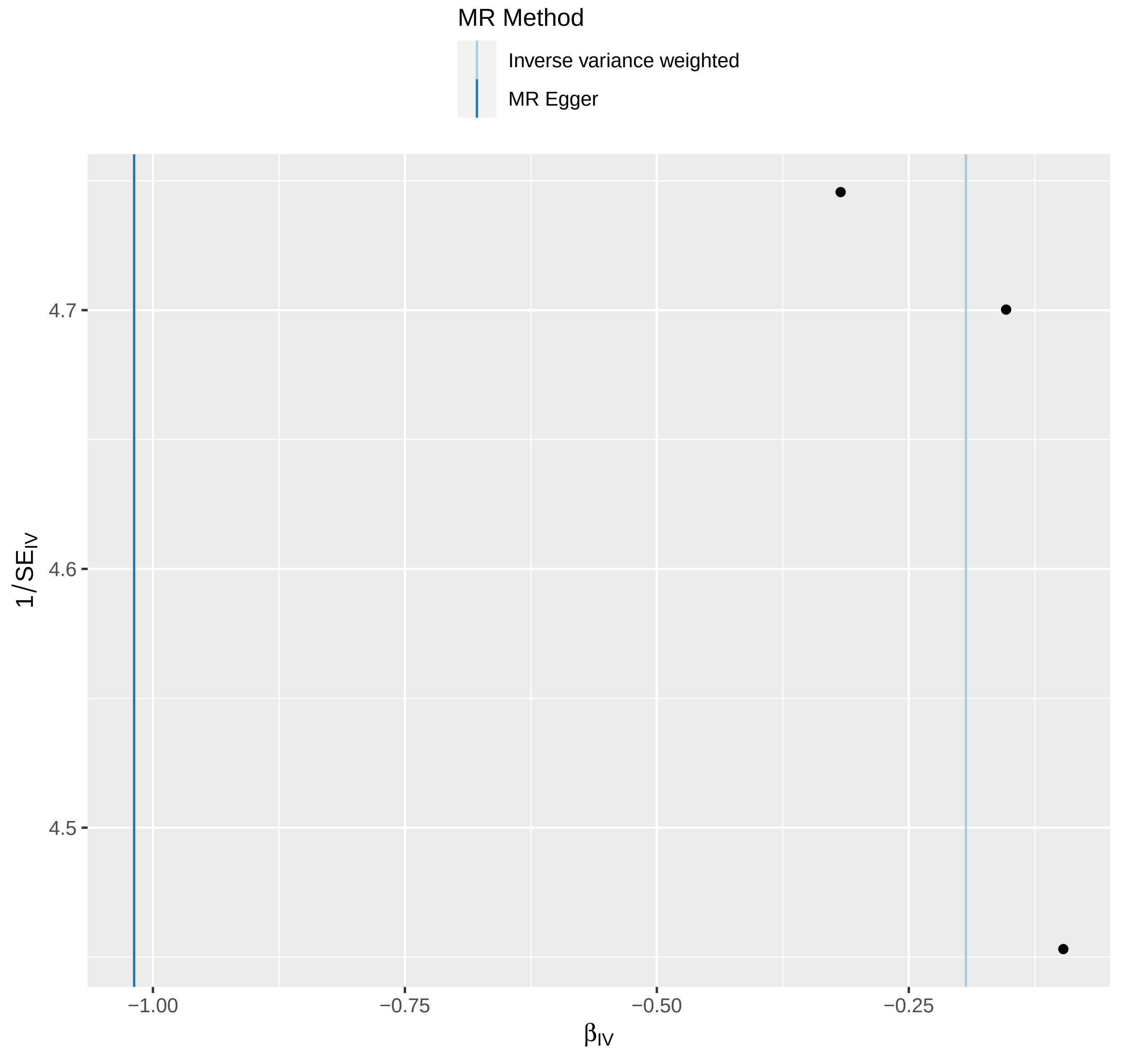

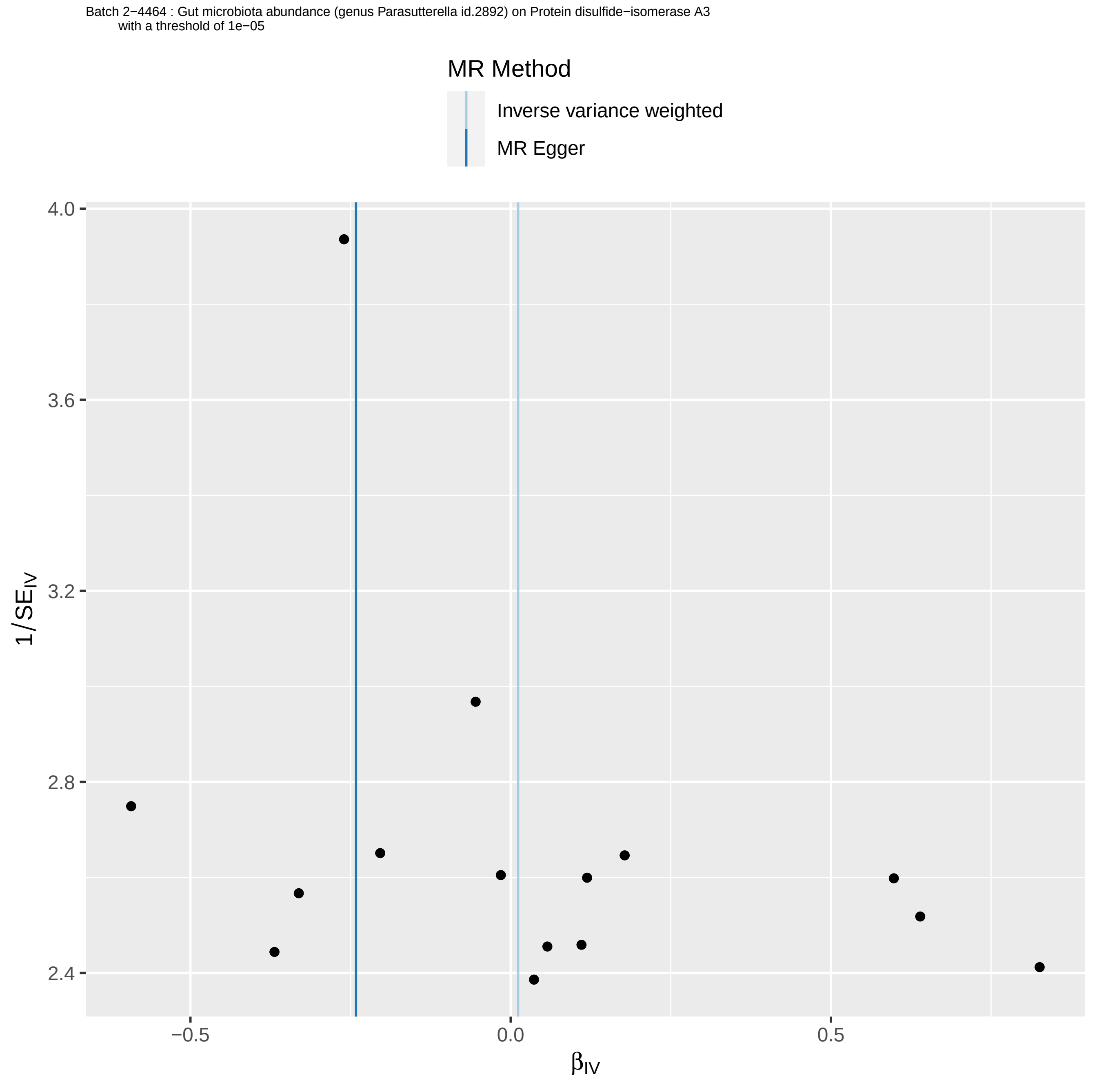

Batch 2−4464 : Gut microbiota abundance (genus Parasutterella id.2892) on Protein disulfide−isomerase A3
with a threshold of 1e−05
MR Method
Inverse variance weighted
MR Egger
4.0
3.6
3.2
2.8
2.4
1/SE$_{IV}$
−0.5
0.0
0.5
β_{IV}

Batch 2−4465 : Gut microbiota abundance (genus Catenibacterium id.2153) on Protein disulfide−isomerase A5
with a threshold of 1e−05

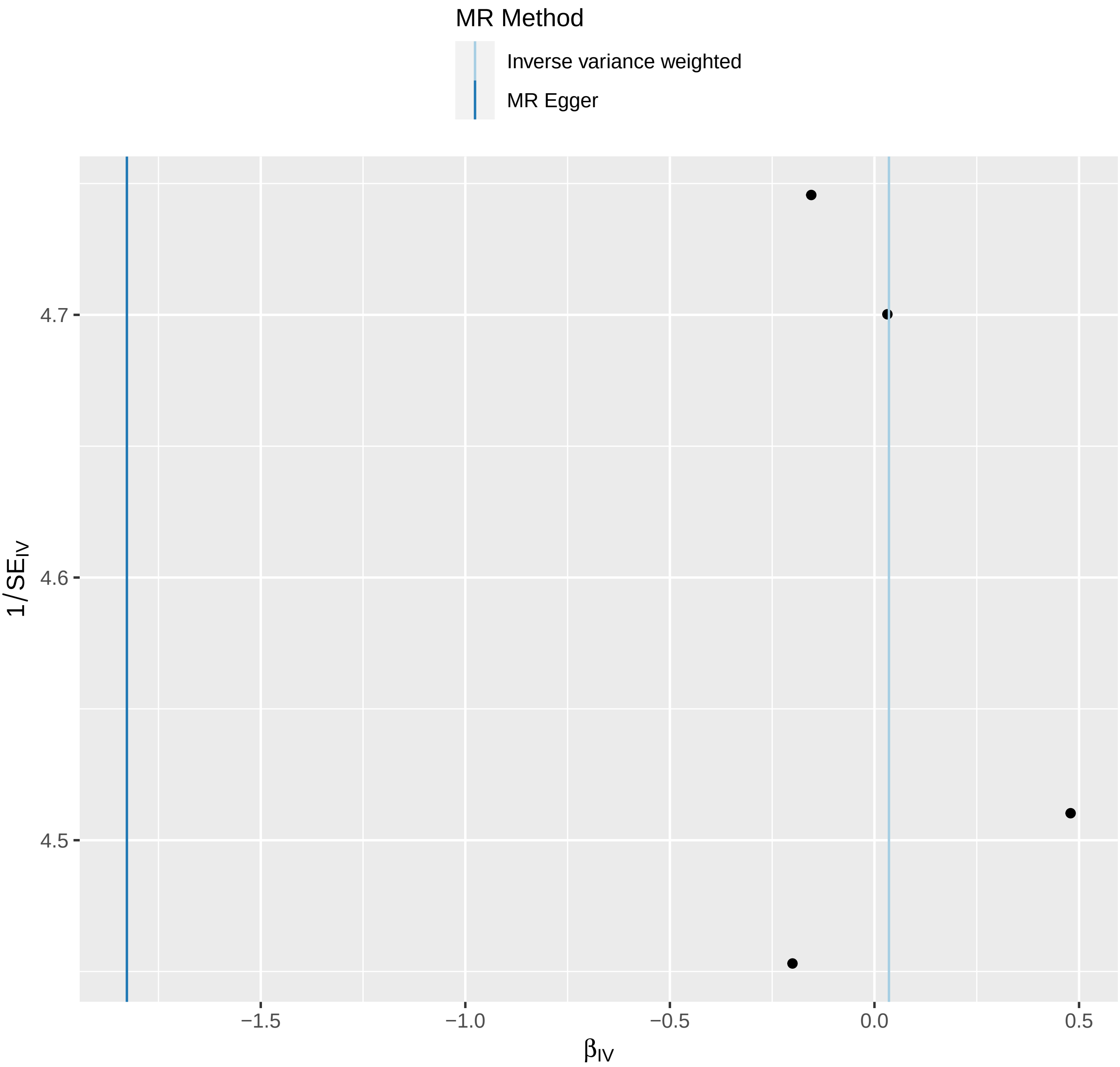

Batch 2−4466 : Gut microbiota abundance (genus Parasutterella id.2892) on Protein disulfide−isomerase A5
with a threshold of 1e−05

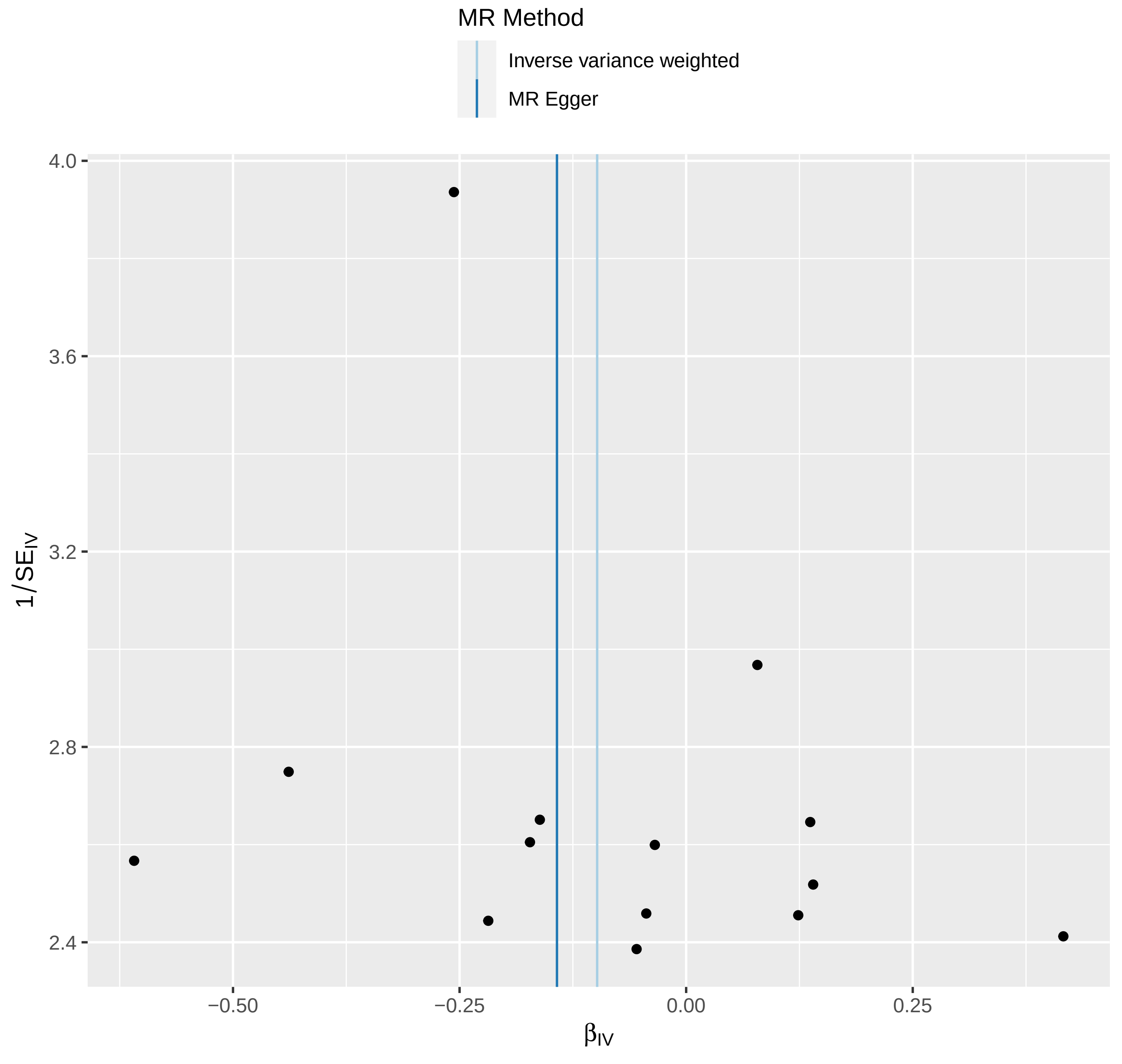

Batch 2−4467 : Gut microbiota abundance (genus Catenibacterium id.2153) on Protein disulfide−isomerase−like protein of the testis
with a threshold of 1e−05

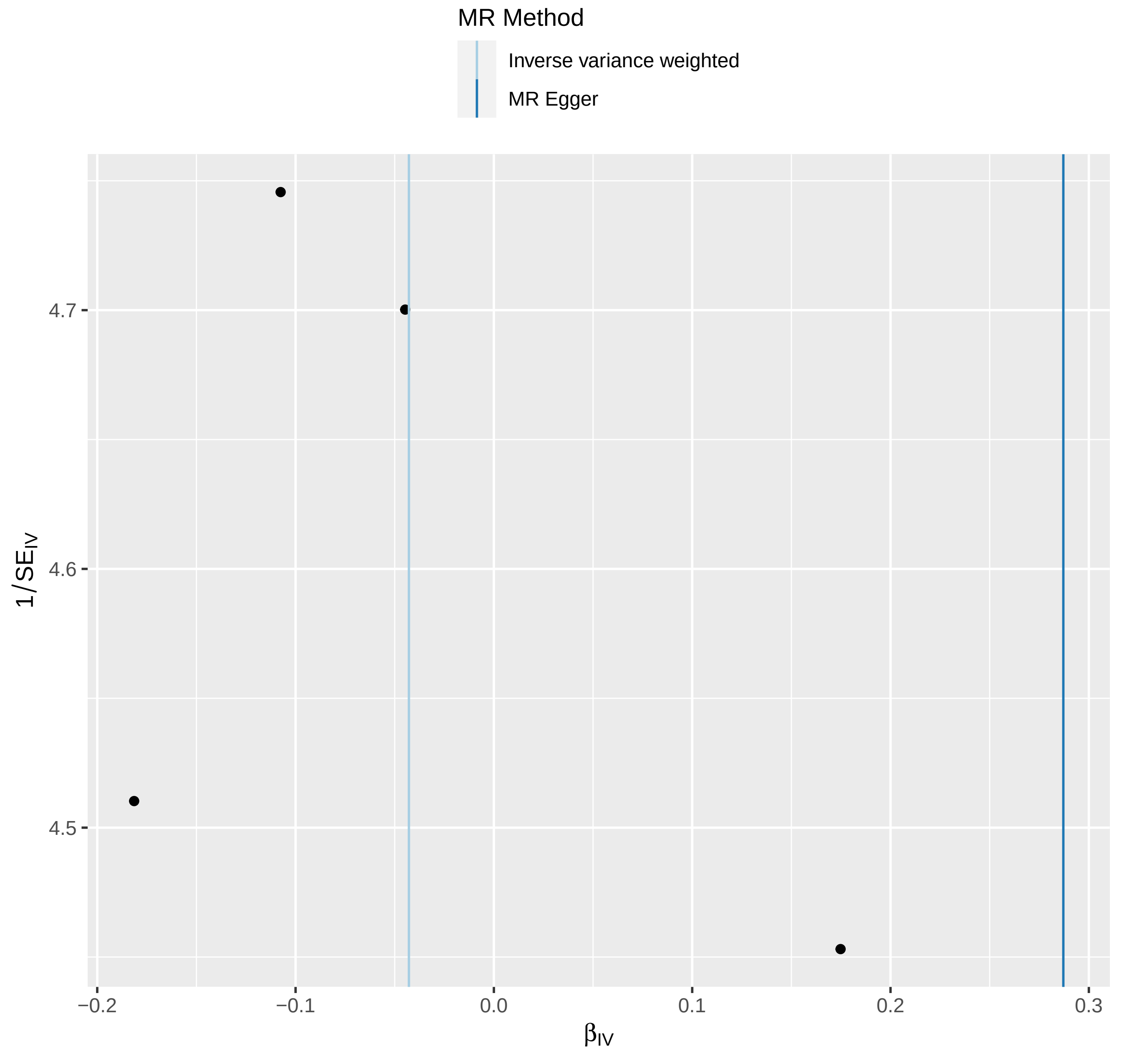

Batch 2−4468 : Gut microbiota abundance (genus Parasutterella id.2892) on Protein disulfide−isomerase−like protein of the testis
with a threshold of 1e−05

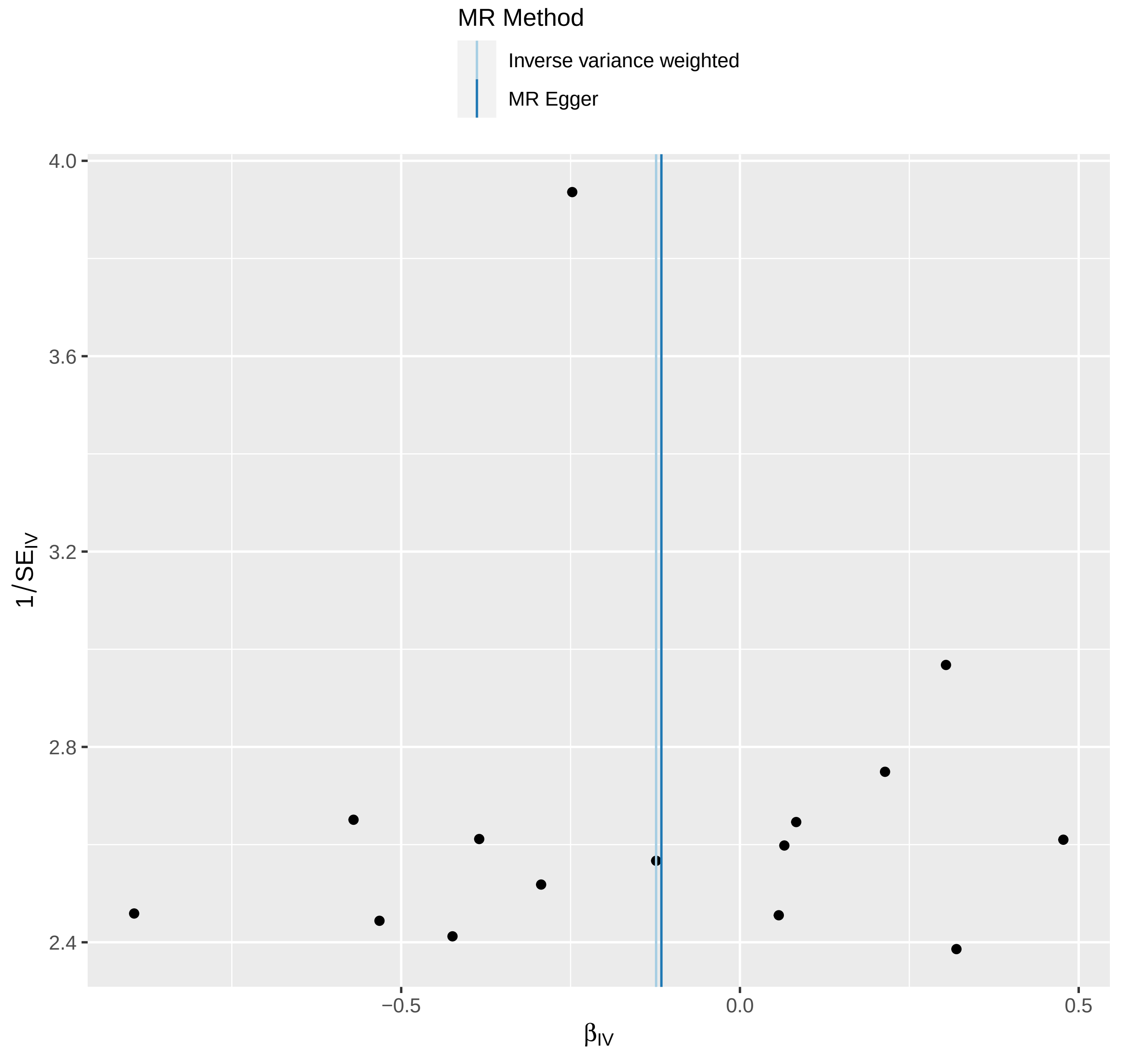

Batch 2−4469 : Gut microbiota abundance (genus Catenibacterium id.2153) on [Pyruvate dehydrogenase (acetyl−transferring)] kinase isozyme 1, mitochondrial
with a threshold of 1e−05

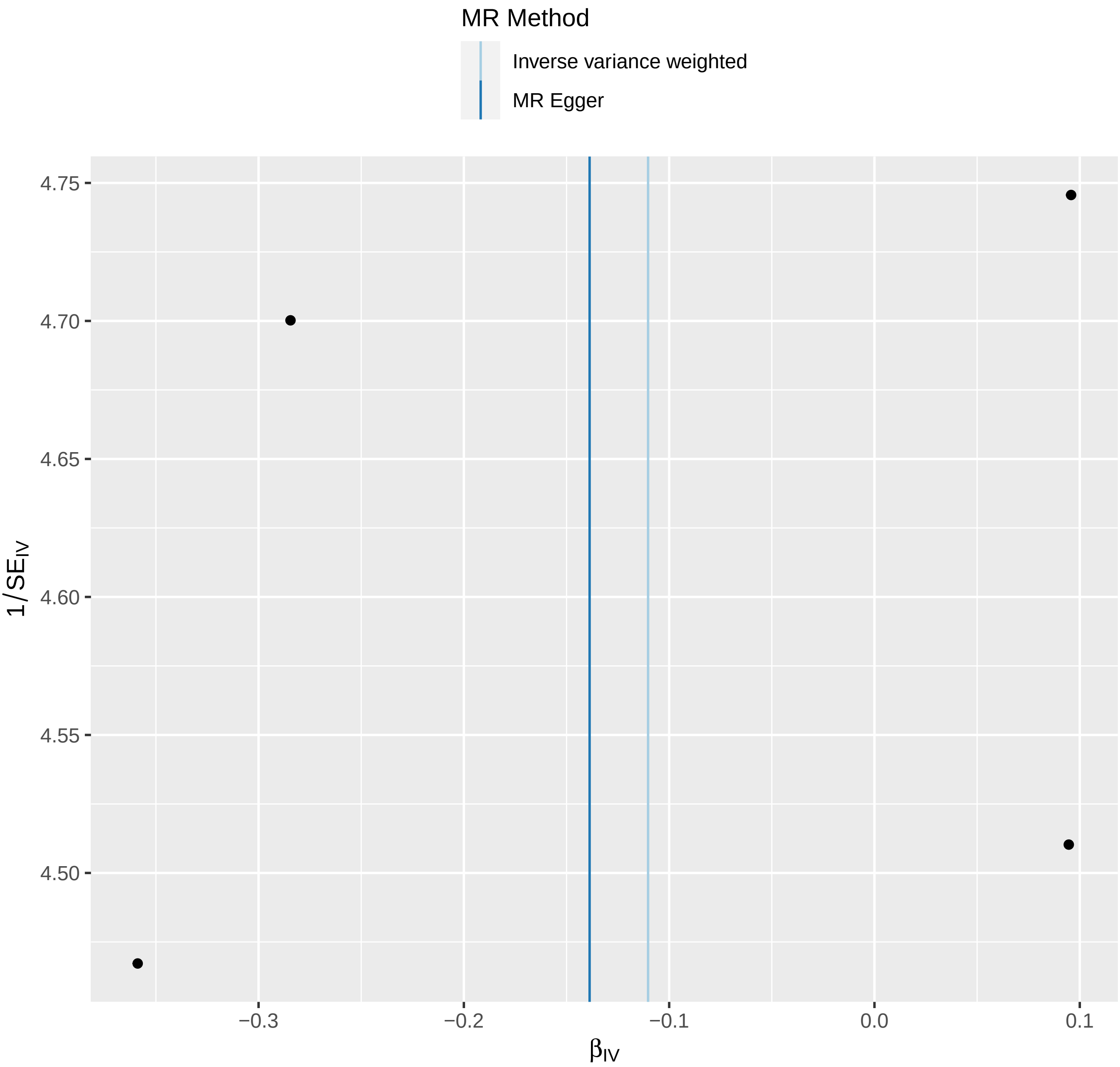

Batch 2−4470 : Gut microbiota abundance (genus Parasutterella id.2892) on [Pyruvate dehydrogenase (acetyl−transferring)] kinase isozyme 1, mitochondrial
with a threshold of 1e−05

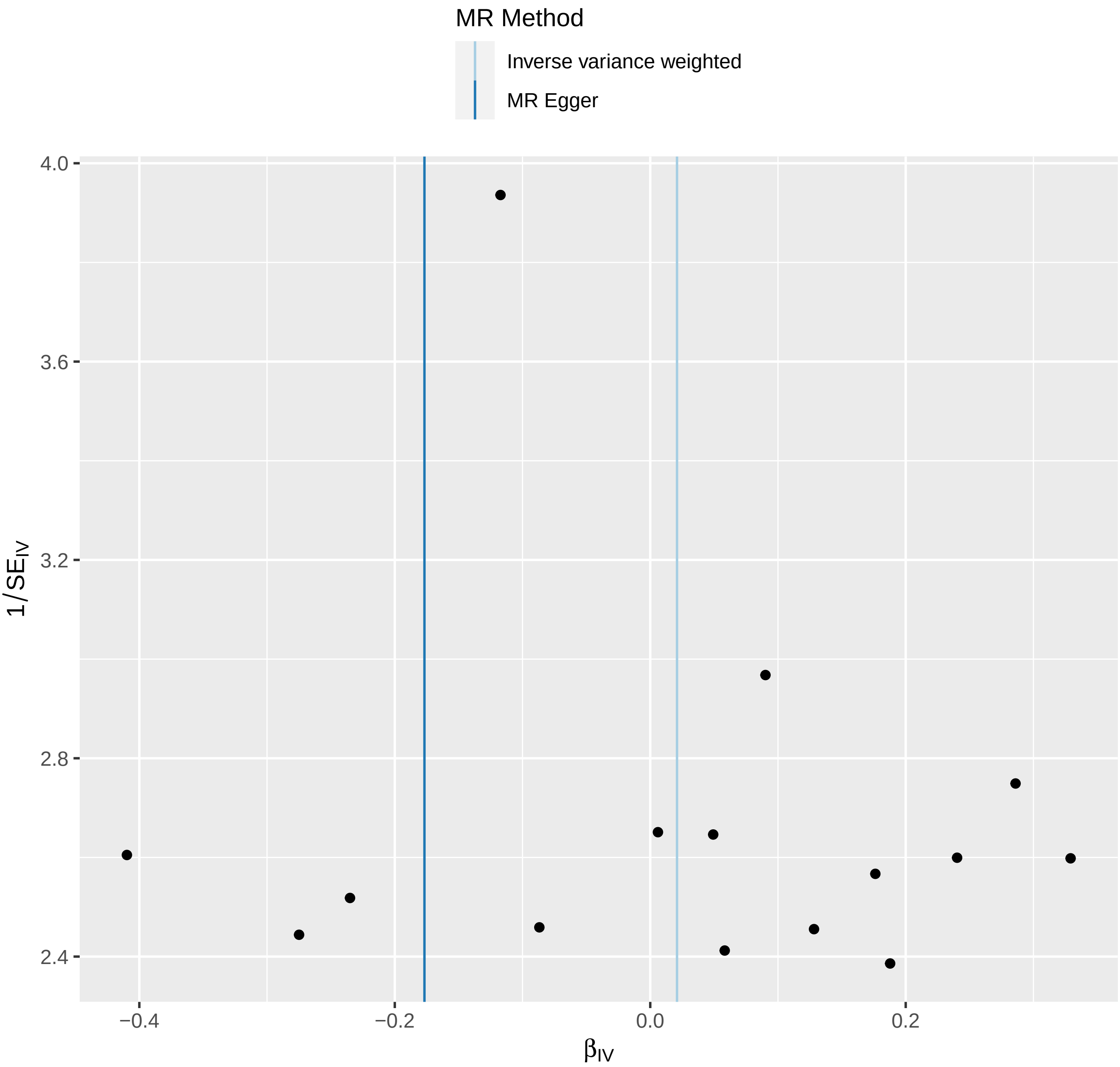

Batch 2−4471 : Gut microbiota abundance (genus Catenibacterium id.2153) on [Pyruvate dehydrogenase (acetyl−transferring)] kinase isozyme 2, mitochondrial with a threshold of 1e−05

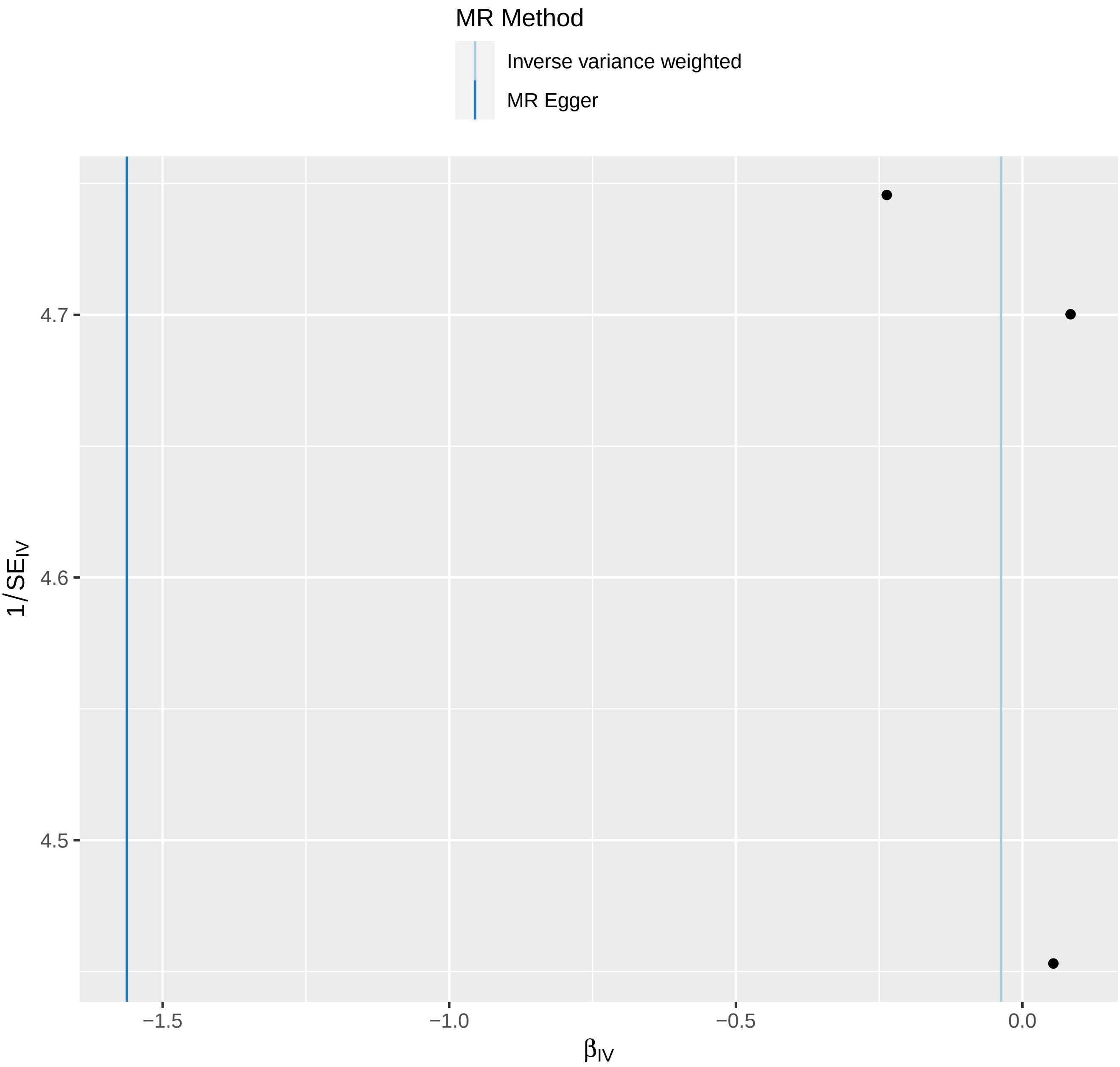

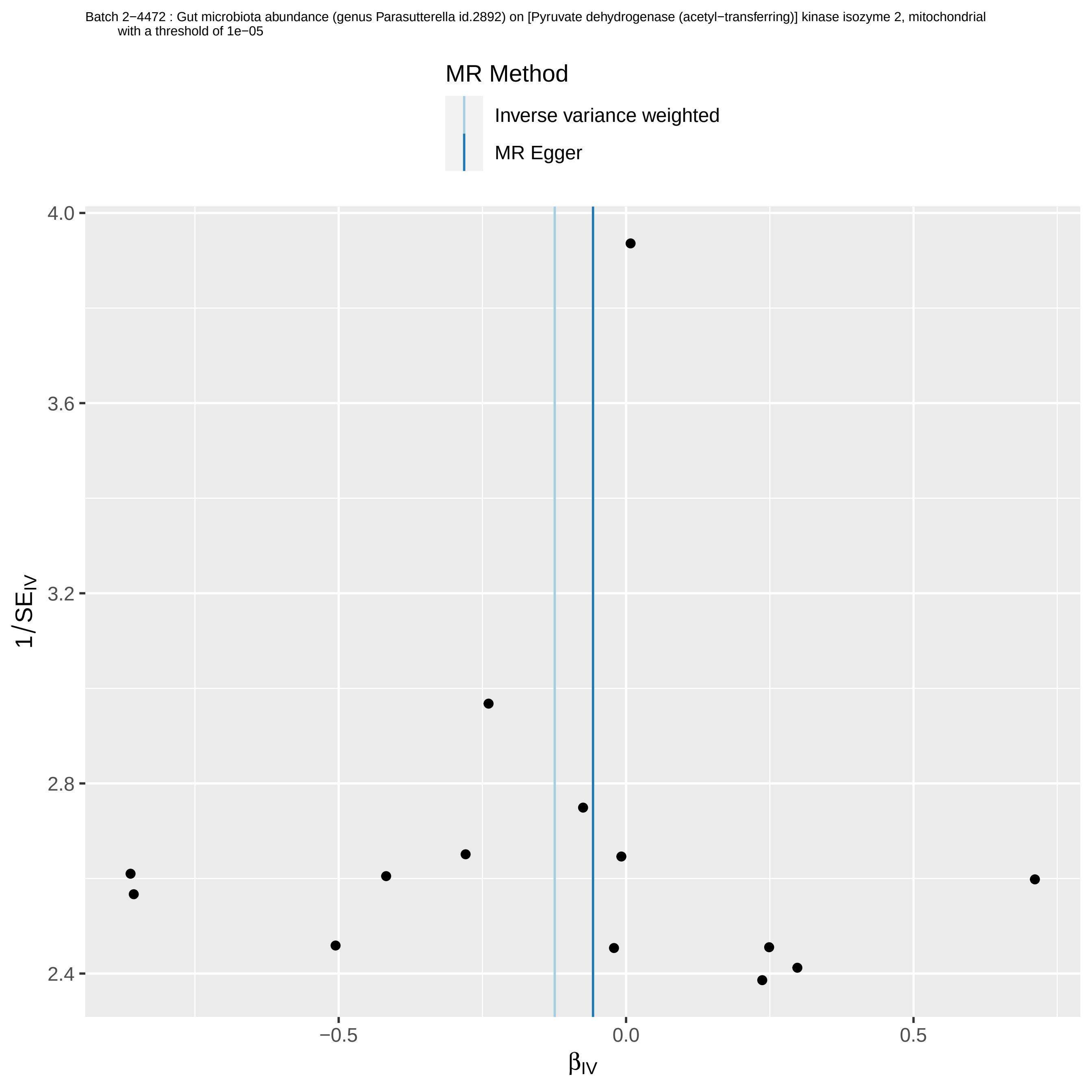
Batch 2−4472 : Gut microbiota abundance (genus Parasutterella id.2892) on [Pyruvate dehydrogenase (acetyl−transferring)] kinase isozyme 2, mitochondrial
with a threshold of 1e−05
MR Method
Inverse variance weighted
MR Egger
4.0
3.6
3.2
2.8
2.4
1/SE_IV
−0.5
0.0
0.5
β_IV

Batch 2−4473 : Gut microbiota abundance (genus Catenibacterium id.2153) on PDZ and LIM domain protein 4 with a threshold of 1e−05

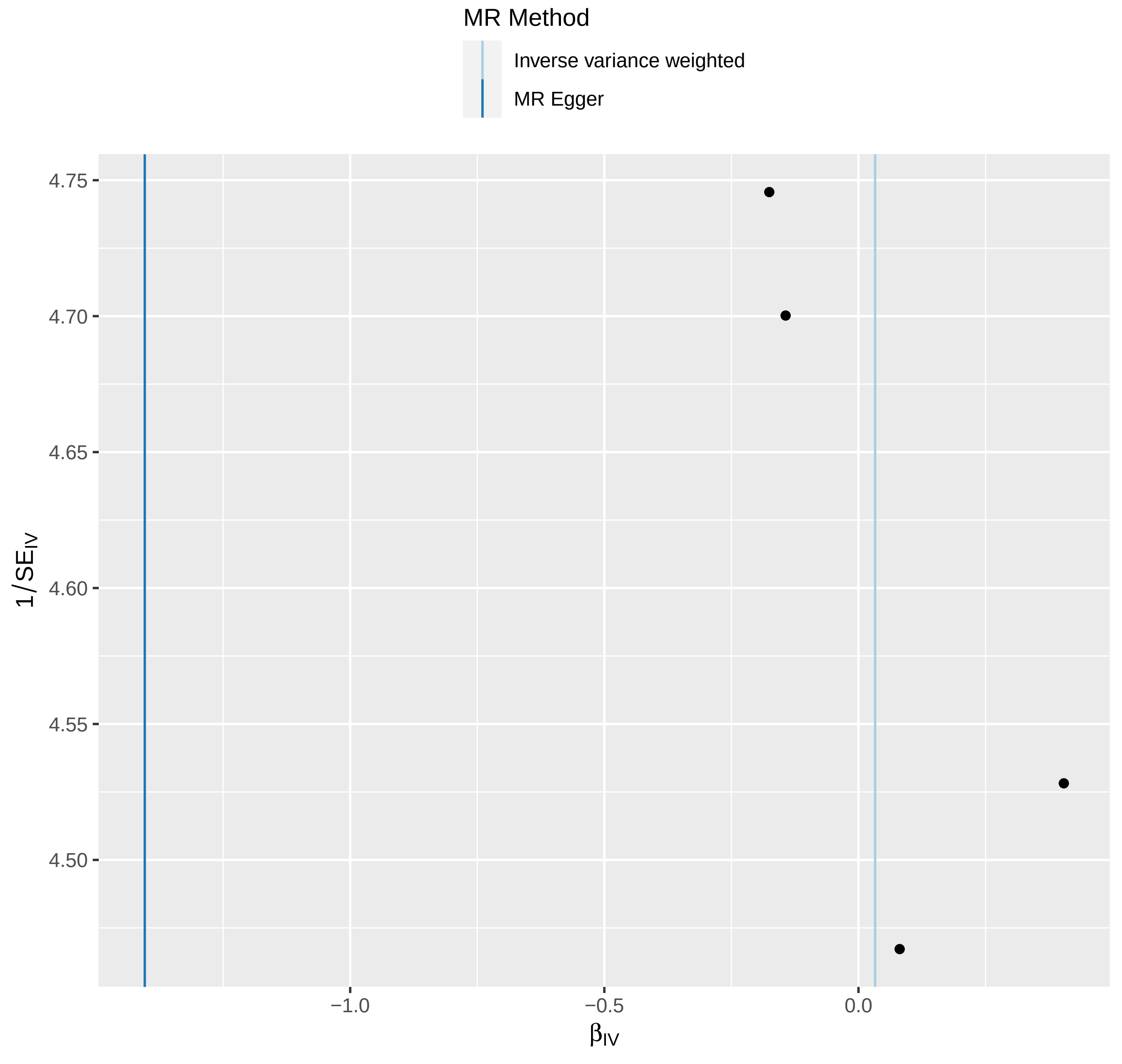

Batch 2−4474 : Gut microbiota abundance (genus Parasutterella id.2892) on PDZ and LIM domain protein 4
with a threshold of 1e−05

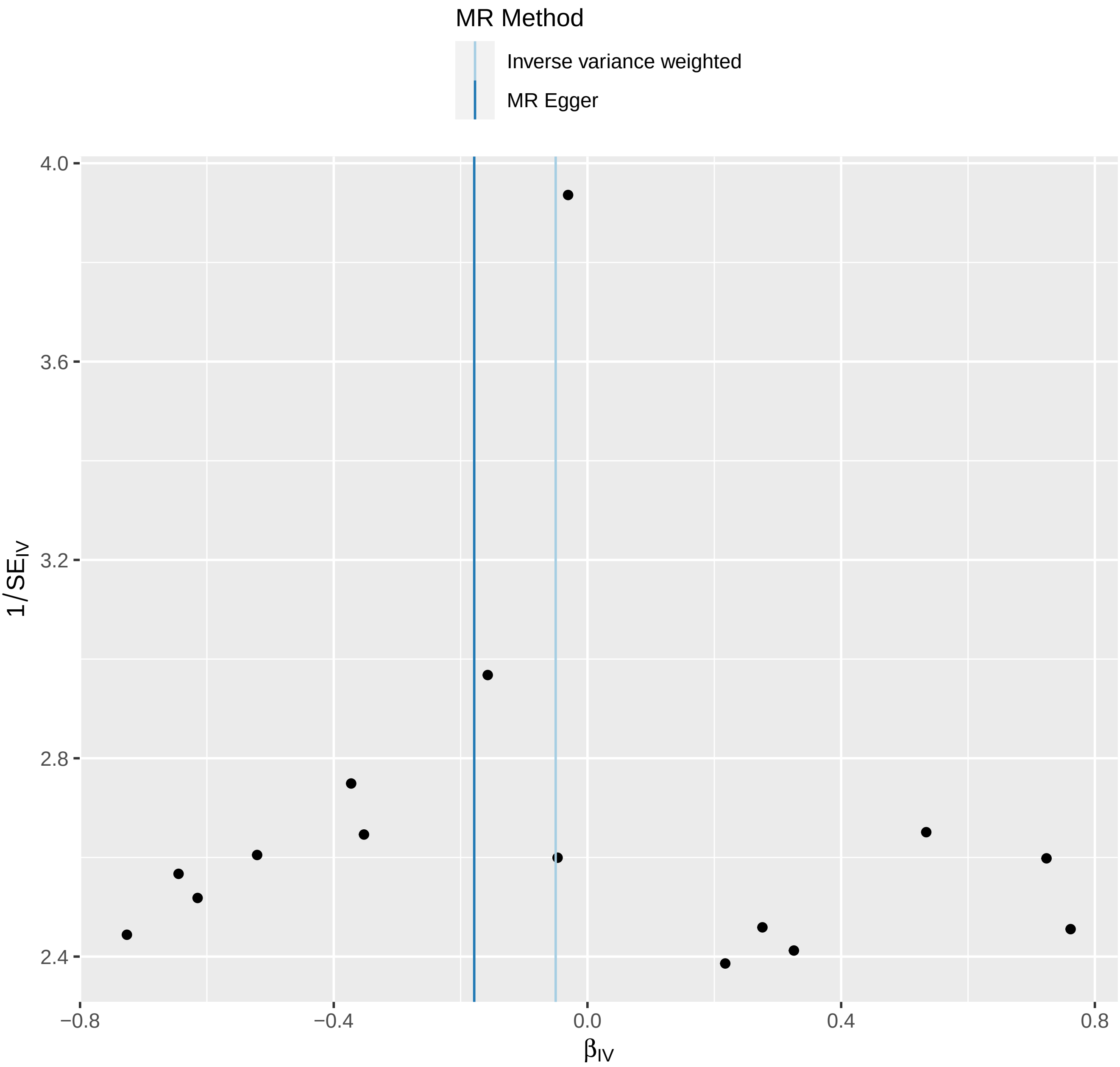

Batch 2−4475 : Gut microbiota abundance (genus Catenibacterium id.2153) on p53 and DNA damage−regulated protein 1
with a threshold of 1e−05

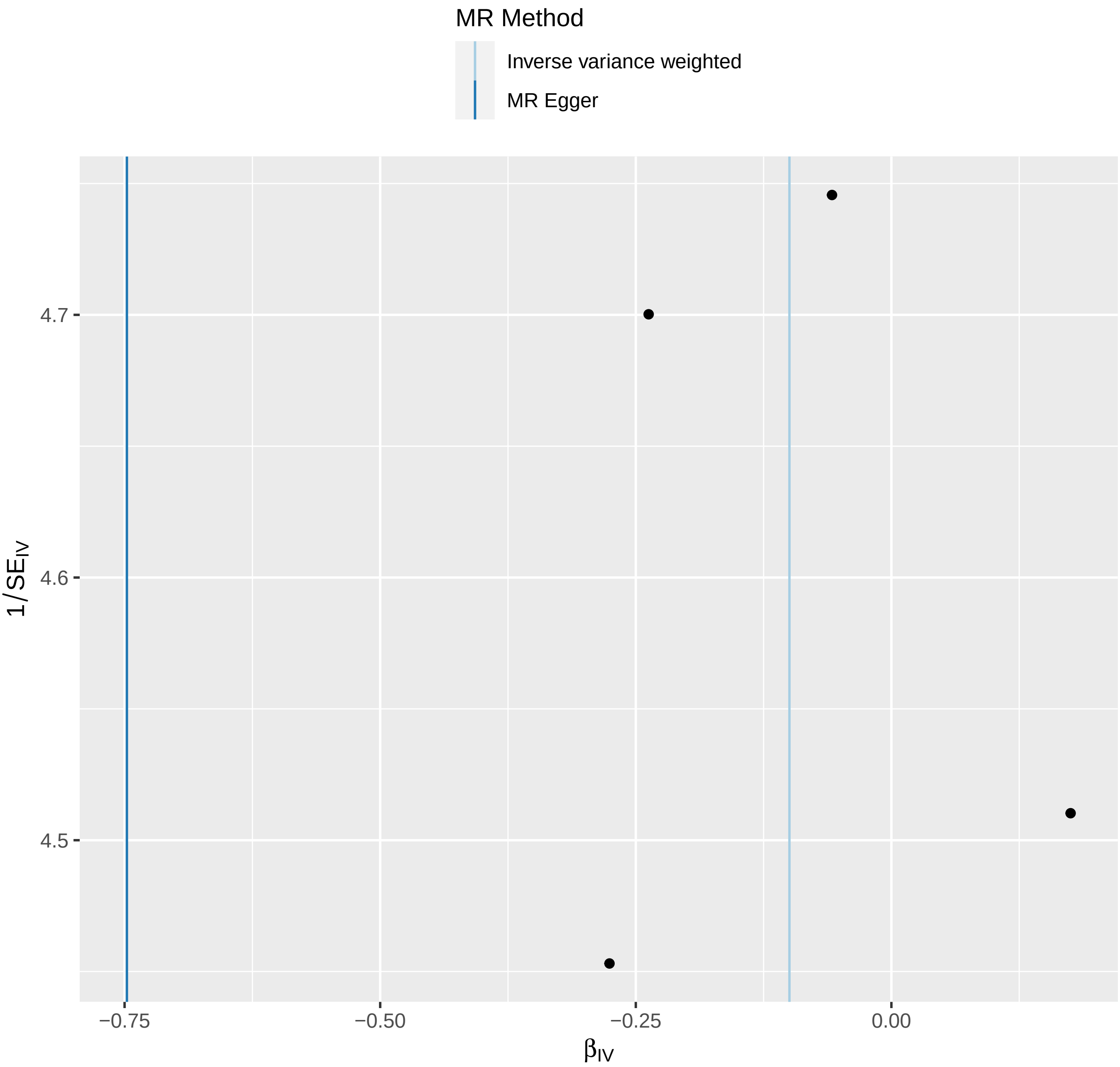

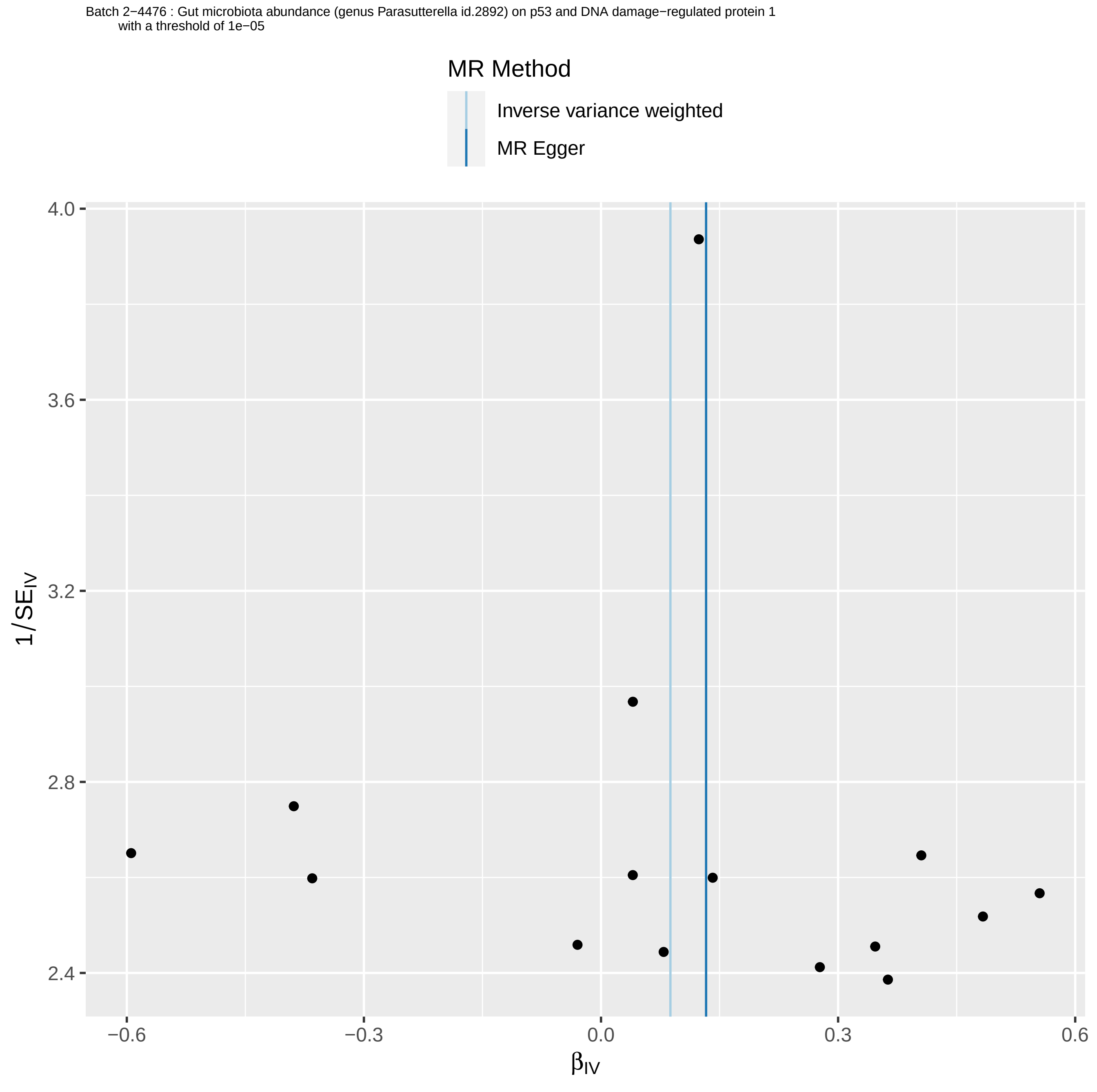
Batch 2−4476 : Gut microbiota abundance (genus Parasutterella id.2892) on p53 and DNA damage−regulated protein 1
with a threshold of 1e−05
MR Method
Inverse variance weighted
MR Egger
4.0
3.6
3.2
2.8
2.4
1/SE_IV
−0.6
−0.3
0.0
0.3
0.6
β_IV

Batch 2−4477 : Gut microbiota abundance (genus Catenibacterium id.2153) on Pyridoxal kinase
with a threshold of 1e−05

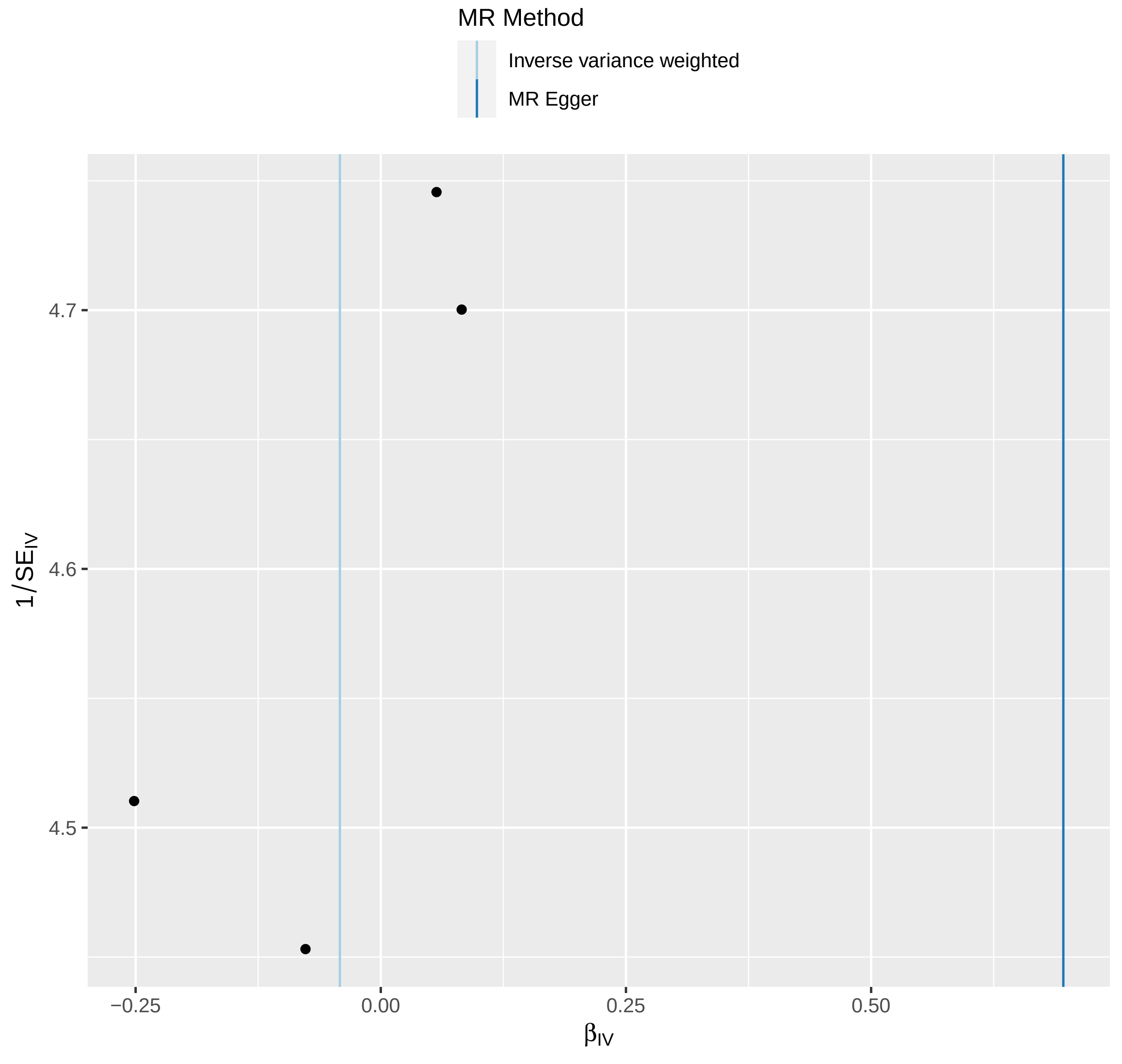

Batch 2−4478 : Gut microbiota abundance (genus Parasutterella id.2892) on Pyridoxal kinase
with a threshold of 1e−05

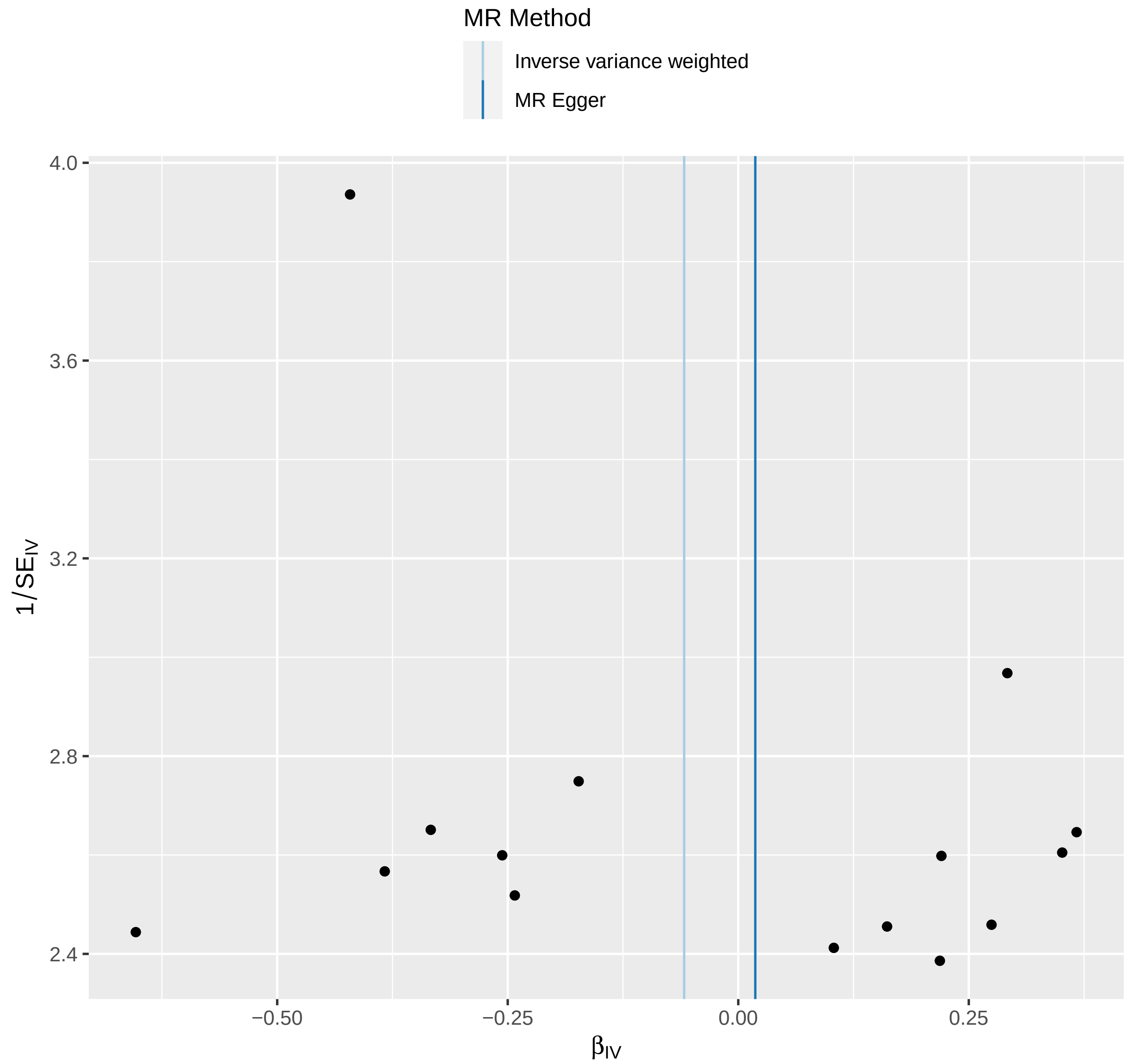

Batch 2−4479 : Gut microbiota abundance (genus Catenibacterium id.2153) on Pyridoxal phosphate phosphatase
with a threshold of 1e−05

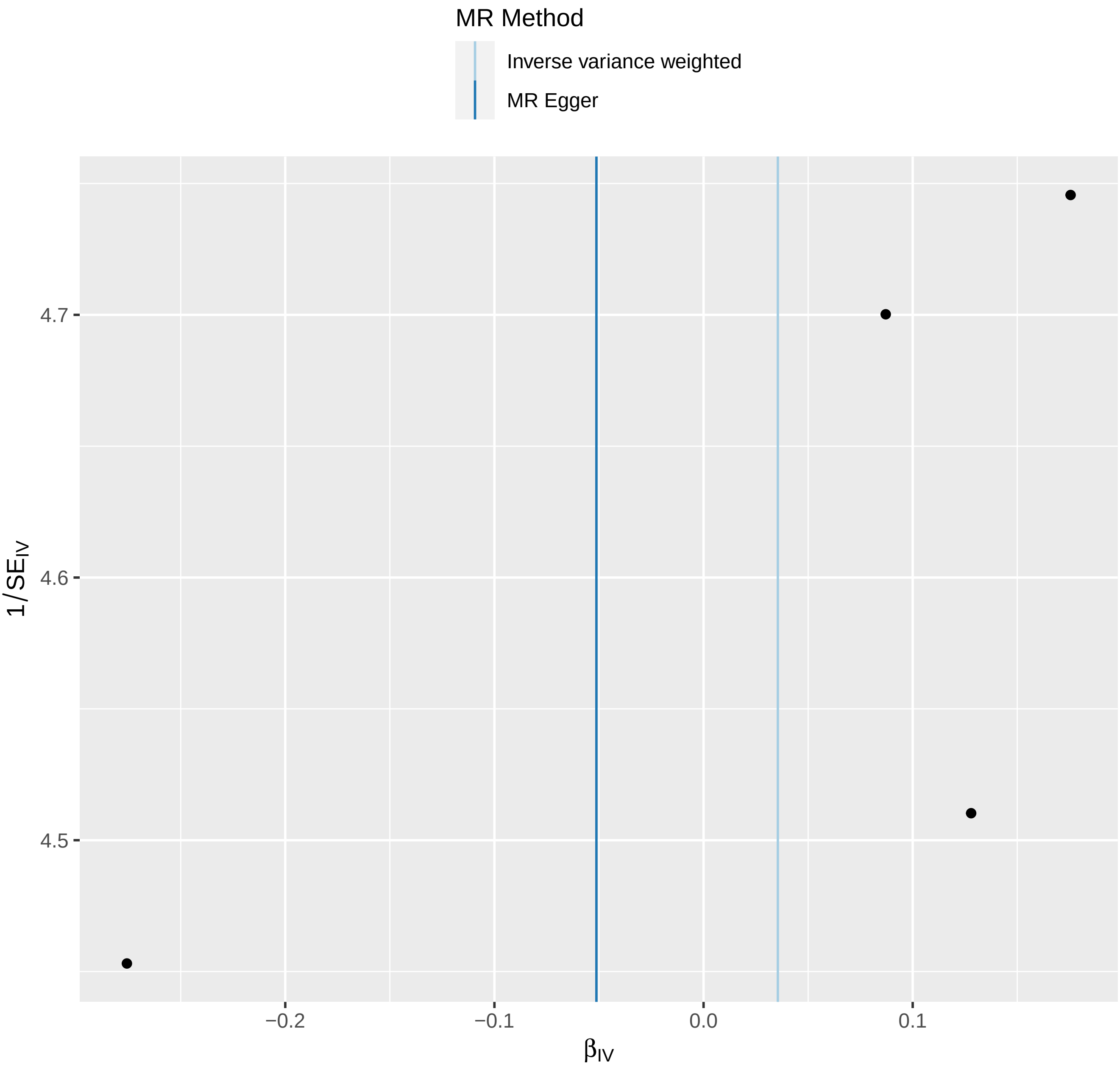

Batch 2−4480 : Gut microbiota abundance (genus Parasutterella id.2892) on Pyridoxal phosphate phosphatase
with a threshold of 1e−05

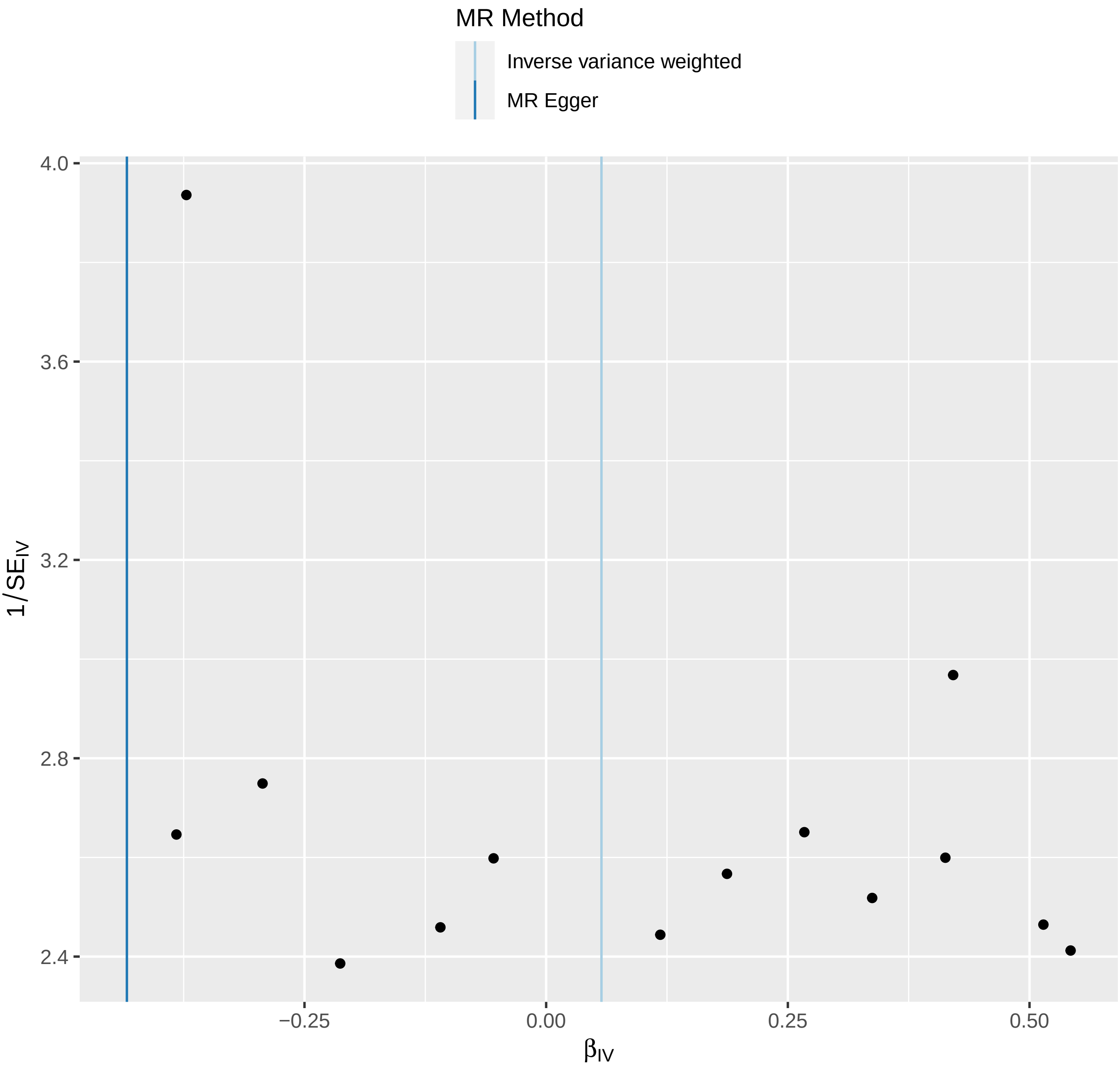

Batch 2−4481 : Gut microbiota abundance (genus Catenibacterium id.2153) on PDZ domain−containing protein 11
with a threshold of 1e−05

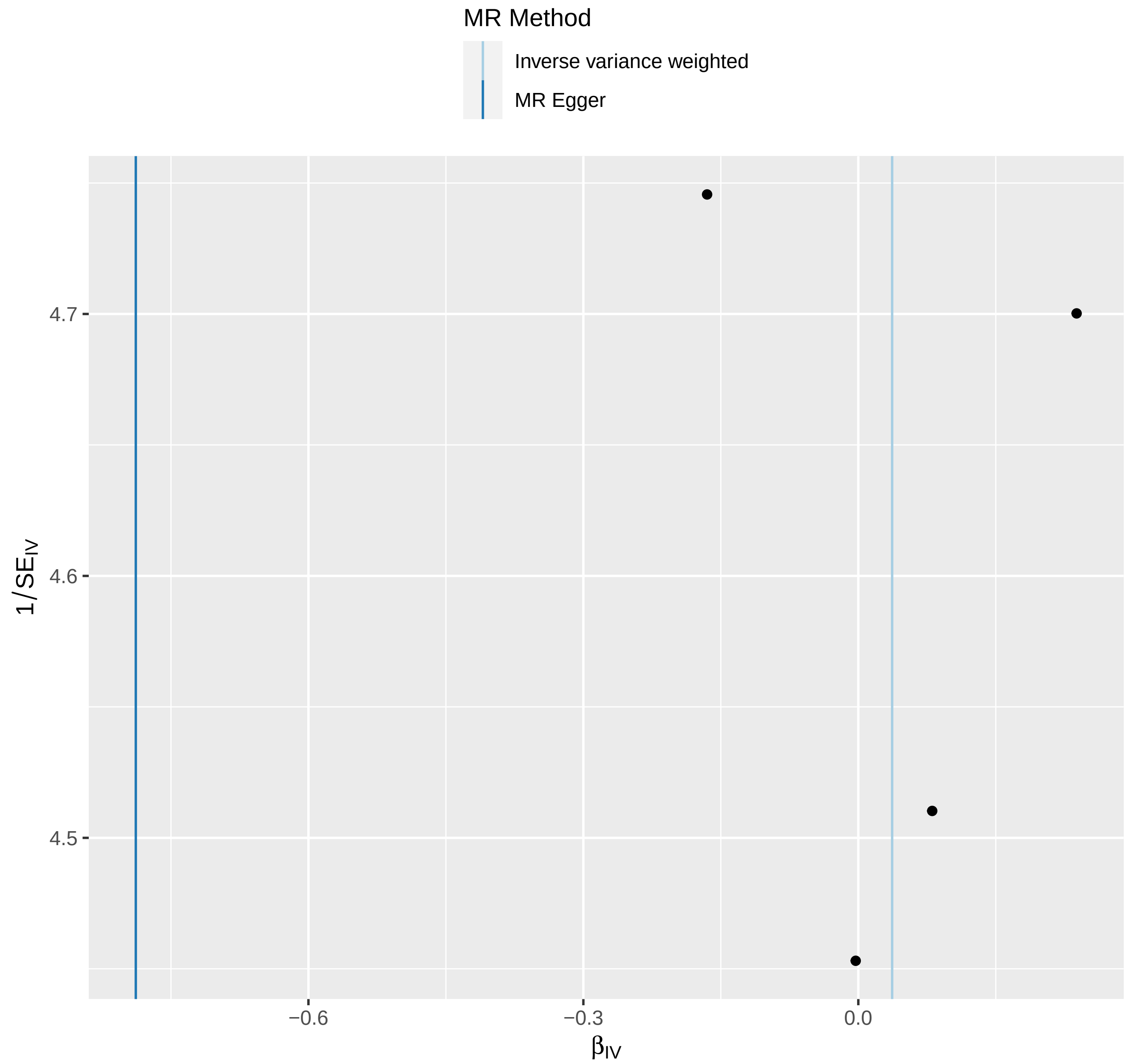

Batch 2−4482 : Gut microbiota abundance (genus Parasutterella id.2892) on PDZ domain−containing protein 11 with a threshold of 1e−05

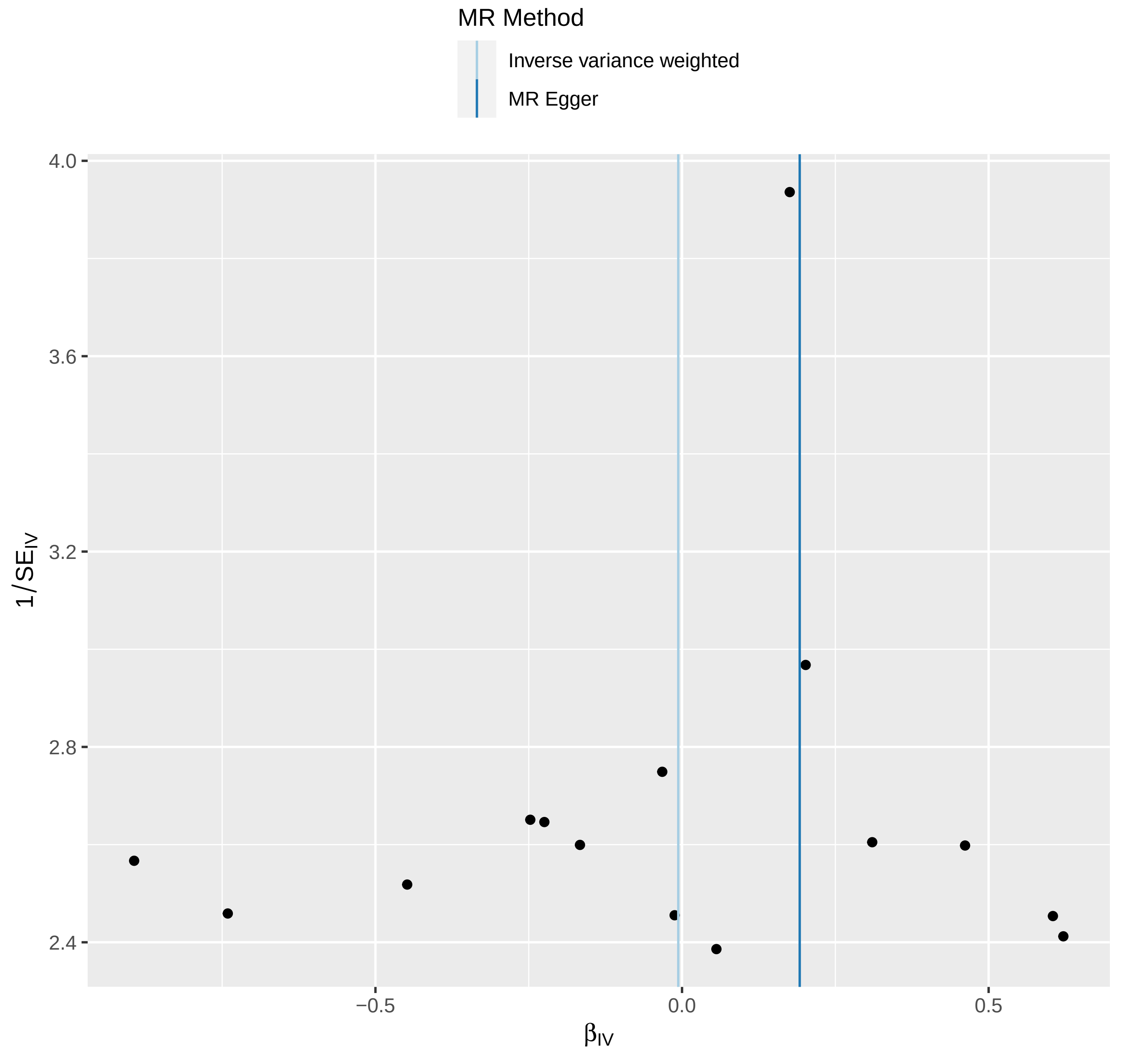

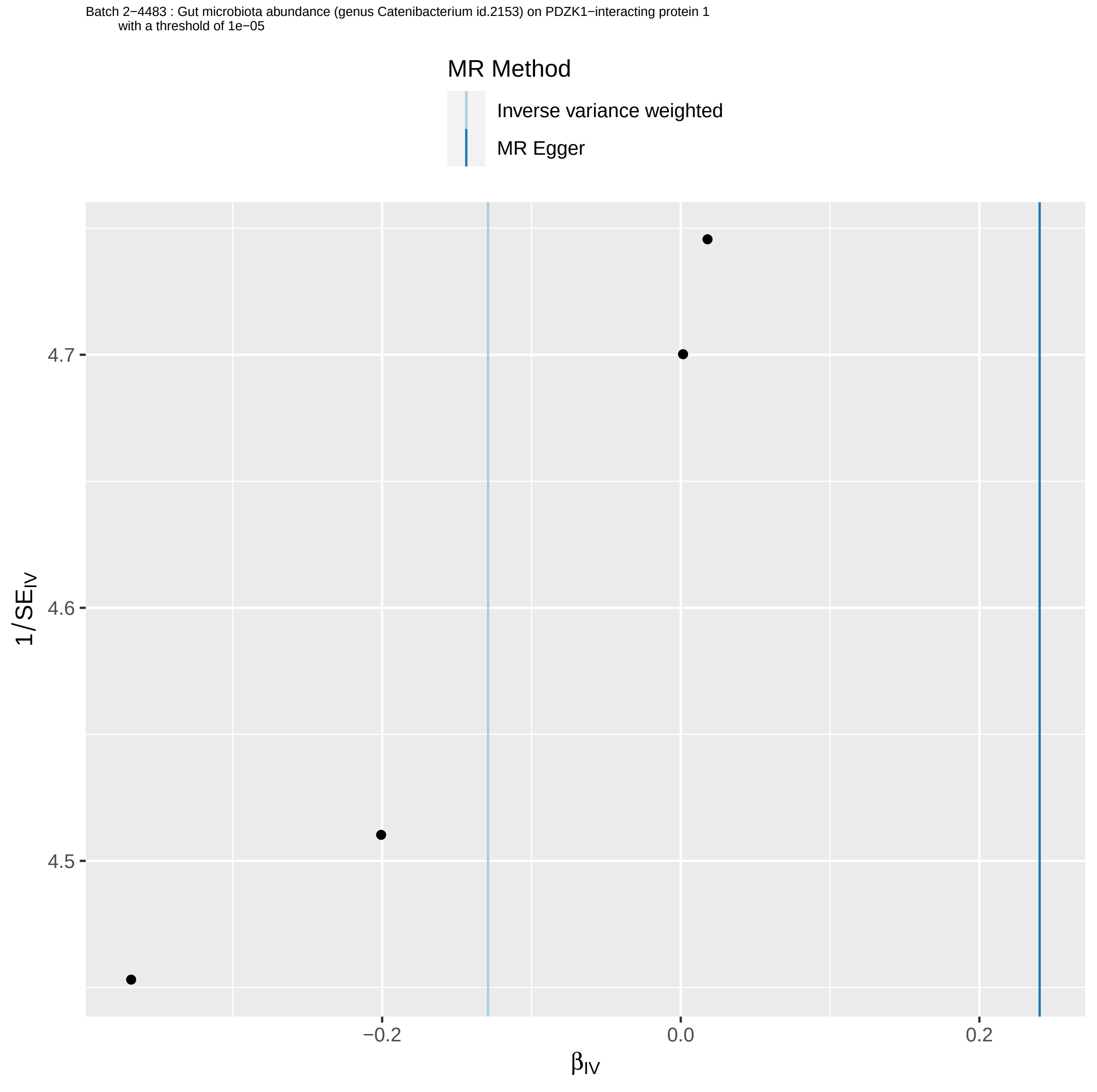

Batch 2−4483 : Gut microbiota abundance (genus Catenibacterium id.2153) on PDZK1−interacting protein 1
with a threshold of 1e−05
MR Method
Inverse variance weighted
MR Egger
4.7
4.6
4.5
1/SE$_{IV}$
−0.2
0.0
0.2
β$_{IV}$

Batch 2−4484 : Gut microbiota abundance (genus Parasutterella id.2892) on PDZK1−interacting protein 1
with a threshold of 1e−05

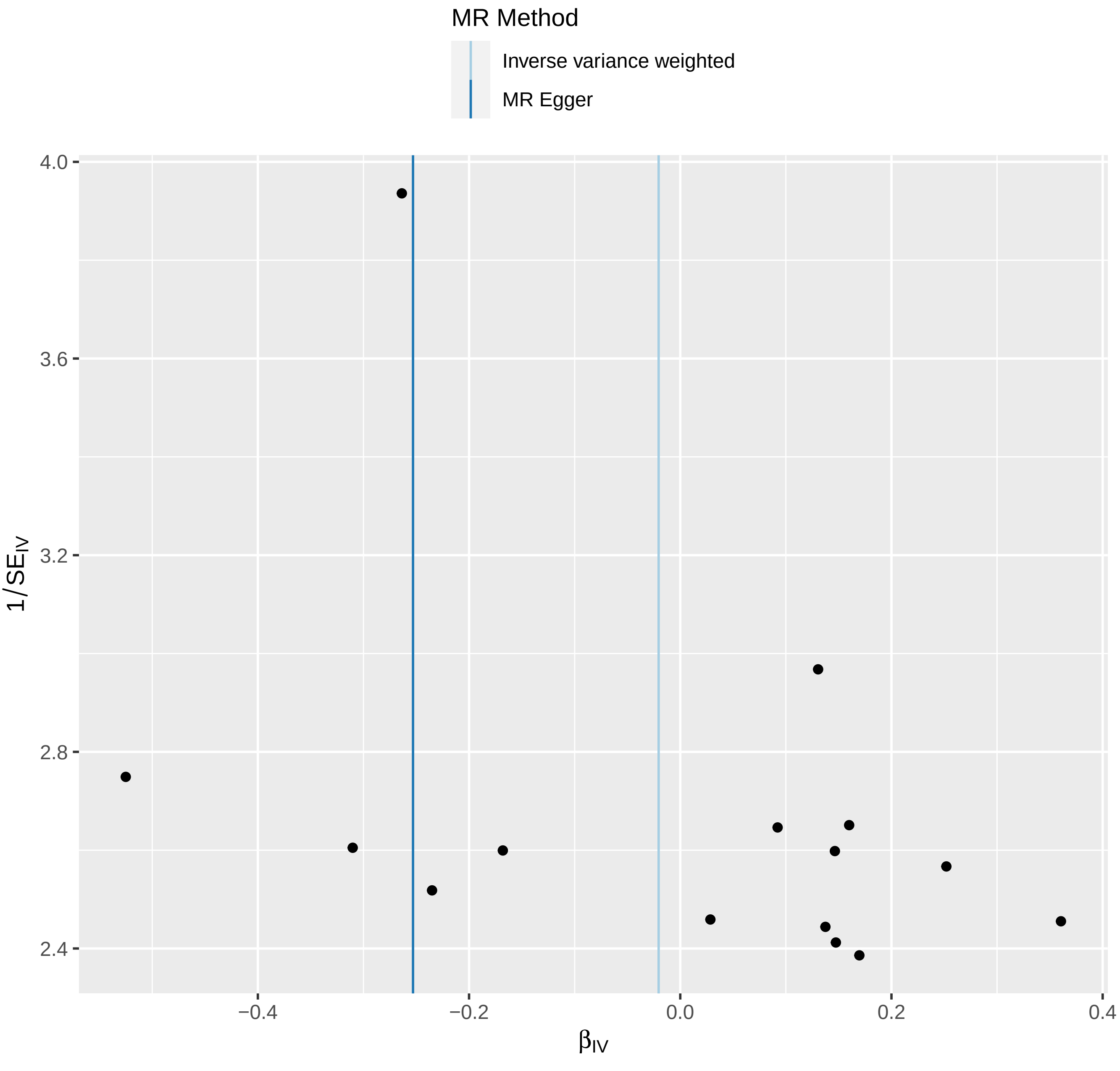

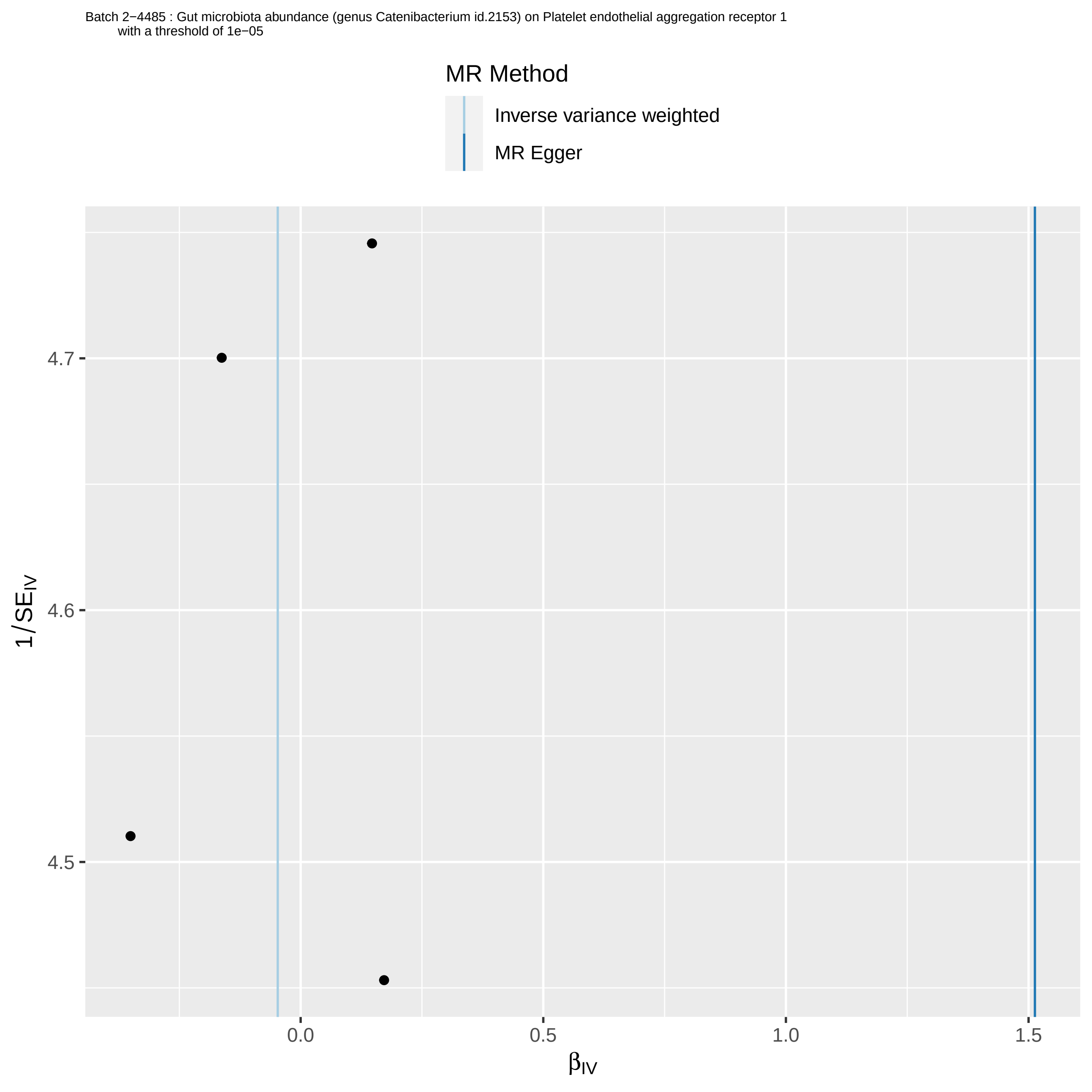

Batch 2−4485 : Gut microbiota abundance (genus Catenibacterium id.2153) on Platelet endothelial aggregation receptor 1
with a threshold of 1e−05
MR Method
Inverse variance weighted
MR Egger
4.7
4.6
4.5
1/SE_IV
0.0
0.5
1.0
1.5
β_IV

Batch 2−4486 : Gut microbiota abundance (genus Parasutterella id.2892) on Platelet endothelial aggregation receptor 1
with a threshold of 1e−05

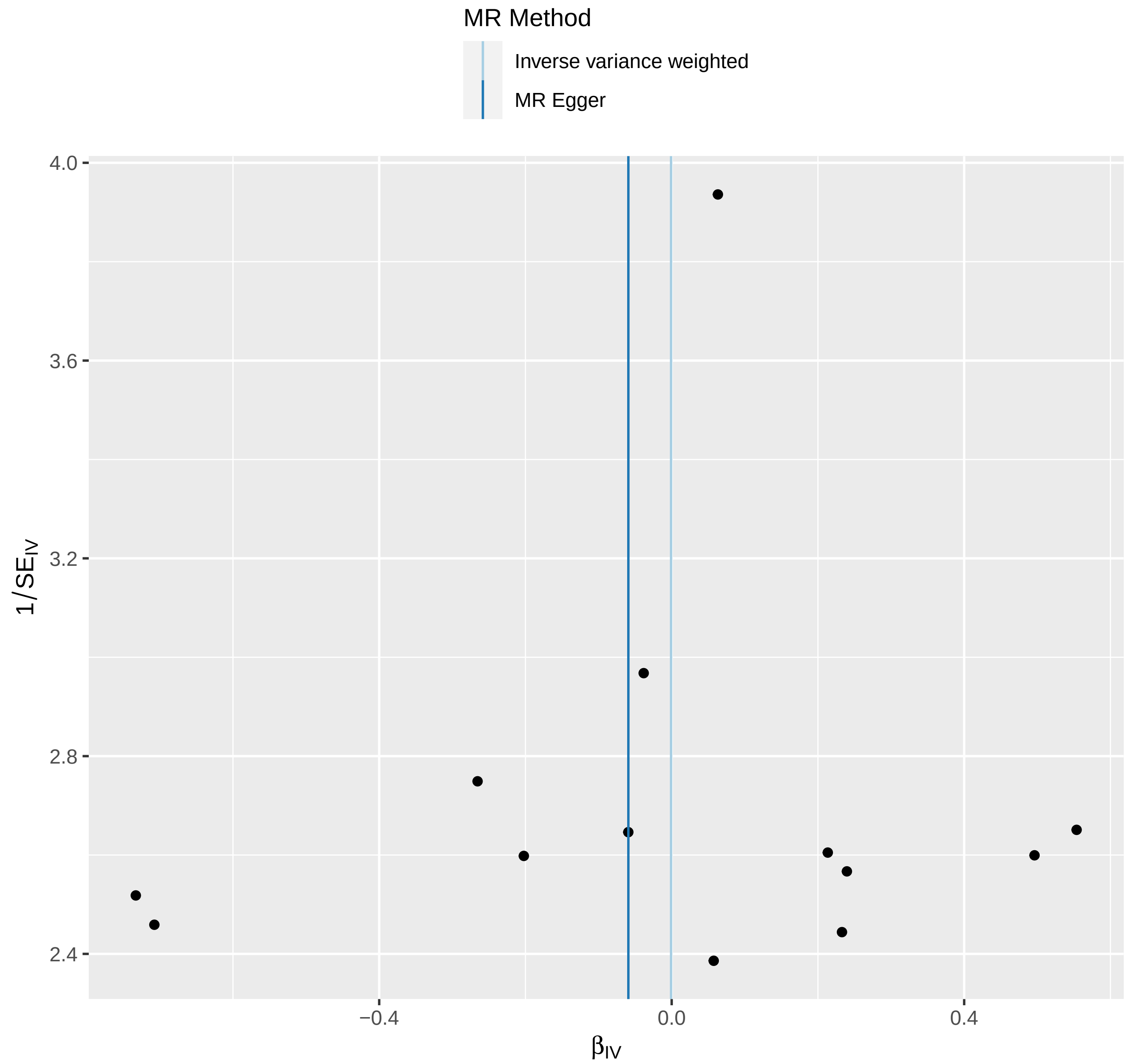

Batch 2−4487 : Gut microbiota abundance (genus Catenibacterium id.2153) on Platelet endothelial aggregation receptor 1
with a threshold of 1e−05

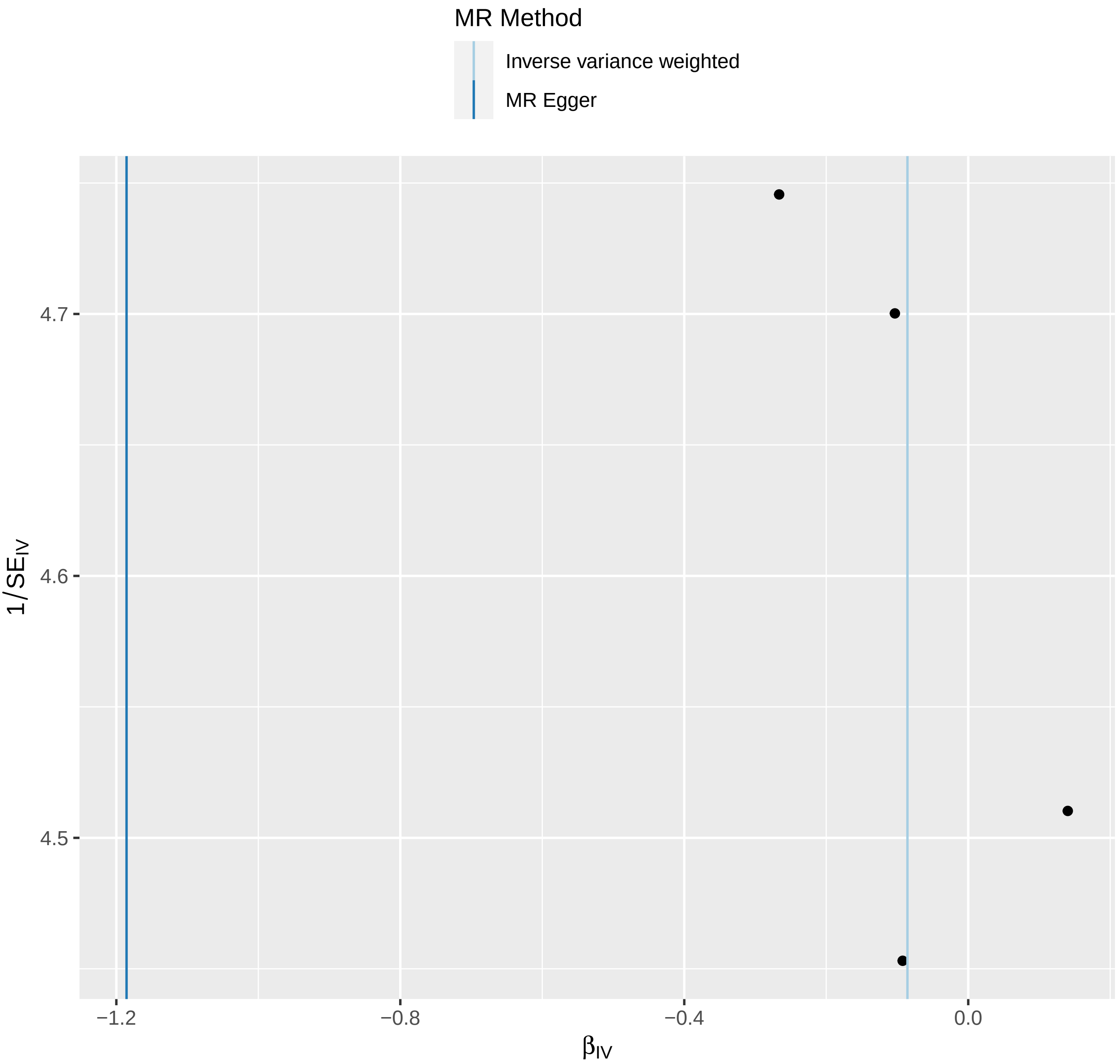

Batch 2−4488 : Gut microbiota abundance (genus Parasutterella id.2892) on Platelet endothelial aggregation receptor 1
with a threshold of 1e−05

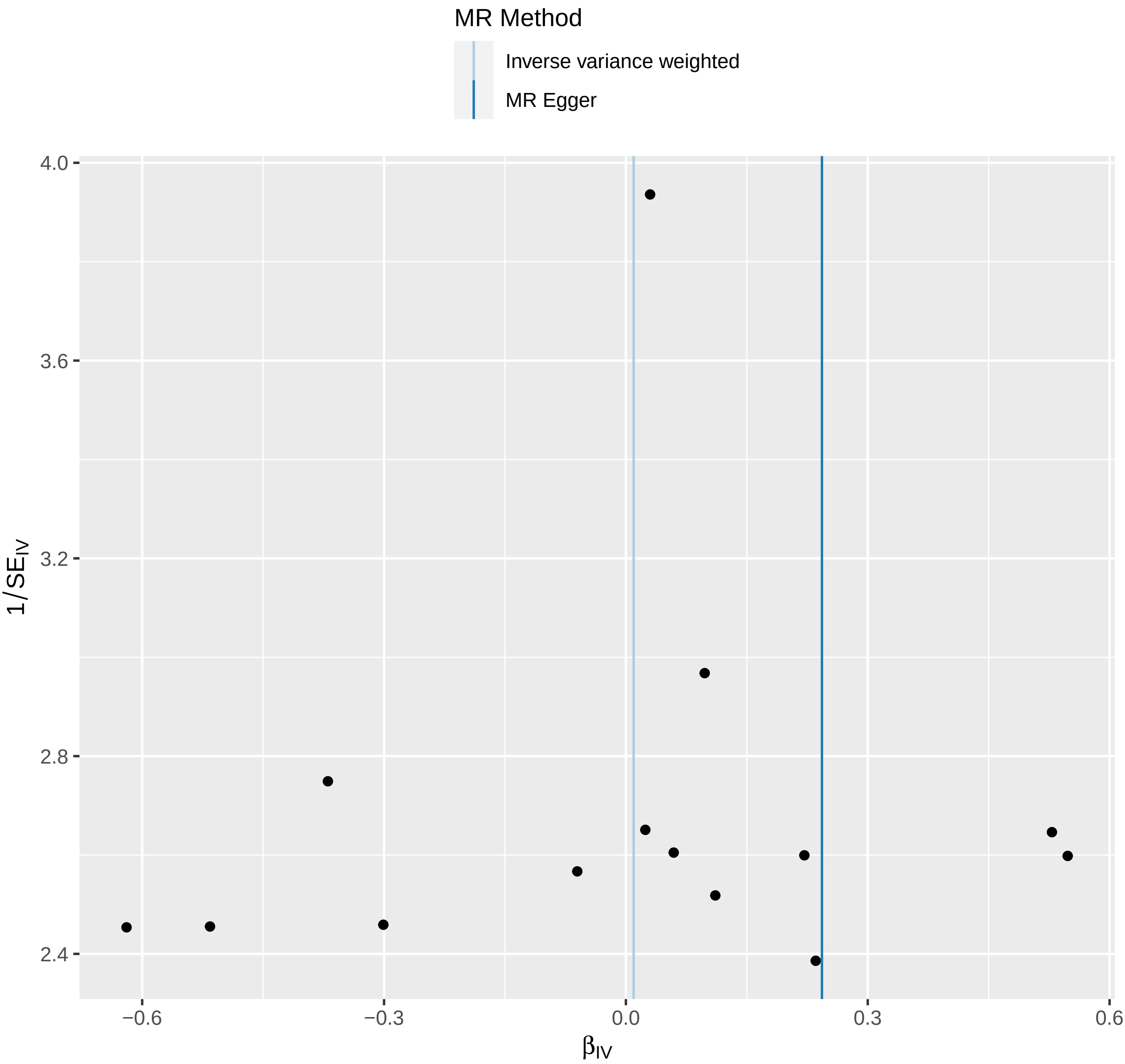

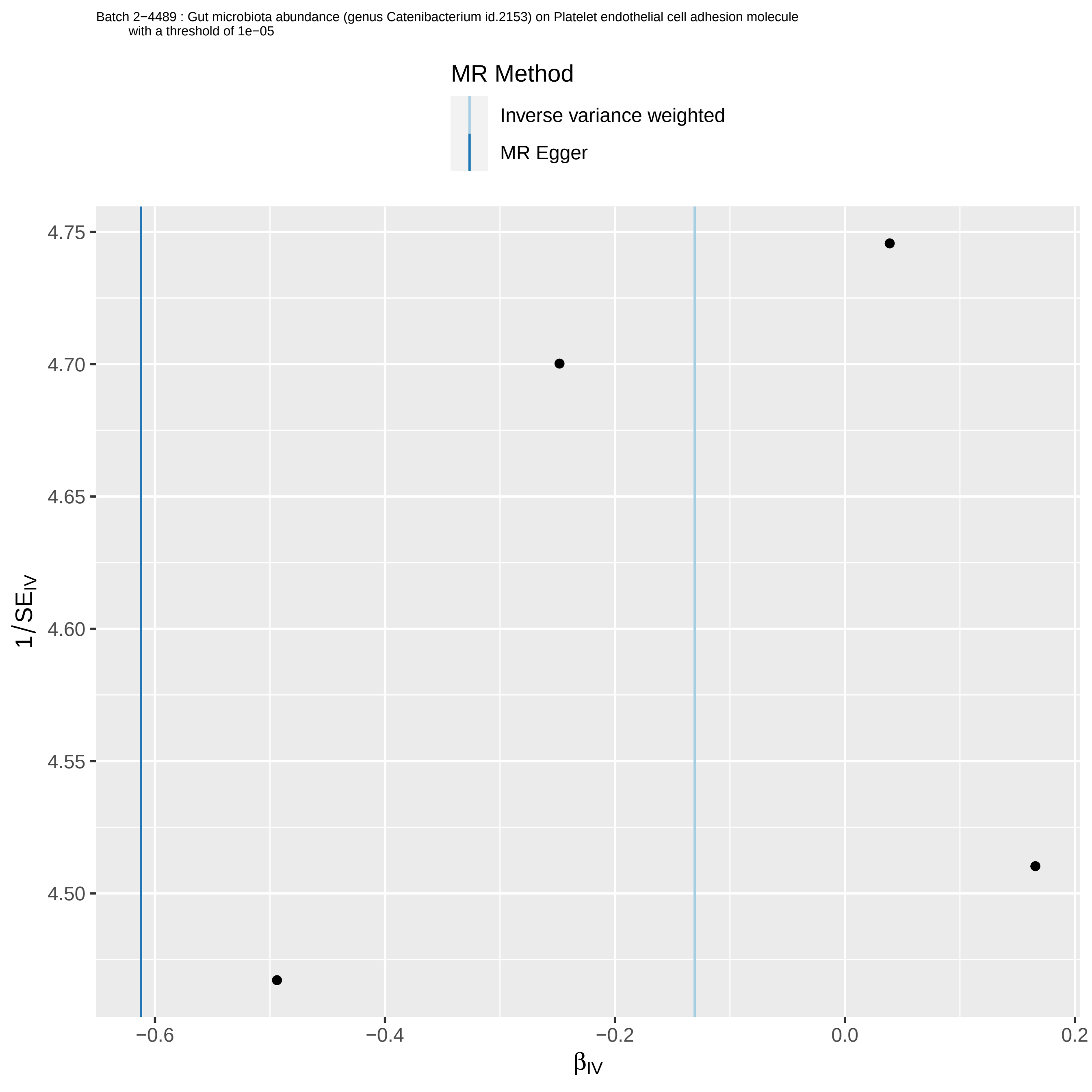
Batch 2−4489 : Gut microbiota abundance (genus Catenibacterium id.2153) on Platelet endothelial cell adhesion molecule
with a threshold of 1e−05
MR Method
Inverse variance weighted
MR Egger
4.75
4.70
4.65
4.60
4.55
4.50
1/SE_IV
−0.6
−0.4
−0.2
0.0
0.2
β_IV

Batch 2−4490 : Gut microbiota abundance (genus Parasutterella id.2892) on Platelet endothelial cell adhesion molecule
with a threshold of 1e−05

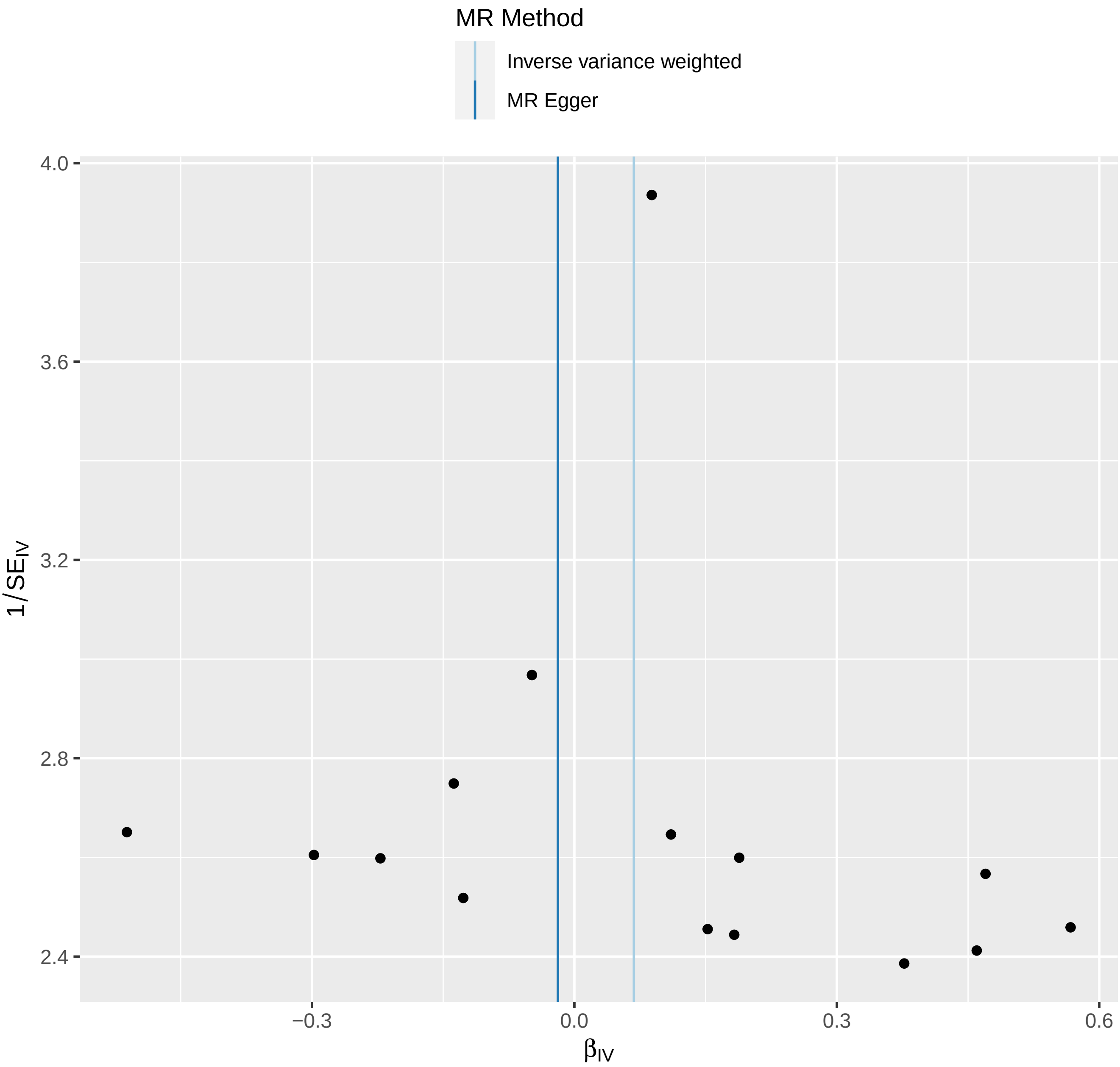

Batch 2−4491 : Gut microbiota abundance (genus Catenibacterium id.2153) on E3 ubiquitin−protein ligase pellino homolog 2 with a threshold of 1e−05

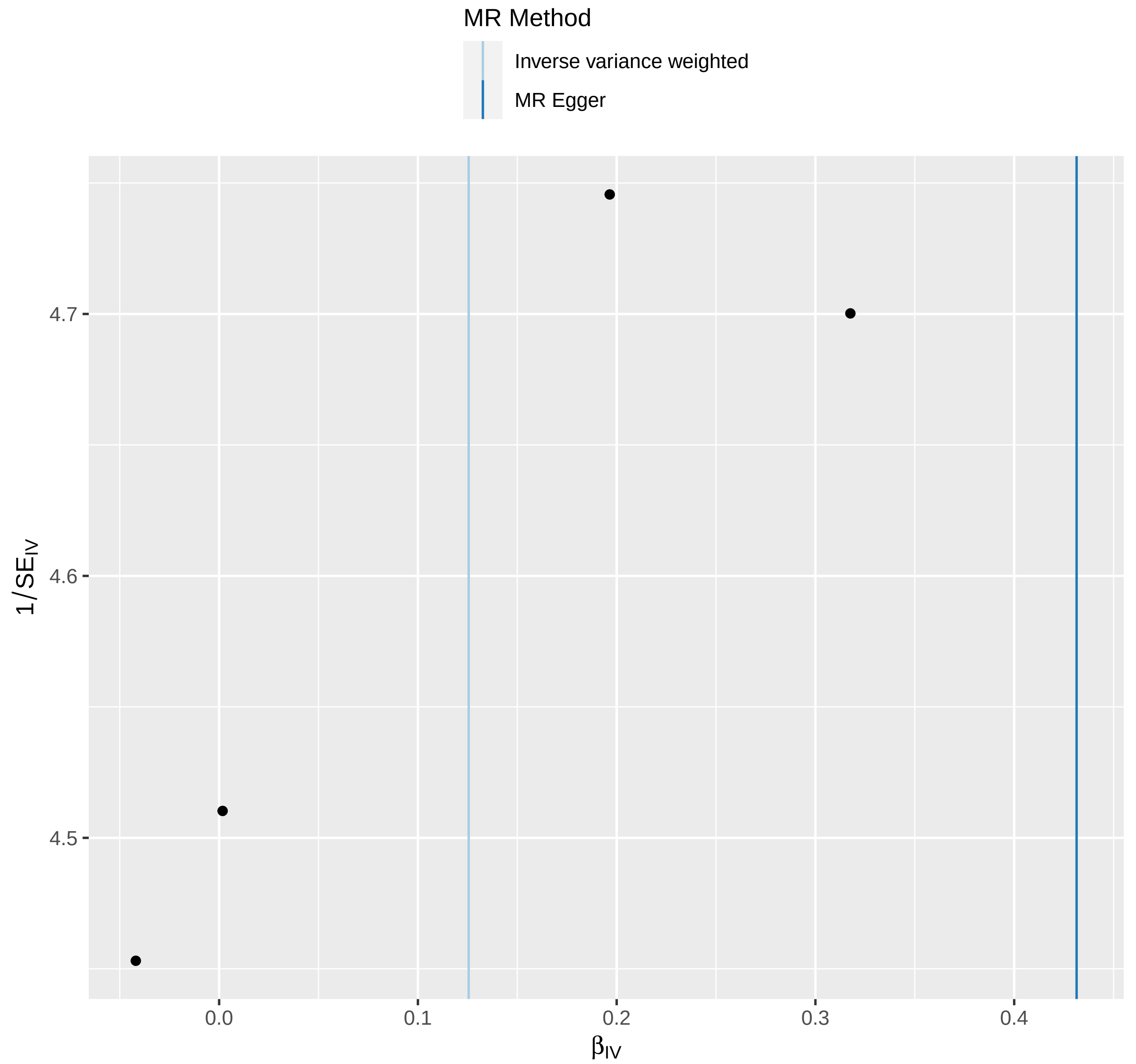

Batch 2−4492 : Gut microbiota abundance (genus Parasutterella id.2892) on E3 ubiquitin−protein ligase pellino homolog 2
with a threshold of 1e−05

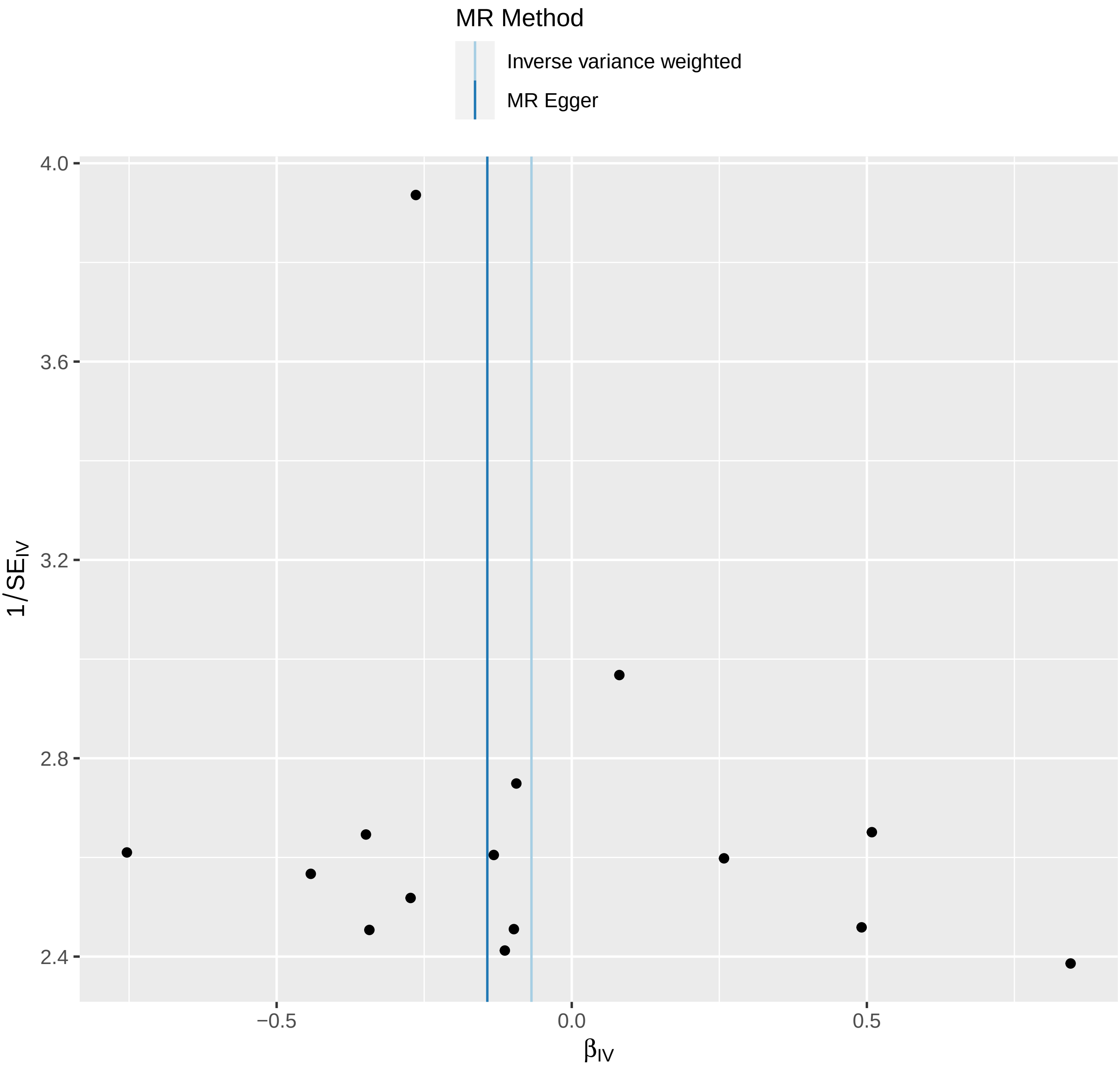

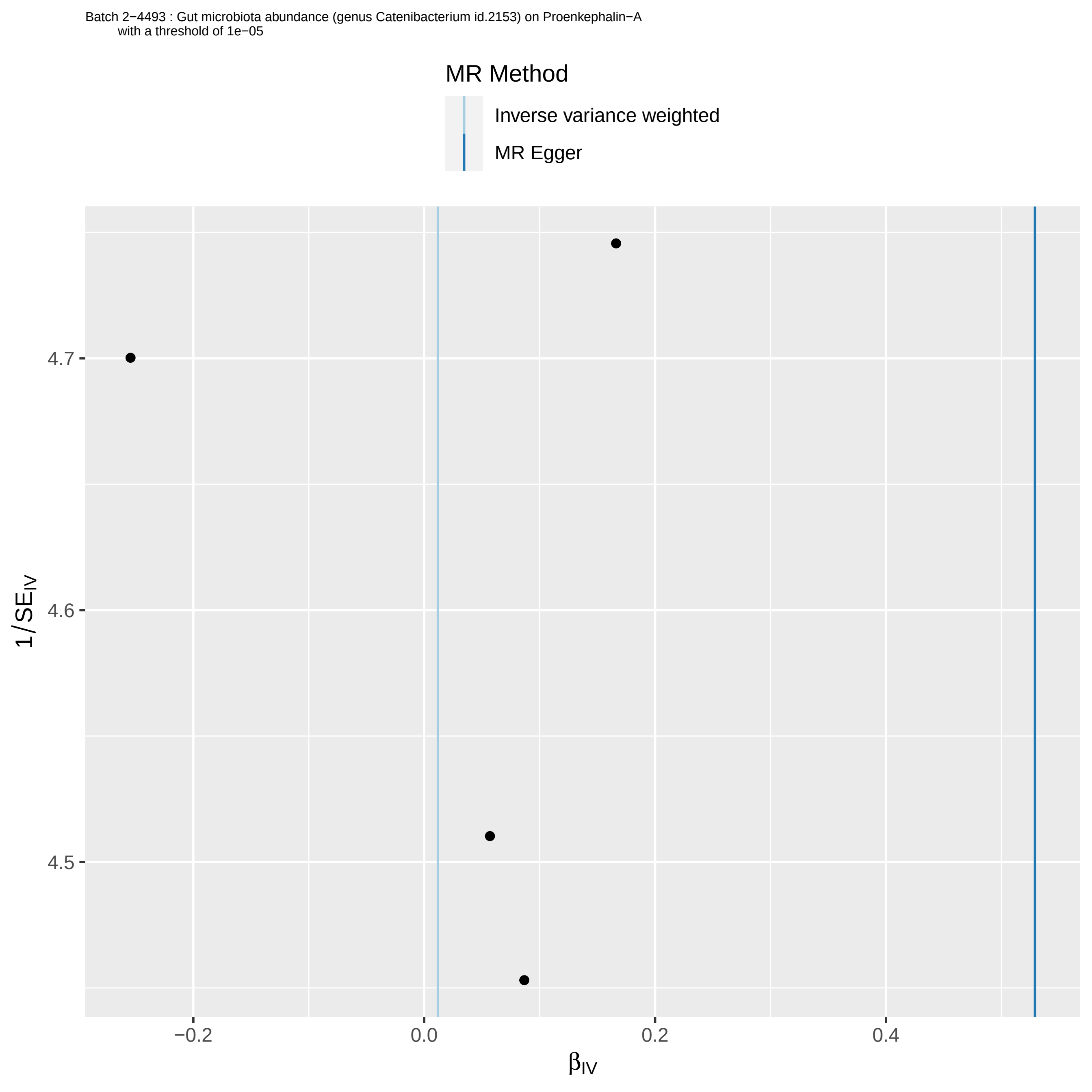
Batch 2−4493 : Gut microbiota abundance (genus Catenibacterium id.2153) on Proenkephalin−A
with a threshold of 1e−05
MR Method
Inverse variance weighted
MR Egger
4.7
4.6
4.5
1/SE_IV
−0.2
0.0
0.2
0.4
β_IV

Batch 2−4494 : Gut microbiota abundance (genus Parasutterella id.2892) on Proenkephalin−A with a threshold of 1e−05

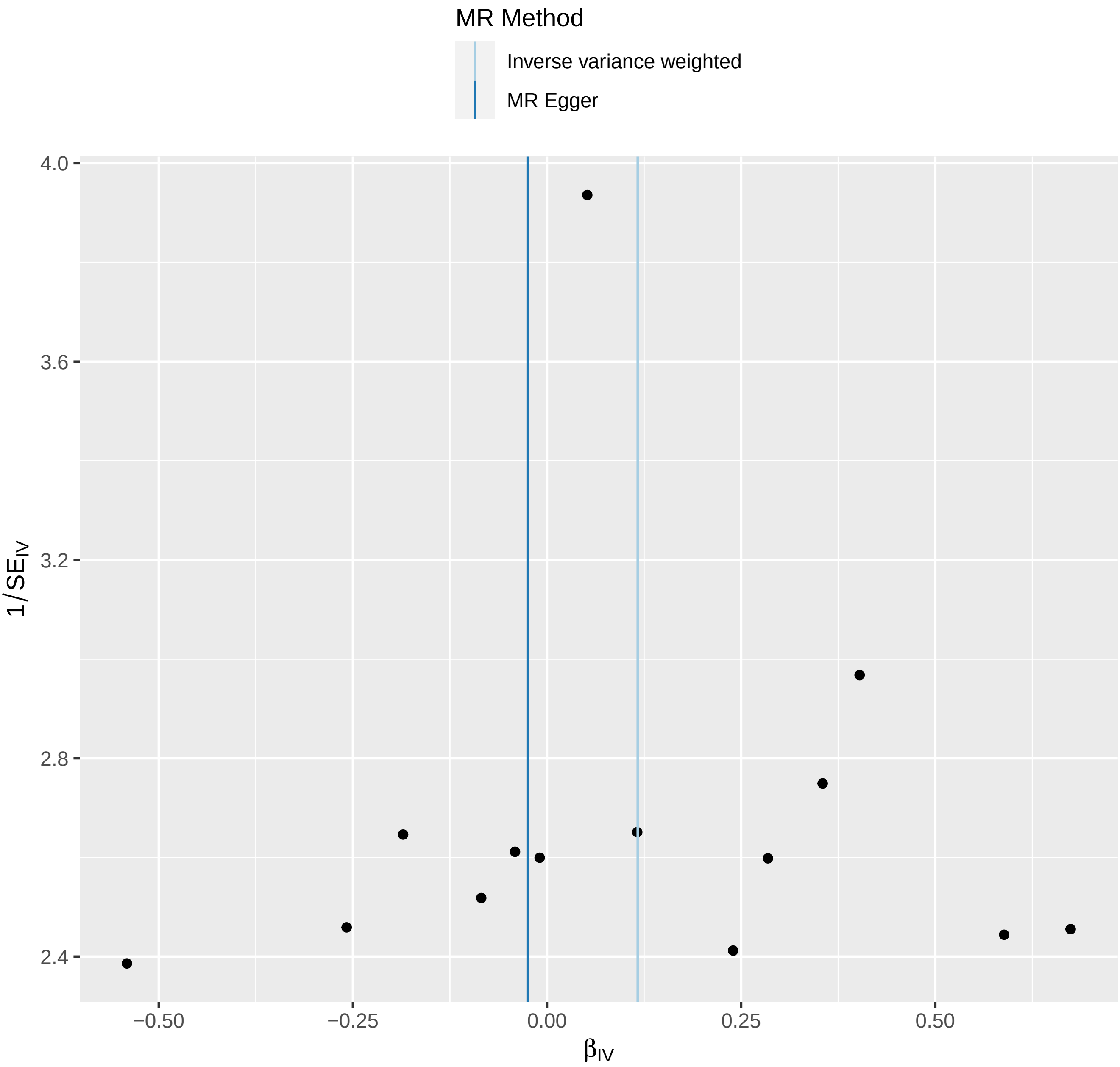

Batch 2−4495 : Gut microbiota abundance (genus Catenibacterium id.2153) on Pescadillo homolog
with a threshold of 1e−05

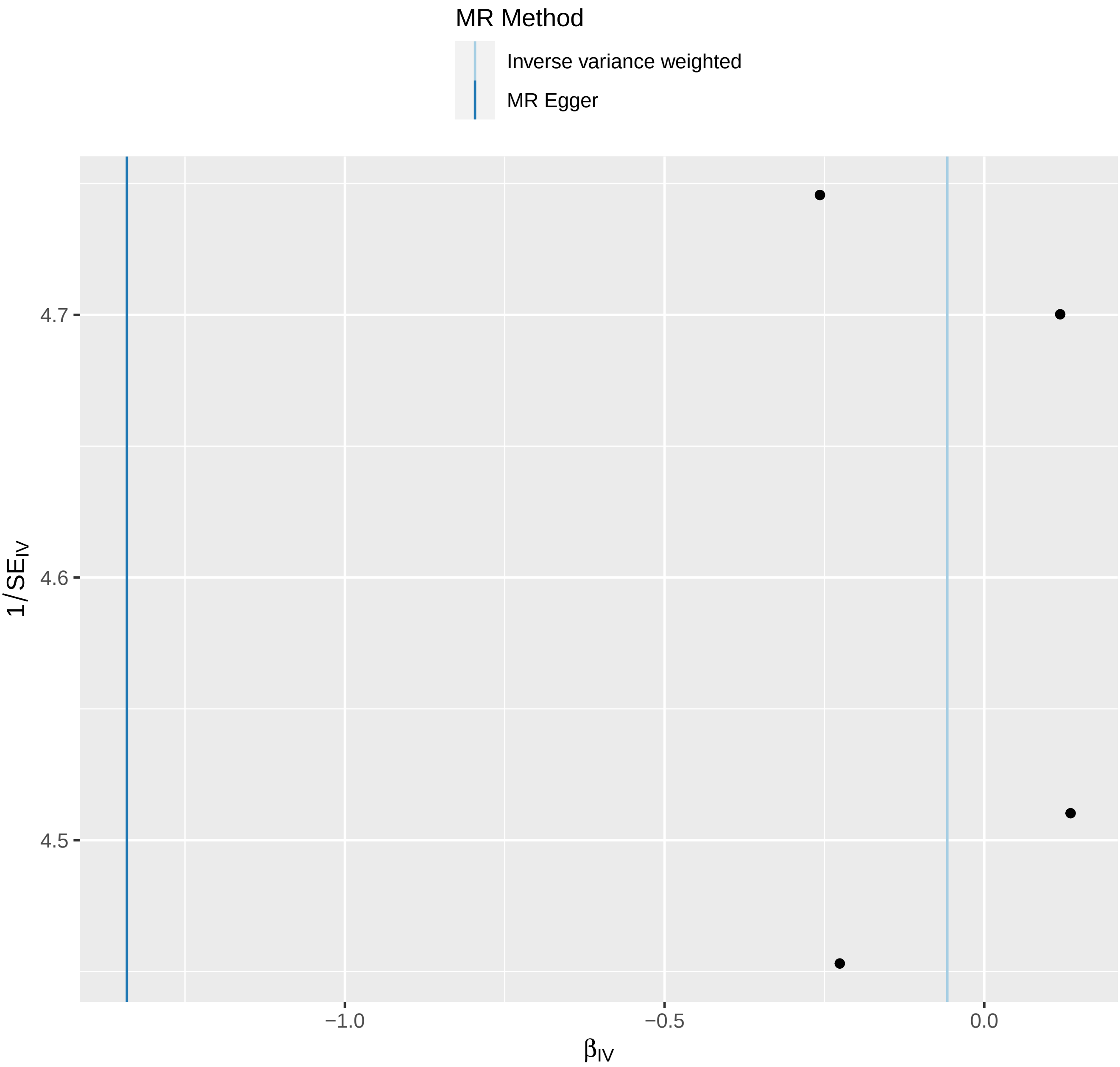

Batch 2−4496 : Gut microbiota abundance (genus Parasutterella id.2892) on Pescadillo homolog
with a threshold of 1e−05

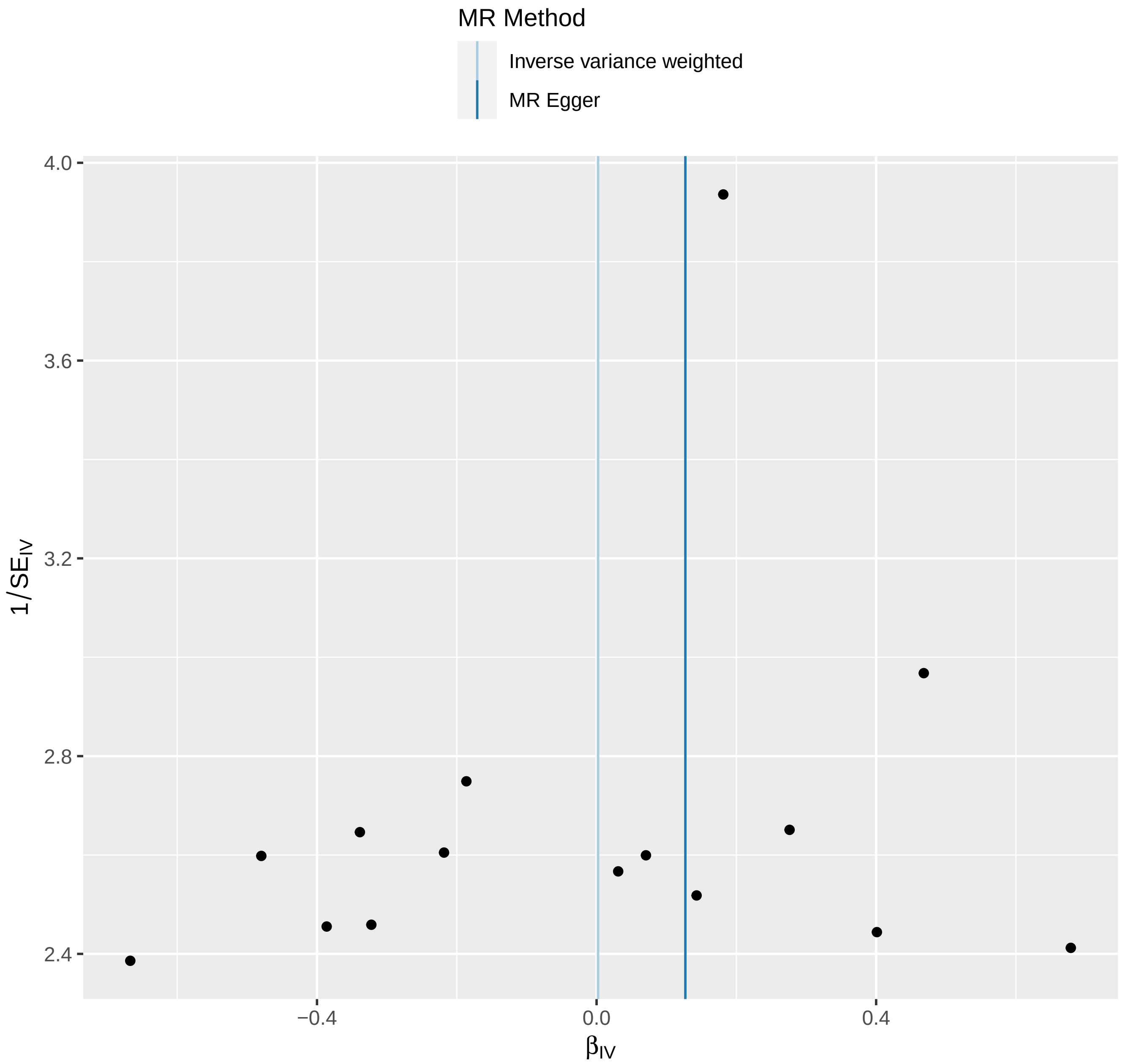

Batch 2−4497 : Gut microbiota abundance (genus Catenibacterium id.2153) on Peroxisomal membrane protein PEX14
with a threshold of 1e−05

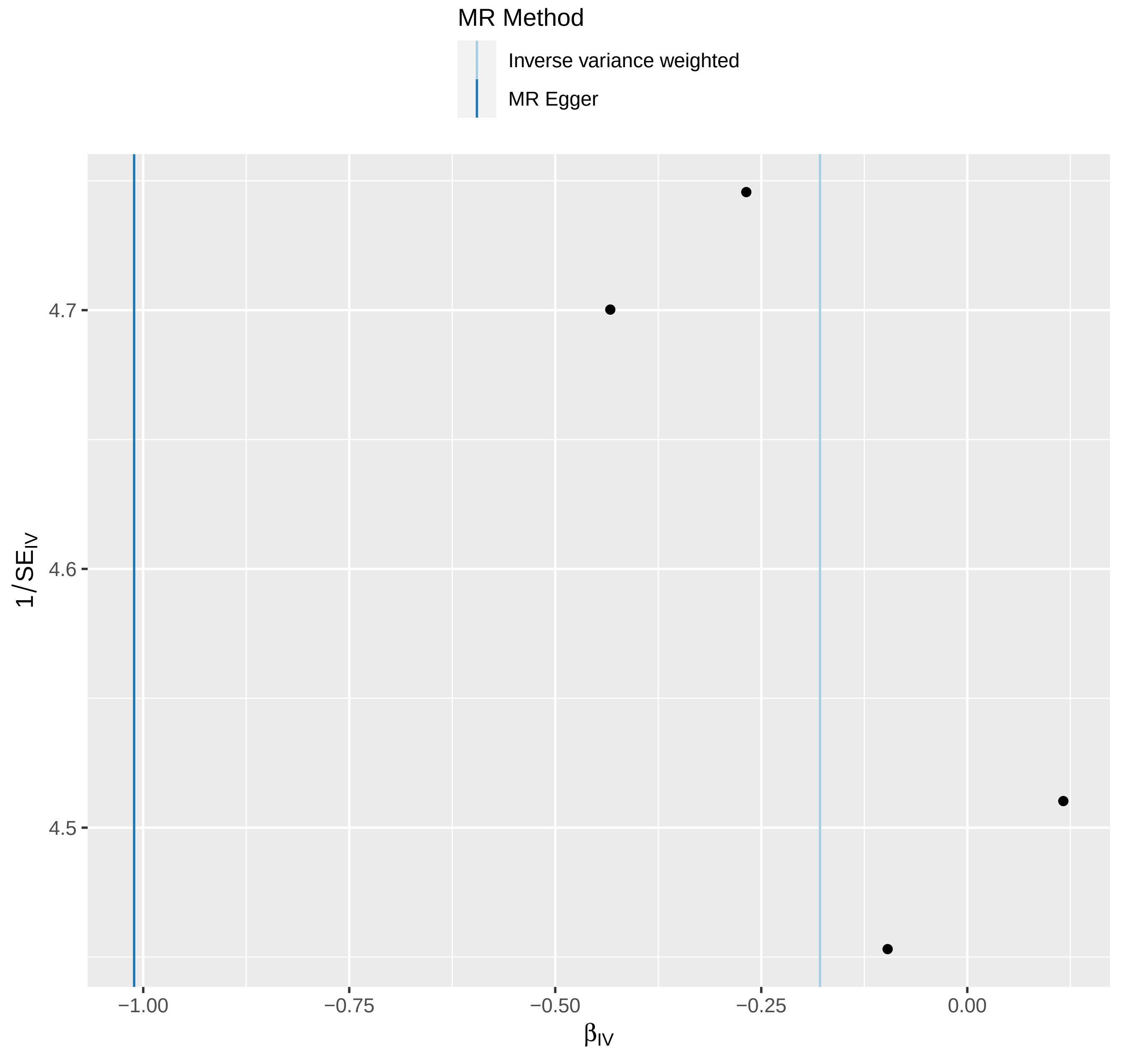

Batch 2−4498 : Gut microbiota abundance (genus Parasutterella id.2892) on Peroxisomal membrane protein PEX14
with a threshold of 1e−05

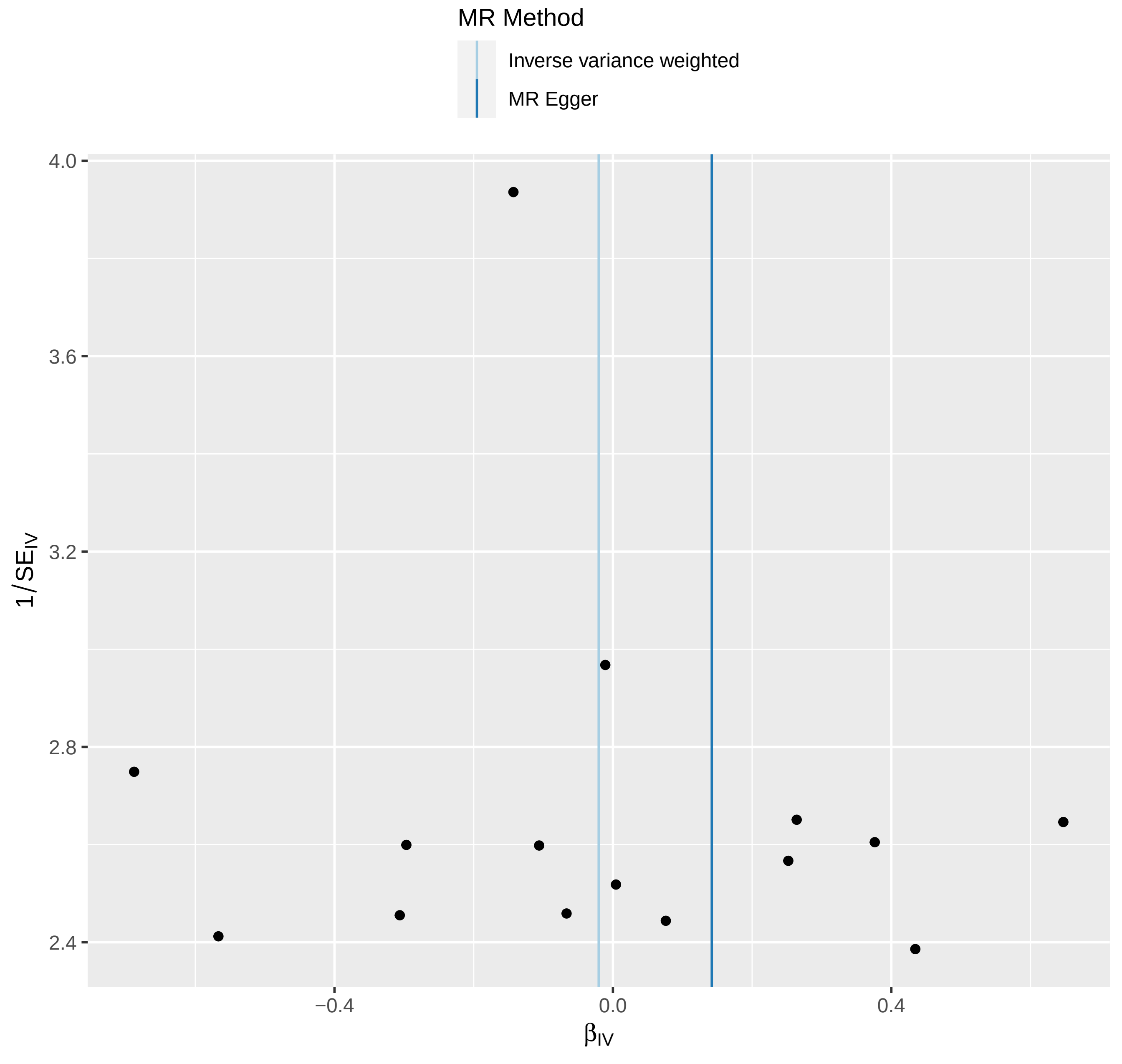

Batch 2−4499 : Gut microbiota abundance (genus Catenibacterium id.2153) on Peroxisomal targeting signal 1 receptor with a threshold of 1e−05

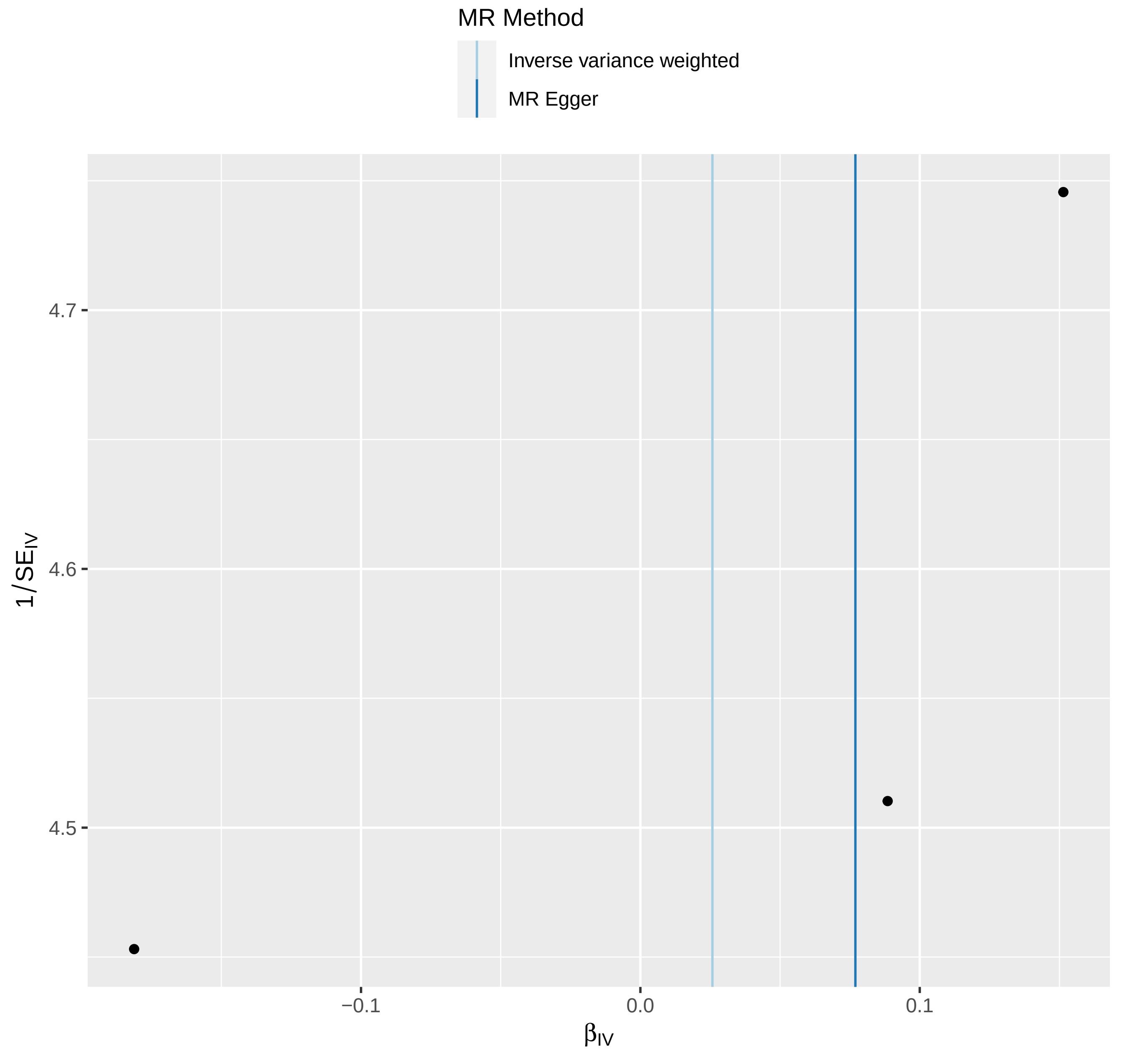

Batch 2−4500 : Gut microbiota abundance (genus Parasutterella id.2892) on Peroxisomal targeting signal 1 receptor
with a threshold of 1e−05

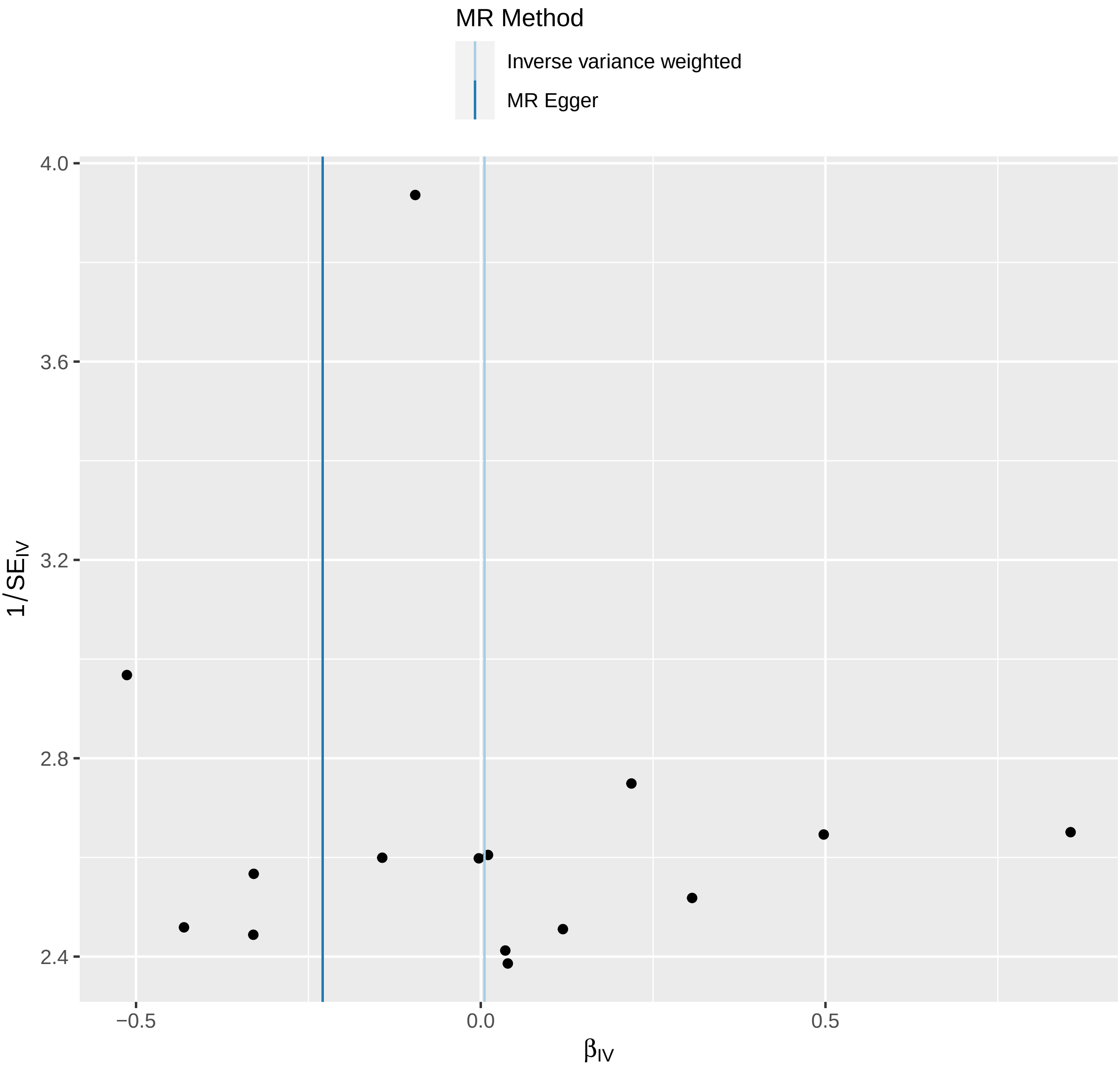

Batch 2−4501 : Gut microbiota abundance (genus Catenibacterium id.2153) on Platelet factor 4 variant
with a threshold of 1e−05

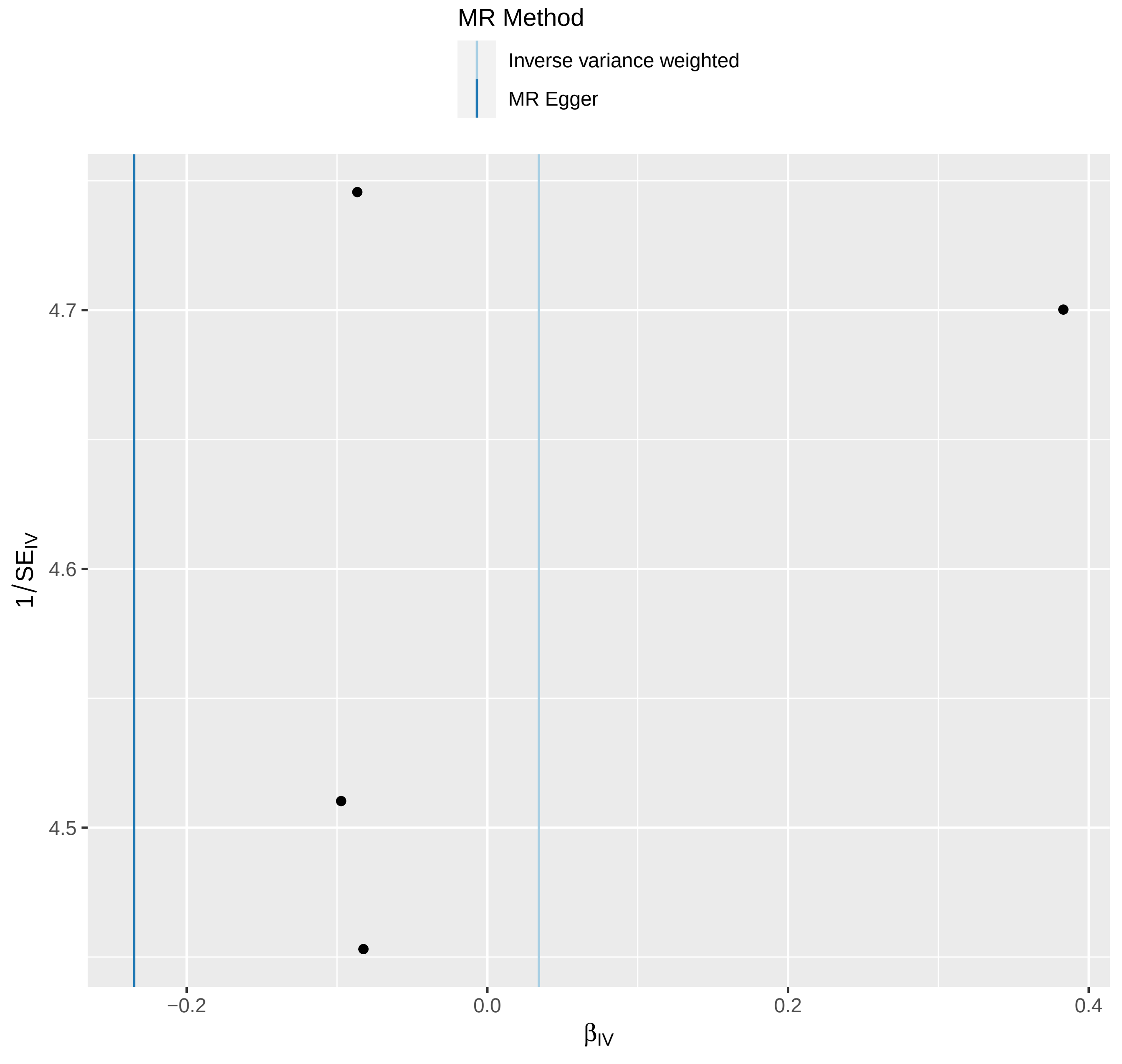

Batch 2−4502 : Gut microbiota abundance (genus Parasutterella id.2892) on Platelet factor 4 variant
with a threshold of 1e−05

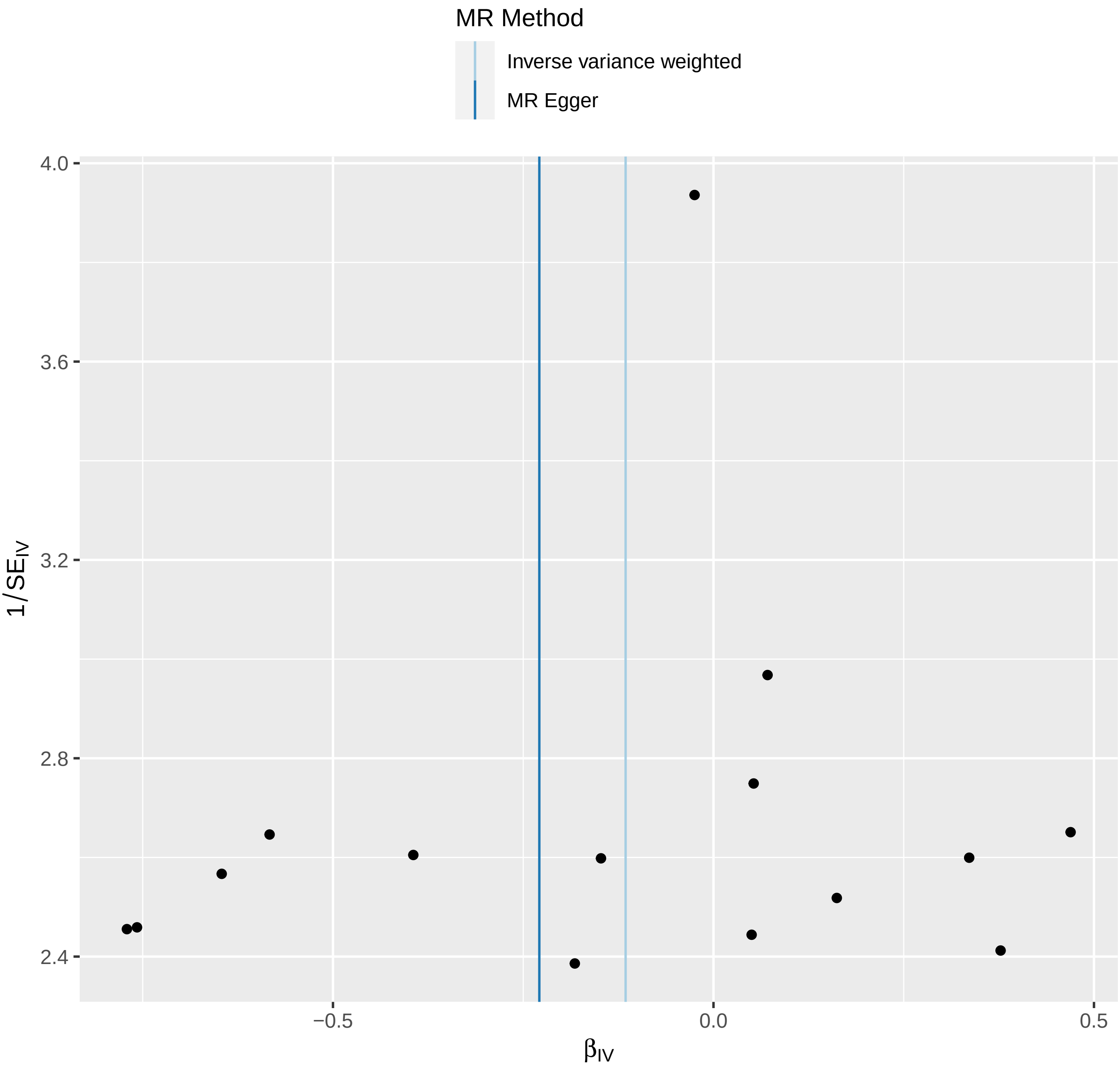

Batch 2−4503 : Gut microbiota abundance (genus Catenibacterium id.2153) on Prefoldin subunit 5
with a threshold of 1e−05

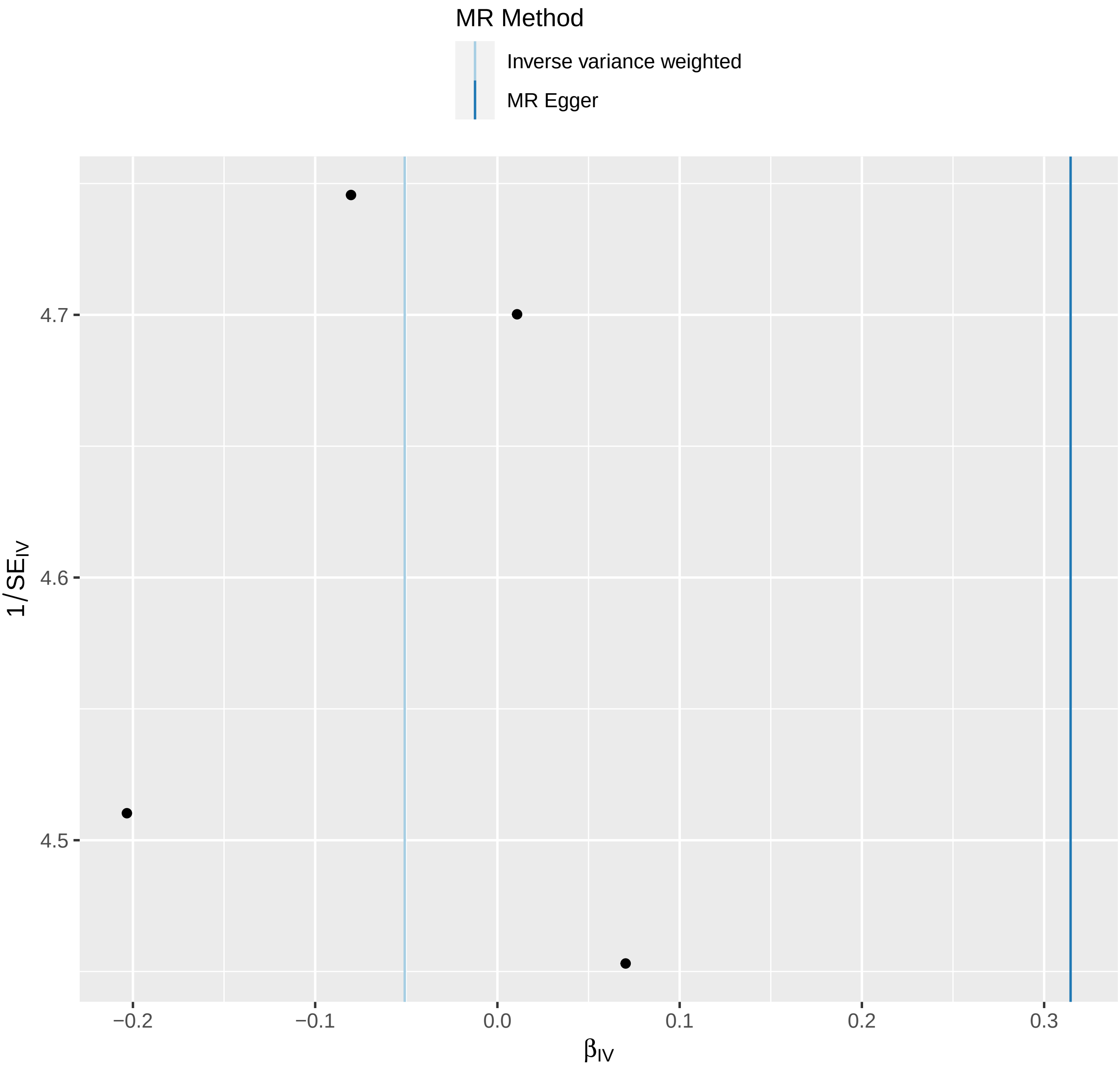

Batch 2−4504 : Gut microbiota abundance (genus Parasutterella id.2892) on Prefoldin subunit 5
with a threshold of 1e−05

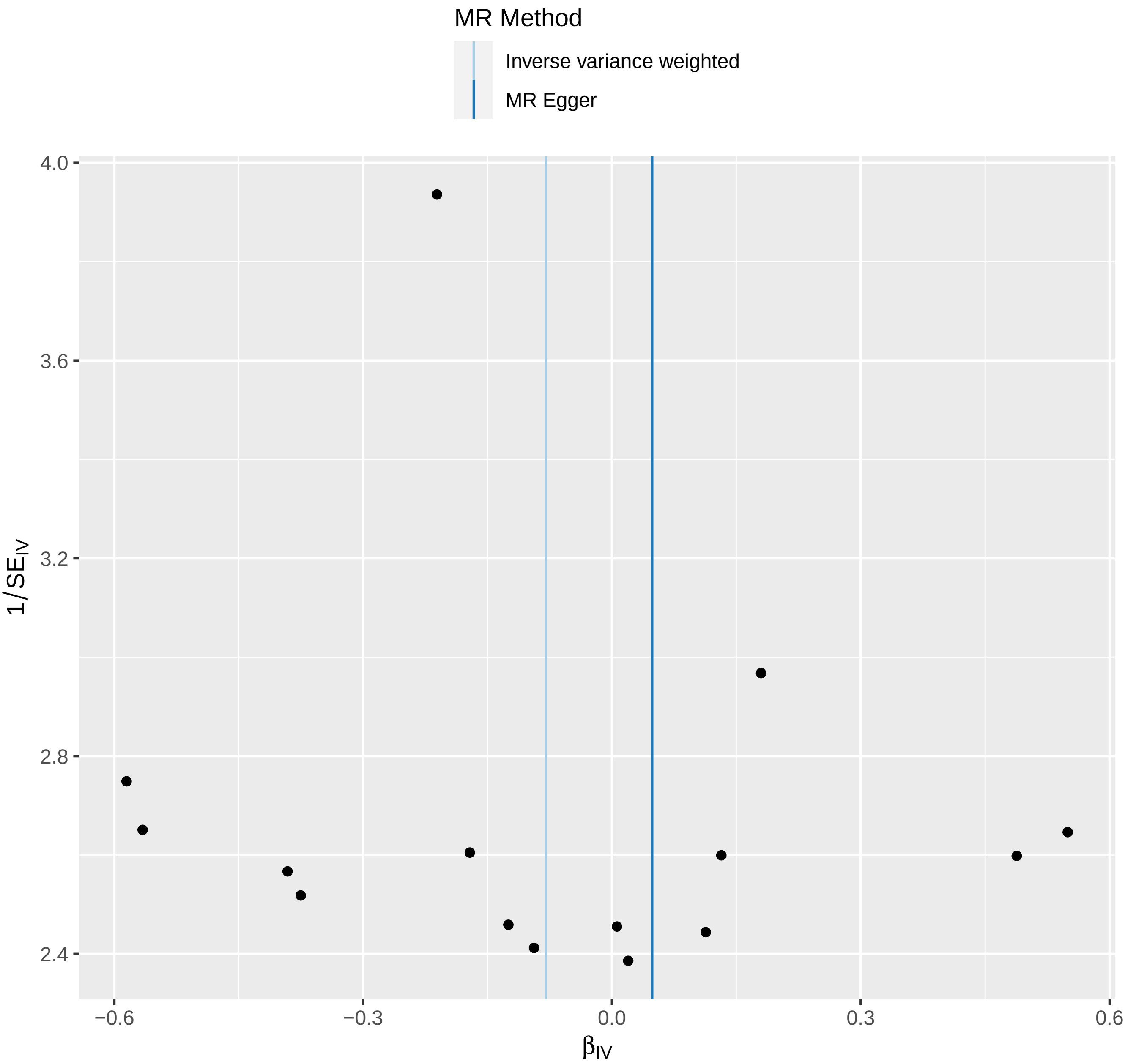

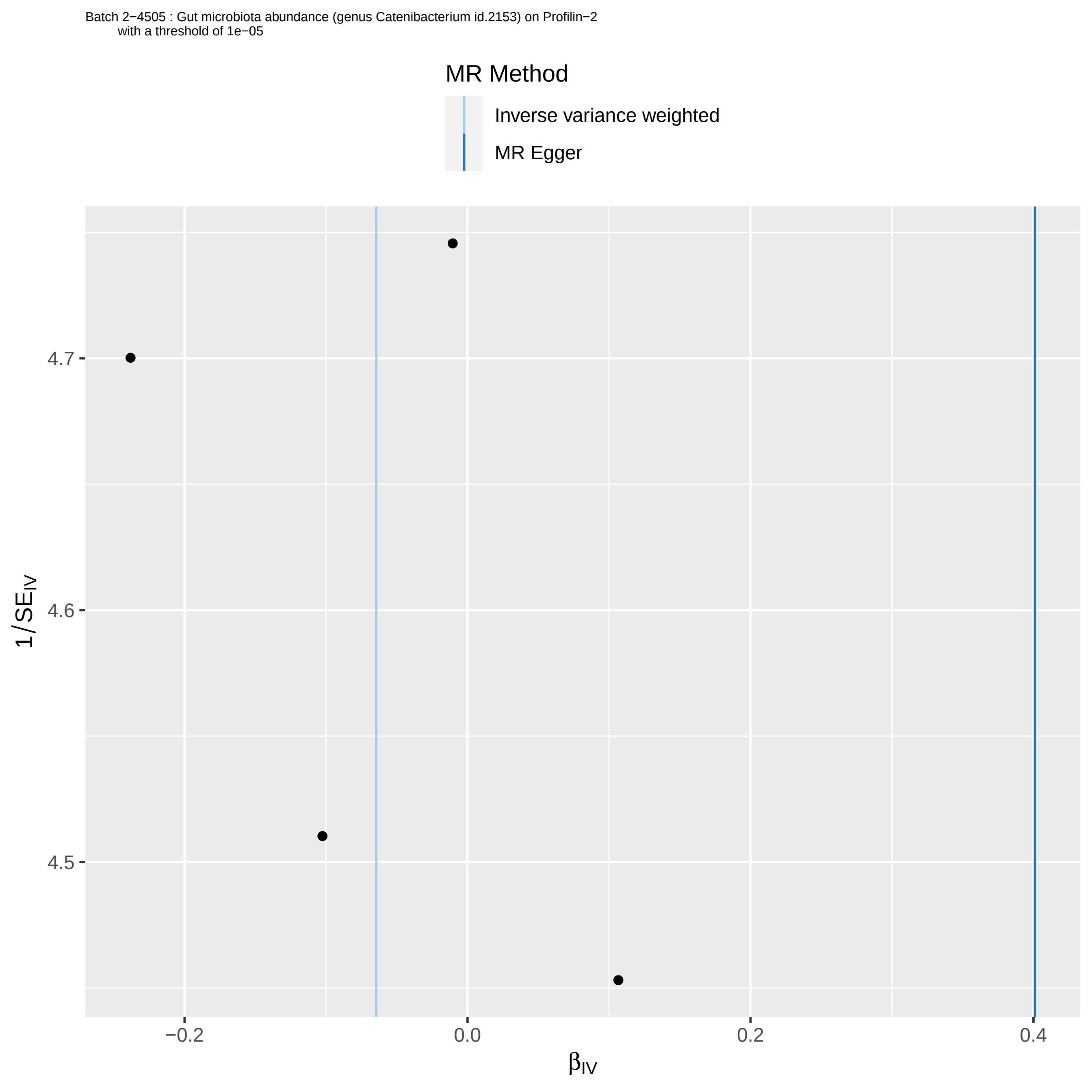
Batch 2−4505 : Gut microbiota abundance (genus Catenibacterium id.2153) on Profilin−2
with a threshold of 1e−05
MR Method
Inverse variance weighted
MR Egger
4.7
4.6
4.5
1/SE_IV
−0.2
0.0
0.2
0.4
β_IV

Batch 2−4506 : Gut microbiota abundance (genus Parasutterella id.2892) on Profilin−2
with a threshold of 1e−05

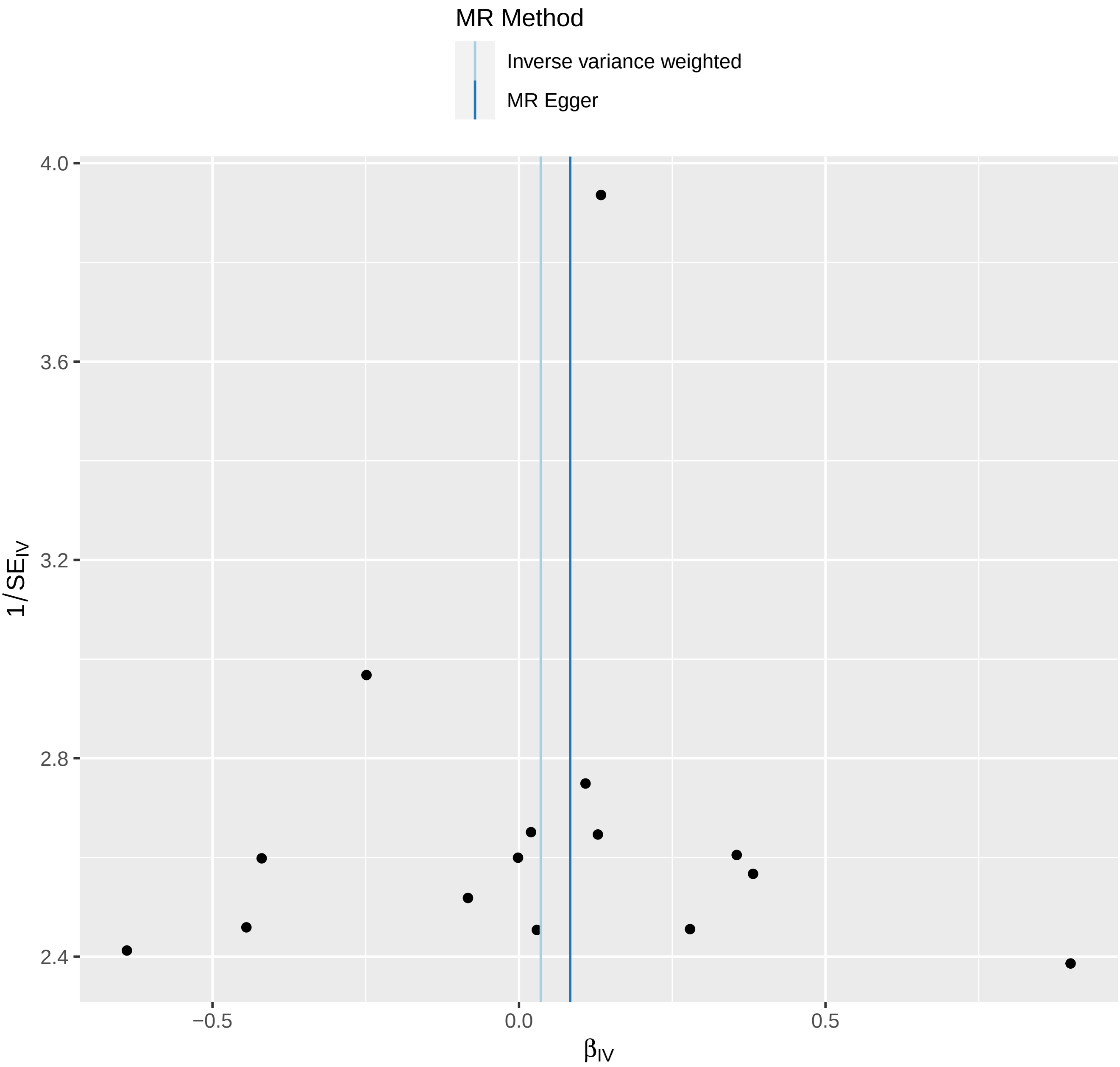

Batch 2−4507 : Gut microbiota abundance (genus Catenibacterium id.2153) on Placenta growth factor
with a threshold of 1e−05

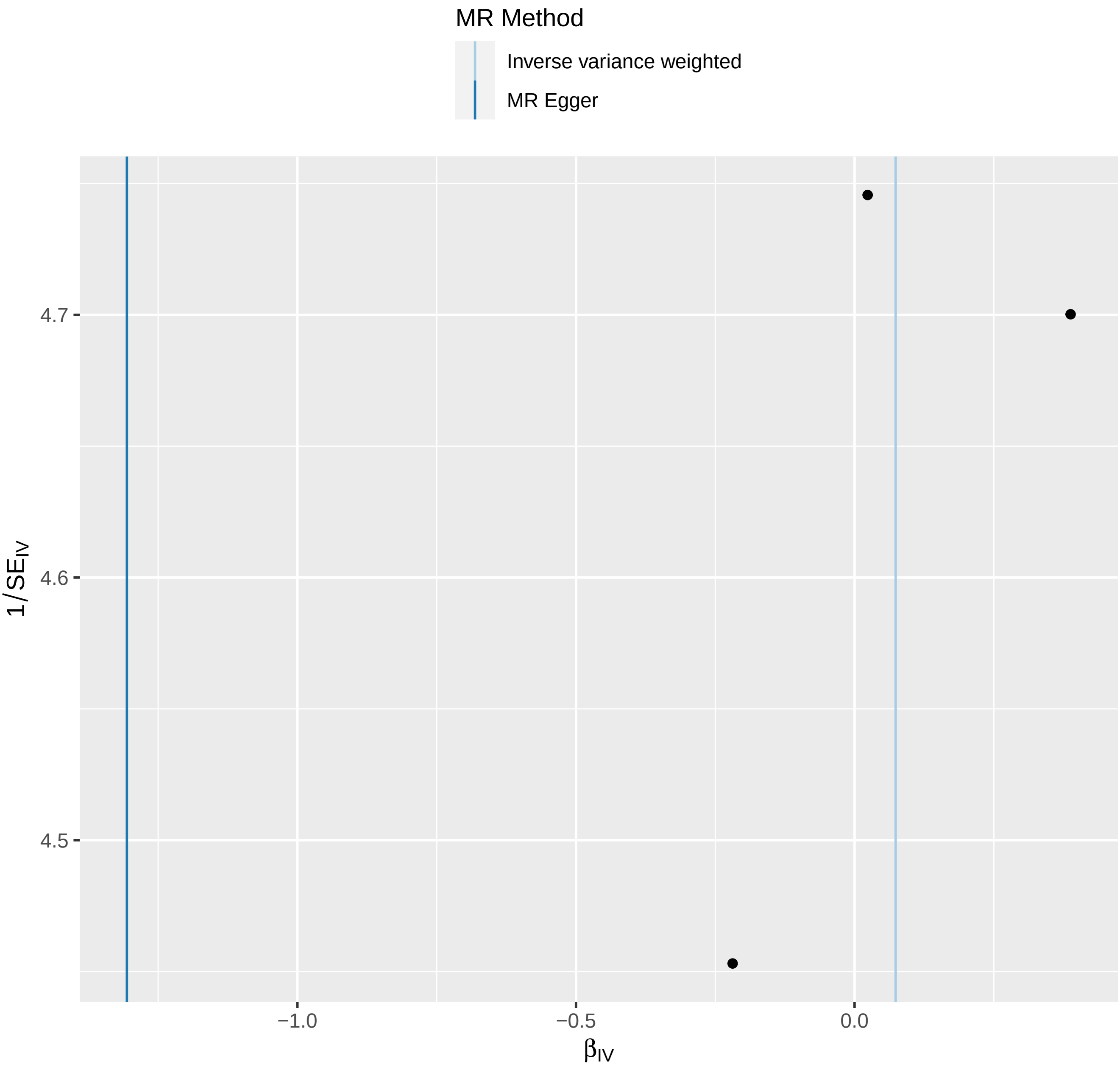

Batch 2−4508 : Gut microbiota abundance (genus Parasutterella id.2892) on Placenta growth factor
with a threshold of 1e−05

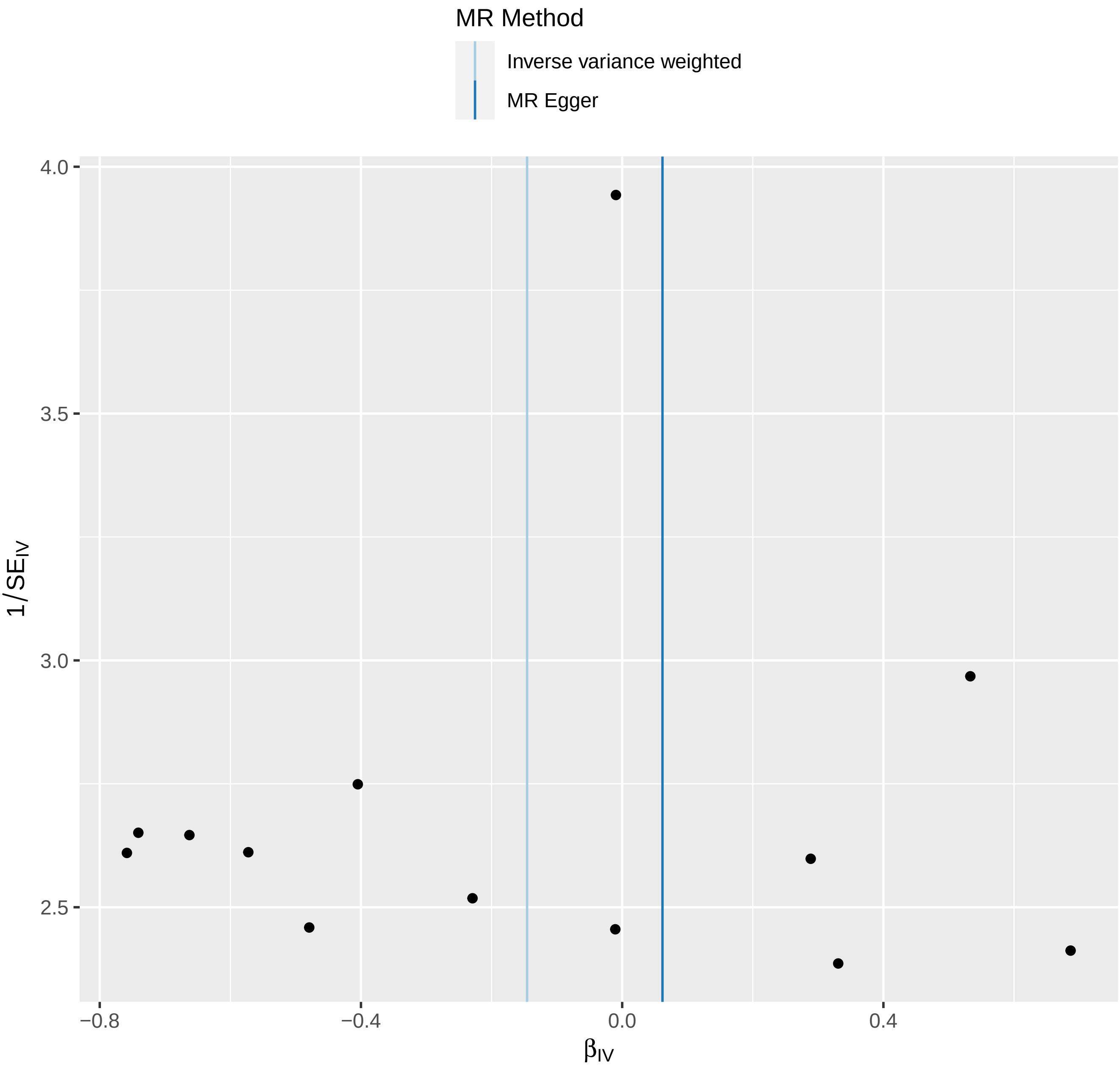

Batch 2−4509 : Gut microbiota abundance (genus Catenibacterium id.2153) on Phosphoglycerate kinase 1
with a threshold of 1e−05

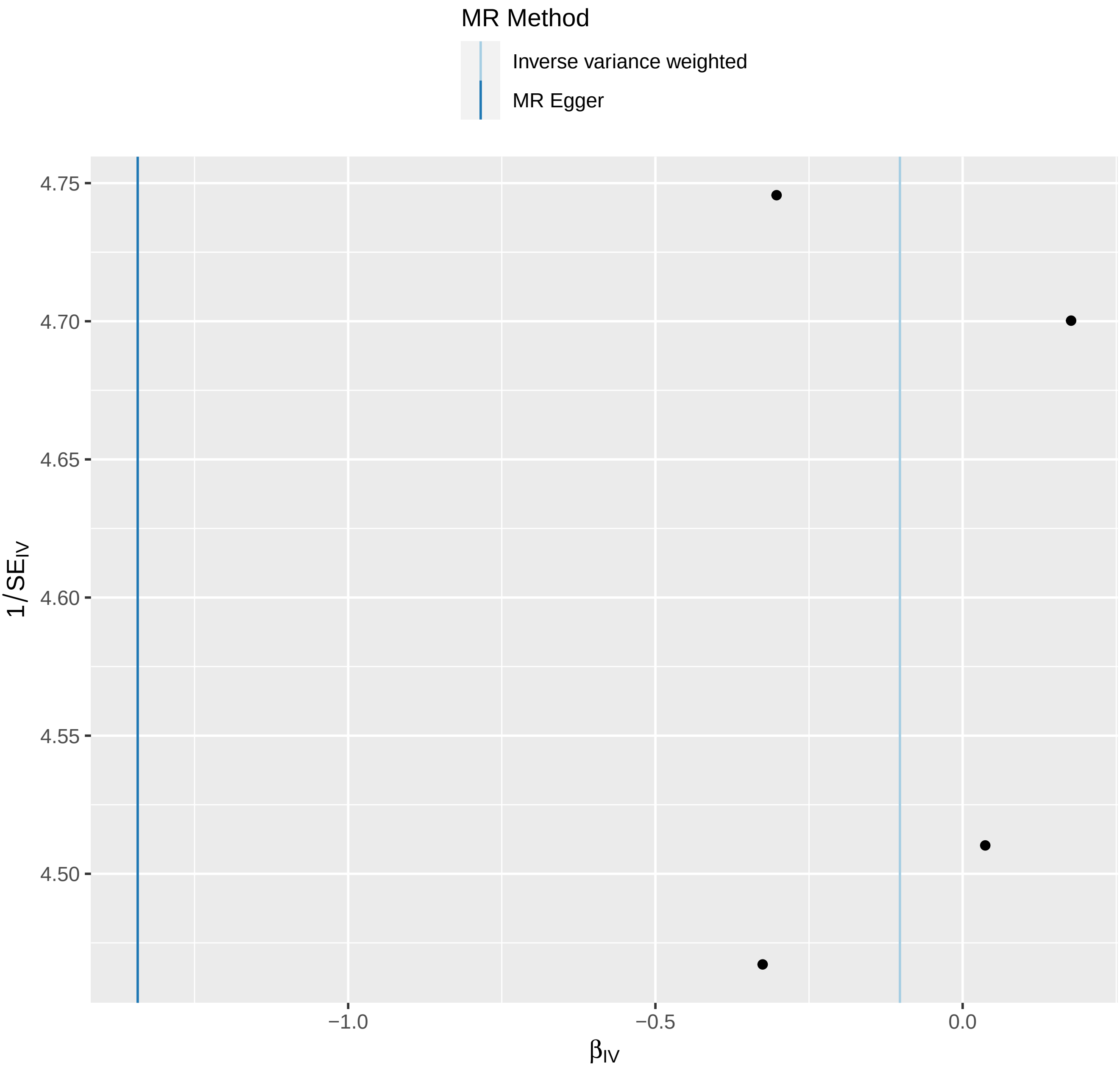

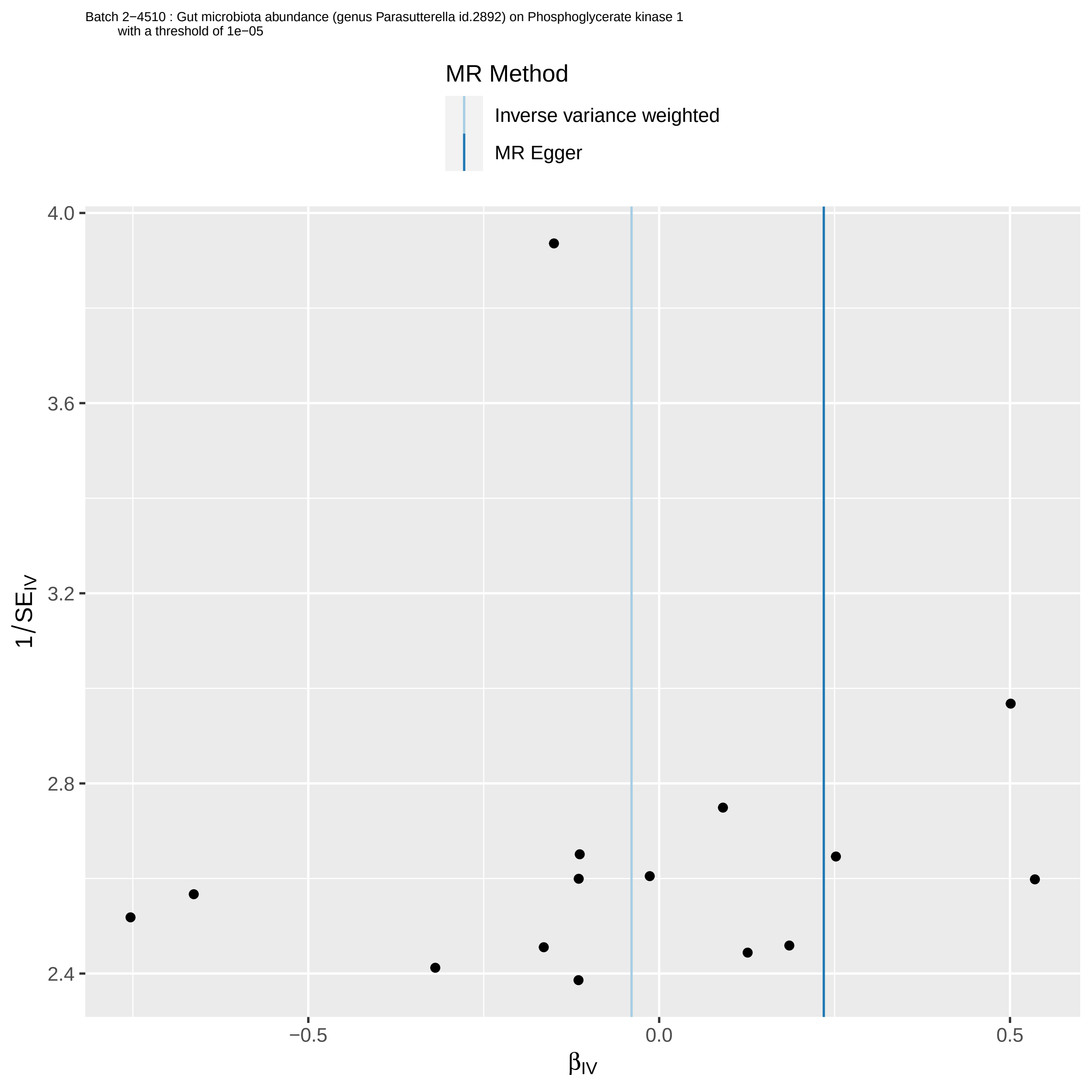

Batch 2−4510 : Gut microbiota abundance (genus Parasutterella id.2892) on Phosphoglycerate kinase 1
with a threshold of 1e−05
MR Method
Inverse variance weighted
MR Egger
4.0
3.6
3.2
2.8
2.4
1/SE$_{IV}$
−0.5
0.0
0.5
β$_{IV}$

Batch 2−4511 : Gut microbiota abundance (genus Catenibacterium id.2153) on Phosphoglycerate kinase 2
with a threshold of 1e−05

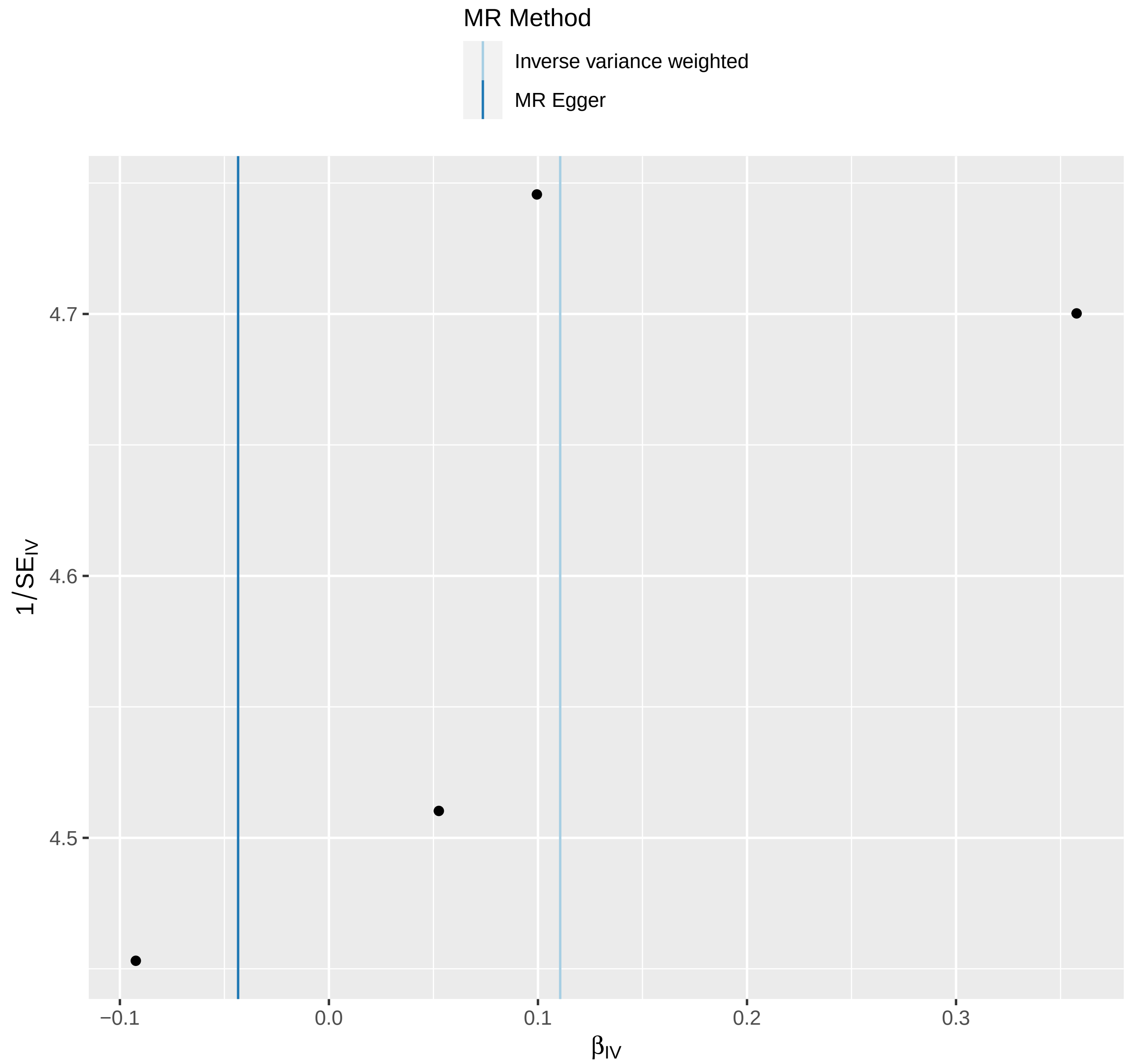

Batch 2−4512 : Gut microbiota abundance (genus Parasutterella id.2892) on Phosphoglycerate kinase 2
with a threshold of 1e−05

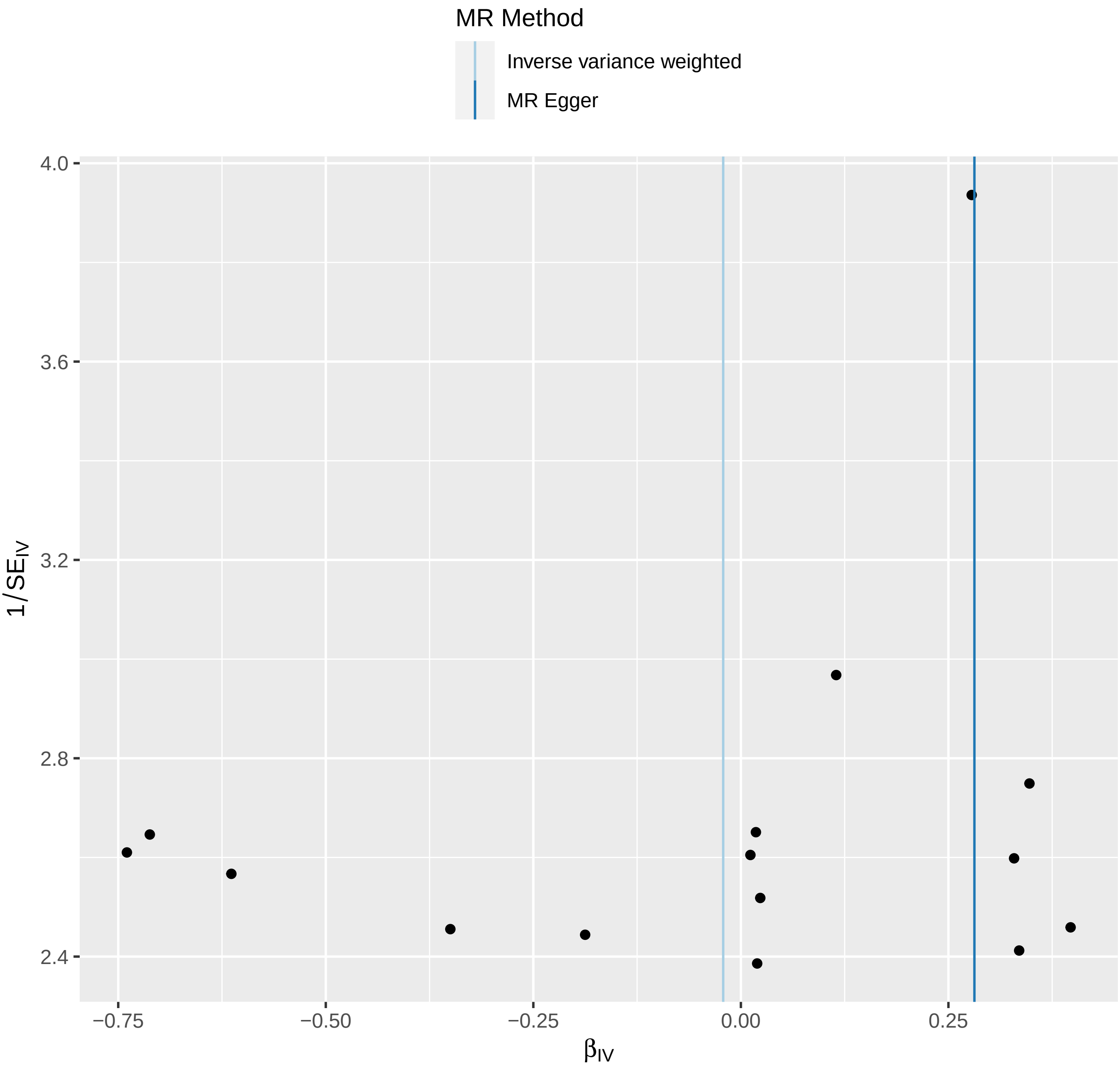

Batch 2−4513 : Gut microbiota abundance (genus Catenibacterium id.2153) on Peptidoglycan recognition protein 1 with a threshold of 1e−05

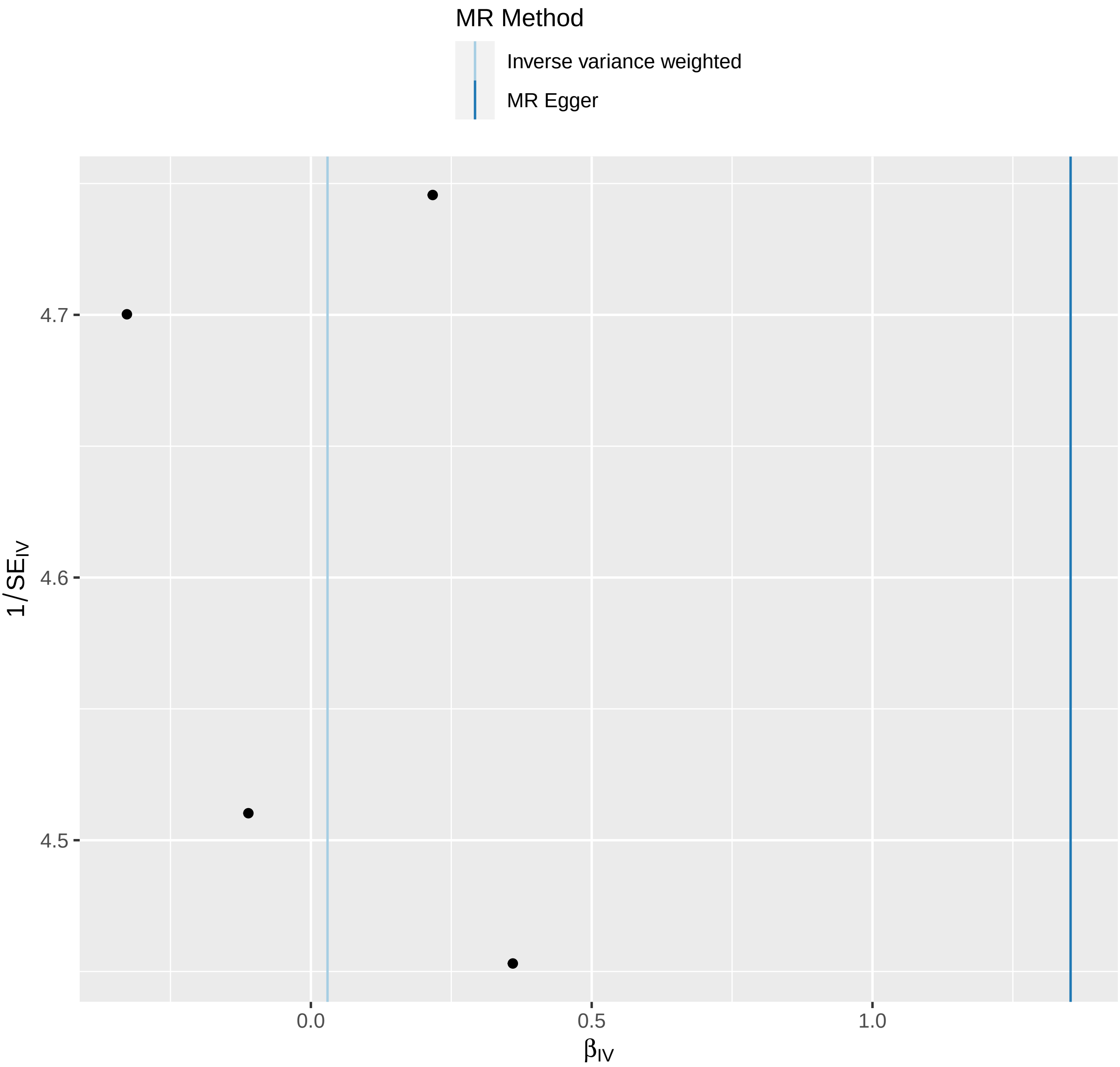

Batch 2−4514 : Gut microbiota abundance (genus Parasutterella id.2892) on Peptidoglycan recognition protein 1
with a threshold of 1e−05

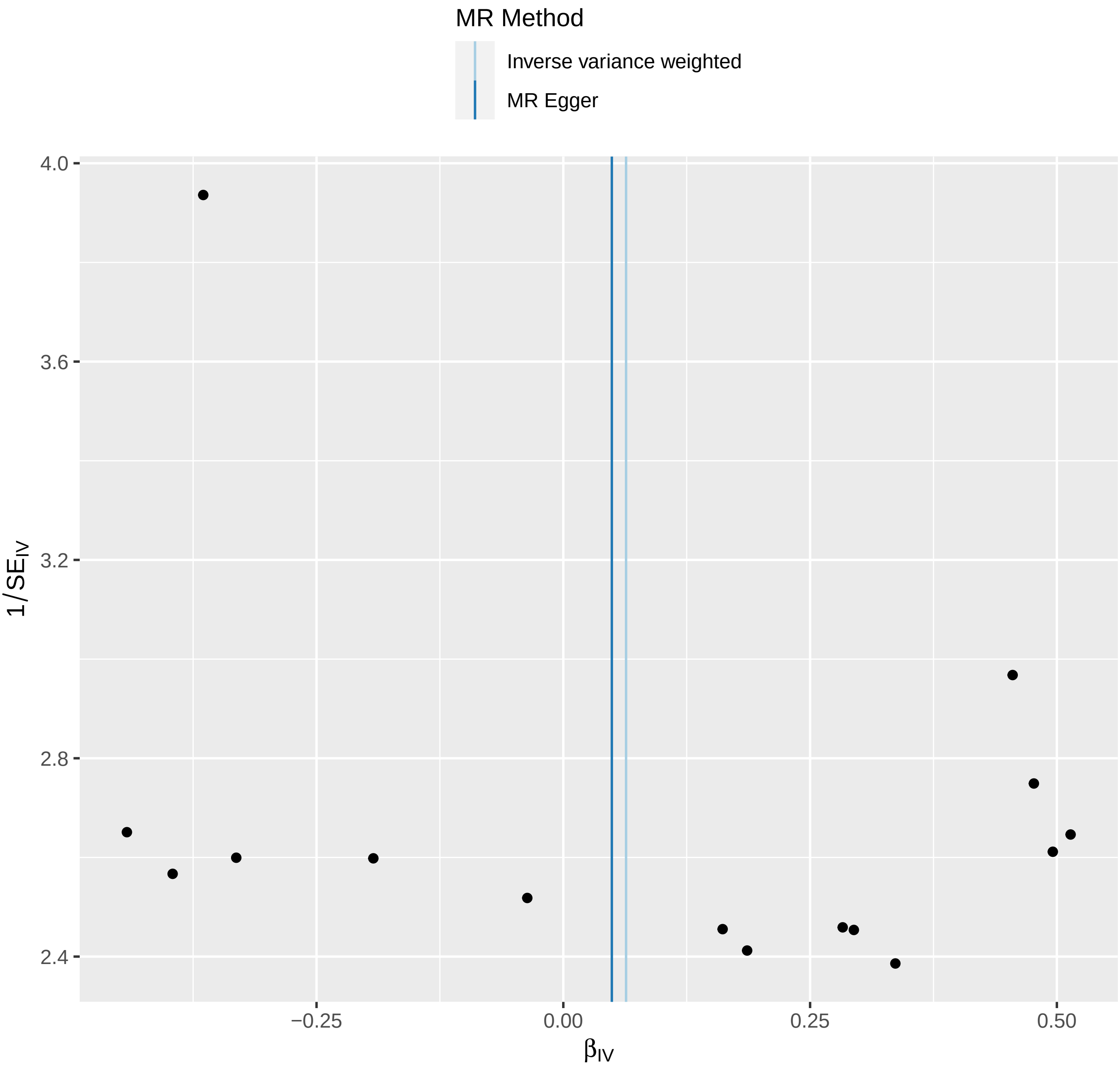

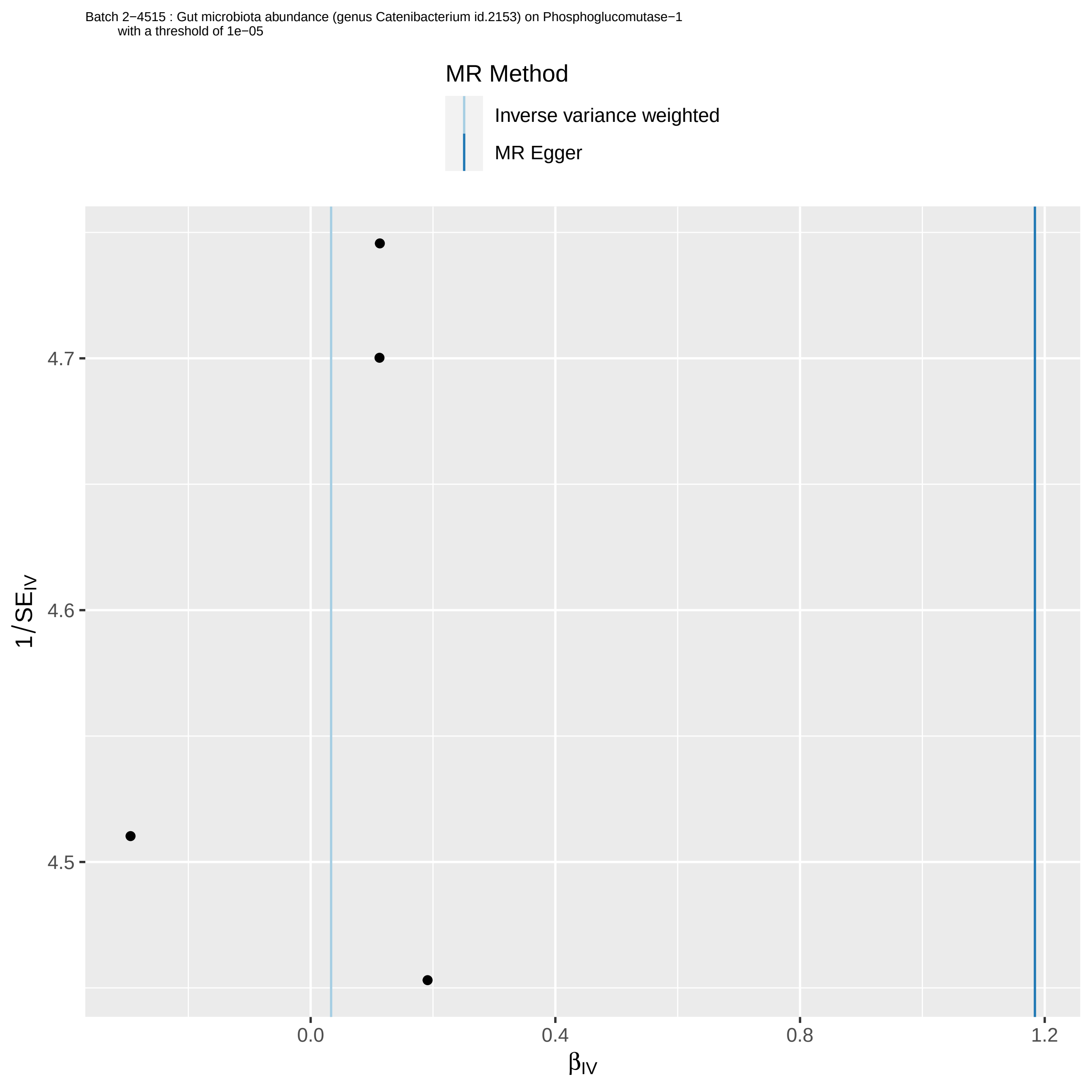
Batch 2−4515 : Gut microbiota abundance (genus Catenibacterium id.2153) on Phosphoglucomutase−1
with a threshold of 1e−05
MR Method
Inverse variance weighted
MR Egger
4.7
4.6
4.5
1/SE_IV
0.0
0.4
0.8
1.2
β_IV

Batch 2−4516 : Gut microbiota abundance (genus Parasutterella id.2892) on Phosphoglucomutase−1
with a threshold of 1e−05

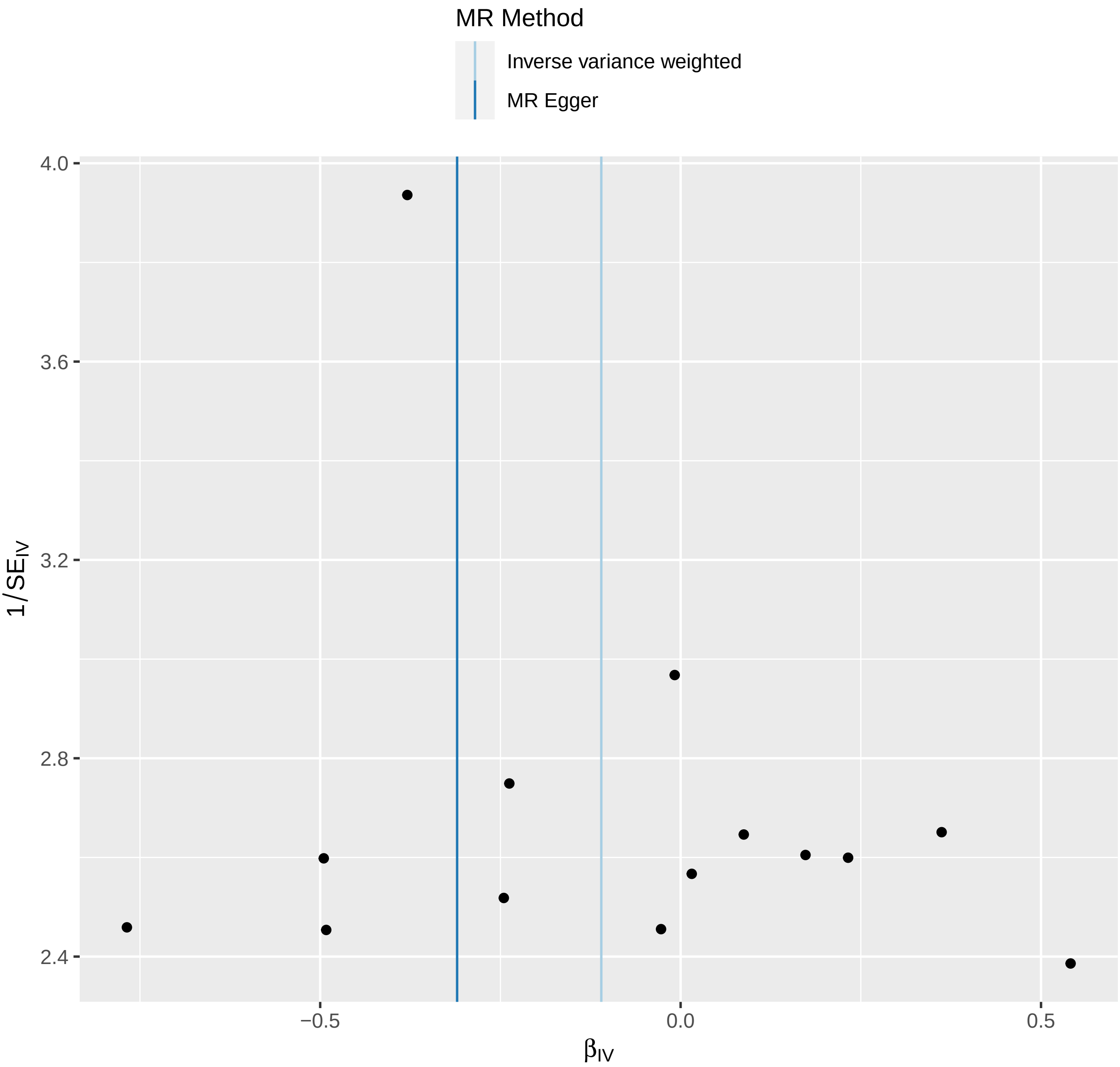

Batch 2−4517 : Gut microbiota abundance (genus Catenibacterium id.2153) on Membrane−associated progesterone receptor component 1 with a threshold of 1e−05

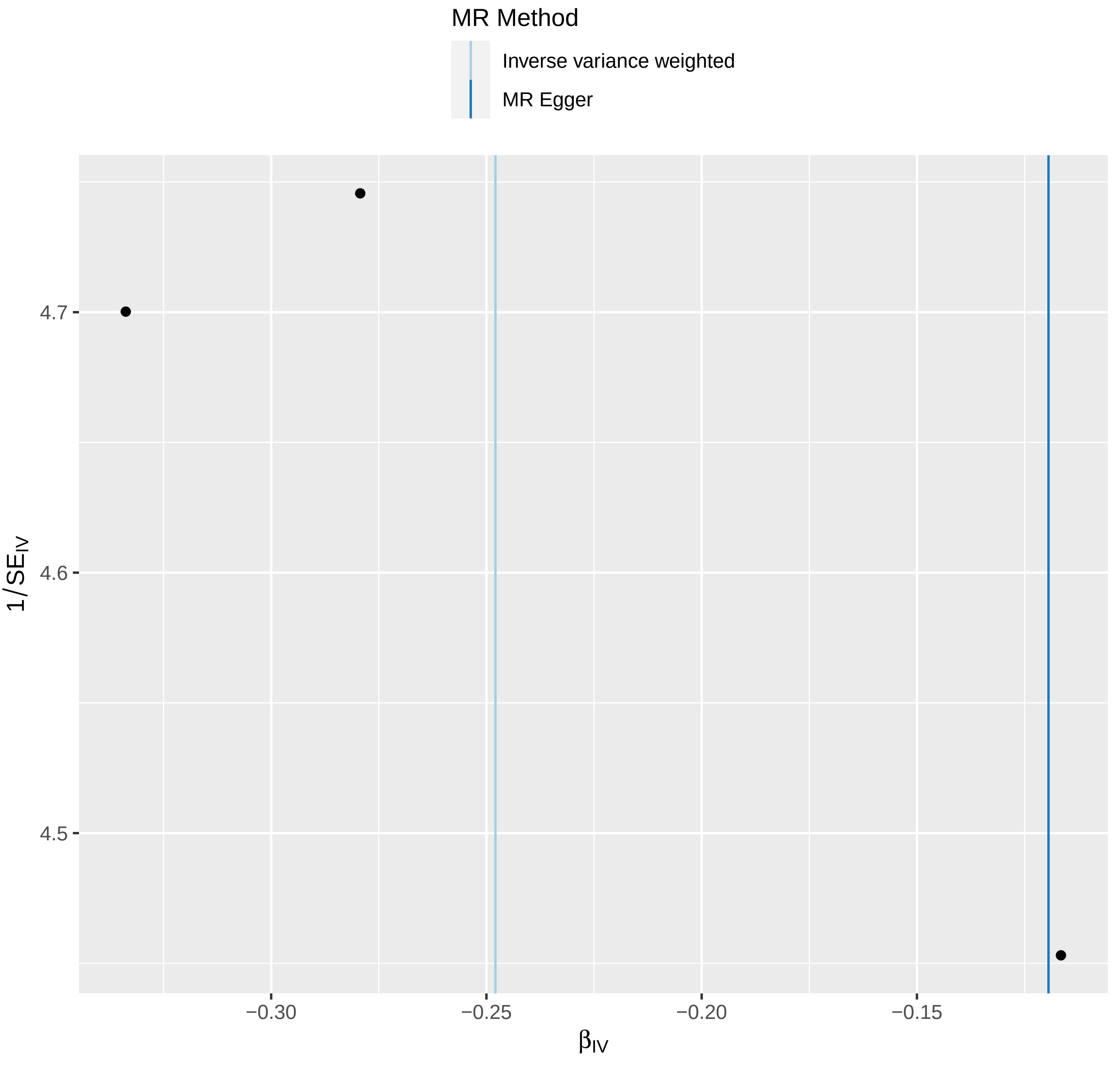

Batch 2−4518 : Gut microbiota abundance (genus Parasutterella id.2892) on Membrane−associated progesterone receptor component 1 with a threshold of 1e−05

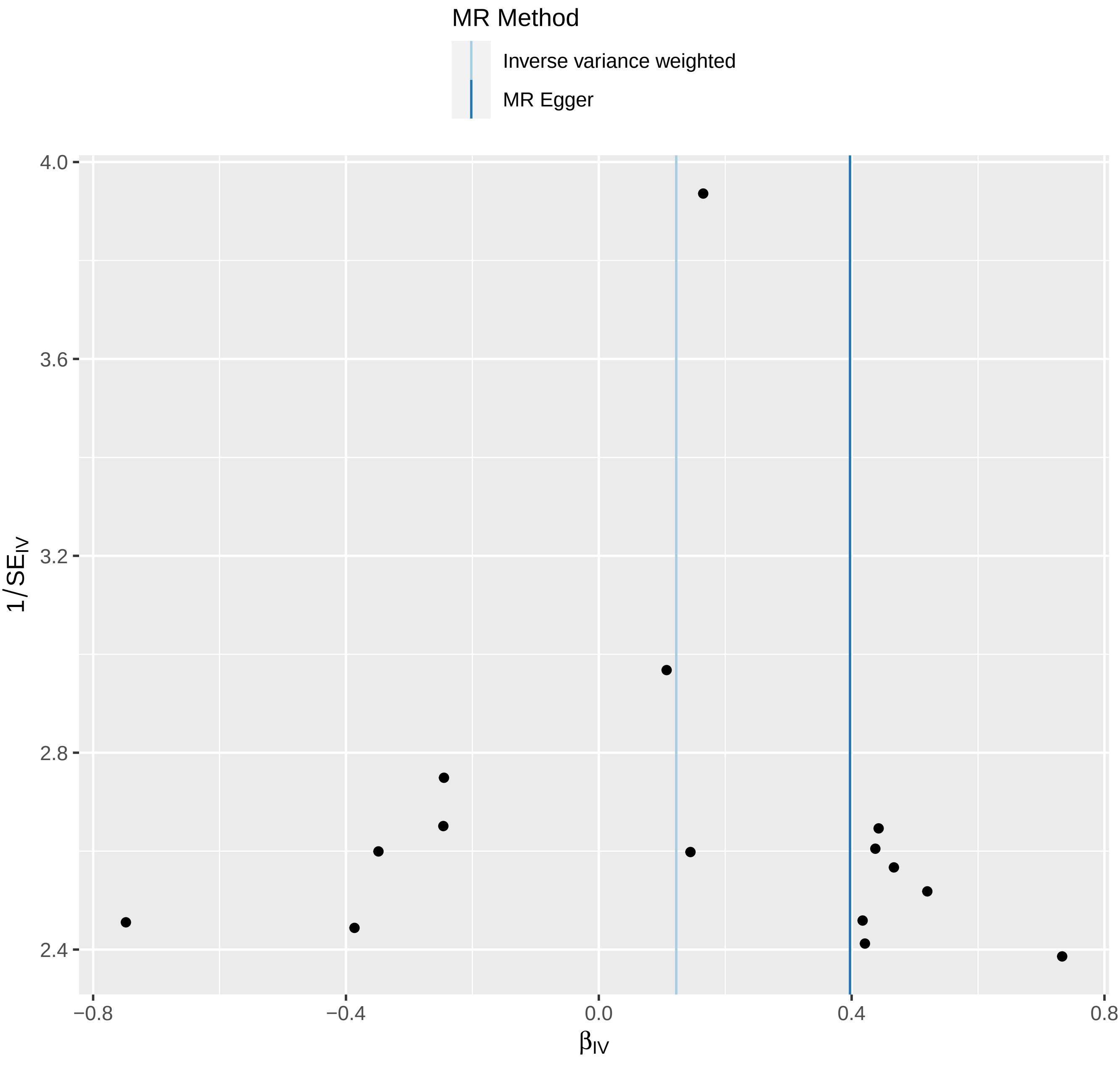

Batch 2−4519 : Gut microbiota abundance (genus Catenibacterium id.2153) on Membrane−associated progesterone receptor component 2 with a threshold of 1e−05

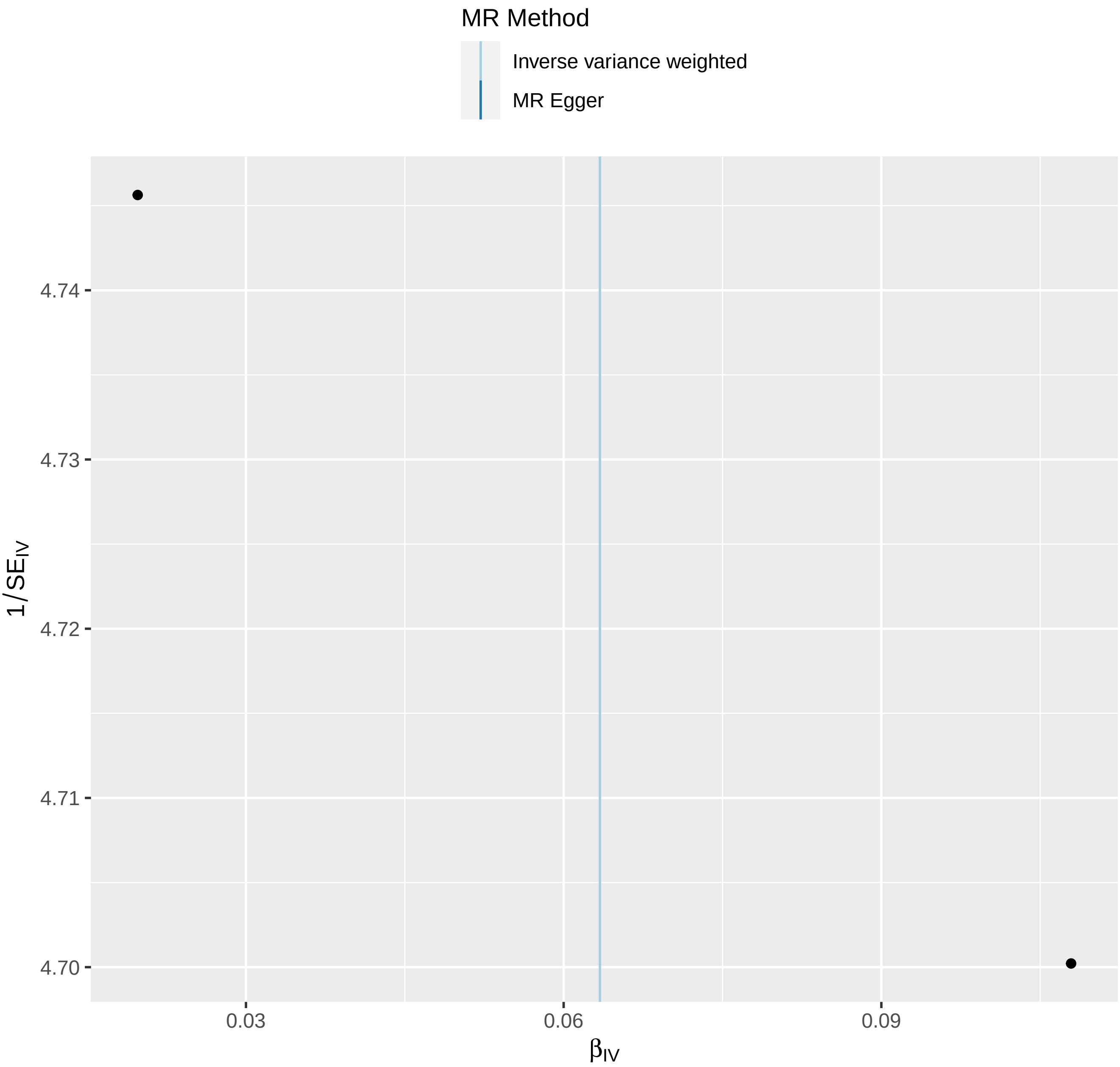

Batch 2−4520 : Gut microbiota abundance (genus Parasutterella id.2892) on Membrane−associated progesterone receptor component 2
with a threshold of 1e−05

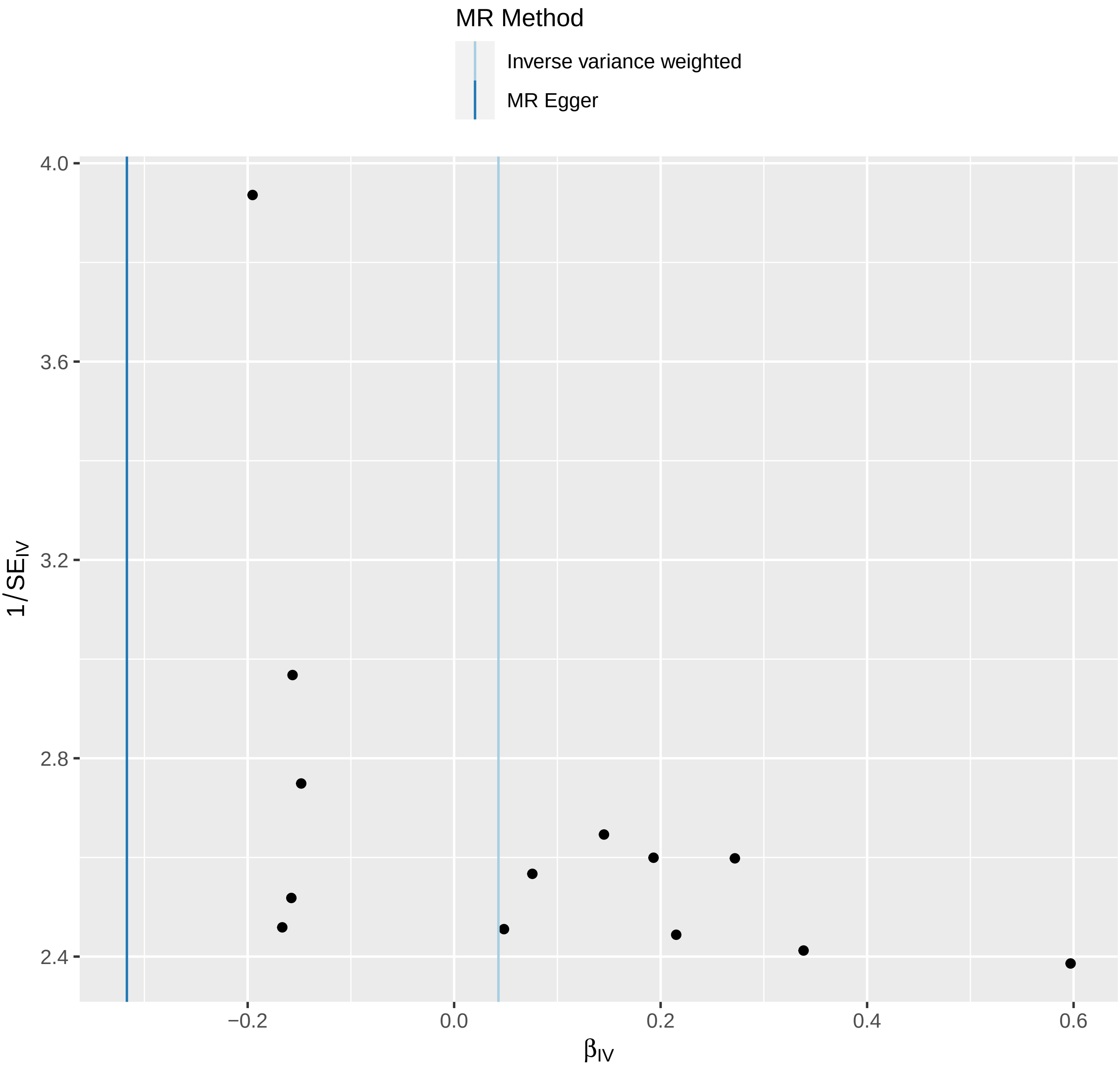

Batch 2−4521 : Gut microbiota abundance (genus Catenibacterium id.2153) on Membrane−associated progesterone receptor component 2 with a threshold of 1e−05

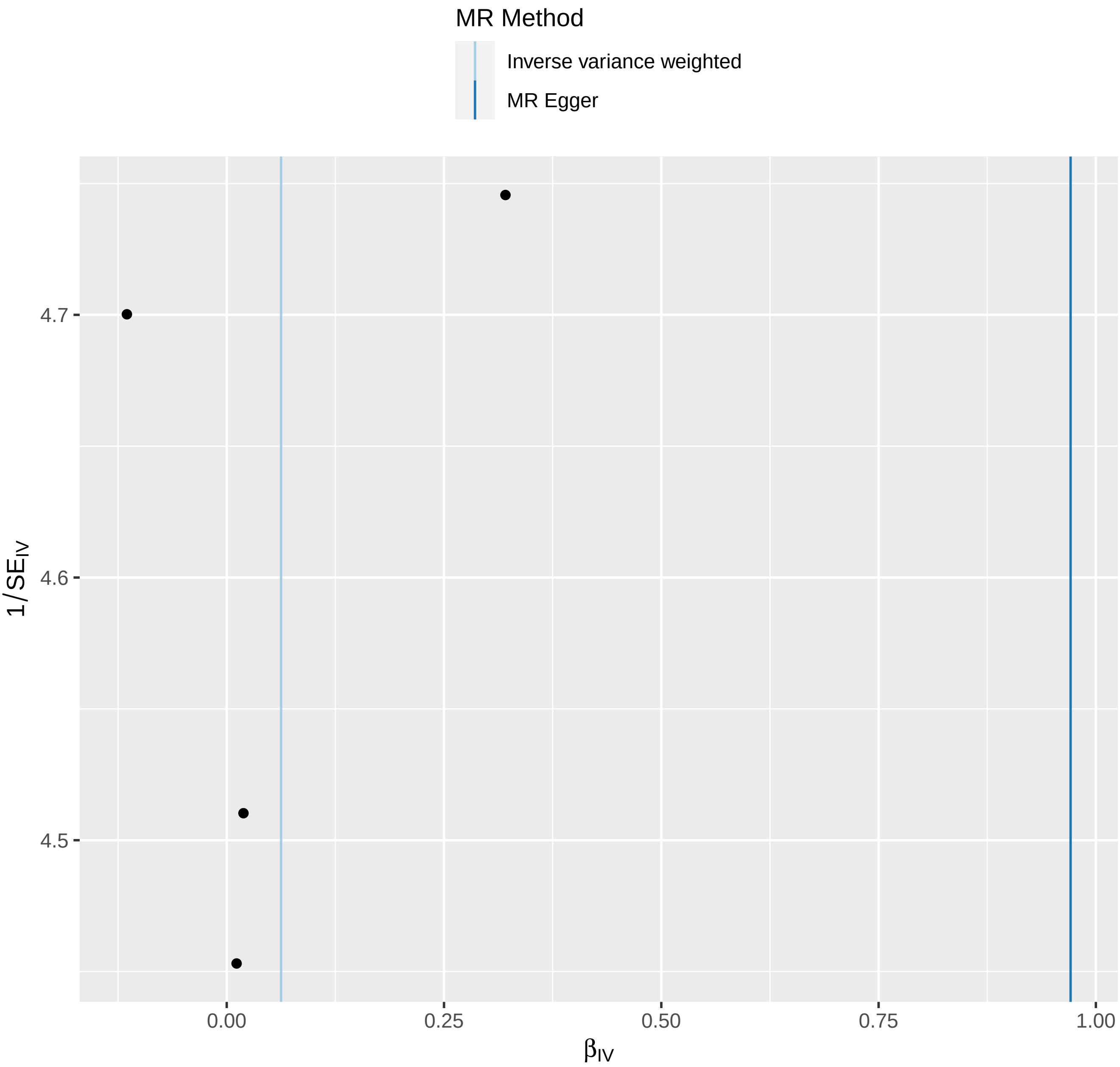

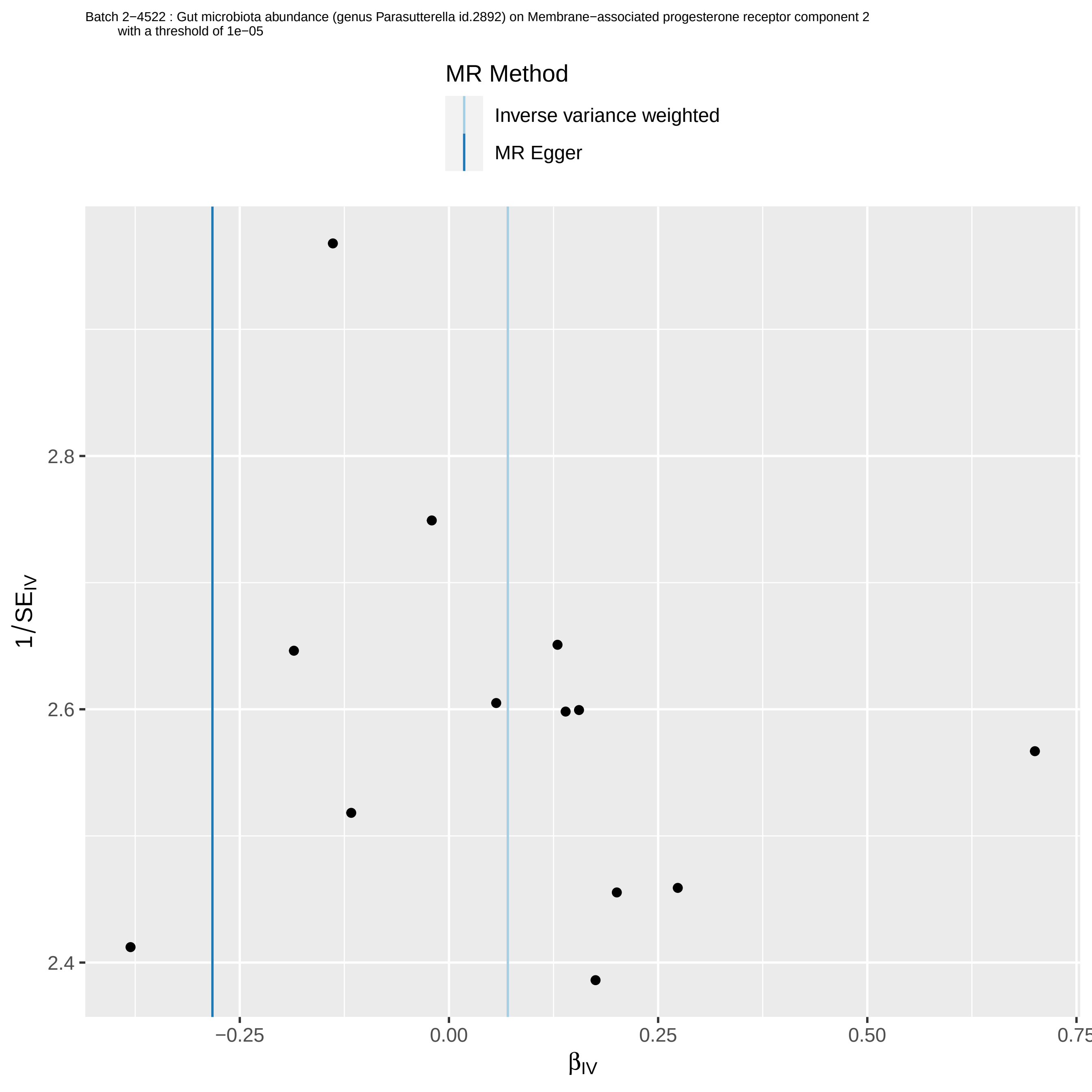

Batch 2−4522 : Gut microbiota abundance (genus Parasutterella id.2892) on Membrane−associated progesterone receptor component 2
with a threshold of 1e−05
MR Method
Inverse variance weighted
MR Egger
2.8
2.6
2.4
1/SE_IV
−0.25
0.00
0.25
0.50
0.75
β_IV

Batch 2−4523 : Gut microbiota abundance (genus Catenibacterium id.2153) on Membrane−associated progesterone receptor component 2 with a threshold of 1e−05

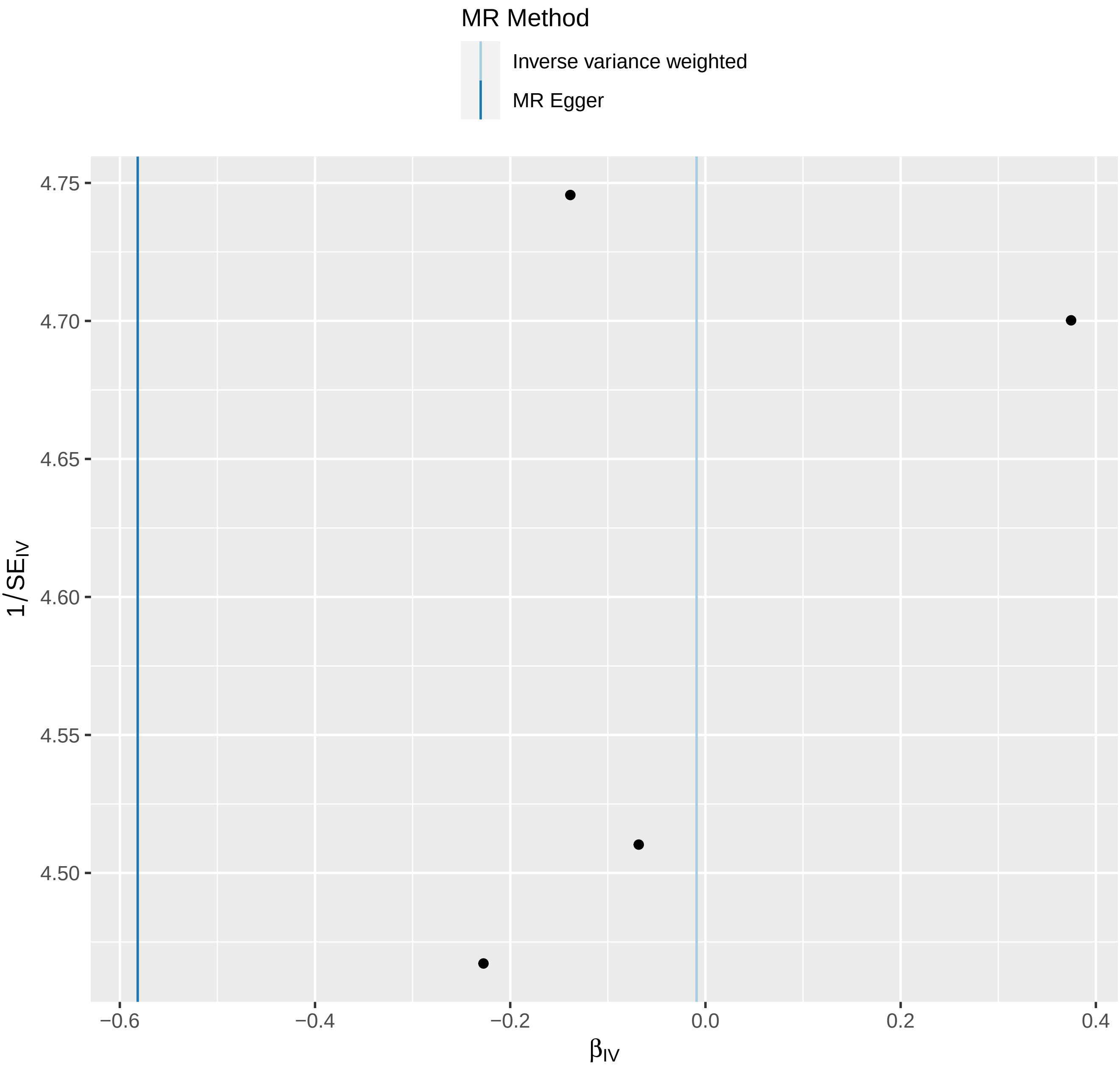

Batch 2−4524 : Gut microbiota abundance (genus Parasutterella id.2892) on Membrane−associated progesterone receptor component 2
with a threshold of 1e−05

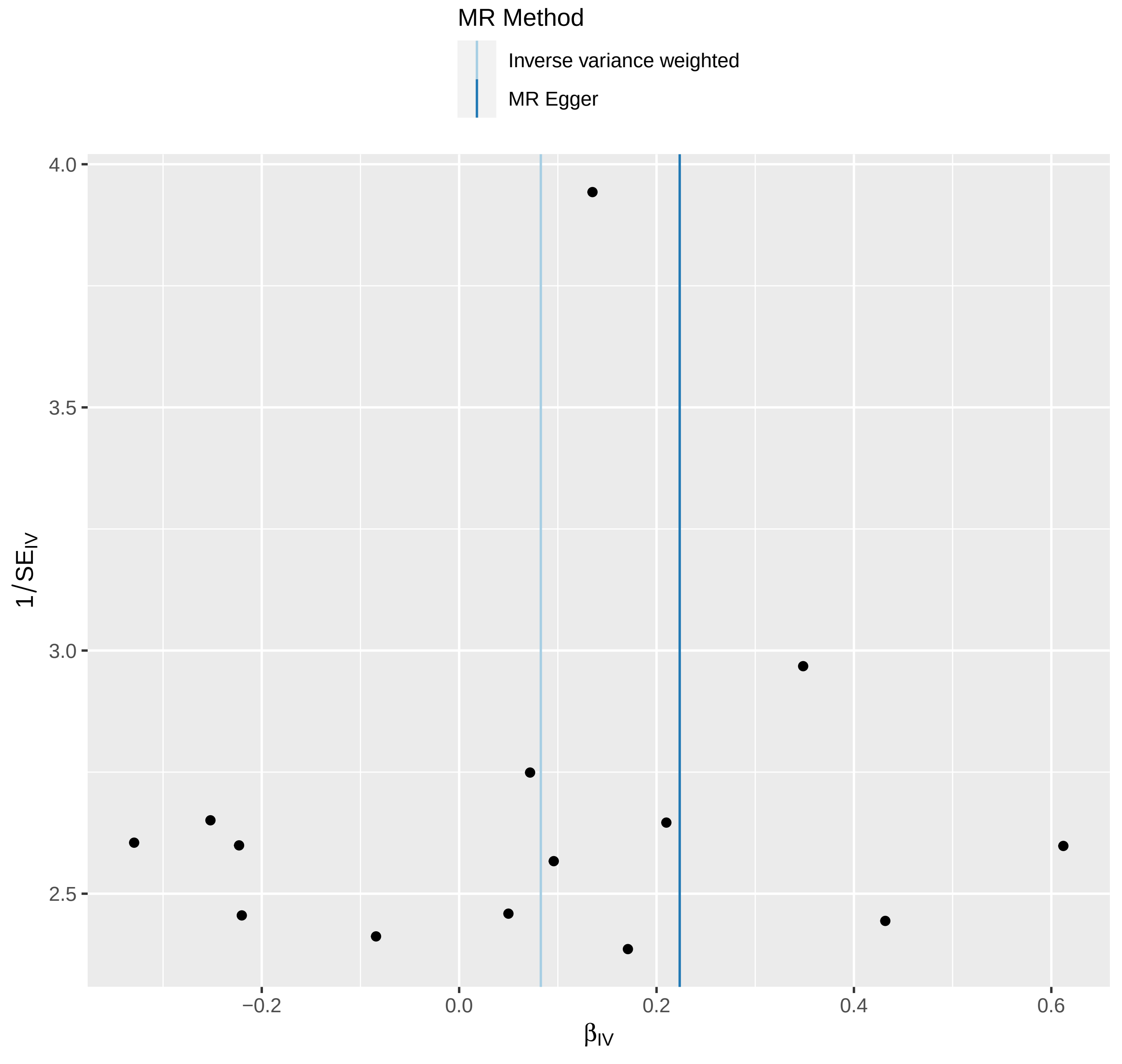

Batch 2−4525 : Gut microbiota abundance (genus Catenibacterium id.2153) on PHD finger protein 3
with a threshold of 1e−05

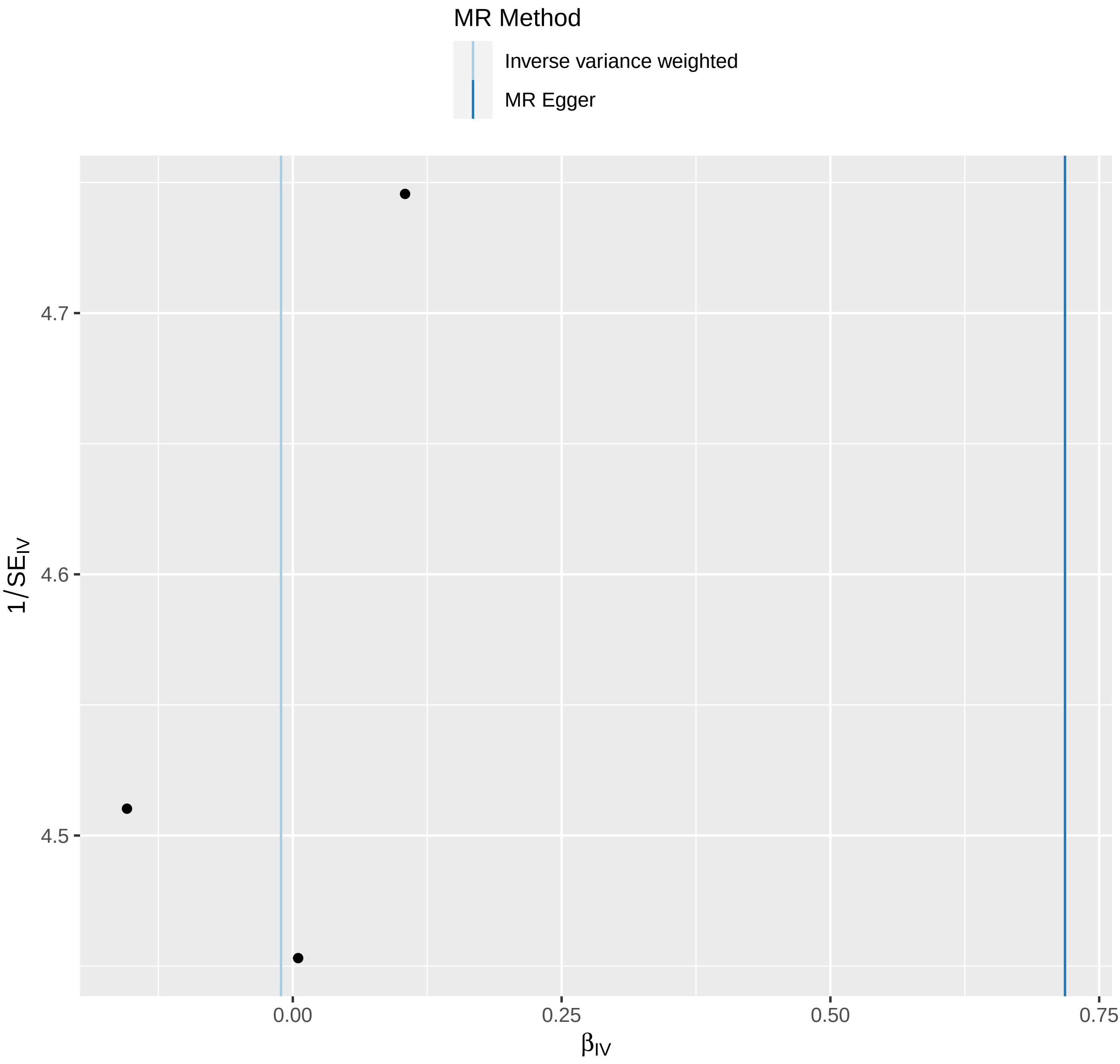

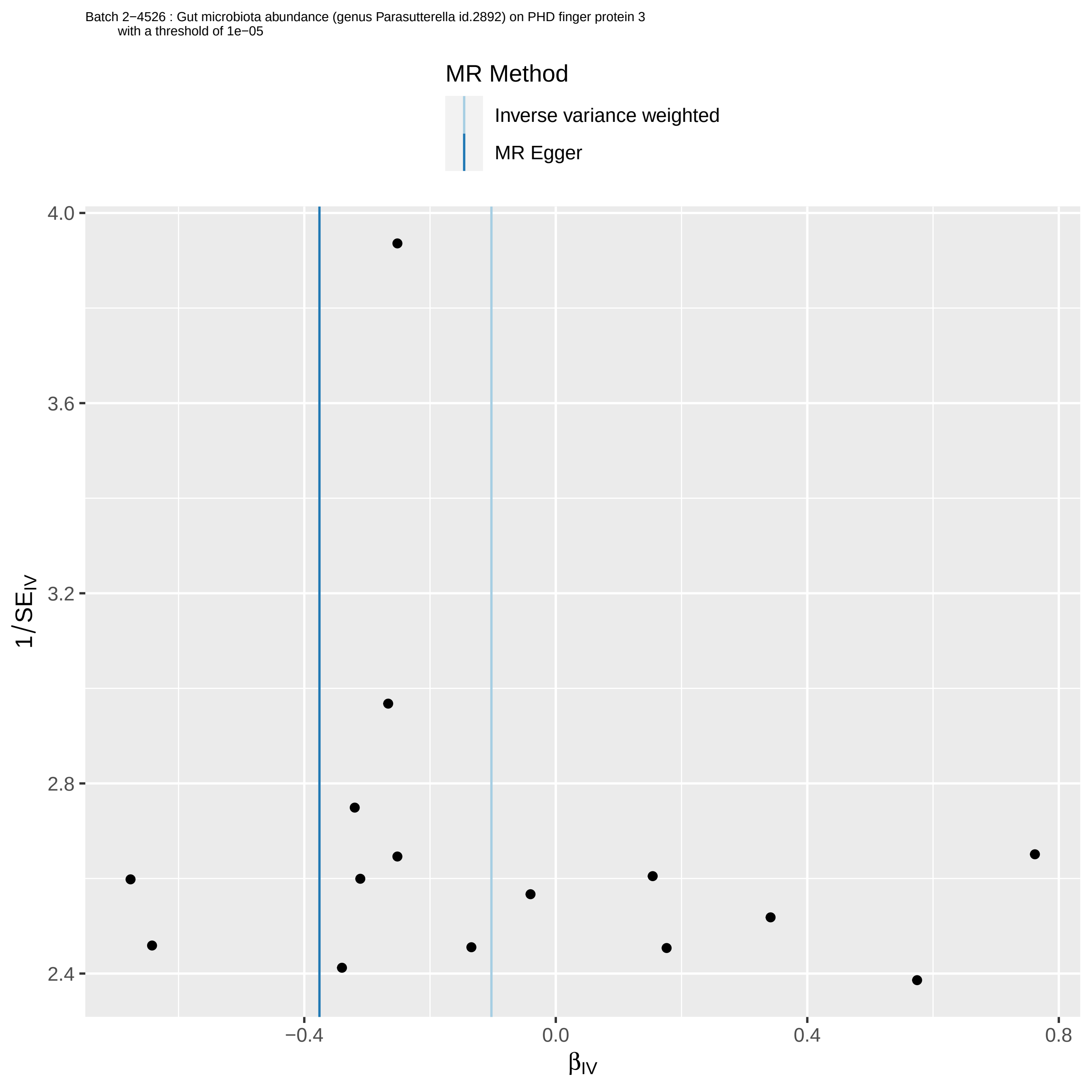
Batch 2−4526 : Gut microbiota abundance (genus Parasutterella id.2892) on PHD finger protein 3
with a threshold of 1e−05
MR Method
Inverse variance weighted
MR Egger
4.0
3.6
3.2
2.8
2.4
1/SE_IV
−0.4
0.0
0.4
0.8
β_IV

Batch 2−4527 : Gut microbiota abundance (genus Catenibacterium id.2153) on Peptidase inhibitor 15
with a threshold of 1e−05

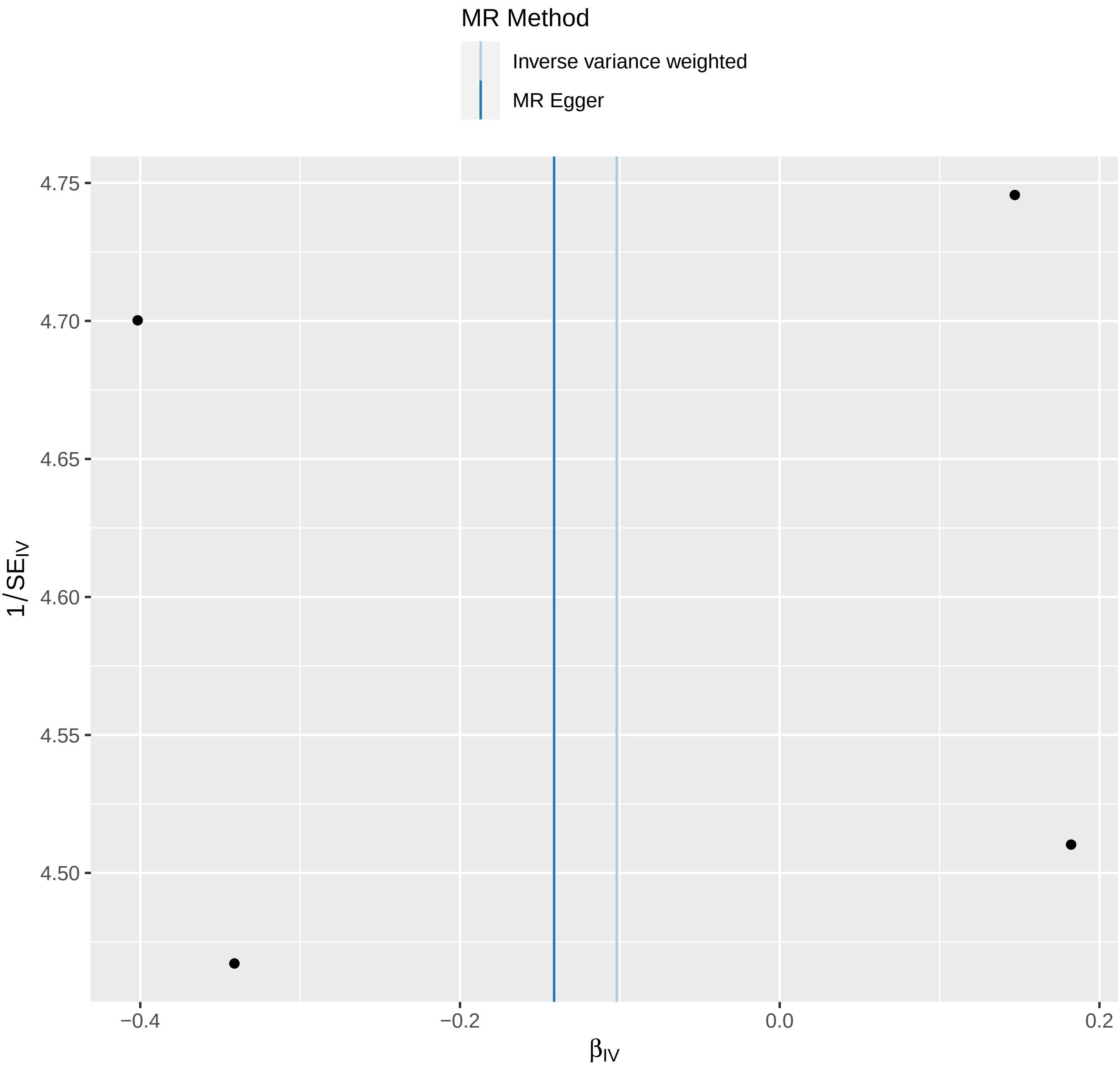

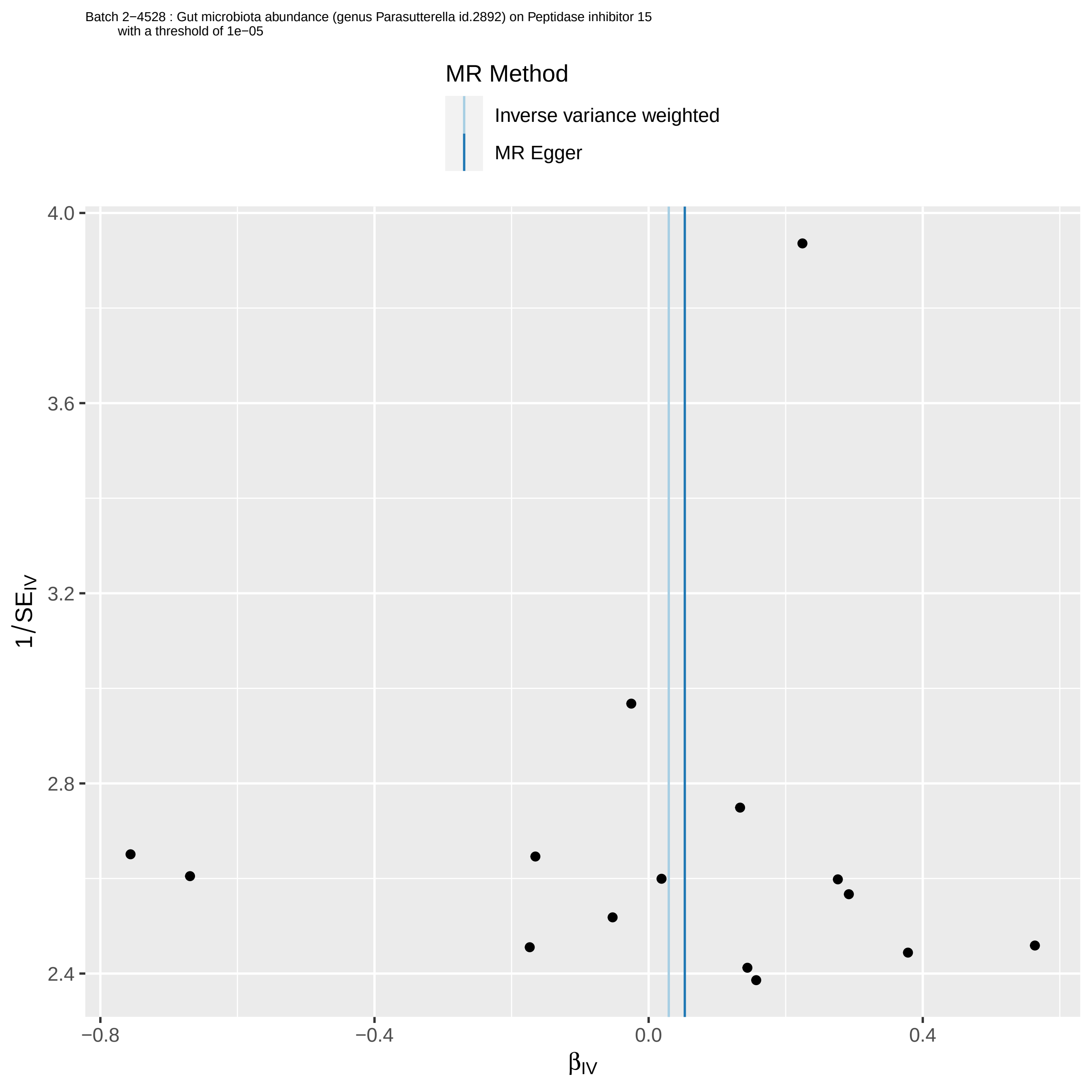

Batch 2−4528 : Gut microbiota abundance (genus Parasutterella id.2892) on Peptidase inhibitor 15
with a threshold of 1e−05
MR Method
Inverse variance weighted
MR Egger
4.0
3.6
3.2
2.8
2.4
1/SE$_{IV}$
−0.8
−0.4
0.0
0.4
β$_{IV}$

Batch 2−4529 : Gut microbiota abundance (genus Catenibacterium id.2153) on Elafin
with a threshold of 1e−05

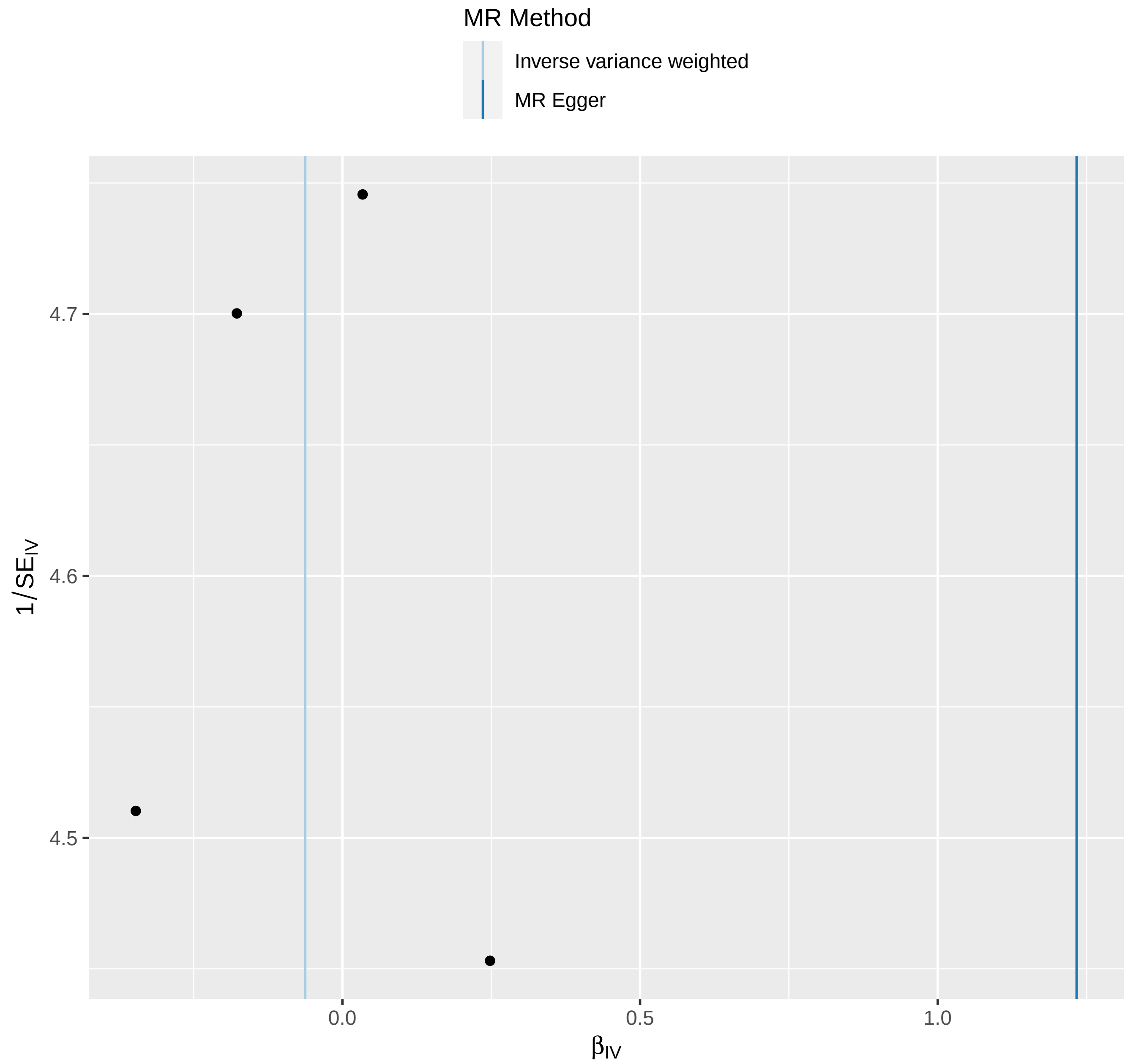

Batch 2−4530 : Gut microbiota abundance (genus Parasutterella id.2892) on Elafin
with a threshold of 1e−05

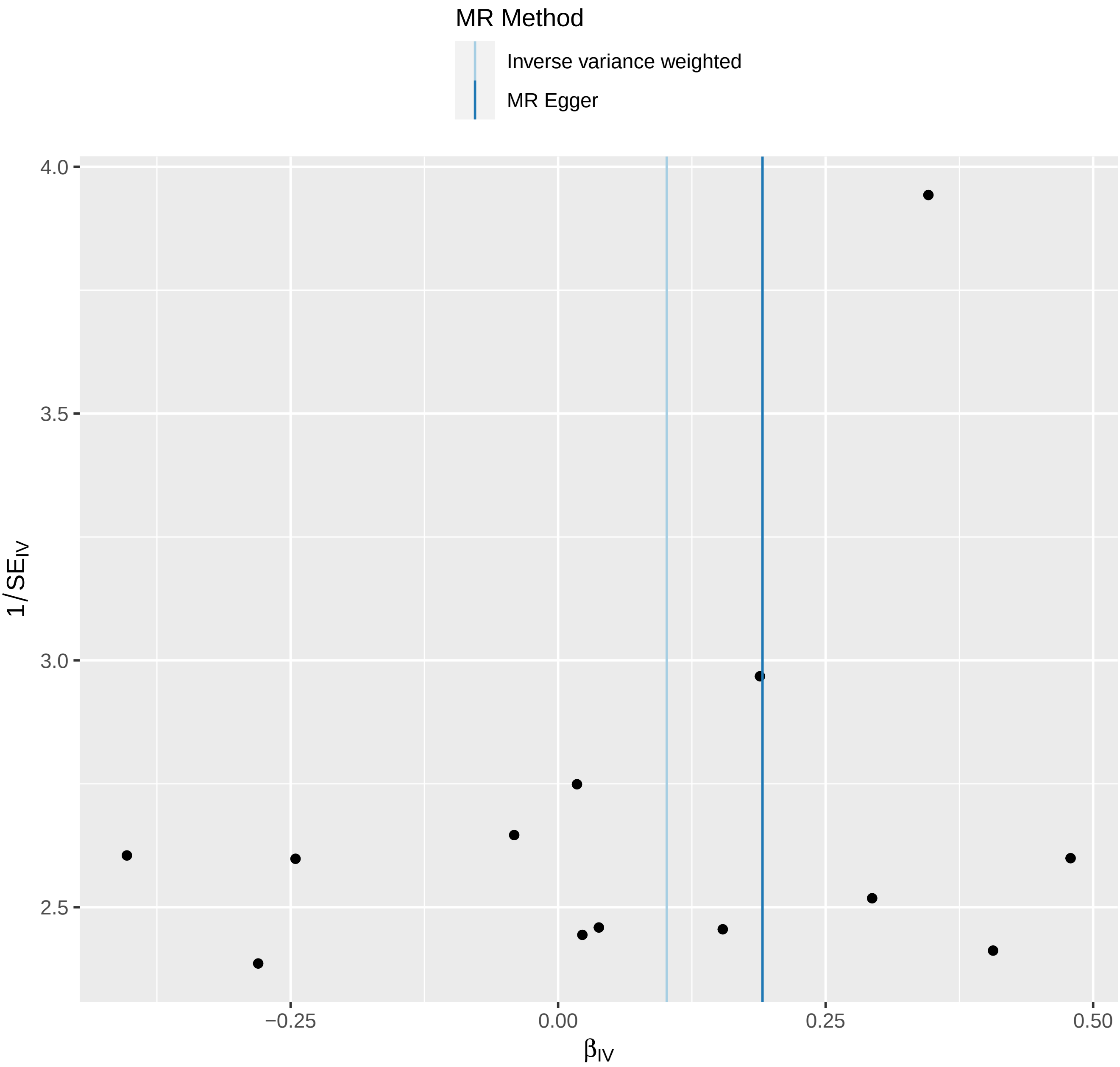

Batch 2−4531 : Gut microbiota abundance (genus Catenibacterium id.2153) on PILR alpha−associated neural protein with a threshold of 1e−05

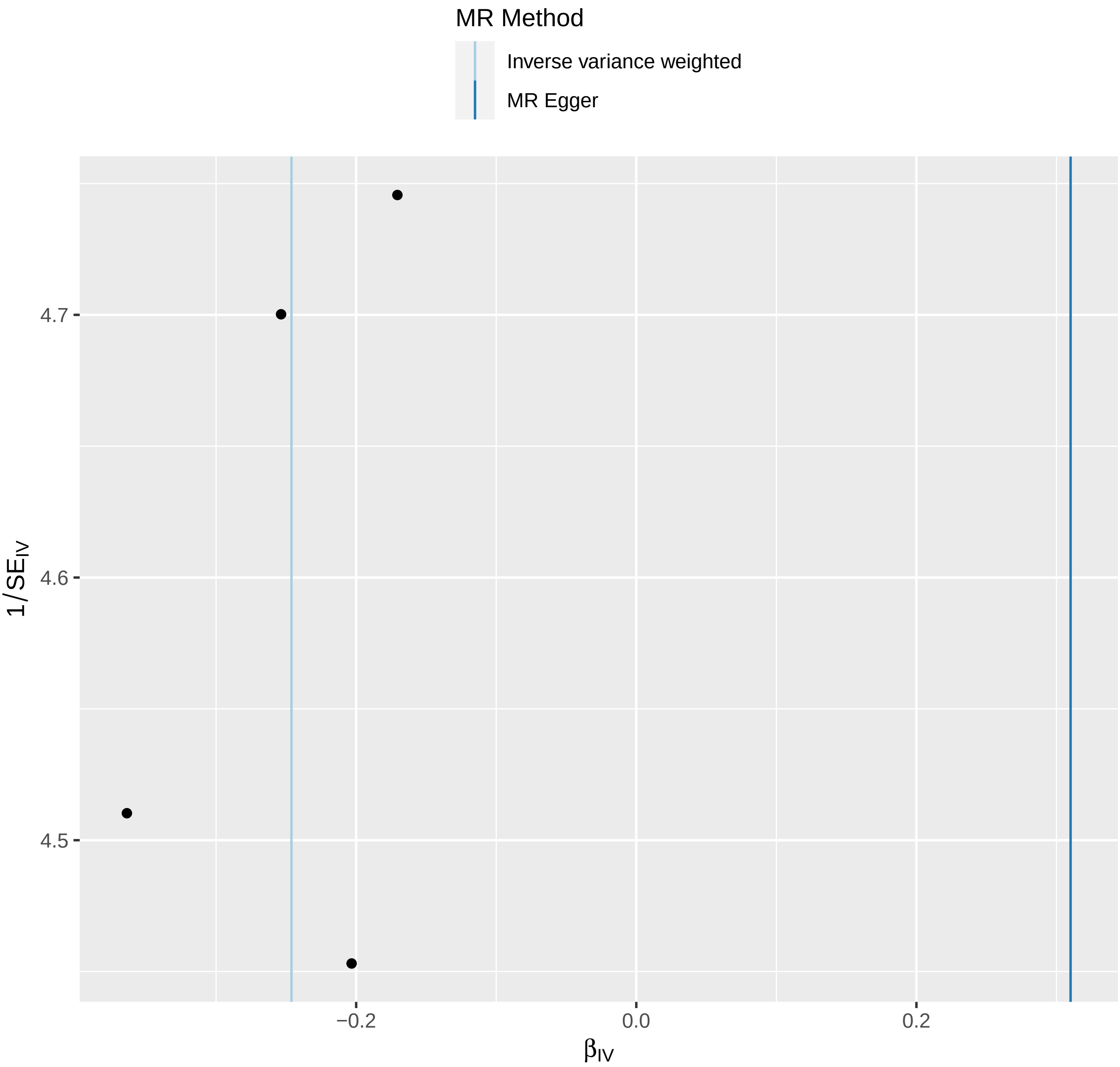

Batch 2−4532 : Gut microbiota abundance (genus Parasutterella id.2892) on PILR alpha−associated neural protein with a threshold of 1e−05

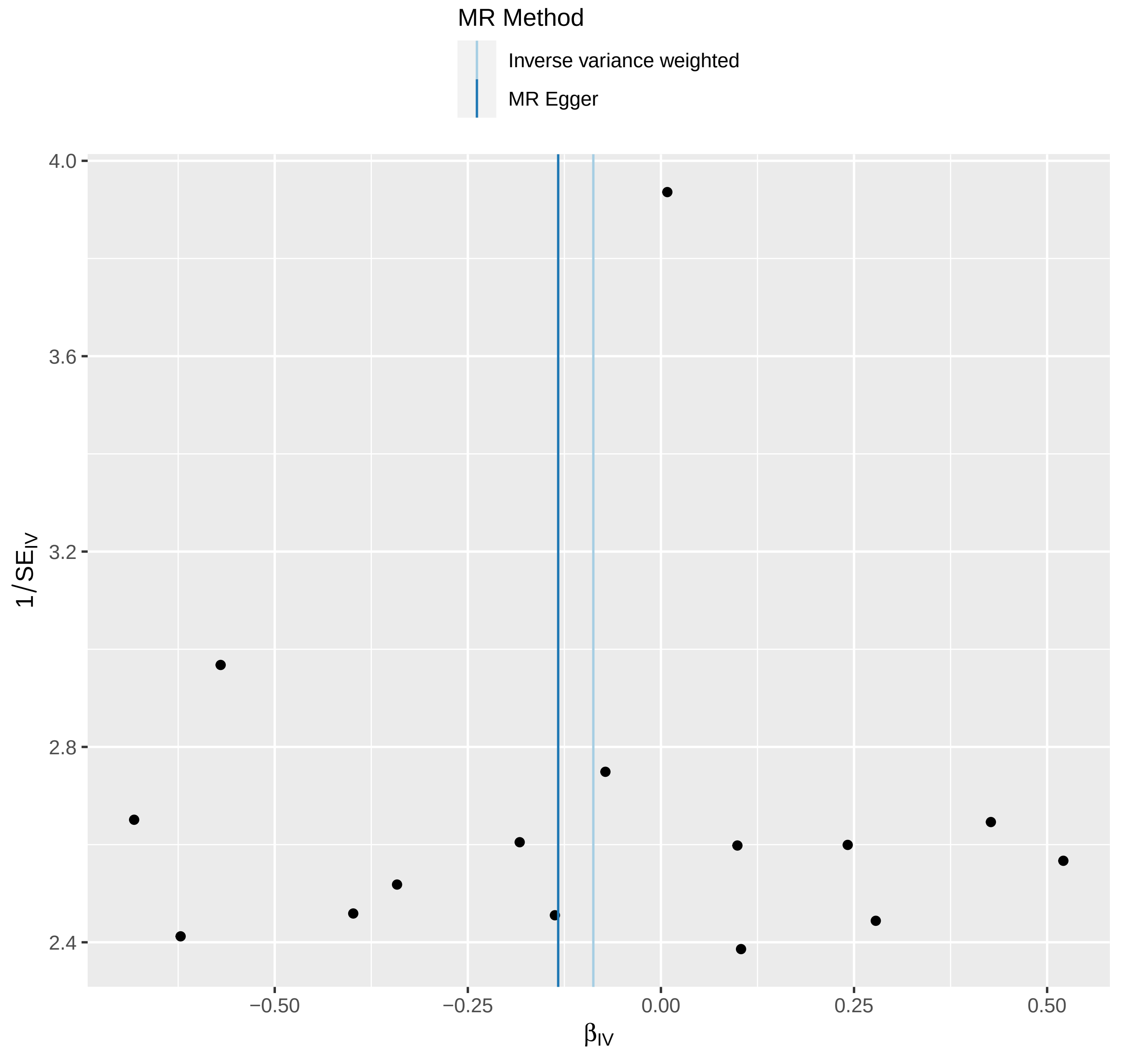

Batch 2−4533 : Gut microbiota abundance (genus Catenibacterium id.2153) on PILR alpha−associated neural protein with a threshold of 1e−05

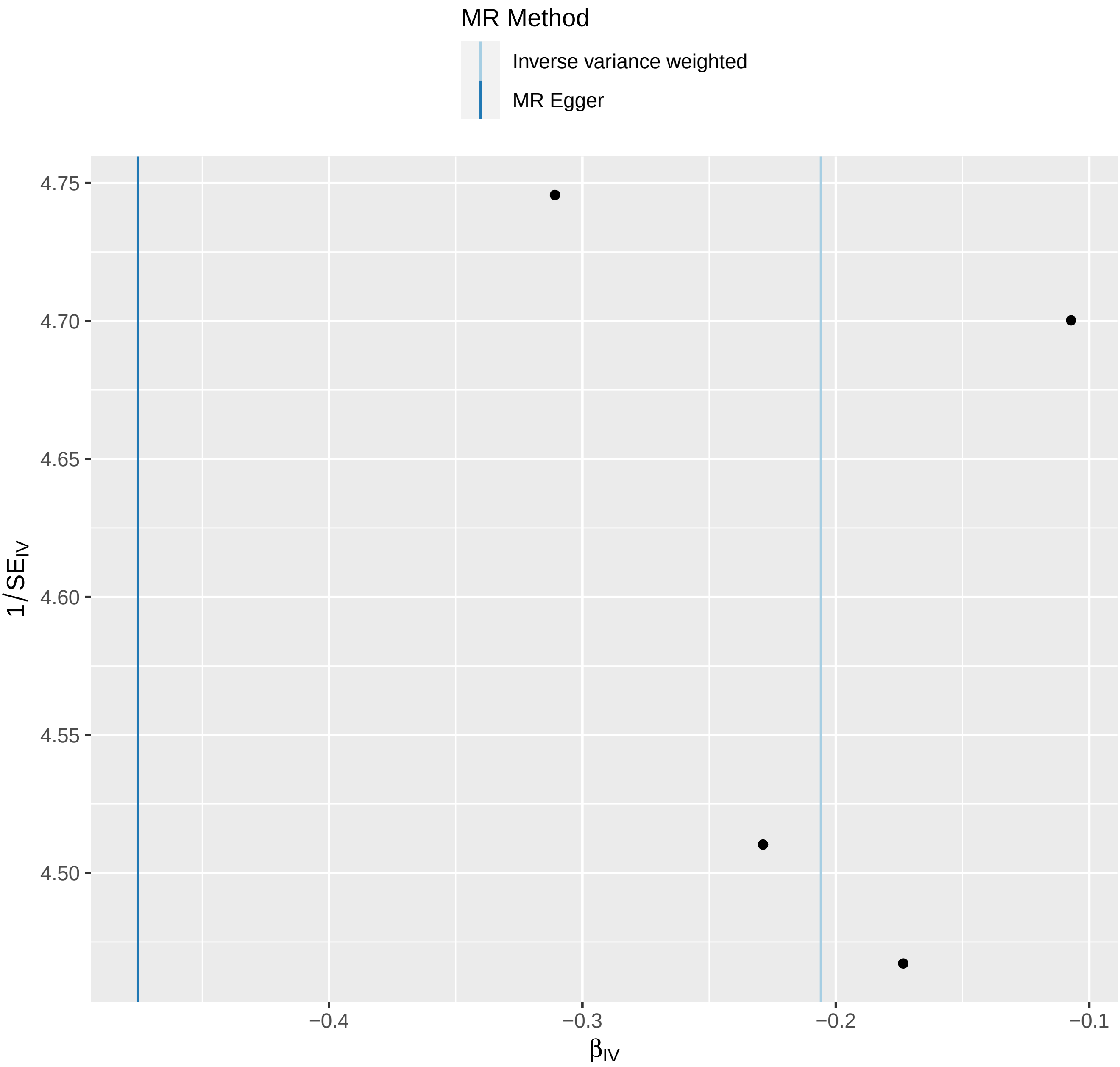

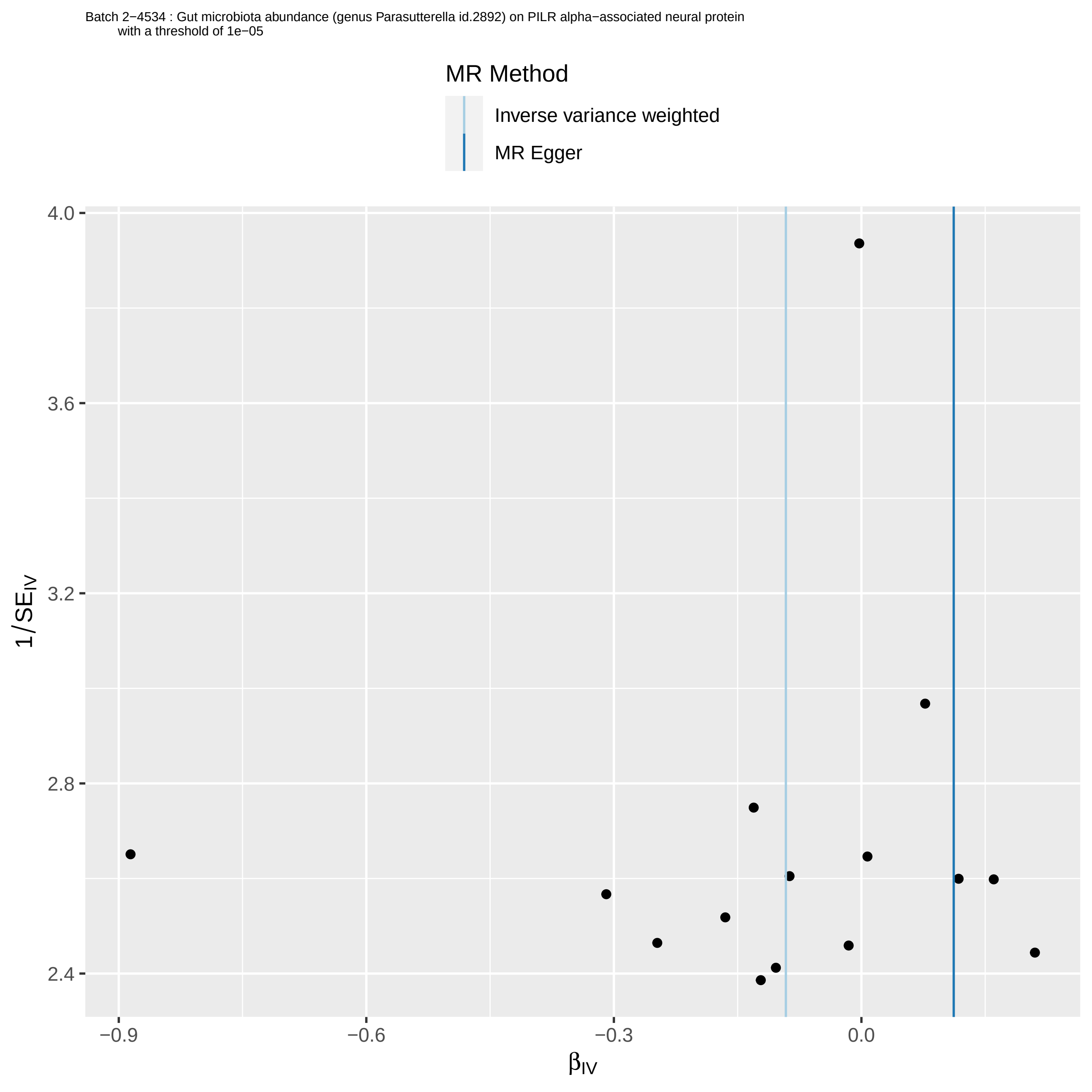
Batch 2−4534 : Gut microbiota abundance (genus Parasutterella id.2892) on PILR alpha−associated neural protein
with a threshold of 1e−05
MR Method
Inverse variance weighted
MR Egger
4.0
3.6
3.2
2.8
2.4
1/SE_IV
−0.9
−0.6
−0.3
0.0
β_IV

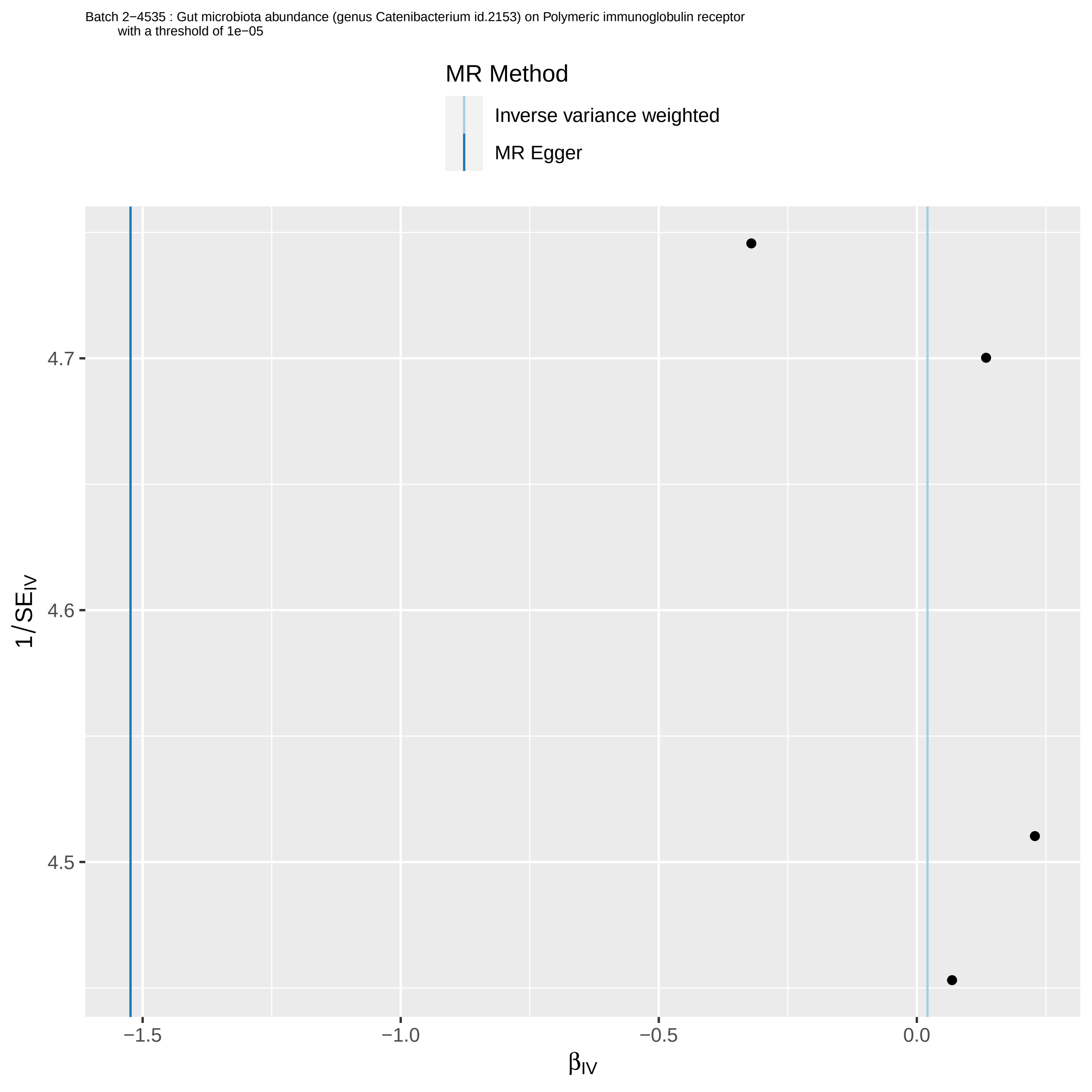
Batch 2−4535 : Gut microbiota abundance (genus Catenibacterium id.2153) on Polymeric immunoglobulin receptor
with a threshold of 1e−05
MR Method
Inverse variance weighted
MR Egger
4.7
4.6
4.5
1/SE_IV
−1.5
−1.0
−0.5
0.0
β_IV

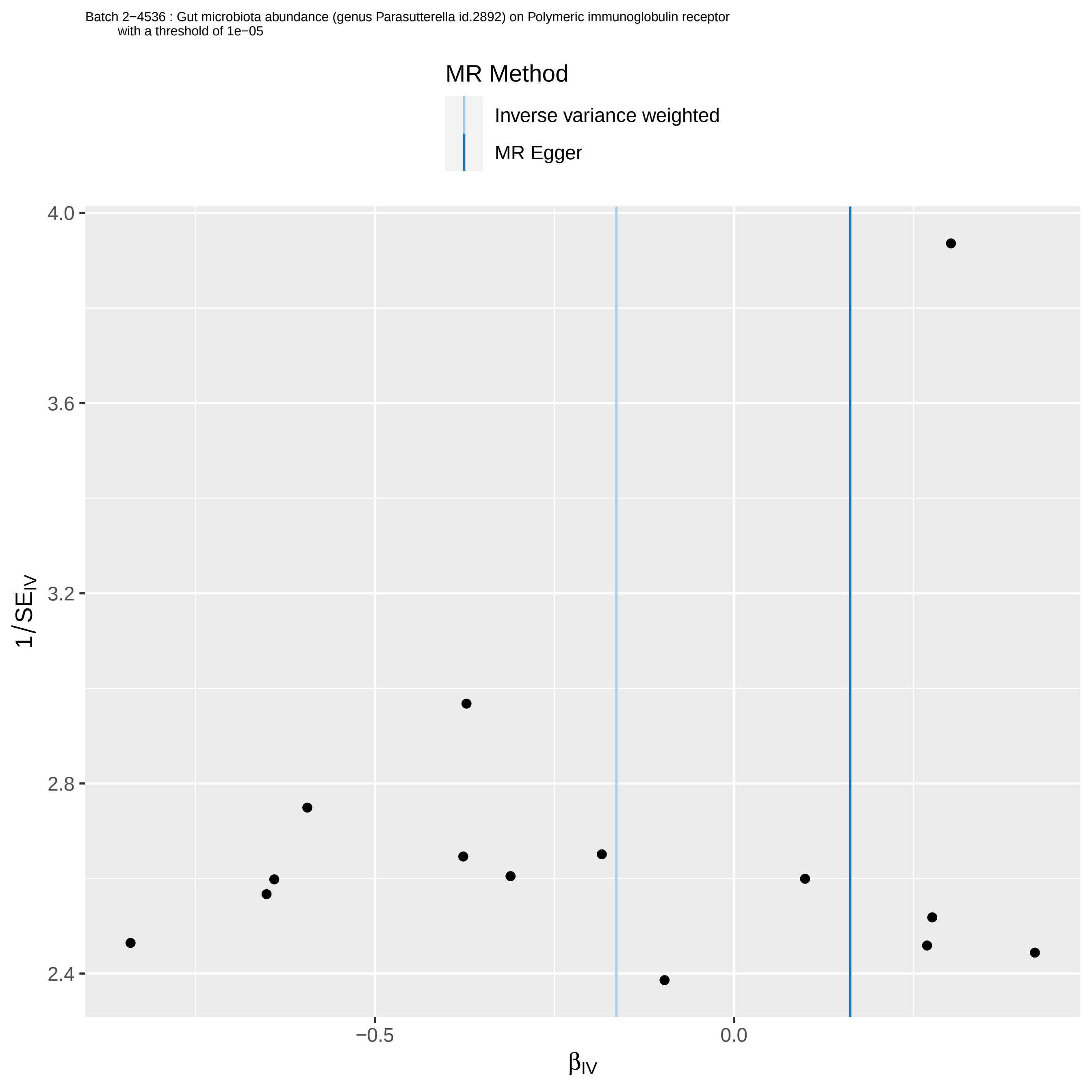

Batch 2−4536 : Gut microbiota abundance (genus Parasutterella id.2892) on Polymeric immunoglobulin receptor
with a threshold of 1e−05
MR Method
Inverse variance weighted
MR Egger
4.0
3.6
3.2
2.8
2.4
1/SE_IV
−0.5
0.0
β_IV

Batch 2−4537 : Gut microbiota abundance (genus Catenibacterium id.2153) on Phosphatidylinositol 4−phosphate 3−kinase C2 domain−containing subunit alpha with a threshold of 1e−05

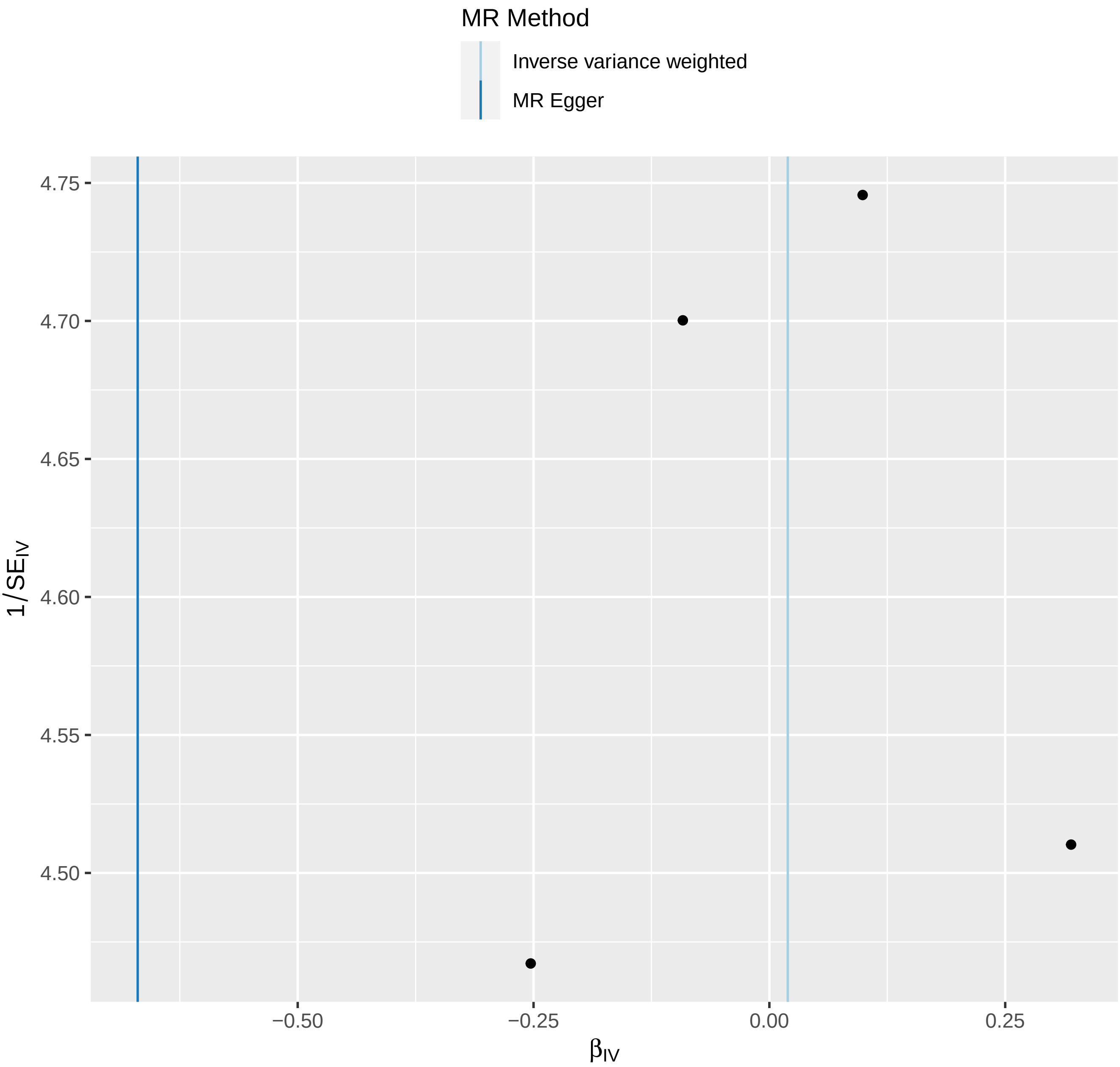

Batch 2−4538 : Gut microbiota abundance (genus Parasutterella id.2892) on Phosphatidylinositol 4−phosphate 3−kinase C2 domain−containing subunit alpha with a threshold of 1e−05

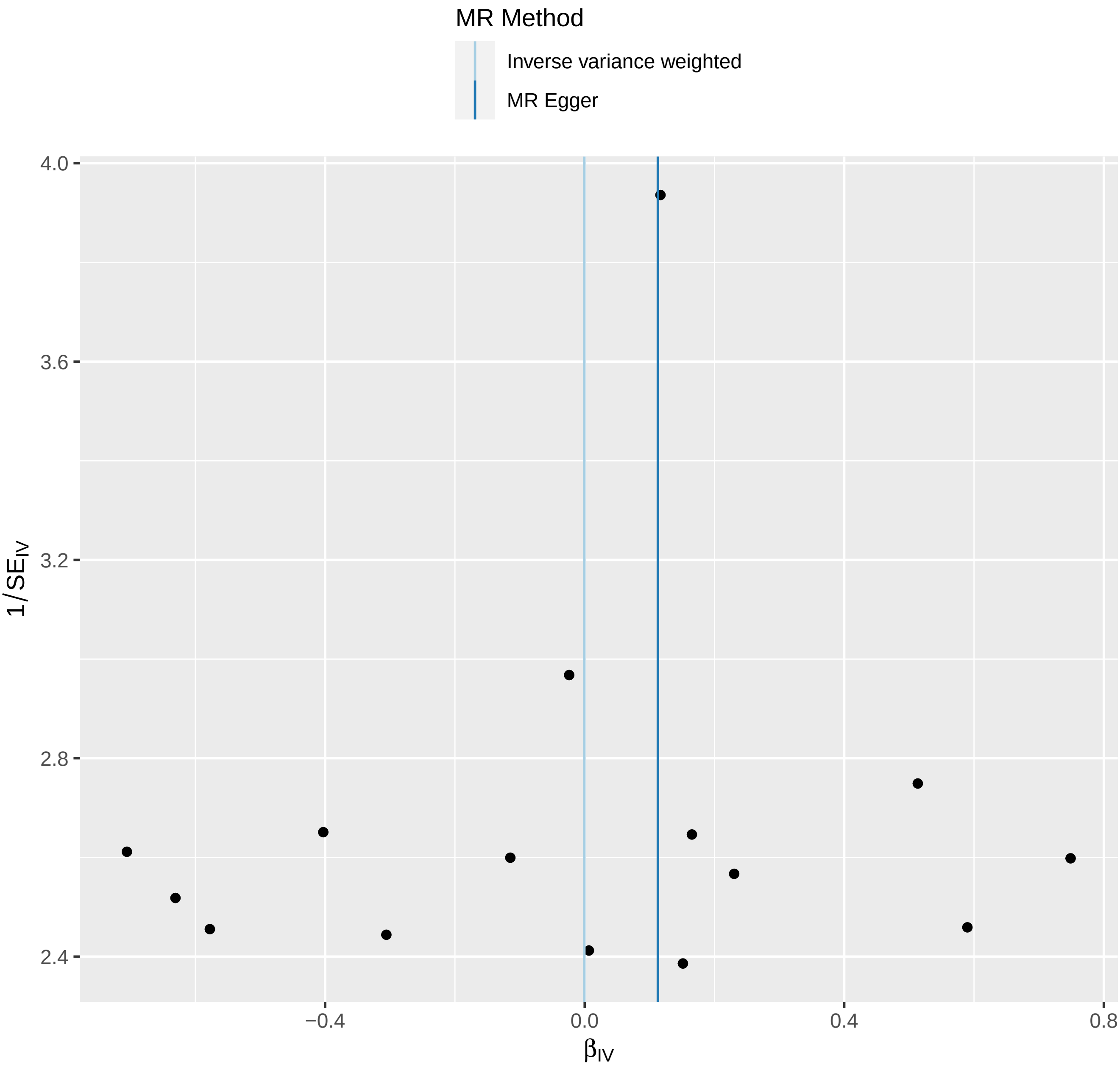

Batch 2−4539 : Gut microbiota abundance (genus Catenibacterium id.2153) on PIK3CA/PIK3R1
with a threshold of 1e−05

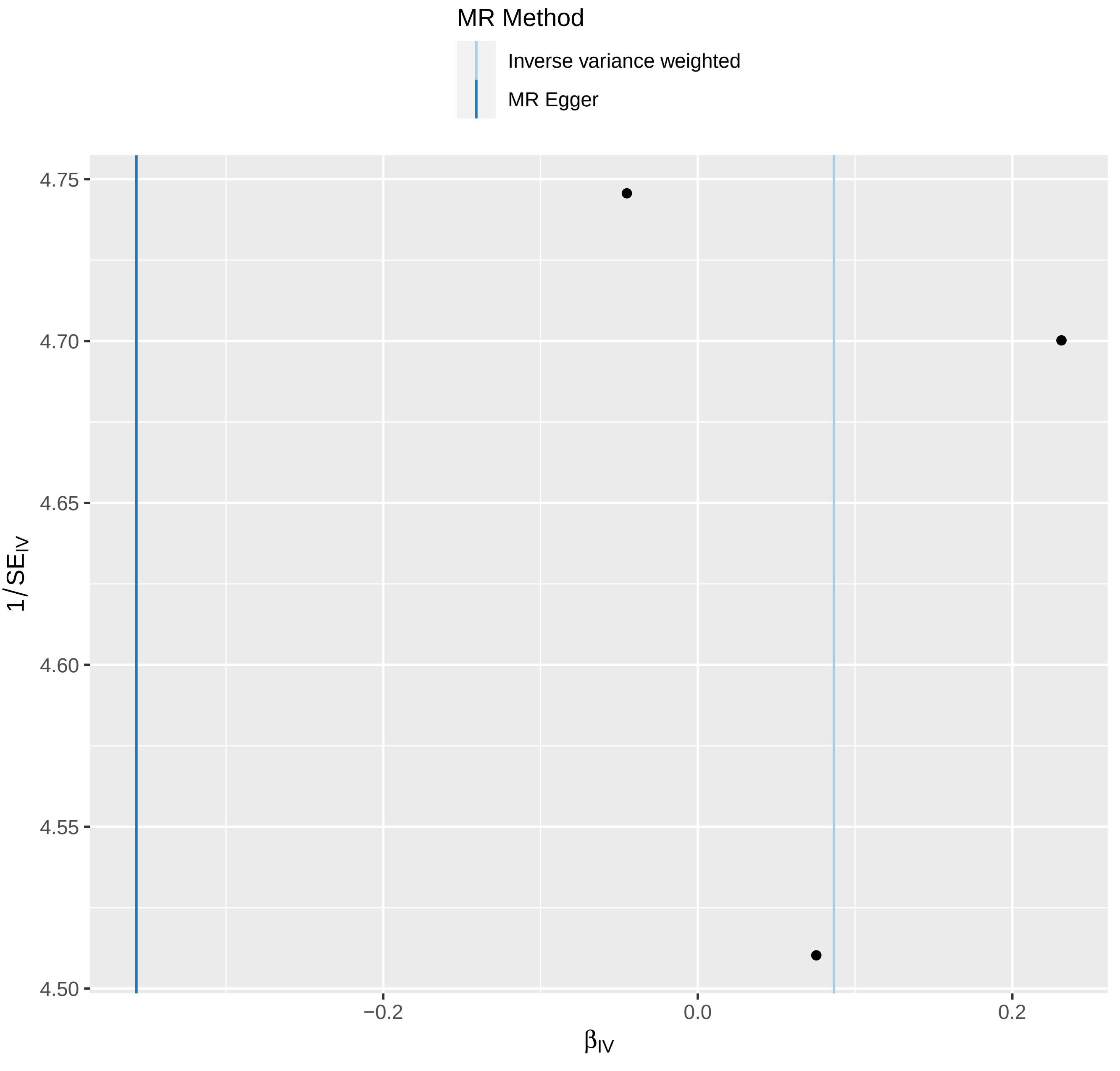

Batch 2−4540 : Gut microbiota abundance (genus Parasutterella id.2892) on PIK3CA/PIK3R1
with a threshold of 1e−05

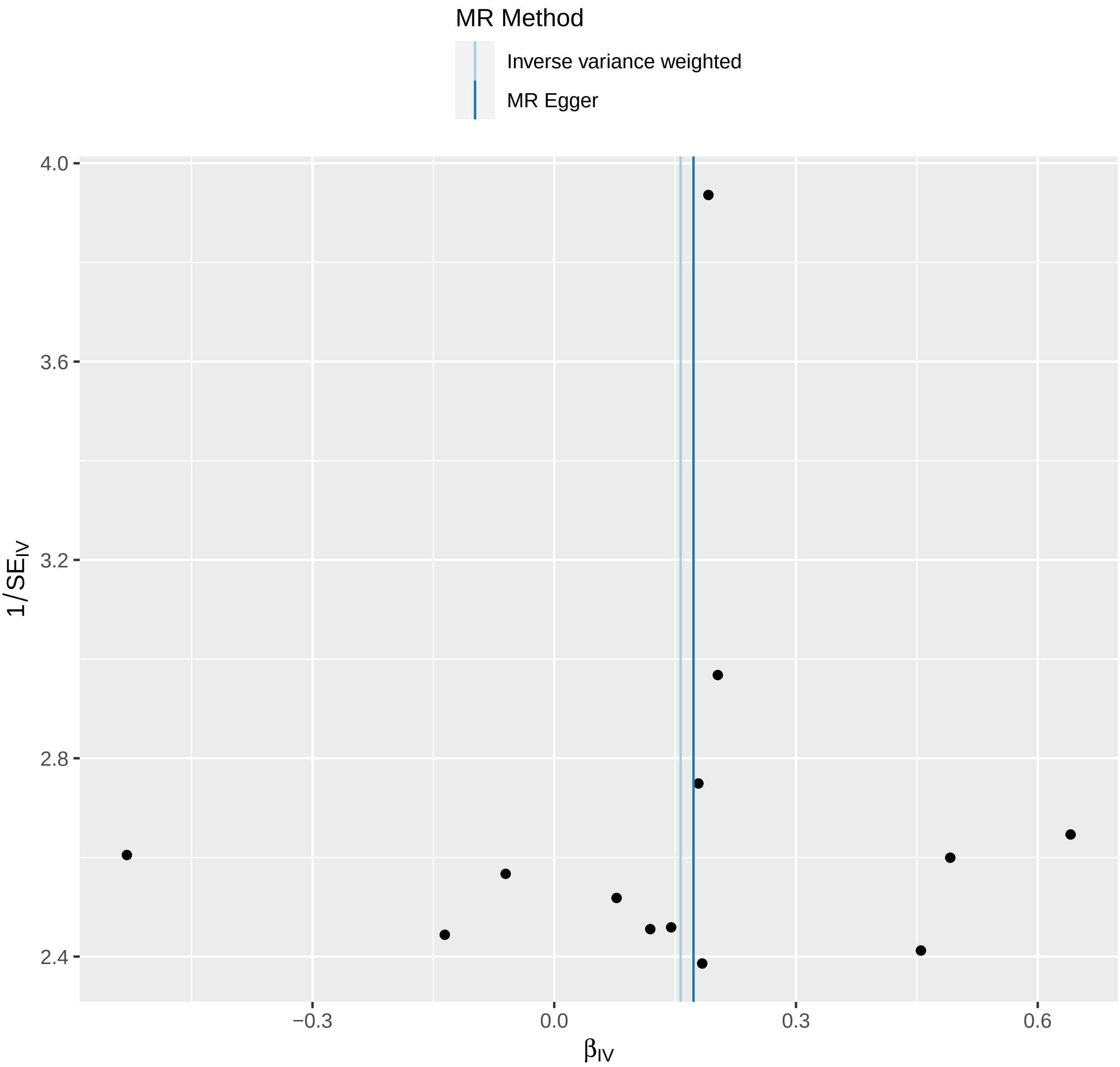

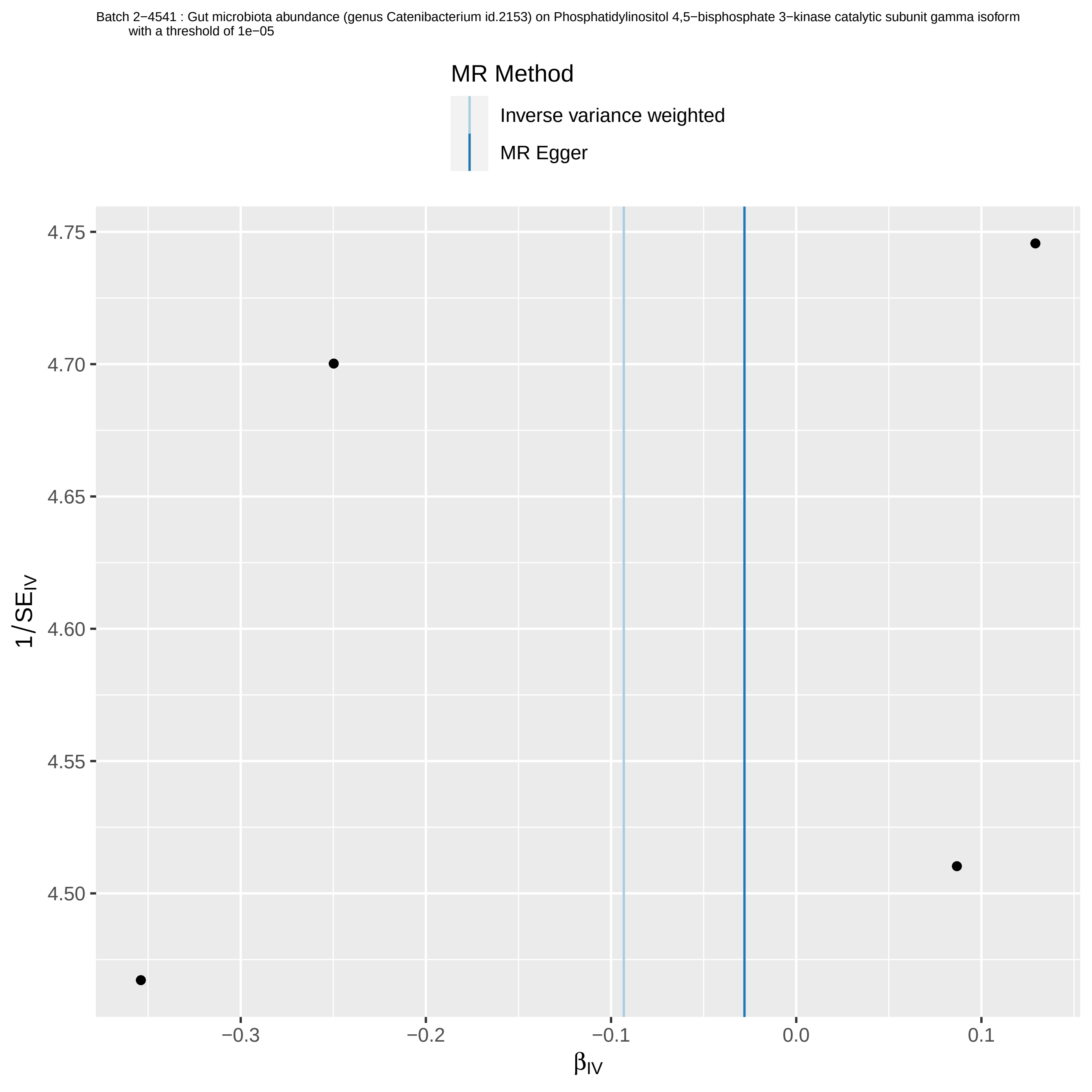
Batch 2−4541 : Gut microbiota abundance (genus Catenibacterium id.2153) on Phosphatidylinositol 4,5−bisphosphate 3−kinase catalytic subunit gamma isoform with a threshold of 1e−05
MR Method
Inverse variance weighted
MR Egger
4.75
4.70
4.65
4.60
4.55
4.50
1/SEIV
−0.3
−0.2
−0.1
0.0
0.1
βIV

Batch 2−4542 : Gut microbiota abundance (genus Parasutterella id.2892) on Phosphatidylinositol 4,5−bisphosphate 3−kinase catalytic subunit gamma isoform with a threshold of 1e−05

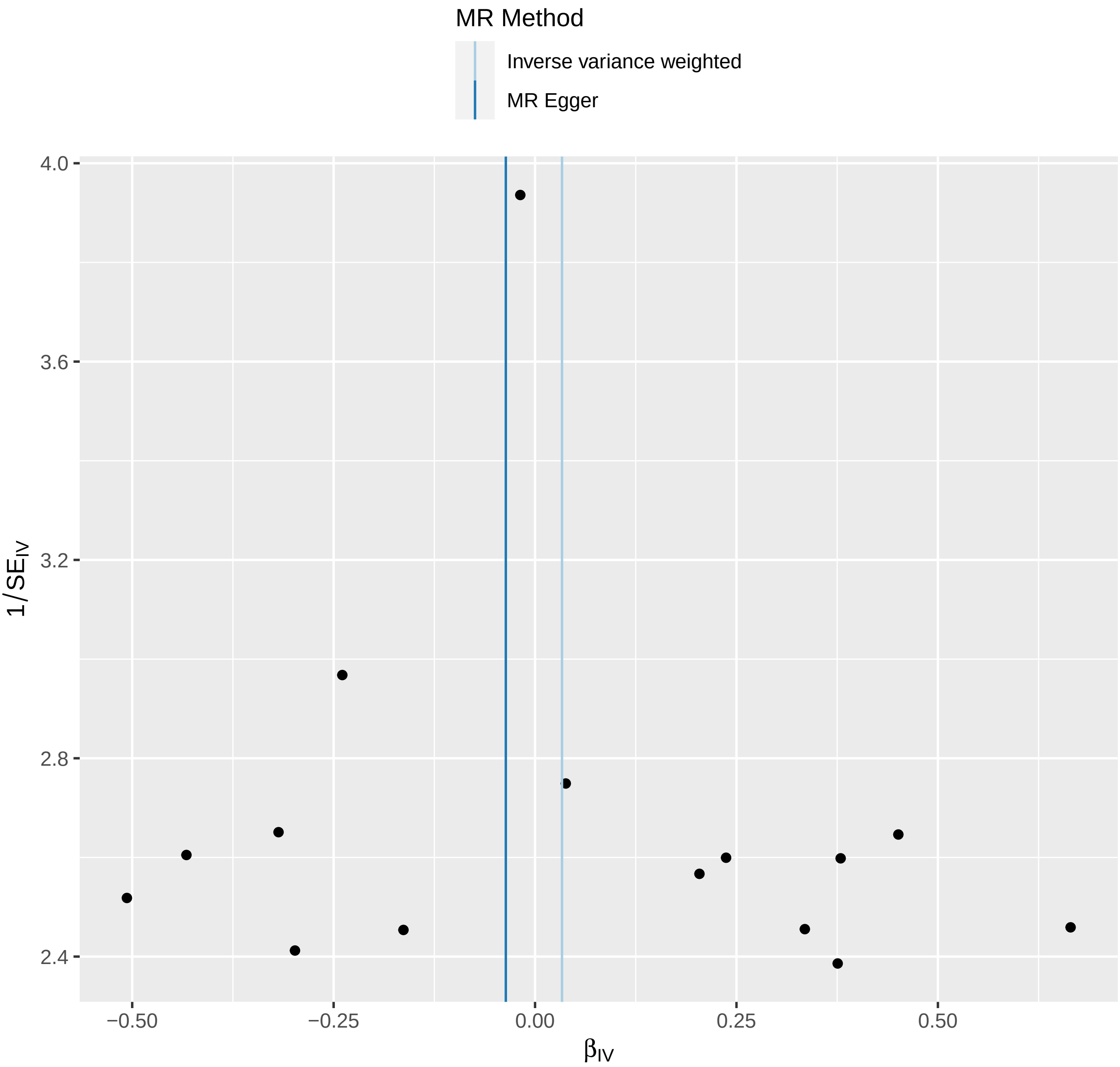

Batch 2−4543 : Gut microbiota abundance (genus Catenibacterium id.2153) on Paired immunoglobulin−like type 2 receptor alpha
with a threshold of 1e−05

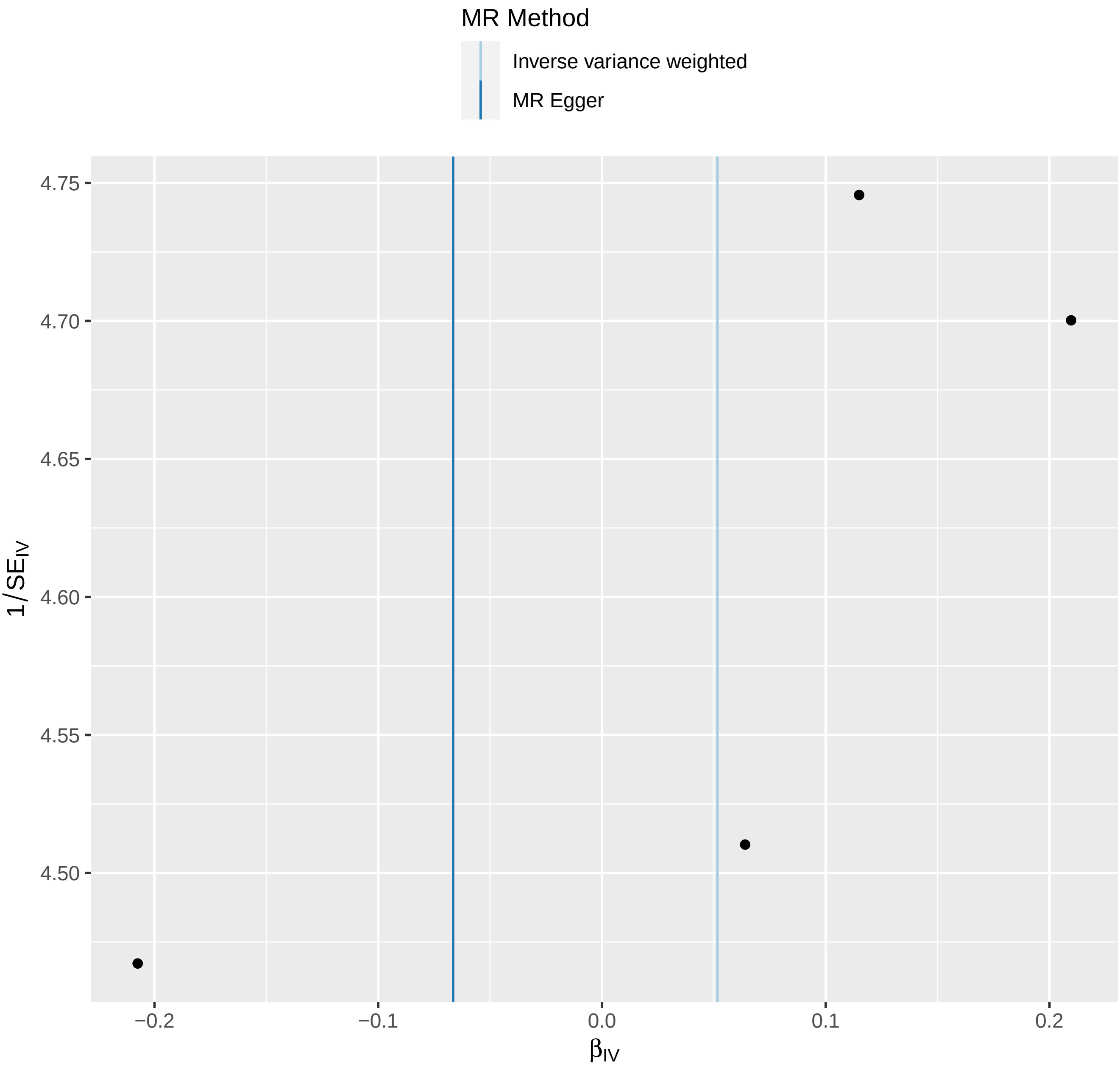

Batch 2−4544 : Gut microbiota abundance (genus Parasutterella id.2892) on Paired immunoglobulin−like type 2 receptor alpha
with a threshold of 1e−05

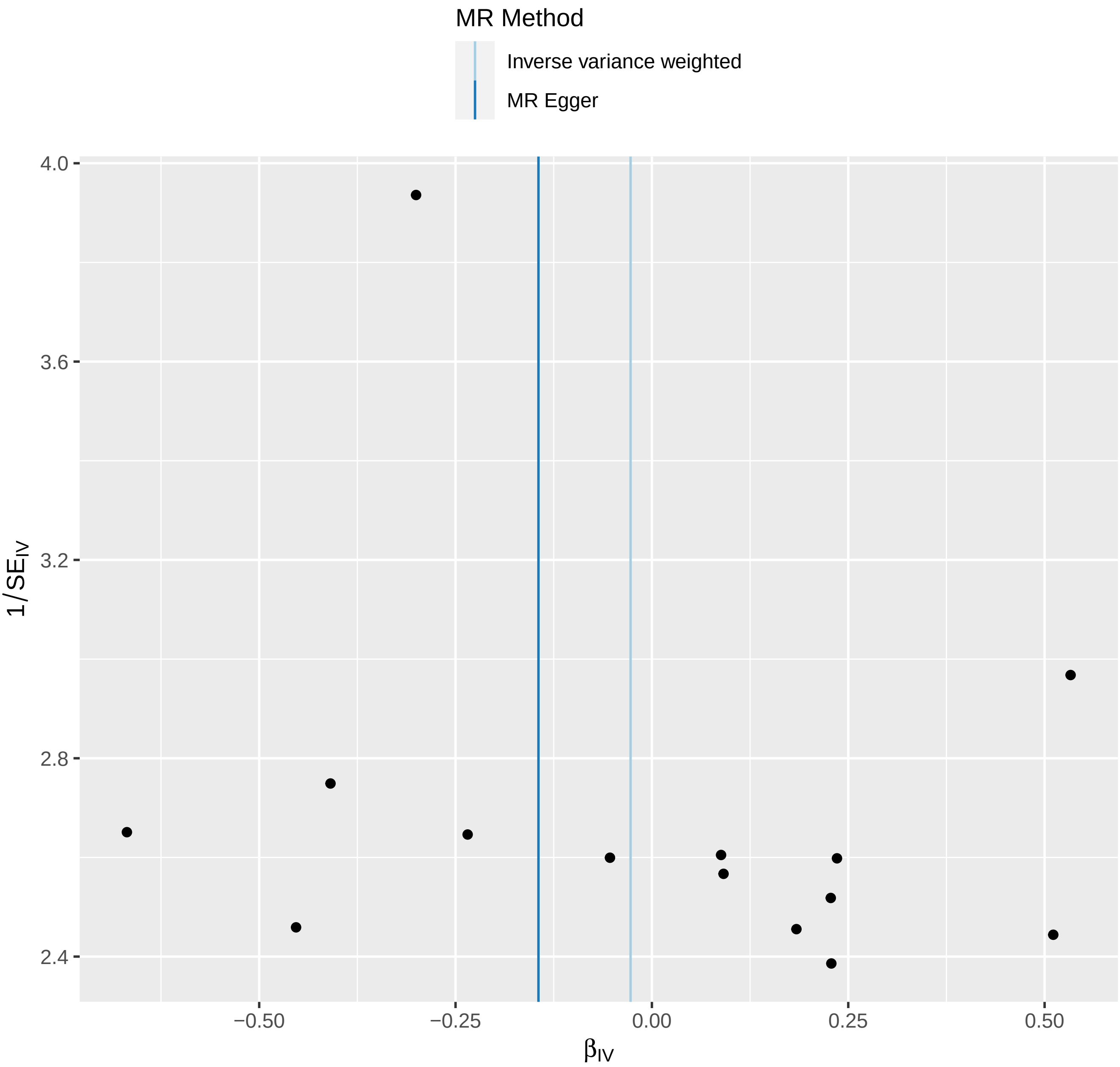

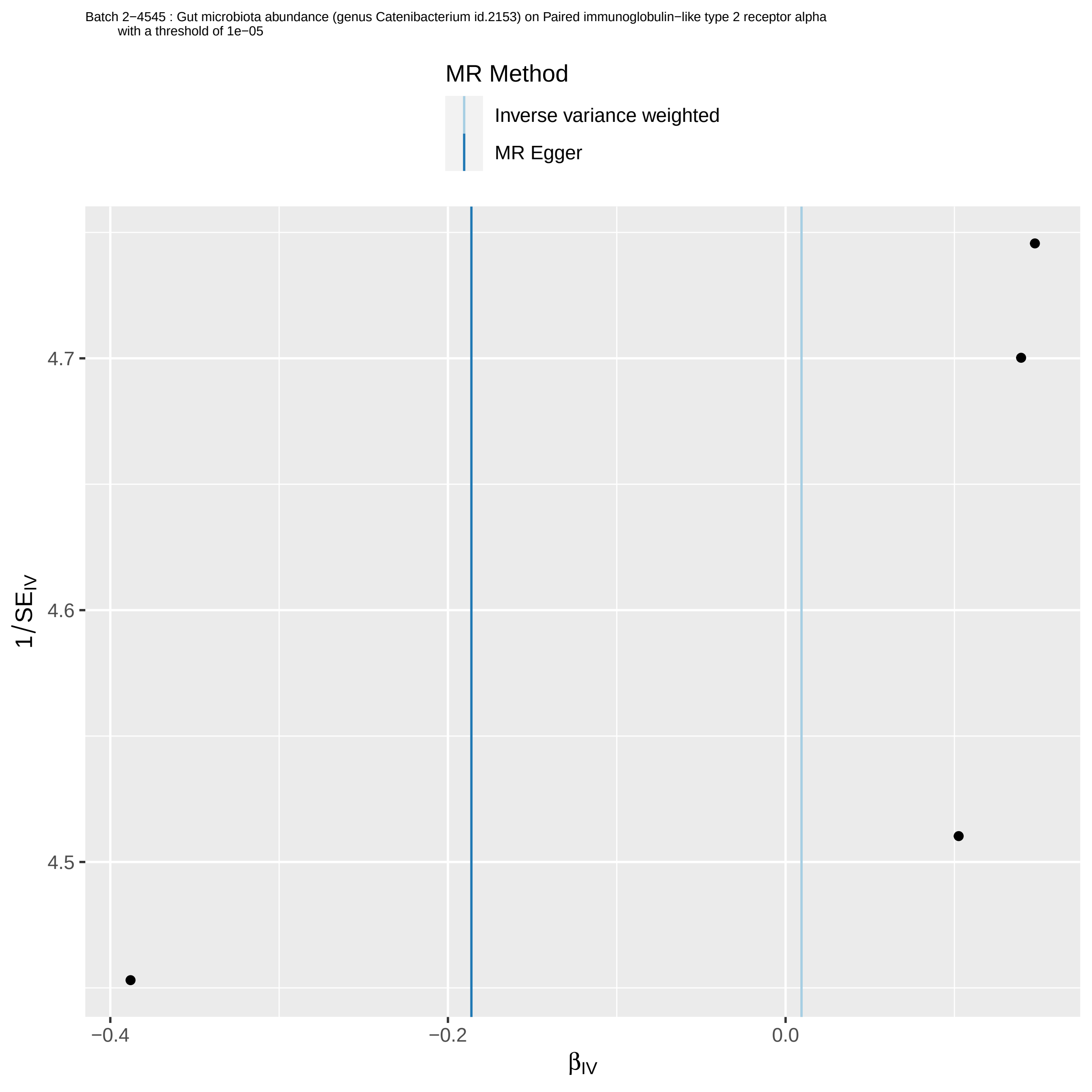
Batch 2−4545 : Gut microbiota abundance (genus Catenibacterium id.2153) on Paired immunoglobulin−like type 2 receptor alpha
with a threshold of 1e−05
MR Method
Inverse variance weighted
MR Egger
4.7
4.6
4.5
1/SE_IV
−0.4
−0.2
0.0
β_IV

Batch 2−4546 : Gut microbiota abundance (genus Parasutterella id.2892) on Paired immunoglobulin−like type 2 receptor alpha with a threshold of 1e−05

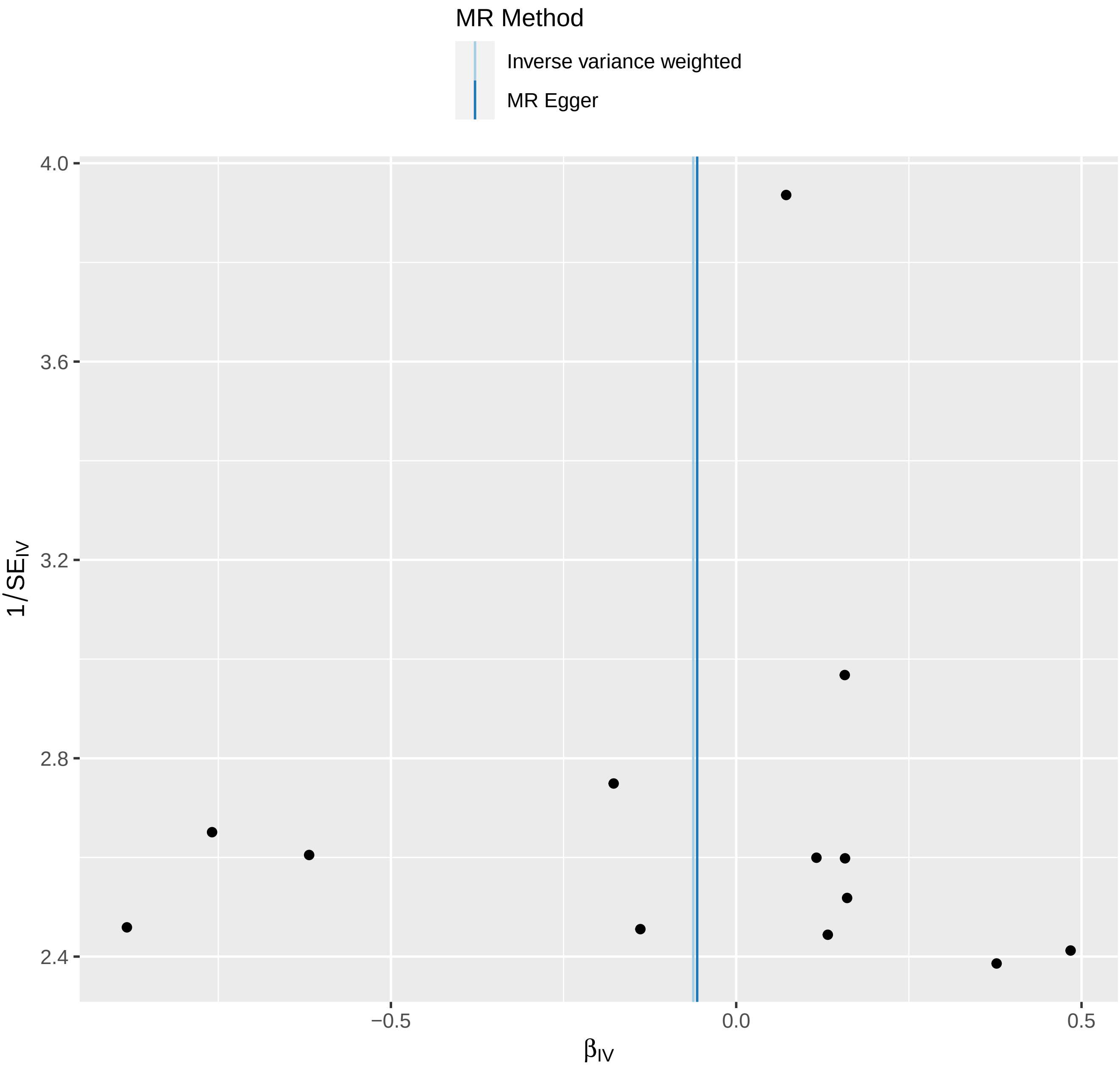

Batch 2−4547 : Gut microbiota abundance (genus Catenibacterium id.2153) on Serine/threonine−protein kinase pim−1
with a threshold of 1e−05

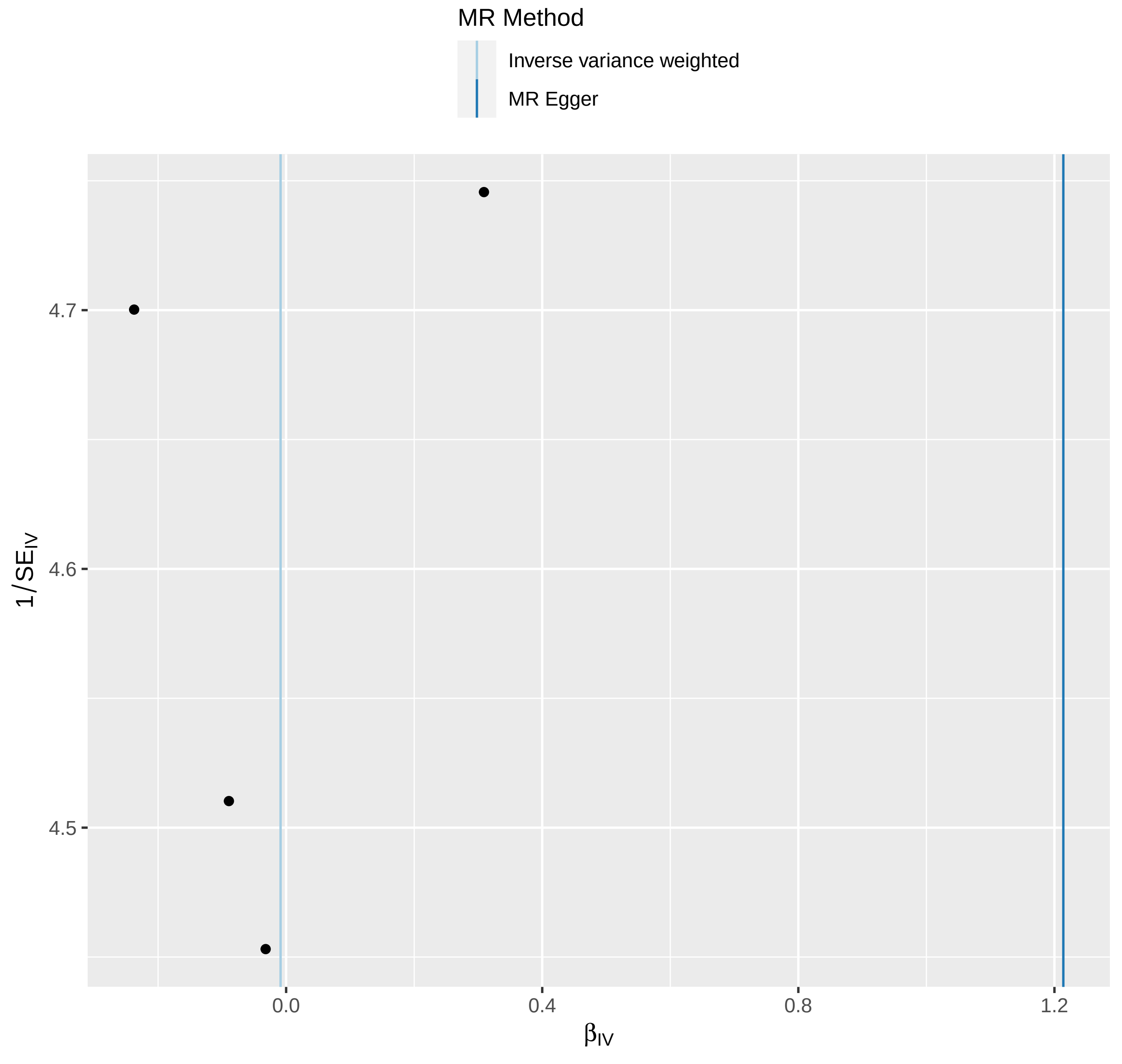

Batch 2−4548 : Gut microbiota abundance (genus Parasutterella id.2892) on Serine/threonine−protein kinase pim−1
with a threshold of 1e−05

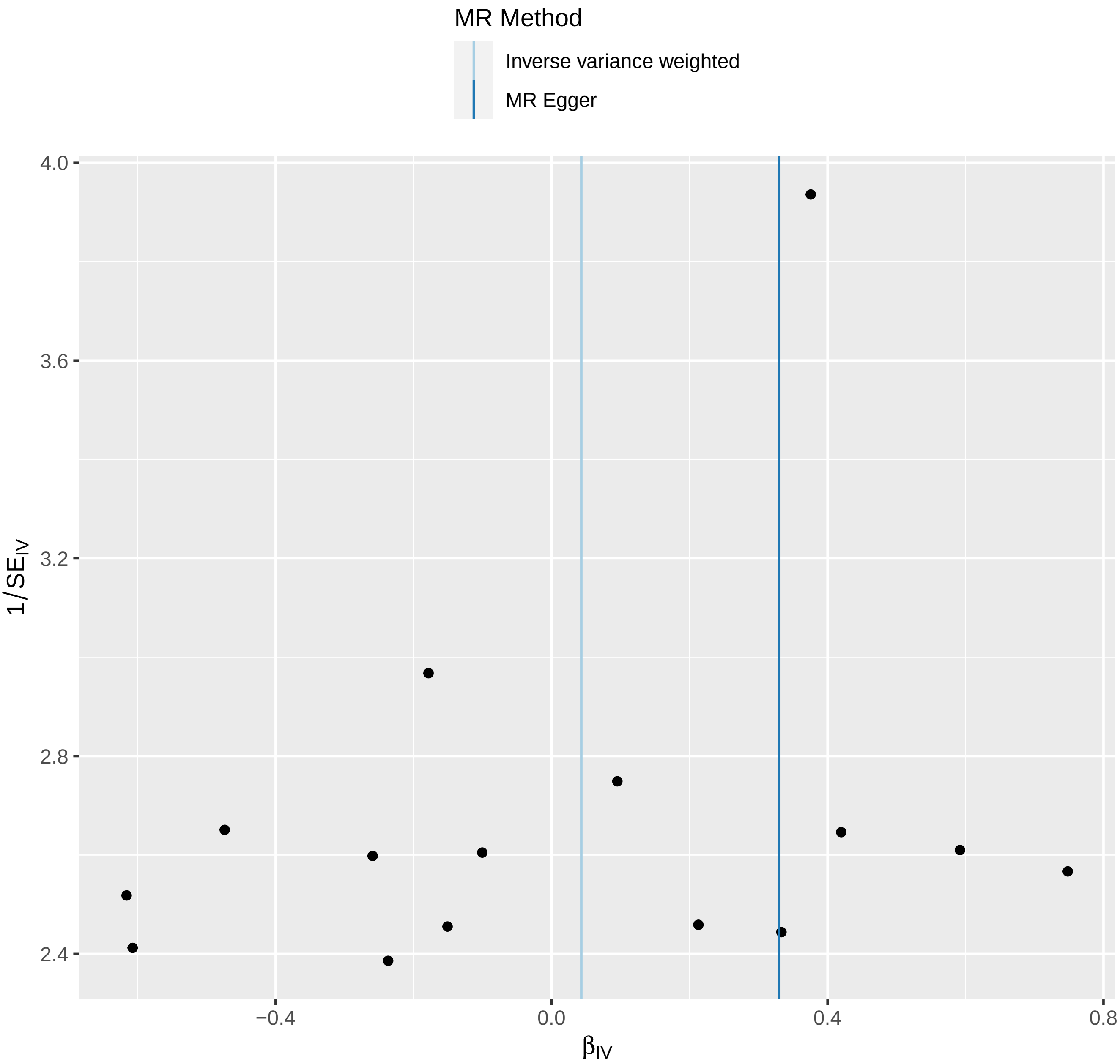

Batch 2−4549 : Gut microbiota abundance (genus Catenibacterium id.2153) on Peptidyl−prolyl cis−trans isomerase NIMA−interacting 4 with a threshold of 1e−05

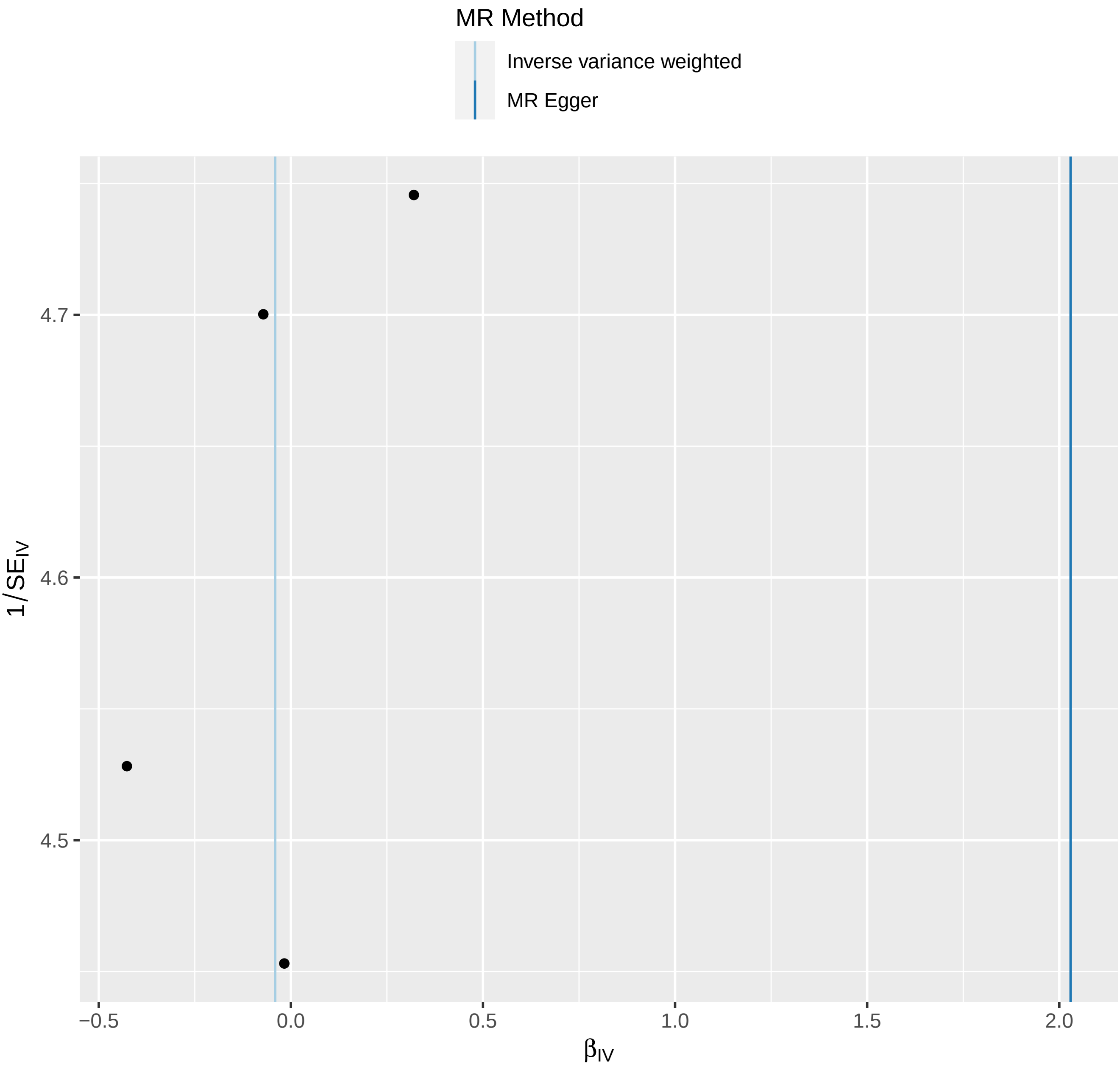

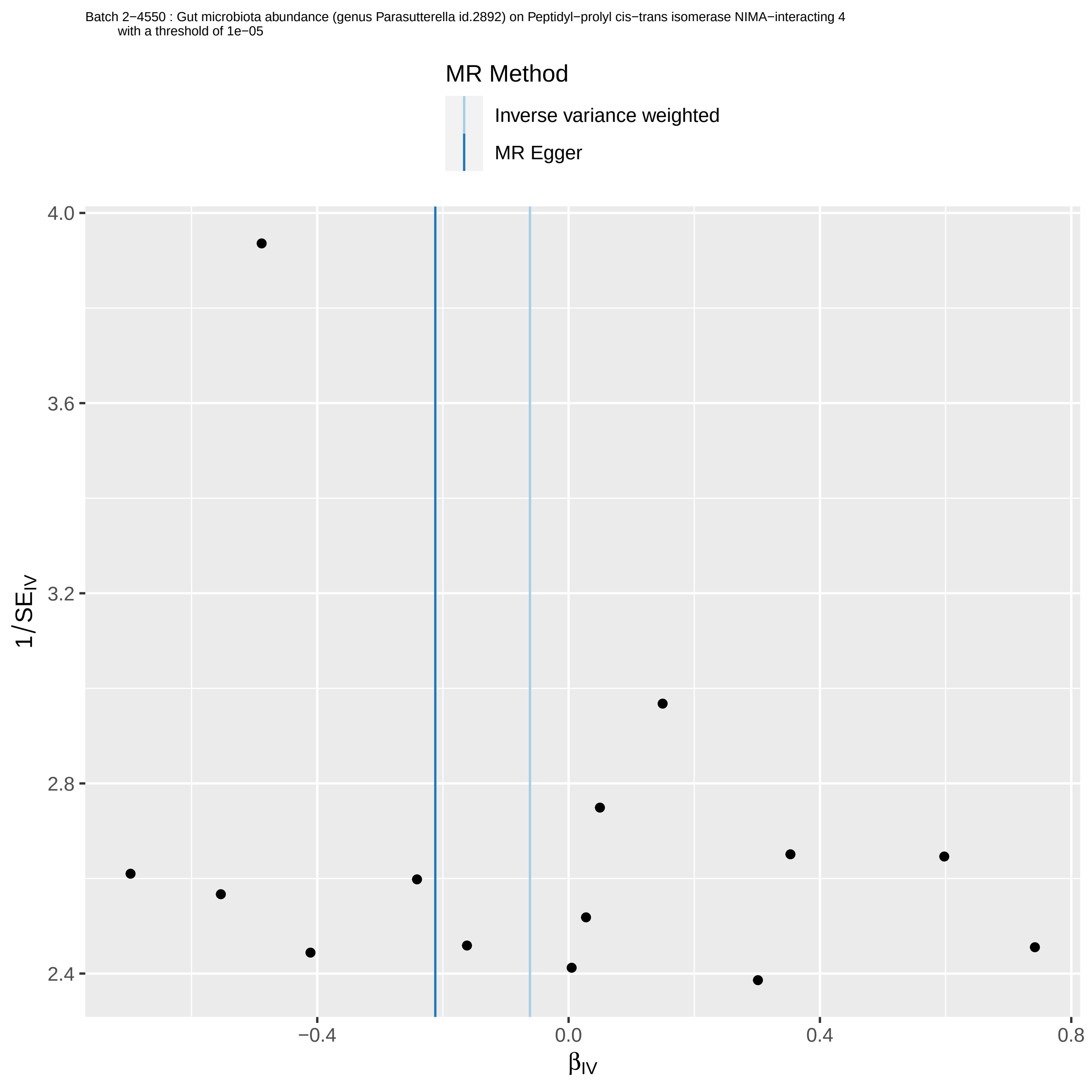
Batch 2−4550 : Gut microbiota abundance (genus Parasutterella id.2892) on Peptidyl−prolyl cis−trans isomerase NIMA−interacting 4
with a threshold of 1e−05
MR Method
Inverse variance weighted
MR Egger
4.0
3.6
3.2
2.8
2.4
1/SE_IV
−0.4
0.0
0.4
0.8
β_IV

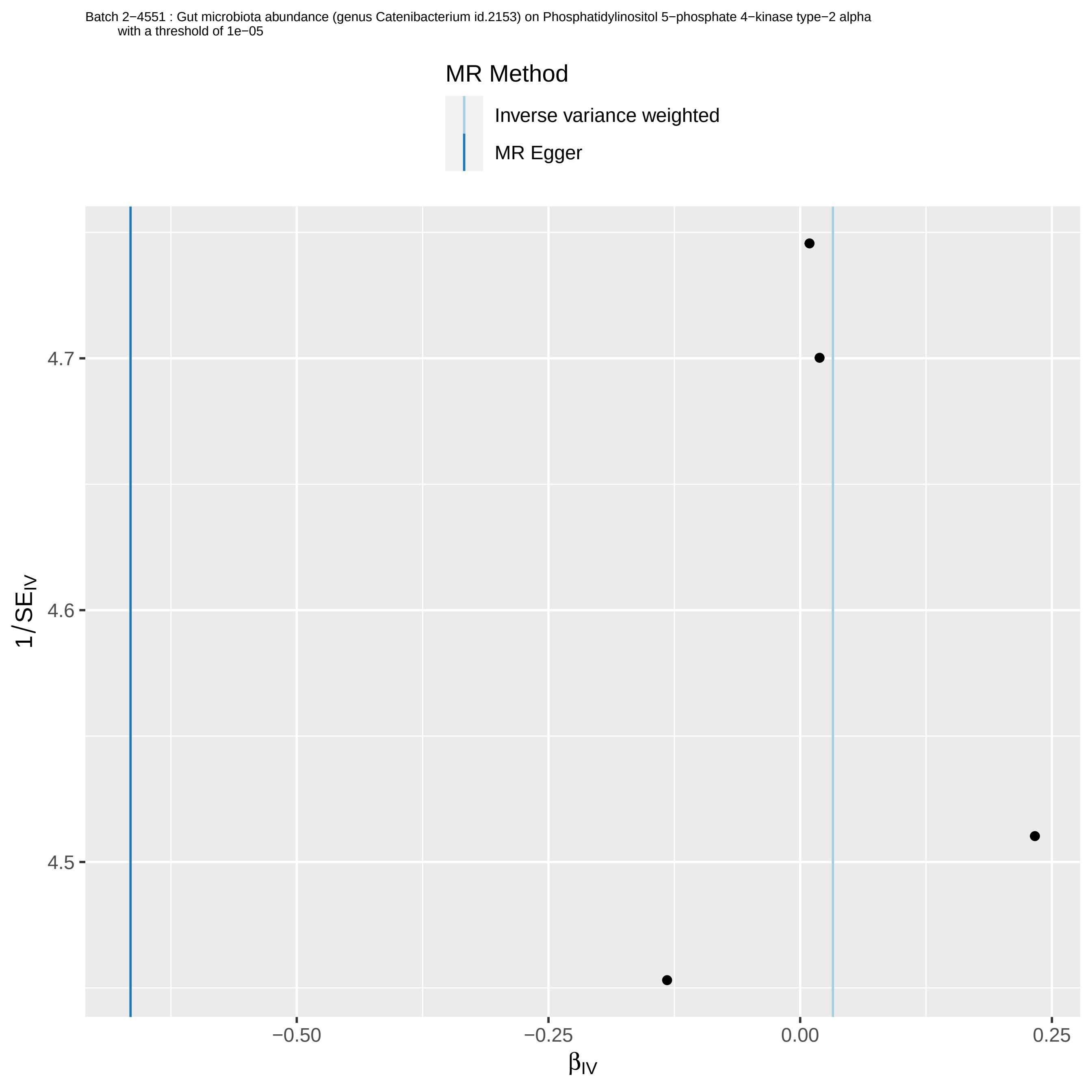
Batch 2−4551 : Gut microbiota abundance (genus Catenibacterium id.2153) on Phosphatidylinositol 5−phosphate 4−kinase type−2 alpha
with a threshold of 1e−05
MR Method
Inverse variance weighted
MR Egger
4.7
4.6
4.5
1/SE_IV
−0.50
−0.25
0.00
0.25
β_IV

Batch 2−4552 : Gut microbiota abundance (genus Parasutterella id.2892) on Phosphatidylinositol 5−phosphate 4−kinase type−2 alpha with a threshold of 1e−05

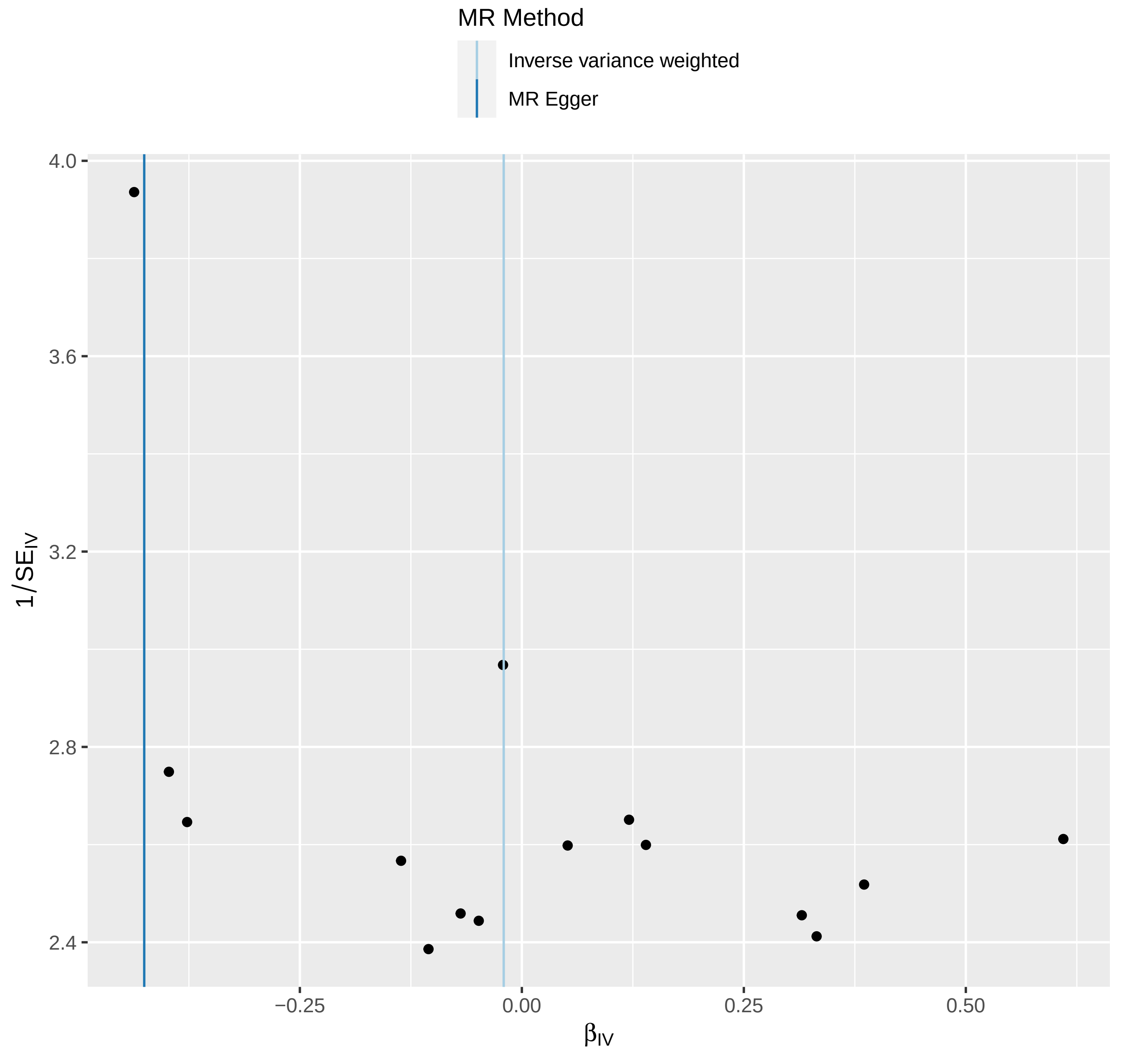

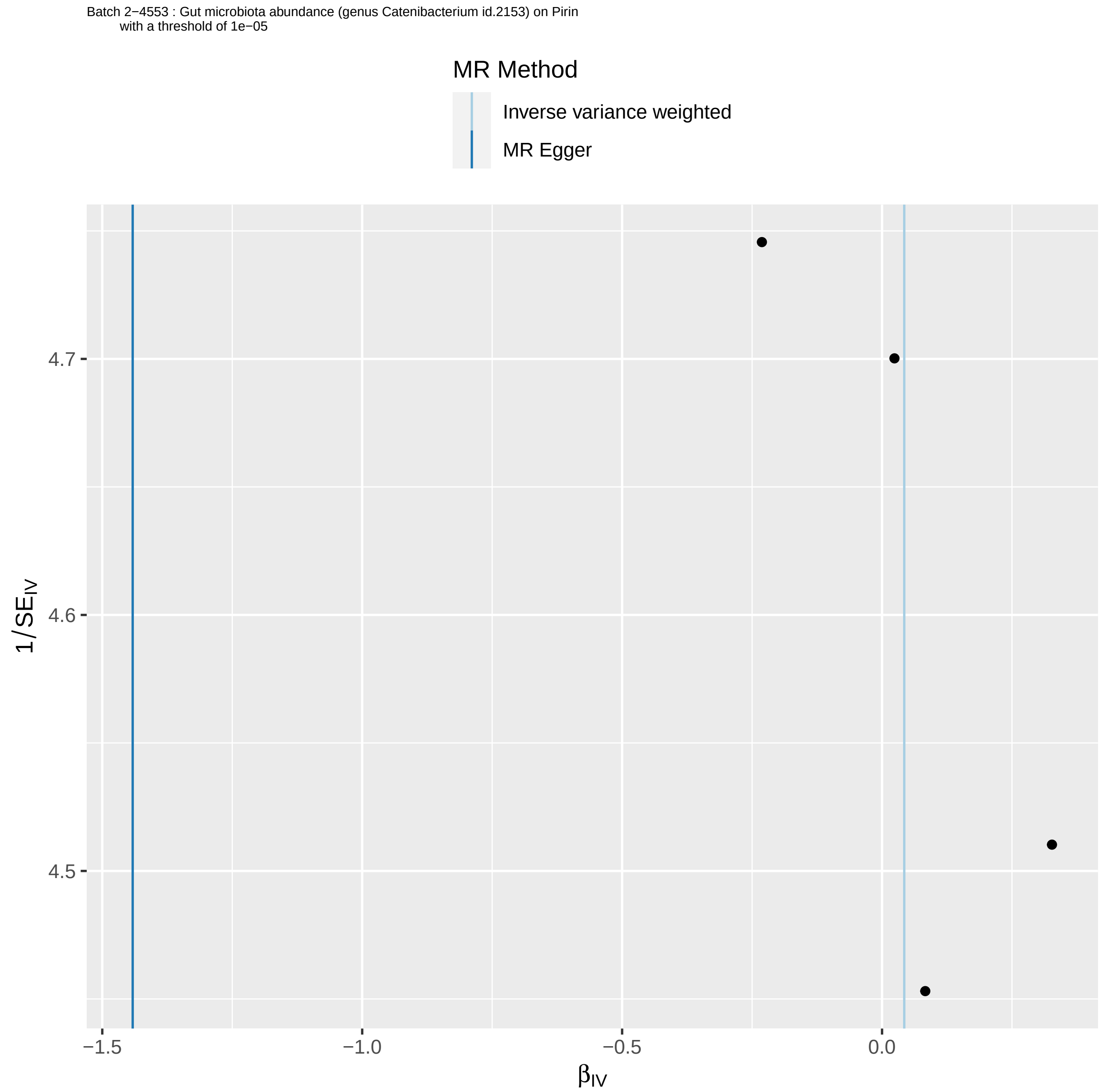
Batch 2−4553 : Gut microbiota abundance (genus Catenibacterium id.2153) on Pirin
with a threshold of 1e−05
MR Method
Inverse variance weighted
MR Egger
4.7
4.6
4.5
1/SE_IV
−1.5
−1.0
−0.5
0.0
β_IV

Batch 2−4554 : Gut microbiota abundance (genus Parasutterella id.2892) on Pirin
with a threshold of 1e−05

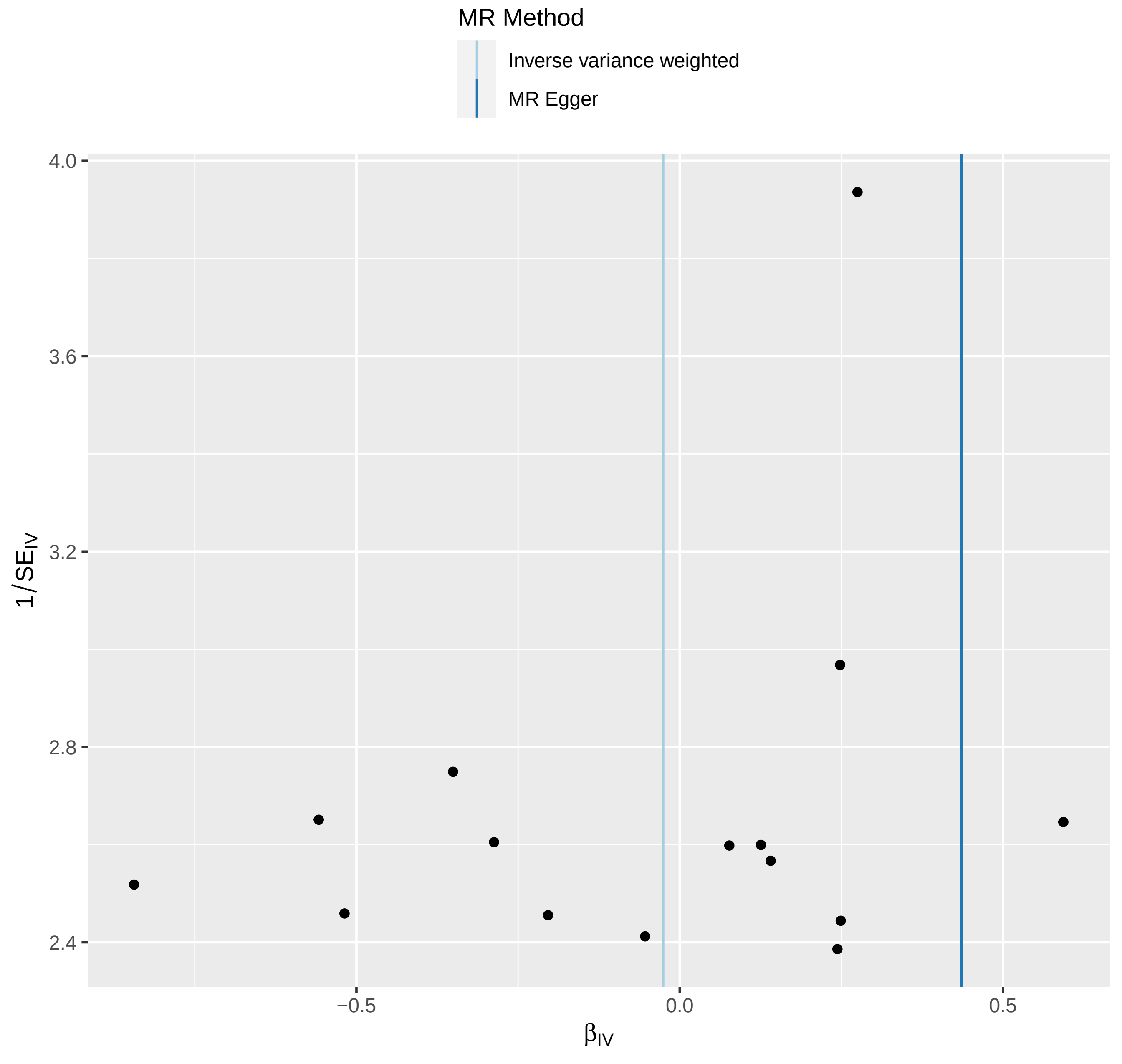

Batch 2−4555 : Gut microbiota abundance (genus Catenibacterium id.2153) on Phosphatidylinositol transfer protein alpha isoform with a threshold of 1e−05

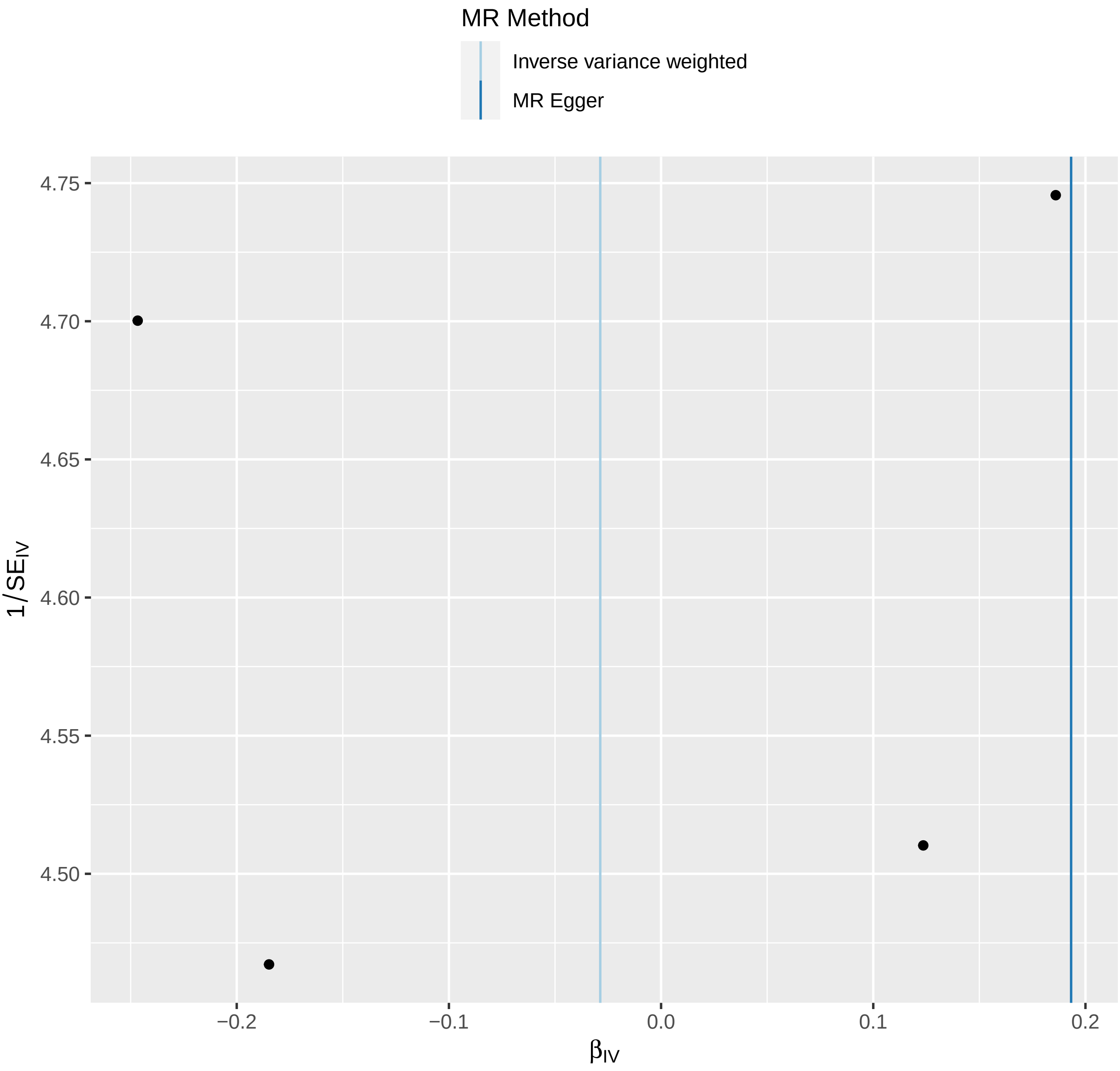

Batch 2−4556 : Gut microbiota abundance (genus Parasutterella id.2892) on Phosphatidylinositol transfer protein alpha isoform
with a threshold of 1e−05

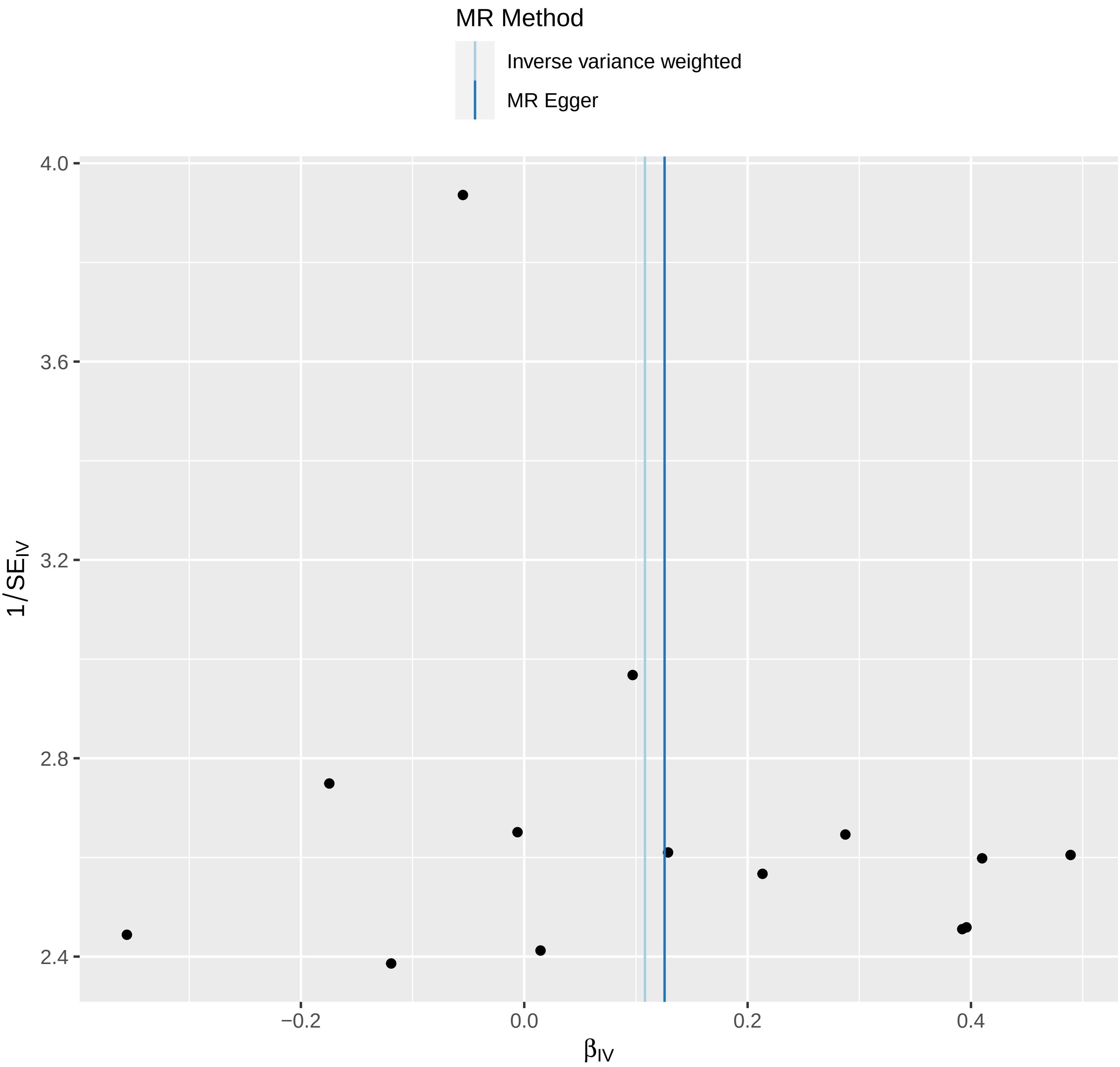

Batch 2−4557 : Gut microbiota abundance (genus Catenibacterium id.2153) on Phosphatidylinositol transfer protein beta isoform with a threshold of 1e−05

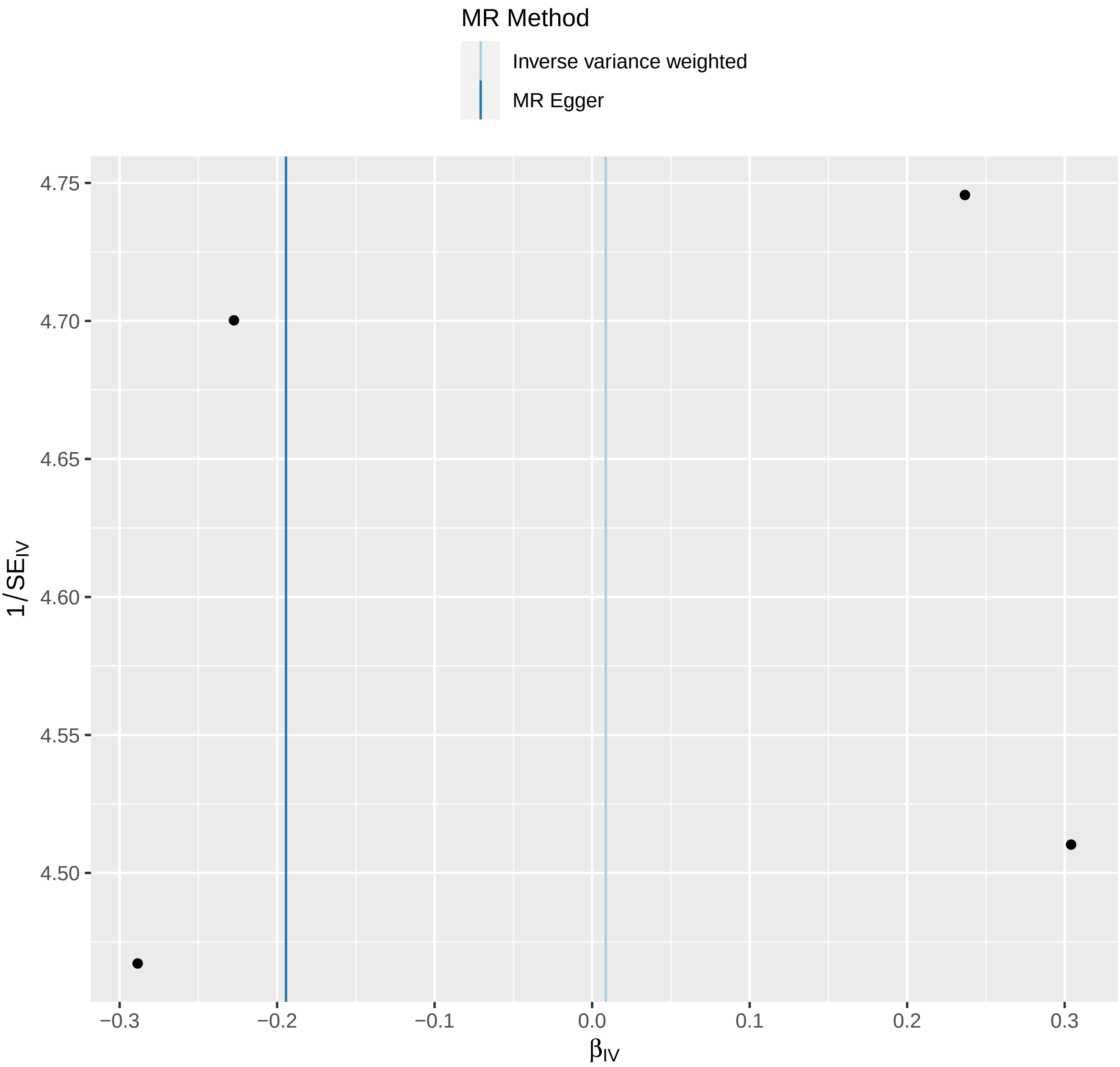

Batch 2−4558 : Gut microbiota abundance (genus Parasutterella id.2892) on Phosphatidylinositol transfer protein beta isoform
with a threshold of 1e−05

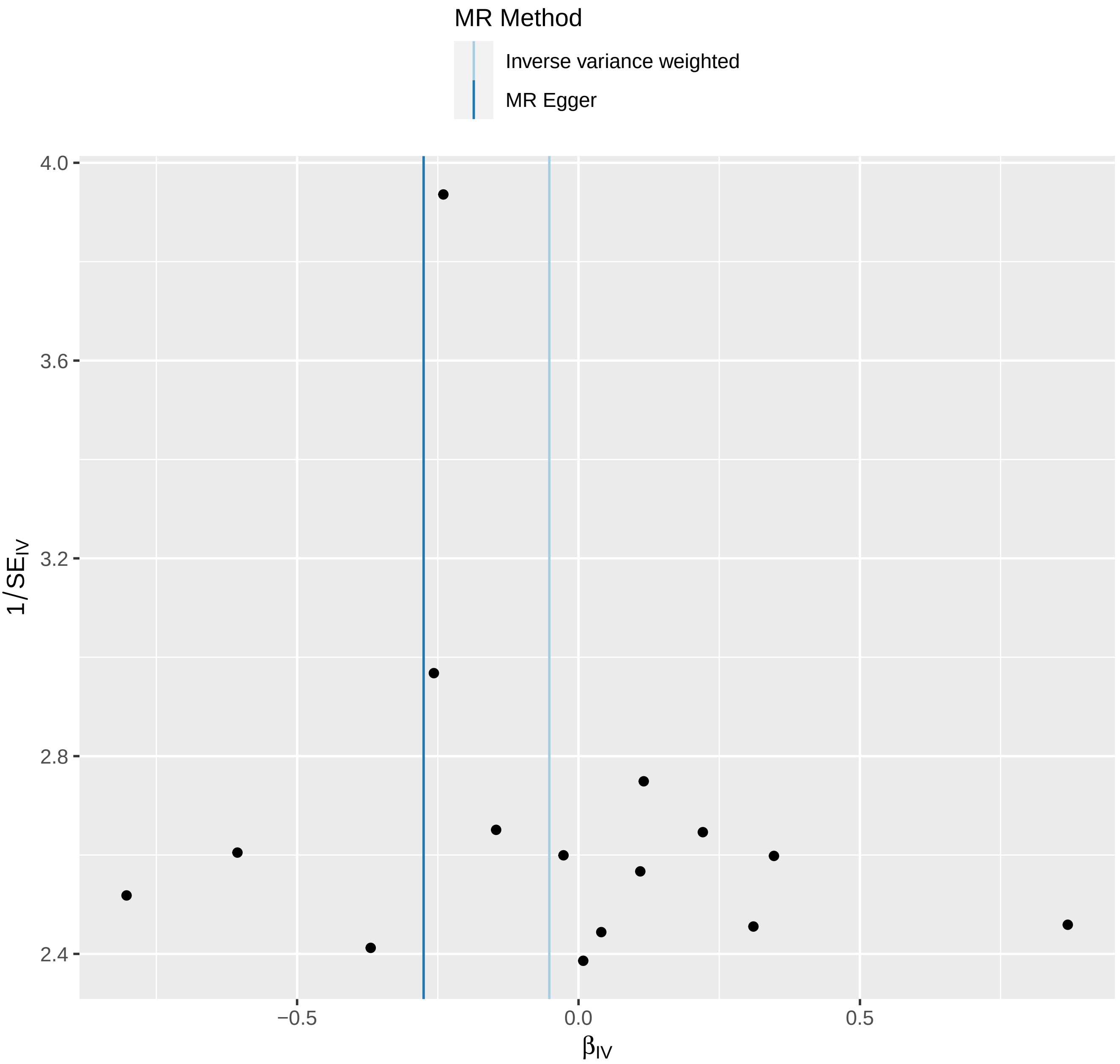

Batch 2−4559 : Gut microbiota abundance (genus Catenibacterium id.2153) on Piwi−like protein 1
with a threshold of 1e−05

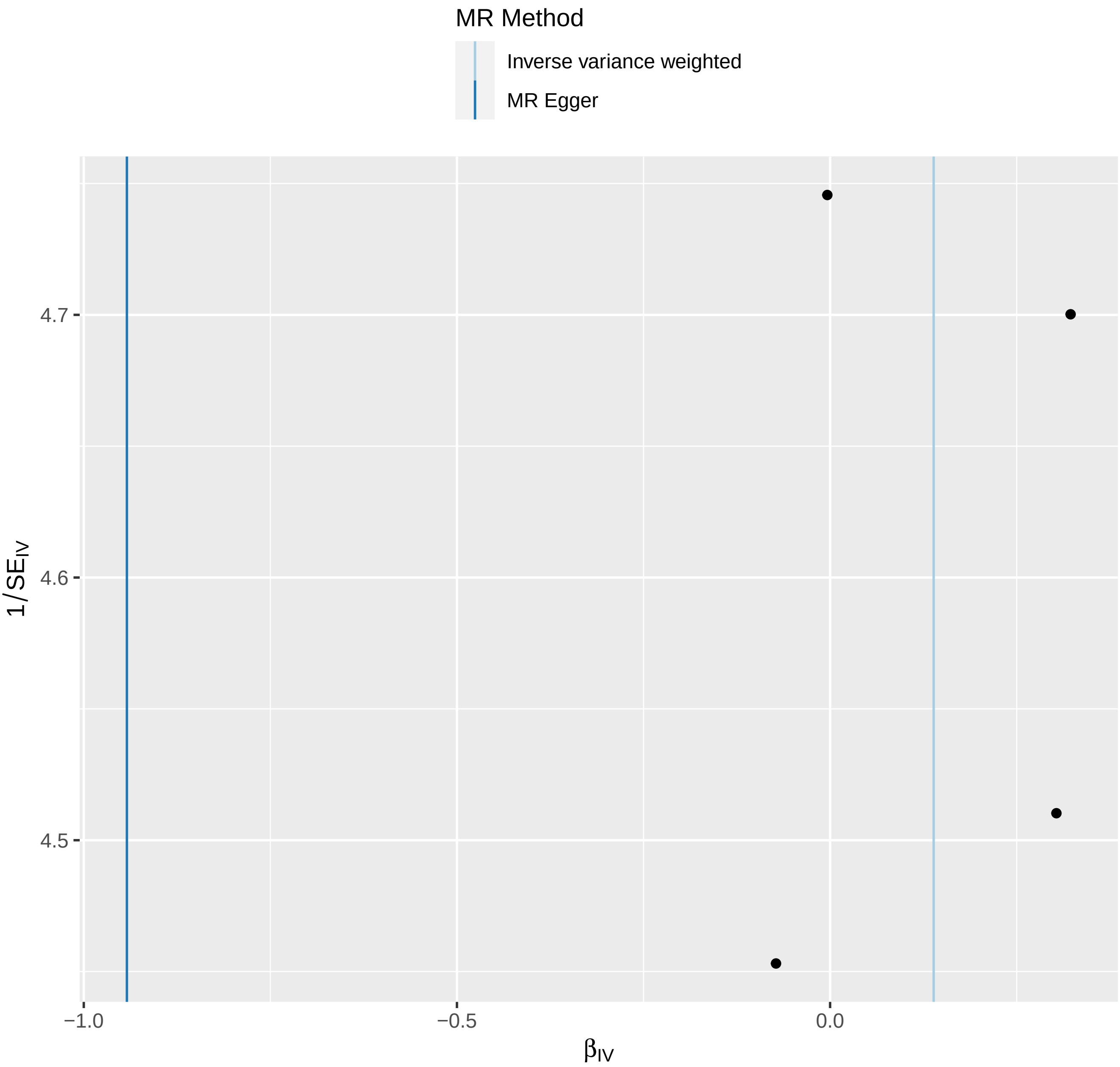

Batch 2−4560 : Gut microbiota abundance (genus Parasutterella id.2892) on Piwi−like protein 1
with a threshold of 1e−05

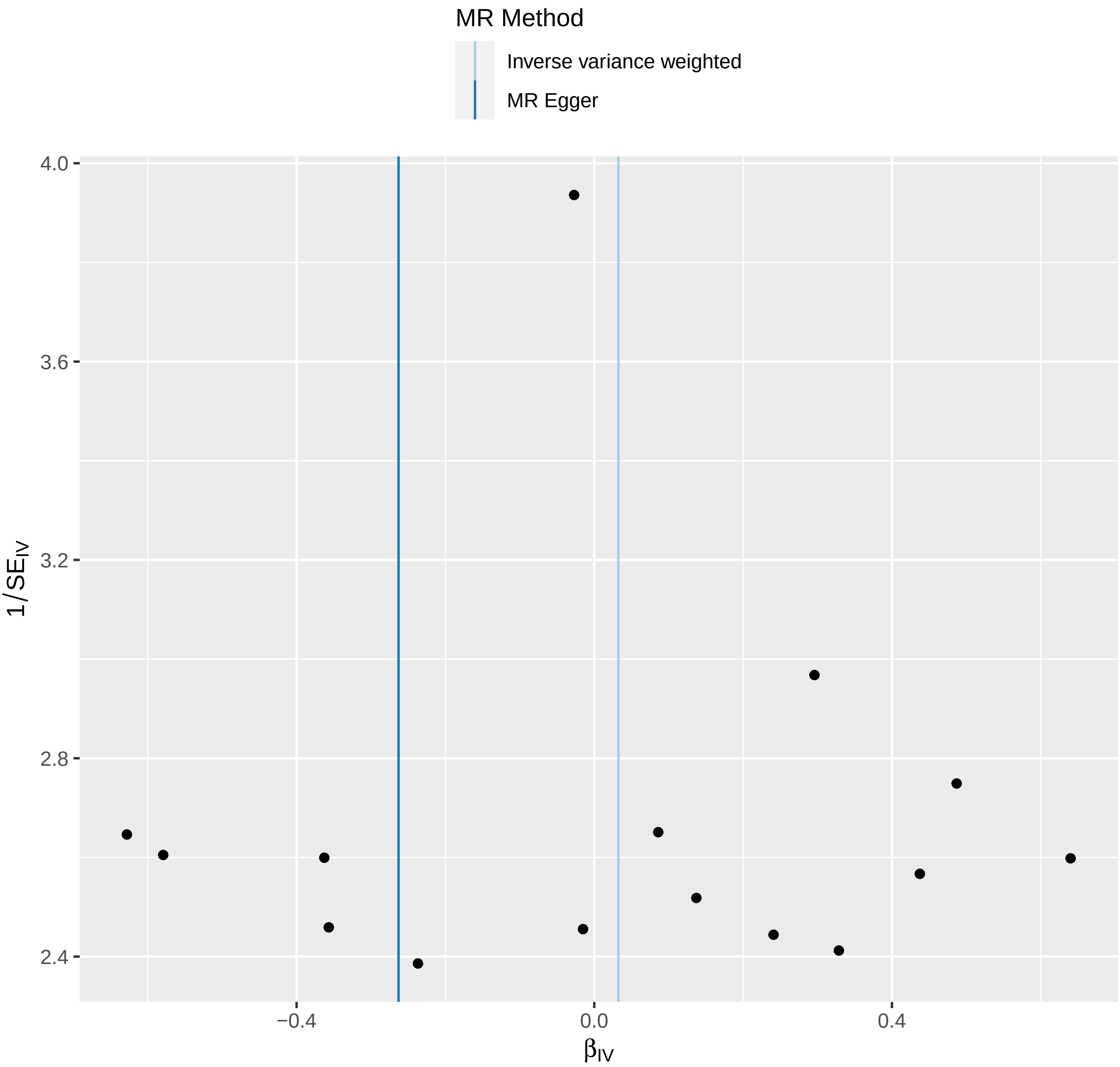

Batch 2−4561 : Gut microbiota abundance (genus Catenibacterium id.2153) on Polycystin−2
with a threshold of 1e−05

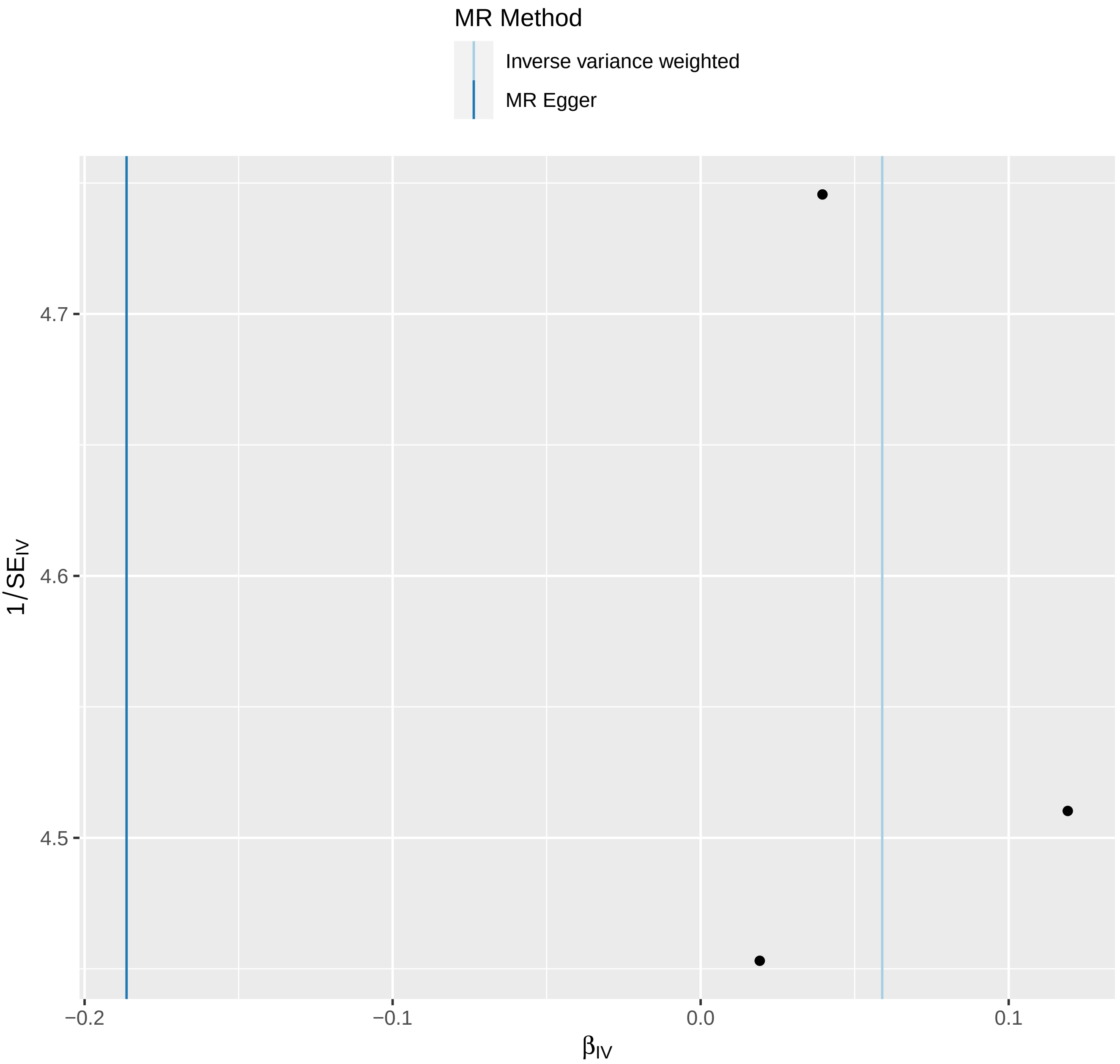

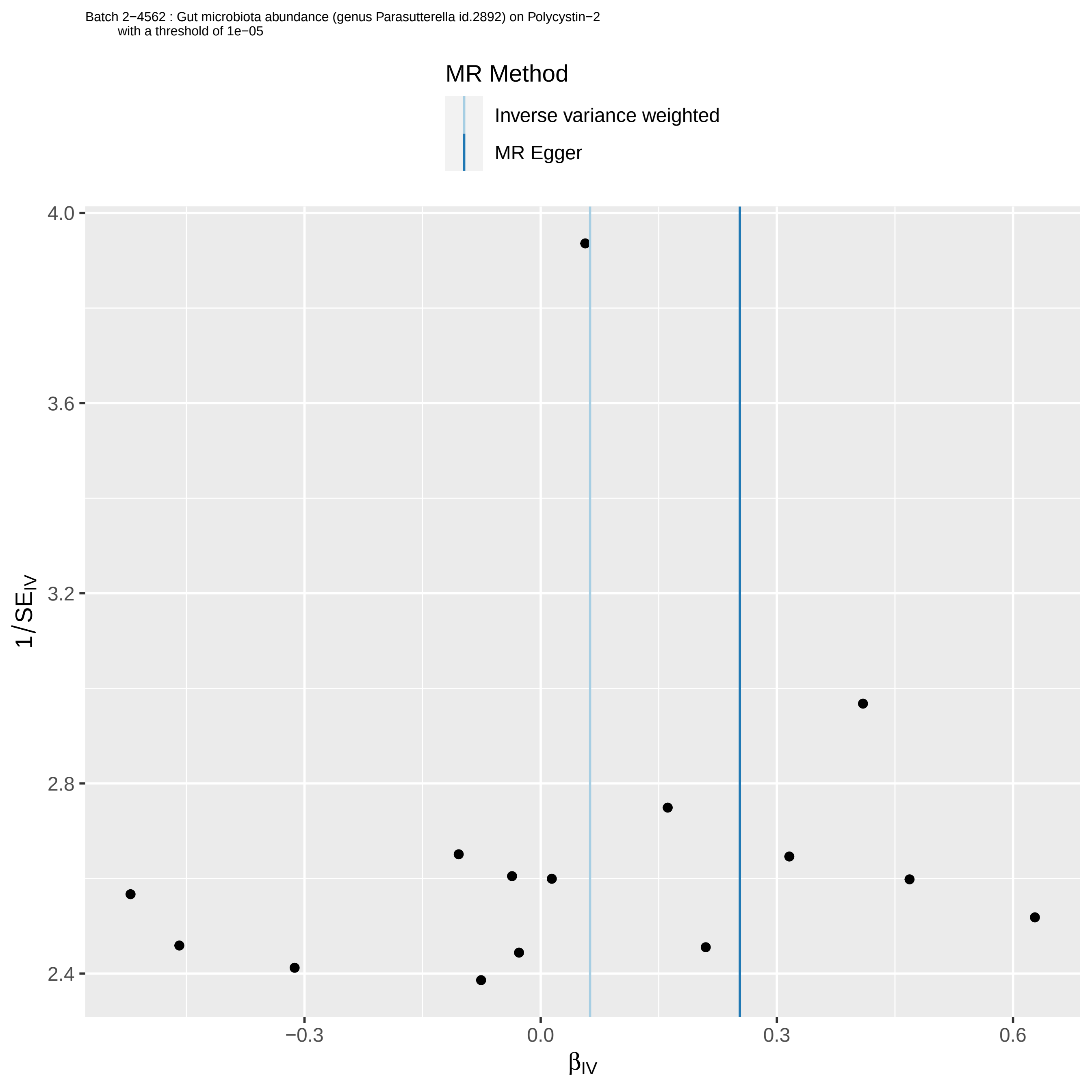

Batch 2−4562 : Gut microbiota abundance (genus Parasutterella id.2892) on Polycystin−2
with a threshold of 1e−05
MR Method
Inverse variance weighted
MR Egger
4.0
3.6
3.2
2.8
2.4
1/SE_IV
−0.3
0.0
0.3
0.6
β_IV

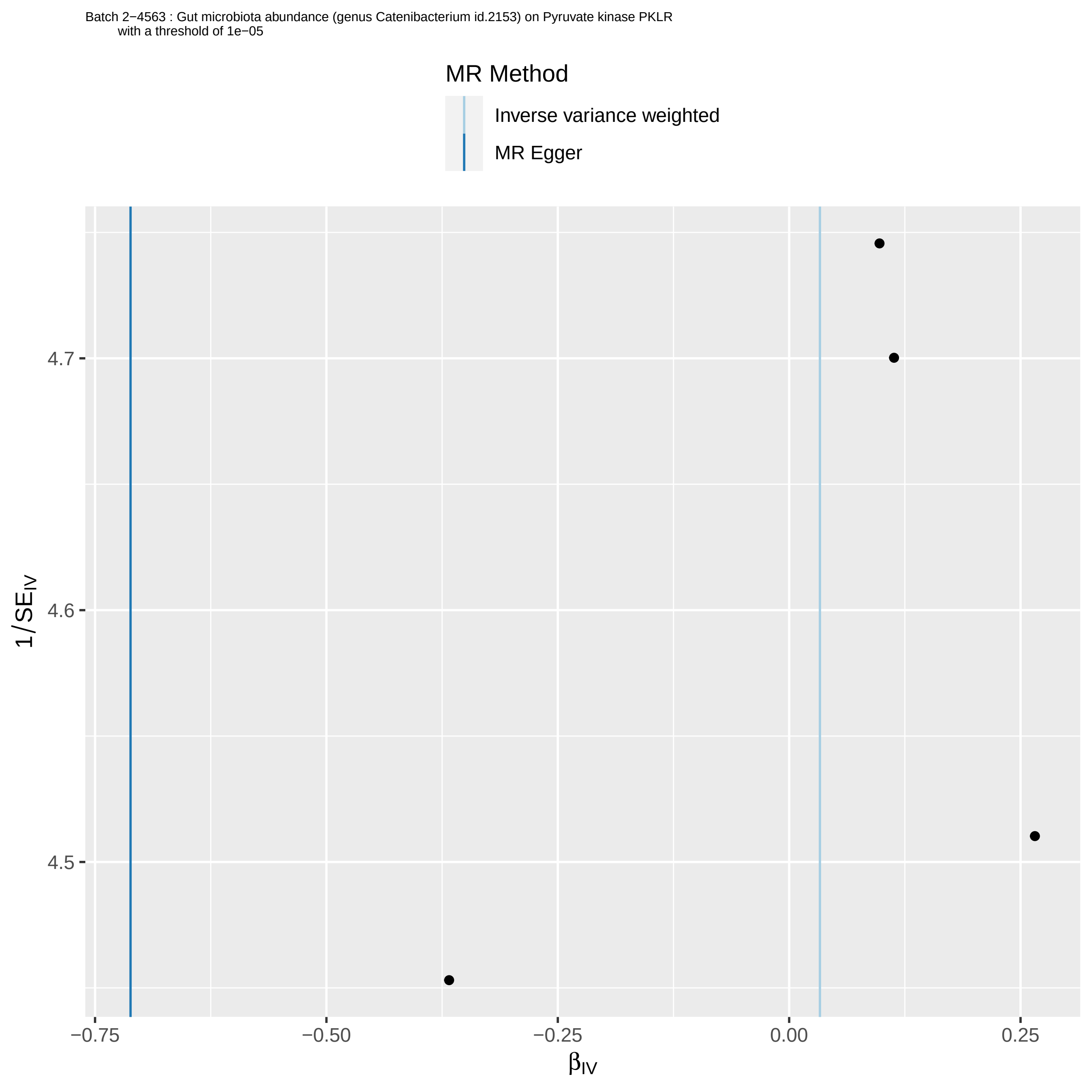

Batch 2−4563 : Gut microbiota abundance (genus Catenibacterium id.2153) on Pyruvate kinase PKLR
with a threshold of 1e−05
MR Method
Inverse variance weighted
MR Egger
4.7
4.6
4.5
1/SE_IV
−0.75
−0.50
−0.25
0.00
0.25
β_IV

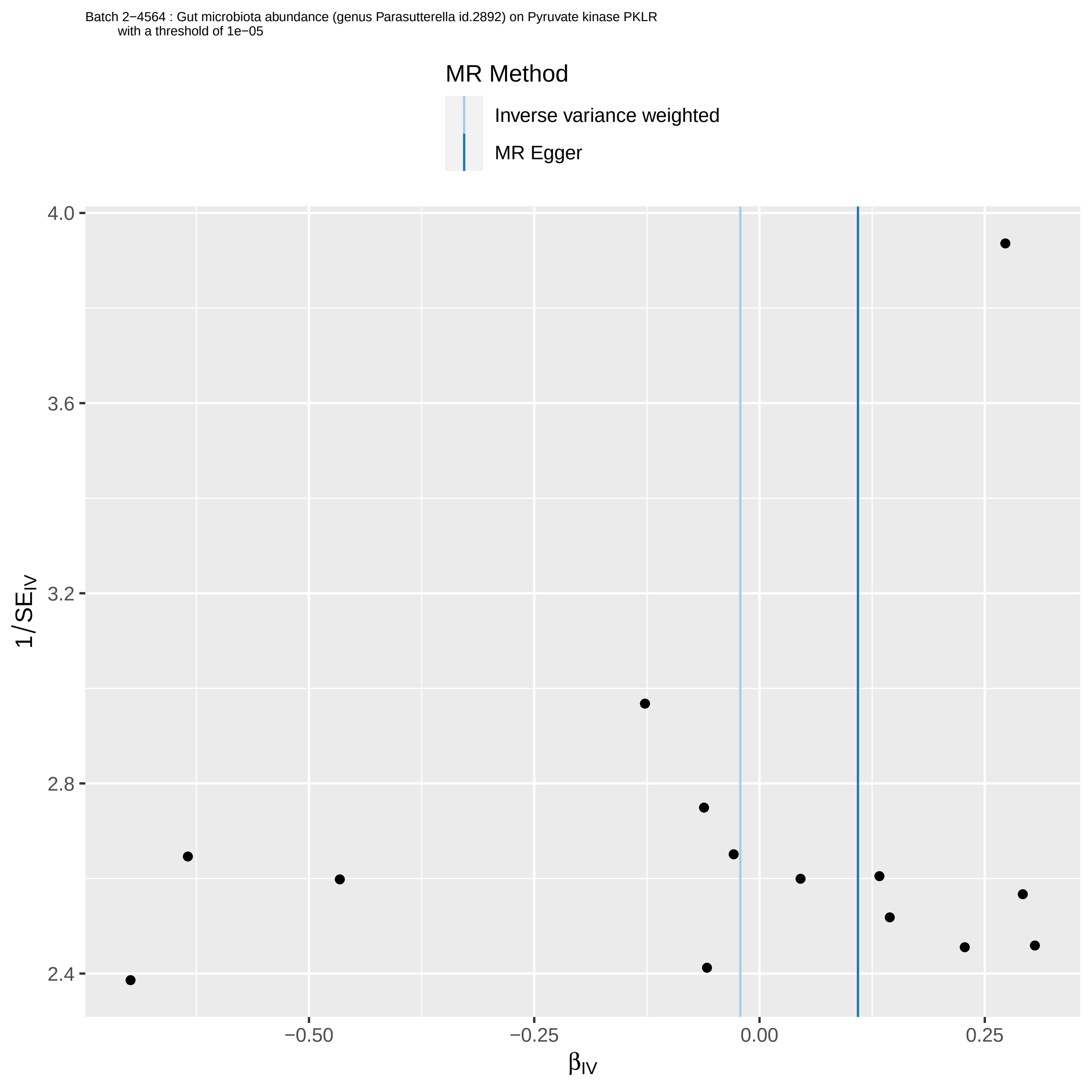
Batch 2−4564 : Gut microbiota abundance (genus Parasutterella id.2892) on Pyruvate kinase PKLR
with a threshold of 1e−05
MR Method
Inverse variance weighted
MR Egger
4.0
3.6
3.2
2.8
2.4
1/SE_IV
−0.50
−0.25
0.00
0.25
β_IV

Batch 2−4565 : Gut microbiota abundance (genus Catenibacterium id.2153) on Group 10 secretory phospholipase A2
with a threshold of 1e−05

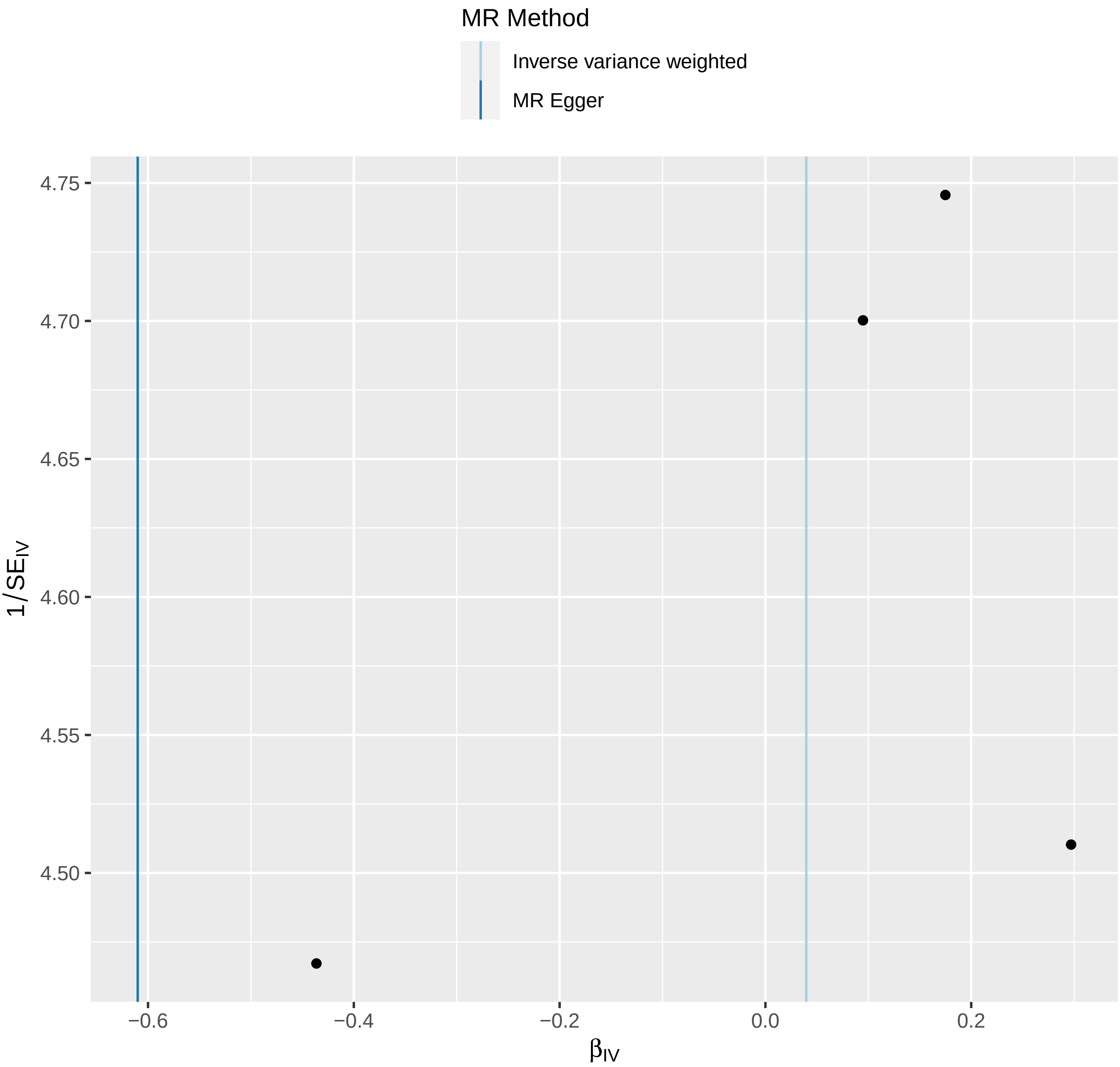

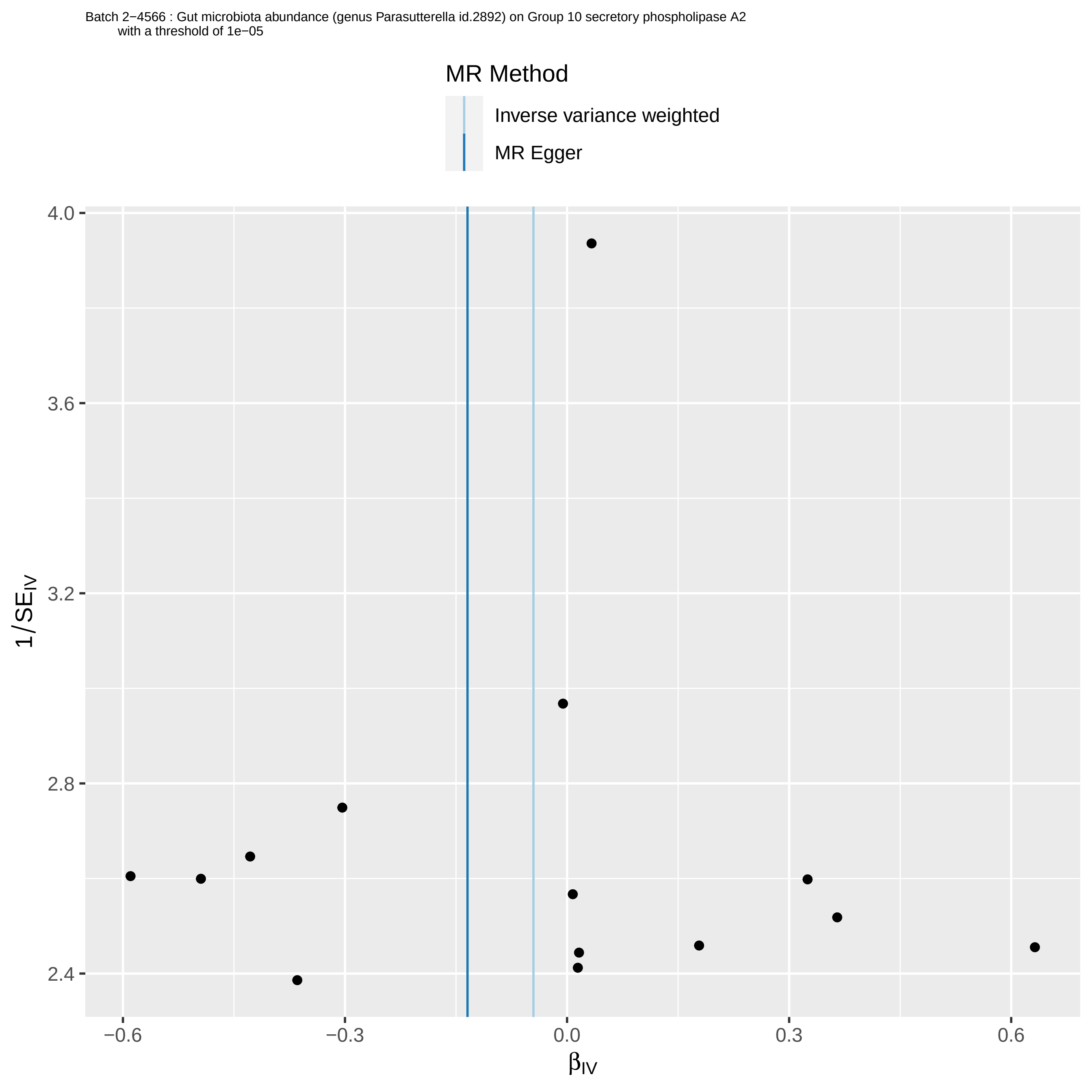

Batch 2−4566 : Gut microbiota abundance (genus Parasutterella id.2892) on Group 10 secretory phospholipase A2
with a threshold of 1e−05
MR Method
Inverse variance weighted
MR Egger
4.0
3.6
3.2
2.8
2.4
1/SE_IV
−0.6
−0.3
0.0
0.3
0.6
β_IV

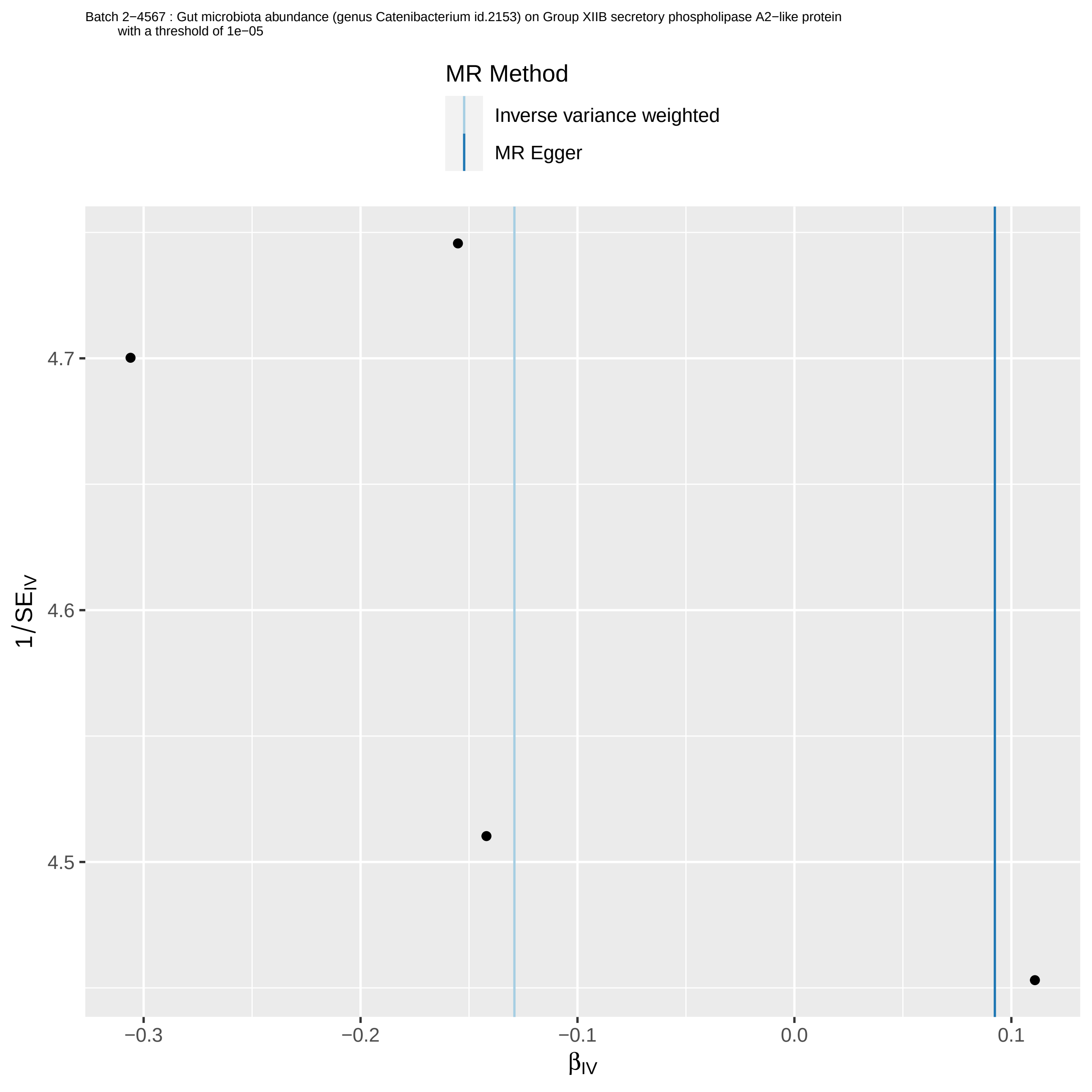
Batch 2−4567 : Gut microbiota abundance (genus Catenibacterium id.2153) on Group XIIB secretory phospholipase A2−like protein
with a threshold of 1e−05
MR Method
Inverse variance weighted
MR Egger
4.7
4.6
4.5
1/SEIV
−0.3
−0.2
−0.1
0.0
0.1
βIV

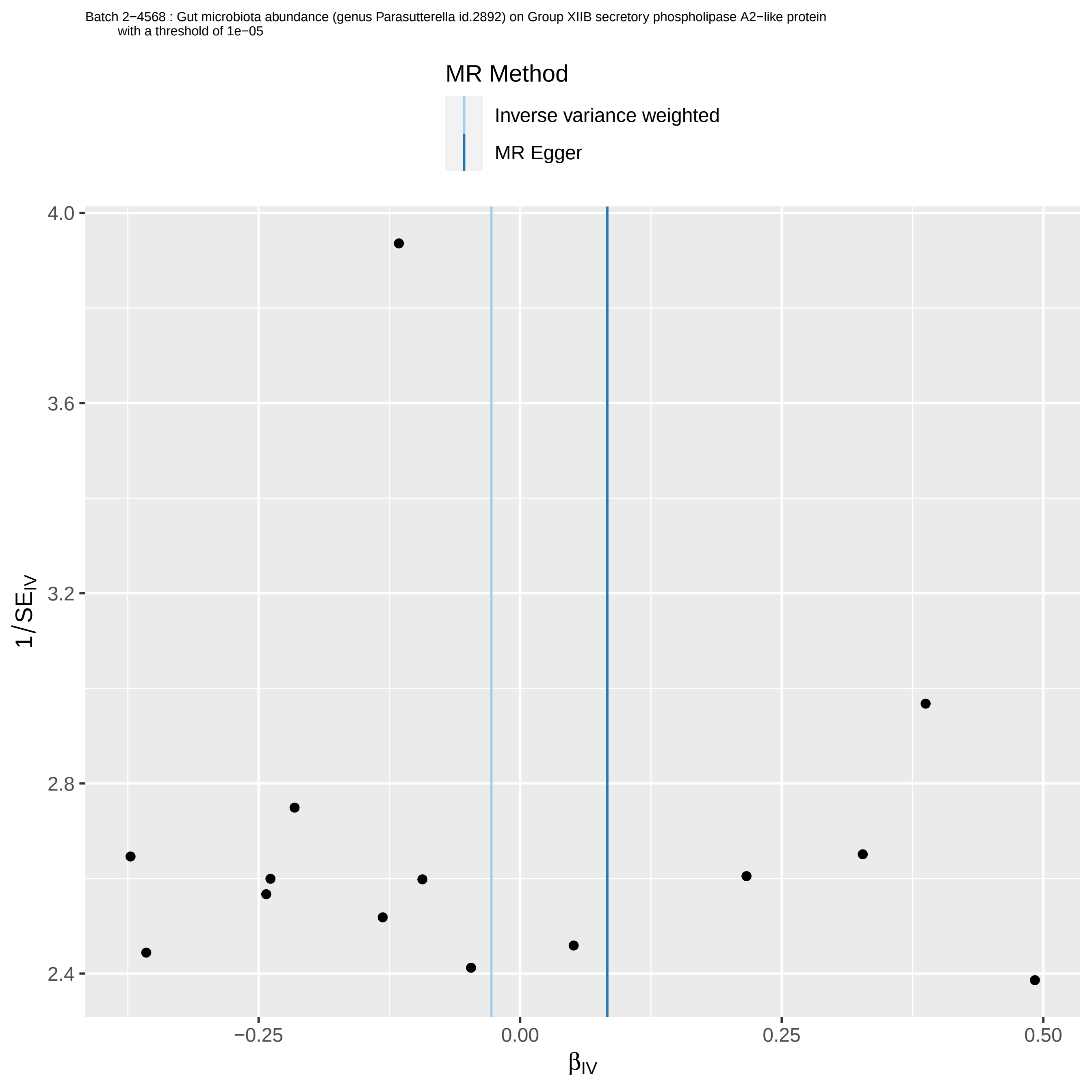
Batch 2−4568 : Gut microbiota abundance (genus Parasutterella id.2892) on Group XIIB secretory phospholipase A2−like protein
with a threshold of 1e−05
MR Method
Inverse variance weighted
MR Egger
4.0
3.6
3.2
2.8
2.4
1/SE_IV
−0.25
0.00
0.25
0.50
β_IV

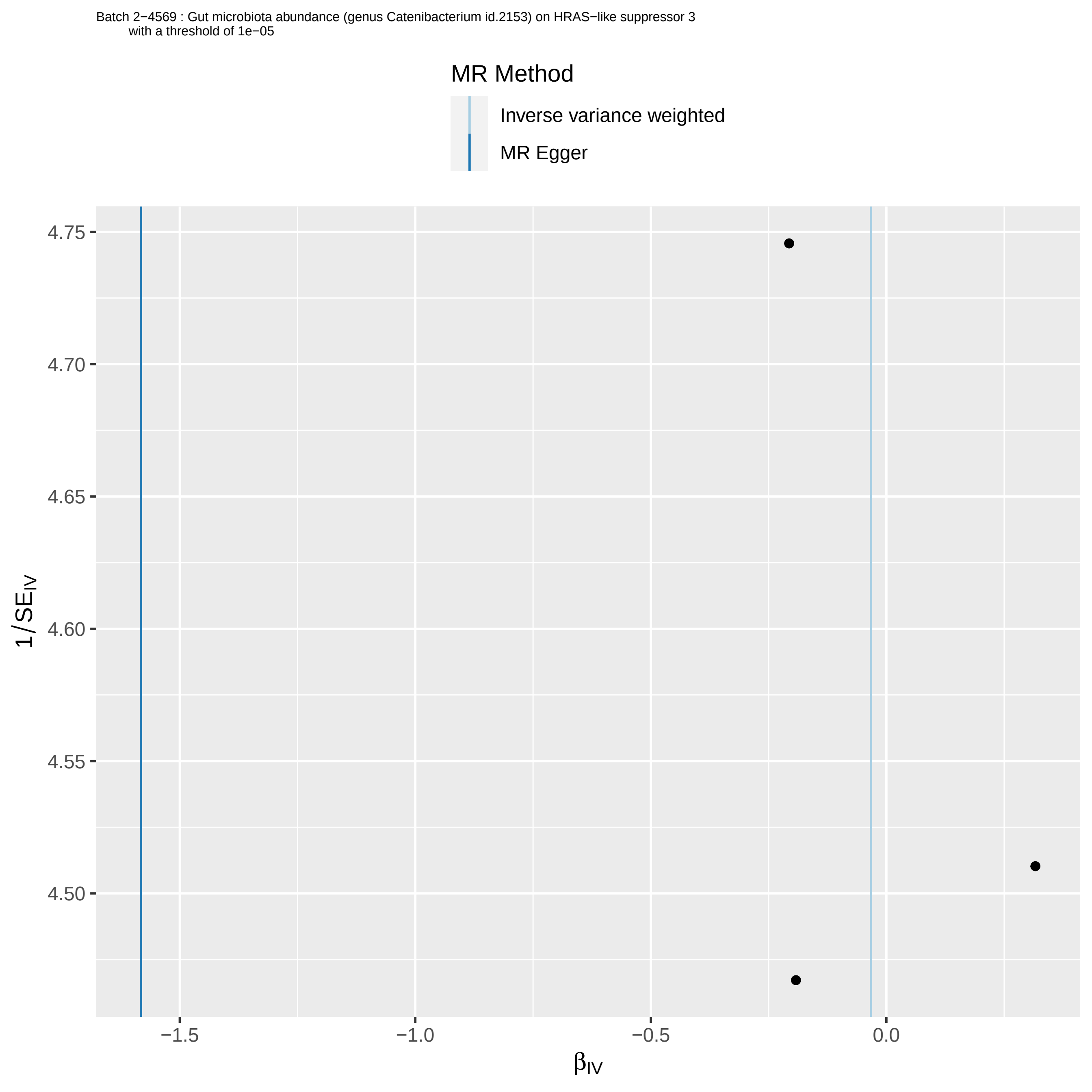
Batch 2−4569 : Gut microbiota abundance (genus Catenibacterium id.2153) on HRAS−like suppressor 3
with a threshold of 1e−05
MR Method
Inverse variance weighted
MR Egger
4.75
4.70
4.65
4.60
4.55
4.50
1/SE_IV
−1.5
−1.0
−0.5
0.0
β_IV

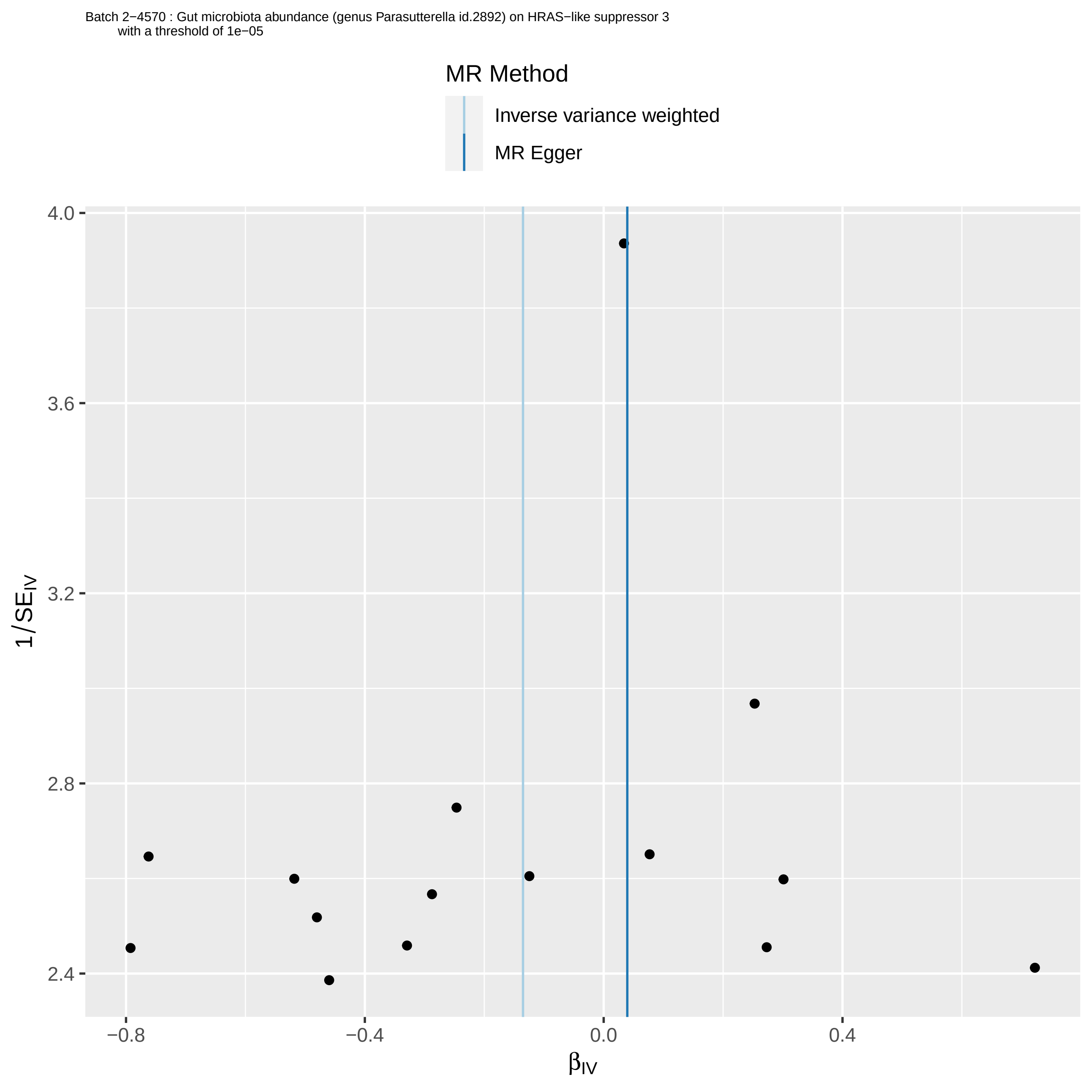
Batch 2−4570 : Gut microbiota abundance (genus Parasutterella id.2892) on HRAS−like suppressor 3
with a threshold of 1e−05
MR Method
Inverse variance weighted
MR Egger
4.0
3.6
3.2
2.8
2.4
1/SE_IV
−0.8
−0.4
0.0
0.4
β_IV

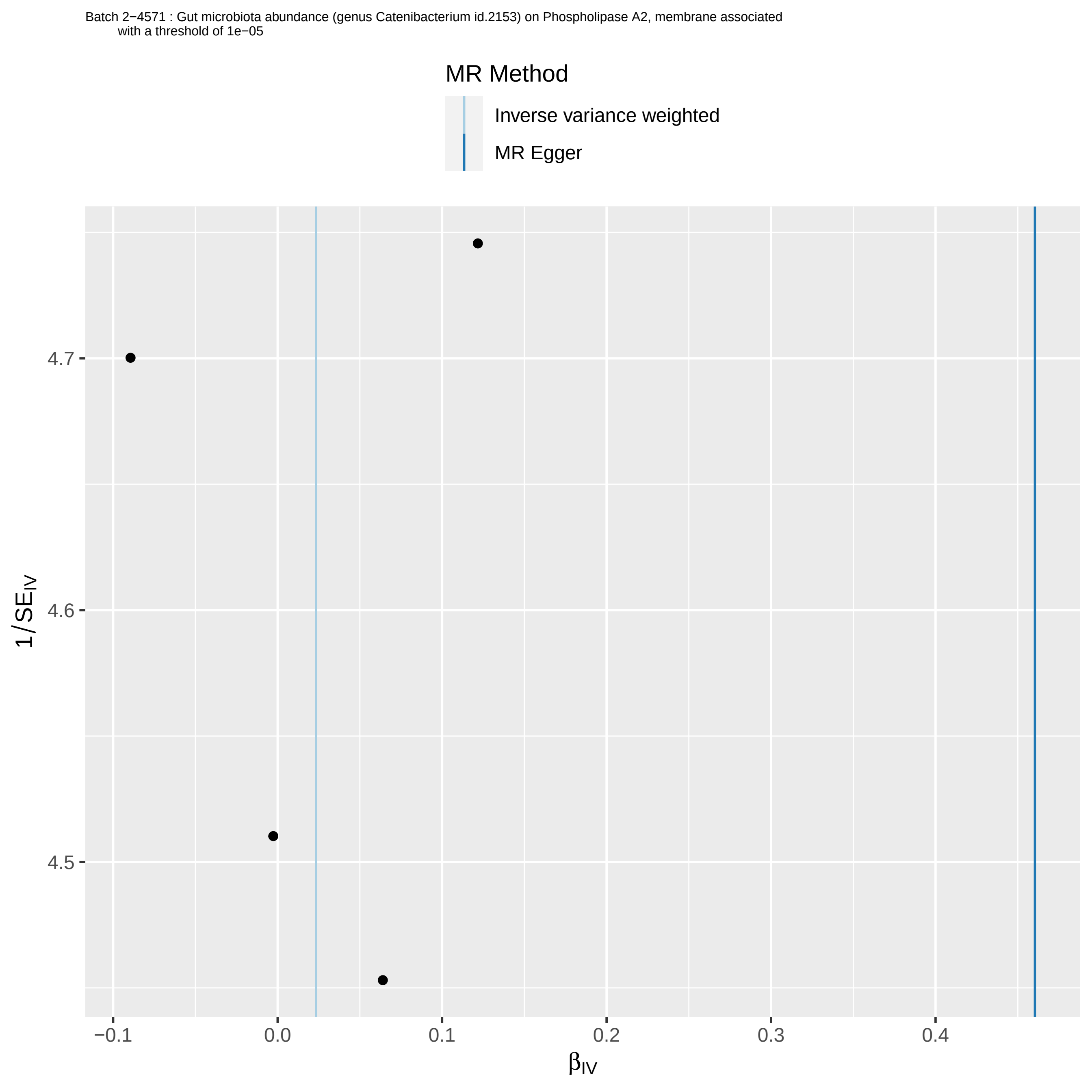

Batch 2−4571 : Gut microbiota abundance (genus Catenibacterium id.2153) on Phospholipase A2, membrane associated with a threshold of 1e−05
MR Method
Inverse variance weighted
MR Egger
4.7
4.6
4.5
1/SE_IV
−0.1
0.0
0.1
0.2
0.3
0.4
β_IV

Batch 2−4572 : Gut microbiota abundance (genus Parasutterella id.2892) on Phospholipase A2, membrane associated
with a threshold of 1e−05

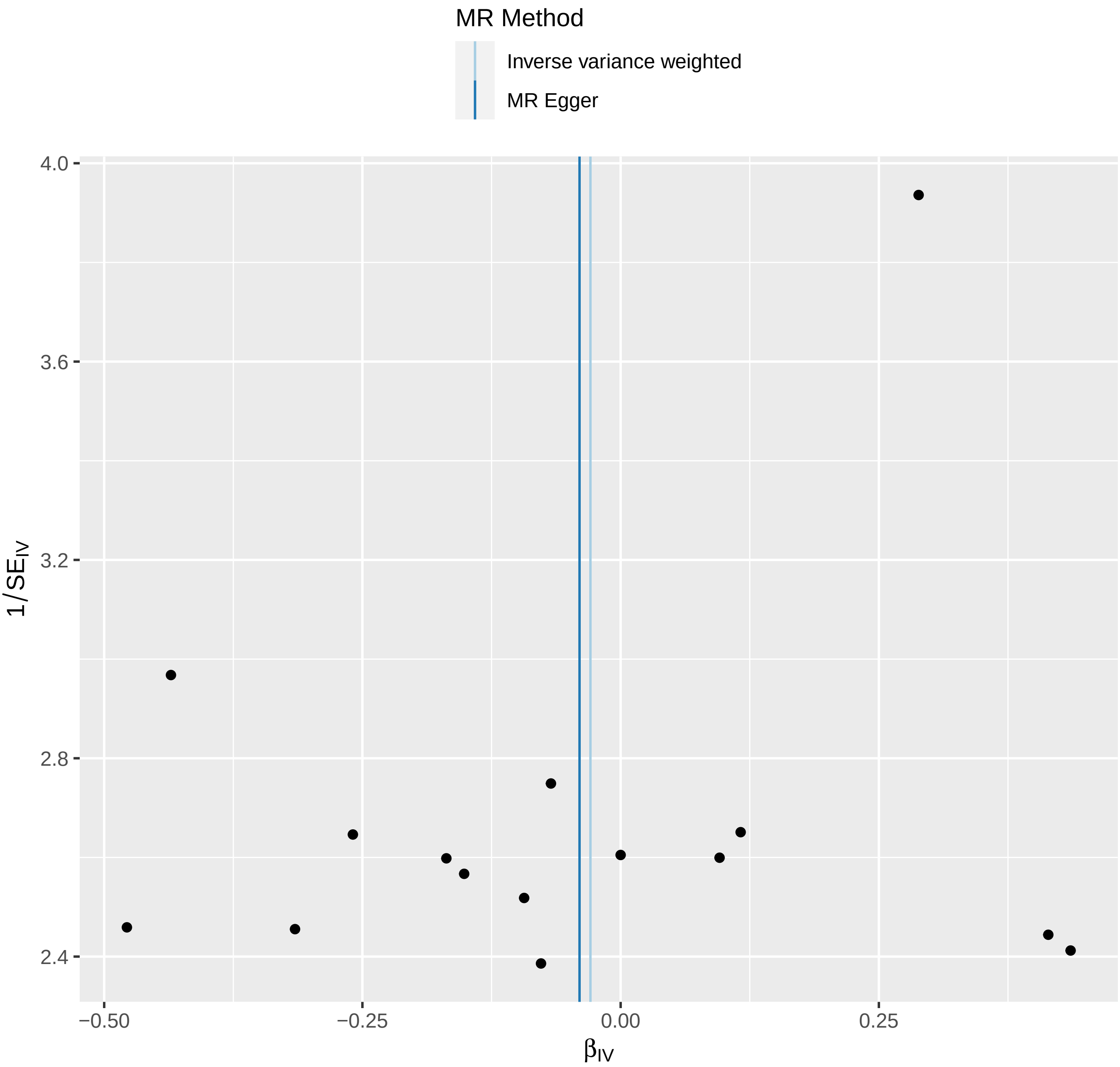

Batch 2−4573 : Gut microbiota abundance (genus Catenibacterium id.2153) on Putative inactive group IIC secretory phospholipase A2
with a threshold of 1e−05

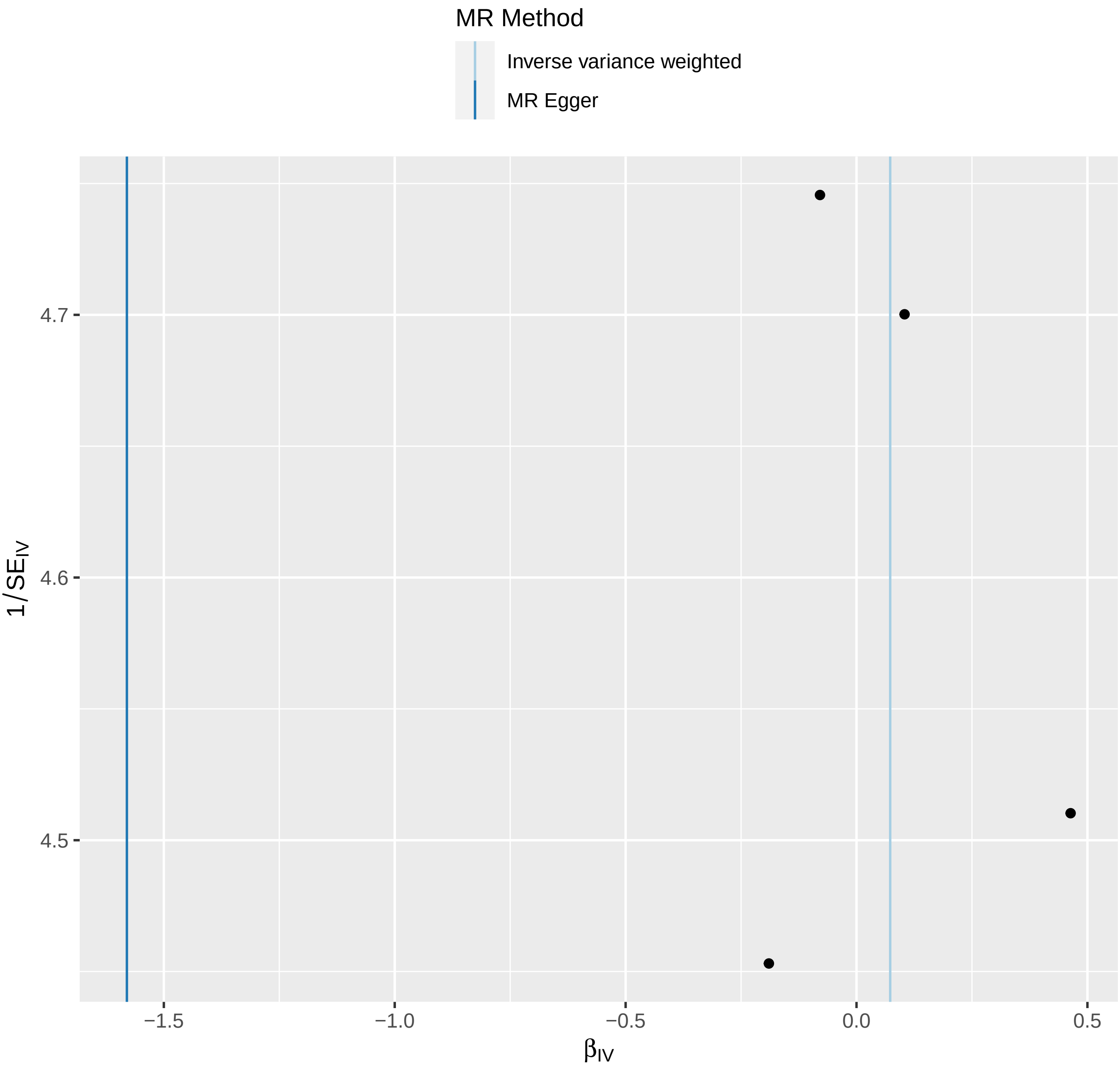

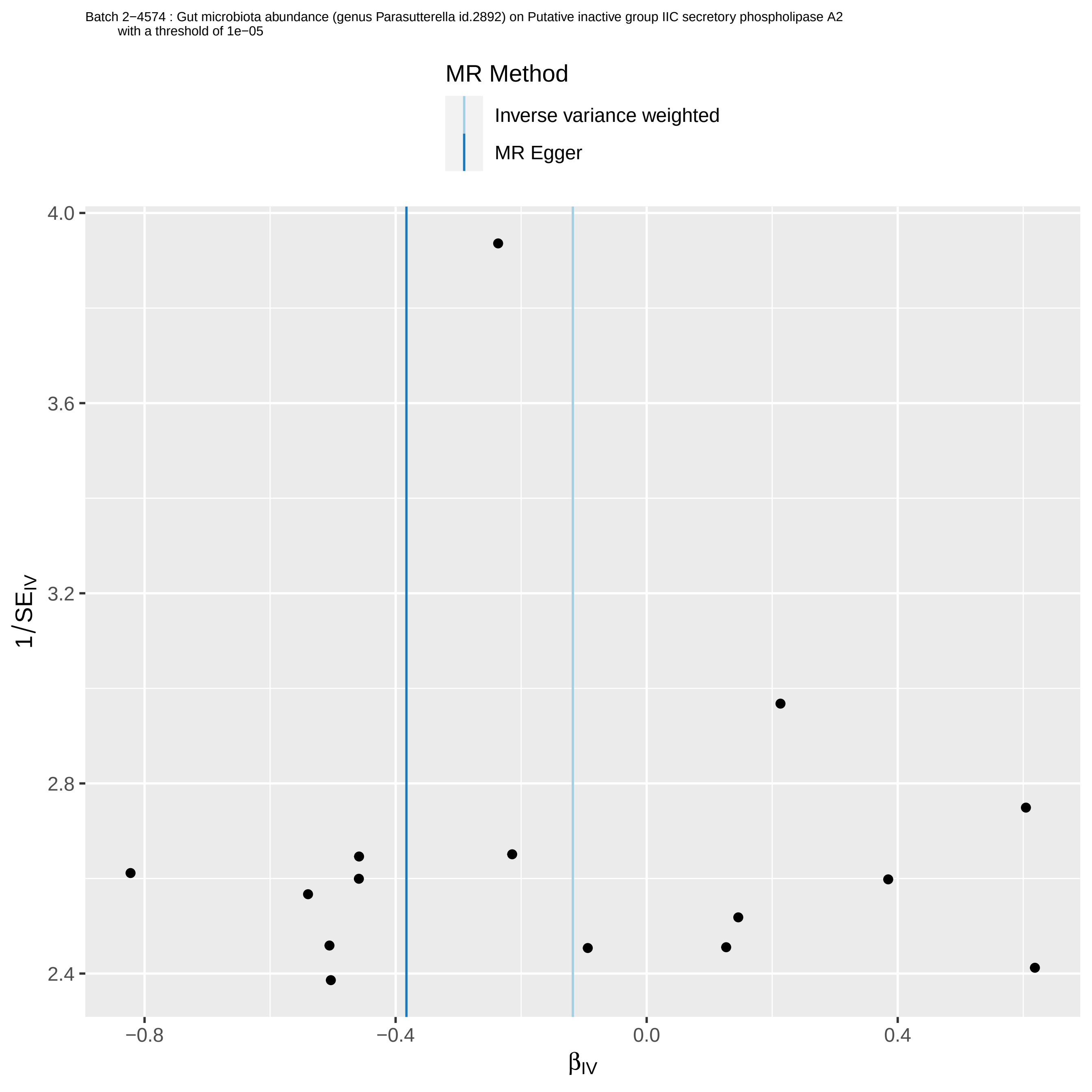
Batch 2−4574 : Gut microbiota abundance (genus Parasutterella id.2892) on Putative inactive group IIC secretory phospholipase A2
with a threshold of 1e−05
MR Method
Inverse variance weighted
MR Egger
4.0
3.6
3.2
2.8
2.4
1/SE$_{IV}$
−0.8
−0.4
0.0
0.4
β_{IV}

Batch 2−4575 : Gut microbiota abundance (genus Catenibacterium id.2153) on Group IIE secretory phospholipase A2
with a threshold of 1e−05

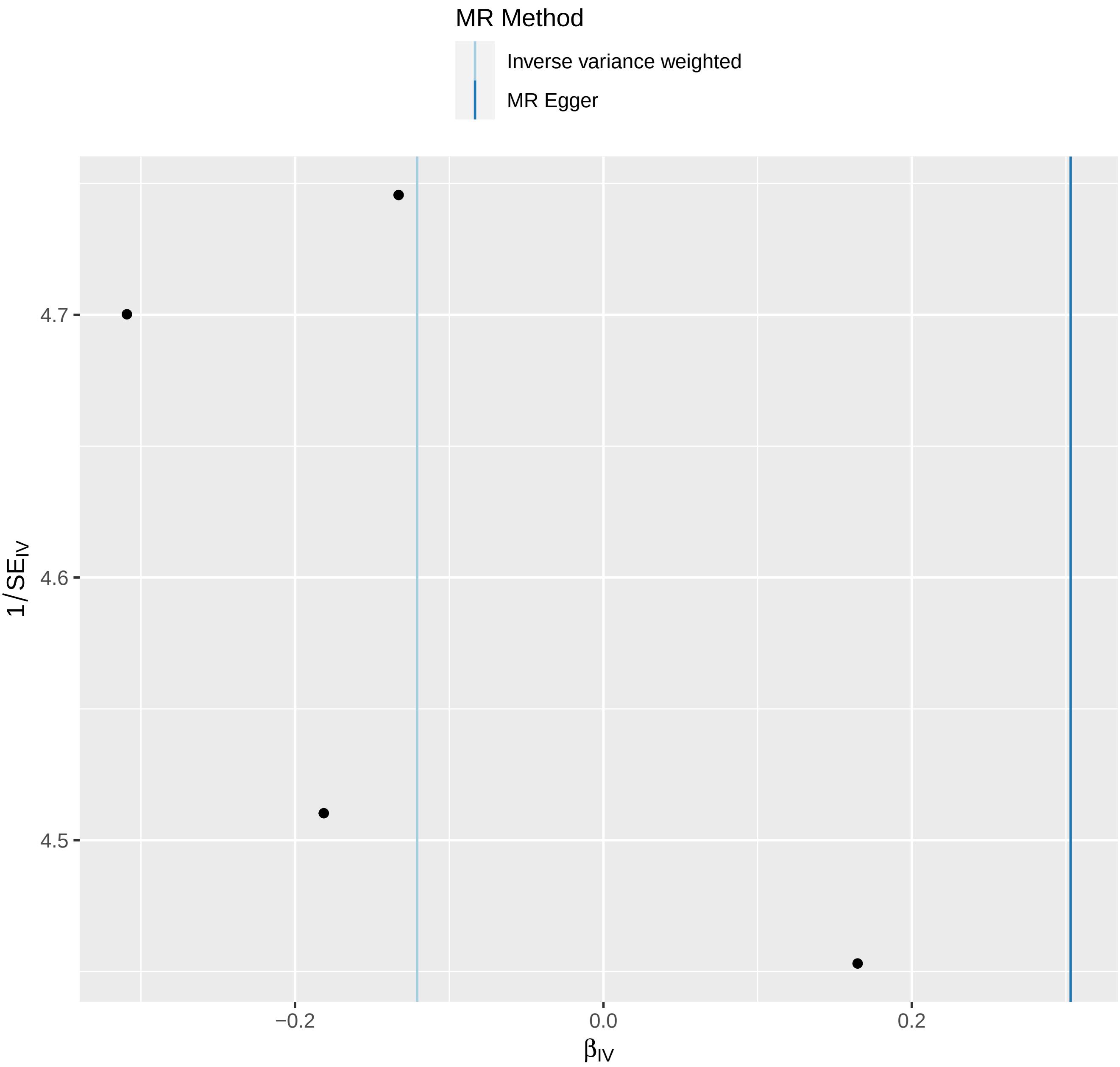

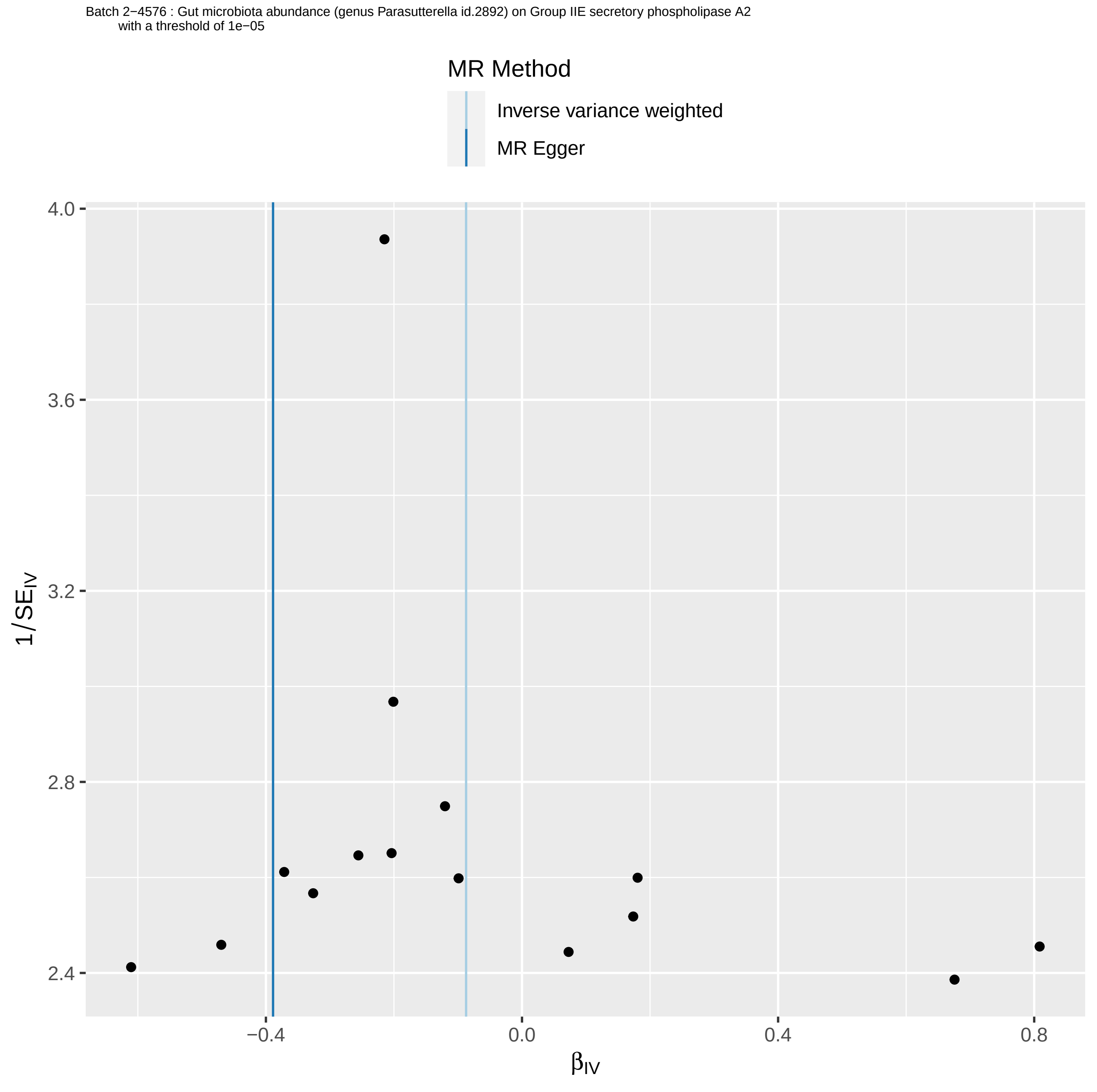
Batch 2−4576 : Gut microbiota abundance (genus Parasutterella id.2892) on Group IIE secretory phospholipase A2
with a threshold of 1e−05
MR Method
Inverse variance weighted
MR Egger
4.0
3.6
3.2
2.8
2.4
1/SE$_{IV}$
−0.4
0.0
0.4
0.8
β_{IV}

Batch 2−4577 : Gut microbiota abundance (genus Catenibacterium id.2153) on Group IIE secretory phospholipase A2
with a threshold of 1e−05

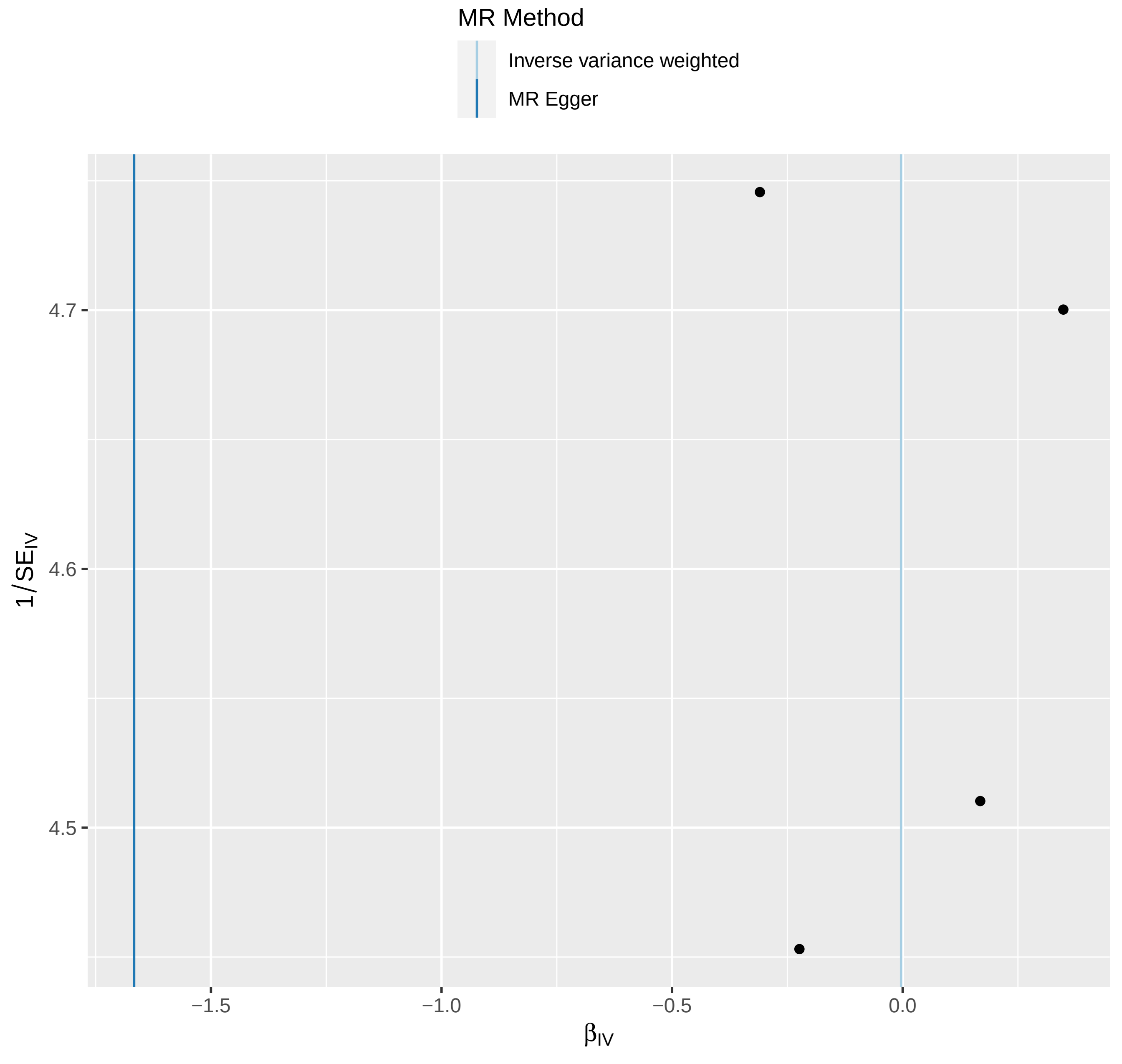

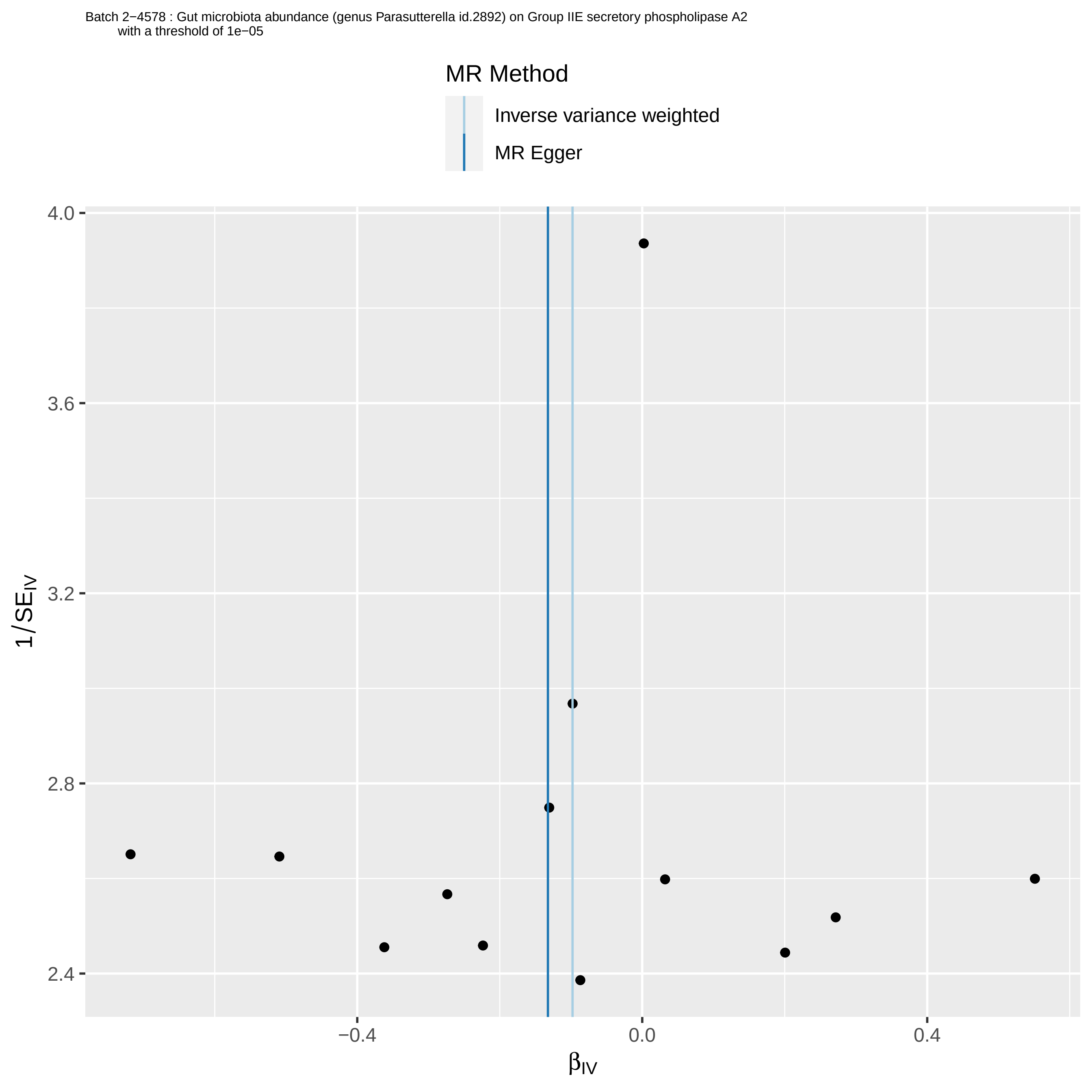

Batch 2−4578 : Gut microbiota abundance (genus Parasutterella id.2892) on Group IIE secretory phospholipase A2
with a threshold of 1e−05
MR Method
Inverse variance weighted
MR Egger
4.0
3.6
3.2
2.8
2.4
1/SE$_{IV}$
−0.4
0.0
0.4
β_{IV}

Batch 2−4579 : Gut microbiota abundance (genus Catenibacterium id.2153) on Calcium−dependent phospholipase A2
with a threshold of 1e−05

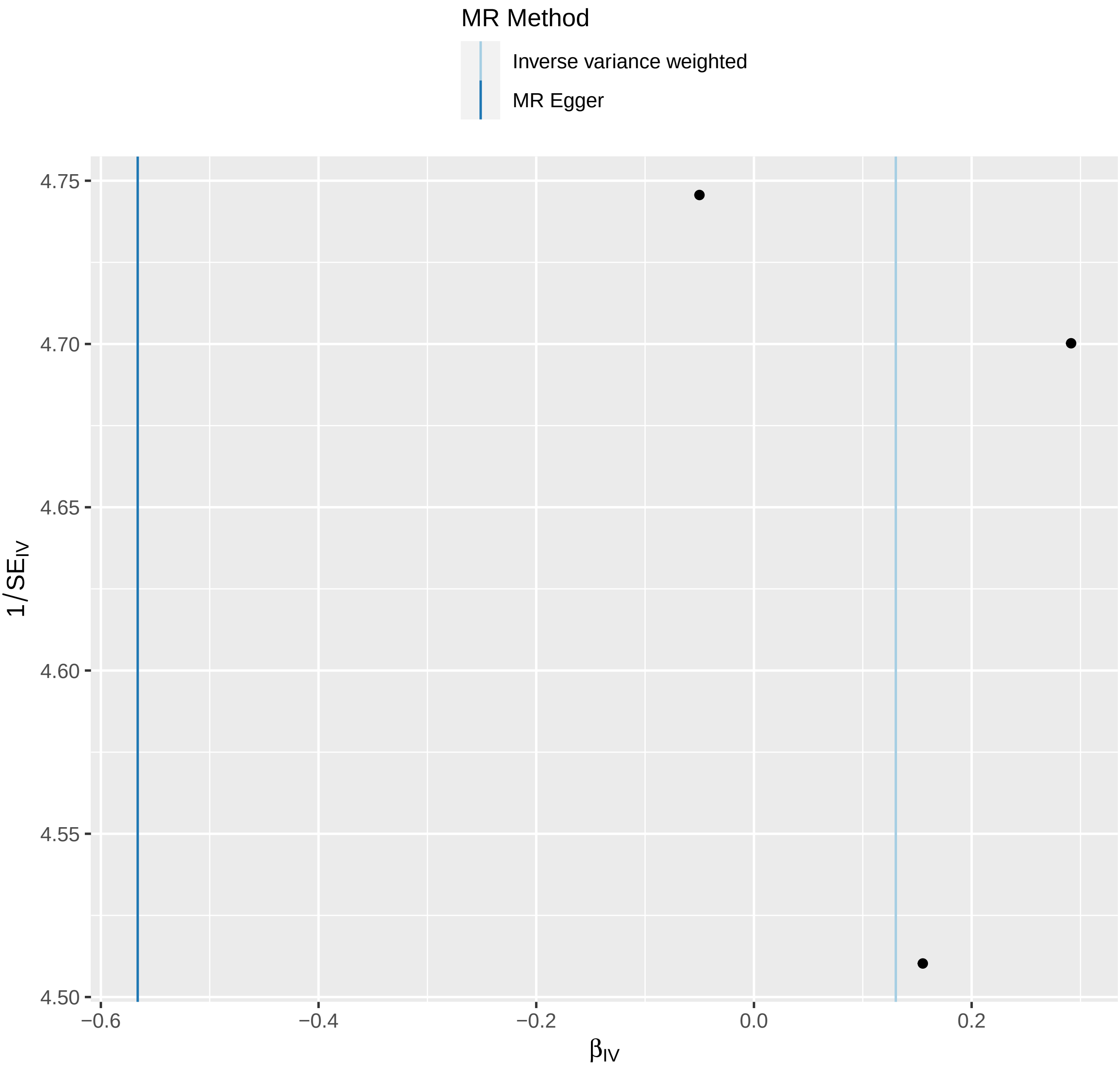

Batch 2−4580 : Gut microbiota abundance (genus Parasutterella id.2892) on Calcium−dependent phospholipase A2
with a threshold of 1e−05

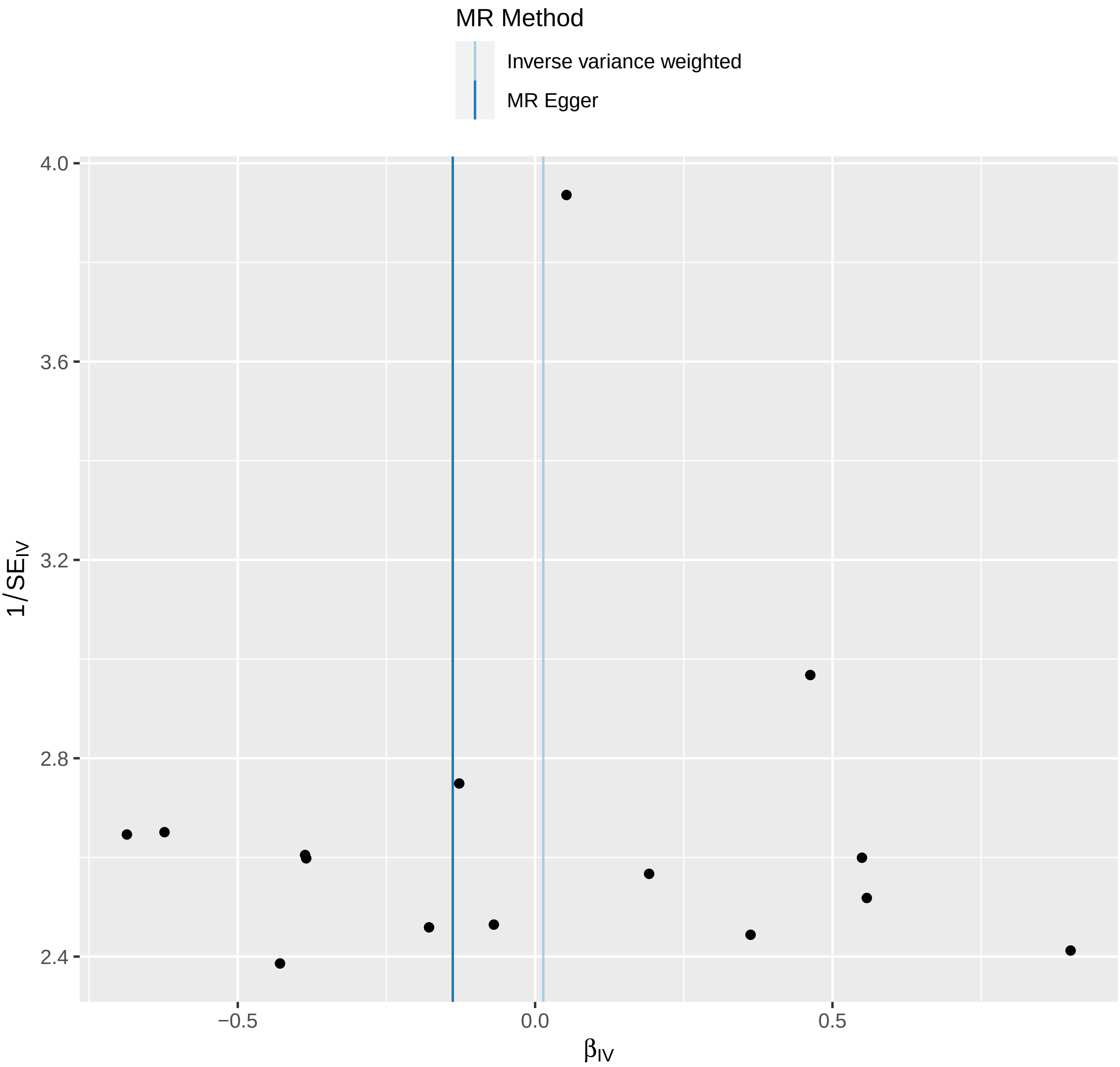

Batch 2−4581 : Gut microbiota abundance (genus Catenibacterium id.2153) on Secretory phospholipase A2 receptor
with a threshold of 1e−05

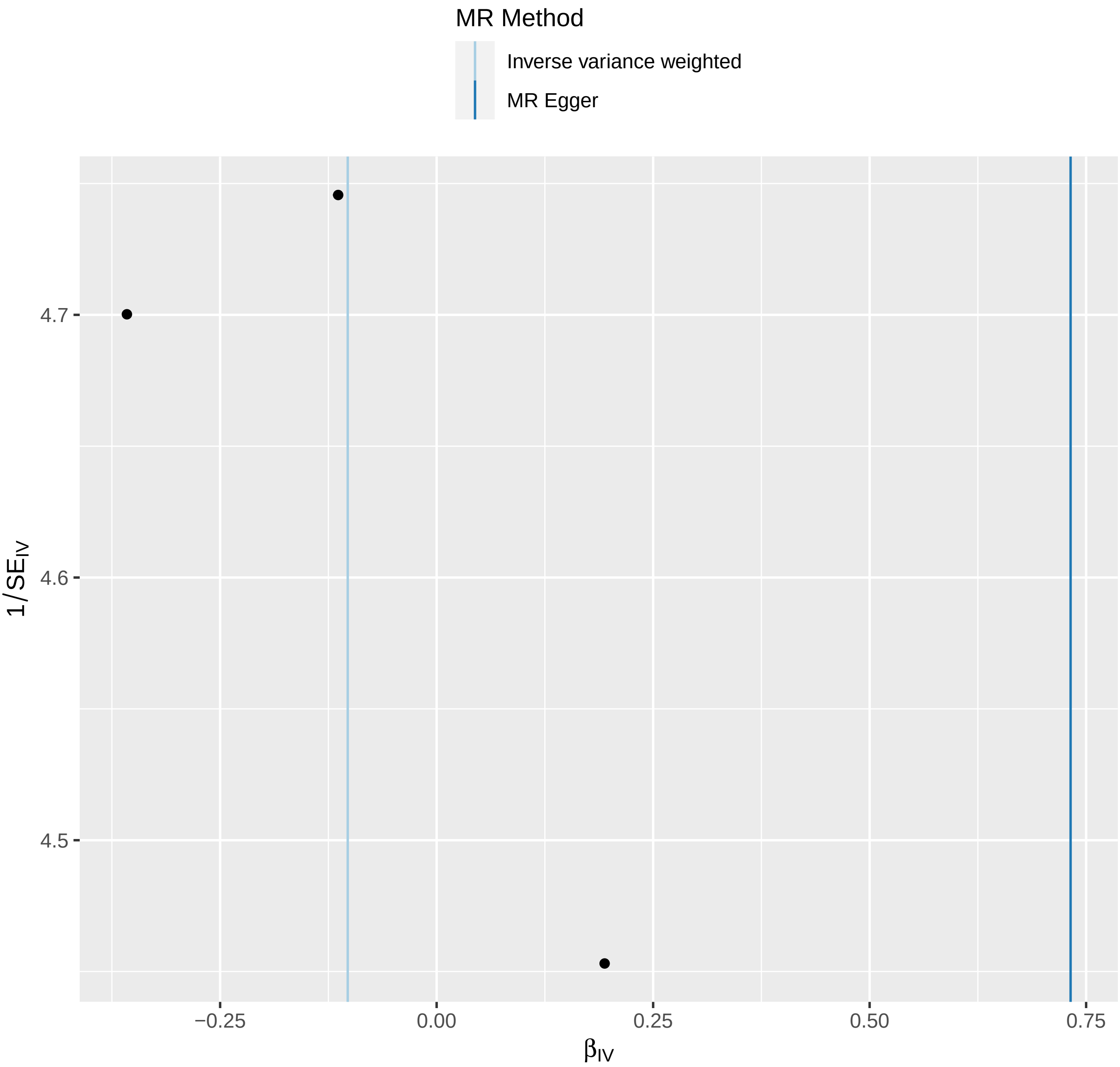

Batch 2−4582 : Gut microbiota abundance (genus Parasutterella id.2892) on Secretory phospholipase A2 receptor
with a threshold of 1e−05

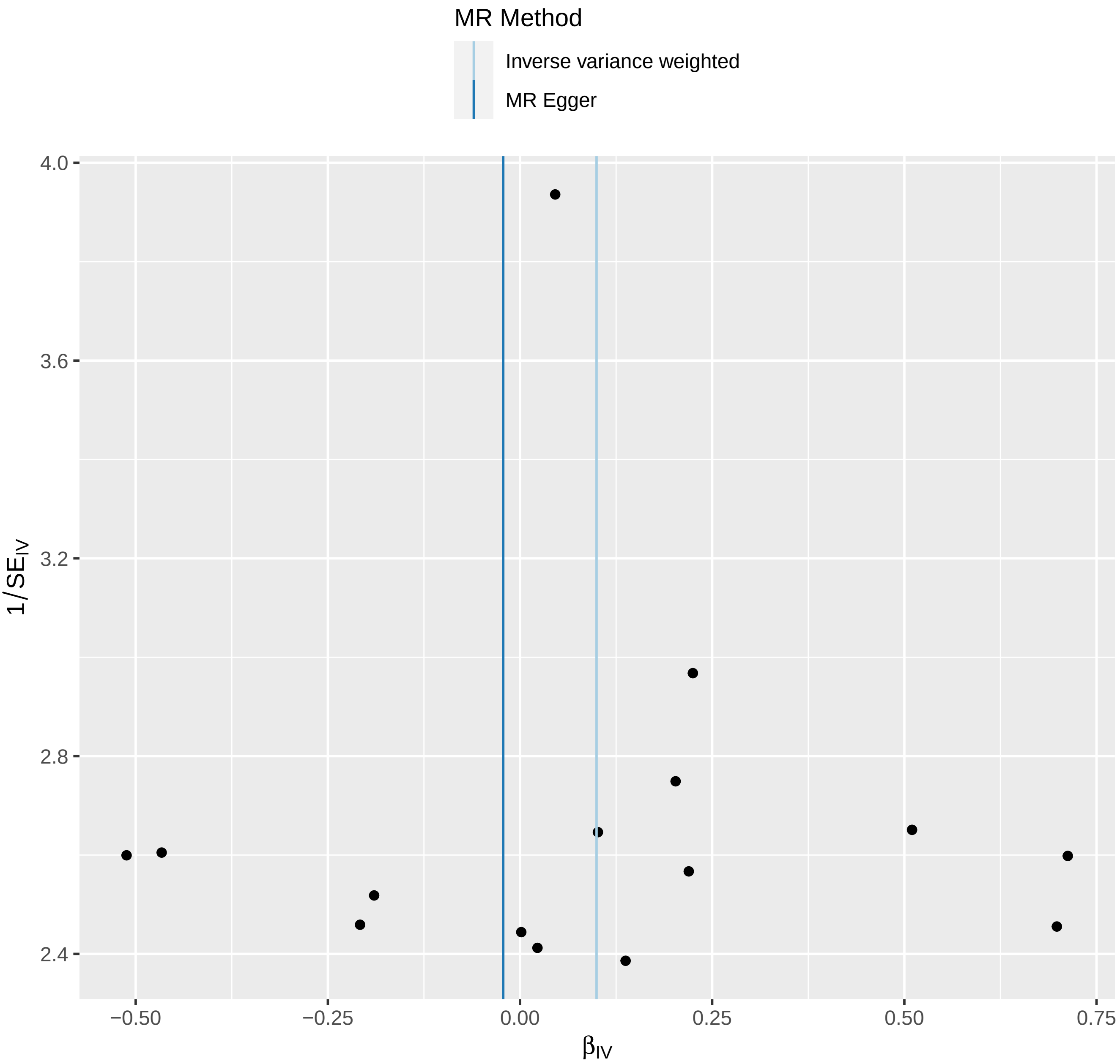

Batch 2−4583 : Gut microbiota abundance (genus Catenibacterium id.2153) on Urokinase−type plasminogen activator
with a threshold of 1e−05

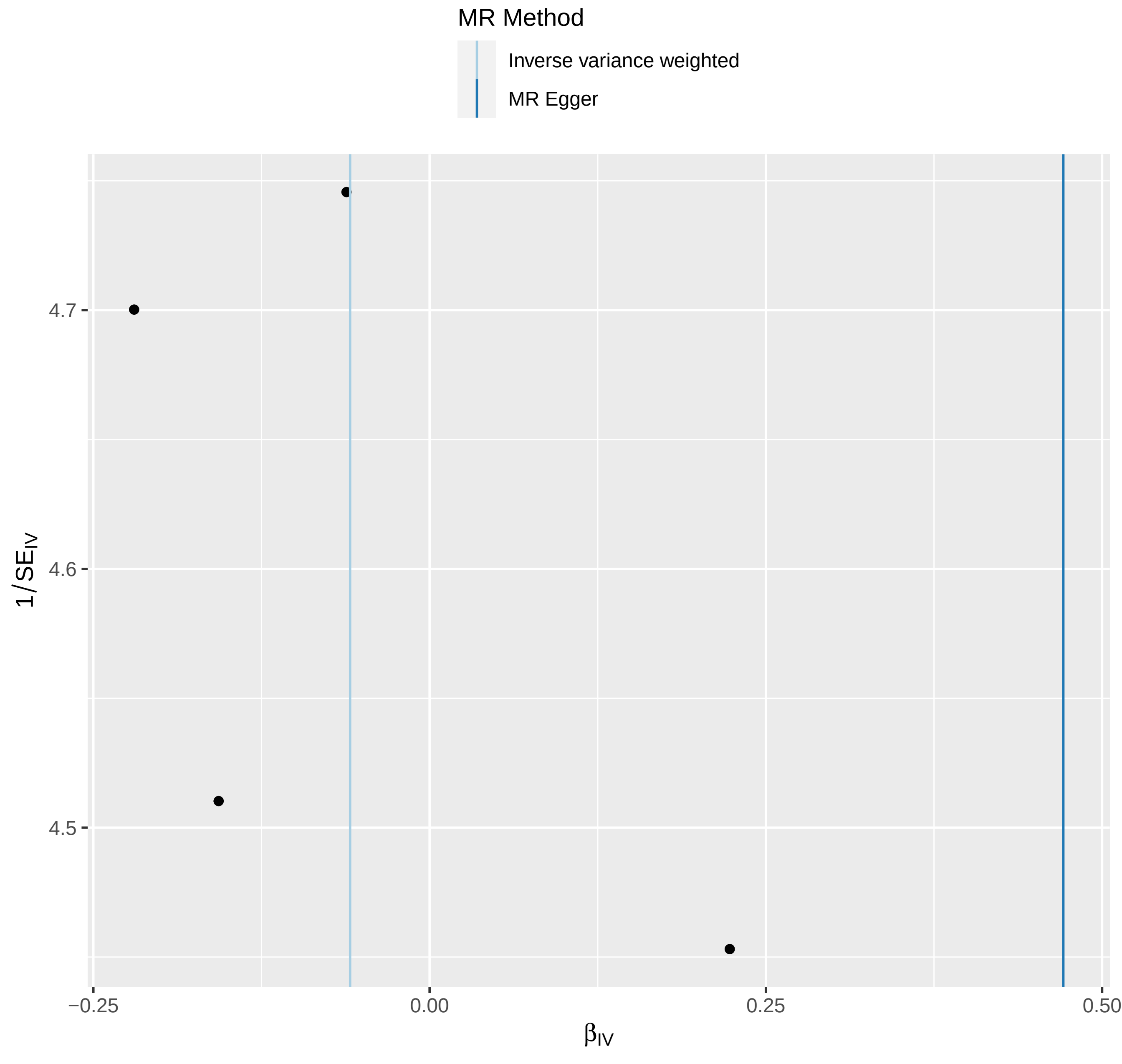

Batch 2−4584 : Gut microbiota abundance (genus Parasutterella id.2892) on Urokinase−type plasminogen activator
with a threshold of 1e−05

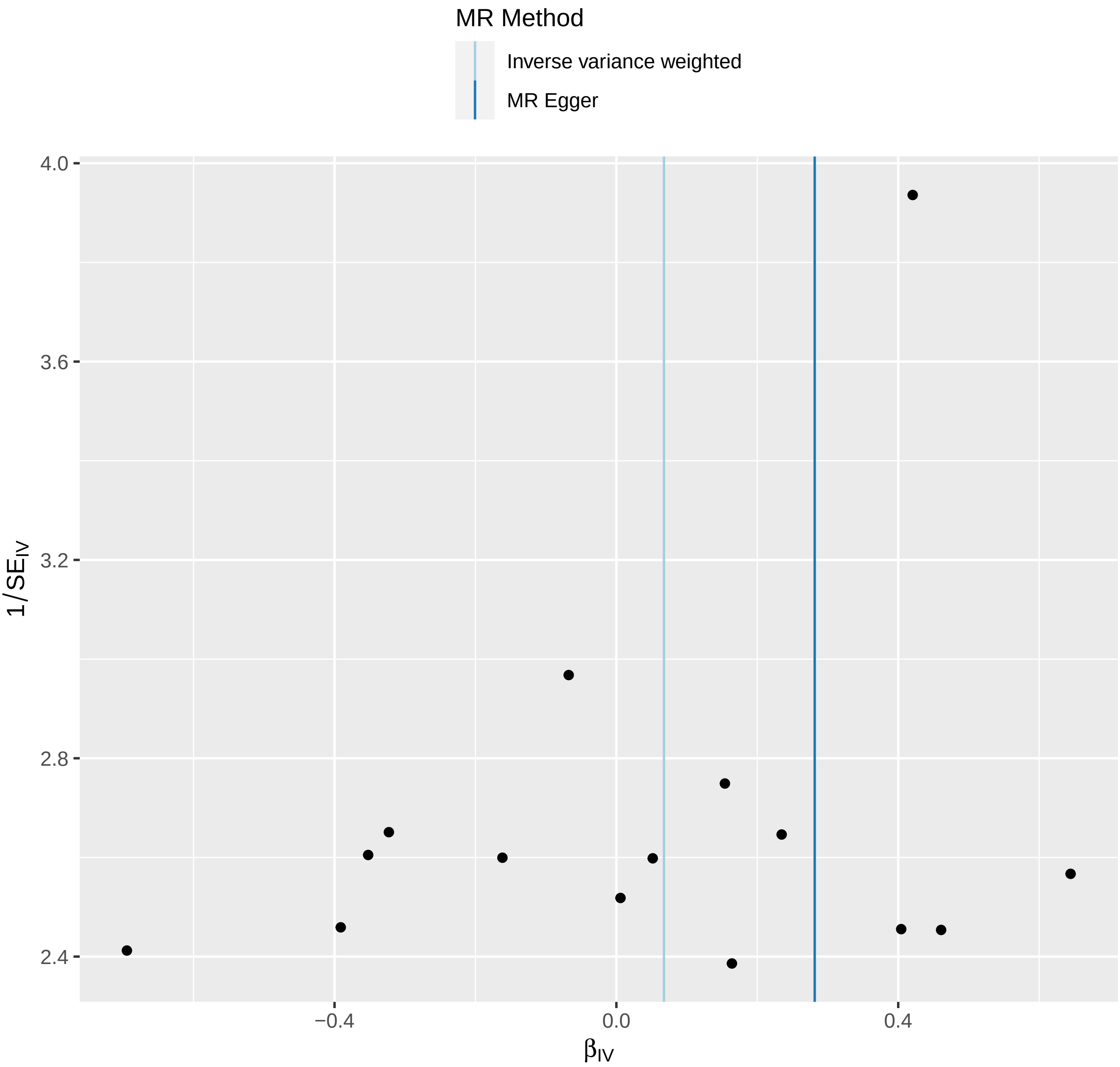

Batch 2−4585 : Gut microbiota abundance (genus Catenibacterium id.2153) on Urokinase plasminogen activator surface receptor with a threshold of 1e−05

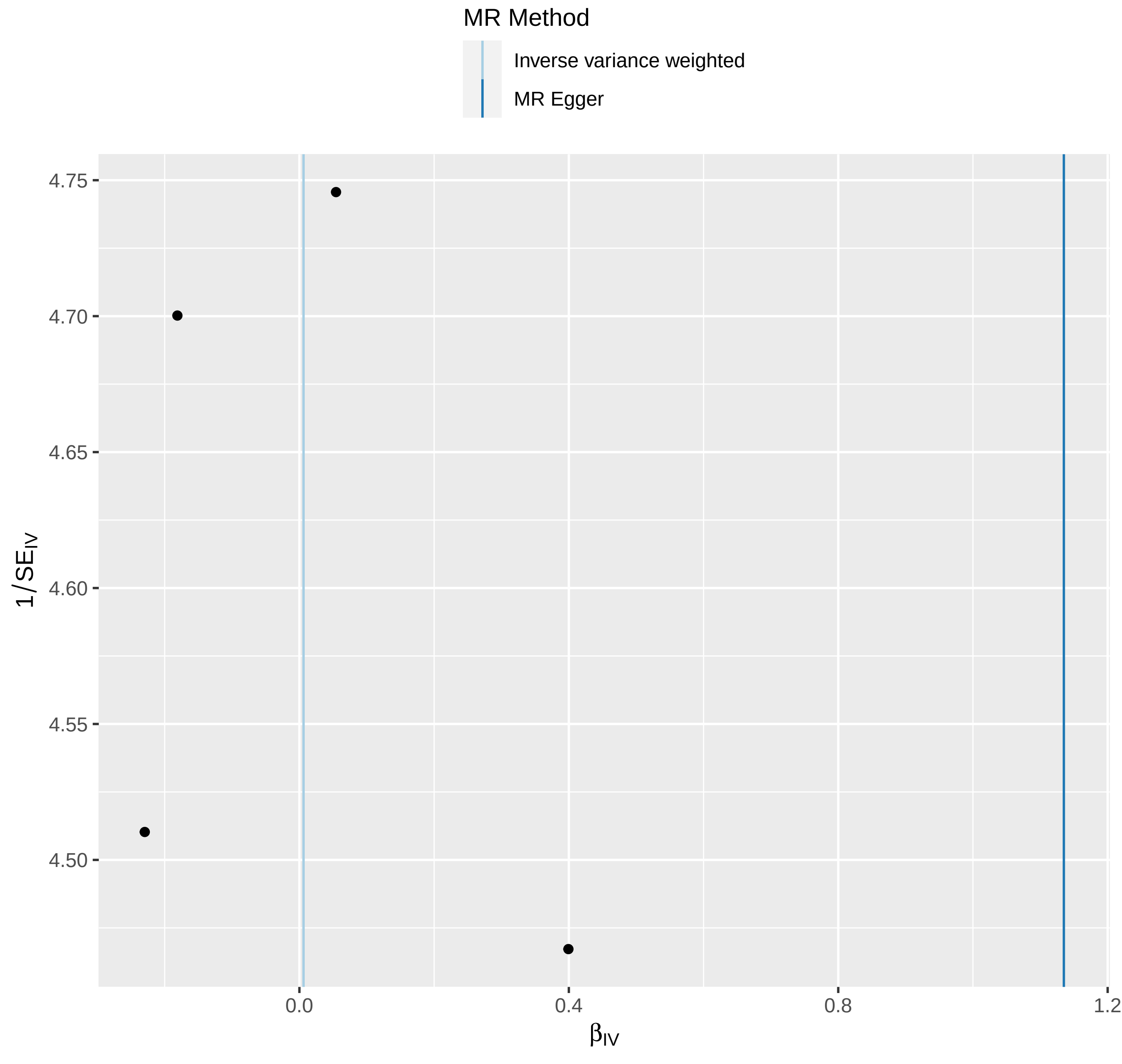

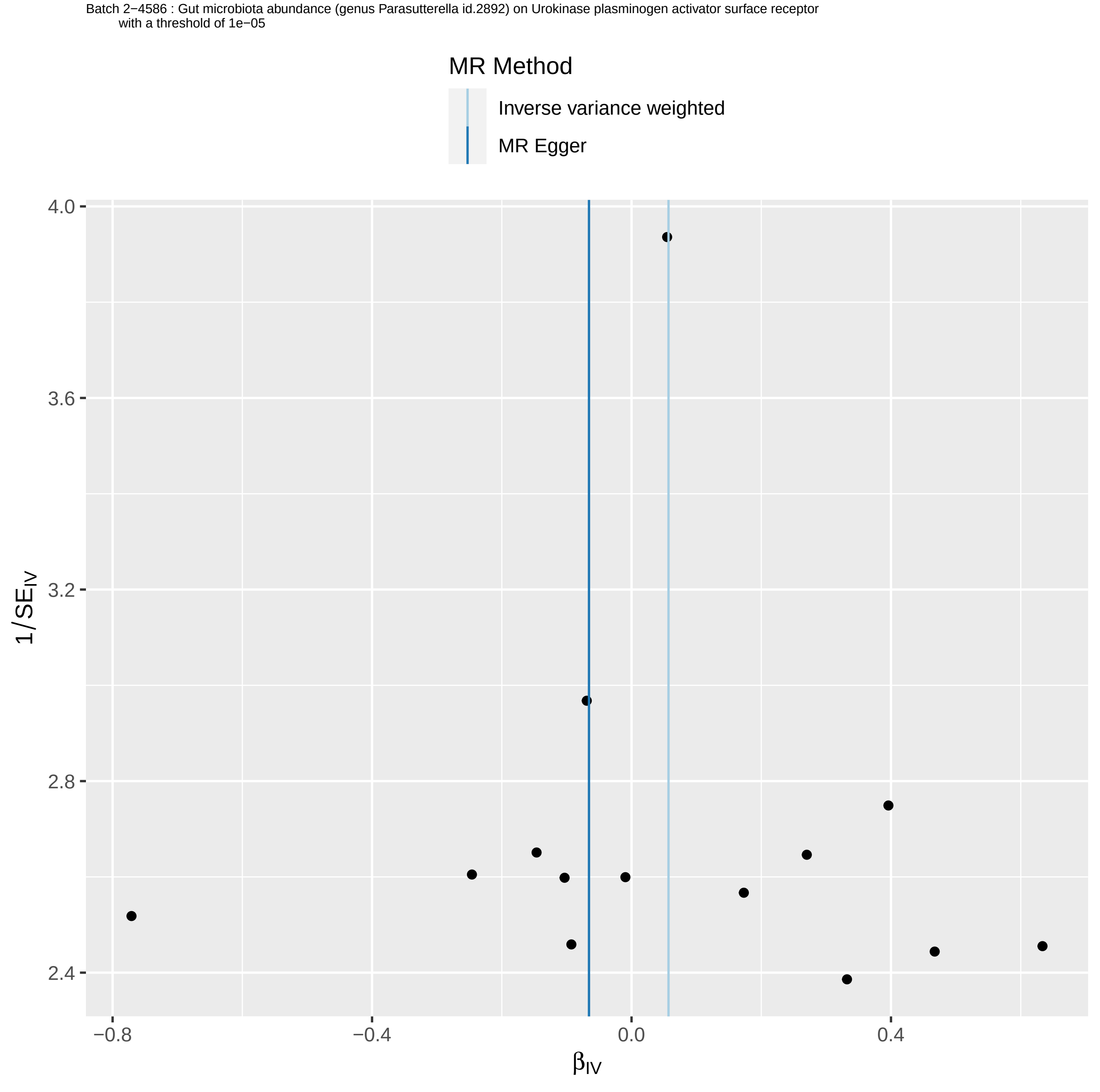
Batch 2−4586 : Gut microbiota abundance (genus Parasutterella id.2892) on Urokinase plasminogen activator surface receptor
with a threshold of 1e−05
MR Method
Inverse variance weighted
MR Egger
4.0
3.6
3.2
2.8
2.4
1/SE_IV
−0.8
−0.4
0.0
0.4
β_IV

Batch 2−4587 : Gut microbiota abundance (genus Catenibacterium id.2153) on Phospholipase B−like 1
with a threshold of 1e−05

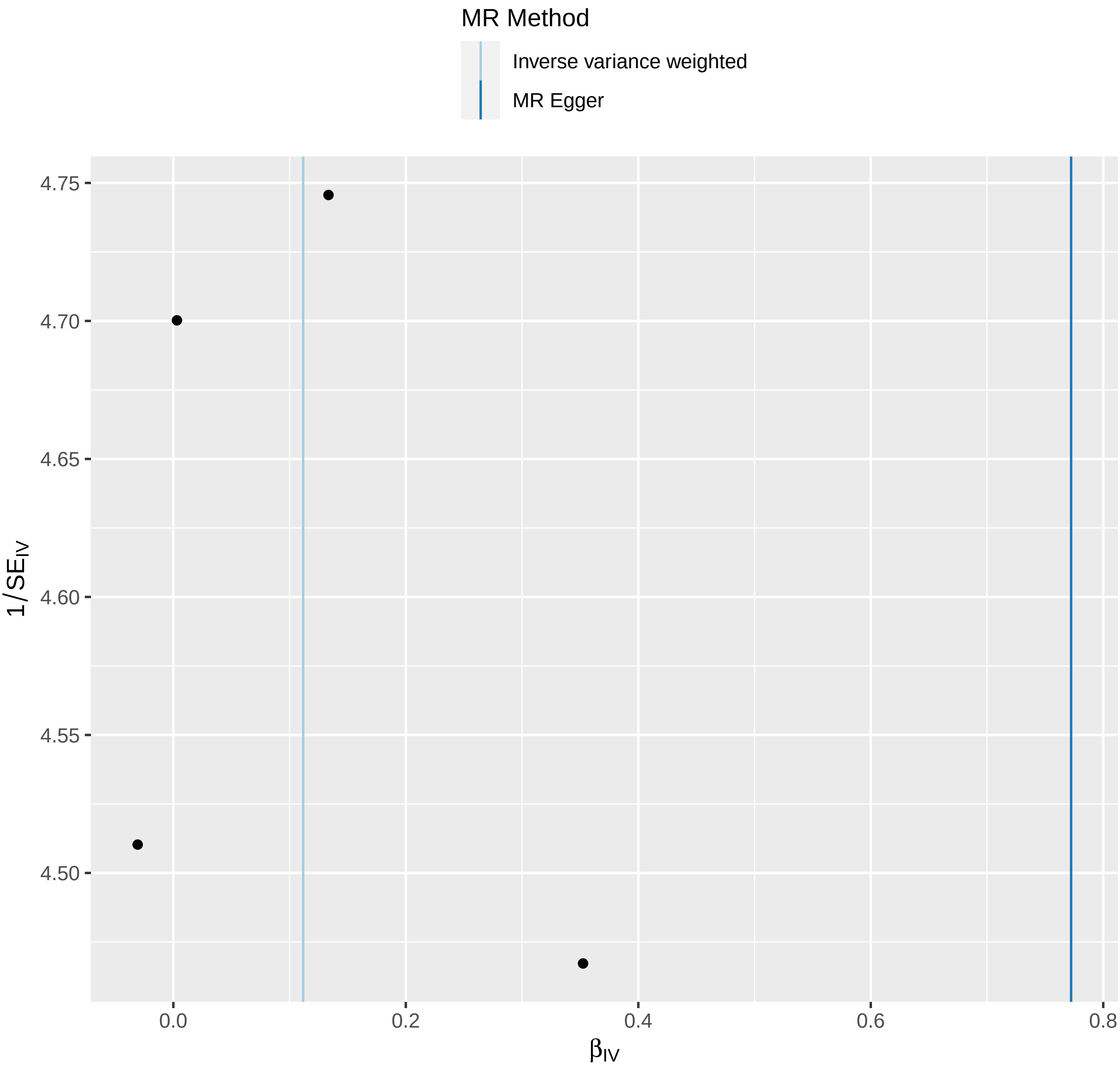

Batch 2−4588 : Gut microbiota abundance (genus Parasutterella id.2892) on Phospholipase B−like 1
with a threshold of 1e−05

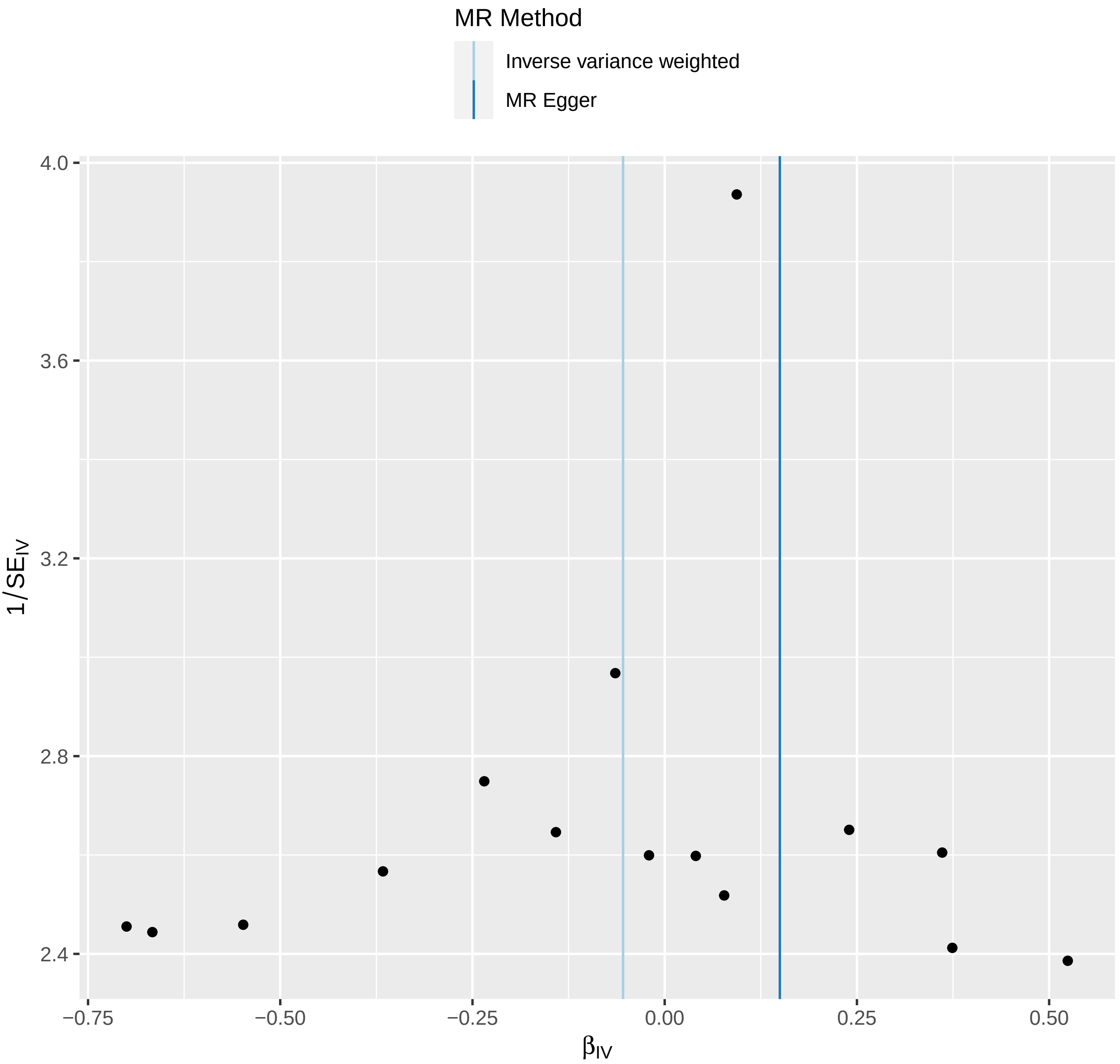

Batch 2−4589 : Gut microbiota abundance (genus Catenibacterium id.2153) on Putative phospholipase B−like 2
with a threshold of 1e−05

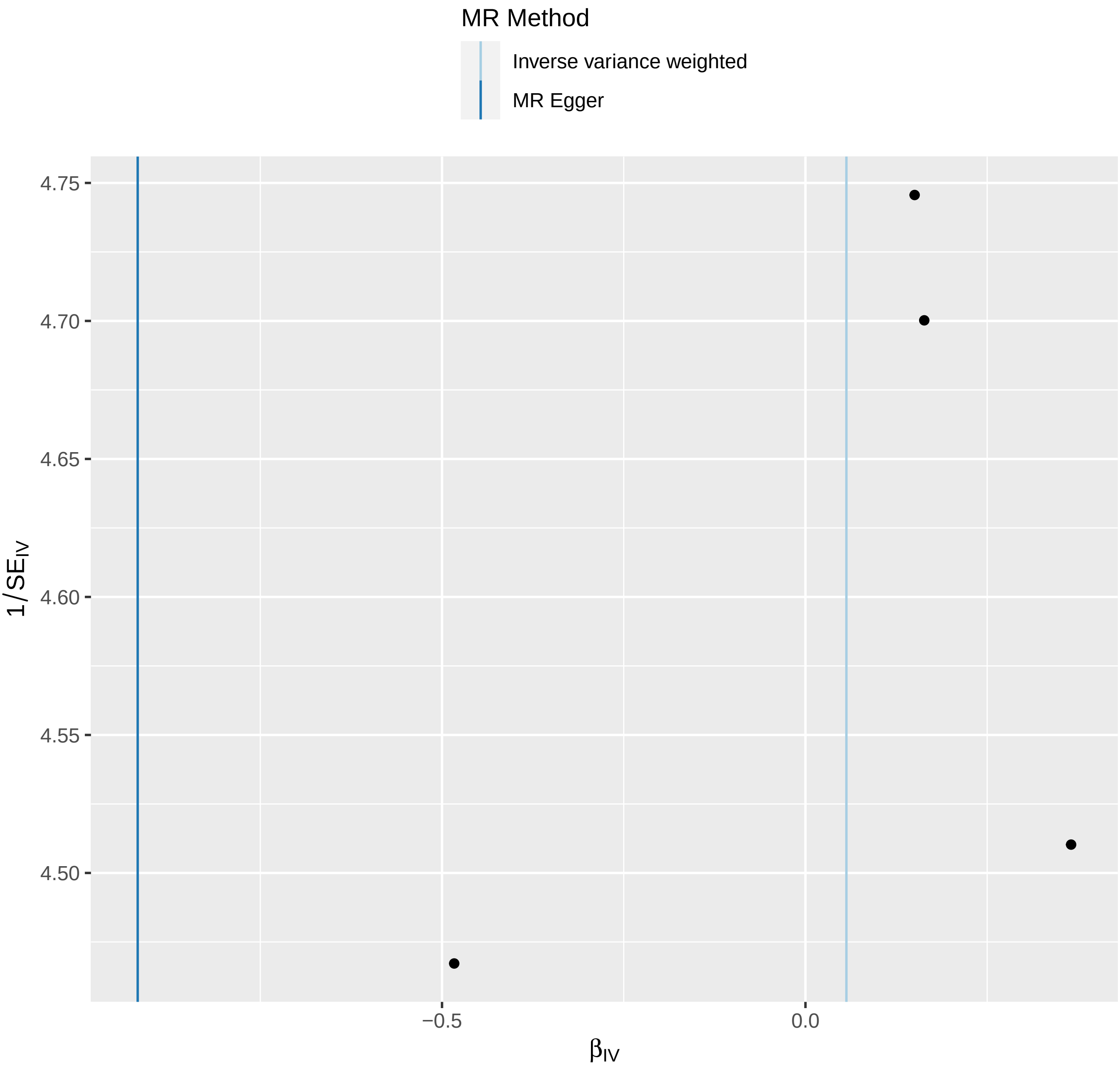

Batch 2−4590 : Gut microbiota abundance (genus Parasutterella id.2892) on Putative phospholipase B−like 2
with a threshold of 1e−05

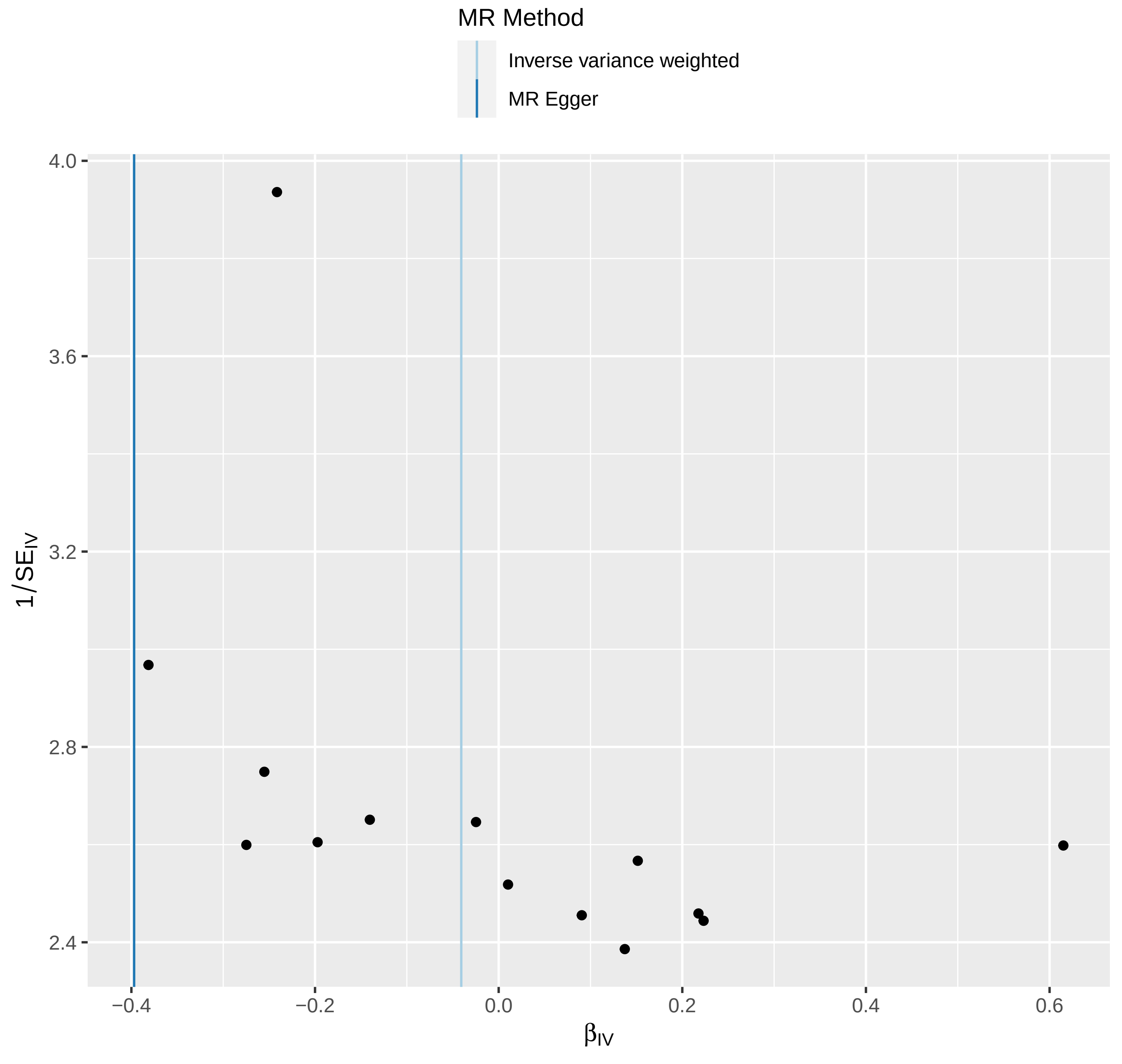

Batch 2−4591 : Gut microbiota abundance (genus Catenibacterium id.2153) on 1−phosphatidylinositol 4,5−bisphosphate phosphodiesterase gamma−1
with a threshold of 1e−05

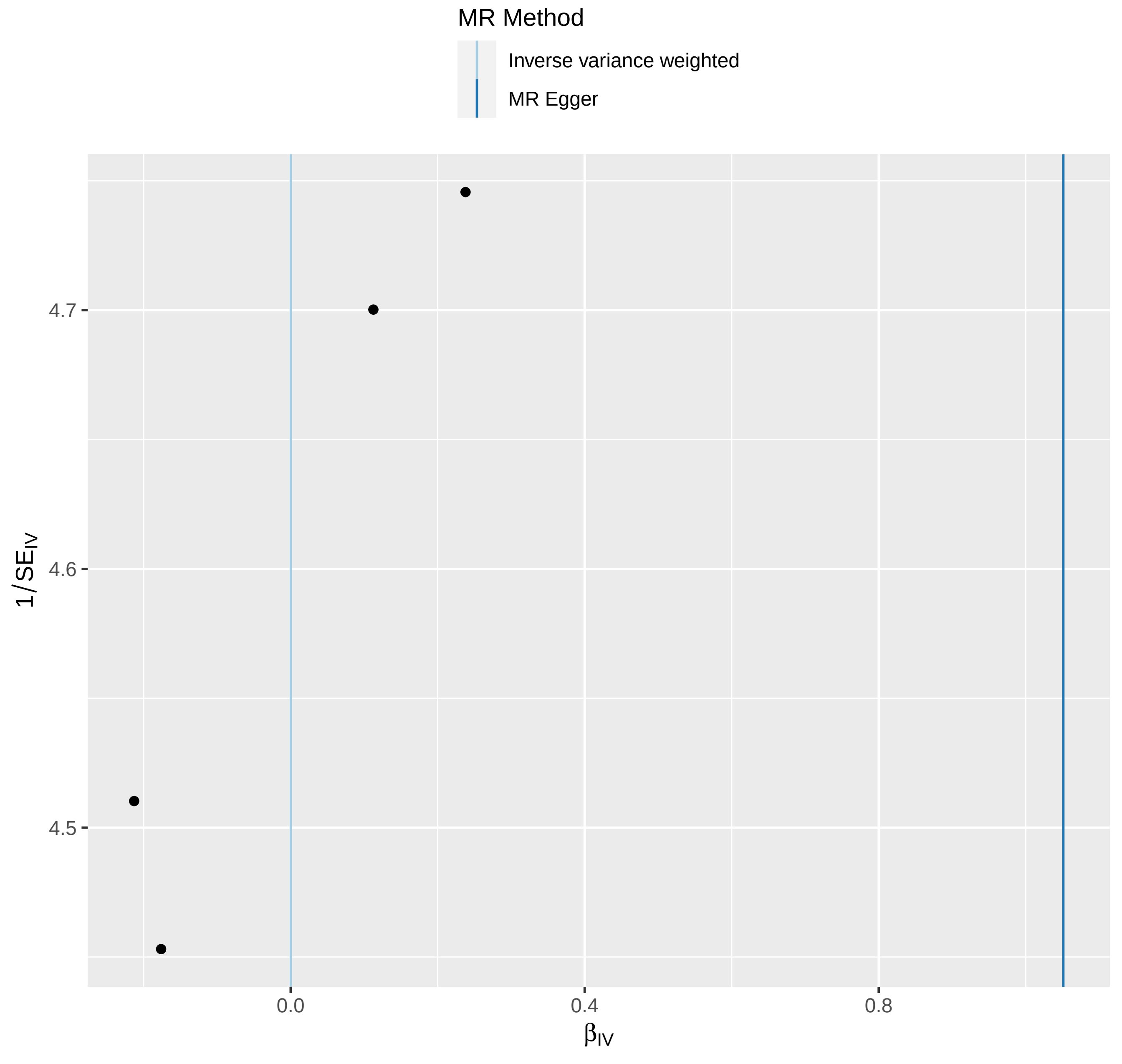

Batch 2−4592 : Gut microbiota abundance (genus Parasutterella id.2892) on 1−phosphatidylinositol 4,5−bisphosphate phosphodiesterase gamma−1 with a threshold of 1e−05

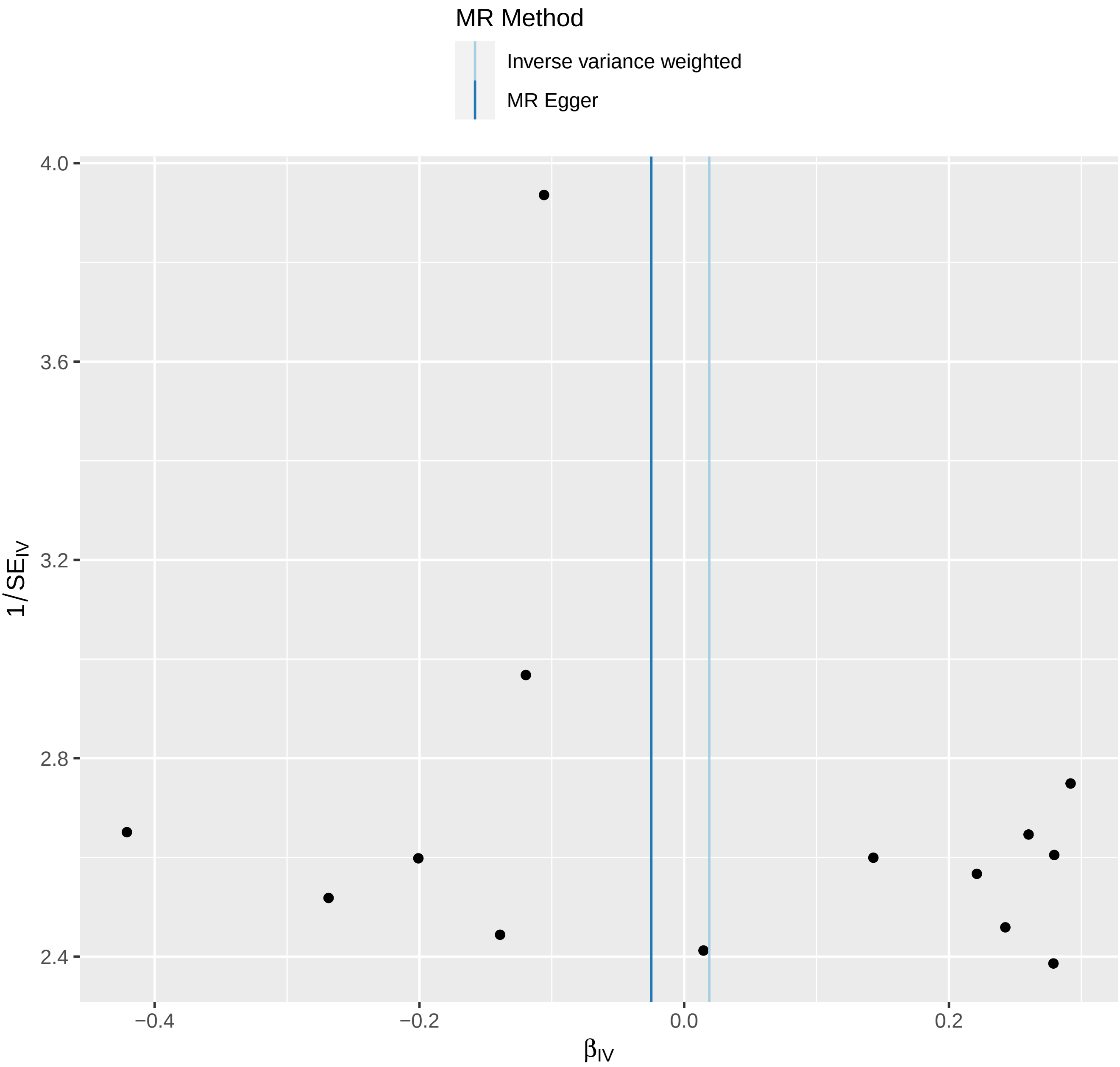

Batch 2−4593 : Gut microbiota abundance (genus Catenibacterium id.2153) on Phospholipase D3
with a threshold of 1e−05

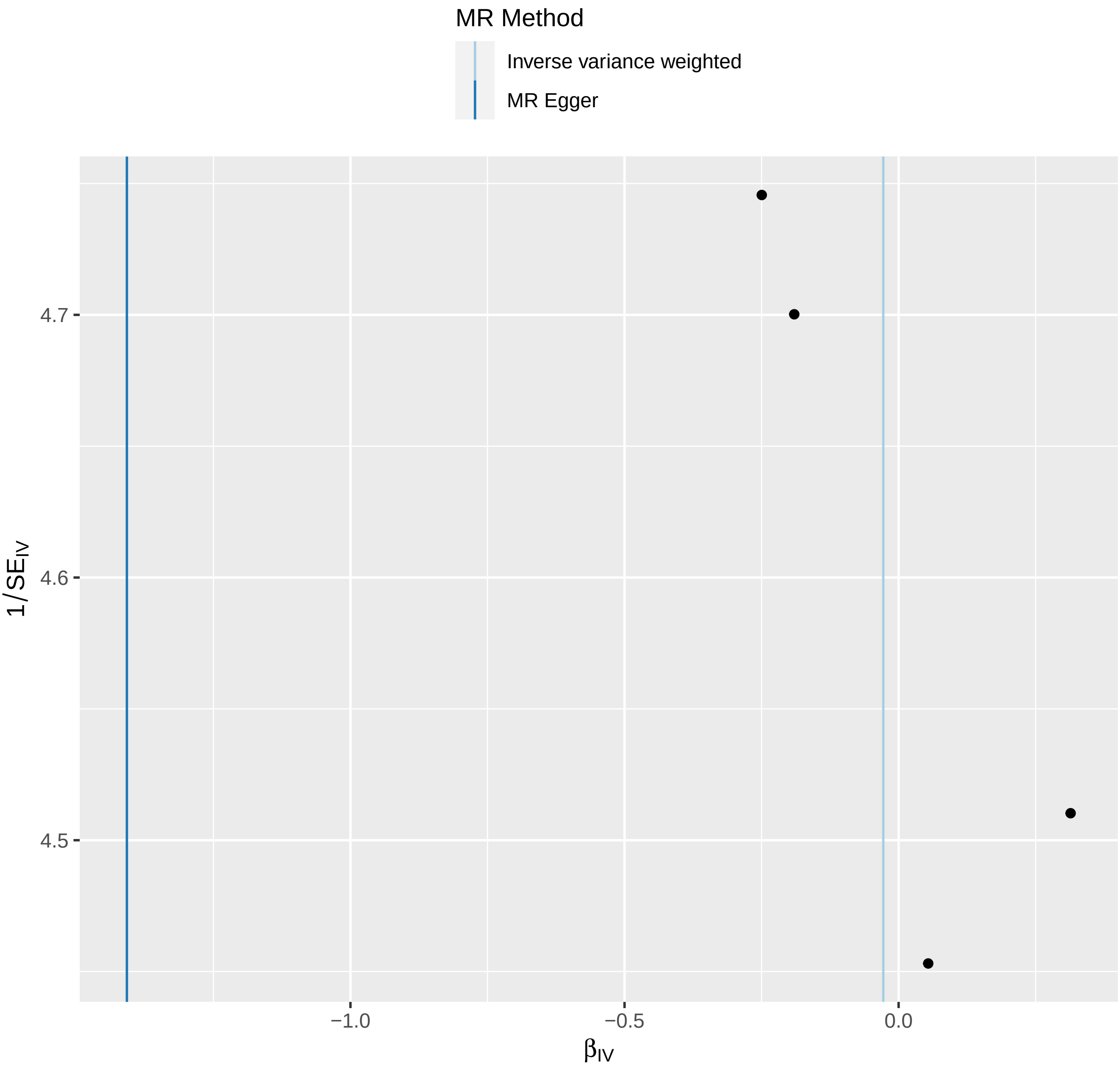

Batch 2−4594 : Gut microbiota abundance (genus Parasutterella id.2892) on Phospholipase D3
with a threshold of 1e−05

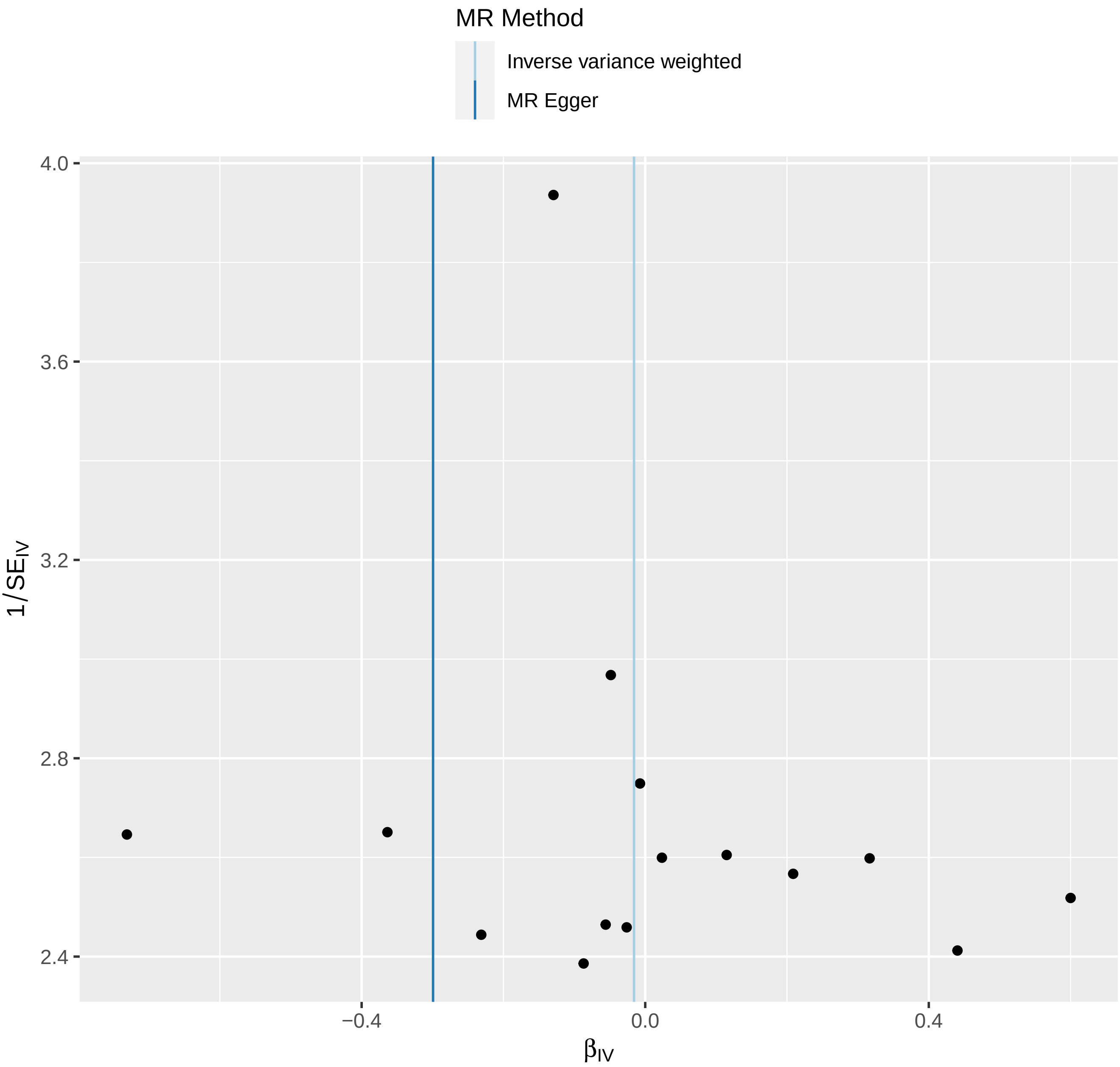

Batch 2−4595 : Gut microbiota abundance (genus Catenibacterium id.2153) on Inactive phospholipase D5
with a threshold of 1e−05

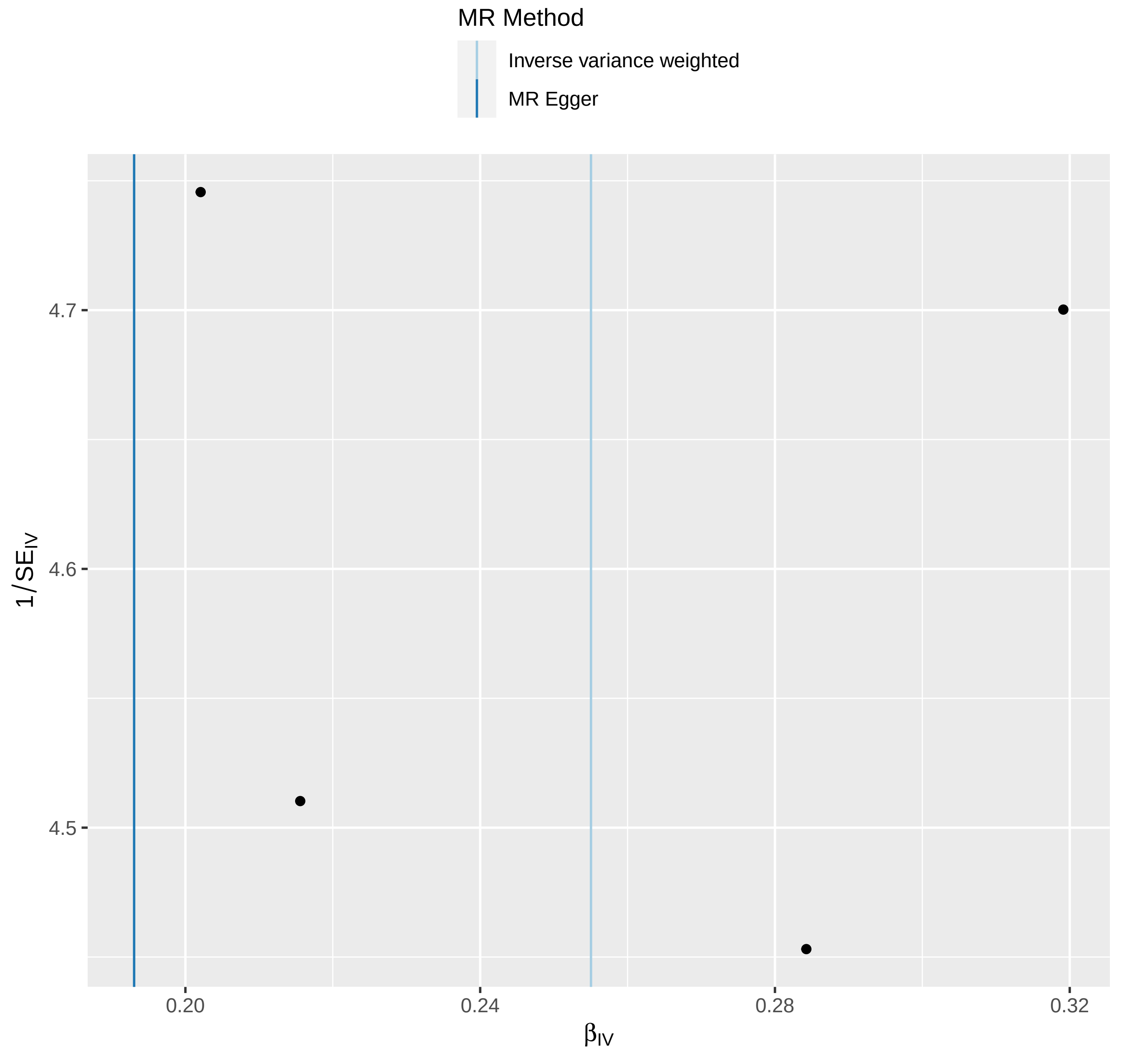

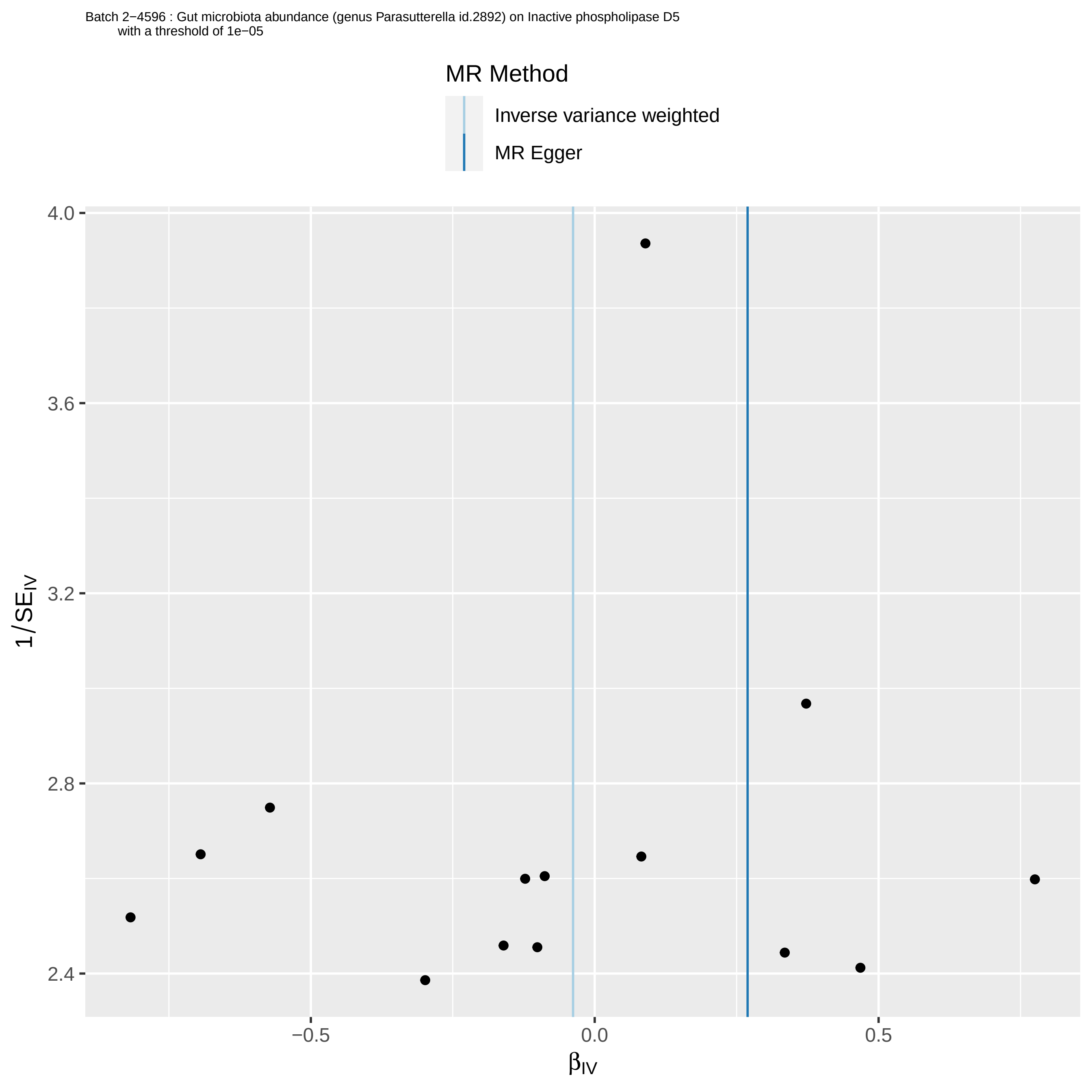

Batch 2−4596 : Gut microbiota abundance (genus Parasutterella id.2892) on Inactive phospholipase D5
with a threshold of 1e−05
MR Method
Inverse variance weighted
MR Egger
4.0
3.6
3.2
2.8
2.4
1/SE_IV
−0.5
0.0
0.5
β_IV

Batch 2−4597 : Gut microbiota abundance (genus Catenibacterium id.2153) on Pleckstrin homology domain−containing family A member 1
with a threshold of 1e−05

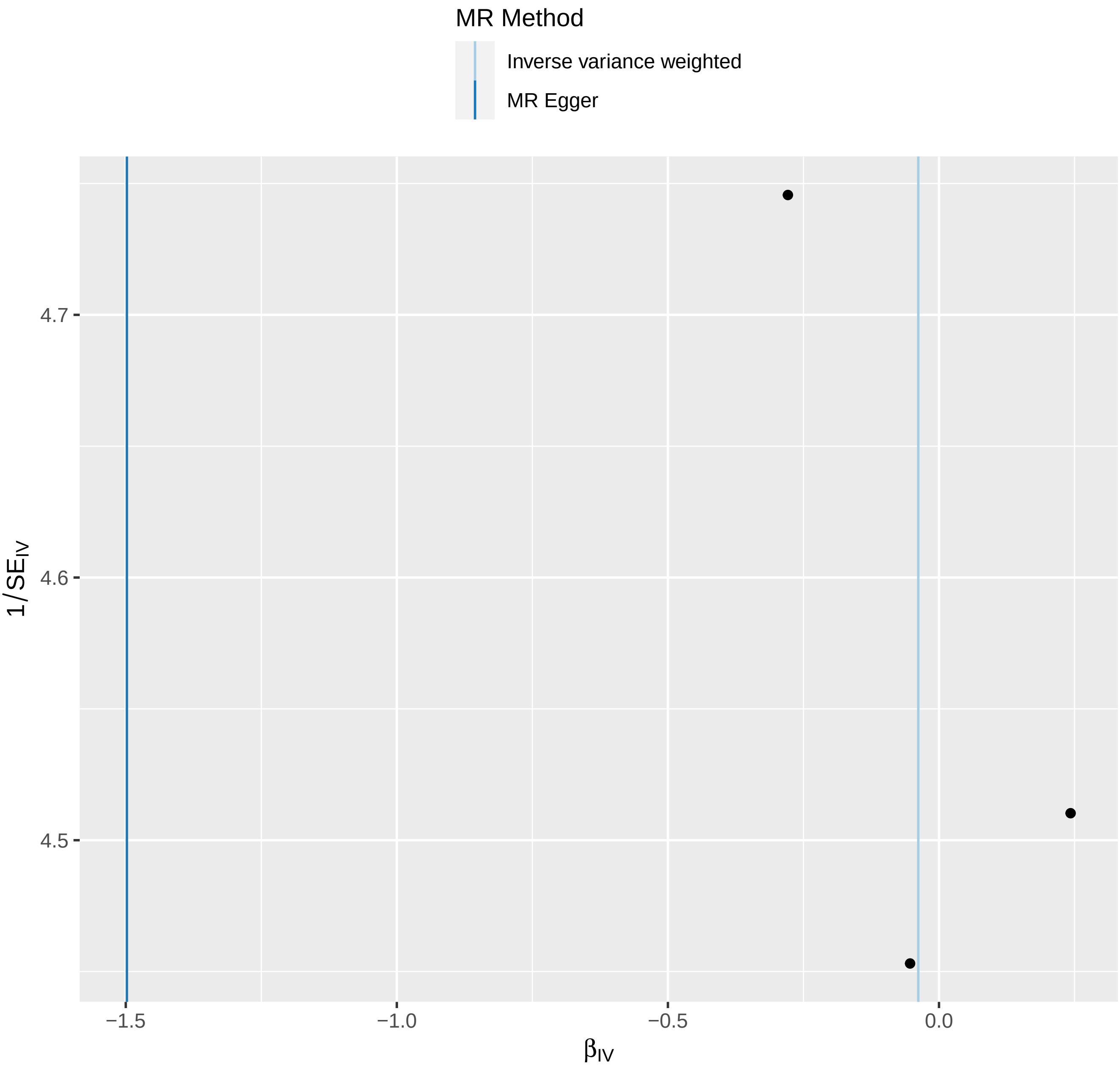

Batch 2−4598 : Gut microbiota abundance (genus Parasutterella id.2892) on Pleckstrin homology domain−containing family A member 1
with a threshold of 1e−05

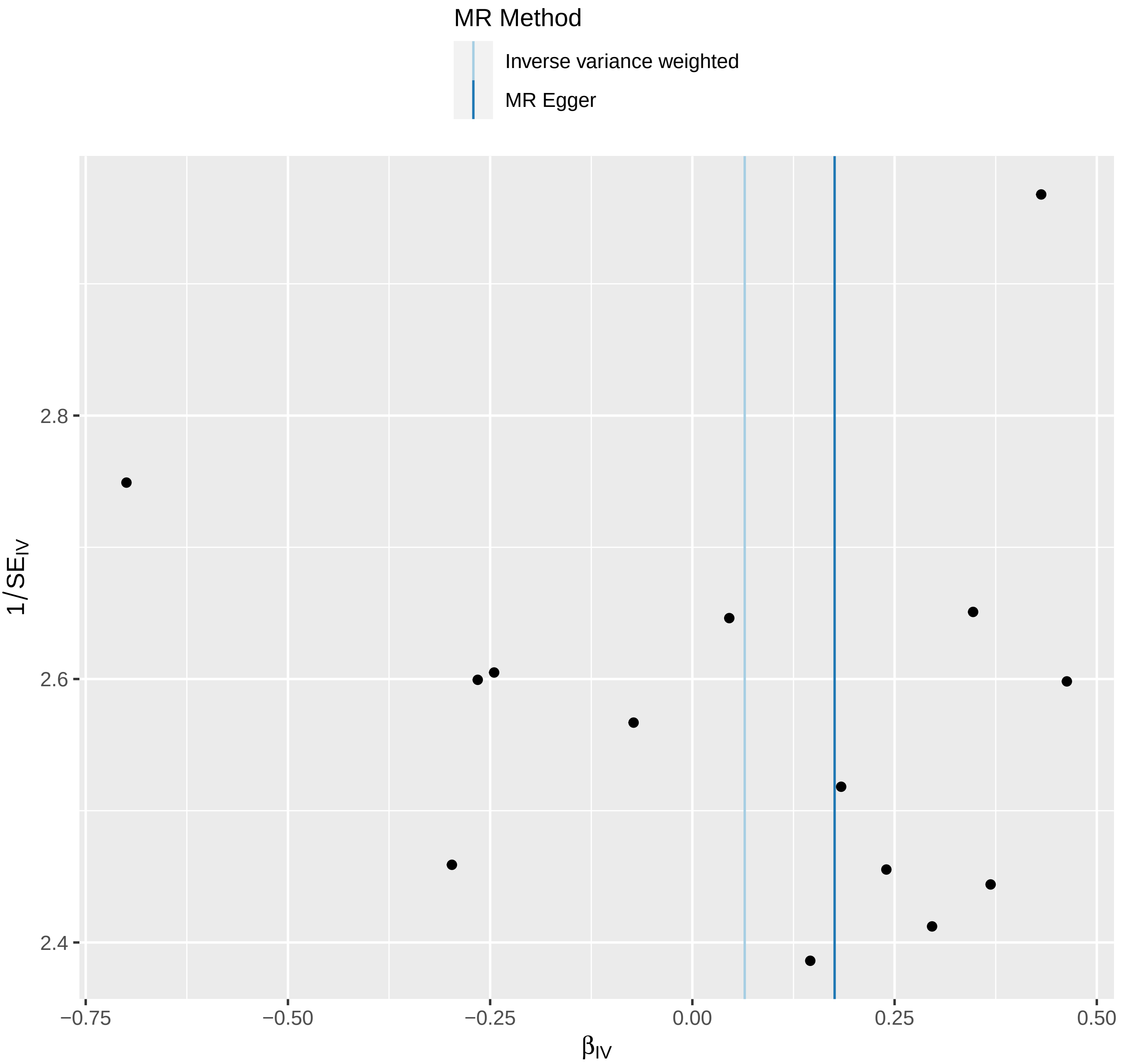

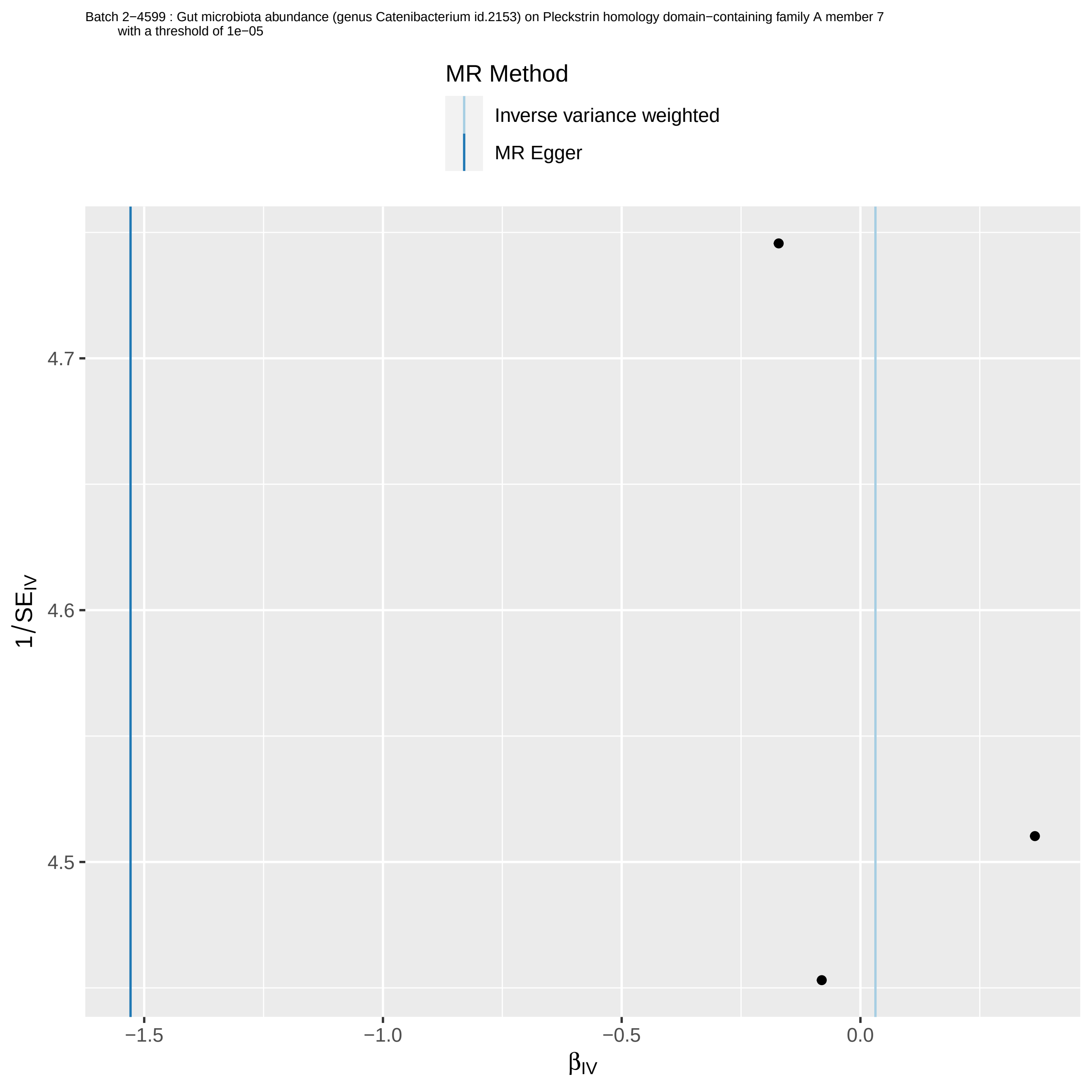
Batch 2−4599 : Gut microbiota abundance (genus Catenibacterium id.2153) on Pleckstrin homology domain−containing family A member 7
with a threshold of 1e−05
MR Method
Inverse variance weighted
MR Egger
4.7
4.6
4.5
1/SE$_{IV}$
−1.5
−1.0
−0.5
0.0
β$_{IV}$

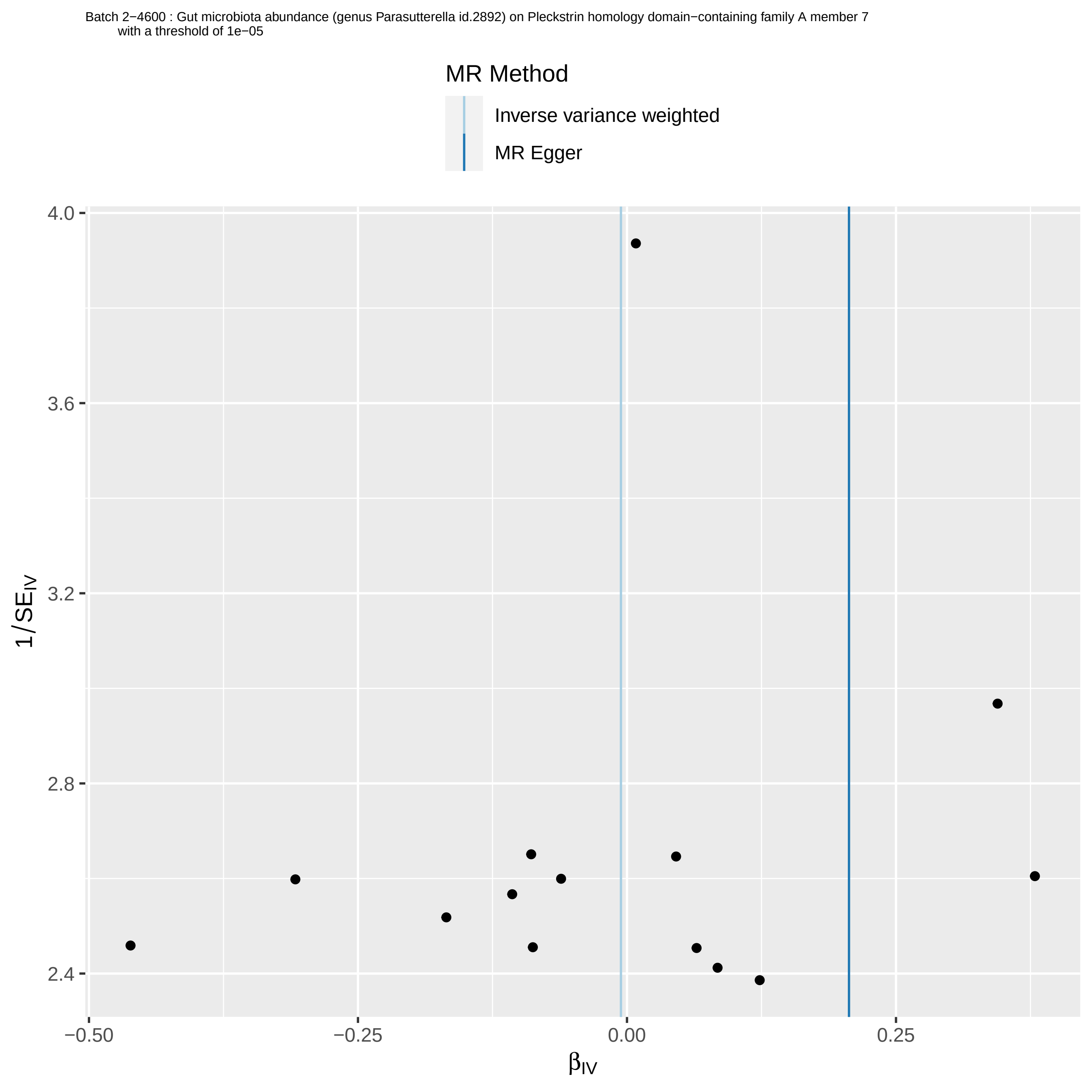
Batch 2−4600 : Gut microbiota abundance (genus Parasutterella id.2892) on Pleckstrin homology domain−containing family A member 7
with a threshold of 1e−05
MR Method
Inverse variance weighted
MR Egger
4.0
3.6
3.2
2.8
2.4
1/SE_IV
−0.50
−0.25
0.00
0.25
β_IV

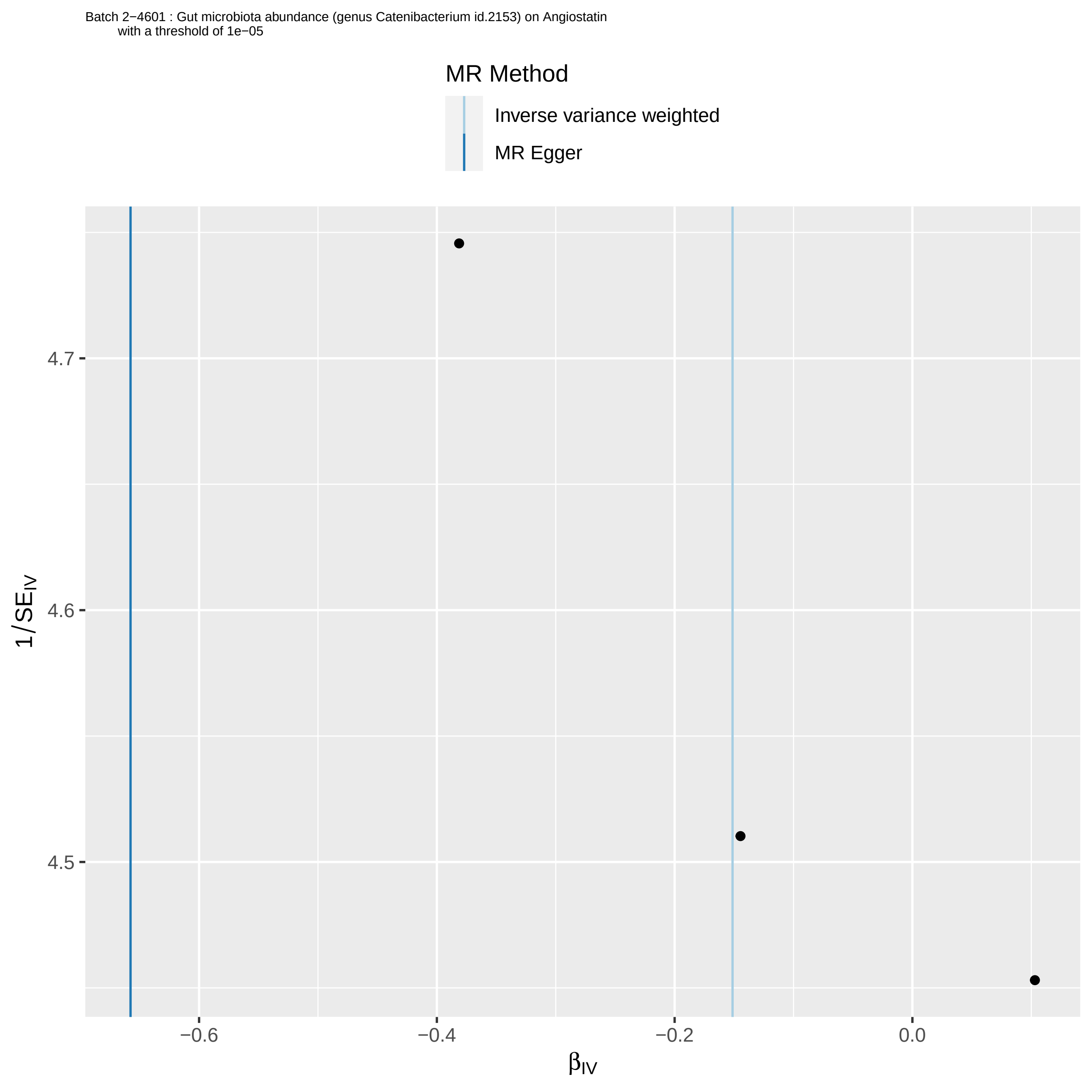
Batch 2−4601 : Gut microbiota abundance (genus Catenibacterium id.2153) on Angiostatin
with a threshold of 1e−05
MR Method
Inverse variance weighted
MR Egger
4.7
4.6
4.5
1/SEIV
−0.6
−0.4
−0.2
0.0
βIV

Batch 2−4602 : Gut microbiota abundance (genus Parasutterella id.2892) on Angiostatin
with a threshold of 1e−05

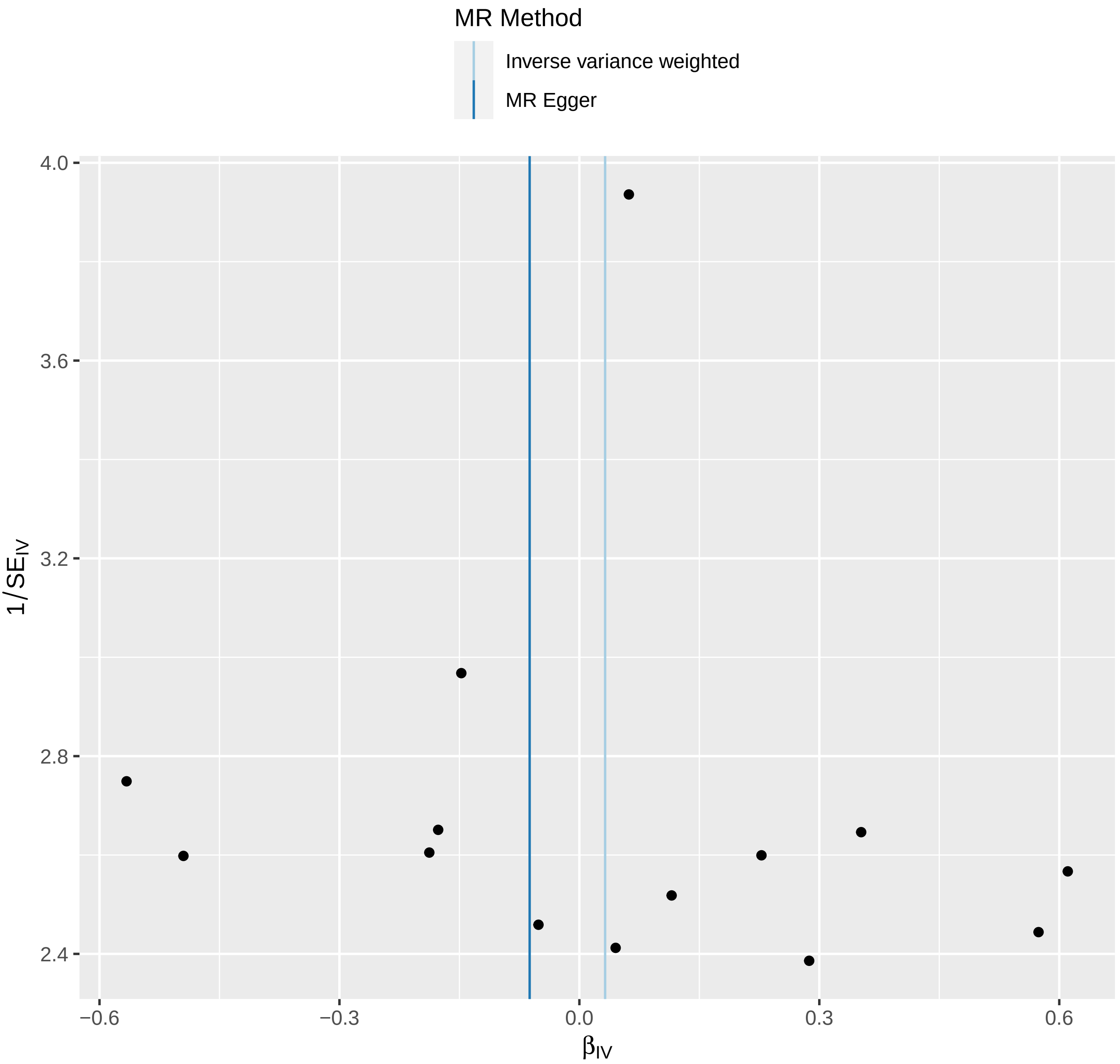

Batch 2−4603 : Gut microbiota abundance (genus Catenibacterium id.2153) on Plasmin
with a threshold of 1e−05

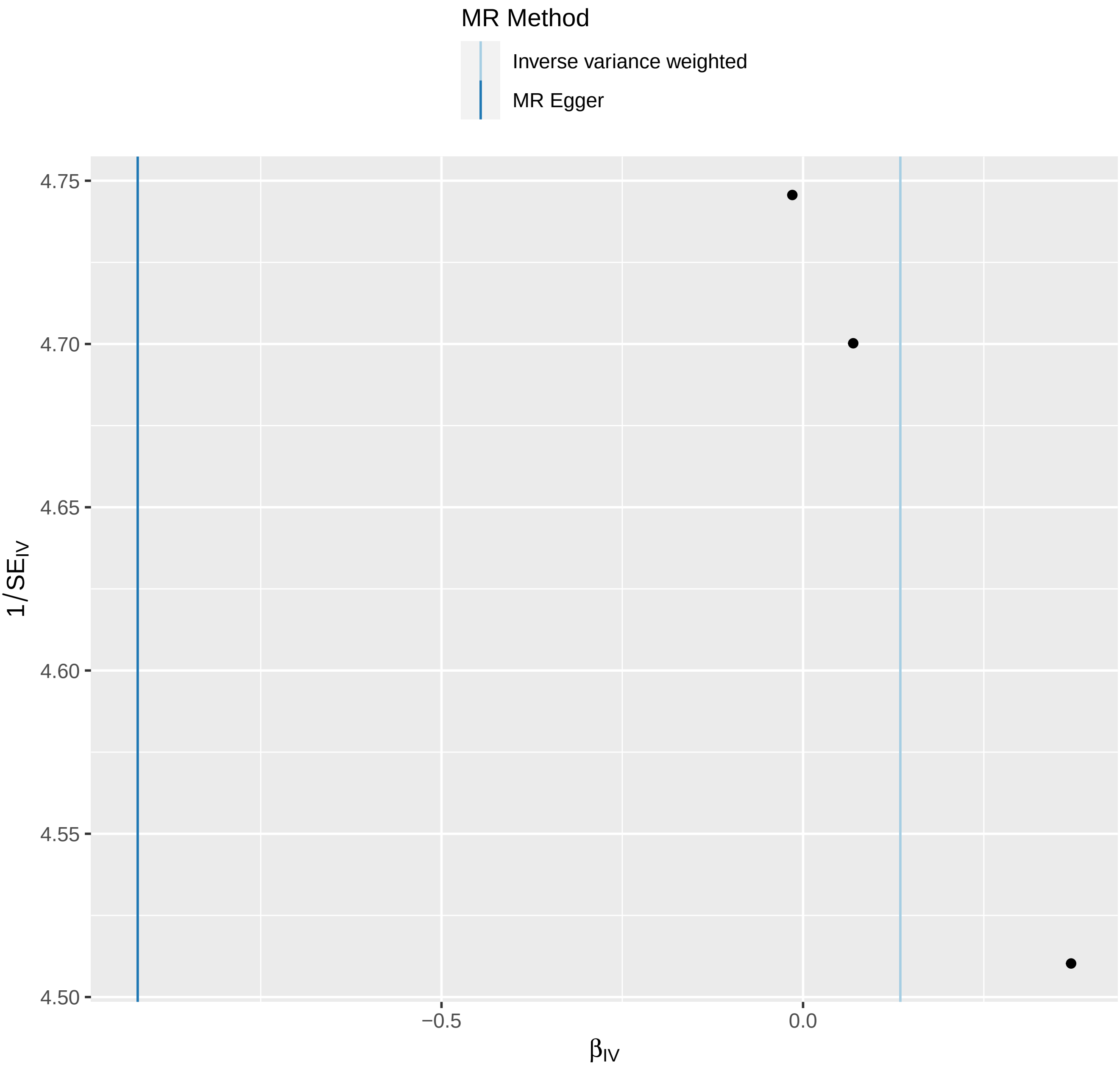

Batch 2−4604 : Gut microbiota abundance (genus Parasutterella id.2892) on Plasmin
with a threshold of 1e−05

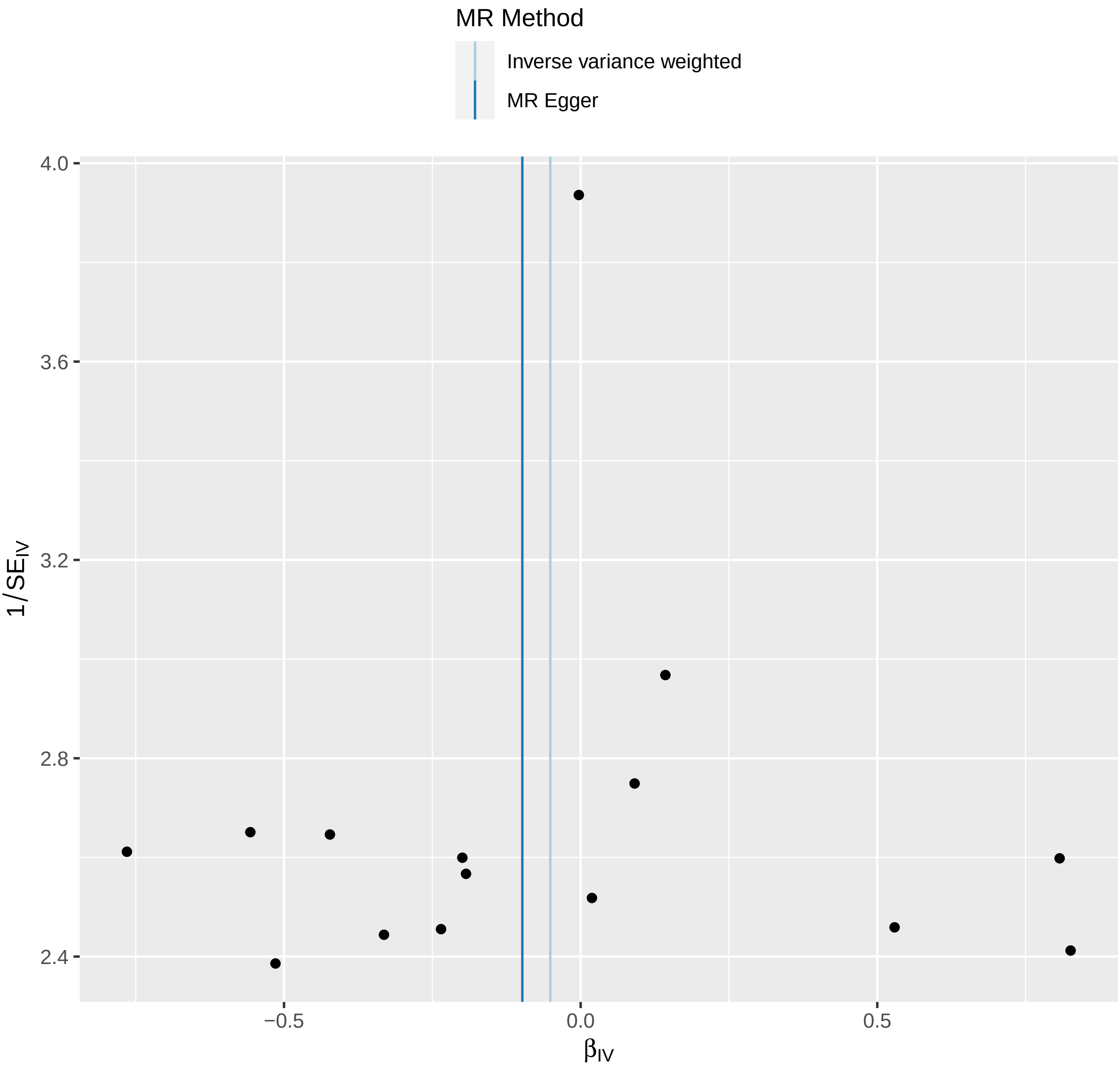

Batch 2−4605 : Gut microbiota abundance (genus Catenibacterium id.2153) on Serine/threonine−protein kinase PLK1
with a threshold of 1e−05

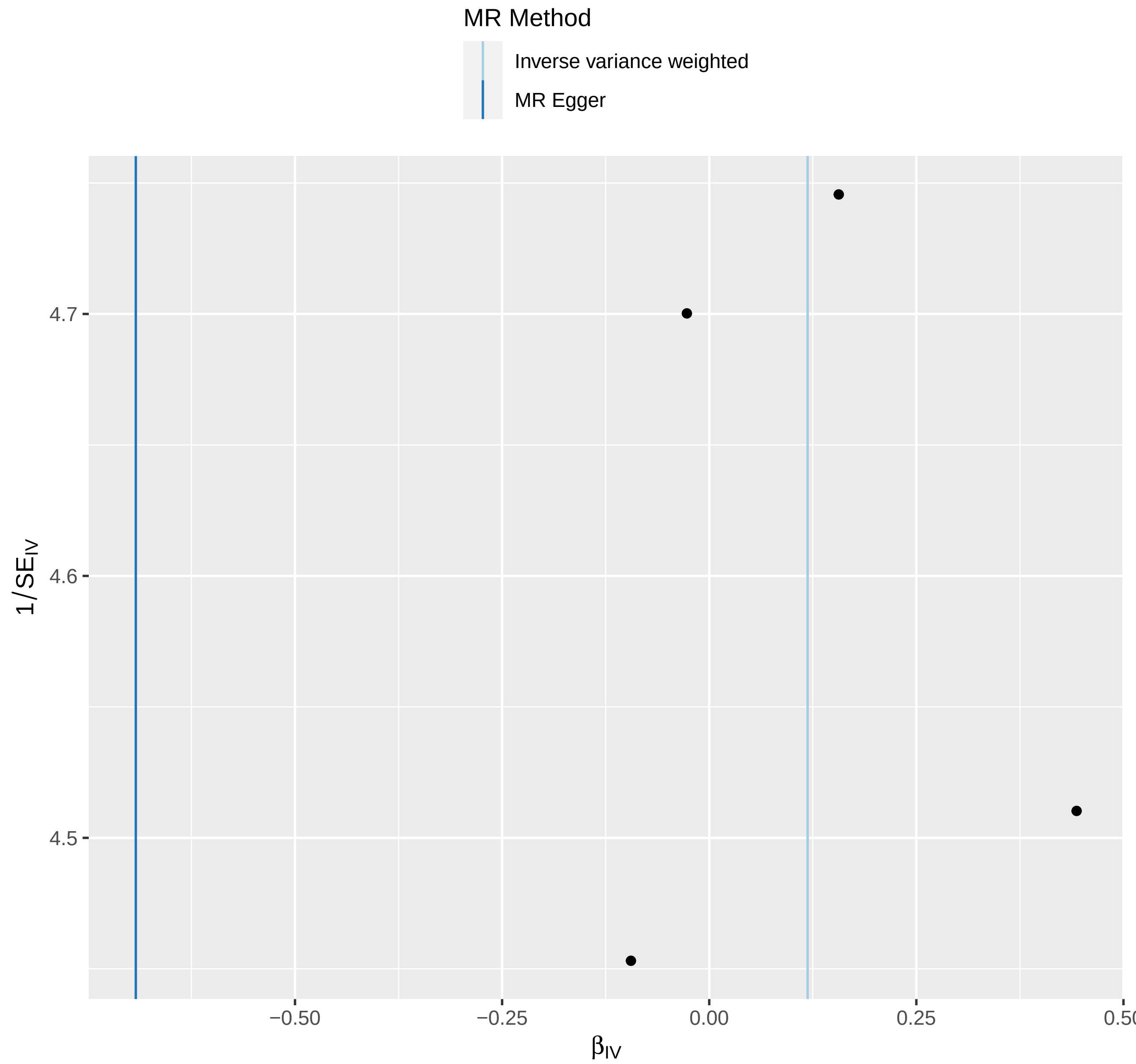

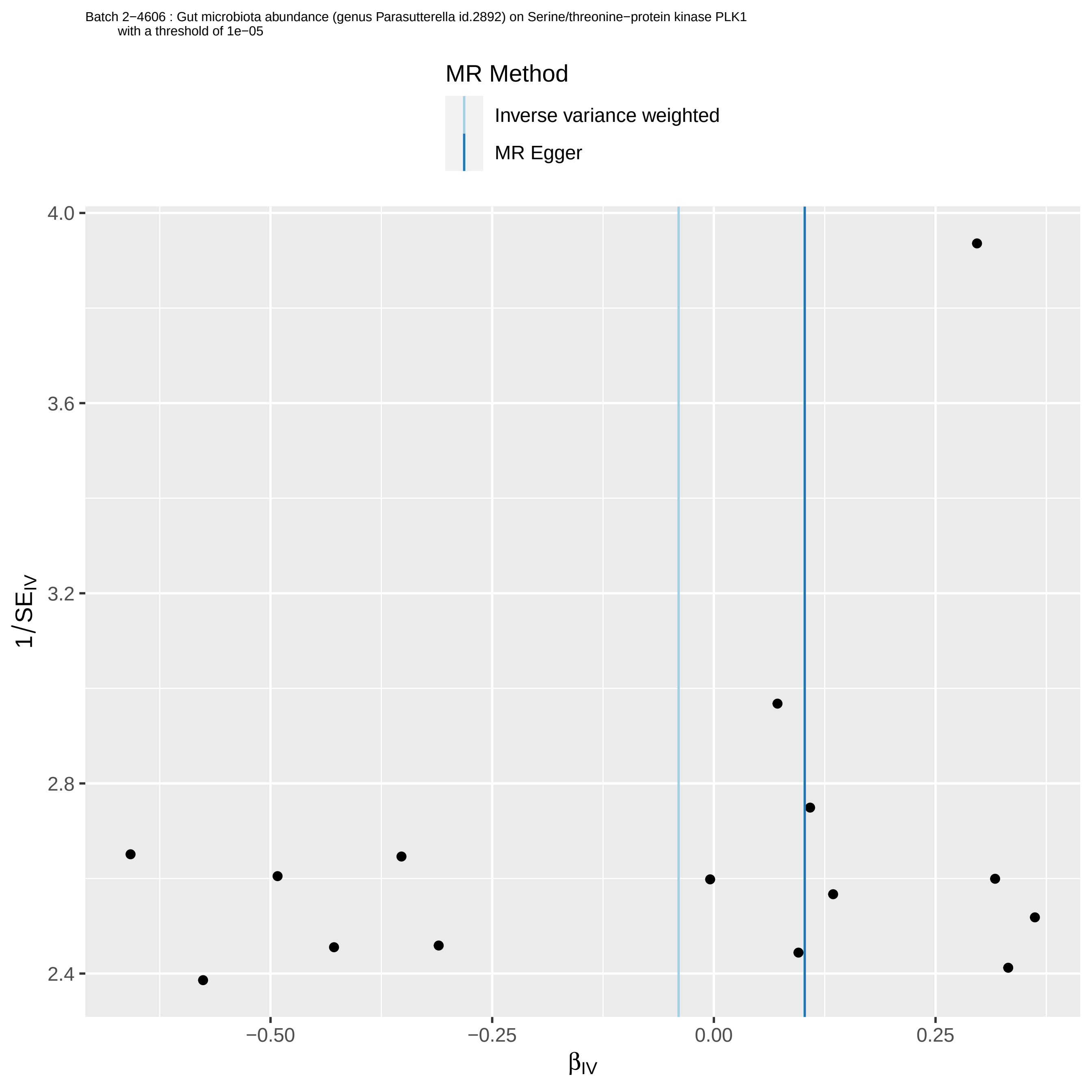
Batch 2−4606 : Gut microbiota abundance (genus Parasutterella id.2892) on Serine/threonine−protein kinase PLK1
with a threshold of 1e−05
MR Method
Inverse variance weighted
MR Egger
4.0
3.6
3.2
2.8
2.4
1/SE_IV
−0.50
−0.25
0.00
0.25
β_IV

Batch 2−4607 : Gut microbiota abundance (genus Catenibacterium id.2153) on Procollagen−lysine,2−oxoglutarate 5−dioxygenase 2
with a threshold of 1e−05

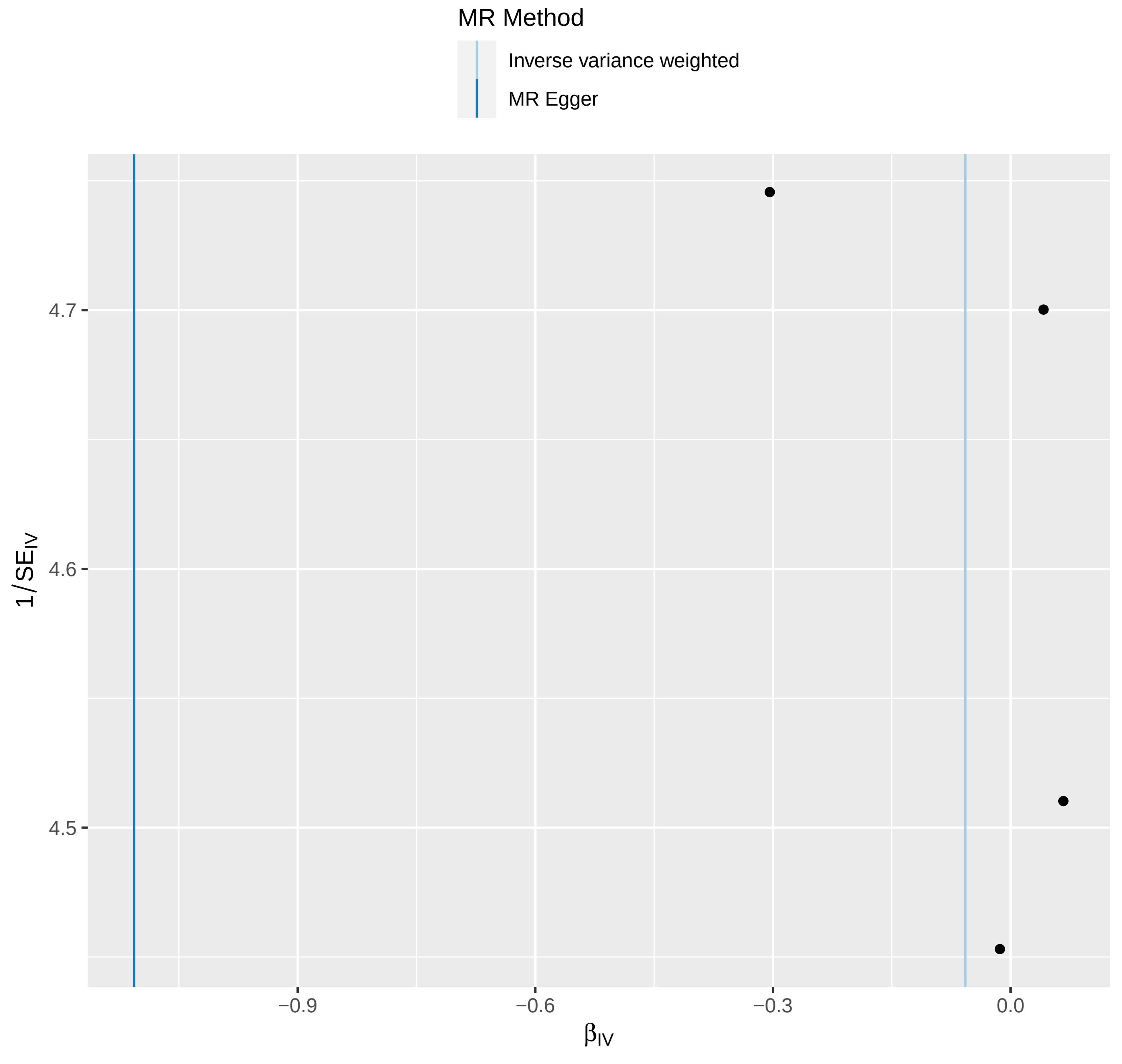

Batch 2−4608 : Gut microbiota abundance (genus Parasutterella id.2892) on Procollagen−lysine,2−oxoglutarate 5−dioxygenase 2
with a threshold of 1e−05

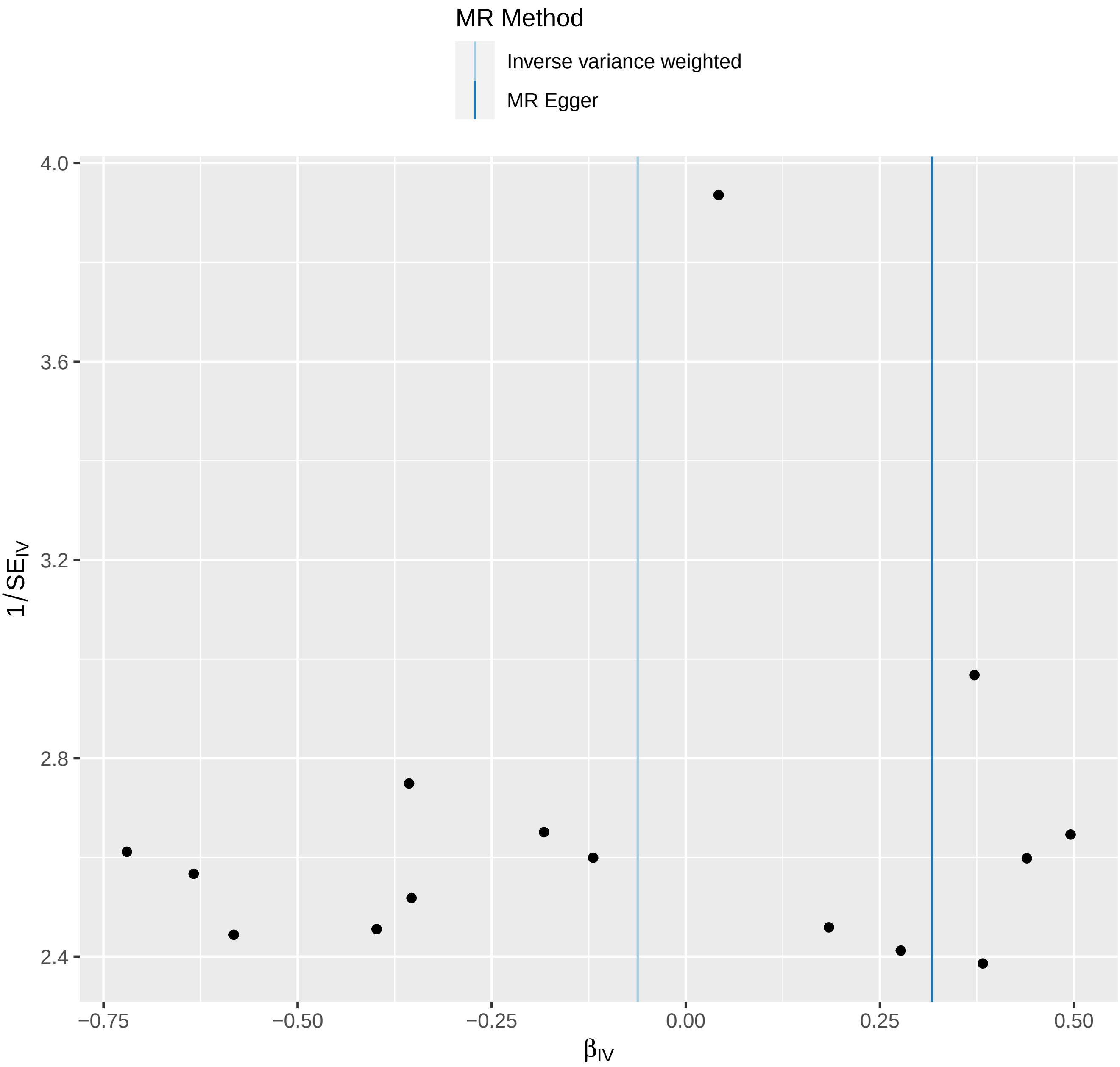

Batch 2−4609 : Gut microbiota abundance (genus Catenibacterium id.2153) on Procollagen−lysine,2−oxoglutarate 5−dioxygenase 3
with a threshold of 1e−05

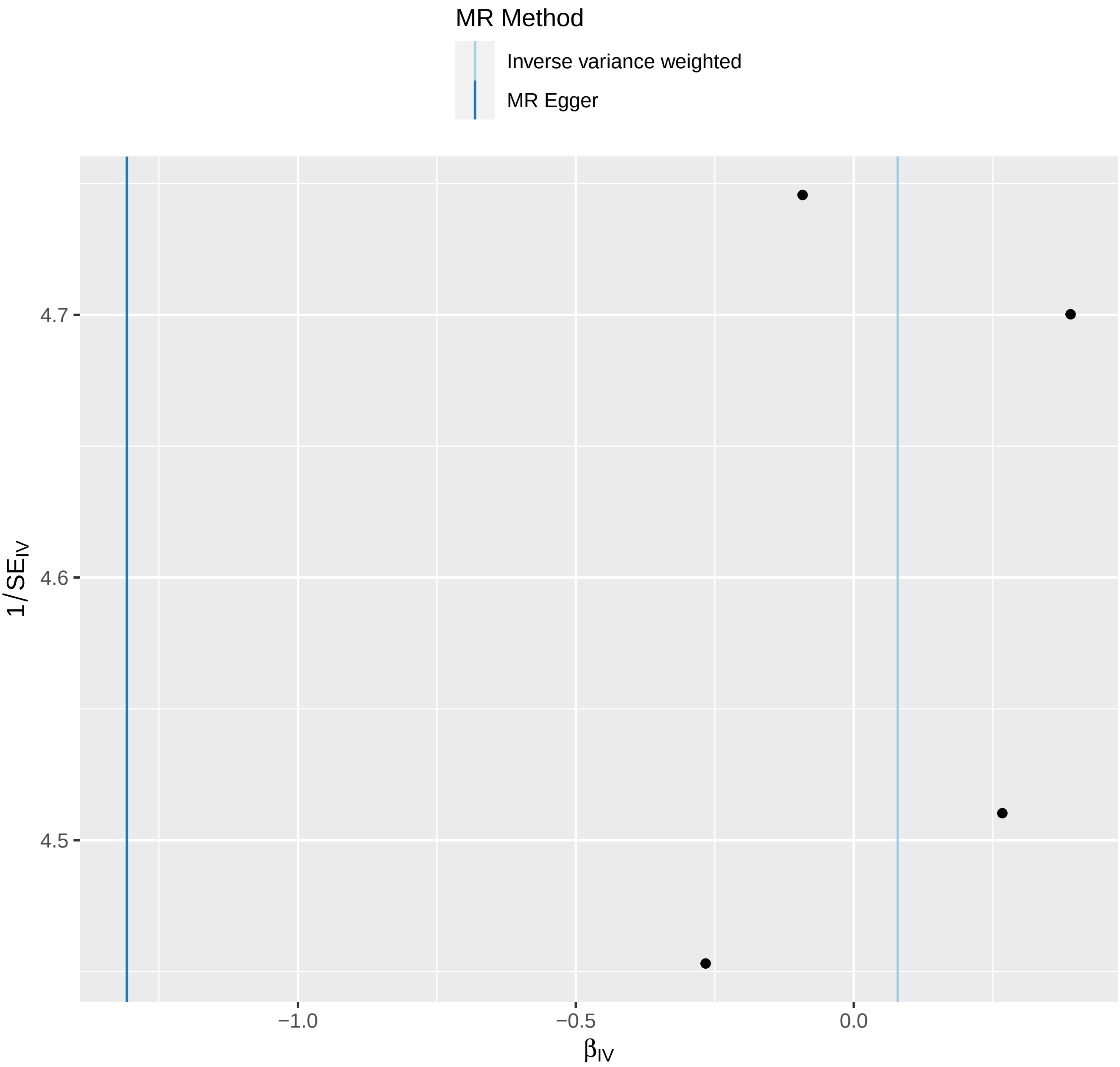

Batch 2−4610 : Gut microbiota abundance (genus Parasutterella id.2892) on Procollagen−lysine,2−oxoglutarate 5−dioxygenase 3
with a threshold of 1e−05

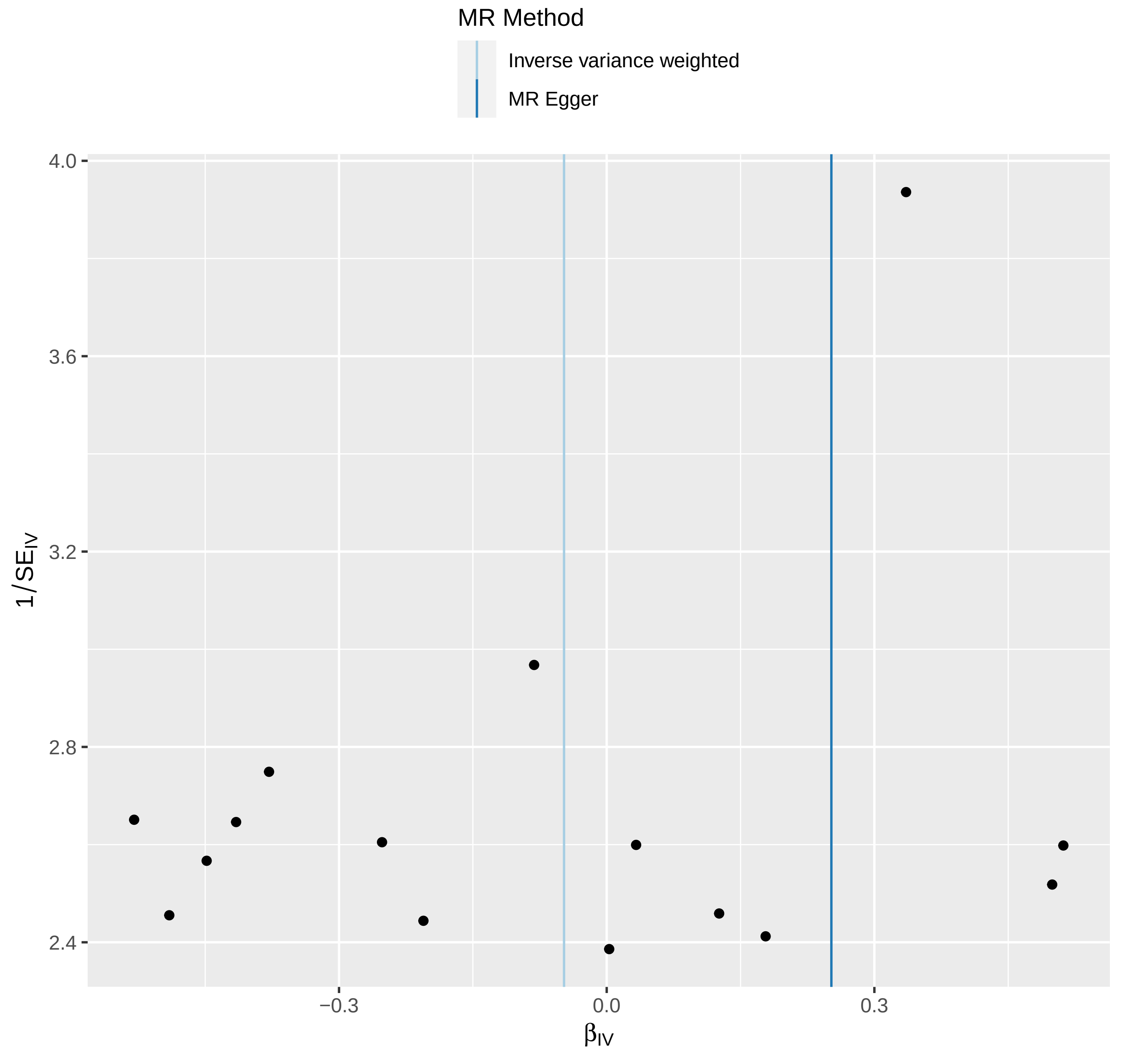

Batch 2−4611 : Gut microbiota abundance (genus Catenibacterium id.2153) on Plastin−1
with a threshold of 1e−05

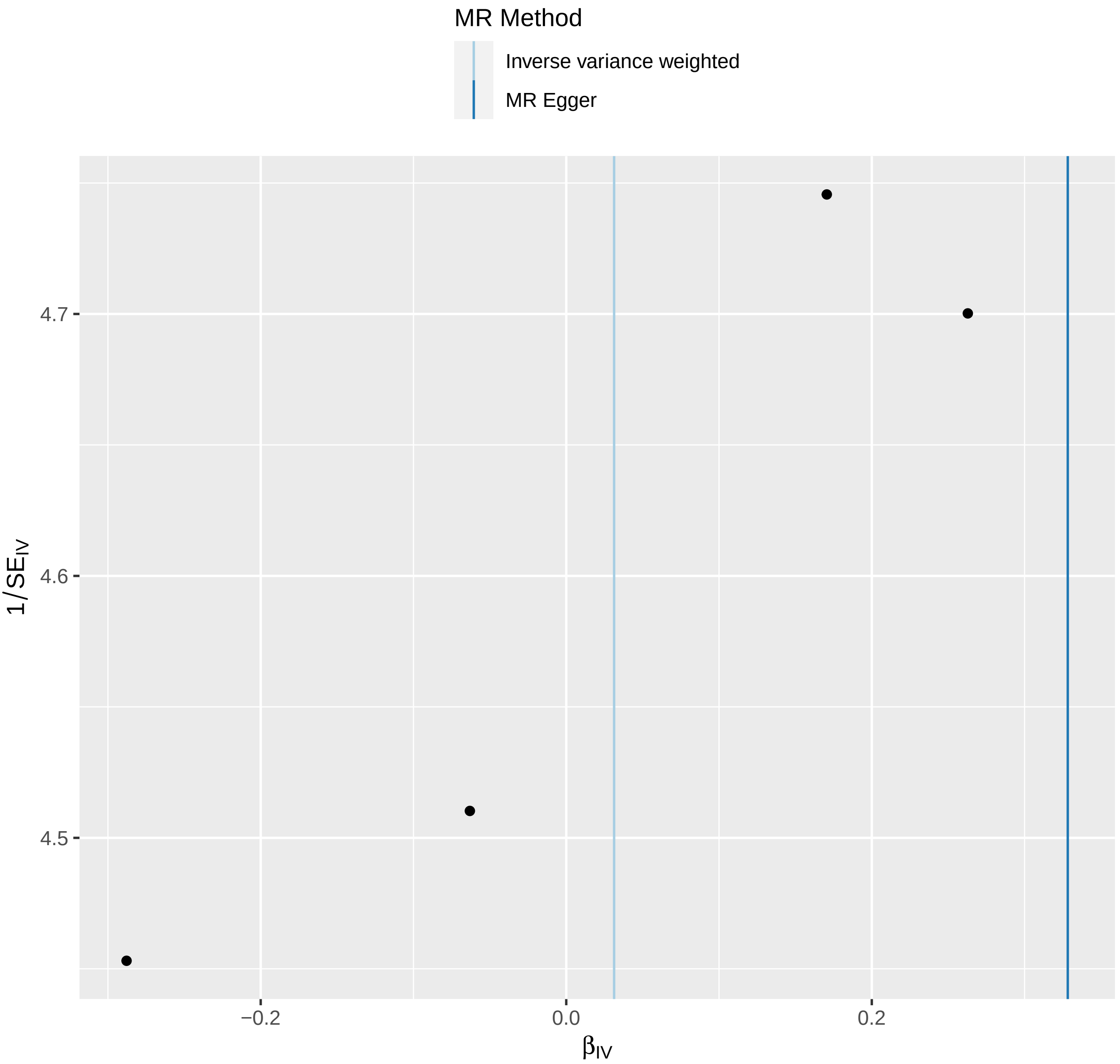

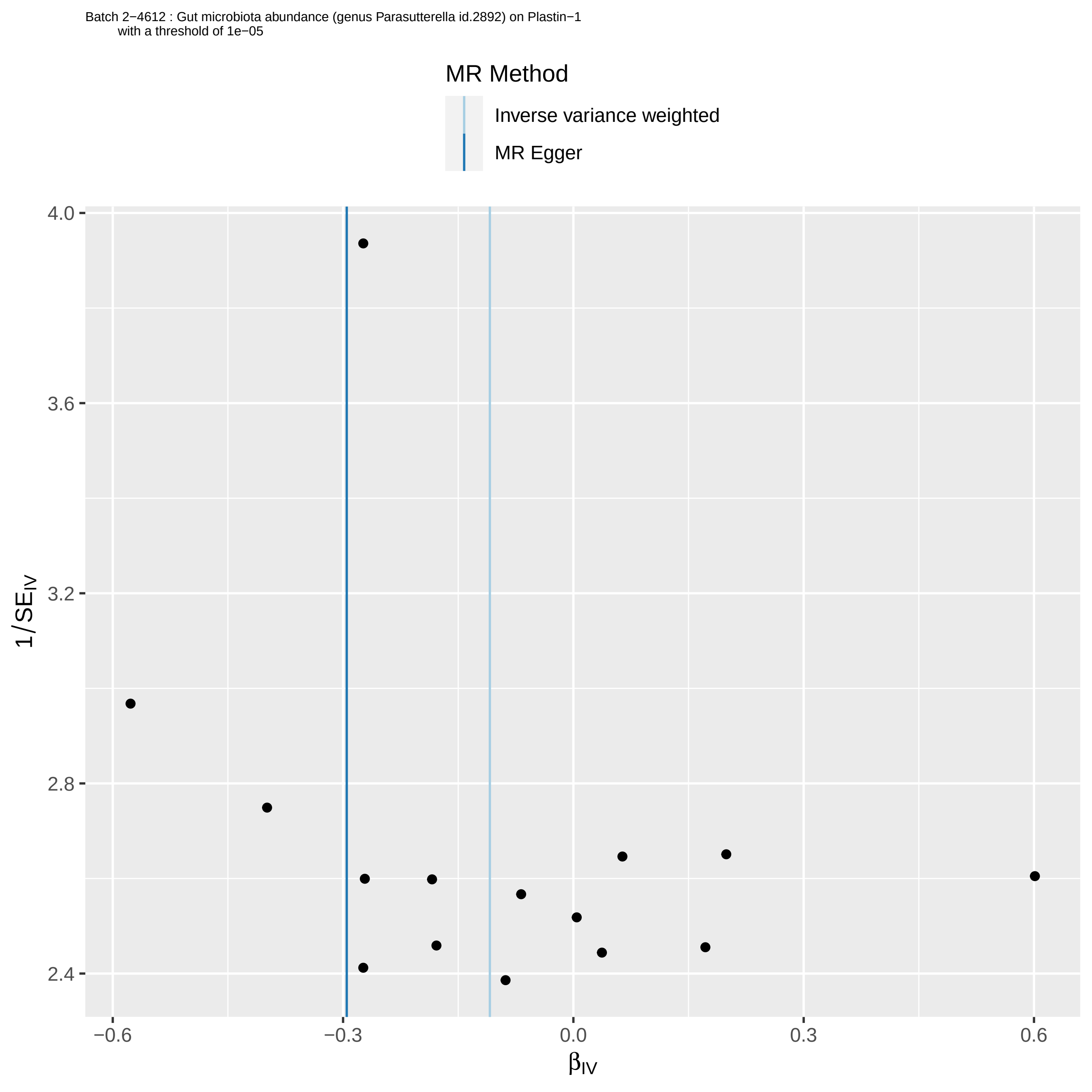

Batch 2−4612 : Gut microbiota abundance (genus Parasutterella id.2892) on Plastin−1
with a threshold of 1e−05
MR Method
Inverse variance weighted
MR Egger
4.0
3.6
3.2
2.8
2.4
1/SE$_{IV}$
−0.6
−0.3
0.0
0.3
0.6
β$_{IV}$

Batch 2−4613 : Gut microbiota abundance (genus Catenibacterium id.2153) on Plexin−A1
with a threshold of 1e−05

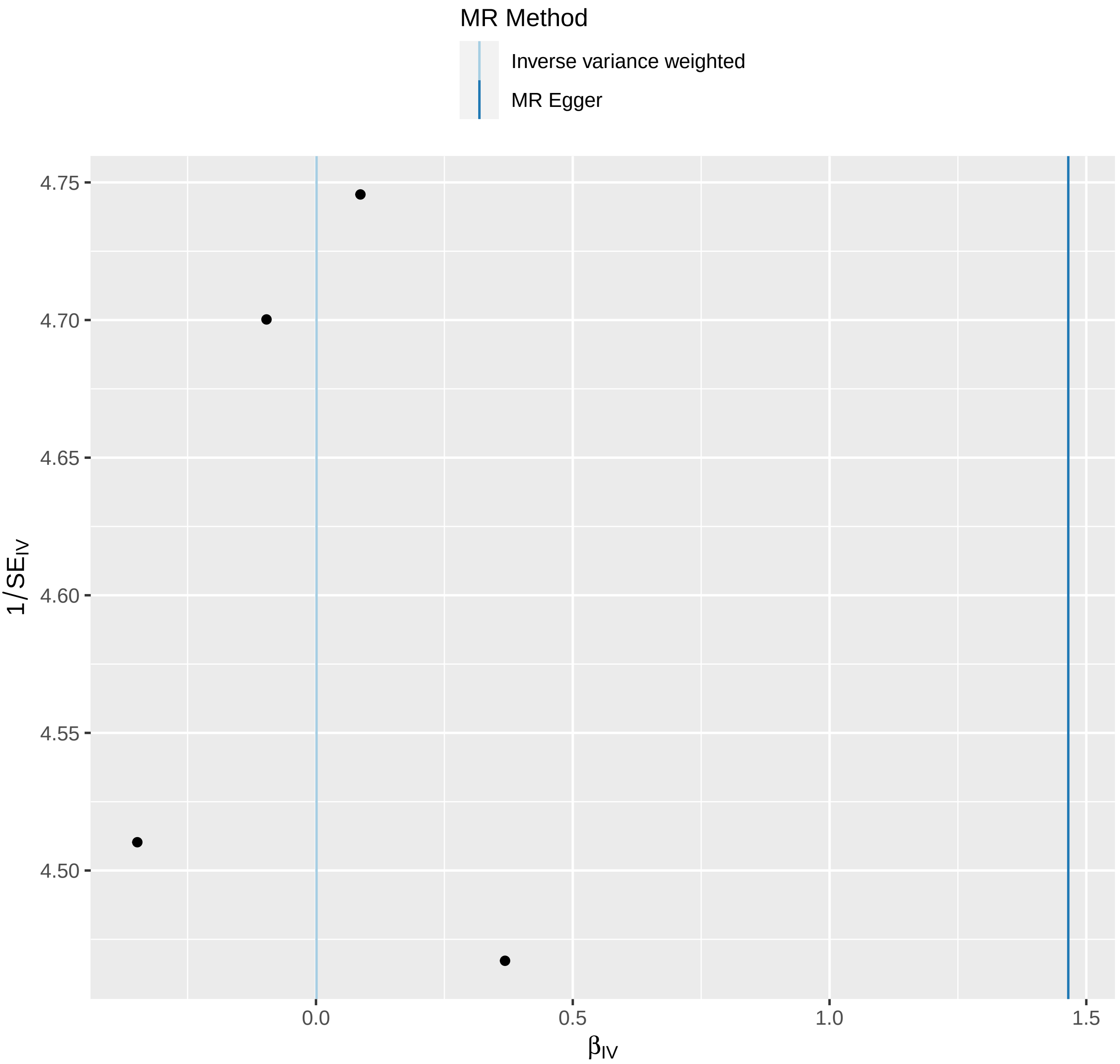

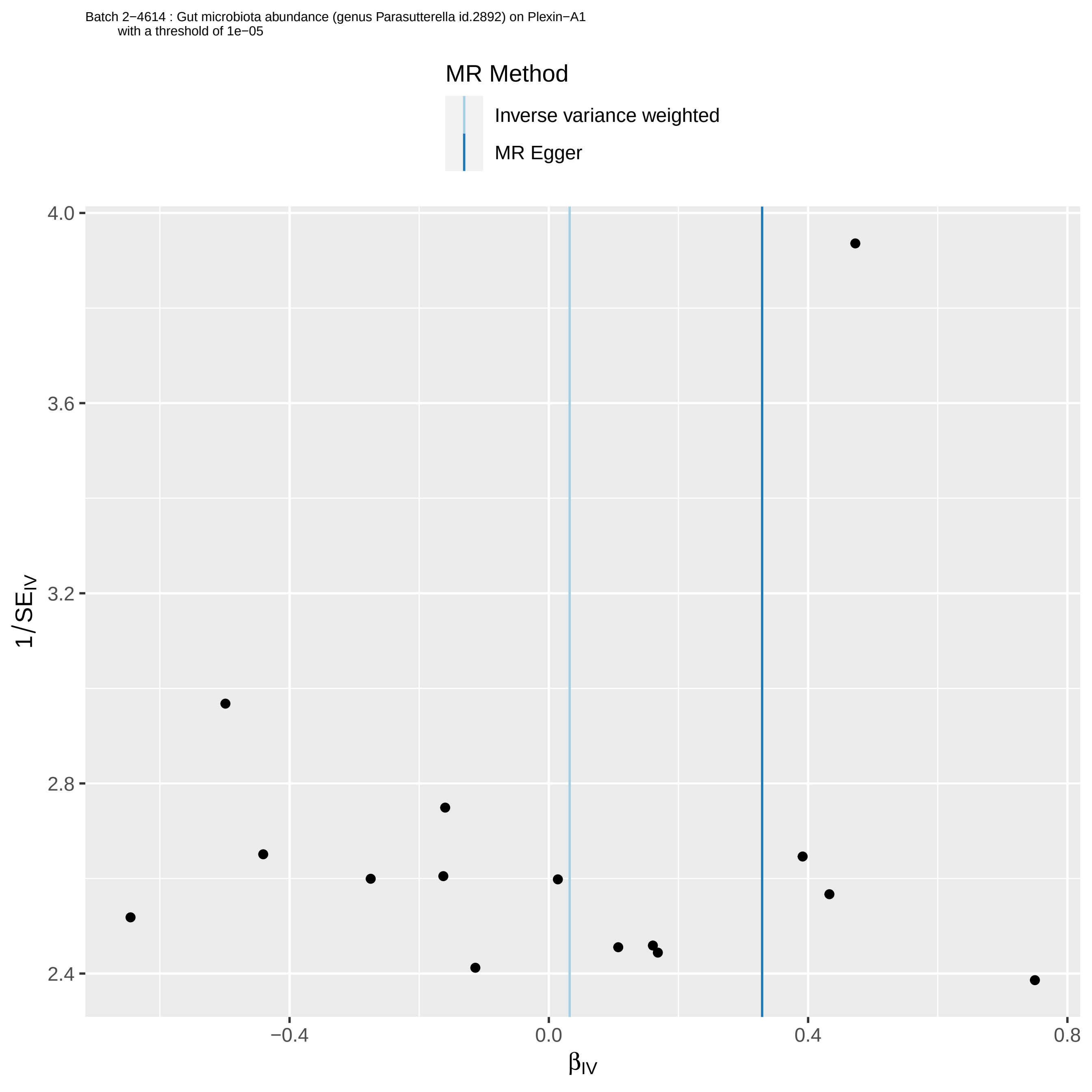

Batch 2−4614 : Gut microbiota abundance (genus Parasutterella id.2892) on Plexin−A1
with a threshold of 1e−05
MR Method
Inverse variance weighted
MR Egger
4.0
3.6
3.2
2.8
2.4
1/SE_IV
−0.4
0.0
0.4
0.8
β_IV

Batch 2−4615 : Gut microbiota abundance (genus Catenibacterium id.2153) on Plexin−B2
with a threshold of 1e−05

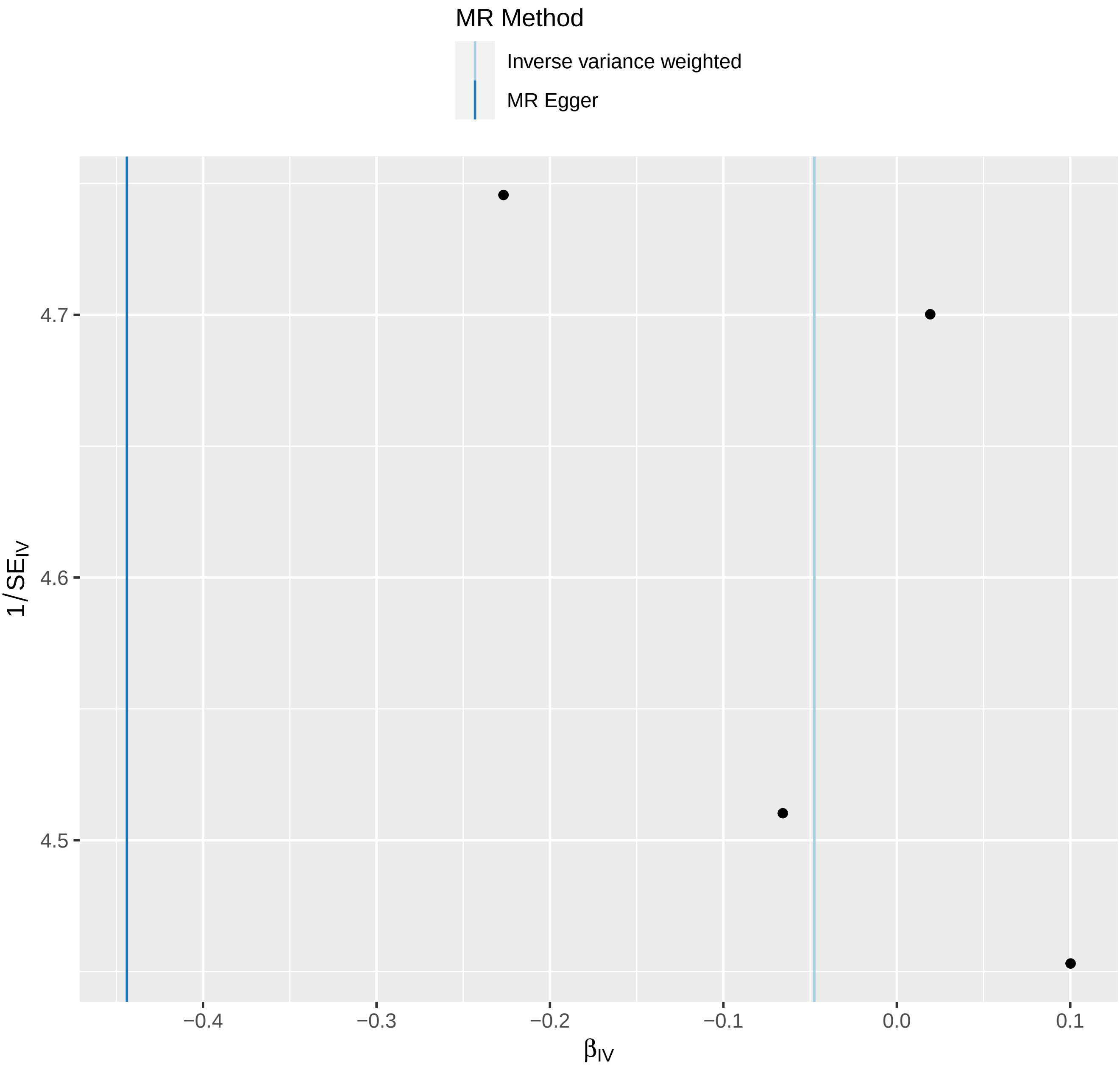

Batch 2−4616 : Gut microbiota abundance (genus Parasutterella id.2892) on Plexin−B2
with a threshold of 1e−05

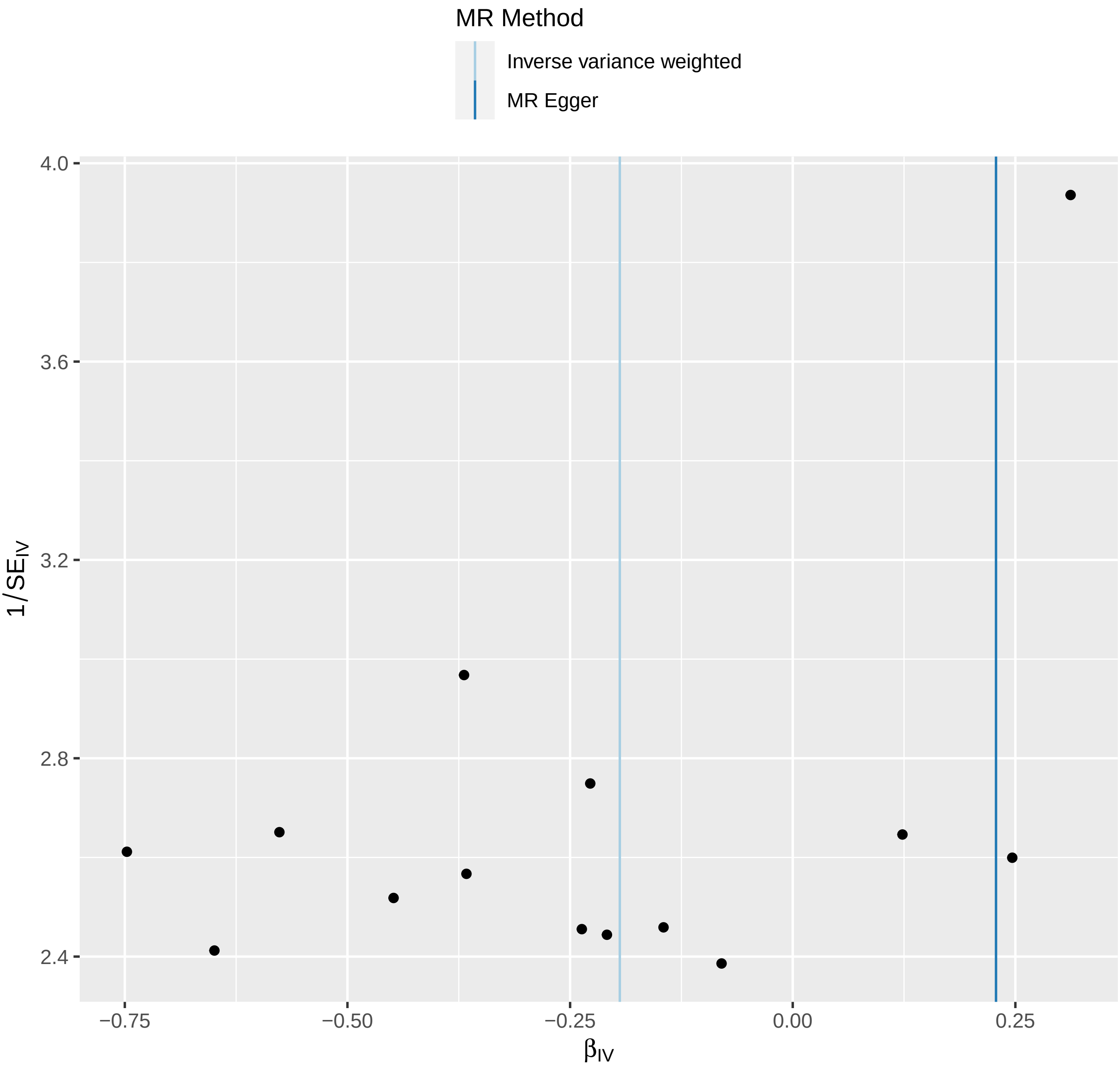

Batch 2−4617 : Gut microbiota abundance (genus Catenibacterium id.2153) on Plexin−C1
with a threshold of 1e−05

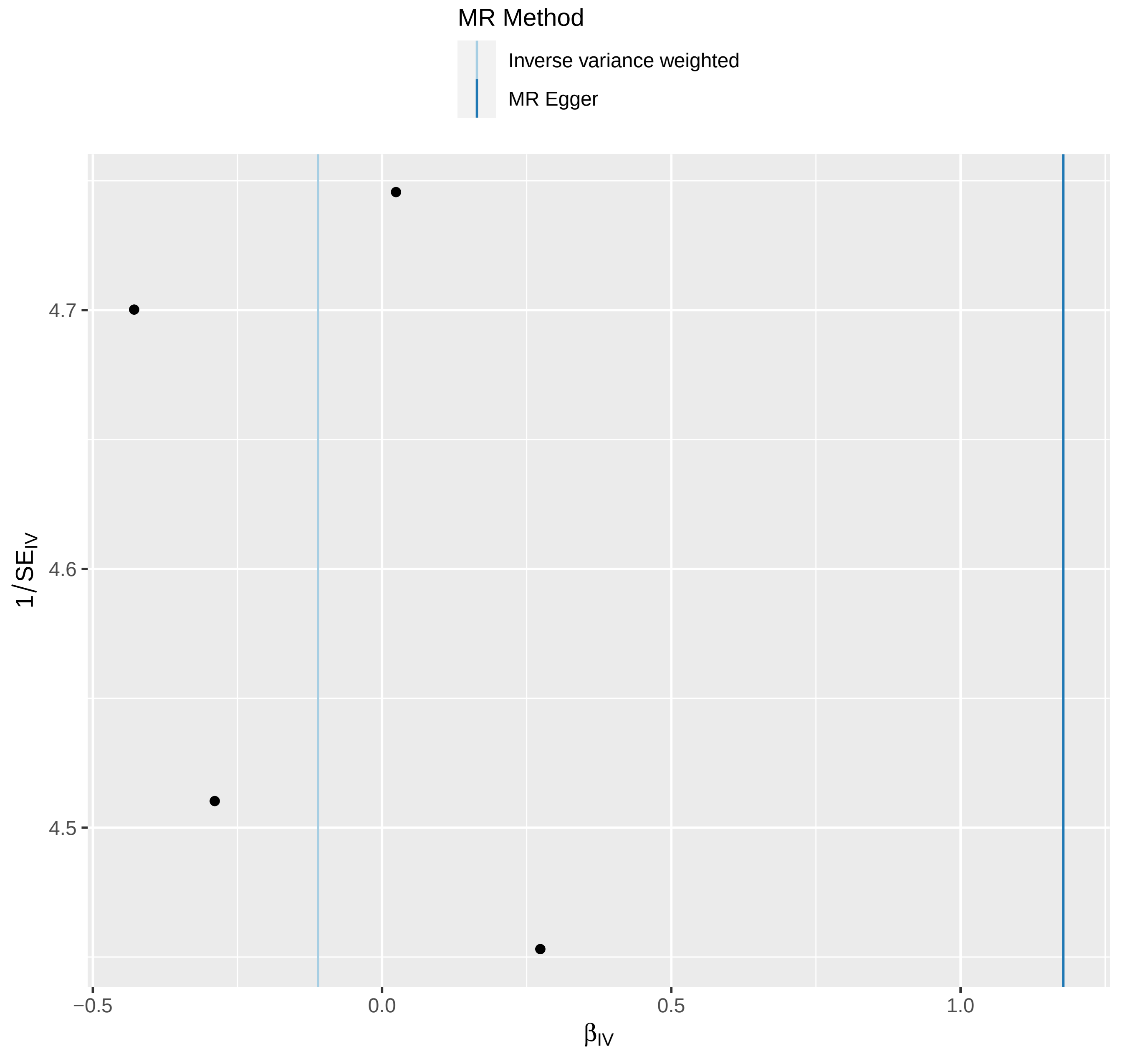

Batch 2−4618 : Gut microbiota abundance (genus Parasutterella id.2892) on Plexin−C1
with a threshold of 1e−05

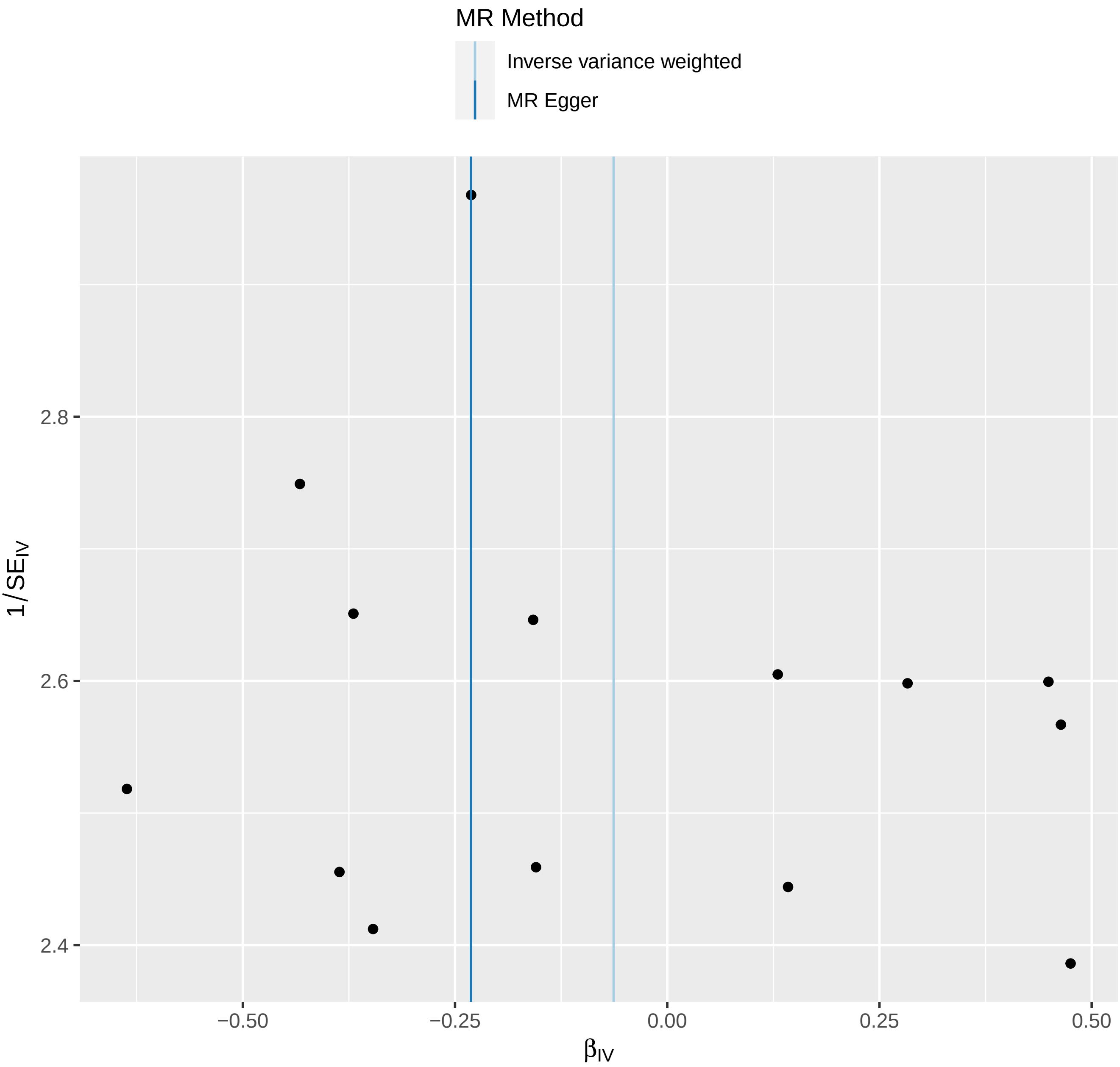

Batch 2−4619 : Gut microbiota abundance (genus Catenibacterium id.2153) on Melanocyte protein PMEL
with a threshold of 1e−05

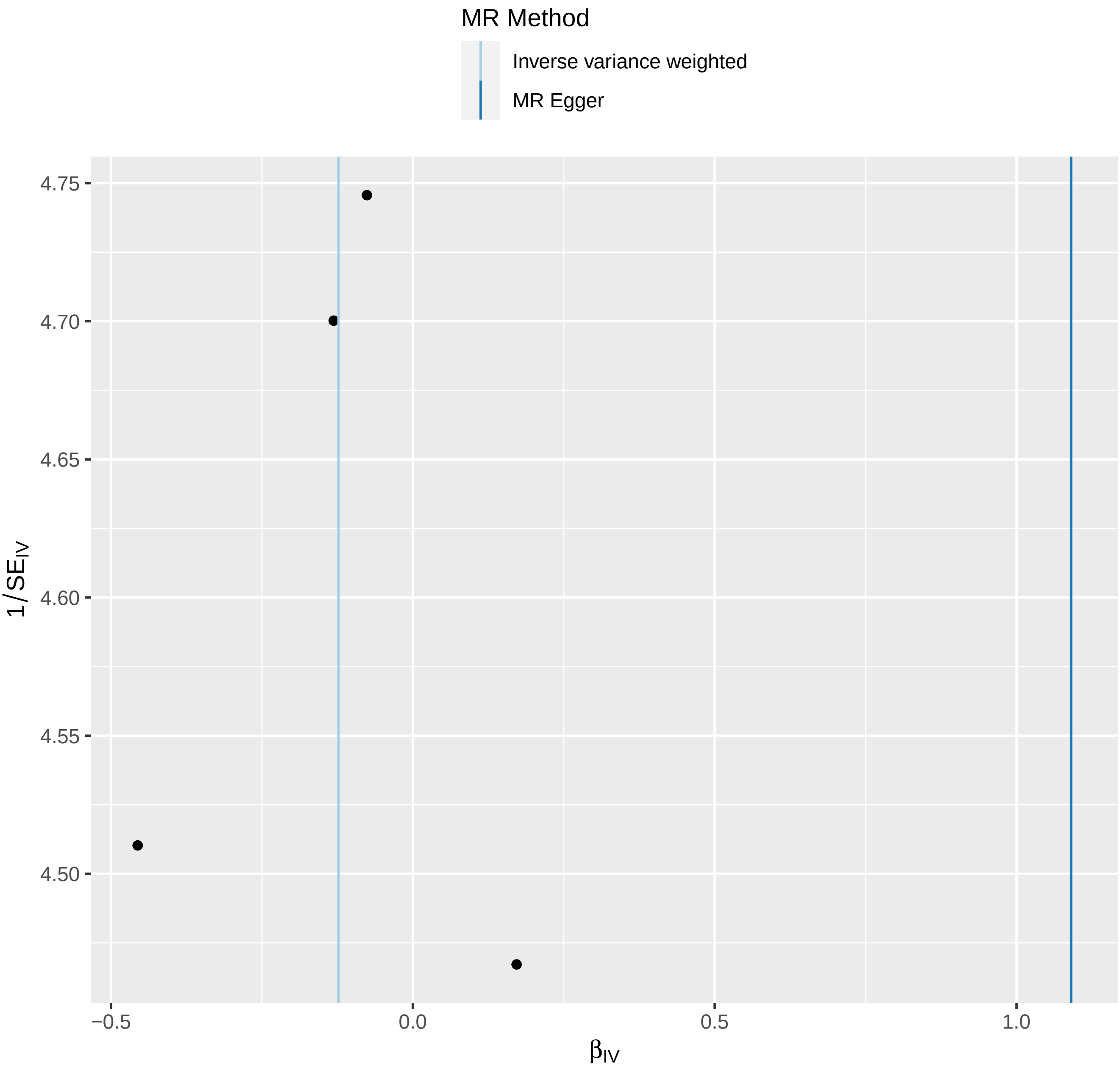

Batch 2−4620 : Gut microbiota abundance (genus Parasutterella id.2892) on Melanocyte protein PMEL
with a threshold of 1e−05

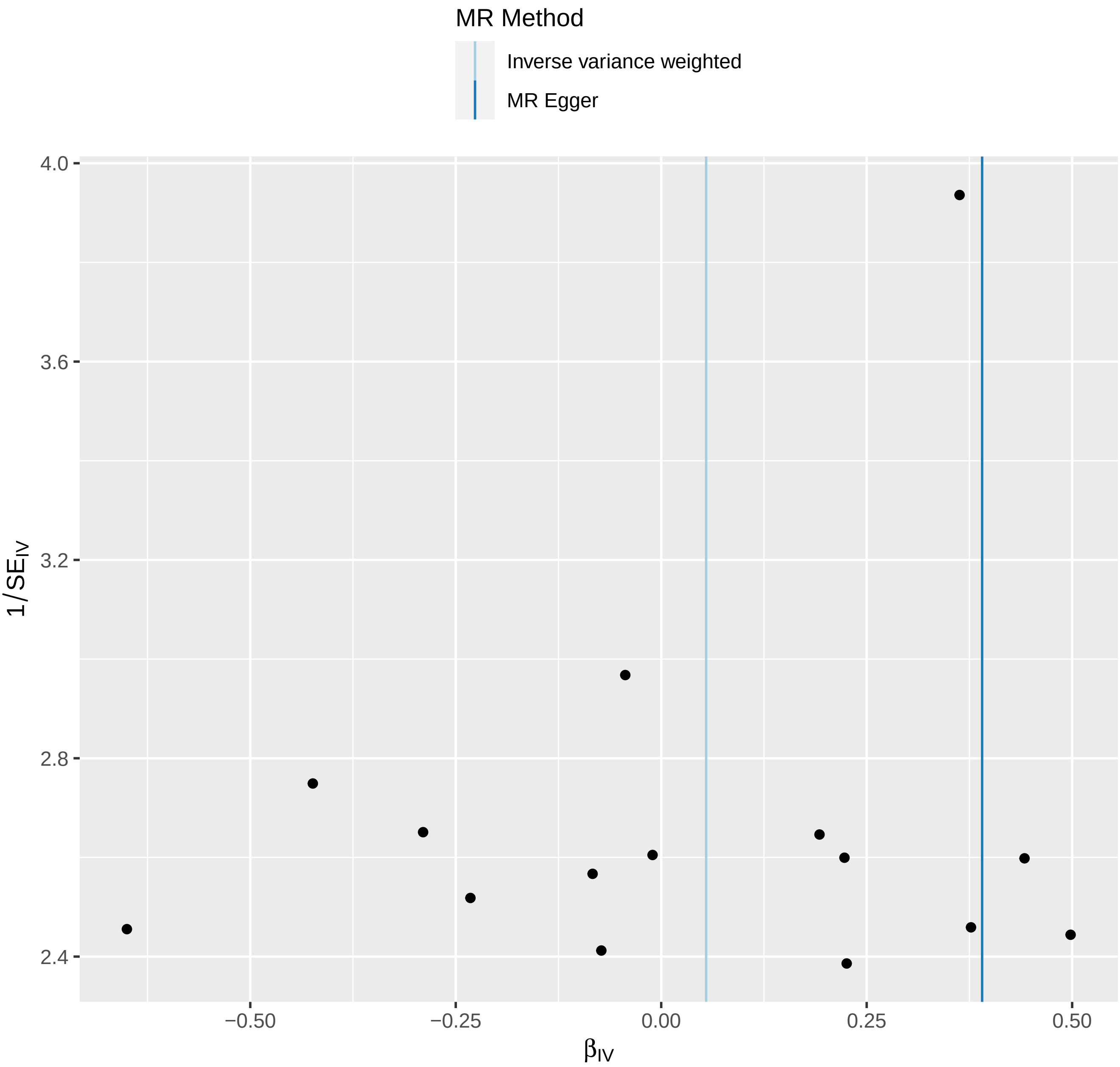

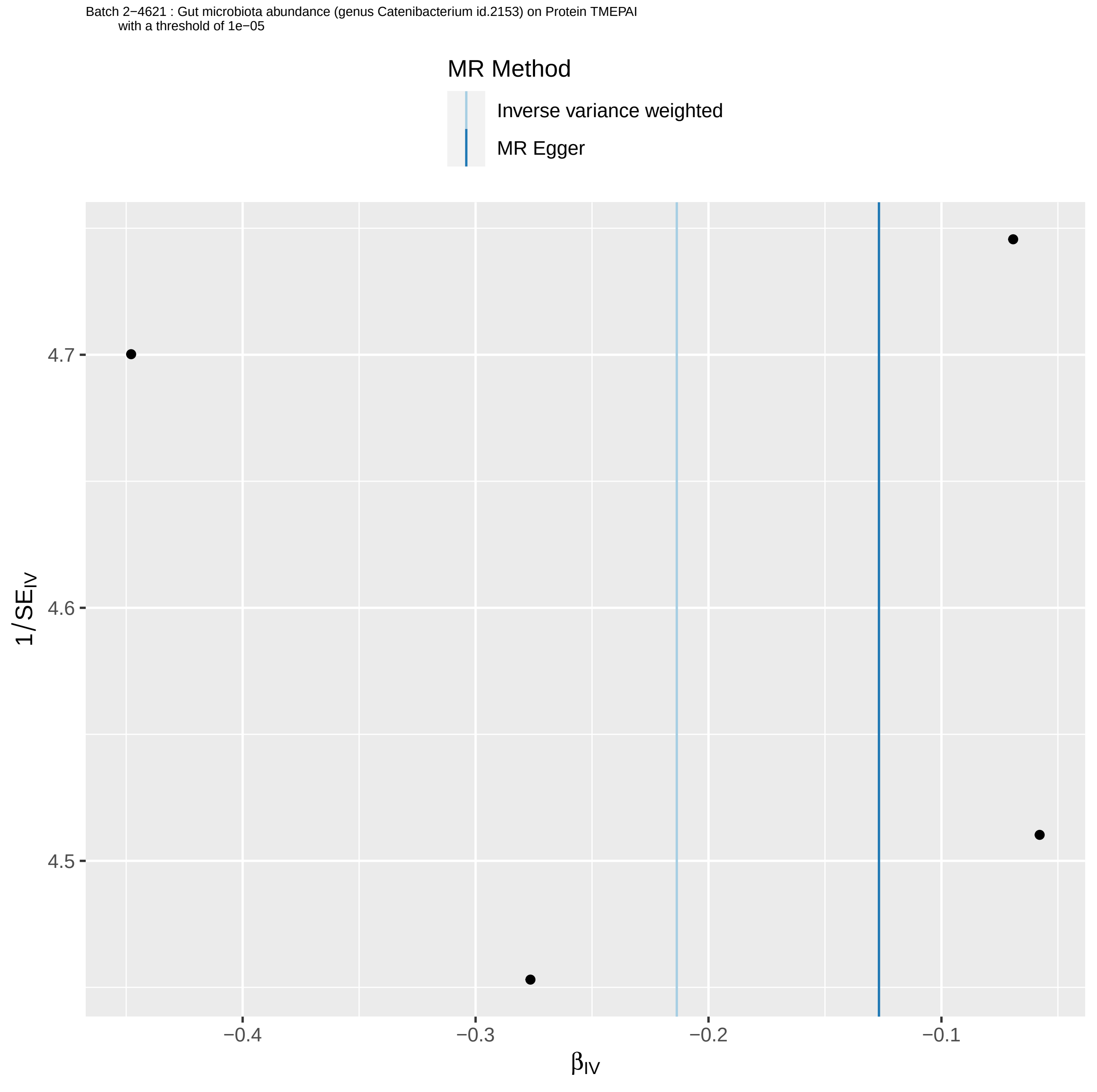

Batch 2−4621 : Gut microbiota abundance (genus Catenibacterium id.2153) on Protein TMEPAI
with a threshold of 1e−05
MR Method
Inverse variance weighted
MR Egger
4.7
4.6
4.5
1/SE_IV
−0.4
−0.3
−0.2
−0.1
β_IV

Batch 2−4622 : Gut microbiota abundance (genus Parasutterella id.2892) on Protein TMEPAI
with a threshold of 1e−05

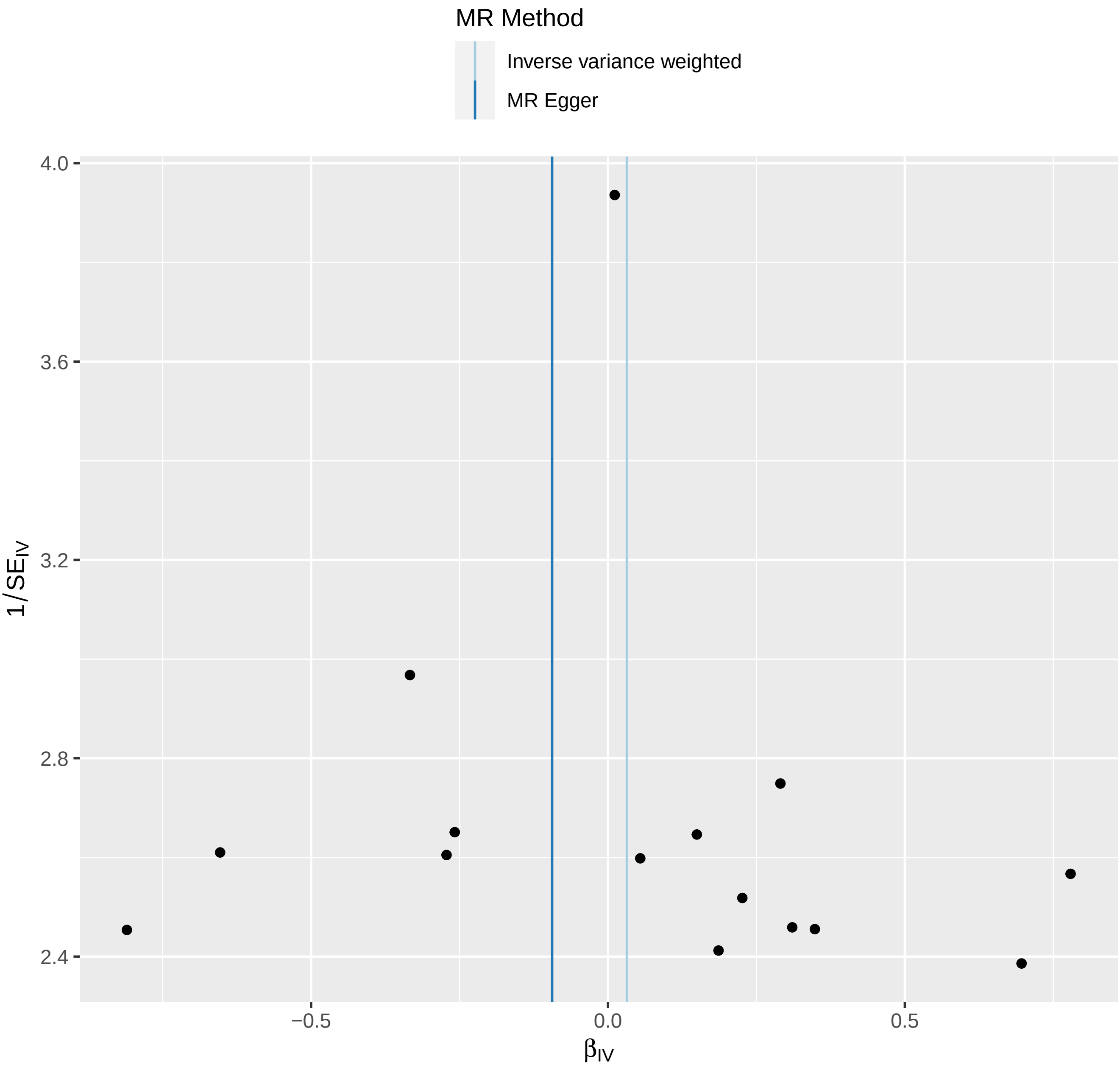

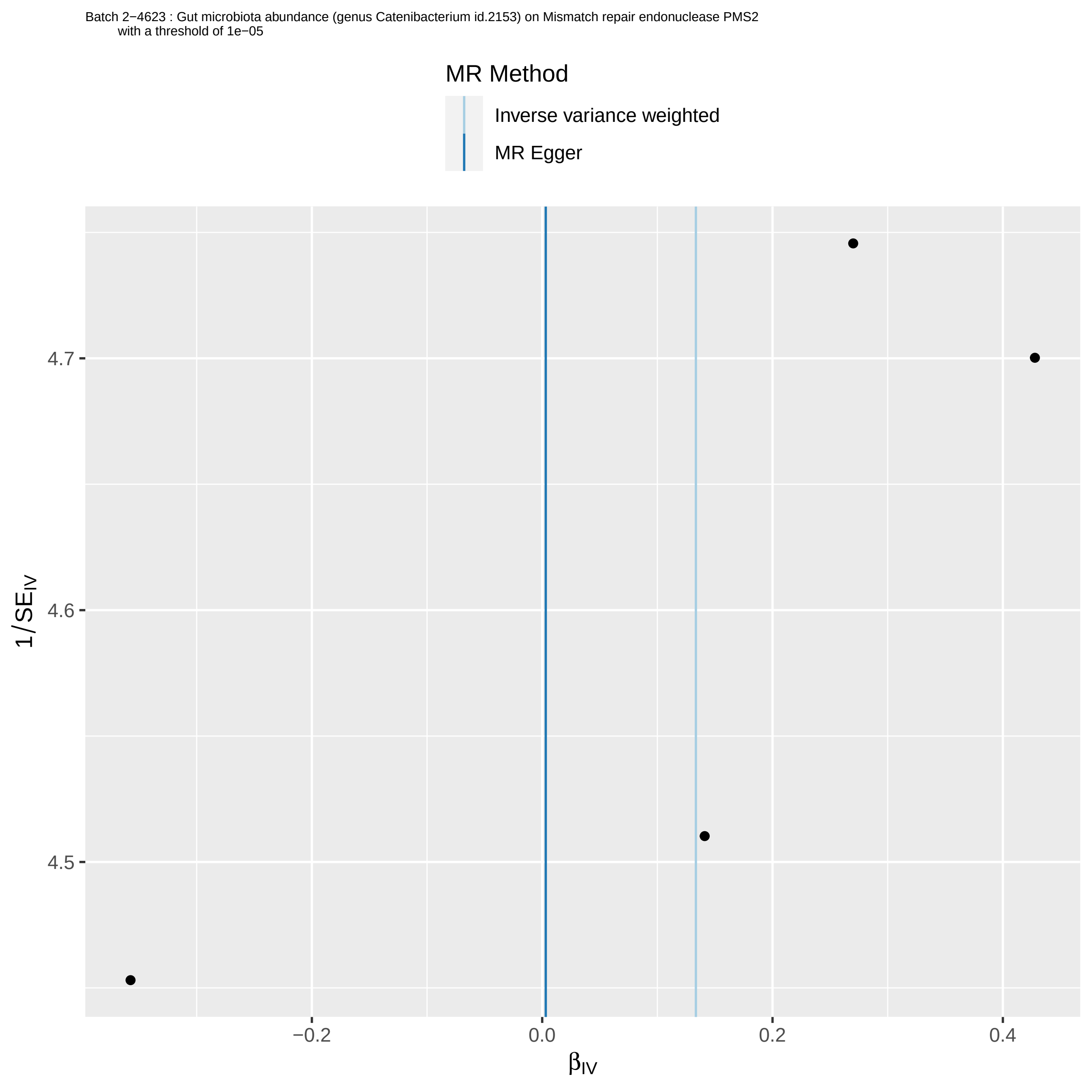

Batch 2−4623 : Gut microbiota abundance (genus Catenibacterium id.2153) on Mismatch repair endonuclease PMS2
with a threshold of 1e−05
MR Method
Inverse variance weighted
MR Egger
4.7
4.6
4.5
1/SE_IV
−0.2
0.0
0.2
0.4
β_IV

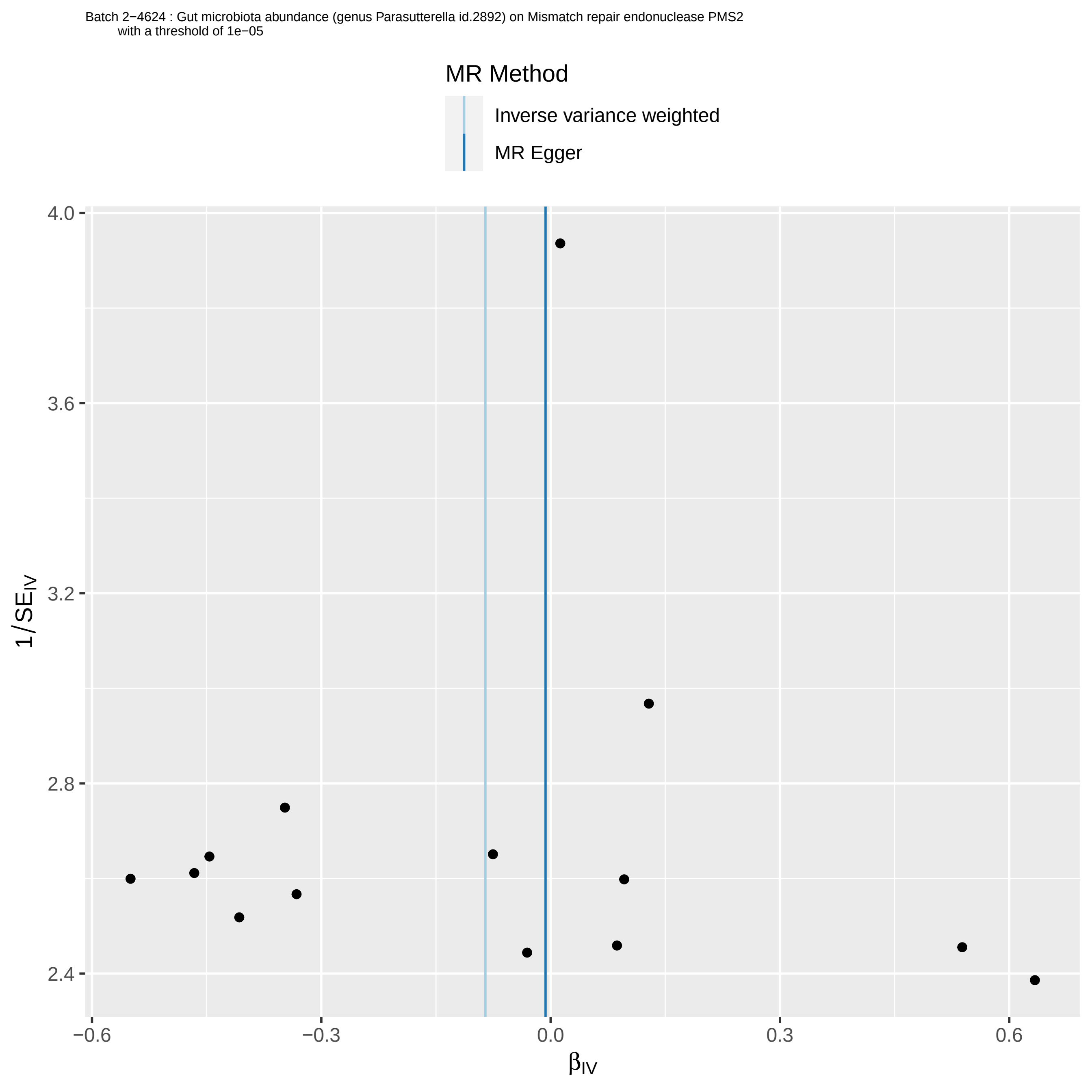
Batch 2−4624 : Gut microbiota abundance (genus Parasutterella id.2892) on Mismatch repair endonuclease PMS2
with a threshold of 1e−05
MR Method
Inverse variance weighted
MR Egger
4.0
3.6
3.2
2.8
2.4
1/SE_IV
−0.6
−0.3
0.0
0.3
0.6
β_IV

Batch 2−4625 : Gut microbiota abundance (genus Catenibacterium id.2153) on Phosphomevalonate kinase
with a threshold of 1e−05

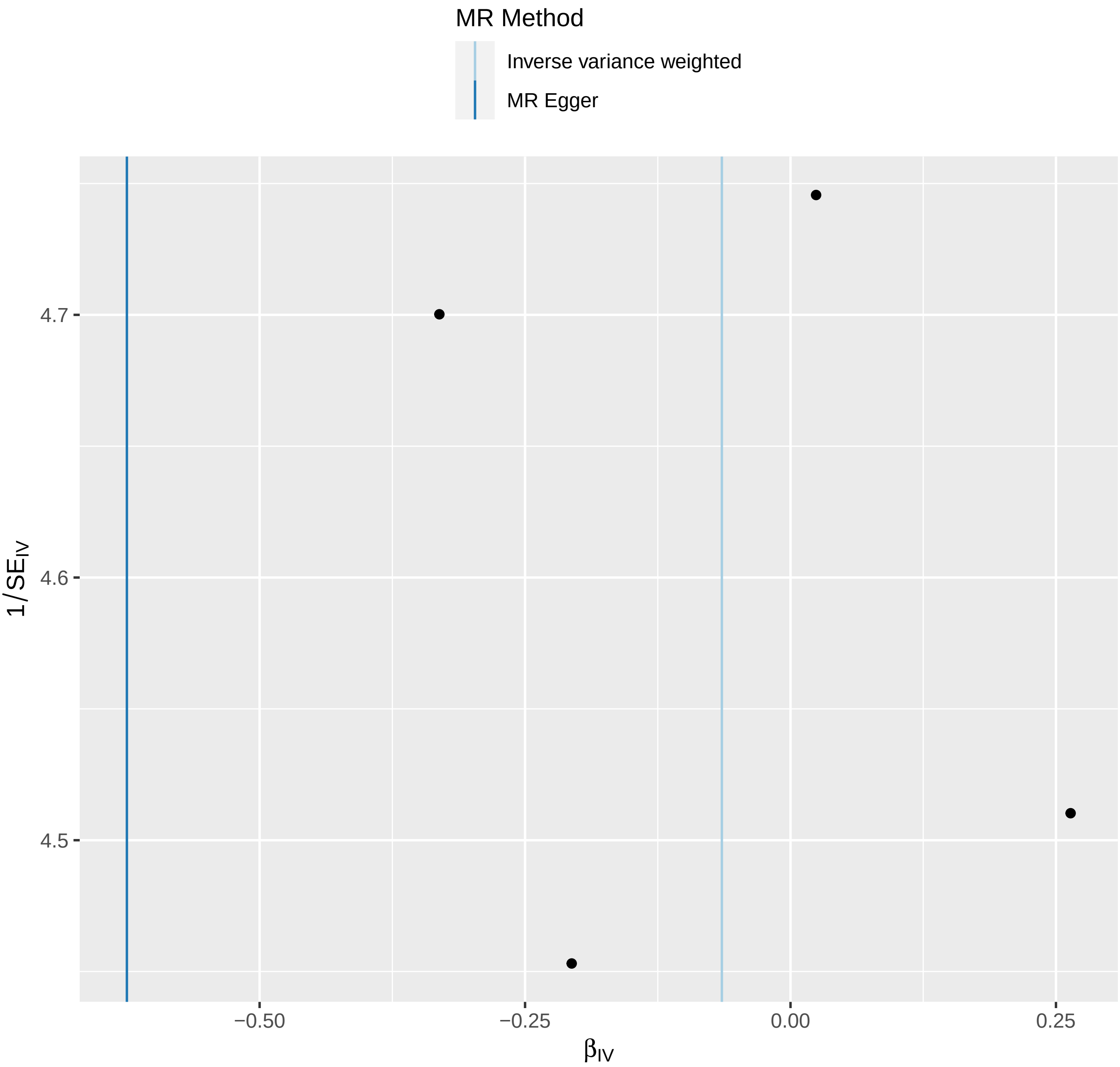

Batch 2−4626 : Gut microbiota abundance (genus Parasutterella id.2892) on Phosphomevalonate kinase
with a threshold of 1e−05

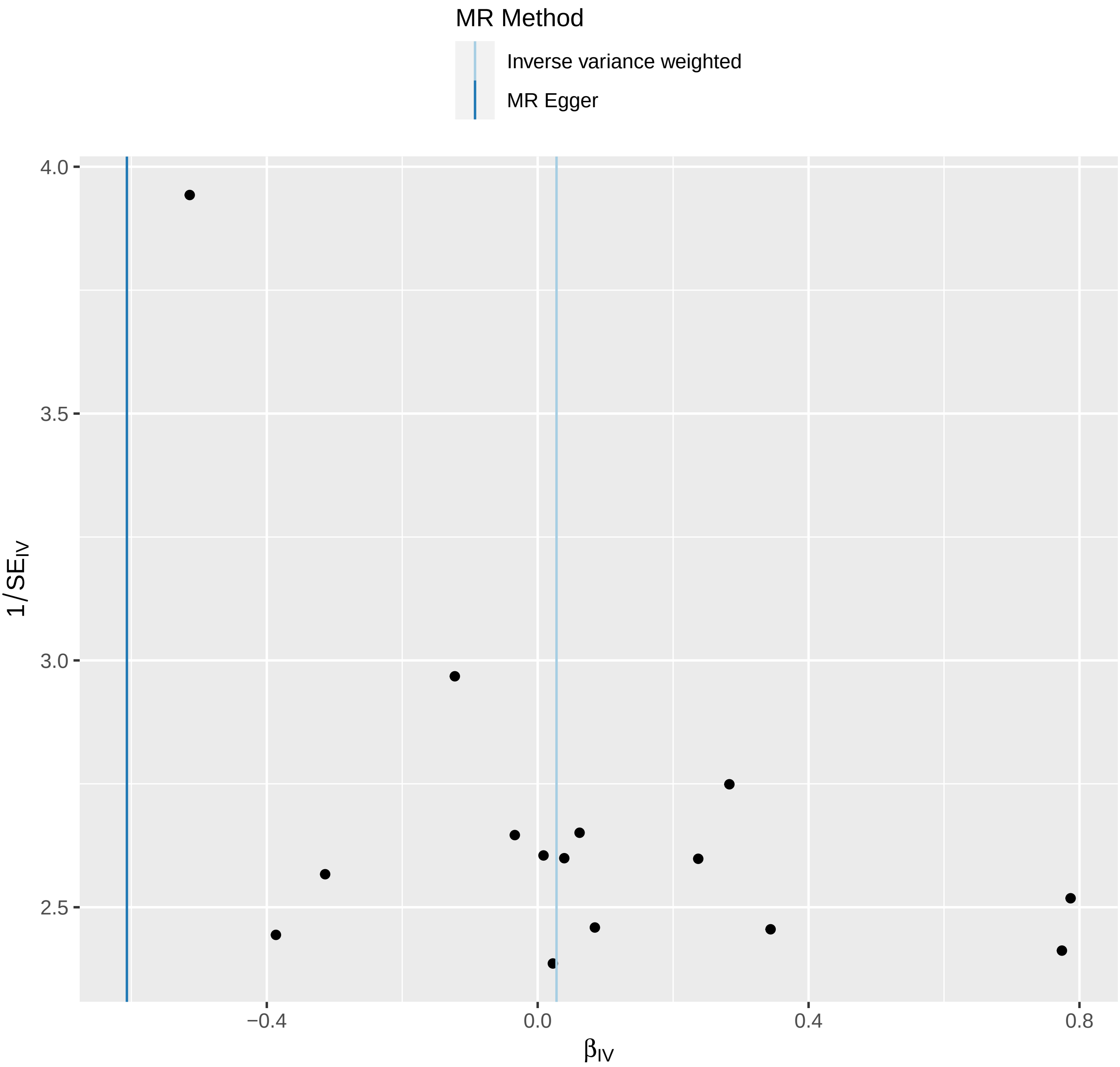

Batch 2−4627 : Gut microbiota abundance (genus Catenibacterium id.2153) on Bifunctional polynucleotide phosphatase/kinase
with a threshold of 1e−05

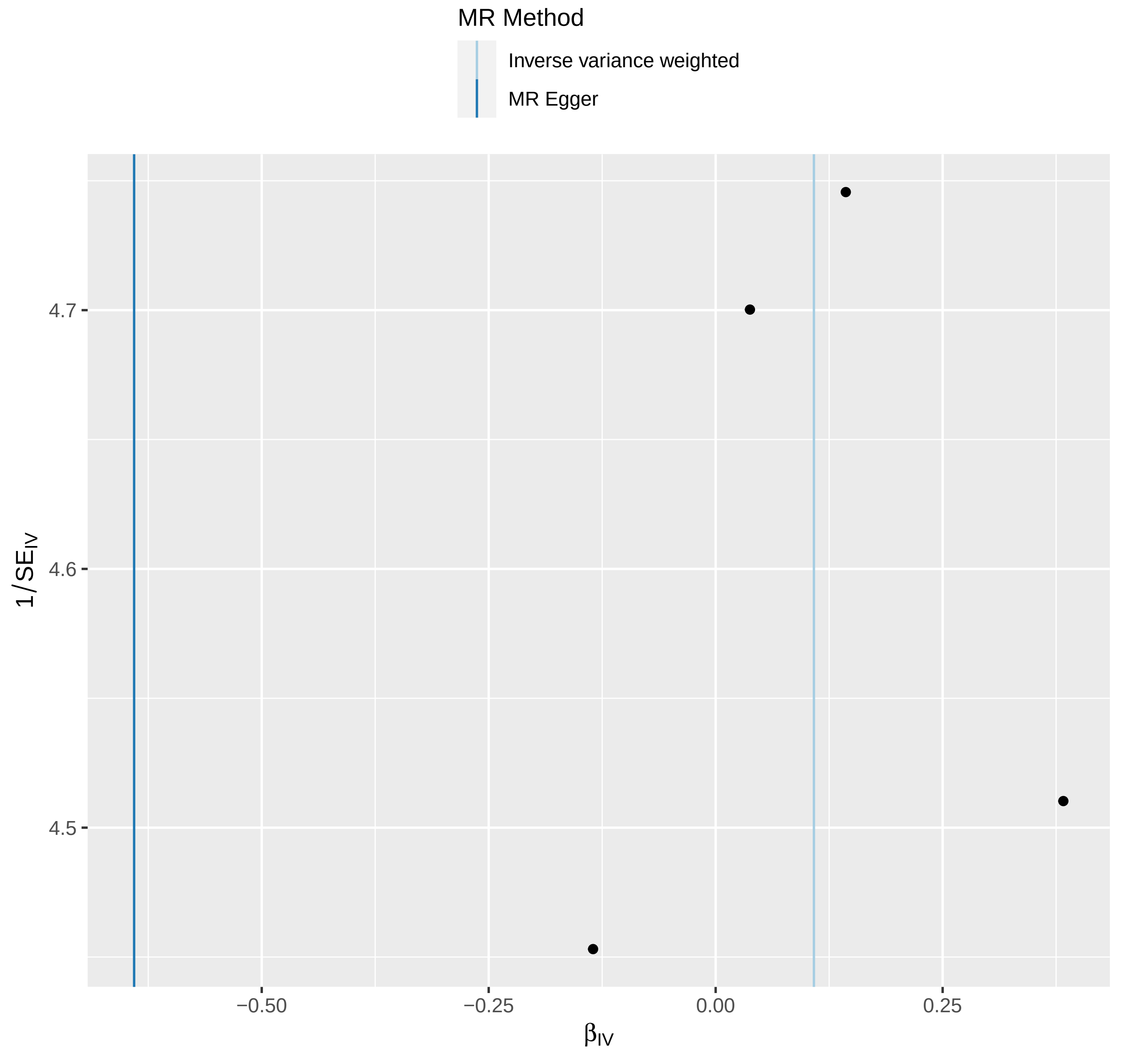

Batch 2−4628 : Gut microbiota abundance (genus Parasutterella id.2892) on Bifunctional polynucleotide phosphatase/kinase
with a threshold of 1e−05

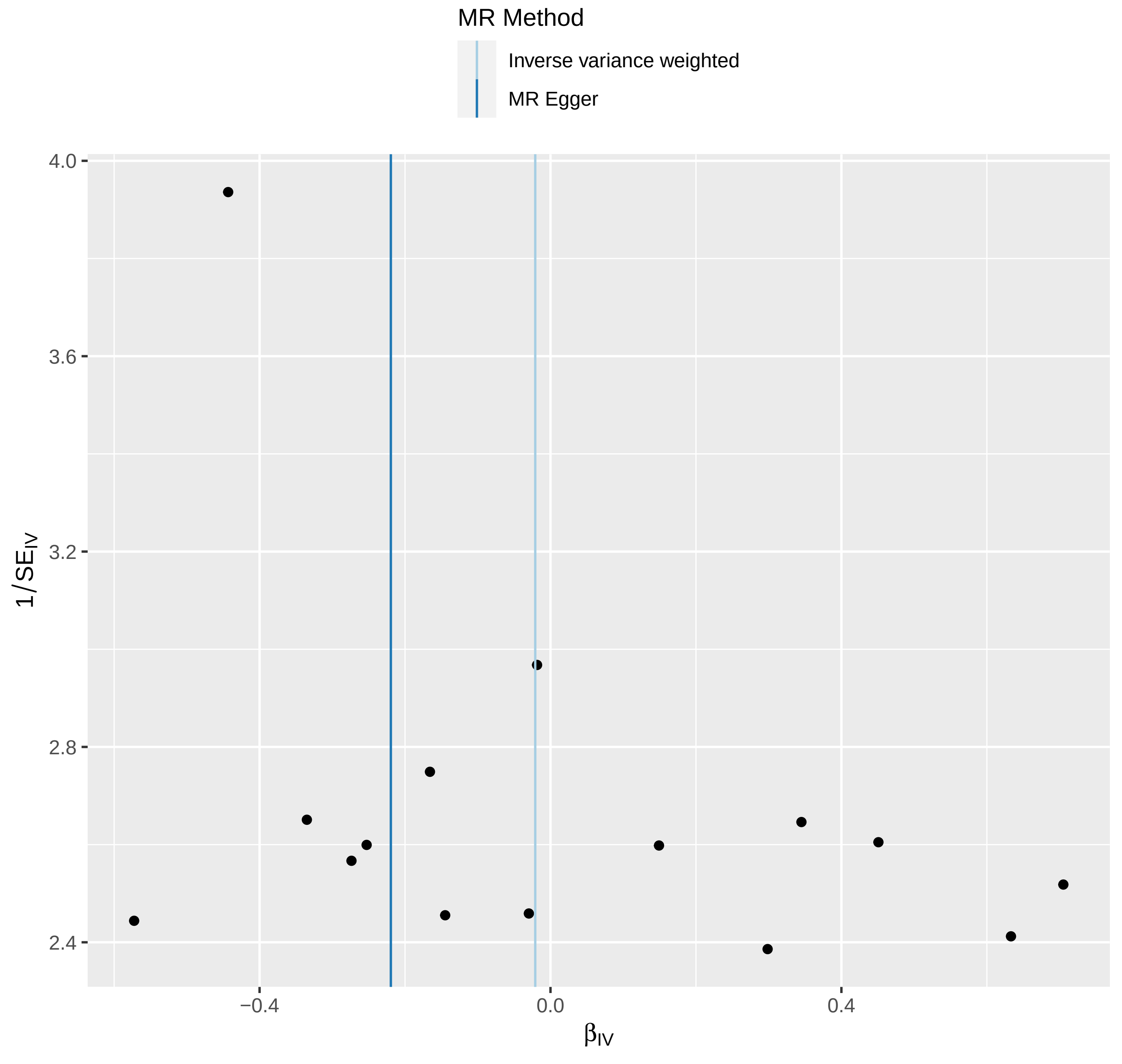

Batch 2−4629 : Gut microbiota abundance (genus Catenibacterium id.2153) on Inactive pancreatic lipase−related protein 1
with a threshold of 1e−05

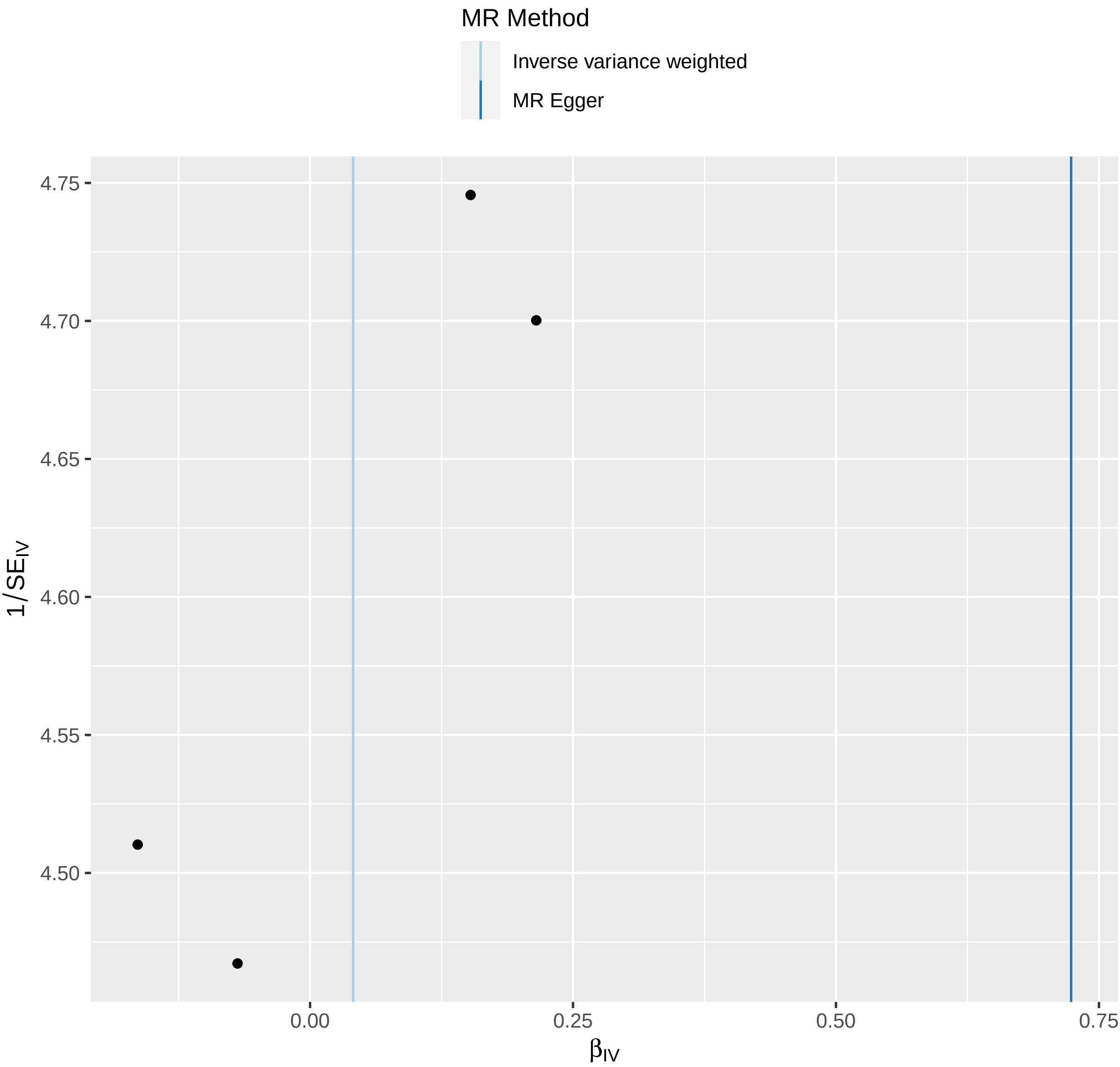

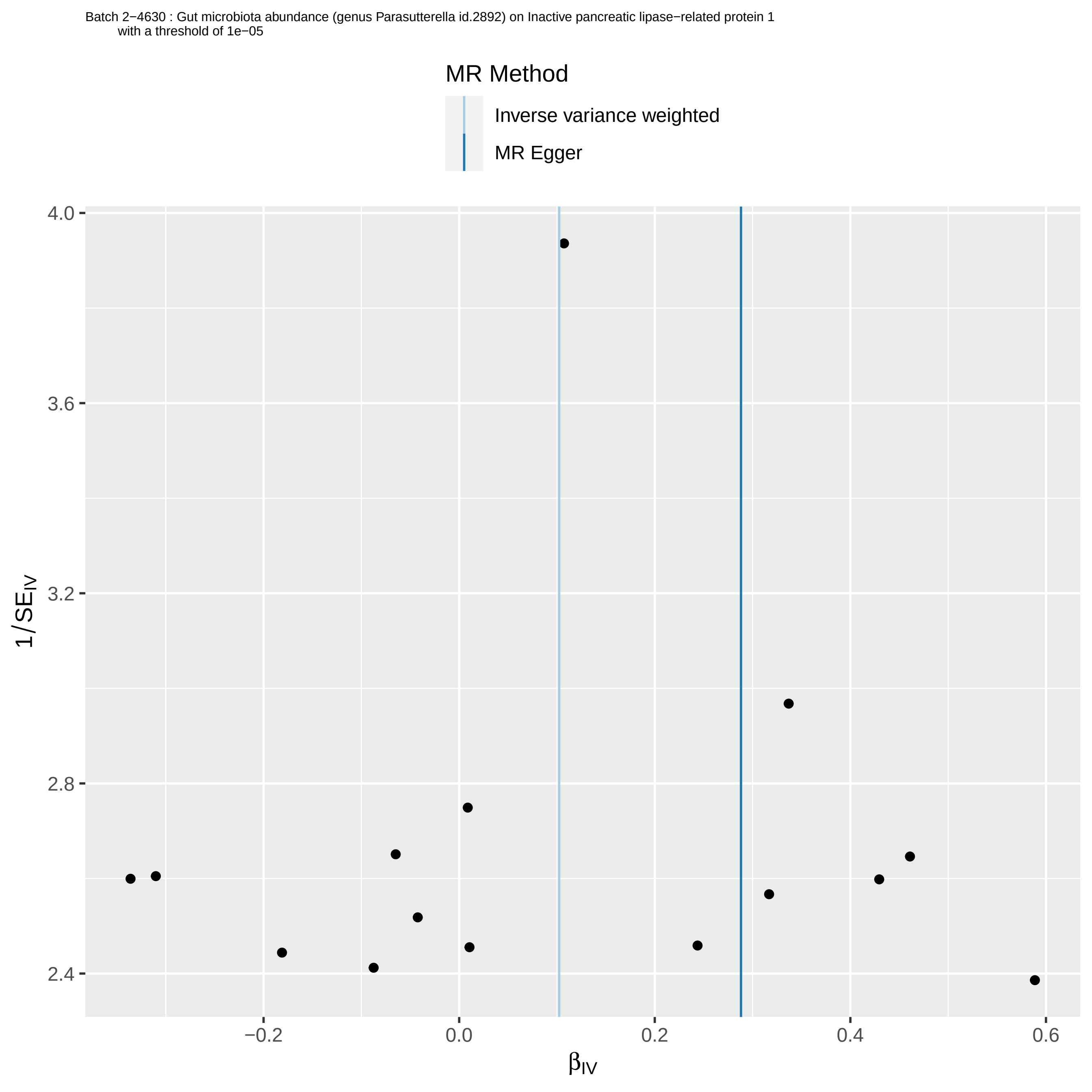

Batch 2−4630 : Gut microbiota abundance (genus Parasutterella id.2892) on Inactive pancreatic lipase−related protein 1
with a threshold of 1e−05
MR Method
Inverse variance weighted
MR Egger
4.0
3.6
3.2
2.8
2.4
1/SE_IV
−0.2
0.0
0.2
0.4
0.6
β_IV

Batch 2−4631 : Gut microbiota abundance (genus Catenibacterium id.2153) on Purine nucleoside phosphorylase
with a threshold of 1e−05

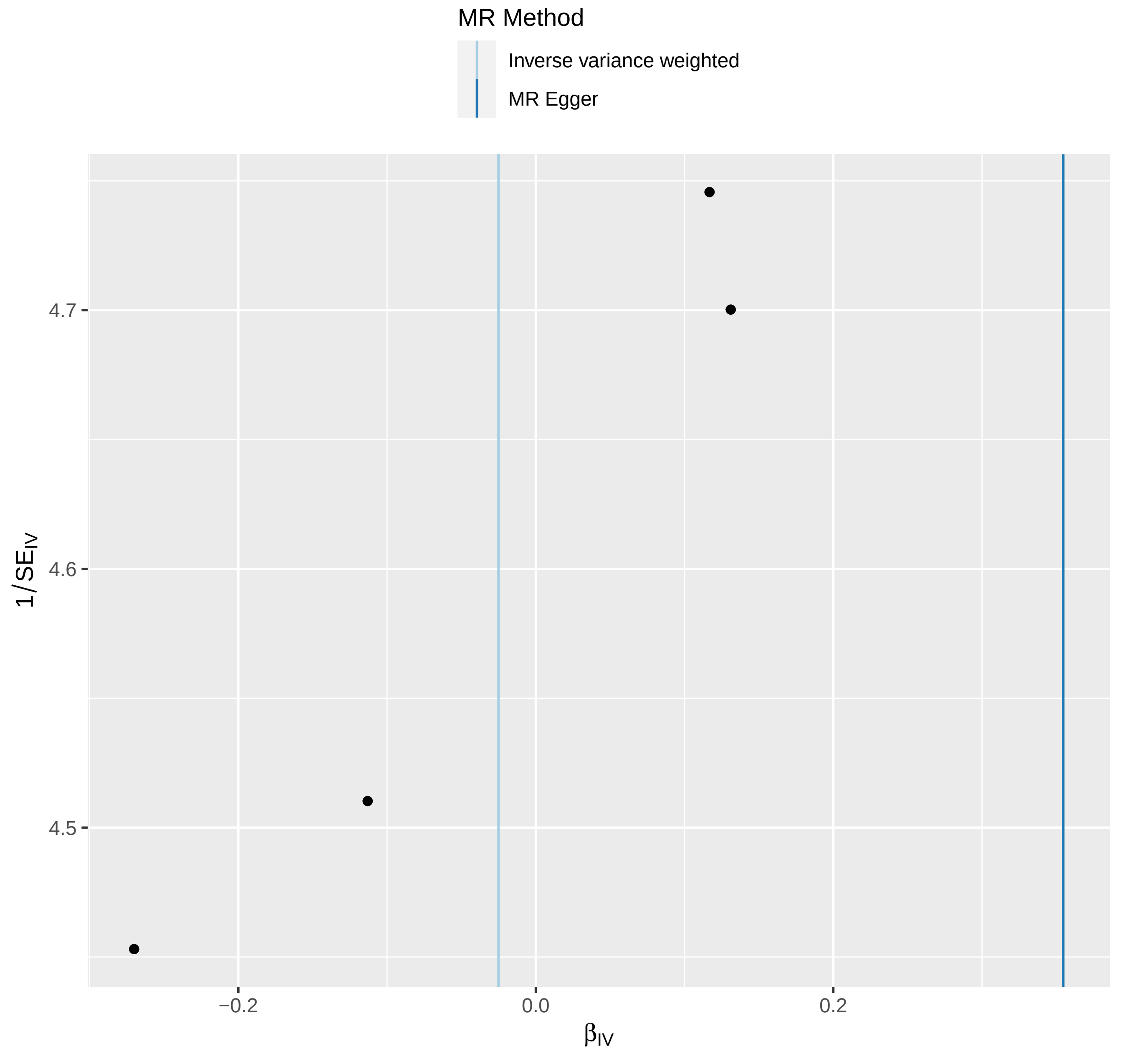

Batch 2−4632 : Gut microbiota abundance (genus Parasutterella id.2892) on Purine nucleoside phosphorylase
with a threshold of 1e−05

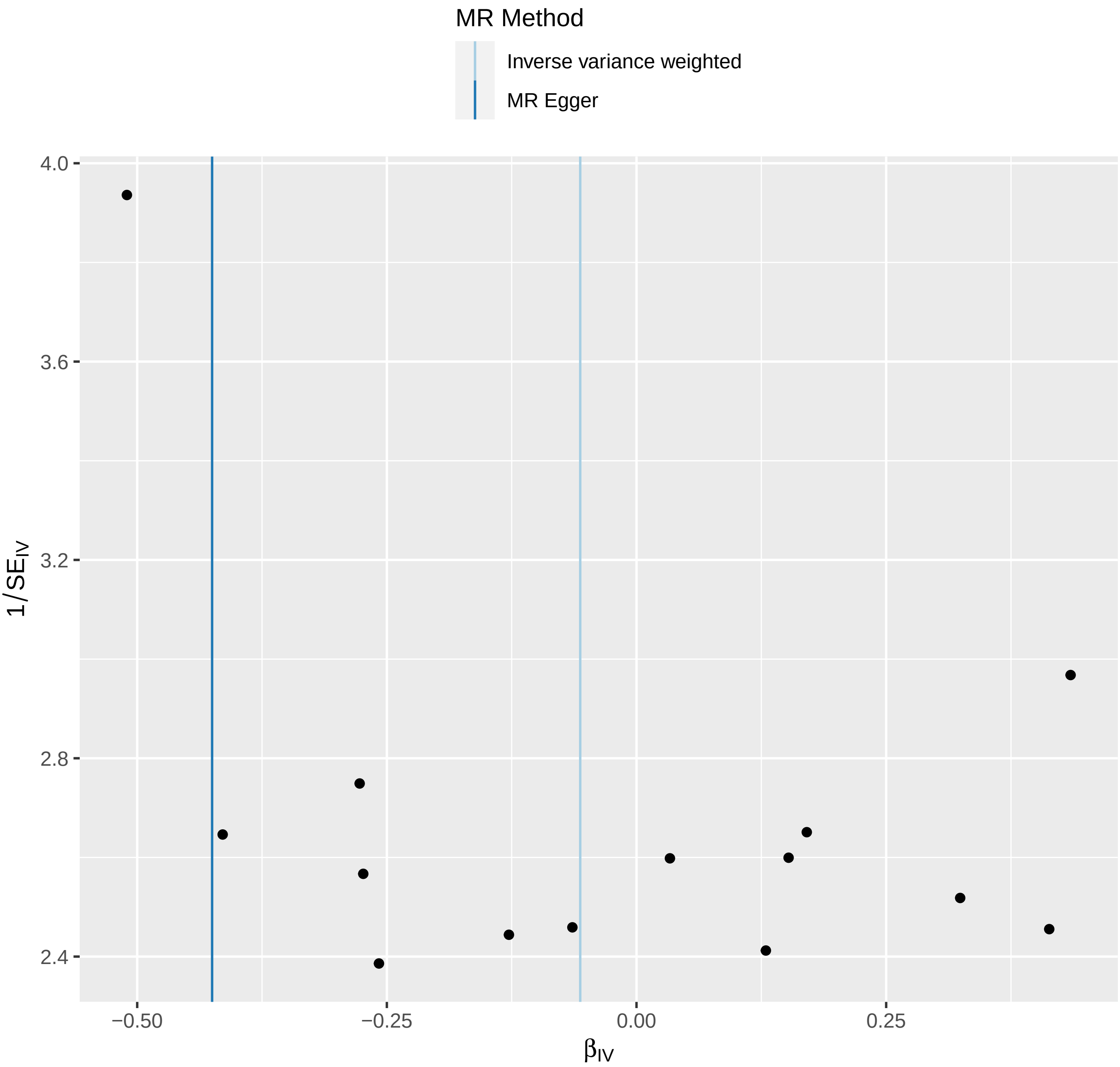

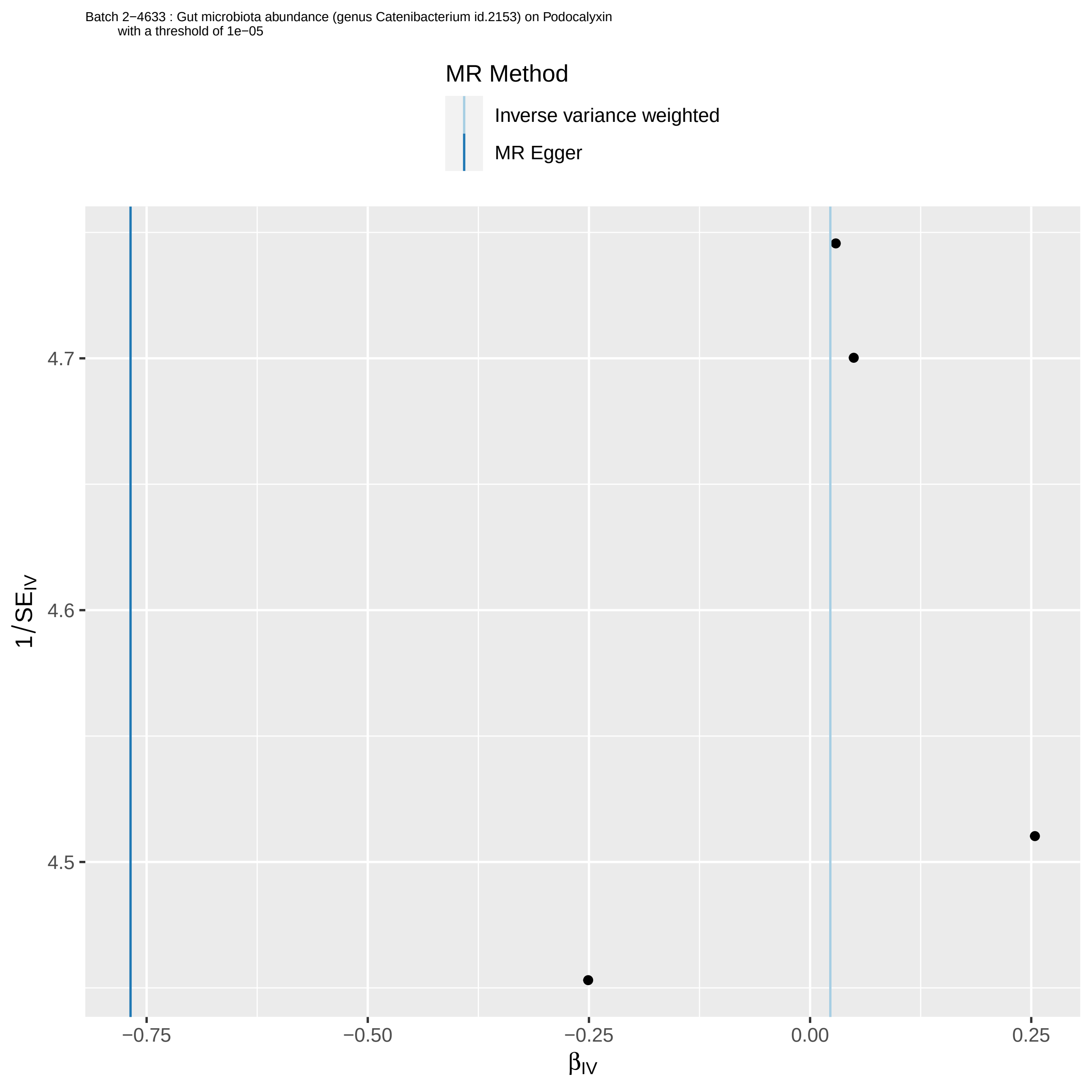

Batch 2−4633 : Gut microbiota abundance (genus Catenibacterium id.2153) on Podocalyxin
with a threshold of 1e−05
MR Method
Inverse variance weighted
MR Egger
4.7
4.6
4.5
1/SE_IV
−0.75
−0.50
−0.25
0.00
0.25
β_IV

Batch 2−4634 : Gut microbiota abundance (genus Parasutterella id.2892) on Podocalyxin
with a threshold of 1e−05

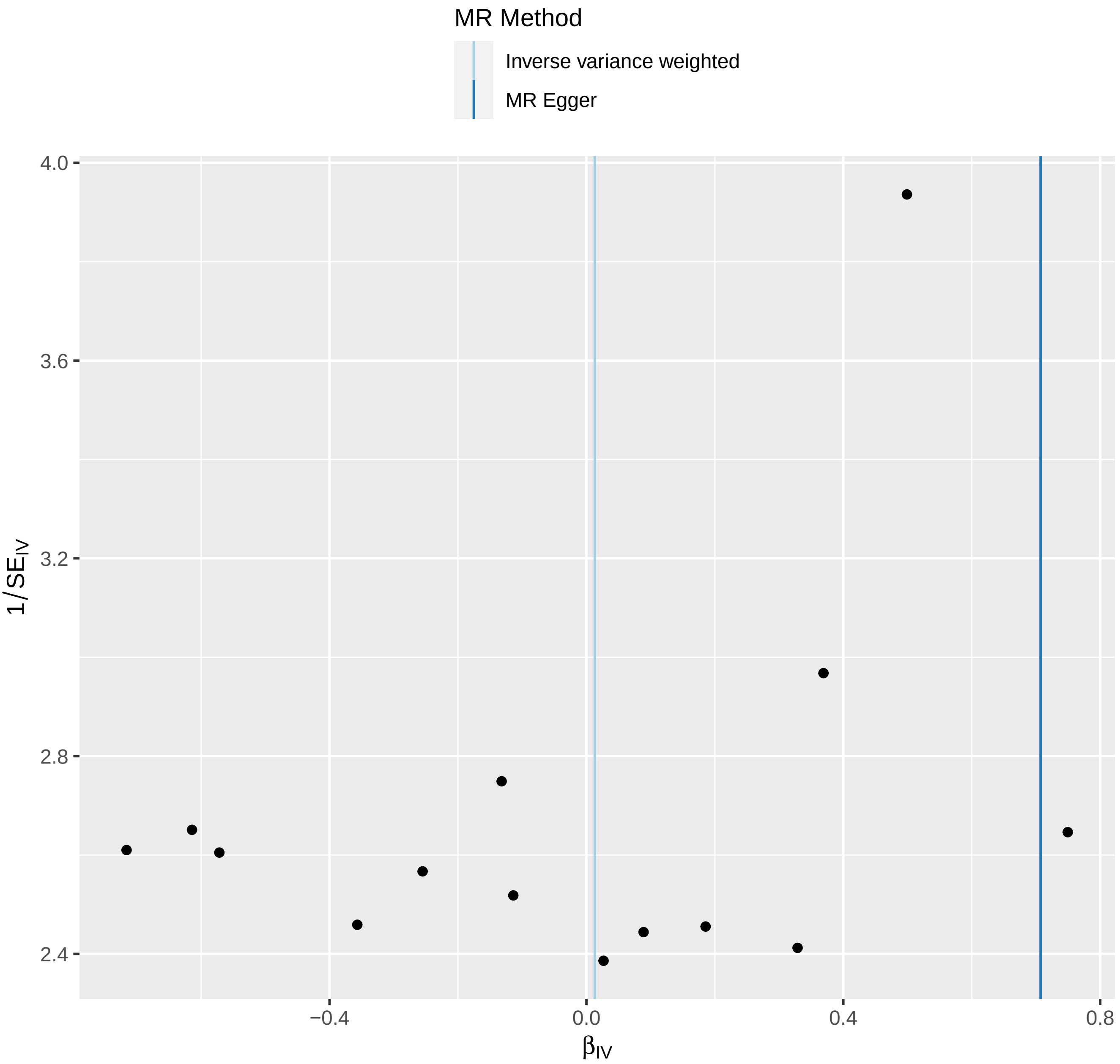

Batch 2−4635 : Gut microbiota abundance (genus Catenibacterium id.2153) on GDP−fucose protein O−fucosyltransferase 1 with a threshold of 1e−05

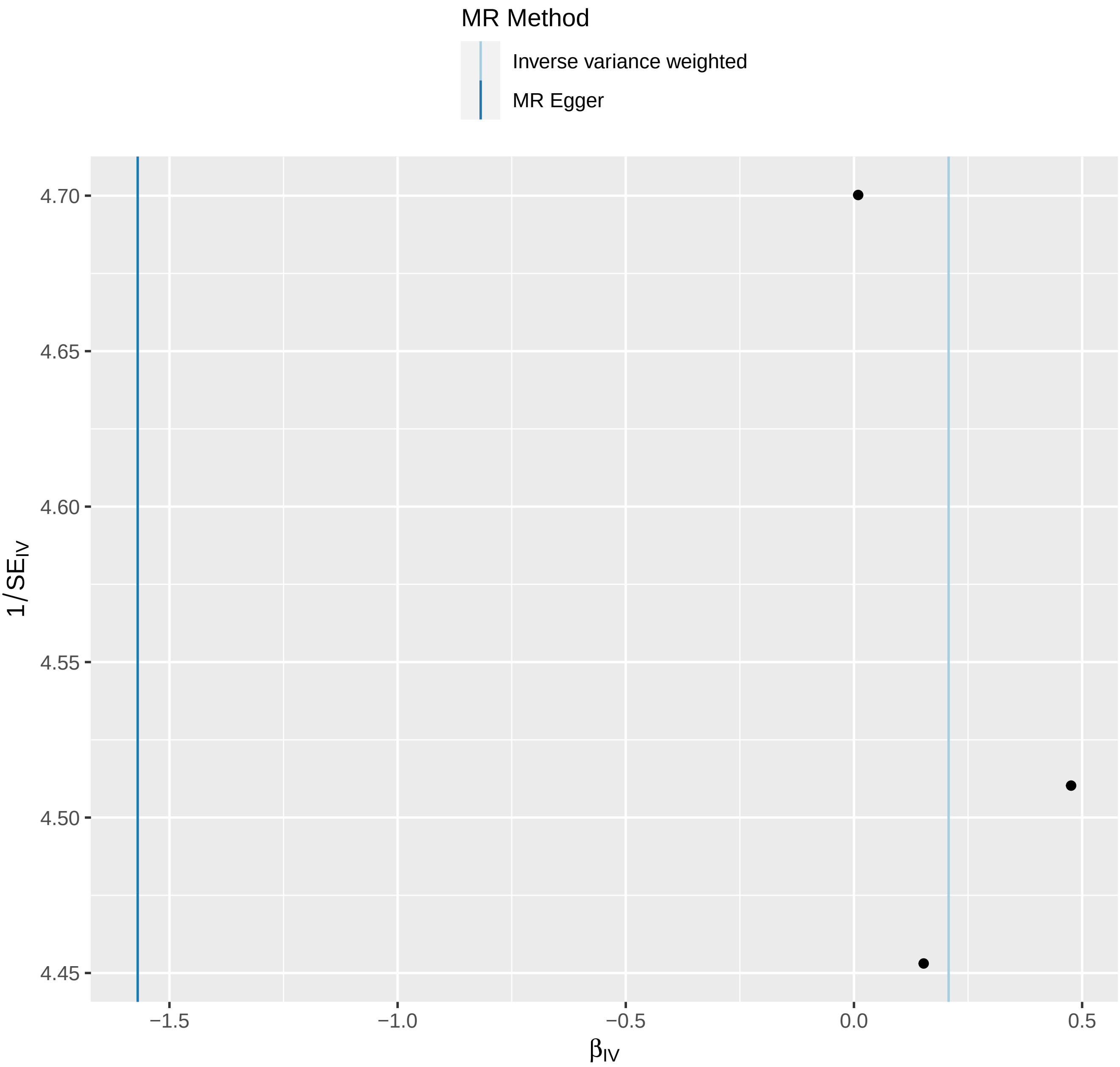

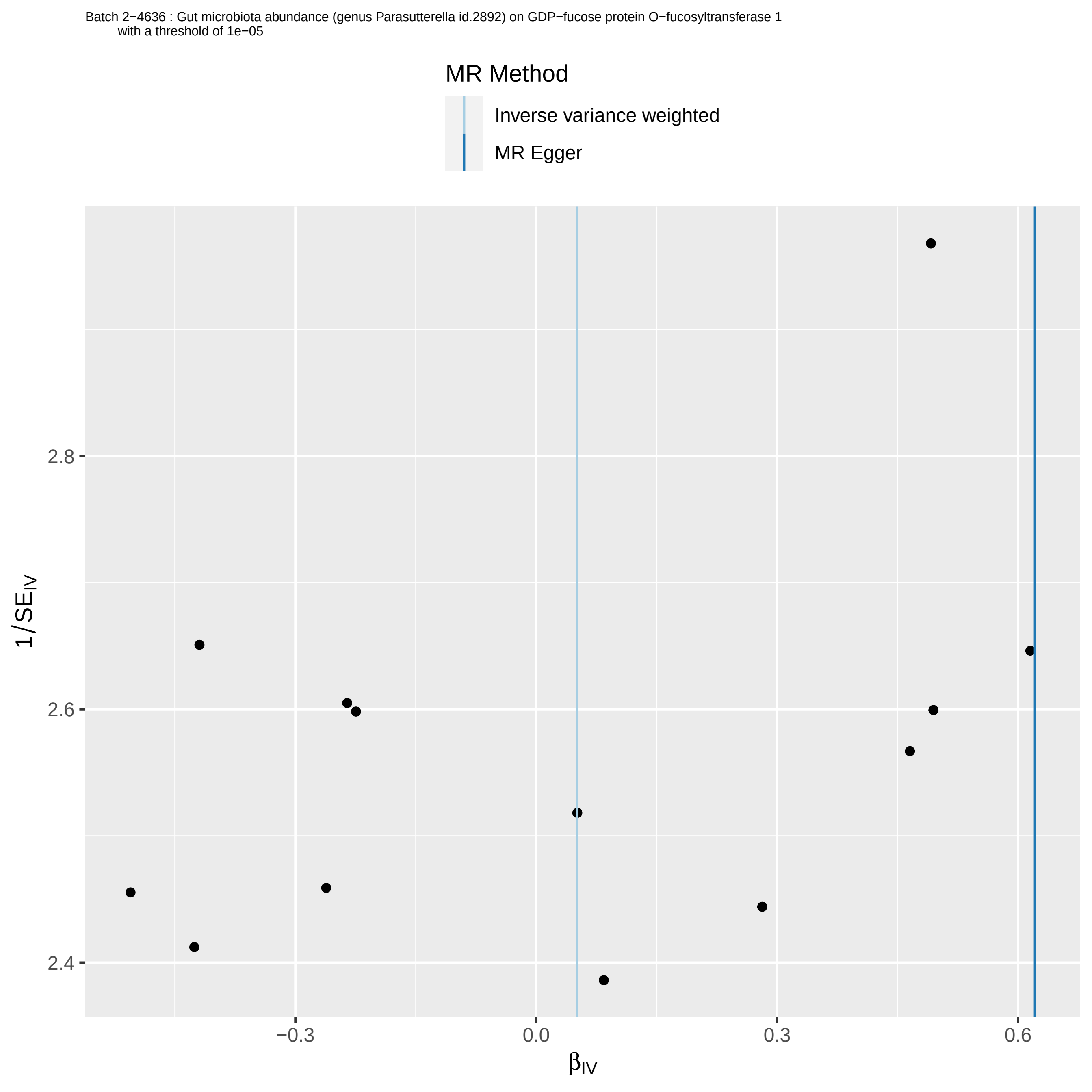

Batch 2−4636 : Gut microbiota abundance (genus Parasutterella id.2892) on GDP−fucose protein O−fucosyltransferase 1
with a threshold of 1e−05
MR Method
Inverse variance weighted
MR Egger
2.8
2.6
2.4
1/SE_IV
−0.3
0.0
0.3
0.6
β_IV

Batch 2−4637 : Gut microbiota abundance (genus Catenibacterium id.2153) on GDP−fucose protein O−fucosyltransferase 2
with a threshold of 1e−05

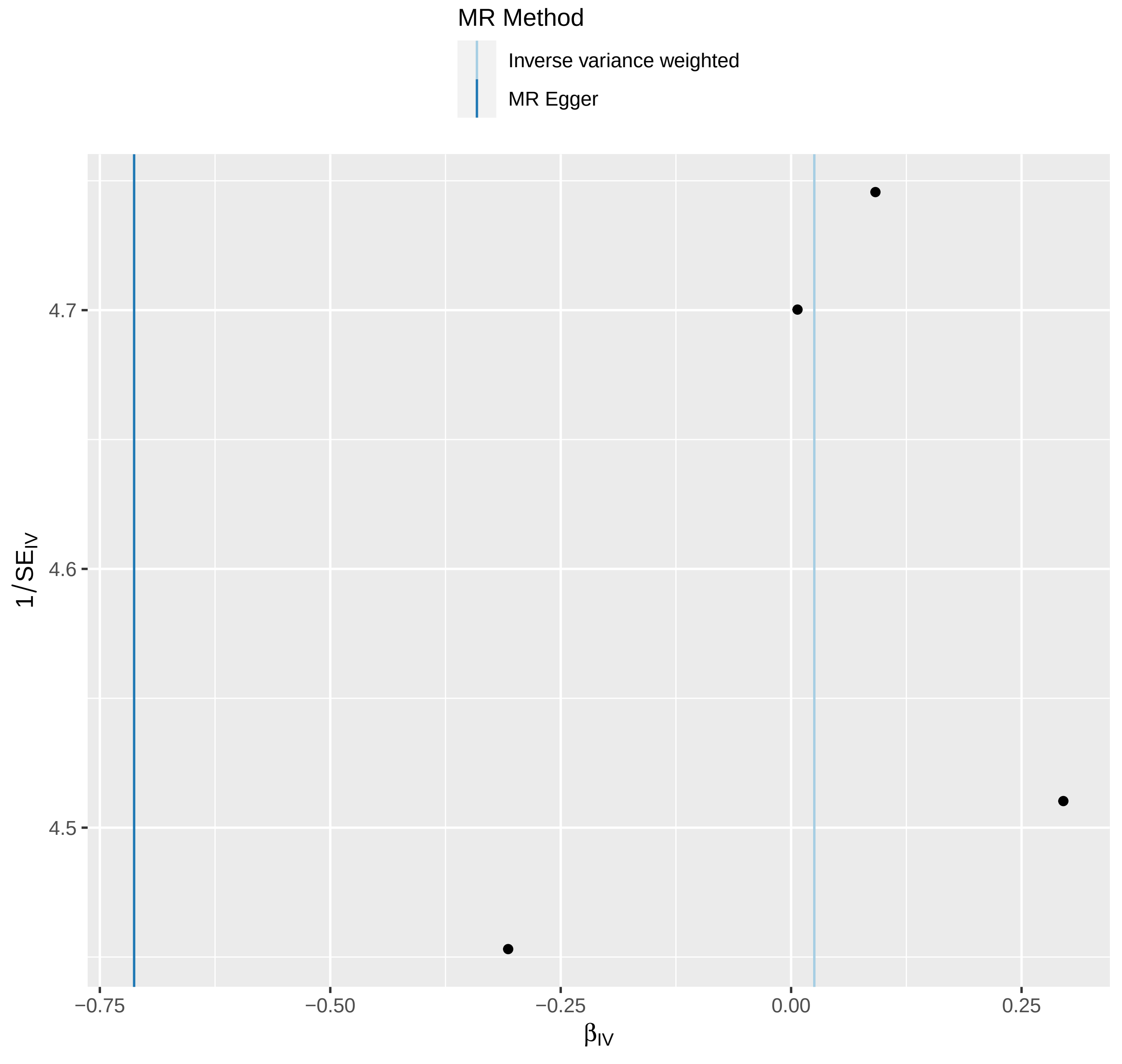

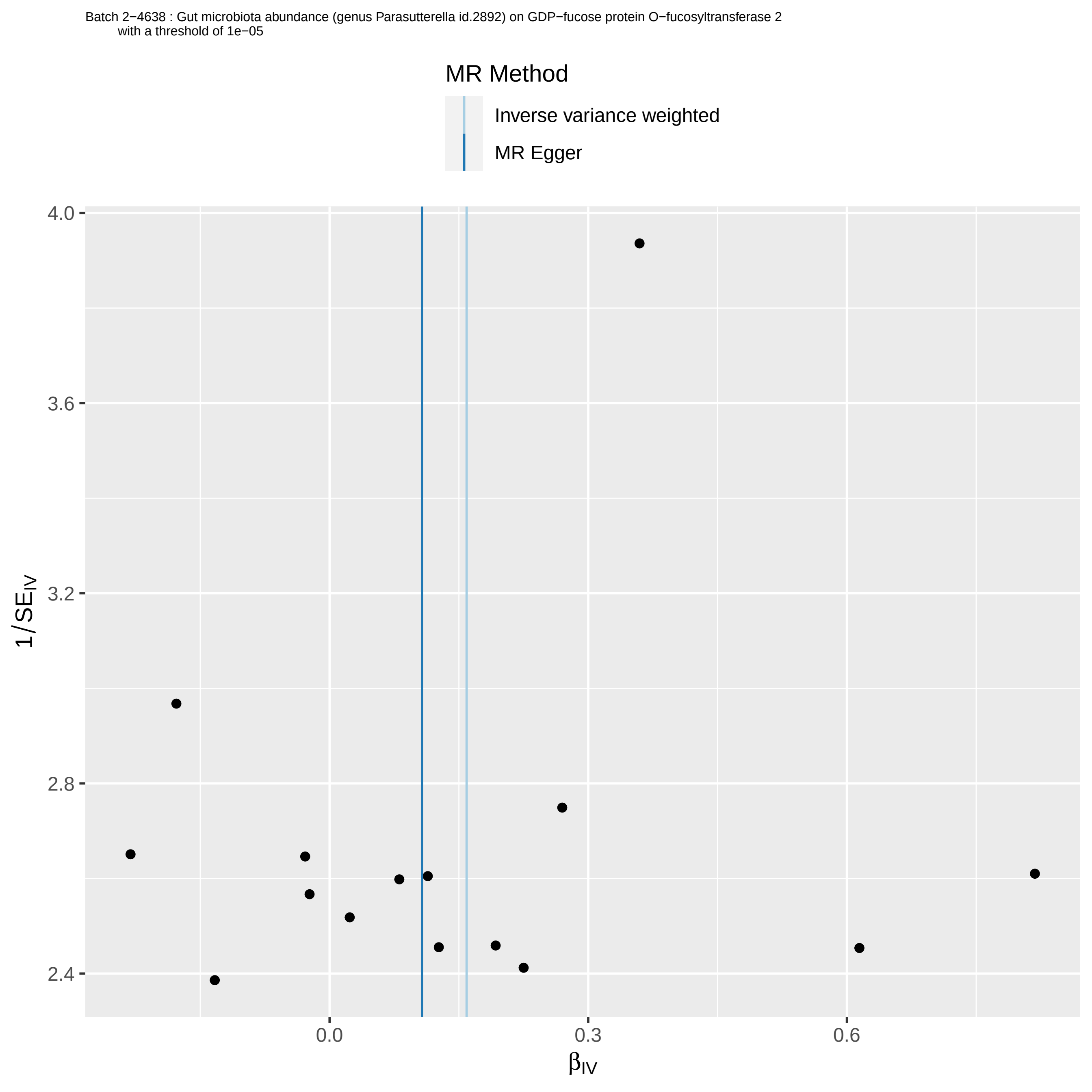
Batch 2−4638 : Gut microbiota abundance (genus Parasutterella id.2892) on GDP−fucose protein O−fucosyltransferase 2
with a threshold of 1e−05
MR Method
Inverse variance weighted
MR Egger
4.0
3.6
3.2
2.8
2.4
1/SE$_{IV}$
0.0
0.3
0.6
β$_{IV}$

Batch 2−4639 : Gut microbiota abundance (genus Catenibacterium id.2153) on Protein O−glucosyltransferase 1
with a threshold of 1e−05

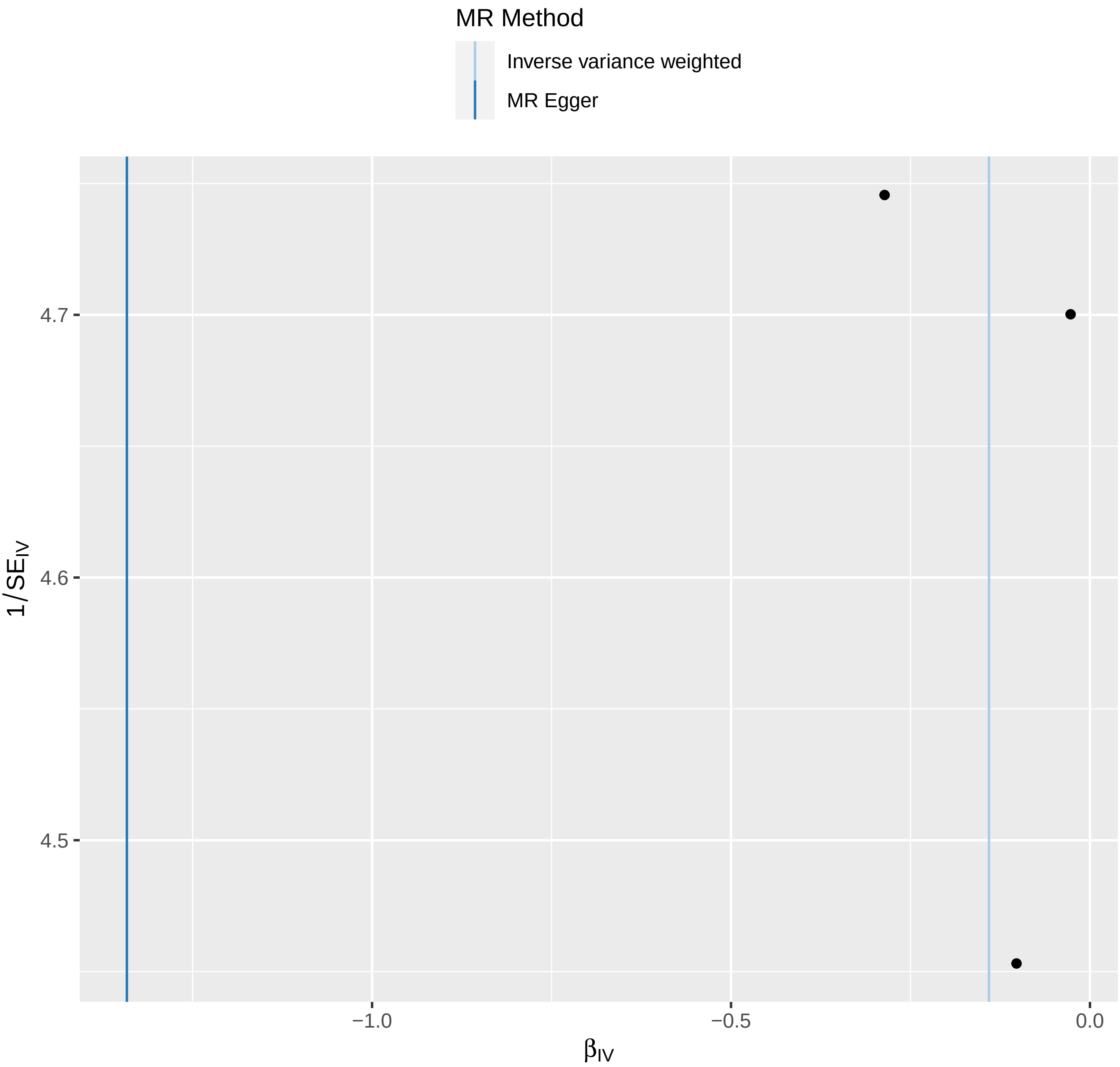

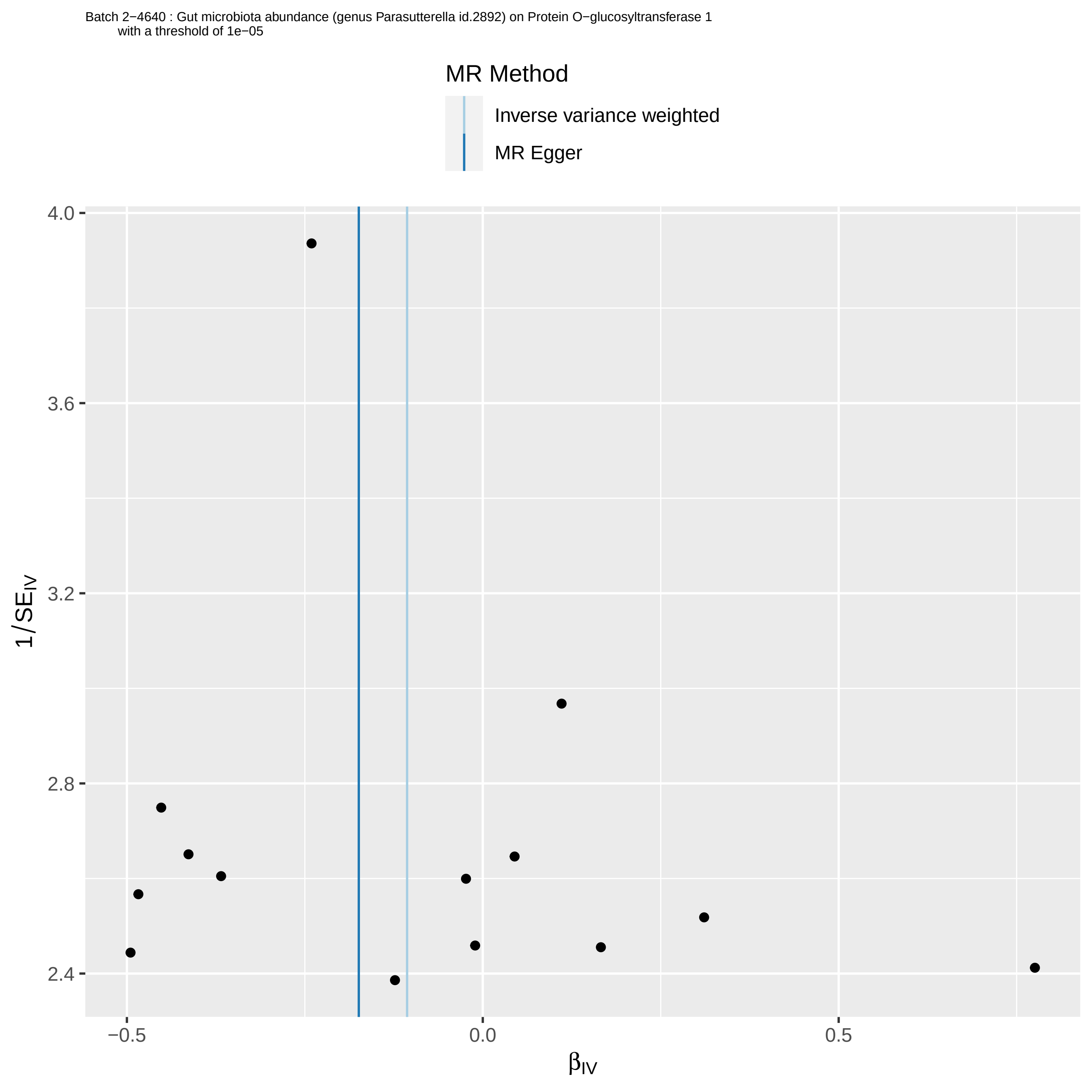

Batch 2−4640 : Gut microbiota abundance (genus Parasutterella id.2892) on Protein O−glucosyltransferase 1
with a threshold of 1e−05
MR Method
Inverse variance weighted
MR Egger
4.0
3.6
3.2
2.8
2.4
1/SE_IV
−0.5
0.0
0.5
β_IV

Batch 2−4641 : Gut microbiota abundance (genus Catenibacterium id.2153) on DNA polymerase eta with a threshold of 1e−05

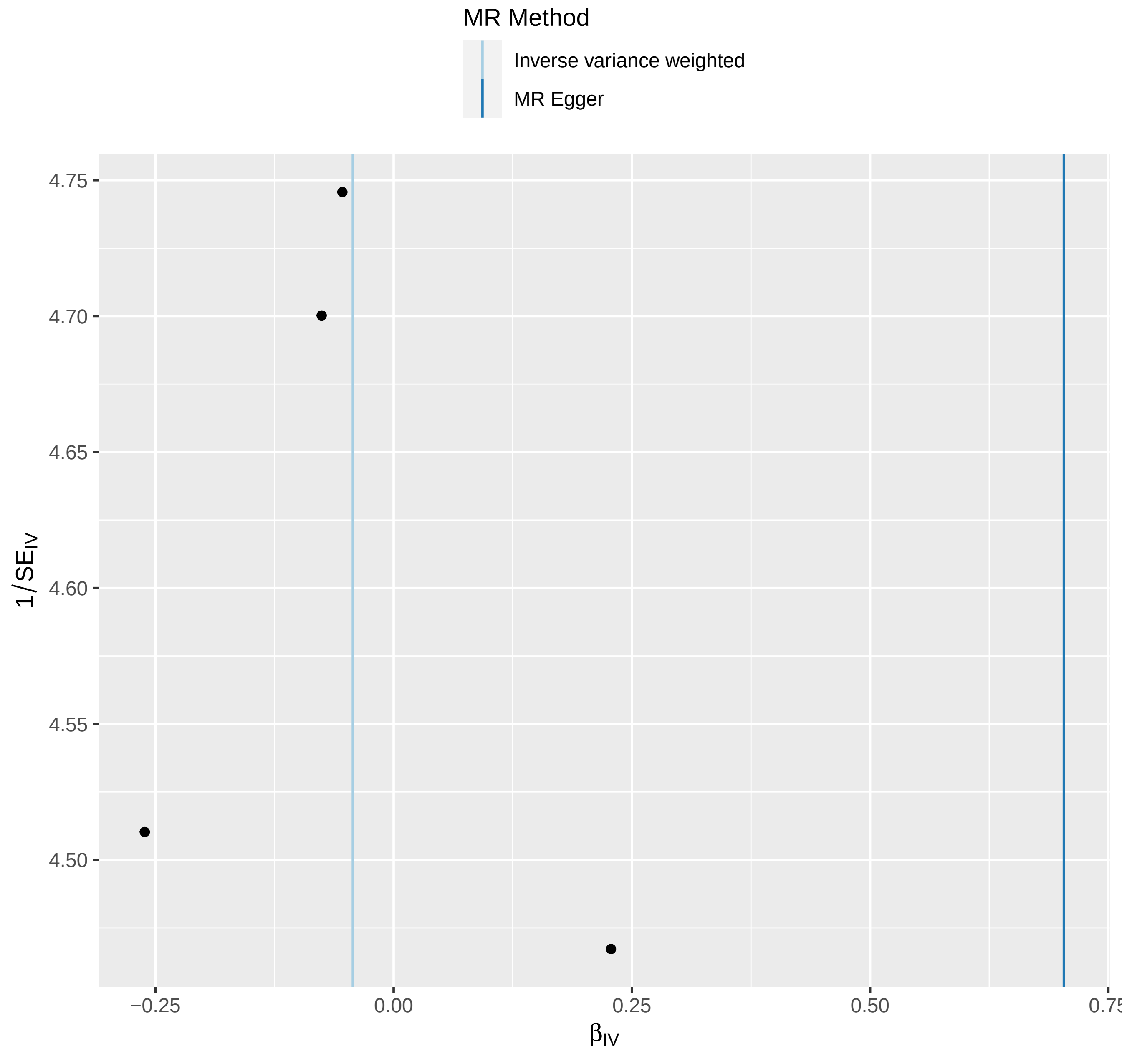

Batch 2−4642 : Gut microbiota abundance (genus Parasutterella id.2892) on DNA polymerase eta
with a threshold of 1e−05

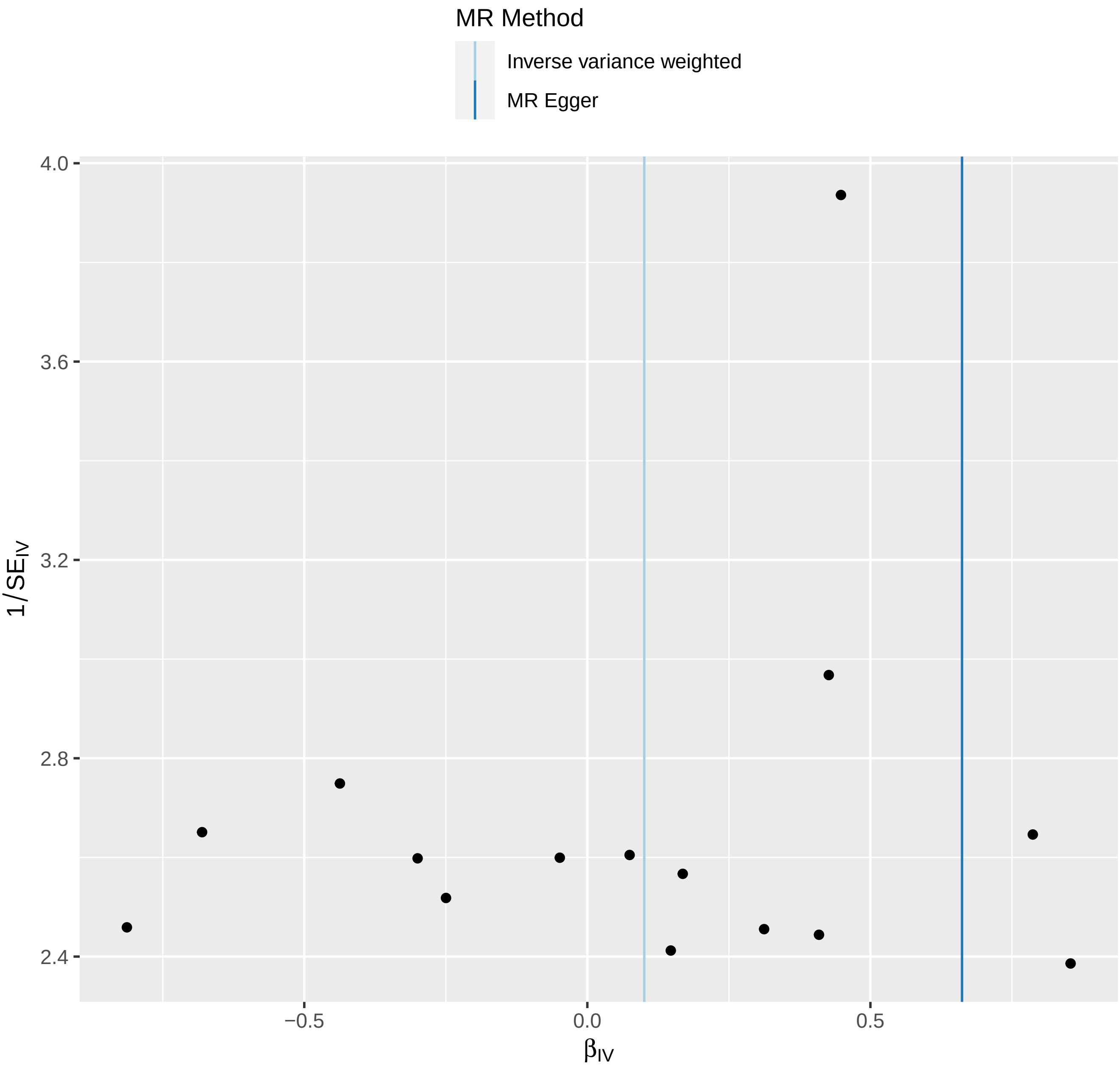

Batch 2−4643 : Gut microbiota abundance (genus Catenibacterium id.2153) on DNA polymerase iota
with a threshold of 1e−05

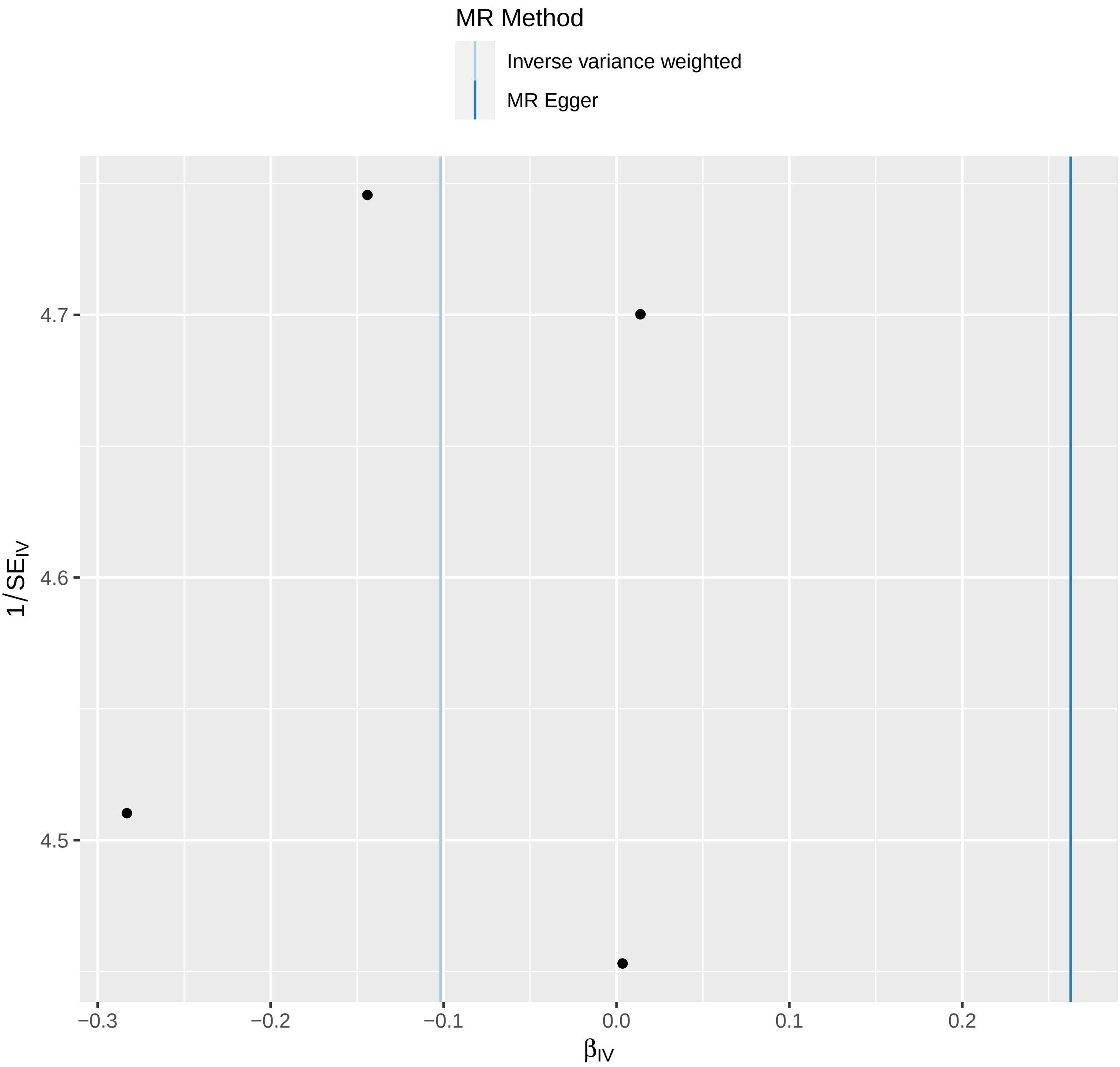

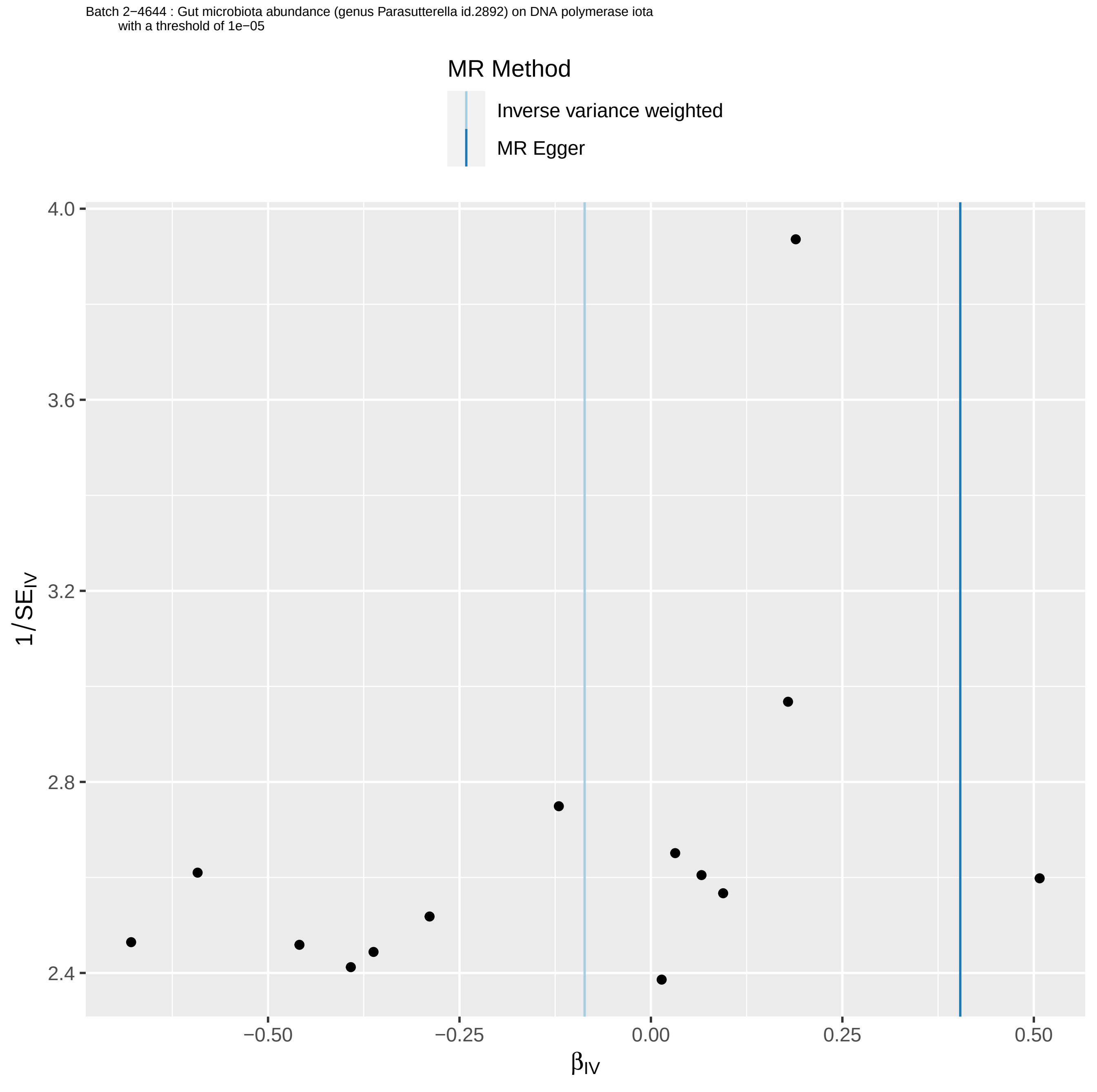
Batch 2−4644 : Gut microbiota abundance (genus Parasutterella id.2892) on DNA polymerase iota
with a threshold of 1e−05
MR Method
Inverse variance weighted
MR Egger
4.0
3.6
3.2
2.8
2.4
1/SE_IV
−0.50
−0.25
0.00
0.25
0.50
β_IV

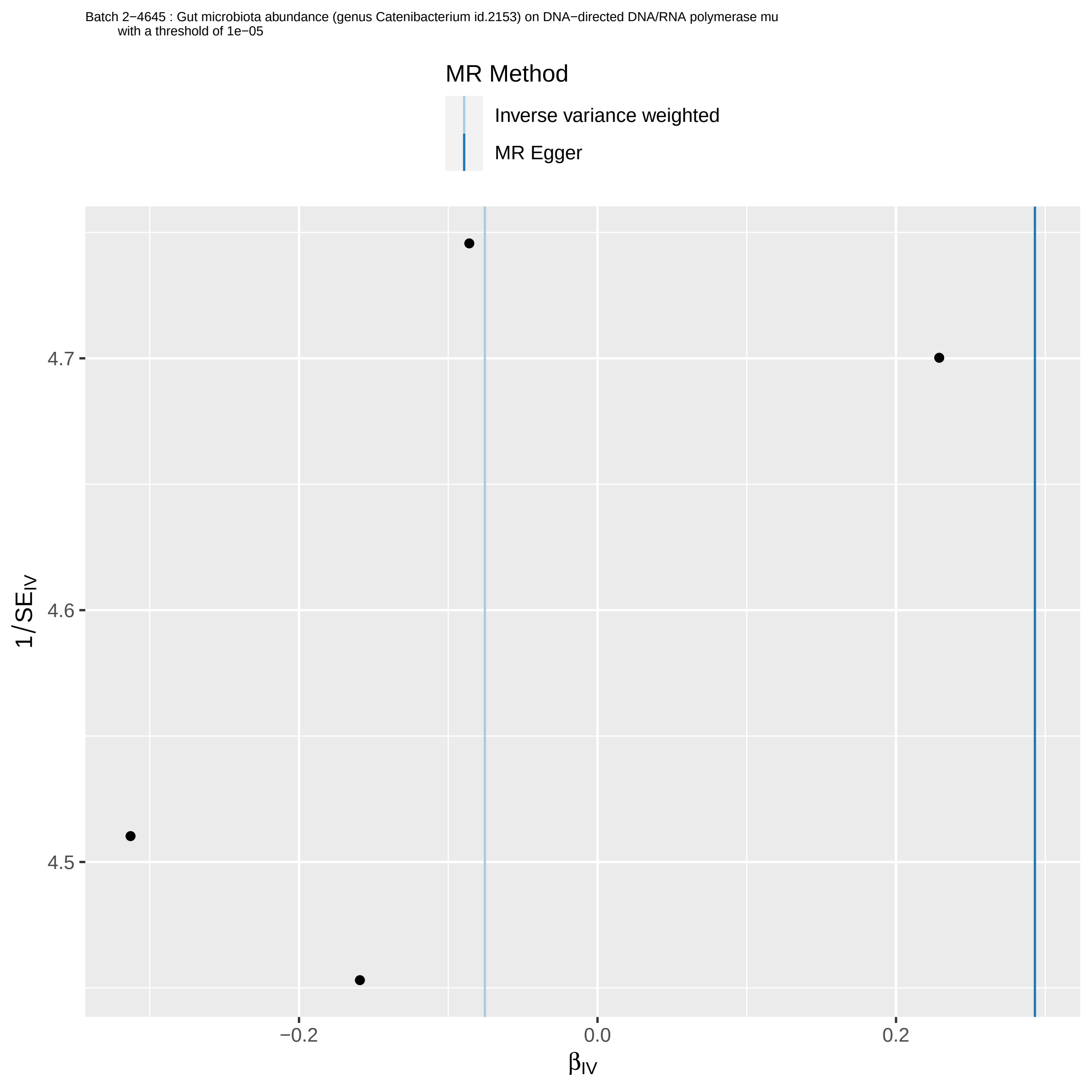

Batch 2−4645 : Gut microbiota abundance (genus Catenibacterium id.2153) on DNA−directed DNA/RNA polymerase mu
with a threshold of 1e−05
MR Method
Inverse variance weighted
MR Egger
4.7
4.6
4.5
1/SEIV
−0.2
0.0
0.2
βIV

Batch 2−4646 : Gut microbiota abundance (genus Parasutterella id.2892) on DNA−directed DNA/RNA polymerase mu
with a threshold of 1e−05

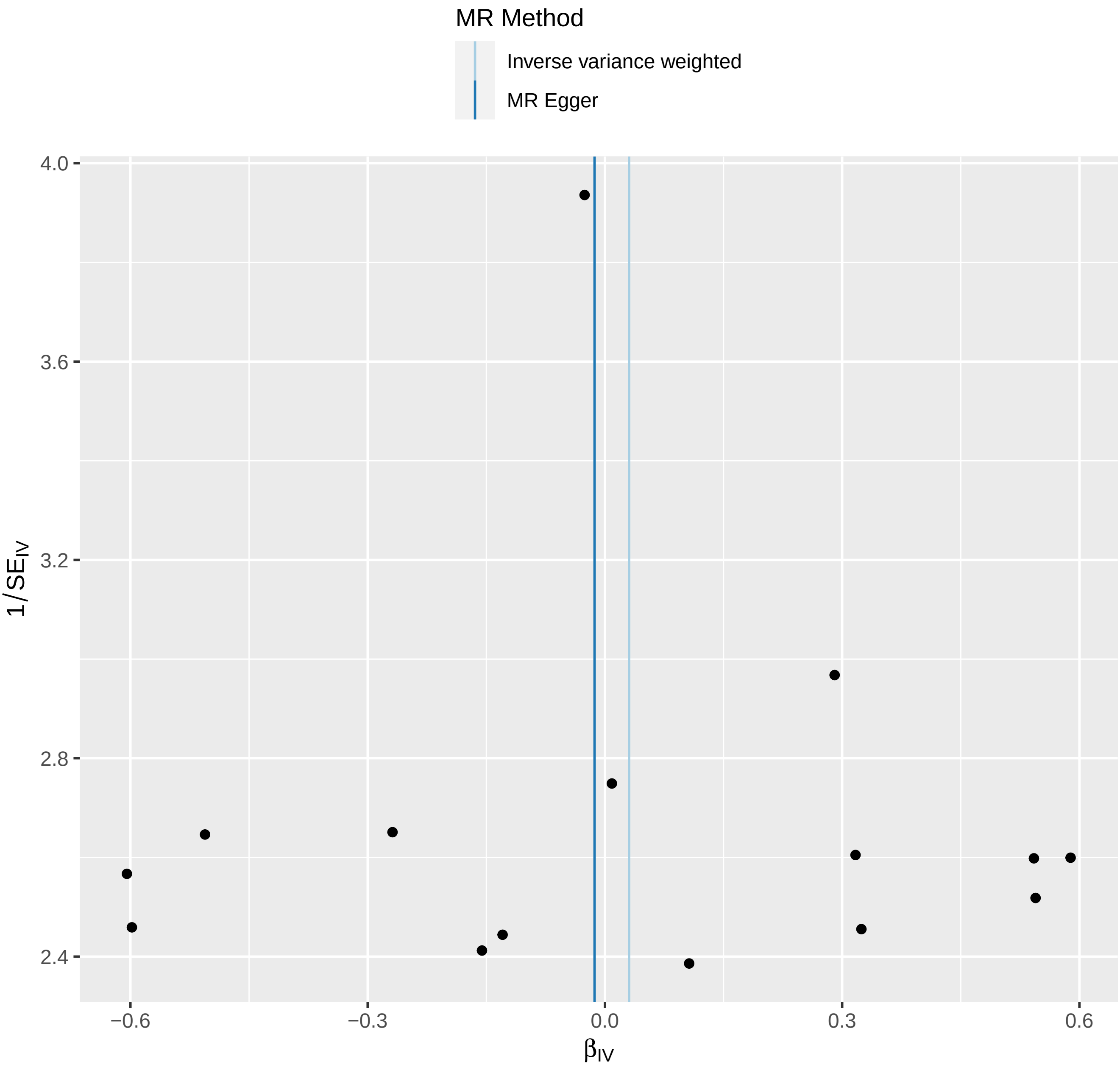

Batch 2−4647 : Gut microbiota abundance (genus Catenibacterium id.2153) on DNA−directed RNA polymerases I and III subunit RPAC1
with a threshold of 1e−05

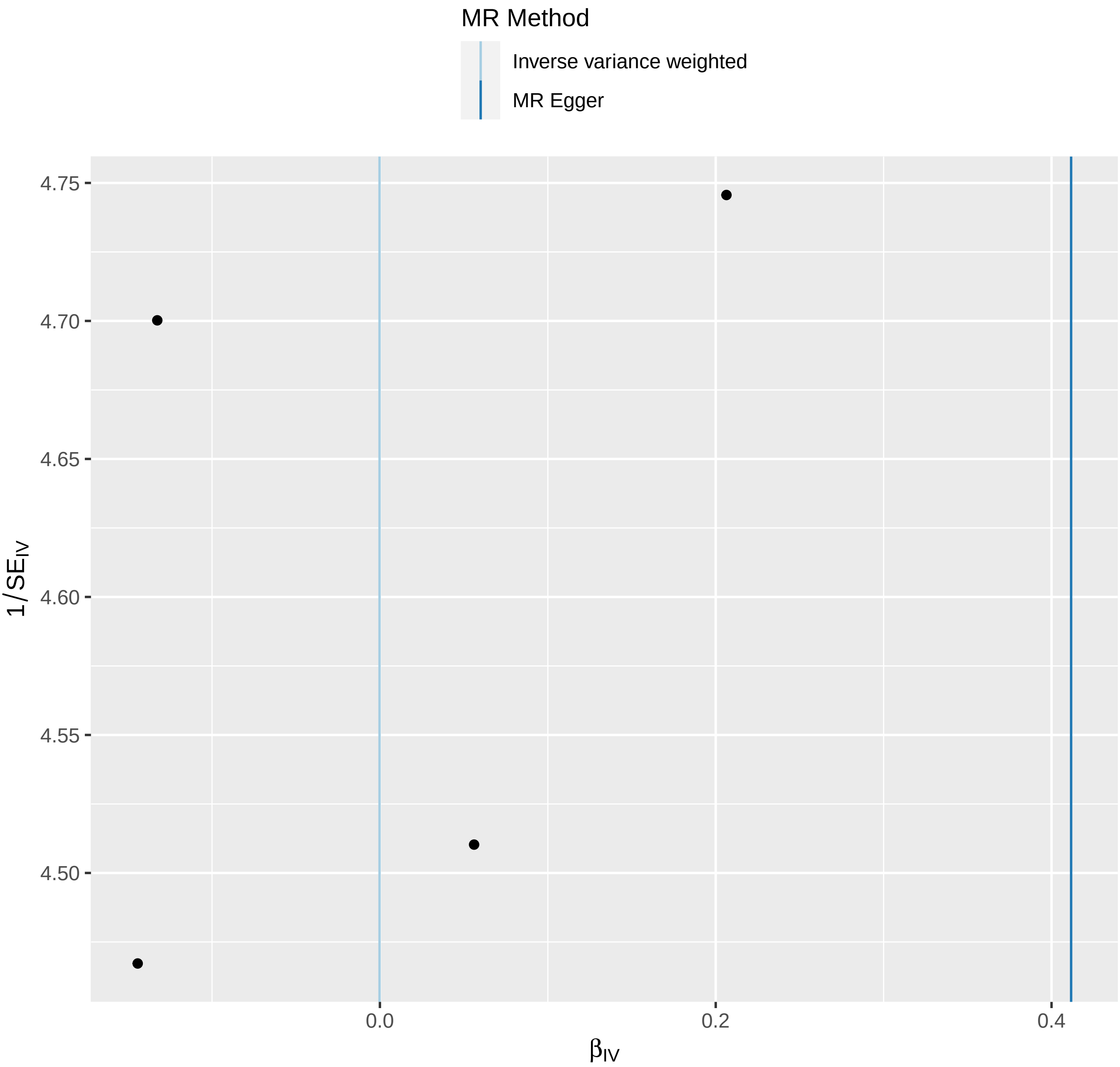

Batch 2−4648 : Gut microbiota abundance (genus Parasutterella id.2892) on DNA−directed RNA polymerases I and III subunit RPAC1
with a threshold of 1e−05

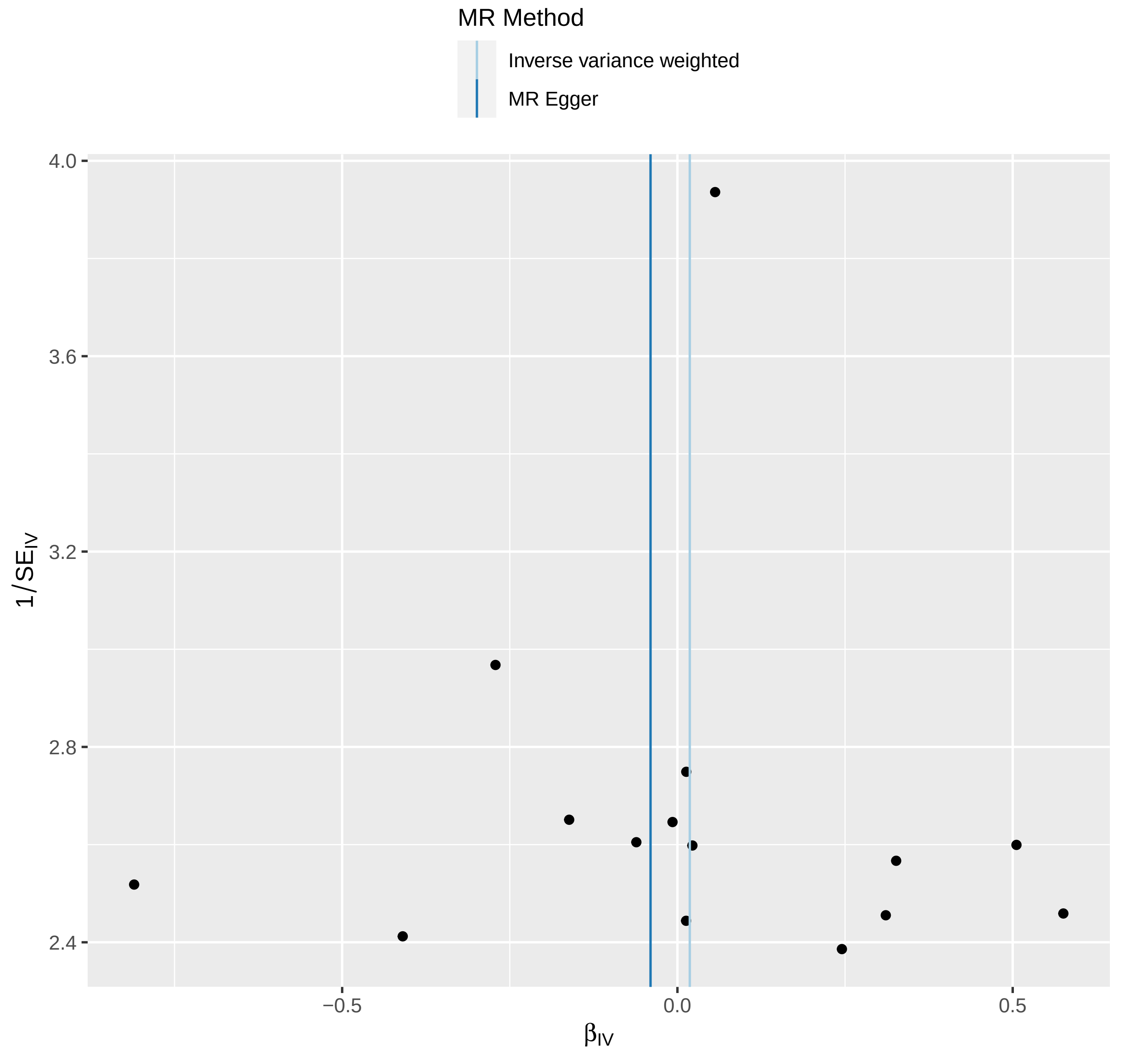

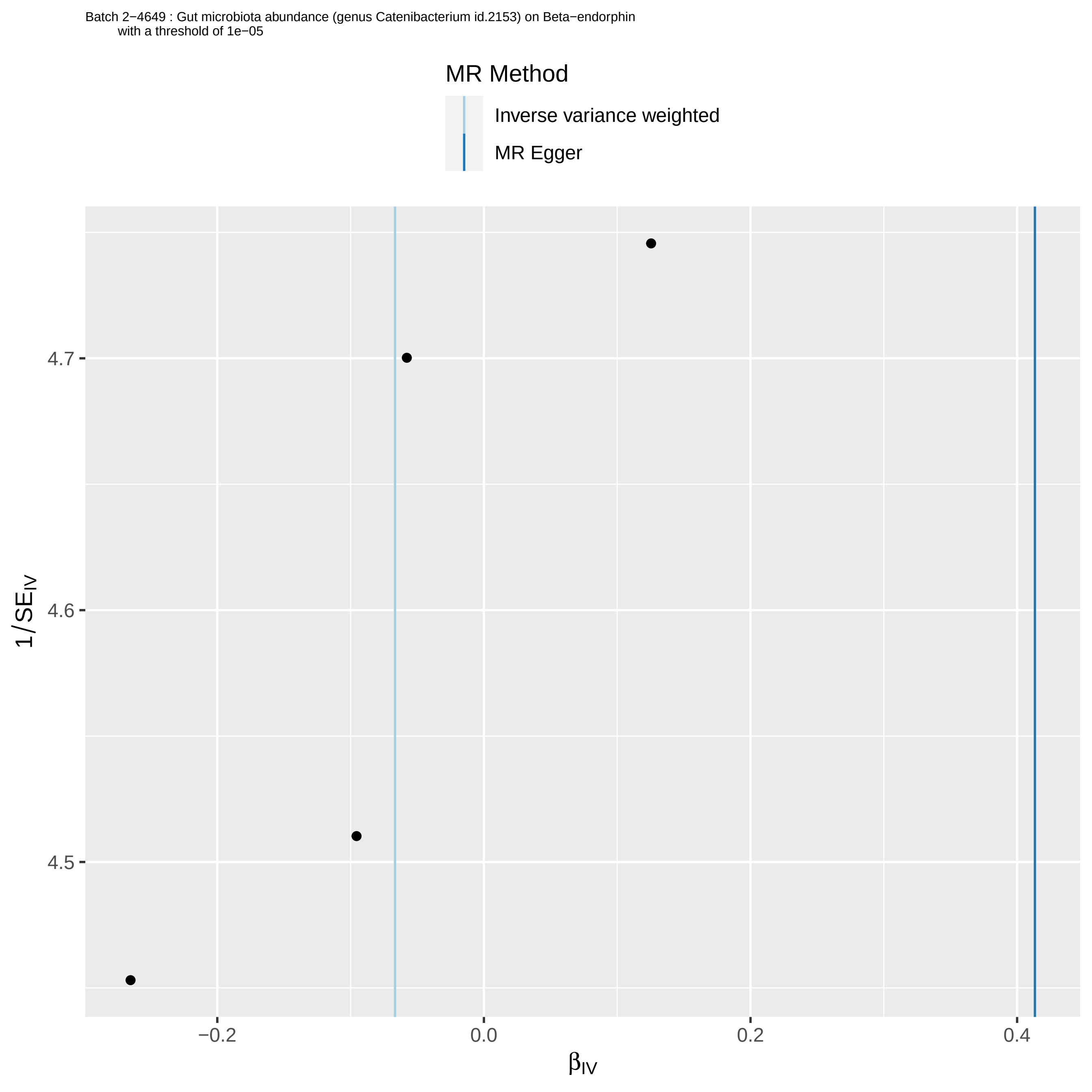
Batch 2−4649 : Gut microbiota abundance (genus Catenibacterium id.2153) on Beta−endorphin
with a threshold of 1e−05
MR Method
Inverse variance weighted
MR Egger
4.7
4.6
4.5
1/SE_IV
−0.2
0.0
0.2
0.4
β_IV

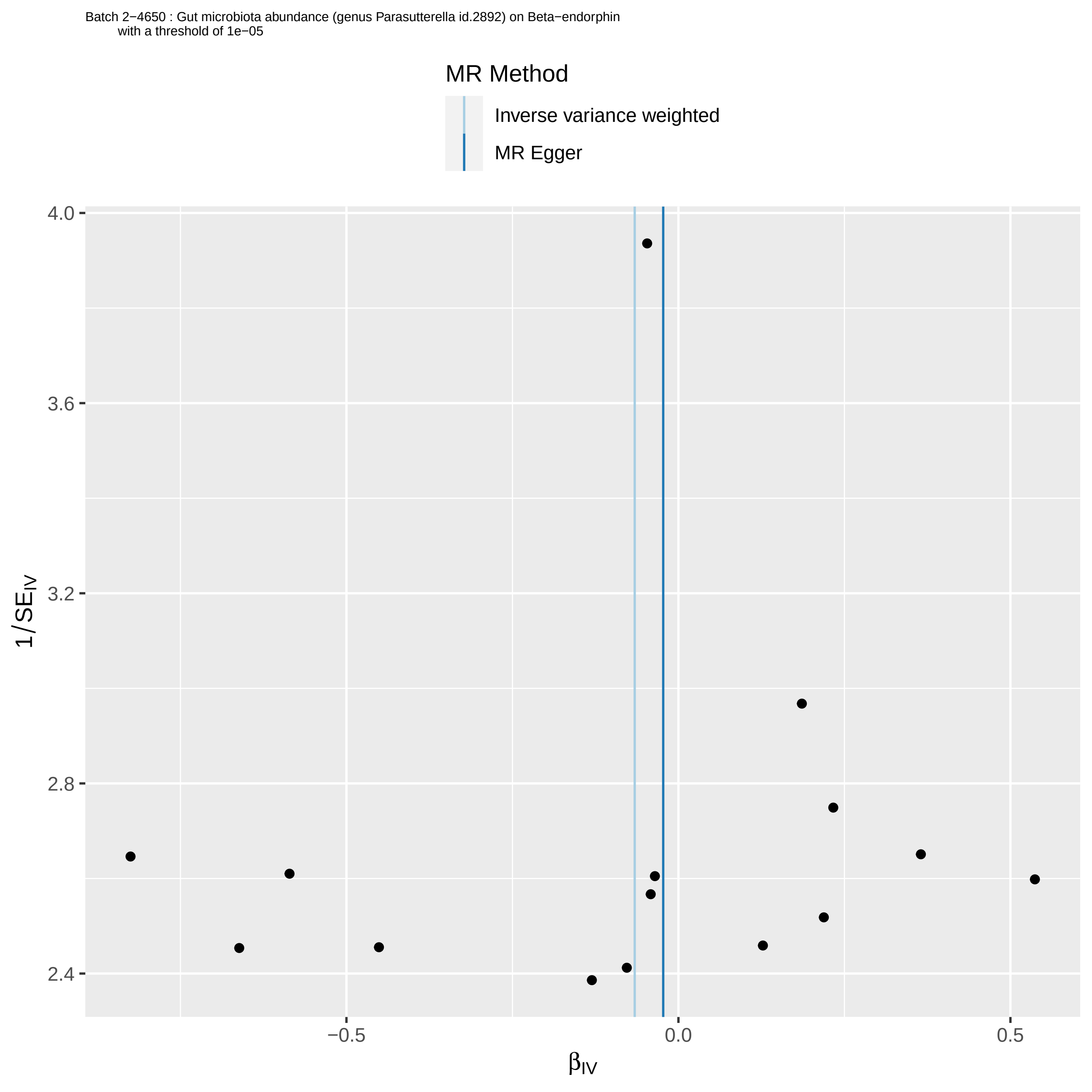

Batch 2−4650 : Gut microbiota abundance (genus Parasutterella id.2892) on Beta−endorphin
with a threshold of 1e−05
MR Method
Inverse variance weighted
MR Egger
4.0
3.6
3.2
2.8
2.4
1/SE_IV
−0.5
0.0
0.5
β_IV

Batch 2−4651 : Gut microbiota abundance (genus Catenibacterium id.2153) on Corticotropin
with a threshold of 1e−05

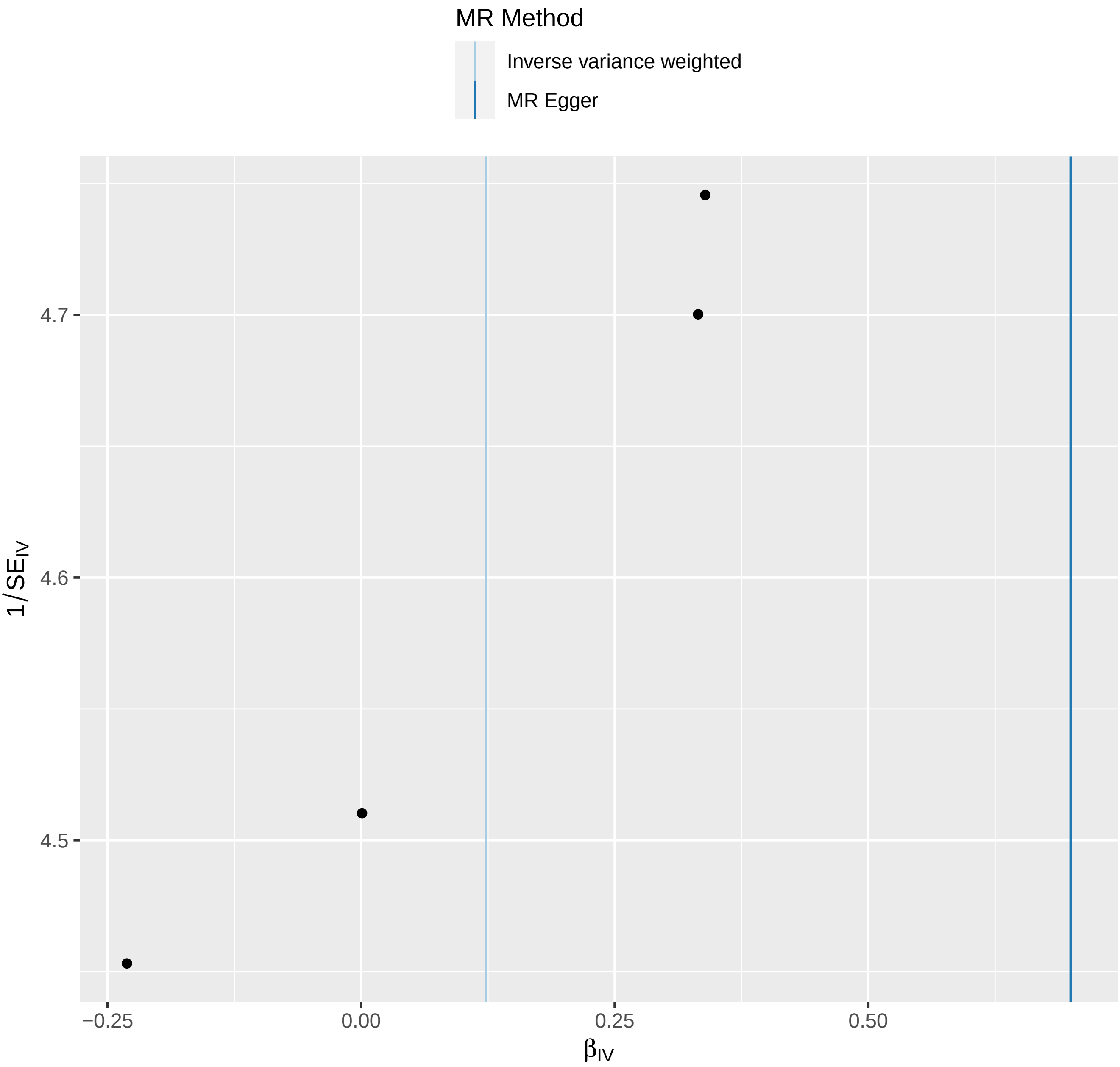

Batch 2−4652 : Gut microbiota abundance (genus Parasutterella id.2892) on Corticotropin
with a threshold of 1e−05

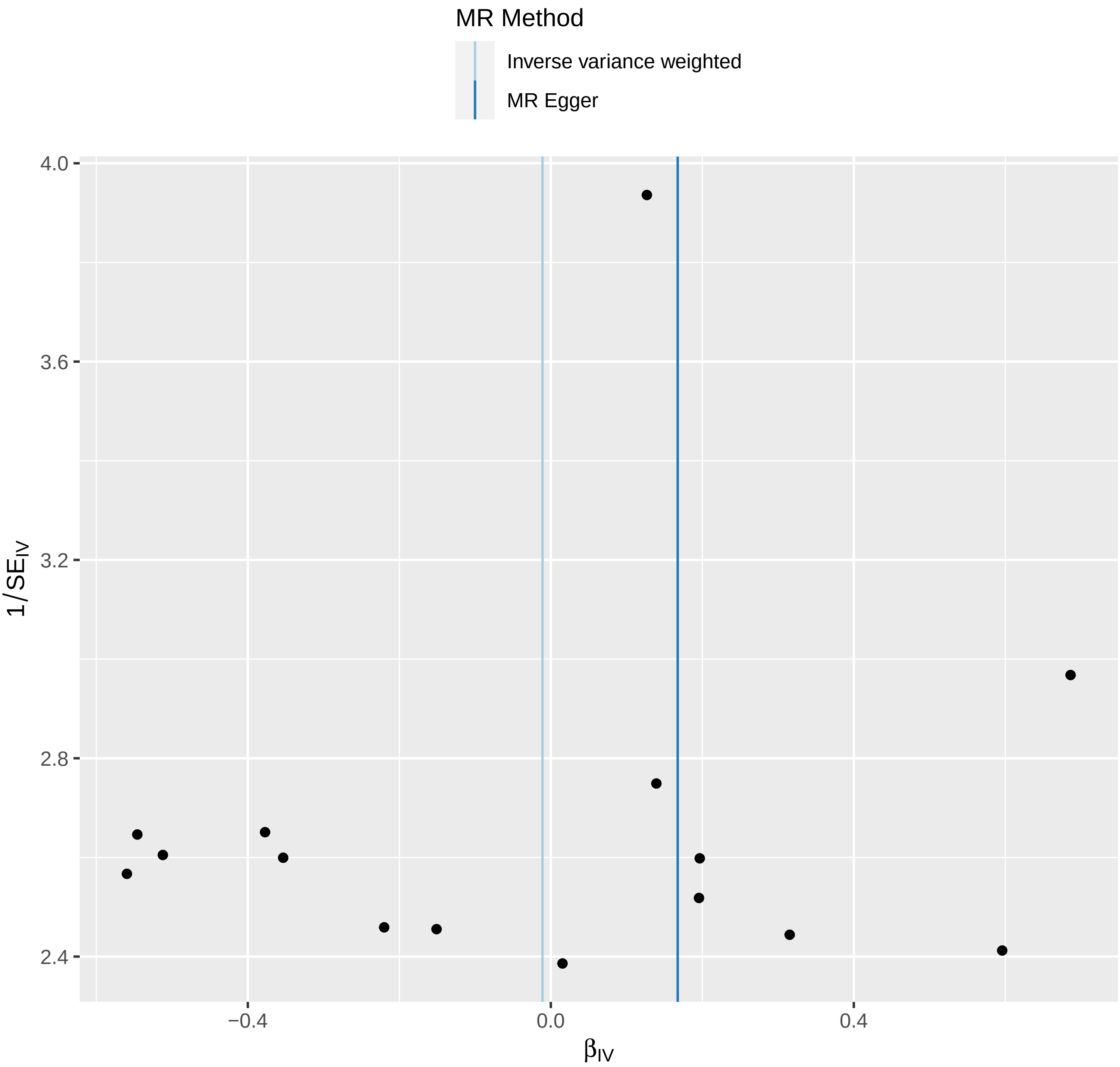

Batch 2−4653 : Gut microbiota abundance (genus Catenibacterium id.2153) on Pro−opiomelanocortin
with a threshold of 1e−05

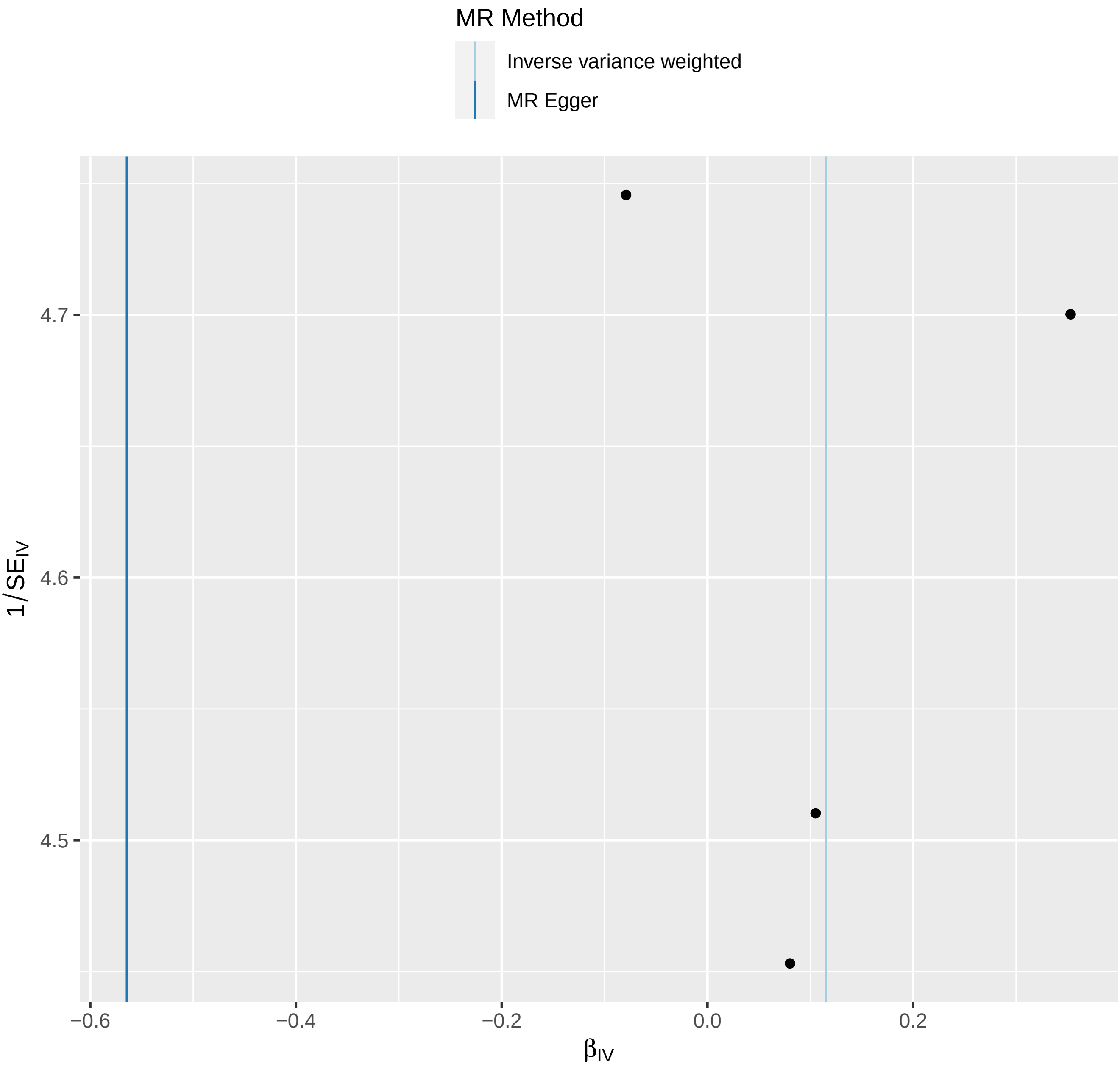

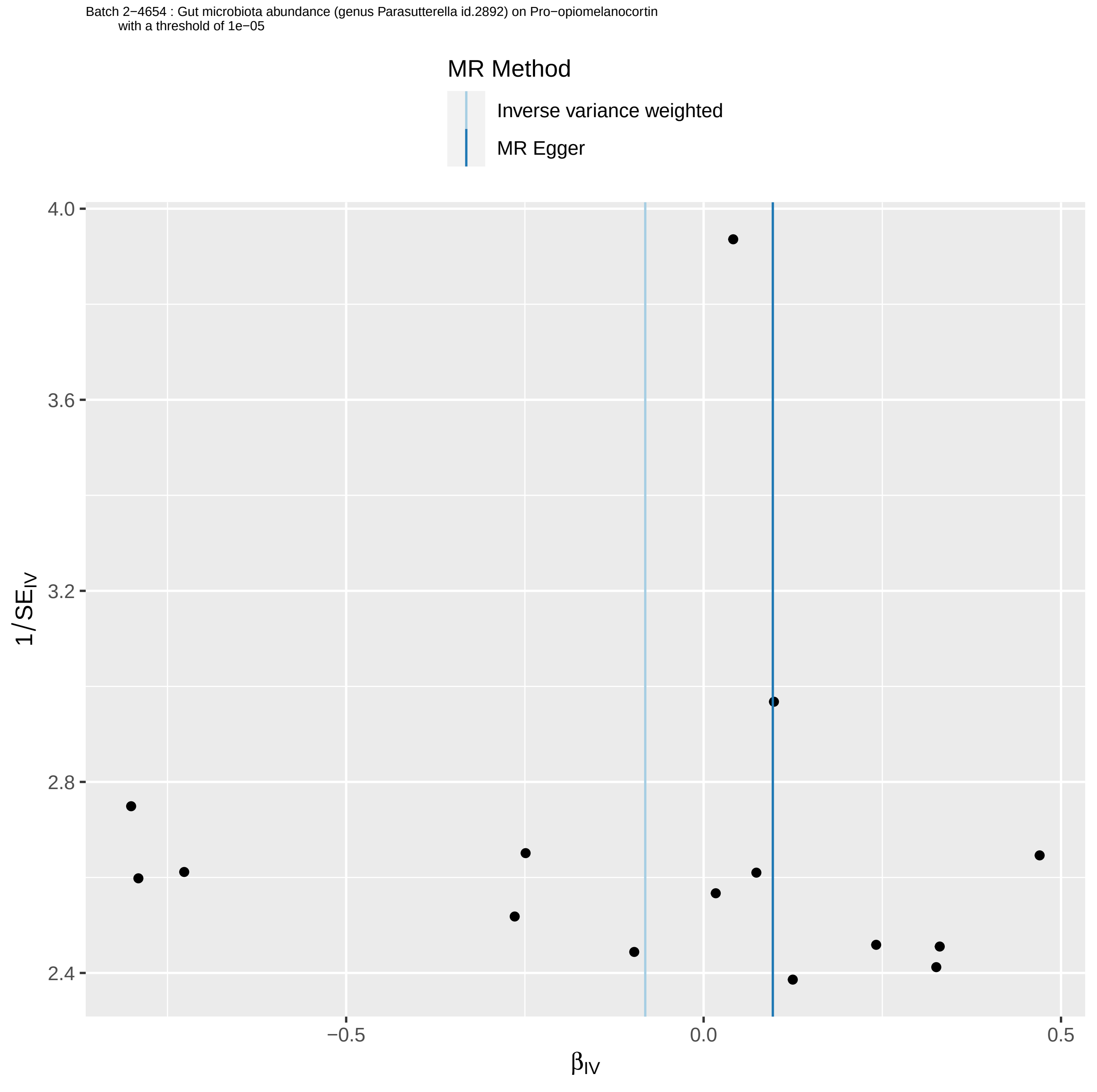

Batch 2−4654 : Gut microbiota abundance (genus Parasutterella id.2892) on Pro−opiomelanocortin
with a threshold of 1e−05
MR Method
Inverse variance weighted
MR Egger
4.0
3.6
3.2
2.8
2.4
1/SE_IV
−0.5
0.0
0.5
β_IV

Batch 2−4655 : Gut microbiota abundance (genus Catenibacterium id.2153) on Protein O−linked−mannose beta−1,2−N−acetylglucosaminyltransferase 1 with a threshold of 1e−05

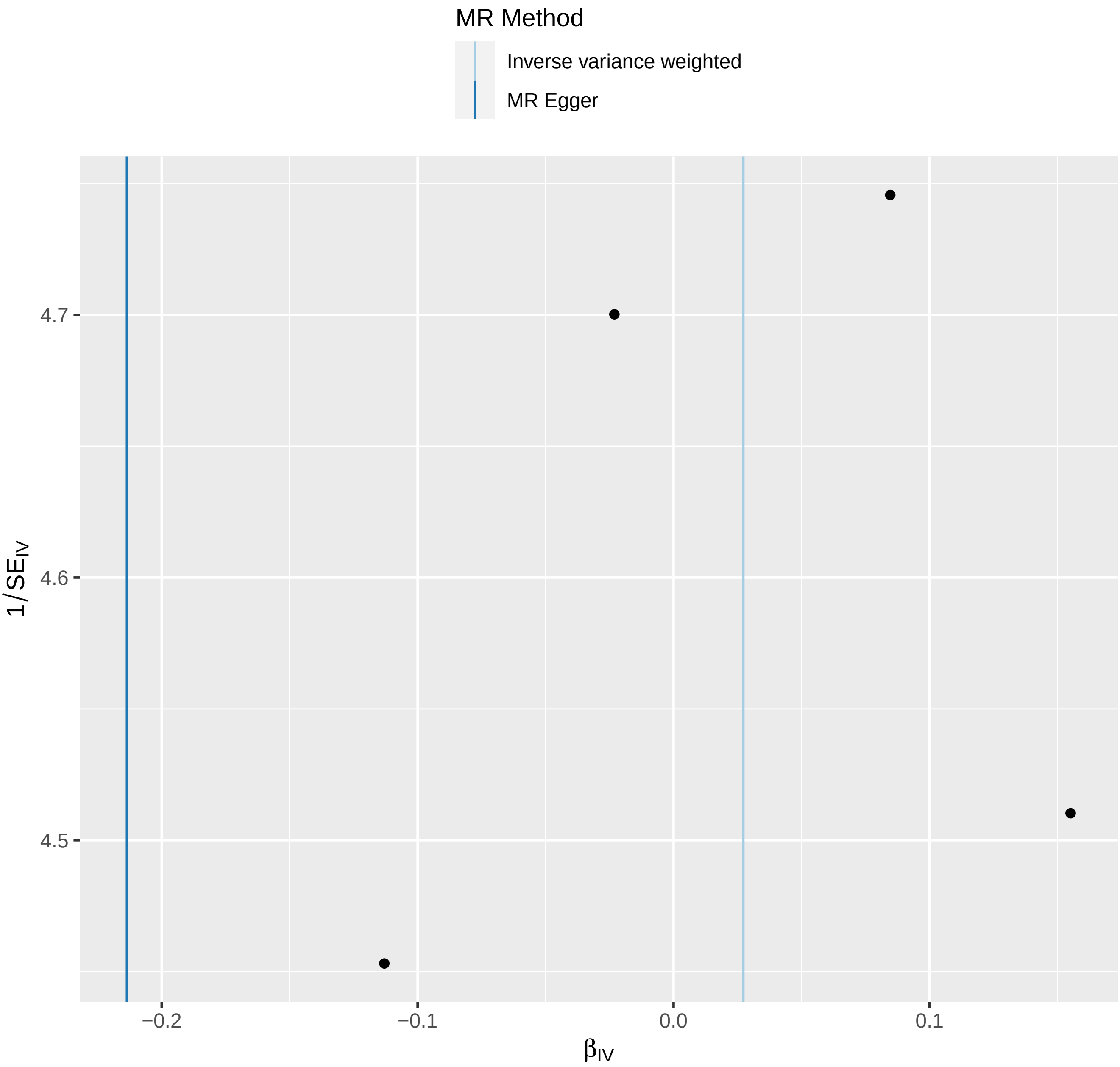

Batch 2−4656 : Gut microbiota abundance (genus Parasutterella id.2892) on Protein O−linked−mannose beta−1,2−N−acetylglucosaminyltransferase 1 with a threshold of 1e−05

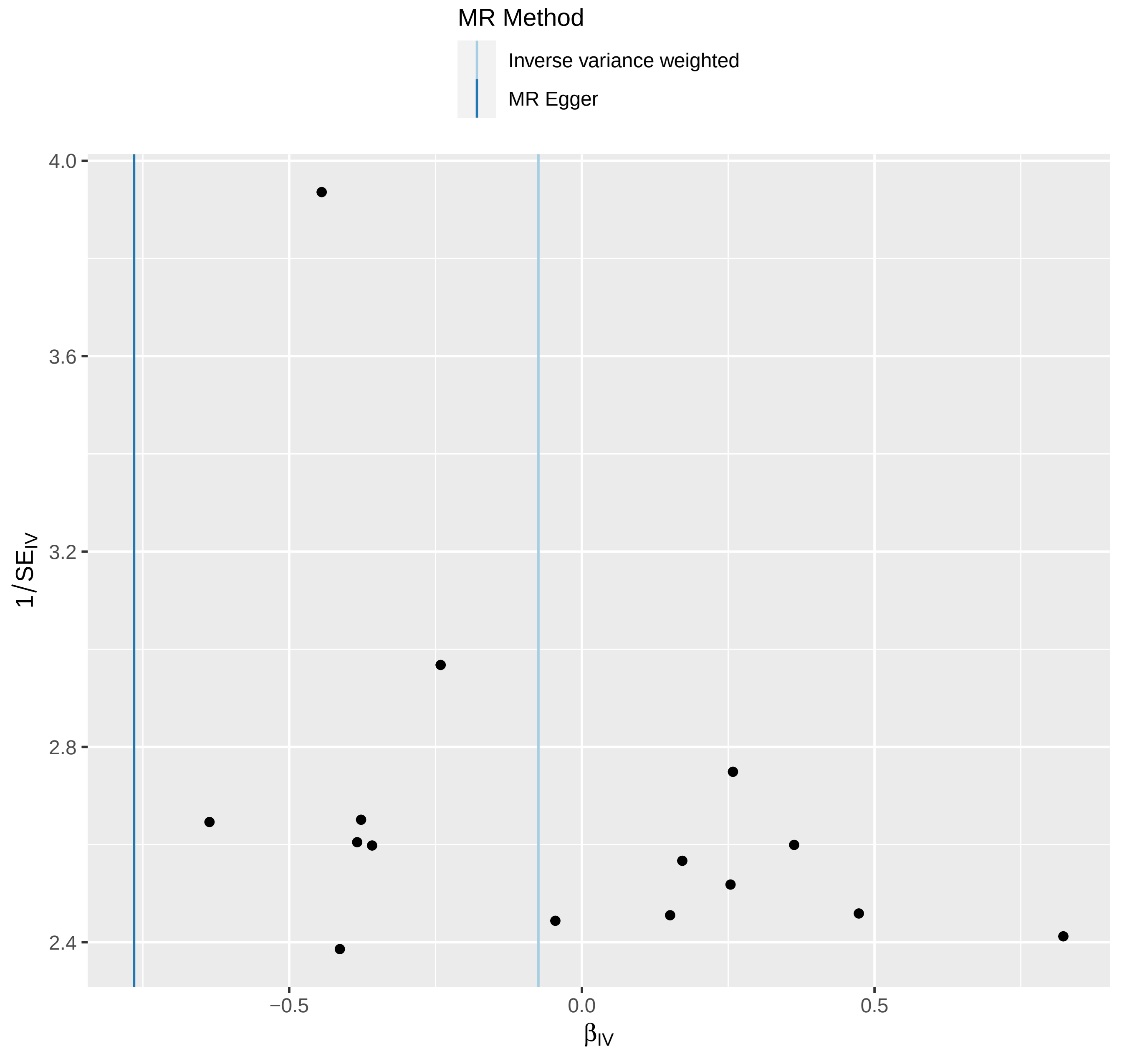

Batch 2−4657 : Gut microbiota abundance (genus Catenibacterium id.2153) on Protein O−linked−mannose beta−1,4−N−acetylglucosaminyltransferase 2 with a threshold of 1e−05

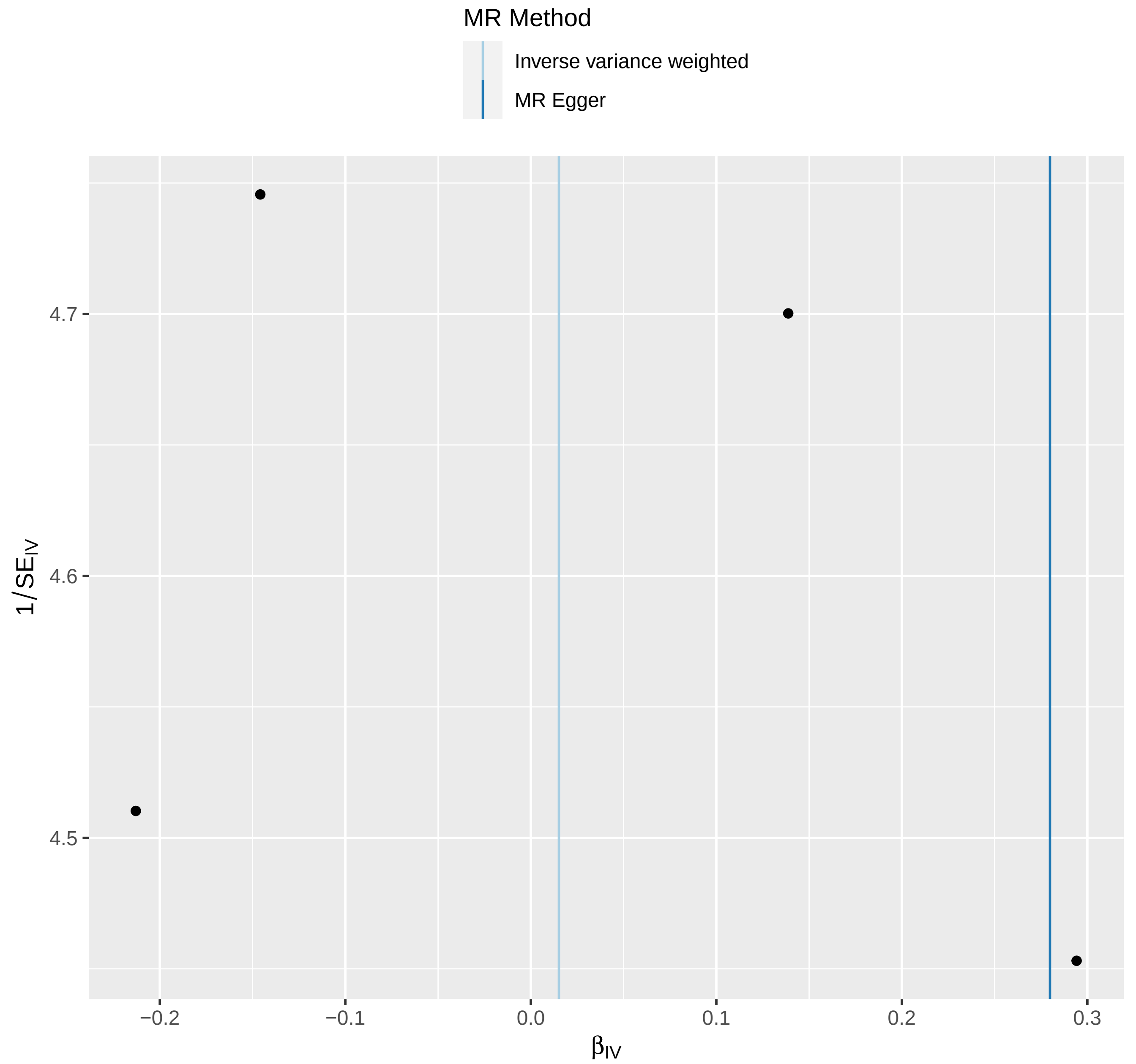

Batch 2−4658 : Gut microbiota abundance (genus Parasutterella id.2892) on Protein O−linked−mannose beta−1,4−N−acetylglucosaminyltransferase 2
with a threshold of 1e−05

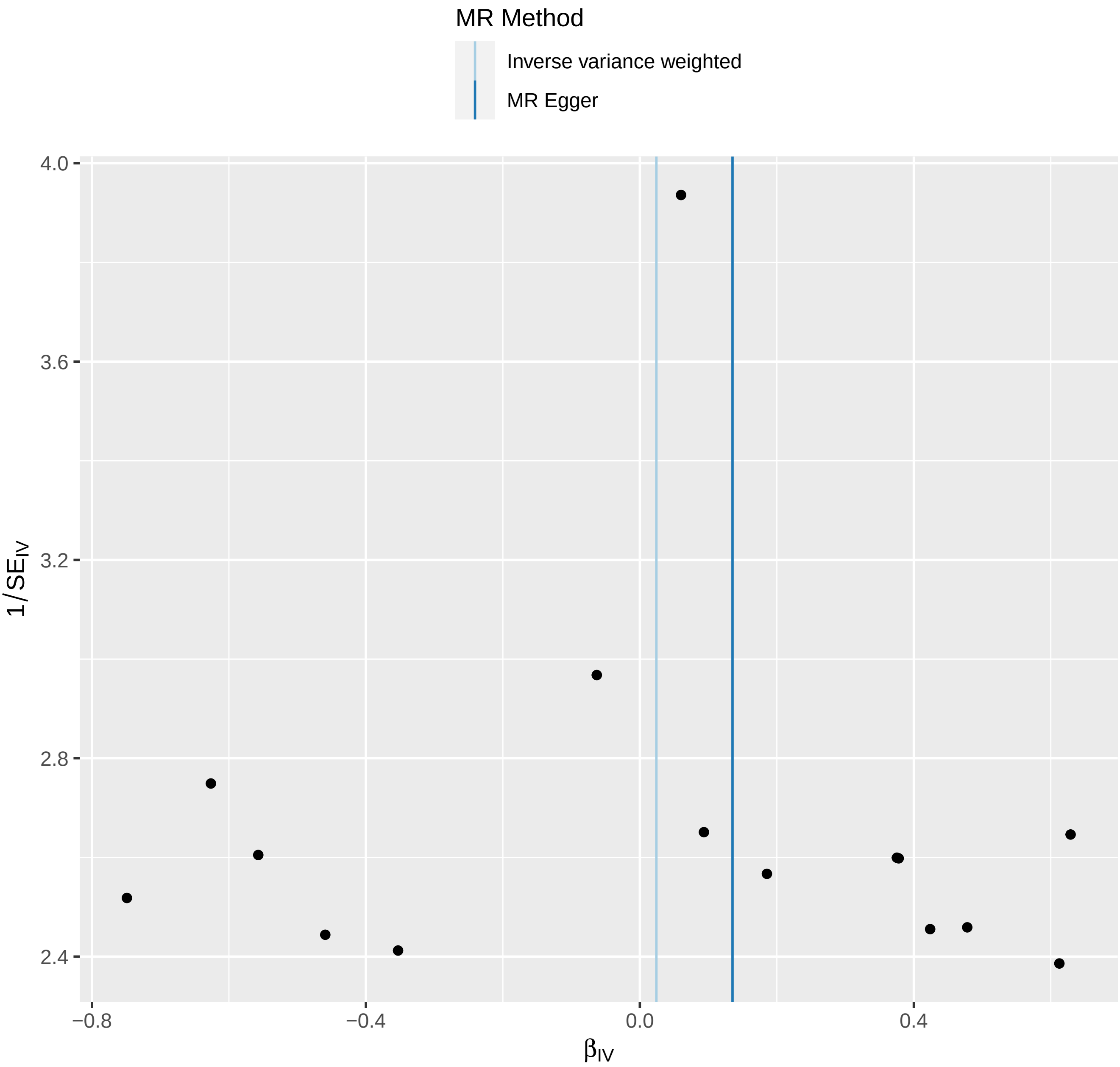

Batch 2−4659 : Gut microbiota abundance (genus Catenibacterium id.2153) on NADPH−−cytochrome P450 reductase
with a threshold of 1e−05

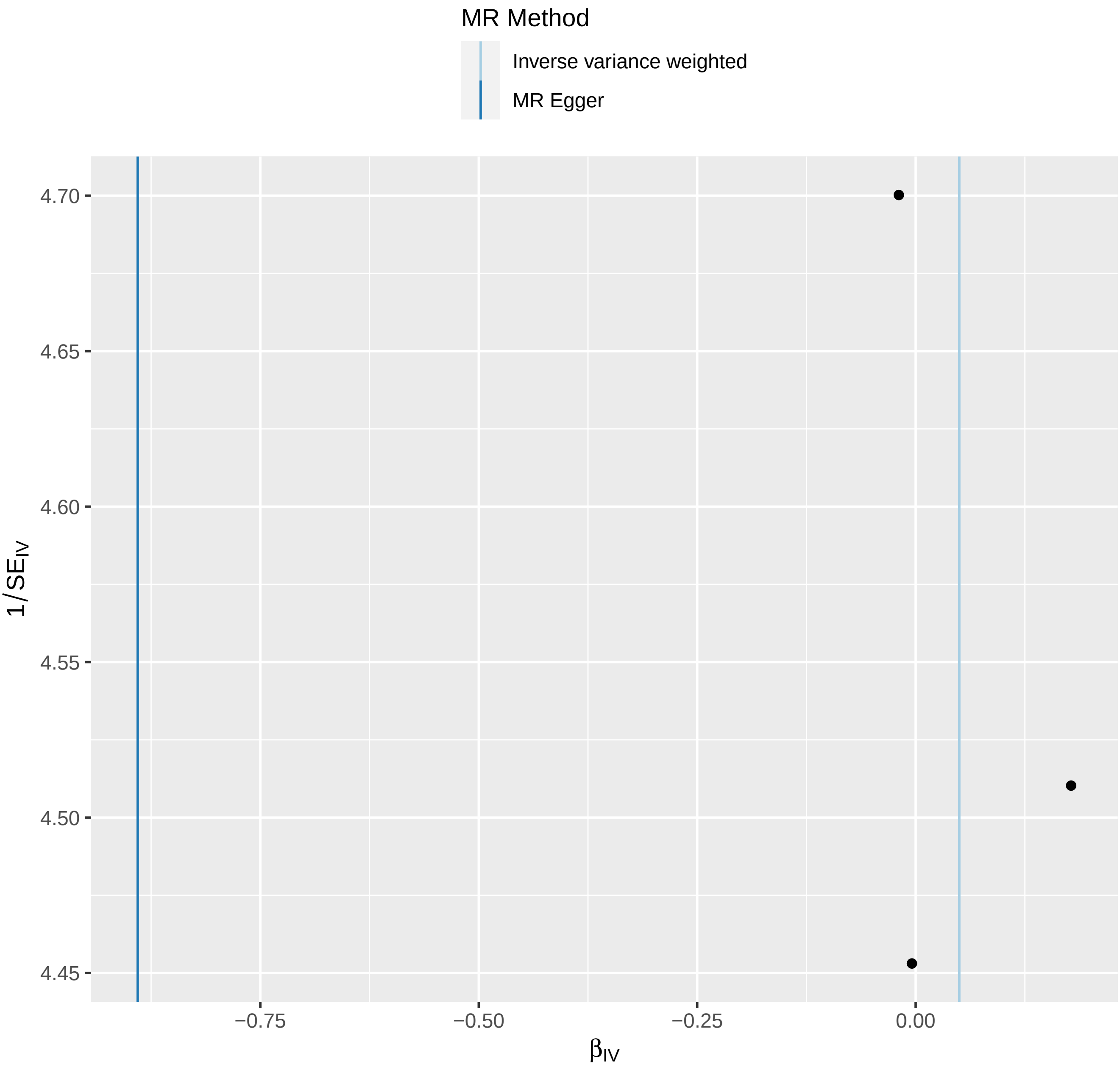

Batch 2−4660 : Gut microbiota abundance (genus Parasutterella id.2892) on NADPH−−cytochrome P450 reductase
with a threshold of 1e−05

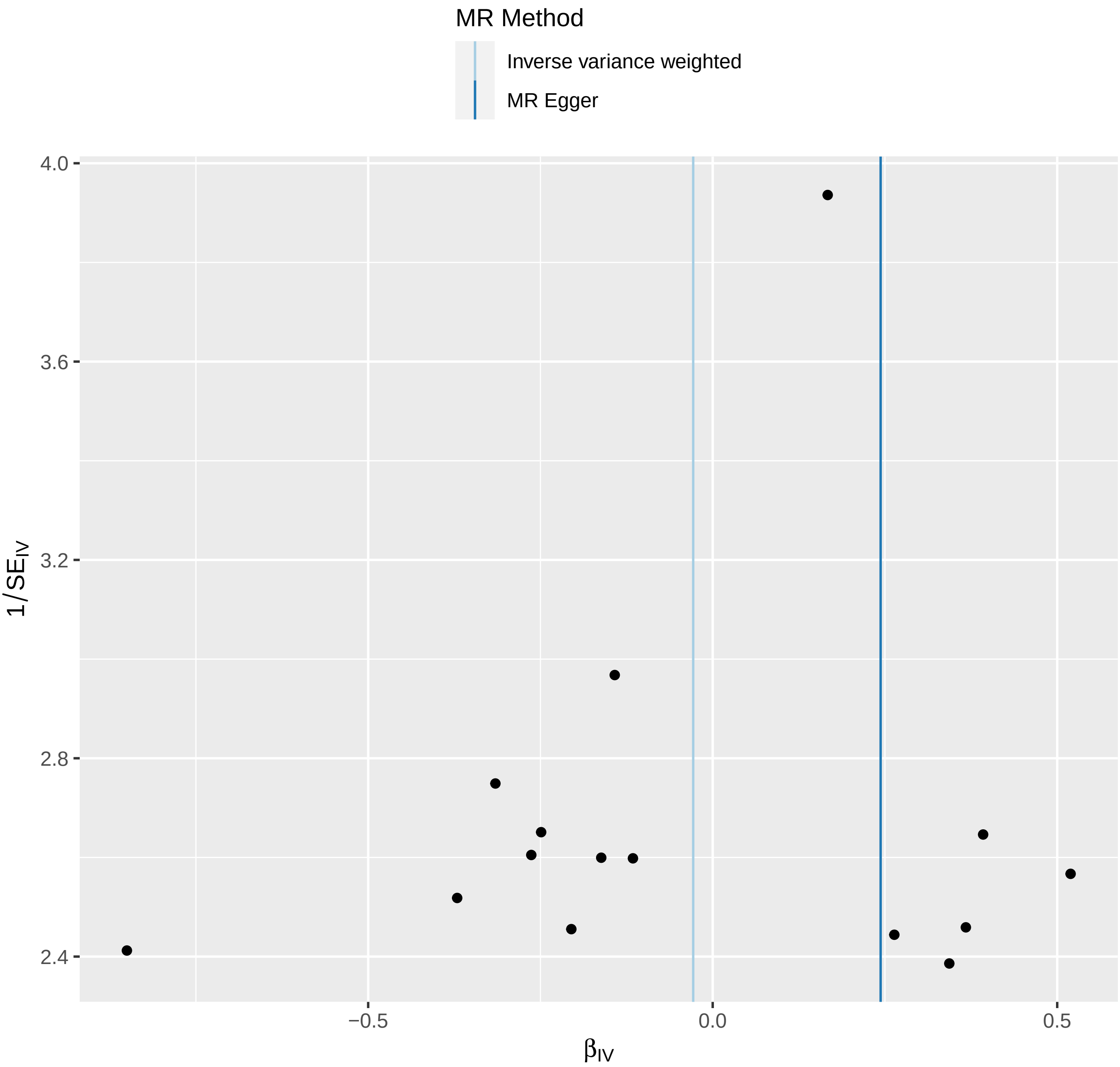

Batch 2−4661 : Gut microbiota abundance (genus Catenibacterium id.2153) on Periostin
with a threshold of 1e−05

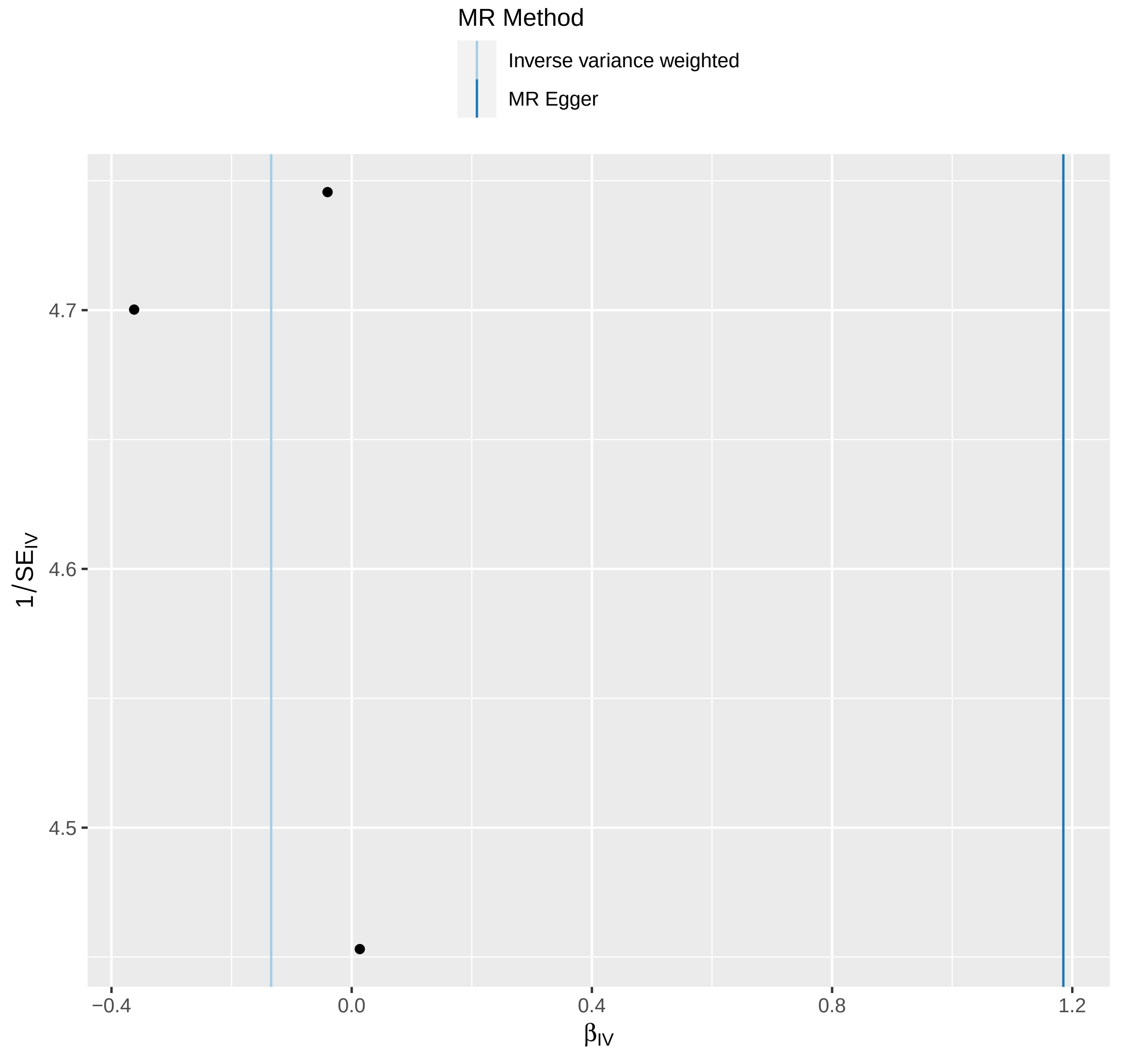

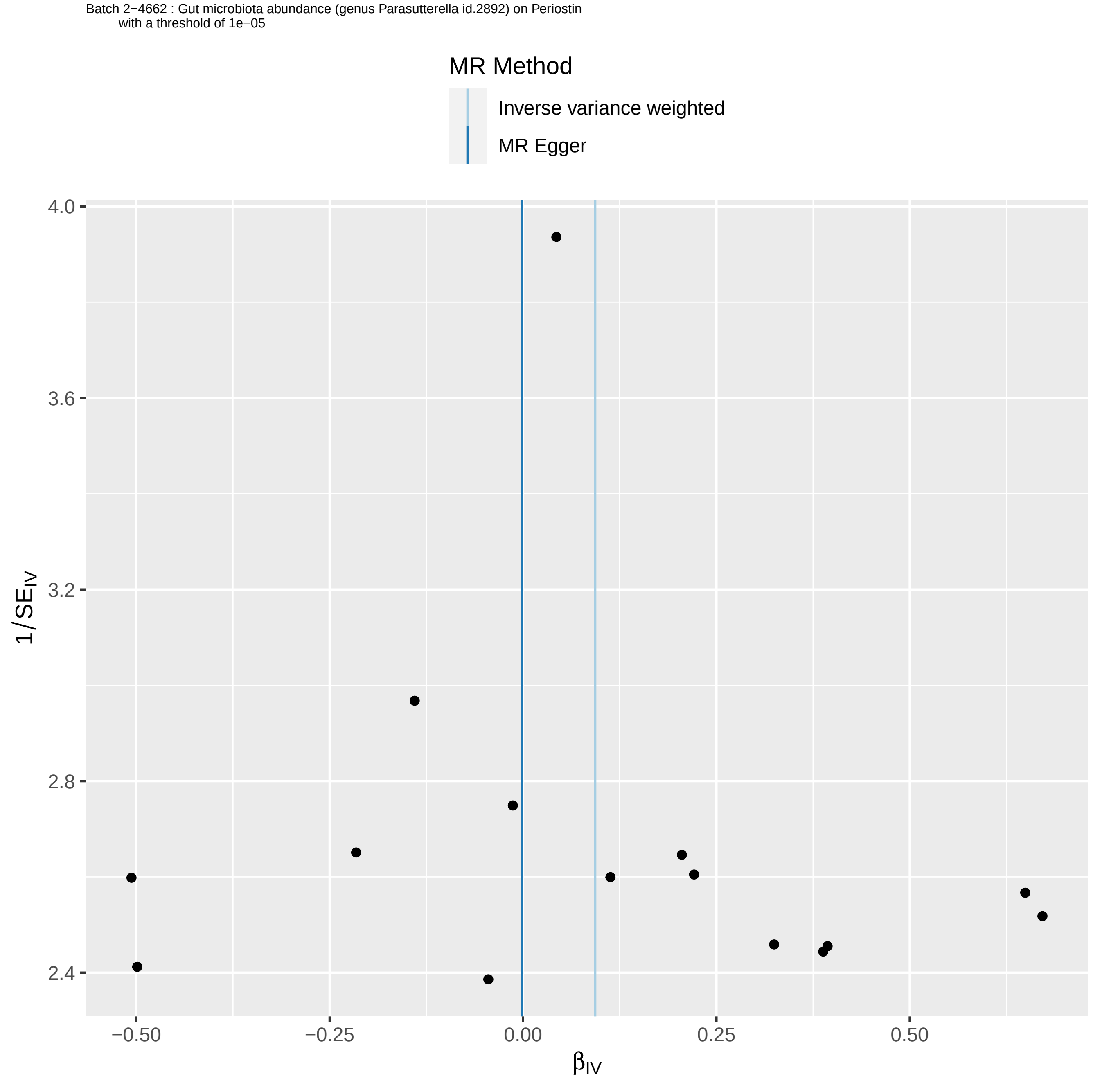
Batch 2−4662 : Gut microbiota abundance (genus Parasutterella id.2892) on Periostin
with a threshold of 1e−05
MR Method
Inverse variance weighted
MR Egger
4.0
3.6
3.2
2.8
2.4
1/SE_IV
−0.50
−0.25
0.00
0.25
0.50
β_IV

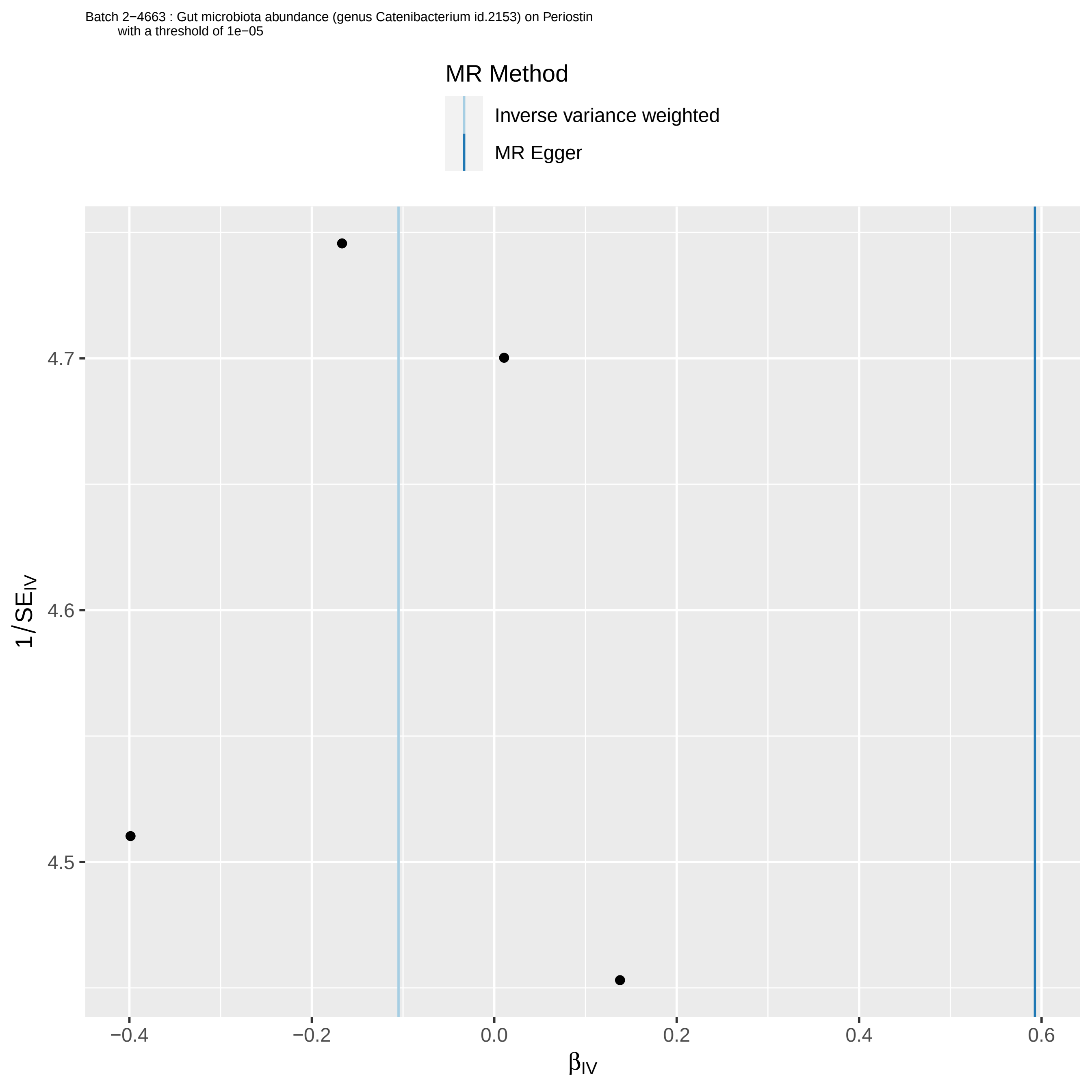

Batch 2−4663 : Gut microbiota abundance (genus Catenibacterium id.2153) on Periostin
with a threshold of 1e−05
MR Method
Inverse variance weighted
MR Egger
4.7
4.6
4.5
1/SE_IV
−0.4
−0.2
0.0
0.2
0.4
0.6
β_IV

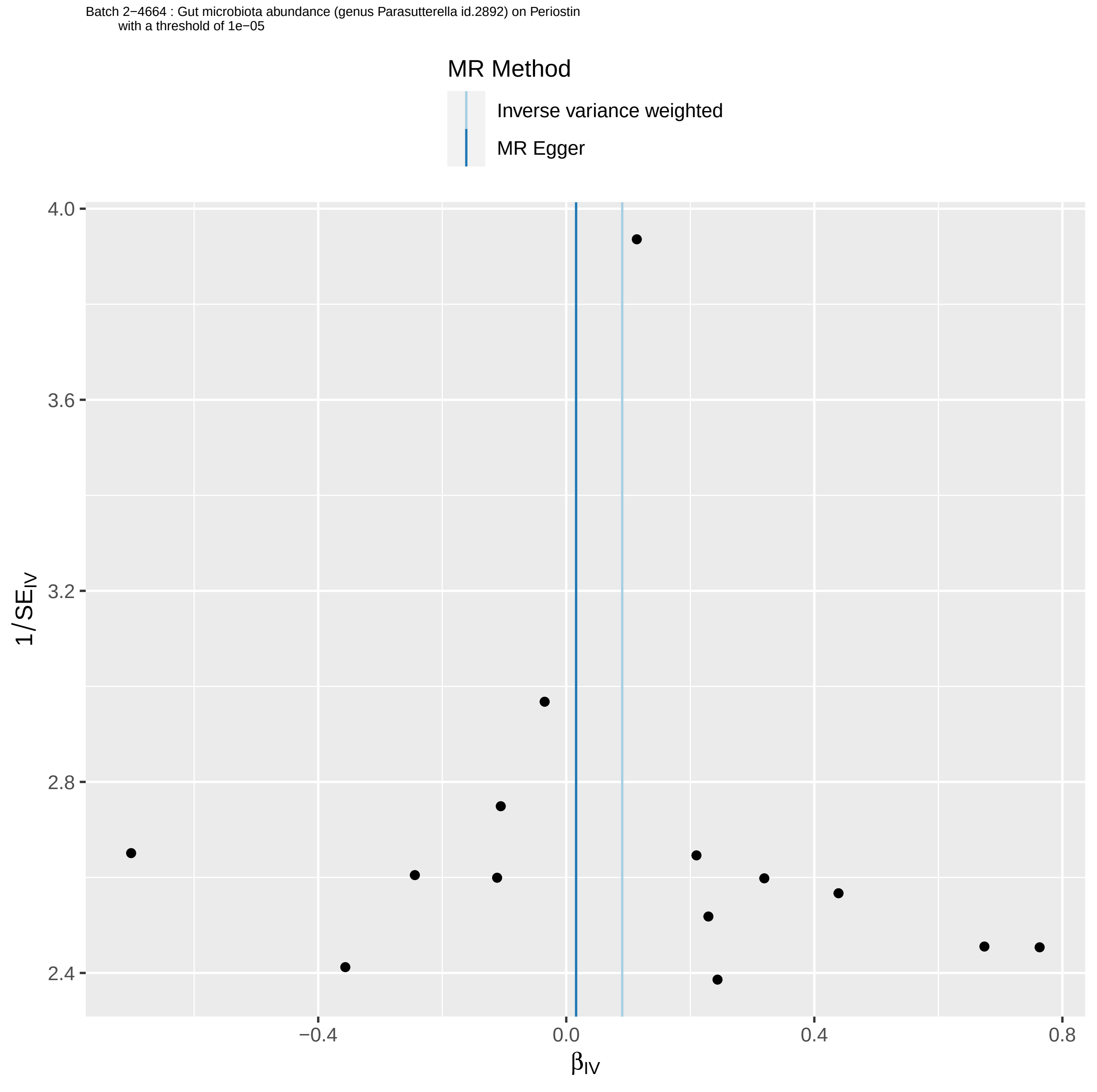
Batch 2−4664 : Gut microbiota abundance (genus Parasutterella id.2892) on Periostin
with a threshold of 1e−05
MR Method
Inverse variance weighted
MR Egger
4.0
3.6
3.2
2.8
2.4
$1/SE_{IV}$
−0.4
0.0
0.4
0.8
β_{IV}

Batch 2−4665 : Gut microbiota abundance (genus Catenibacterium id.2153) on Periostin
with a threshold of 1e−05

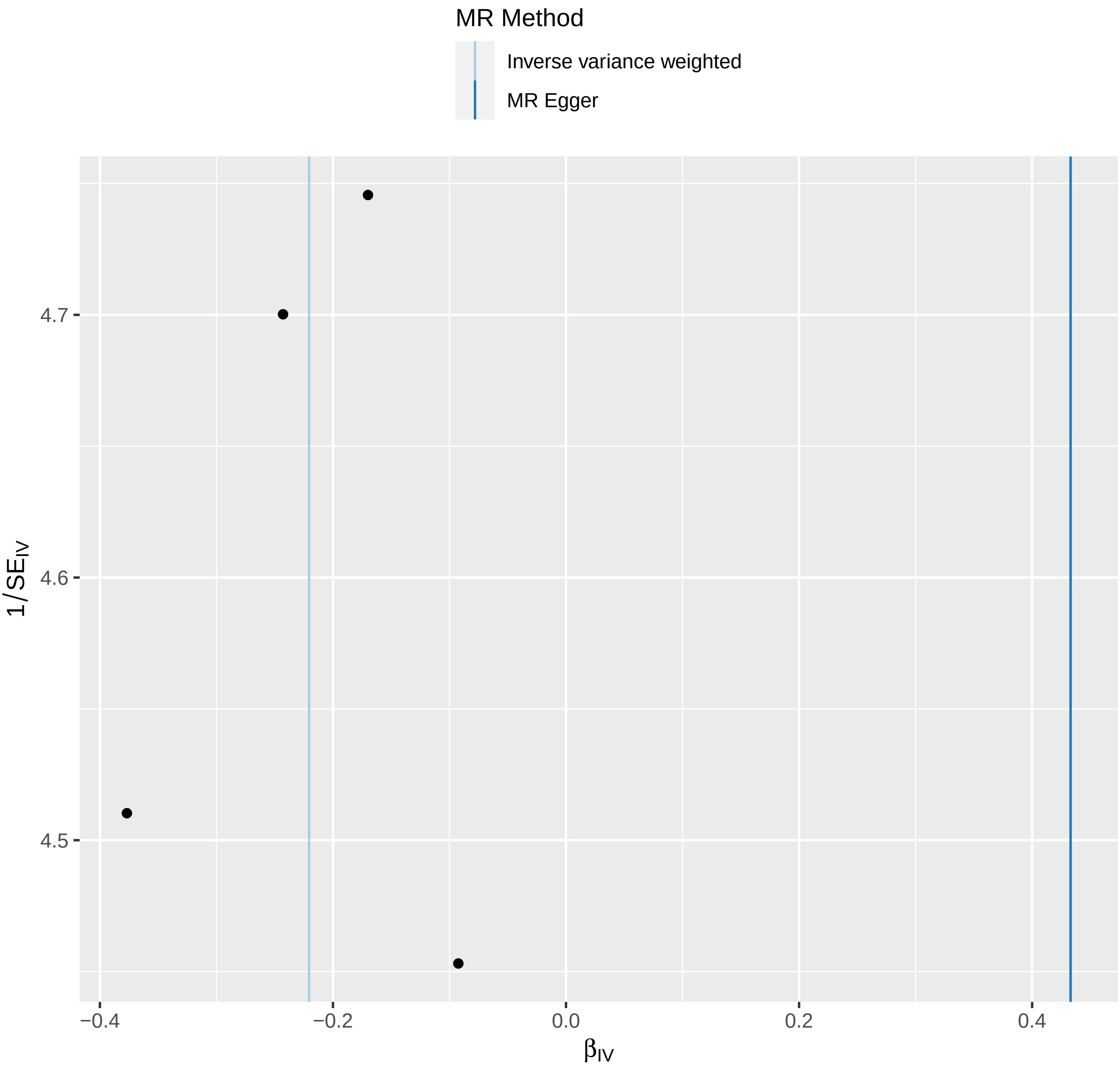

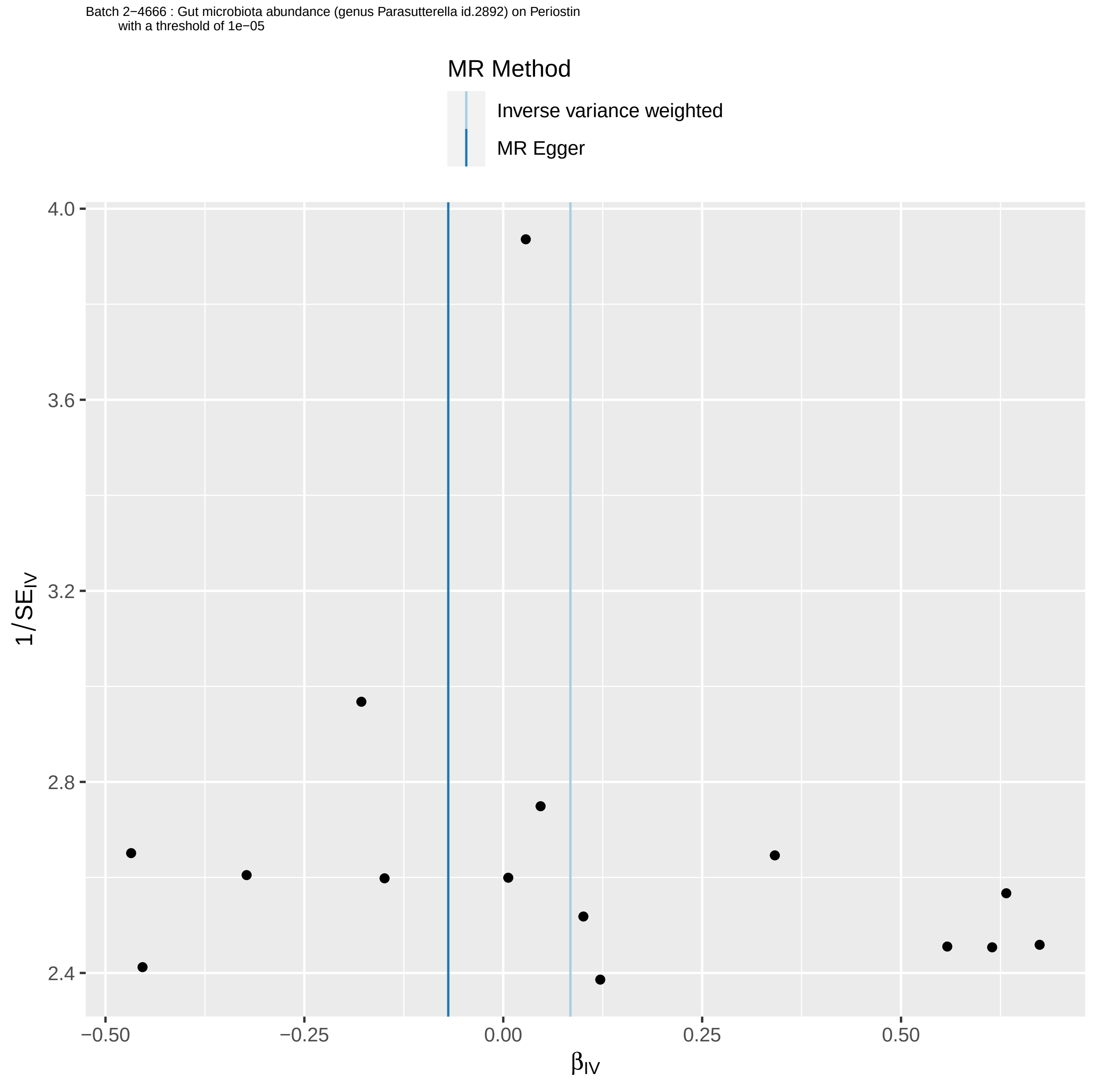

Batch 2−4666 : Gut microbiota abundance (genus Parasutterella id.2892) on Periostin
with a threshold of 1e−05
MR Method
Inverse variance weighted
MR Egger
4.0
3.6
3.2
2.8
2.4
1/SE_IV
−0.50
−0.25
0.00
0.25
0.50
β_IV

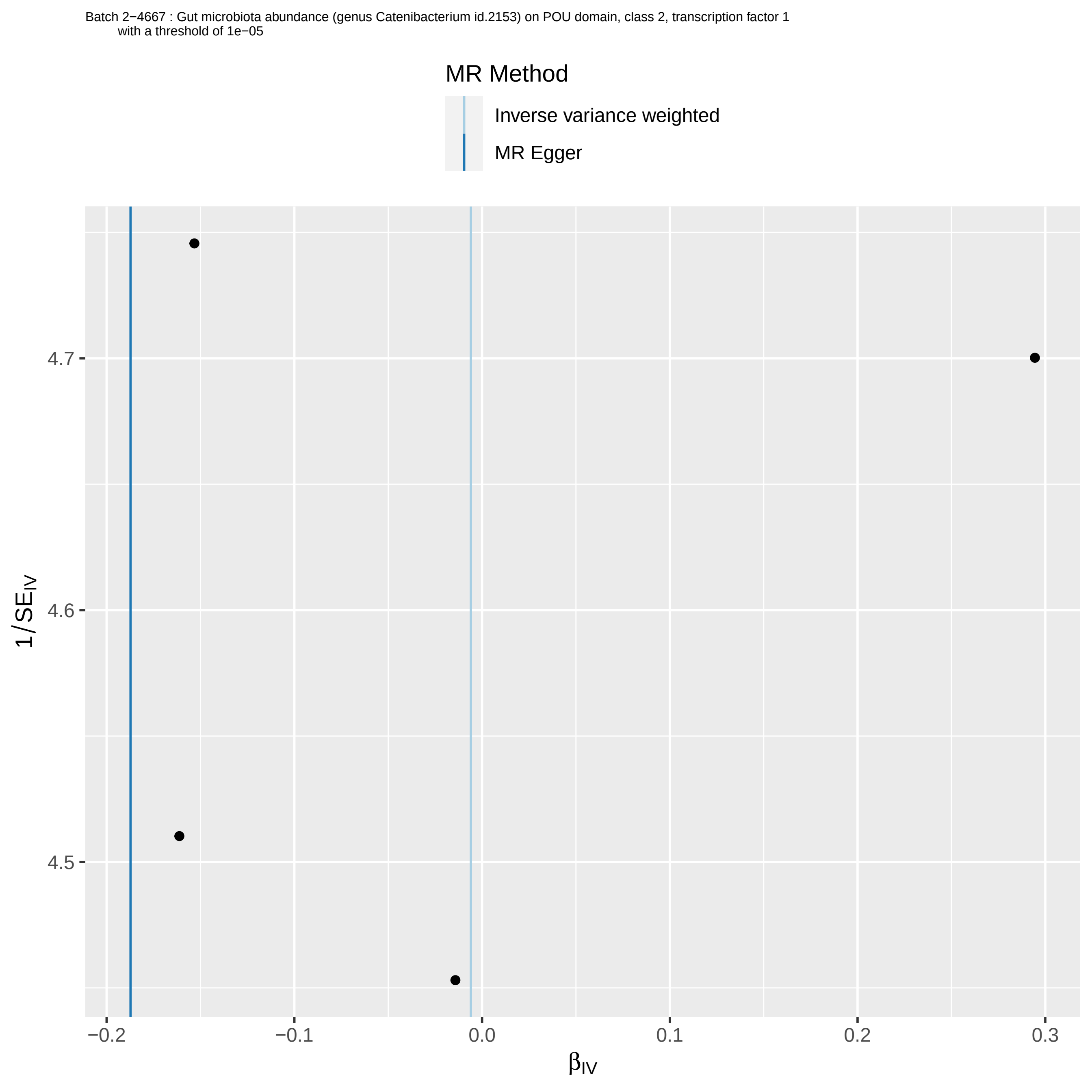

Batch 2−4667 : Gut microbiota abundance (genus Catenibacterium id.2153) on POU domain, class 2, transcription factor 1
with a threshold of 1e−05
MR Method
Inverse variance weighted
MR Egger
4.7
4.6
4.5
1/SE_IV
−0.2
−0.1
0.0
0.1
0.2
0.3
β_IV

Batch 2−4668 : Gut microbiota abundance (genus Parasutterella id.2892) on POU domain, class 2, transcription factor 1
with a threshold of 1e−05

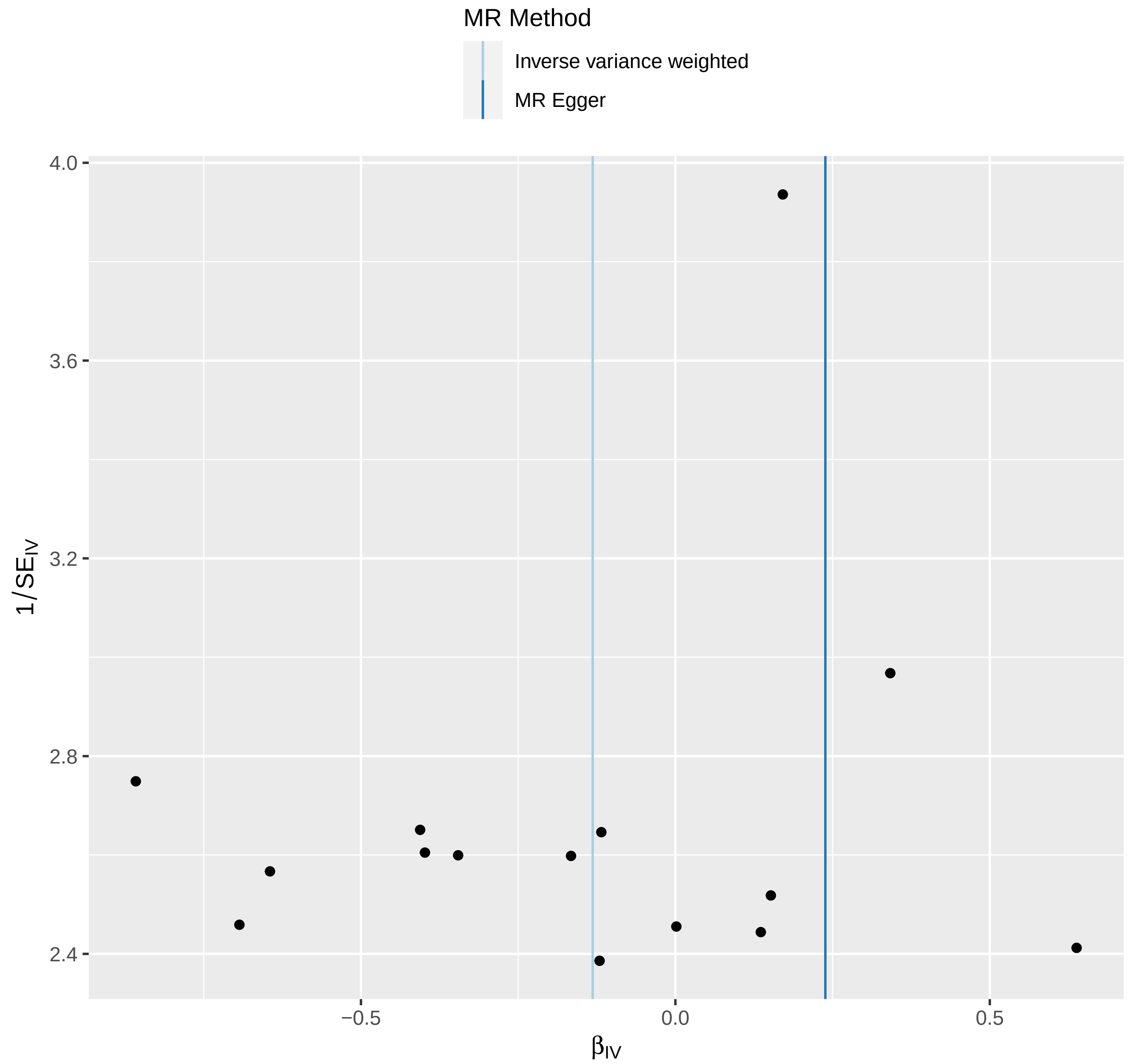

Batch 2−4669 : Gut microbiota abundance (genus Catenibacterium id.2153) on Phosphatidate phosphatase PPAPDC1A
with a threshold of 1e−05

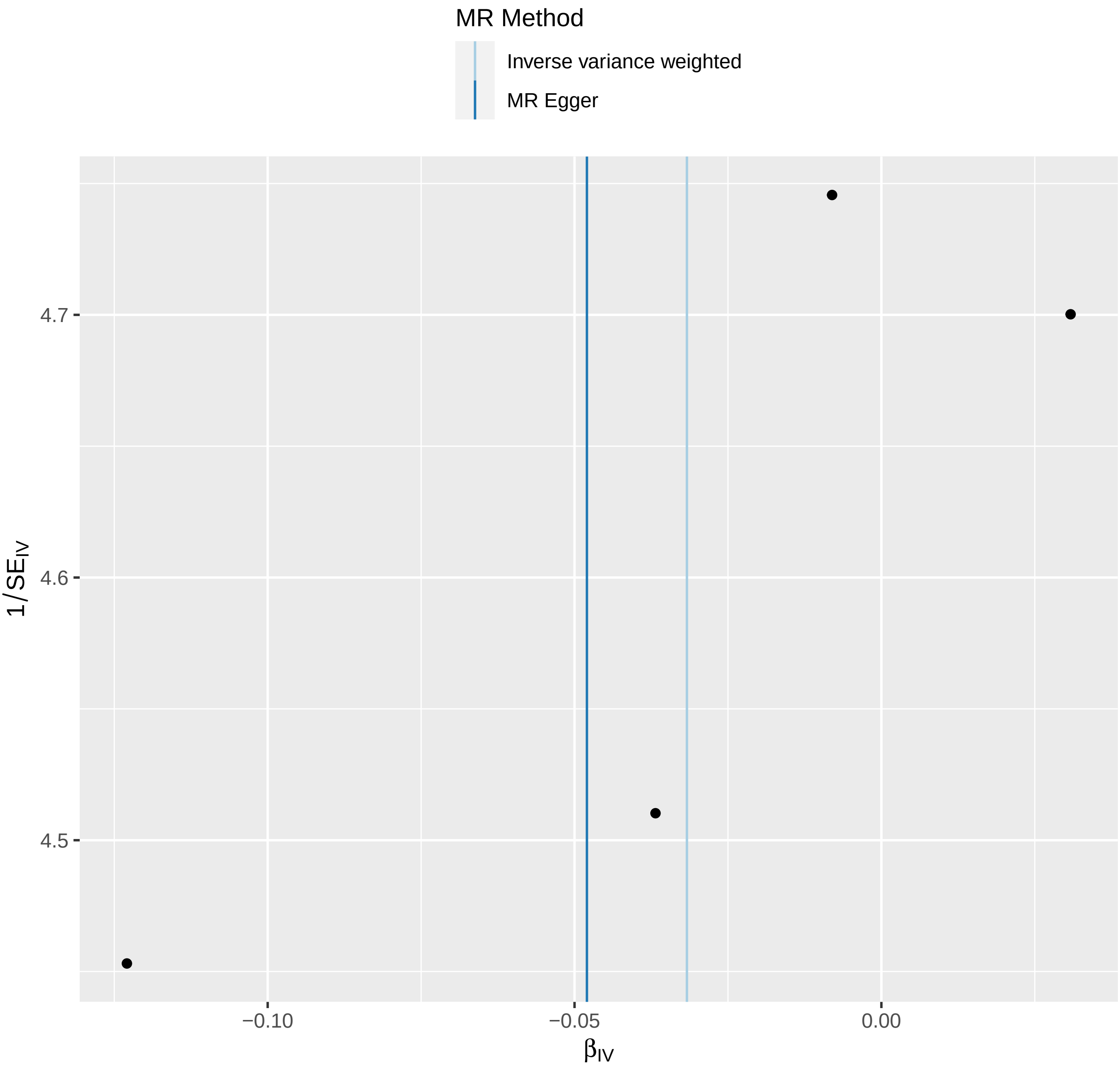

Batch 2−4670 : Gut microbiota abundance (genus Parasutterella id.2892) on Phosphatidate phosphatase PPAPDC1A
with a threshold of 1e−05

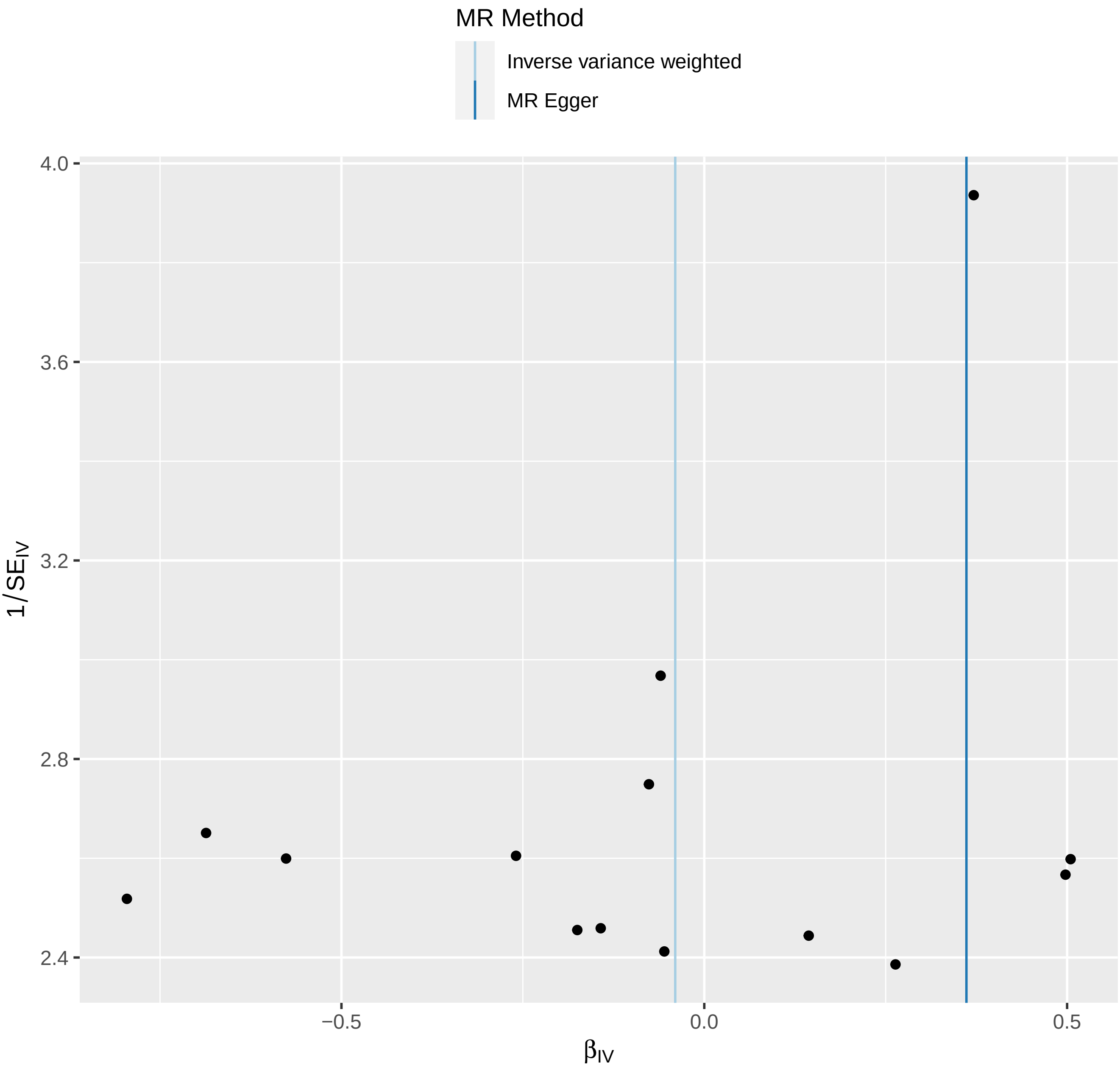

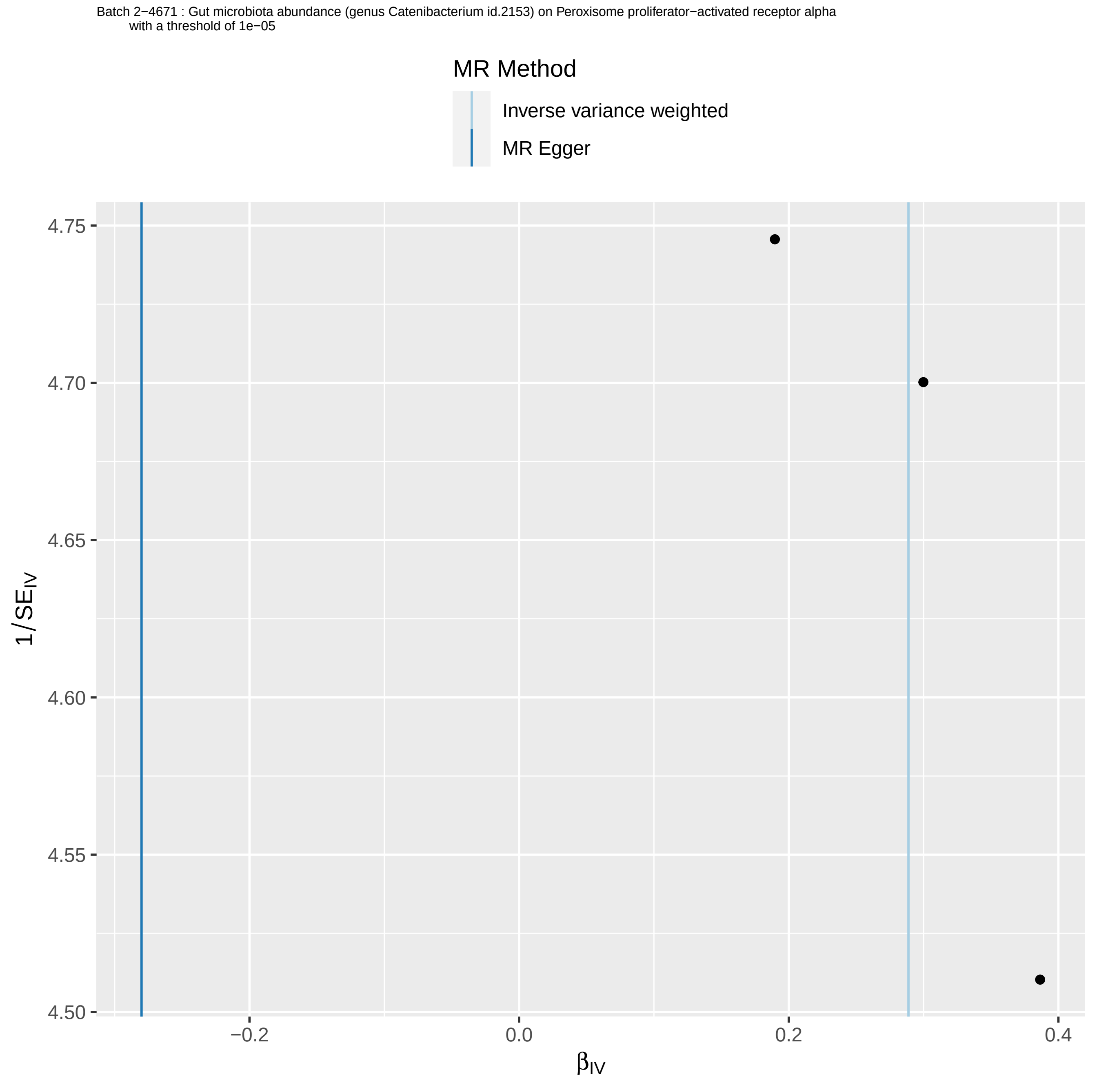

Batch 2−4671 : Gut microbiota abundance (genus Catenibacterium id.2153) on Peroxisome proliferator−activated receptor alpha
with a threshold of 1e−05
MR Method
Inverse variance weighted
MR Egger
4.75
4.70
4.65
4.60
4.55
4.50
1/SE IV
−0.2
0.0
0.2
0.4
β IV

Batch 2−4672 : Gut microbiota abundance (genus Parasutterella id.2892) on Peroxisome proliferator−activated receptor alpha with a threshold of 1e−05

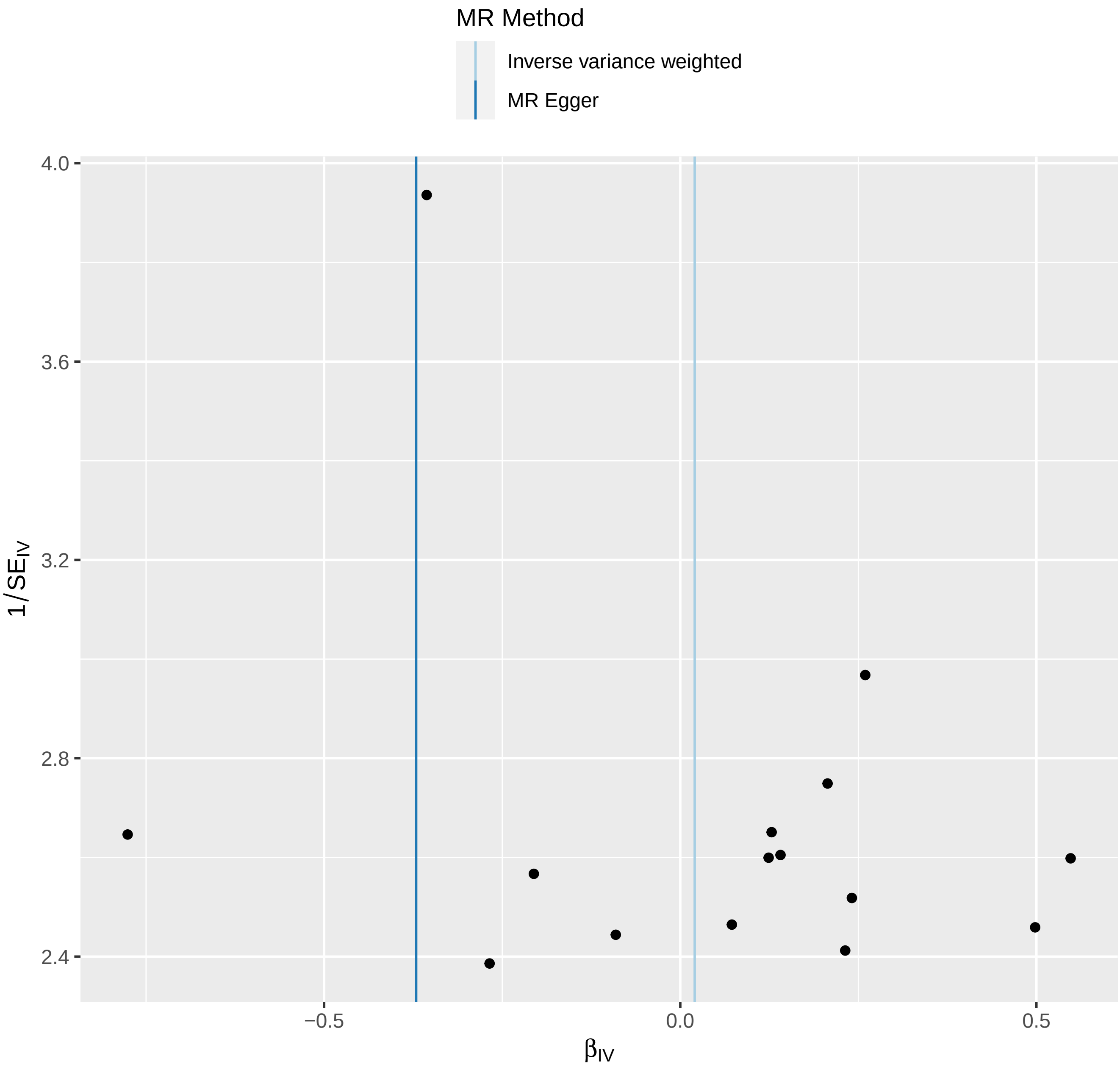

Batch 2−4673 : Gut microbiota abundance (genus Catenibacterium id.2153) on Neutrophil−activating peptide 2
with a threshold of 1e−05

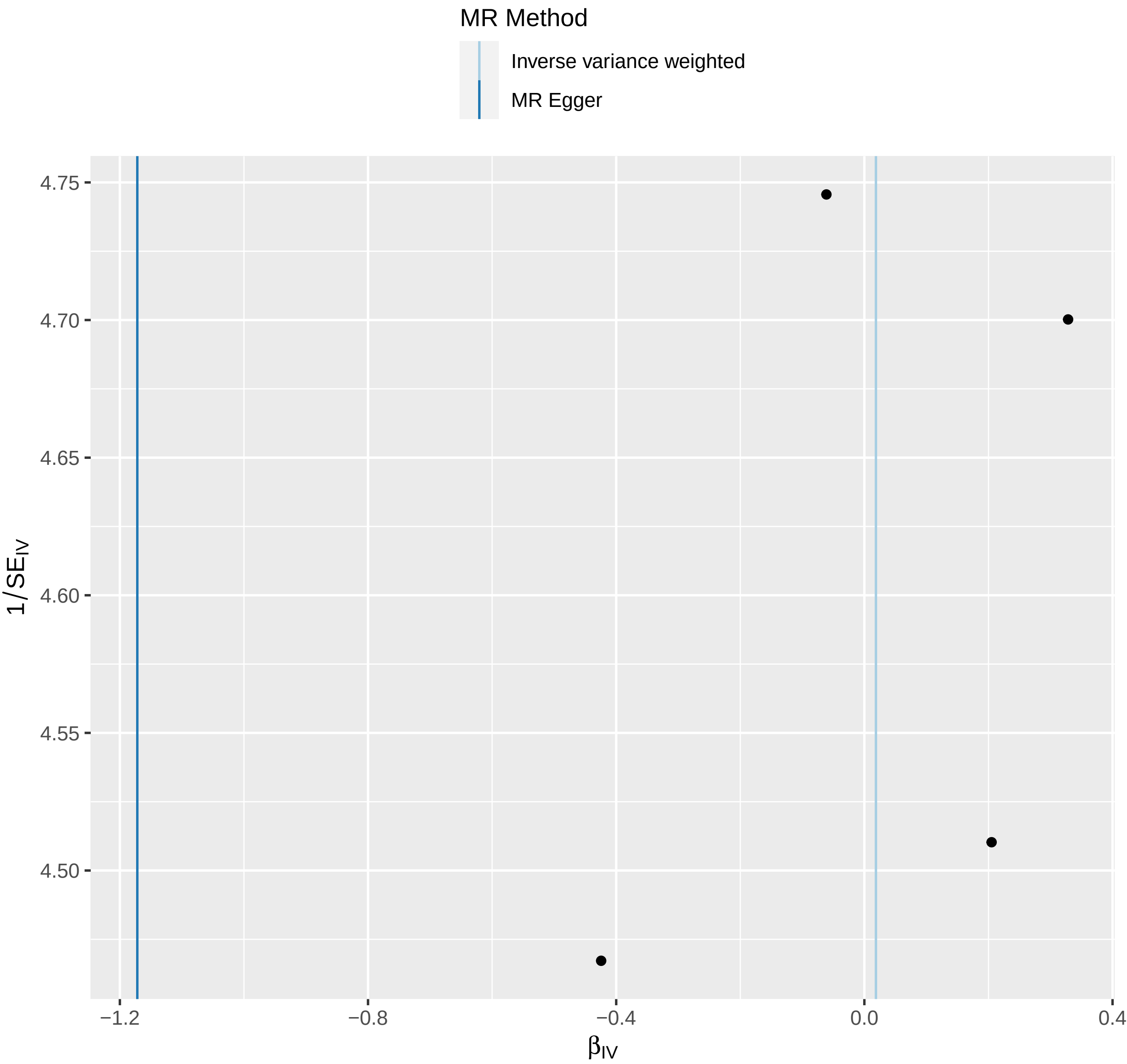

Batch 2−4674 : Gut microbiota abundance (genus Parasutterella id.2892) on Neutrophil−activating peptide 2
with a threshold of 1e−05

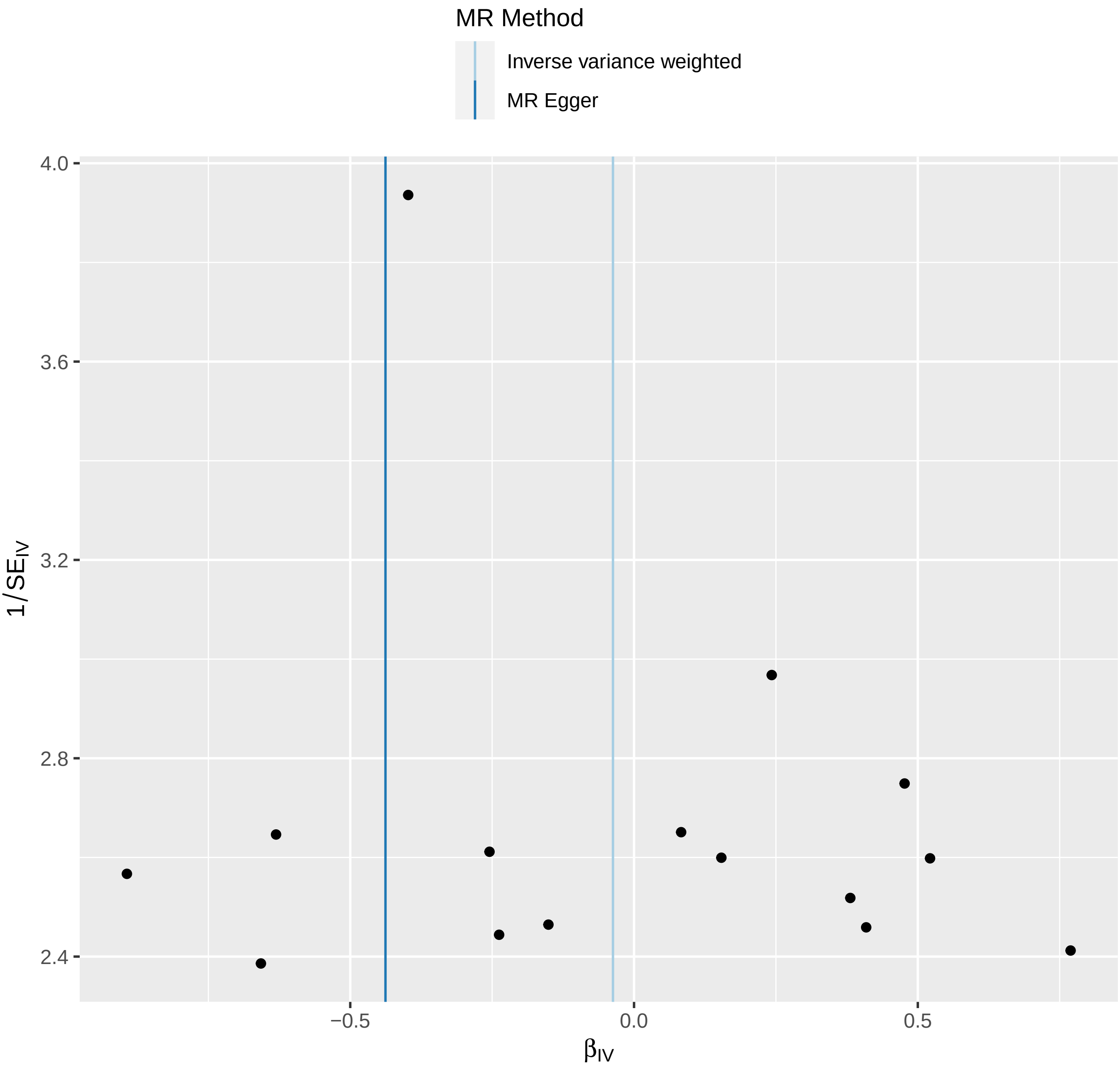

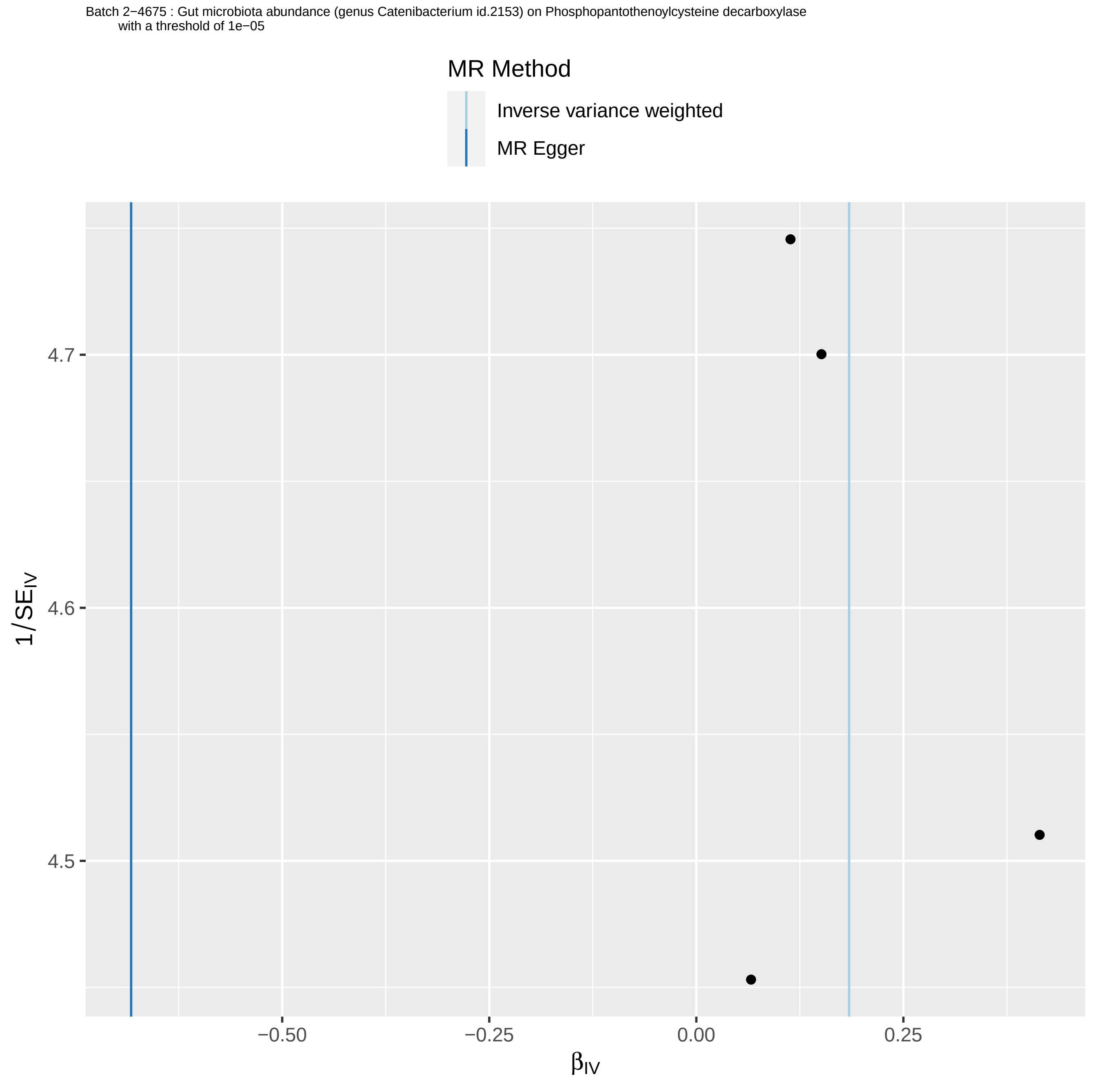
Batch 2−4675 : Gut microbiota abundance (genus Catenibacterium id.2153) on Phosphopantothenoylcysteine decarboxylase
with a threshold of 1e−05
MR Method
Inverse variance weighted
MR Egger
4.7
4.6
4.5
1/SE_IV
−0.50
−0.25
0.00
0.25
β_IV

Batch 2−4676 : Gut microbiota abundance (genus Parasutterella id.2892) on Phosphopantothenoylcysteine decarboxylase with a threshold of 1e−05

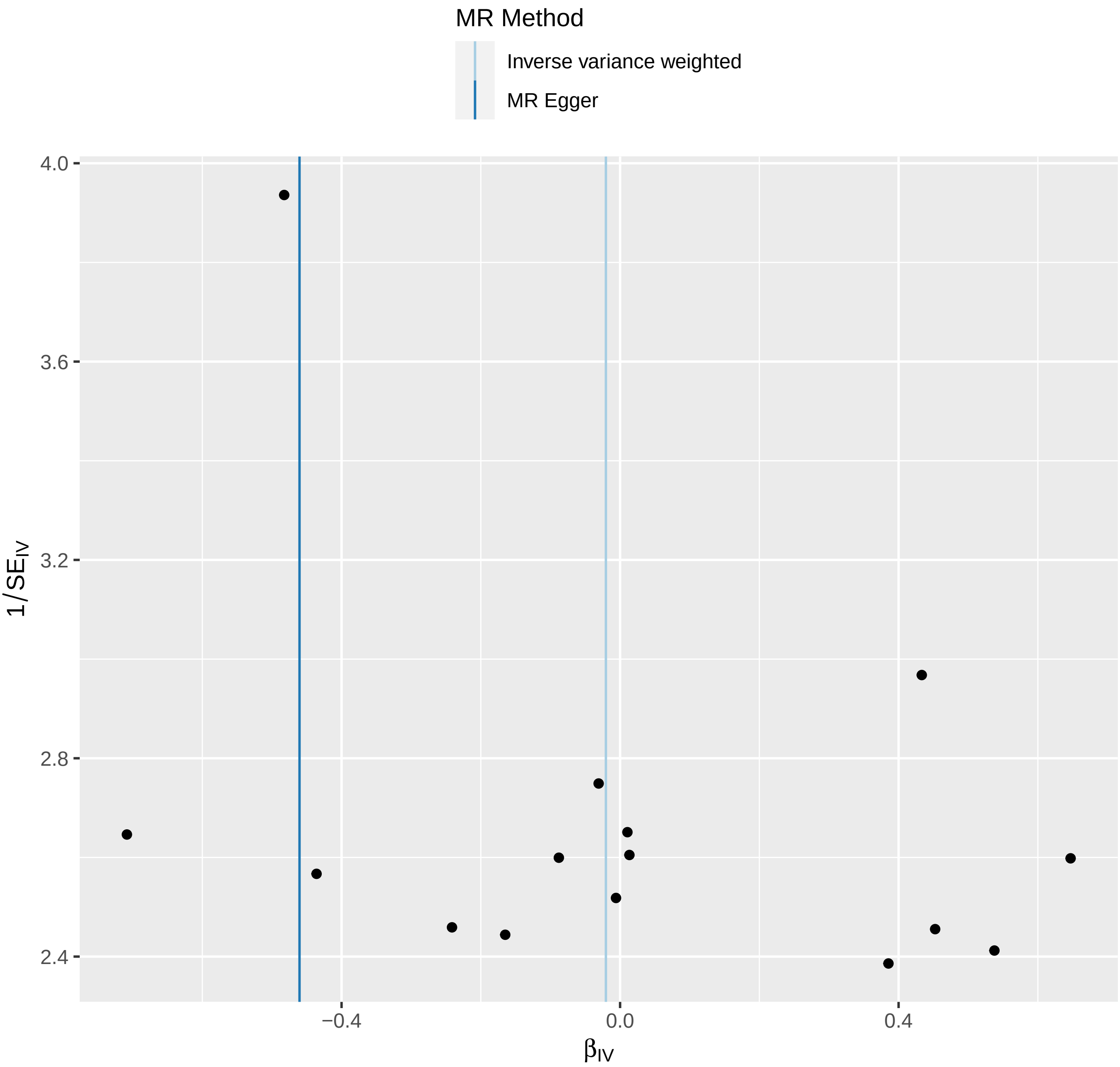

Batch 2−4677 : Gut microbiota abundance (genus Catenibacterium id.2153) on Peptidyl−prolyl cis−trans isomerase A with a threshold of 1e−05

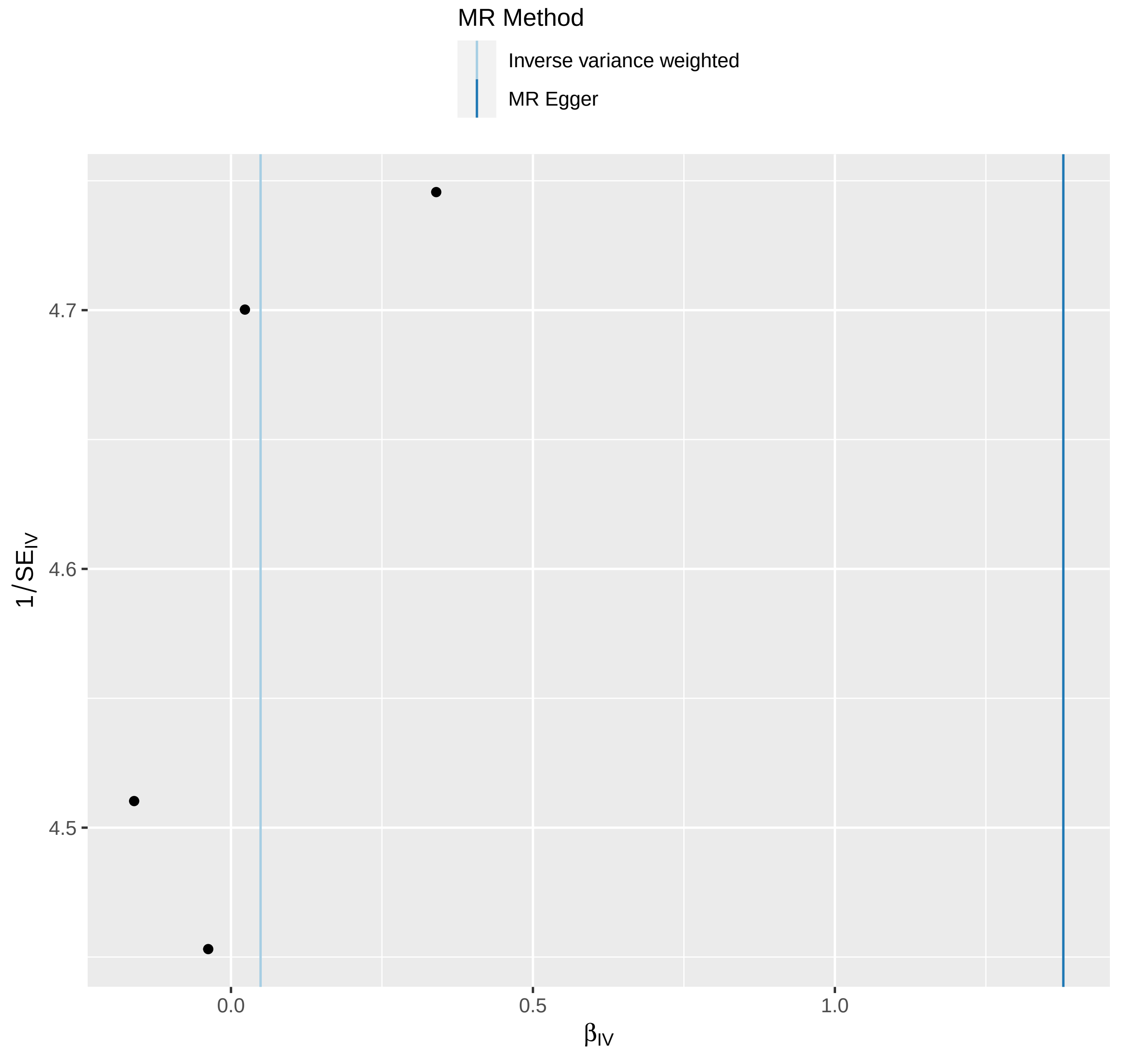

Batch 2−4678 : Gut microbiota abundance (genus Parasutterella id.2892) on Peptidyl−prolyl cis−trans isomerase A
with a threshold of 1e−05

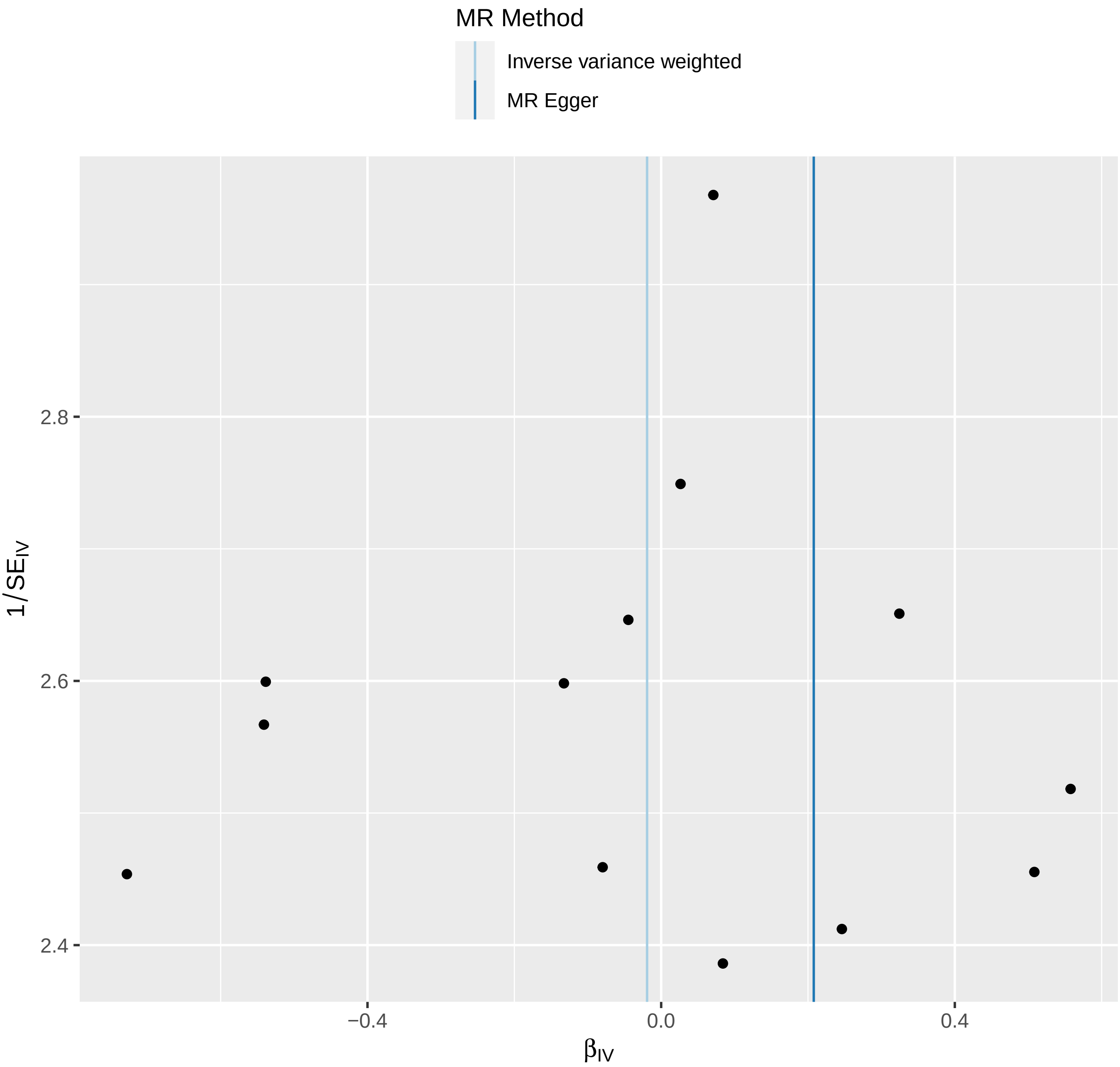

Batch 2−4679 : Gut microbiota abundance (genus Catenibacterium id.2153) on Peptidyl−prolyl cis−trans isomerase B
with a threshold of 1e−05

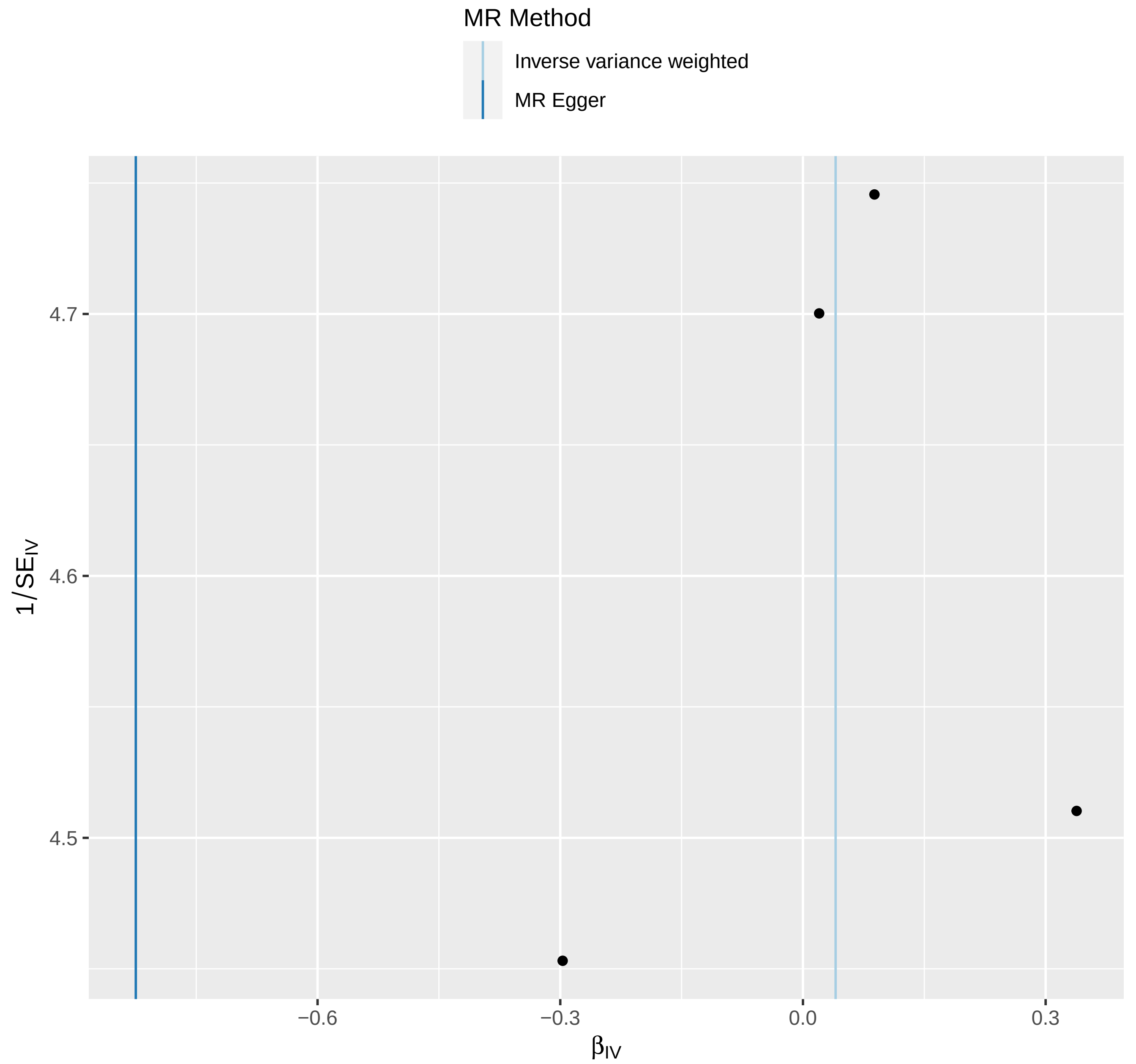

Batch 2−4680 : Gut microbiota abundance (genus Parasutterella id.2892) on Peptidyl−prolyl cis−trans isomerase B
with a threshold of 1e−05

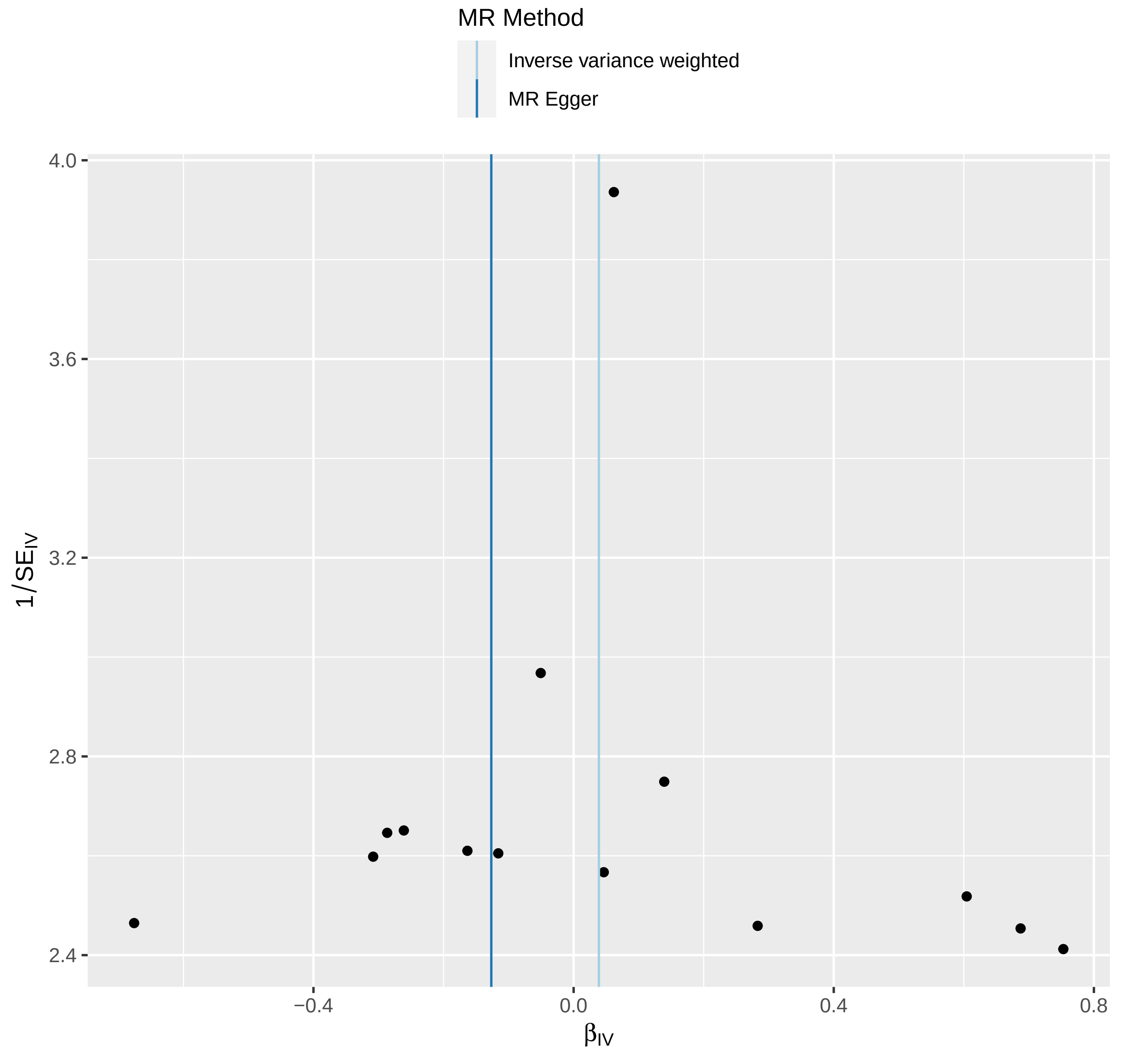

Batch 2−4681 : Gut microbiota abundance (genus Catenibacterium id.2153) on Peptidyl−prolyl cis−trans isomerase E
with a threshold of 1e−05

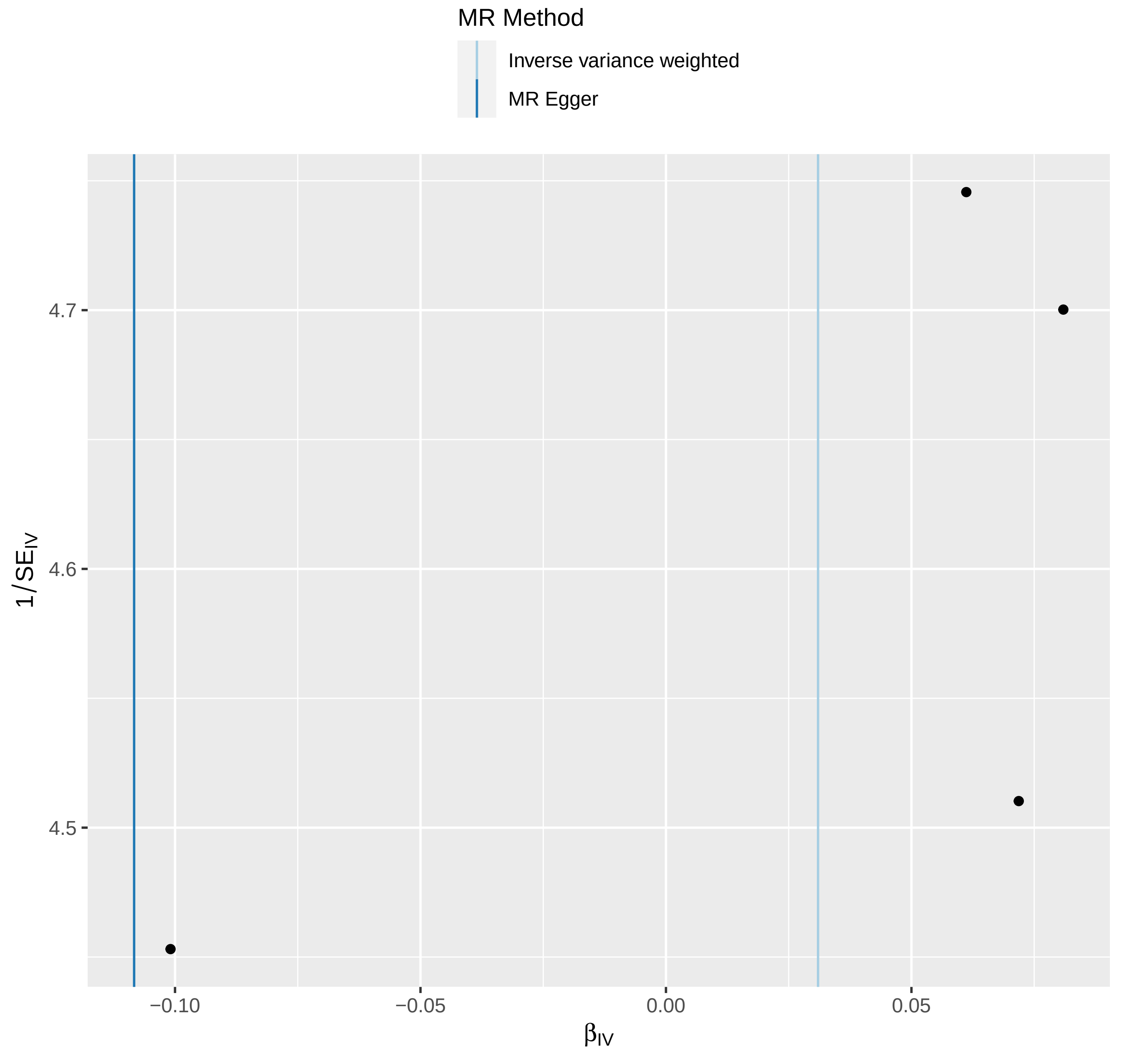

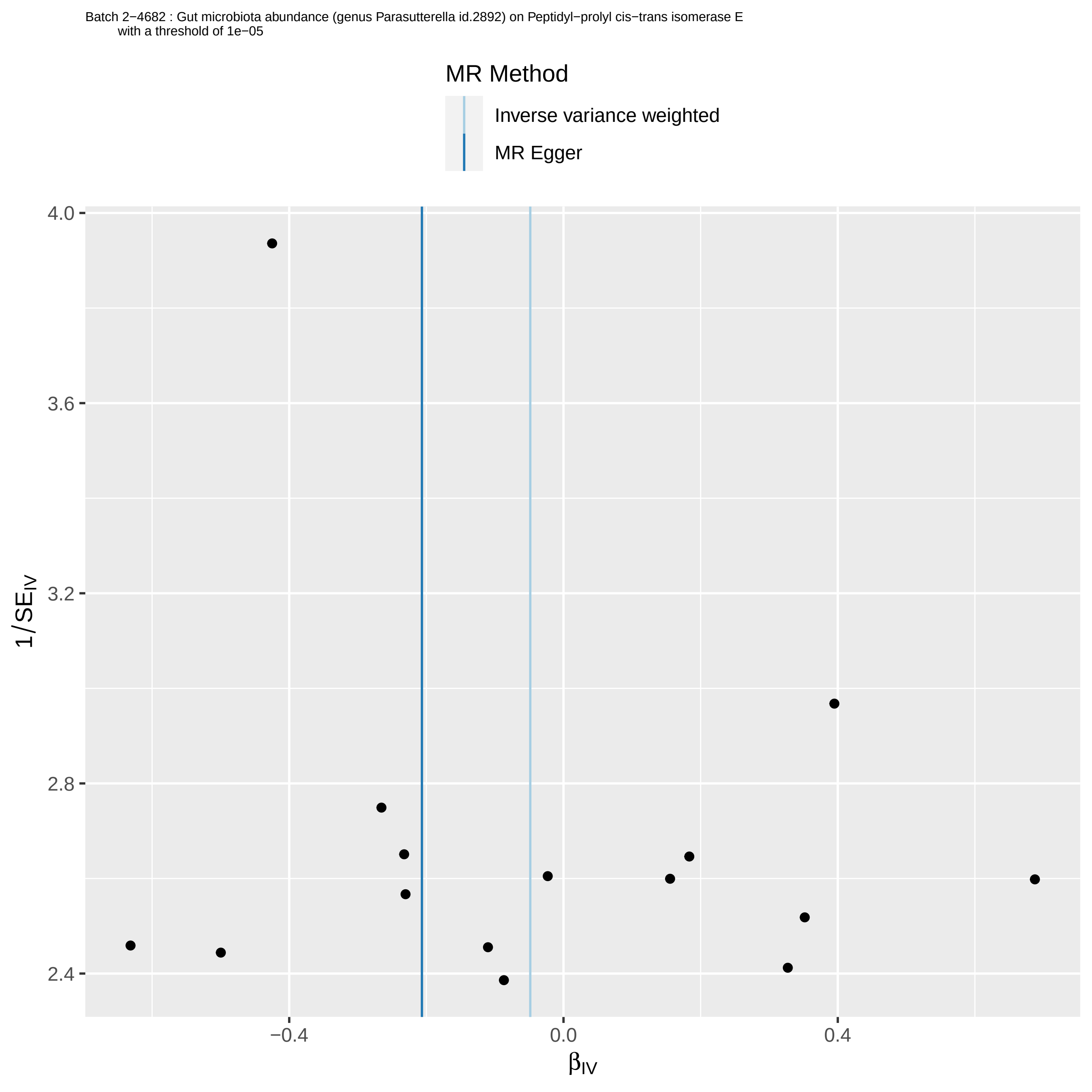
Batch 2−4682 : Gut microbiota abundance (genus Parasutterella id.2892) on Peptidyl−prolyl cis−trans isomerase E
with a threshold of 1e−05
MR Method
Inverse variance weighted
MR Egger
4.0
3.6
3.2
2.8
2.4
1/SE_IV
−0.4
0.0
0.4
β_IV

Batch 2−4683 : Gut microbiota abundance (genus Catenibacterium id.2153) on Peptidyl−prolyl cis−trans isomerase H
with a threshold of 1e−05

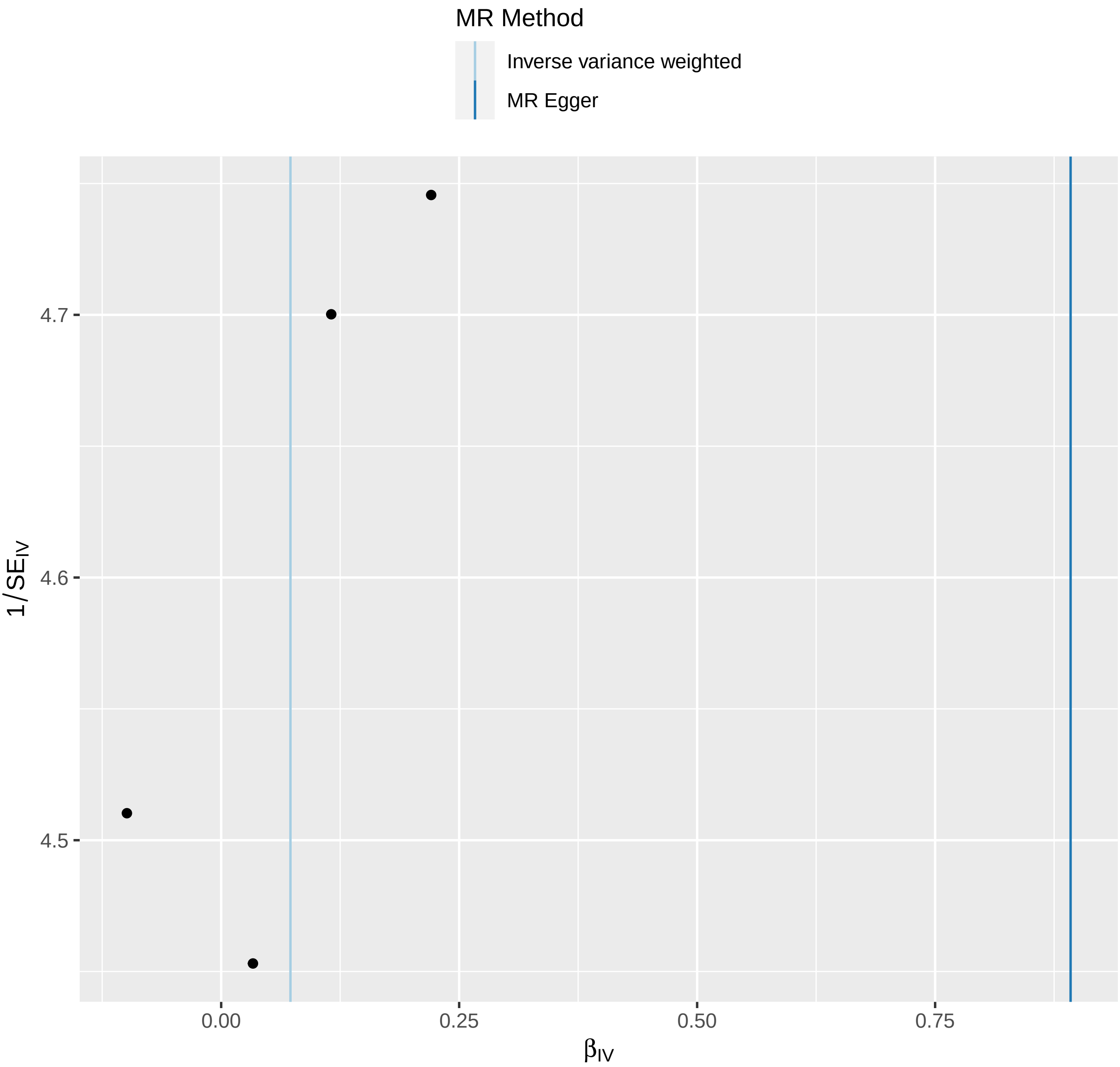

Batch 2−4684 : Gut microbiota abundance (genus Parasutterella id.2892) on Peptidyl−prolyl cis−trans isomerase H
with a threshold of 1e−05

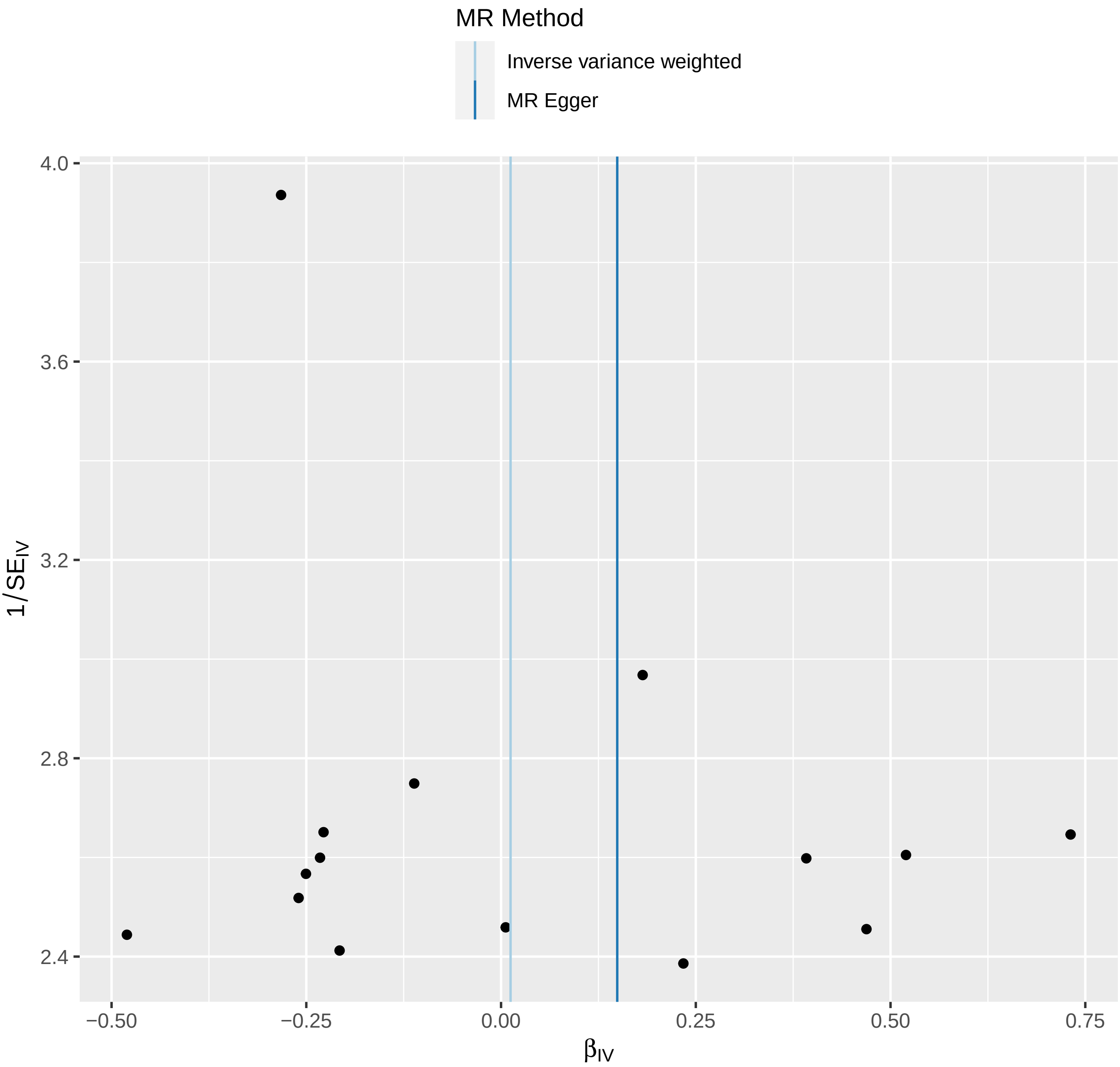

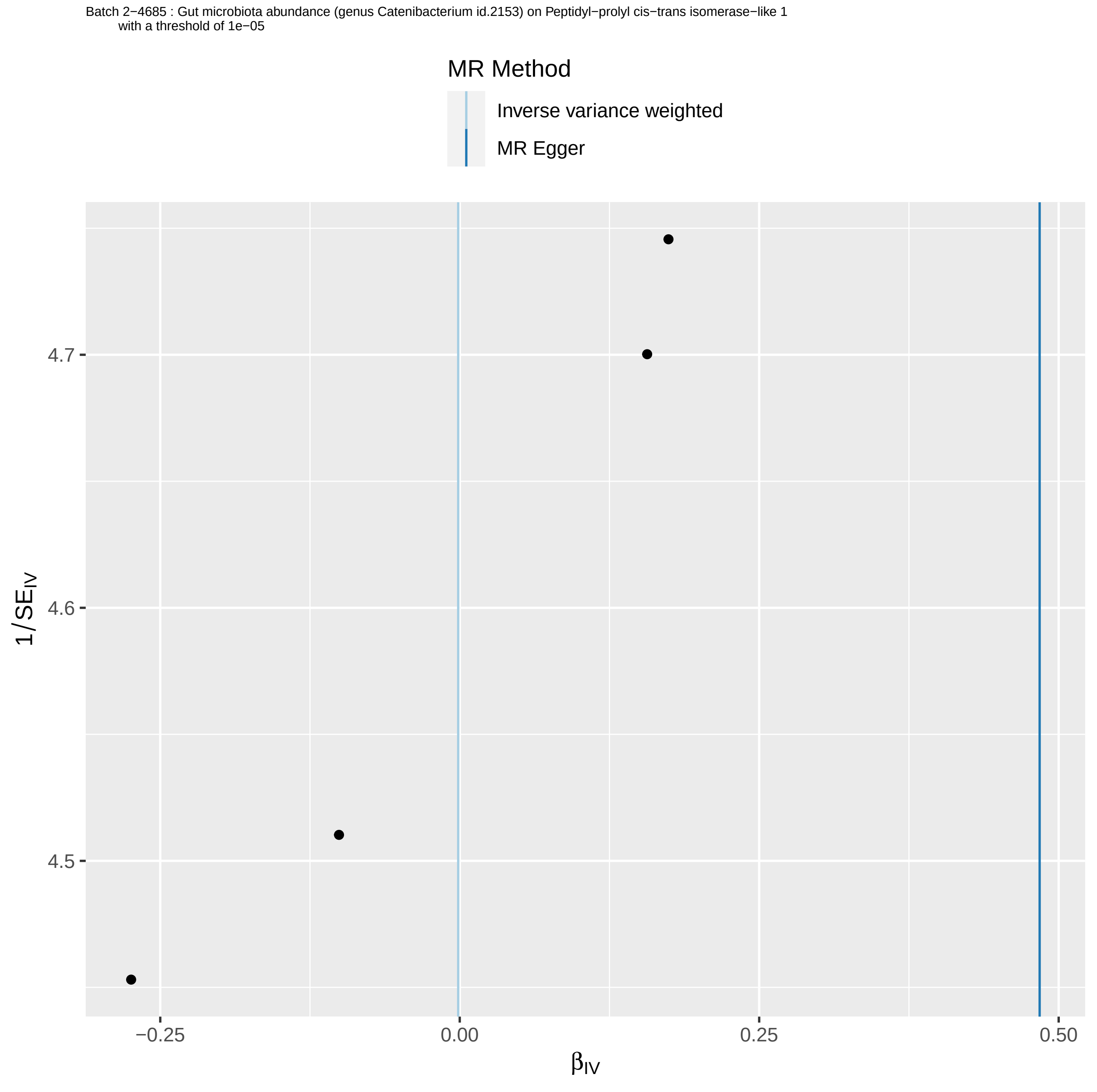
Batch 2−4685 : Gut microbiota abundance (genus Catenibacterium id.2153) on Peptidyl−prolyl cis−trans isomerase−like 1
with a threshold of 1e−05
MR Method
Inverse variance weighted
MR Egger
4.7
4.6
4.5
$1/SE_{IV}$
−0.25
0.00
0.25
0.50
β_{IV}

Batch 2−4686 : Gut microbiota abundance (genus Parasutterella id.2892) on Peptidyl−prolyl cis−trans isomerase−like 1
with a threshold of 1e−05

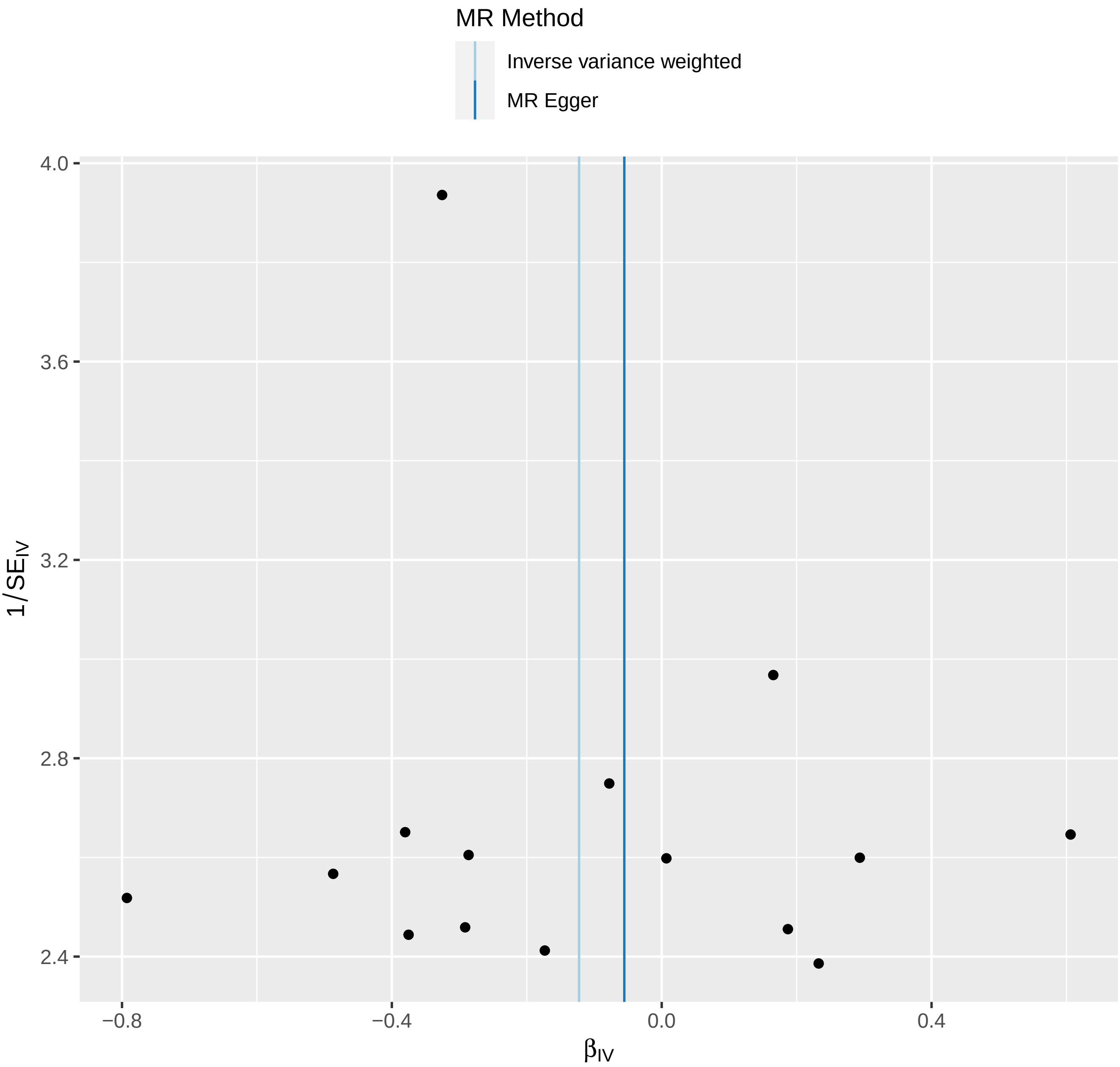

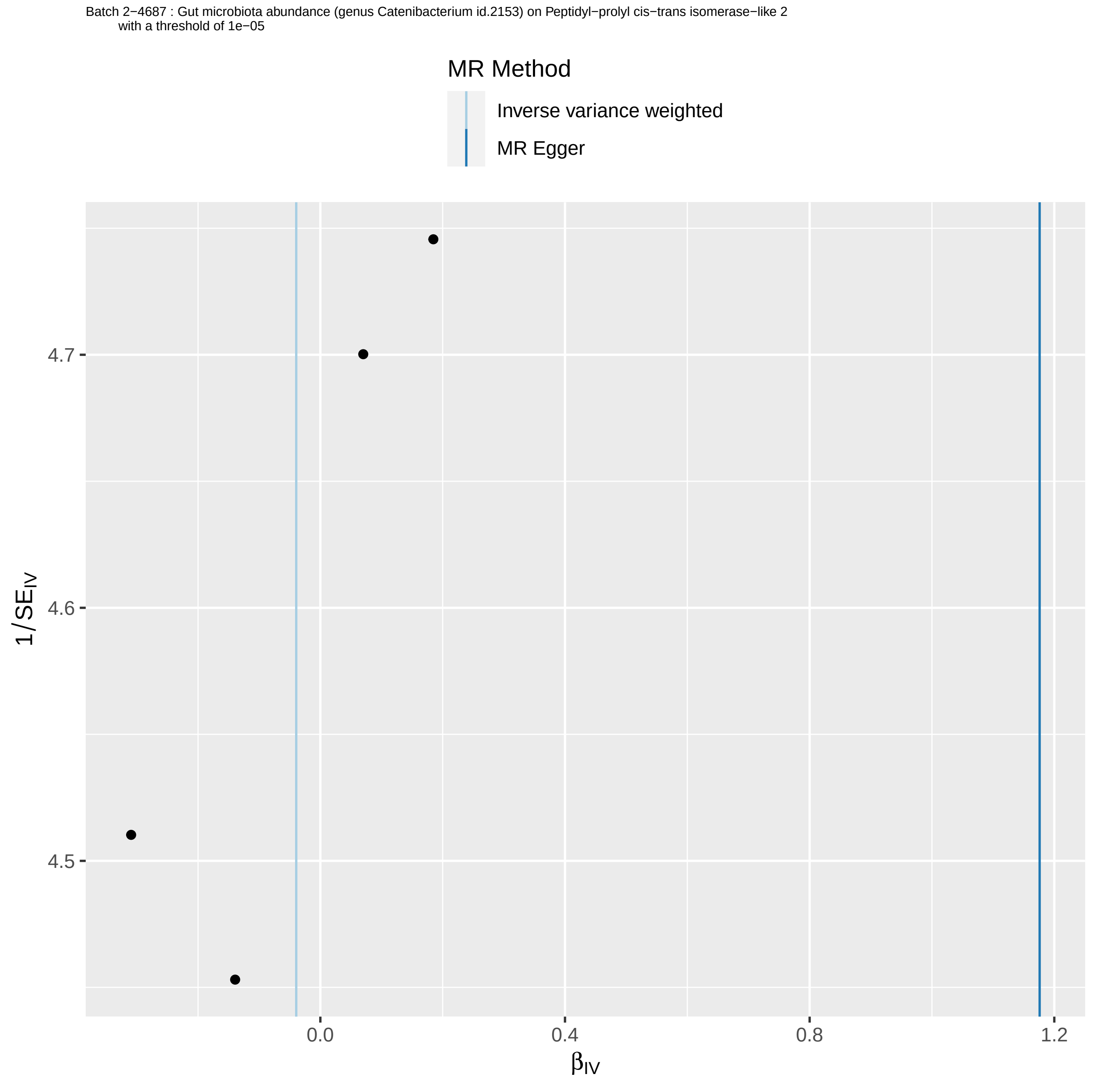
Batch 2−4687 : Gut microbiota abundance (genus Catenibacterium id.2153) on Peptidyl−prolyl cis−trans isomerase−like 2
with a threshold of 1e−05
MR Method
Inverse variance weighted
MR Egger
4.7
4.6
4.5
1/SE$_{IV}$
0.0
0.4
0.8
1.2
β_{IV}

Batch 2−4688 : Gut microbiota abundance (genus Parasutterella id.2892) on Peptidyl−prolyl cis−trans isomerase−like 2 with a threshold of 1e−05

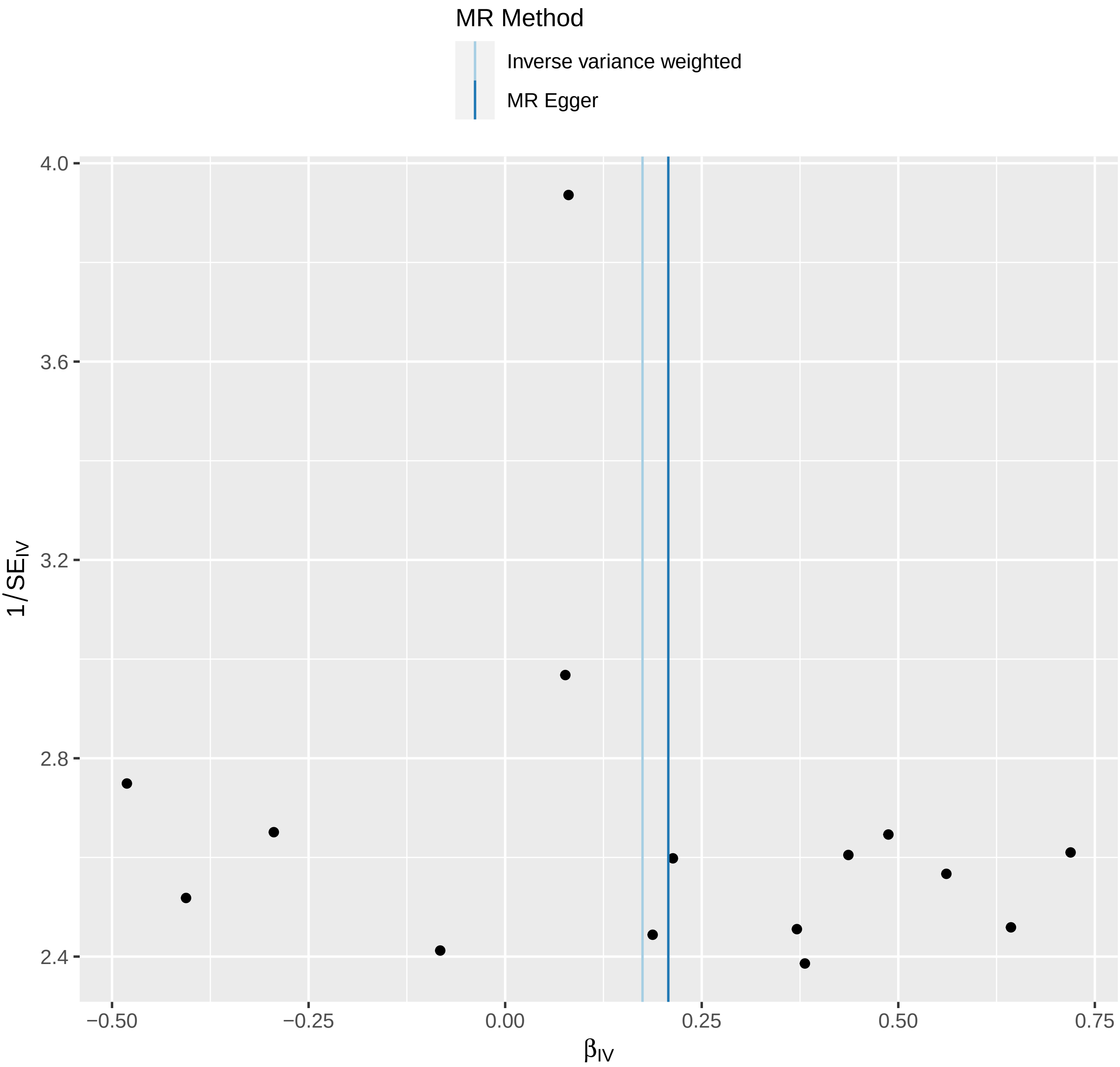

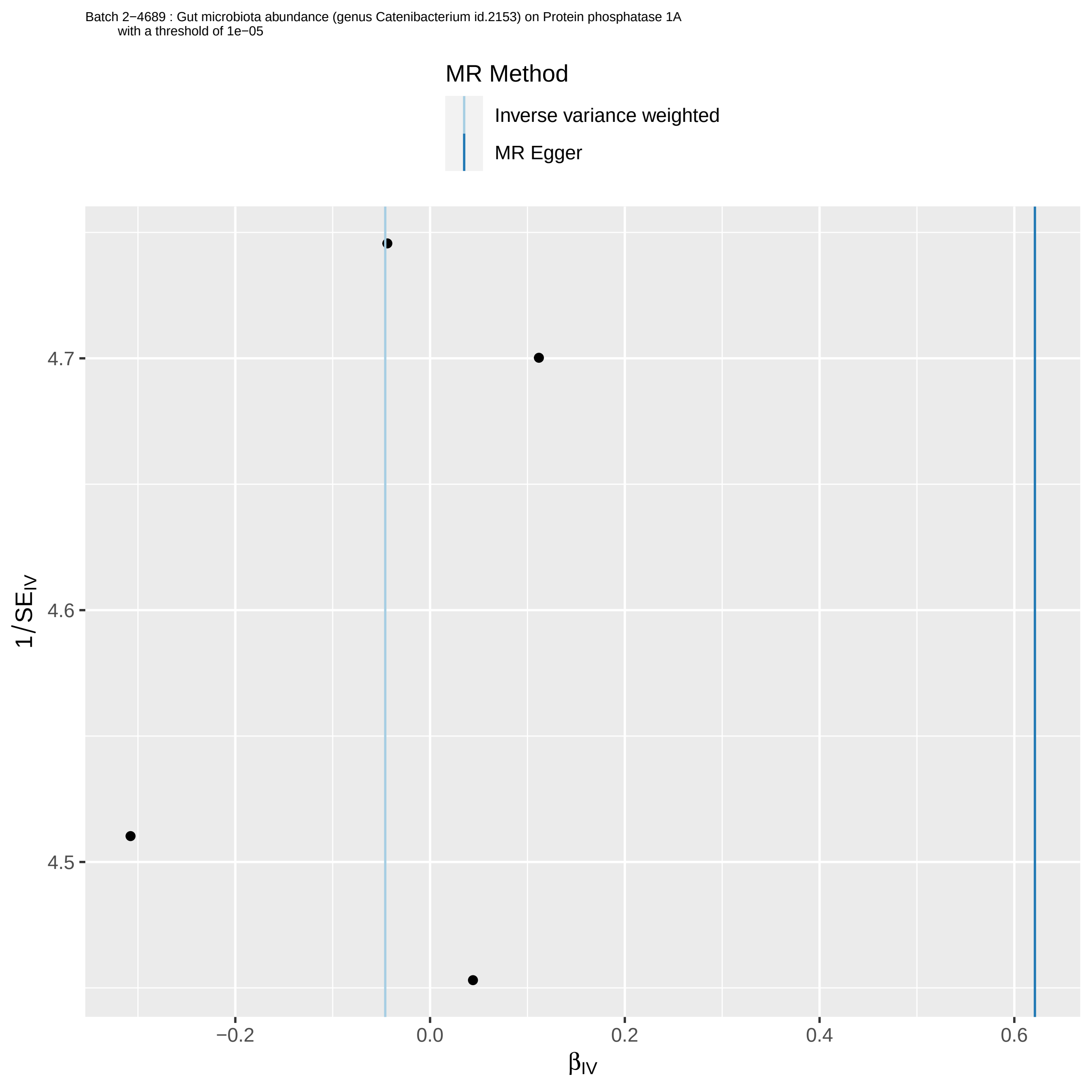
Batch 2−4689 : Gut microbiota abundance (genus Catenibacterium id.2153) on Protein phosphatase 1A
with a threshold of 1e−05
MR Method
Inverse variance weighted
MR Egger
4.7
4.6
4.5
1/SE$_{IV}$
−0.2
0.0
0.2
0.4
0.6
β_{IV}

Batch 2−4690 : Gut microbiota abundance (genus Parasutterella id.2892) on Protein phosphatase 1A
with a threshold of 1e−05

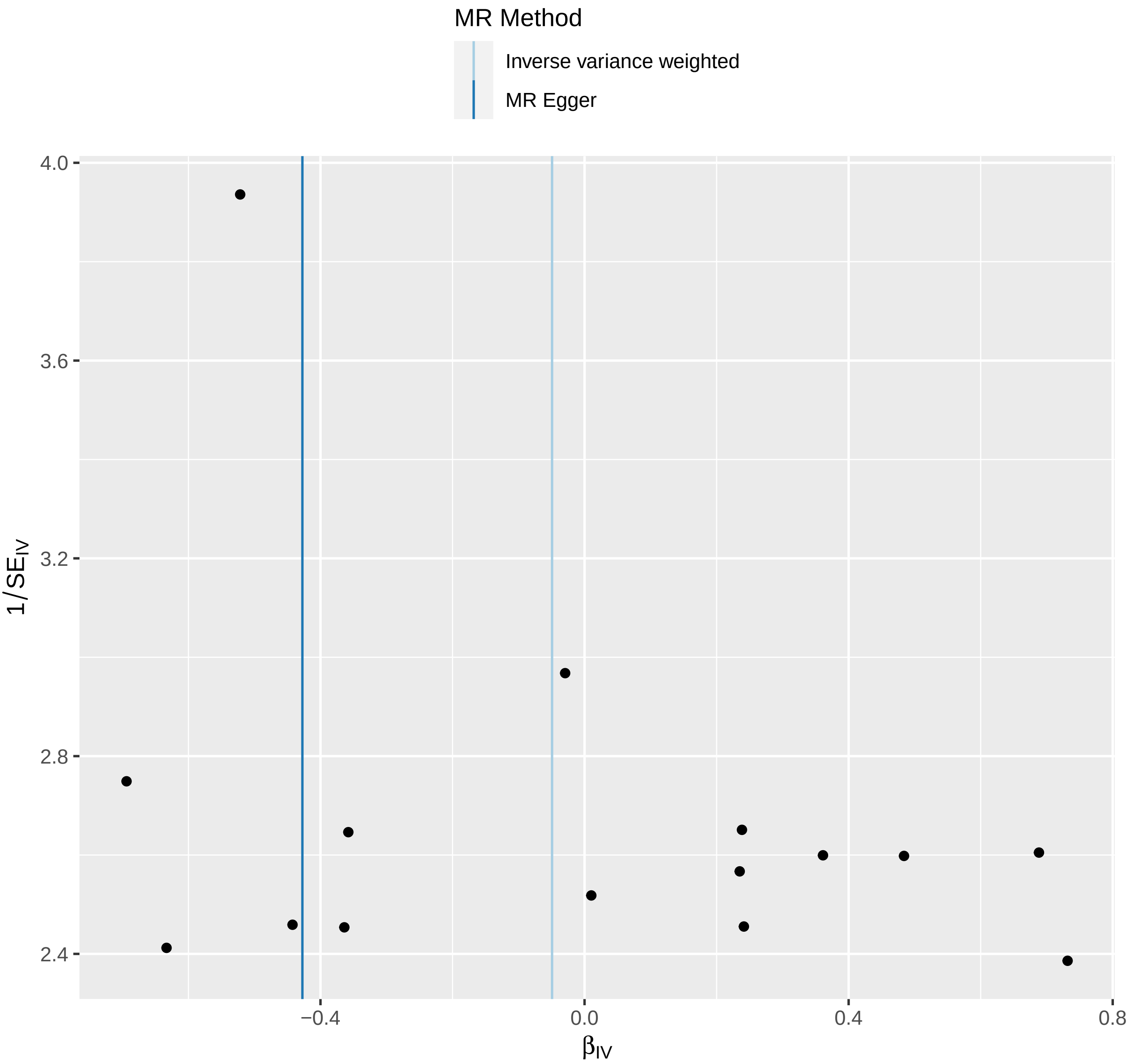

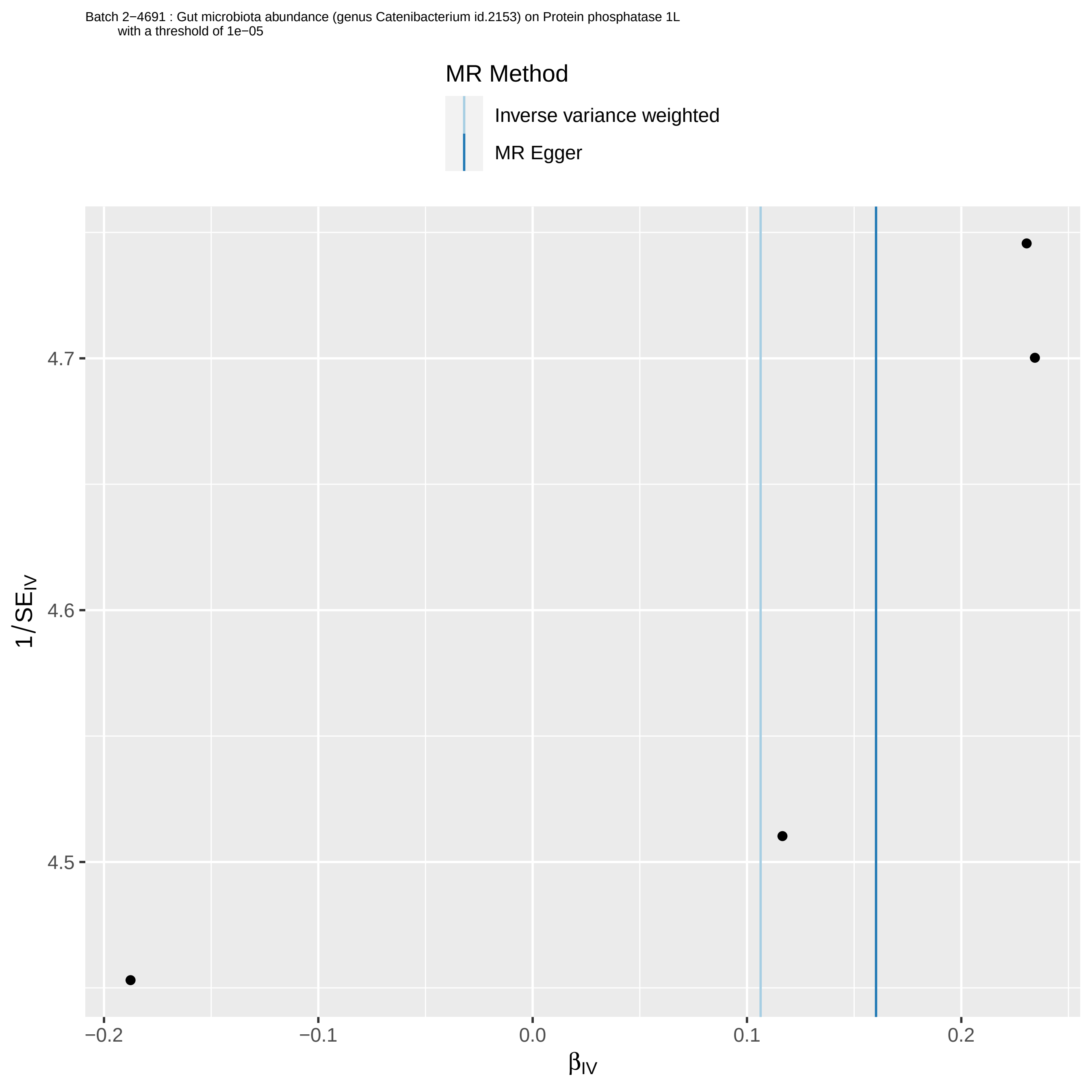
Batch 2−4691 : Gut microbiota abundance (genus Catenibacterium id.2153) on Protein phosphatase 1L
with a threshold of 1e−05
MR Method
Inverse variance weighted
MR Egger
4.7
4.6
4.5
1/SE$_{IV}$
−0.2
−0.1
0.0
0.1
0.2
β$_{IV}$

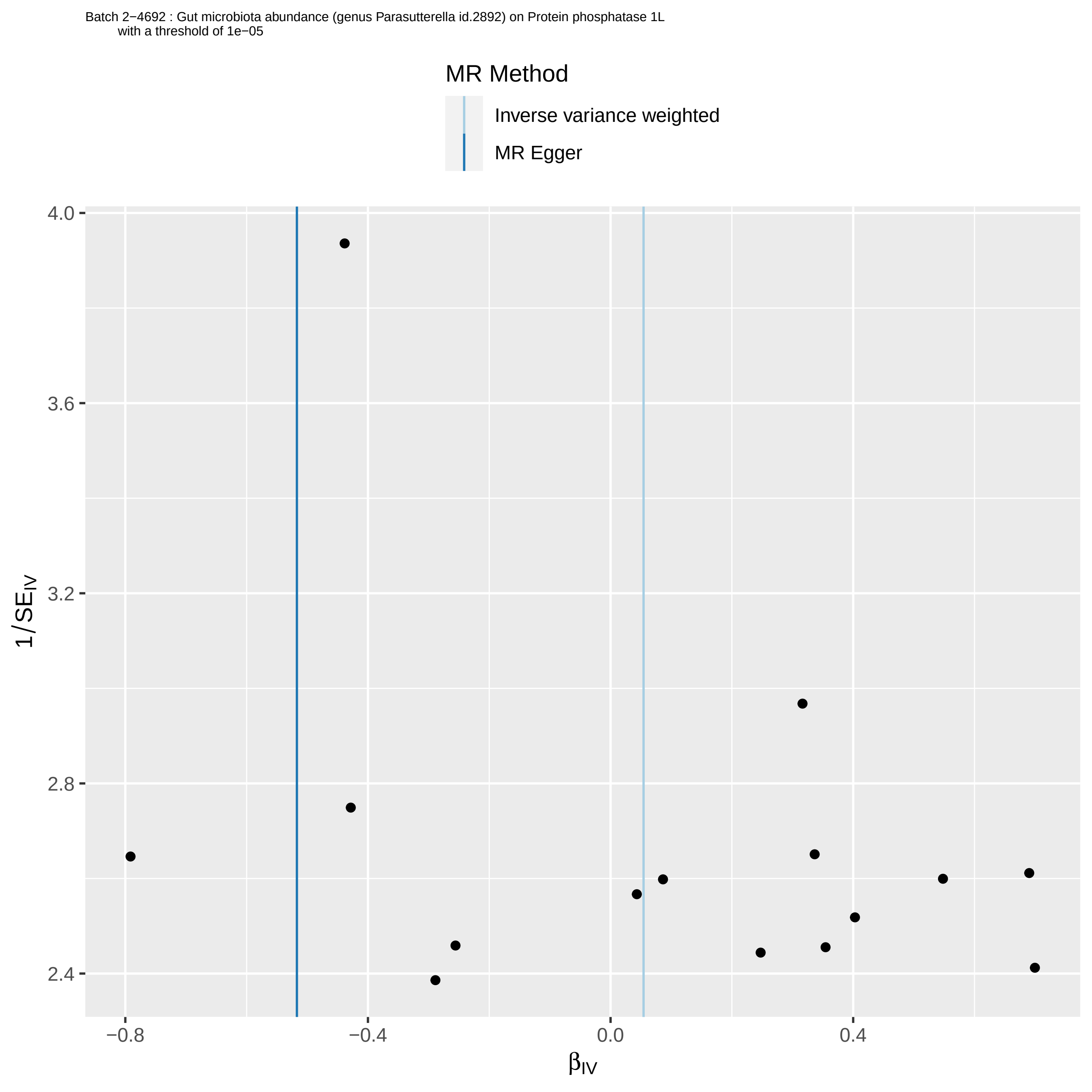

Batch 2−4692 : Gut microbiota abundance (genus Parasutterella id.2892) on Protein phosphatase 1L
with a threshold of 1e−05
MR Method
Inverse variance weighted
MR Egger
4.0
3.6
3.2
2.8
2.4
1/SE_IV
−0.8
−0.4
0.0
0.4
β_IV

Batch 2−4693 : Gut microbiota abundance (genus Catenibacterium id.2153) on Protein phosphatase 1 regulatory subunit 3B with a threshold of 1e−05

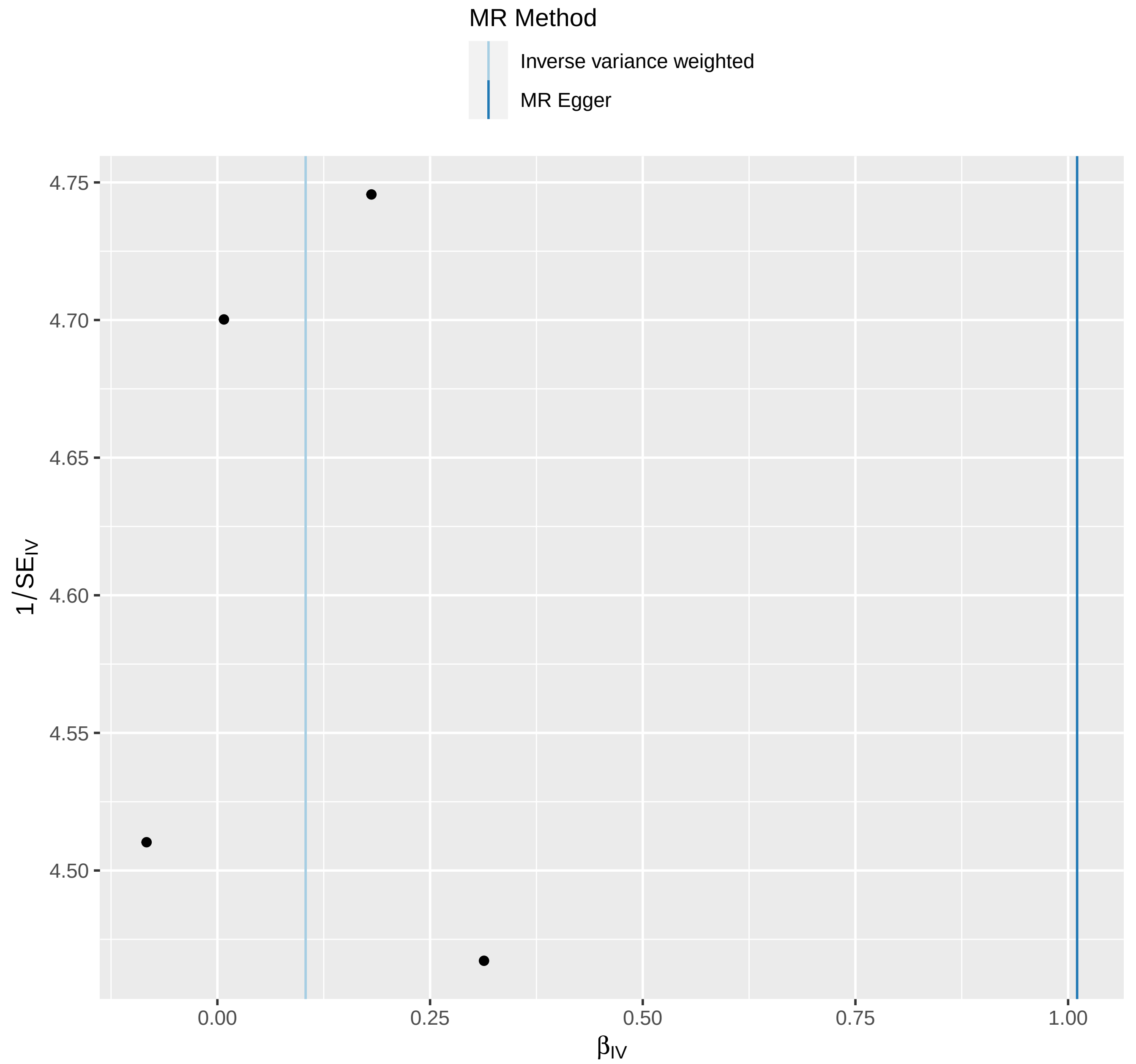

Batch 2−4694 : Gut microbiota abundance (genus Parasutterella id.2892) on Protein phosphatase 1 regulatory subunit 3B
with a threshold of 1e−05

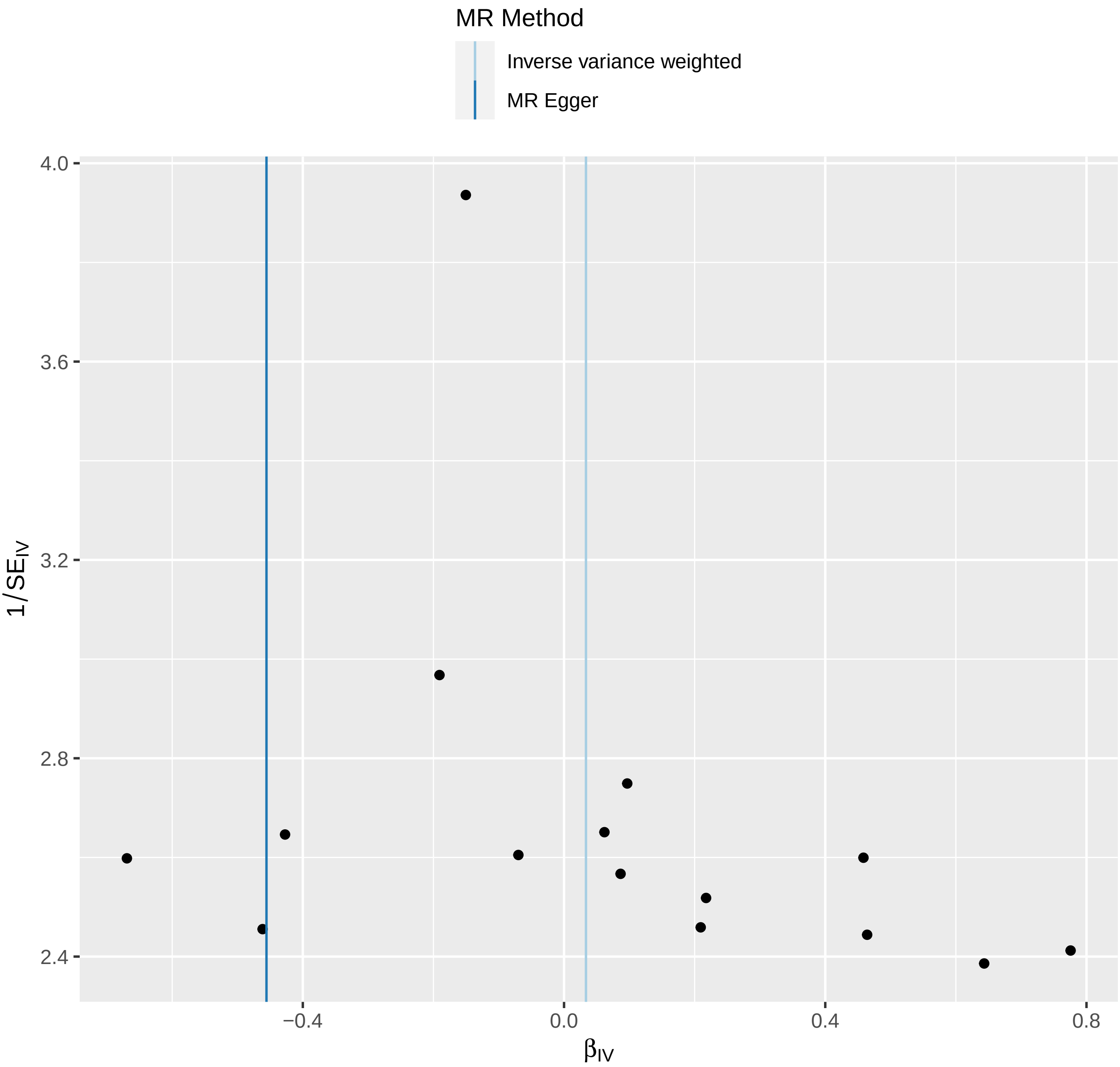

Batch 2−4695 : Gut microbiota abundance (genus Catenibacterium id.2153) on Serine/threonine−protein phosphatase 2A 65 kDa regulatory subunit A alpha isoform with a threshold of 1e−05

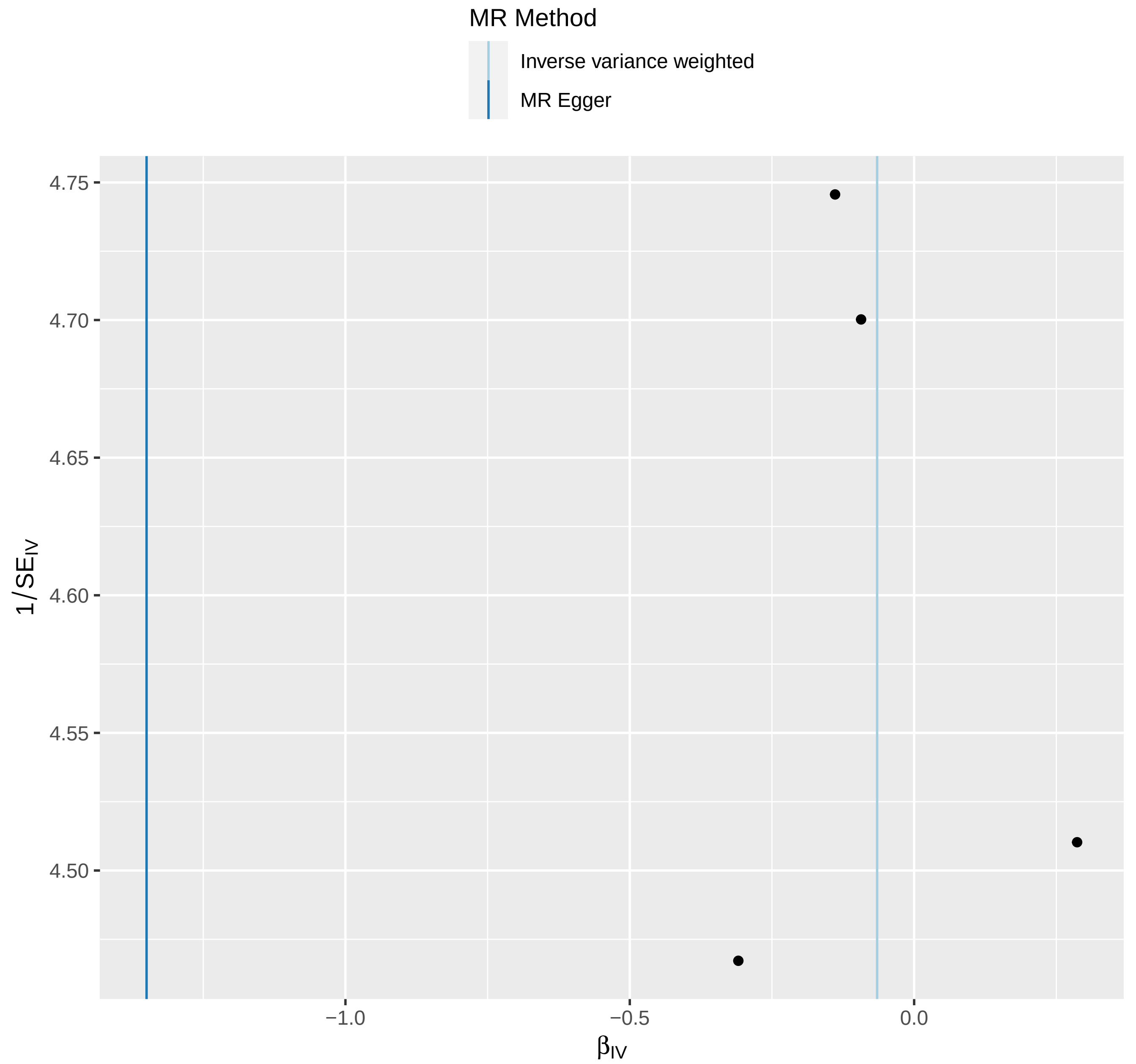

Batch 2−4696 : Gut microbiota abundance (genus Parasutterella id.2892) on Serine/threonine−protein phosphatase 2A 65 kDa regulatory subunit A alpha isoform
with a threshold of 1e−05

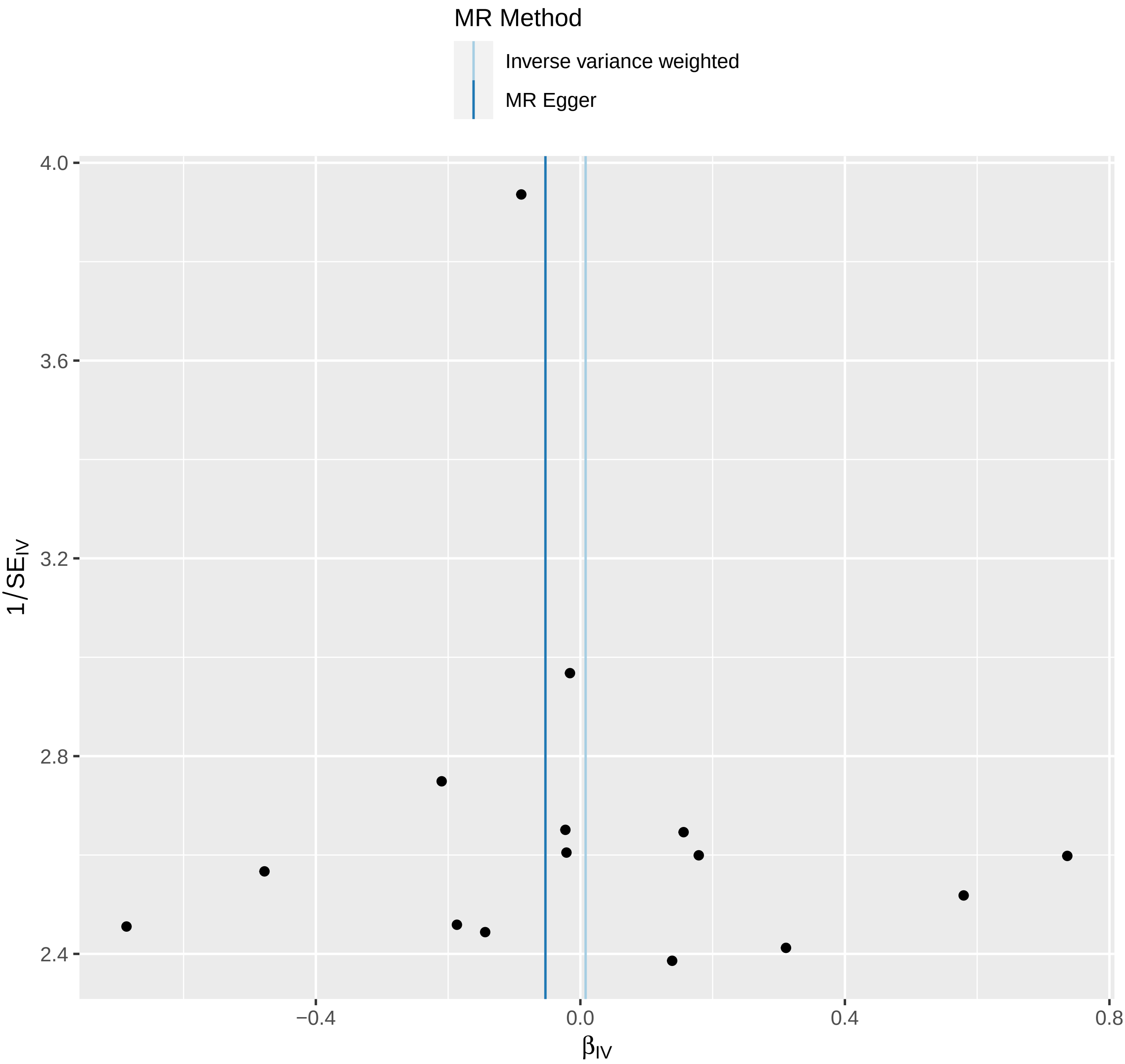

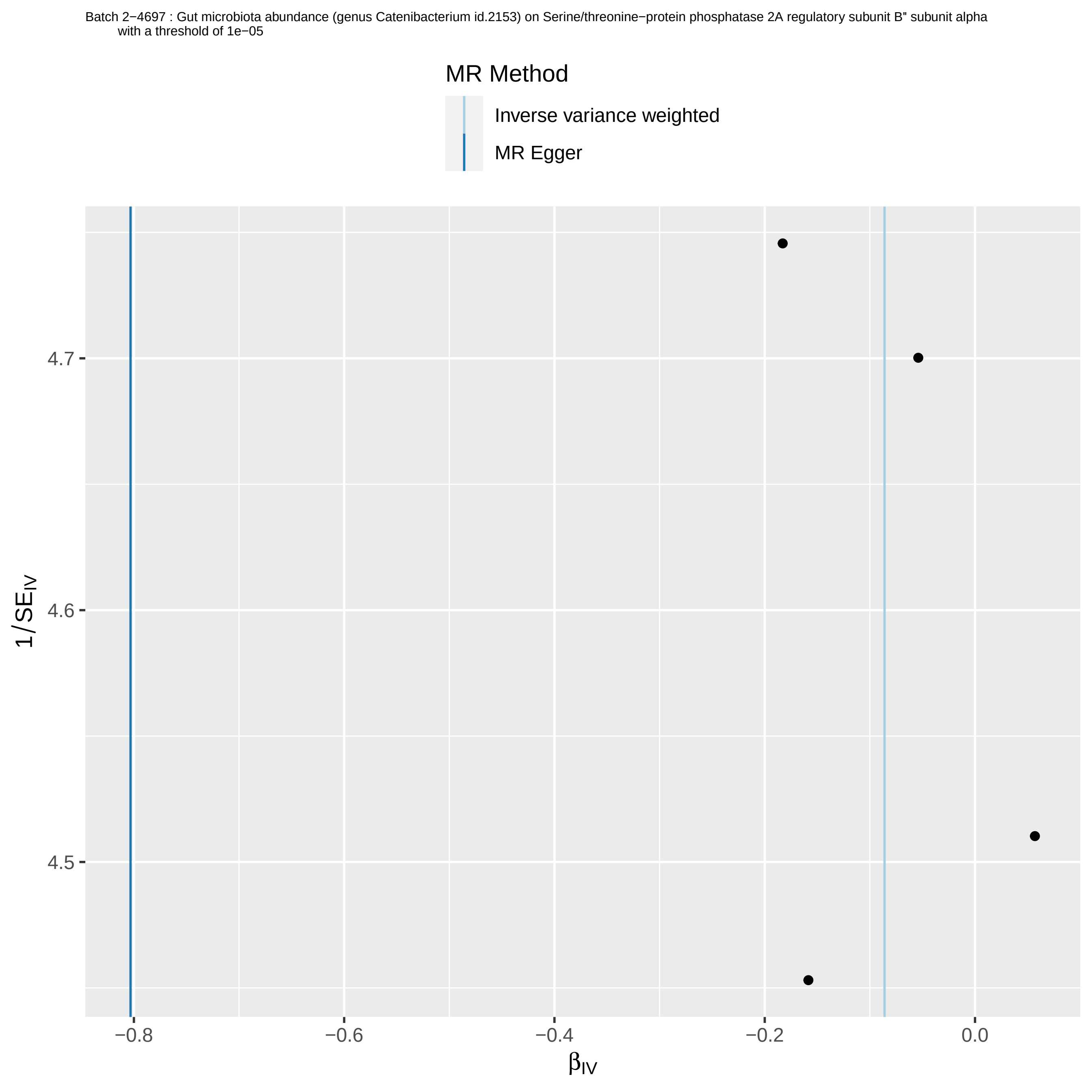
Batch 2−4697 : Gut microbiota abundance (genus Catenibacterium id.2153) on Serine/threonine−protein phosphatase 2A regulatory subunit B" subunit alpha with a threshold of 1e−05
MR Method
Inverse variance weighted
MR Egger
4.7
4.6
4.5
1/SE_IV
−0.8
−0.6
−0.4
−0.2
0.0
β_IV

Batch 2−4698 : Gut microbiota abundance (genus Parasutterella id.2892) on Serine/threonine−protein phosphatase 2A regulatory subunit B" subunit alpha with a threshold of 1e−05

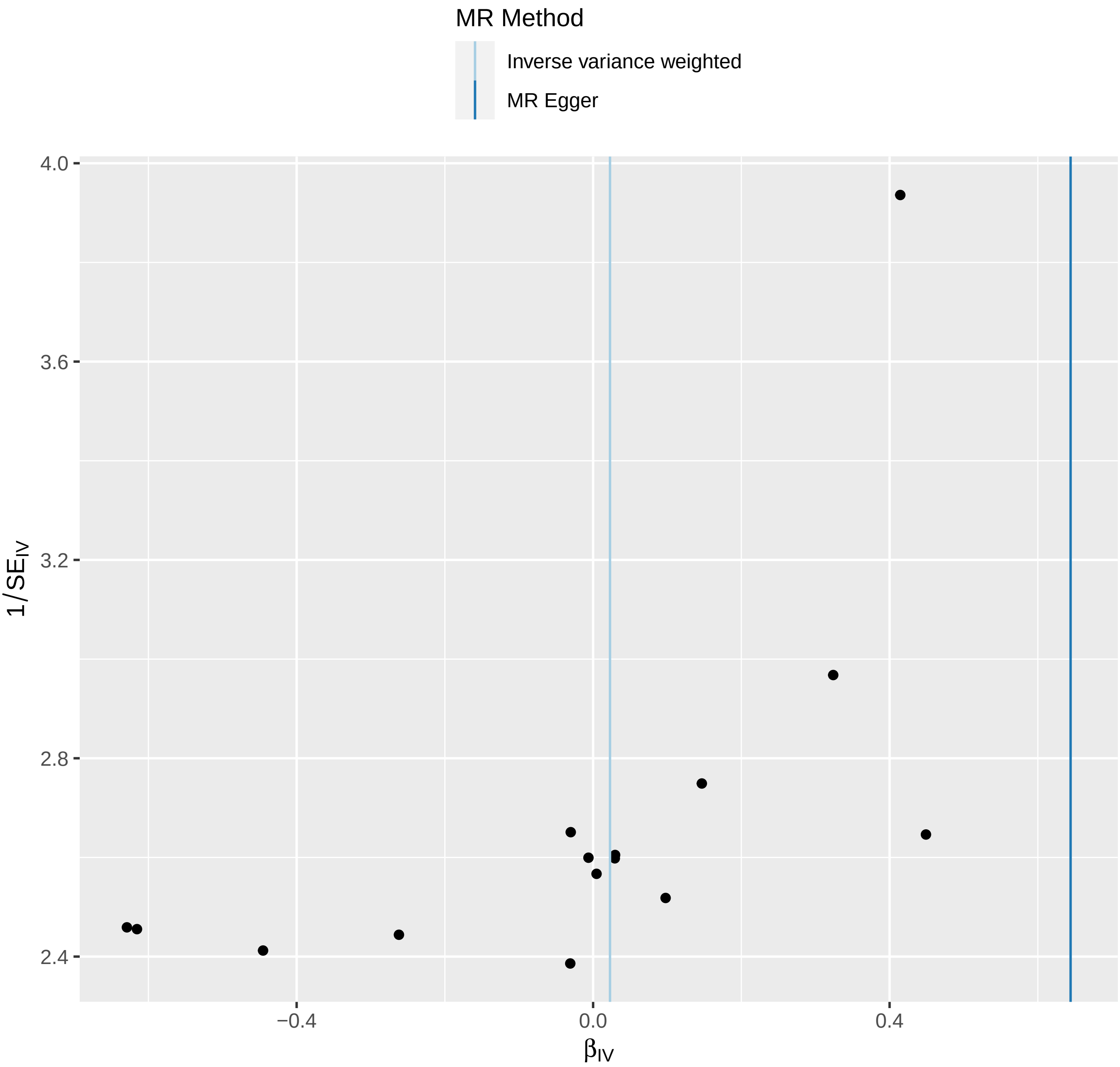

Batch 2−4699 : Gut microbiota abundance (genus Catenibacterium id.2153) on Calcineurin subunit B type 1
with a threshold of 1e−05

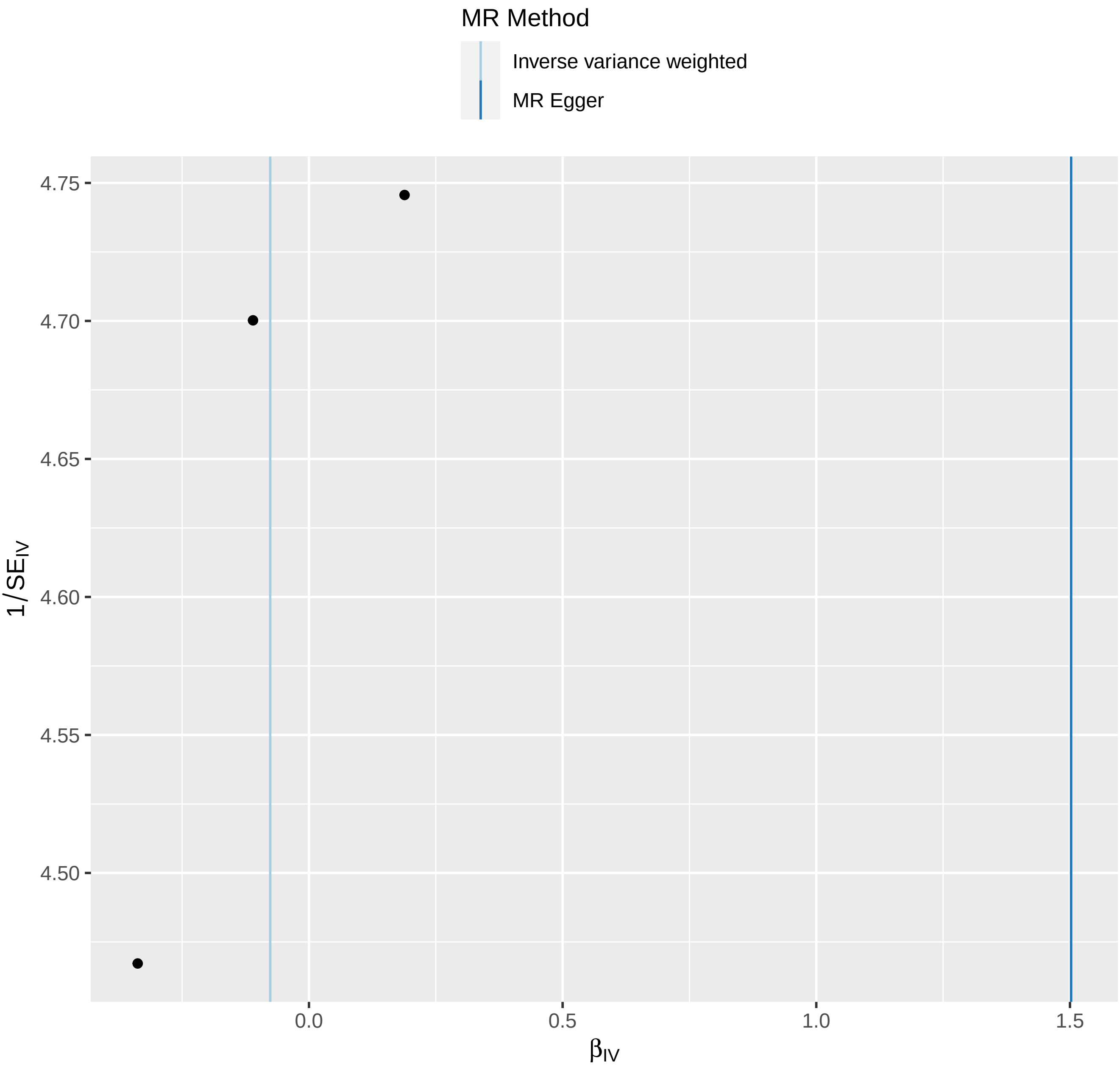

Batch 2−4700 : Gut microbiota abundance (genus Parasutterella id.2892) on Calcineurin subunit B type 1
with a threshold of 1e−05

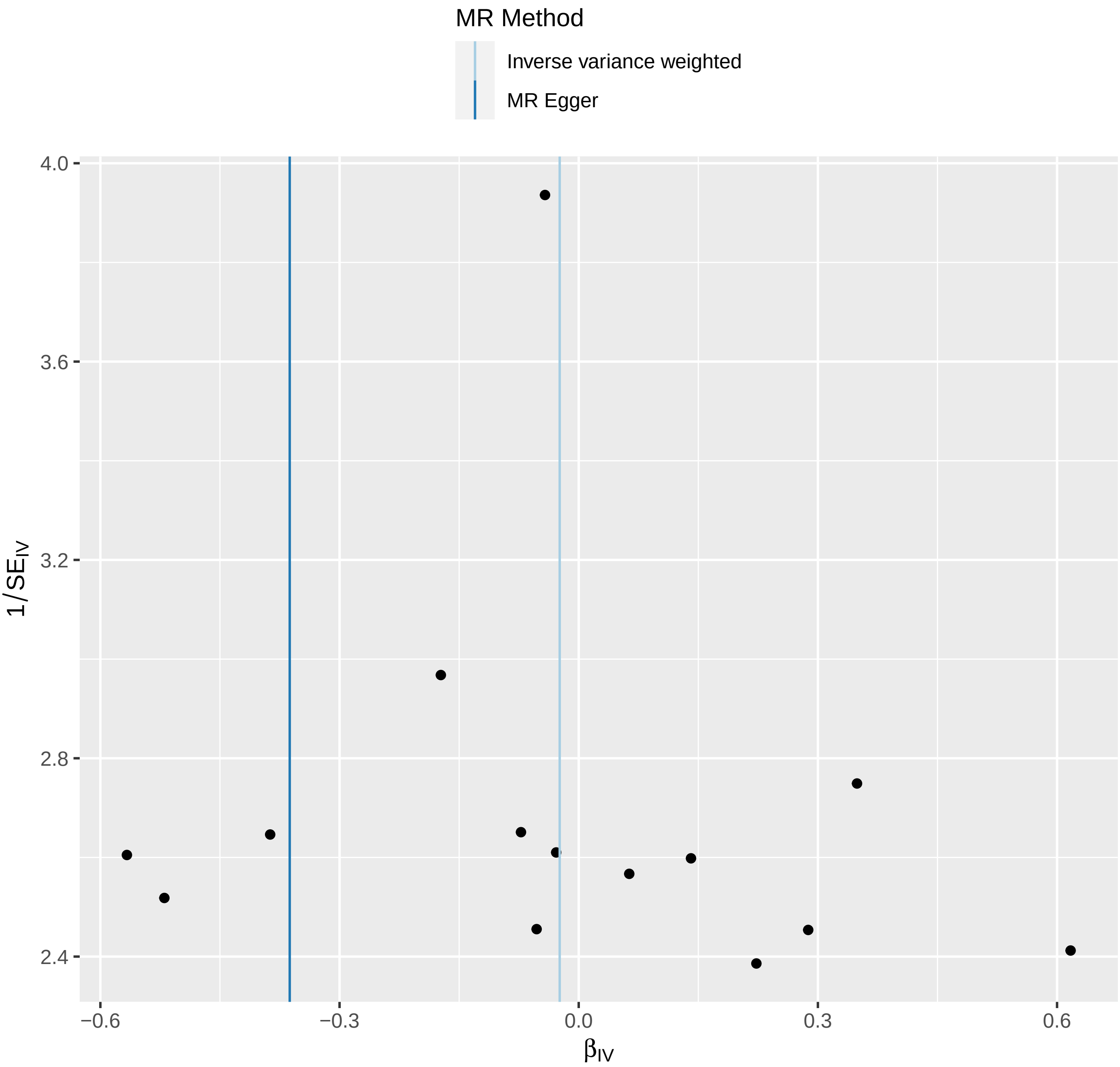

Batch 2−4701 : Gut microbiota abundance (genus Catenibacterium id.2153) on Palmitoyl−protein thioesterase 1 with a threshold of 1e−05

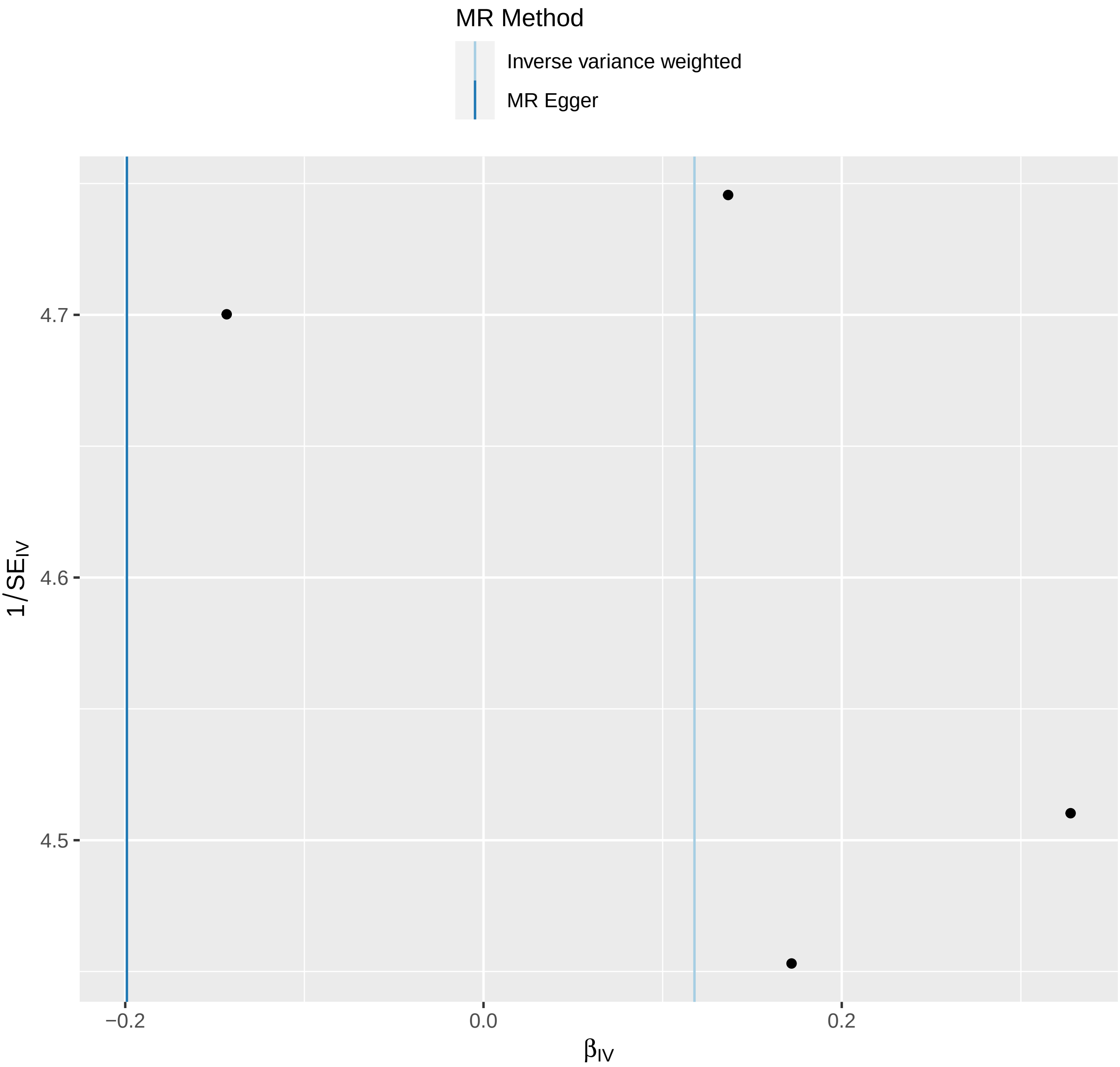

Batch 2−4702 : Gut microbiota abundance (genus Parasutterella id.2892) on Palmitoyl−protein thioesterase 1
with a threshold of 1e−05

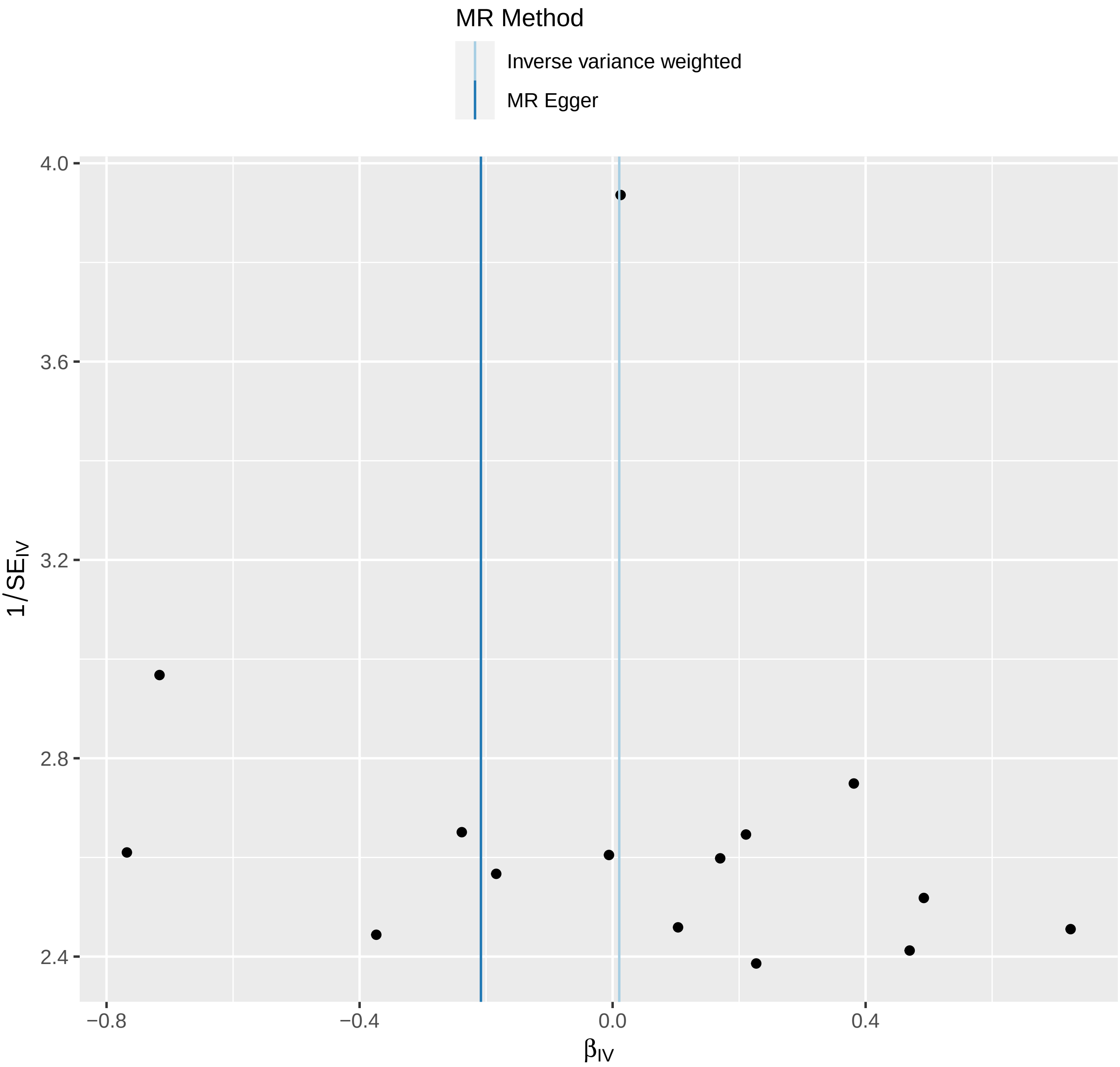

Batch 2−4703 : Gut microbiota abundance (genus Catenibacterium id.2153) on Pancreatic hormone
with a threshold of 1e−05

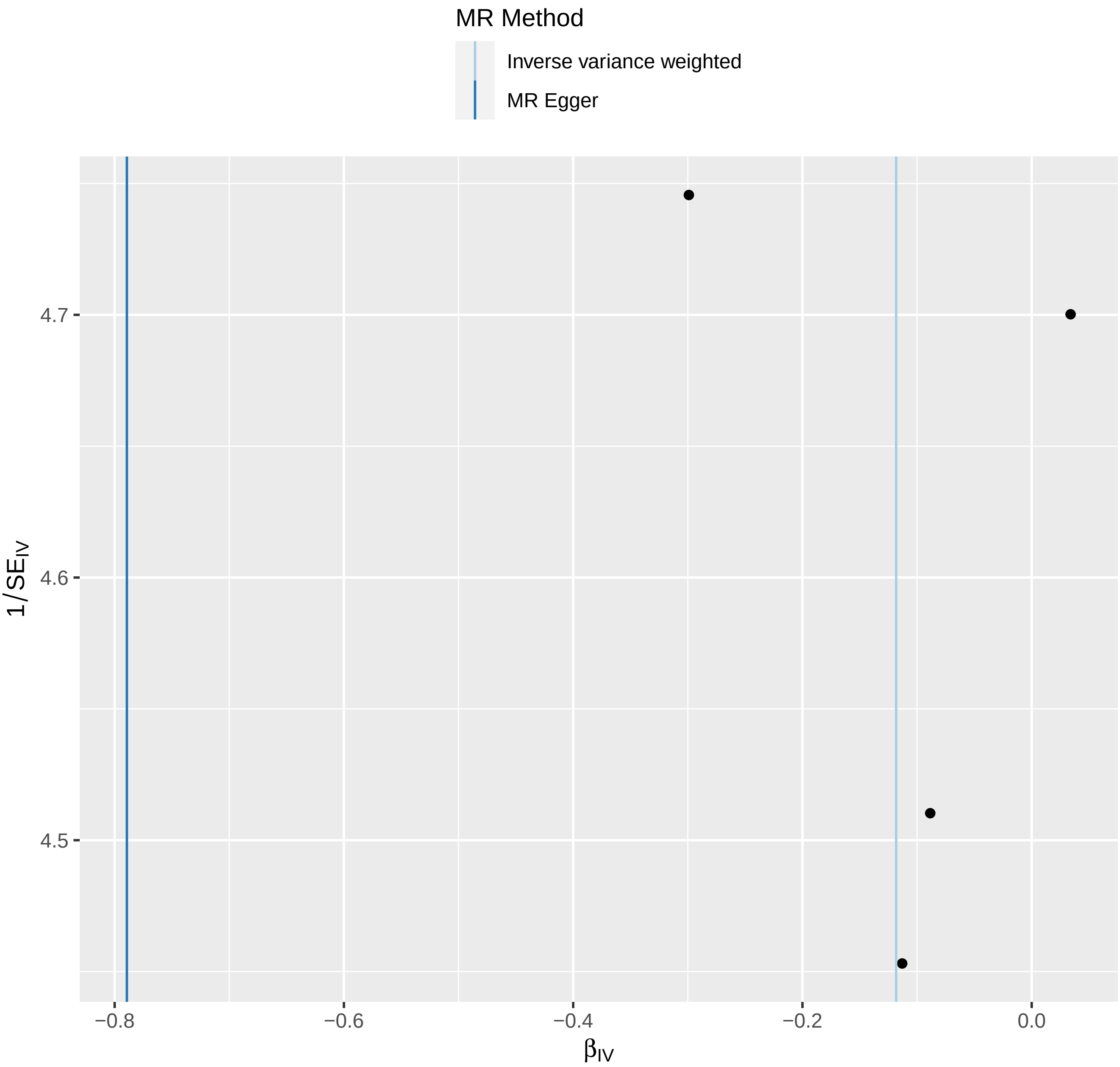

Batch 2−4704 : Gut microbiota abundance (genus Parasutterella id.2892) on Pancreatic hormone
with a threshold of 1e−05

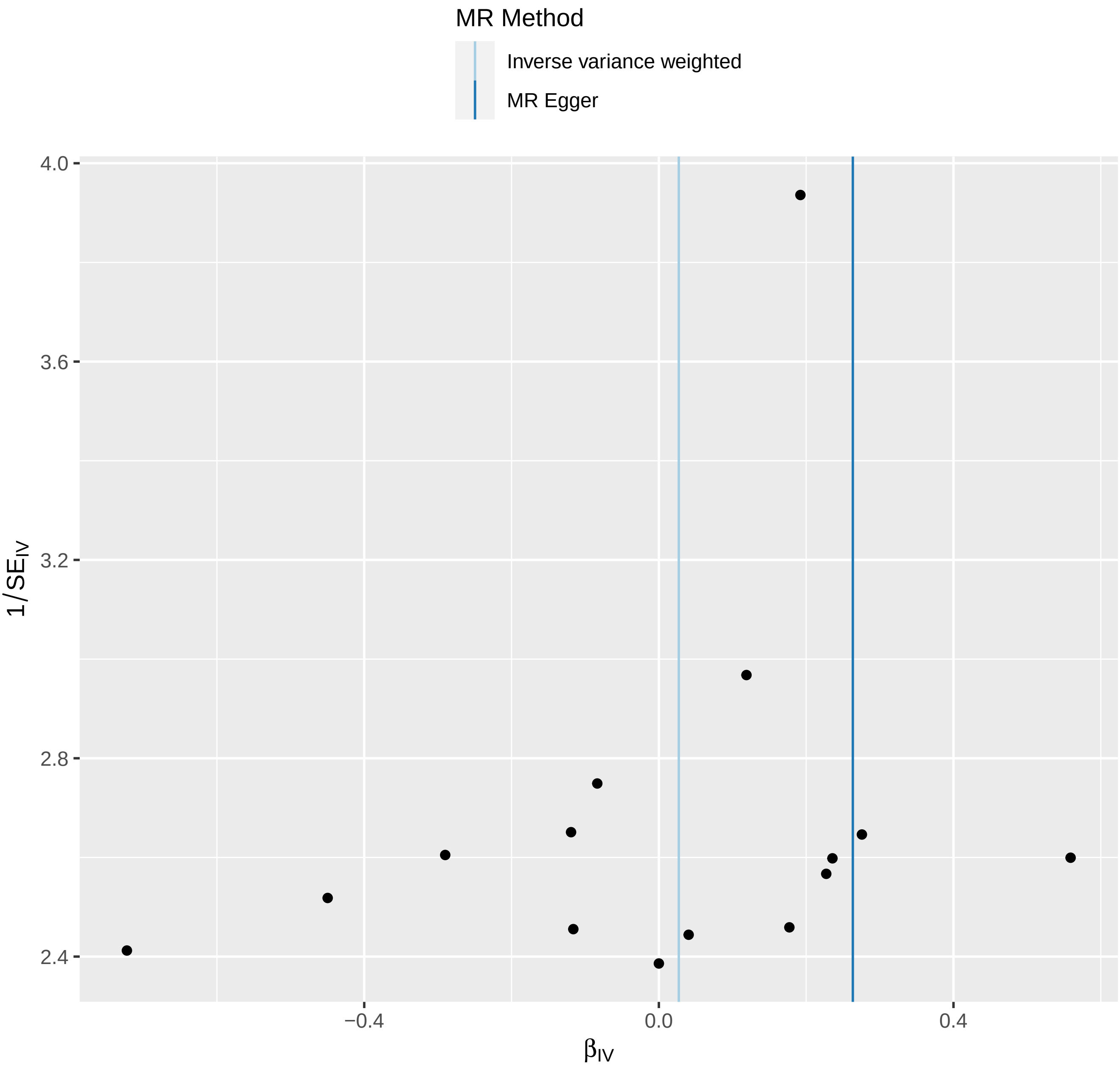

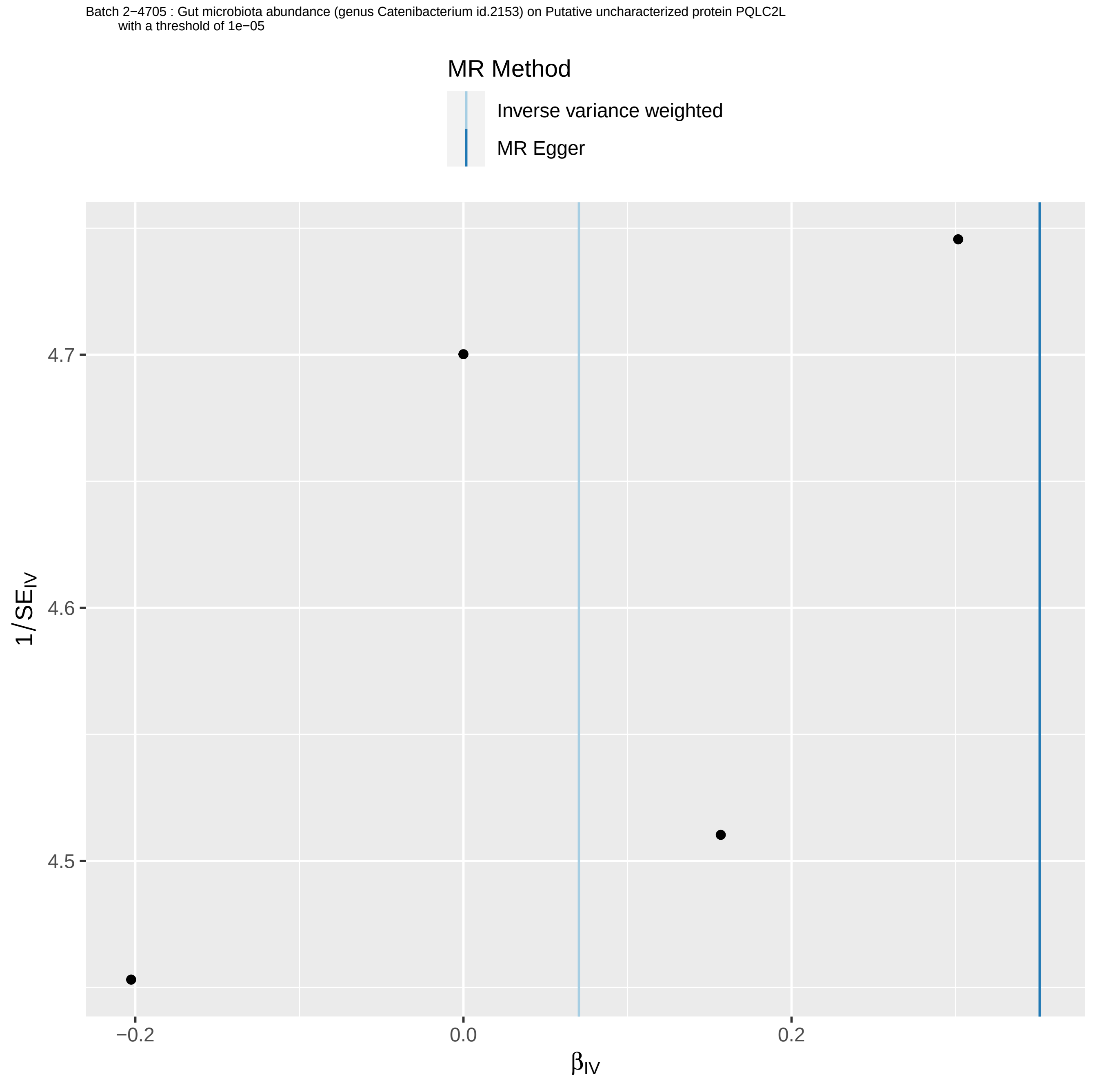

Batch 2−4705 : Gut microbiota abundance (genus Catenibacterium id.2153) on Putative uncharacterized protein PQLC2L
with a threshold of 1e−05
MR Method
Inverse variance weighted
MR Egger
4.7
4.6
4.5
1/SE$_{IV}$
−0.2
0.0
0.2
β_{IV}

Batch 2−4706 : Gut microbiota abundance (genus Parasutterella id.2892) on Putative uncharacterized protein PQLC2L
with a threshold of 1e−05

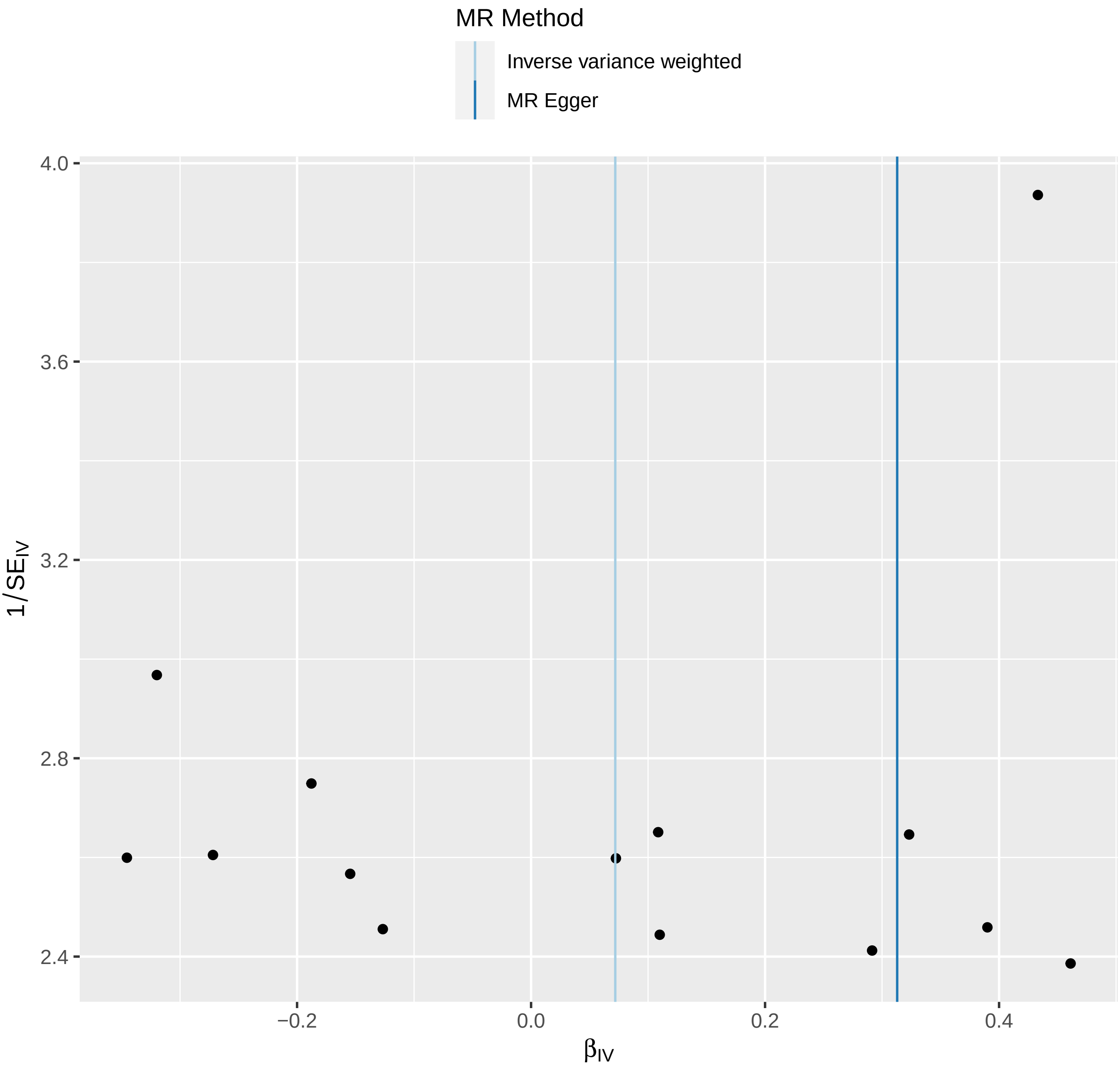

Batch 2−4707 : Gut microbiota abundance (genus Catenibacterium id.2153) on Basic salivary proline−rich protein 4
with a threshold of 1e−05

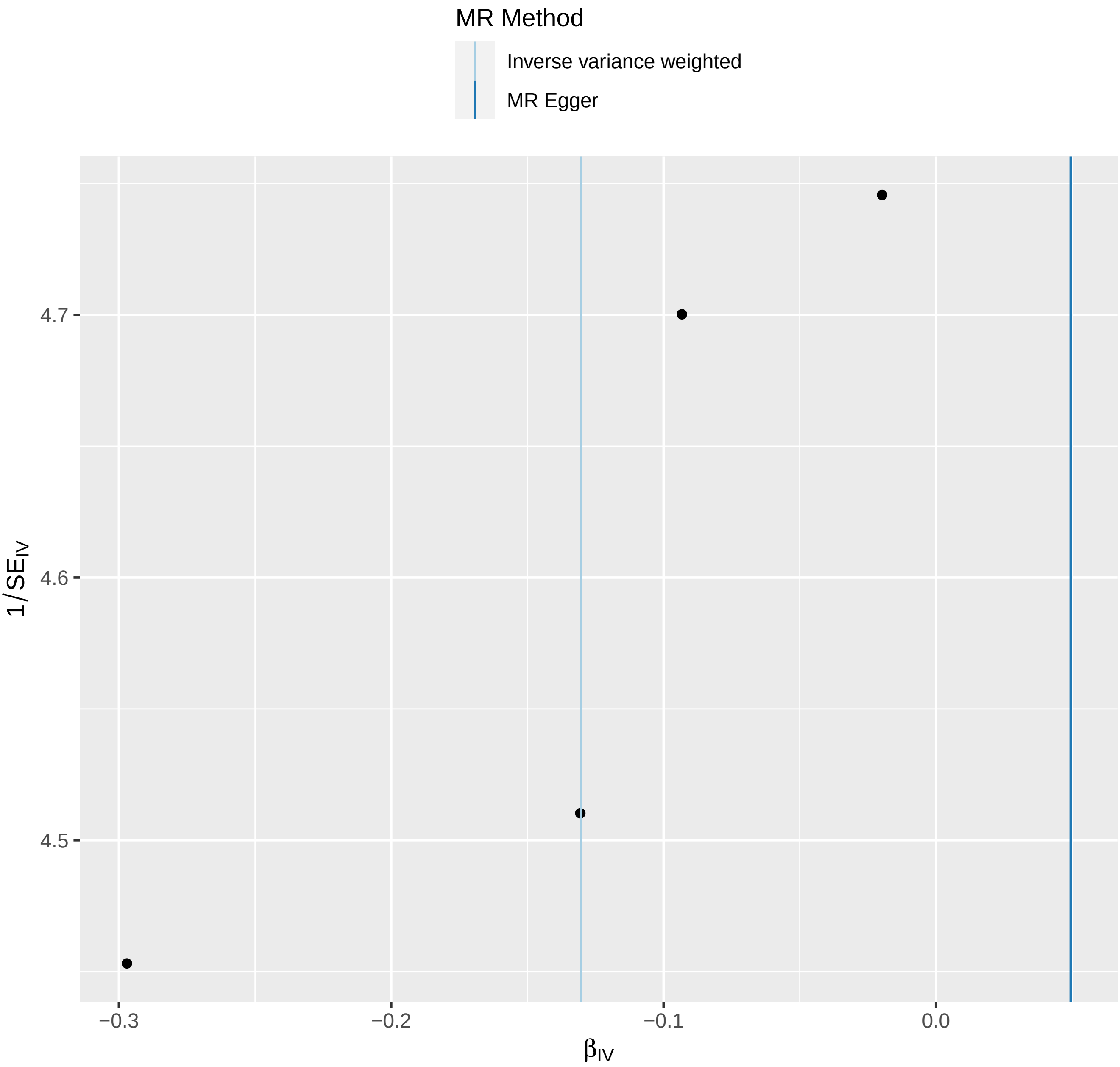

Batch 2−4708 : Gut microbiota abundance (genus Parasutterella id.2892) on Basic salivary proline−rich protein 4
with a threshold of 1e−05

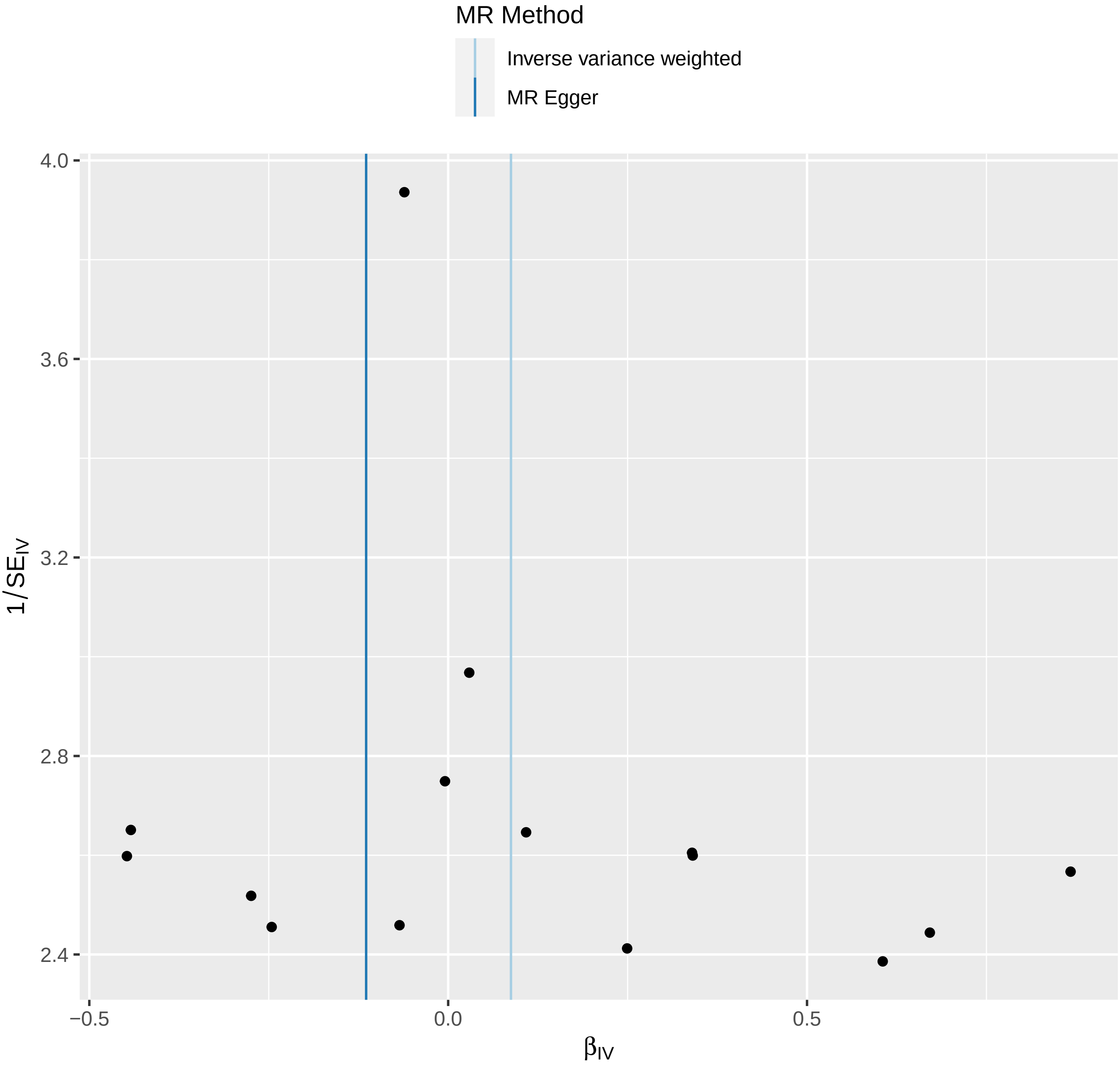

Batch 2−4709 : Gut microbiota abundance (genus Catenibacterium id.2153) on Protein regulator of cytokinesis 1
with a threshold of 1e−05

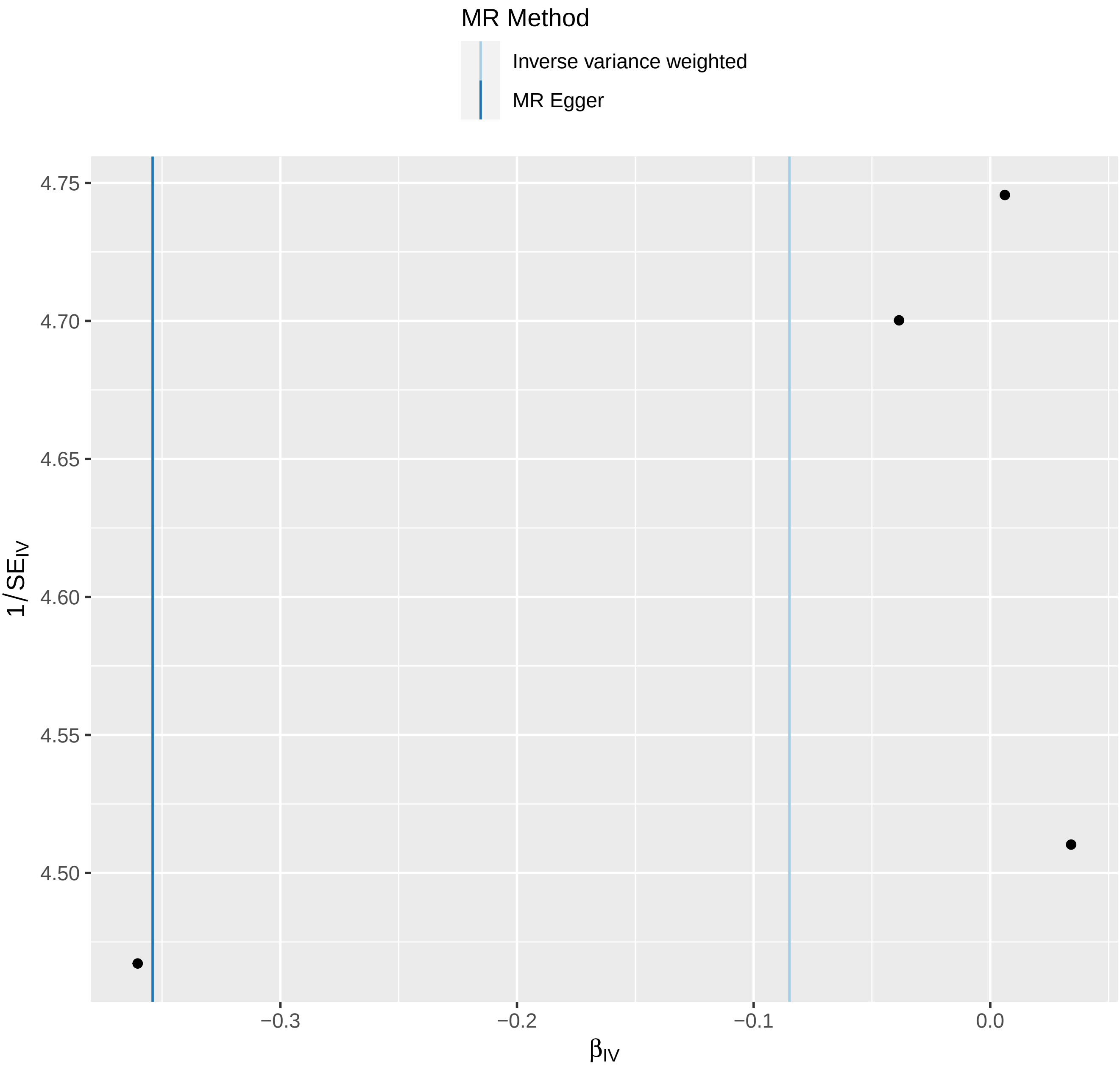

Batch 2−4710 : Gut microbiota abundance (genus Parasutterella id.2892) on Protein regulator of cytokinesis 1
with a threshold of 1e−05

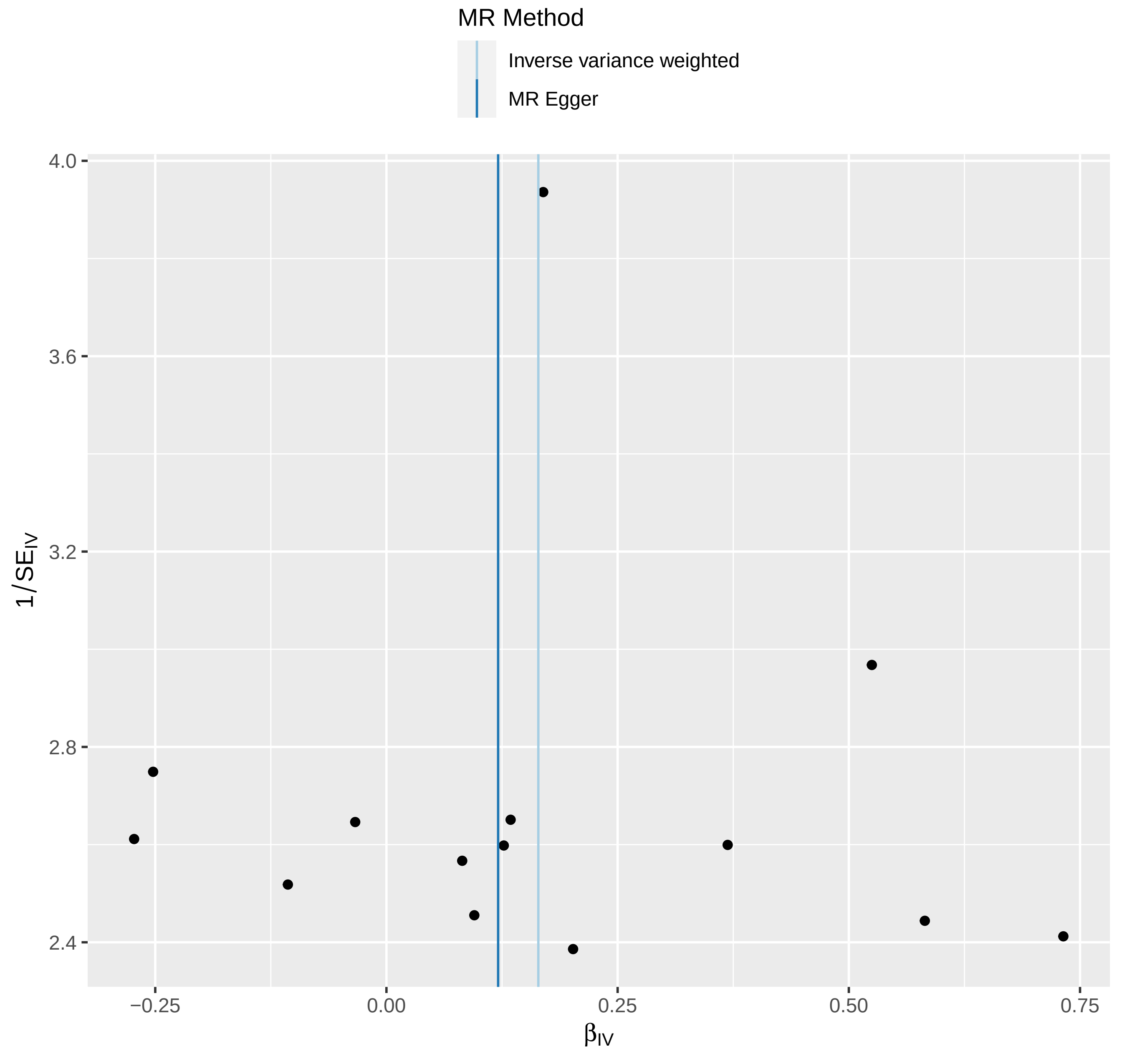

Batch 2−4711 : Gut microbiota abundance (genus Catenibacterium id.2153) on Lysosomal Pro−X carboxypeptidase
with a threshold of 1e−05

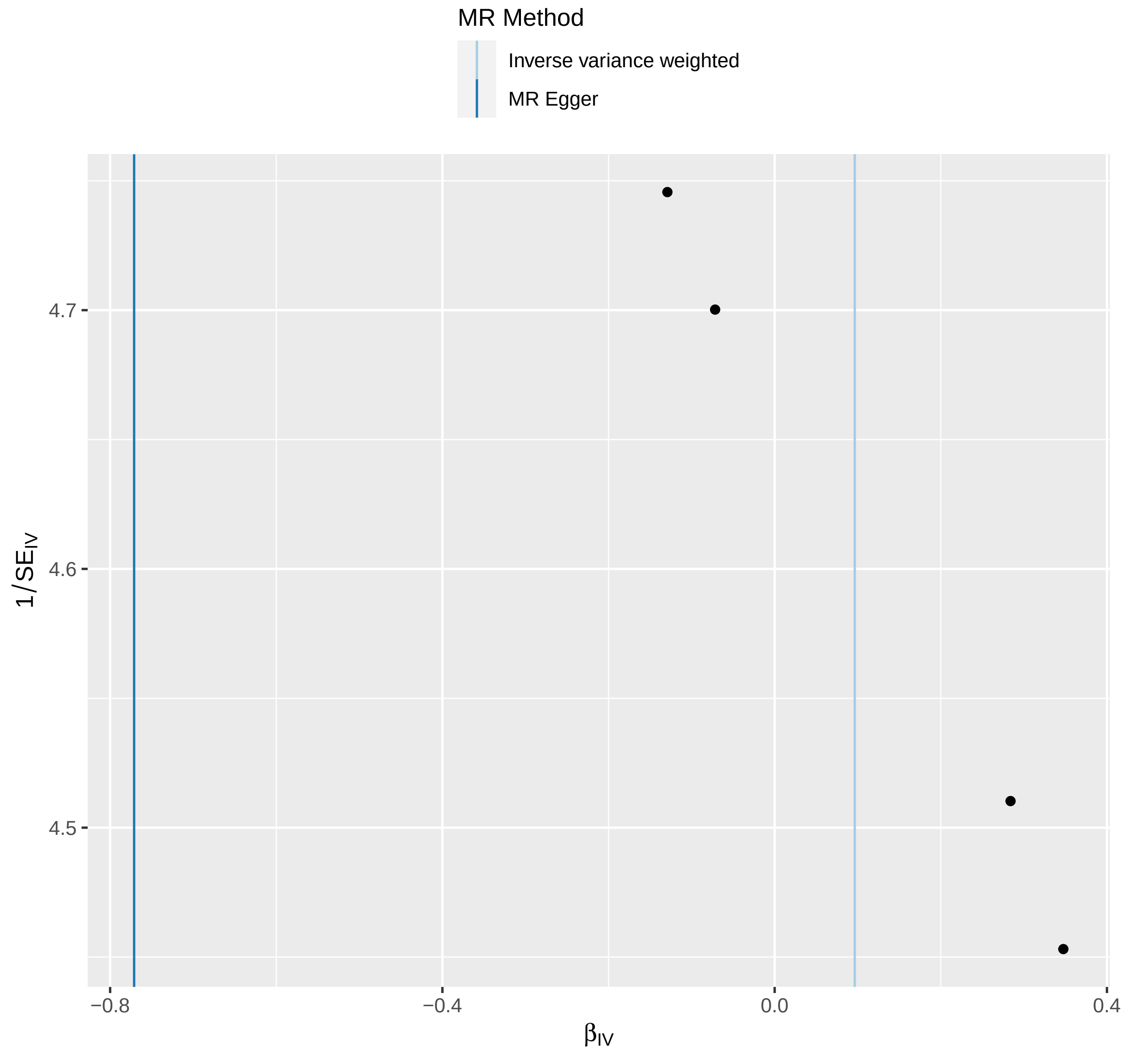

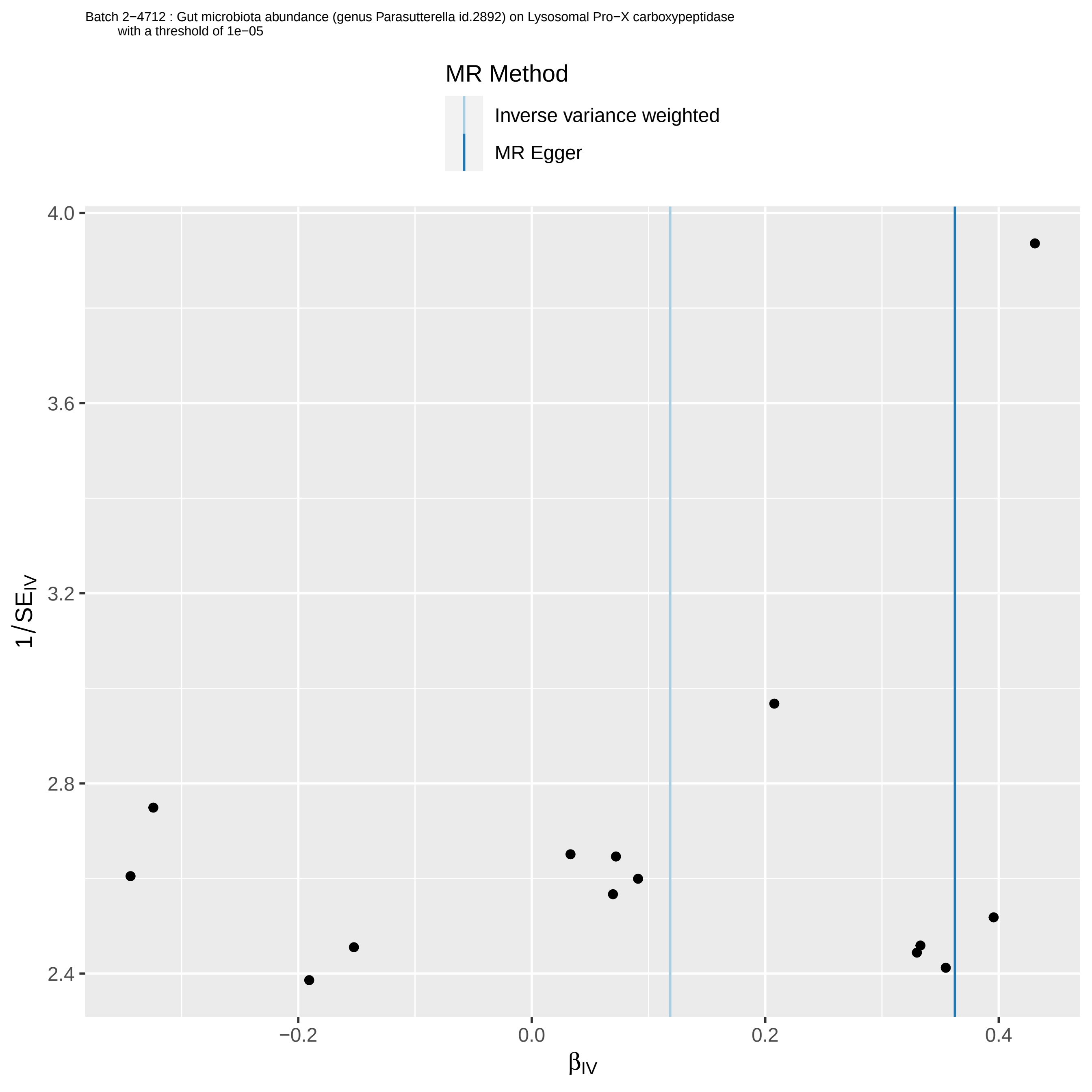
Batch 2−4712 : Gut microbiota abundance (genus Parasutterella id.2892) on Lysosomal Pro−X carboxypeptidase
with a threshold of 1e−05
MR Method
Inverse variance weighted
MR Egger
4.0
3.6
3.2
2.8
2.4
1/SE_IV
−0.2
0.0
0.2
0.4
β_IV

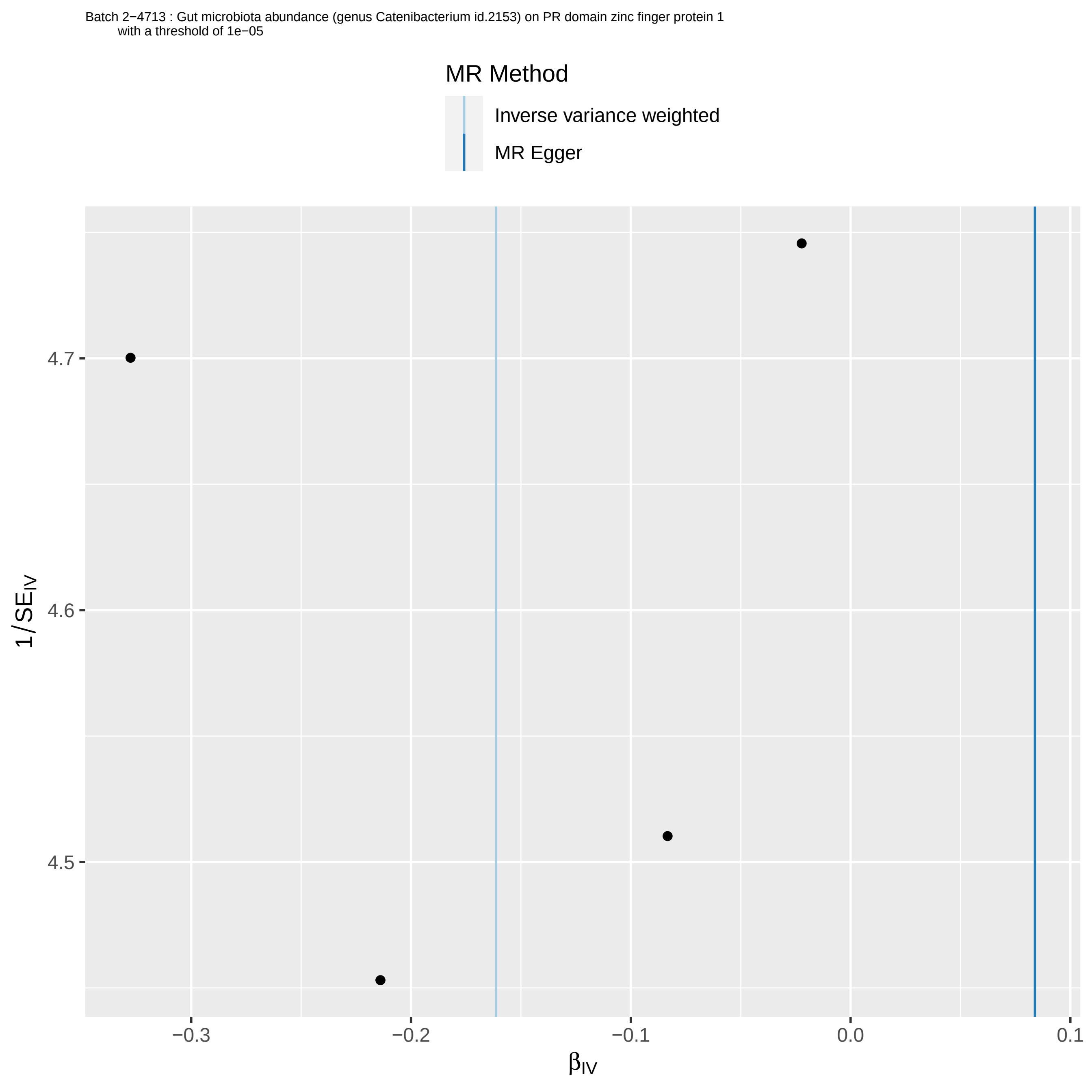

Batch 2−4713 : Gut microbiota abundance (genus Catenibacterium id.2153) on PR domain zinc finger protein 1
with a threshold of 1e−05
MR Method
Inverse variance weighted
MR Egger
4.7
4.6
4.5
1/SE$_{IV}$
−0.3
−0.2
−0.1
0.0
0.1
β$_{IV}$

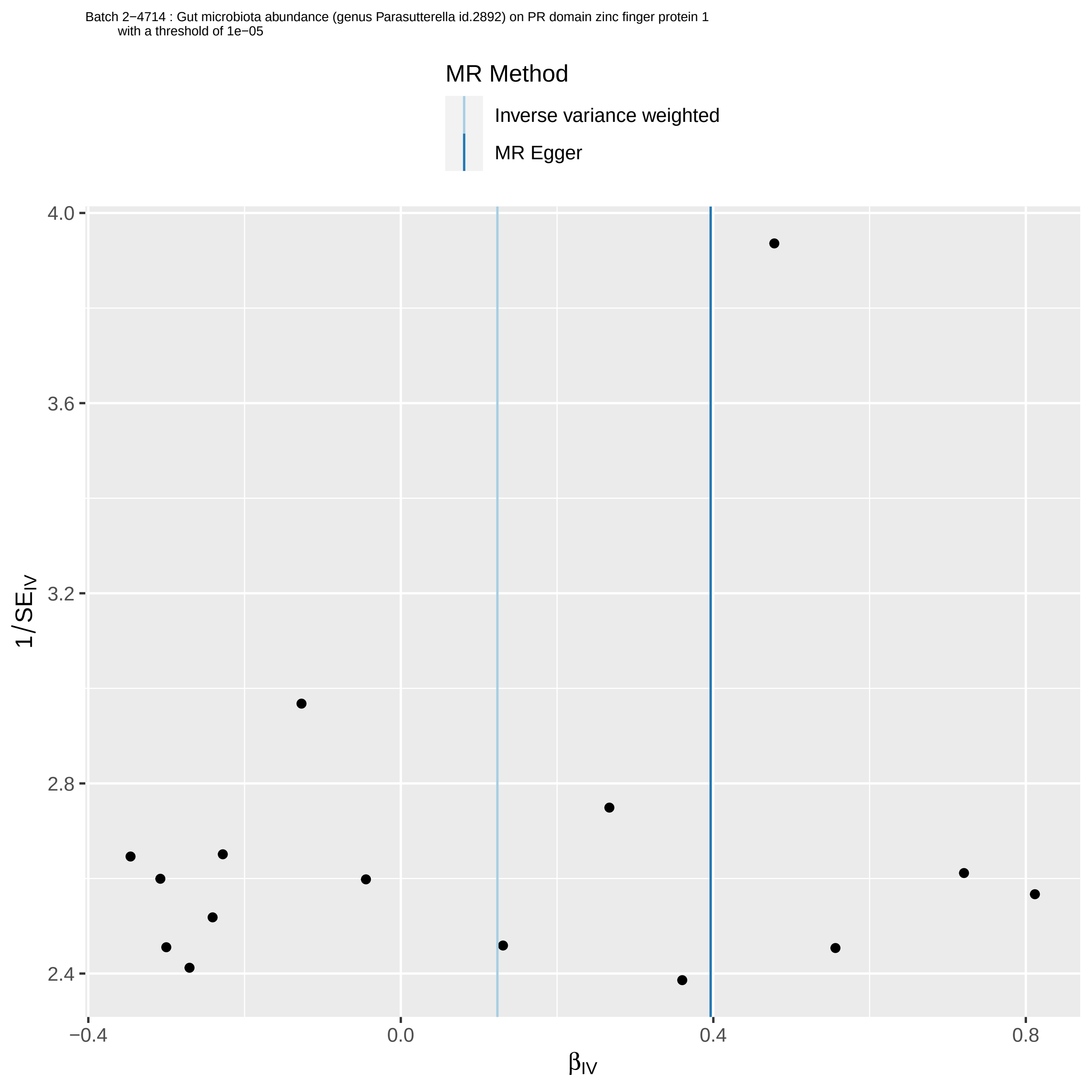

Batch 2−4714 : Gut microbiota abundance (genus Parasutterella id.2892) on PR domain zinc finger protein 1
with a threshold of 1e−05
MR Method
Inverse variance weighted
MR Egger
4.0
3.6
3.2
2.8
2.4
1/SE_IV
−0.4
0.0
0.4
0.8
β_IV

Batch 2−4715 : Gut microbiota abundance (genus Catenibacterium id.2153) on PR domain zinc finger protein 4
with a threshold of 1e−05

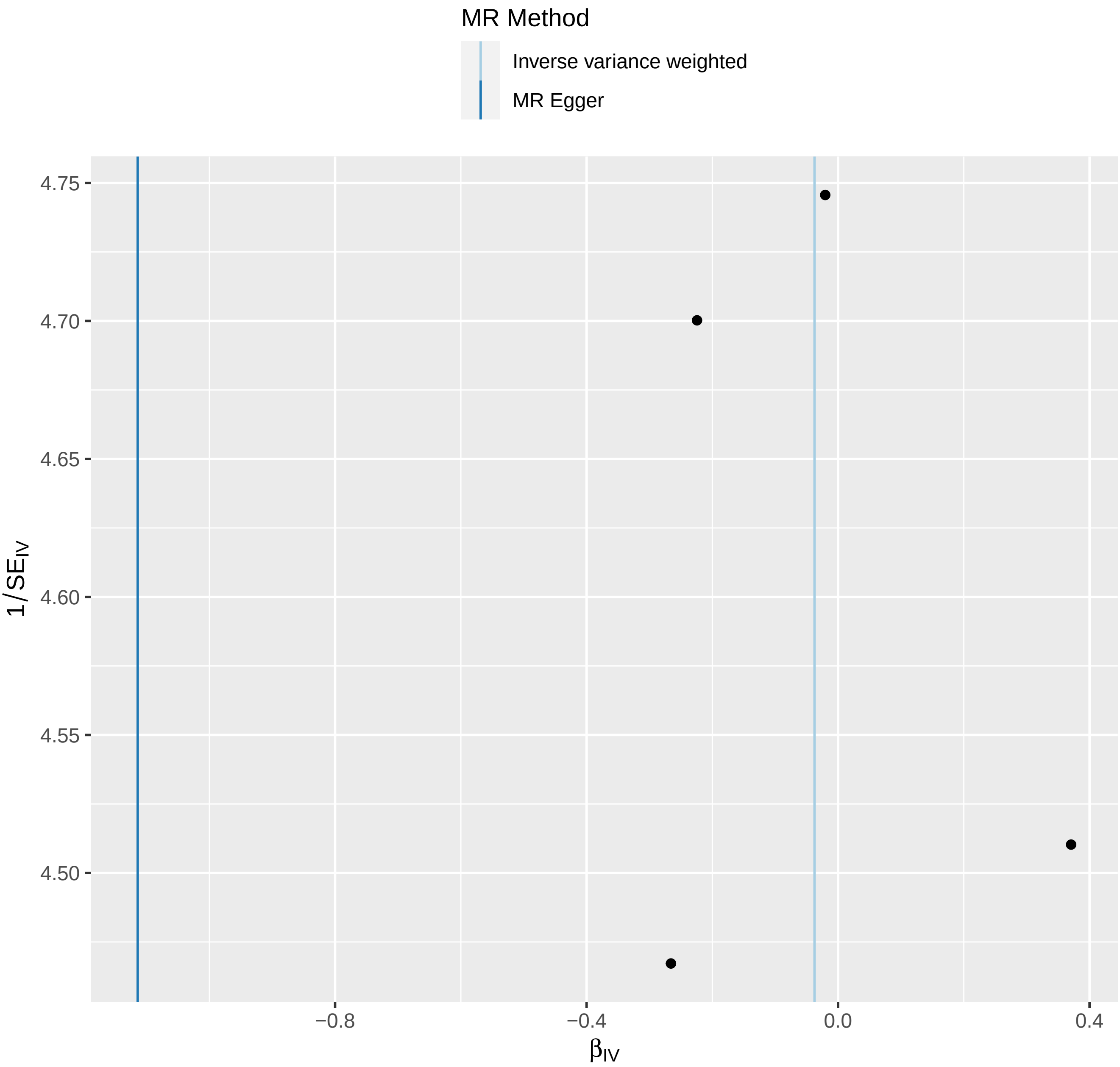

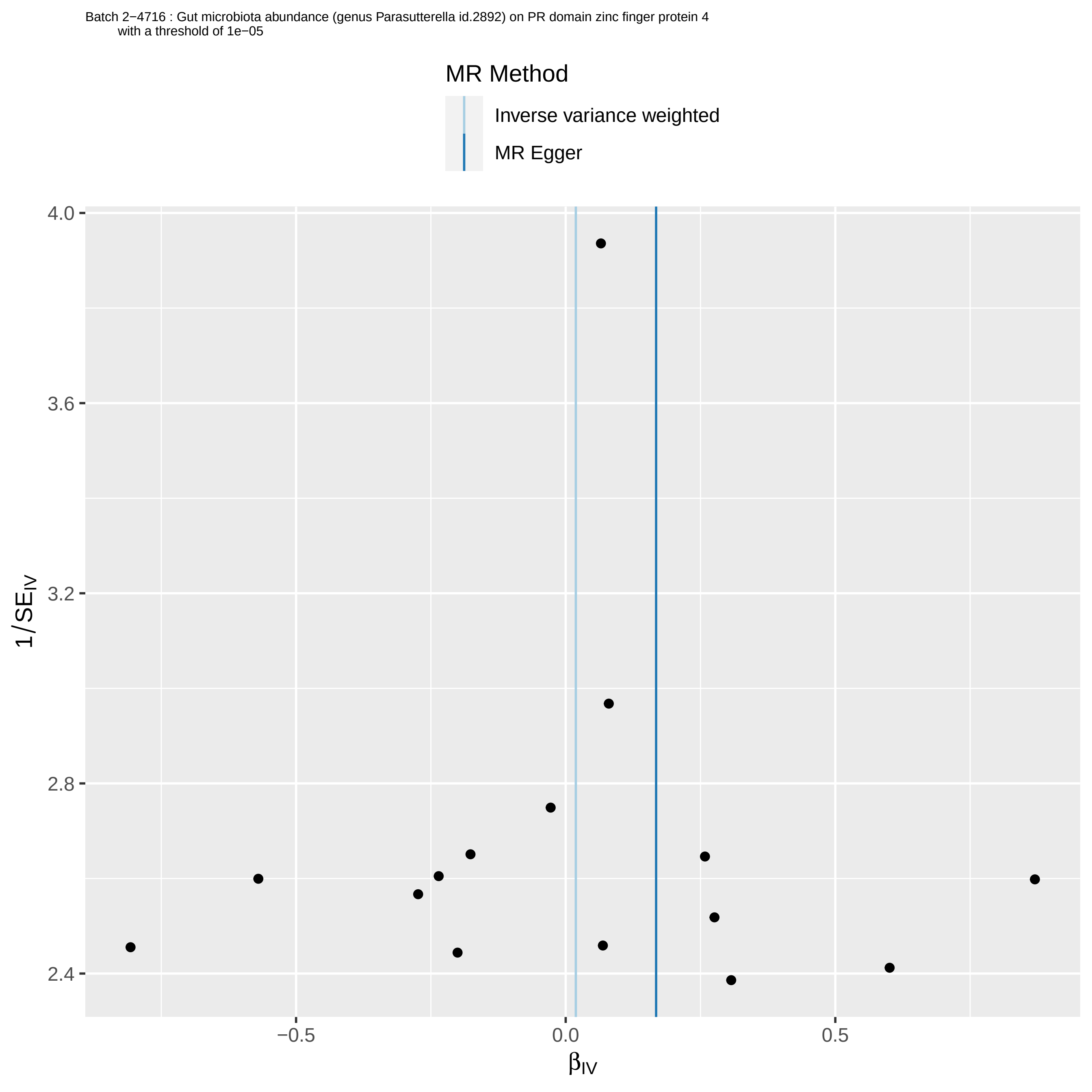

Batch 2−4716 : Gut microbiota abundance (genus Parasutterella id.2892) on PR domain zinc finger protein 4
with a threshold of 1e−05
MR Method
Inverse variance weighted
MR Egger
4.0
3.6
3.2
2.8
2.4
1/SE_IV
−0.5
0.0
0.5
β_IV

Batch 2−4717 : Gut microbiota abundance (genus Catenibacterium id.2153) on Peroxiredoxin−1
with a threshold of 1e−05

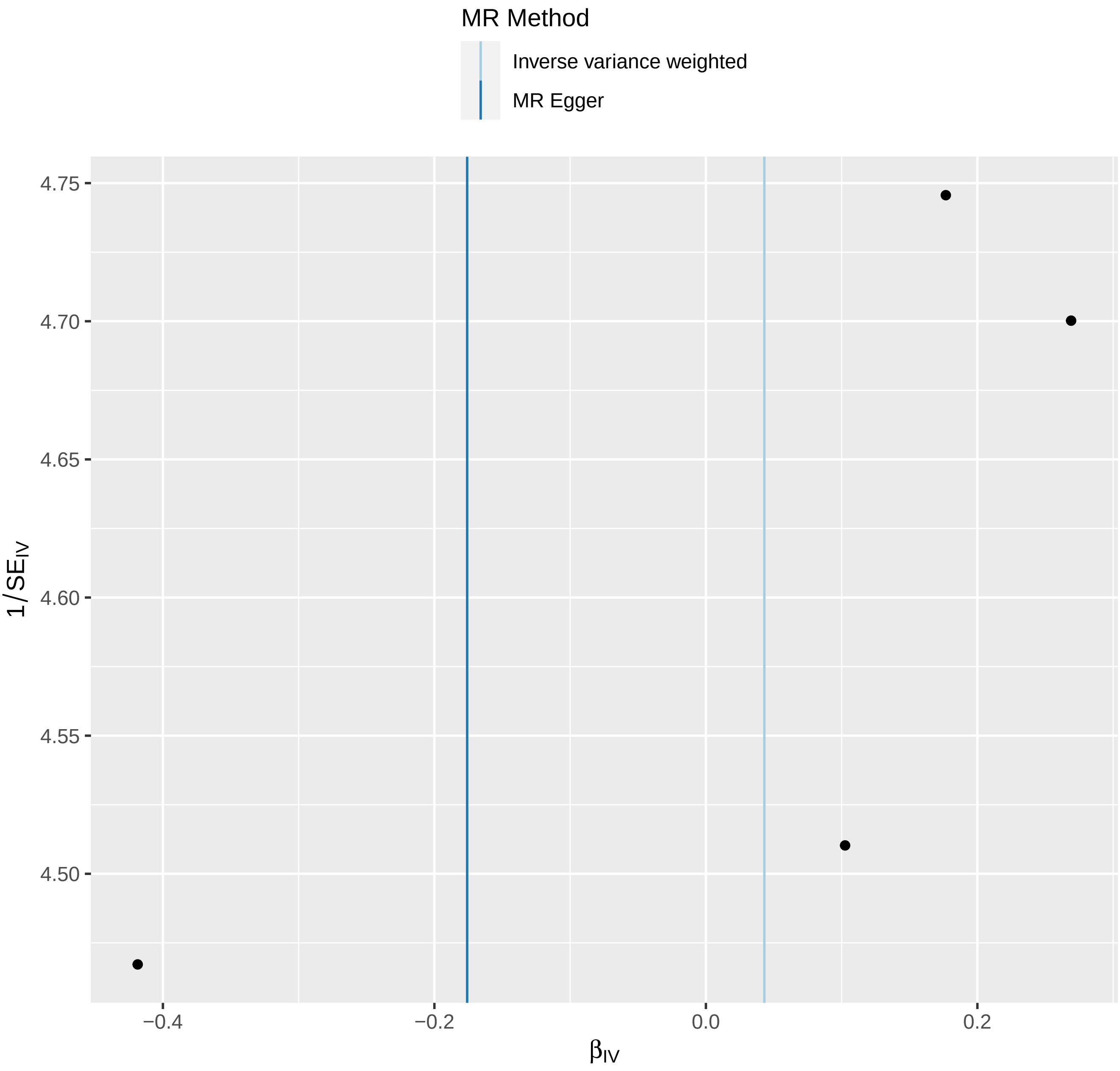

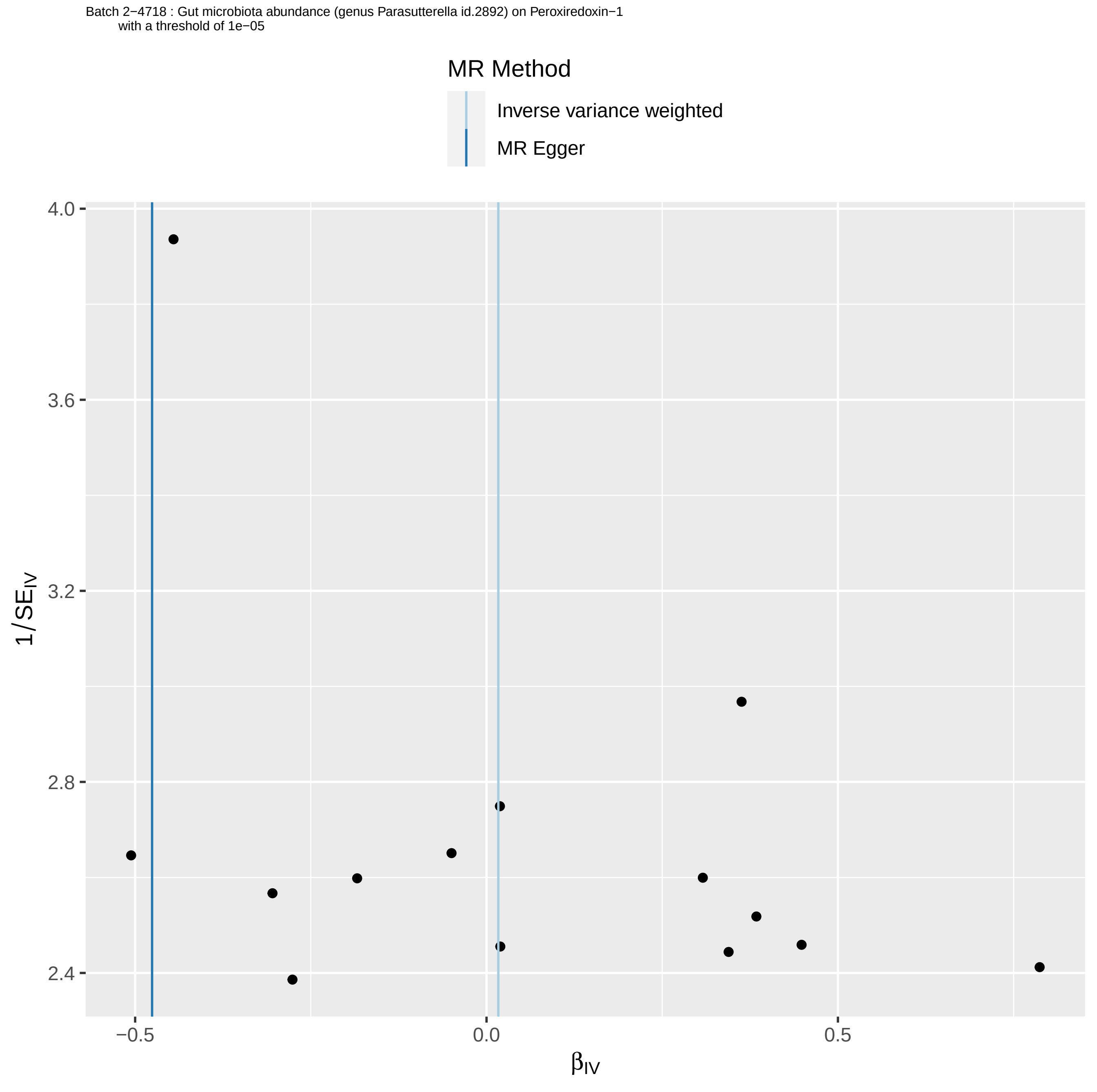
Batch 2−4718 : Gut microbiota abundance (genus Parasutterella id.2892) on Peroxiredoxin−1
with a threshold of 1e−05
MR Method
Inverse variance weighted
MR Egger
4.0
3.6
3.2
2.8
2.4
1/SE$_{IV}$
−0.5
0.0
0.5
β$_{IV}$

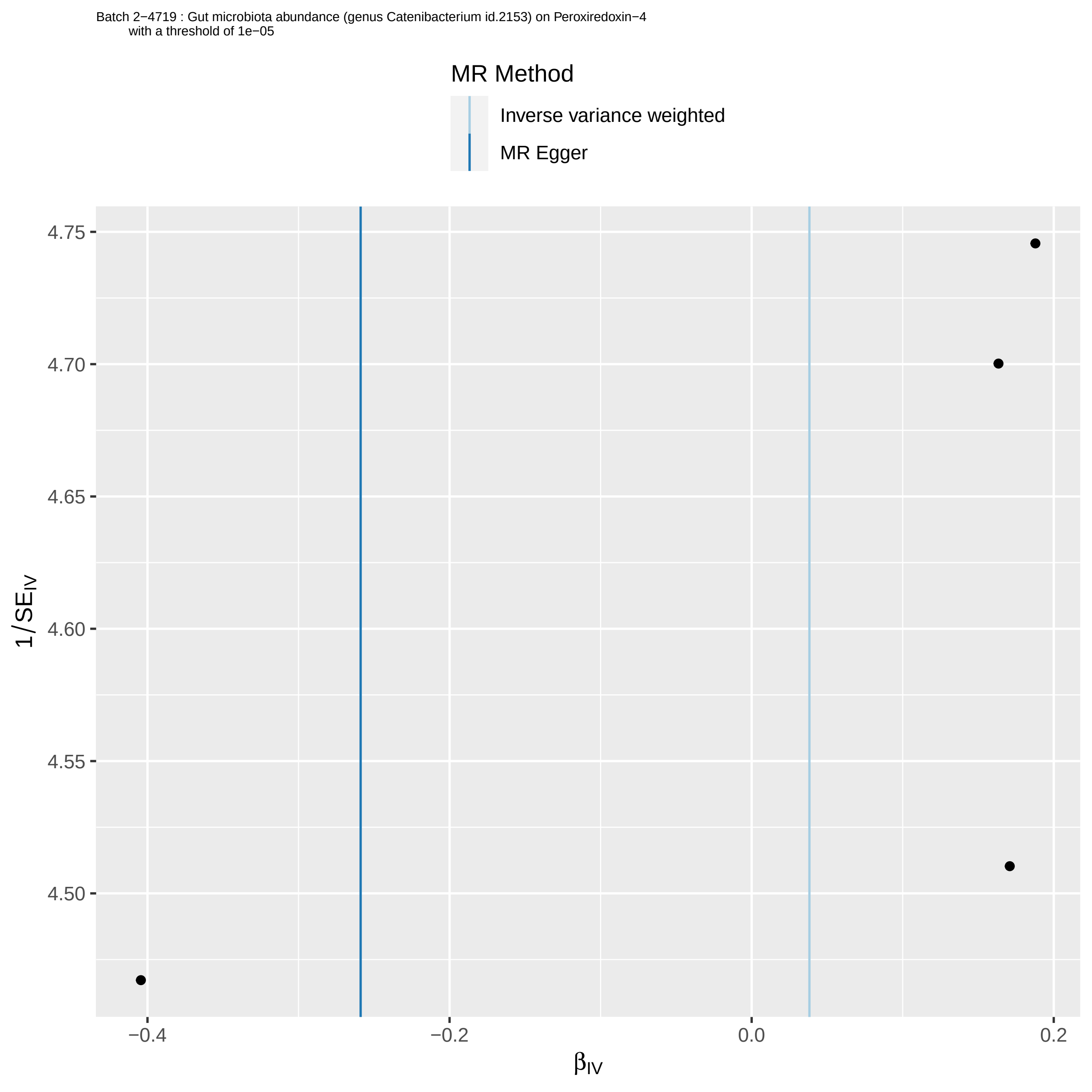
Batch 2−4719 : Gut microbiota abundance (genus Catenibacterium id.2153) on Peroxiredoxin−4
with a threshold of 1e−05
MR Method
Inverse variance weighted
MR Egger
4.75
4.70
4.65
4.60
4.55
4.50
1/SE_IV
−0.4
−0.2
0.0
0.2
β_IV

Batch 2−4720 : Gut microbiota abundance (genus Parasutterella id.2892) on Peroxiredoxin−4
with a threshold of 1e−05

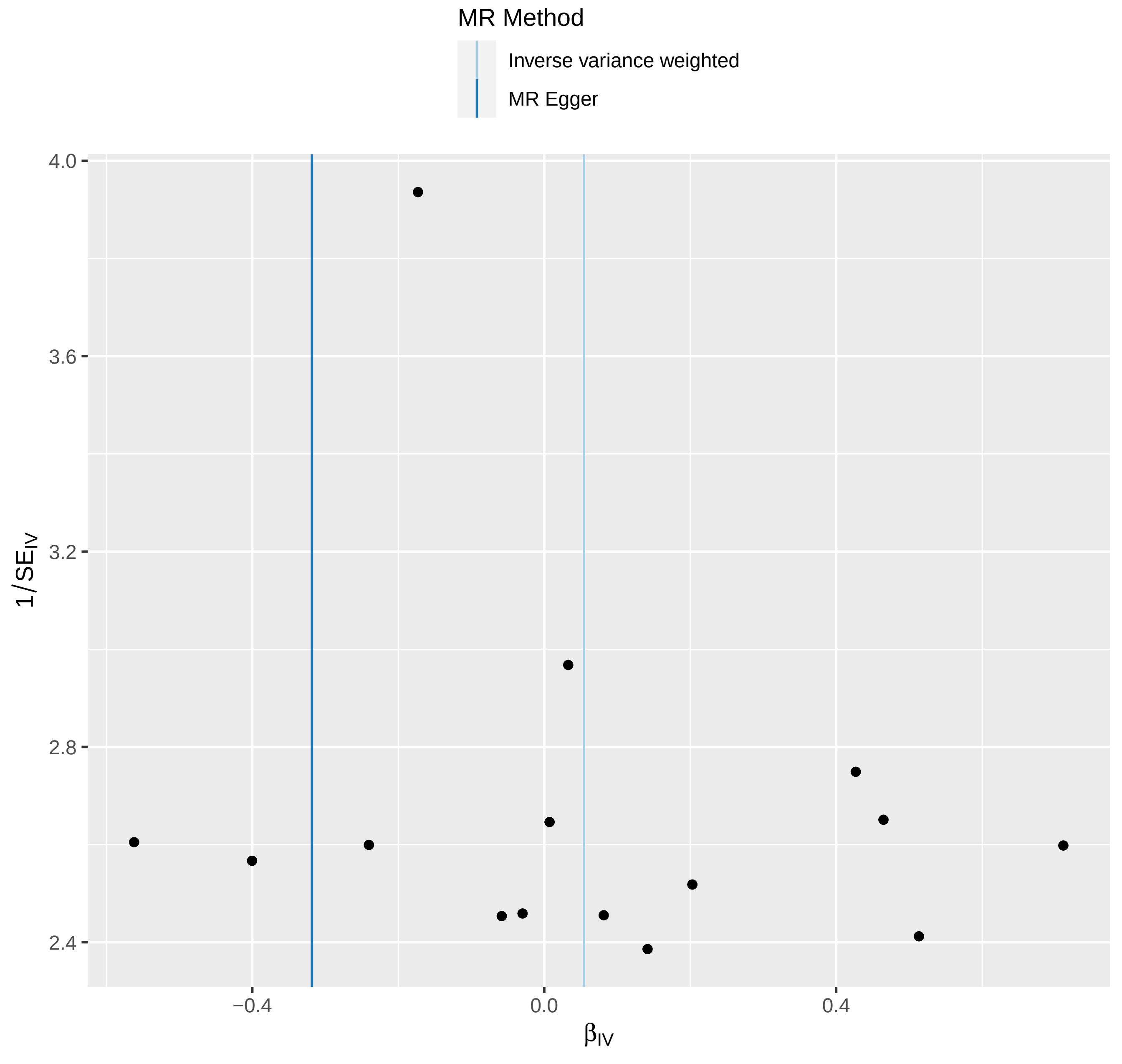

Batch 2−4721 : Gut microbiota abundance (genus Catenibacterium id.2153) on Prolargin
with a threshold of 1e−05

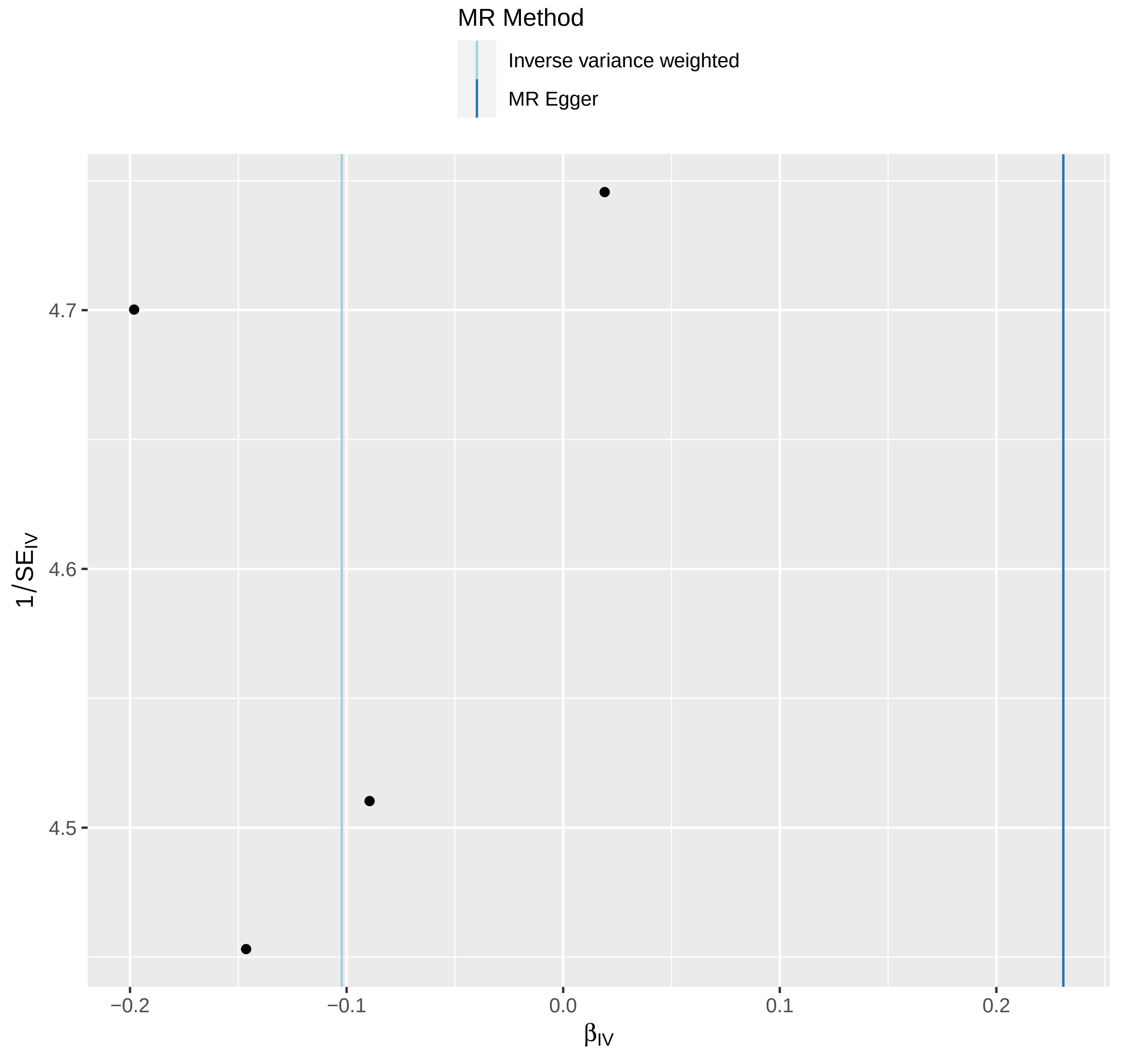

Batch 2−4722 : Gut microbiota abundance (genus Parasutterella id.2892) on Prolargin
with a threshold of 1e−05

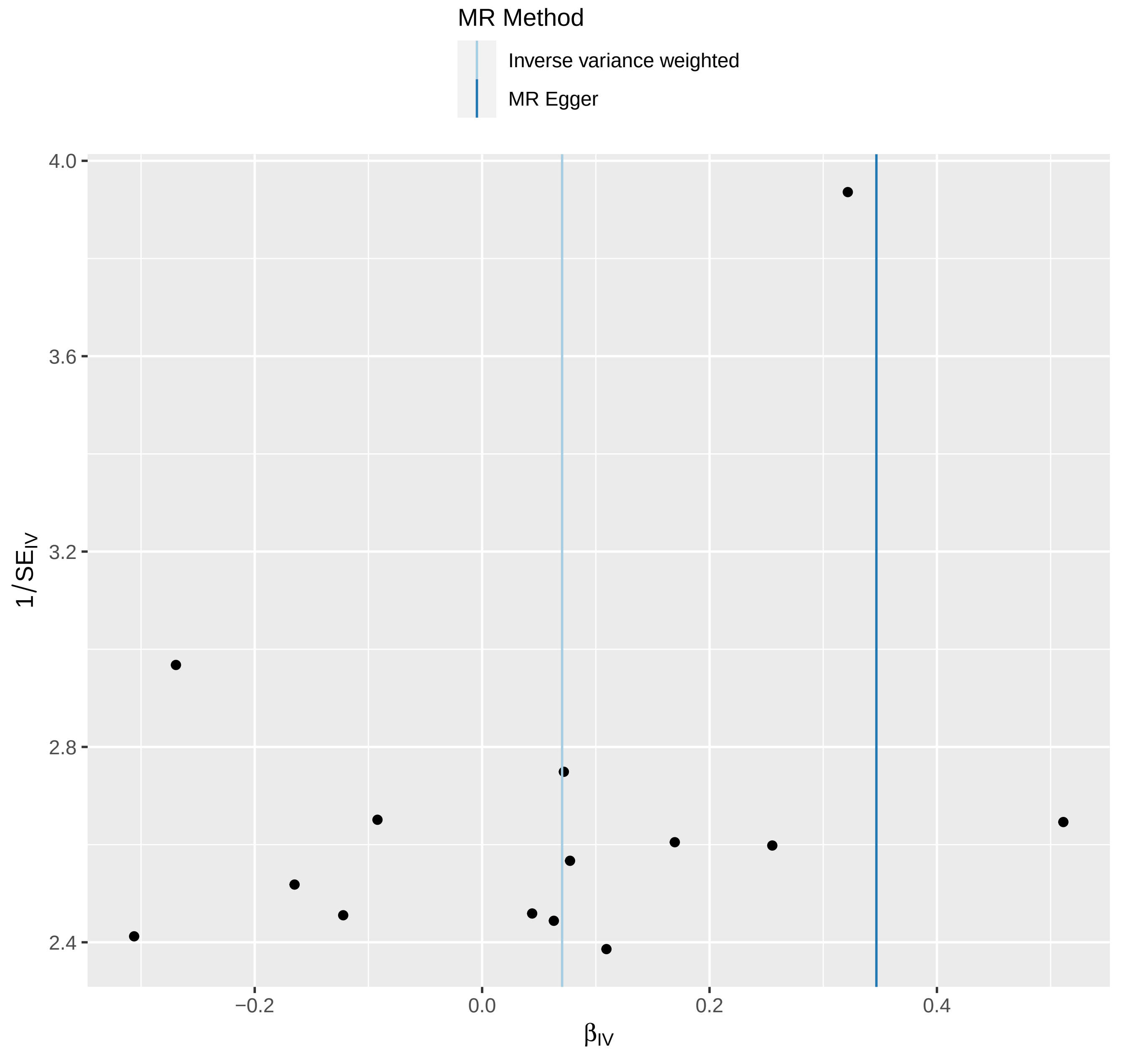

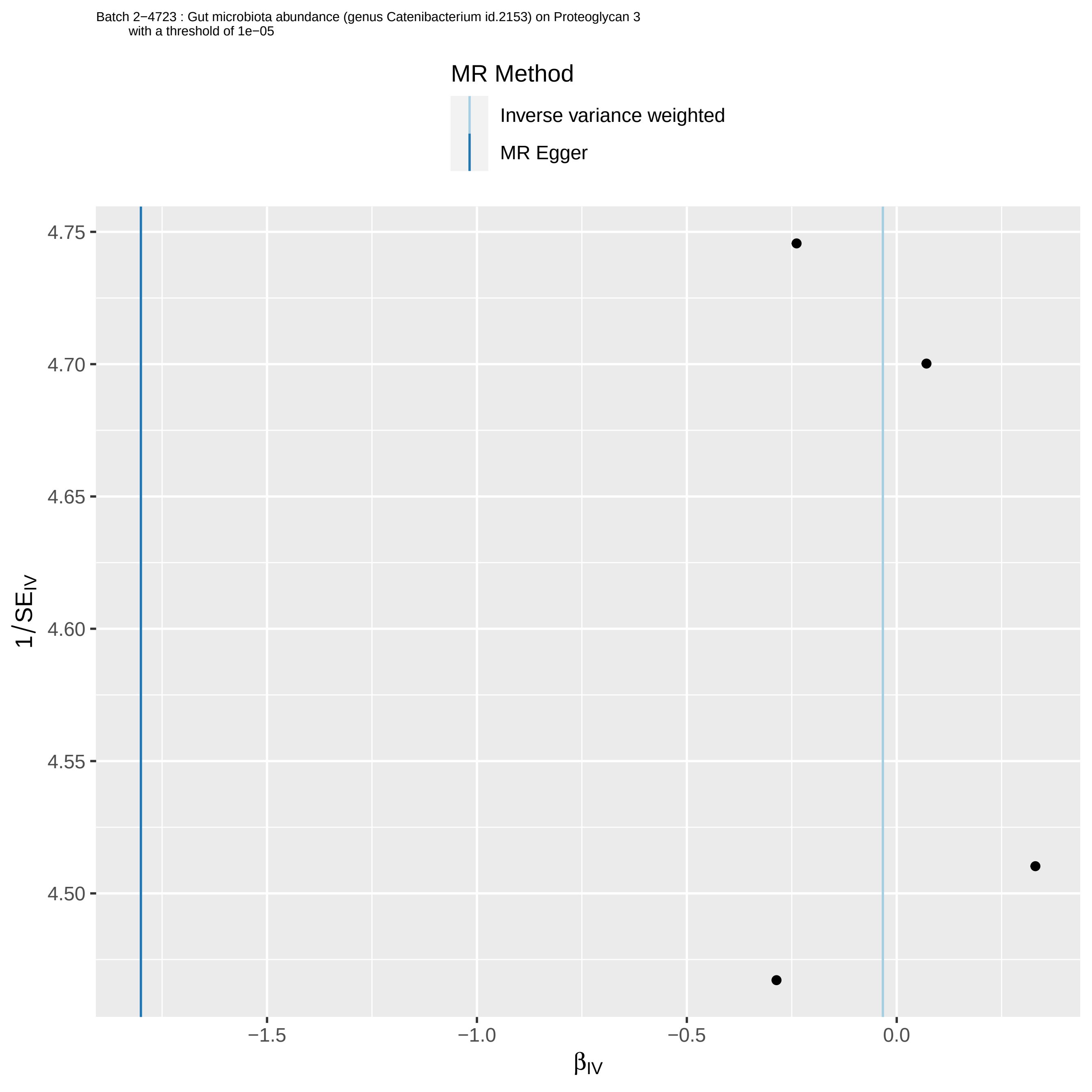
Batch 2−4723 : Gut microbiota abundance (genus Catenibacterium id.2153) on Proteoglycan 3
with a threshold of 1e−05
MR Method
Inverse variance weighted
MR Egger
4.75
4.70
4.65
4.60
4.55
4.50
1/SE_IV
−1.5
−1.0
−0.5
0.0
β_IV

Batch 2−4724 : Gut microbiota abundance (genus Parasutterella id.2892) on Proteoglycan 3
with a threshold of 1e−05

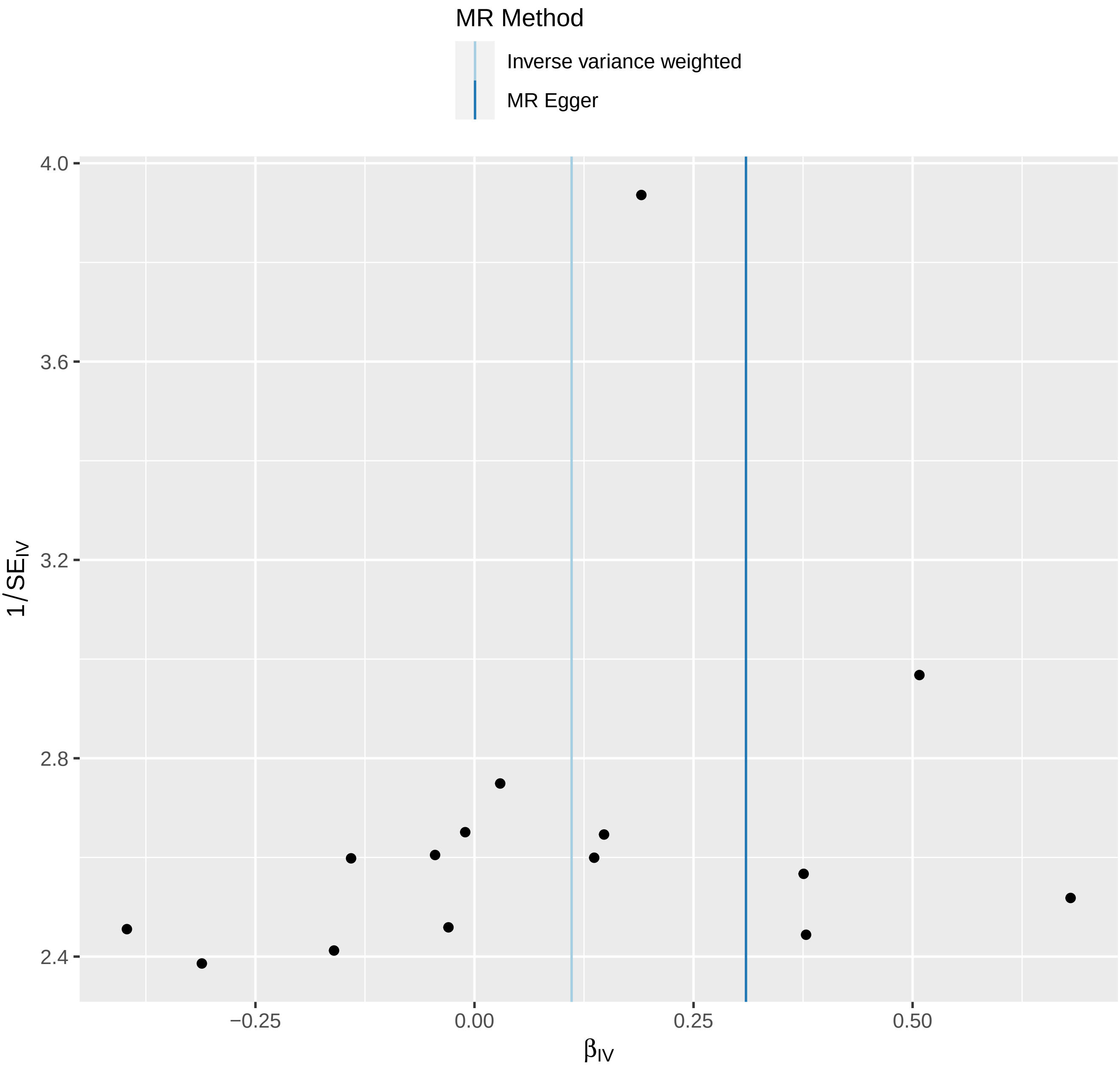

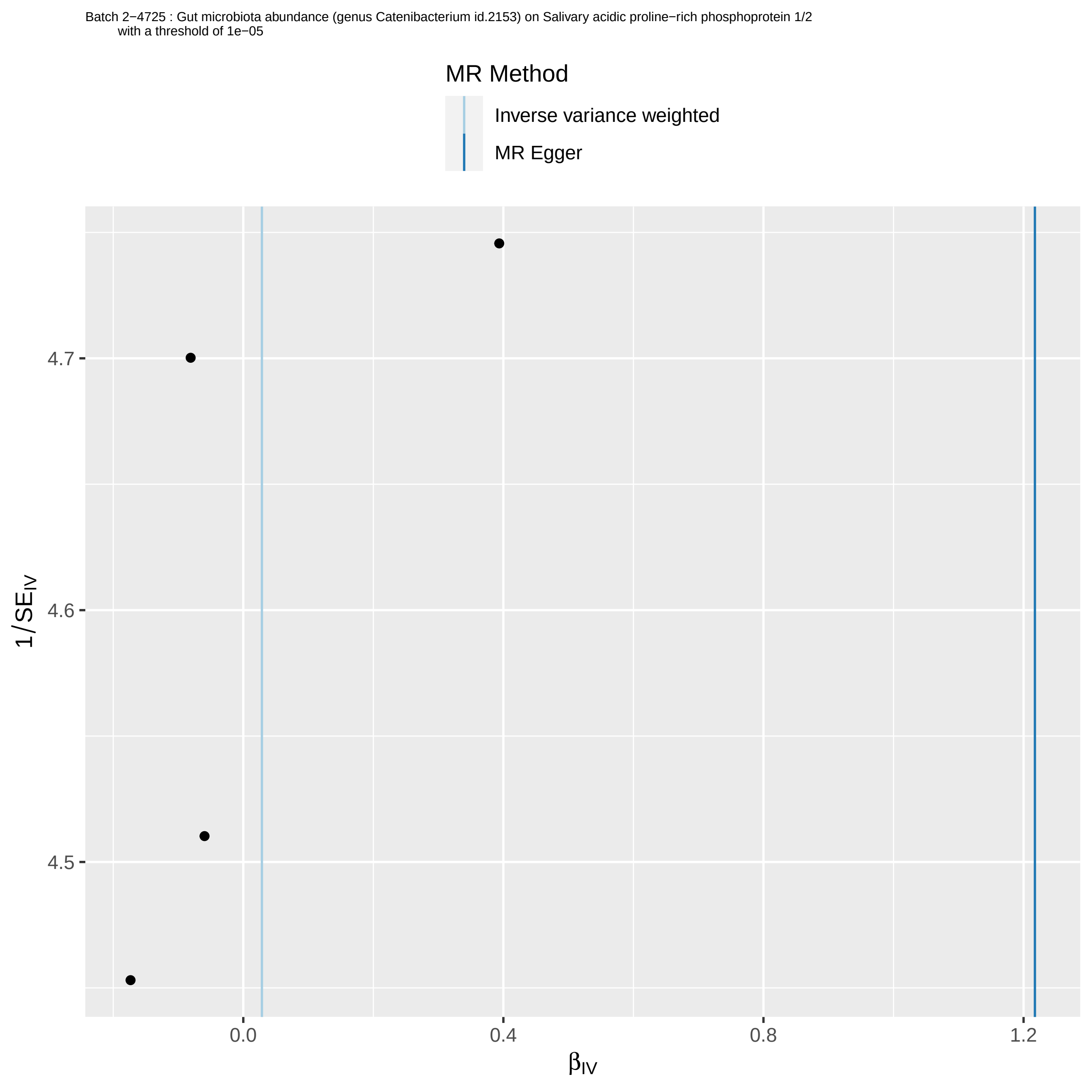
Batch 2−4725 : Gut microbiota abundance (genus Catenibacterium id.2153) on Salivary acidic proline−rich phosphoprotein 1/2
with a threshold of 1e−05
MR Method
Inverse variance weighted
MR Egger
4.7
4.6
4.5
1/SE_IV
0.0
0.4
0.8
1.2
β_IV

Batch 2−4726 : Gut microbiota abundance (genus Parasutterella id.2892) on Salivary acidic proline−rich phosphoprotein 1/2
with a threshold of 1e−05

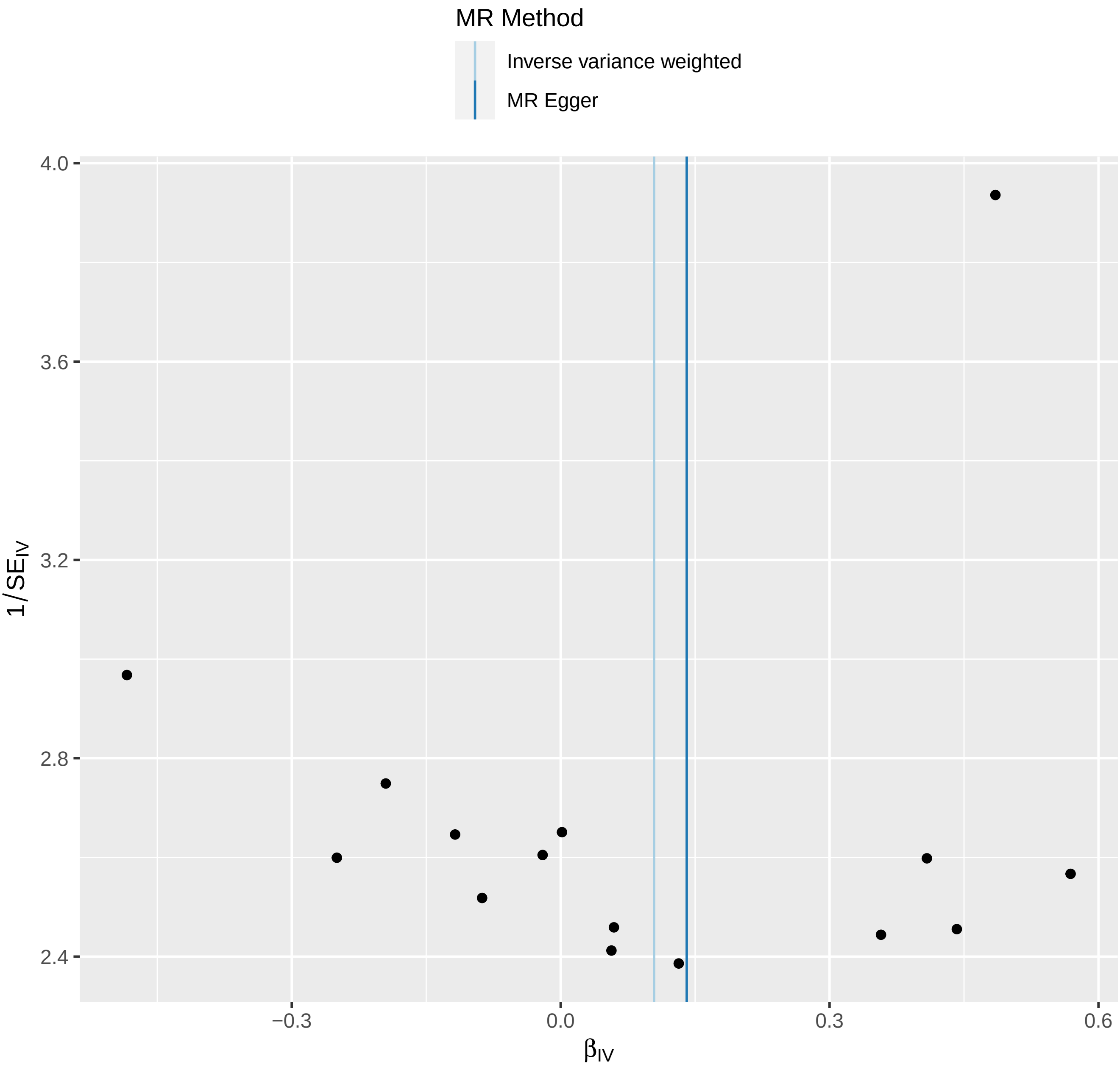

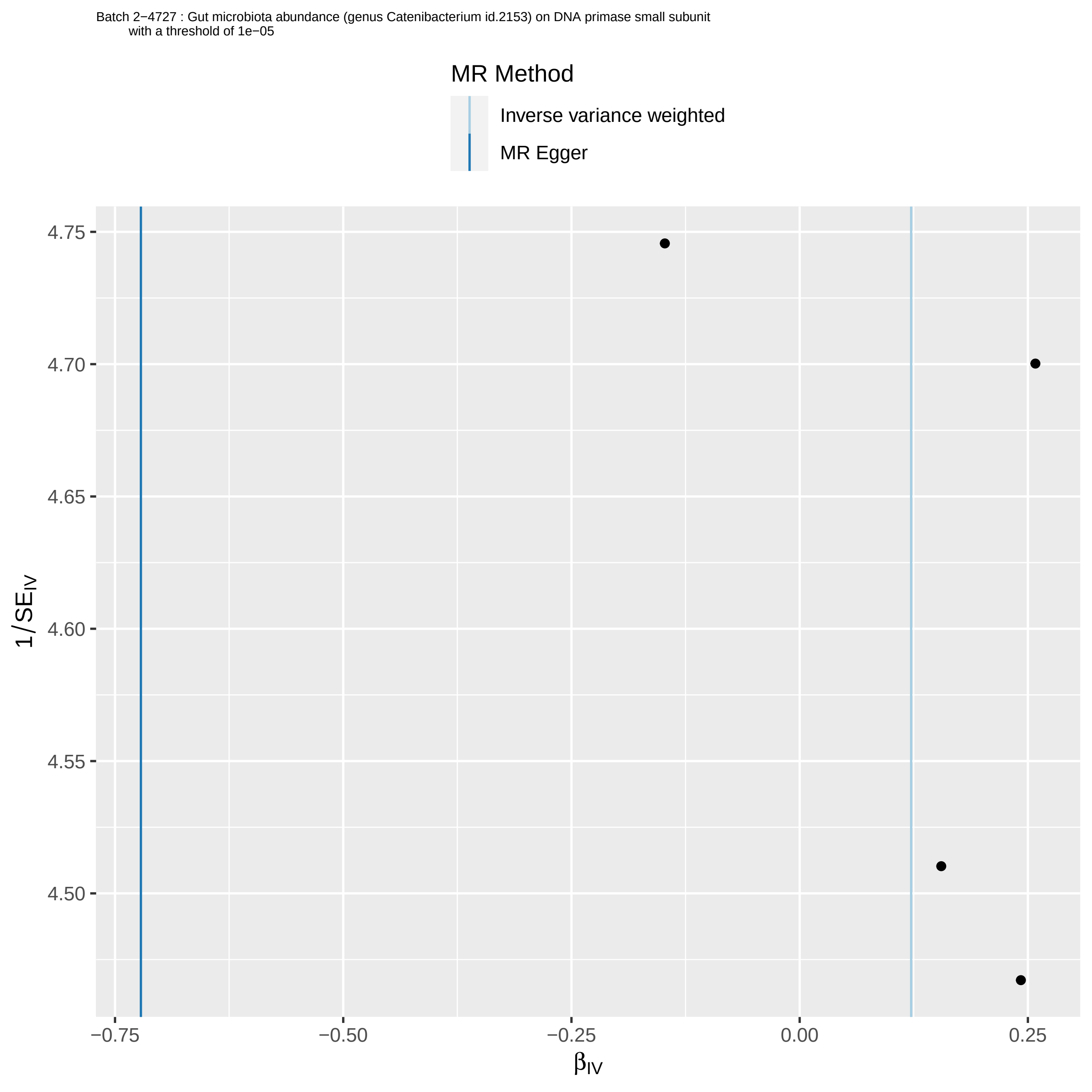

Batch 2−4727 : Gut microbiota abundance (genus Catenibacterium id.2153) on DNA primase small subunit
with a threshold of 1e−05
MR Method
Inverse variance weighted
MR Egger
4.75
4.70
4.65
4.60
4.55
4.50
1/SE_IV
−0.75
−0.50
−0.25
0.00
0.25
β_IV

Batch 2−4728 : Gut microbiota abundance (genus Parasutterella id.2892) on DNA primase small subunit
with a threshold of 1e−05

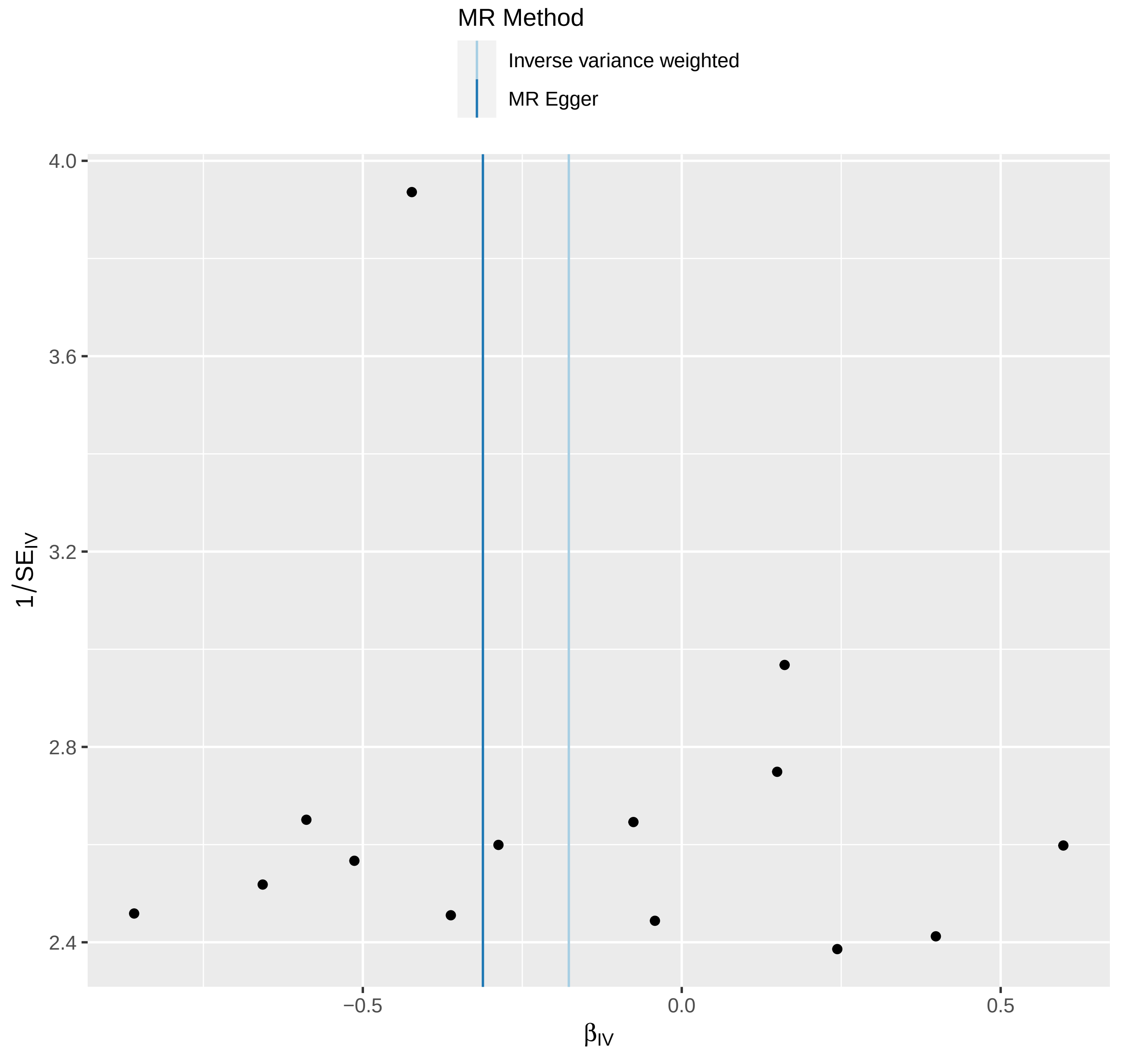

Batch 2−4729 : Gut microbiota abundance (genus Catenibacterium id.2153) on AMP Kinase (alpha1beta1gamma1)
with a threshold of 1e−05

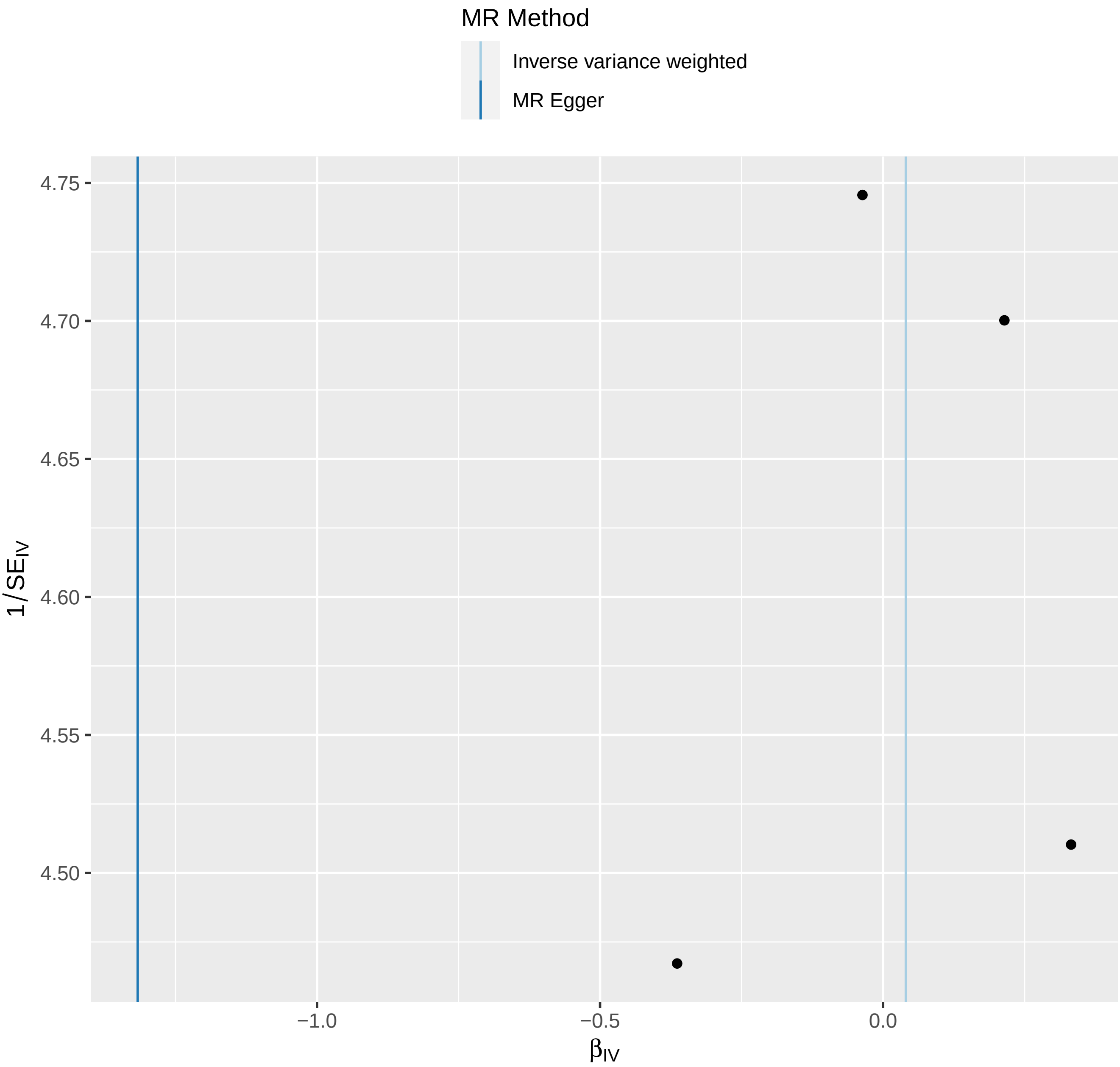

Batch 2−4730 : Gut microbiota abundance (genus Parasutterella id.2892) on AMP Kinase (alpha1beta1gamma1) with a threshold of 1e−05

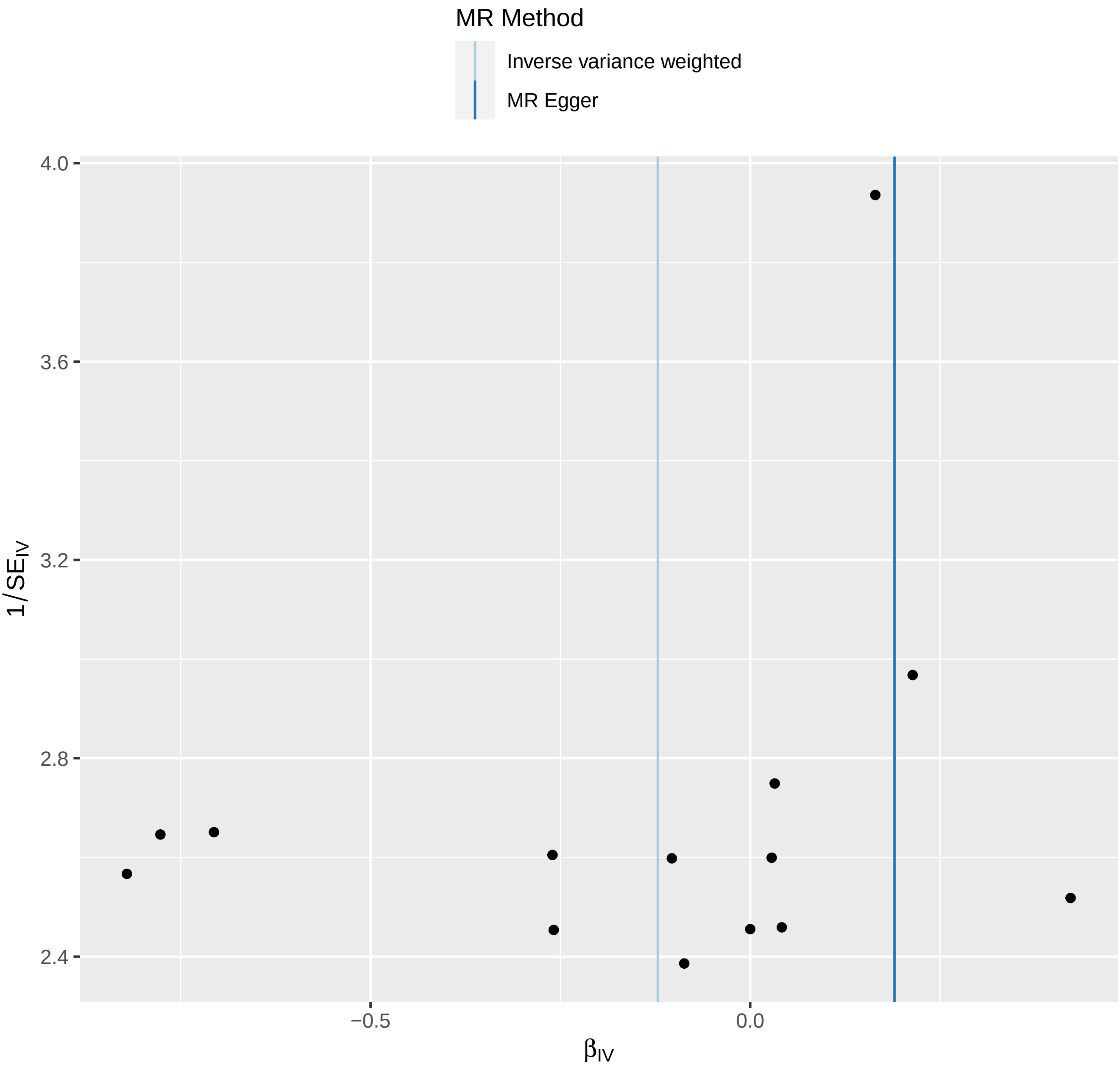

Batch 2−4731 : Gut microbiota abundance (genus Catenibacterium id.2153) on AMP Kinase (alpha2beta2gamma1)
with a threshold of 1e−05

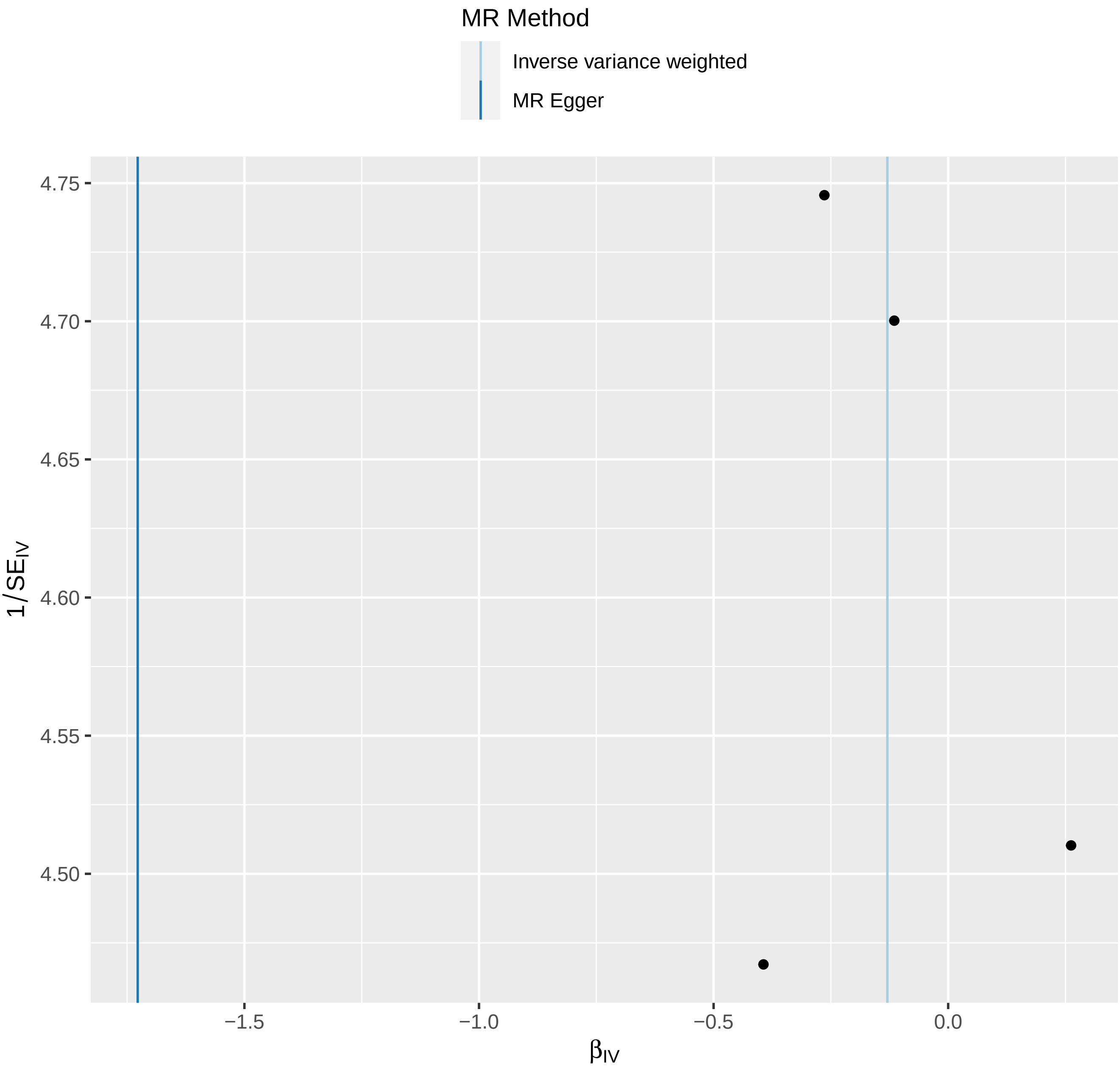

Batch 2−4732 : Gut microbiota abundance (genus Parasutterella id.2892) on AMP Kinase (alpha2beta2gamma1)
with a threshold of 1e−05

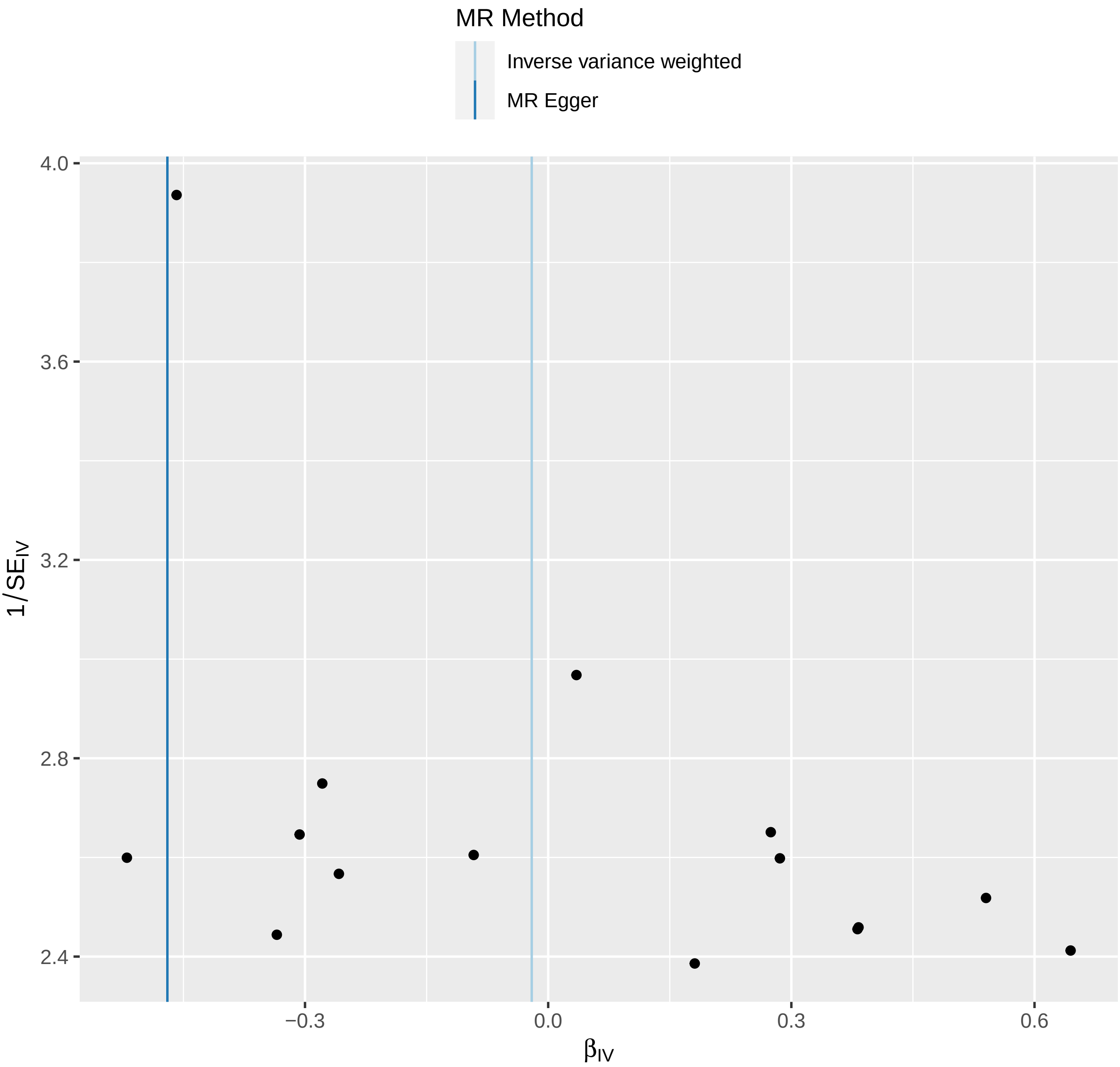

Batch 2−4733 : Gut microbiota abundance (genus Catenibacterium id.2153) on cAMP−dependent protein kinase catalytic subunit alpha
with a threshold of 1e−05

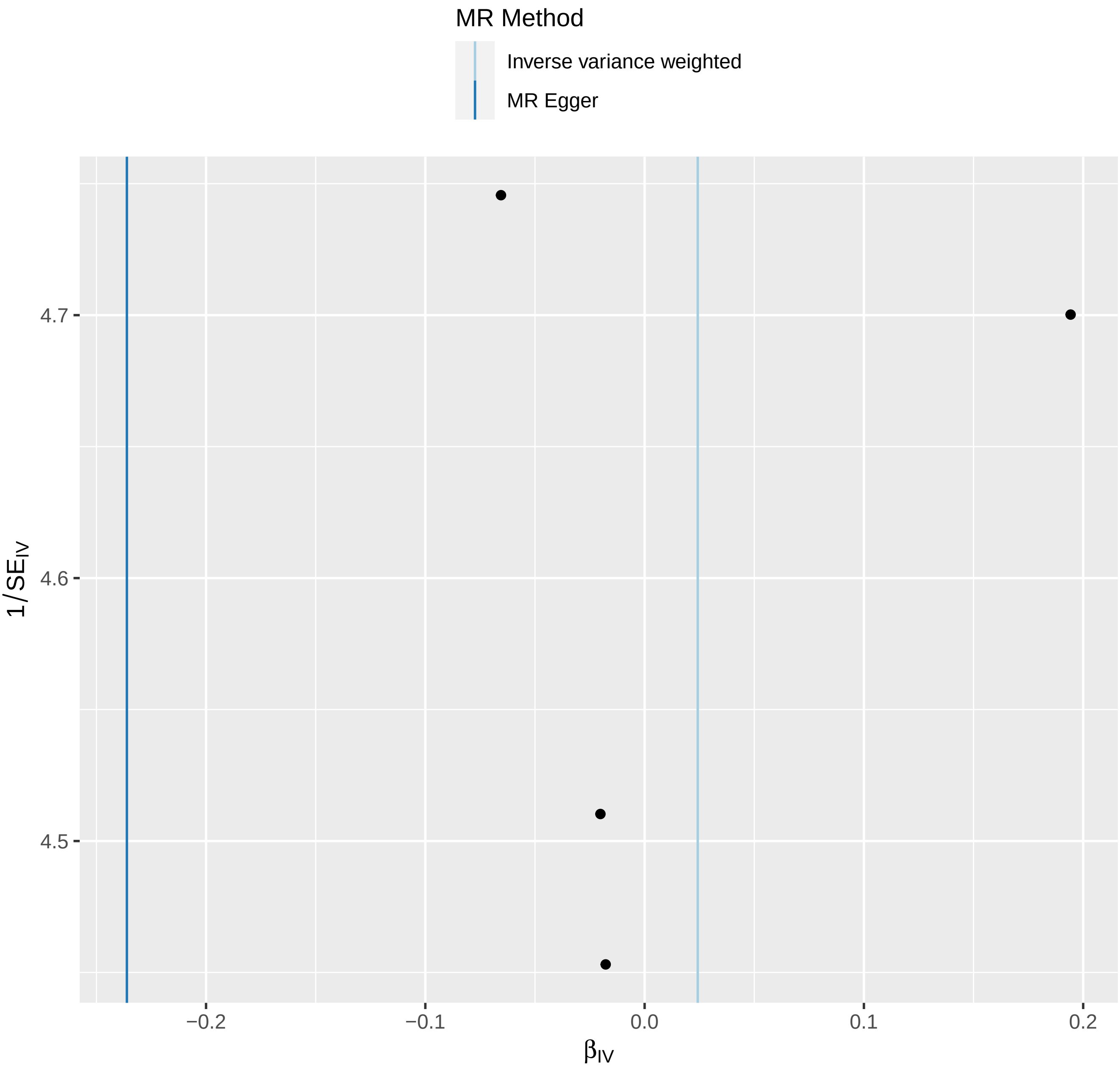

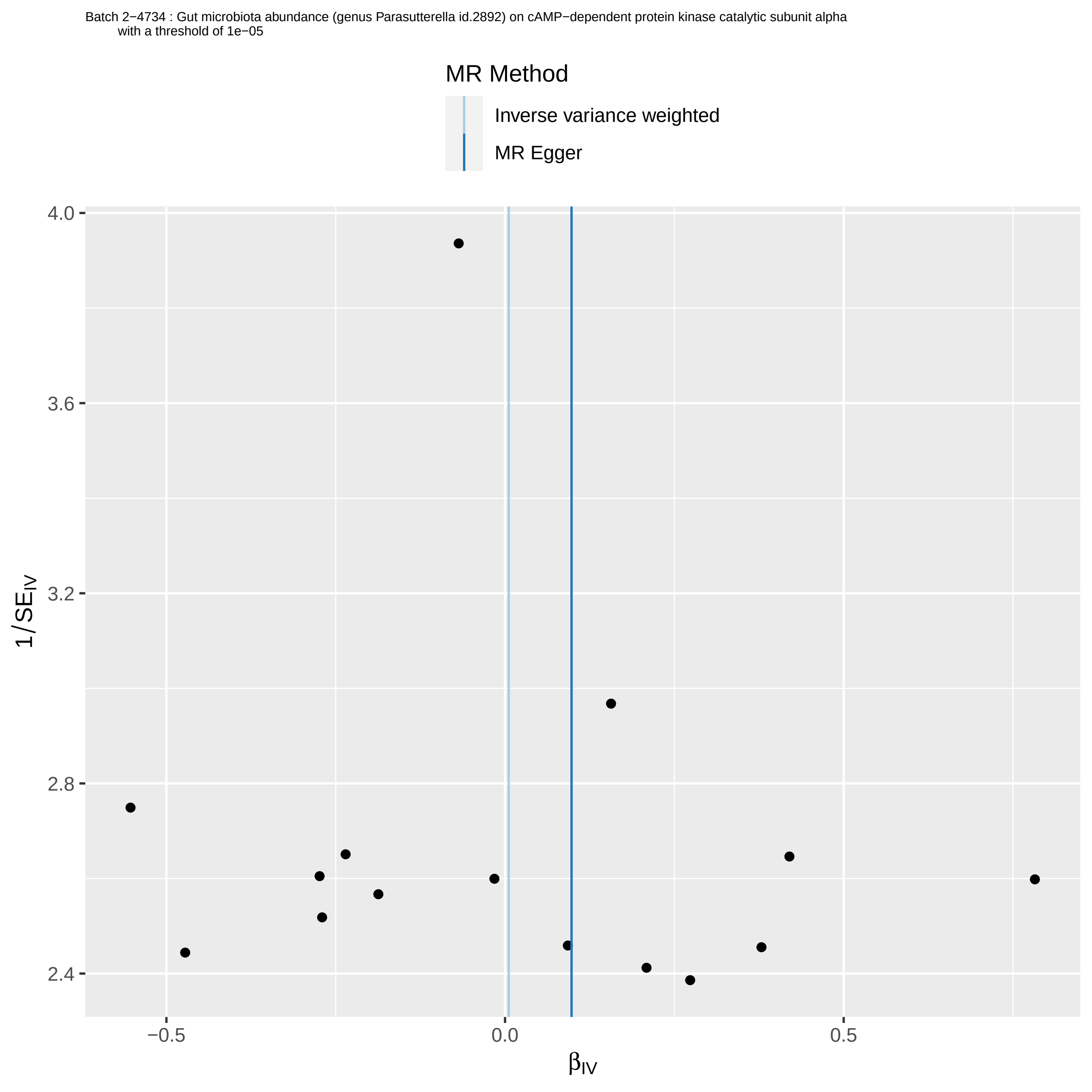
Batch 2−4734 : Gut microbiota abundance (genus Parasutterella id.2892) on cAMP−dependent protein kinase catalytic subunit alpha
with a threshold of 1e−05
MR Method
Inverse variance weighted
MR Egger
4.0
3.6
3.2
2.8
2.4
1/SE_IV
−0.5
0.0
0.5
β_IV

Batch 2−4735 : Gut microbiota abundance (genus Catenibacterium id.2153) on cAMP−dependent protein kinase type I−beta regulatory subunit with a threshold of 1e−05

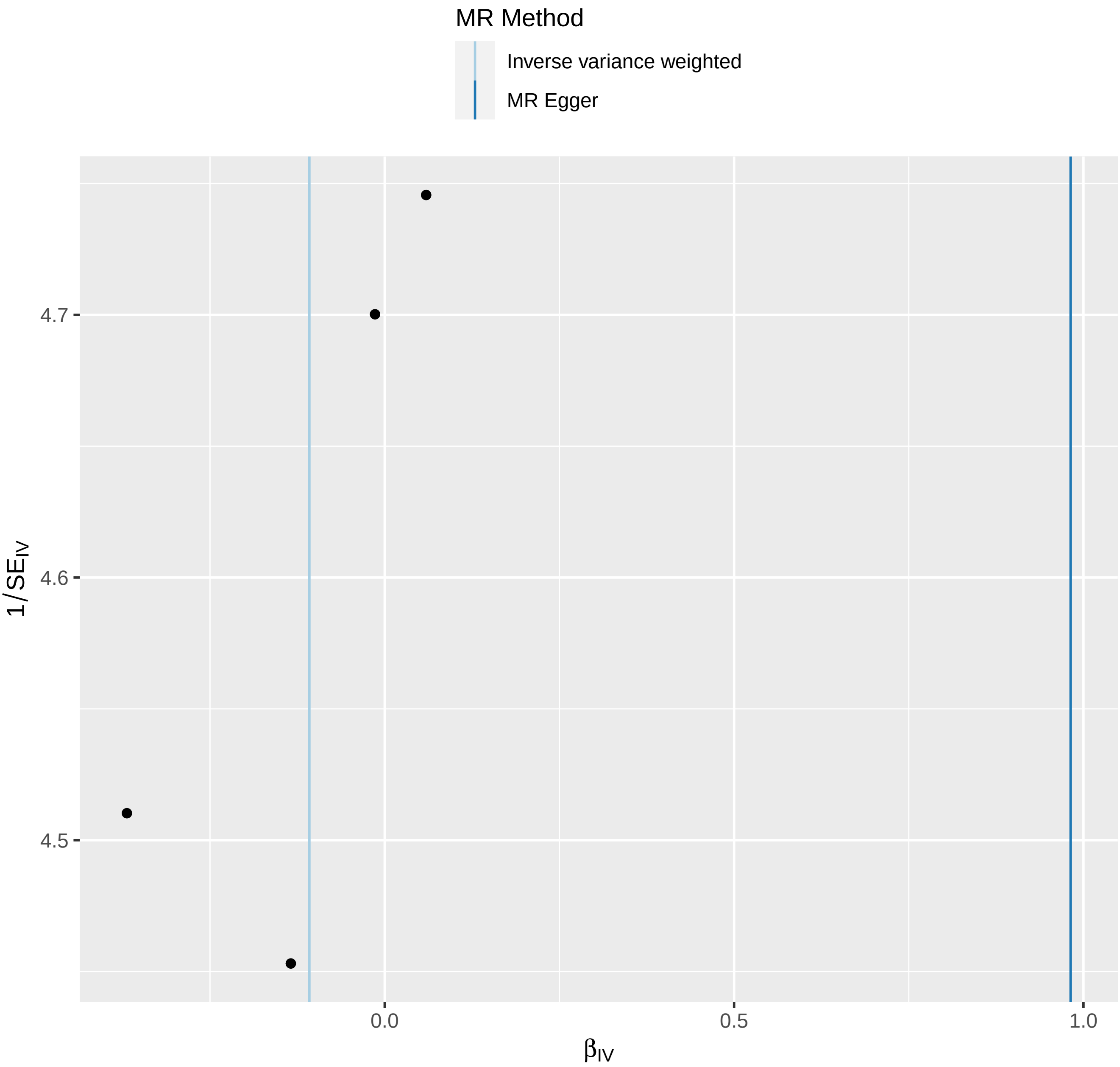

Batch 2−4736 : Gut microbiota abundance (genus Parasutterella id.2892) on cAMP−dependent protein kinase type I−beta regulatory subunit
with a threshold of 1e−05

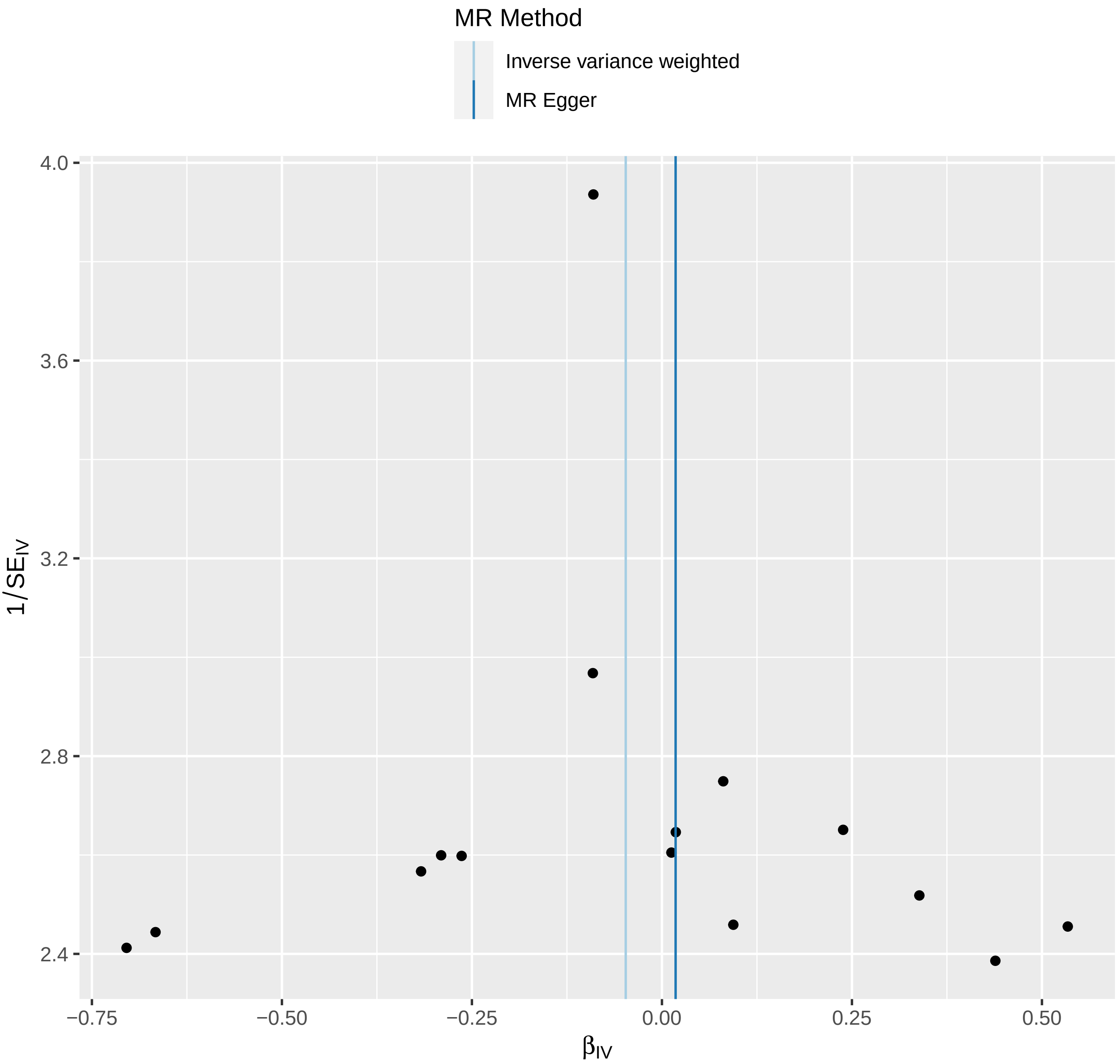

Batch 2−4737 : Gut microbiota abundance (genus Catenibacterium id.2153) on Protein kinase C alpha type
with a threshold of 1e−05

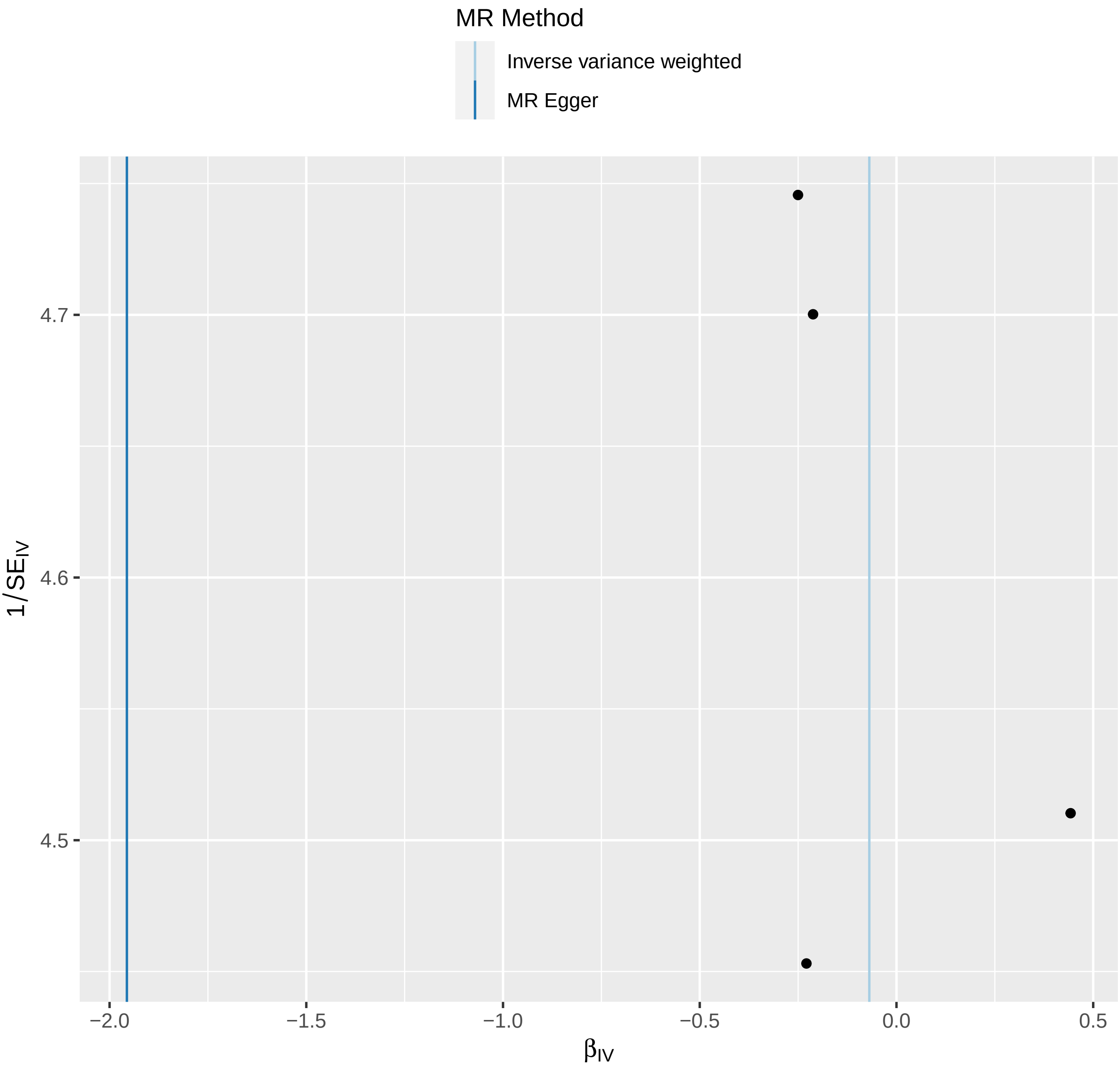

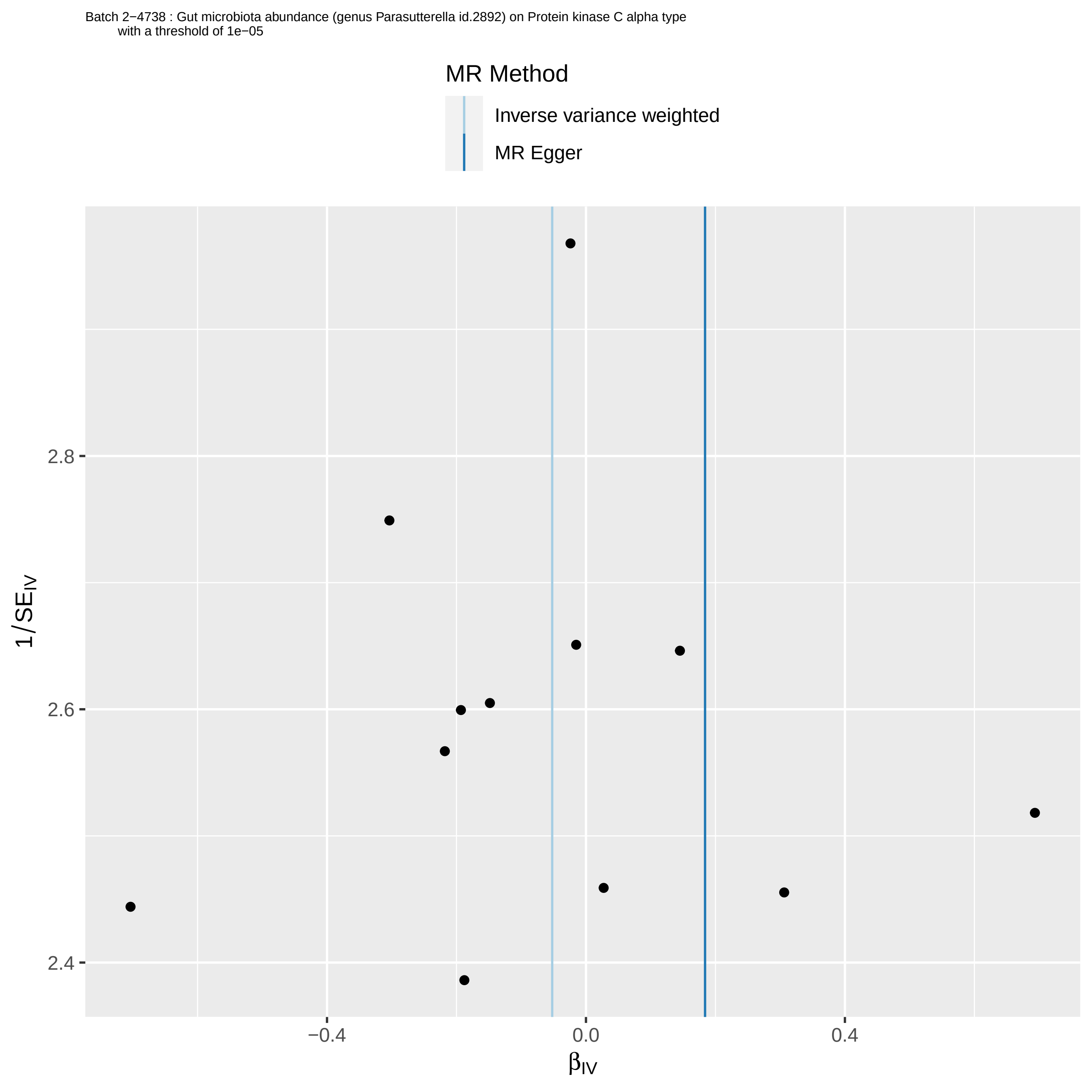

Batch 2−4738 : Gut microbiota abundance (genus Parasutterella id.2892) on Protein kinase C alpha type
with a threshold of 1e−05
MR Method
Inverse variance weighted
MR Egger
2.8
2.6
2.4
1/SE_IV
−0.4
0.0
0.4
β_IV

Batch 2−4739 : Gut microbiota abundance (genus Catenibacterium id.2153) on Protein kinase C beta type (splice variant beta−II) with a threshold of 1e−05

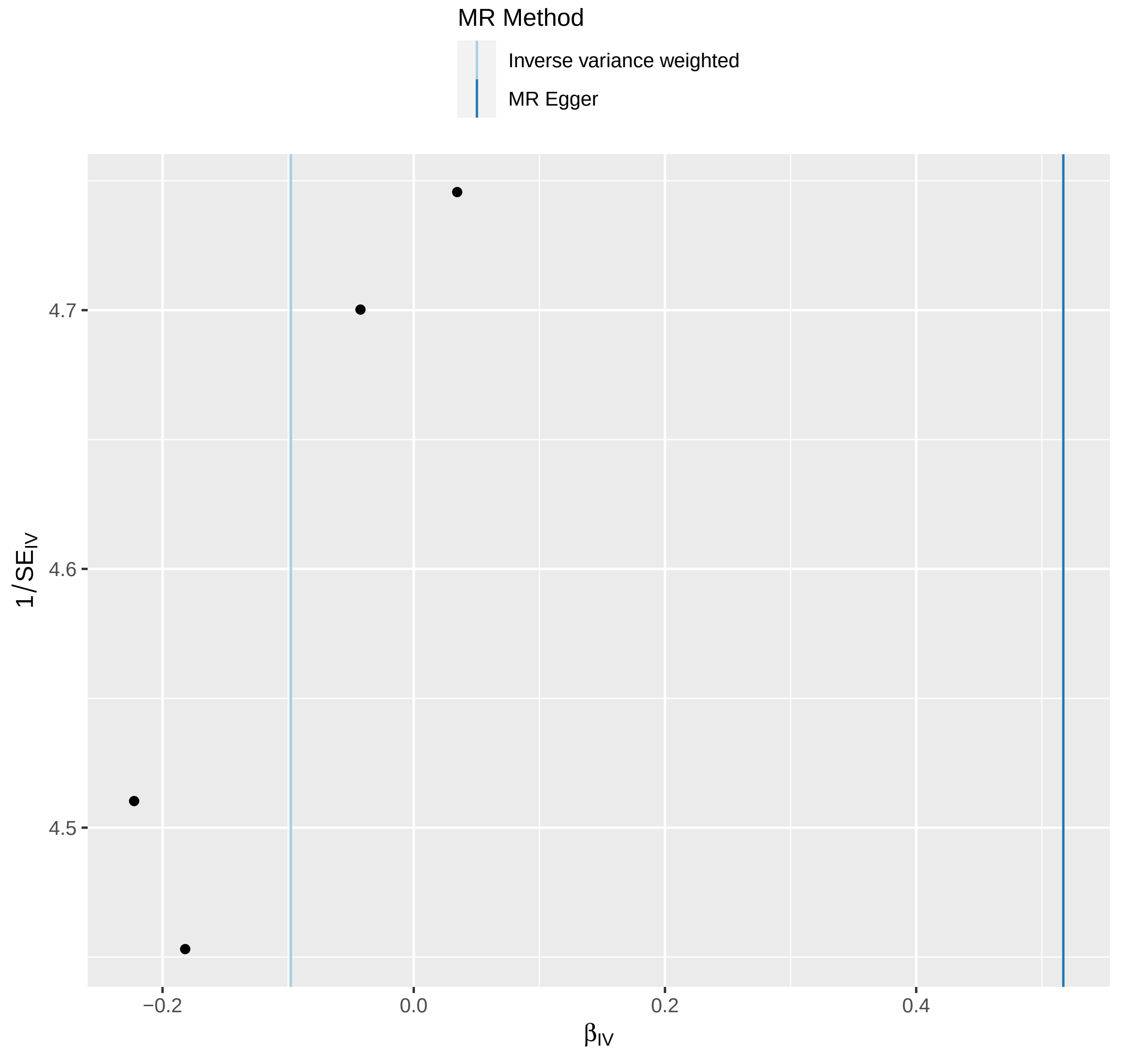

Batch 2−4740 : Gut microbiota abundance (genus Parasutterella id.2892) on Protein kinase C beta type (splice variant beta−II) with a threshold of 1e−05

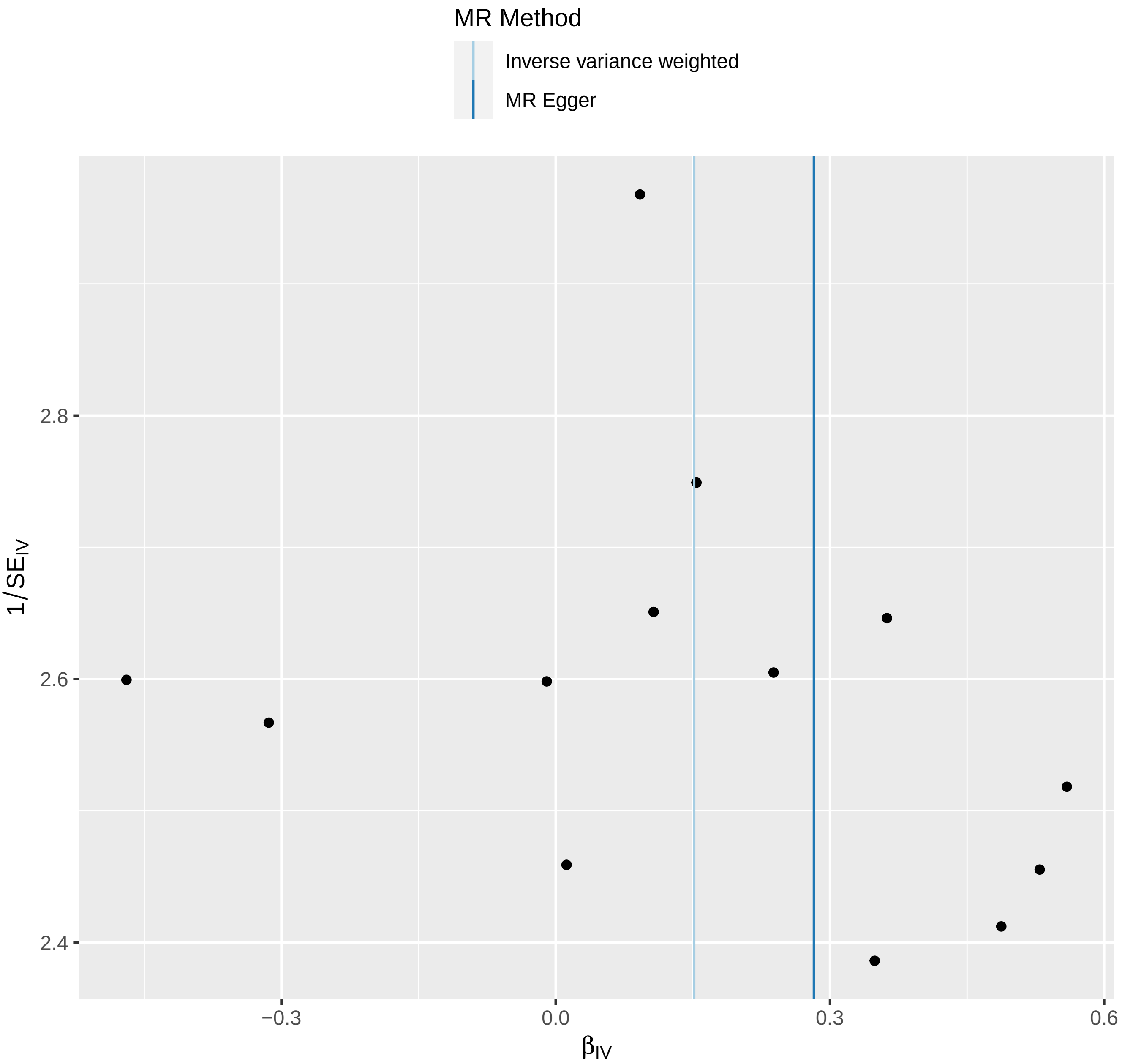

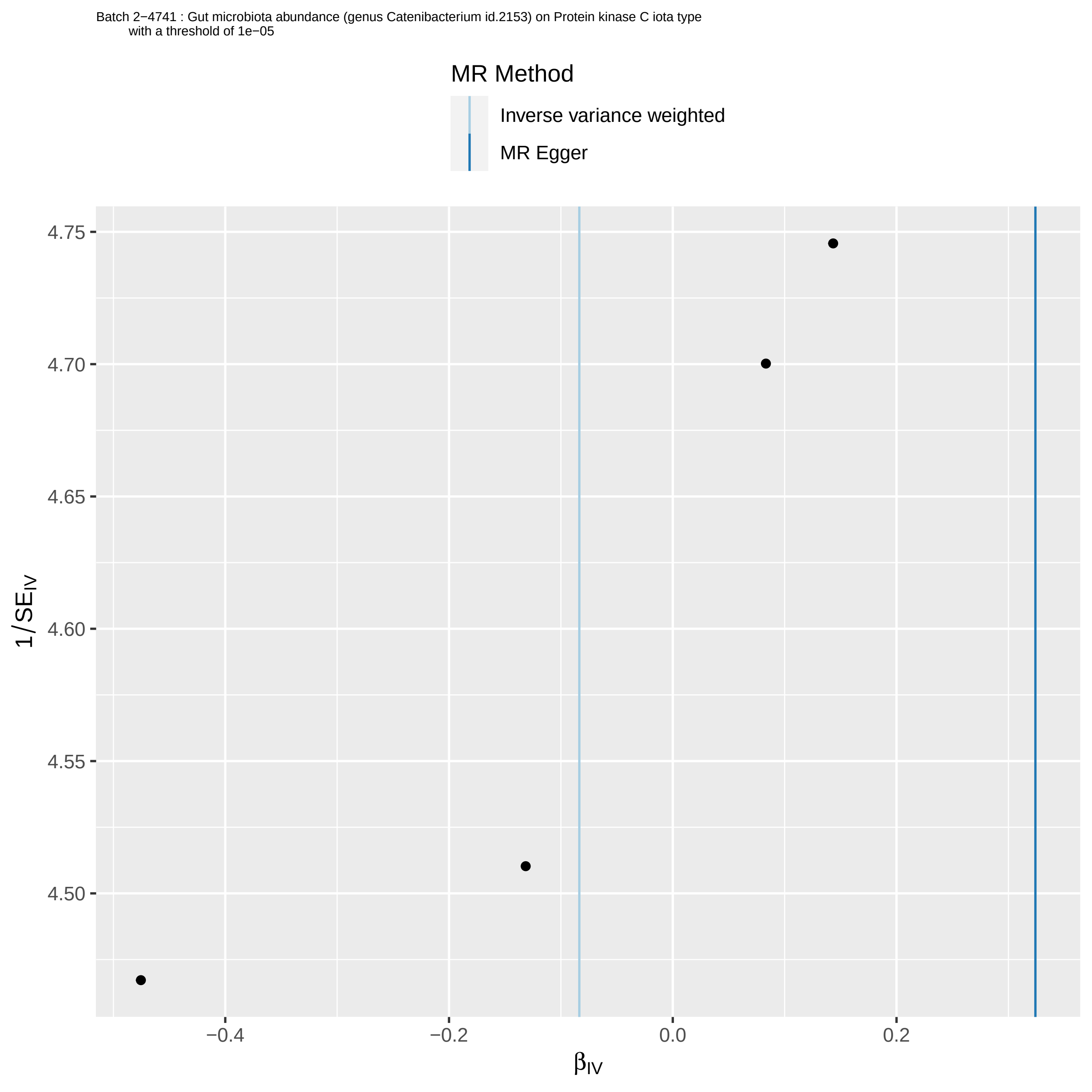
Batch 2−4741 : Gut microbiota abundance (genus Catenibacterium id.2153) on Protein kinase C iota type
with a threshold of 1e−05
MR Method
Inverse variance weighted
MR Egger
4.75
4.70
4.65
4.60
4.55
4.50
1/SE_IV
−0.4
−0.2
0.0
0.2
β_IV

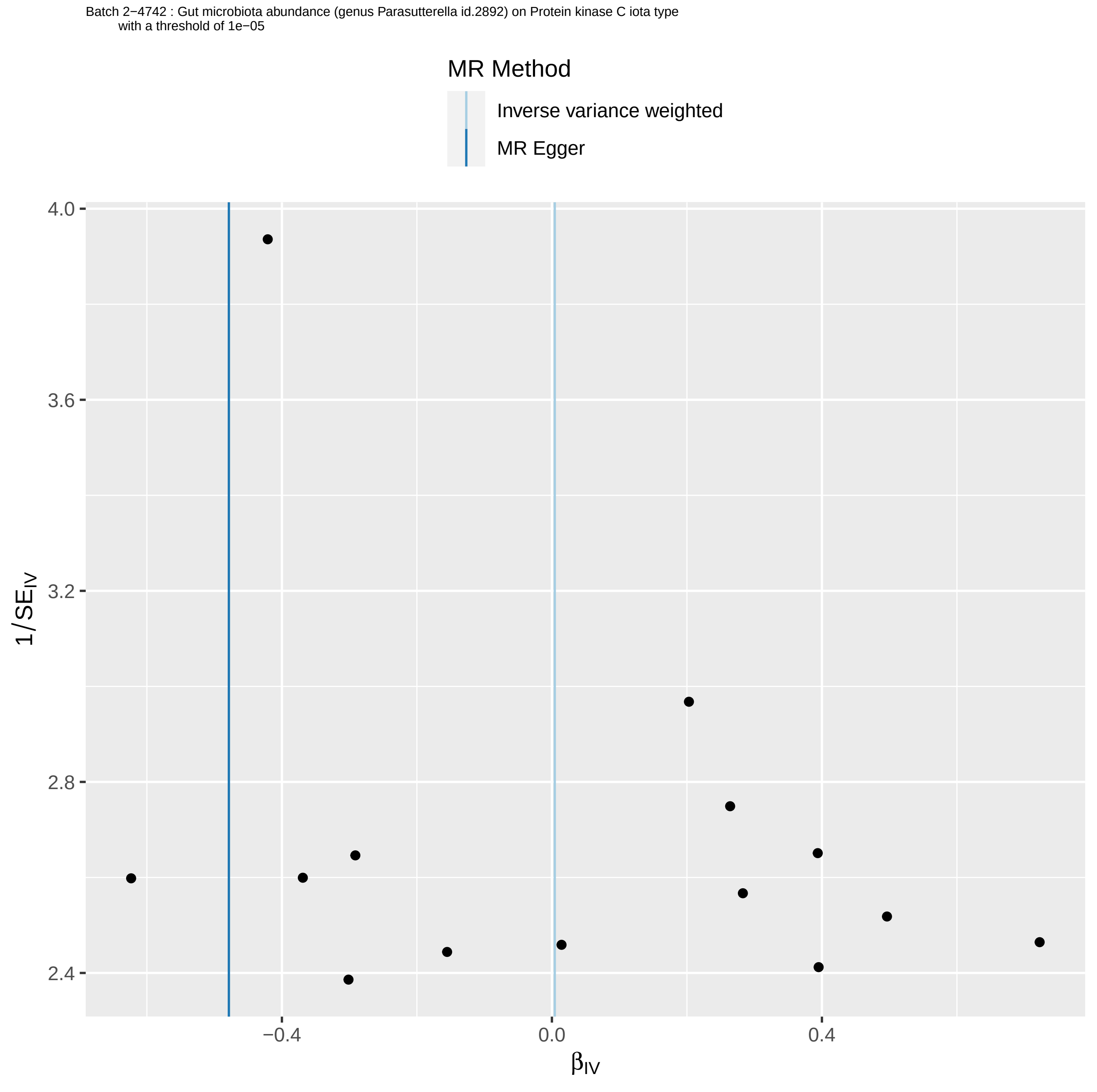
Batch 2−4742 : Gut microbiota abundance (genus Parasutterella id.2892) on Protein kinase C iota type
with a threshold of 1e−05
MR Method
Inverse variance weighted
MR Egger
4.0
3.6
3.2
2.8
2.4
1/SE$_{IV}$
−0.4
0.0
0.4
β$_{IV}$

Batch 2−4743 : Gut microbiota abundance (genus Catenibacterium id.2153) on Glucosidase 2 subunit beta
with a threshold of 1e−05

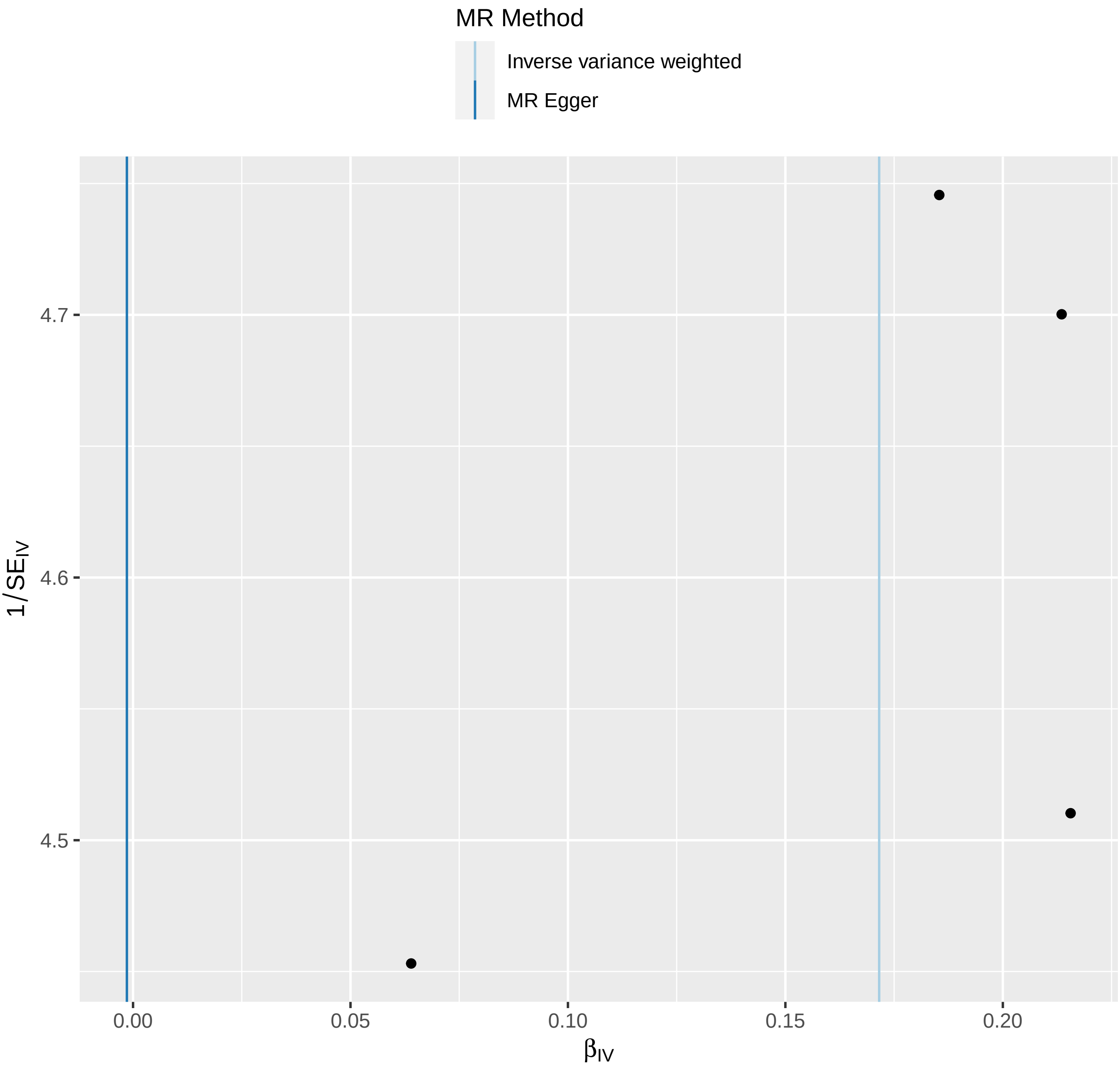

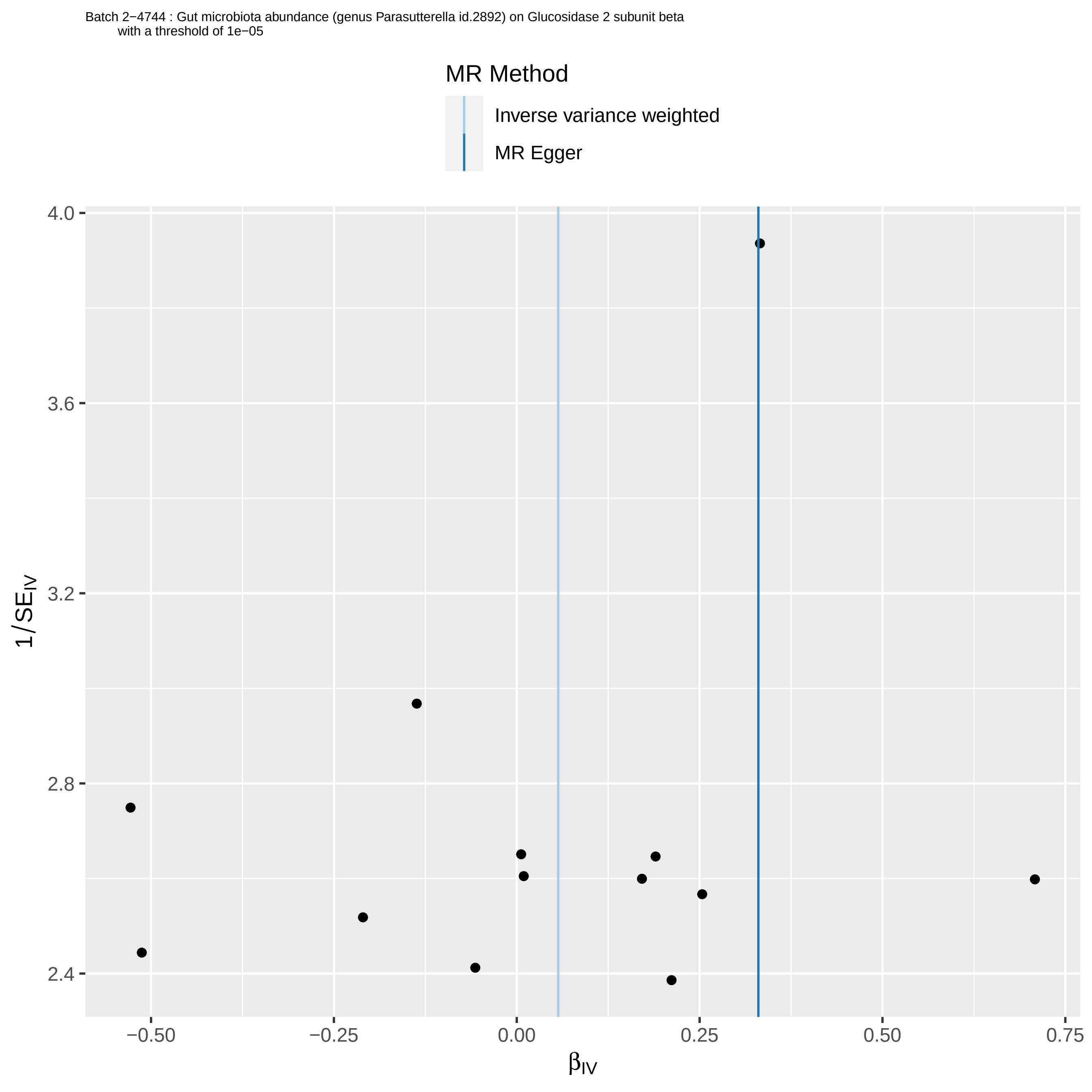

Batch 2−4744 : Gut microbiota abundance (genus Parasutterella id.2892) on Glucosidase 2 subunit beta
with a threshold of 1e−05
MR Method
Inverse variance weighted
MR Egger
4.0
3.6
3.2
2.8
2.4
1/SE_IV
−0.50
−0.25
0.00
0.25
0.50
0.75
β_IV

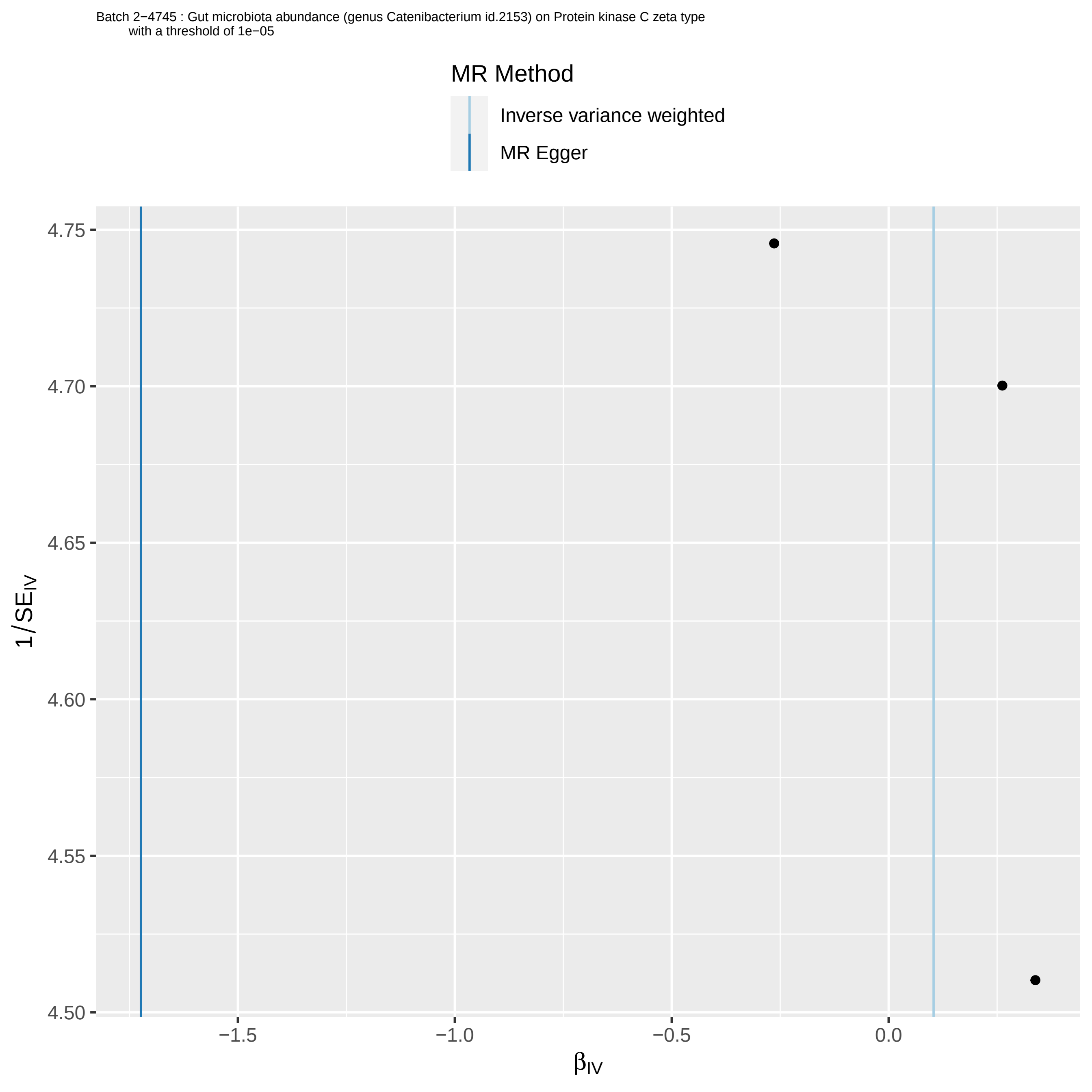
Batch 2−4745 : Gut microbiota abundance (genus Catenibacterium id.2153) on Protein kinase C zeta type
with a threshold of 1e−05
MR Method
Inverse variance weighted
MR Egger
4.75
4.70
4.65
4.60
4.55
4.50
1/SE_IV
−1.5
−1.0
−0.5
0.0
β_IV

Batch 2−4746 : Gut microbiota abundance (genus Parasutterella id.2892) on Protein kinase C zeta type
with a threshold of 1e−05

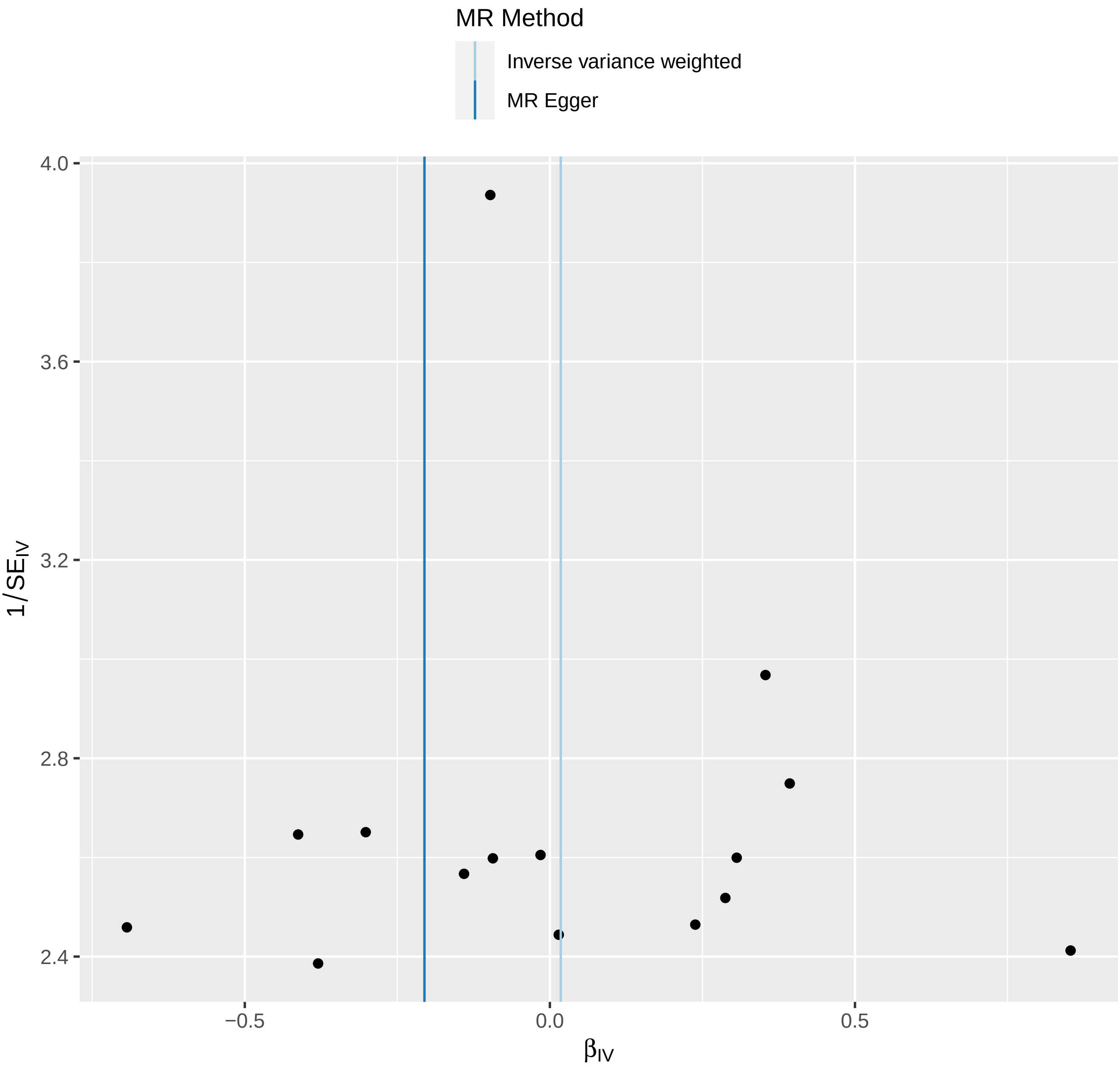

Batch 2−4747 : Gut microbiota abundance (genus Catenibacterium id.2153) on cGMP−dependent protein kinase 1, beta isozyme
with a threshold of 1e−05

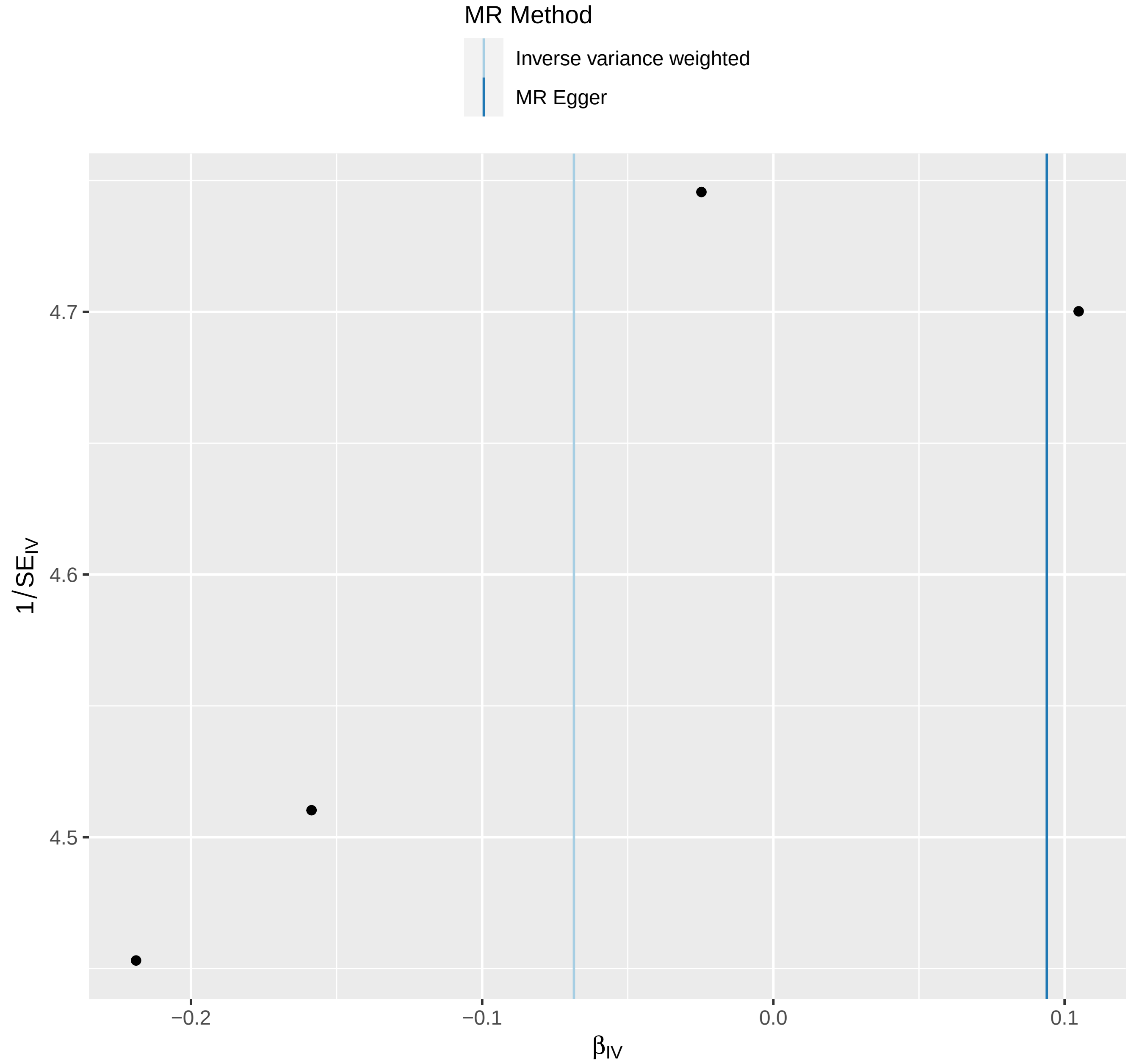

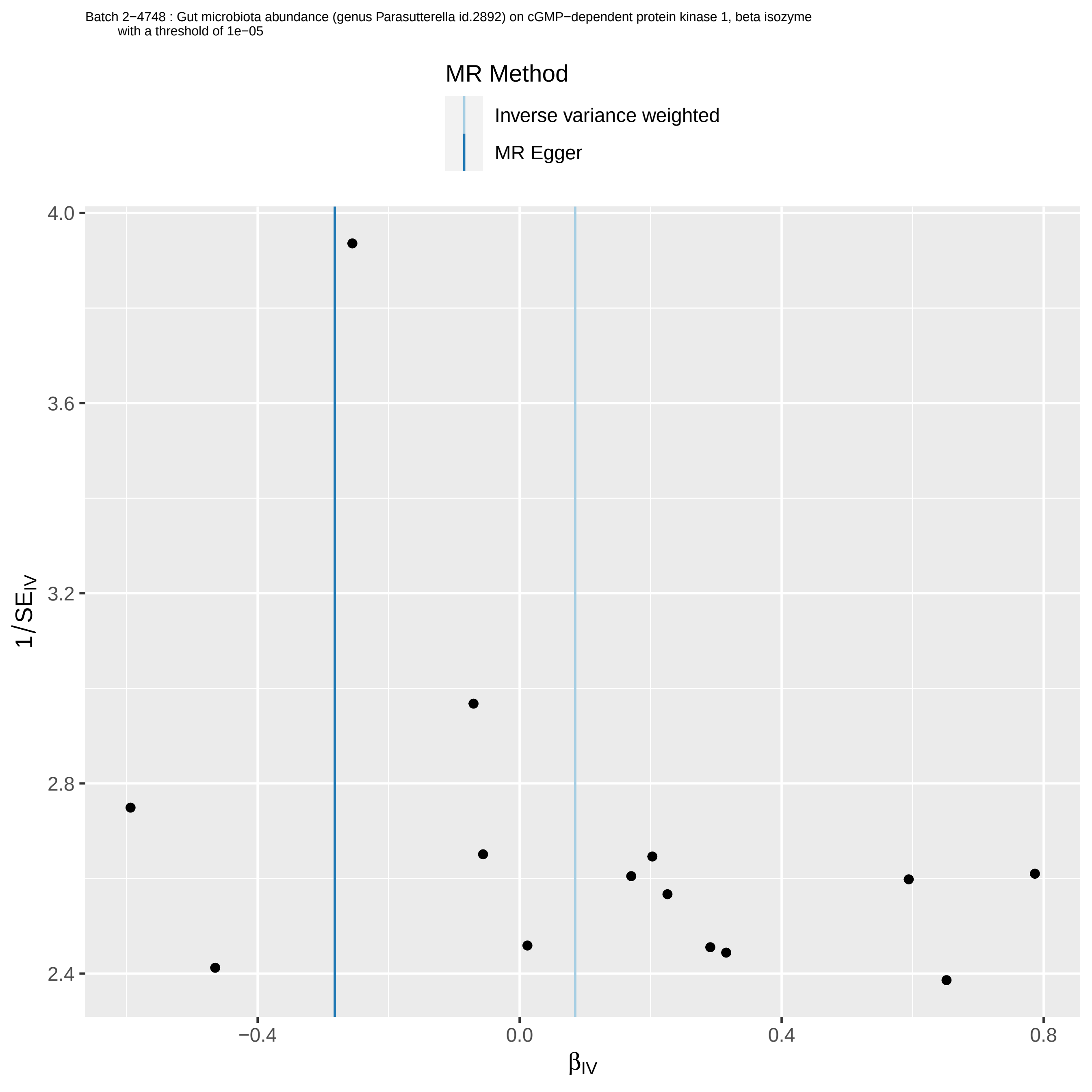
Batch 2−4748 : Gut microbiota abundance (genus Parasutterella id.2892) on cGMP−dependent protein kinase 1, beta isozyme
with a threshold of 1e−05
MR Method
Inverse variance weighted
MR Egger
4.0
3.6
3.2
2.8
2.4
1/SE_IV
−0.4
0.0
0.4
0.8
β_IV

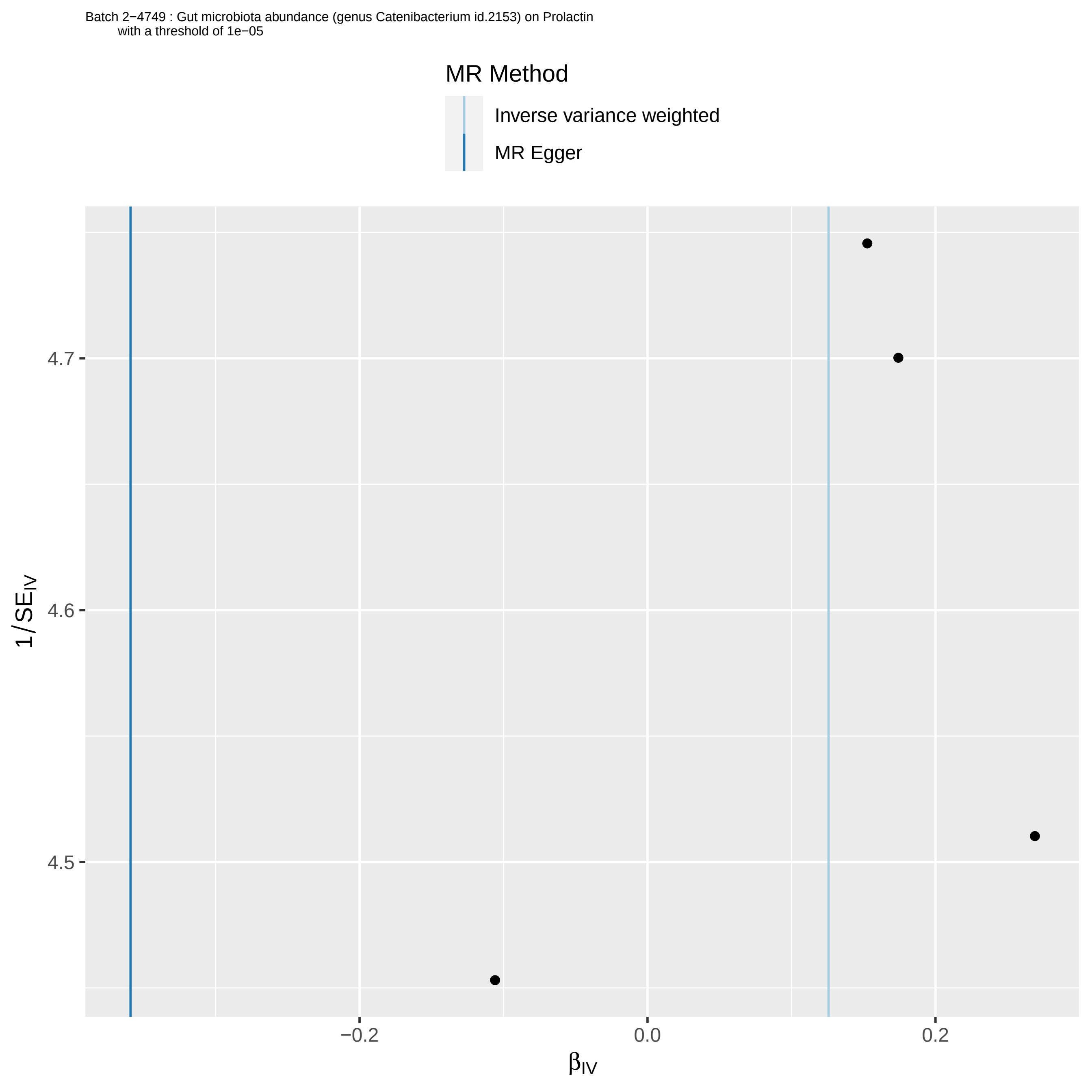
Batch 2−4749 : Gut microbiota abundance (genus Catenibacterium id.2153) on Prolactin
with a threshold of 1e−05
MR Method
Inverse variance weighted
MR Egger
4.7
4.6
4.5
1/SE$_{IV}$
−0.2
0.0
0.2
β$_{IV}$

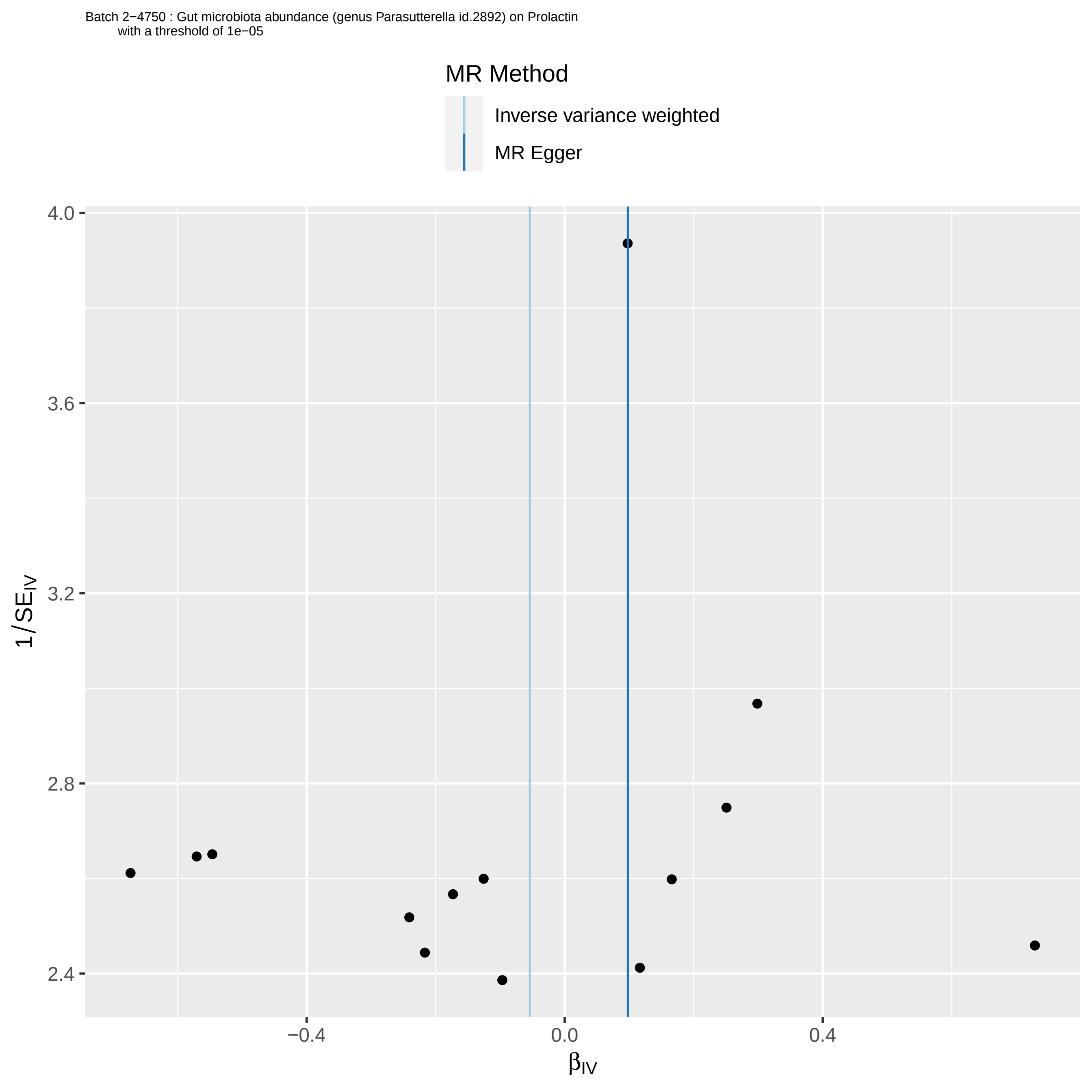
Batch 2−4750 : Gut microbiota abundance (genus Parasutterella id.2892) on Prolactin
with a threshold of 1e−05
MR Method
Inverse variance weighted
MR Egger
4.0
3.6
3.2
2.8
2.4
1/SE_IV
−0.4
0.0
0.4
β_IV

Batch 2−4751 : Gut microbiota abundance (genus Catenibacterium id.2153) on Prolactin−releasing peptide
with a threshold of 1e−05

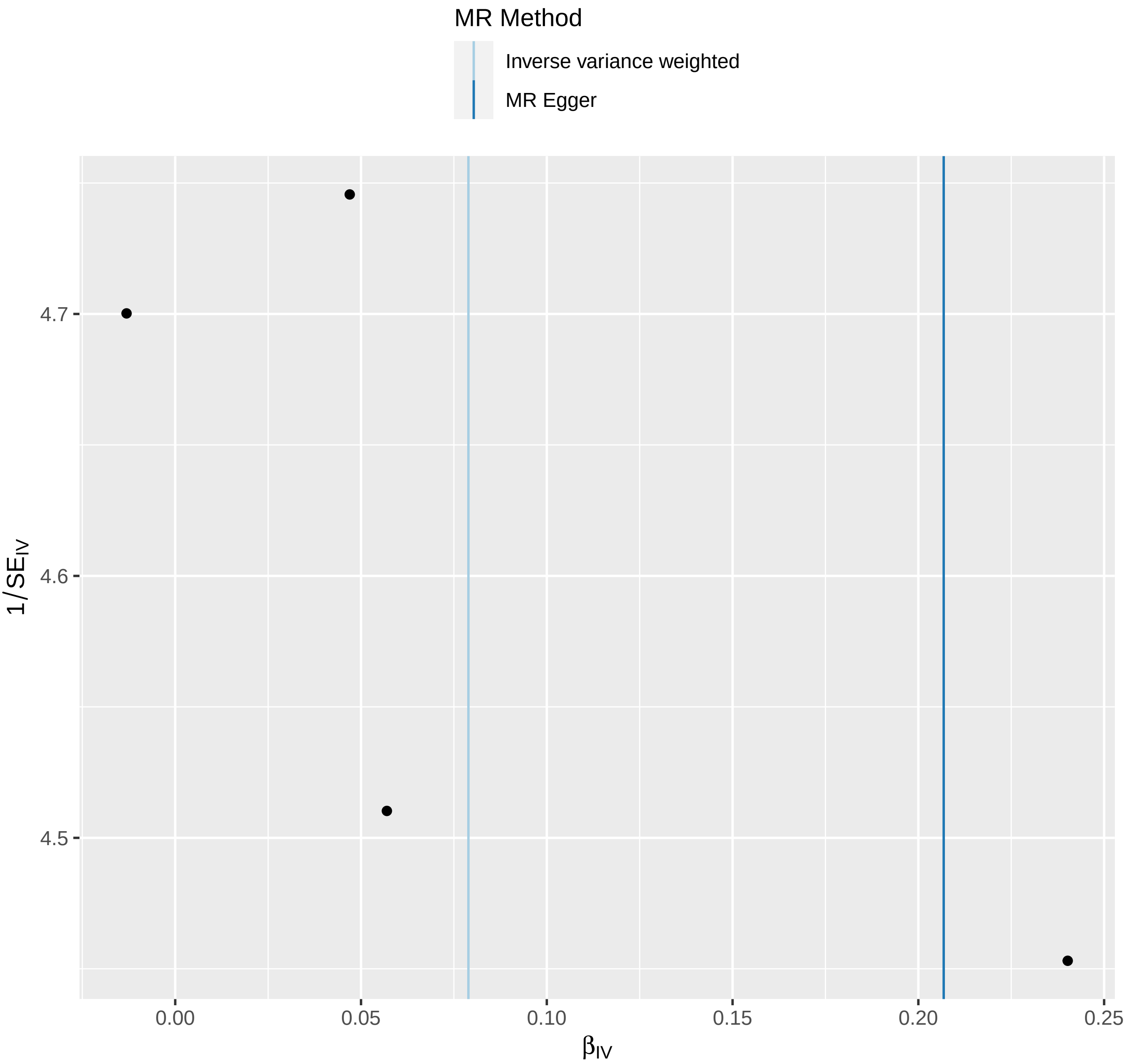

Batch 2−4752 : Gut microbiota abundance (genus Parasutterella id.2892) on Prolactin−releasing peptide
with a threshold of 1e−05

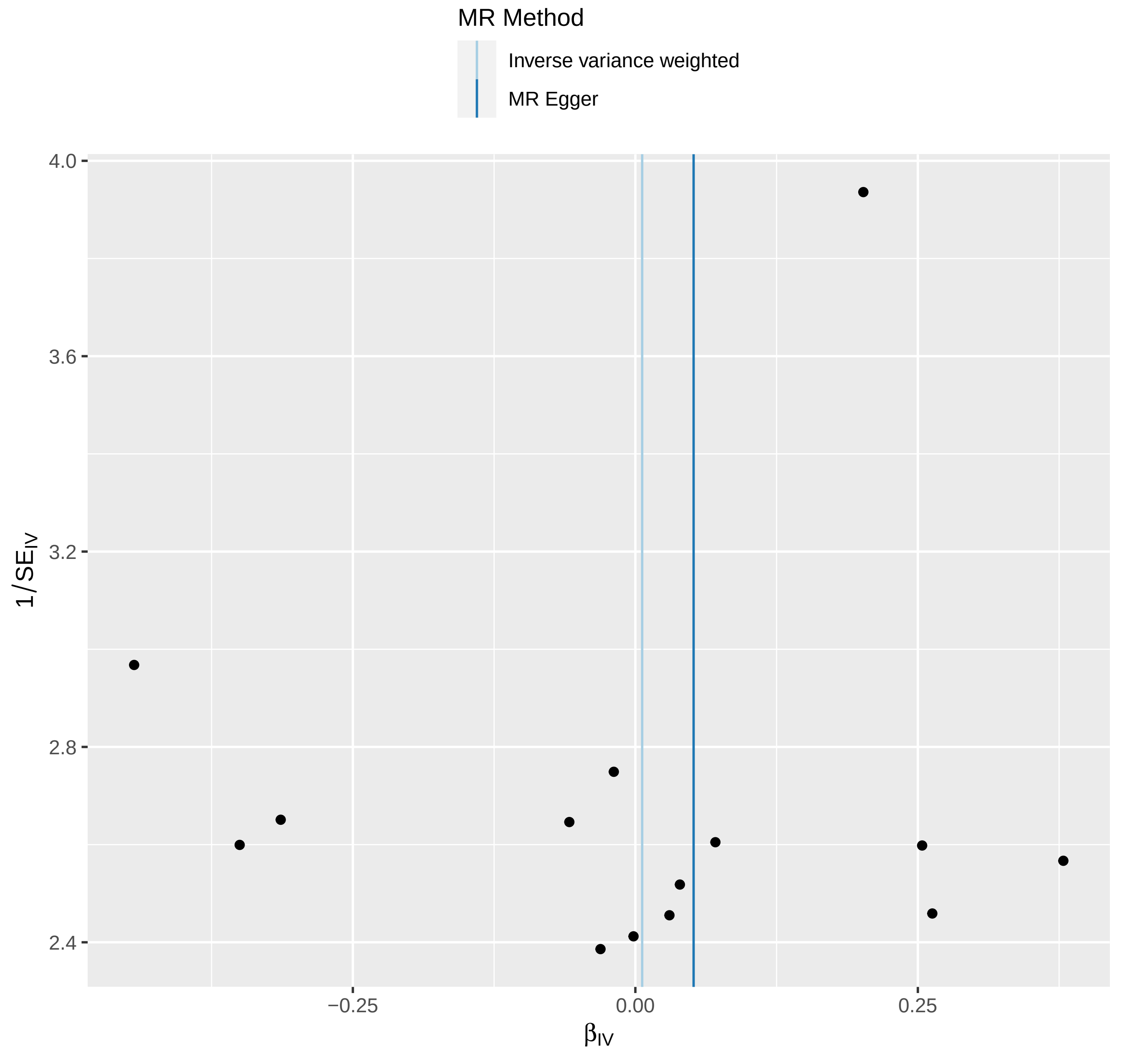

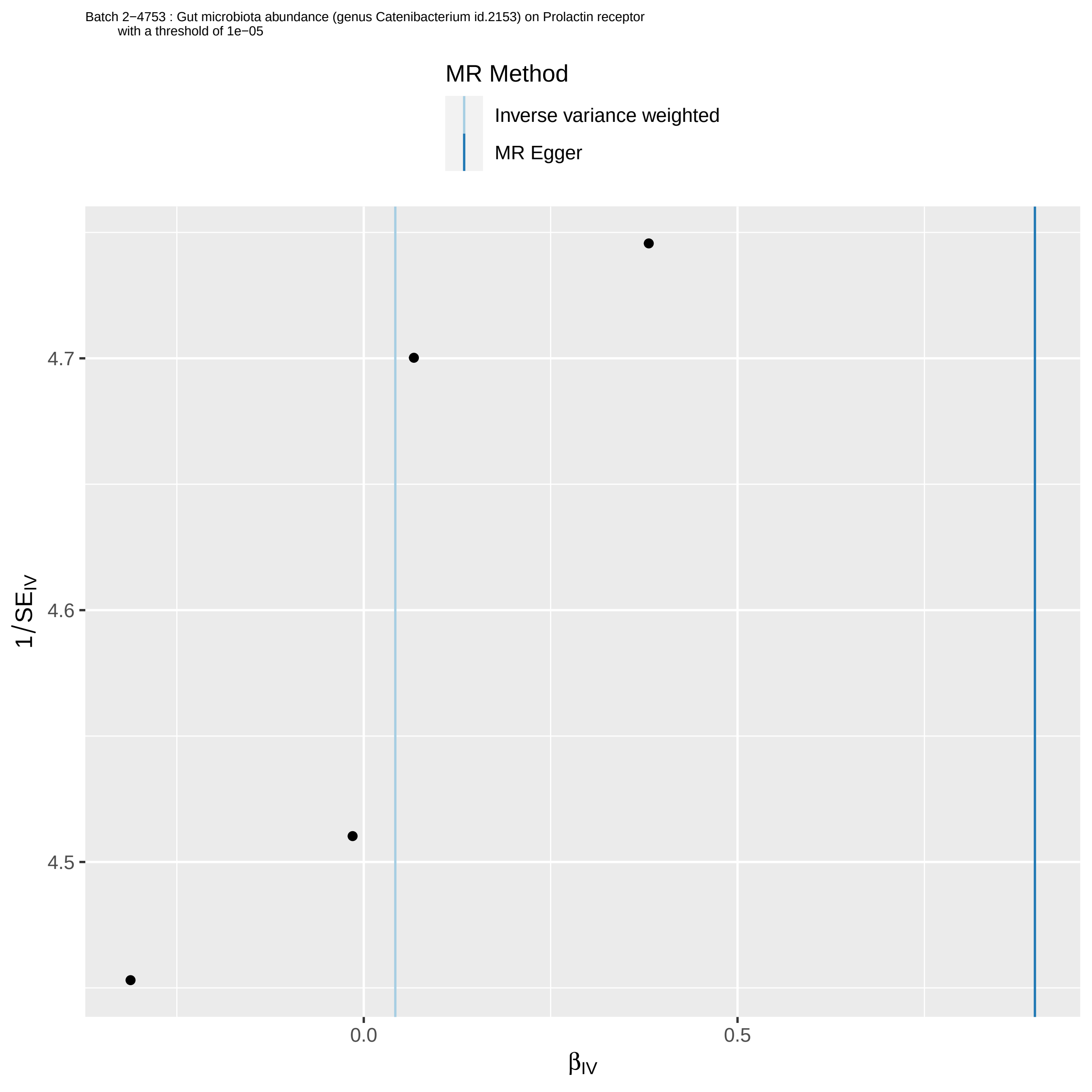

Batch 2−4753 : Gut microbiota abundance (genus Catenibacterium id.2153) on Prolactin receptor
with a threshold of 1e−05
MR Method
Inverse variance weighted
MR Egger
4.7
4.6
4.5
$1/SE_{IV}$
0.0
0.5
β_{IV}

Batch 2−4754 : Gut microbiota abundance (genus Parasutterella id.2892) on Prolactin receptor
with a threshold of 1e−05

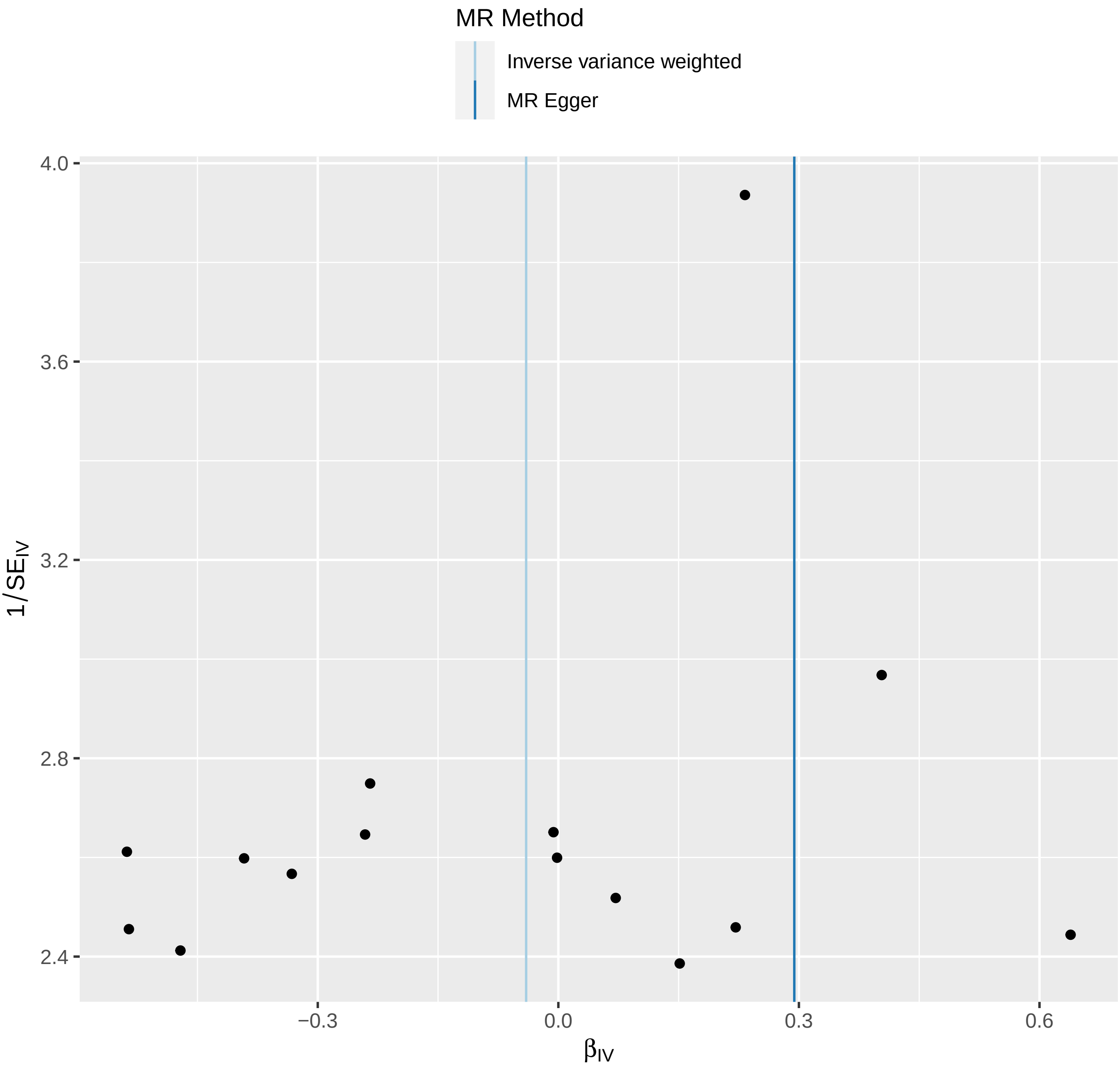

Batch 2−4755 : Gut microbiota abundance (genus Catenibacterium id.2153) on Protein arginine N−methyltransferase 3
with a threshold of 1e−05

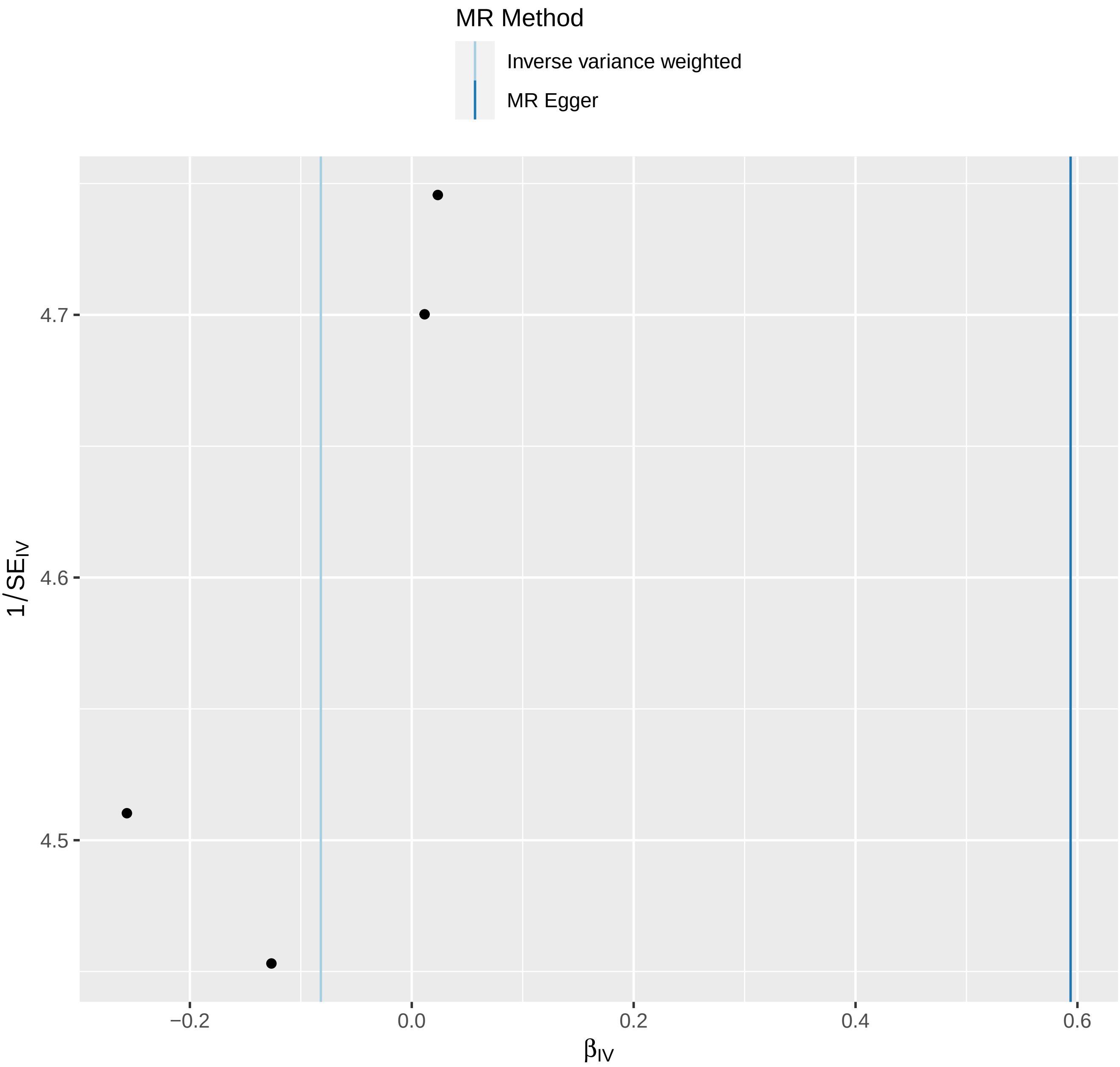

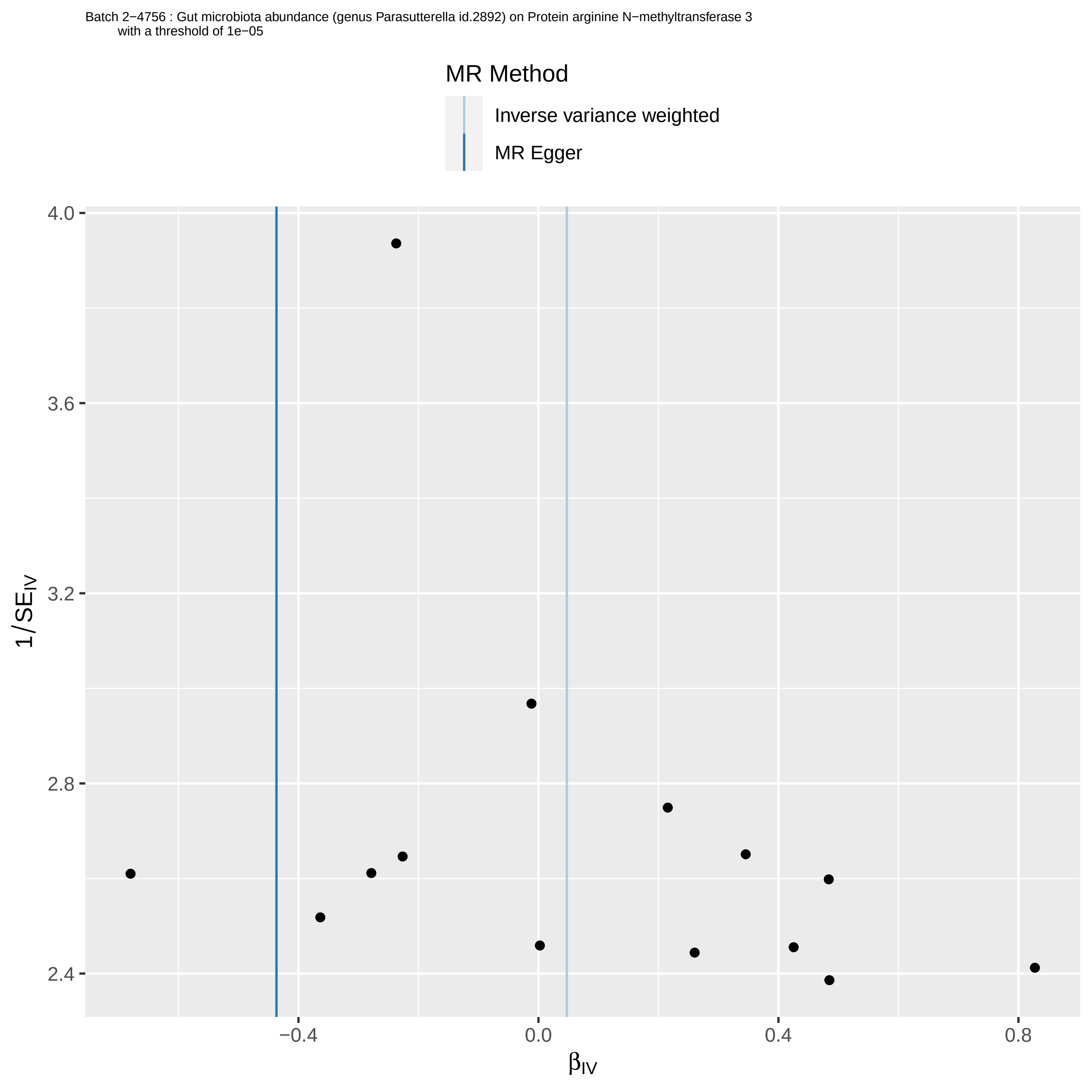
Batch 2−4756 : Gut microbiota abundance (genus Parasutterella id.2892) on Protein arginine N−methyltransferase 3
with a threshold of 1e−05
MR Method
Inverse variance weighted
MR Egger
4.0
3.6
3.2
2.8
2.4
1/SE_IV
−0.4
0.0
0.4
0.8
β_IV

Batch 2−4757 : Gut microbiota abundance (genus Catenibacterium id.2153) on Major prion protein
with a threshold of 1e−05

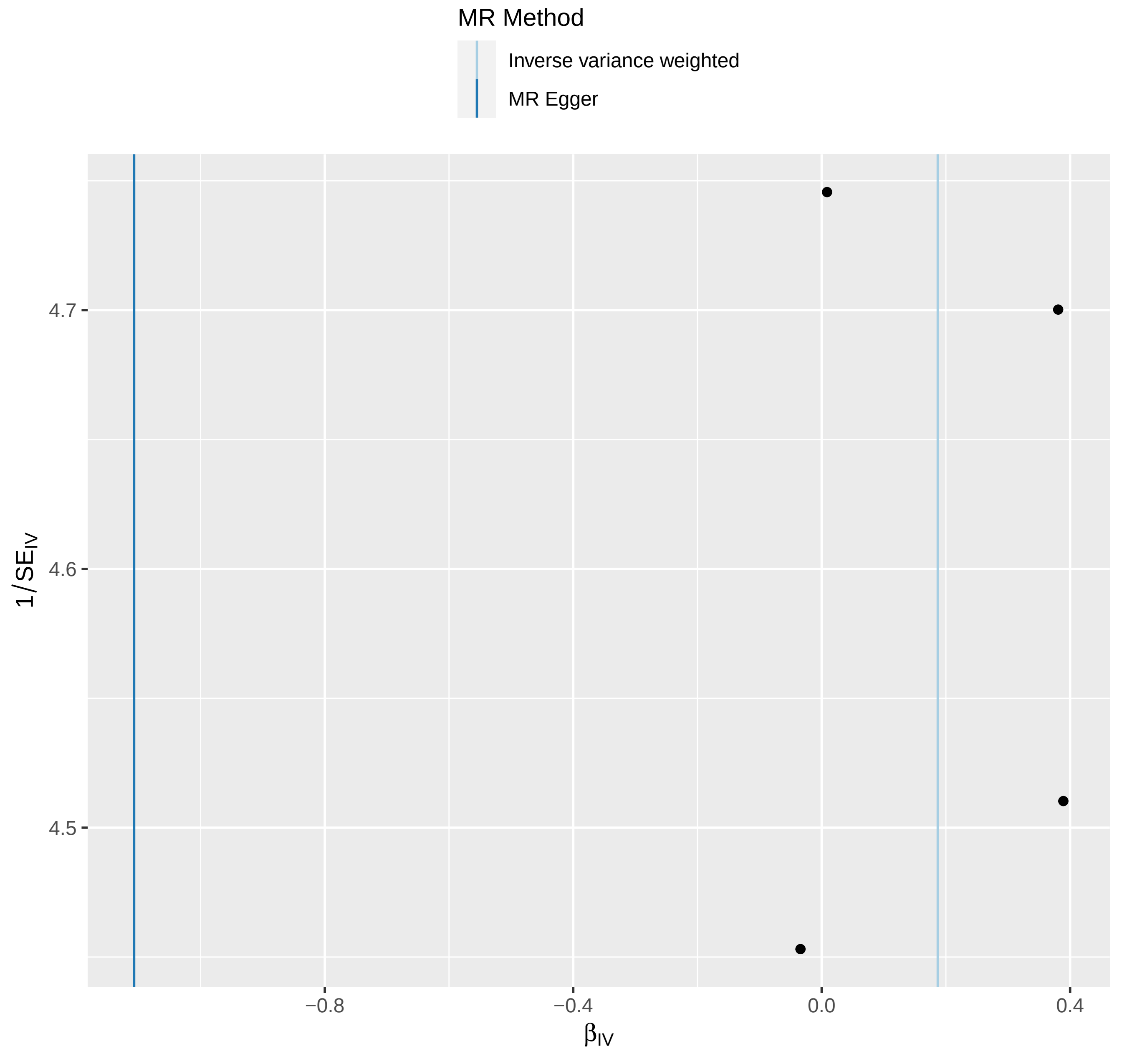

Batch 2−4758 : Gut microbiota abundance (genus Parasutterella id.2892) on Major prion protein
with a threshold of 1e−05

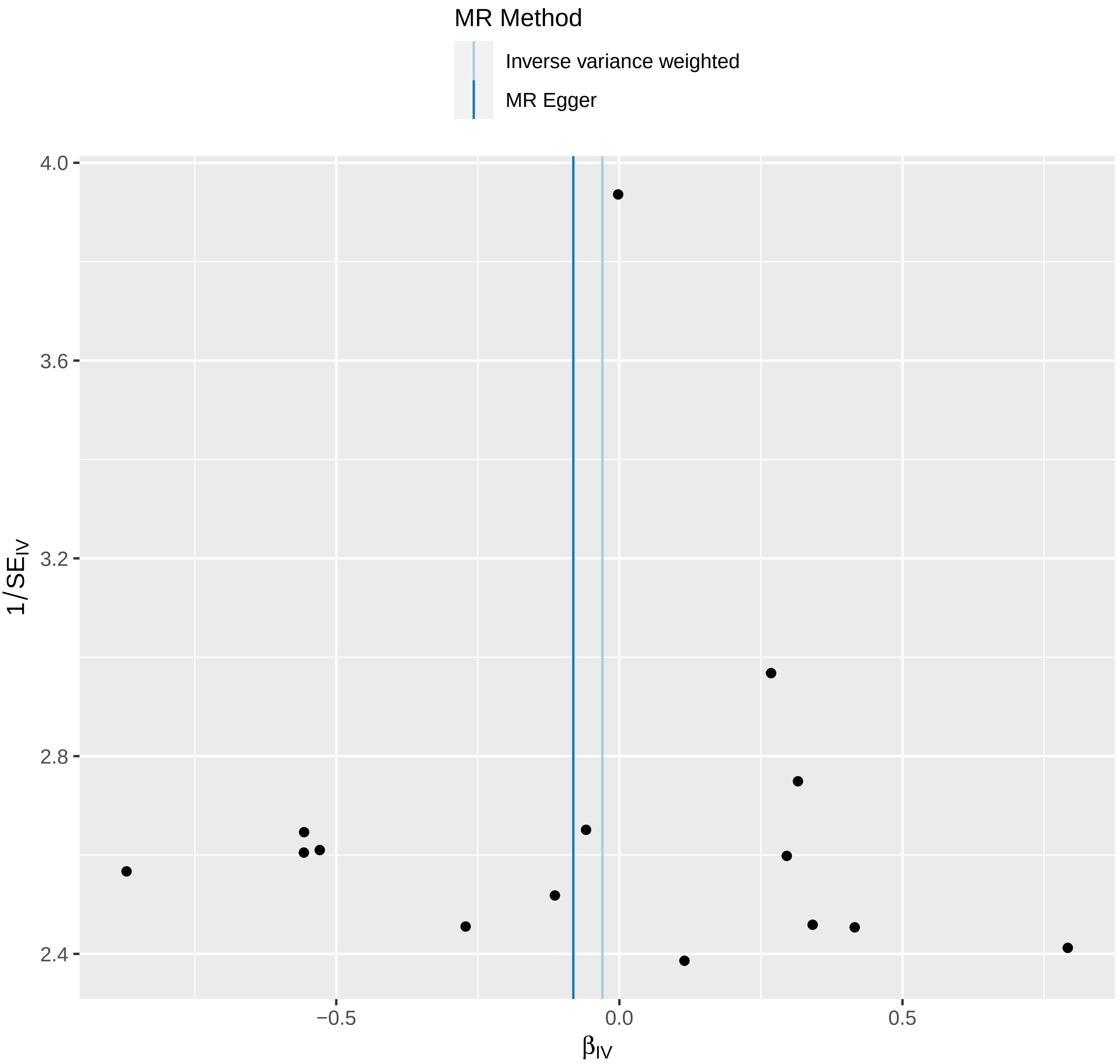

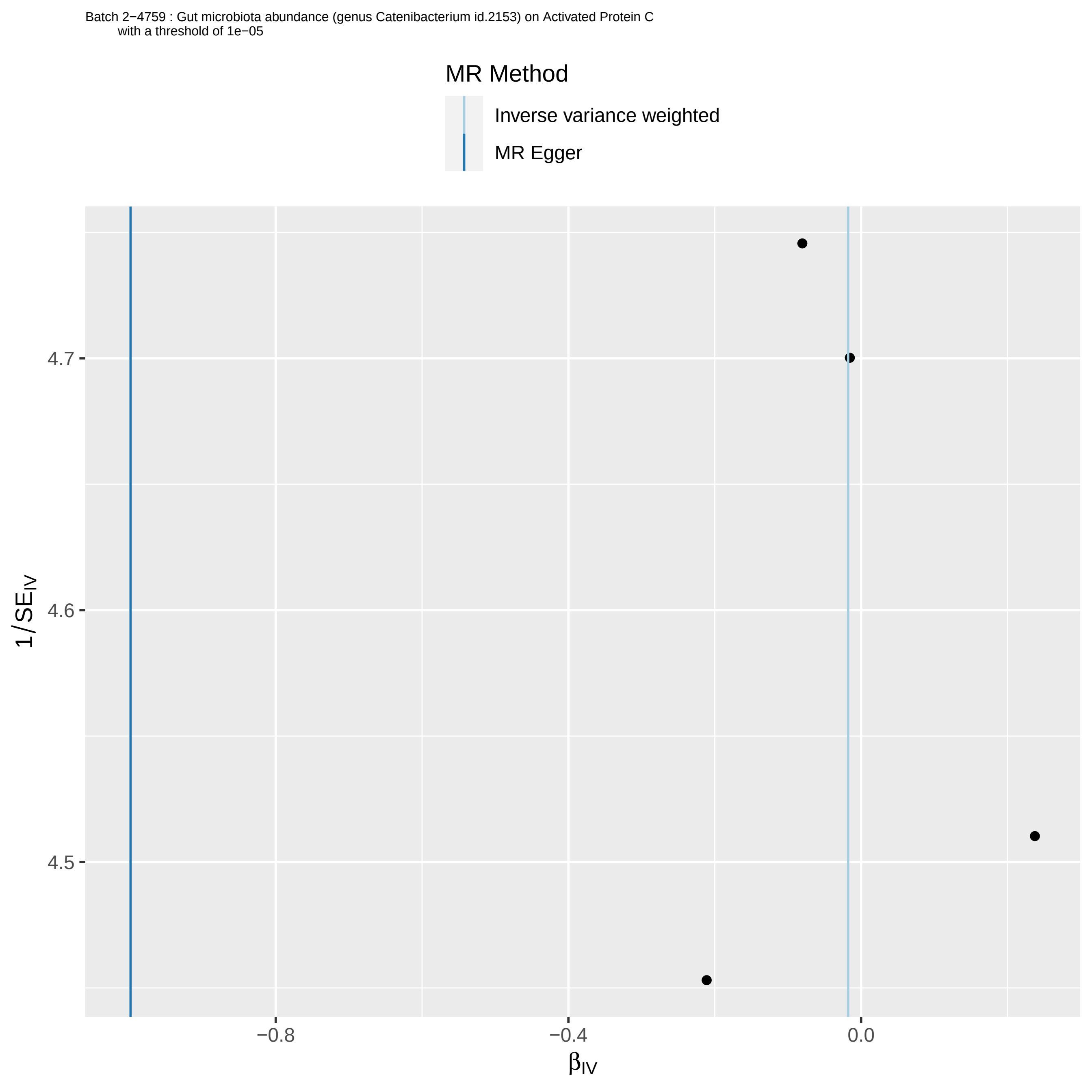
Batch 2−4759 : Gut microbiota abundance (genus Catenibacterium id.2153) on Activated Protein C
with a threshold of 1e−05
MR Method
Inverse variance weighted
MR Egger
4.7
4.6
4.5
1/SE_IV
−0.8
−0.4
0.0
β_IV

Batch 2−4760 : Gut microbiota abundance (genus Parasutterella id.2892) on Activated Protein C
with a threshold of 1e−05

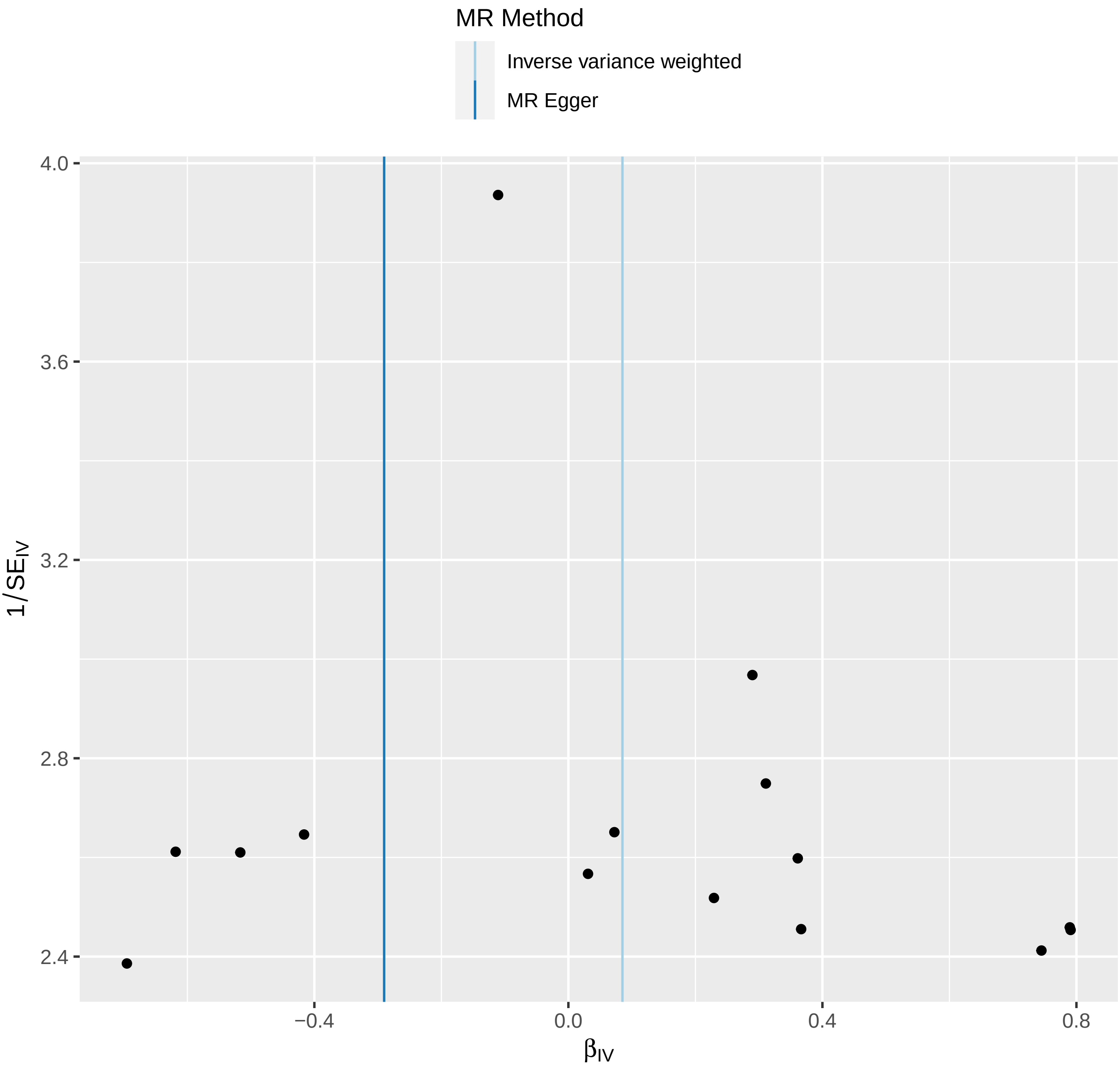

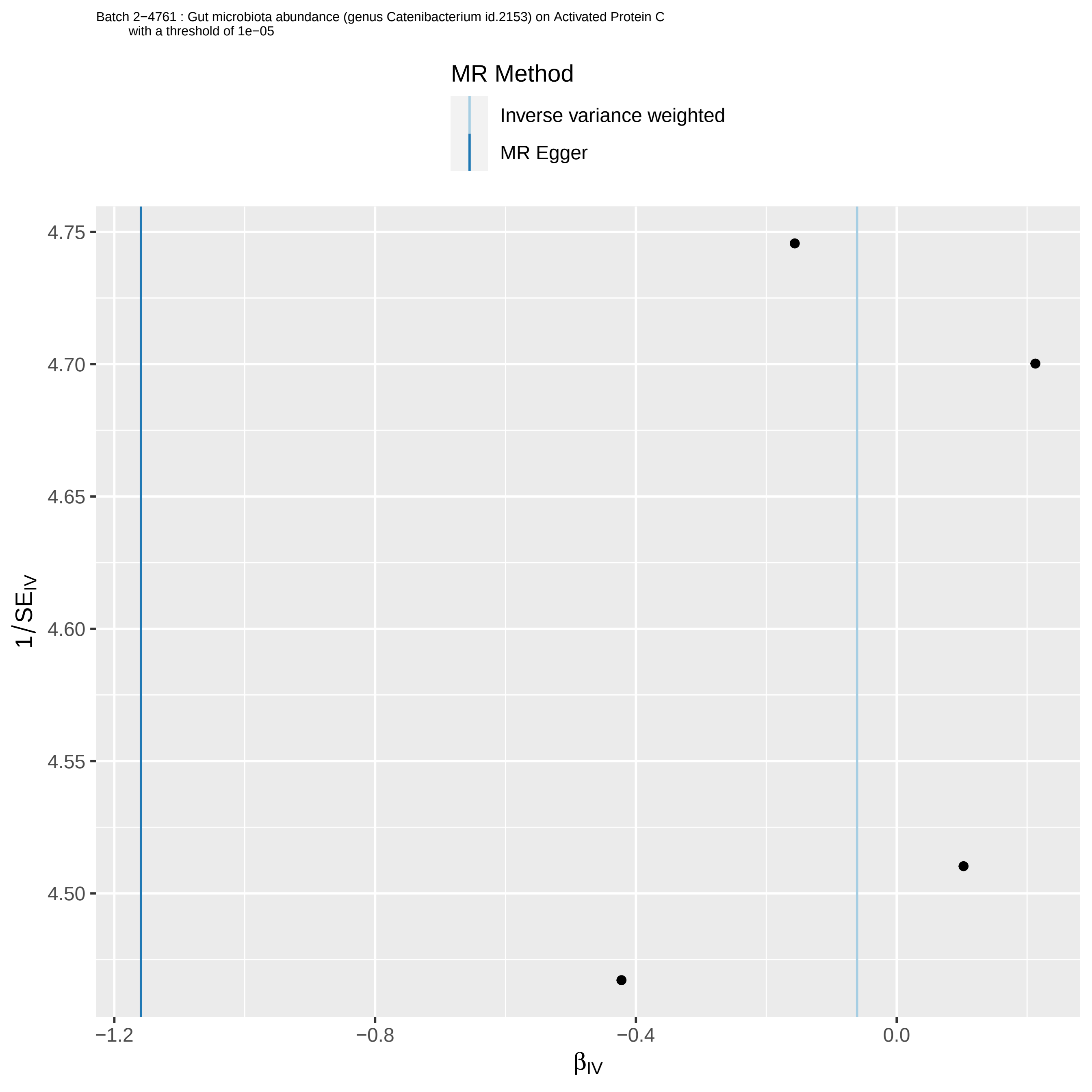

Batch 2−4761 : Gut microbiota abundance (genus Catenibacterium id.2153) on Activated Protein C
with a threshold of 1e−05
MR Method
Inverse variance weighted
MR Egger
4.75
4.70
4.65
4.60
4.55
4.50
1/SE_IV
−1.2
−0.8
−0.4
0.0
β_IV

Batch 2−4762 : Gut microbiota abundance (genus Parasutterella id.2892) on Activated Protein C
with a threshold of 1e−05

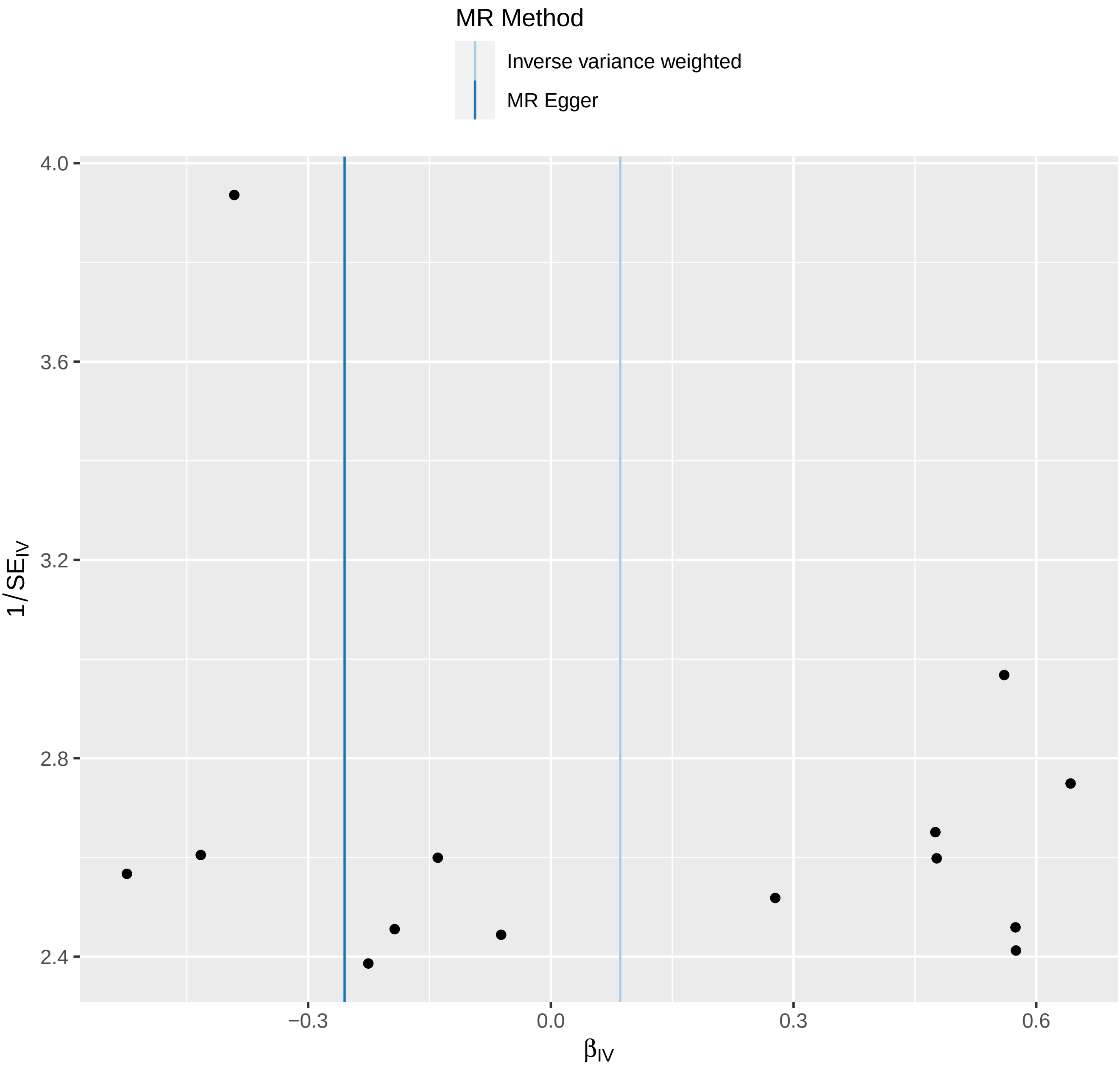

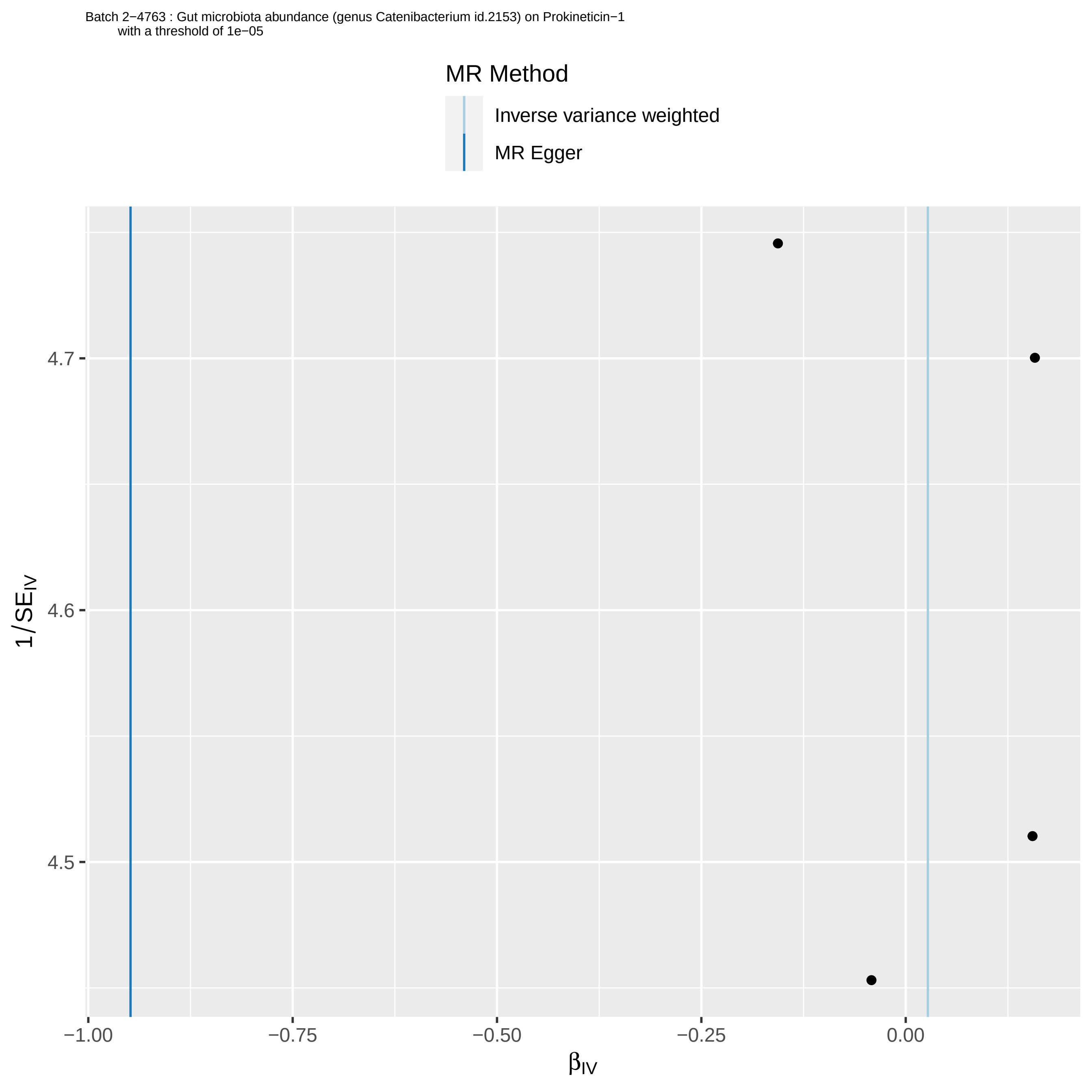

Batch 2−4763 : Gut microbiota abundance (genus Catenibacterium id.2153) on Prokineticin−1
with a threshold of 1e−05
MR Method
Inverse variance weighted
MR Egger
4.7
4.6
4.5
1/SE_IV
−1.00
−0.75
−0.50
−0.25
0.00
β_IV

Batch 2−4764 : Gut microbiota abundance (genus Parasutterella id.2892) on Prokineticin−1
with a threshold of 1e−05

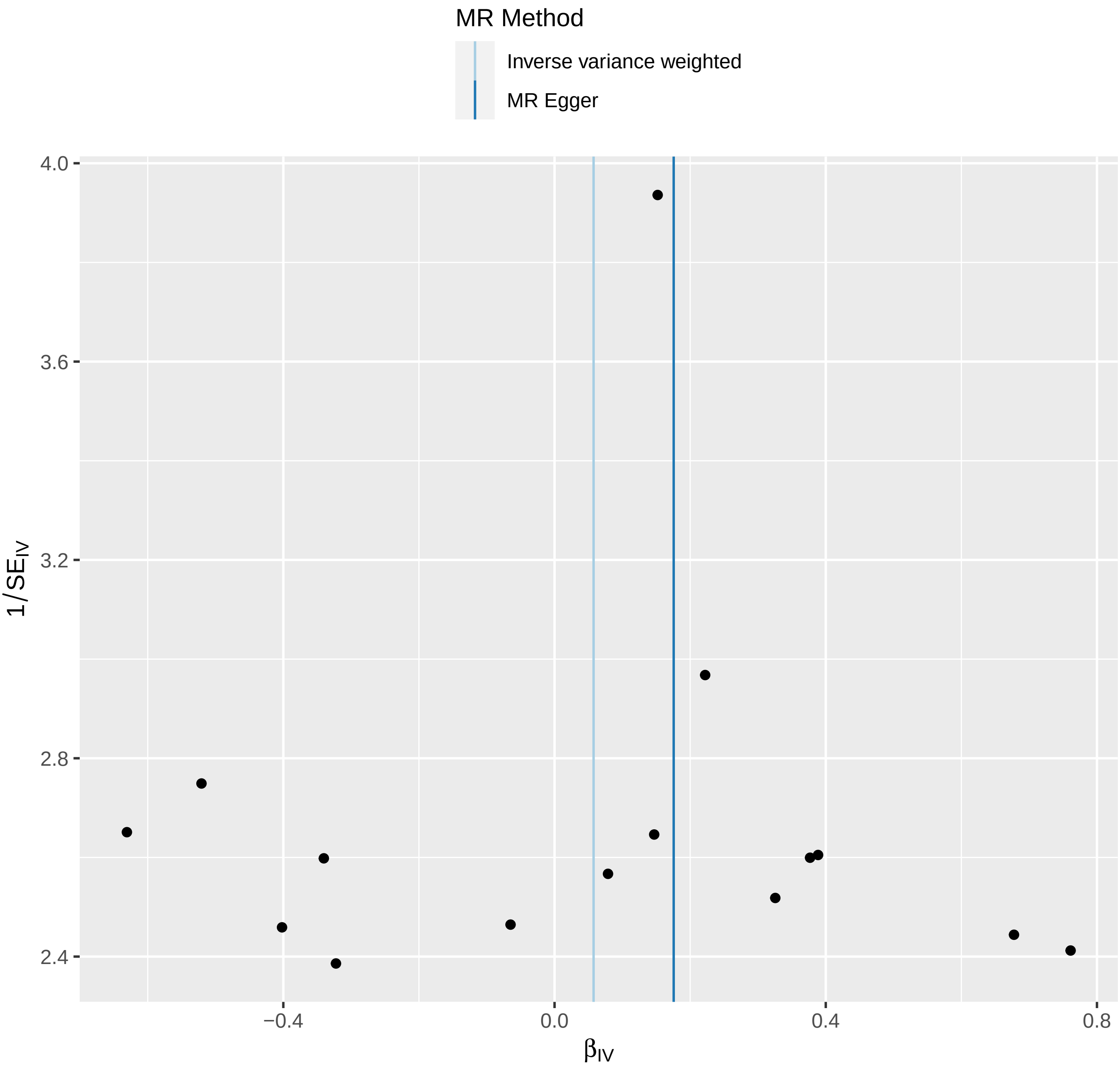

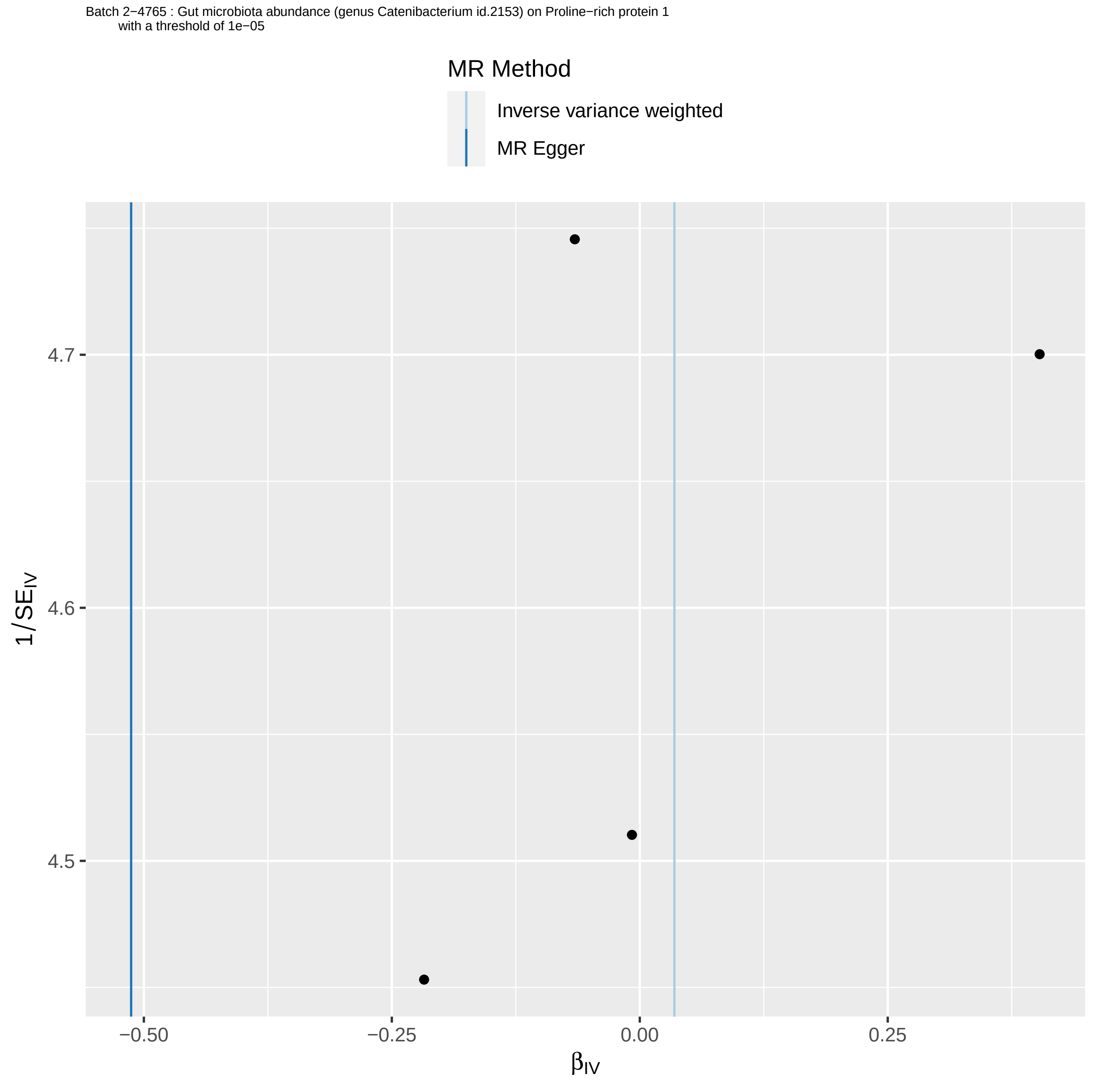
Batch 2−4765 : Gut microbiota abundance (genus Catenibacterium id.2153) on Proline−rich protein 1
with a threshold of 1e−05
MR Method
Inverse variance weighted
MR Egger
4.7
4.6
4.5
1/SE_IV
−0.50
−0.25
0.00
0.25
β_IV

Batch 2−4766 : Gut microbiota abundance (genus Parasutterella id.2892) on Proline−rich protein 1
with a threshold of 1e−05

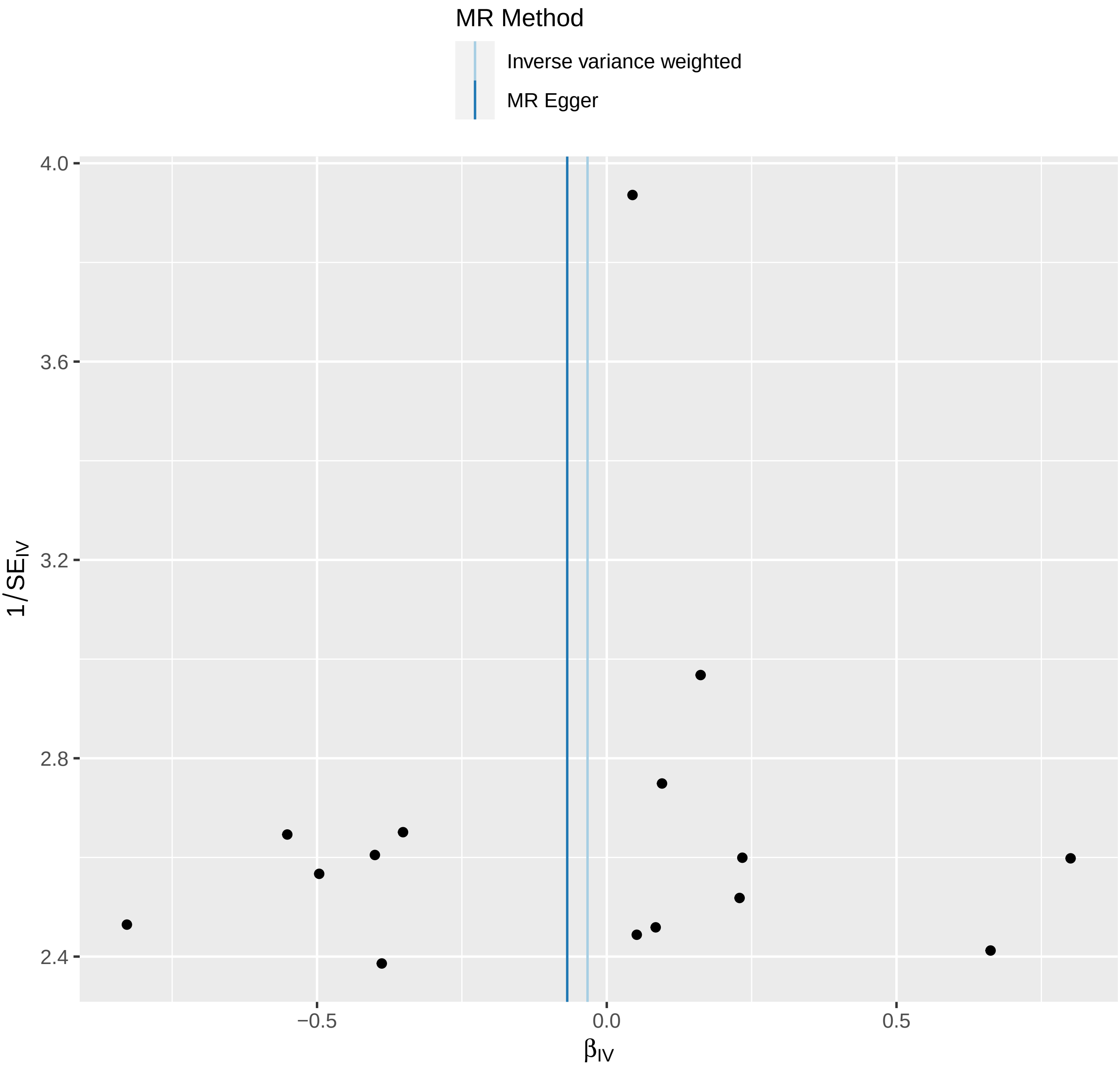

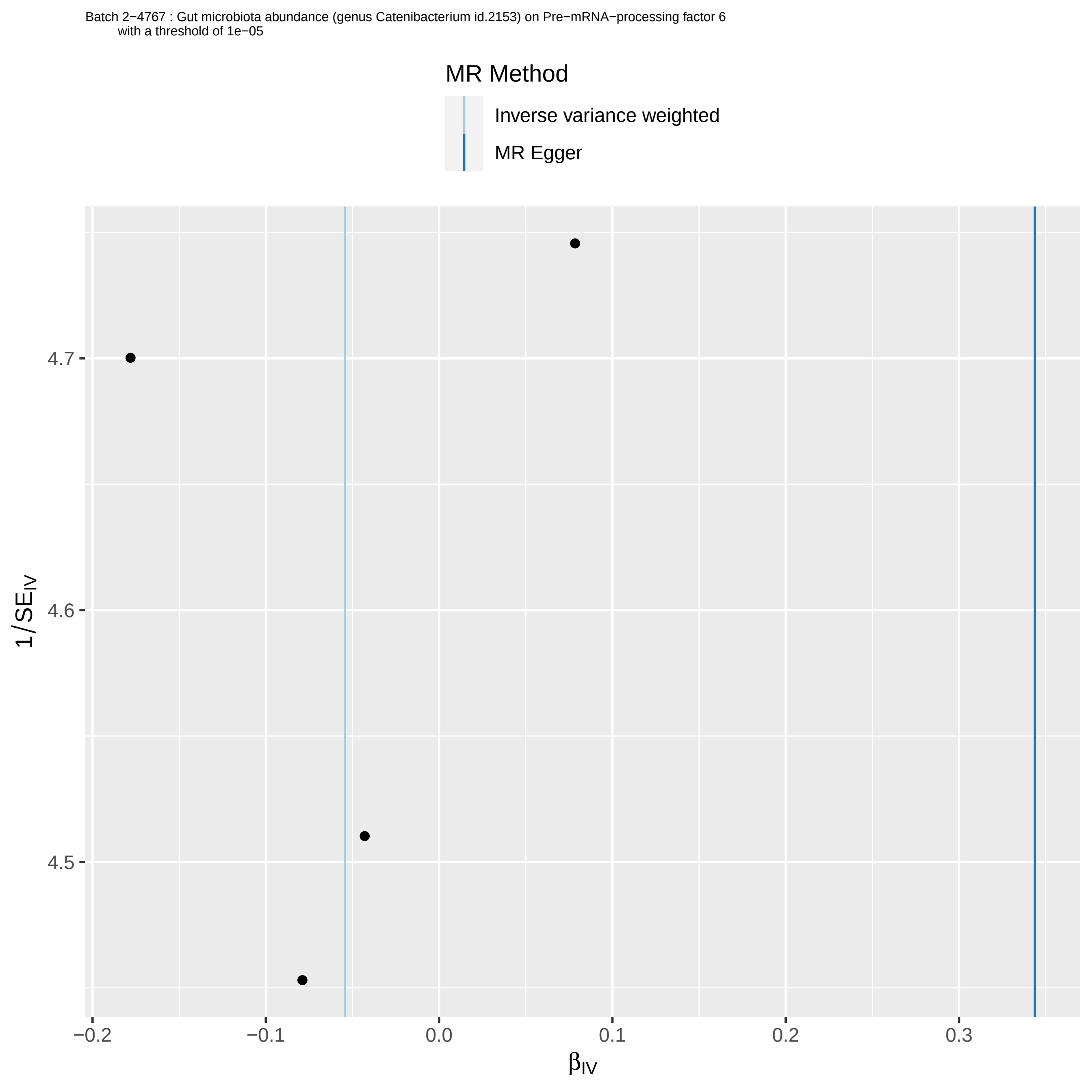

Batch 2−4767 : Gut microbiota abundance (genus Catenibacterium id.2153) on Pre−mRNA−processing factor 6
with a threshold of 1e−05
MR Method
Inverse variance weighted
MR Egger
4.7
4.6
4.5
1/SE_IV
−0.2
−0.1
0.0
0.1
0.2
0.3
β_IV

Batch 2−4768 : Gut microbiota abundance (genus Parasutterella id.2892) on Pre−mRNA−processing factor 6
with a threshold of 1e−05

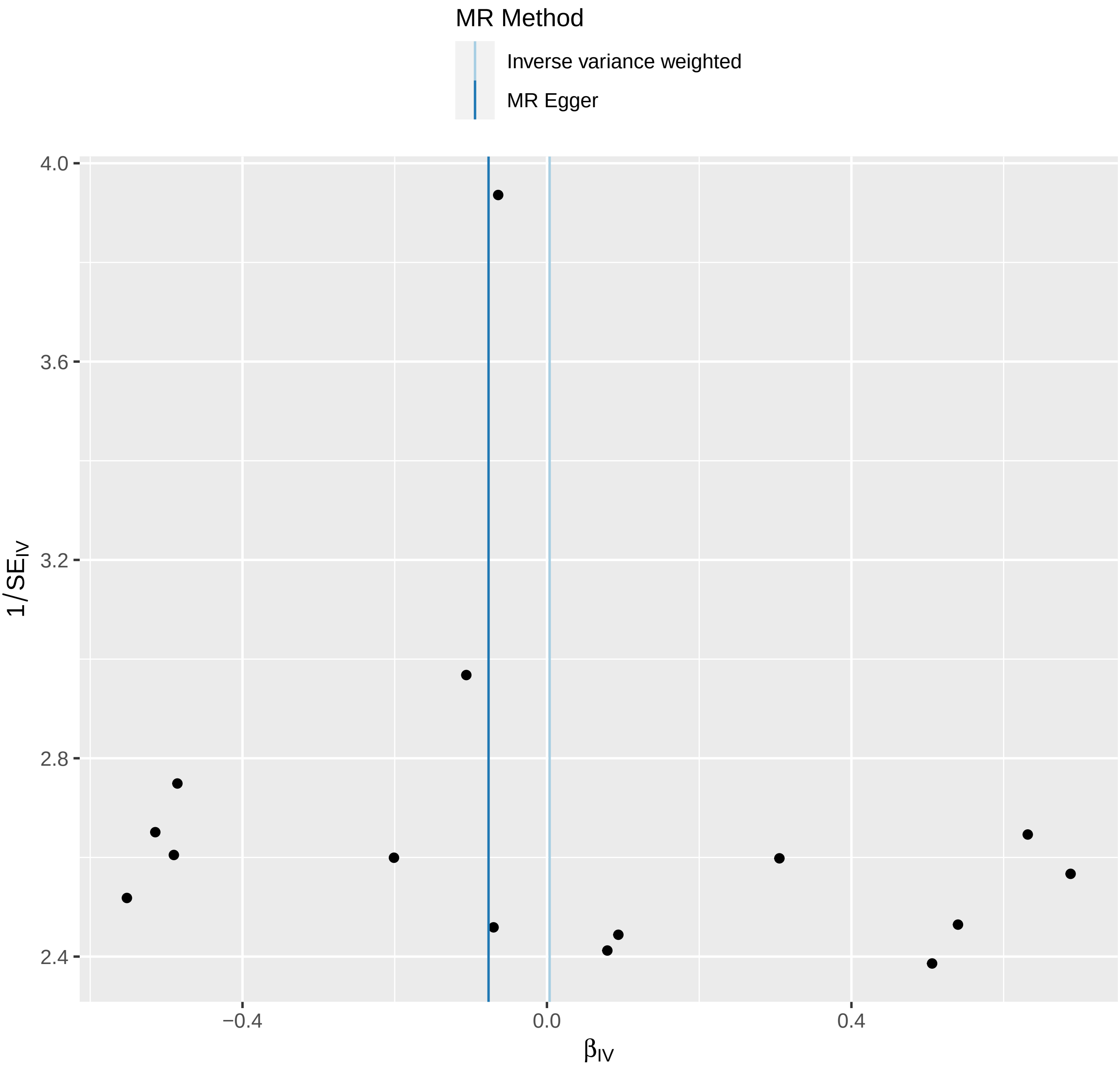

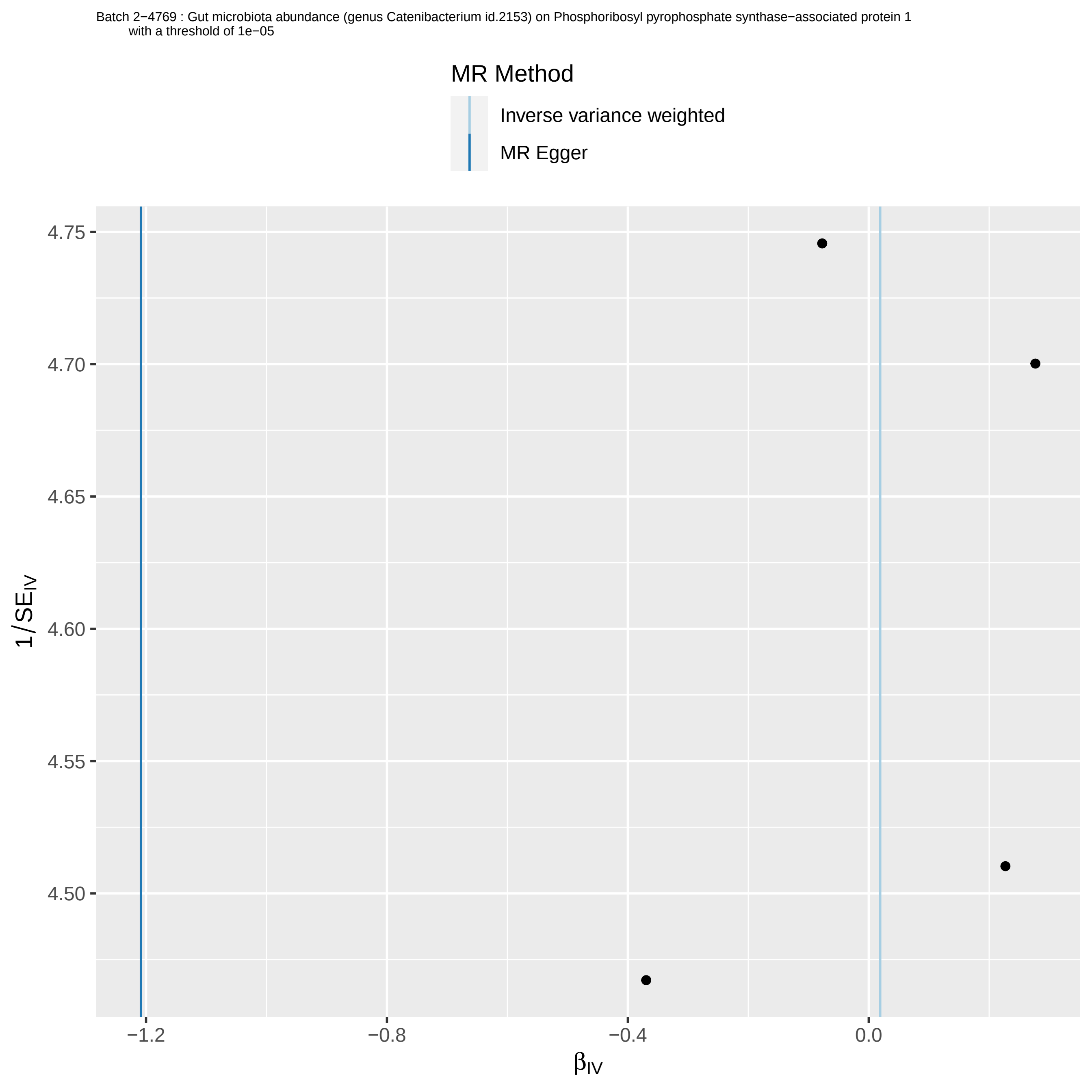
Batch 2−4769 : Gut microbiota abundance (genus Catenibacterium id.2153) on Phosphoribosyl pyrophosphate synthase−associated protein 1
with a threshold of 1e−05
MR Method
Inverse variance weighted
MR Egger
4.75
4.70
4.65
4.60
4.55
4.50
1/SE_IV
−1.2
−0.8
−0.4
0.0
β_IV

Batch 2−4770 : Gut microbiota abundance (genus Parasutterella id.2892) on Phosphoribosyl pyrophosphate synthase−associated protein 1
with a threshold of 1e−05

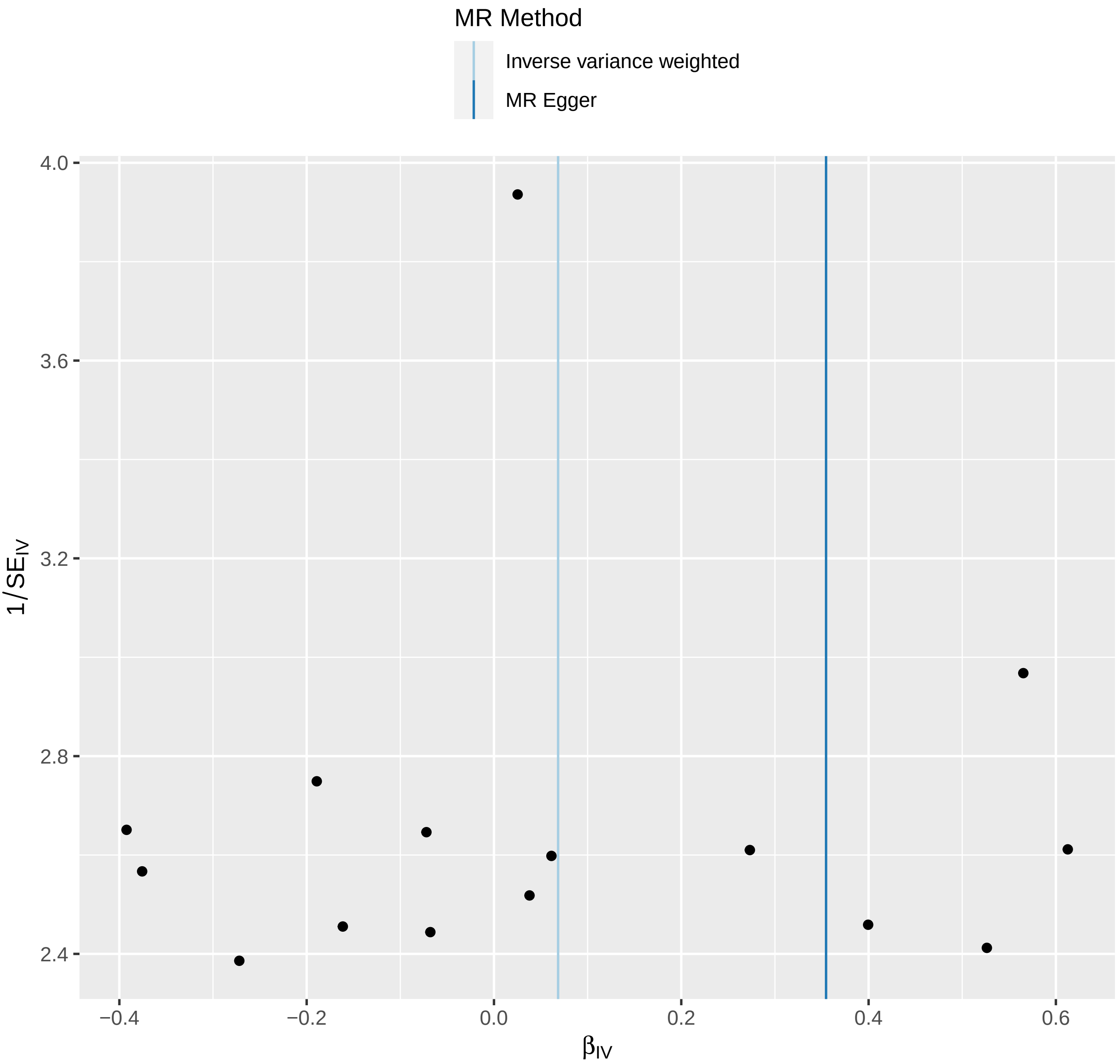

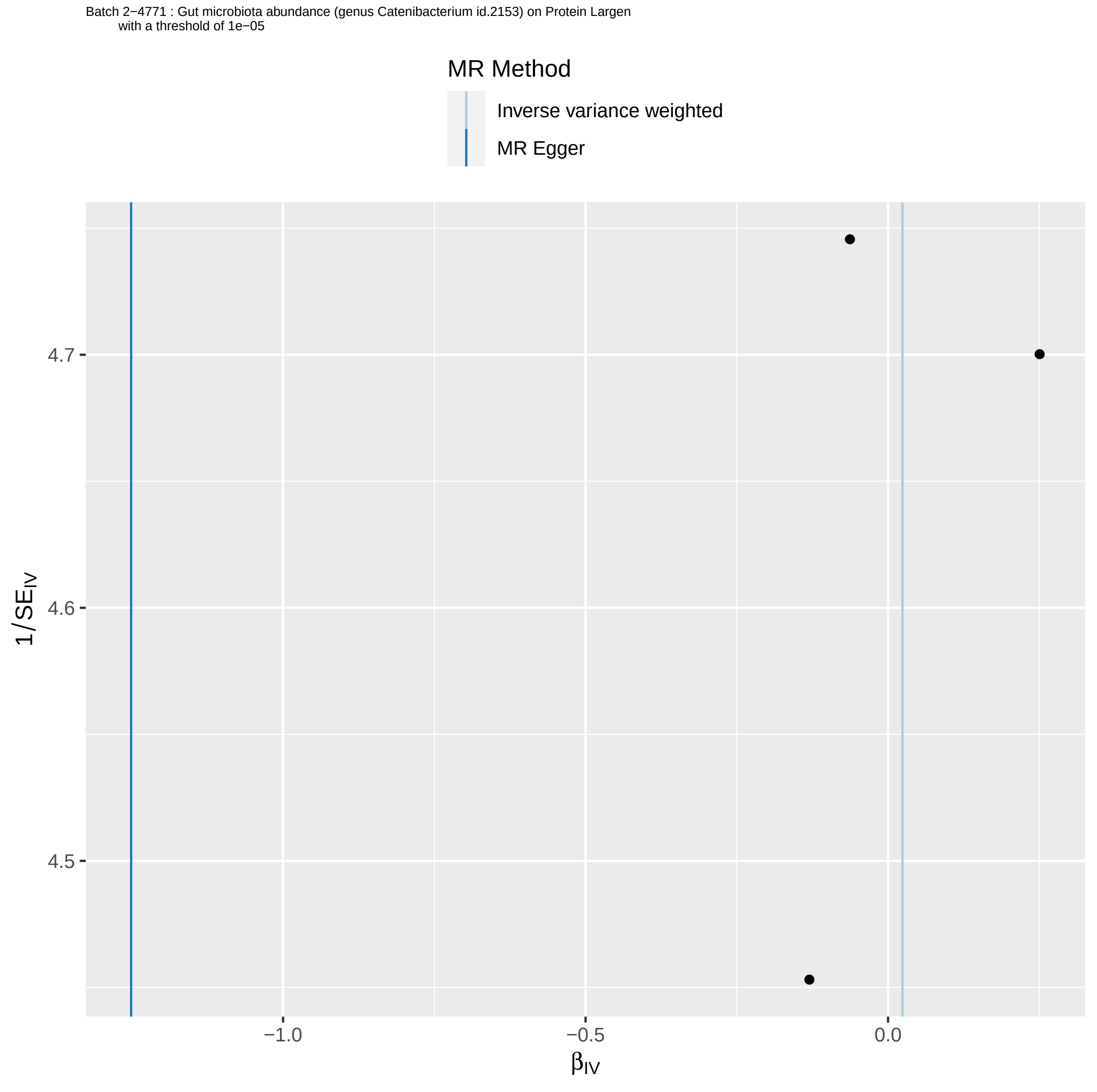

Batch 2−4771 : Gut microbiota abundance (genus Catenibacterium id.2153) on Protein Largen
with a threshold of 1e−05
MR Method
Inverse variance weighted
MR Egger
4.7
4.6
4.5
1/SE_IV
−1.0
−0.5
0.0
β_IV

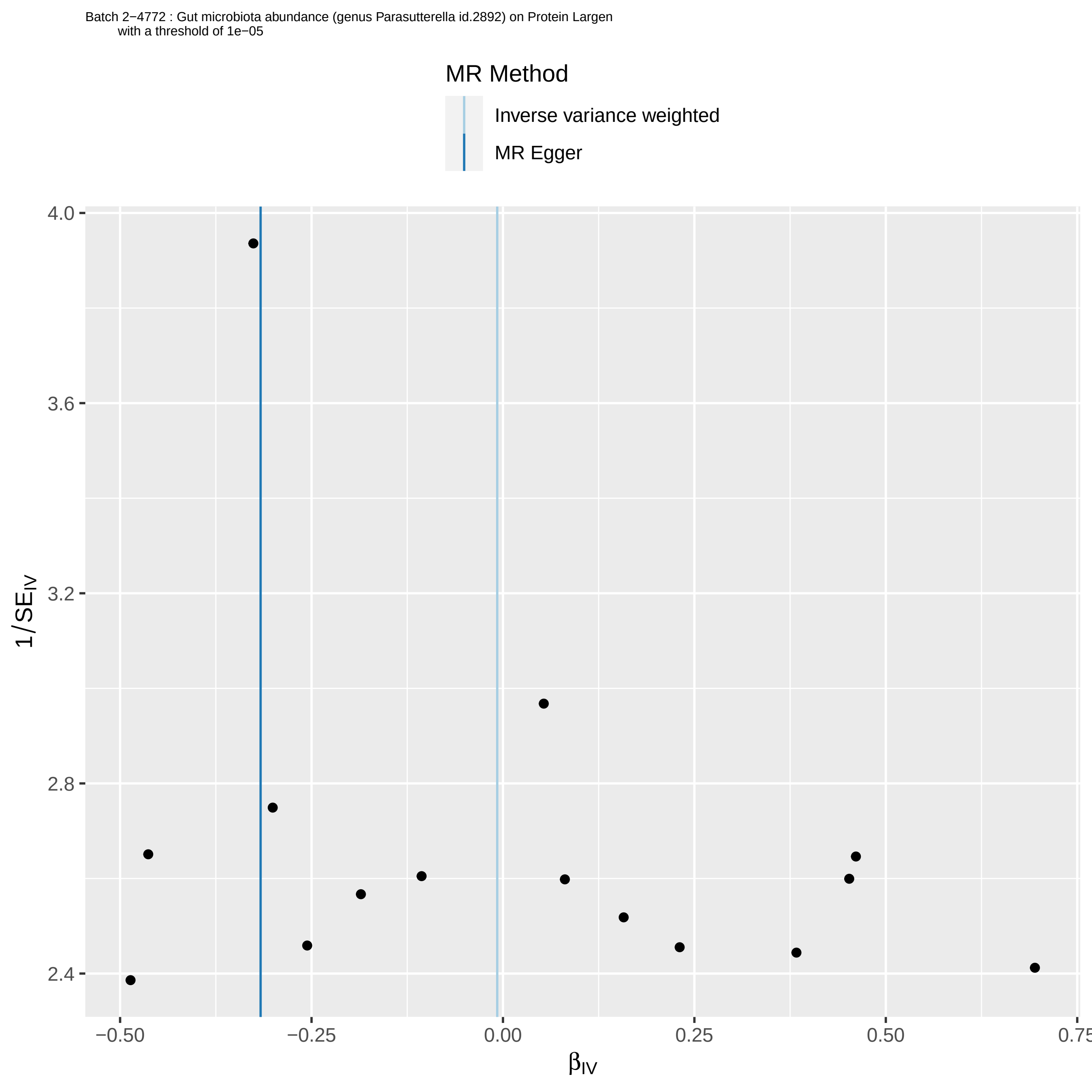

Batch 2−4772 : Gut microbiota abundance (genus Parasutterella id.2892) on Protein Largen
with a threshold of 1e−05
MR Method
Inverse variance weighted
MR Egger
4.0
3.6
3.2
2.8
2.4
1/SE$_{IV}$
−0.50
−0.25
0.00
0.25
0.50
0.75
β$_{IV}$

Batch 2−4773 : Gut microbiota abundance (genus Catenibacterium id.2153) on Transmembrane gamma−carboxyglutamic acid protein 1
with a threshold of 1e−05

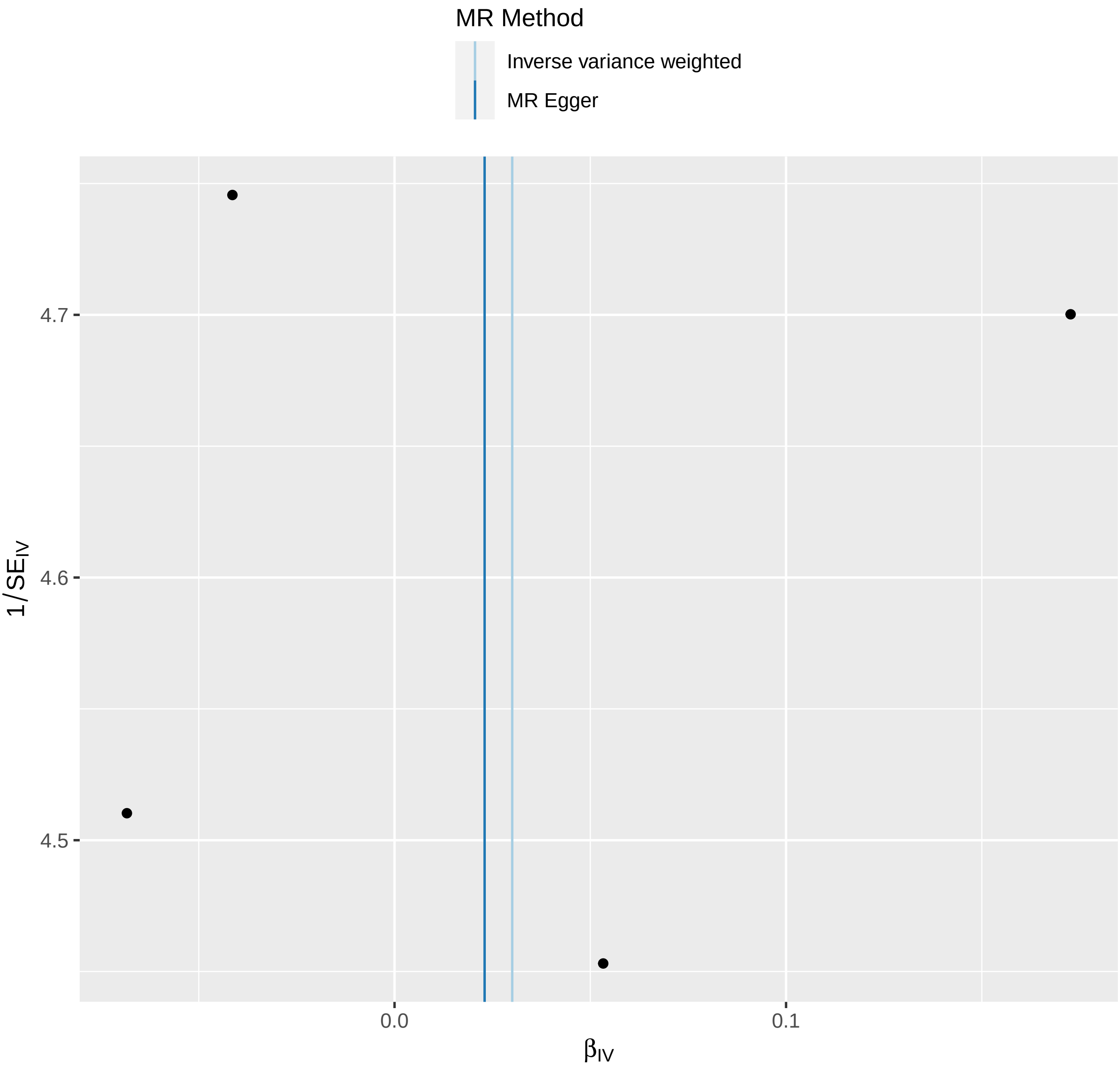

Batch 2−4774 : Gut microbiota abundance (genus Parasutterella id.2892) on Transmembrane gamma−carboxyglutamic acid protein 1
with a threshold of 1e−05

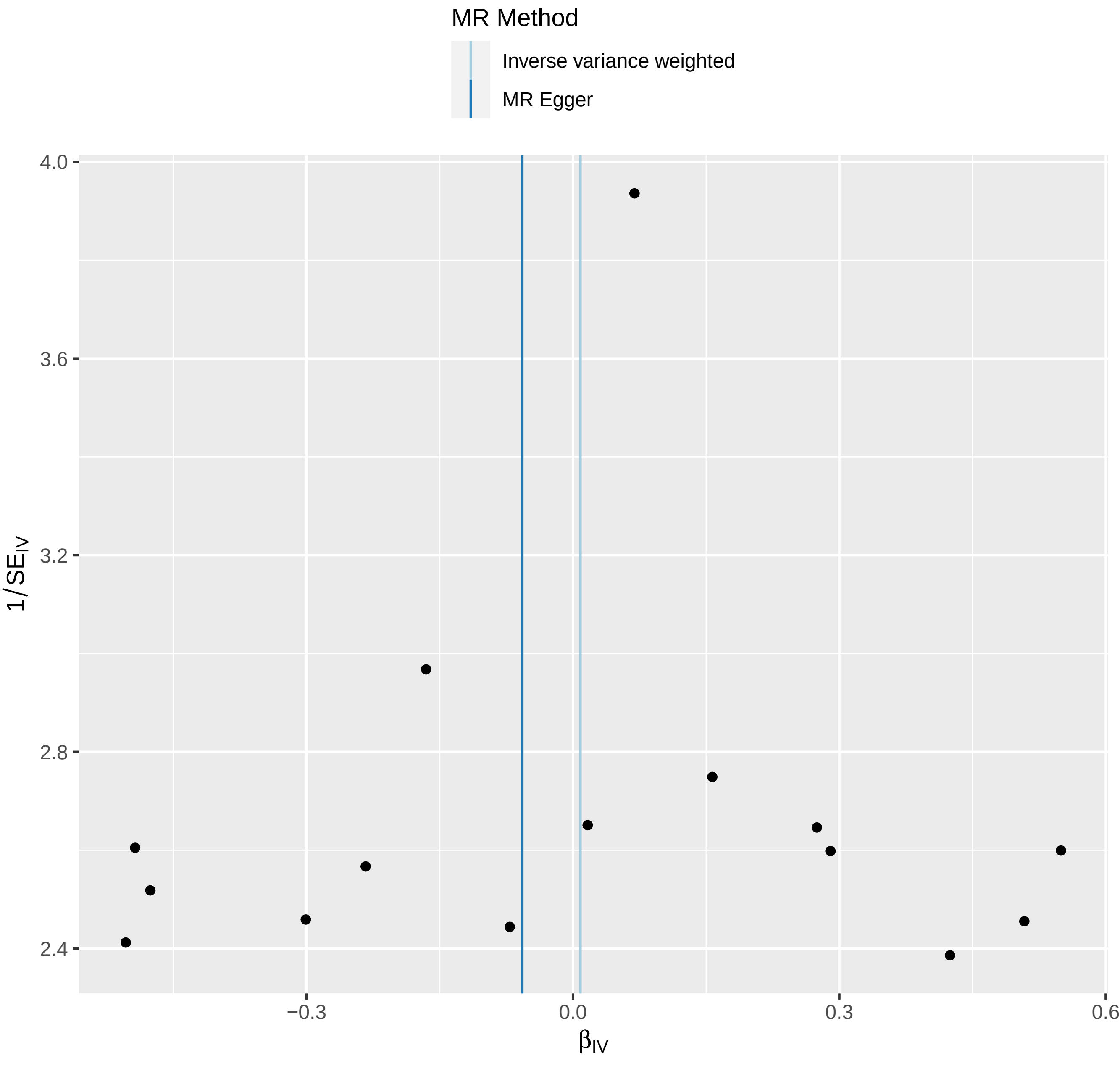

Batch 2−4775 : Gut microbiota abundance (genus Catenibacterium id.2153) on Transmembrane gamma−carboxyglutamic acid protein 1 with a threshold of 1e−05

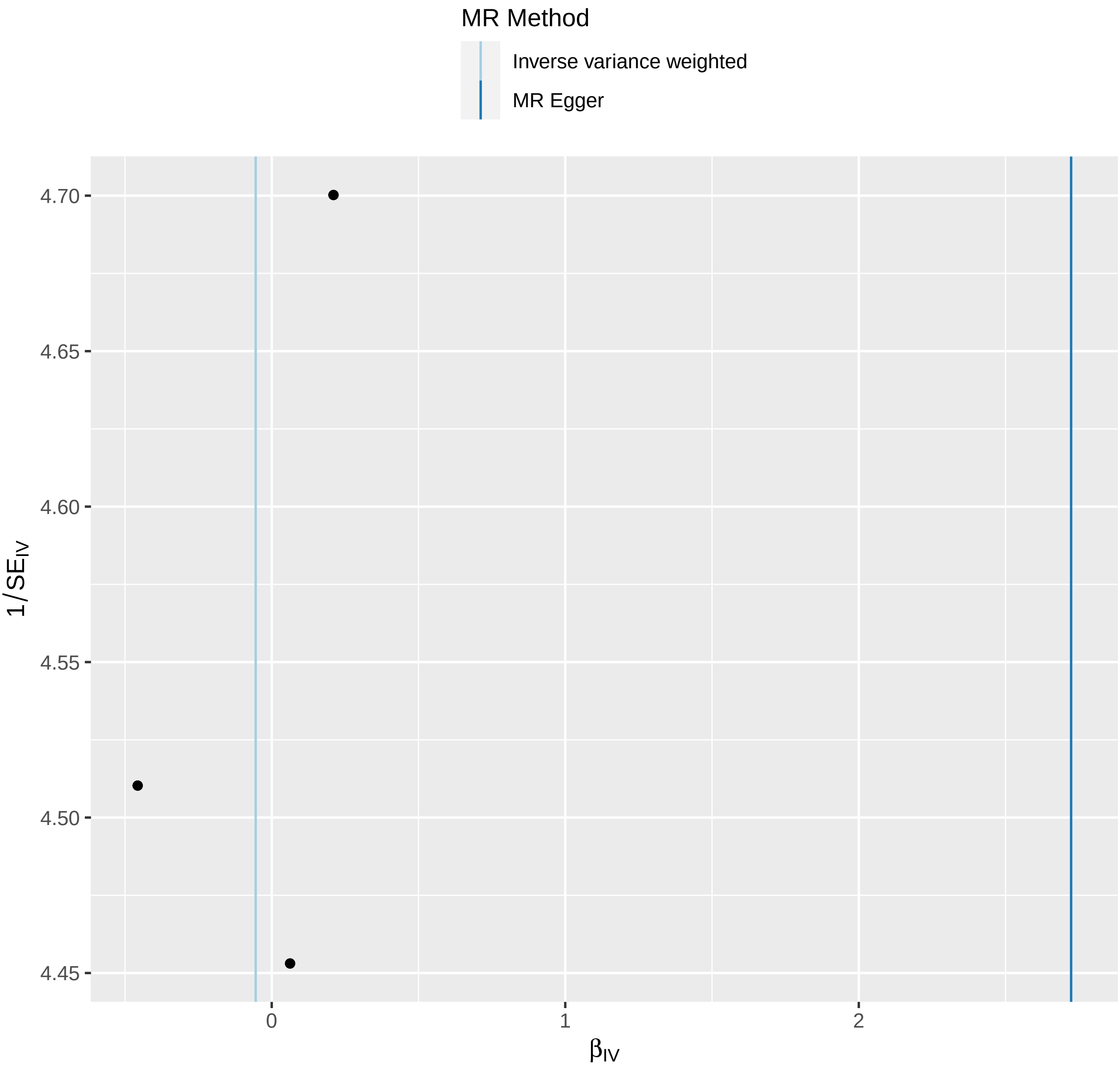

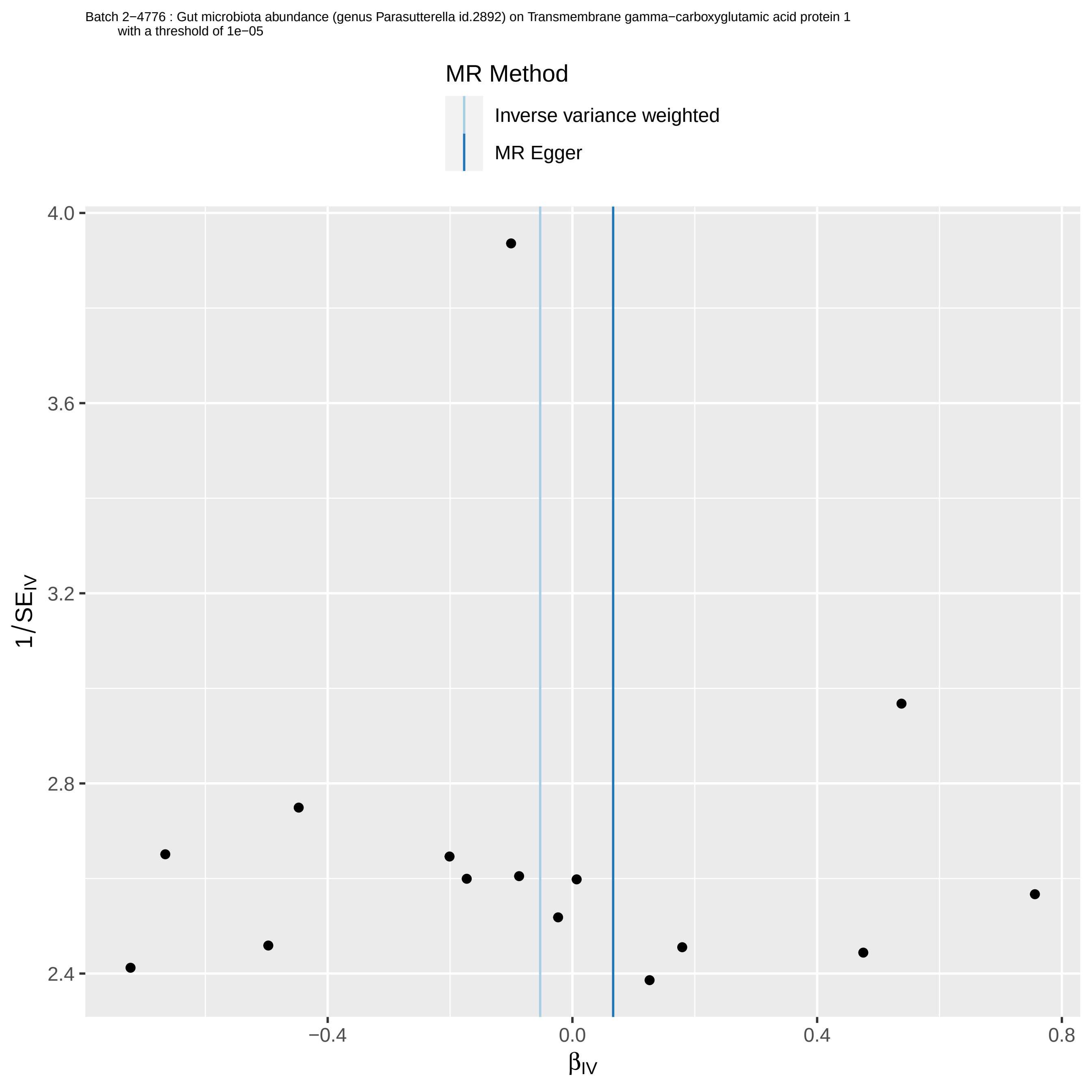

Batch 2−4776 : Gut microbiota abundance (genus Parasutterella id.2892) on Transmembrane gamma−carboxyglutamic acid protein 1
with a threshold of 1e−05
MR Method
Inverse variance weighted
MR Egger
4.0
3.6
3.2
2.8
2.4
1/SE_IV
−0.4
0.0
0.4
0.8
β_IV

Batch 2−4777 : Gut microbiota abundance (genus Catenibacterium id.2153) on Transmembrane gamma−carboxyglutamic acid protein 4
with a threshold of 1e−05

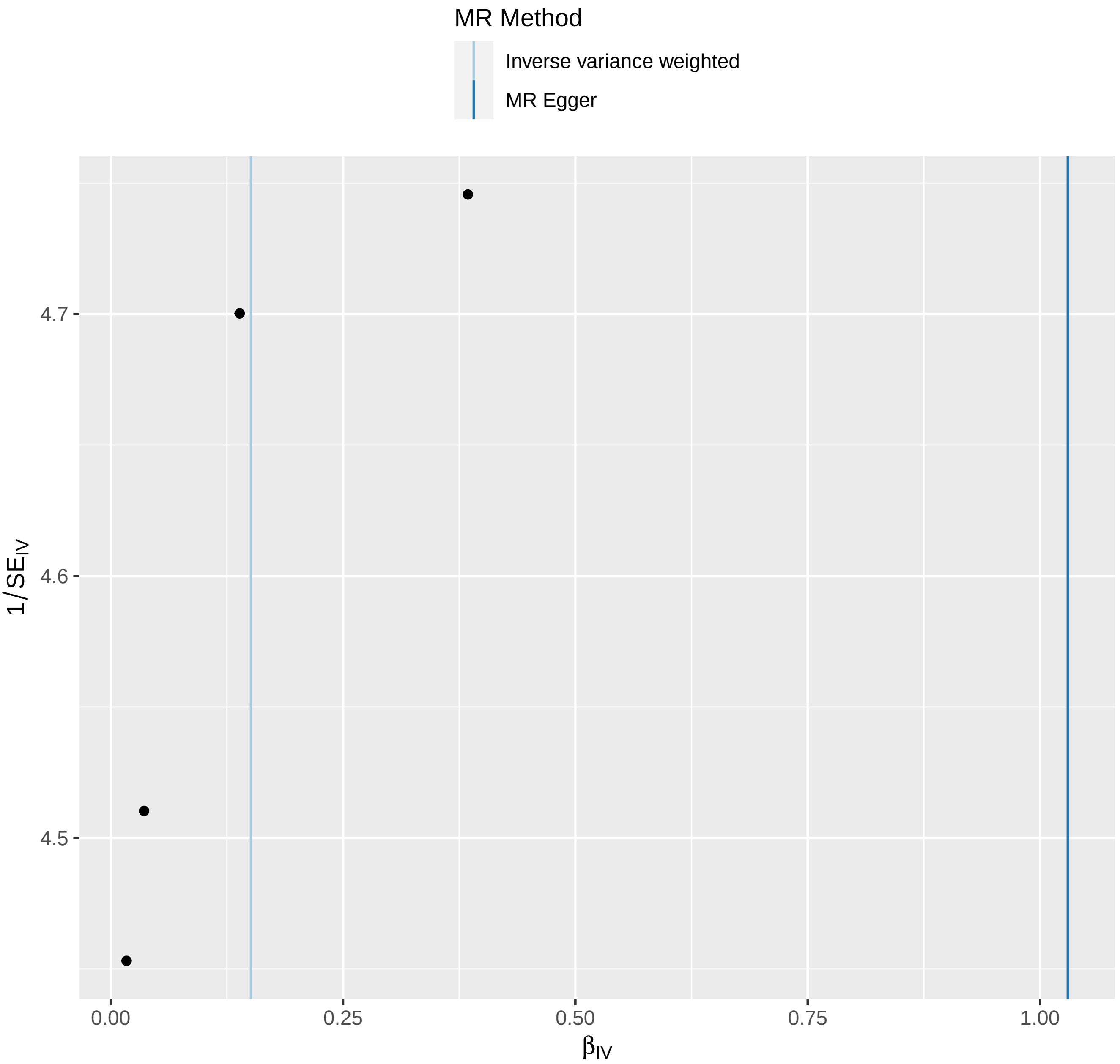

Batch 2−4778 : Gut microbiota abundance (genus Parasutterella id.2892) on Transmembrane gamma−carboxyglutamic acid protein 4
with a threshold of 1e−05

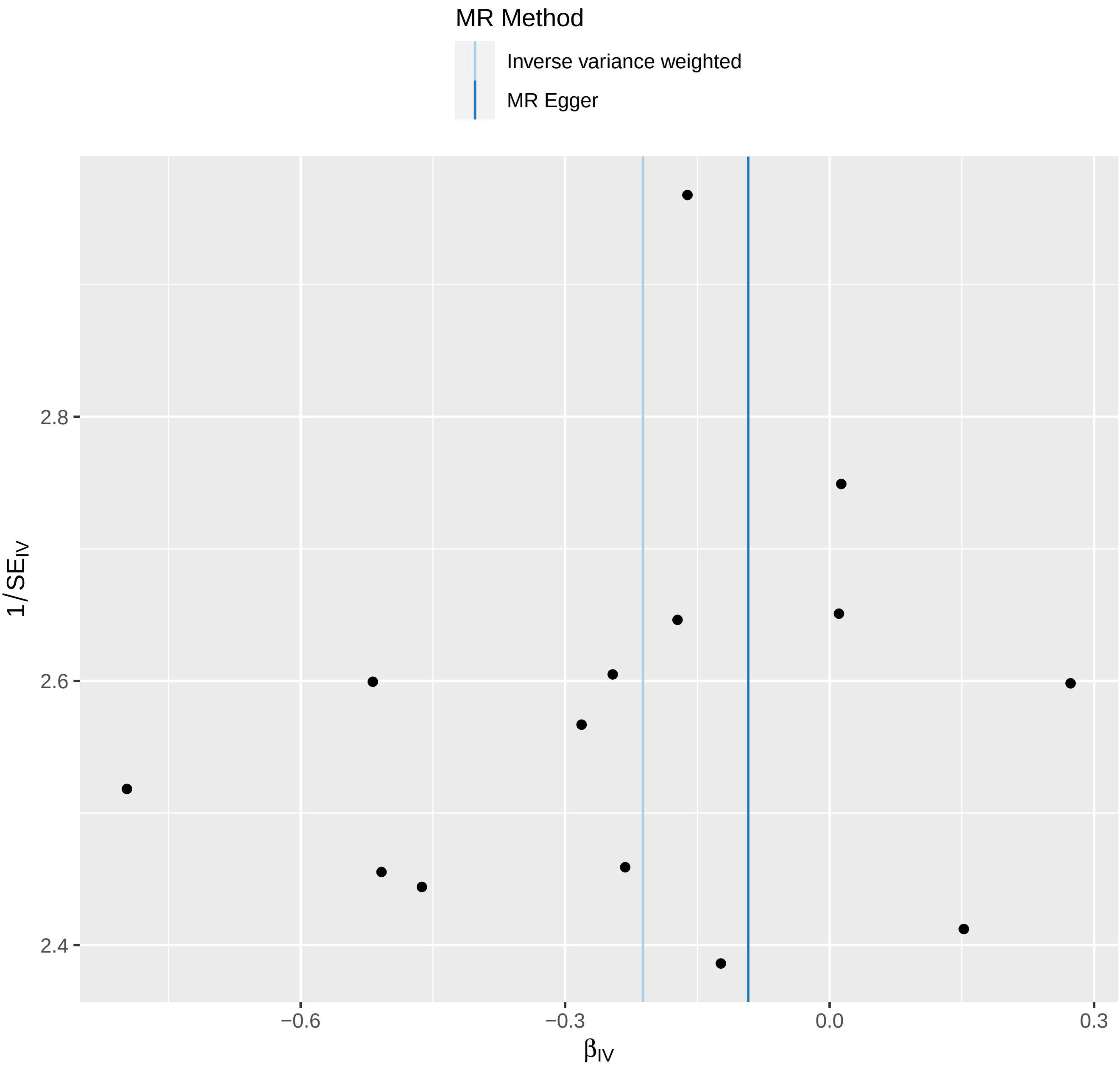

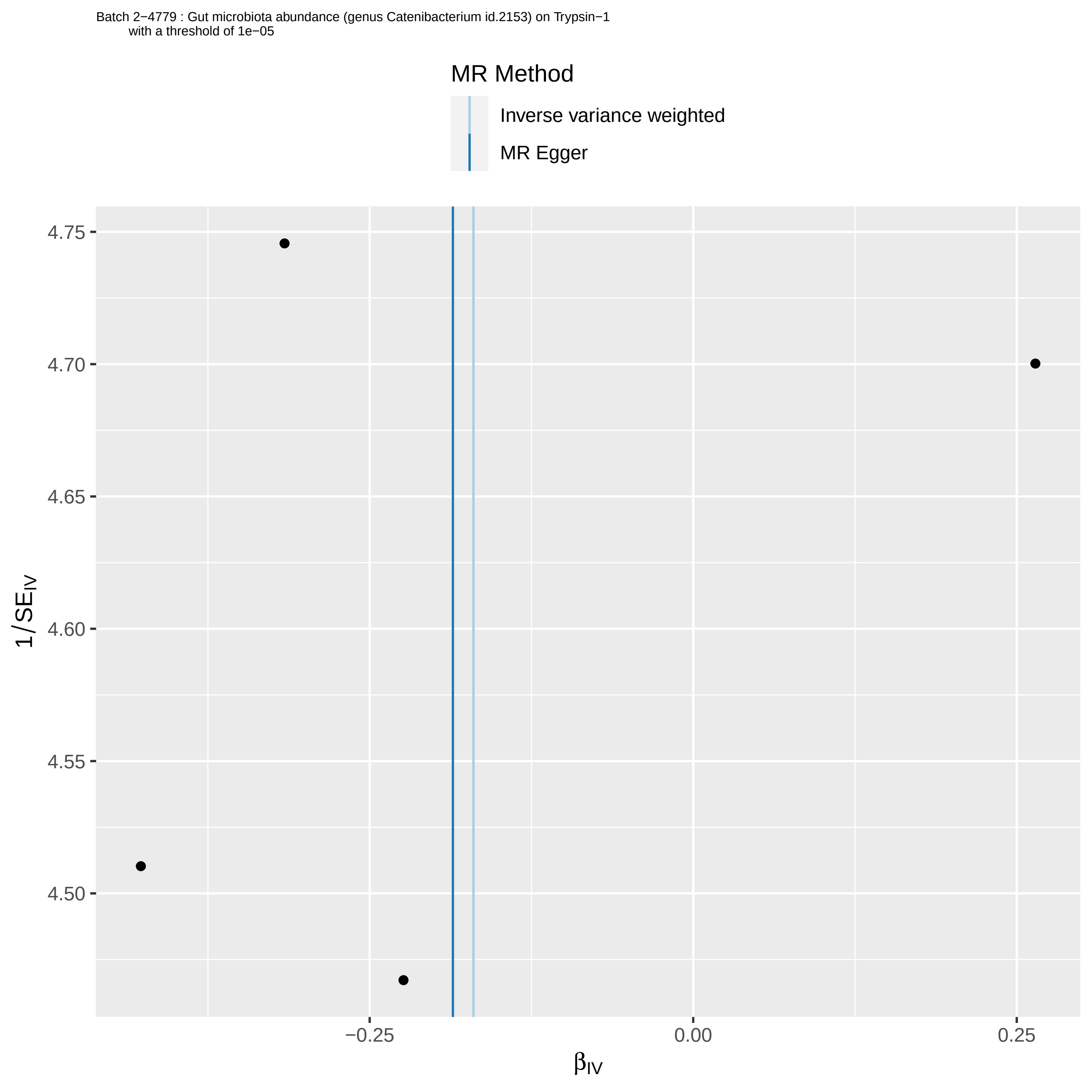

Batch 2−4779 : Gut microbiota abundance (genus Catenibacterium id.2153) on Trypsin−1
with a threshold of 1e−05
MR Method
Inverse variance weighted
MR Egger
4.75
4.70
4.65
4.60
4.55
4.50
1/SE$_{IV}$
−0.25
0.00
0.25
β$_{IV}$

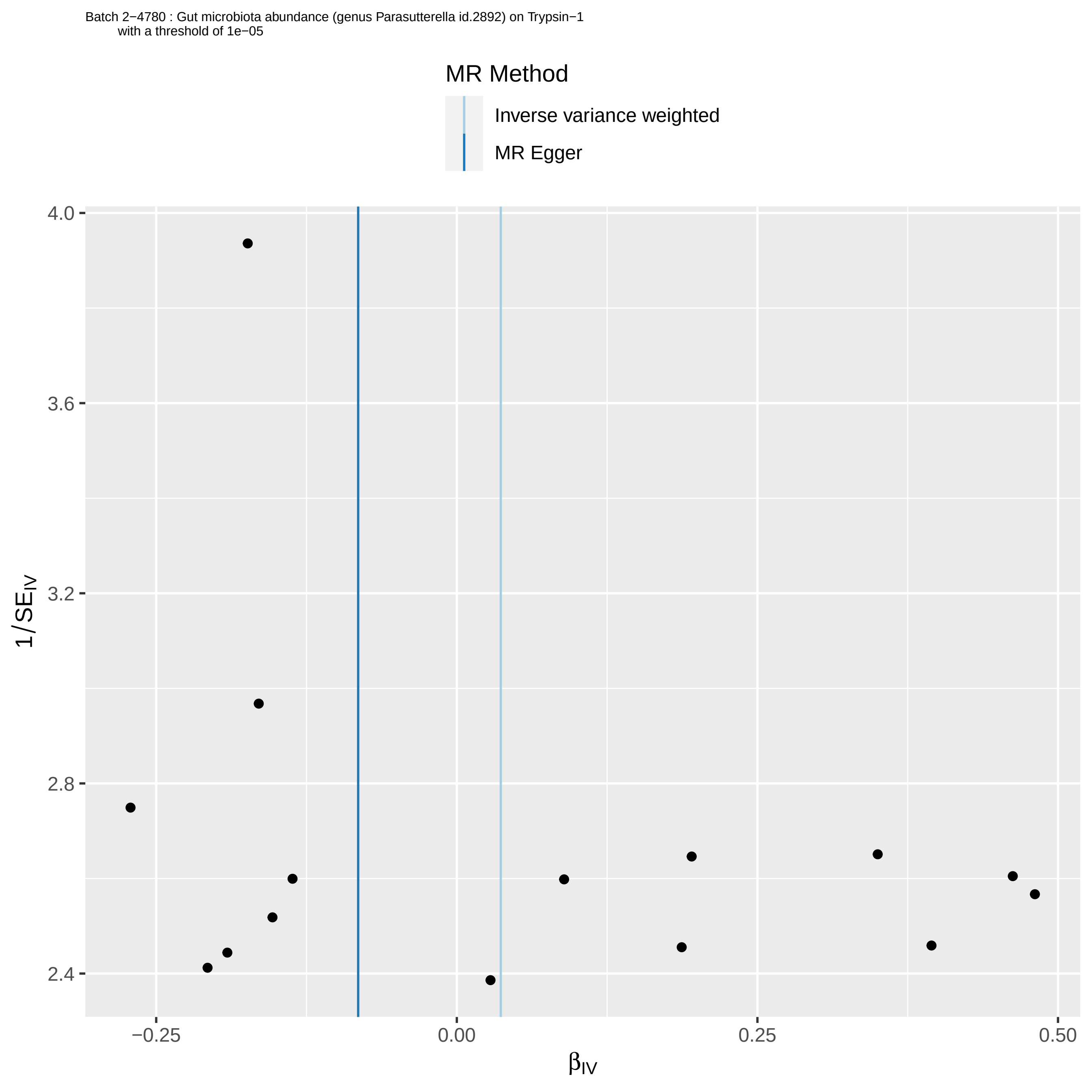

Batch 2−4780 : Gut microbiota abundance (genus Parasutterella id.2892) on Trypsin−1
with a threshold of 1e−05
MR Method
Inverse variance weighted
MR Egger
4.0
3.6
3.2
2.8
2.4
1/SE_IV
−0.25
0.00
0.25
0.50
β_IV

Batch 2−4781 : Gut microbiota abundance (genus Catenibacterium id.2153) on Brain−specific serine protease 4
with a threshold of 1e−05

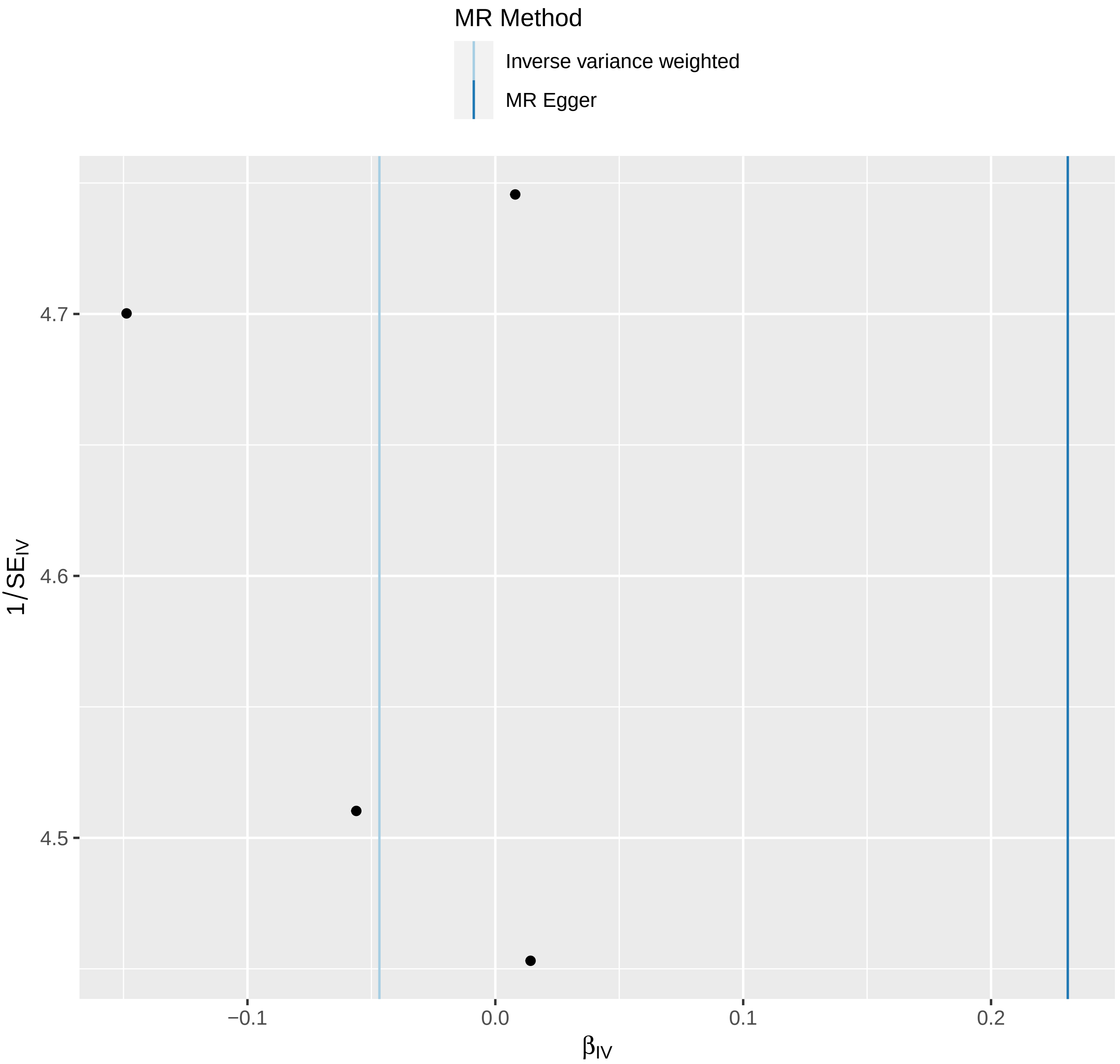

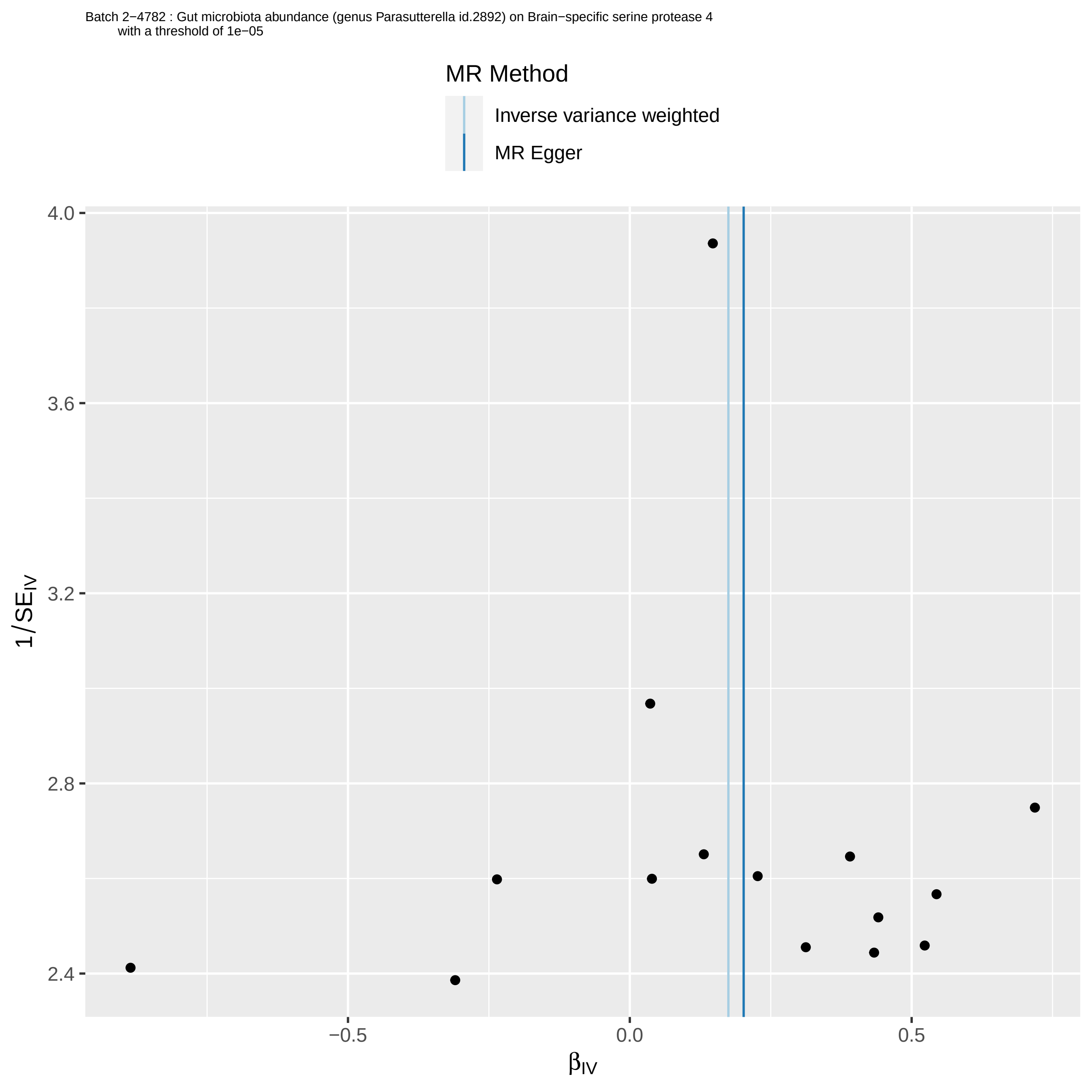

Batch 2−4782 : Gut microbiota abundance (genus Parasutterella id.2892) on Brain−specific serine protease 4
with a threshold of 1e−05
MR Method
Inverse variance weighted
MR Egger
4.0
3.6
3.2
2.8
2.4
1/SE_IV
−0.5
0.0
0.5
β_IV

Batch 2−4783 : Gut microbiota abundance (genus Catenibacterium id.2153) on Trypsin−2
with a threshold of 1e−05

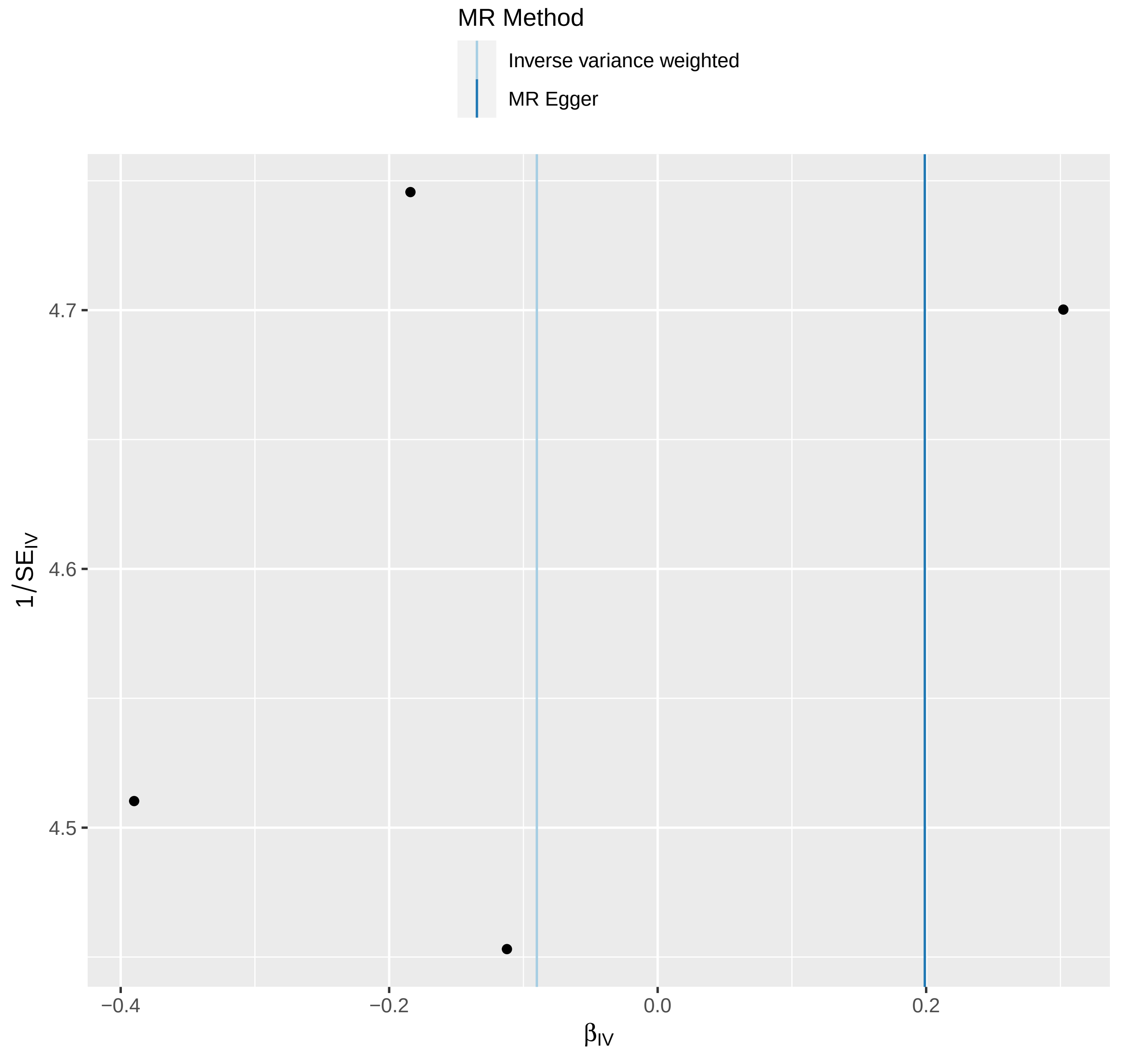

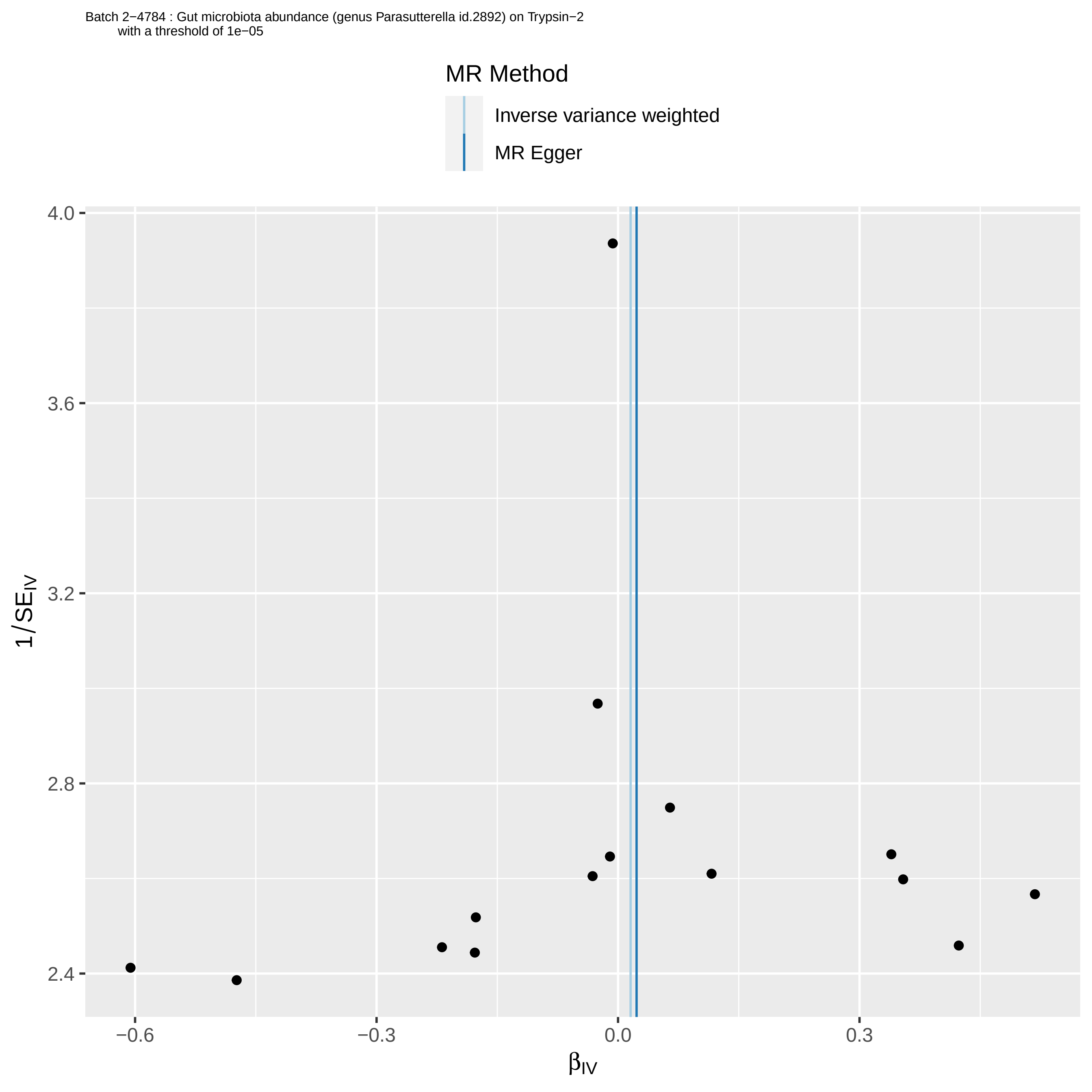
Batch 2−4784 : Gut microbiota abundance (genus Parasutterella id.2892) on Trypsin−2
with a threshold of 1e−05
MR Method
Inverse variance weighted
MR Egger
4.0
3.6
3.2
2.8
2.4
1/SE_IV
−0.6
−0.3
0.0
0.3
β_IV

Batch 2−4785 : Gut microbiota abundance (genus Catenibacterium id.2153) on Trypsin−3
with a threshold of 1e−05

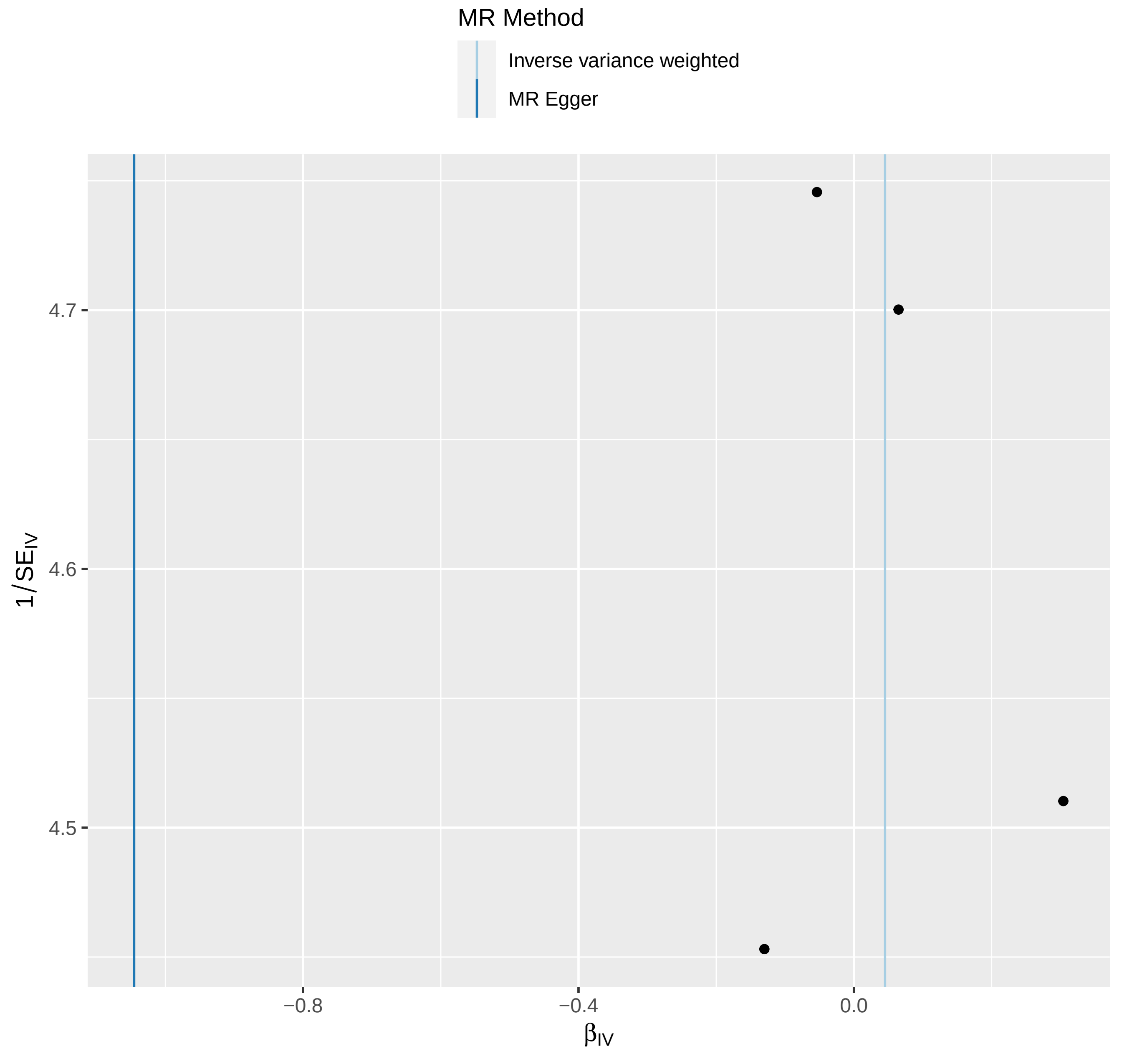

Batch 2−4786 : Gut microbiota abundance (genus Parasutterella id.2892) on Trypsin−3
with a threshold of 1e−05

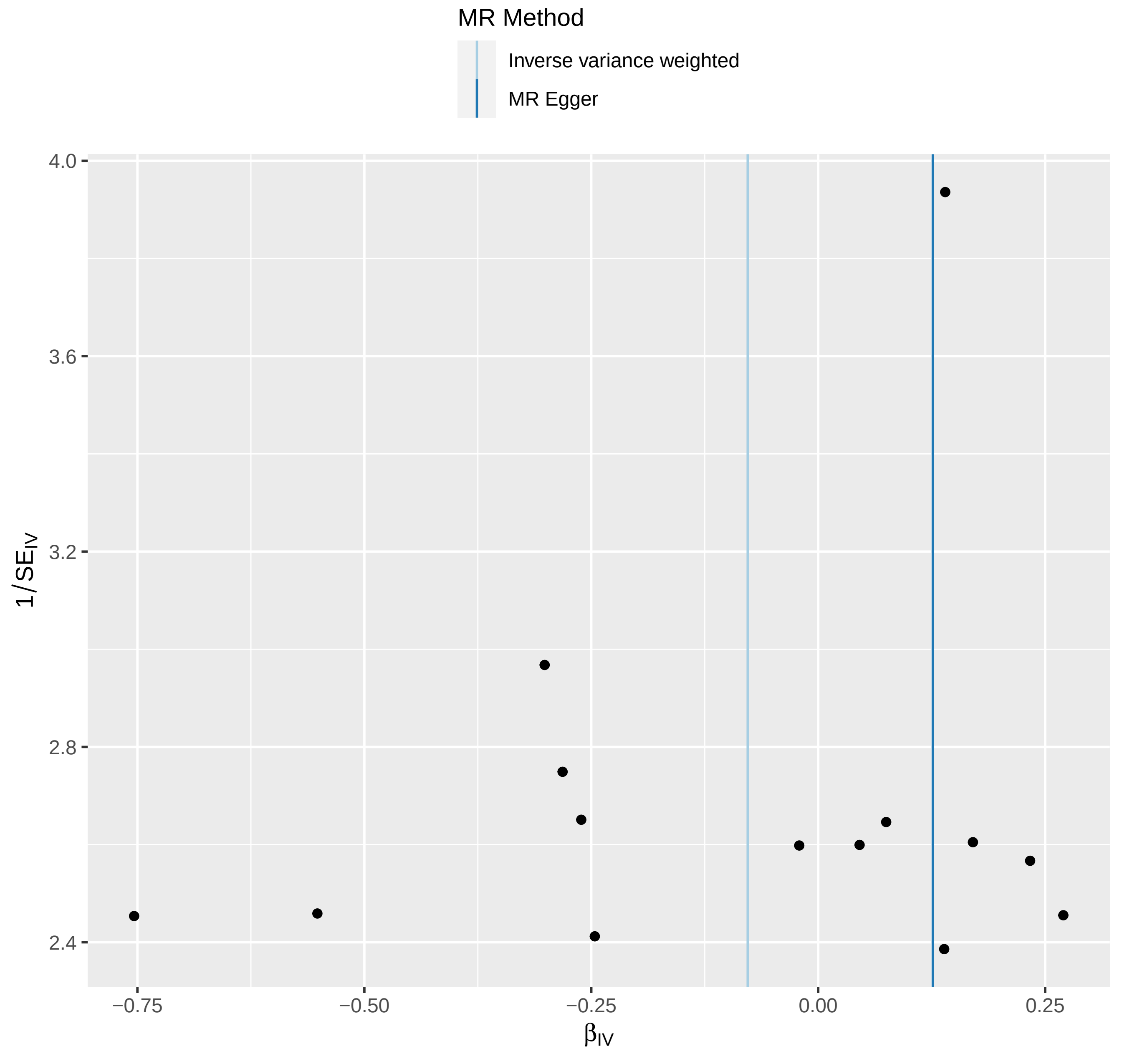

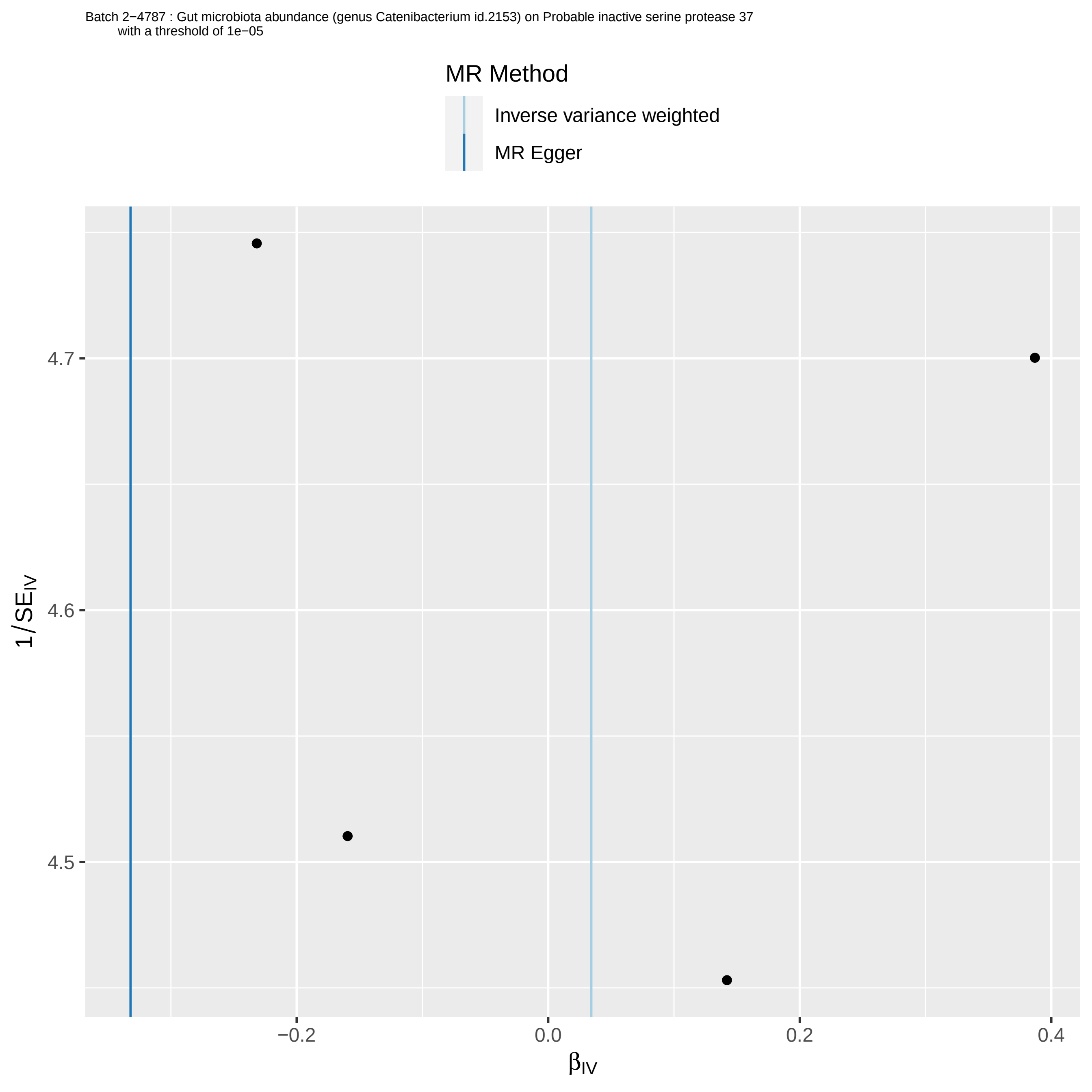

Batch 2−4787 : Gut microbiota abundance (genus Catenibacterium id.2153) on Probable inactive serine protease 37
with a threshold of 1e−05
MR Method
Inverse variance weighted
MR Egger
4.7
4.6
4.5
1/SE_IV
−0.2
0.0
0.2
0.4
β_IV

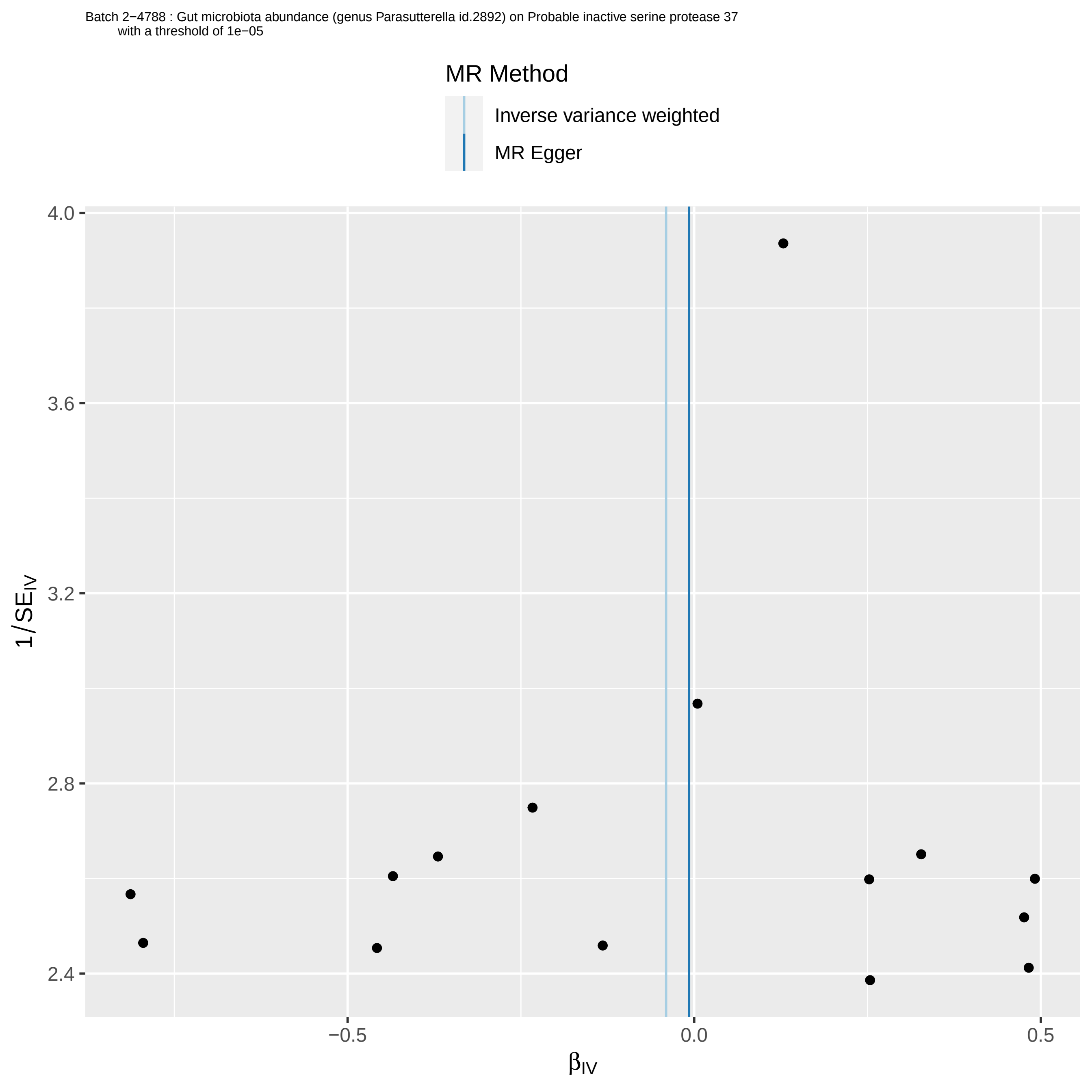
Batch 2−4788 : Gut microbiota abundance (genus Parasutterella id.2892) on Probable inactive serine protease 37
with a threshold of 1e−05
MR Method
Inverse variance weighted
MR Egger
4.0
3.6
3.2
2.8
2.4
$1/SE_{IV}$
−0.5
0.0
0.5
β_{IV}

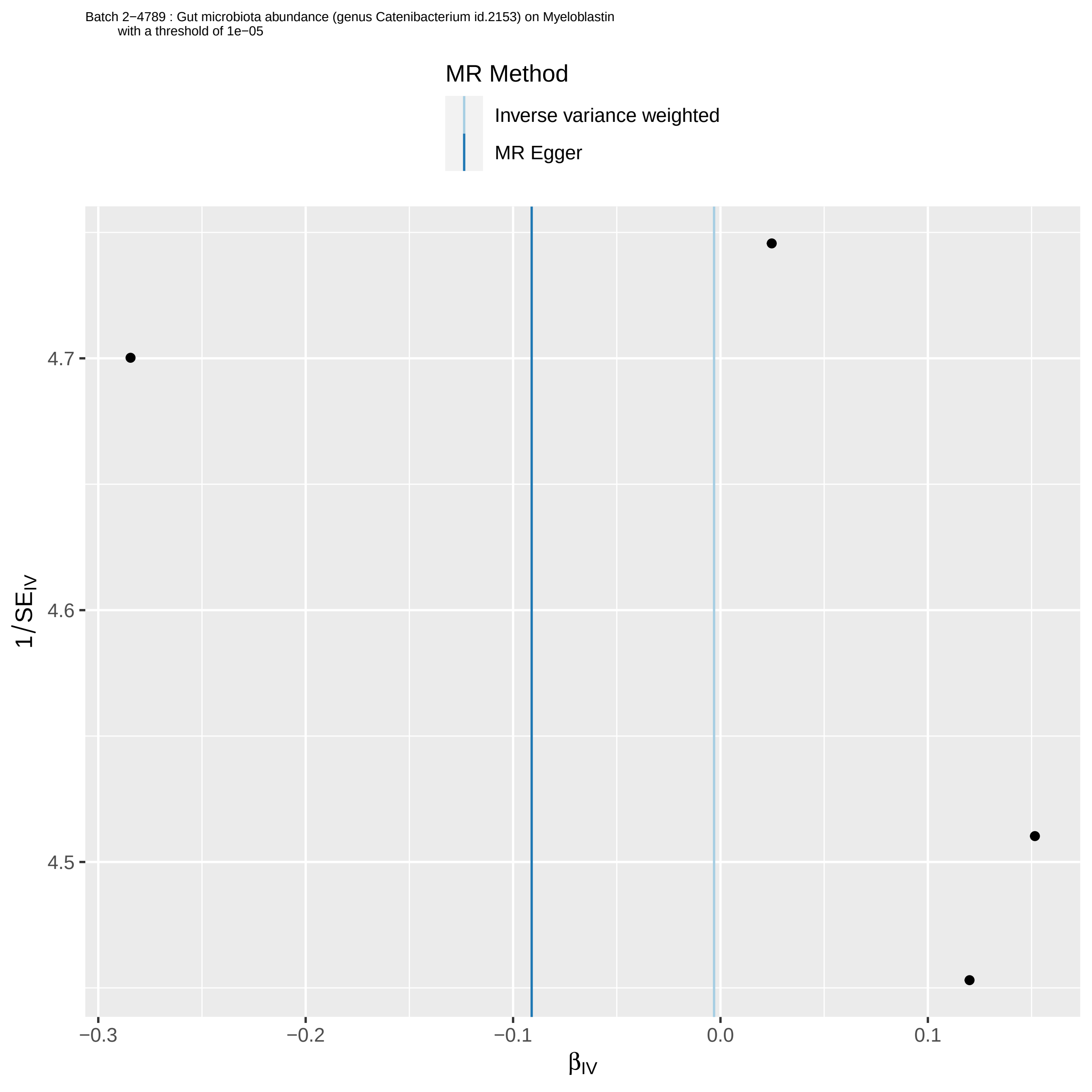
Batch 2−4789 : Gut microbiota abundance (genus Catenibacterium id.2153) on Myeloblastin
with a threshold of 1e−05
MR Method
Inverse variance weighted
MR Egger
4.7
4.6
4.5
$1/SE_{IV}$
−0.3
−0.2
−0.1
0.0
0.1
β_{IV}

Batch 2−4790 : Gut microbiota abundance (genus Parasutterella id.2892) on Myeloblastin
with a threshold of 1e−05

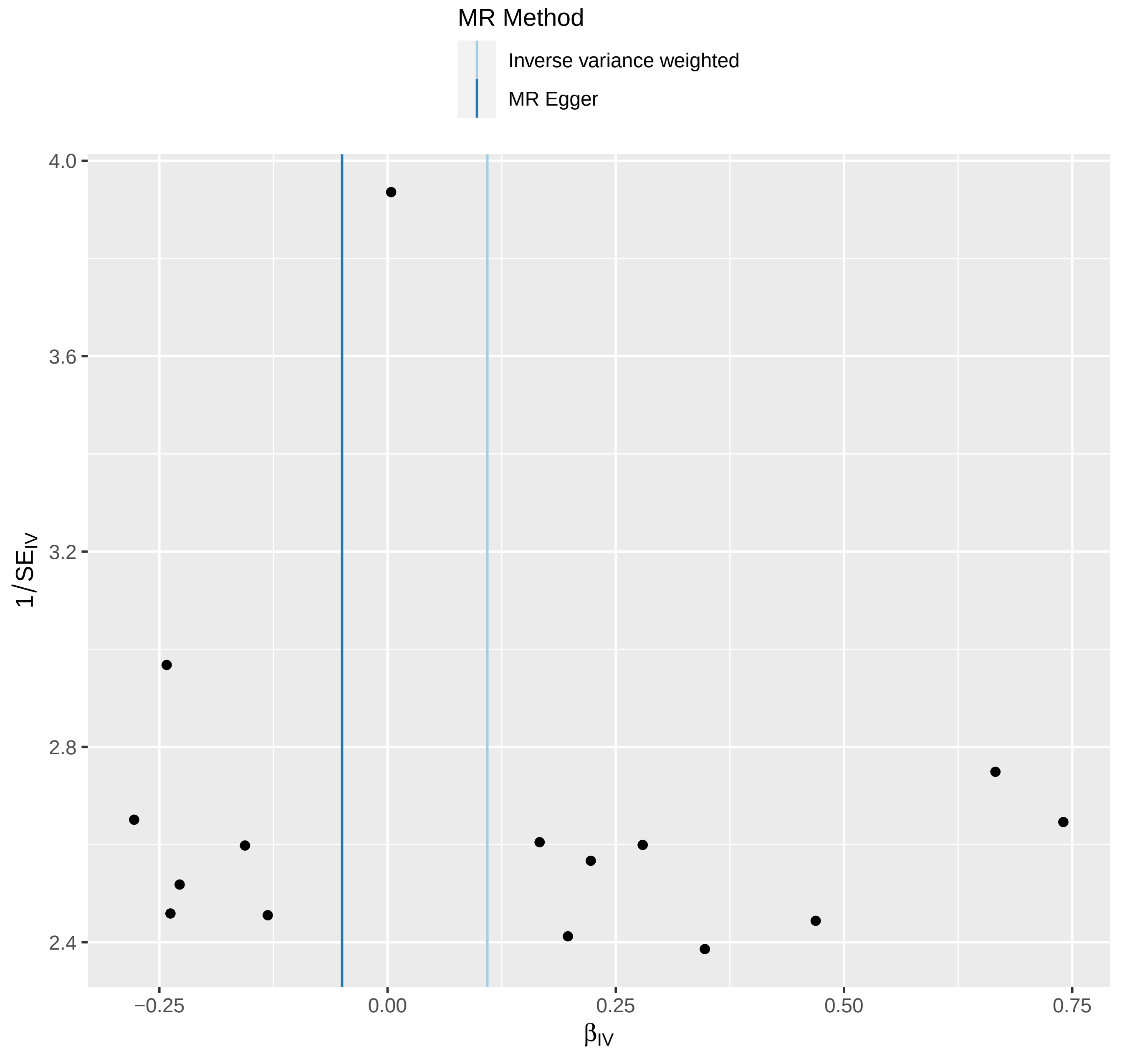

Batch 2−4791 : Gut microbiota abundance (genus Catenibacterium id.2153) on Myeloblastin
with a threshold of 1e−05

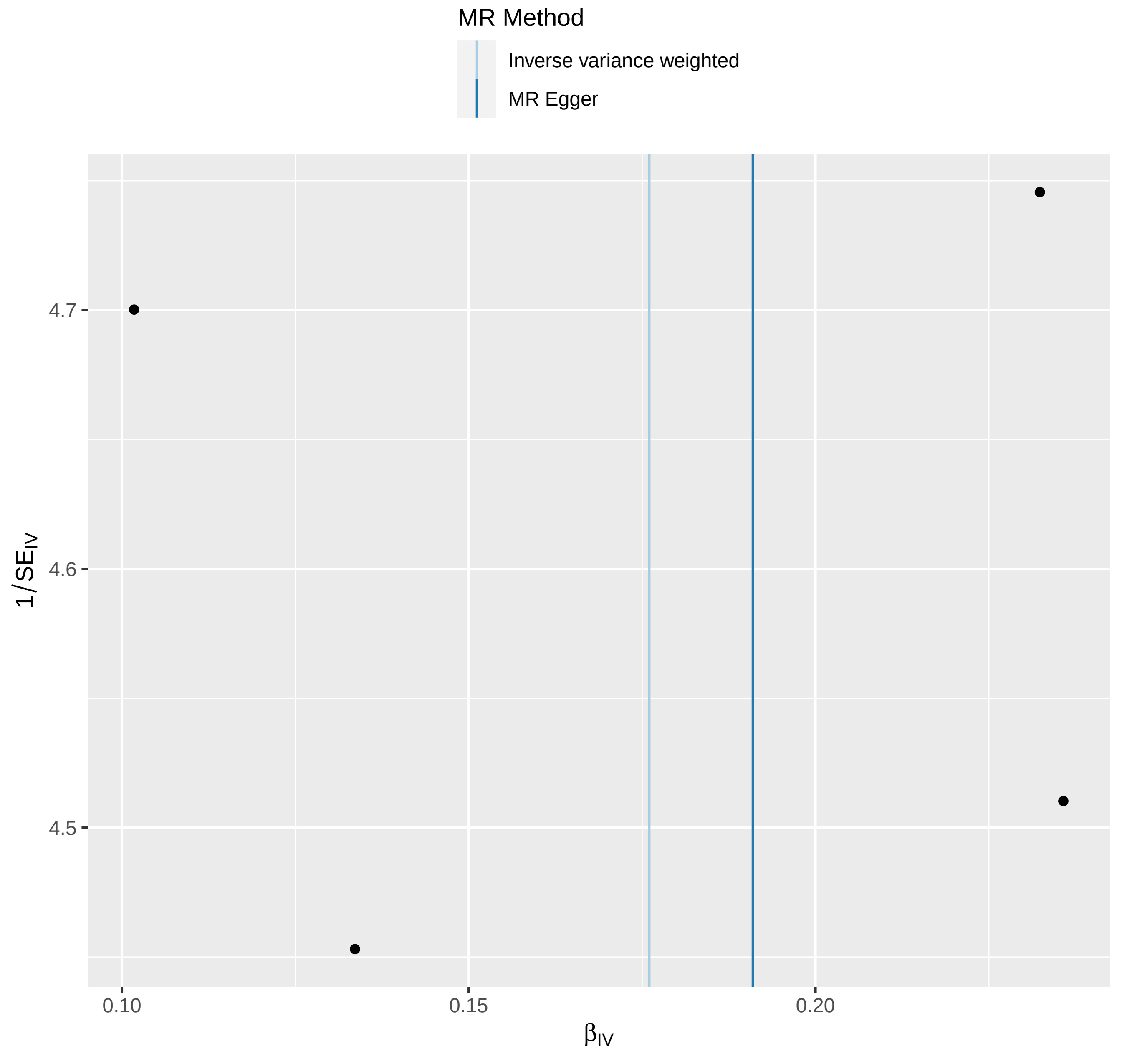

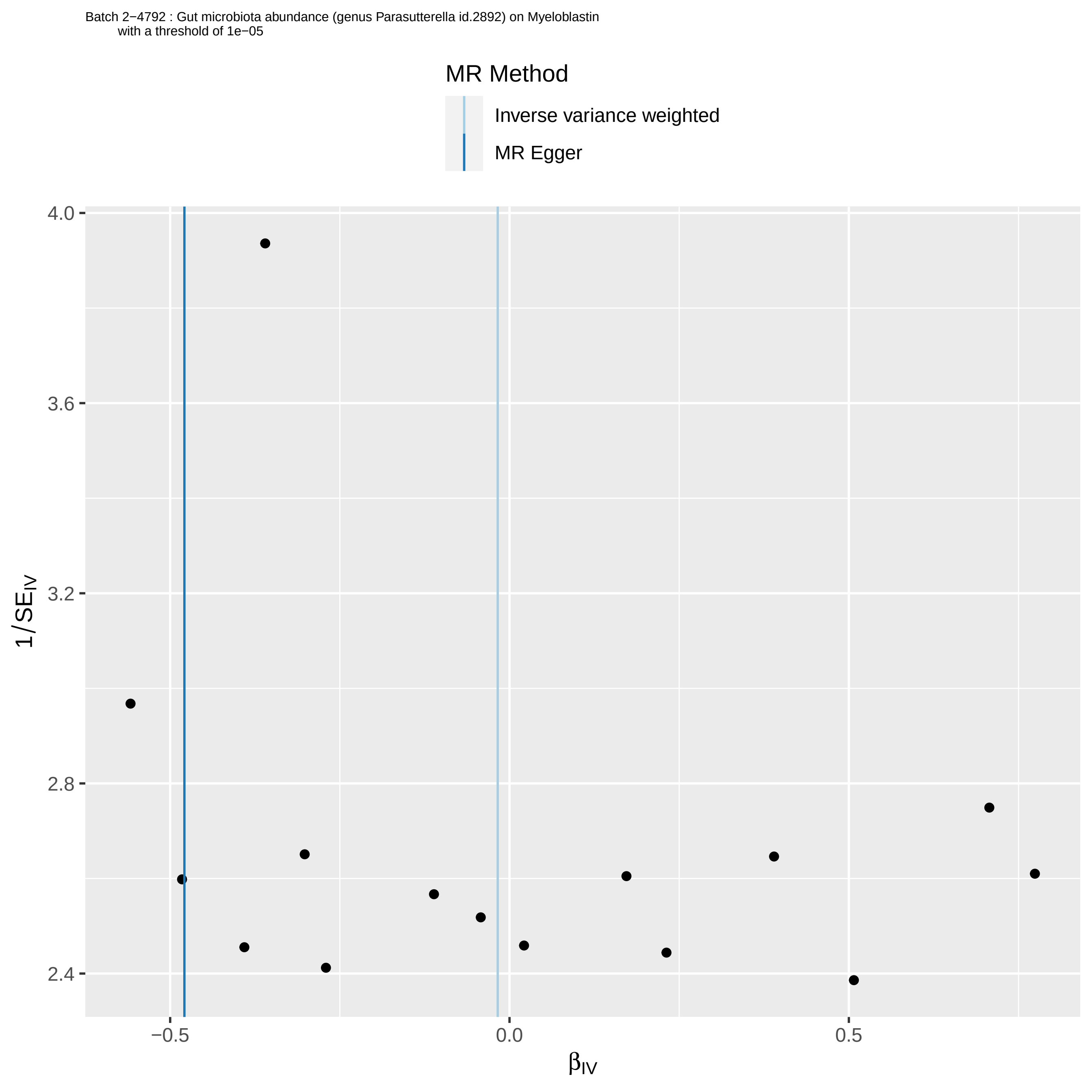
Batch 2−4792 : Gut microbiota abundance (genus Parasutterella id.2892) on Myeloblastin
with a threshold of 1e−05
MR Method
Inverse variance weighted
MR Egger
4.0
3.6
3.2
2.8
2.4
1/SE$_{IV}$
−0.5
0.0
0.5
β$_{IV}$

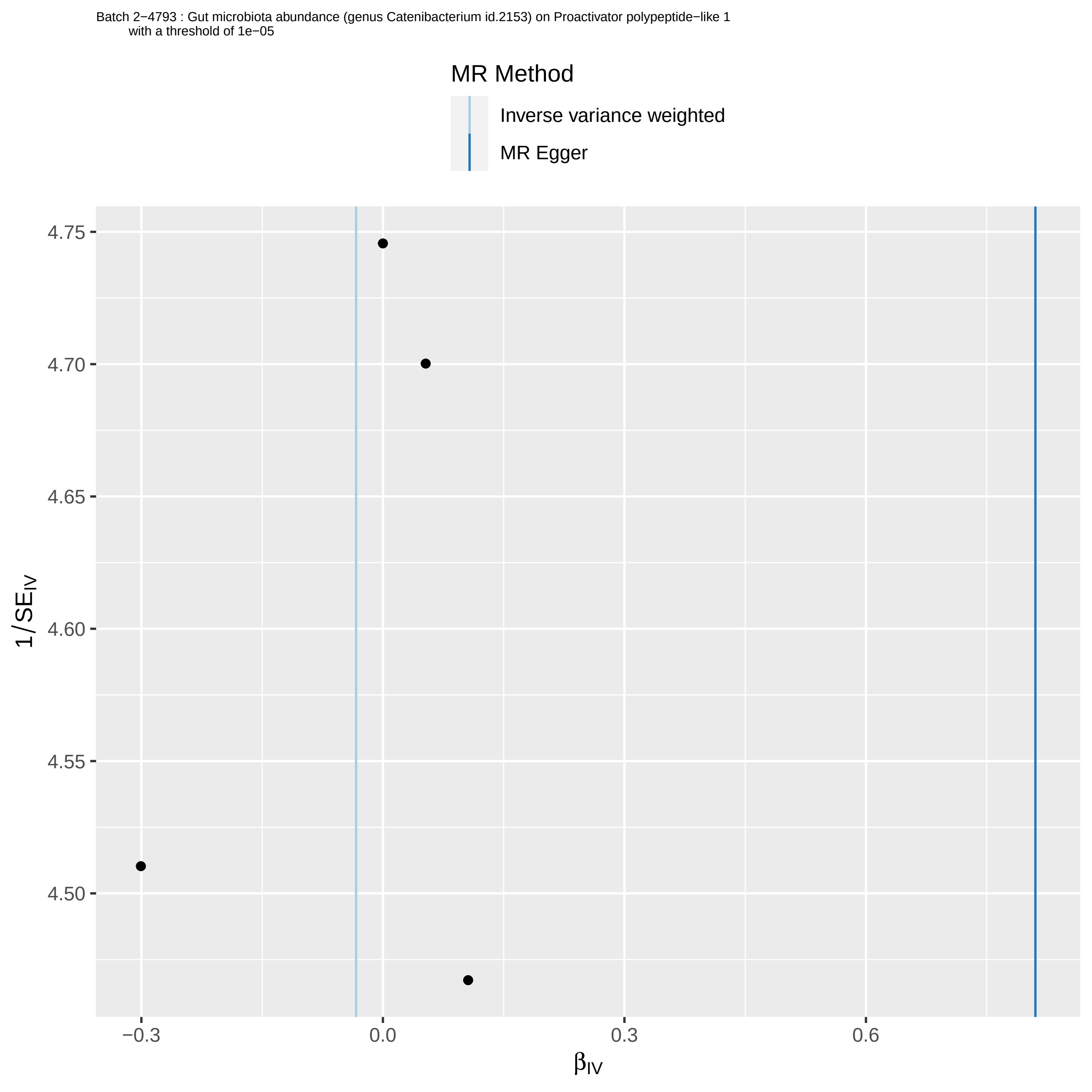

Batch 2−4793 : Gut microbiota abundance (genus Catenibacterium id.2153) on Proactivator polypeptide−like 1
with a threshold of 1e−05
MR Method
Inverse variance weighted
MR Egger
4.75
4.70
4.65
4.60
4.55
4.50
1/SE_IV
−0.3
0.0
0.3
0.6
β_IV

Batch 2−4794 : Gut microbiota abundance (genus Parasutterella id.2892) on Proactivator polypeptide−like 1
with a threshold of 1e−05

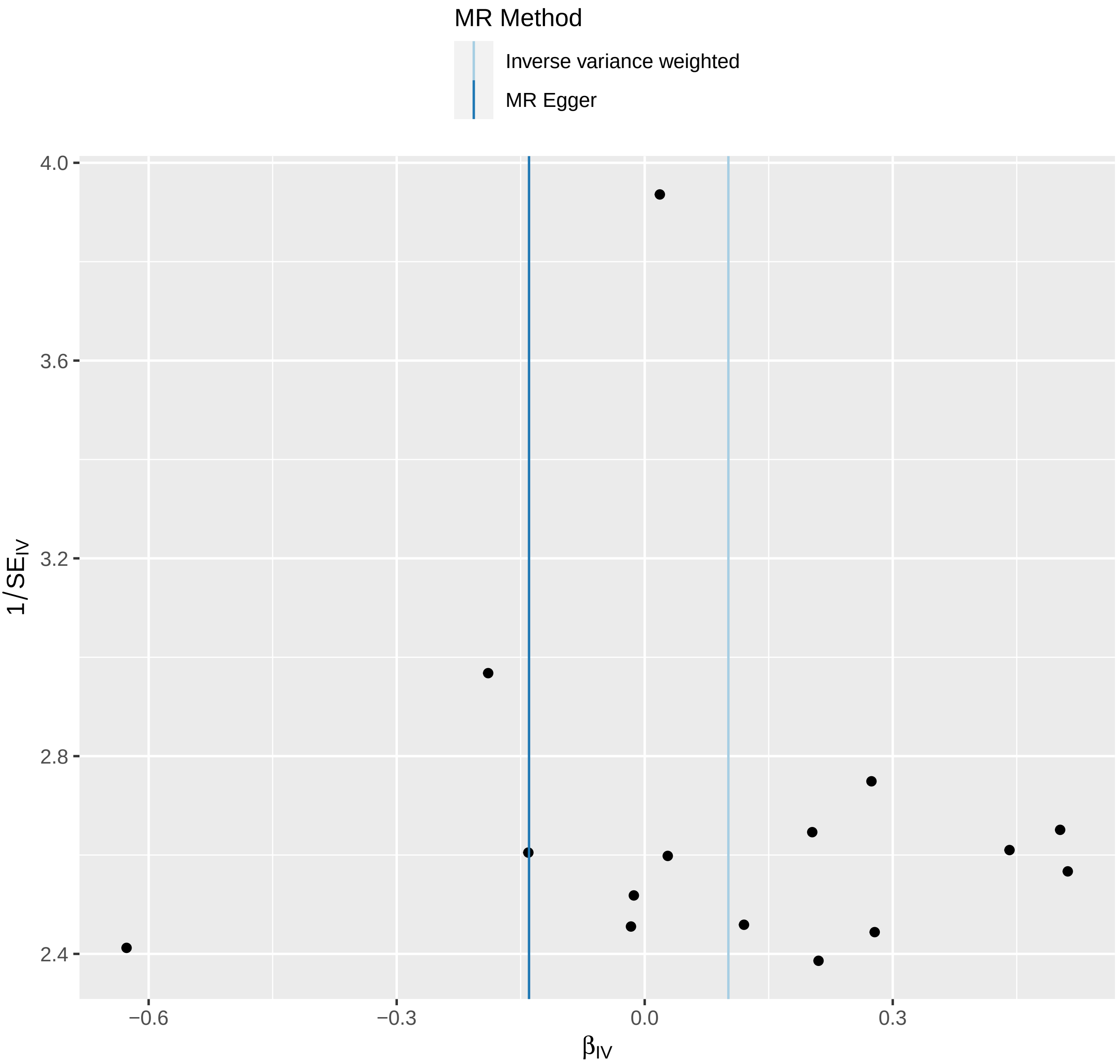

Batch 2−4795 : Gut microbiota abundance (genus Catenibacterium id.2153) on PH and SEC7 domain−containing protein 1
with a threshold of 1e−05

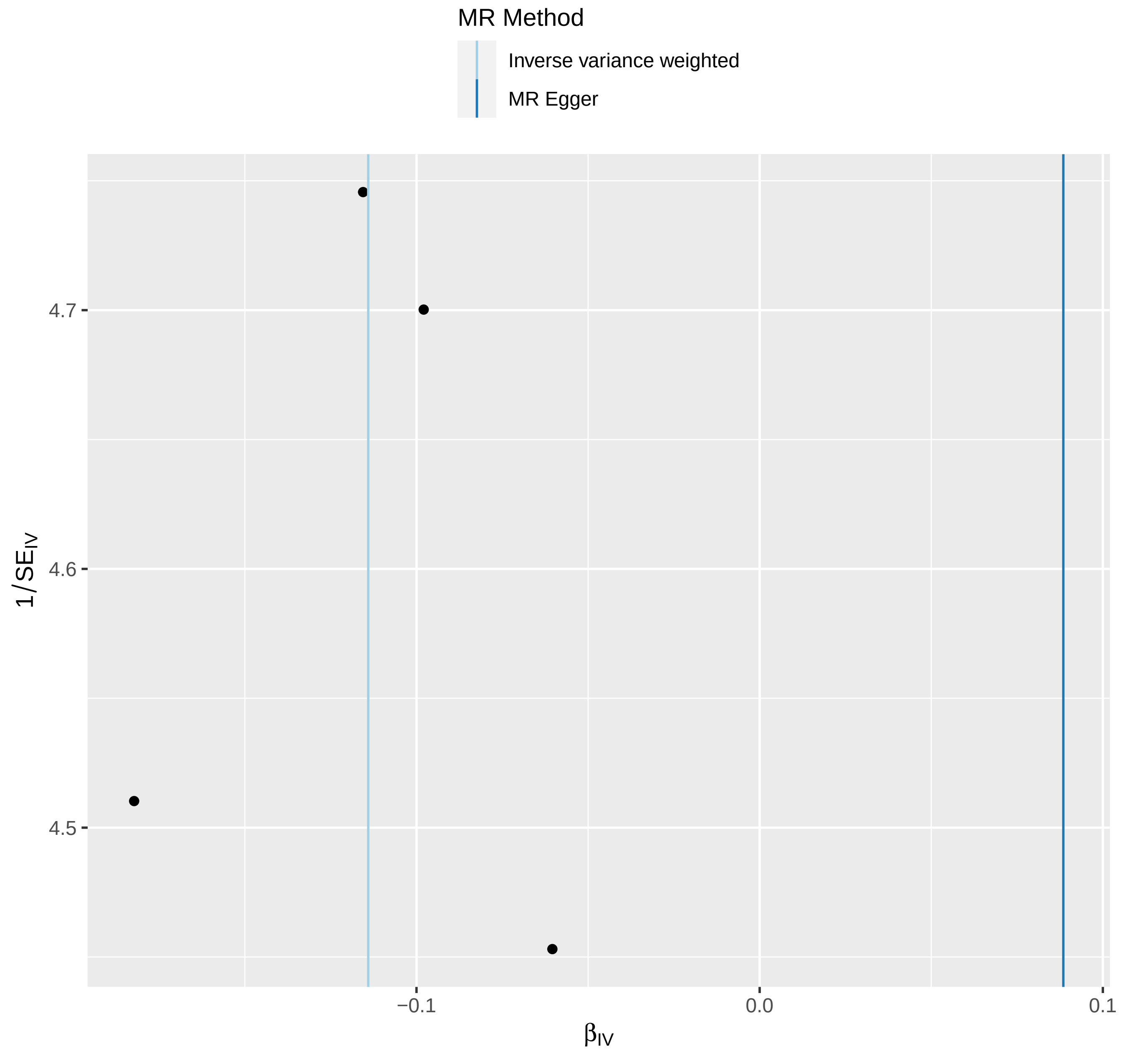

Batch 2−4796 : Gut microbiota abundance (genus Parasutterella id.2892) on PH and SEC7 domain−containing protein 1 with a threshold of 1e−05

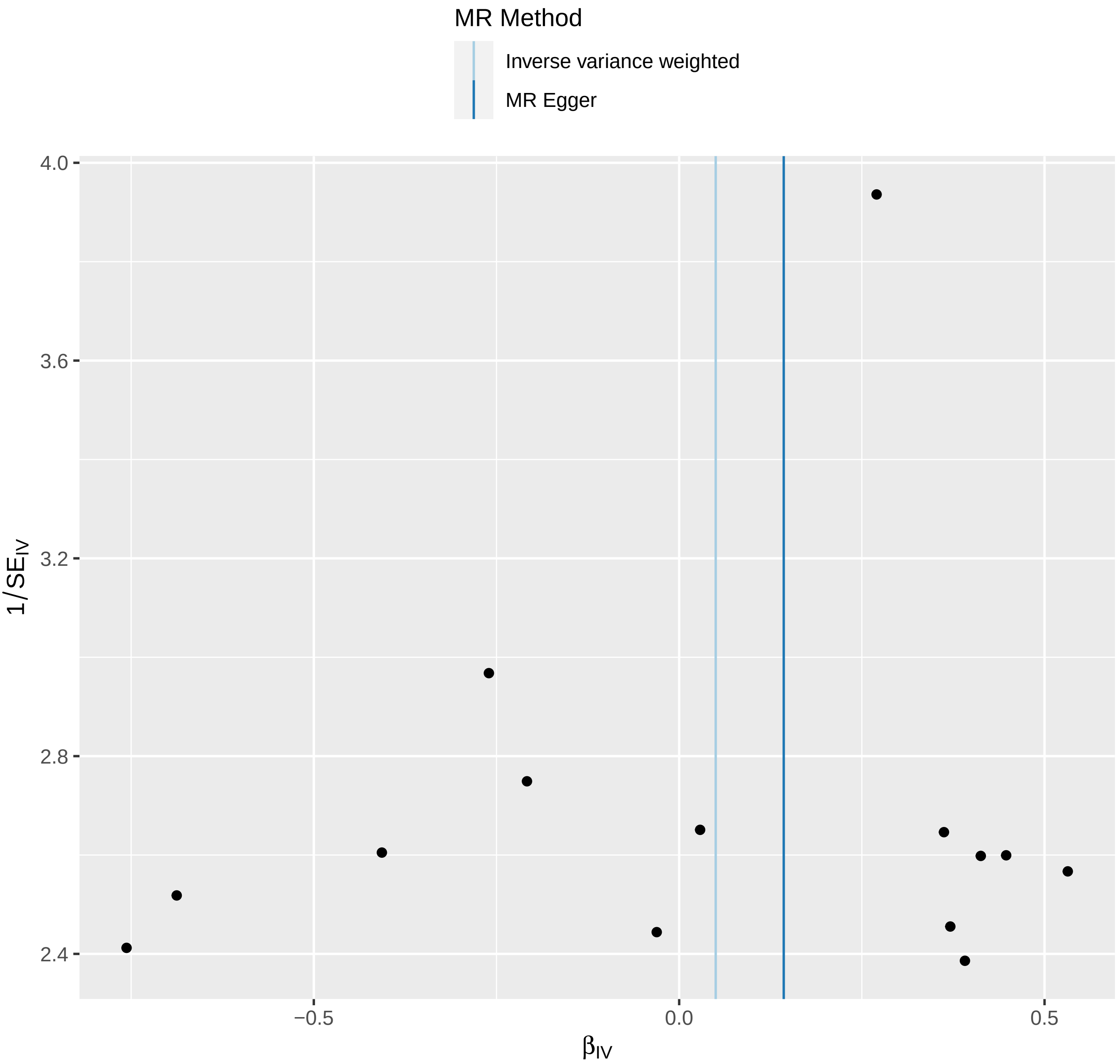

Batch 2−4797 : Gut microbiota abundance (genus Catenibacterium id.2153) on PH and SEC7 domain−containing protein 2
with a threshold of 1e−05

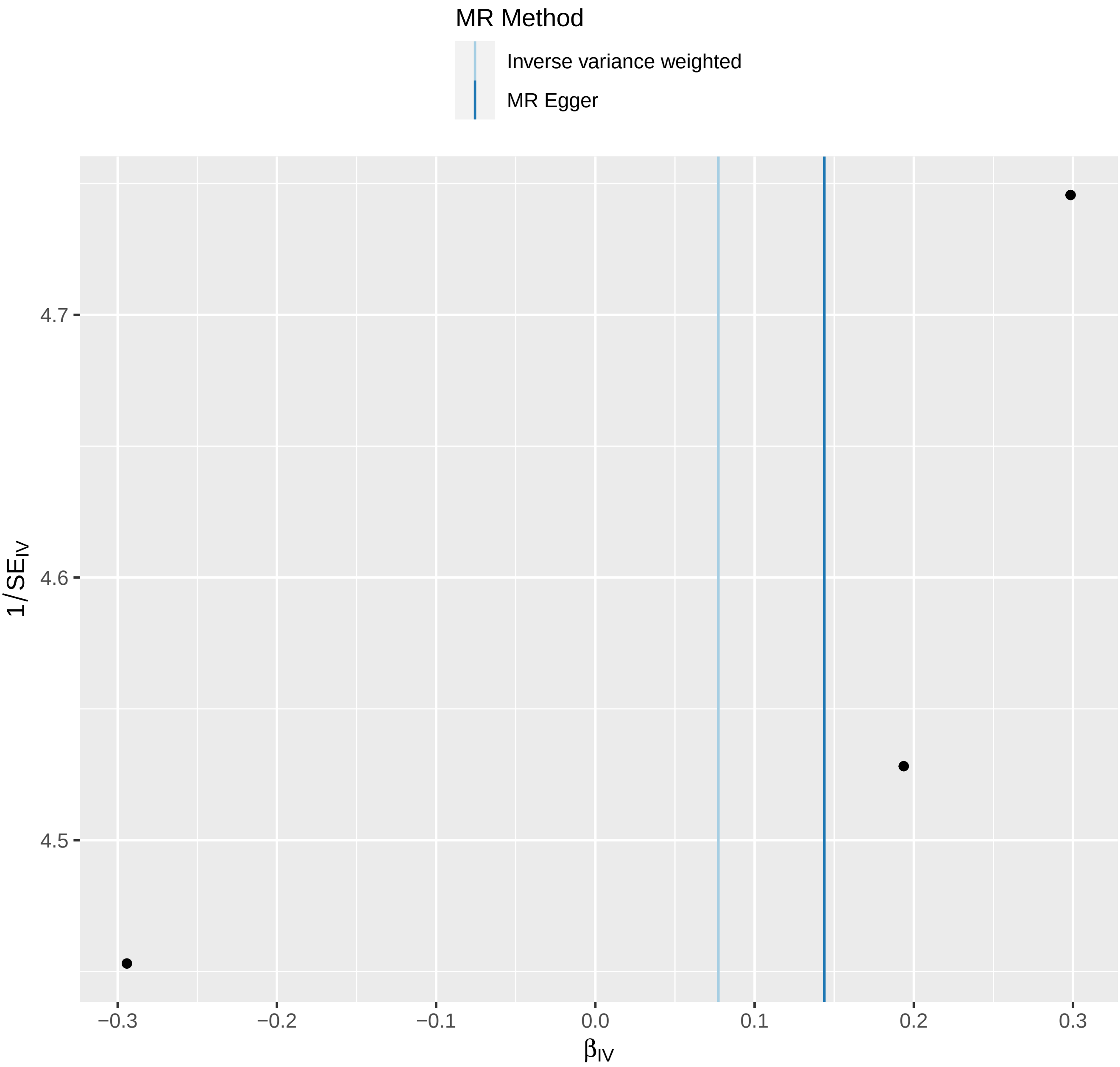

Batch 2−4798 : Gut microbiota abundance (genus Parasutterella id.2892) on PH and SEC7 domain−containing protein 2
with a threshold of 1e−05

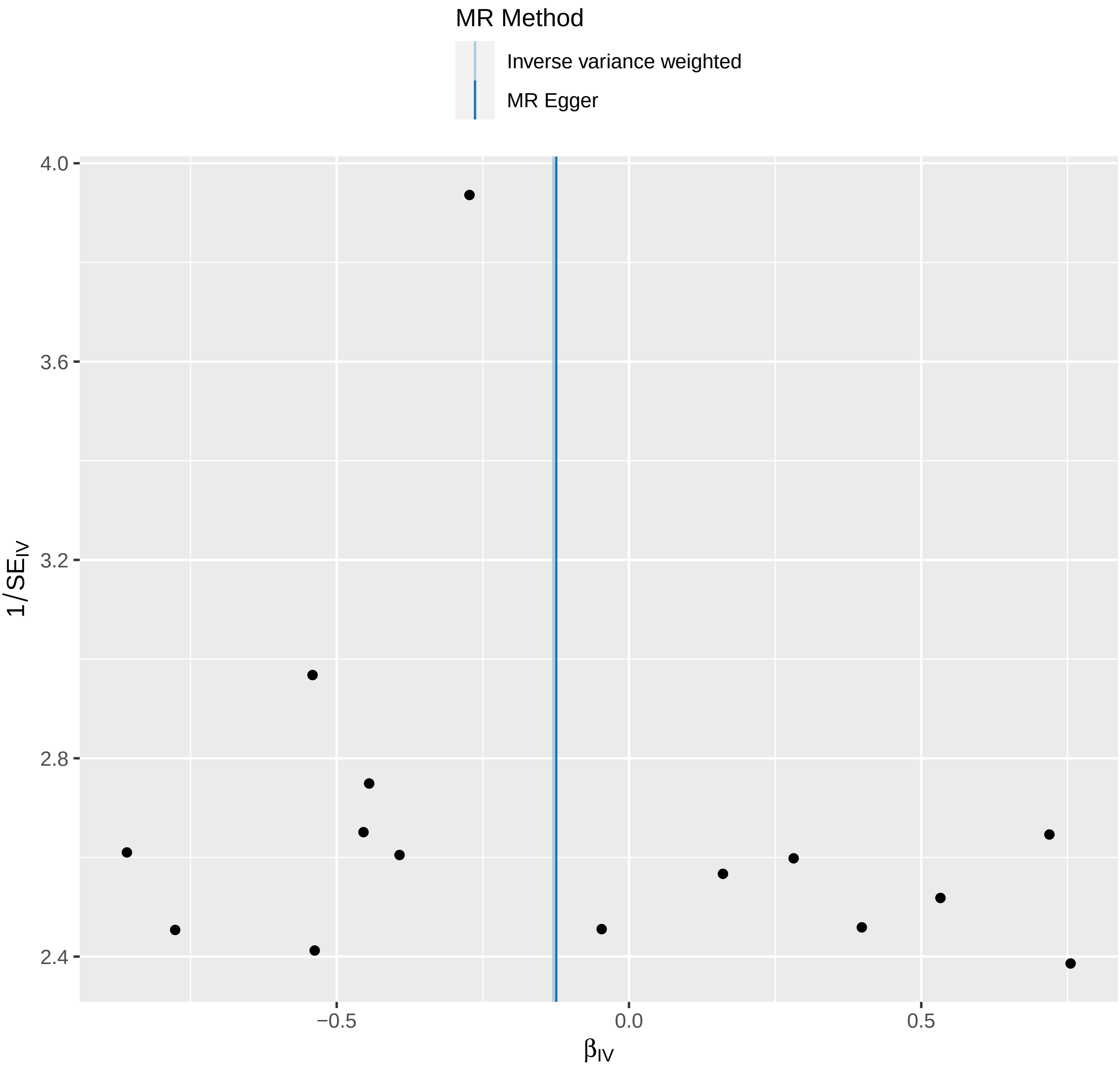

Batch 2−4799 : Gut microbiota abundance (genus Catenibacterium id.2153) on Pregnancy−specific beta−1−glycoprotein 11
with a threshold of 1e−05

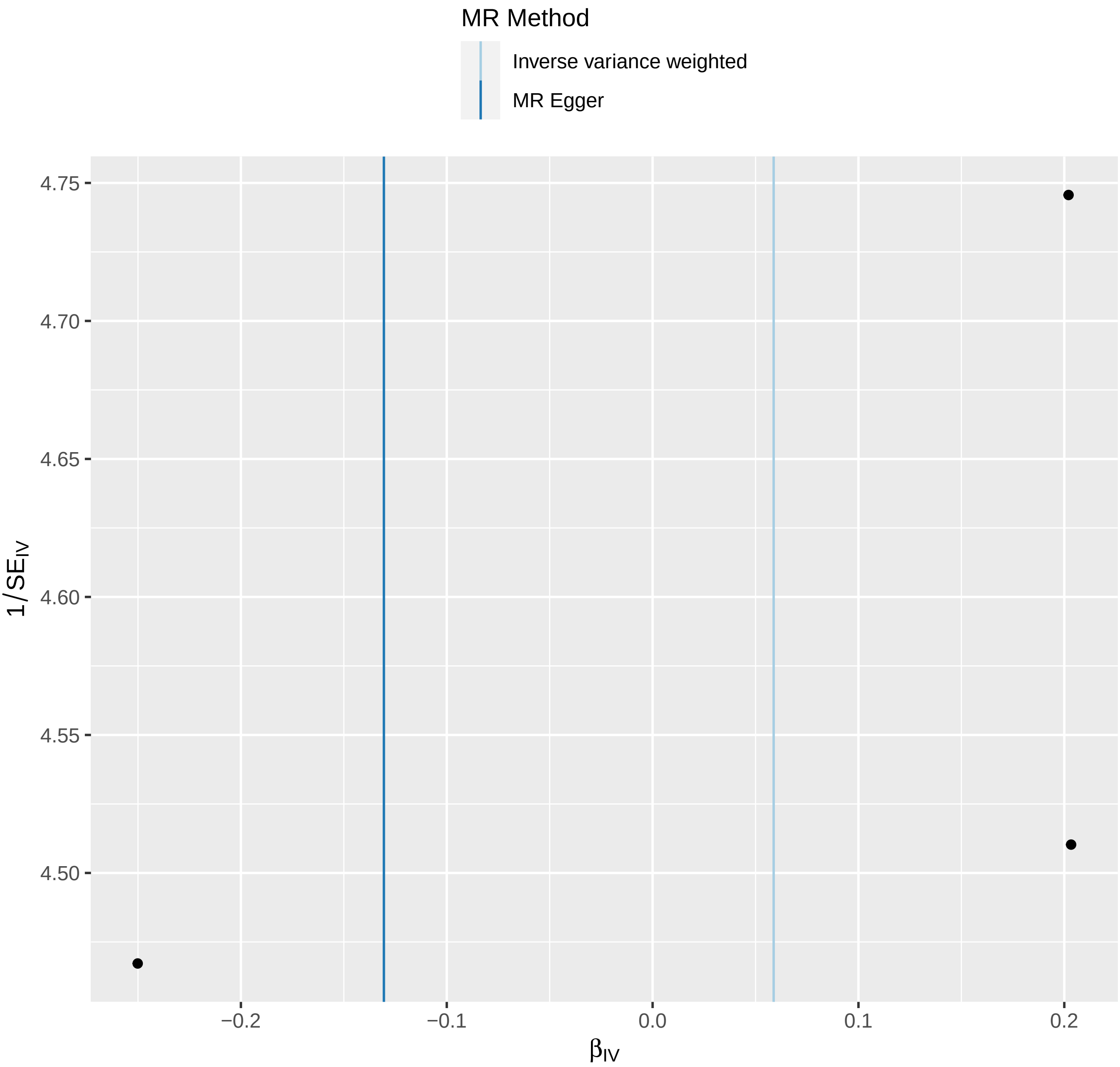

Batch 2−4800 : Gut microbiota abundance (genus Parasutterella id.2892) on Pregnancy−specific beta−1−glycoprotein 11
with a threshold of 1e−05

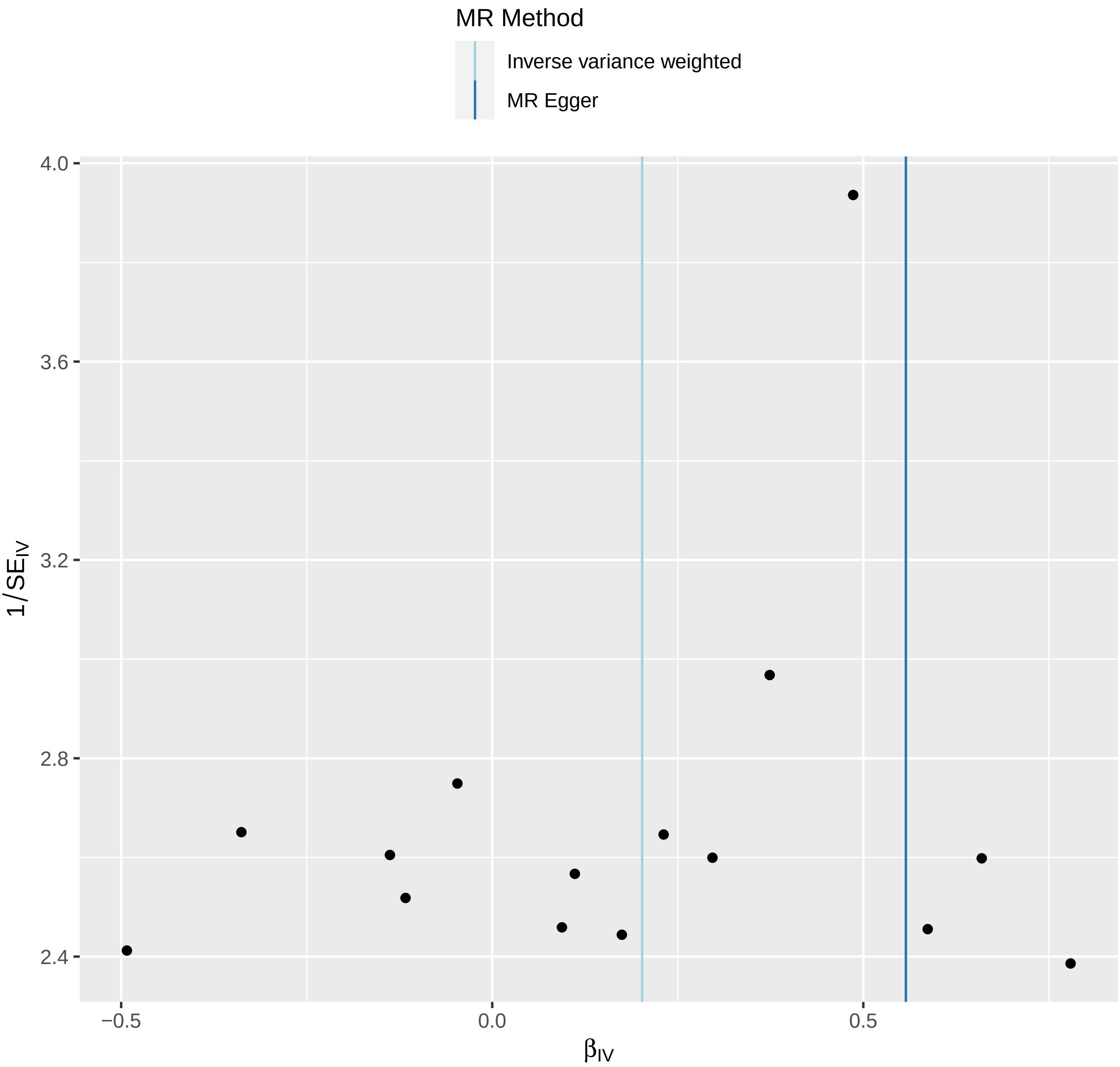

Batch 2−4801 : Gut microbiota abundance (genus Catenibacterium id.2153) on Pregnancy−specific beta−1−glycoprotein 2
with a threshold of 1e−05

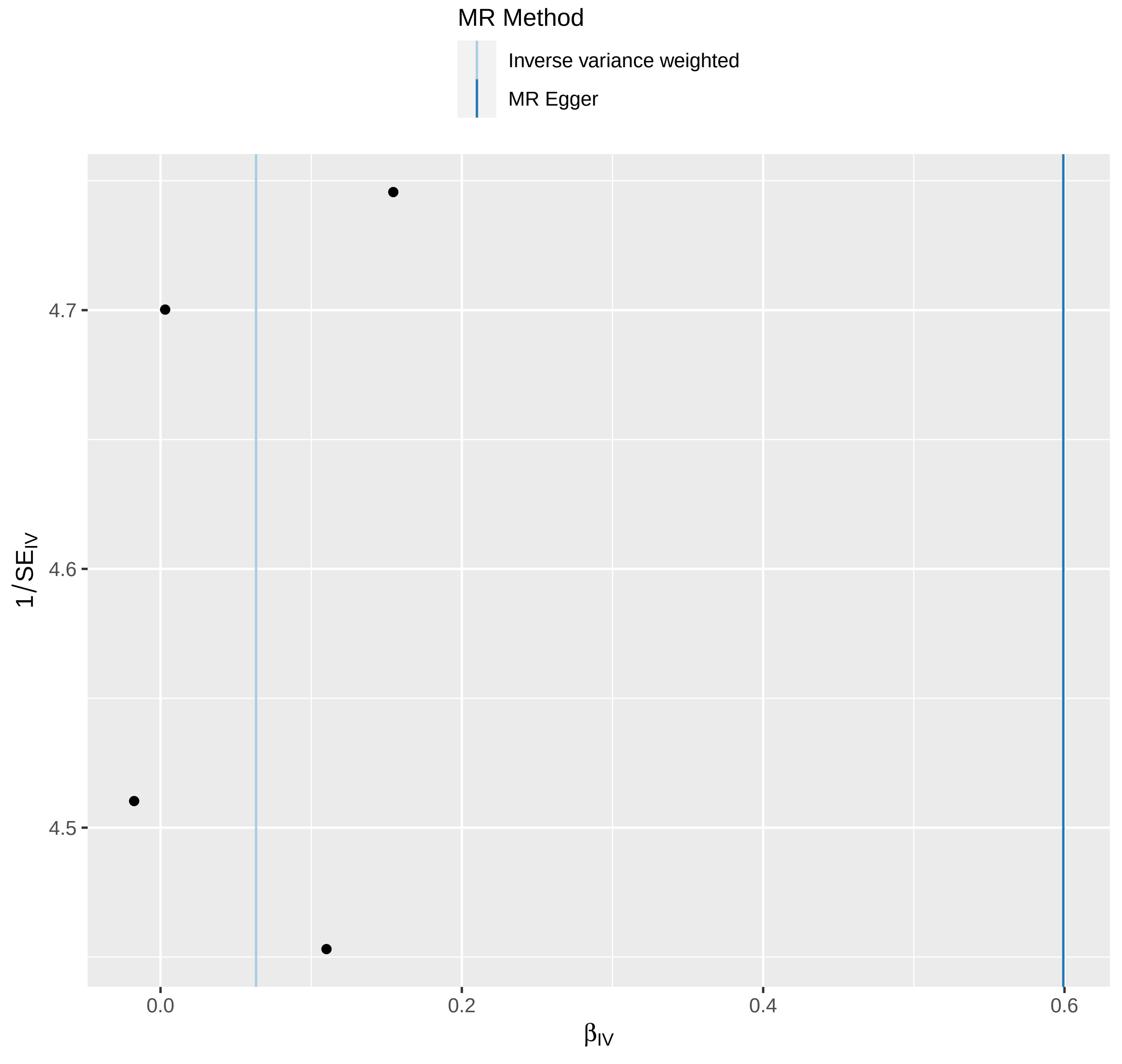

Batch 2−4802 : Gut microbiota abundance (genus Parasutterella id.2892) on Pregnancy−specific beta−1−glycoprotein 2
with a threshold of 1e−05

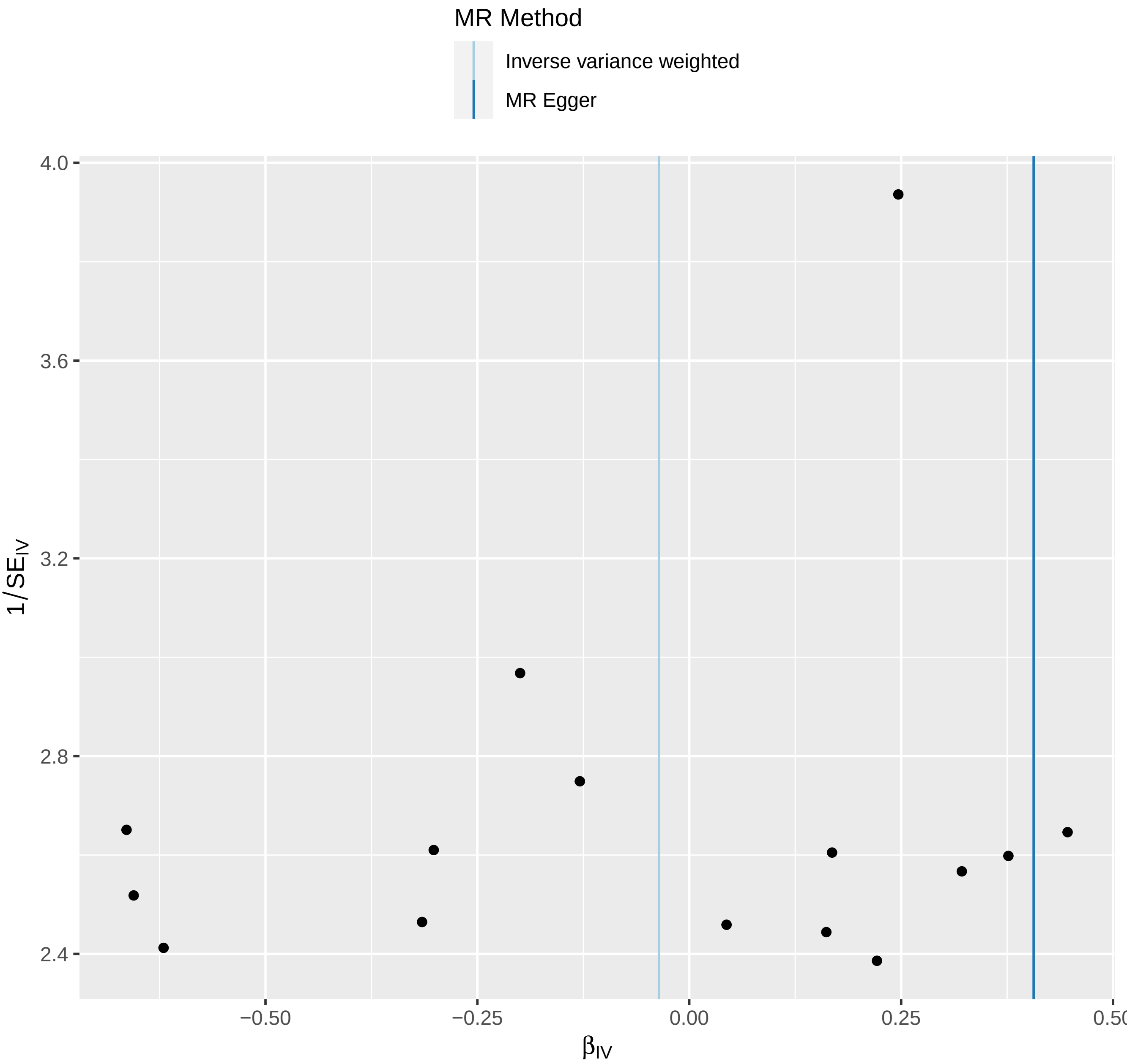

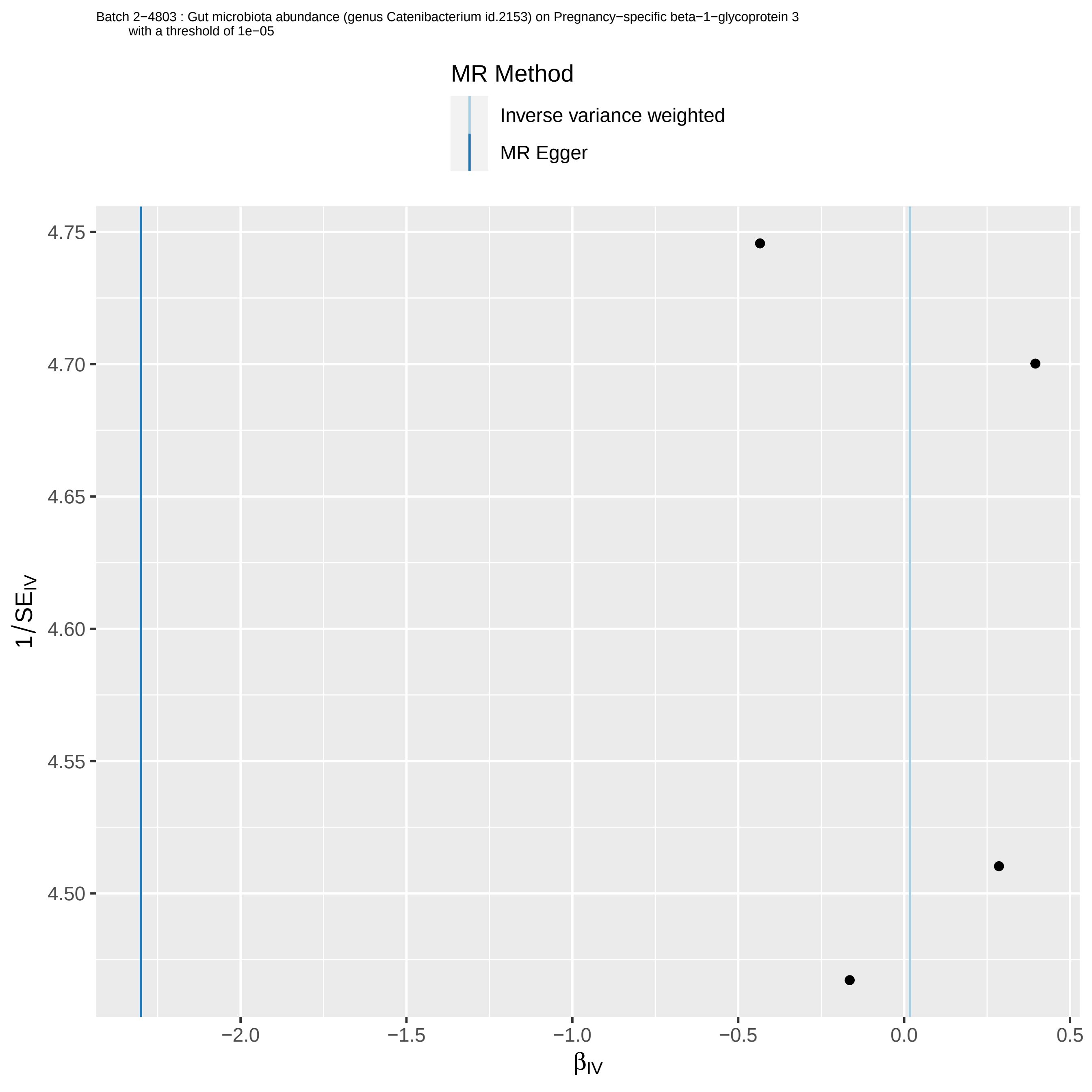
Batch 2−4803 : Gut microbiota abundance (genus Catenibacterium id.2153) on Pregnancy−specific beta−1−glycoprotein 3
with a threshold of 1e−05
MR Method
Inverse variance weighted
MR Egger
4.75
4.70
4.65
4.60
4.55
4.50
1/SEIV
−2.0
−1.5
−1.0
−0.5
0.0
0.5
βIV

Batch 2−4804 : Gut microbiota abundance (genus Parasutterella id.2892) on Pregnancy−specific beta−1−glycoprotein 3
with a threshold of 1e−05

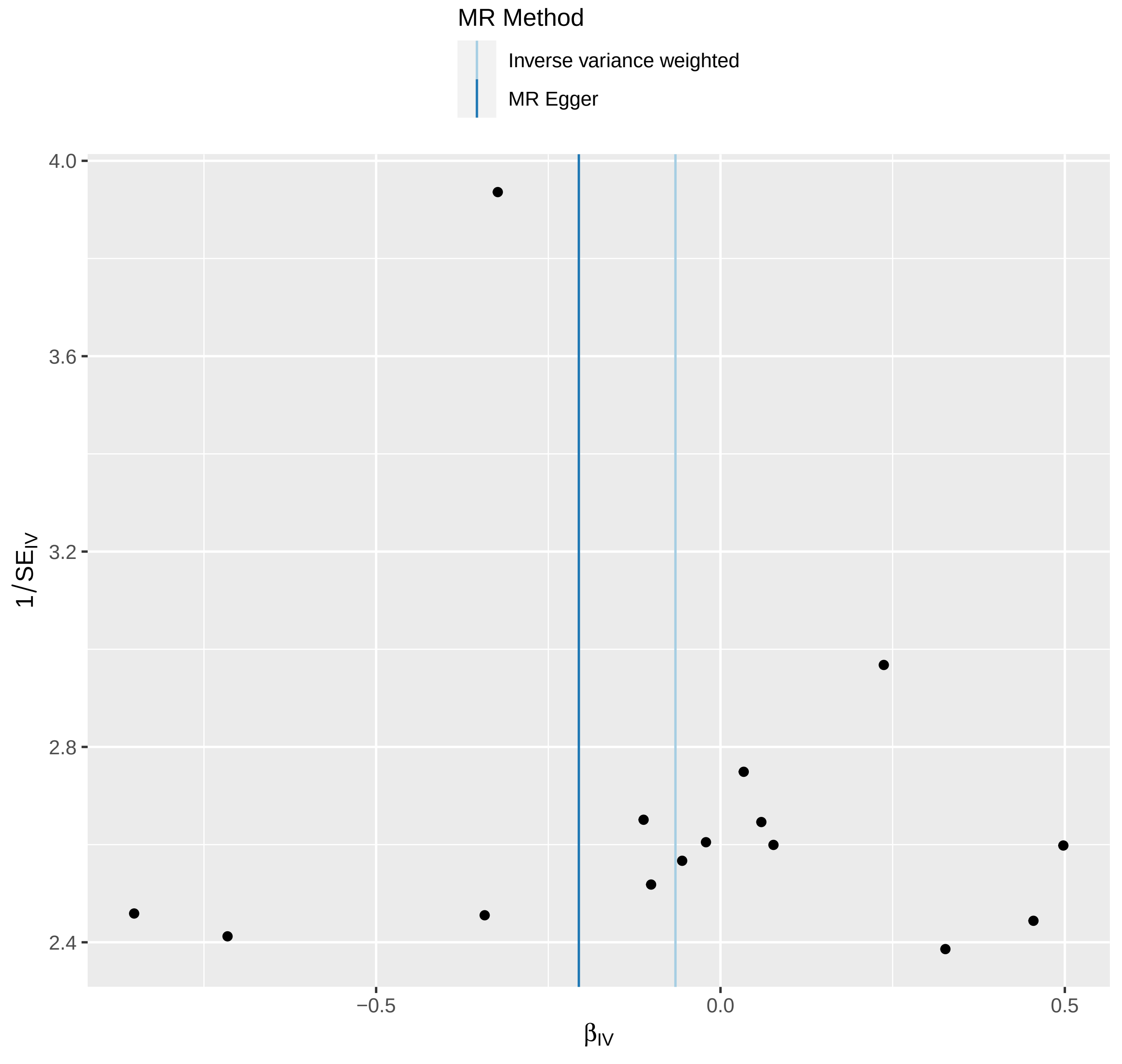

Batch 2−4805 : Gut microbiota abundance (genus Catenibacterium id.2153) on Pregnancy−specific beta−1−glycoprotein 4
with a threshold of 1e−05

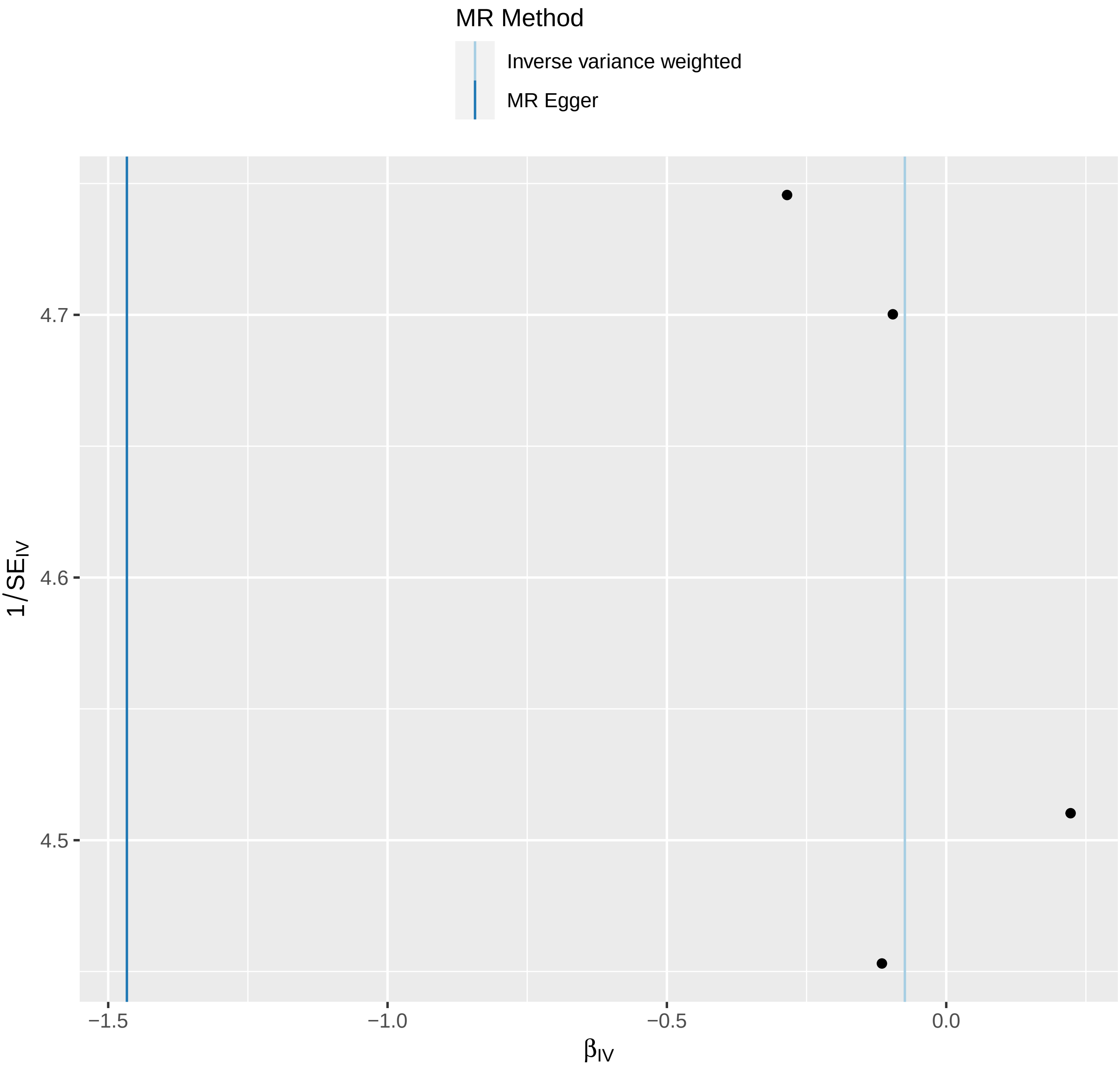

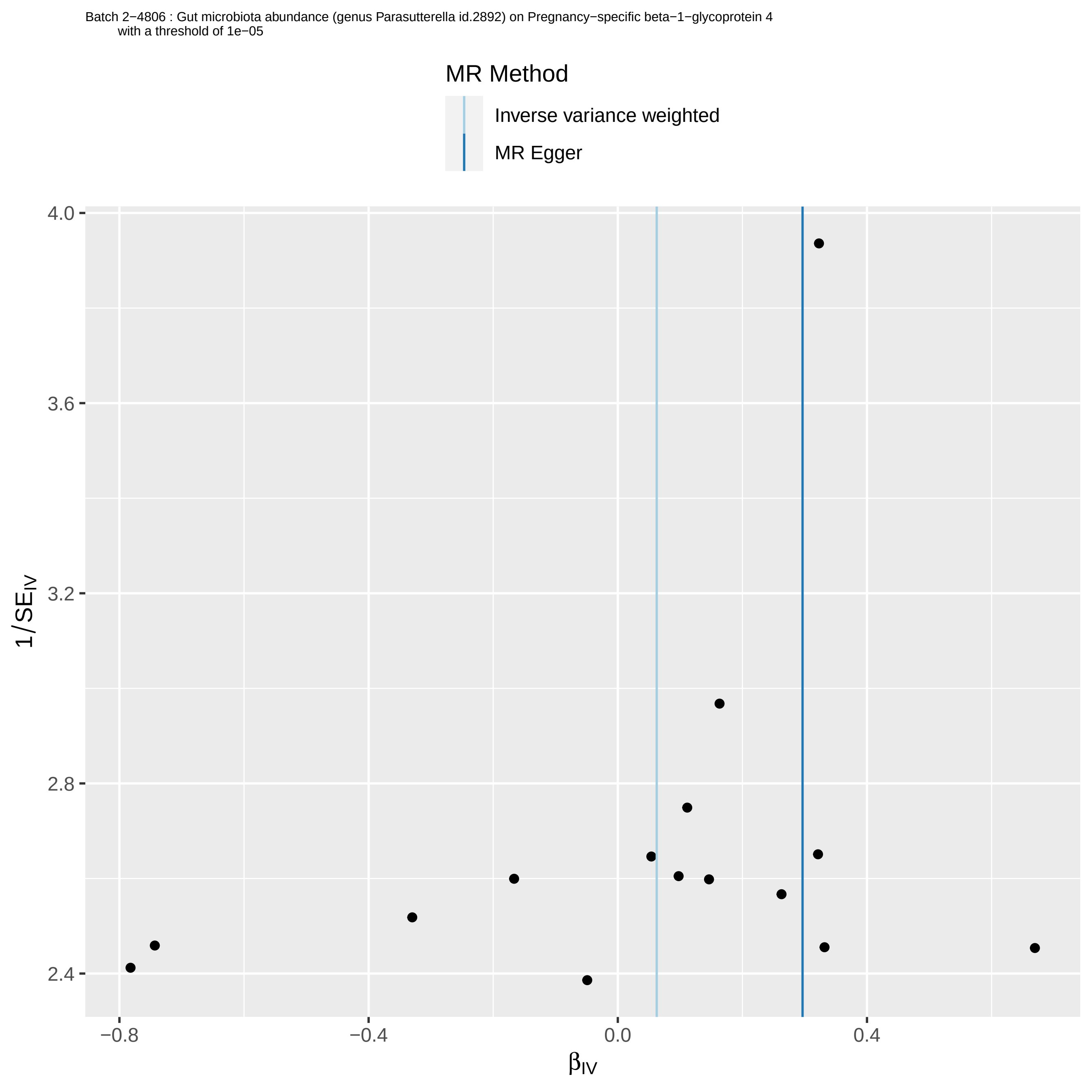
Batch 2−4806 : Gut microbiota abundance (genus Parasutterella id.2892) on Pregnancy−specific beta−1−glycoprotein 4
with a threshold of 1e−05
MR Method
Inverse variance weighted
MR Egger
4.0
3.6
3.2
2.8
2.4
1/SE$_{IV}$
−0.8
−0.4
0.0
0.4
β$_{IV}$

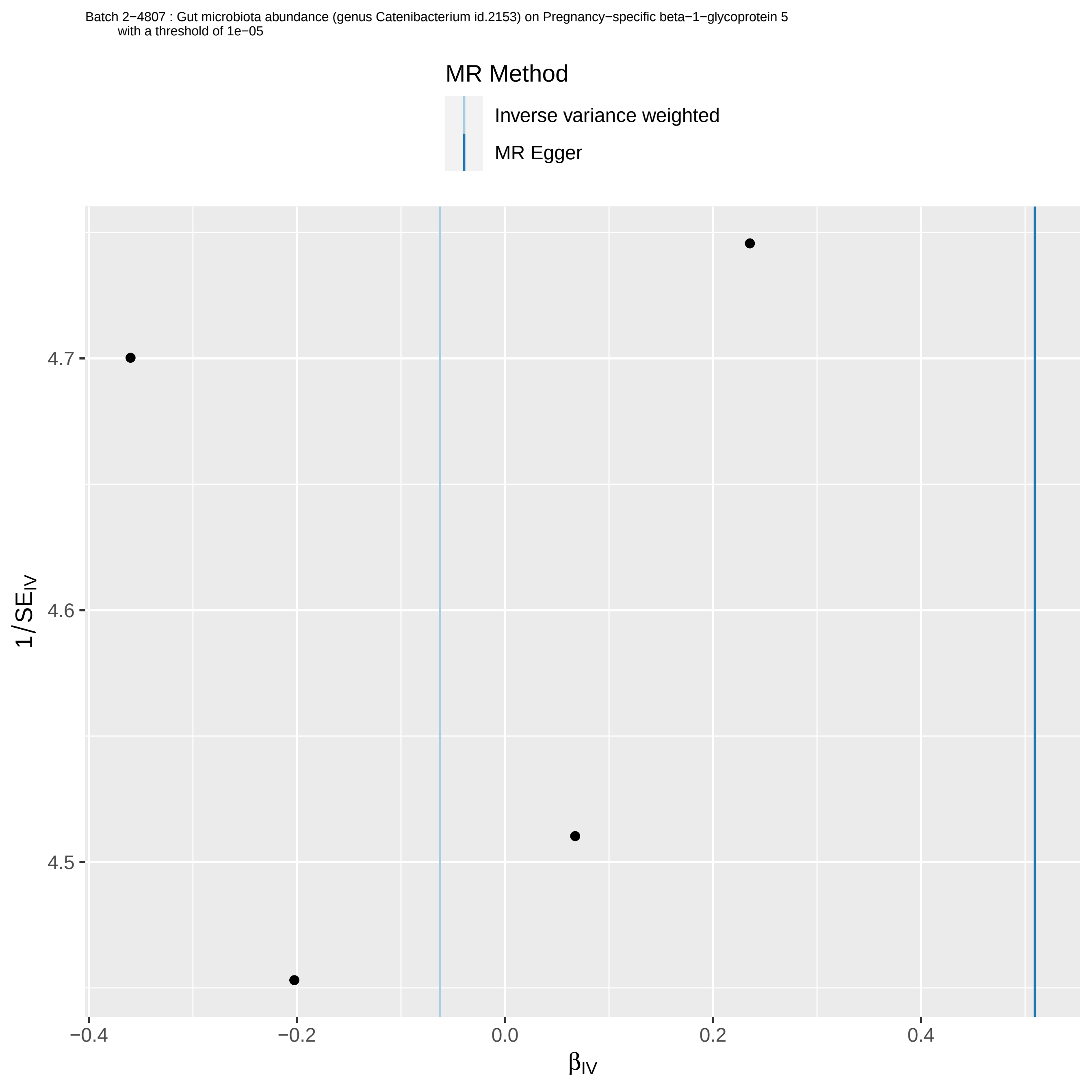
Batch 2−4807 : Gut microbiota abundance (genus Catenibacterium id.2153) on Pregnancy−specific beta−1−glycoprotein 5
with a threshold of 1e−05
MR Method
Inverse variance weighted
MR Egger
4.7
4.6
4.5
1/SE_IV
−0.4
−0.2
0.0
0.2
0.4
β_IV

Batch 2−4808 : Gut microbiota abundance (genus Parasutterella id.2892) on Pregnancy−specific beta−1−glycoprotein 5
with a threshold of 1e−05

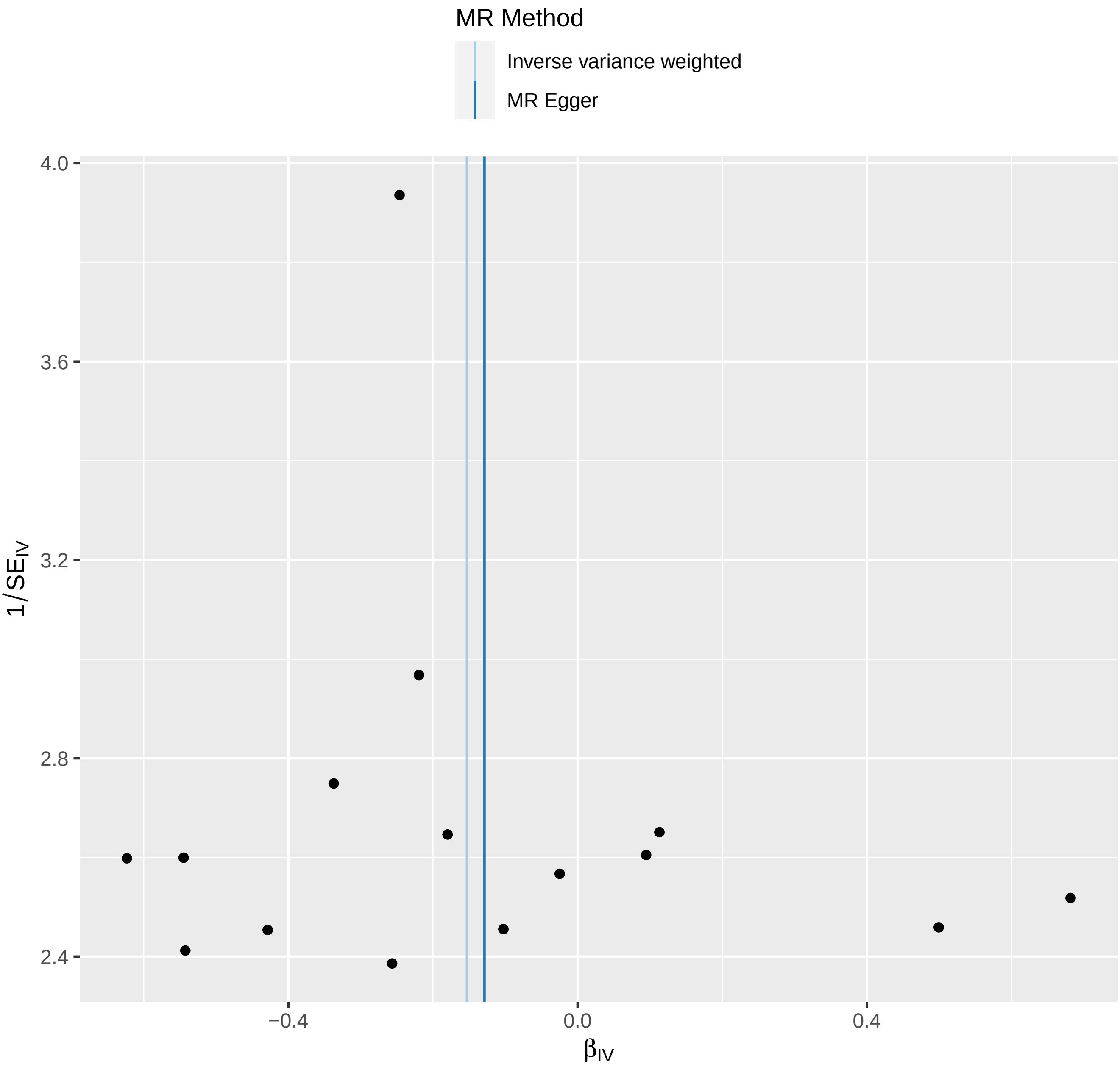

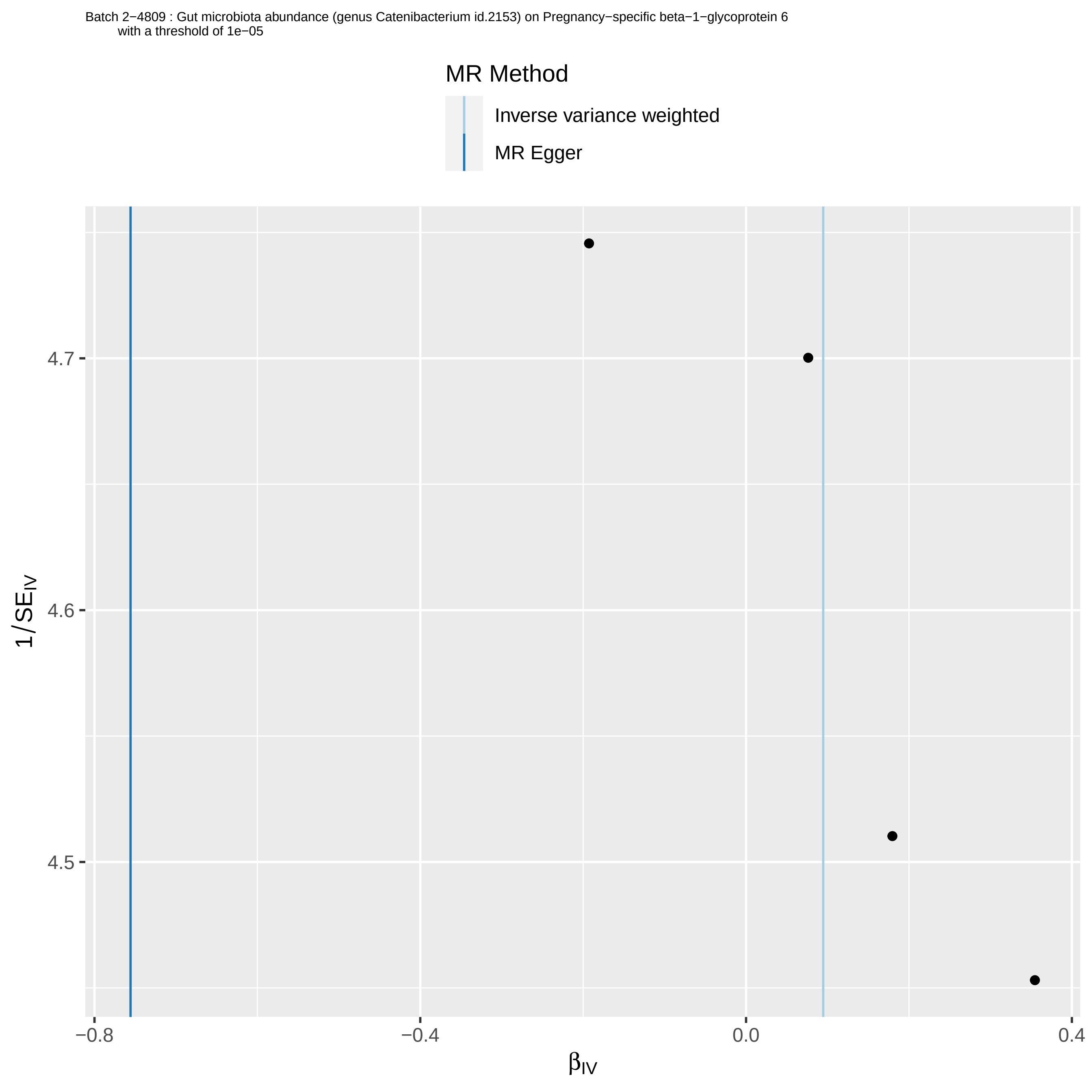

Batch 2−4809 : Gut microbiota abundance (genus Catenibacterium id.2153) on Pregnancy−specific beta−1−glycoprotein 6
with a threshold of 1e−05
MR Method
Inverse variance weighted
MR Egger
4.7
4.6
4.5
1/SE_IV
−0.8
−0.4
0.0
0.4
β_IV

Batch 2−4810 : Gut microbiota abundance (genus Parasutterella id.2892) on Pregnancy−specific beta−1−glycoprotein 6 with a threshold of 1e−05

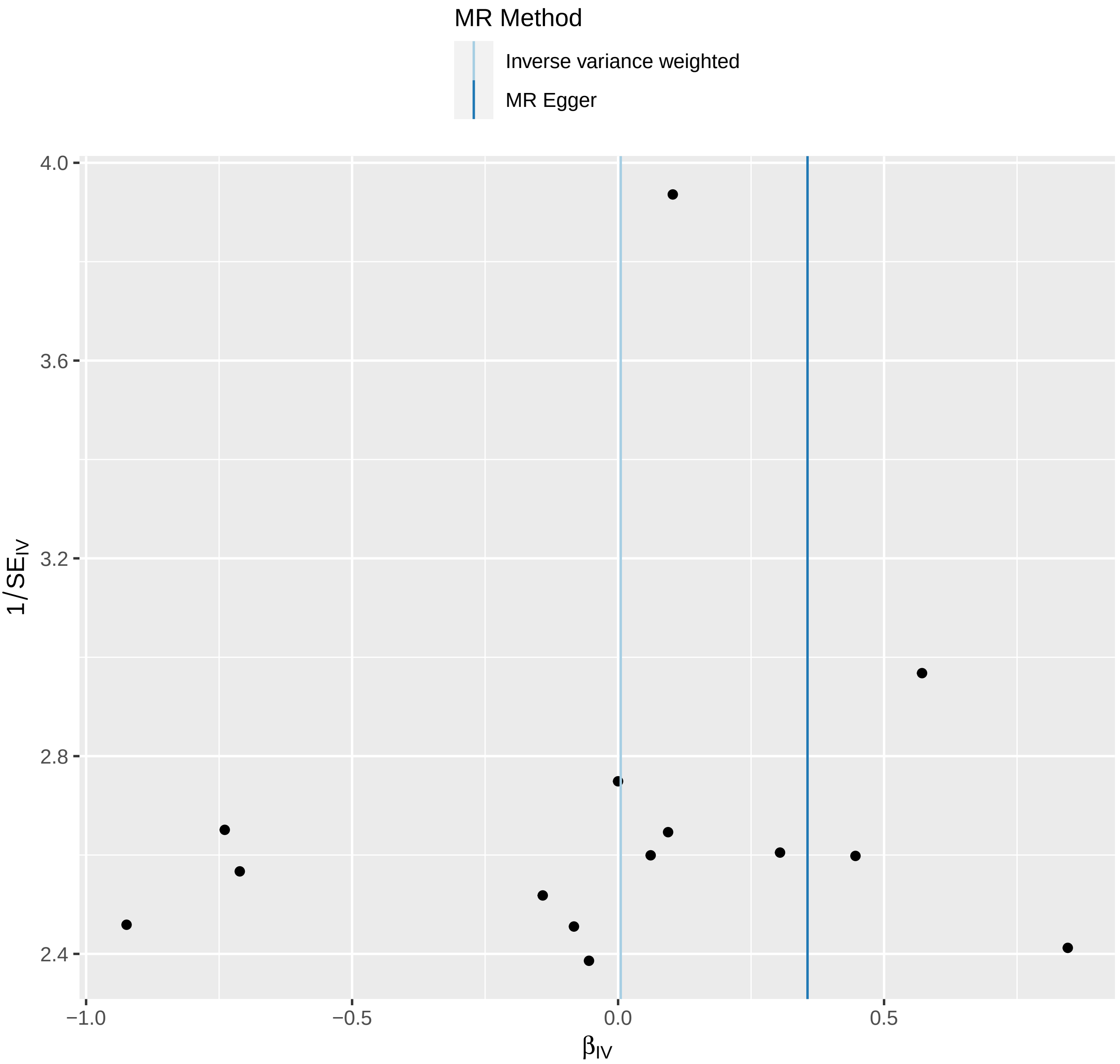

Batch 2−4811 : Gut microbiota abundance (genus Catenibacterium id.2153) on Putative pregnancy−specific beta−1−glycoprotein 7
with a threshold of 1e−05

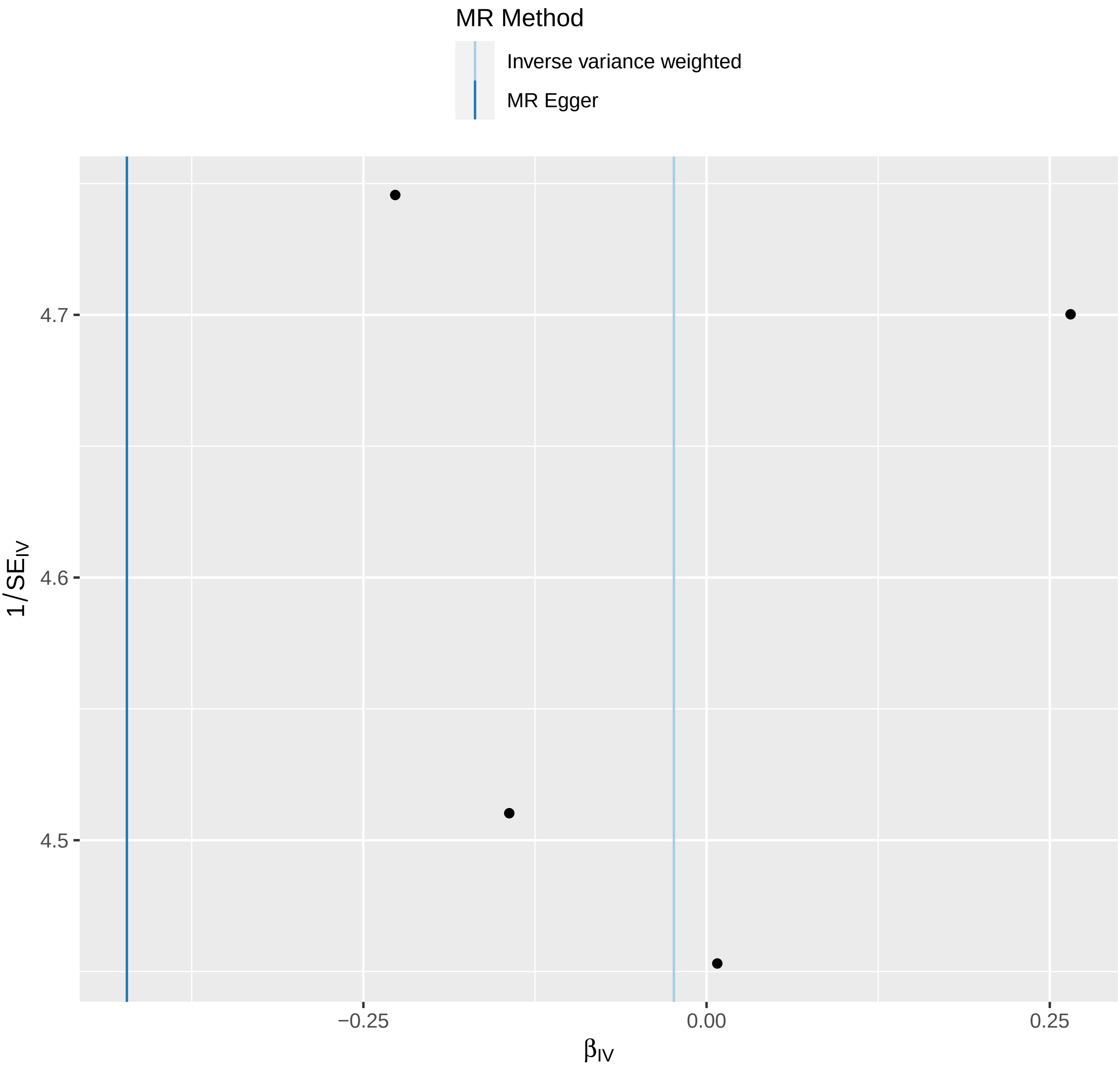

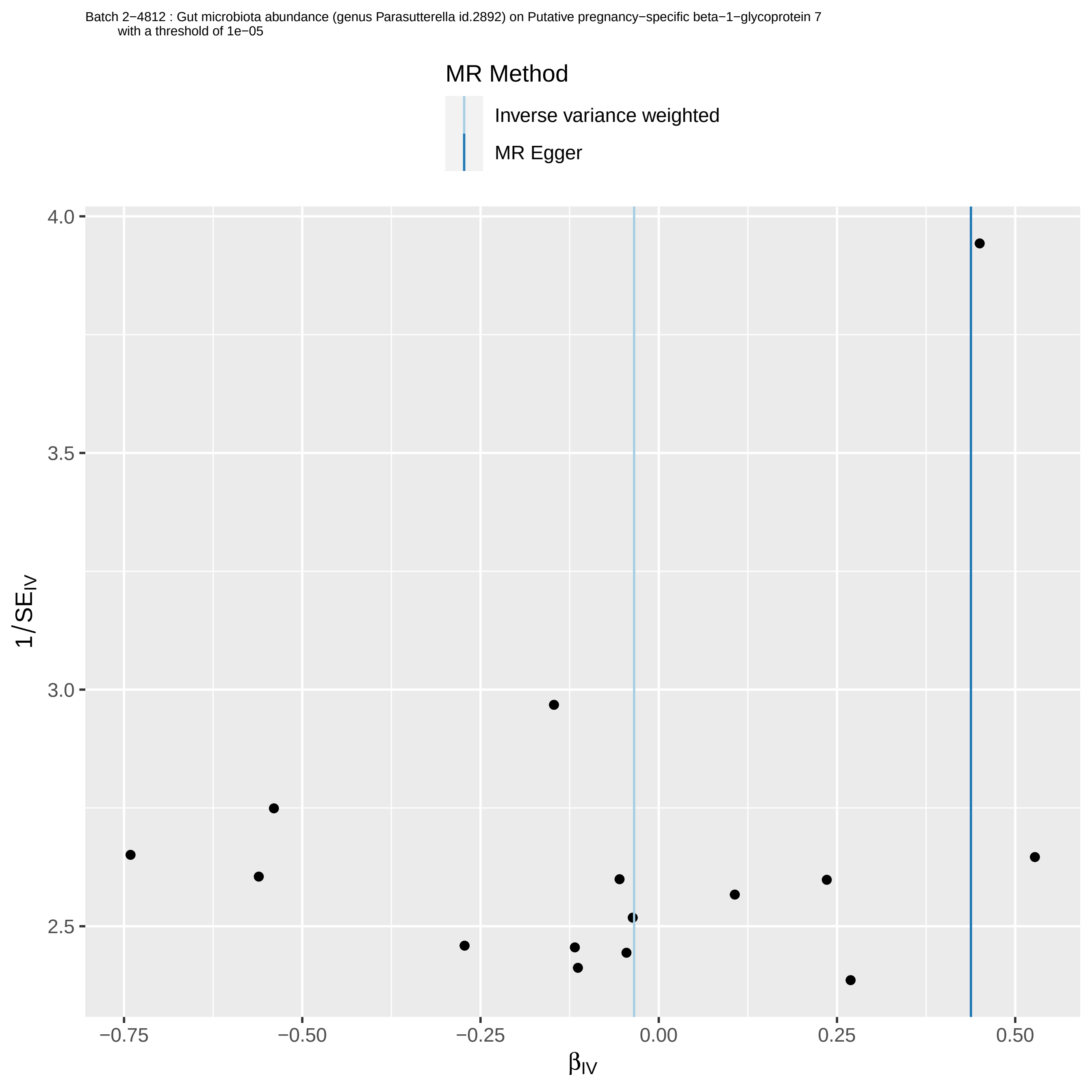

Batch 2−4812 : Gut microbiota abundance (genus Parasutterella id.2892) on Putative pregnancy−specific beta−1−glycoprotein 7
with a threshold of 1e−05
MR Method
Inverse variance weighted
MR Egger
4.0
3.5
3.0
2.5
1/SE_IV
−0.75
−0.50
−0.25
0.00
0.25
0.50
β_IV

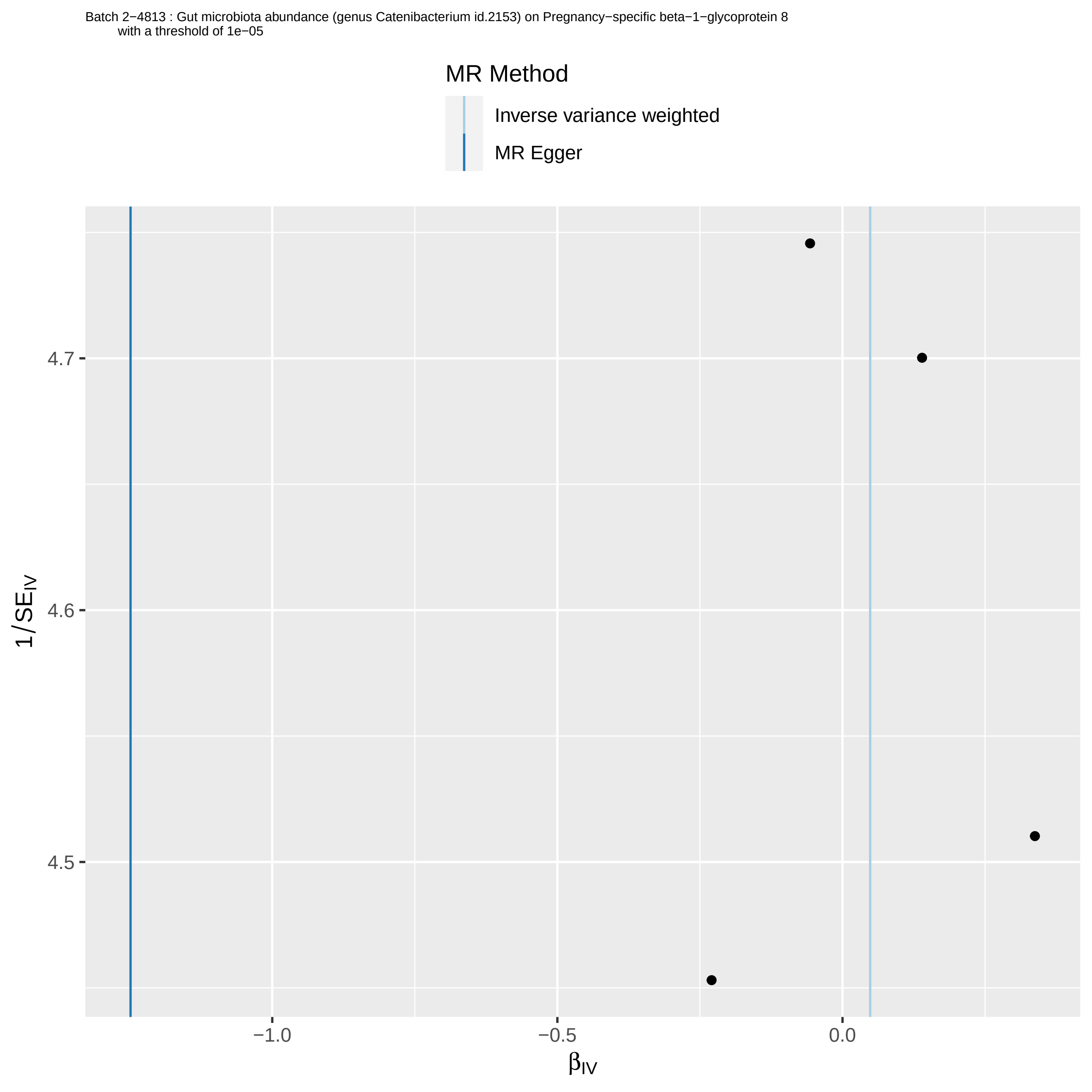

Batch 2−4813 : Gut microbiota abundance (genus Catenibacterium id.2153) on Pregnancy−specific beta−1−glycoprotein 8
with a threshold of 1e−05
MR Method
Inverse variance weighted
MR Egger
4.7
4.6
4.5
1/SE_IV
−1.0
−0.5
0.0
β_IV

Batch 2−4814 : Gut microbiota abundance (genus Parasutterella id.2892) on Pregnancy−specific beta−1−glycoprotein 8 with a threshold of 1e−05

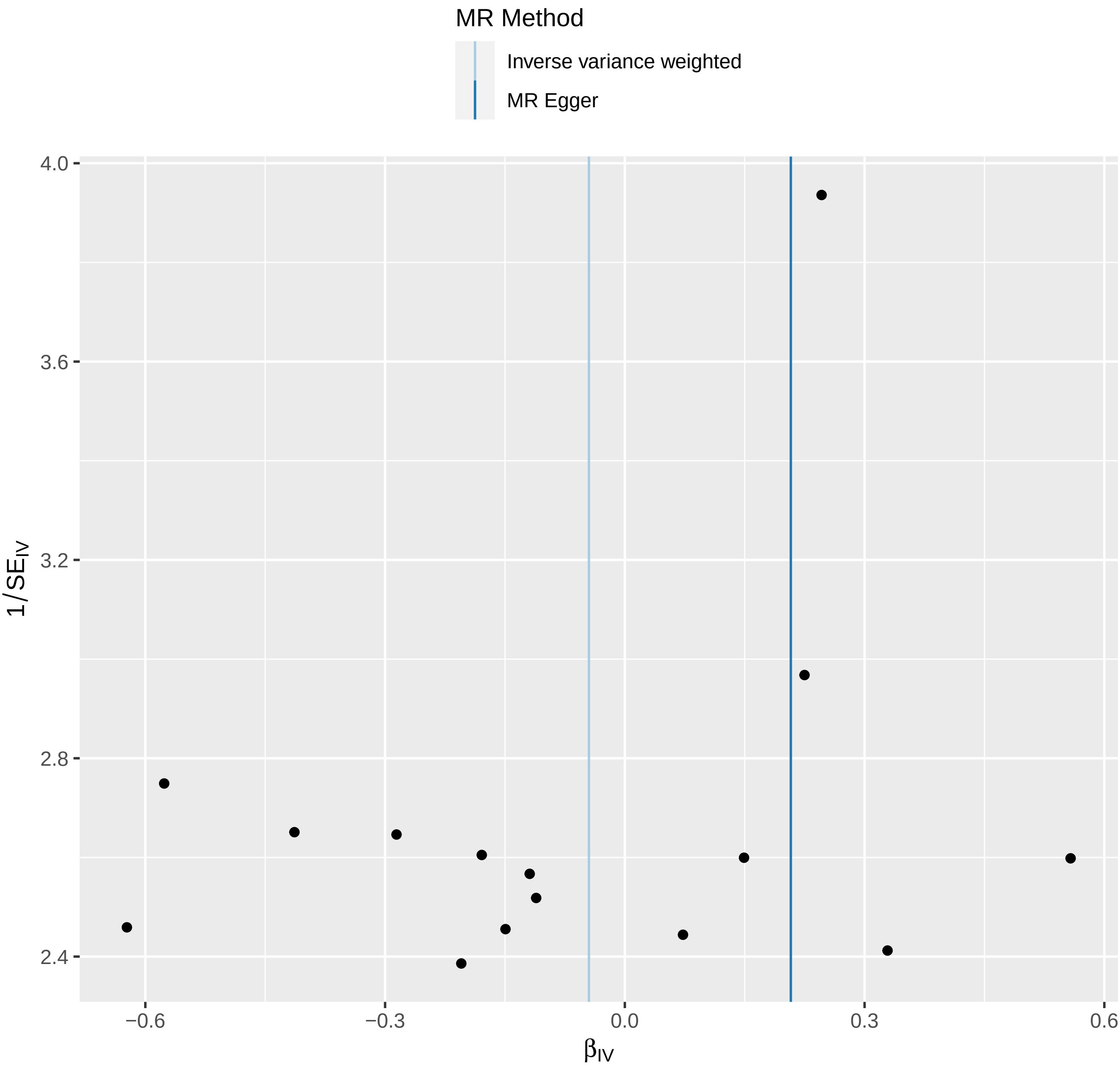

Batch 2−4815 : Gut microbiota abundance (genus Catenibacterium id.2153) on Pregnancy−specific beta−1−glycoprotein 9
with a threshold of 1e−05

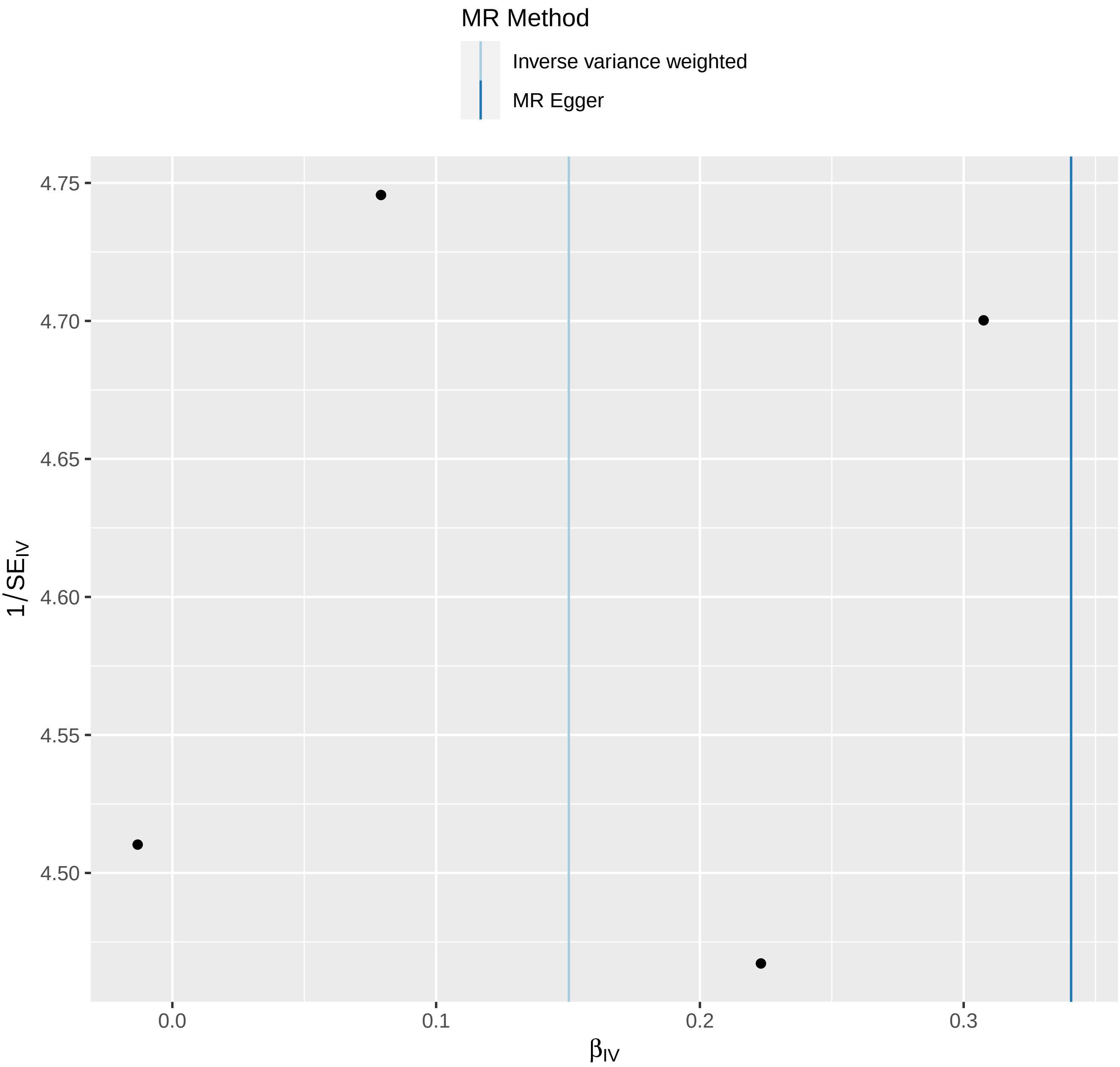

Batch 2−4816 : Gut microbiota abundance (genus Parasutterella id.2892) on Pregnancy−specific beta−1−glycoprotein 9
with a threshold of 1e−05

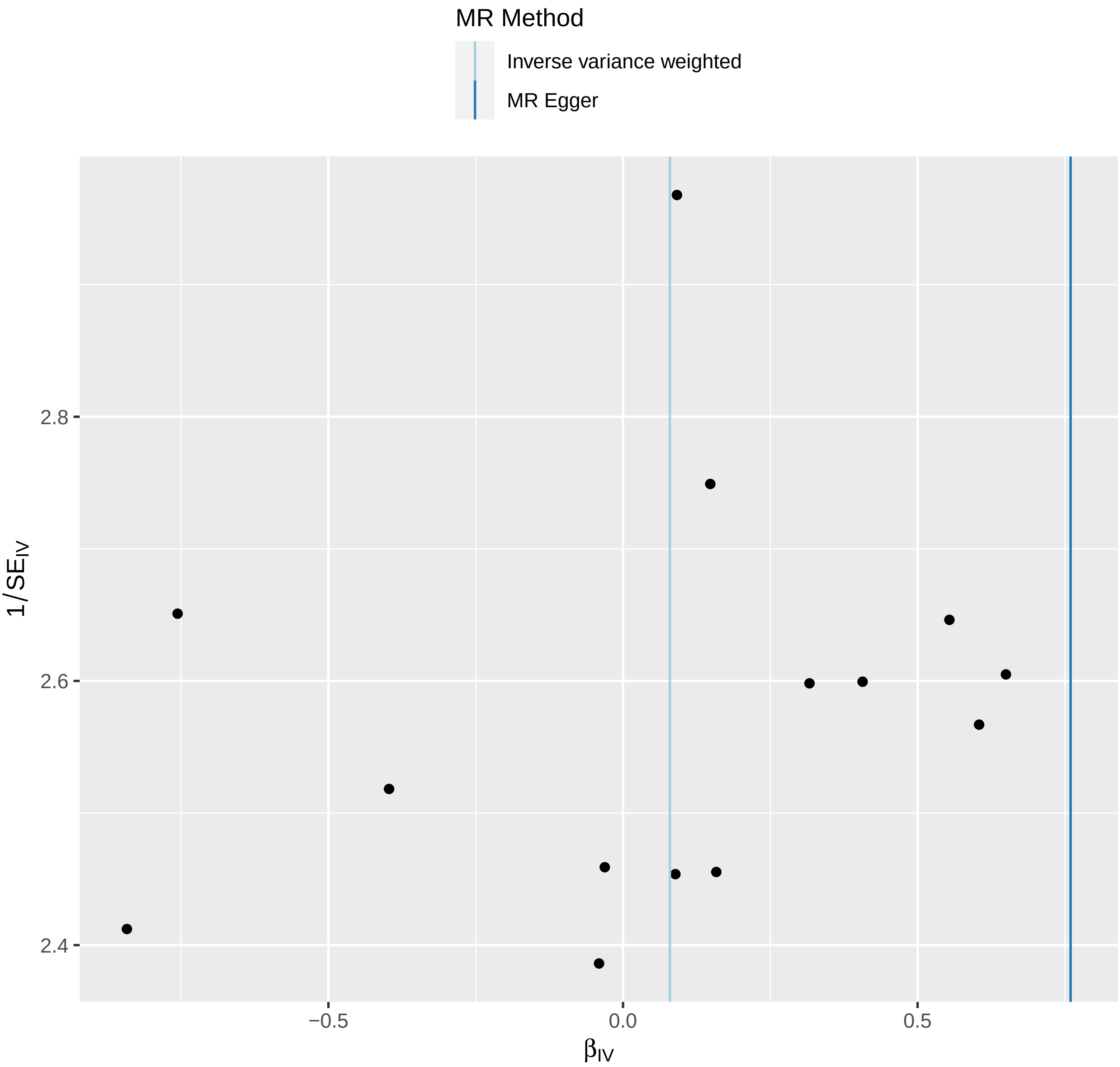

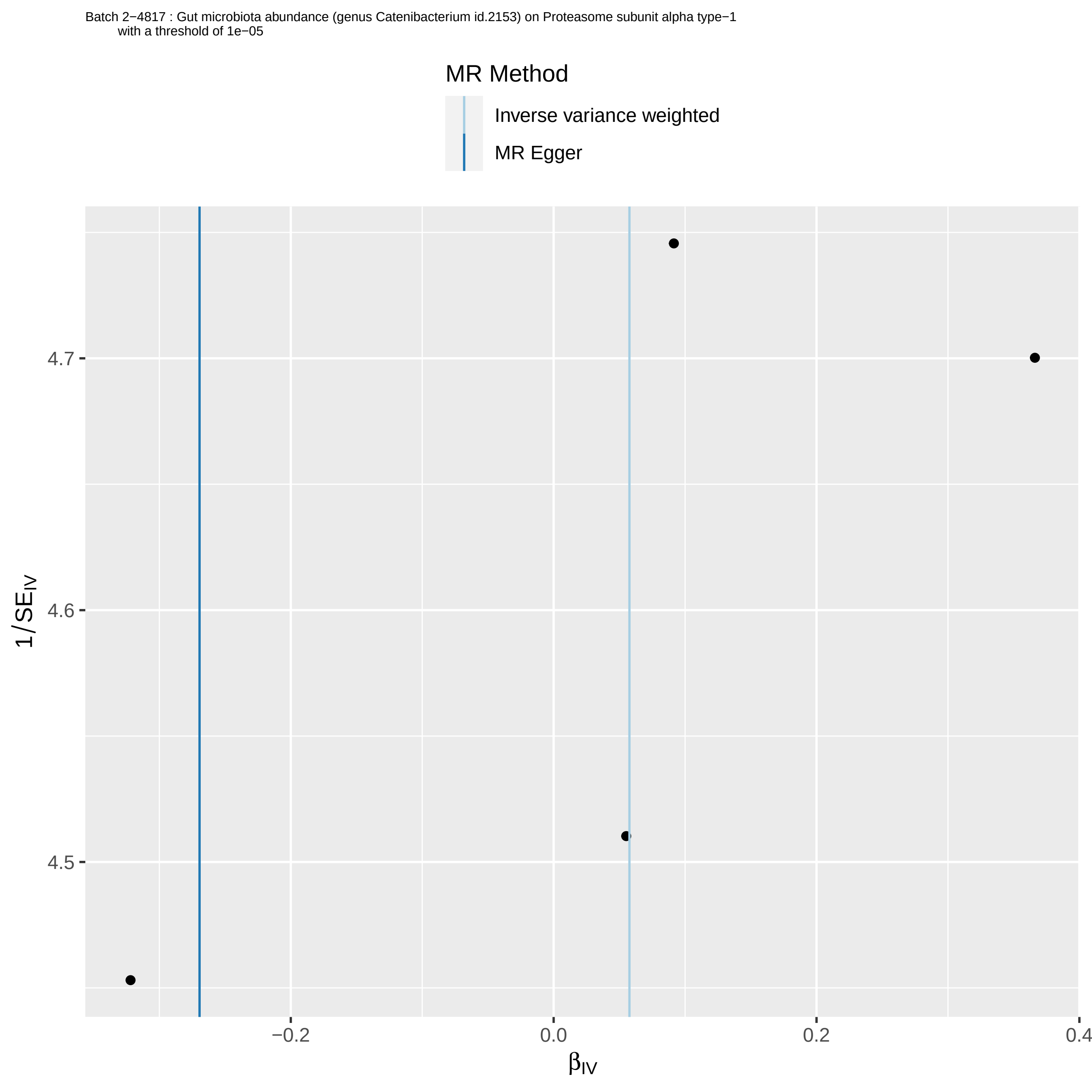
Batch 2−4817 : Gut microbiota abundance (genus Catenibacterium id.2153) on Proteasome subunit alpha type−1
with a threshold of 1e−05
MR Method
Inverse variance weighted
MR Egger
4.7
4.6
4.5
1/SE_IV
−0.2
0.0
0.2
0.4
β_IV

Batch 2−4818 : Gut microbiota abundance (genus Parasutterella id.2892) on Proteasome subunit alpha type−1
with a threshold of 1e−05

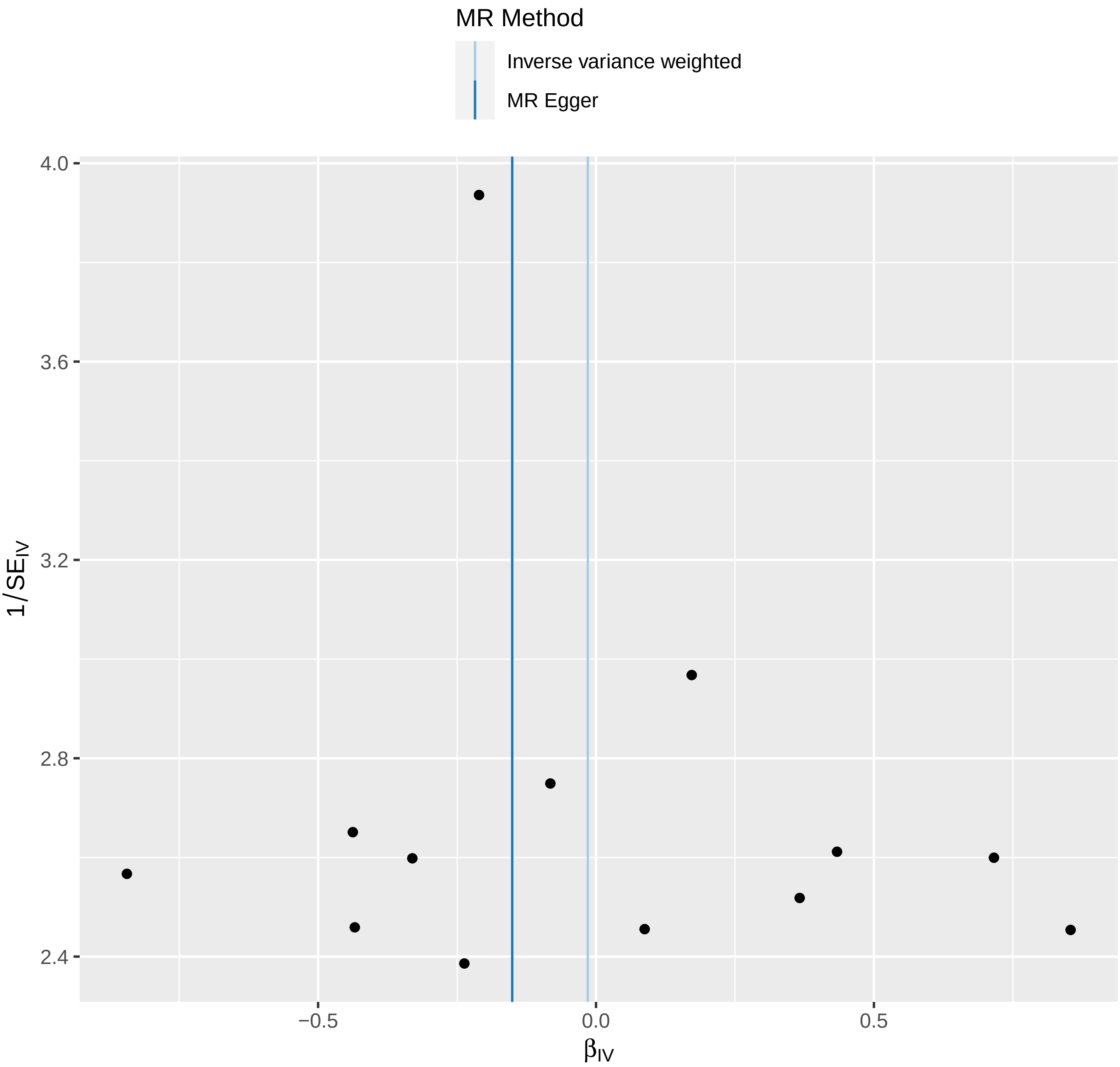

Batch 2−4819 : Gut microbiota abundance (genus Catenibacterium id.2153) on Proteasome subunit alpha type−1
with a threshold of 1e−05

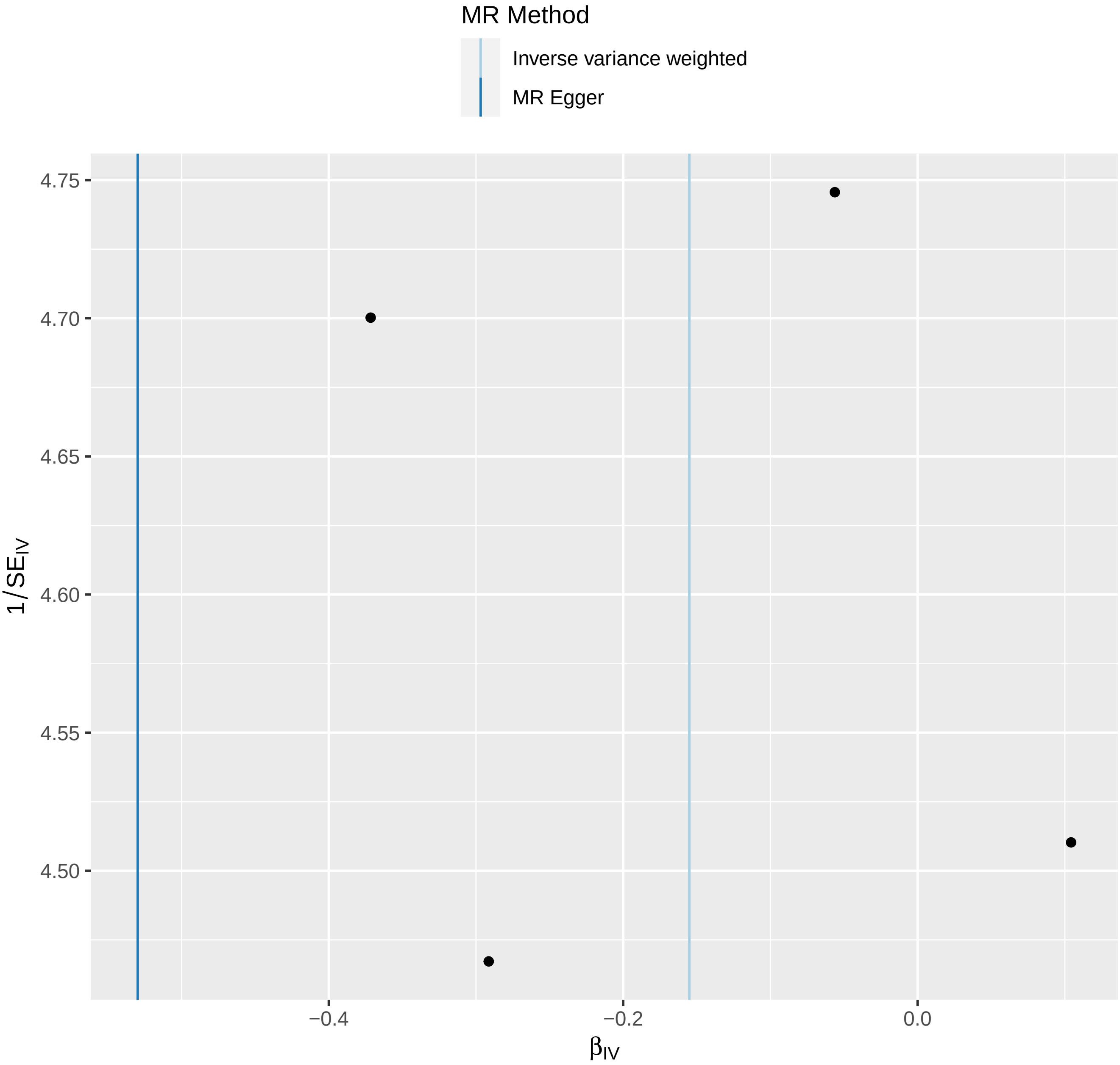

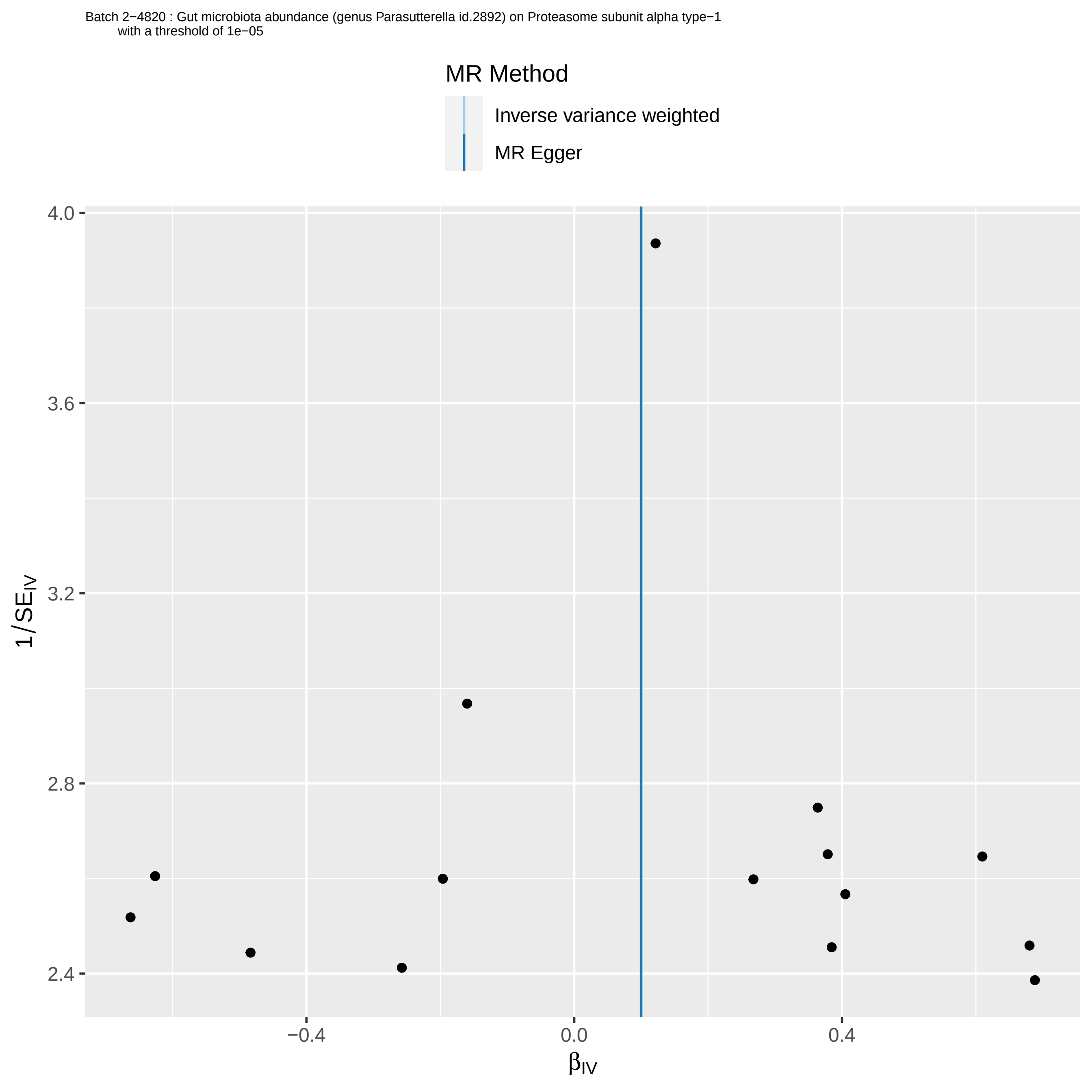

Batch 2−4820 : Gut microbiota abundance (genus Parasutterella id.2892) on Proteasome subunit alpha type−1
with a threshold of 1e−05
MR Method
Inverse variance weighted
MR Egger
4.0
3.6
3.2
2.8
2.4
1/SE_IV
−0.4
0.0
0.4
β_IV

Batch 2−4821 : Gut microbiota abundance (genus Catenibacterium id.2153) on Proteasome subunit alpha type−2
with a threshold of 1e−05

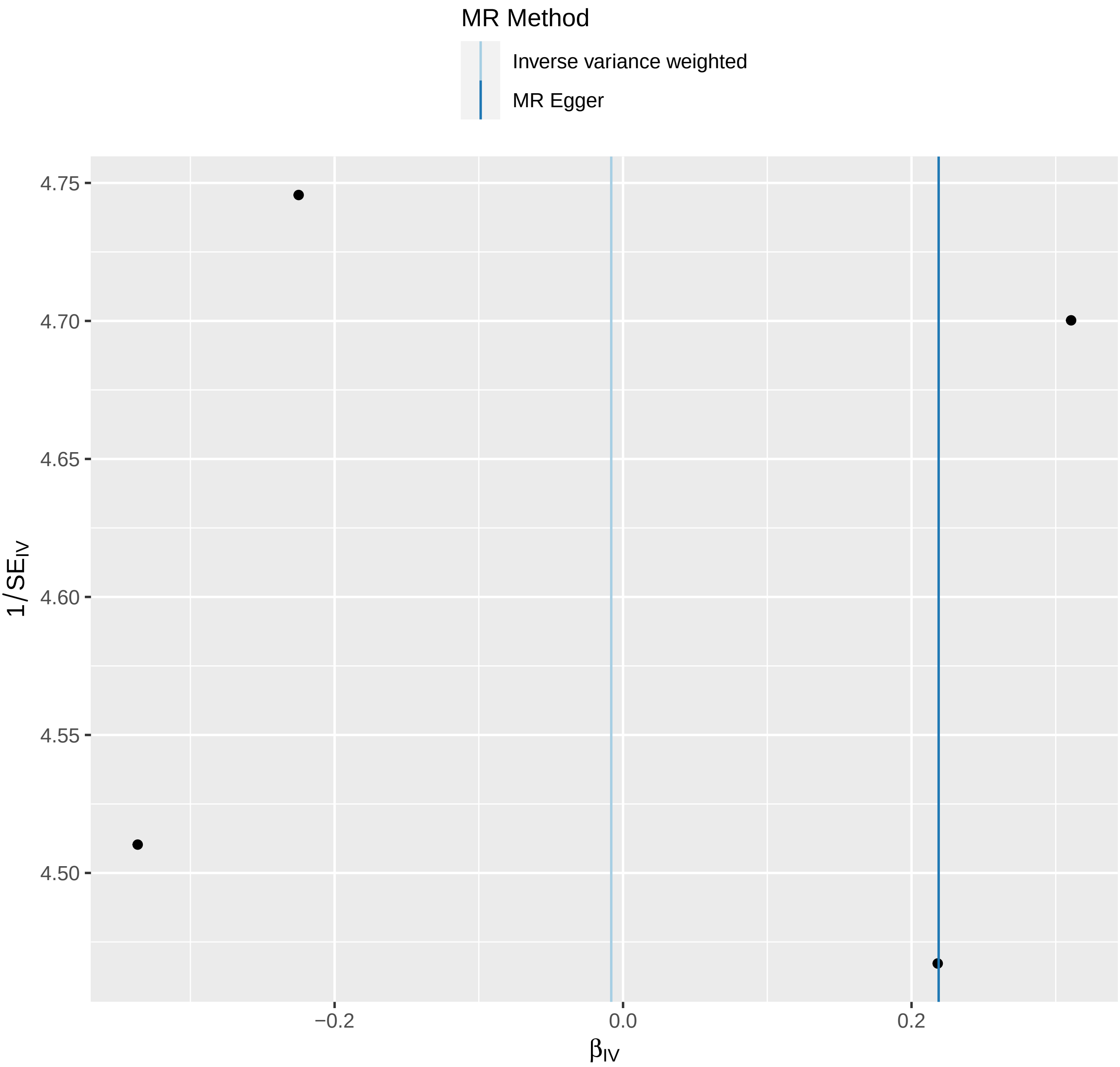

Batch 2−4822 : Gut microbiota abundance (genus Parasutterella id.2892) on Proteasome subunit alpha type−2
with a threshold of 1e−05

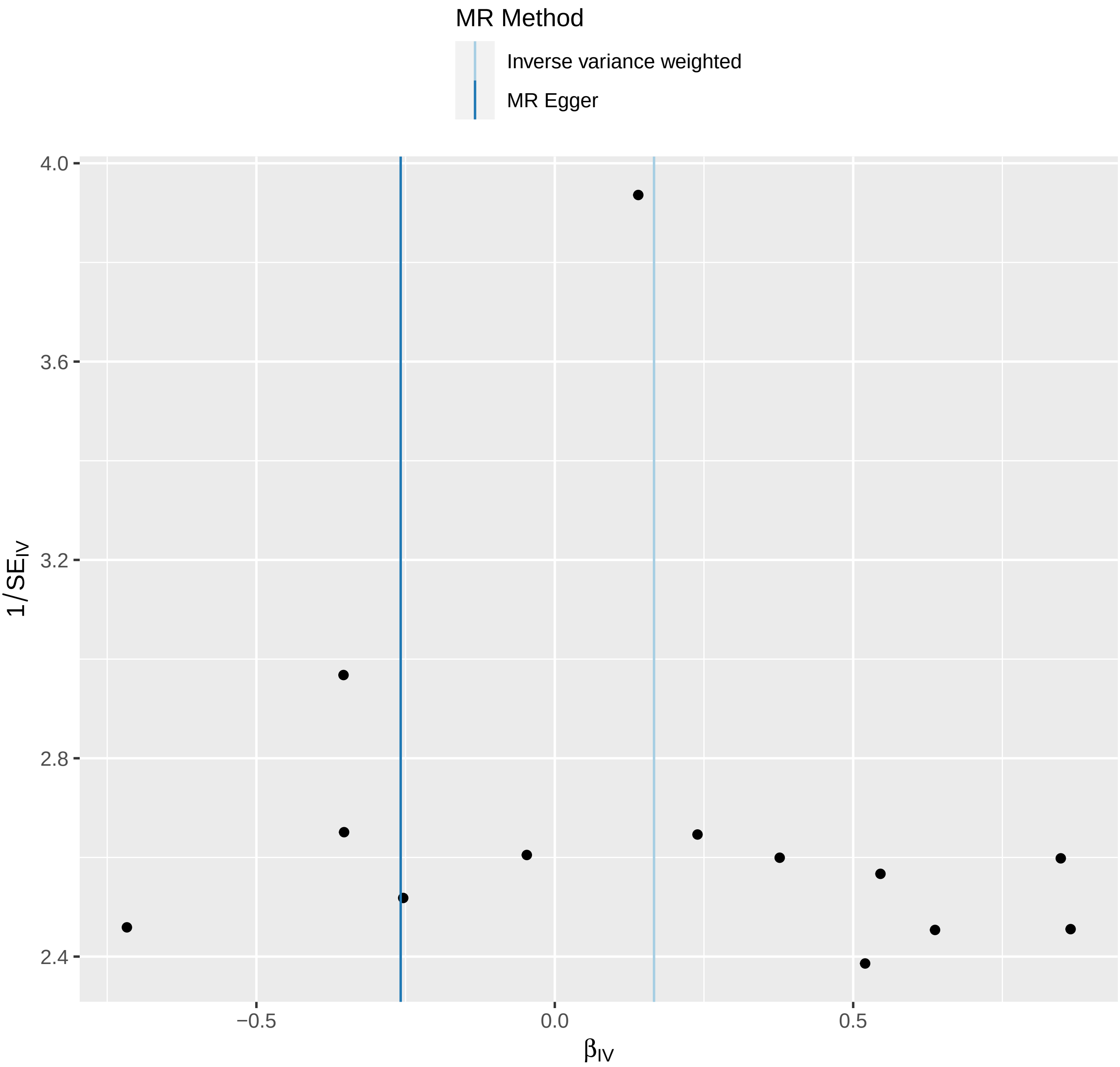

Batch 2−4823 : Gut microbiota abundance (genus Catenibacterium id.2153) on Proteasome subunit alpha type−4
with a threshold of 1e−05

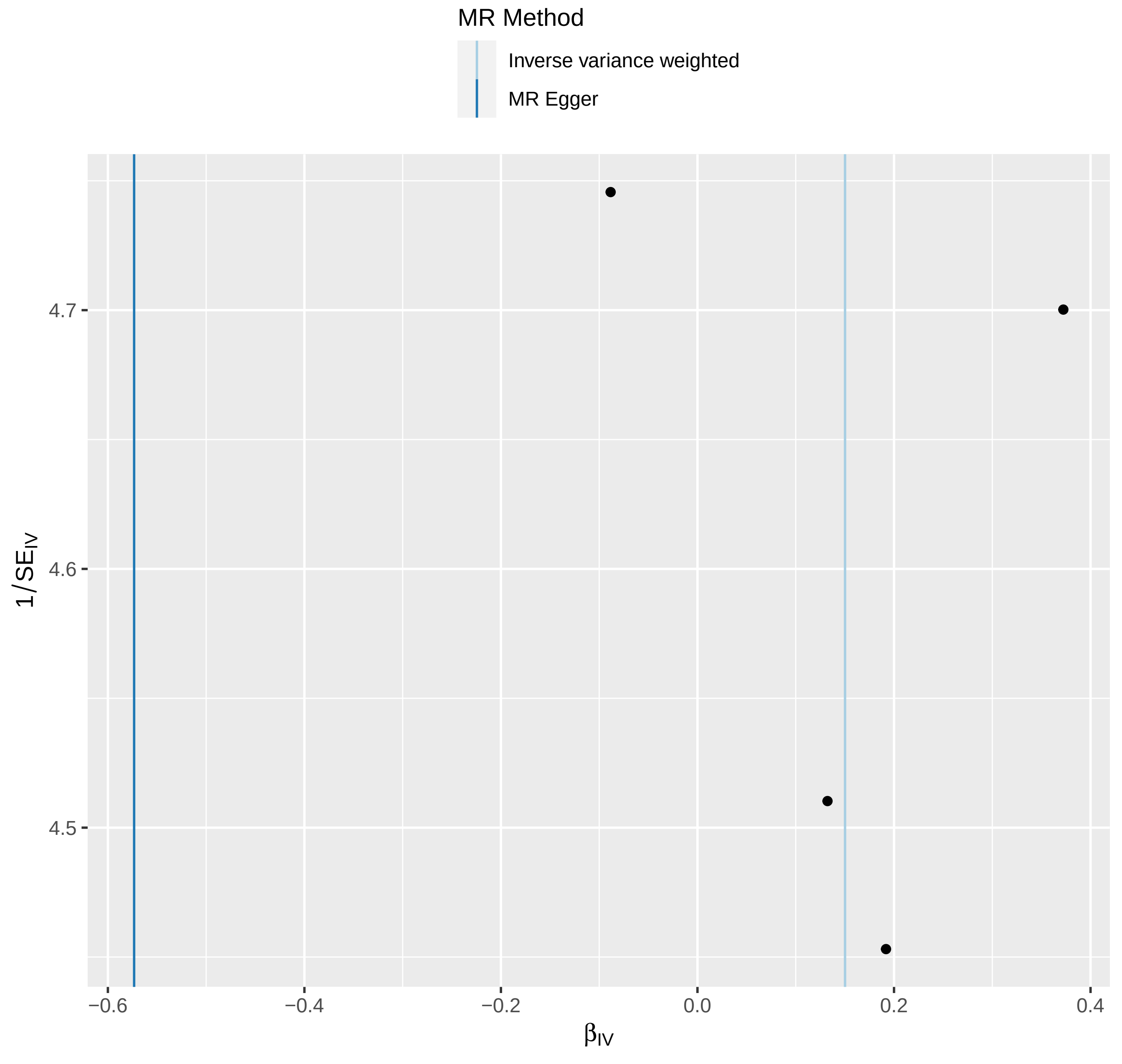

Batch 2−4824 : Gut microbiota abundance (genus Parasutterella id.2892) on Proteasome subunit alpha type−4
with a threshold of 1e−05

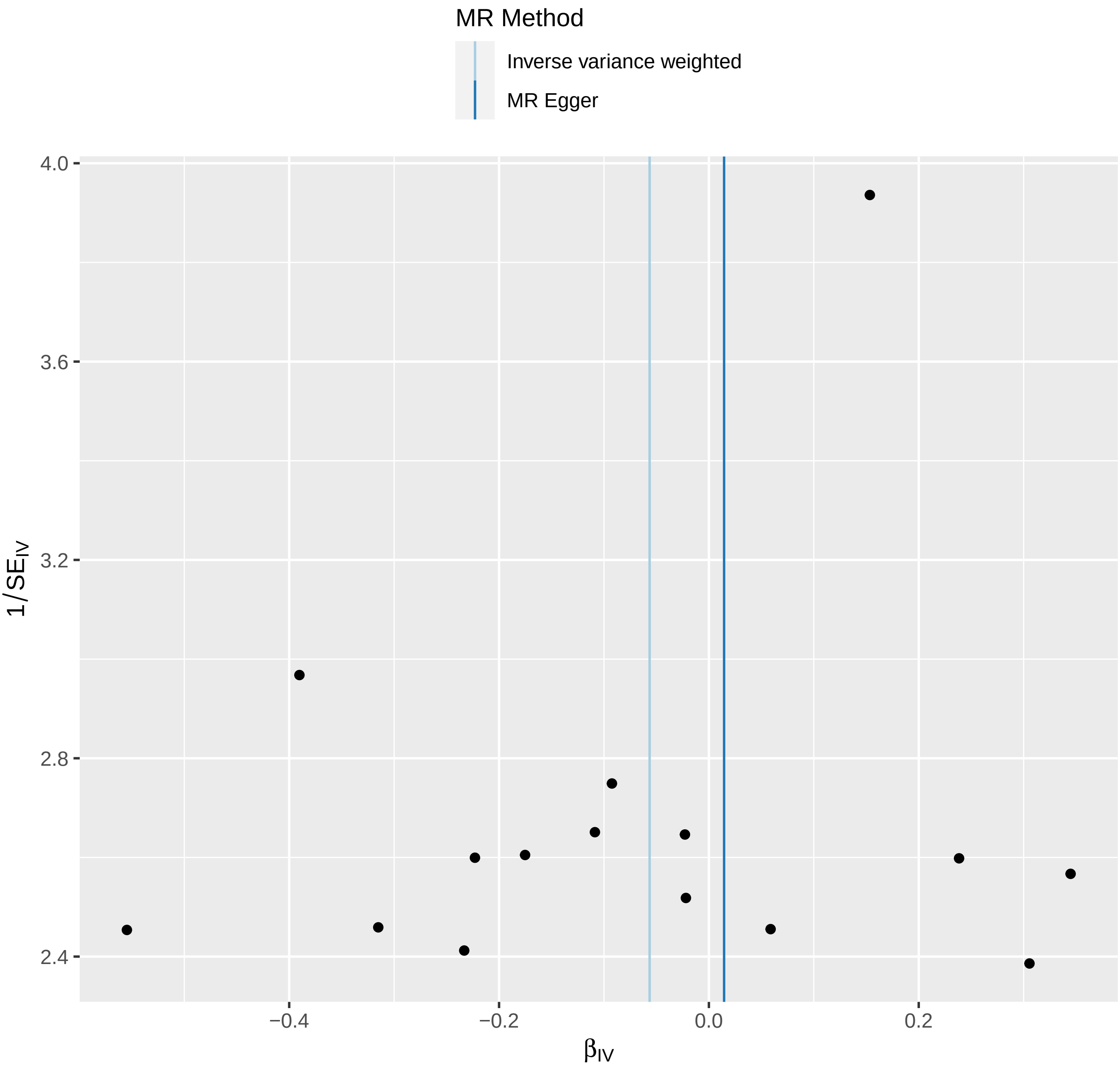

Batch 2−4825 : Gut microbiota abundance (genus Catenibacterium id.2153) on Proteasome subunit alpha type−6
with a threshold of 1e−05

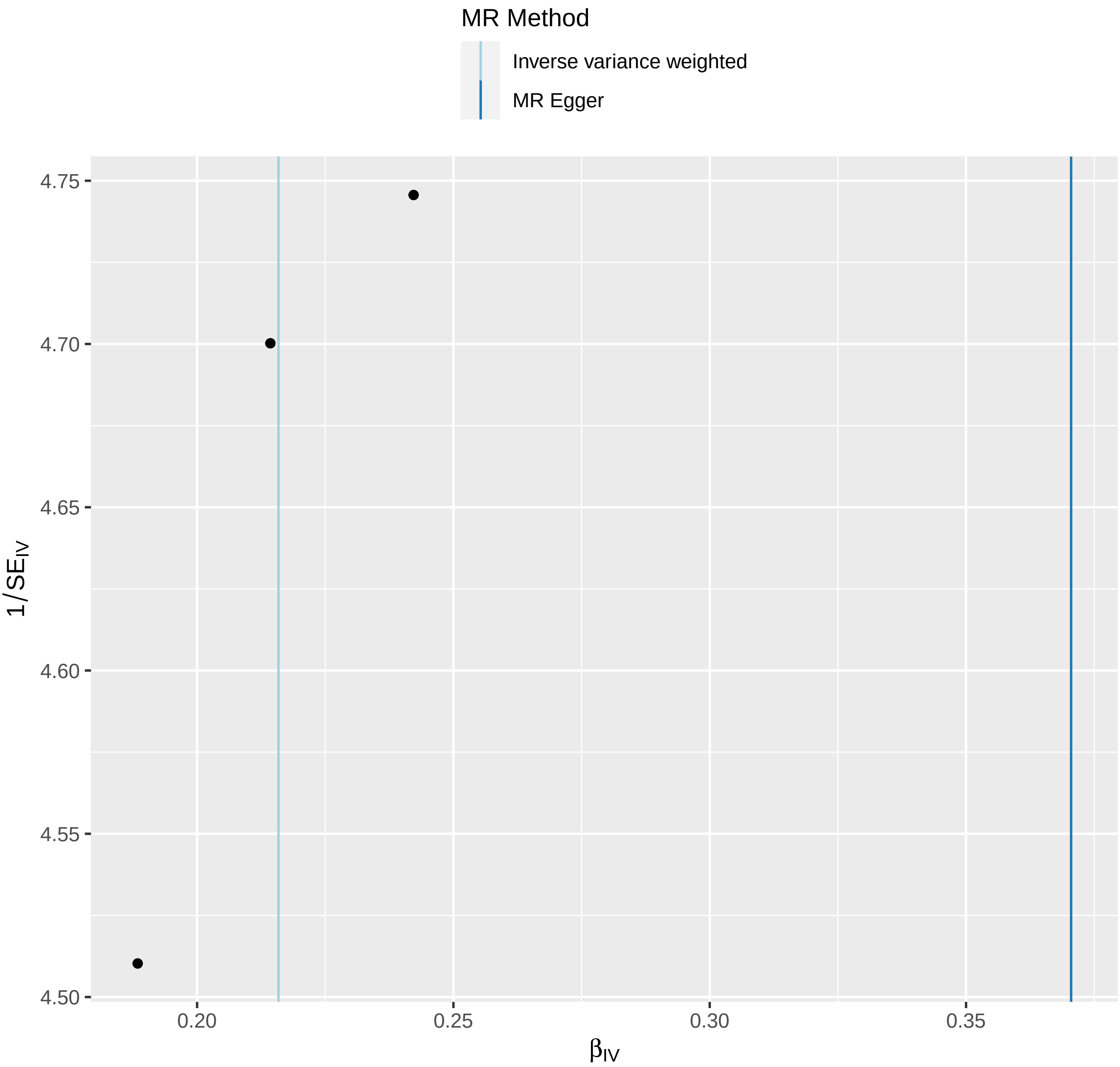

Batch 2−4826 : Gut microbiota abundance (genus Parasutterella id.2892) on Proteasome subunit alpha type−6
with a threshold of 1e−05

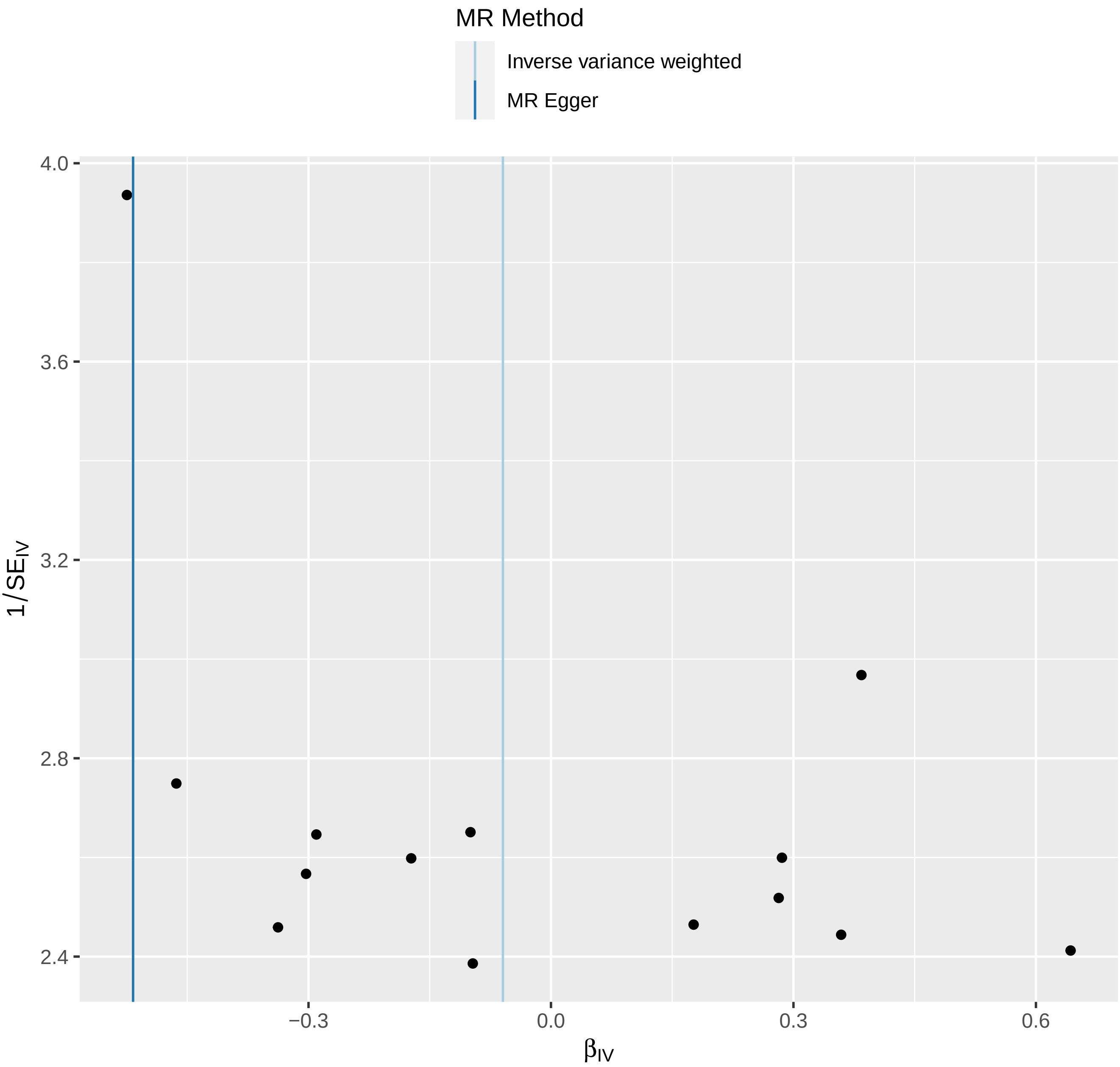

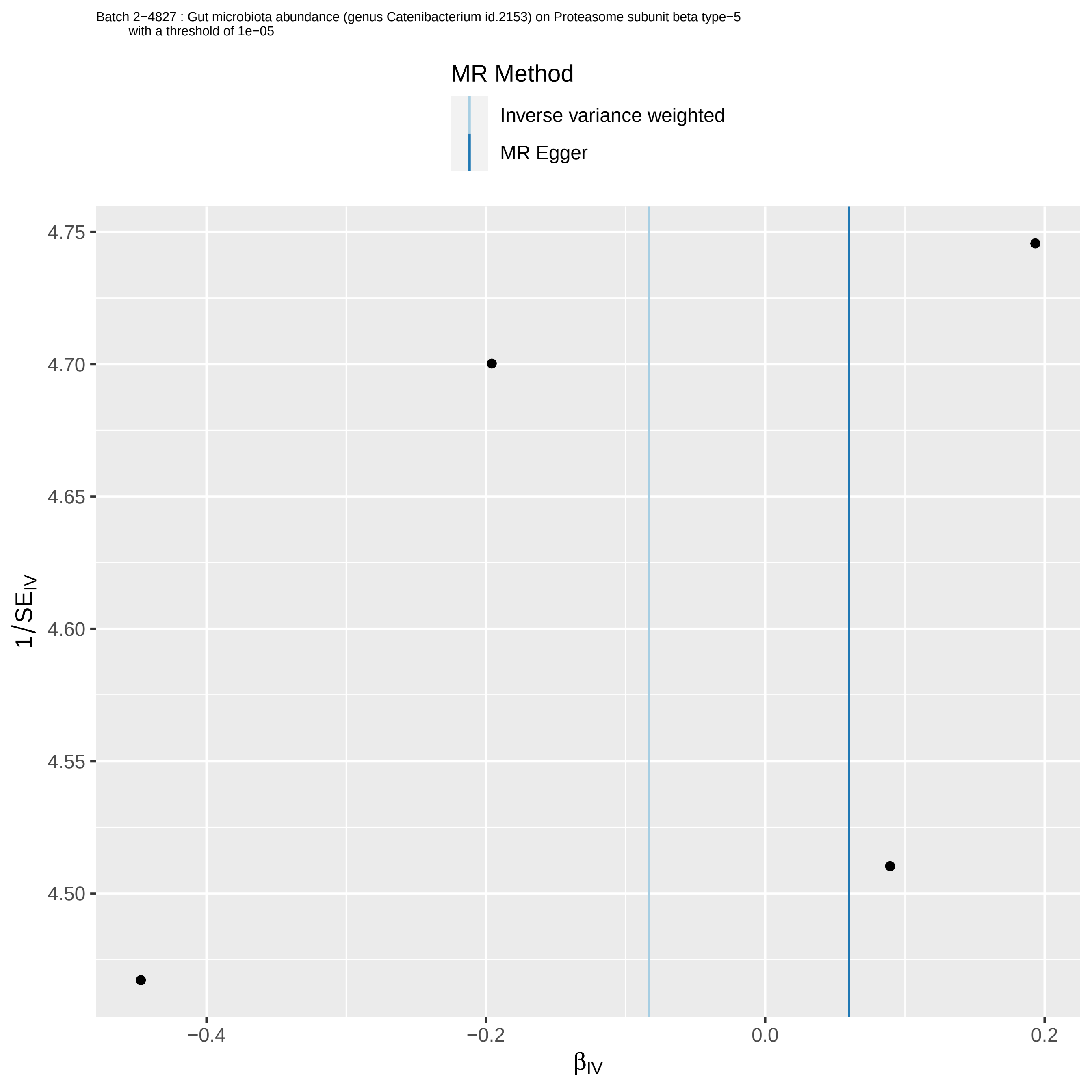

Batch 2−4827 : Gut microbiota abundance (genus Catenibacterium id.2153) on Proteasome subunit beta type−5
with a threshold of 1e−05
MR Method
Inverse variance weighted
MR Egger
4.75
4.70
4.65
4.60
4.55
4.50
1/SE_IV
−0.4
−0.2
0.0
0.2
β_IV

Batch 2−4828 : Gut microbiota abundance (genus Parasutterella id.2892) on Proteasome subunit beta type−5
with a threshold of 1e−05

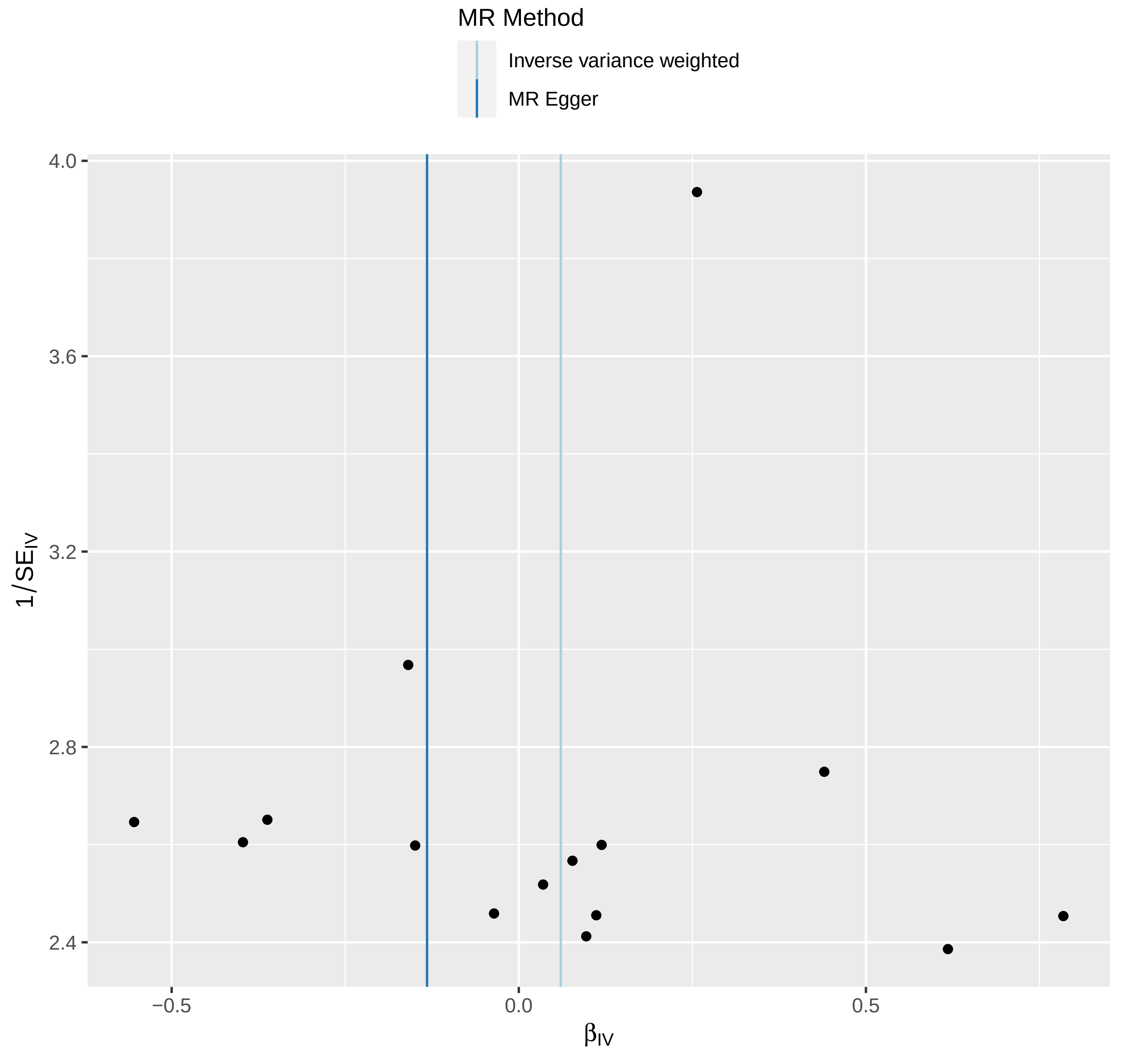

Batch 2−4829 : Gut microbiota abundance (genus Catenibacterium id.2153) on 26S proteasome non−ATPase regulatory subunit 11 with a threshold of 1e−05

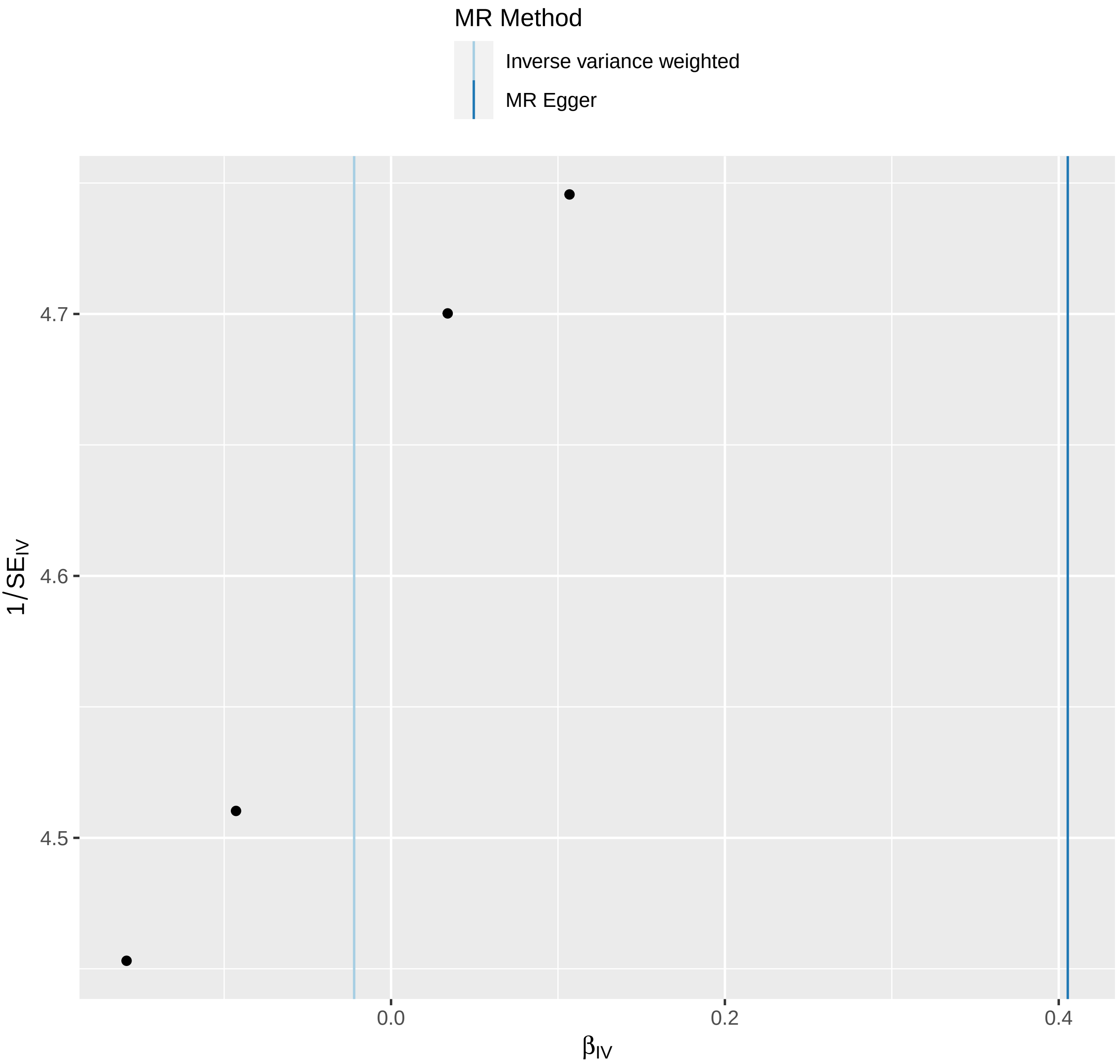

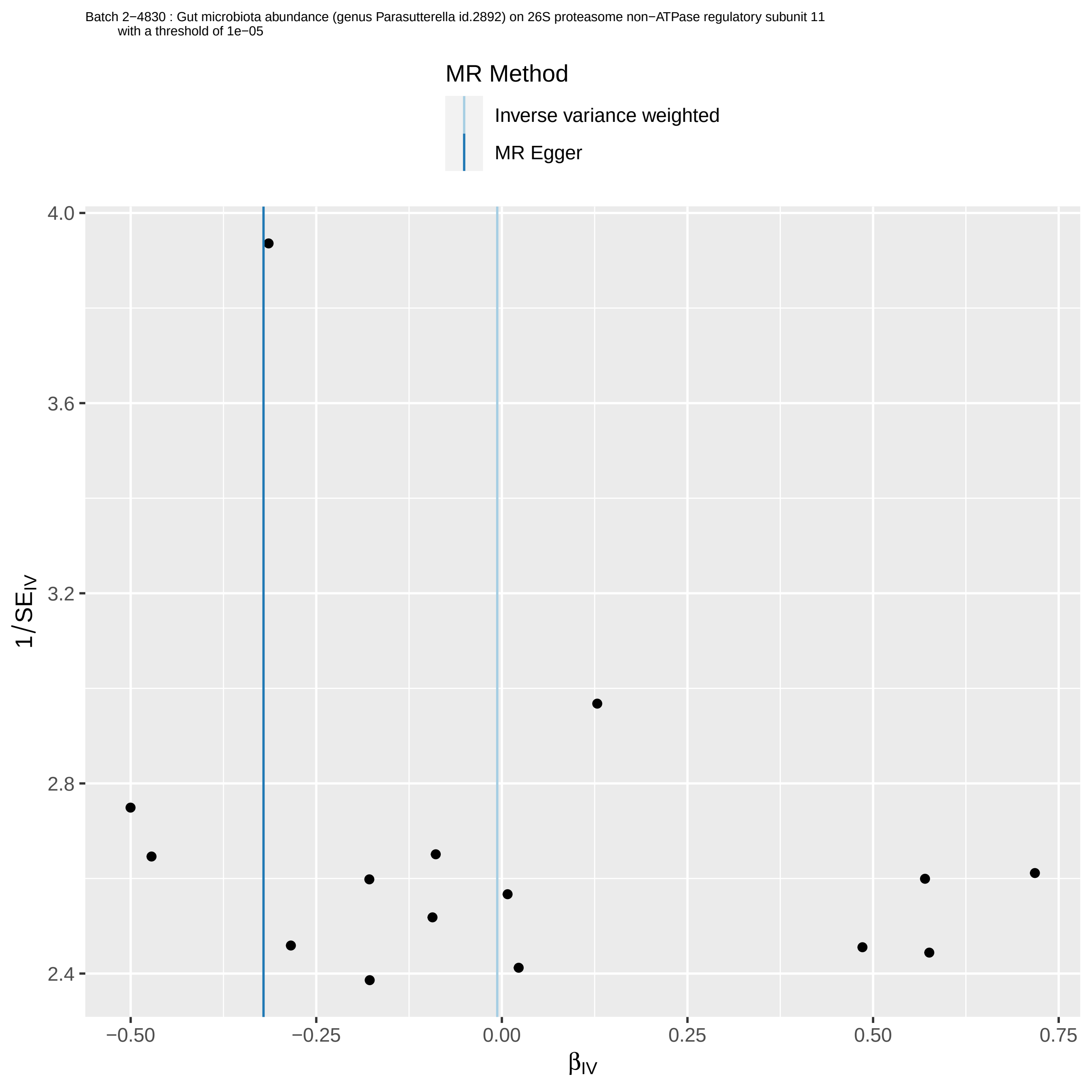
Batch 2−4830 : Gut microbiota abundance (genus Parasutterella id.2892) on 26S proteasome non−ATPase regulatory subunit 11
with a threshold of 1e−05
MR Method
Inverse variance weighted
MR Egger
4.0
3.6
3.2
2.8
2.4
1/SE$_{IV}$
−0.50
−0.25
0.00
0.25
0.50
0.75
β$_{IV}$

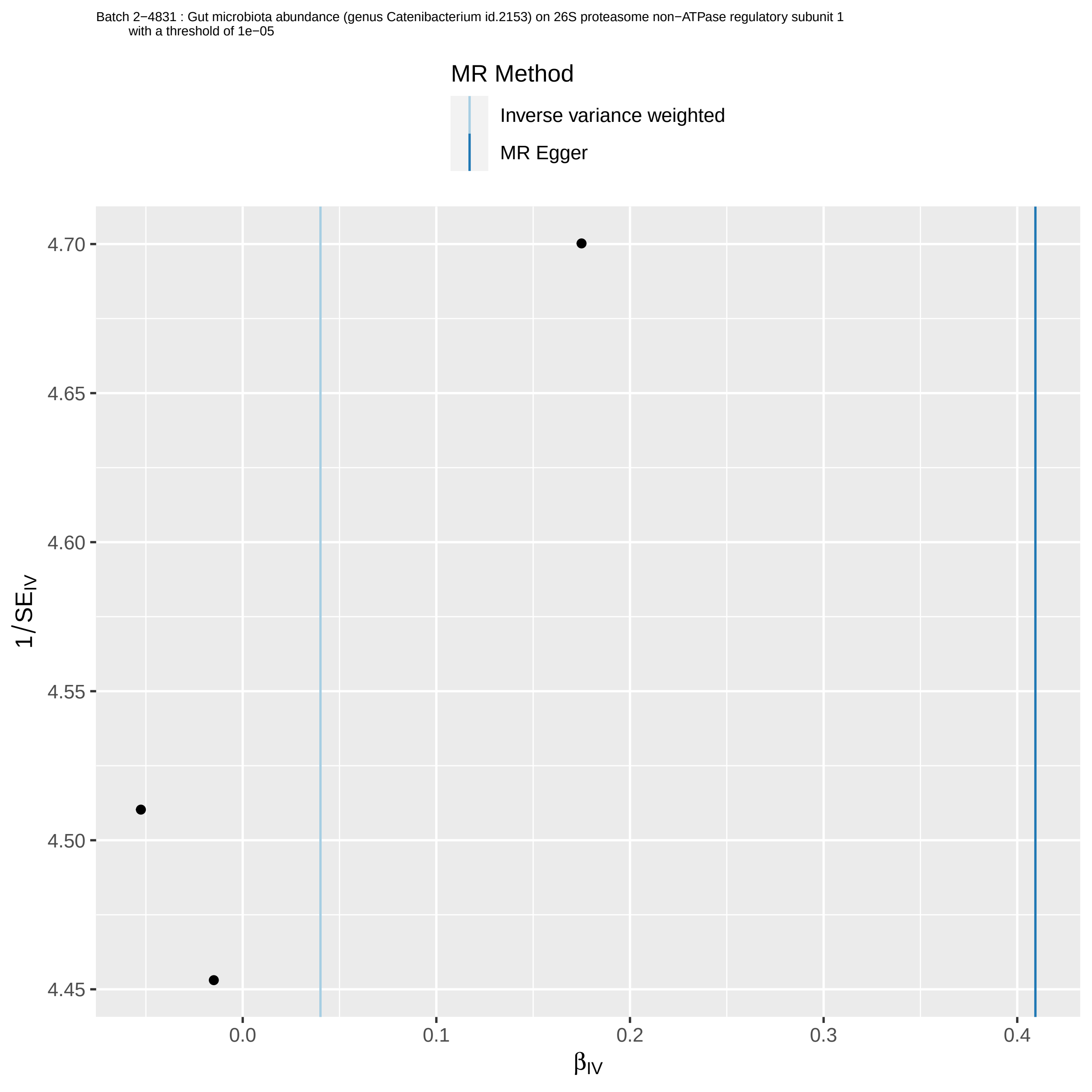

Batch 2−4831 : Gut microbiota abundance (genus Catenibacterium id.2153) on 26S proteasome non−ATPase regulatory subunit 1
with a threshold of 1e−05
MR Method
Inverse variance weighted
MR Egger
4.70
4.65
4.60
4.55
4.50
4.45
1/SE_IV
0.0
0.1
0.2
0.3
0.4
β_IV

Batch 2−4832 : Gut microbiota abundance (genus Parasutterella id.2892) on 26S proteasome non−ATPase regulatory subunit 1
with a threshold of 1e−05

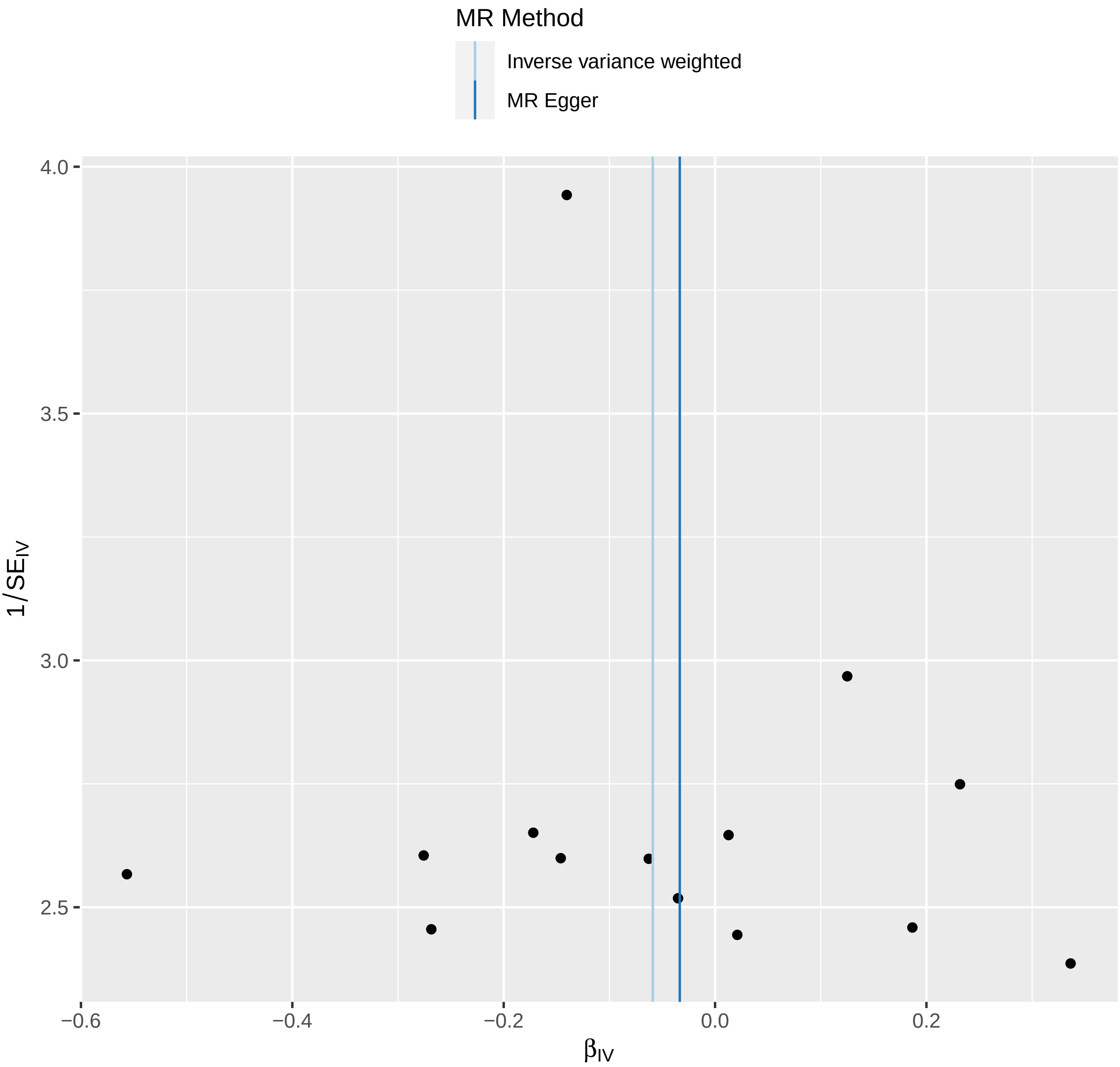

Batch 2−4833 : Gut microbiota abundance (genus Catenibacterium id.2153) on 26S proteasome non−ATPase regulatory subunit 4
with a threshold of 1e−05

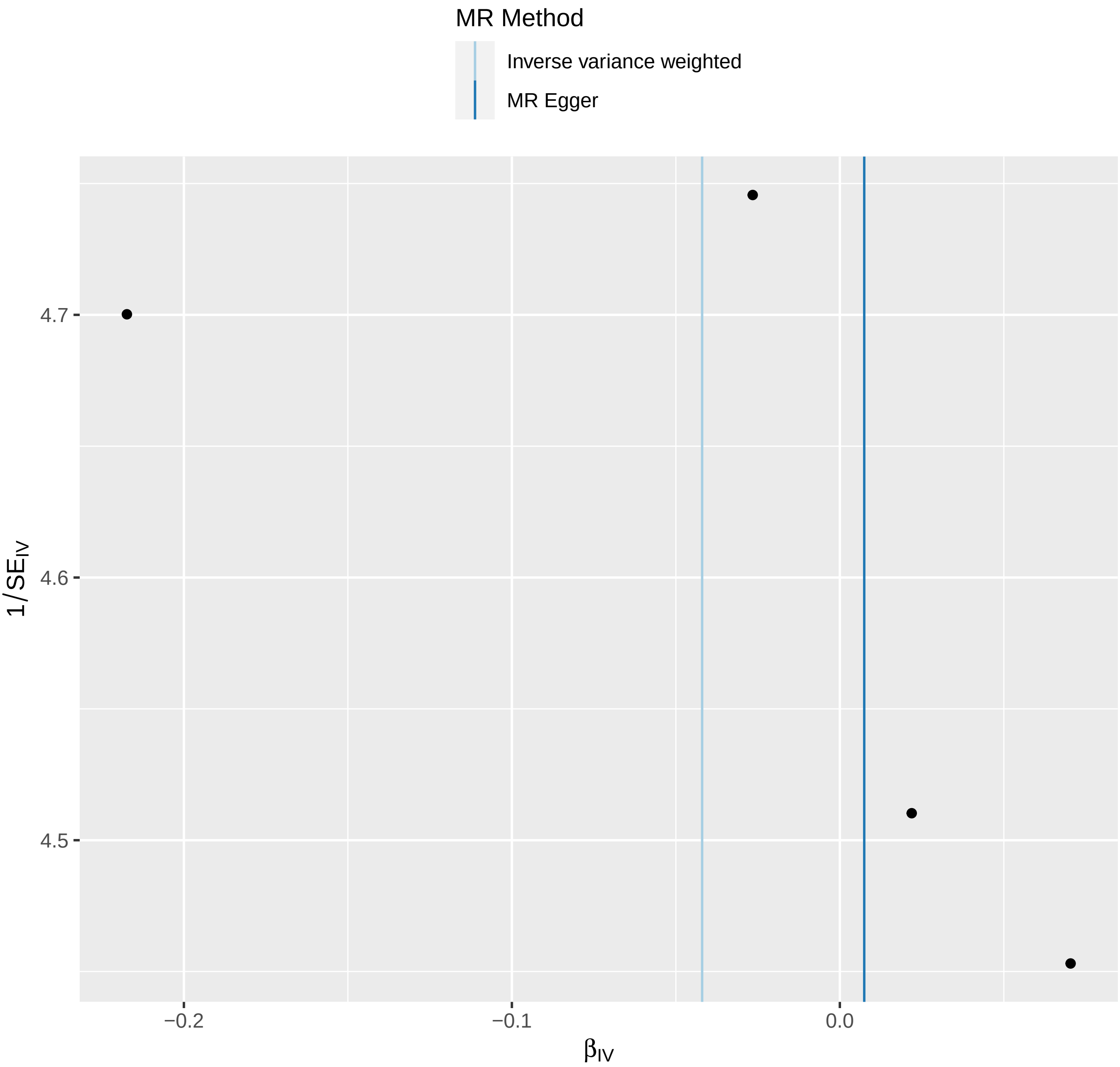

Batch 2−4834 : Gut microbiota abundance (genus Parasutterella id.2892) on 26S proteasome non−ATPase regulatory subunit 4
with a threshold of 1e−05

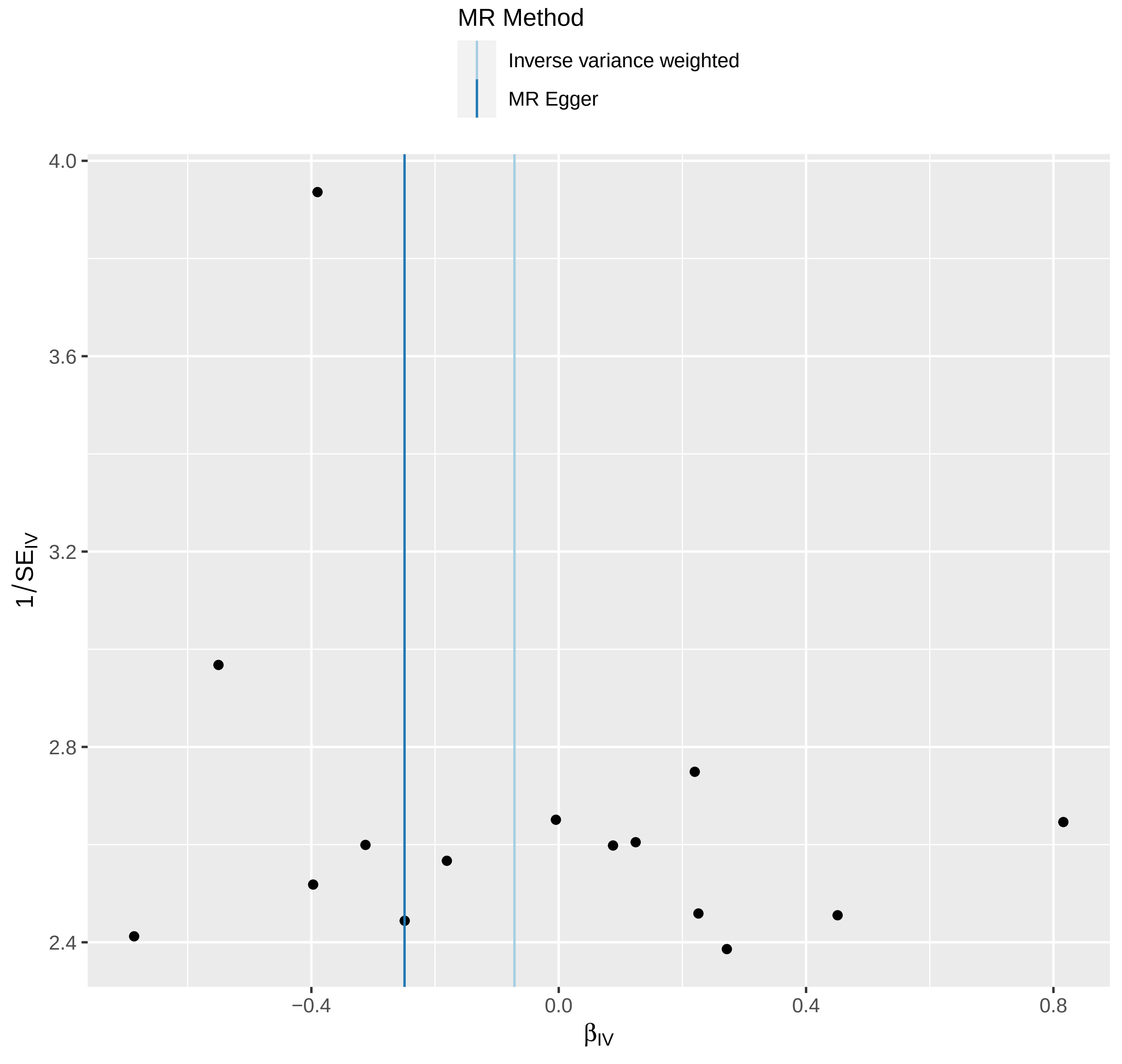

Batch 2−4835 : Gut microbiota abundance (genus Catenibacterium id.2153) on 26S proteasome non−ATPase regulatory subunit 5 with a threshold of 1e−05

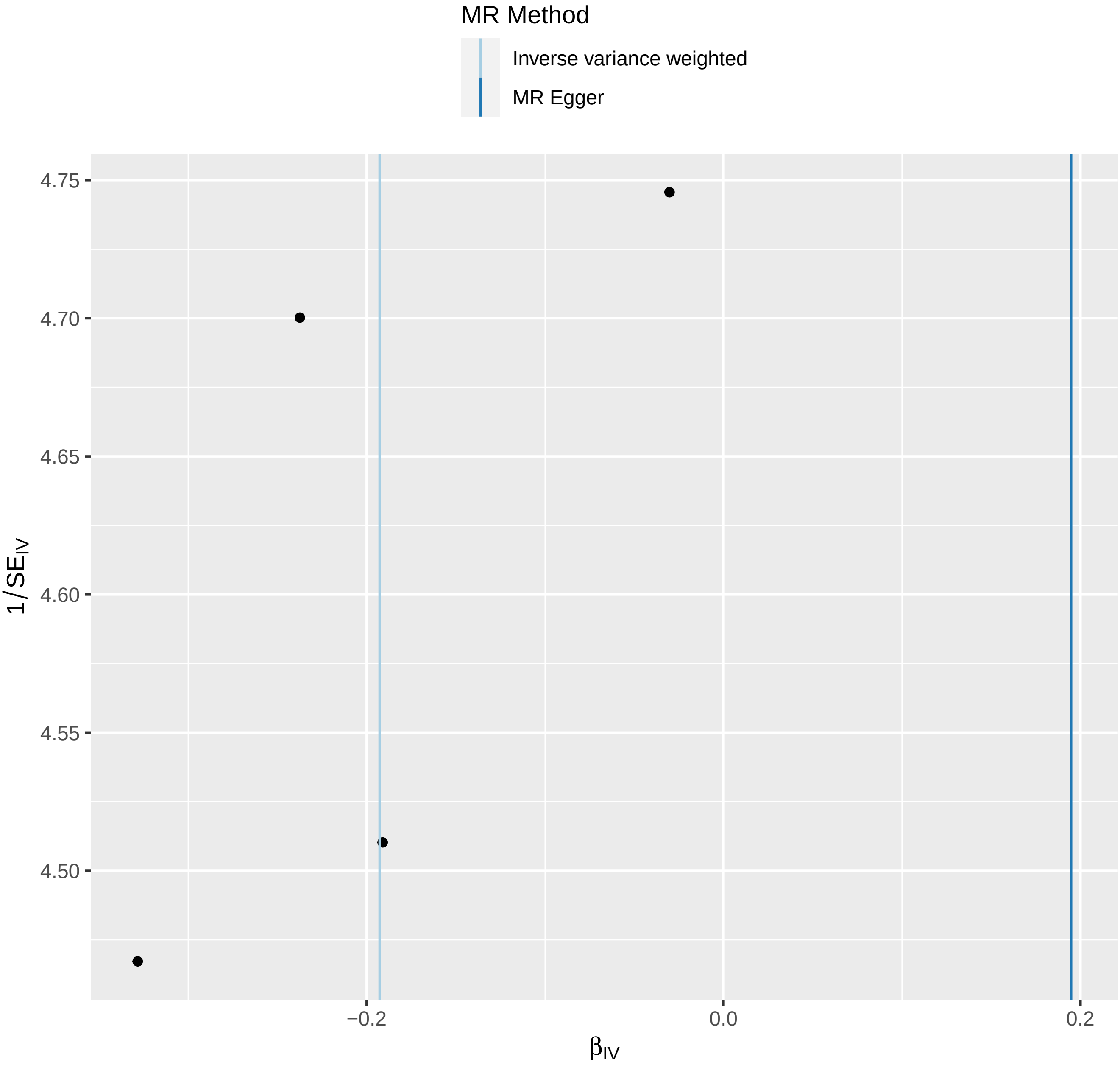

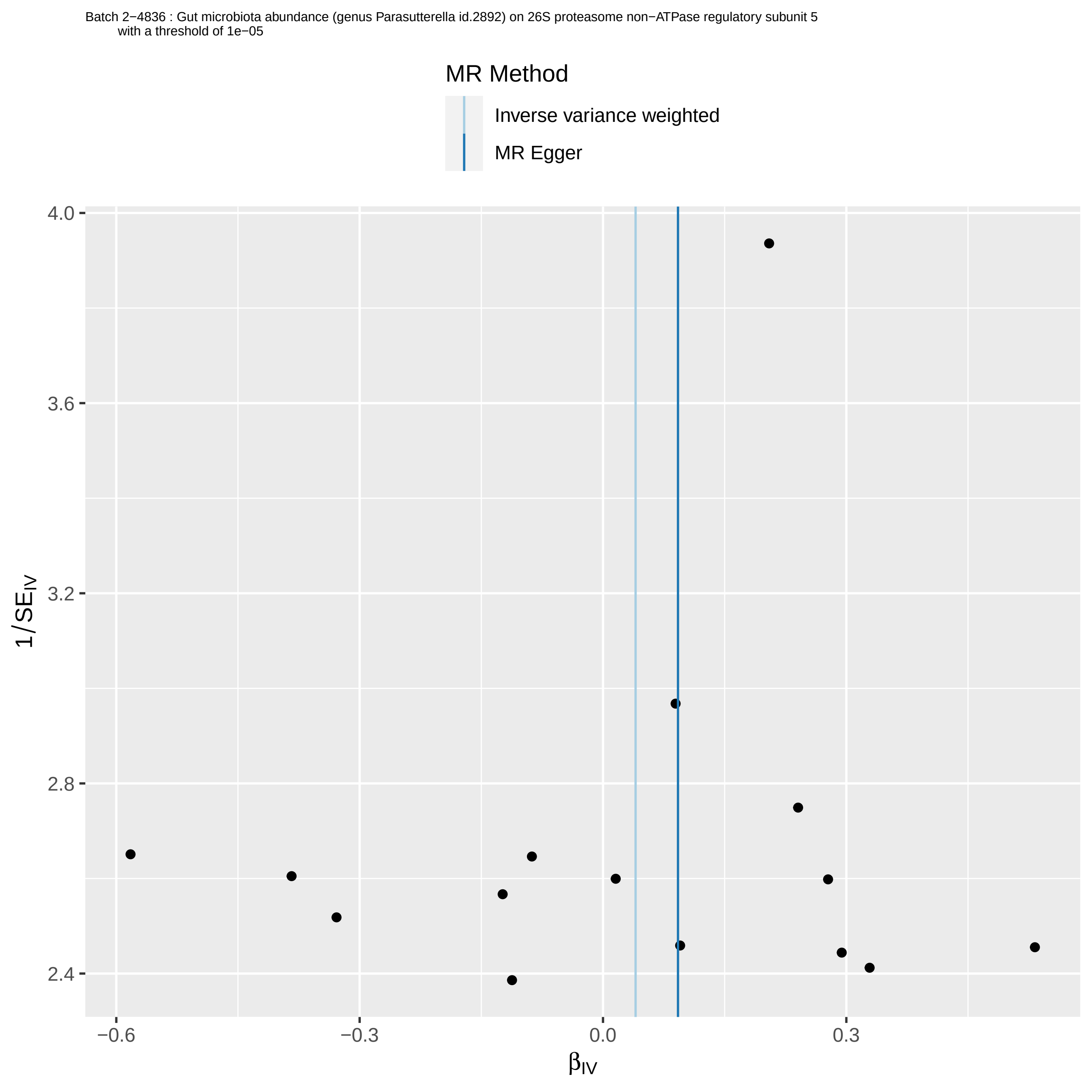

Batch 2−4836 : Gut microbiota abundance (genus Parasutterella id.2892) on 26S proteasome non−ATPase regulatory subunit 5
with a threshold of 1e−05
MR Method
Inverse variance weighted
MR Egger
4.0
3.6
3.2
2.8
2.4
1/SE_IV
−0.6
−0.3
0.0
0.3
β_IV

Batch 2−4837 : Gut microbiota abundance (genus Catenibacterium id.2153) on 26S proteasome non−ATPase regulatory subunit 7
with a threshold of 1e−05

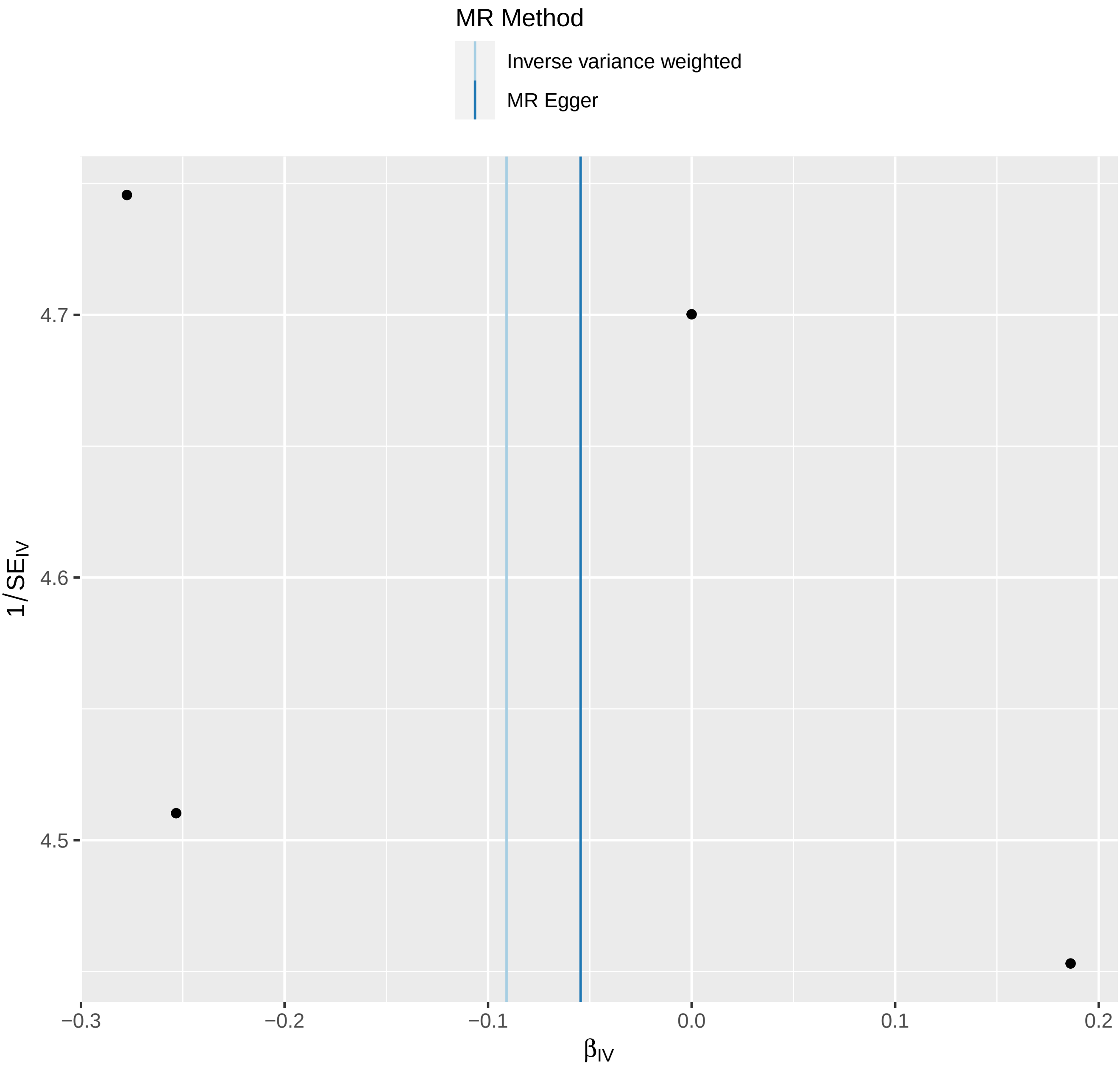

Batch 2−4838 : Gut microbiota abundance (genus Parasutterella id.2892) on 26S proteasome non−ATPase regulatory subunit 7 with a threshold of 1e−05

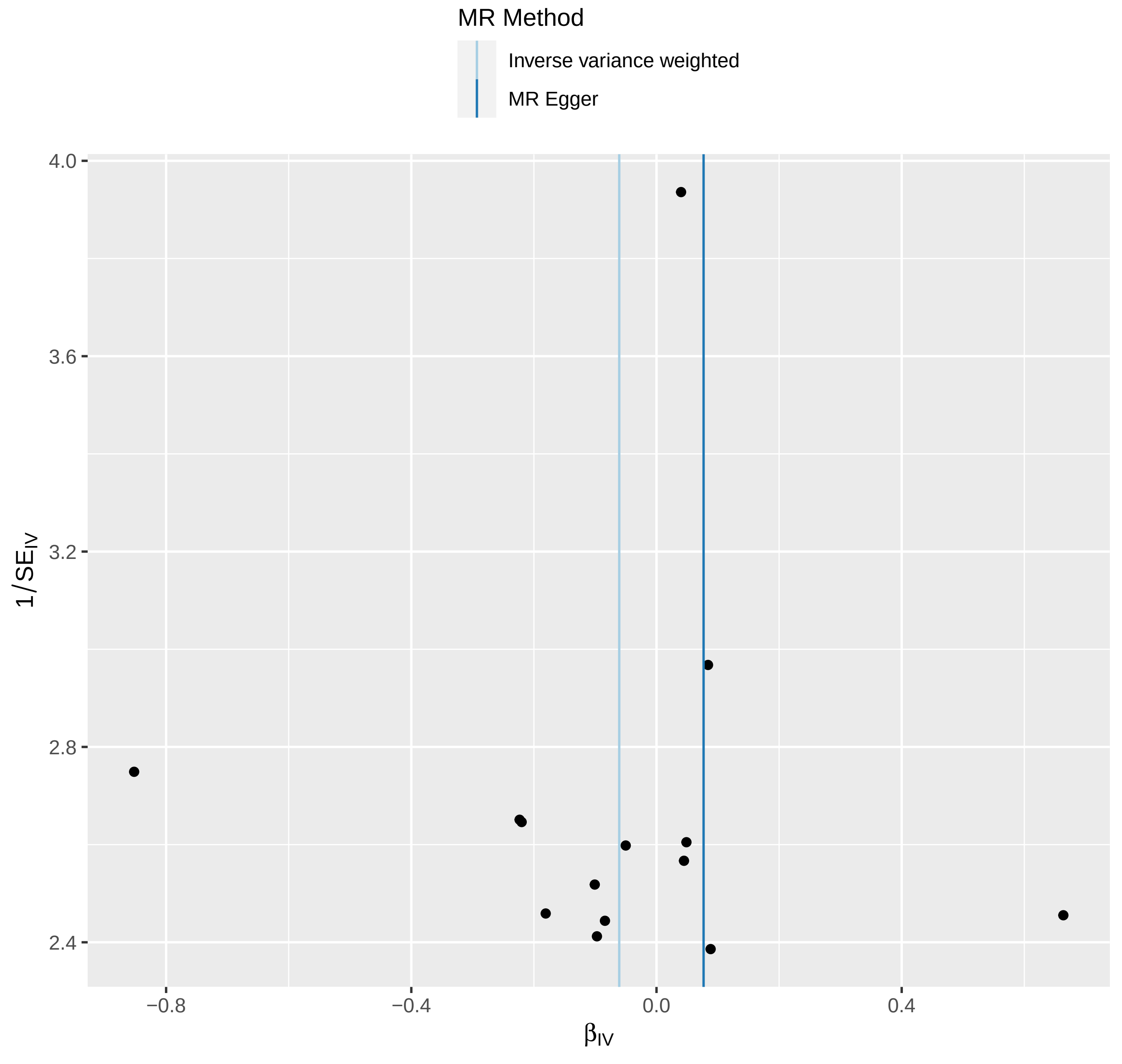

Batch 2−4839 : Gut microbiota abundance (genus Catenibacterium id.2153) on Proteasome activator complex subunit 1
with a threshold of 1e−05

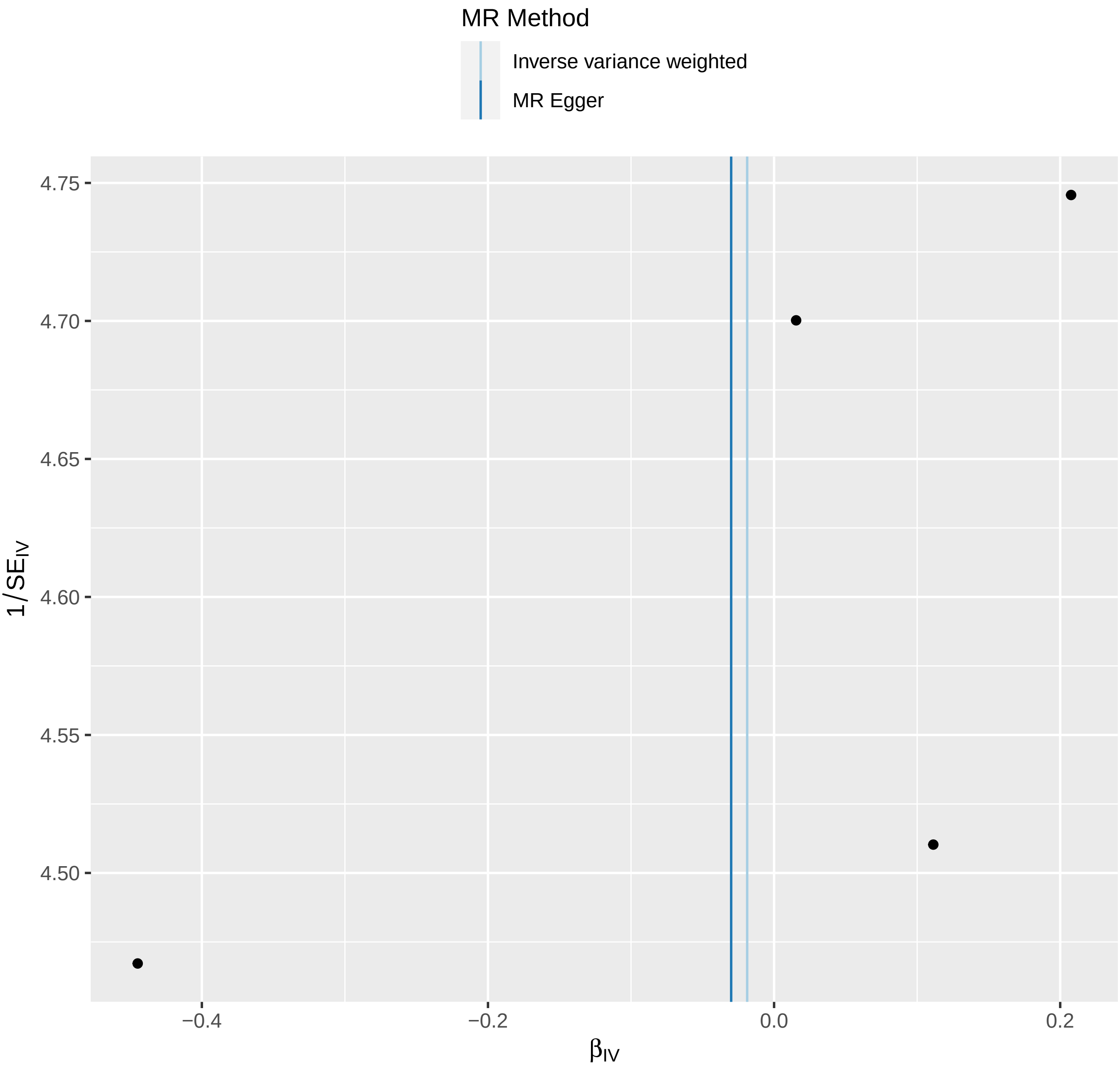

Batch 2−4840 : Gut microbiota abundance (genus Parasutterella id.2892) on Proteasome activator complex subunit 1
with a threshold of 1e−05

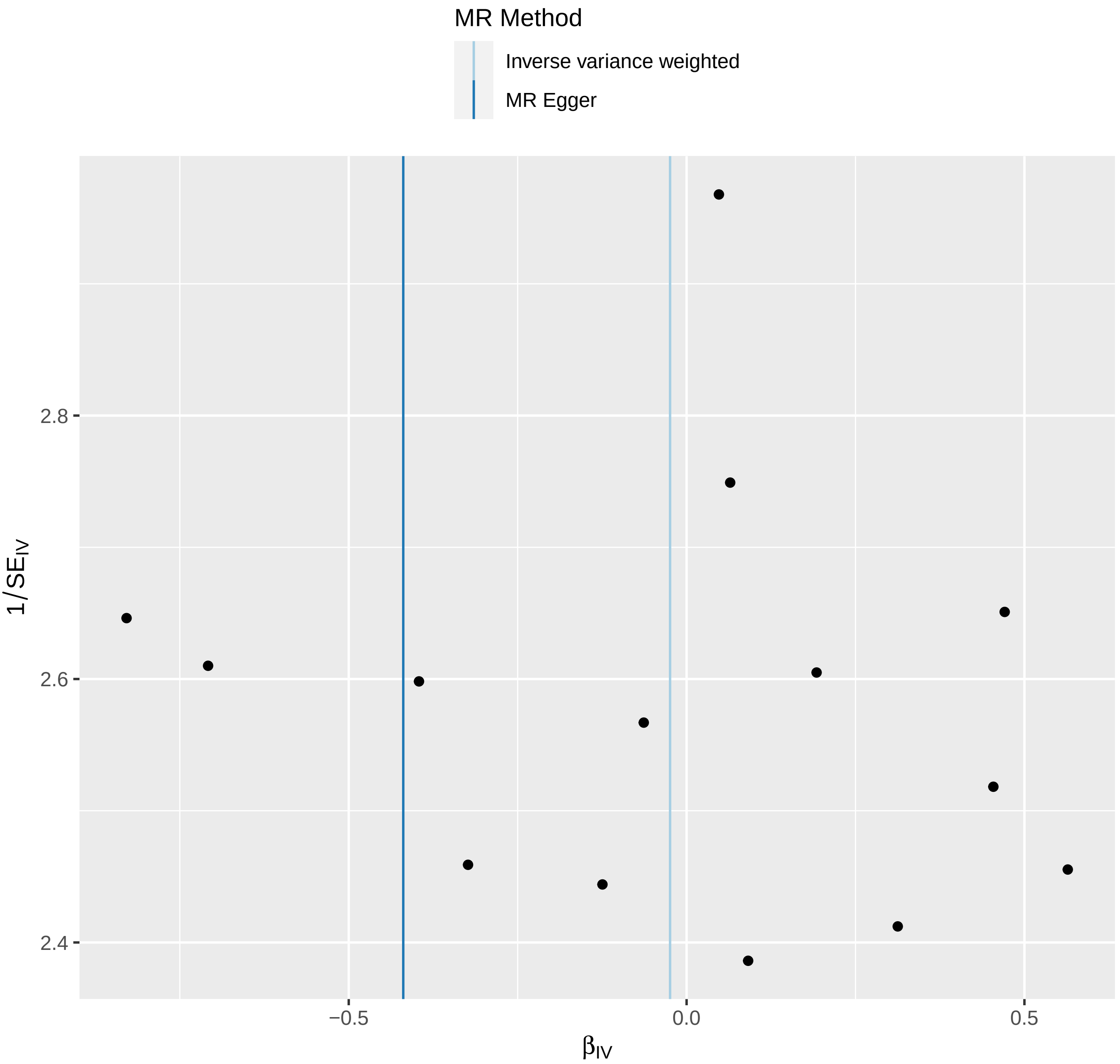

Batch 2−4841 : Gut microbiota abundance (genus Catenibacterium id.2153) on Proteasome activator complex subunit 3
with a threshold of 1e−05

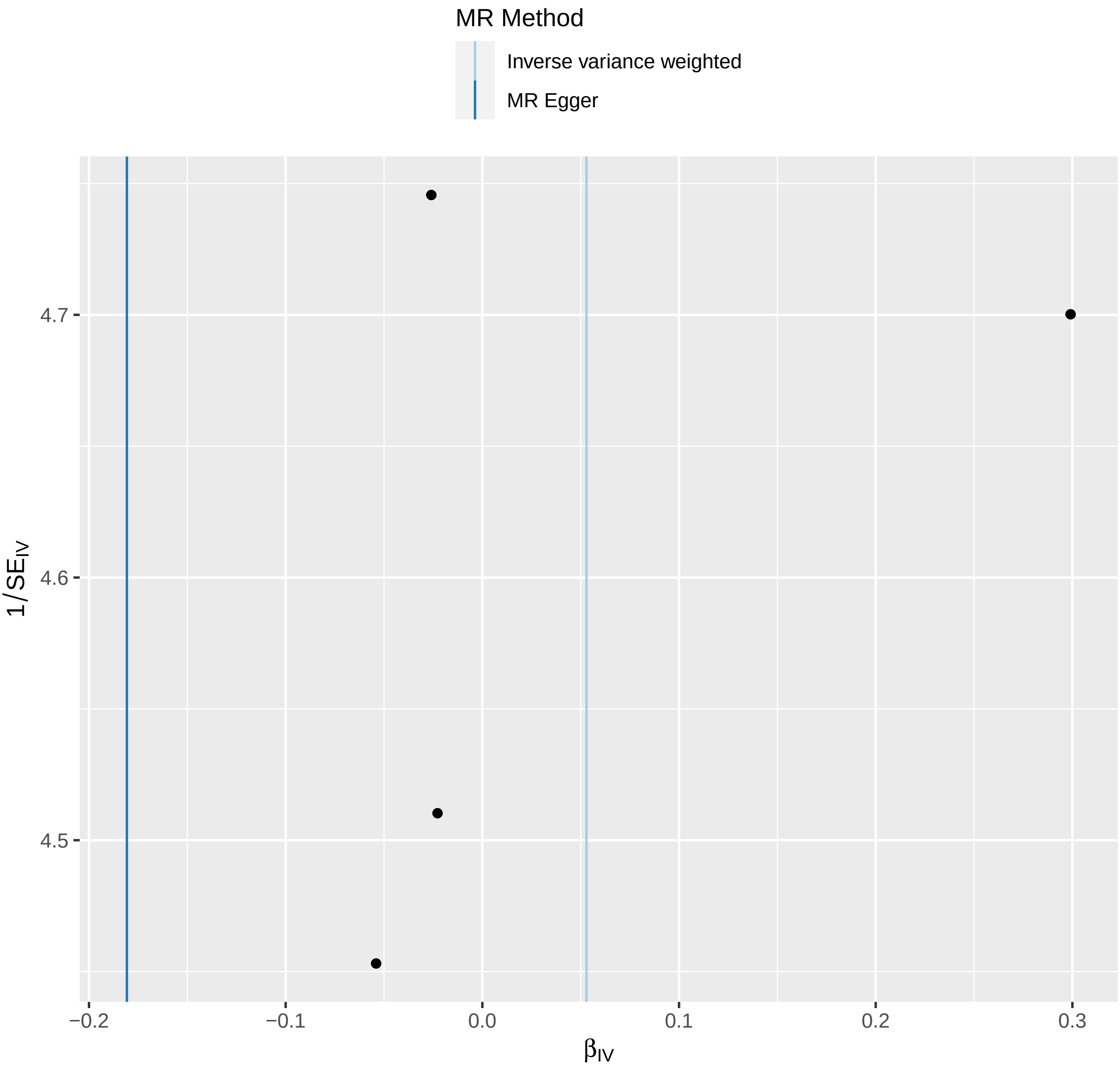

Batch 2−4842 : Gut microbiota abundance (genus Parasutterella id.2892) on Proteasome activator complex subunit 3 with a threshold of 1e−05

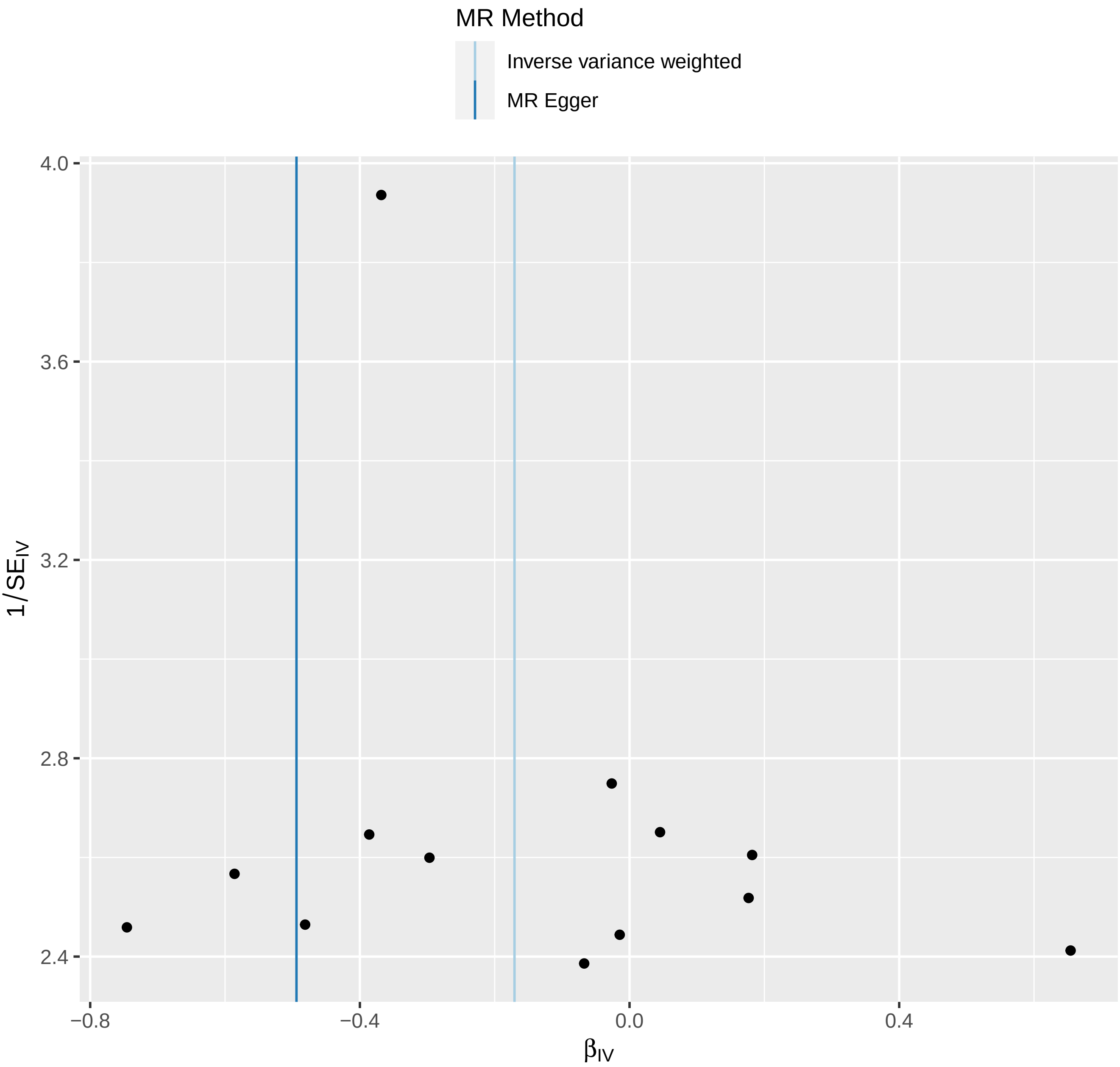

Batch 2−4843 : Gut microbiota abundance (genus Catenibacterium id.2153) on Proteasome assembly chaperone 3
with a threshold of 1e−05

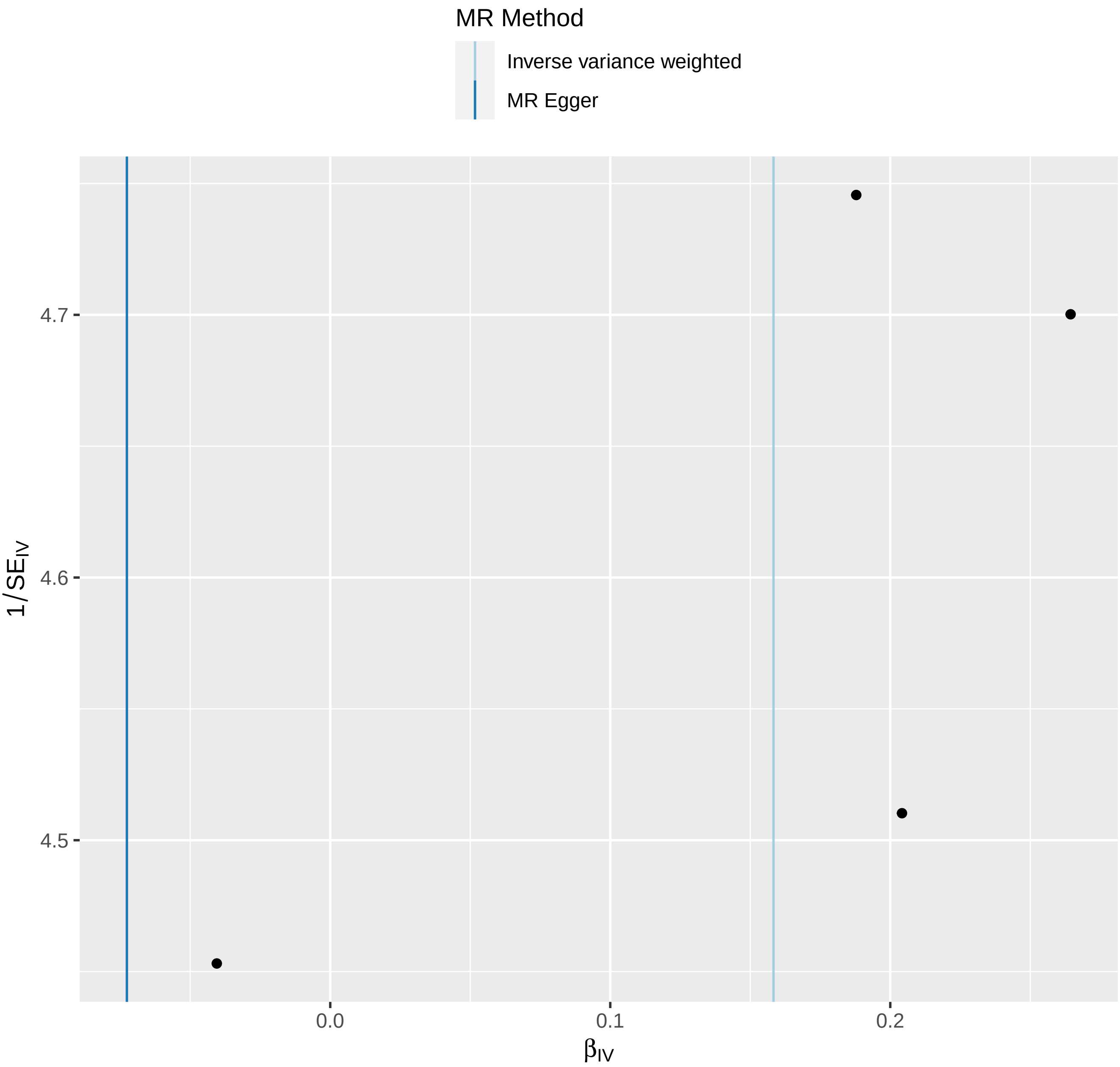

Batch 2−4844 : Gut microbiota abundance (genus Parasutterella id.2892) on Proteasome assembly chaperone 3
with a threshold of 1e−05

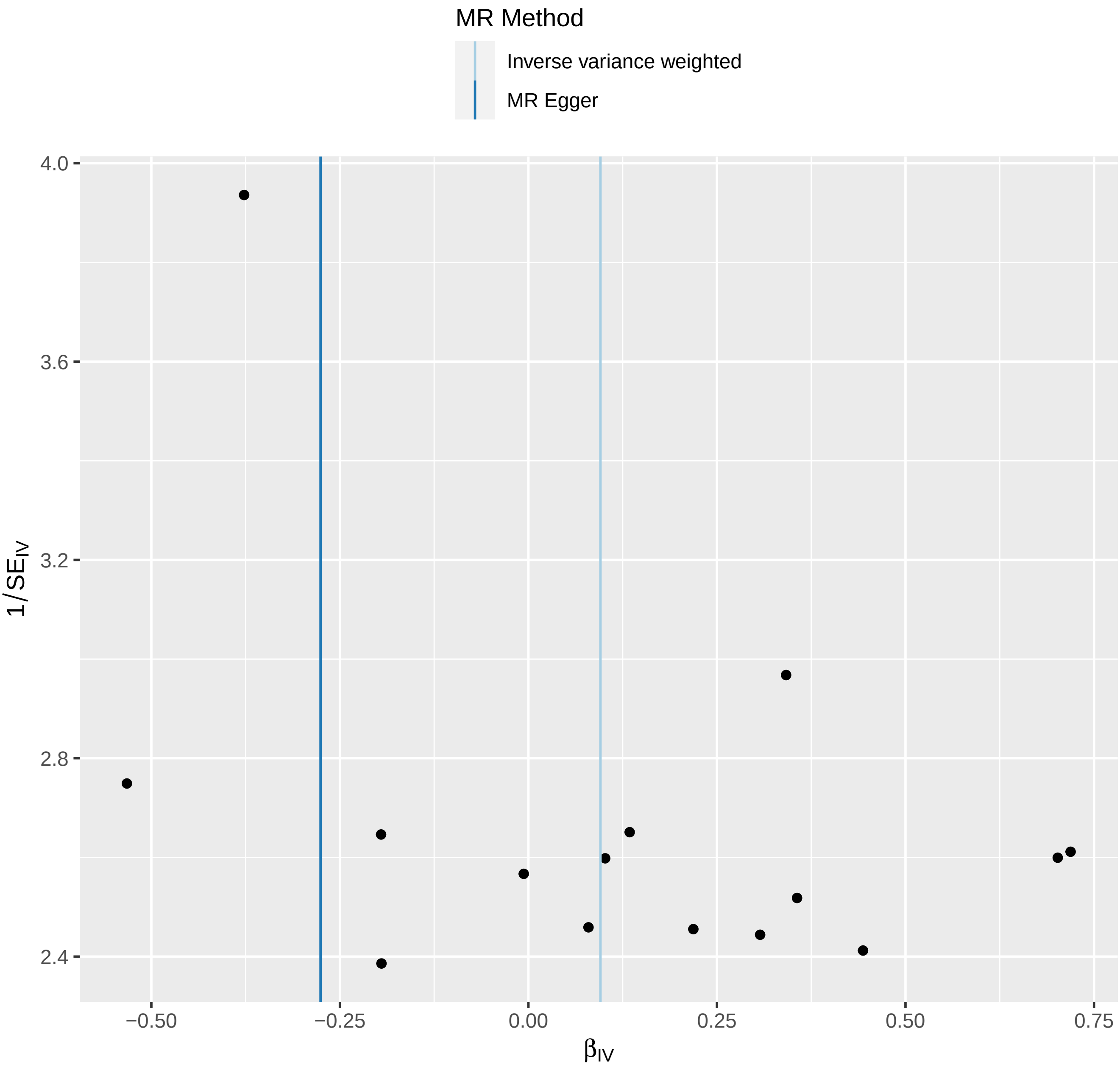

Batch 2−4845 : Gut microbiota abundance (genus Catenibacterium id.2153) on Persephin
with a threshold of 1e−05

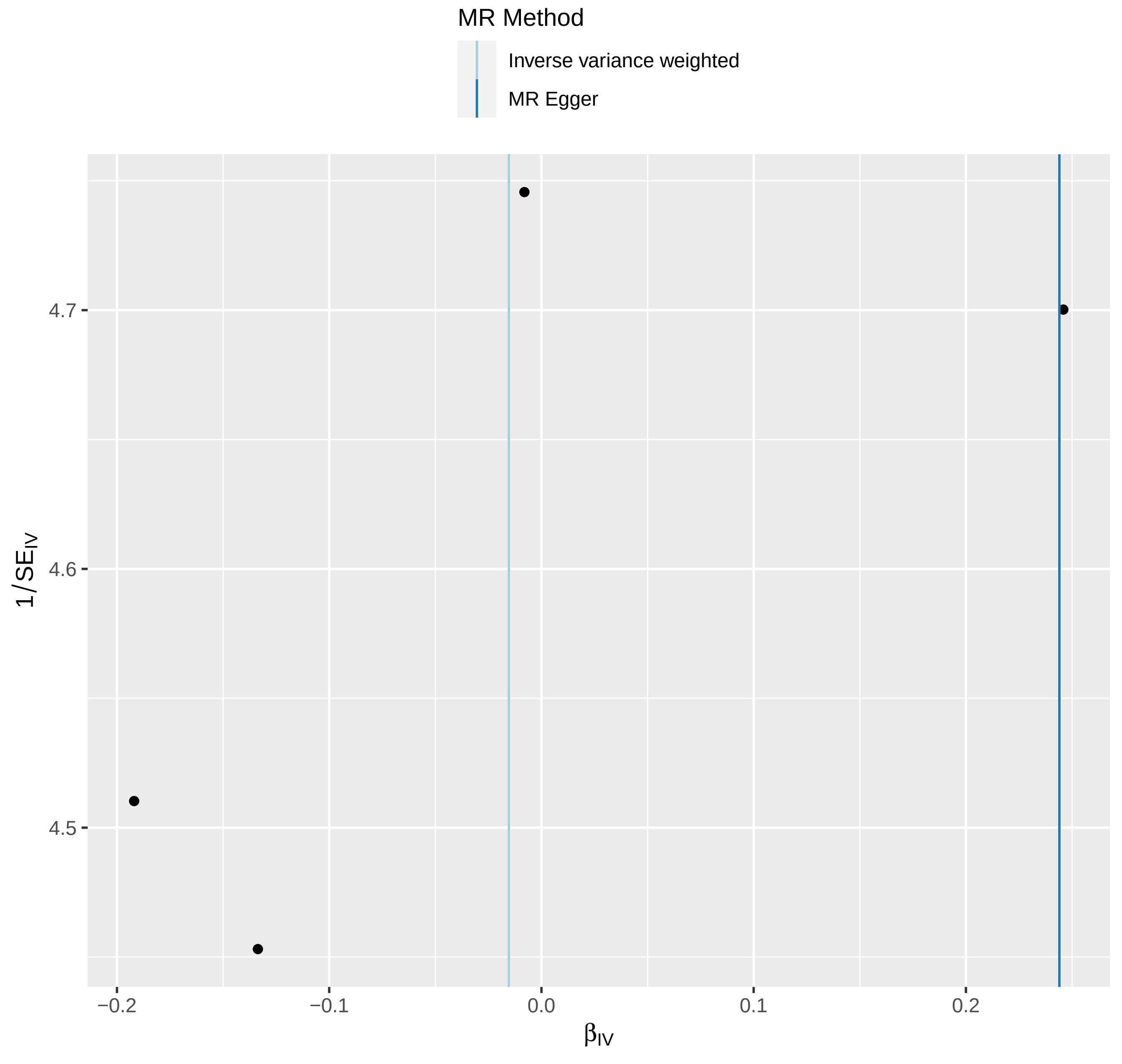

Batch 2−4846 : Gut microbiota abundance (genus Parasutterella id.2892) on Persephin
with a threshold of 1e−05

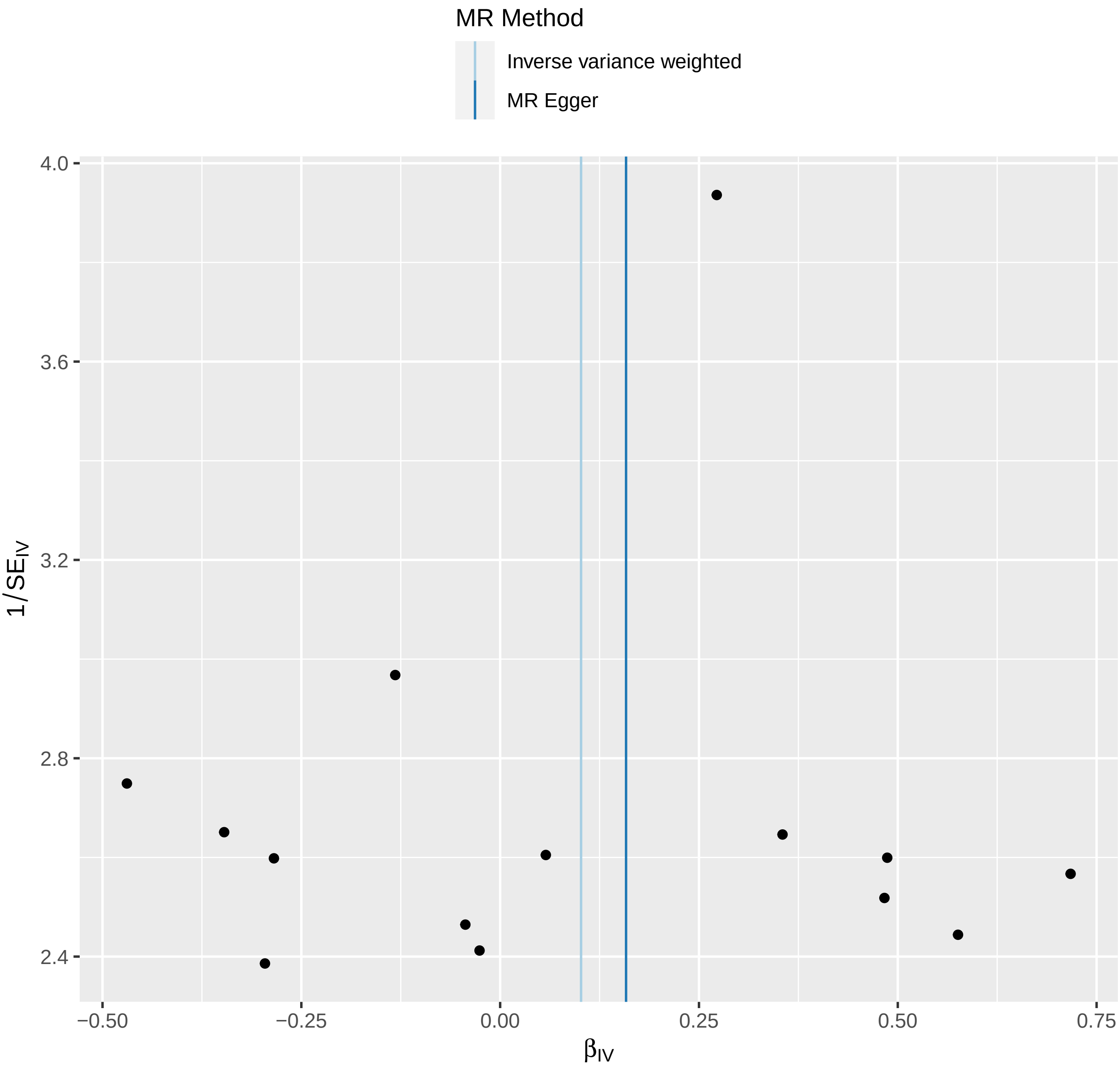

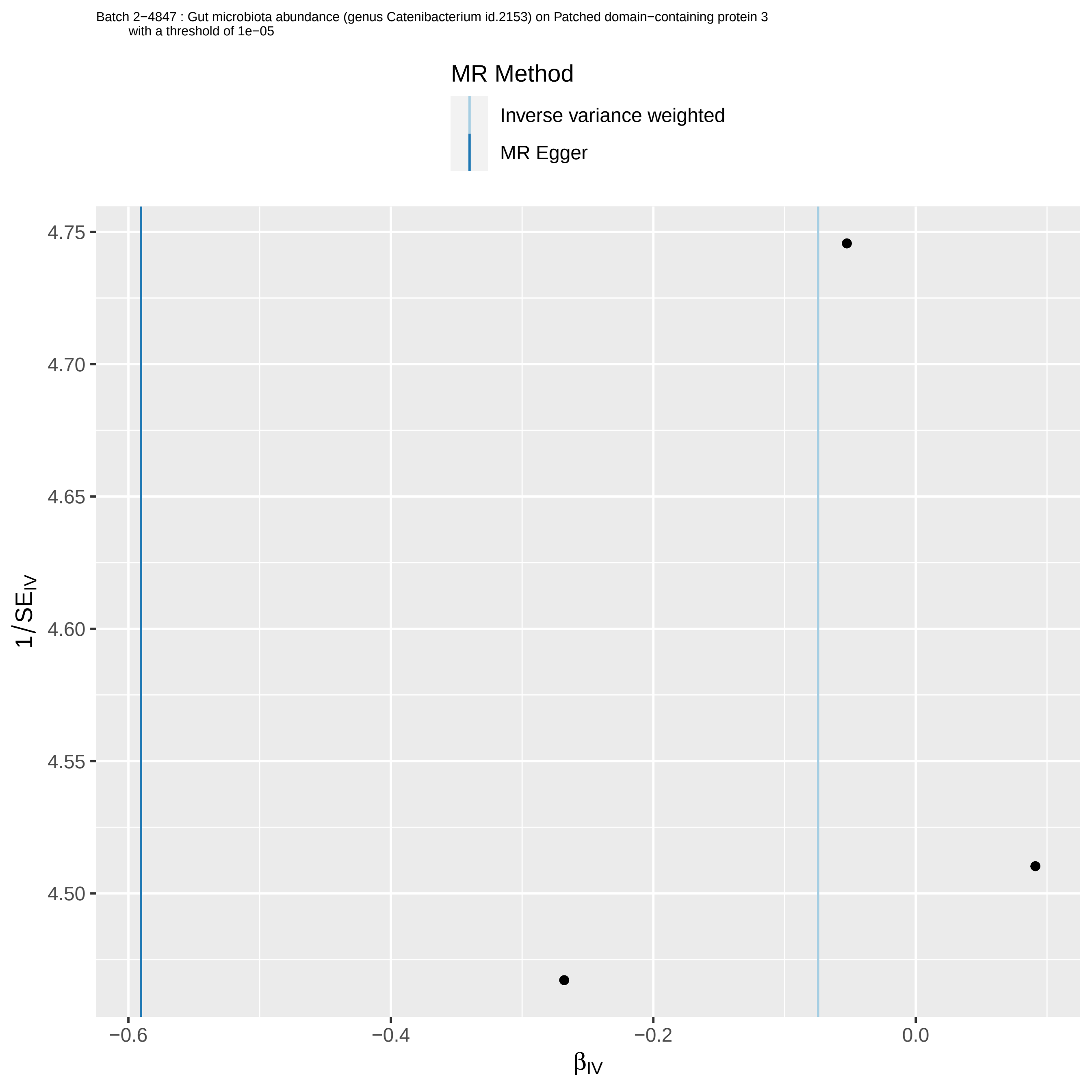

Batch 2−4847 : Gut microbiota abundance (genus Catenibacterium id.2153) on Patched domain−containing protein 3
with a threshold of 1e−05
MR Method
Inverse variance weighted
MR Egger
4.75
4.70
4.65
4.60
4.55
4.50
$1/SE_{IV}$
−0.6
−0.4
−0.2
0.0
β_{IV}

Batch 2−4848 : Gut microbiota abundance (genus Parasutterella id.2892) on Patched domain−containing protein 3
with a threshold of 1e−05

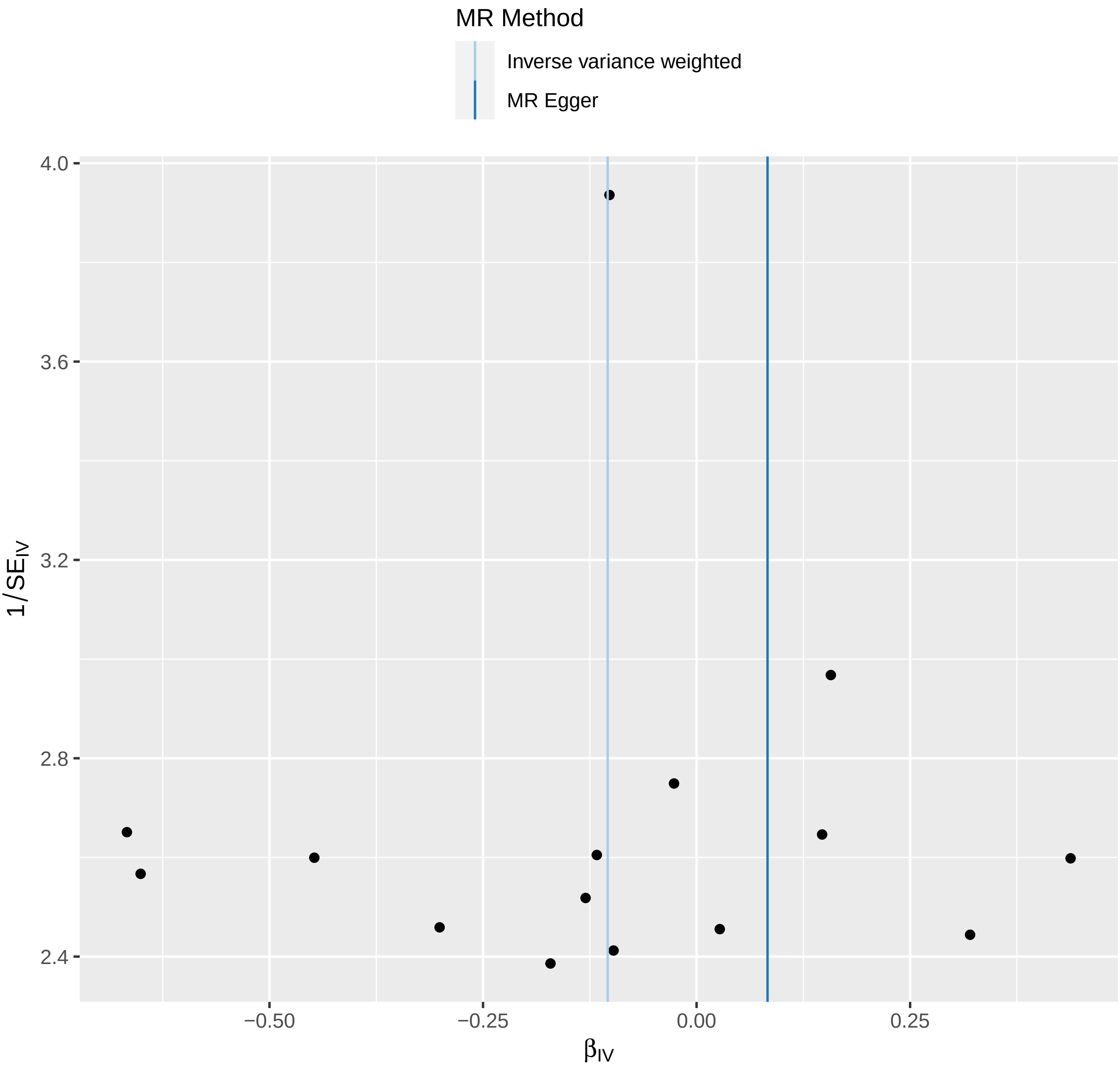

Batch 2−4849 : Gut microbiota abundance (genus Catenibacterium id.2153) on Phosphatidylinositol 3,4,5−trisphosphate 3−phosphatase and dual−specificity protein phospha
with a threshold of 1e−05

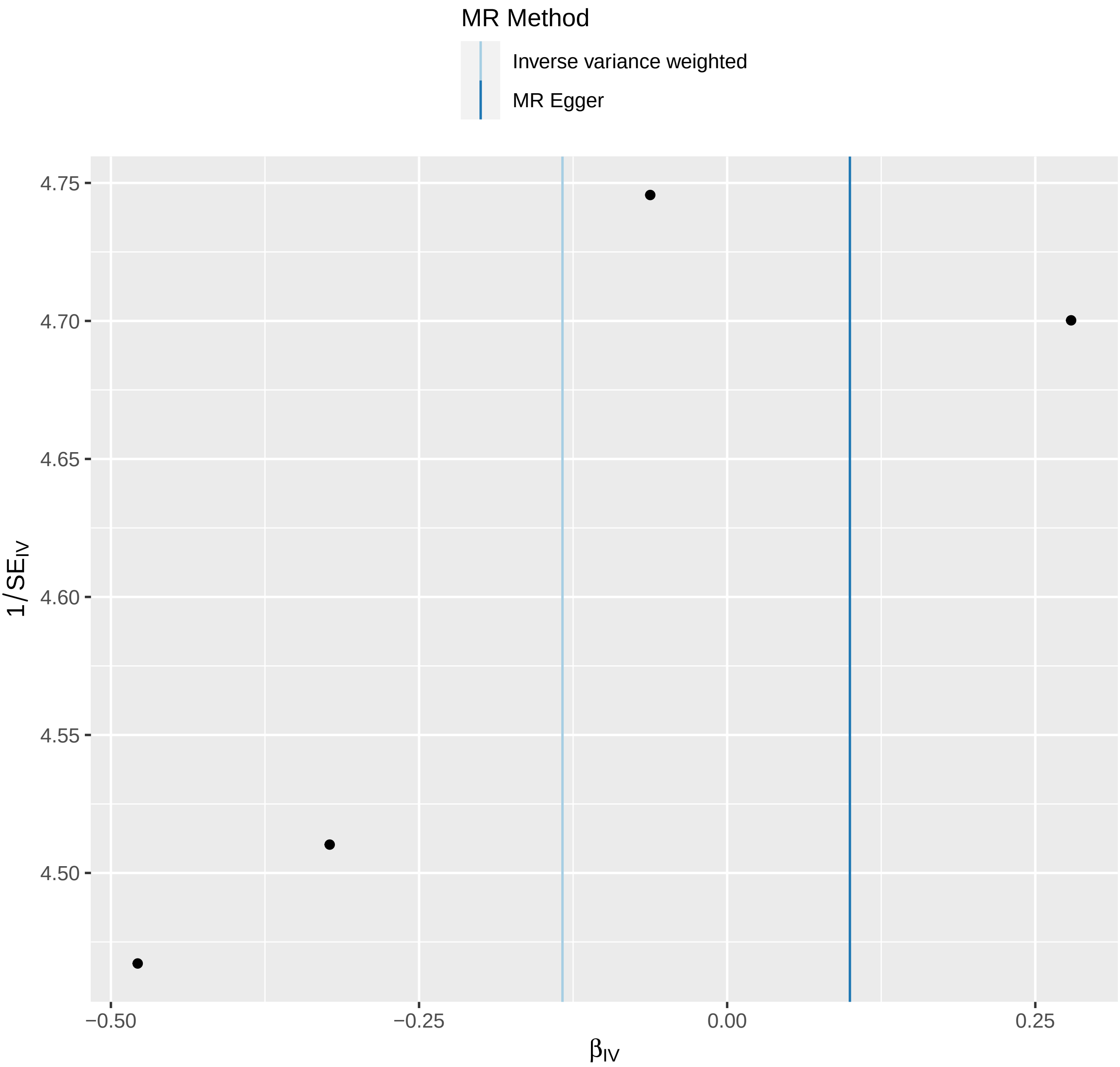

Batch 2−4850 : Gut microbiota abundance (genus Parasutterella id.2892) on Phosphatidylinositol 3,4,5−trisphosphate 3−phosphatase and dual−specificity protein phosphatase PTEN
with a threshold of 1e−05

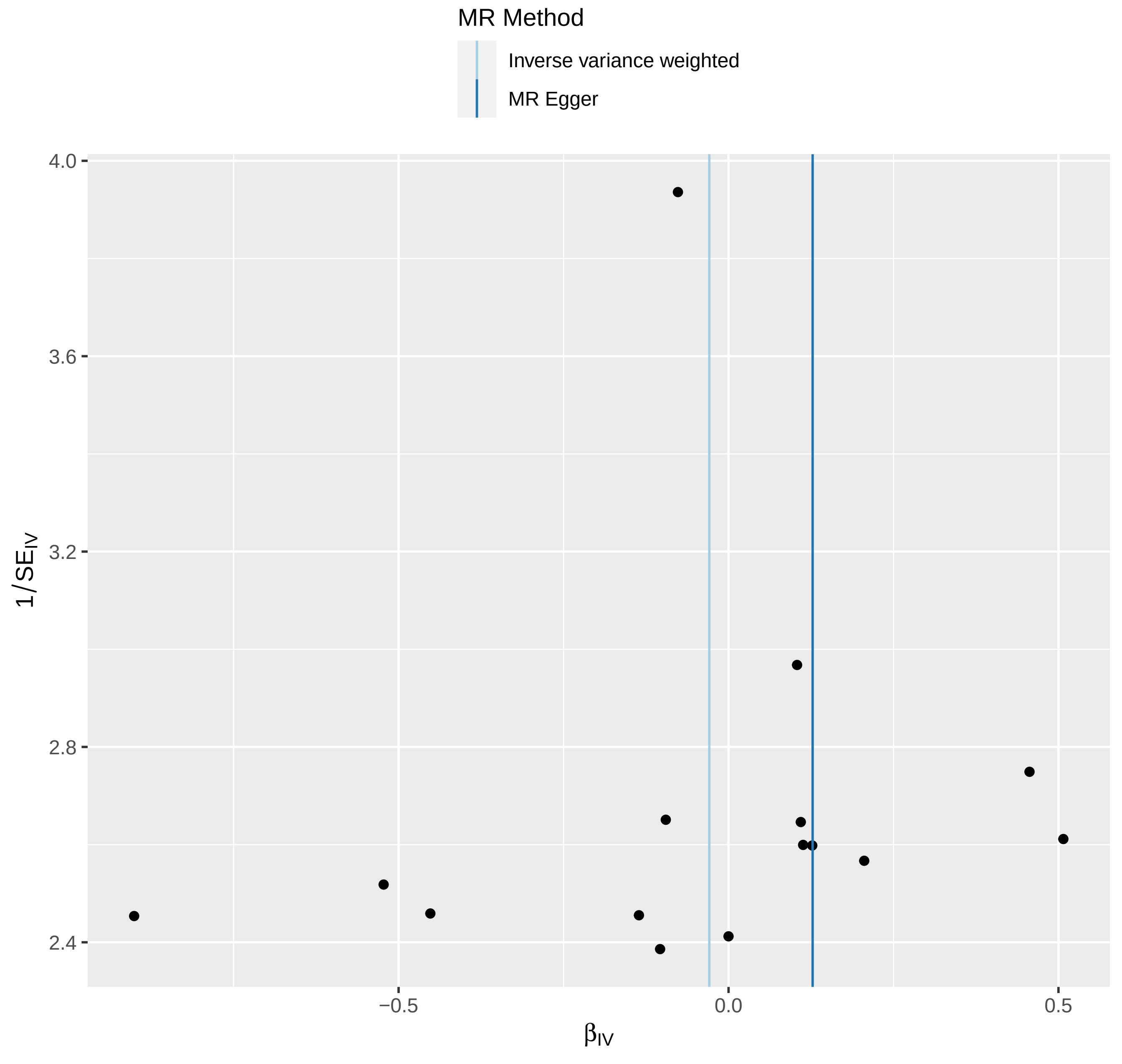

Batch 2−4851 : Gut microbiota abundance (genus Catenibacterium id.2153) on Prostaglandin−H2 D−isomerase
with a threshold of 1e−05

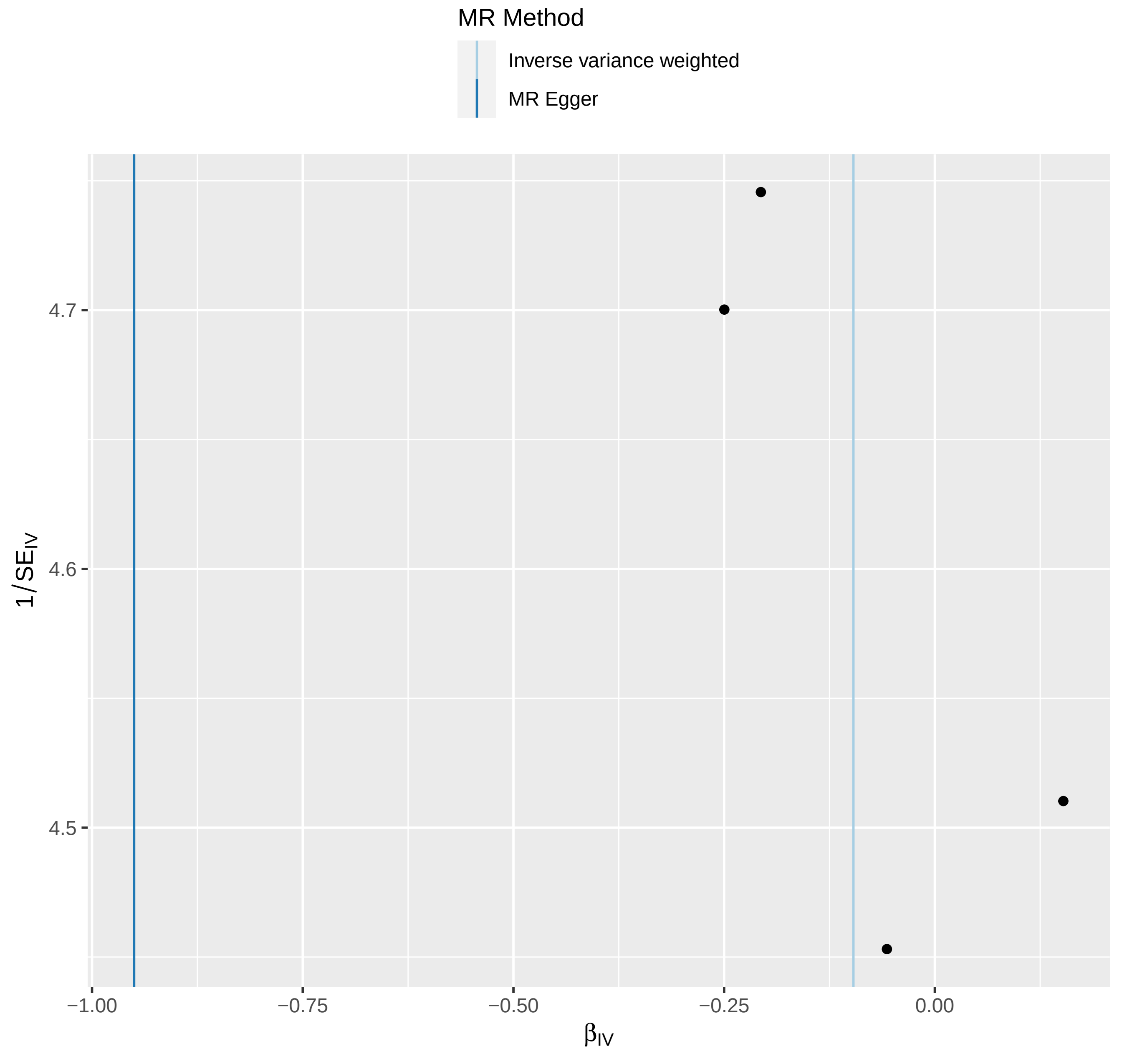

Batch 2−4852 : Gut microbiota abundance (genus Parasutterella id.2892) on Prostaglandin−H2 D−isomerase
with a threshold of 1e−05

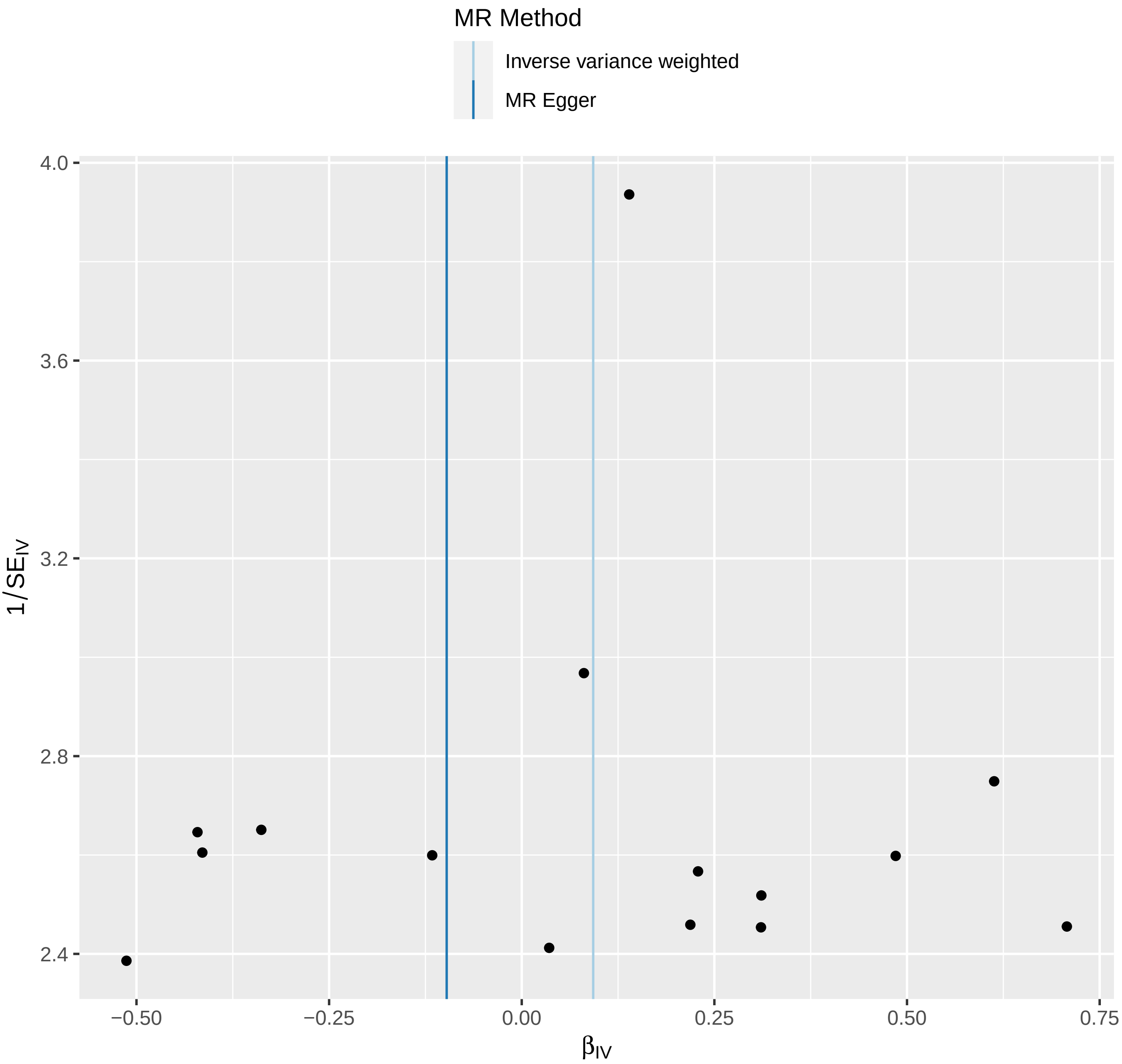

Batch 2−4853 : Gut microbiota abundance (genus Catenibacterium id.2153) on Prostaglandin F2 receptor negative regulator
with a threshold of 1e−05

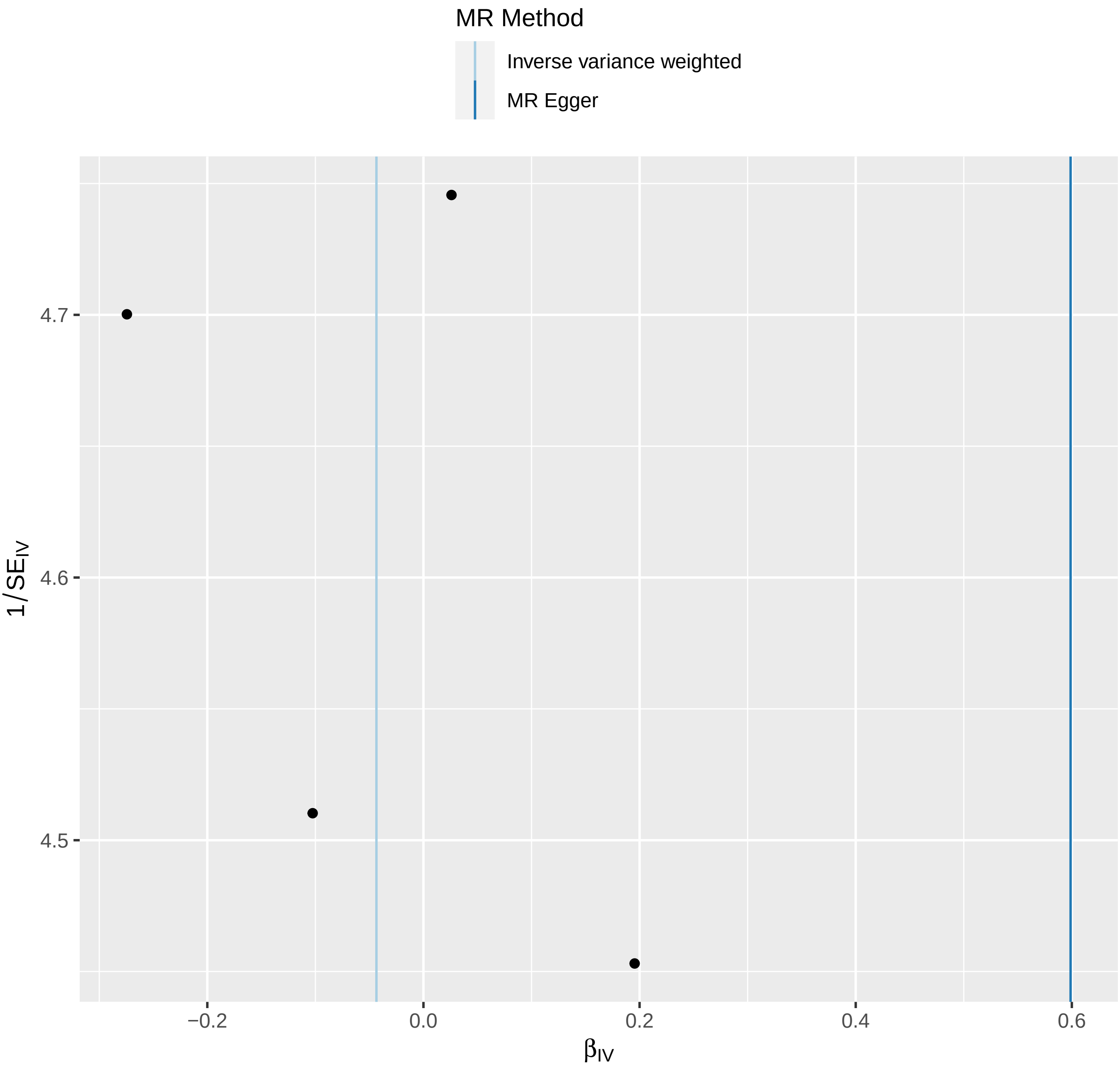

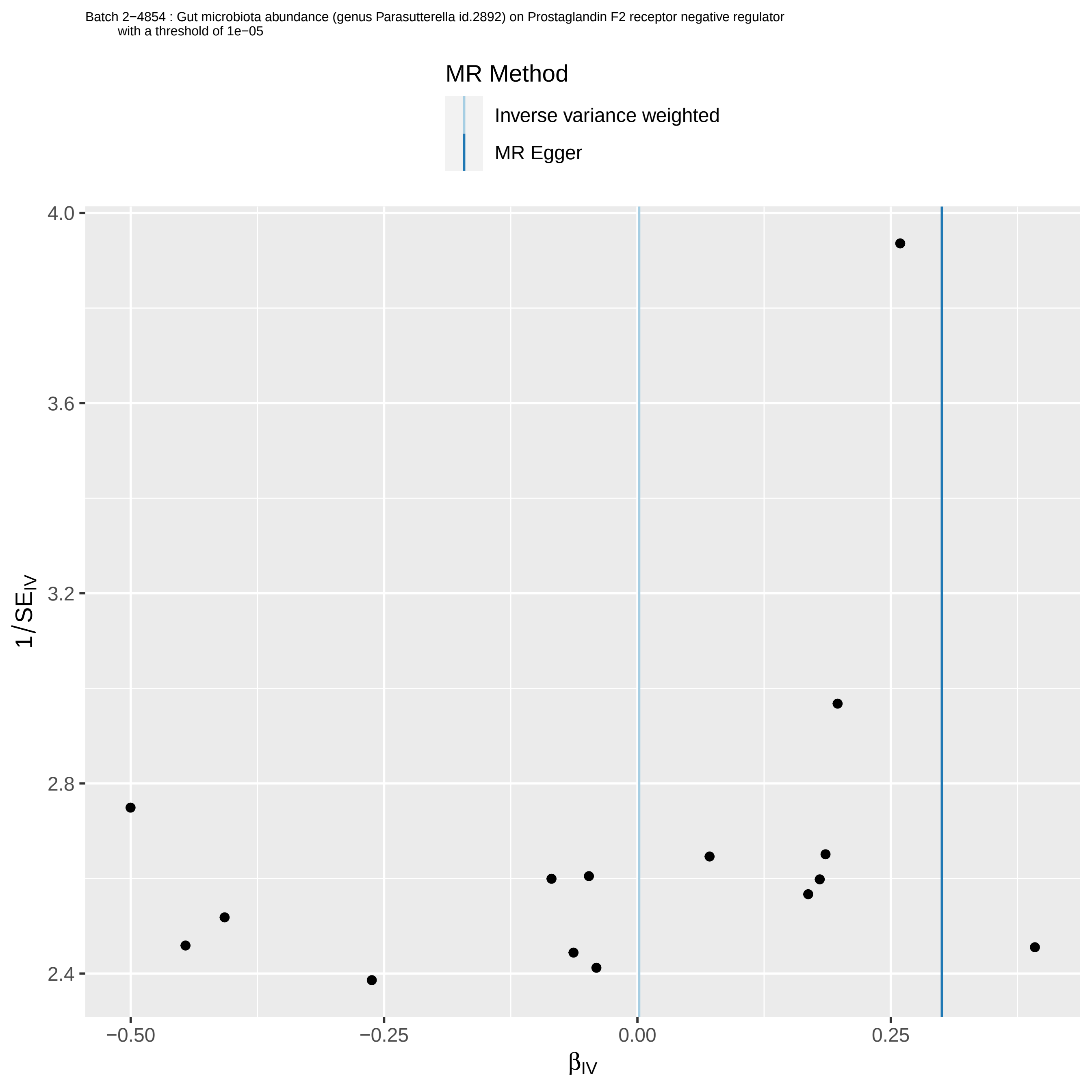
Batch 2−4854 : Gut microbiota abundance (genus Parasutterella id.2892) on Prostaglandin F2 receptor negative regulator
with a threshold of 1e−05
MR Method
Inverse variance weighted
MR Egger
4.0
3.6
3.2
2.8
2.4
$1/SE_{IV}$
−0.50
−0.25
0.00
0.25
β_{IV}

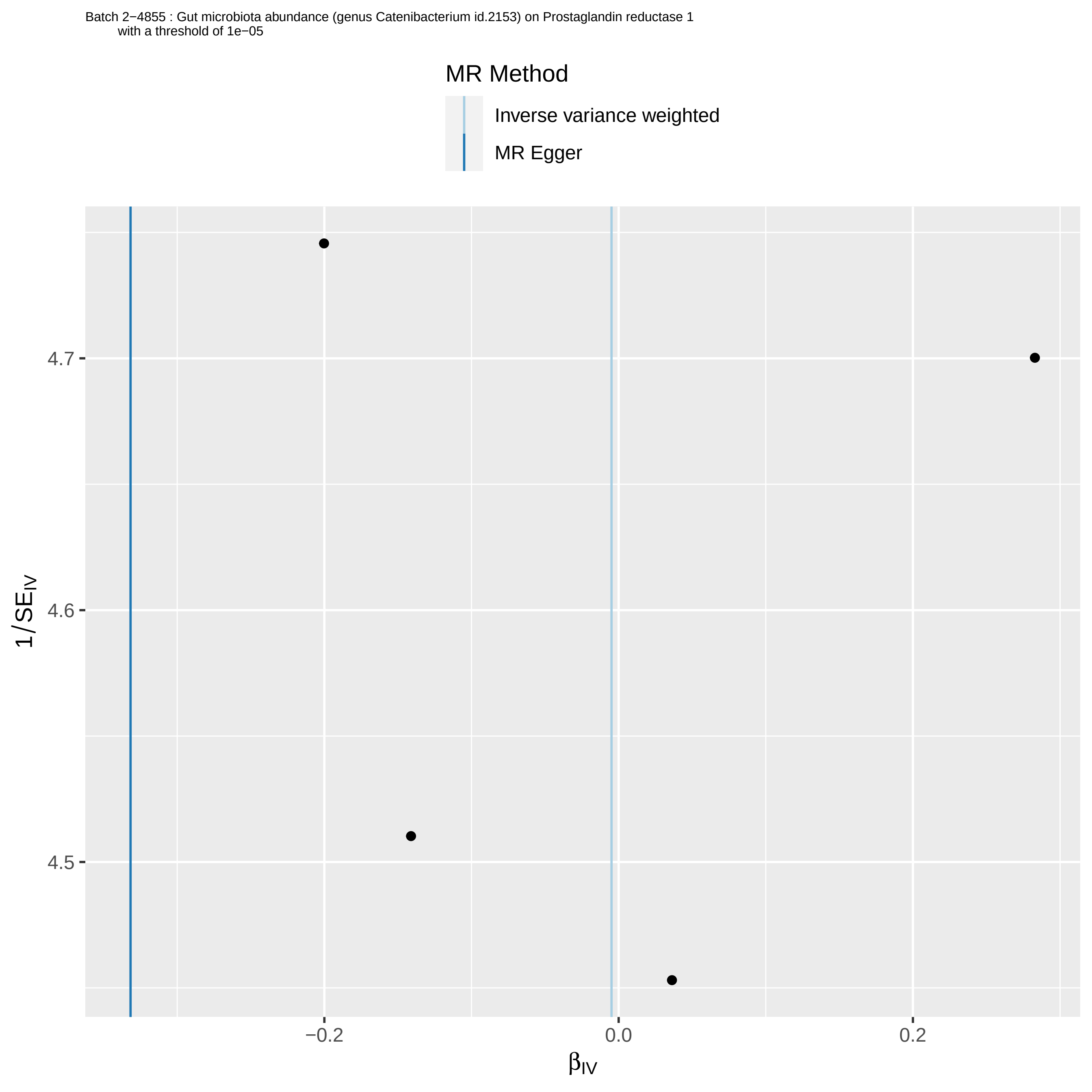

Batch 2−4855 : Gut microbiota abundance (genus Catenibacterium id.2153) on Prostaglandin reductase 1
with a threshold of 1e−05
MR Method
Inverse variance weighted
MR Egger
4.7
4.6
4.5
1/SE_IV
−0.2
0.0
0.2
β_IV

Batch 2−4856 : Gut microbiota abundance (genus Parasutterella id.2892) on Prostaglandin reductase 1
with a threshold of 1e−05

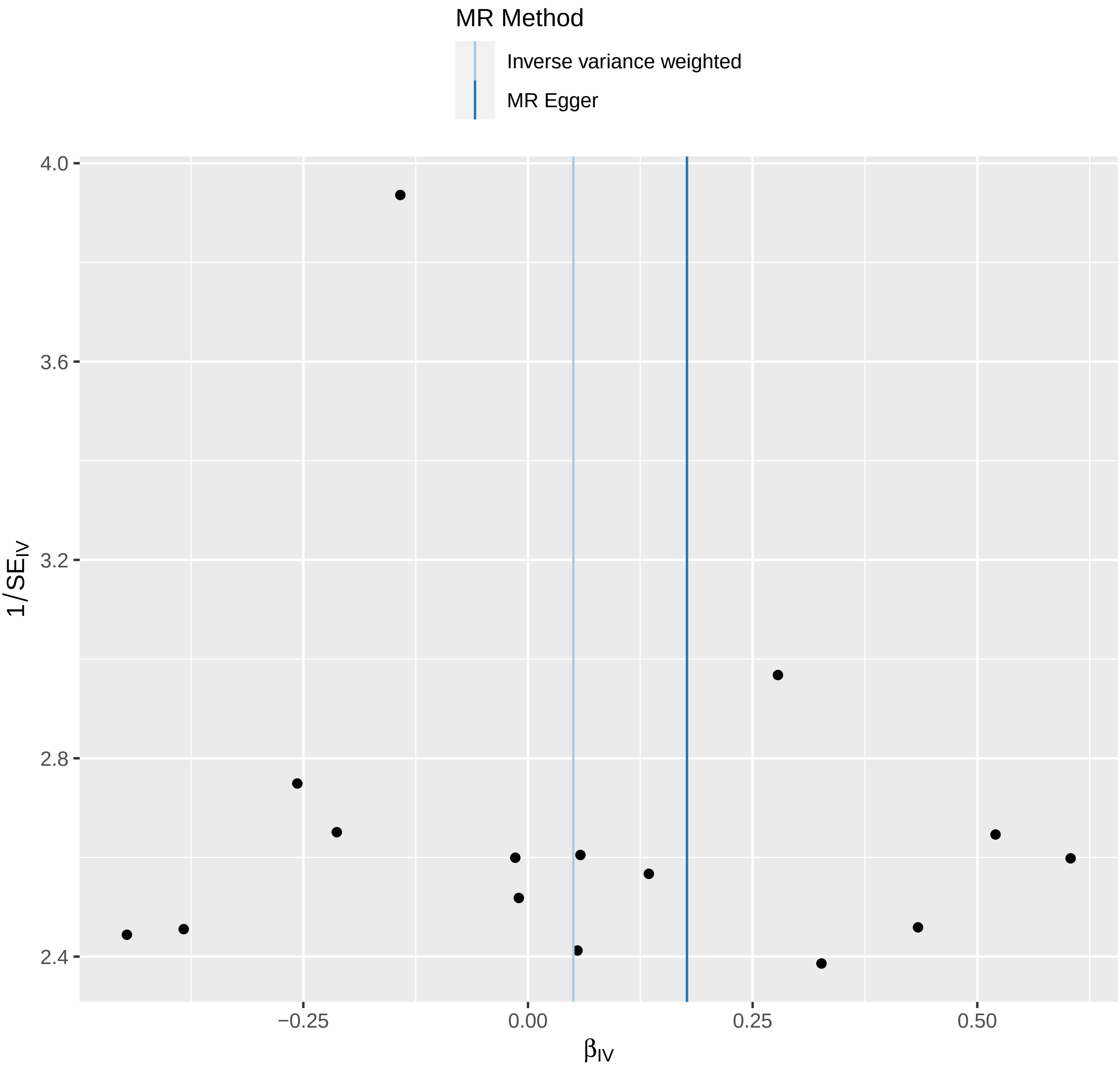

Batch 2−4857 : Gut microbiota abundance (genus Catenibacterium id.2153) on Parathyroid hormone/parathyroid hormone−related peptide receptor with a threshold of 1e−05

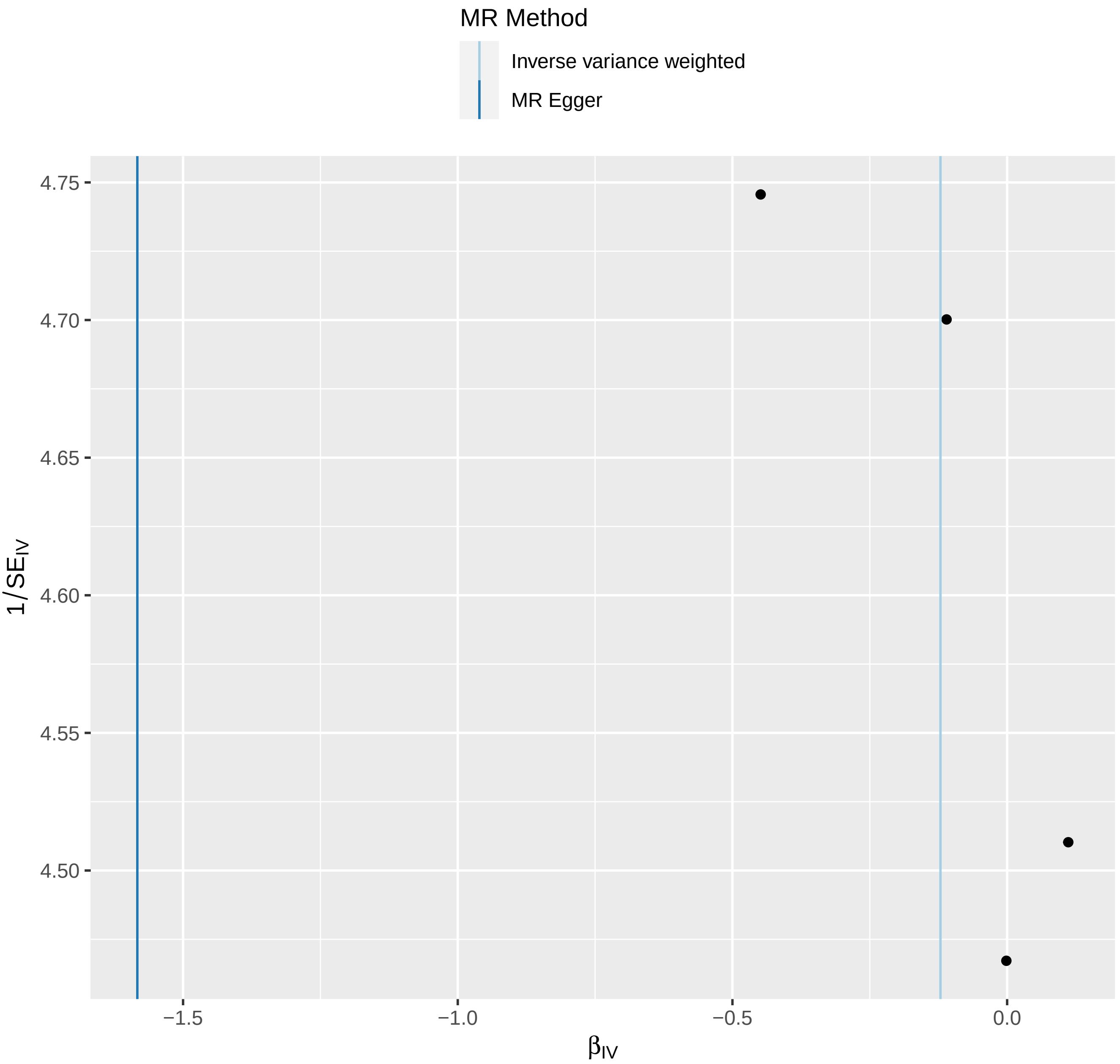

Batch 2−4858 : Gut microbiota abundance (genus Parasutterella id.2892) on Parathyroid hormone/parathyroid hormone−related peptide receptor with a threshold of 1e−05

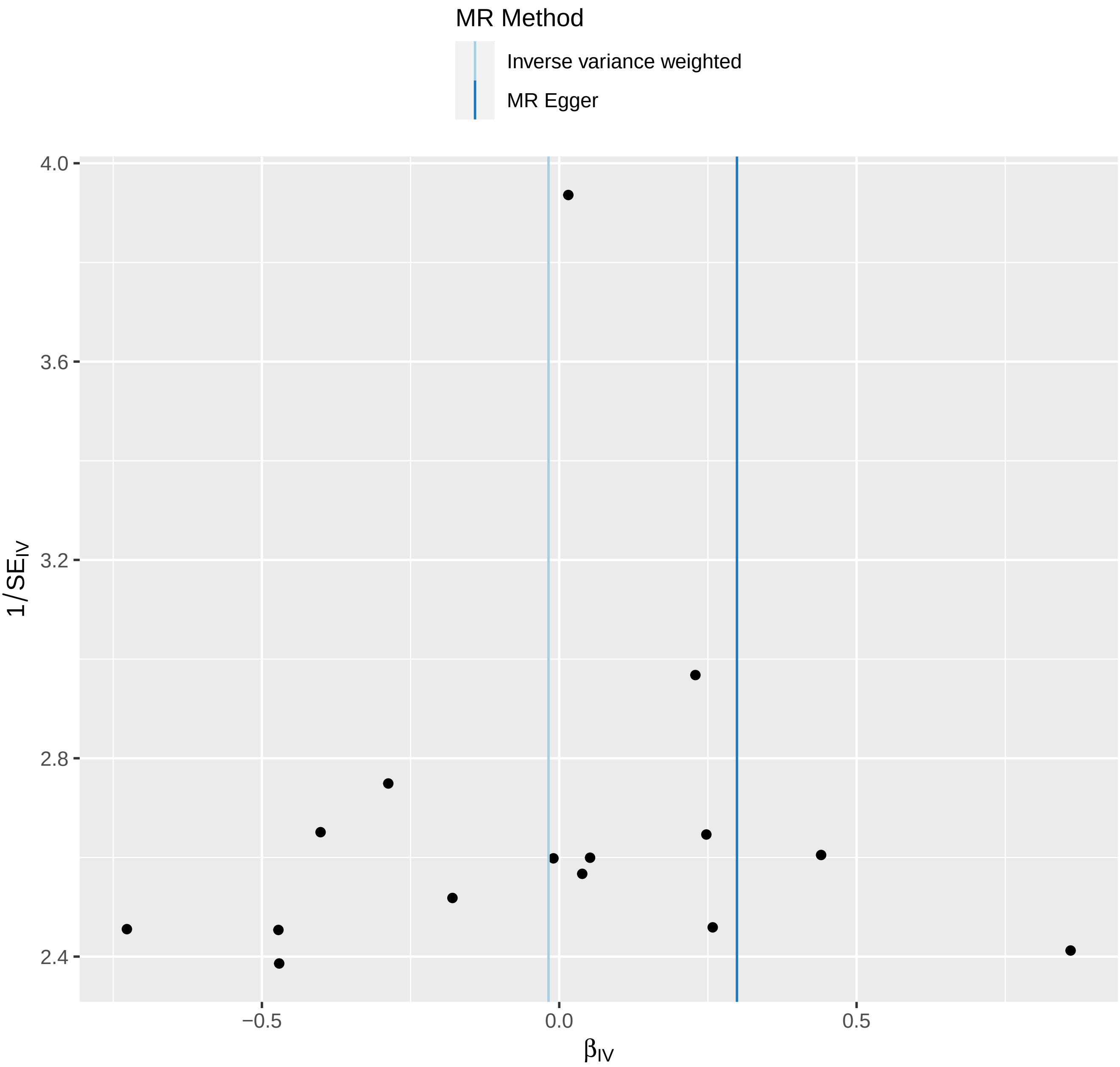

Batch 2−4859 : Gut microbiota abundance (genus Catenibacterium id.2153) on Tuberoinfundibular peptide of 39 residues with a threshold of 1e−05

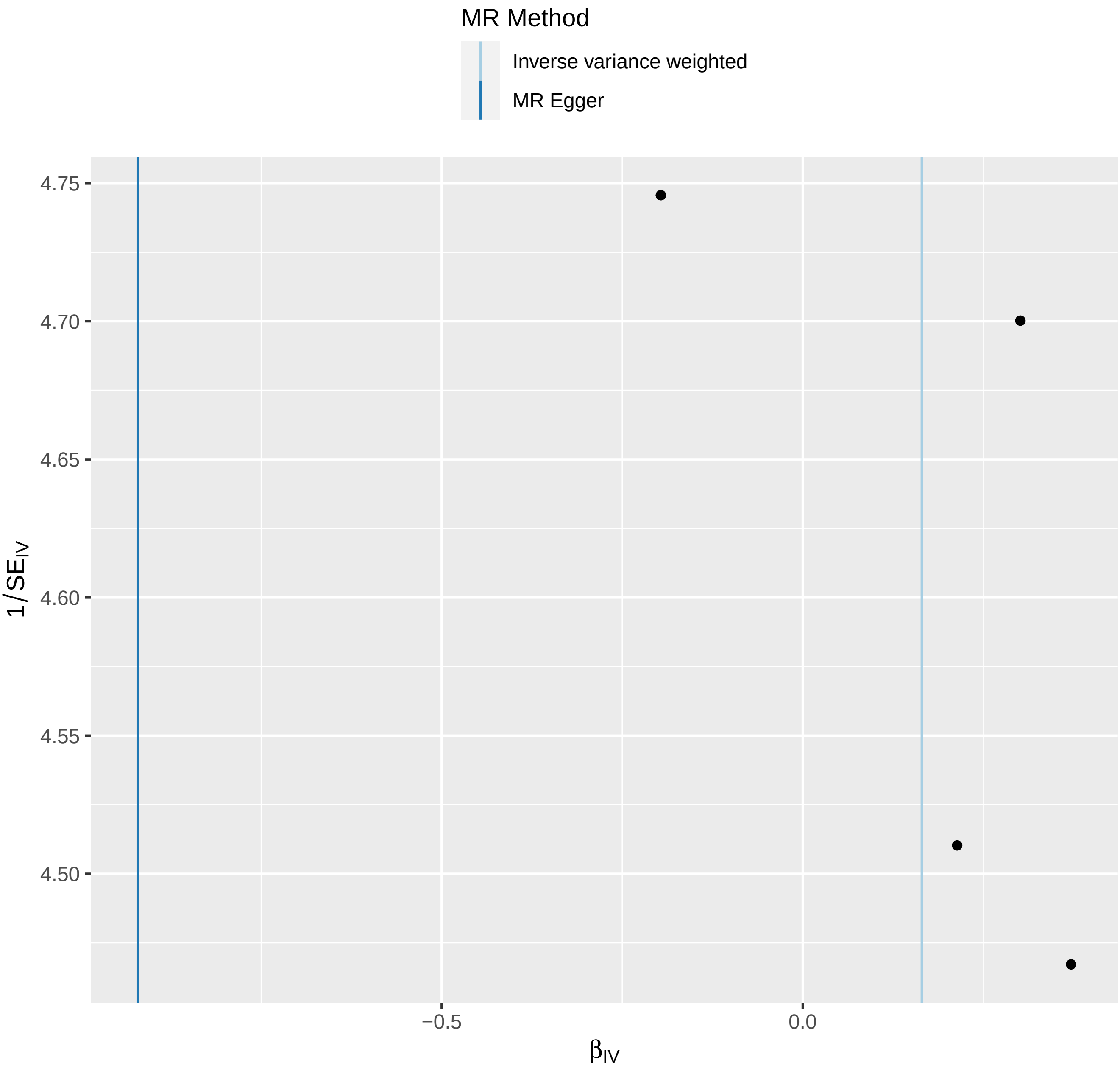

Batch 2−4860 : Gut microbiota abundance (genus Parasutterella id.2892) on Tuberoinfundibular peptide of 39 residues
with a threshold of 1e−05

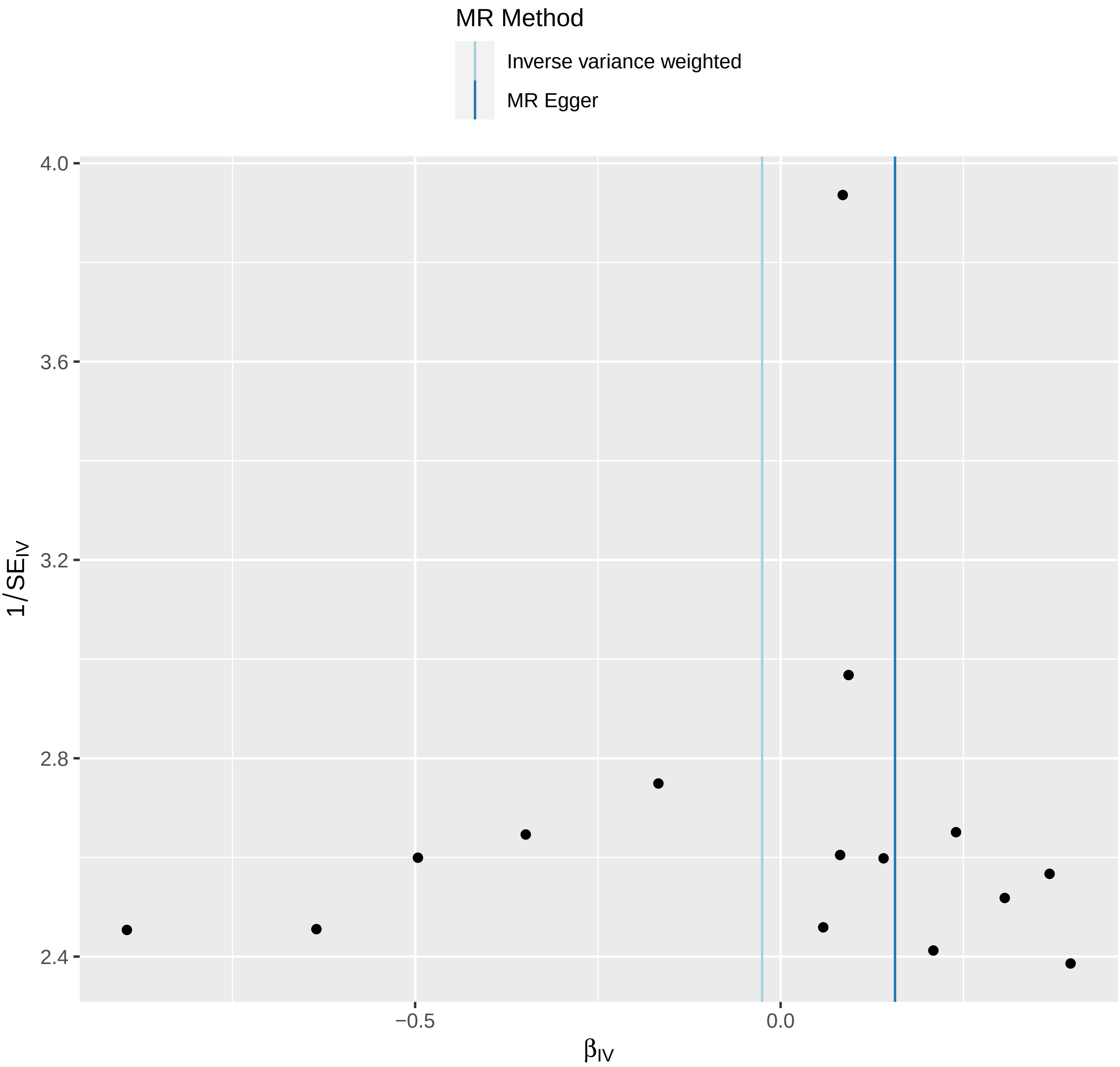

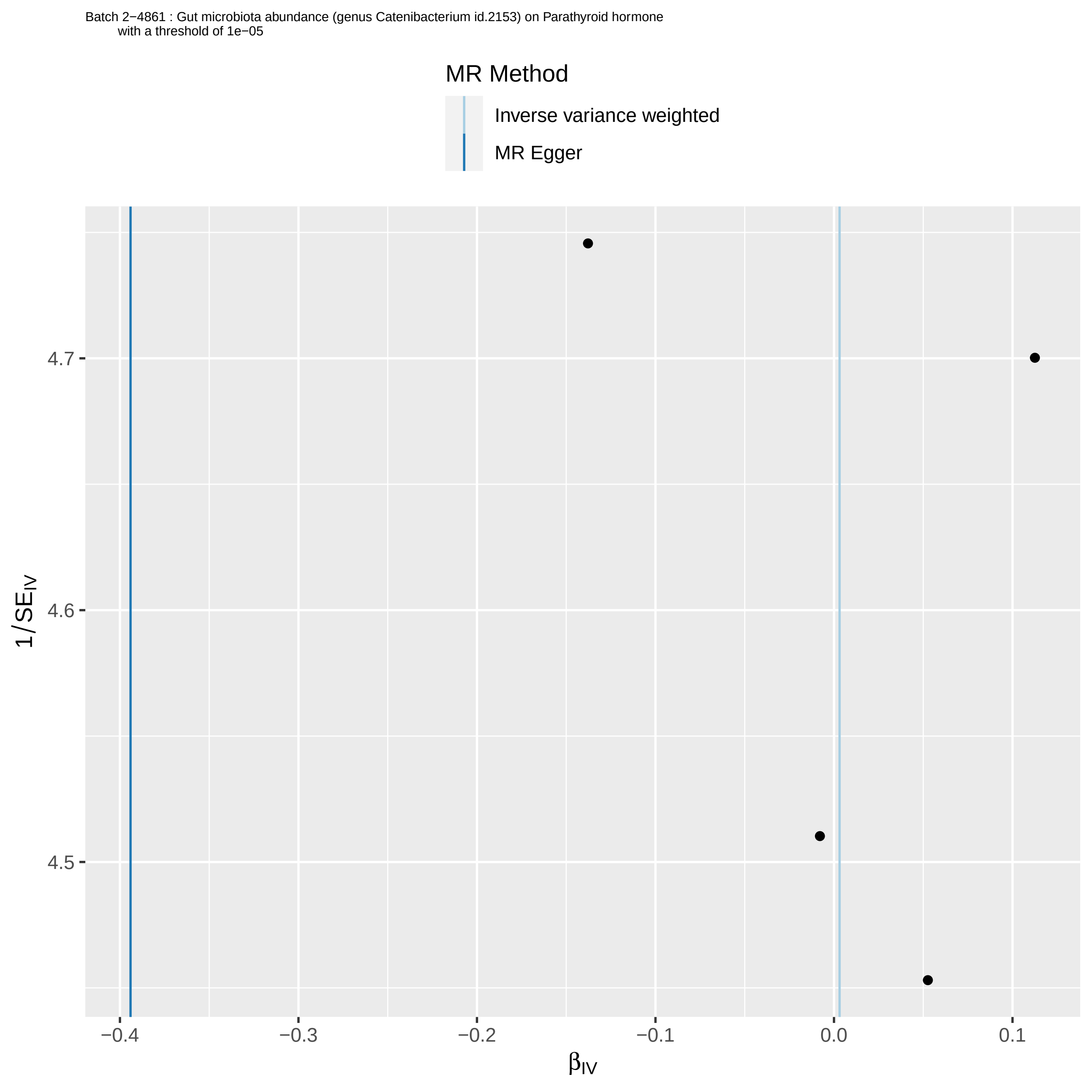
Batch 2−4861 : Gut microbiota abundance (genus Catenibacterium id.2153) on Parathyroid hormone
with a threshold of 1e−05
MR Method
Inverse variance weighted
MR Egger
4.7
4.6
4.5
1/SE_IV
−0.4
−0.3
−0.2
−0.1
0.0
0.1
β_IV

Batch 2−4862 : Gut microbiota abundance (genus Parasutterella id.2892) on Parathyroid hormone
with a threshold of 1e−05

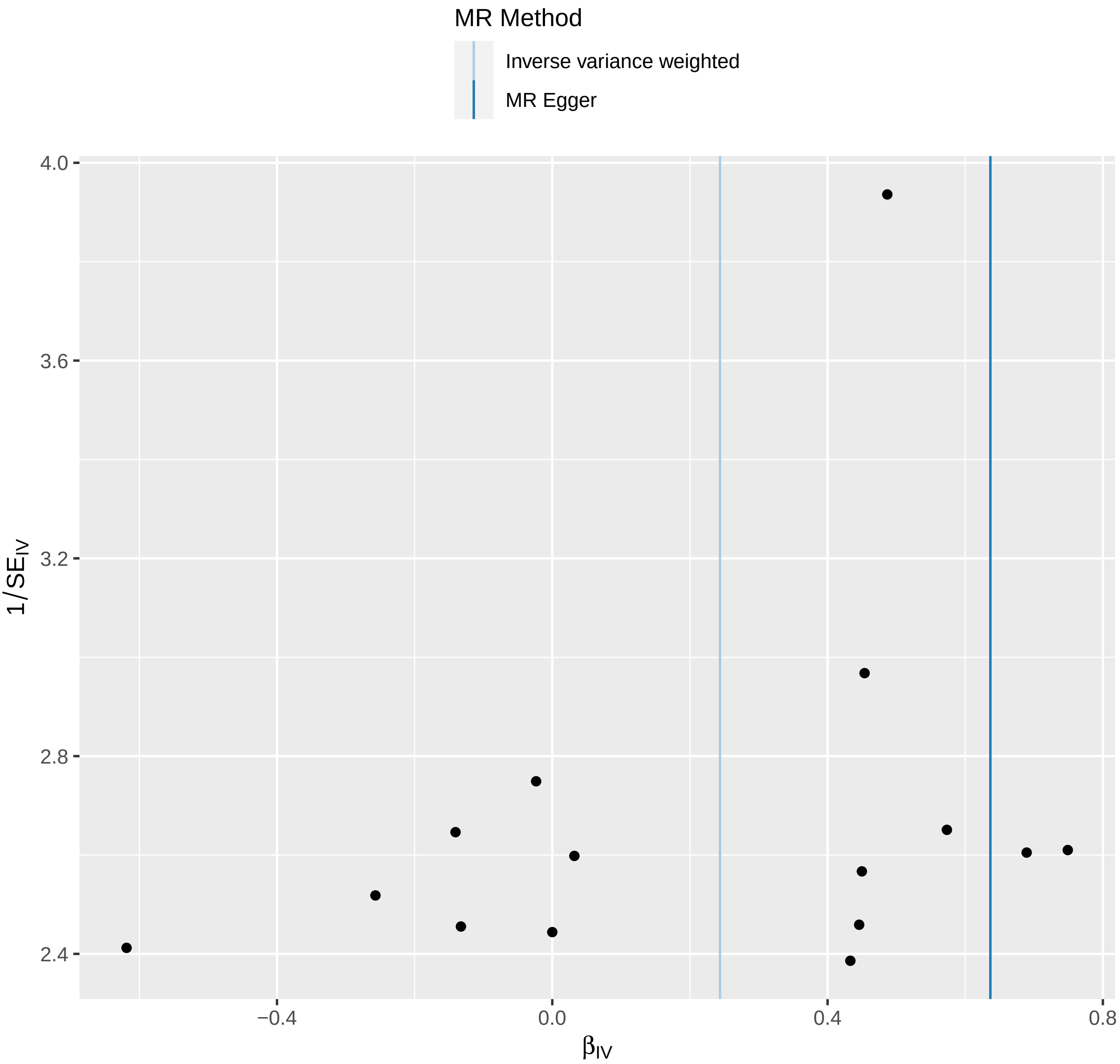

Batch 2−4863 : Gut microbiota abundance (genus Catenibacterium id.2153) on Parathyroid hormone−related protein
with a threshold of 1e−05

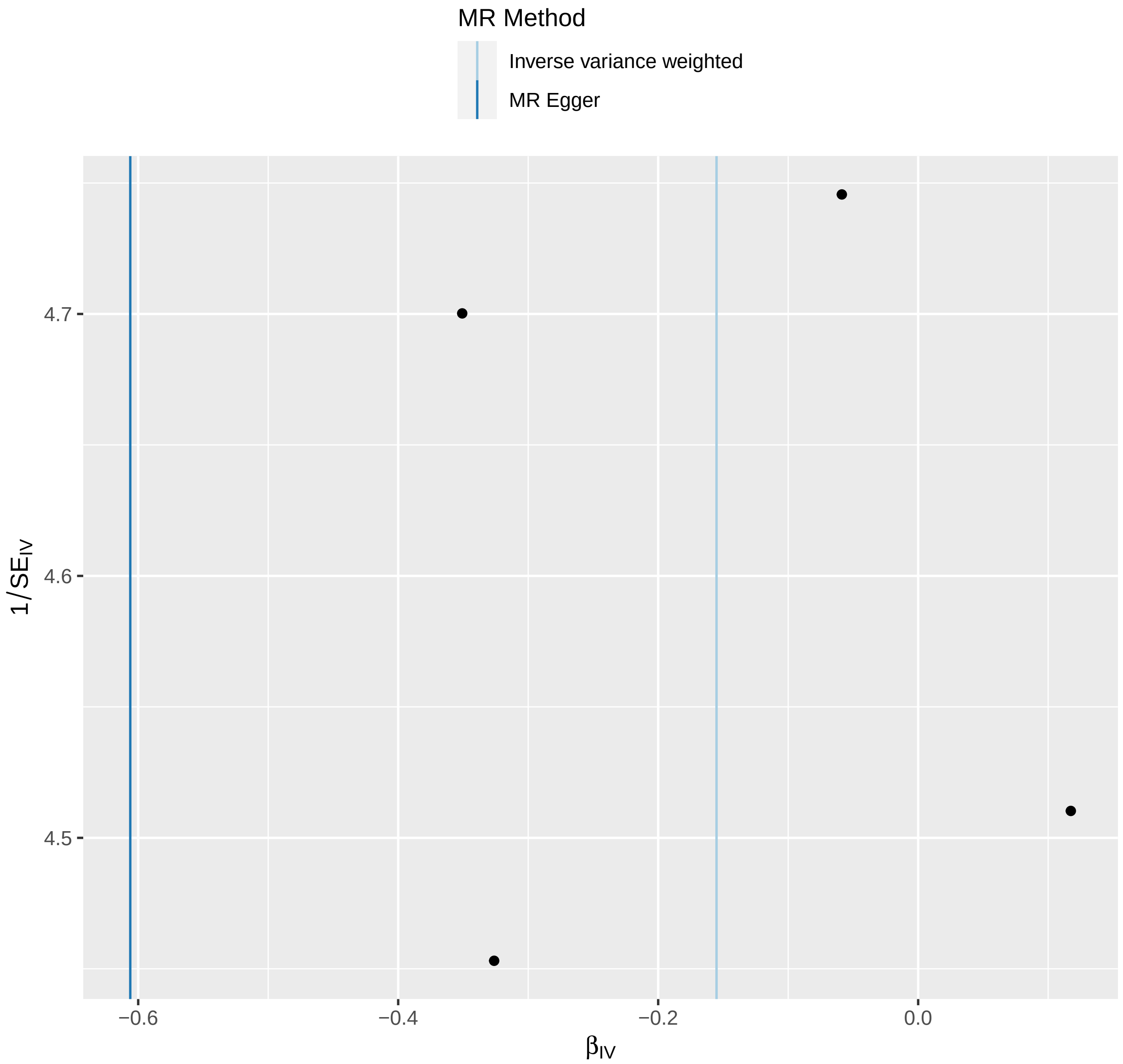

Batch 2−4864 : Gut microbiota abundance (genus Parasutterella id.2892) on Parathyroid hormone−related protein with a threshold of 1e−05

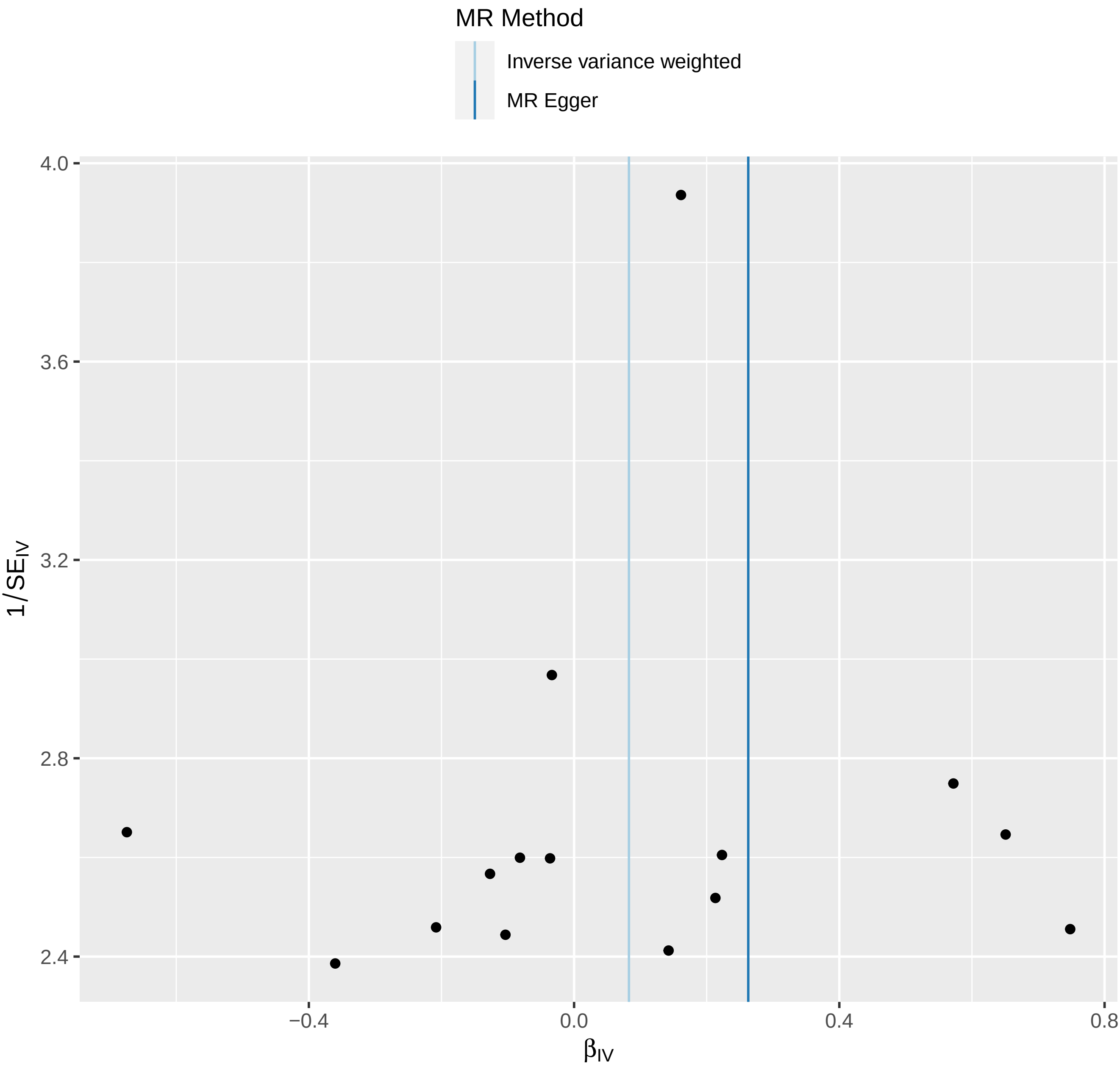

Batch 2−4865 : Gut microbiota abundance (genus Catenibacterium id.2153) on Protein−tyrosine kinase 2−beta
with a threshold of 1e−05

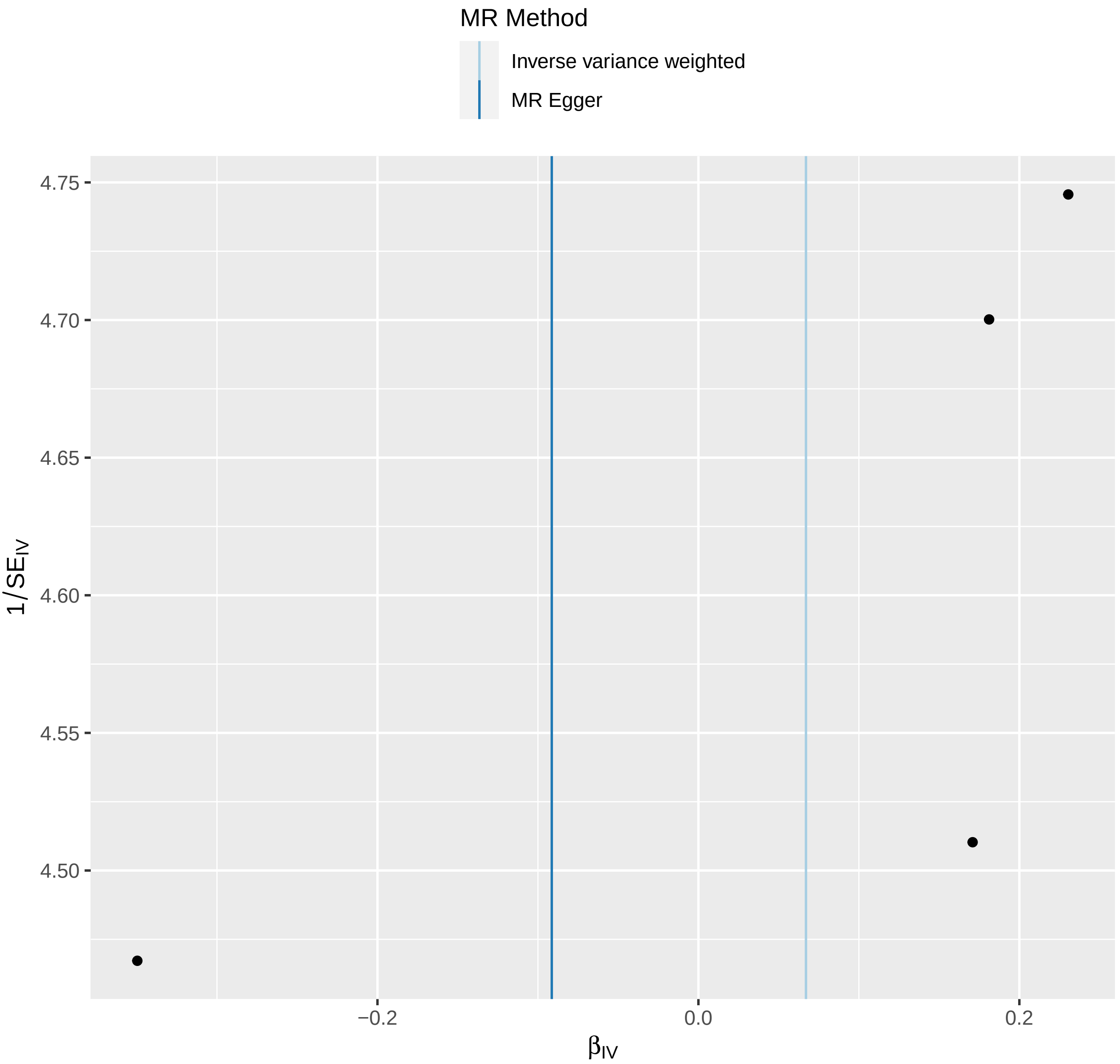

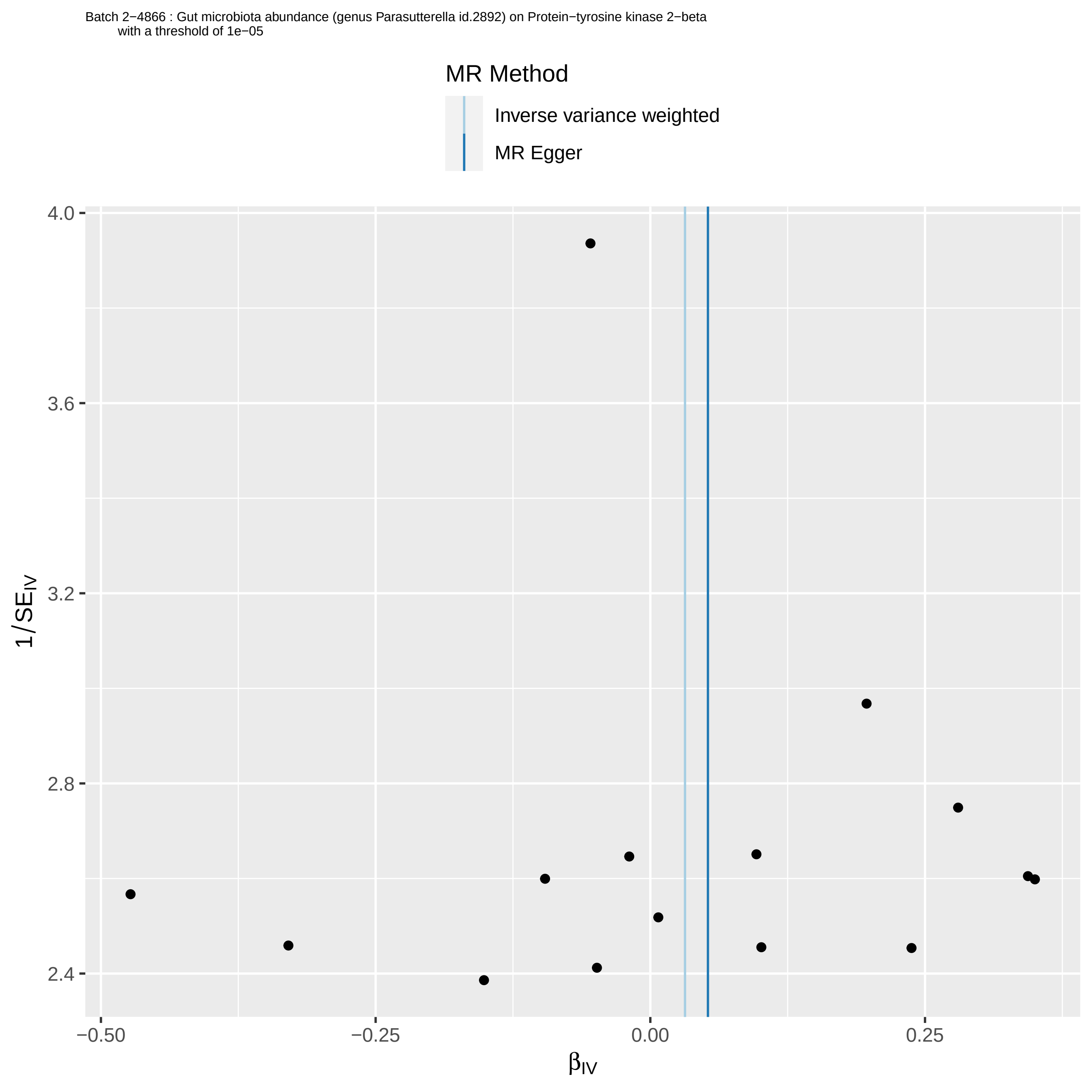

Batch 2−4866 : Gut microbiota abundance (genus Parasutterella id.2892) on Protein−tyrosine kinase 2−beta
with a threshold of 1e−05
MR Method
Inverse variance weighted
MR Egger
4.0
3.6
3.2
2.8
2.4
1/SE_IV
−0.50
−0.25
0.00
0.25
β_IV

Batch 2−4867 : Gut microbiota abundance (genus Catenibacterium id.2153) on Protein−tyrosine kinase 6
with a threshold of 1e−05

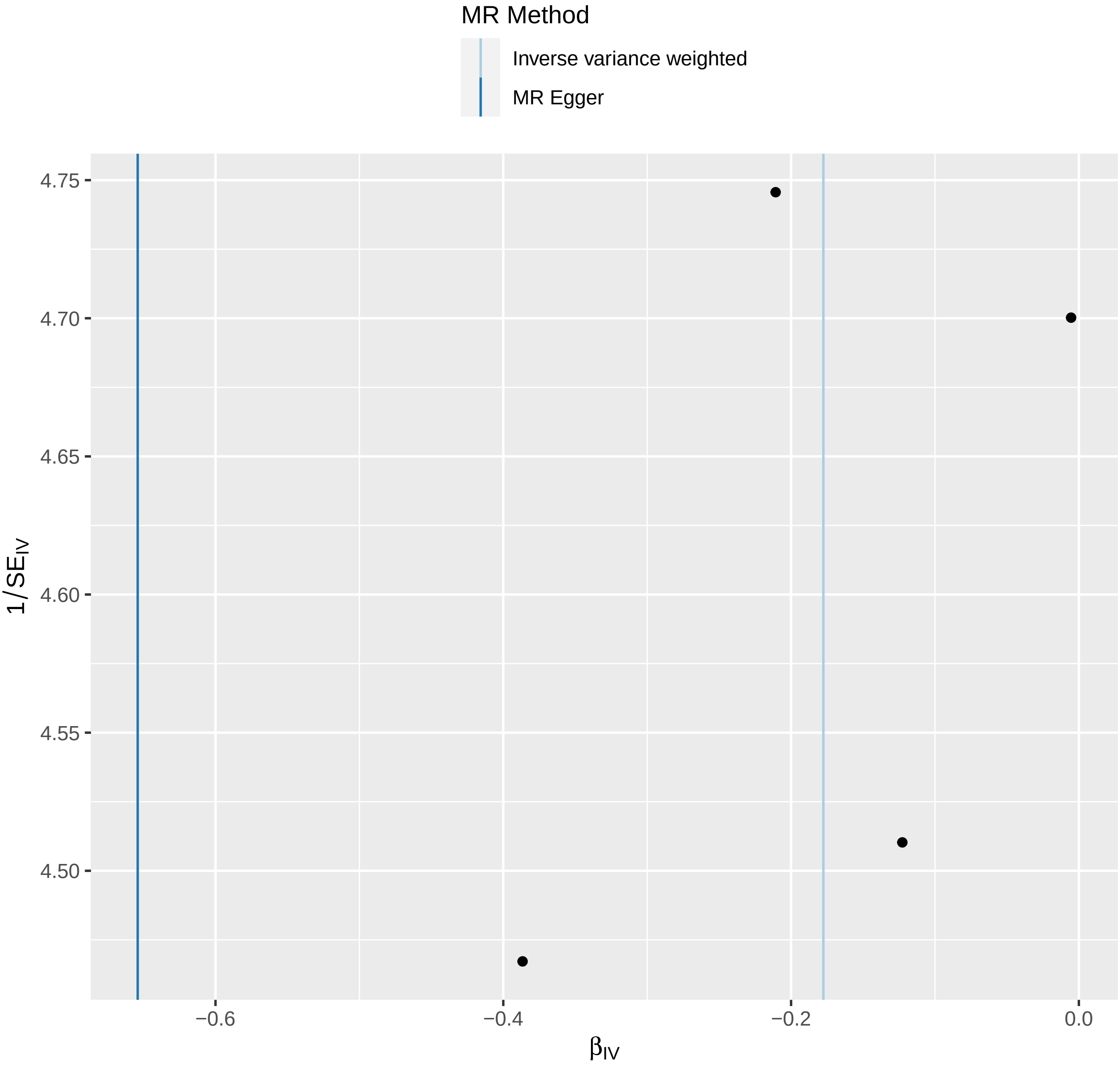

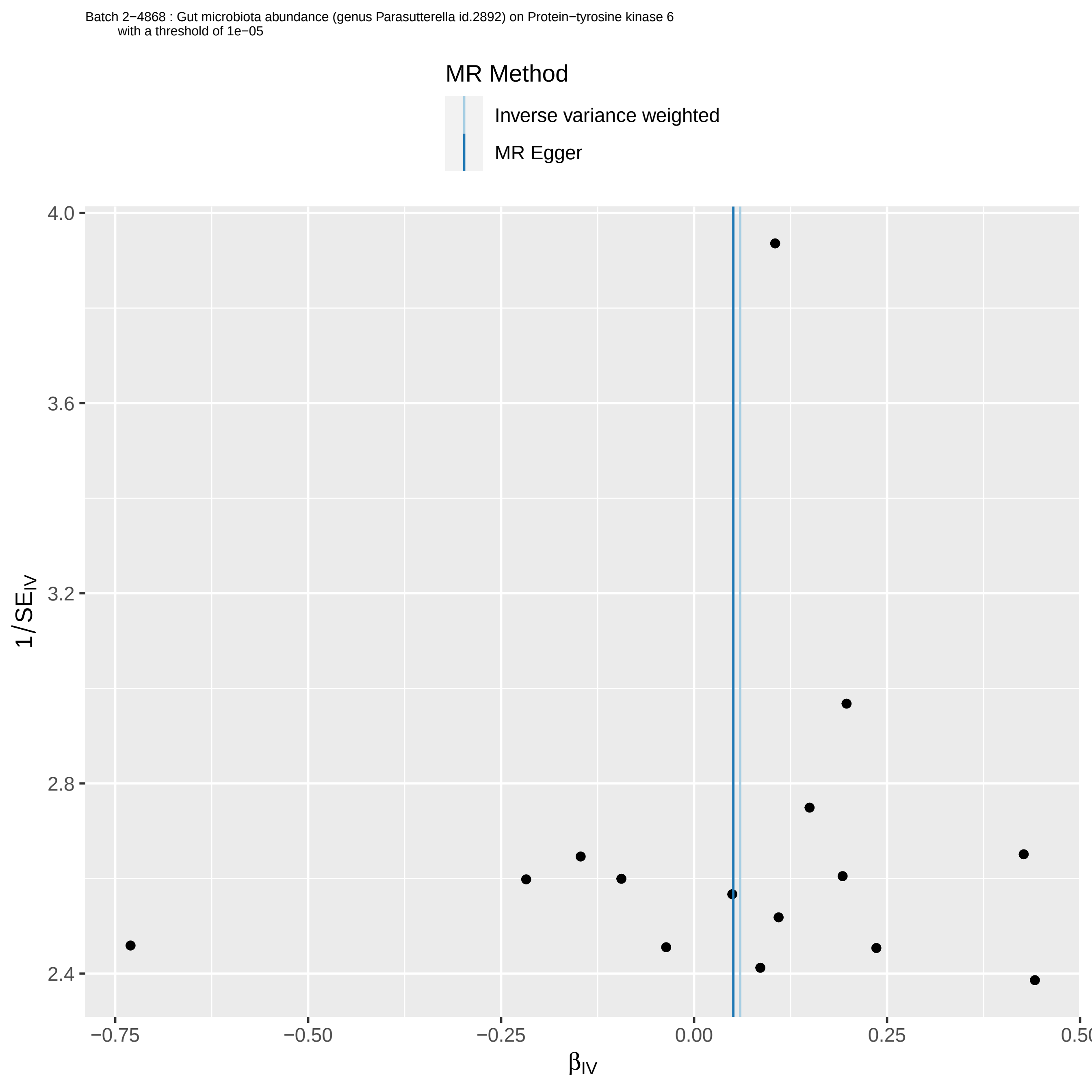
Batch 2−4868 : Gut microbiota abundance (genus Parasutterella id.2892) on Protein−tyrosine kinase 6
with a threshold of 1e−05
MR Method
Inverse variance weighted
MR Egger
4.0
3.6
3.2
2.8
2.4
1/SE_IV
−0.75
−0.50
−0.25
0.00
0.25
0.50
β_IV

Batch 2−4869 : Gut microbiota abundance (genus Catenibacterium id.2153) on Pleiotrophin
with a threshold of 1e−05

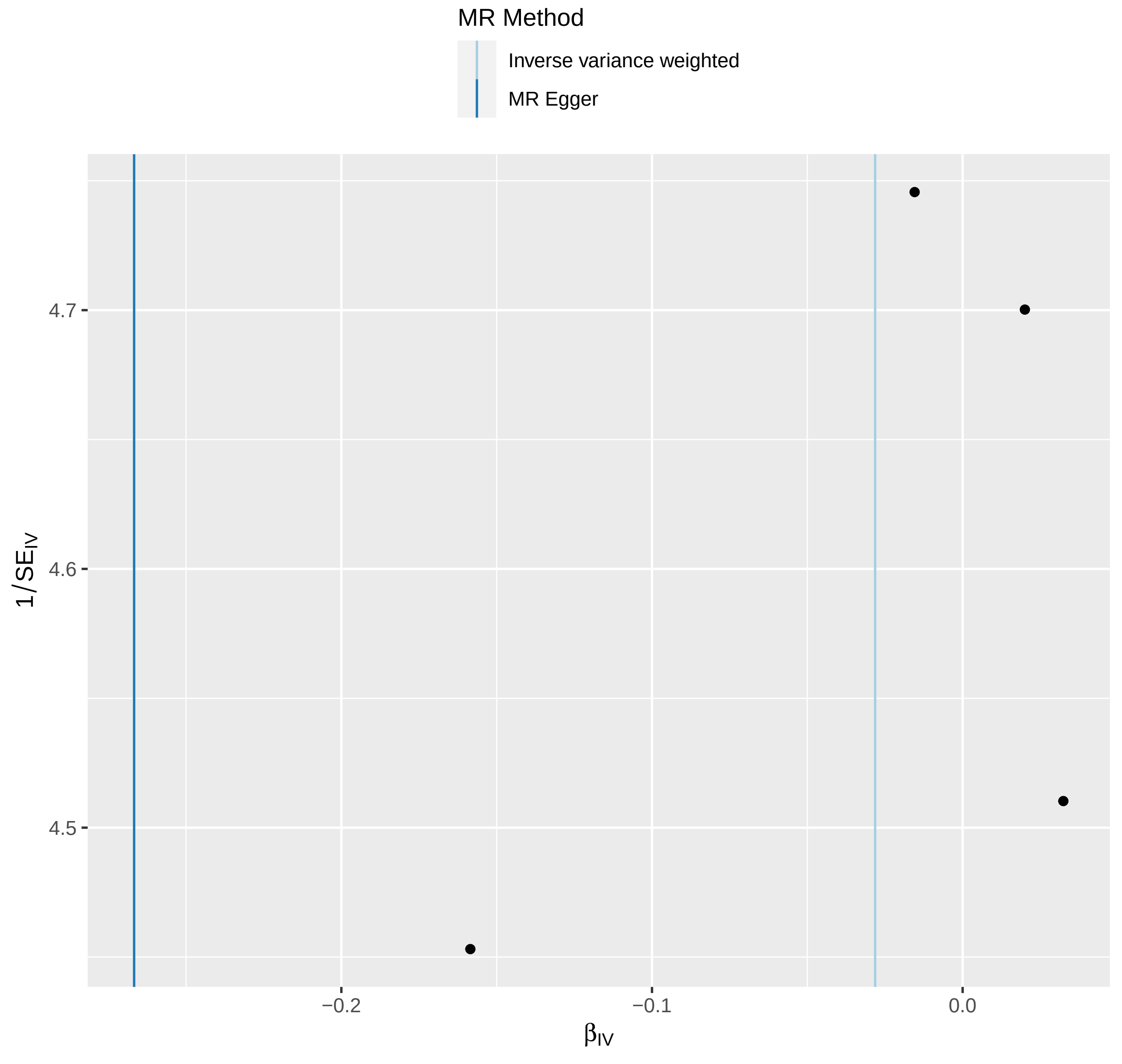

Batch 2−4870 : Gut microbiota abundance (genus Parasutterella id.2892) on Pleiotrophin
with a threshold of 1e−05

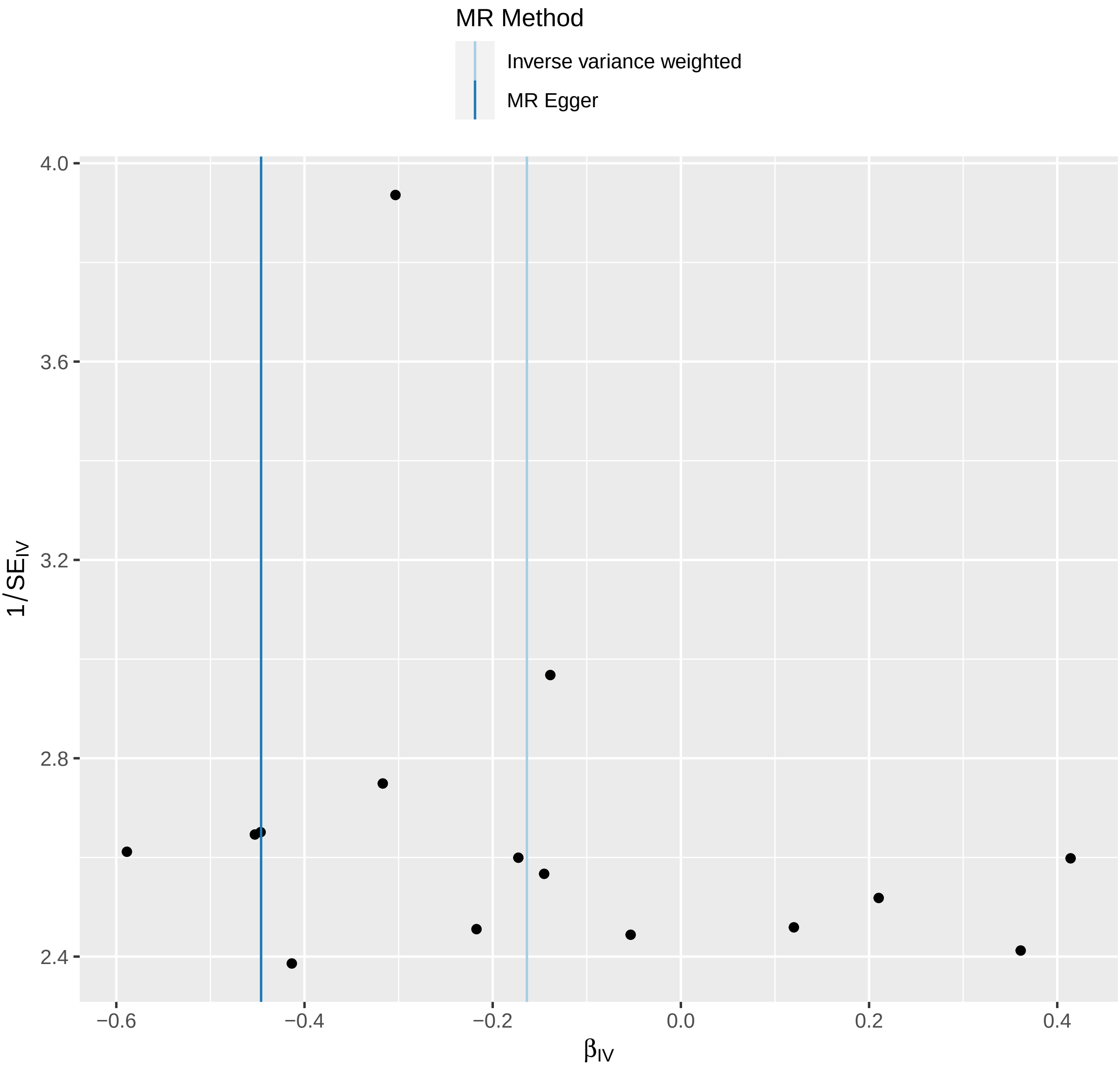

Batch 2−4871 : Gut microbiota abundance (genus Catenibacterium id.2153) on Protein tyrosine phosphatase type IVA 2
with a threshold of 1e−05

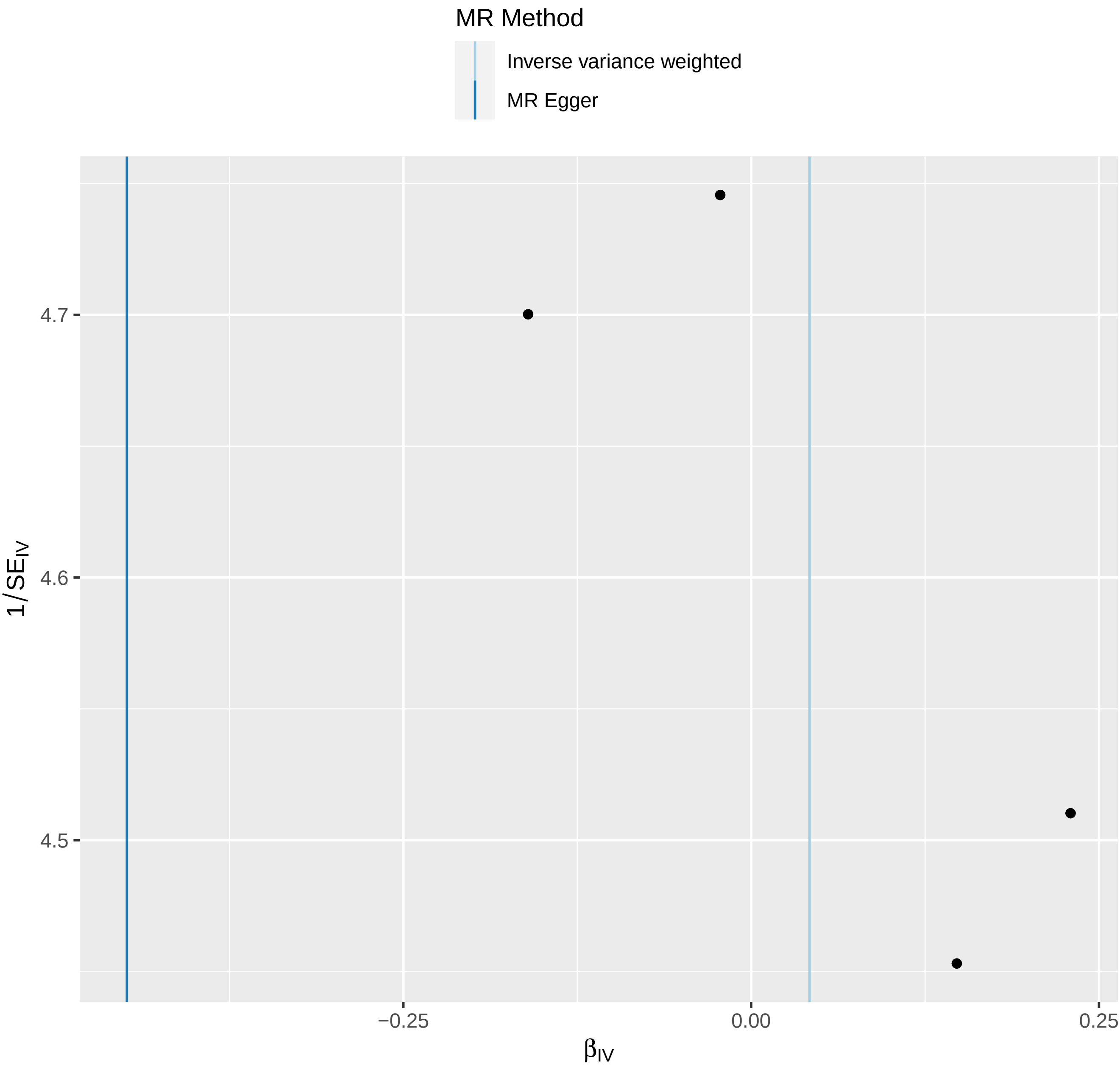

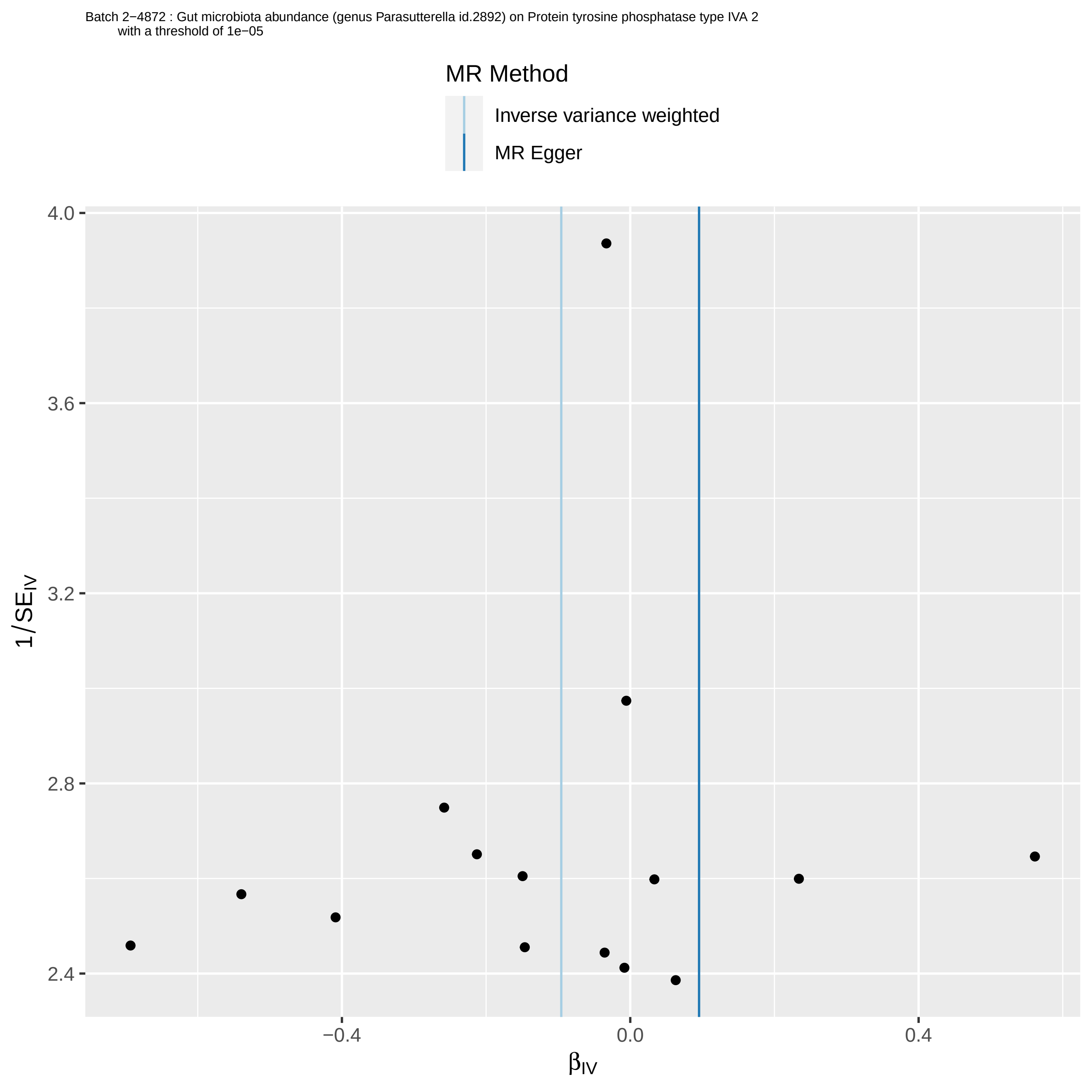
Batch 2−4872 : Gut microbiota abundance (genus Parasutterella id.2892) on Protein tyrosine phosphatase type IVA 2
with a threshold of 1e−05
MR Method
Inverse variance weighted
MR Egger
4.0
3.6
3.2
2.8
2.4
1/SE$_{IV}$
−0.4
0.0
0.4
β$_{IV}$

Batch 2−4873 : Gut microbiota abundance (genus Catenibacterium id.2153) on Tyrosine−protein phosphatase non−receptor type 11 with a threshold of 1e−05

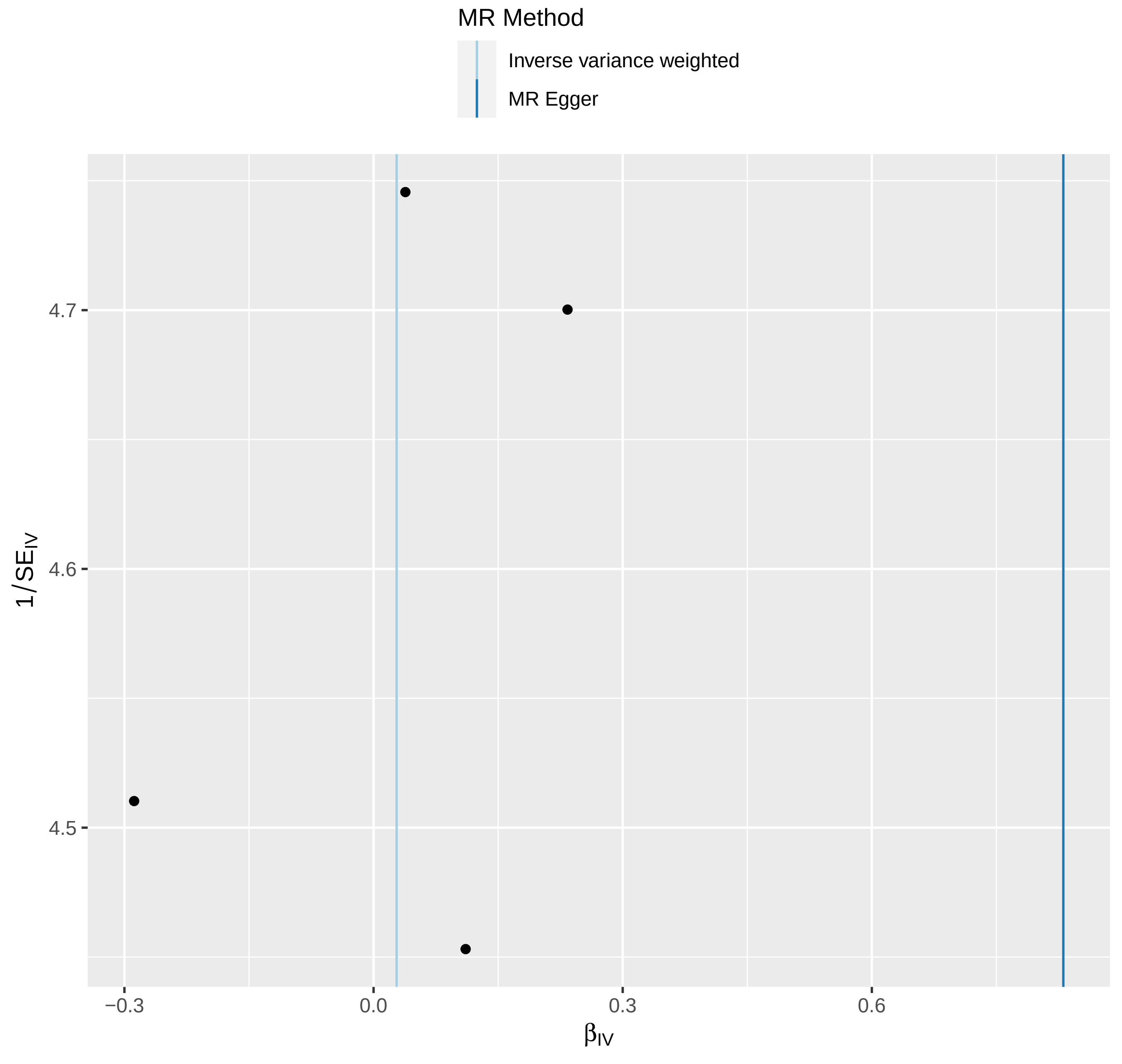

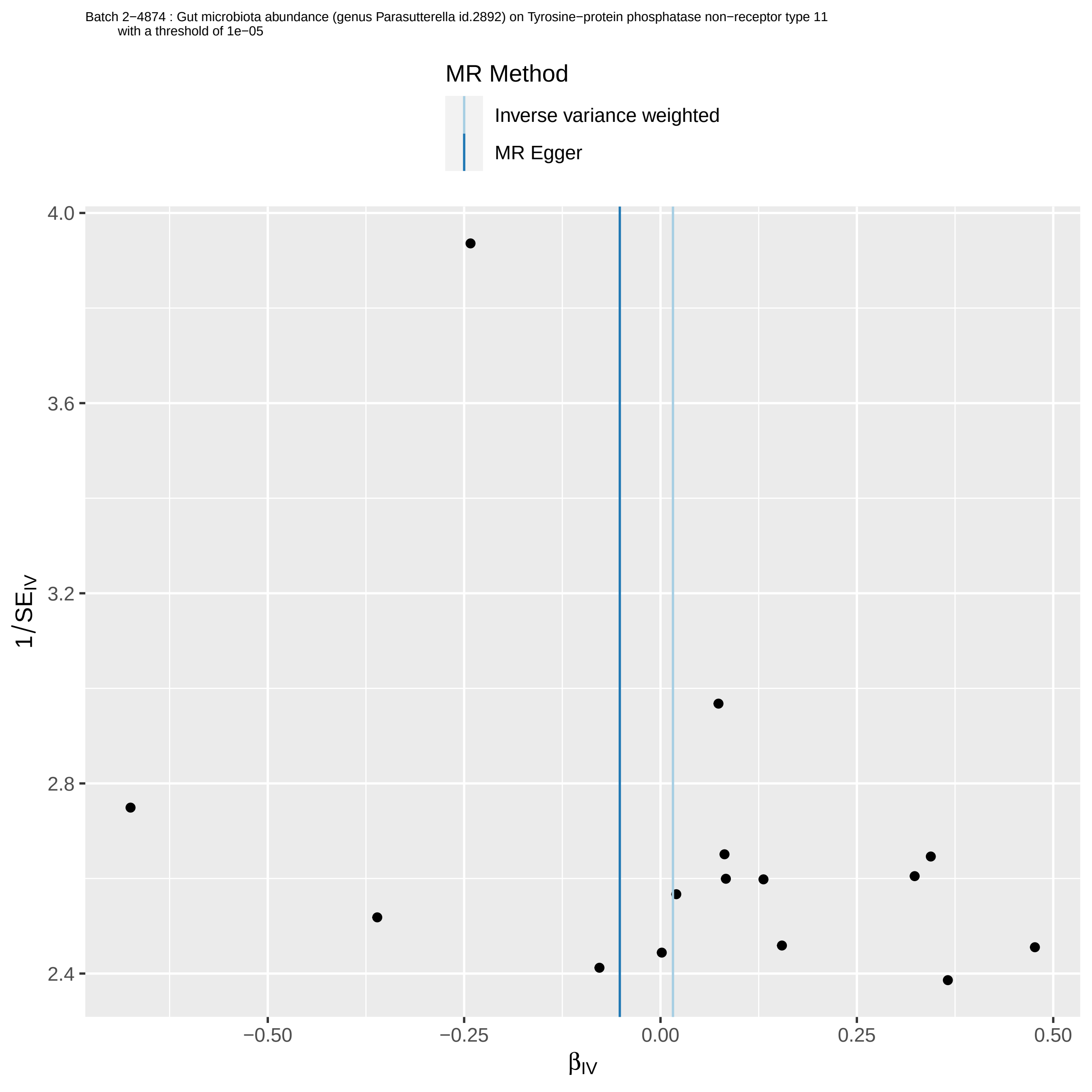
Batch 2−4874 : Gut microbiota abundance (genus Parasutterella id.2892) on Tyrosine−protein phosphatase non−receptor type 11
with a threshold of 1e−05
MR Method
Inverse variance weighted
MR Egger
4.0
3.6
3.2
2.8
2.4
1/SE_IV
−0.50
−0.25
0.00
0.25
0.50
β_IV

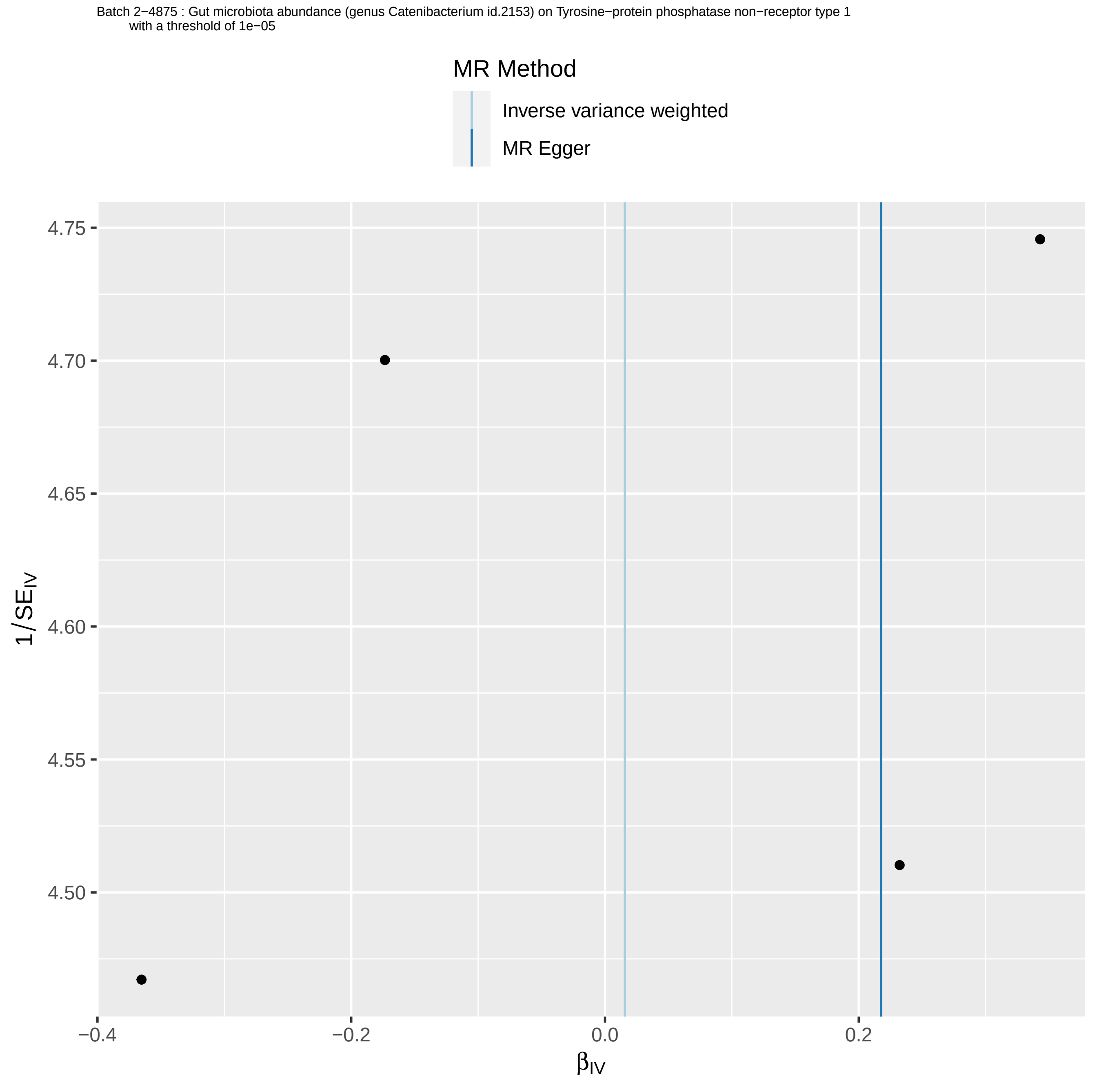

Batch 2−4875 : Gut microbiota abundance (genus Catenibacterium id.2153) on Tyrosine−protein phosphatase non−receptor type 1 with a threshold of 1e−05
MR Method
Inverse variance weighted
MR Egger
4.75
4.70
4.65
4.60
4.55
4.50
$1/SE_{IV}$
−0.4
−0.2
0.0
0.2
β_{IV}

Batch 2−4876 : Gut microbiota abundance (genus Parasutterella id.2892) on Tyrosine−protein phosphatase non−receptor type 1
with a threshold of 1e−05

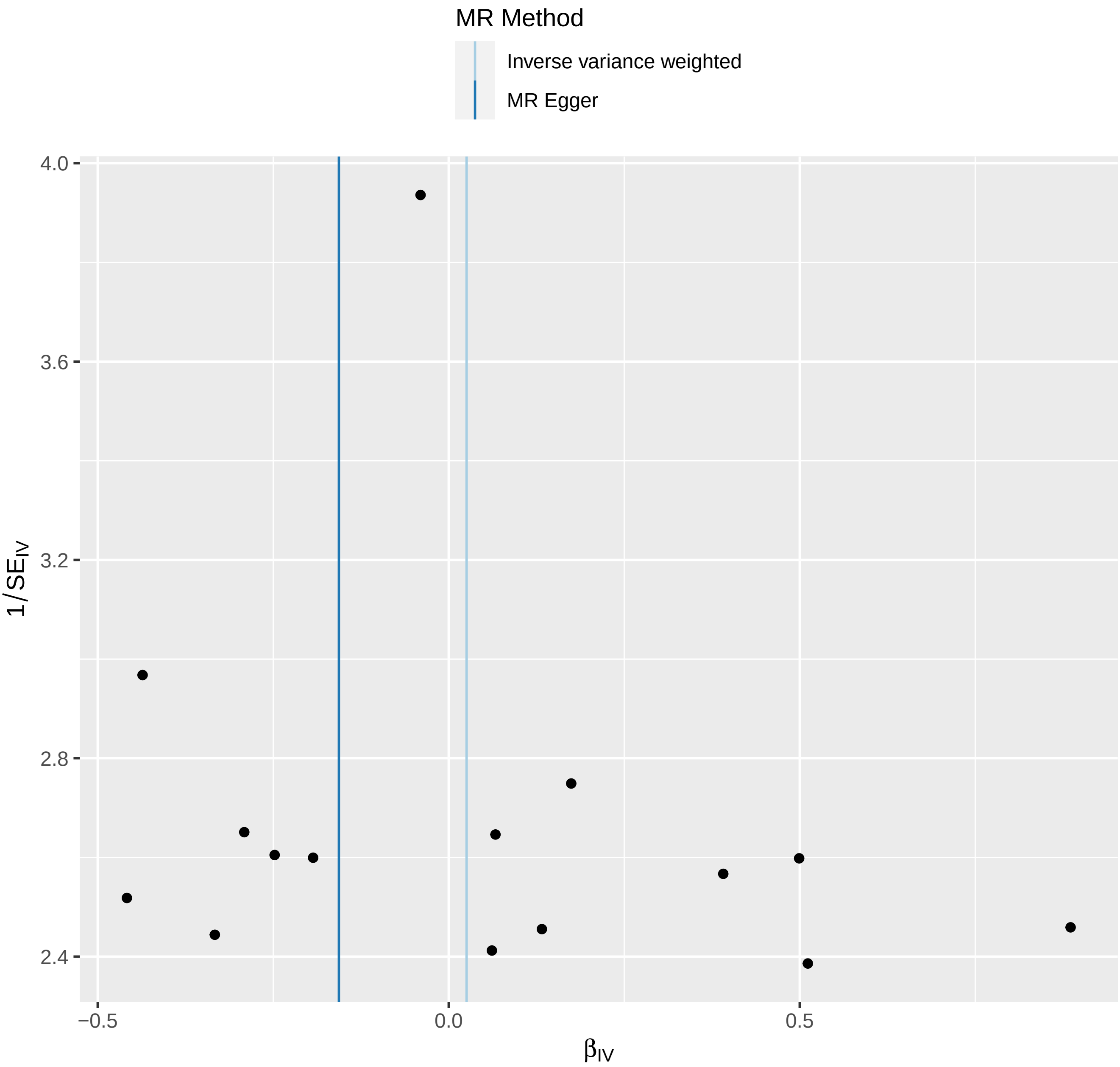

Batch 2−4877 : Gut microbiota abundance (genus Catenibacterium id.2153) on Tyrosine−protein phosphatase non−receptor type 2
with a threshold of 1e−05

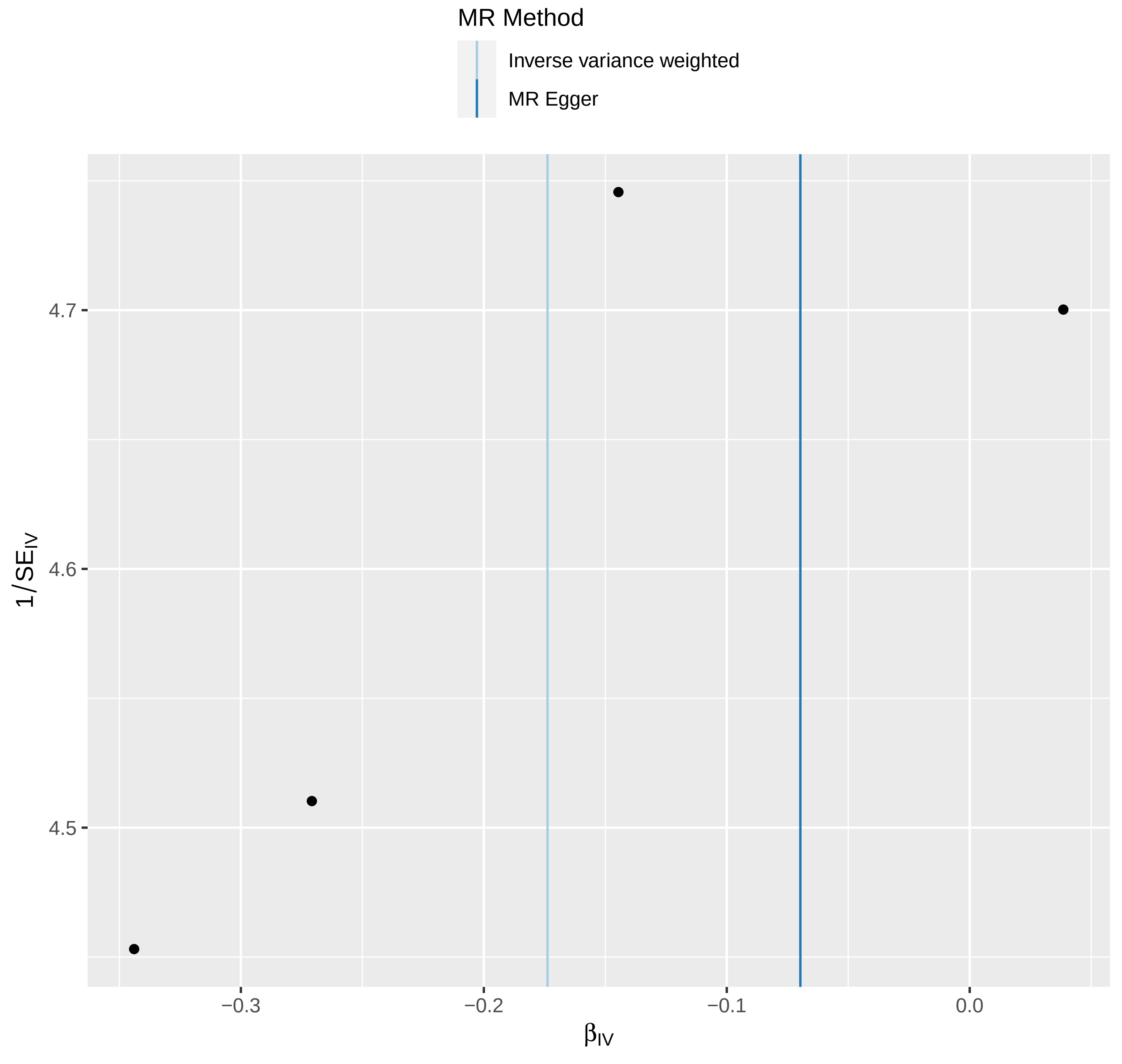

Batch 2−4878 : Gut microbiota abundance (genus Parasutterella id.2892) on Tyrosine−protein phosphatase non−receptor type 2
with a threshold of 1e−05

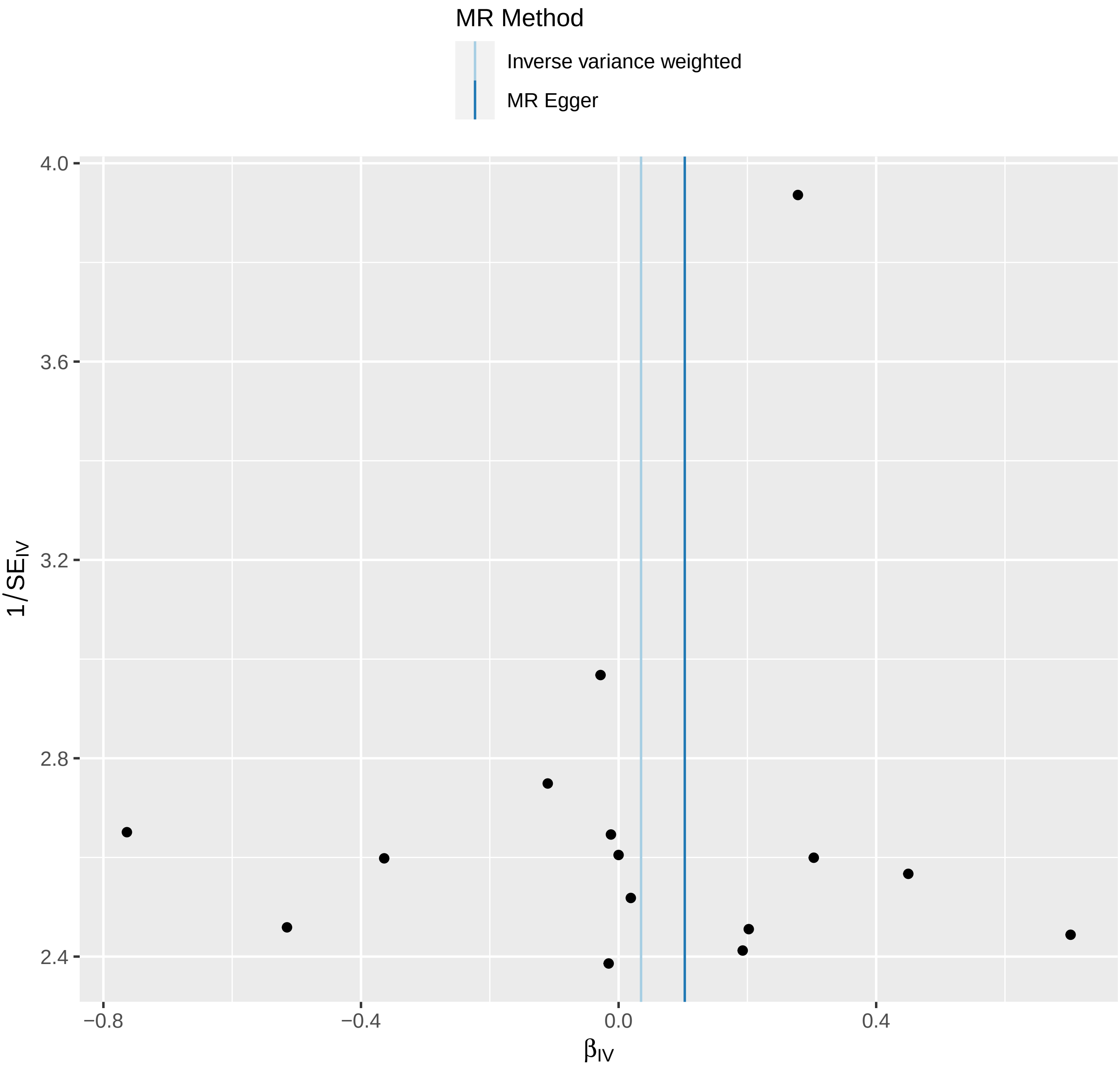

Batch 2−4879 : Gut microbiota abundance (genus Catenibacterium id.2153) on Tyrosine−protein phosphatase non−receptor type 4
with a threshold of 1e−05

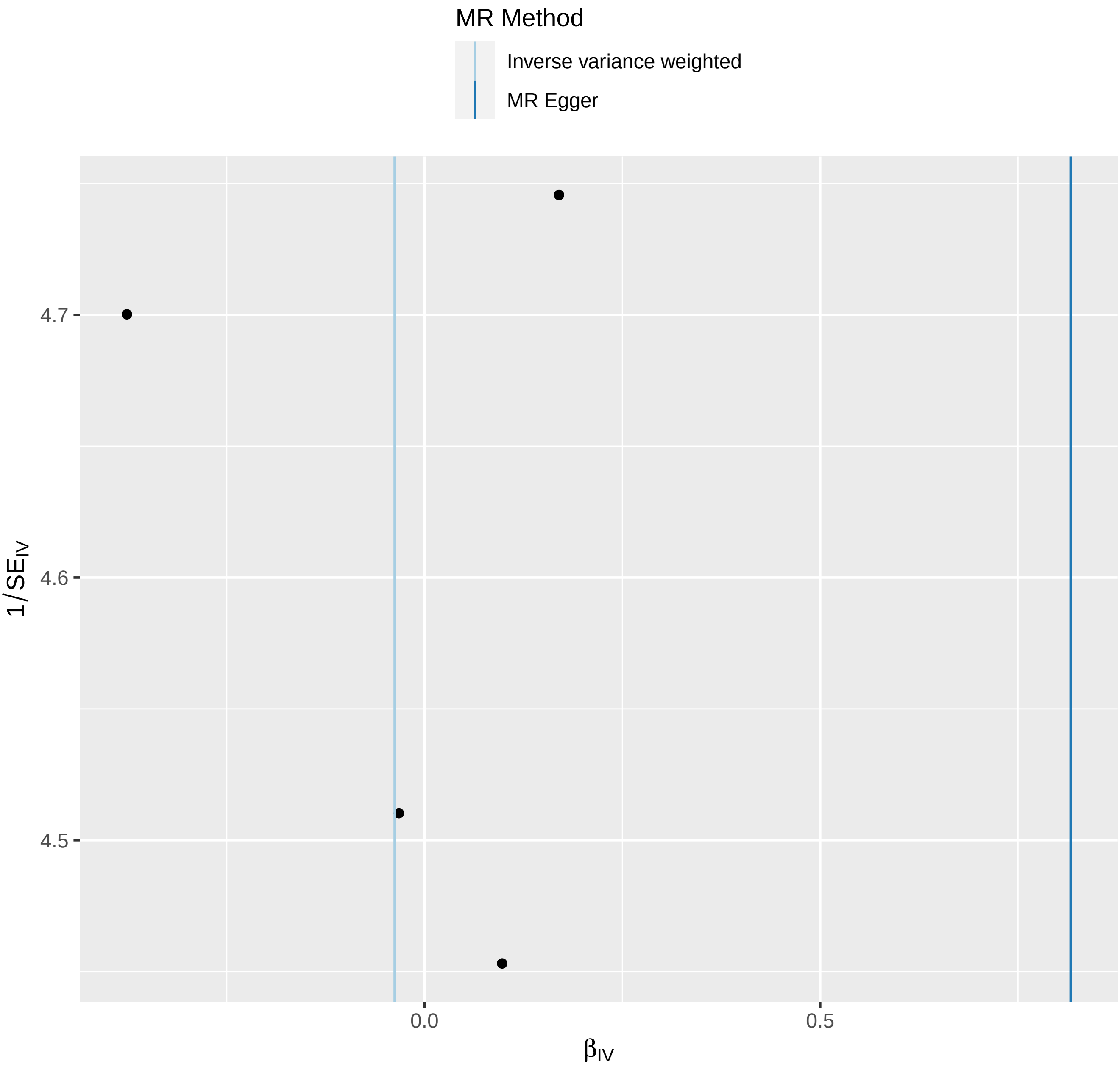

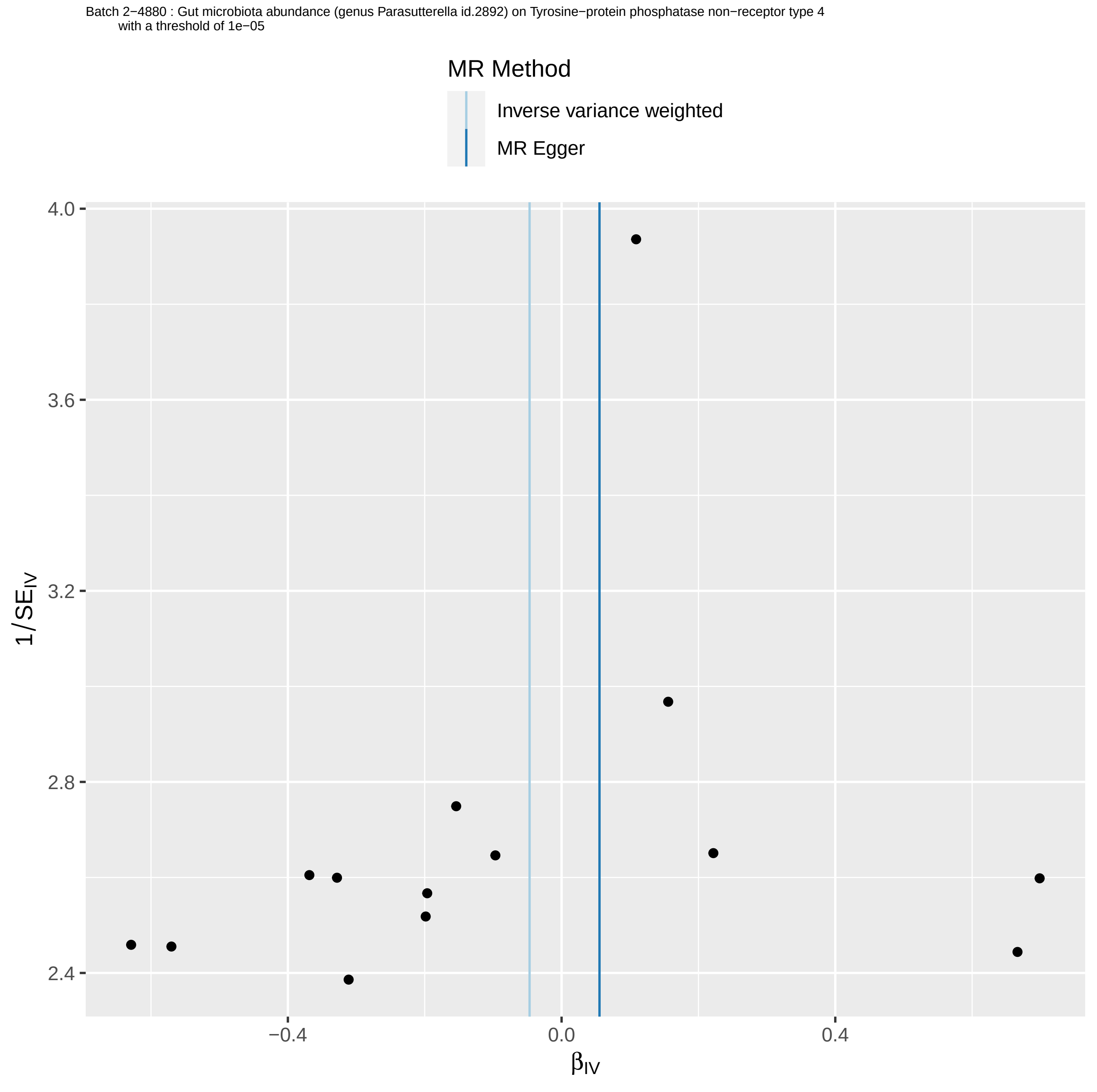

Batch 2−4880 : Gut microbiota abundance (genus Parasutterella id.2892) on Tyrosine−protein phosphatase non−receptor type 4
with a threshold of 1e−05
MR Method
Inverse variance weighted
MR Egger
4.0
3.6
3.2
2.8
2.4
1/SE_IV
−0.4
0.0
0.4
β_IV

Batch 2−4881 : Gut microbiota abundance (genus Catenibacterium id.2153) on Tyrosine−protein phosphatase non−receptor type 7
with a threshold of 1e−05

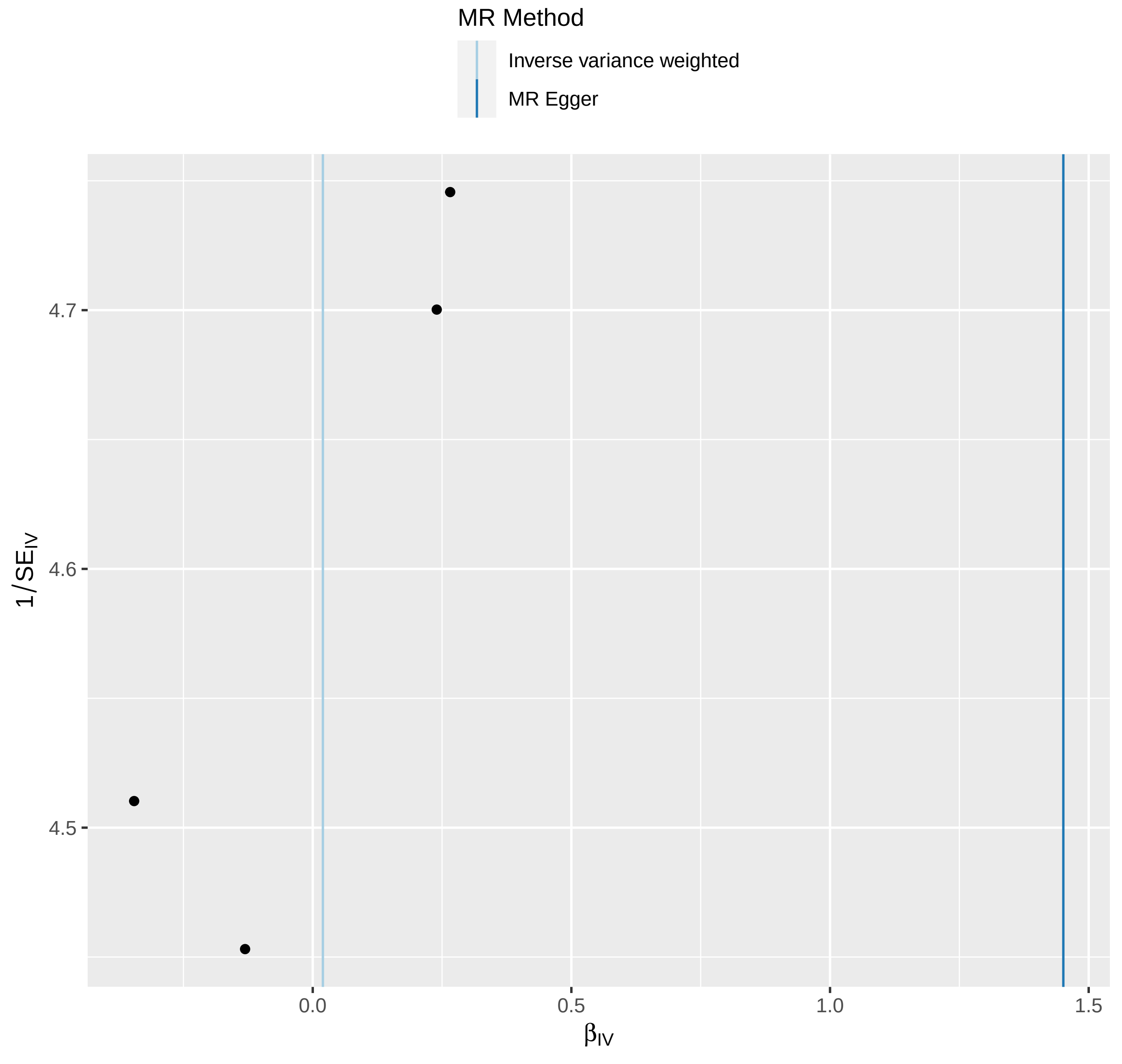

Batch 2−4882 : Gut microbiota abundance (genus Parasutterella id.2892) on Tyrosine−protein phosphatase non−receptor type 7
with a threshold of 1e−05

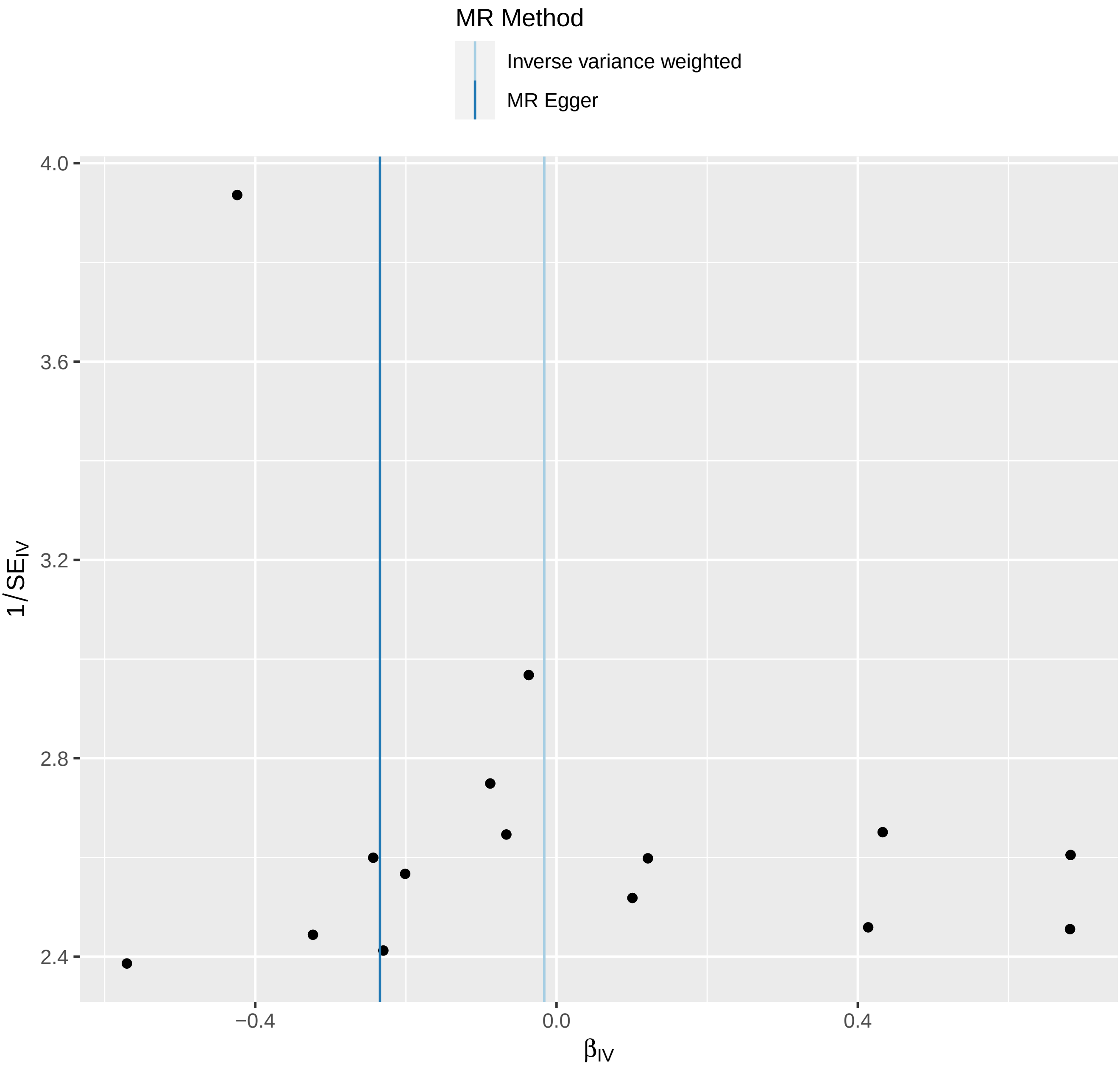

Batch 2−4883 : Gut microbiota abundance (genus Catenibacterium id.2153) on Tyrosine−protein phosphatase non−receptor type 9 with a threshold of 1e−05

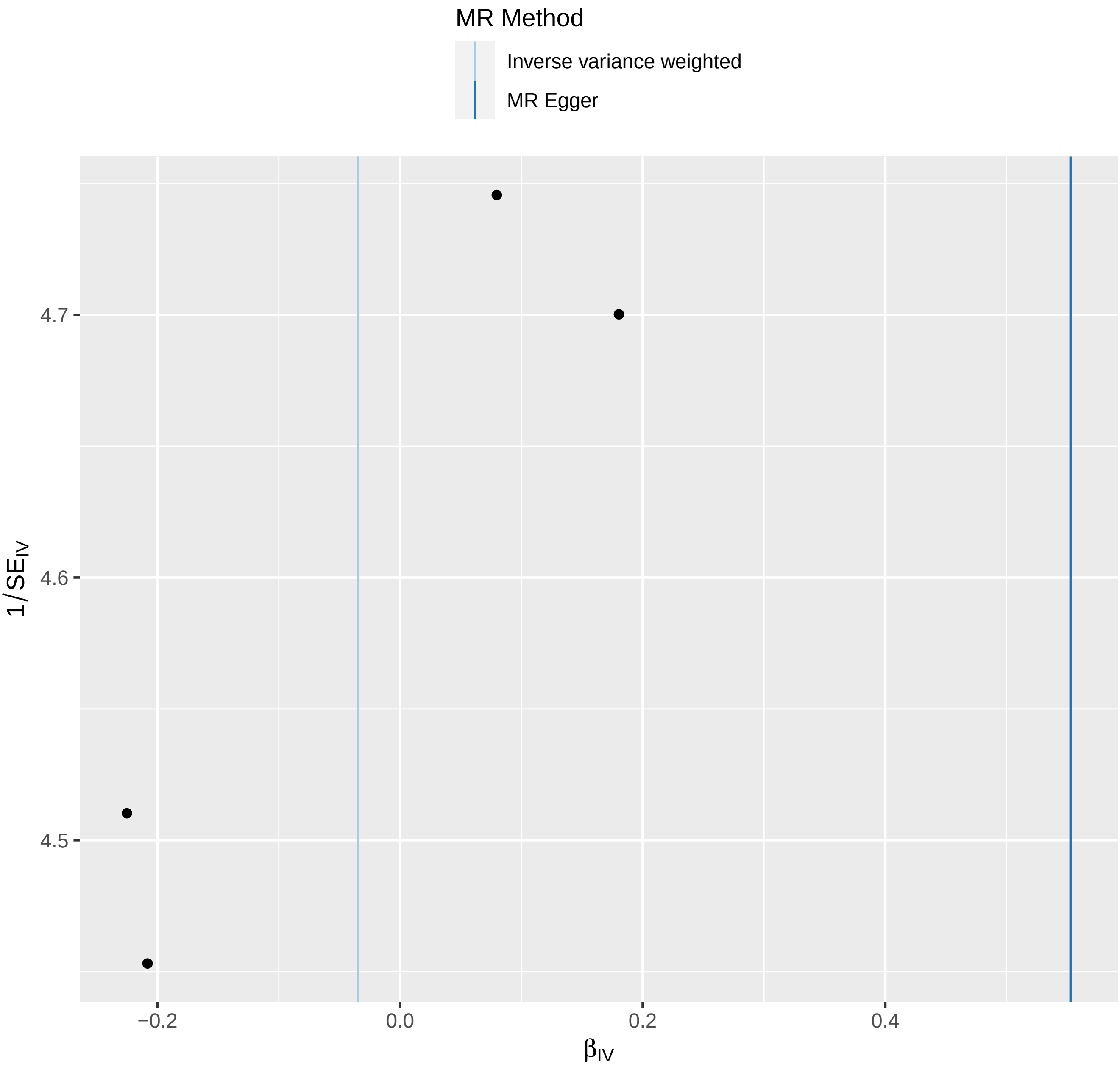

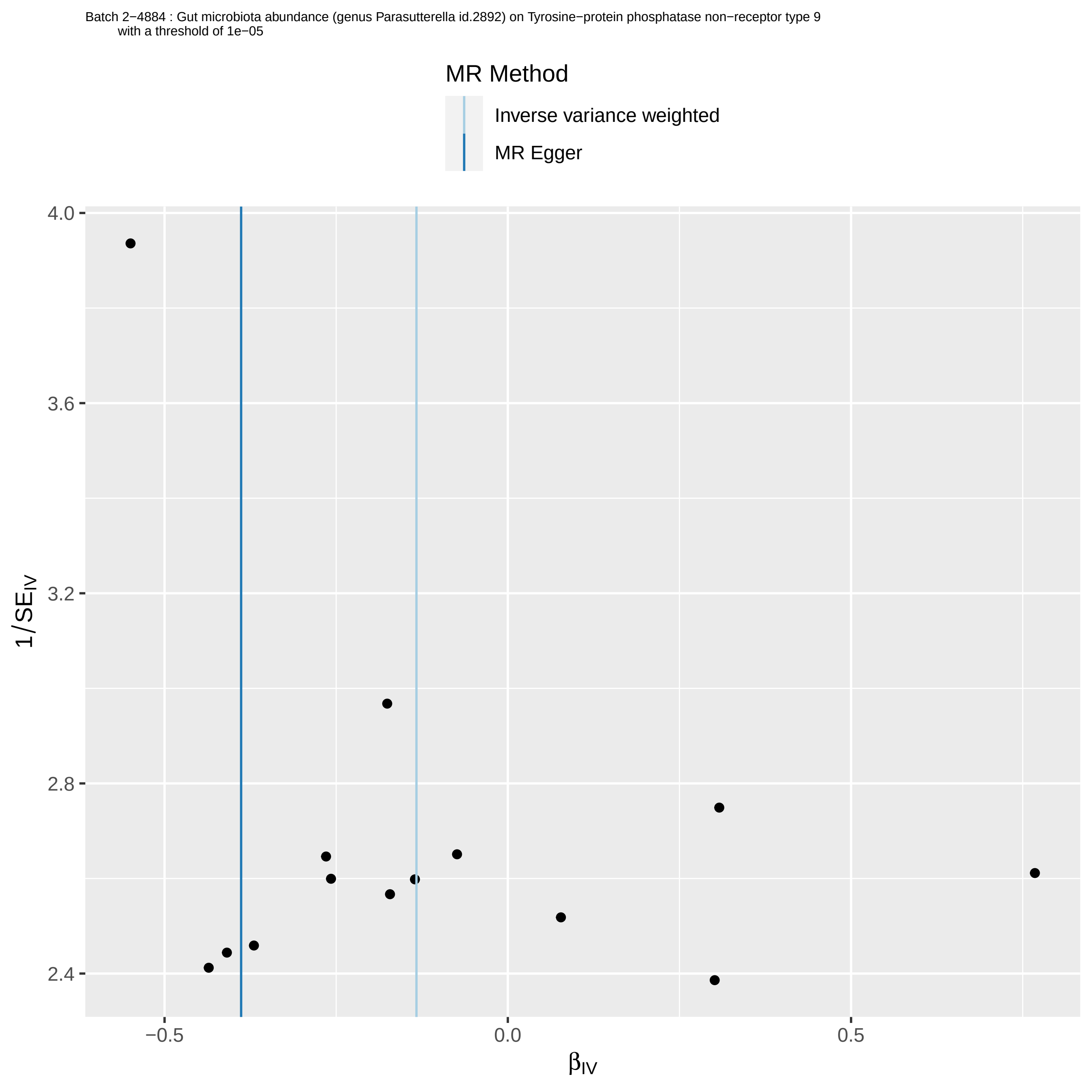
Batch 2−4884 : Gut microbiota abundance (genus Parasutterella id.2892) on Tyrosine−protein phosphatase non−receptor type 9
with a threshold of 1e−05
MR Method
Inverse variance weighted
MR Egger
4.0
3.6
3.2
2.8
2.4
1/SE_IV
−0.5
0.0
0.5
β_IV

Batch 2−4885 : Gut microbiota abundance (genus Catenibacterium id.2153) on Receptor−type tyrosine−protein phosphatase delta
with a threshold of 1e−05

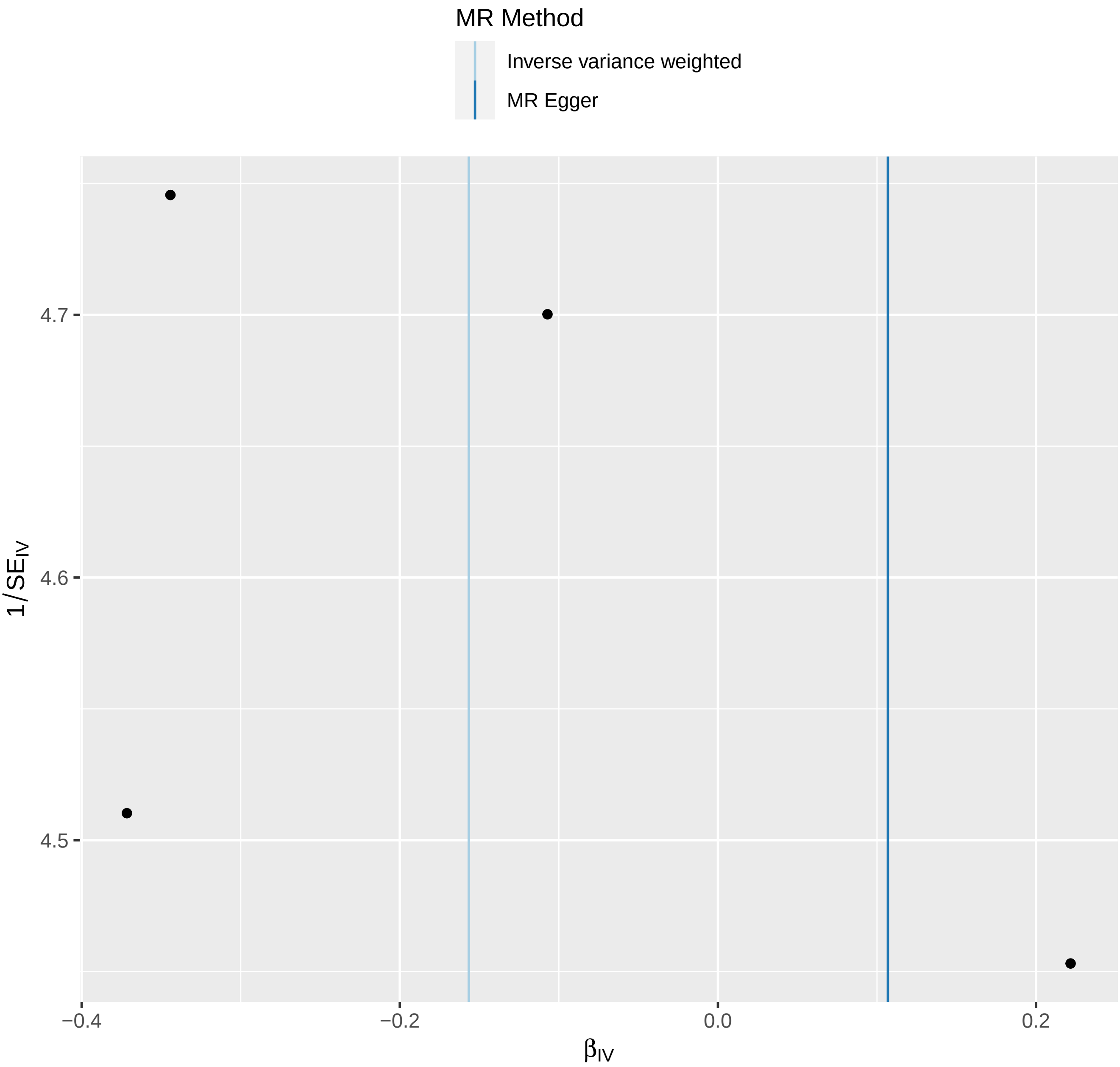

Batch 2−4886 : Gut microbiota abundance (genus Parasutterella id.2892) on Receptor−type tyrosine−protein phosphatase delta
with a threshold of 1e−05

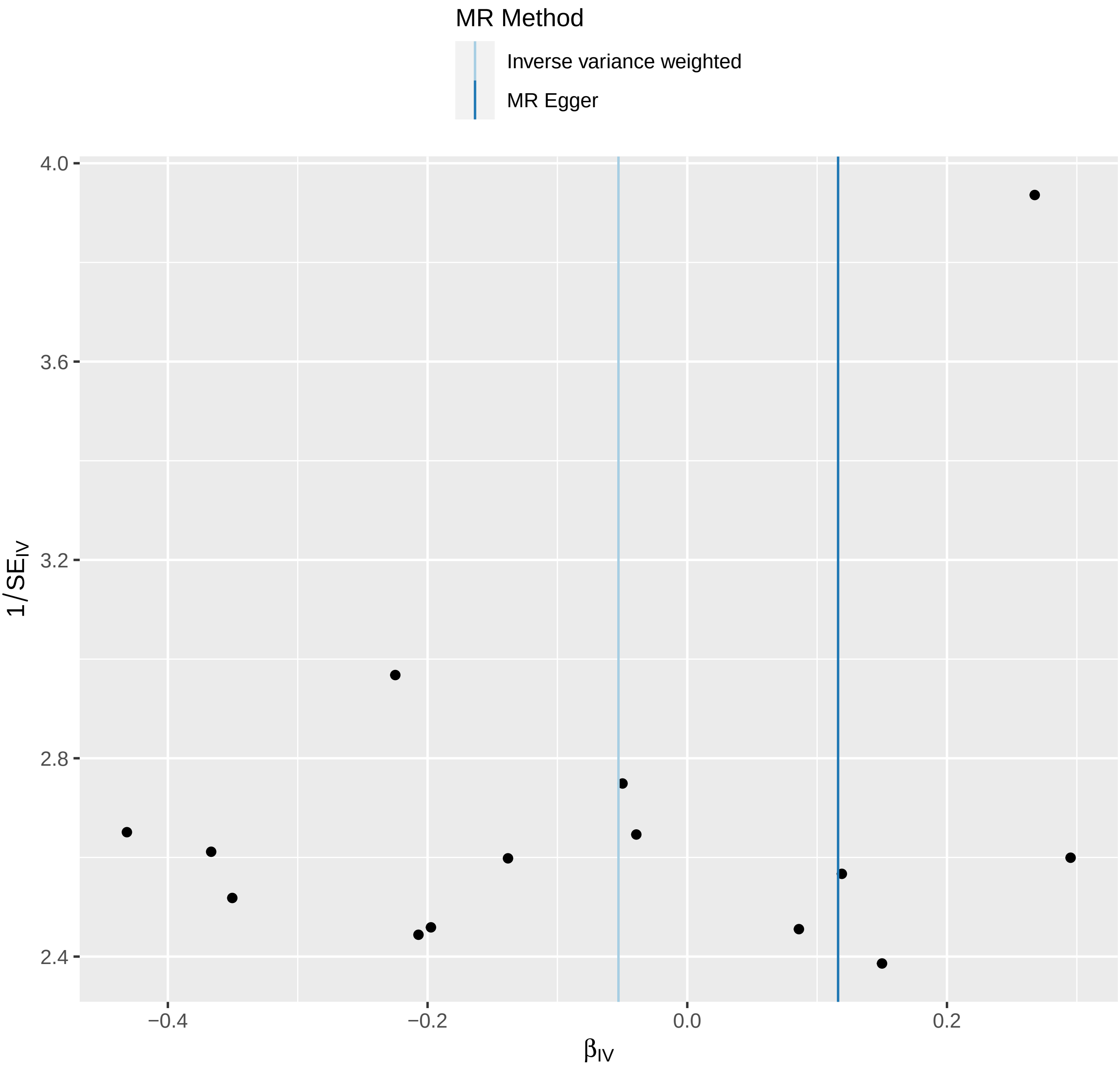

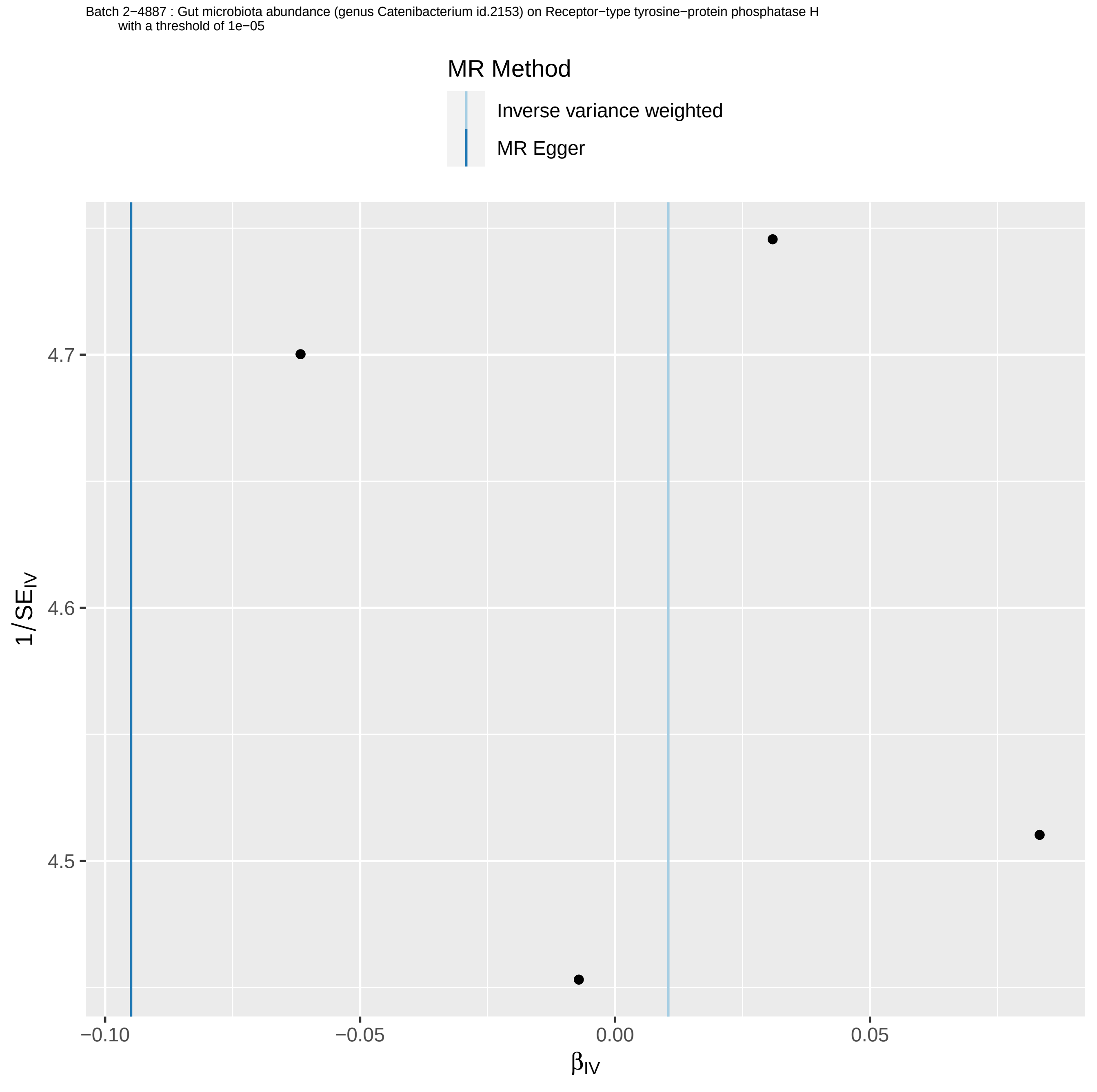
Batch 2−4887 : Gut microbiota abundance (genus Catenibacterium id.2153) on Receptor−type tyrosine−protein phosphatase H
with a threshold of 1e−05
MR Method
Inverse variance weighted
MR Egger
4.7
4.6
4.5
1/SEIV
−0.10
−0.05
0.00
0.05
βIV

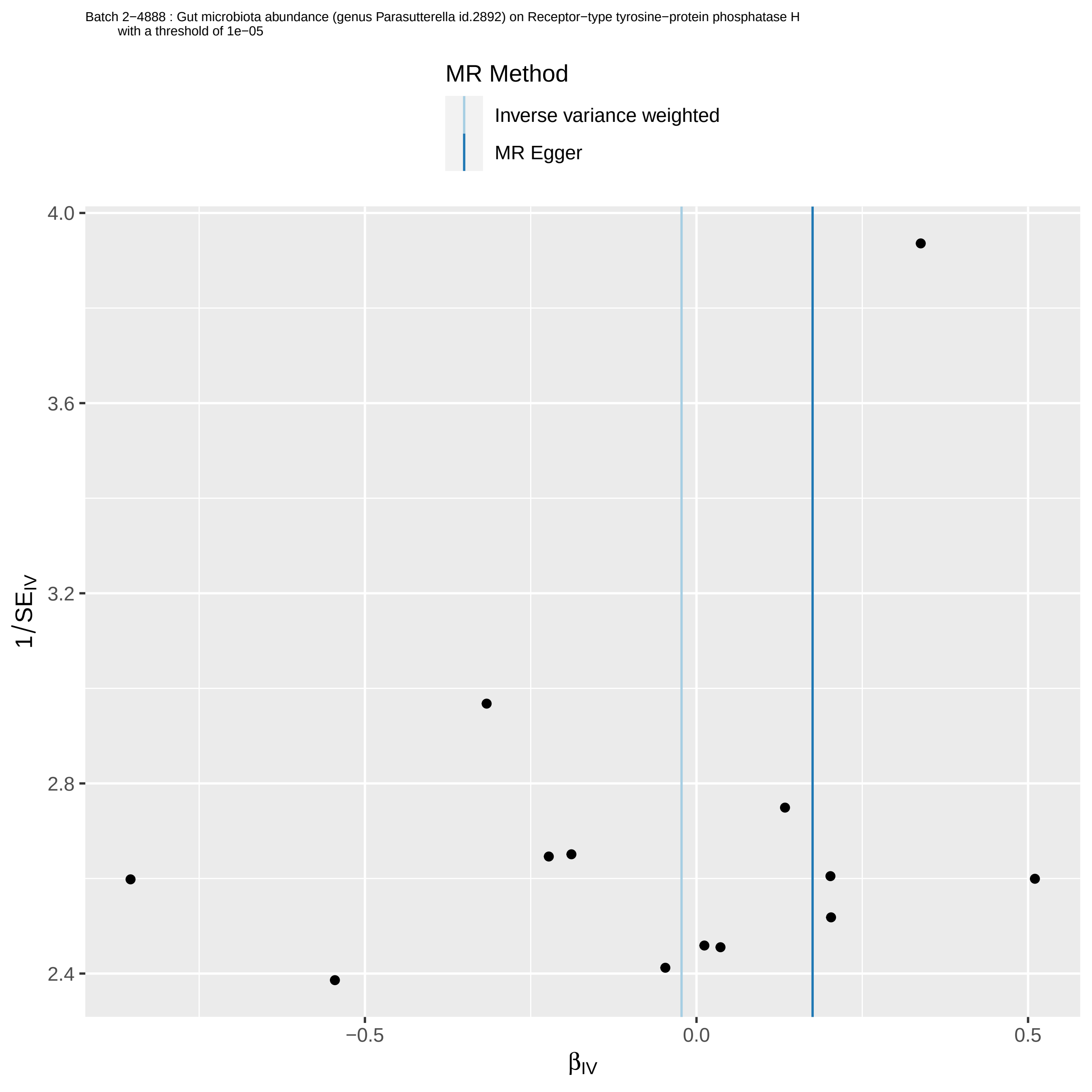

Batch 2−4888 : Gut microbiota abundance (genus Parasutterella id.2892) on Receptor−type tyrosine−protein phosphatase H
with a threshold of 1e−05
MR Method
Inverse variance weighted
MR Egger
4.0
3.6
3.2
2.8
2.4
1/SE_IV
−0.5
0.0
0.5
β_IV

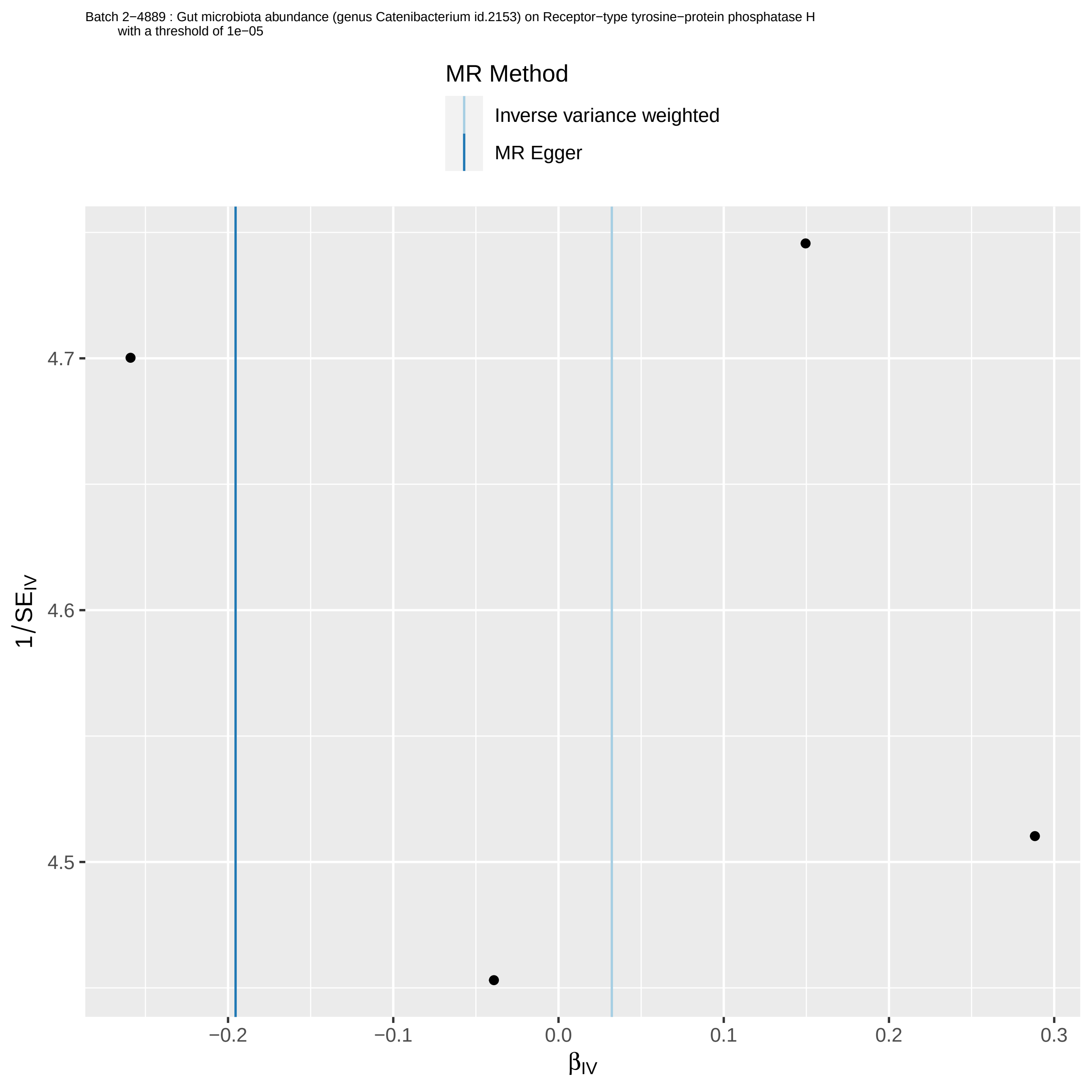

Batch 2−4889 : Gut microbiota abundance (genus Catenibacterium id.2153) on Receptor−type tyrosine−protein phosphatase H
with a threshold of 1e−05
MR Method
Inverse variance weighted
MR Egger
4.7
4.6
4.5
1/SE_IV
−0.2
−0.1
0.0
0.1
0.2
0.3
β_IV

Batch 2−4890 : Gut microbiota abundance (genus Parasutterella id.2892) on Receptor−type tyrosine−protein phosphatase H
with a threshold of 1e−05

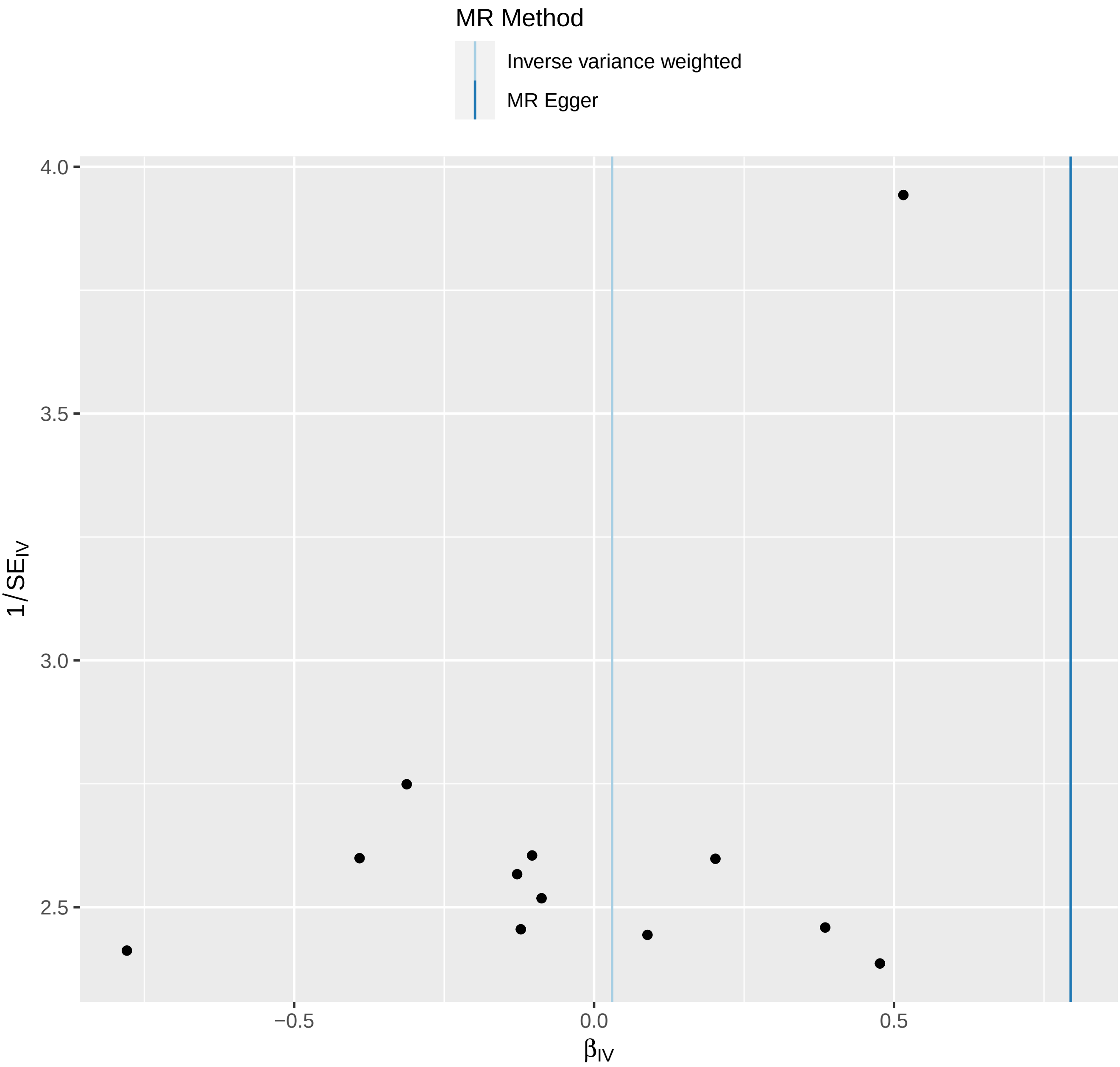

Batch 2−4891 : Gut microbiota abundance (genus Catenibacterium id.2153) on Receptor−type tyrosine−protein phosphatase eta
with a threshold of 1e−05

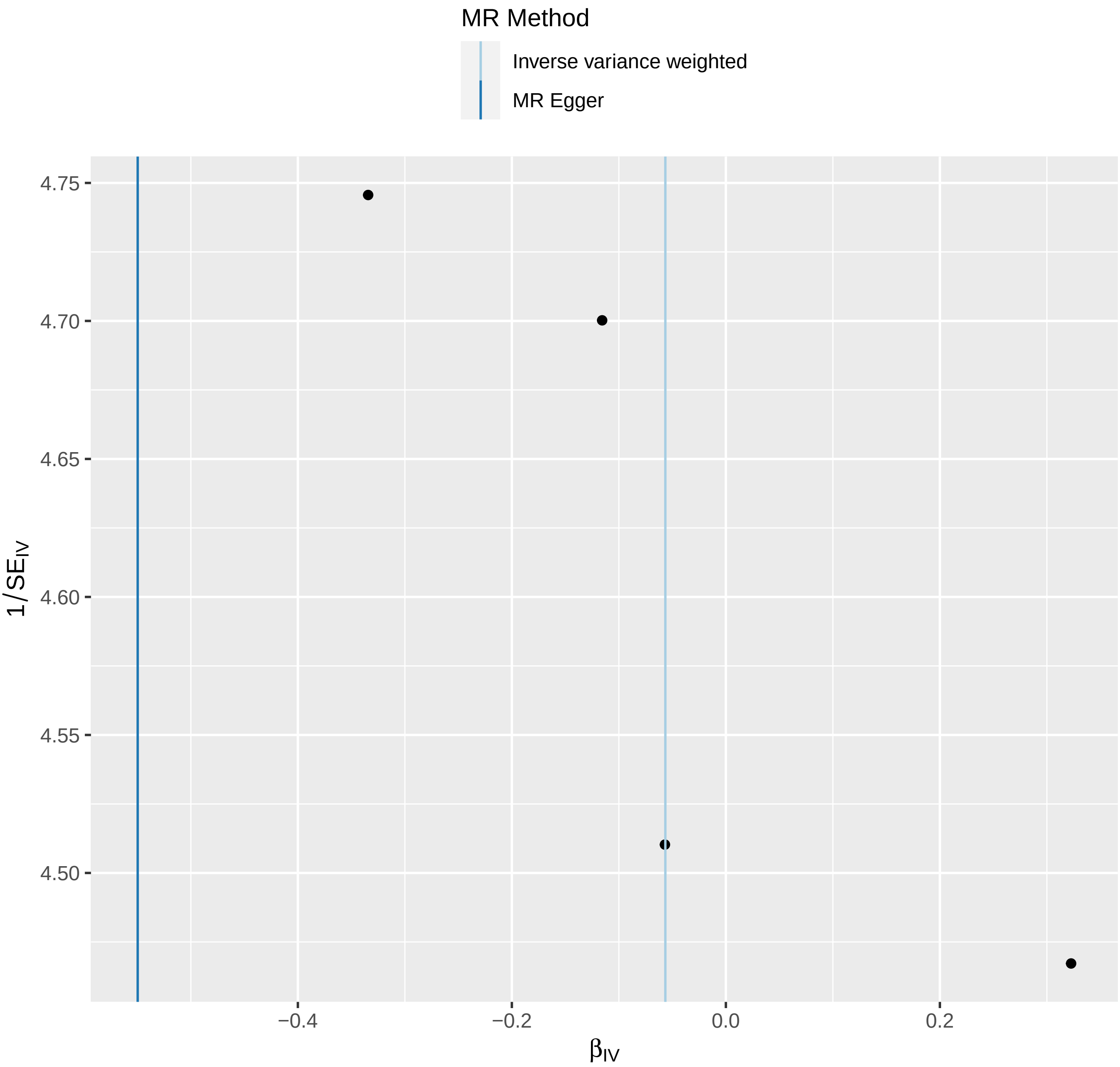

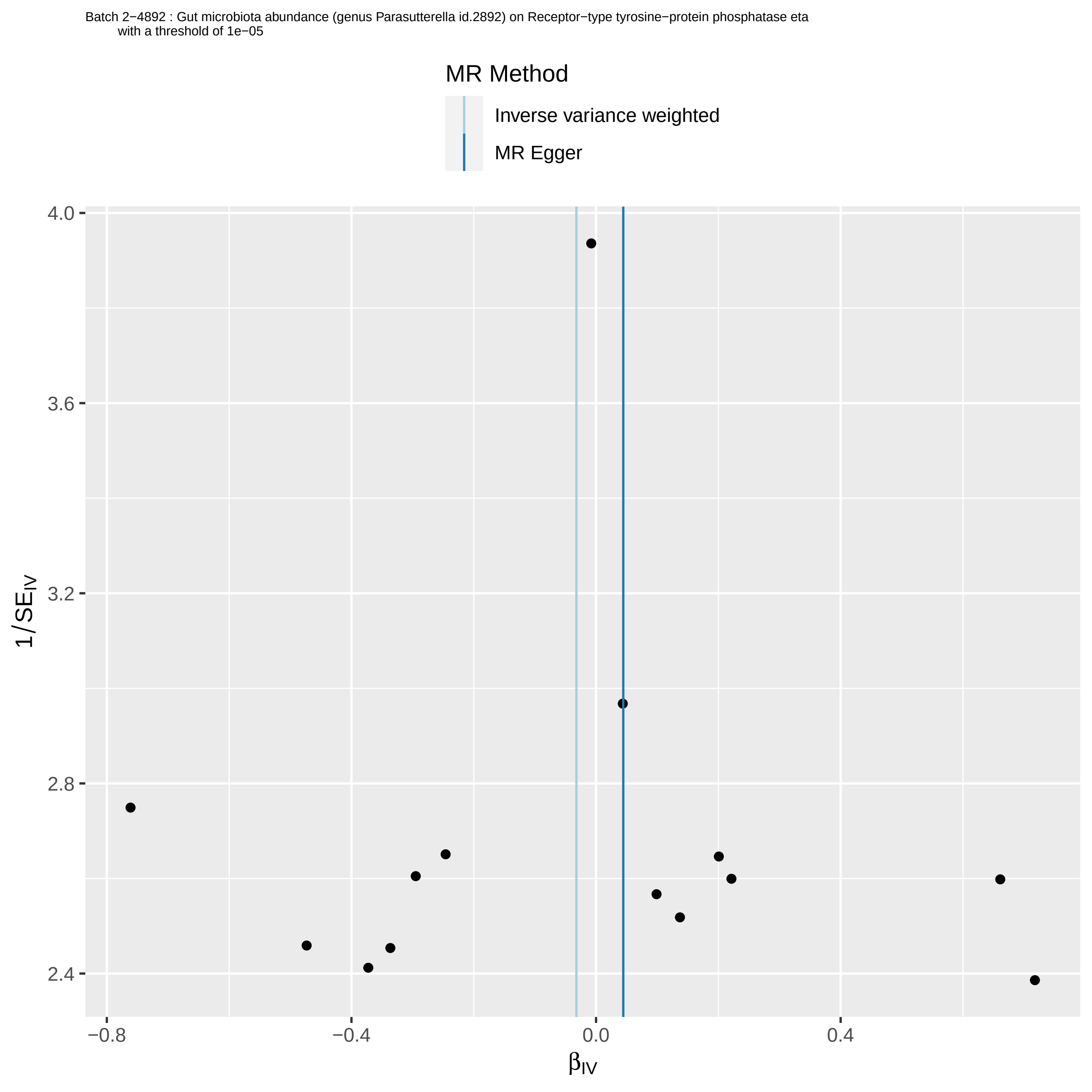
Batch 2−4892 : Gut microbiota abundance (genus Parasutterella id.2892) on Receptor−type tyrosine−protein phosphatase eta
with a threshold of 1e−05
MR Method
Inverse variance weighted
MR Egger
4.0
3.6
3.2
2.8
2.4
1/SE_IV
−0.8
−0.4
0.0
0.4
β_IV

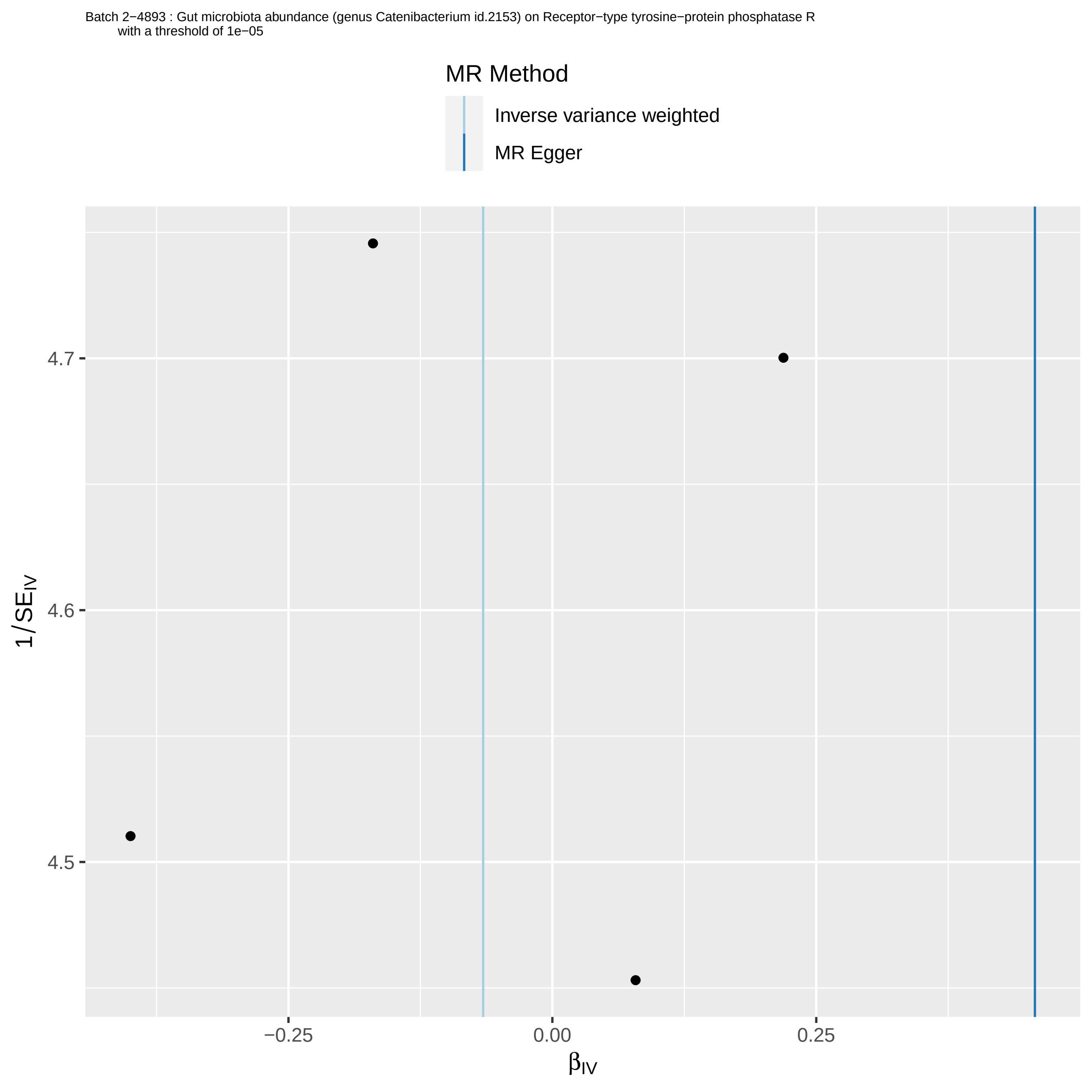

Batch 2−4893 : Gut microbiota abundance (genus Catenibacterium id.2153) on Receptor−type tyrosine−protein phosphatase R
with a threshold of 1e−05
MR Method
Inverse variance weighted
MR Egger
4.7
4.6
4.5
1/SE_IV
−0.25
0.00
0.25
β_IV

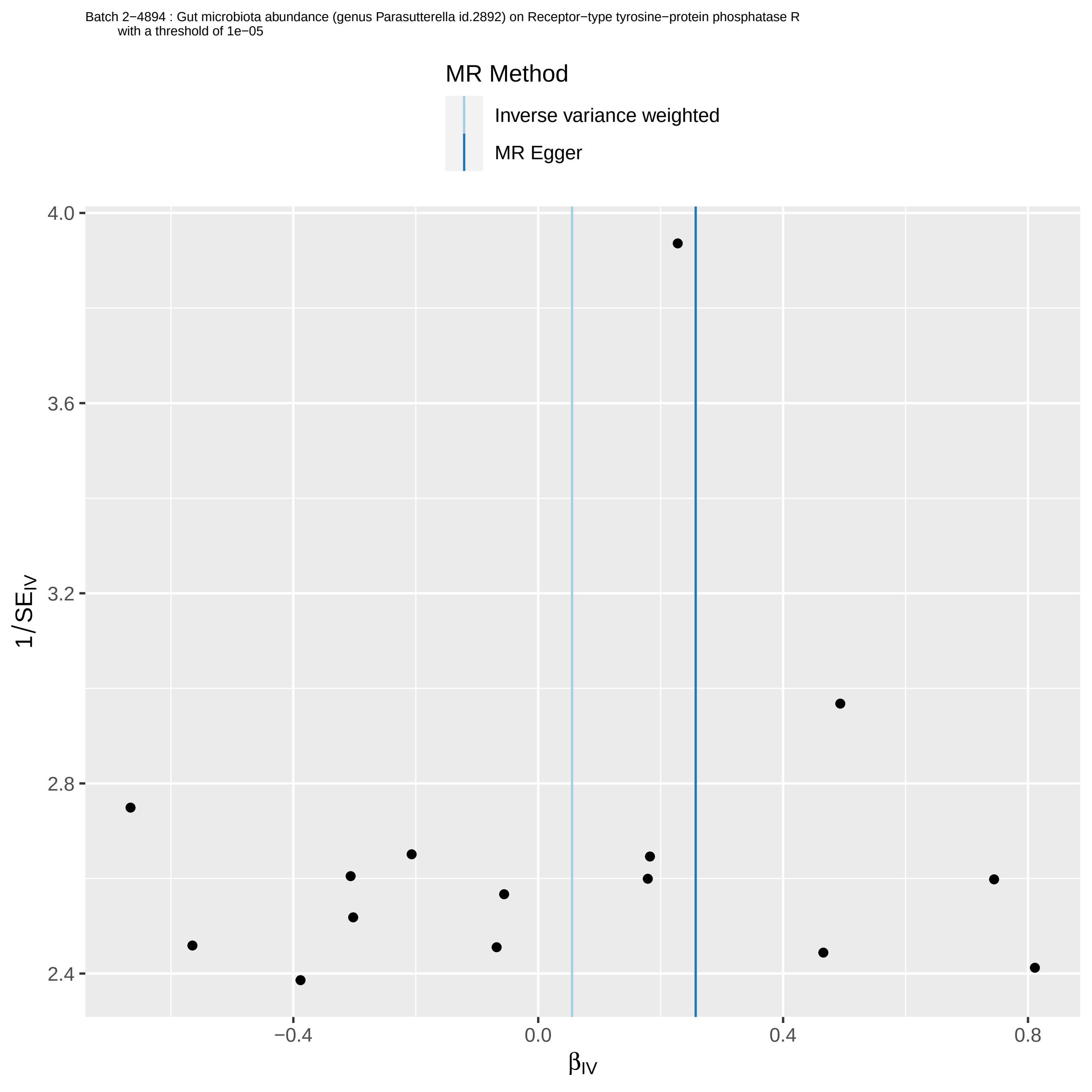

Batch 2−4894 : Gut microbiota abundance (genus Parasutterella id.2892) on Receptor−type tyrosine−protein phosphatase R
with a threshold of 1e−05
MR Method
Inverse variance weighted
MR Egger
4.0
3.6
3.2
2.8
2.4
1/SE_IV
−0.4
0.0
0.4
0.8
β_IV

Batch 2−4895 : Gut microbiota abundance (genus Catenibacterium id.2153) on Receptor−type tyrosine−protein phosphatase S
with a threshold of 1e−05

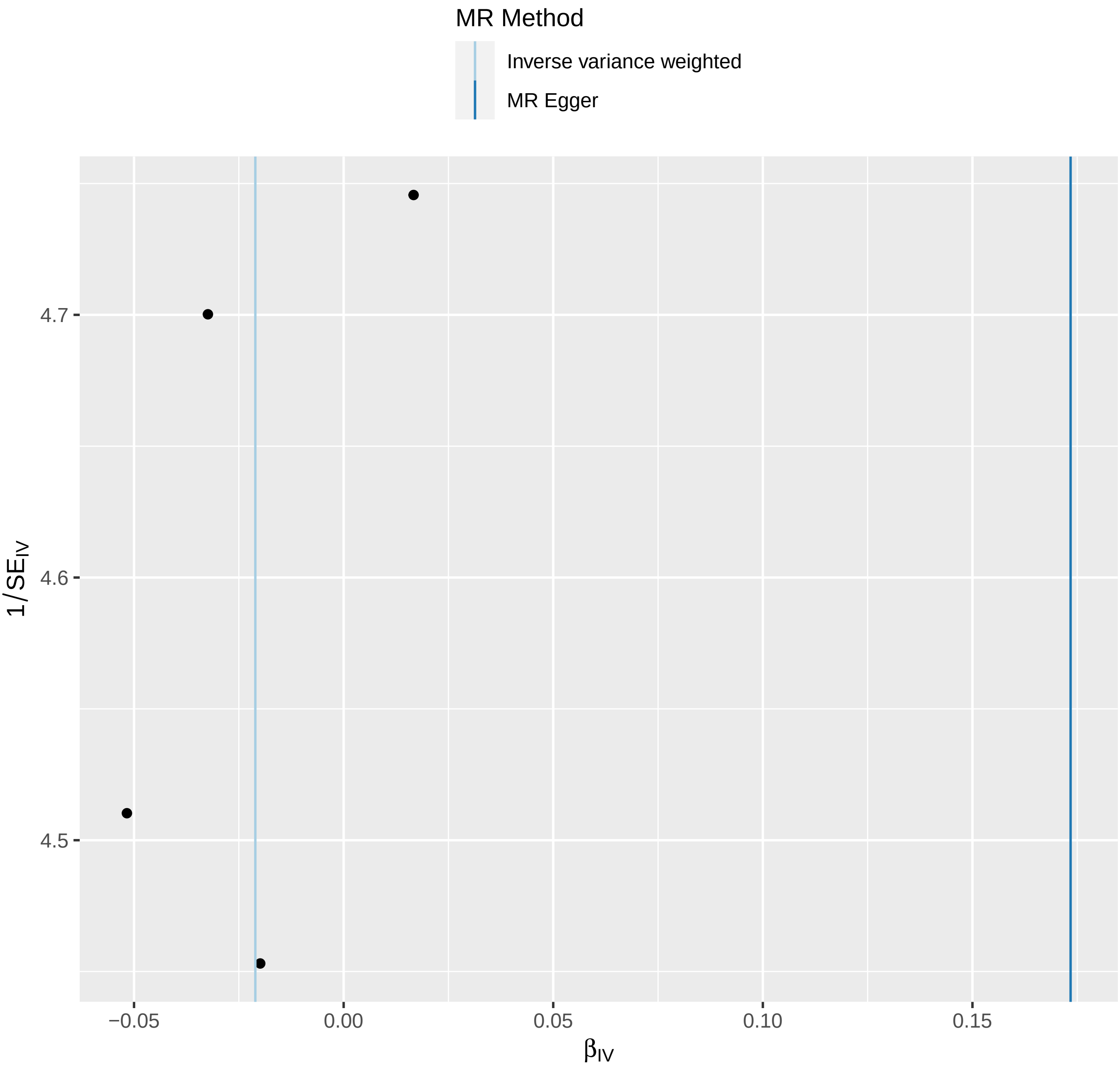

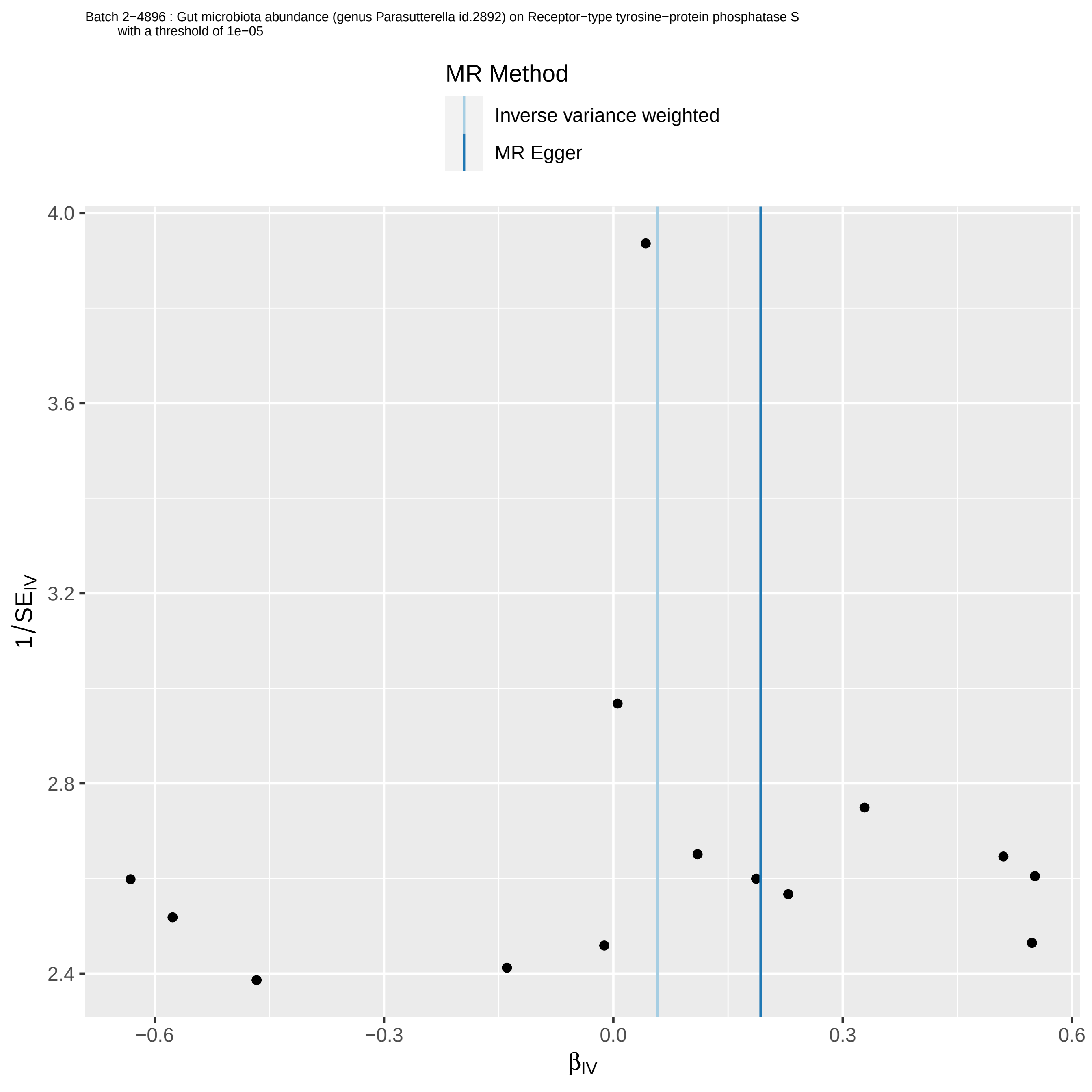
Batch 2−4896 : Gut microbiota abundance (genus Parasutterella id.2892) on Receptor−type tyrosine−protein phosphatase S
with a threshold of 1e−05
MR Method
Inverse variance weighted
MR Egger
4.0
3.6
3.2
2.8
2.4
1/SE_IV
−0.6
−0.3
0.0
0.3
0.6
β_IV

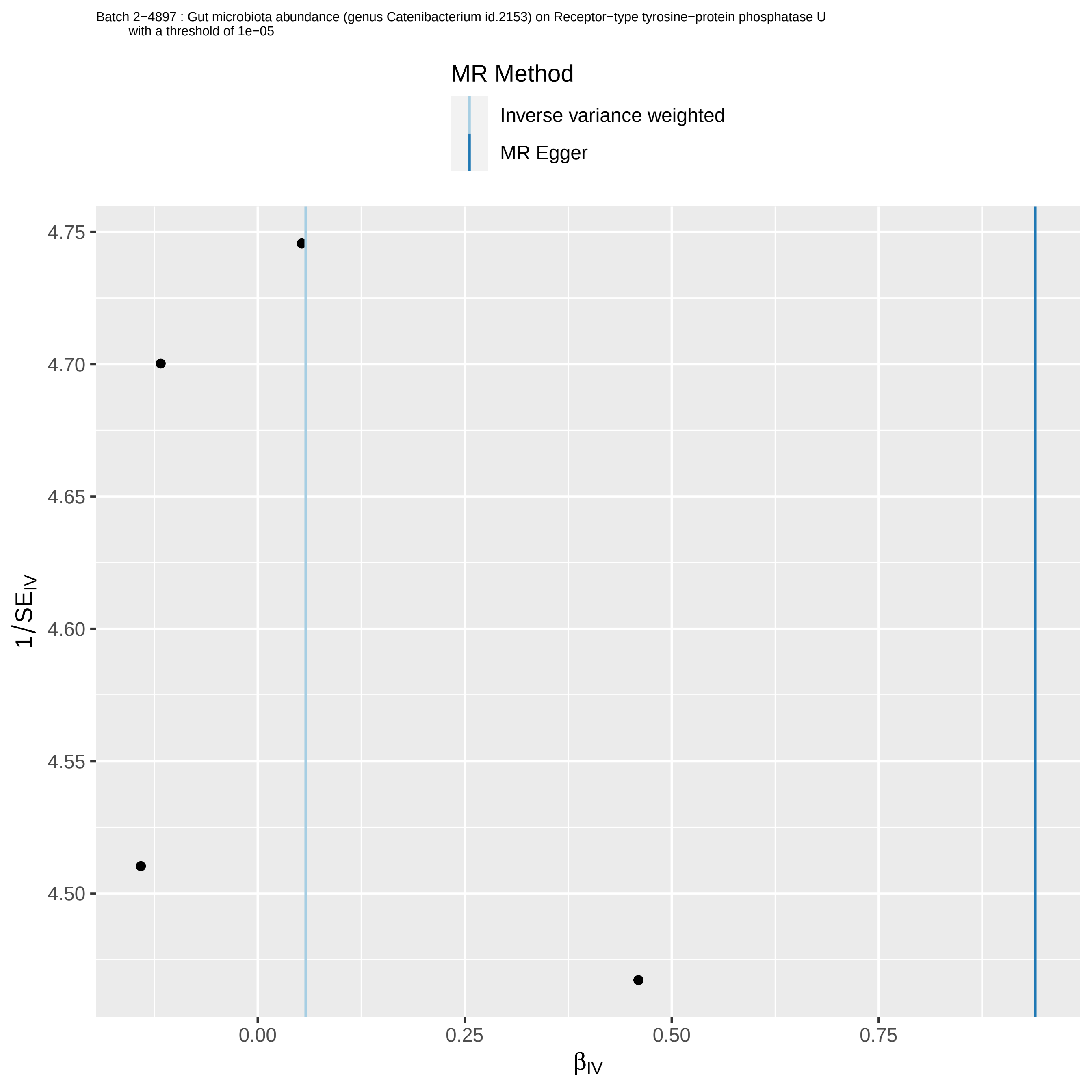
Batch 2−4897 : Gut microbiota abundance (genus Catenibacterium id.2153) on Receptor−type tyrosine−protein phosphatase U
with a threshold of 1e−05
MR Method
Inverse variance weighted
MR Egger
4.75
4.70
4.65
4.60
4.55
4.50
1/SE_IV
0.00
0.25
0.50
0.75
β_IV

Batch 2−4898 : Gut microbiota abundance (genus Parasutterella id.2892) on Receptor−type tyrosine−protein phosphatase U
with a threshold of 1e−05

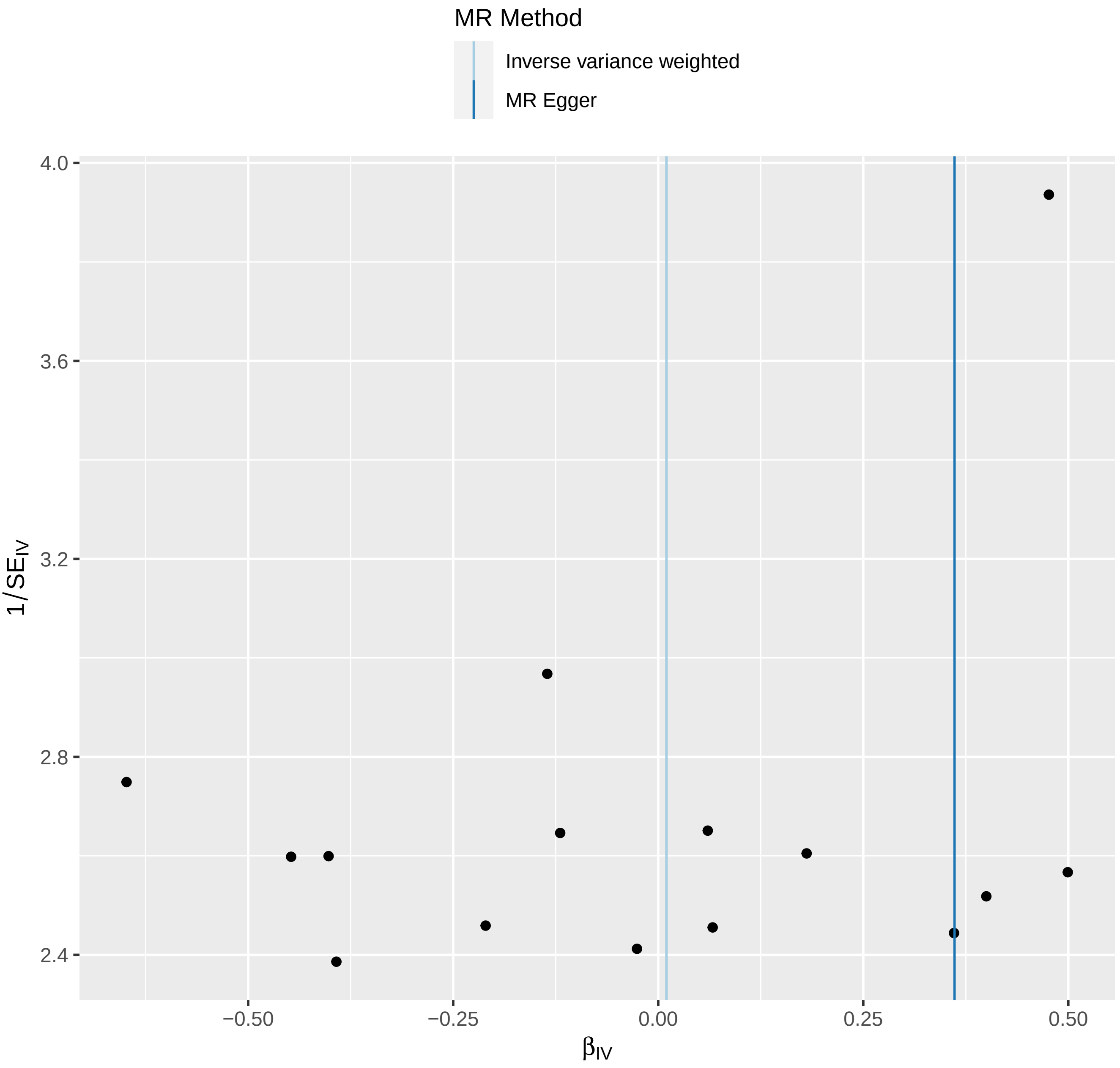

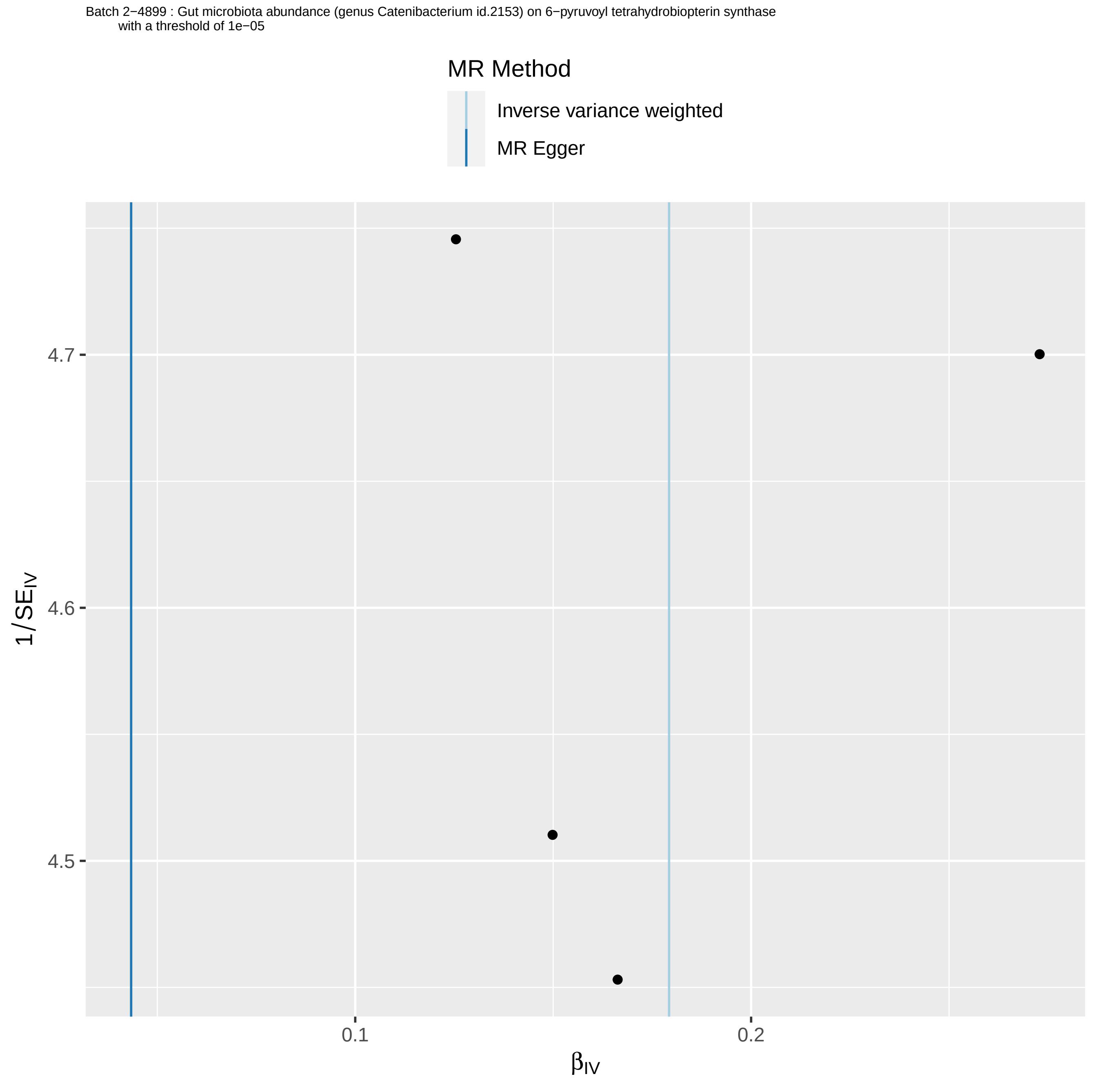
Batch 2−4899 : Gut microbiota abundance (genus Catenibacterium id.2153) on 6−pyruvoyl tetrahydrobiopterin synthase
with a threshold of 1e−05
MR Method
Inverse variance weighted
MR Egger
4.7
4.6
4.5
1/SE_IV
0.1
0.2
β_IV

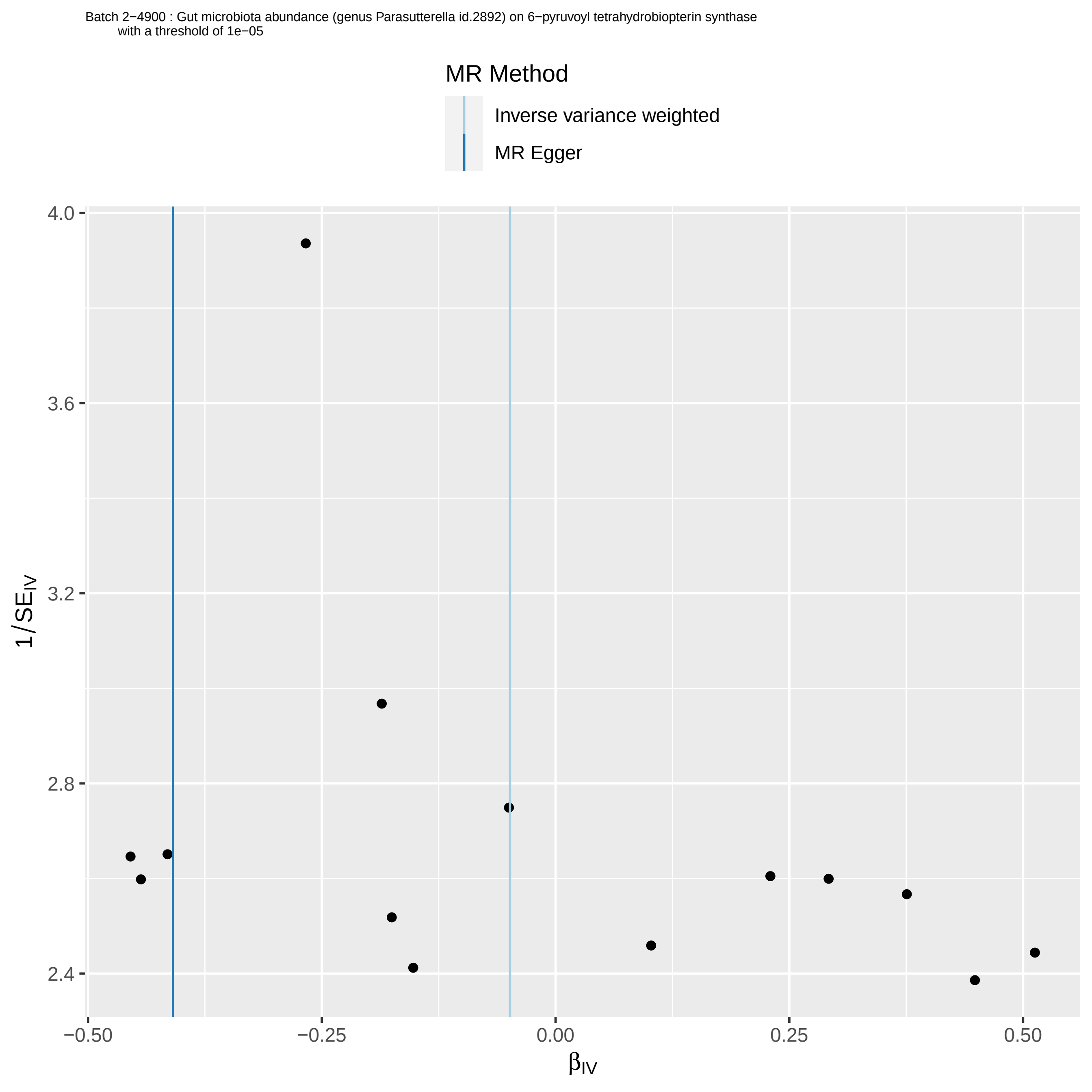

Batch 2−4900 : Gut microbiota abundance (genus Parasutterella id.2892) on 6−pyruvoyl tetrahydrobiopterin synthase
with a threshold of 1e−05
MR Method
Inverse variance weighted
MR Egger
4.0
3.6
3.2
2.8
2.4
1/SE_IV
−0.50
−0.25
0.00
0.25
0.50
β_IV

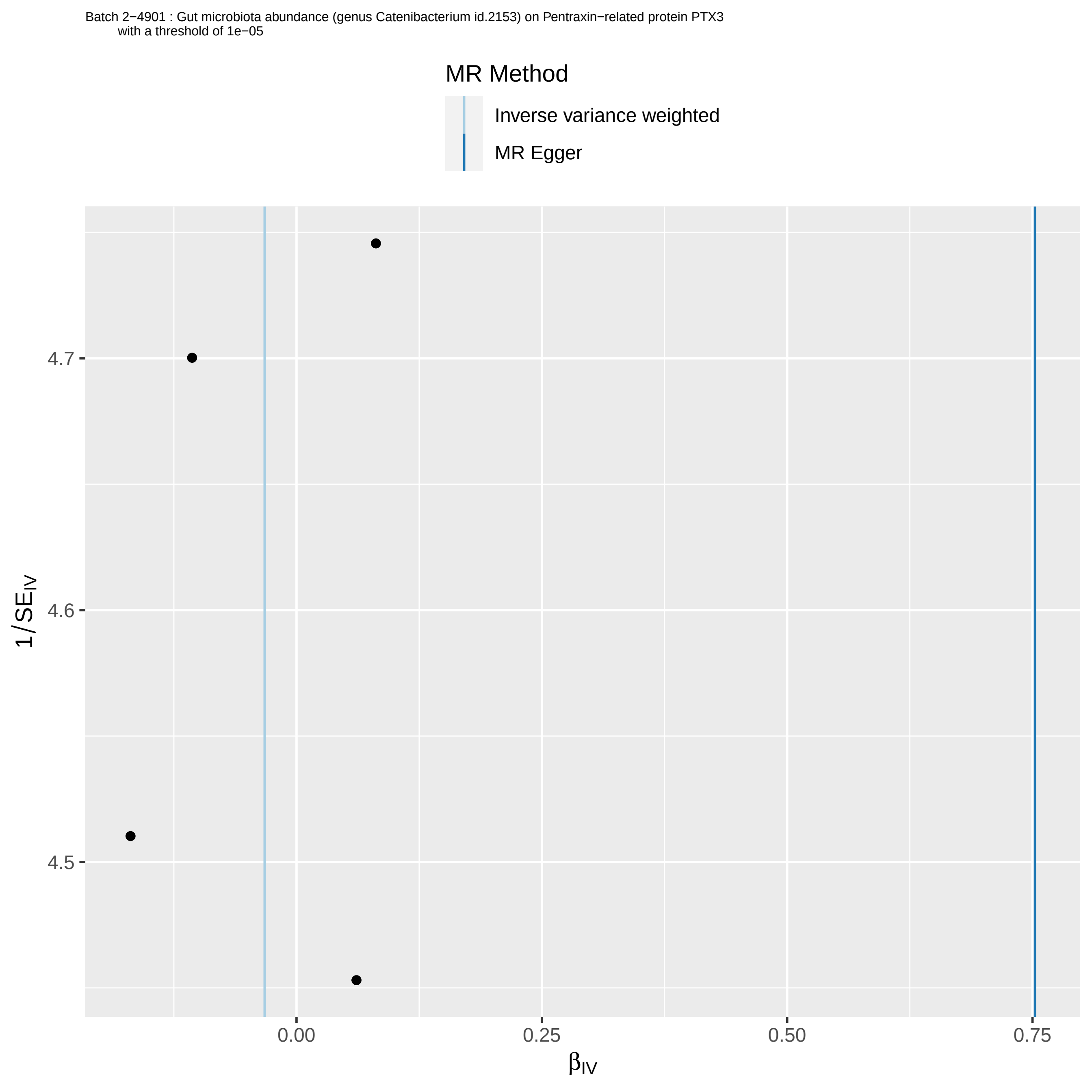

Batch 2−4901 : Gut microbiota abundance (genus Catenibacterium id.2153) on Pentraxin−related protein PTX3
with a threshold of 1e−05
MR Method
Inverse variance weighted
MR Egger
4.7
4.6
4.5
1/SE_IV
0.00
0.25
0.50
0.75
β_IV

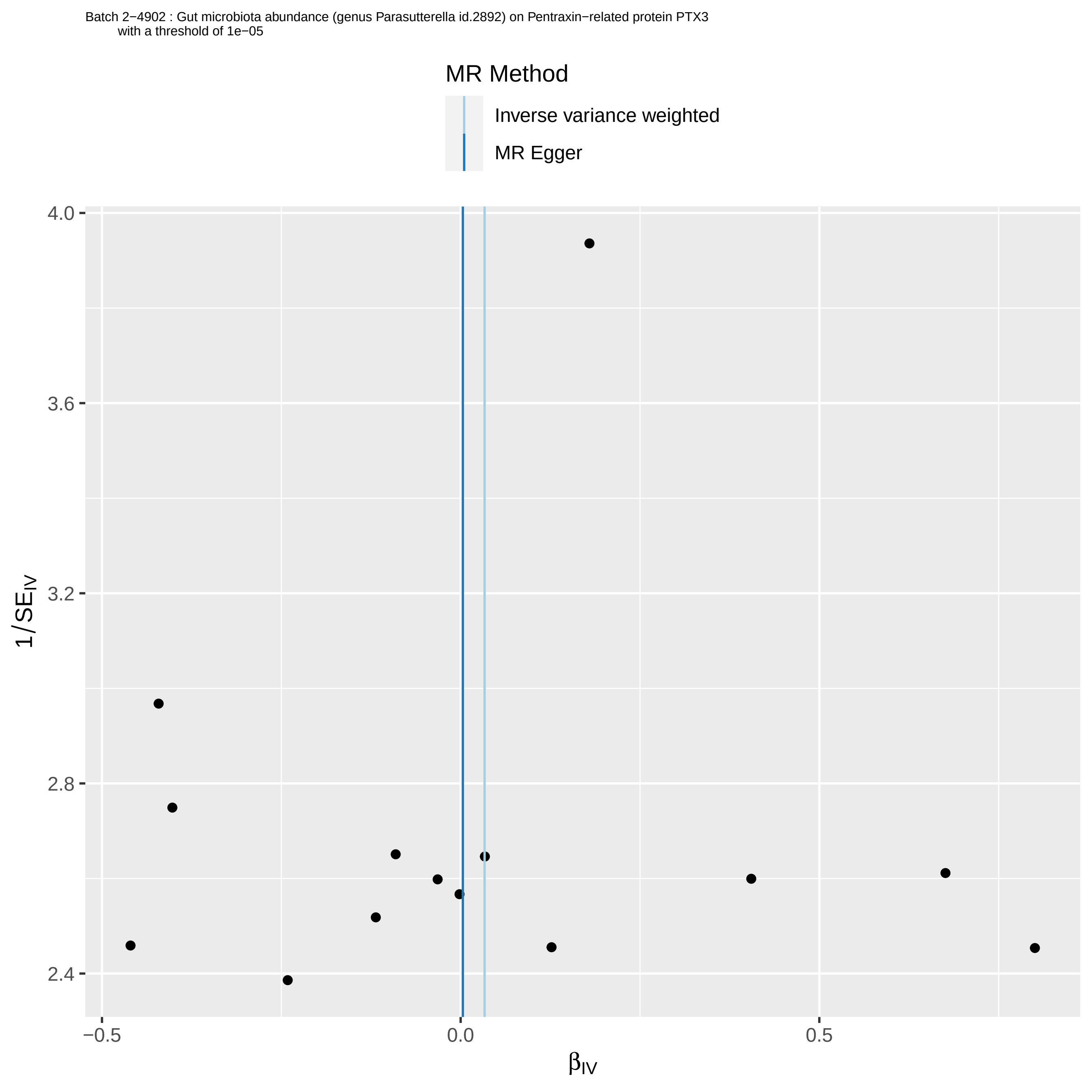
Batch 2−4902 : Gut microbiota abundance (genus Parasutterella id.2892) on Pentraxin−related protein PTX3
with a threshold of 1e−05
MR Method
Inverse variance weighted
MR Egger
4.0
3.6
3.2
2.8
2.4
1/SE$_{IV}$
−0.5
0.0
0.5
β$_{IV}$

Batch 2−4903 : Gut microbiota abundance (genus Catenibacterium id.2153) on Poly(U)−binding−splicing factor PUF60
with a threshold of 1e−05

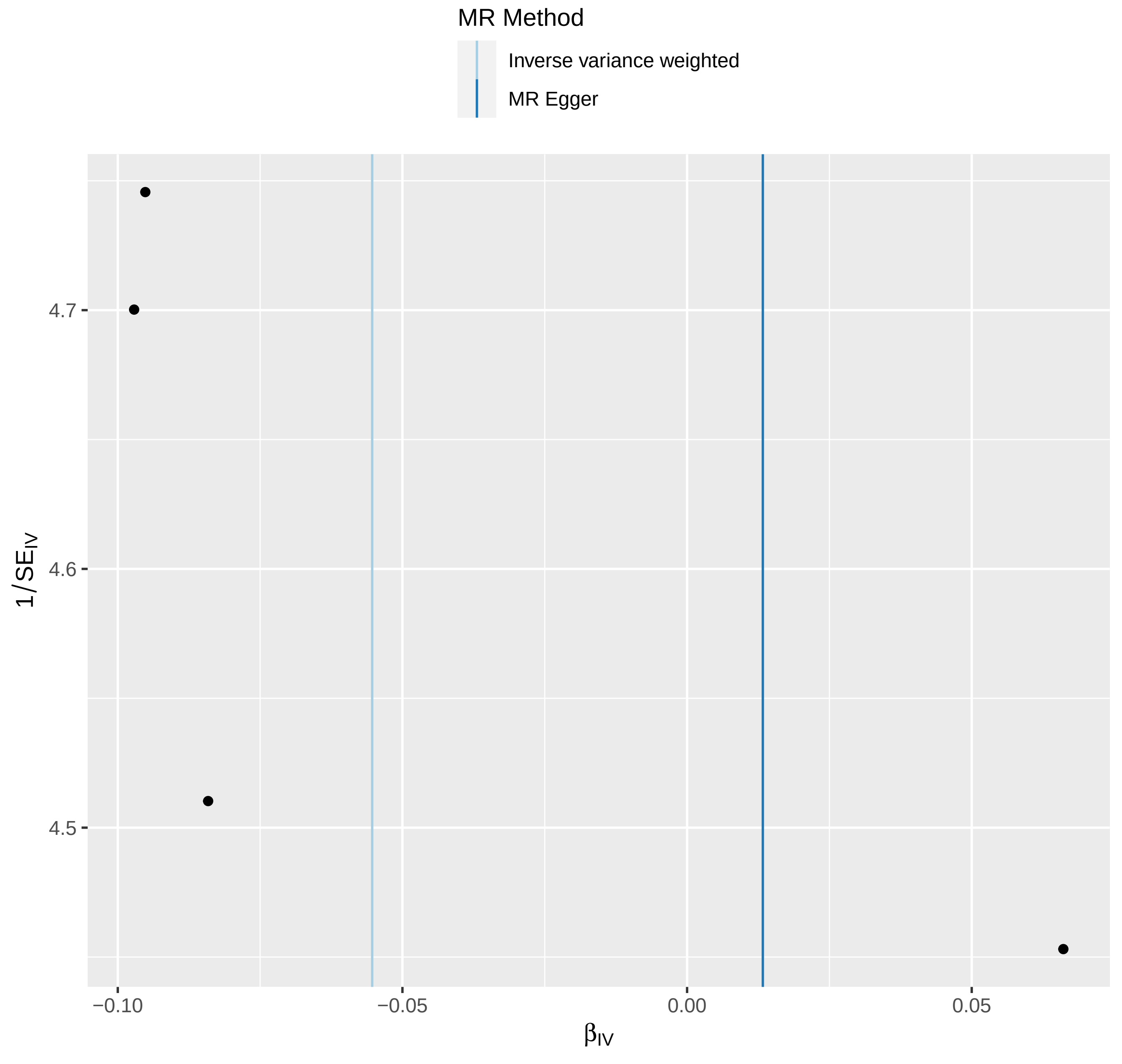

Batch 2−4904 : Gut microbiota abundance (genus Parasutterella id.2892) on Poly(U)−binding−splicing factor PUF60
with a threshold of 1e−05

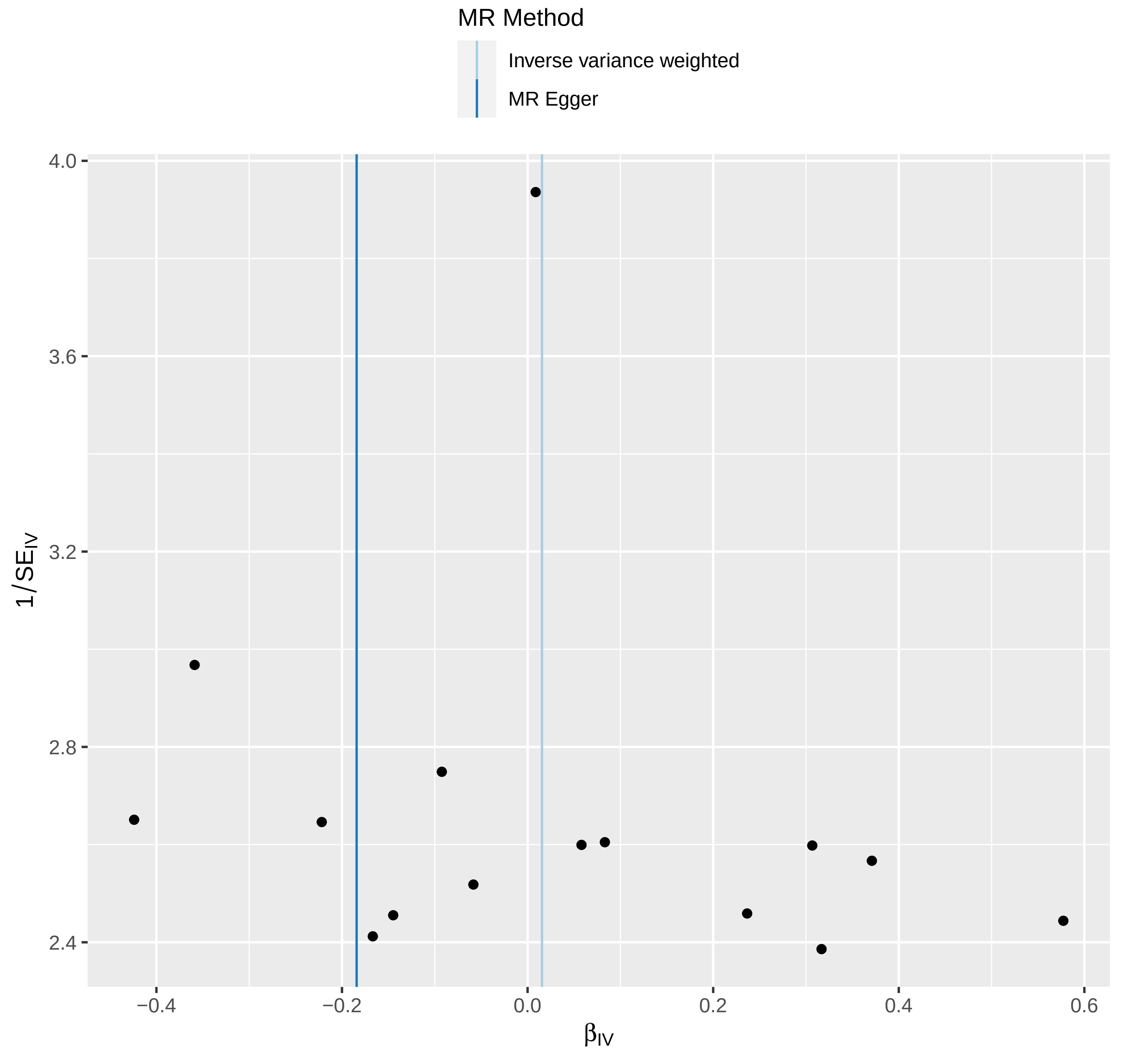

Batch 2−4905 : Gut microbiota abundance (genus Catenibacterium id.2153) on Transcriptional activator protein Pur−alpha
with a threshold of 1e−05

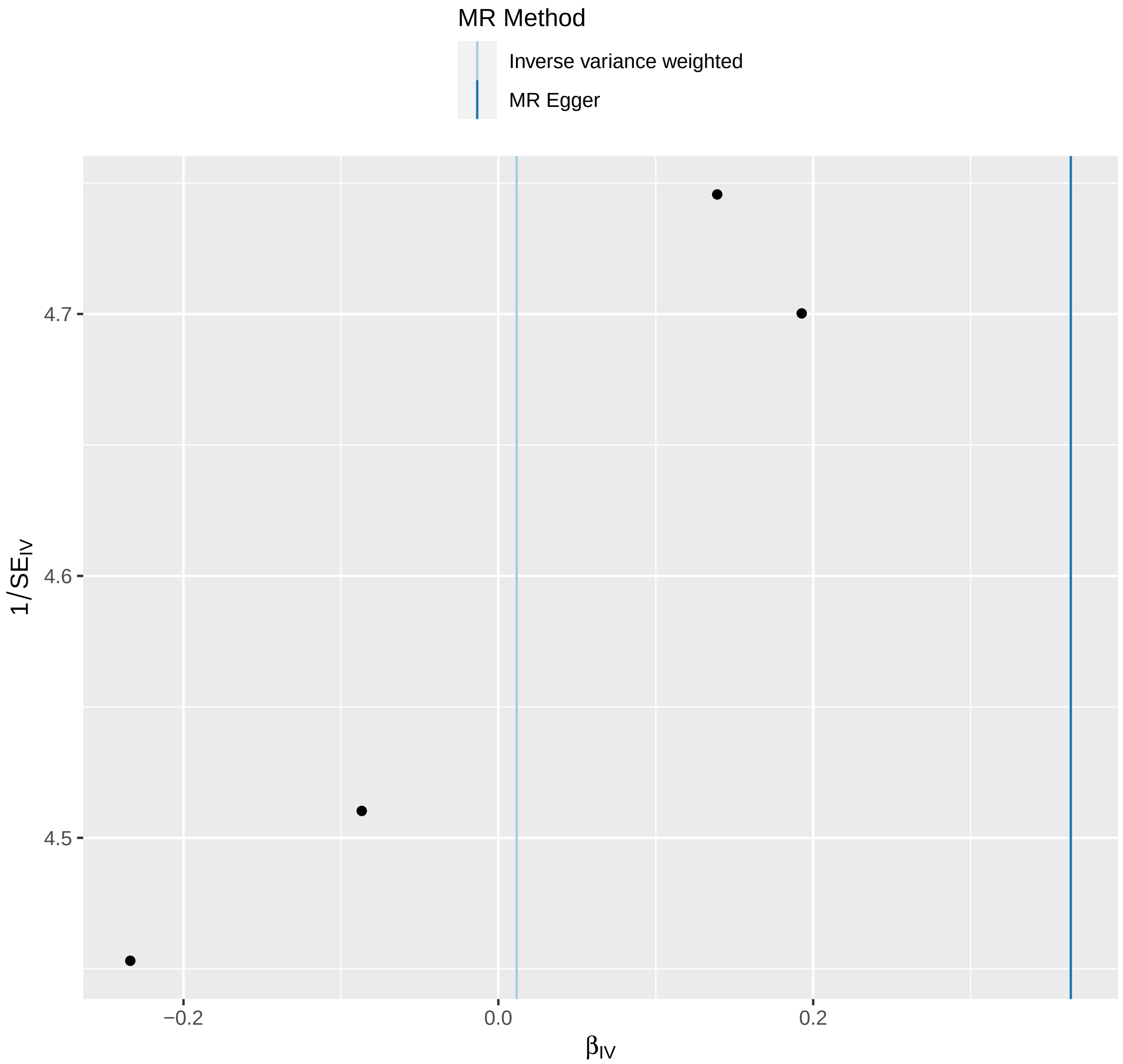

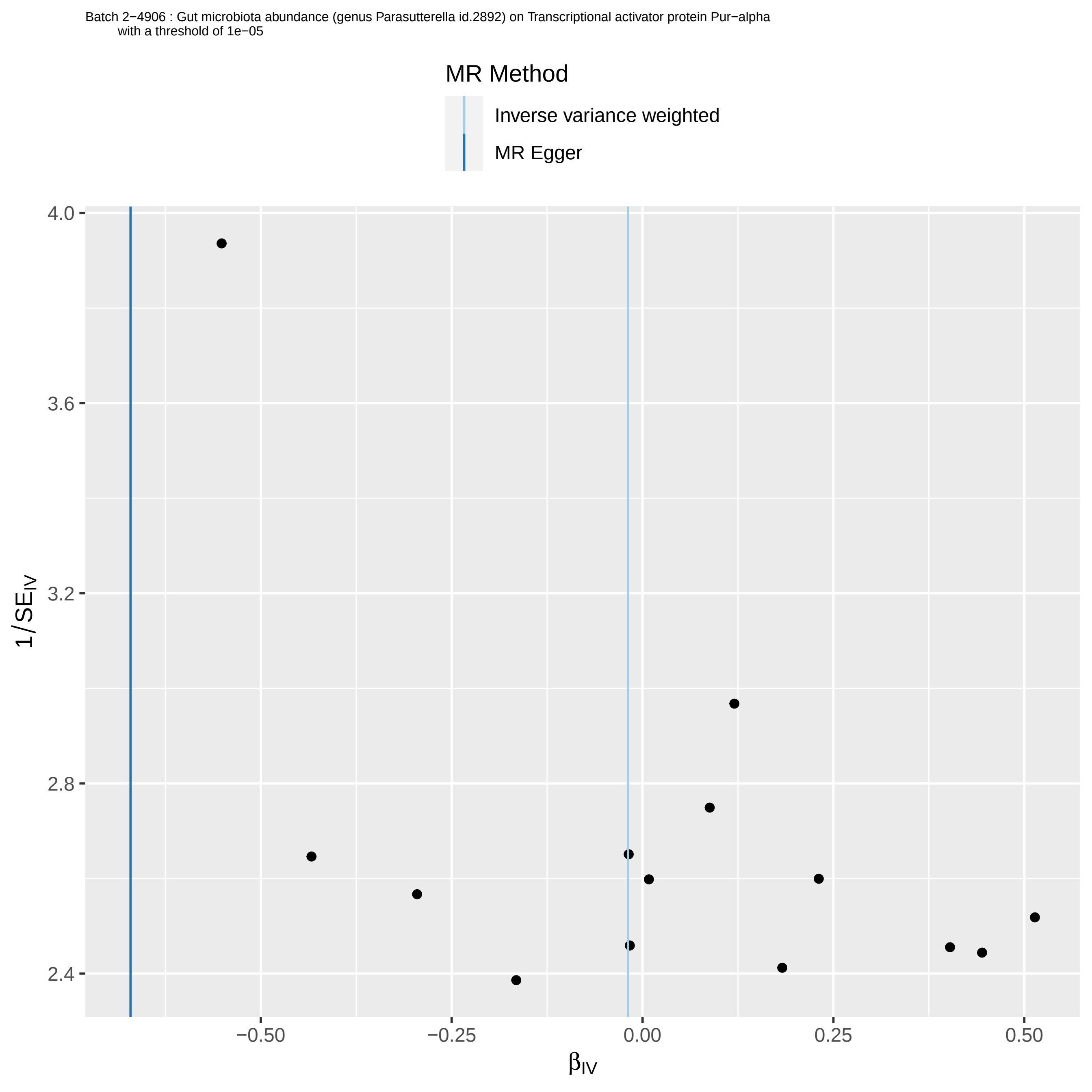

Batch 2−4906 : Gut microbiota abundance (genus Parasutterella id.2892) on Transcriptional activator protein Pur−alpha
with a threshold of 1e−05
MR Method
Inverse variance weighted
MR Egger
4.0
3.6
3.2
2.8
2.4
1/SE$_{IV}$
−0.50
−0.25
0.00
0.25
0.50
β$_{IV}$

Batch 2−4907 : Gut microbiota abundance (genus Catenibacterium id.2153) on tRNA pseudouridine synthase A, mitochondrial
with a threshold of 1e−05

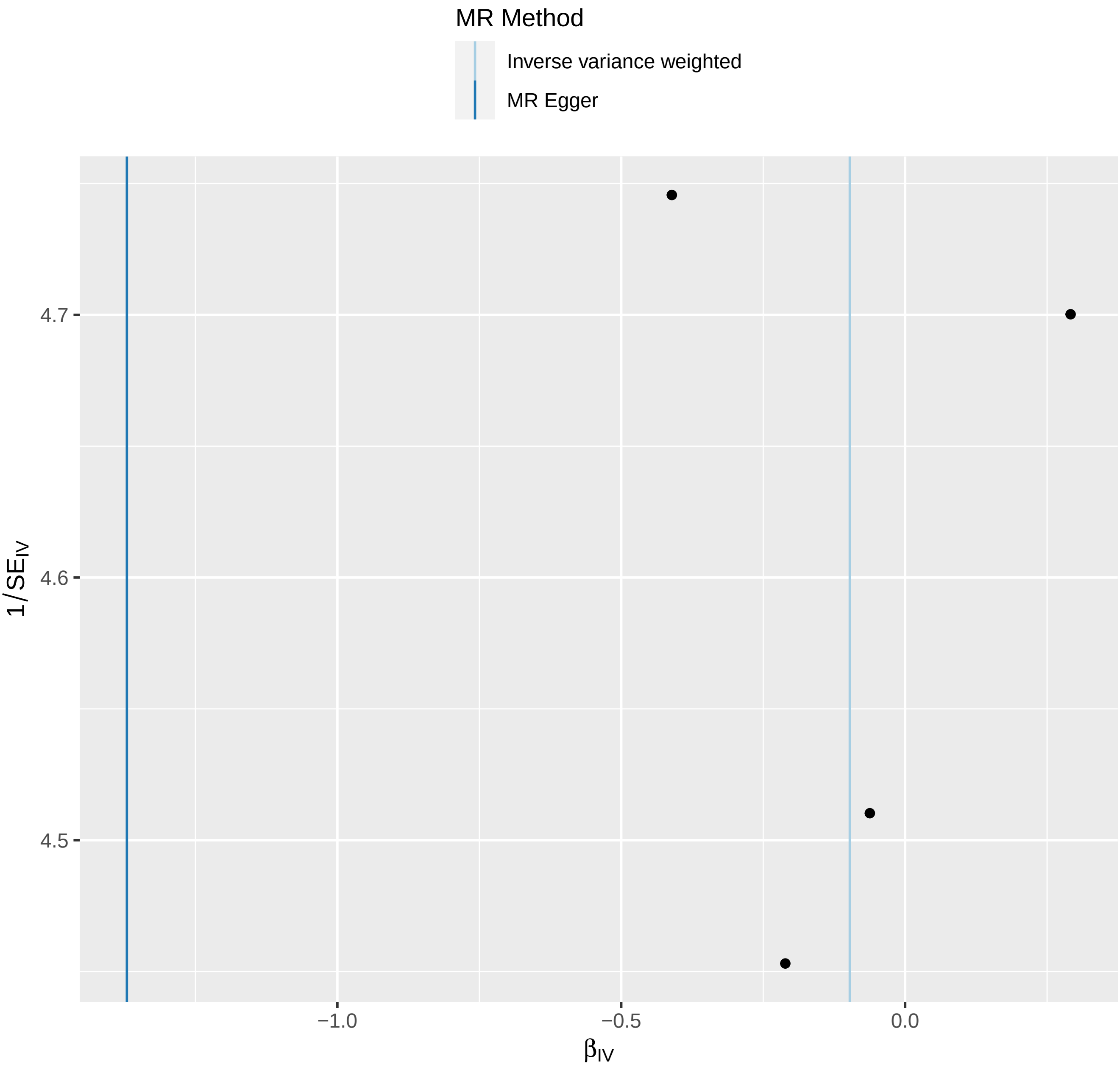

Batch 2−4908 : Gut microbiota abundance (genus Parasutterella id.2892) on tRNA pseudouridine synthase A, mitochondrial
with a threshold of 1e−05

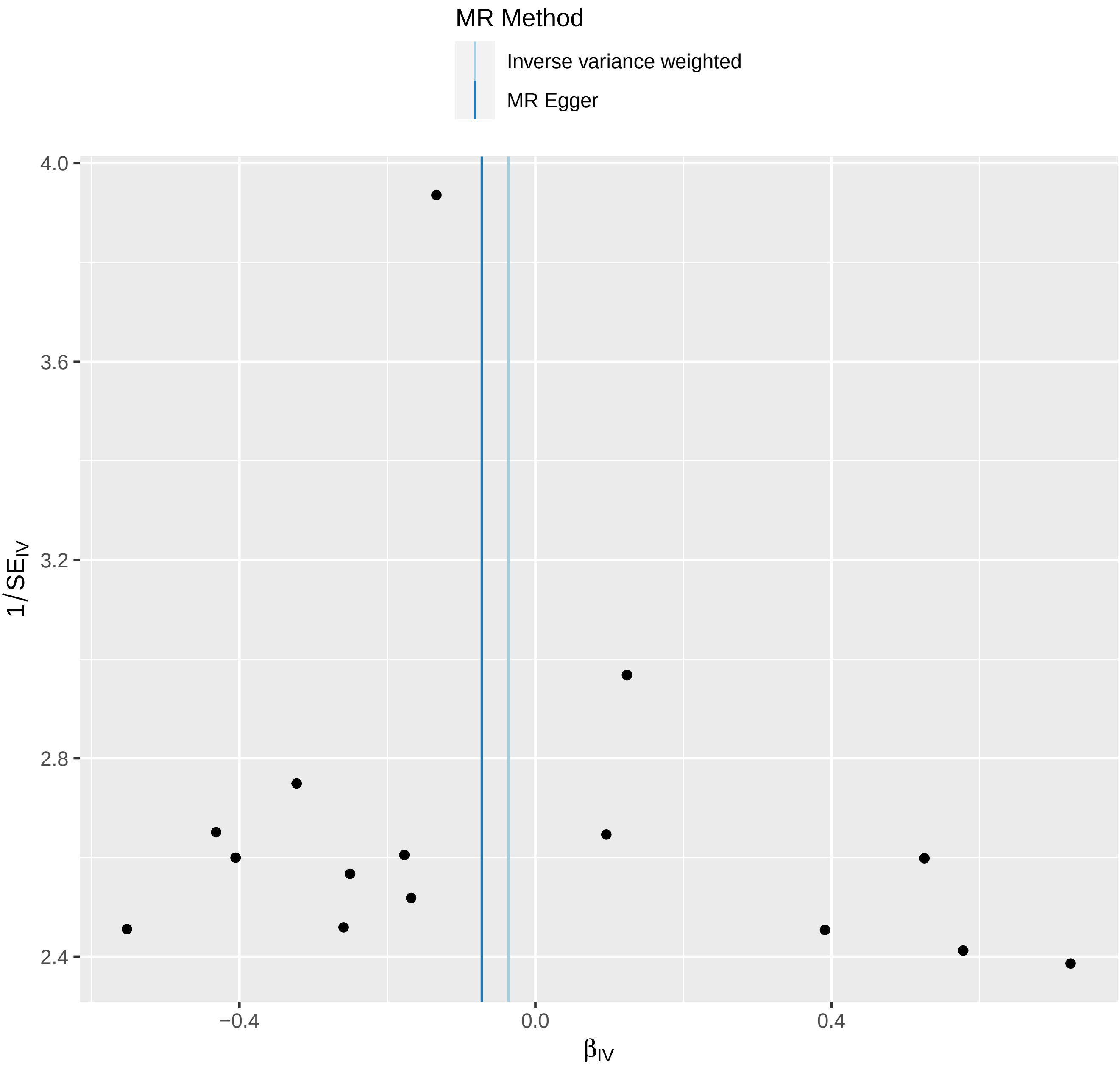

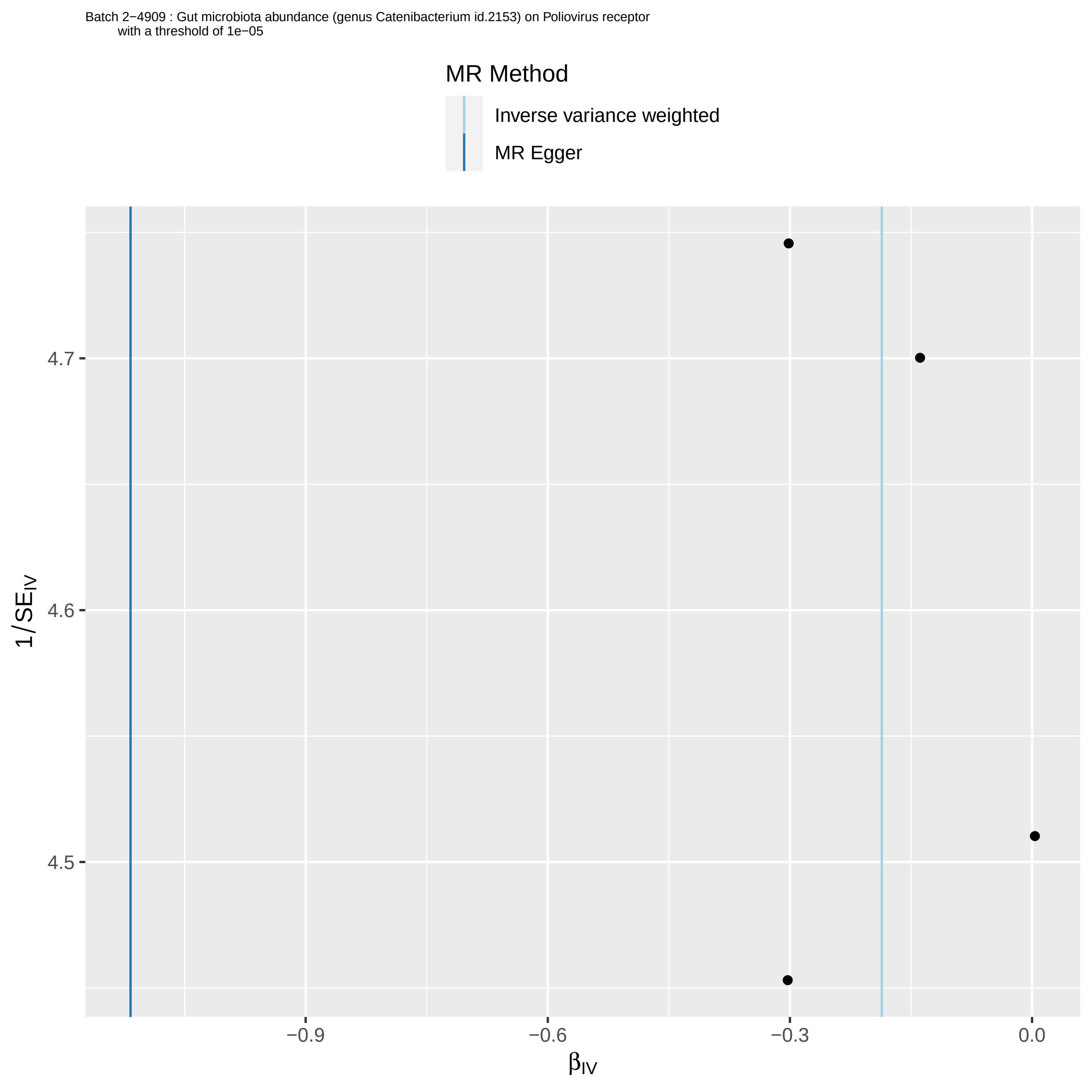

Batch 2−4909 : Gut microbiota abundance (genus Catenibacterium id.2153) on Poliovirus receptor
with a threshold of 1e−05
MR Method
Inverse variance weighted
MR Egger
4.7
4.6
4.5
1/SE_IV
−0.9
−0.6
−0.3
0.0
β_IV

Batch 2−4910 : Gut microbiota abundance (genus Parasutterella id.2892) on Poliovirus receptor
with a threshold of 1e−05

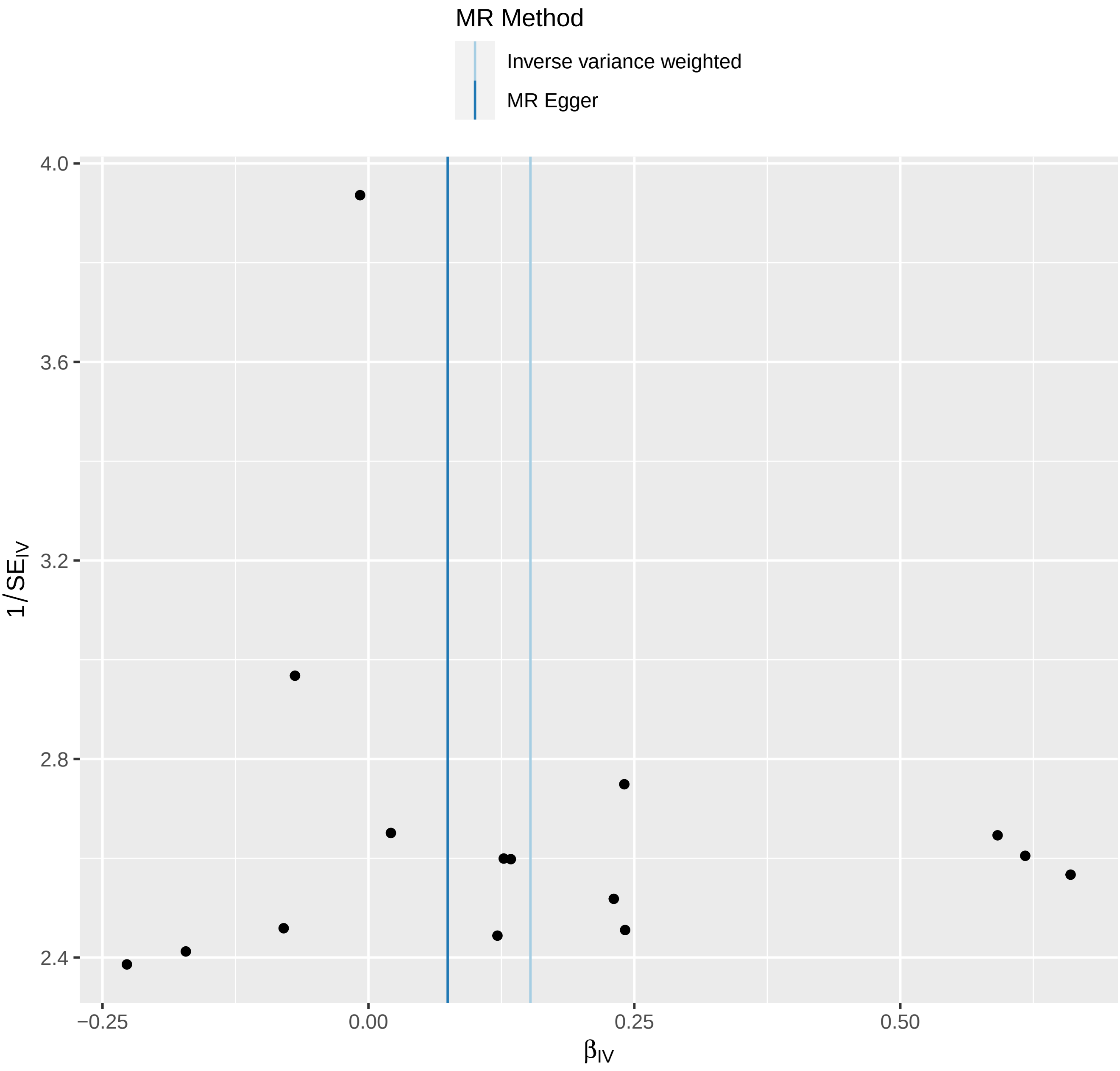

Batch 2−4911 : Gut microbiota abundance (genus Catenibacterium id.2153) on Transmembrane protein PVRIG
with a threshold of 1e−05

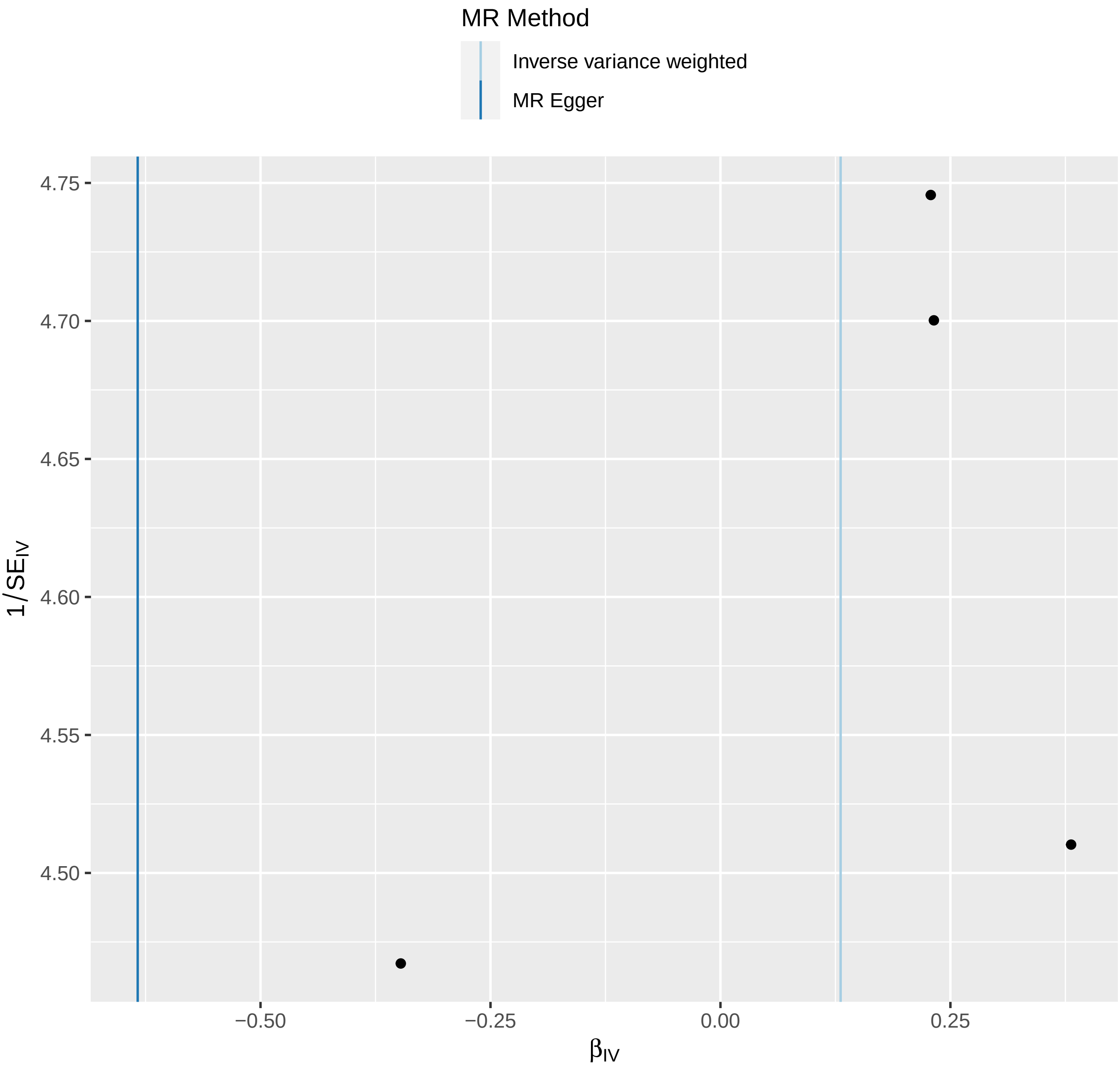

Batch 2−4912 : Gut microbiota abundance (genus Parasutterella id.2892) on Transmembrane protein PVRIG
with a threshold of 1e−05

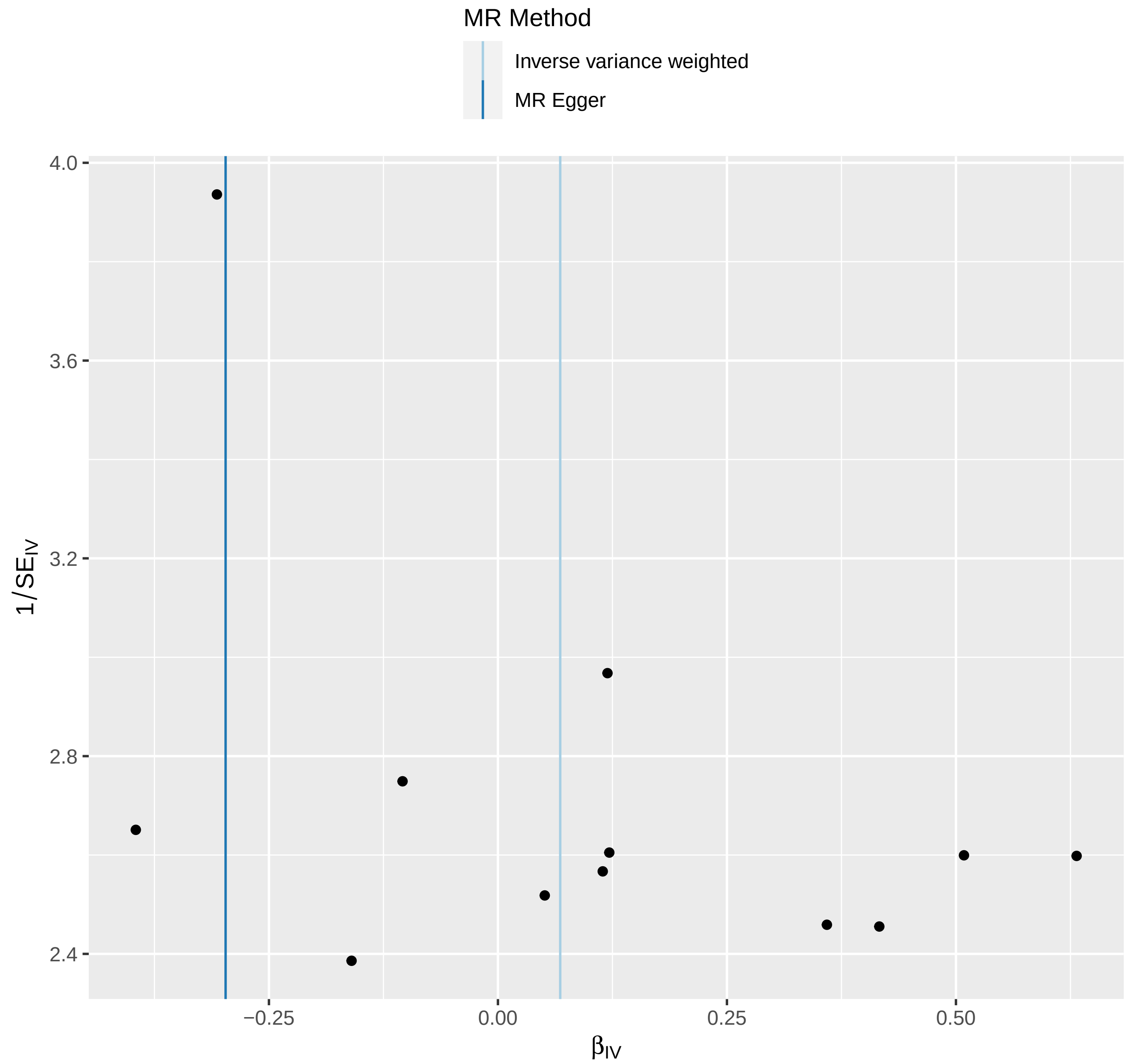

Batch 2−4913 : Gut microbiota abundance (genus Catenibacterium id.2153) on Nectin−2
with a threshold of 1e−05

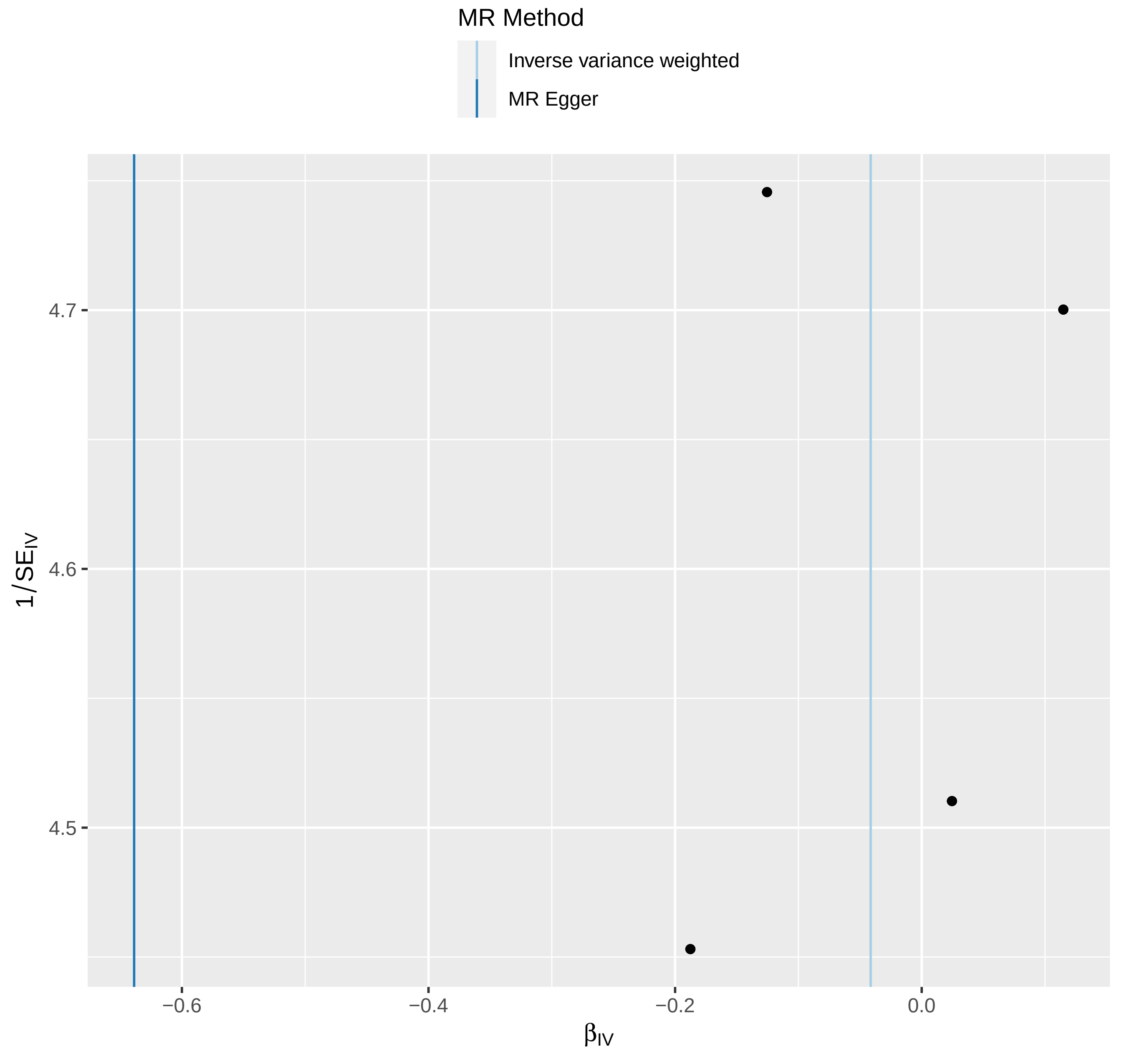

Batch 2−4914 : Gut microbiota abundance (genus Parasutterella id.2892) on Nectin−2
with a threshold of 1e−05

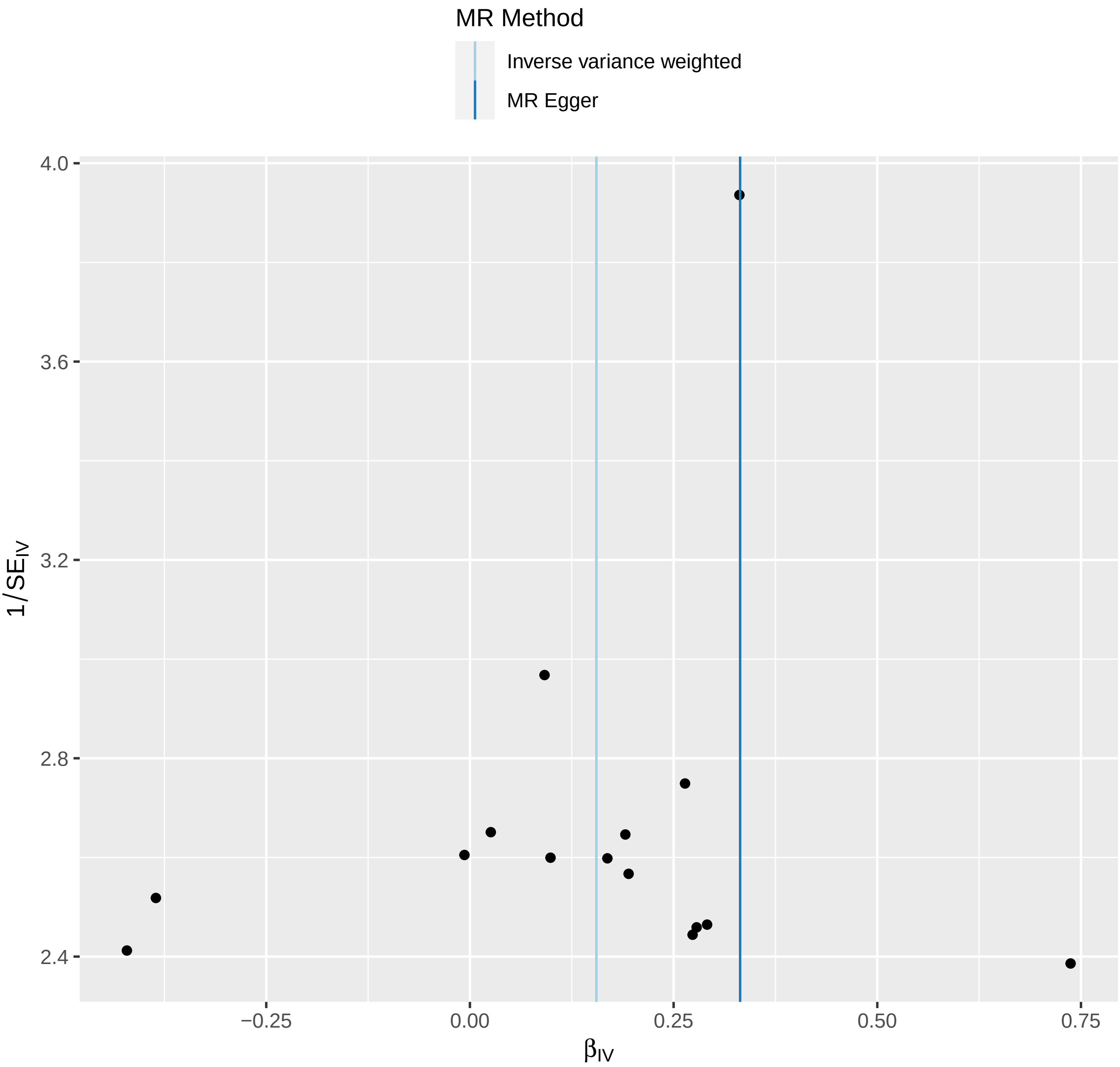

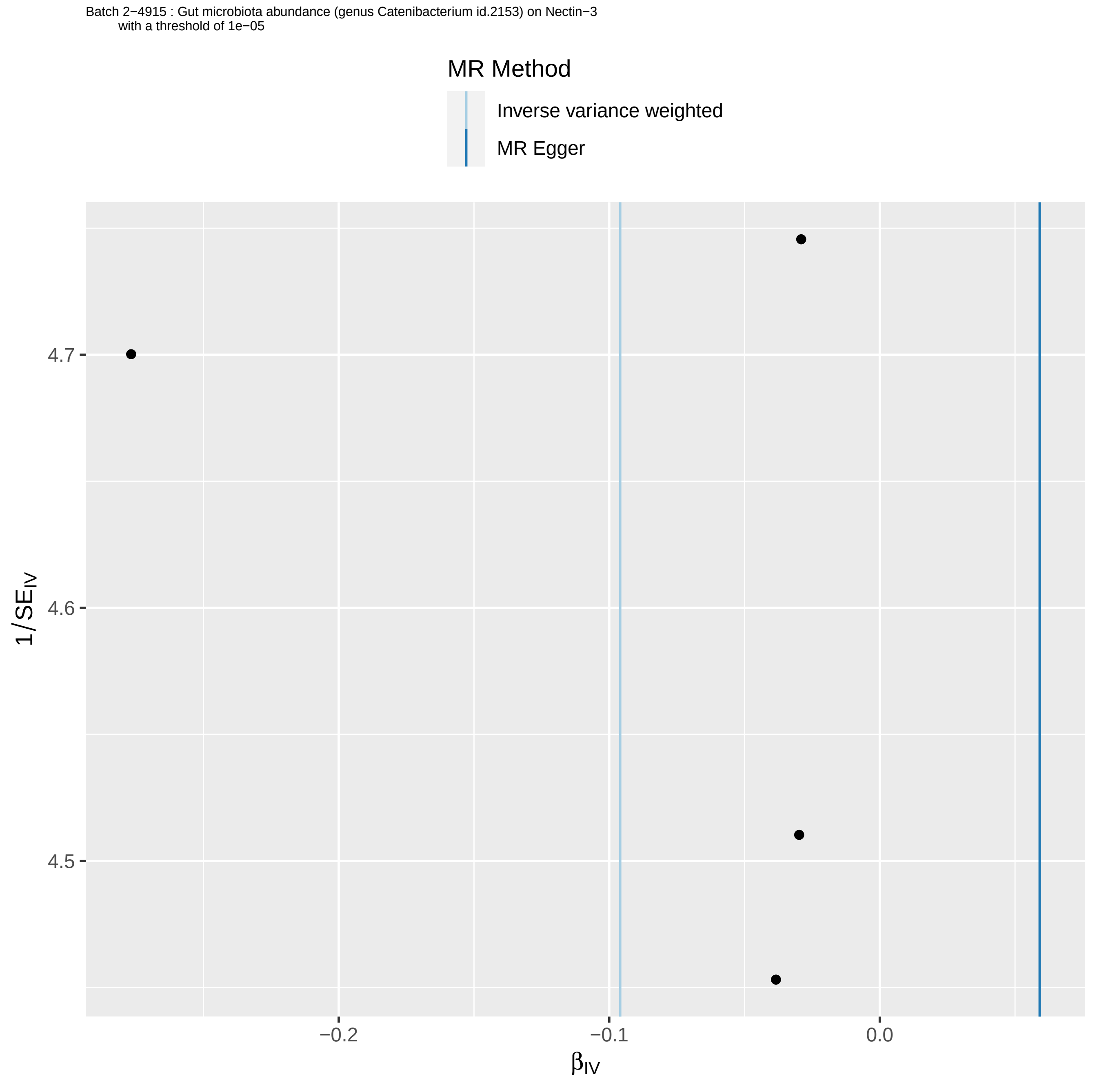
Batch 2−4915 : Gut microbiota abundance (genus Catenibacterium id.2153) on Nectin−3
with a threshold of 1e−05
MR Method
Inverse variance weighted
MR Egger
4.7
4.6
4.5
1/SE_IV
−0.2
−0.1
0.0
β_IV

Batch 2−4916 : Gut microbiota abundance (genus Parasutterella id.2892) on Nectin−3
with a threshold of 1e−05

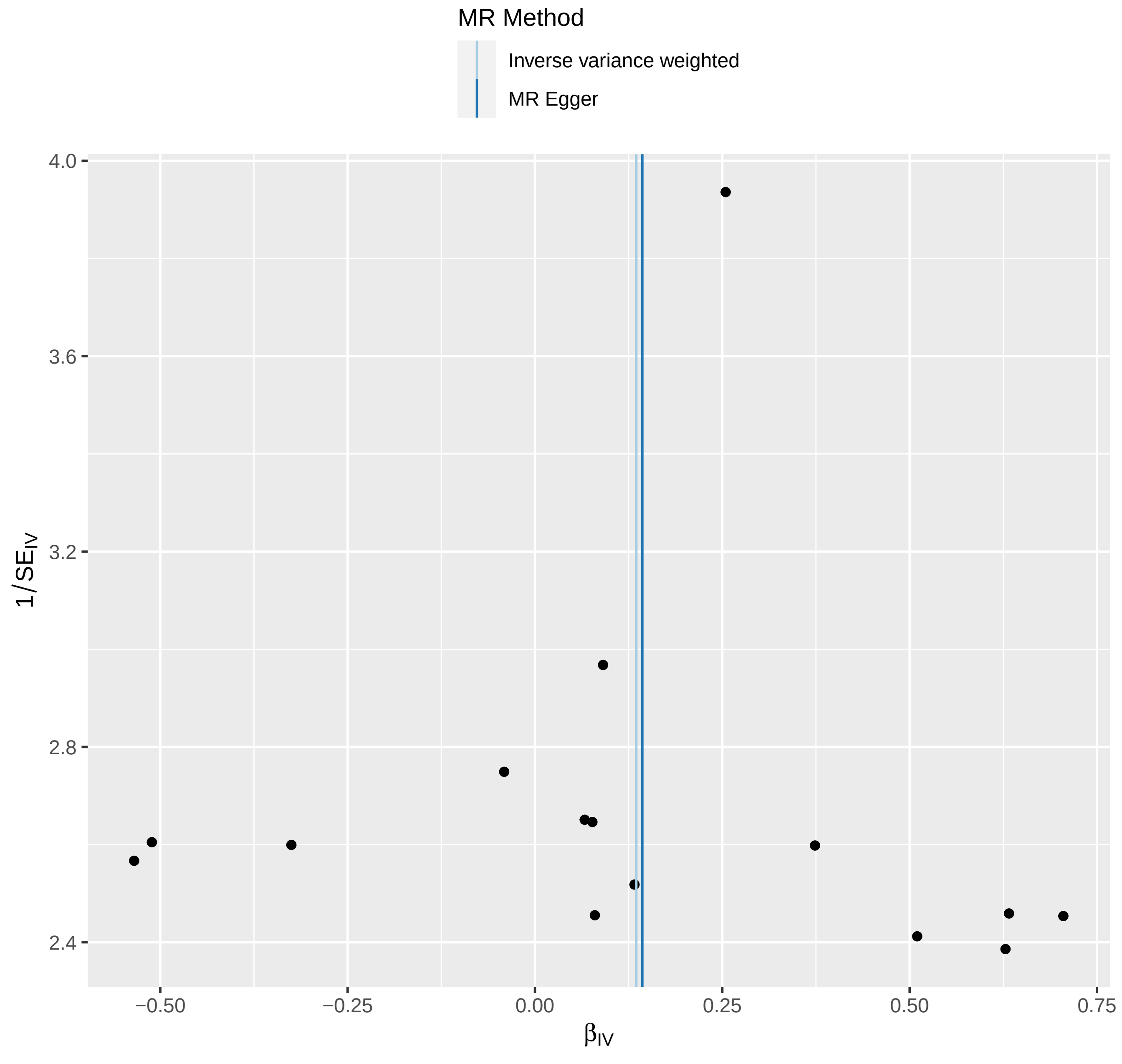

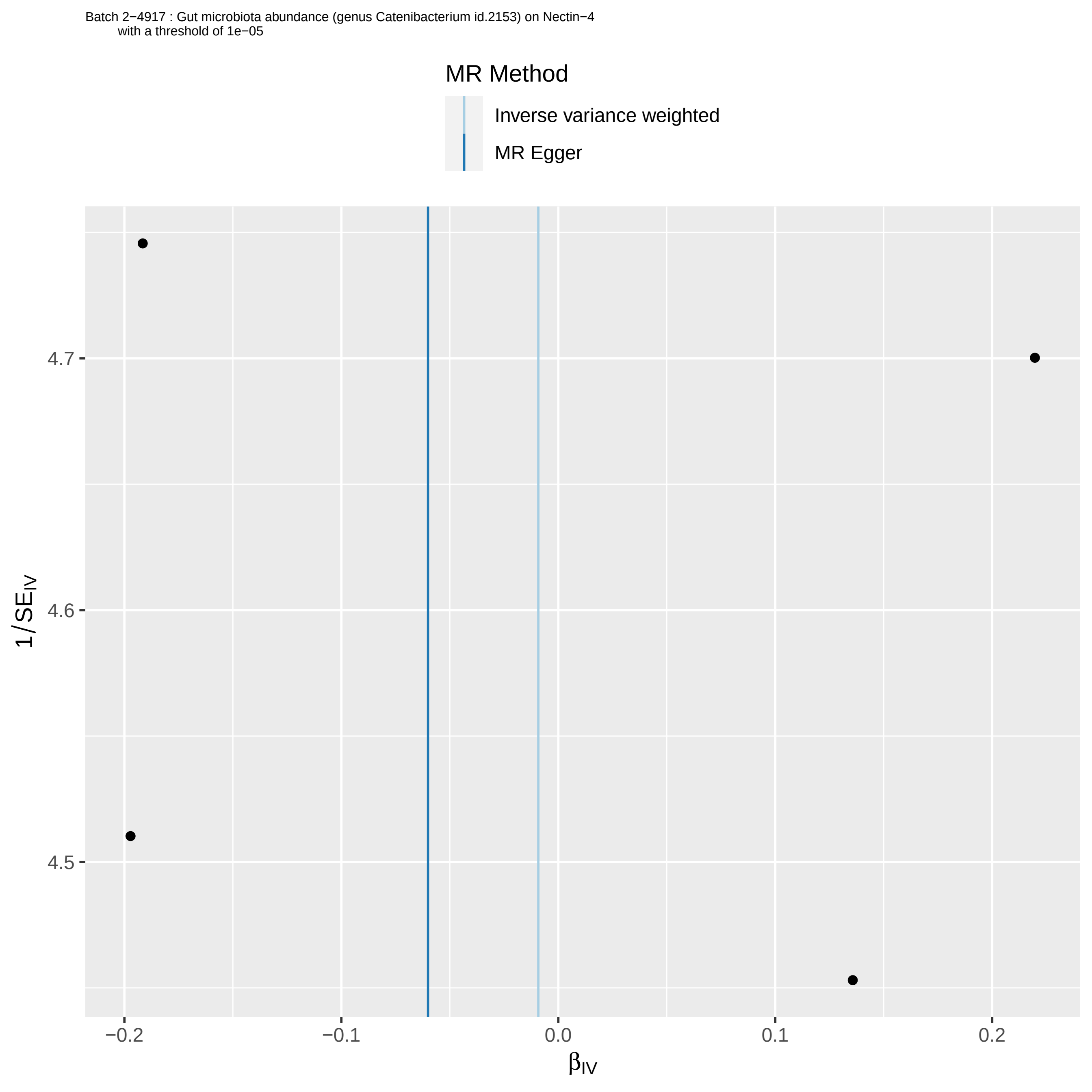
Batch 2−4917 : Gut microbiota abundance (genus Catenibacterium id.2153) on Nectin−4
with a threshold of 1e−05
MR Method
Inverse variance weighted
MR Egger
4.7
4.6
4.5
1/SE_IV
−0.2
−0.1
0.0
0.1
0.2
β_IV

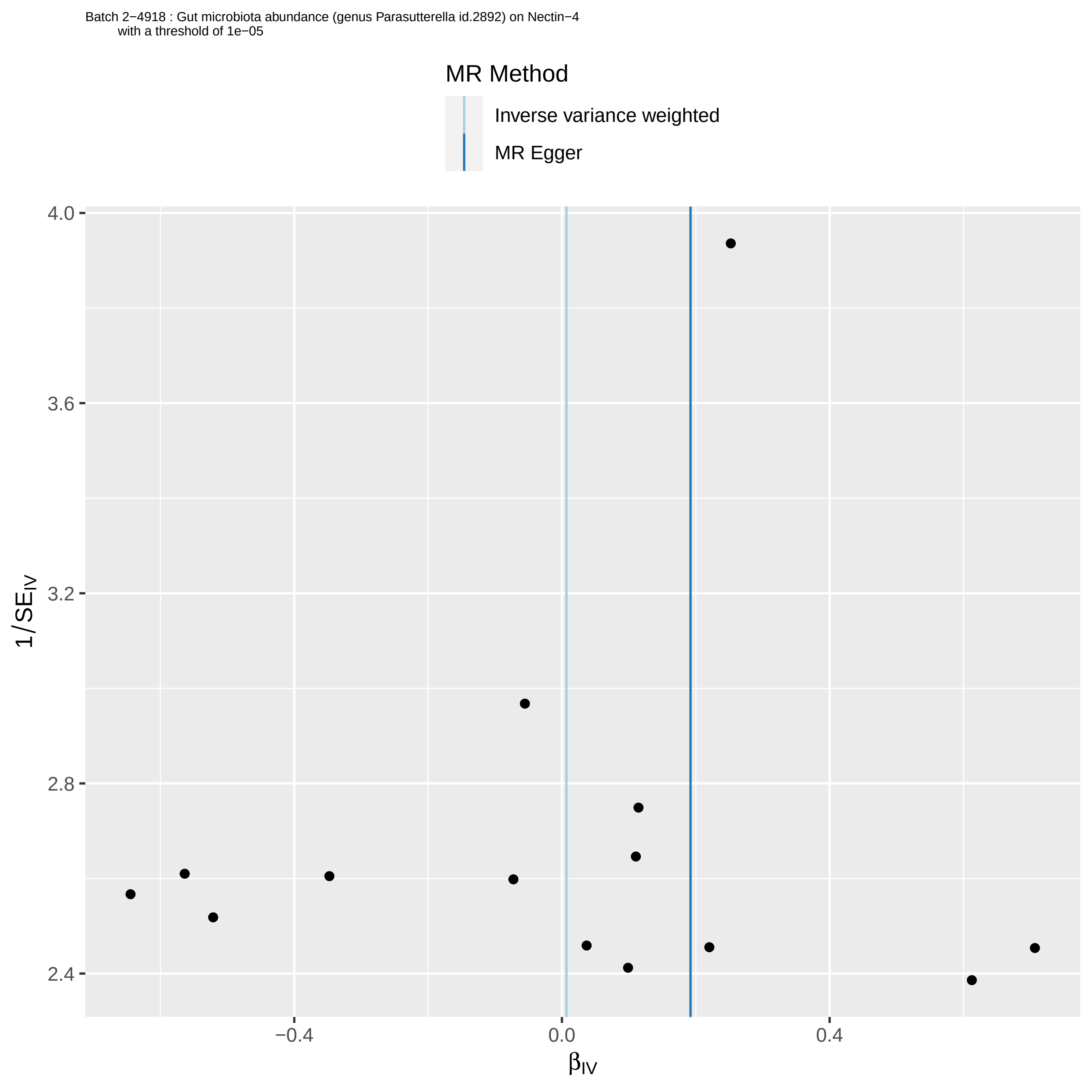
Batch 2−4918 : Gut microbiota abundance (genus Parasutterella id.2892) on Nectin−4
with a threshold of 1e−05
MR Method
Inverse variance weighted
MR Egger
4.0
3.6
3.2
2.8
2.4
1/SE$_{IV}$
−0.4
0.0
0.4
β$_{IV}$

Batch 2−4919 : Gut microbiota abundance (genus Catenibacterium id.2153) on Peroxidasin−like protein
with a threshold of 1e−05

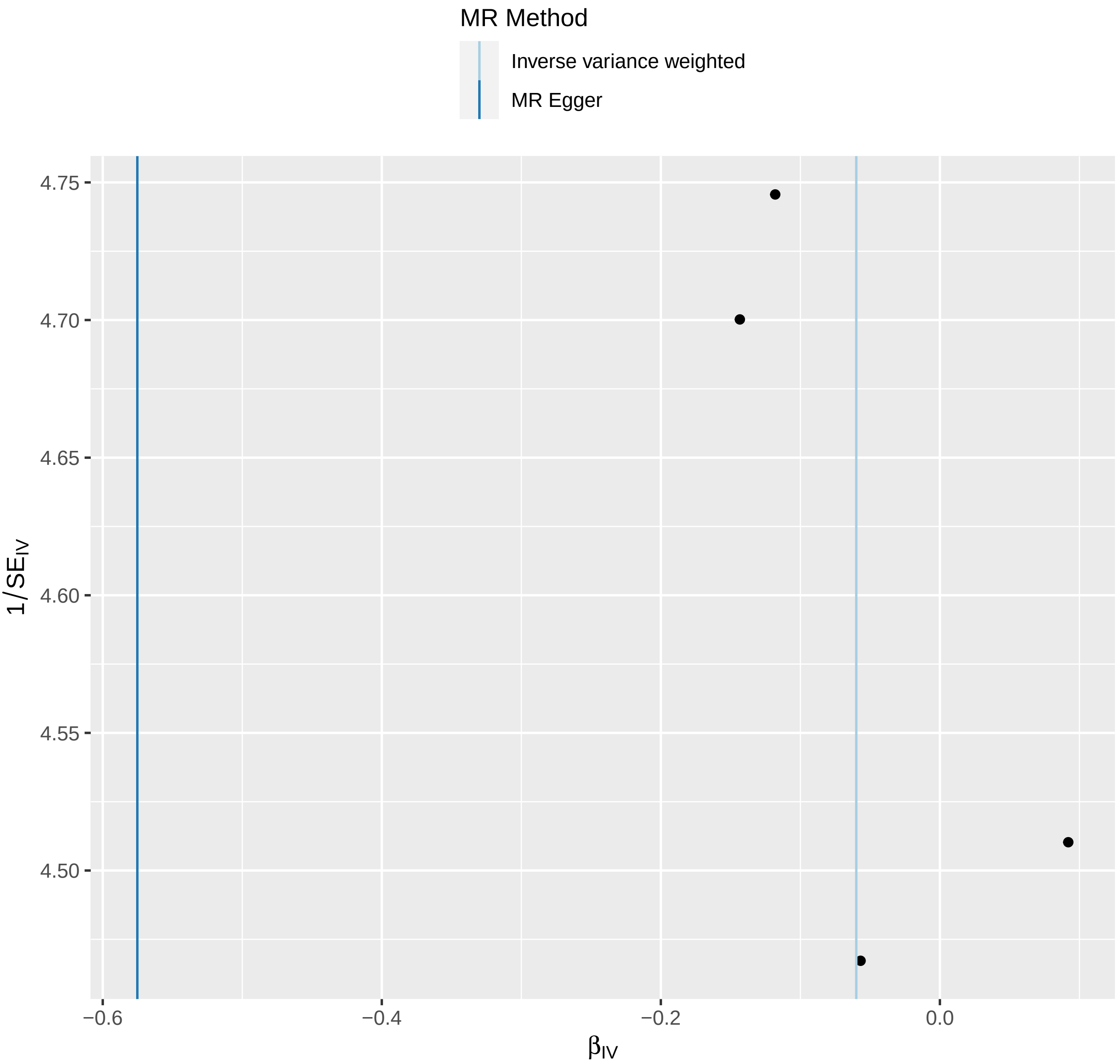

Batch 2−4920 : Gut microbiota abundance (genus Parasutterella id.2892) on Peroxidasin−like protein
with a threshold of 1e−05

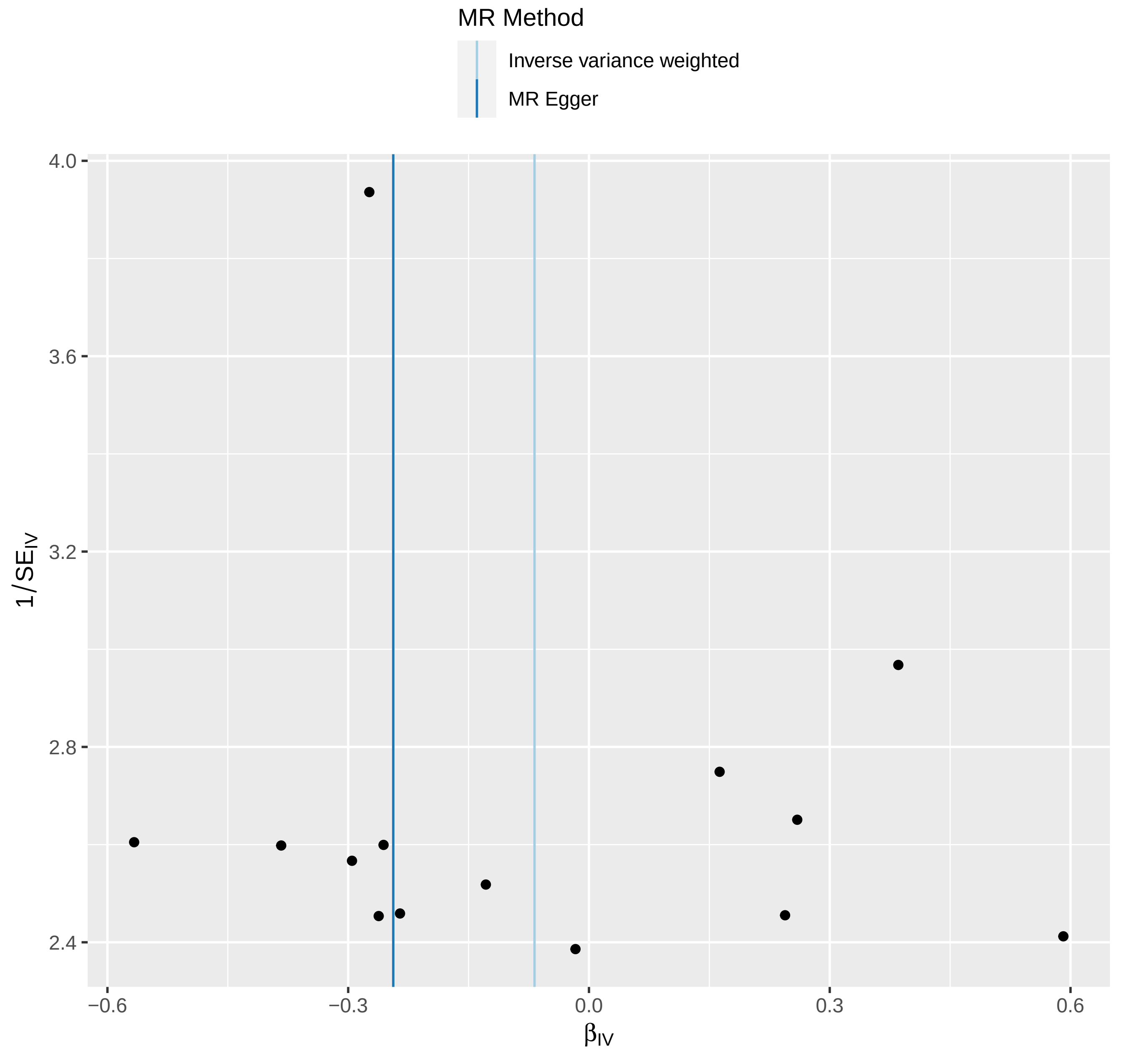

Batch 2−4921 : Gut microbiota abundance (genus Catenibacterium id.2153) on Pyrin domain−containing protein 1
with a threshold of 1e−05

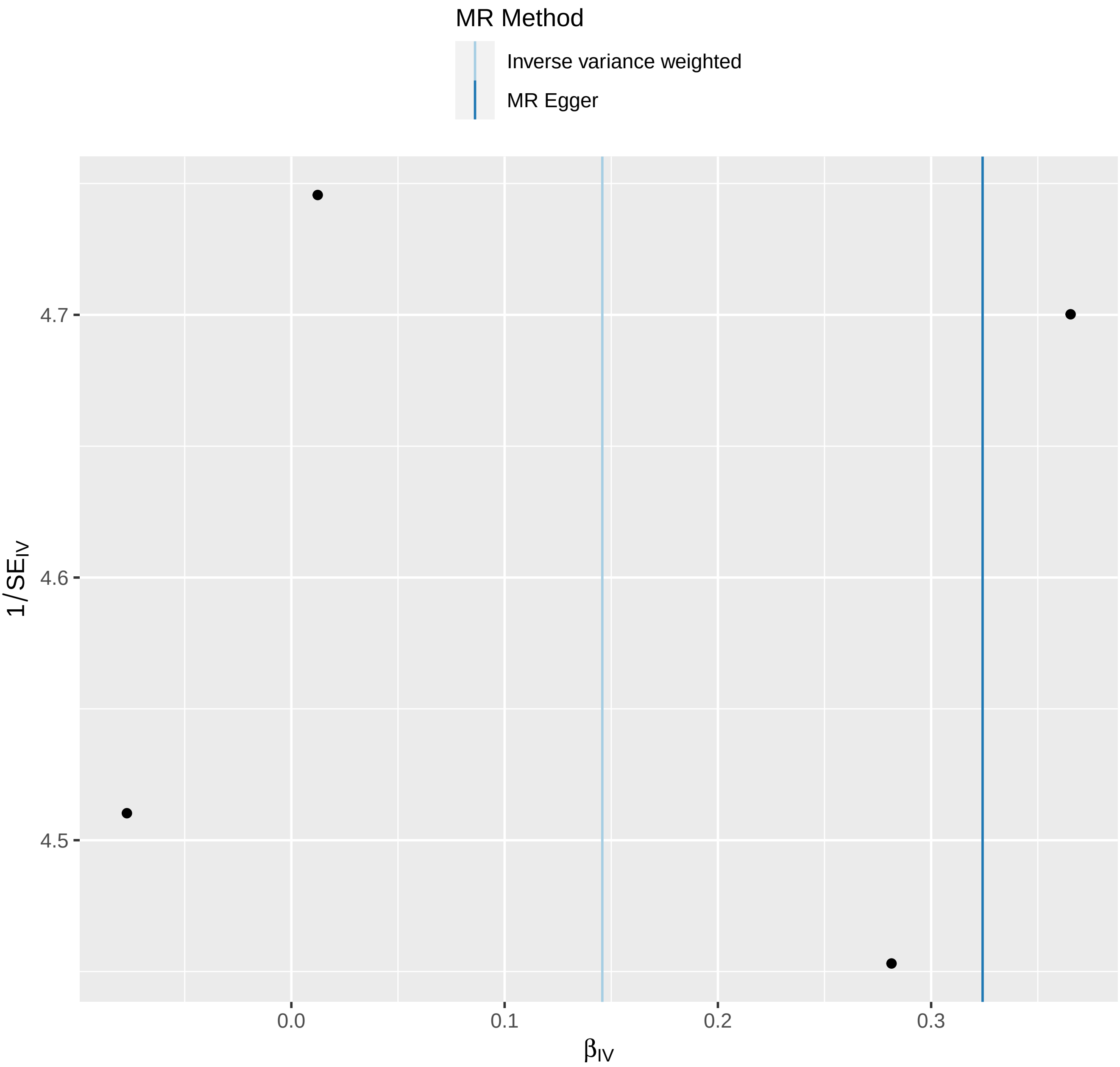

Batch 2−4922 : Gut microbiota abundance (genus Parasutterella id.2892) on Pyrin domain−containing protein 1
with a threshold of 1e−05

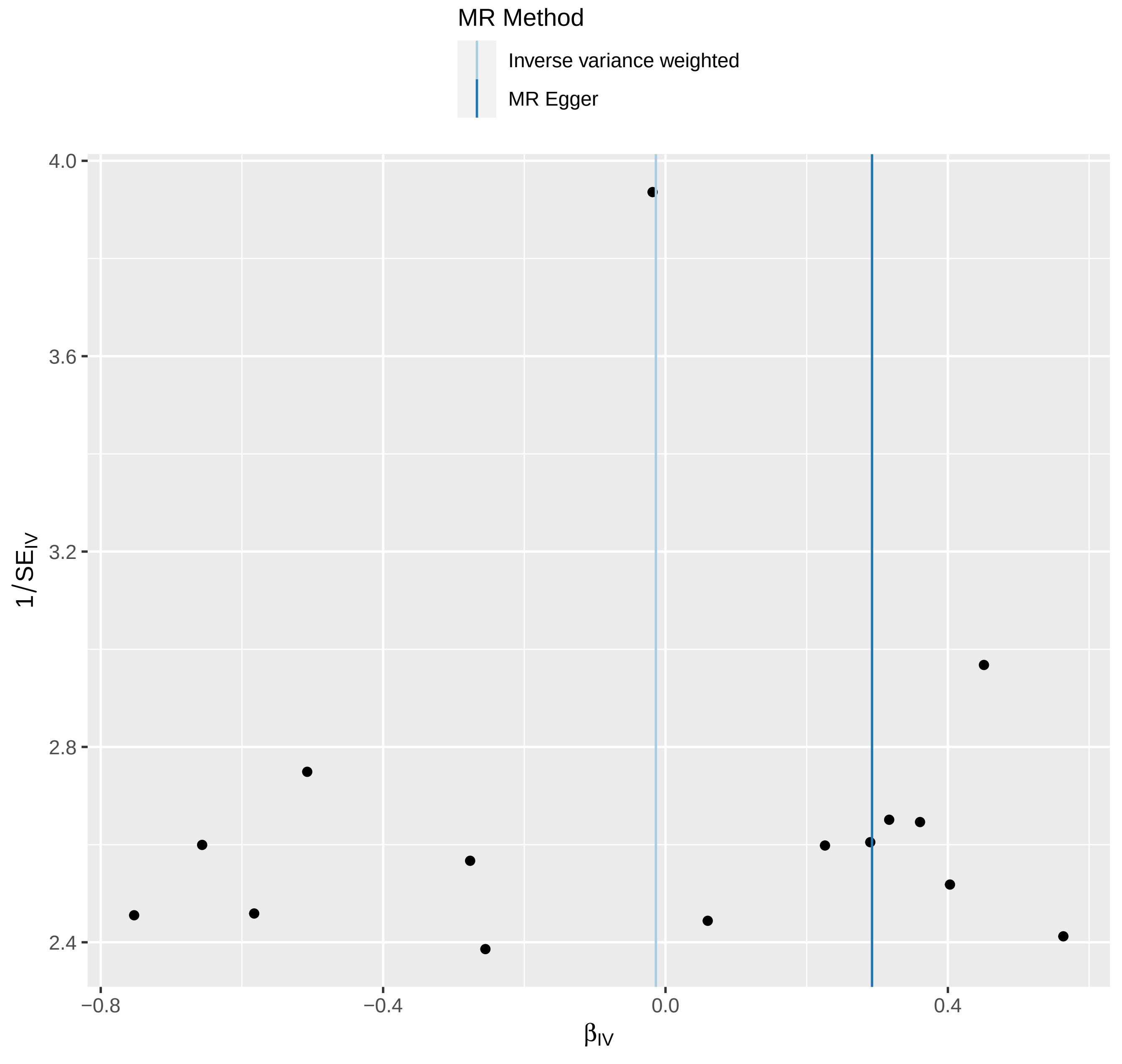

Batch 2−4923 : Gut microbiota abundance (genus Catenibacterium id.2153) on Glycogen phosphorylase, liver form
with a threshold of 1e−05

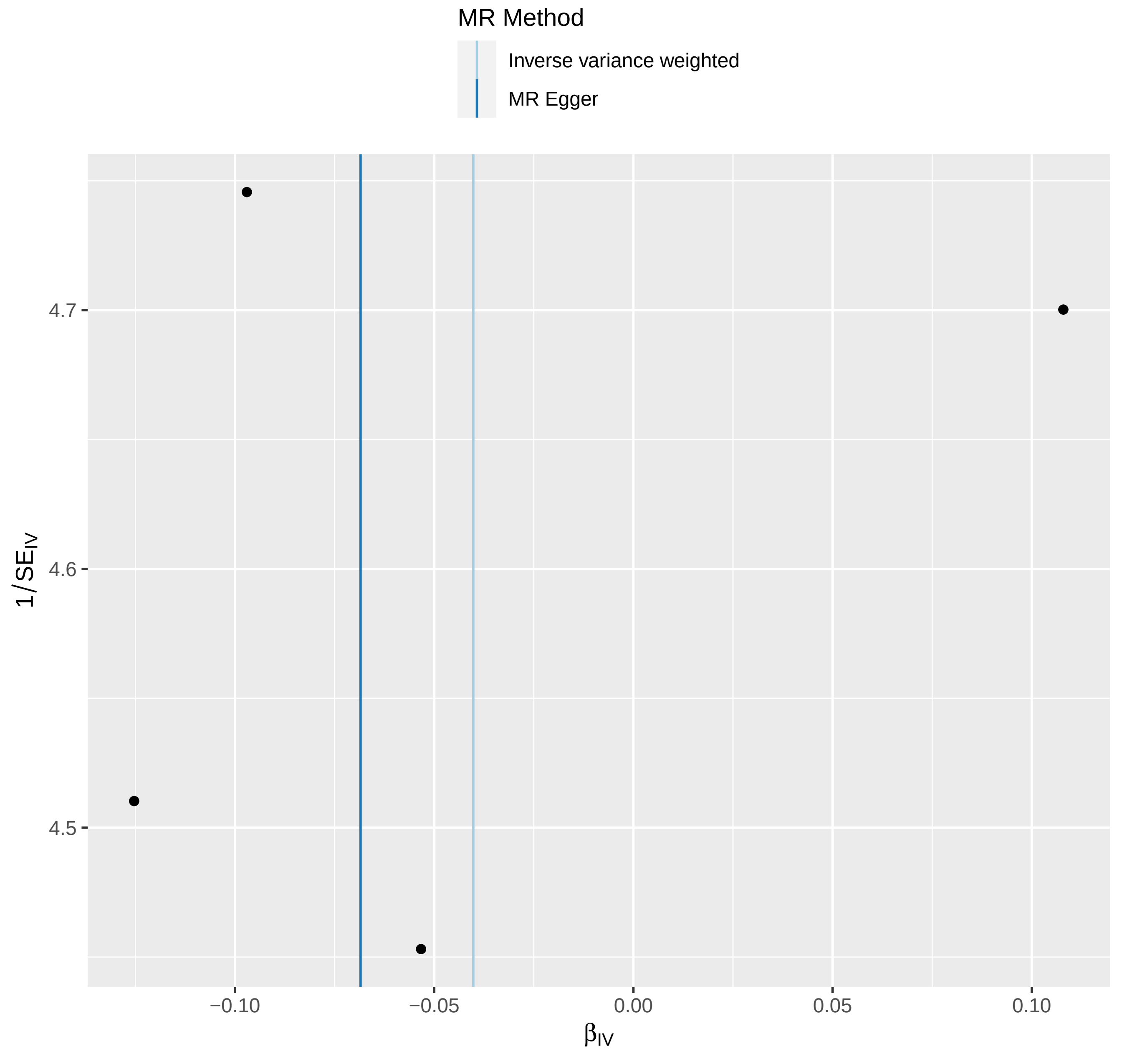

Batch 2−4924 : Gut microbiota abundance (genus Parasutterella id.2892) on Glycogen phosphorylase, liver form
with a threshold of 1e−05

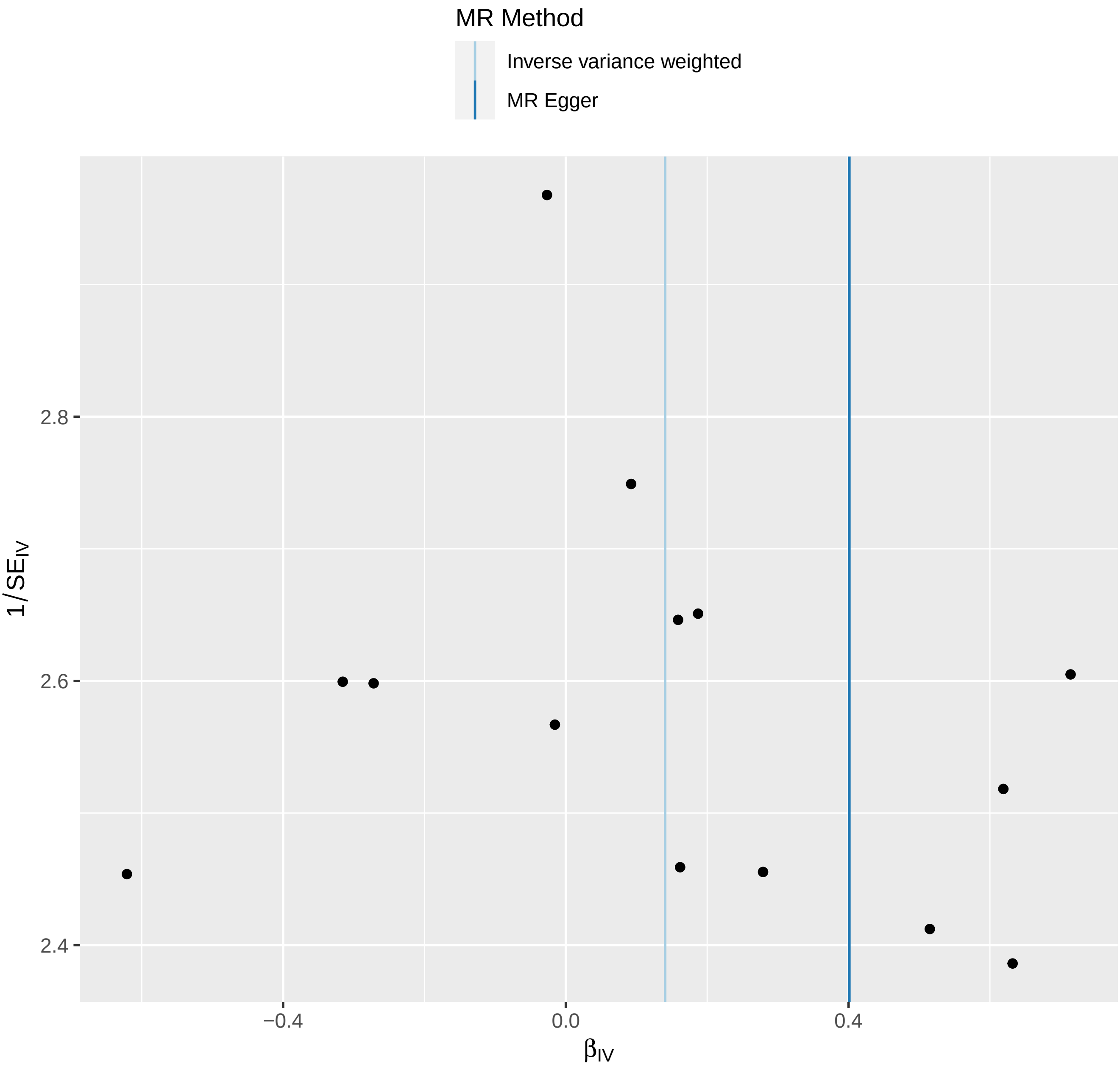

Batch 2−4925 : Gut microbiota abundance (genus Catenibacterium id.2153) on Peptide YY
with a threshold of 1e−05

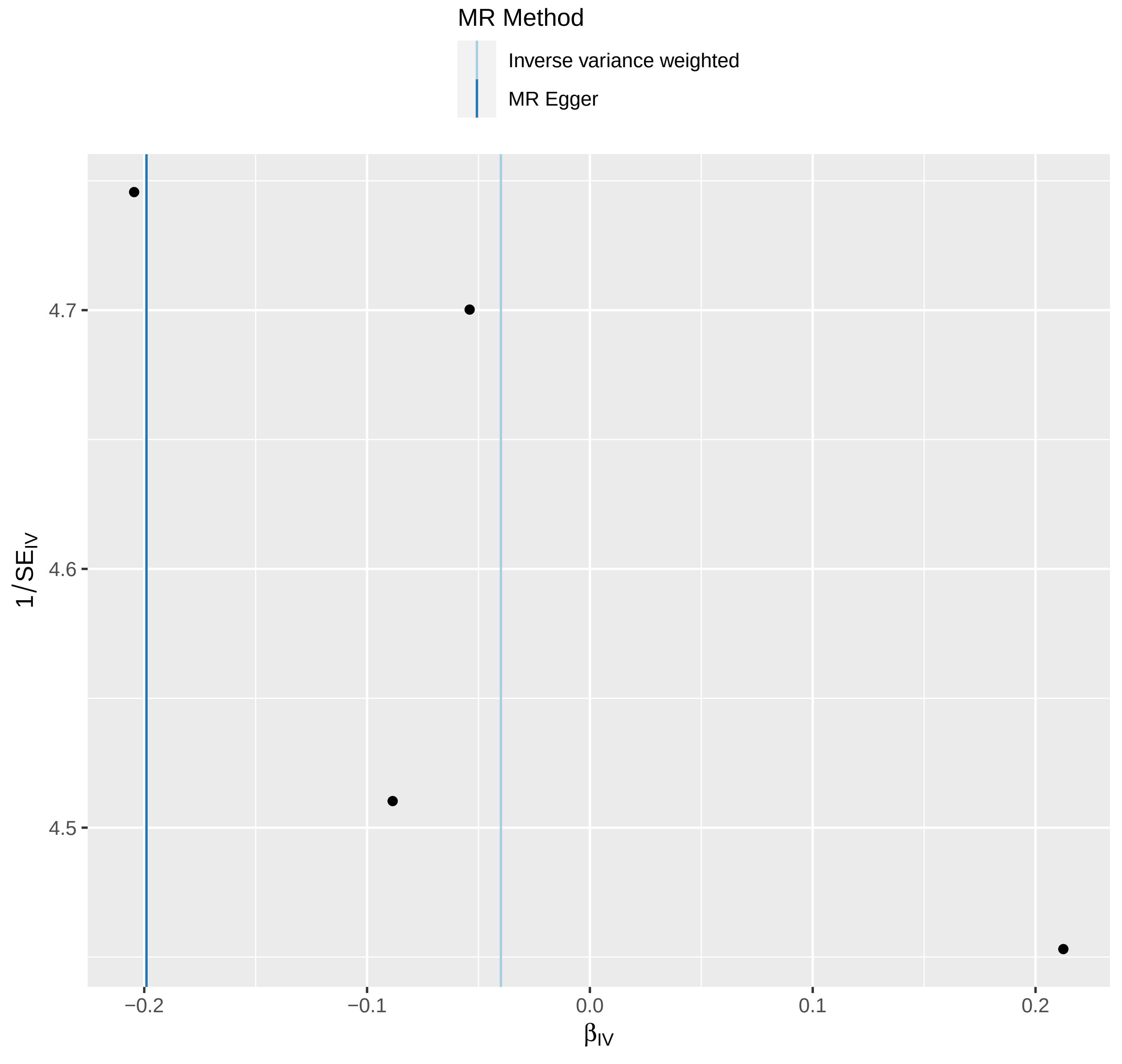

Batch 2−4926 : Gut microbiota abundance (genus Parasutterella id.2892) on Peptide YY
with a threshold of 1e−05

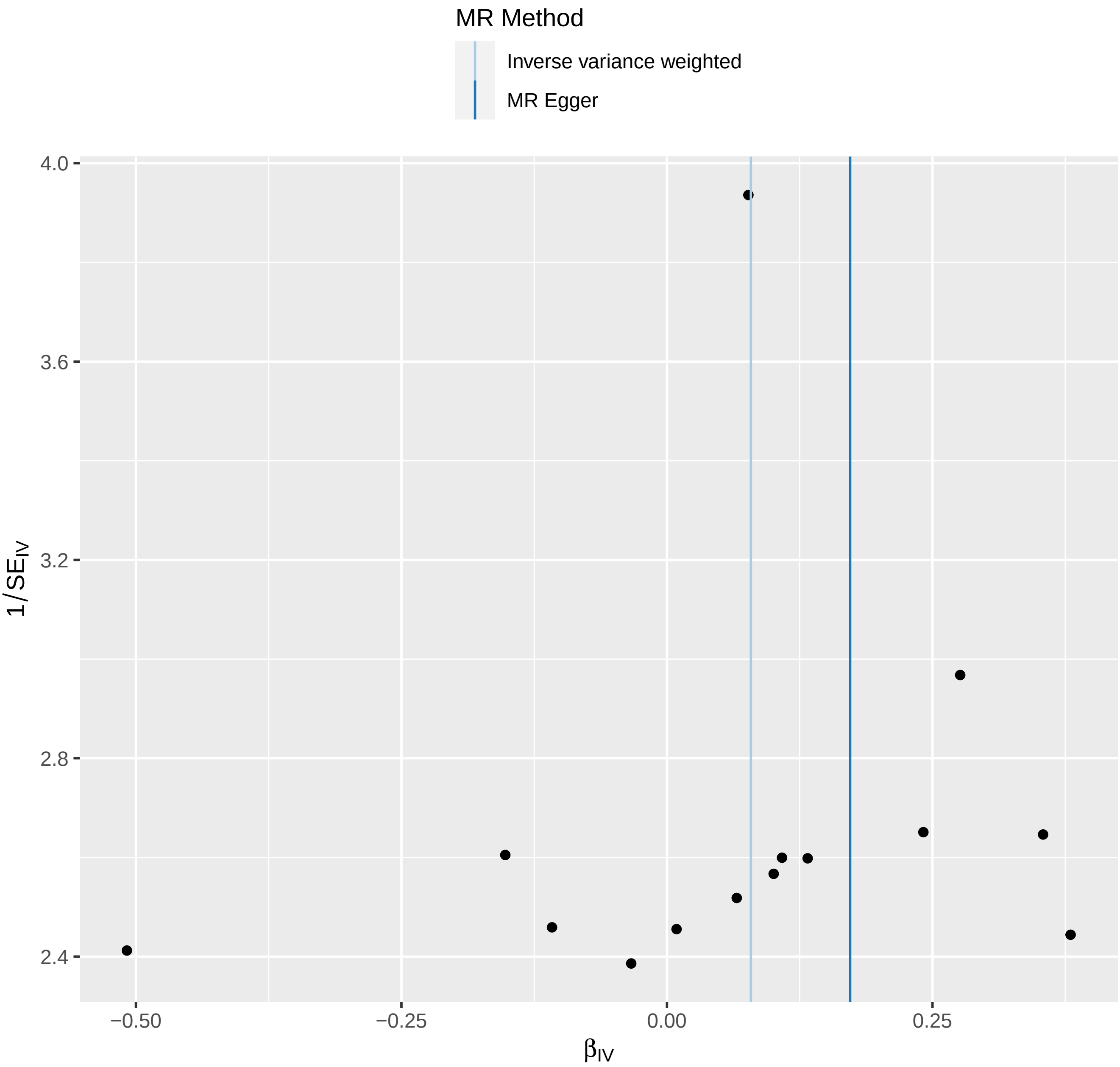

Batch 2−4927 : Gut microbiota abundance (genus Catenibacterium id.2153) on Dihydropteridine reductase
with a threshold of 1e−05

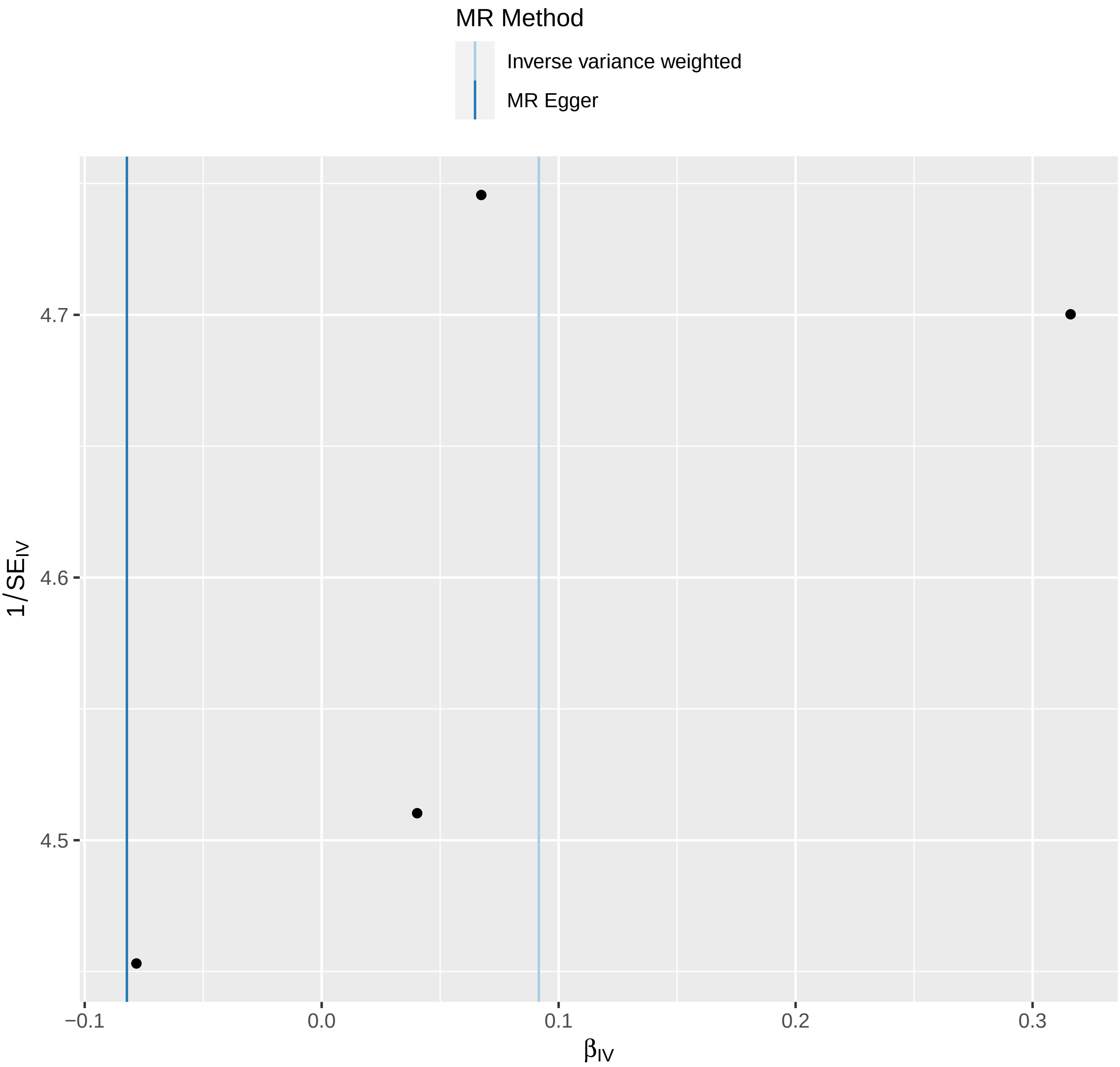

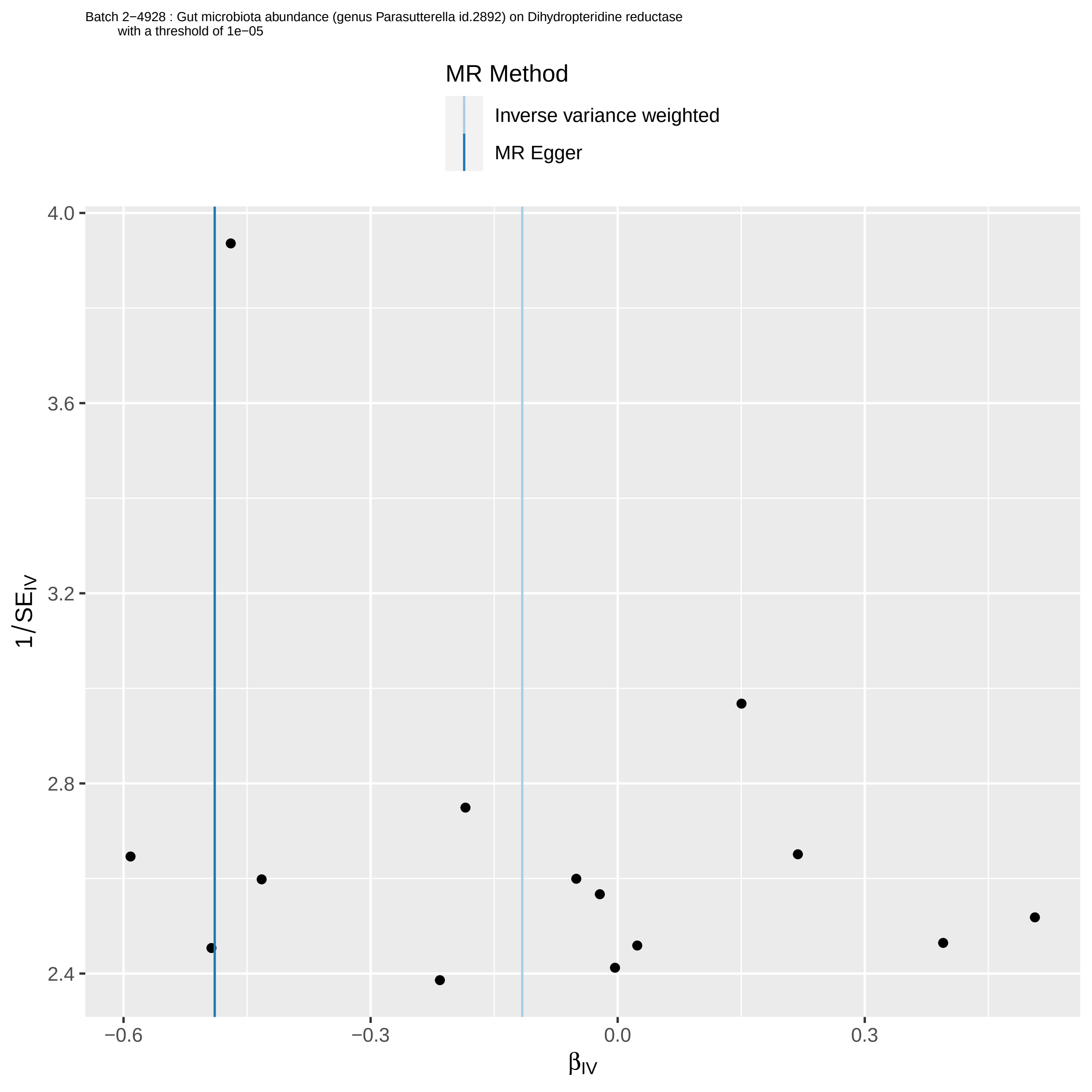

Batch 2−4928 : Gut microbiota abundance (genus Parasutterella id.2892) on Dihydropteridine reductase
with a threshold of 1e−05
MR Method
Inverse variance weighted
MR Egger
4.0
3.6
3.2
2.8
2.4
1/SE_IV
−0.6
−0.3
0.0
0.3
β_IV

Batch 2−4929 : Gut microbiota abundance (genus Catenibacterium id.2153) on Glutaminyl−peptide cyclotransferase
with a threshold of 1e−05

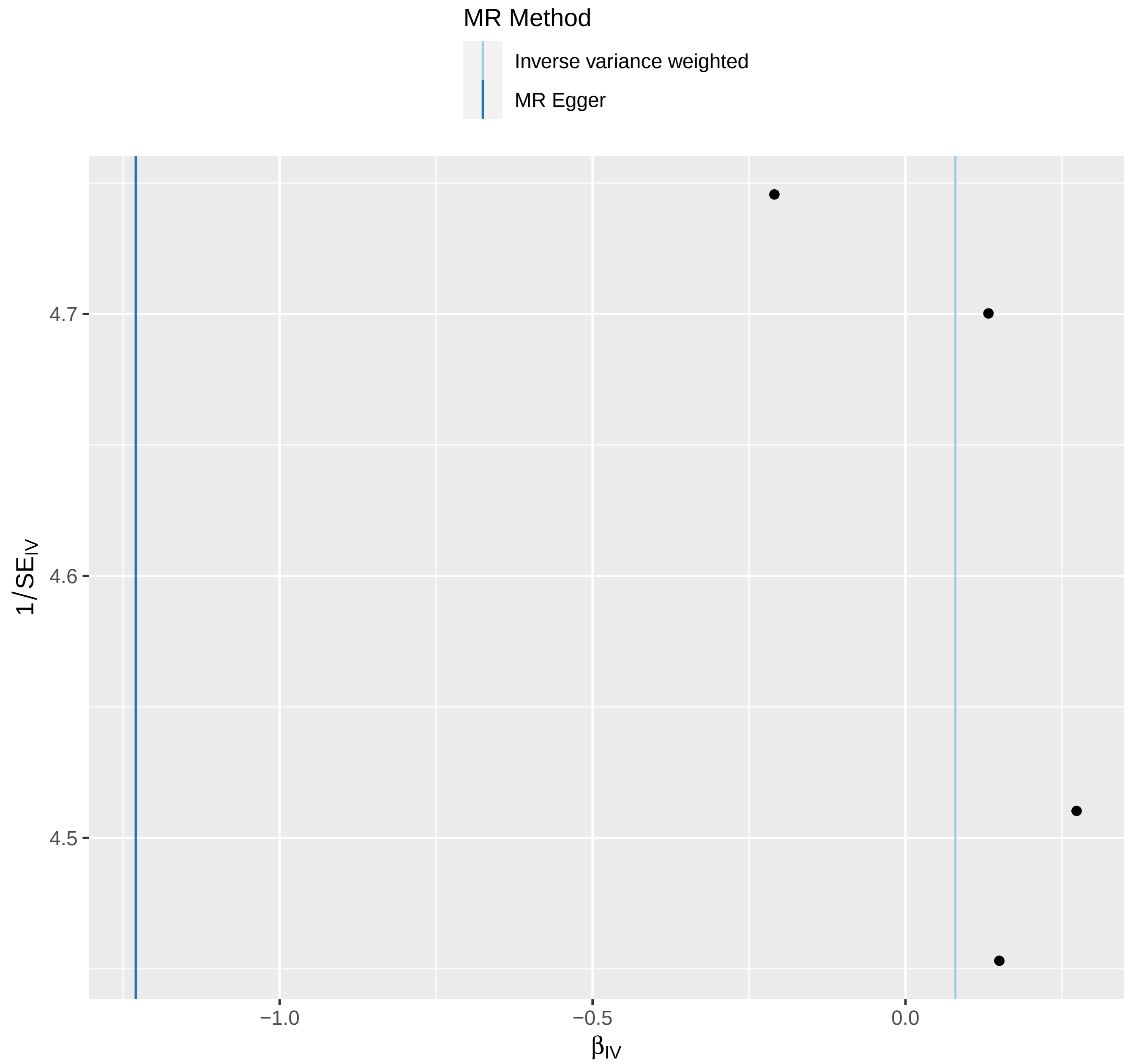

Batch 2−4930 : Gut microbiota abundance (genus Parasutterella id.2892) on Glutaminyl−peptide cyclotransferase
with a threshold of 1e−05

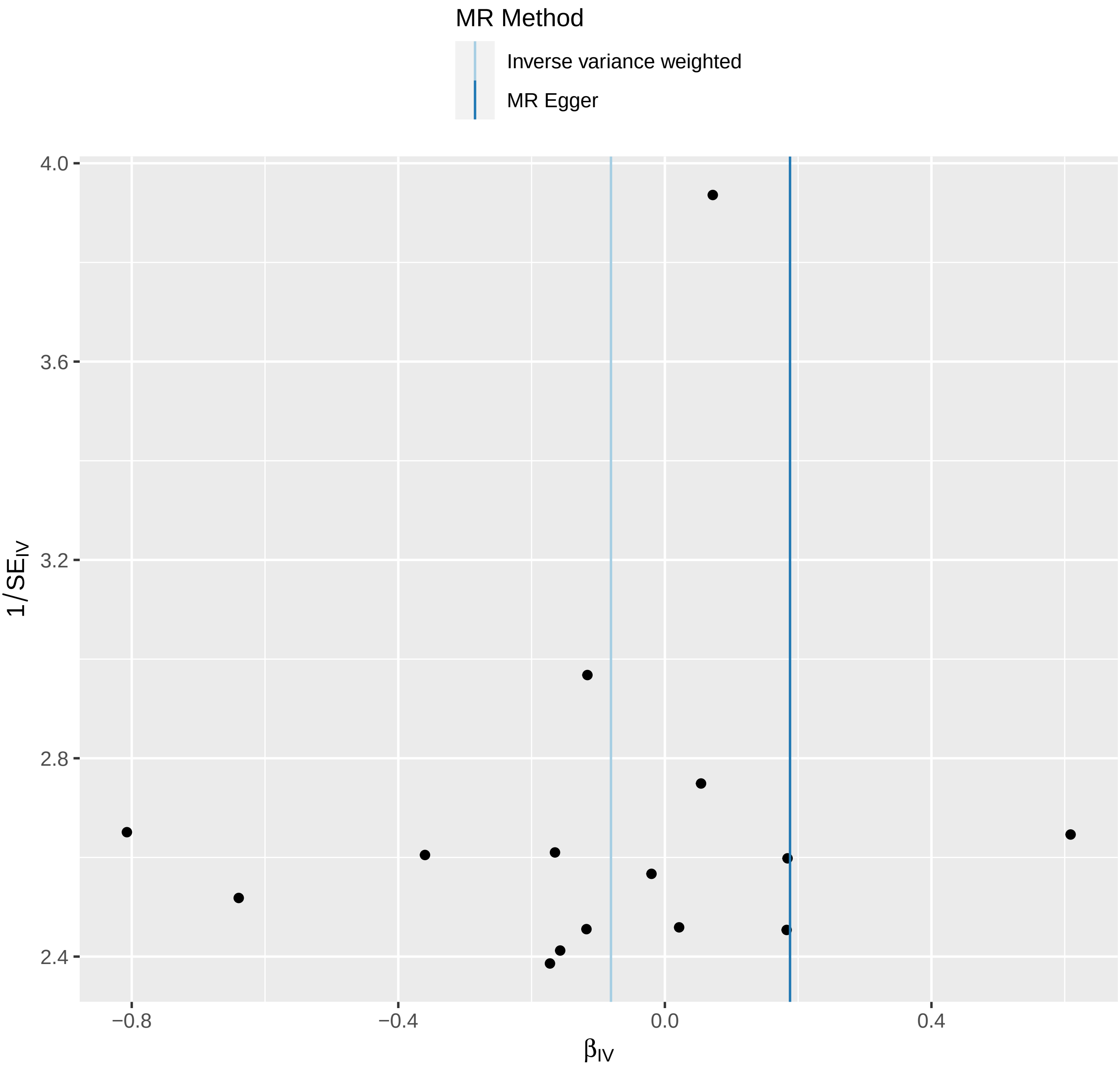

Batch 2−4931 : Gut microbiota abundance (genus Catenibacterium id.2153) on Glutaminyl−peptide cyclotransferase−like protein
with a threshold of 1e−05

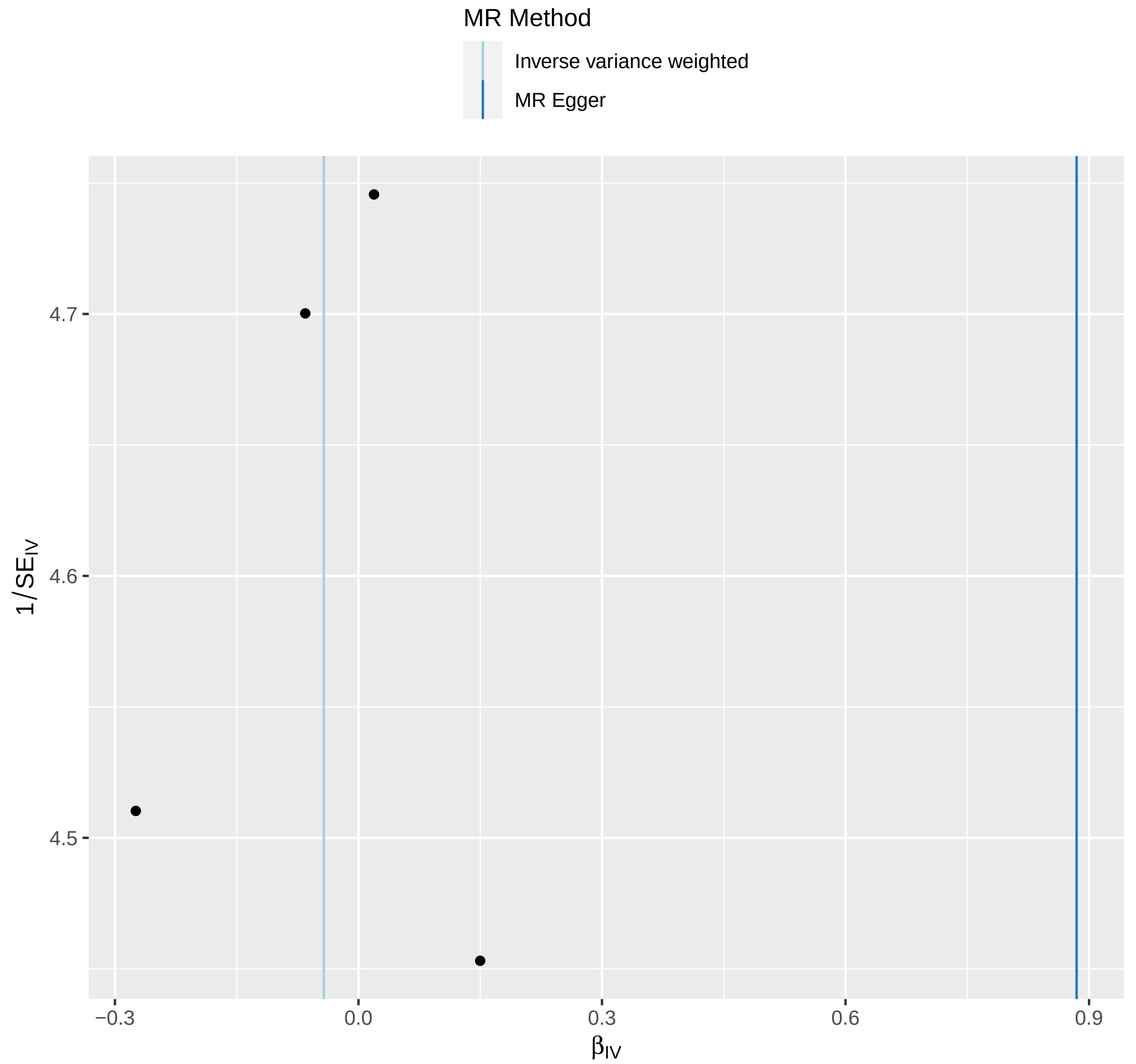

Batch 2−4932 : Gut microbiota abundance (genus Parasutterella id.2892) on Glutaminyl−peptide cyclotransferase−like protein
with a threshold of 1e−05

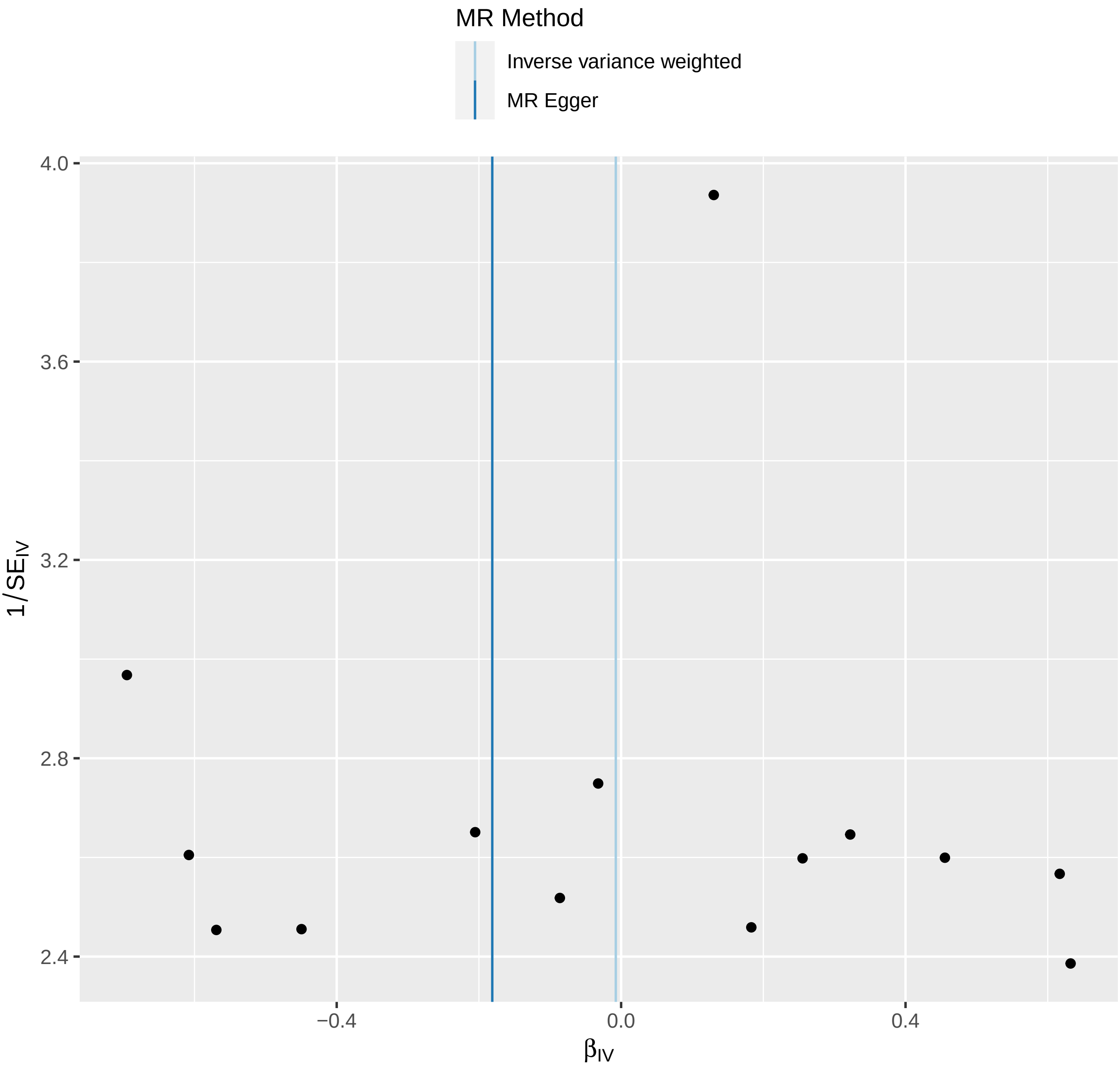

Batch 2−4933 : Gut microbiota abundance (genus Catenibacterium id.2153) on Orexigenic neuropeptide QRFP
with a threshold of 1e−05

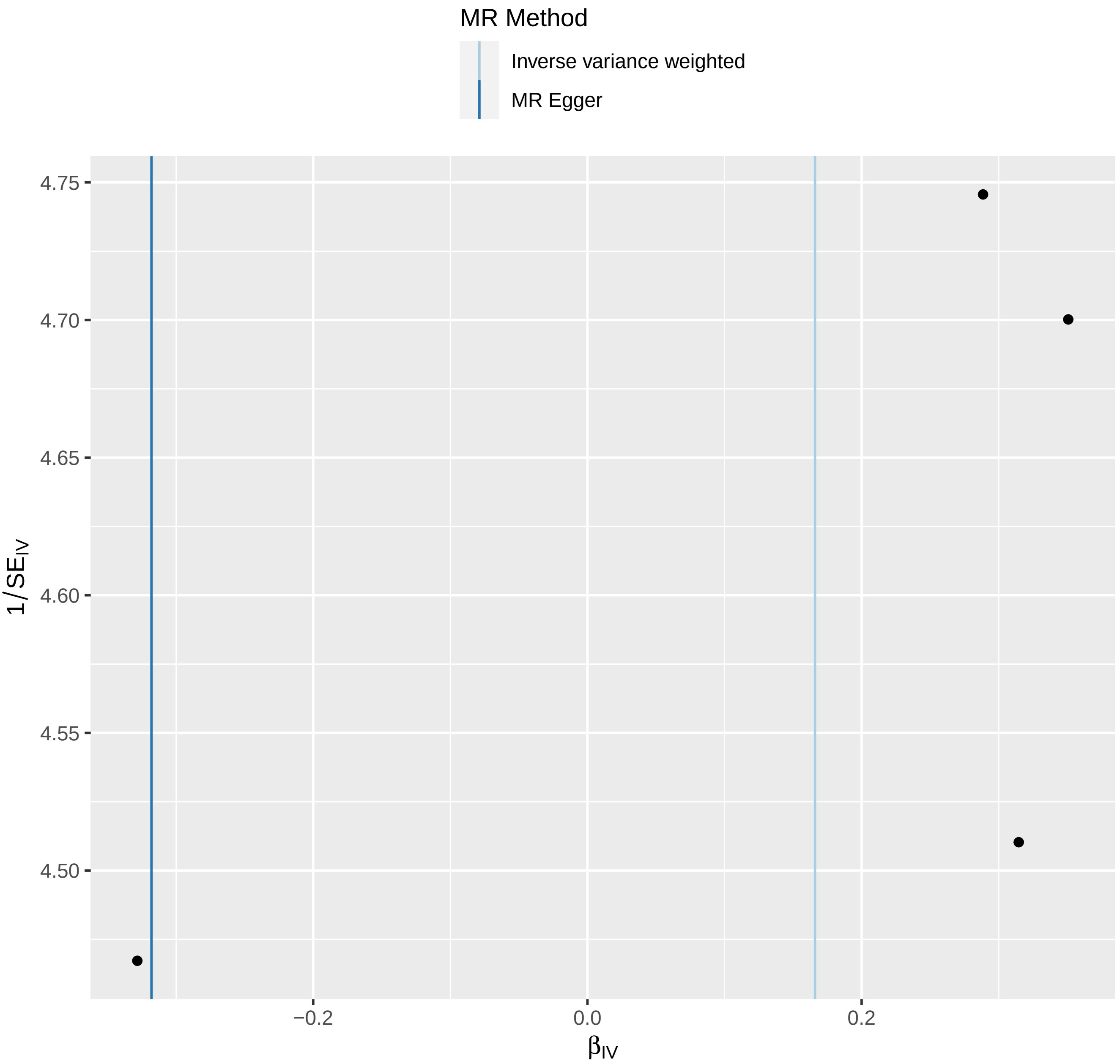

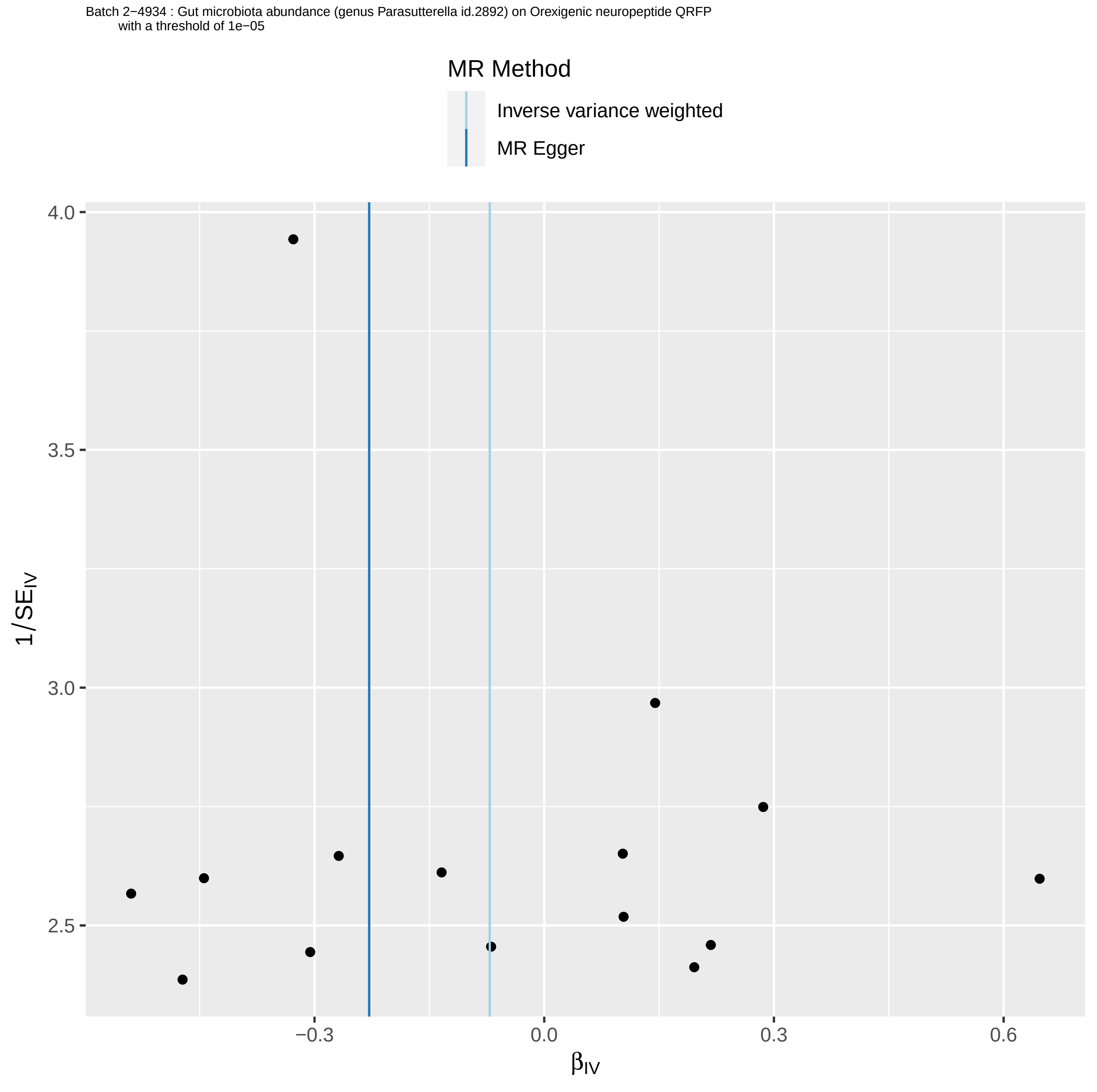

Batch 2−4934 : Gut microbiota abundance (genus Parasutterella id.2892) on Orexigenic neuropeptide QRFP
with a threshold of 1e−05
MR Method
Inverse variance weighted
MR Egger
4.0
3.5
3.0
2.5
1/SE_IV
−0.3
0.0
0.3
0.6
β_IV

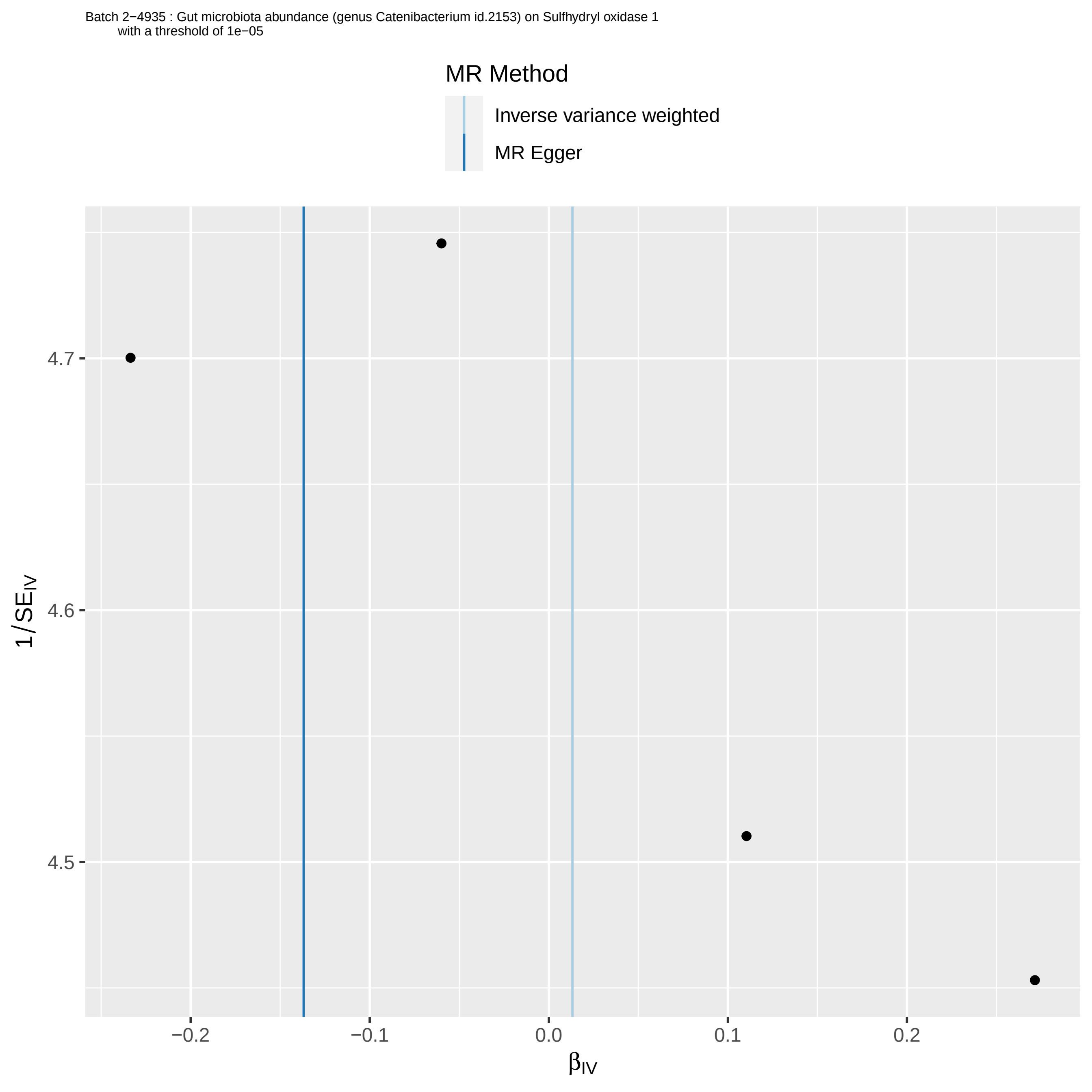

Batch 2−4935 : Gut microbiota abundance (genus Catenibacterium id.2153) on Sulfhydryl oxidase 1
with a threshold of 1e−05
MR Method
Inverse variance weighted
MR Egger
4.7
4.6
4.5
1/SE_IV
−0.2
−0.1
0.0
0.1
0.2
β_IV

Batch 2−4936 : Gut microbiota abundance (genus Parasutterella id.2892) on Sulfhydryl oxidase 1
with a threshold of 1e−05

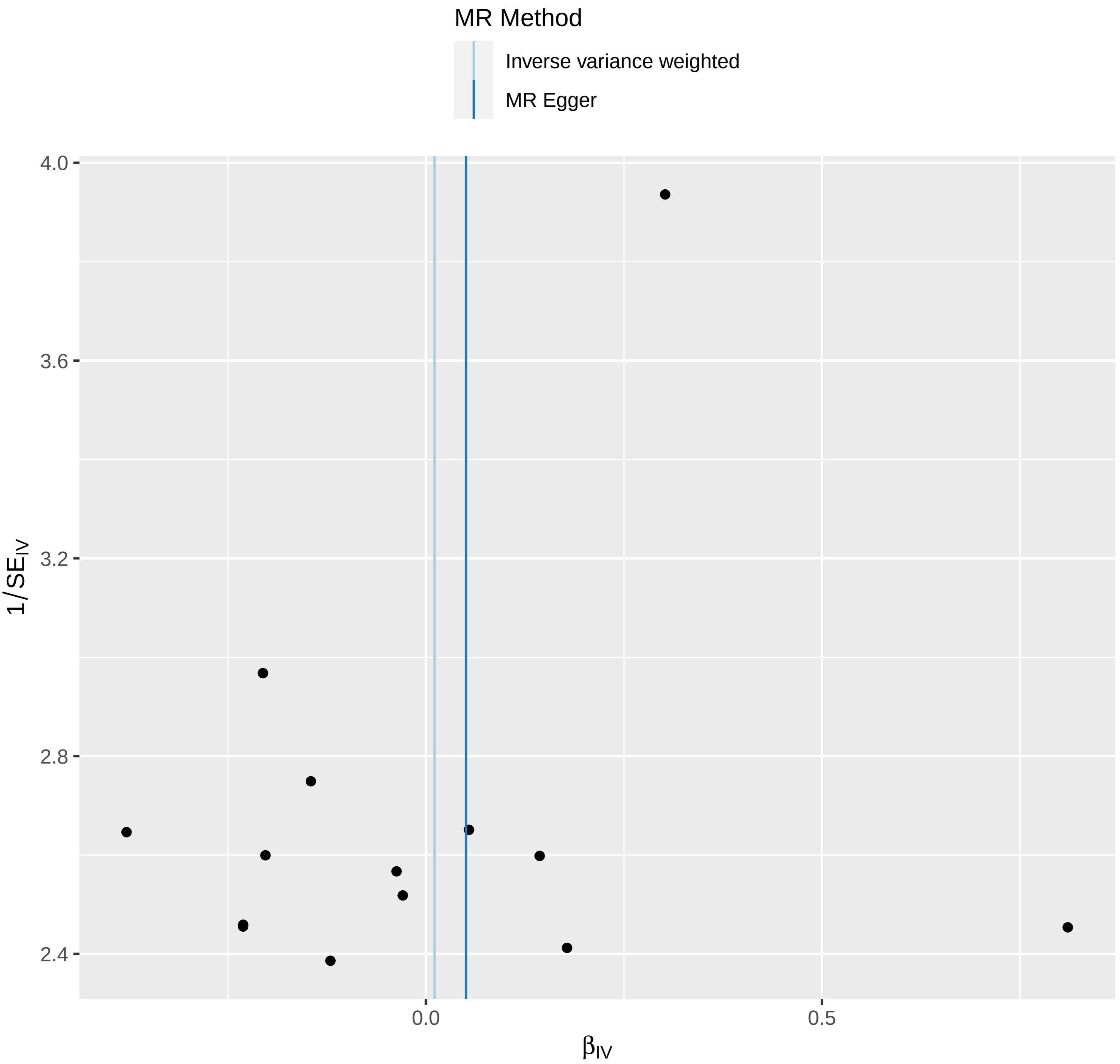

Batch 2−4937 : Gut microbiota abundance (genus Catenibacterium id.2153) on Sulfhydryl oxidase 1
with a threshold of 1e−05

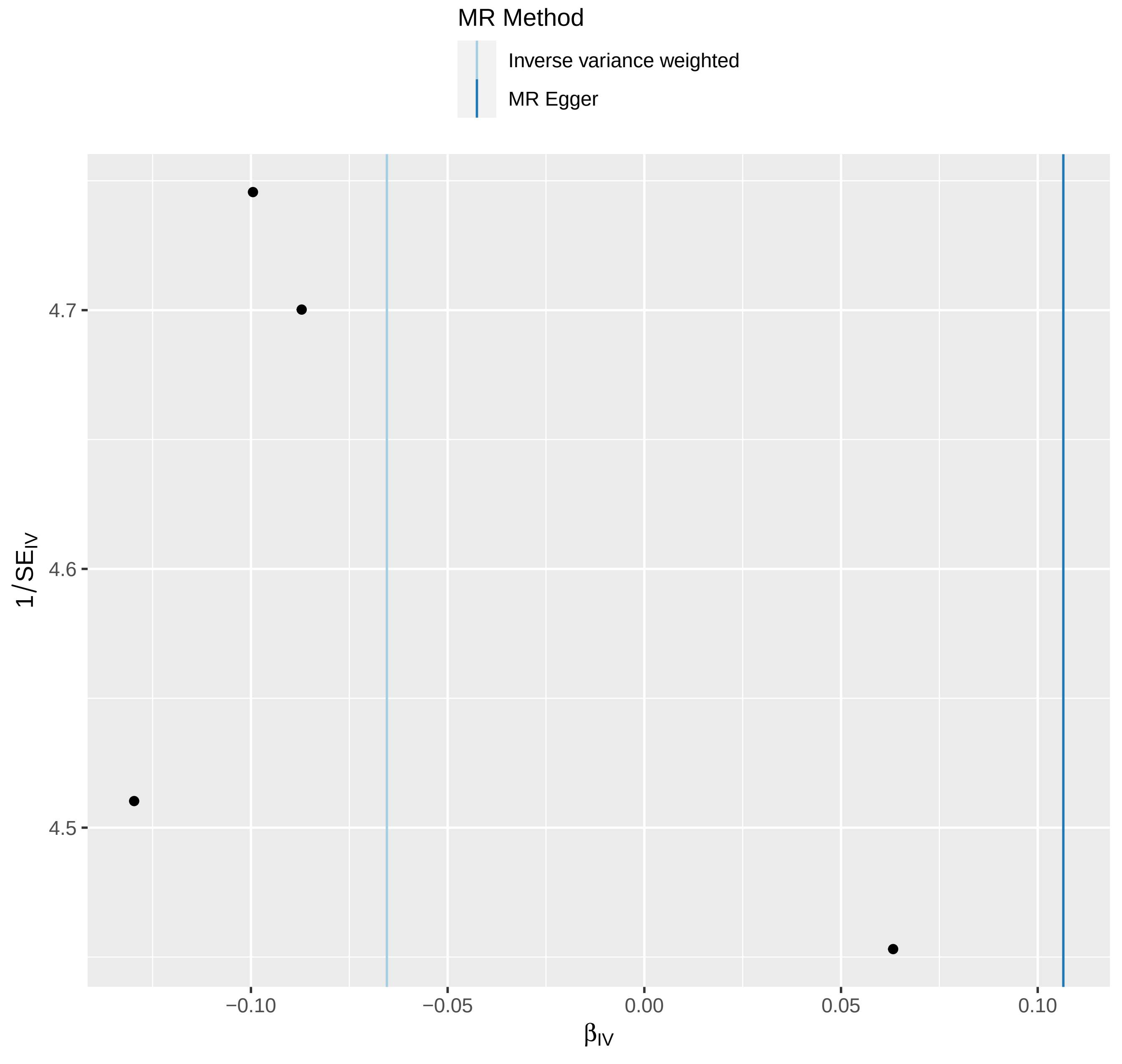

Batch 2−4938 : Gut microbiota abundance (genus Parasutterella id.2892) on Sulfhydryl oxidase 1
with a threshold of 1e−05

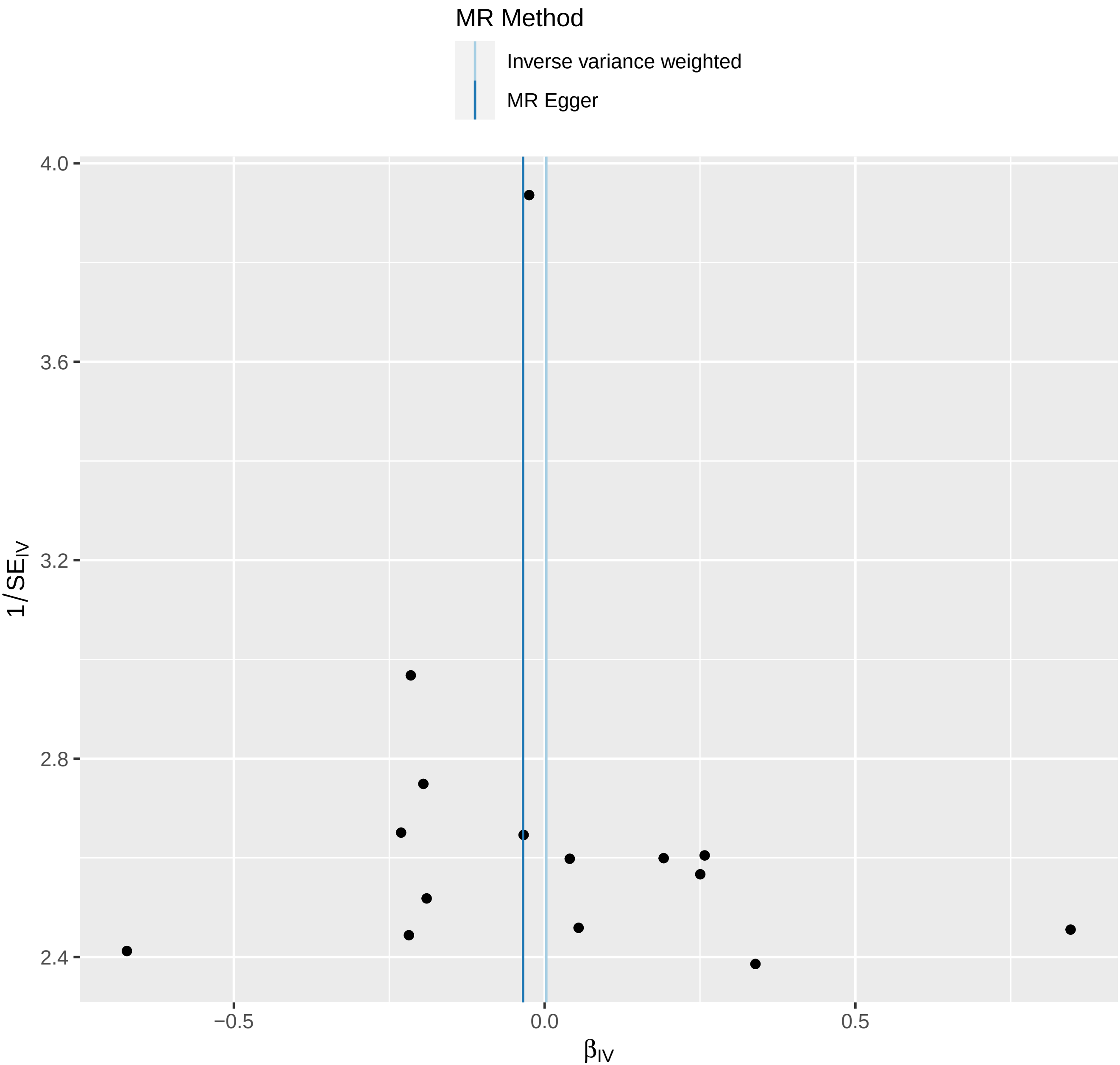

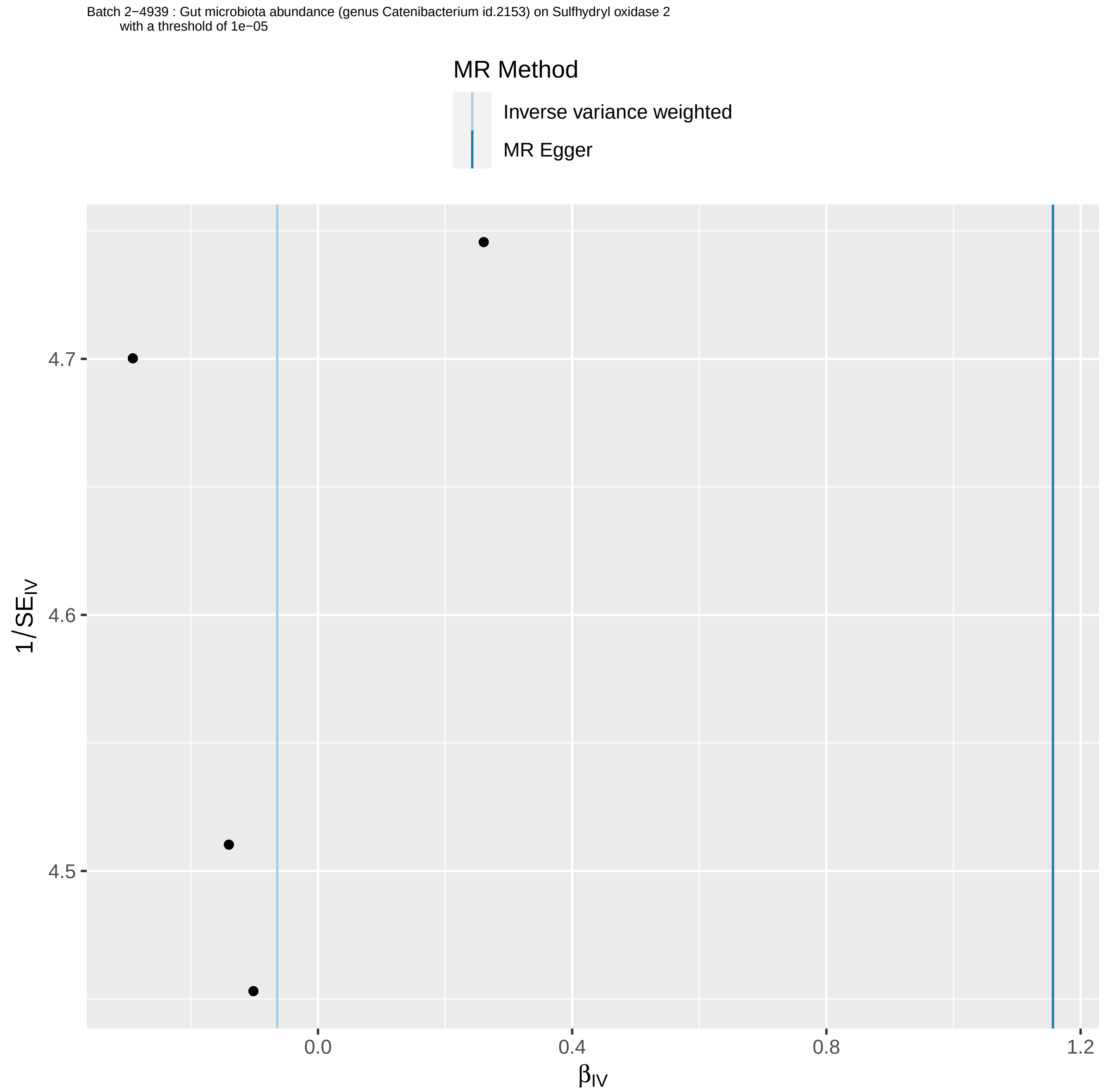
Batch 2−4939 : Gut microbiota abundance (genus Catenibacterium id.2153) on Sulfhydryl oxidase 2
with a threshold of 1e−05
MR Method
Inverse variance weighted
MR Egger
4.7
4.6
4.5
1/SE_IV
0.0
0.4
0.8
1.2
β_IV

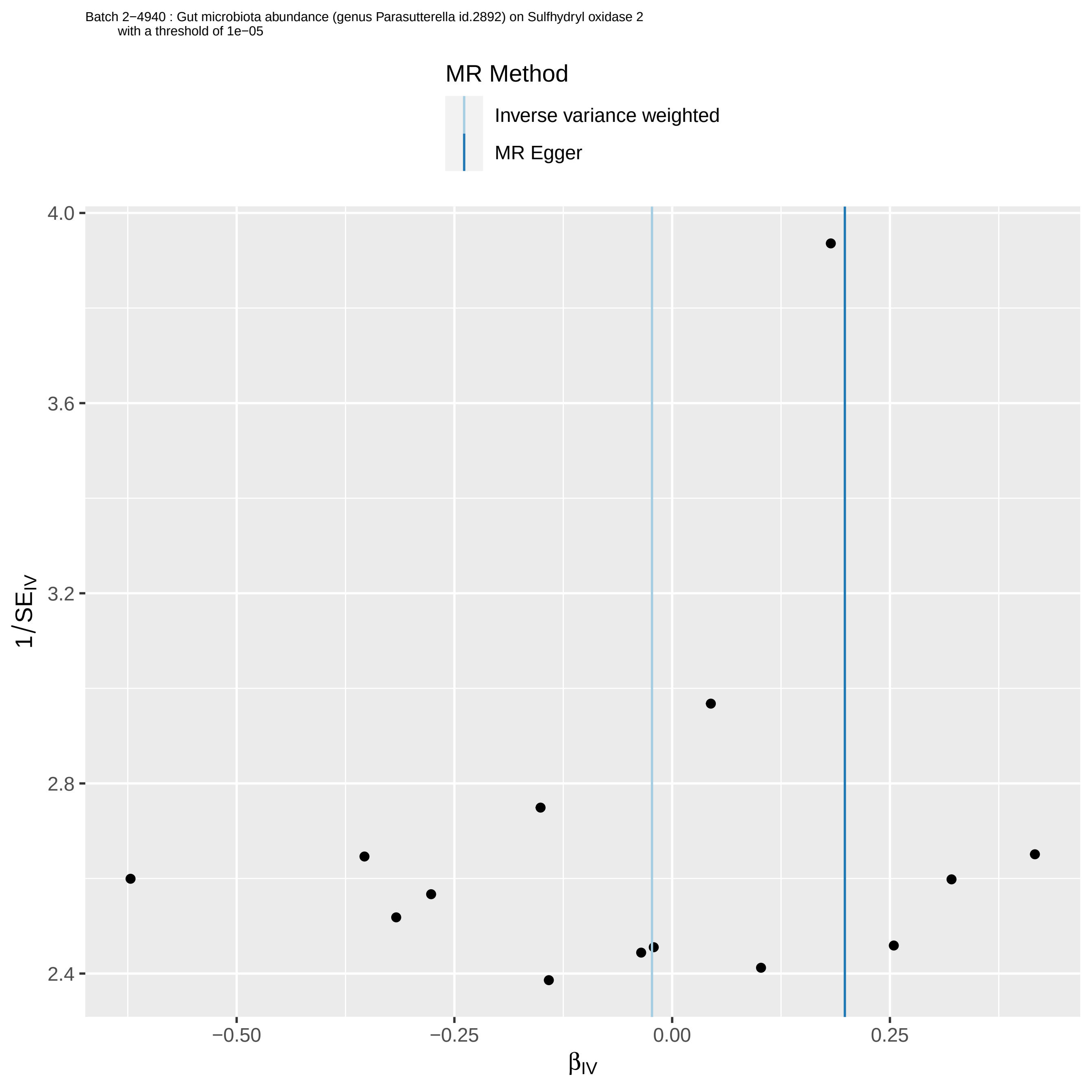
Batch 2−4940 : Gut microbiota abundance (genus Parasutterella id.2892) on Sulfhydryl oxidase 2
with a threshold of 1e−05
MR Method
Inverse variance weighted
MR Egger
4.0
3.6
3.2
2.8
2.4
1/SE_IV
−0.50
−0.25
0.00
0.25
β_IV

Batch 2−4941 : Gut microbiota abundance (genus Catenibacterium id.2153) on Ras−related protein Rab−14
with a threshold of 1e−05

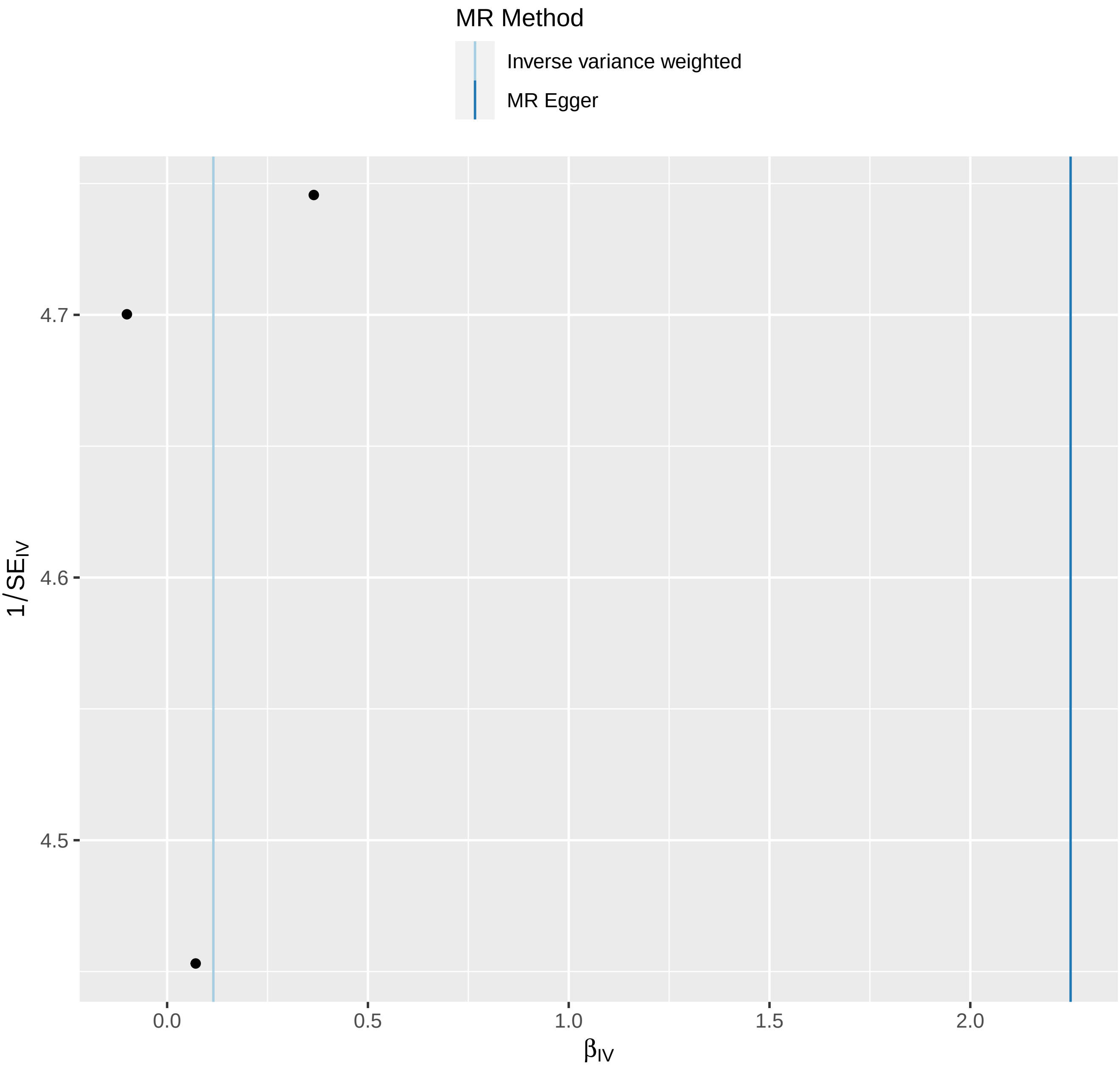

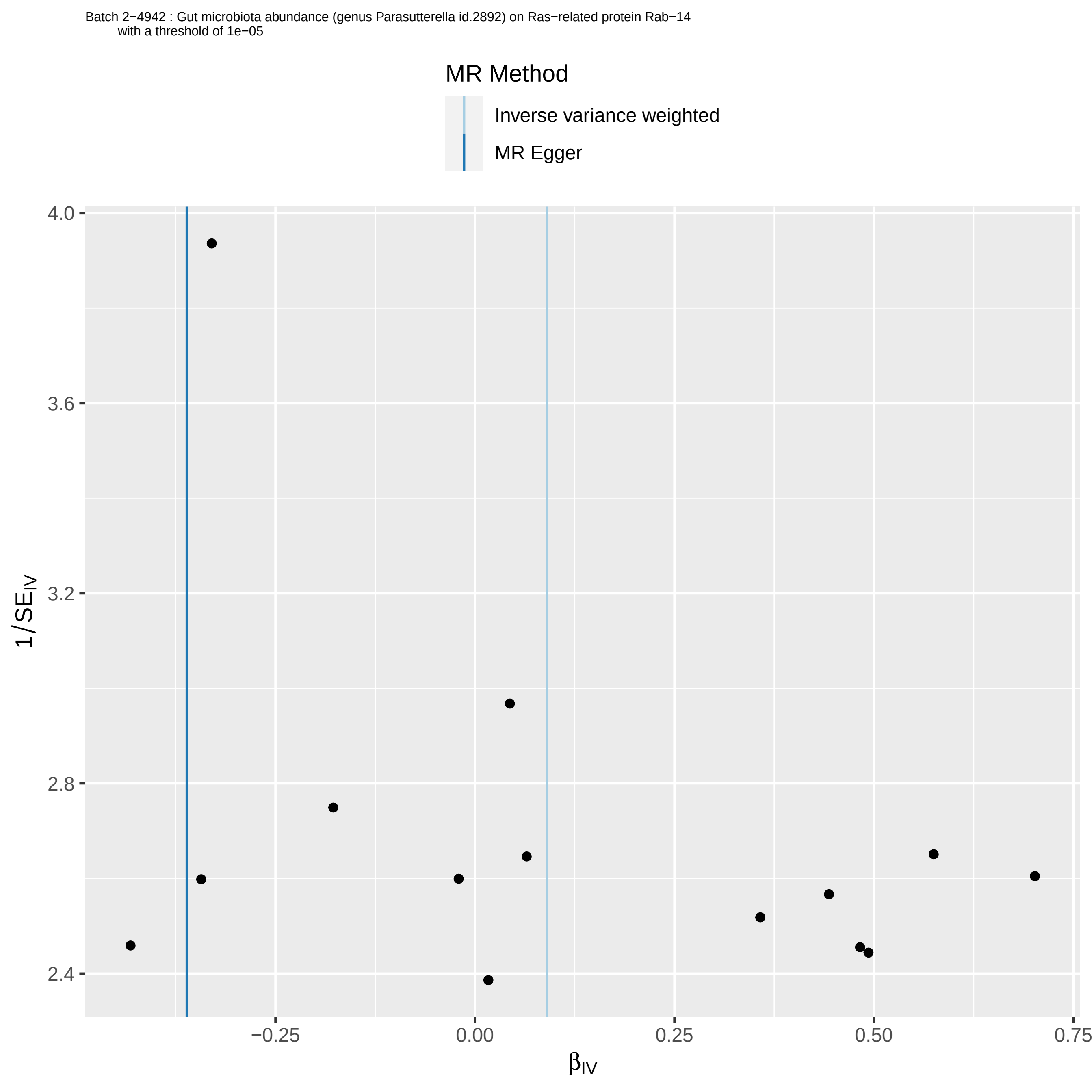

Batch 2−4942 : Gut microbiota abundance (genus Parasutterella id.2892) on Ras−related protein Rab−14
with a threshold of 1e−05
MR Method
Inverse variance weighted
MR Egger
4.0
3.6
3.2
2.8
2.4
1/SE_IV
−0.25
0.00
0.25
0.50
0.75
β_IV

Batch 2−4943 : Gut microbiota abundance (genus Catenibacterium id.2153) on Ras−related protein Rab−22A
with a threshold of 1e−05

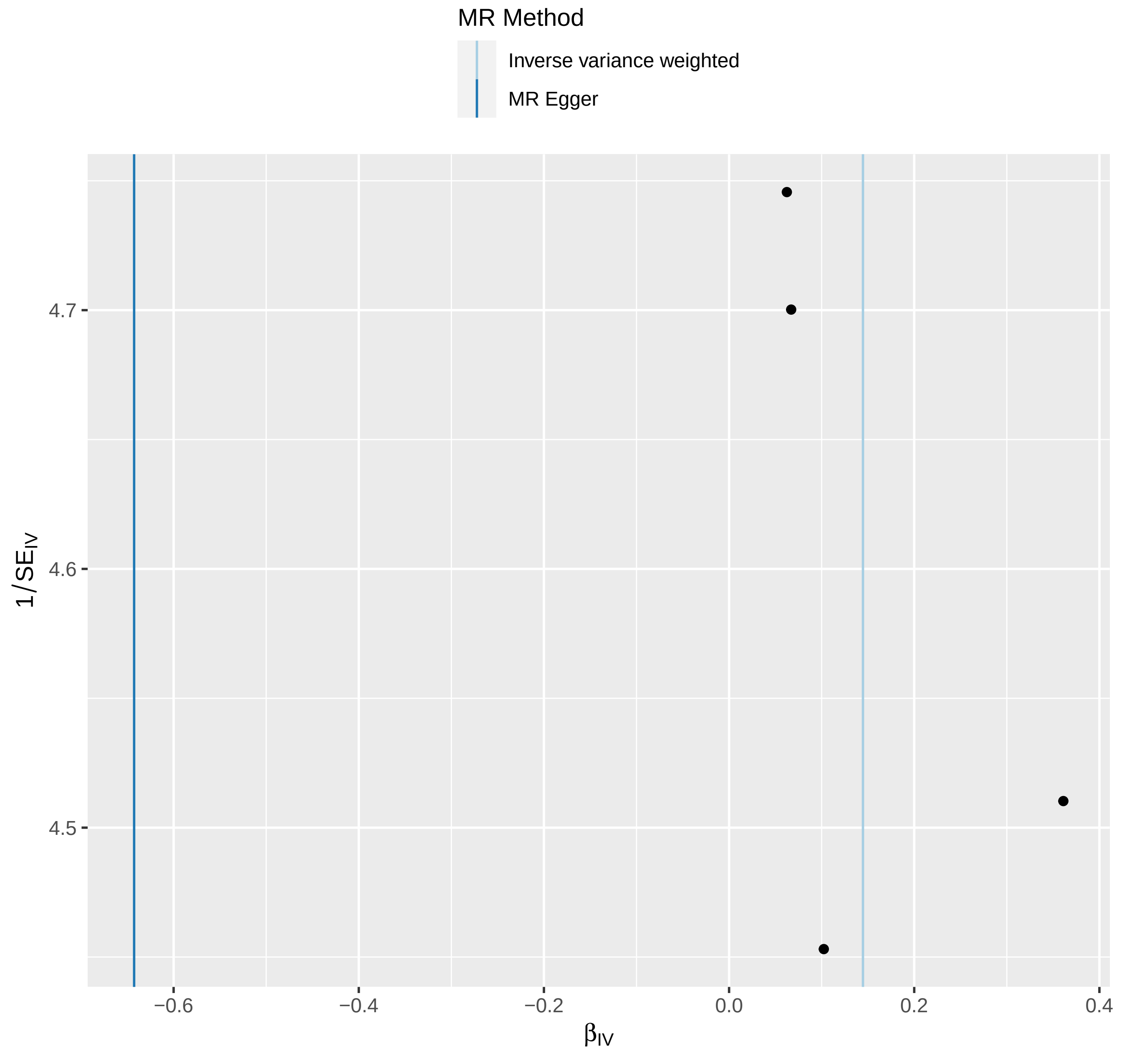

Batch 2−4944 : Gut microbiota abundance (genus Parasutterella id.2892) on Ras−related protein Rab−22A
with a threshold of 1e−05

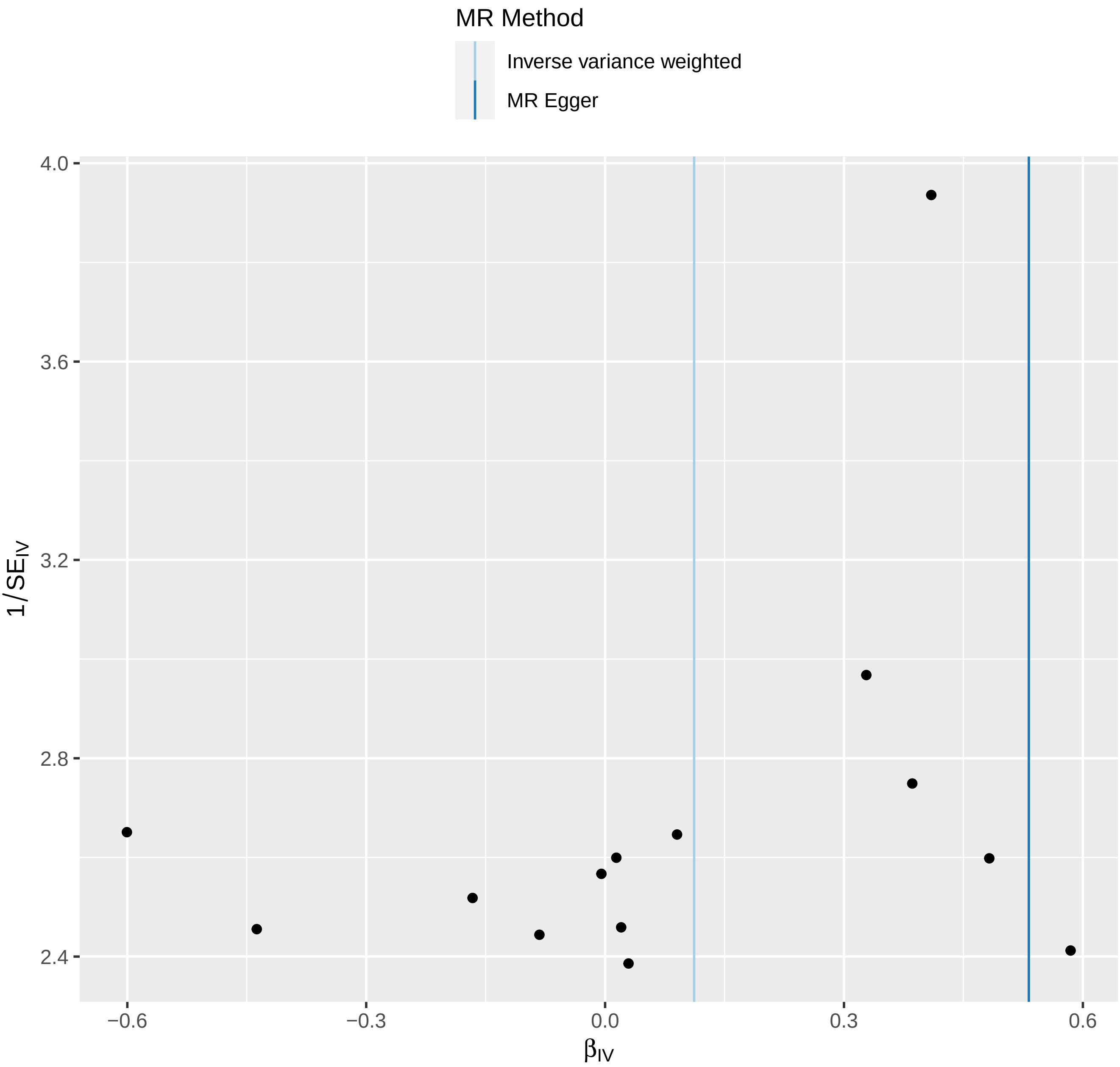

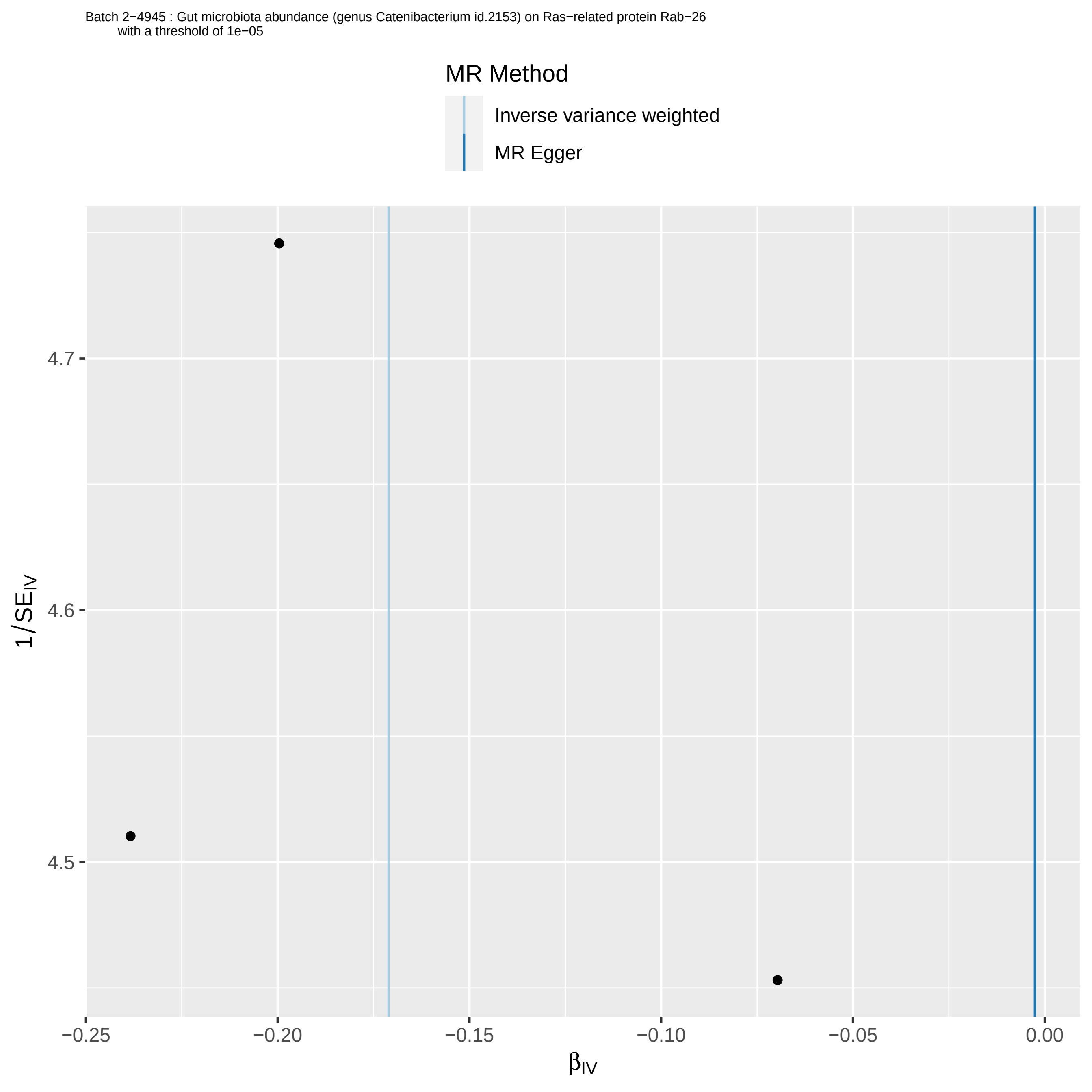
Batch 2−4945 : Gut microbiota abundance (genus Catenibacterium id.2153) on Ras−related protein Rab−26
with a threshold of 1e−05
MR Method
Inverse variance weighted
MR Egger
4.7
4.6
4.5
1/SE$_{IV}$
−0.25
−0.20
−0.15
−0.10
−0.05
0.00
β$_{IV}$

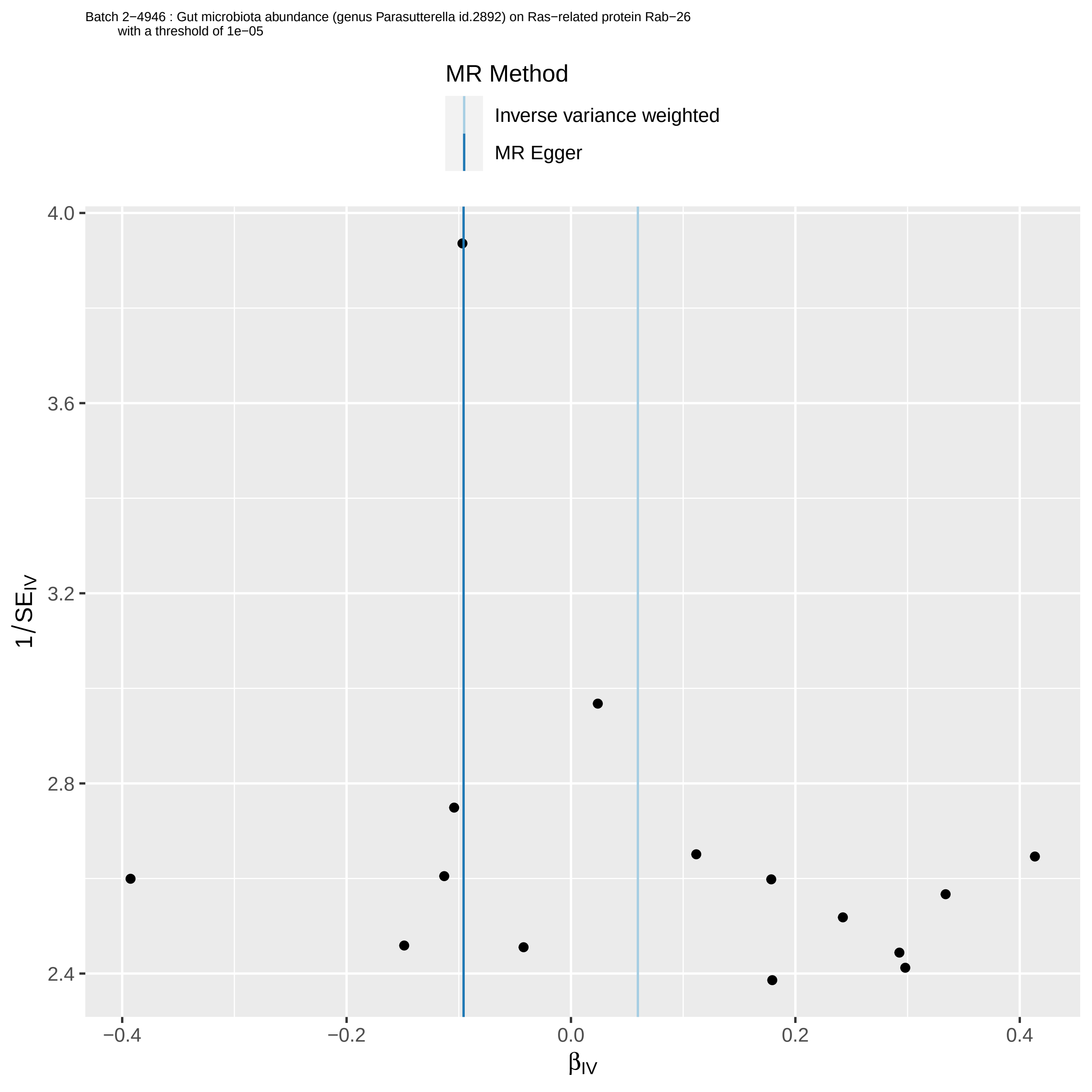
Batch 2−4946 : Gut microbiota abundance (genus Parasutterella id.2892) on Ras−related protein Rab−26
with a threshold of 1e−05
MR Method
Inverse variance weighted
MR Egger
4.0
3.6
3.2
2.8
2.4
1/SE_IV
−0.4
−0.2
0.0
0.2
0.4
β_IV

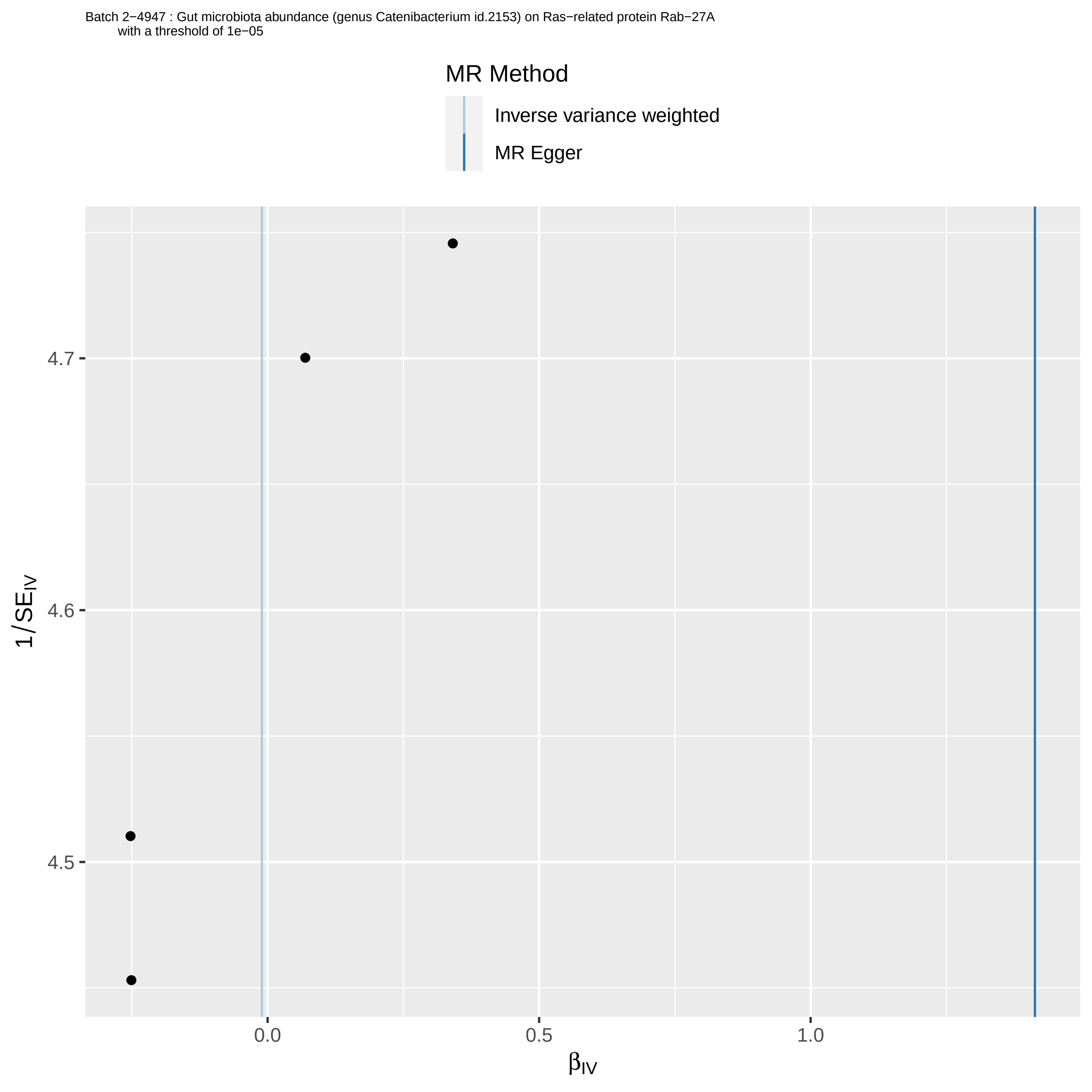
Batch 2−4947 : Gut microbiota abundance (genus Catenibacterium id.2153) on Ras−related protein Rab−27A
with a threshold of 1e−05
MR Method
Inverse variance weighted
MR Egger
4.7
4.6
4.5
1/SE$_{IV}$
0.0
0.5
1.0
β_{IV}

Batch 2−4948 : Gut microbiota abundance (genus Parasutterella id.2892) on Ras−related protein Rab−27A
with a threshold of 1e−05

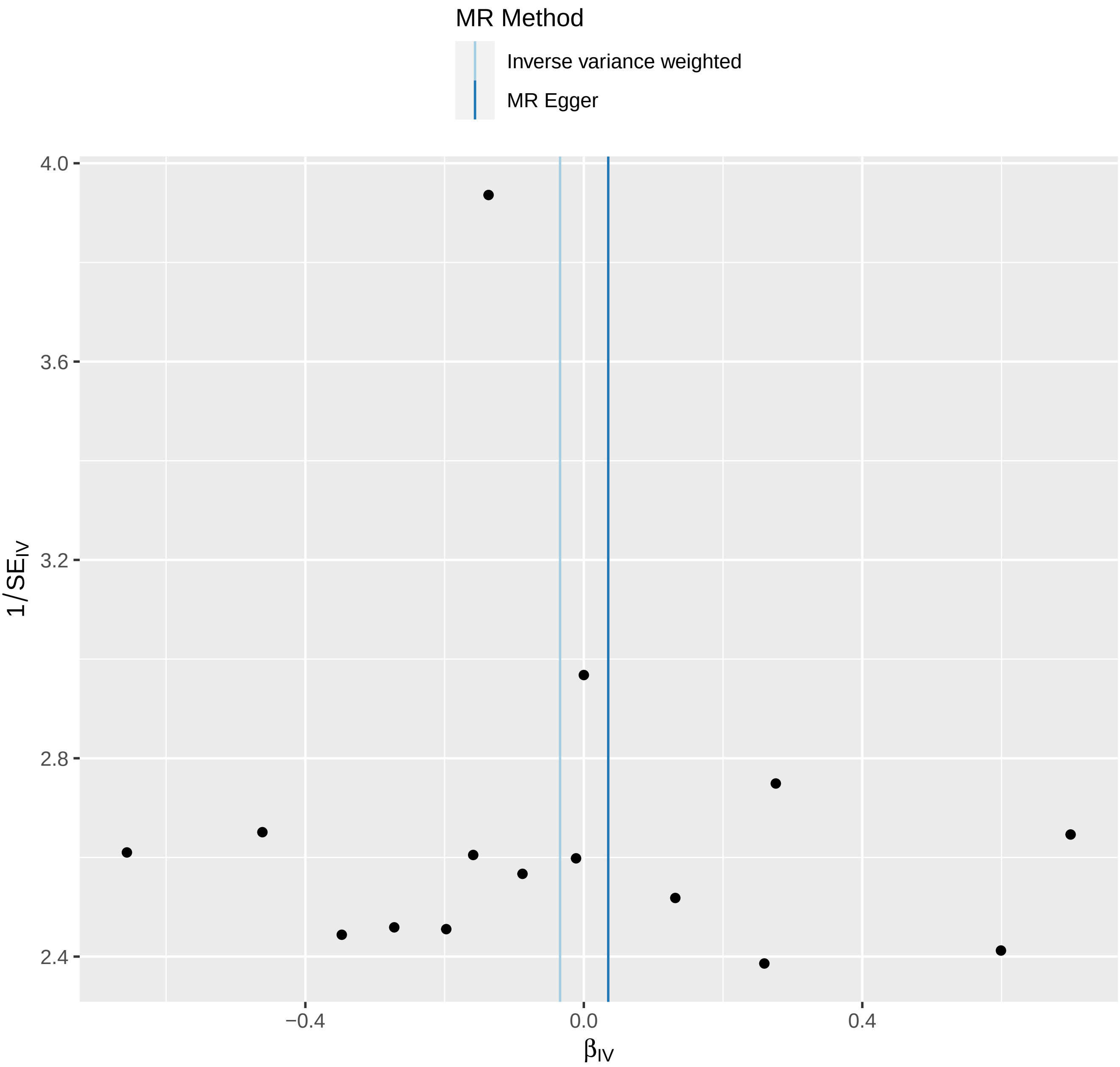

Batch 2−4949 : Gut microbiota abundance (genus Catenibacterium id.2153) on Ras−related protein Rab−27B
with a threshold of 1e−05

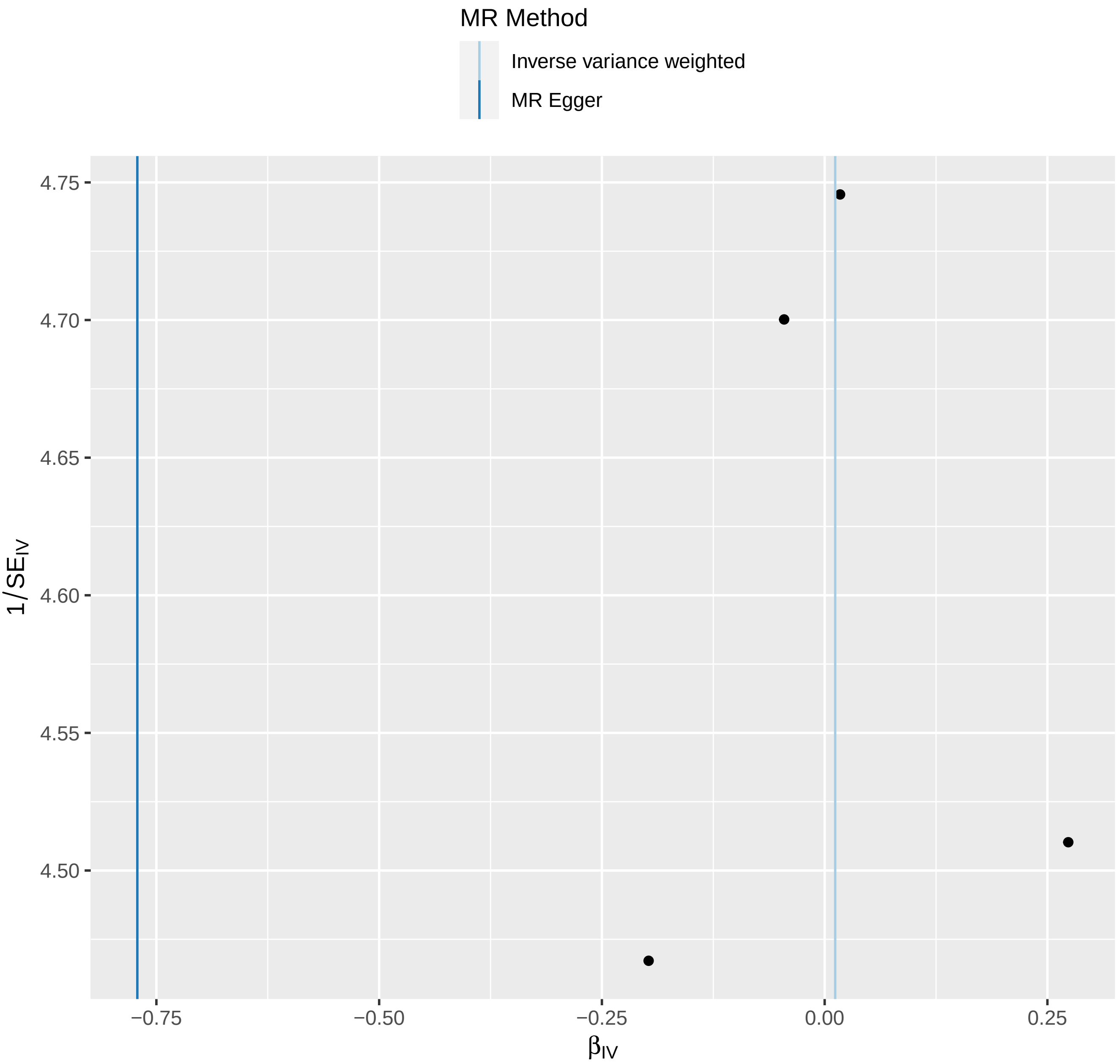

Batch 2−4950 : Gut microbiota abundance (genus Parasutterella id.2892) on Ras−related protein Rab−27B
with a threshold of 1e−05

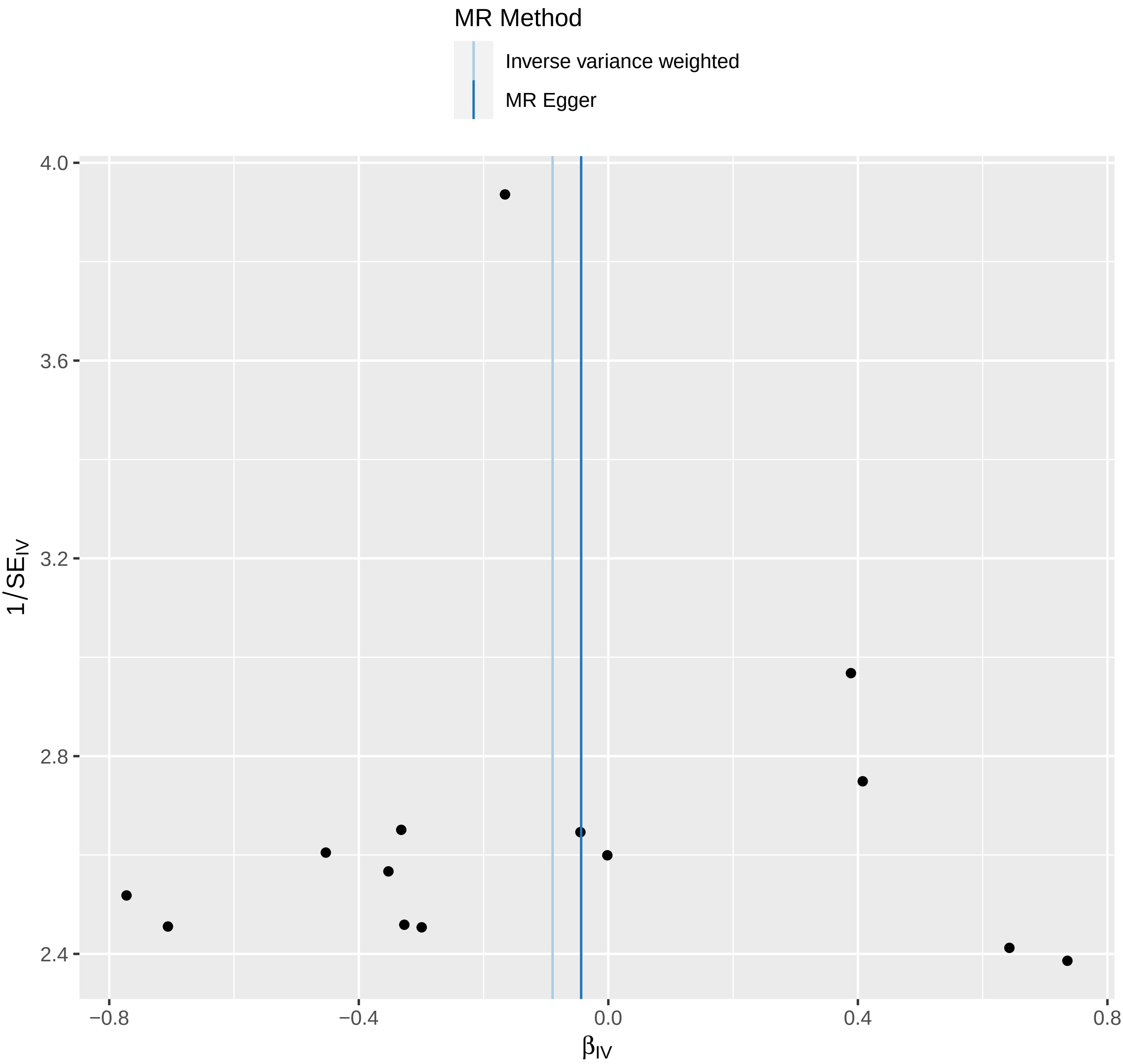

Batch 2−4951 : Gut microbiota abundance (genus Catenibacterium id.2153) on Ras−related protein Rab−35
with a threshold of 1e−05

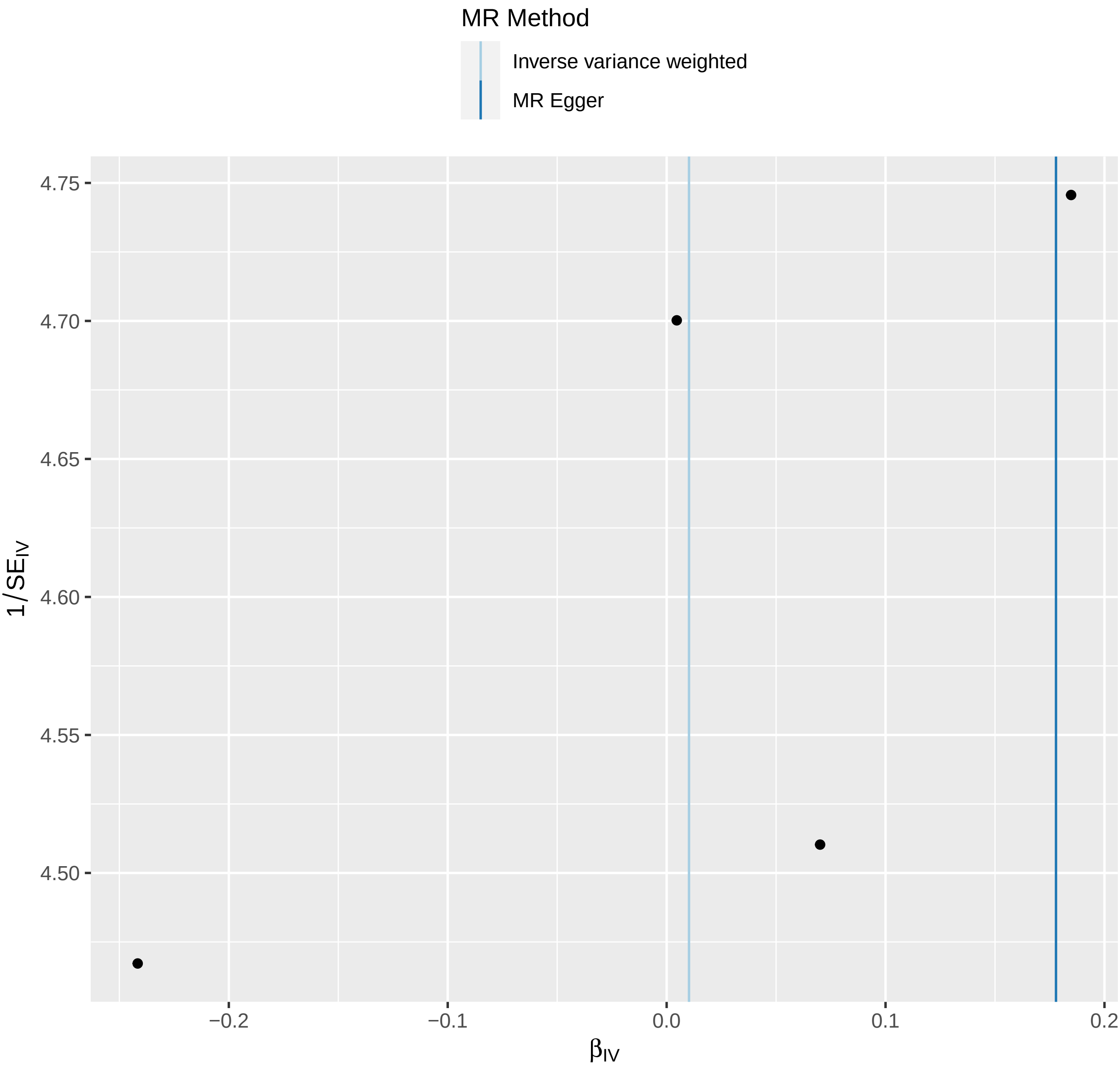

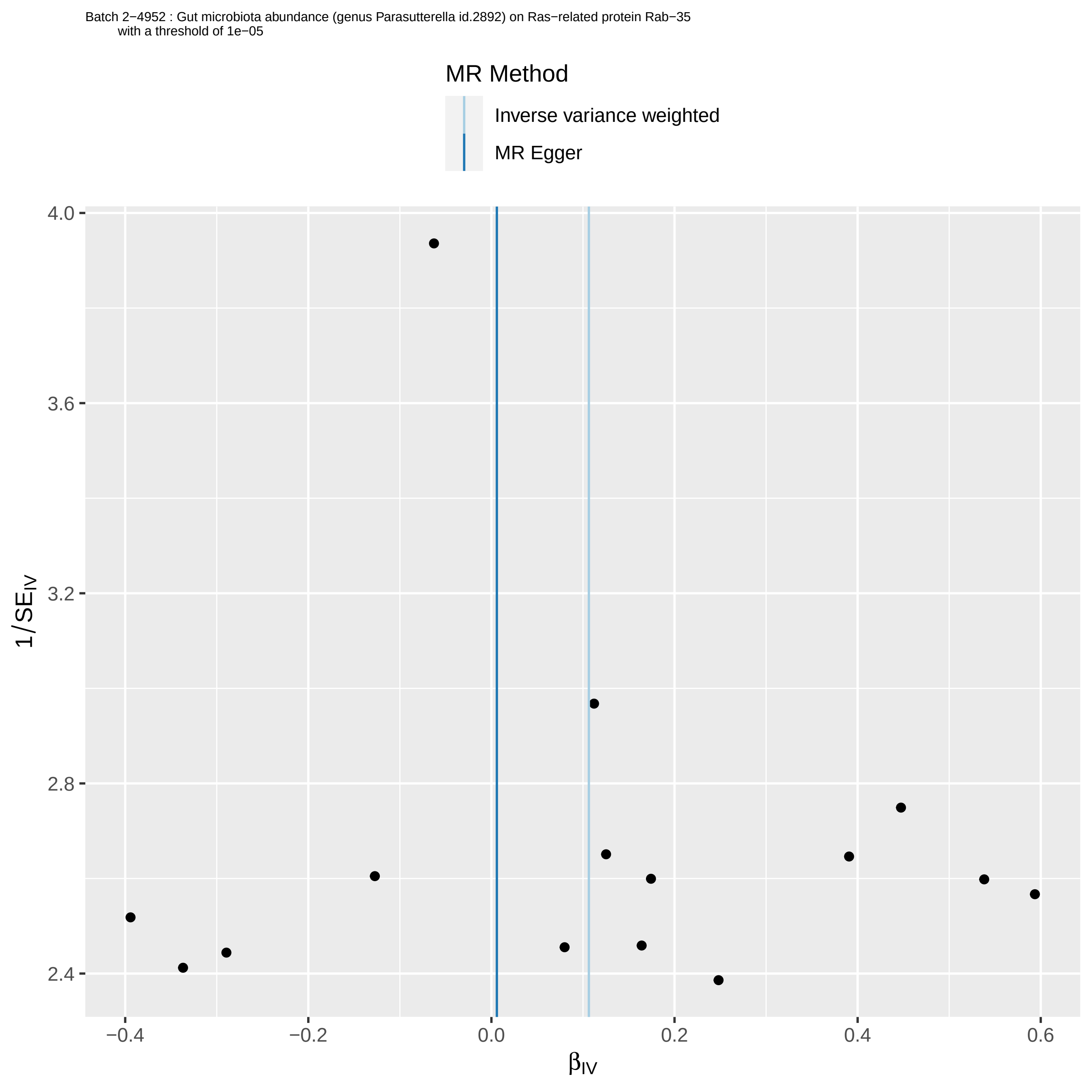
Batch 2−4952 : Gut microbiota abundance (genus Parasutterella id.2892) on Ras−related protein Rab−35
with a threshold of 1e−05
MR Method
Inverse variance weighted
MR Egger
4.0
3.6
3.2
2.8
2.4
1/SE_IV
−0.4
−0.2
0.0
0.2
0.4
0.6
β_IV

Batch 2−4953 : Gut microbiota abundance (genus Catenibacterium id.2153) on Ras−related protein Rab−39B with a threshold of 1e−05

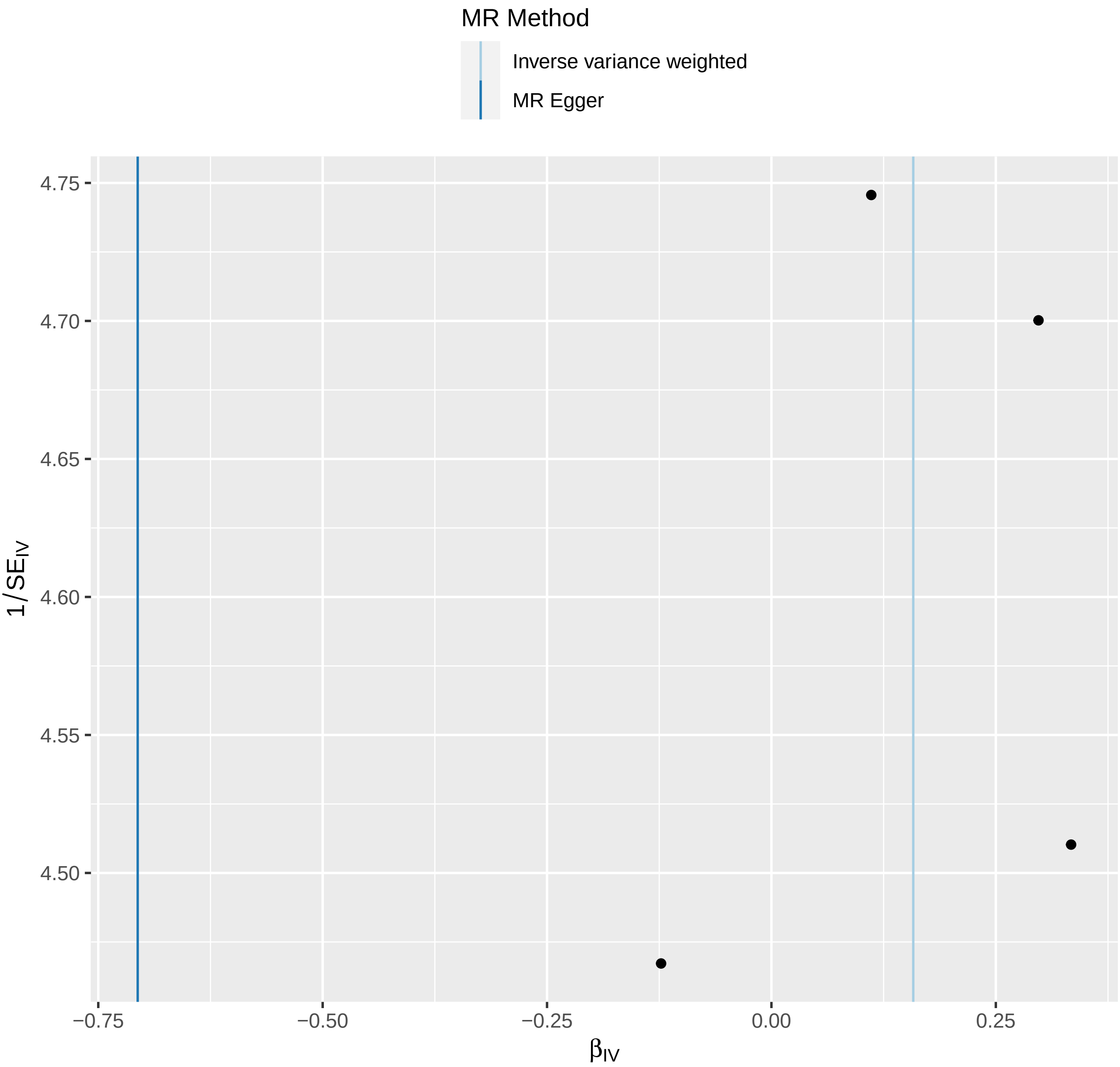

Batch 2−4954 : Gut microbiota abundance (genus Parasutterella id.2892) on Ras−related protein Rab−39B
with a threshold of 1e−05

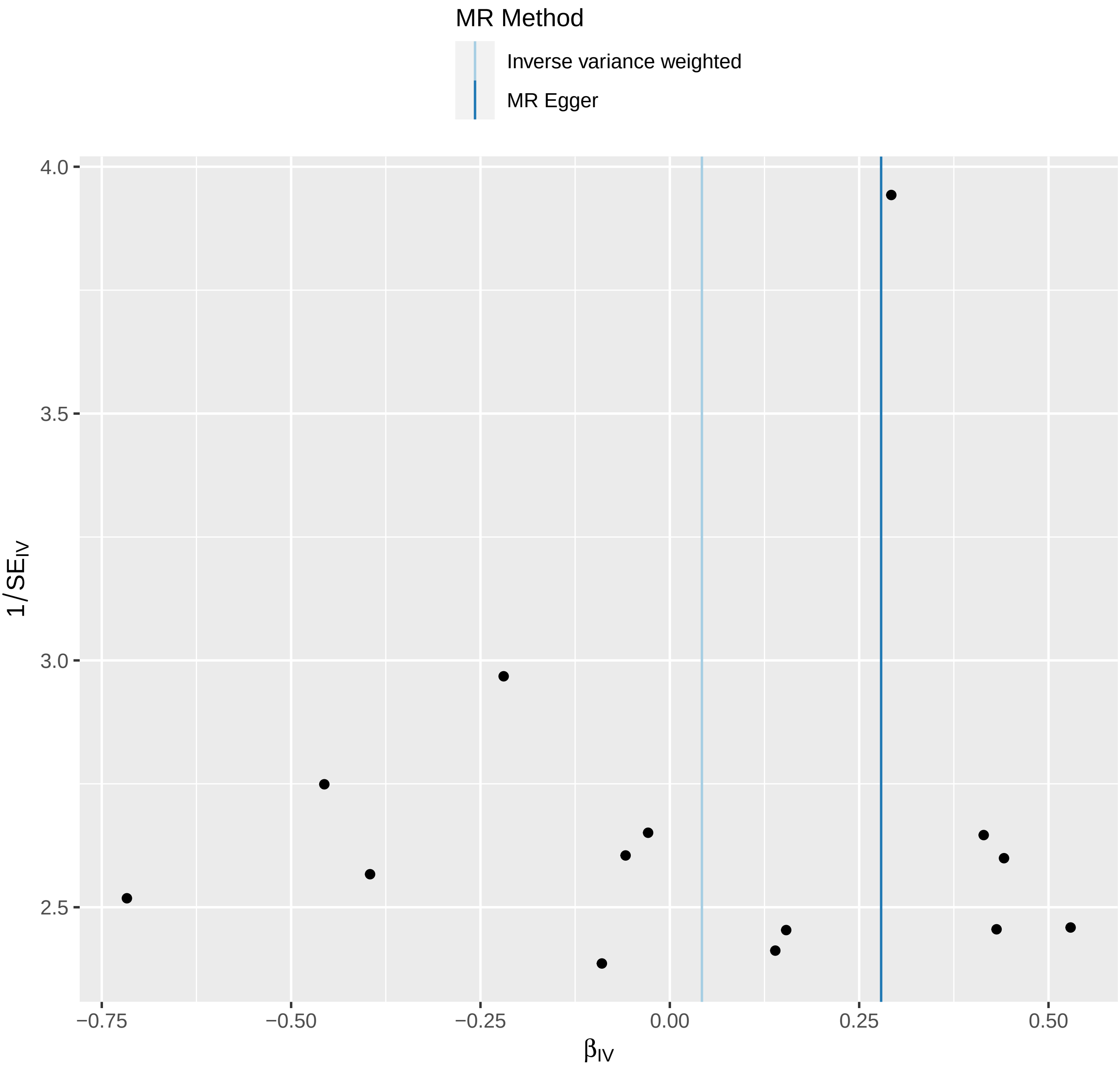

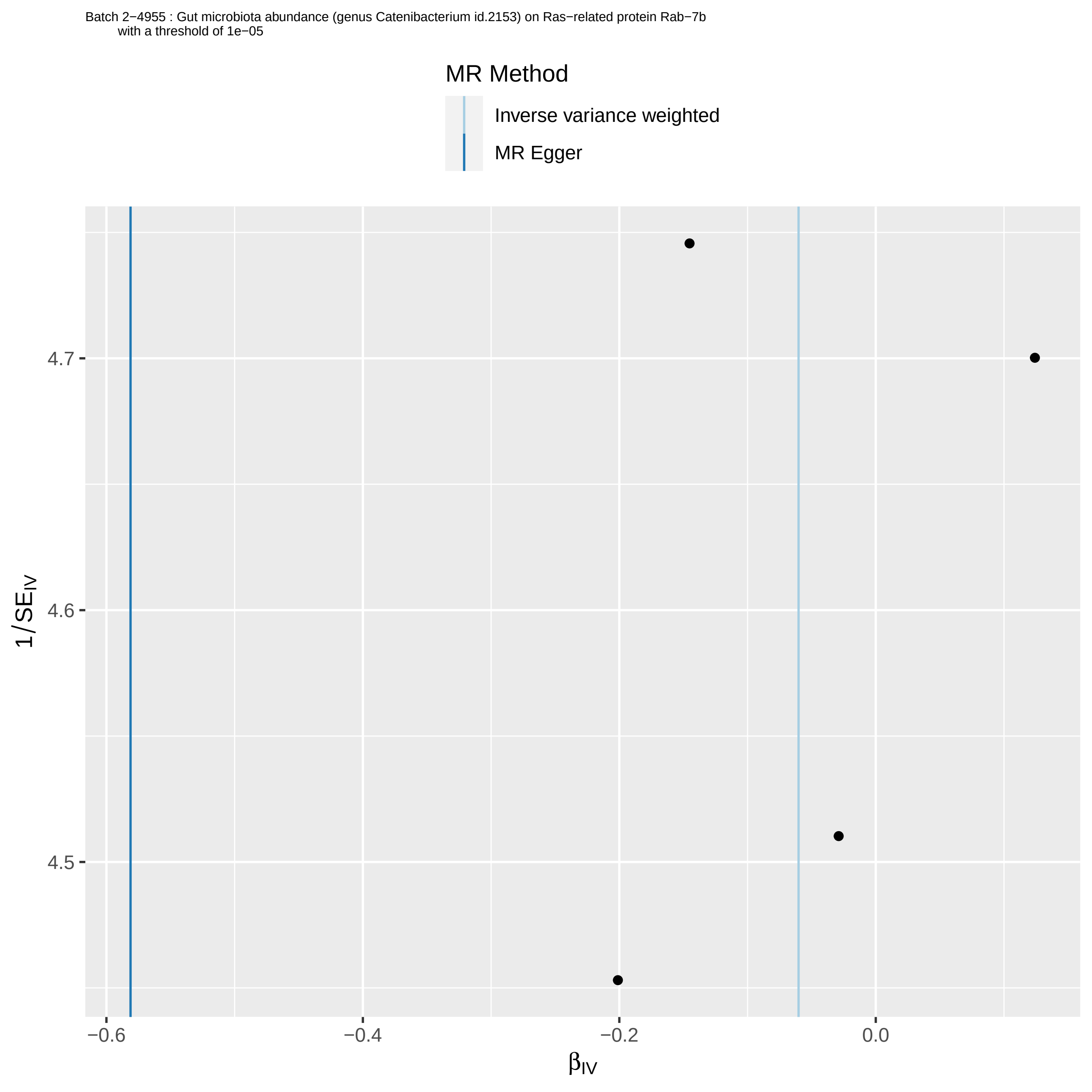
Batch 2−4955 : Gut microbiota abundance (genus Catenibacterium id.2153) on Ras−related protein Rab−7b
with a threshold of 1e−05
MR Method
Inverse variance weighted
MR Egger
4.7
4.6
4.5
1/SE_IV
−0.6
−0.4
−0.2
0.0
β_IV

Batch 2−4956 : Gut microbiota abundance (genus Parasutterella id.2892) on Ras−related protein Rab−7b
with a threshold of 1e−05

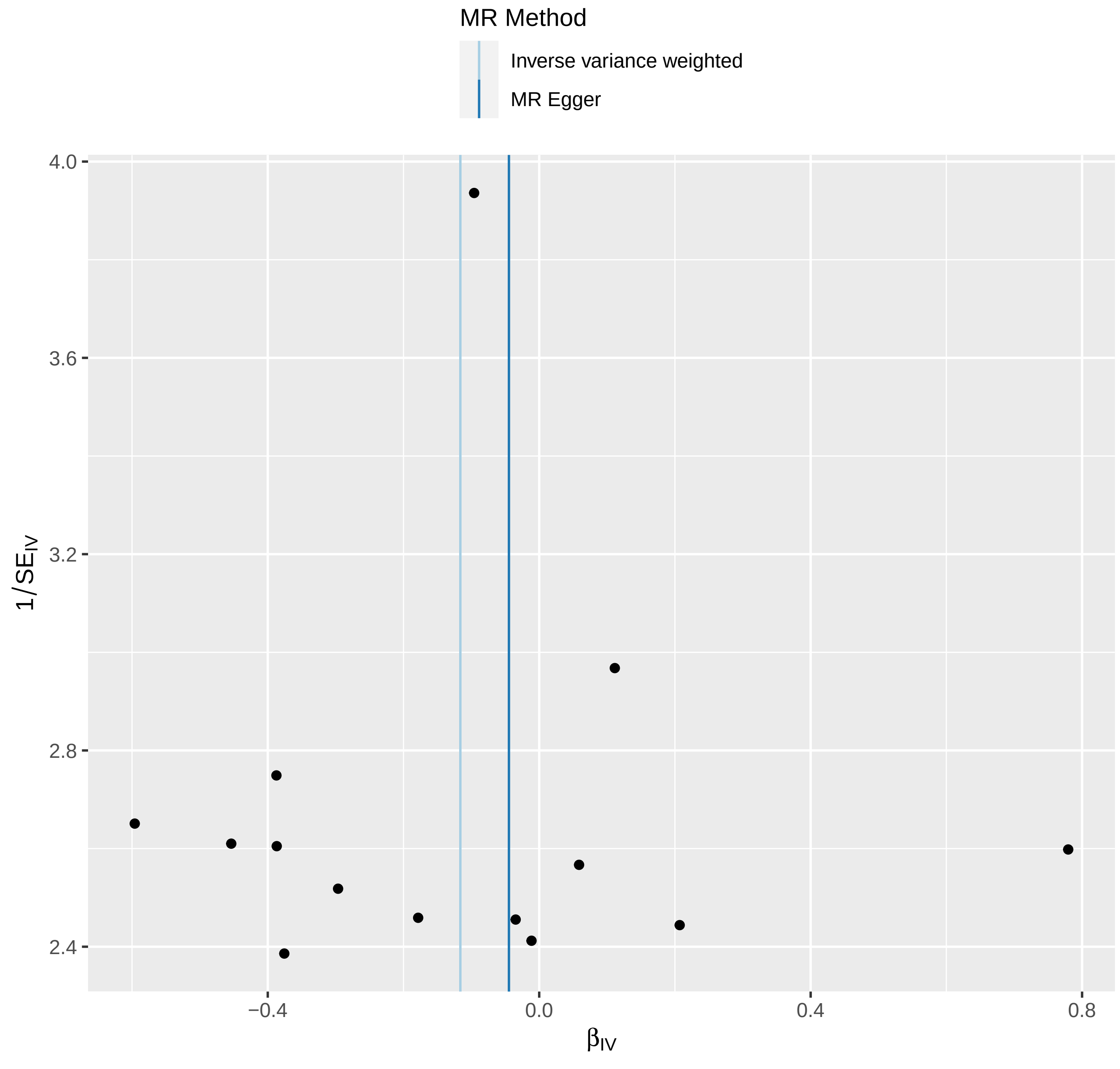

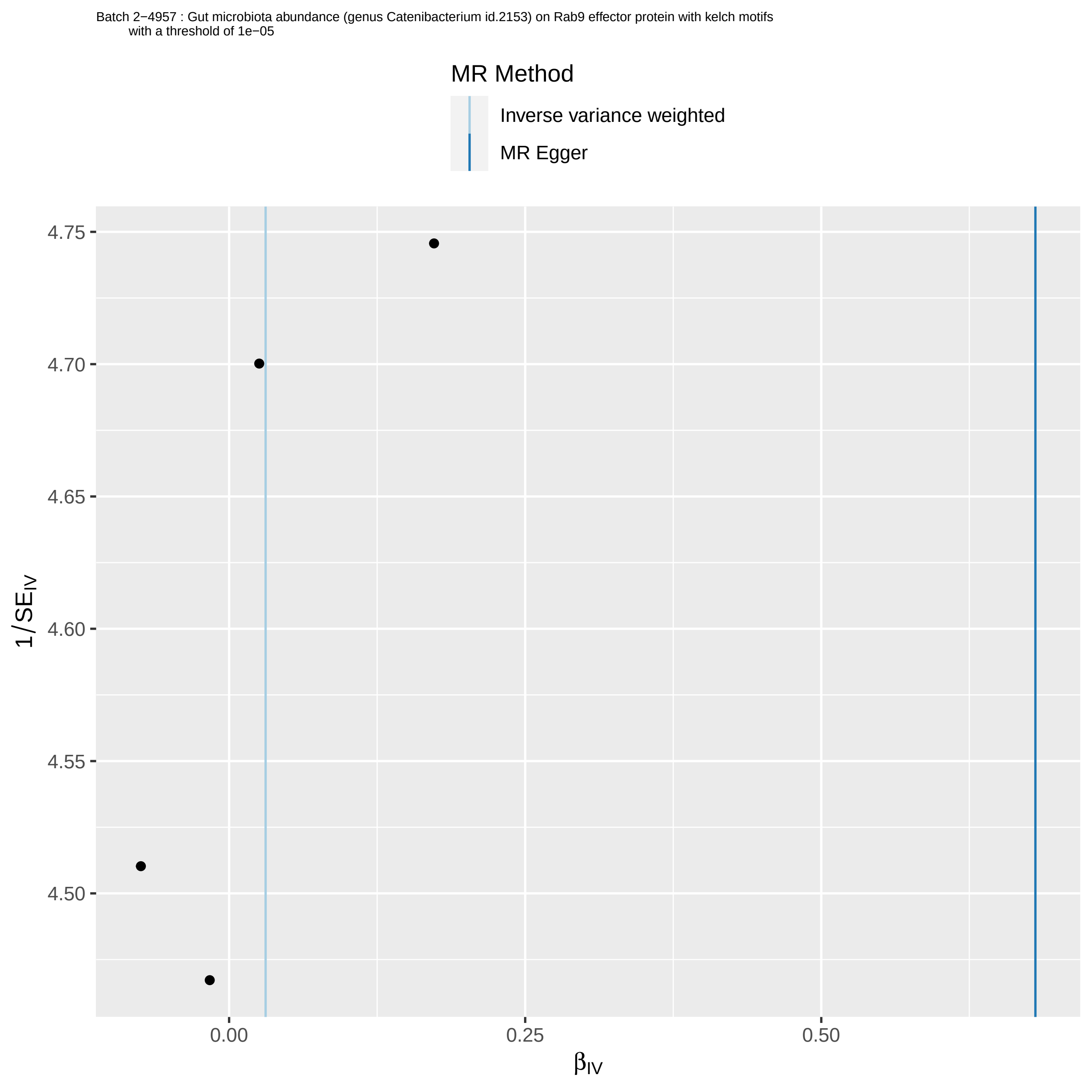

Batch 2−4957 : Gut microbiota abundance (genus Catenibacterium id.2153) on Rab9 effector protein with kelch motifs
with a threshold of 1e−05
MR Method
Inverse variance weighted
MR Egger
4.75
4.70
4.65
4.60
4.55
4.50
1/SE_IV
0.00
0.25
0.50
β_IV

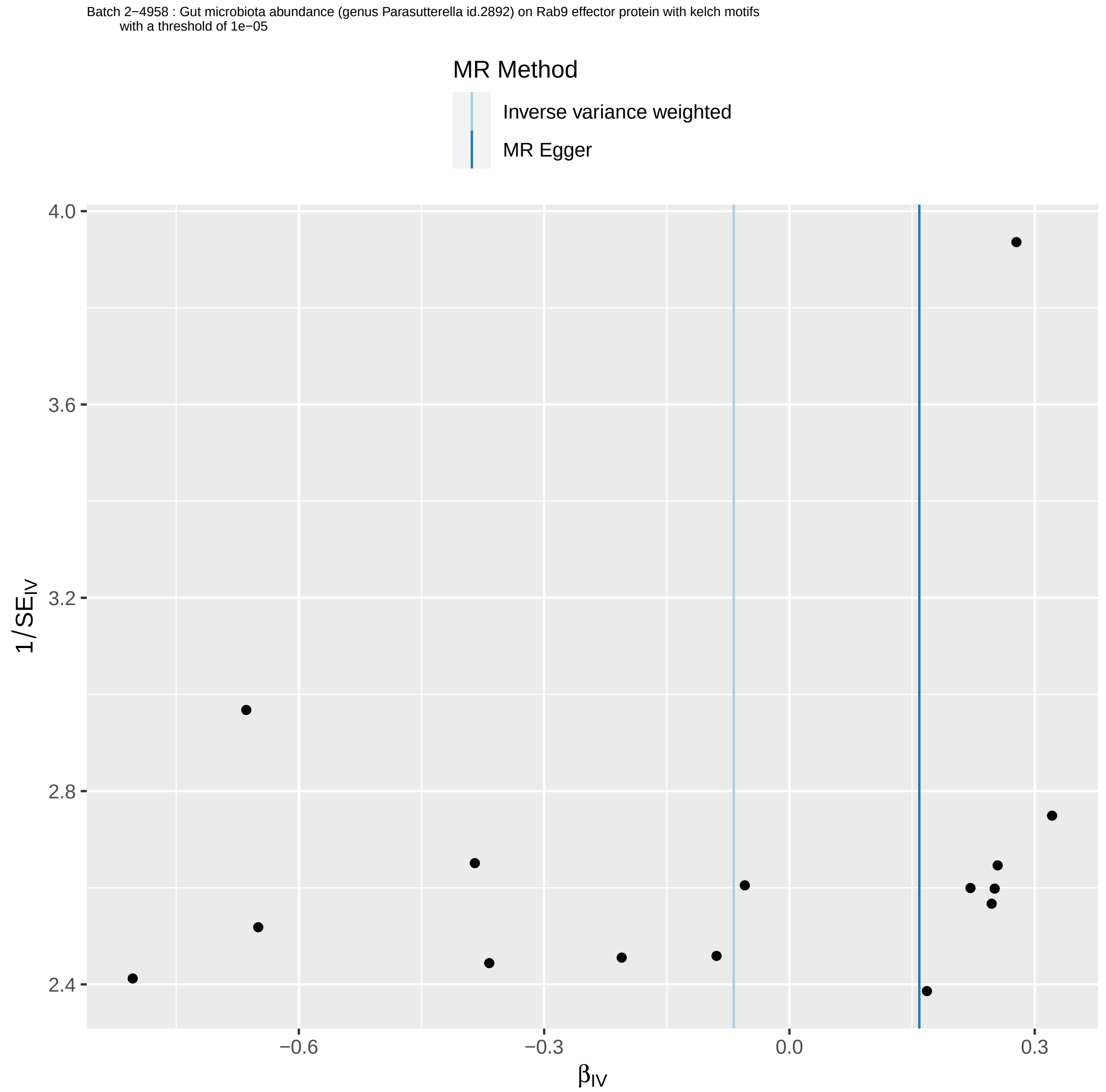
Batch 2−4958 : Gut microbiota abundance (genus Parasutterella id.2892) on Rab9 effector protein with kelch motifs
with a threshold of 1e−05
MR Method
Inverse variance weighted
MR Egger
4.0
3.6
3.2
2.8
2.4
1/SE_IV
−0.6
−0.3
0.0
0.3
β_IV

Batch 2−4959 : Gut microbiota abundance (genus Catenibacterium id.2153) on Ras−related C3 botulinum toxin substrate 3 with a threshold of 1e−05

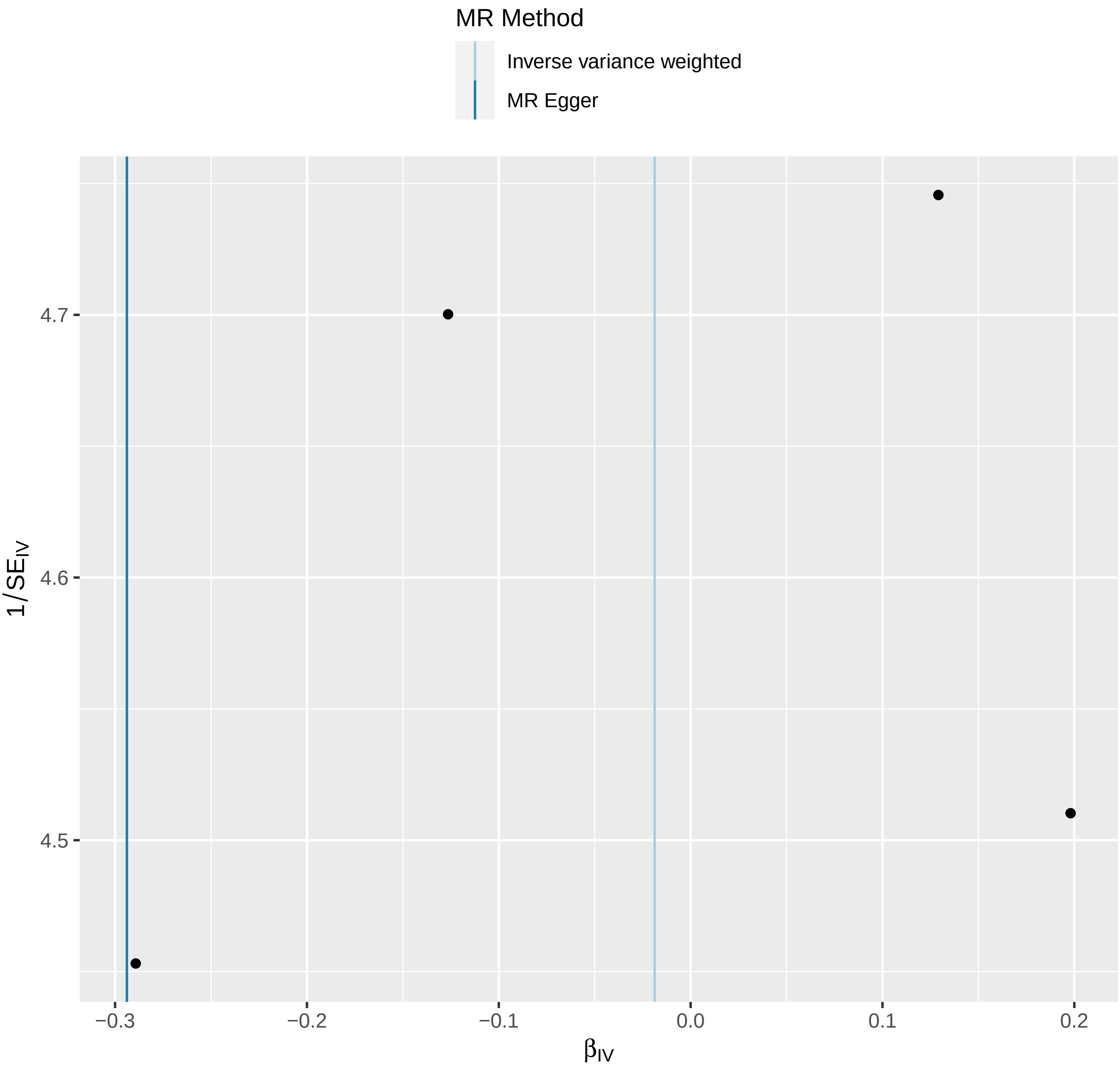

Batch 2−4960 : Gut microbiota abundance (genus Parasutterella id.2892) on Ras−related C3 botulinum toxin substrate 3 with a threshold of 1e−05

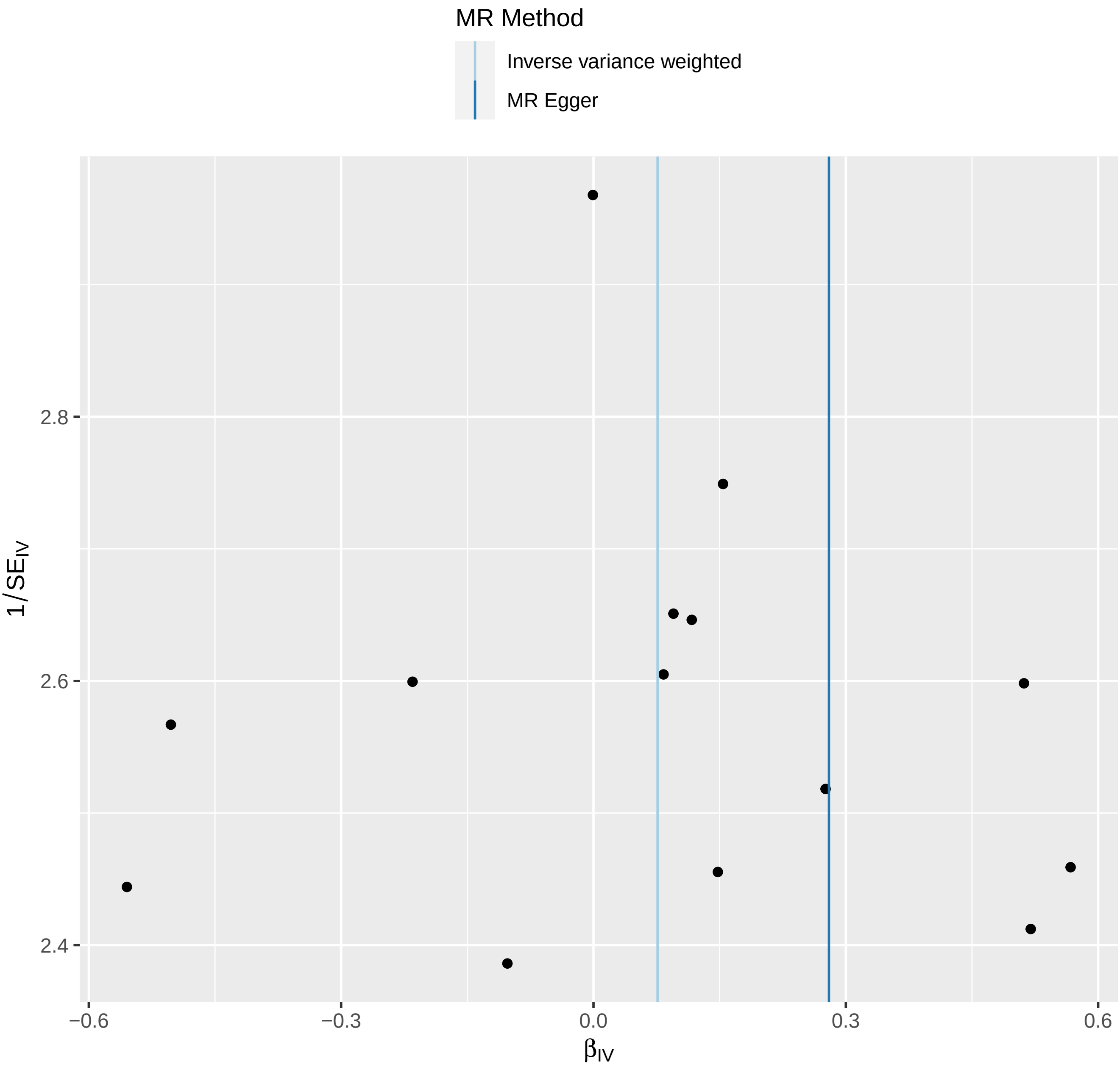

Batch 2−4961 : Gut microbiota abundance (genus Catenibacterium id.2153) on Rac GTPase−activating protein 1
with a threshold of 1e−05

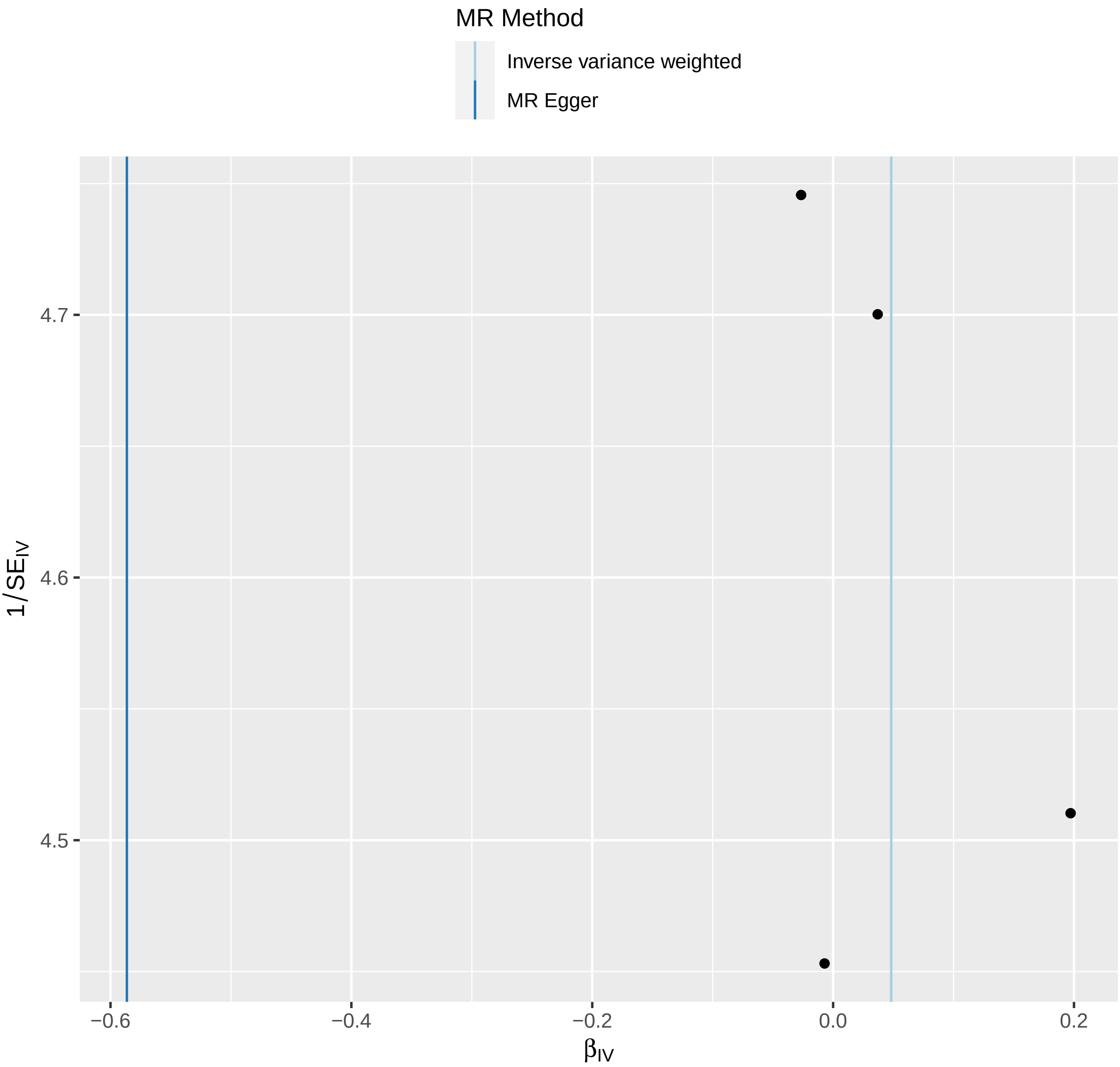

Batch 2−4962 : Gut microbiota abundance (genus Parasutterella id.2892) on Rac GTPase−activating protein 1
with a threshold of 1e−05

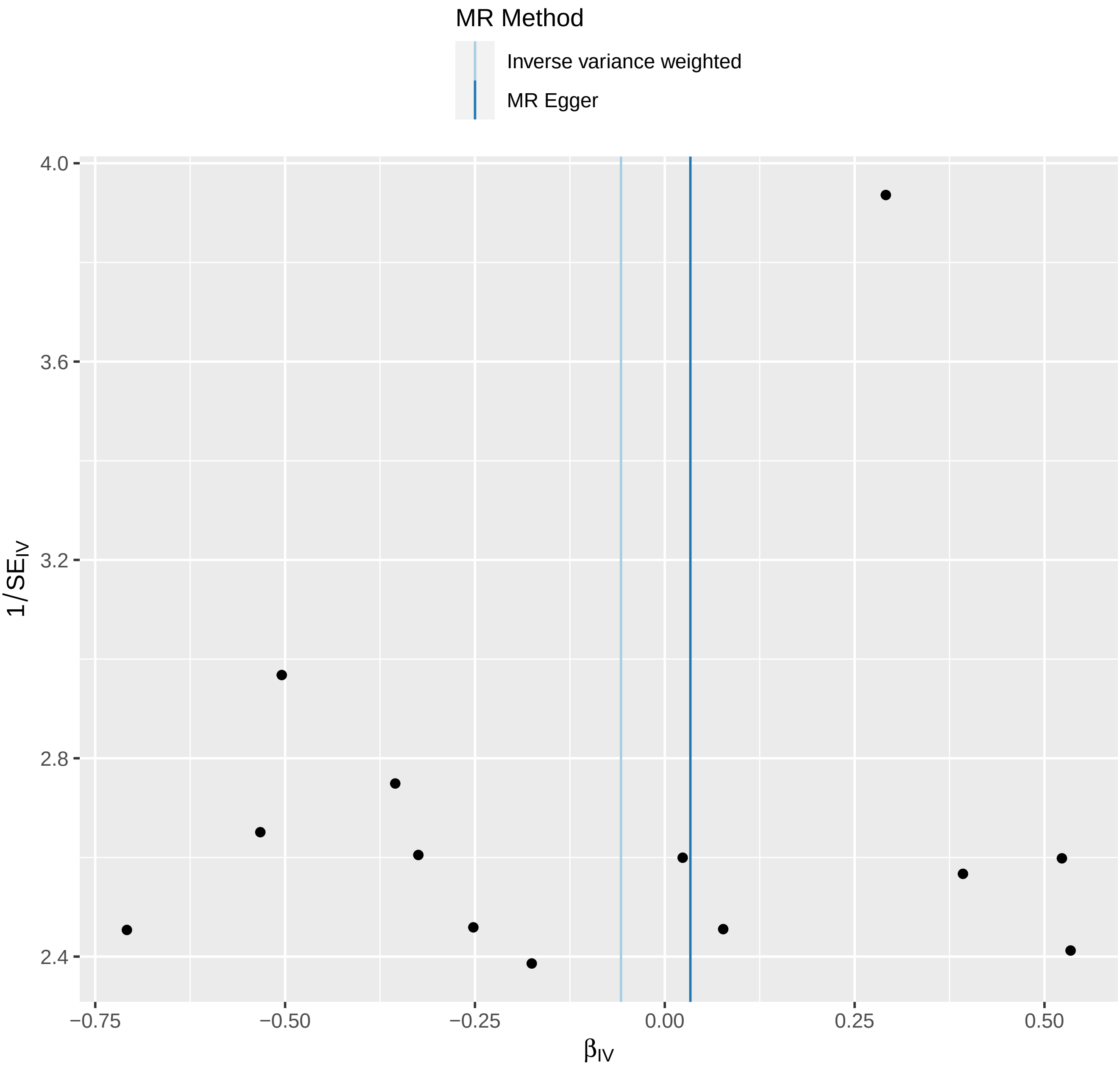

Batch 2−4963 : Gut microbiota abundance (genus Catenibacterium id.2153) on Cell cycle checkpoint protein RAD1
with a threshold of 1e−05

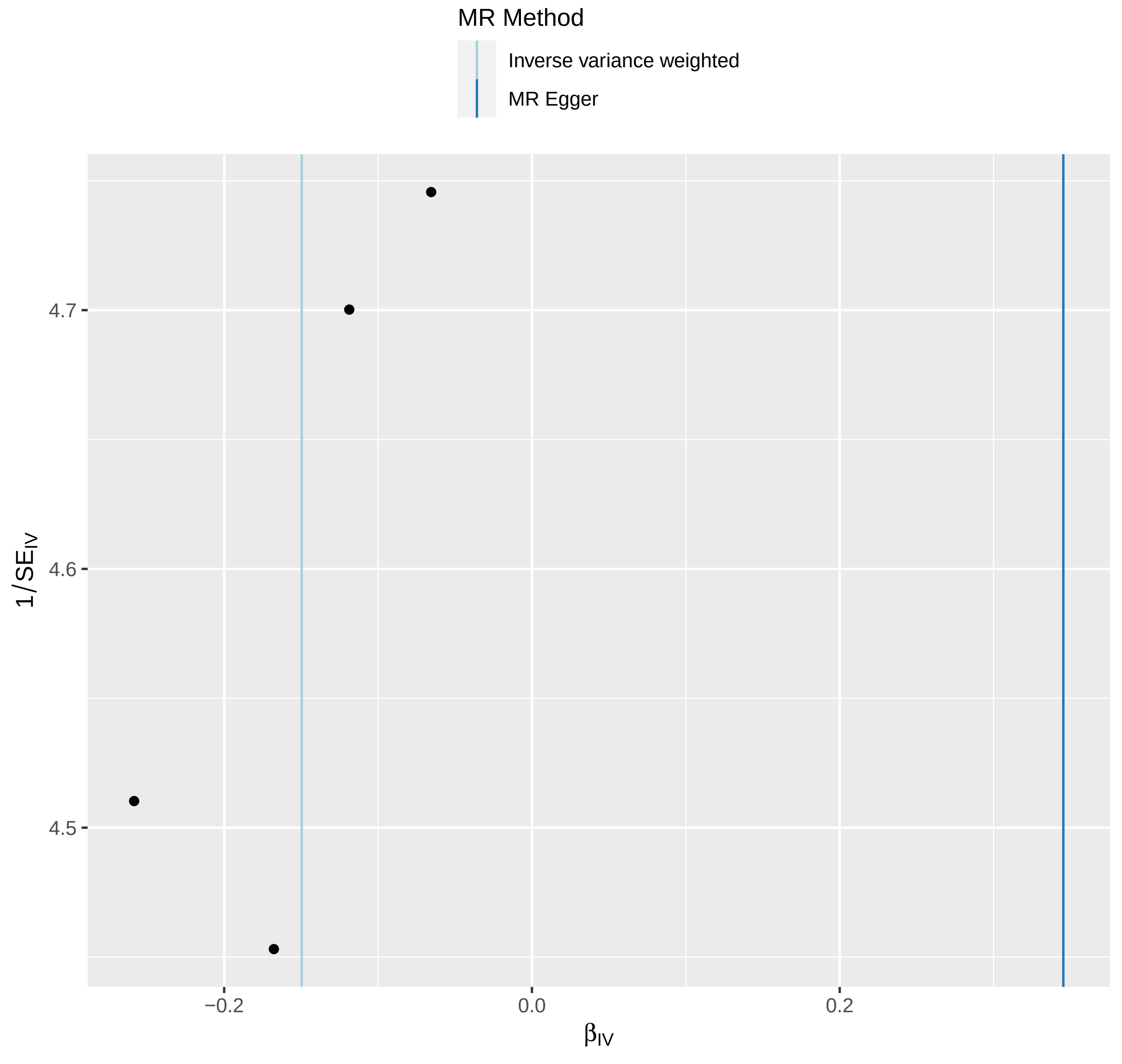

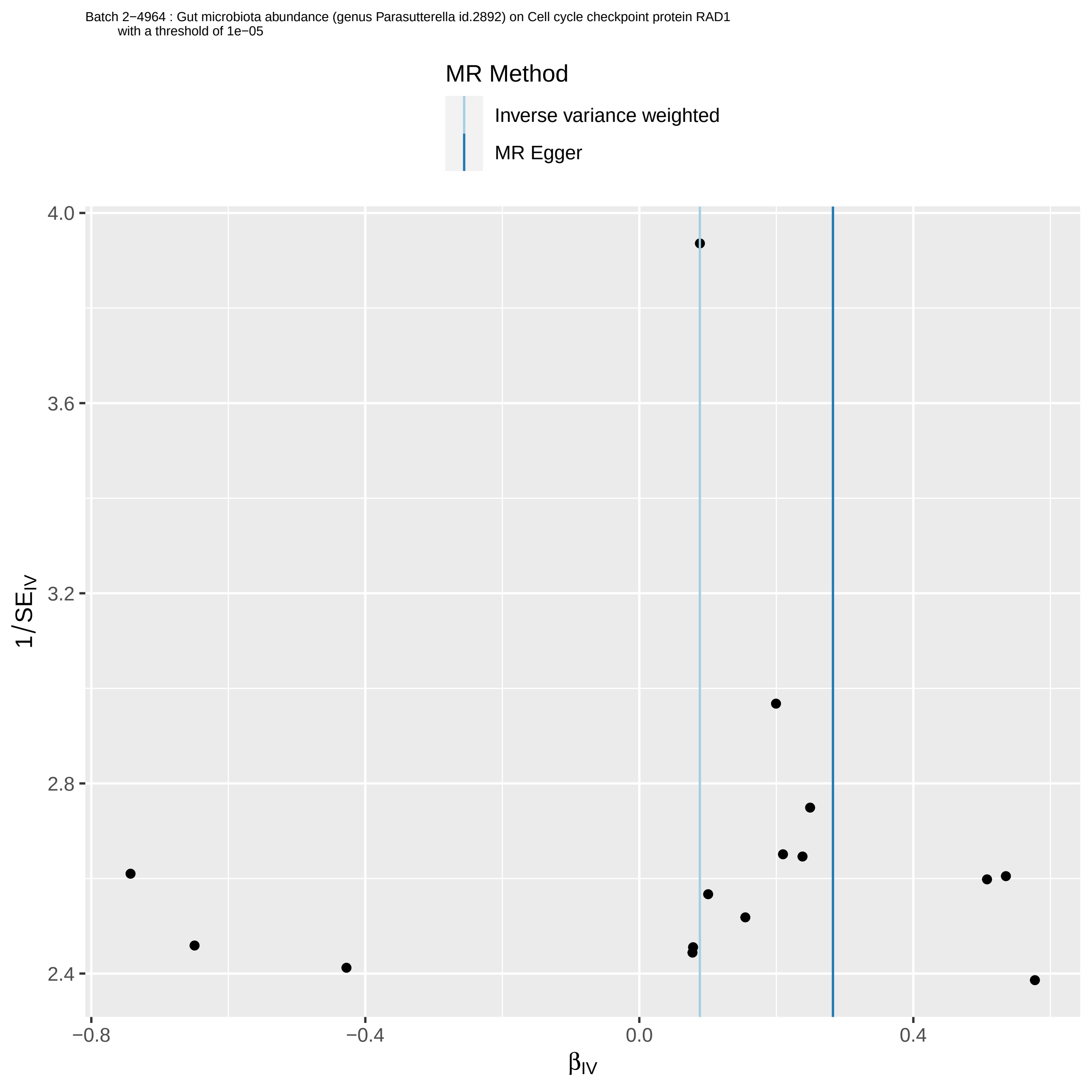
Batch 2−4964 : Gut microbiota abundance (genus Parasutterella id.2892) on Cell cycle checkpoint protein RAD1
with a threshold of 1e−05
MR Method
Inverse variance weighted
MR Egger
4.0
3.6
3.2
2.8
2.4
1/SE$_{IV}$
−0.8
−0.4
0.0
0.4
β$_{IV}$

Batch 2−4965 : Gut microbiota abundance (genus Catenibacterium id.2153) on UV excision repair protein RAD23 homolog A
with a threshold of 1e−05

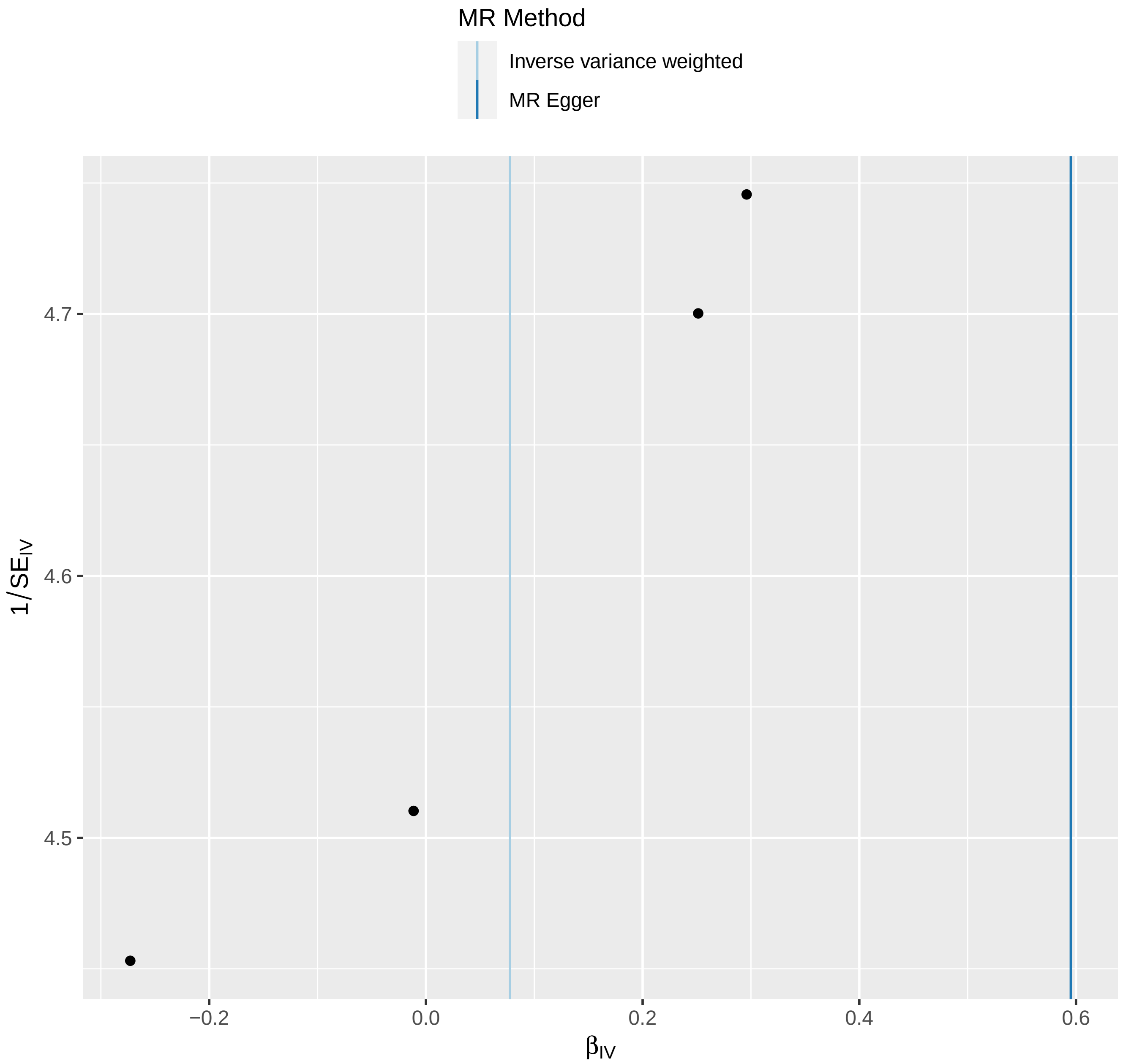

Batch 2−4966 : Gut microbiota abundance (genus Parasutterella id.2892) on UV excision repair protein RAD23 homolog A with a threshold of 1e−05

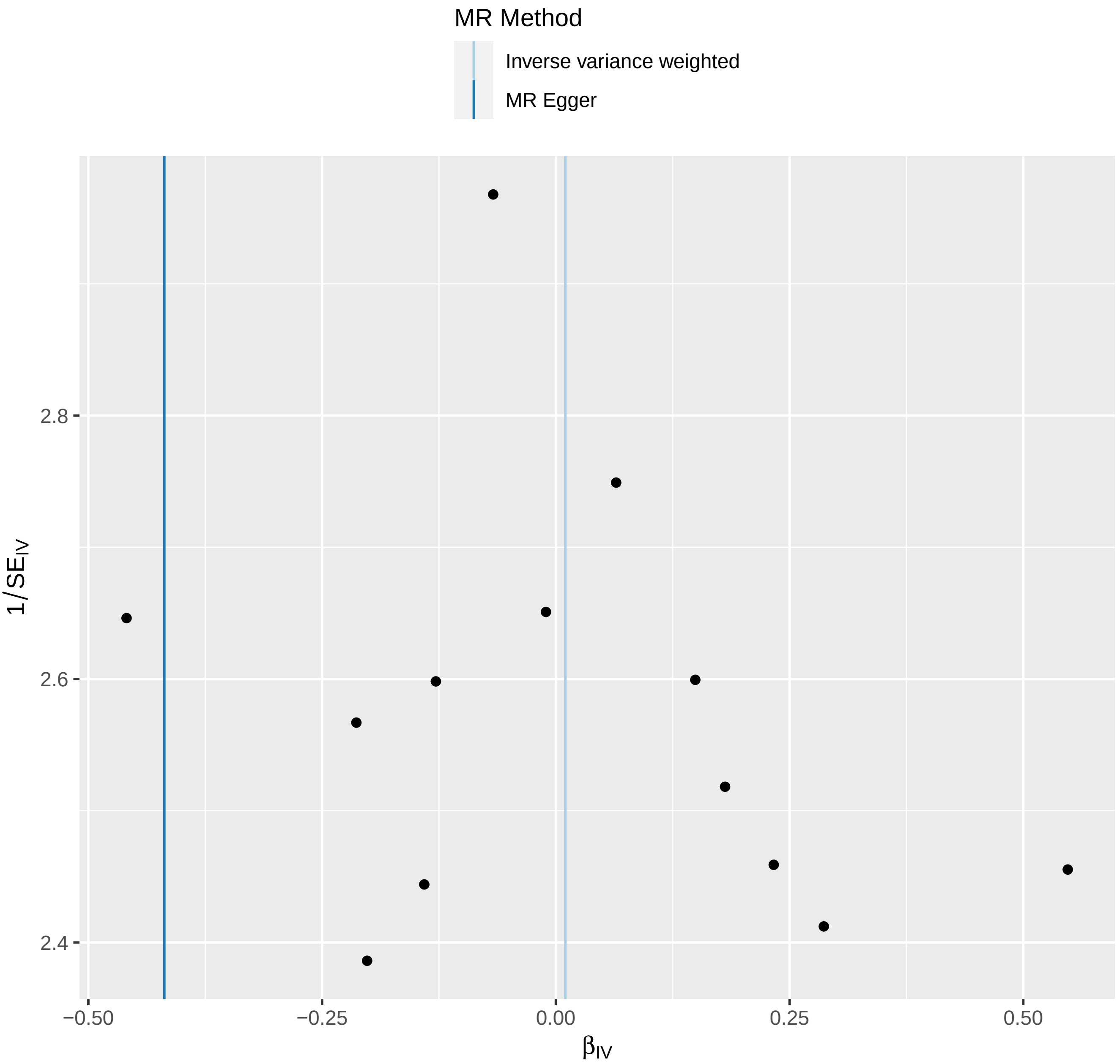

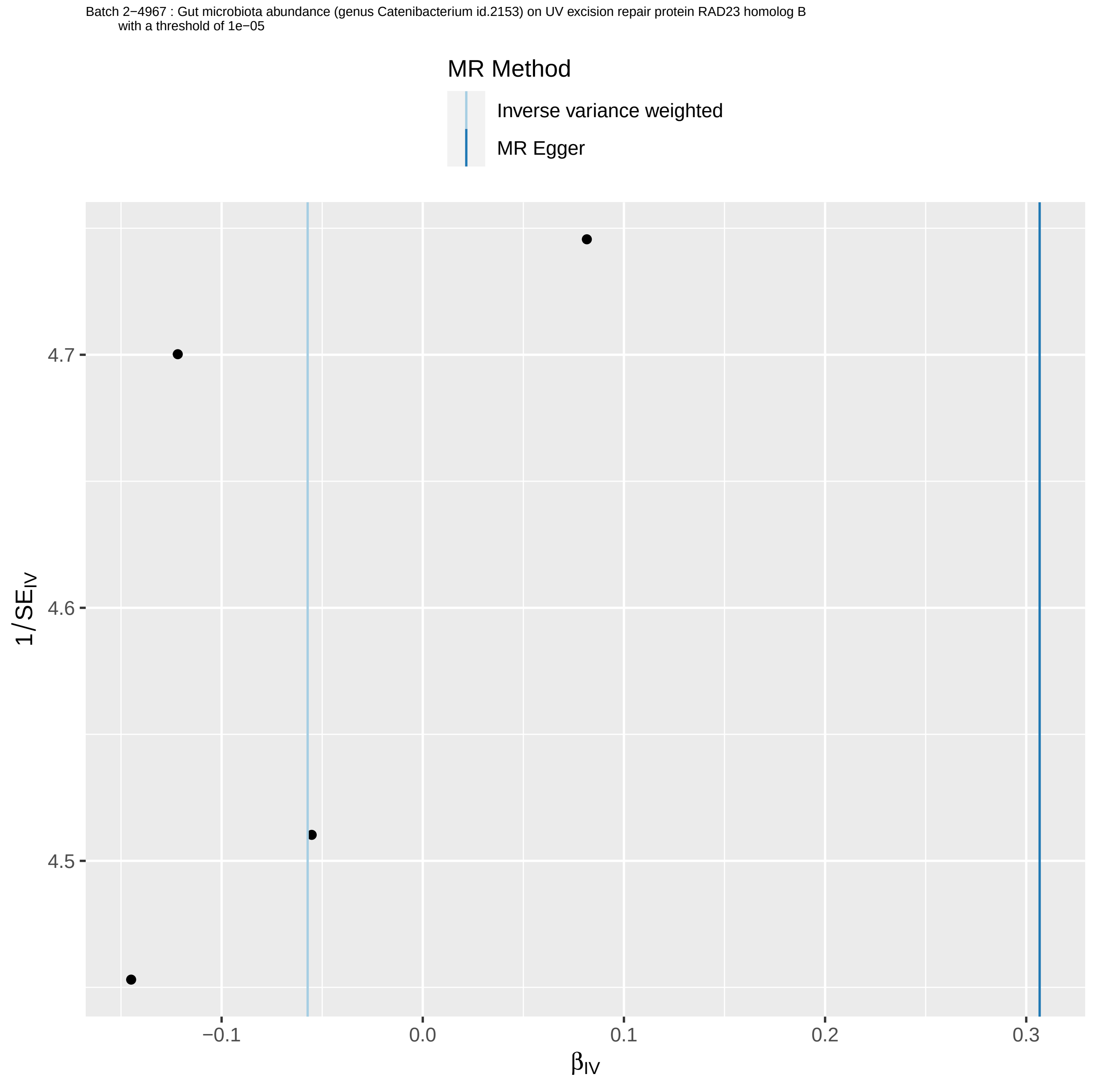
Batch 2−4967 : Gut microbiota abundance (genus Catenibacterium id.2153) on UV excision repair protein RAD23 homolog B
with a threshold of 1e−05
MR Method
Inverse variance weighted
MR Egger
4.7
4.6
4.5
1/SE_IV
−0.1
0.0
0.1
0.2
0.3
β_IV

Batch 2−4968 : Gut microbiota abundance (genus Parasutterella id.2892) on UV excision repair protein RAD23 homolog B
with a threshold of 1e−05

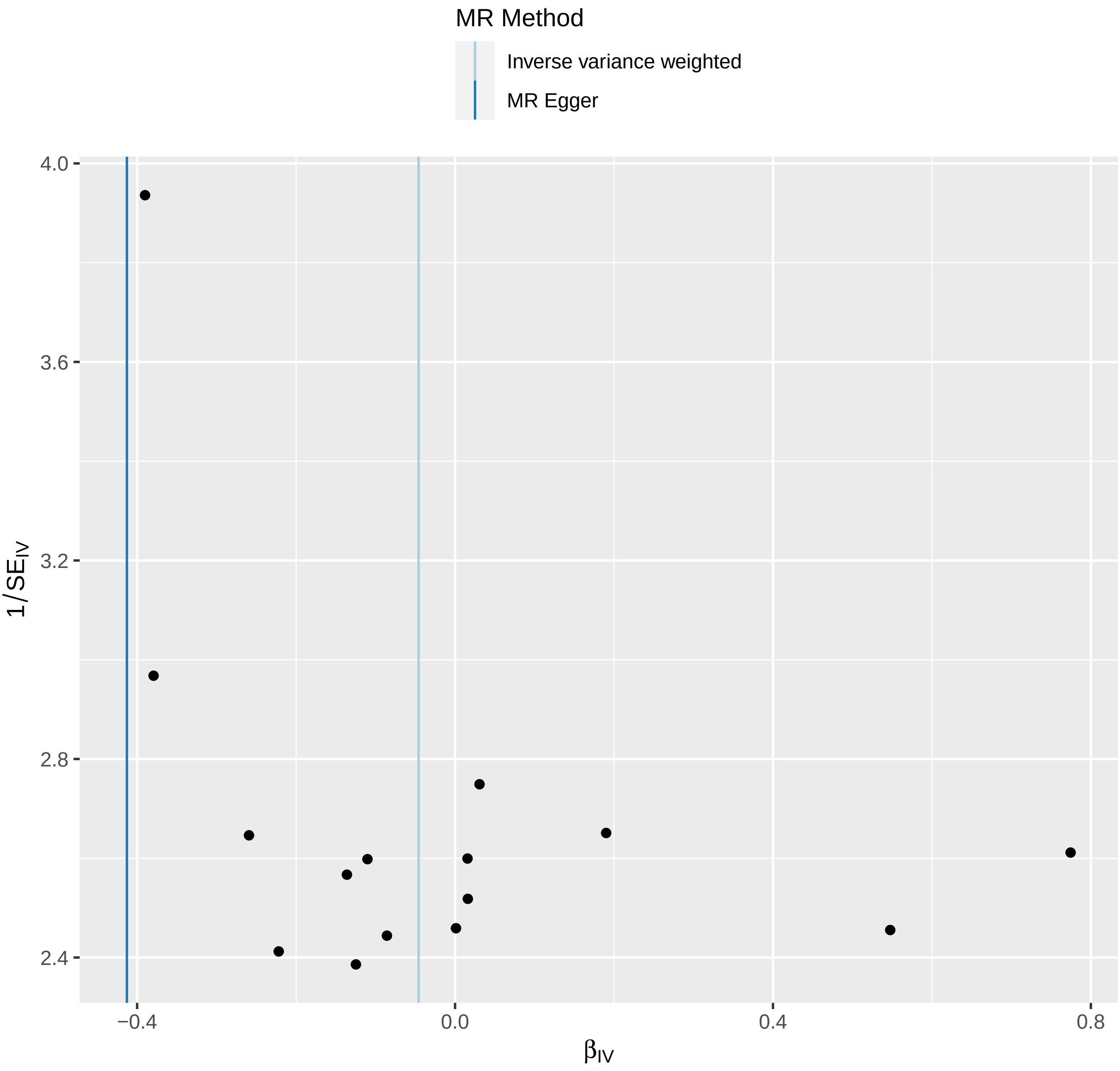

Batch 2−4969 : Gut microbiota abundance (genus Catenibacterium id.2153) on DNA repair protein RAD51 homolog 1
with a threshold of 1e−05

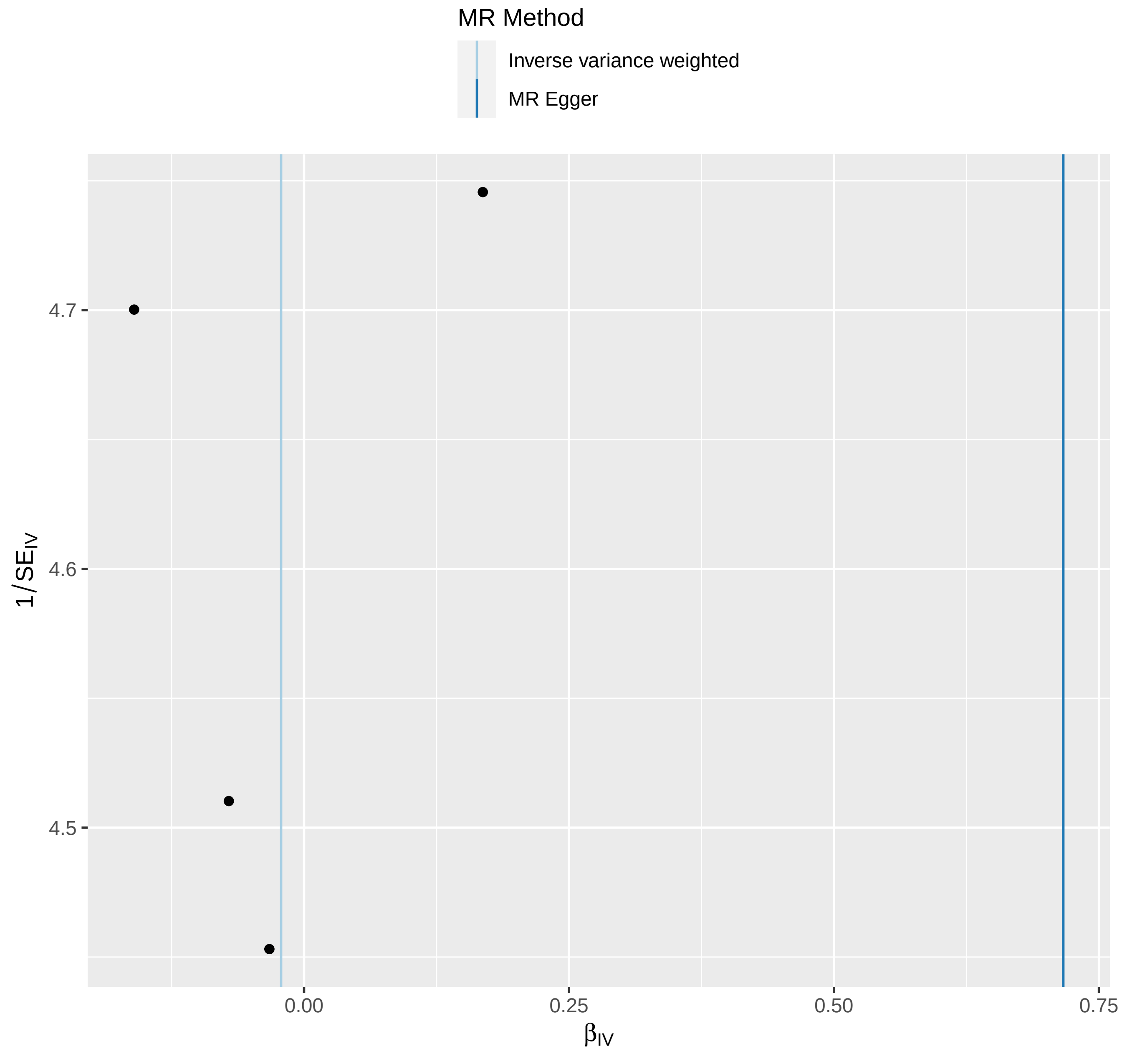

Batch 2−4970 : Gut microbiota abundance (genus Parasutterella id.2892) on DNA repair protein RAD51 homolog 1
with a threshold of 1e−05

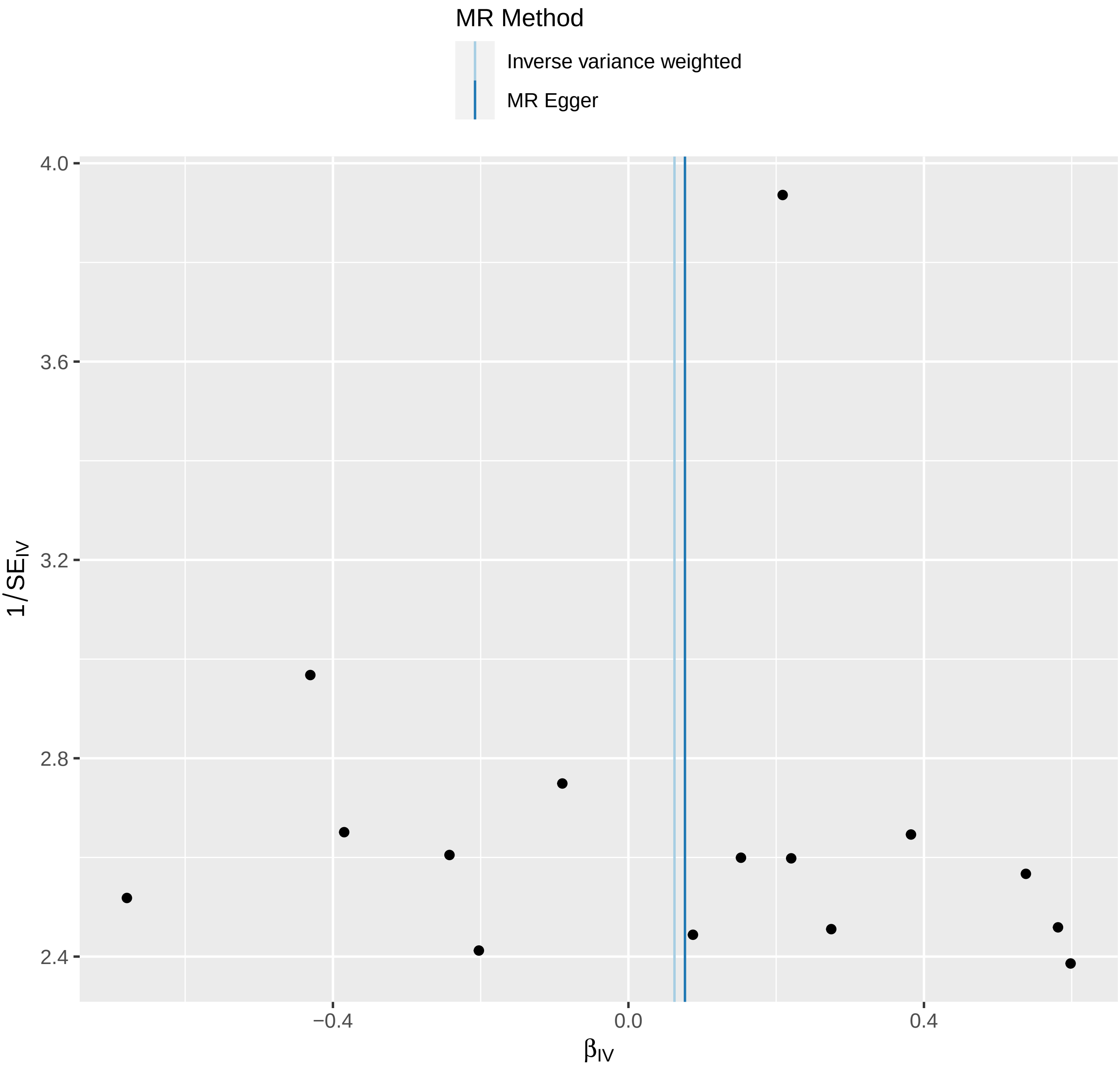

Batch 2−4971 : Gut microbiota abundance (genus Catenibacterium id.2153) on DNA repair protein RAD51 homolog 4
with a threshold of 1e−05

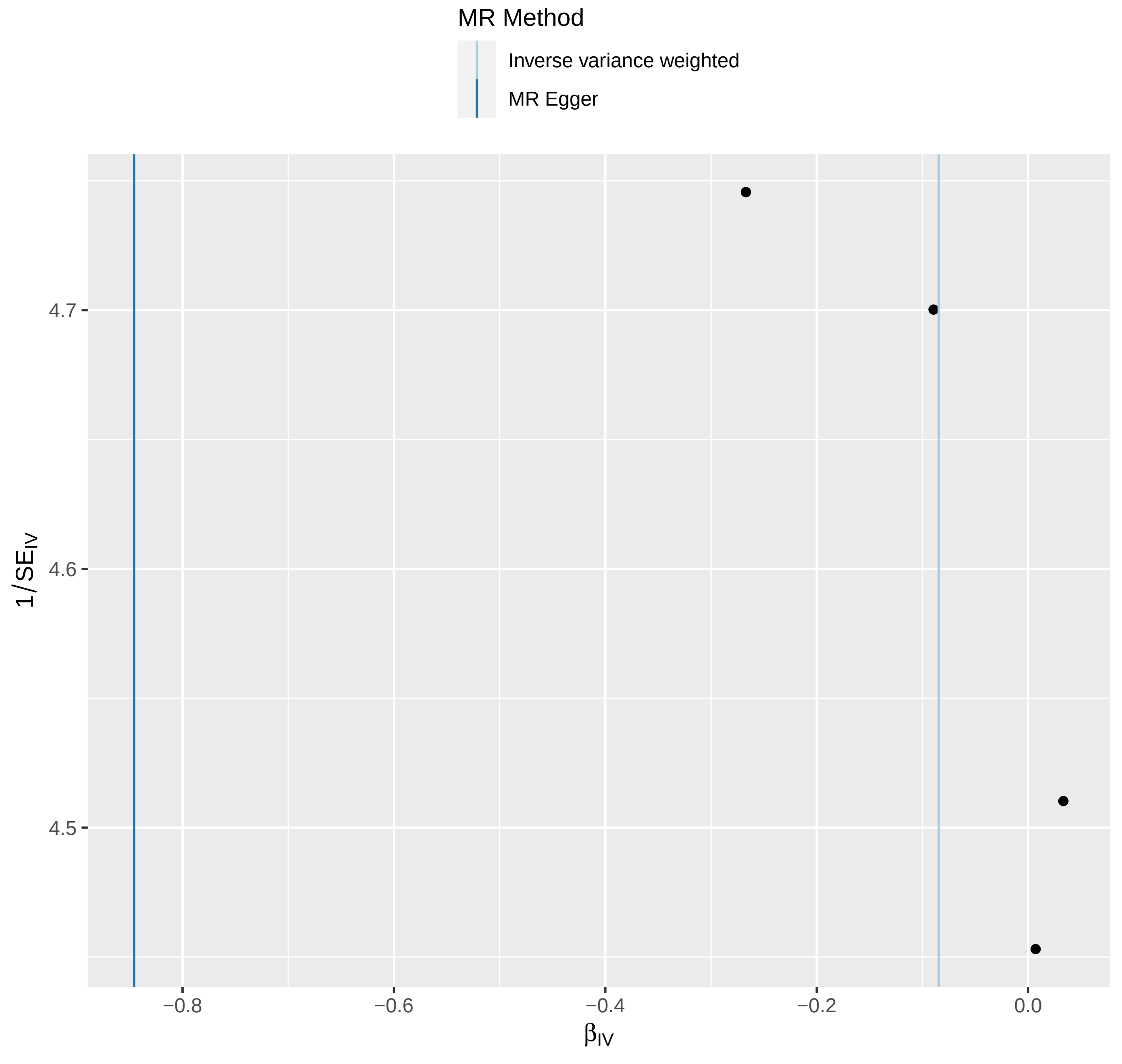

Batch 2−4972 : Gut microbiota abundance (genus Parasutterella id.2892) on DNA repair protein RAD51 homolog 4
with a threshold of 1e−05

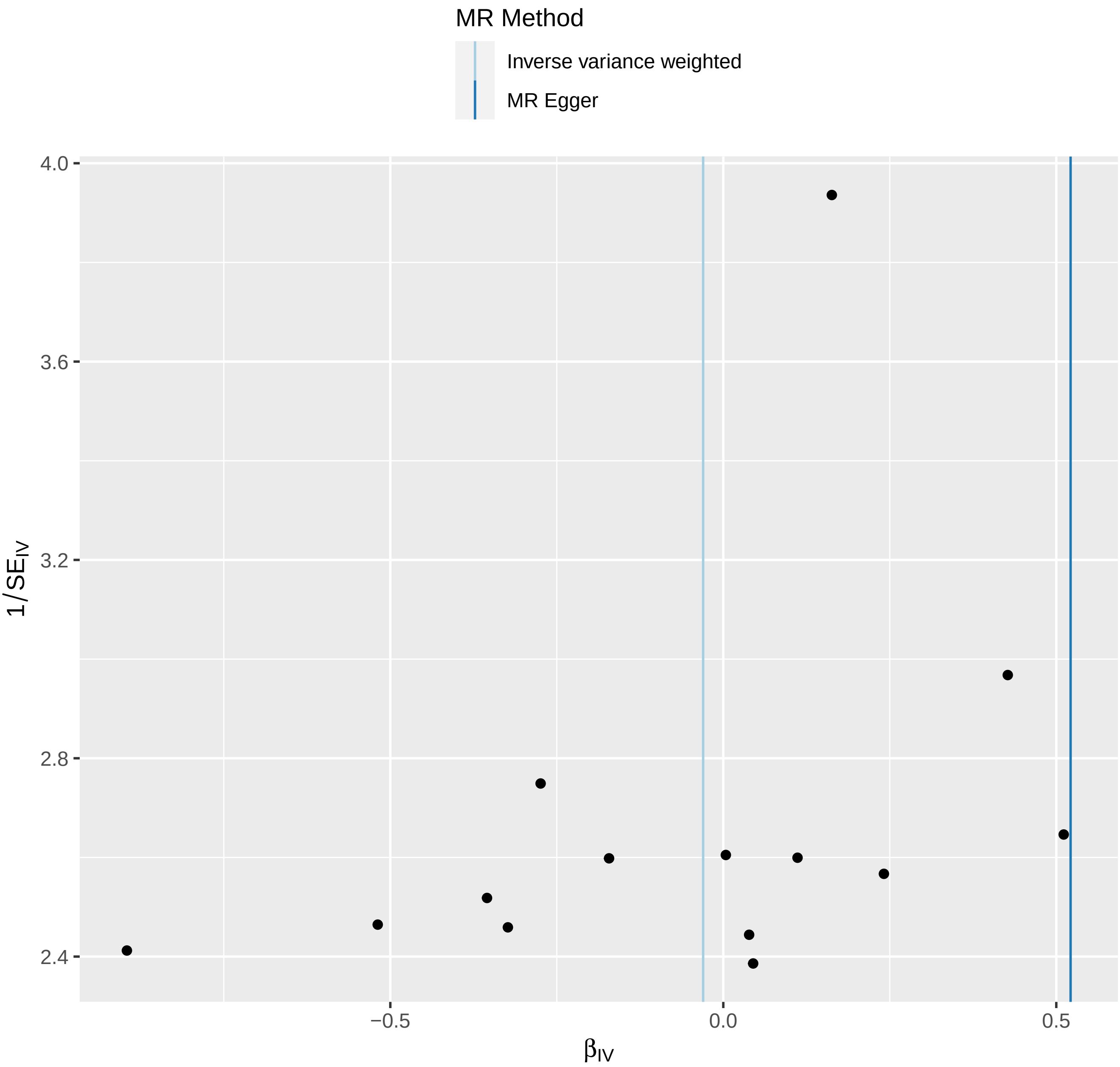

Batch 2−4973 : Gut microbiota abundance (genus Catenibacterium id.2153) on NKG2D ligand 4
with a threshold of 1e−05

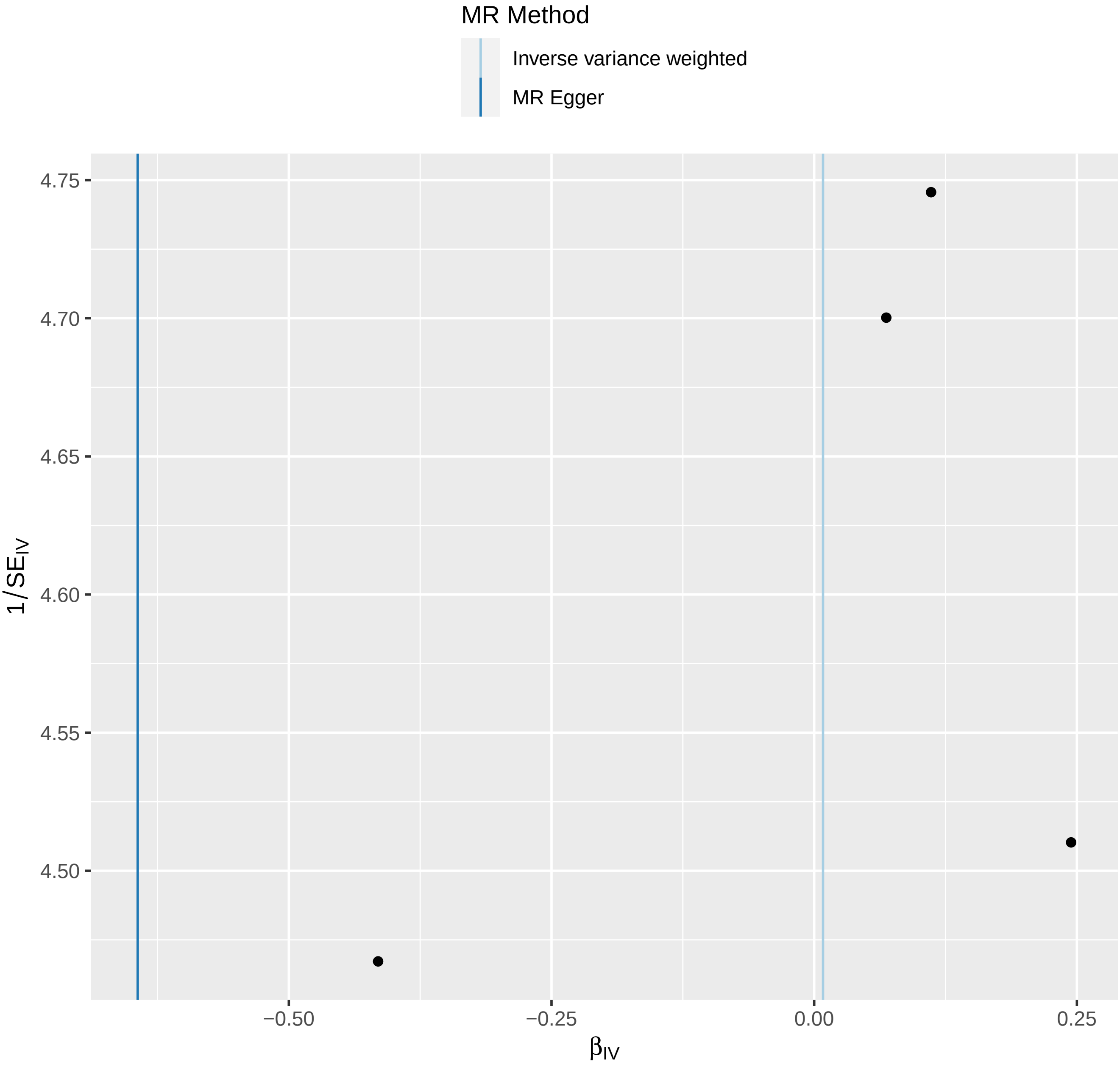

Batch 2−4974 : Gut microbiota abundance (genus Parasutterella id.2892) on NKG2D ligand 4
with a threshold of 1e−05

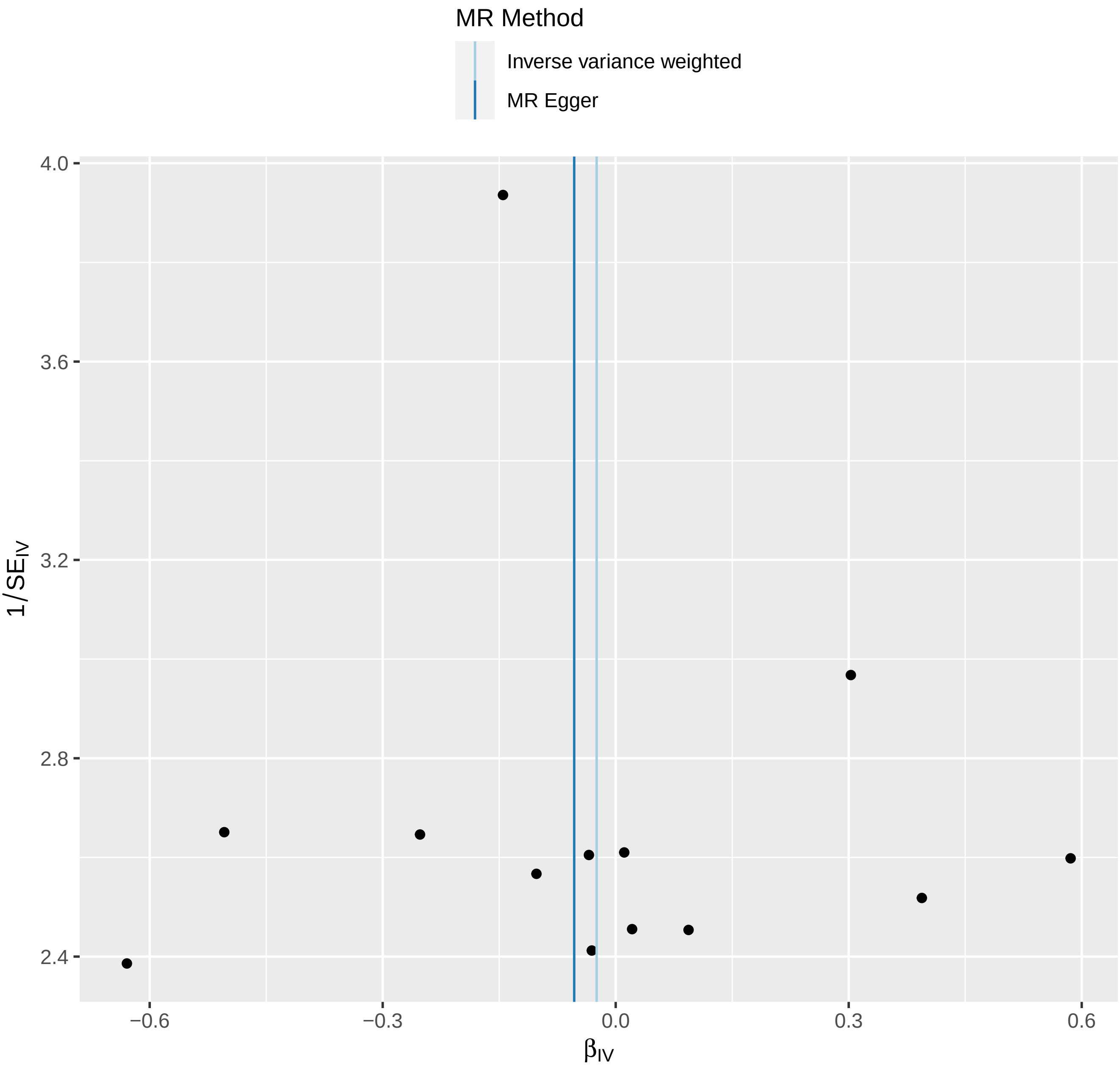

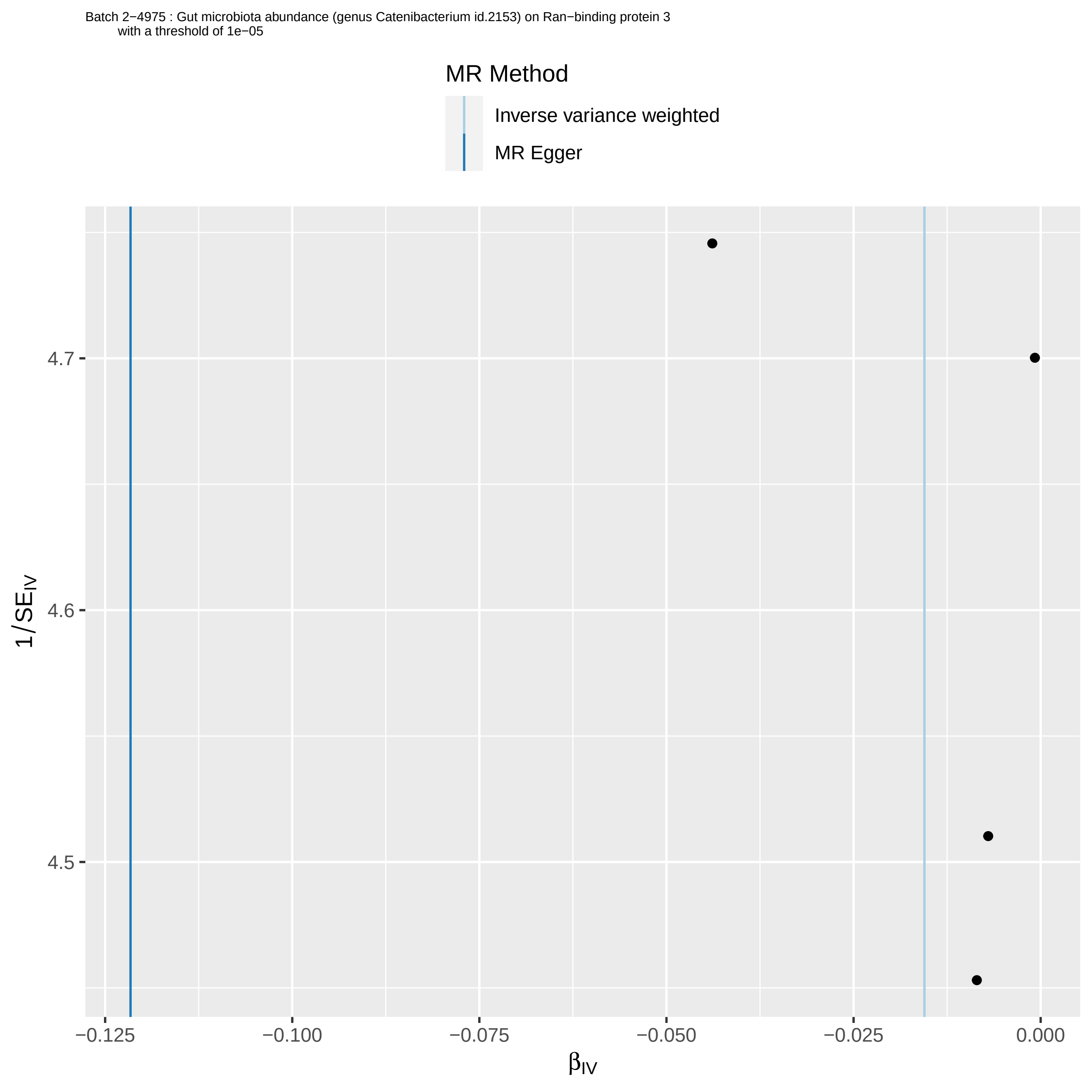

Batch 2−4975 : Gut microbiota abundance (genus Catenibacterium id.2153) on Ran−binding protein 3
with a threshold of 1e−05
MR Method
Inverse variance weighted
MR Egger
4.7
4.6
4.5
1/SE_IV
−0.125
−0.100
−0.075
−0.050
−0.025
0.000
β_IV

Batch 2−4976 : Gut microbiota abundance (genus Parasutterella id.2892) on Ran−binding protein 3
with a threshold of 1e−05

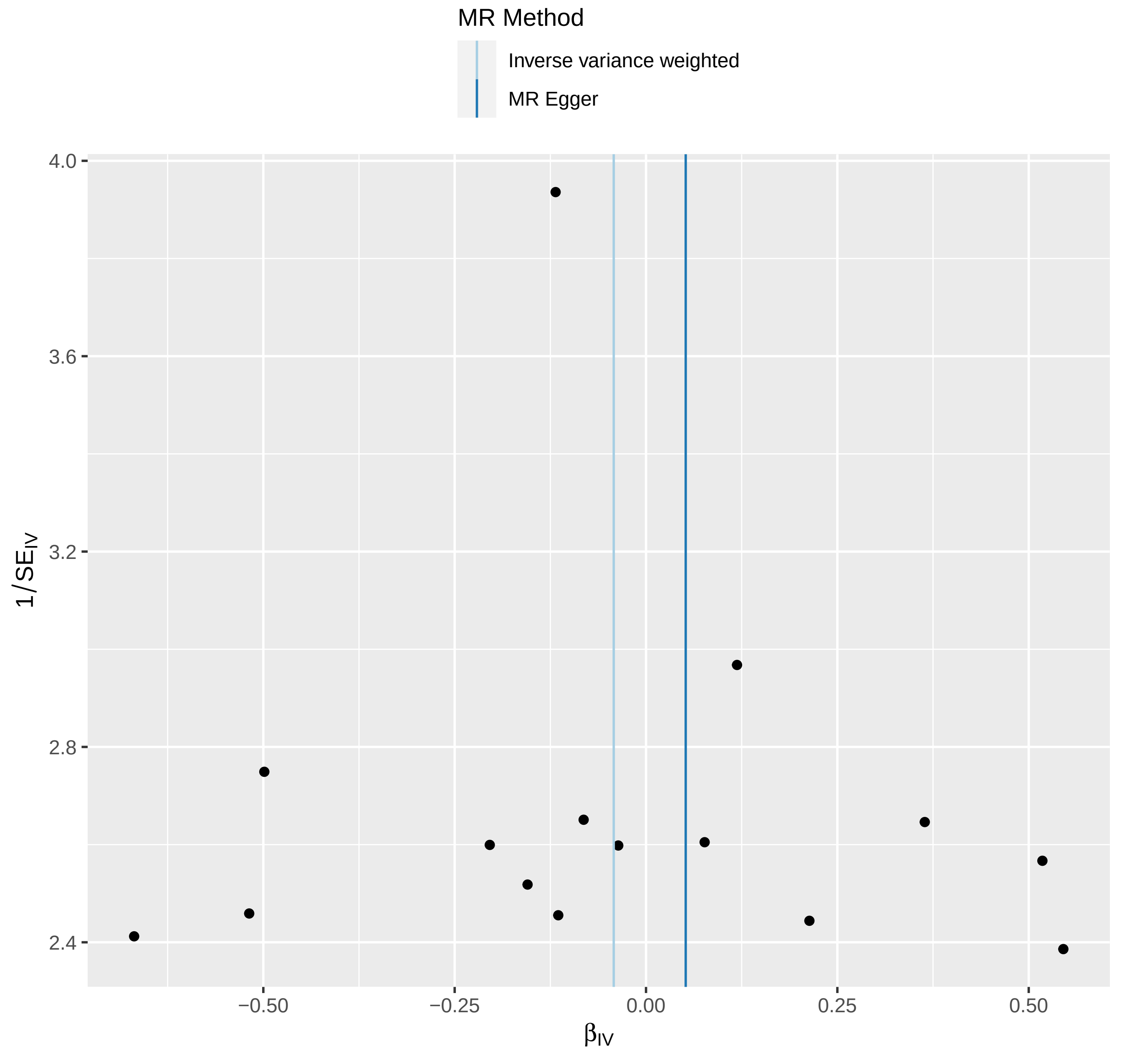

Batch 2−4977 : Gut microbiota abundance (genus Catenibacterium id.2153) on Rap1 GTPase−activating protein 1 with a threshold of 1e−05

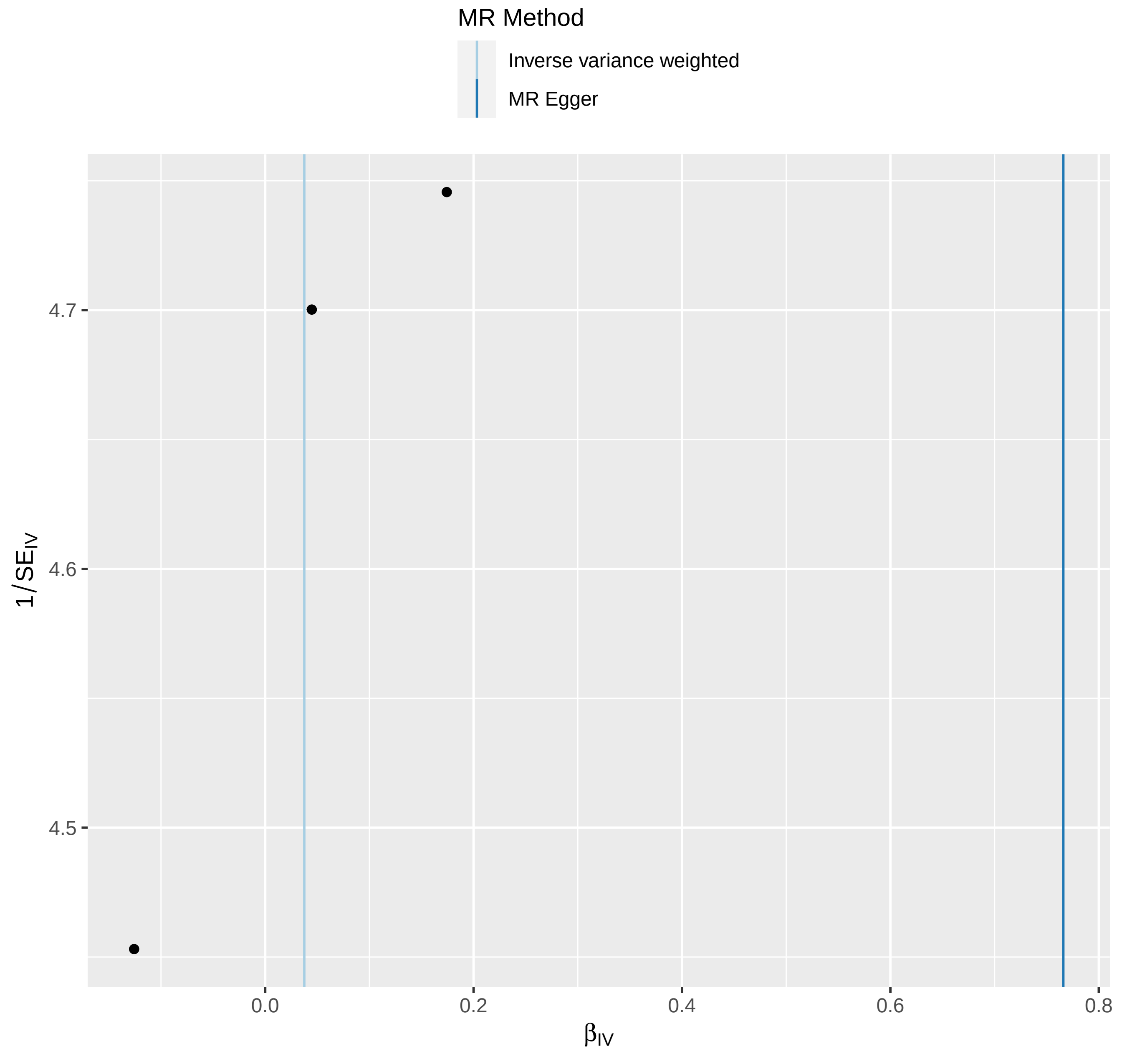

Batch 2−4978 : Gut microbiota abundance (genus Parasutterella id.2892) on Rap1 GTPase−activating protein 1
with a threshold of 1e−05

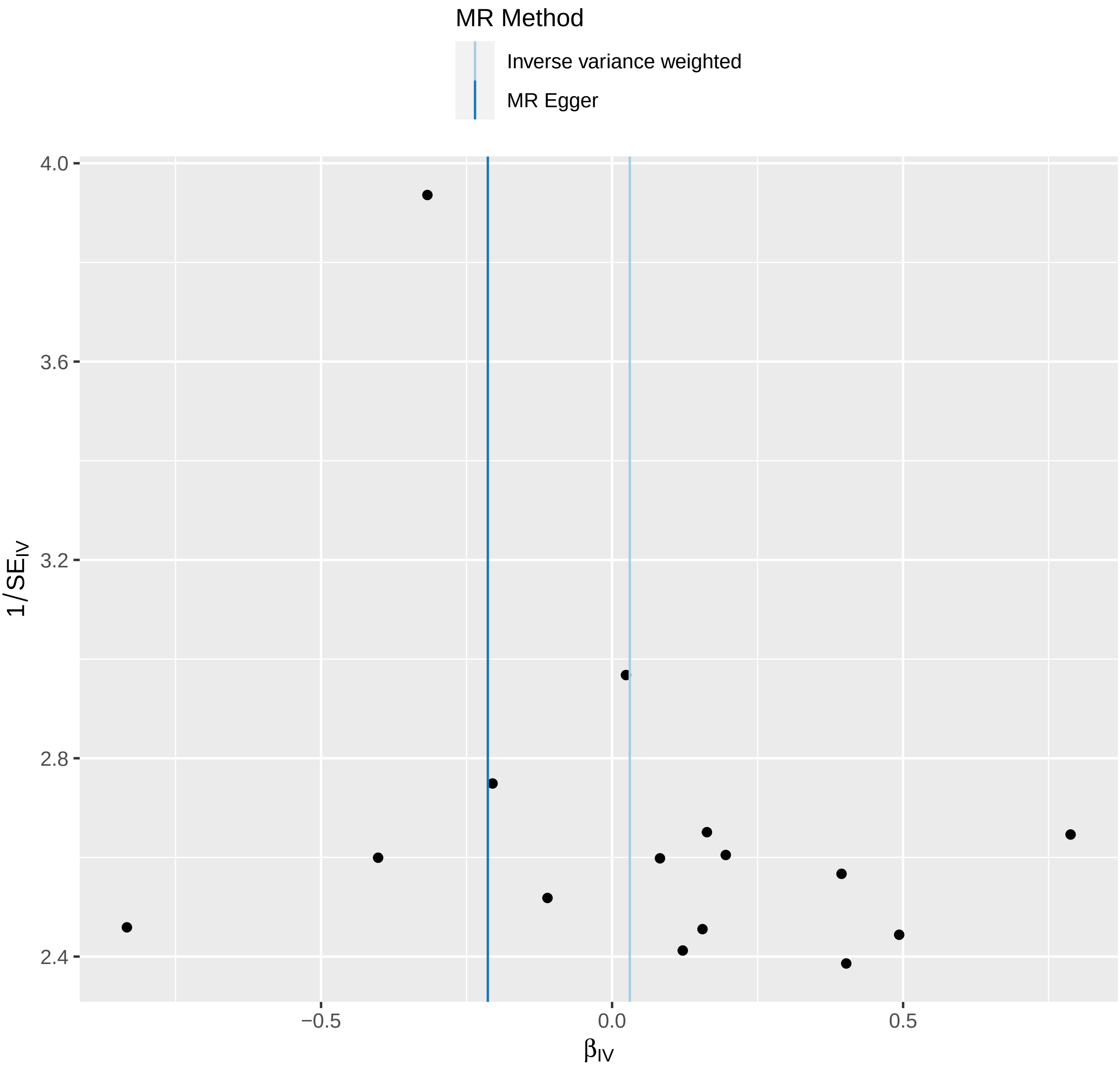

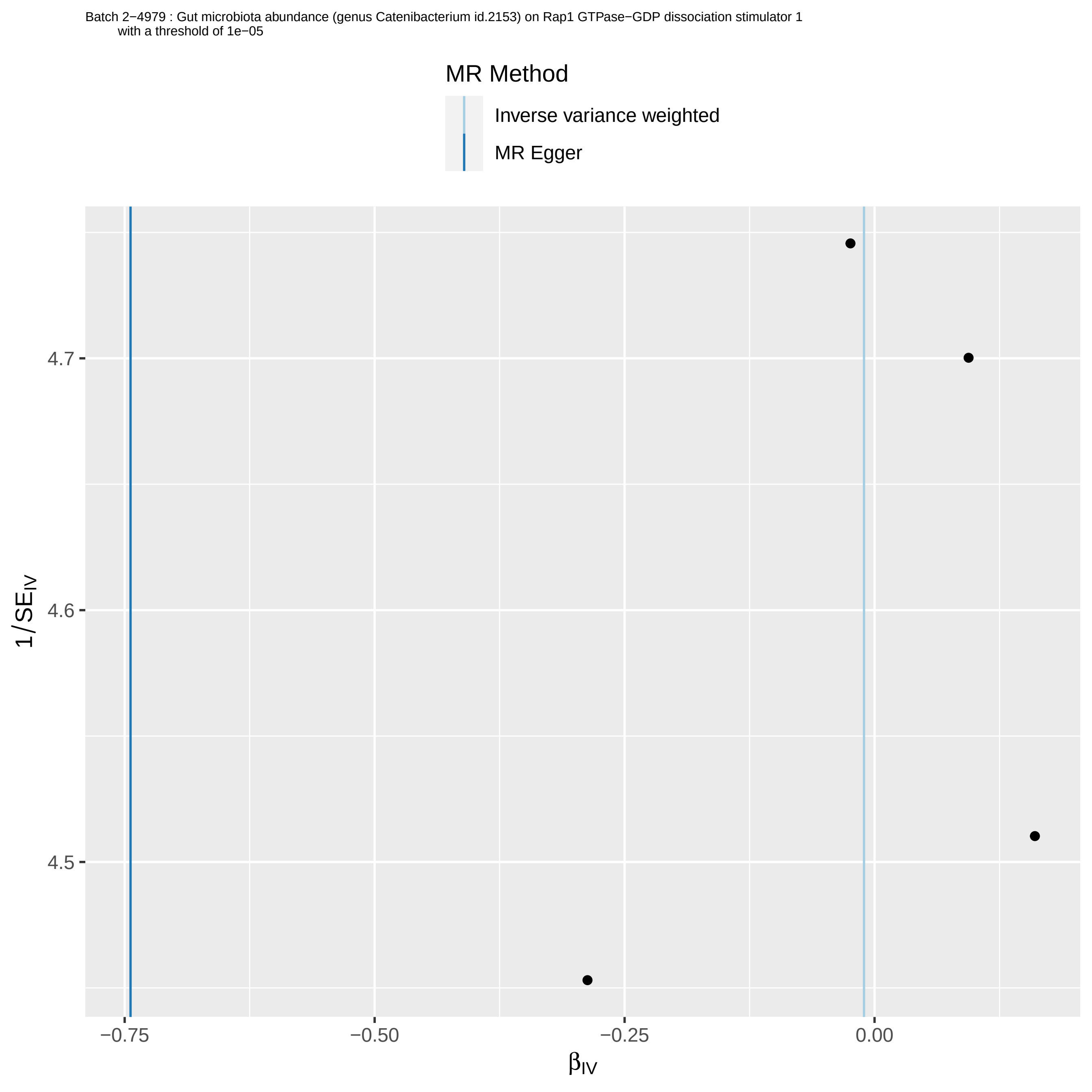
Batch 2−4979 : Gut microbiota abundance (genus Catenibacterium id.2153) on Rap1 GTPase−GDP dissociation stimulator 1
with a threshold of 1e−05
MR Method
Inverse variance weighted
MR Egger
4.7
4.6
4.5
1/SE_IV
−0.75
−0.50
−0.25
0.00
β_IV

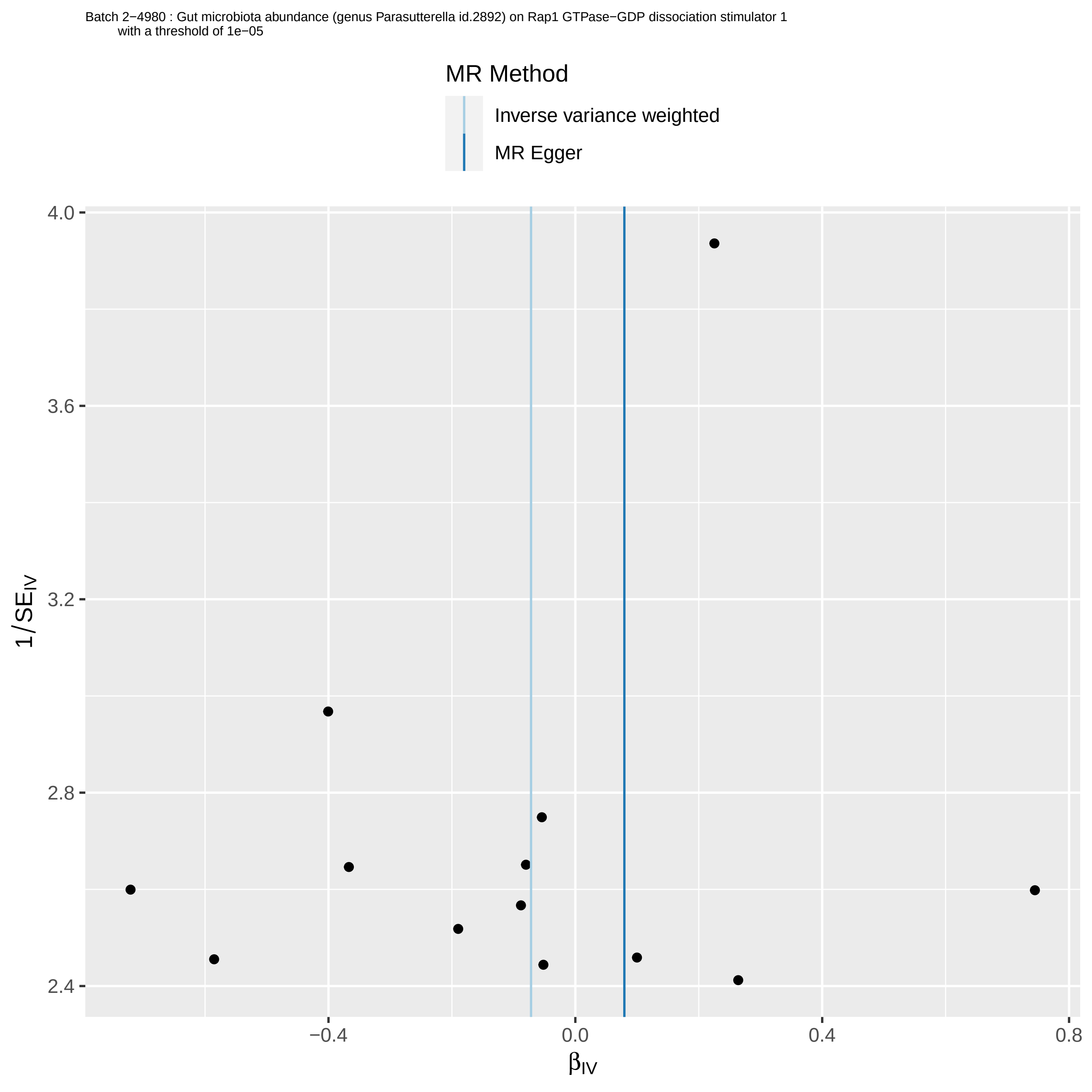
Batch 2−4980 : Gut microbiota abundance (genus Parasutterella id.2892) on Rap1 GTPase−GDP dissociation stimulator 1
with a threshold of 1e−05
MR Method
Inverse variance weighted
MR Egger
4.0
3.6
3.2
2.8
2.4
1/SE_IV
−0.4
0.0
0.4
0.8
β_IV

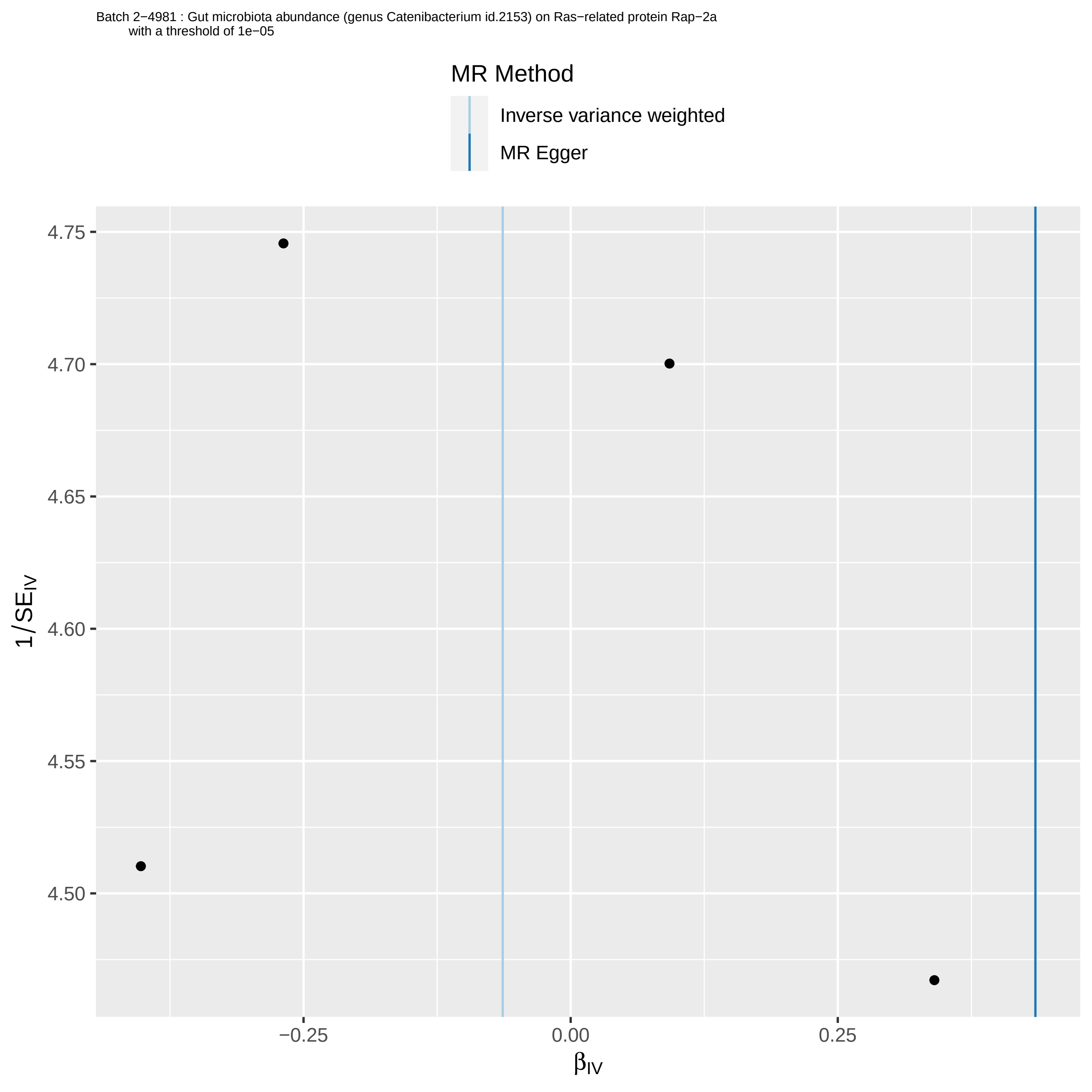
Batch 2−4981 : Gut microbiota abundance (genus Catenibacterium id.2153) on Ras−related protein Rap−2a
with a threshold of 1e−05
MR Method
Inverse variance weighted
MR Egger
4.75
4.70
4.65
4.60
4.55
4.50
1/SE_IV
−0.25
0.00
0.25
β_IV

Batch 2−4982 : Gut microbiota abundance (genus Parasutterella id.2892) on Ras−related protein Rap−2a
with a threshold of 1e−05

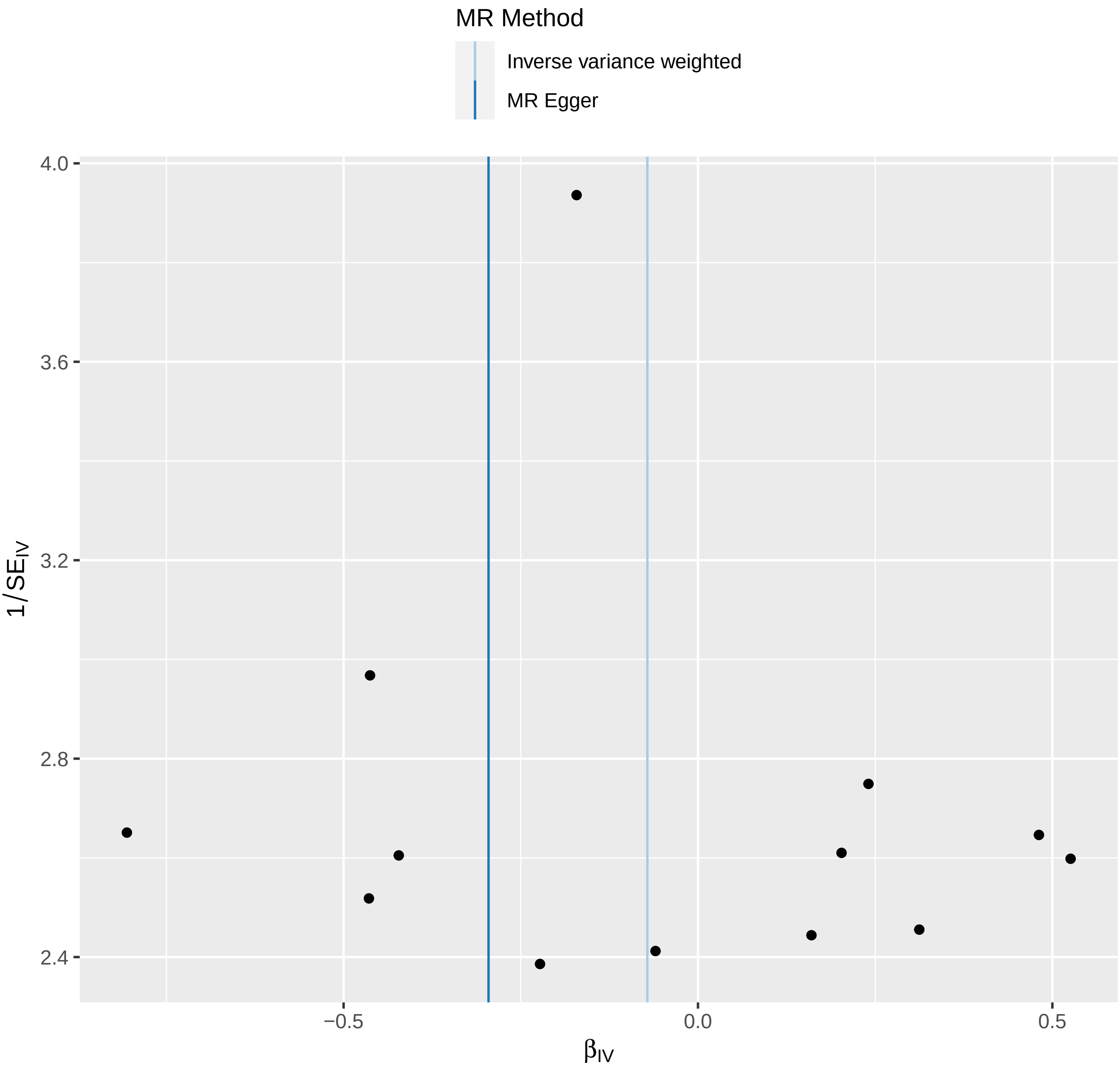

Batch 2−4983 : Gut microbiota abundance (genus Catenibacterium id.2153) on Rap guanine nucleotide exchange factor 5
with a threshold of 1e−05

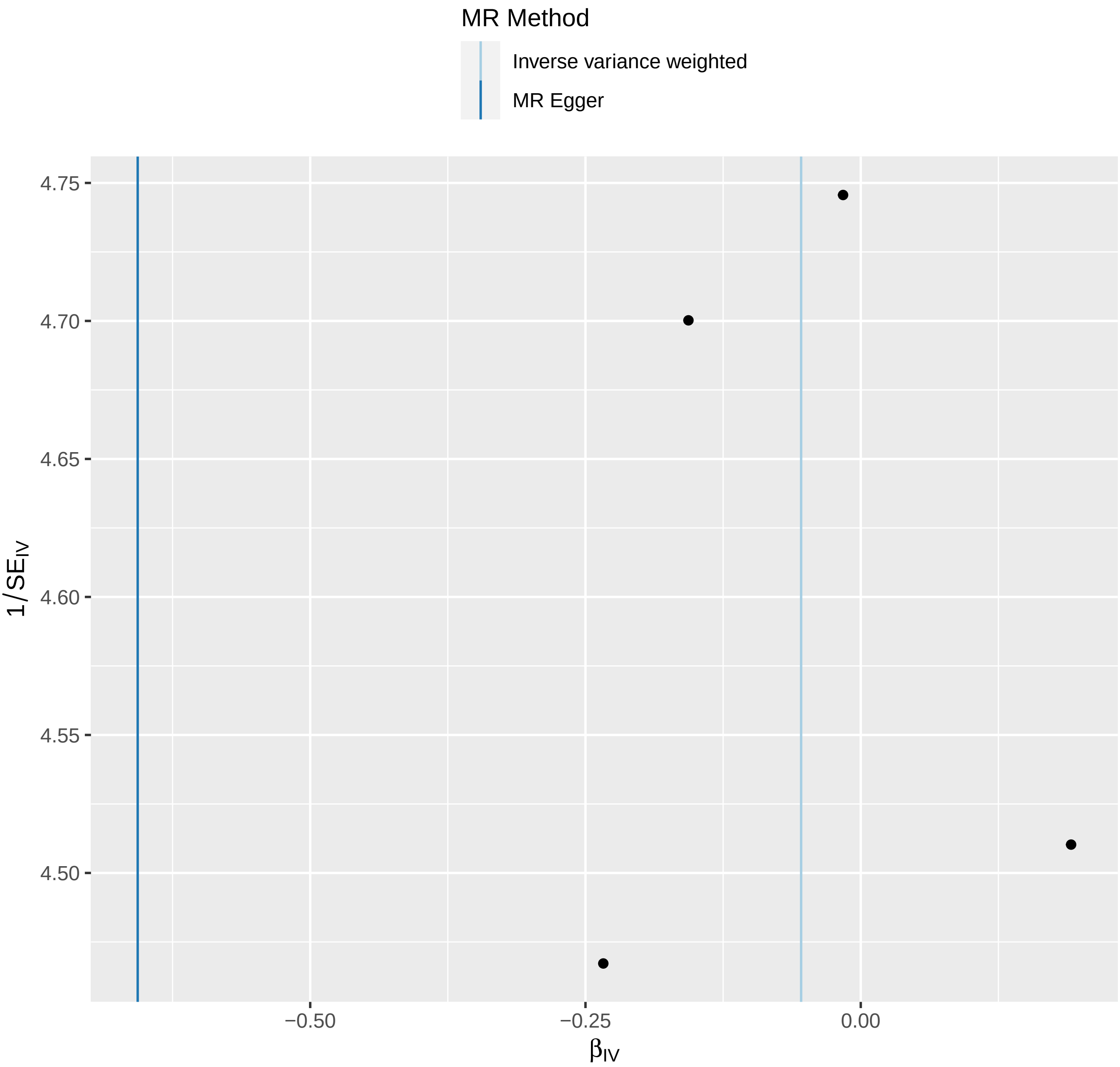

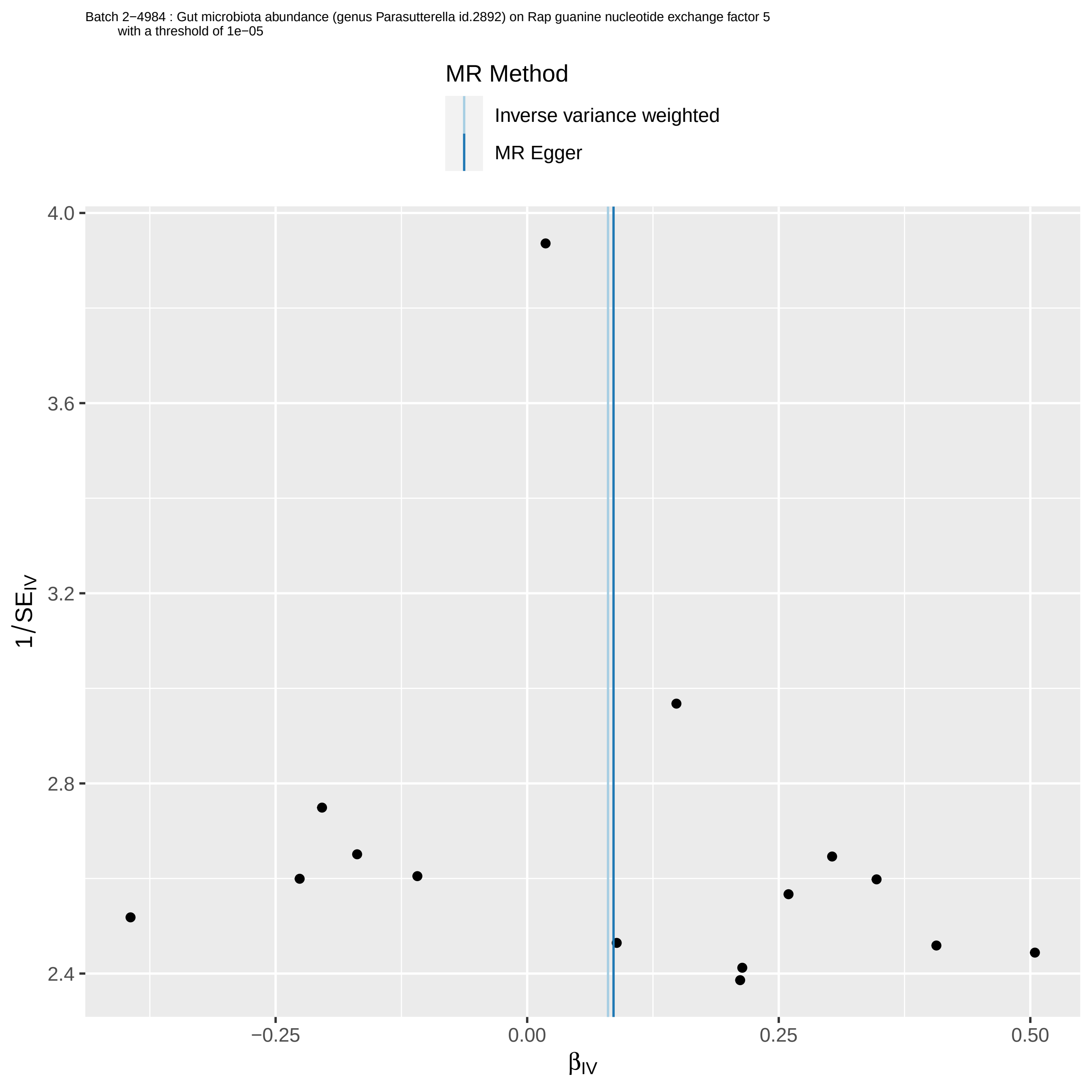
Batch 2−4984 : Gut microbiota abundance (genus Parasutterella id.2892) on Rap guanine nucleotide exchange factor 5
with a threshold of 1e−05
MR Method
Inverse variance weighted
MR Egger
4.0
3.6
3.2
2.8
2.4
1/SE_IV
−0.25
0.00
0.25
0.50
β_IV

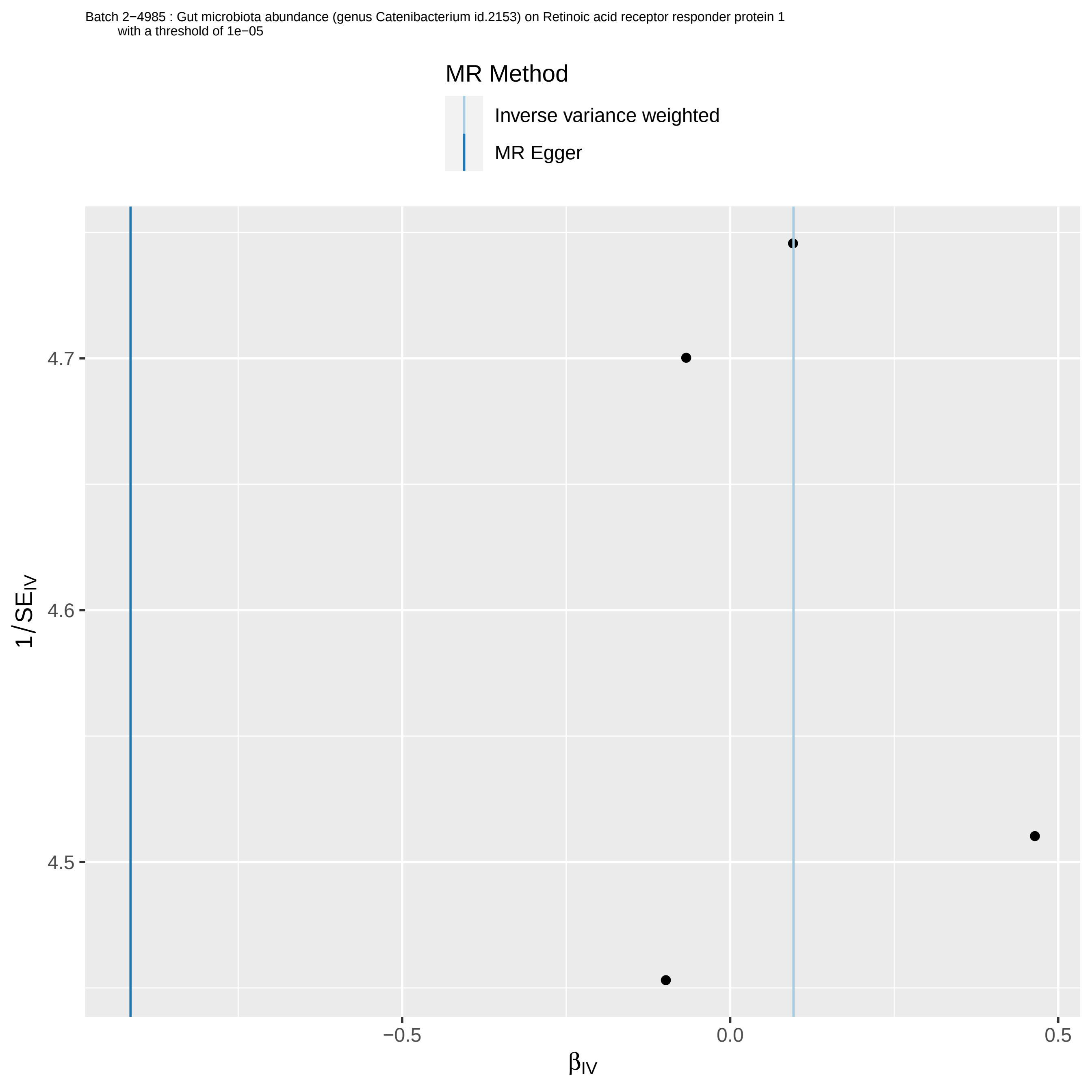
Batch 2−4985 : Gut microbiota abundance (genus Catenibacterium id.2153) on Retinoic acid receptor responder protein 1
with a threshold of 1e−05
MR Method
Inverse variance weighted
MR Egger
4.7
4.6
4.5
1/SE_IV
−0.5
0.0
0.5
β_IV

Batch 2−4986 : Gut microbiota abundance (genus Parasutterella id.2892) on Retinoic acid receptor responder protein 1 with a threshold of 1e−05

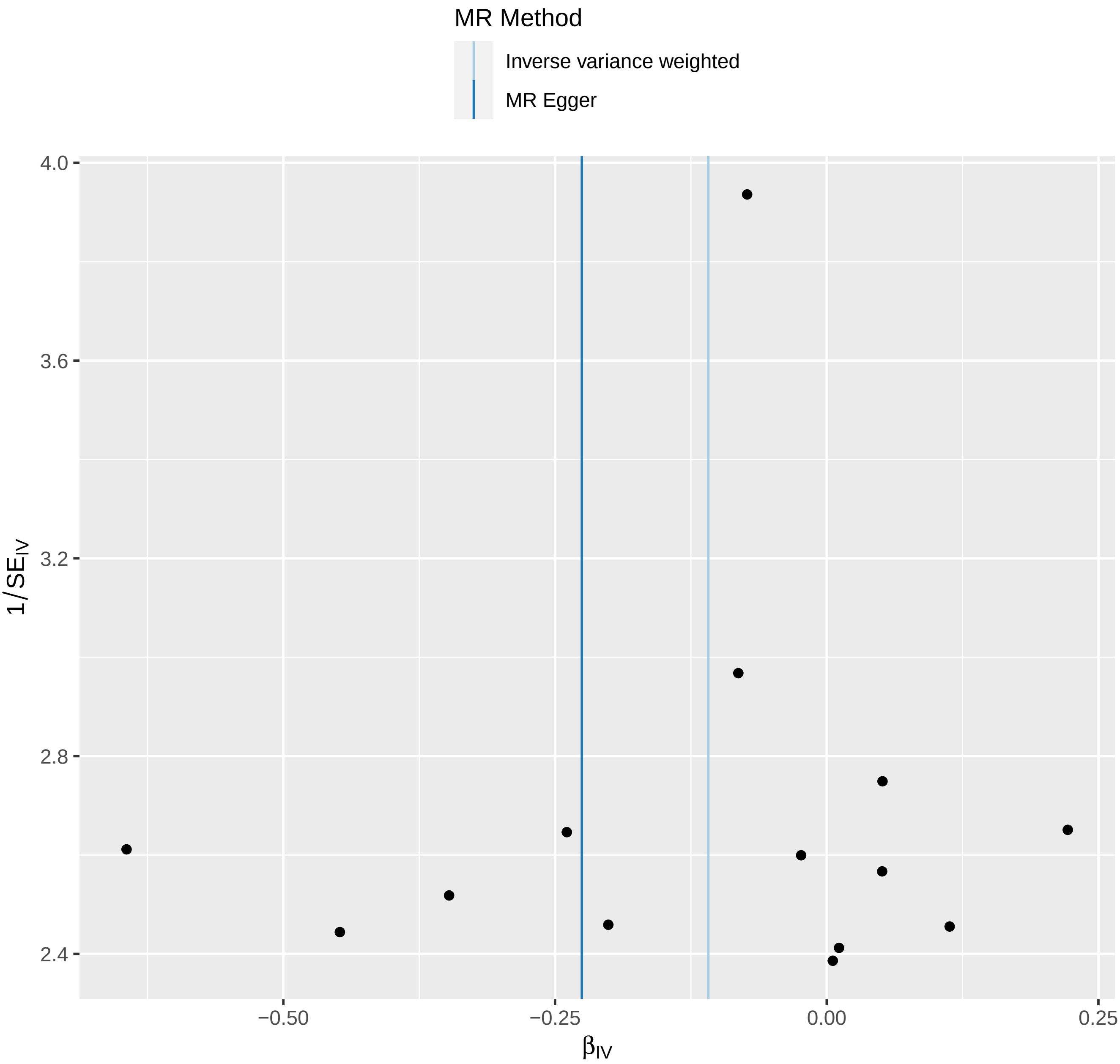

Batch 2−4987 : Gut microbiota abundance (genus Catenibacterium id.2153) on Retinoic acid receptor responder protein 3 with a threshold of 1e−05

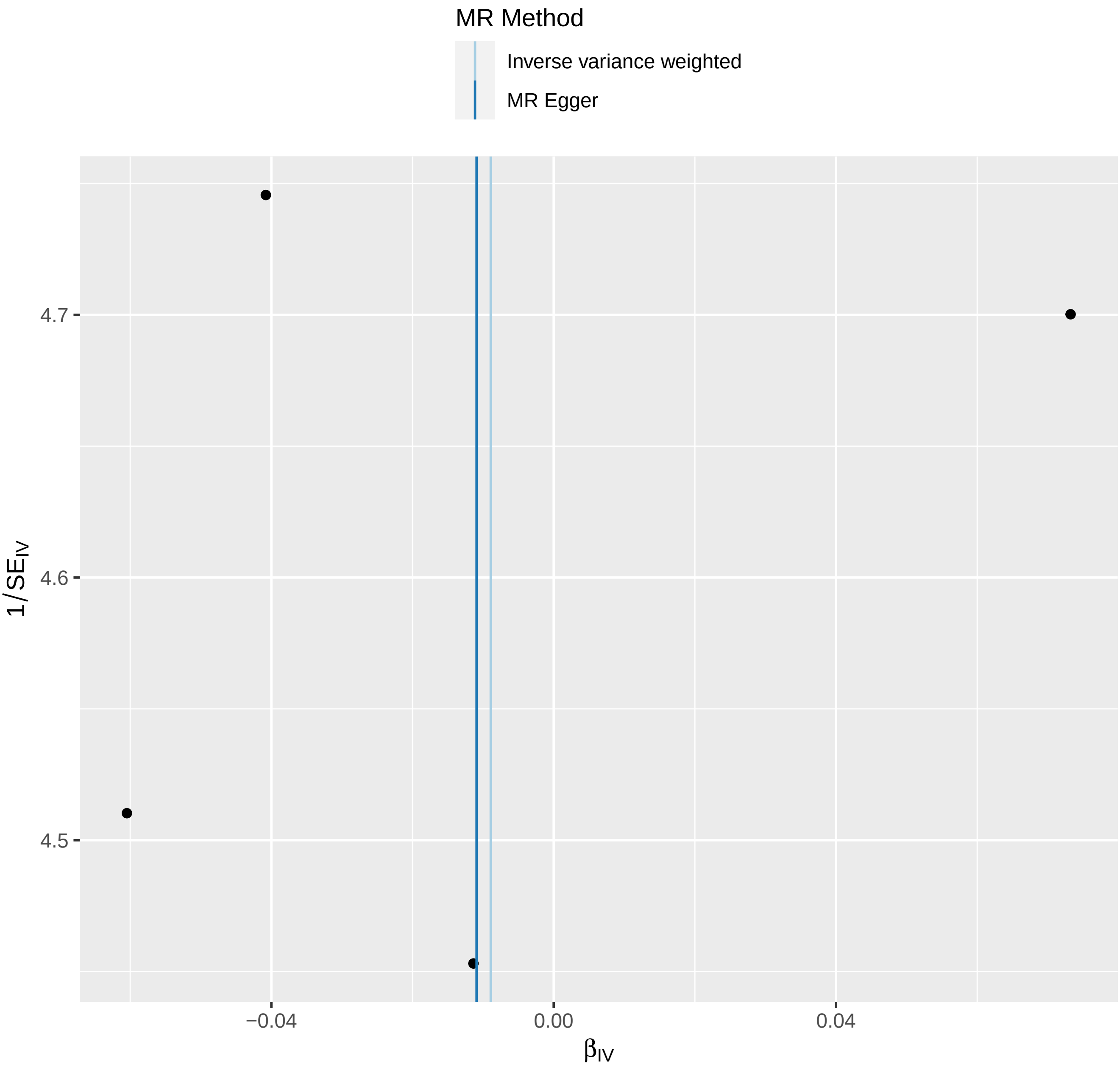

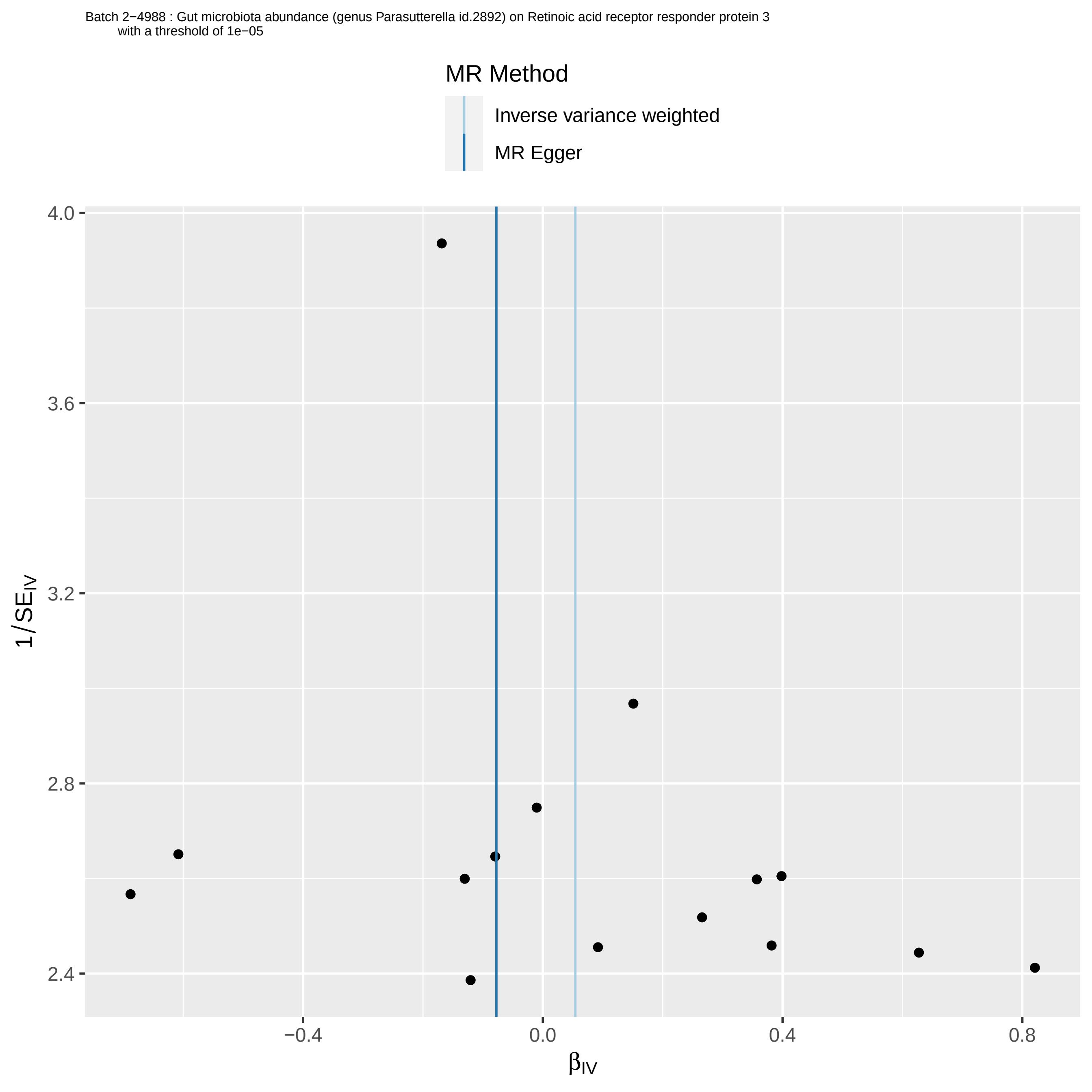

Batch 2−4988 : Gut microbiota abundance (genus Parasutterella id.2892) on Retinoic acid receptor responder protein 3
with a threshold of 1e−05
MR Method
Inverse variance weighted
MR Egger
4.0
3.6
3.2
2.8
2.4
1/SE_IV
−0.4
0.0
0.4
0.8
β_IV

Batch 2−4989 : Gut microbiota abundance (genus Catenibacterium id.2153) on Ras GTPase−activating protein 1
with a threshold of 1e−05

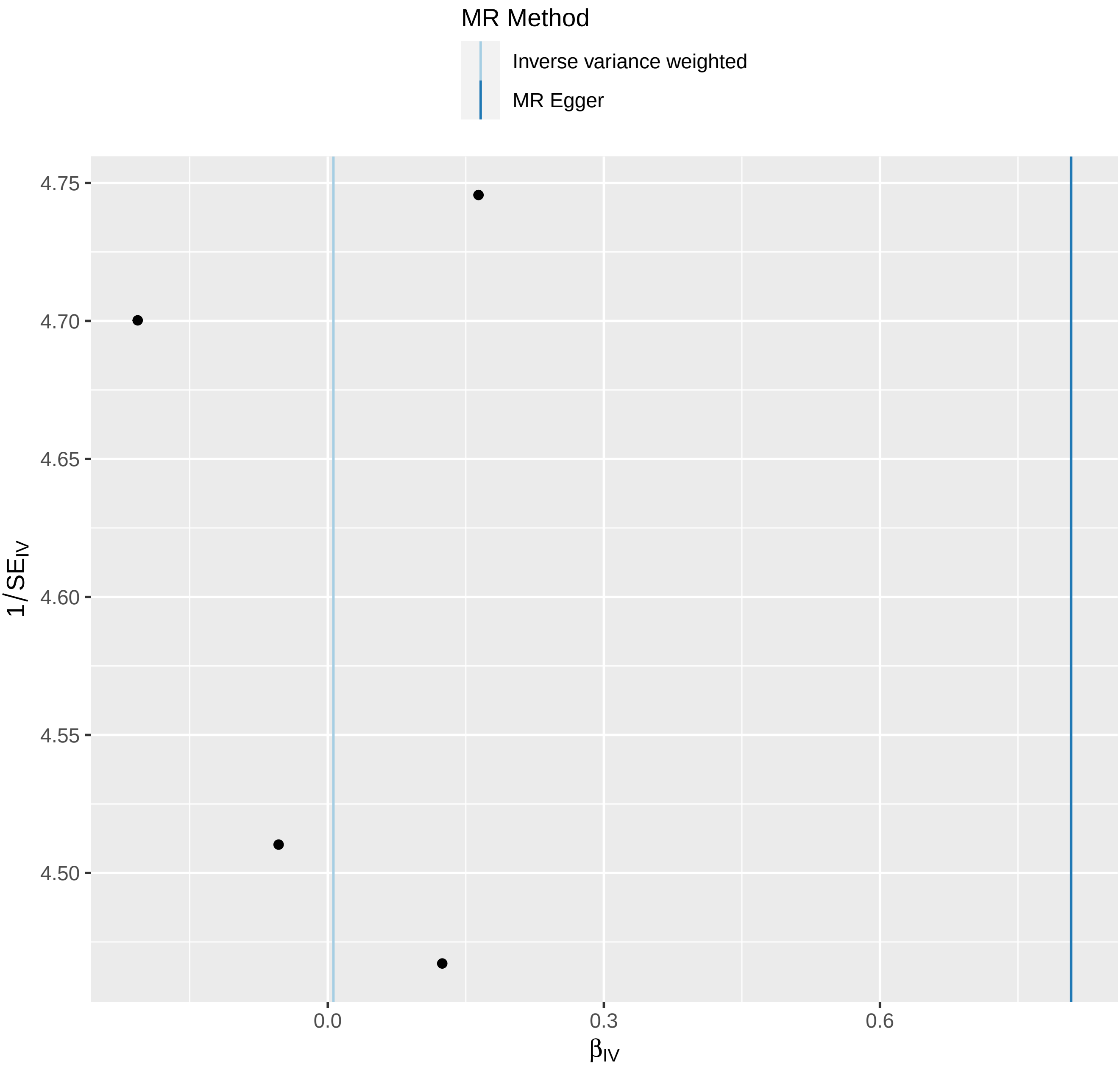

Batch 2−4990 : Gut microbiota abundance (genus Parasutterella id.2892) on Ras GTPase−activating protein 1
with a threshold of 1e−05

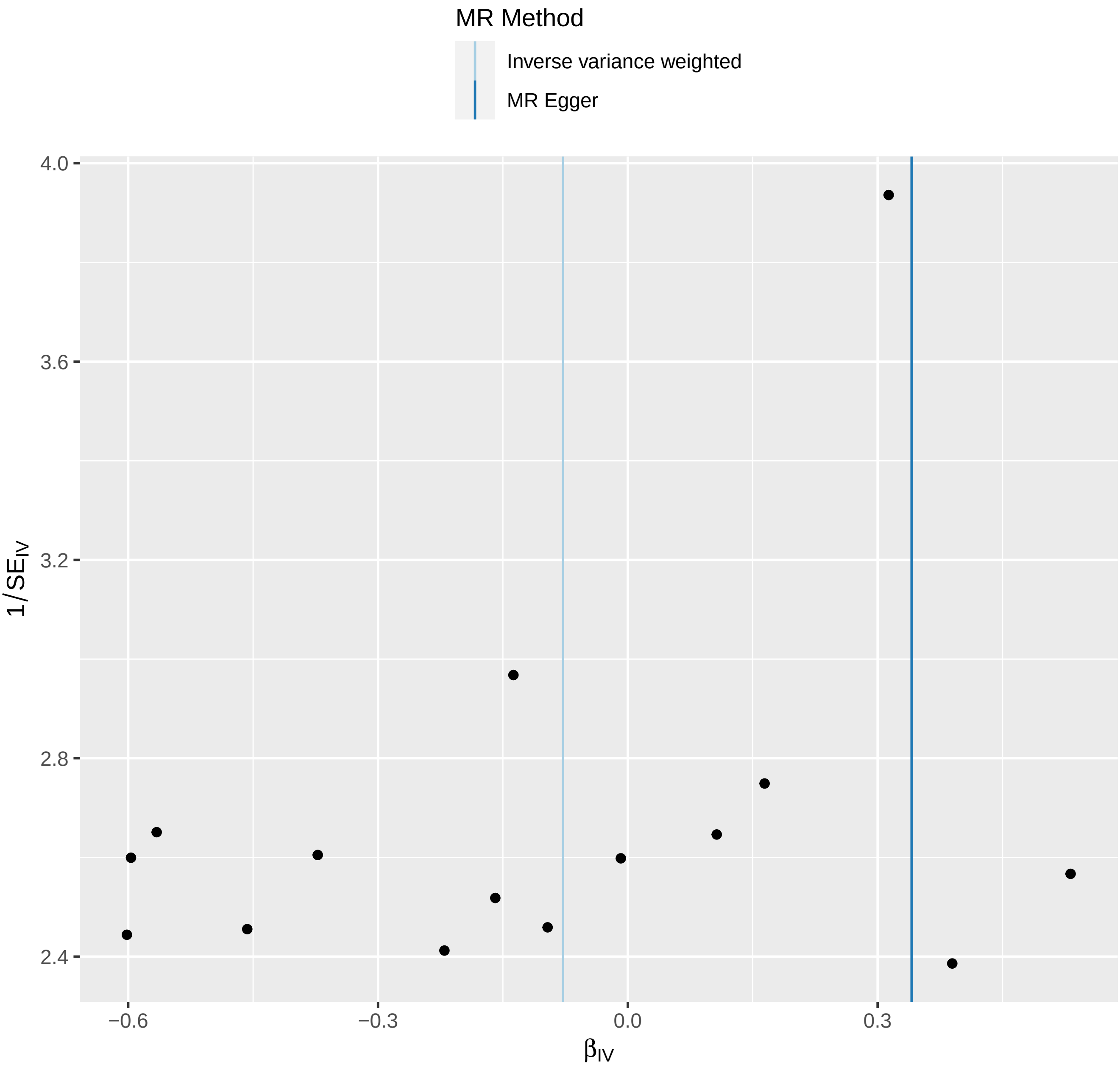

Batch 2−4991 : Gut microbiota abundance (genus Catenibacterium id.2153) on Retinoblastoma−associated protein
with a threshold of 1e−05

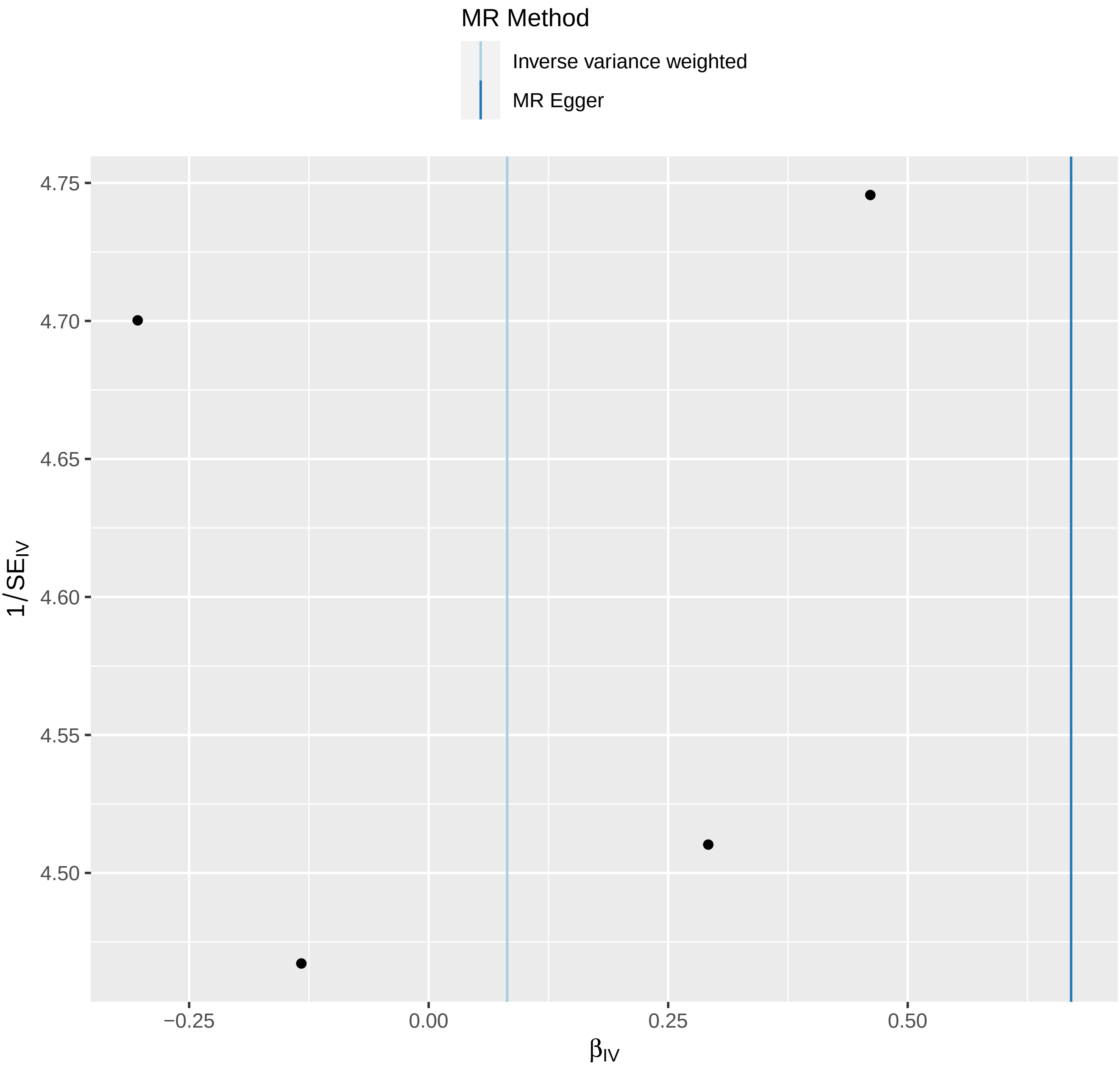

Batch 2−4992 : Gut microbiota abundance (genus Parasutterella id.2892) on Retinoblastoma−associated protein
with a threshold of 1e−05

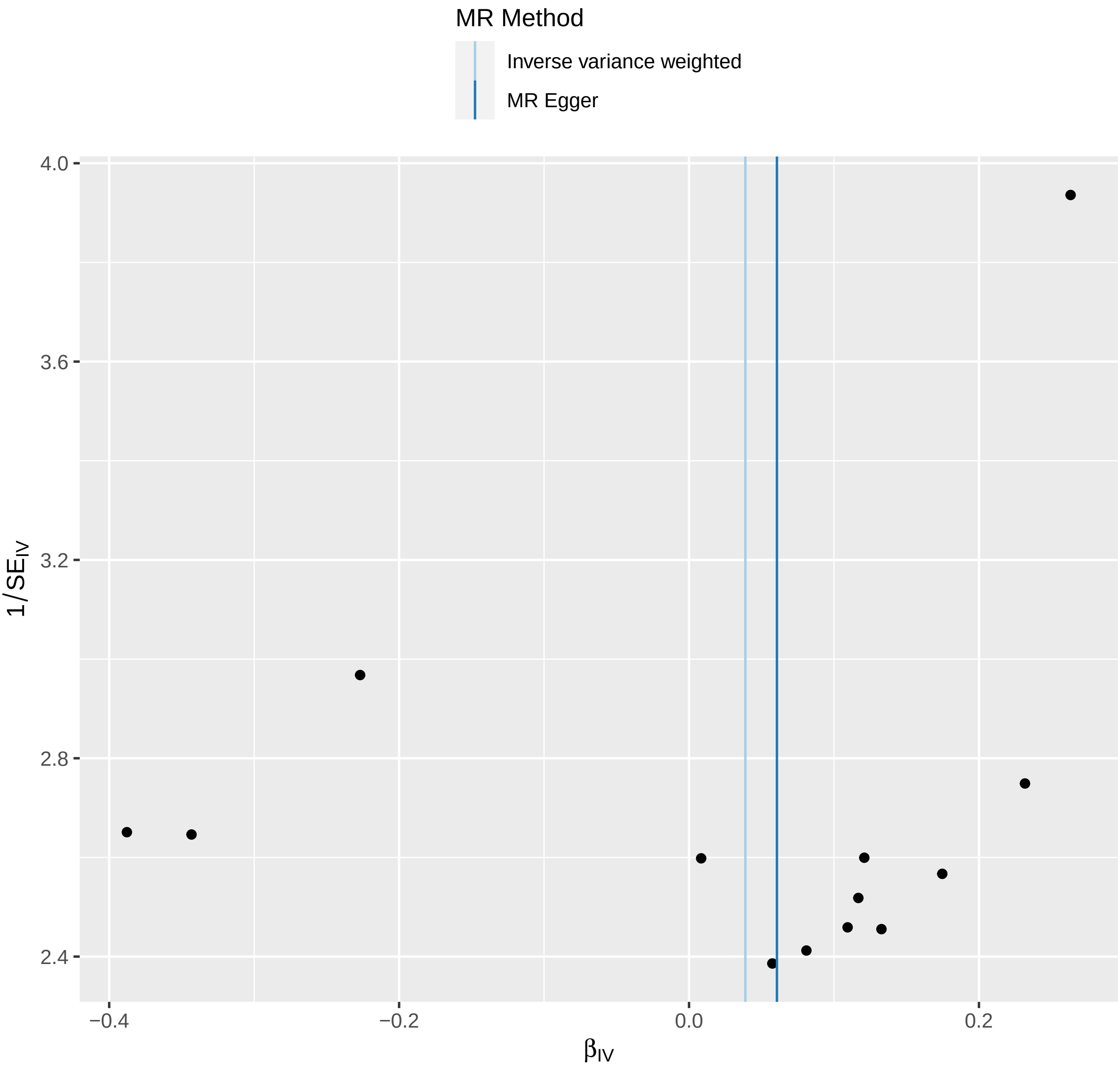

Batch 2−4993 : Gut microbiota abundance (genus Catenibacterium id.2153) on Retinoblastoma−binding protein 5
with a threshold of 1e−05

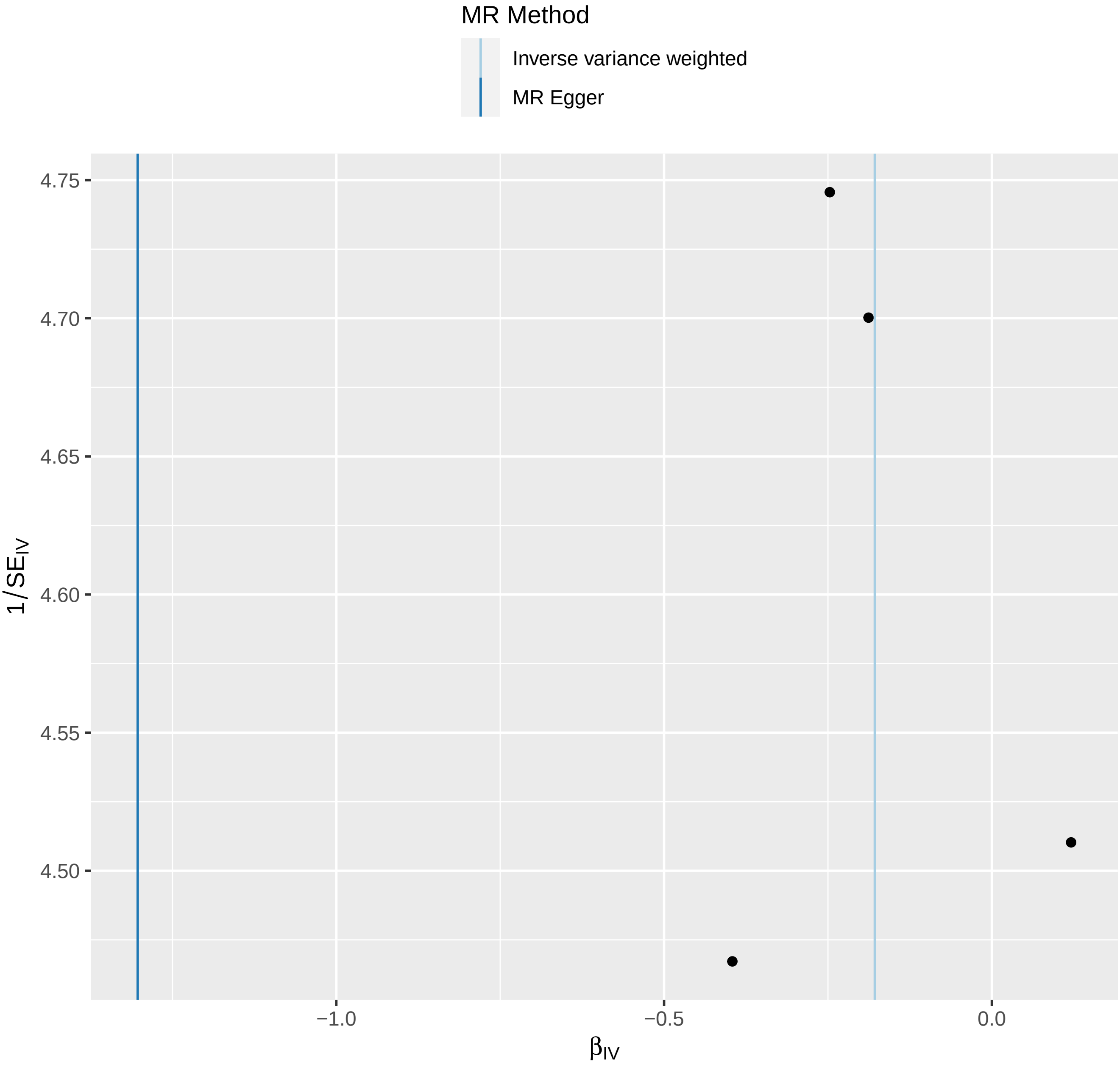

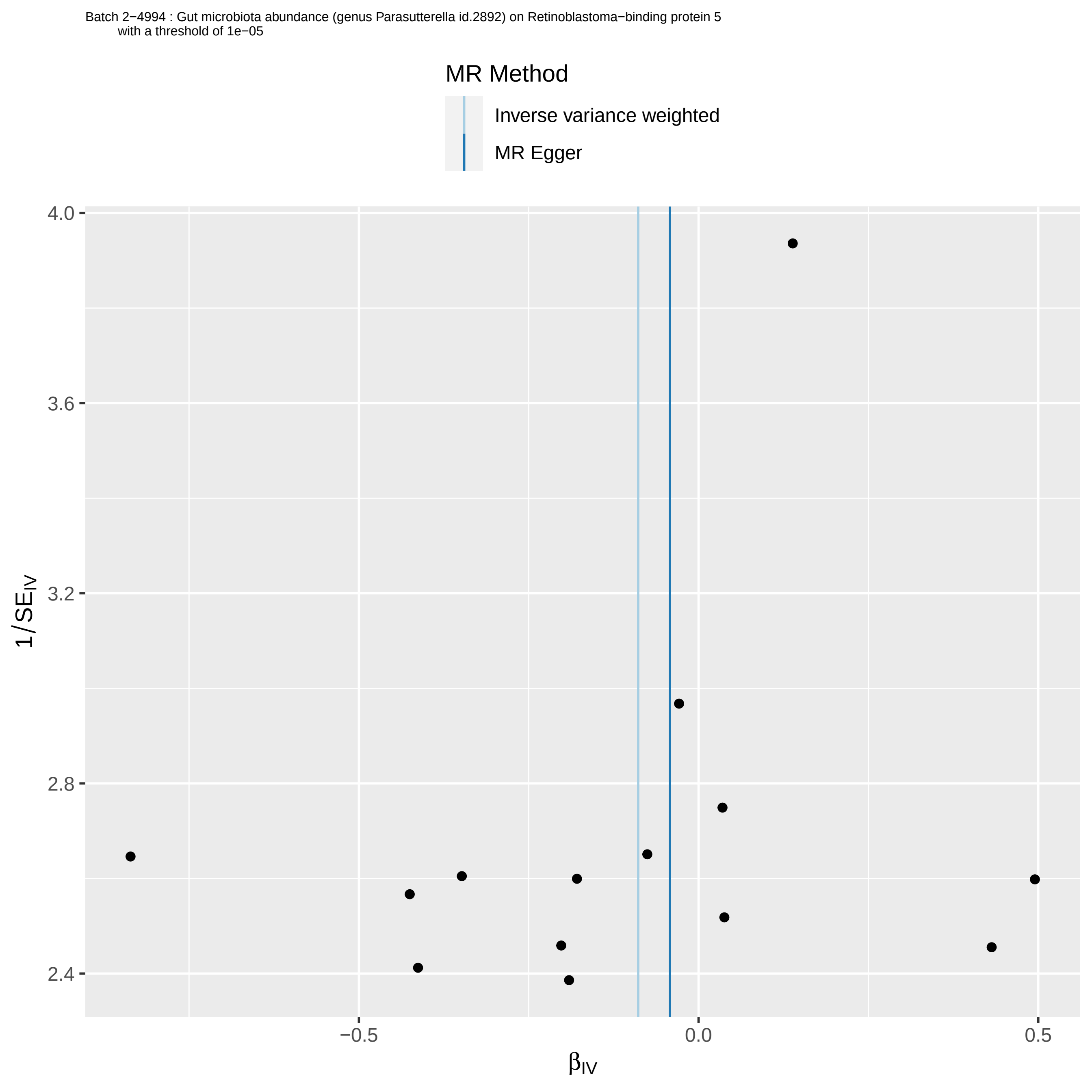
Batch 2−4994 : Gut microbiota abundance (genus Parasutterella id.2892) on Retinoblastoma−binding protein 5
with a threshold of 1e−05
MR Method
Inverse variance weighted
MR Egger
4.0
3.6
3.2
2.8
2.4
1/SE_IV
−0.5
0.0
0.5
β_IV

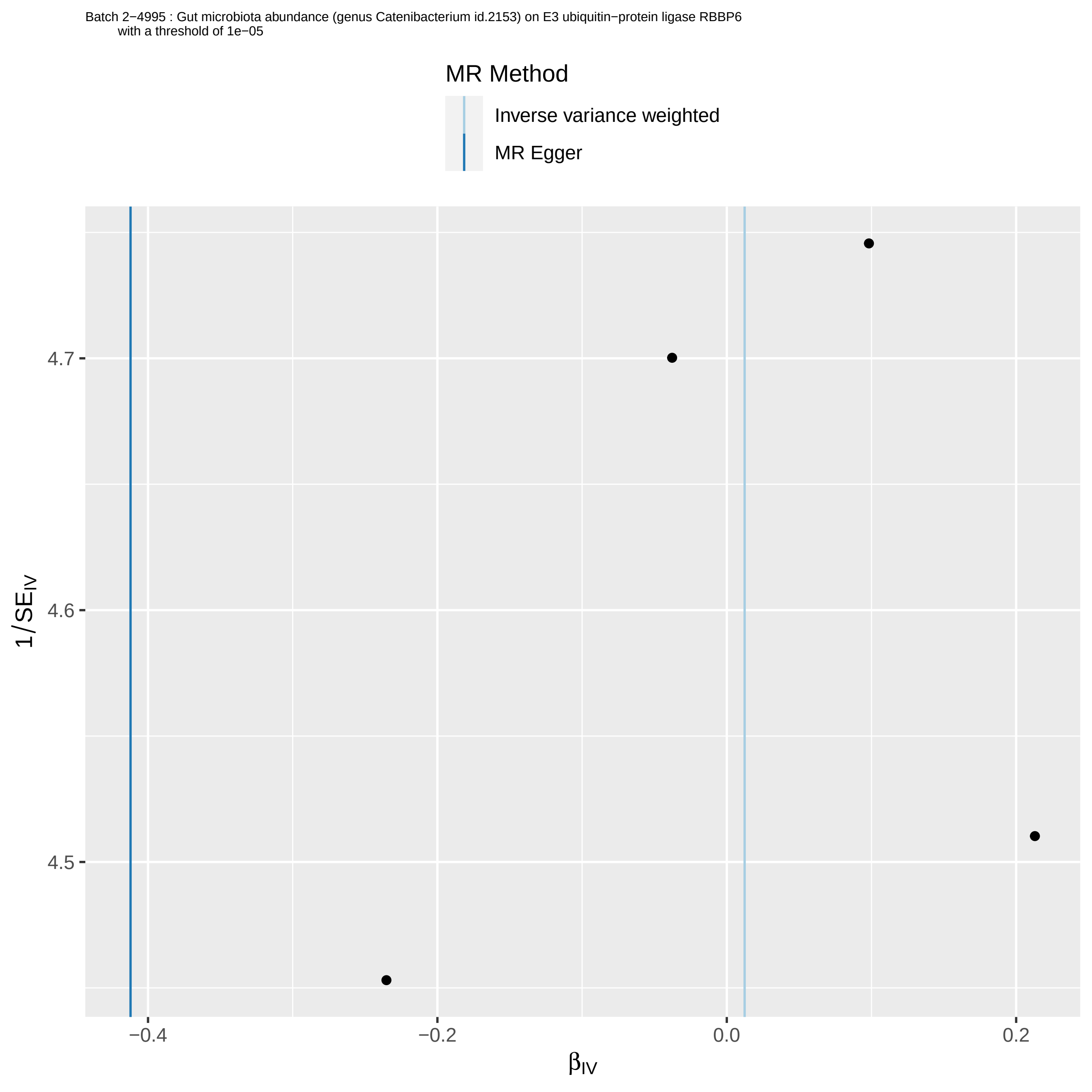
Batch 2−4995 : Gut microbiota abundance (genus Catenibacterium id.2153) on E3 ubiquitin−protein ligase RBBP6
with a threshold of 1e−05
MR Method
Inverse variance weighted
MR Egger
4.7
4.6
4.5
1/SE_IV
−0.4
−0.2
0.0
0.2
β_IV

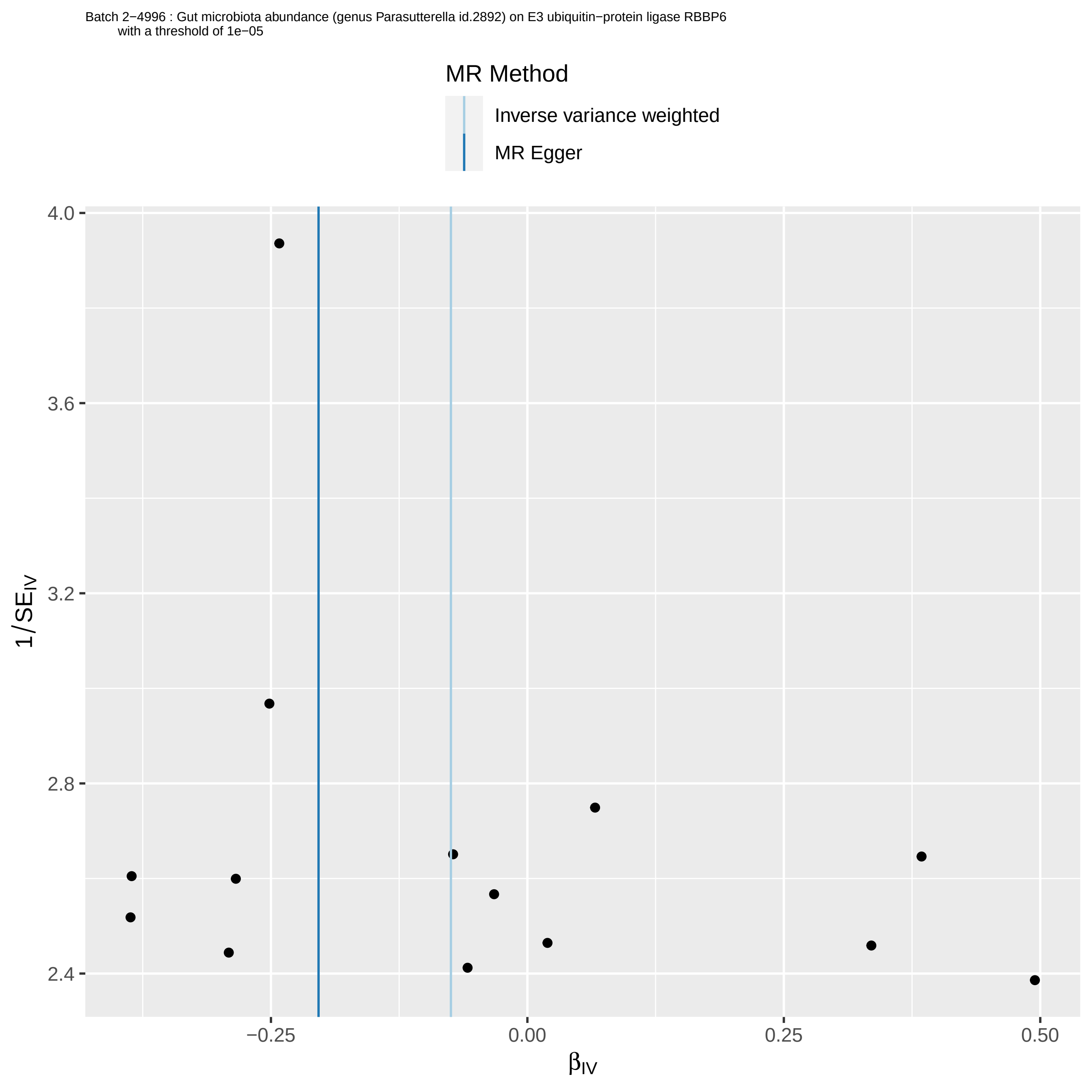

Batch 2−4996 : Gut microbiota abundance (genus Parasutterella id.2892) on E3 ubiquitin−protein ligase RBBP6
with a threshold of 1e−05
MR Method
Inverse variance weighted
MR Egger
4.0
3.6
3.2
2.8
2.4
1/SE_IV
−0.25
0.00
0.25
0.50
β_IV

Batch 2−4997 : Gut microbiota abundance (genus Catenibacterium id.2153) on RNA binding protein fox−1 homolog 2
with a threshold of 1e−05

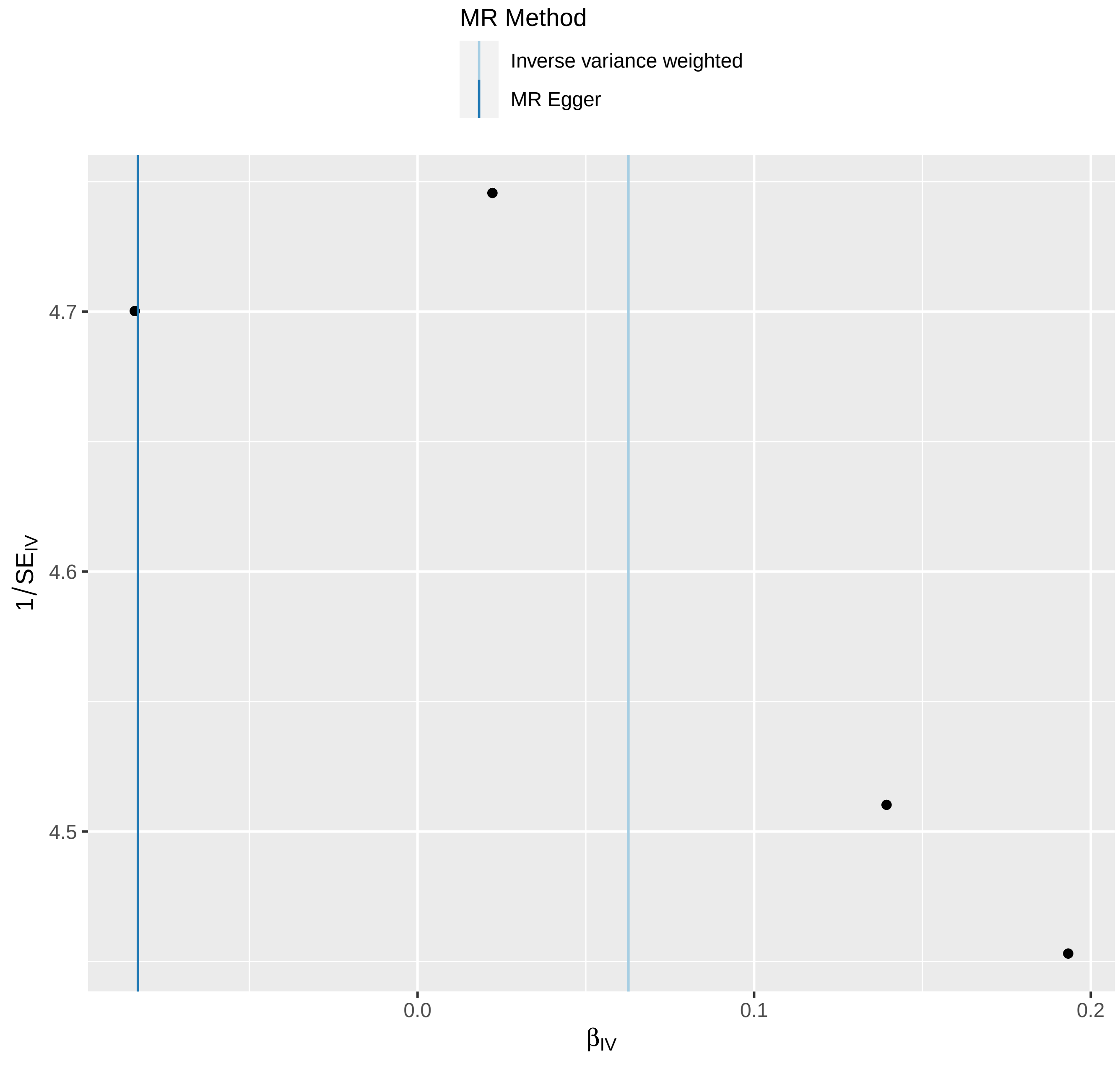

Batch 2−4998 : Gut microbiota abundance (genus Parasutterella id.2892) on RNA binding protein fox−1 homolog 2
with a threshold of 1e−05

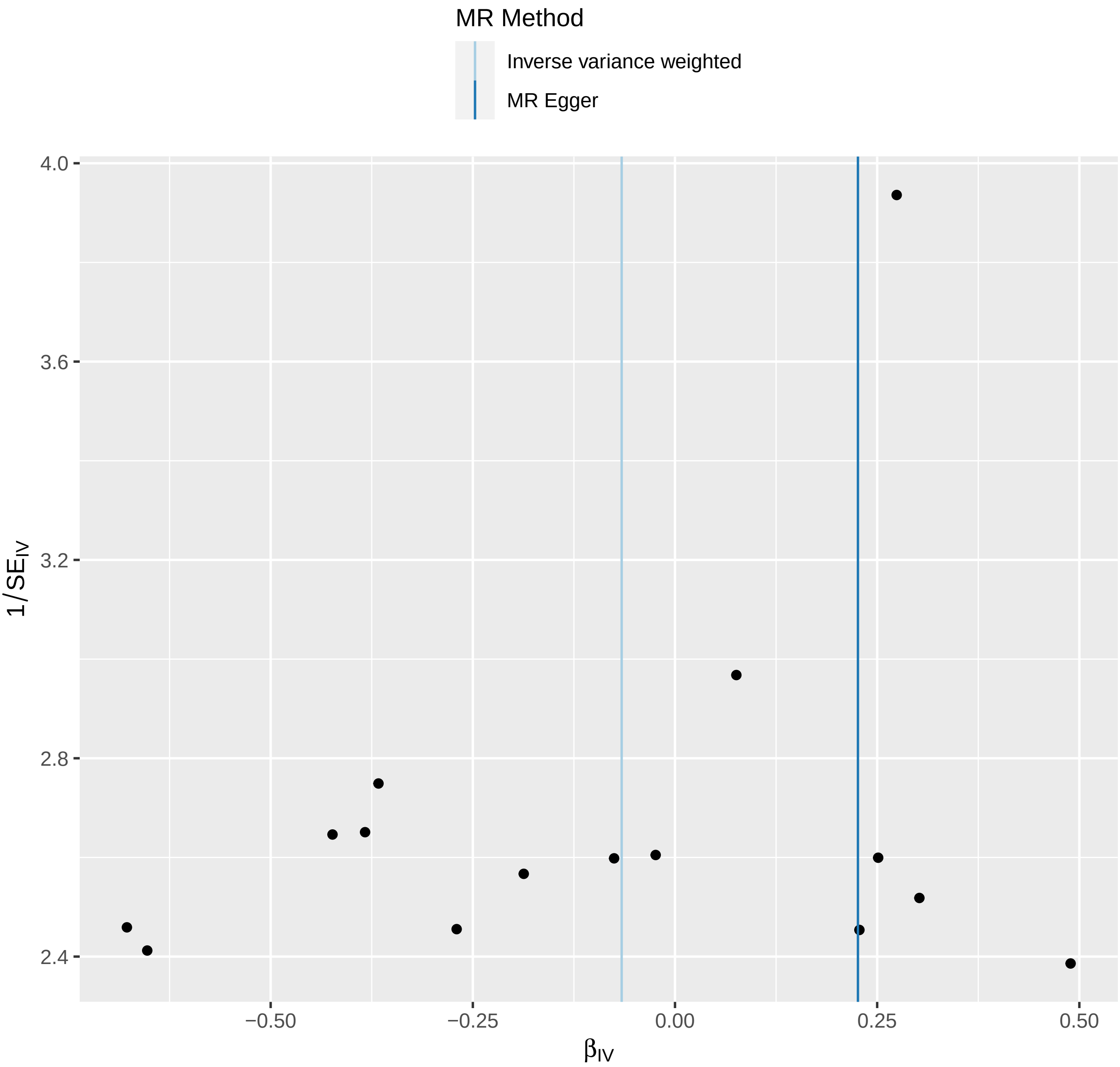

Batch 2−4999 : Gut microbiota abundance (genus Catenibacterium id.2153) on Retinoblastoma−like protein 1
with a threshold of 1e−05

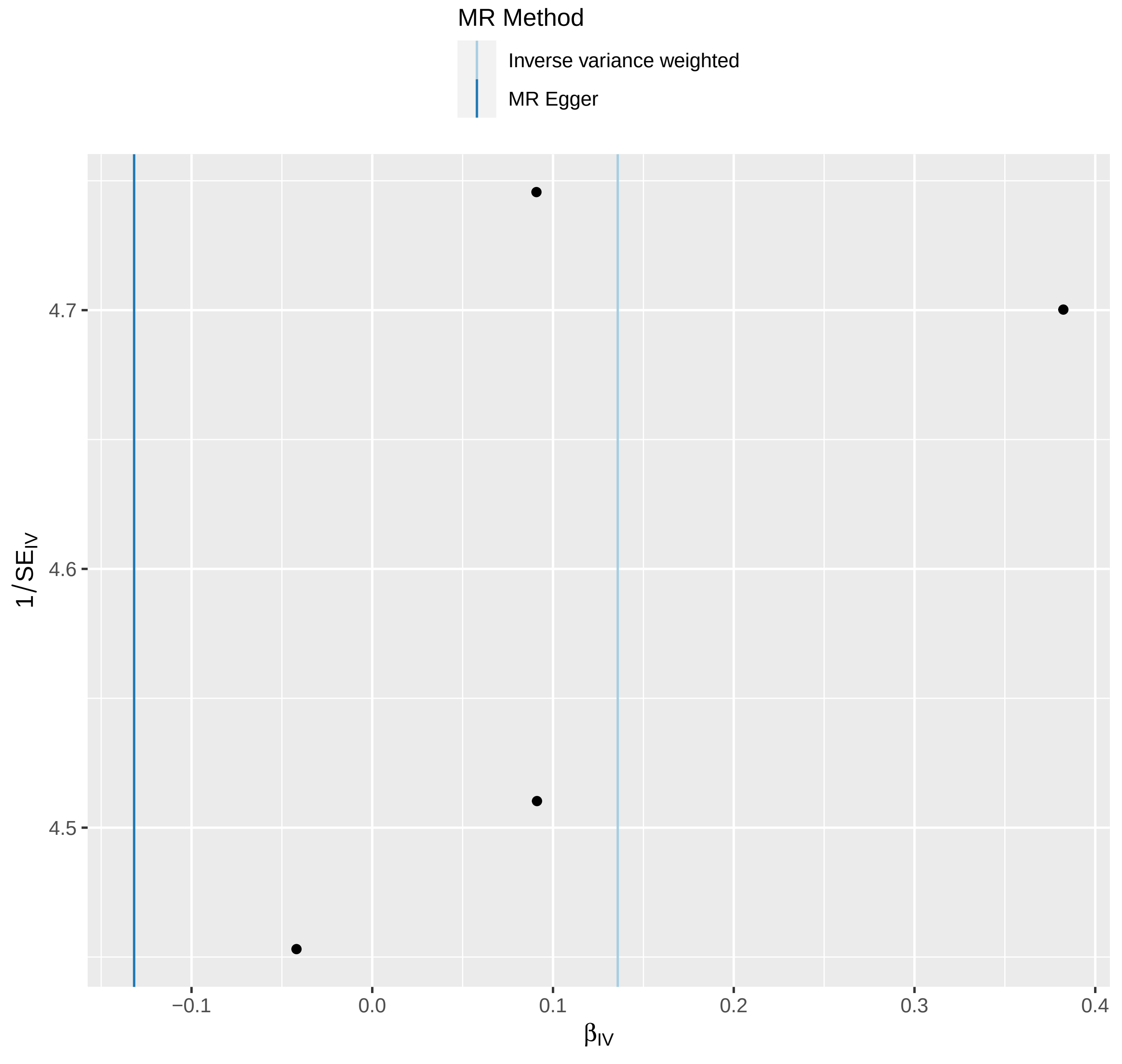

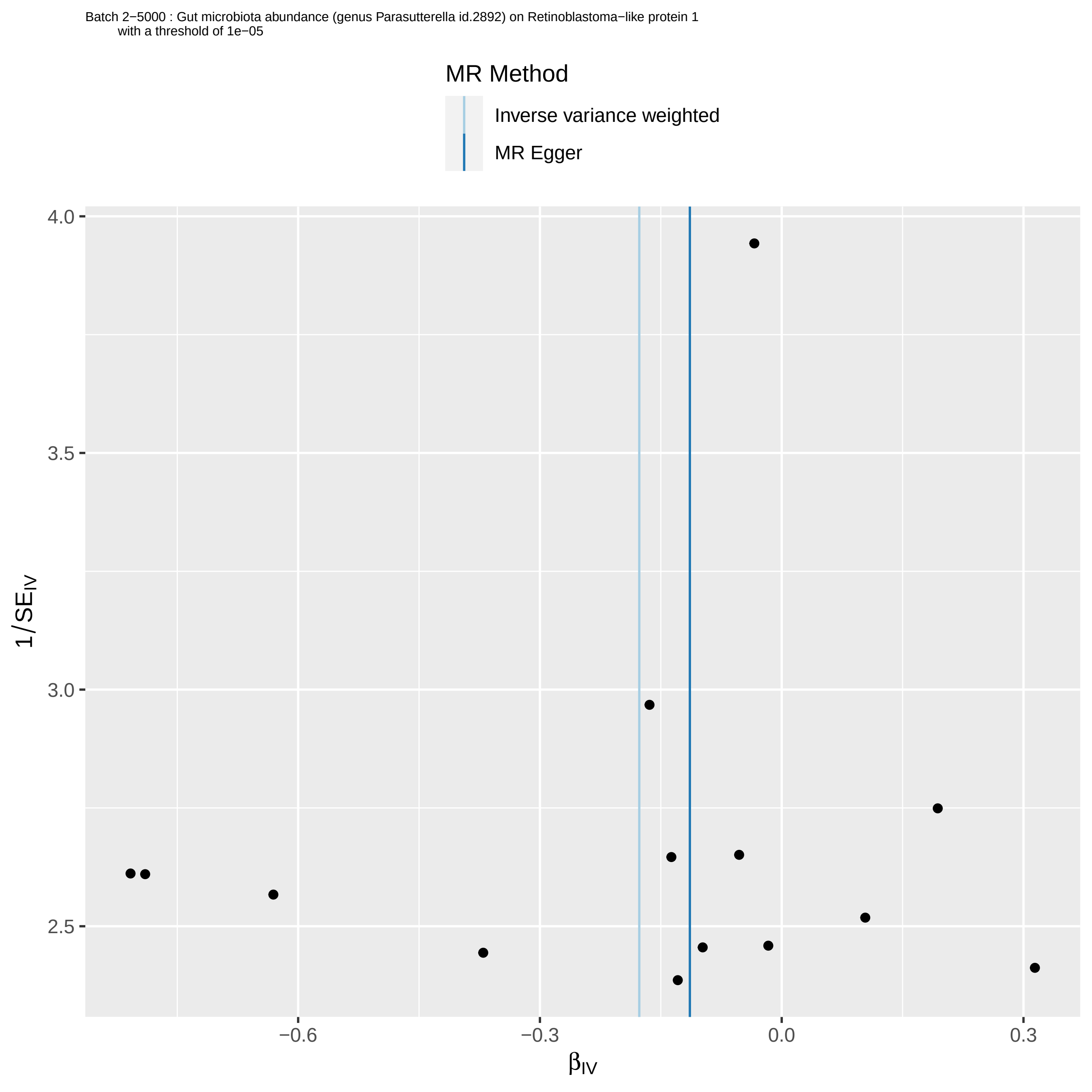

Batch 2−5000 : Gut microbiota abundance (genus Parasutterella id.2892) on Retinoblastoma−like protein 1
with a threshold of 1e−05
MR Method
Inverse variance weighted
MR Egger
4.0
3.5
3.0
2.5
1/SE_IV
−0.6
−0.3
0.0
0.3
β_IV

Batch 2−5001 : Gut microbiota abundance (genus Catenibacterium id.2153) on Probable RNA−binding protein 19
with a threshold of 1e−05

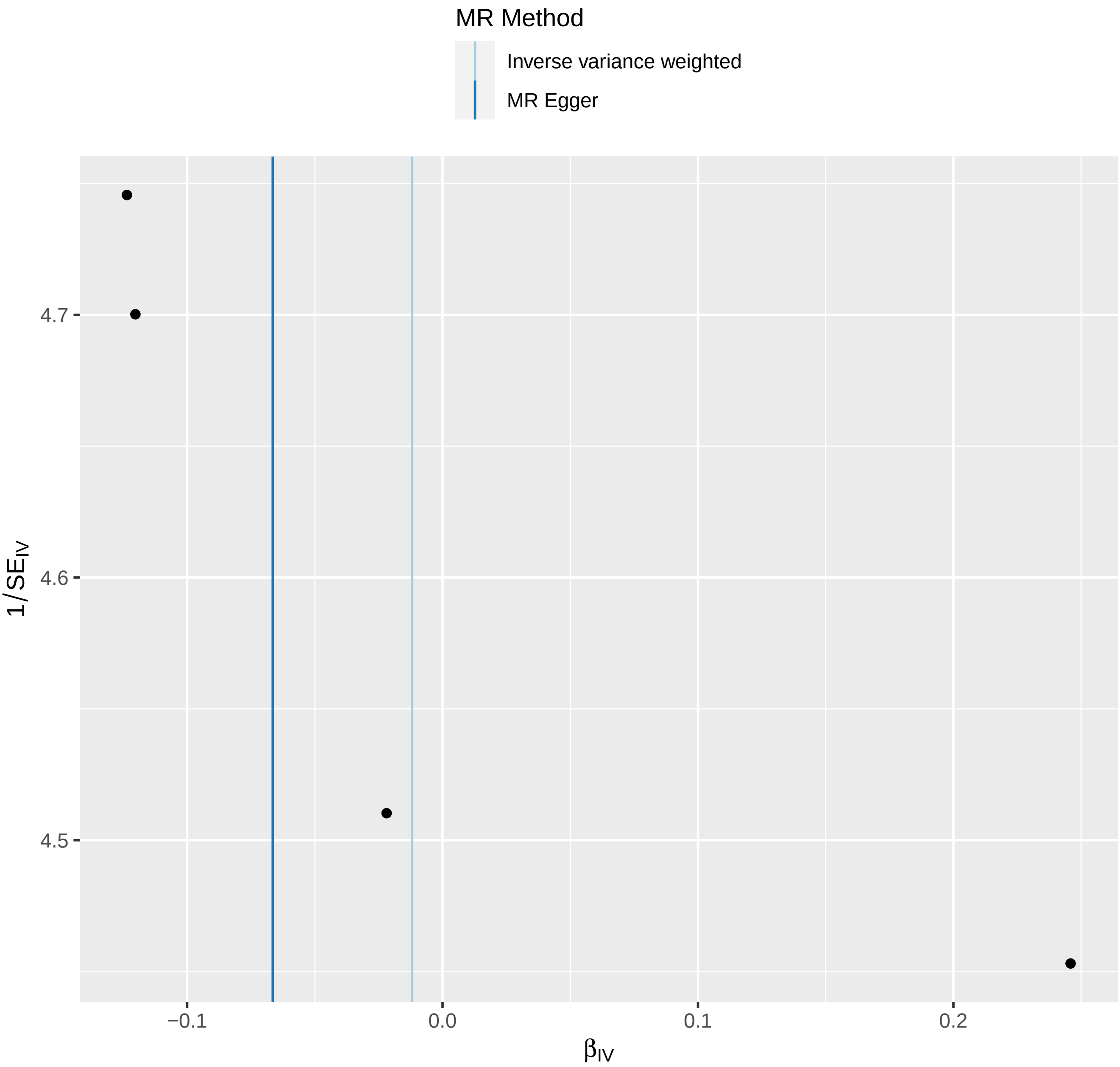

Batch 2−5002 : Gut microbiota abundance (genus Parasutterella id.2892) on Probable RNA−binding protein 19
with a threshold of 1e−05

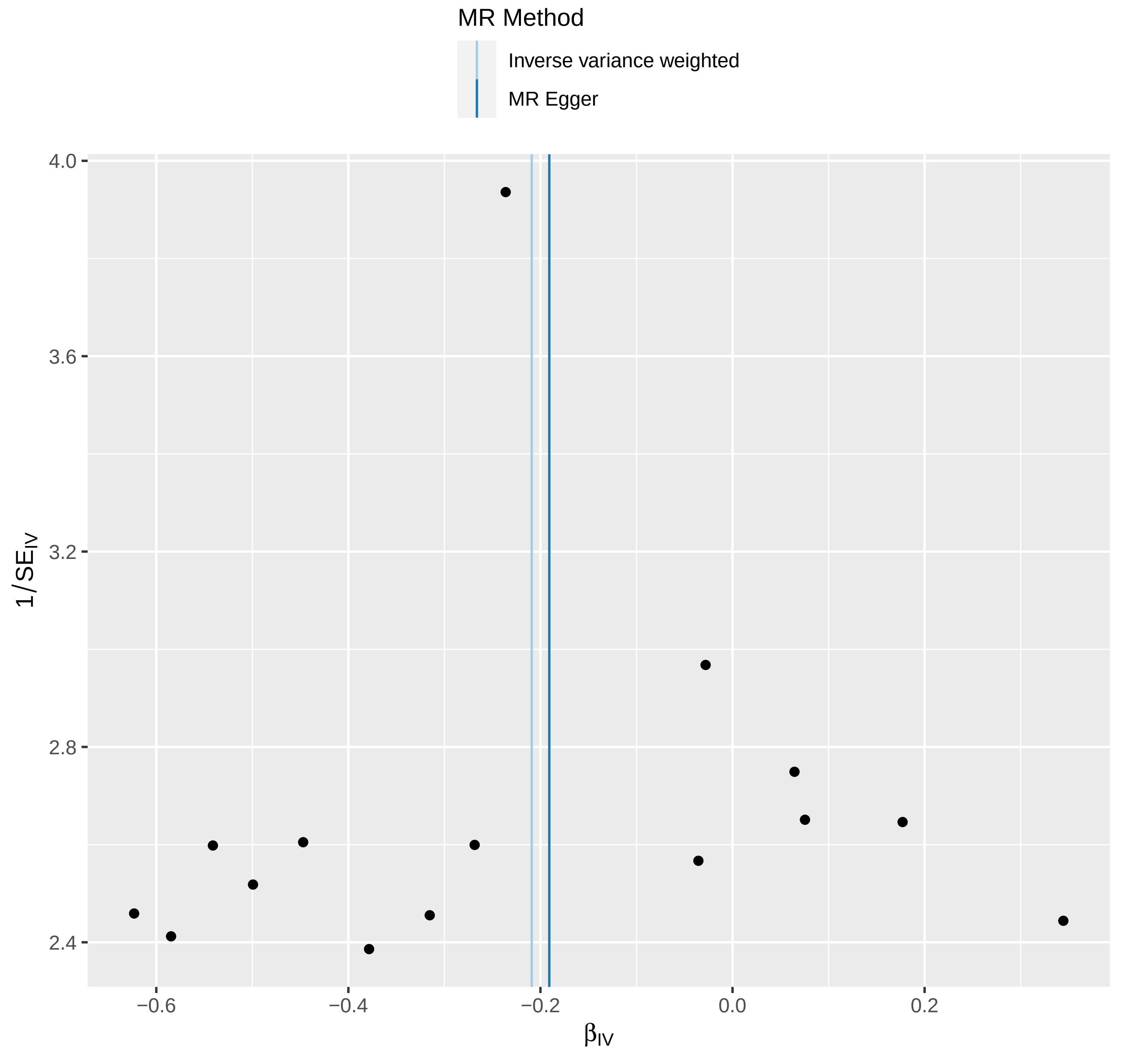

Batch 2−5003 : Gut microbiota abundance (genus Catenibacterium id.2153) on Probable RNA−binding protein 23
with a threshold of 1e−05

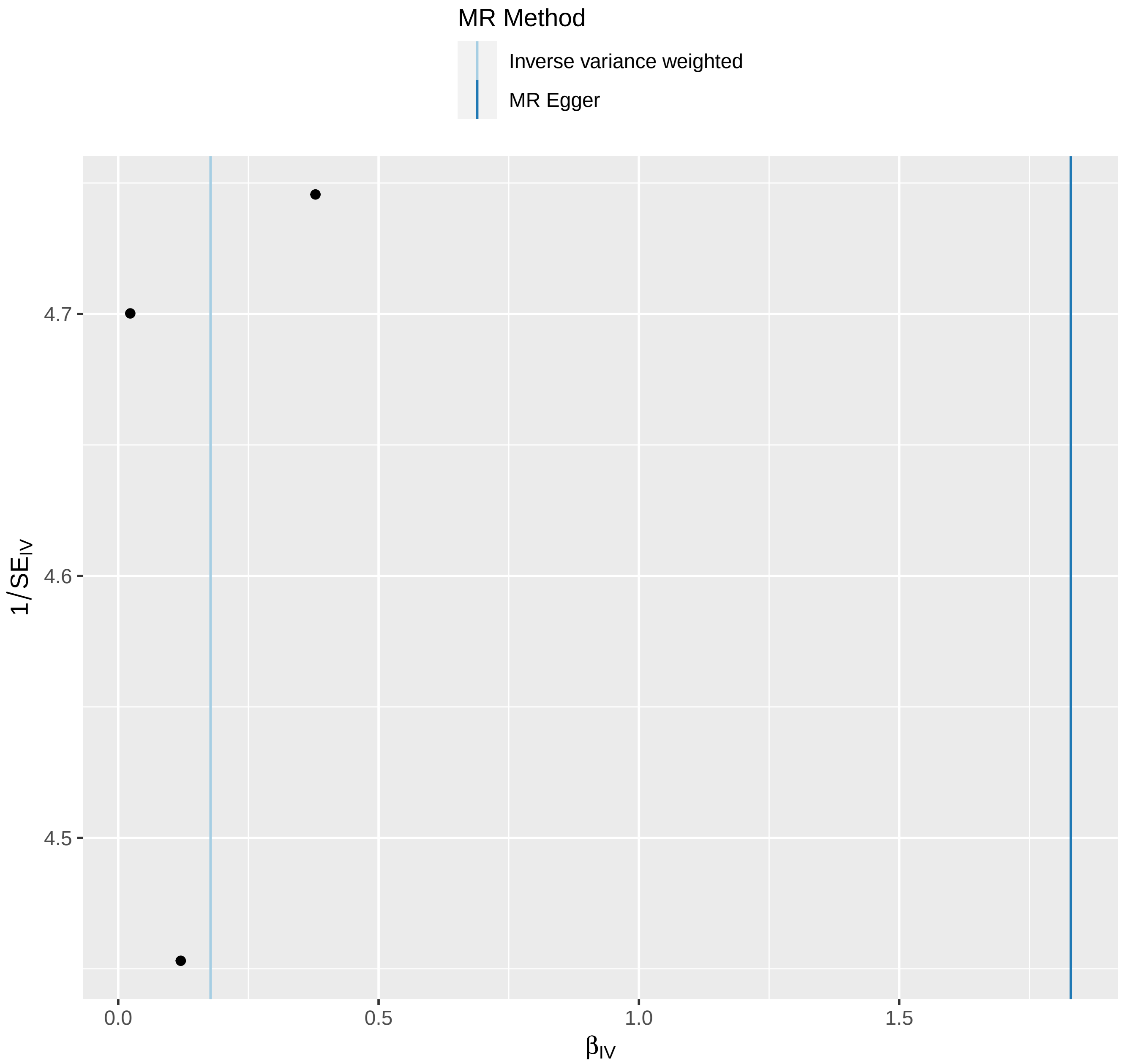

Batch 2−5004 : Gut microbiota abundance (genus Parasutterella id.2892) on Probable RNA−binding protein 23
with a threshold of 1e−05

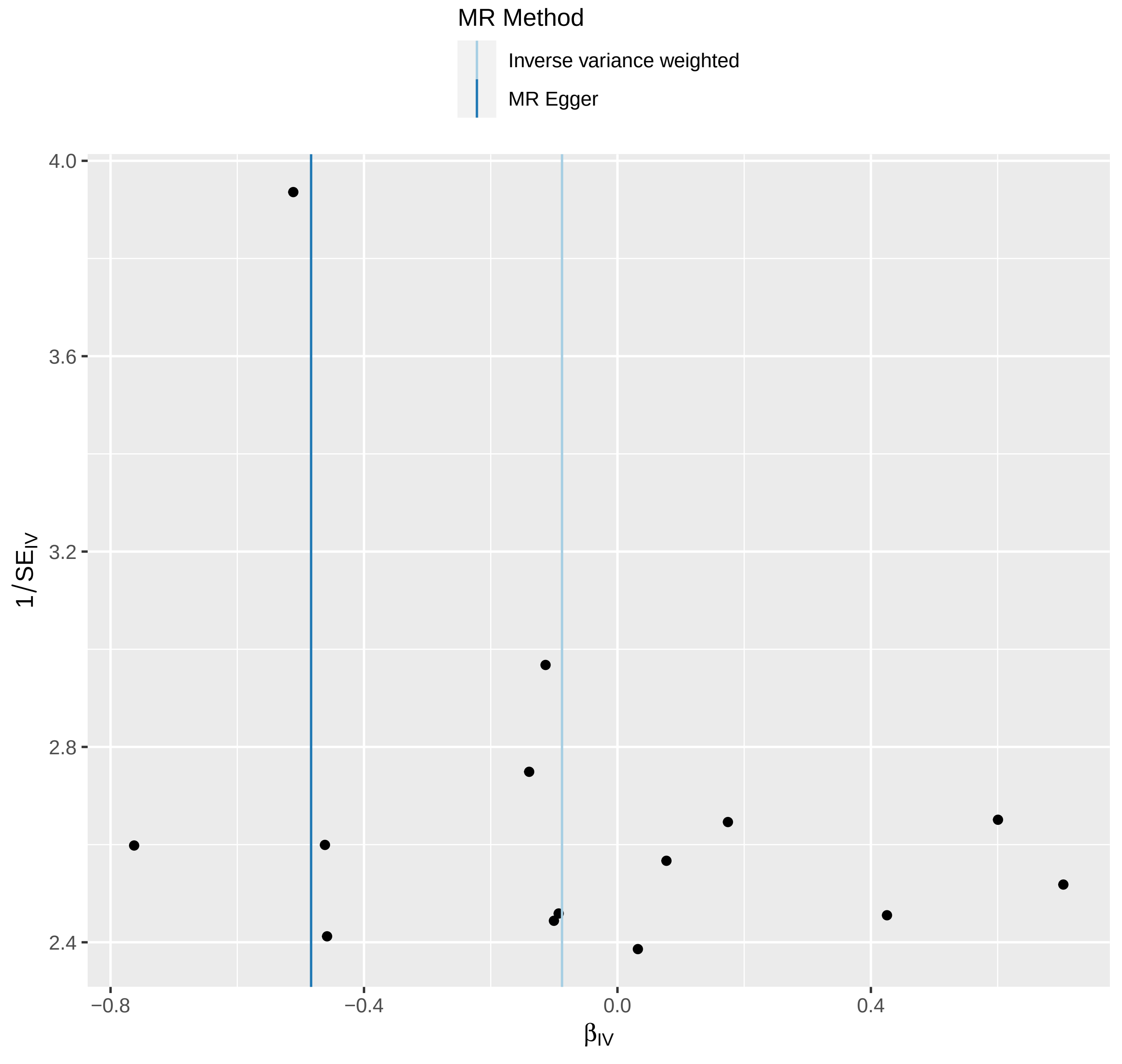

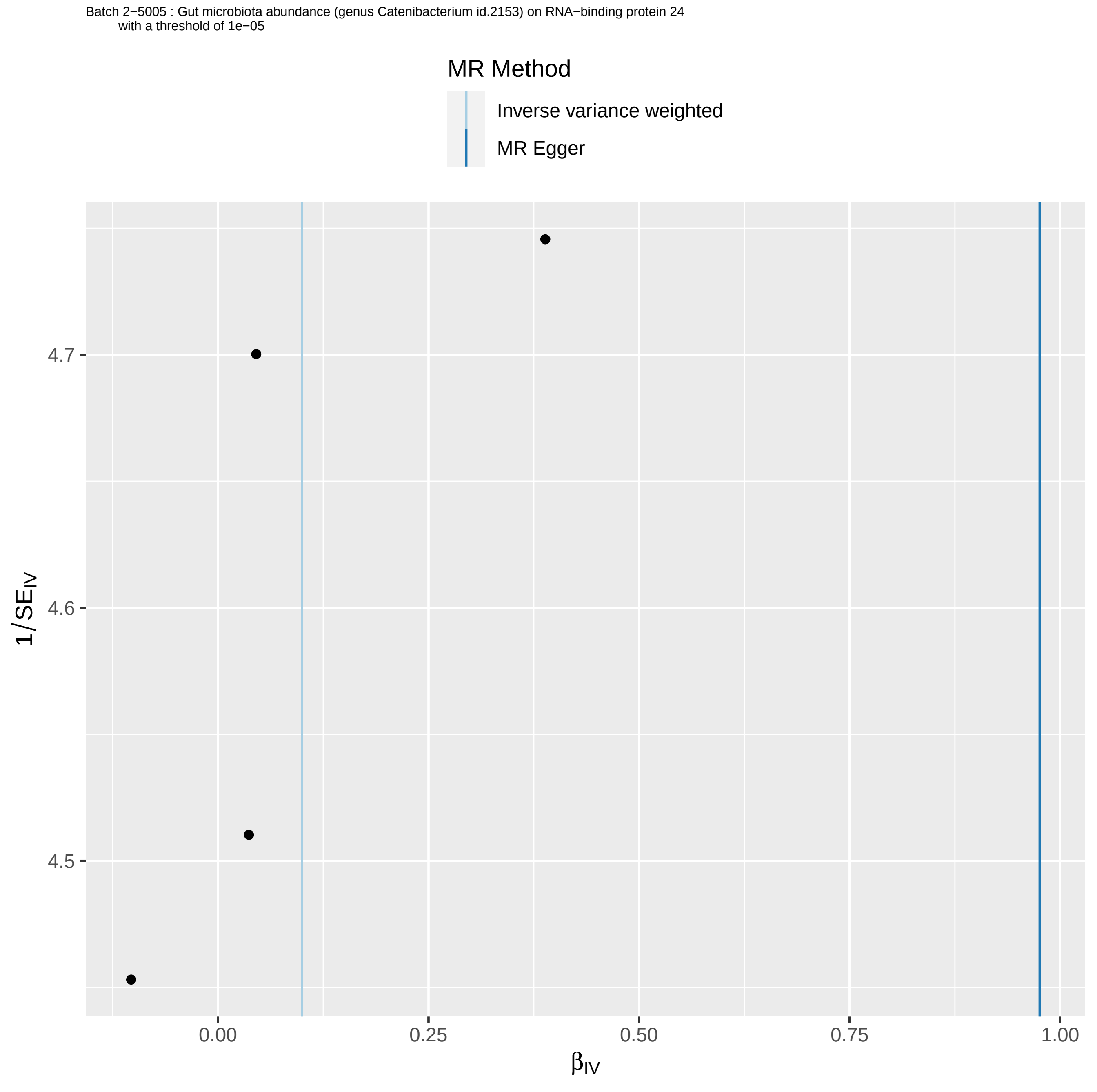

Batch 2−5005 : Gut microbiota abundance (genus Catenibacterium id.2153) on RNA−binding protein 24
with a threshold of 1e−05
MR Method
Inverse variance weighted
MR Egger
4.7
4.6
4.5
1/SE_IV
0.00
0.25
0.50
0.75
1.00
β_IV

Batch 2−5006 : Gut microbiota abundance (genus Parasutterella id.2892) on RNA−binding protein 24
with a threshold of 1e−05

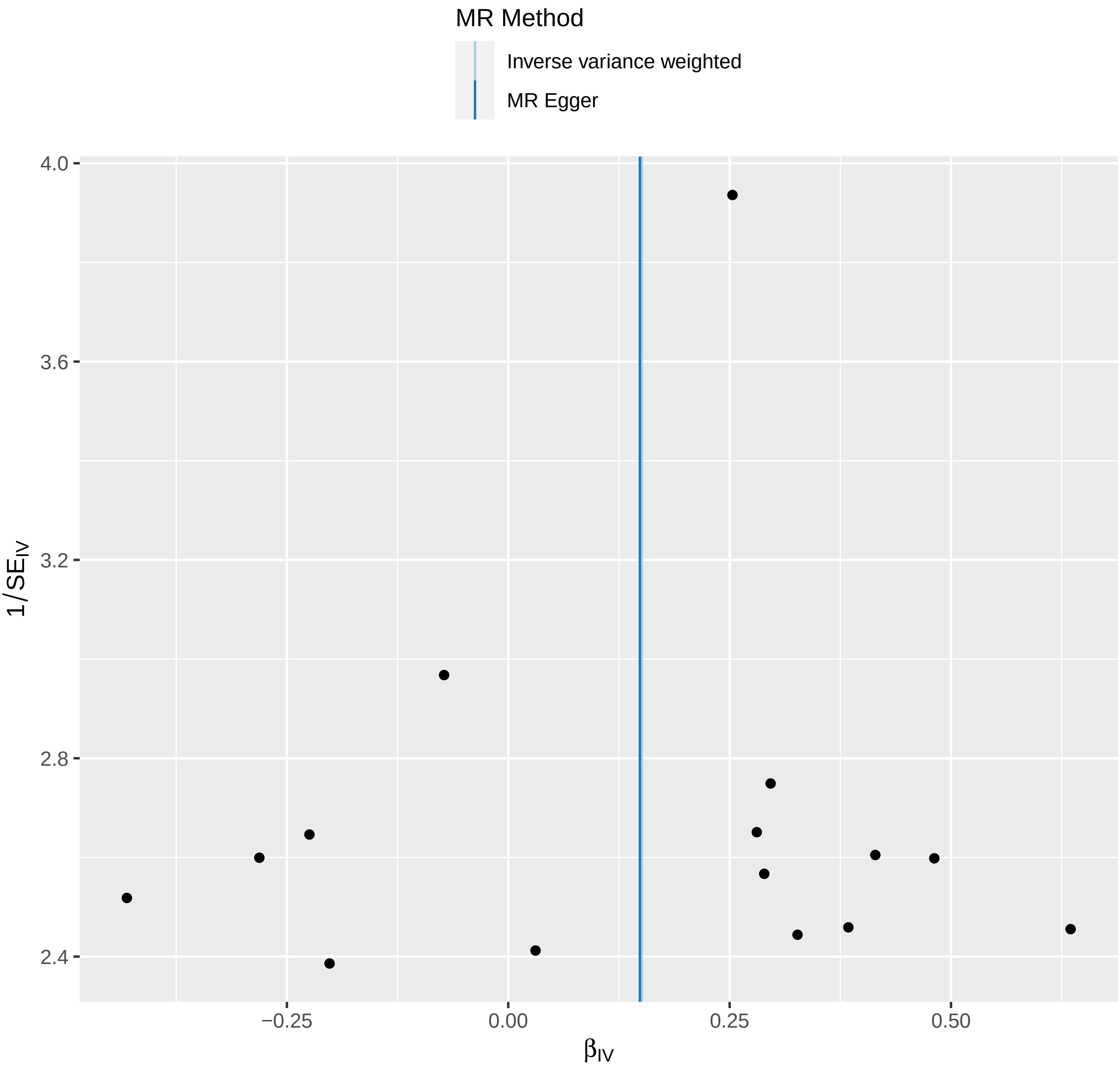

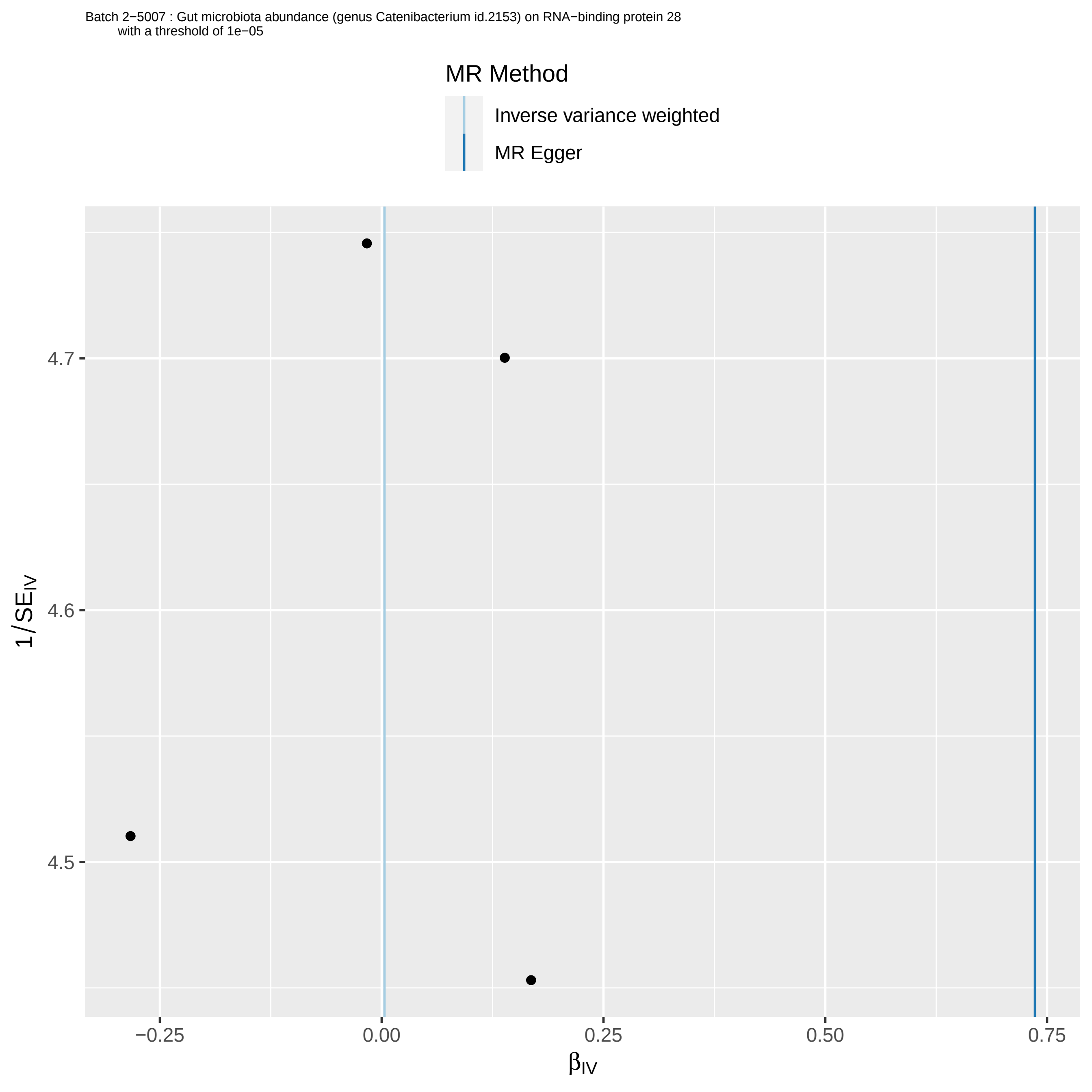

Batch 2−5007 : Gut microbiota abundance (genus Catenibacterium id.2153) on RNA−binding protein 28
with a threshold of 1e−05
MR Method
Inverse variance weighted
MR Egger
4.7
4.6
4.5
1/SE_IV
−0.25
0.00
0.25
0.50
0.75
β_IV

Batch 2−5008 : Gut microbiota abundance (genus Parasutterella id.2892) on RNA−binding protein 28
with a threshold of 1e−05

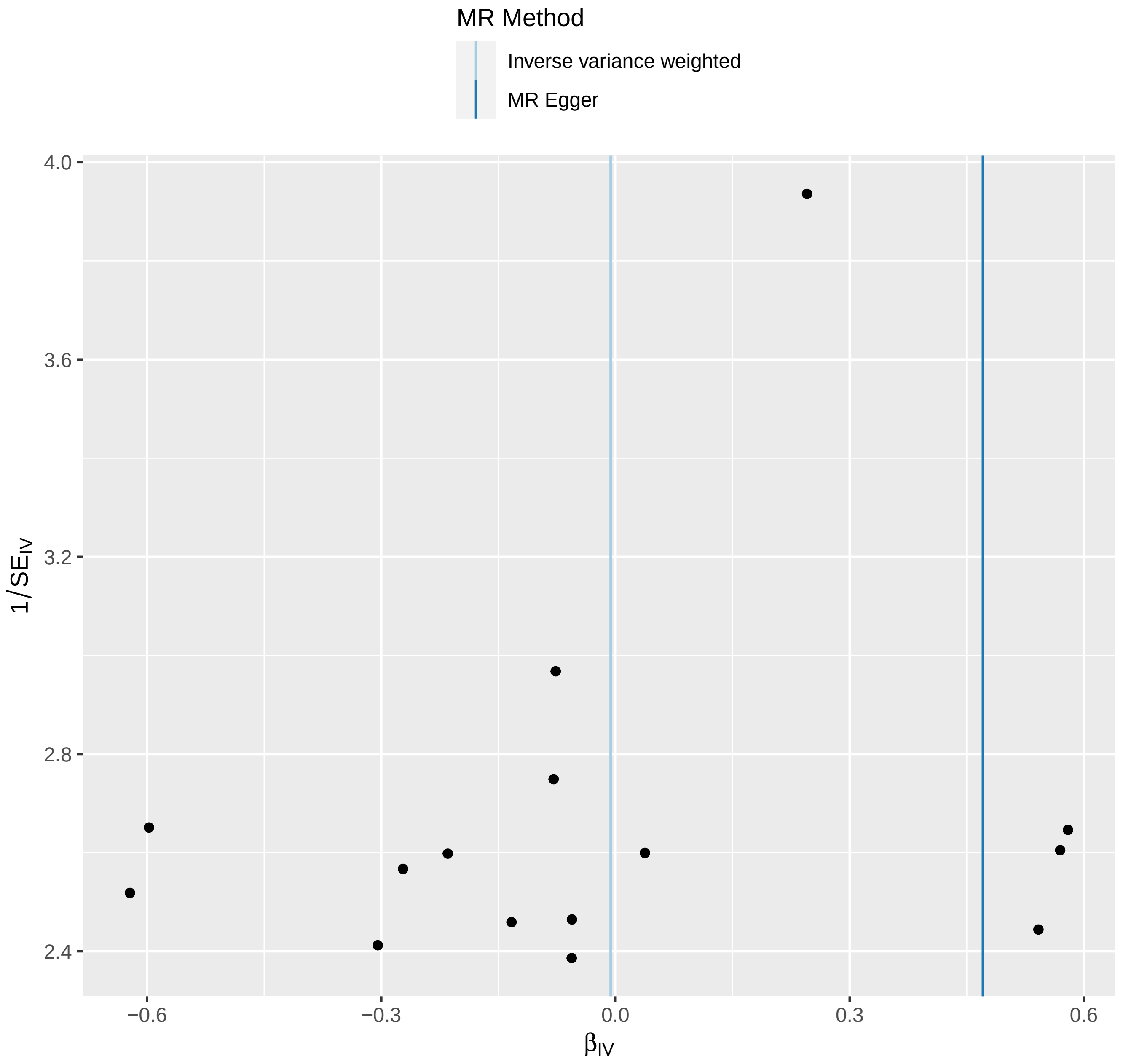

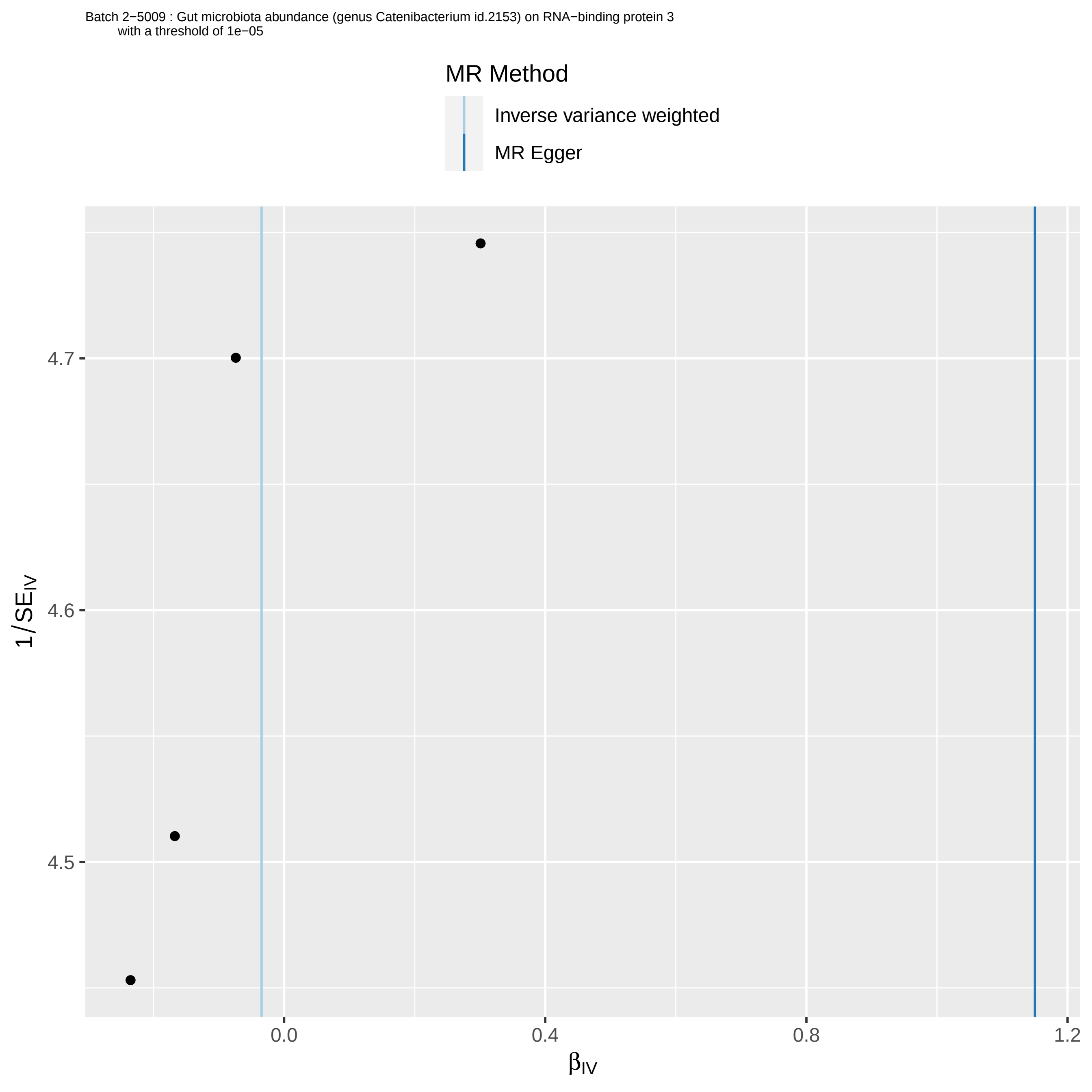
Batch 2−5009 : Gut microbiota abundance (genus Catenibacterium id.2153) on RNA−binding protein 3
with a threshold of 1e−05
MR Method
Inverse variance weighted
MR Egger
4.7
4.6
4.5
1/SE_IV
0.0
0.4
0.8
1.2
β_IV

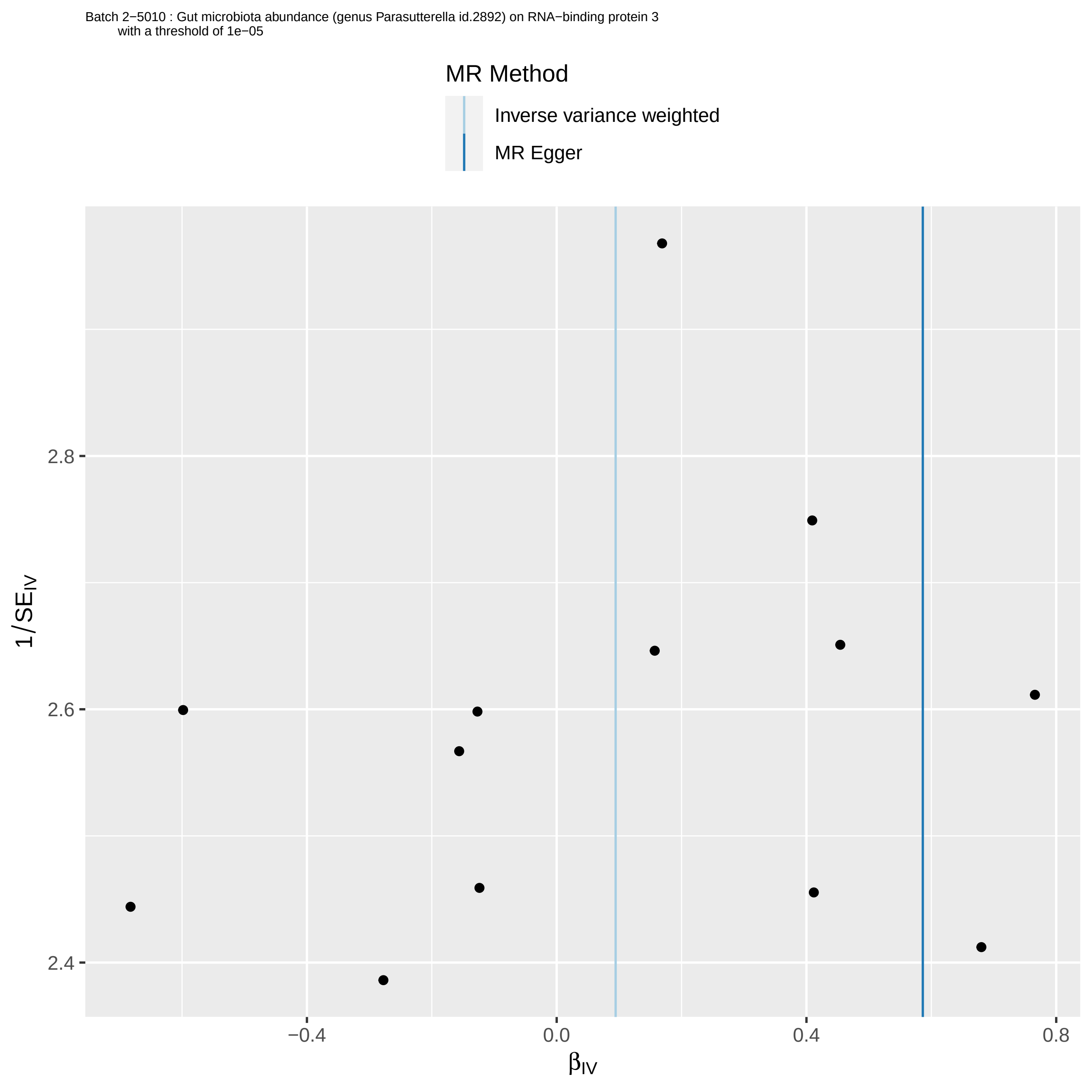
Batch 2−5010 : Gut microbiota abundance (genus Parasutterella id.2892) on RNA−binding protein 3
with a threshold of 1e−05
MR Method
Inverse variance weighted
MR Egger
2.8
2.6
2.4
1/SE$_{IV}$
−0.4
0.0
0.4
0.8
β_{IV}

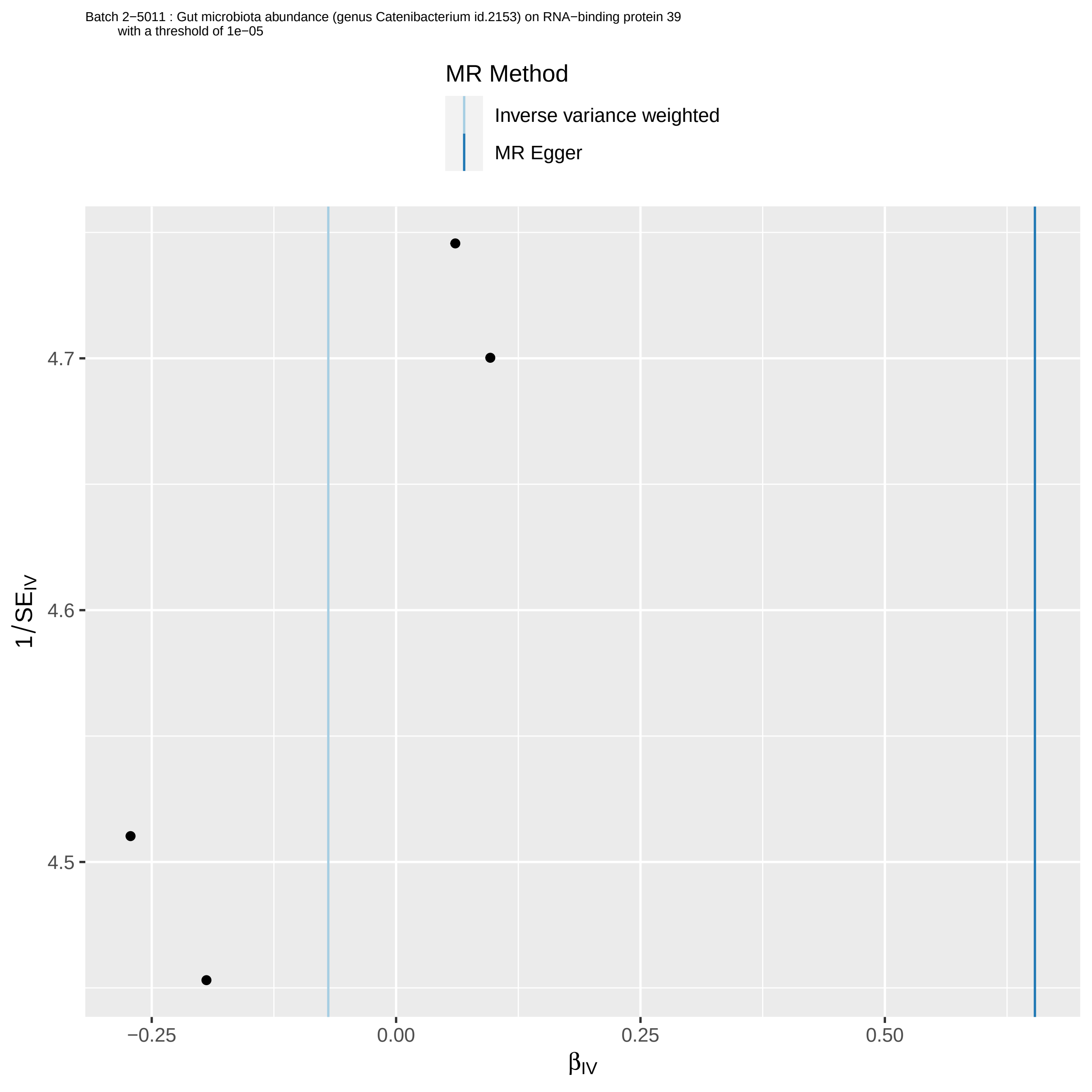
Batch 2−5011 : Gut microbiota abundance (genus Catenibacterium id.2153) on RNA−binding protein 39
with a threshold of 1e−05
MR Method
Inverse variance weighted
MR Egger
4.7
4.6
4.5
1/SE_IV
−0.25
0.00
0.25
0.50
β_IV

Batch 2−5012 : Gut microbiota abundance (genus Parasutterella id.2892) on RNA−binding protein 39
with a threshold of 1e−05

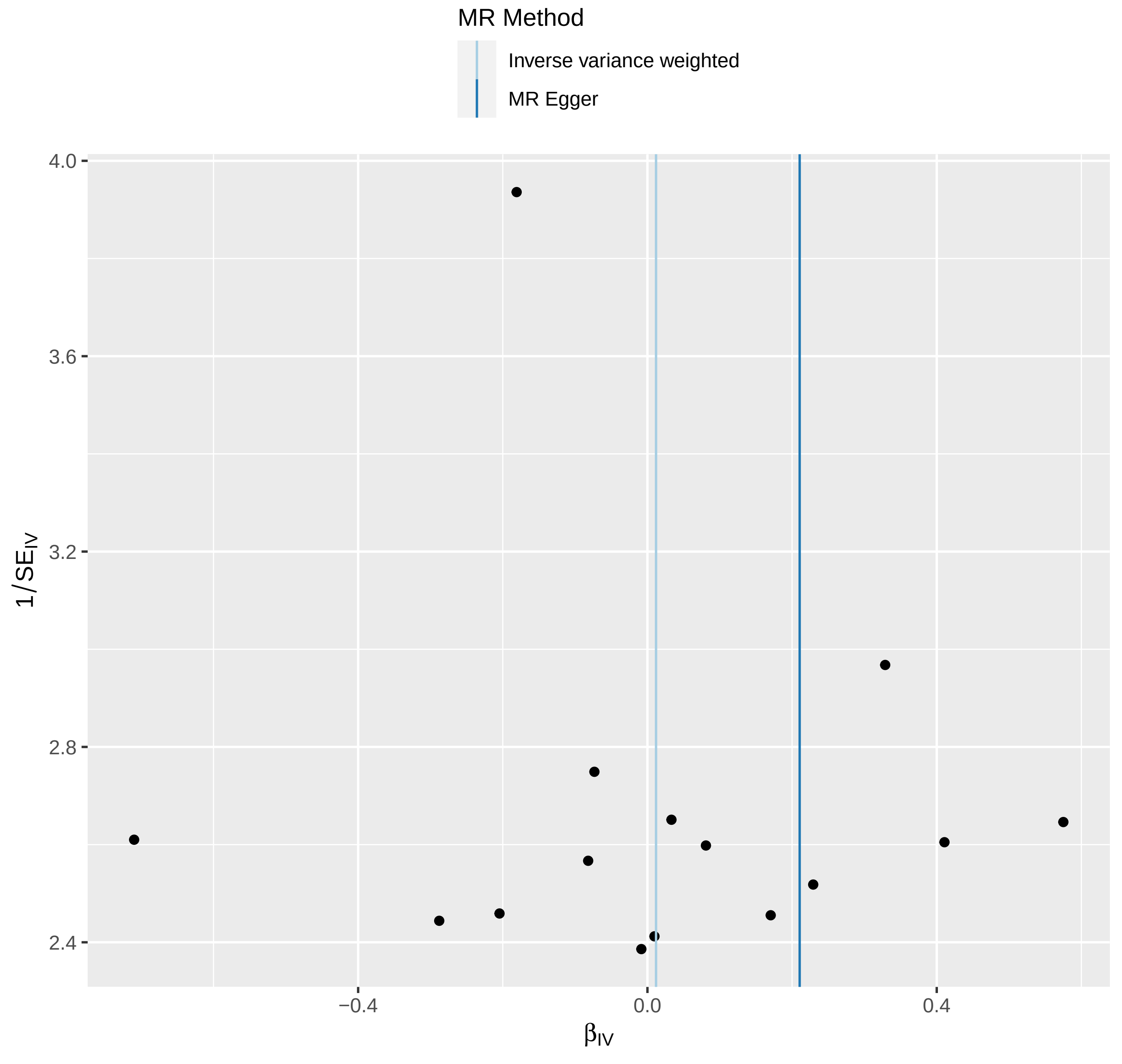

Batch 2−5013 : Gut microbiota abundance (genus Catenibacterium id.2153) on Retinol−binding protein 4
with a threshold of 1e−05

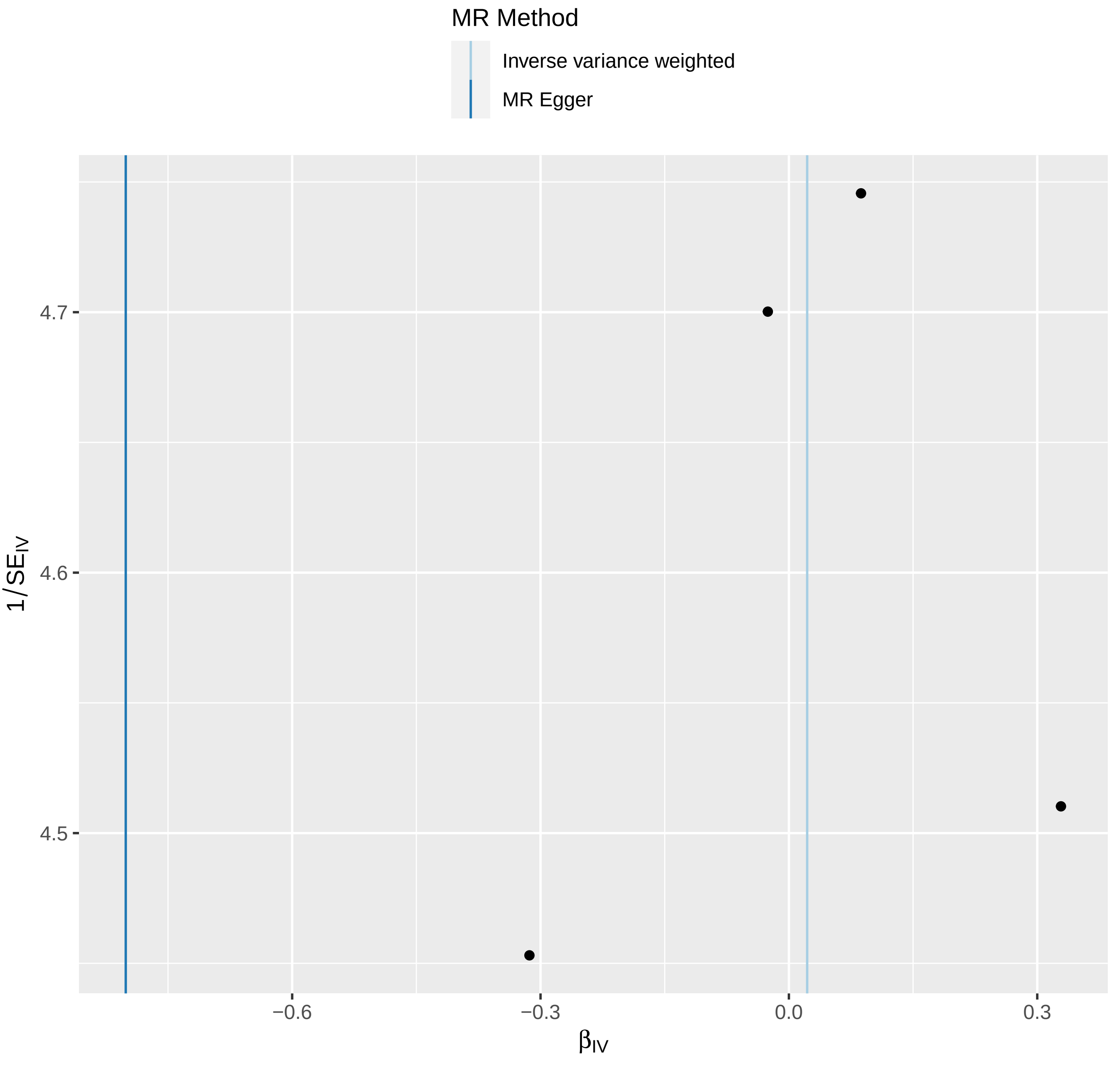

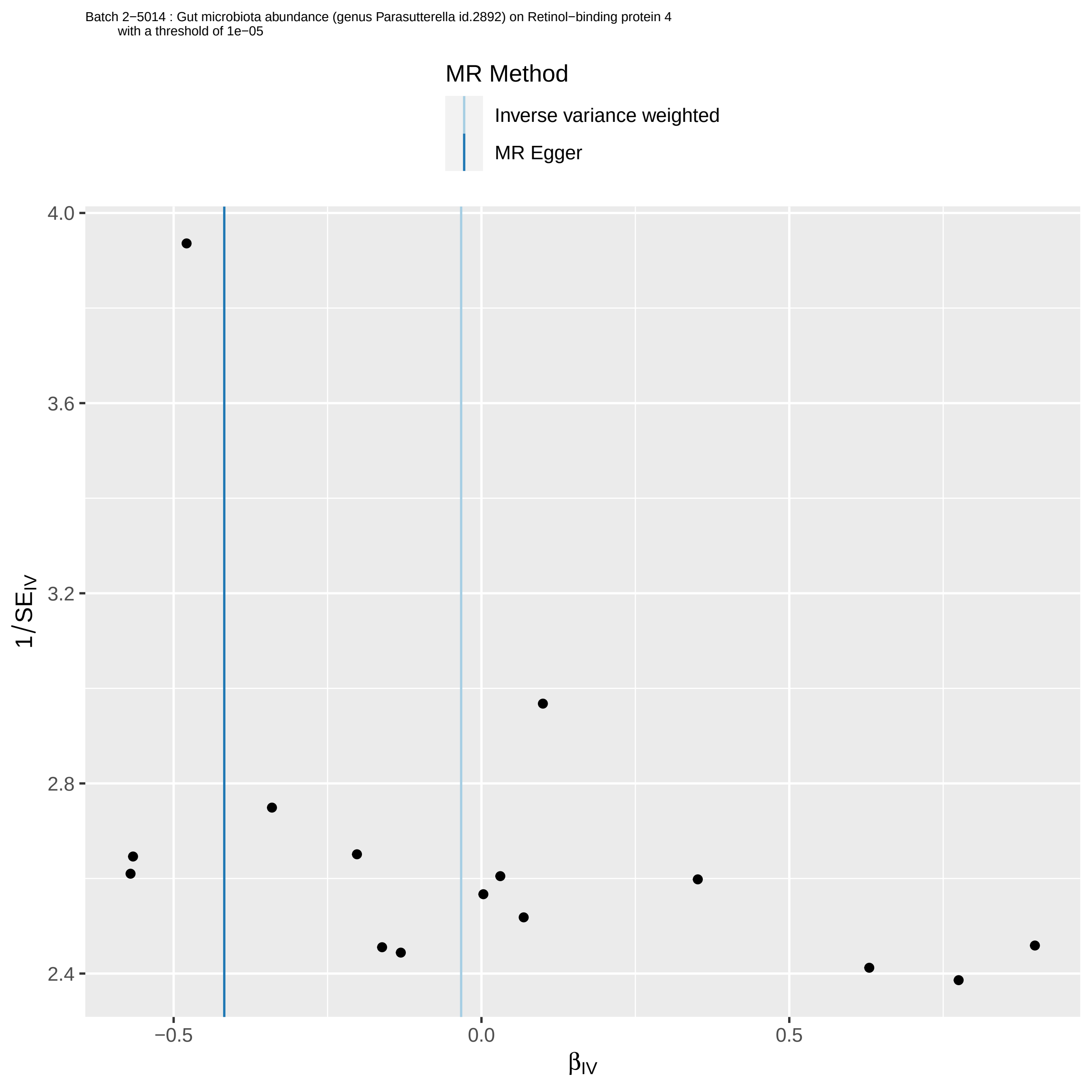
Batch 2−5014 : Gut microbiota abundance (genus Parasutterella id.2892) on Retinol−binding protein 4
with a threshold of 1e−05
MR Method
Inverse variance weighted
MR Egger
4.0
3.6
3.2
2.8
2.4
1/SE_IV
−0.5
0.0
0.5
β_IV

Batch 2−5015 : Gut microbiota abundance (genus Catenibacterium id.2153) on Retinoid−binding protein 7
with a threshold of 1e−05

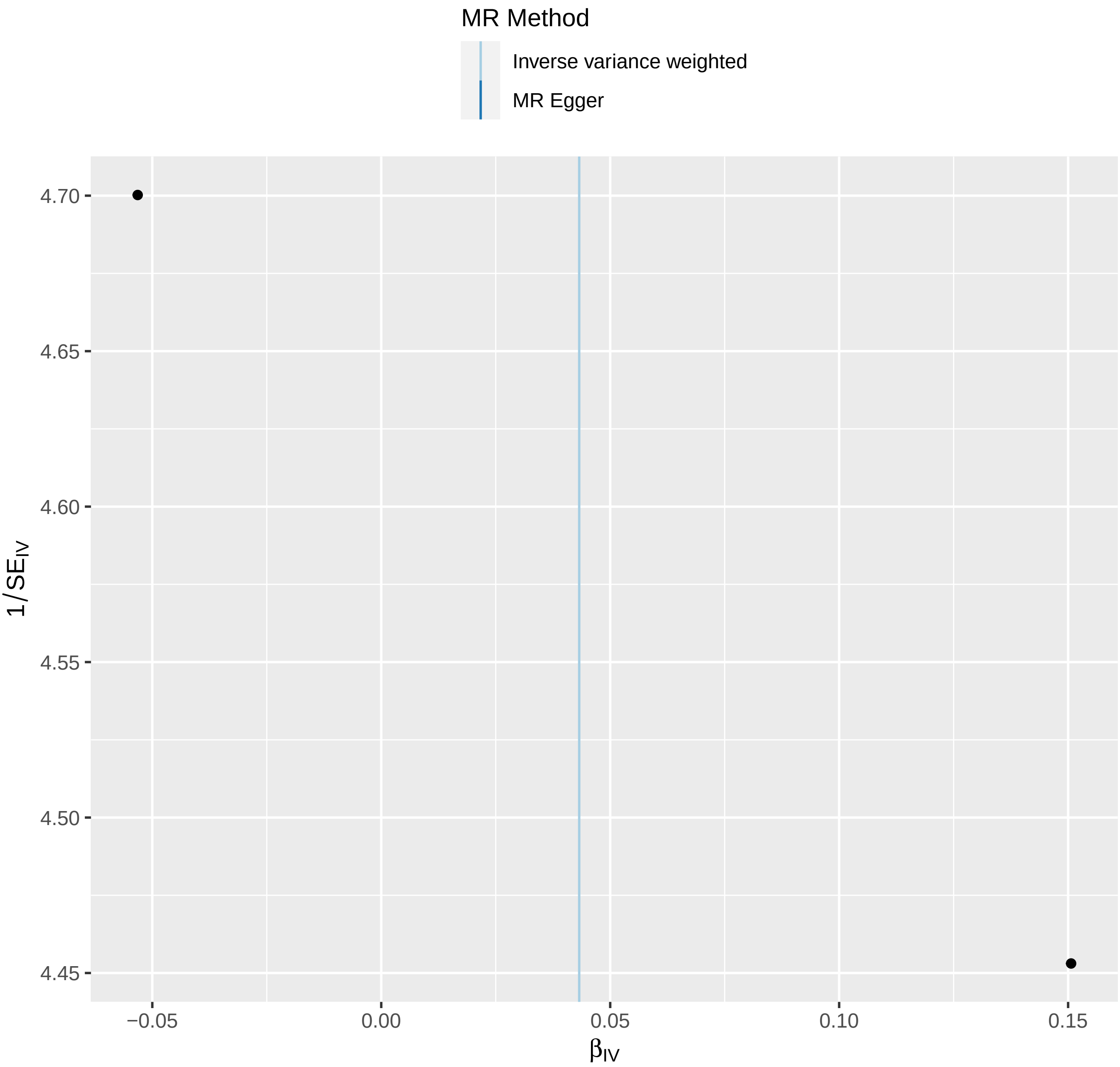

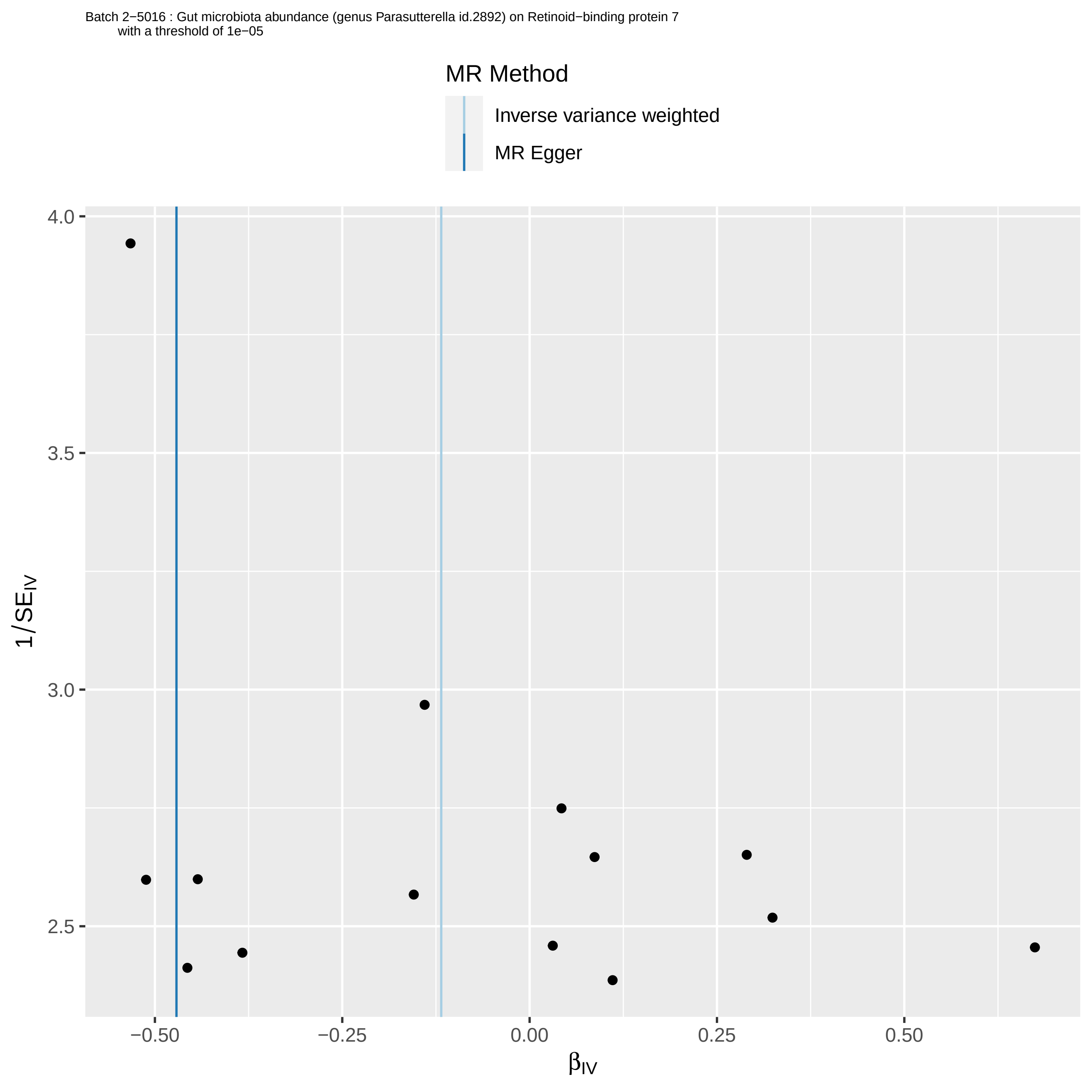

Batch 2−5016 : Gut microbiota abundance (genus Parasutterella id.2892) on Retinoid−binding protein 7
with a threshold of 1e−05
MR Method
Inverse variance weighted
MR Egger
4.0
3.5
3.0
2.5
$1/SE_{IV}$
−0.50
−0.25
0.00
0.25
0.50
β_{IV}

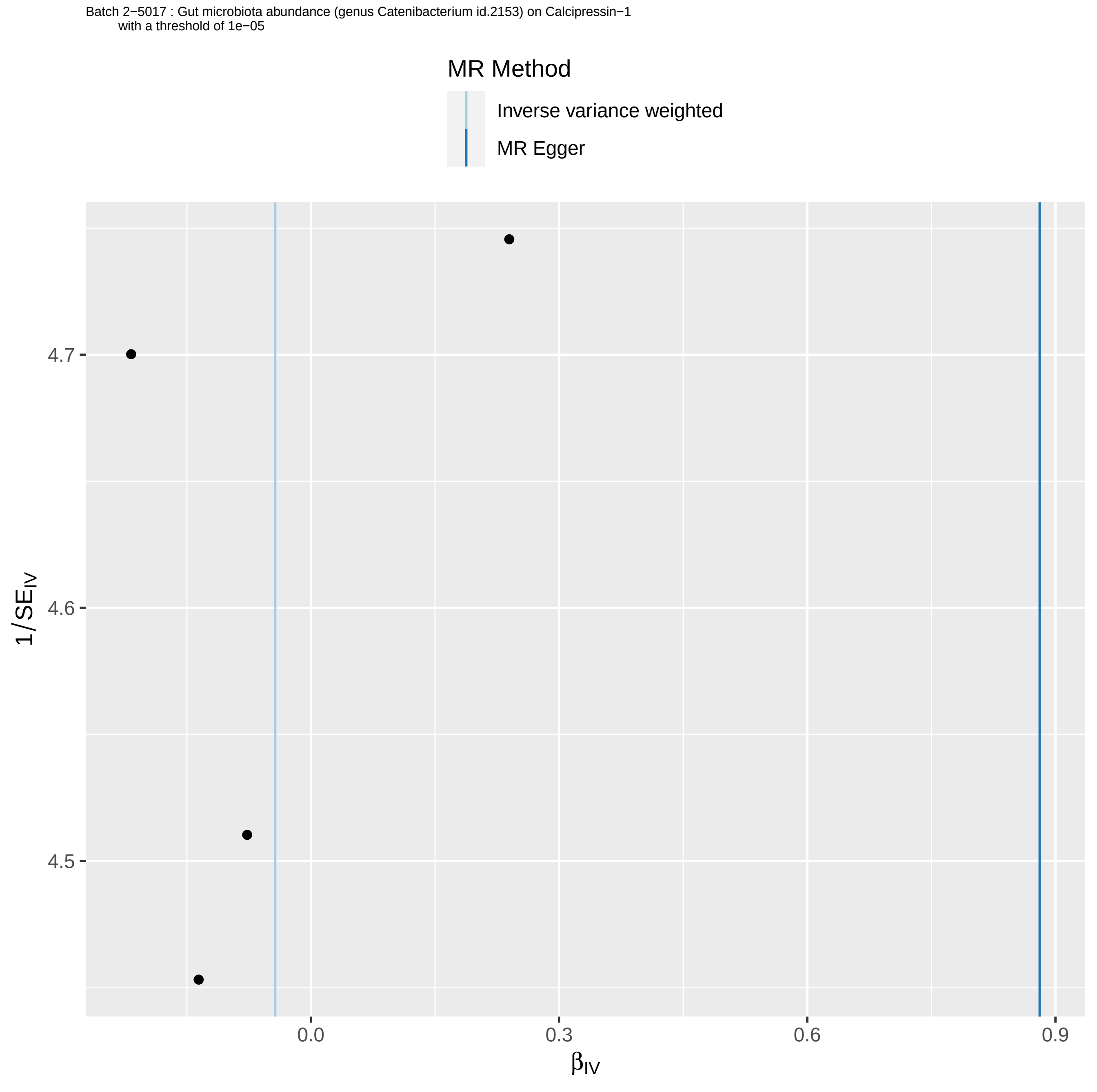
Batch 2−5017 : Gut microbiota abundance (genus Catenibacterium id.2153) on Calcipressin−1
with a threshold of 1e−05
MR Method
Inverse variance weighted
MR Egger
4.7
4.6
4.5
$1/SE_{IV}$
0.0
0.3
0.6
0.9
β_{IV}

Batch 2−5018 : Gut microbiota abundance (genus Parasutterella id.2892) on Calcipressin−1
with a threshold of 1e−05

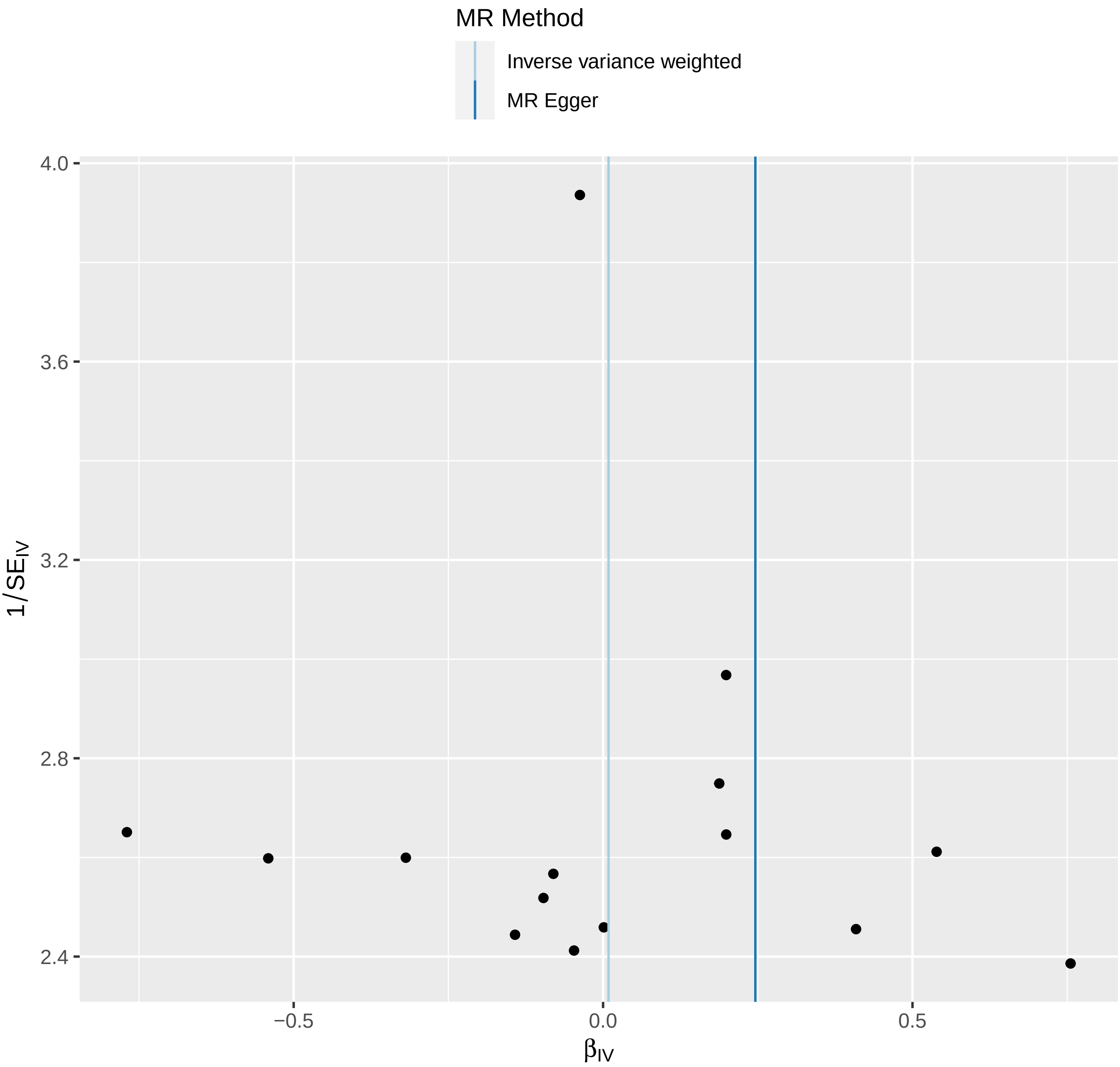

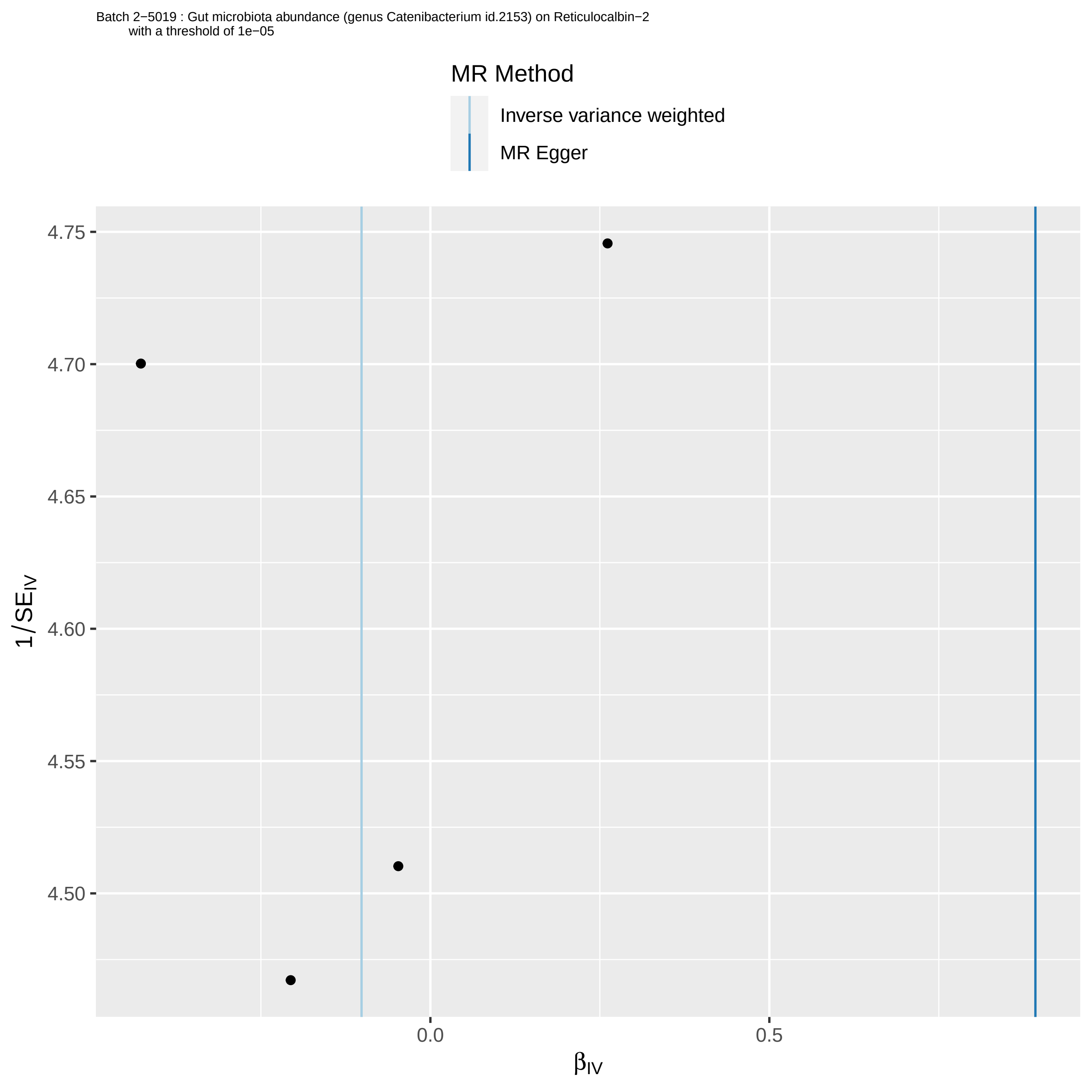
Batch 2−5019 : Gut microbiota abundance (genus Catenibacterium id.2153) on Reticulocalbin−2
with a threshold of 1e−05
MR Method
Inverse variance weighted
MR Egger
4.75
4.70
4.65
4.60
4.55
4.50
1/SE_IV
0.0
0.5
β_IV

Batch 2−5020 : Gut microbiota abundance (genus Parasutterella id.2892) on Reticulocalbin−2
with a threshold of 1e−05

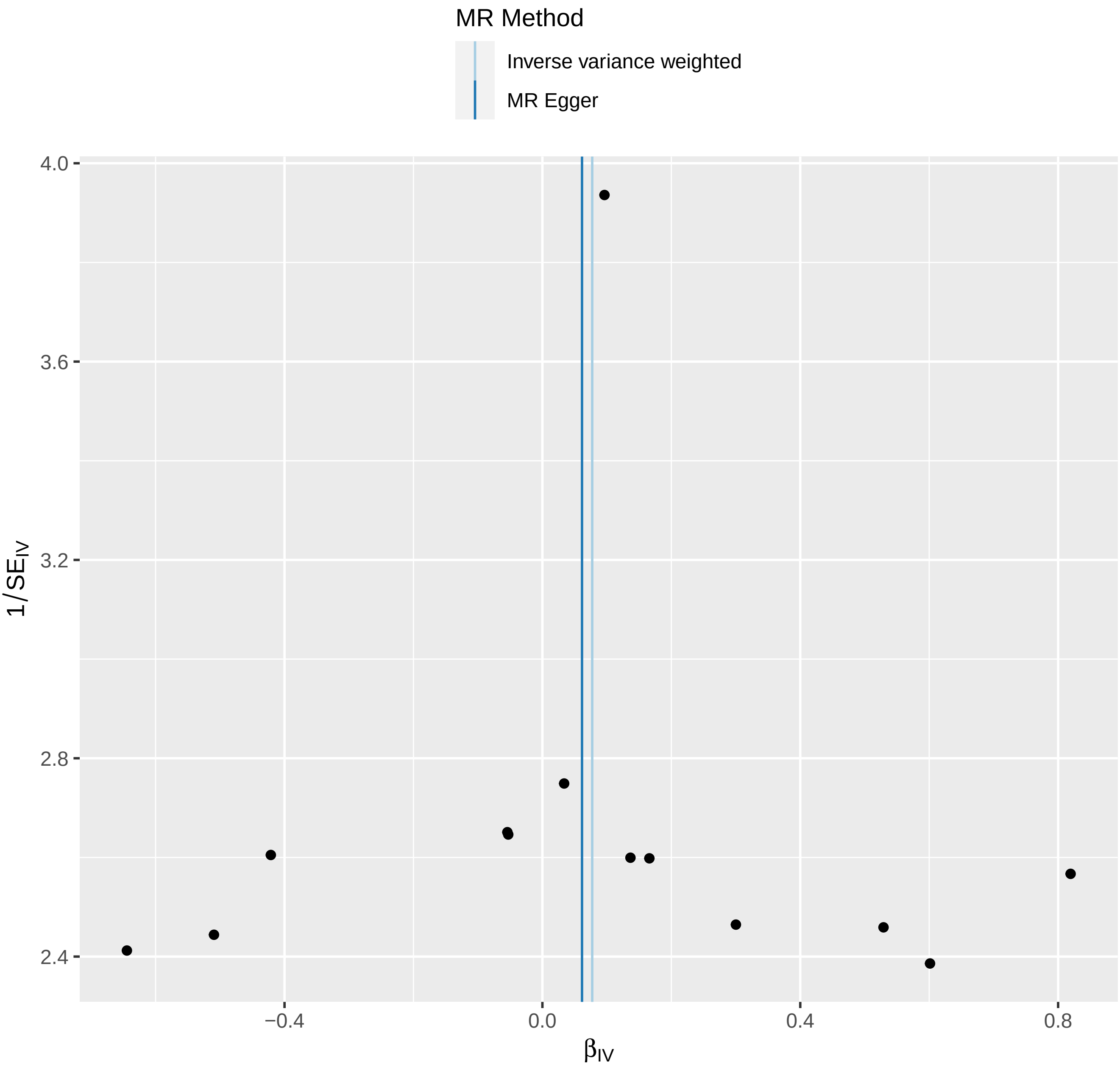

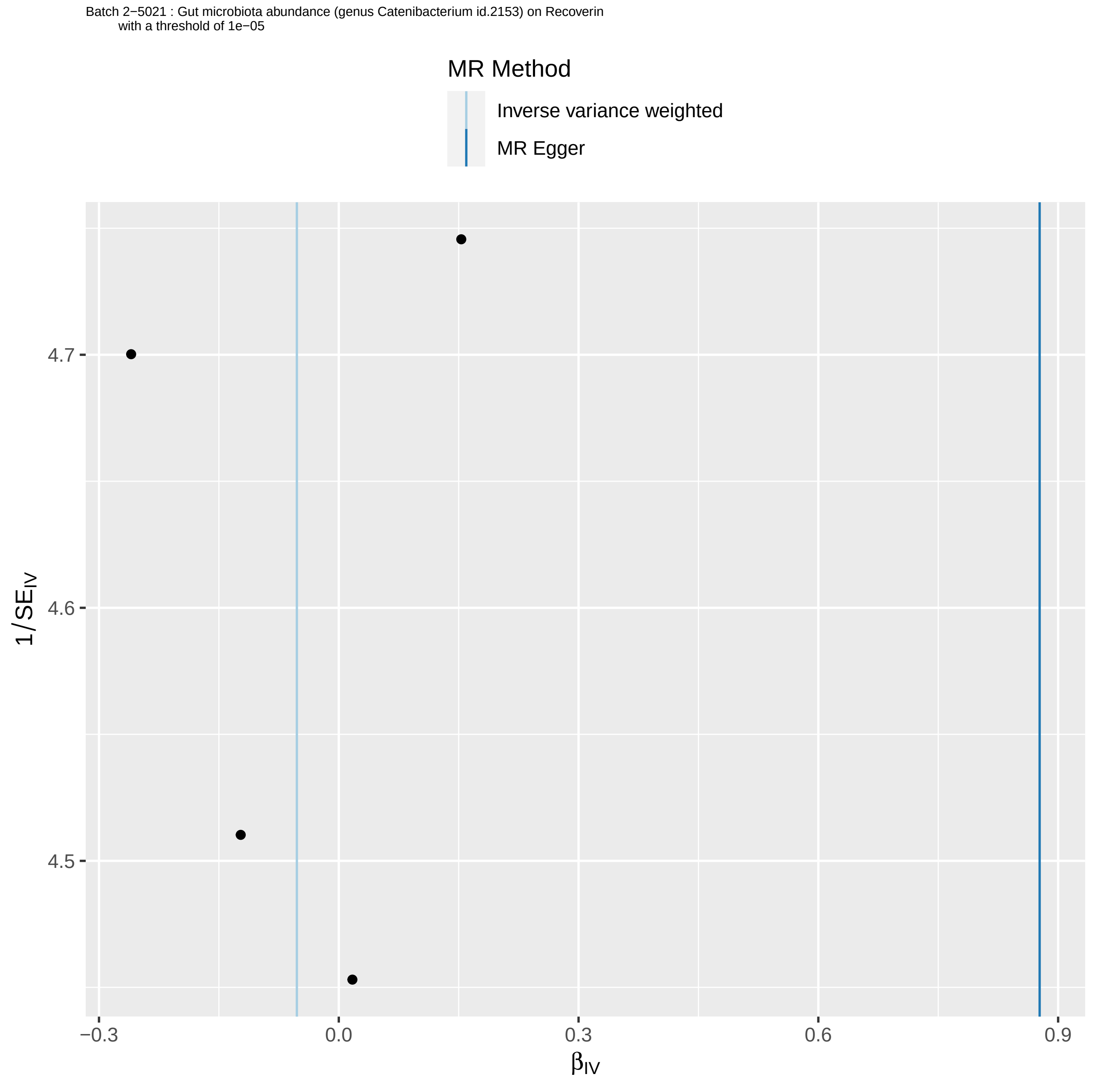

Batch 2−5021 : Gut microbiota abundance (genus Catenibacterium id.2153) on Recoverin
with a threshold of 1e−05
MR Method
Inverse variance weighted
MR Egger
4.7
4.6
4.5
1/SE_IV
−0.3
0.0
0.3
0.6
0.9
β_IV

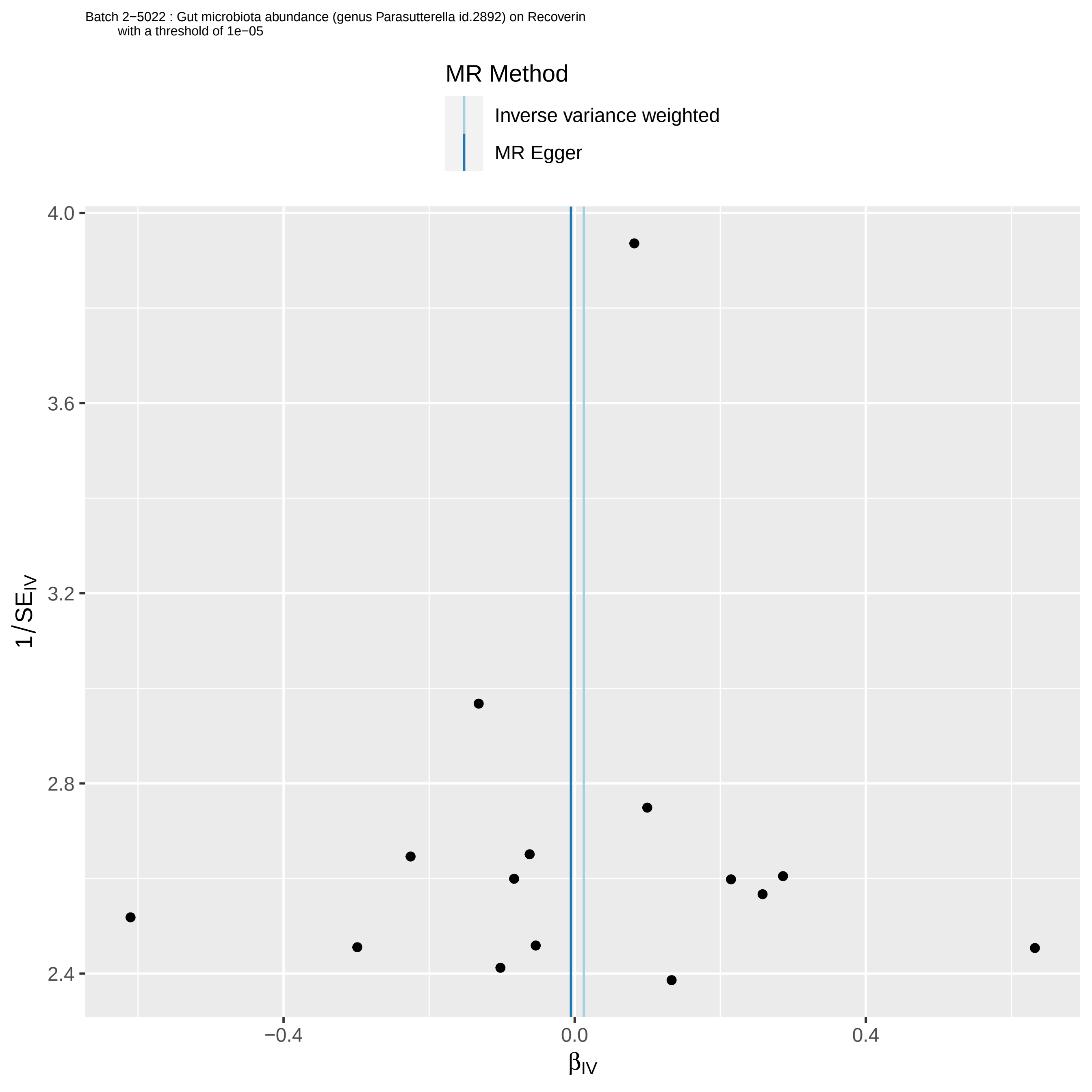
Batch 2−5022 : Gut microbiota abundance (genus Parasutterella id.2892) on Recoverin
with a threshold of 1e−05
MR Method
Inverse variance weighted
MR Egger
4.0
3.6
3.2
2.8
2.4
$1/SE_{IV}$
−0.4
0.0
0.4
β_{IV}

Batch 2−5023 : Gut microbiota abundance (genus Catenibacterium id.2153) on Retinol dehydrogenase 16
with a threshold of 1e−05

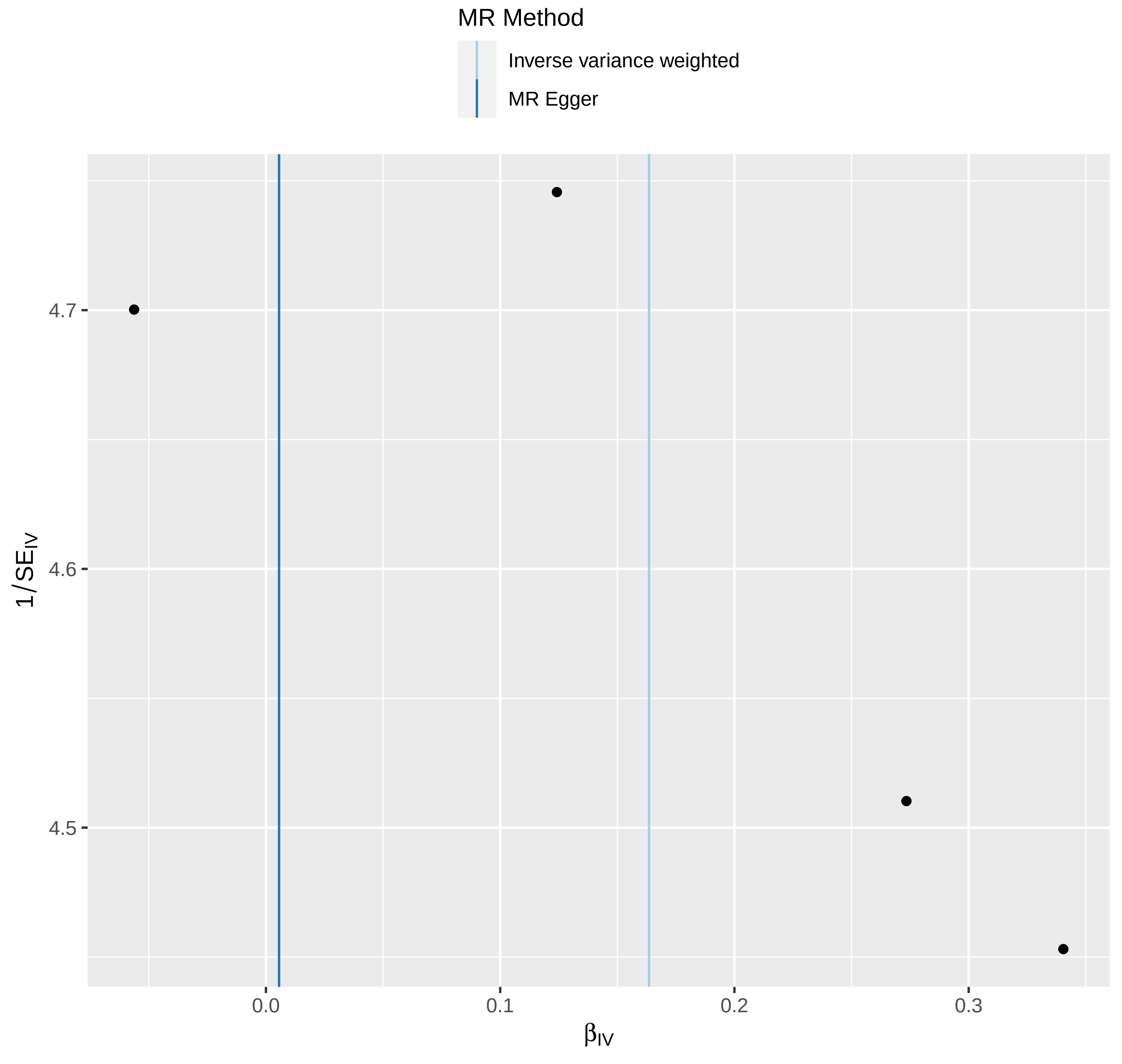

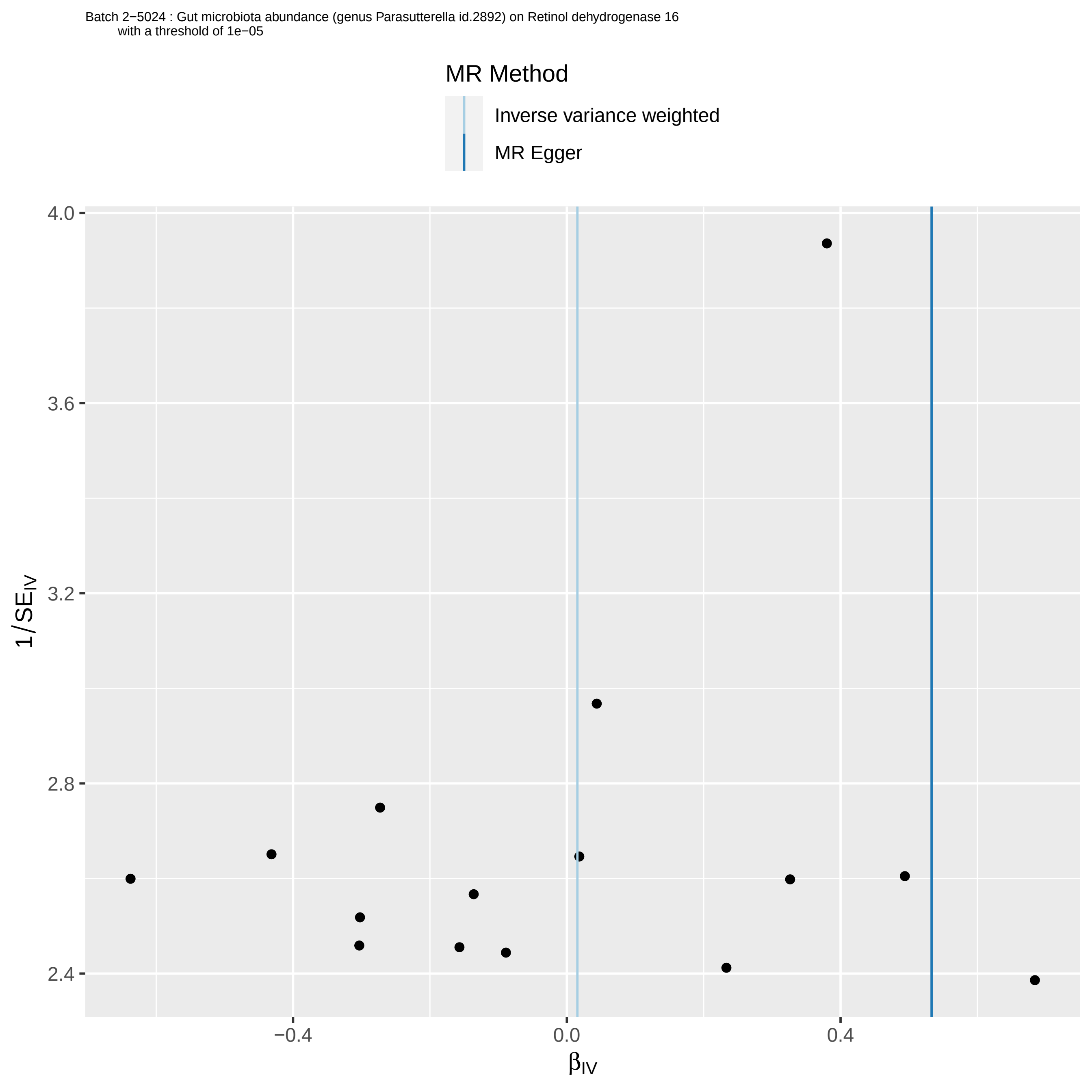
Batch 2−5024 : Gut microbiota abundance (genus Parasutterella id.2892) on Retinol dehydrogenase 16
with a threshold of 1e−05
MR Method
Inverse variance weighted
MR Egger
4.0
3.6
3.2
2.8
2.4
1/SE_IV
−0.4
0.0
0.4
β_IV

Batch 2−5025 : Gut microbiota abundance (genus Catenibacterium id.2153) on ATP−dependent DNA helicase Q1
with a threshold of 1e−05

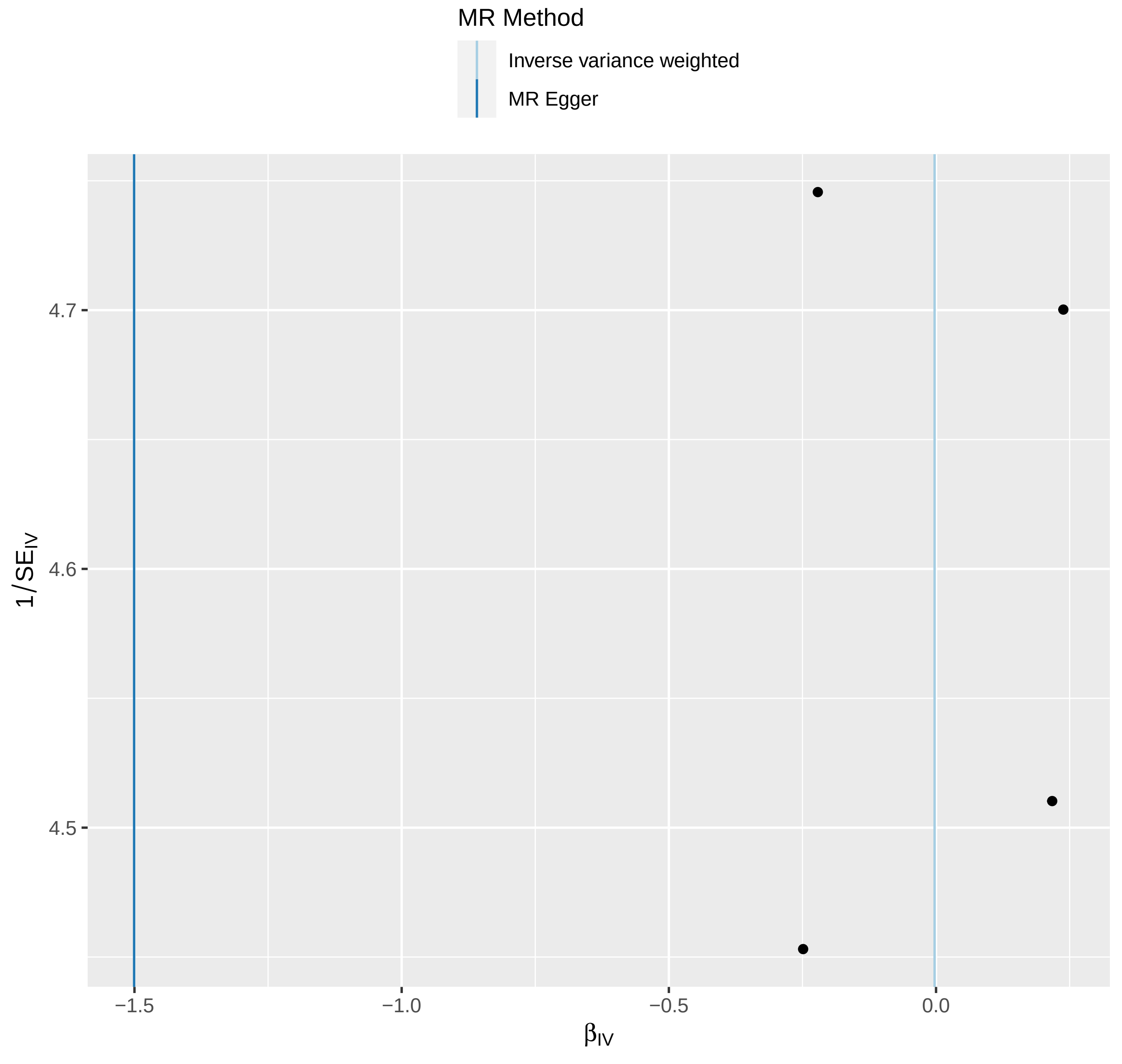

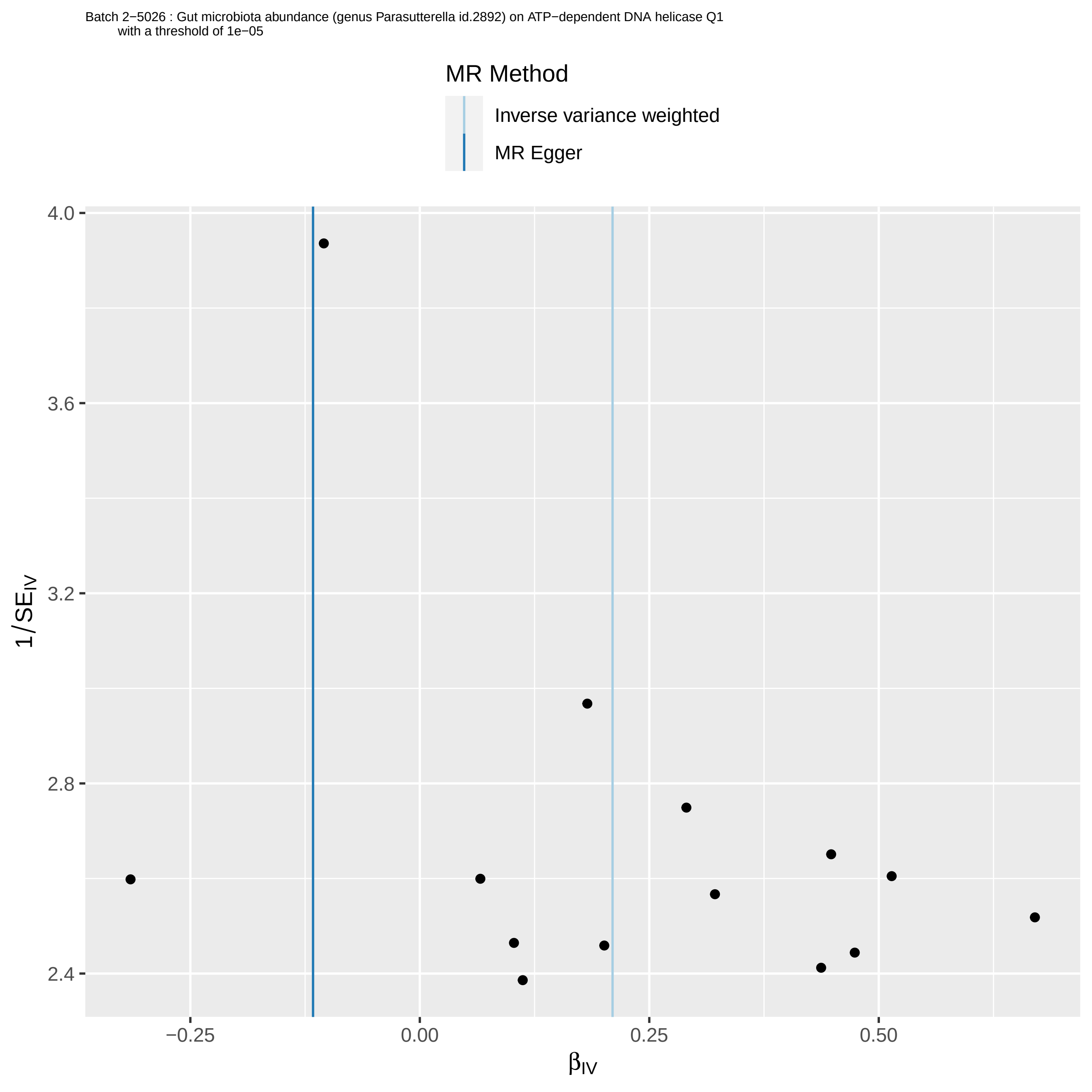

Batch 2−5026 : Gut microbiota abundance (genus Parasutterella id.2892) on ATP−dependent DNA helicase Q1
with a threshold of 1e−05
MR Method
Inverse variance weighted
MR Egger
4.0
3.6
3.2
2.8
2.4
1/SE_IV
−0.25
0.00
0.25
0.50
β_IV

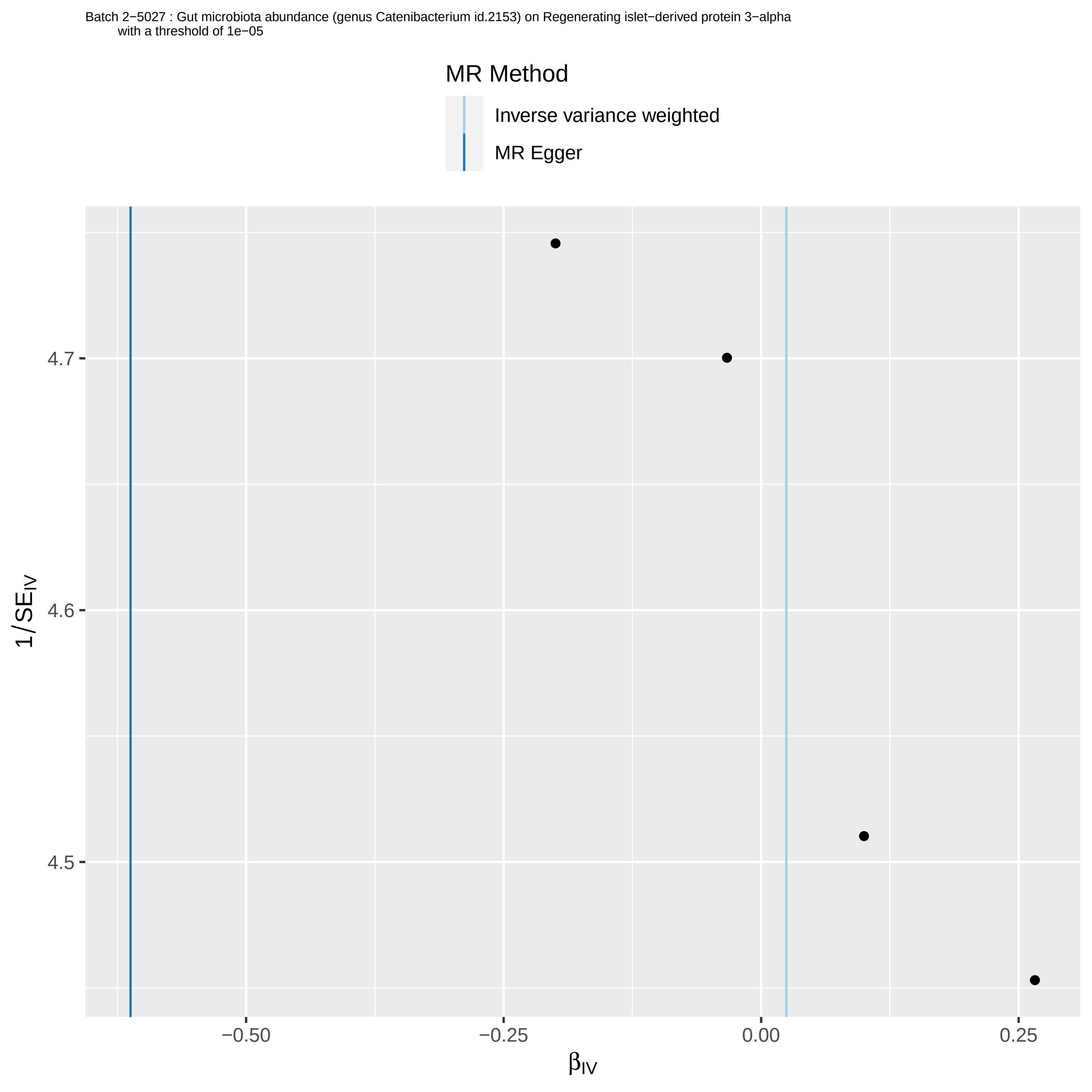

Batch 2−5027 : Gut microbiota abundance (genus Catenibacterium id.2153) on Regenerating islet−derived protein 3−alpha
with a threshold of 1e−05
MR Method
Inverse variance weighted
MR Egger
4.7
4.6
4.5
1/SE_IV
−0.50
−0.25
0.00
0.25
β_IV

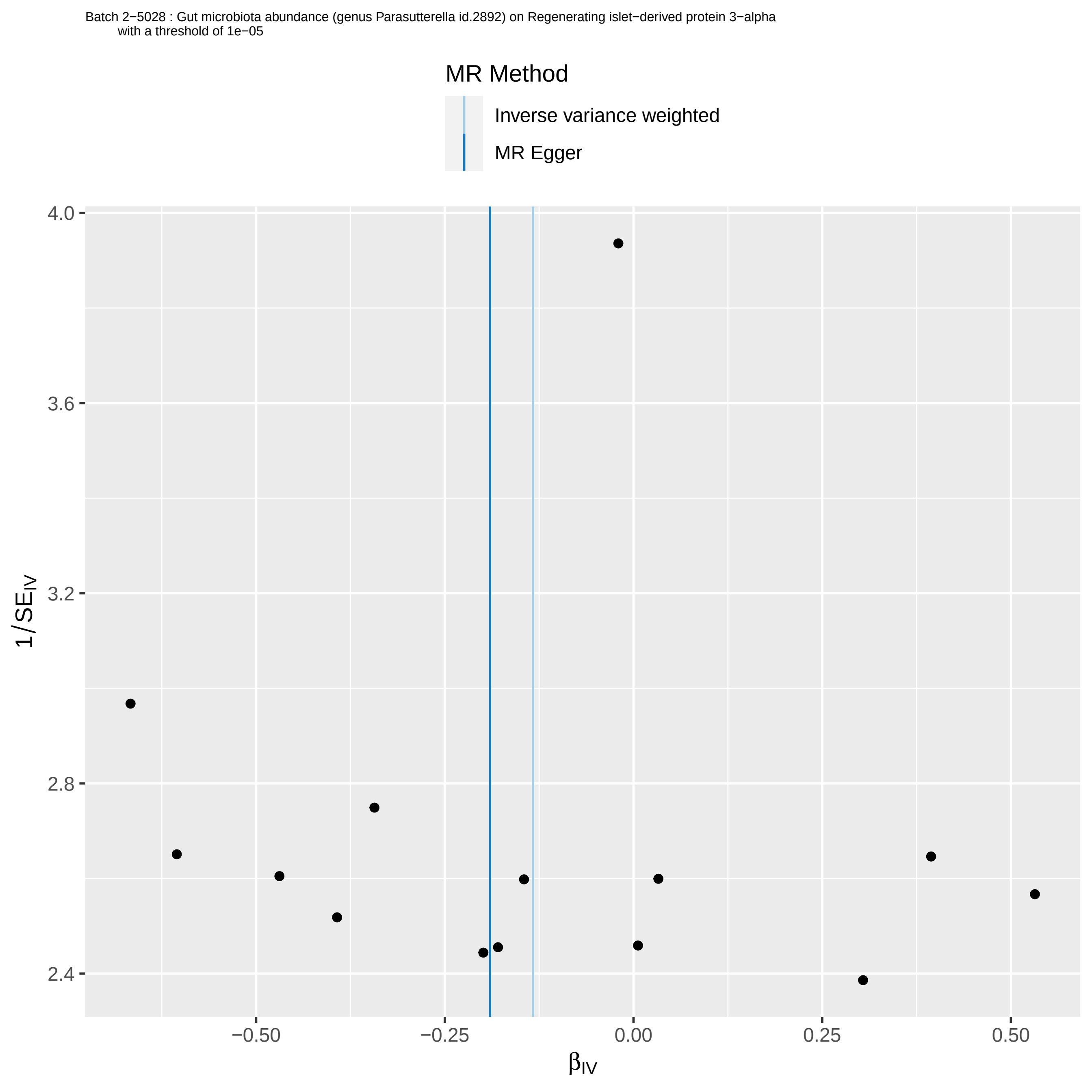

Batch 2−5028 : Gut microbiota abundance (genus Parasutterella id.2892) on Regenerating islet−derived protein 3−alpha
with a threshold of 1e−05
MR Method
Inverse variance weighted
MR Egger
4.0
3.6
3.2
2.8
2.4
1/SE_IV
−0.50
−0.25
0.00
0.25
0.50
β_IV

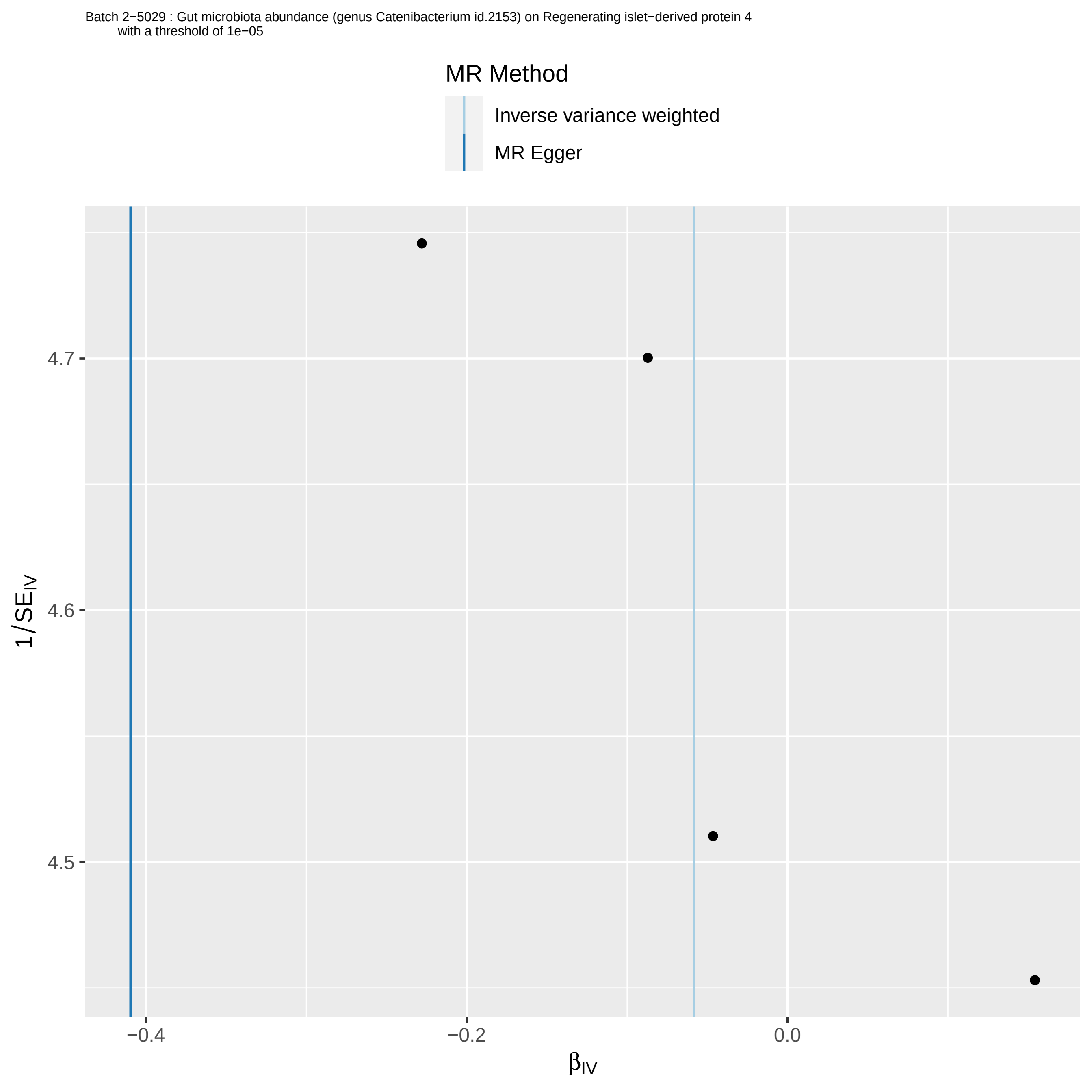
Batch 2−5029 : Gut microbiota abundance (genus Catenibacterium id.2153) on Regenerating islet−derived protein 4
with a threshold of 1e−05
MR Method
Inverse variance weighted
MR Egger
4.7
4.6
4.5
1/SE$_{IV}$
−0.4
−0.2
0.0
β_{IV}

Batch 2−5030 : Gut microbiota abundance (genus Parasutterella id.2892) on Regenerating islet−derived protein 4
with a threshold of 1e−05

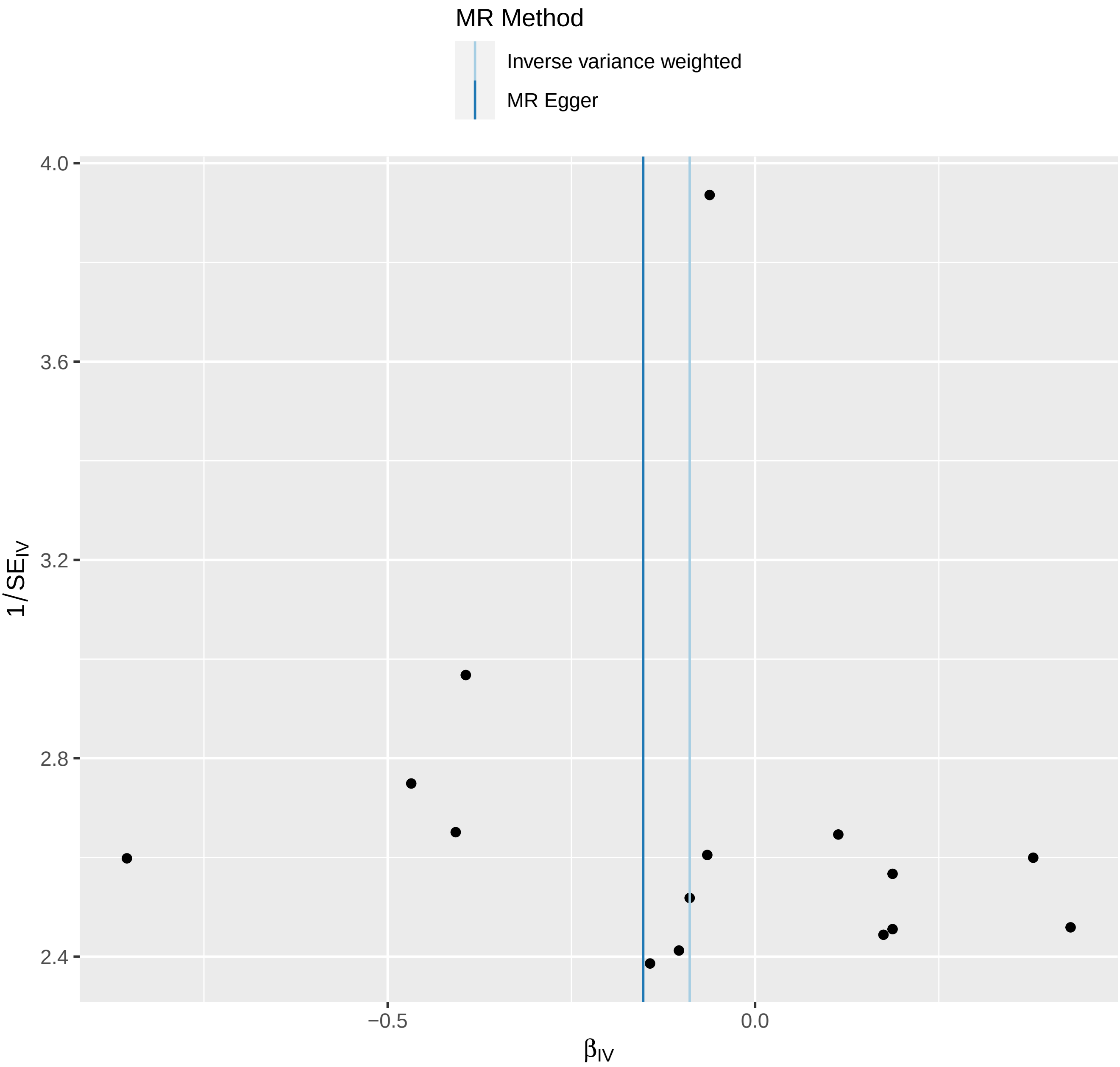

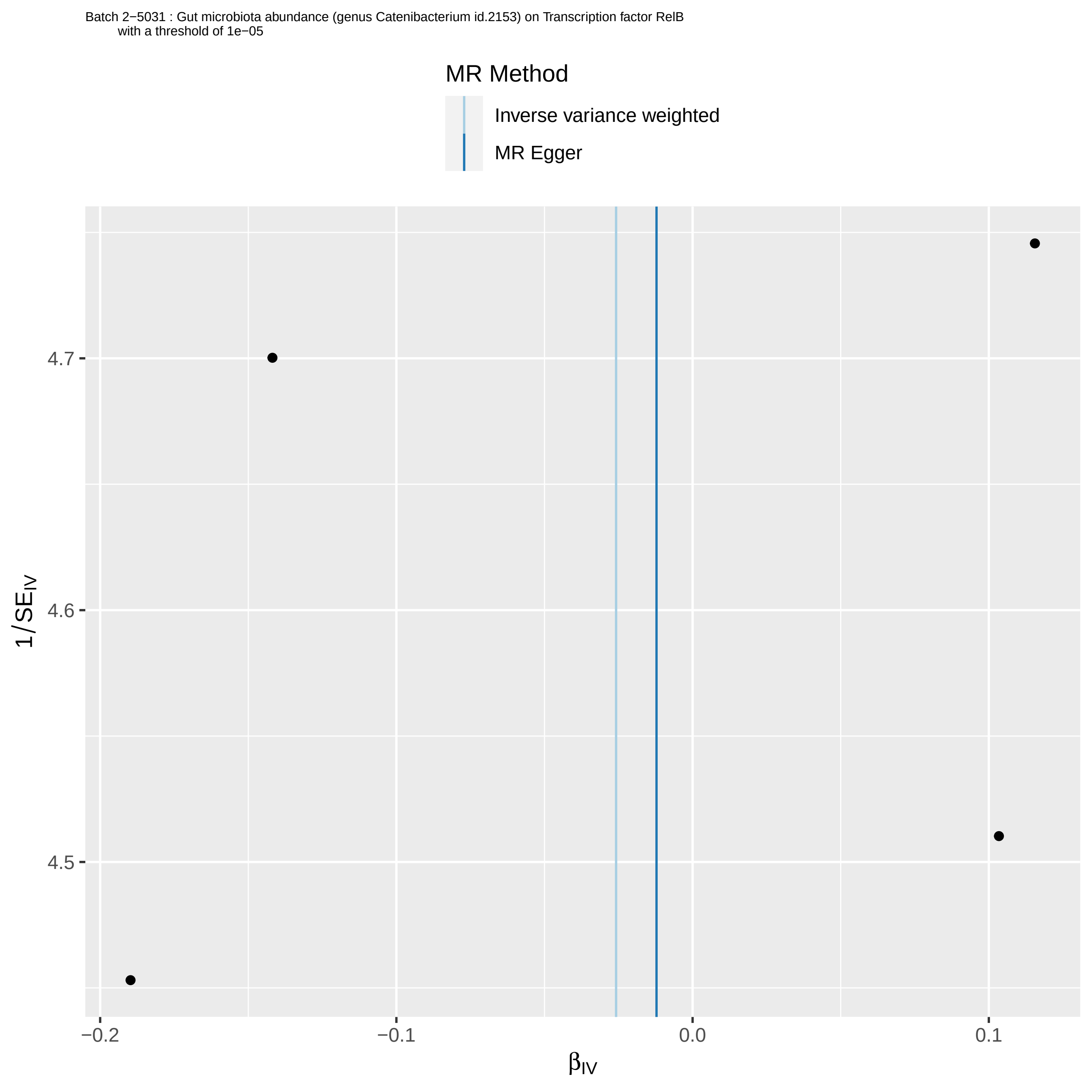
Batch 2−5031 : Gut microbiota abundance (genus Catenibacterium id.2153) on Transcription factor RelB
with a threshold of 1e−05
MR Method
Inverse variance weighted
MR Egger
4.7
4.6
4.5
1/SE_IV
−0.2
−0.1
0.0
0.1
β_IV

Batch 2−5032 : Gut microbiota abundance (genus Parasutterella id.2892) on Transcription factor RelB with a threshold of 1e−05

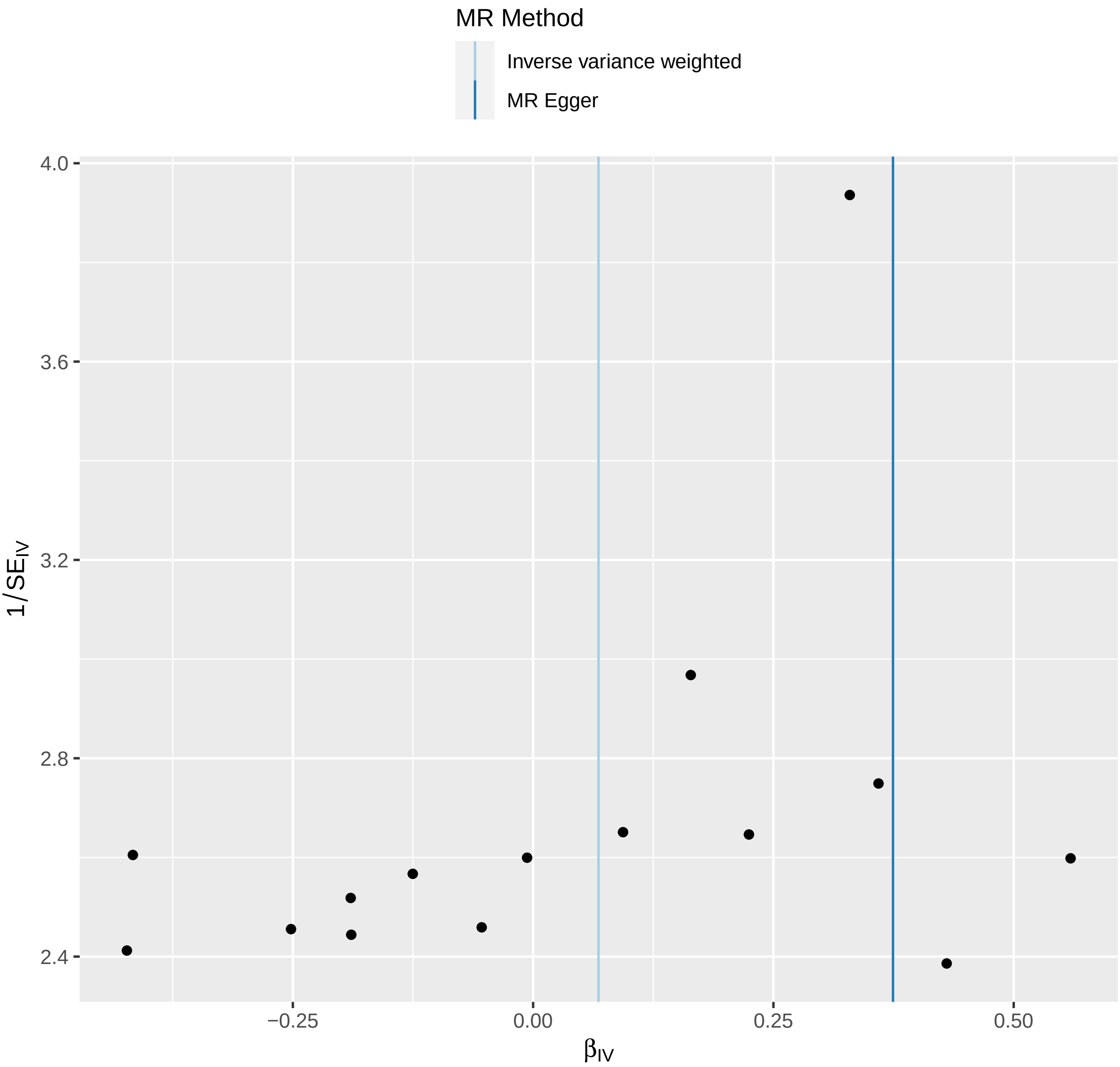

Batch 2−5033 : Gut microbiota abundance (genus Catenibacterium id.2153) on RELT−like protein 1
with a threshold of 1e−05

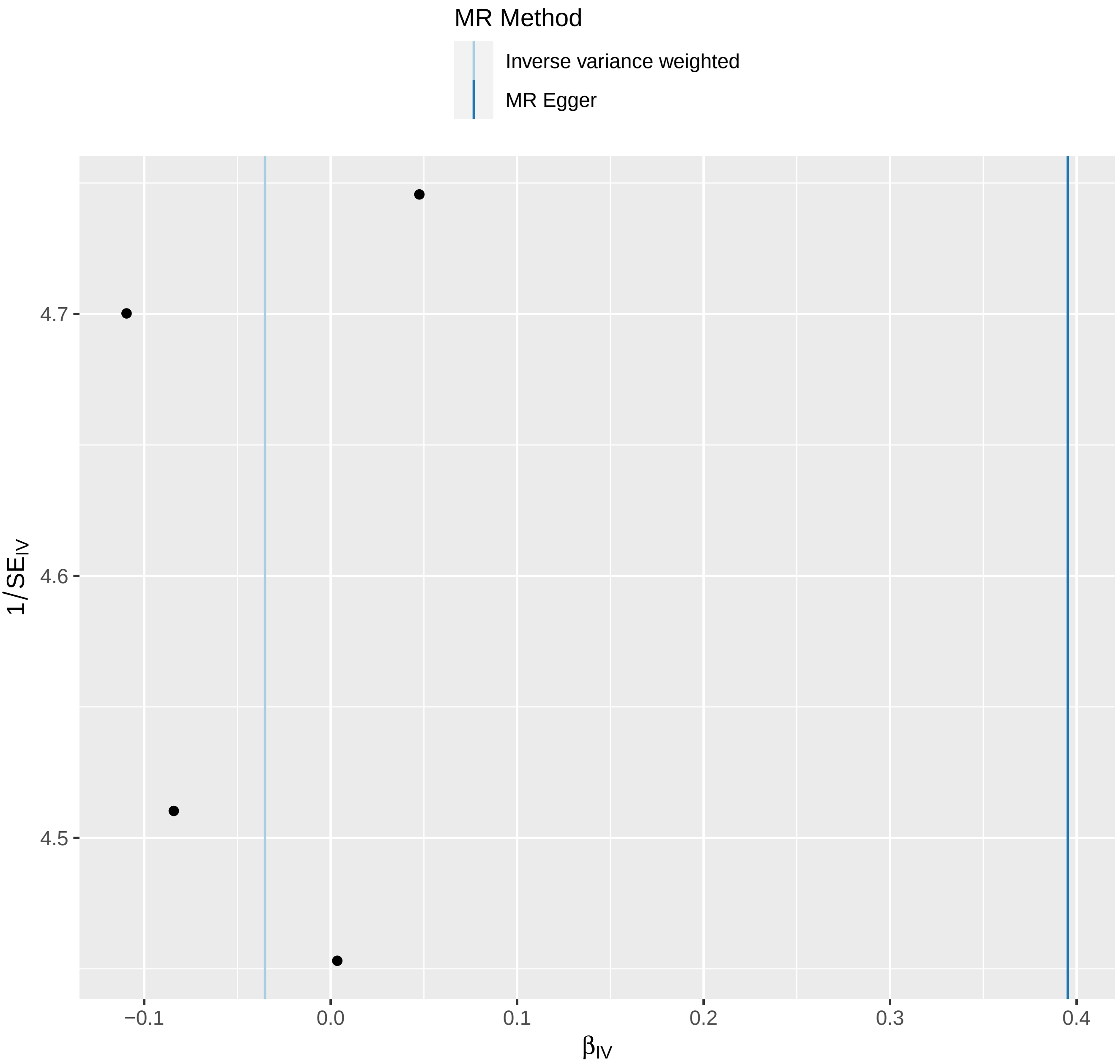

Batch 2−5034 : Gut microbiota abundance (genus Parasutterella id.2892) on RELT−like protein 1
with a threshold of 1e−05

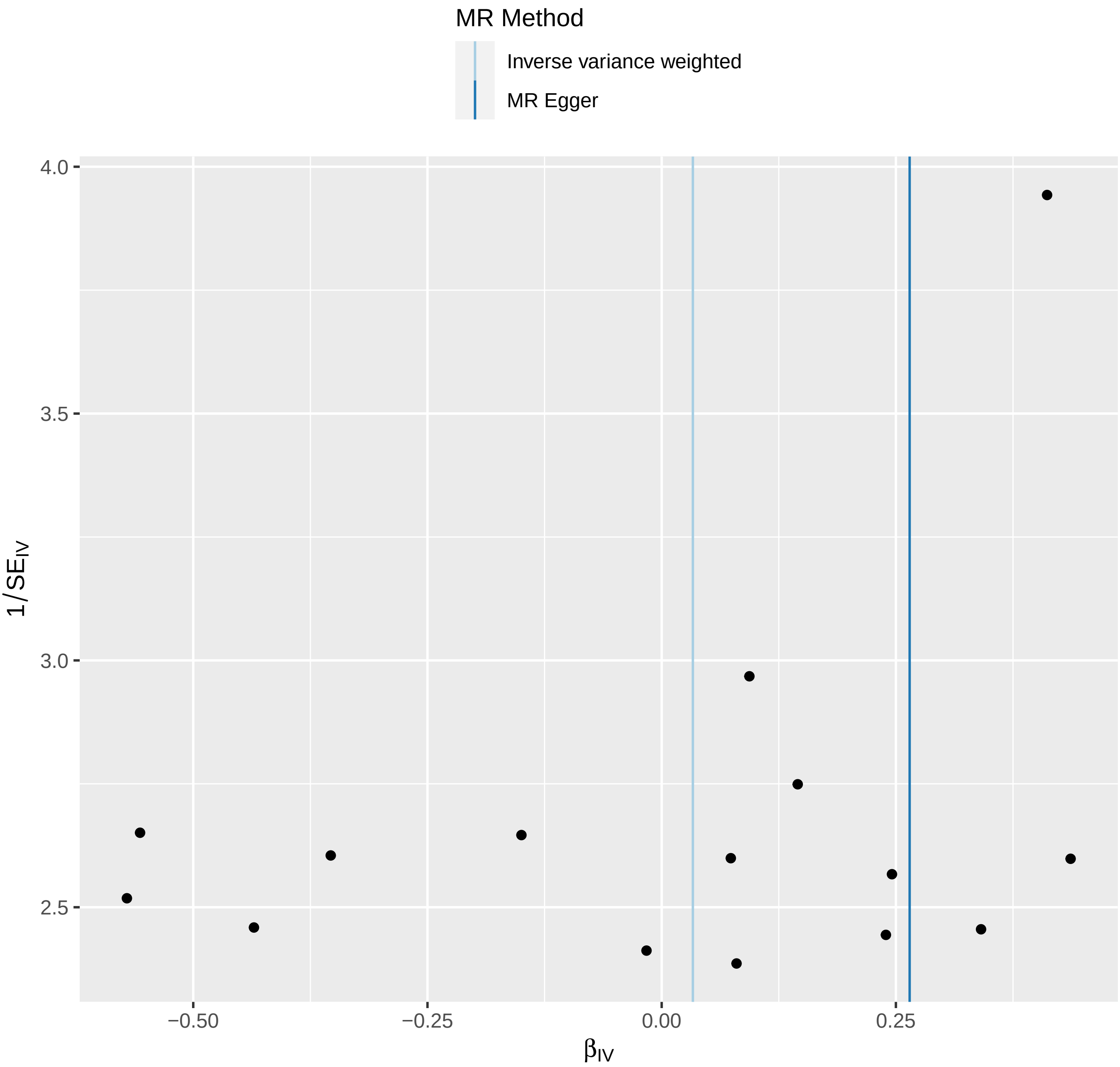

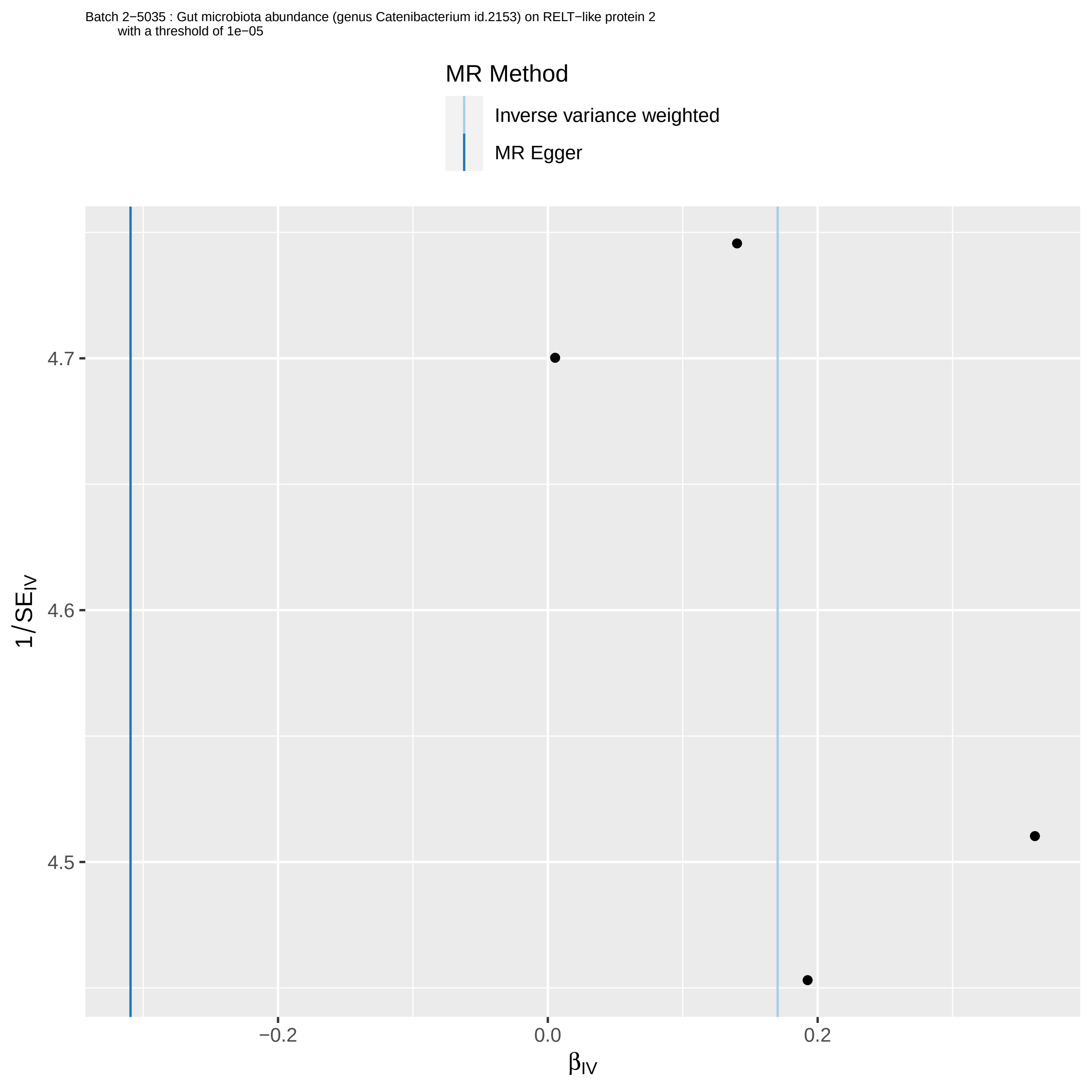

Batch 2−5035 : Gut microbiota abundance (genus Catenibacterium id.2153) on RELT−like protein 2
with a threshold of 1e−05
MR Method
Inverse variance weighted
MR Egger
4.7
4.6
4.5
1/SE_IV
−0.2
0.0
0.2
β_IV

Batch 2−5036 : Gut microbiota abundance (genus Parasutterella id.2892) on RELT−like protein 2
with a threshold of 1e−05

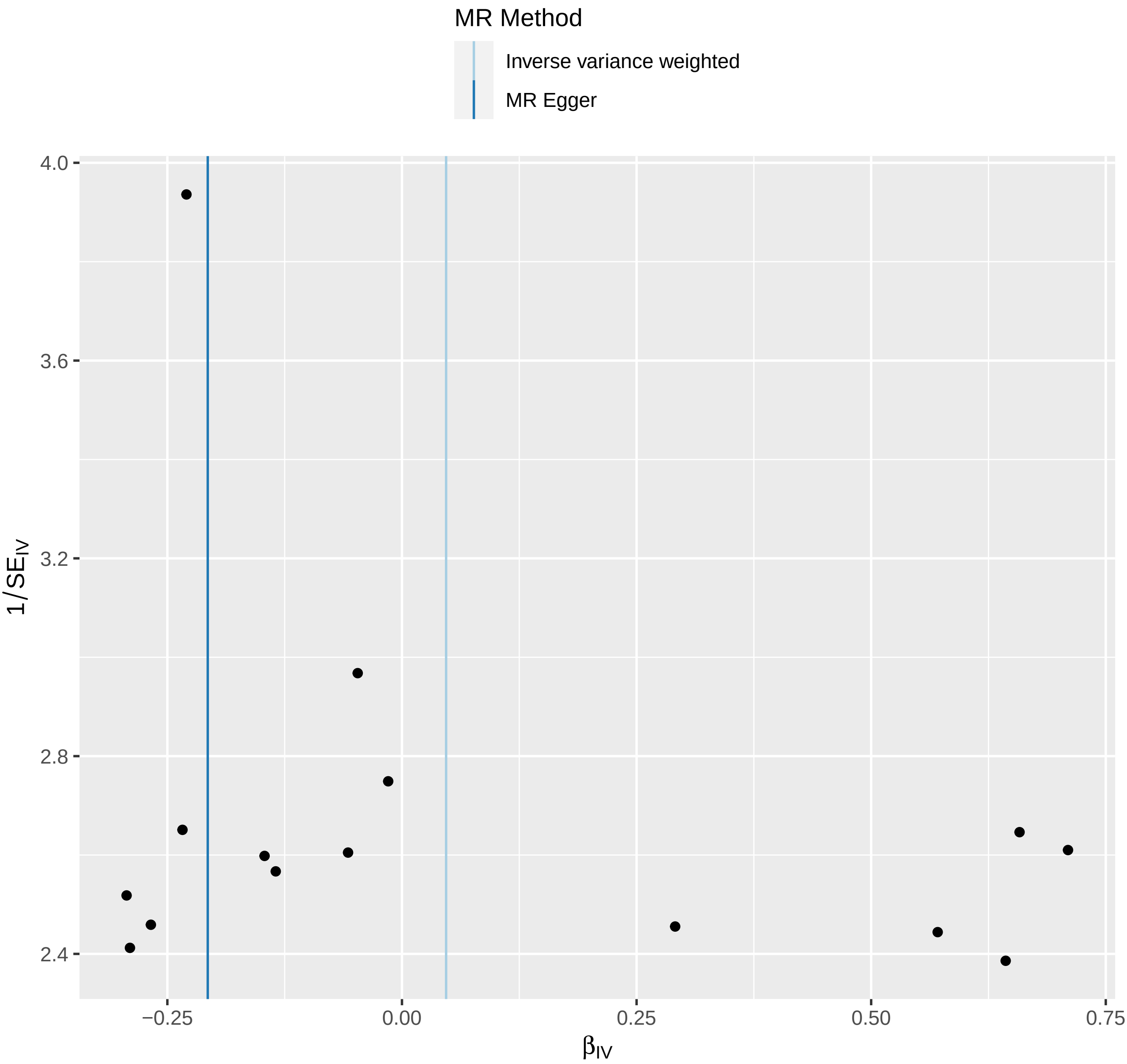

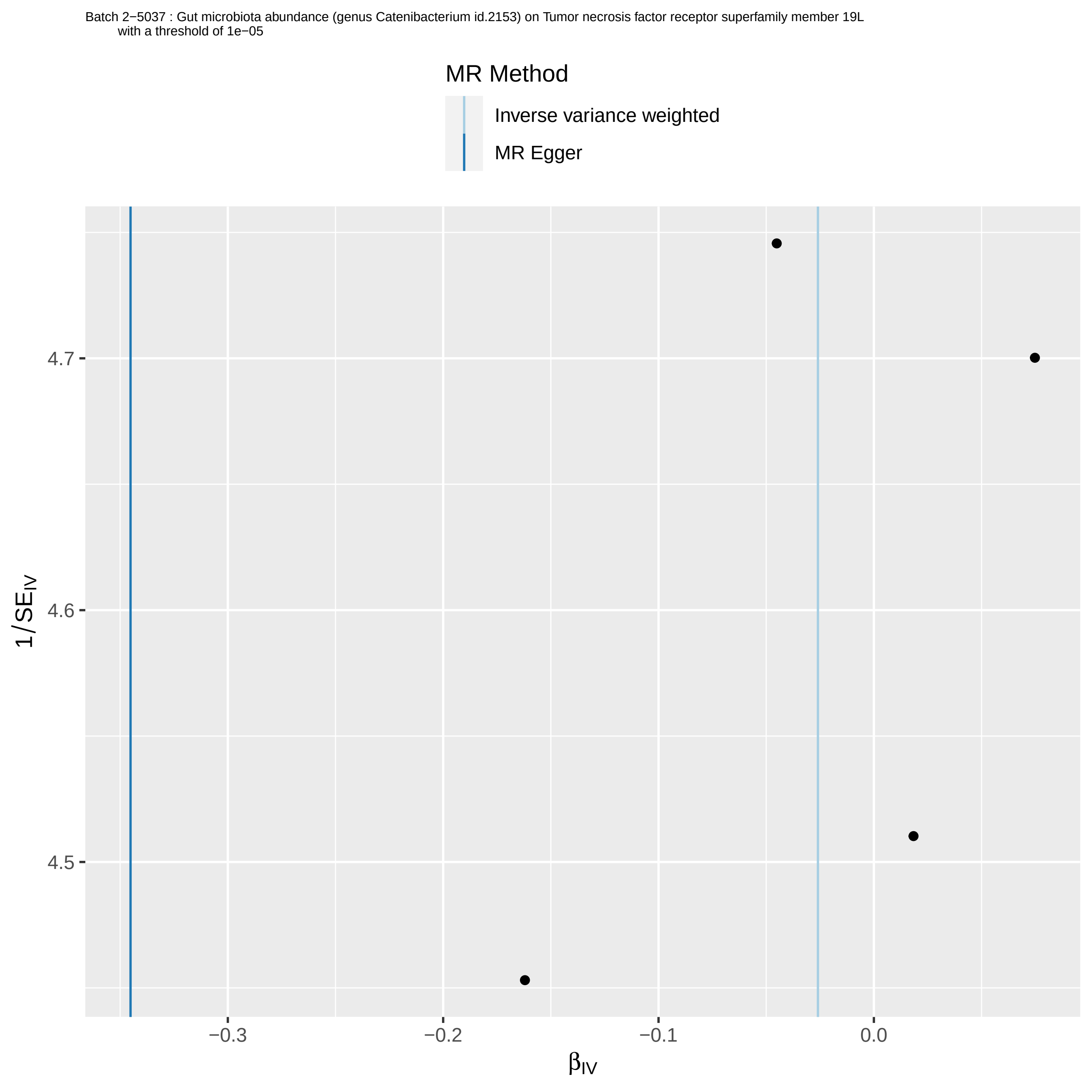
Batch 2−5037 : Gut microbiota abundance (genus Catenibacterium id.2153) on Tumor necrosis factor receptor superfamily member 19L
with a threshold of 1e−05
MR Method
Inverse variance weighted
MR Egger
4.7
4.6
4.5
1/SE$_{IV}$
−0.3
−0.2
−0.1
0.0
β_{IV}

Batch 2−5038 : Gut microbiota abundance (genus Parasutterella id.2892) on Tumor necrosis factor receptor superfamily member 19L
with a threshold of 1e−05

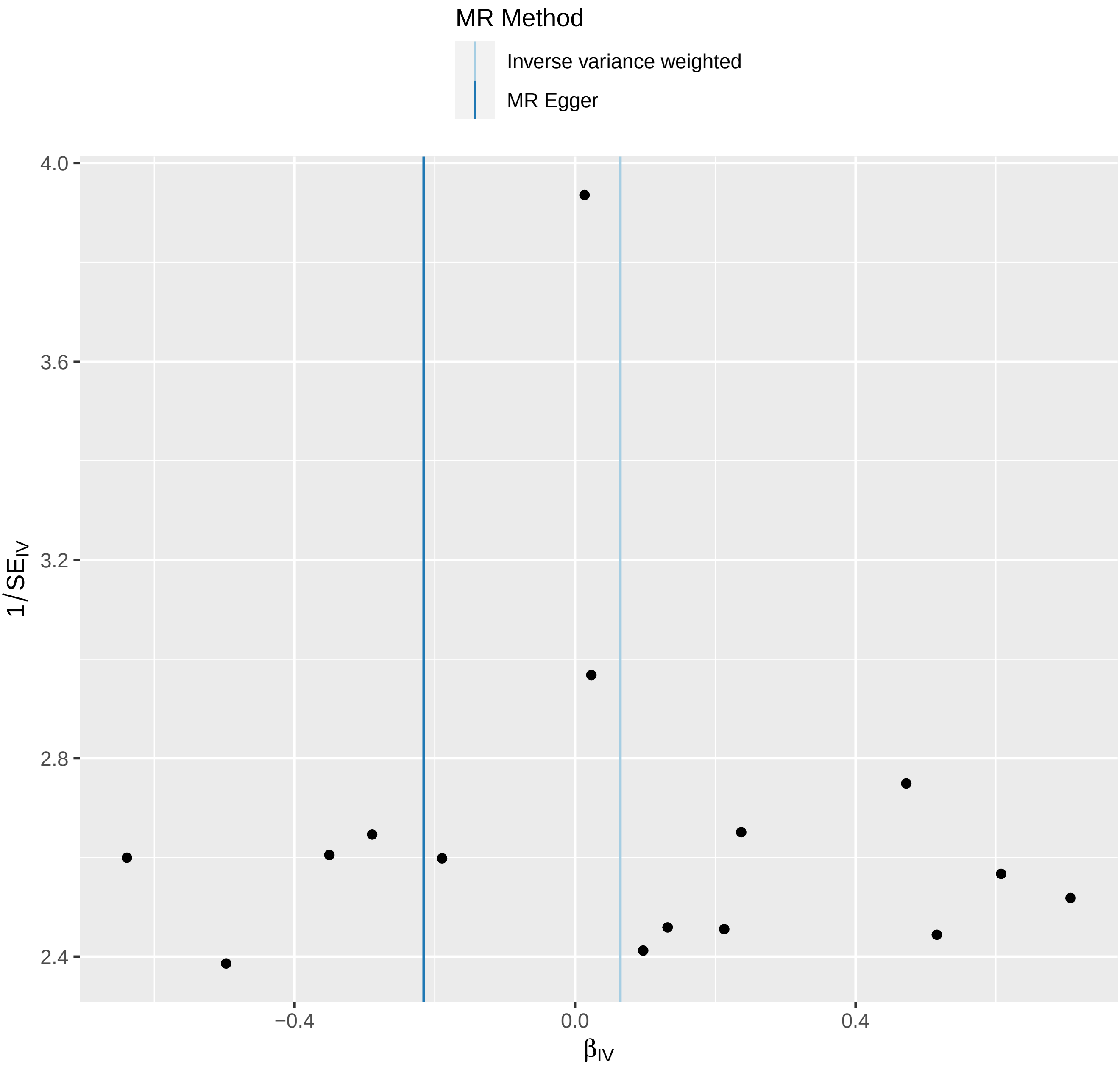

Batch 2−5039 : Gut microbiota abundance (genus Catenibacterium id.2153) on Tumor necrosis factor receptor superfamily member 19L with a threshold of 1e−05

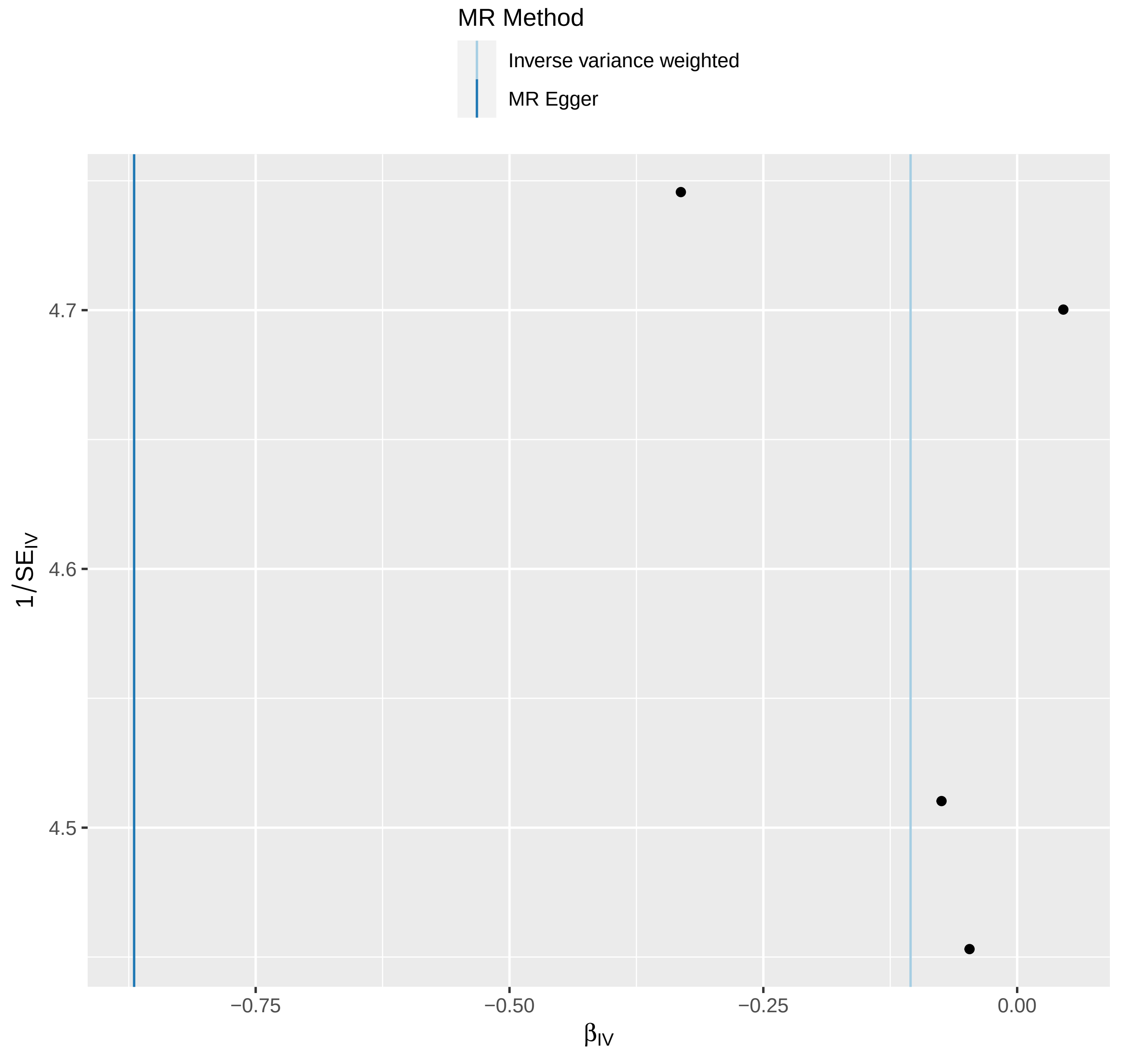

Batch 2−5040 : Gut microbiota abundance (genus Parasutterella id.2892) on Tumor necrosis factor receptor superfamily member 19L
with a threshold of 1e−05

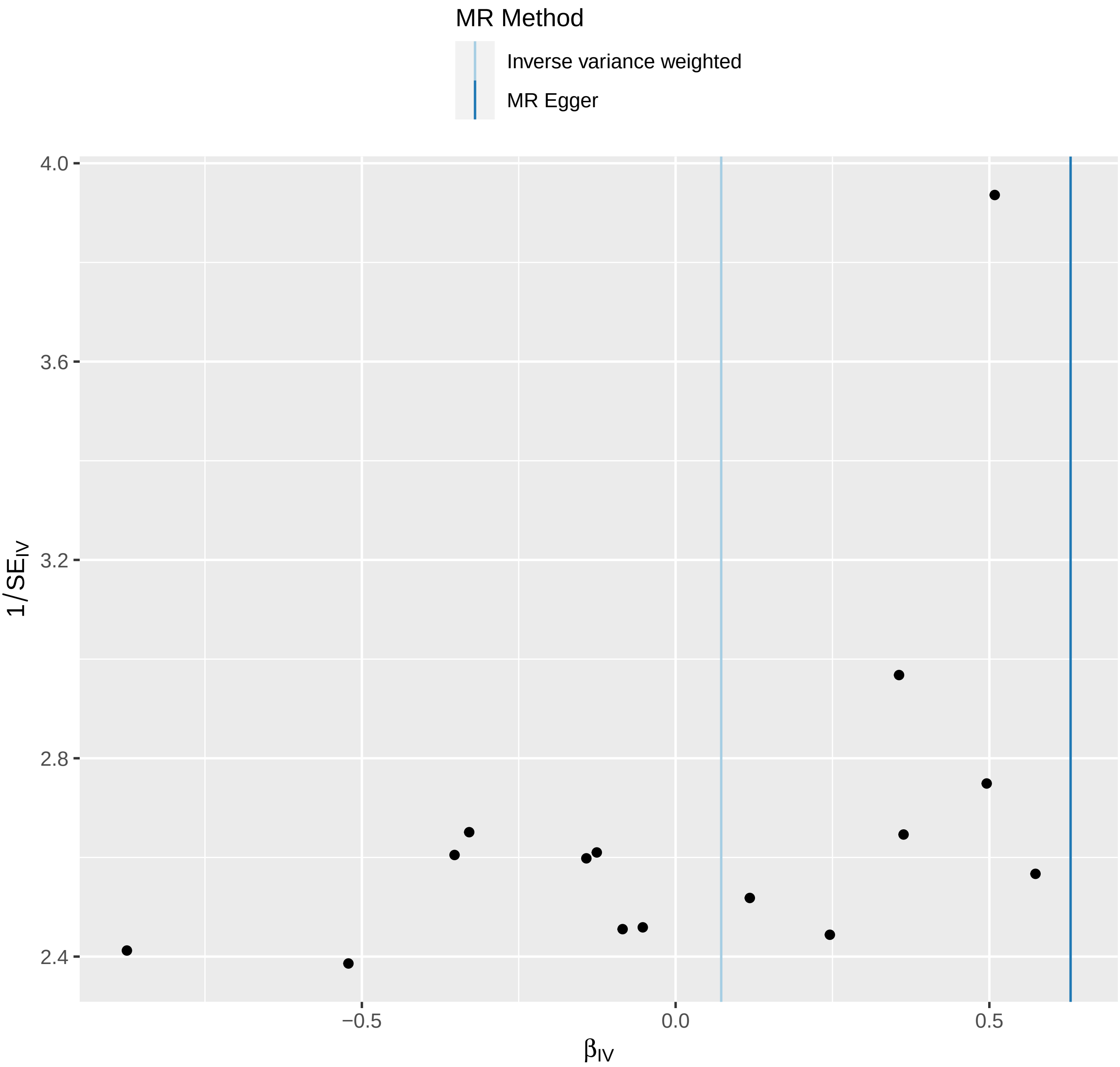

Batch 2−5041 : Gut microbiota abundance (genus Catenibacterium id.2153) on Renin
with a threshold of 1e−05

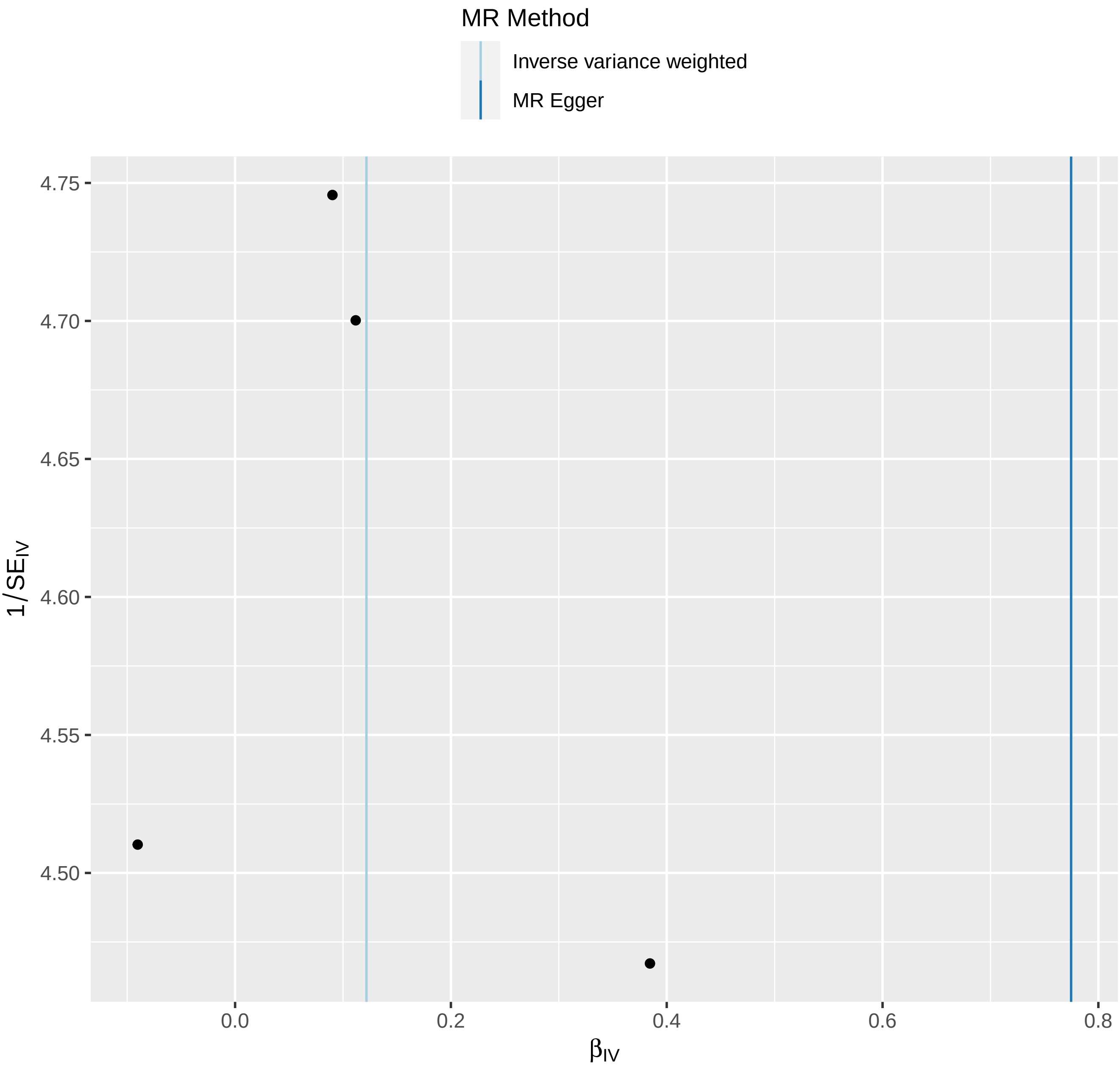

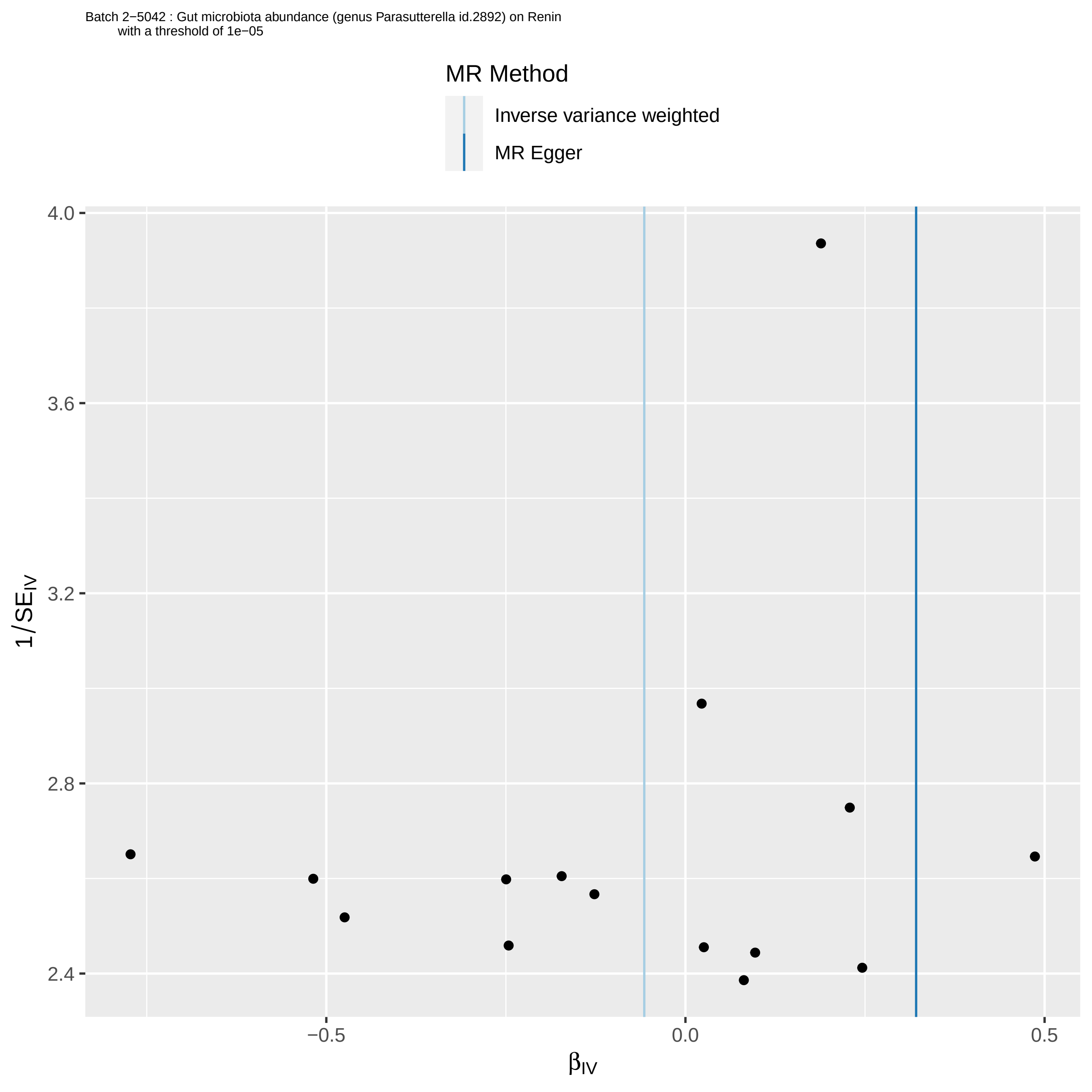

Batch 2−5042 : Gut microbiota abundance (genus Parasutterella id.2892) on Renin
with a threshold of 1e−05
MR Method
Inverse variance weighted
MR Egger
4.0
3.6
3.2
2.8
2.4
1/SE_IV
−0.5
0.0
0.5
β_IV

Batch 2−5043 : Gut microbiota abundance (genus Catenibacterium id.2153) on Replication initiator 1
with a threshold of 1e−05

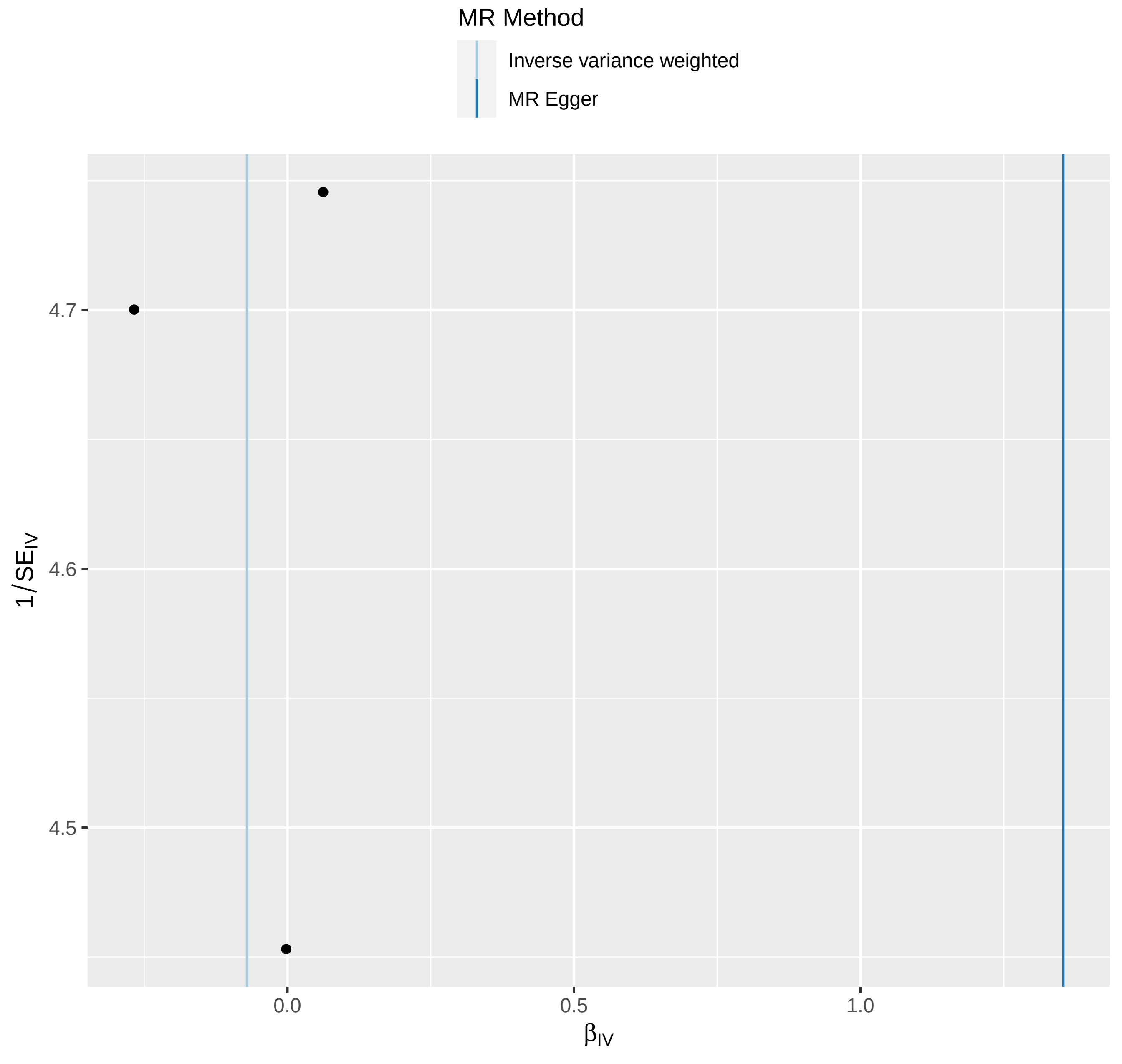

Batch 2−5044 : Gut microbiota abundance (genus Parasutterella id.2892) on Replication initiator 1
with a threshold of 1e−05

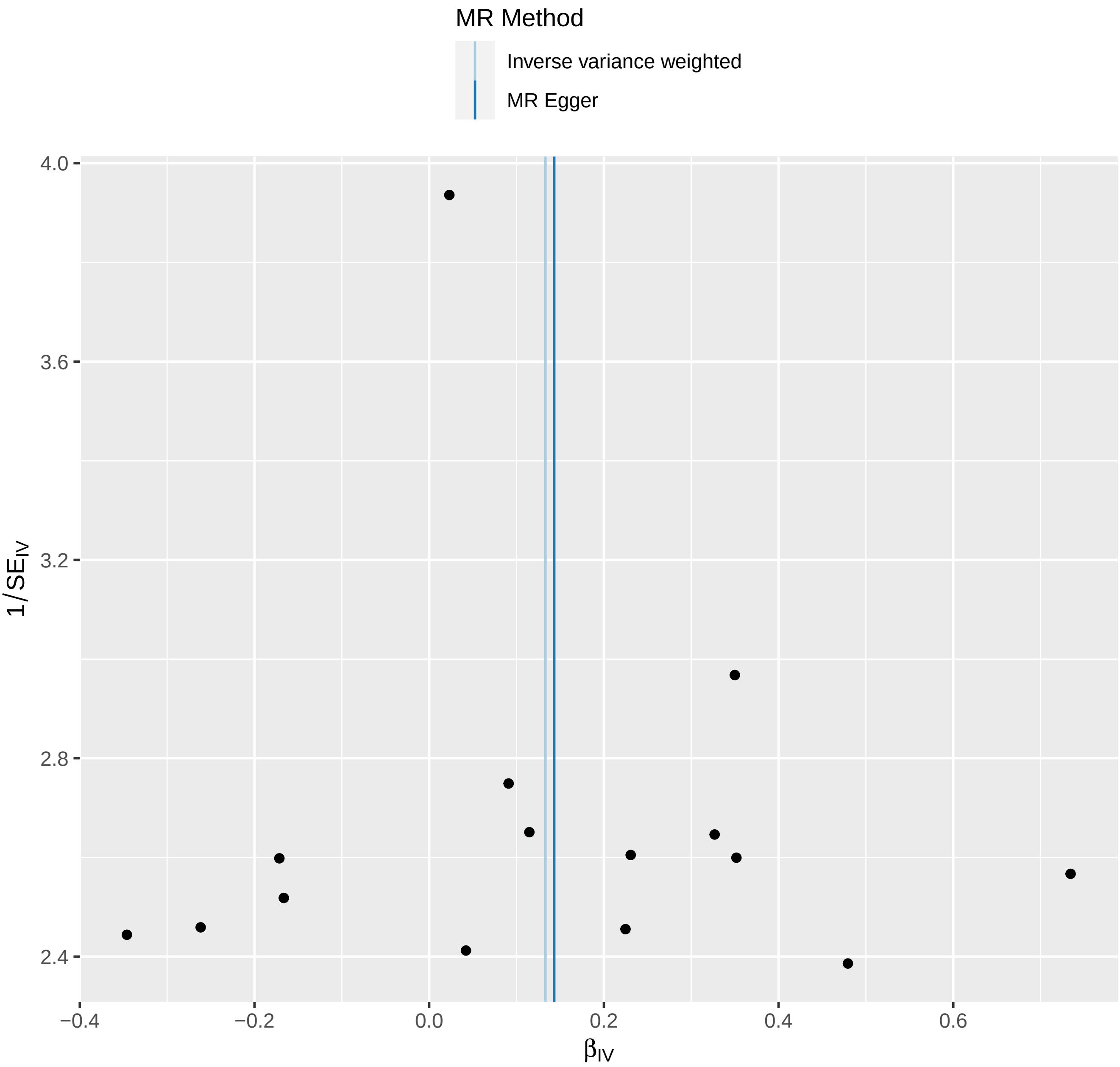

Batch 2−5045 : Gut microbiota abundance (genus Catenibacterium id.2153) on Proto−oncogene tyrosine−protein kinase receptor Ret
with a threshold of 1e−05

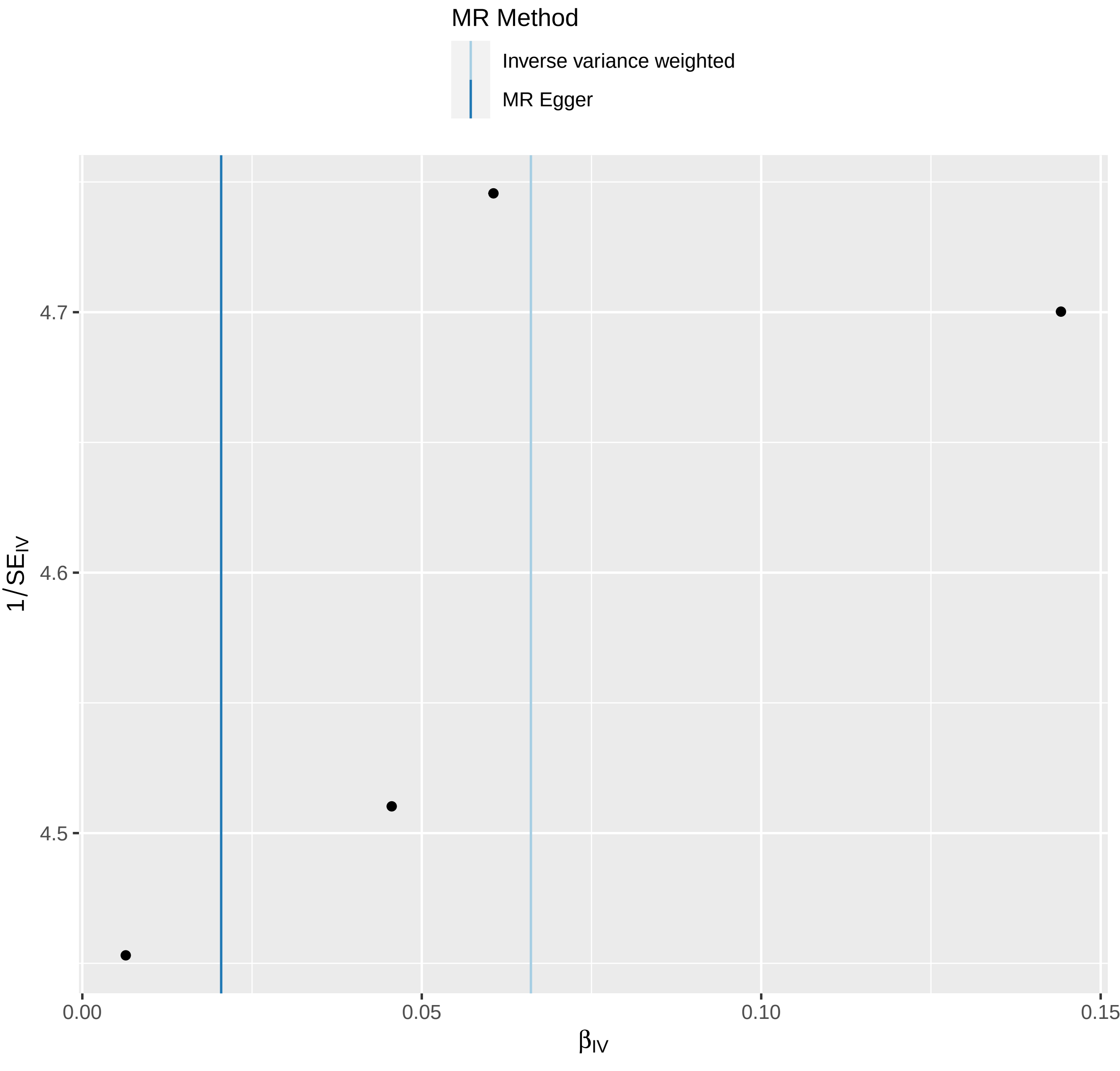

Batch 2−5046 : Gut microbiota abundance (genus Parasutterella id.2892) on Proto−oncogene tyrosine−protein kinase receptor Ret
with a threshold of 1e−05

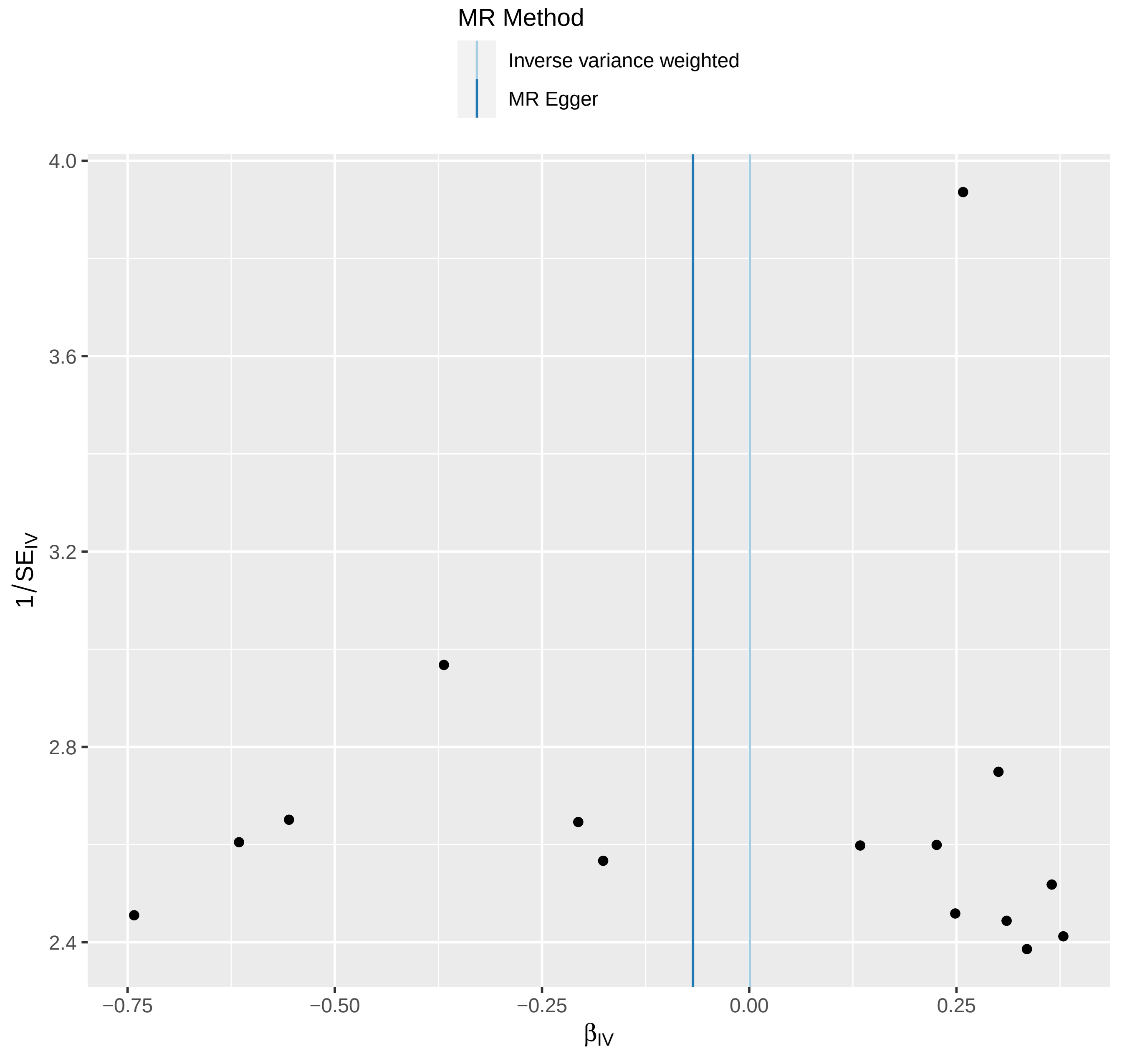

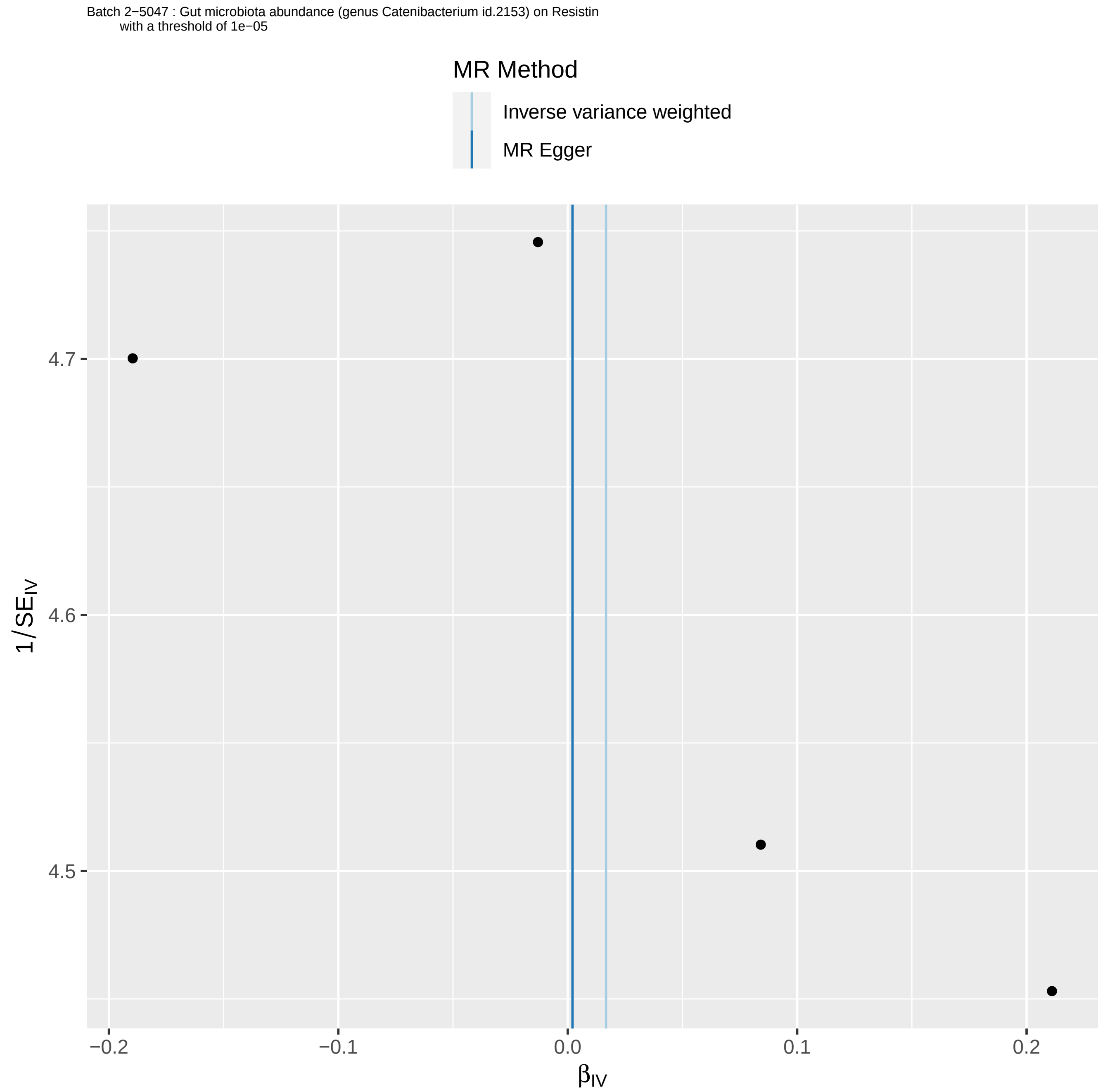

Batch 2−5047 : Gut microbiota abundance (genus Catenibacterium id.2153) on Resistin
with a threshold of 1e−05
MR Method
Inverse variance weighted
MR Egger
4.7
4.6
4.5
1/SE_IV
−0.2
−0.1
0.0
0.1
0.2
β_IV

Batch 2−5048 : Gut microbiota abundance (genus Parasutterella id.2892) on Resistin
with a threshold of 1e−05

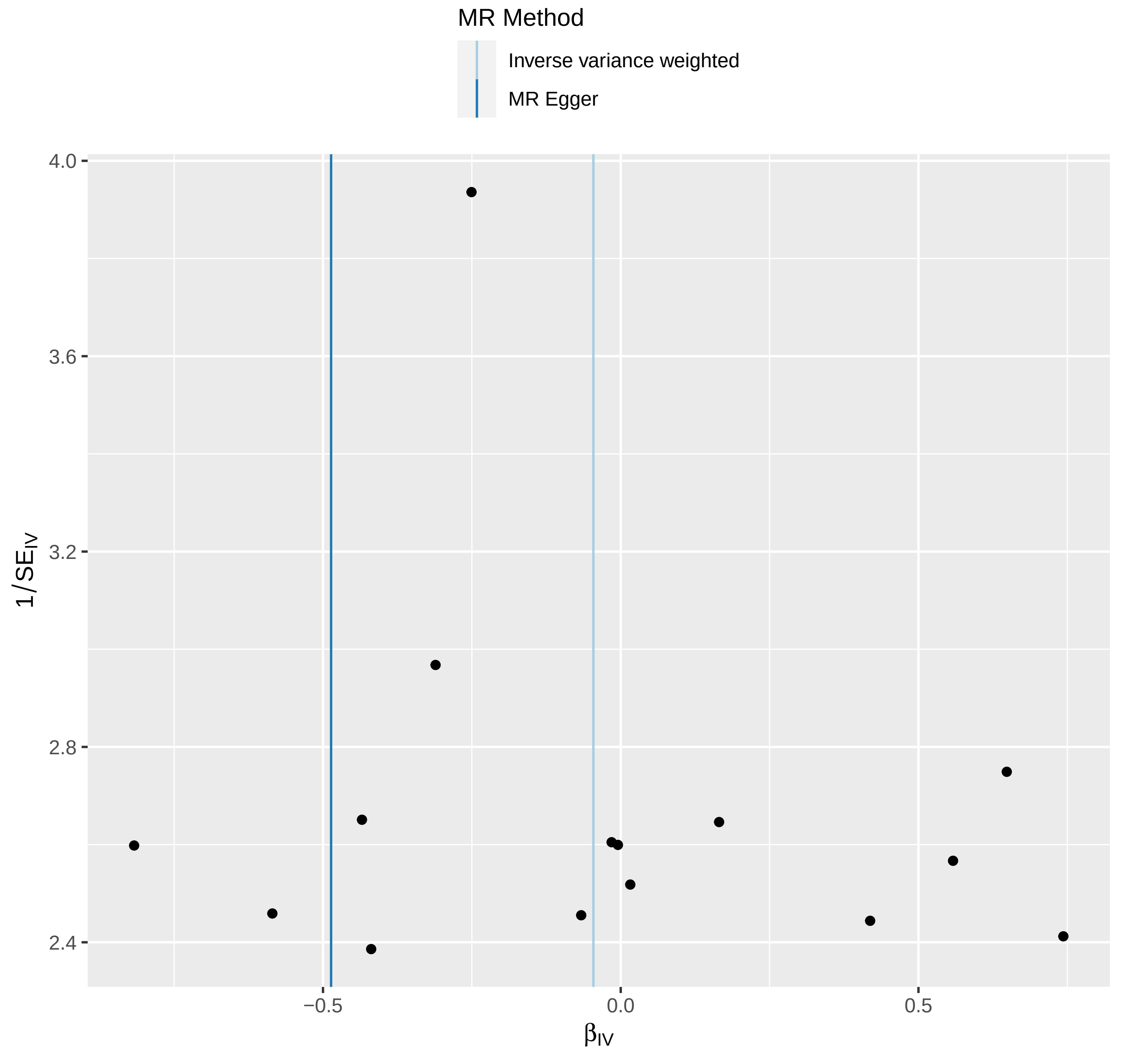

Batch 2−5049 : Gut microbiota abundance (genus Catenibacterium id.2153) on All−trans−retinol 13,14−reductase with a threshold of 1e−05

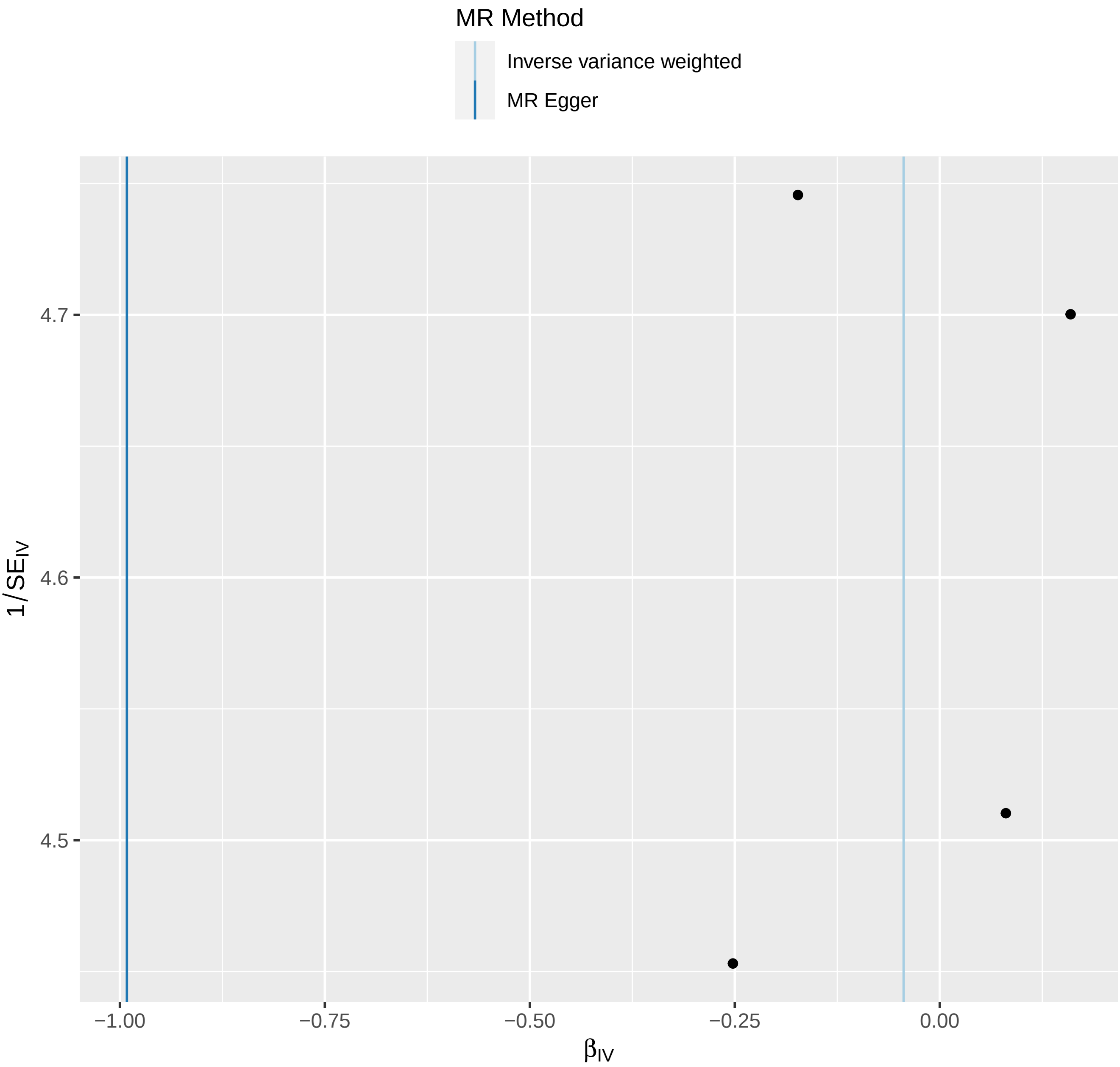

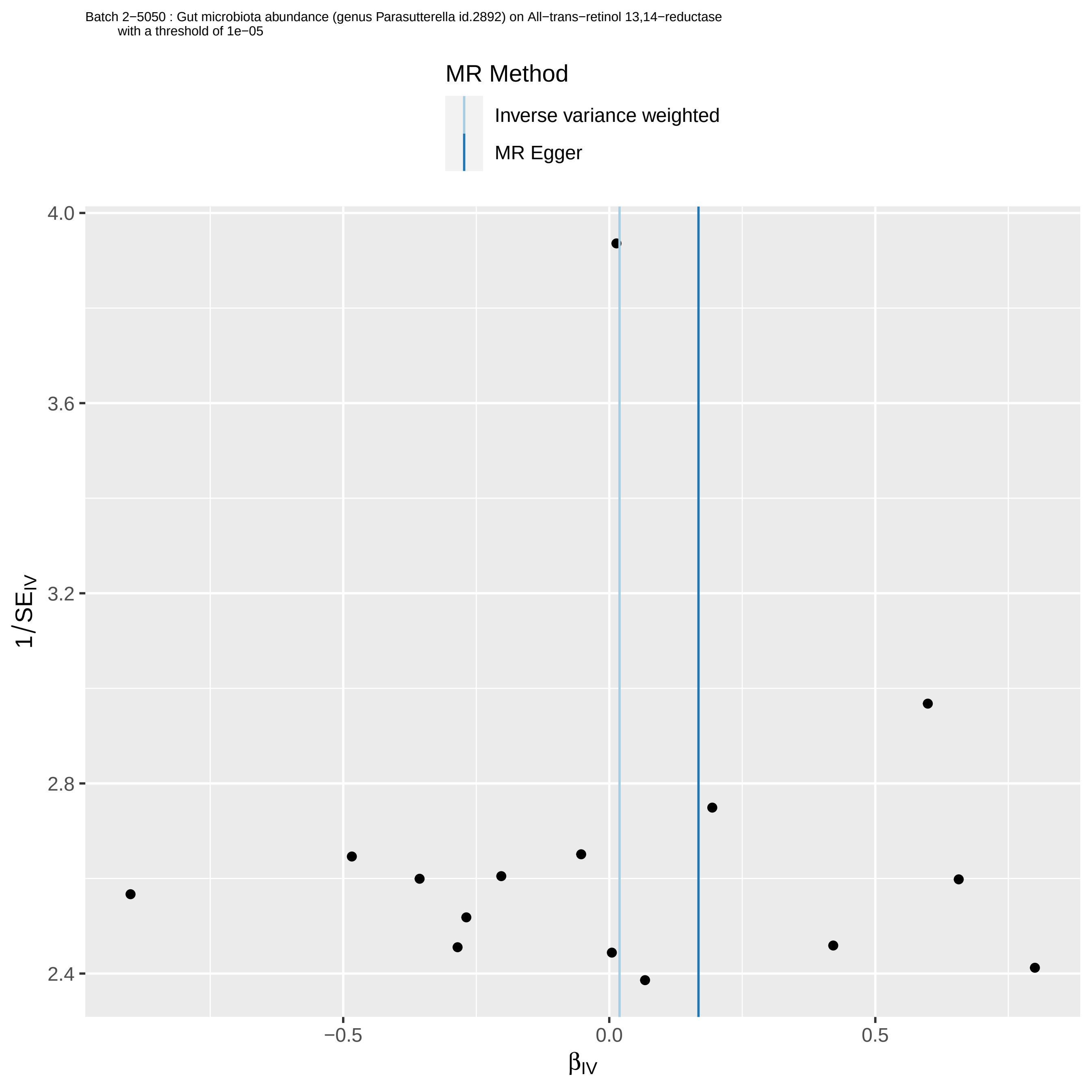
Batch 2−5050 : Gut microbiota abundance (genus Parasutterella id.2892) on All−trans−retinol 13,14−reductase
with a threshold of 1e−05
MR Method
Inverse variance weighted
MR Egger
4.0
3.6
3.2
2.8
2.4
1/SE_IV
−0.5
0.0
0.5
β_IV

Batch 2−5051 : Gut microbiota abundance (genus Catenibacterium id.2153) on Oligoribonuclease, mitochondrial
with a threshold of 1e−05

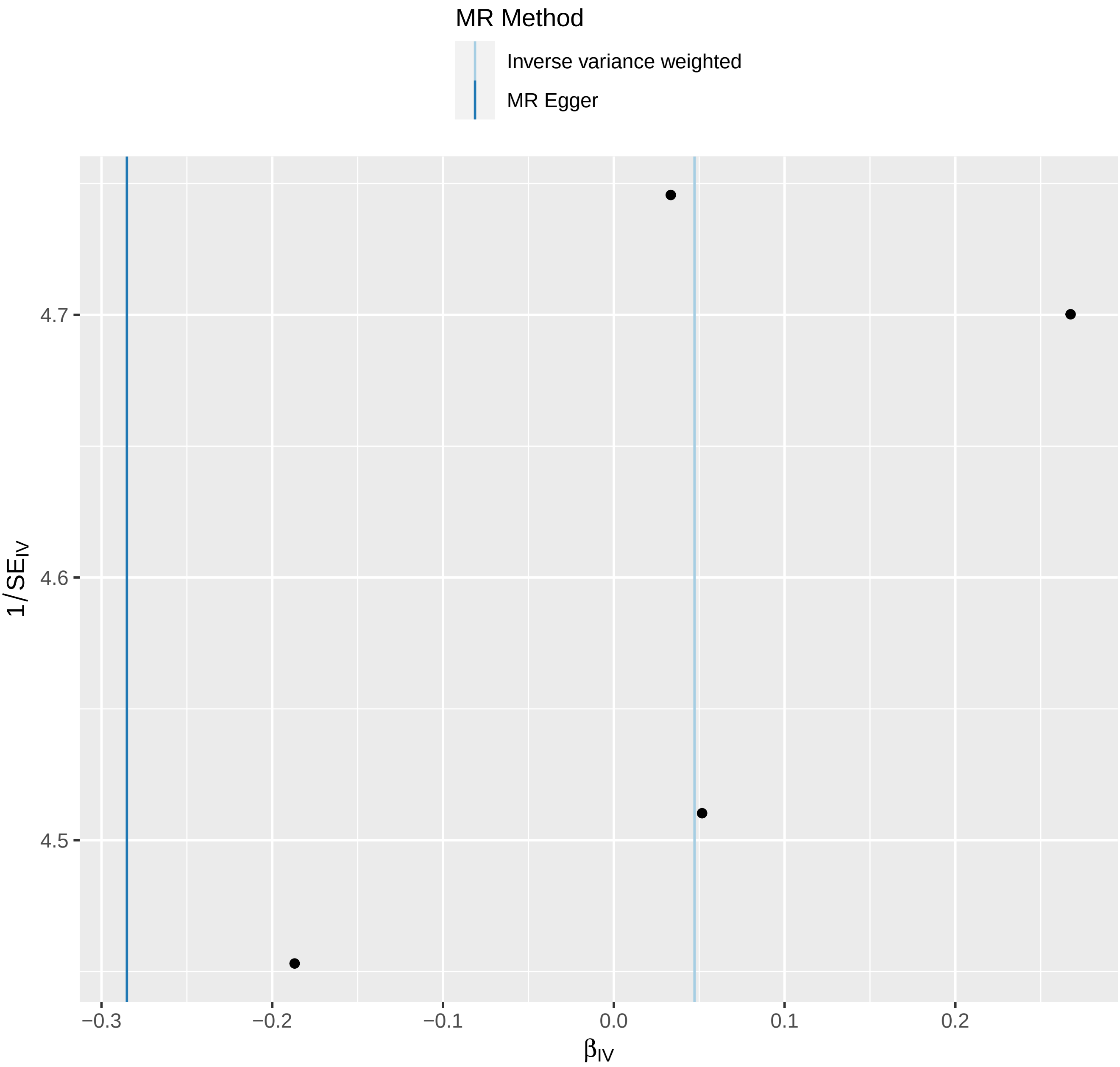

Batch 2−5052 : Gut microbiota abundance (genus Parasutterella id.2892) on Oligoribonuclease, mitochondrial
with a threshold of 1e−05

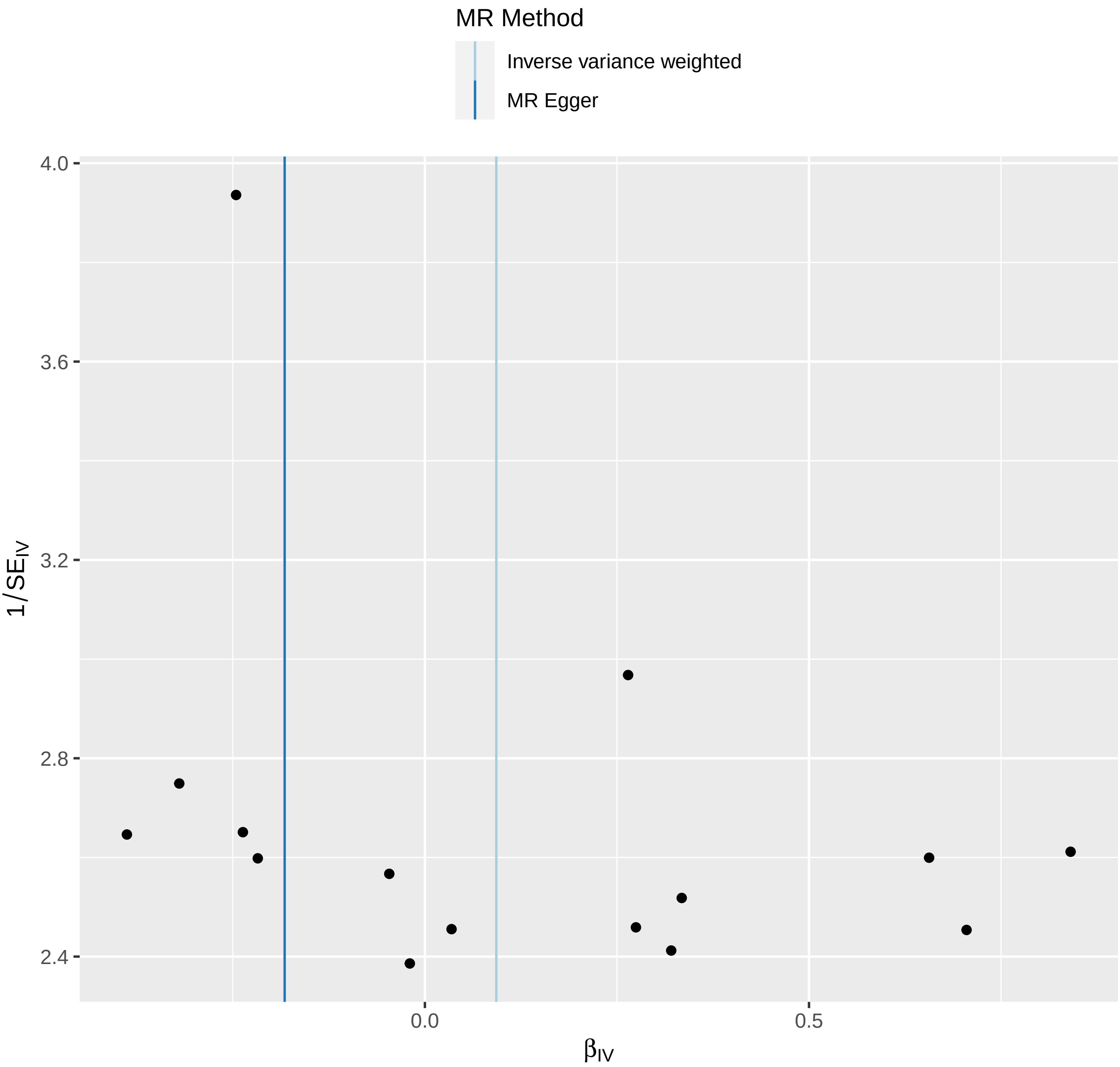

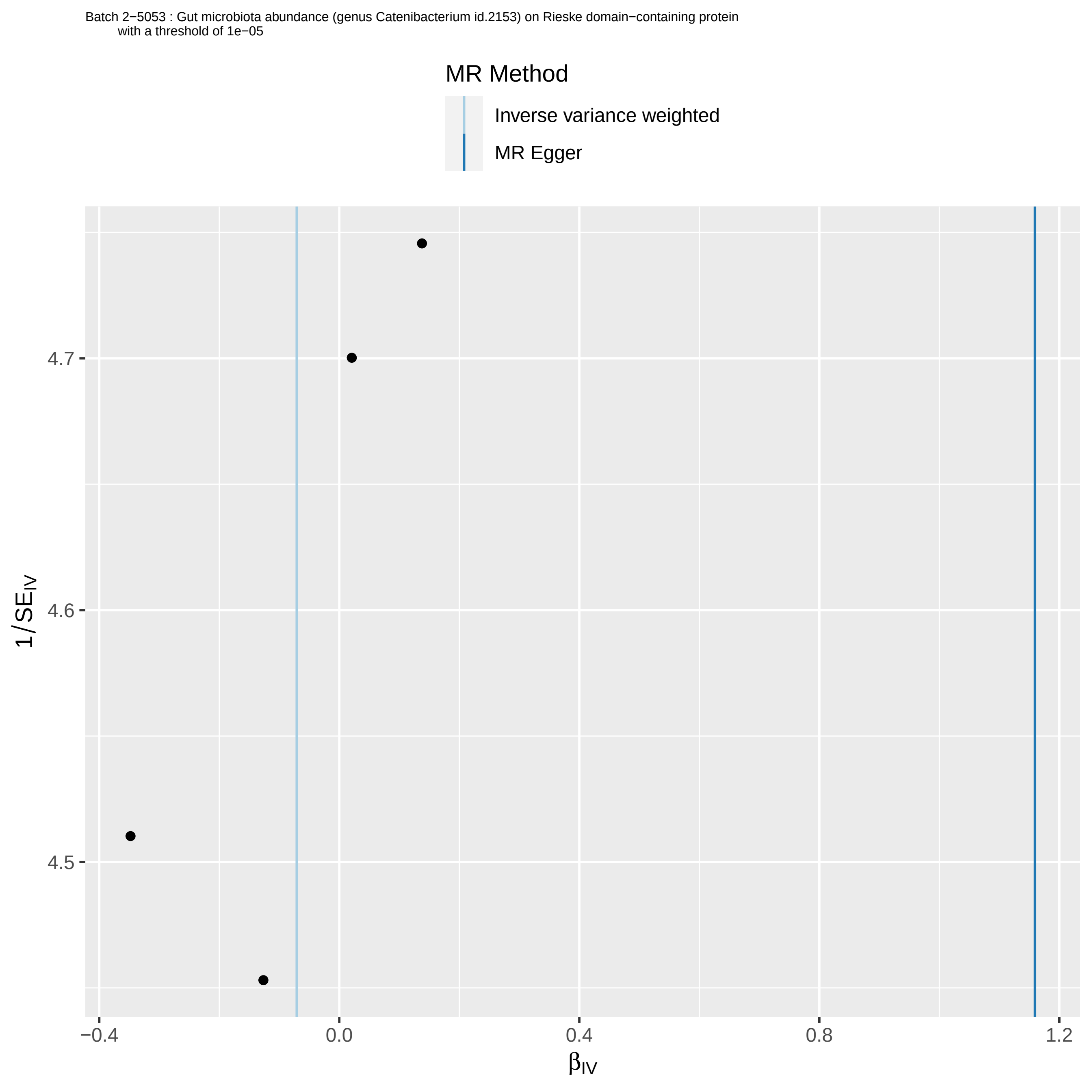
Batch 2−5053 : Gut microbiota abundance (genus Catenibacterium id.2153) on Rieske domain−containing protein
with a threshold of 1e−05
MR Method
Inverse variance weighted
MR Egger
4.7
4.6
4.5
$1/SE_{IV}$
−0.4
0.0
0.4
0.8
1.2
β_{IV}

Batch 2−5054 : Gut microbiota abundance (genus Parasutterella id.2892) on Rieske domain−containing protein
with a threshold of 1e−05

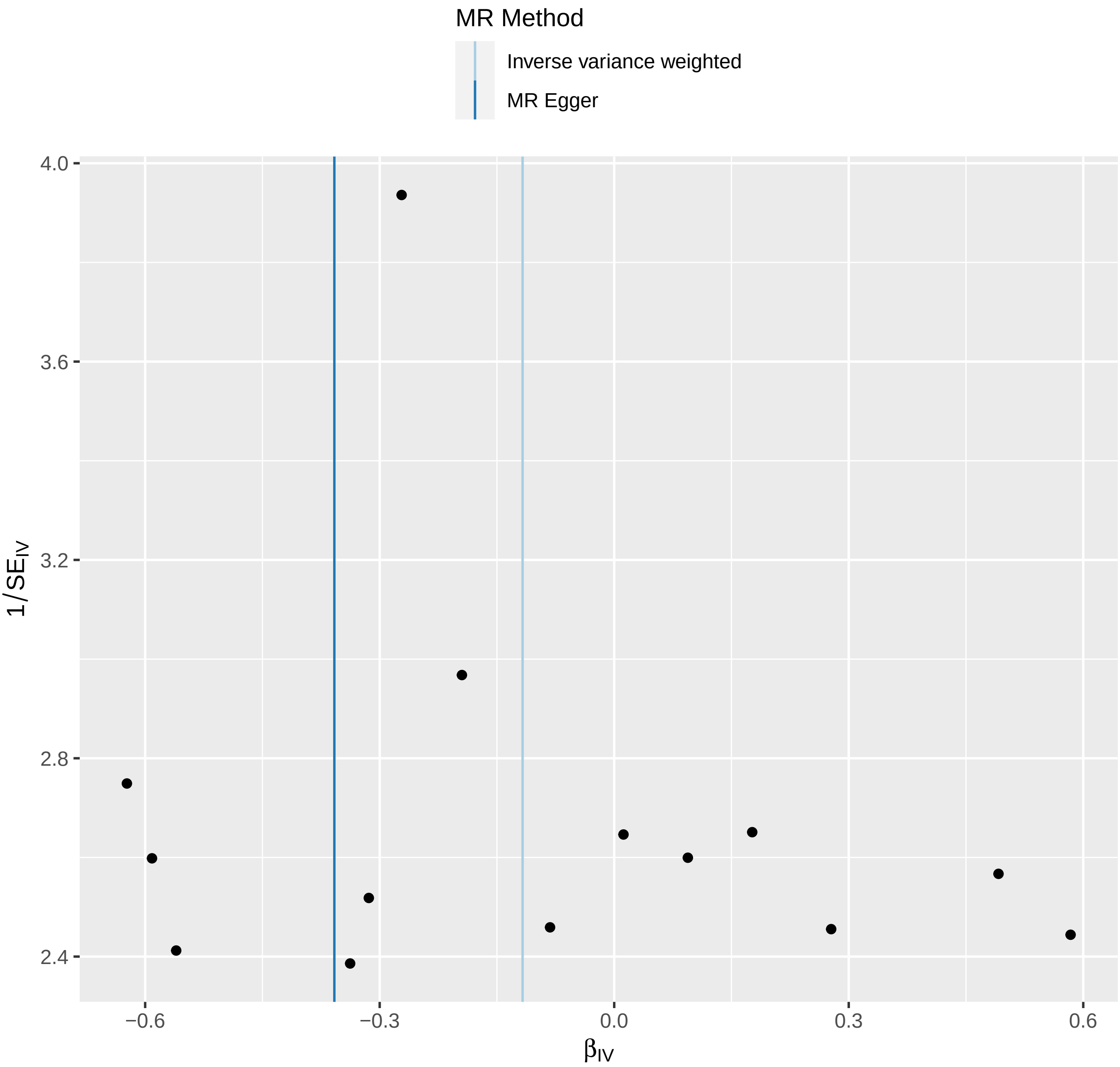

Batch 2−5055 : Gut microbiota abundance (genus Catenibacterium id.2153) on E3 ubiquitin−protein ligase rififylin
with a threshold of 1e−05

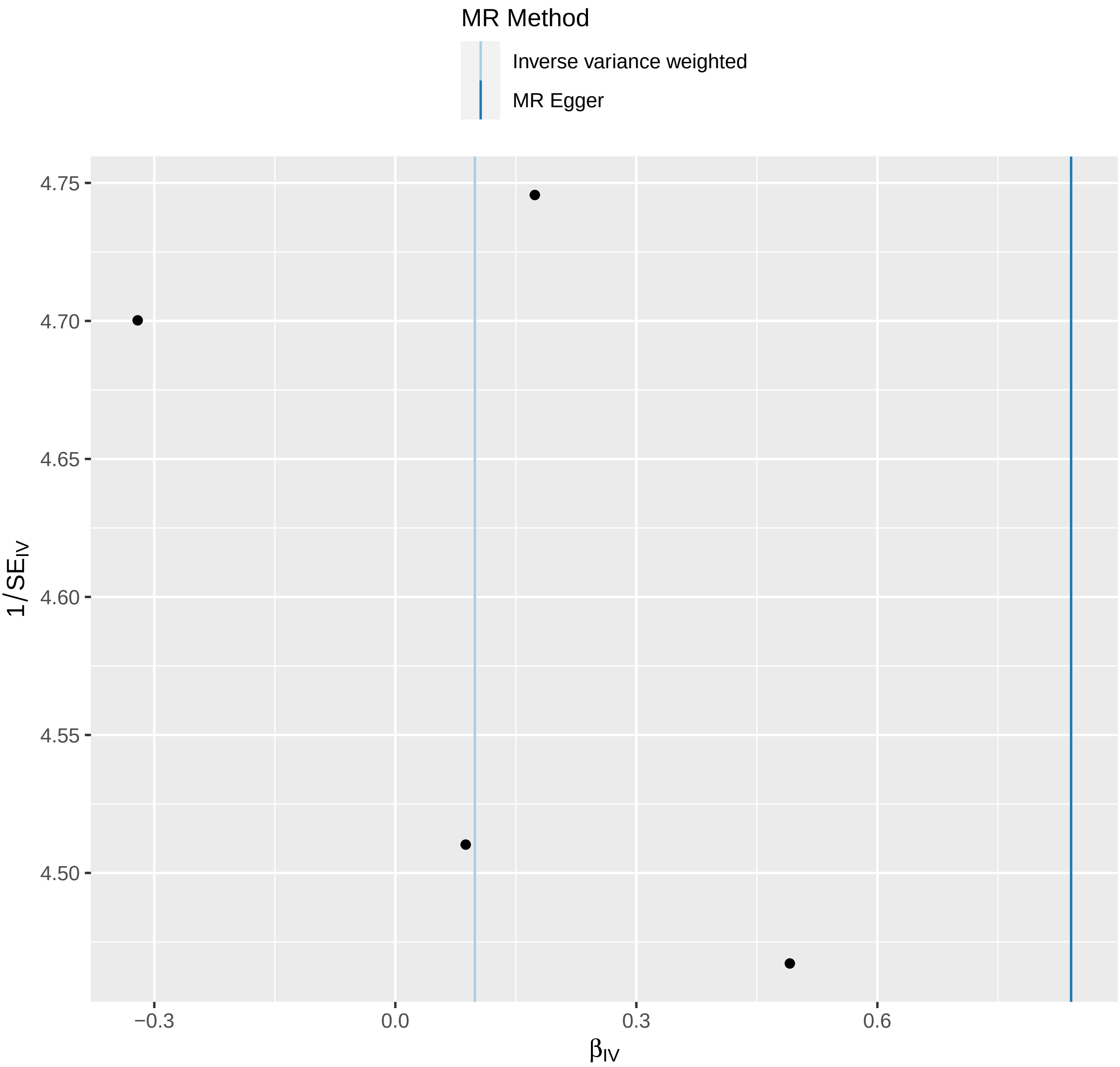

Batch 2−5056 : Gut microbiota abundance (genus Parasutterella id.2892) on E3 ubiquitin−protein ligase rififylin with a threshold of 1e−05

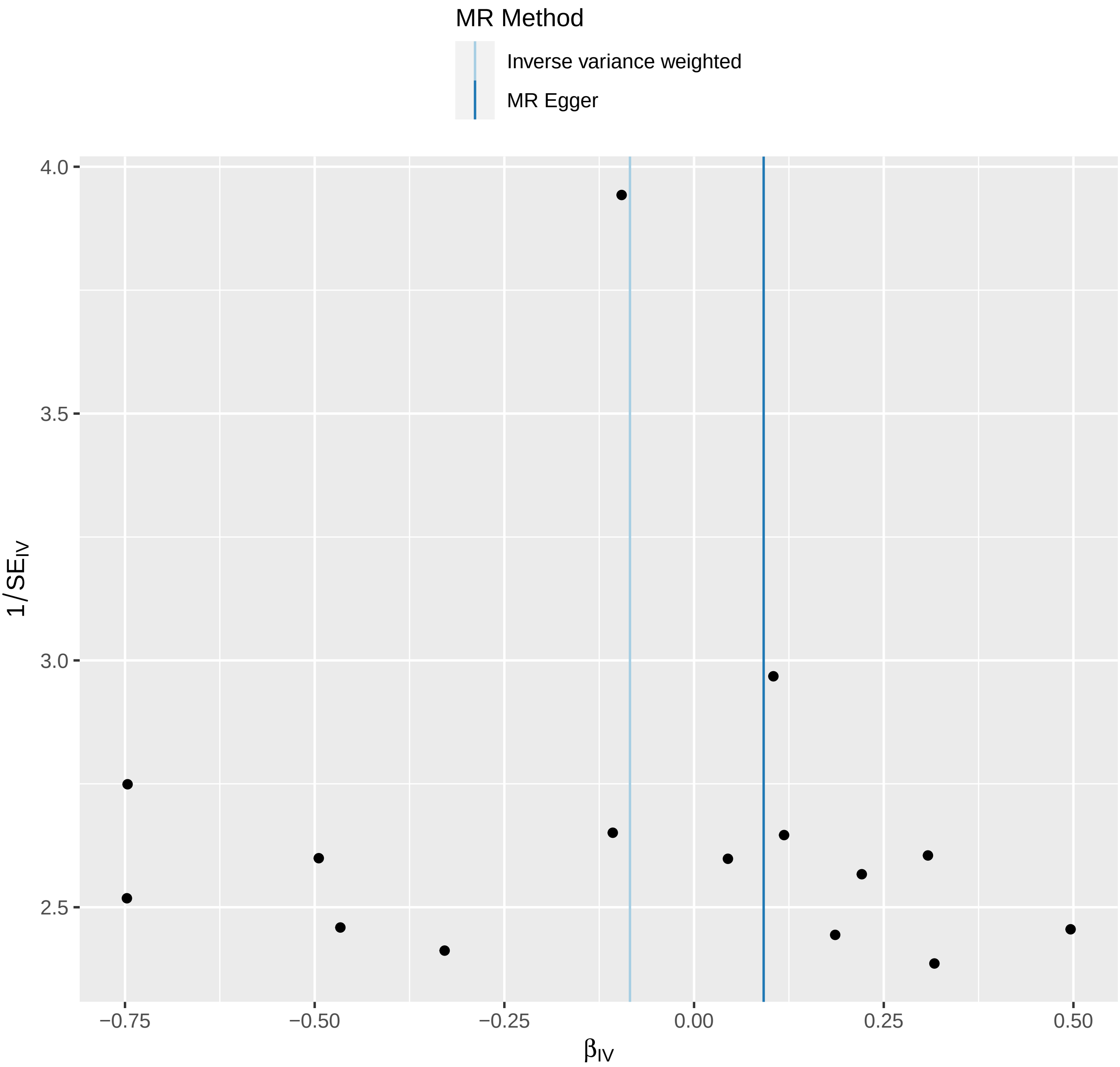

Batch 2−5057 : Gut microbiota abundance (genus Catenibacterium id.2153) on Riboflavin kinase
with a threshold of 1e−05

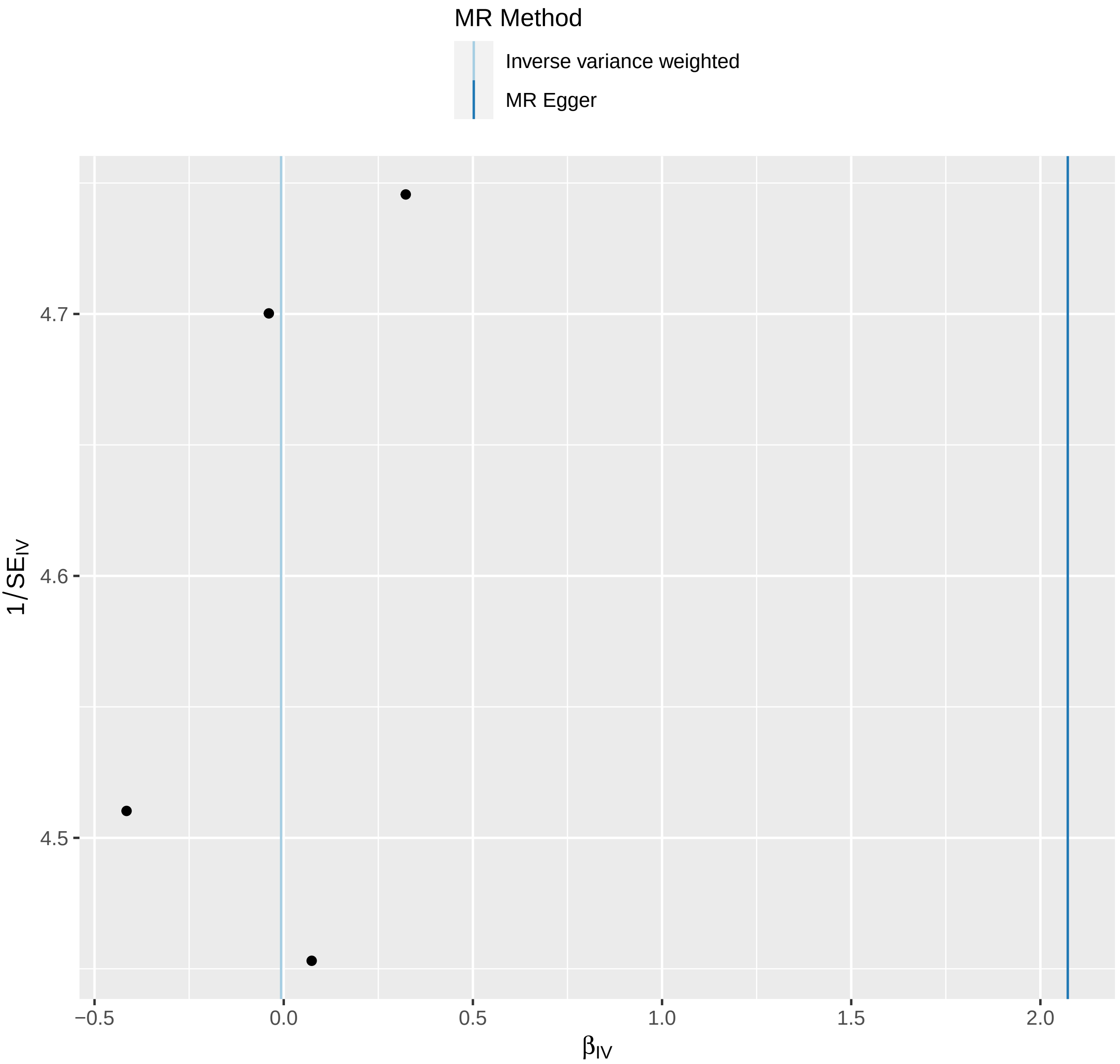

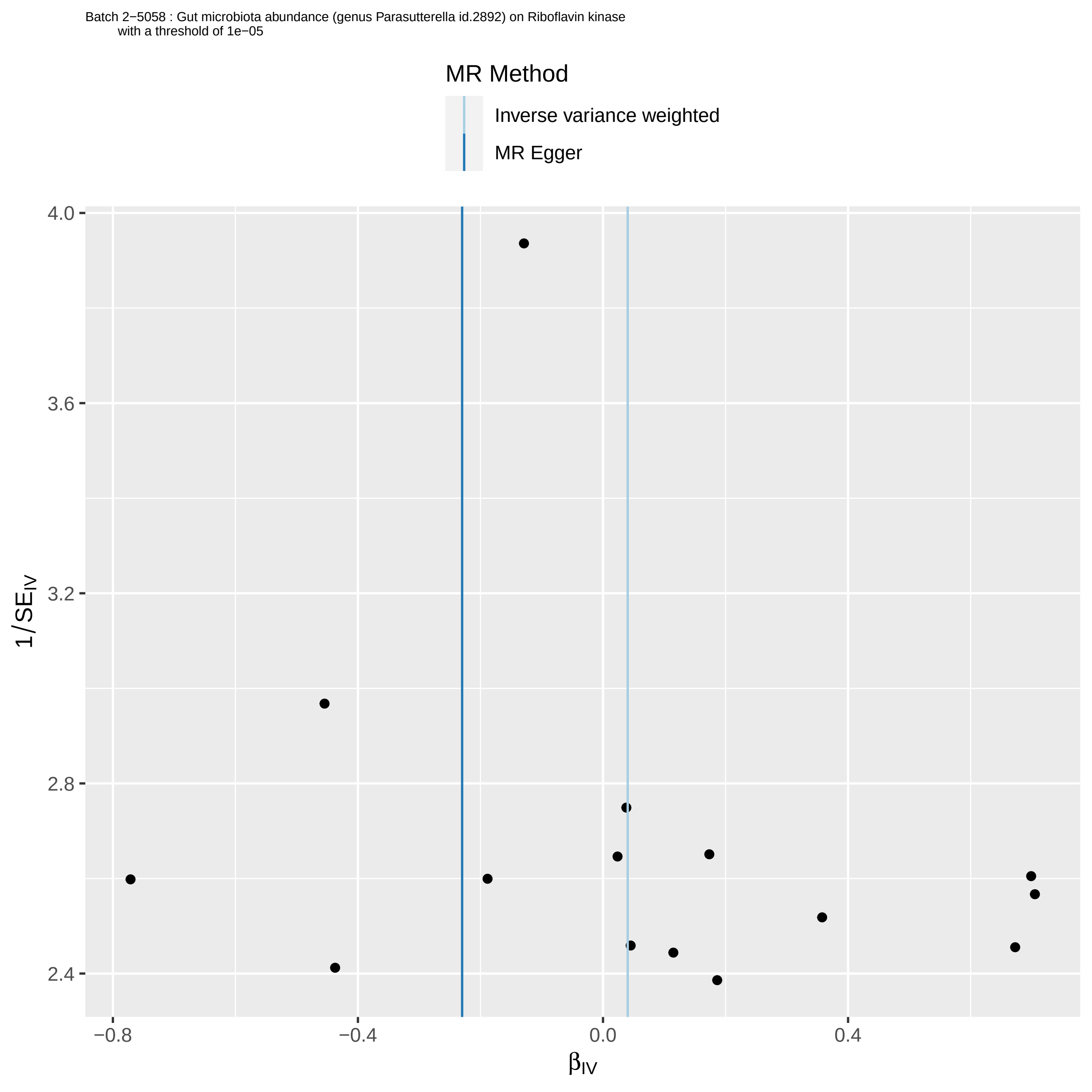
Batch 2−5058 : Gut microbiota abundance (genus Parasutterella id.2892) on Riboflavin kinase
with a threshold of 1e−05
MR Method
Inverse variance weighted
MR Egger
4.0
3.6
3.2
2.8
2.4
1/SE$_{IV}$
−0.8
−0.4
0.0
0.4
β$_{IV}$

Batch 2−5059 : Gut microbiota abundance (genus Catenibacterium id.2153) on Beta−1,3−N−acetylglucosaminyltransferase radical fringe with a threshold of 1e−05

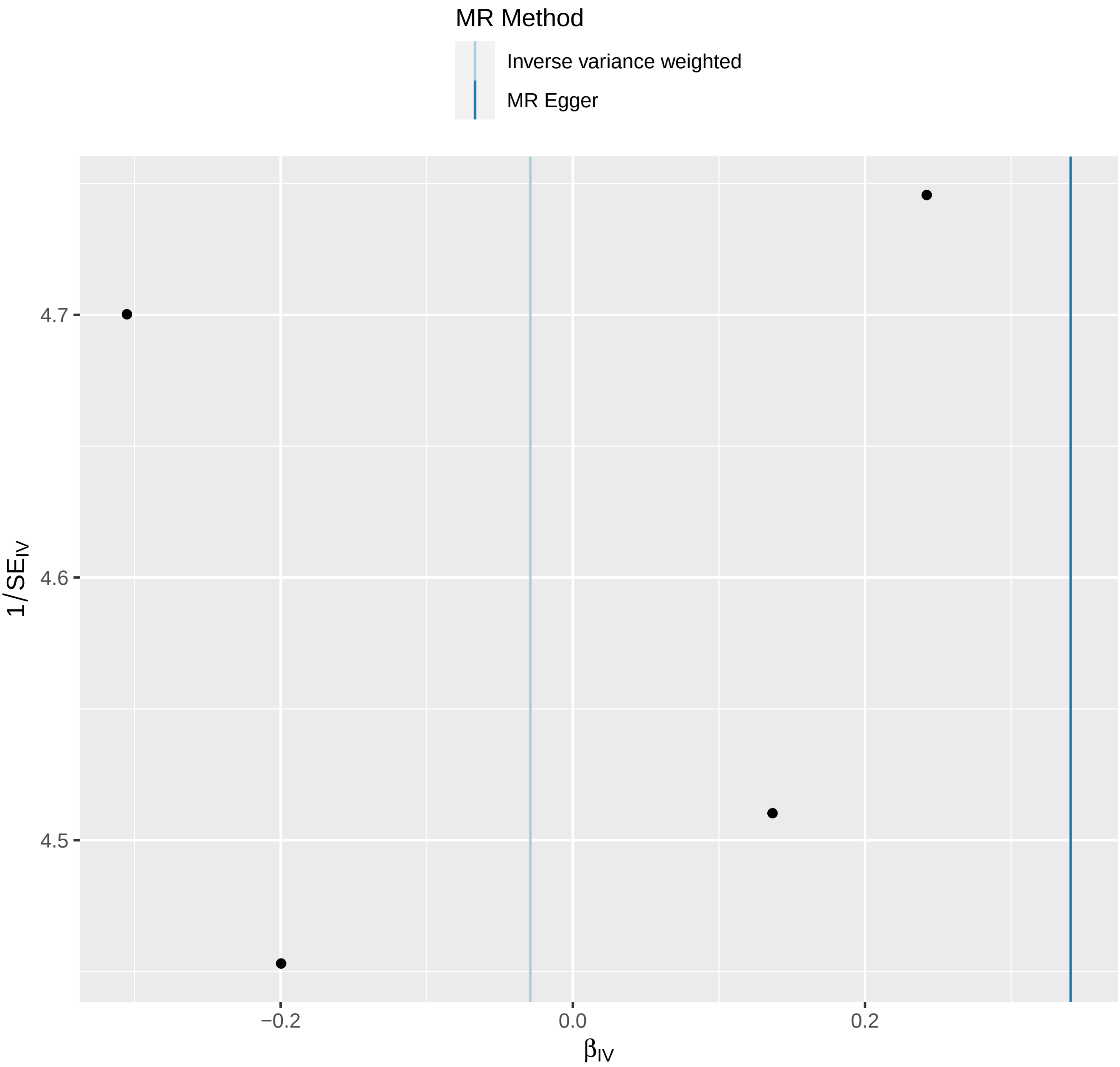

Batch 2−5060 : Gut microbiota abundance (genus Parasutterella id.2892) on Beta−1,3−N−acetylglucosaminyltransferase radical fringe with a threshold of 1e−05

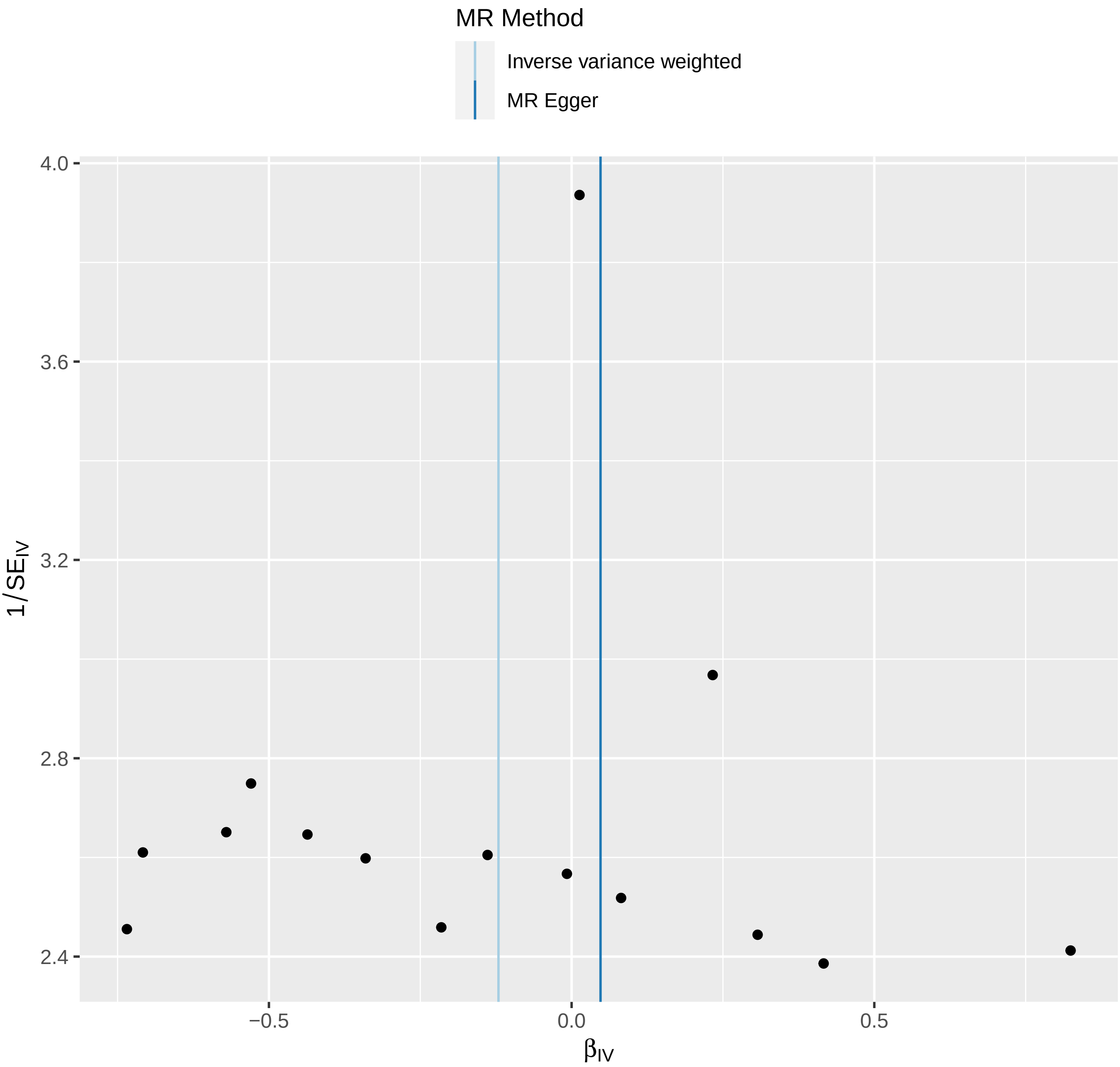

Batch 2−5061 : Gut microbiota abundance (genus Catenibacterium id.2153) on Regulatory factor X−associated protein with a threshold of 1e−05

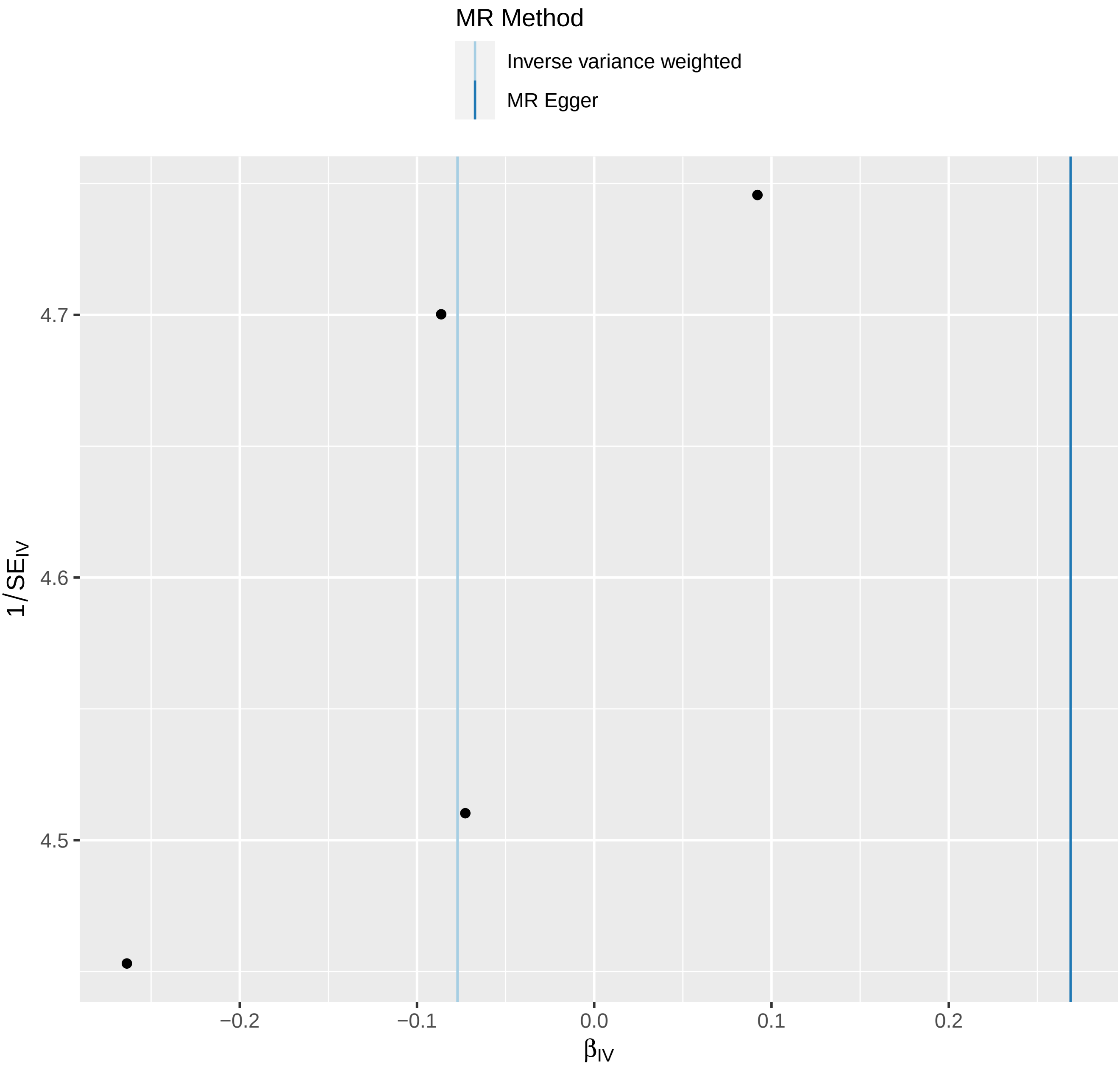

Batch 2−5062 : Gut microbiota abundance (genus Parasutterella id.2892) on Regulatory factor X−associated protein
with a threshold of 1e−05

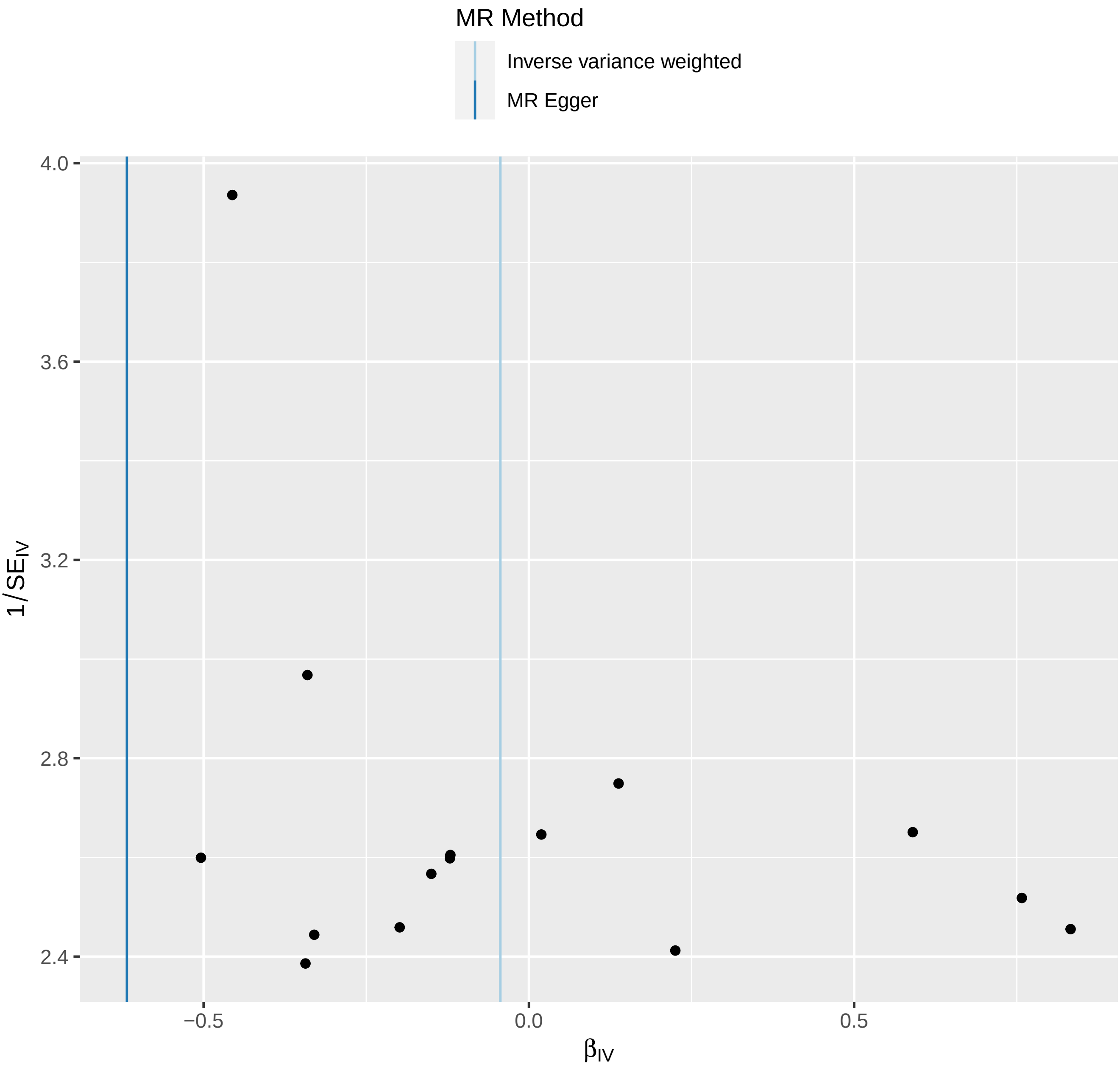

Batch 2−5063 : Gut microbiota abundance (genus Catenibacterium id.2153) on Repulsive guidance molecule A
with a threshold of 1e−05

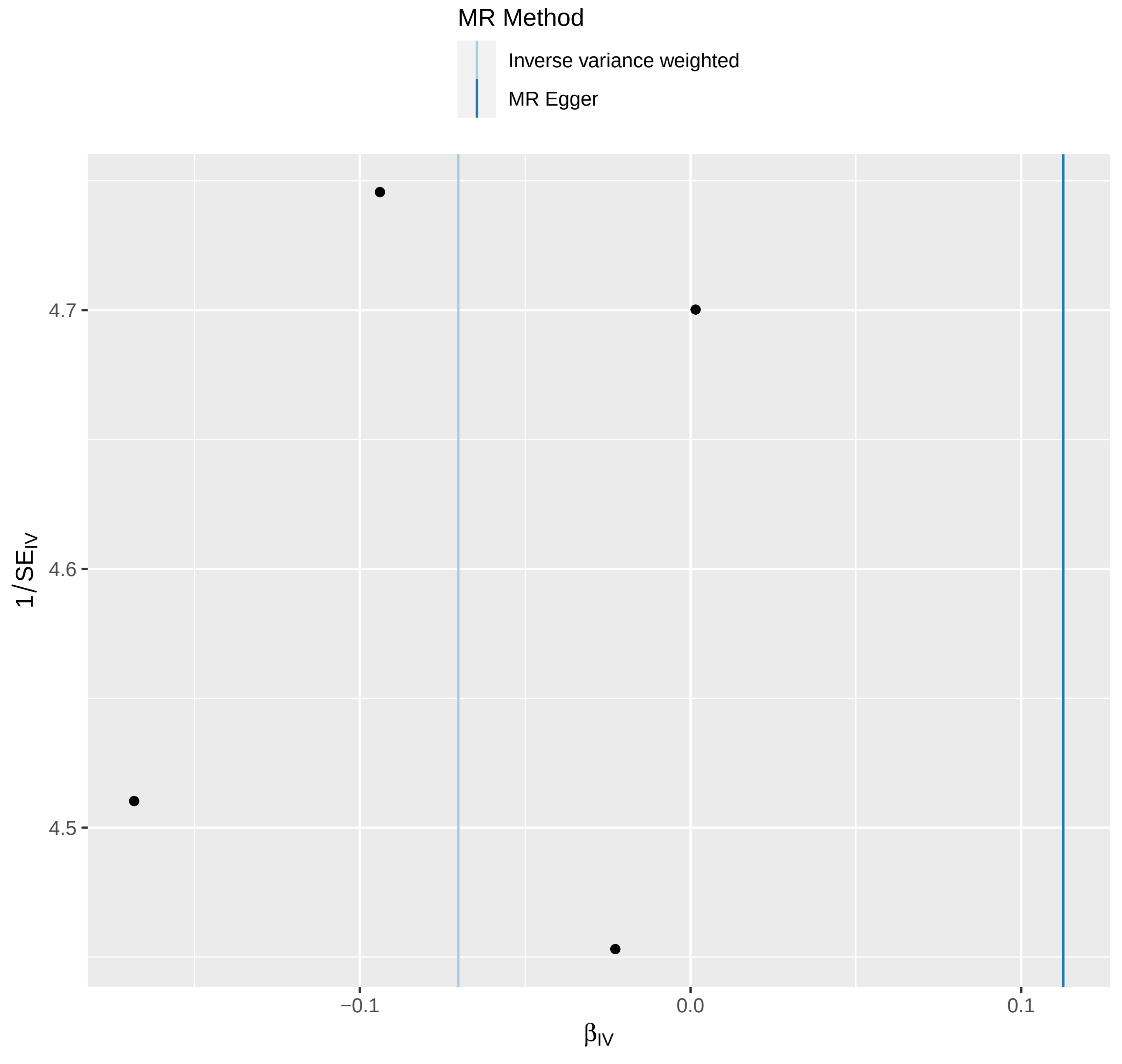

Batch 2−5064 : Gut microbiota abundance (genus Parasutterella id.2892) on Repulsive guidance molecule A
with a threshold of 1e−05

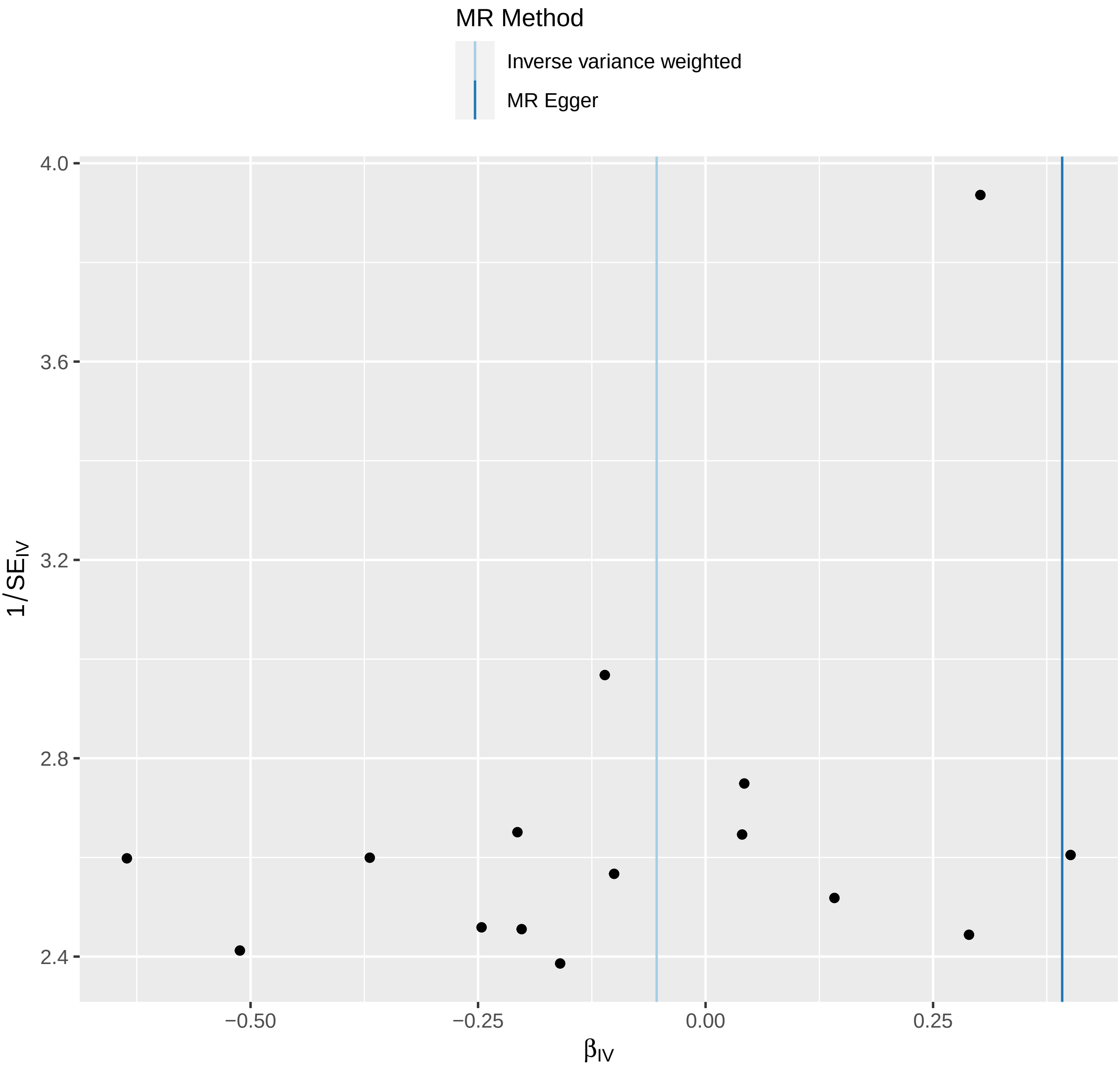

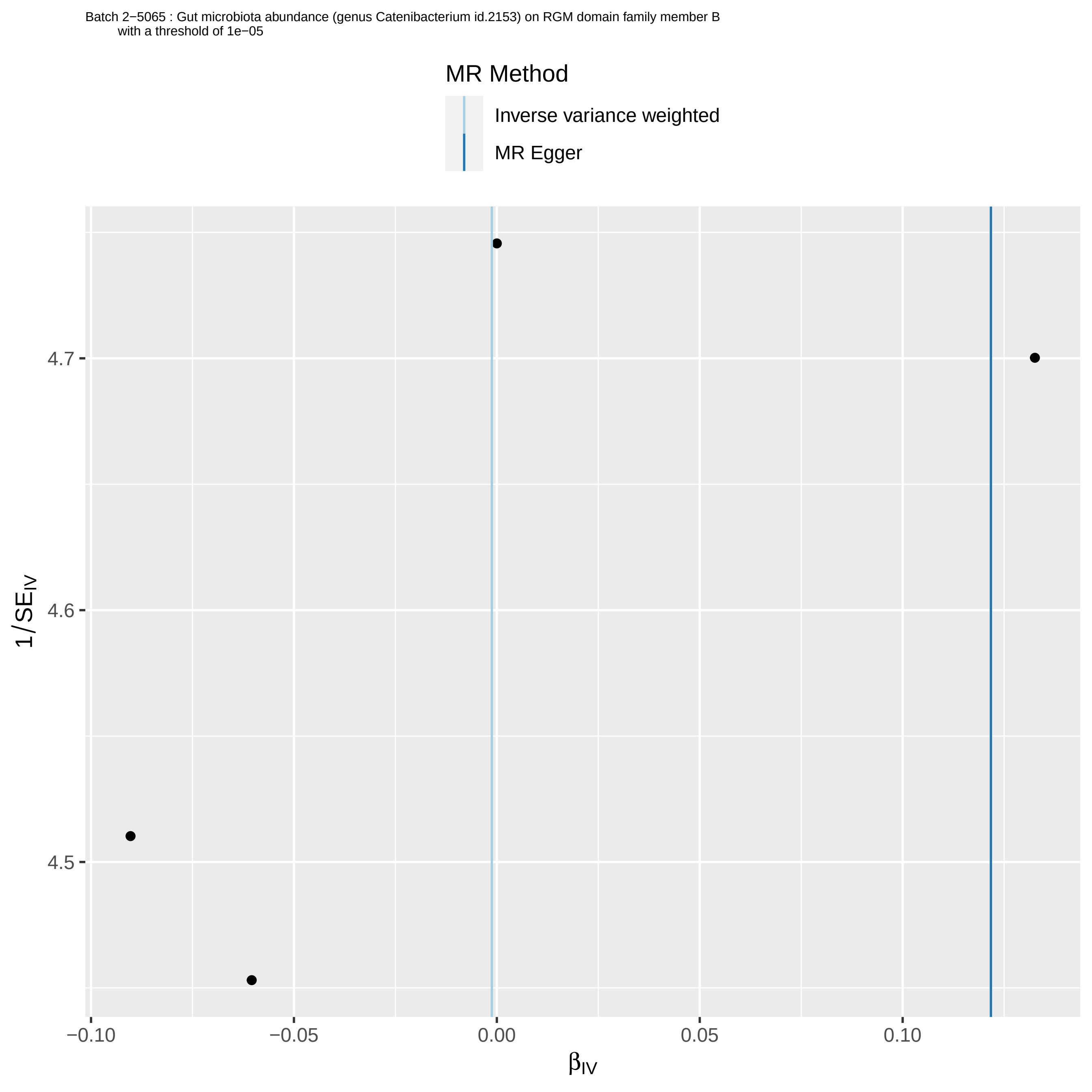
Batch 2−5065 : Gut microbiota abundance (genus Catenibacterium id.2153) on RGM domain family member B
with a threshold of 1e−05
MR Method
Inverse variance weighted
MR Egger
4.7
4.6
4.5
1/SE$_{IV}$
−0.10
−0.05
0.00
0.05
0.10
β$_{IV}$

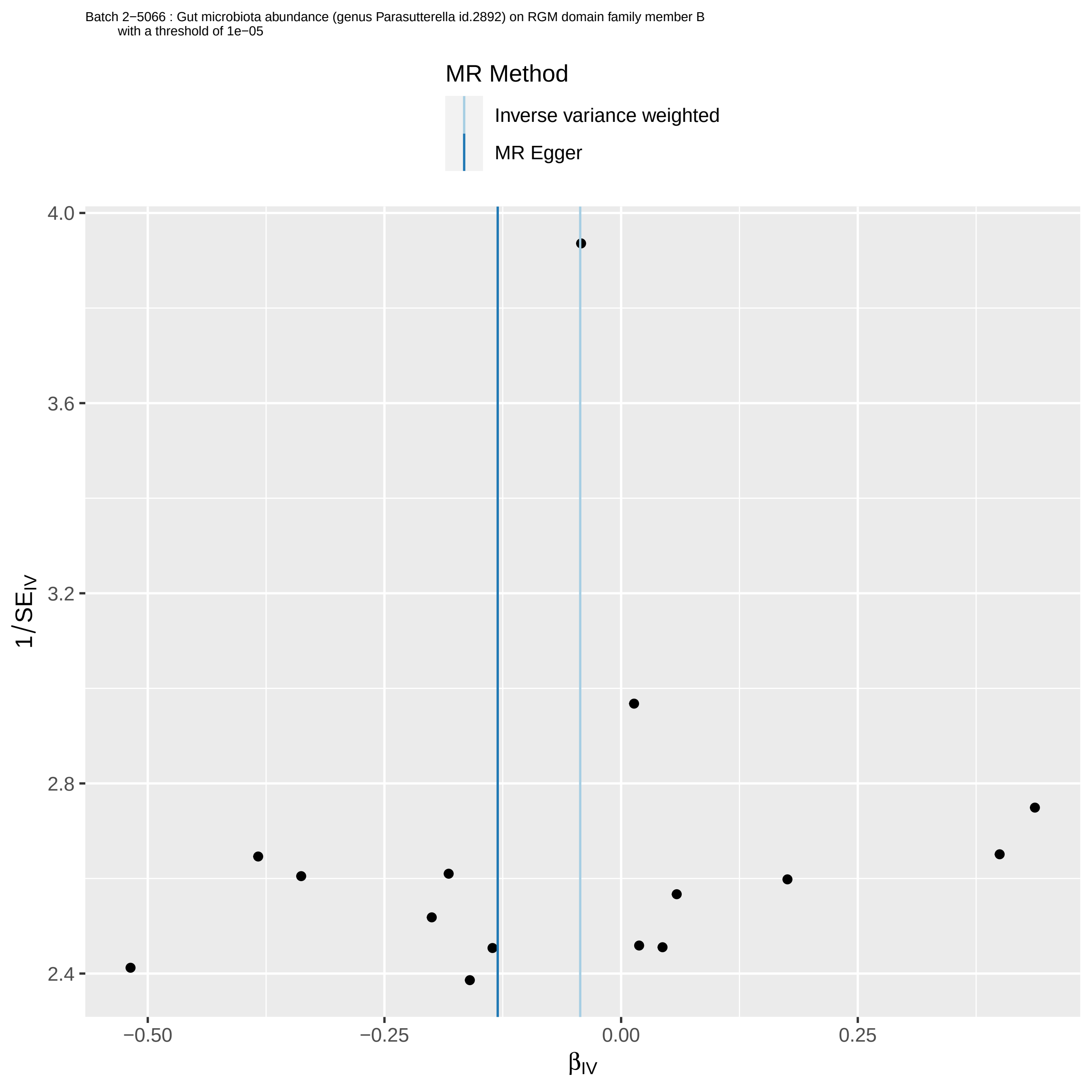

Batch 2−5066 : Gut microbiota abundance (genus Parasutterella id.2892) on RGM domain family member B
with a threshold of 1e−05
MR Method
Inverse variance weighted
MR Egger
4.0
3.6
3.2
2.8
2.4
1/SE_IV
−0.50
−0.25
0.00
0.25
β_IV

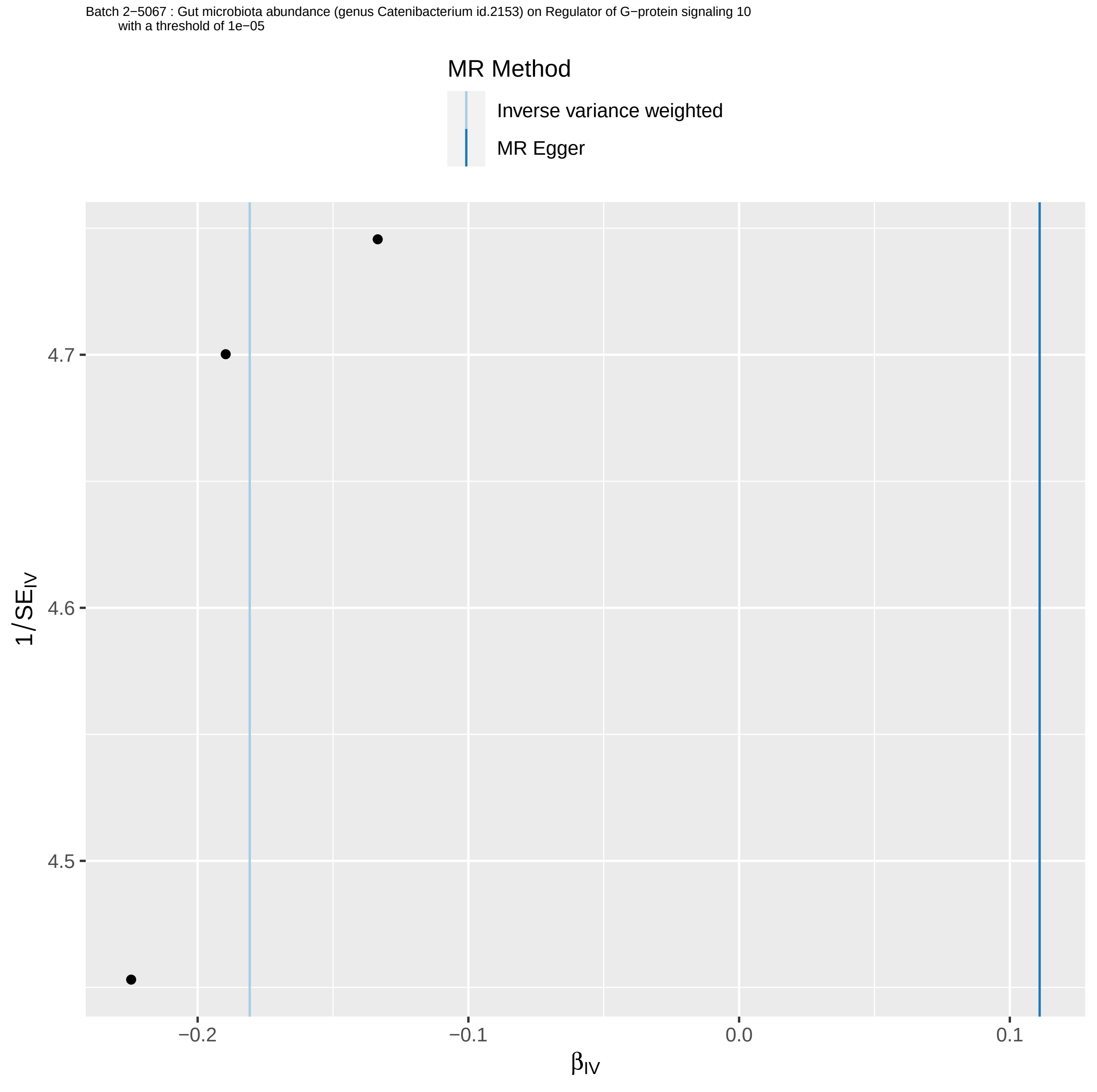
Batch 2−5067 : Gut microbiota abundance (genus Catenibacterium id.2153) on Regulator of G−protein signaling 10
with a threshold of 1e−05
MR Method
Inverse variance weighted
MR Egger
4.7
4.6
4.5
1/SE_IV
−0.2
−0.1
0.0
0.1
β_IV

Batch 2−5068 : Gut microbiota abundance (genus Parasutterella id.2892) on Regulator of G−protein signaling 10
with a threshold of 1e−05

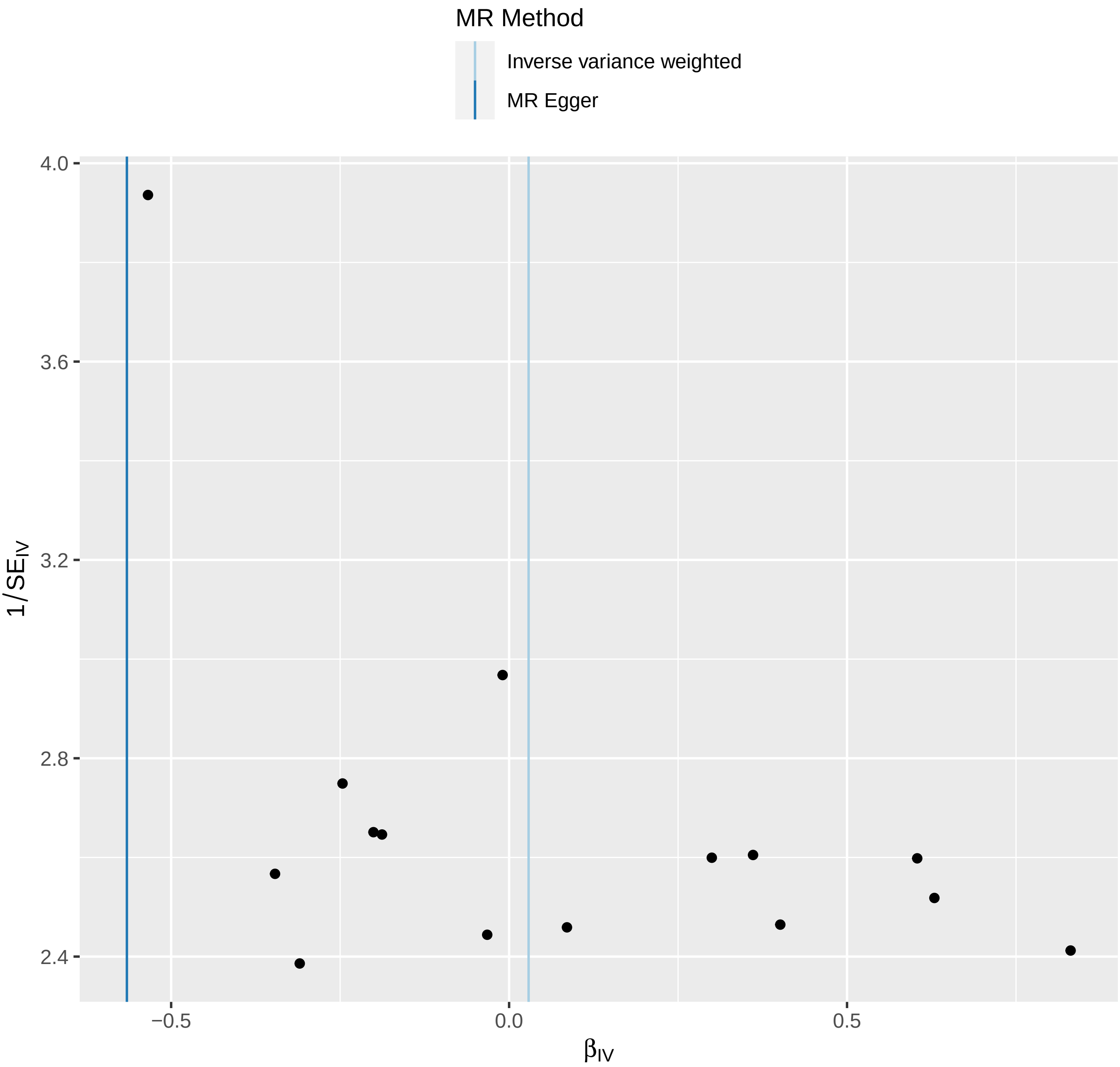

Batch 2−5069 : Gut microbiota abundance (genus Catenibacterium id.2153) on Regulator of G−protein signaling 18
with a threshold of 1e−05

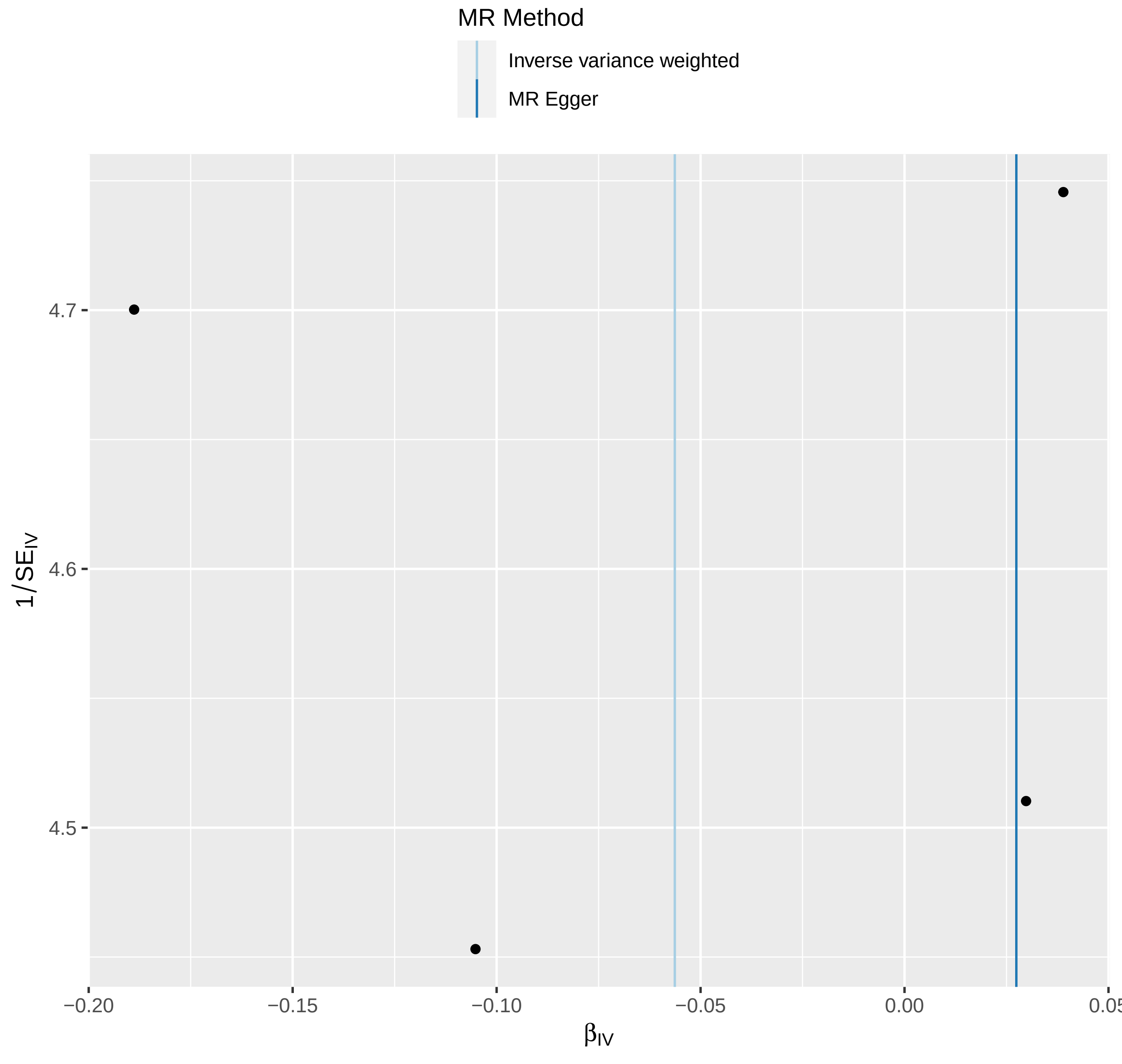

Batch 2−5070 : Gut microbiota abundance (genus Parasutterella id.2892) on Regulator of G−protein signaling 18 with a threshold of 1e−05

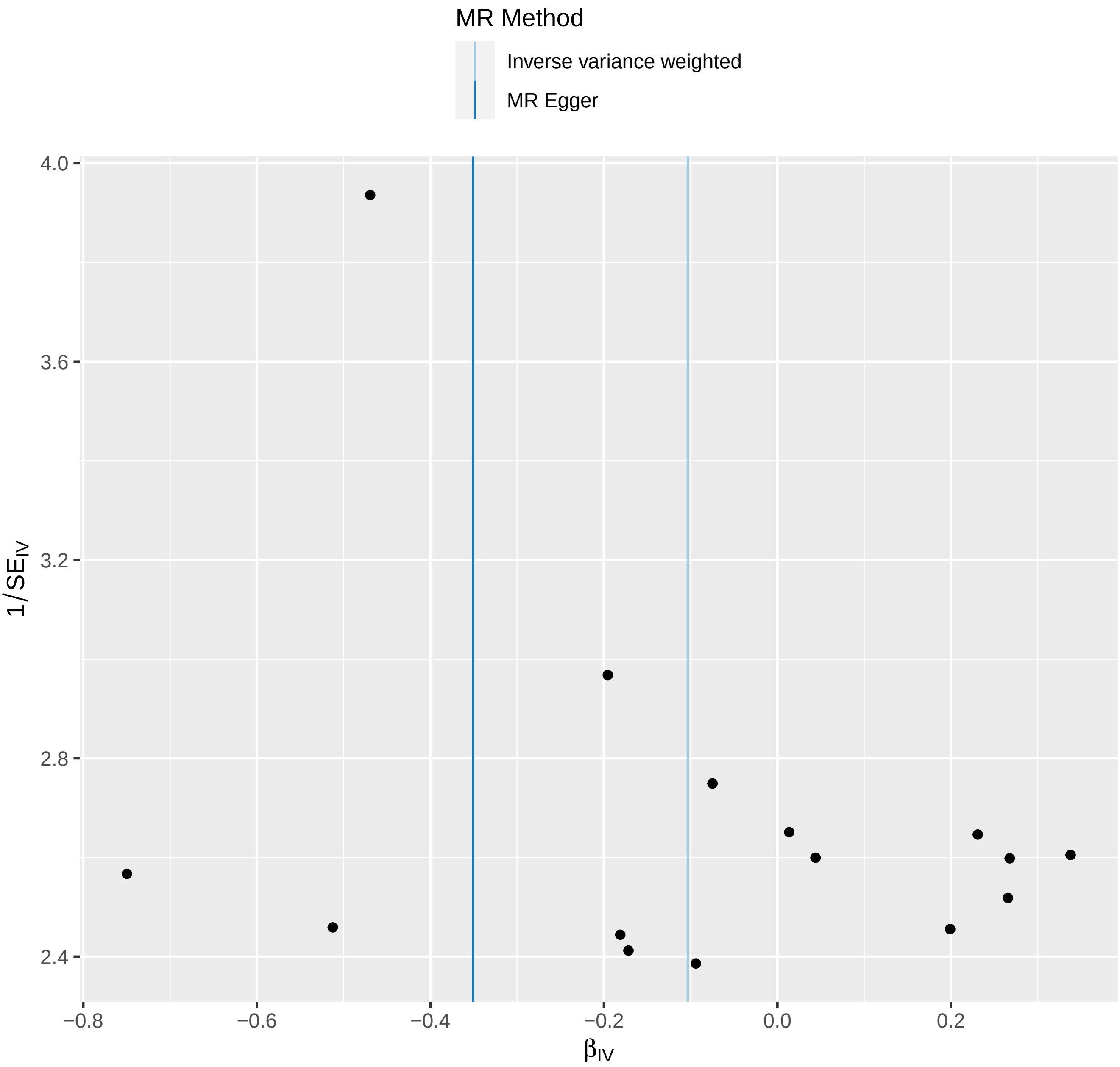

Batch 2−5071 : Gut microbiota abundance (genus Catenibacterium id.2153) on Regulator of G−protein signaling 19
with a threshold of 1e−05

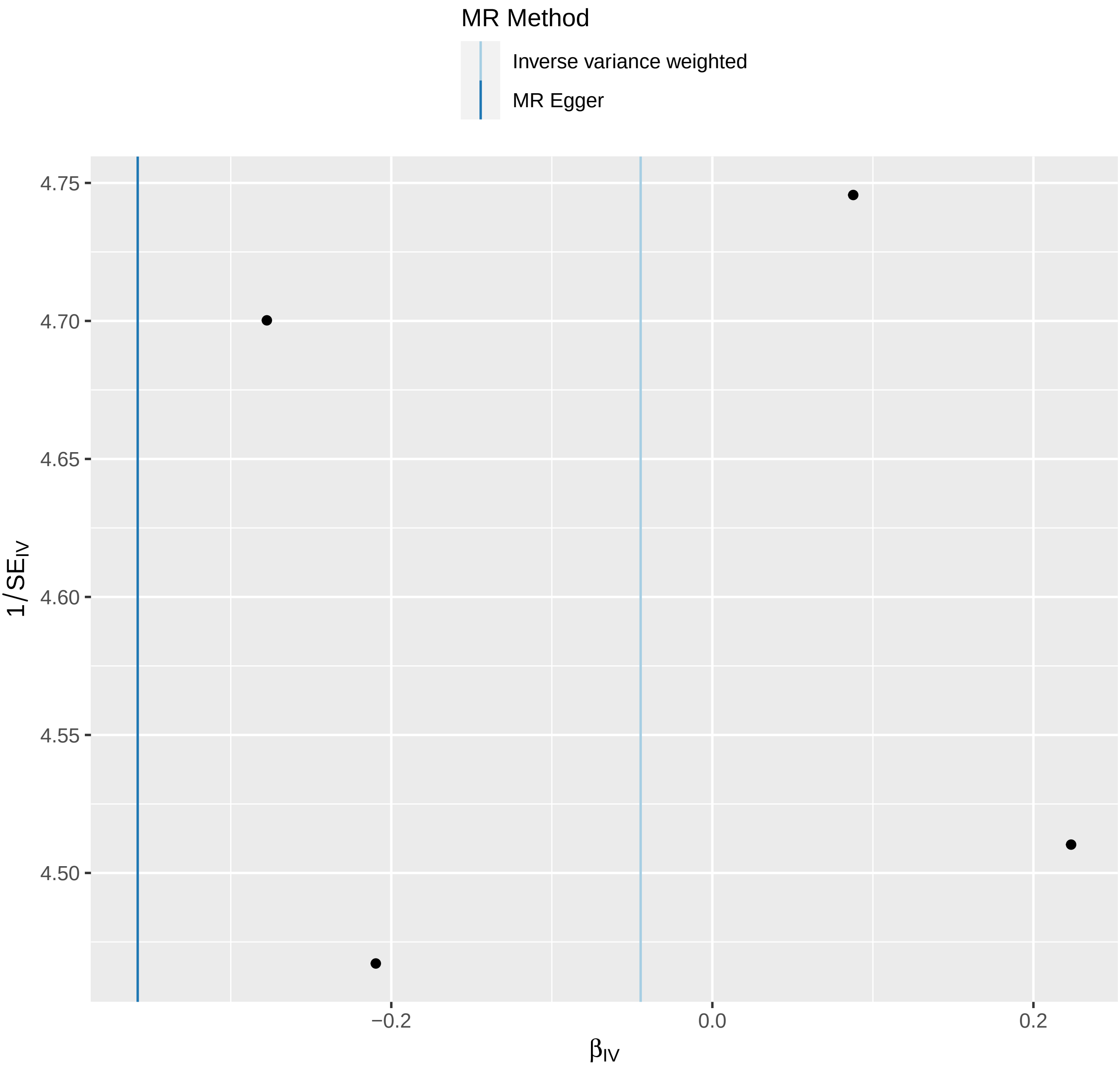

Batch 2−5072 : Gut microbiota abundance (genus Parasutterella id.2892) on Regulator of G−protein signaling 19
with a threshold of 1e−05

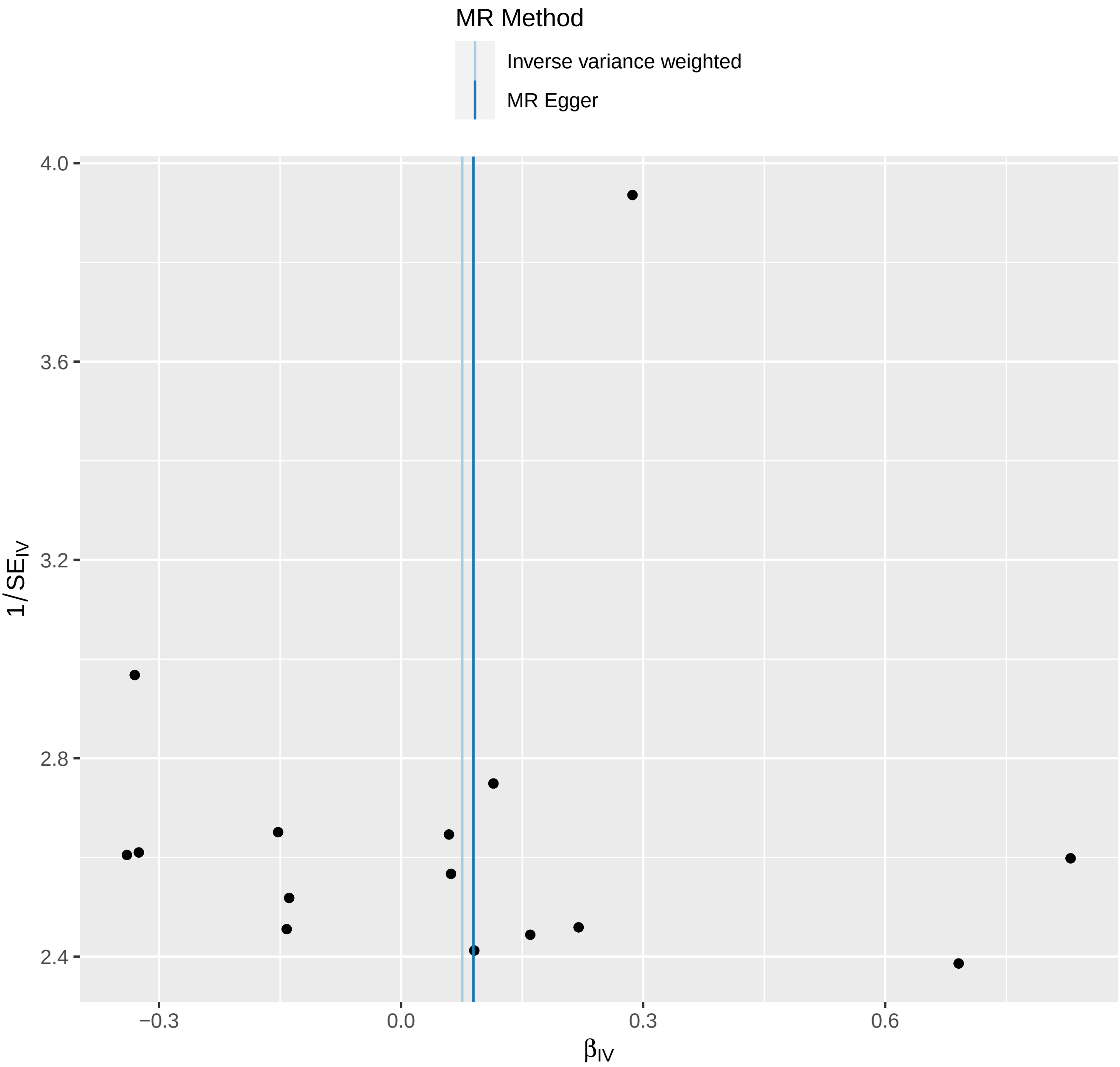

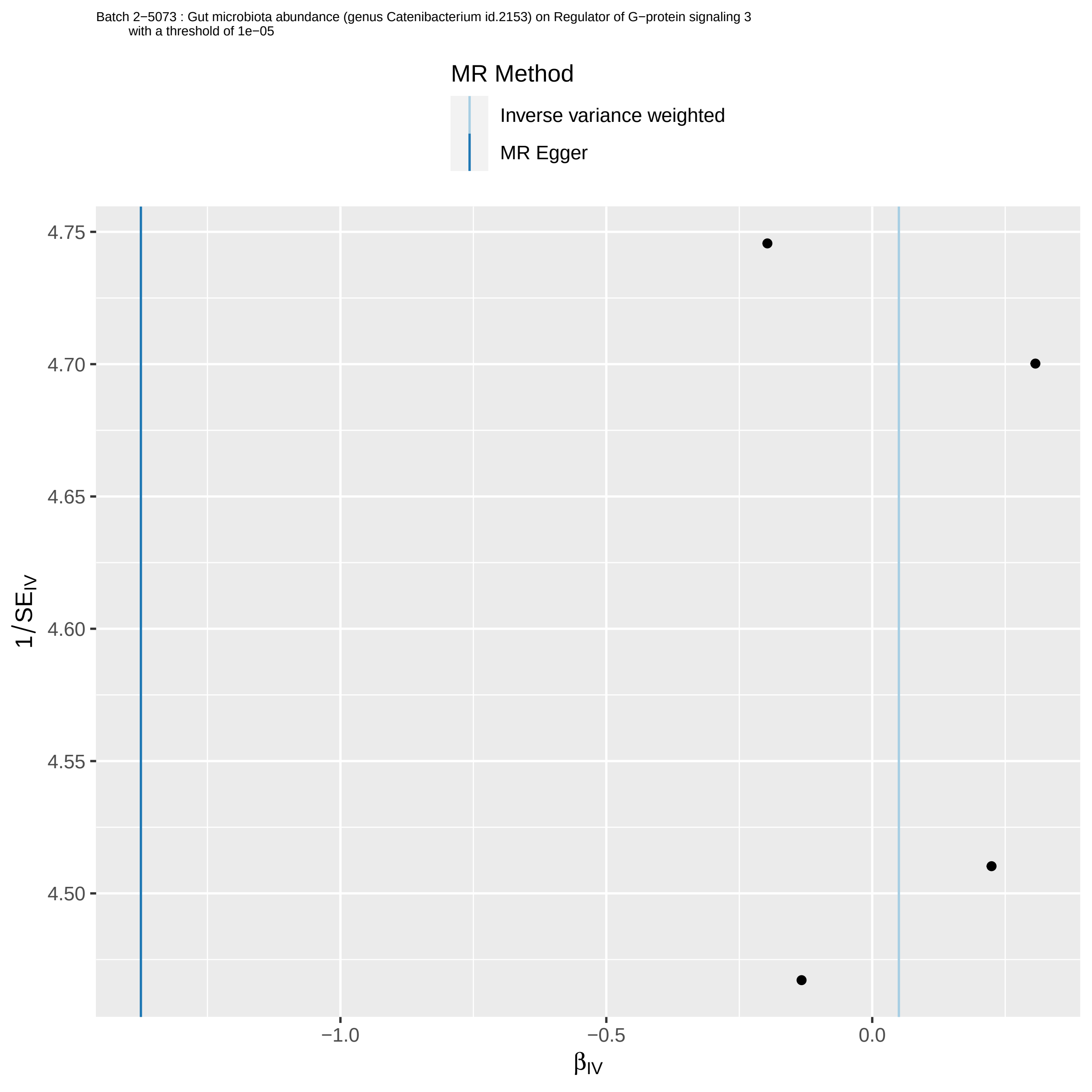
Batch 2−5073 : Gut microbiota abundance (genus Catenibacterium id.2153) on Regulator of G−protein signaling 3
with a threshold of 1e−05
MR Method
Inverse variance weighted
MR Egger
4.75
4.70
4.65
4.60
4.55
4.50
1/SE_IV
−1.0
−0.5
0.0
β_IV

Batch 2−5074 : Gut microbiota abundance (genus Parasutterella id.2892) on Regulator of G−protein signaling 3
with a threshold of 1e−05

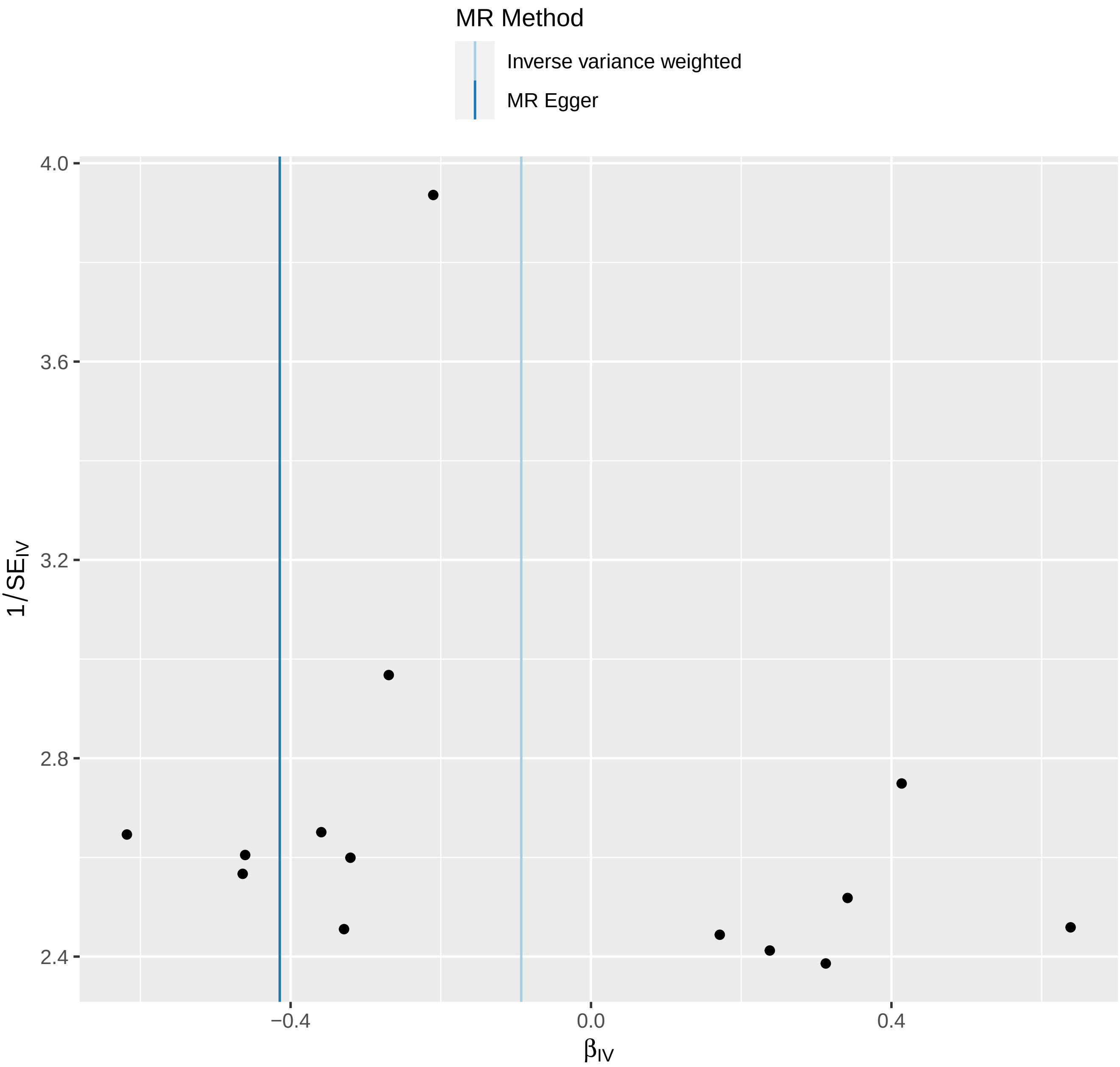

Batch 2−5075 : Gut microbiota abundance (genus Catenibacterium id.2153) on Regulator of G−protein signaling 8
with a threshold of 1e−05

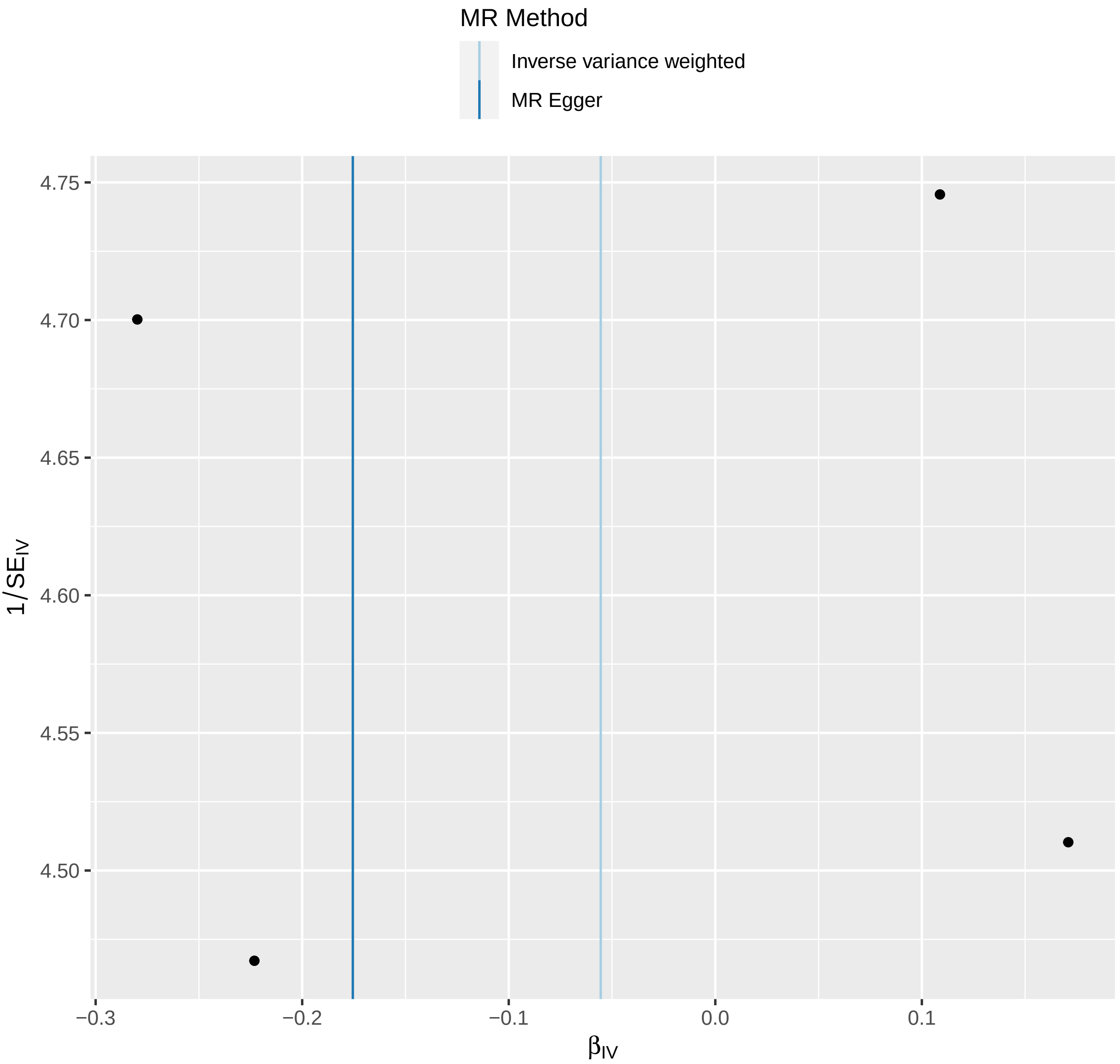

Batch 2−5076 : Gut microbiota abundance (genus Parasutterella id.2892) on Regulator of G−protein signaling 8
with a threshold of 1e−05

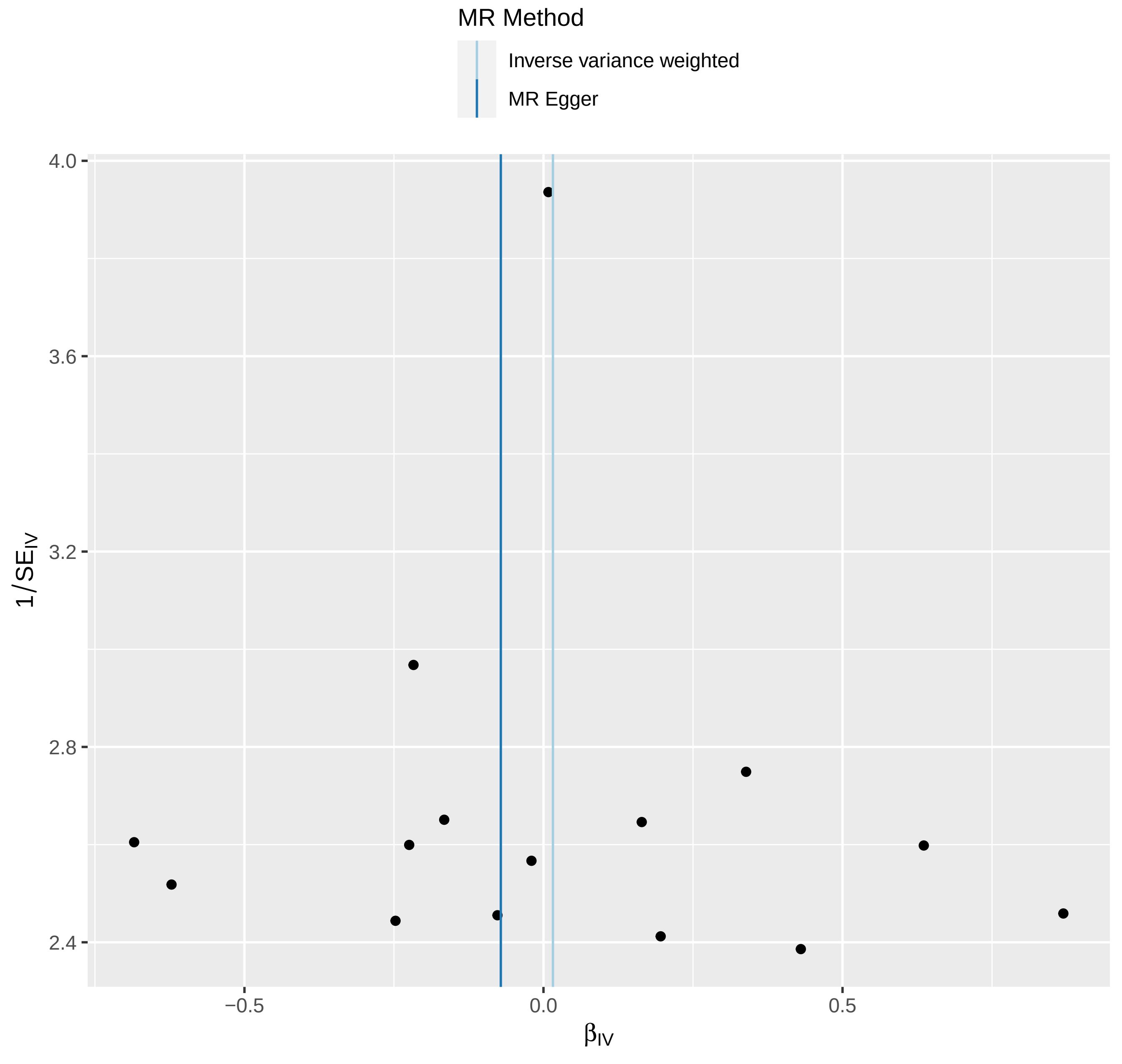

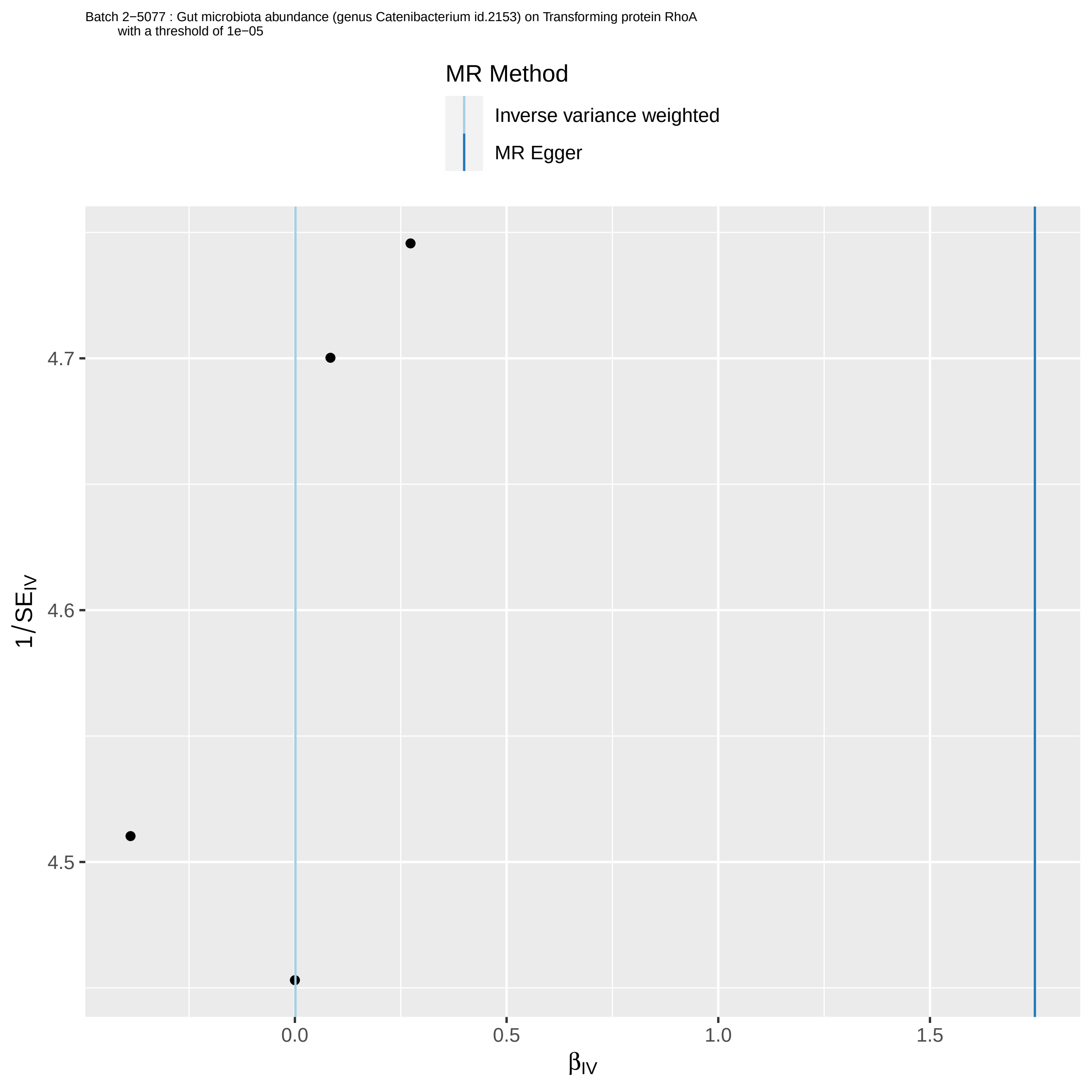
Batch 2−5077 : Gut microbiota abundance (genus Catenibacterium id.2153) on Transforming protein RhoA
with a threshold of 1e−05
MR Method
Inverse variance weighted
MR Egger
4.7
4.6
4.5
1/SE_IV
0.0
0.5
1.0
1.5
β_IV

Batch 2−5078 : Gut microbiota abundance (genus Parasutterella id.2892) on Transforming protein RhoA
with a threshold of 1e−05

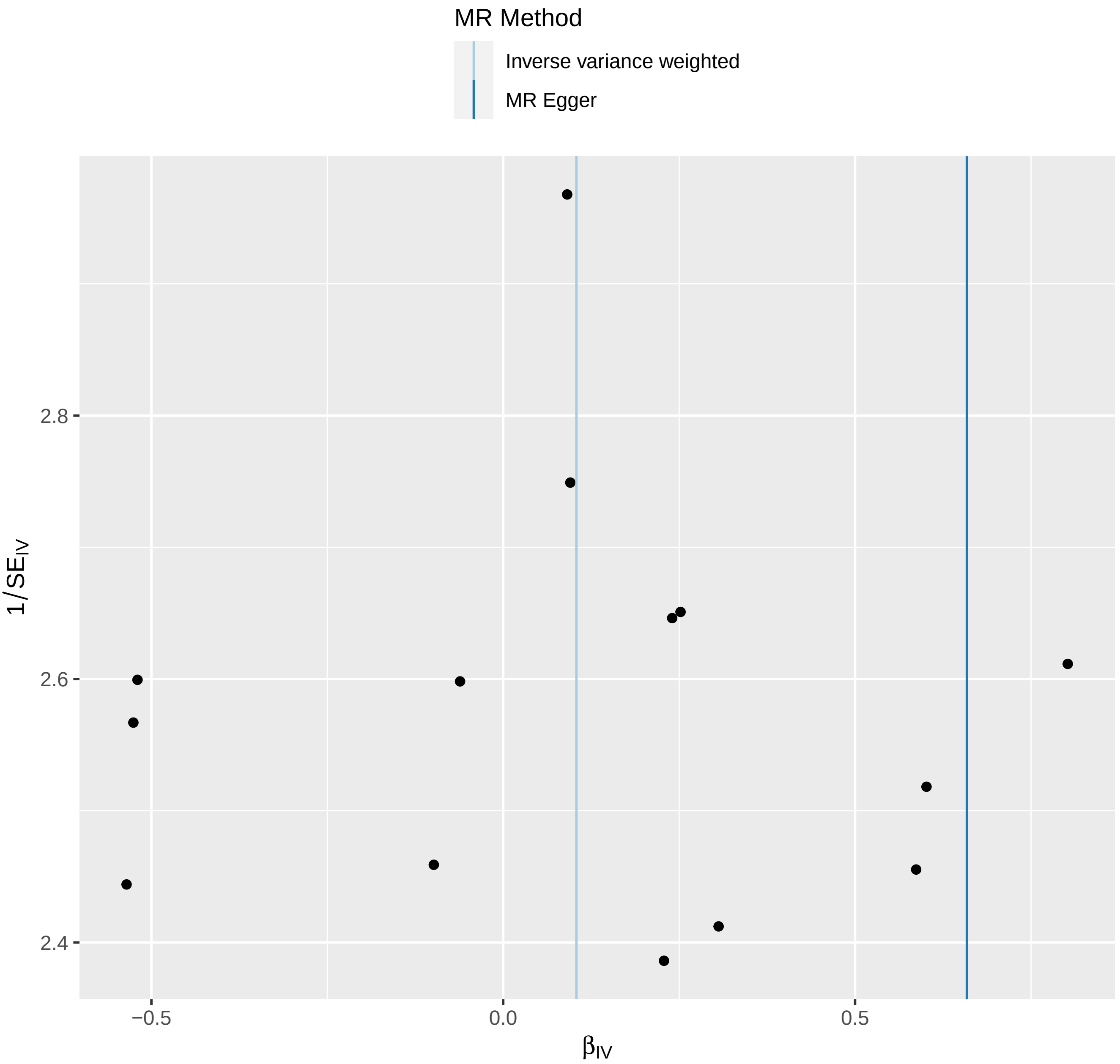

Batch 2−5079 : Gut microbiota abundance (genus Catenibacterium id.2153) on Rho−related GTP−binding protein RhoD
with a threshold of 1e−05

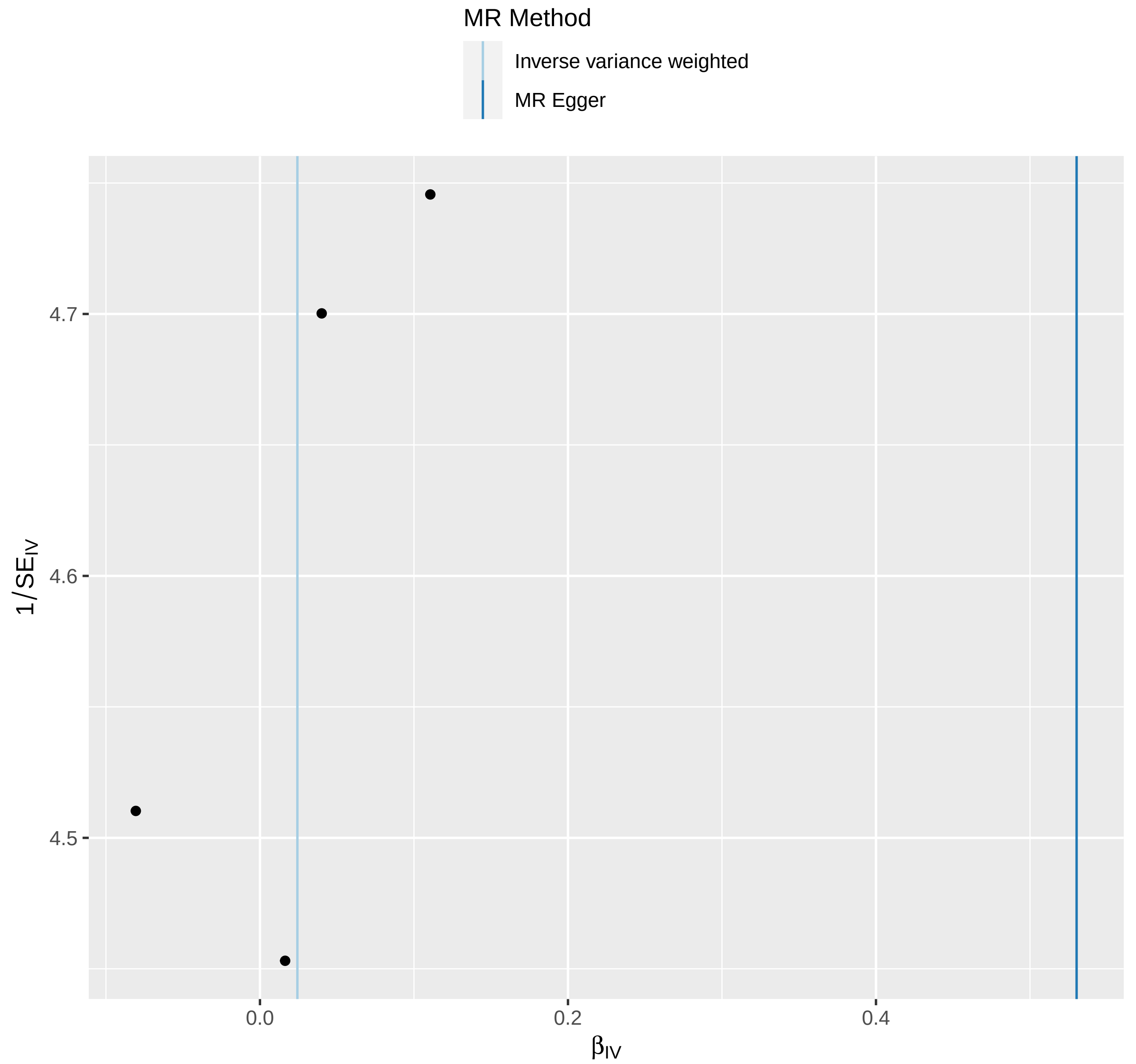

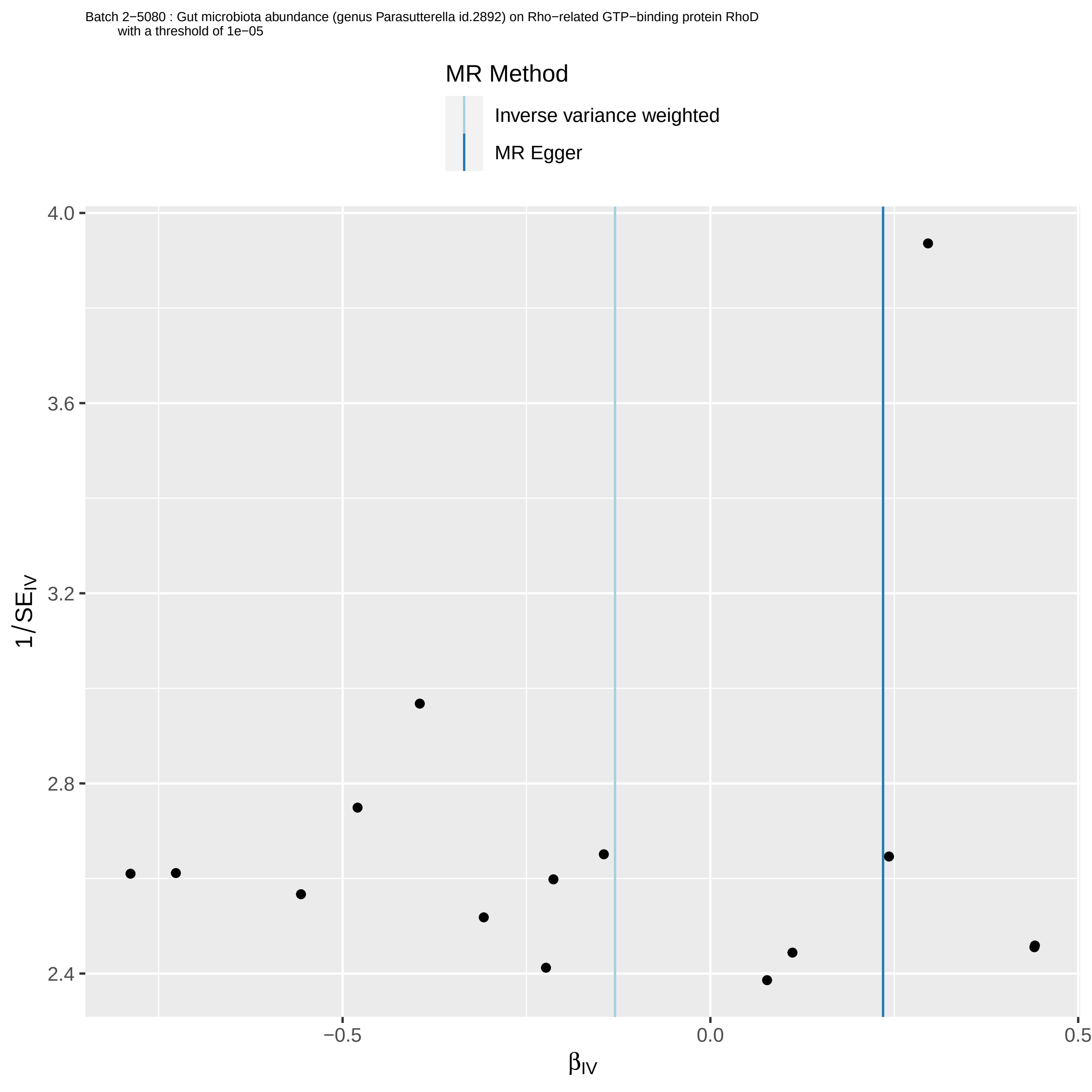
Batch 2−5080 : Gut microbiota abundance (genus Parasutterella id.2892) on Rho−related GTP−binding protein RhoD
with a threshold of 1e−05
MR Method
Inverse variance weighted
MR Egger
4.0
3.6
3.2
2.8
2.4
1/SE_IV
−0.5
0.0
0.5
β_IV

Batch 2−5081 : Gut microbiota abundance (genus Catenibacterium id.2153) on Rho−related GTP−binding protein RhoG with a threshold of 1e−05

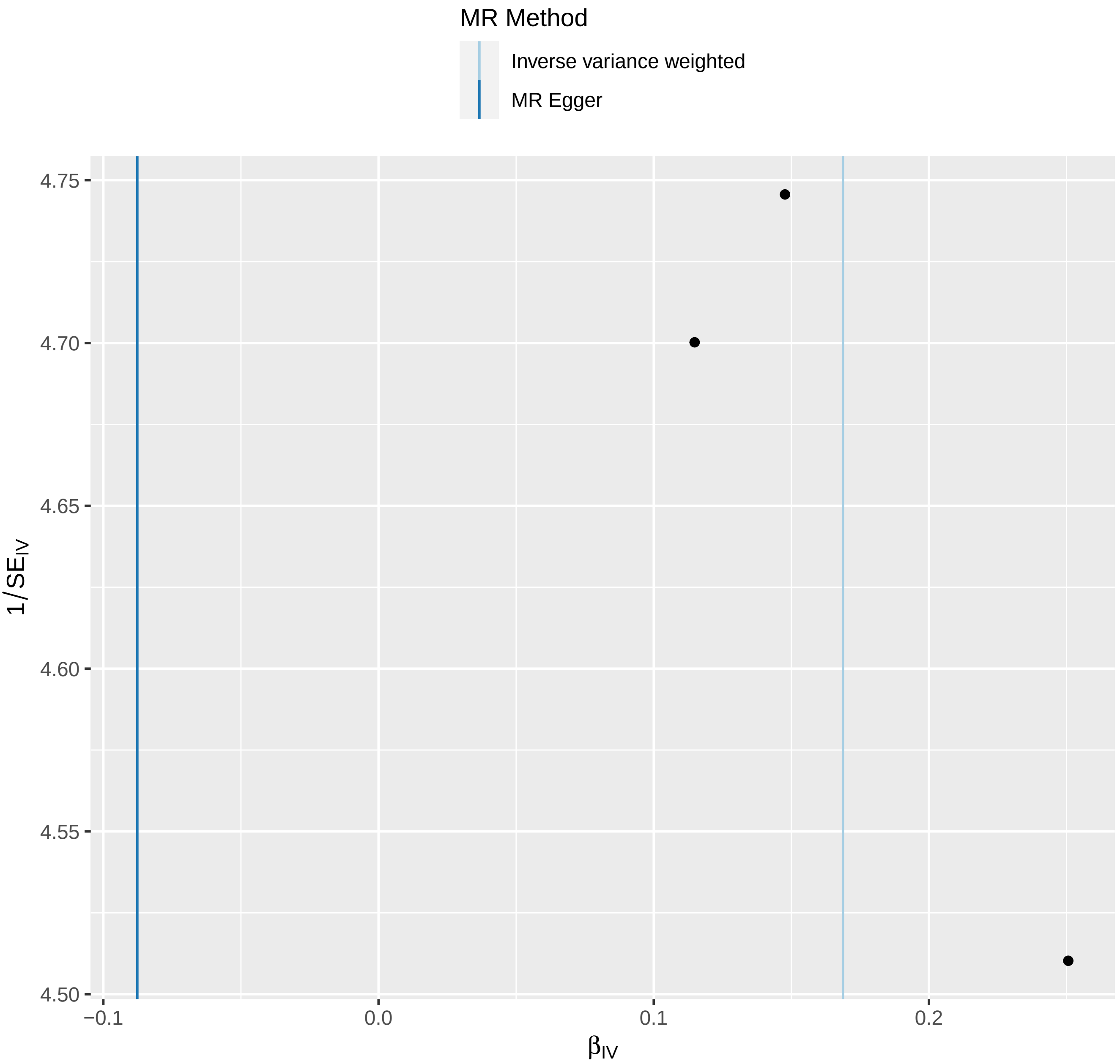

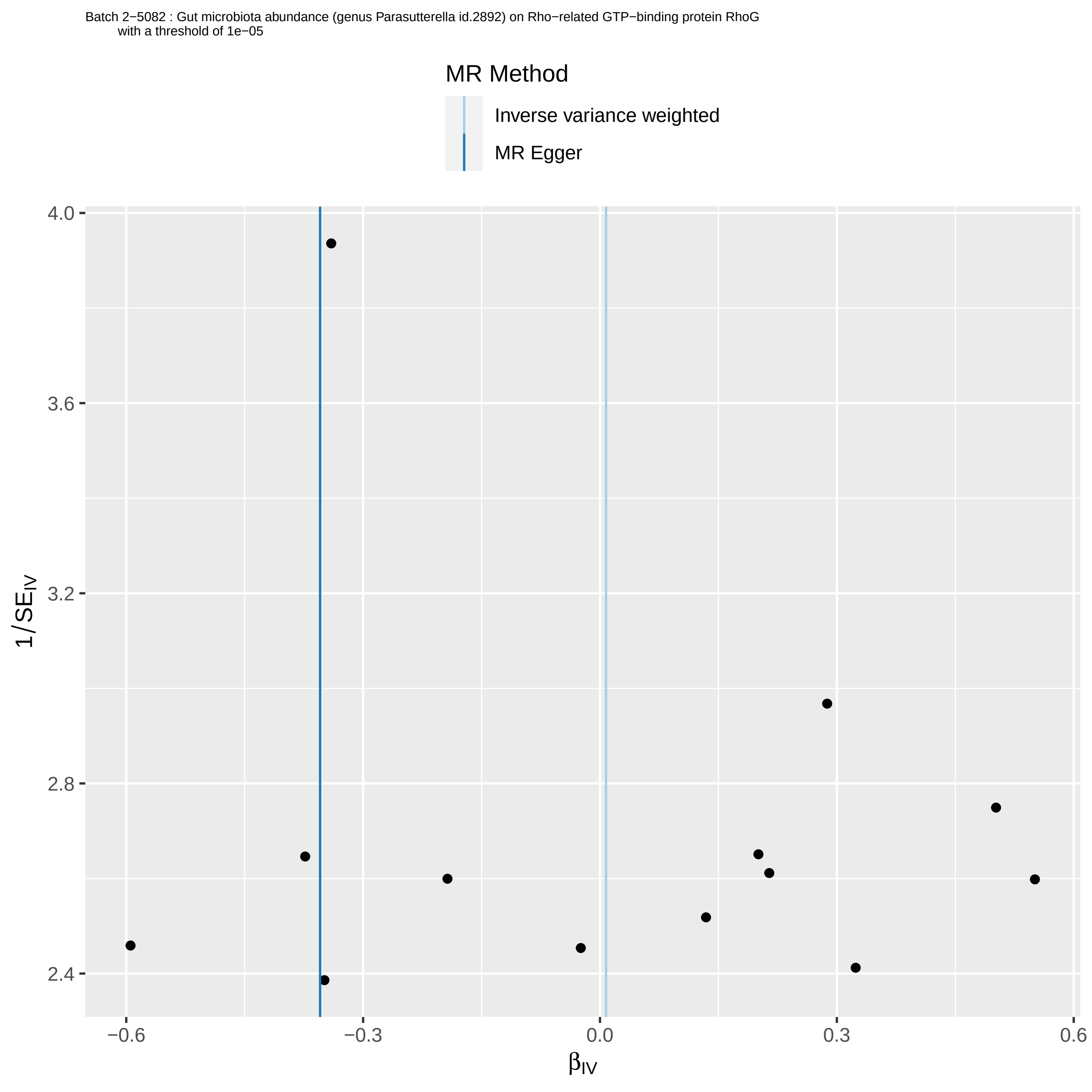

Batch 2−5082 : Gut microbiota abundance (genus Parasutterella id.2892) on Rho−related GTP−binding protein RhoG
with a threshold of 1e−05
MR Method
Inverse variance weighted
MR Egger
4.0
3.6
3.2
2.8
2.4
1/SE_IV
−0.6
−0.3
0.0
0.3
0.6
β_IV

Batch 2−5083 : Gut microbiota abundance (genus Catenibacterium id.2153) on Rhophilin−2
with a threshold of 1e−05

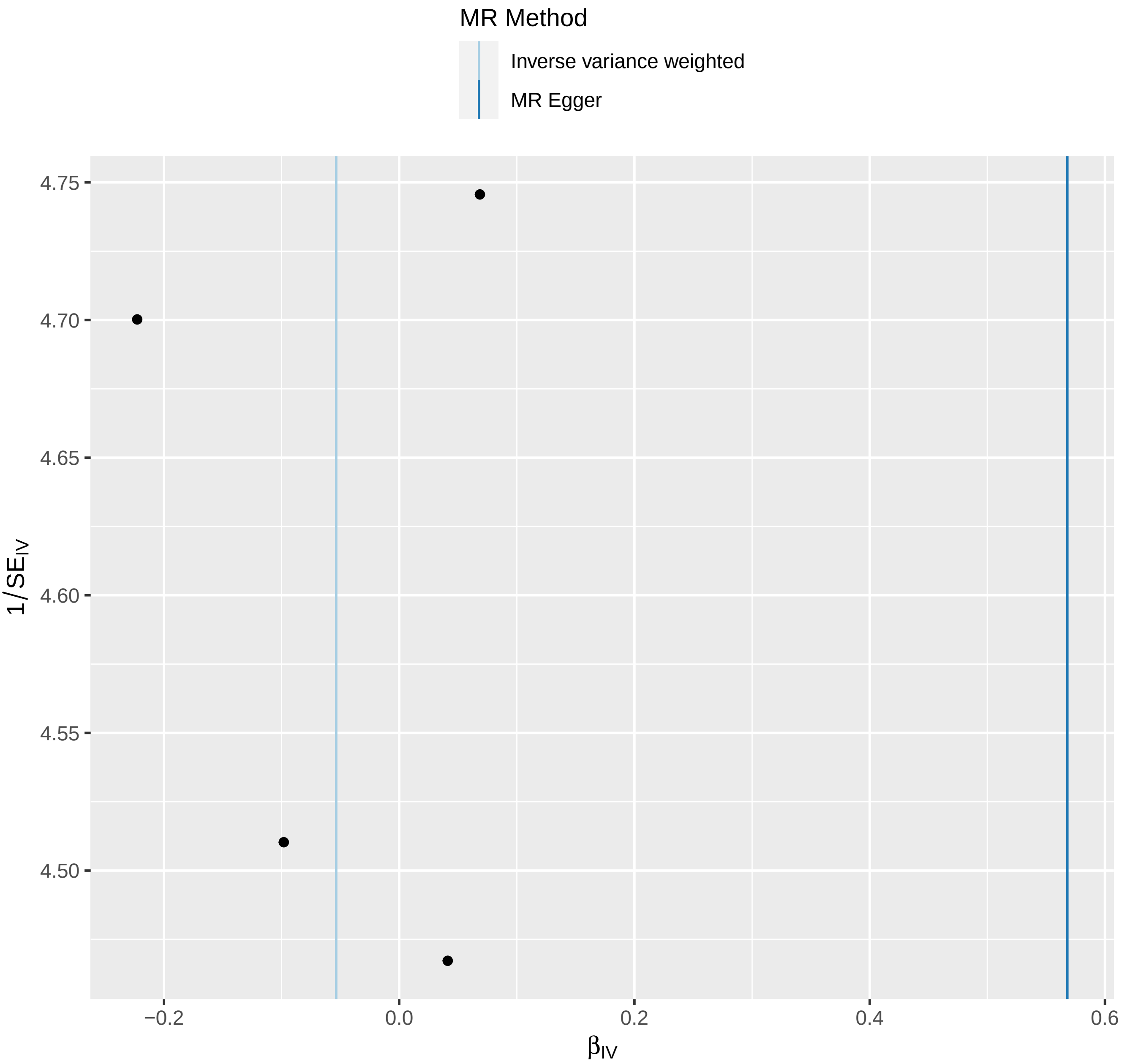

Batch 2−5084 : Gut microbiota abundance (genus Parasutterella id.2892) on Rhophilin−2
with a threshold of 1e−05

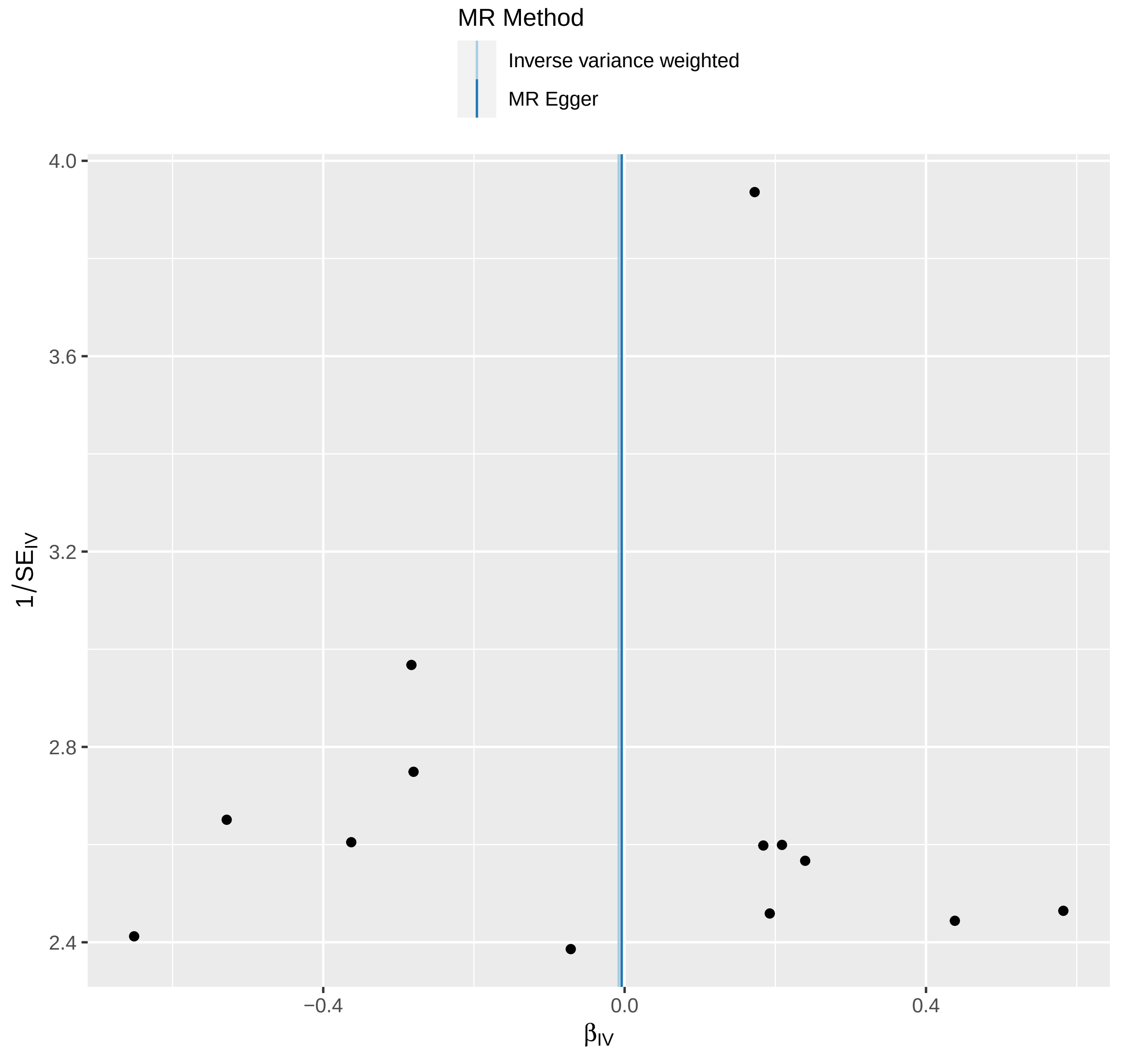

Batch 2−5085 : Gut microbiota abundance (genus Catenibacterium id.2153) on Protein RIC−3
with a threshold of 1e−05

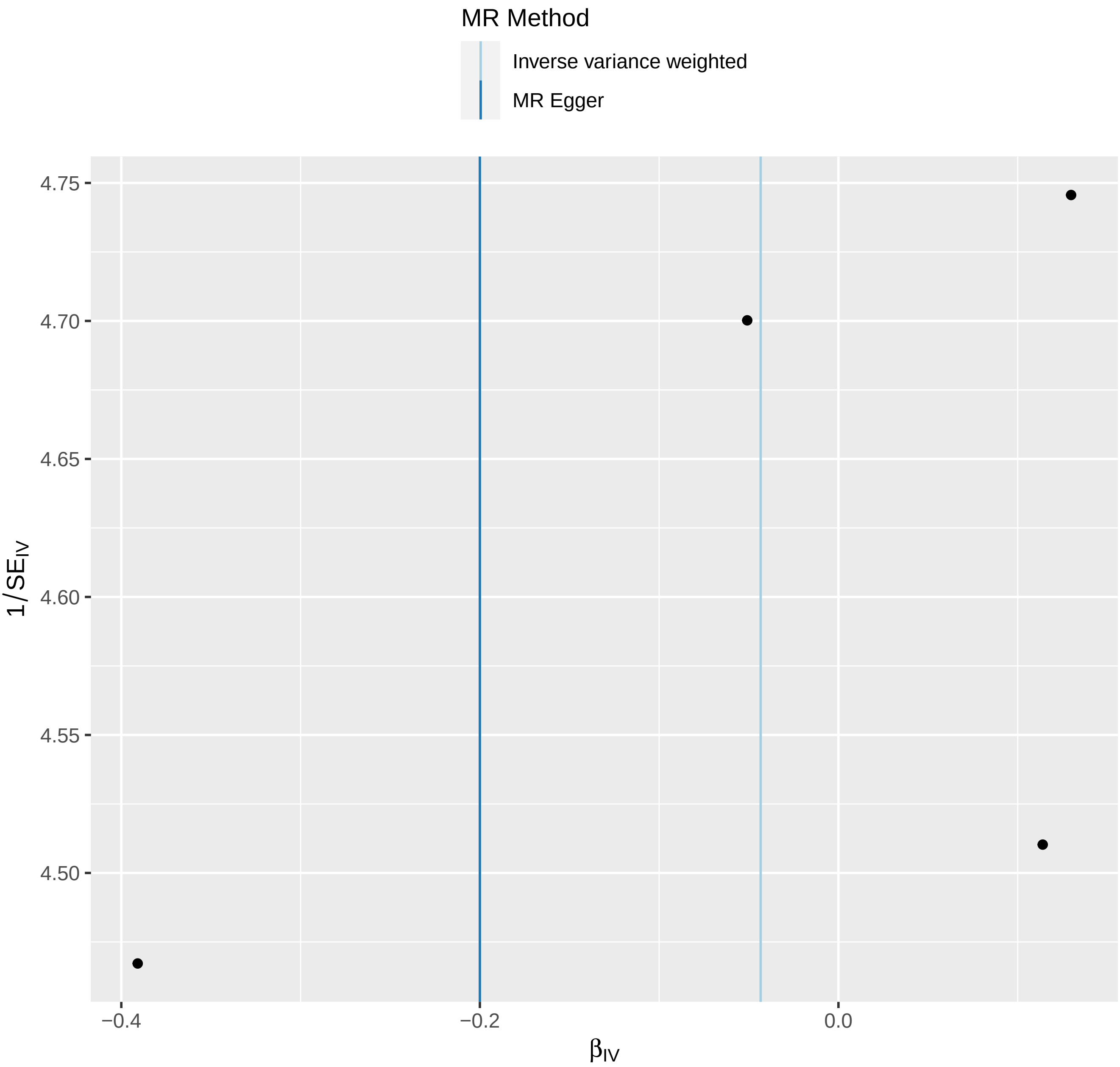

Batch 2−5086 : Gut microbiota abundance (genus Parasutterella id.2892) on Protein RIC−3
with a threshold of 1e−05

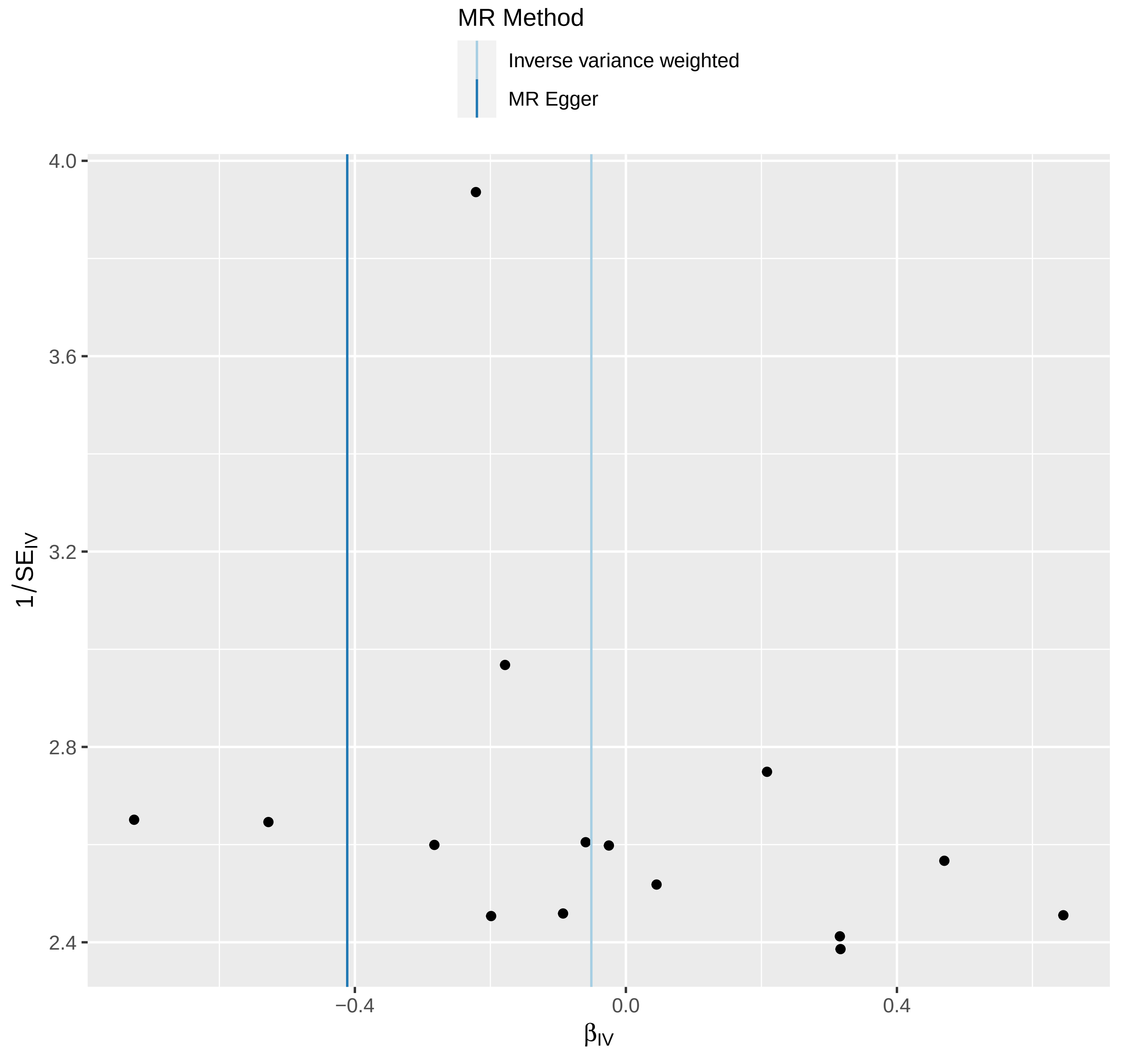

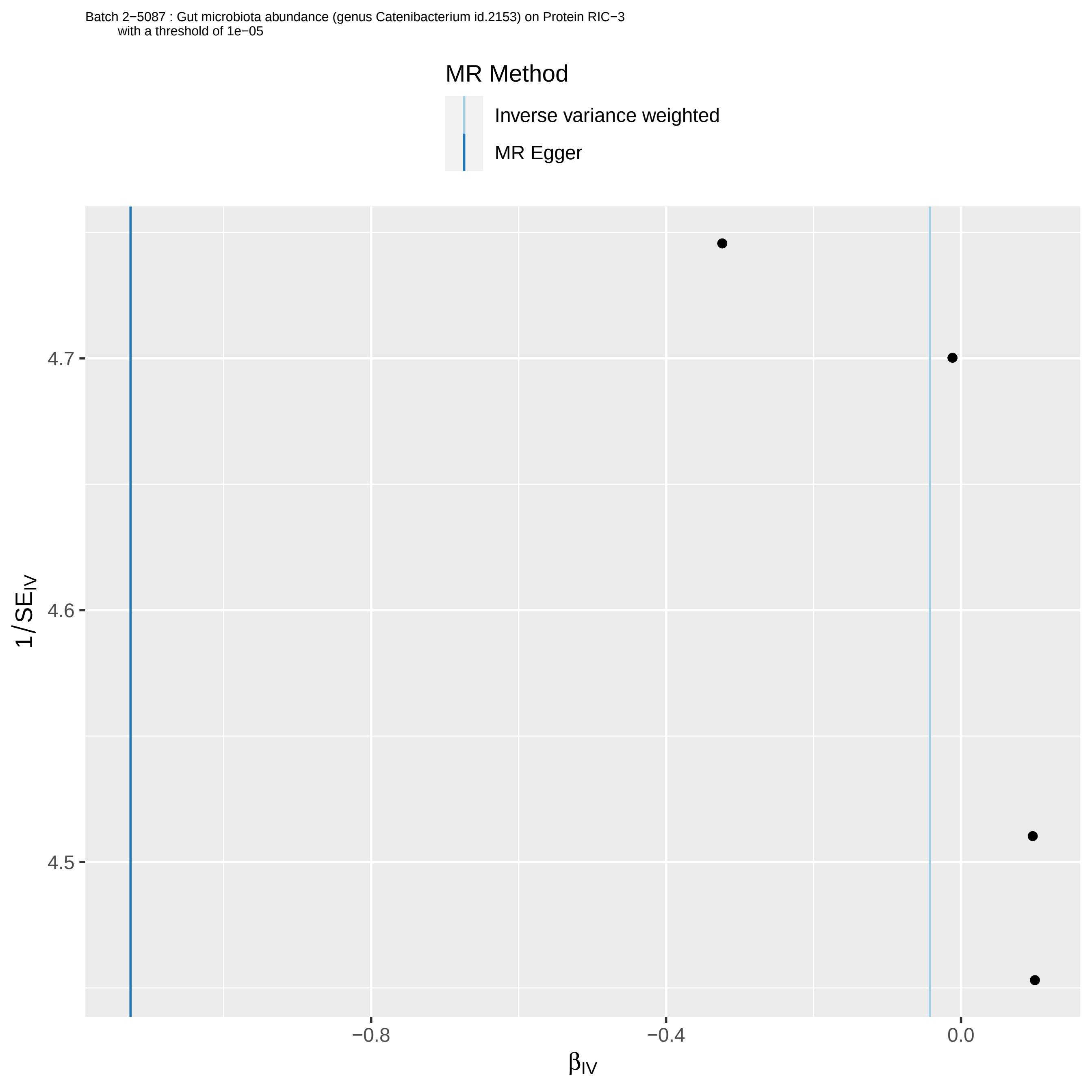

Batch 2−5087 : Gut microbiota abundance (genus Catenibacterium id.2153) on Protein RIC−3
with a threshold of 1e−05
MR Method
Inverse variance weighted
MR Egger
4.7
4.6
4.5
1/SE_IV
−0.8
−0.4
0.0
β_IV

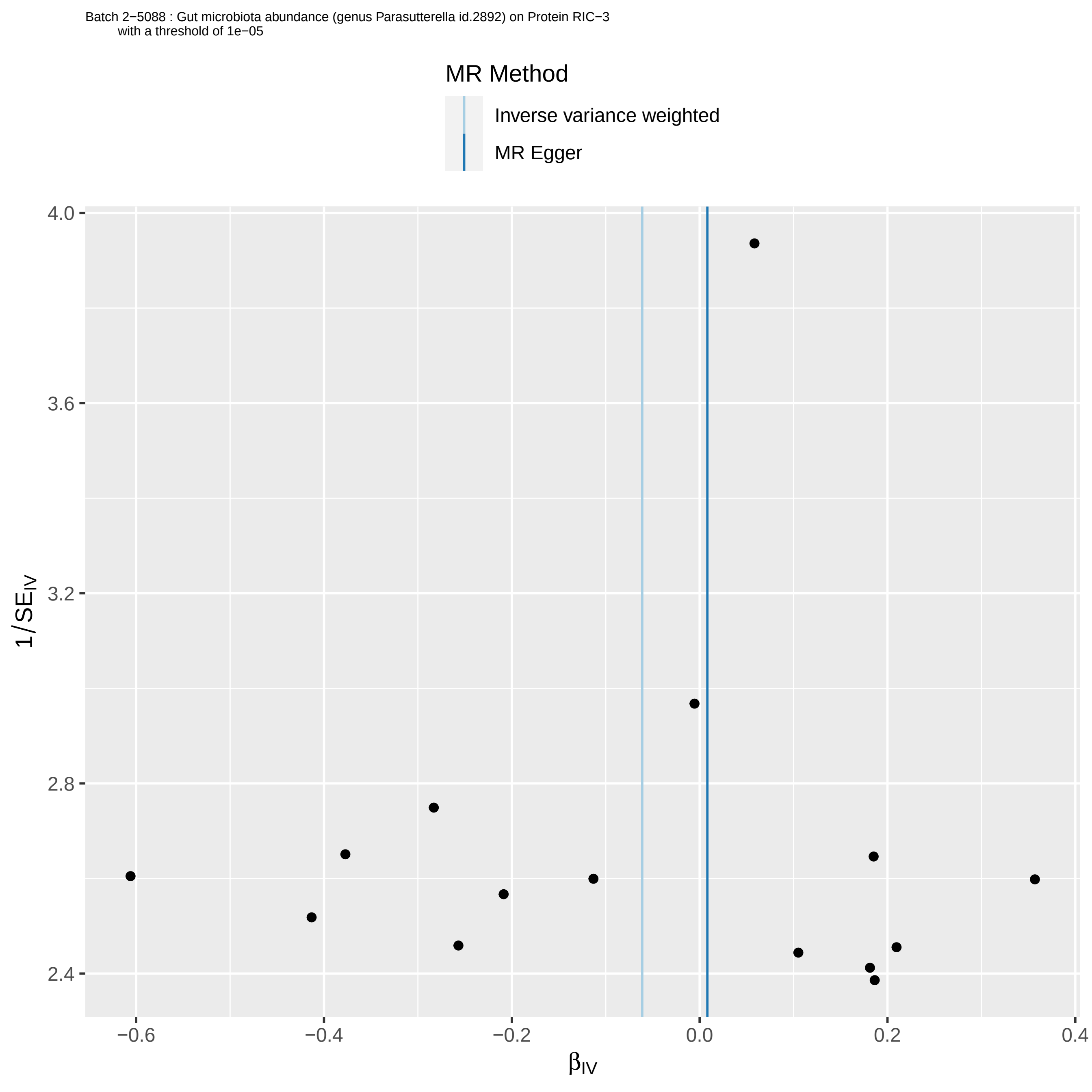

Batch 2−5088 : Gut microbiota abundance (genus Parasutterella id.2892) on Protein RIC−3
with a threshold of 1e−05
MR Method
Inverse variance weighted
MR Egger
4.0
3.6
3.2
2.8
2.4
1/SE$_{IV}$
−0.6
−0.4
−0.2
0.0
0.2
0.4
β$_{IV}$

Batch 2−5089 : Gut microbiota abundance (genus Catenibacterium id.2153) on Receptor−interacting serine/threonine−protein kinase 2
with a threshold of 1e−05

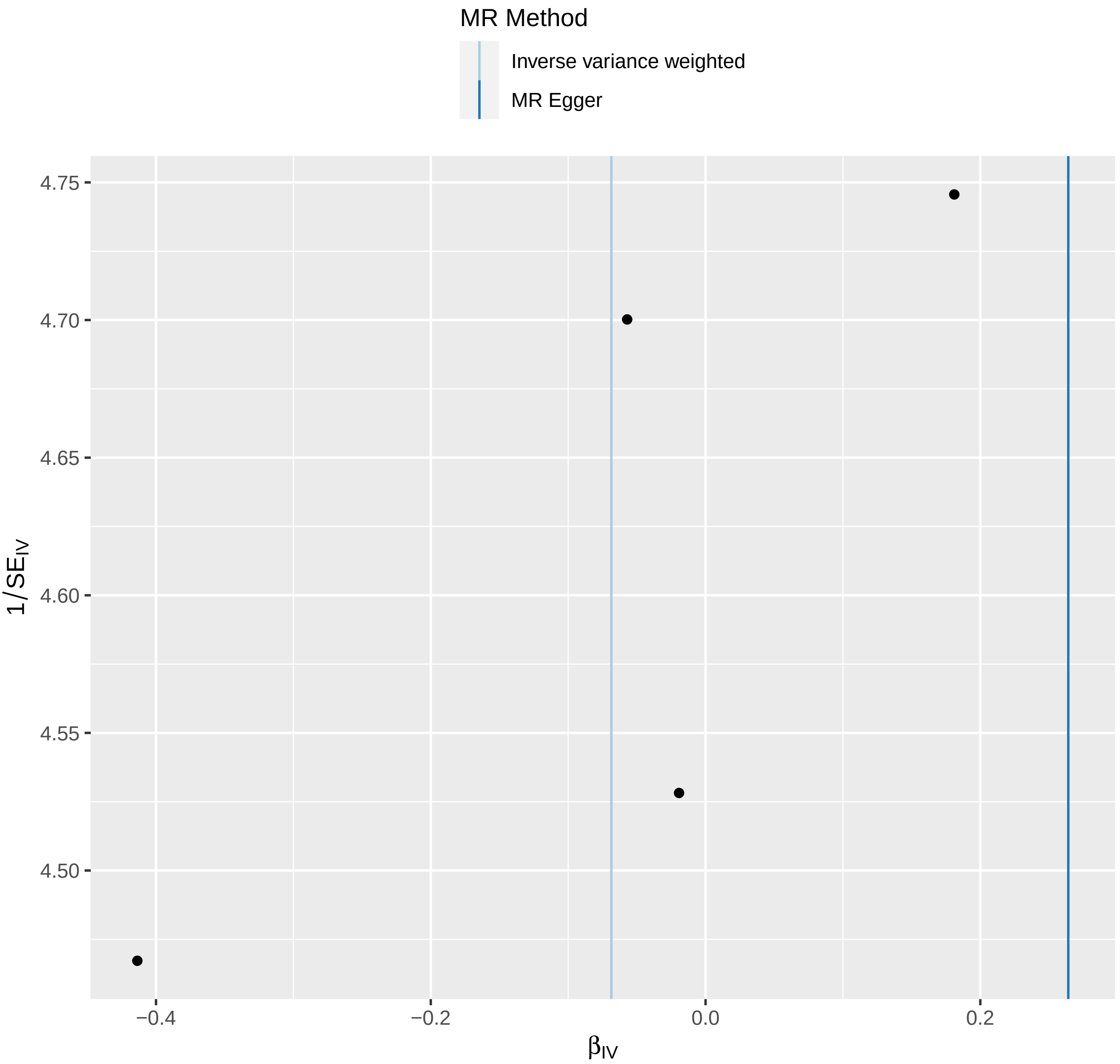

Batch 2−5090 : Gut microbiota abundance (genus Parasutterella id.2892) on Receptor−interacting serine/threonine−protein kinase 2 with a threshold of 1e−05

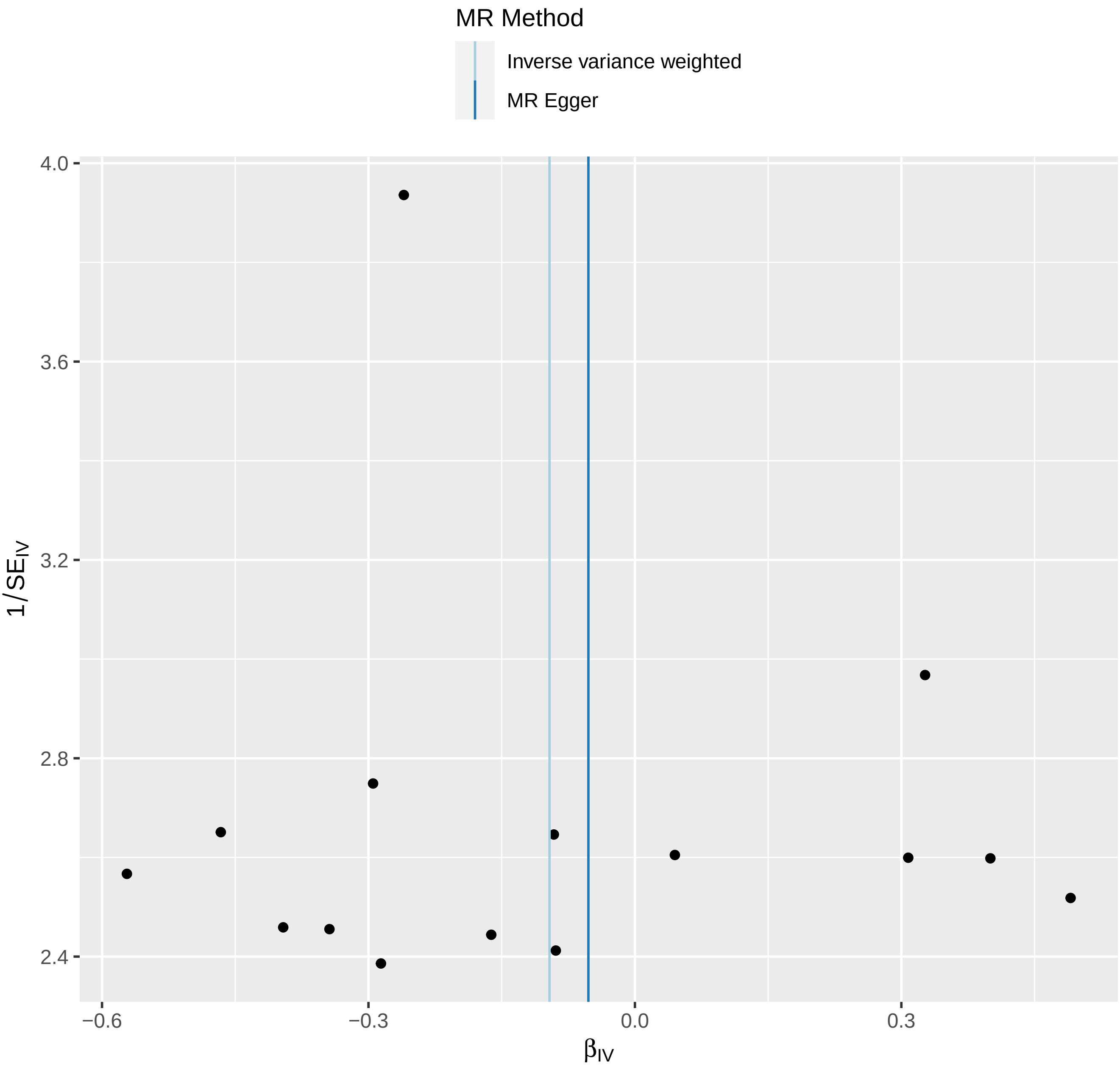

Batch 2−5091 : Gut microbiota abundance (genus Catenibacterium id.2153) on Receptor−interacting serine/threonine−protein kinase 2
with a threshold of 1e−05

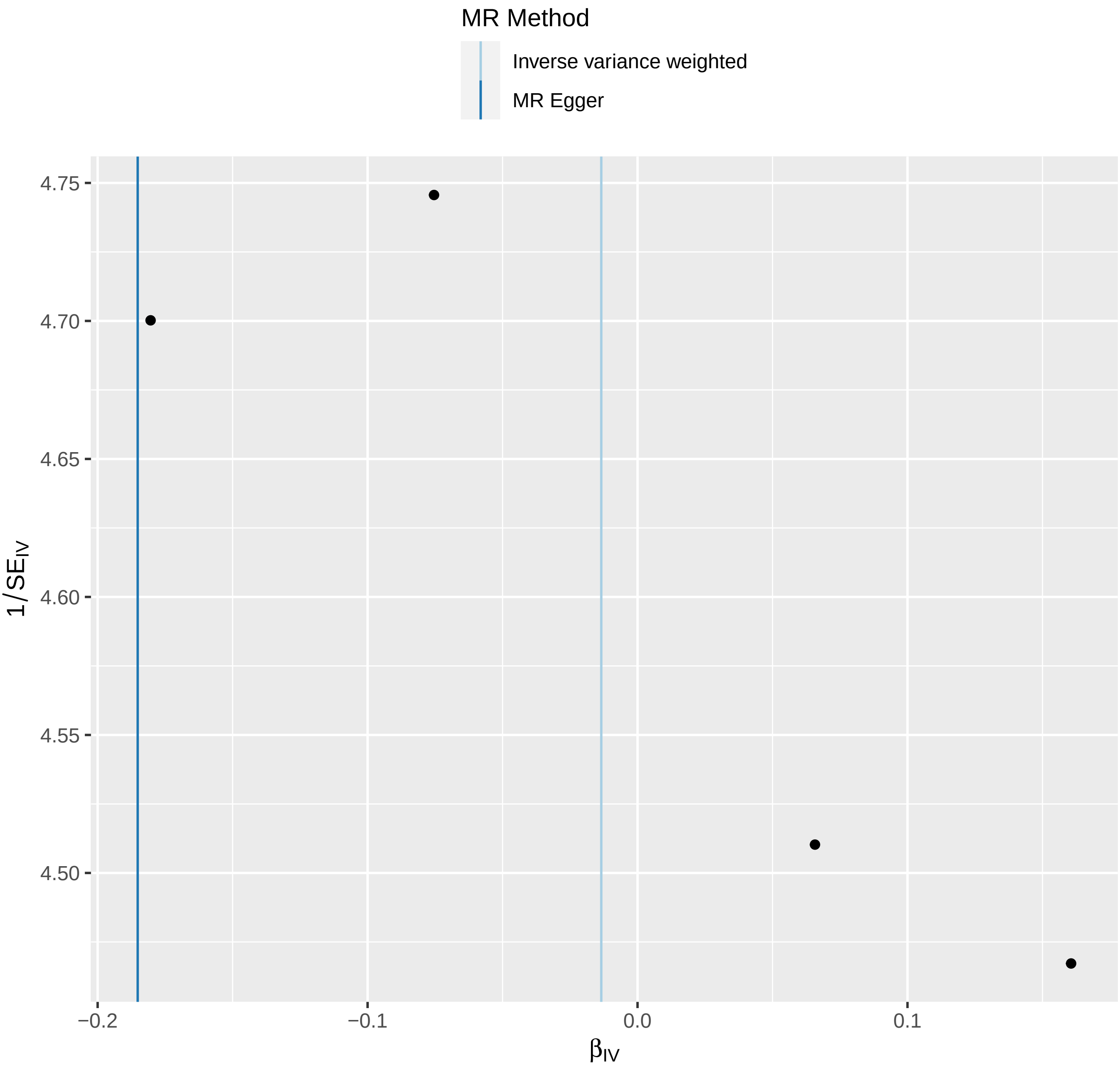

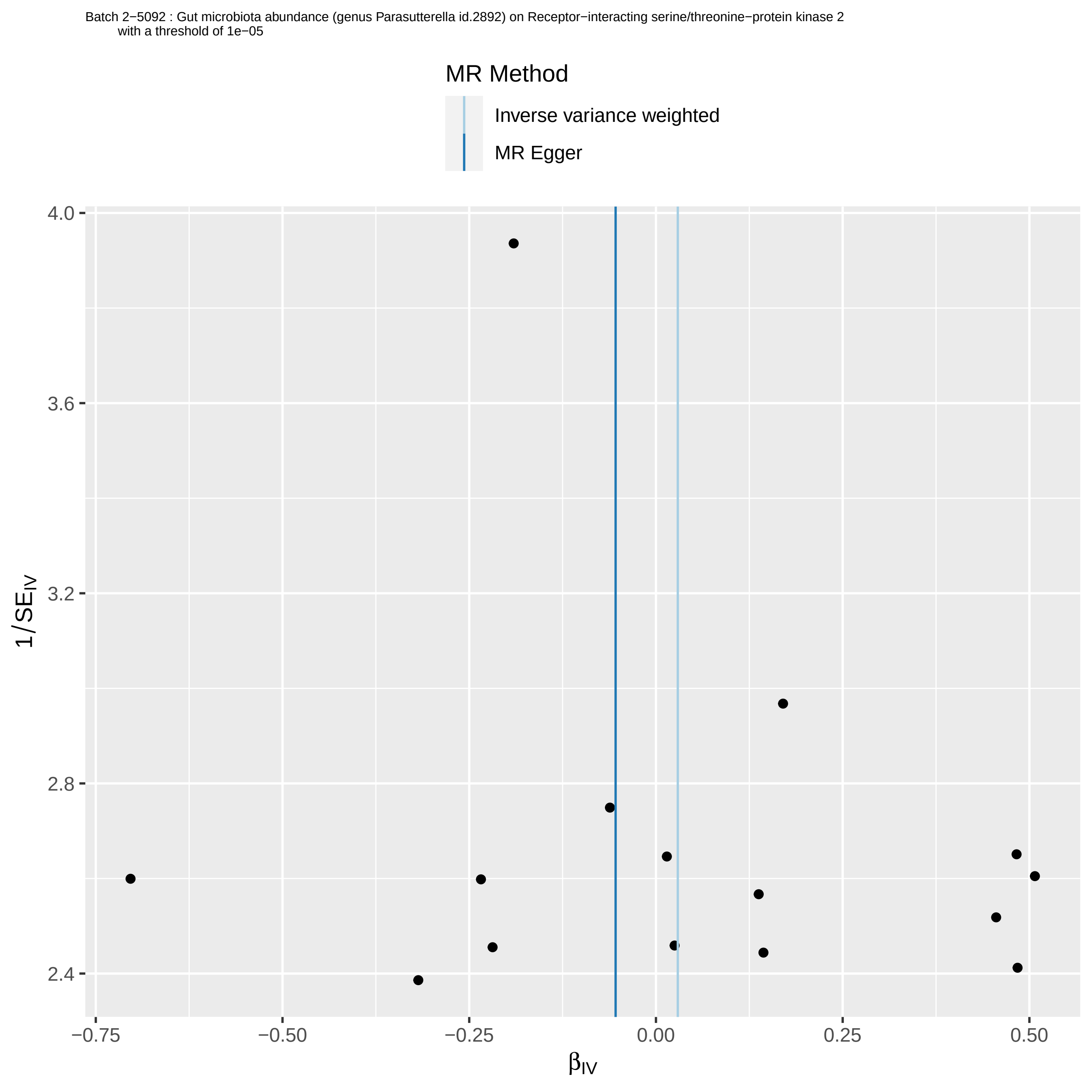
Batch 2−5092 : Gut microbiota abundance (genus Parasutterella id.2892) on Receptor−interacting serine/threonine−protein kinase 2
with a threshold of 1e−05
MR Method
Inverse variance weighted
MR Egger
4.0
3.6
3.2
2.8
2.4
1/SE_IV
−0.75
−0.50
−0.25
0.00
0.25
0.50
β_IV

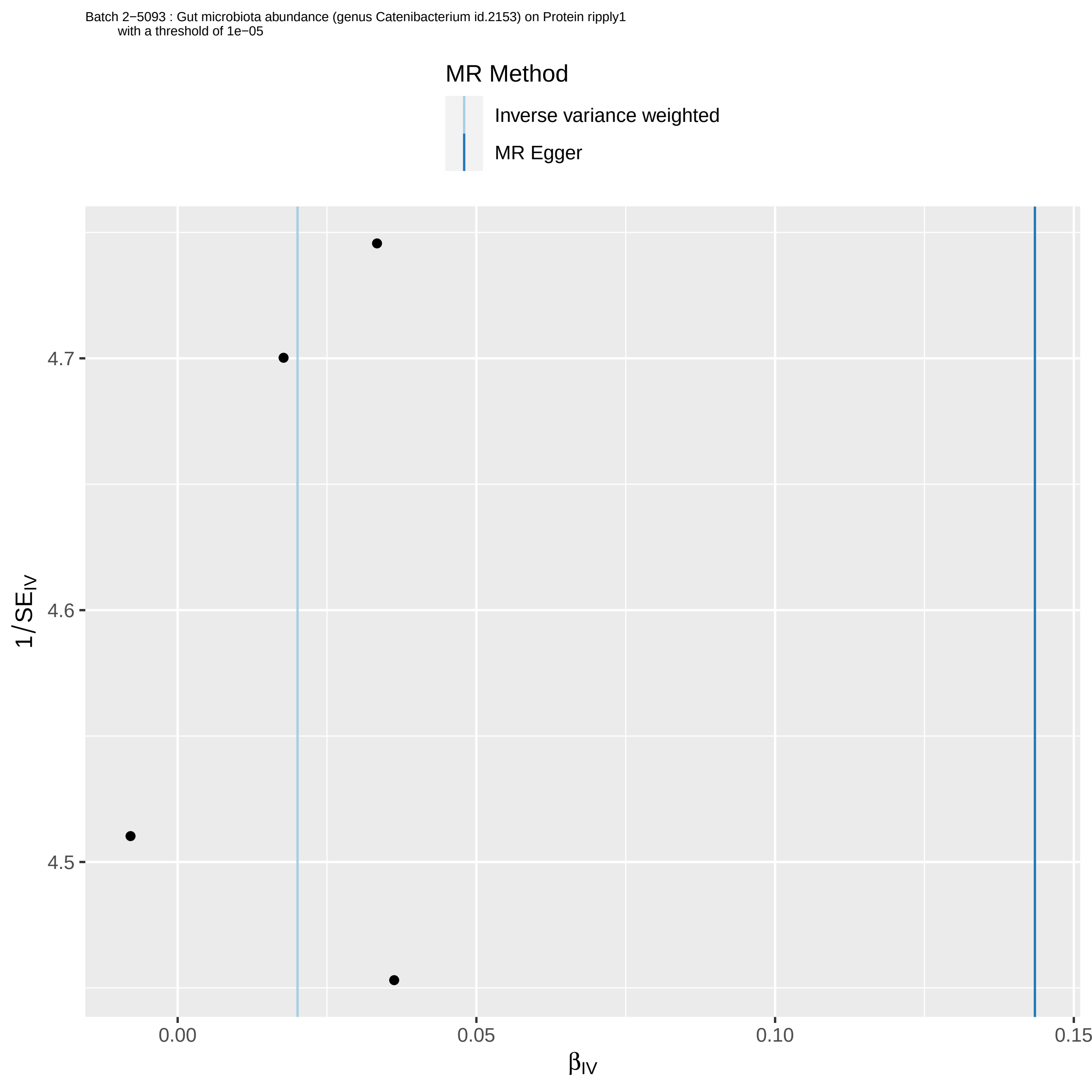
Batch 2−5093 : Gut microbiota abundance (genus Catenibacterium id.2153) on Protein ripply1
with a threshold of 1e−05
MR Method
Inverse variance weighted
MR Egger
4.7
4.6
4.5
1/SE_IV
0.00
0.05
0.10
0.15
β_IV

Batch 2−5094 : Gut microbiota abundance (genus Parasutterella id.2892) on Protein ripply1
with a threshold of 1e−05

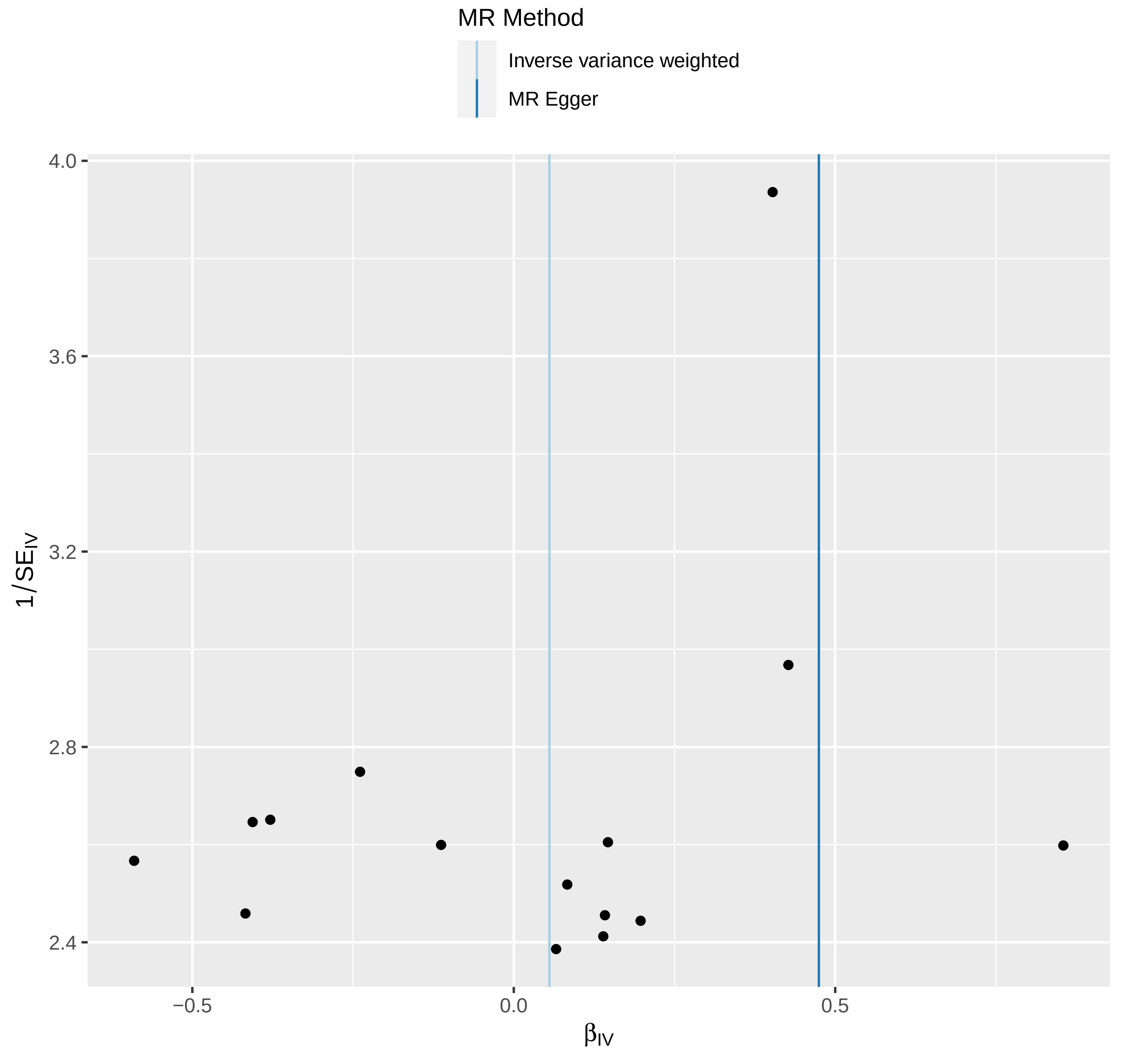

Batch 2−5095 : Gut microbiota abundance (genus Catenibacterium id.2153) on Retinaldehyde−binding protein 1
with a threshold of 1e−05

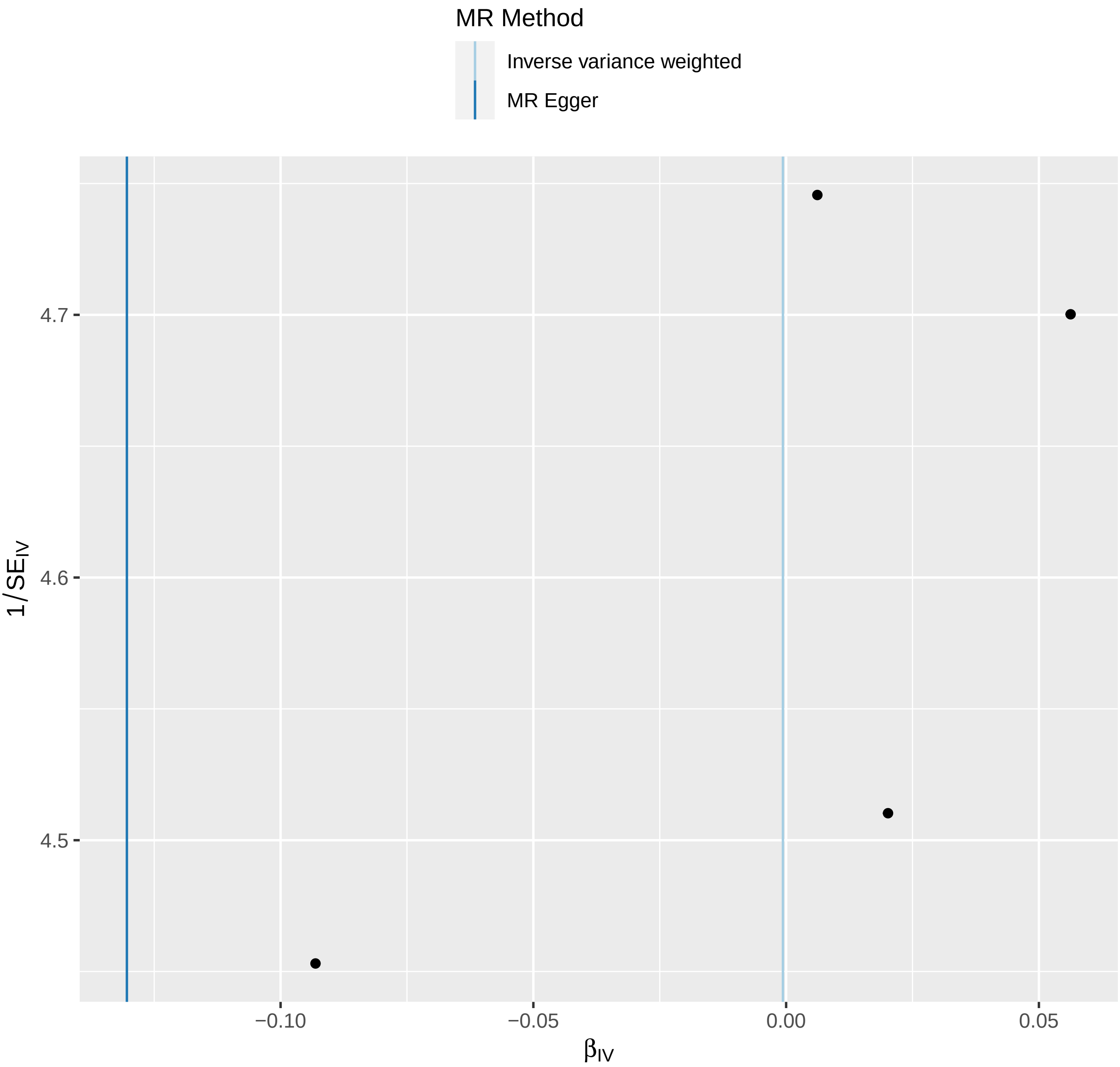

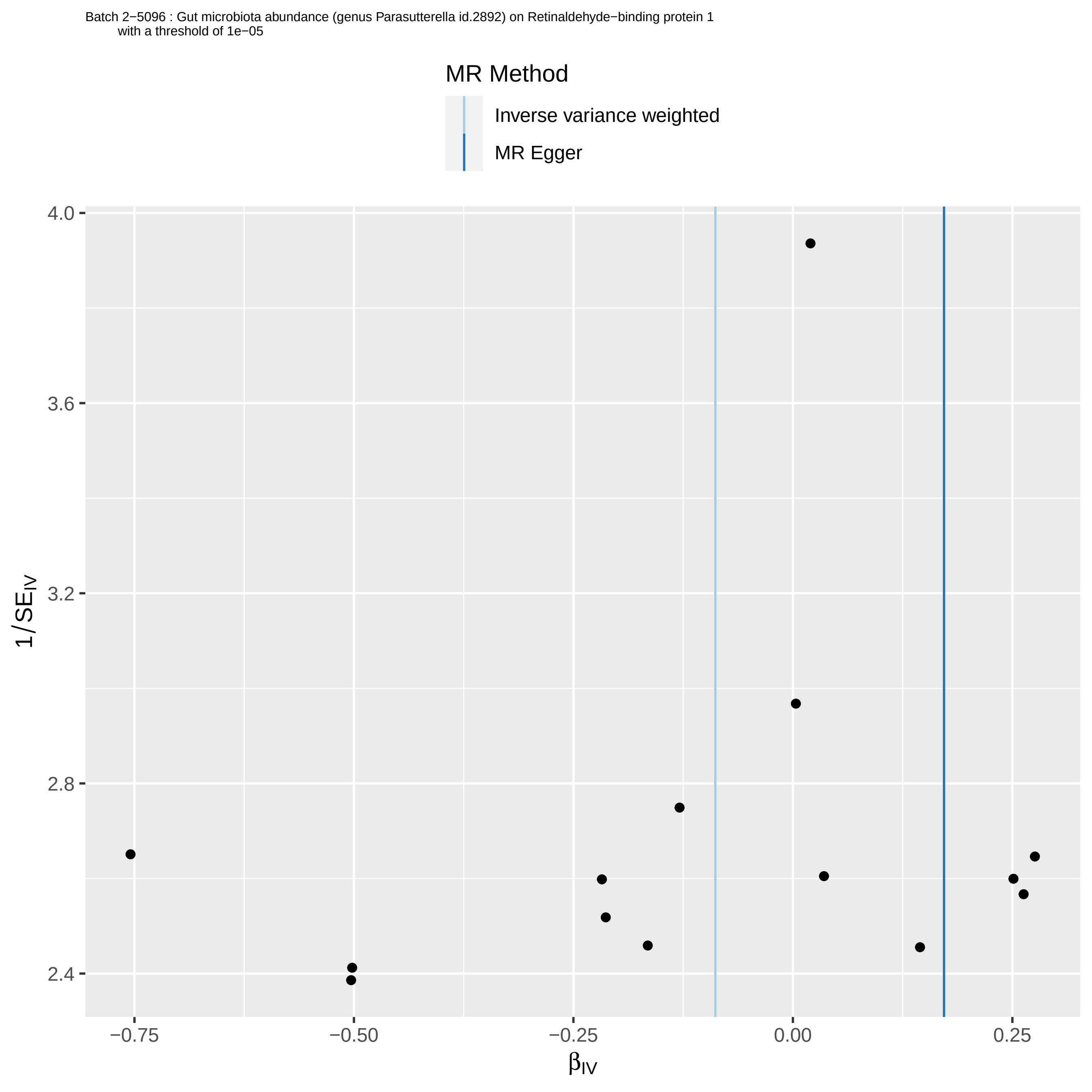
Batch 2−5096 : Gut microbiota abundance (genus Parasutterella id.2892) on Retinaldehyde−binding protein 1
with a threshold of 1e−05
MR Method
Inverse variance weighted
MR Egger
4.0
3.6
3.2
2.8
2.4
1/SE_IV
−0.75
−0.50
−0.25
0.00
0.25
β_IV

Batch 2−5097 : Gut microbiota abundance (genus Catenibacterium id.2153) on Regulator of microtubule dynamics protein 1
with a threshold of 1e−05

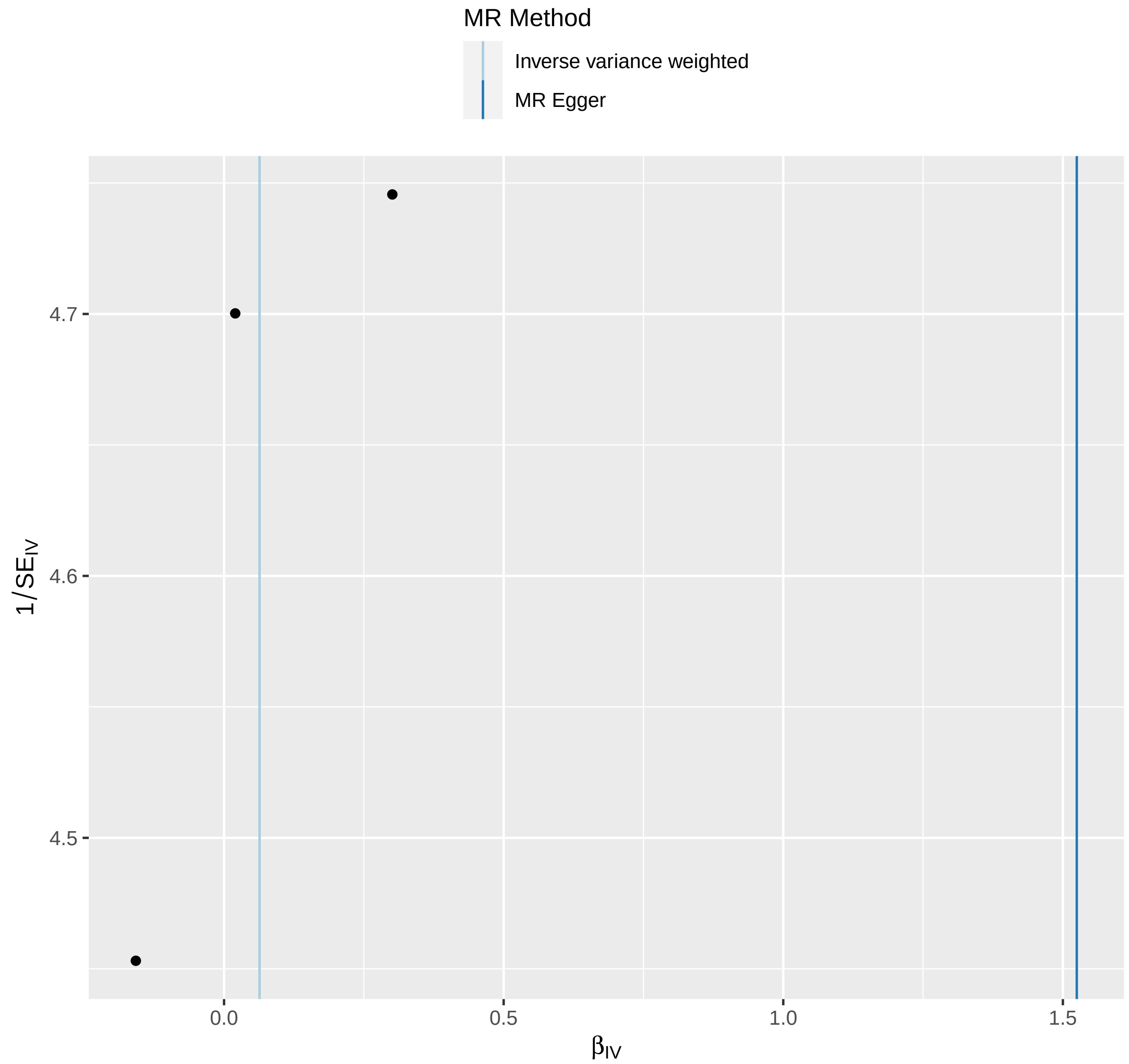

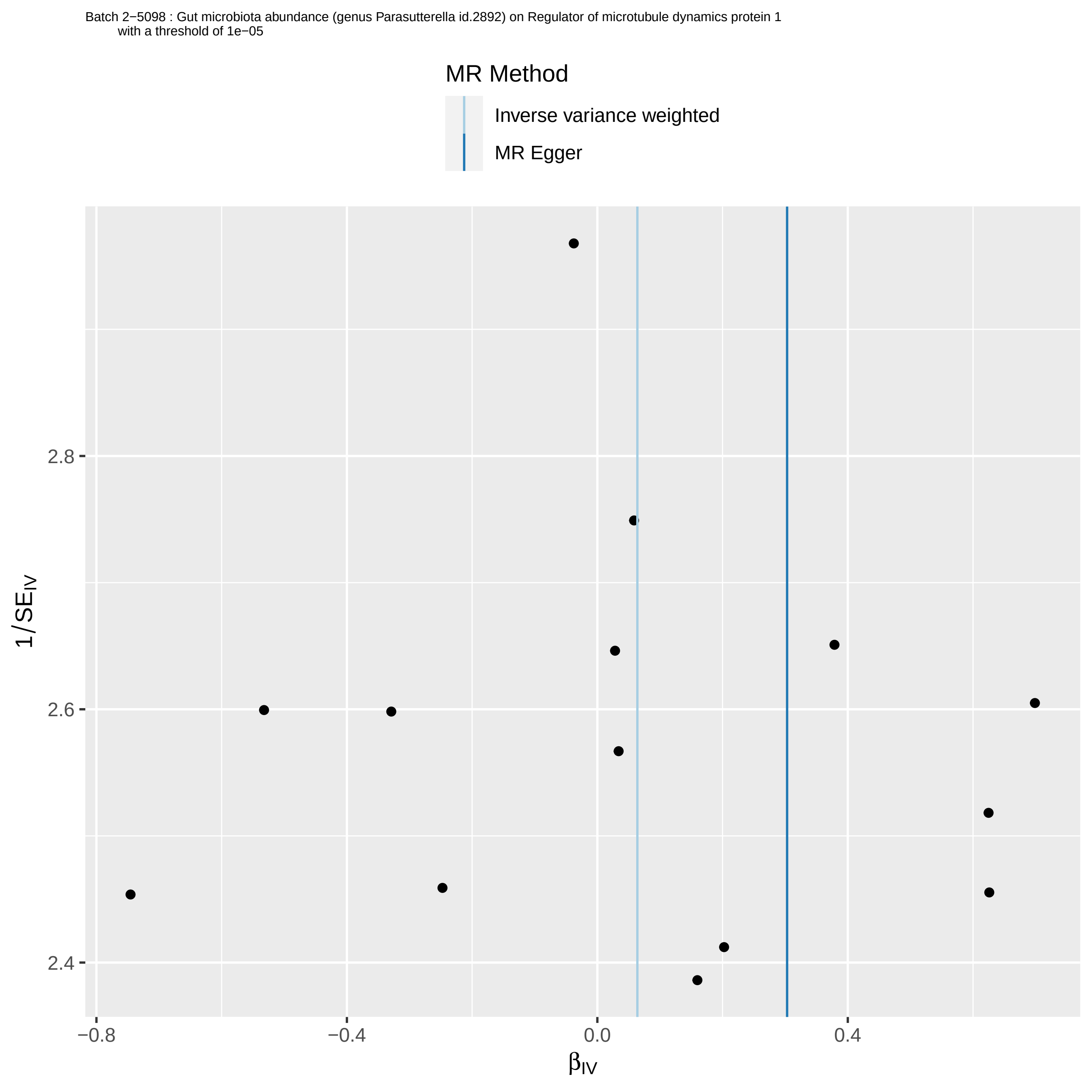

Batch 2−5098 : Gut microbiota abundance (genus Parasutterella id.2892) on Regulator of microtubule dynamics protein 1
with a threshold of 1e−05
MR Method
Inverse variance weighted
MR Egger
2.8
2.6
2.4
1/SE_IV
−0.8
−0.4
0.0
0.4
β_IV

Batch 2−5099 : Gut microbiota abundance (genus Catenibacterium id.2153) on Regulator of microtubule dynamics protein 3
with a threshold of 1e−05

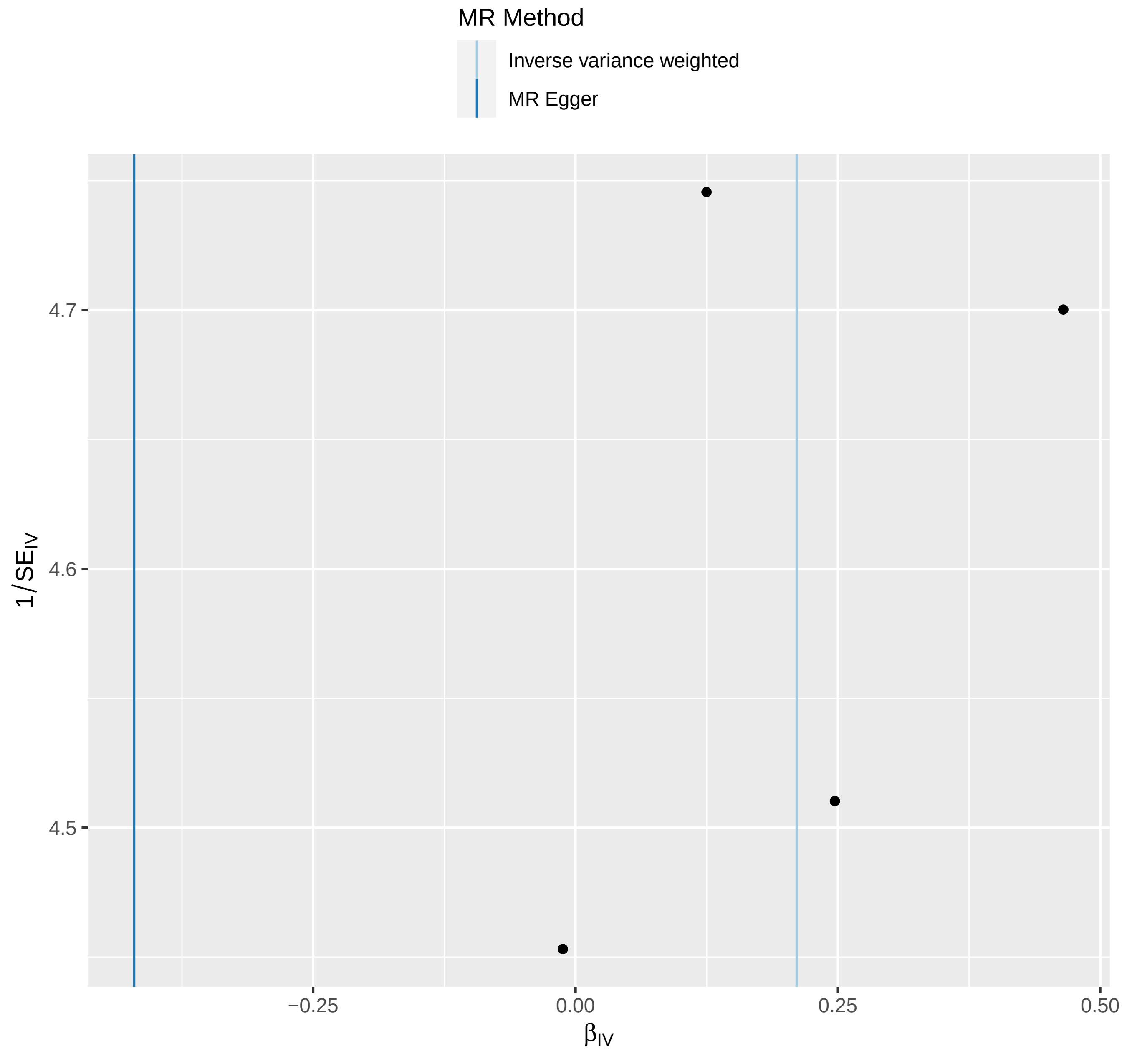

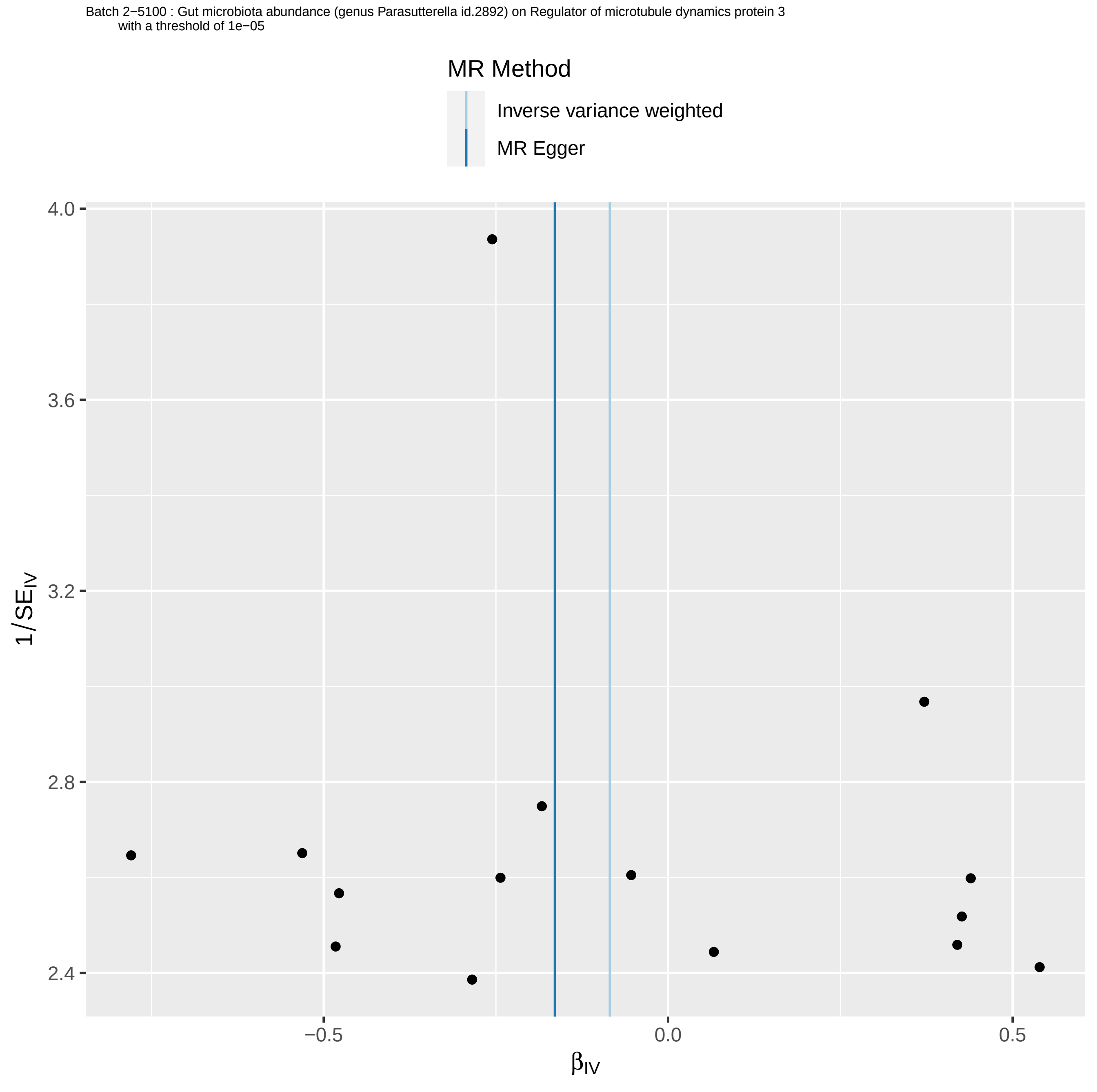
Batch 2−5100 : Gut microbiota abundance (genus Parasutterella id.2892) on Regulator of microtubule dynamics protein 3
with a threshold of 1e−05
MR Method
Inverse variance weighted
MR Egger
4.0
3.6
3.2
2.8
2.4
1/SE_IV
−0.5
0.0
0.5
β_IV

Batch 2−5101 : Gut microbiota abundance (genus Catenibacterium id.2153) on RecQ−mediated genome instability protein 1
with a threshold of 1e−05

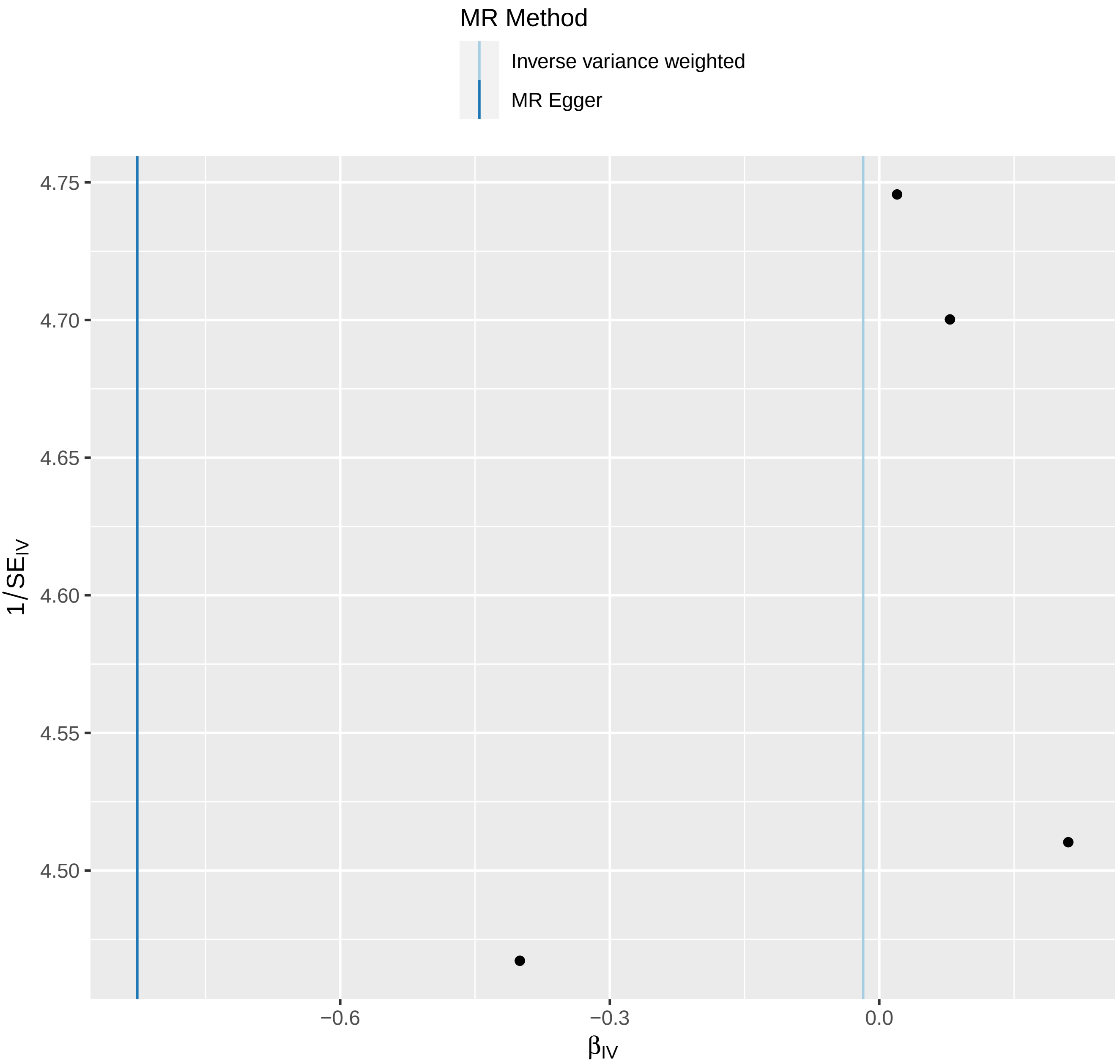

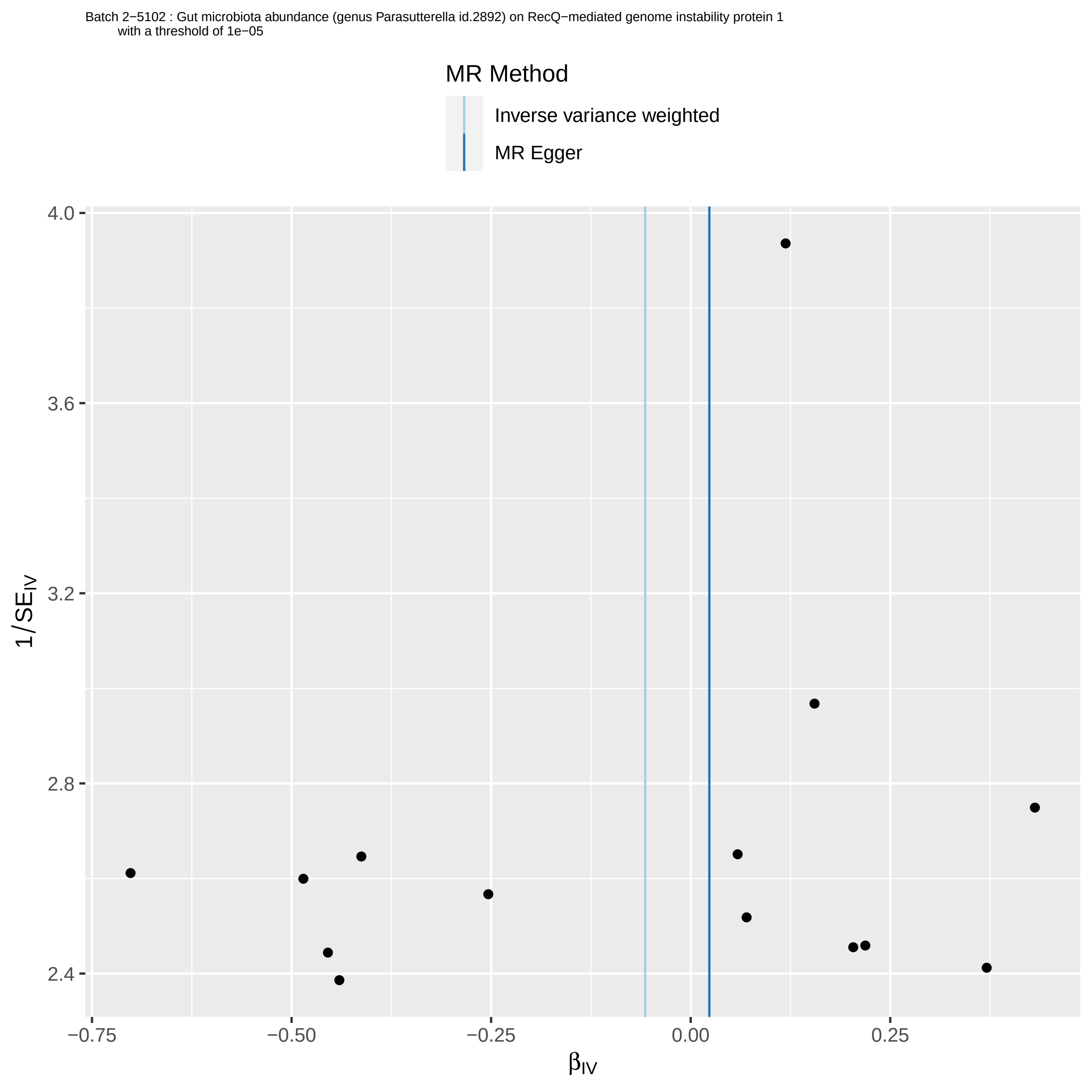
Batch 2−5102 : Gut microbiota abundance (genus Parasutterella id.2892) on RecQ−mediated genome instability protein 1
with a threshold of 1e−05
MR Method
Inverse variance weighted
MR Egger
4.0
3.6
3.2
2.8
2.4
1/SE_IV
−0.75
−0.50
−0.25
0.00
0.25
β_IV

Batch 2−5103 : Gut microbiota abundance (genus Catenibacterium id.2153) on Inactive ribonuclease−like protein 10
with a threshold of 1e−05

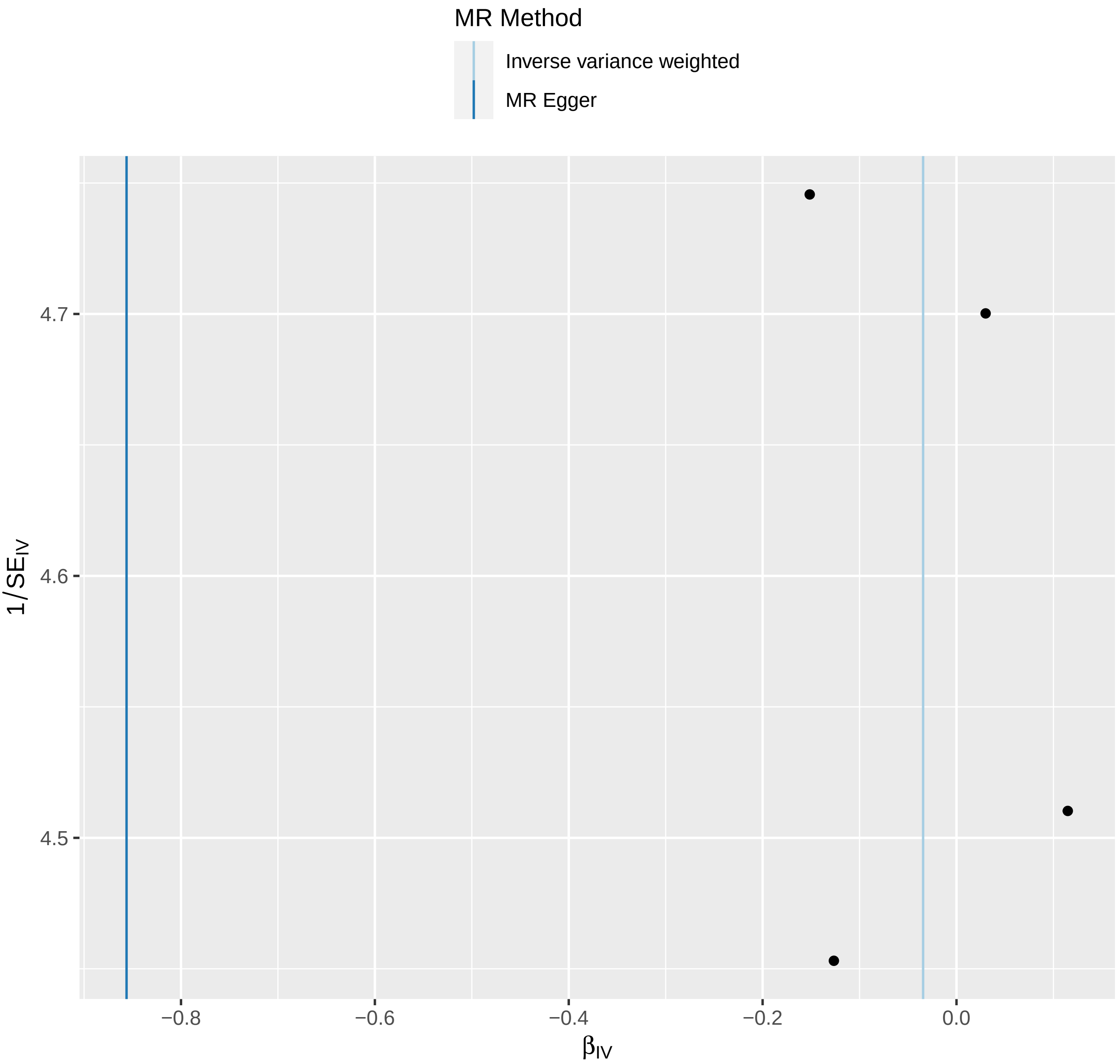

Batch 2−5104 : Gut microbiota abundance (genus Parasutterella id.2892) on Inactive ribonuclease−like protein 10
with a threshold of 1e−05

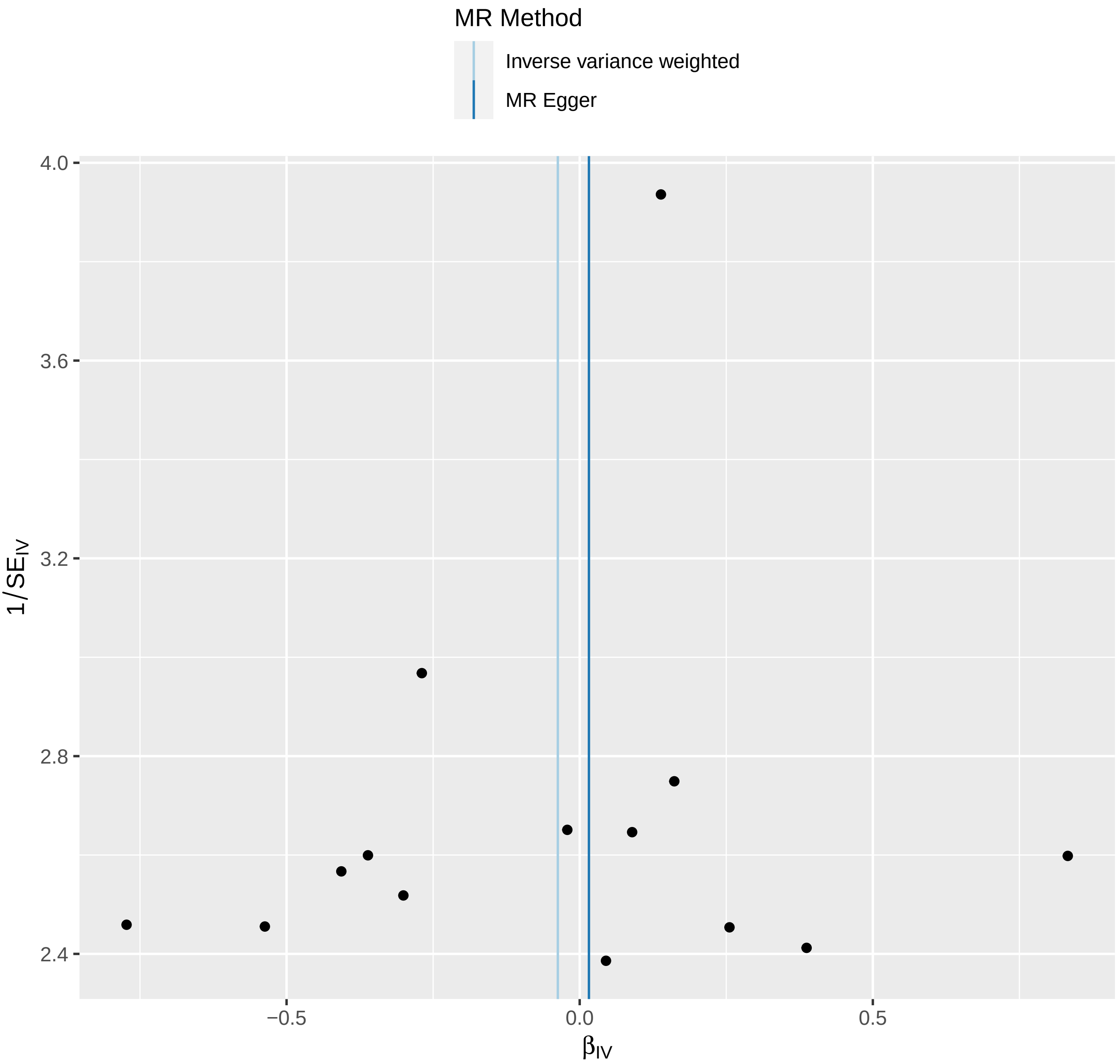

Batch 2−5105 : Gut microbiota abundance (genus Catenibacterium id.2153) on Probable inactive ribonuclease−like protein 13
with a threshold of 1e−05

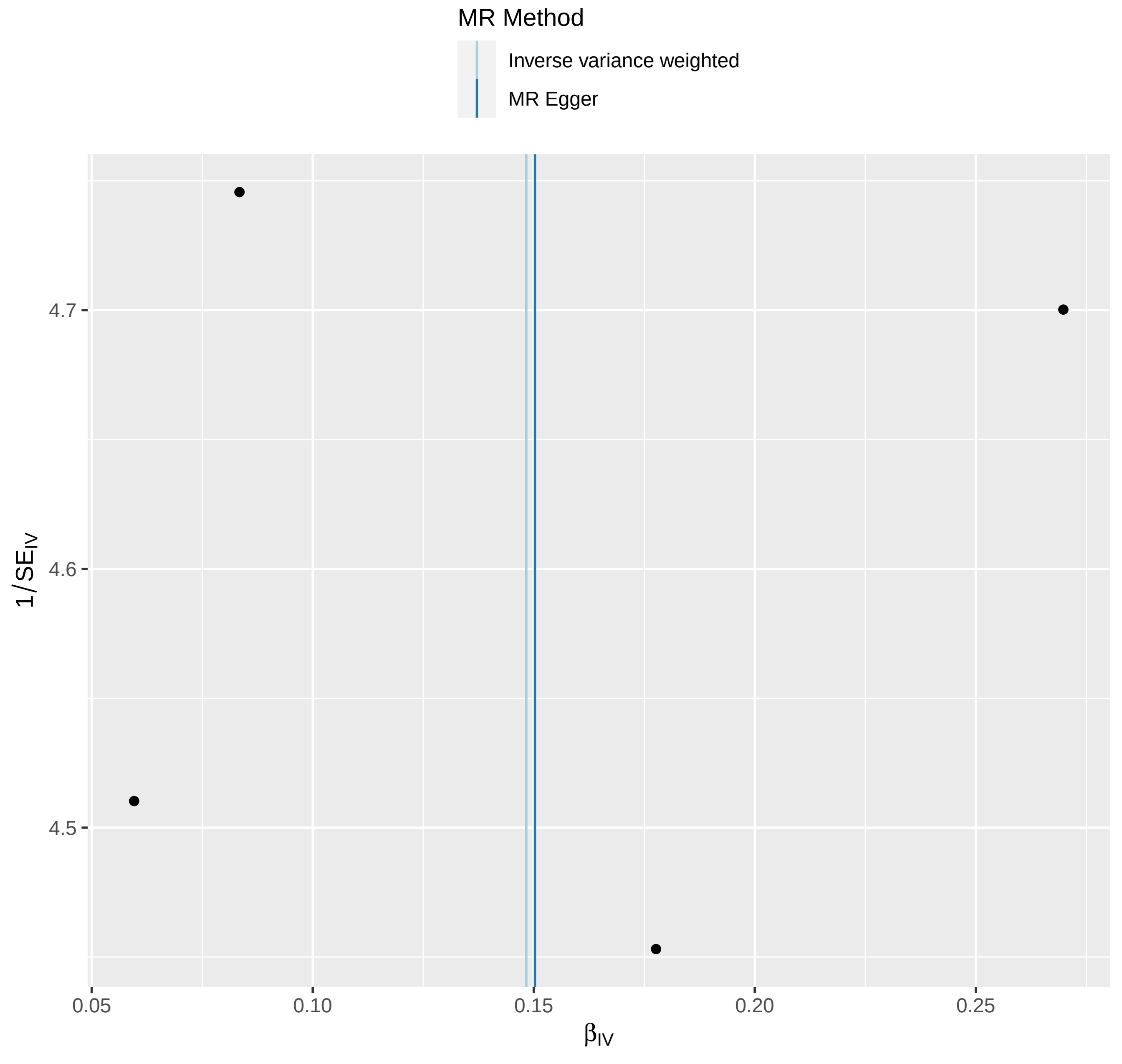

Batch 2−5106 : Gut microbiota abundance (genus Parasutterella id.2892) on Probable inactive ribonuclease−like protein 13
with a threshold of 1e−05

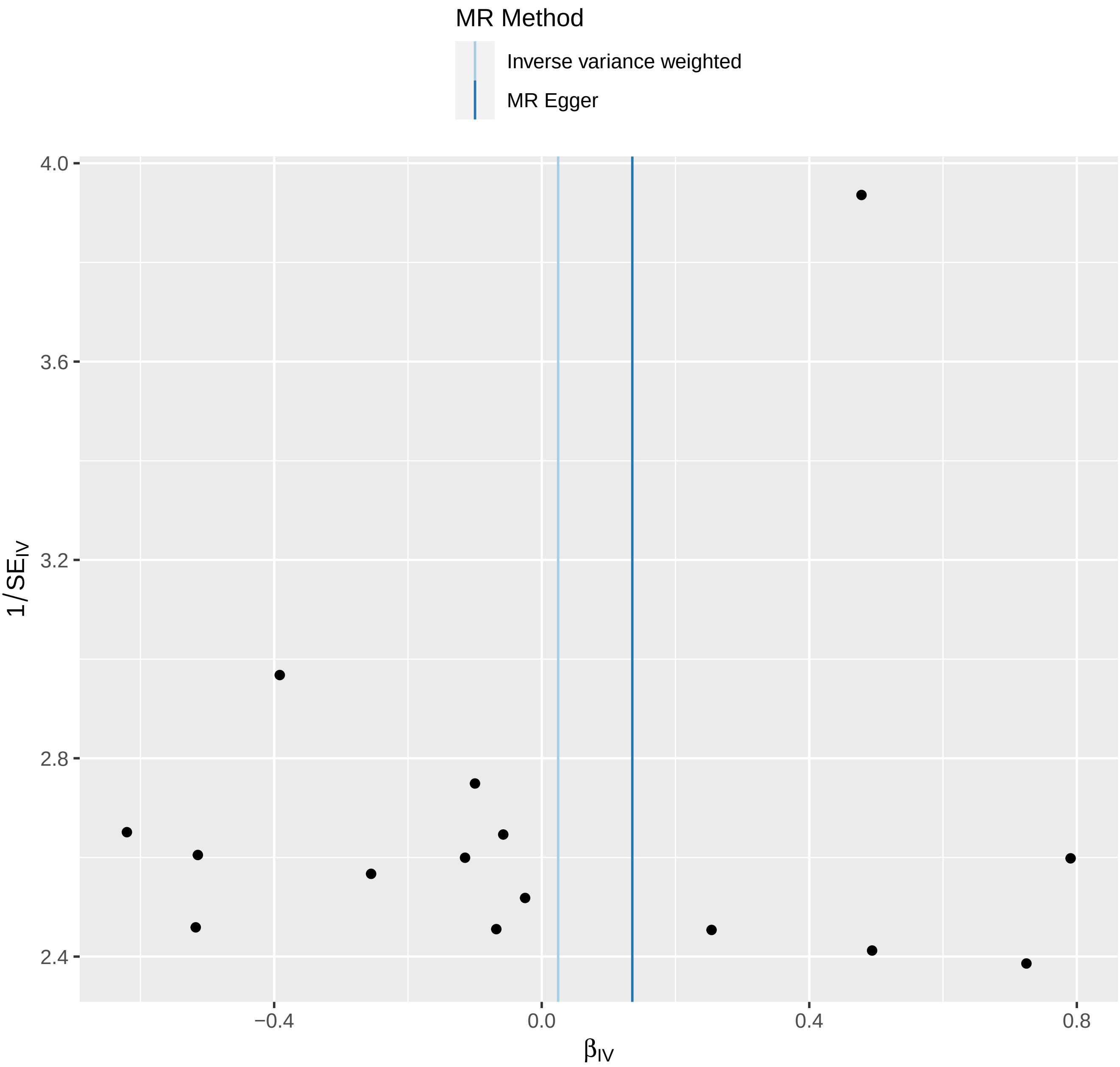

Batch 2−5107 : Gut microbiota abundance (genus Catenibacterium id.2153) on Ribonuclease pancreatic
with a threshold of 1e−05

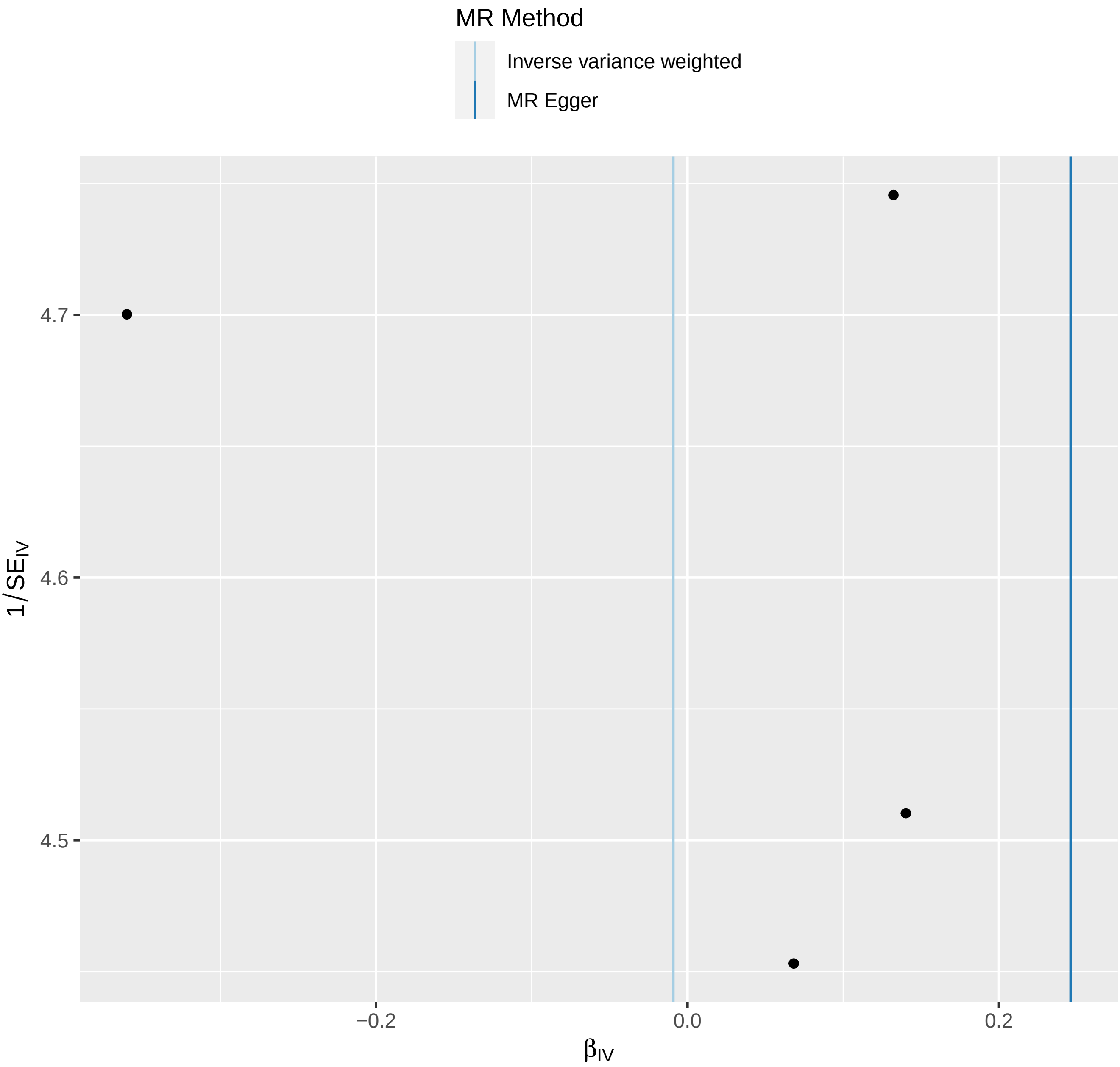

Batch 2−5108 : Gut microbiota abundance (genus Parasutterella id.2892) on Ribonuclease pancreatic
with a threshold of 1e−05

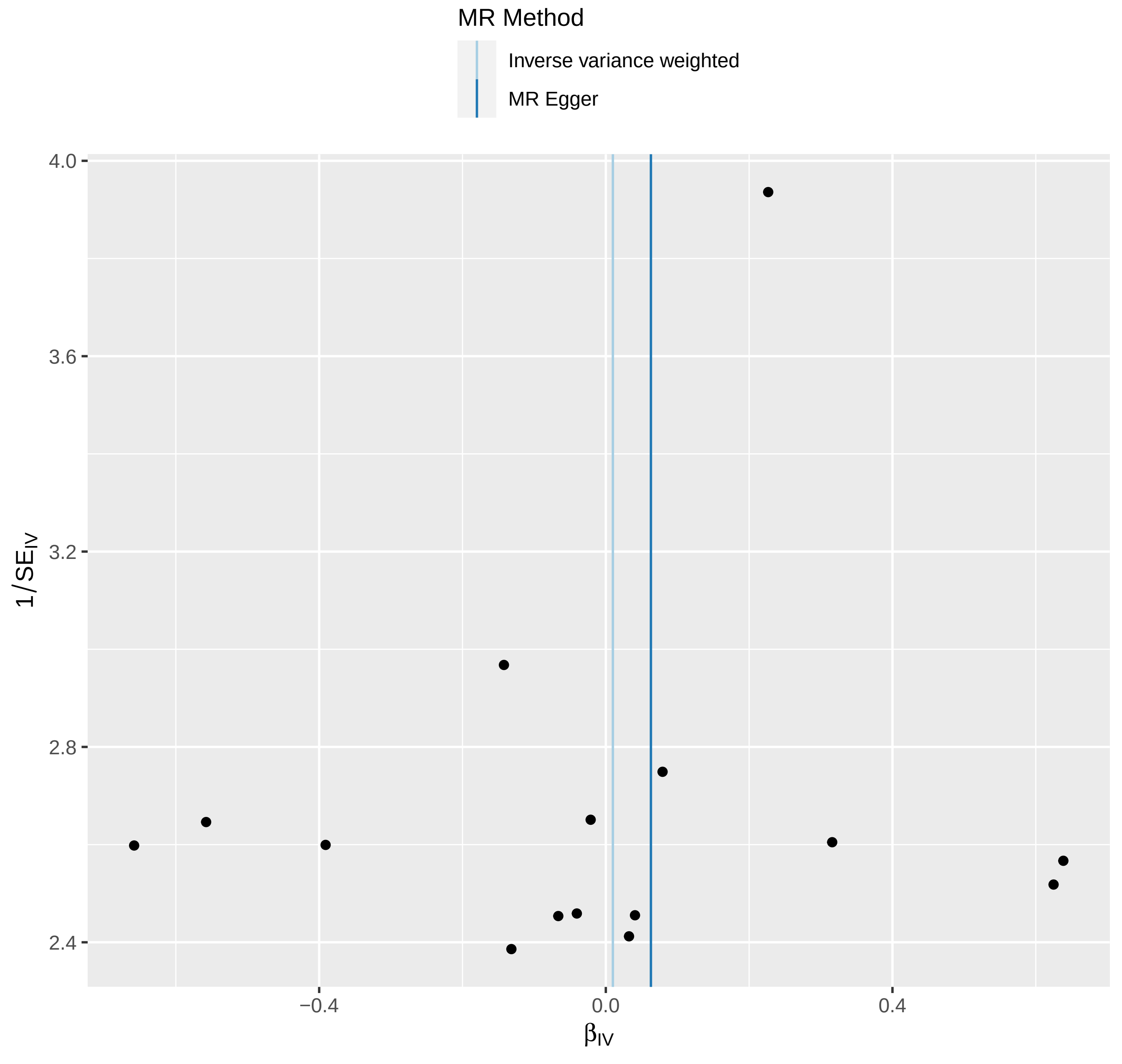

Batch 2−5109 : Gut microbiota abundance (genus Catenibacterium id.2153) on Non−secretory ribonuclease
with a threshold of 1e−05

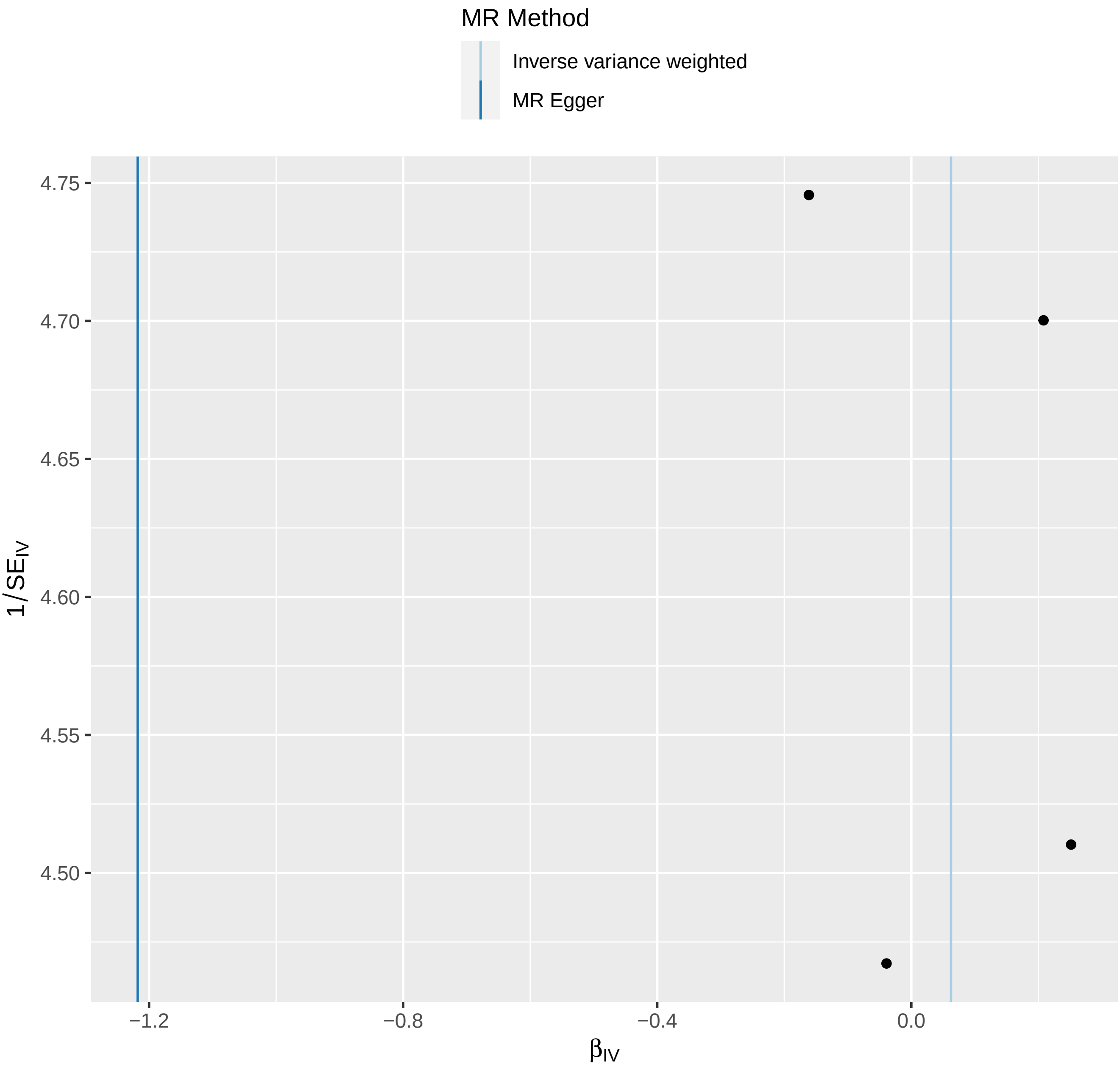

Batch 2−5110 : Gut microbiota abundance (genus Parasutterella id.2892) on Non−secretory ribonuclease
with a threshold of 1e−05

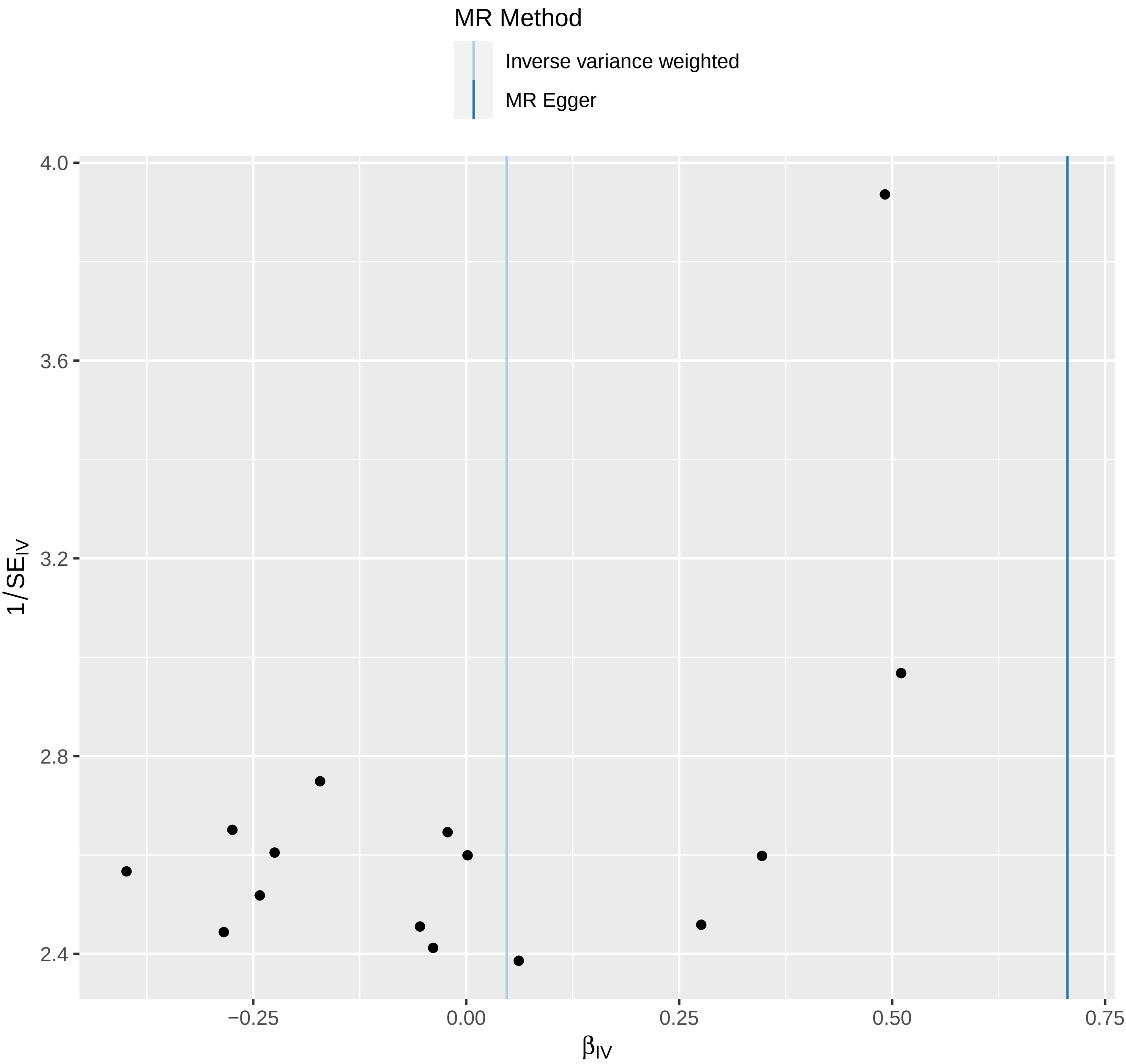

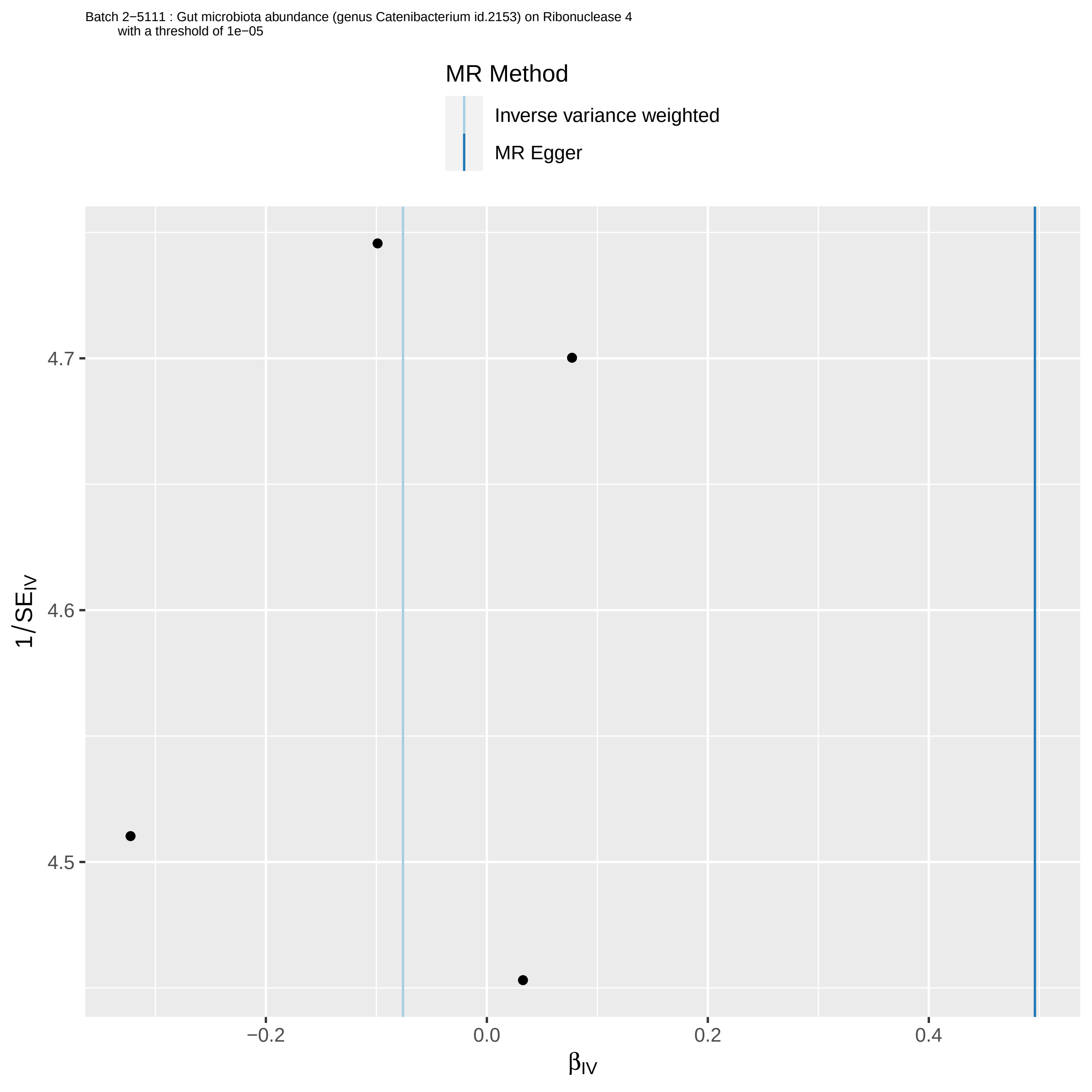

Batch 2−5111 : Gut microbiota abundance (genus Catenibacterium id.2153) on Ribonuclease 4
with a threshold of 1e−05
MR Method
Inverse variance weighted
MR Egger
4.7
4.6
4.5
$1/SE_{IV}$
−0.2
0.0
0.2
0.4
β_{IV}

Batch 2−5112 : Gut microbiota abundance (genus Parasutterella id.2892) on Ribonuclease 4
with a threshold of 1e−05

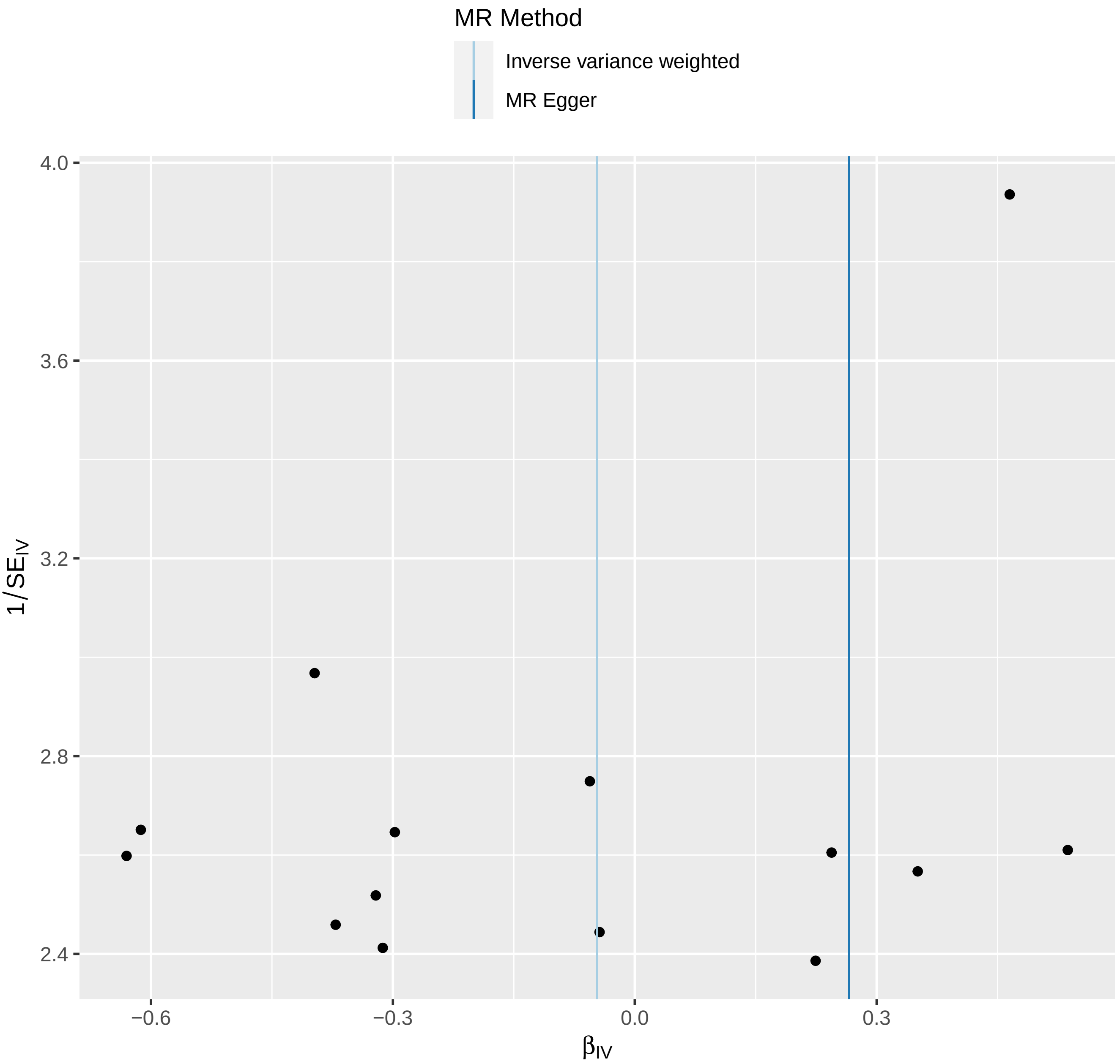

Batch 2−5113 : Gut microbiota abundance (genus Catenibacterium id.2153) on Ribonuclease K6
with a threshold of 1e−05

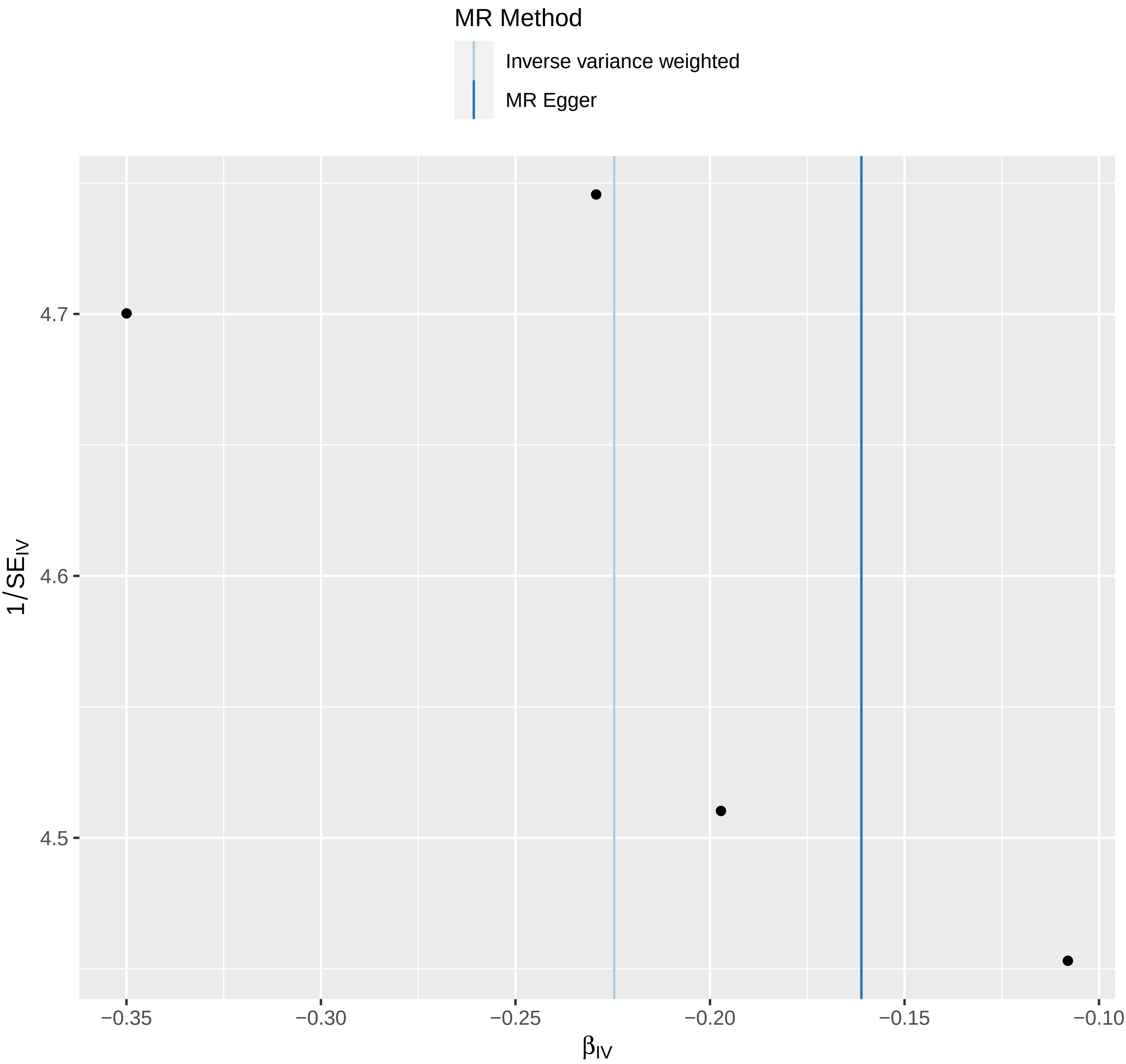

Batch 2−5114 : Gut microbiota abundance (genus Parasutterella id.2892) on Ribonuclease K6
with a threshold of 1e−05

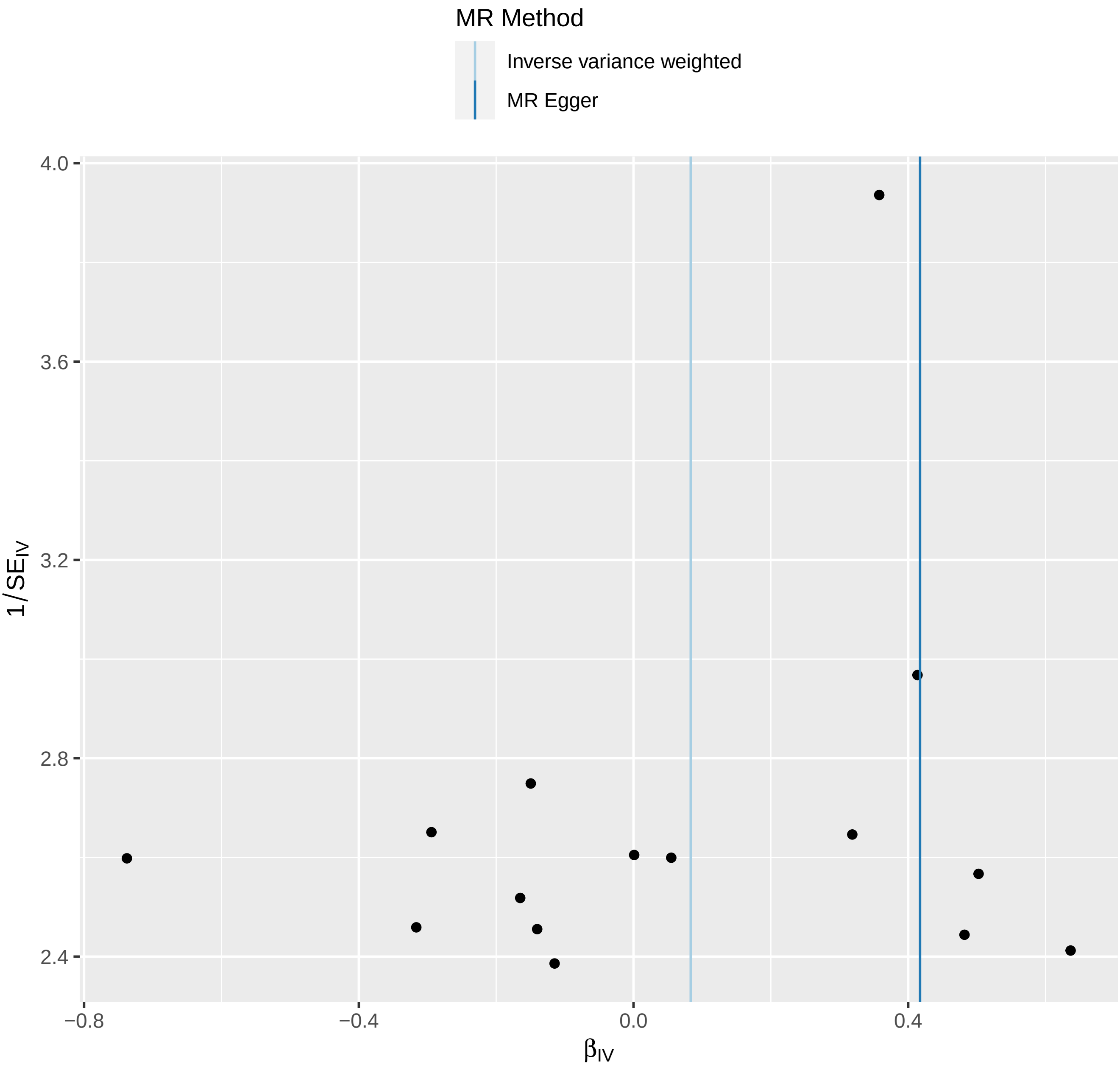

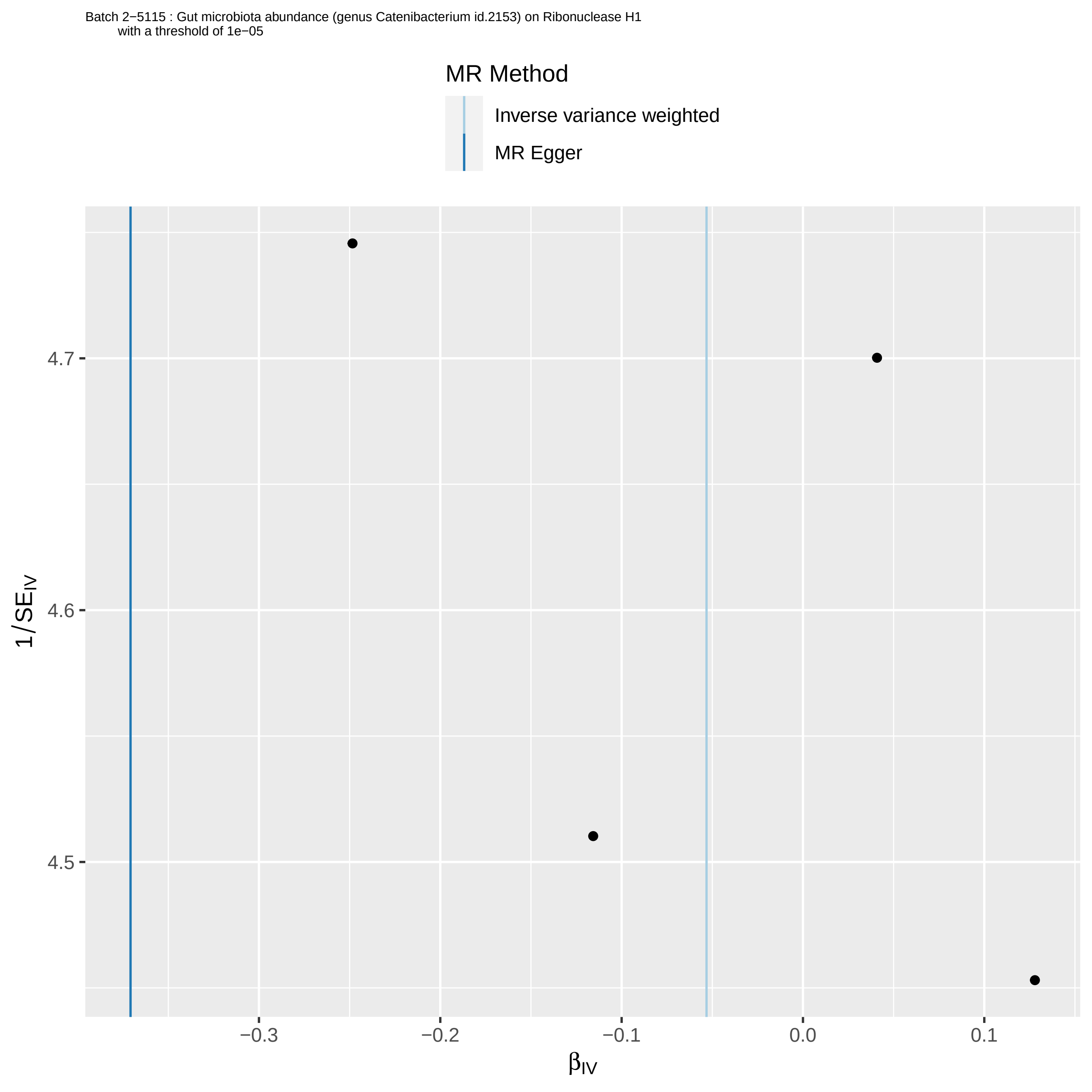
Batch 2−5115 : Gut microbiota abundance (genus Catenibacterium id.2153) on Ribonuclease H1
with a threshold of 1e−05
MR Method
Inverse variance weighted
MR Egger
4.7
4.6
4.5
1/SE_IV
−0.3
−0.2
−0.1
0.0
0.1
β_IV

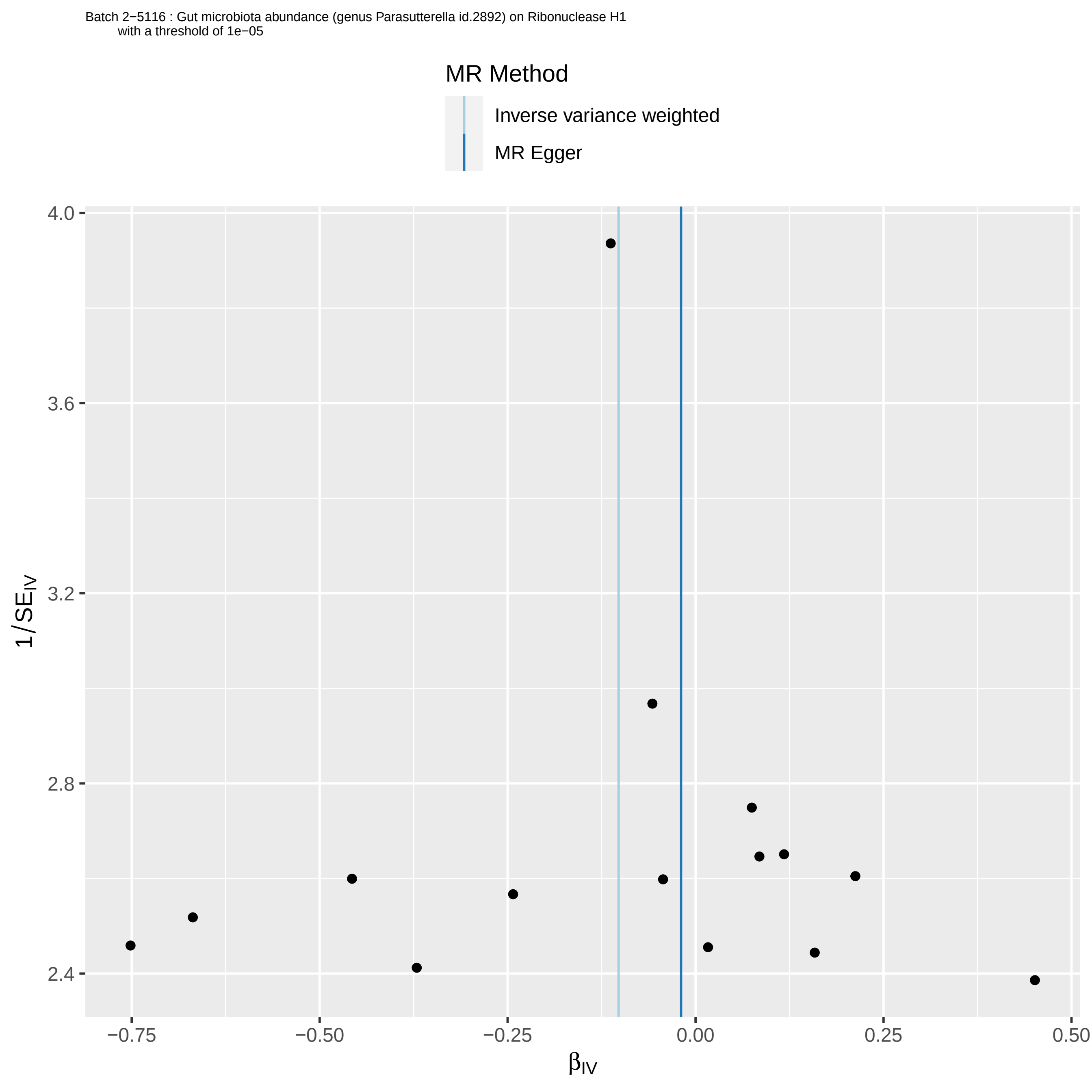

Batch 2−5116 : Gut microbiota abundance (genus Parasutterella id.2892) on Ribonuclease H1
with a threshold of 1e−05
MR Method
Inverse variance weighted
MR Egger
4.0
3.6
3.2
2.8
2.4
1/SE_IV
−0.75
−0.50
−0.25
0.00
0.25
0.50
β_IV

Batch 2−5117 : Gut microbiota abundance (genus Catenibacterium id.2153) on Rho−related GTP−binding protein Rho6
with a threshold of 1e−05

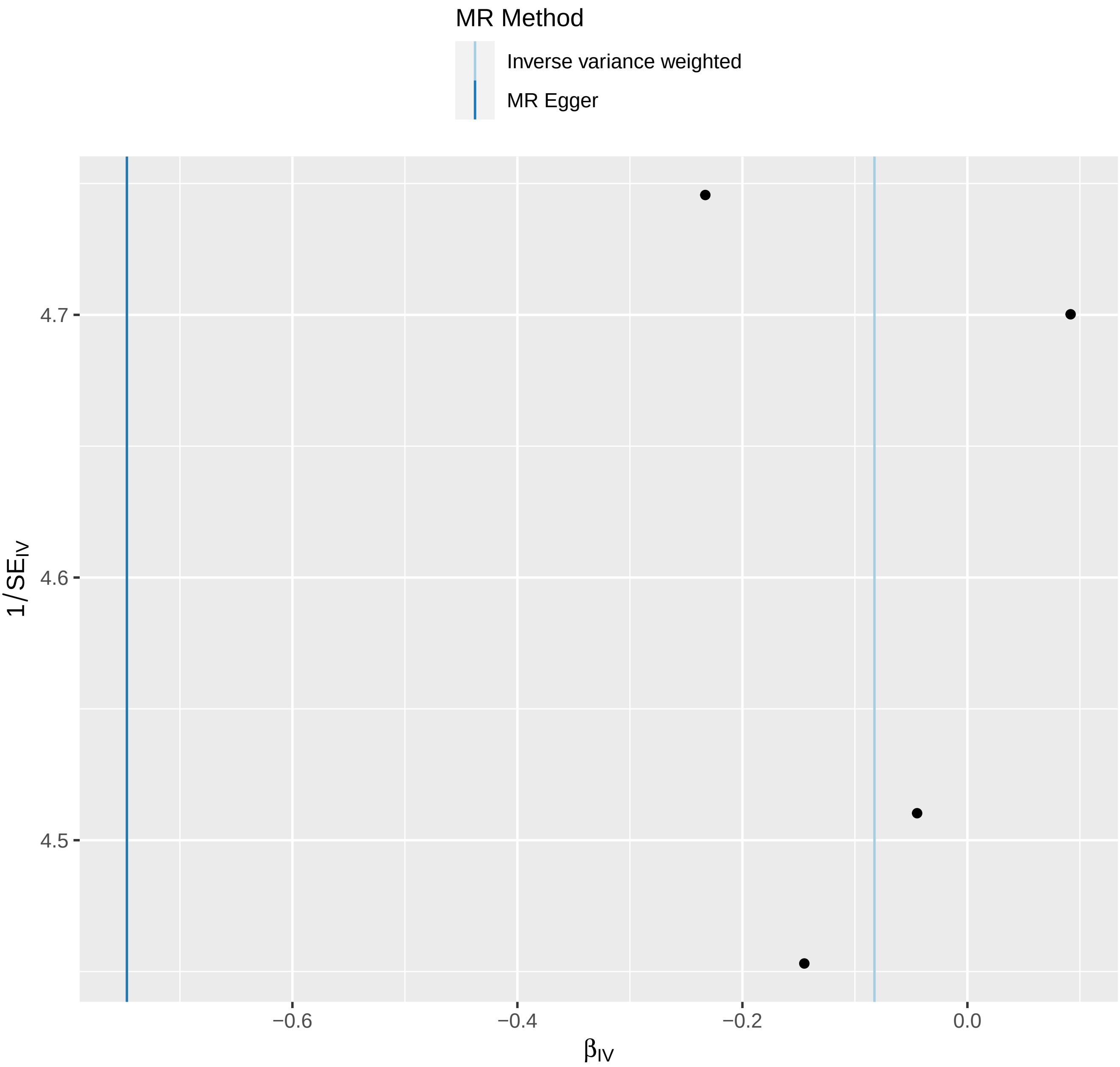

Batch 2−5118 : Gut microbiota abundance (genus Parasutterella id.2892) on Rho−related GTP−binding protein Rho6
with a threshold of 1e−05

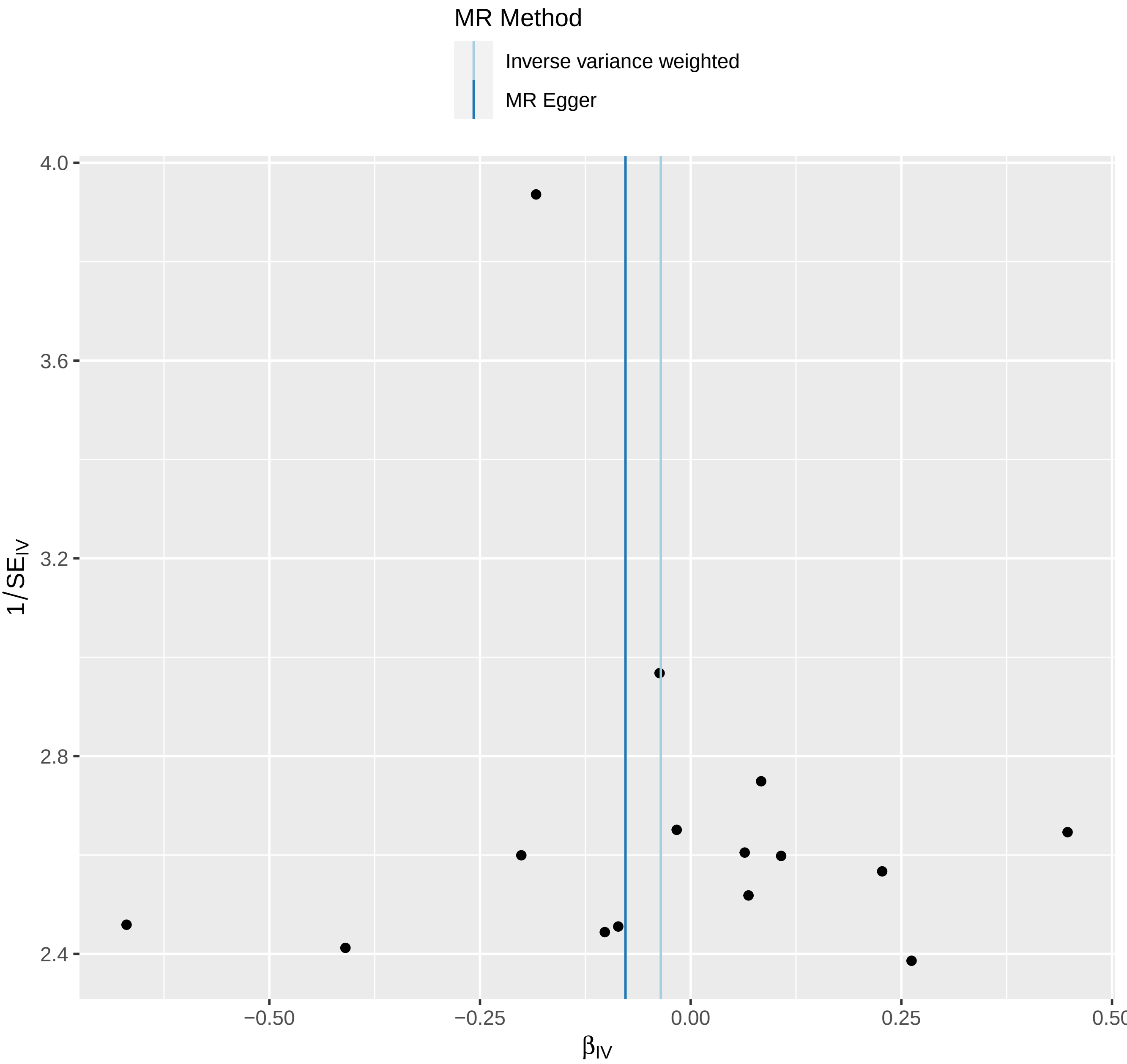

Batch 2−5119 : Gut microbiota abundance (genus Catenibacterium id.2153) on E3 ubiquitin−protein ligase RNF114
with a threshold of 1e−05

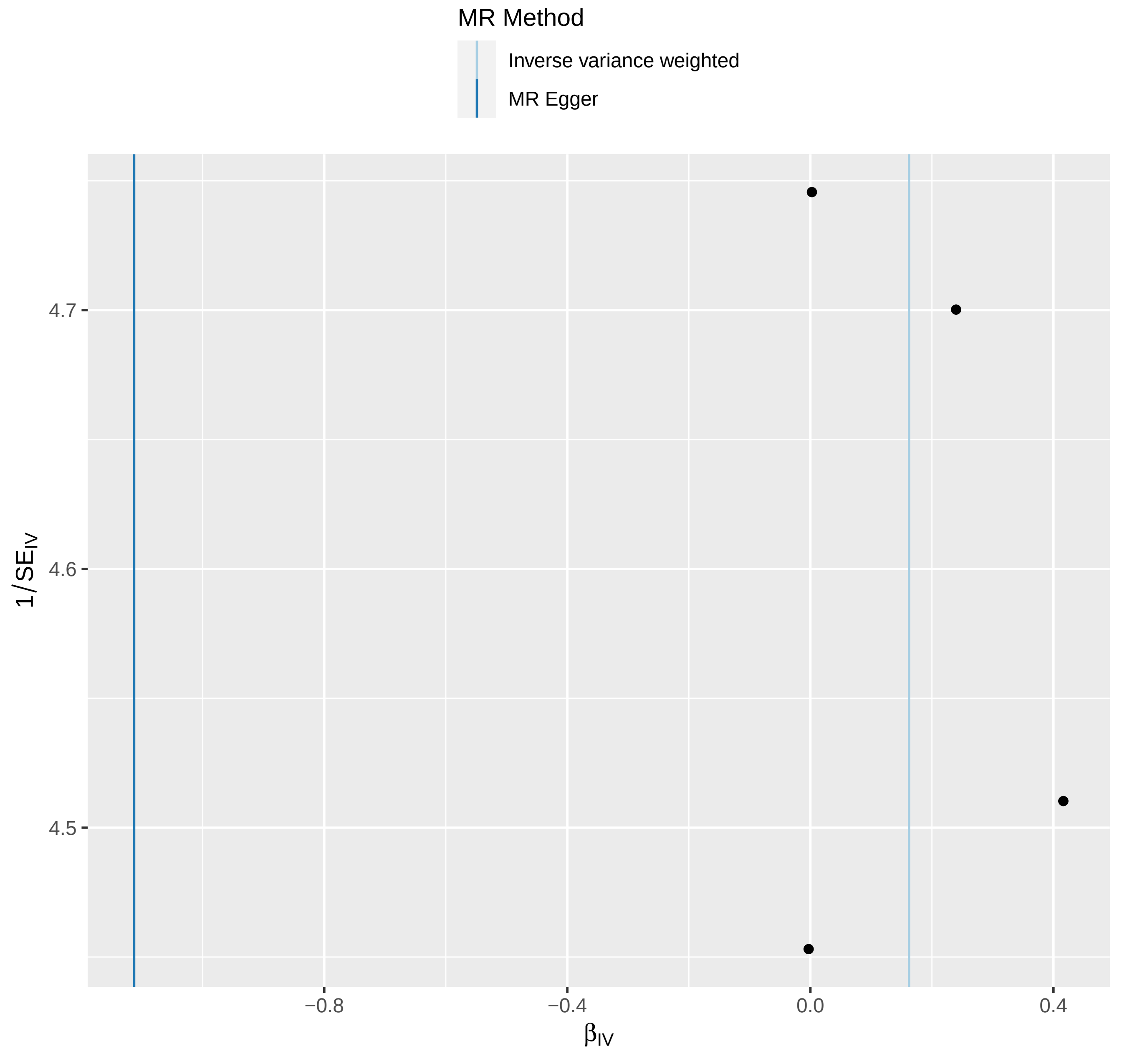

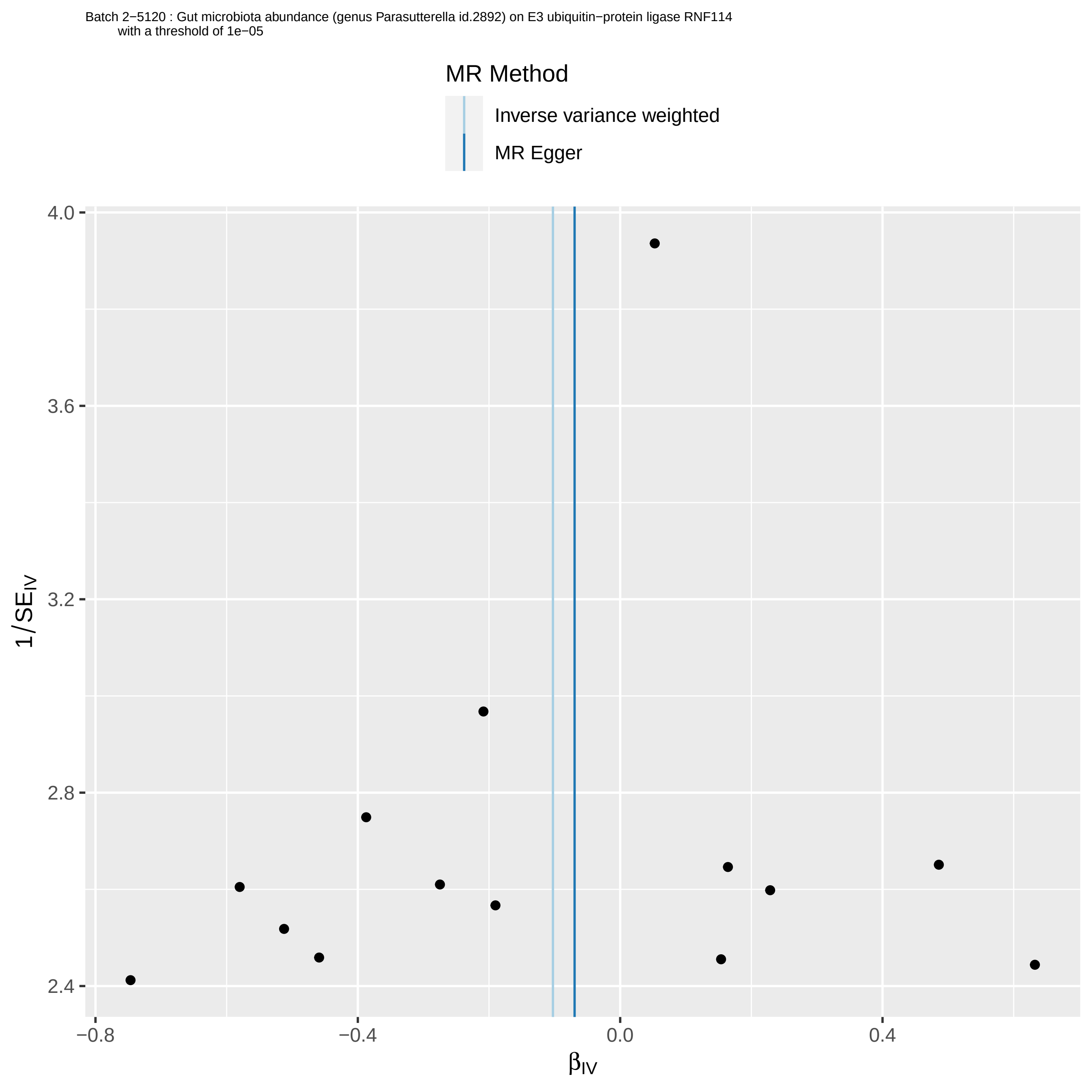
Batch 2−5120 : Gut microbiota abundance (genus Parasutterella id.2892) on E3 ubiquitin−protein ligase RNF114
with a threshold of 1e−05
MR Method
Inverse variance weighted
MR Egger
4.0
3.6
3.2
2.8
2.4
1/SE_IV
−0.8
−0.4
0.0
0.4
β_IV

Batch 2−5121 : Gut microbiota abundance (genus Catenibacterium id.2153) on RING finger protein 122
with a threshold of 1e−05

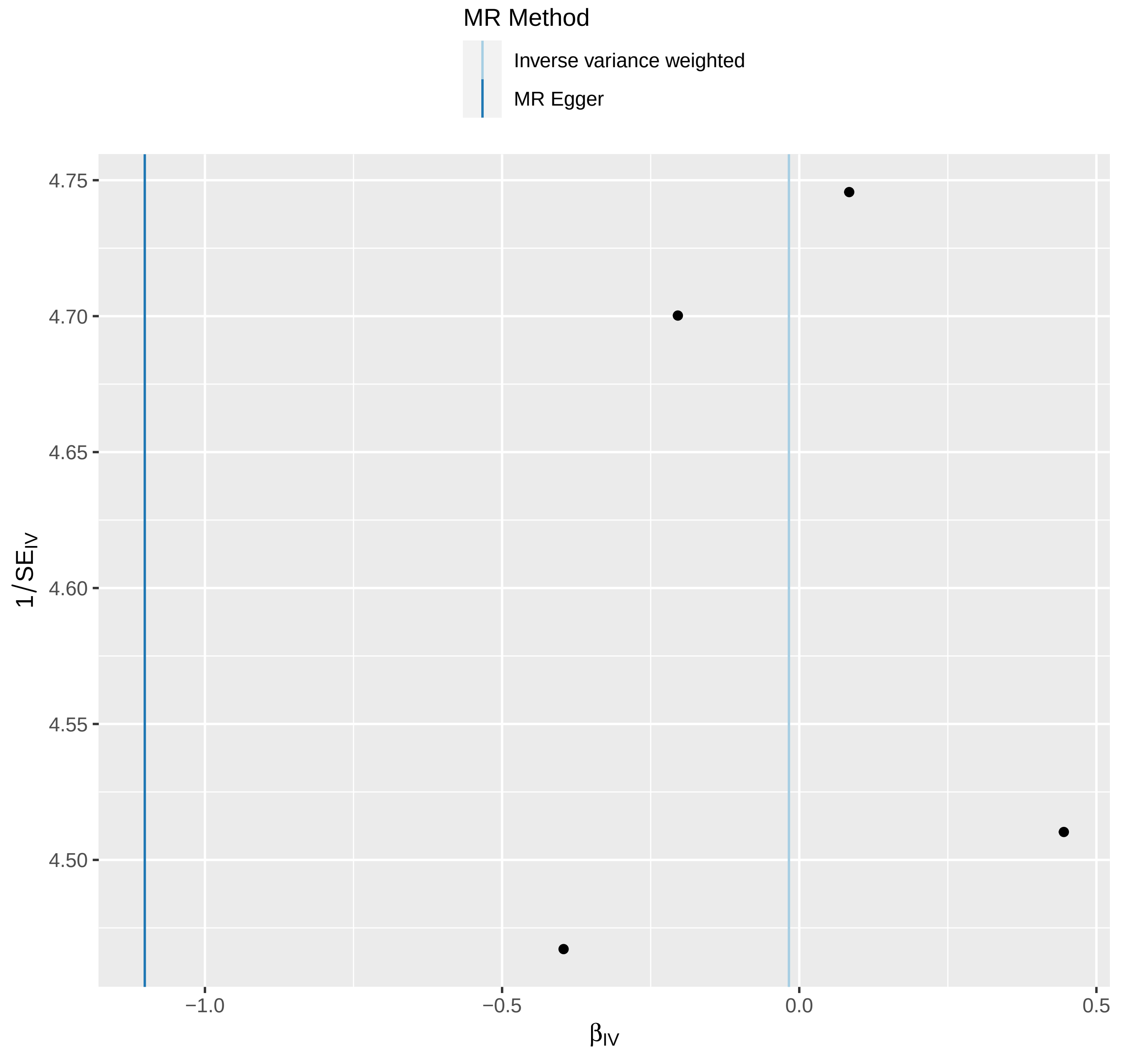

Batch 2−5122 : Gut microbiota abundance (genus Parasutterella id.2892) on RING finger protein 122
with a threshold of 1e−05

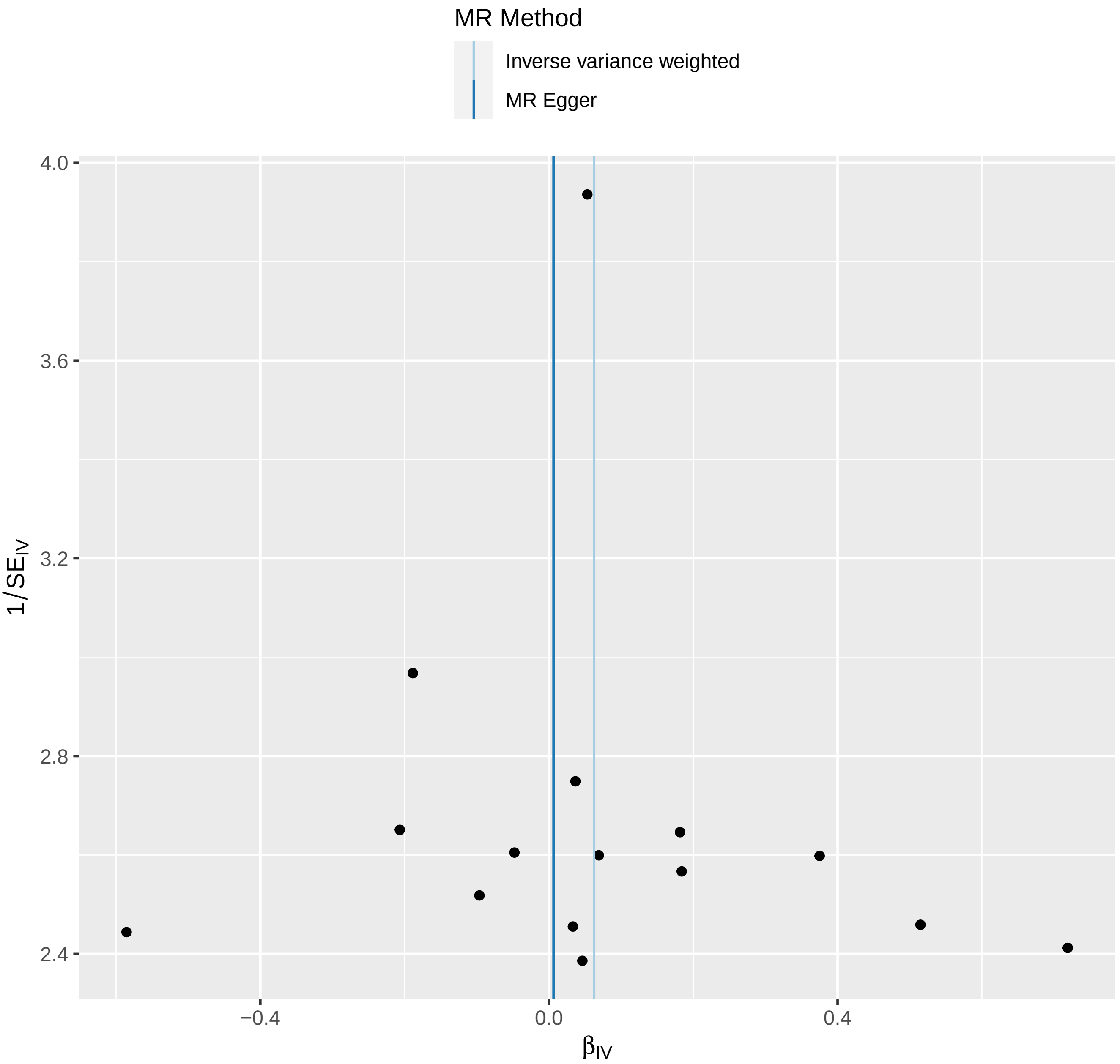

Batch 2−5123 : Gut microbiota abundance (genus Catenibacterium id.2153) on E3 ubiquitin−protein ligase RNF128 with a threshold of 1e−05

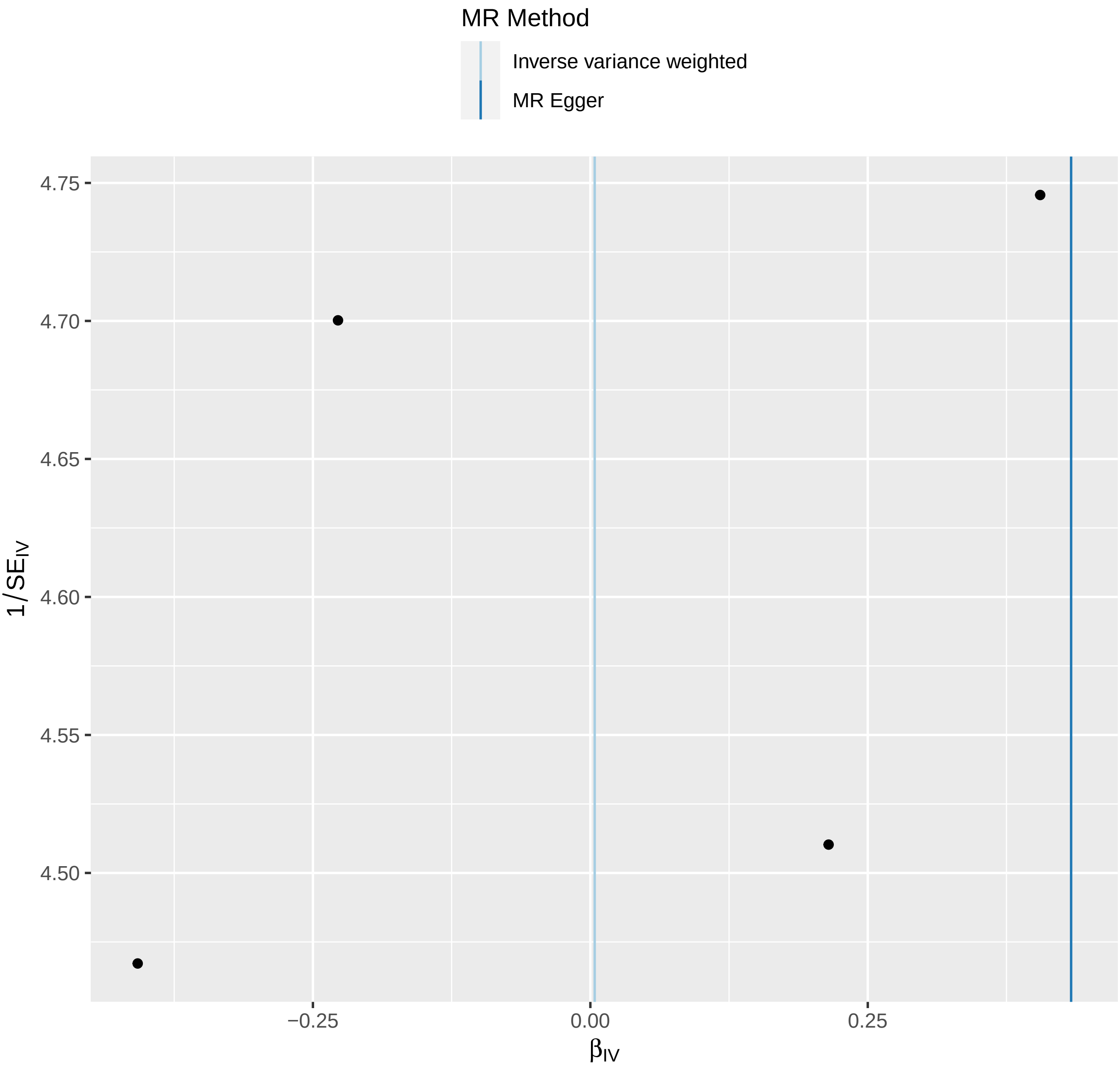

Batch 2−5124 : Gut microbiota abundance (genus Parasutterella id.2892) on E3 ubiquitin−protein ligase RNF128
with a threshold of 1e−05

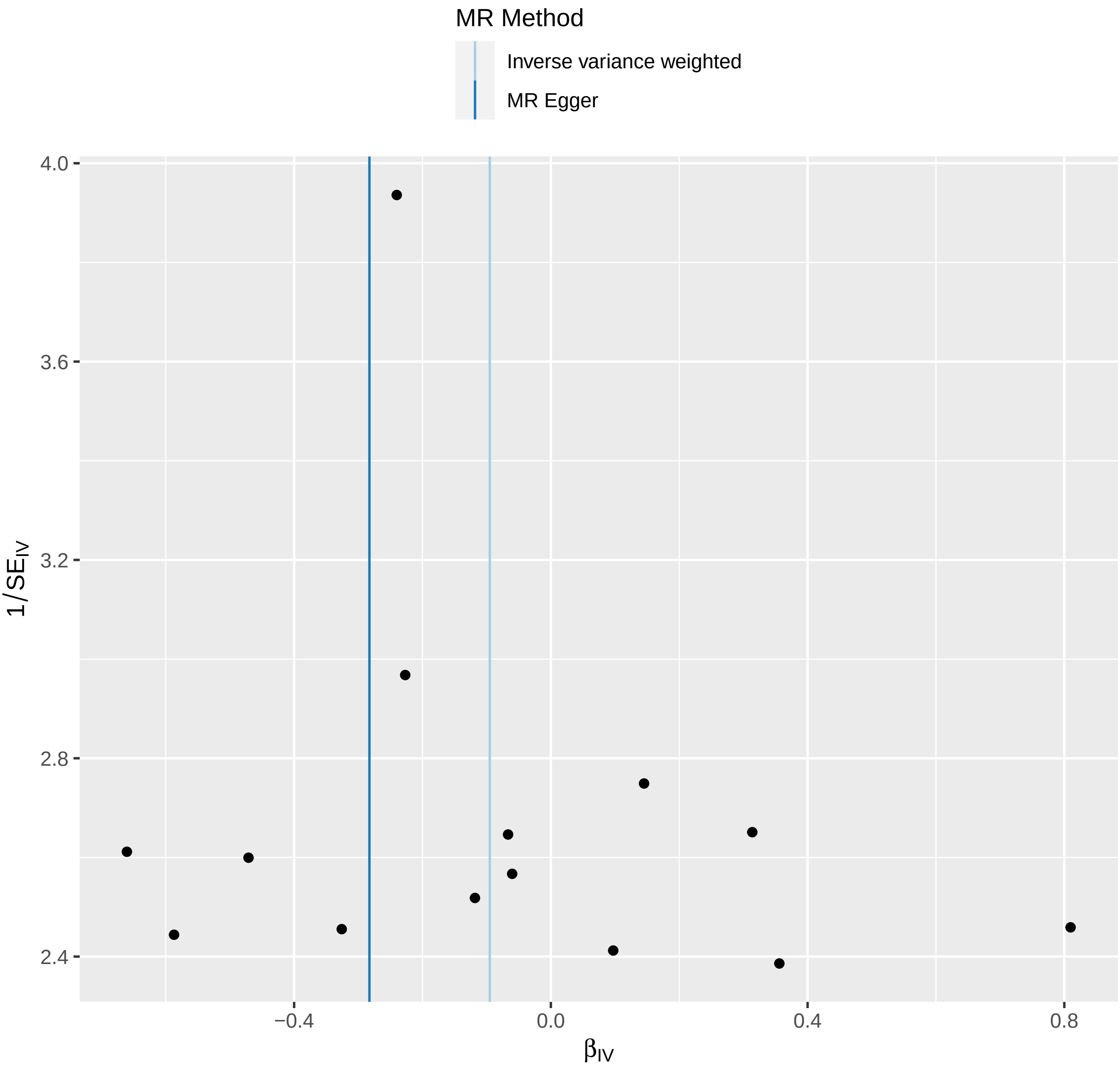

Batch 2−5125 : Gut microbiota abundance (genus Catenibacterium id.2153) on E3 ubiquitin−protein ligase RNF13
with a threshold of 1e−05

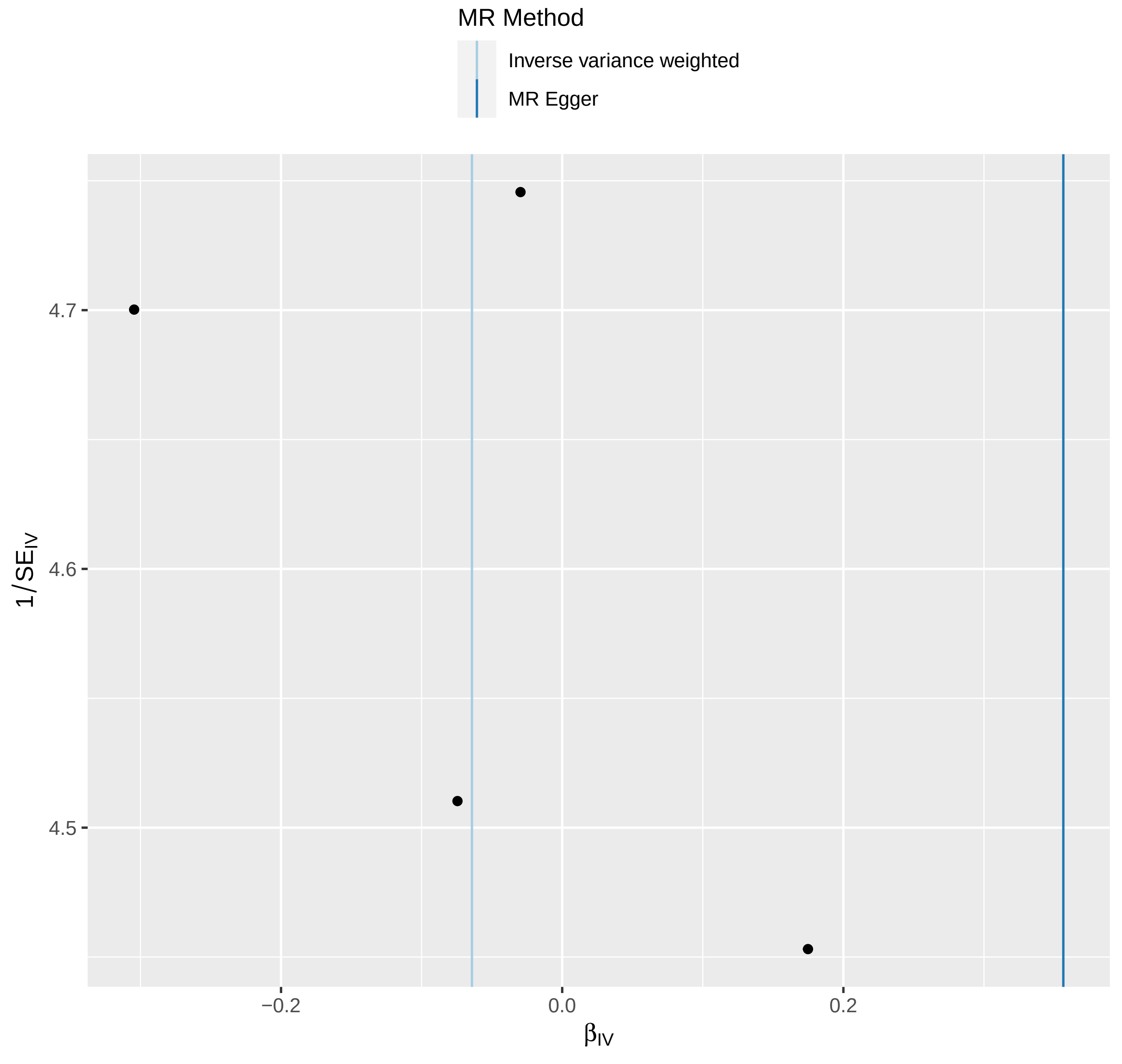

Batch 2−5126 : Gut microbiota abundance (genus Parasutterella id.2892) on E3 ubiquitin−protein ligase RNF13
with a threshold of 1e−05

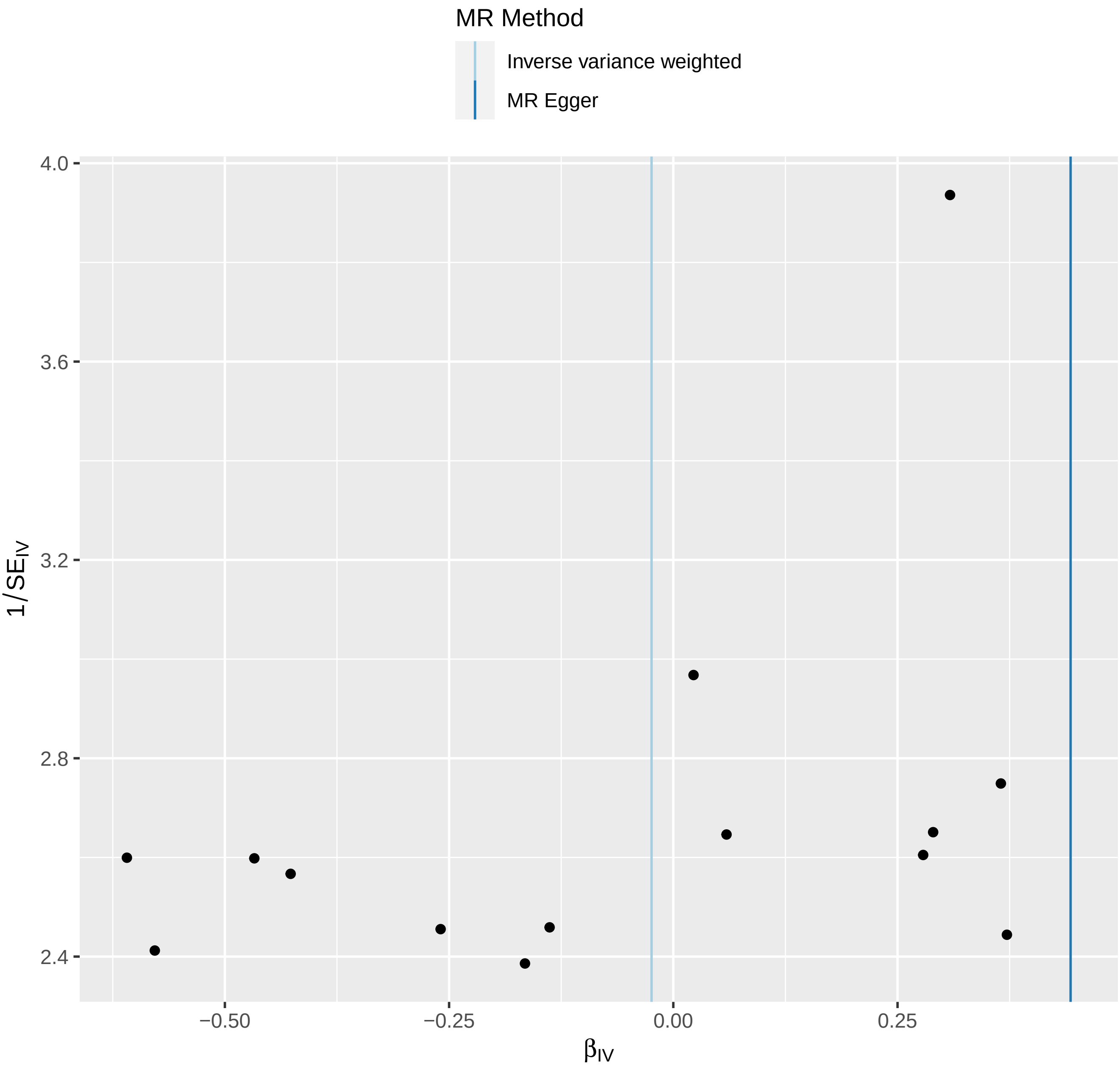

Batch 2−5127 : Gut microbiota abundance (genus Catenibacterium id.2153) on RING finger protein 148
with a threshold of 1e−05

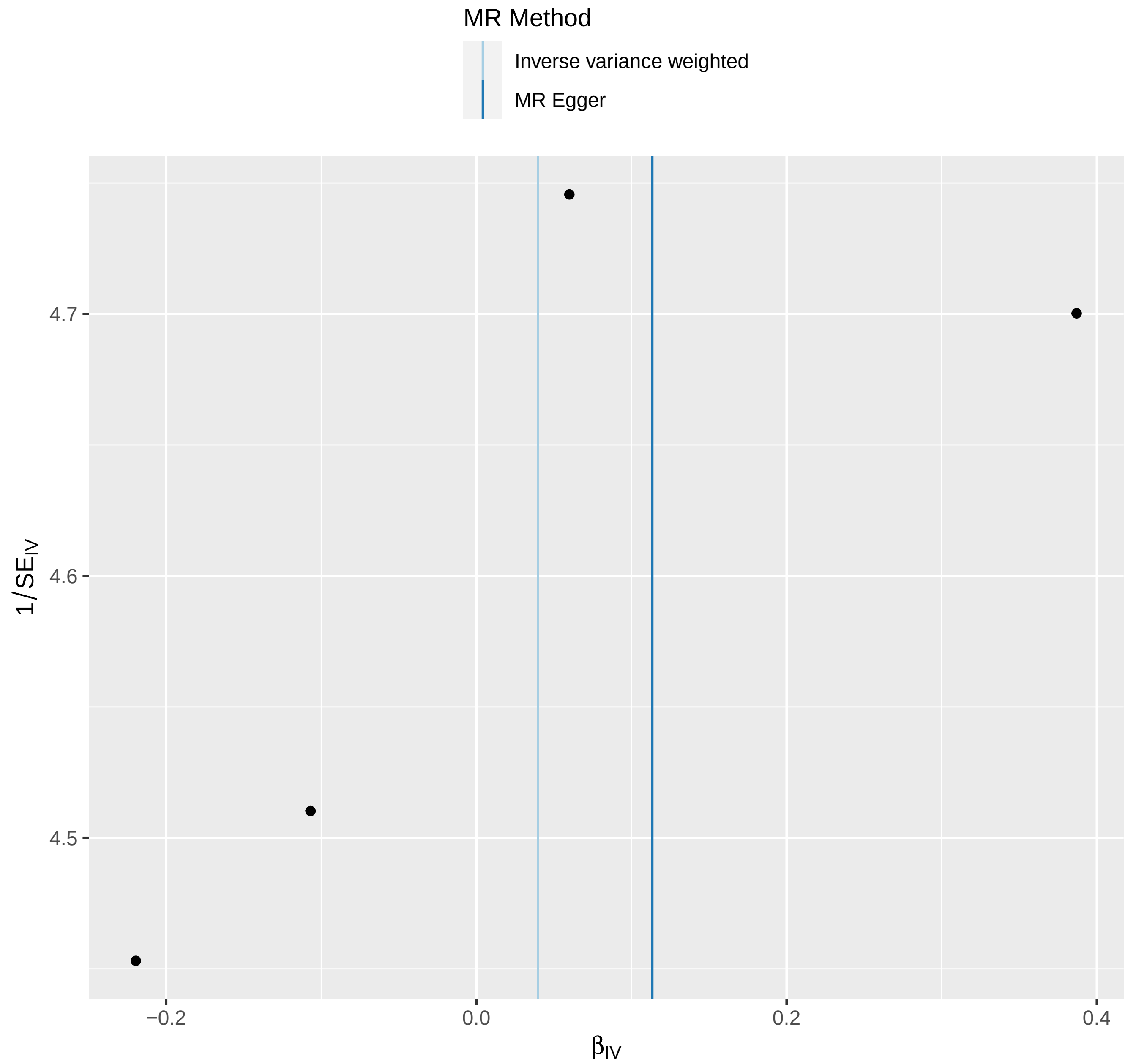

Batch 2−5128 : Gut microbiota abundance (genus Parasutterella id.2892) on RING finger protein 148
with a threshold of 1e−05

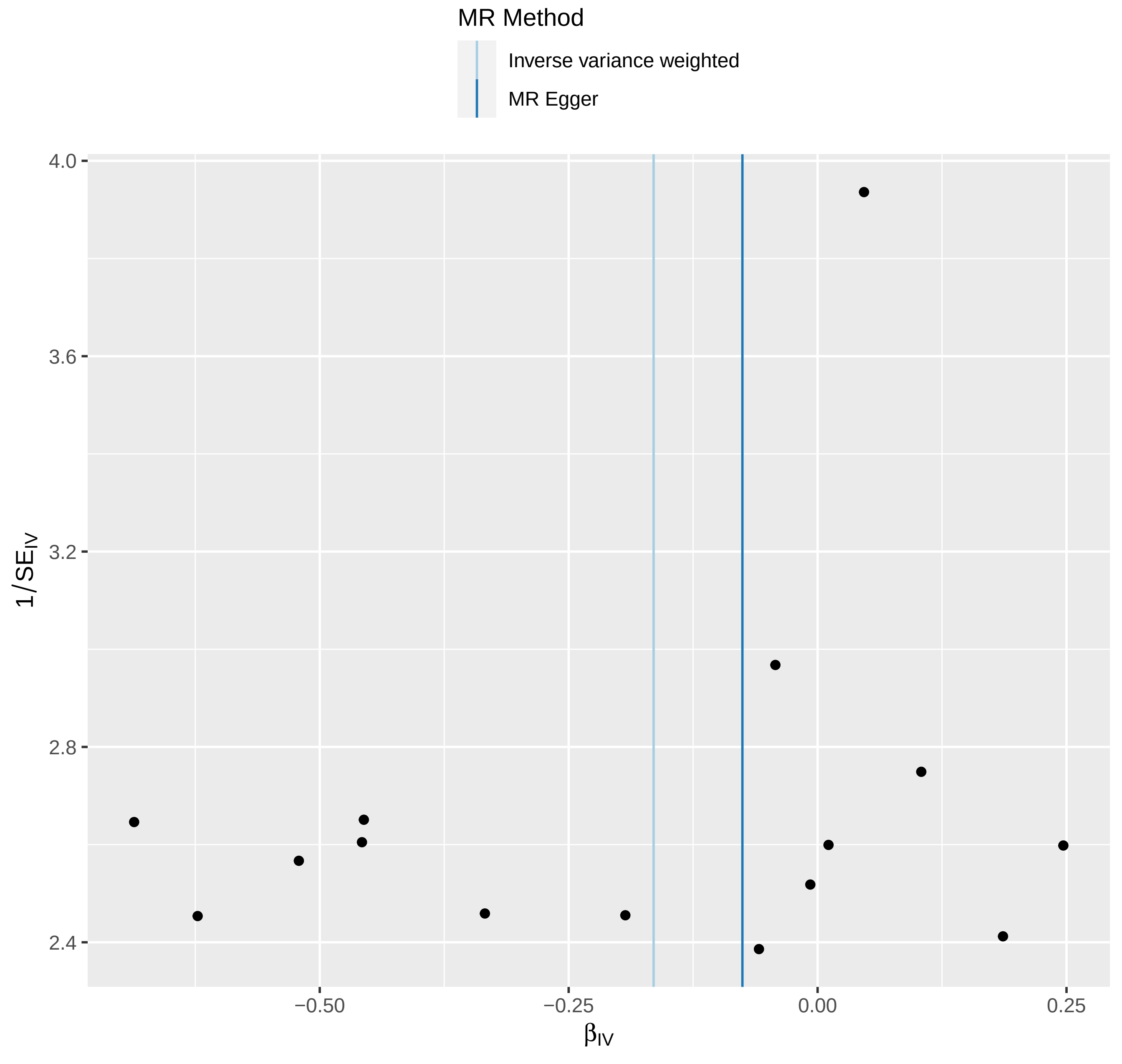

Batch 2−5129 : Gut microbiota abundance (genus Catenibacterium id.2153) on RING finger protein 148
with a threshold of 1e−05

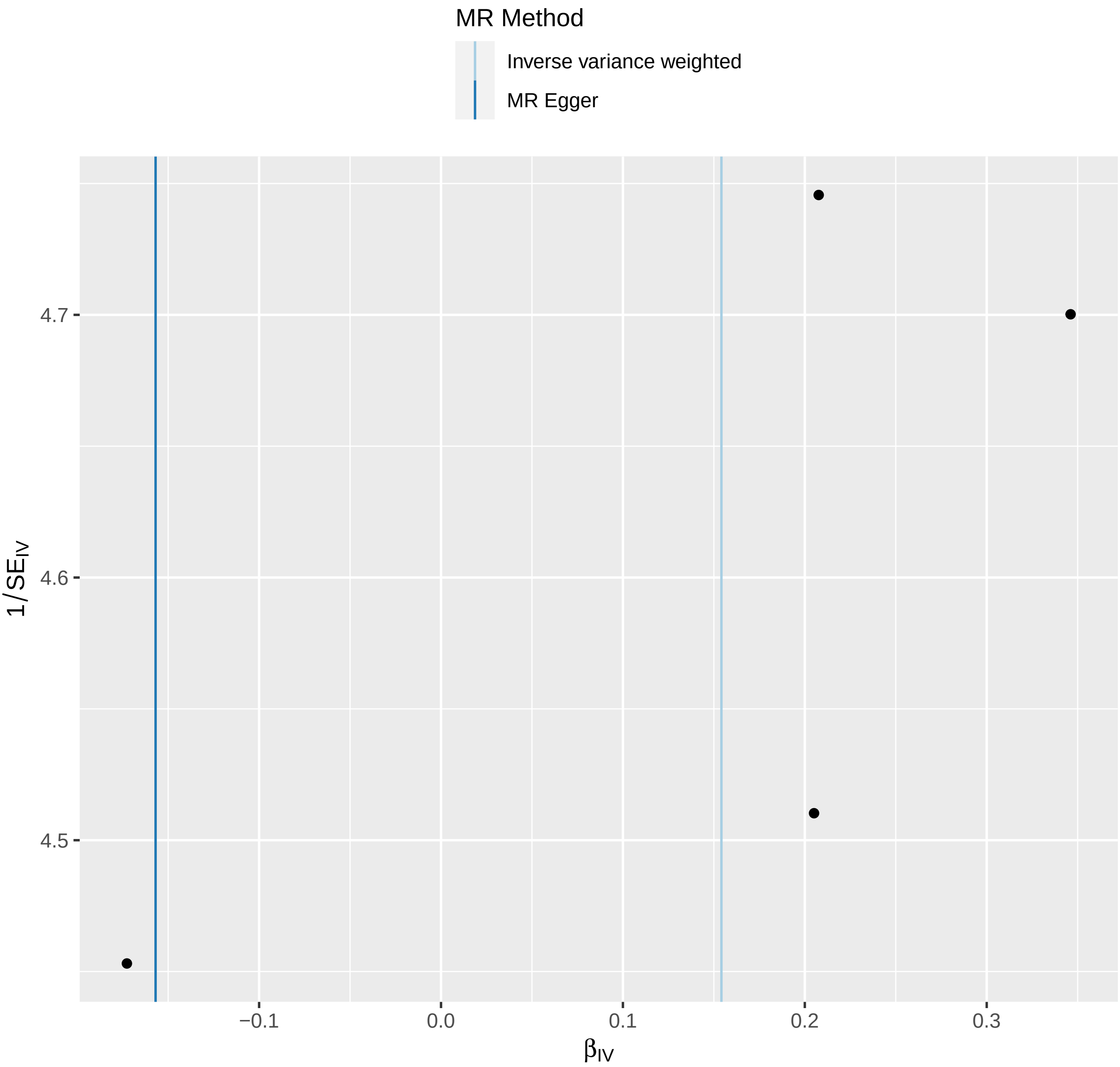

Batch 2−5130 : Gut microbiota abundance (genus Parasutterella id.2892) on RING finger protein 148
with a threshold of 1e−05

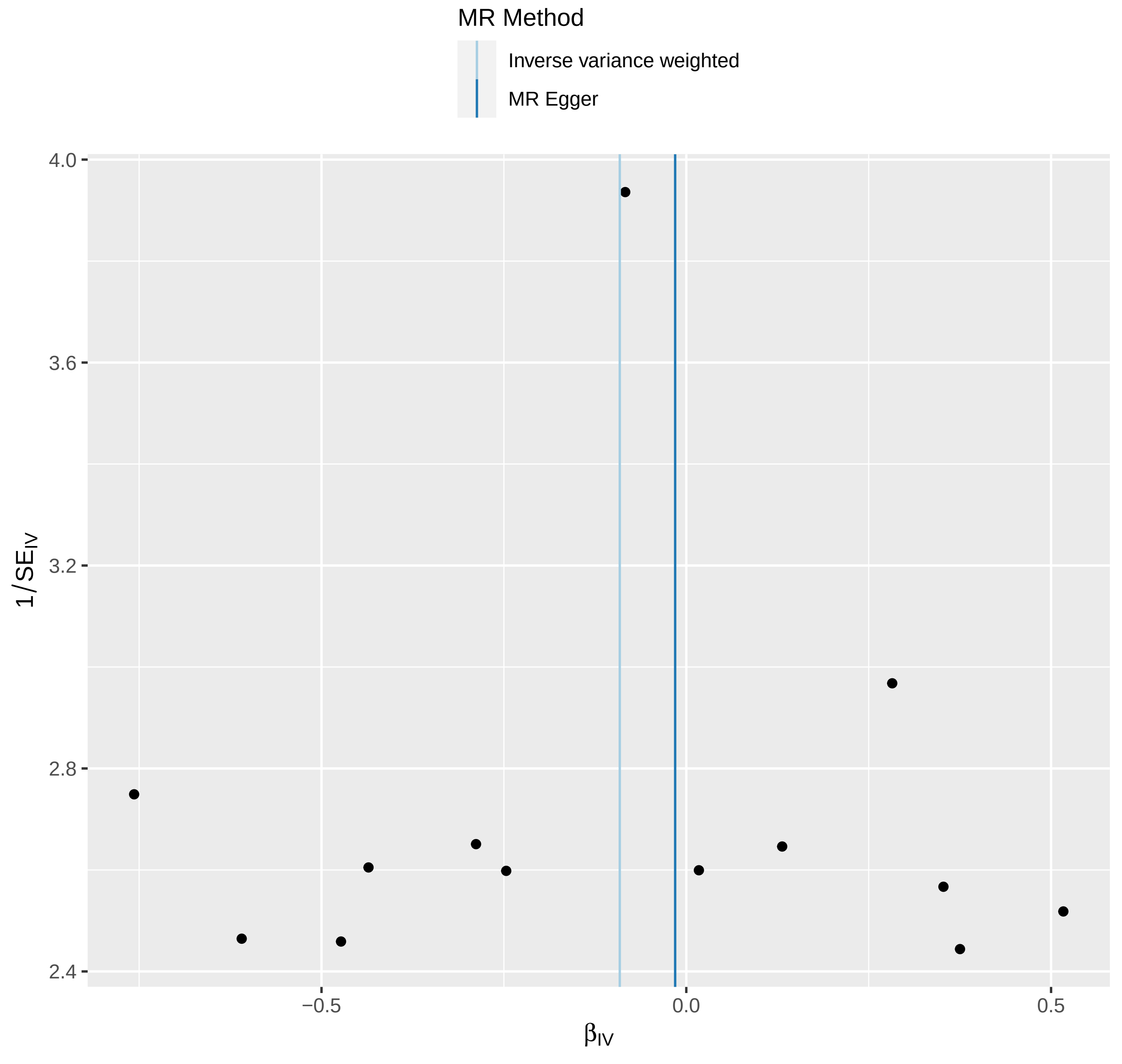

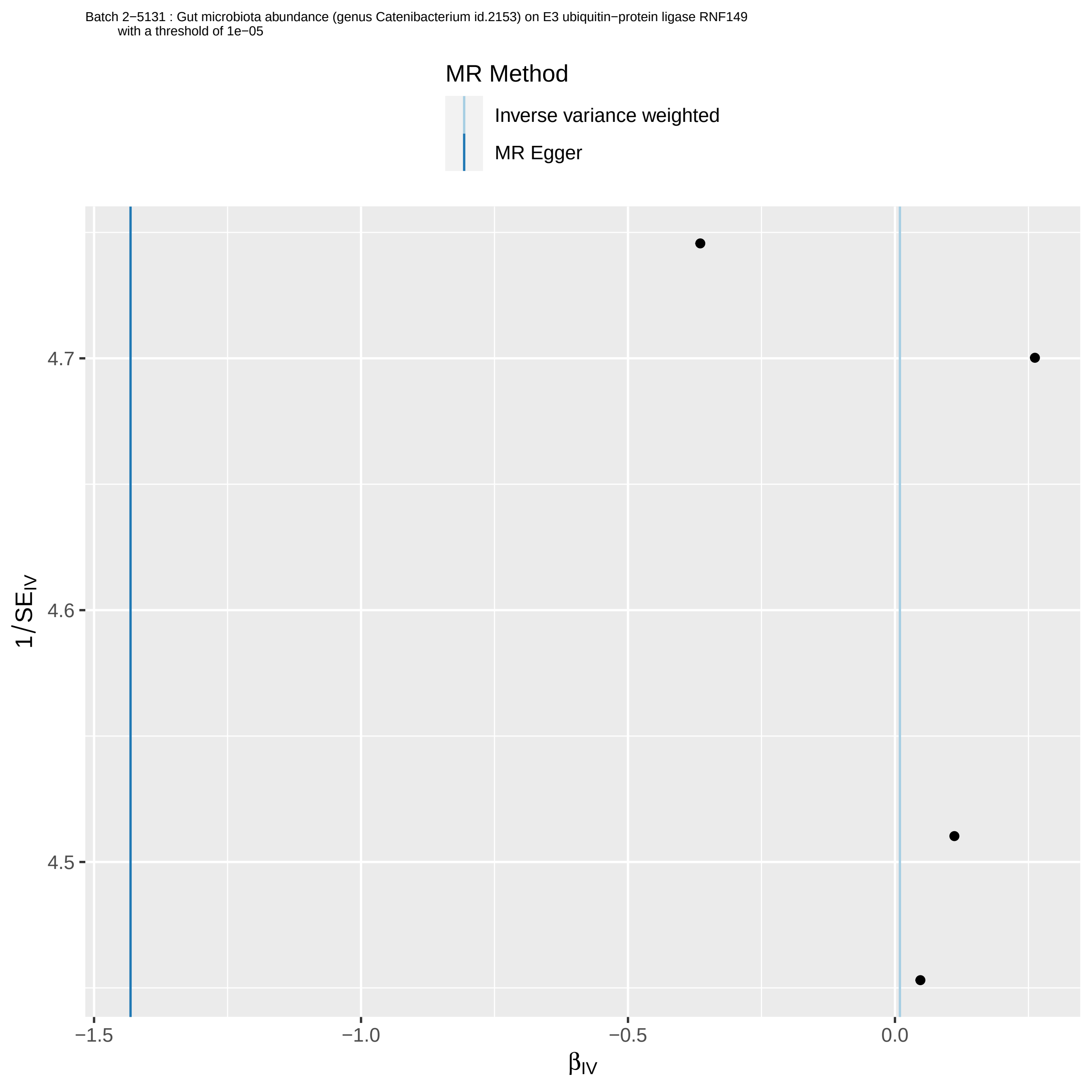
Batch 2−5131 : Gut microbiota abundance (genus Catenibacterium id.2153) on E3 ubiquitin−protein ligase RNF149
with a threshold of 1e−05
MR Method
Inverse variance weighted
MR Egger
4.7
4.6
4.5
1/SE_IV
−1.5
−1.0
−0.5
0.0
β_IV

Batch 2−5132 : Gut microbiota abundance (genus Parasutterella id.2892) on E3 ubiquitin−protein ligase RNF149
with a threshold of 1e−05

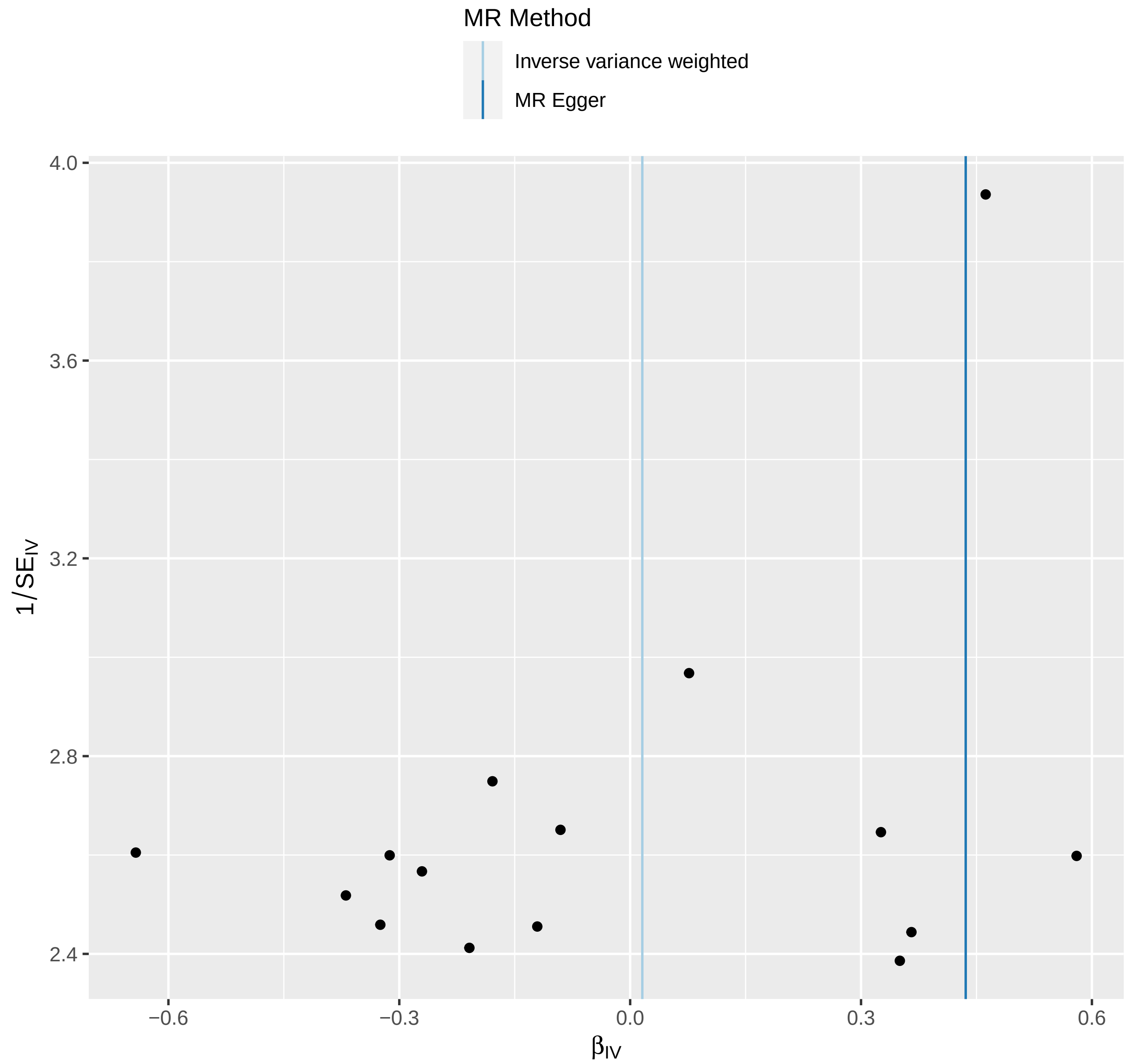

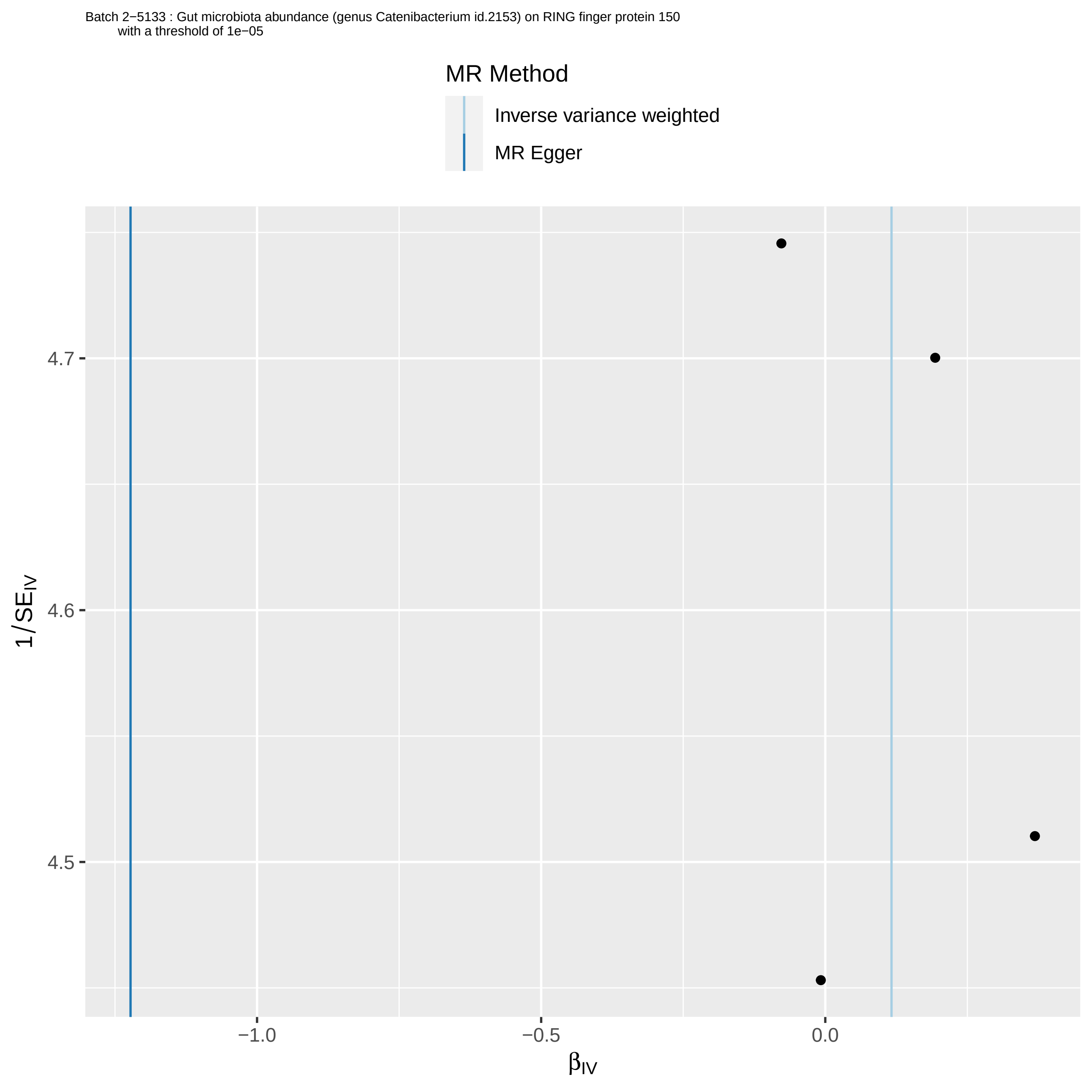
Batch 2−5133 : Gut microbiota abundance (genus Catenibacterium id.2153) on RING finger protein 150
with a threshold of 1e−05
MR Method
Inverse variance weighted
MR Egger
4.7
4.6
4.5
$1/SE_{IV}$
−1.0
−0.5
0.0
β_{IV}

Batch 2−5134 : Gut microbiota abundance (genus Parasutterella id.2892) on RING finger protein 150
with a threshold of 1e−05

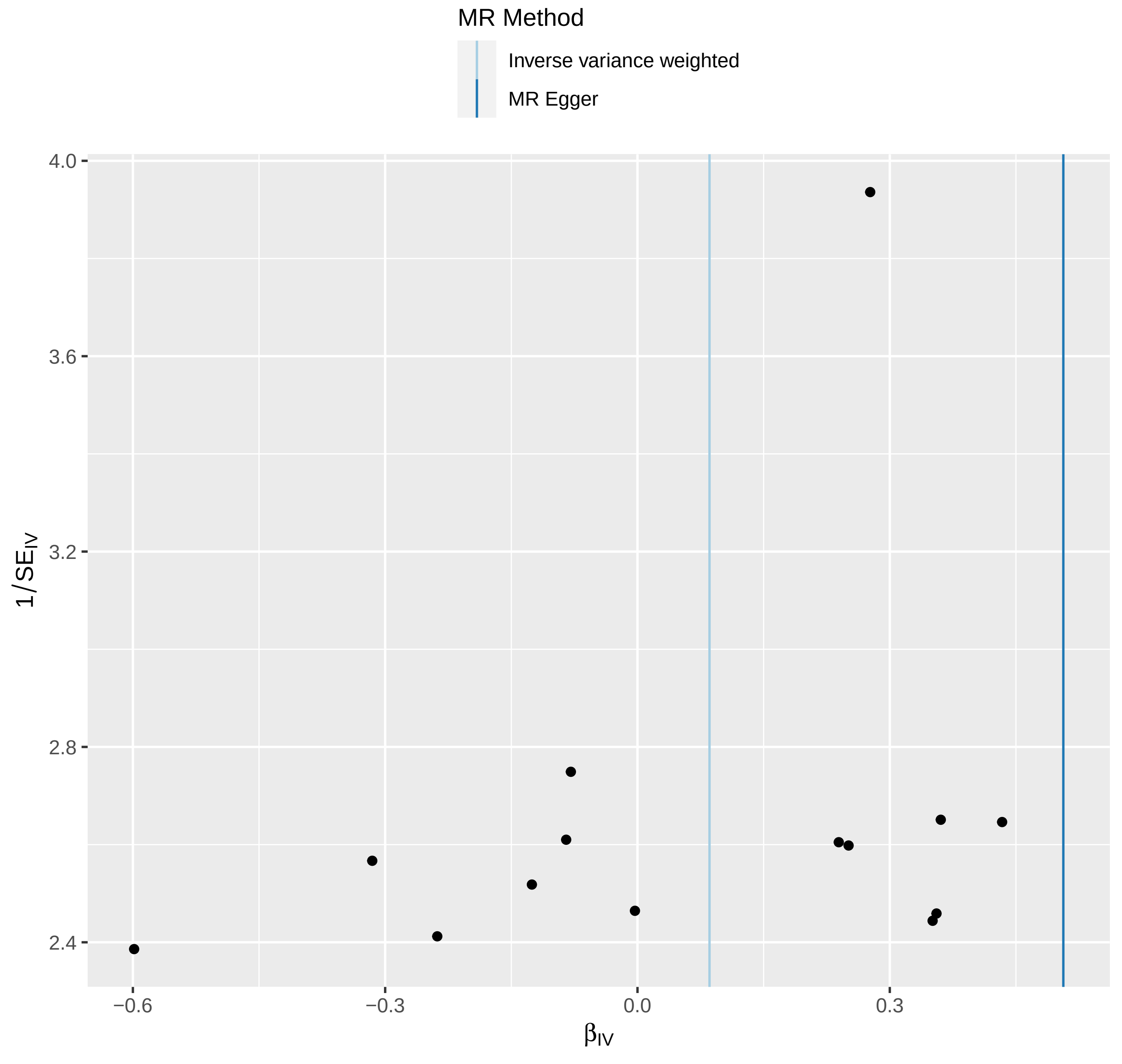

Batch 2−5135 : Gut microbiota abundance (genus Catenibacterium id.2153) on RING finger protein 165
with a threshold of 1e−05

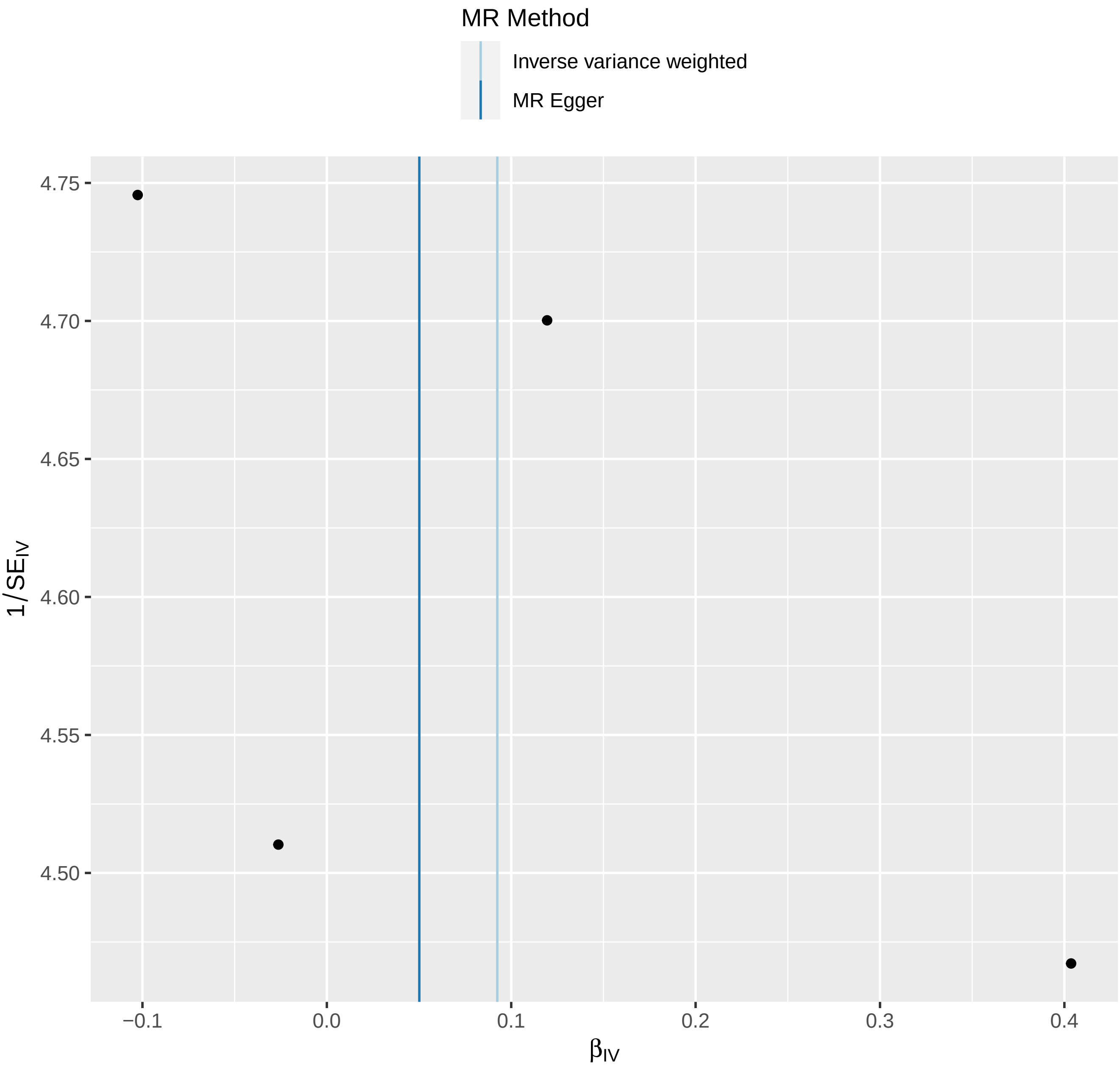

Batch 2−5136 : Gut microbiota abundance (genus Parasutterella id.2892) on RING finger protein 165
with a threshold of 1e−05

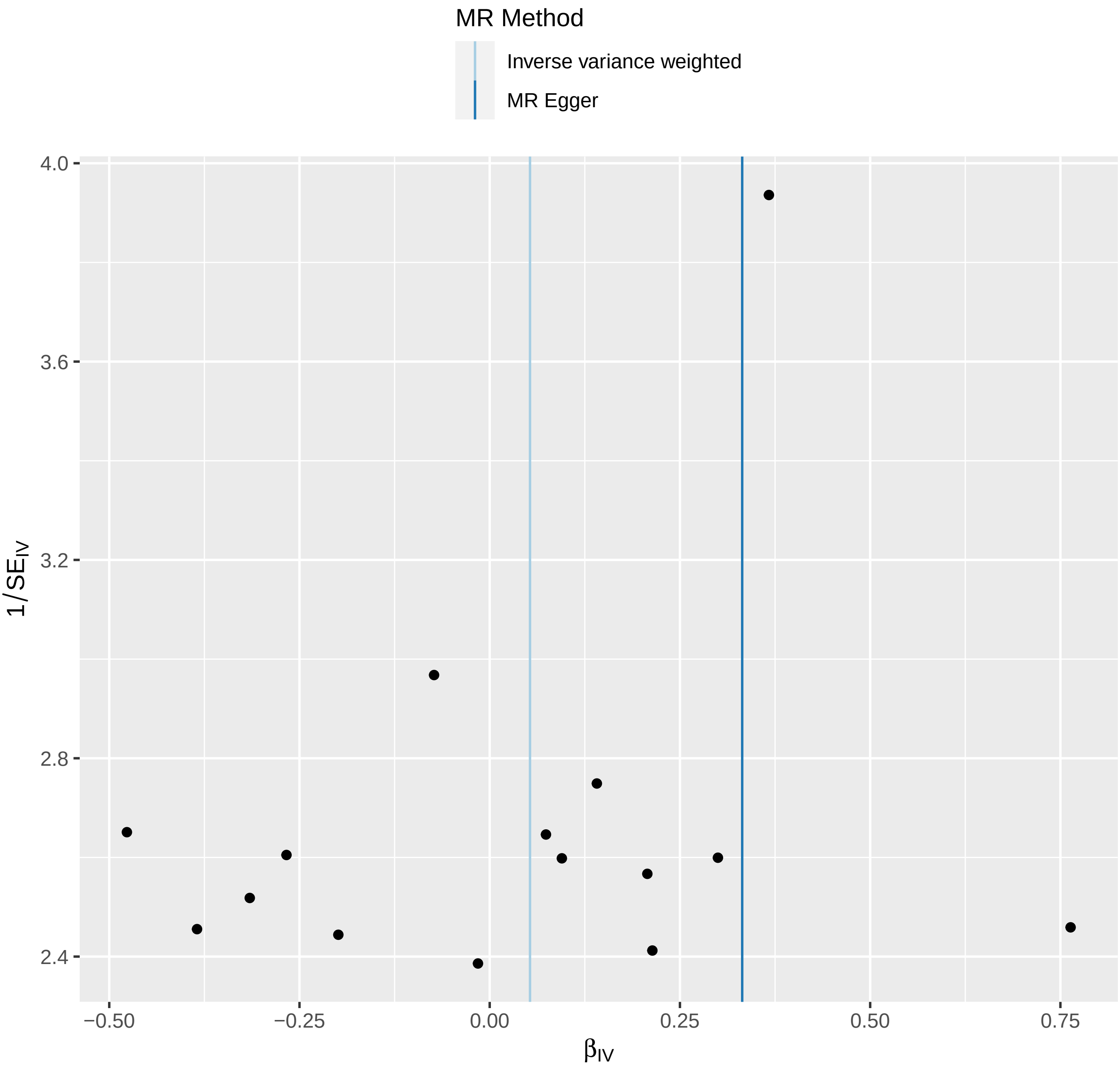

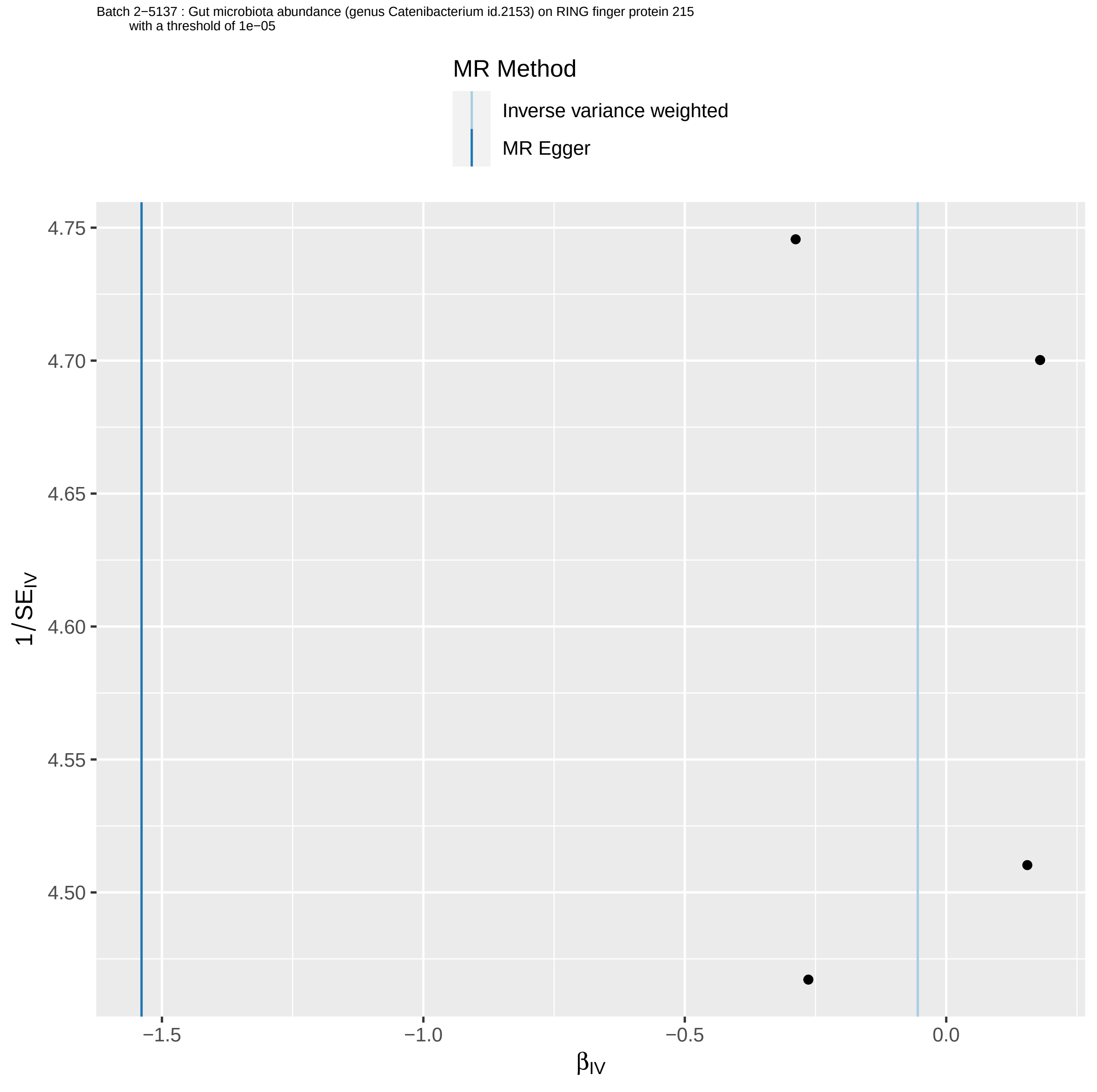

Batch 2−5137 : Gut microbiota abundance (genus Catenibacterium id.2153) on RING finger protein 215
with a threshold of 1e−05
MR Method
Inverse variance weighted
MR Egger
4.75
4.70
4.65
4.60
4.55
4.50
1/SE_IV
−1.5
−1.0
−0.5
0.0
β_IV

Batch 2−5138 : Gut microbiota abundance (genus Parasutterella id.2892) on RING finger protein 215
with a threshold of 1e−05

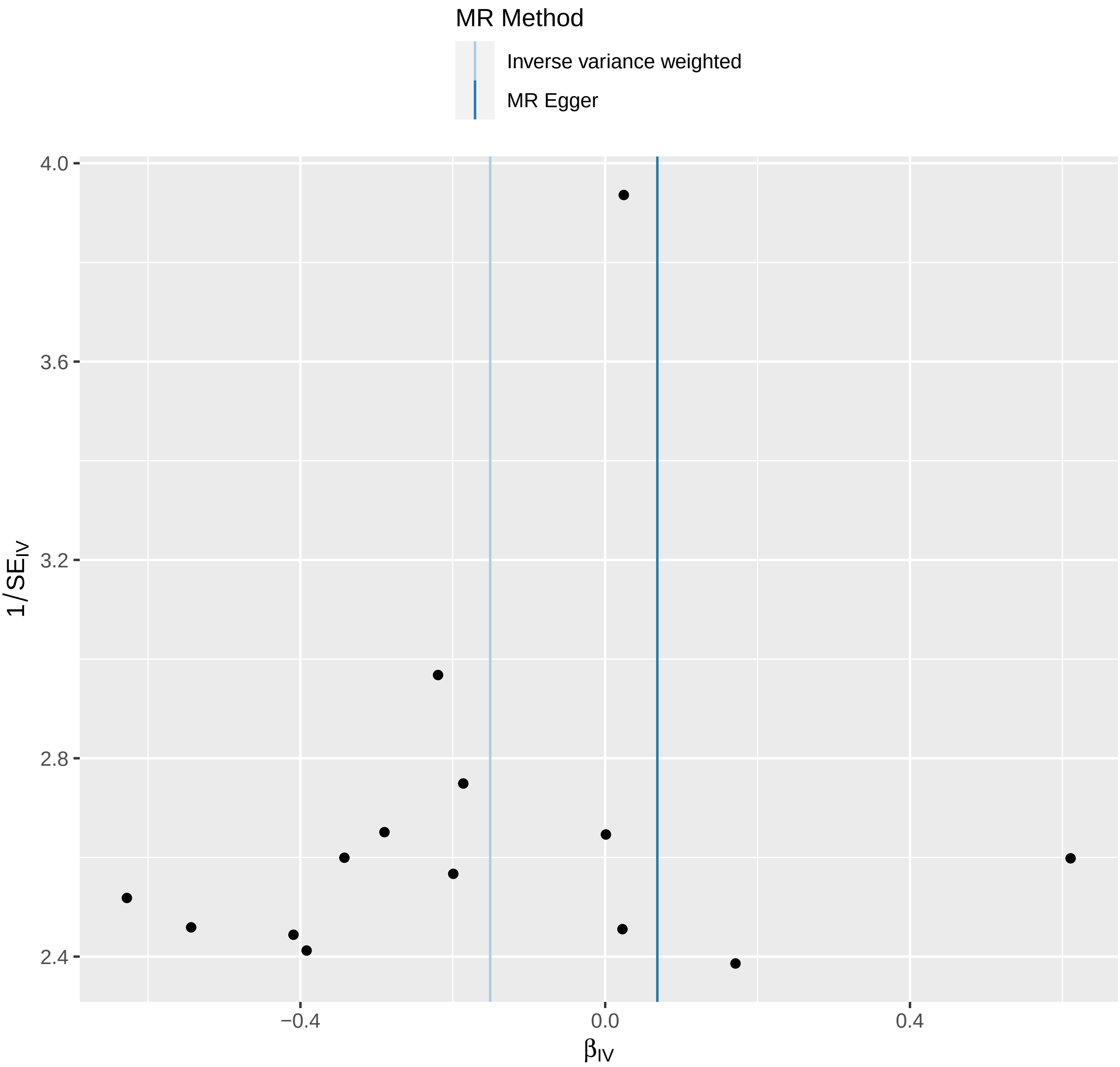

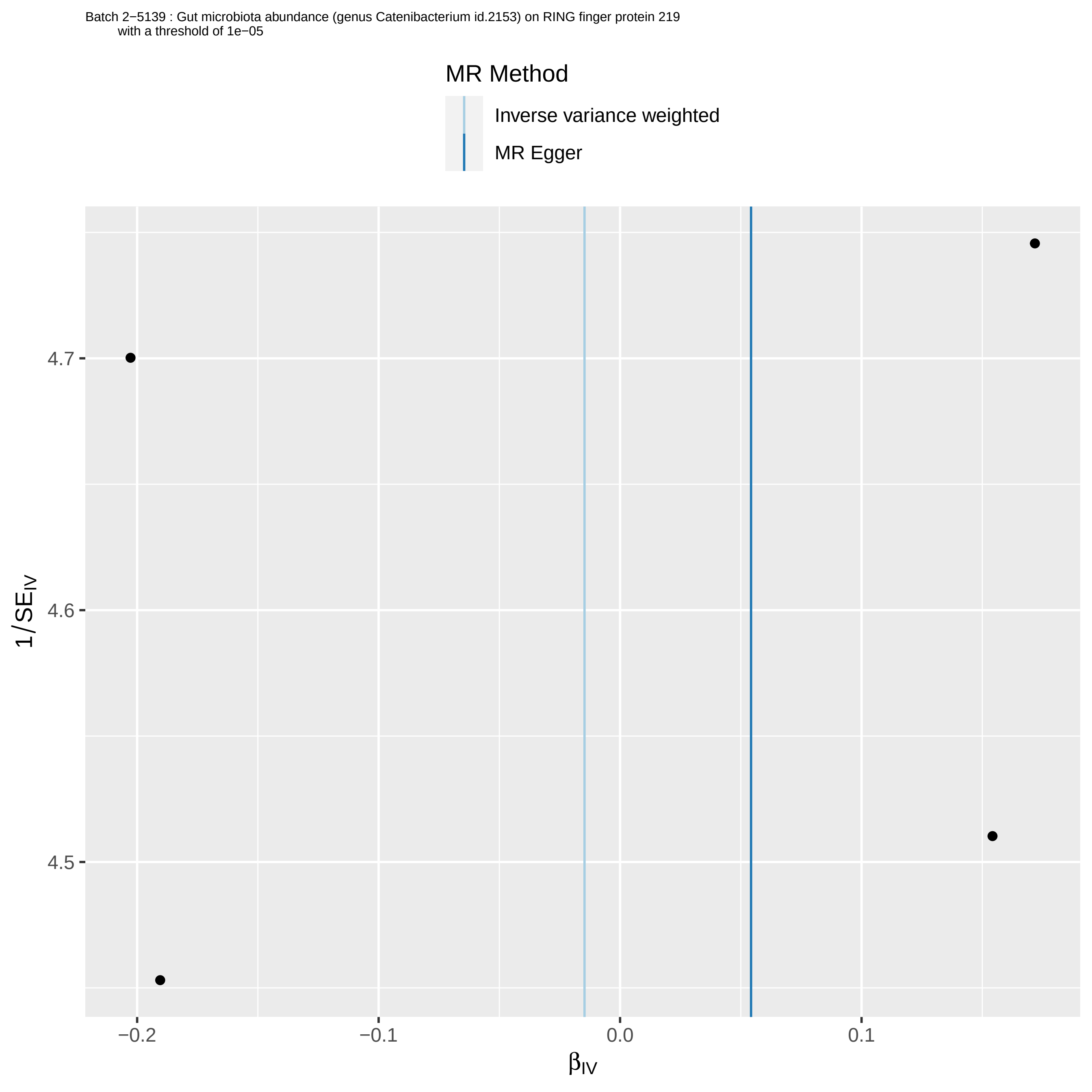
Batch 2−5139 : Gut microbiota abundance (genus Catenibacterium id.2153) on RING finger protein 219
with a threshold of 1e−05
MR Method
Inverse variance weighted
MR Egger
4.7
4.6
4.5
1/SE_IV
−0.2
−0.1
0.0
0.1
β_IV

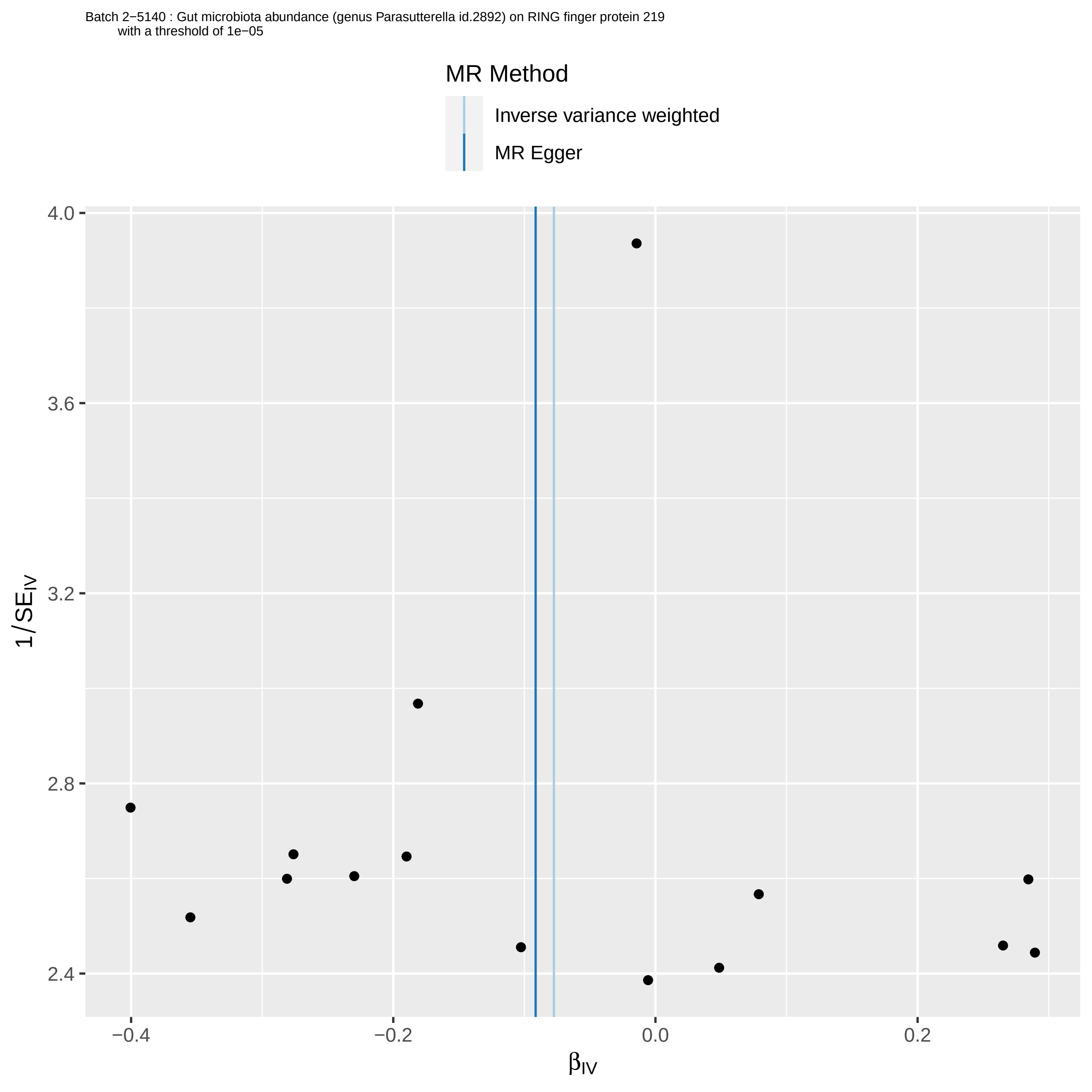

Batch 2−5140 : Gut microbiota abundance (genus Parasutterella id.2892) on RING finger protein 219
with a threshold of 1e−05
MR Method
Inverse variance weighted
MR Egger
4.0
3.6
3.2
2.8
2.4
1/SE_IV
−0.4
−0.2
0.0
0.2
β_IV

Batch 2−5141 : Gut microbiota abundance (genus Catenibacterium id.2153) on E3 ubiquitin−protein ligase RNF34
with a threshold of 1e−05

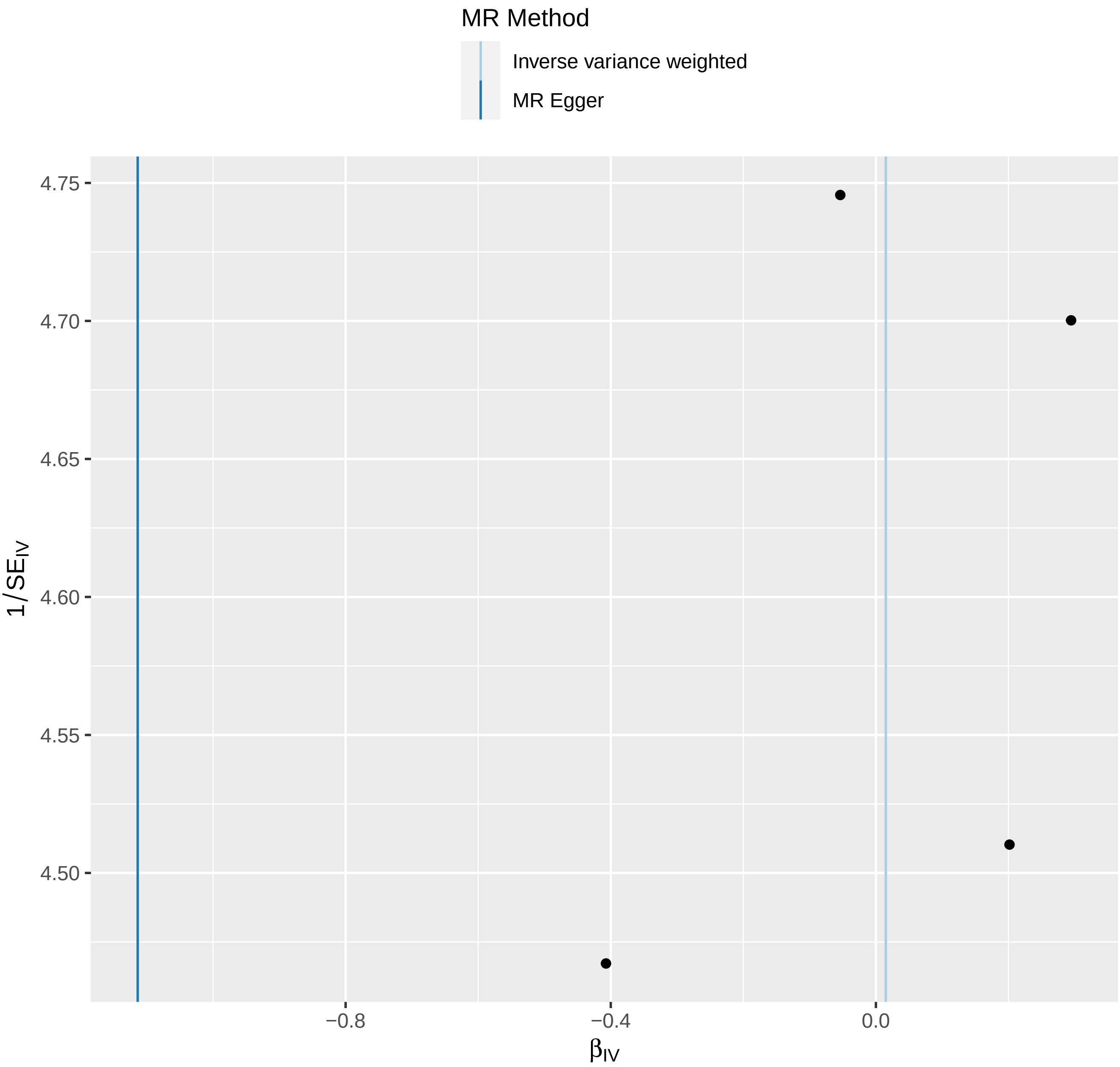

Batch 2−5142 : Gut microbiota abundance (genus Parasutterella id.2892) on E3 ubiquitin−protein ligase RNF34
with a threshold of 1e−05

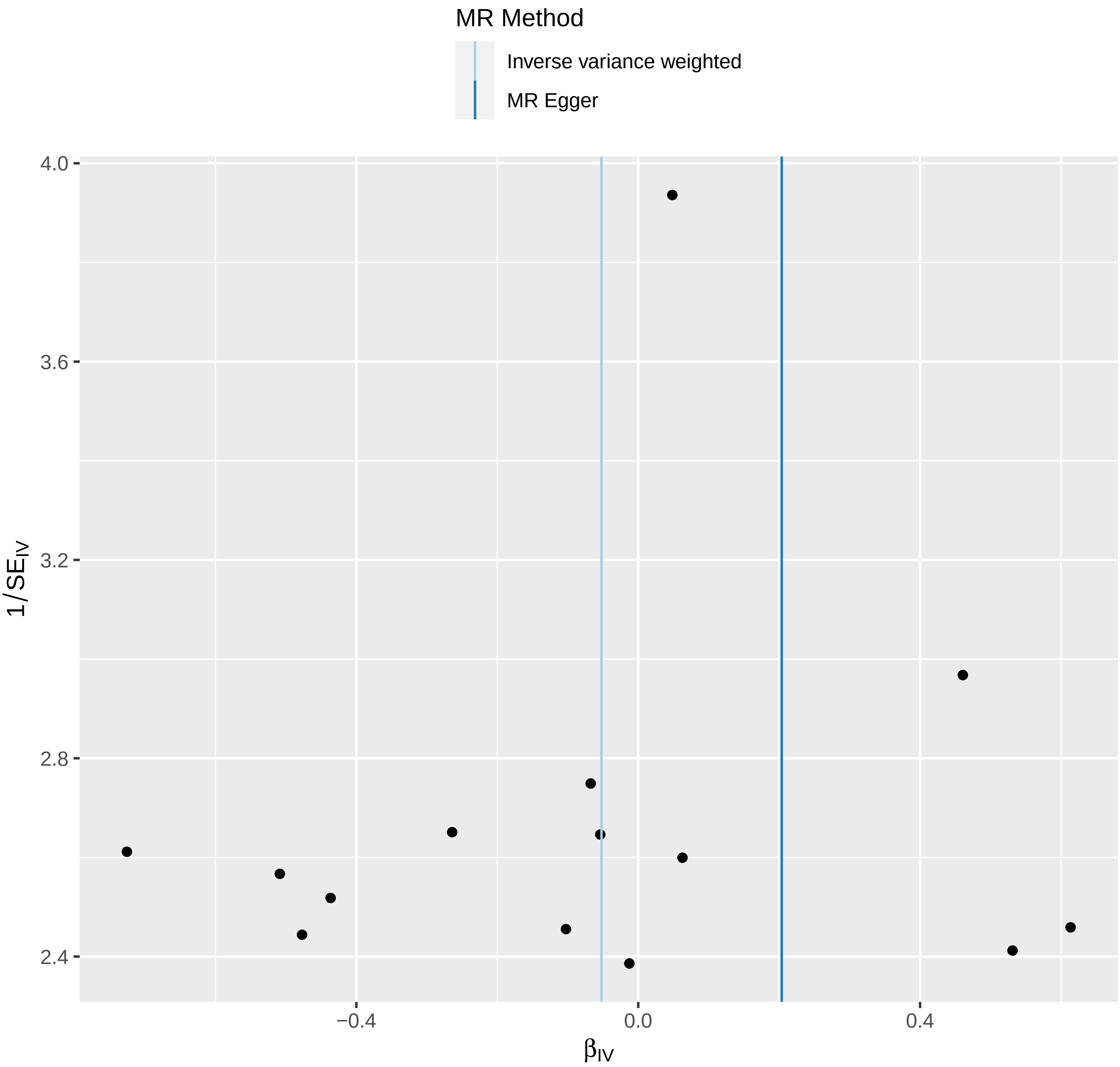

Batch 2−5143 : Gut microbiota abundance (genus Catenibacterium id.2153) on E3 ubiquitin−protein ligase RNF43
with a threshold of 1e−05

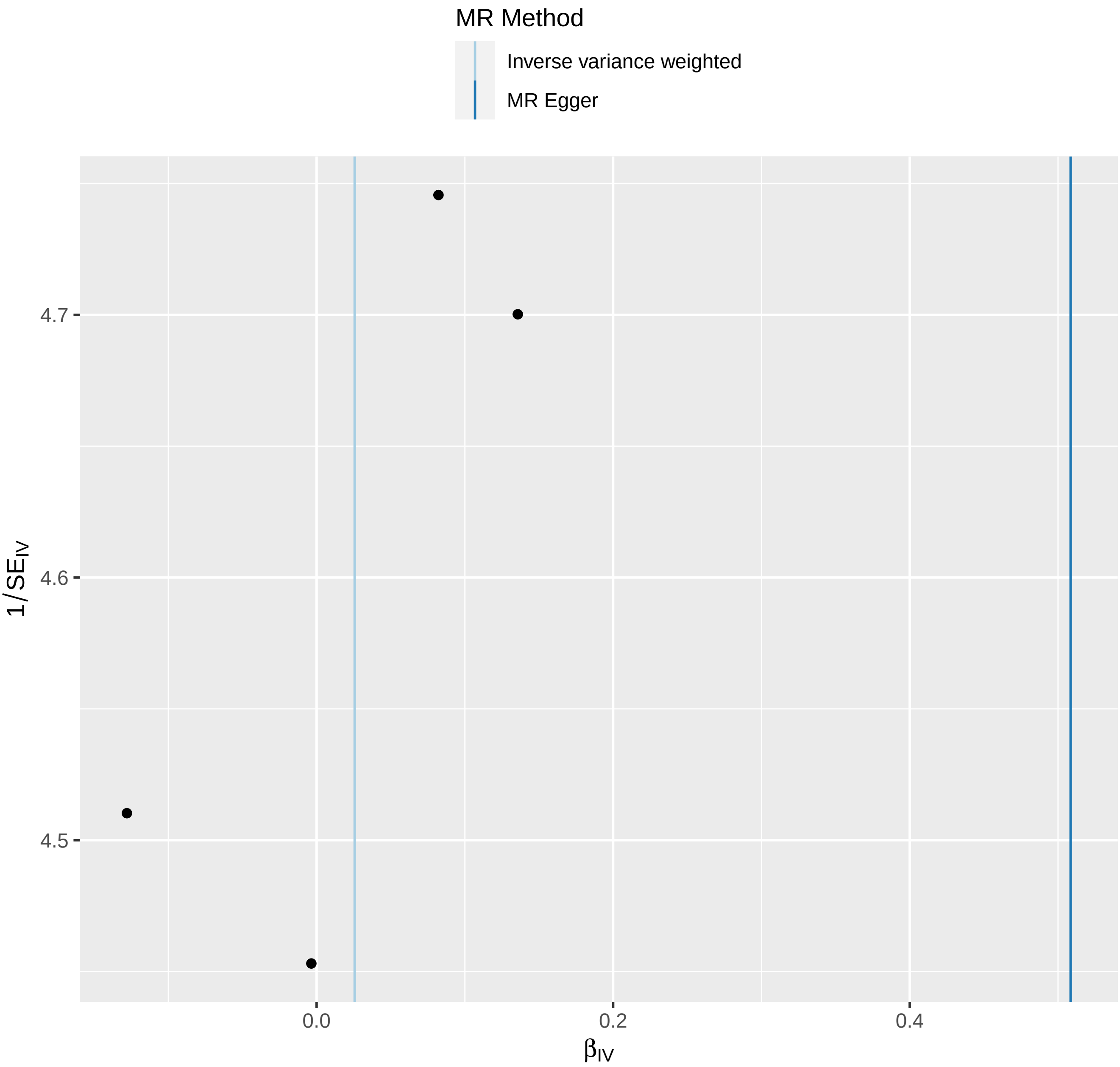

Batch 2−5144 : Gut microbiota abundance (genus Parasutterella id.2892) on E3 ubiquitin−protein ligase RNF43
with a threshold of 1e−05

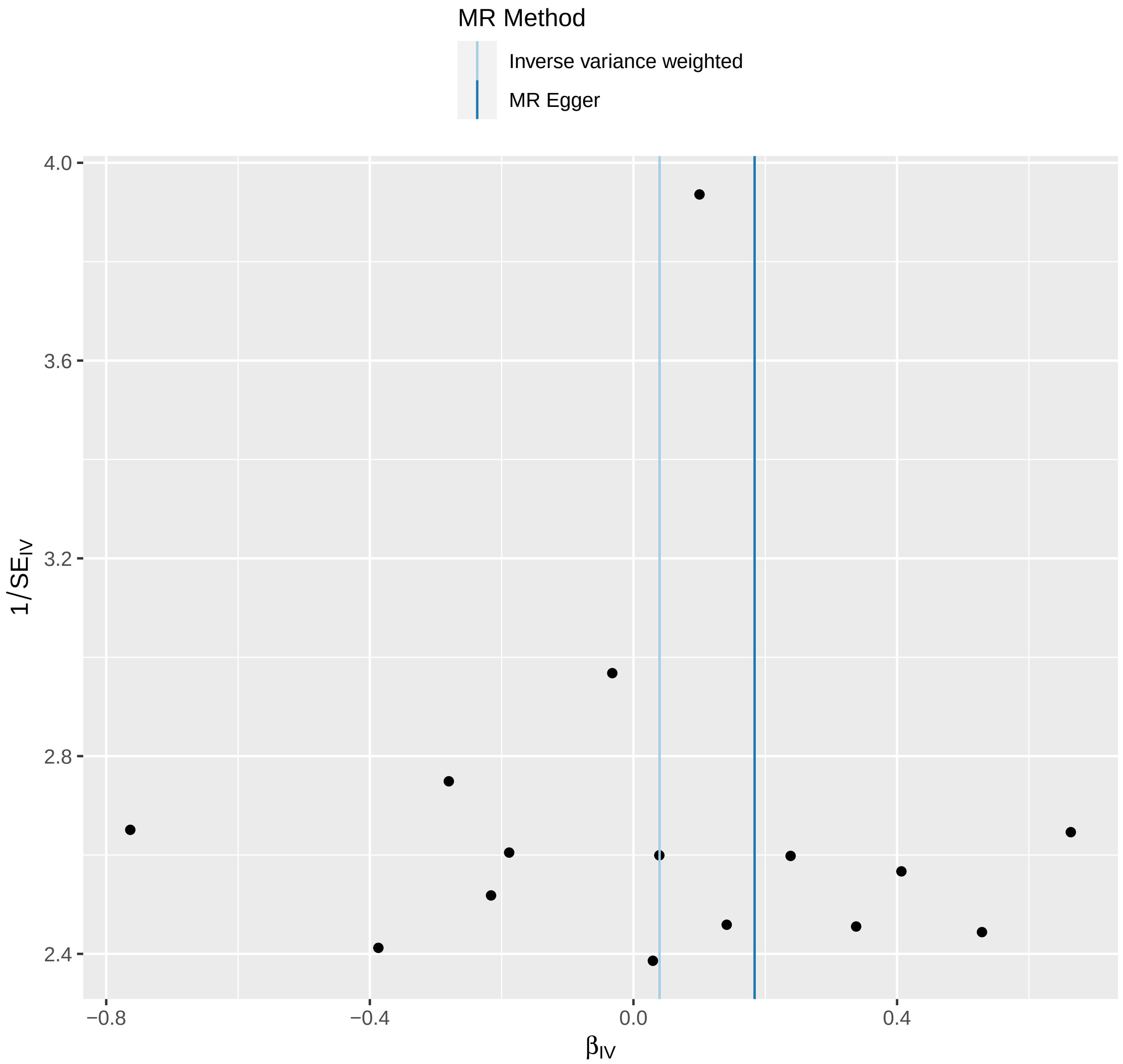

Batch 2−5145 : Gut microbiota abundance (genus Catenibacterium id.2153) on E3 ubiquitin−protein ligase RNF8
with a threshold of 1e−05

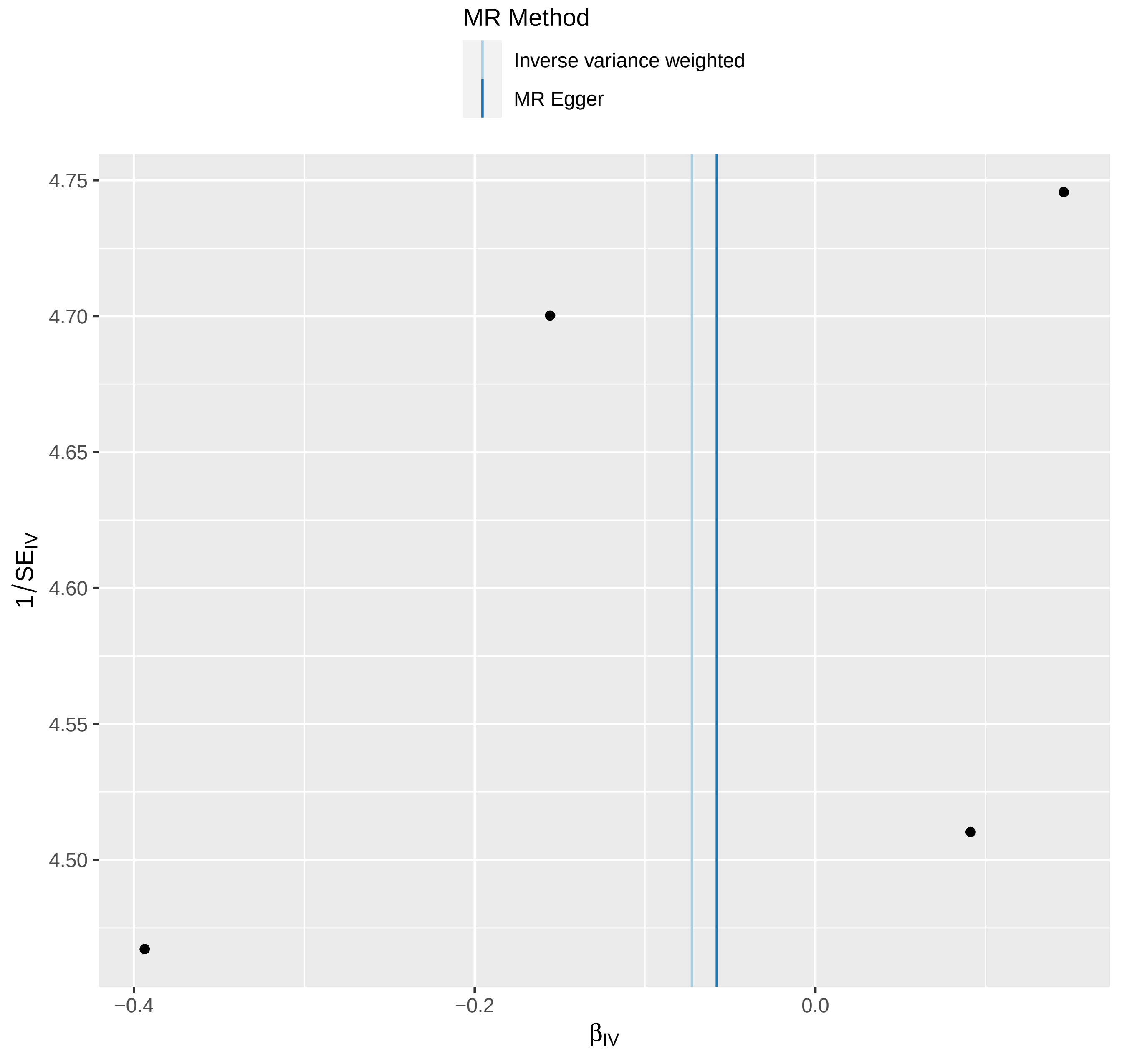

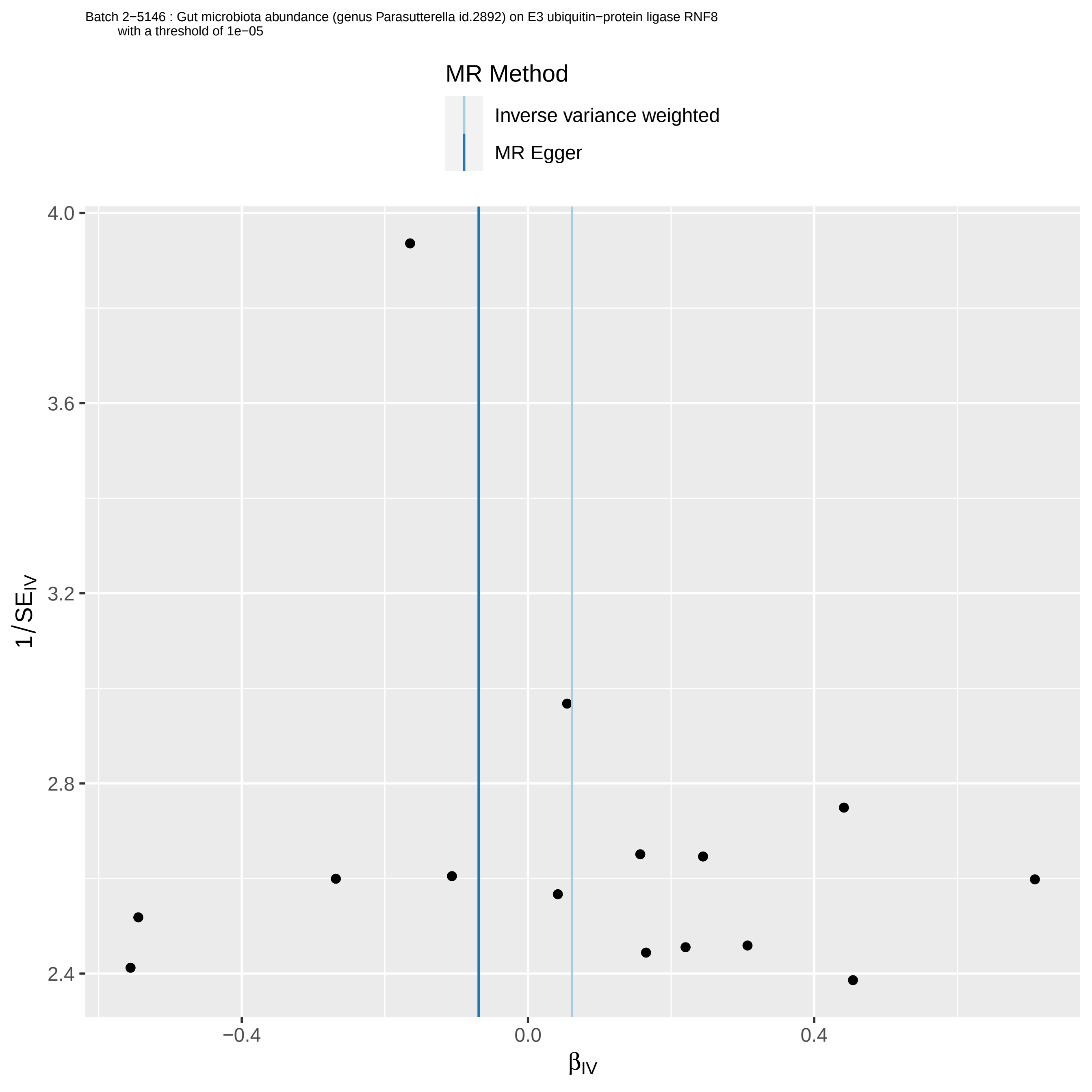
Batch 2−5146 : Gut microbiota abundance (genus Parasutterella id.2892) on E3 ubiquitin−protein ligase RNF8
with a threshold of 1e−05
MR Method
Inverse variance weighted
MR Egger
4.0
3.6
3.2
2.8
2.4
1/SE_IV
−0.4
0.0
0.4
β_IV

Batch 2−5147 : Gut microbiota abundance (genus Catenibacterium id.2153) on mRNA−capping enzyme
with a threshold of 1e−05

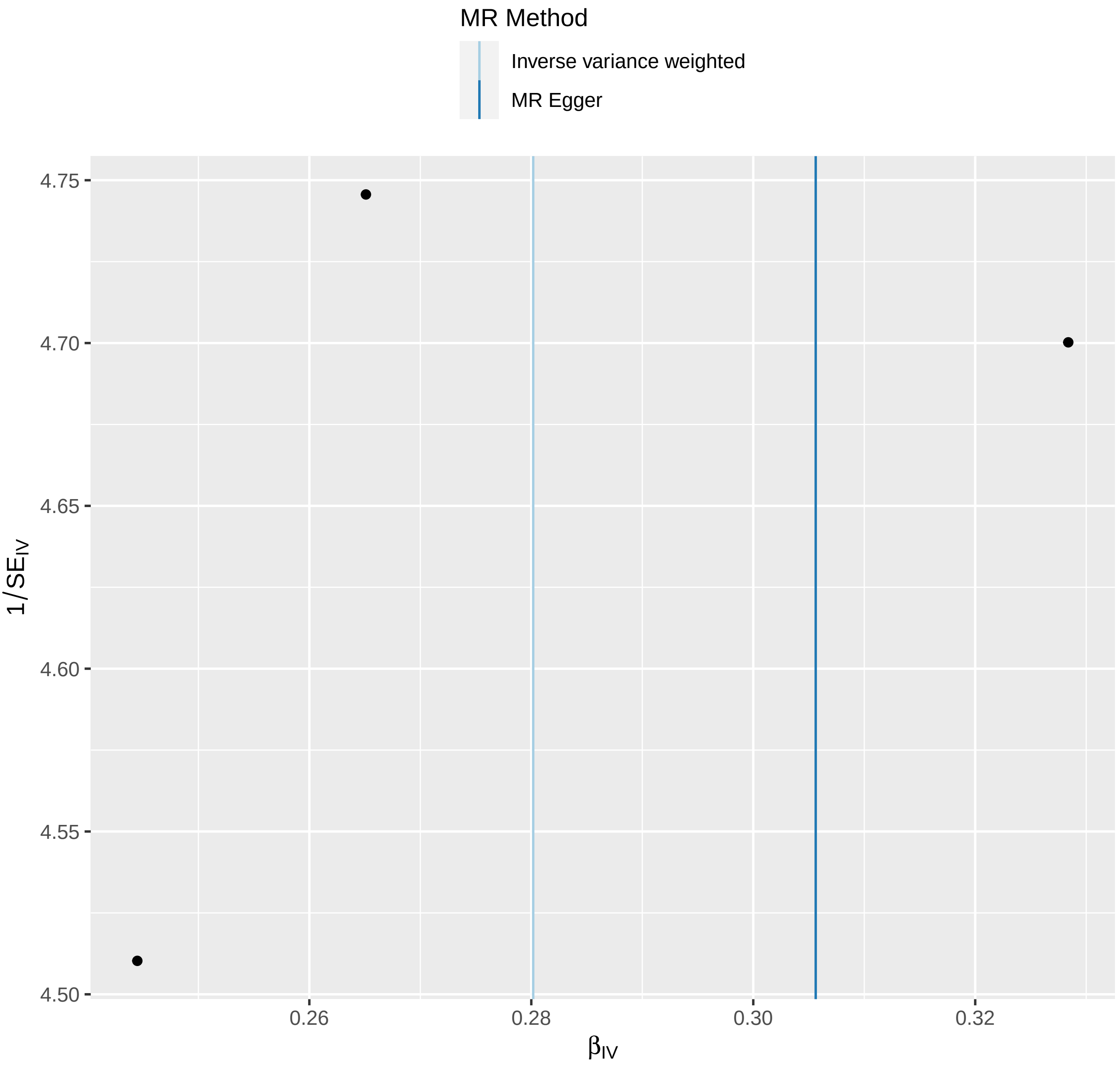

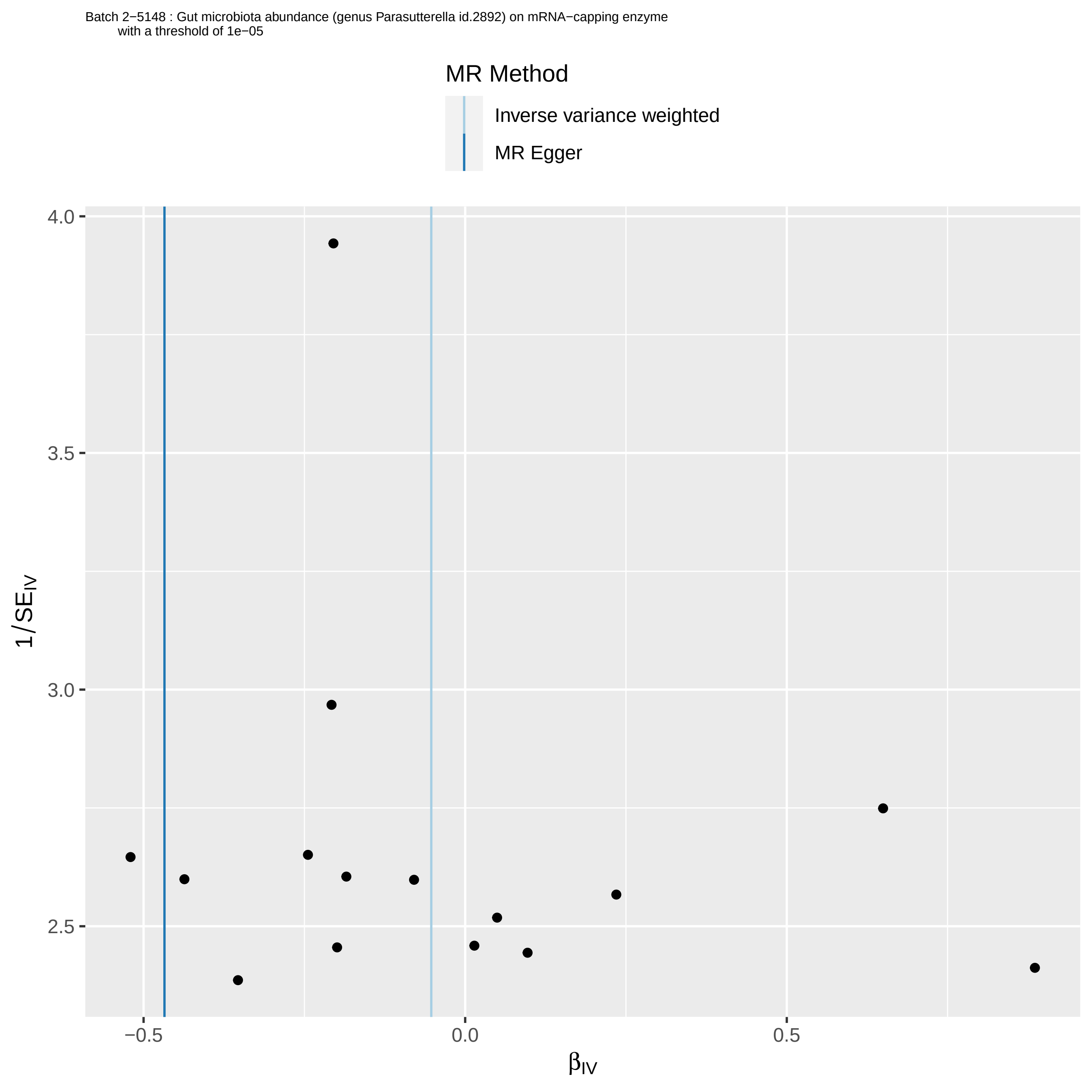

Batch 2−5148 : Gut microbiota abundance (genus Parasutterella id.2892) on mRNA−capping enzyme
with a threshold of 1e−05
MR Method
Inverse variance weighted
MR Egger
4.0
3.5
3.0
2.5
1/SE$_{IV}$
−0.5
0.0
0.5
β_{IV}

Batch 2−5149 : Gut microbiota abundance (genus Catenibacterium id.2153) on rRNA methyltransferase 3, mitochondrial
with a threshold of 1e−05

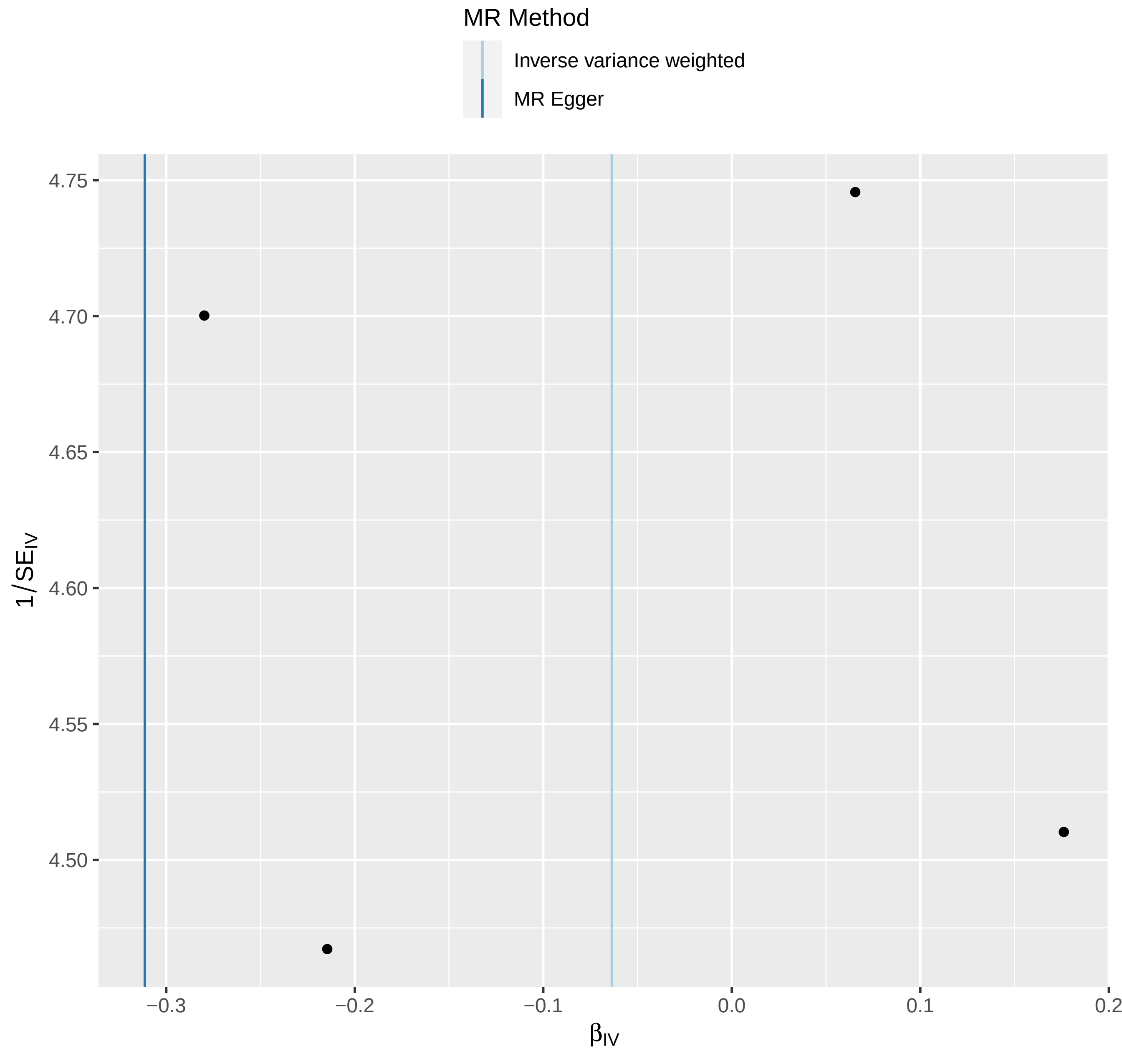

Batch 2−5150 : Gut microbiota abundance (genus Parasutterella id.2892) on rRNA methyltransferase 3, mitochondrial
with a threshold of 1e−05

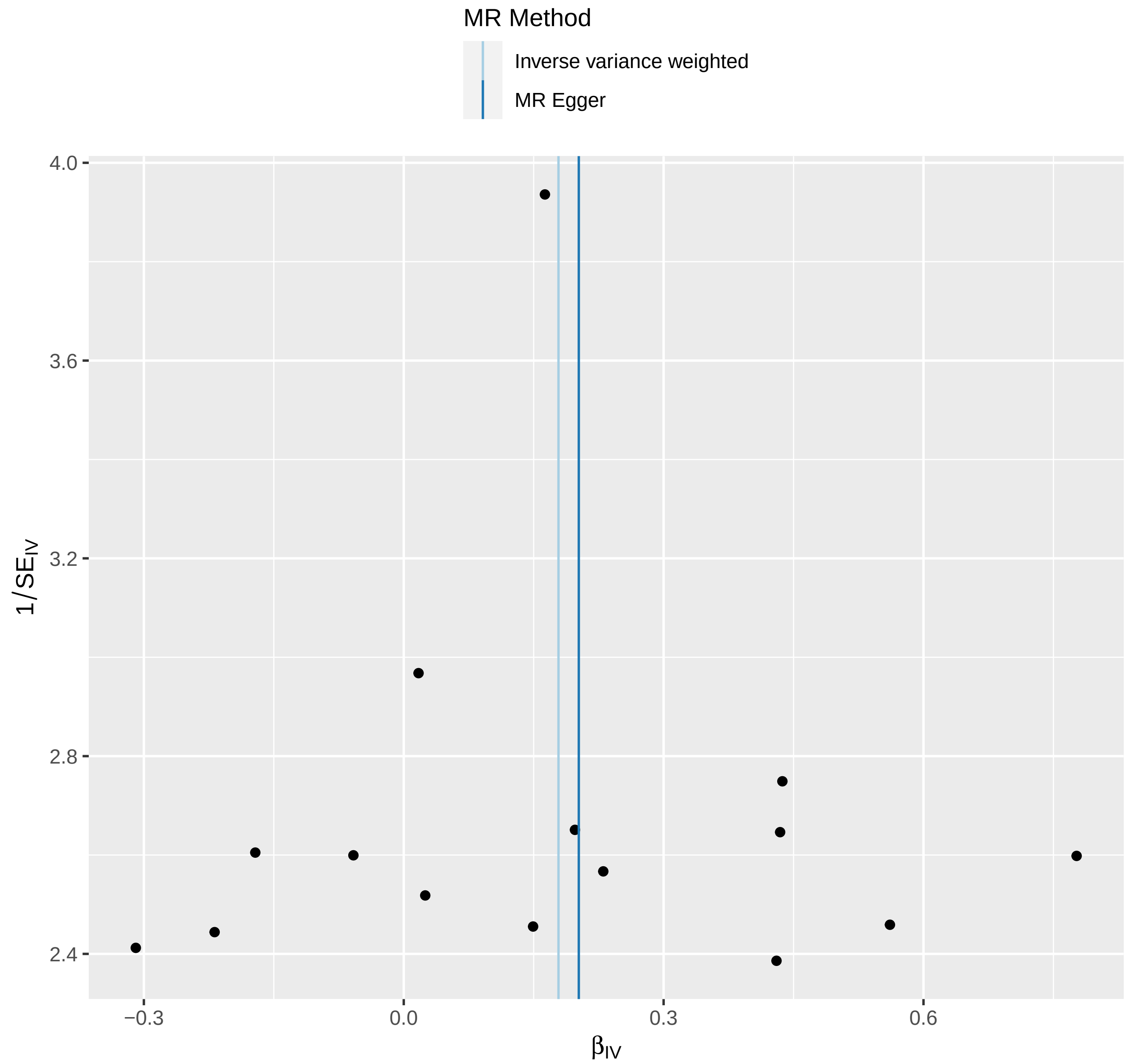

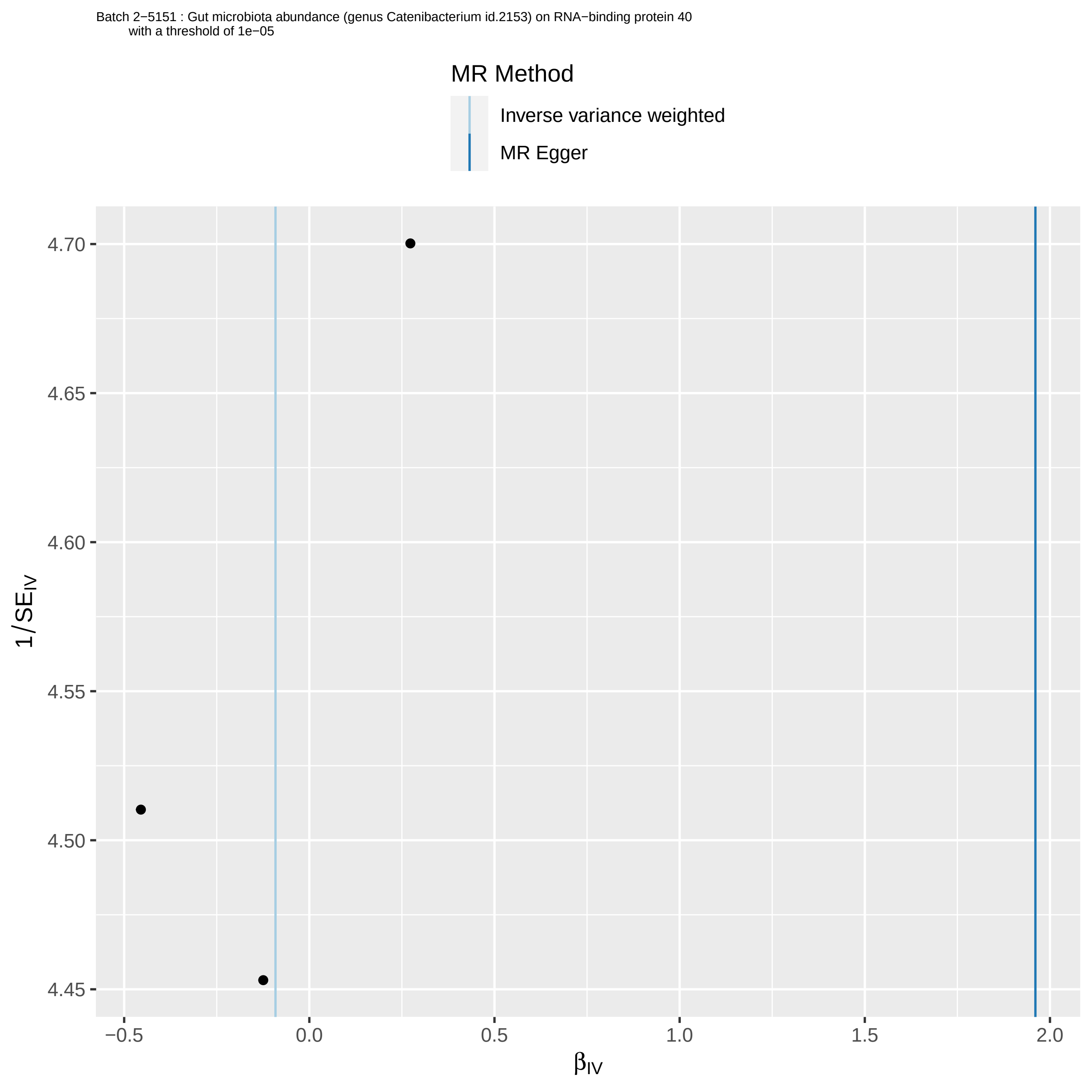
Batch 2−5151 : Gut microbiota abundance (genus Catenibacterium id.2153) on RNA−binding protein 40
with a threshold of 1e−05
MR Method
Inverse variance weighted
MR Egger
4.70
4.65
4.60
4.55
4.50
4.45
1/SE_IV
−0.5
0.0
0.5
1.0
1.5
2.0
β_IV

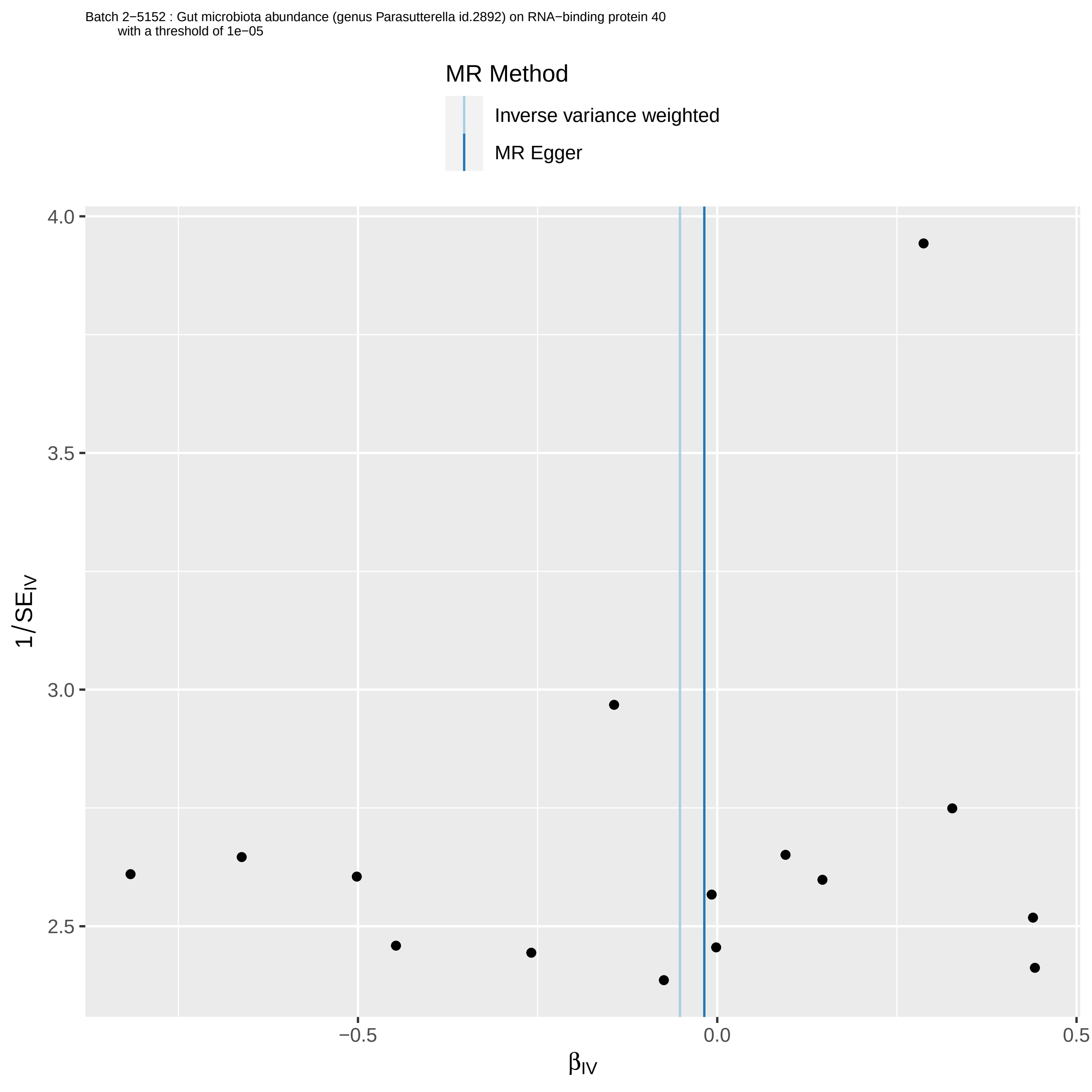
Batch 2−5152 : Gut microbiota abundance (genus Parasutterella id.2892) on RNA−binding protein 40
with a threshold of 1e−05
MR Method
Inverse variance weighted
MR Egger
4.0
3.5
3.0
2.5
1/SE_IV
−0.5
0.0
0.5
β_IV

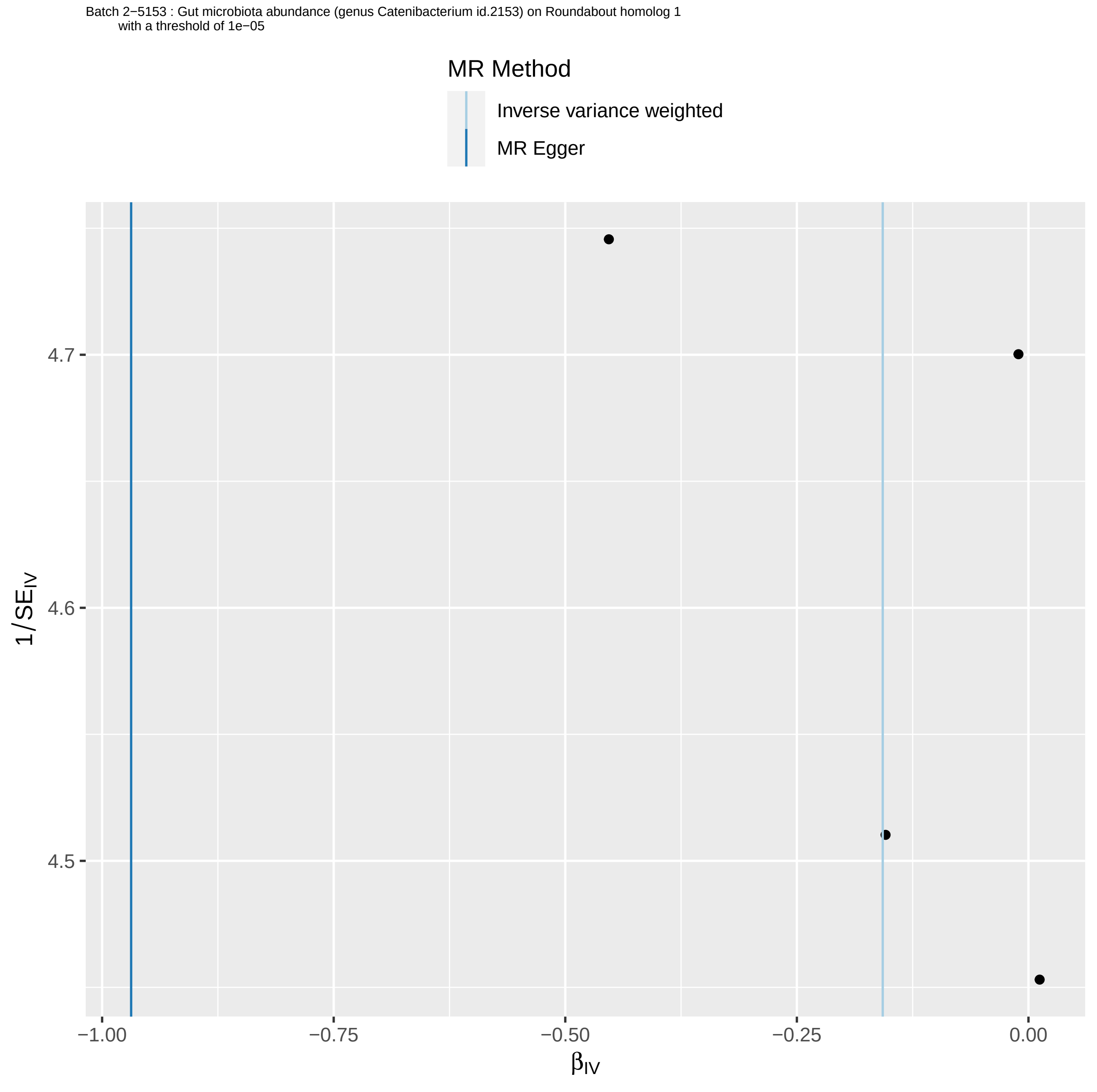

Batch 2−5153 : Gut microbiota abundance (genus Catenibacterium id.2153) on Roundabout homolog 1
with a threshold of 1e−05
MR Method
Inverse variance weighted
MR Egger
4.7
4.6
4.5
1/SE_IV
−1.00
−0.75
−0.50
−0.25
0.00
β_IV

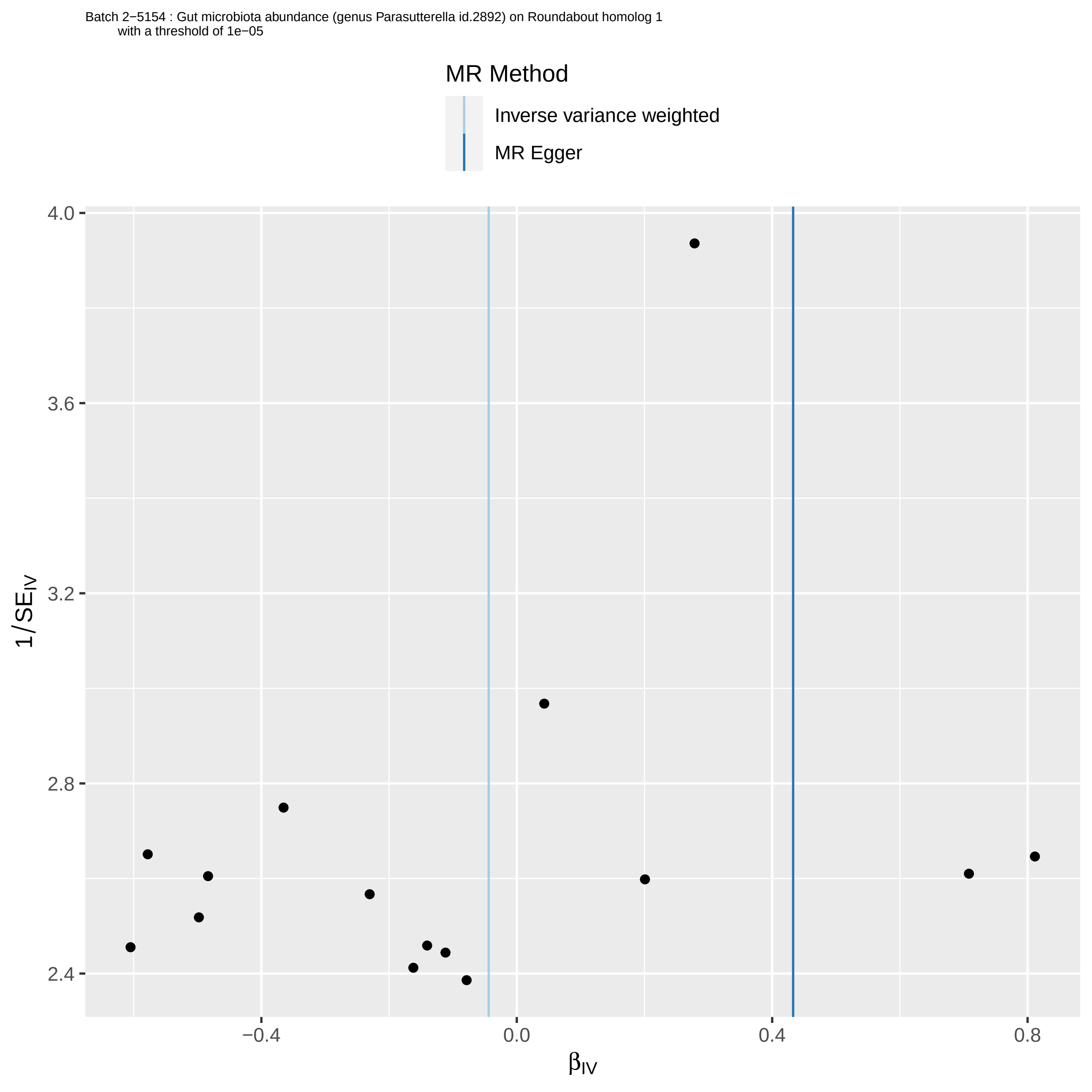
Batch 2−5154 : Gut microbiota abundance (genus Parasutterella id.2892) on Roundabout homolog 1
with a threshold of 1e−05
MR Method
Inverse variance weighted
MR Egger
4.0
3.6
3.2
2.8
2.4
1/SE_IV
−0.4
0.0
0.4
0.8
β_IV

Batch 2−5155 : Gut microbiota abundance (genus Catenibacterium id.2153) on Roundabout homolog 2
with a threshold of 1e−05

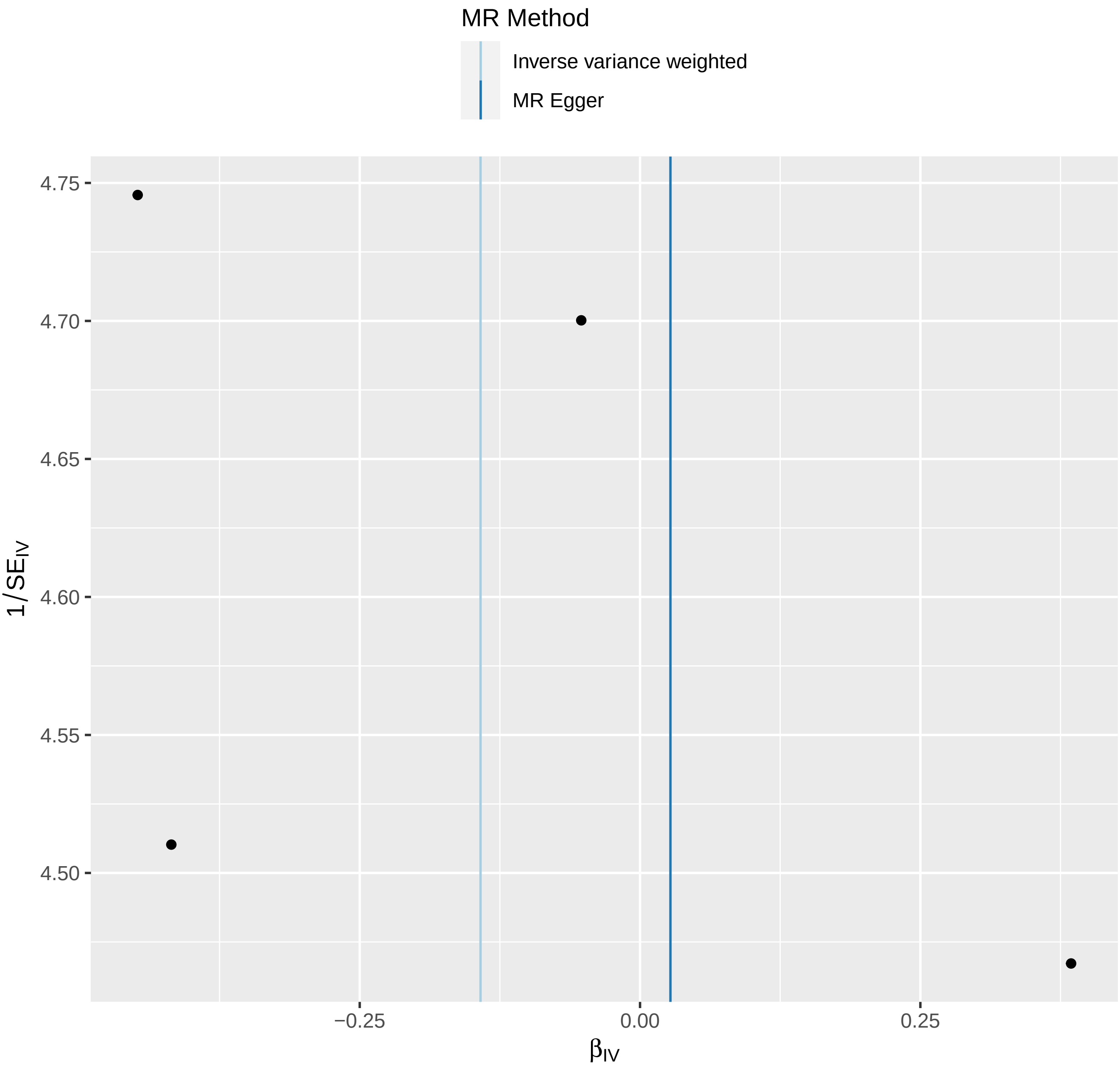

Batch 2−5156 : Gut microbiota abundance (genus Parasutterella id.2892) on Roundabout homolog 2
with a threshold of 1e−05

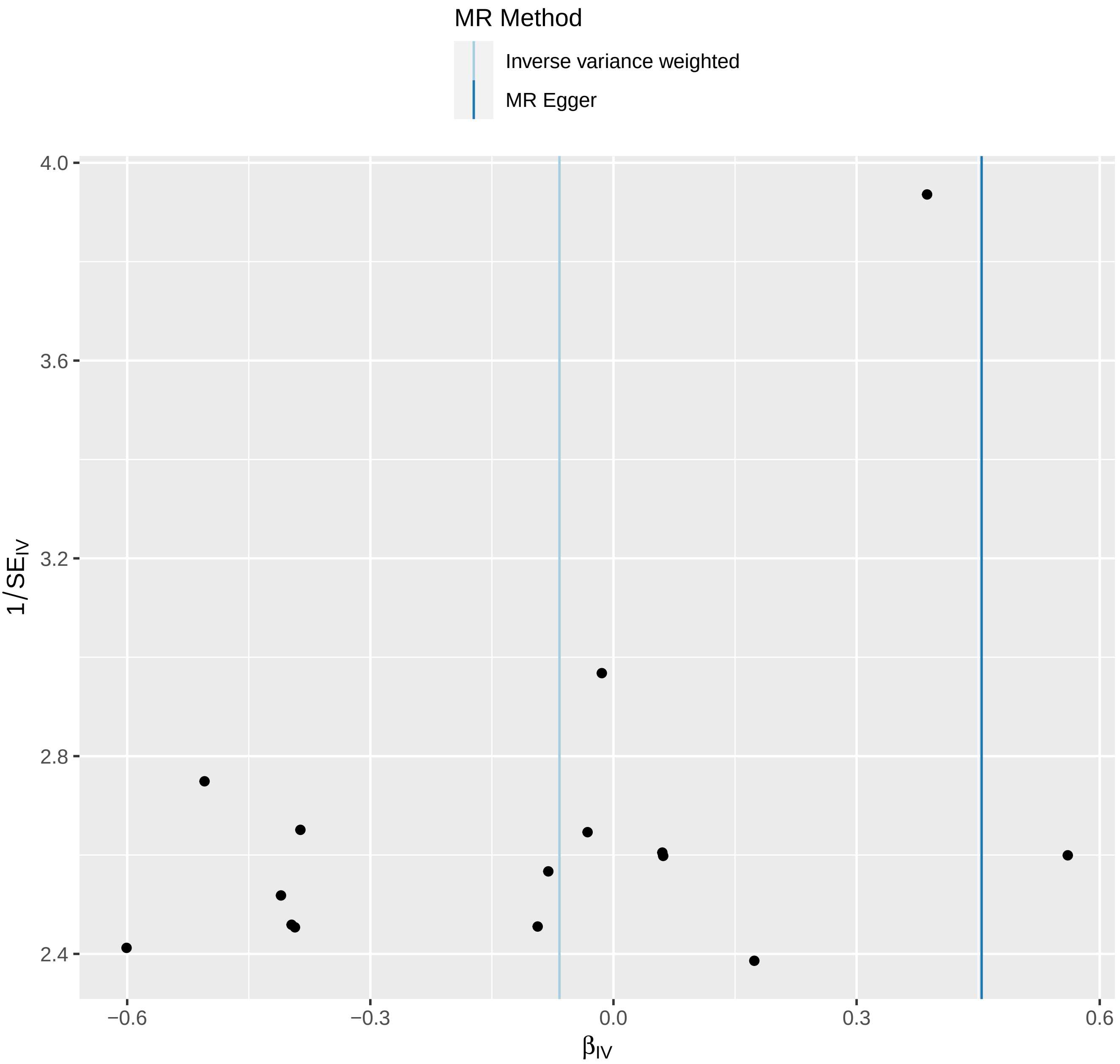

Batch 2−5157 : Gut microbiota abundance (genus Catenibacterium id.2153) on Roundabout homolog 3
with a threshold of 1e−05

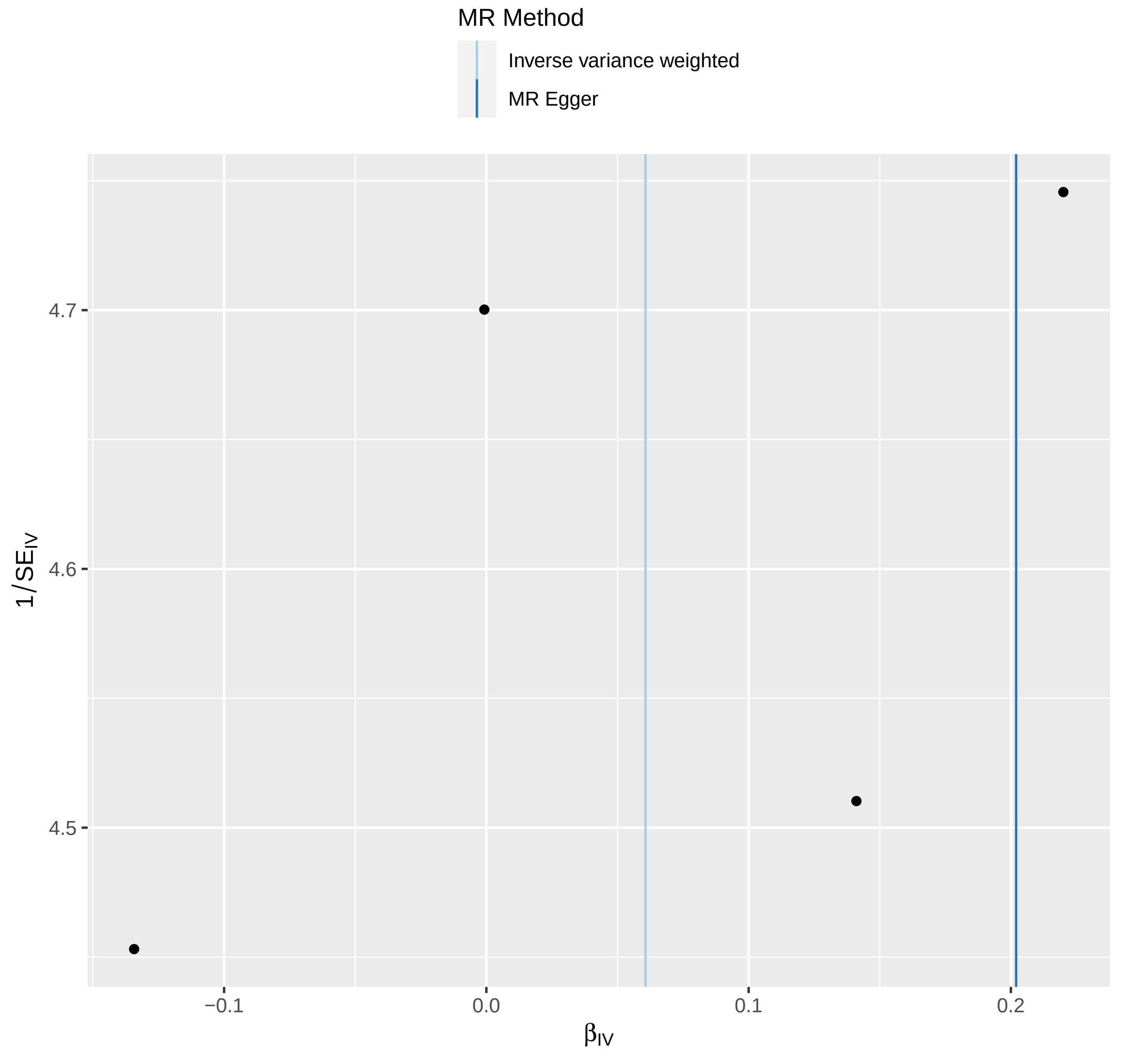

Batch 2−5158 : Gut microbiota abundance (genus Parasutterella id.2892) on Roundabout homolog 3
with a threshold of 1e−05

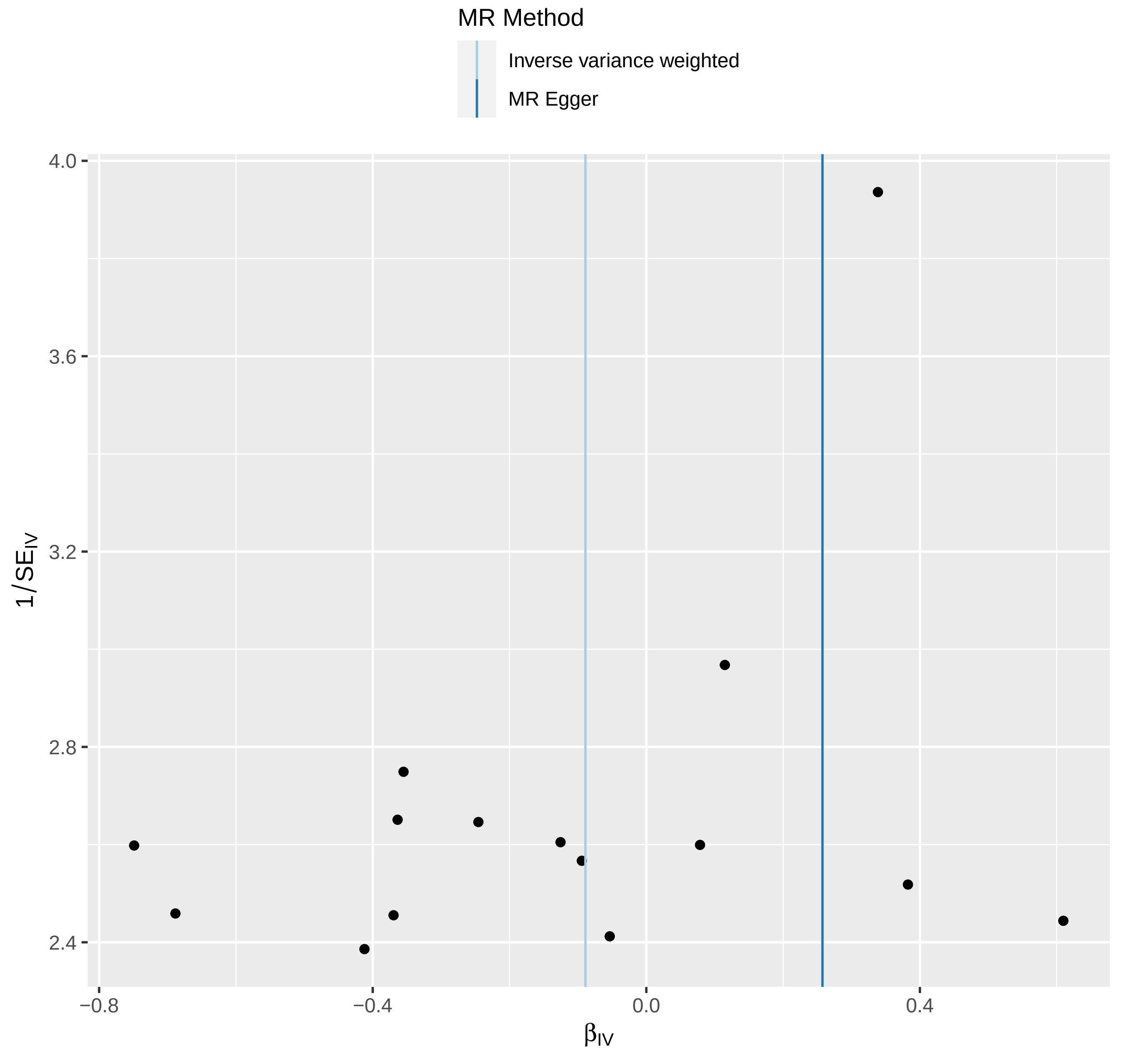

Batch 2−5159 : Gut microbiota abundance (genus Catenibacterium id.2153) on Rho−associated protein kinase 2
with a threshold of 1e−05

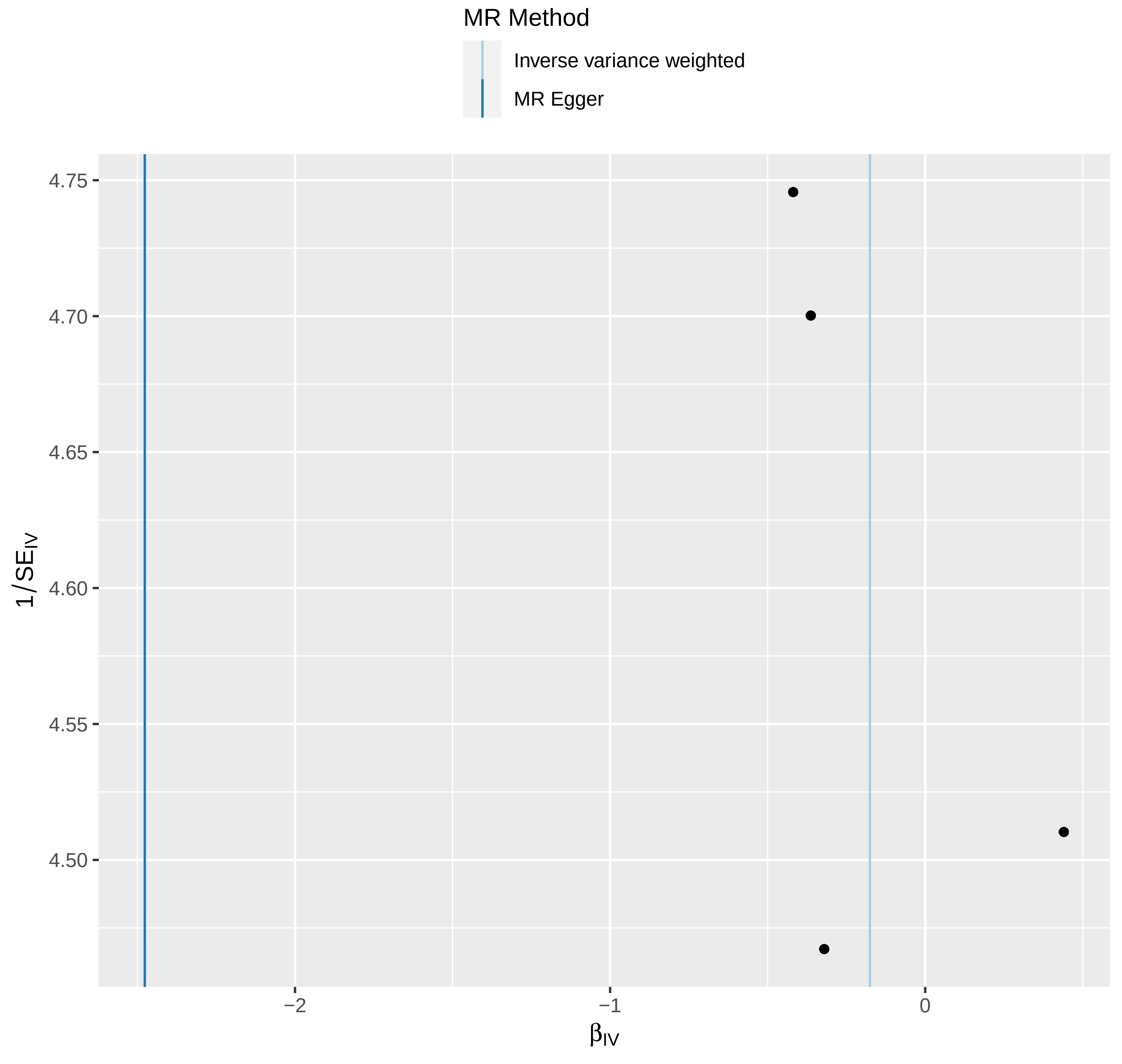

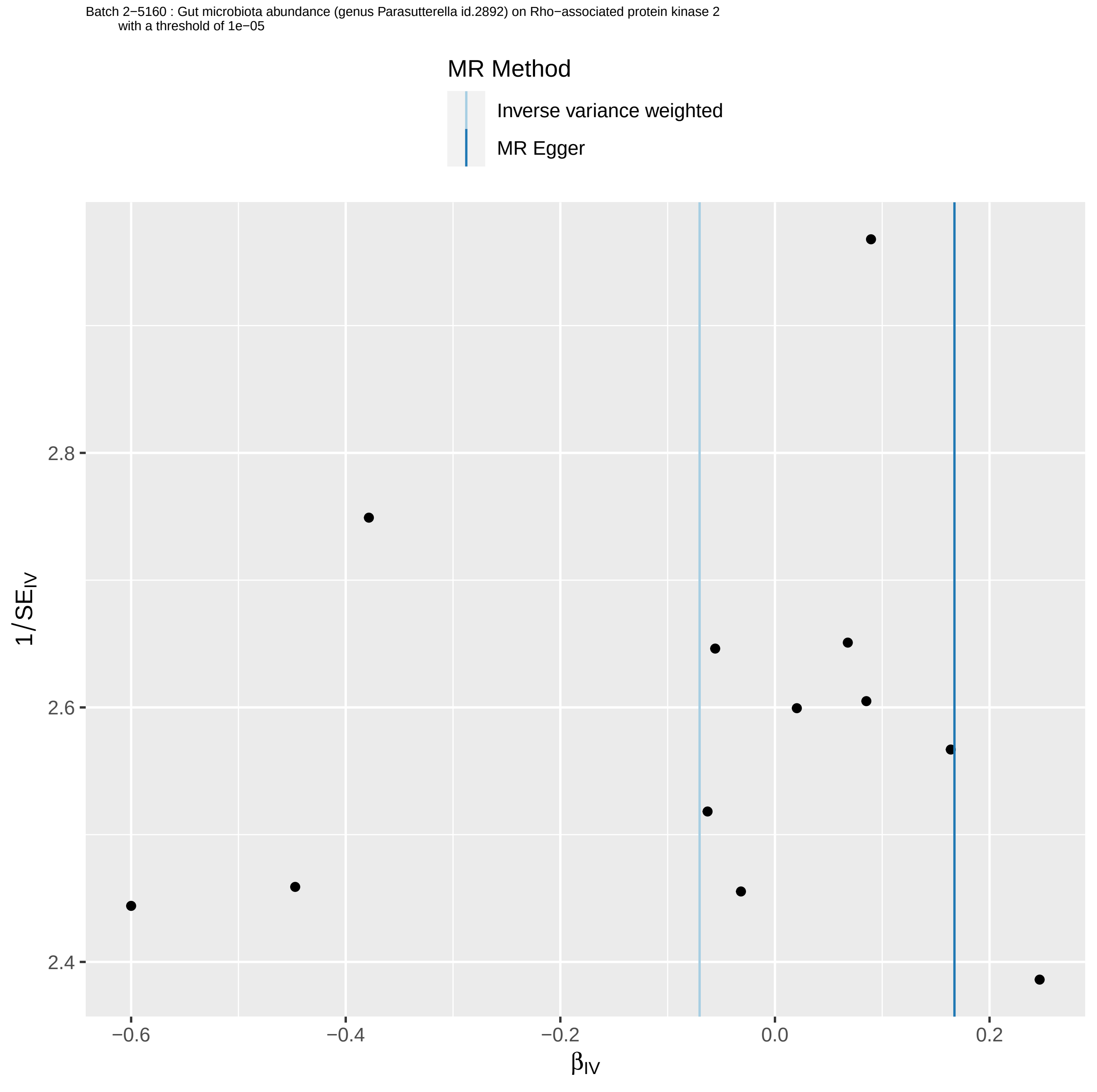
Batch 2−5160 : Gut microbiota abundance (genus Parasutterella id.2892) on Rho−associated protein kinase 2
with a threshold of 1e−05
MR Method
Inverse variance weighted
MR Egger
2.8
2.6
2.4
1/SE_IV
−0.6
−0.4
−0.2
0.0
0.2
β_IV

Batch 2−5161 : Gut microbiota abundance (genus Catenibacterium id.2153) on Tyrosine−protein kinase transmembrane receptor ROR1 with a threshold of 1e−05

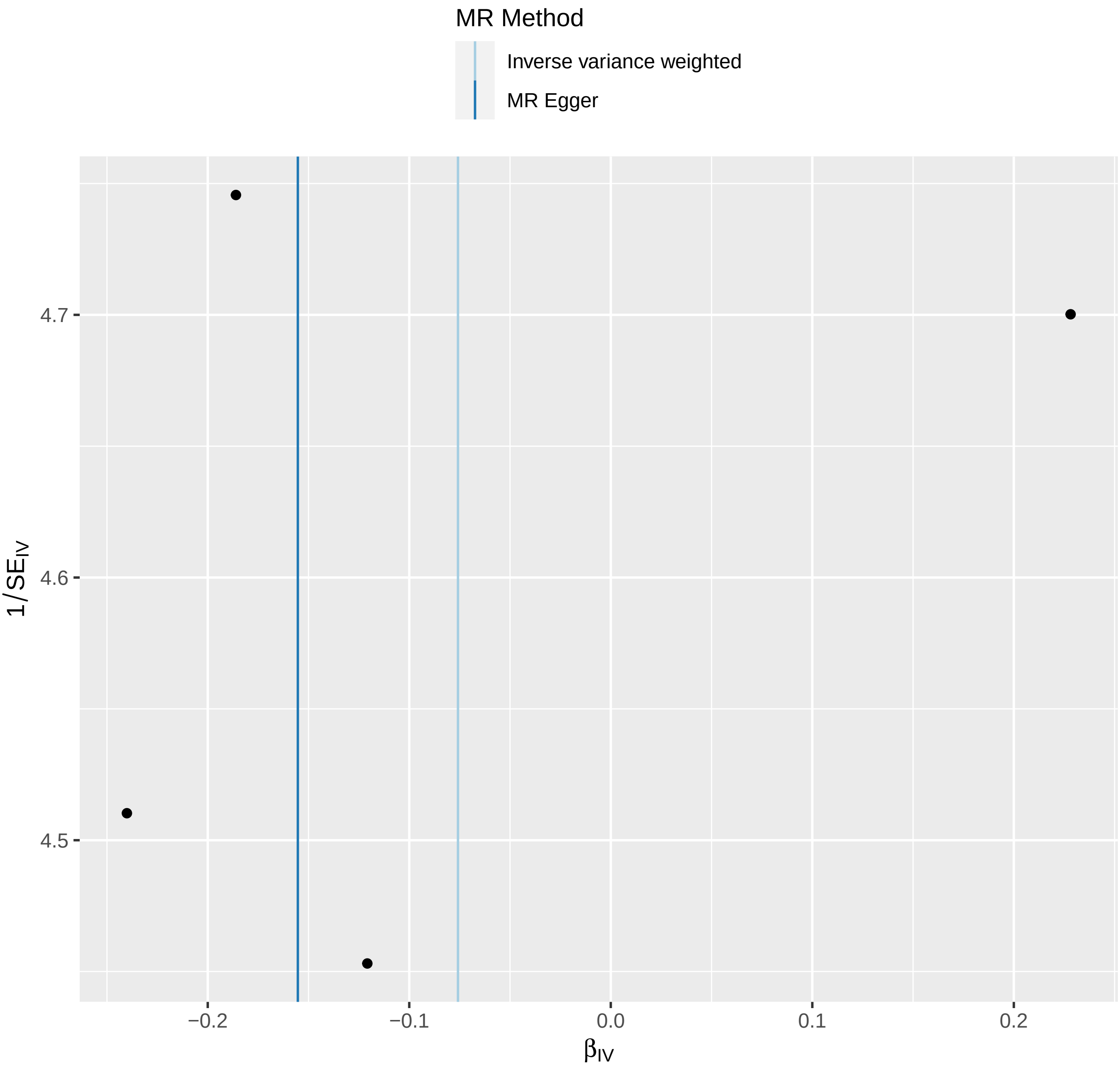

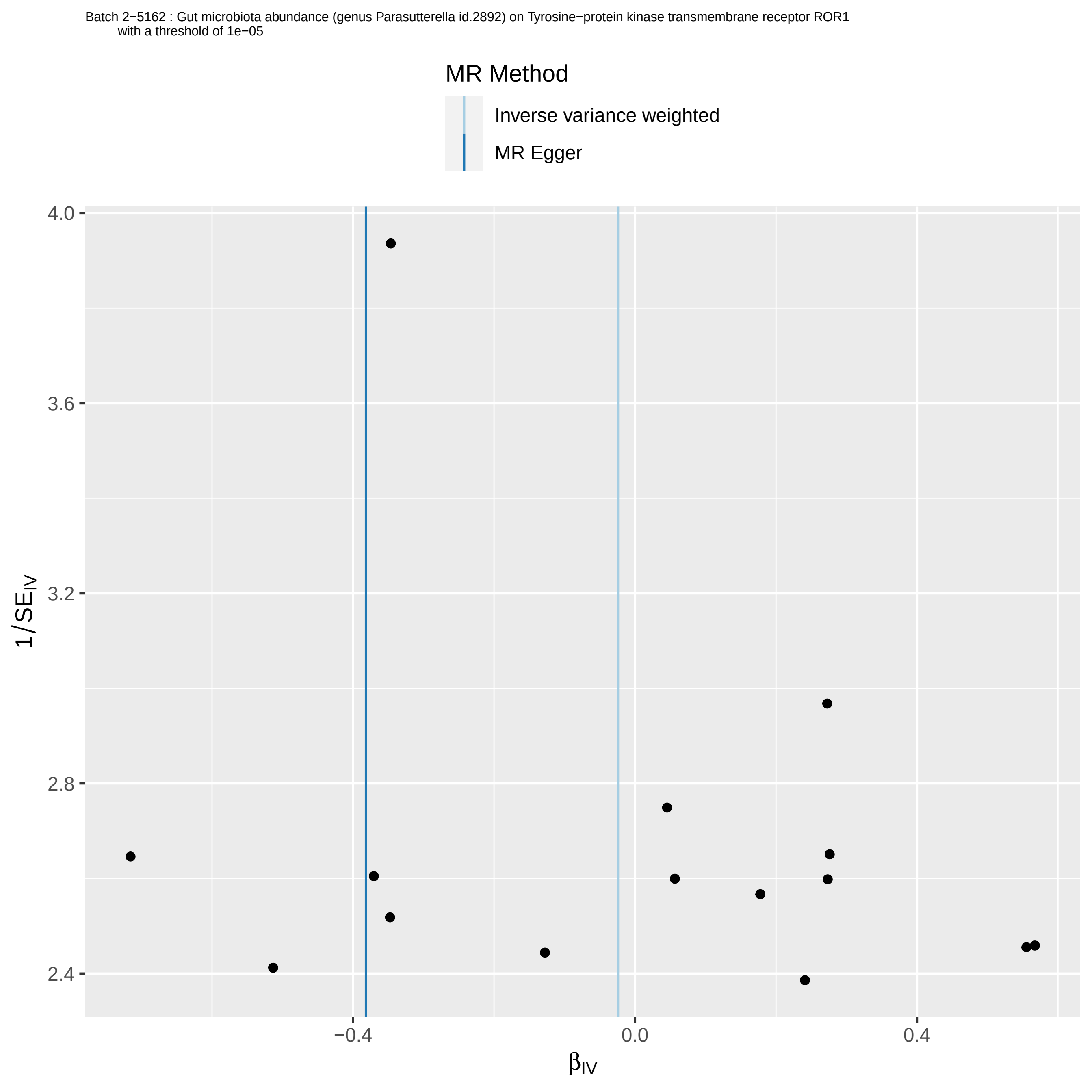

Batch 2−5162 : Gut microbiota abundance (genus Parasutterella id.2892) on Tyrosine−protein kinase transmembrane receptor ROR1
with a threshold of 1e−05
MR Method
Inverse variance weighted
MR Egger
4.0
3.6
3.2
2.8
2.4
1/SE_IV
−0.4
0.0
0.4
β_IV

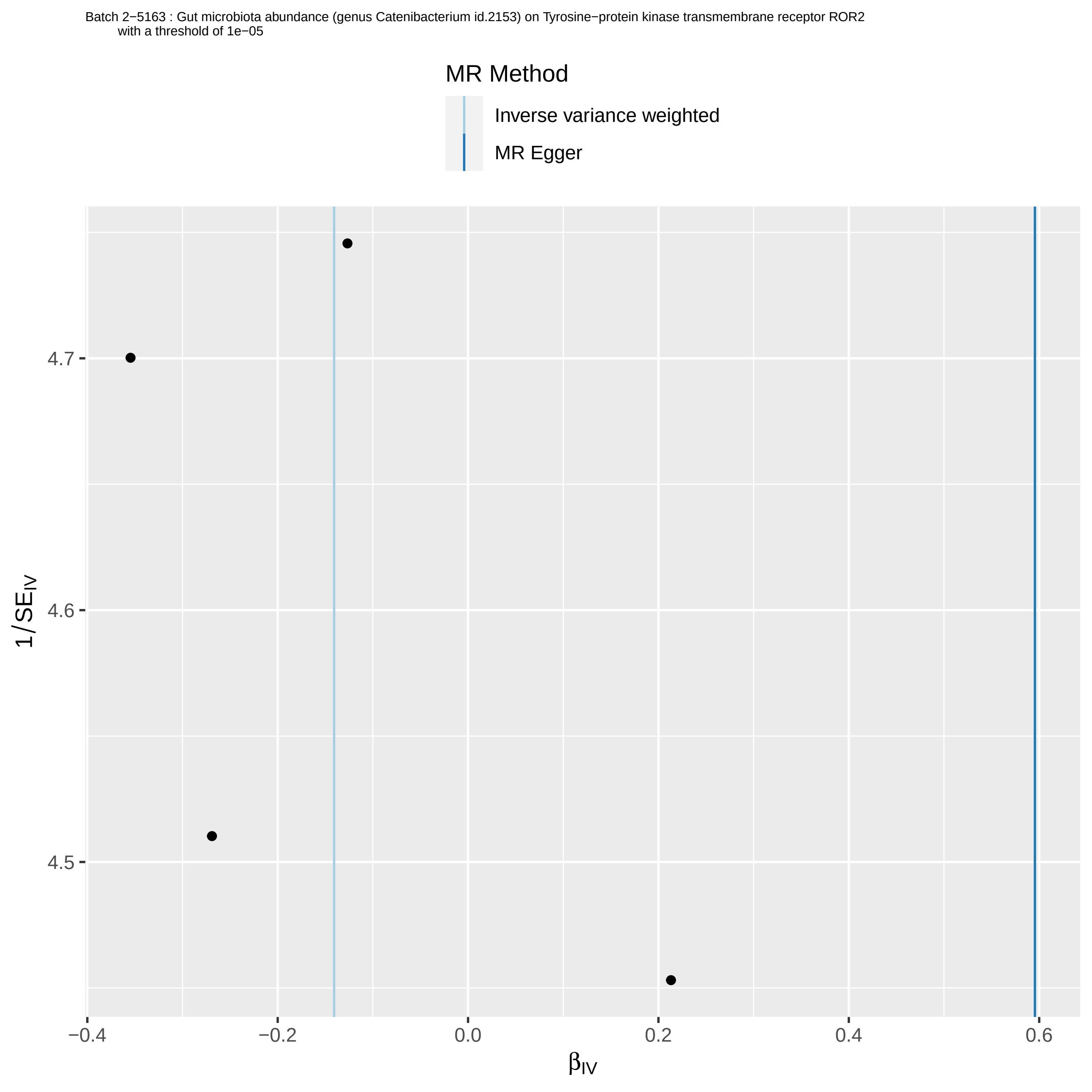

Batch 2−5163 : Gut microbiota abundance (genus Catenibacterium id.2153) on Tyrosine−protein kinase transmembrane receptor ROR2
with a threshold of 1e−05
MR Method
Inverse variance weighted
MR Egger
4.7
4.6
4.5
1/SE_IV
−0.4
−0.2
0.0
0.2
0.4
0.6
β_IV

Batch 2−5164 : Gut microbiota abundance (genus Parasutterella id.2892) on Tyrosine−protein kinase transmembrane receptor ROR2
with a threshold of 1e−05

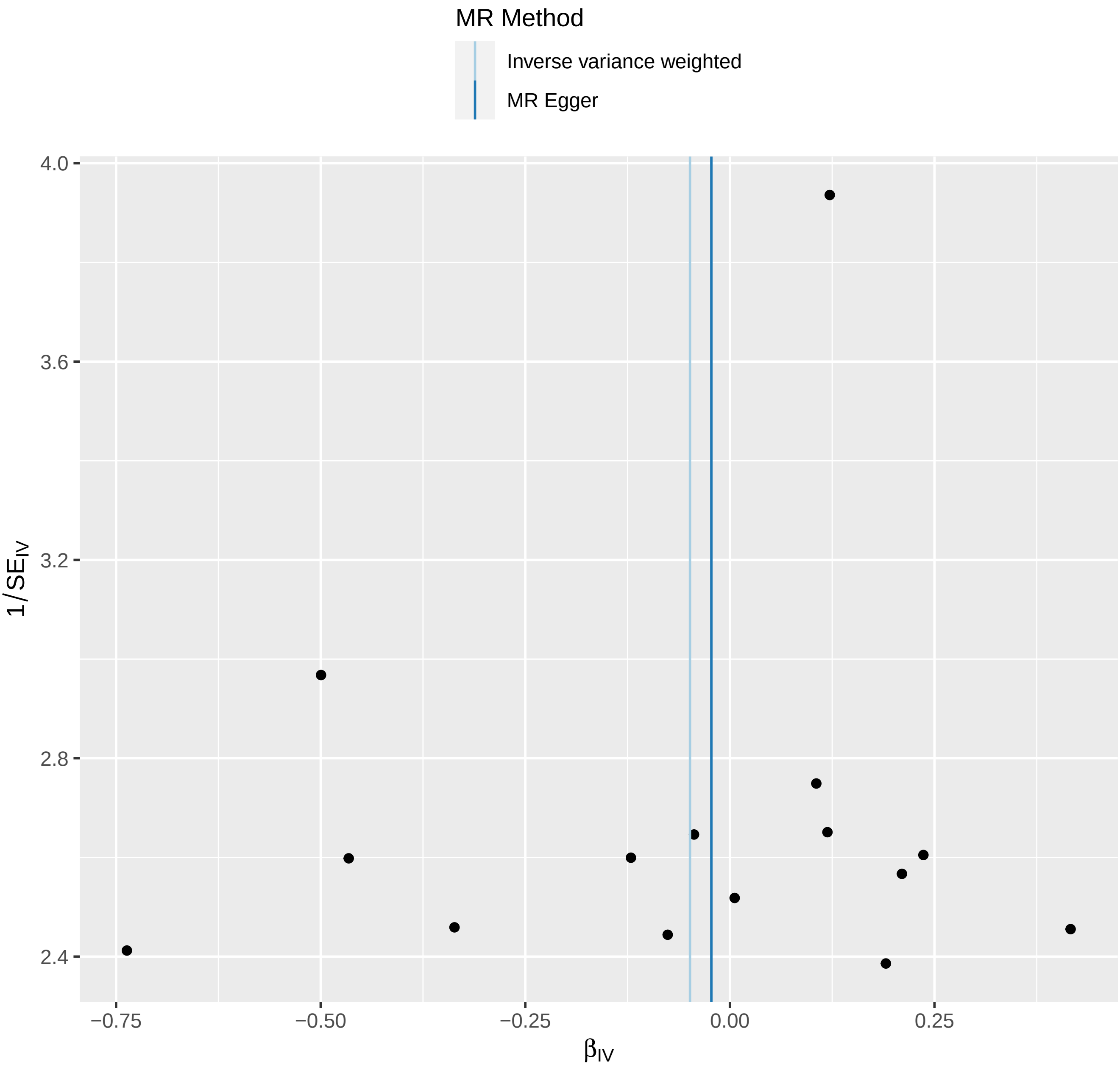

Batch 2−5165 : Gut microbiota abundance (genus Catenibacterium id.2153) on Nuclear receptor ROR−beta
with a threshold of 1e−05

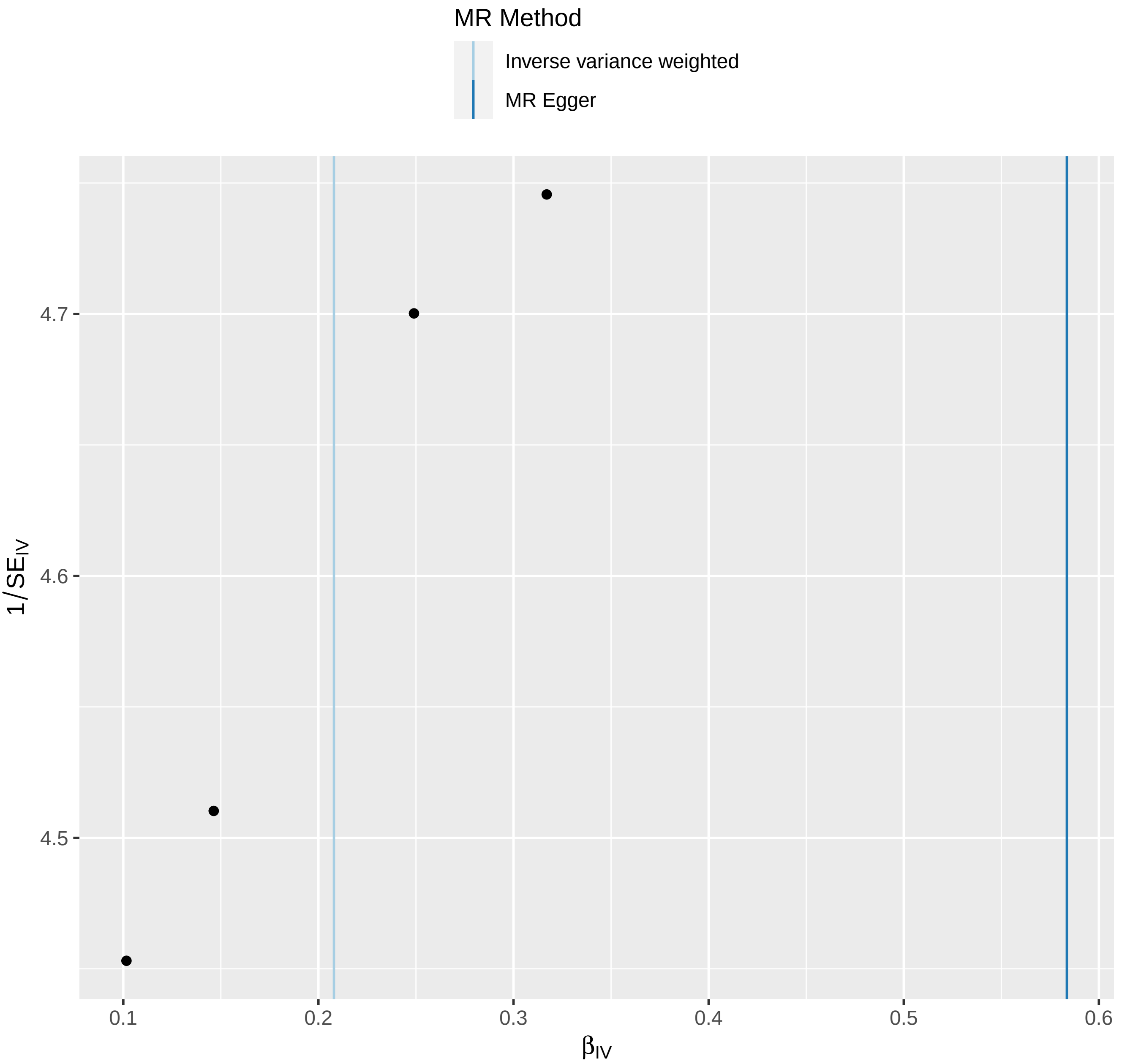

Batch 2−5166 : Gut microbiota abundance (genus Parasutterella id.2892) on Nuclear receptor ROR−beta
with a threshold of 1e−05

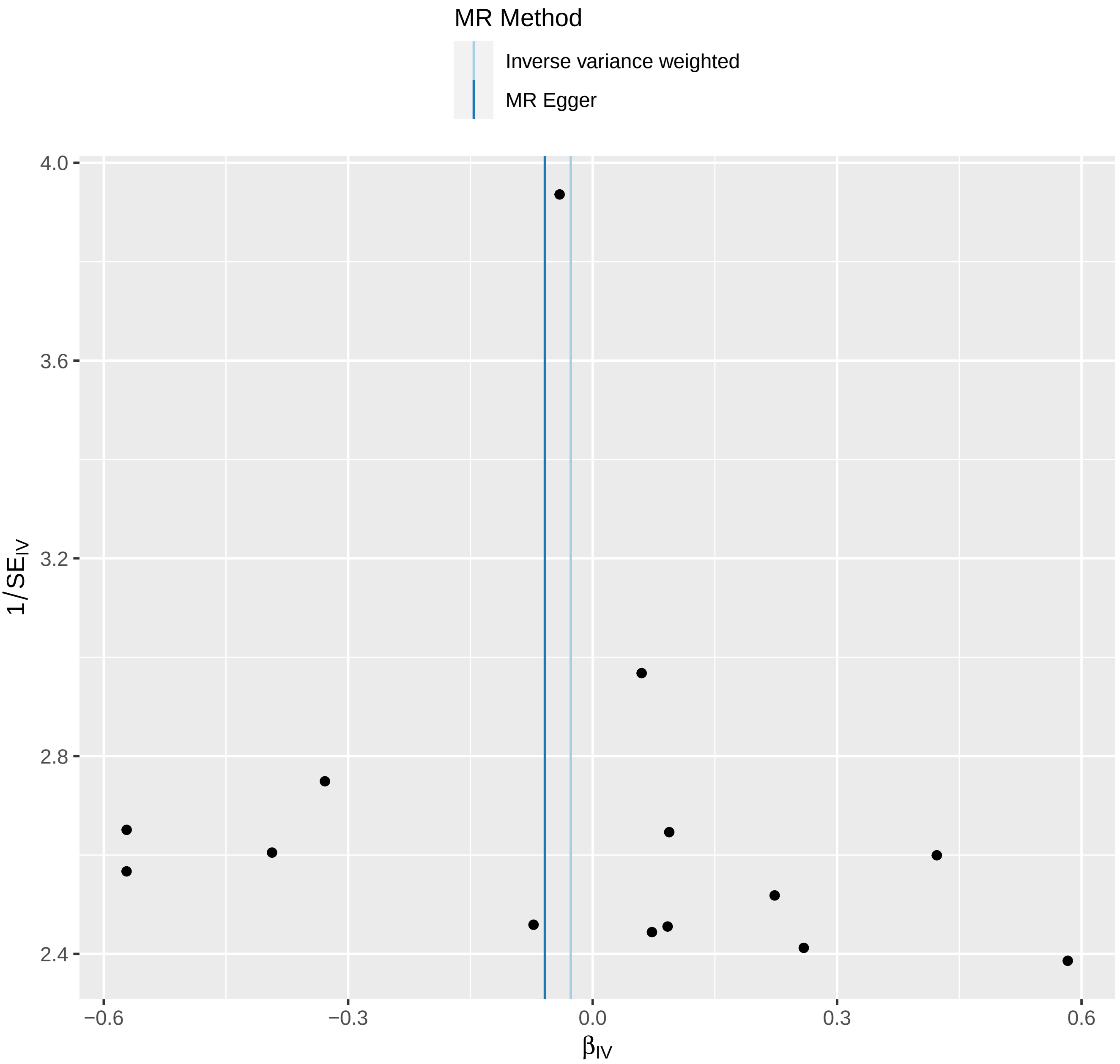

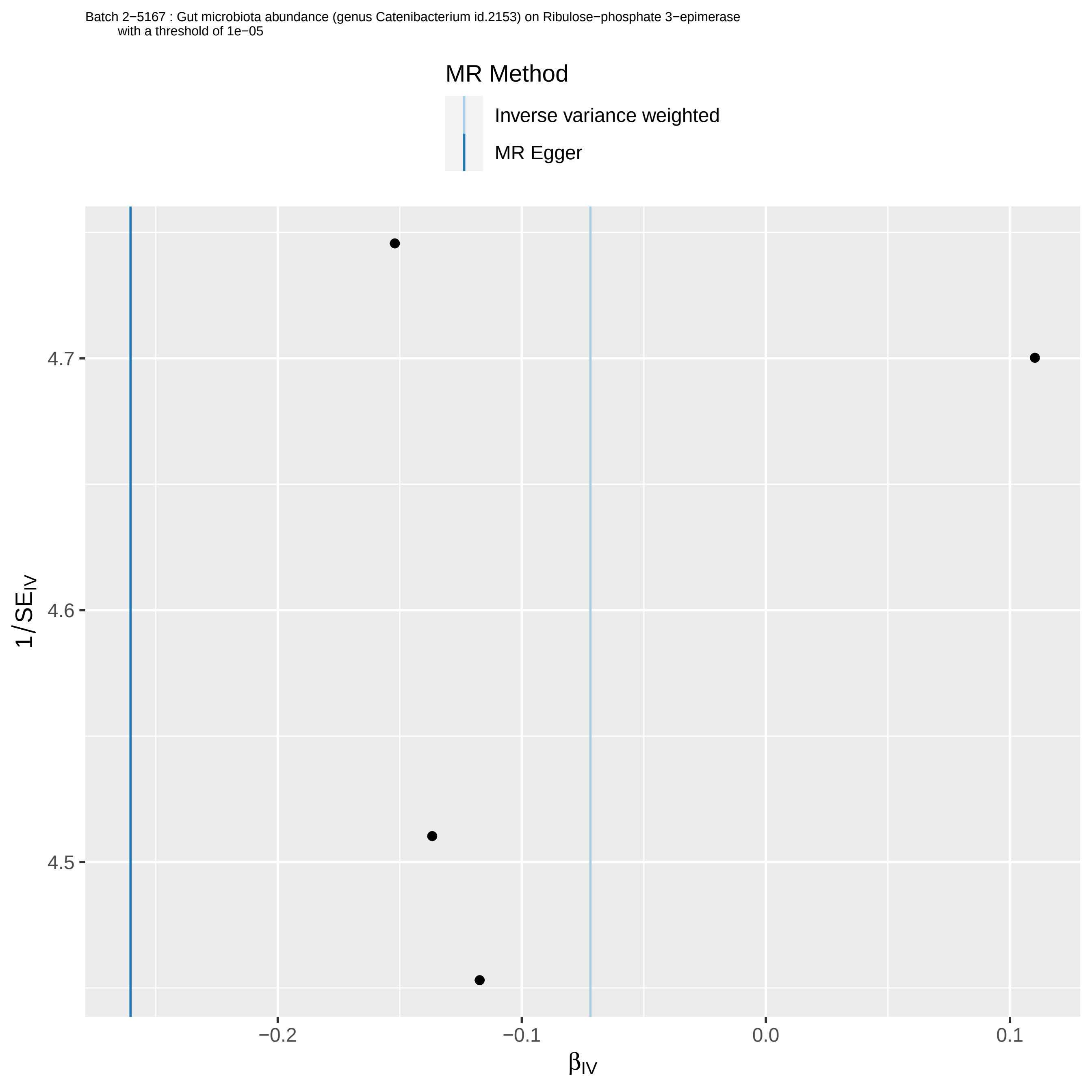

Batch 2−5167 : Gut microbiota abundance (genus Catenibacterium id.2153) on Ribulose−phosphate 3−epimerase
with a threshold of 1e−05
MR Method
Inverse variance weighted
MR Egger
4.7
4.6
4.5
1/SE_IV
−0.2
−0.1
0.0
0.1
β_IV

Batch 2−5168 : Gut microbiota abundance (genus Parasutterella id.2892) on Ribulose−phosphate 3−epimerase
with a threshold of 1e−05

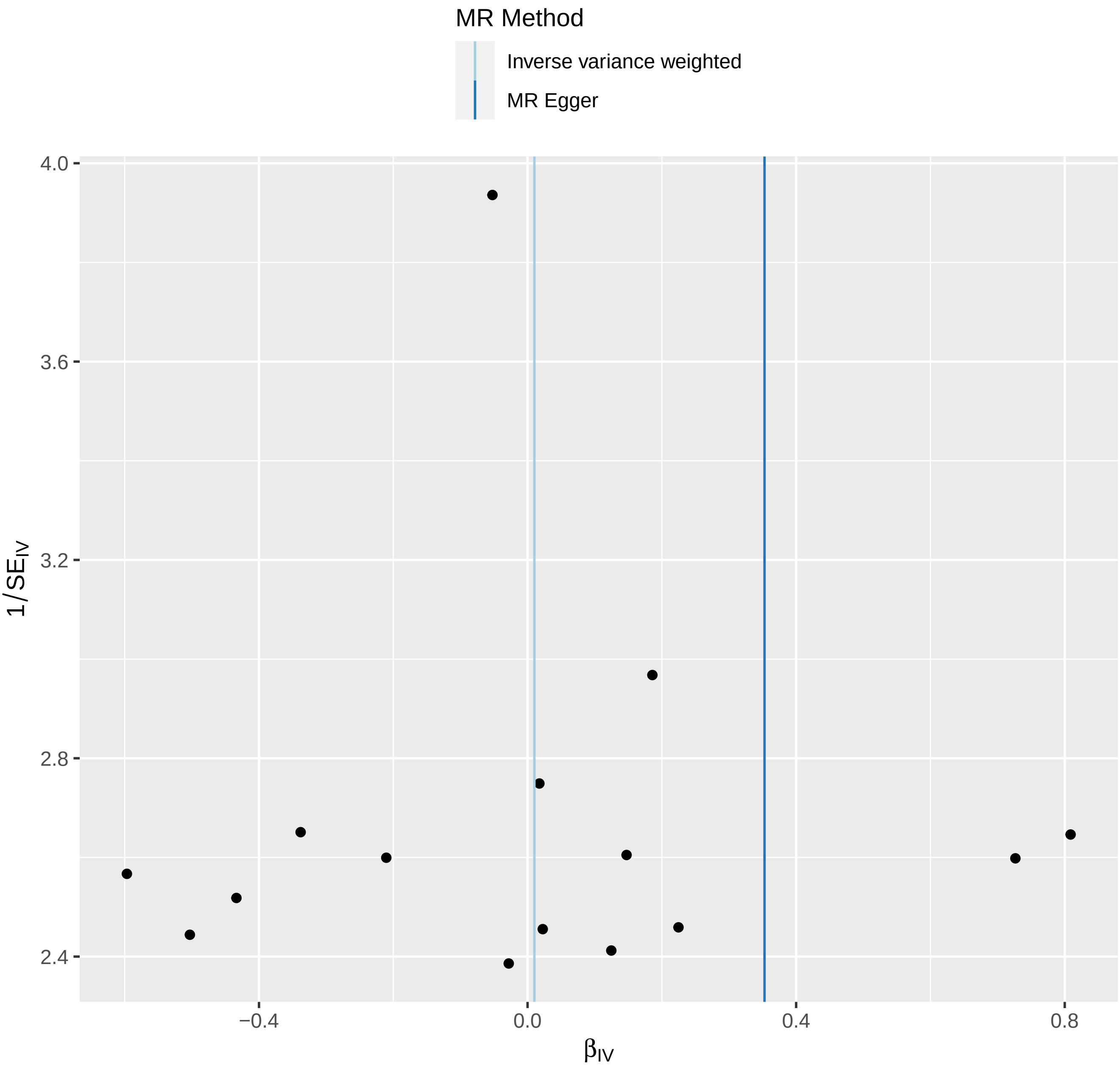

Batch 2−5169 : Gut microbiota abundance (genus Catenibacterium id.2153) on Ribose−5−phosphate isomerase with a threshold of 1e−05

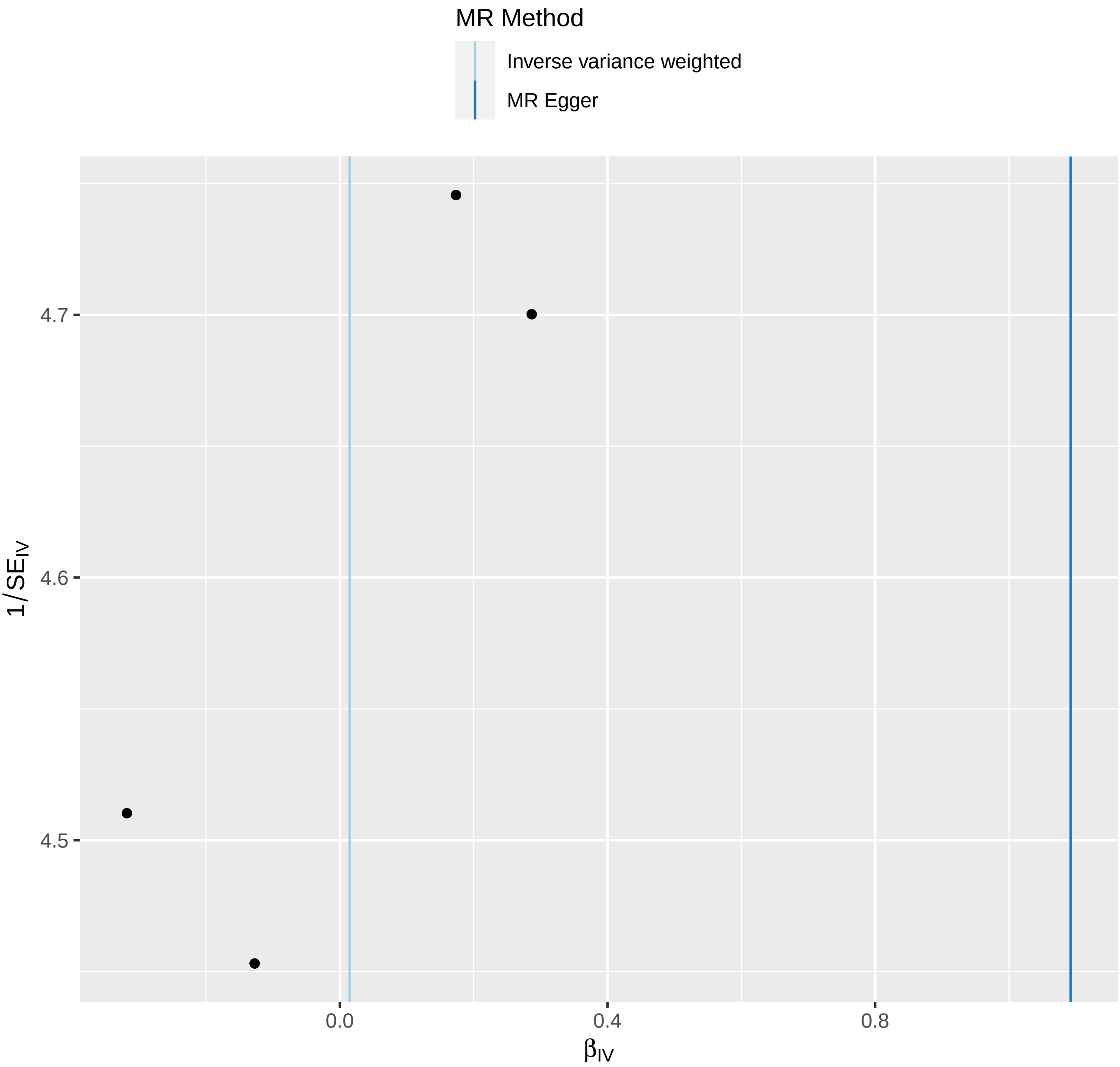

Batch 2−5170 : Gut microbiota abundance (genus Parasutterella id.2892) on Ribose−5−phosphate isomerase
with a threshold of 1e−05

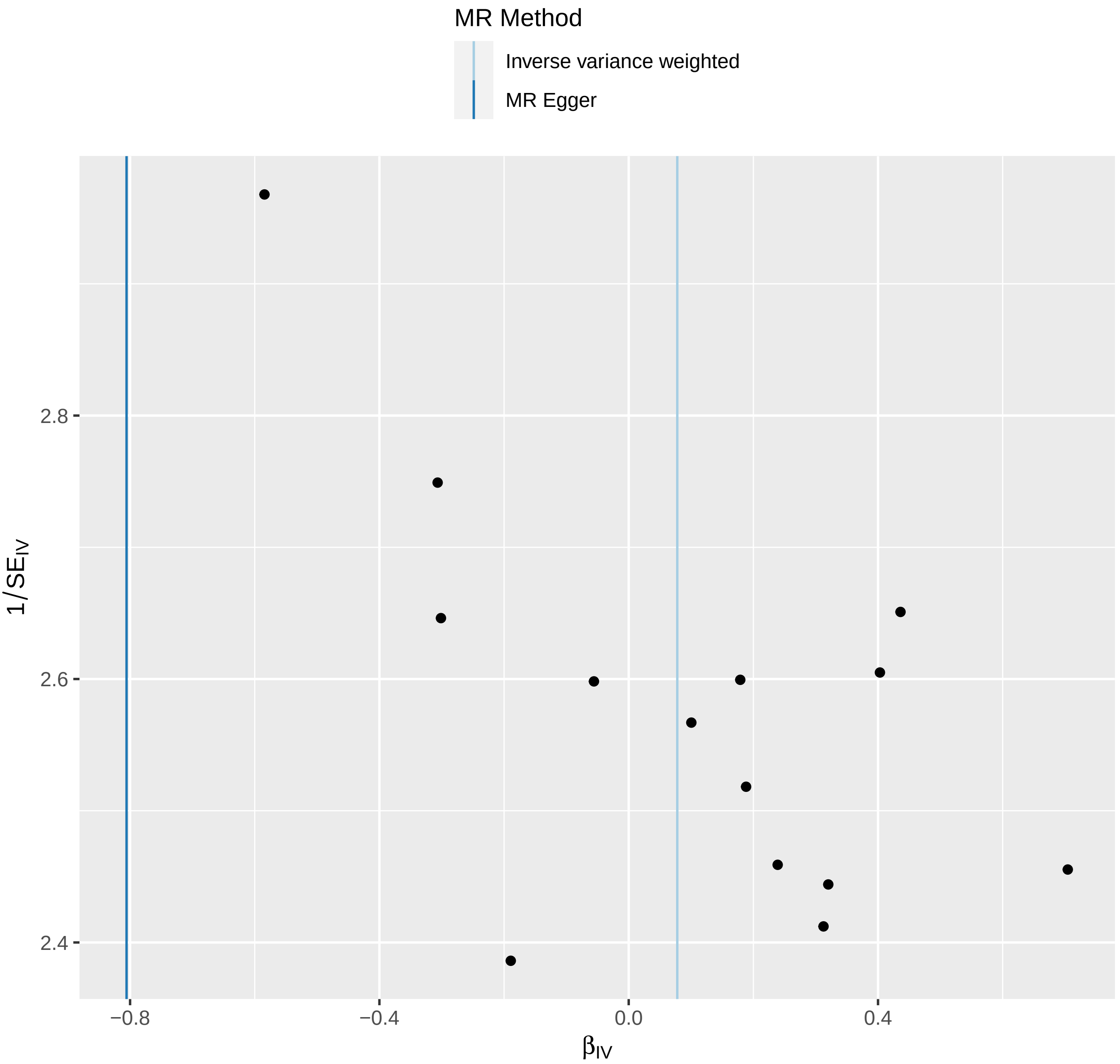

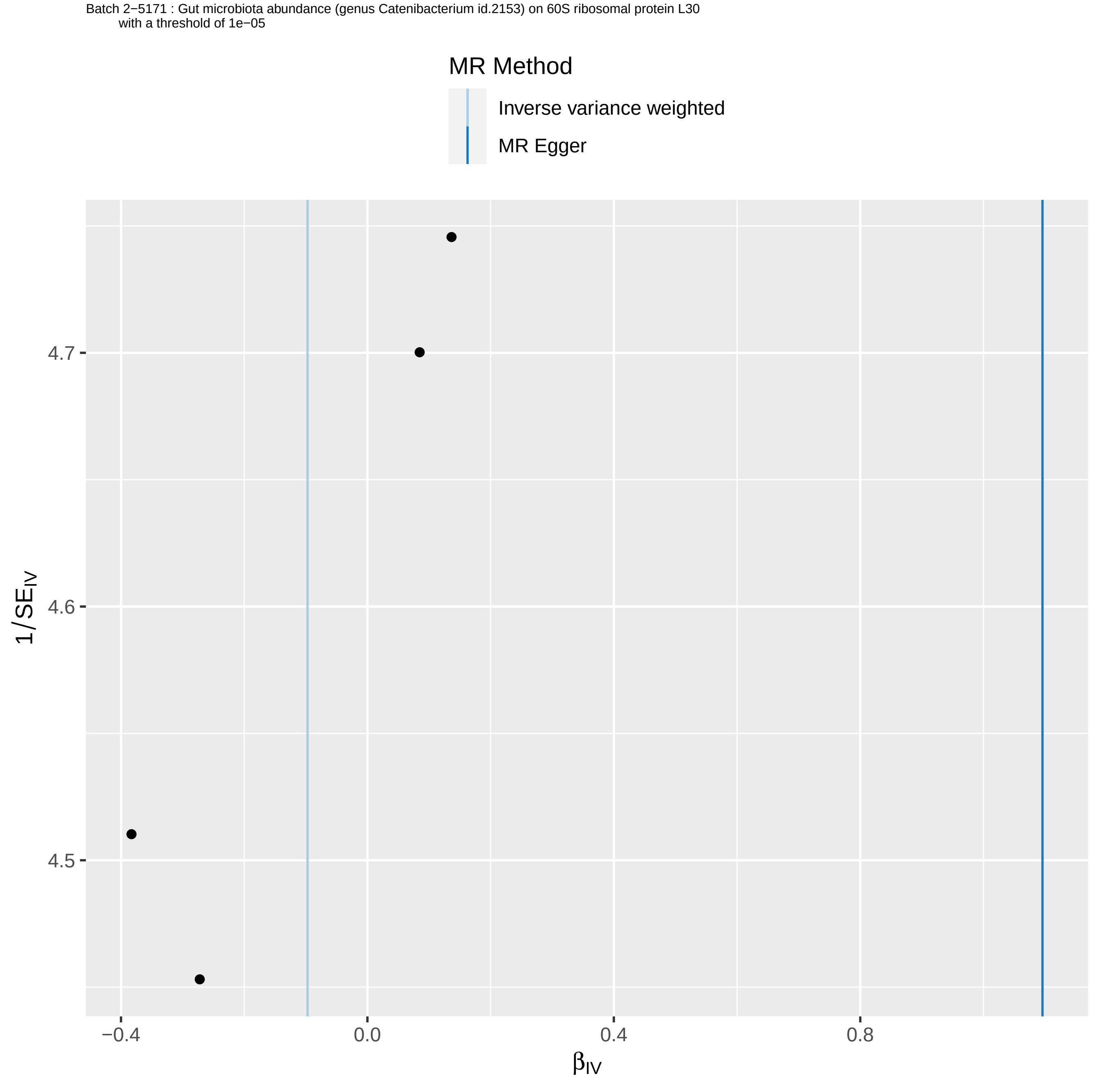
Batch 2−5171 : Gut microbiota abundance (genus Catenibacterium id.2153) on 60S ribosomal protein L30
with a threshold of 1e−05
MR Method
Inverse variance weighted
MR Egger
4.7
4.6
4.5
1/SE_IV
−0.4
0.0
0.4
0.8
β_IV

Batch 2−5172 : Gut microbiota abundance (genus Parasutterella id.2892) on 60S ribosomal protein L30
with a threshold of 1e−05

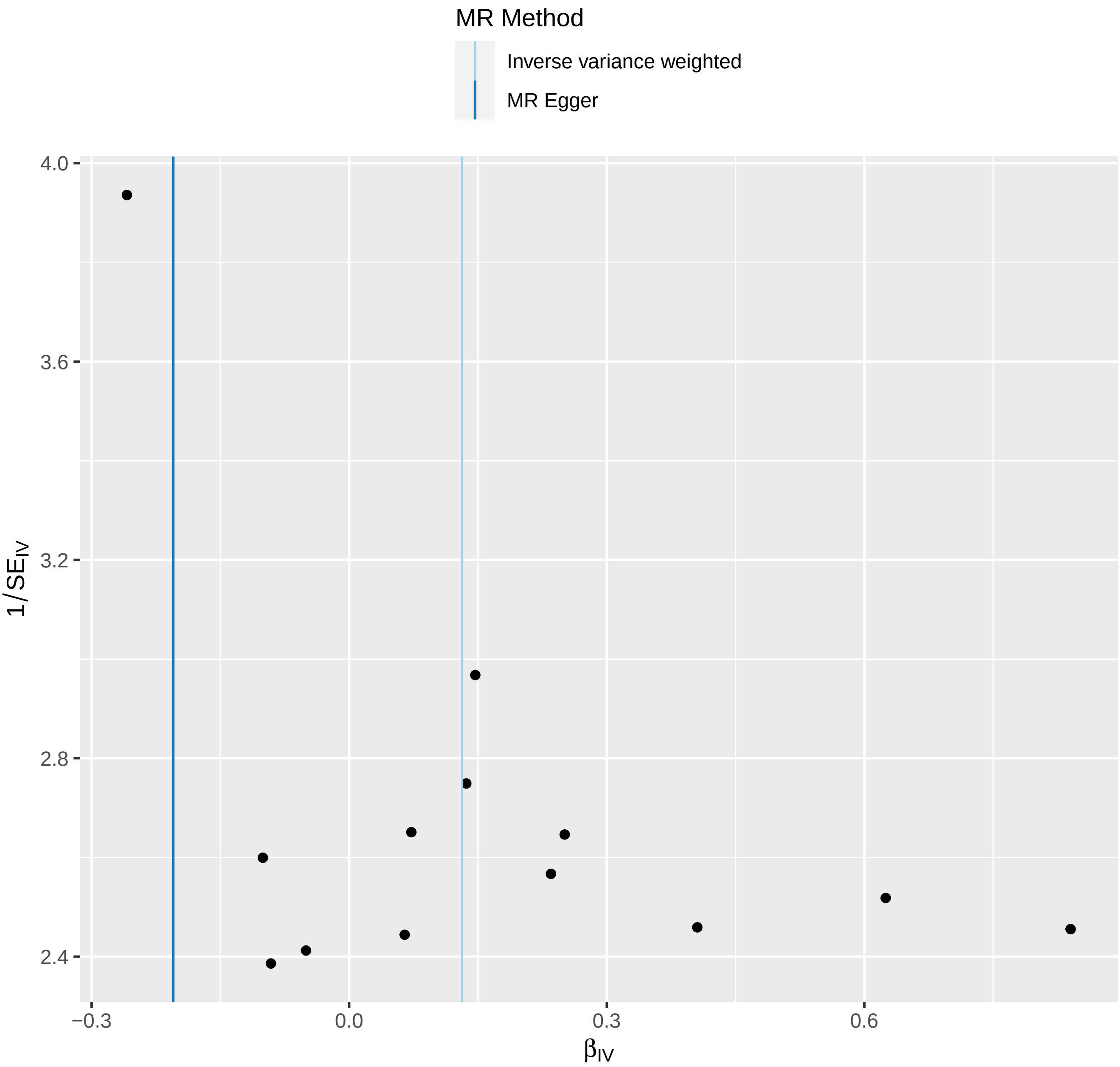

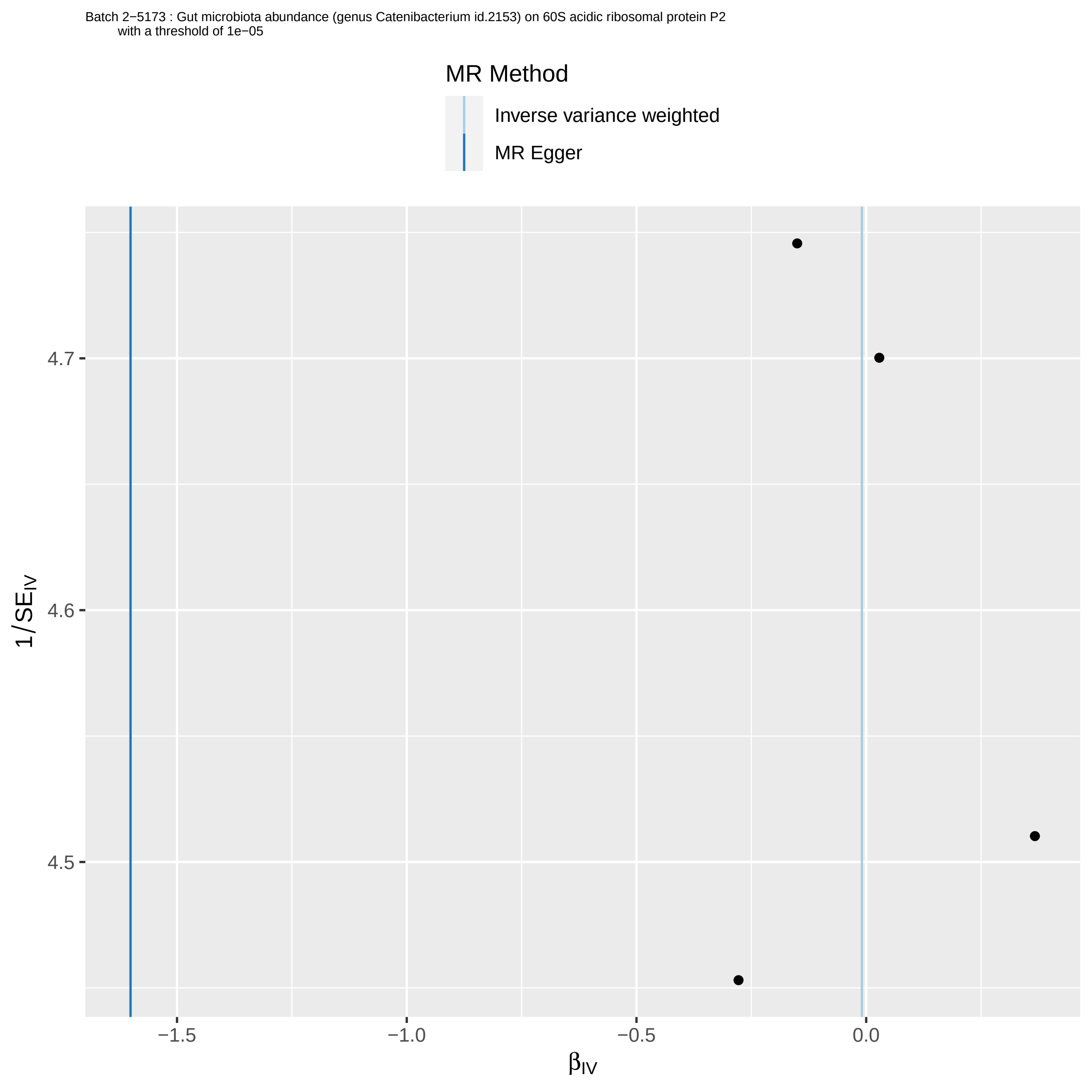

Batch 2−5173 : Gut microbiota abundance (genus Catenibacterium id.2153) on 60S acidic ribosomal protein P2
with a threshold of 1e−05
MR Method
Inverse variance weighted
MR Egger
4.7
4.6
4.5
1/SE_IV
−1.5
−1.0
−0.5
0.0
β_IV

Batch 2−5174 : Gut microbiota abundance (genus Parasutterella id.2892) on 60S acidic ribosomal protein P2
with a threshold of 1e−05

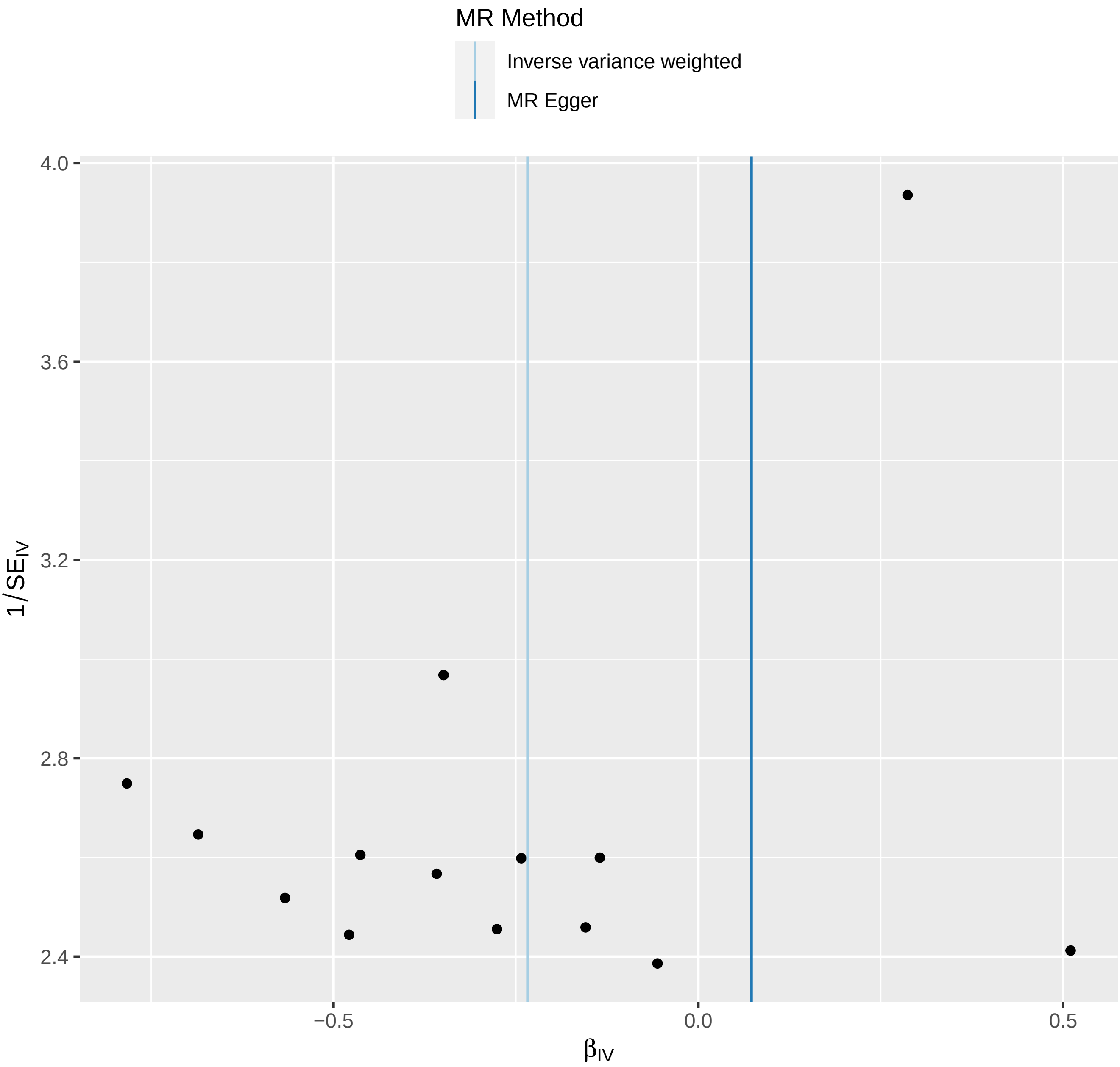

Batch 2−5175 : Gut microbiota abundance (genus Catenibacterium id.2153) on Dolichyl−diphosphooligosaccharide−−protein glycosyltransferase subunit 1
with a threshold of 1e−05

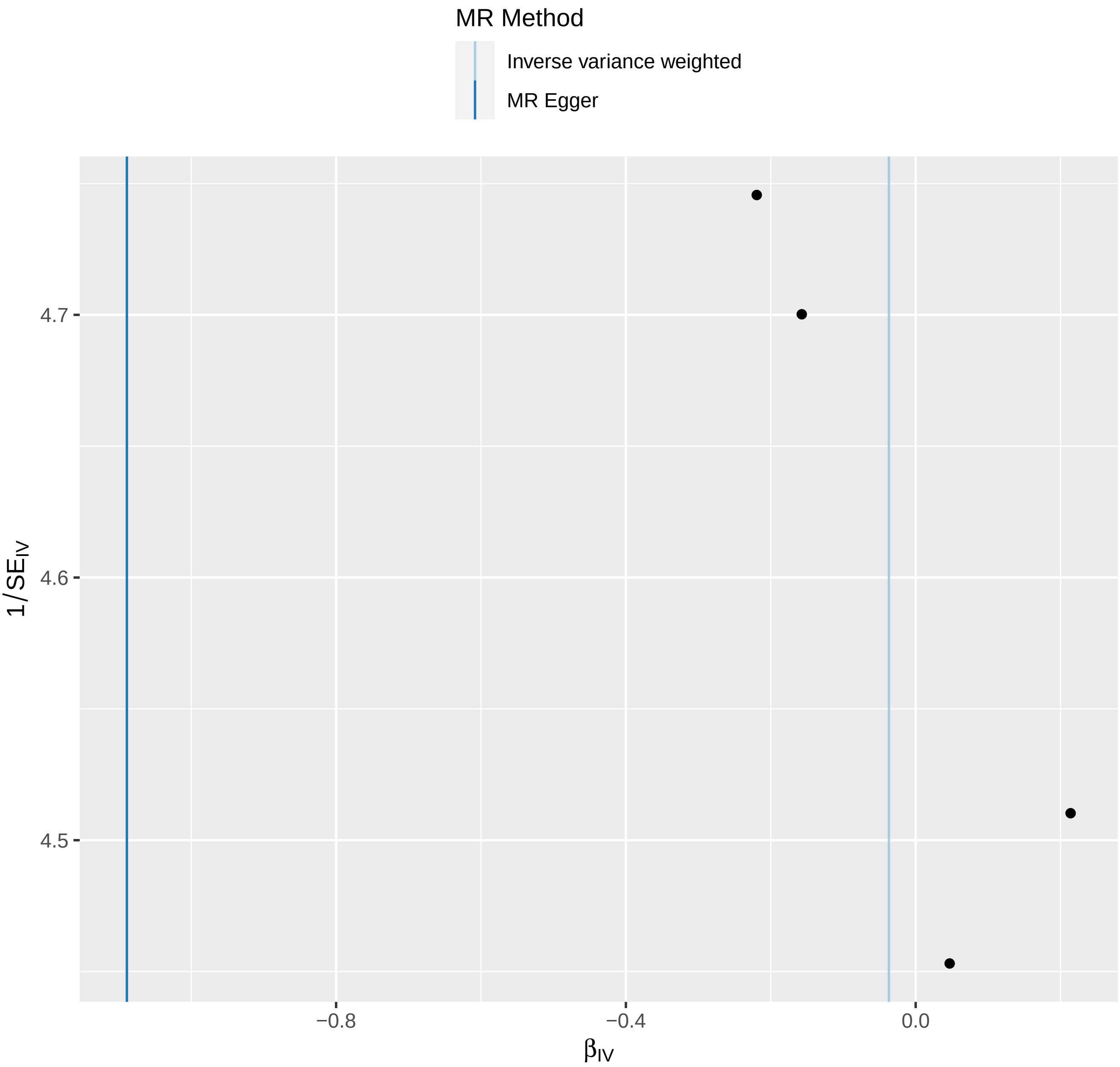

Batch 2−5176 : Gut microbiota abundance (genus Parasutterella id.2892) on Dolichyl−diphosphooligosaccharide−−protein glycosyltransferase subunit 1
with a threshold of 1e−05

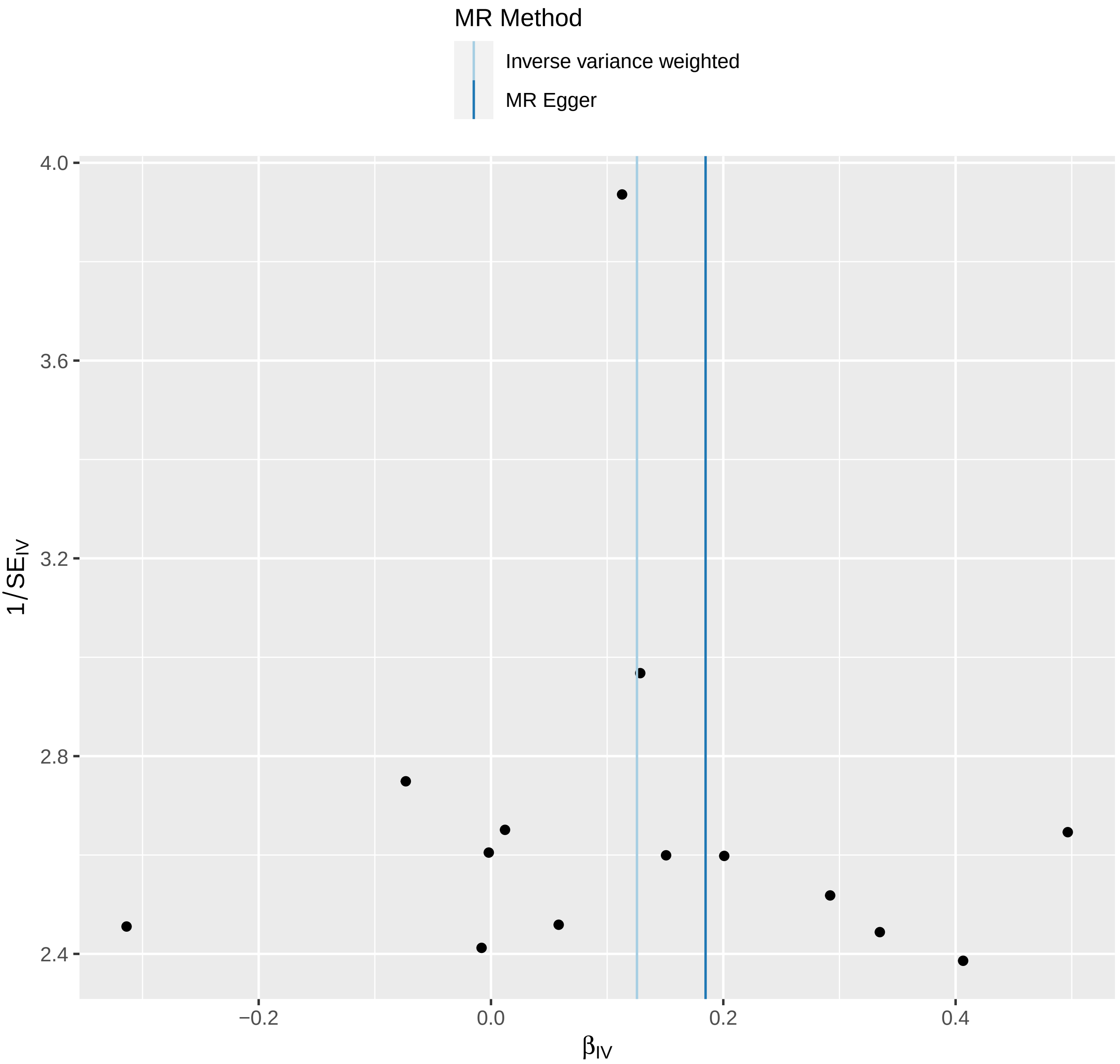

Batch 2−5177 : Gut microbiota abundance (genus Catenibacterium id.2153) on Dolichyl−diphosphooligosaccharide−−protein glycosyltransferase subunit 1 with a threshold of 1e−05

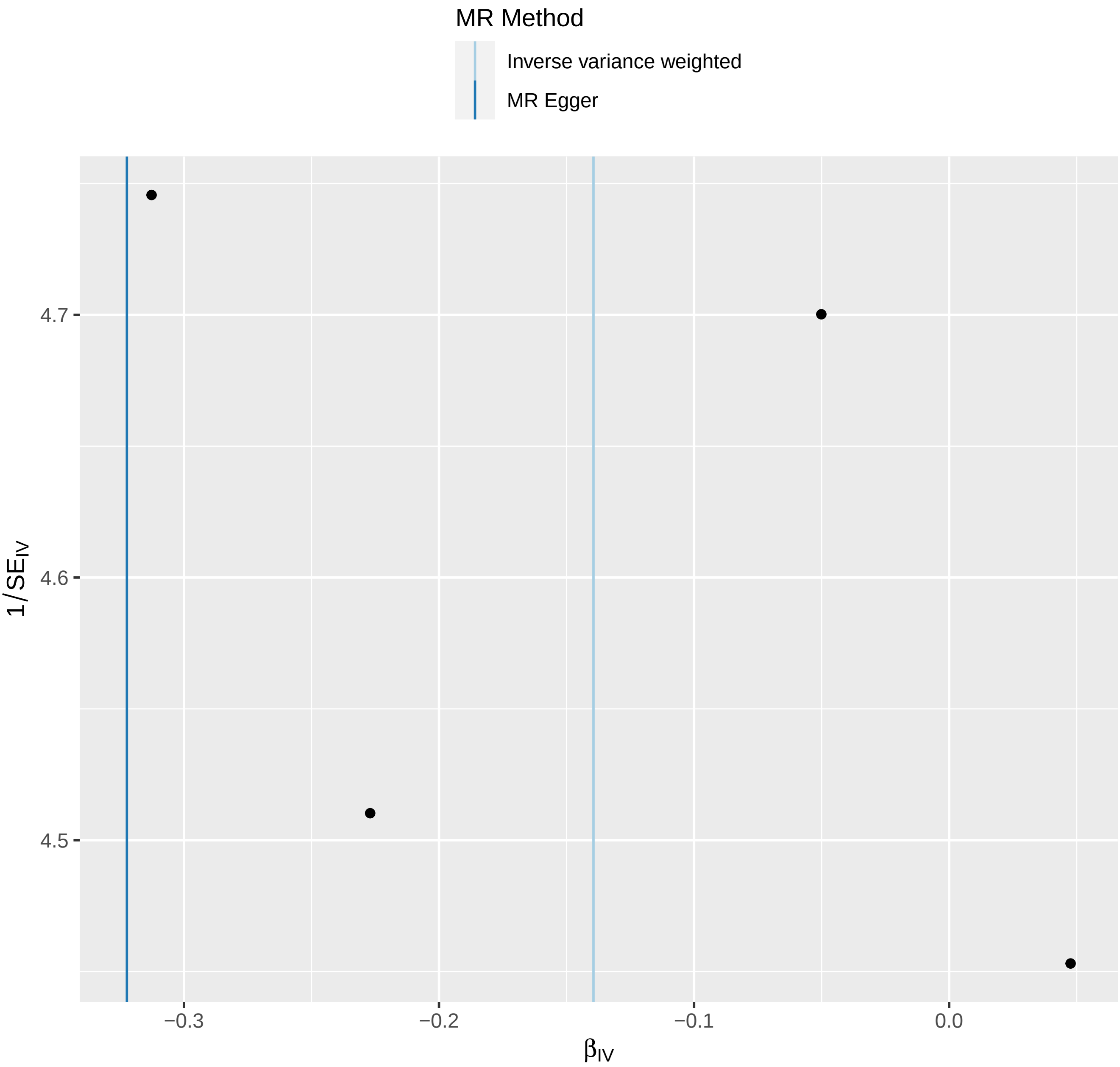

Batch 2−5178 : Gut microbiota abundance (genus Parasutterella id.2892) on Dolichyl−diphosphooligosaccharide−−protein glycosyltransferase subunit 1 with a threshold of 1e−05

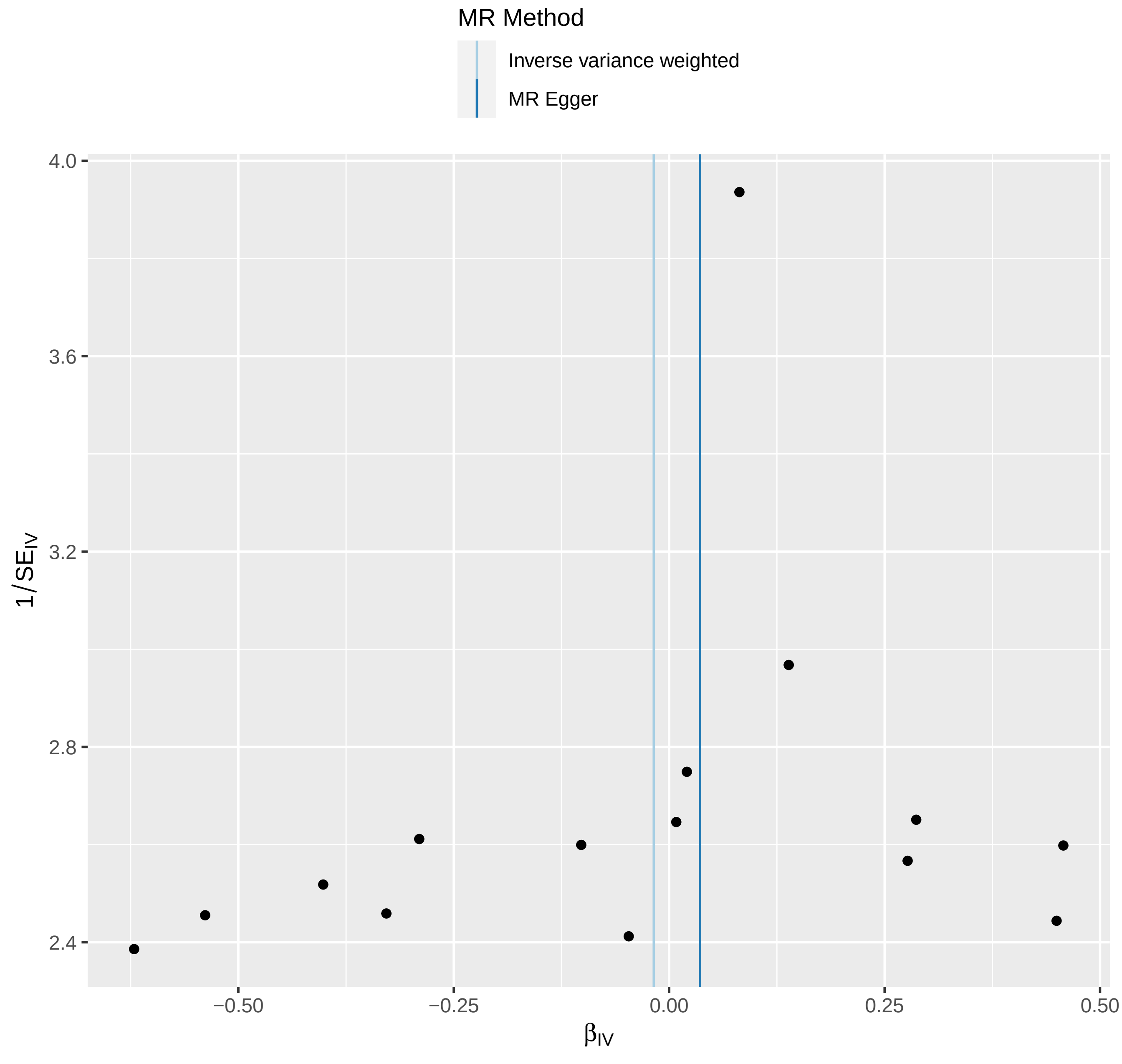

Batch 2−5179 : Gut microbiota abundance (genus Catenibacterium id.2153) on Regulation of nuclear pre−mRNA domain−containing protein 1A with a threshold of 1e−05

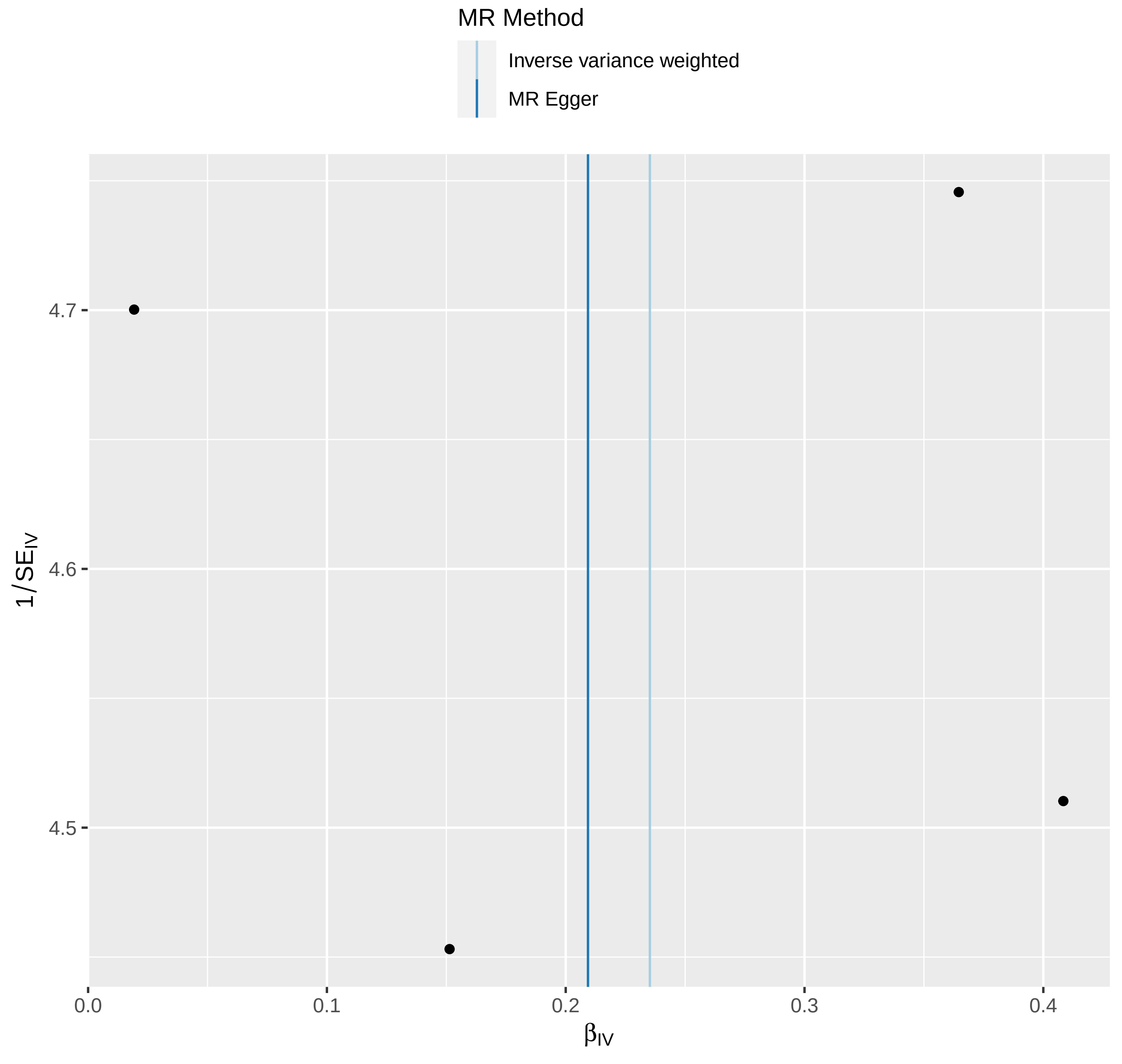

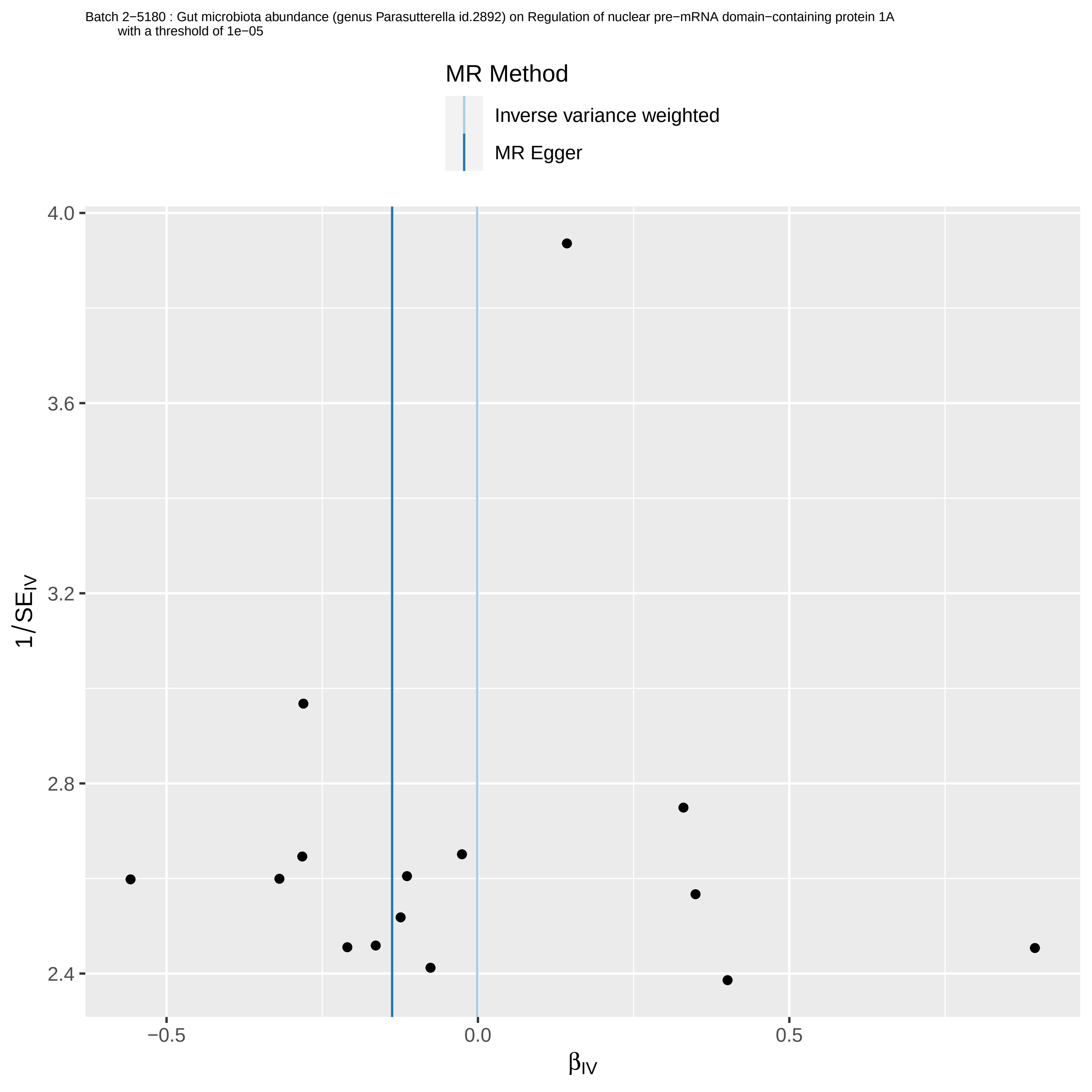
Batch 2−5180 : Gut microbiota abundance (genus Parasutterella id.2892) on Regulation of nuclear pre−mRNA domain−containing protein 1A
with a threshold of 1e−05
MR Method
Inverse variance weighted
MR Egger
4.0
3.6
3.2
2.8
2.4
1/SE$_{IV}$
−0.5
0.0
0.5
β$_{IV}$

Batch 2−5181 : Gut microbiota abundance (genus Catenibacterium id.2153) on Ubiquitin+1, truncated mutation for UbB with a threshold of 1e−05

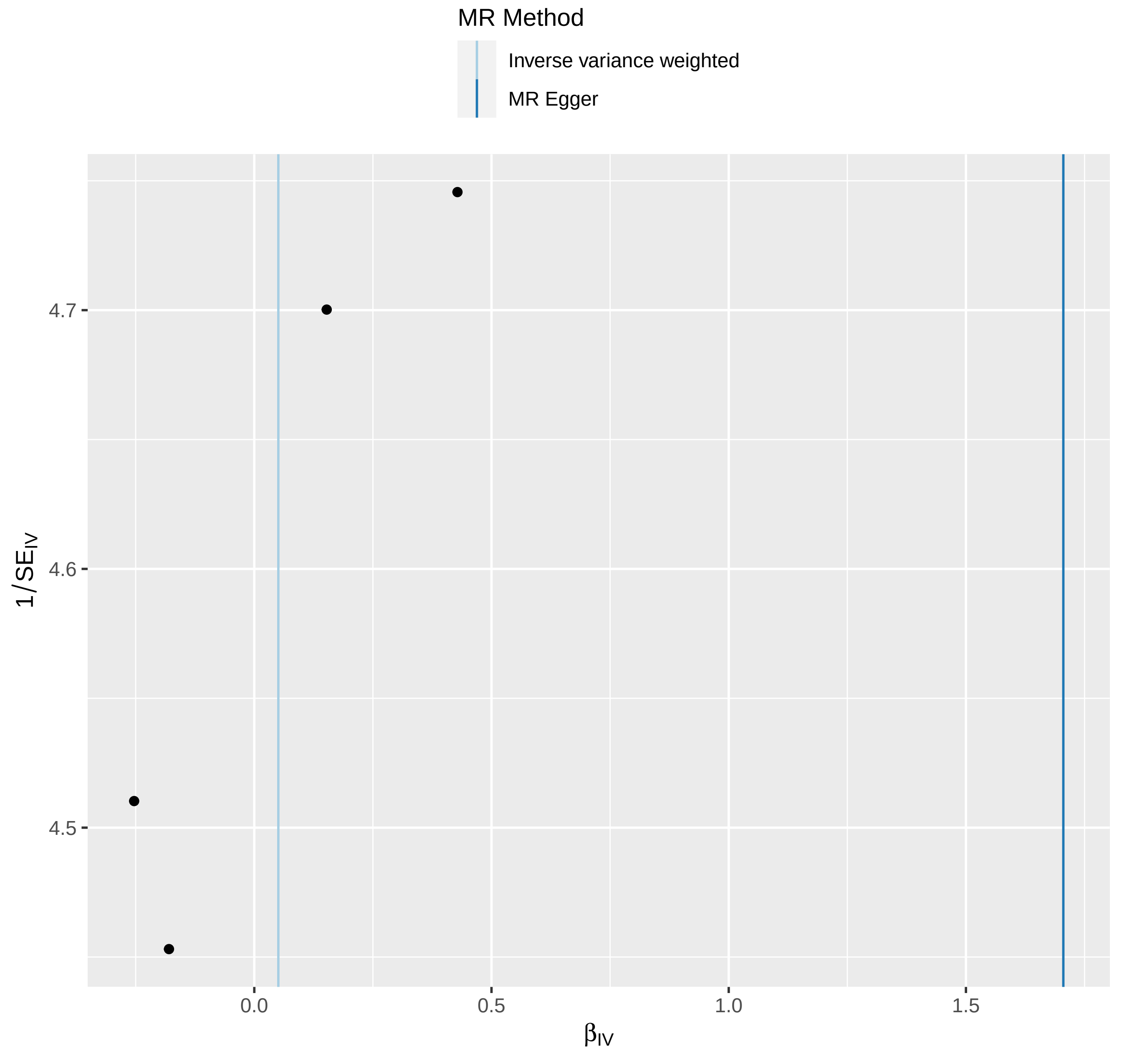

Batch 2−5182 : Gut microbiota abundance (genus Parasutterella id.2892) on Ubiquitin+1, truncated mutation for UbB with a threshold of 1e−05

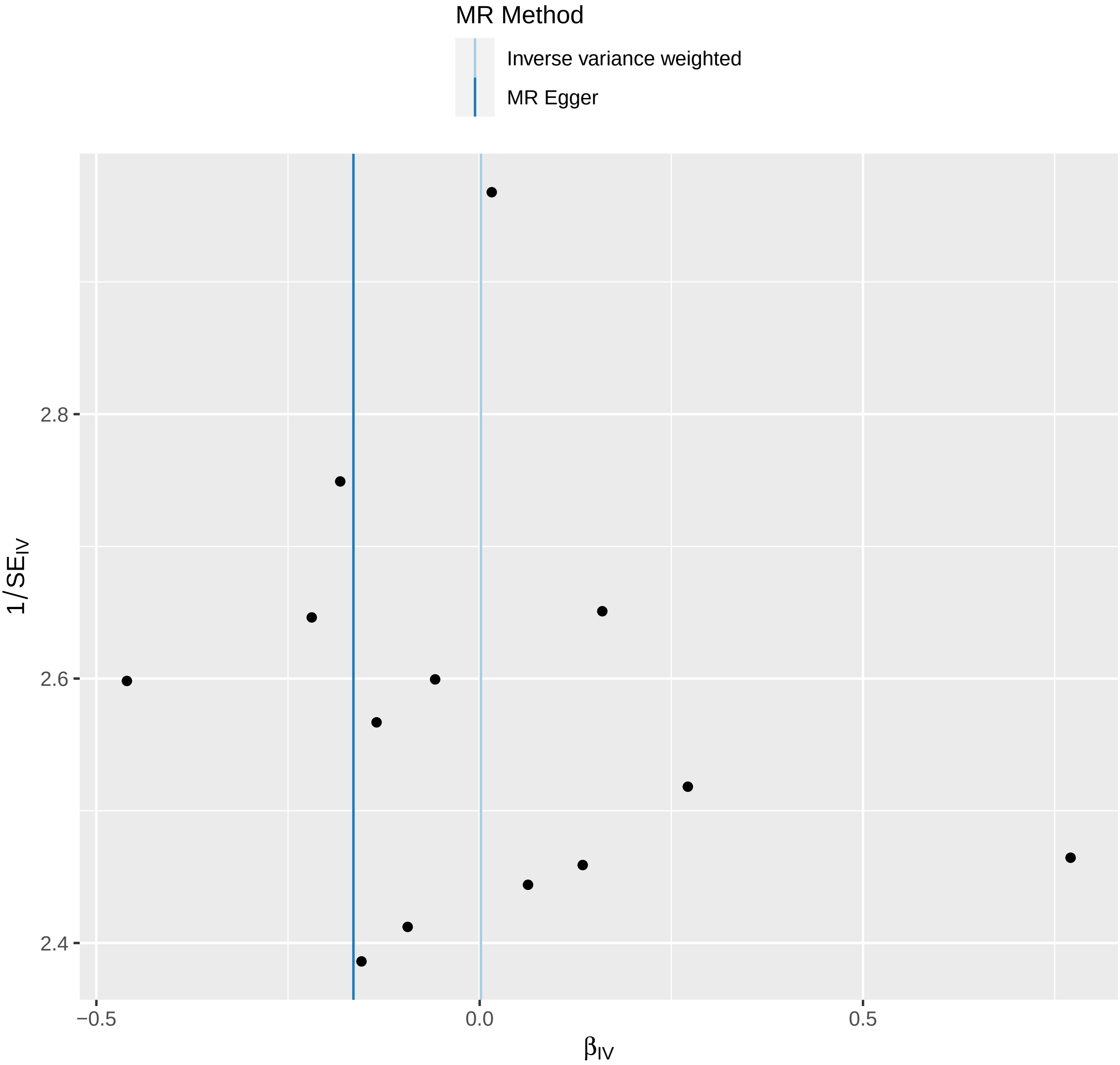

Batch 2−5183 : Gut microbiota abundance (genus Catenibacterium id.2153) on Ubiquitin
with a threshold of 1e−05

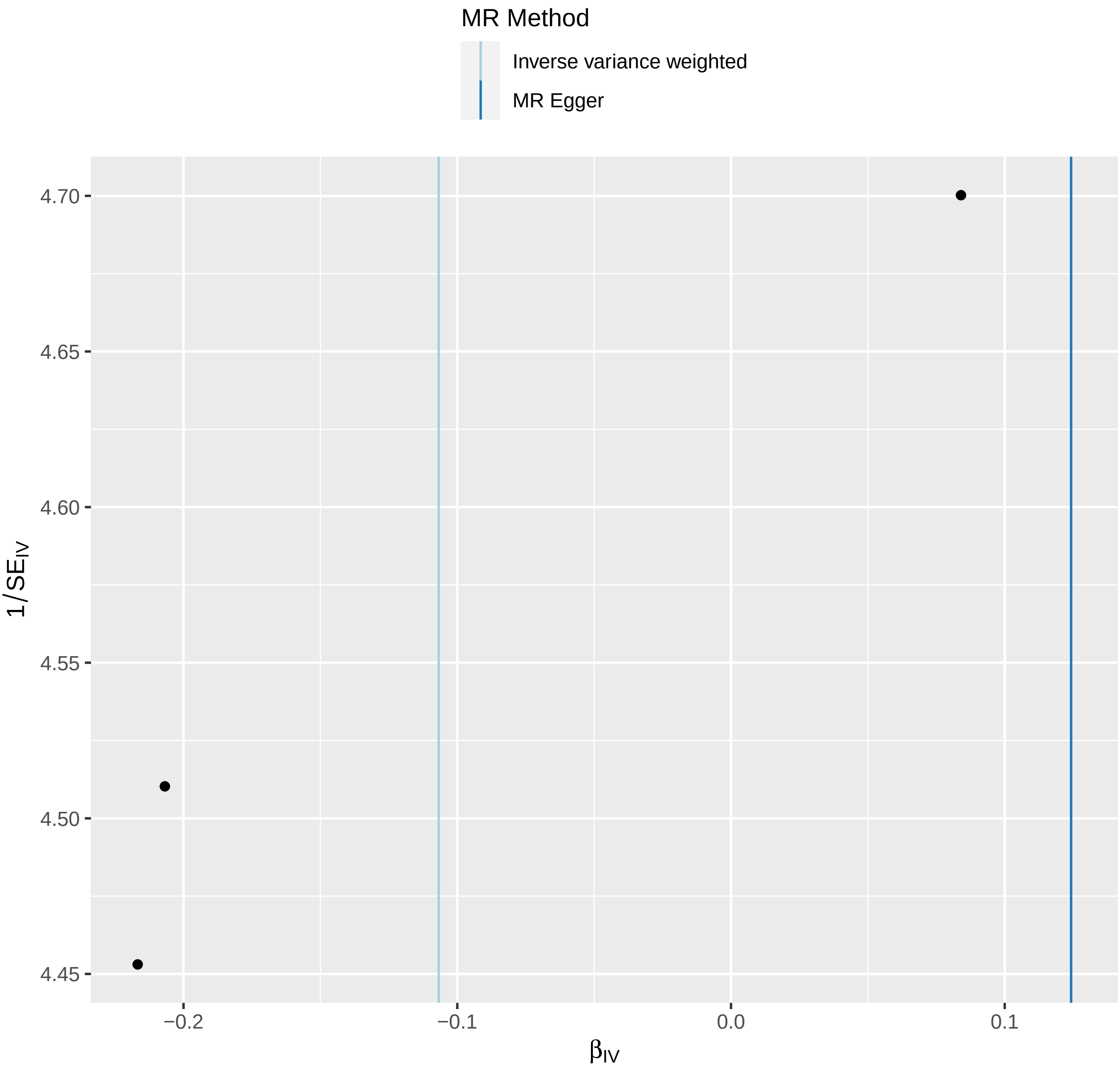

Batch 2−5184 : Gut microbiota abundance (genus Parasutterella id.2892) on Ubiquitin
with a threshold of 1e−05

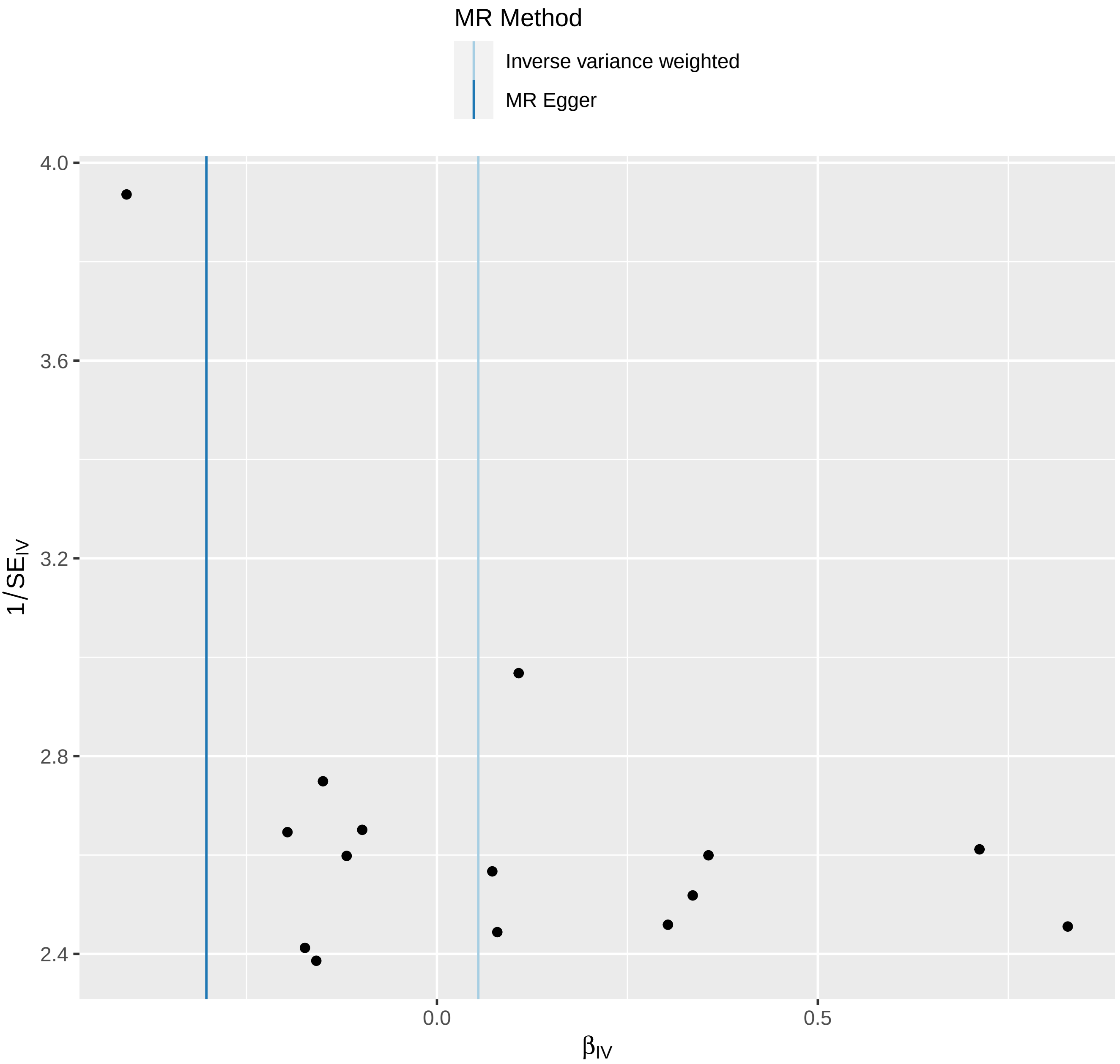

Batch 2−5185 : Gut microbiota abundance (genus Catenibacterium id.2153) on 40S ribosomal protein S3
with a threshold of 1e−05

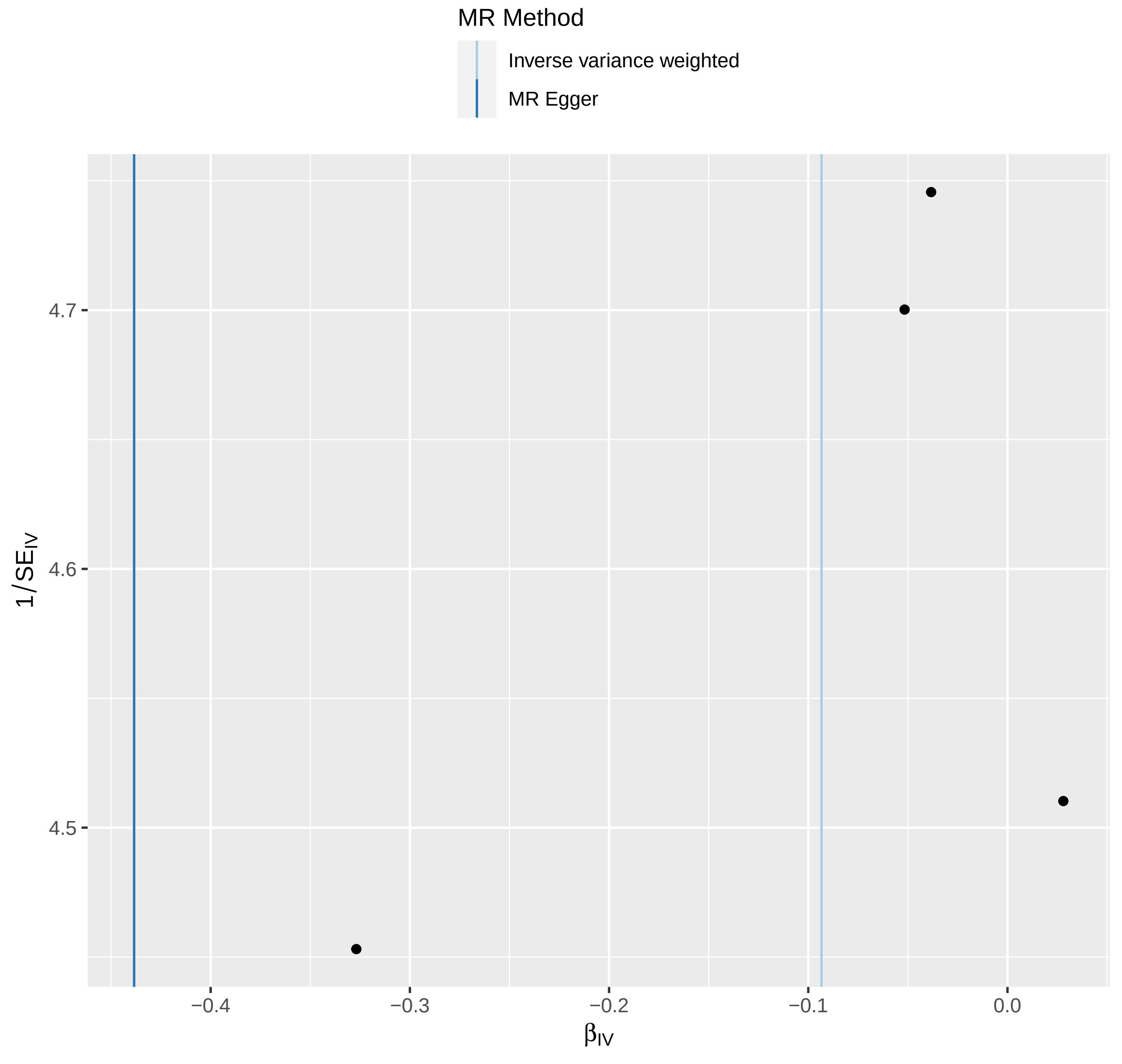

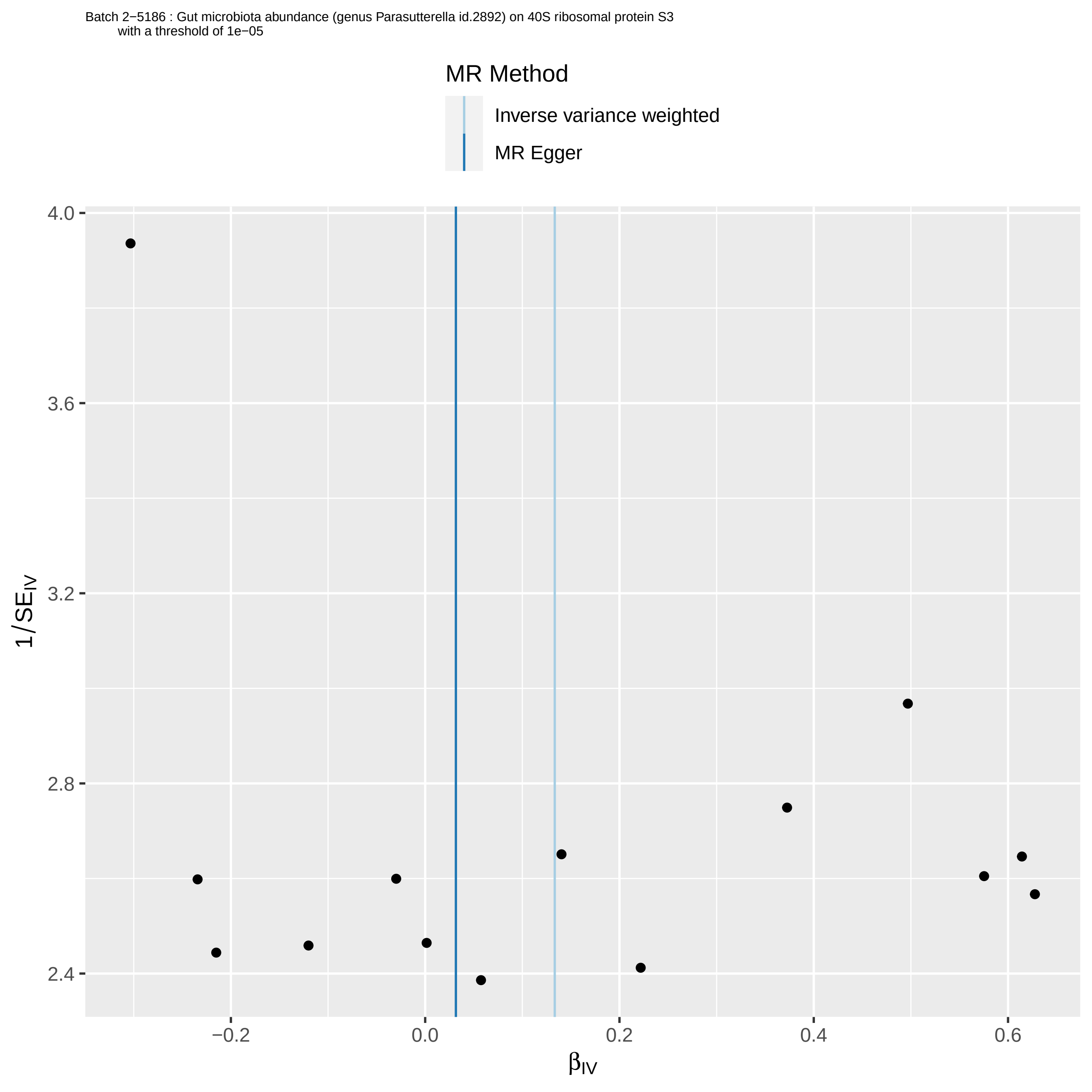

Batch 2−5186 : Gut microbiota abundance (genus Parasutterella id.2892) on 40S ribosomal protein S3
with a threshold of 1e−05
MR Method
Inverse variance weighted
MR Egger
4.0
3.6
3.2
2.8
2.4
1/SE_IV
−0.2
0.0
0.2
0.4
0.6
β_IV

Batch 2−5187 : Gut microbiota abundance (genus Catenibacterium id.2153) on 40S ribosomal protein S3a
with a threshold of 1e−05

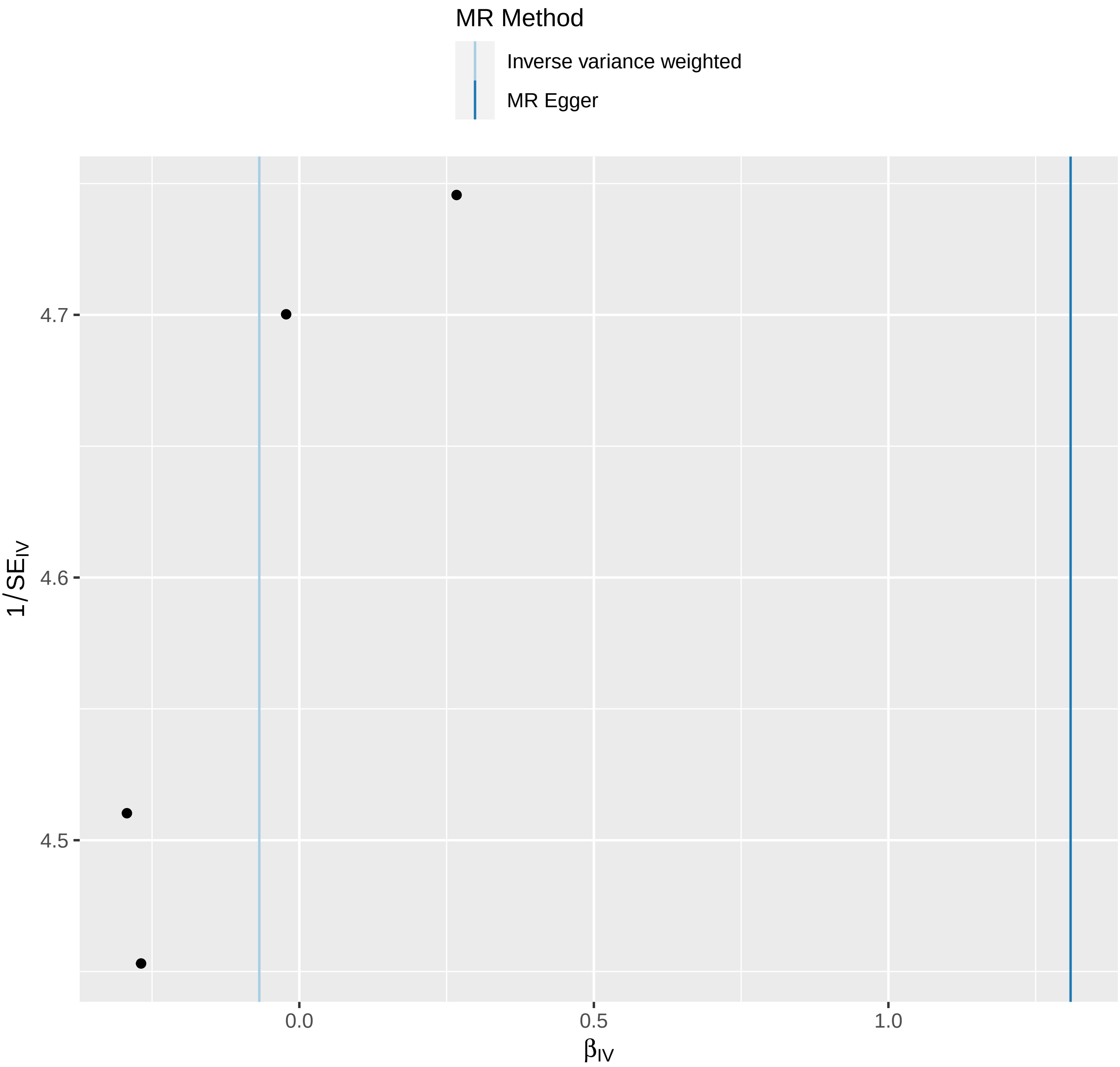

Batch 2−5188 : Gut microbiota abundance (genus Parasutterella id.2892) on 40S ribosomal protein S3a
with a threshold of 1e−05

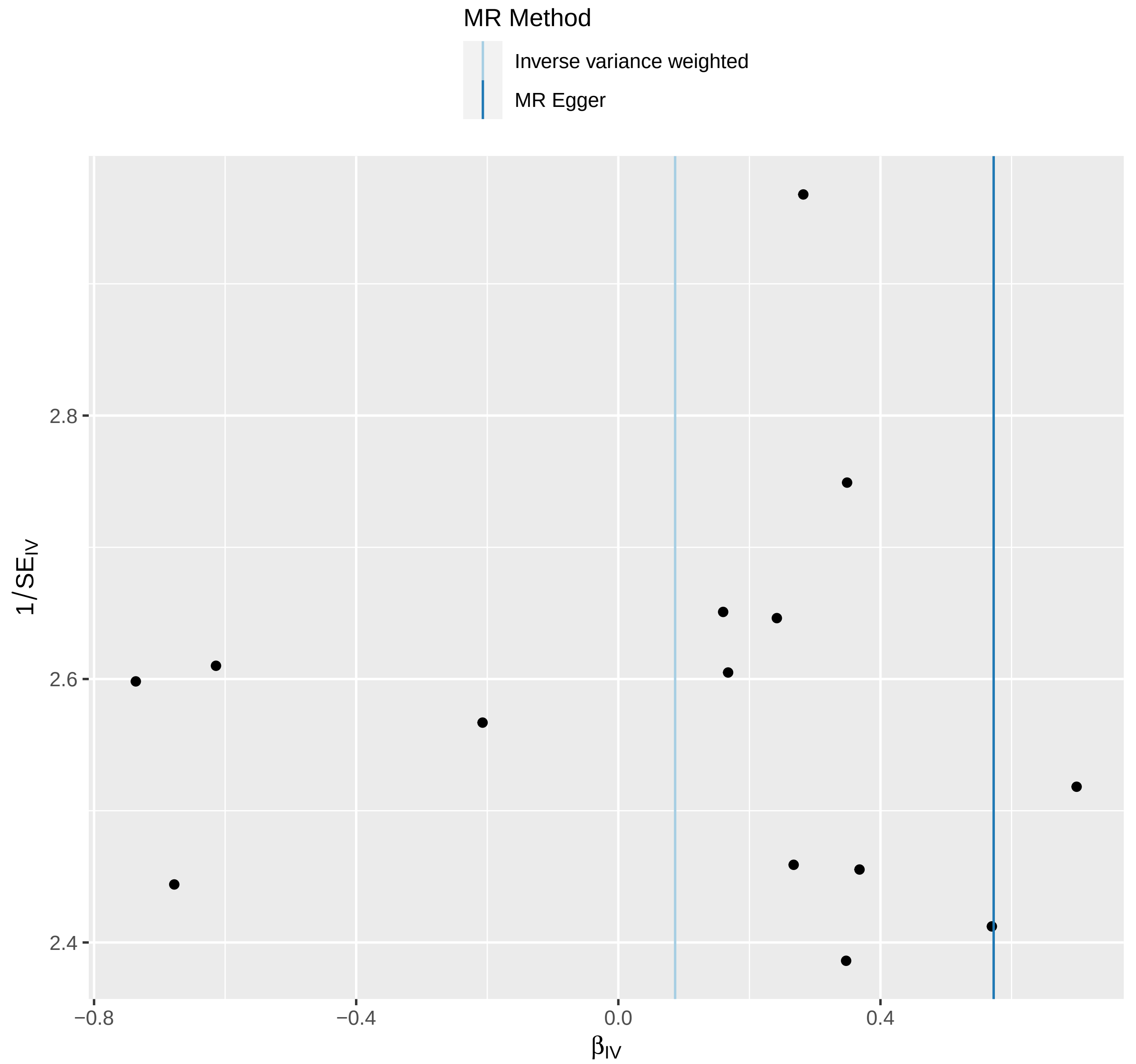

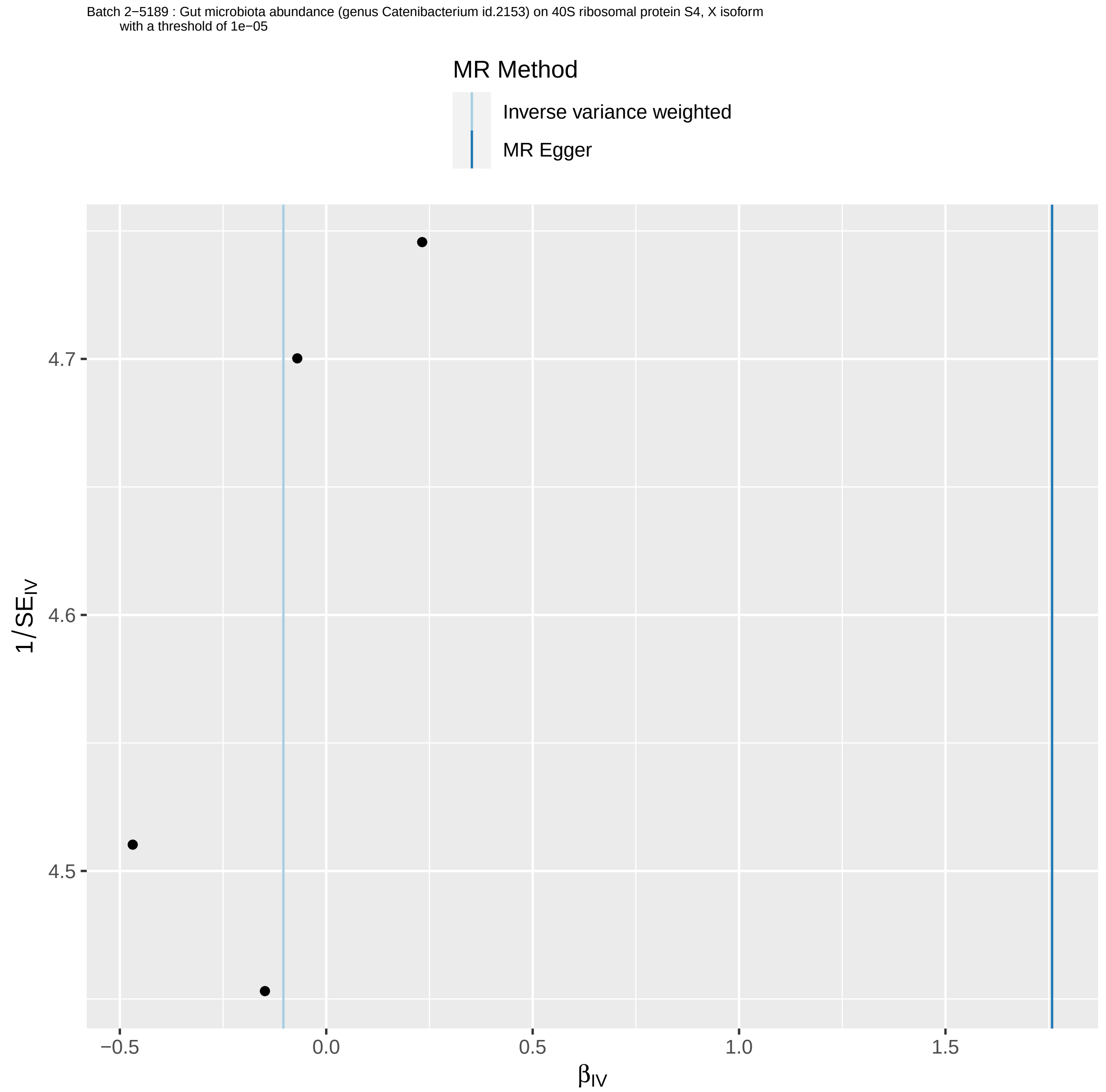

Batch 2−5189 : Gut microbiota abundance (genus Catenibacterium id.2153) on 40S ribosomal protein S4, X isoform
with a threshold of 1e−05
MR Method
Inverse variance weighted
MR Egger
4.7
4.6
4.5
1/SE_IV
−0.5
0.0
0.5
1.0
1.5
β_IV

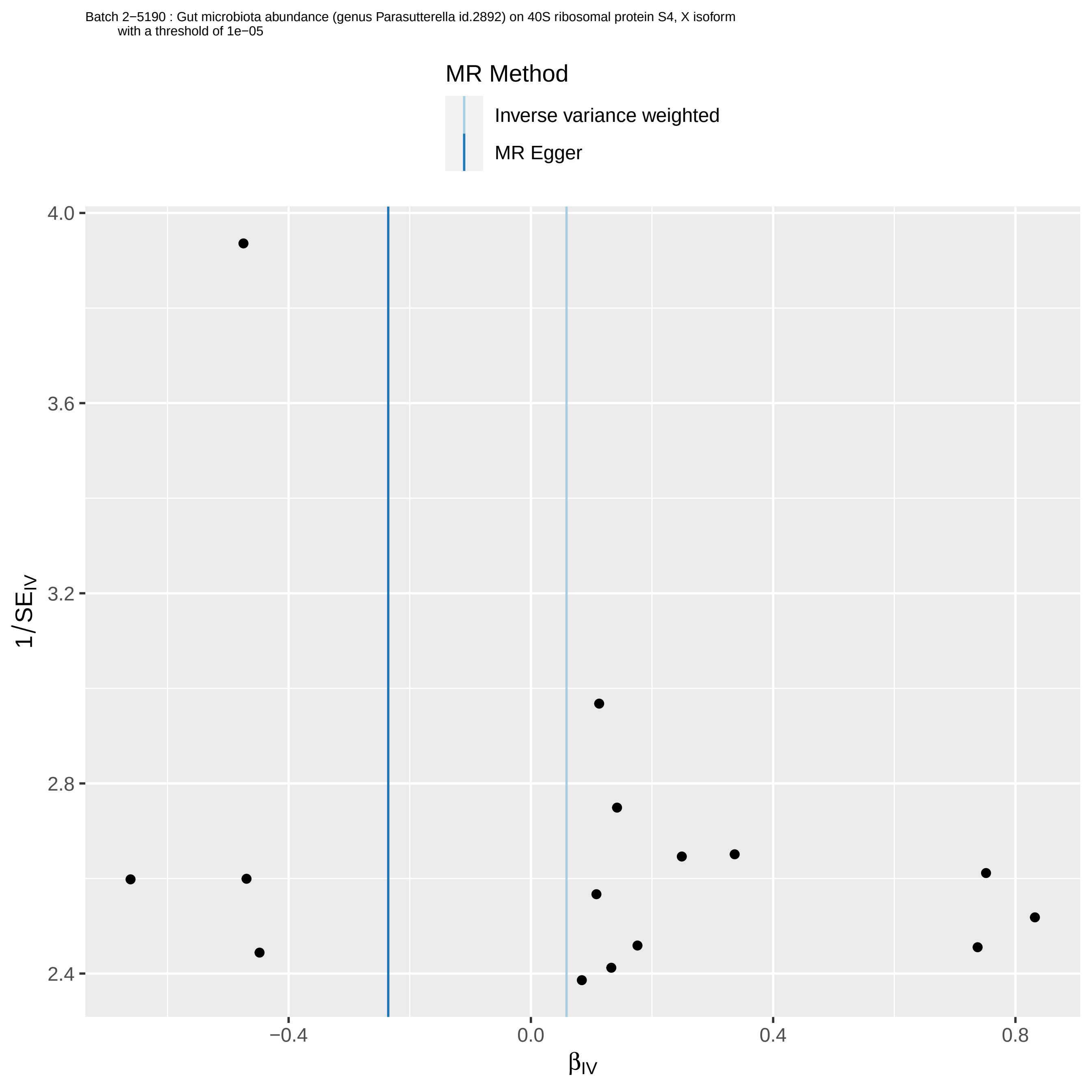

Batch 2−5190 : Gut microbiota abundance (genus Parasutterella id.2892) on 40S ribosomal protein S4, X isoform
with a threshold of 1e−05
MR Method
Inverse variance weighted
MR Egger
4.0
3.6
3.2
2.8
2.4
1/SE_IV
−0.4
0.0
0.4
0.8
β_IV

Batch 2−5191 : Gut microbiota abundance (genus Catenibacterium id.2153) on Ribosomal protein S6 kinase alpha−6
with a threshold of 1e−05

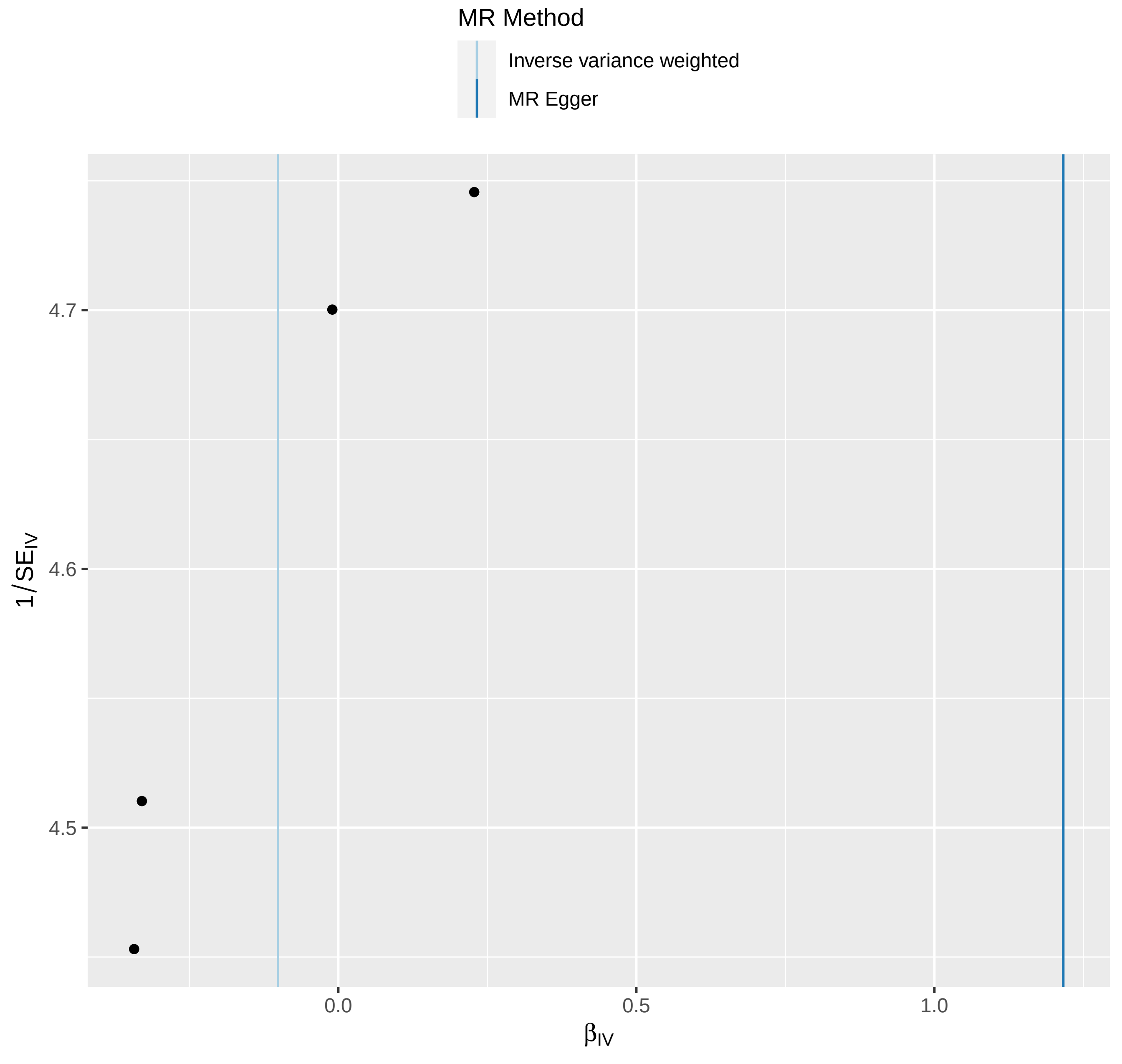

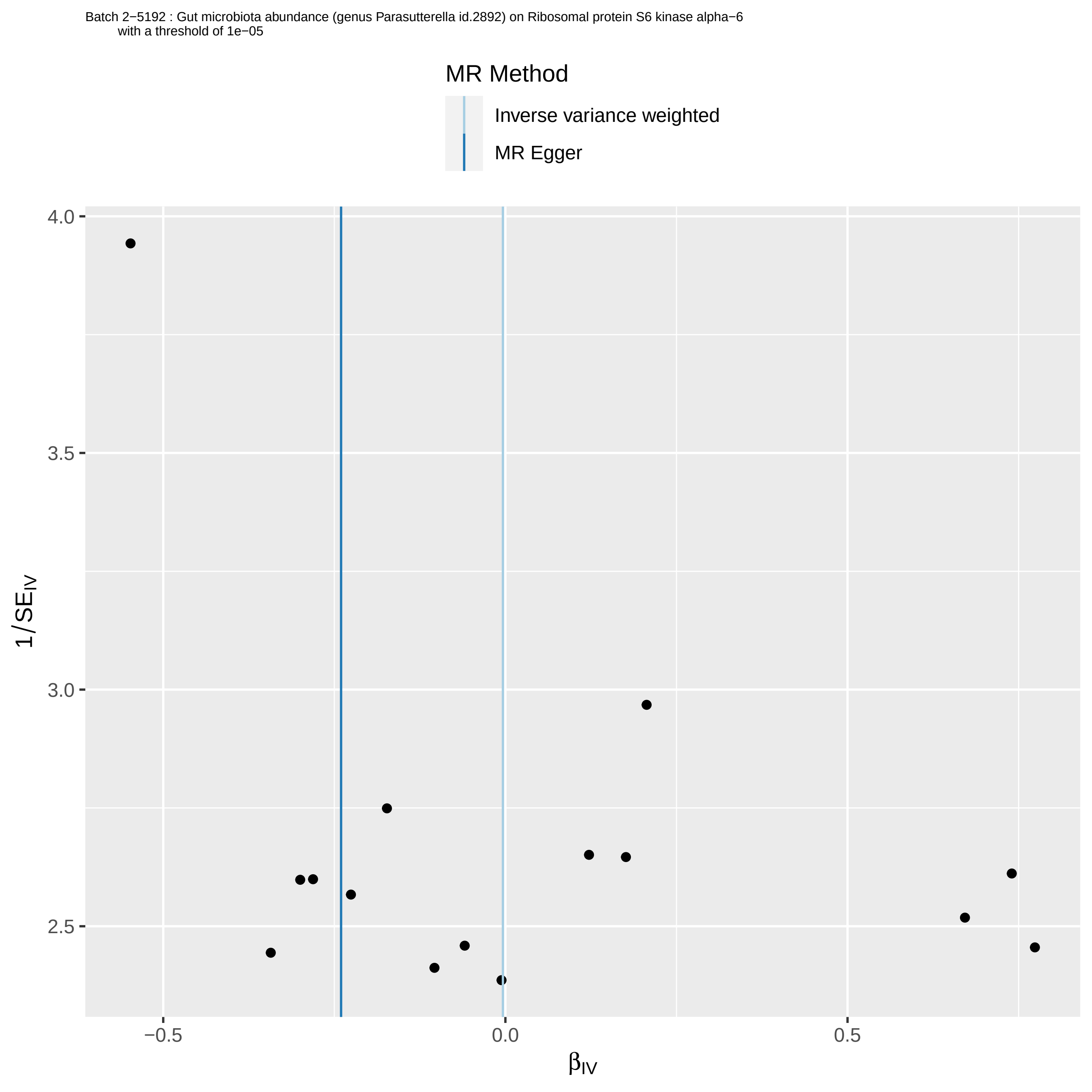

Batch 2−5192 : Gut microbiota abundance (genus Parasutterella id.2892) on Ribosomal protein S6 kinase alpha−6
with a threshold of 1e−05
MR Method
Inverse variance weighted
MR Egger
4.0
3.5
3.0
2.5
1/SE_IV
−0.5
0.0
0.5
β_IV

Batch 2−5193 : Gut microbiota abundance (genus Catenibacterium id.2153) on 40S ribosomal protein S7
with a threshold of 1e−05

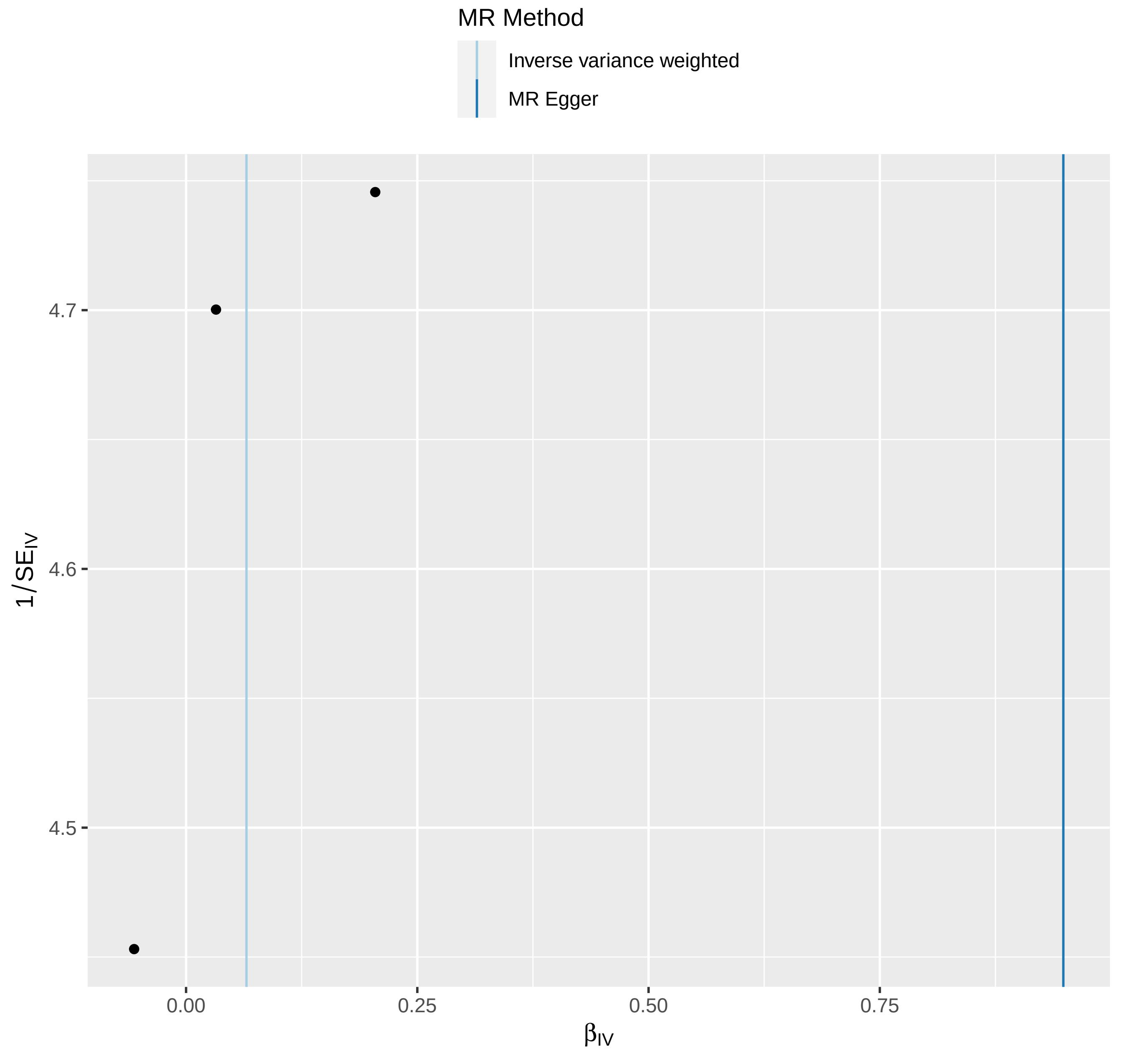

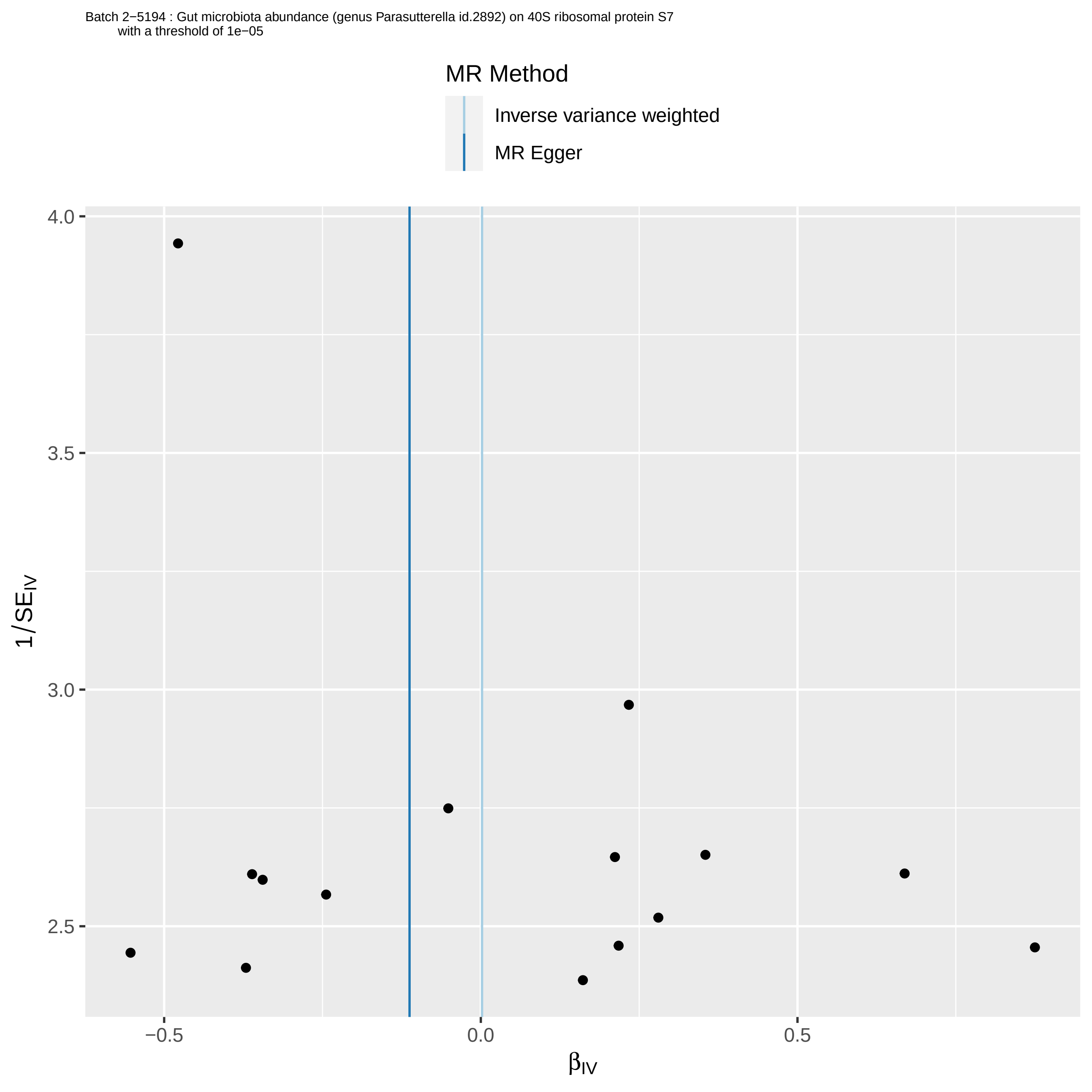

Batch 2−5194 : Gut microbiota abundance (genus Parasutterella id.2892) on 40S ribosomal protein S7
with a threshold of 1e−05
MR Method
Inverse variance weighted
MR Egger
4.0
3.5
3.0
2.5
1/SE$_{IV}$
−0.5
0.0
0.5
β$_{IV}$

Batch 2−5195 : Gut microbiota abundance (genus Catenibacterium id.2153) on 40S ribosomal protein SA
with a threshold of 1e−05

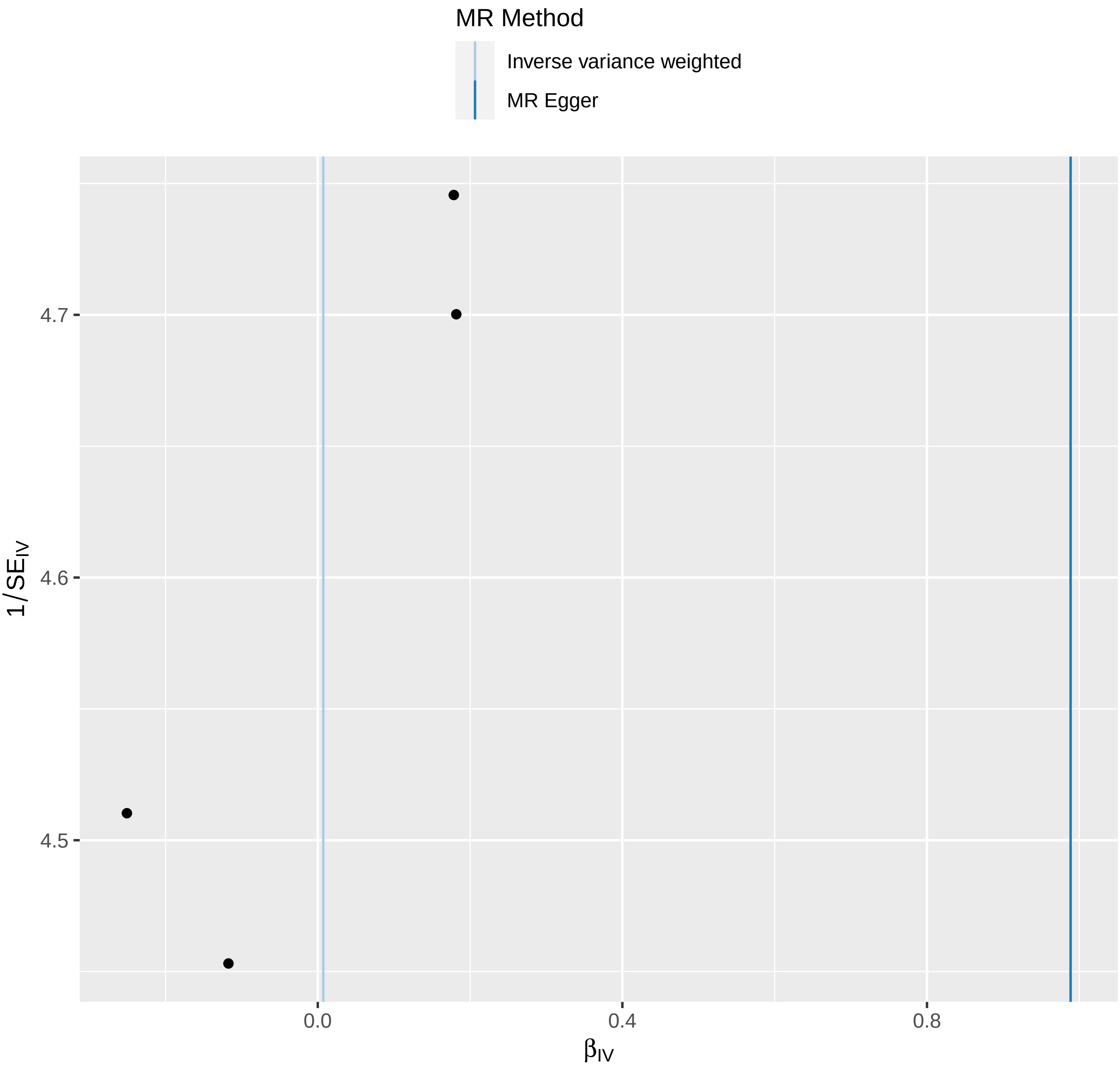

Batch 2−5196 : Gut microbiota abundance (genus Parasutterella id.2892) on 40S ribosomal protein SA
with a threshold of 1e−05

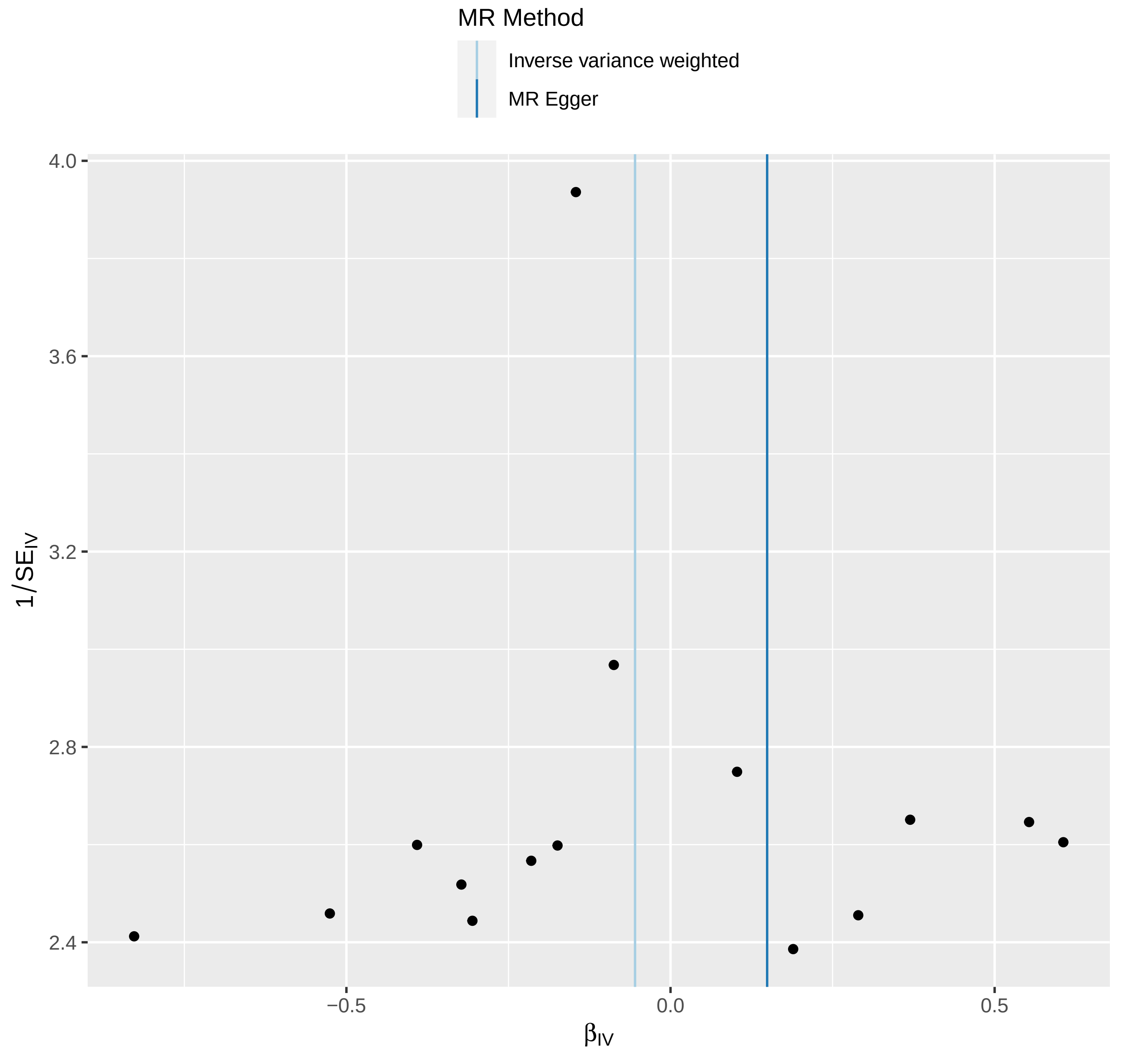

Batch 2−5197 : Gut microbiota abundance (genus Catenibacterium id.2153) on Cell differentiation protein RCD1 homolog
with a threshold of 1e−05

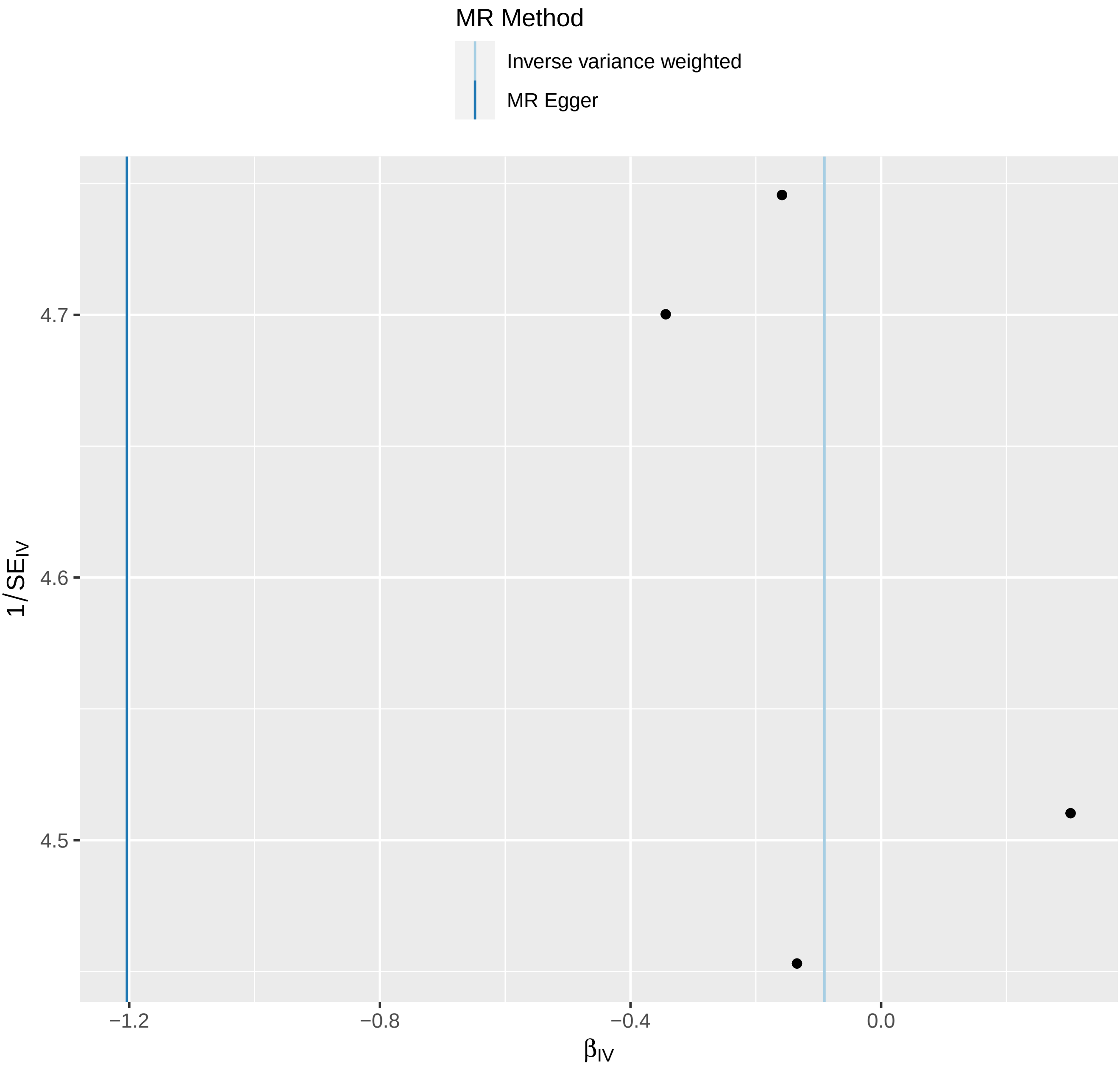

Batch 2−5198 : Gut microbiota abundance (genus Parasutterella id.2892) on Cell differentiation protein RCD1 homolog
with a threshold of 1e−05

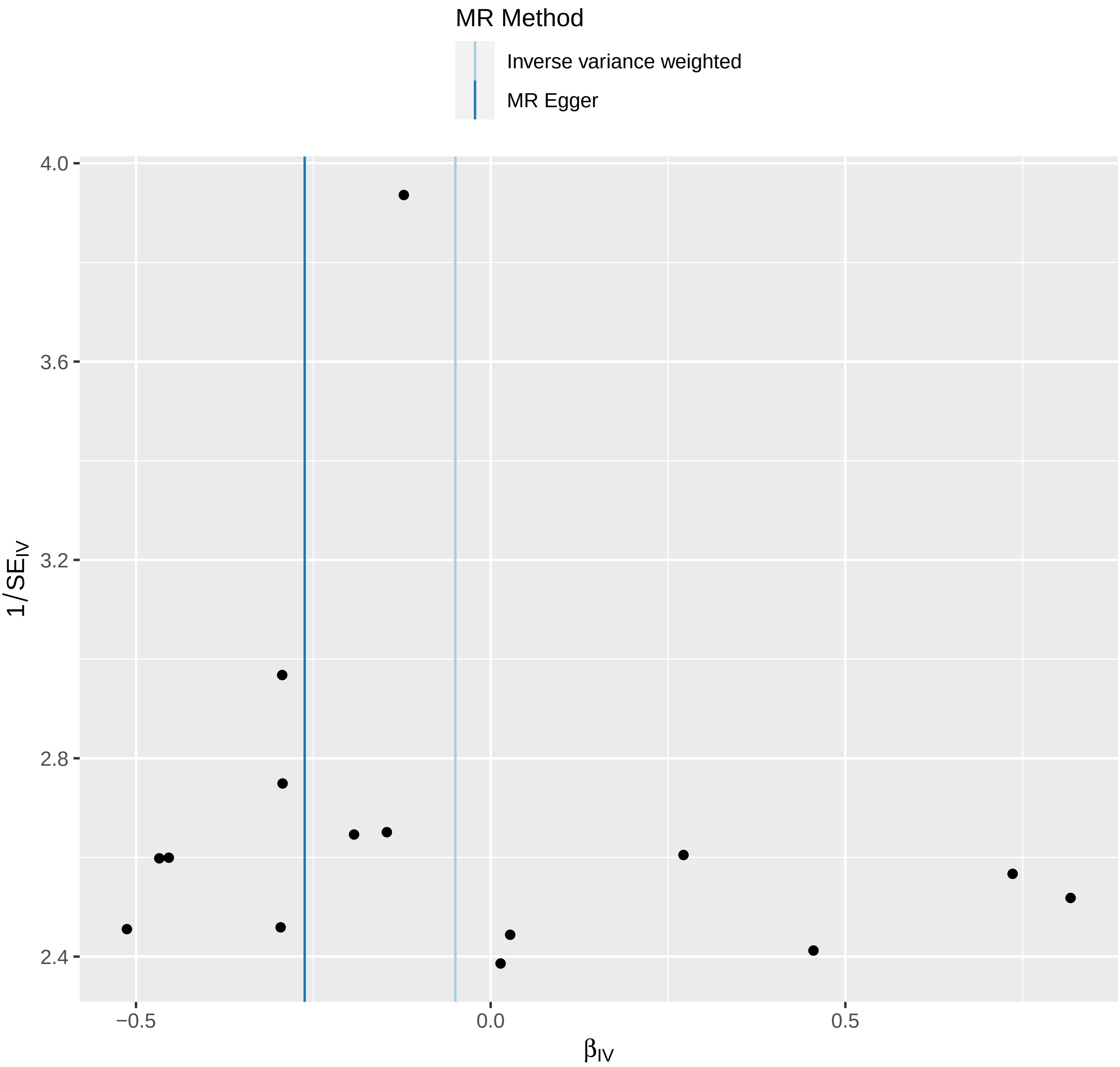

Batch 2−5199 : Gut microbiota abundance (genus Catenibacterium id.2153) on Ribosome−binding protein 1
with a threshold of 1e−05

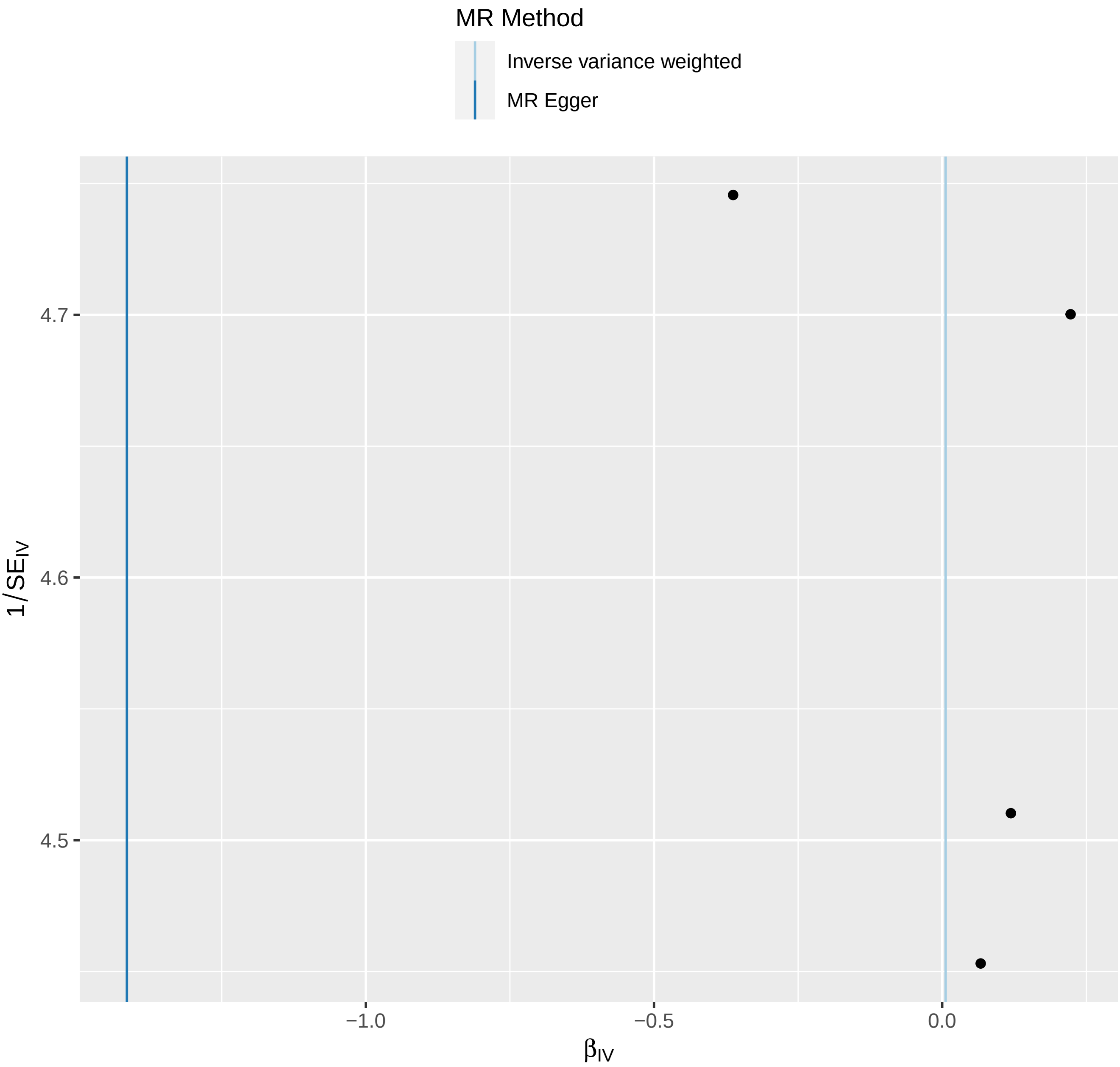

Batch 2−5200 : Gut microbiota abundance (genus Parasutterella id.2892) on Ribosome−binding protein 1
with a threshold of 1e−05

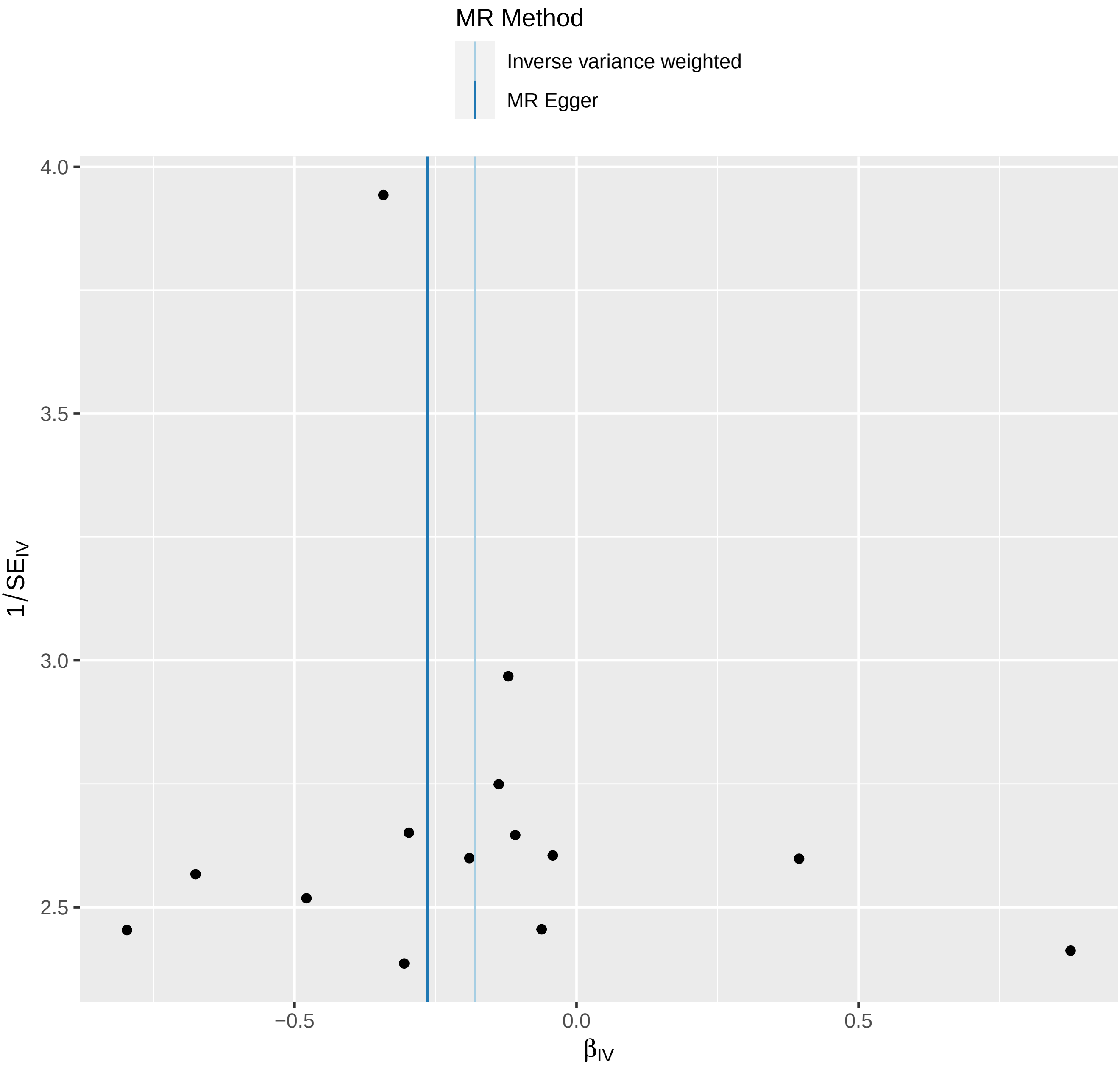

Batch 2−5201 : Gut microbiota abundance (genus Catenibacterium id.2153) on Ribonucleoside−diphosphate reductase large subunit with a threshold of 1e−05

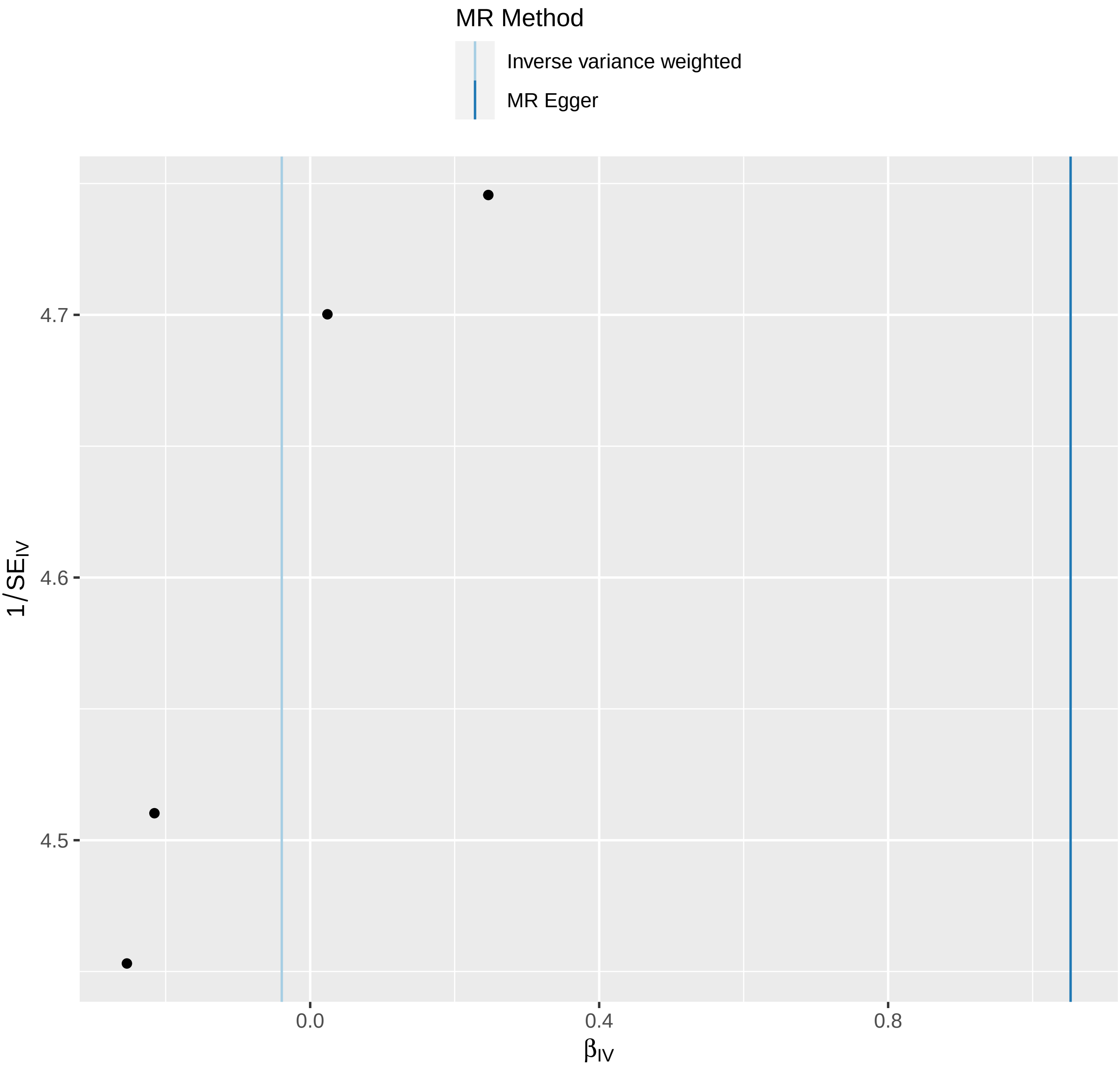

Batch 2−5202 : Gut microbiota abundance (genus Parasutterella id.2892) on Ribonucleoside−diphosphate reductase large subunit with a threshold of 1e−05

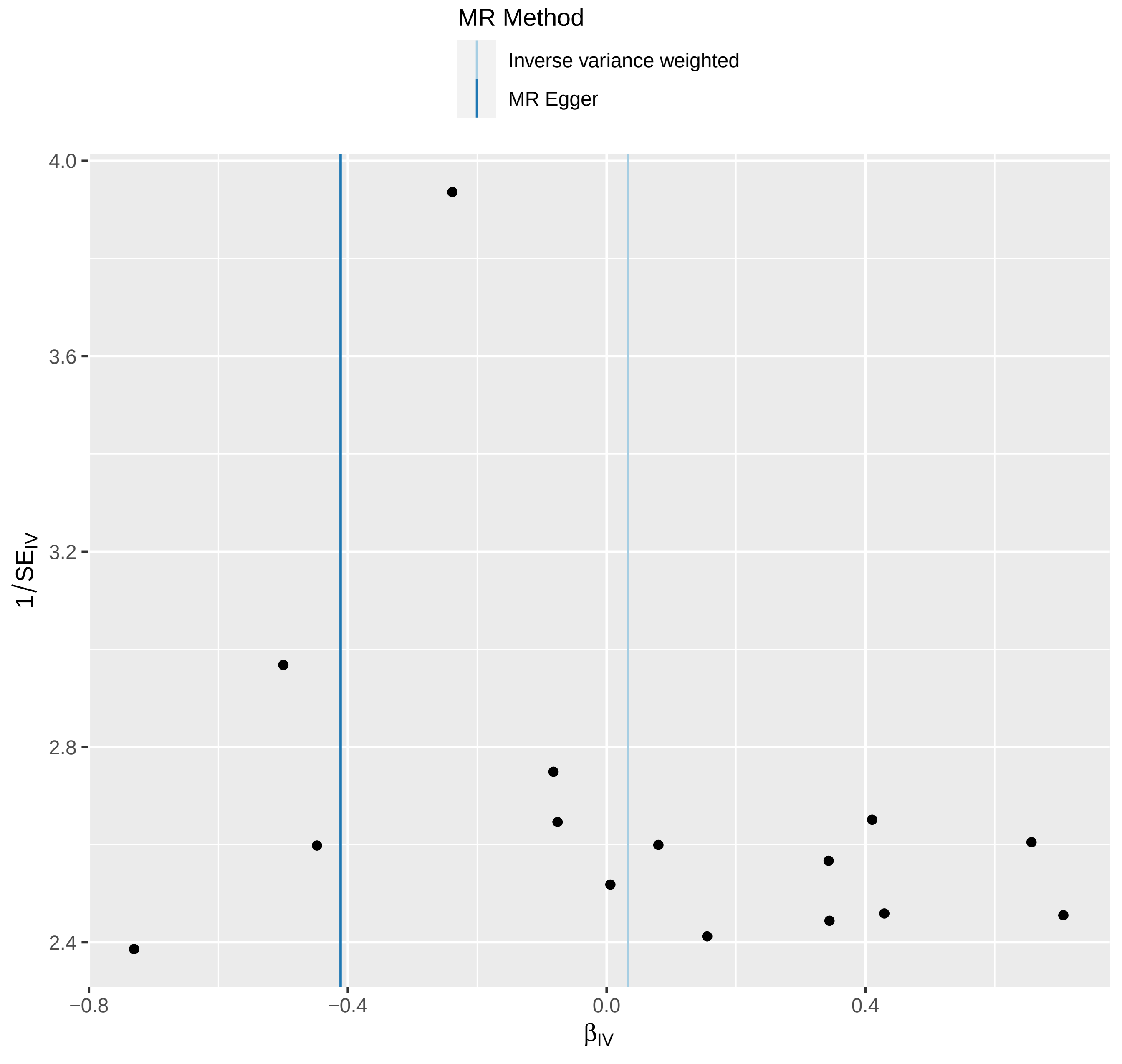

Batch 2−5203 : Gut microbiota abundance (genus Catenibacterium id.2153) on Ribonucleoside−diphosphate reductase subunit M2 B
with a threshold of 1e−05

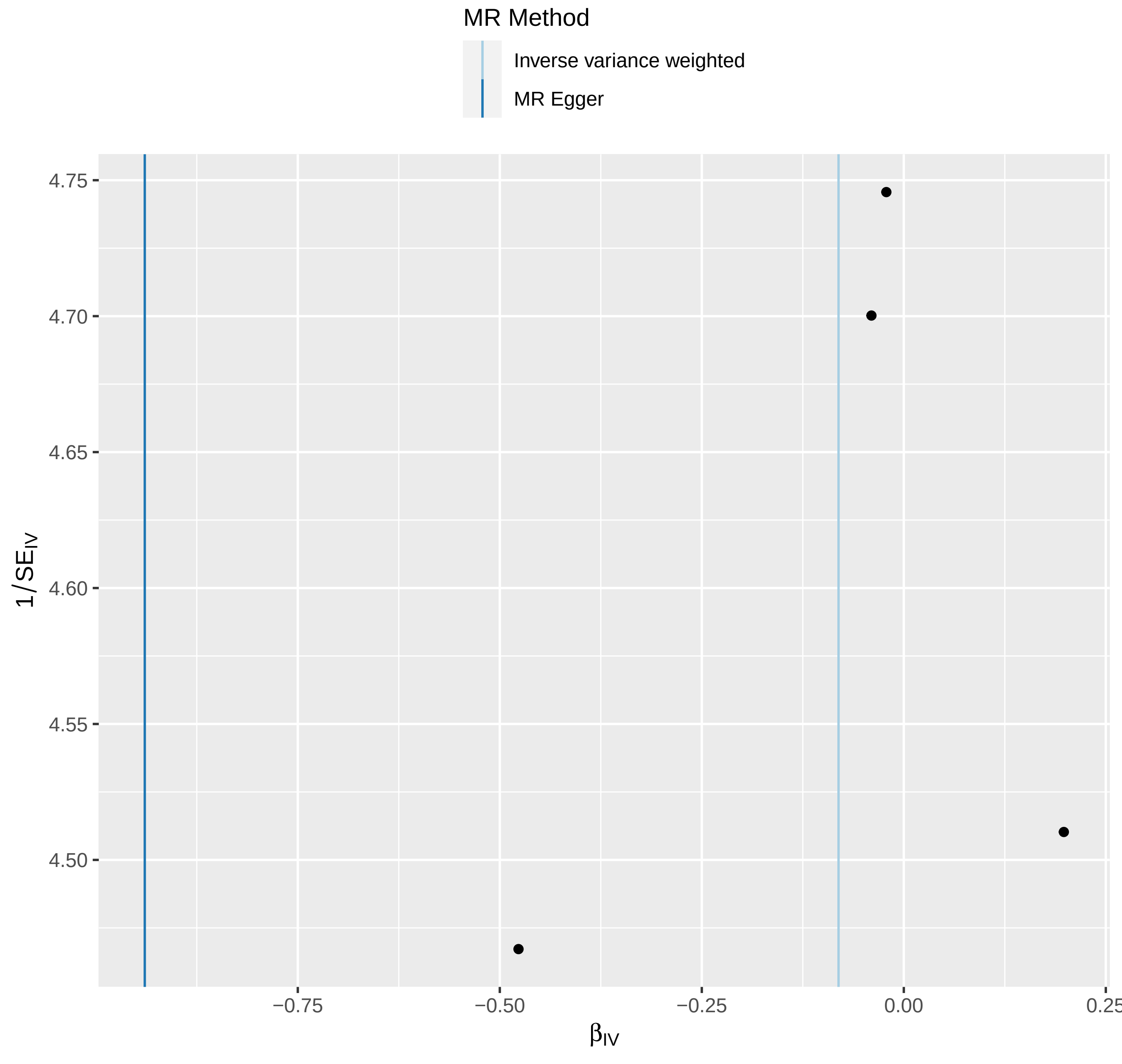

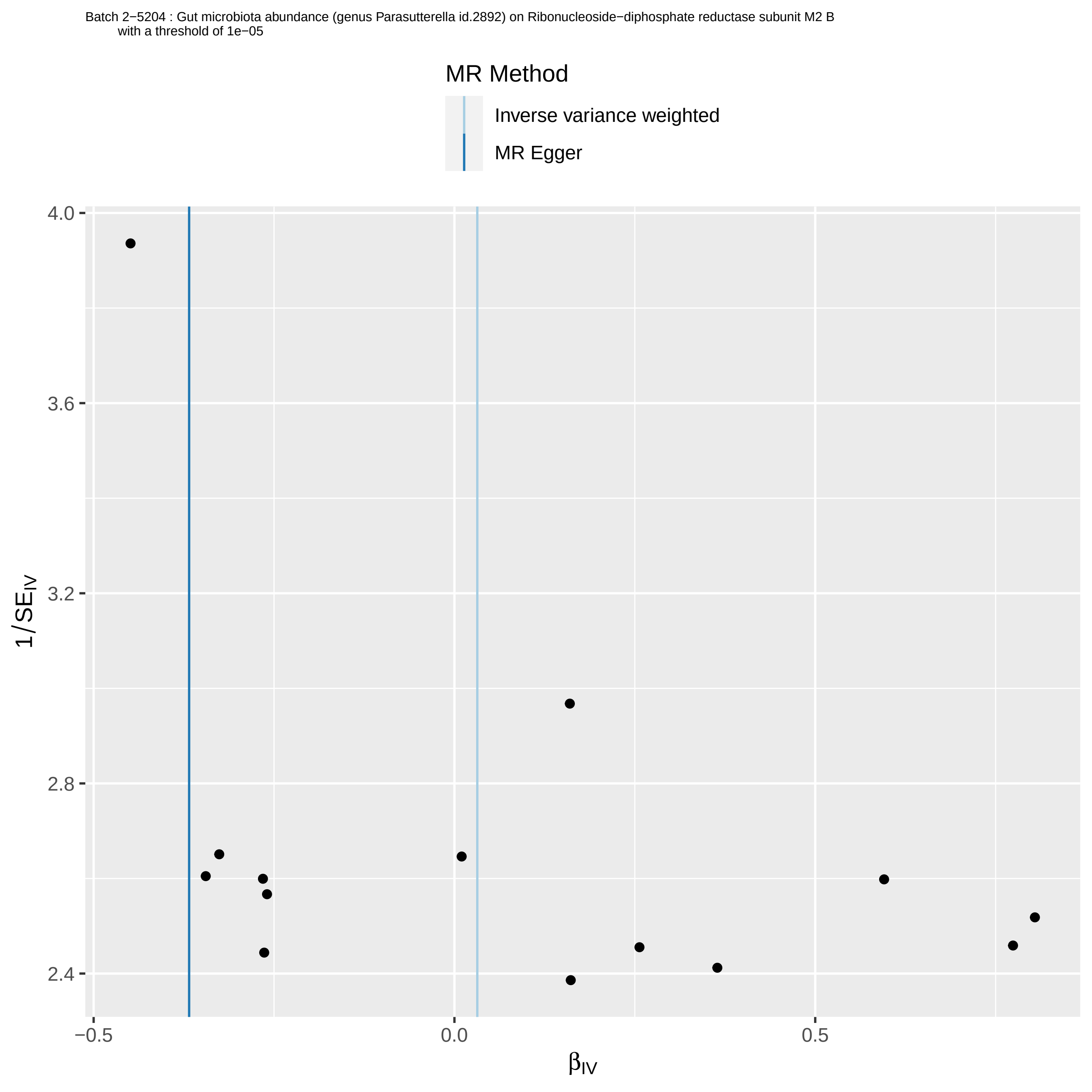
Batch 2−5204 : Gut microbiota abundance (genus Parasutterella id.2892) on Ribonucleoside−diphosphate reductase subunit M2 B
with a threshold of 1e−05
MR Method
Inverse variance weighted
MR Egger
4.0
3.6
3.2
2.8
2.4
1/SE$_{IV}$
−0.5
0.0
0.5
β_{IV}

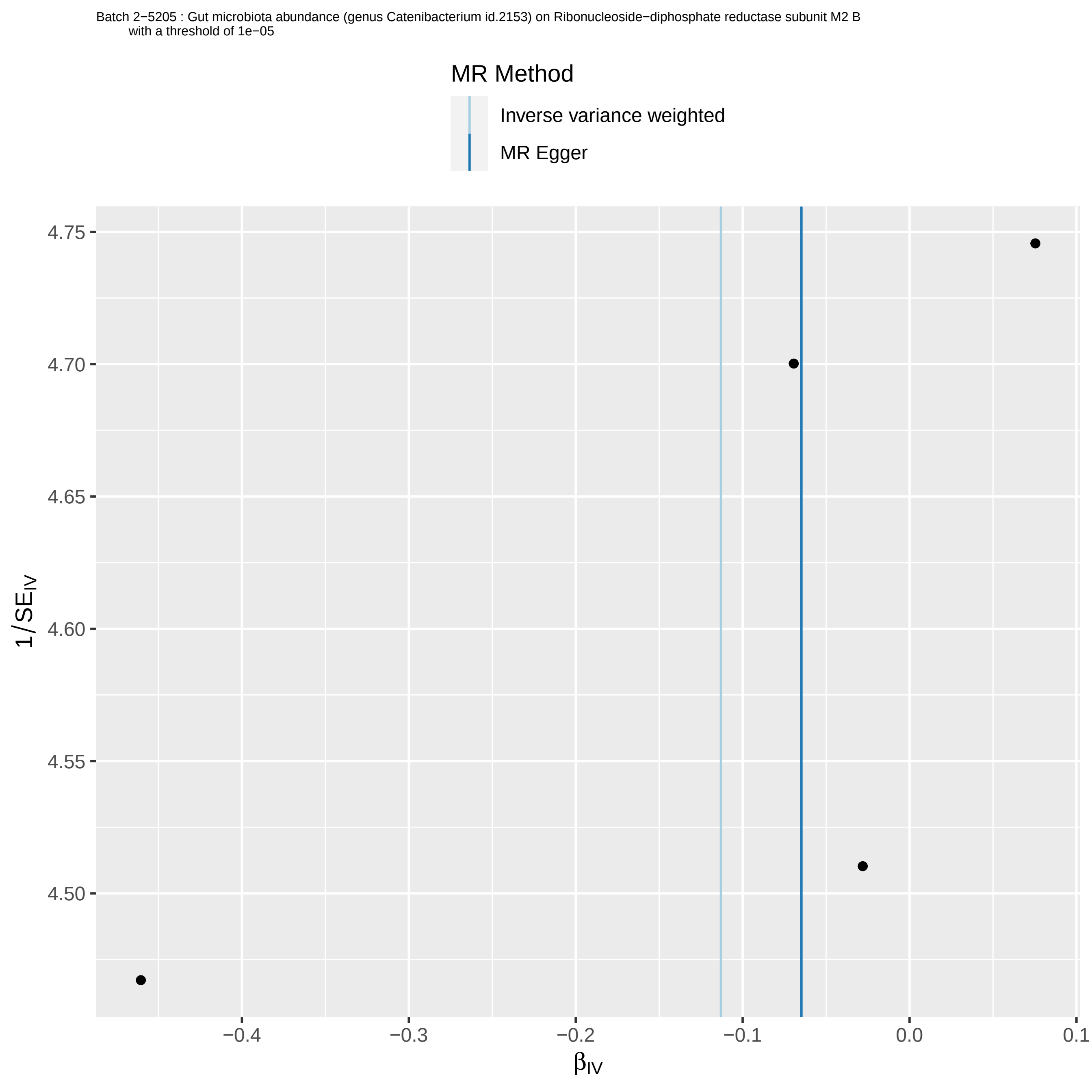

Batch 2−5205 : Gut microbiota abundance (genus Catenibacterium id.2153) on Ribonucleoside−diphosphate reductase subunit M2 B
with a threshold of 1e−05
MR Method
Inverse variance weighted
MR Egger
4.75
4.70
4.65
4.60
4.55
4.50
1/SE_IV
−0.4
−0.3
−0.2
−0.1
0.0
0.1
β_IV

Batch 2−5206 : Gut microbiota abundance (genus Parasutterella id.2892) on Ribonucleoside−diphosphate reductase subunit M2 B
with a threshold of 1e−05

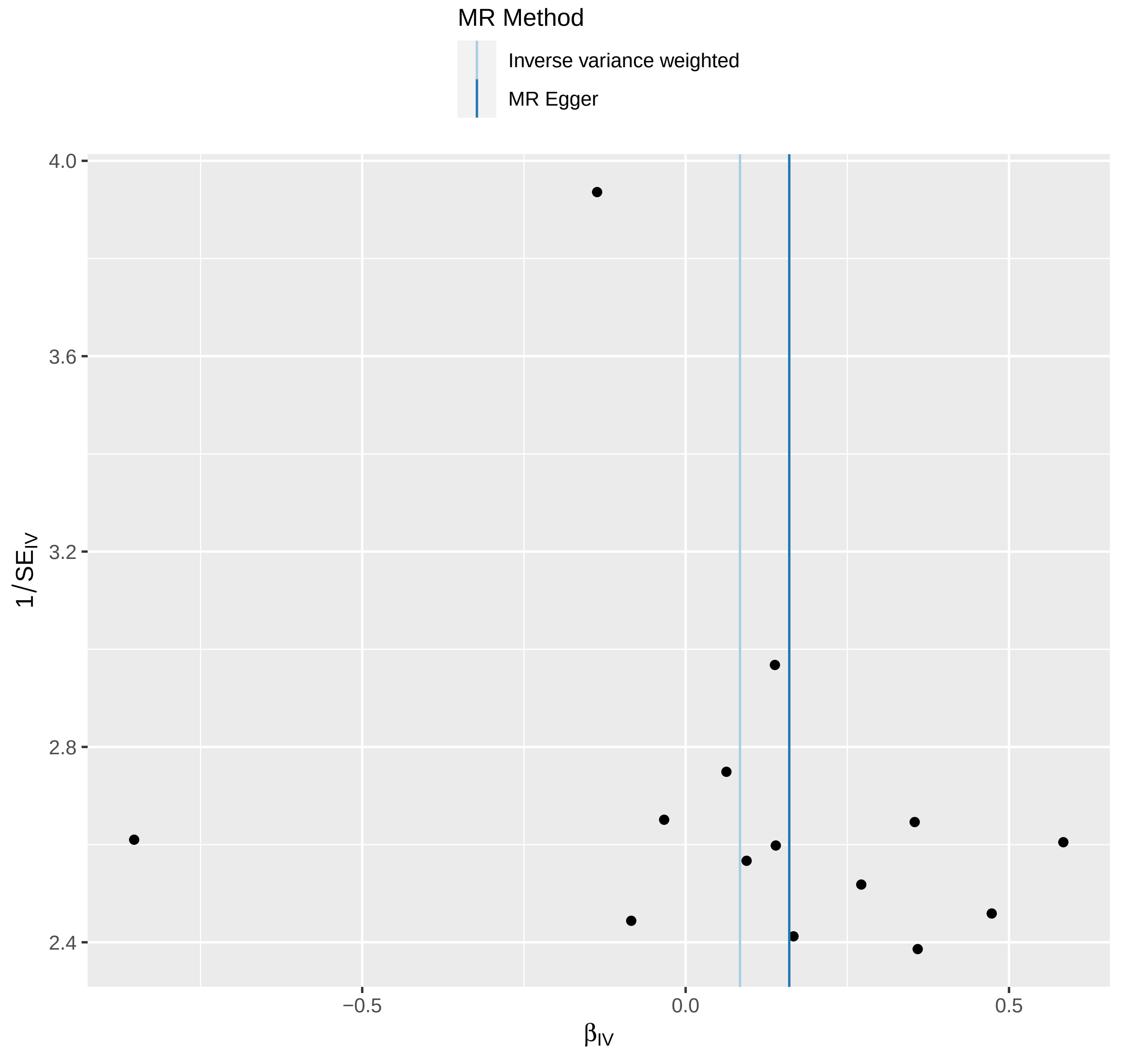

Batch 2−5207 : Gut microbiota abundance (genus Catenibacterium id.2153) on Retinoschisin
with a threshold of 1e−05

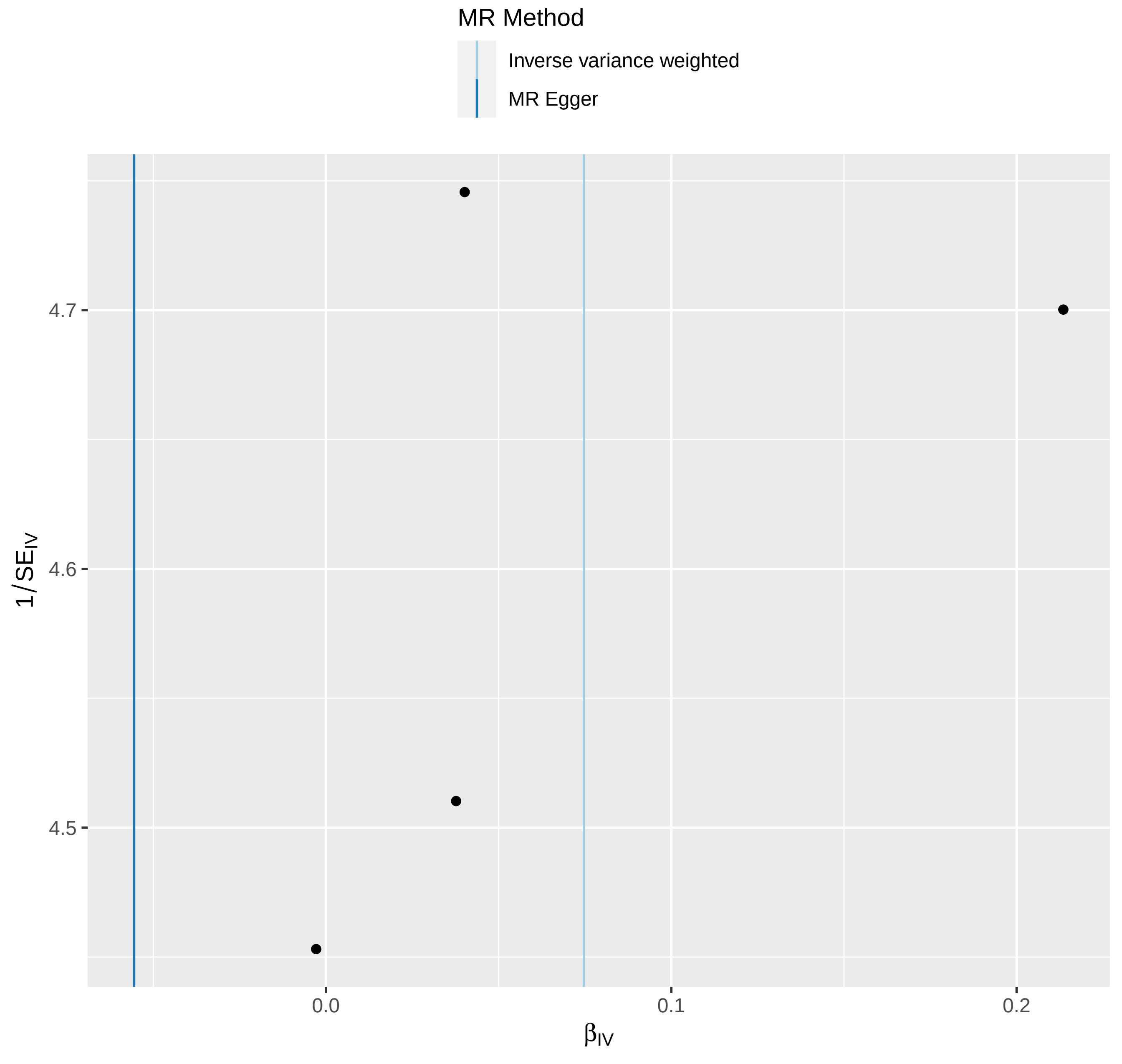

Batch 2−5208 : Gut microbiota abundance (genus Parasutterella id.2892) on Retinoschisin
with a threshold of 1e−05

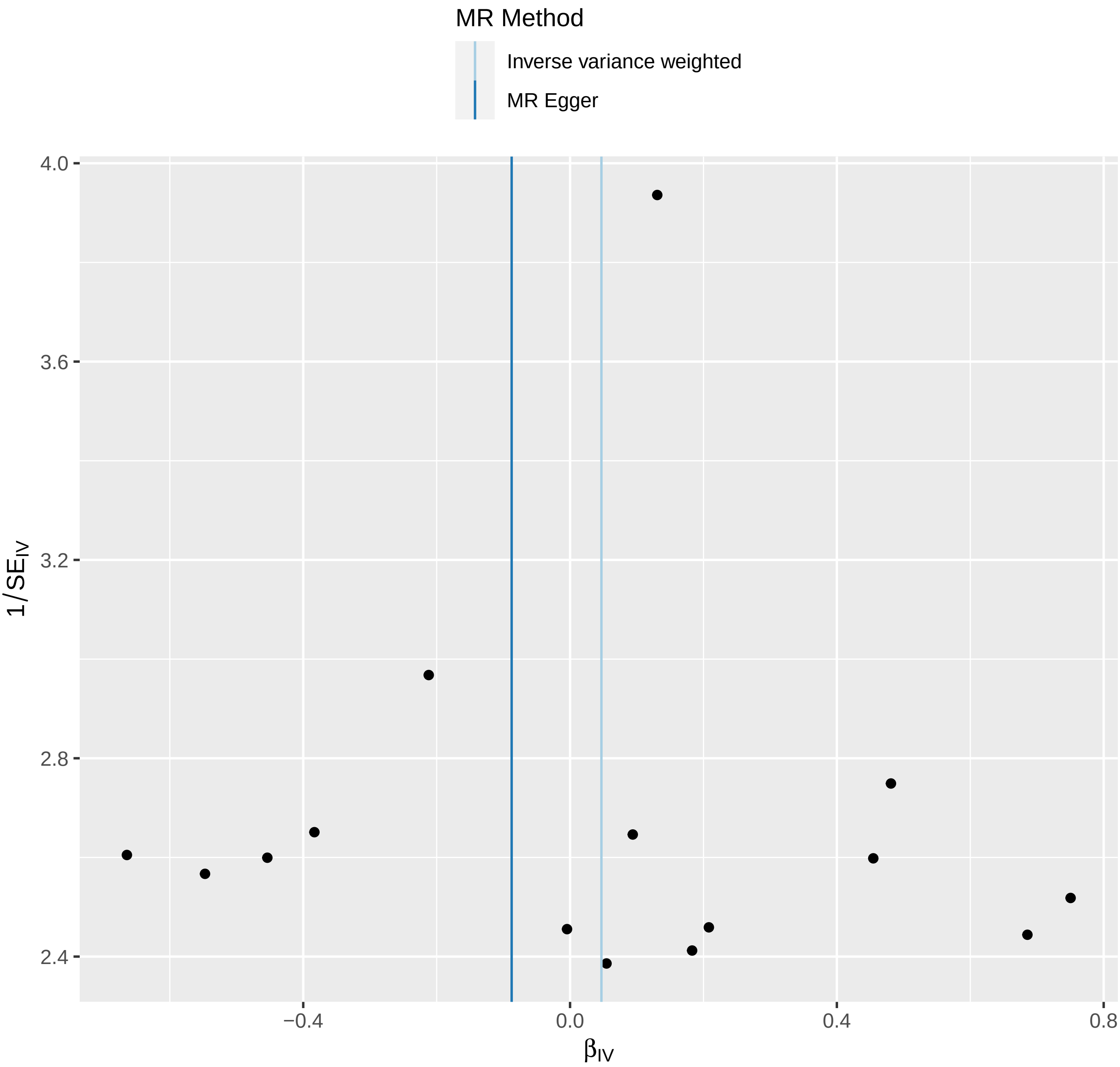

Batch 2−5209 : Gut microbiota abundance (genus Catenibacterium id.2153) on R−spondin−3
with a threshold of 1e−05

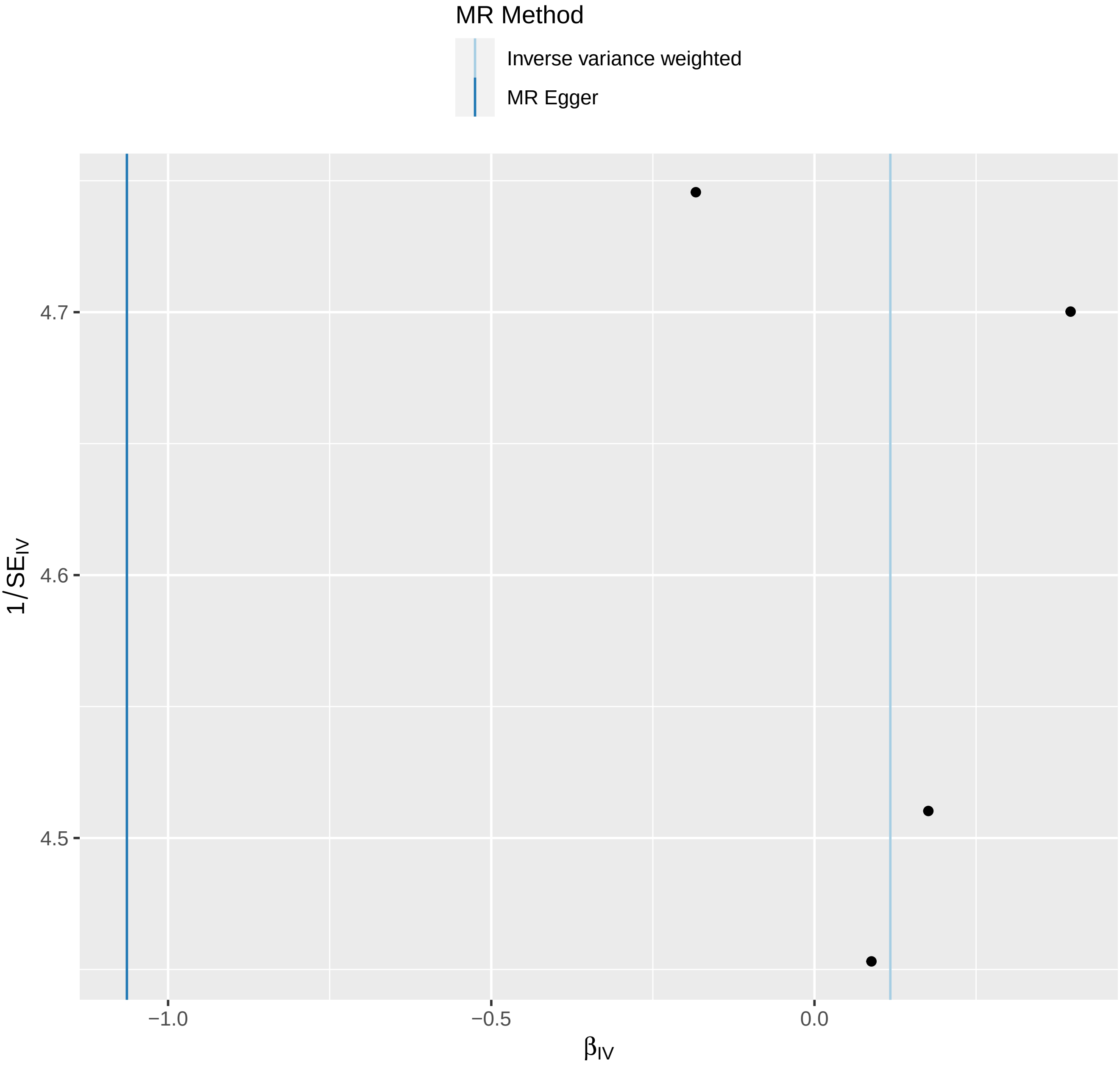

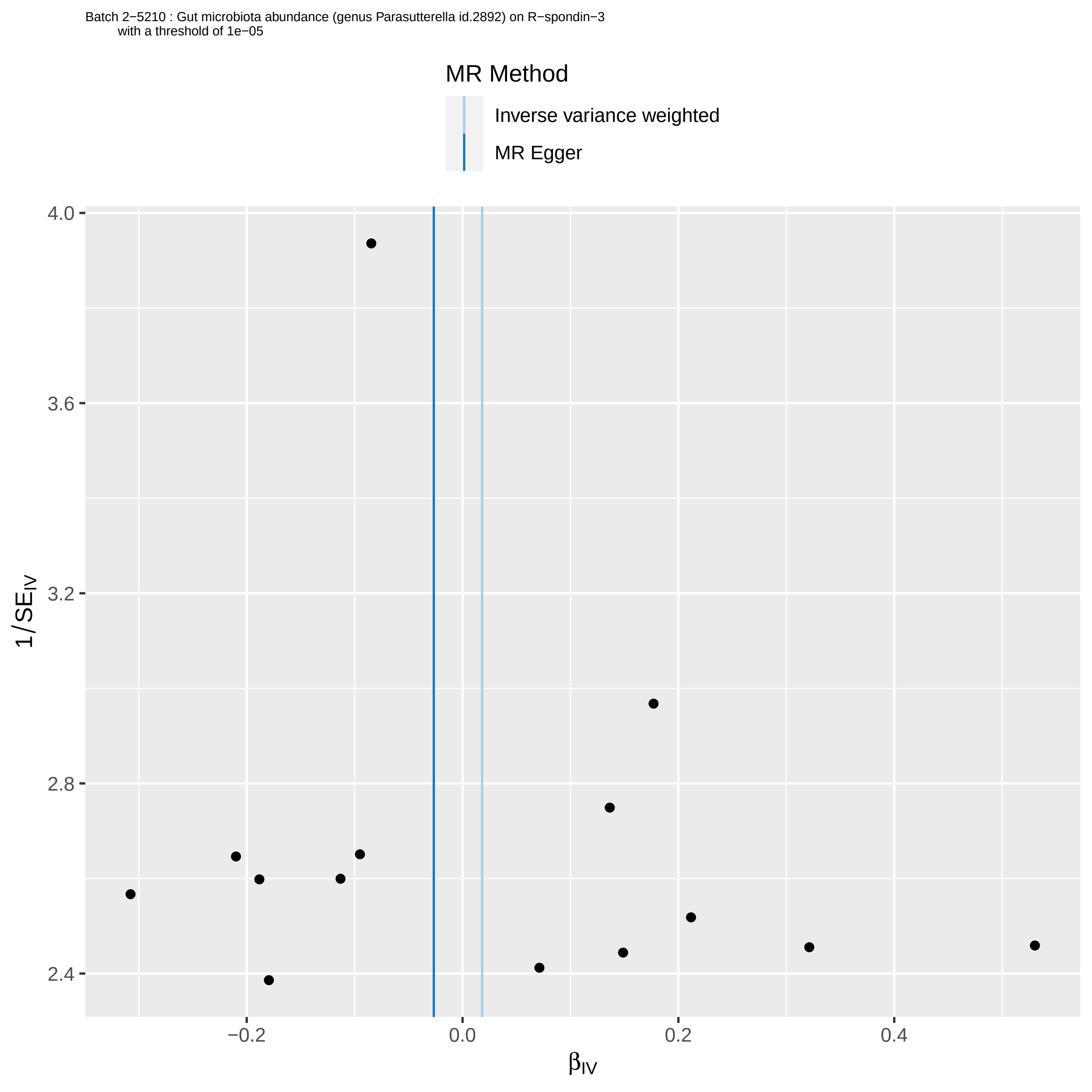
Batch 2−5210 : Gut microbiota abundance (genus Parasutterella id.2892) on R−spondin−3
with a threshold of 1e−05
MR Method
Inverse variance weighted
MR Egger
4.0
3.6
3.2
2.8
2.4
1/SE_IV
−0.2
0.0
0.2
0.4
β_IV

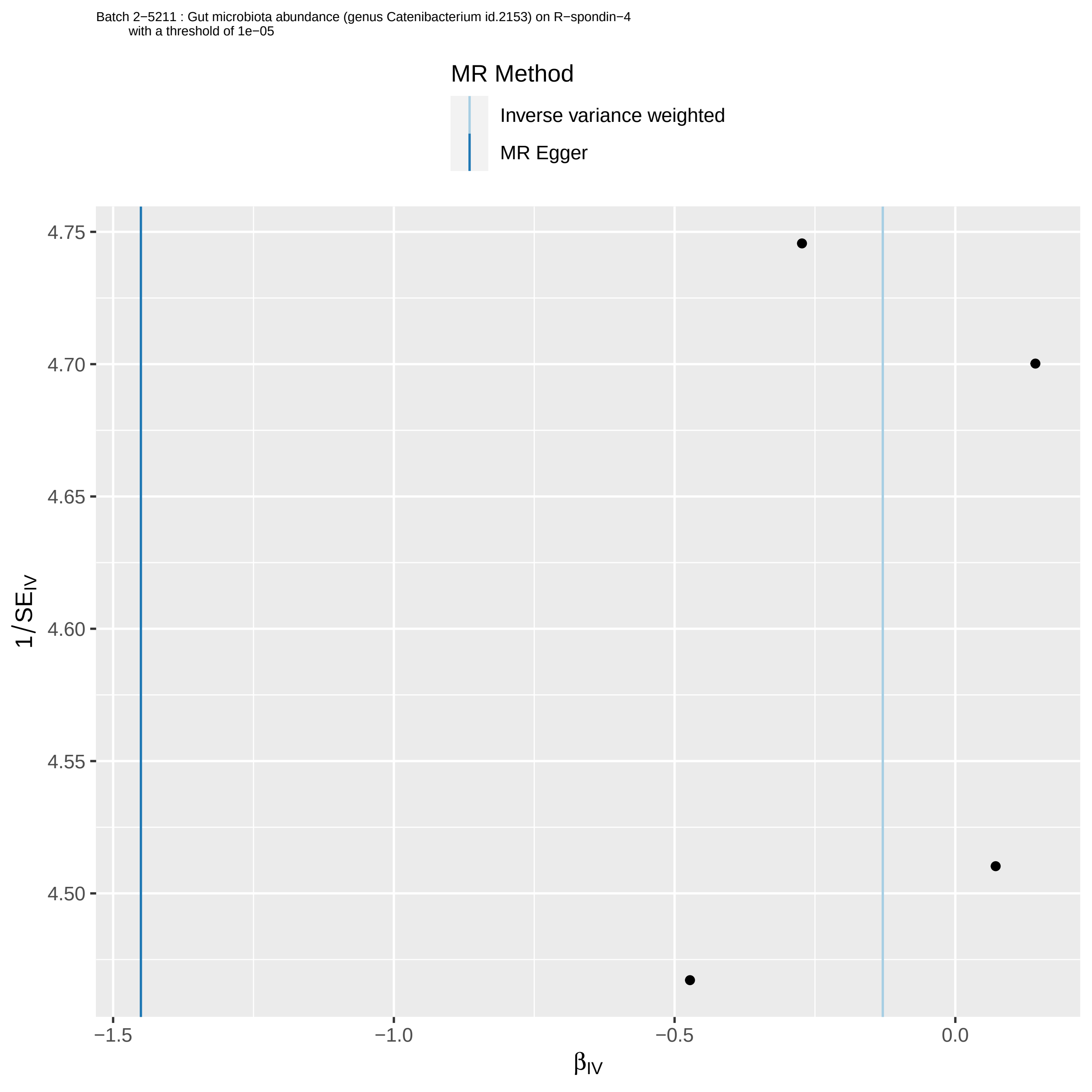
Batch 2−5211 : Gut microbiota abundance (genus Catenibacterium id.2153) on R−spondin−4
with a threshold of 1e−05
MR Method
Inverse variance weighted
MR Egger
4.75
4.70
4.65
4.60
4.55
4.50
1/SE$_{IV}$
−1.5
−1.0
−0.5
0.0
β$_{IV}$

Batch 2−5212 : Gut microbiota abundance (genus Parasutterella id.2892) on R−spondin−4
with a threshold of 1e−05

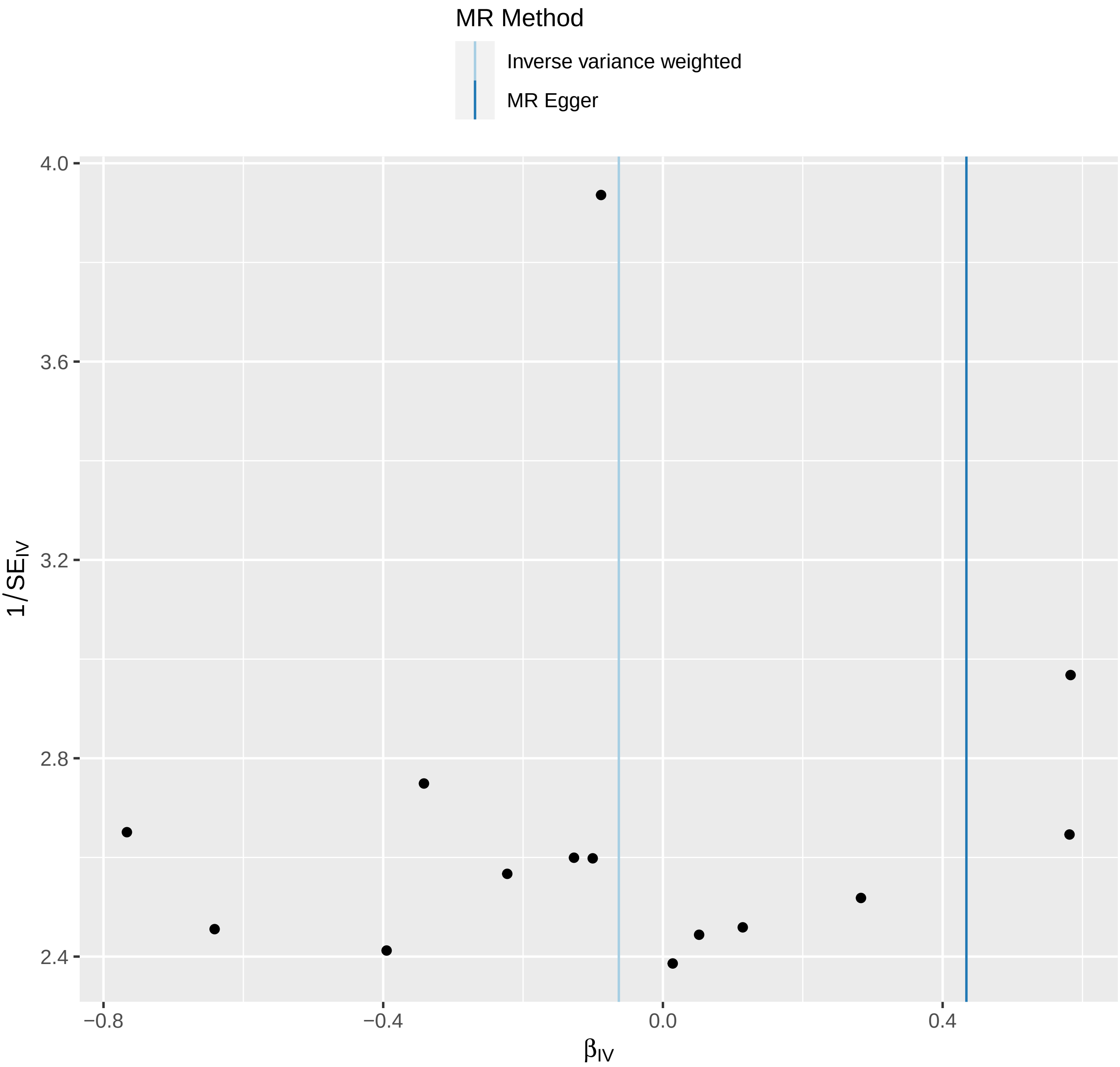

Batch 2−5213 : Gut microbiota abundance (genus Catenibacterium id.2153) on Arginine/serine−rich protein 1
with a threshold of 1e−05

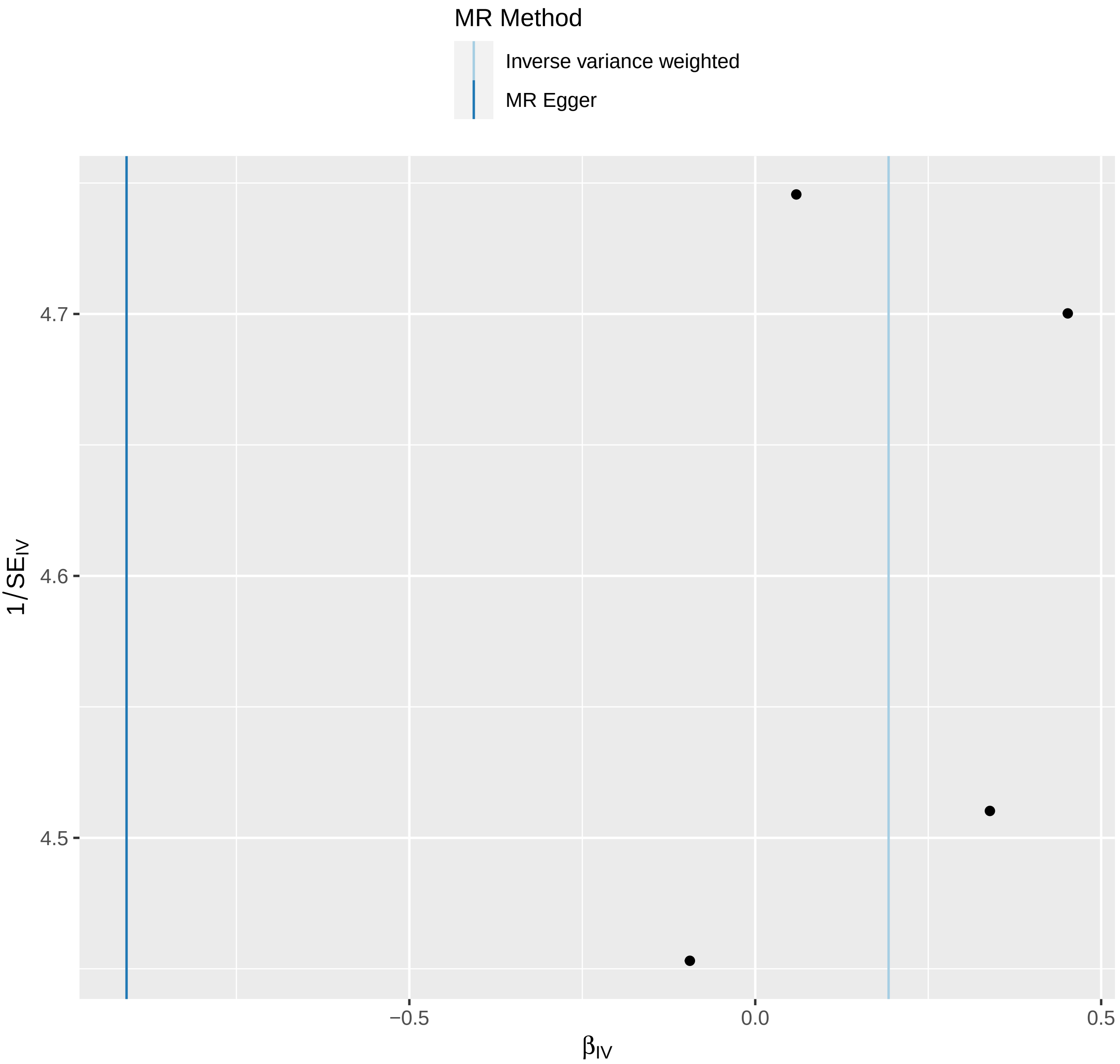

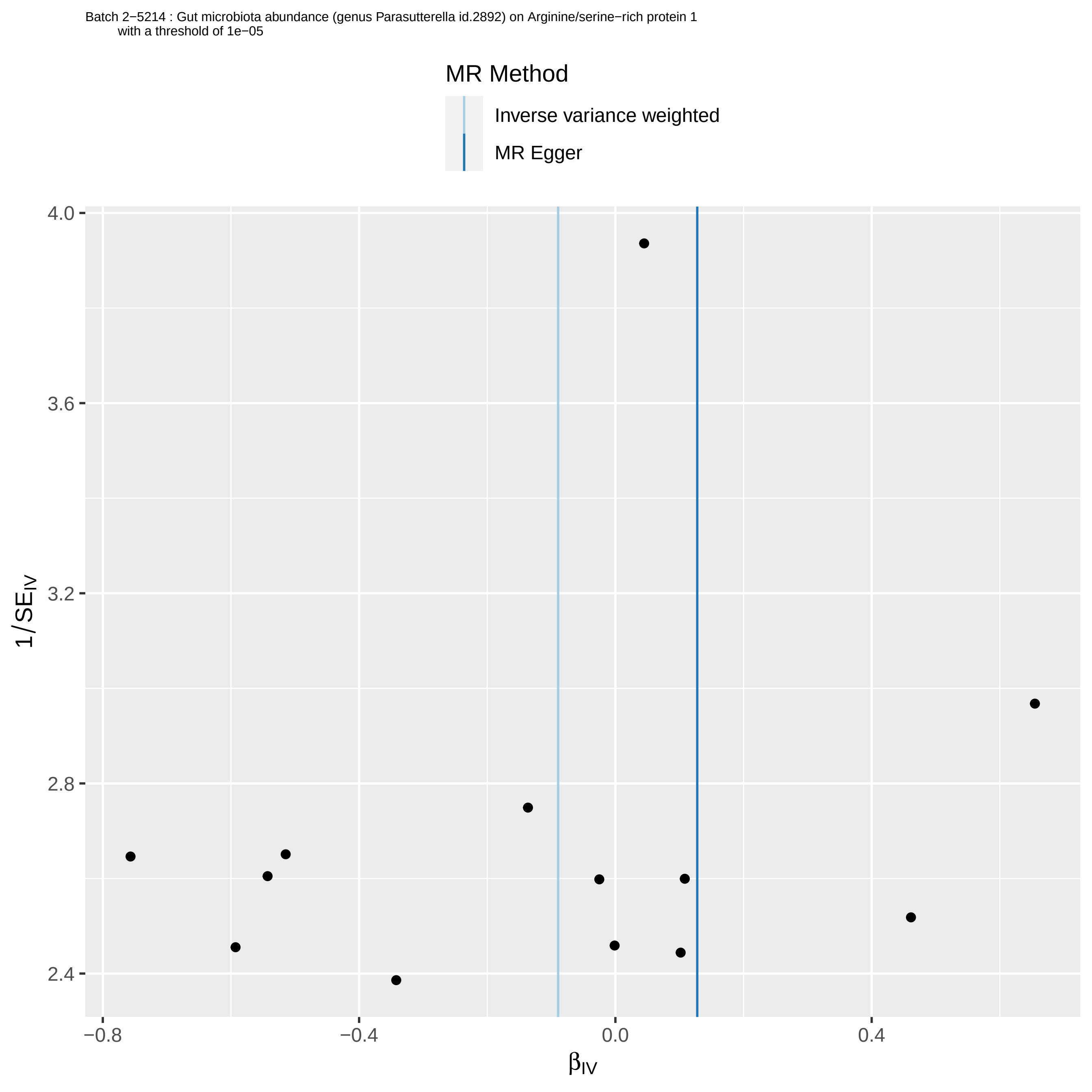
Batch 2−5214 : Gut microbiota abundance (genus Parasutterella id.2892) on Arginine/serine−rich protein 1
with a threshold of 1e−05
MR Method
Inverse variance weighted
MR Egger
4.0
3.6
3.2
2.8
2.4
1/SE_IV
−0.8
−0.4
0.0
0.4
β_IV

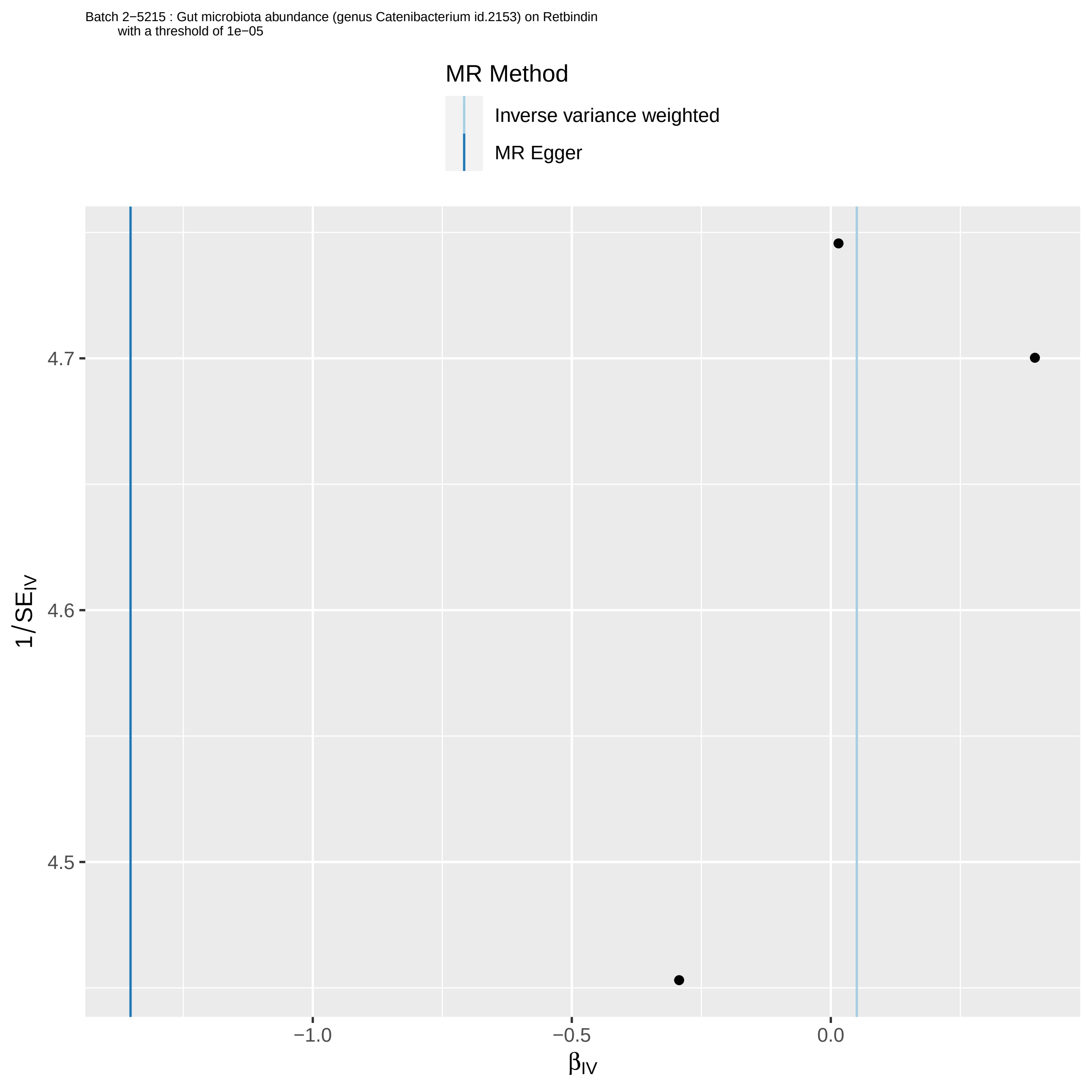
Batch 2−5215 : Gut microbiota abundance (genus Catenibacterium id.2153) on Retbindin
with a threshold of 1e−05
MR Method
Inverse variance weighted
MR Egger
4.7
4.6
4.5
$1/SE_{IV}$
−1.0
−0.5
0.0
β_{IV}

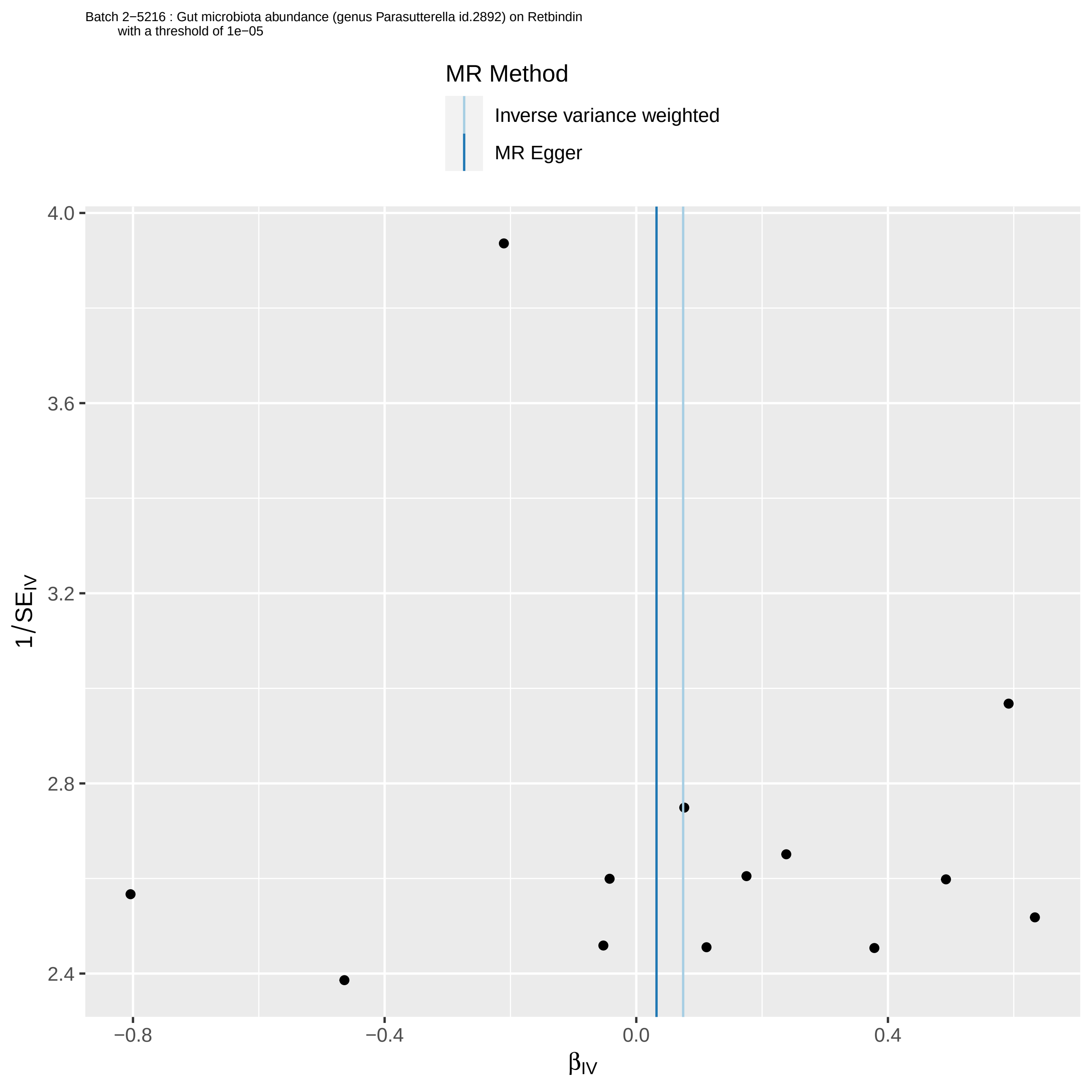
Batch 2−5216 : Gut microbiota abundance (genus Parasutterella id.2892) on Retbindin
with a threshold of 1e−05
MR Method
Inverse variance weighted
MR Egger
1/SE_IV
4.0
3.6
3.2
2.8
2.4
−0.8
−0.4
0.0
0.4
β_IV

Batch 2−5217 : Gut microbiota abundance (genus Catenibacterium id.2153) on Reticulon−4 receptor
with a threshold of 1e−05

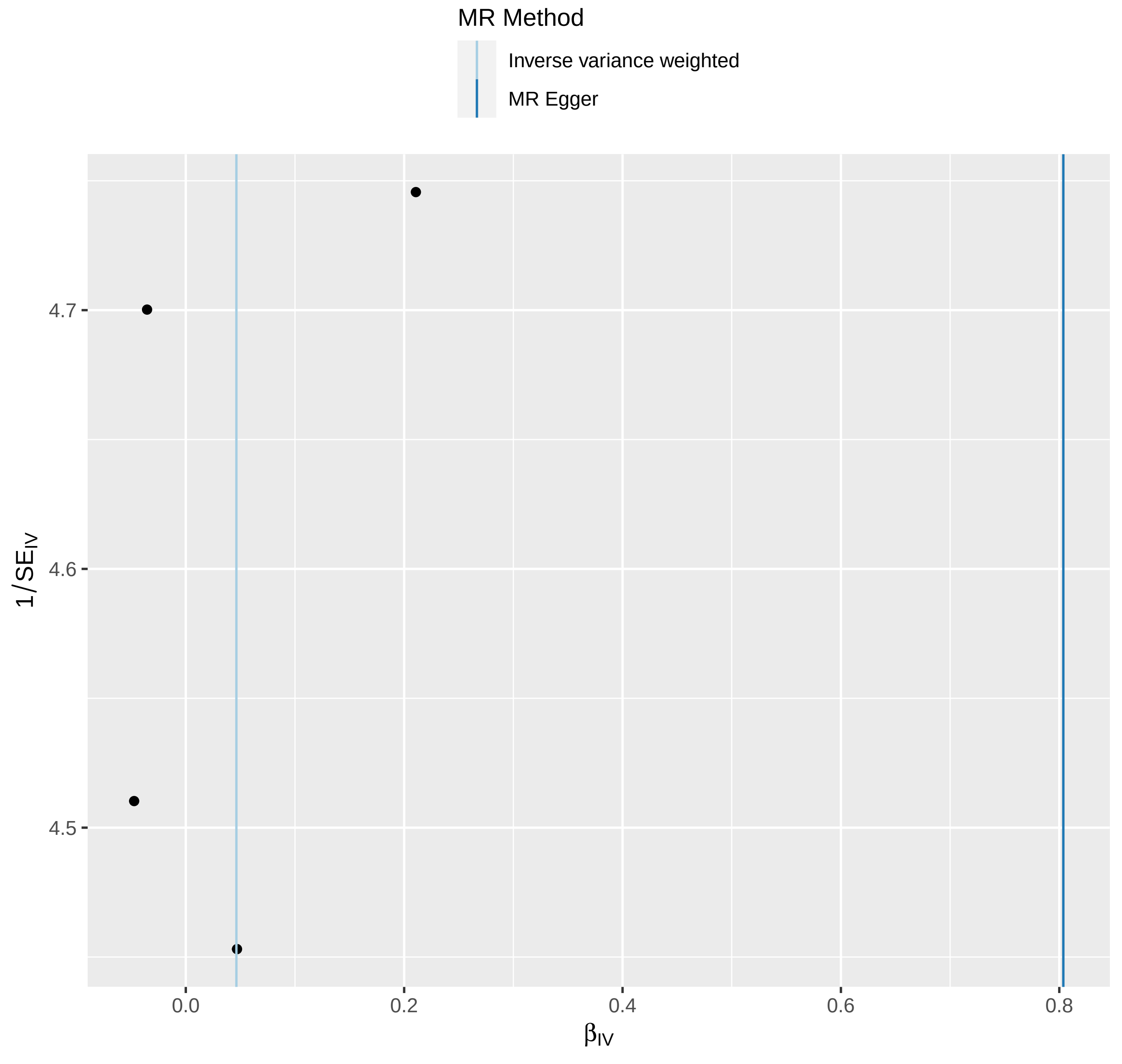

Batch 2−5218 : Gut microbiota abundance (genus Parasutterella id.2892) on Reticulon−4 receptor
with a threshold of 1e−05

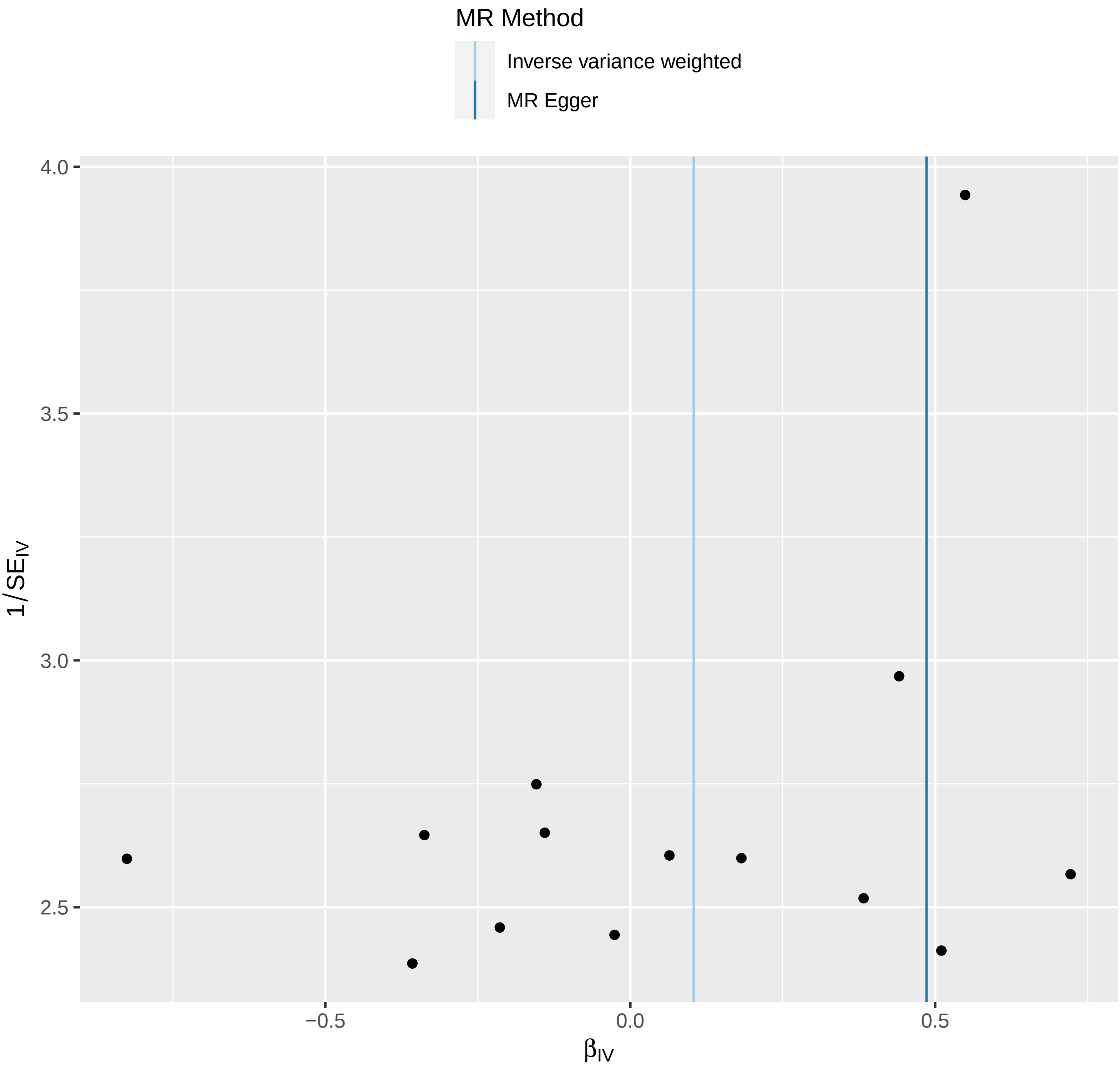

Batch 2−5219 : Gut microbiota abundance (genus Catenibacterium id.2153) on RUN and FYVE domain−containing protein 1
with a threshold of 1e−05

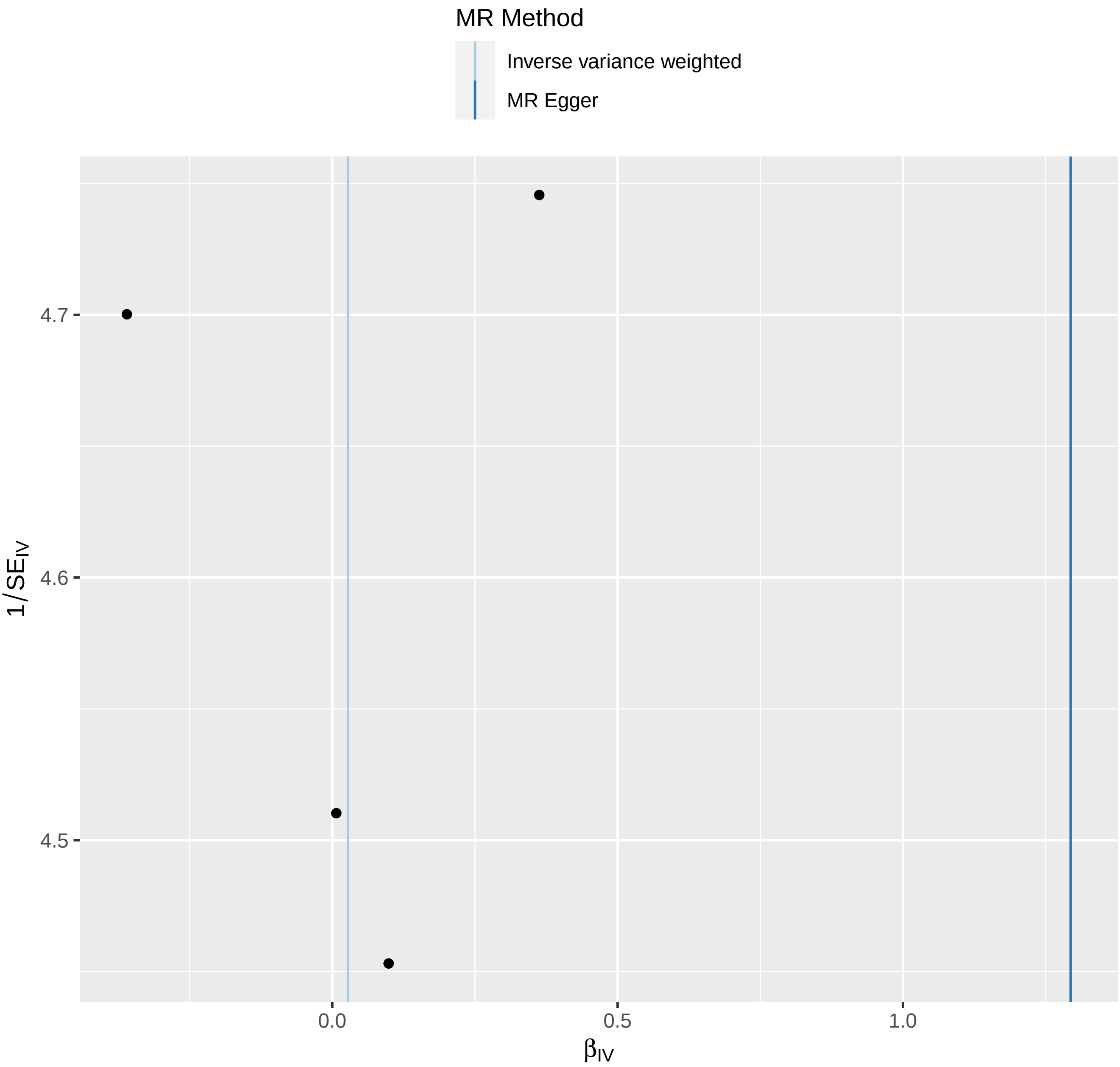

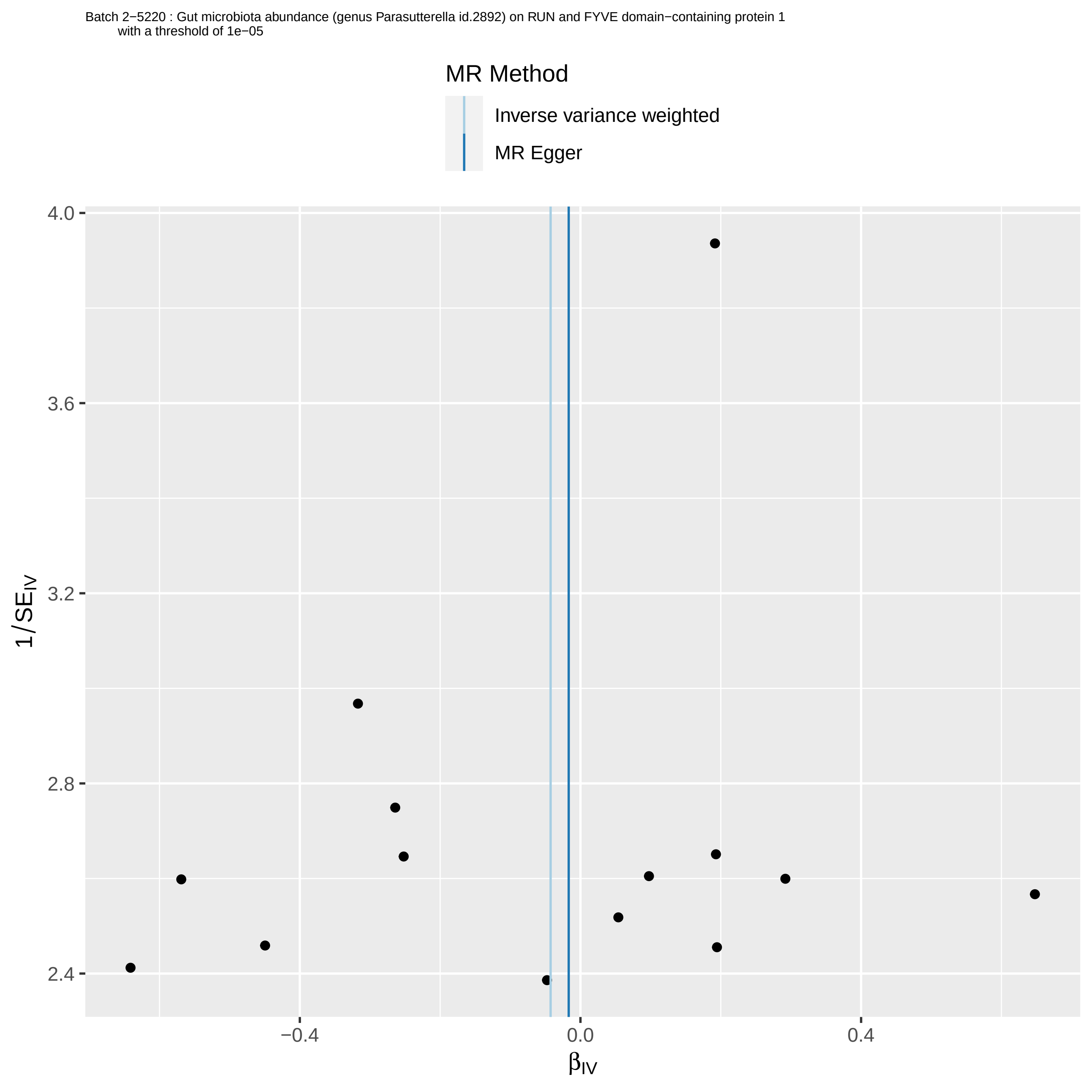

Batch 2−5220 : Gut microbiota abundance (genus Parasutterella id.2892) on RUN and FYVE domain−containing protein 1
with a threshold of 1e−05
MR Method
Inverse variance weighted
MR Egger
4.0
3.6
3.2
2.8
2.4
1/SE_IV
−0.4
0.0
0.4
β_IV

Batch 2−5221 : Gut microbiota abundance (genus Catenibacterium id.2153) on Runt−related transcription factor 3
with a threshold of 1e−05

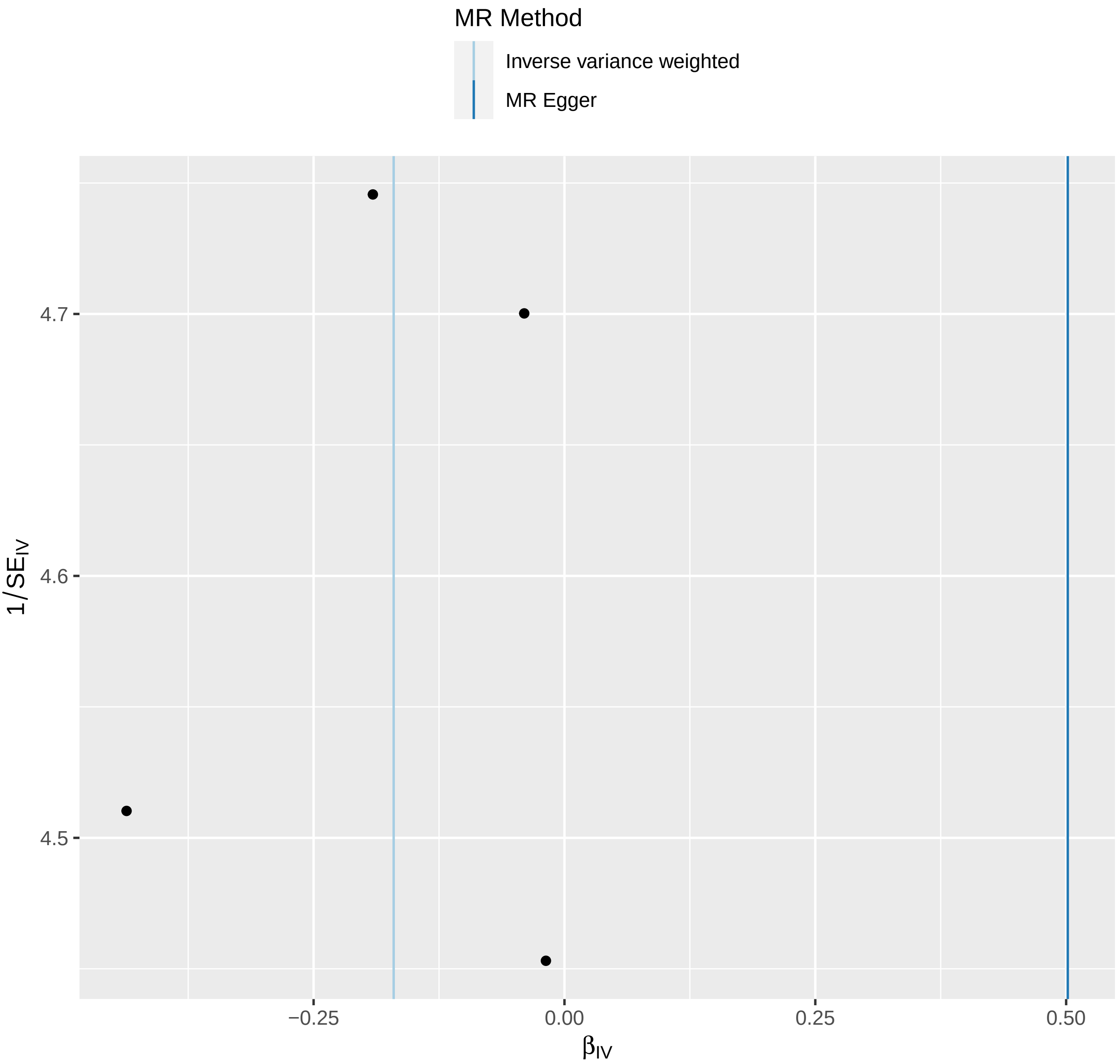

Batch 2−5222 : Gut microbiota abundance (genus Parasutterella id.2892) on Runt−related transcription factor 3
with a threshold of 1e−05

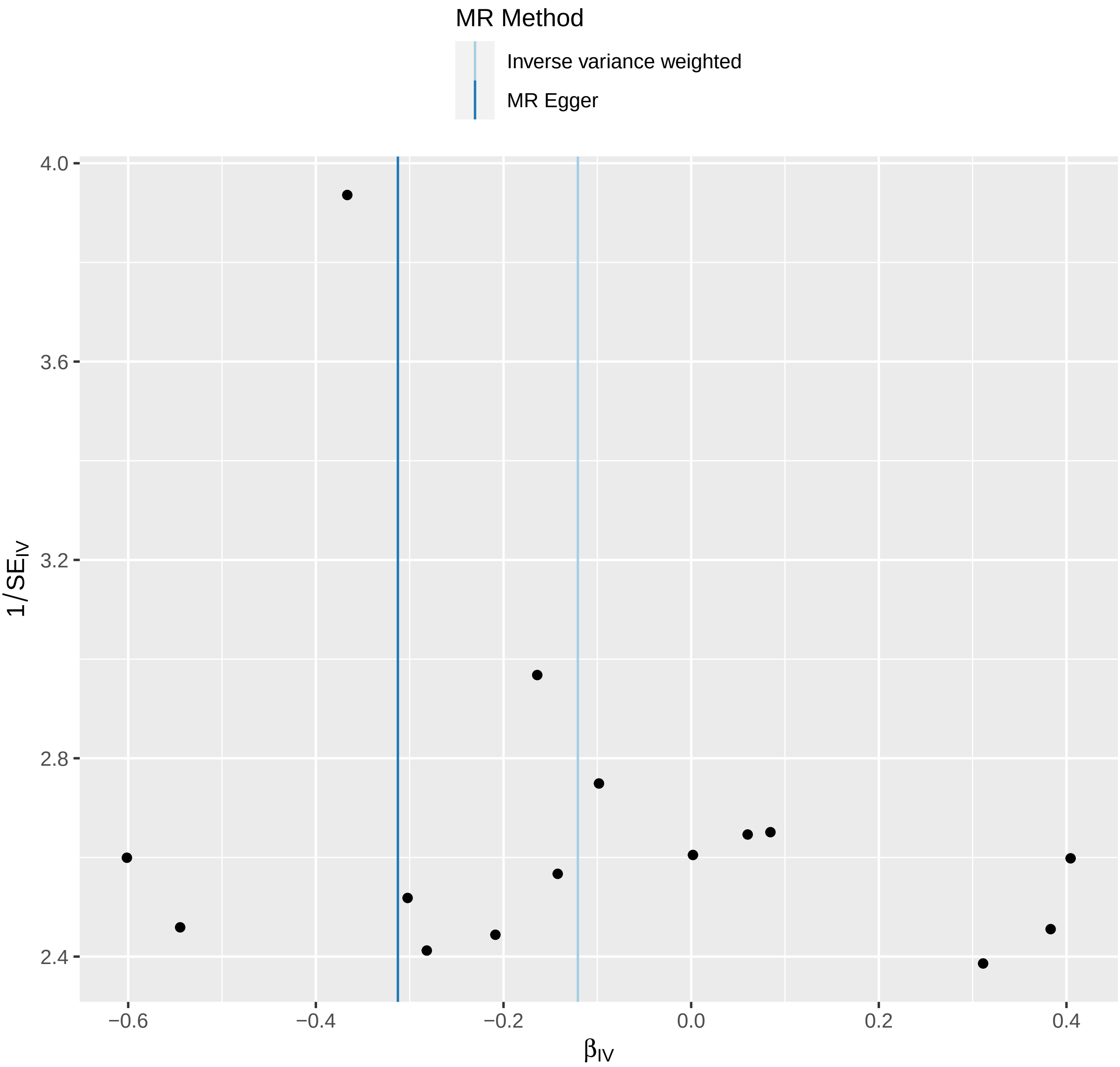

Batch 2−5223 : Gut microbiota abundance (genus Catenibacterium id.2153) on Relaxin receptor 1
with a threshold of 1e−05

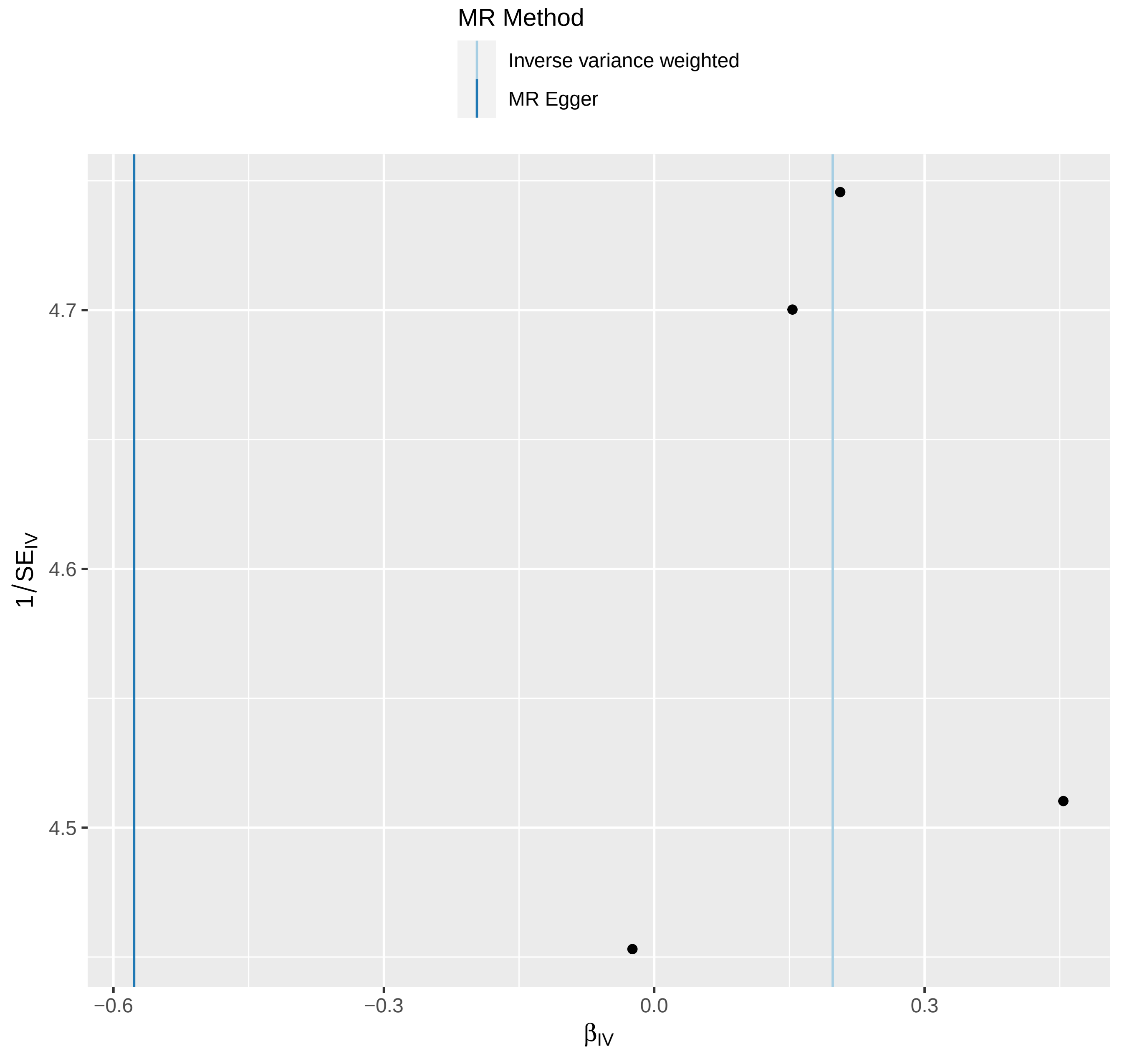

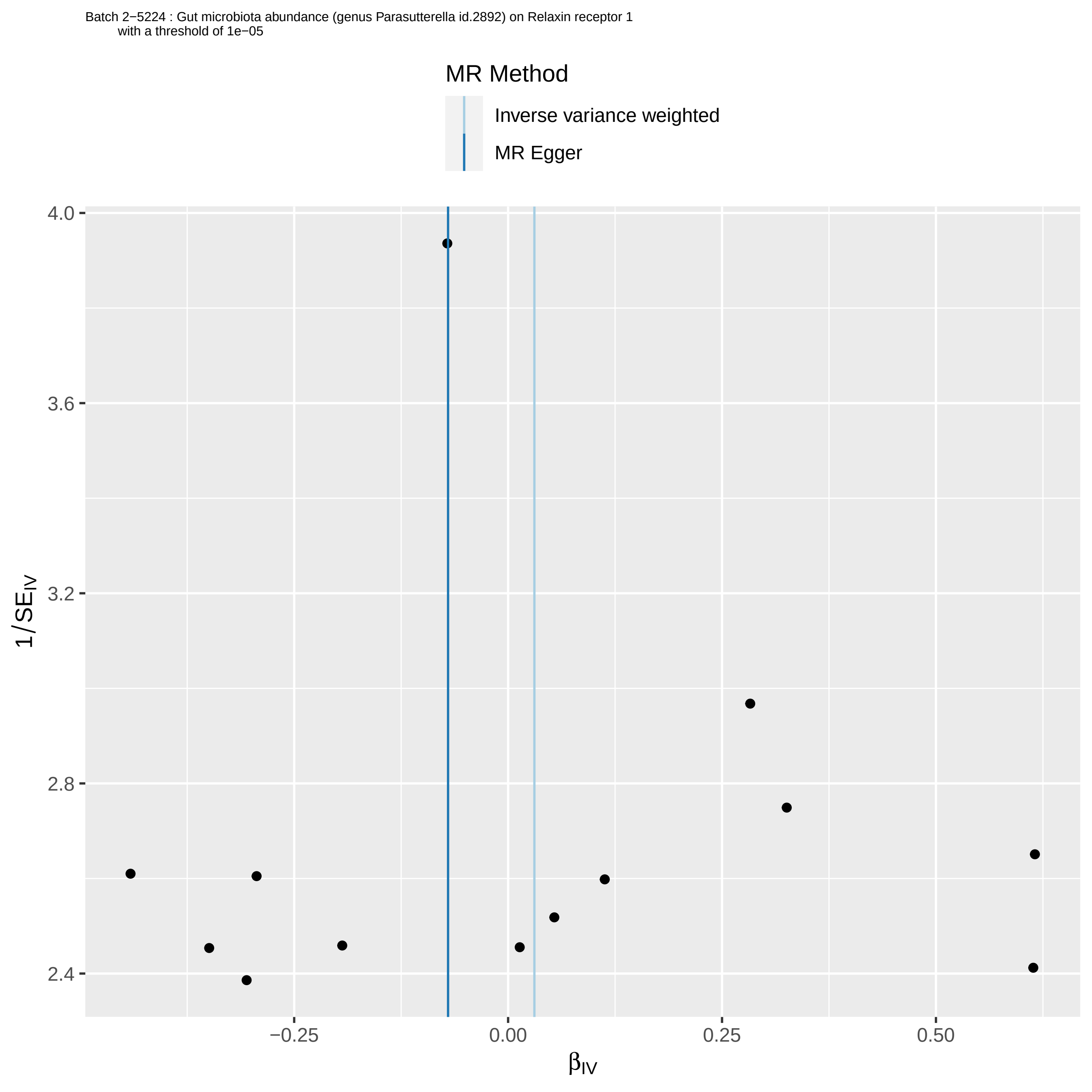
Batch 2−5224 : Gut microbiota abundance (genus Parasutterella id.2892) on Relaxin receptor 1
with a threshold of 1e−05
MR Method
Inverse variance weighted
MR Egger
4.0
3.6
3.2
2.8
2.4
1/SE$_{IV}$
−0.25
0.00
0.25
0.50
β$_{IV}$

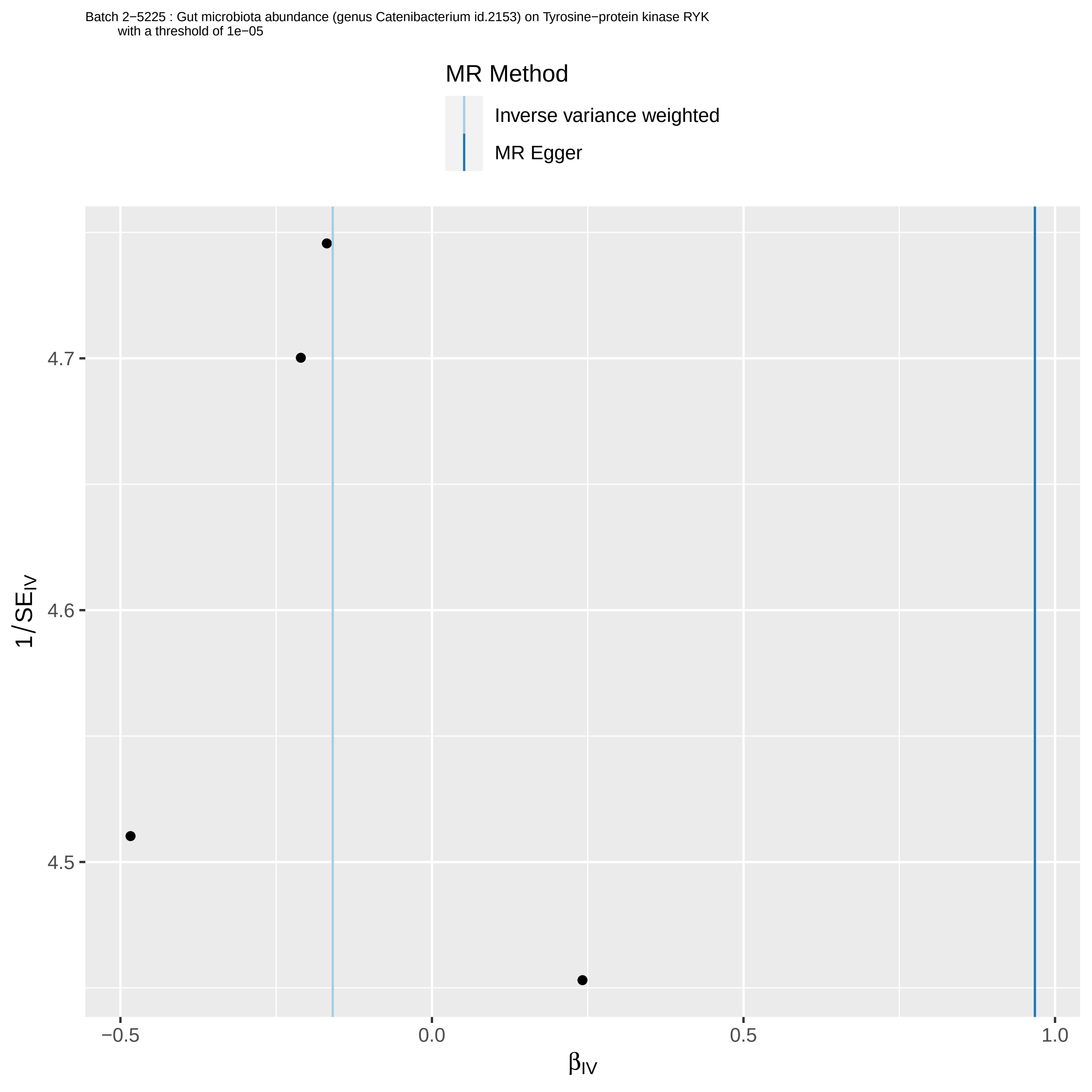
Batch 2−5225 : Gut microbiota abundance (genus Catenibacterium id.2153) on Tyrosine−protein kinase RYK
with a threshold of 1e−05
MR Method
Inverse variance weighted
MR Egger
4.7
4.6
4.5
1/SE_IV
−0.5
0.0
0.5
1.0
β_IV

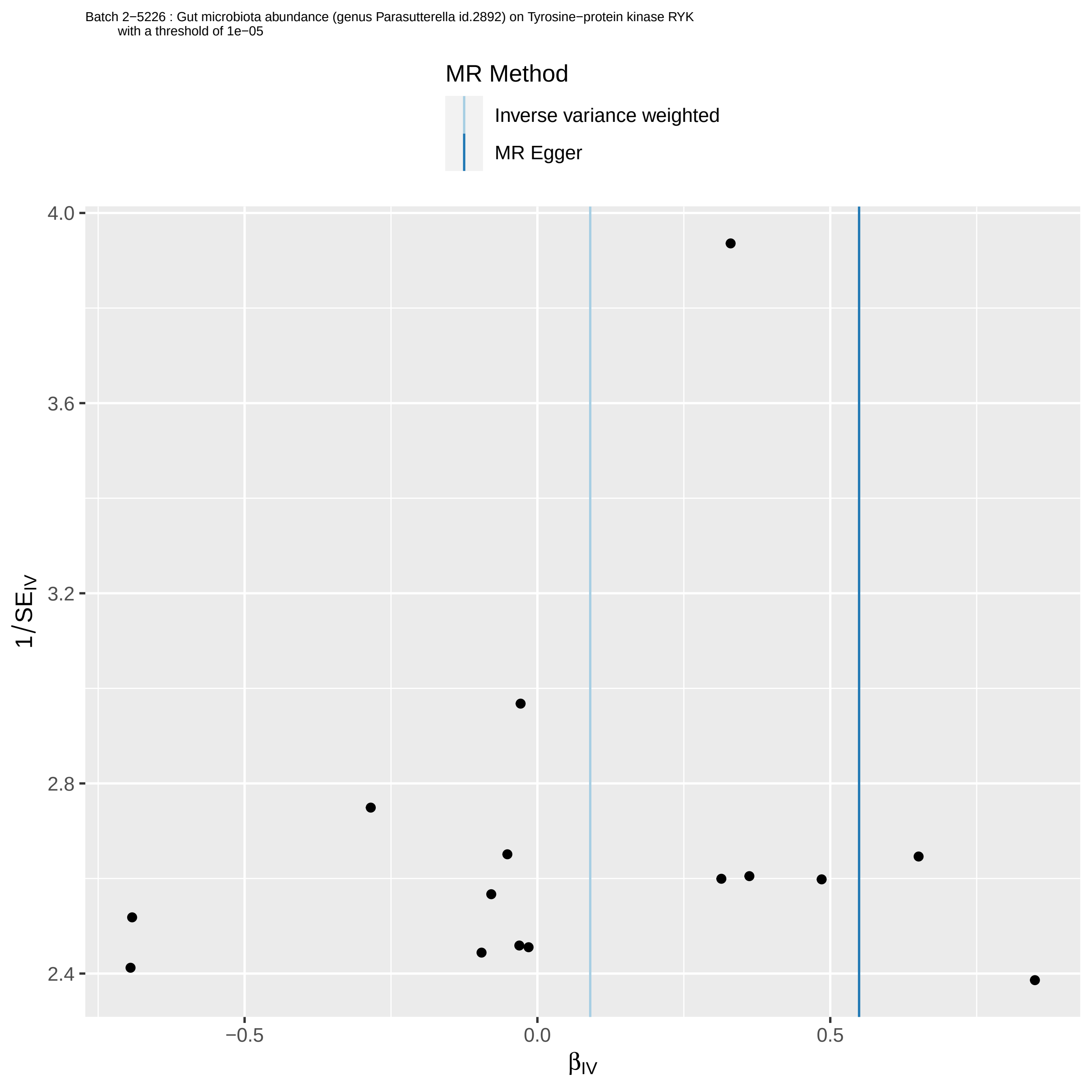
Batch 2−5226 : Gut microbiota abundance (genus Parasutterella id.2892) on Tyrosine−protein kinase RYK
with a threshold of 1e−05
MR Method
Inverse variance weighted
MR Egger
4.0
3.6
3.2
2.8
2.4
1/SE_IV
−0.5
0.0
0.5
β_IV

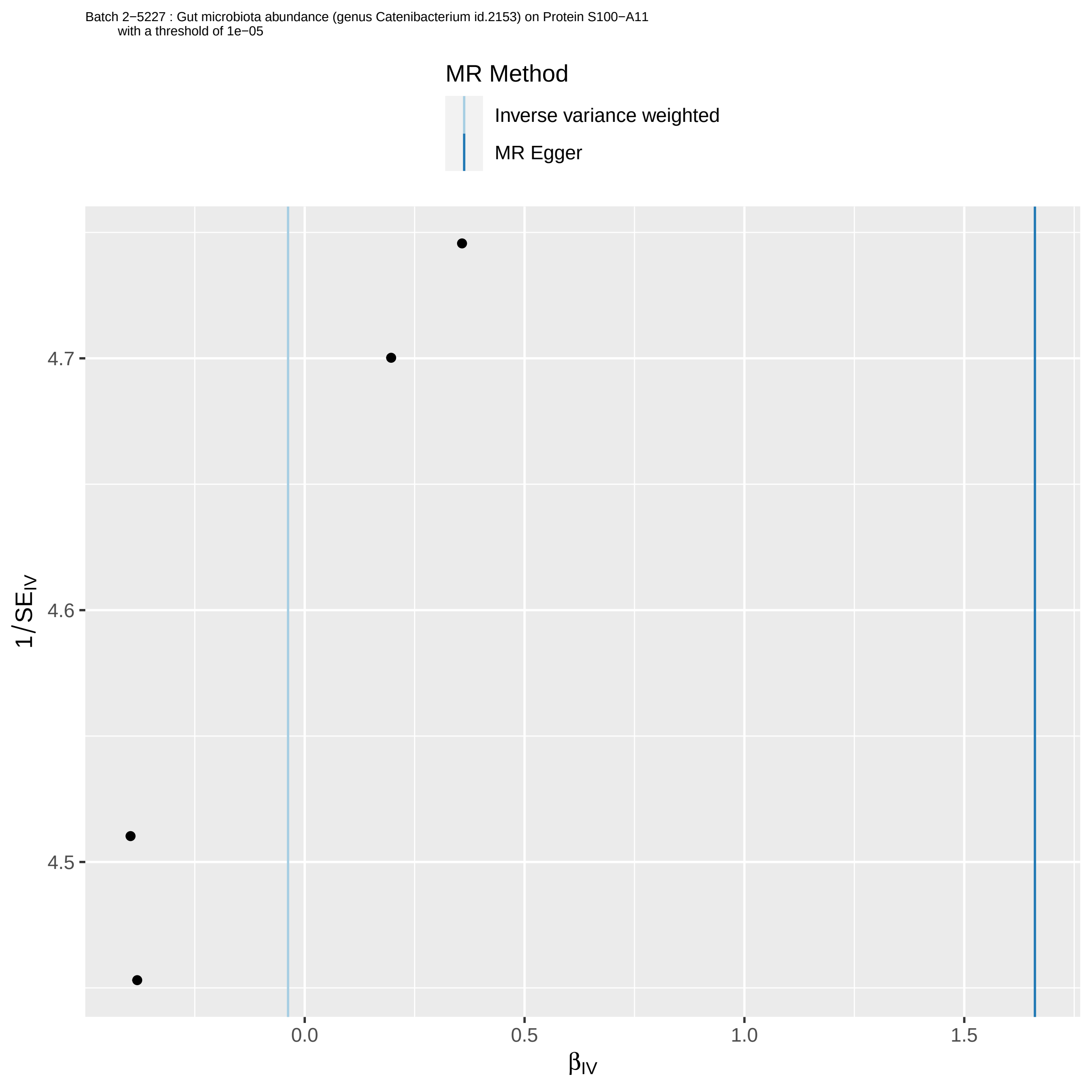
Batch 2−5227 : Gut microbiota abundance (genus Catenibacterium id.2153) on Protein S100−A11
with a threshold of 1e−05
MR Method
Inverse variance weighted
MR Egger
4.7
4.6
4.5
1/SE_IV
0.0
0.5
1.0
1.5
β_IV

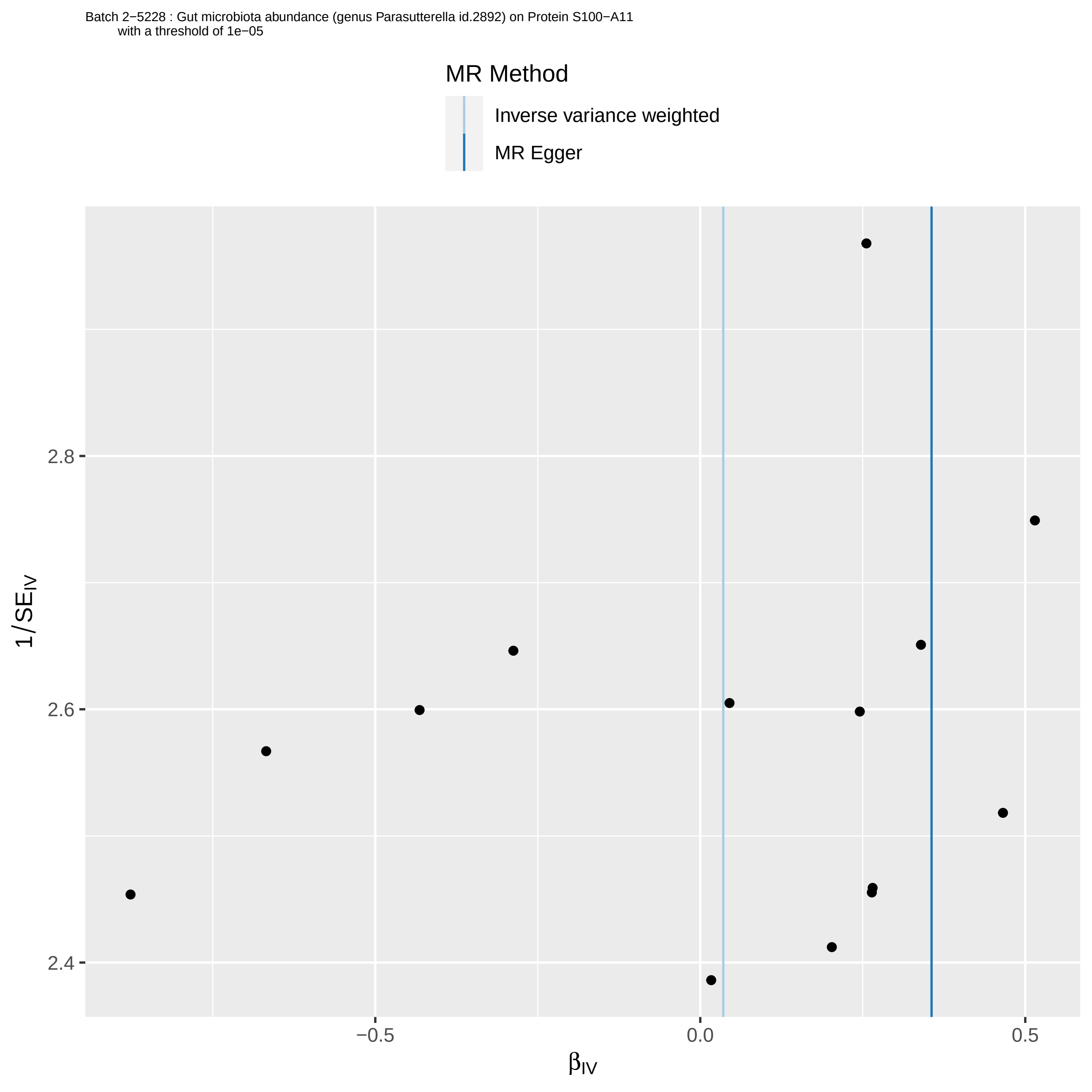

Batch 2−5228 : Gut microbiota abundance (genus Parasutterella id.2892) on Protein S100−A11
with a threshold of 1e−05
MR Method
Inverse variance weighted
MR Egger
2.8
2.6
2.4
1/SE_IV
−0.5
0.0
0.5
β_IV

Batch 2−5229 : Gut microbiota abundance (genus Catenibacterium id.2153) on Protein S100−A12
with a threshold of 1e−05

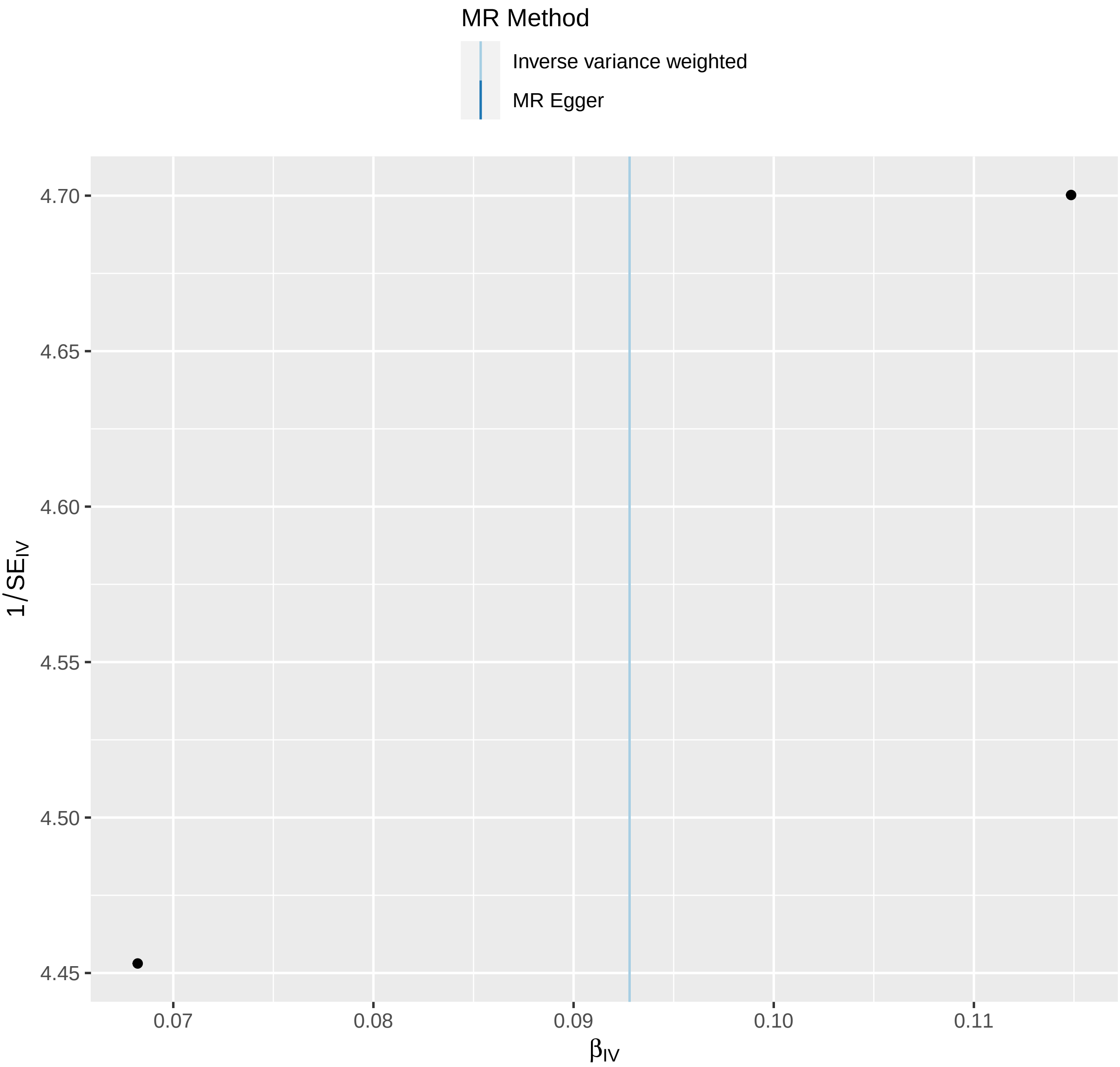

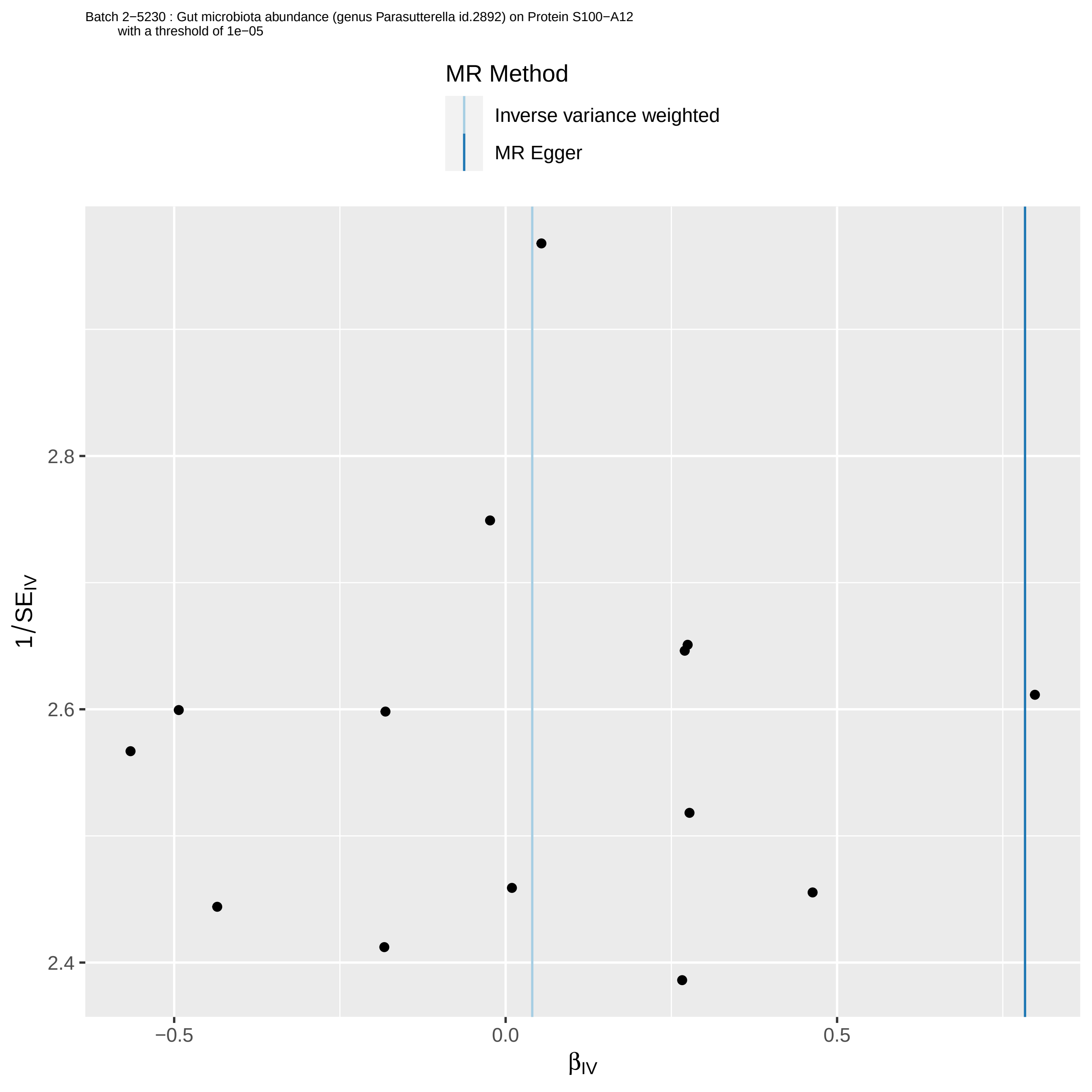

Batch 2−5230 : Gut microbiota abundance (genus Parasutterella id.2892) on Protein S100−A12
with a threshold of 1e−05
MR Method
Inverse variance weighted
MR Egger
2.8
2.6
2.4
1/SE_IV
−0.5
0.0
0.5
β_IV

Batch 2−5231 : Gut microbiota abundance (genus Catenibacterium id.2153) on Protein S100−A13
with a threshold of 1e−05

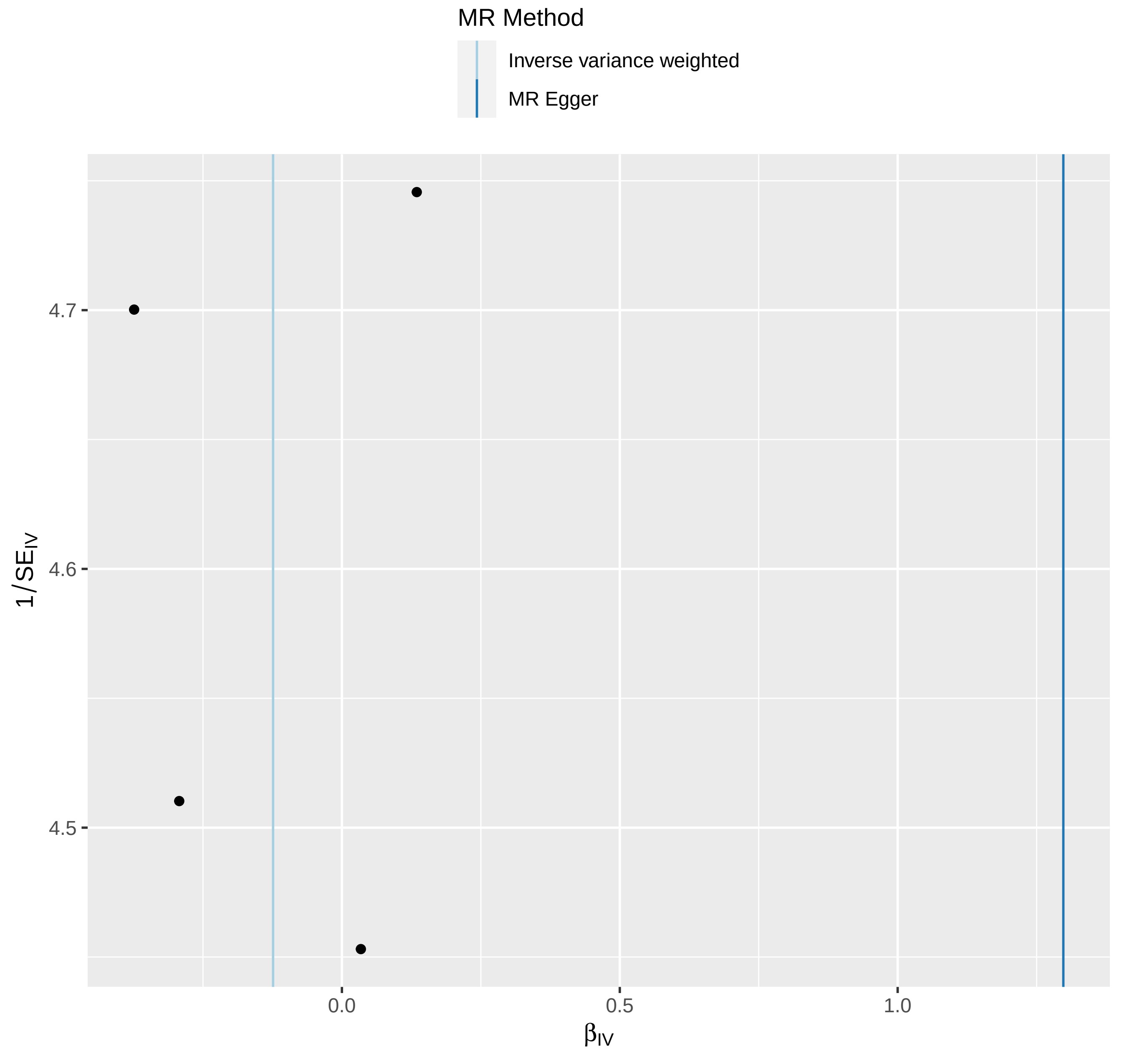

Batch 2−5232 : Gut microbiota abundance (genus Parasutterella id.2892) on Protein S100−A13
with a threshold of 1e−05

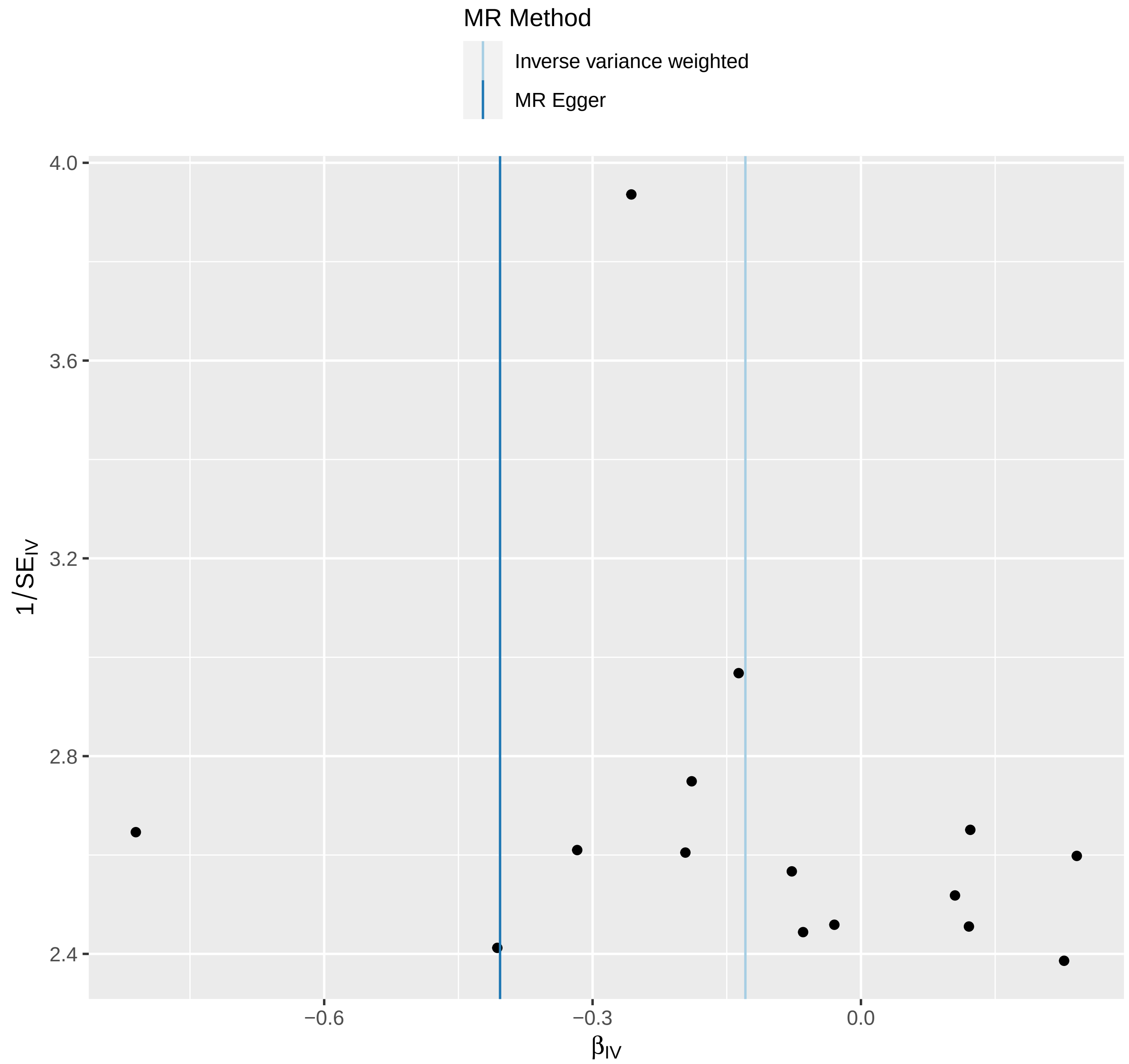

Batch 2−5233 : Gut microbiota abundance (genus Catenibacterium id.2153) on Protein S100−A2
with a threshold of 1e−05

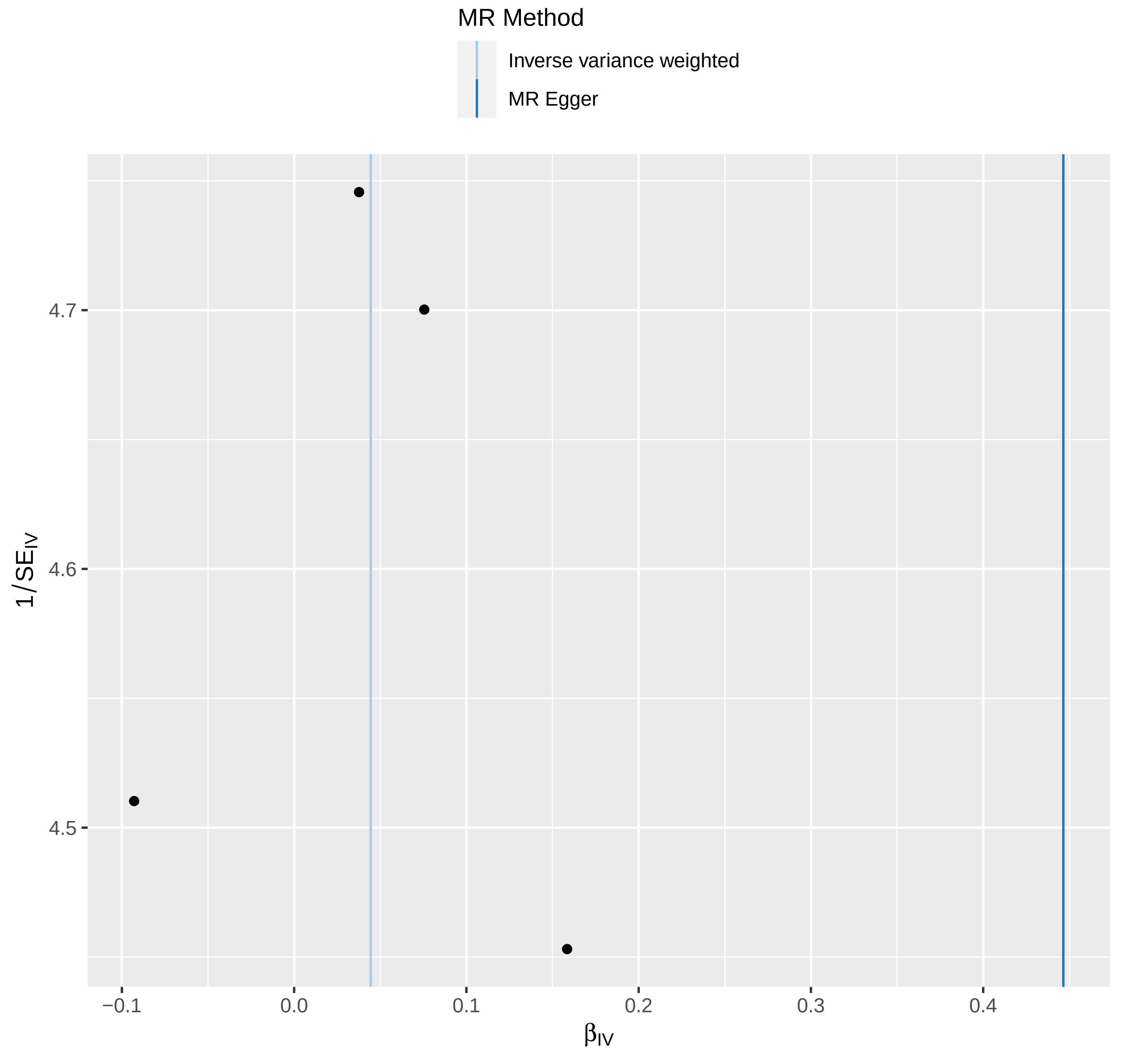

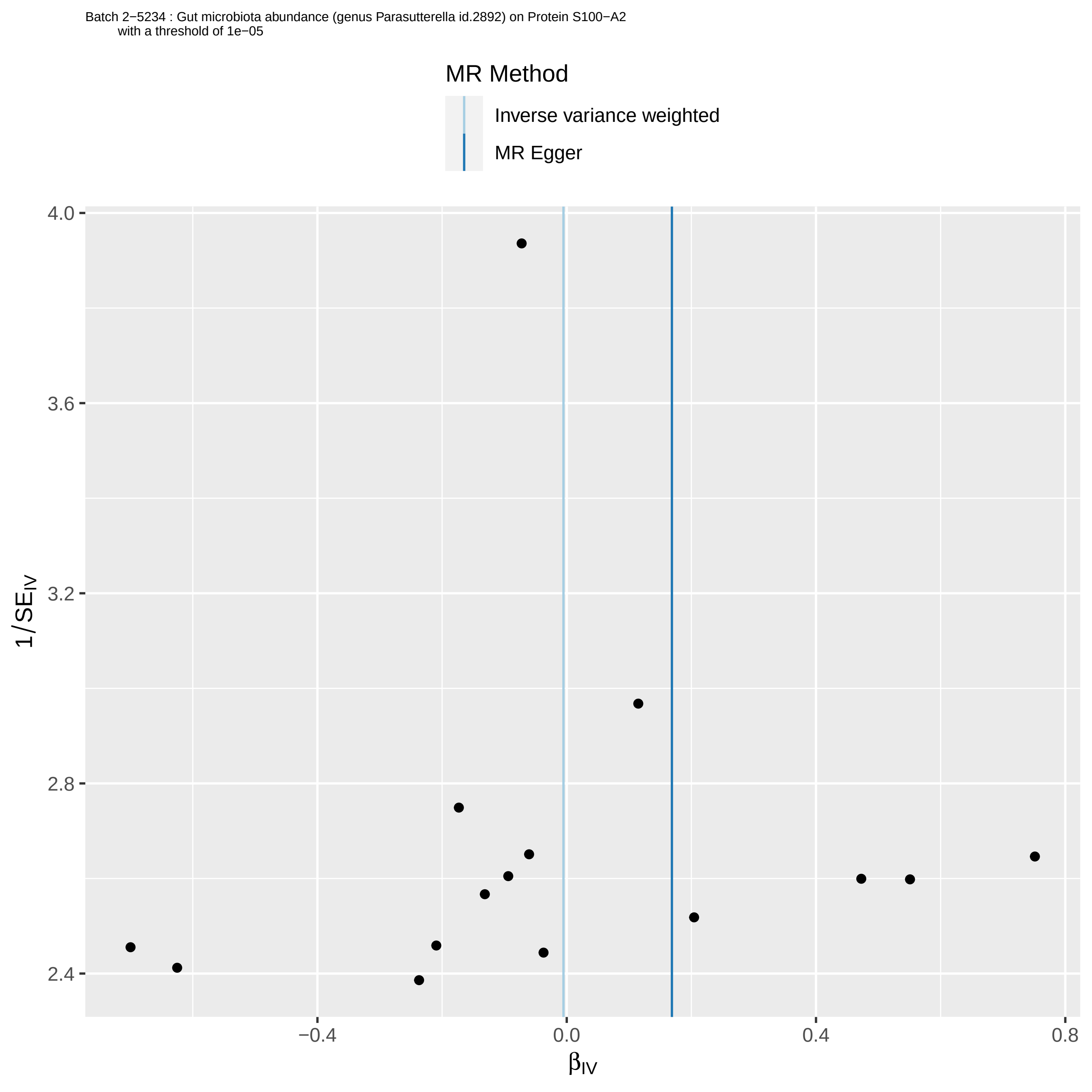

Batch 2−5234 : Gut microbiota abundance (genus Parasutterella id.2892) on Protein S100−A2
with a threshold of 1e−05
MR Method
Inverse variance weighted
MR Egger
4.0
3.6
3.2
2.8
2.4
1/SE_IV
−0.4
0.0
0.4
0.8
β_IV

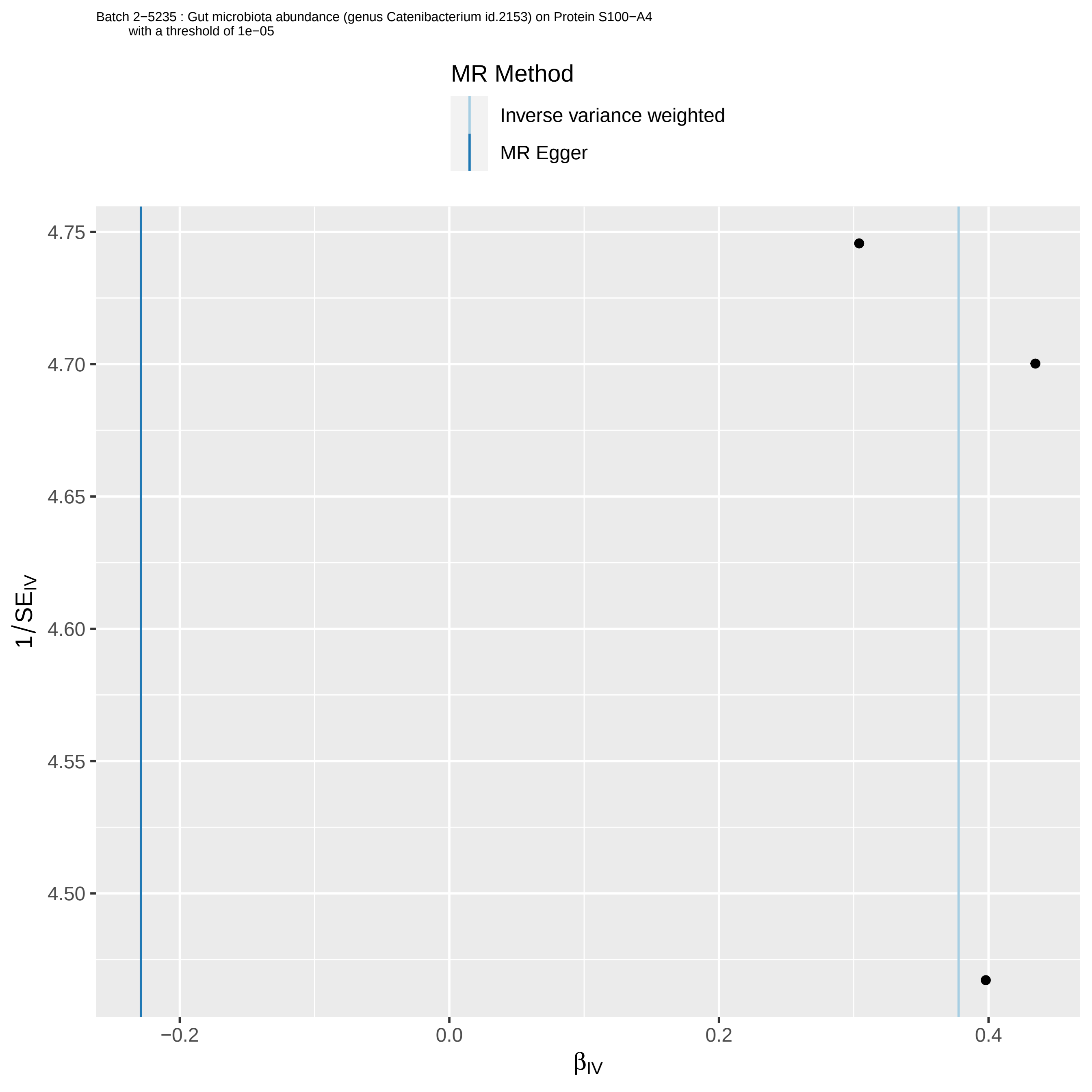
Batch 2−5235 : Gut microbiota abundance (genus Catenibacterium id.2153) on Protein S100−A4
with a threshold of 1e−05
MR Method
Inverse variance weighted
MR Egger
4.75
4.70
4.65
4.60
4.55
4.50
1/SE_IV
−0.2
0.0
0.2
0.4
β_IV

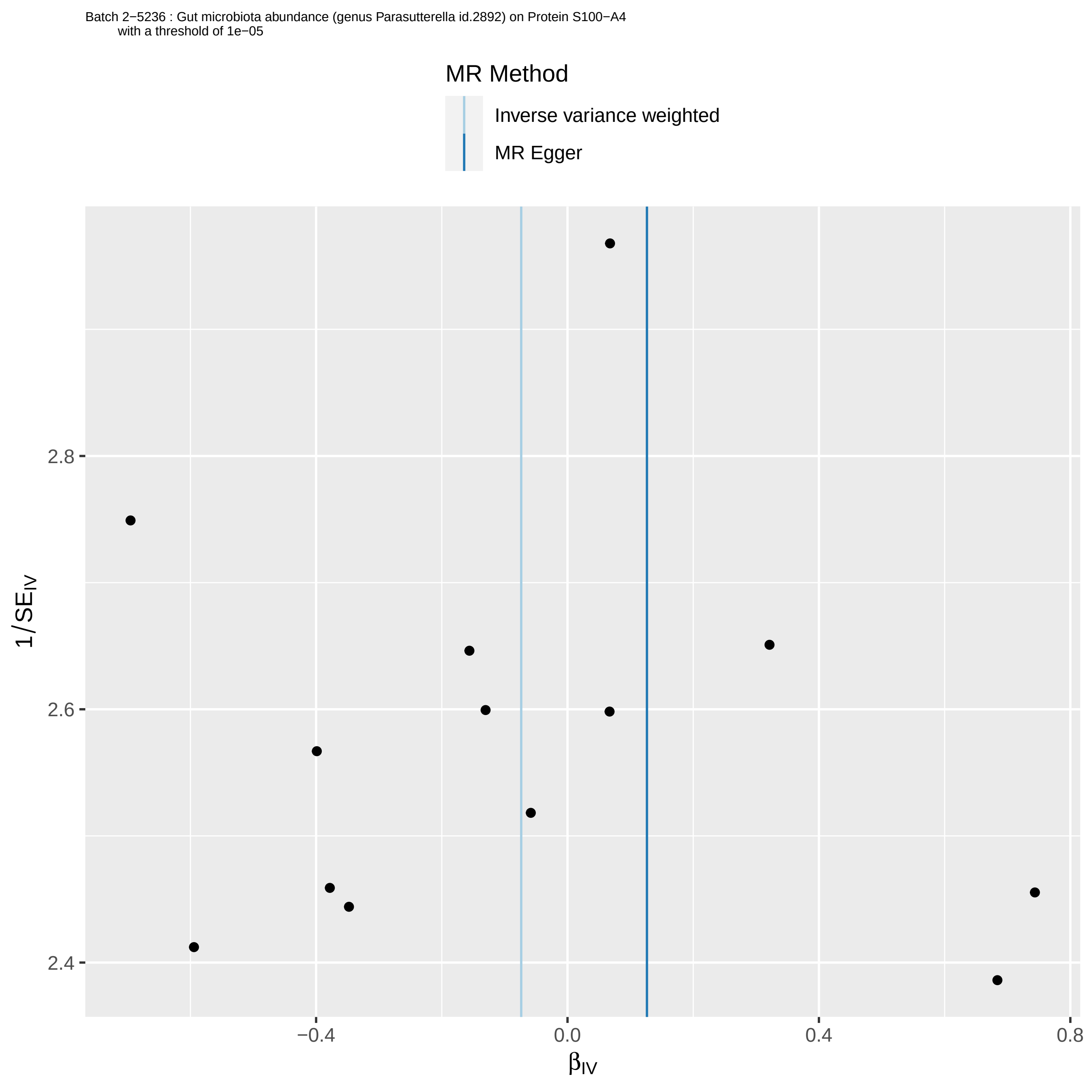

Batch 2−5236 : Gut microbiota abundance (genus Parasutterella id.2892) on Protein S100−A4
with a threshold of 1e−05
MR Method
Inverse variance weighted
MR Egger
2.8
2.6
2.4
1/SE_IV
−0.4
0.0
0.4
0.8
β_IV

Batch 2−5237 : Gut microbiota abundance (genus Catenibacterium id.2153) on Protein S100−A5
with a threshold of 1e−05

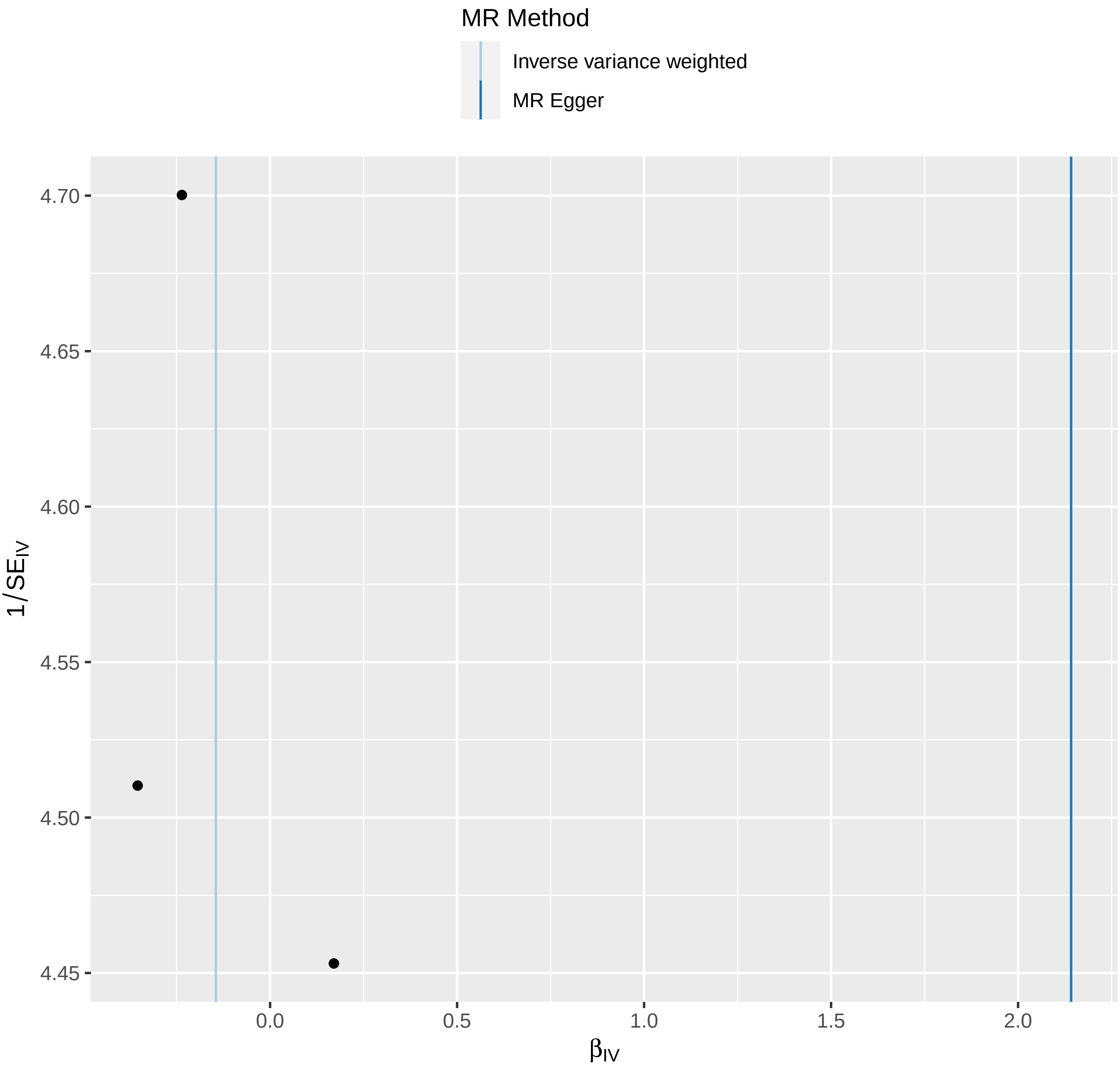

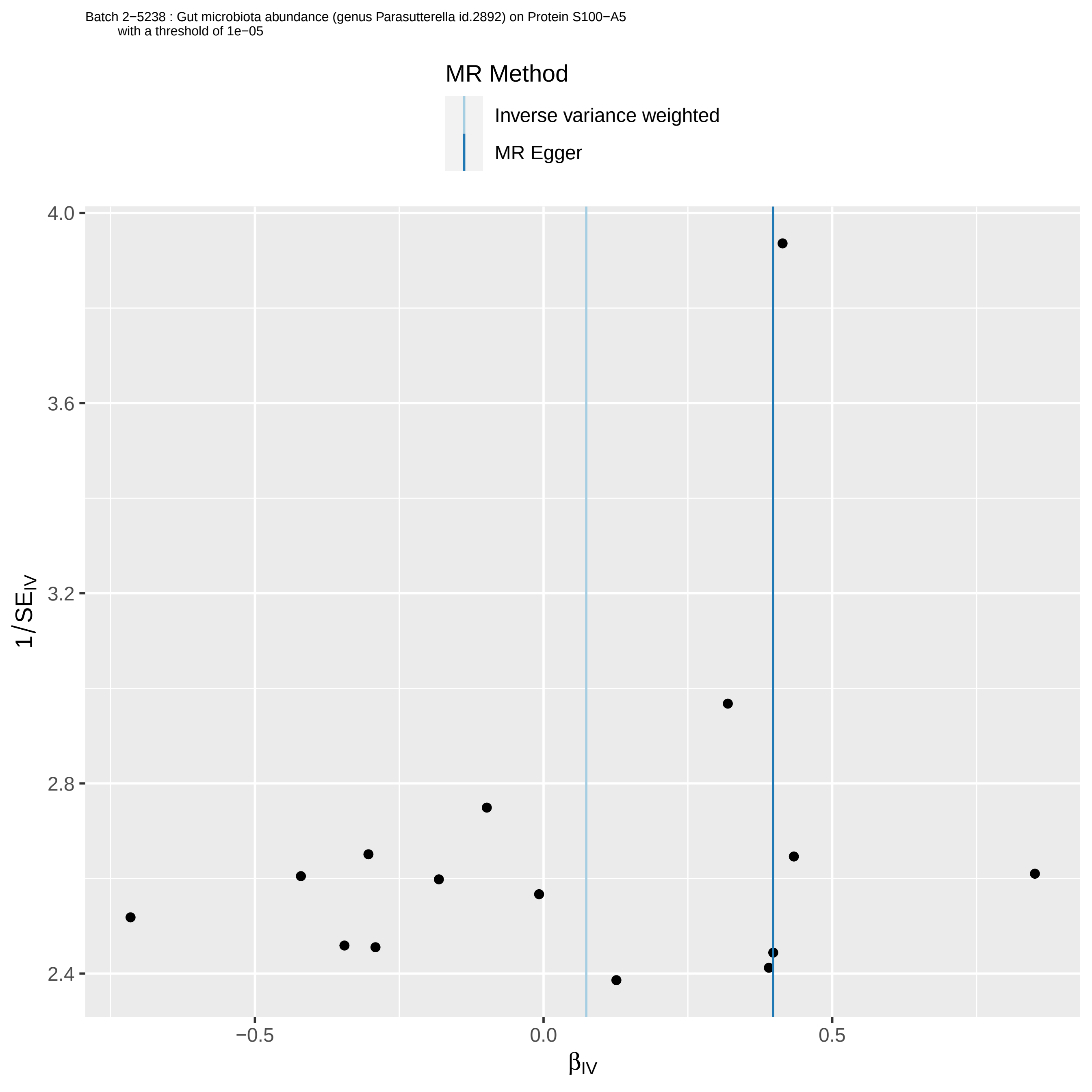

Batch 2−5238 : Gut microbiota abundance (genus Parasutterella id.2892) on Protein S100−A5
with a threshold of 1e−05
MR Method
Inverse variance weighted
MR Egger
4.0
3.6
3.2
2.8
2.4
1/SE_IV
−0.5
0.0
0.5
β_IV

Batch 2−5239 : Gut microbiota abundance (genus Catenibacterium id.2153) on Protein S100−A6
with a threshold of 1e−05

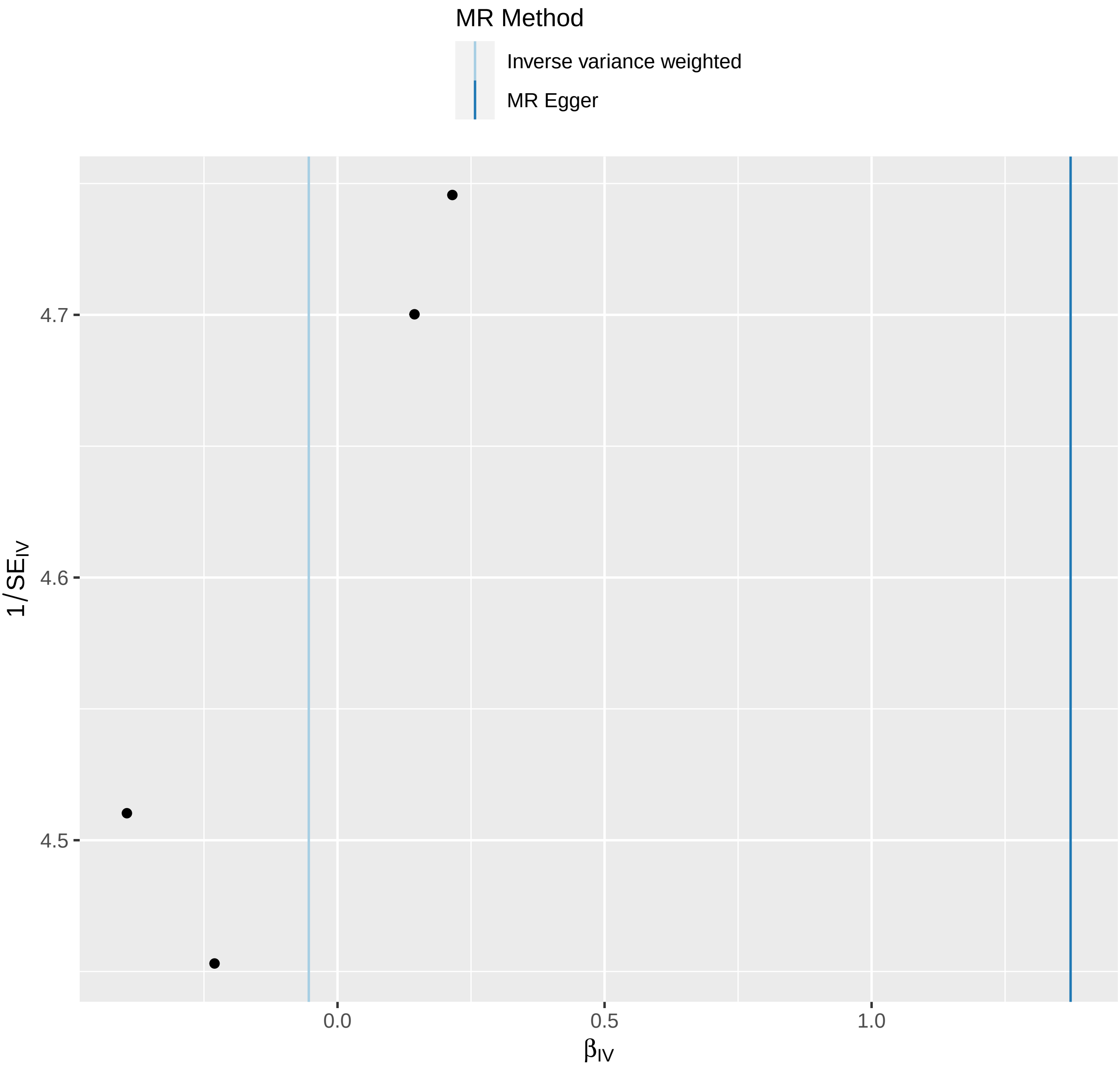

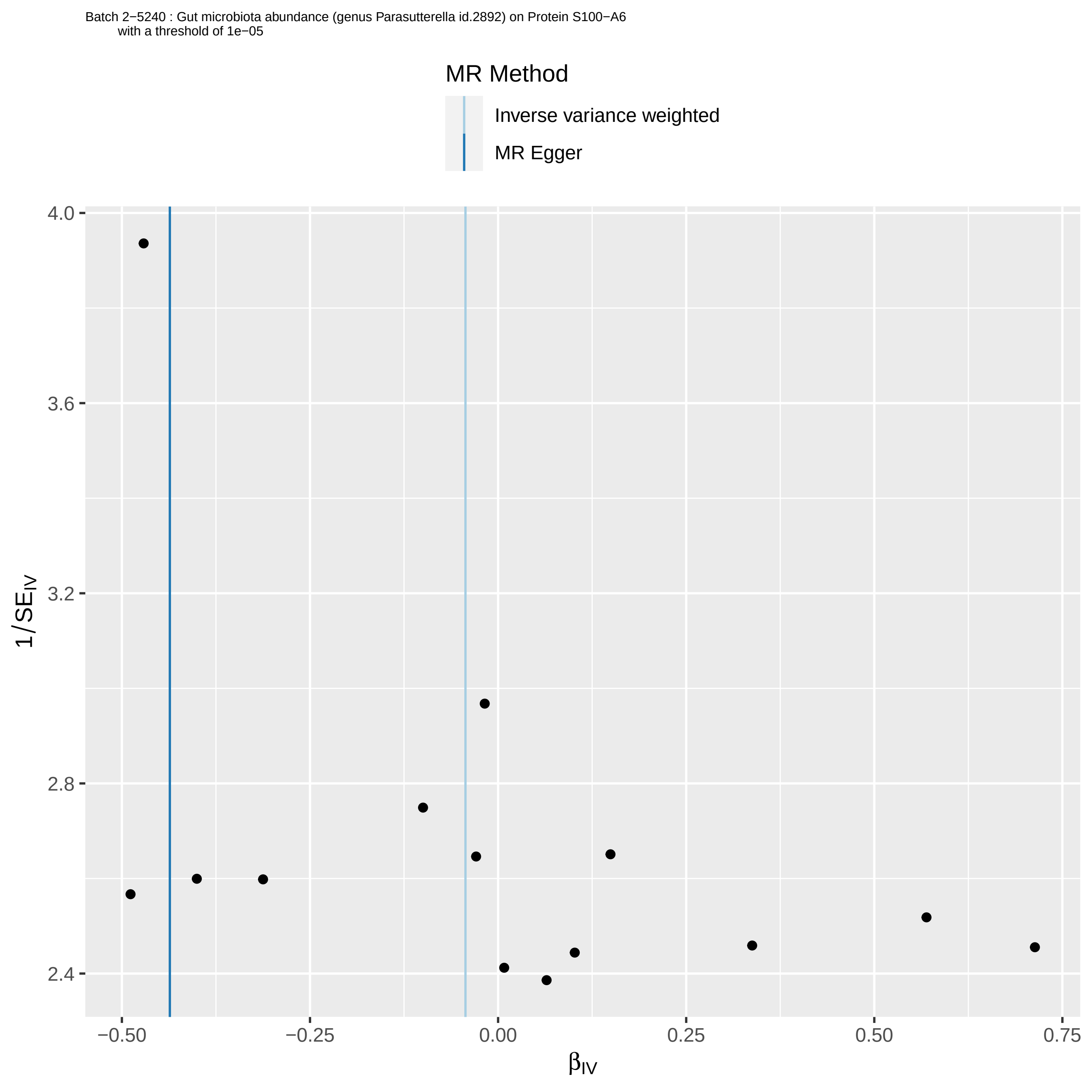
Batch 2−5240 : Gut microbiota abundance (genus Parasutterella id.2892) on Protein S100−A6
with a threshold of 1e−05
MR Method
Inverse variance weighted
MR Egger
4.0
3.6
3.2
2.8
2.4
1/SE_IV
−0.50
−0.25
0.00
0.25
0.50
0.75
β_IV

Batch 2−5241 : Gut microbiota abundance (genus Catenibacterium id.2153) on Protein S100−A7
with a threshold of 1e−05

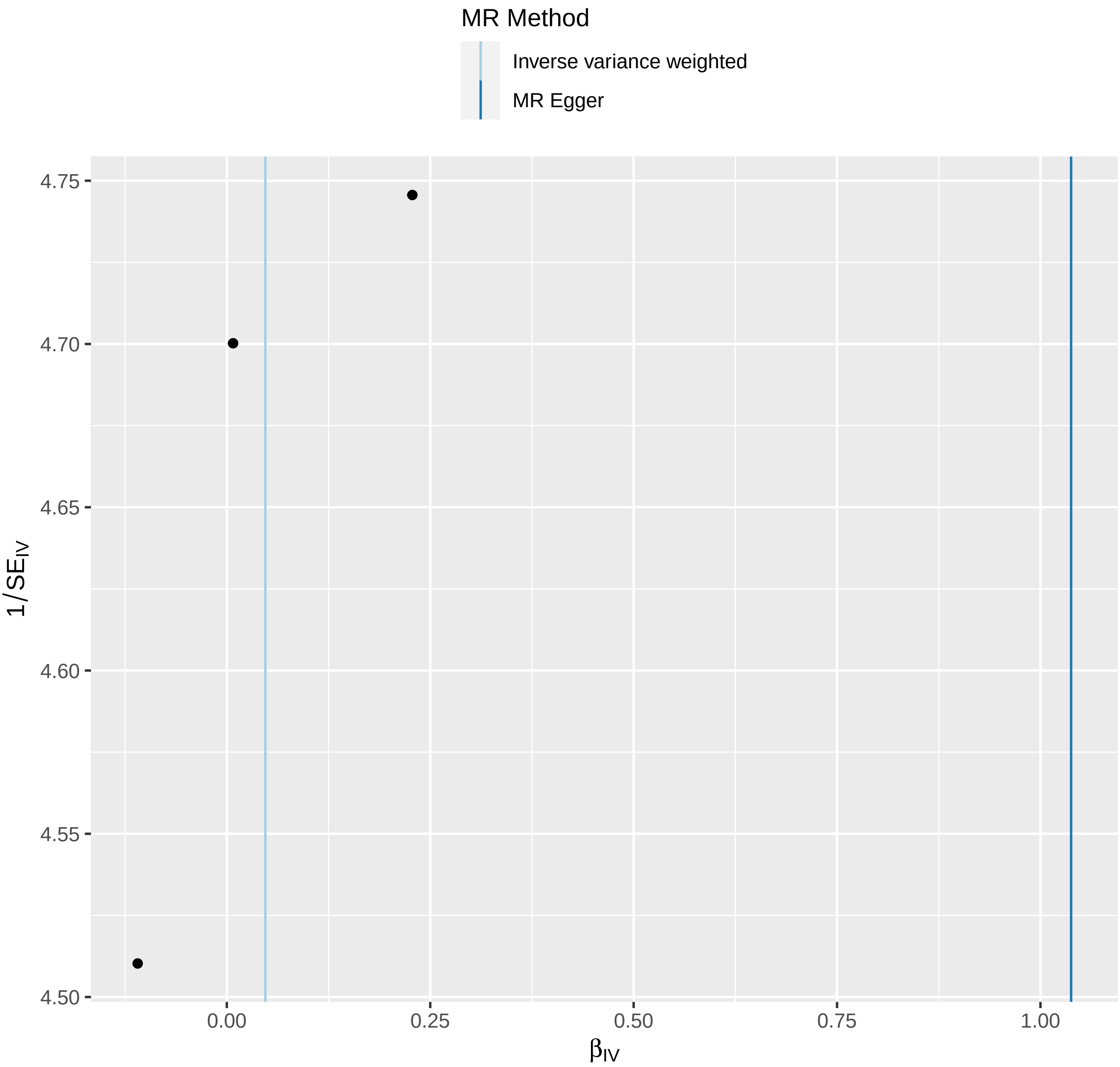

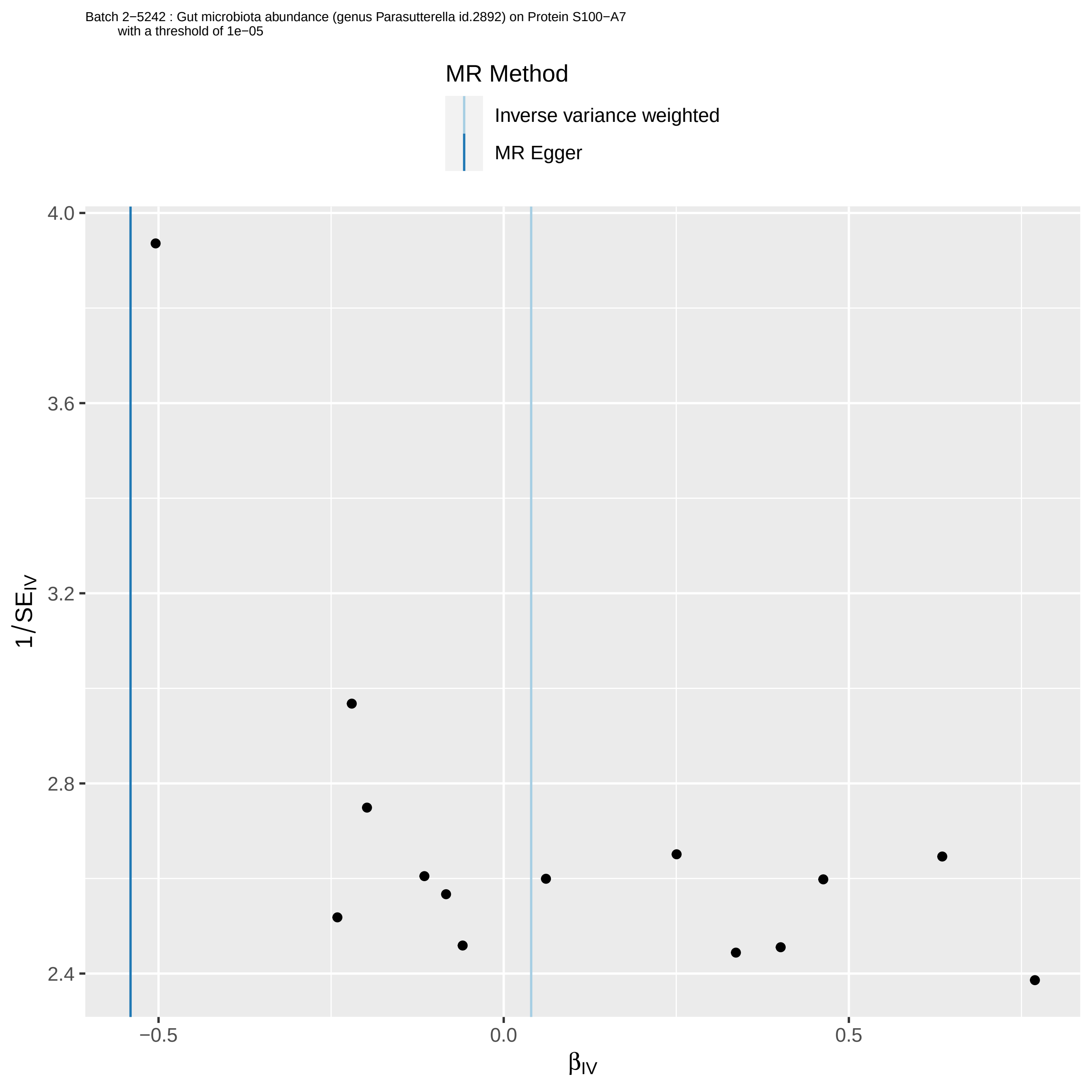
Batch 2−5242 : Gut microbiota abundance (genus Parasutterella id.2892) on Protein S100−A7
with a threshold of 1e−05
MR Method
Inverse variance weighted
MR Egger
4.0
3.6
3.2
2.8
2.4
1/SE_IV
−0.5
0.0
0.5
β_IV

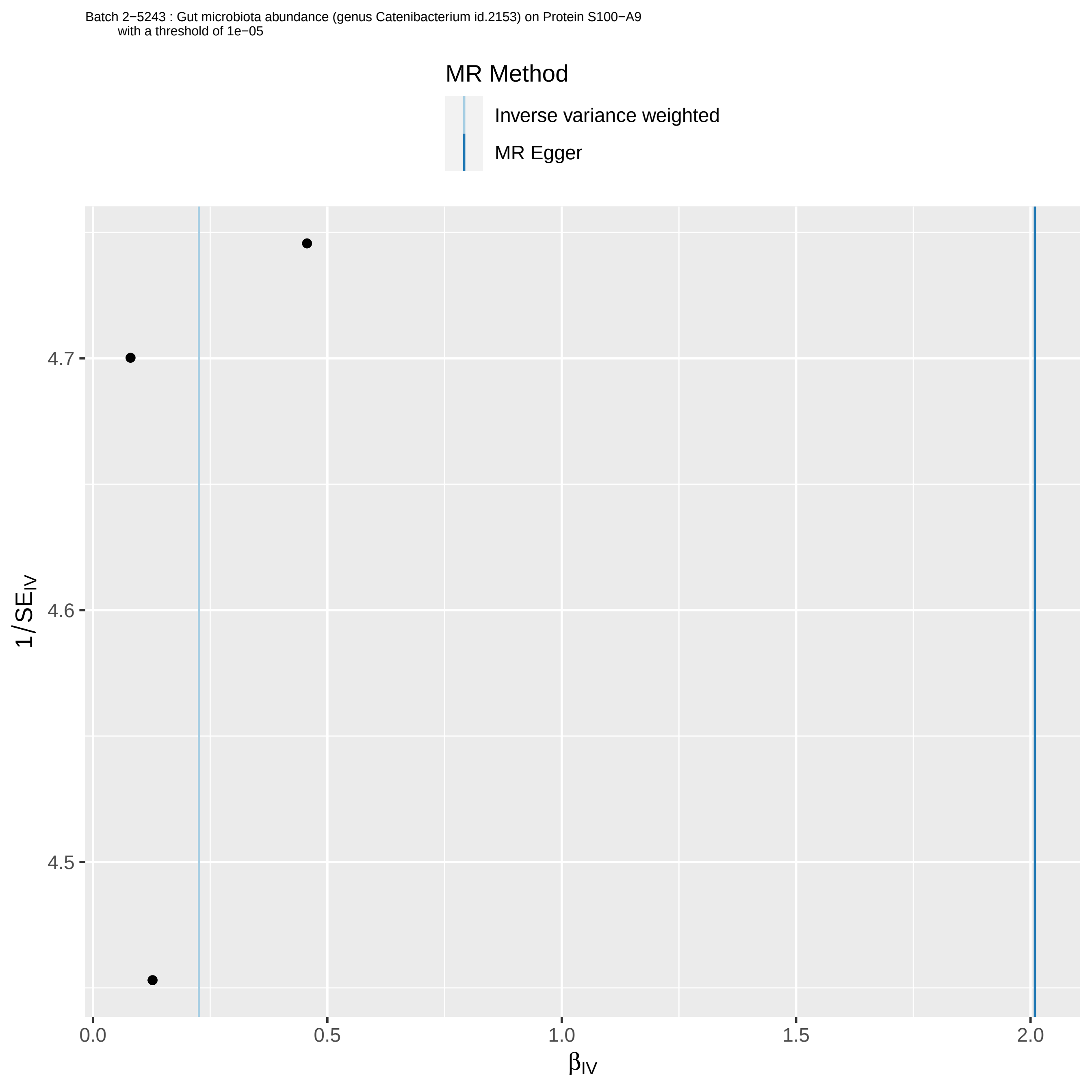

Batch 2−5243 : Gut microbiota abundance (genus Catenibacterium id.2153) on Protein S100−A9
with a threshold of 1e−05
MR Method
Inverse variance weighted
MR Egger
4.7
4.6
4.5
1/SE$_{IV}$
0.0
0.5
1.0
1.5
2.0
β$_{IV}$

Batch 2−5244 : Gut microbiota abundance (genus Parasutterella id.2892) on Protein S100−A9
with a threshold of 1e−05

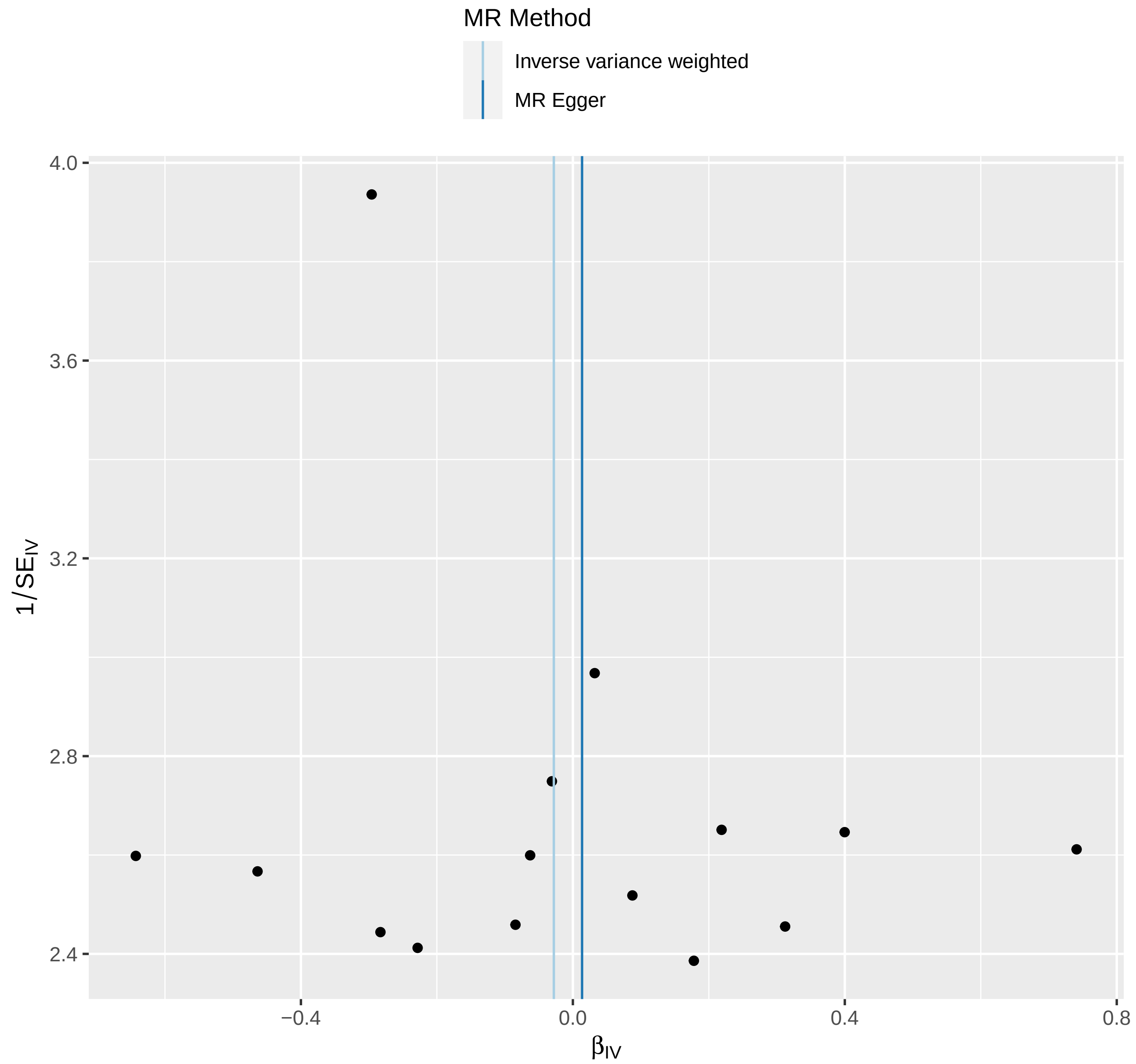

Batch 2−5245 : Gut microbiota abundance (genus Catenibacterium id.2153) on Serum amyloid A−1 protein
with a threshold of 1e−05

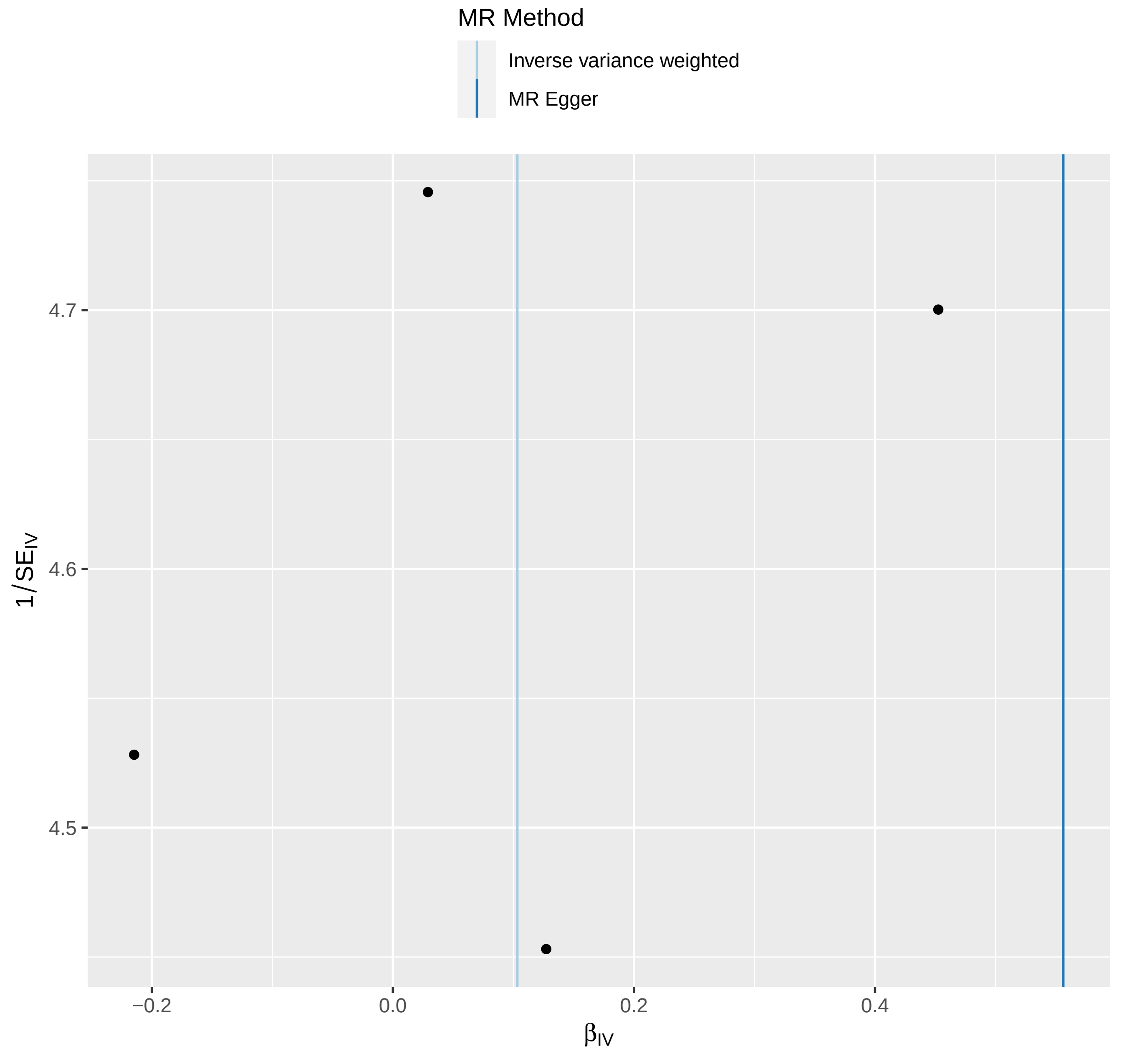

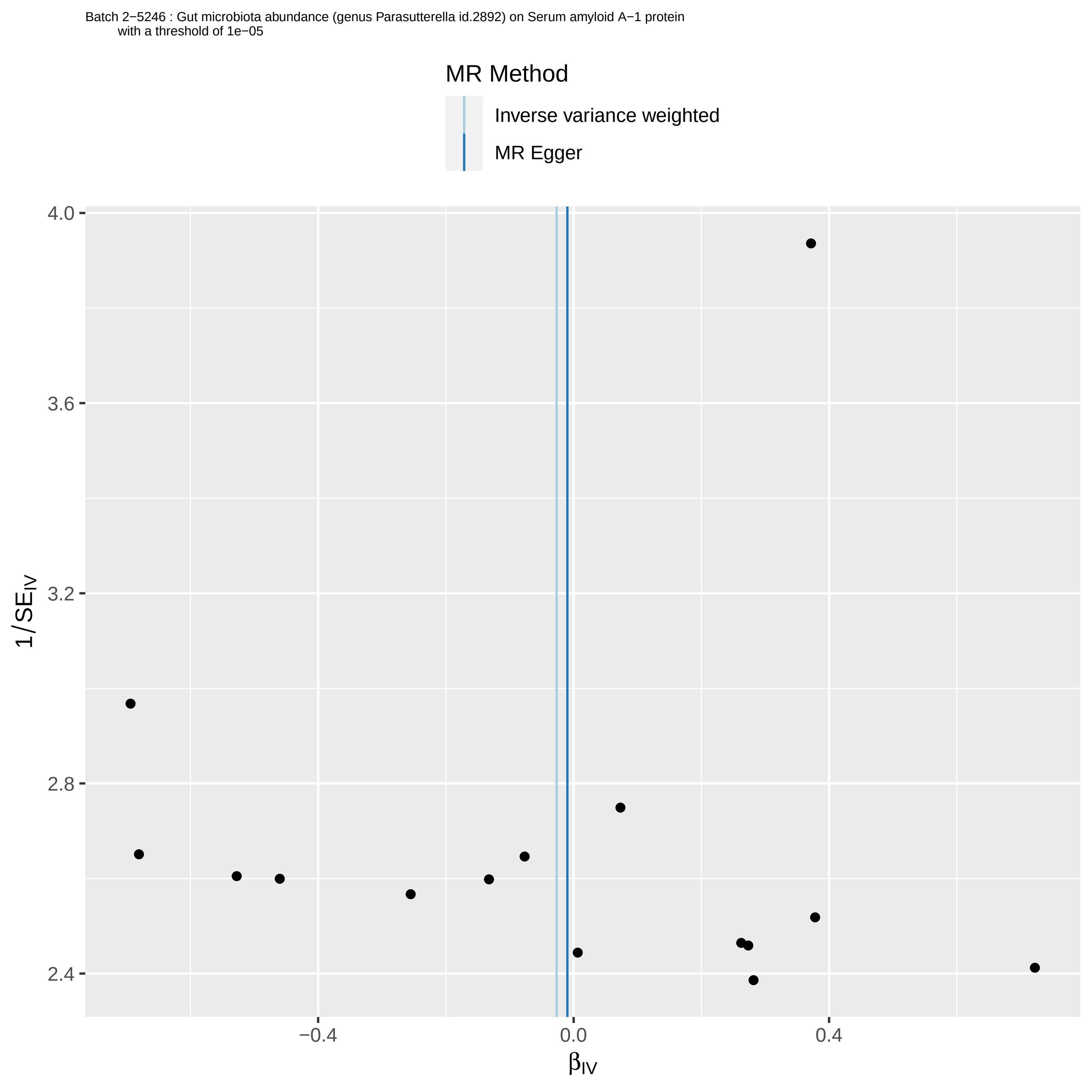
Batch 2−5246 : Gut microbiota abundance (genus Parasutterella id.2892) on Serum amyloid A−1 protein
with a threshold of 1e−05
MR Method
Inverse variance weighted
MR Egger
4.0
3.6
3.2
2.8
2.4
1/SE_IV
−0.4
0.0
0.4
β_IV

Batch 2−5247 : Gut microbiota abundance (genus Catenibacterium id.2153) on Deoxynucleoside triphosphate triphosphohydrolase SAMHD1
with a threshold of 1e−05

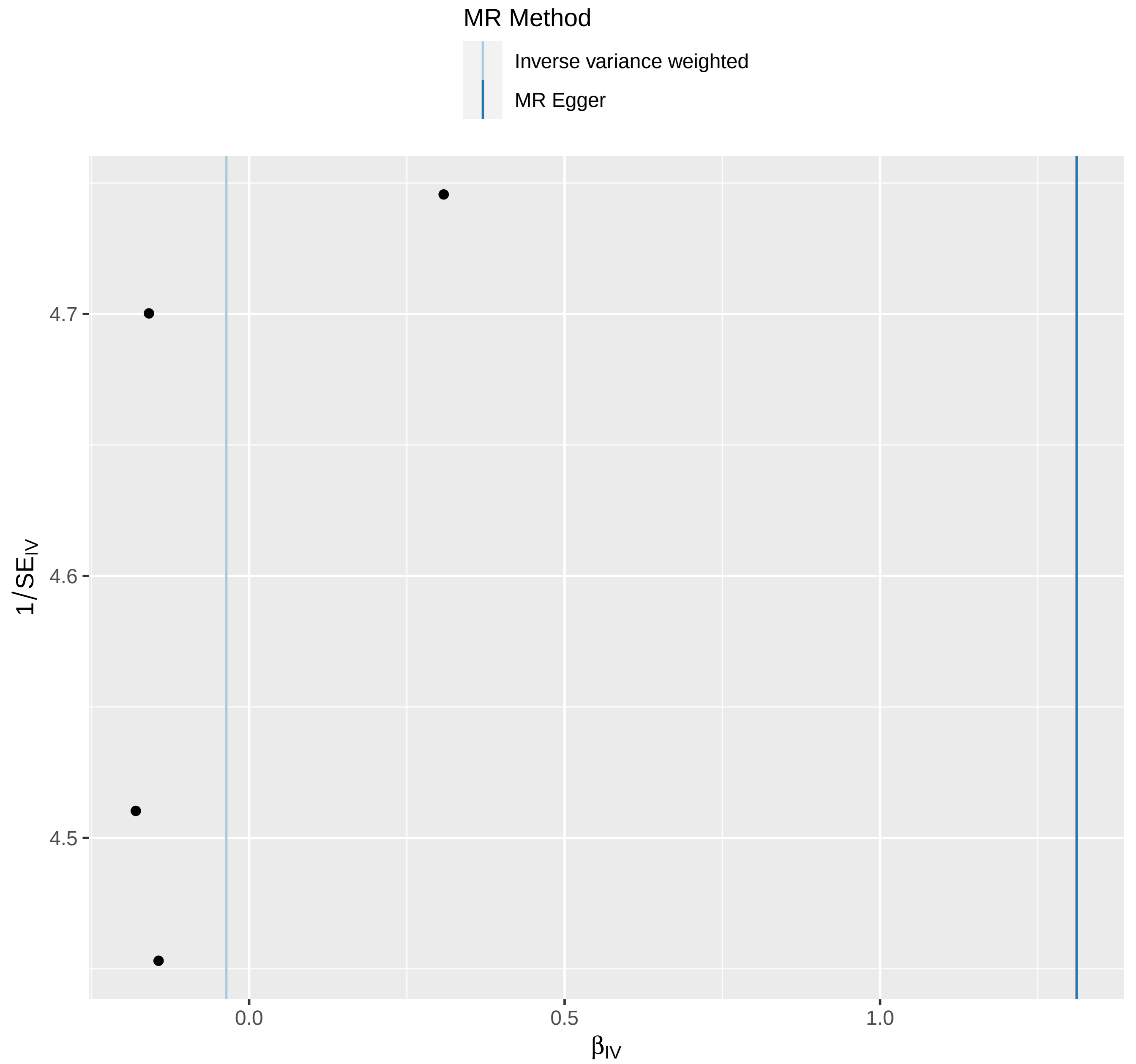

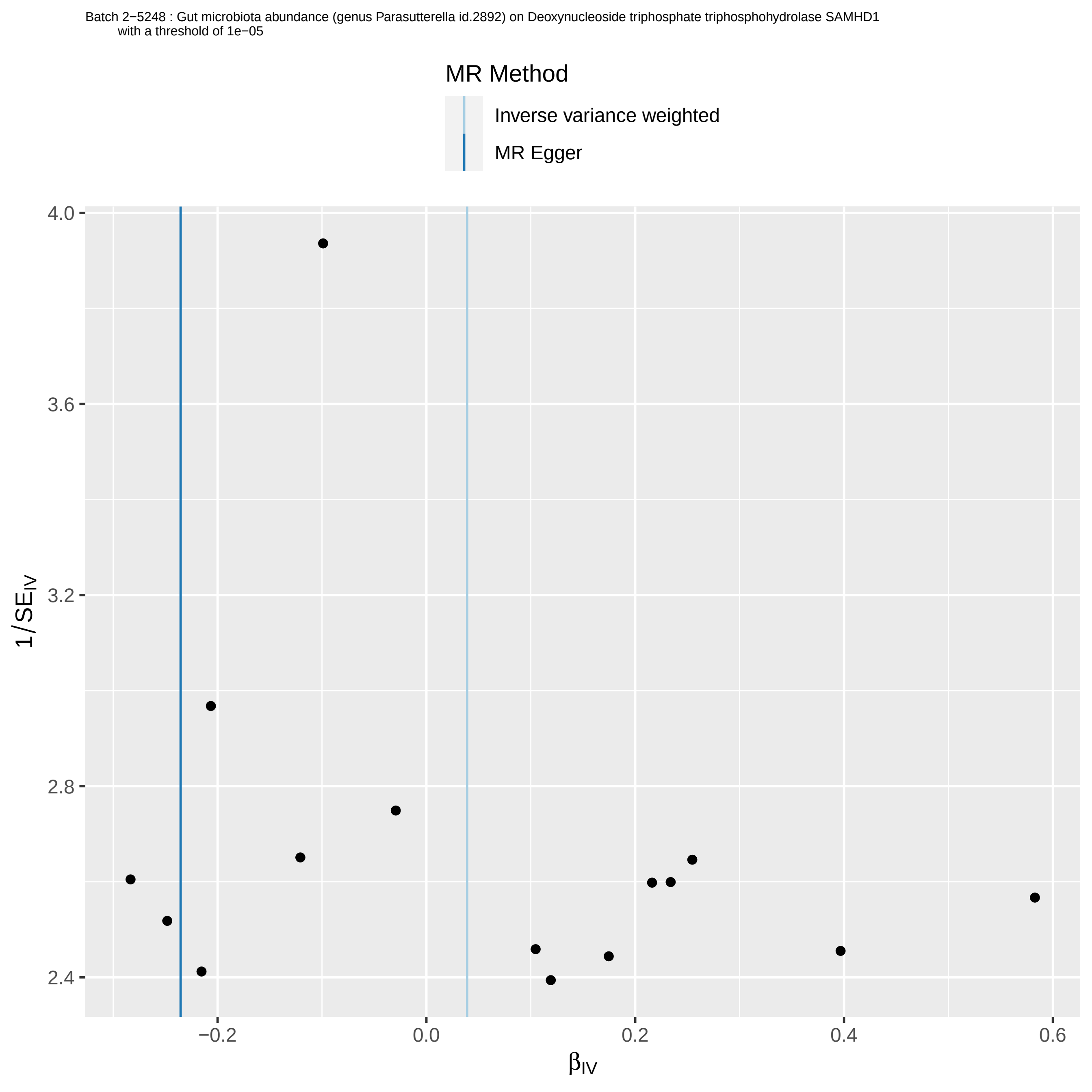

Batch 2−5248 : Gut microbiota abundance (genus Parasutterella id.2892) on Deoxynucleoside triphosphate triphosphohydrolase SAMHD1
with a threshold of 1e−05
MR Method
Inverse variance weighted
MR Egger
4.0
3.6
3.2
2.8
2.4
1/SE_IV
−0.2
0.0
0.2
0.4
0.6
β_IV

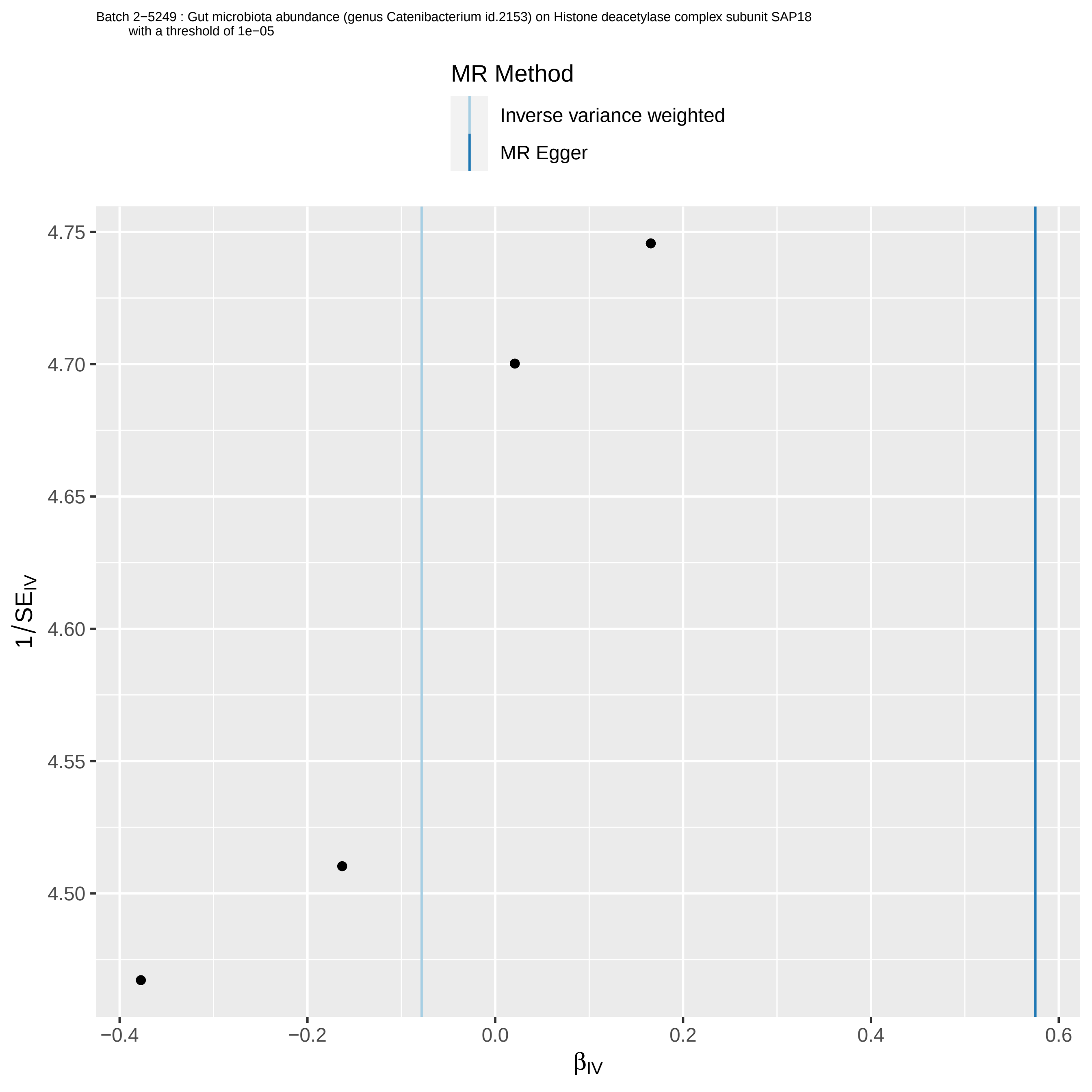
Batch 2−5249 : Gut microbiota abundance (genus Catenibacterium id.2153) on Histone deacetylase complex subunit SAP18
with a threshold of 1e−05
MR Method
Inverse variance weighted
MR Egger
4.75
4.70
4.65
4.60
4.55
4.50
1/SE$_{IV}$
−0.4
−0.2
0.0
0.2
0.4
0.6
β$_{IV}$

Batch 2−5250 : Gut microbiota abundance (genus Parasutterella id.2892) on Histone deacetylase complex subunit SAP18
with a threshold of 1e−05

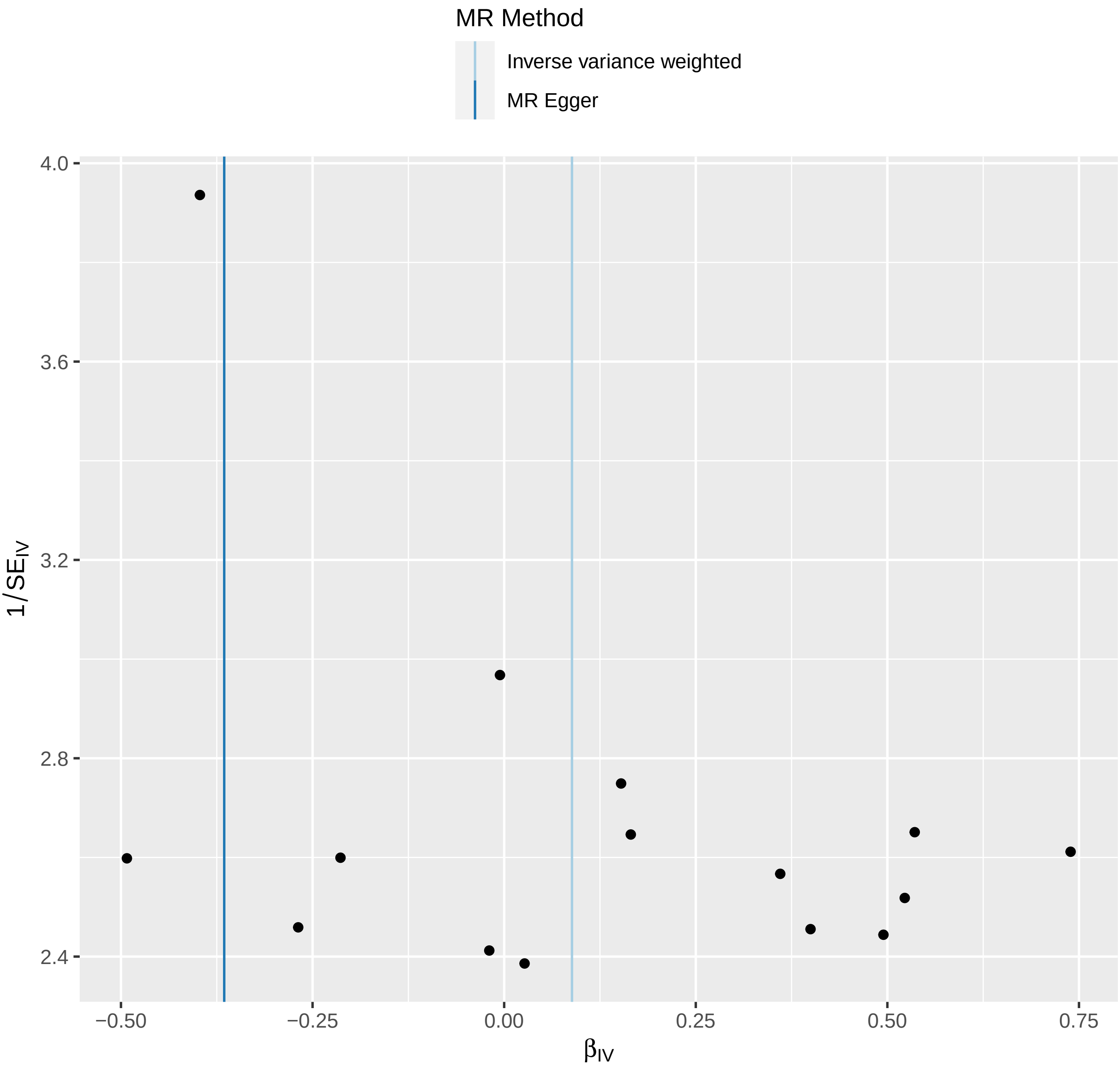

Batch 2−5251 : Gut microbiota abundance (genus Catenibacterium id.2153) on Histone deacetylase complex subunit SAP30
with a threshold of 1e−05

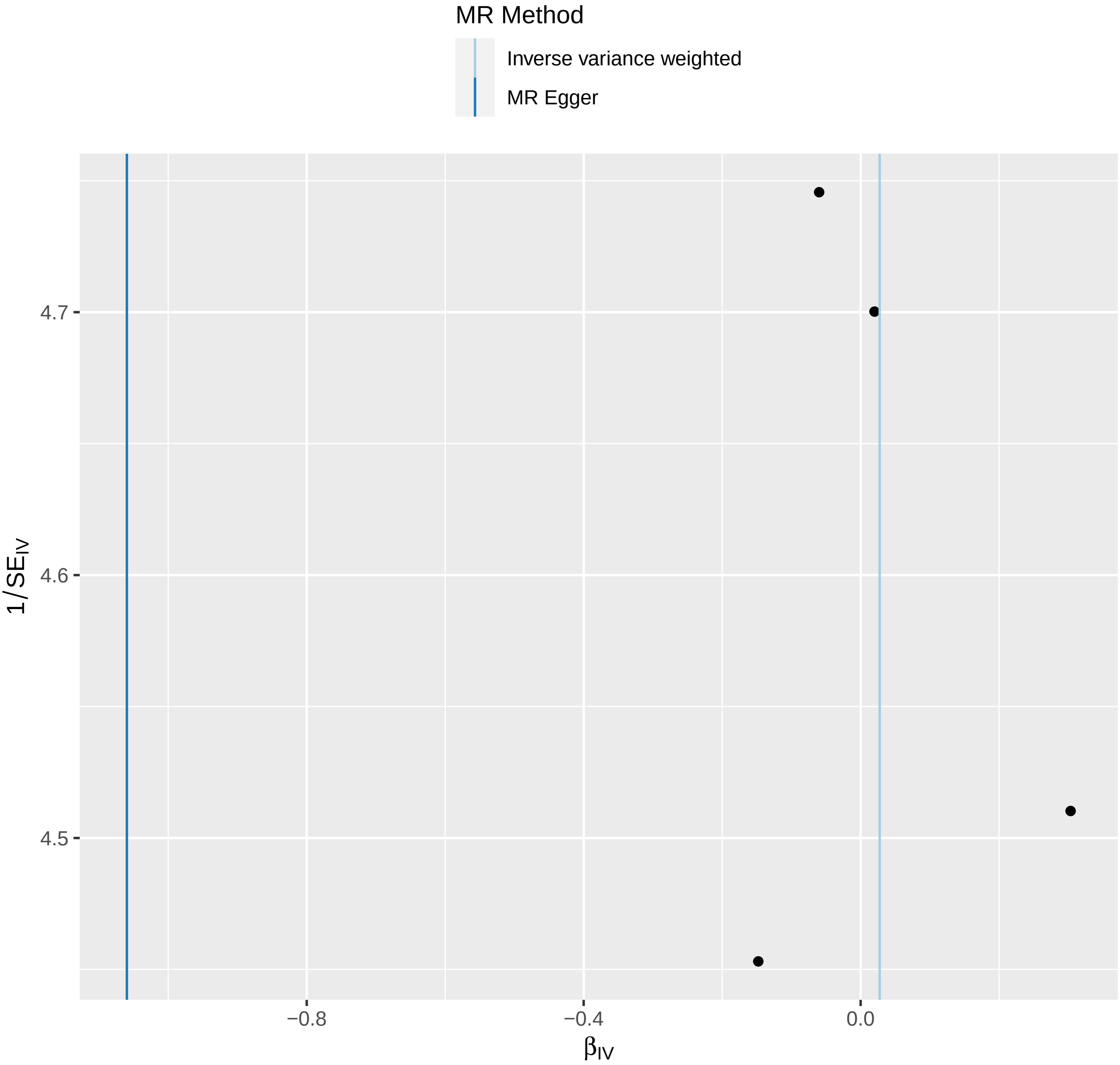

Batch 2−5252 : Gut microbiota abundance (genus Parasutterella id.2892) on Histone deacetylase complex subunit SAP30
with a threshold of 1e−05

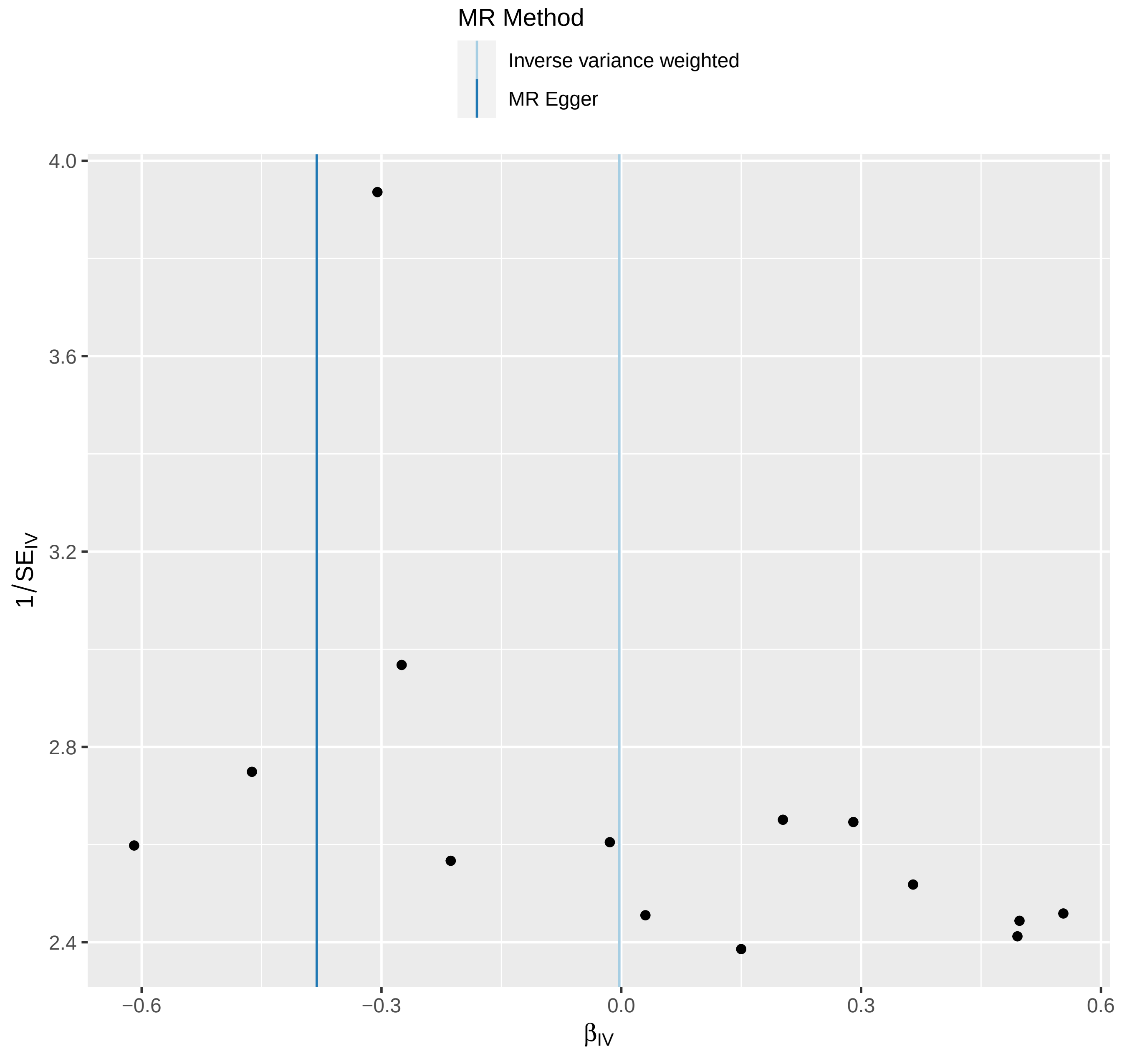

Batch 2−5253 : Gut microbiota abundance (genus Catenibacterium id.2153) on Serine−−tRNA ligase, mitochondrial
with a threshold of 1e−05

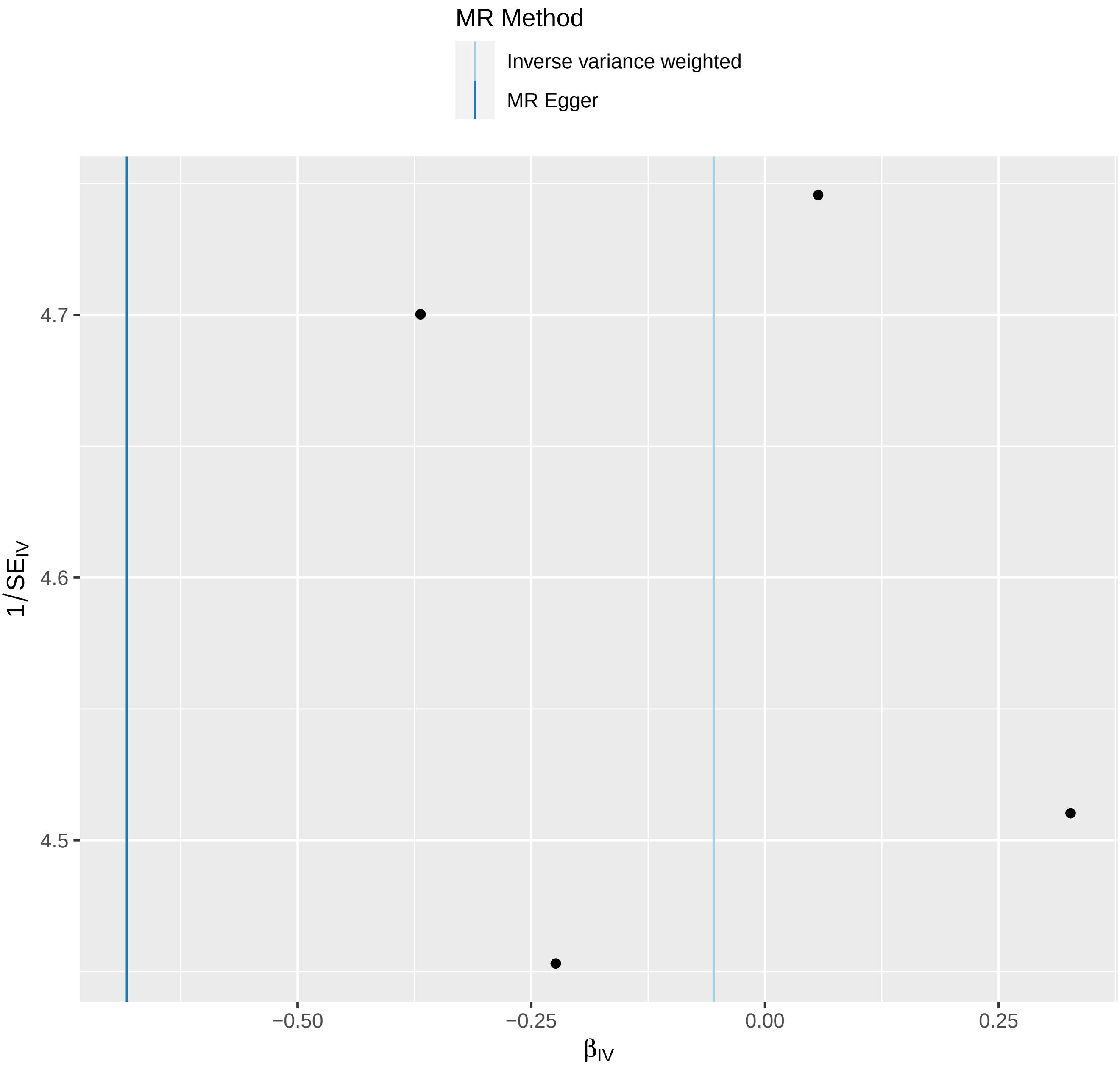

Batch 2−5254 : Gut microbiota abundance (genus Parasutterella id.2892) on Serine−−tRNA ligase, mitochondrial
with a threshold of 1e−05

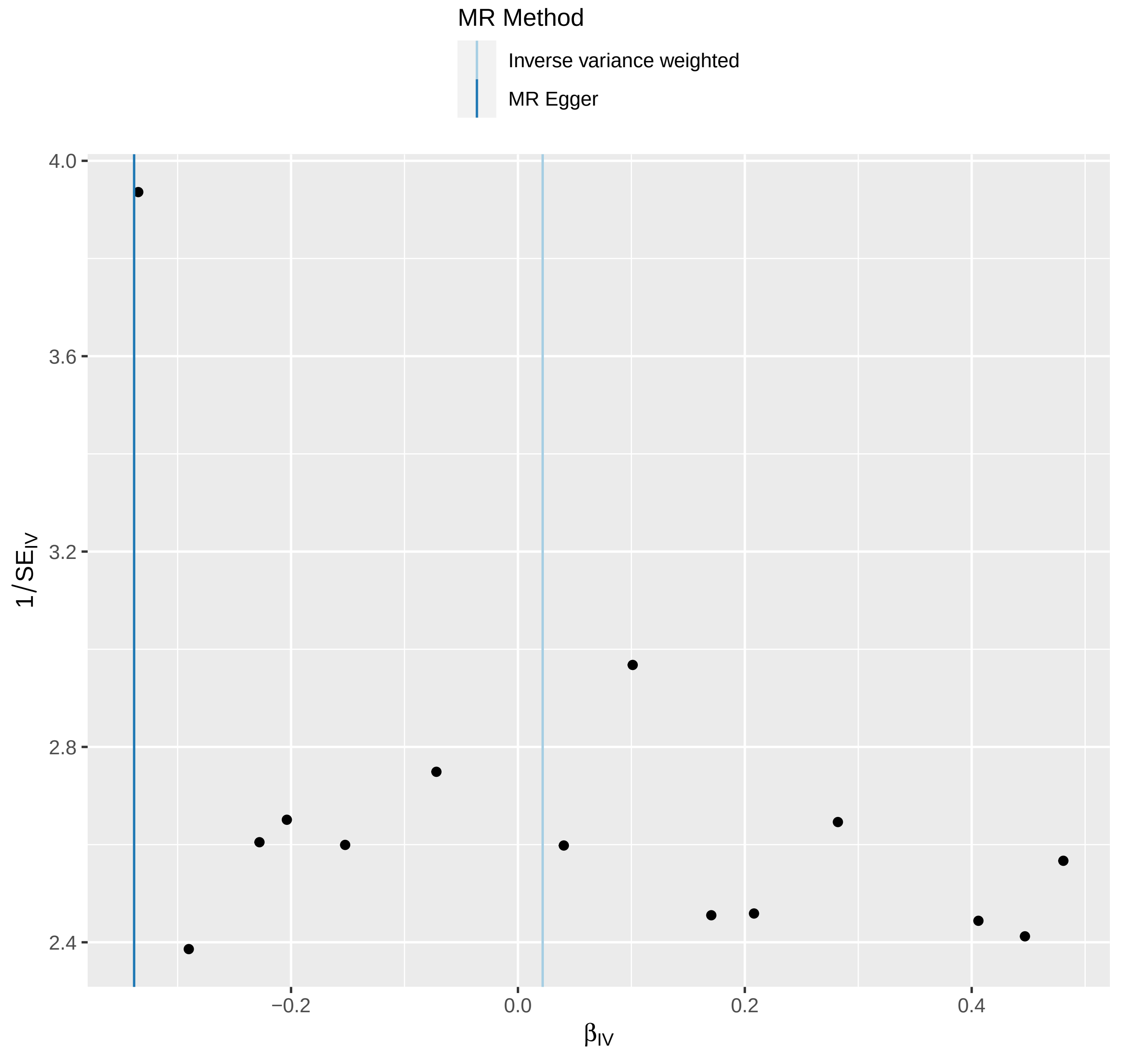

Batch 2−5255 : Gut microbiota abundance (genus Catenibacterium id.2153) on Diamine acetyltransferase 1
with a threshold of 1e−05

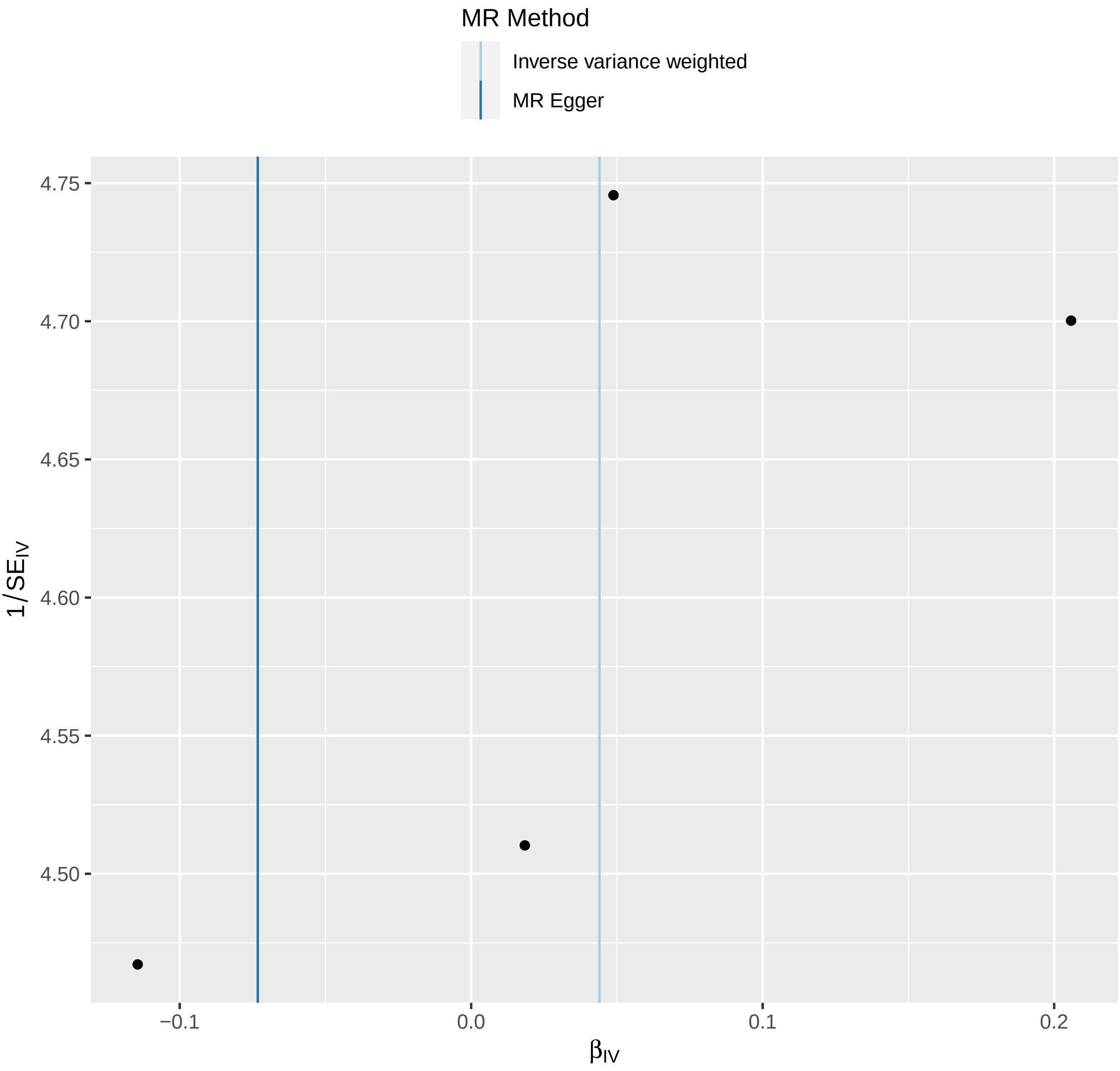

Batch 2−5256 : Gut microbiota abundance (genus Parasutterella id.2892) on Diamine acetyltransferase 1
with a threshold of 1e−05

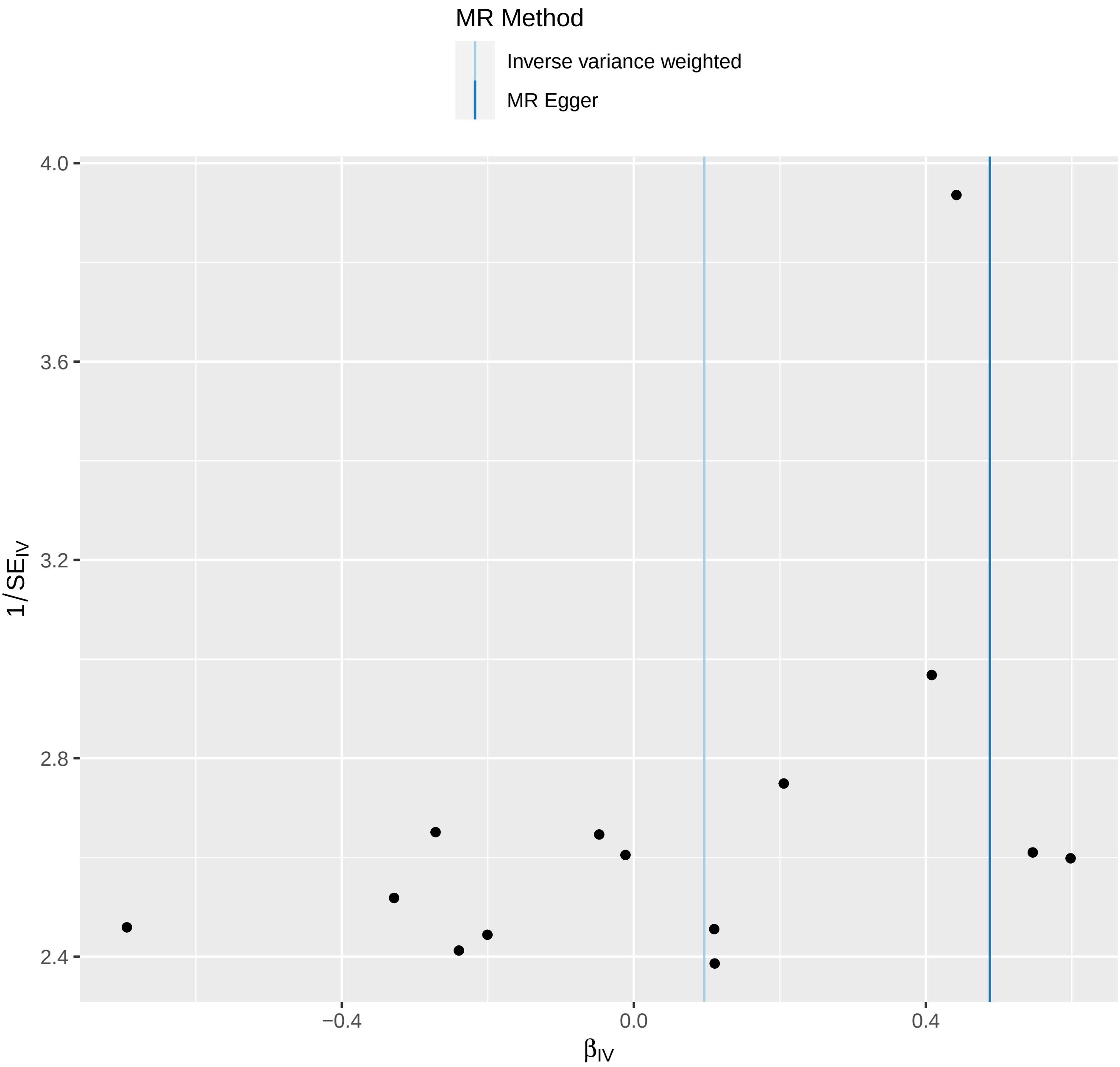

Batch 2−5257 : Gut microbiota abundance (genus Catenibacterium id.2153) on Diamine acetyltransferase 2
with a threshold of 1e−05

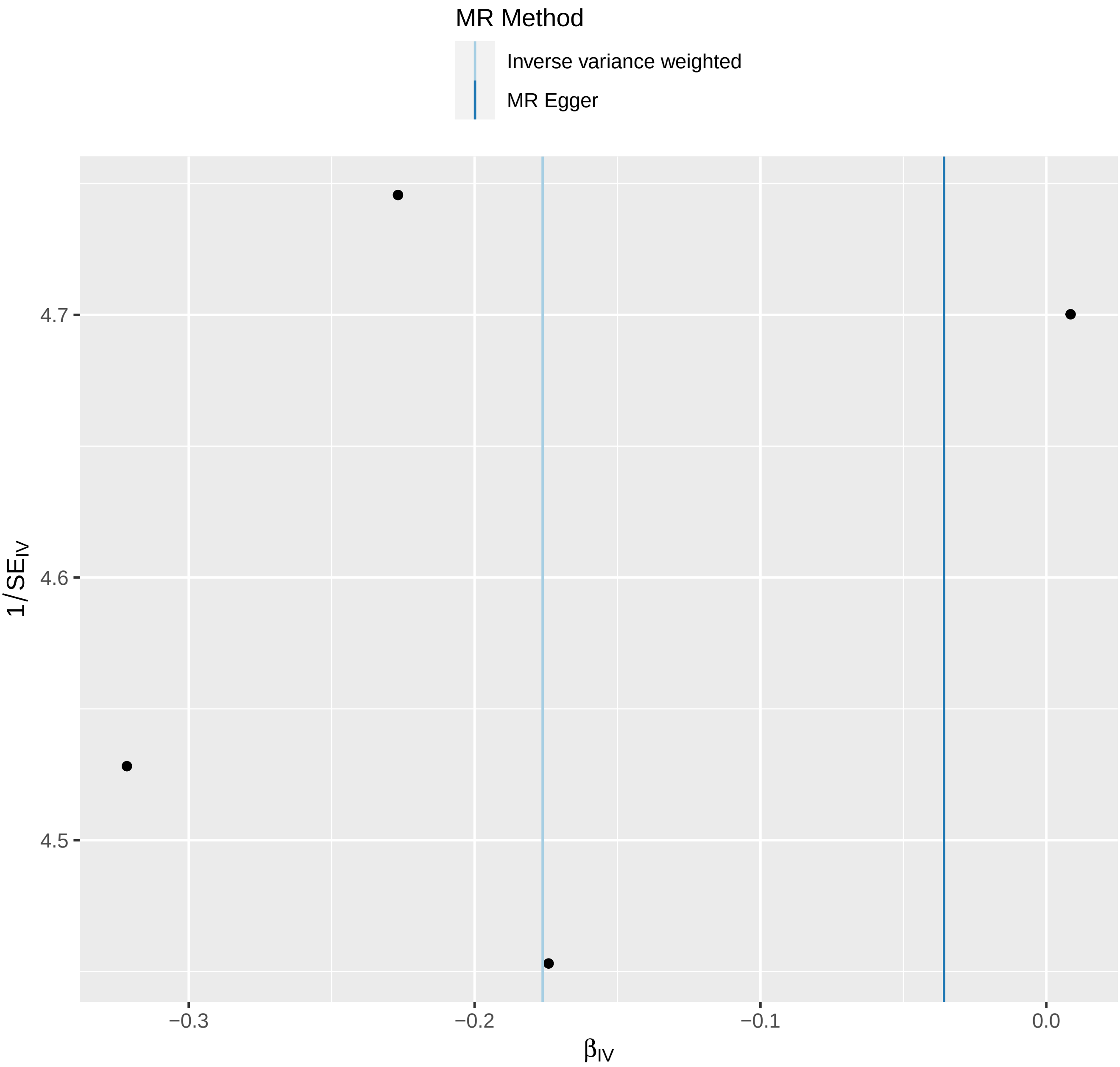

Batch 2−5258 : Gut microbiota abundance (genus Parasutterella id.2892) on Diamine acetyltransferase 2
with a threshold of 1e−05

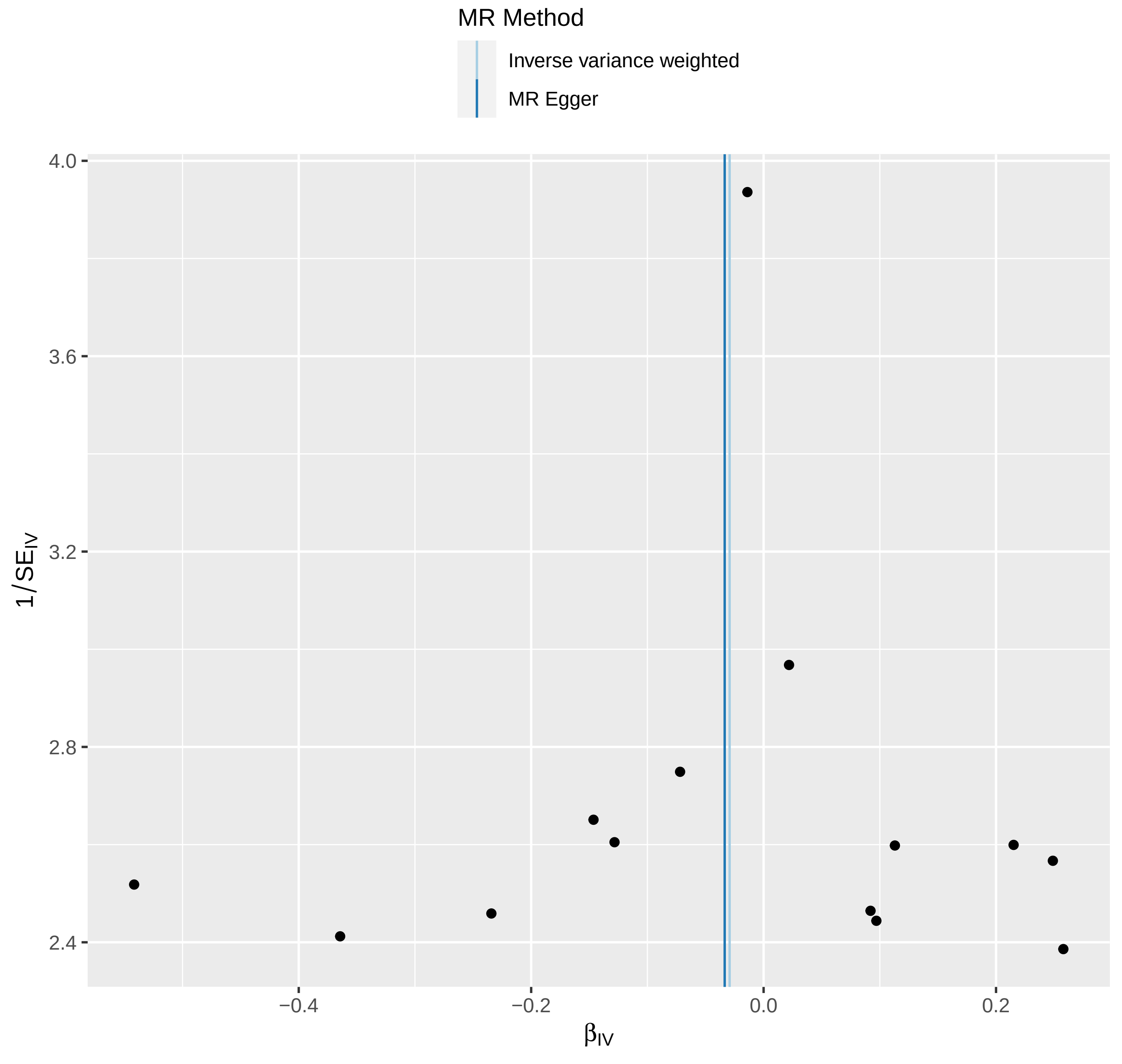

Batch 2−5259 : Gut microbiota abundance (genus Catenibacterium id.2153) on DNA−binding protein SATB1
with a threshold of 1e−05

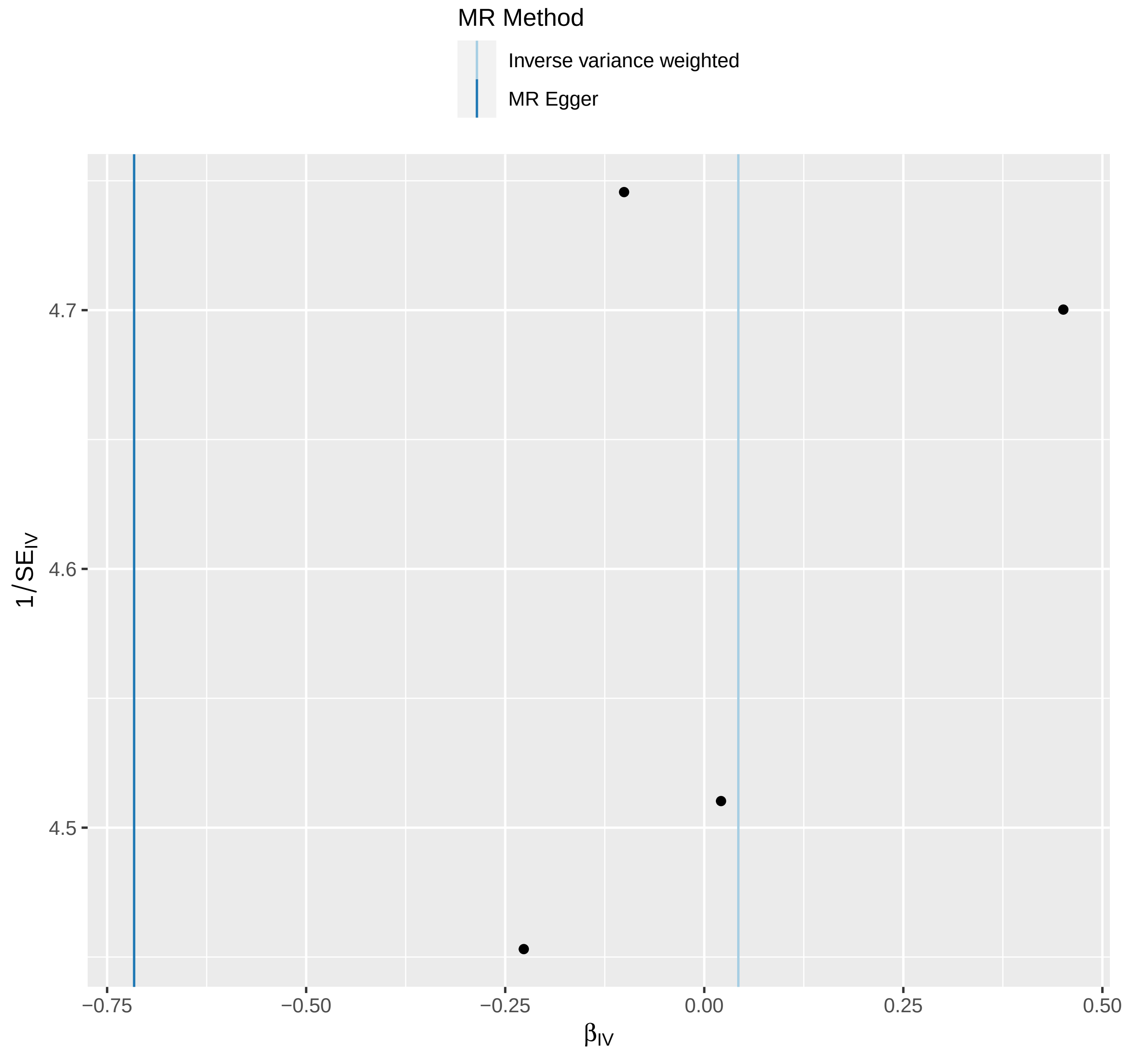

Batch 2−5260 : Gut microbiota abundance (genus Parasutterella id.2892) on DNA−binding protein SATB1
with a threshold of 1e−05

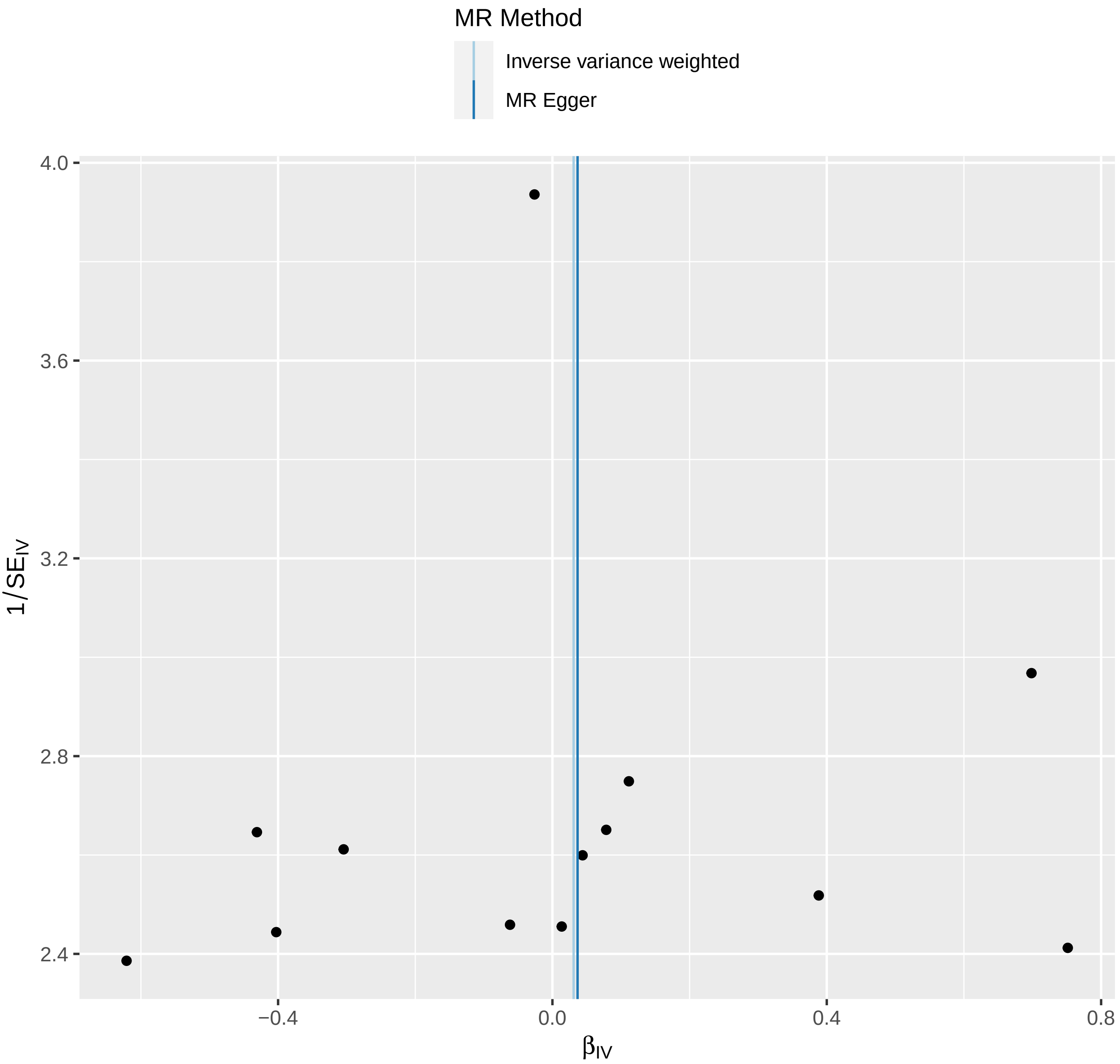

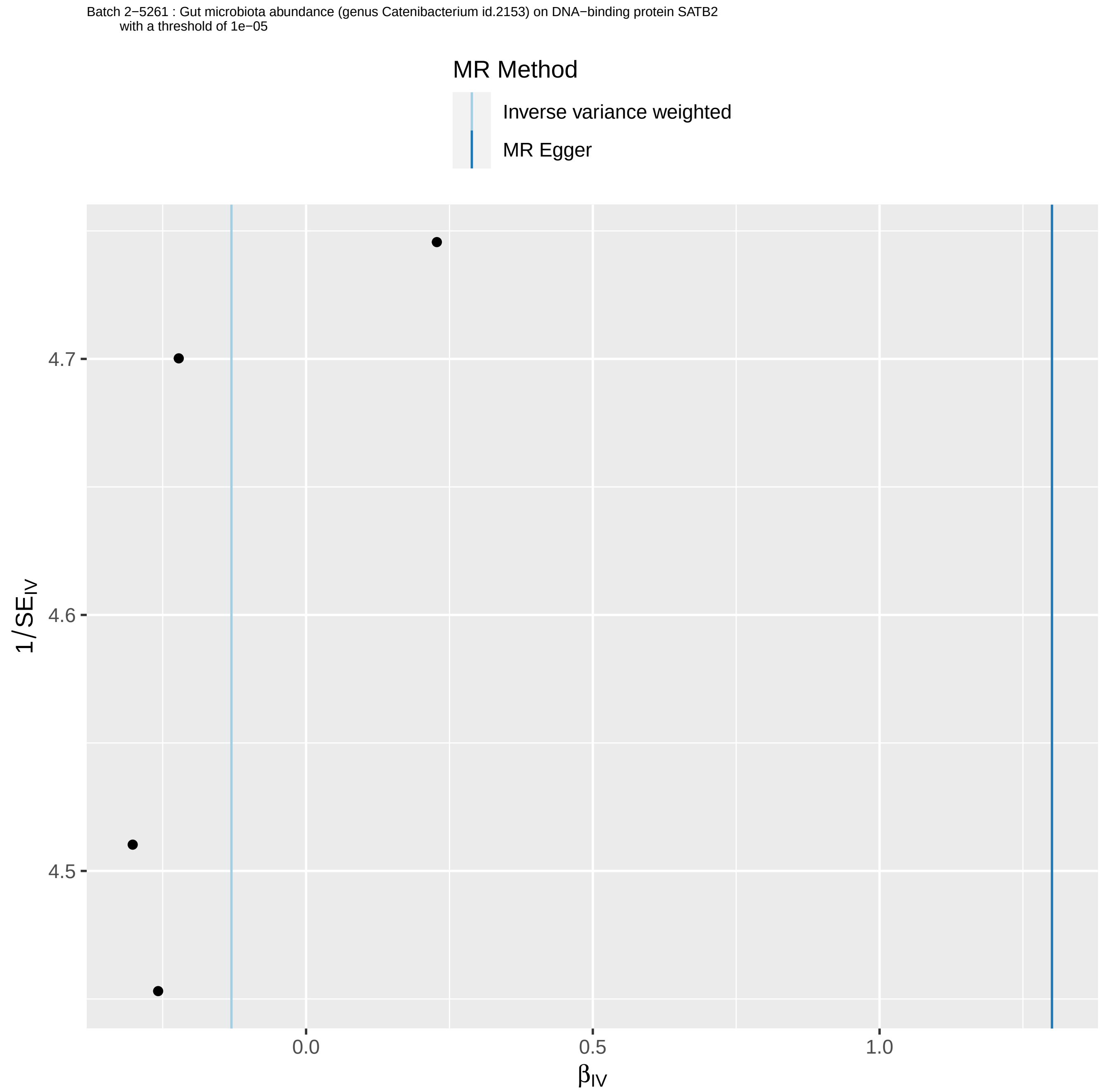

Batch 2−5261 : Gut microbiota abundance (genus Catenibacterium id.2153) on DNA−binding protein SATB2
with a threshold of 1e−05
MR Method
Inverse variance weighted
MR Egger
4.7
4.6
4.5
1/SE_IV
0.0
0.5
1.0
β_IV

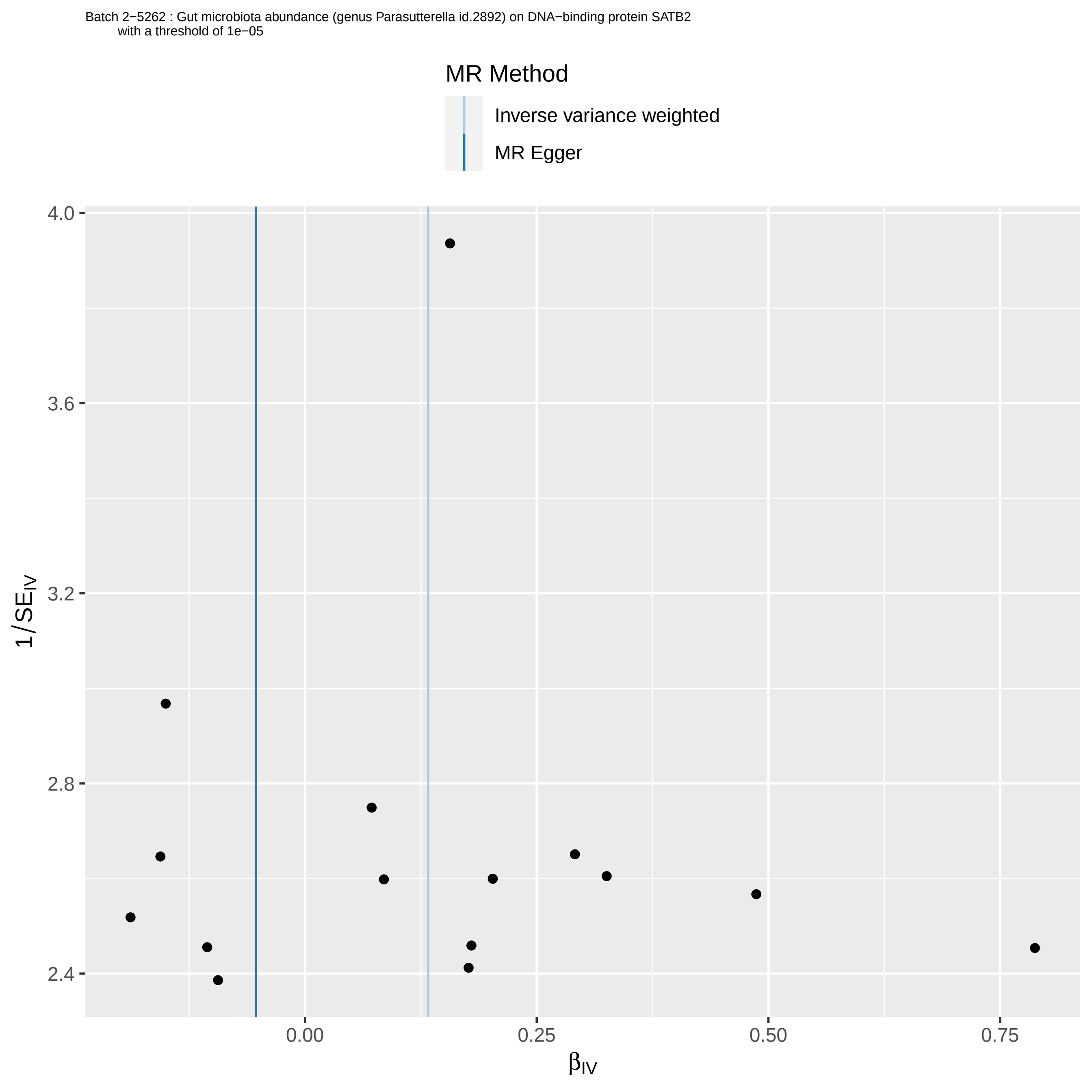
Batch 2−5262 : Gut microbiota abundance (genus Parasutterella id.2892) on DNA−binding protein SATB2
with a threshold of 1e−05
MR Method
Inverse variance weighted
MR Egger
4.0
3.6
3.2
2.8
2.4
1/SE_IV
0.00
0.25
0.50
0.75
β_IV

Batch 2−5263 : Gut microbiota abundance (genus Catenibacterium id.2153) on SAYSvFN domain−containing protein 1
with a threshold of 1e−05

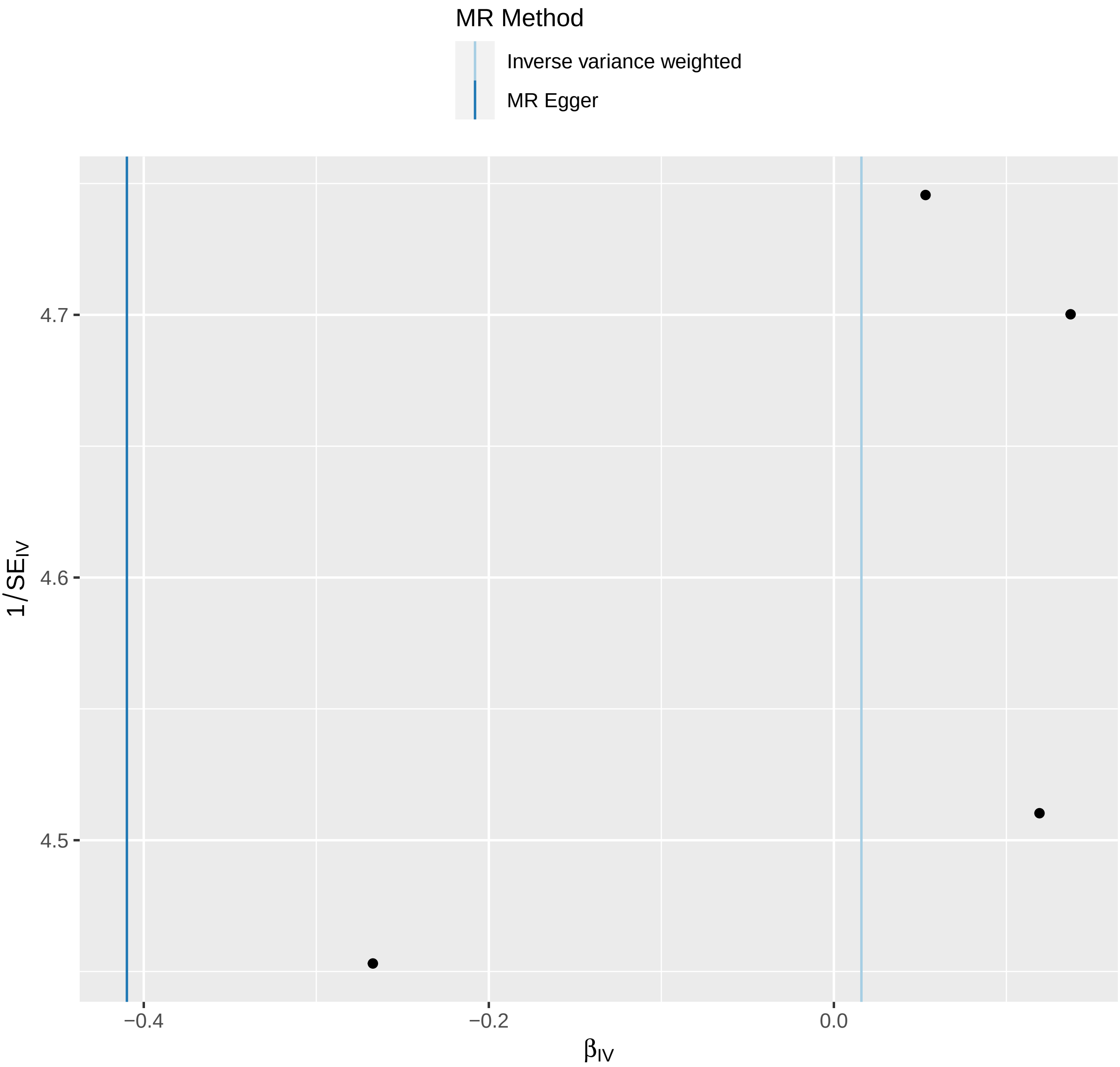

Batch 2−5264 : Gut microbiota abundance (genus Parasutterella id.2892) on SAYSvFN domain−containing protein 1
with a threshold of 1e−05

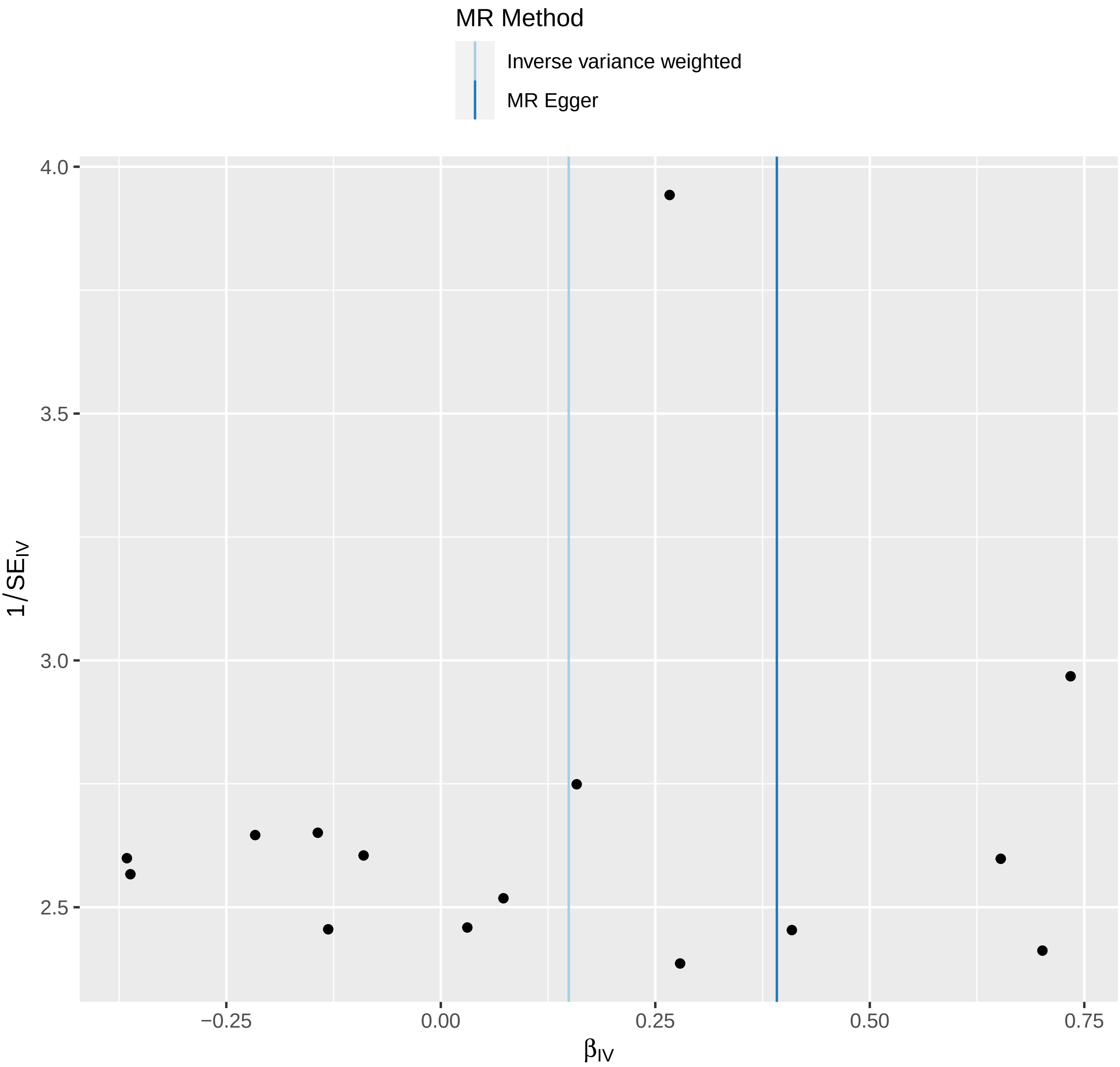

Batch 2−5265 : Gut microbiota abundance (genus Catenibacterium id.2153) on Suprabasin
with a threshold of 1e−05

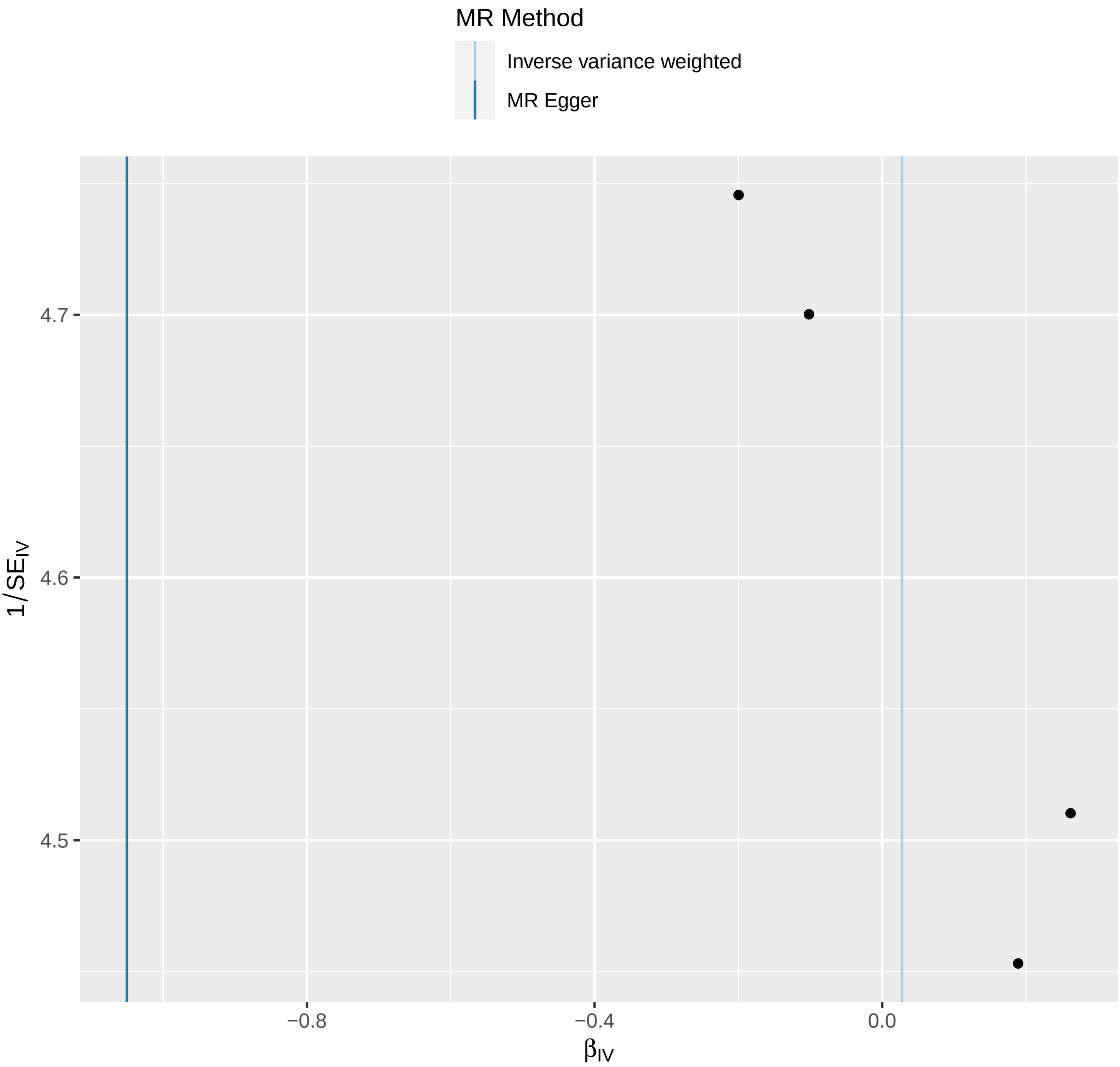

Batch 2−5266 : Gut microbiota abundance (genus Parasutterella id.2892) on Suprabasin
with a threshold of 1e−05

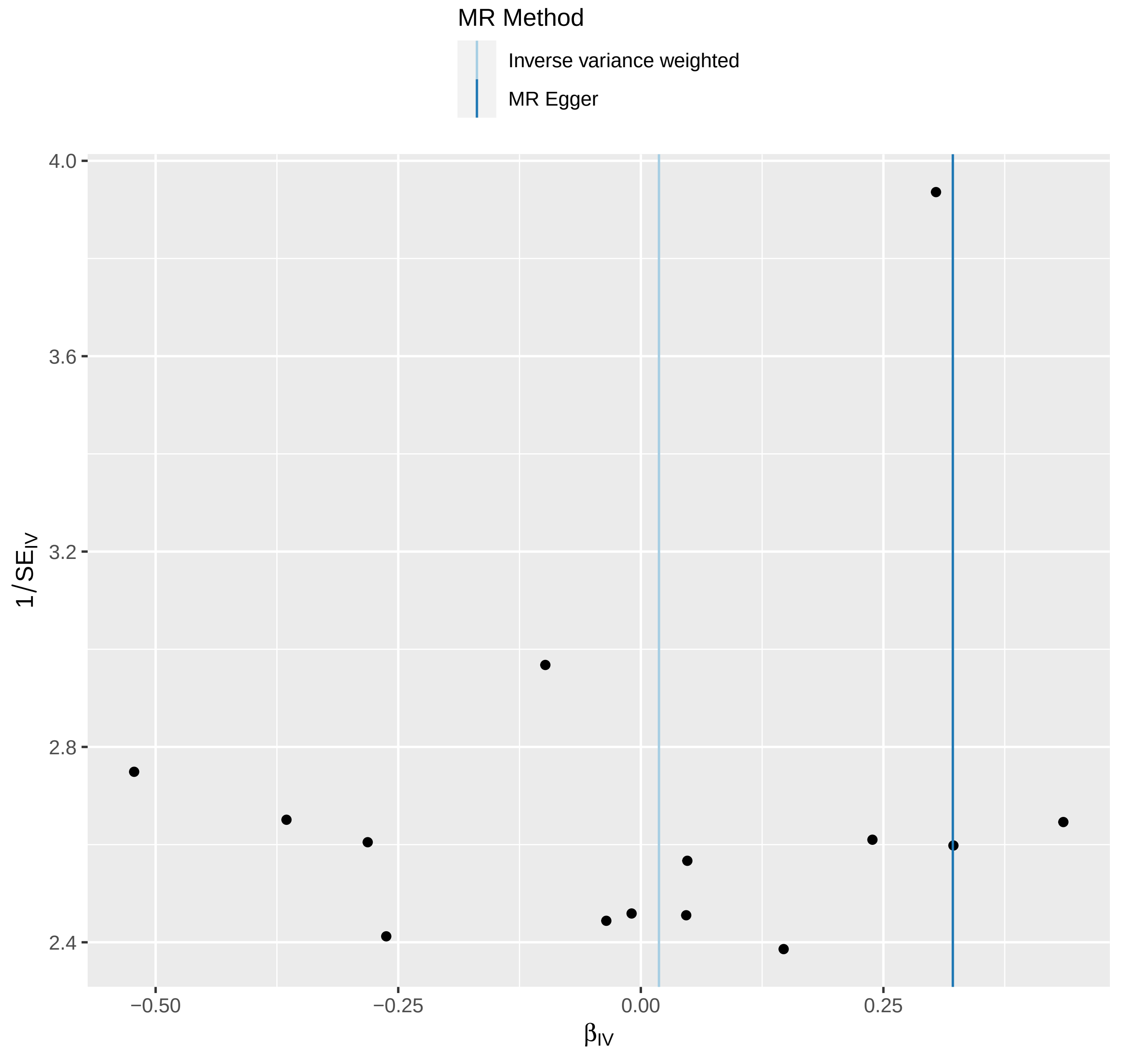

Batch 2−5267 : Gut microbiota abundance (genus Catenibacterium id.2153) on Secretory carrier−associated membrane protein 5
with a threshold of 1e−05

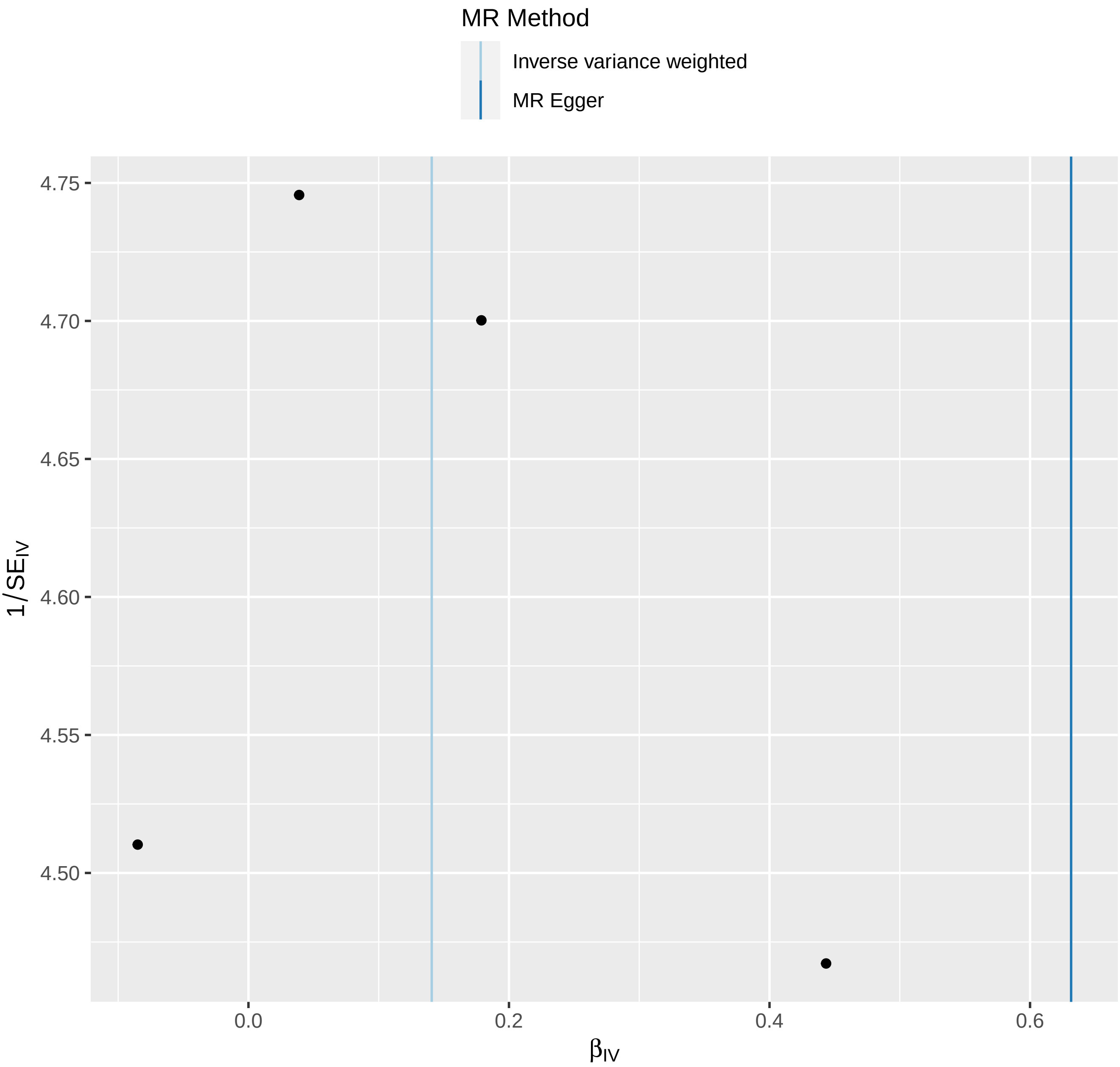

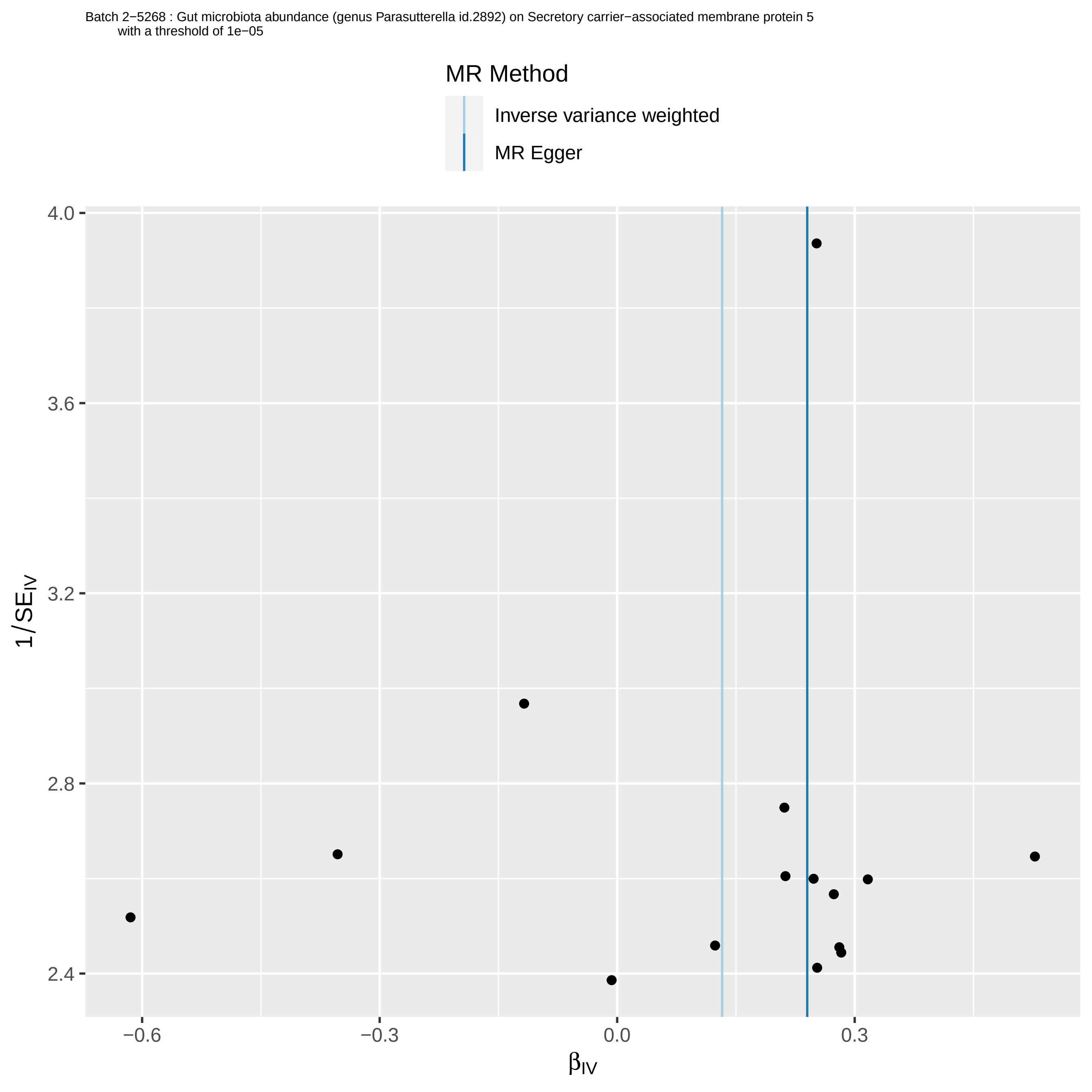

Batch 2−5268 : Gut microbiota abundance (genus Parasutterella id.2892) on Secretory carrier−associated membrane protein 5
with a threshold of 1e−05
MR Method
Inverse variance weighted
MR Egger
4.0
3.6
3.2
2.8
2.4
1/SE_IV
−0.6
−0.3
0.0
0.3
β_IV

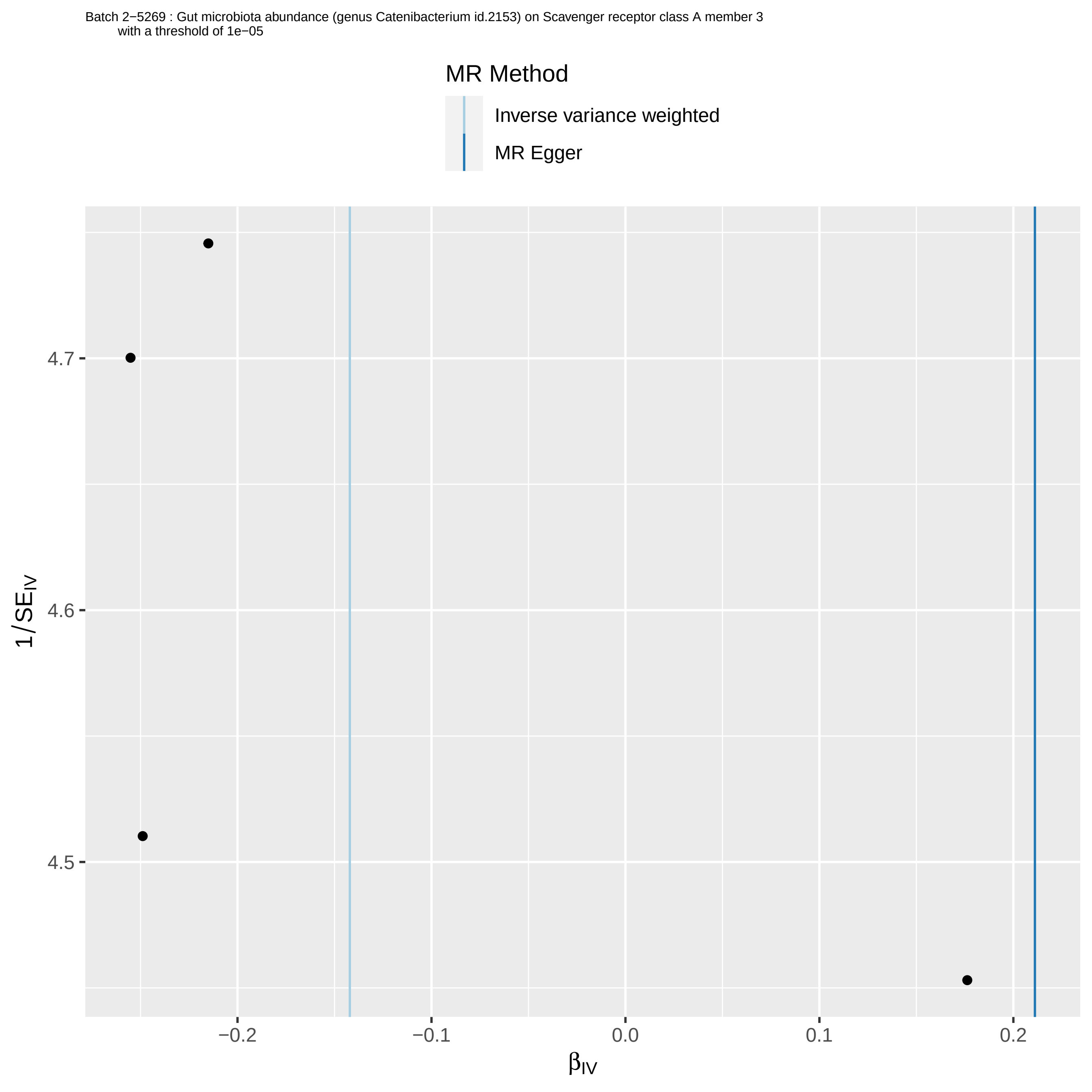

Batch 2−5269 : Gut microbiota abundance (genus Catenibacterium id.2153) on Scavenger receptor class A member 3
with a threshold of 1e−05
MR Method
Inverse variance weighted
MR Egger
4.7
4.6
4.5
1/SE_IV
−0.2
−0.1
0.0
0.1
0.2
β_IV

Batch 2−5270 : Gut microbiota abundance (genus Parasutterella id.2892) on Scavenger receptor class A member 3
with a threshold of 1e−05

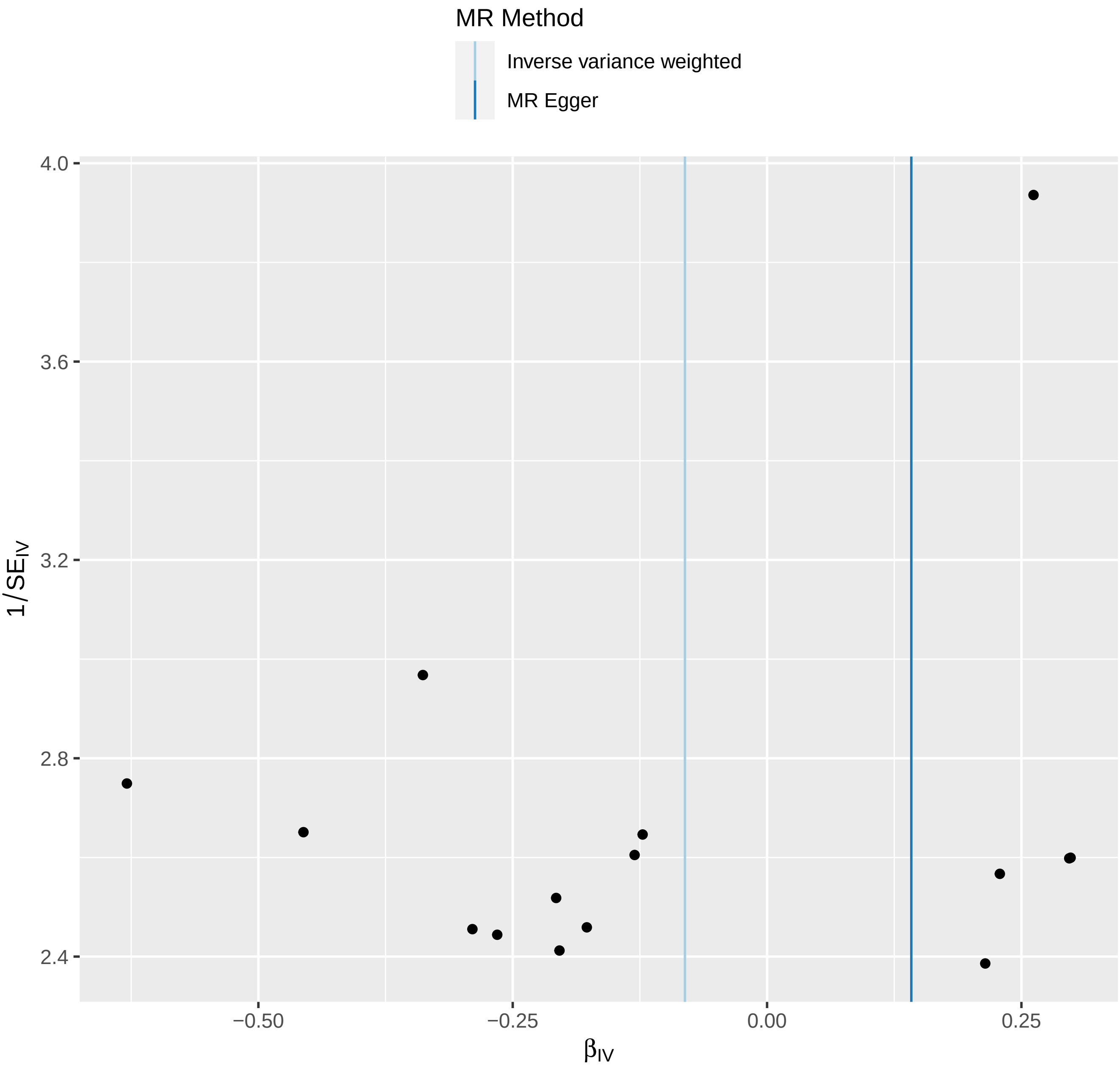

Batch 2−5271 : Gut microbiota abundance (genus Catenibacterium id.2153) on Scavenger receptor class A member 3
with a threshold of 1e−05

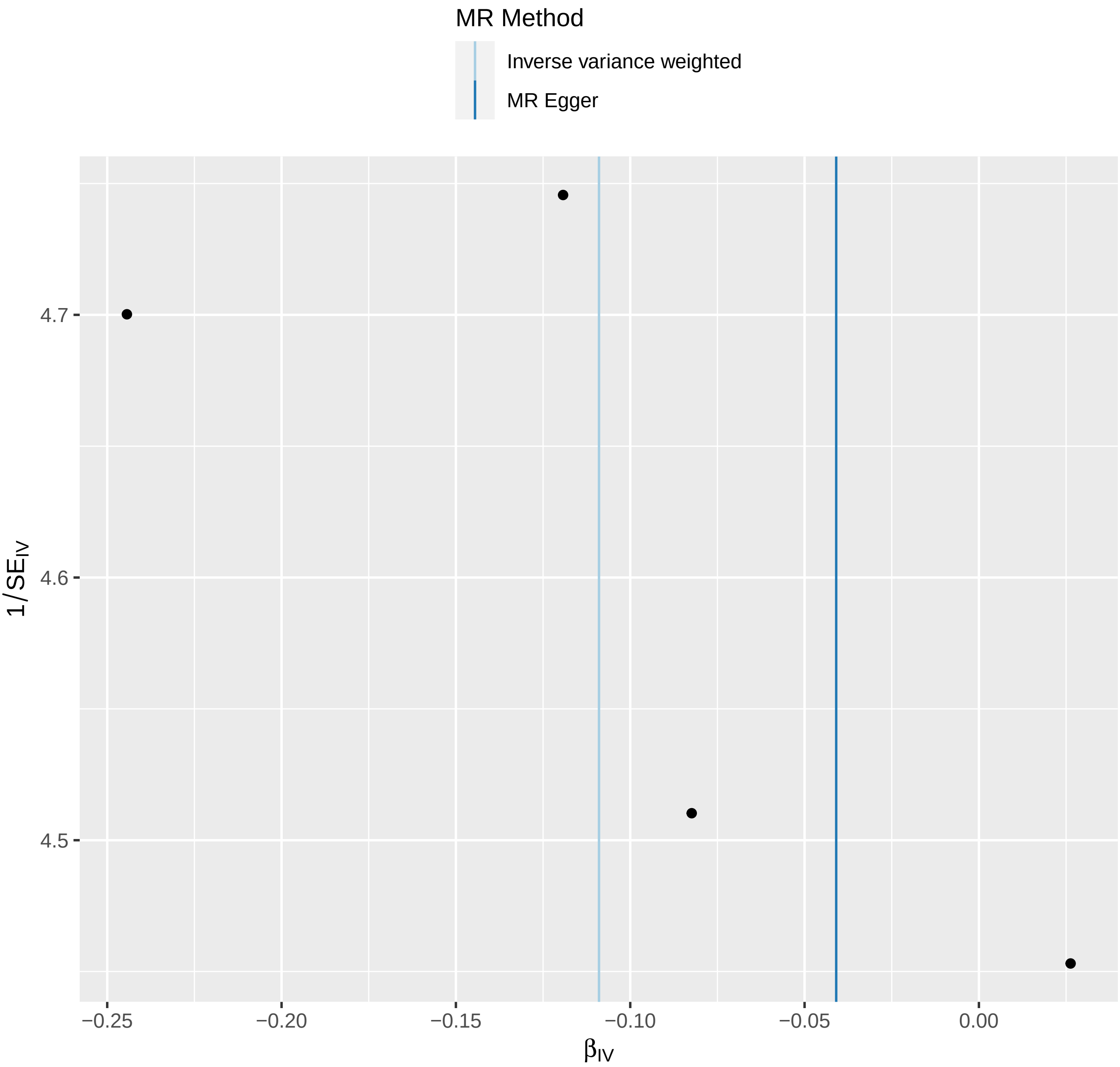

Batch 2−5272 : Gut microbiota abundance (genus Parasutterella id.2892) on Scavenger receptor class A member 3
with a threshold of 1e−05

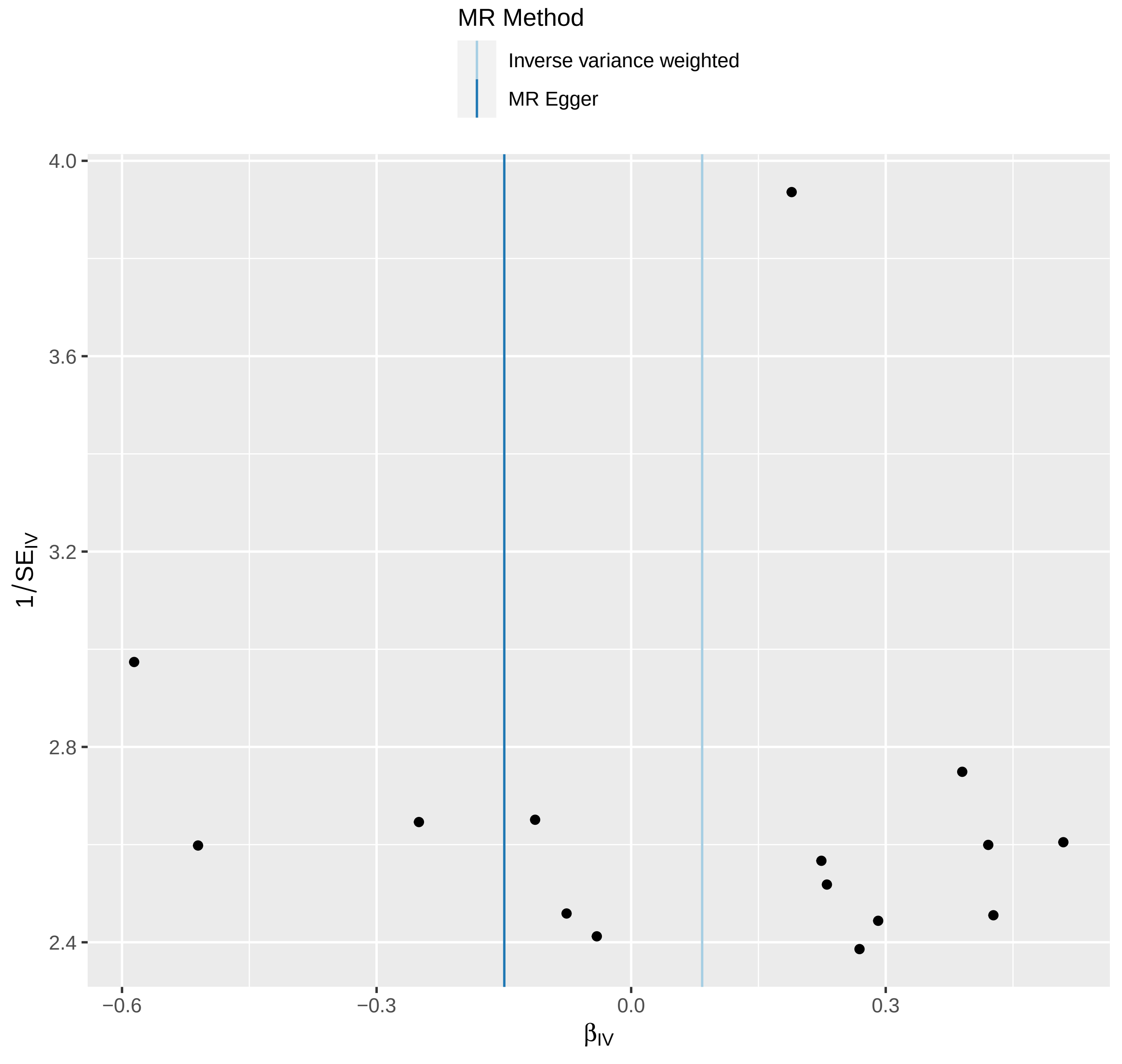

Batch 2−5273 : Gut microbiota abundance (genus Catenibacterium id.2153) on Scavenger receptor class A member 3
with a threshold of 1e−05

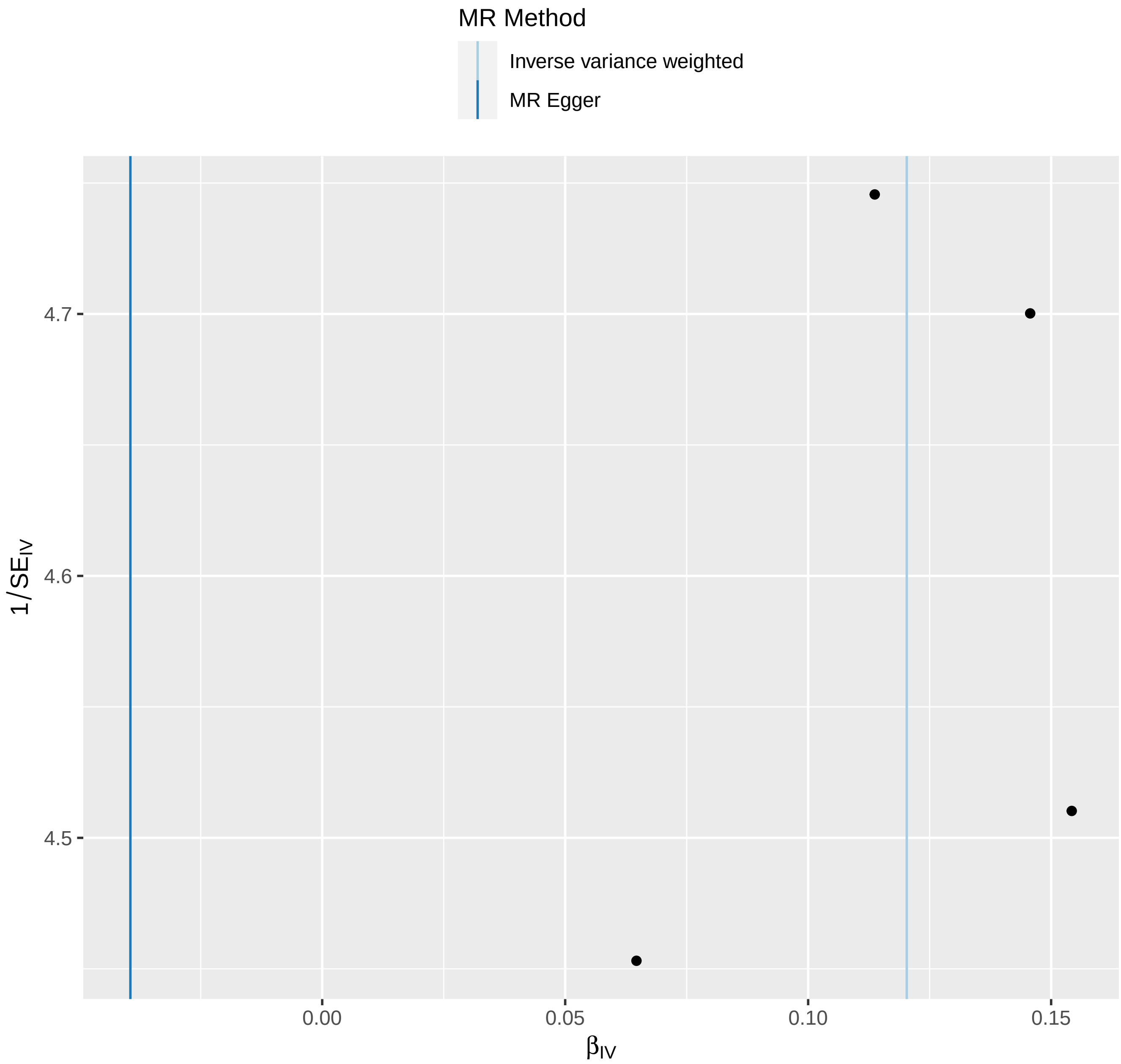

Batch 2−5274 : Gut microbiota abundance (genus Parasutterella id.2892) on Scavenger receptor class A member 3
with a threshold of 1e−05

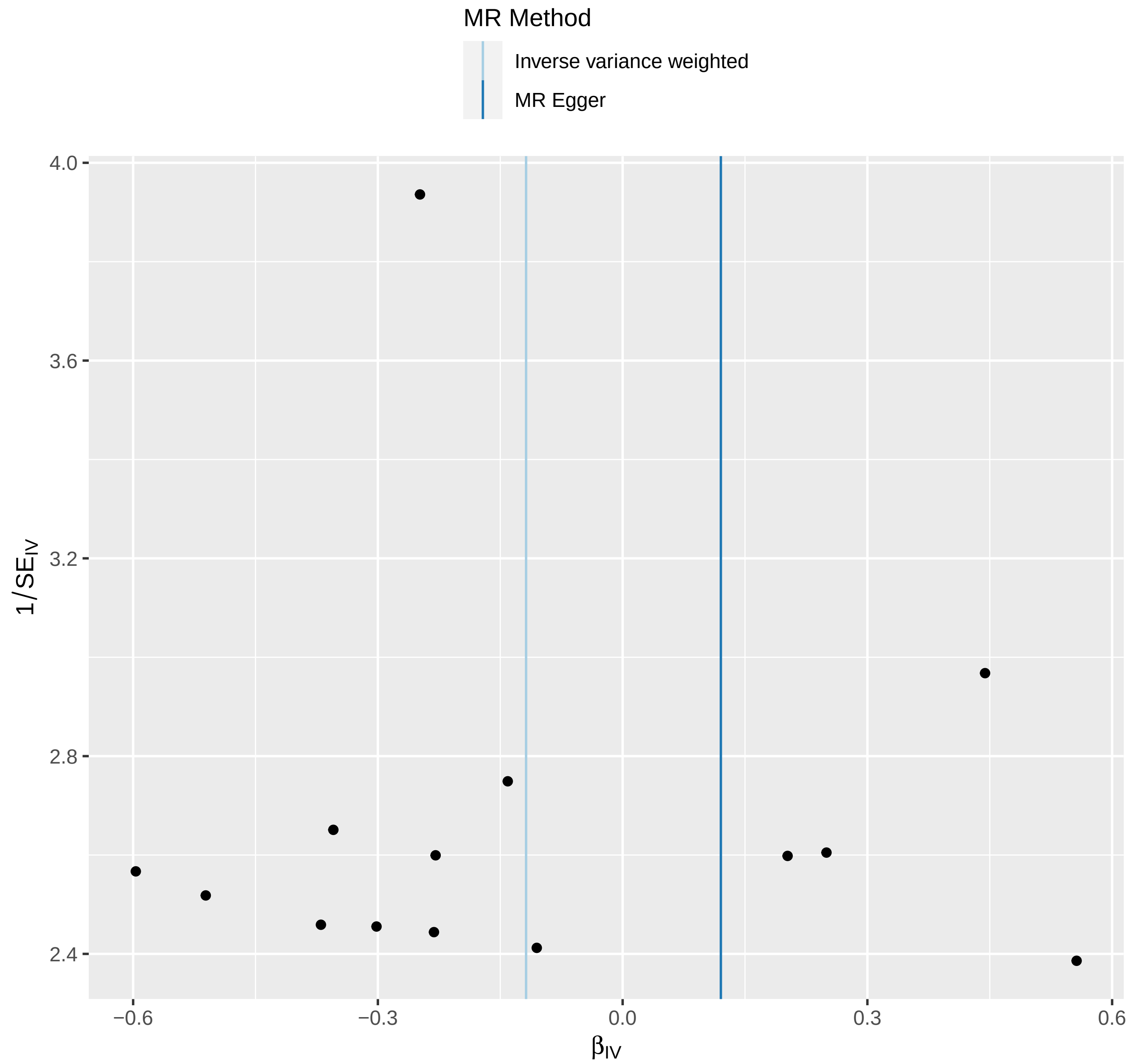

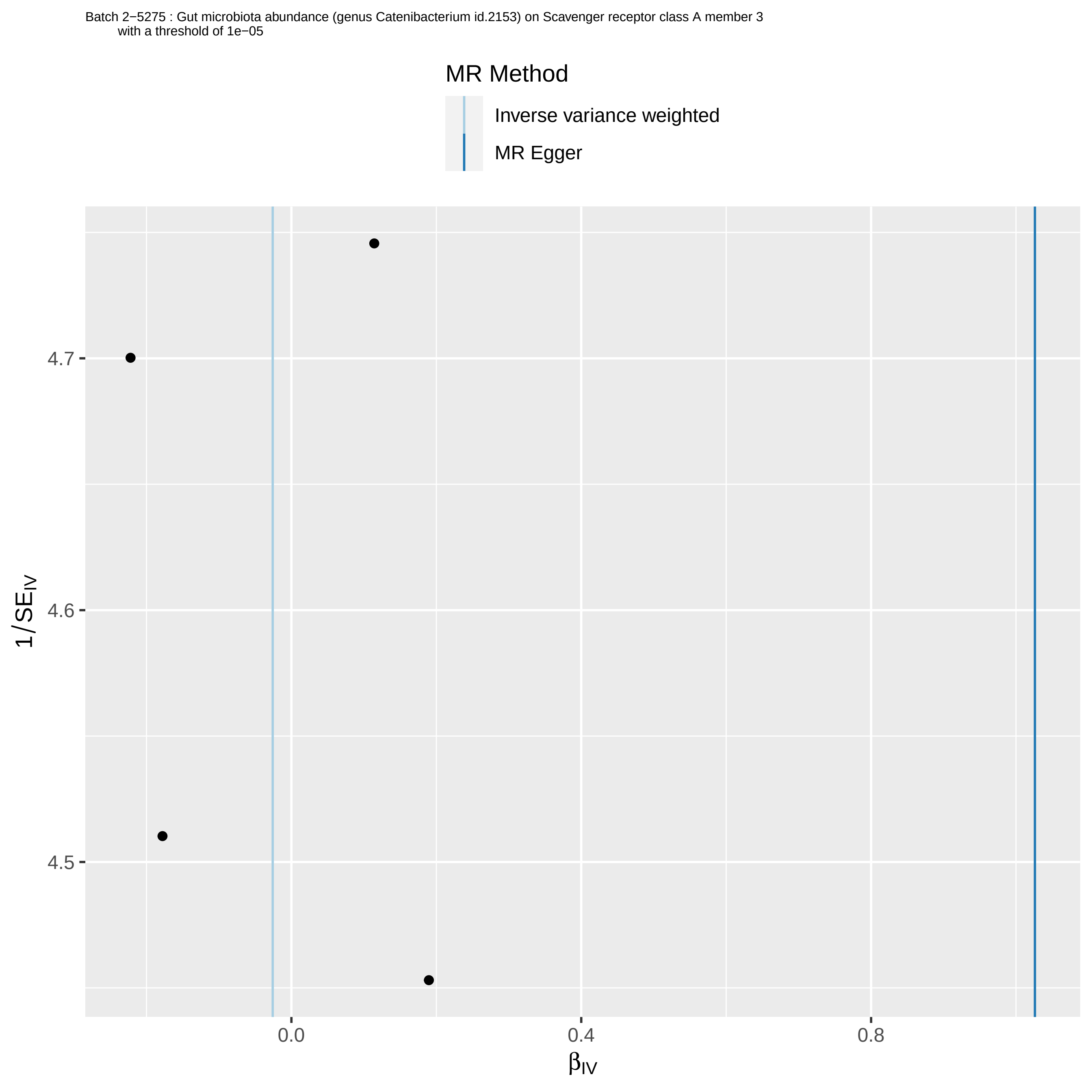
Batch 2−5275 : Gut microbiota abundance (genus Catenibacterium id.2153) on Scavenger receptor class A member 3
with a threshold of 1e−05
MR Method
Inverse variance weighted
MR Egger
4.7
4.6
4.5
1/SE_IV
0.0
0.4
0.8
β_IV

Batch 2−5276 : Gut microbiota abundance (genus Parasutterella id.2892) on Scavenger receptor class A member 3
with a threshold of 1e−05

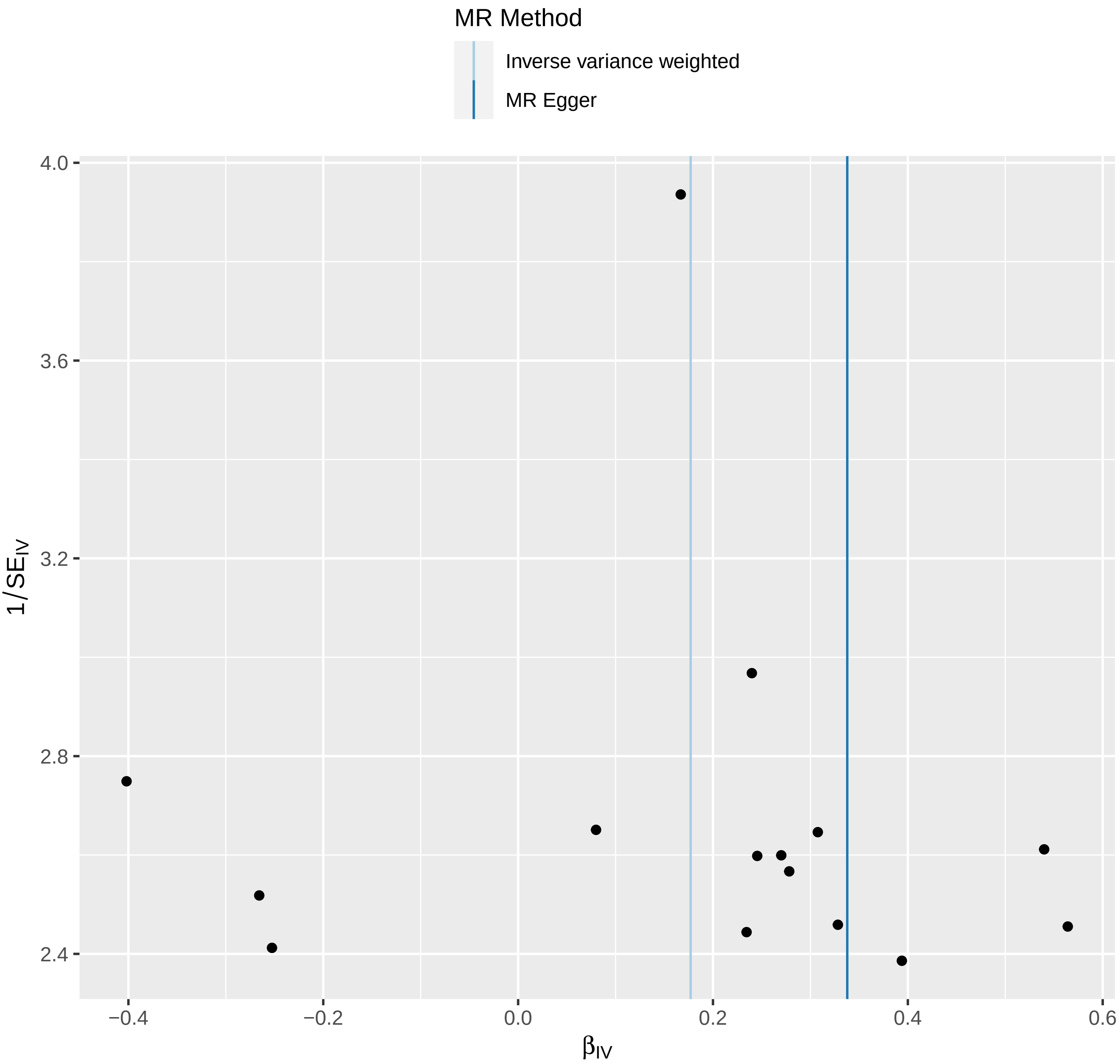

Batch 2−5277 : Gut microbiota abundance (genus Catenibacterium id.2153) on Scavenger receptor class A member 5
with a threshold of 1e−05

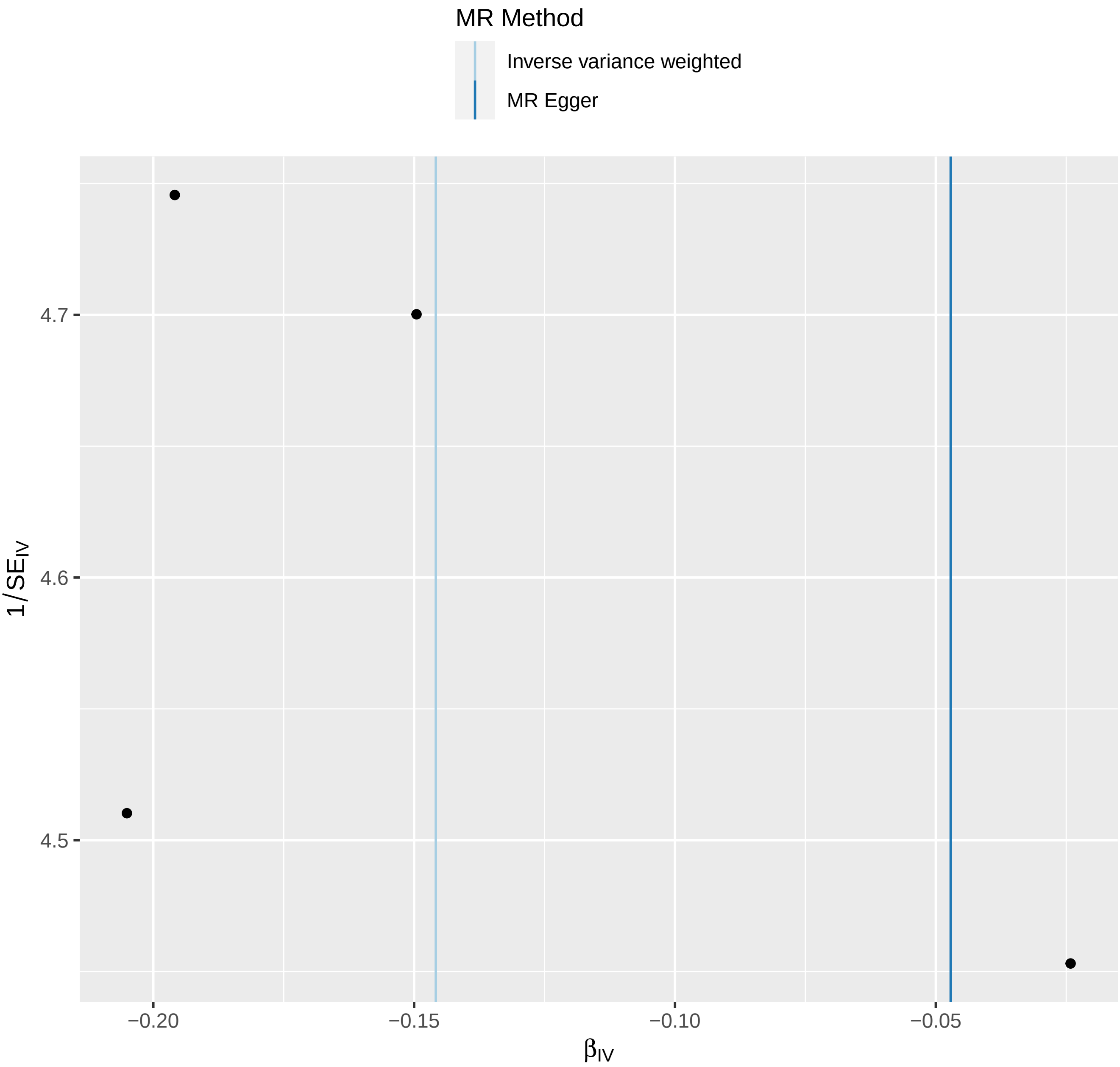

Batch 2−5278 : Gut microbiota abundance (genus Parasutterella id.2892) on Scavenger receptor class A member 5
with a threshold of 1e−05

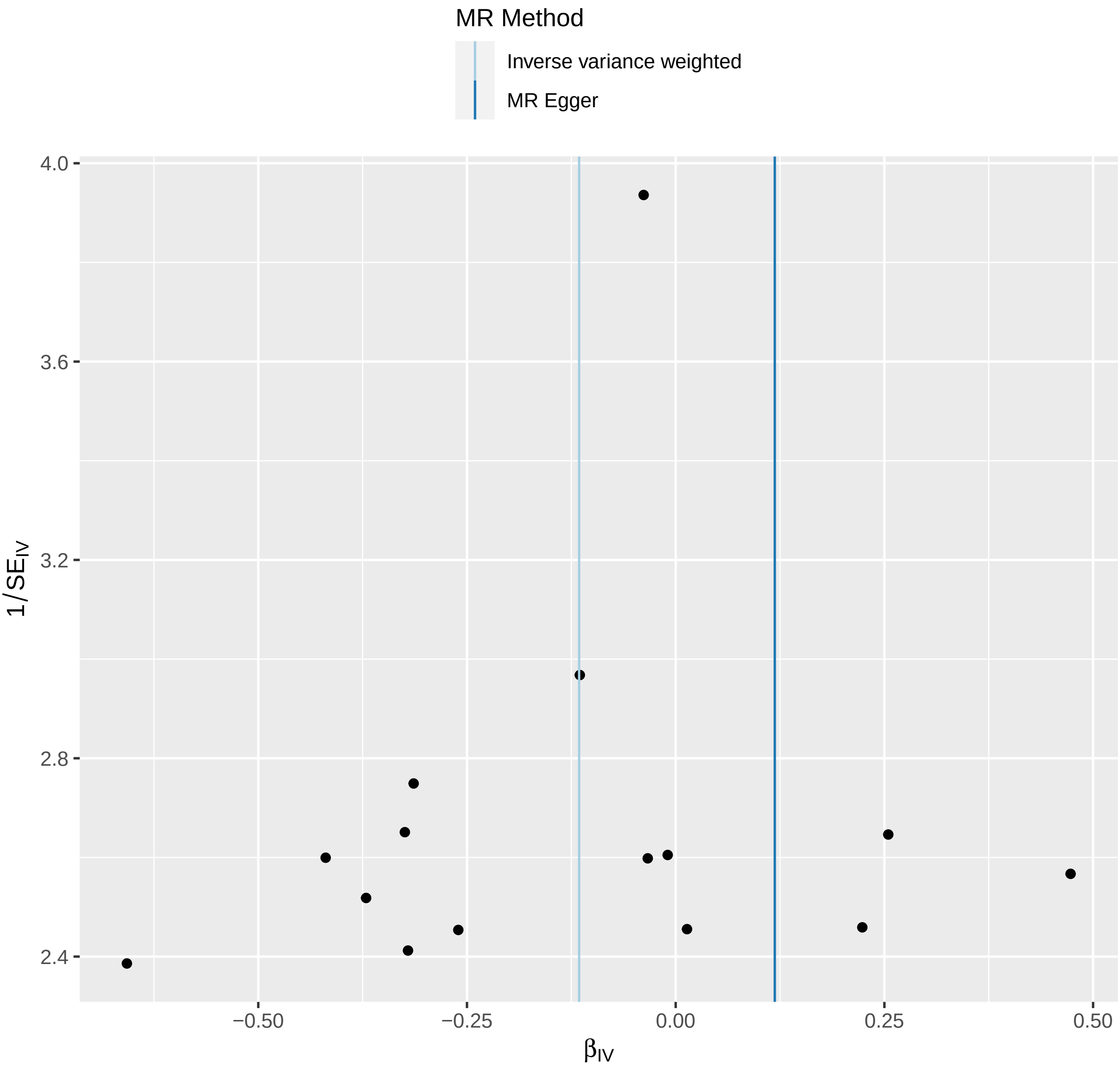

Batch 2−5279 : Gut microbiota abundance (genus Catenibacterium id.2153) on Lysosome membrane protein 2
with a threshold of 1e−05

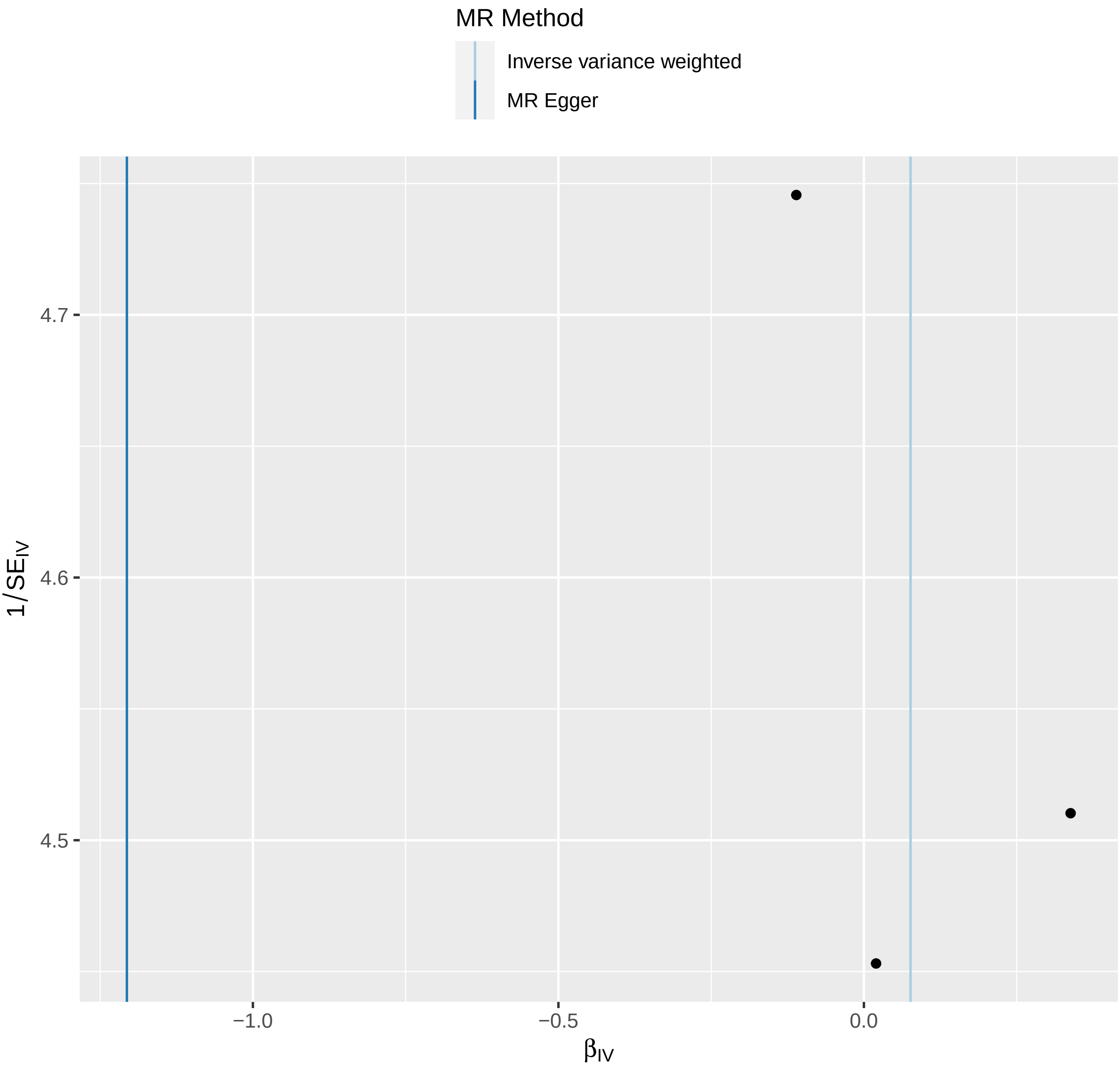

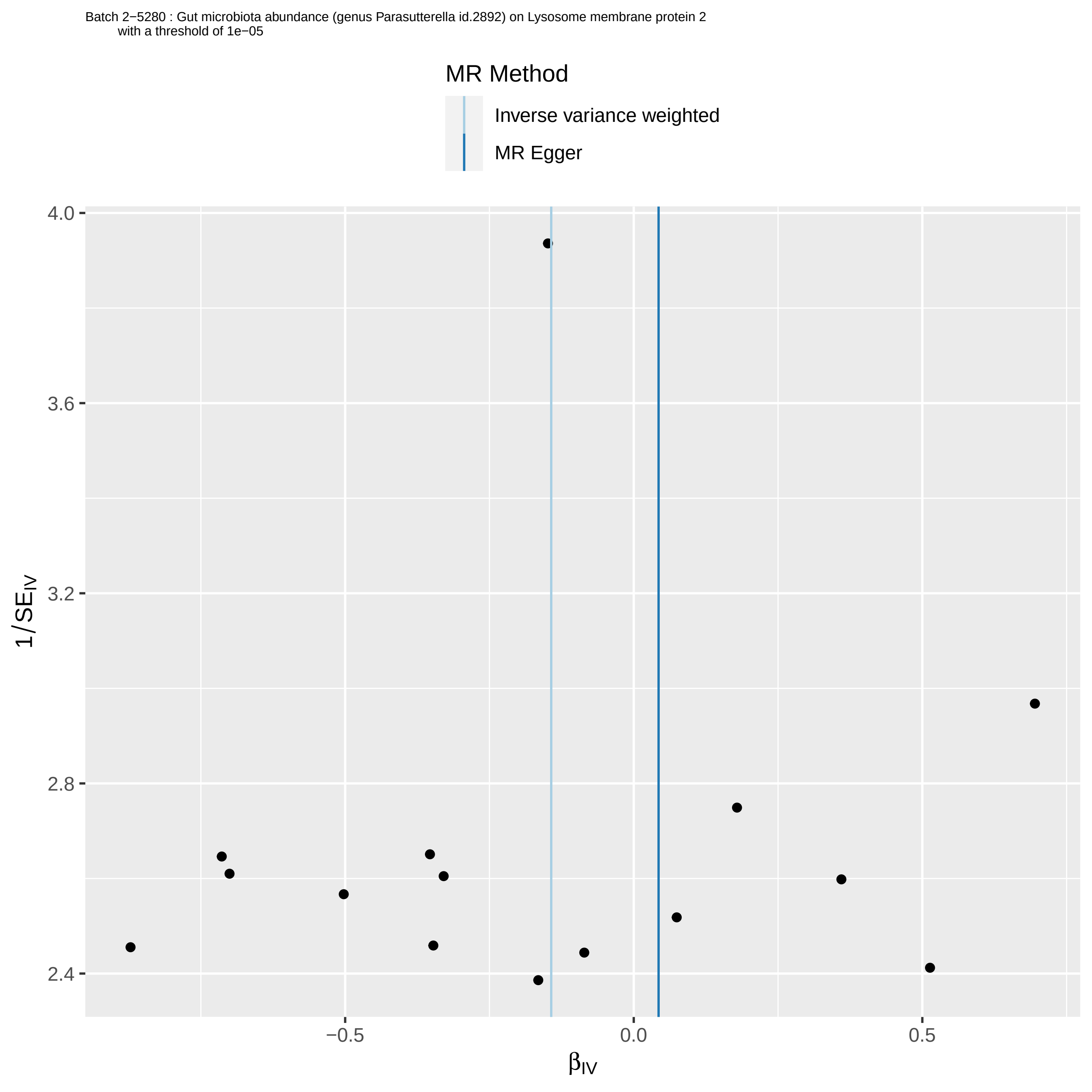

Batch 2−5280 : Gut microbiota abundance (genus Parasutterella id.2892) on Lysosome membrane protein 2
with a threshold of 1e−05
MR Method
Inverse variance weighted
MR Egger
4.0
3.6
3.2
2.8
2.4
1/SE_IV
−0.5
0.0
0.5
β_IV

Batch 2−5281 : Gut microbiota abundance (genus Catenibacterium id.2153) on Scavenger receptor class F member 2
with a threshold of 1e−05

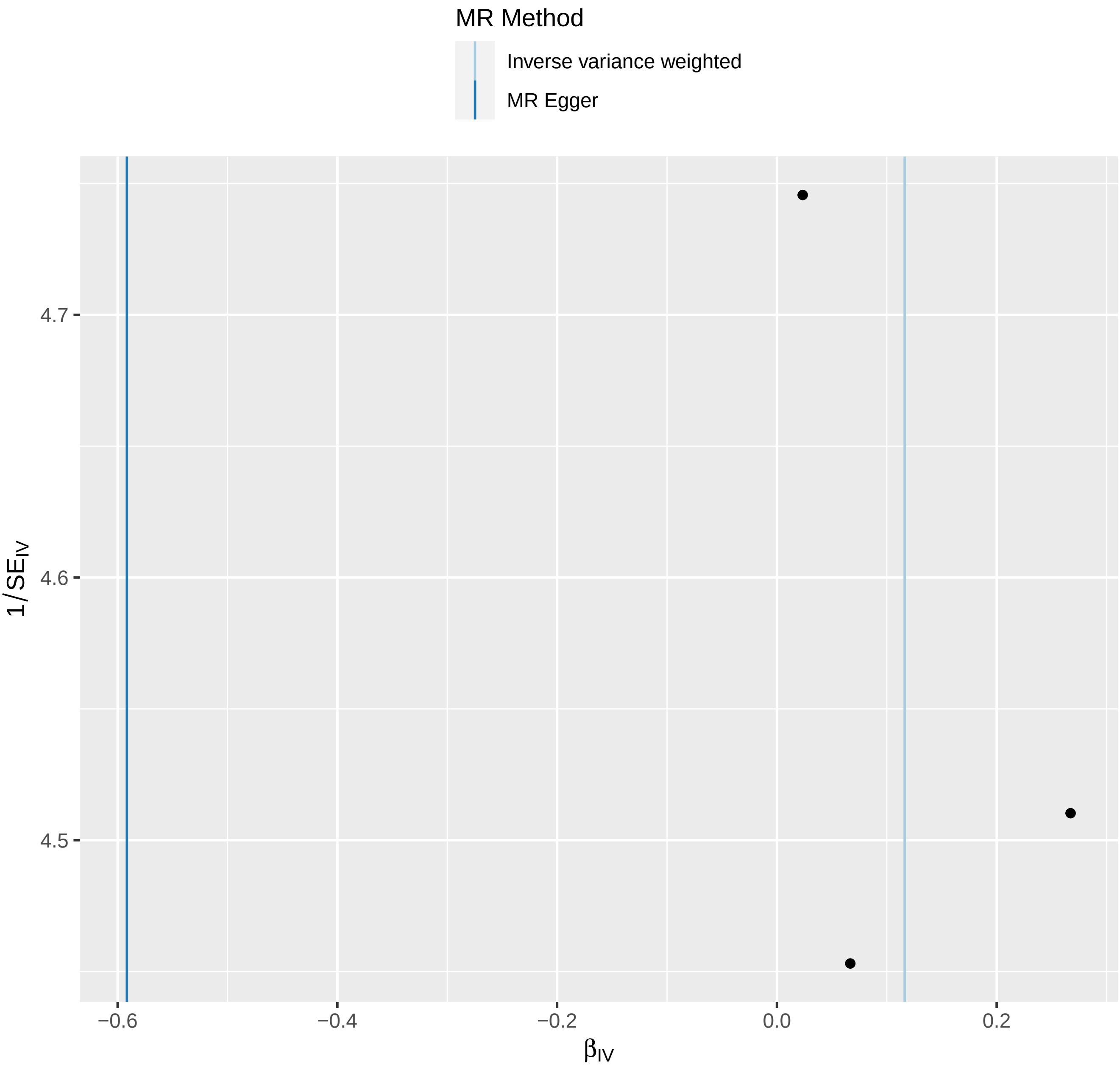

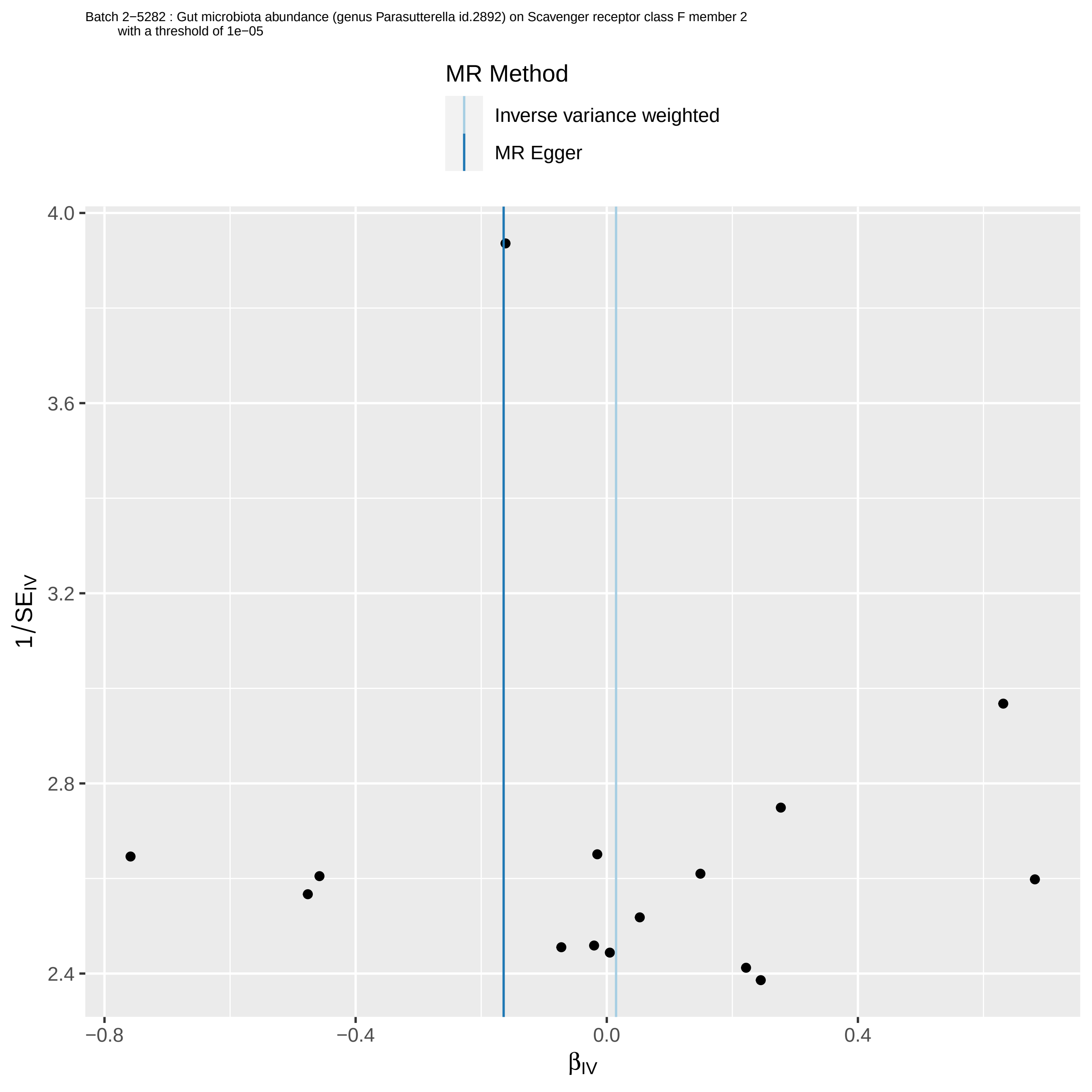

Batch 2−5282 : Gut microbiota abundance (genus Parasutterella id.2892) on Scavenger receptor class F member 2
with a threshold of 1e−05
MR Method
Inverse variance weighted
MR Egger
4.0
3.6
3.2
2.8
2.4
1/SE_IV
−0.8
−0.4
0.0
0.4
β_IV

Batch 2−5283 : Gut microbiota abundance (genus Catenibacterium id.2153) on Scavenger receptor class F member 2
with a threshold of 1e−05

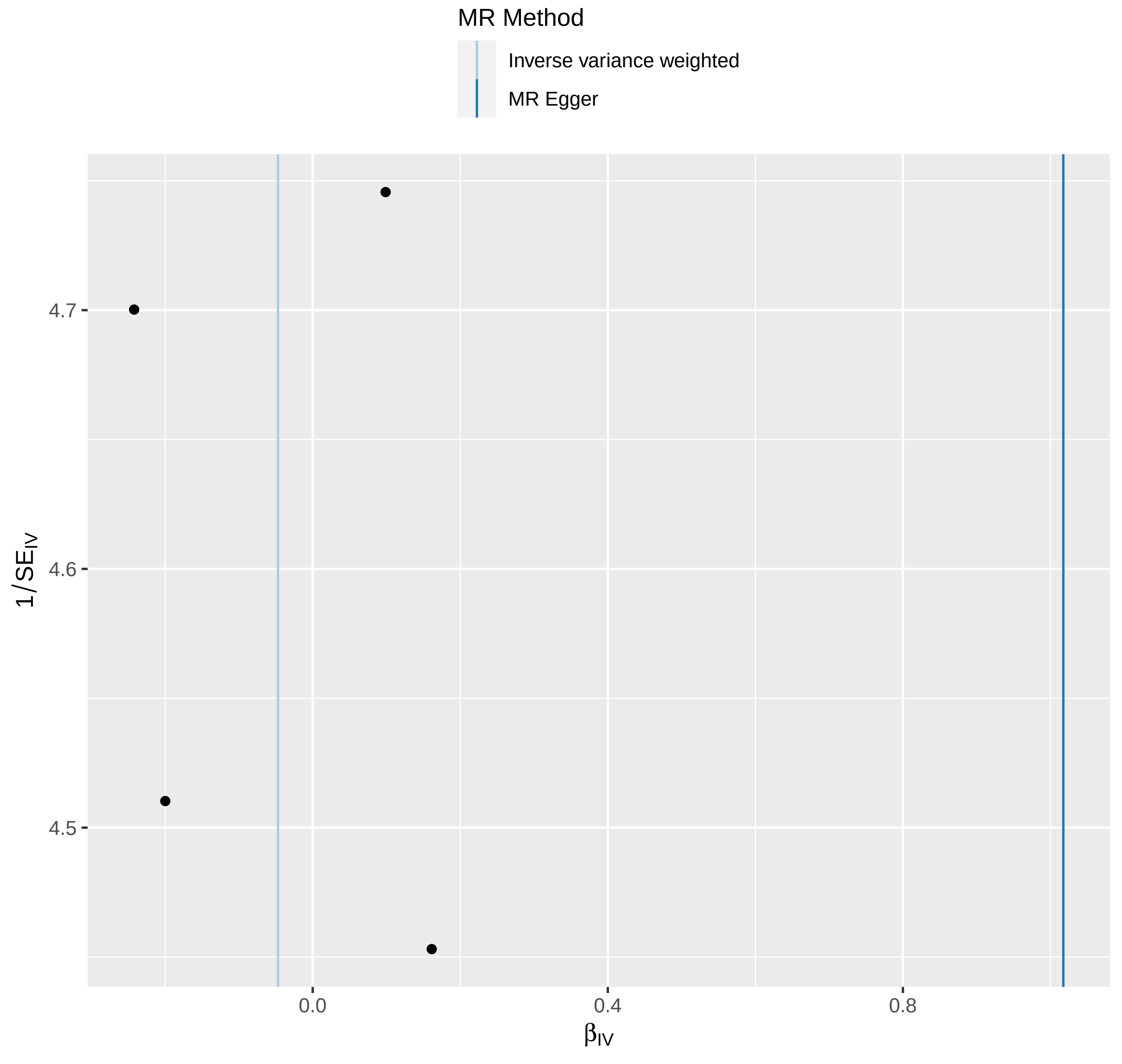

Batch 2−5284 : Gut microbiota abundance (genus Parasutterella id.2892) on Scavenger receptor class F member 2
with a threshold of 1e−05

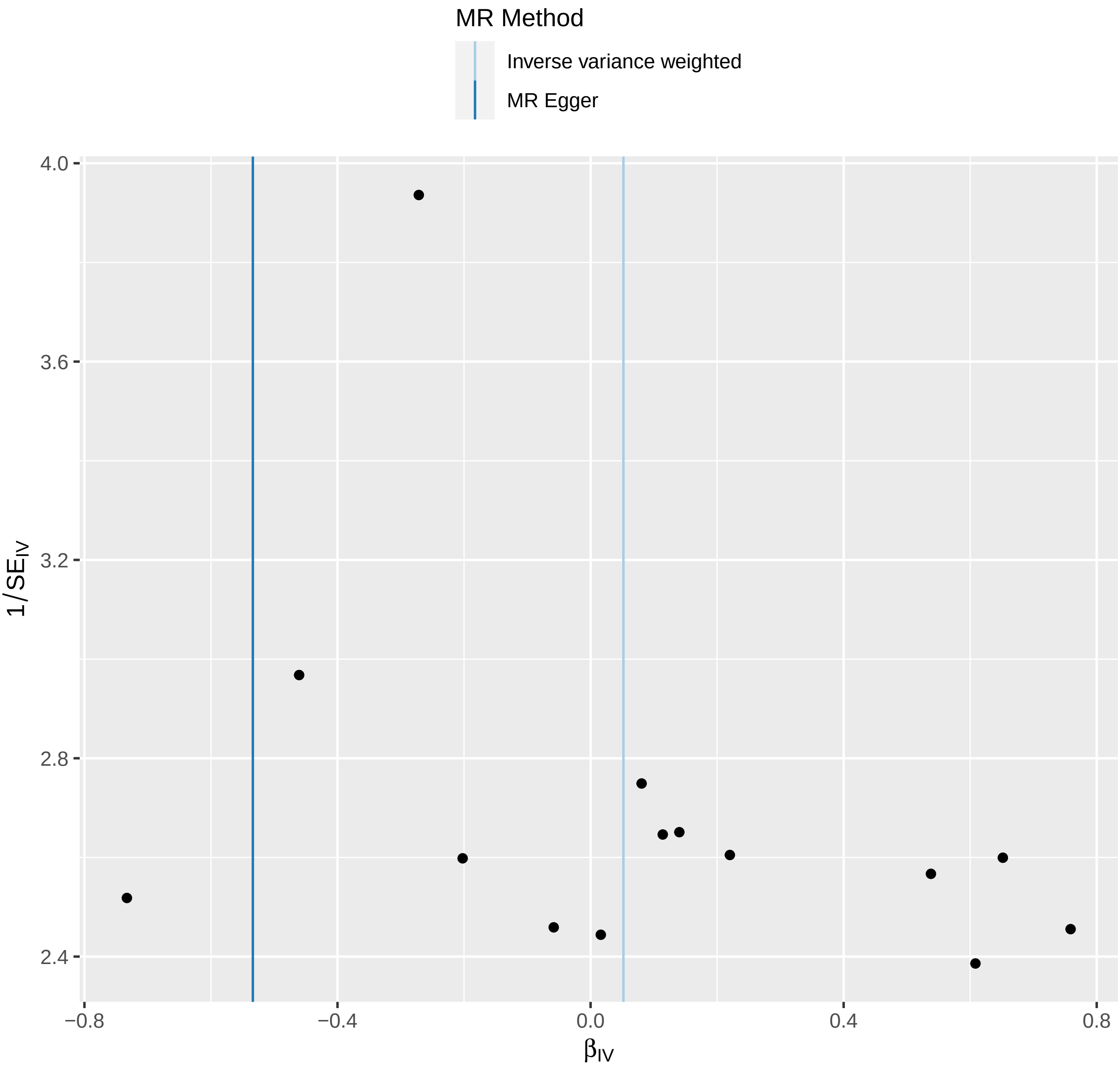

Batch 2−5285 : Gut microbiota abundance (genus Catenibacterium id.2153) on Scavenger receptor class F member 2
with a threshold of 1e−05

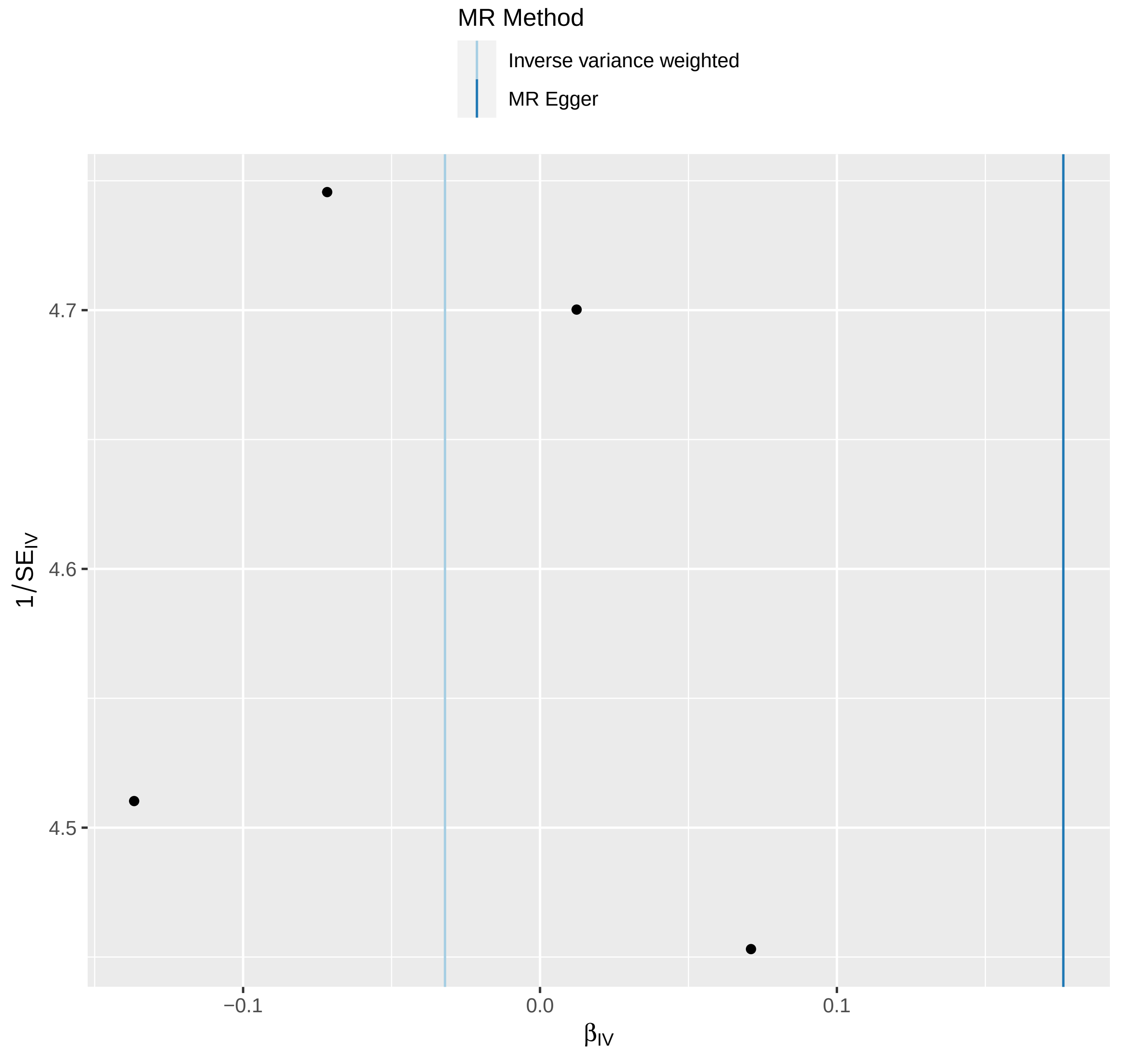

Batch 2−5286 : Gut microbiota abundance (genus Parasutterella id.2892) on Scavenger receptor class F member 2 with a threshold of 1e−05

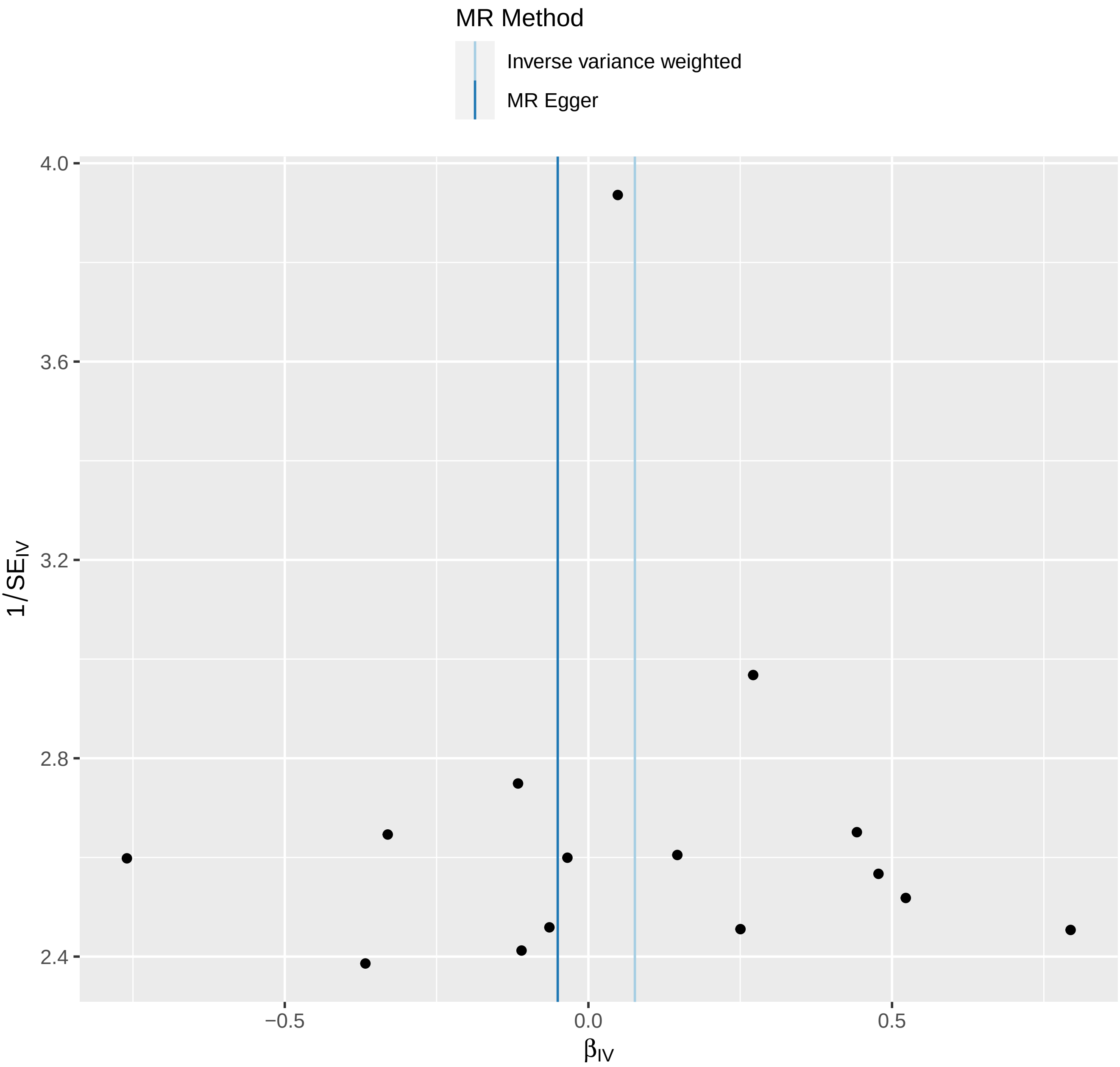

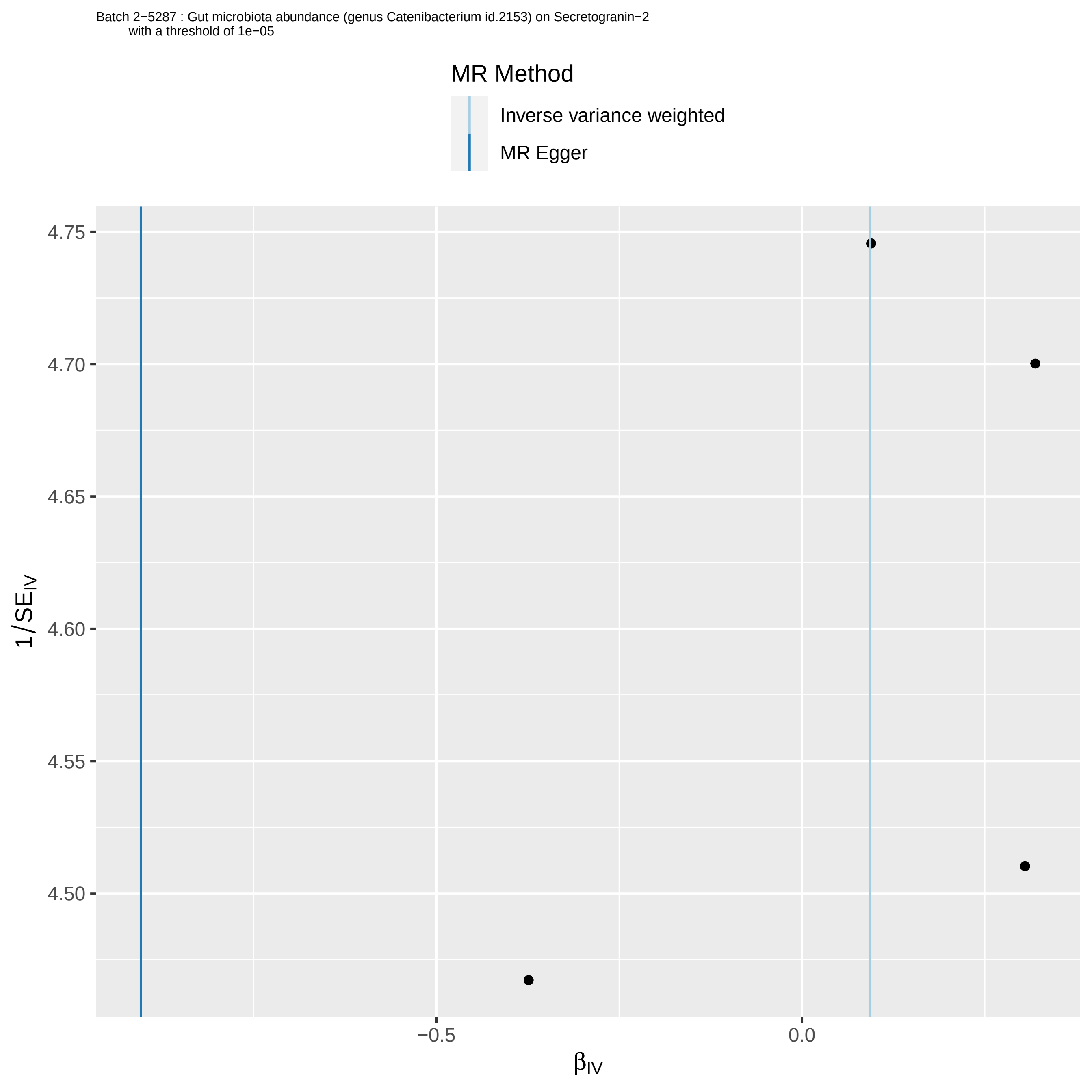
Batch 2−5287 : Gut microbiota abundance (genus Catenibacterium id.2153) on Secretogranin−2
with a threshold of 1e−05
MR Method
Inverse variance weighted
MR Egger
4.75
4.70
4.65
4.60
4.55
4.50
1/SE_IV
−0.5
0.0
β_IV

Batch 2−5288 : Gut microbiota abundance (genus Parasutterella id.2892) on Secretogranin−2
with a threshold of 1e−05

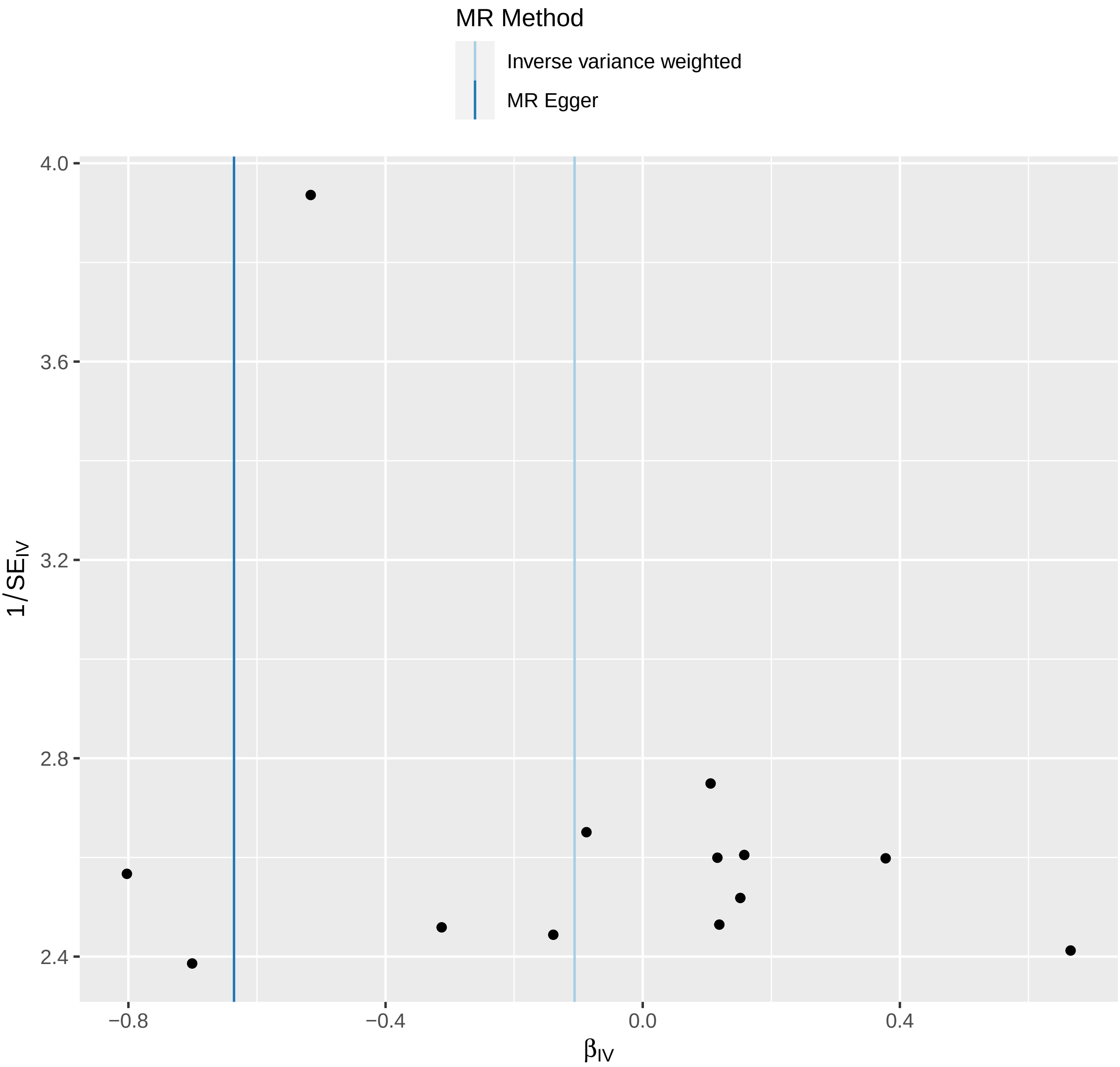

Batch 2−5289 : Gut microbiota abundance (genus Catenibacterium id.2153) on Secretogranin−3
with a threshold of 1e−05

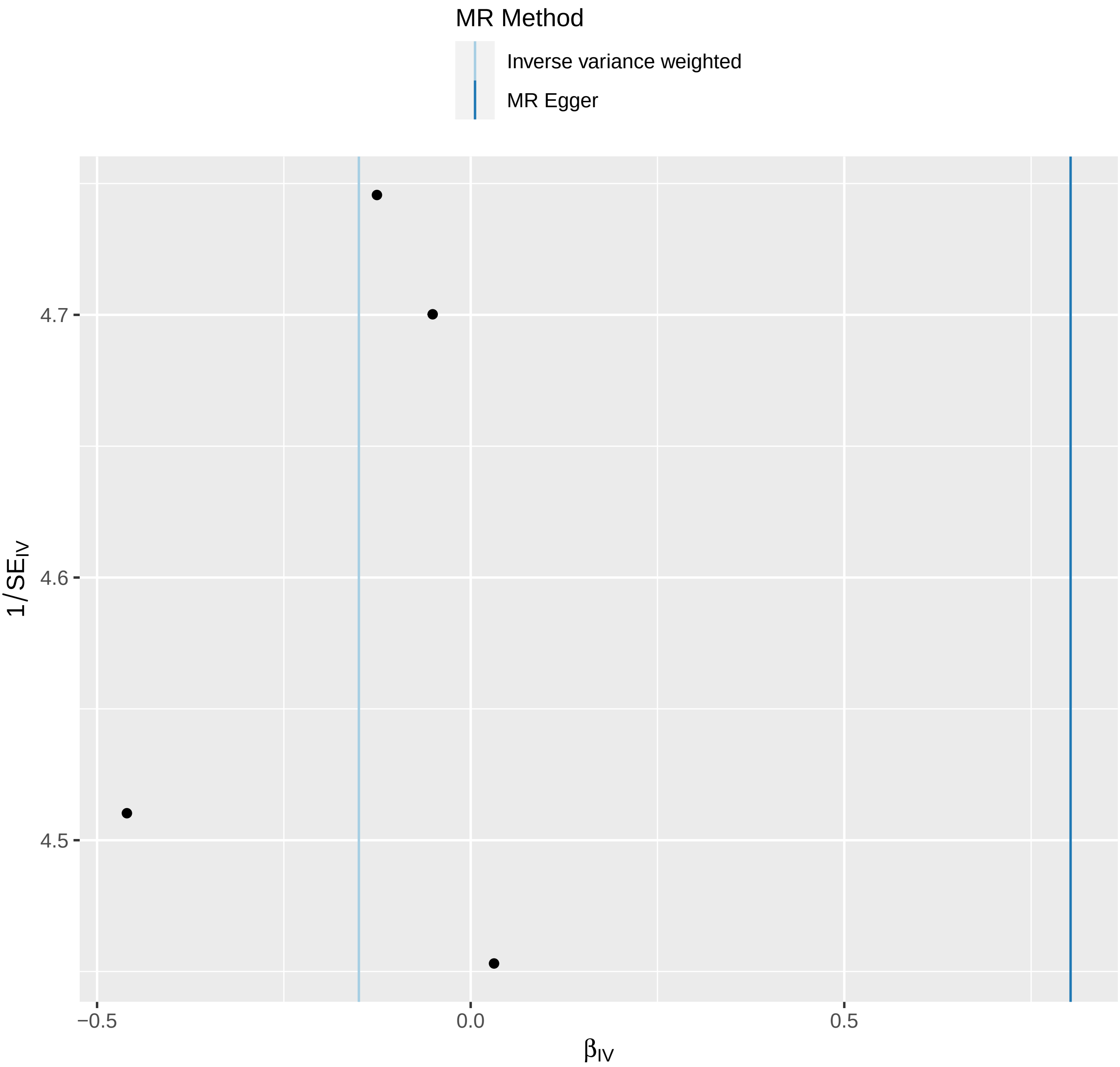

Batch 2−5290 : Gut microbiota abundance (genus Parasutterella id.2892) on Secretogranin−3
with a threshold of 1e−05

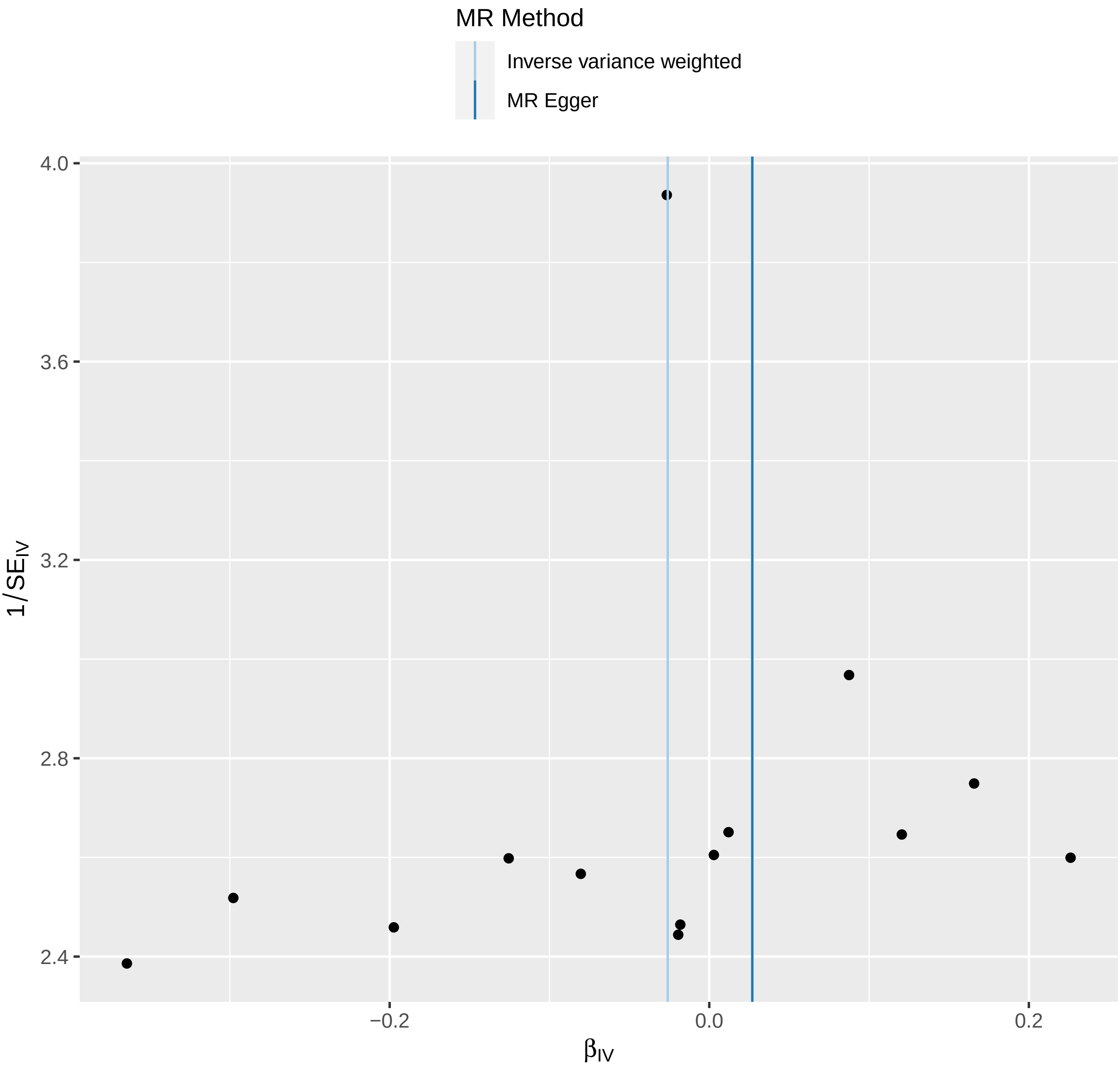

Batch 2−5291 : Gut microbiota abundance (genus Catenibacterium id.2153) on Secretoglobin family 1C member 1
with a threshold of 1e−05

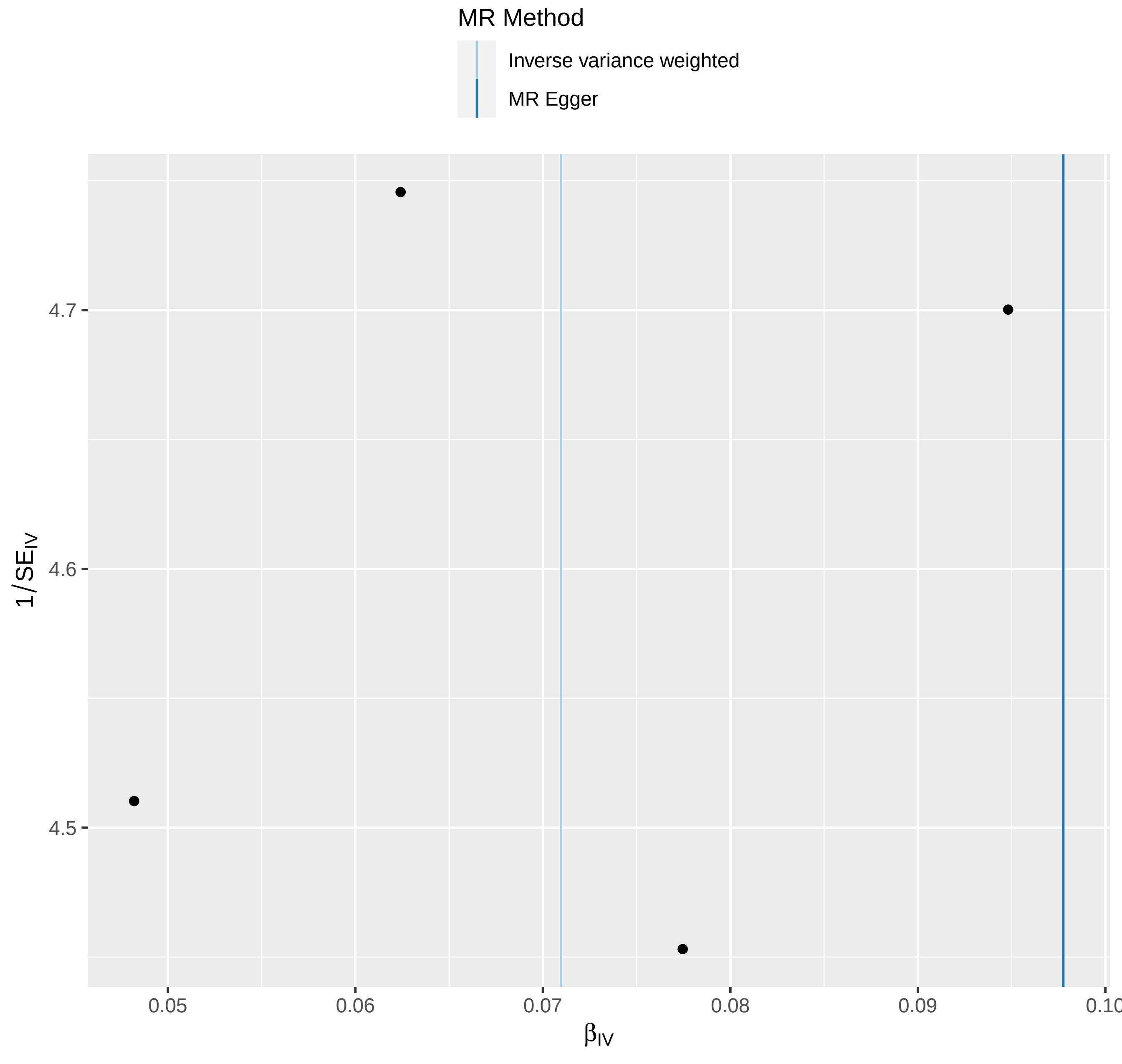

Batch 2−5292 : Gut microbiota abundance (genus Parasutterella id.2892) on Secretoglobin family 1C member 1
with a threshold of 1e−05

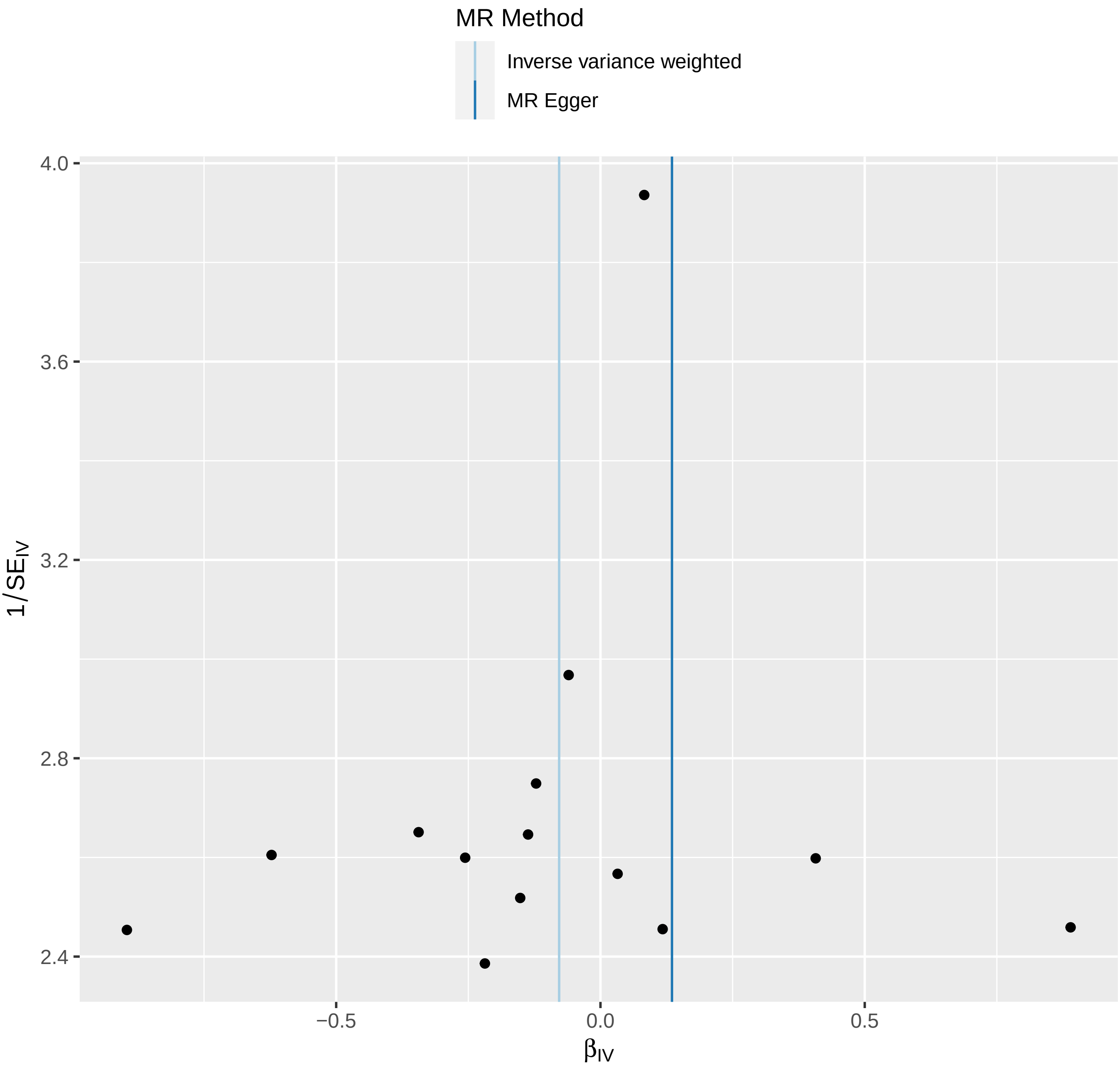

Batch 2−5293 : Gut microbiota abundance (genus Catenibacterium id.2153) on Secretoglobin family 1D member 2 with a threshold of 1e−05

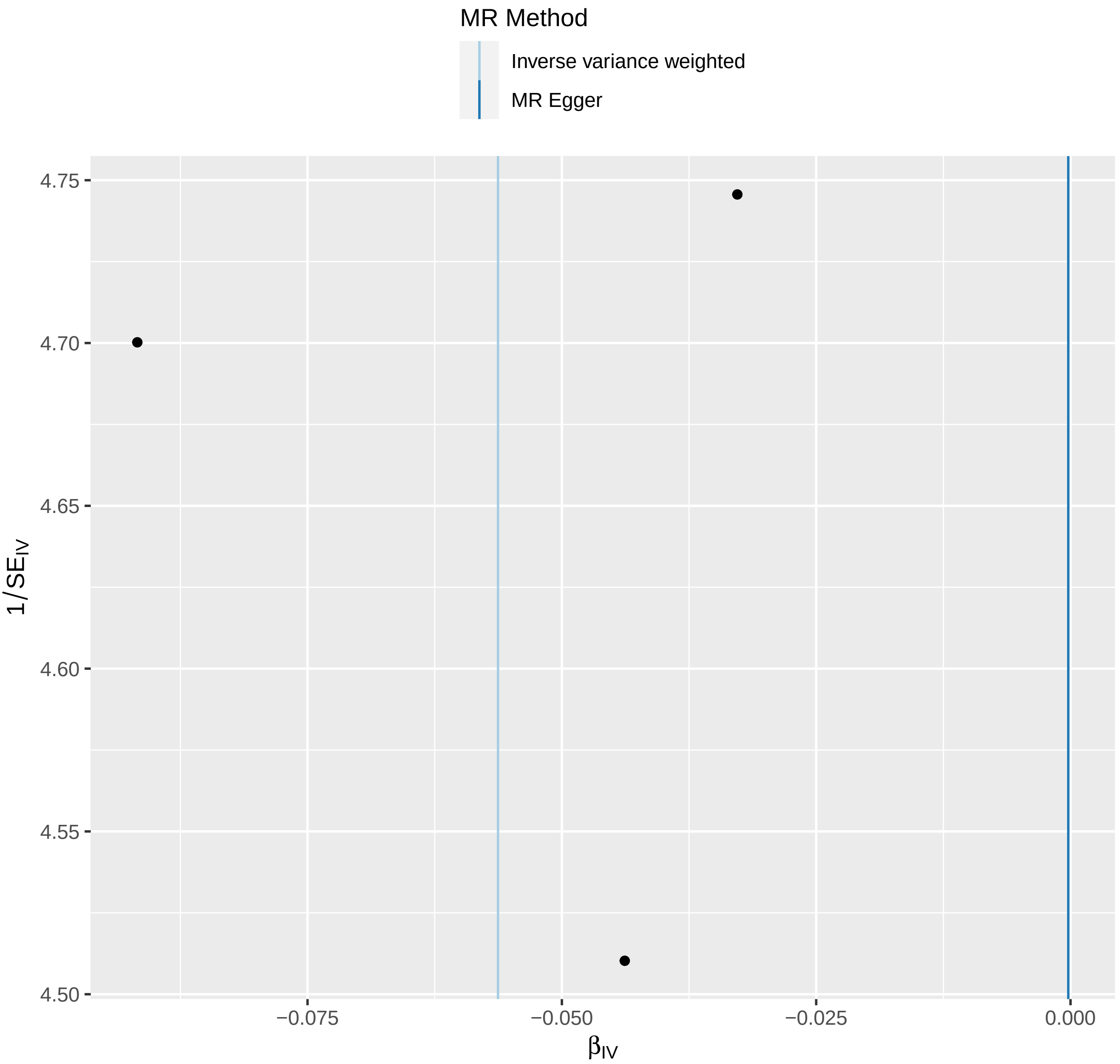

Batch 2−5294 : Gut microbiota abundance (genus Parasutterella id.2892) on Secretoglobin family 1D member 2
with a threshold of 1e−05

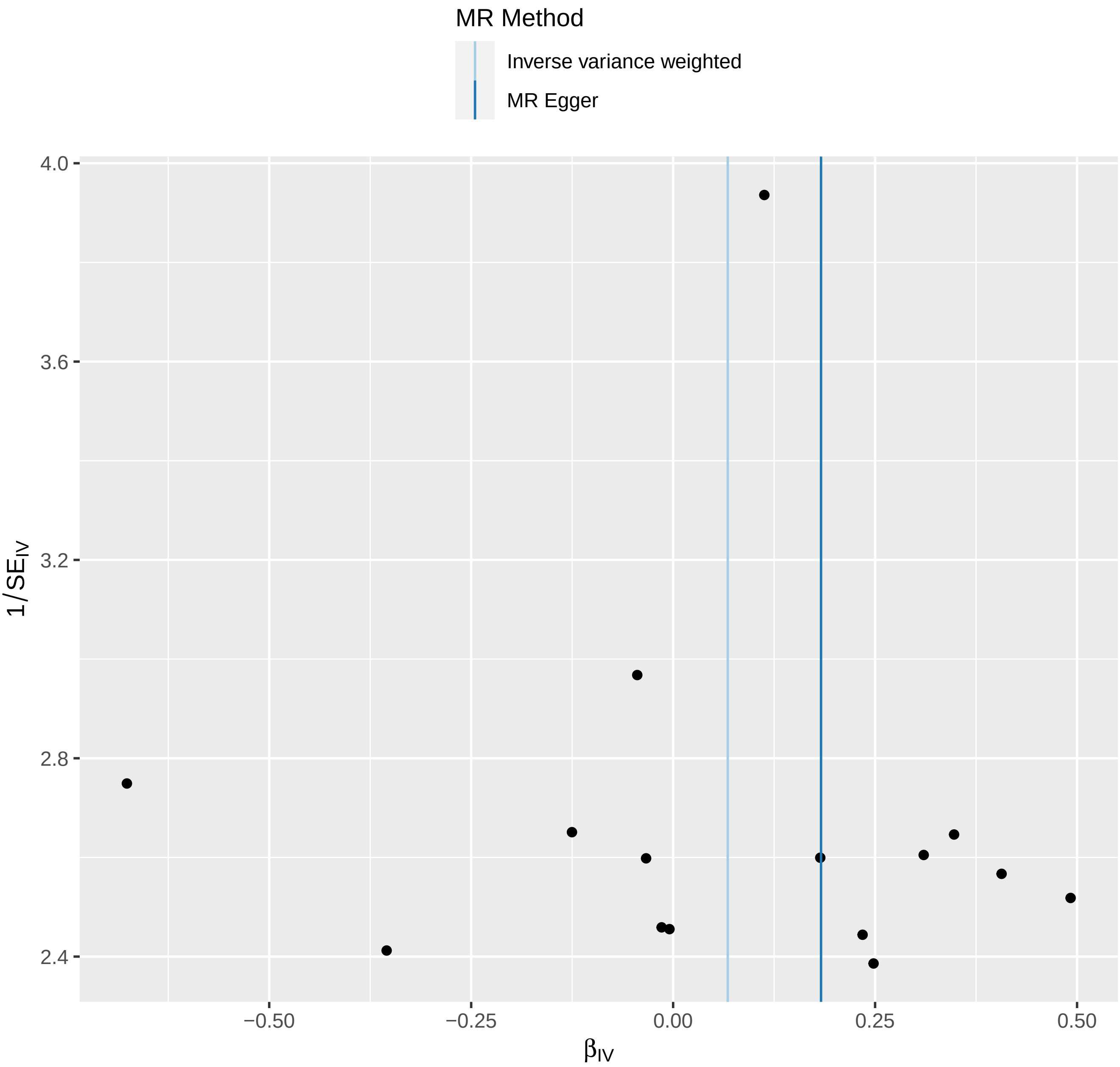

Batch 2−5295 : Gut microbiota abundance (genus Catenibacterium id.2153) on Mammaglobin−B
with a threshold of 1e−05

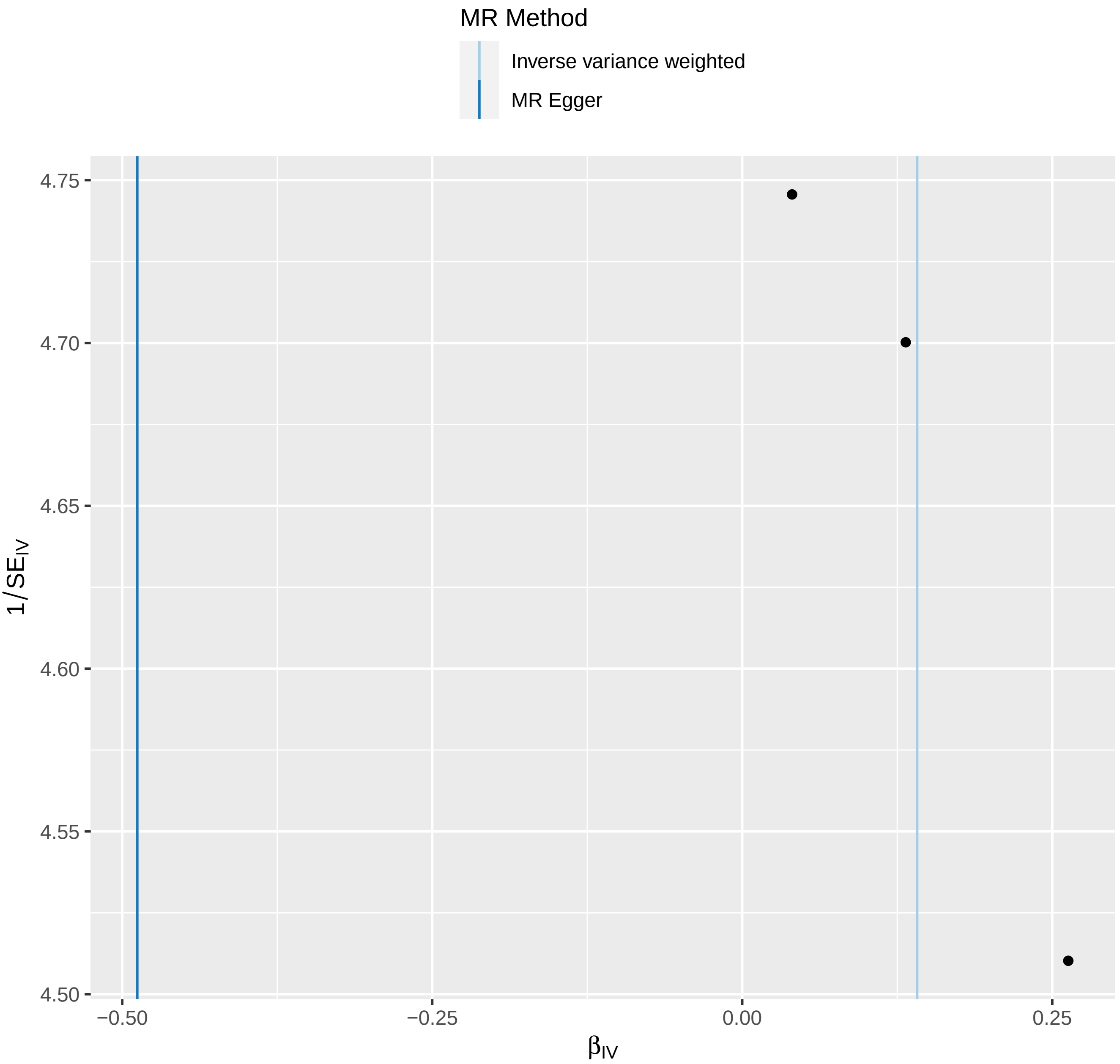

Batch 2−5296 : Gut microbiota abundance (genus Parasutterella id.2892) on Mammaglobin−B
with a threshold of 1e−05

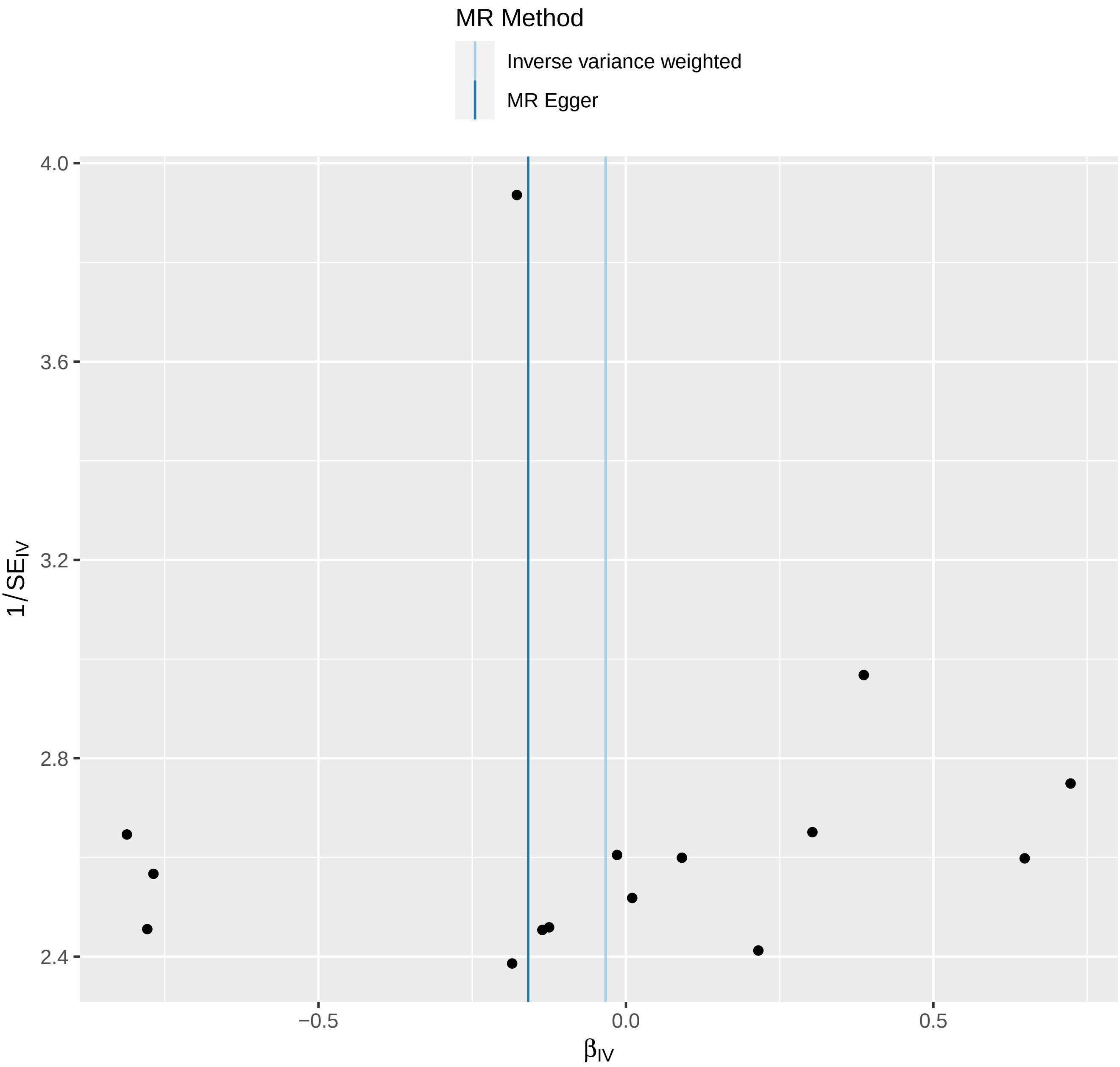

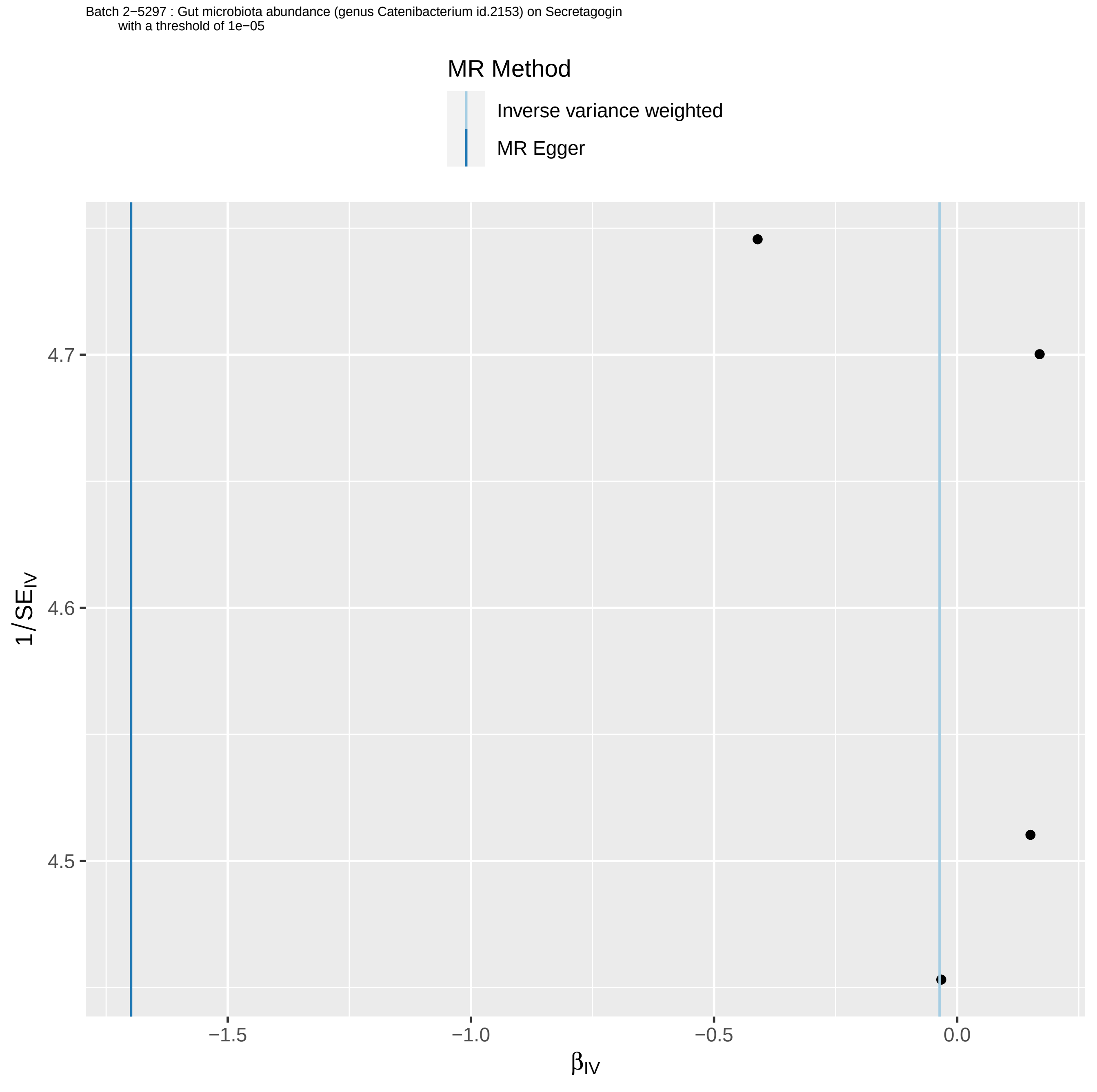
Batch 2−5297 : Gut microbiota abundance (genus Catenibacterium id.2153) on Secretagogin
with a threshold of 1e−05
MR Method
Inverse variance weighted
MR Egger
4.7
4.6
4.5
1/SE_IV
−1.5
−1.0
−0.5
0.0
β_IV

Batch 2−5298 : Gut microbiota abundance (genus Parasutterella id.2892) on Secretagogin
with a threshold of 1e−05

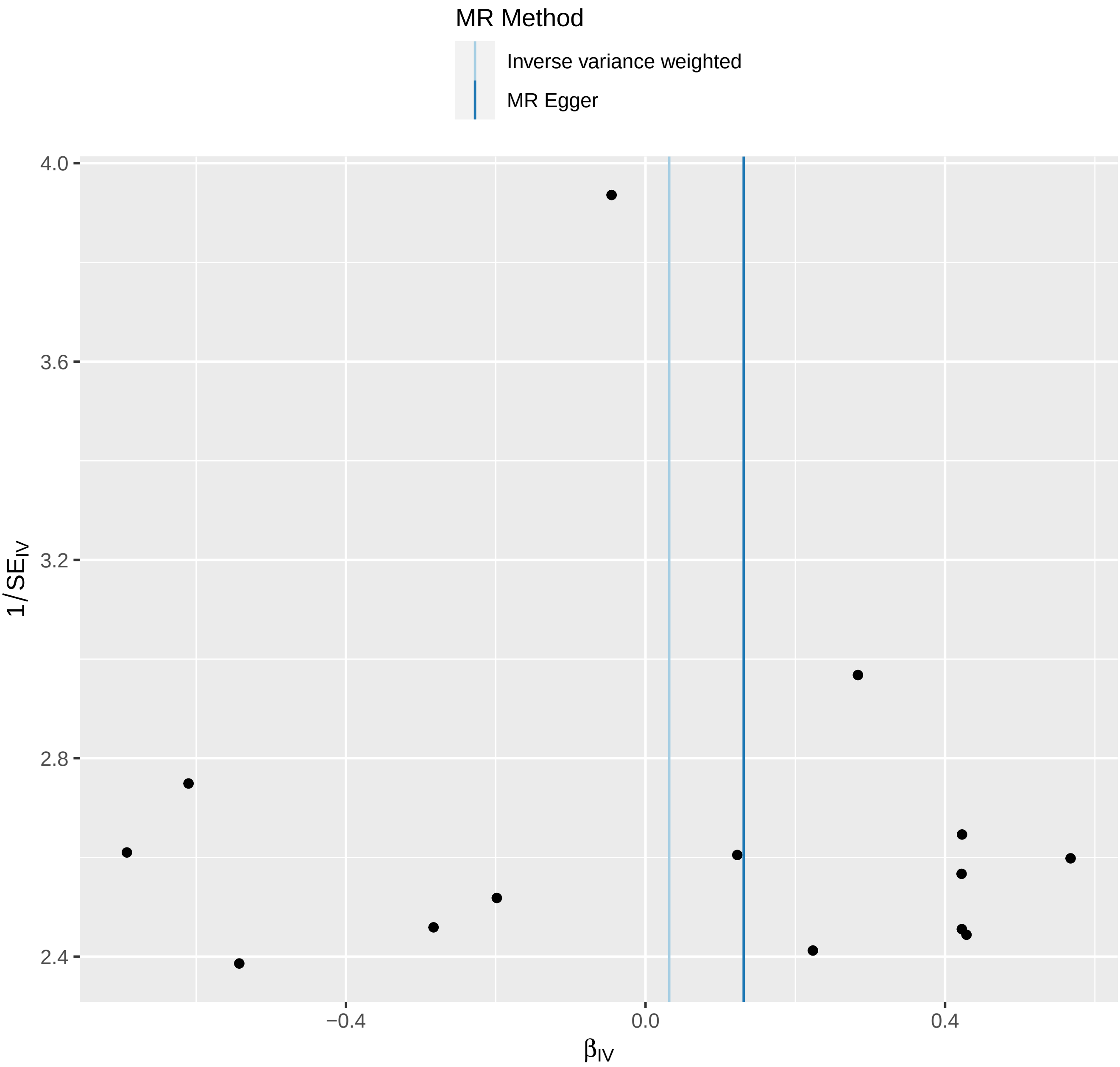

Batch 2−5299 : Gut microbiota abundance (genus Catenibacterium id.2153) on Adseverin
with a threshold of 1e−05

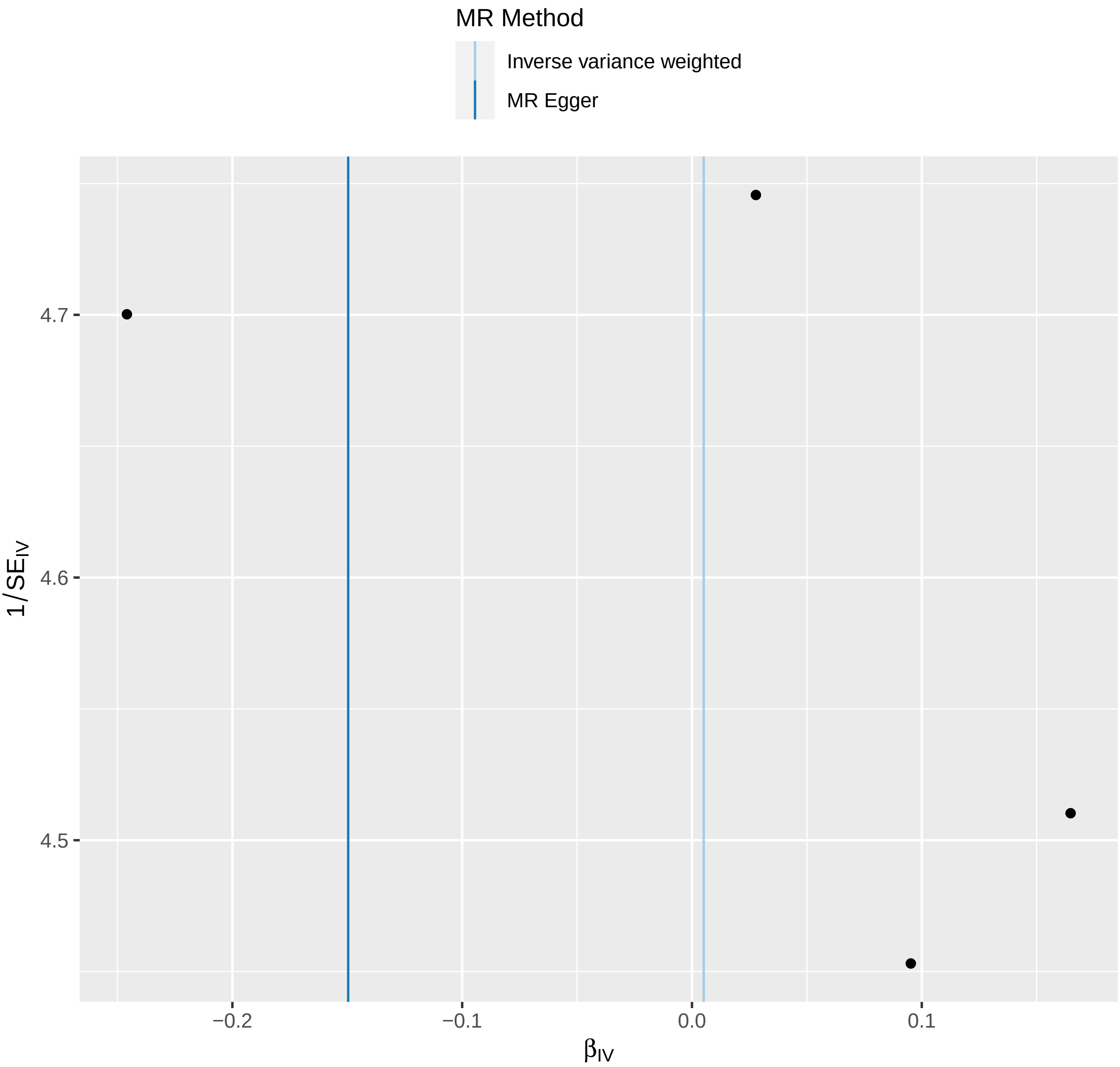

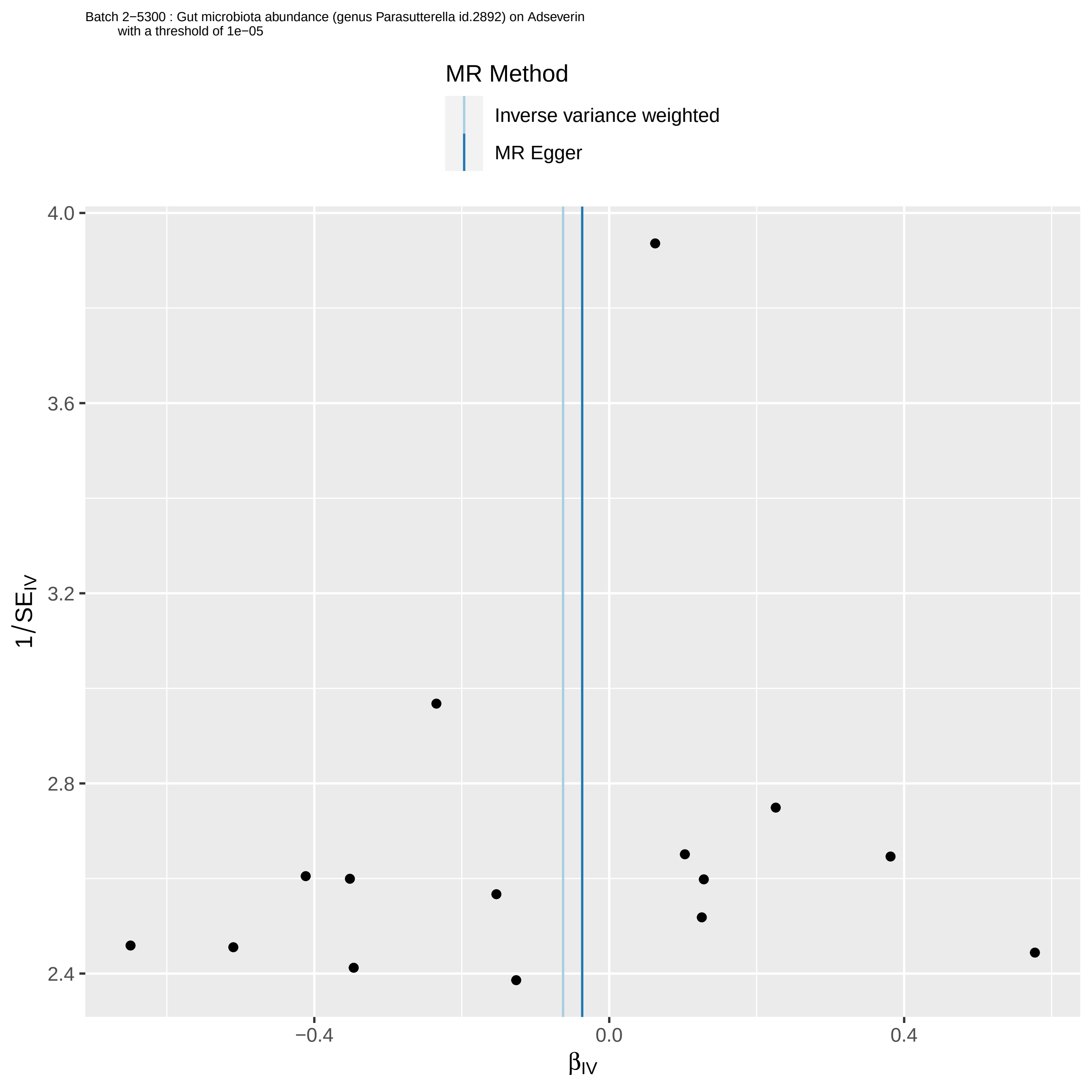
Batch 2−5300 : Gut microbiota abundance (genus Parasutterella id.2892) on Adseverin
with a threshold of 1e−05
MR Method
Inverse variance weighted
MR Egger
4.0
3.6
3.2
2.8
2.4
1/SE$_{IV}$
−0.4
0.0
0.4
β$_{IV}$

Batch 2−5301 : Gut microbiota abundance (genus Catenibacterium id.2153) on Polycomb protein SCMH1
with a threshold of 1e−05

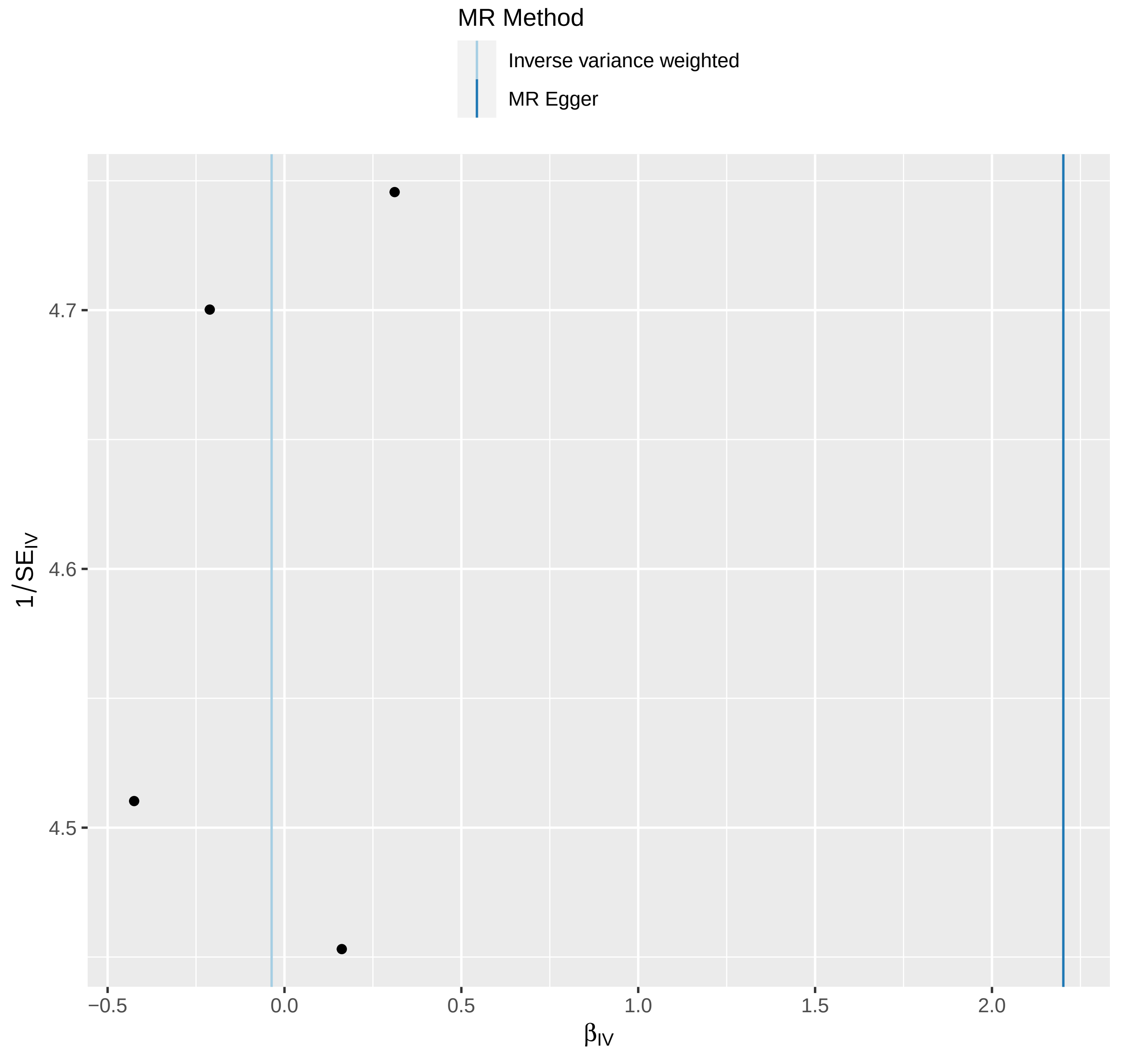

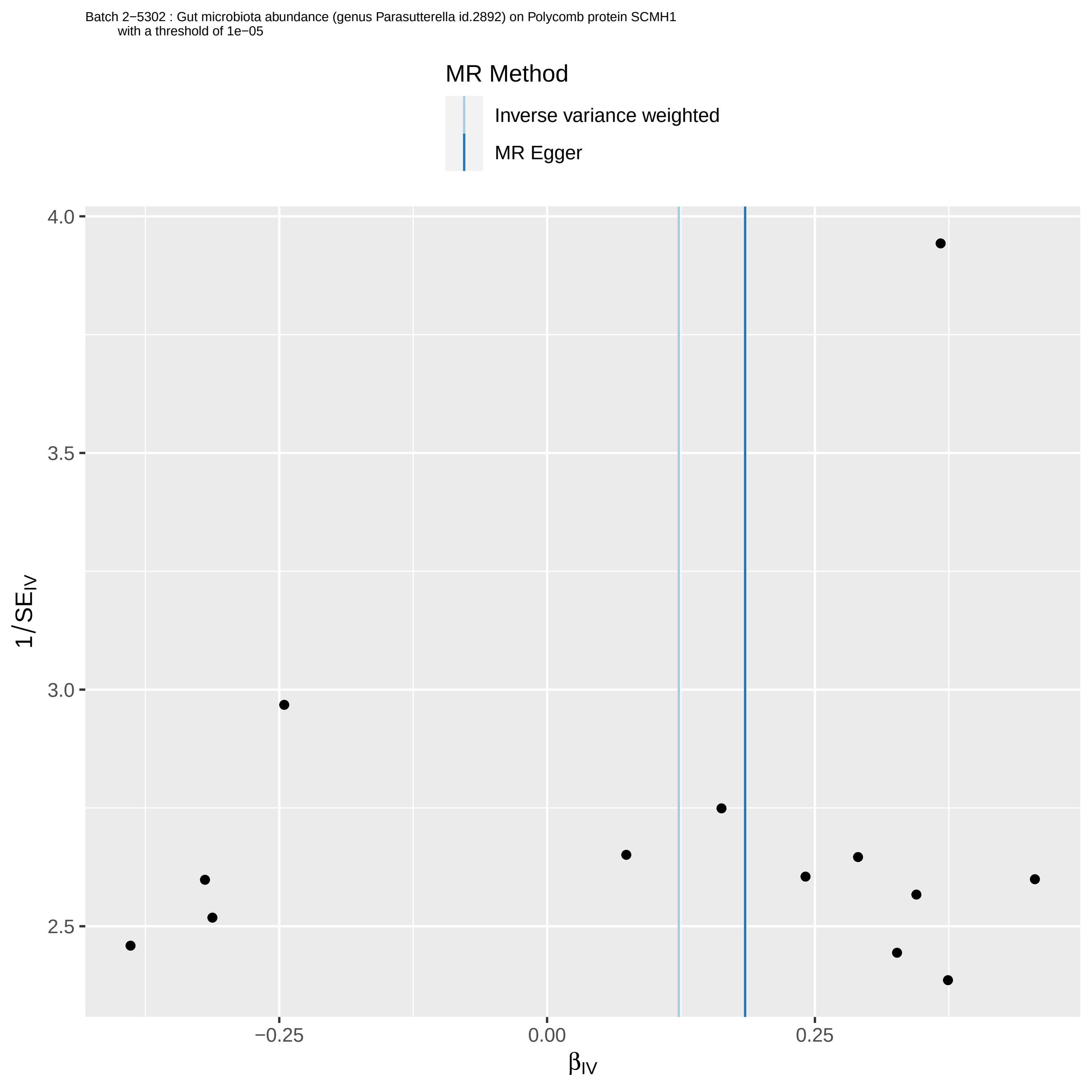
Batch 2−5302 : Gut microbiota abundance (genus Parasutterella id.2892) on Polycomb protein SCMH1
with a threshold of 1e−05
MR Method
Inverse variance weighted
MR Egger
4.0
3.5
3.0
2.5
1/SE$_{IV}$
−0.25
0.00
0.25
β$_{IV}$

Batch 2−5303 : Gut microbiota abundance (genus Catenibacterium id.2153) on Sodium channel subunit beta−2 with a threshold of 1e−05

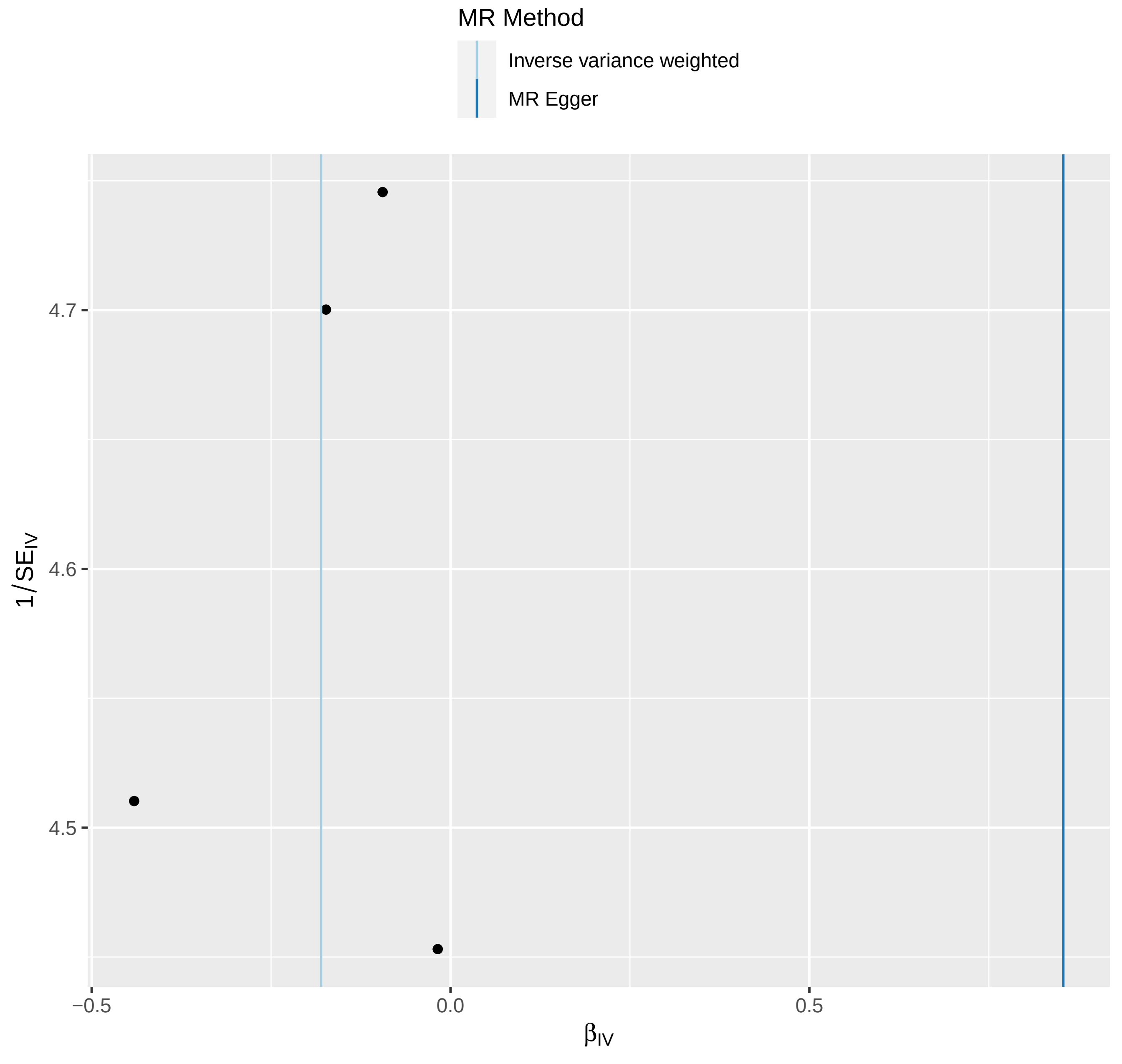

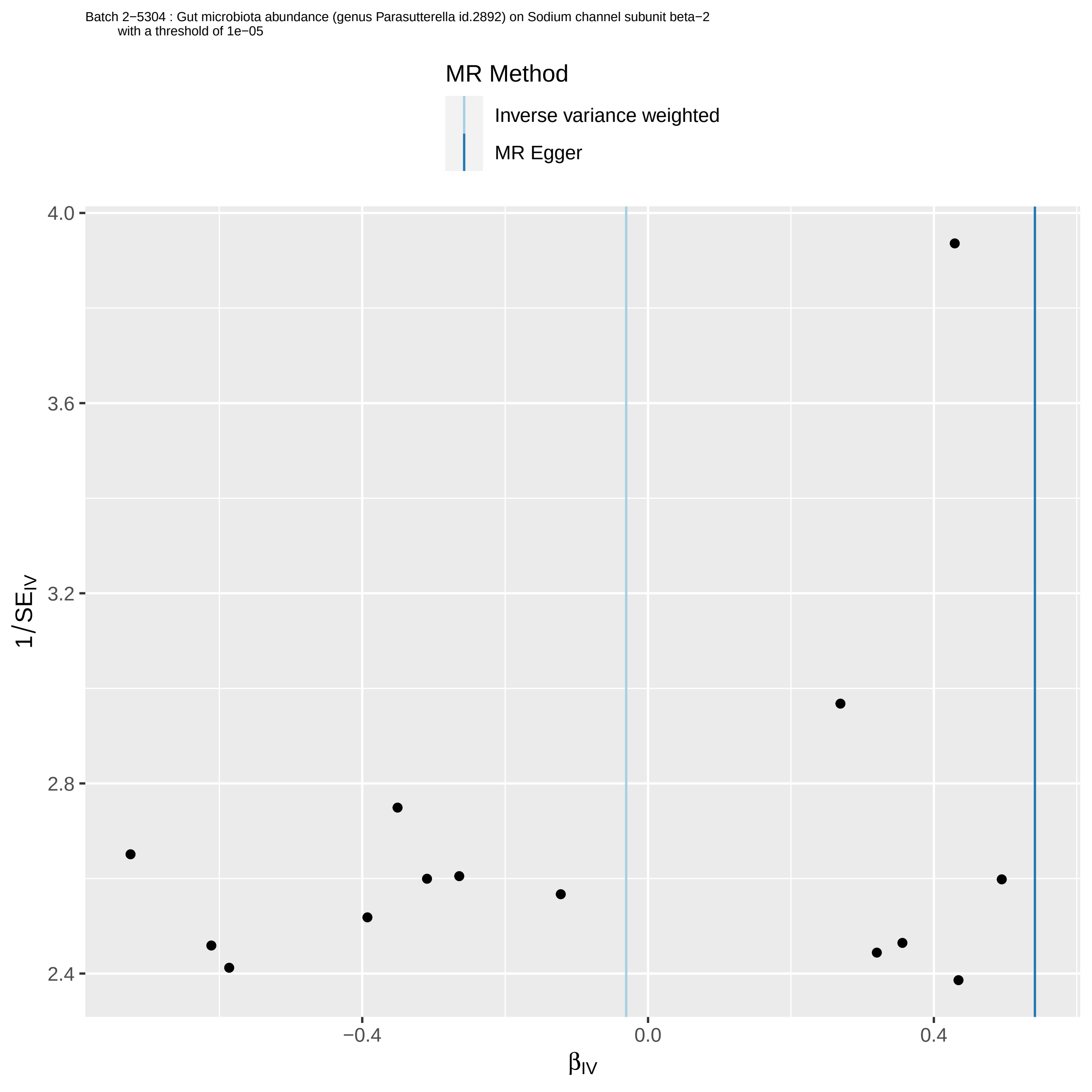

Batch 2−5304 : Gut microbiota abundance (genus Parasutterella id.2892) on Sodium channel subunit beta−2
with a threshold of 1e−05
MR Method
Inverse variance weighted
MR Egger
4.0
3.6
3.2
2.8
2.4
1/SE_IV
−0.4
0.0
0.4
β_IV

Batch 2−5305 : Gut microbiota abundance (genus Catenibacterium id.2153) on Protein SCO1 homolog, mitochondrial
with a threshold of 1e−05

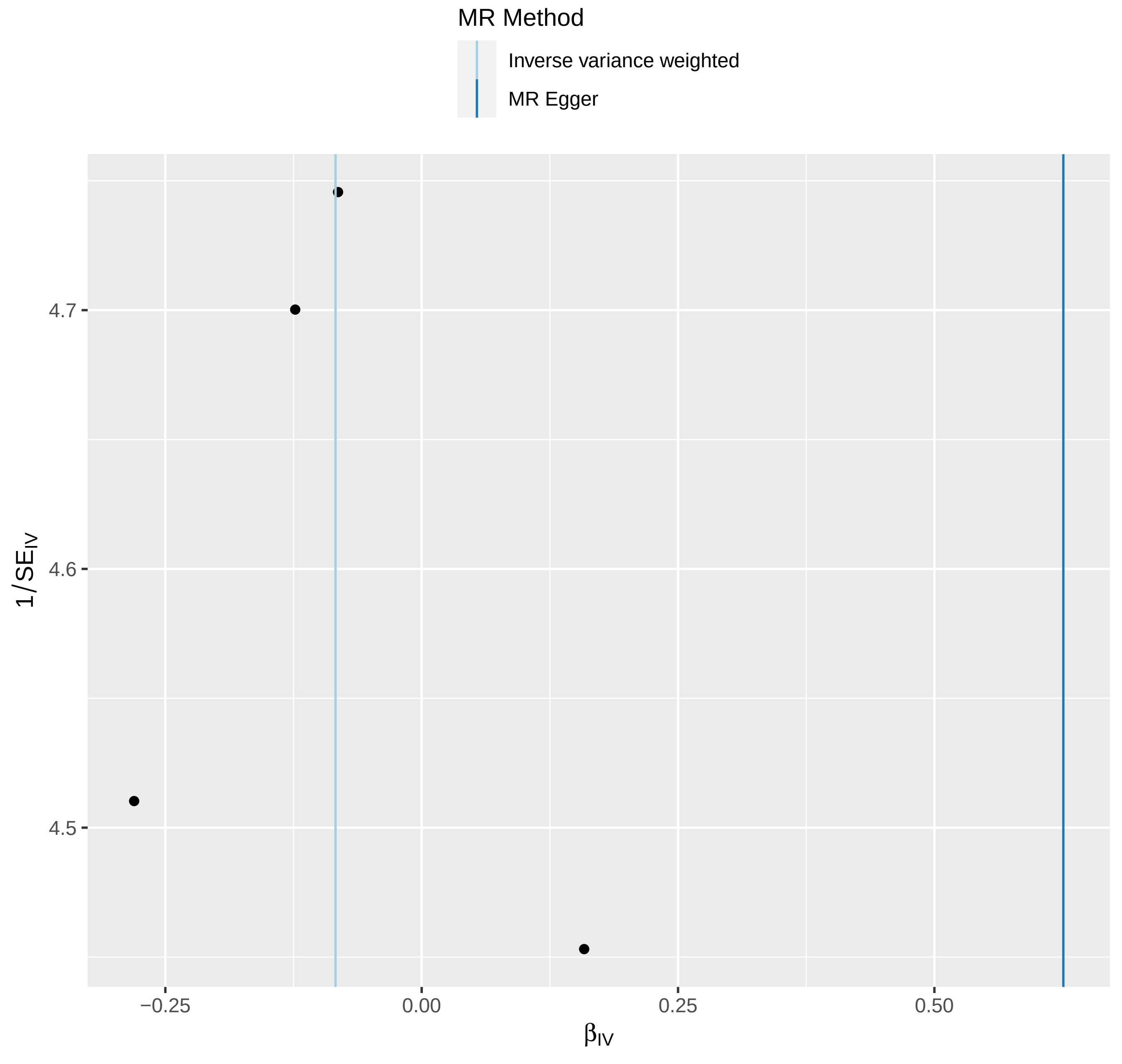

Batch 2−5306 : Gut microbiota abundance (genus Parasutterella id.2892) on Protein SCO1 homolog, mitochondrial
with a threshold of 1e−05

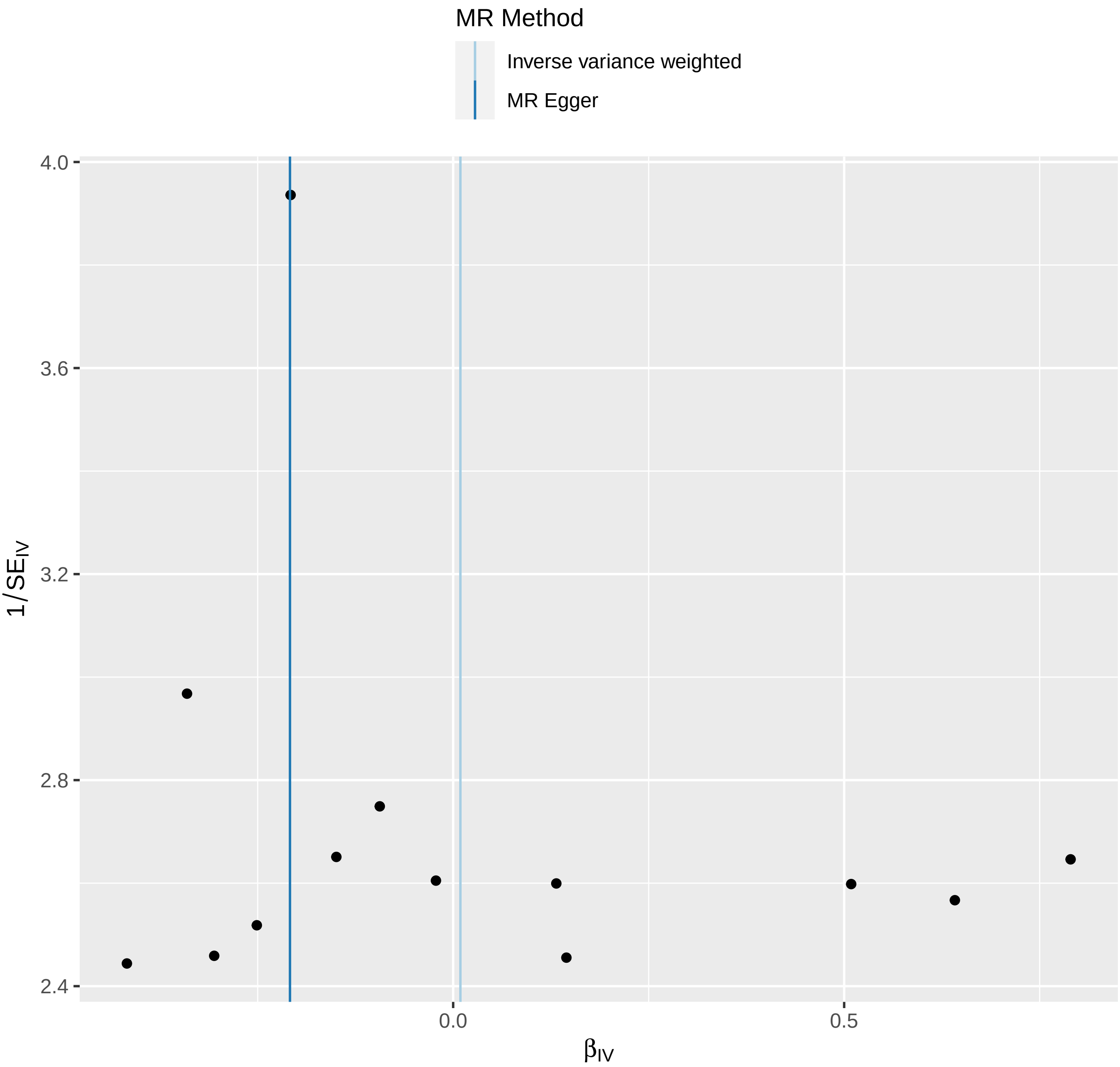

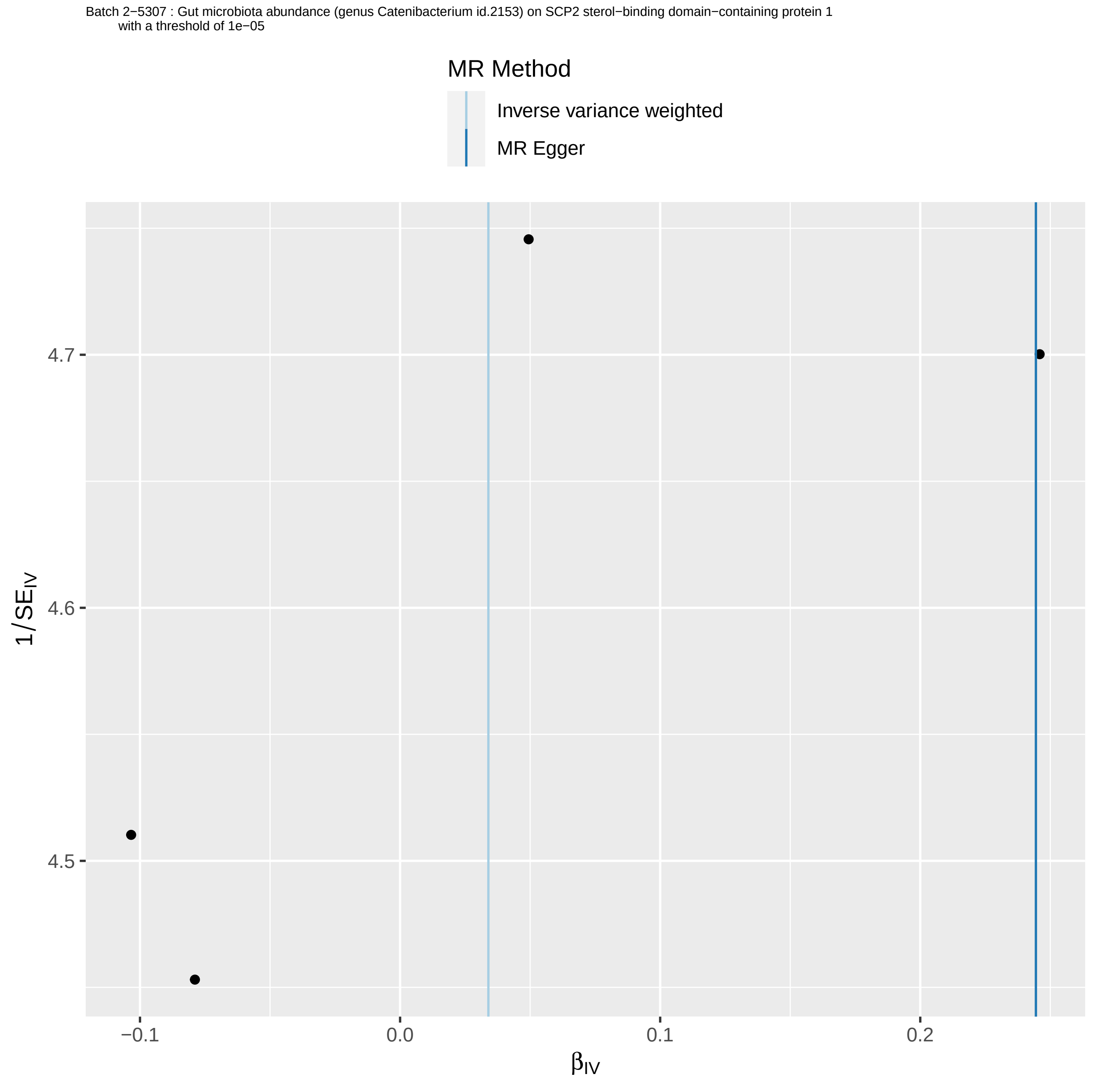
Batch 2−5307 : Gut microbiota abundance (genus Catenibacterium id.2153) on SCP2 sterol−binding domain−containing protein 1
with a threshold of 1e−05
MR Method
Inverse variance weighted
MR Egger
4.7
4.6
4.5
1/SE_IV
−0.1
0.0
0.1
0.2
β_IV

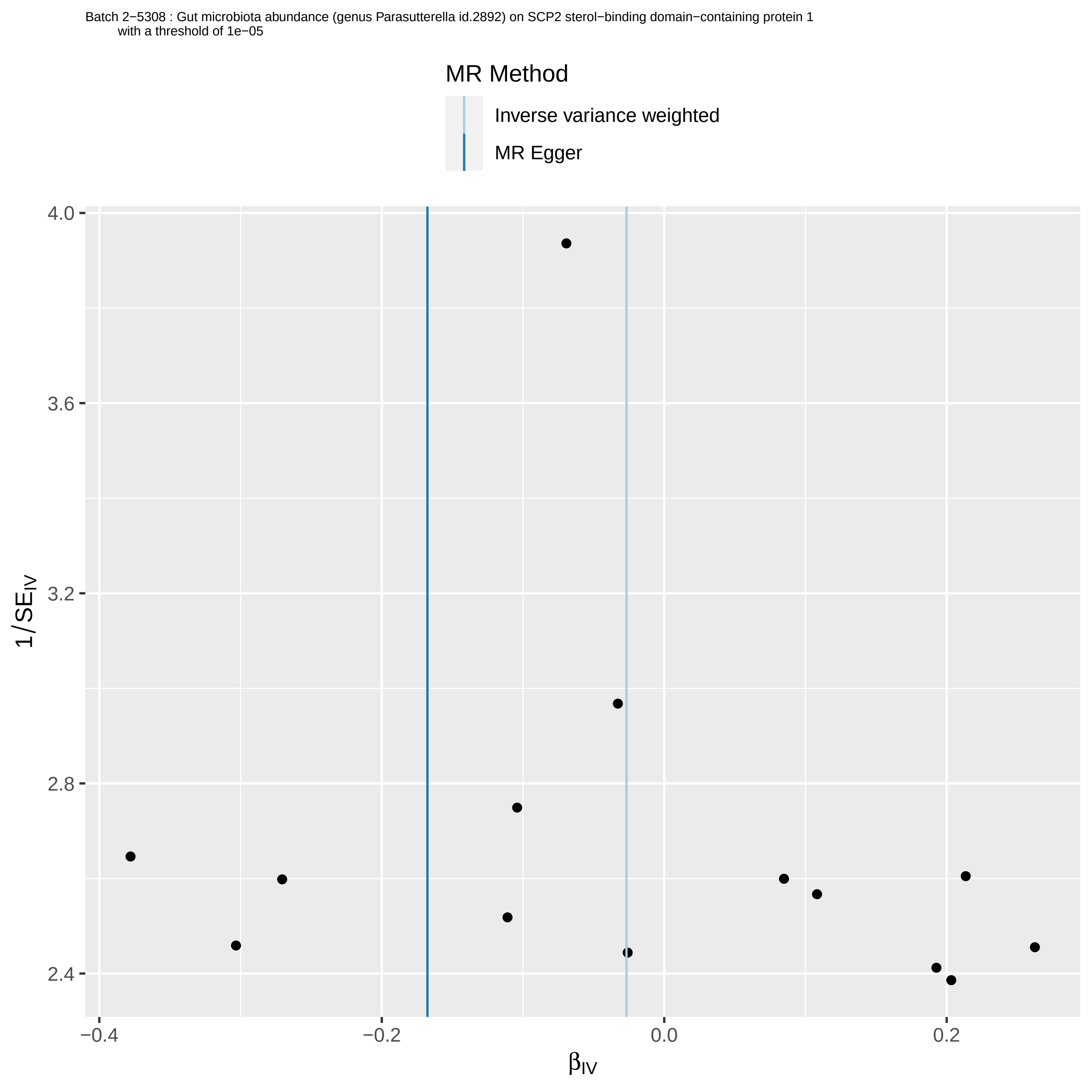

Batch 2−5308 : Gut microbiota abundance (genus Parasutterella id.2892) on SCP2 sterol−binding domain−containing protein 1
with a threshold of 1e−05
MR Method
Inverse variance weighted
MR Egger
4.0
3.6
3.2
2.8
2.4
1/SE_IV
−0.4
−0.2
0.0
0.2
β_IV

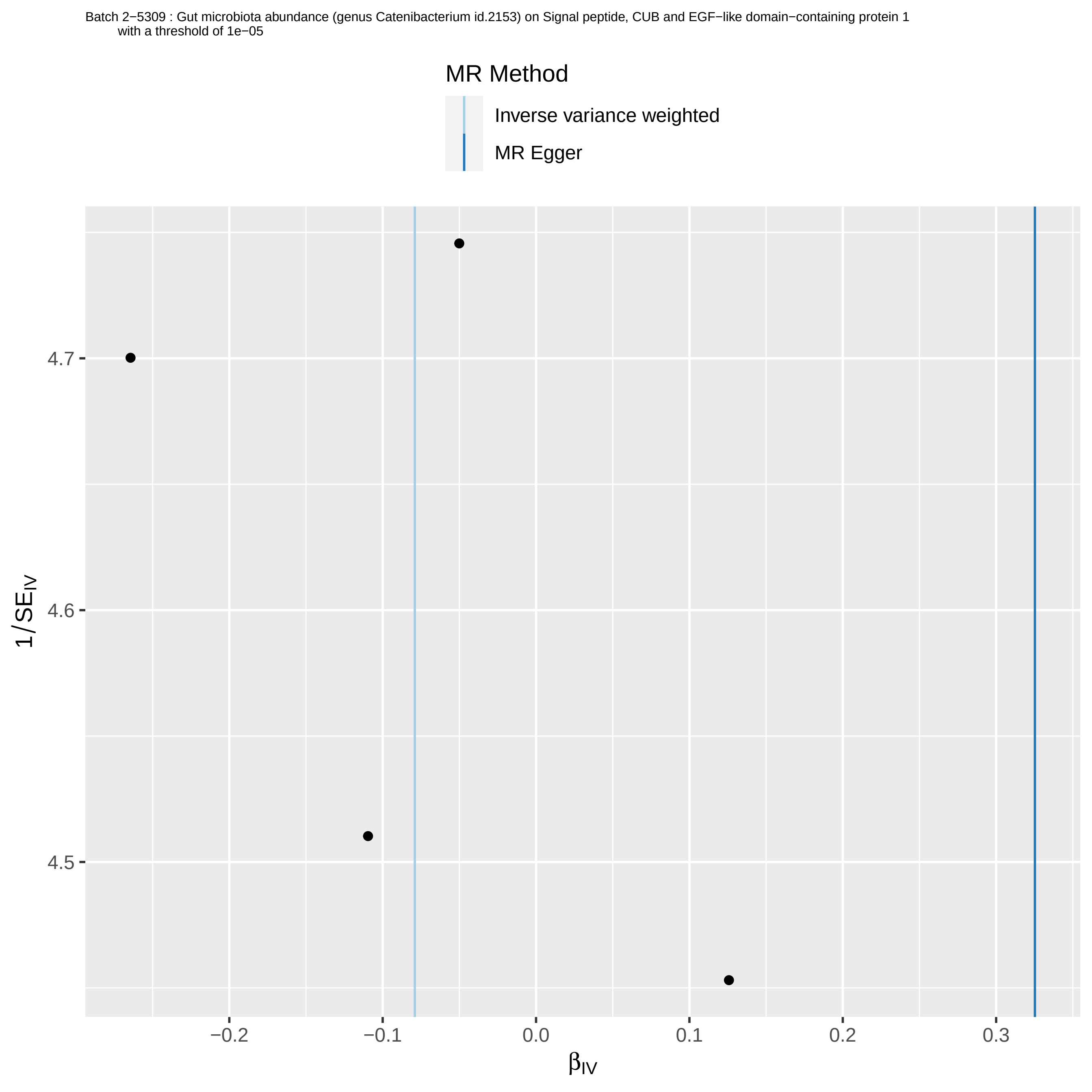

Batch 2−5309 : Gut microbiota abundance (genus Catenibacterium id.2153) on Signal peptide, CUB and EGF−like domain−containing protein 1
with a threshold of 1e−05
MR Method
Inverse variance weighted
MR Egger
4.7
4.6
4.5
$1/SE_{IV}$
−0.2
−0.1
0.0
0.1
0.2
0.3
β_{IV}

Batch 2−5310 : Gut microbiota abundance (genus Parasutterella id.2892) on Signal peptide, CUB and EGF−like domain−containing protein 1 with a threshold of 1e−05

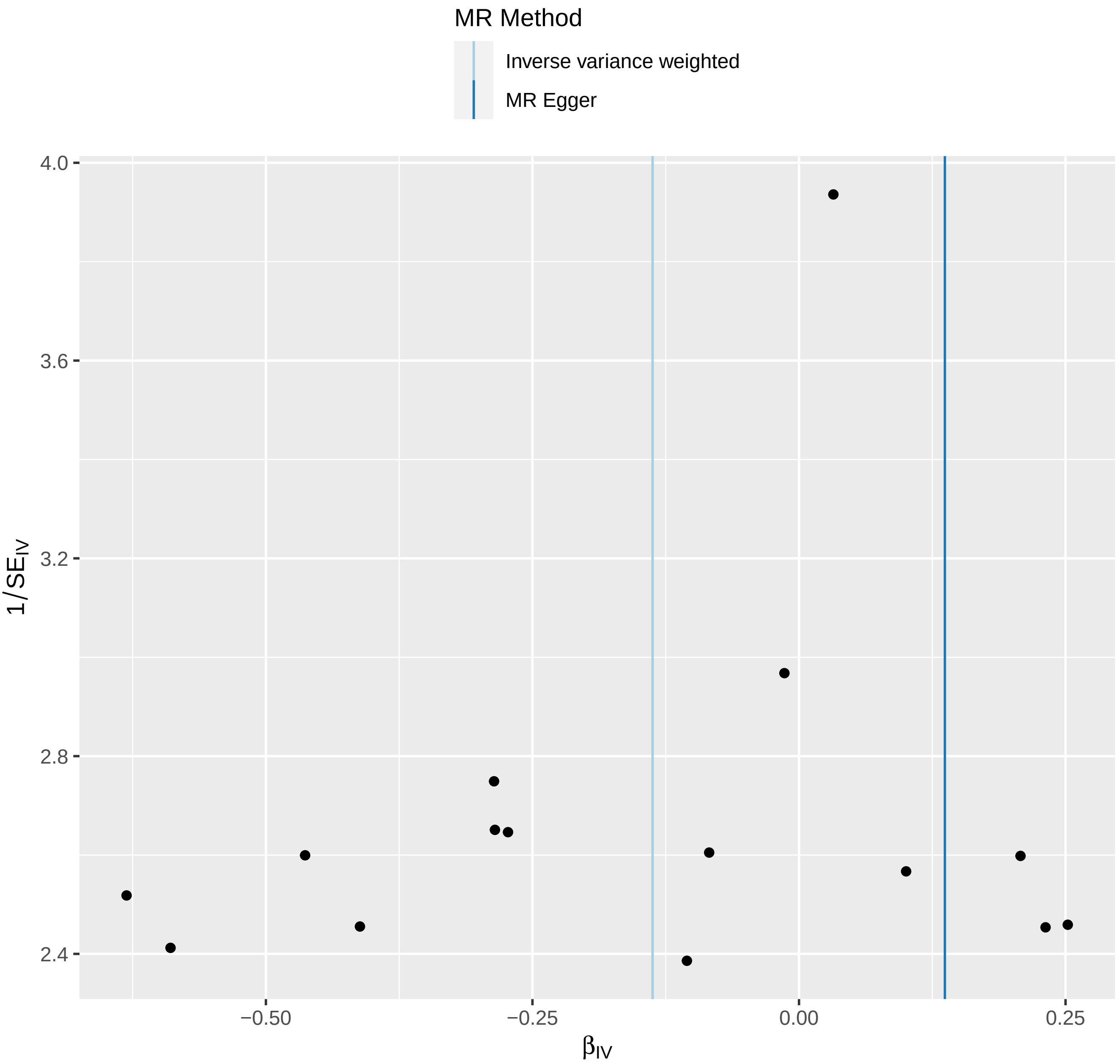

Batch 2−5311 : Gut microbiota abundance (genus Catenibacterium id.2153) on Stromal cell−derived factor 2−like protein 1
with a threshold of 1e−05

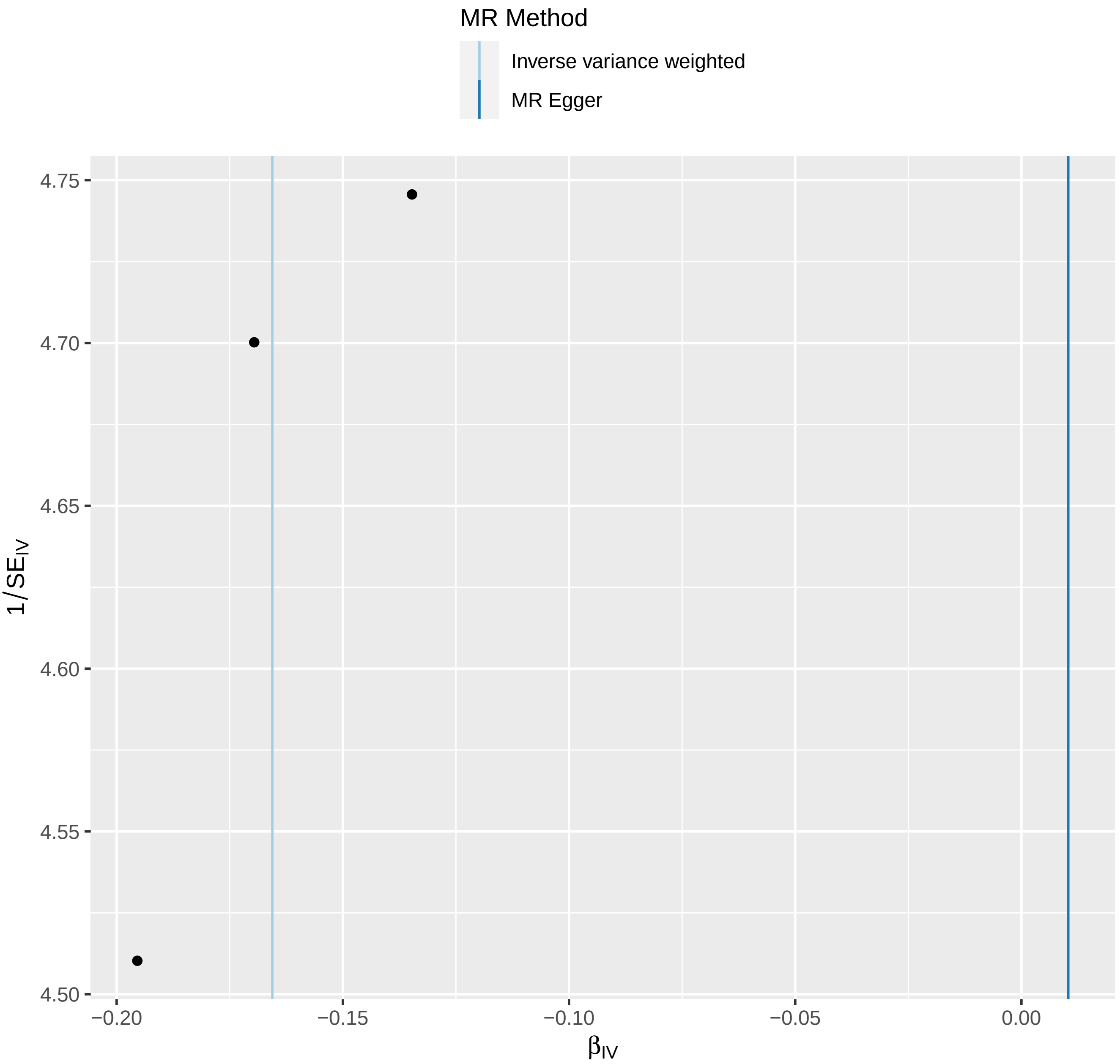

Batch 2−5312 : Gut microbiota abundance (genus Parasutterella id.2892) on Stromal cell−derived factor 2−like protein 1 with a threshold of 1e−05

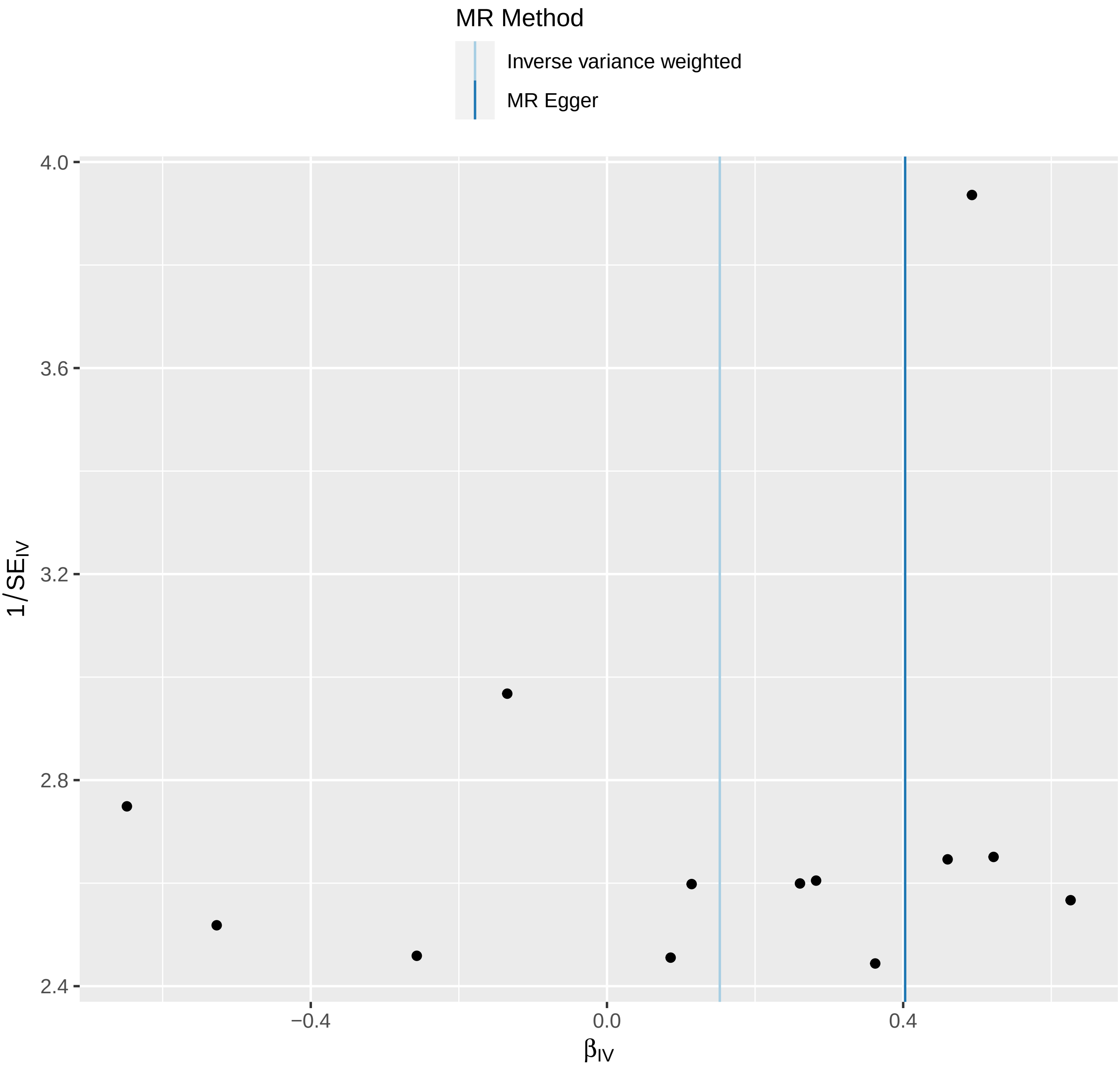

Batch 2−5313 : Gut microbiota abundance (genus Catenibacterium id.2153) on Succinate dehydrogenase assembly factor 2, mitochondrial with a threshold of 1e−05

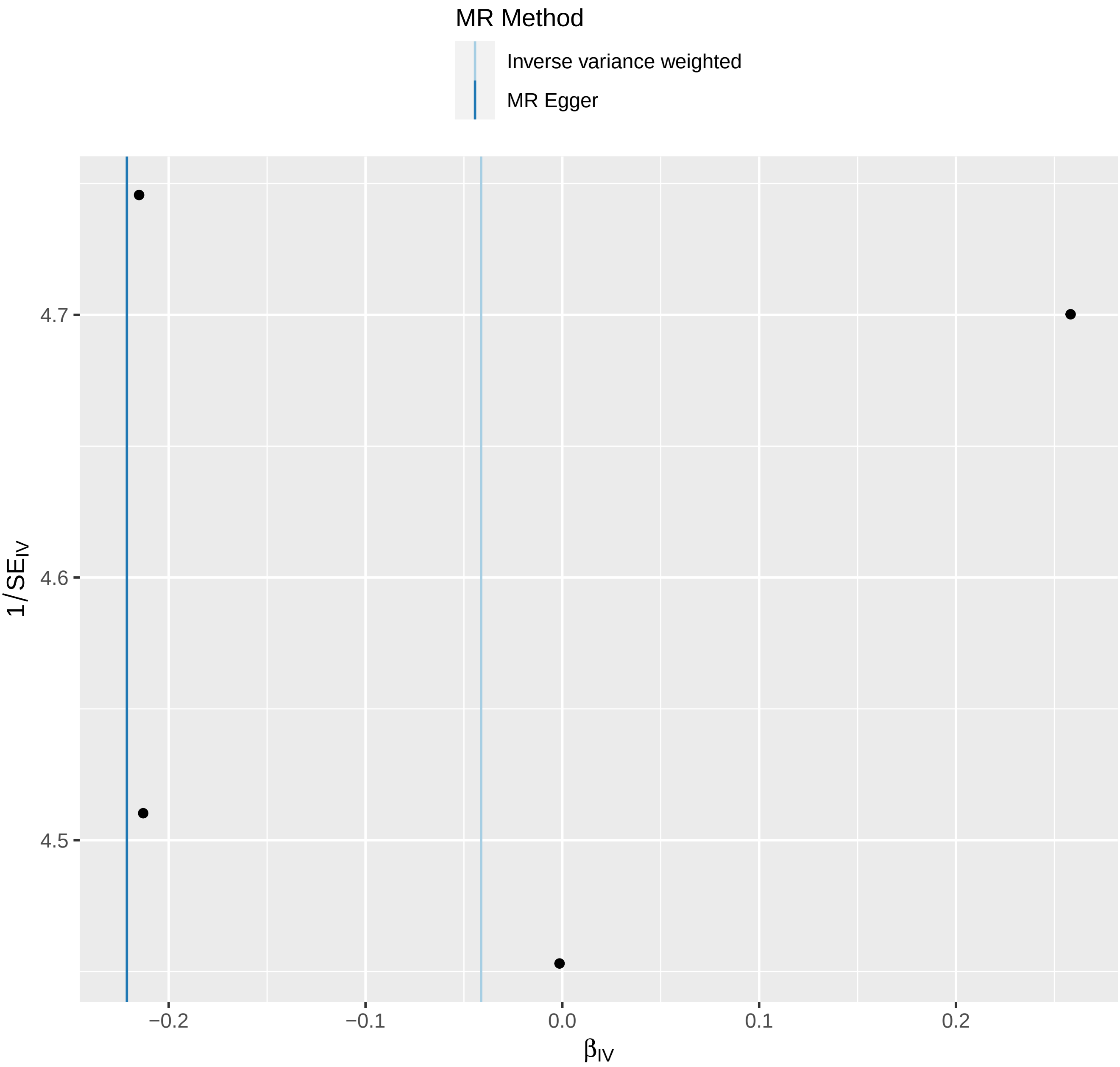

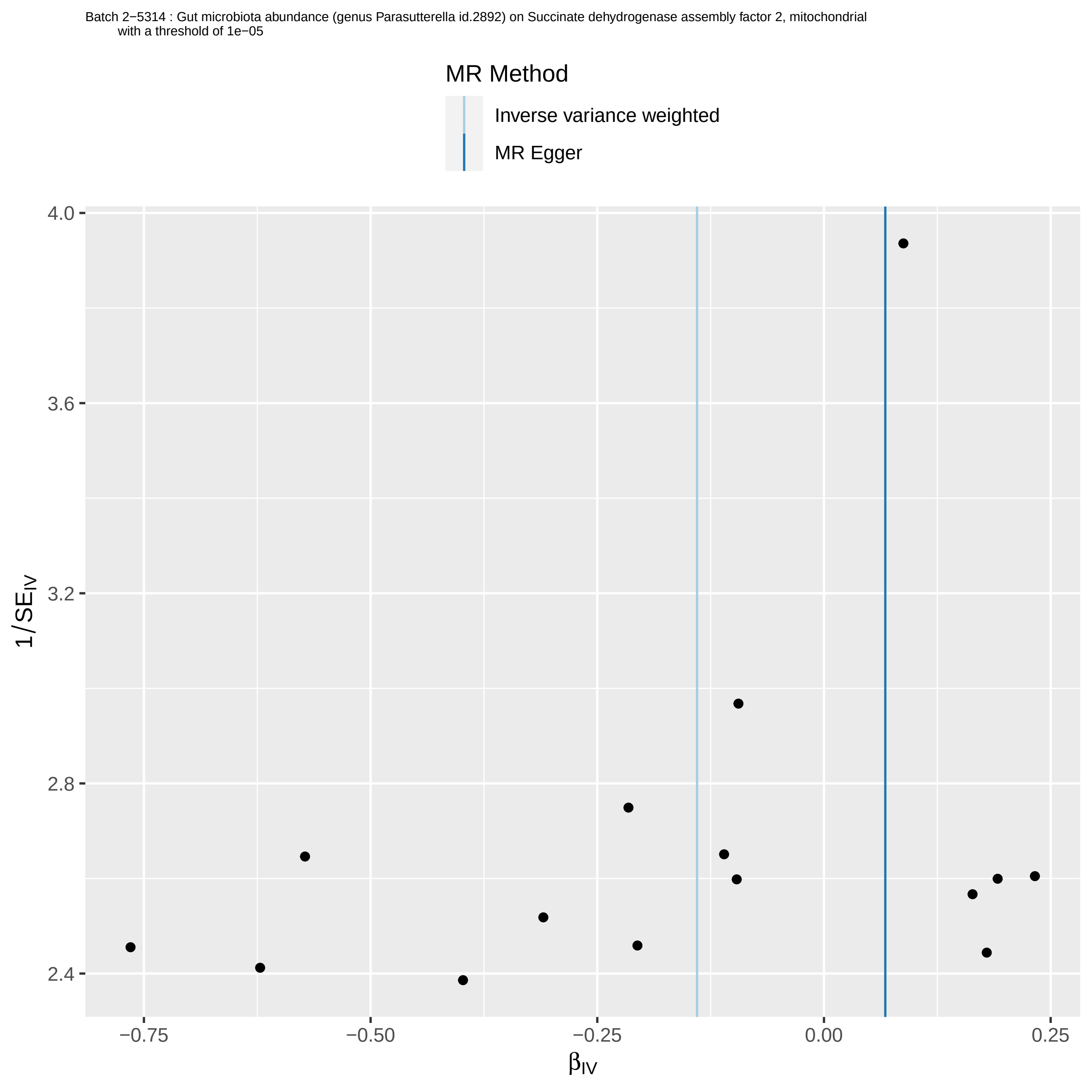
Batch 2−5314 : Gut microbiota abundance (genus Parasutterella id.2892) on Succinate dehydrogenase assembly factor 2, mitochondrial
with a threshold of 1e−05
MR Method
Inverse variance weighted
MR Egger
4.0
3.6
3.2
2.8
2.4
1/SE_IV
−0.75
−0.50
−0.25
0.00
0.25
β_IV

Batch 2−5315 : Gut microbiota abundance (genus Catenibacterium id.2153) on Signal peptidase complex catalytic subunit SEC11C
with a threshold of 1e−05

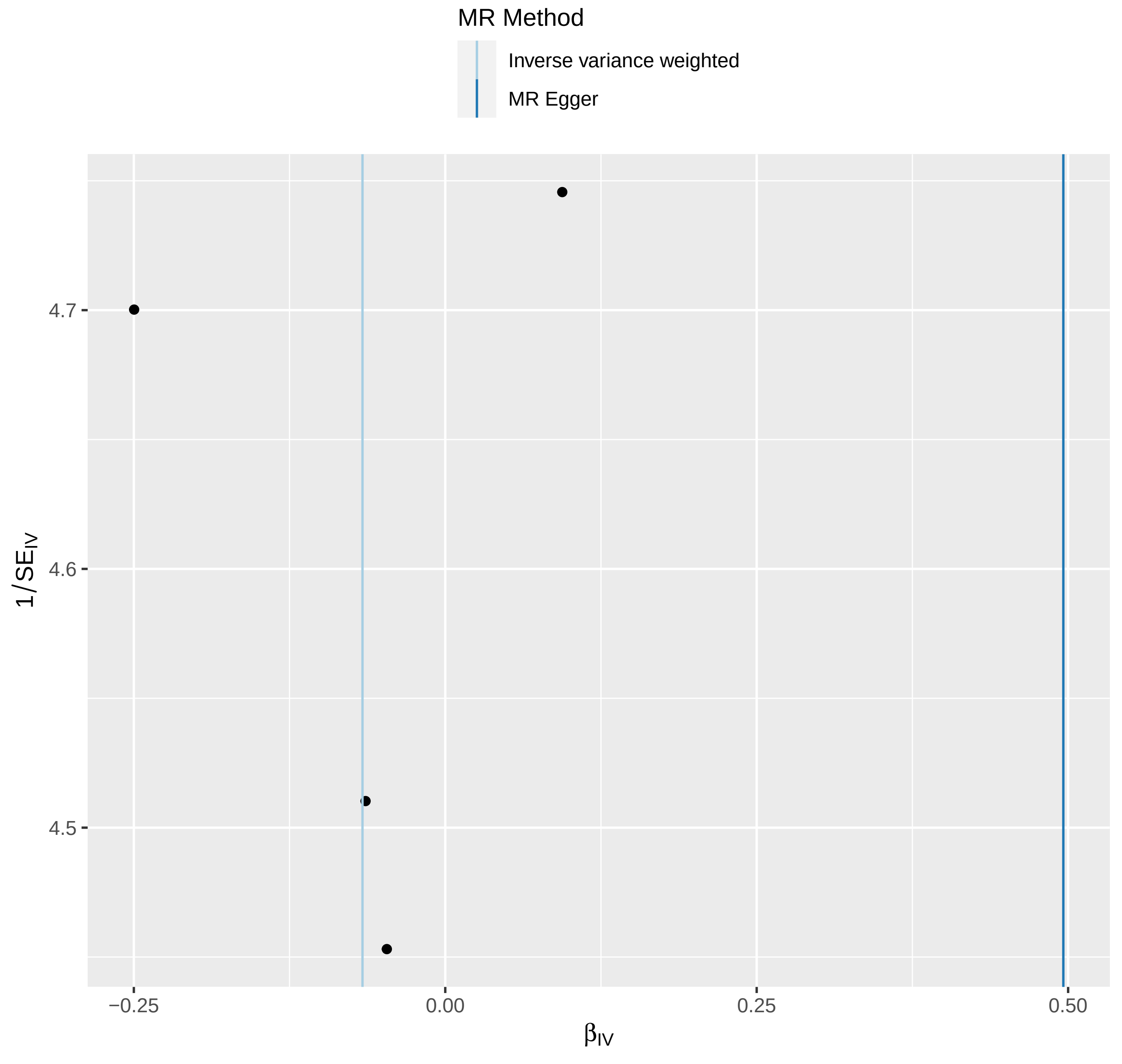

Batch 2−5316 : Gut microbiota abundance (genus Parasutterella id.2892) on Signal peptidase complex catalytic subunit SEC11C
with a threshold of 1e−05

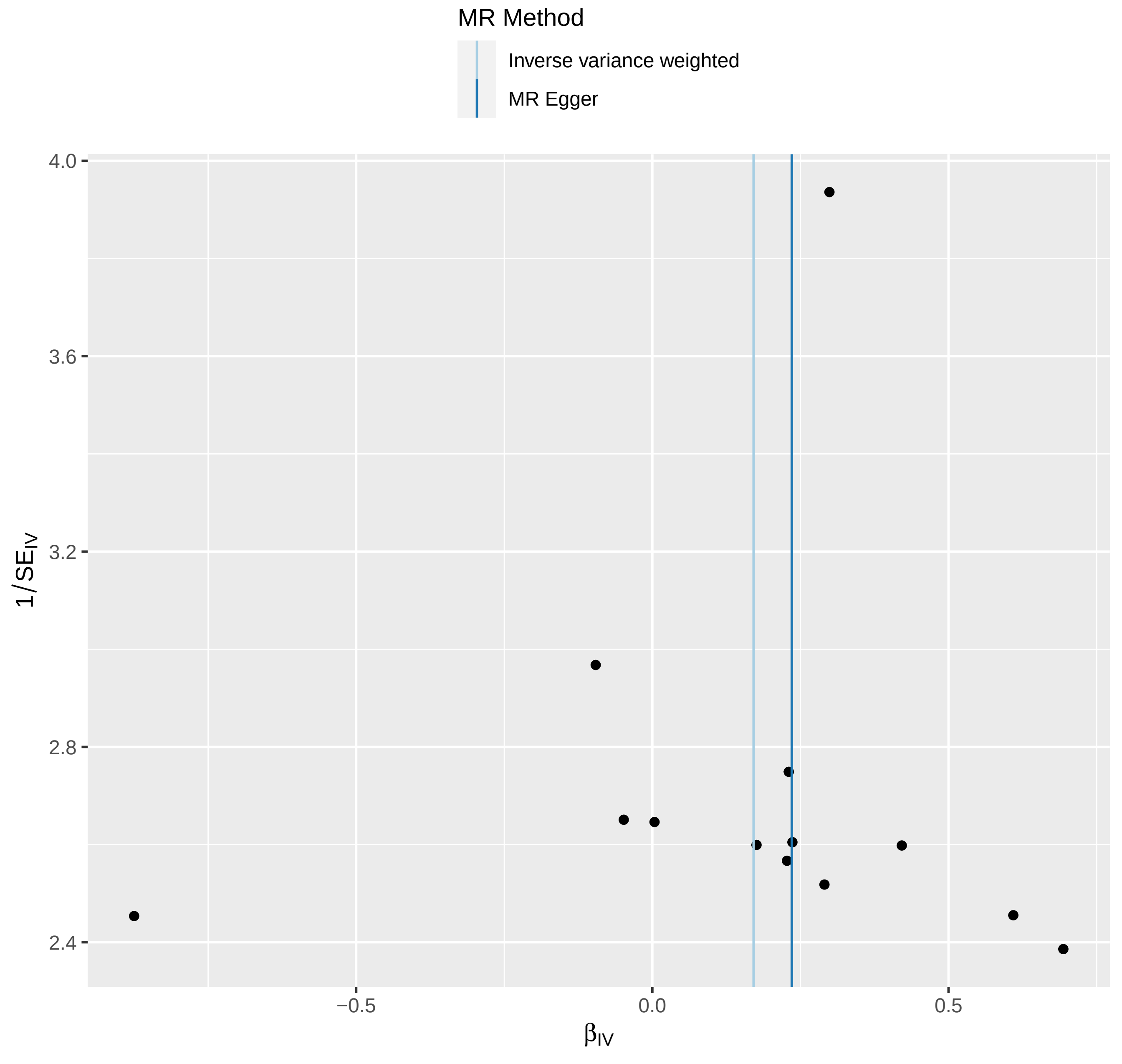

Batch 2−5317 : Gut microbiota abundance (genus Catenibacterium id.2153) on Protein SEC13 homolog
with a threshold of 1e−05

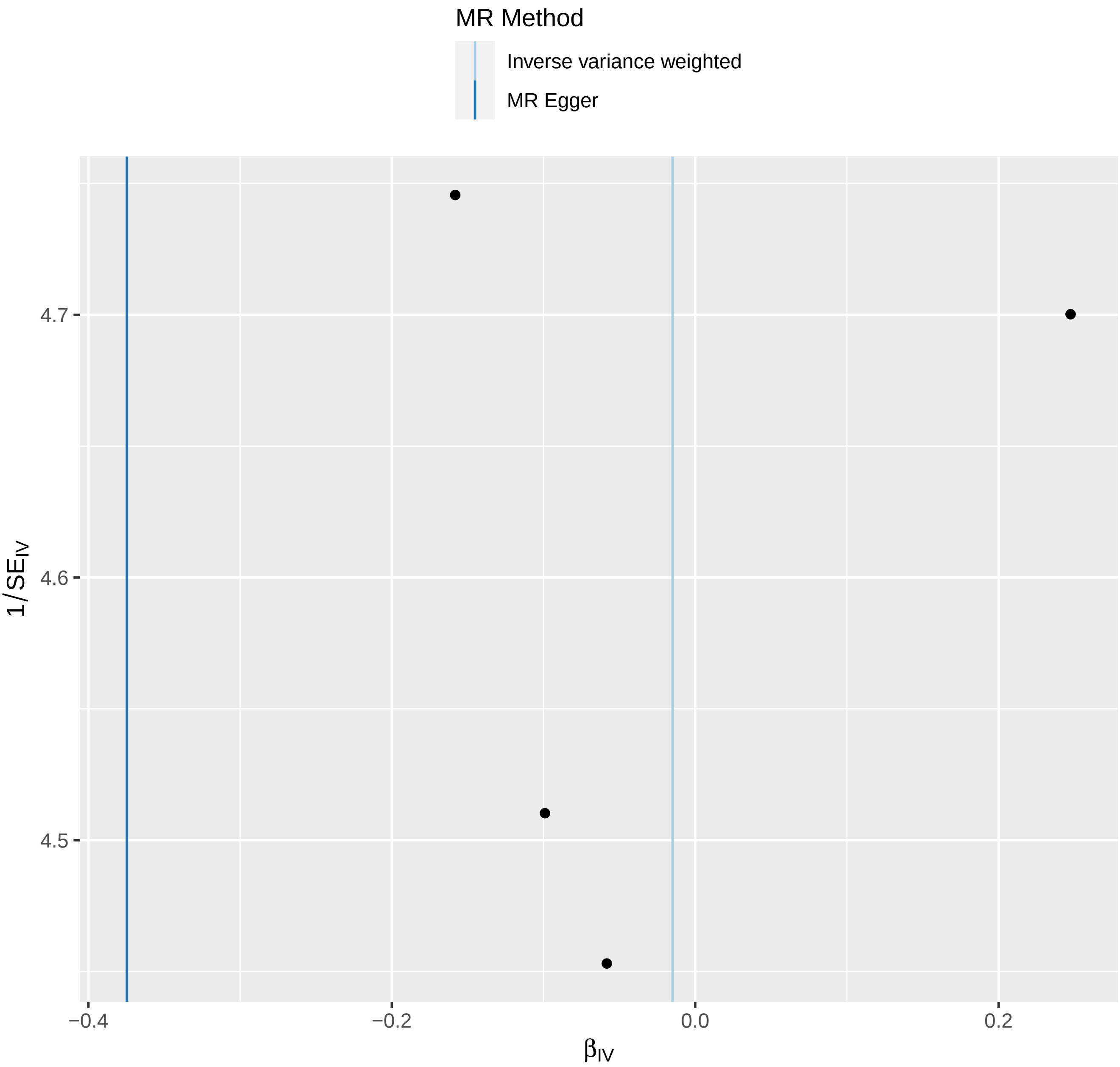

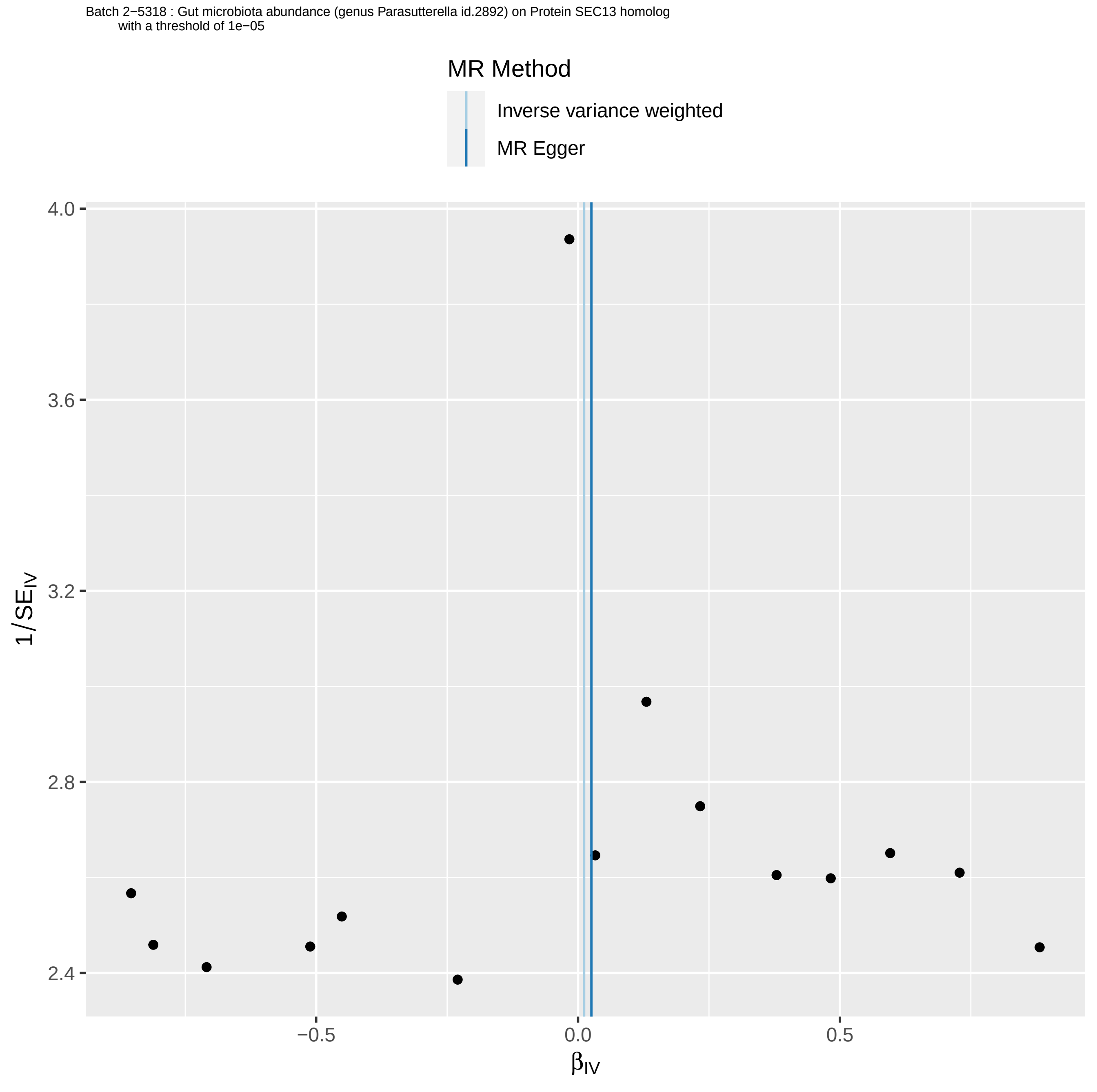

Batch 2−5318 : Gut microbiota abundance (genus Parasutterella id.2892) on Protein SEC13 homolog
with a threshold of 1e−05
MR Method
Inverse variance weighted
MR Egger
4.0
3.6
3.2
2.8
2.4
1/SE_IV
−0.5
0.0
0.5
β_IV

Batch 2−5319 : Gut microbiota abundance (genus Catenibacterium id.2153) on Vesicle−trafficking protein SEC22a with a threshold of 1e−05

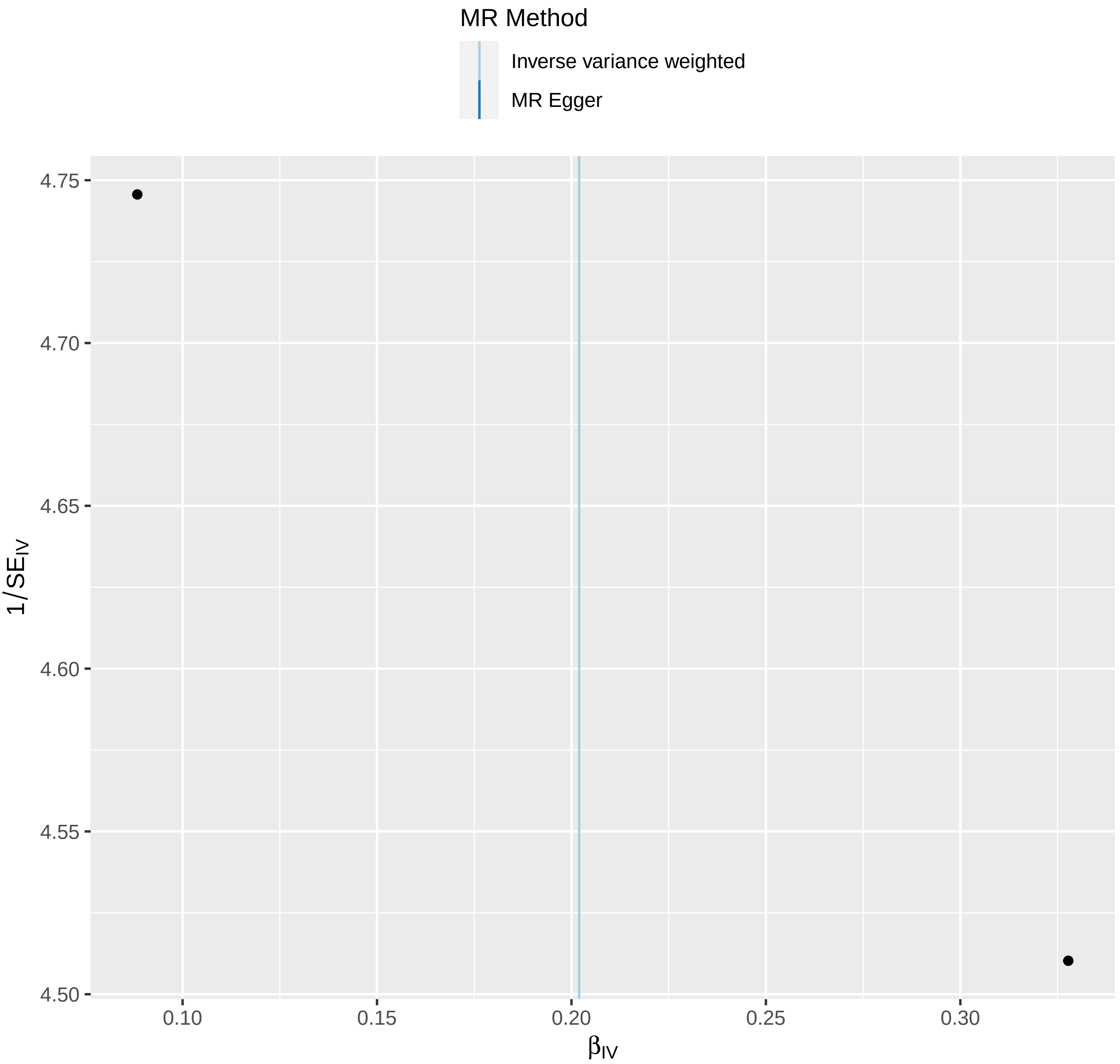

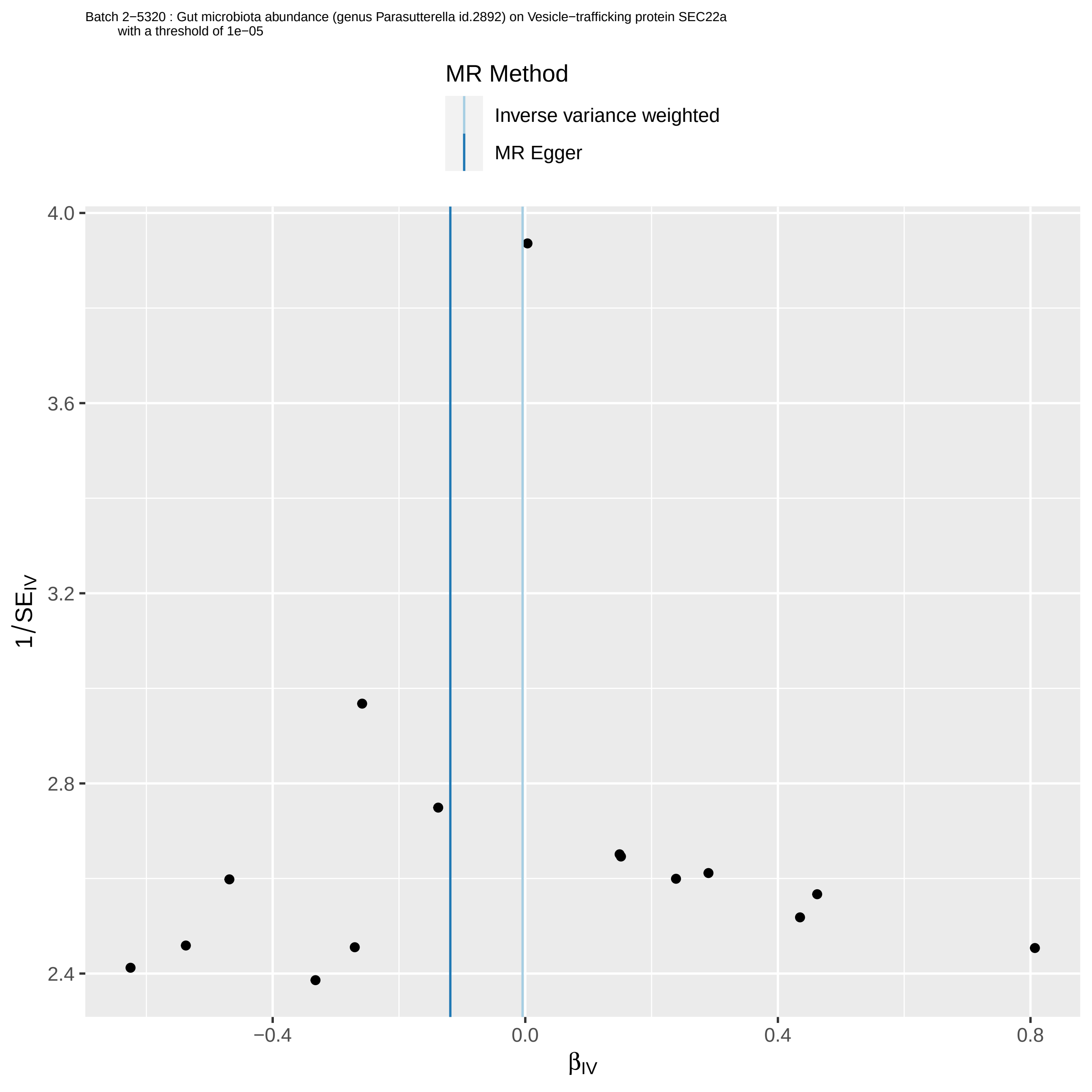

Batch 2−5320 : Gut microbiota abundance (genus Parasutterella id.2892) on Vesicle−trafficking protein SEC22a
with a threshold of 1e−05
MR Method
Inverse variance weighted
MR Egger
4.0
3.6
3.2
2.8
2.4
1/SE_IV
−0.4
0.0
0.4
0.8
β_IV

Batch 2−5321 : Gut microbiota abundance (genus Catenibacterium id.2153) on Protein transport protein Sec61 subunit beta
with a threshold of 1e−05

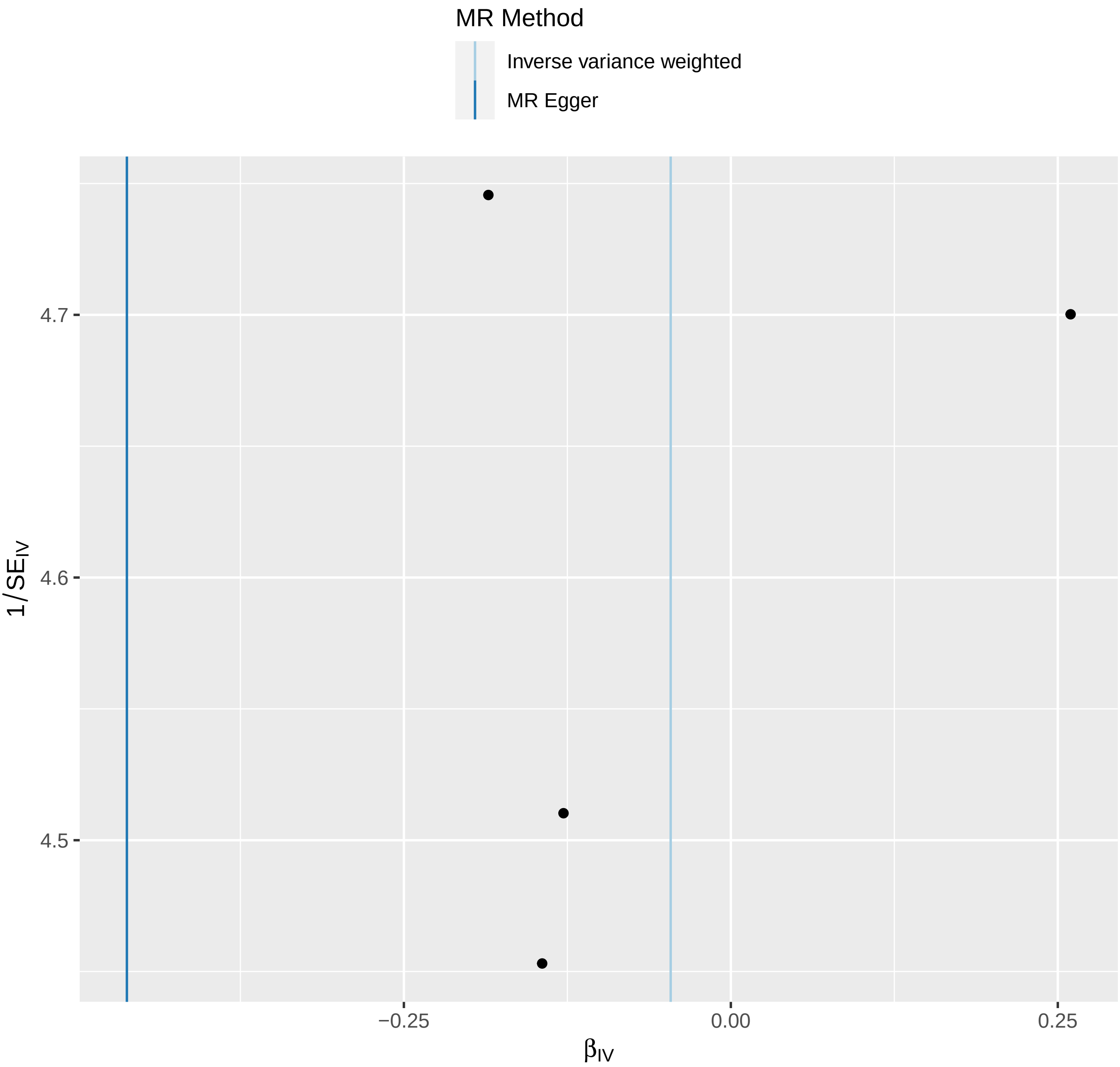

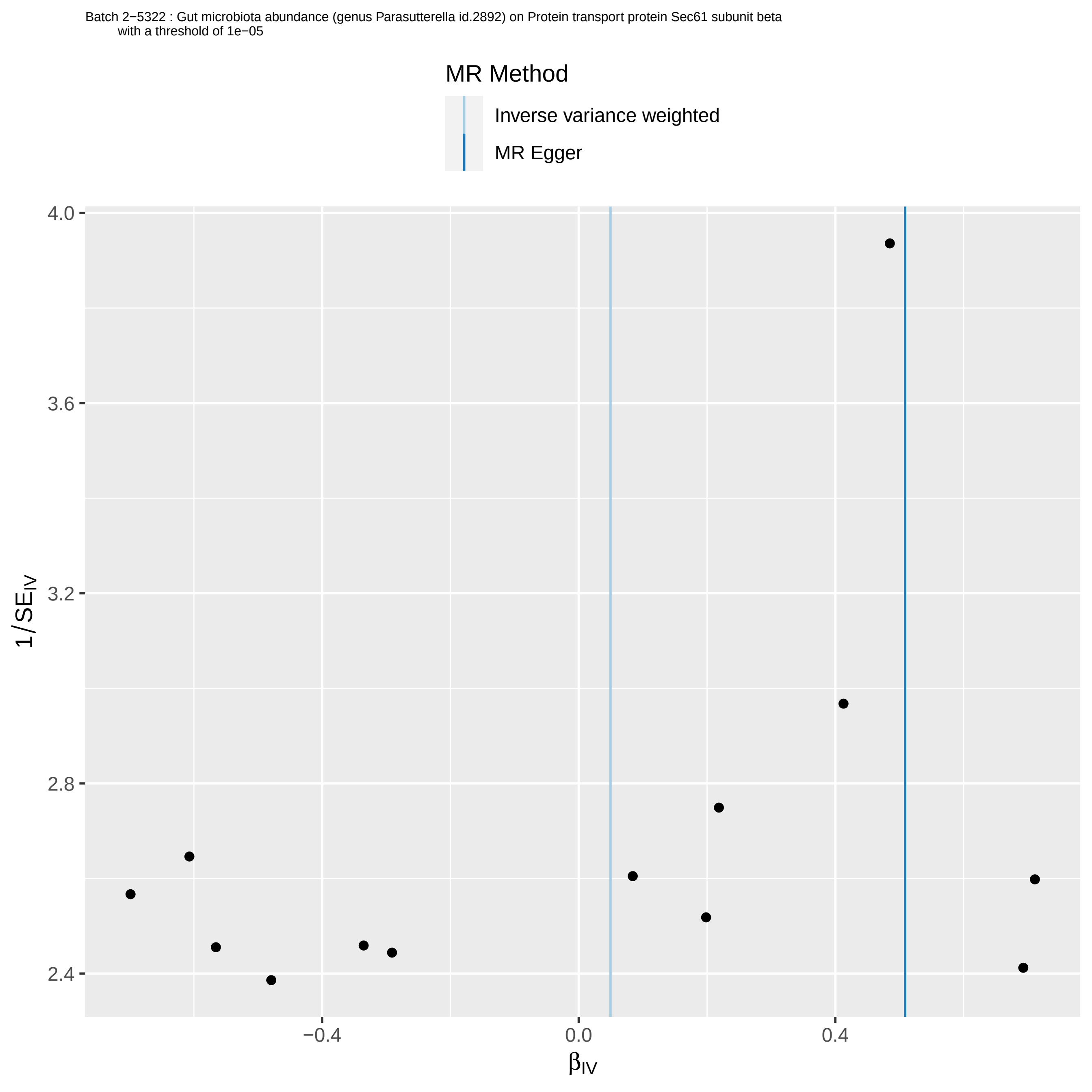
Batch 2−5322 : Gut microbiota abundance (genus Parasutterella id.2892) on Protein transport protein Sec61 subunit beta
with a threshold of 1e−05
MR Method
Inverse variance weighted
MR Egger
4.0
3.6
3.2
2.8
2.4
$1/SE_{IV}$
−0.4
0.0
0.4
β_{IV}

Batch 2−5323 : Gut microbiota abundance (genus Catenibacterium id.2153) on Protein transport protein Sec61 subunit gamma
with a threshold of 1e−05

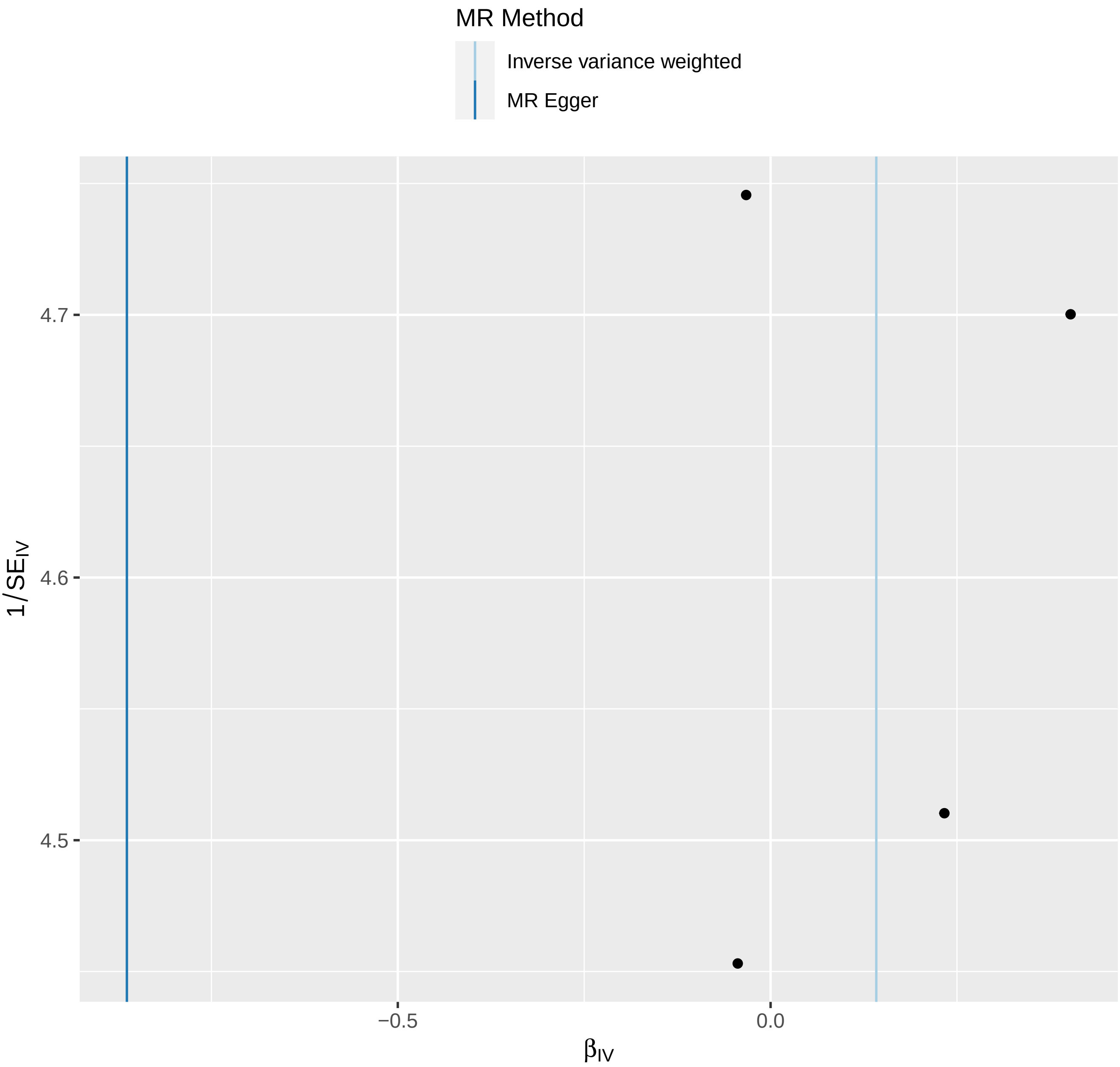

Batch 2−5324 : Gut microbiota abundance (genus Parasutterella id.2892) on Protein transport protein Sec61 subunit gamma with a threshold of 1e−05

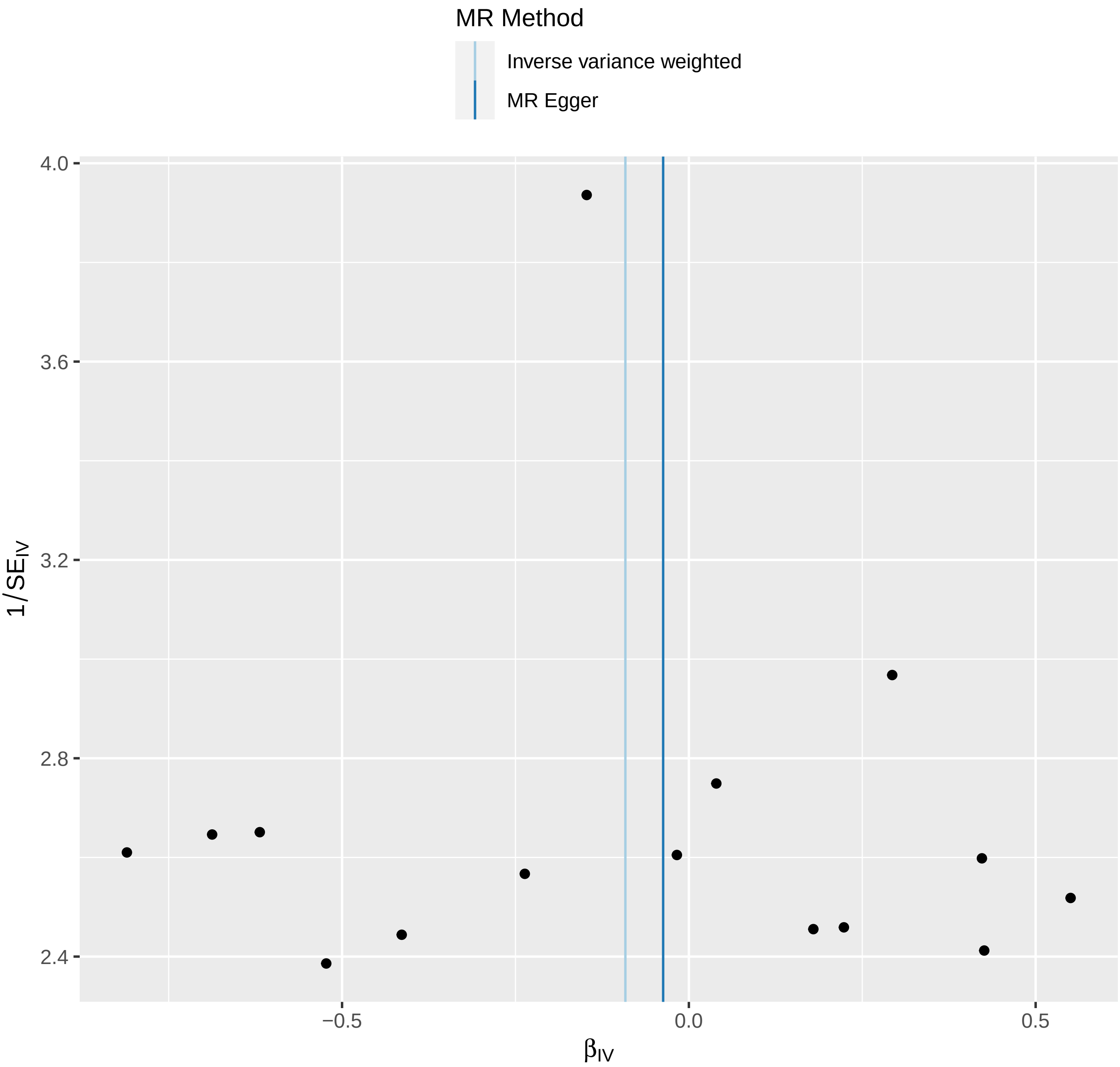

Batch 2−5325 : Gut microbiota abundance (genus Catenibacterium id.2153) on Secreted and transmembrane protein 1
with a threshold of 1e−05

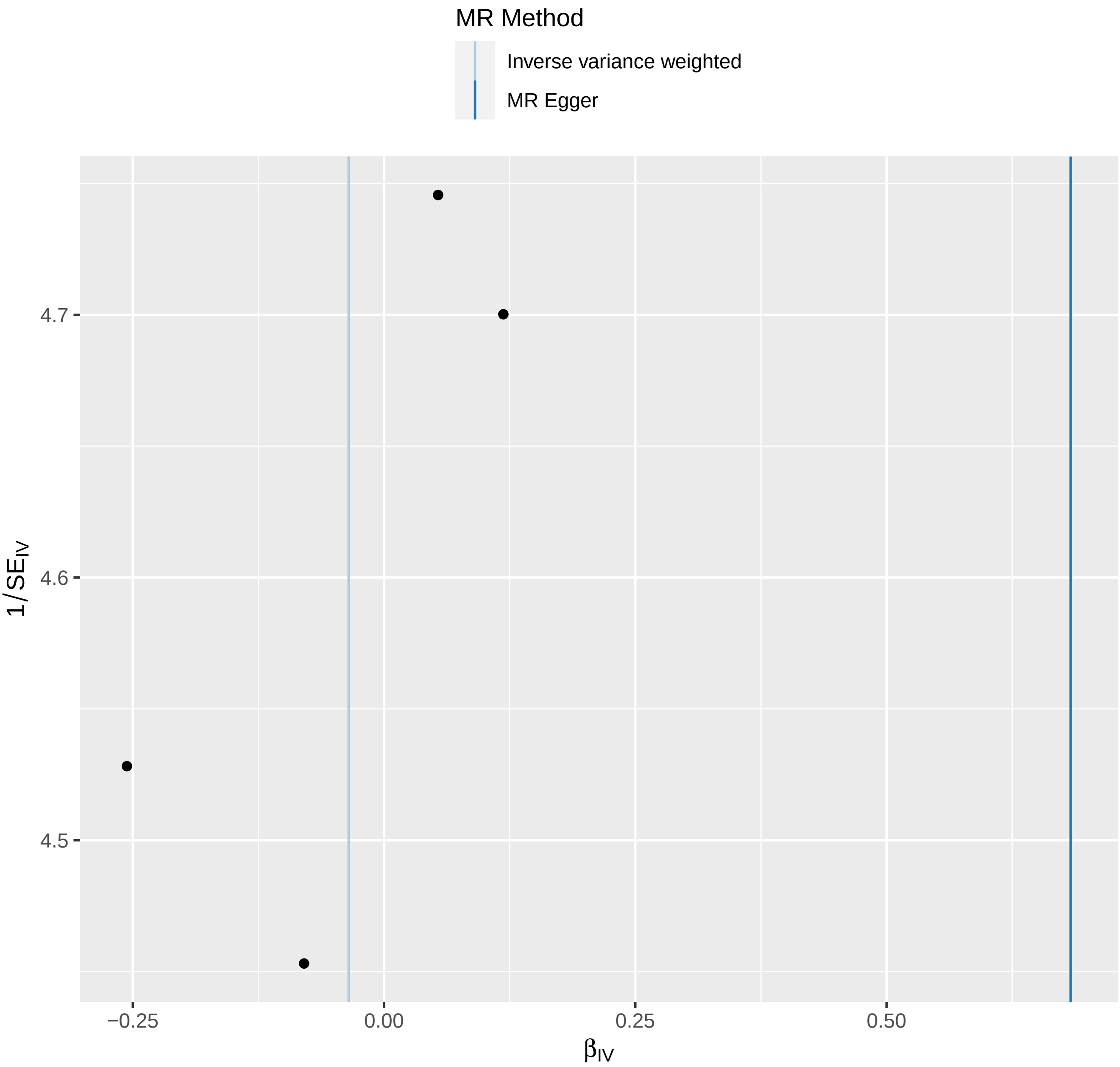

Batch 2−5326 : Gut microbiota abundance (genus Parasutterella id.2892) on Secreted and transmembrane protein 1
with a threshold of 1e−05

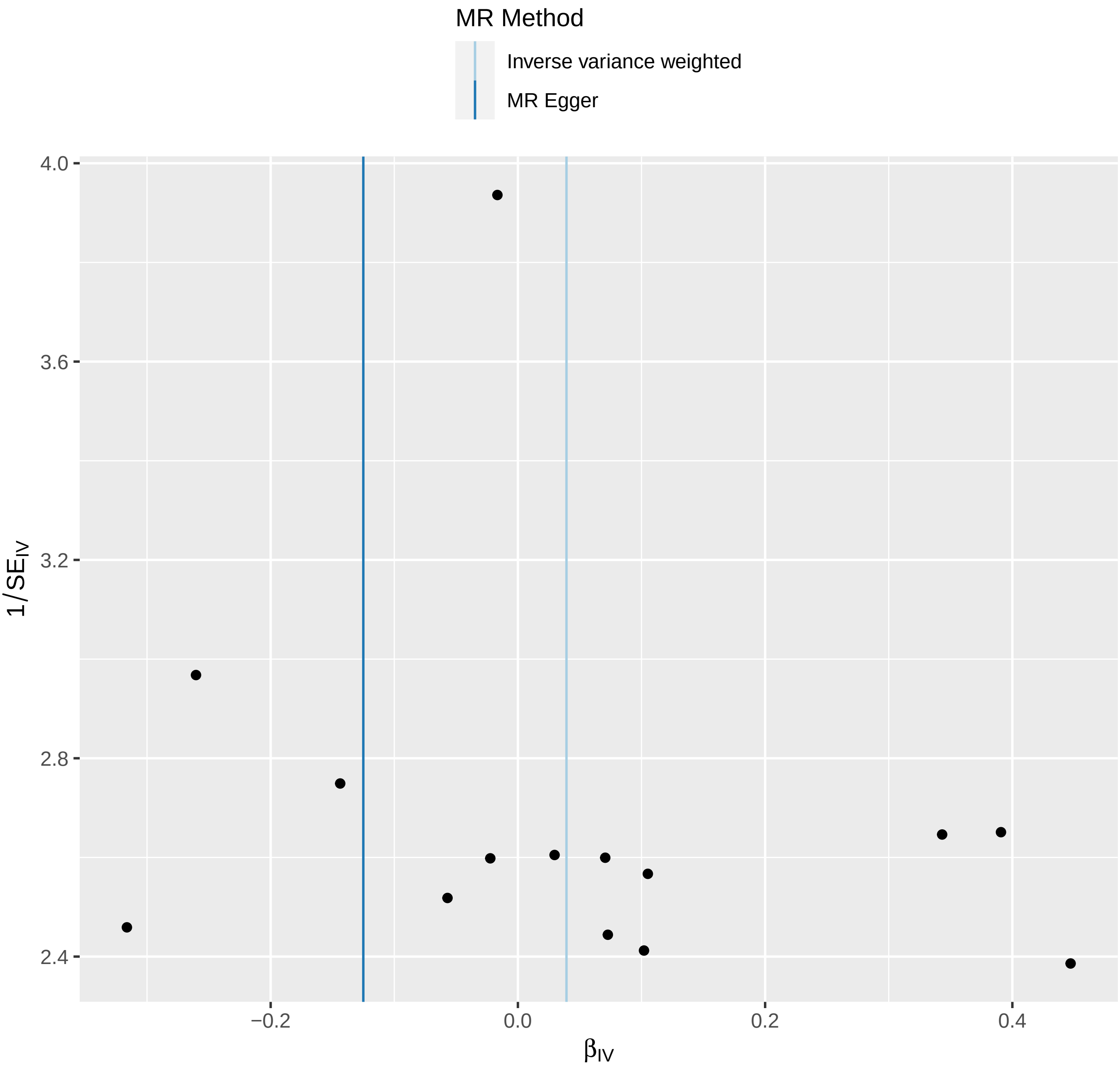

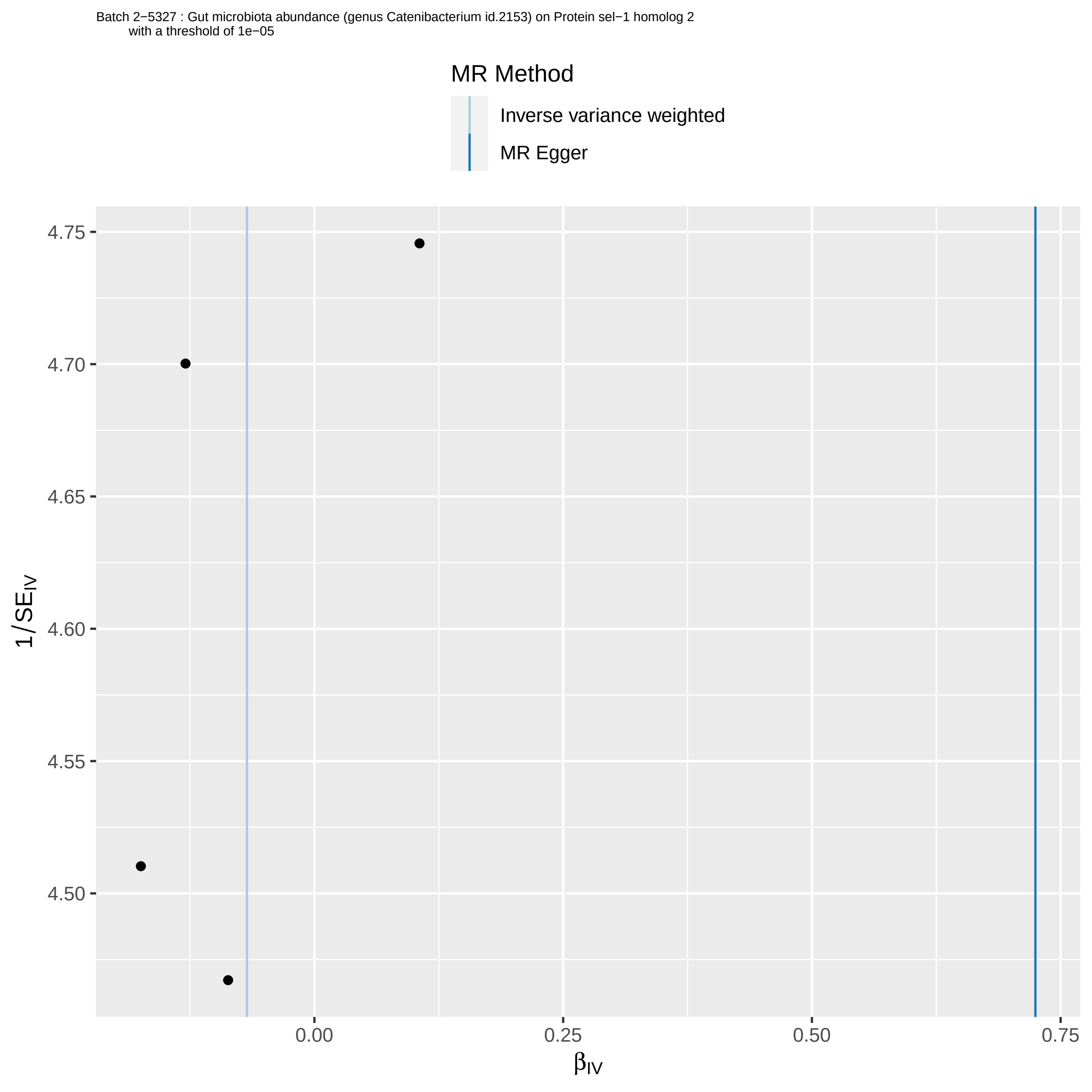
Batch 2−5327 : Gut microbiota abundance (genus Catenibacterium id.2153) on Protein sel−1 homolog 2
with a threshold of 1e−05
MR Method
Inverse variance weighted
MR Egger
4.75
4.70
4.65
4.60
4.55
4.50
1/SE_IV
0.00
0.25
0.50
0.75
β_IV

Batch 2−5328 : Gut microbiota abundance (genus Parasutterella id.2892) on Protein sel−1 homolog 2
with a threshold of 1e−05

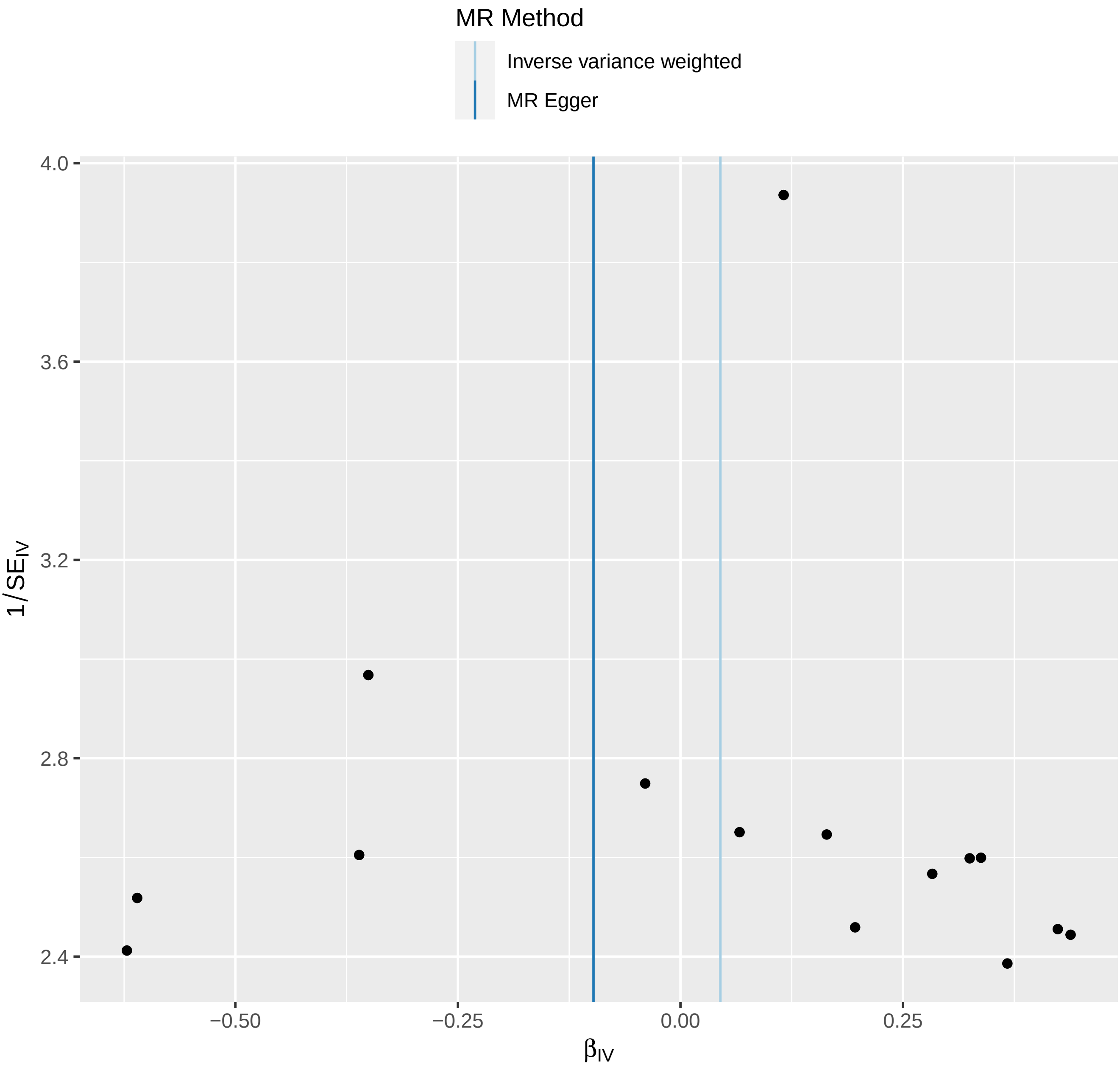

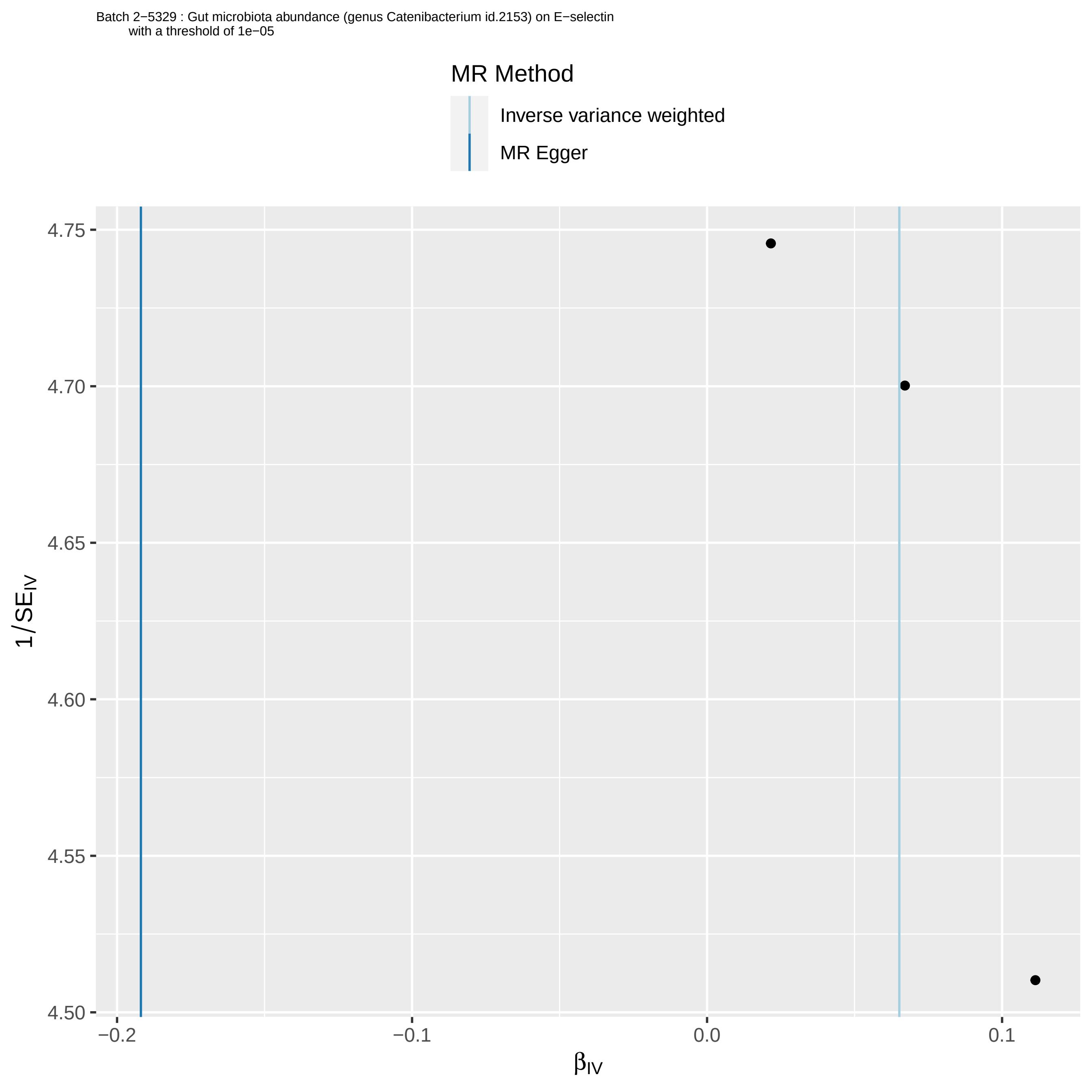

Batch 2−5329 : Gut microbiota abundance (genus Catenibacterium id.2153) on E−selectin
with a threshold of 1e−05
MR Method
Inverse variance weighted
MR Egger
4.75
4.70
4.65
4.60
4.55
4.50
1/SE_IV
−0.2
−0.1
0.0
0.1
β_IV

Batch 2−5330 : Gut microbiota abundance (genus Parasutterella id.2892) on E−selectin
with a threshold of 1e−05

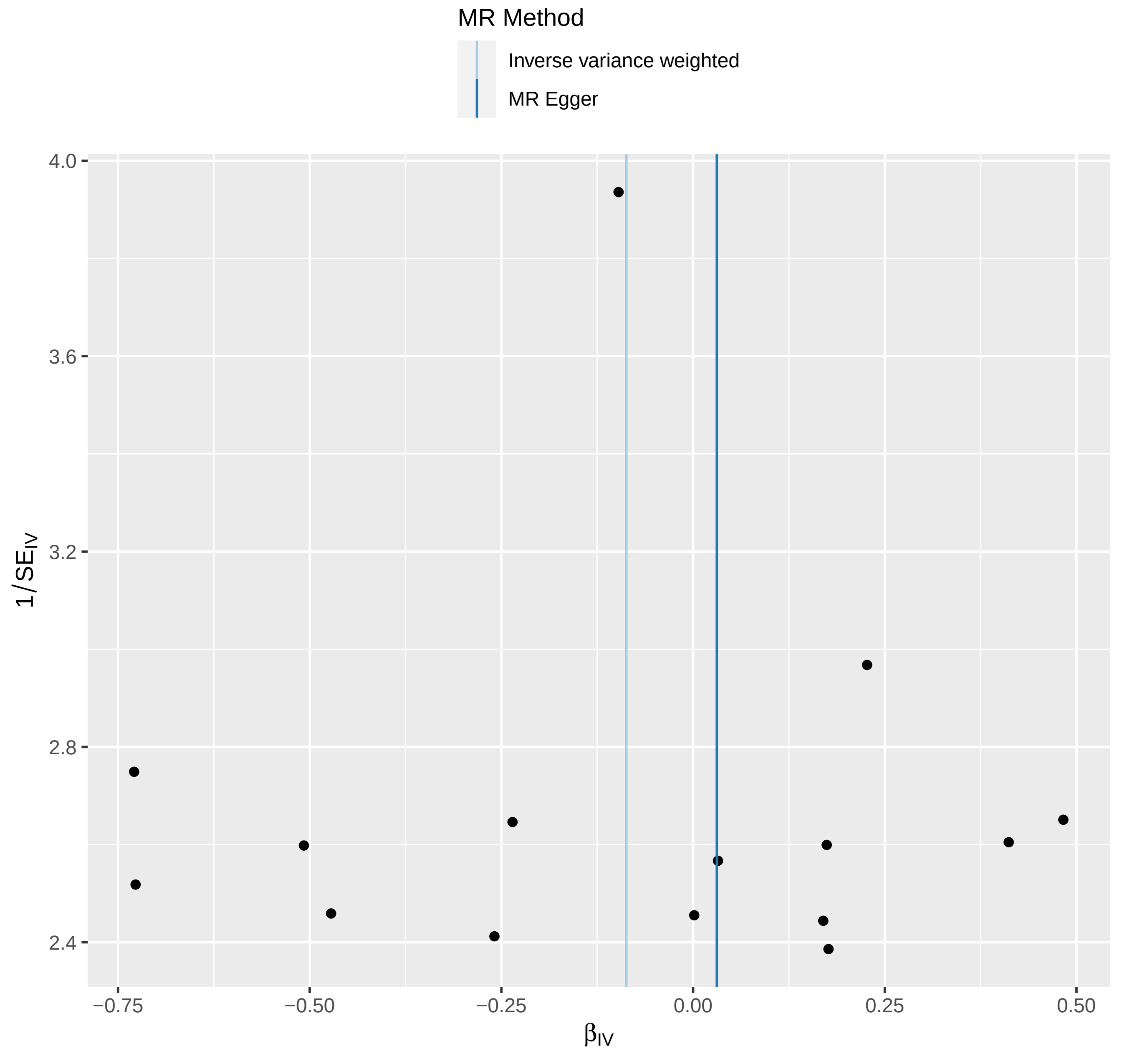

Batch 2−5331 : Gut microbiota abundance (genus Catenibacterium id.2153) on L−Selectin
with a threshold of 1e−05

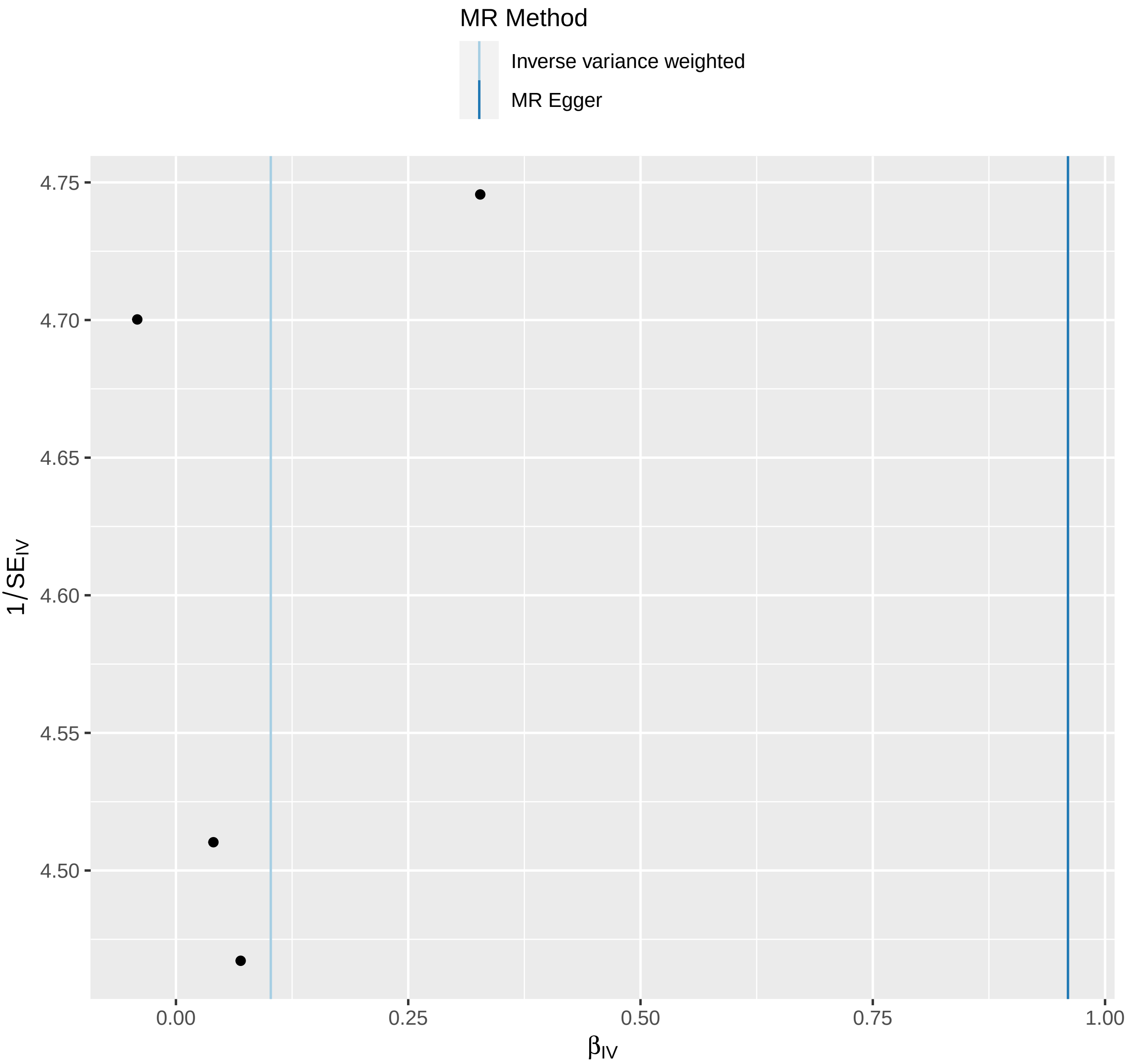

Batch 2−5332 : Gut microbiota abundance (genus Parasutterella id.2892) on L−Selectin
with a threshold of 1e−05

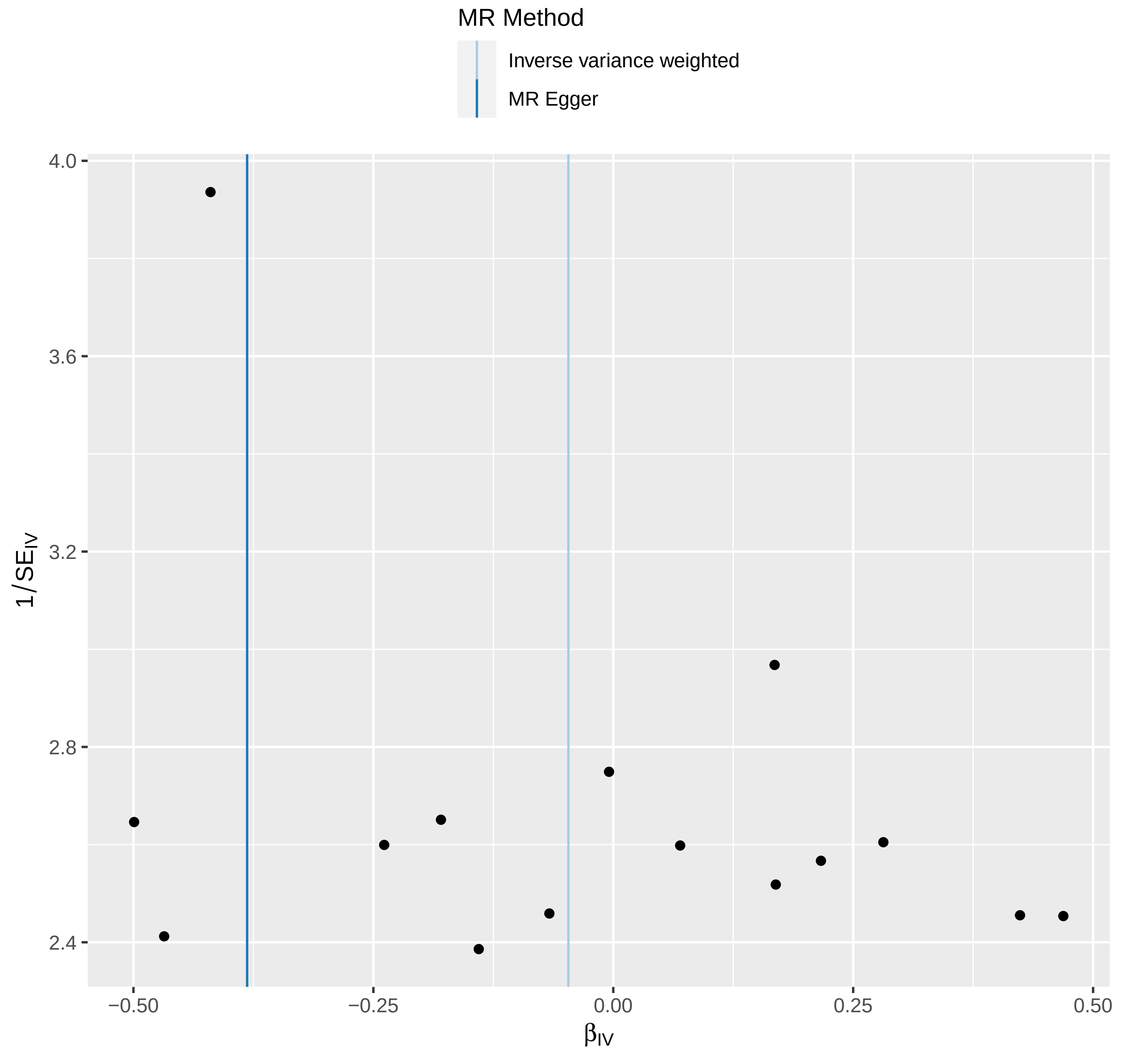

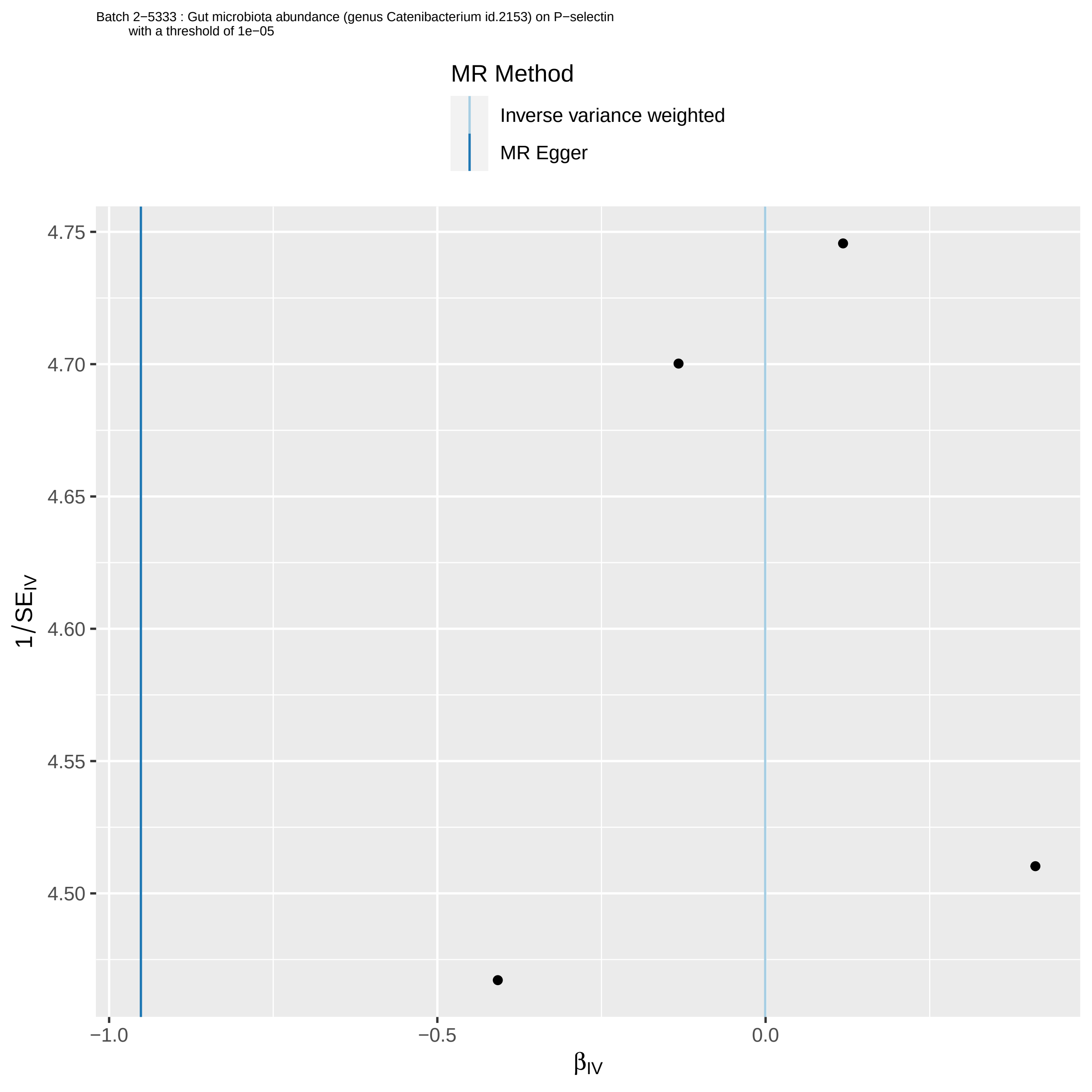
Batch 2−5333 : Gut microbiota abundance (genus Catenibacterium id.2153) on P−selectin
with a threshold of 1e−05
MR Method
Inverse variance weighted
MR Egger
4.75
4.70
4.65
4.60
4.55
4.50
1/SE_IV
−1.0
−0.5
0.0
β_IV

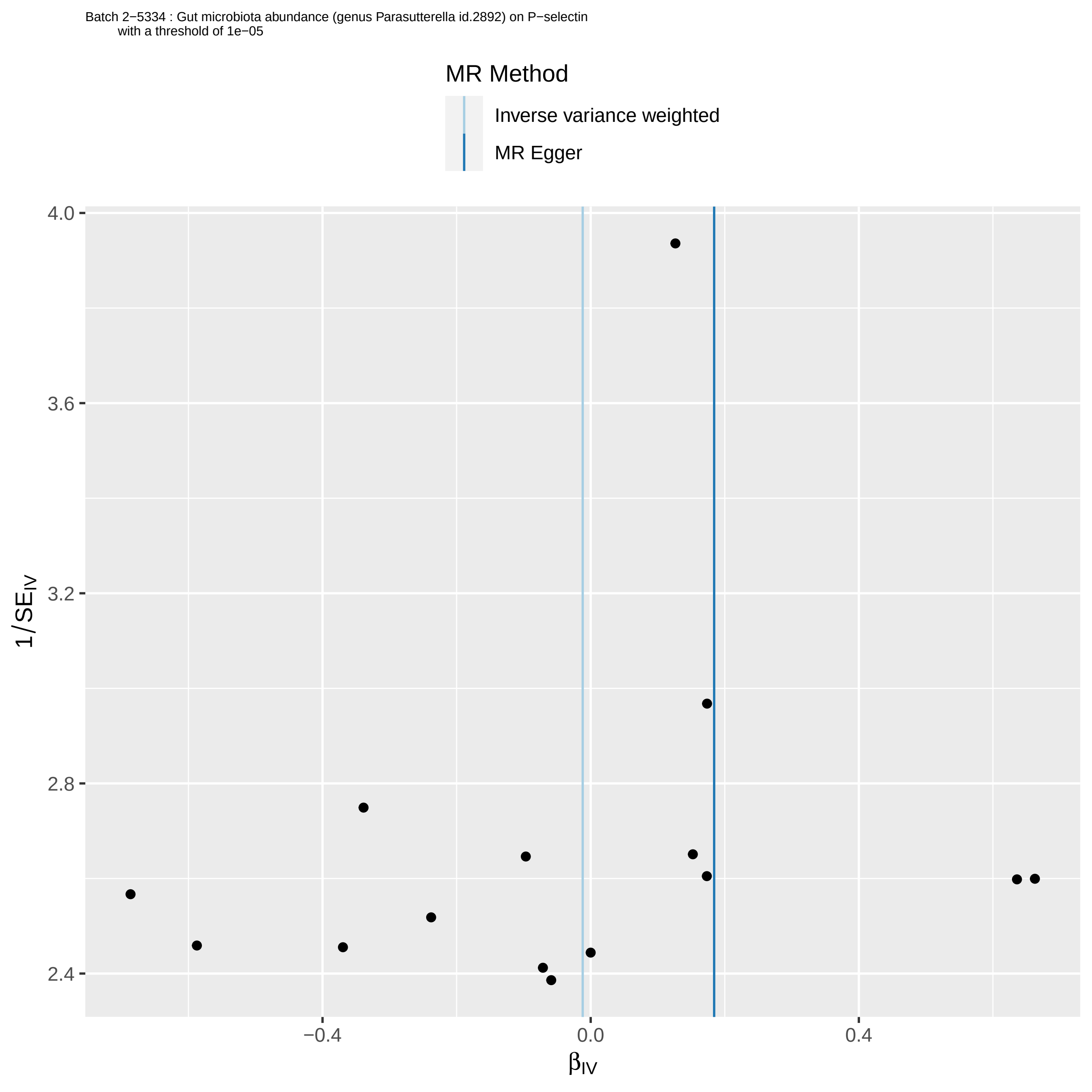
Batch 2−5334 : Gut microbiota abundance (genus Parasutterella id.2892) on P−selectin
with a threshold of 1e−05
MR Method
Inverse variance weighted
MR Egger
4.0
3.6
3.2
2.8
2.4
1/SE_IV
−0.4
0.0
0.4
β_IV

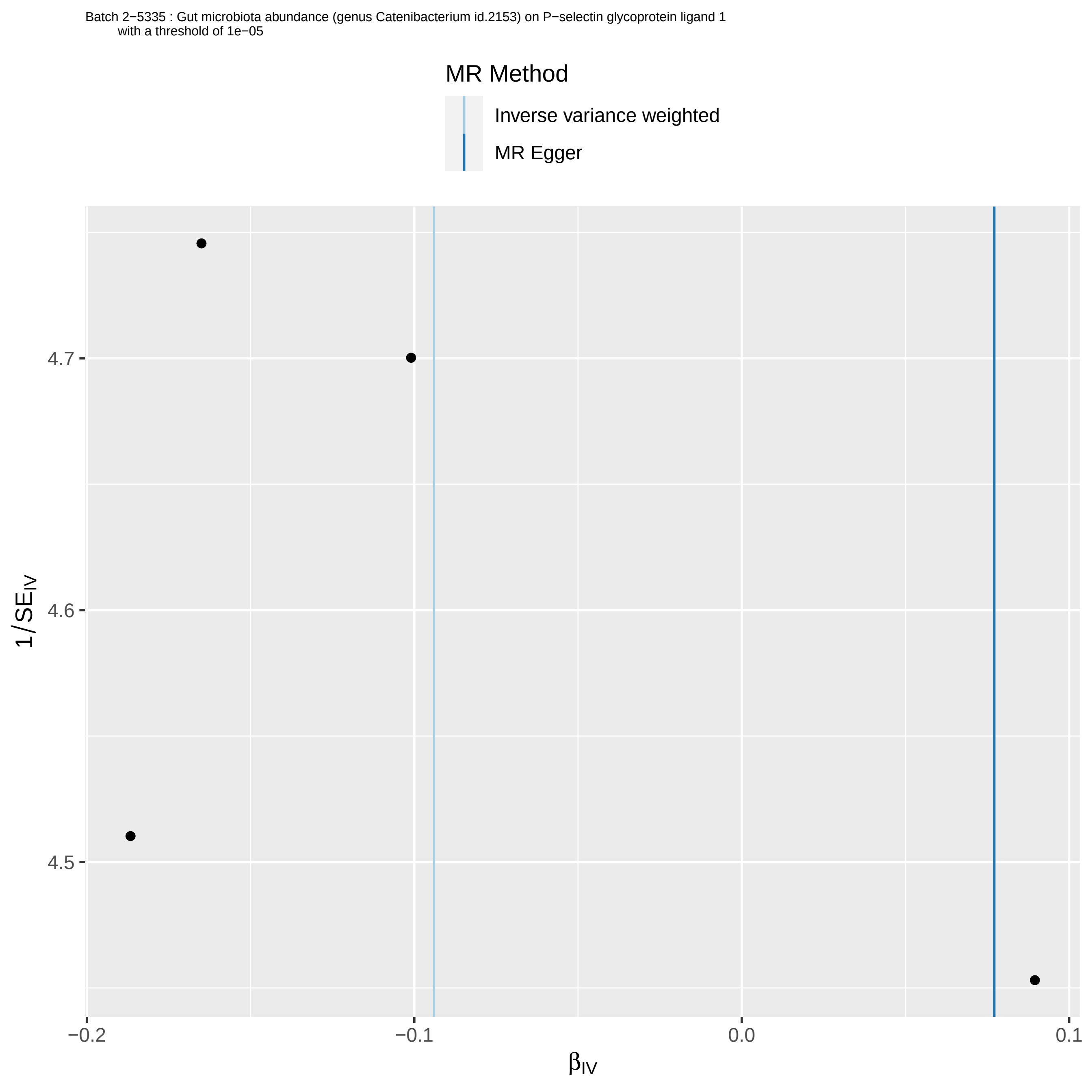
Batch 2−5335 : Gut microbiota abundance (genus Catenibacterium id.2153) on P−selectin glycoprotein ligand 1
with a threshold of 1e−05
MR Method
Inverse variance weighted
MR Egger
4.7
4.6
4.5
1/SE_IV
−0.2
−0.1
0.0
0.1
β_IV

Batch 2−5336 : Gut microbiota abundance (genus Parasutterella id.2892) on P−selectin glycoprotein ligand 1
with a threshold of 1e−05

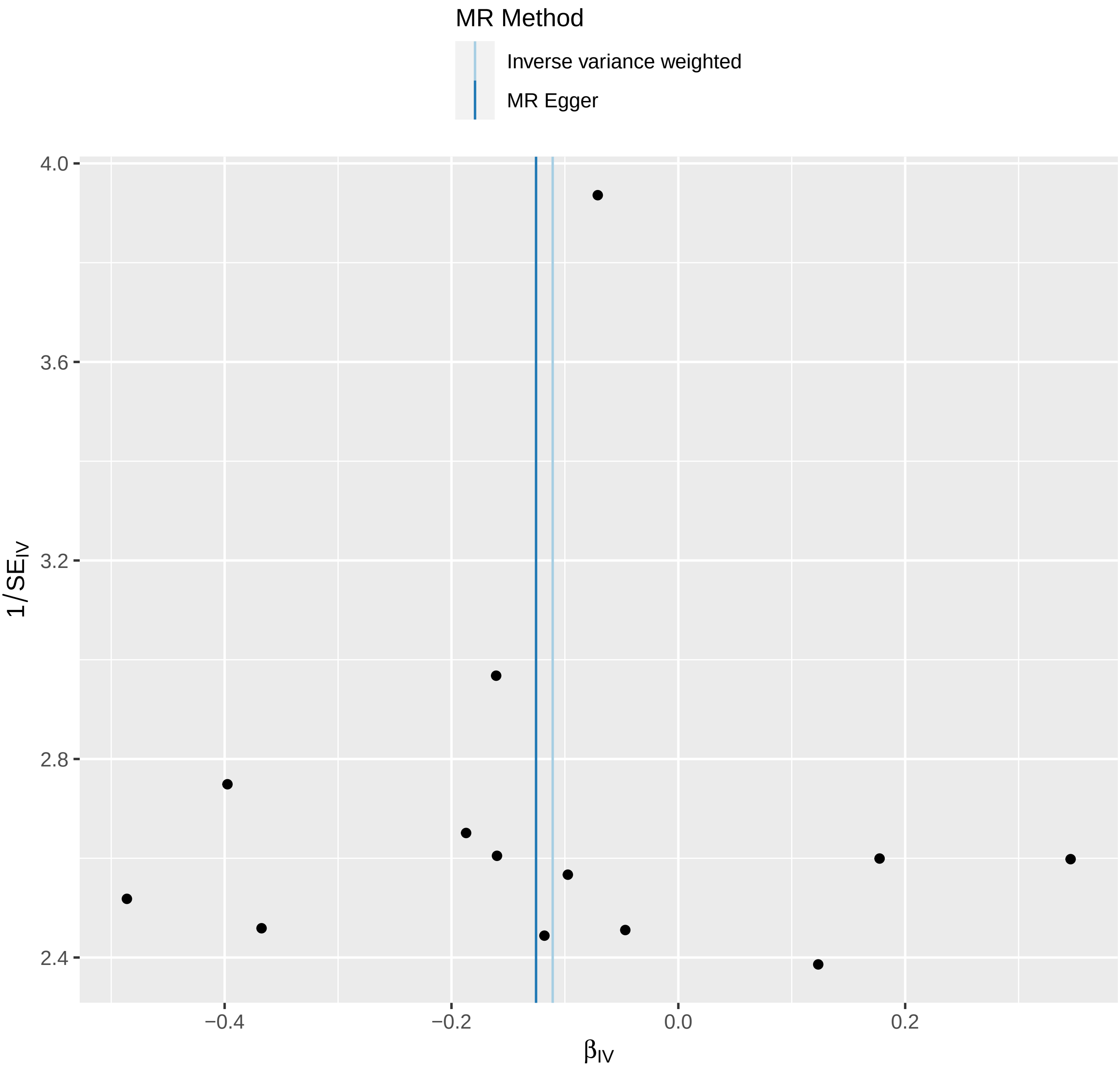

Batch 2−5337 : Gut microbiota abundance (genus Catenibacterium id.2153) on Semaphorin−3A
with a threshold of 1e−05

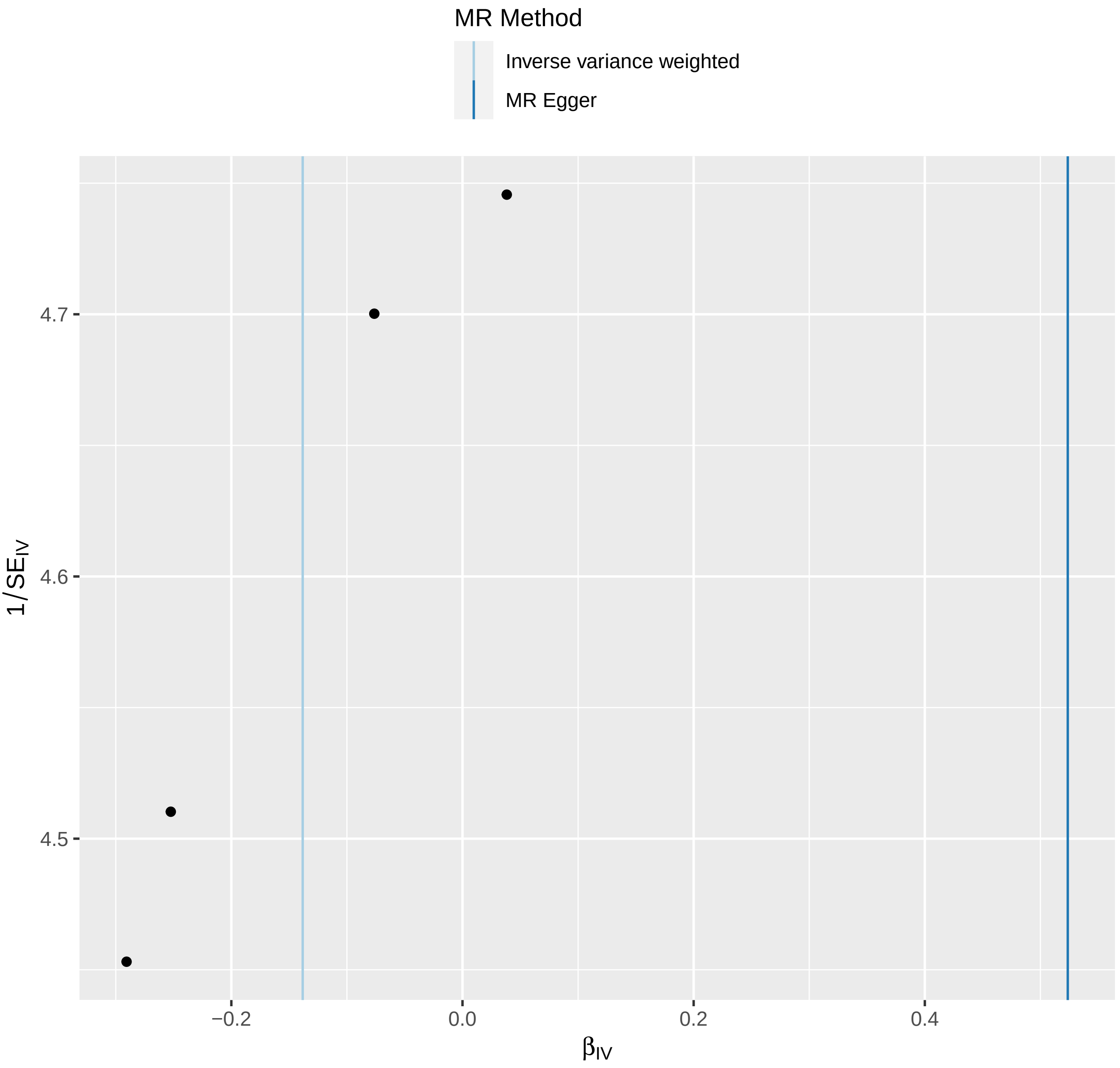

Batch 2−5338 : Gut microbiota abundance (genus Parasutterella id.2892) on Semaphorin−3A
with a threshold of 1e−05

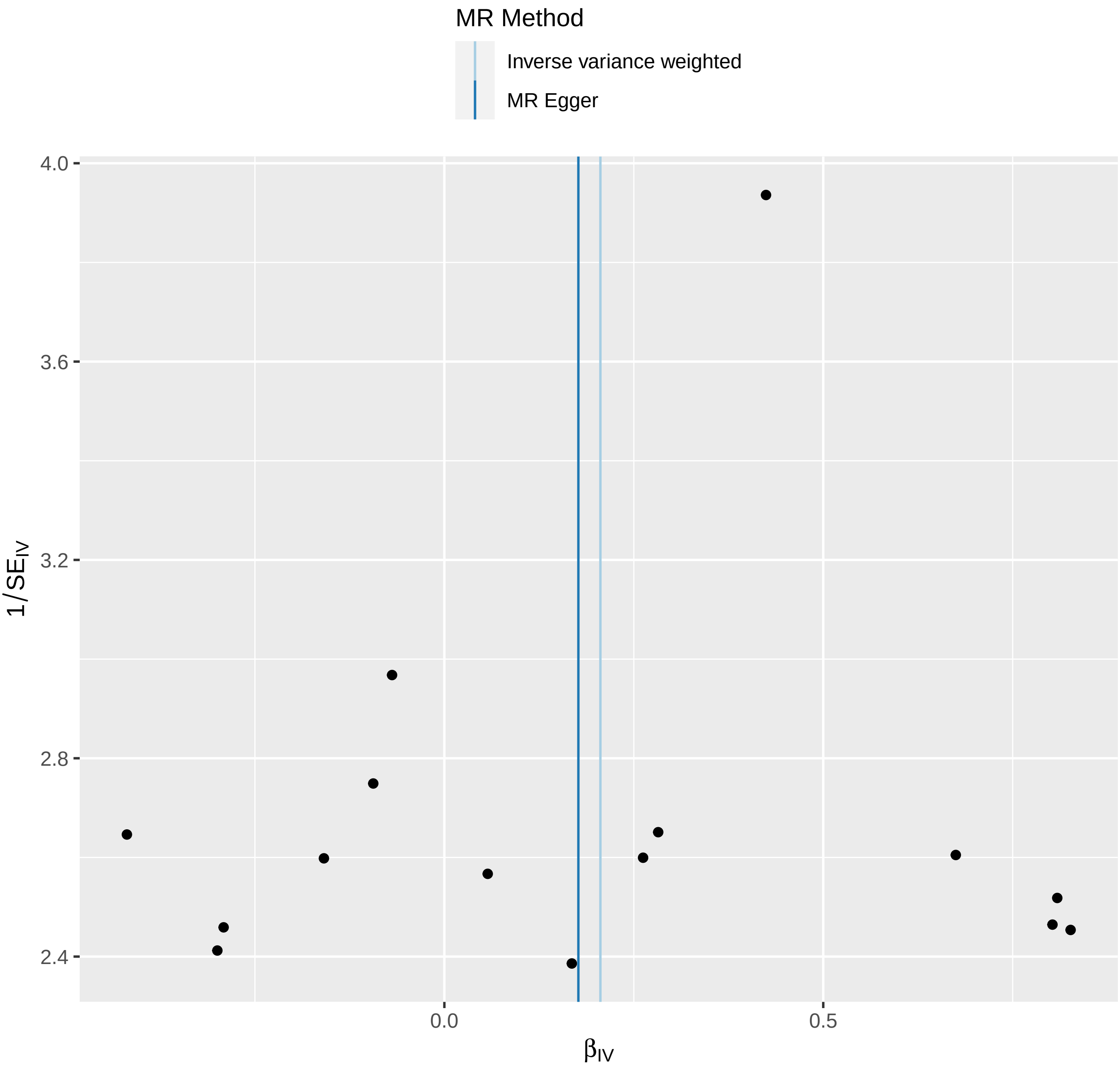

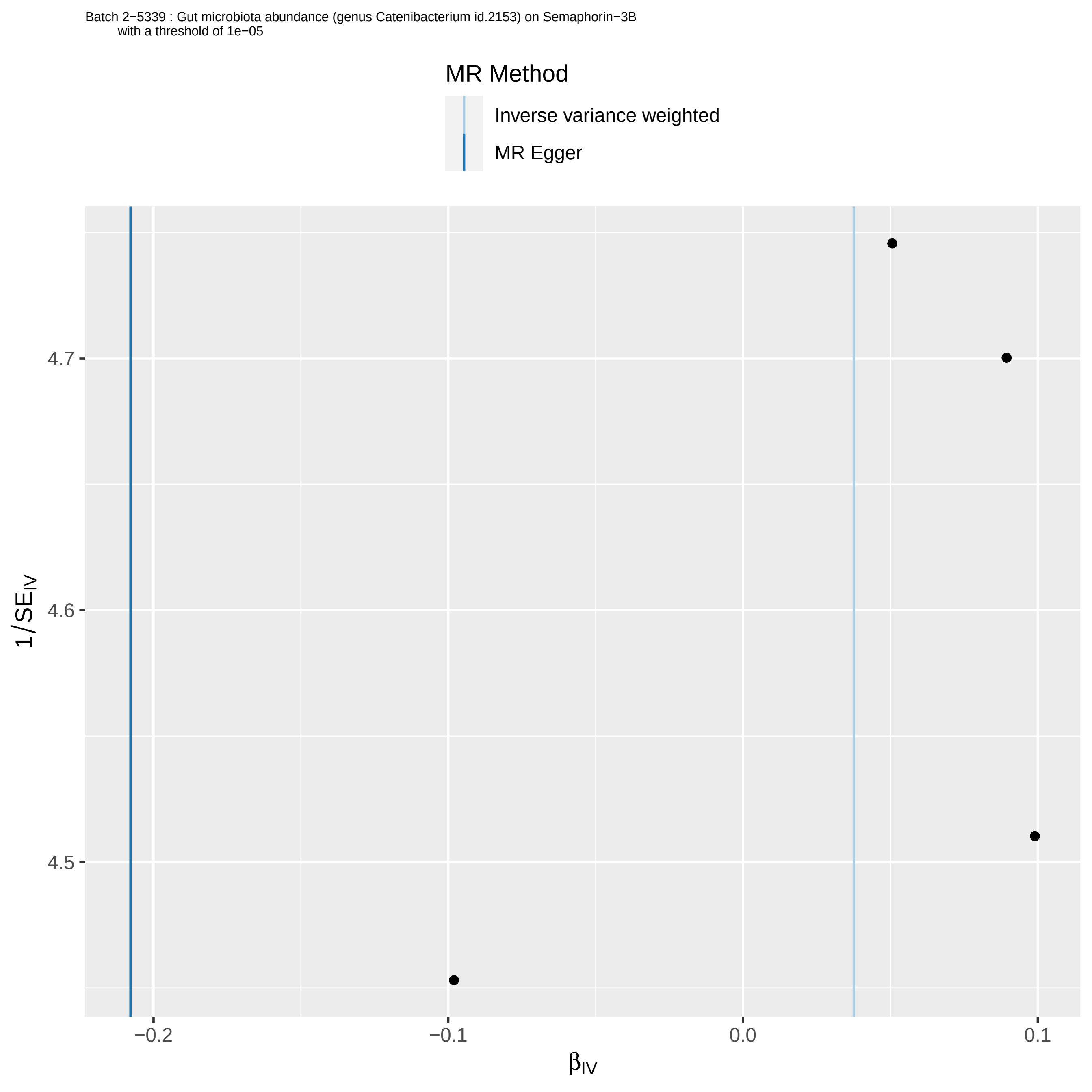

Batch 2−5339 : Gut microbiota abundance (genus Catenibacterium id.2153) on Semaphorin−3B
with a threshold of 1e−05
MR Method
Inverse variance weighted
MR Egger
1/SE_IV
4.7
4.6
4.5
−0.2
−0.1
0.0
0.1
β_IV

Batch 2−5340 : Gut microbiota abundance (genus Parasutterella id.2892) on Semaphorin−3B
with a threshold of 1e−05

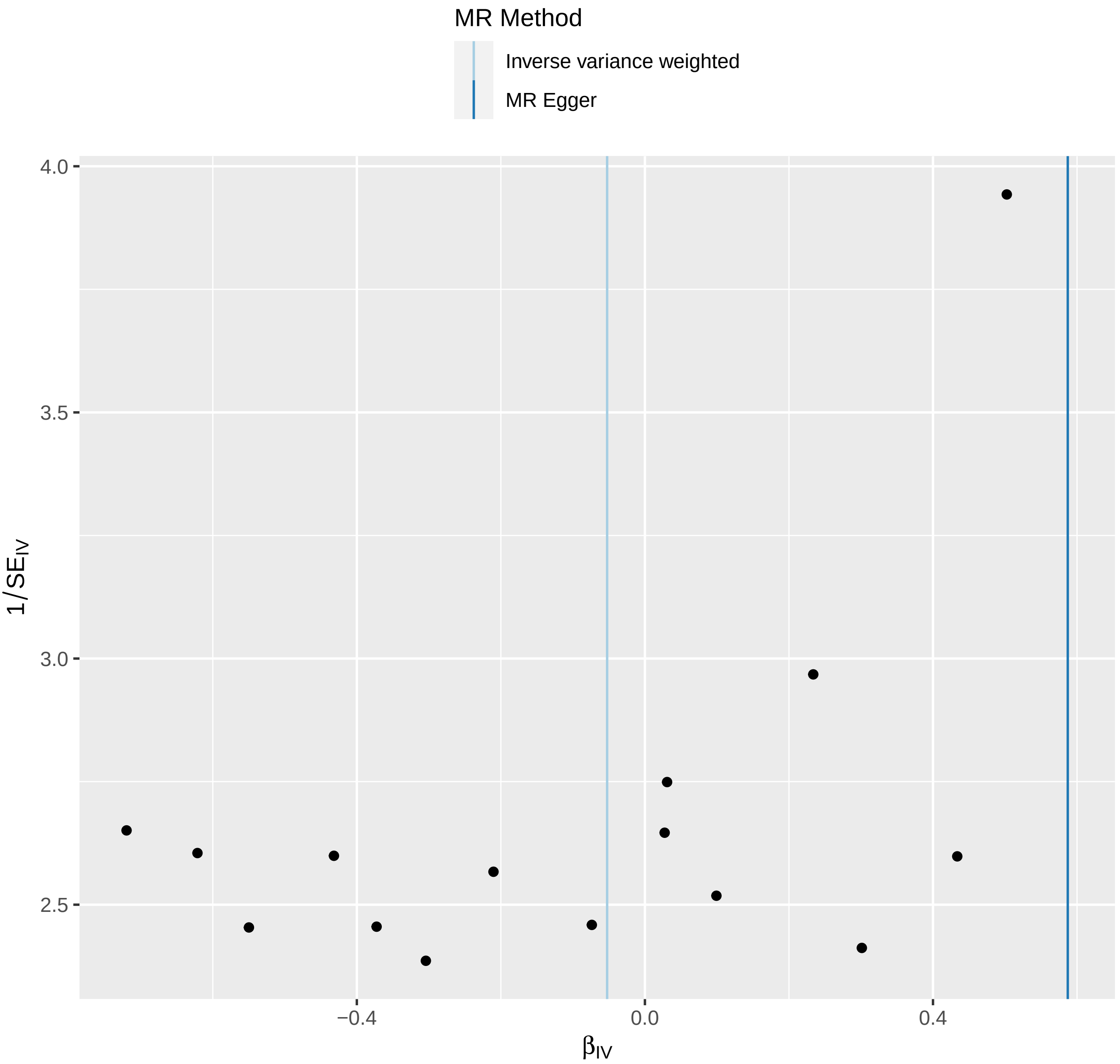

Batch 2−5341 : Gut microbiota abundance (genus Catenibacterium id.2153) on Semaphorin−3C
with a threshold of 1e−05

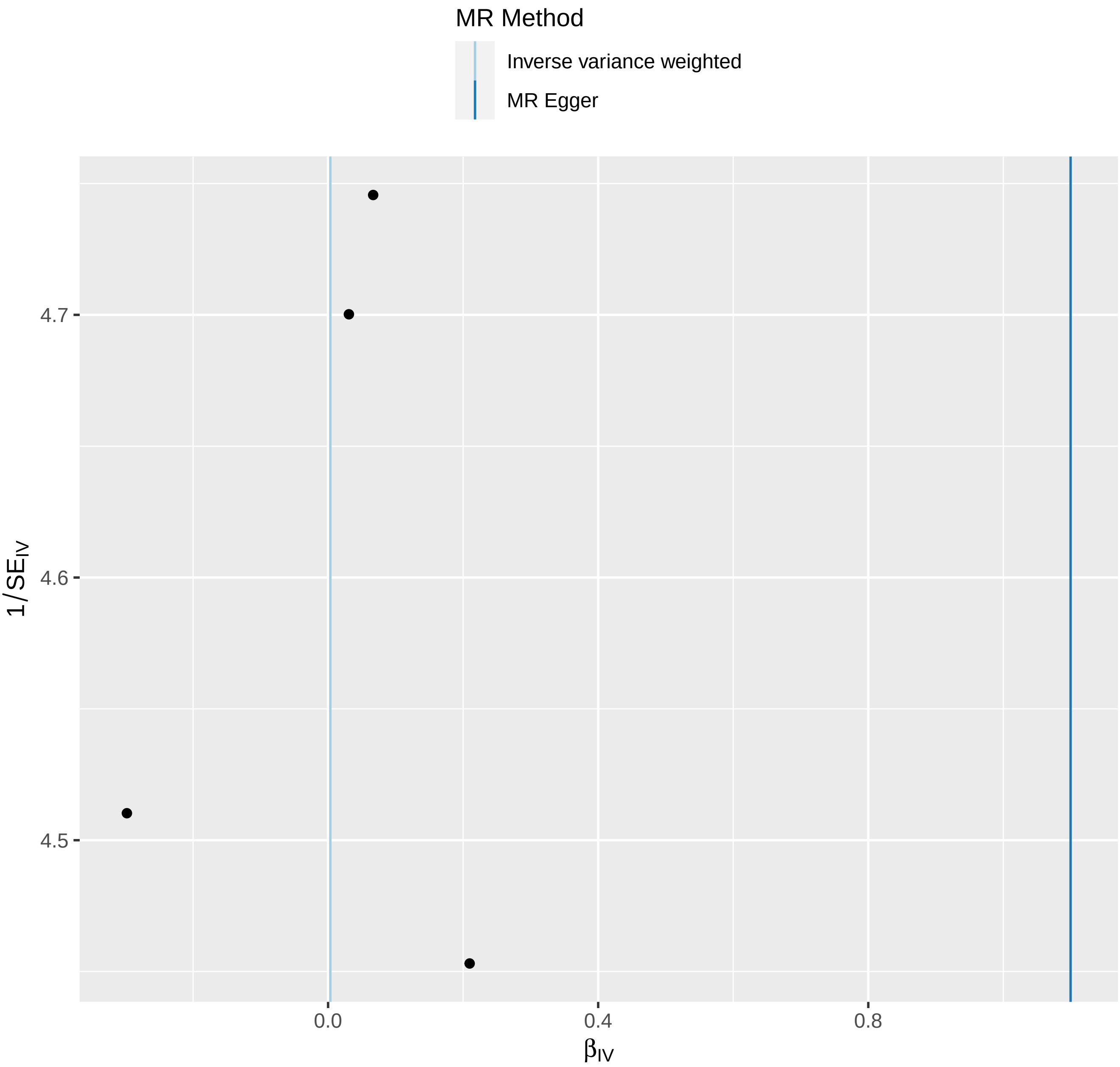

Batch 2−5342 : Gut microbiota abundance (genus Parasutterella id.2892) on Semaphorin−3C
with a threshold of 1e−05

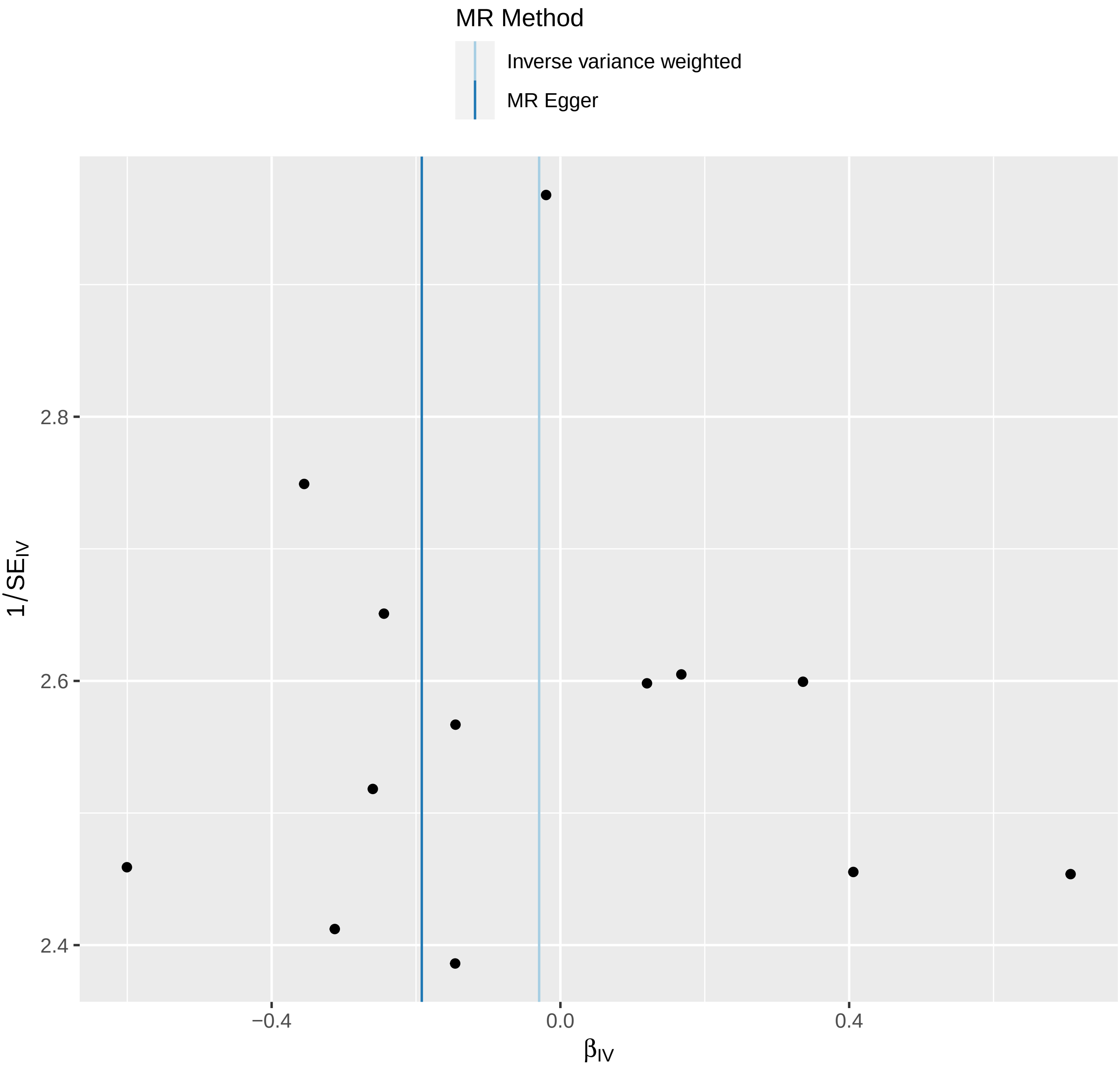

Batch 2−5343 : Gut microbiota abundance (genus Catenibacterium id.2153) on Semaphorin−3E
with a threshold of 1e−05

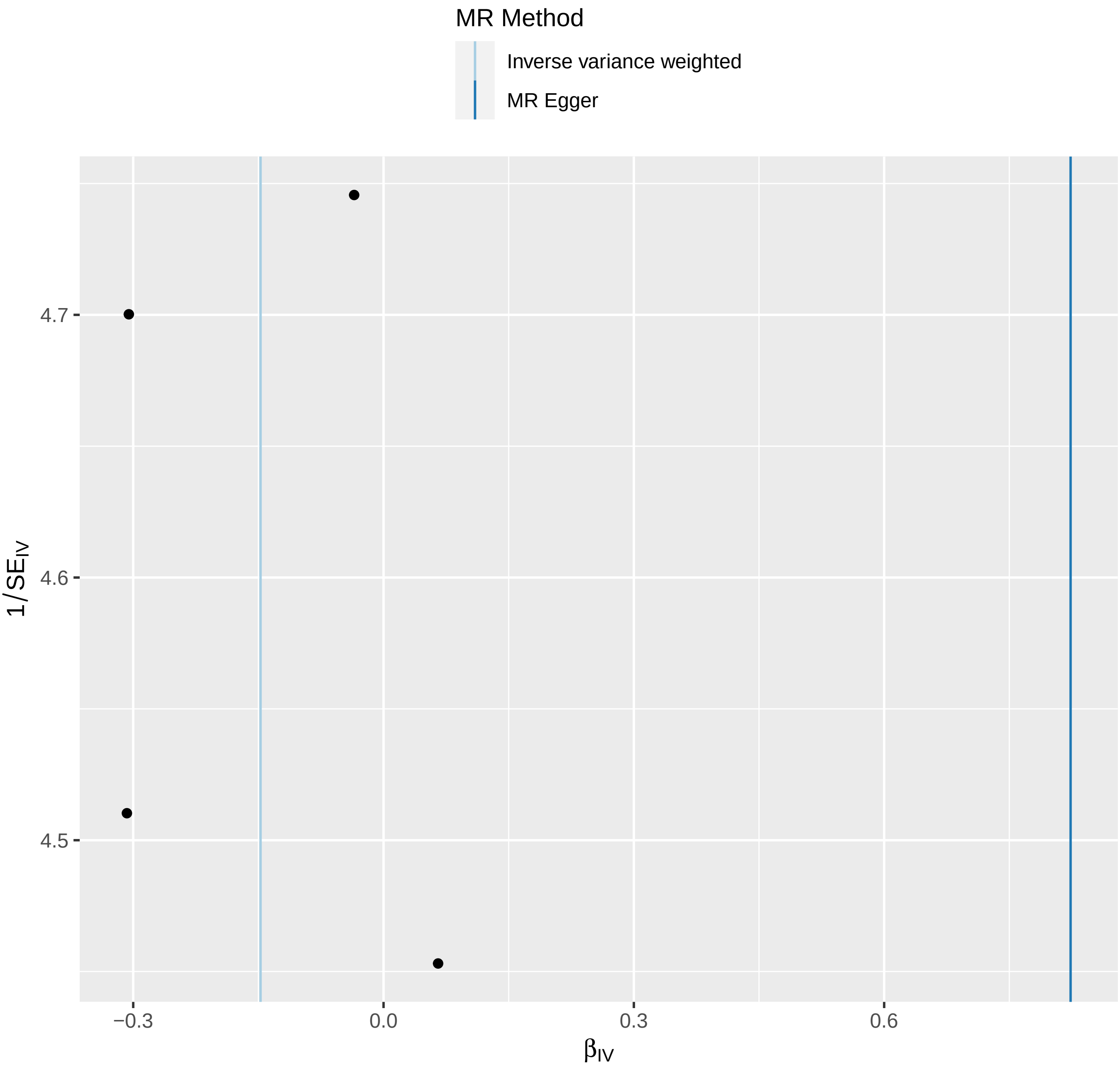

Batch 2−5344 : Gut microbiota abundance (genus Parasutterella id.2892) on Semaphorin−3E
with a threshold of 1e−05

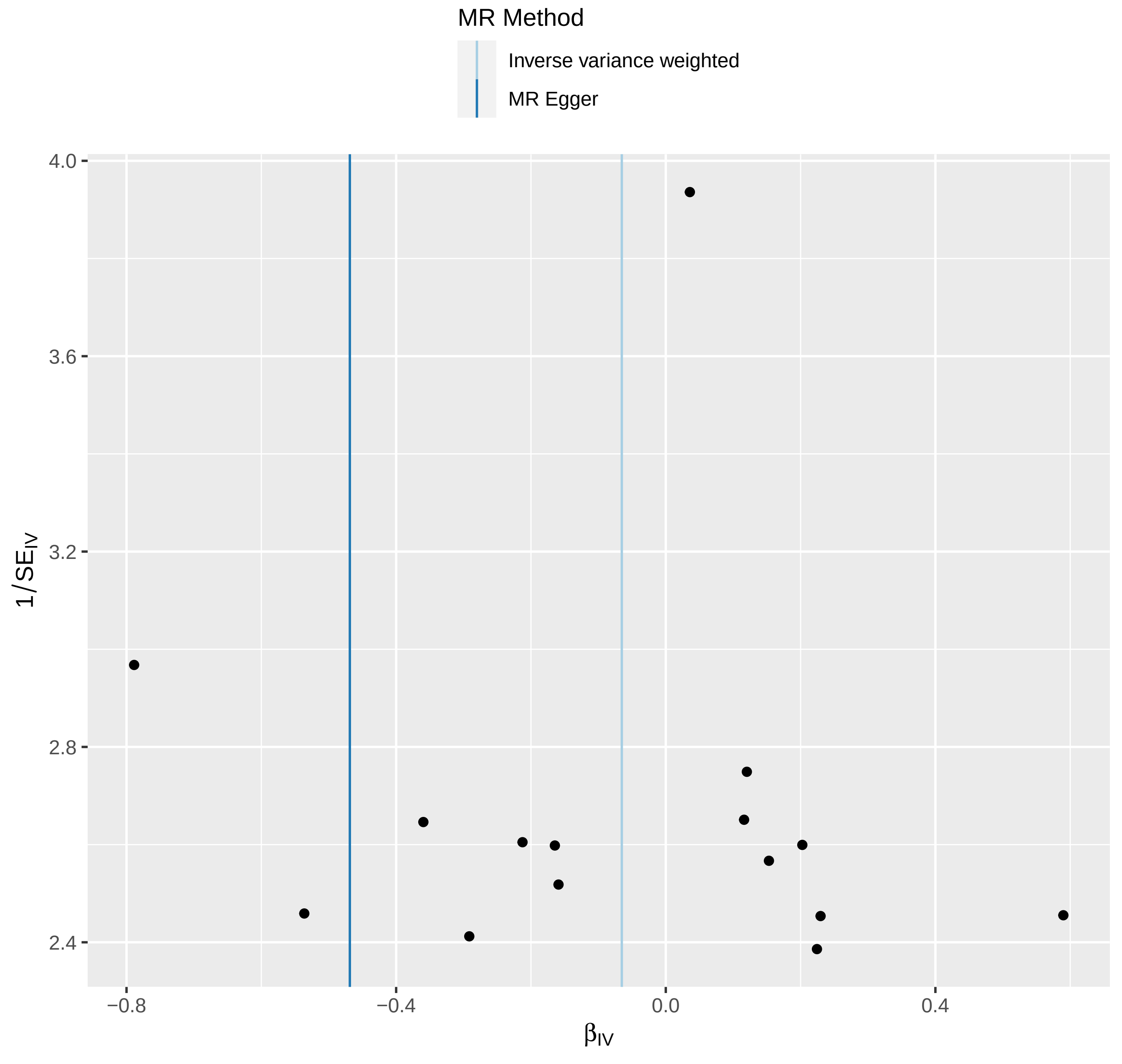

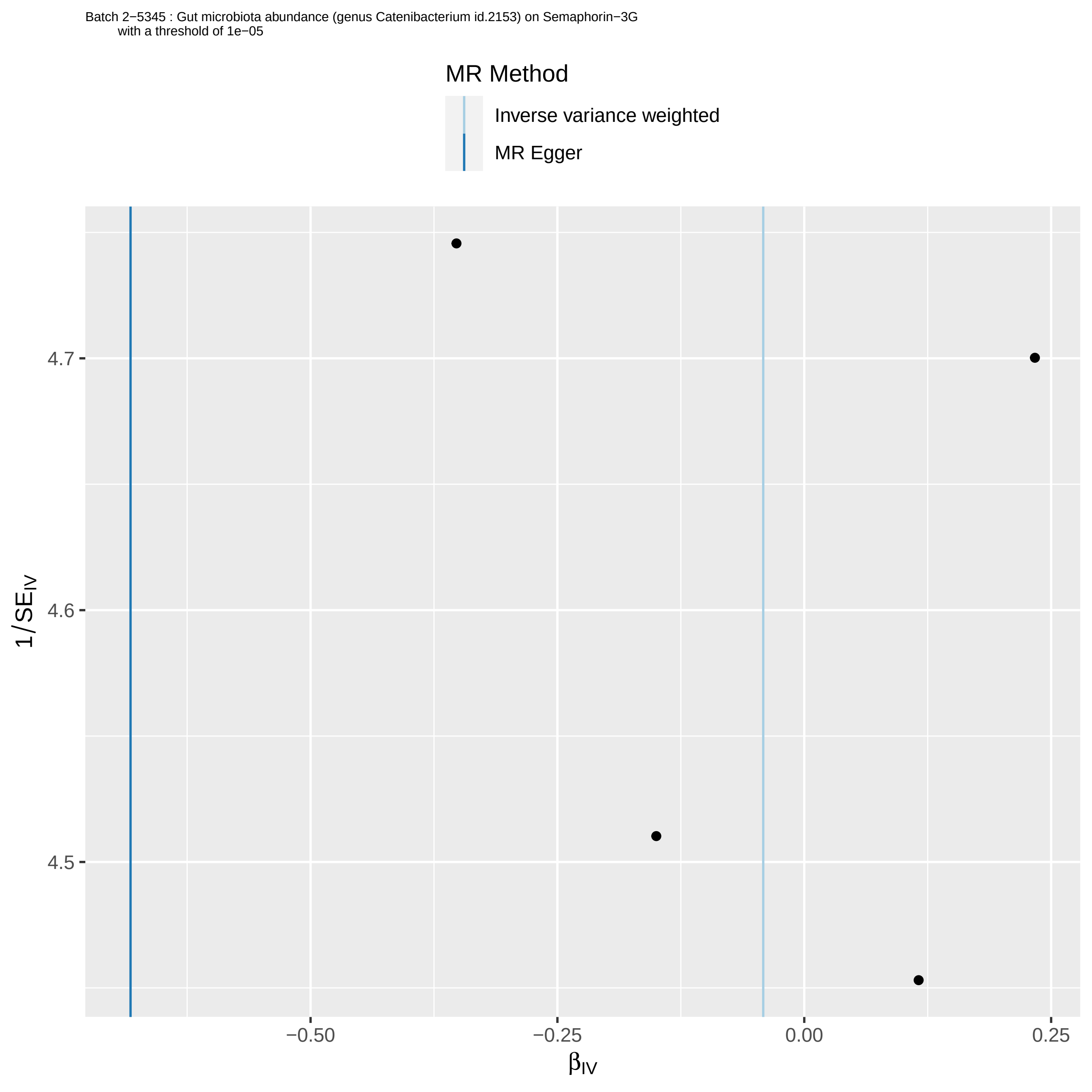

Batch 2−5345 : Gut microbiota abundance (genus Catenibacterium id.2153) on Semaphorin−3G
with a threshold of 1e−05
MR Method
Inverse variance weighted
MR Egger
4.7
4.6
4.5
$1/SE_{IV}$
−0.50
−0.25
0.00
0.25
β_{IV}

Batch 2−5346 : Gut microbiota abundance (genus Parasutterella id.2892) on Semaphorin−3G
with a threshold of 1e−05

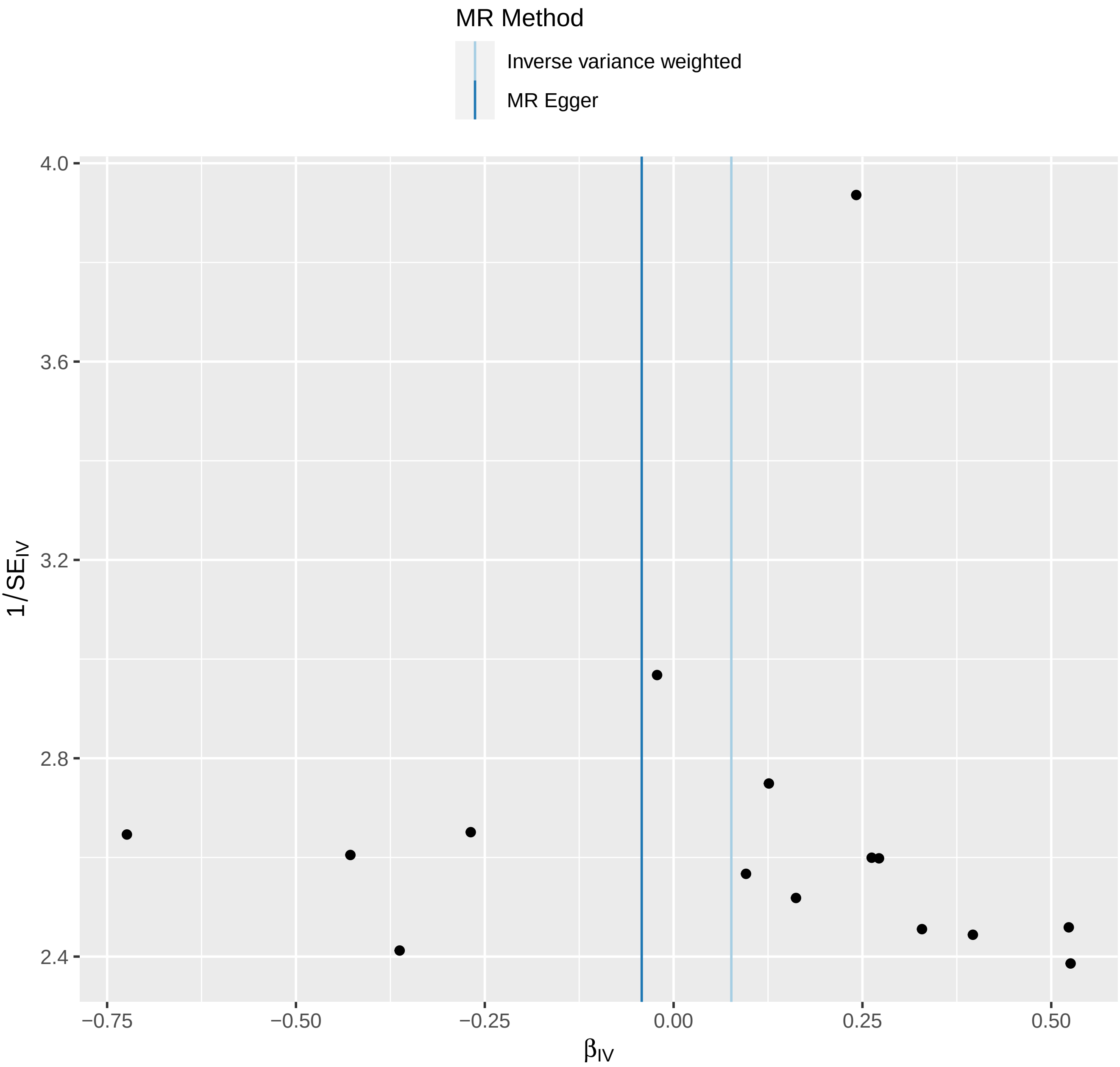

Batch 2−5347 : Gut microbiota abundance (genus Catenibacterium id.2153) on Semaphorin−4C
with a threshold of 1e−05

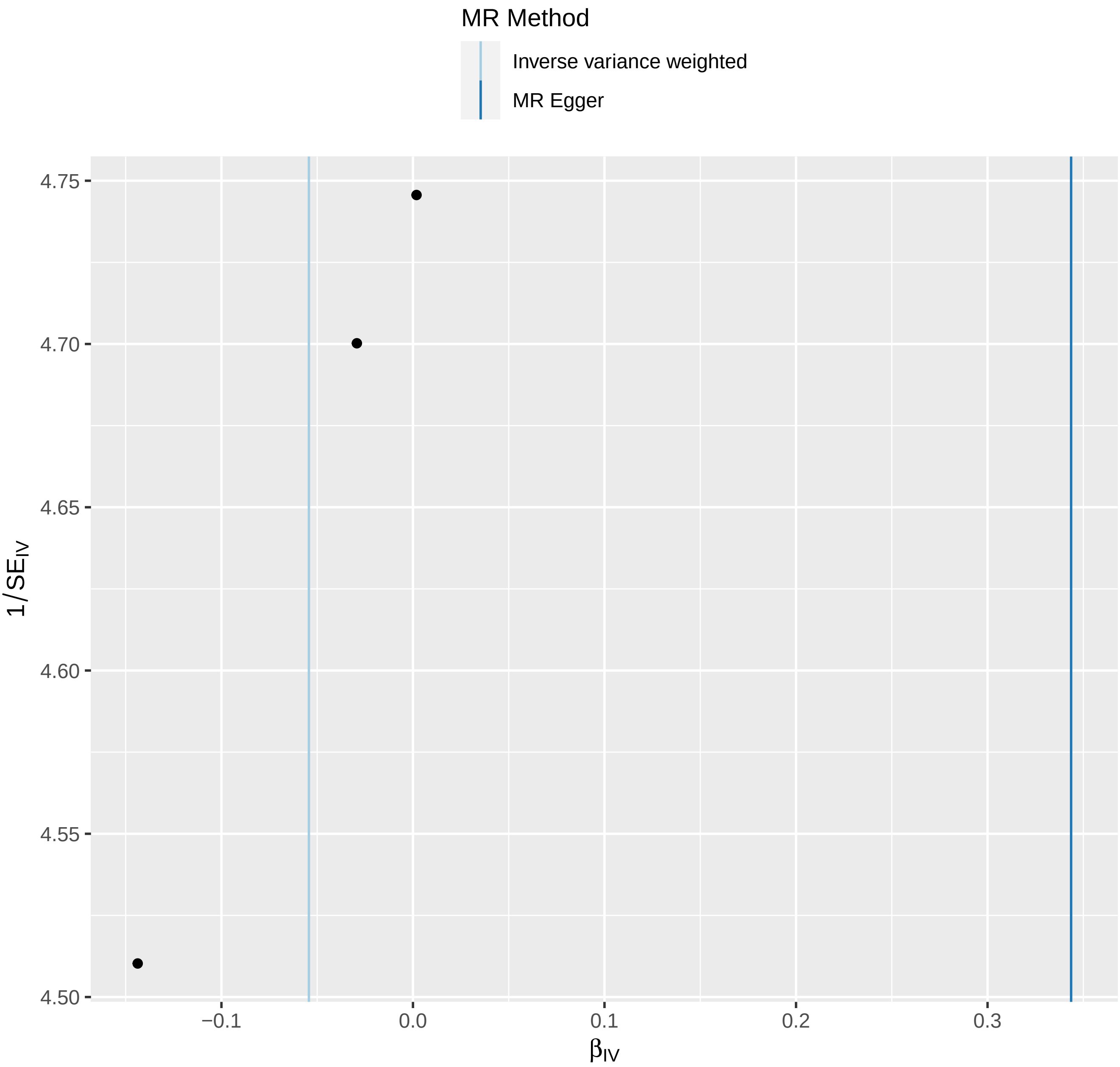

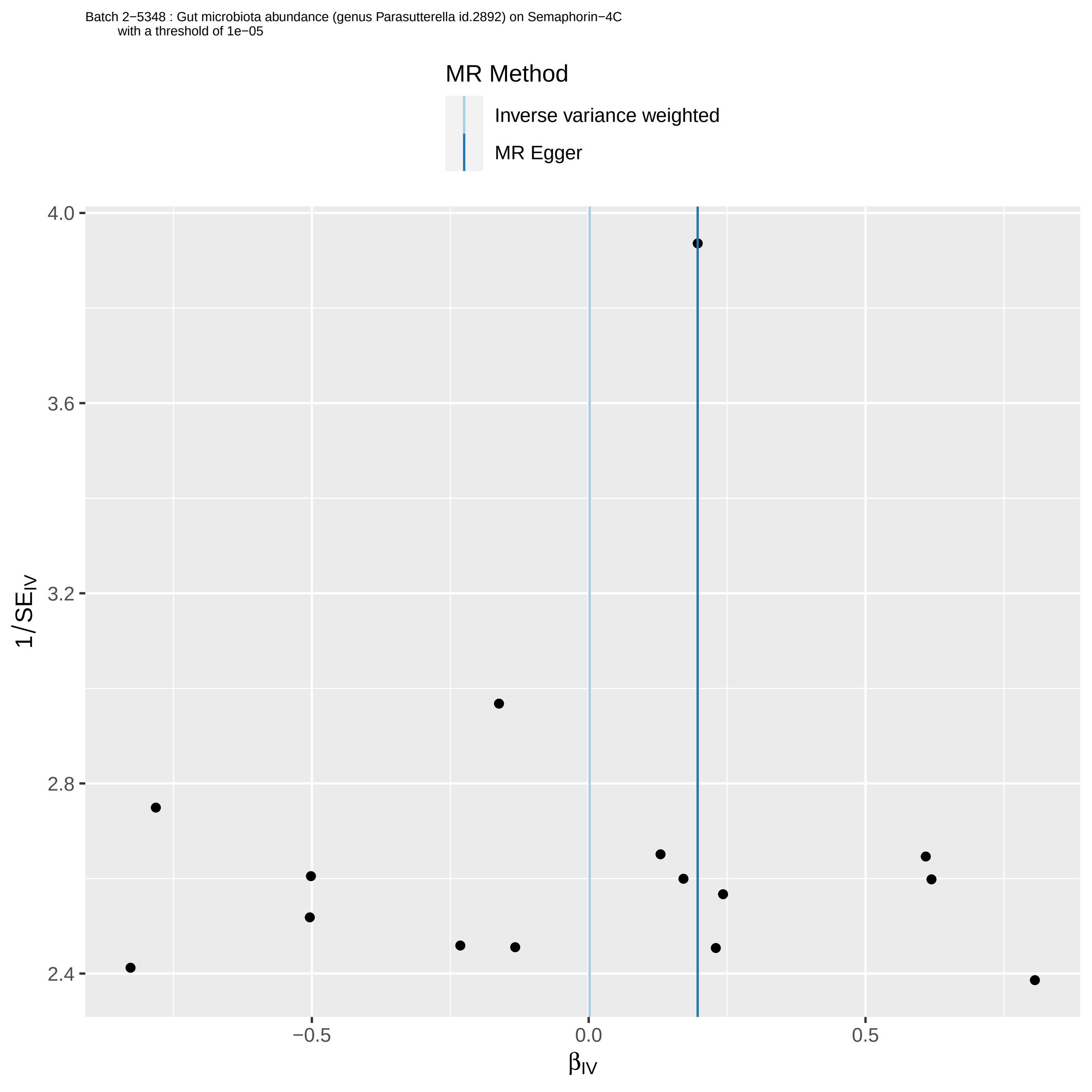

Batch 2−5348 : Gut microbiota abundance (genus Parasutterella id.2892) on Semaphorin−4C
with a threshold of 1e−05
MR Method
Inverse variance weighted
MR Egger
4.0
3.6
3.2
2.8
2.4
1/SE_IV
−0.5
0.0
0.5
β_IV

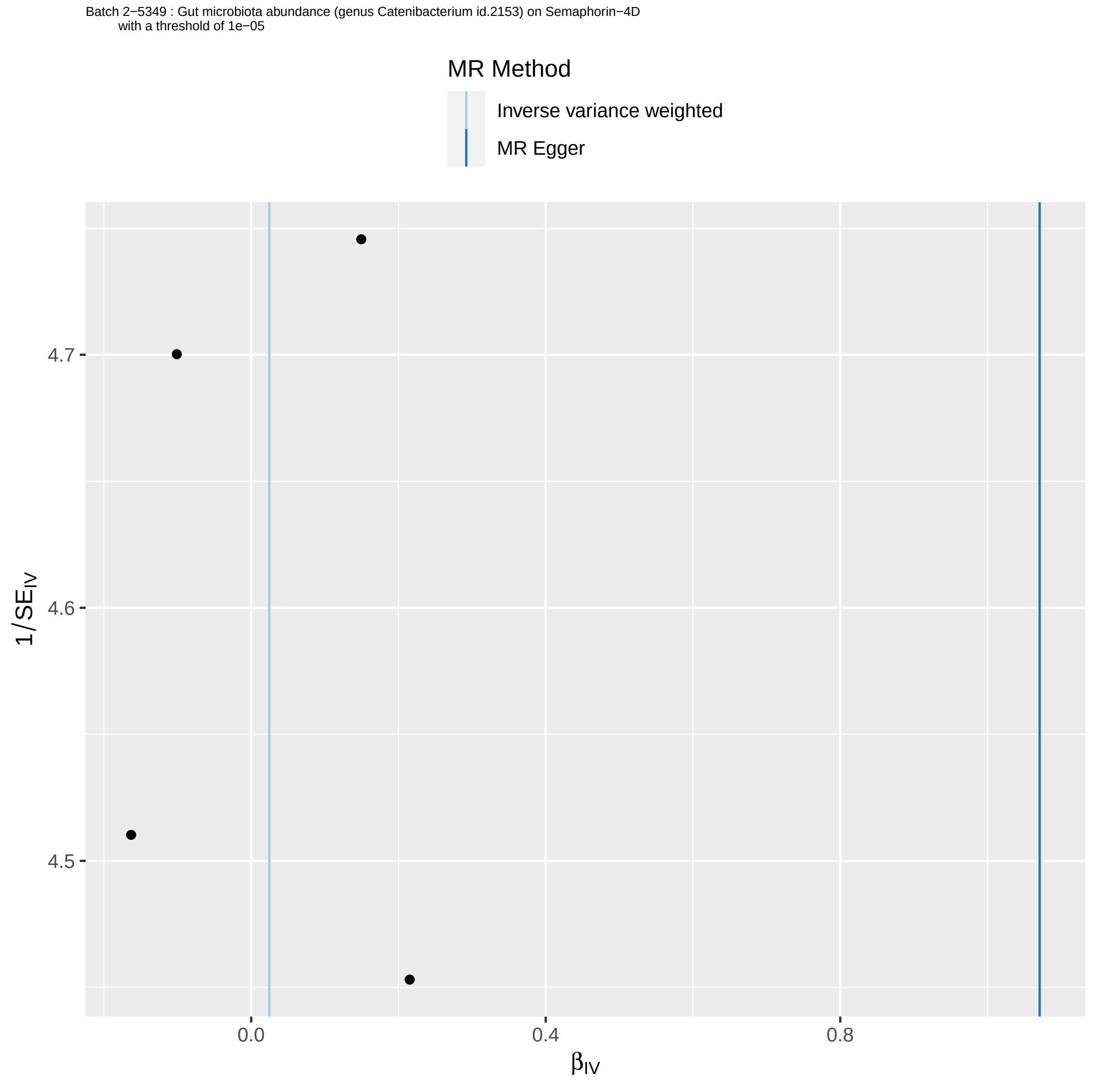
Batch 2−5349 : Gut microbiota abundance (genus Catenibacterium id.2153) on Semaphorin−4D
with a threshold of 1e−05
MR Method
Inverse variance weighted
MR Egger
4.7
4.6
4.5
0.0
0.4
0.8
1/SE_IV
β_IV

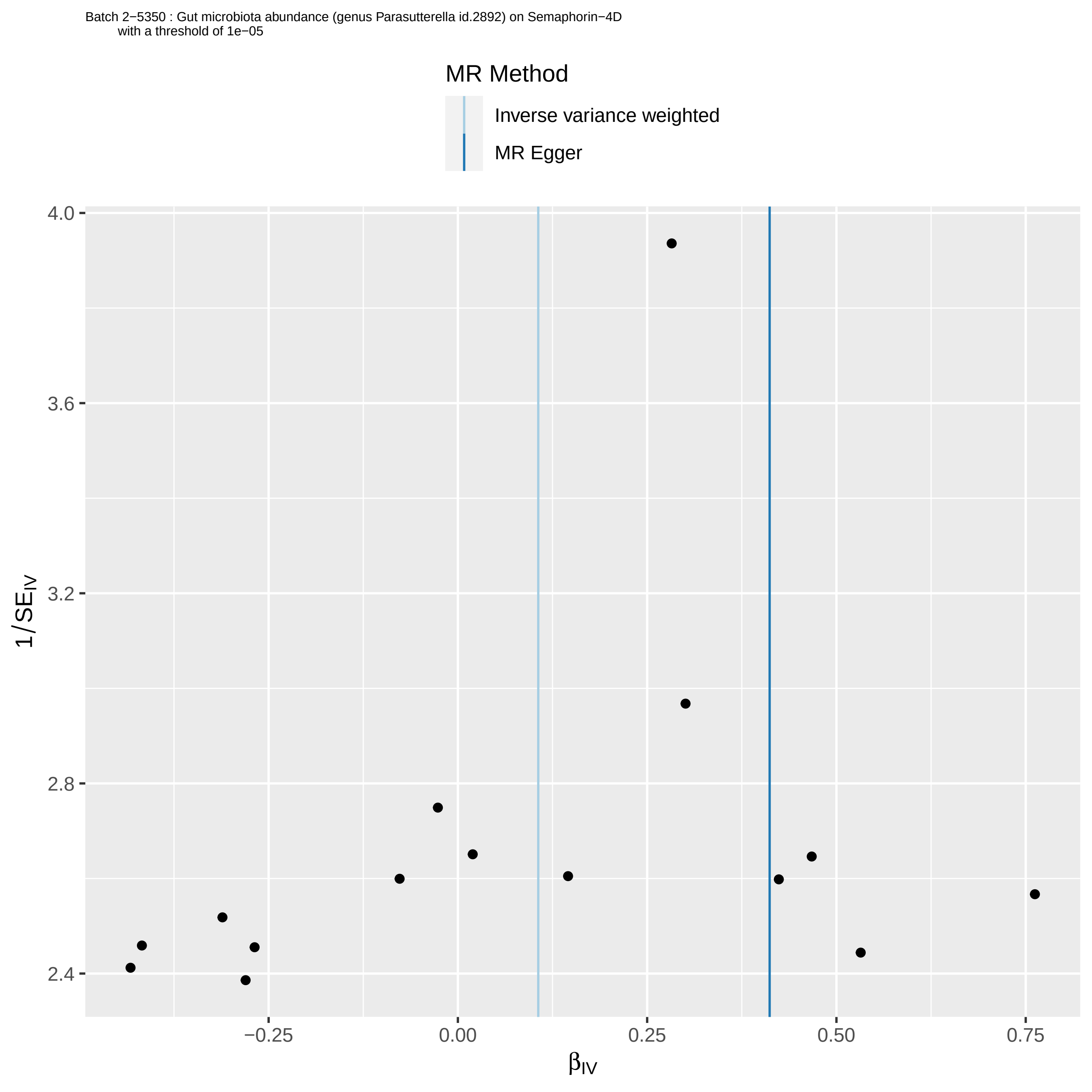

Batch 2−5350 : Gut microbiota abundance (genus Parasutterella id.2892) on Semaphorin−4D
with a threshold of 1e−05
MR Method
Inverse variance weighted
MR Egger
4.0
3.6
3.2
2.8
2.4
1/SE_IV
−0.25
0.00
0.25
0.50
0.75
β_IV

Batch 2−5351 : Gut microbiota abundance (genus Catenibacterium id.2153) on Semaphorin−5A
with a threshold of 1e−05

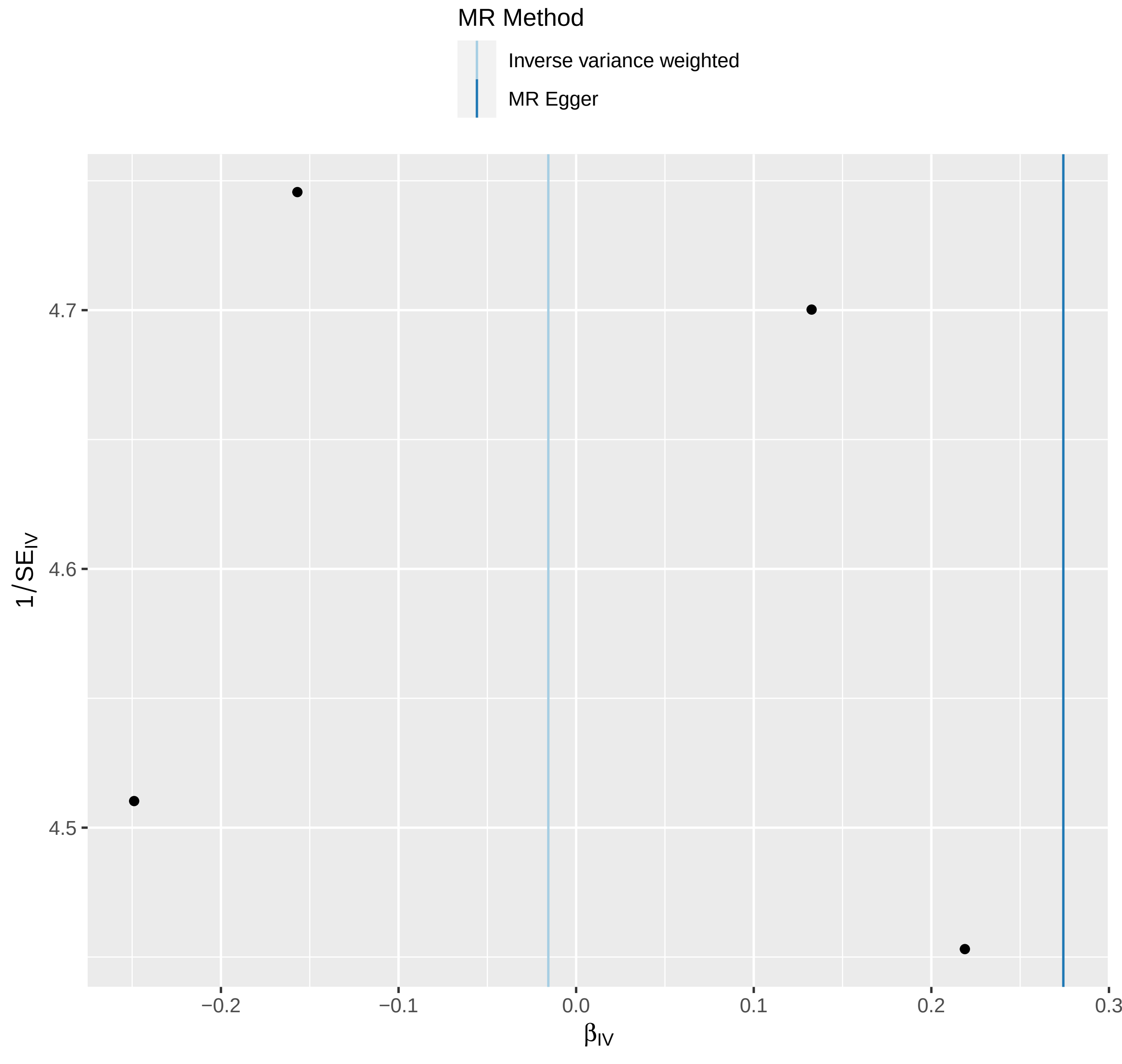

Batch 2−5352 : Gut microbiota abundance (genus Parasutterella id.2892) on Semaphorin−5A
with a threshold of 1e−05

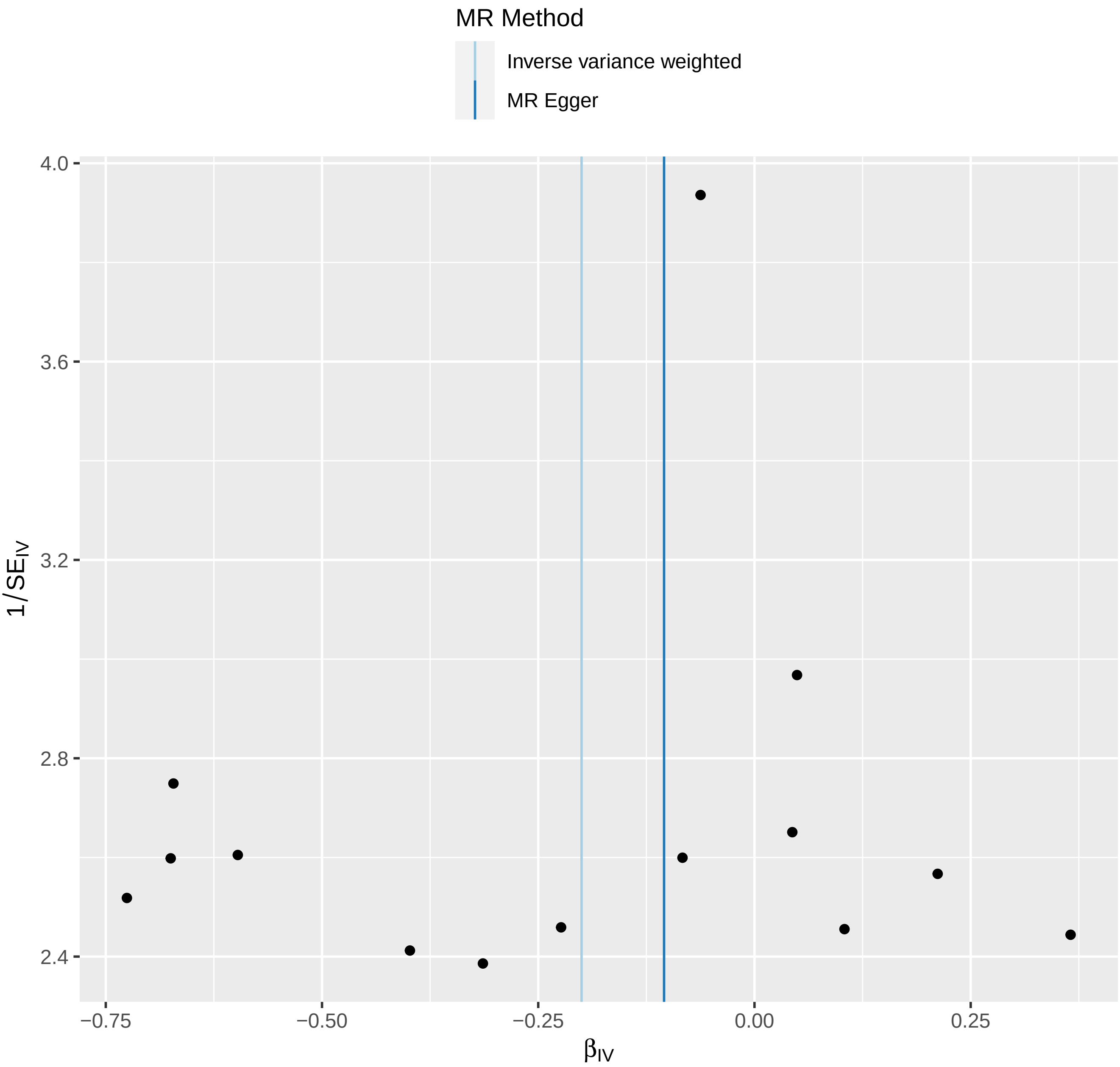

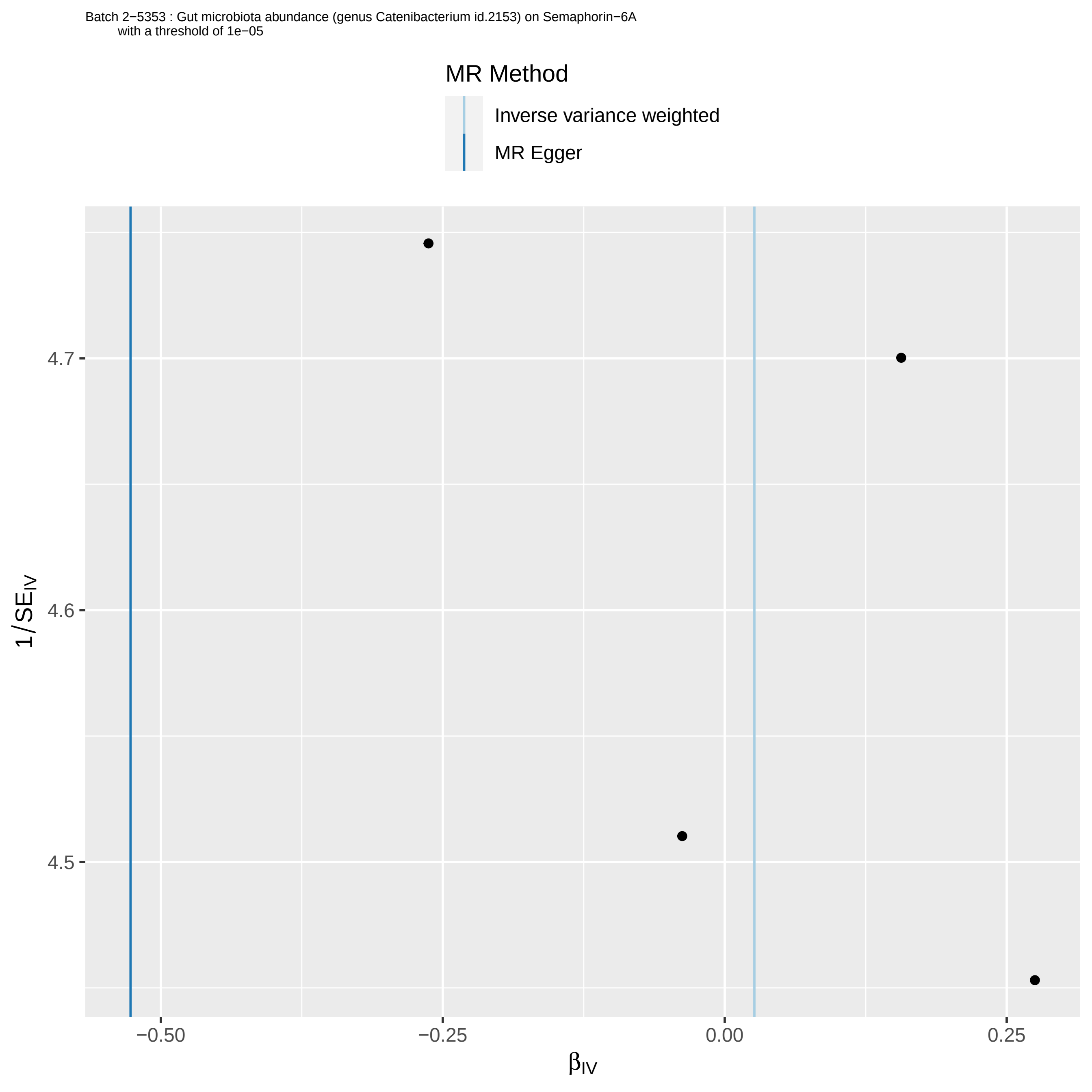

Batch 2−5353 : Gut microbiota abundance (genus Catenibacterium id.2153) on Semaphorin−6A
with a threshold of 1e−05
MR Method
Inverse variance weighted
MR Egger
4.7
4.6
4.5
1/SE_IV
−0.50
−0.25
0.00
0.25
β_IV

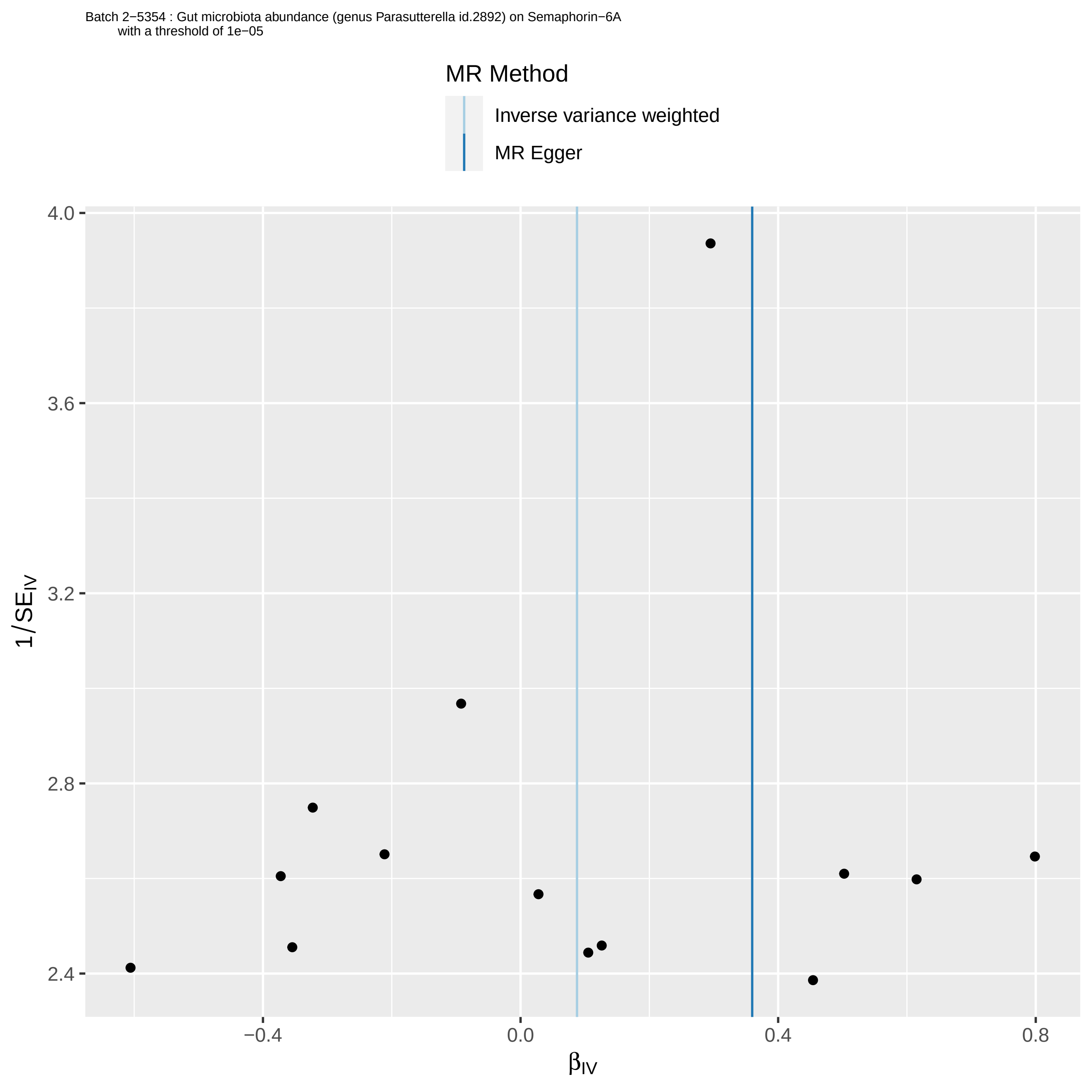

Batch 2−5354 : Gut microbiota abundance (genus Parasutterella id.2892) on Semaphorin−6A
with a threshold of 1e−05
MR Method
Inverse variance weighted
MR Egger
4.0
3.6
3.2
2.8
2.4
1/SE_IV
−0.4
0.0
0.4
0.8
β_IV

Batch 2−5355 : Gut microbiota abundance (genus Catenibacterium id.2153) on Semaphorin−6A
with a threshold of 1e−05

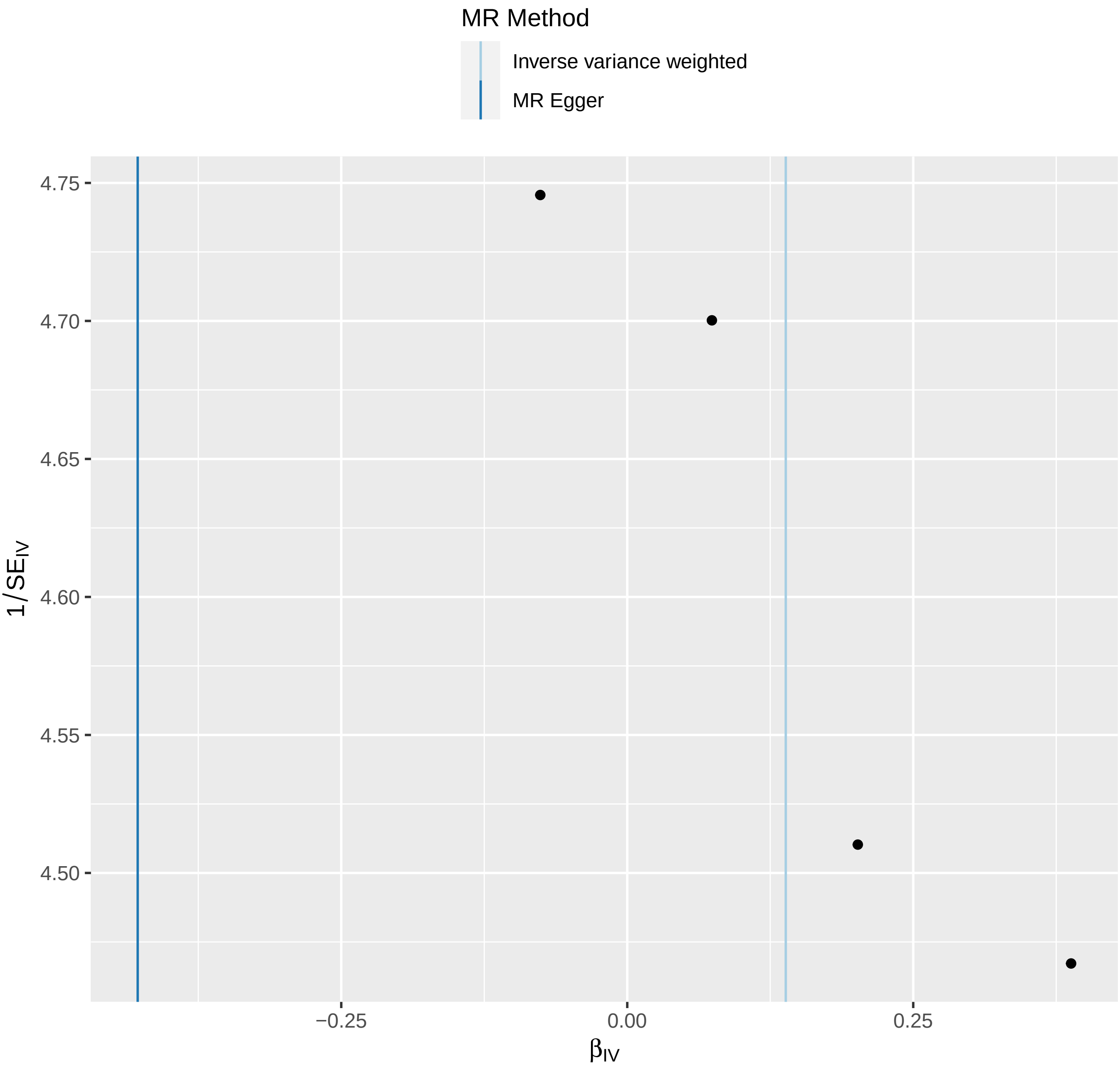

Batch 2−5356 : Gut microbiota abundance (genus Parasutterella id.2892) on Semaphorin−6A
with a threshold of 1e−05

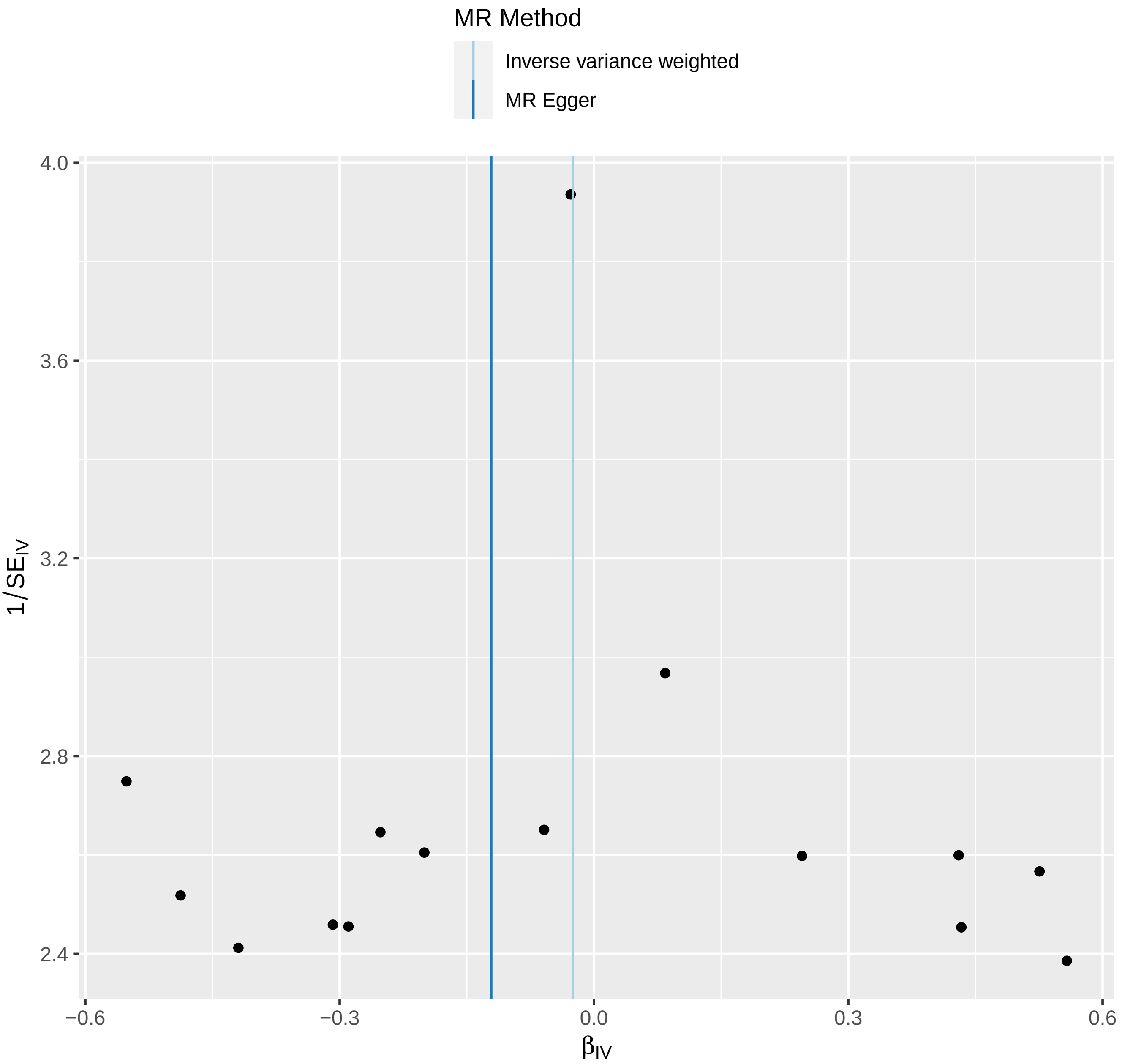

Batch 2−5357 : Gut microbiota abundance (genus Catenibacterium id.2153) on Semaphorin−6C
with a threshold of 1e−05

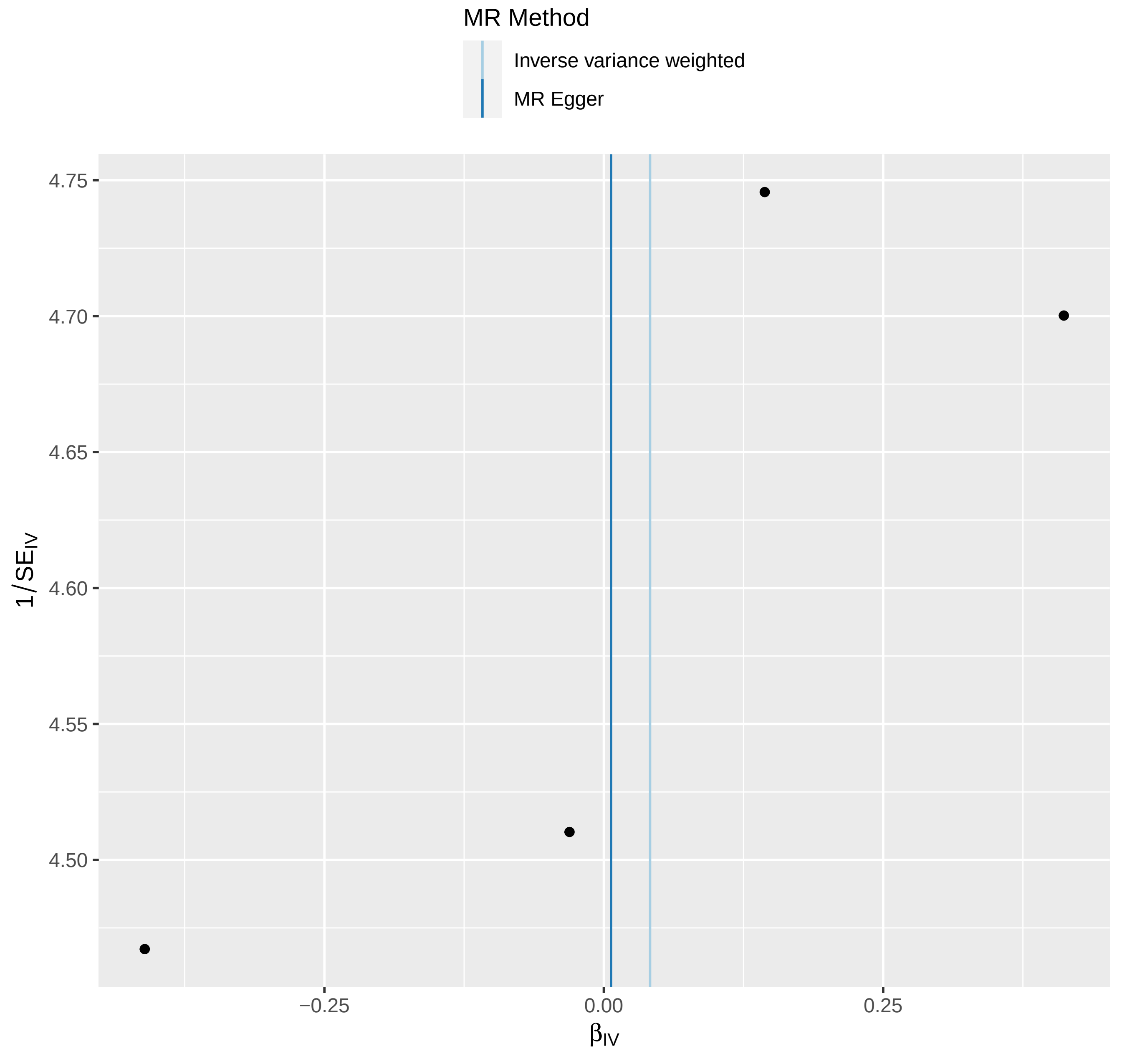

Batch 2−5358 : Gut microbiota abundance (genus Parasutterella id.2892) on Semaphorin−6C
with a threshold of 1e−05

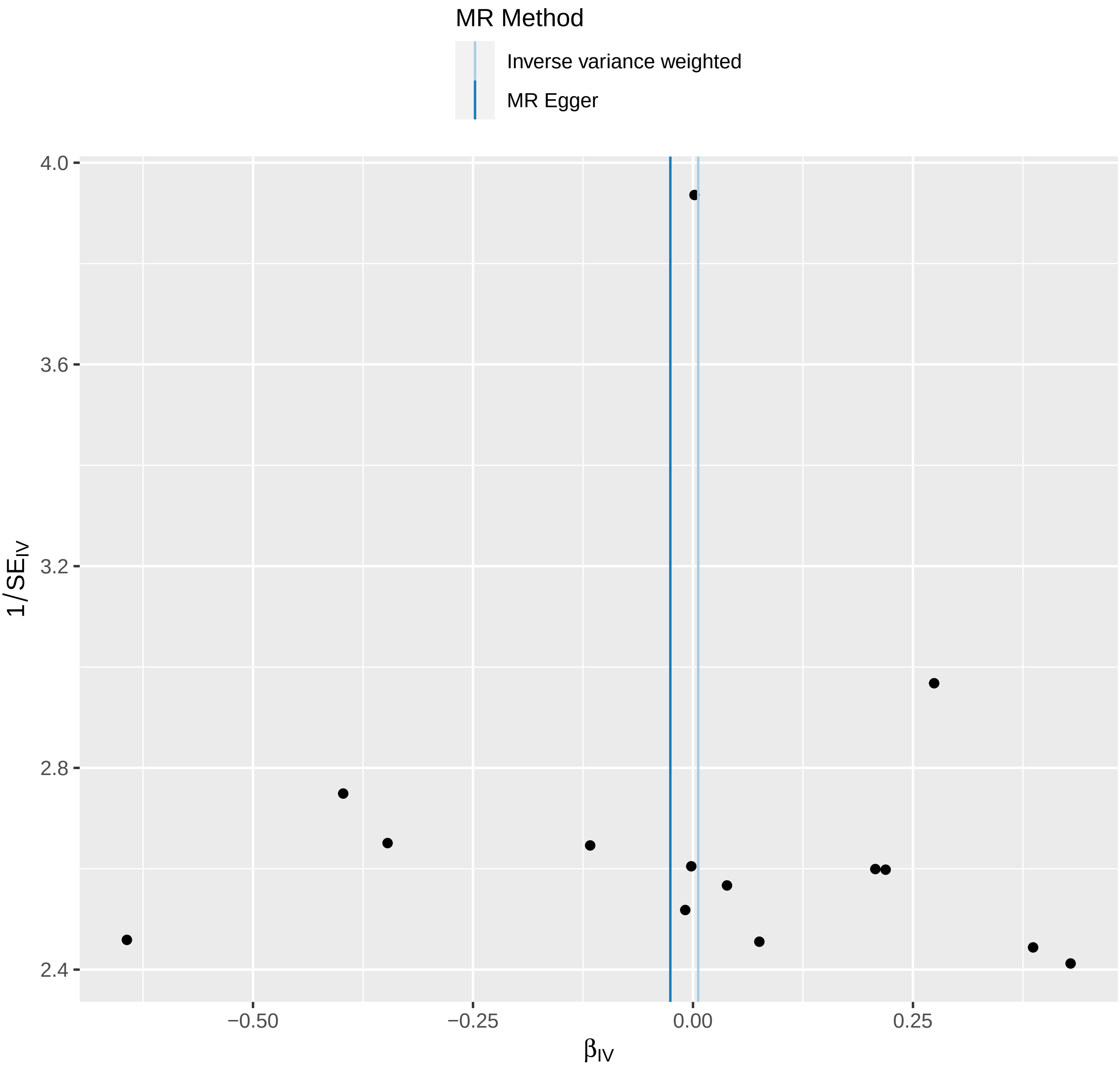

Batch 2−5359 : Gut microbiota abundance (genus Catenibacterium id.2153) on Semaphorin−7A
with a threshold of 1e−05

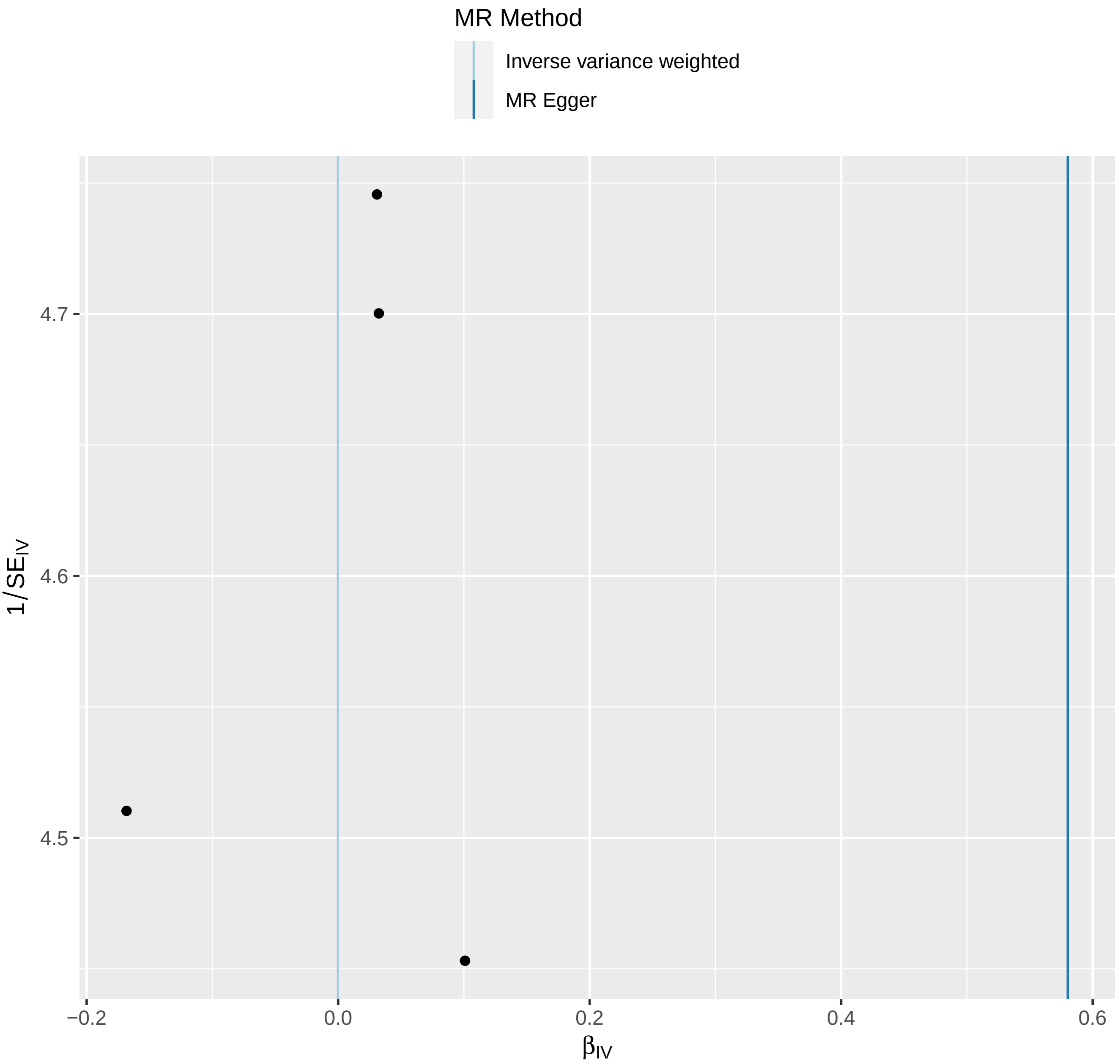

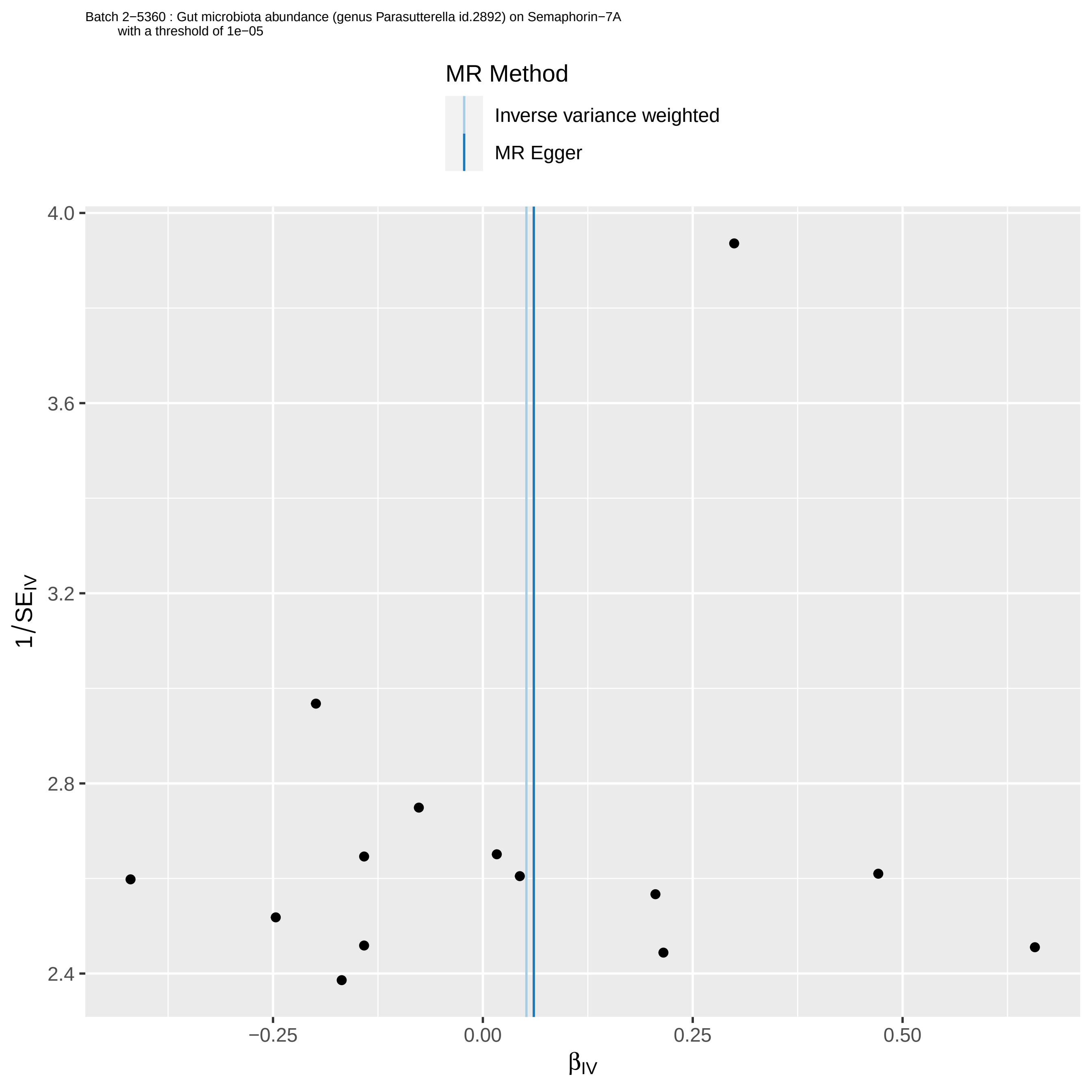

Batch 2−5360 : Gut microbiota abundance (genus Parasutterella id.2892) on Semaphorin−7A
with a threshold of 1e−05
MR Method
Inverse variance weighted
MR Egger
4.0
3.6
3.2
2.8
2.4
1/SE_IV
−0.25
0.00
0.25
0.50
β_IV

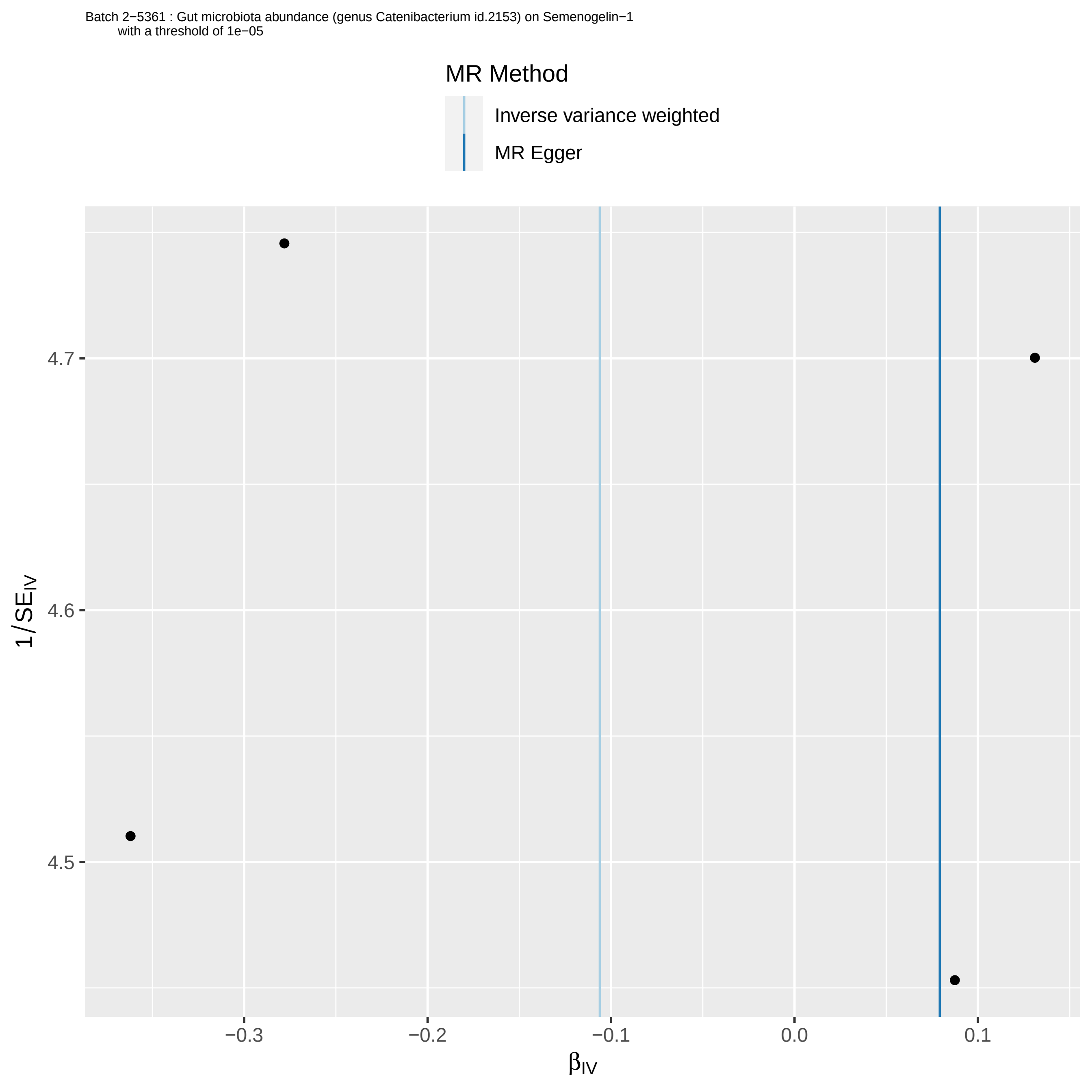

Batch 2−5361 : Gut microbiota abundance (genus Catenibacterium id.2153) on Semenogelin−1
with a threshold of 1e−05
MR Method
Inverse variance weighted
MR Egger
4.7
4.6
4.5
1/SE_IV
−0.3
−0.2
−0.1
0.0
0.1
β_IV

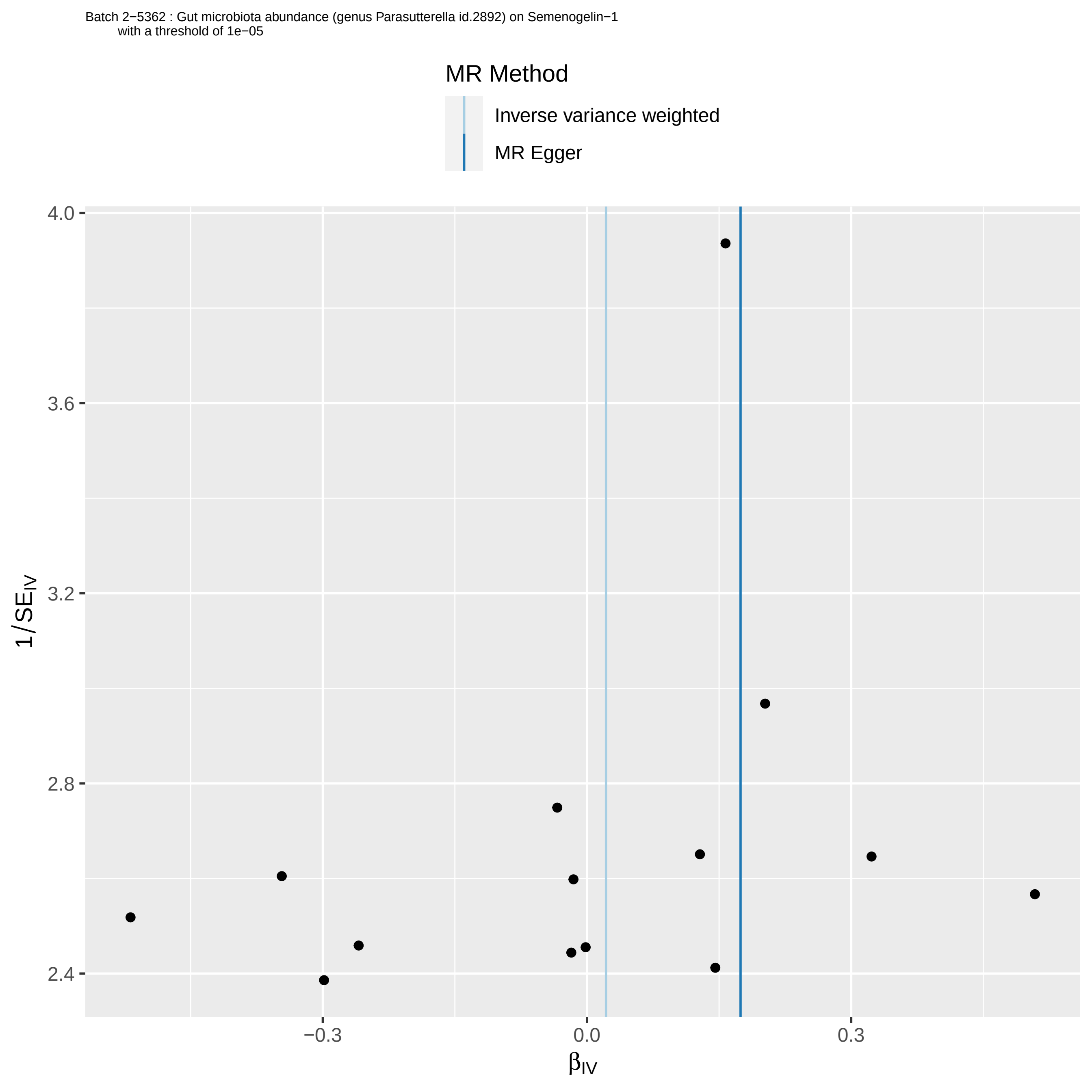

Batch 2−5362 : Gut microbiota abundance (genus Parasutterella id.2892) on Semenogelin−1
with a threshold of 1e−05
MR Method
Inverse variance weighted
MR Egger
4.0
3.6
3.2
2.8
2.4
1/SE_IV
−0.3
0.0
0.3
β_IV

Batch 2−5363 : Gut microbiota abundance (genus Catenibacterium id.2153) on Semenogelin−2
with a threshold of 1e−05

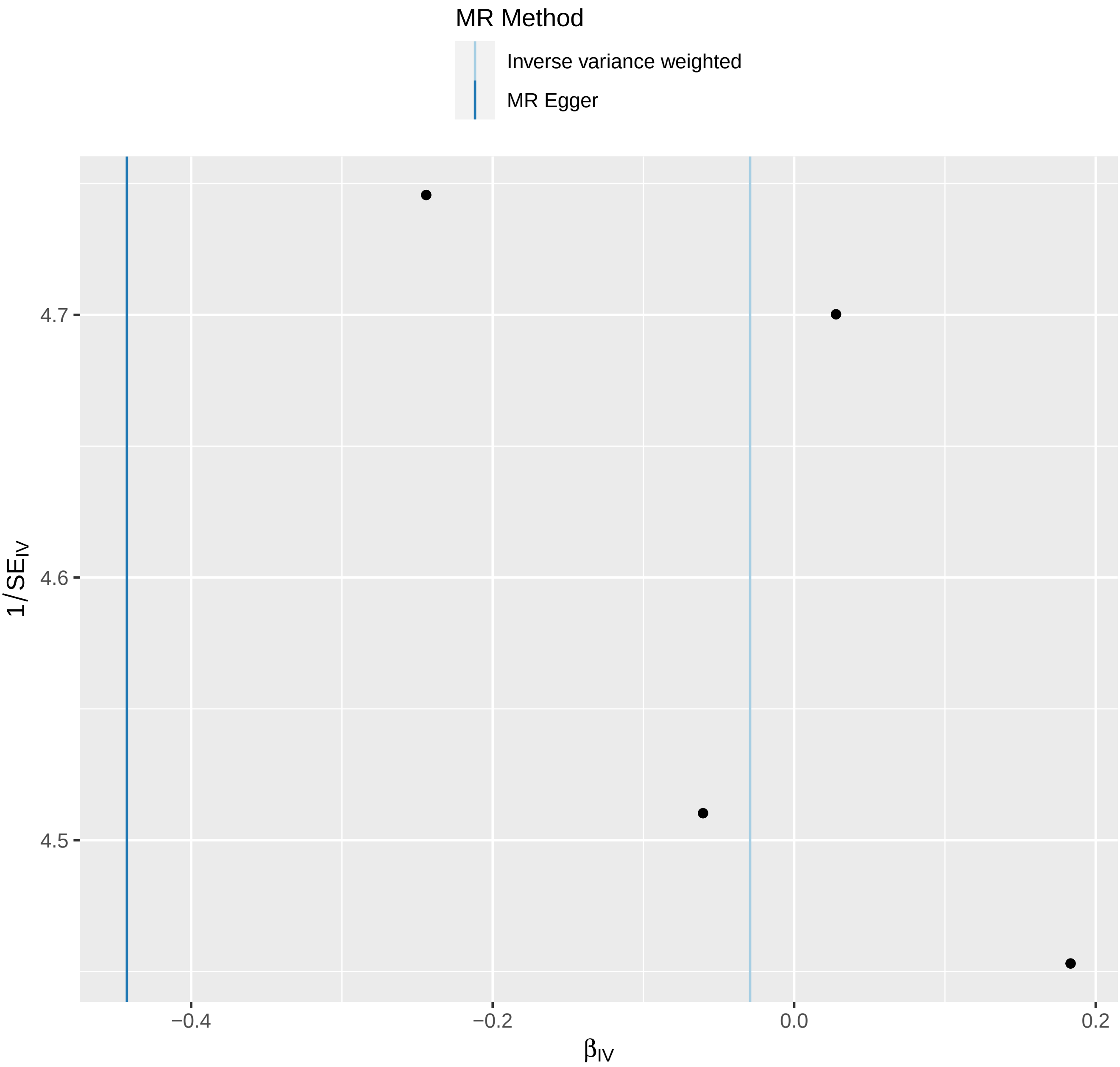

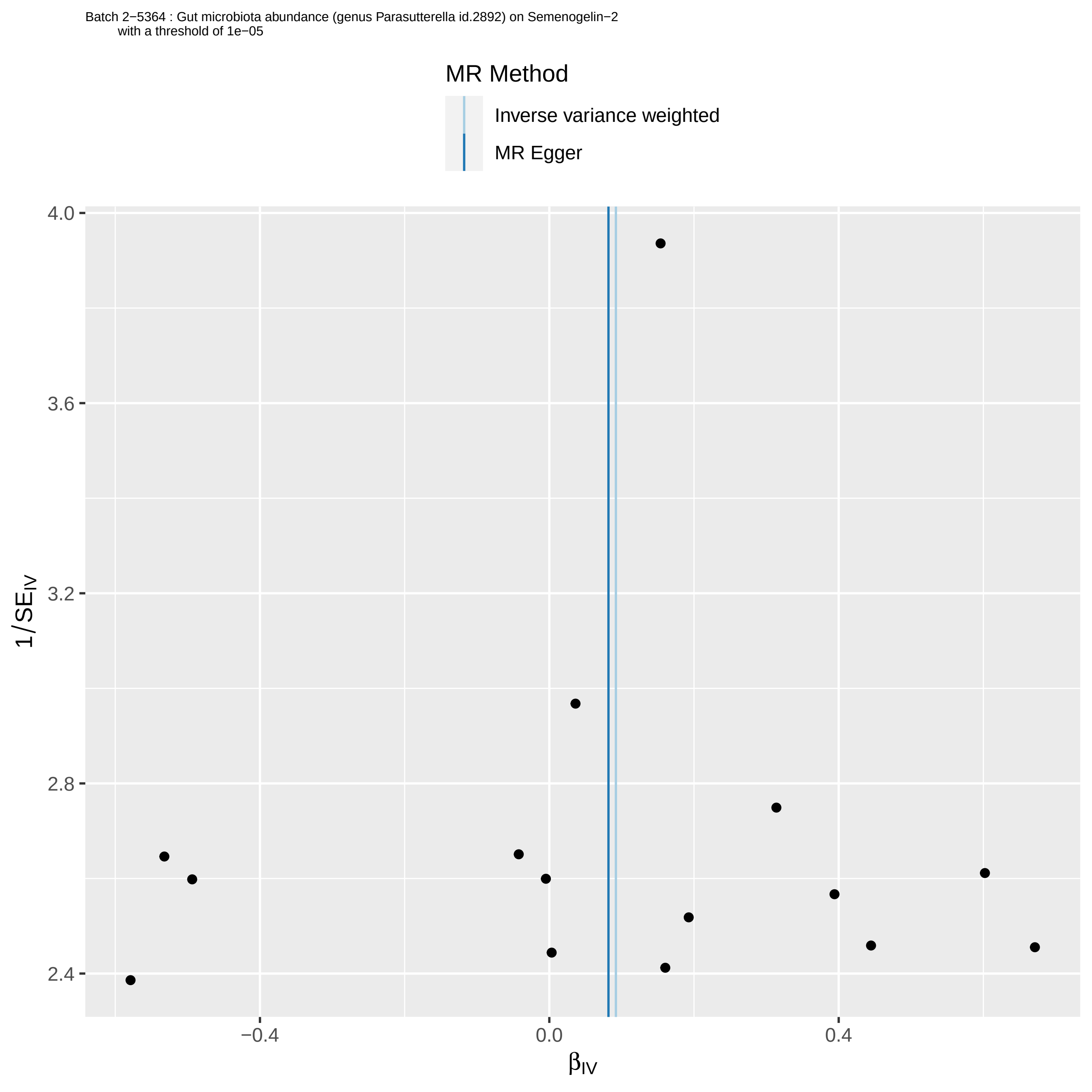
Batch 2−5364 : Gut microbiota abundance (genus Parasutterella id.2892) on Semenogelin−2
with a threshold of 1e−05
MR Method
Inverse variance weighted
MR Egger
4.0
3.6
3.2
2.8
2.4
1/SE$_{IV}$
−0.4
0.0
0.4
β$_{IV}$

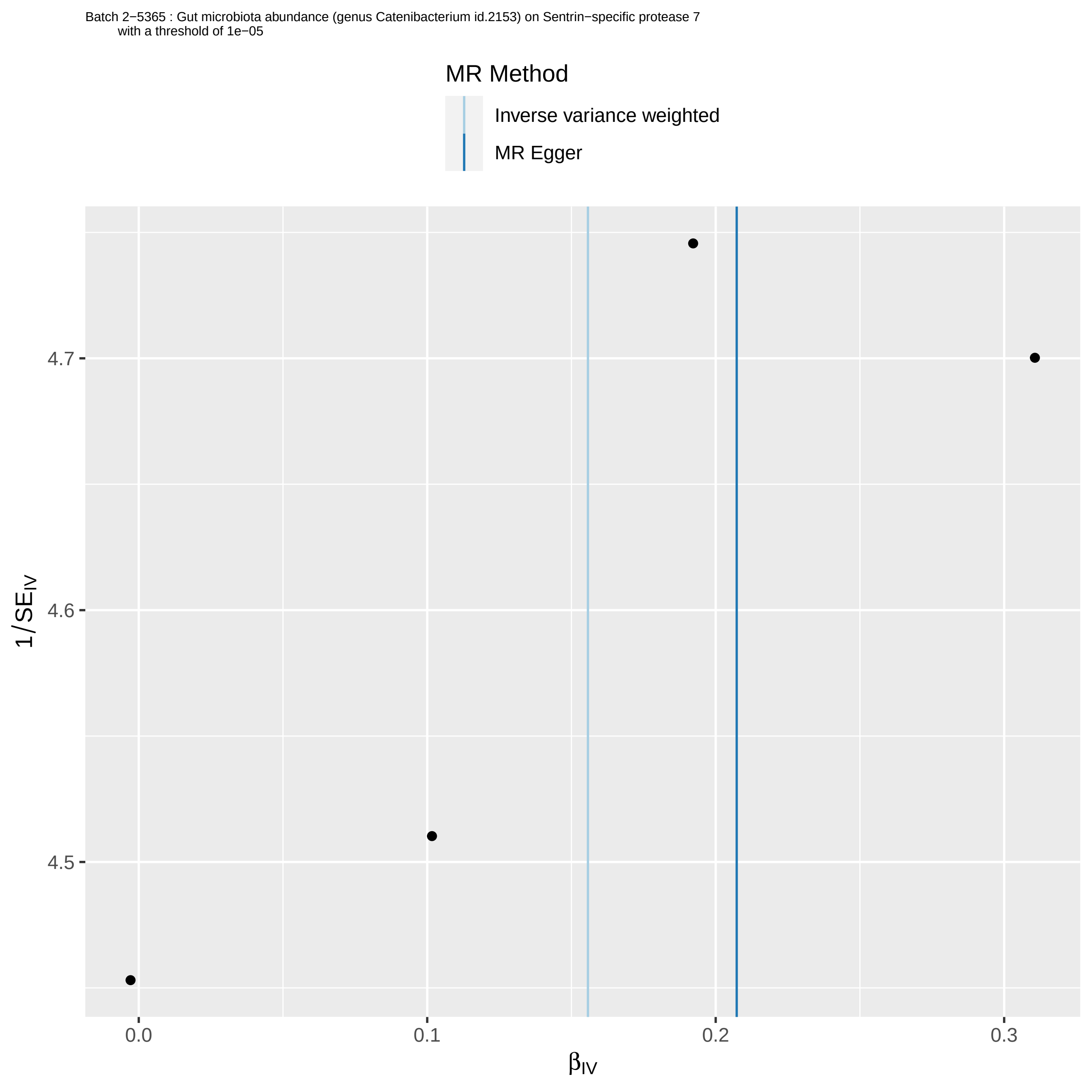
Batch 2−5365 : Gut microbiota abundance (genus Catenibacterium id.2153) on Sentrin−specific protease 7
with a threshold of 1e−05
MR Method
Inverse variance weighted
MR Egger
4.7
4.6
4.5
1/SE_IV
0.0
0.1
0.2
0.3
β_IV

Batch 2−5366 : Gut microbiota abundance (genus Parasutterella id.2892) on Sentrin−specific protease 7
with a threshold of 1e−05

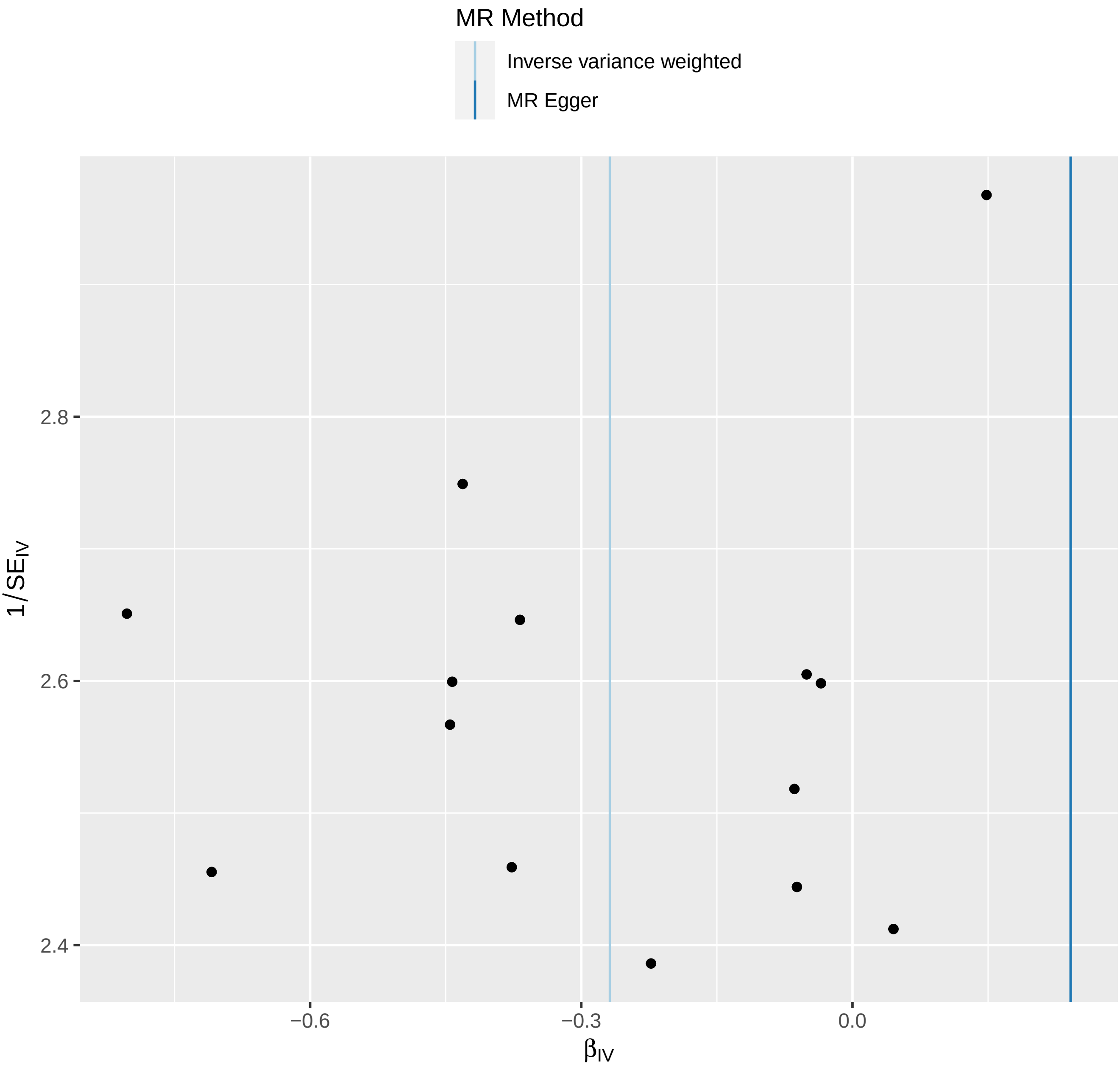

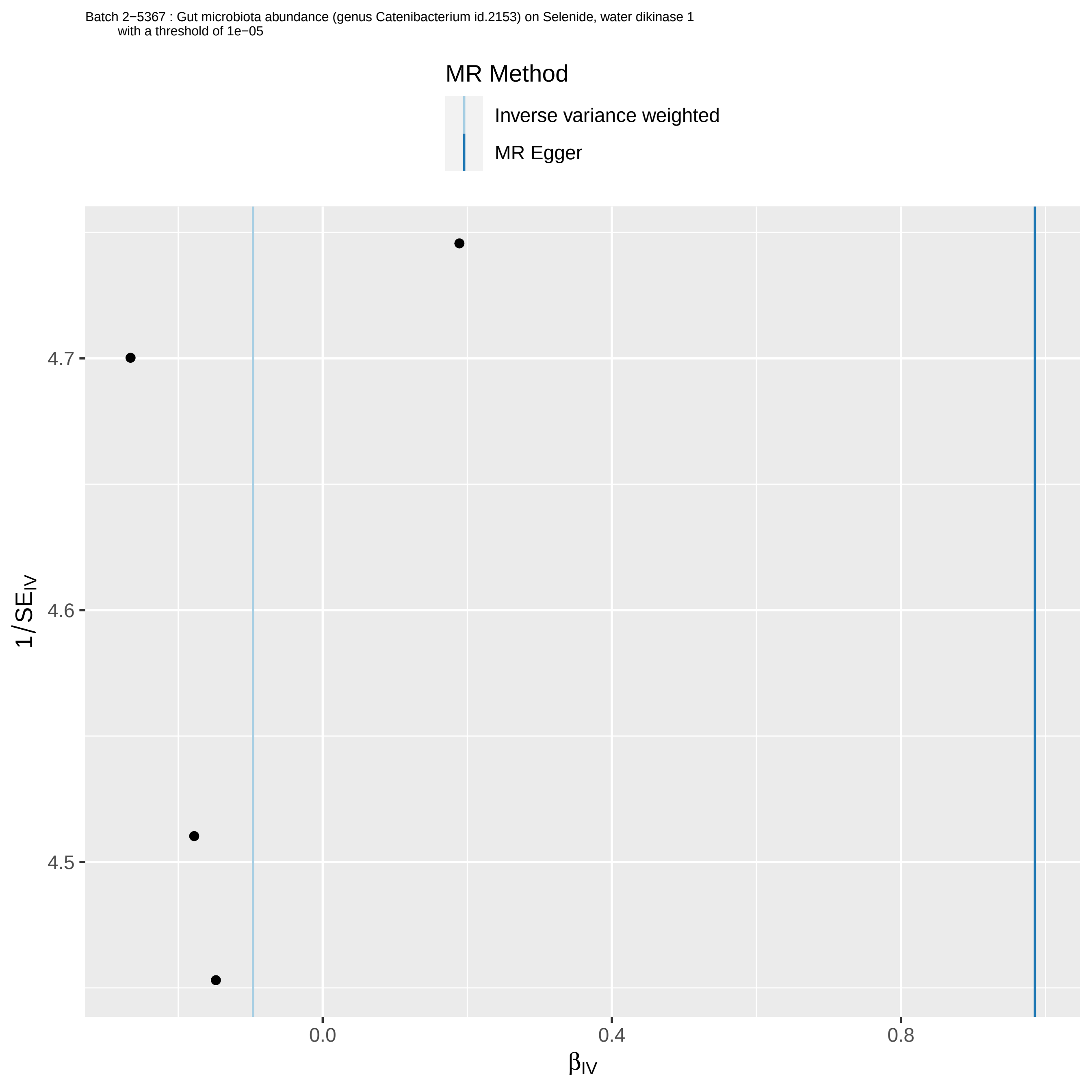
Batch 2−5367 : Gut microbiota abundance (genus Catenibacterium id.2153) on Selenide, water dikinase 1
with a threshold of 1e−05
MR Method
Inverse variance weighted
MR Egger
4.7
4.6
4.5
1/SE_IV
0.0
0.4
0.8
β_IV

Batch 2−5368 : Gut microbiota abundance (genus Parasutterella id.2892) on Selenide, water dikinase 1
with a threshold of 1e−05

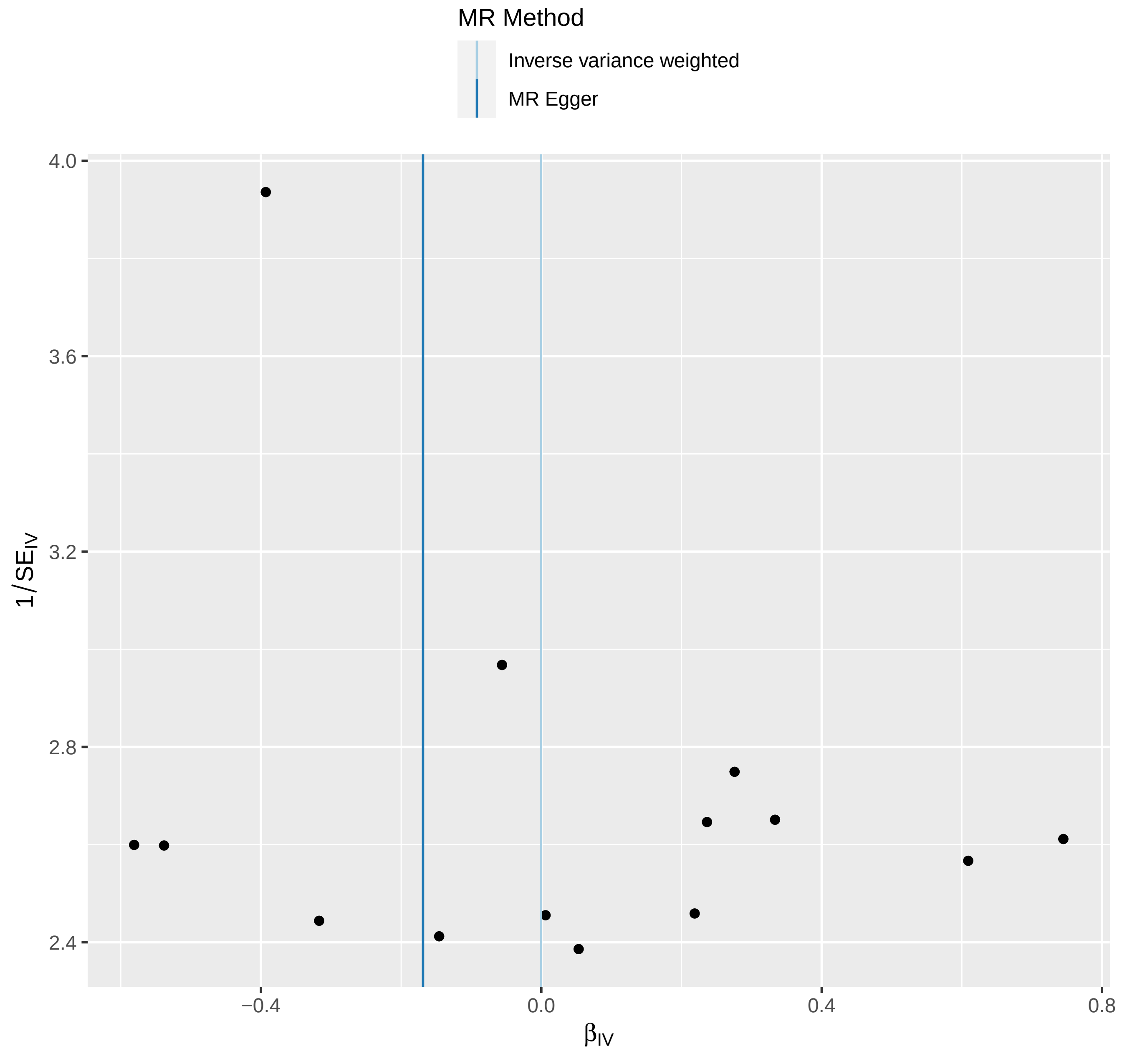

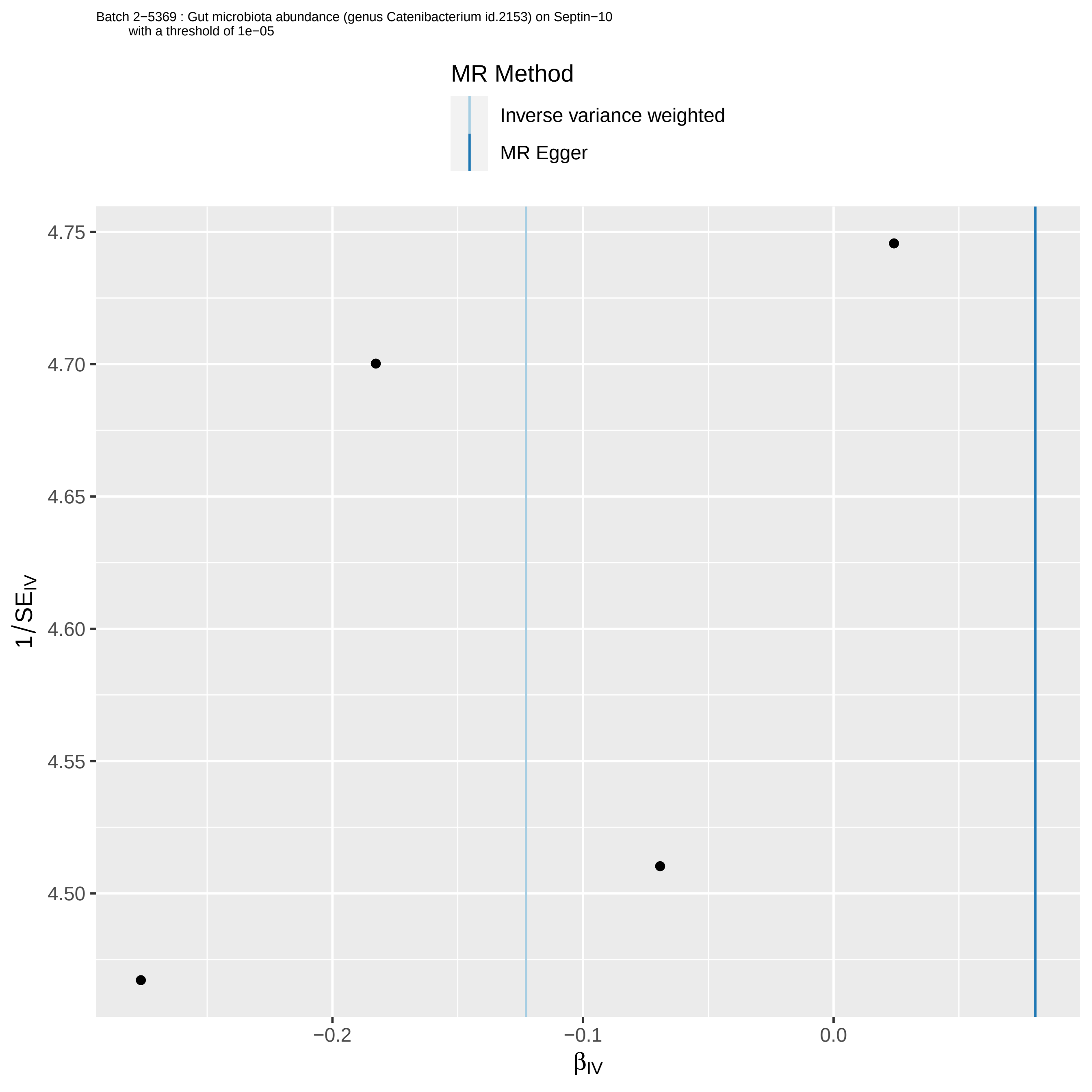
Batch 2−5369 : Gut microbiota abundance (genus Catenibacterium id.2153) on Septin−10
with a threshold of 1e−05
MR Method
Inverse variance weighted
MR Egger
4.75
4.70
4.65
4.60
4.55
4.50
1/SE_IV
−0.2
−0.1
0.0
β_IV

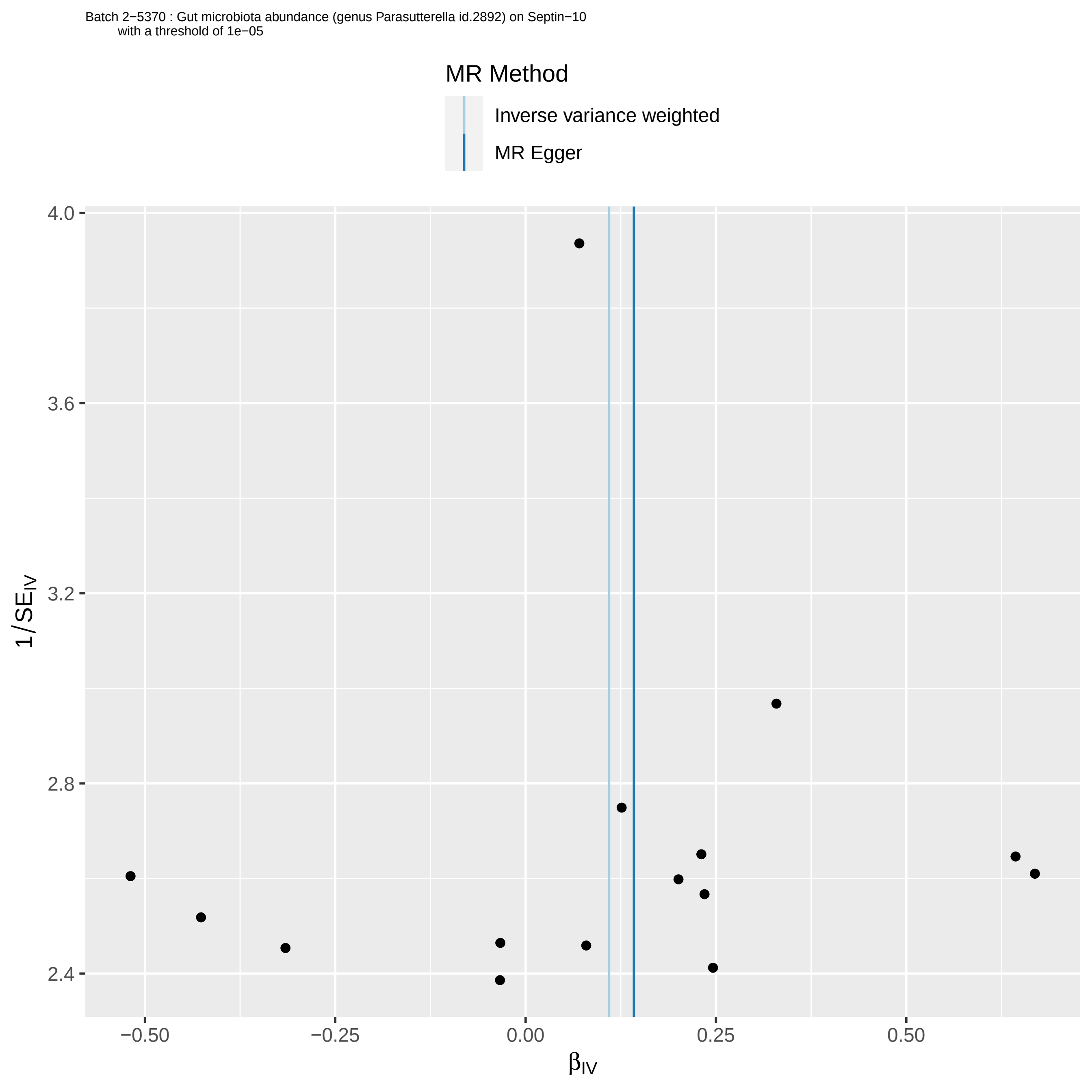

Batch 2−5370 : Gut microbiota abundance (genus Parasutterella id.2892) on Septin−10
with a threshold of 1e−05
MR Method
Inverse variance weighted
MR Egger
4.0
3.6
3.2
2.8
2.4
1/SE_IV
−0.50
−0.25
0.00
0.25
0.50
β_IV

Batch 2−5371 : Gut microbiota abundance (genus Catenibacterium id.2153) on Protein SERAC1
with a threshold of 1e−05

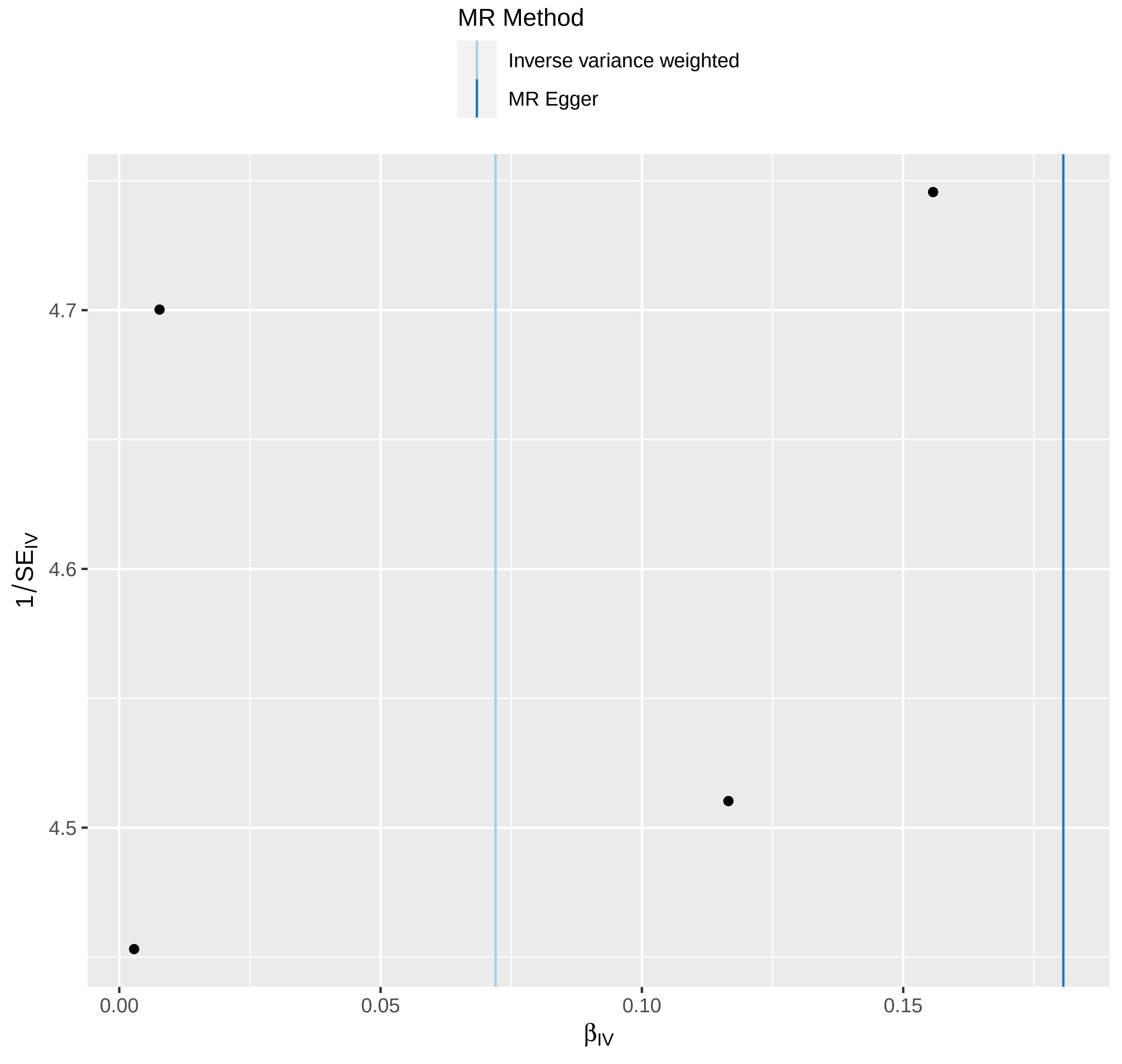

Batch 2−5372 : Gut microbiota abundance (genus Parasutterella id.2892) on Protein SERAC1
with a threshold of 1e−05

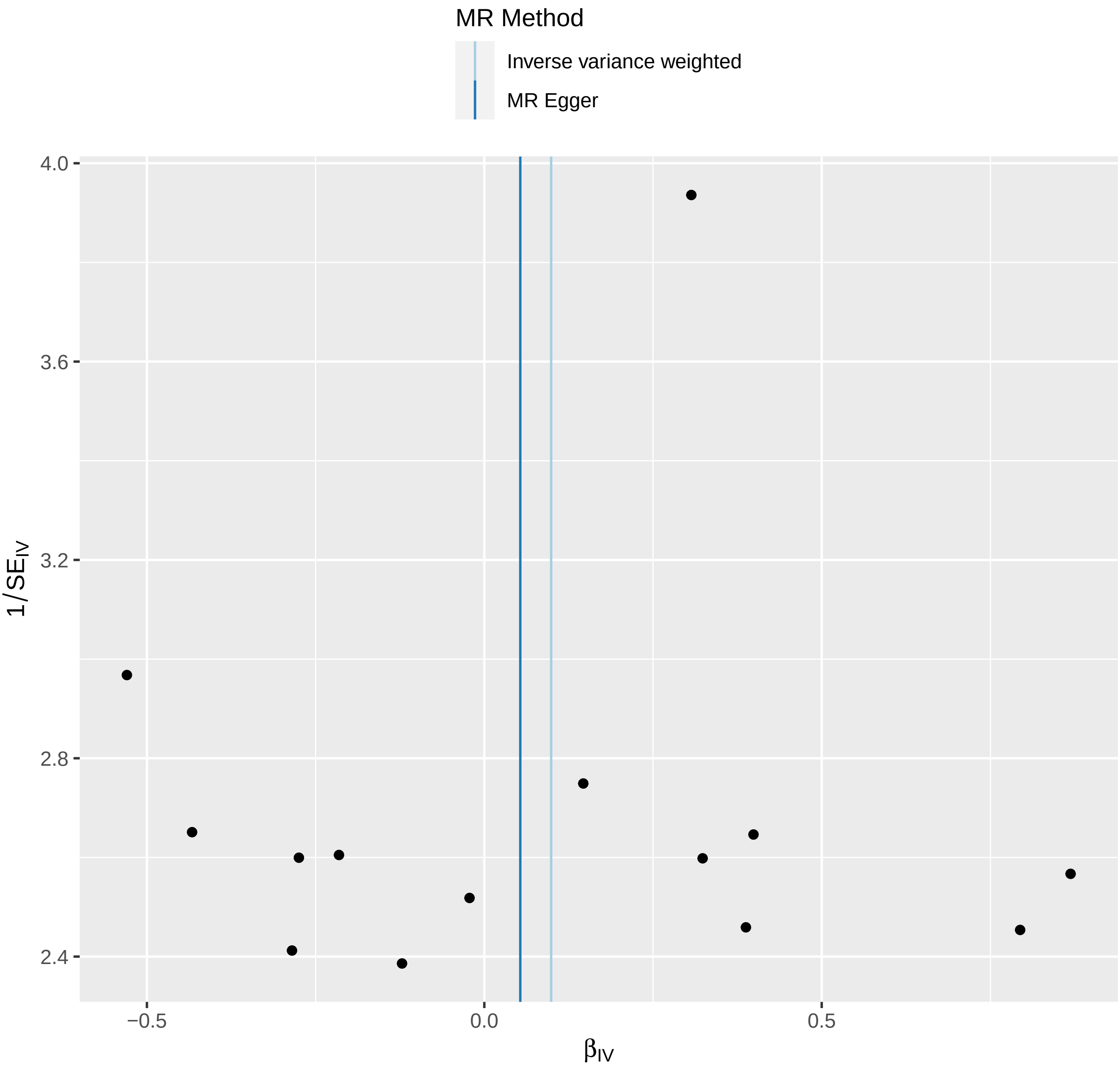

Batch 2−5373 : Gut microbiota abundance (genus Catenibacterium id.2153) on Small EDRK−rich factor 1
with a threshold of 1e−05

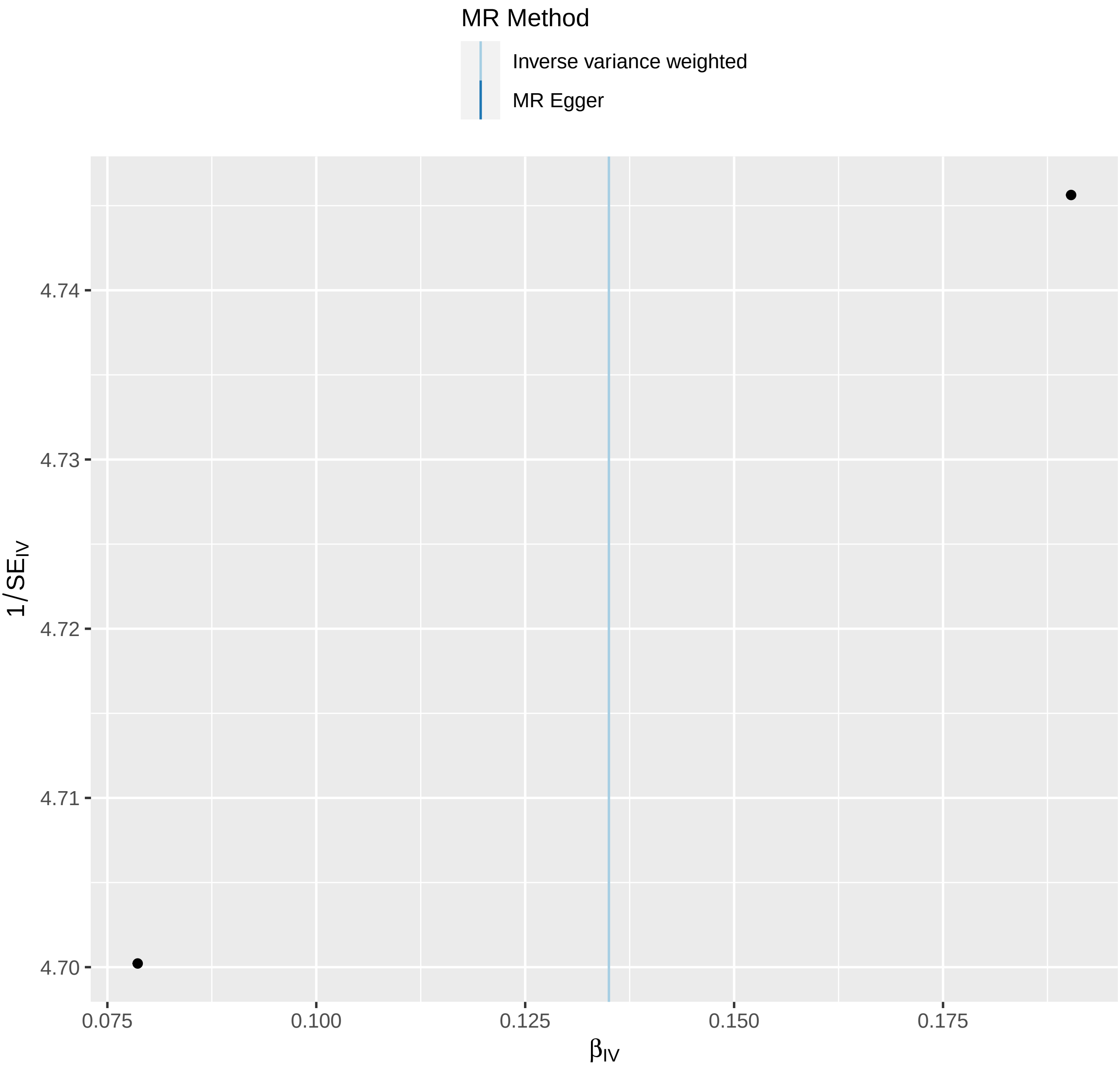

Batch 2−5374 : Gut microbiota abundance (genus Parasutterella id.2892) on Small EDRK−rich factor 1
with a threshold of 1e−05

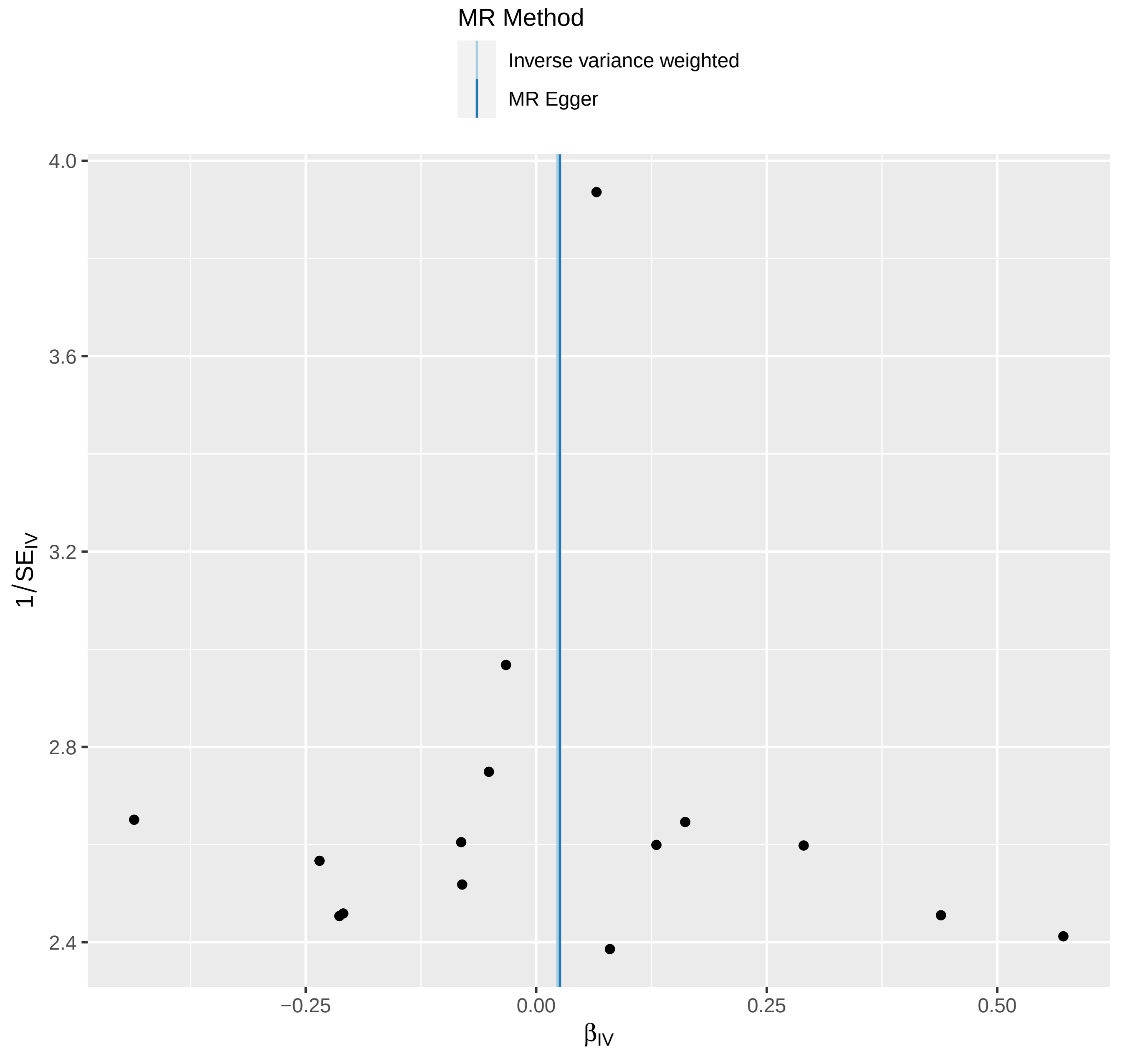

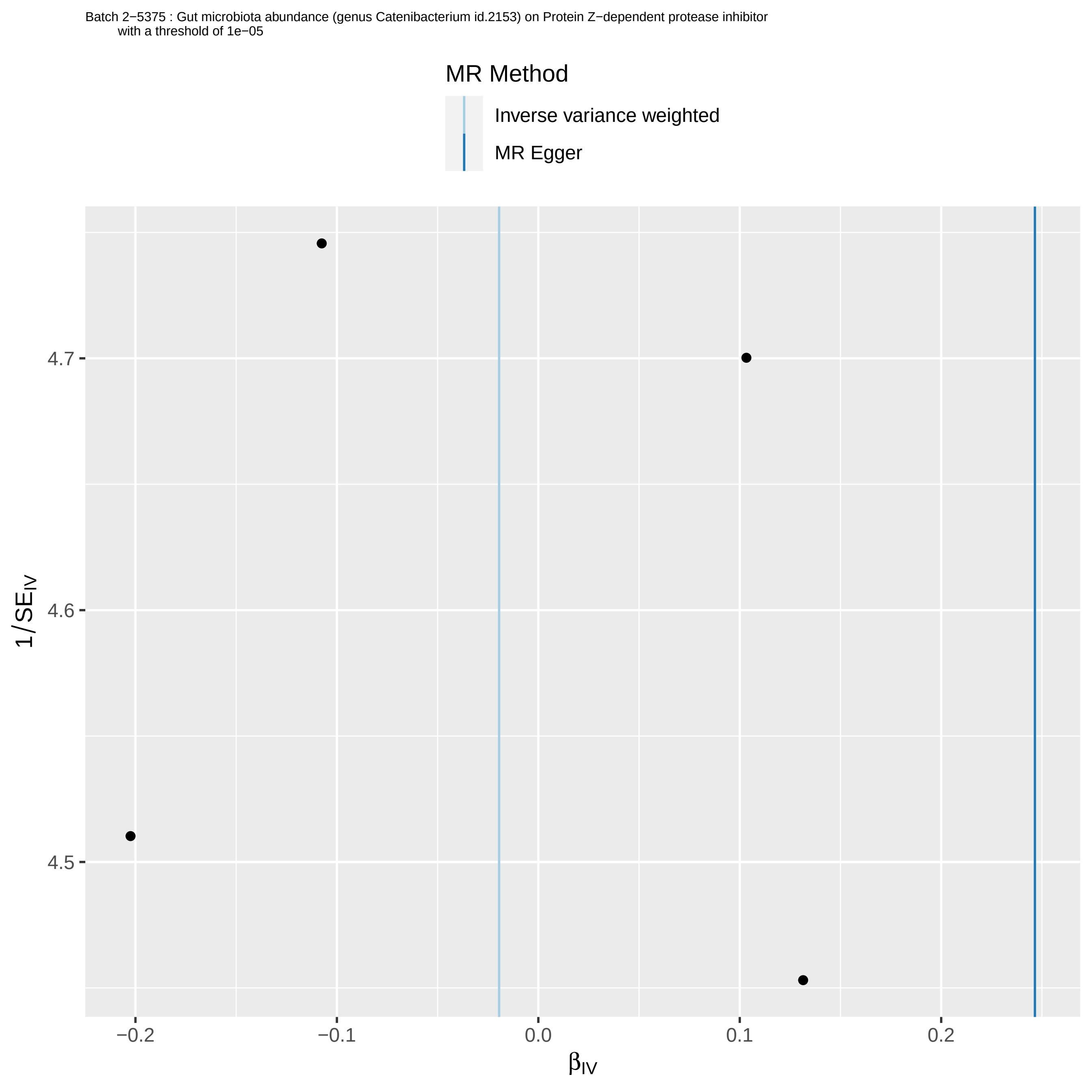

Batch 2−5375 : Gut microbiota abundance (genus Catenibacterium id.2153) on Protein Z−dependent protease inhibitor
with a threshold of 1e−05
MR Method
Inverse variance weighted
MR Egger
4.7
4.6
4.5
1/SE_IV
−0.2
−0.1
0.0
0.1
0.2
β_IV

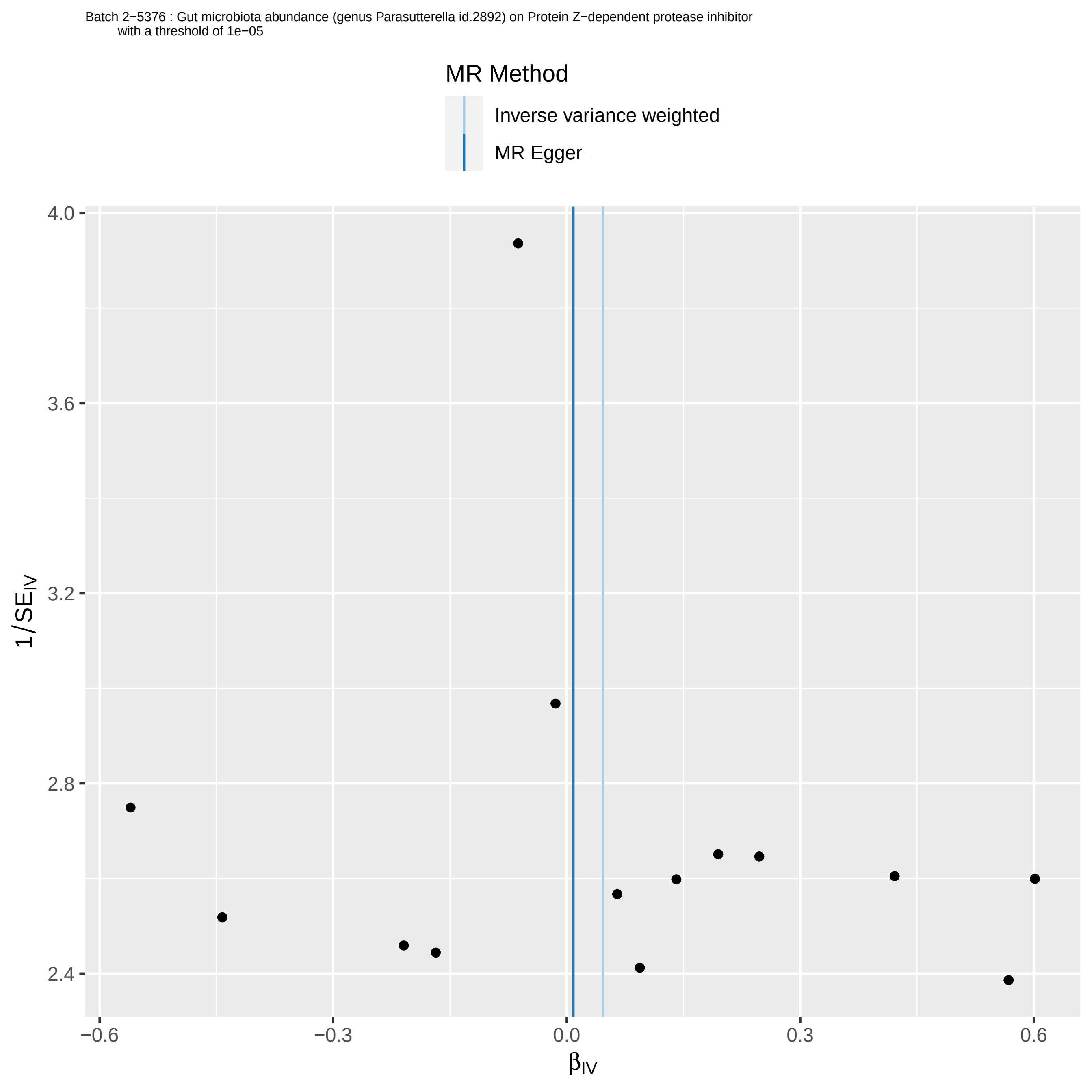

Batch 2−5376 : Gut microbiota abundance (genus Parasutterella id.2892) on Protein Z−dependent protease inhibitor
with a threshold of 1e−05
MR Method
Inverse variance weighted
MR Egger
4.0
3.6
3.2
2.8
2.4
1/SE_IV
−0.6
−0.3
0.0
0.3
0.6
β_IV

Batch 2−5377 : Gut microbiota abundance (genus Catenibacterium id.2153) on Protein Z−dependent protease inhibitor with a threshold of 1e−05

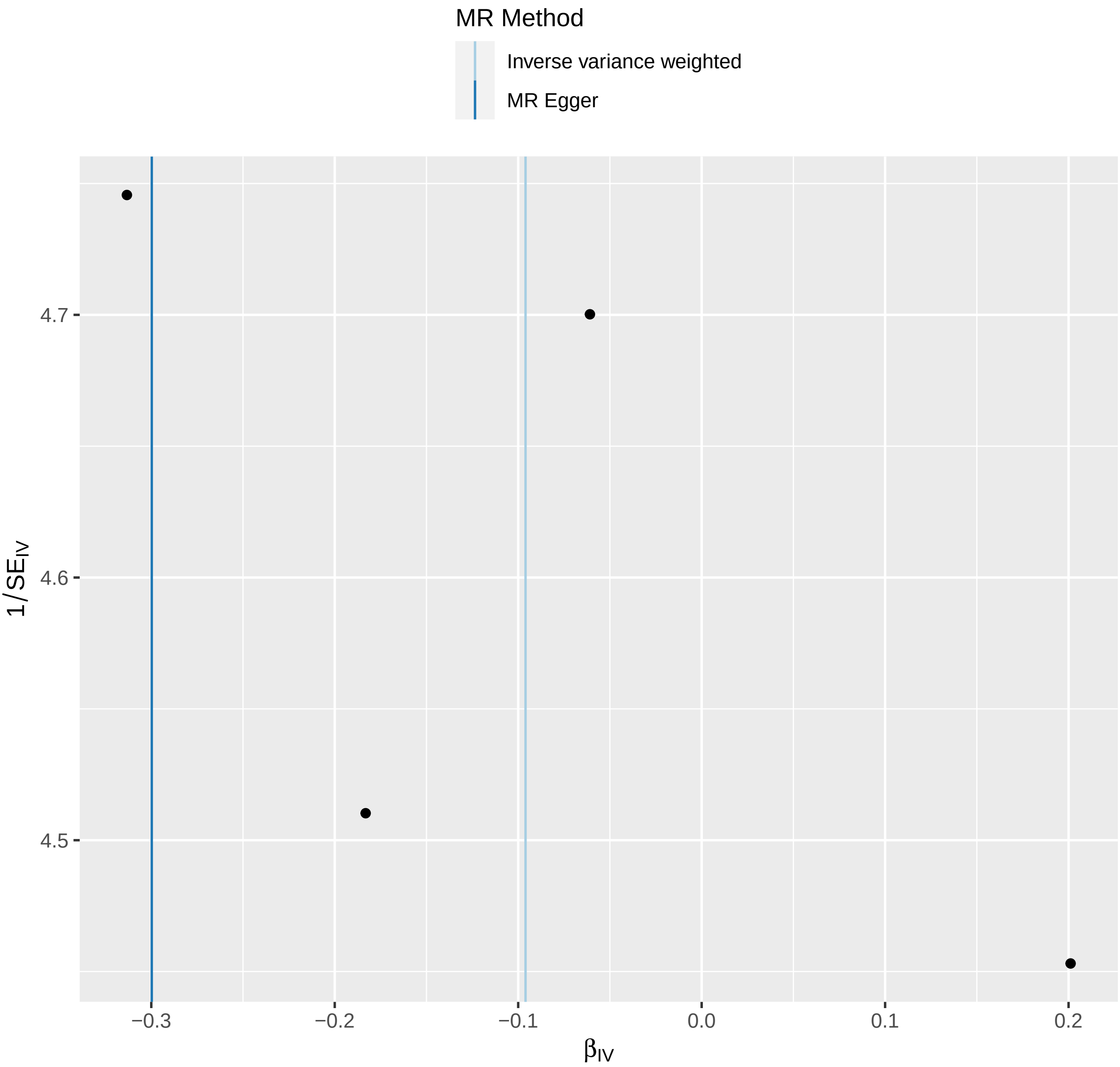

Batch 2−5378 : Gut microbiota abundance (genus Parasutterella id.2892) on Protein Z−dependent protease inhibitor with a threshold of 1e−05

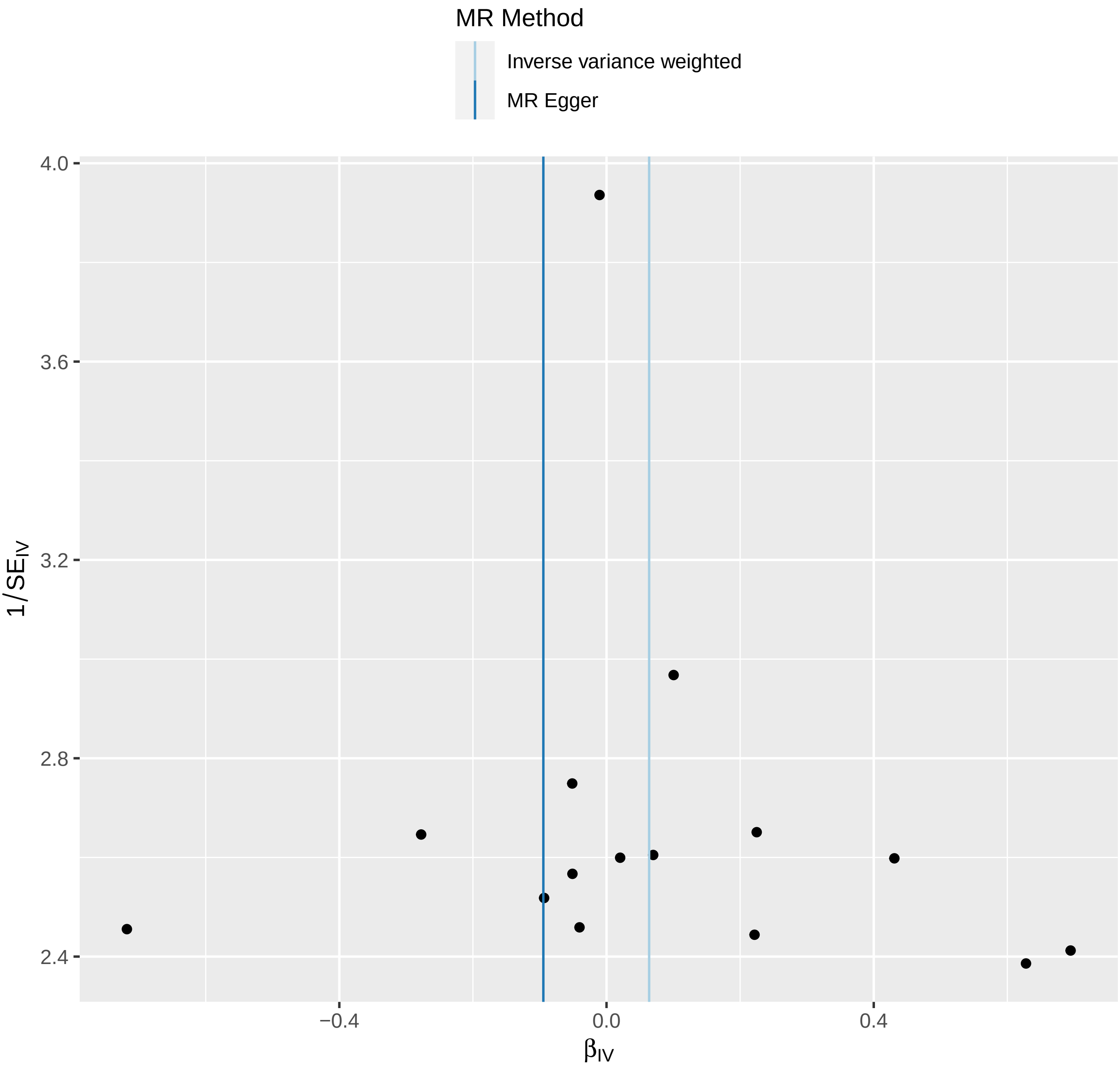

Batch 2−5379 : Gut microbiota abundance (genus Catenibacterium id.2153) on Serpin A12
with a threshold of 1e−05

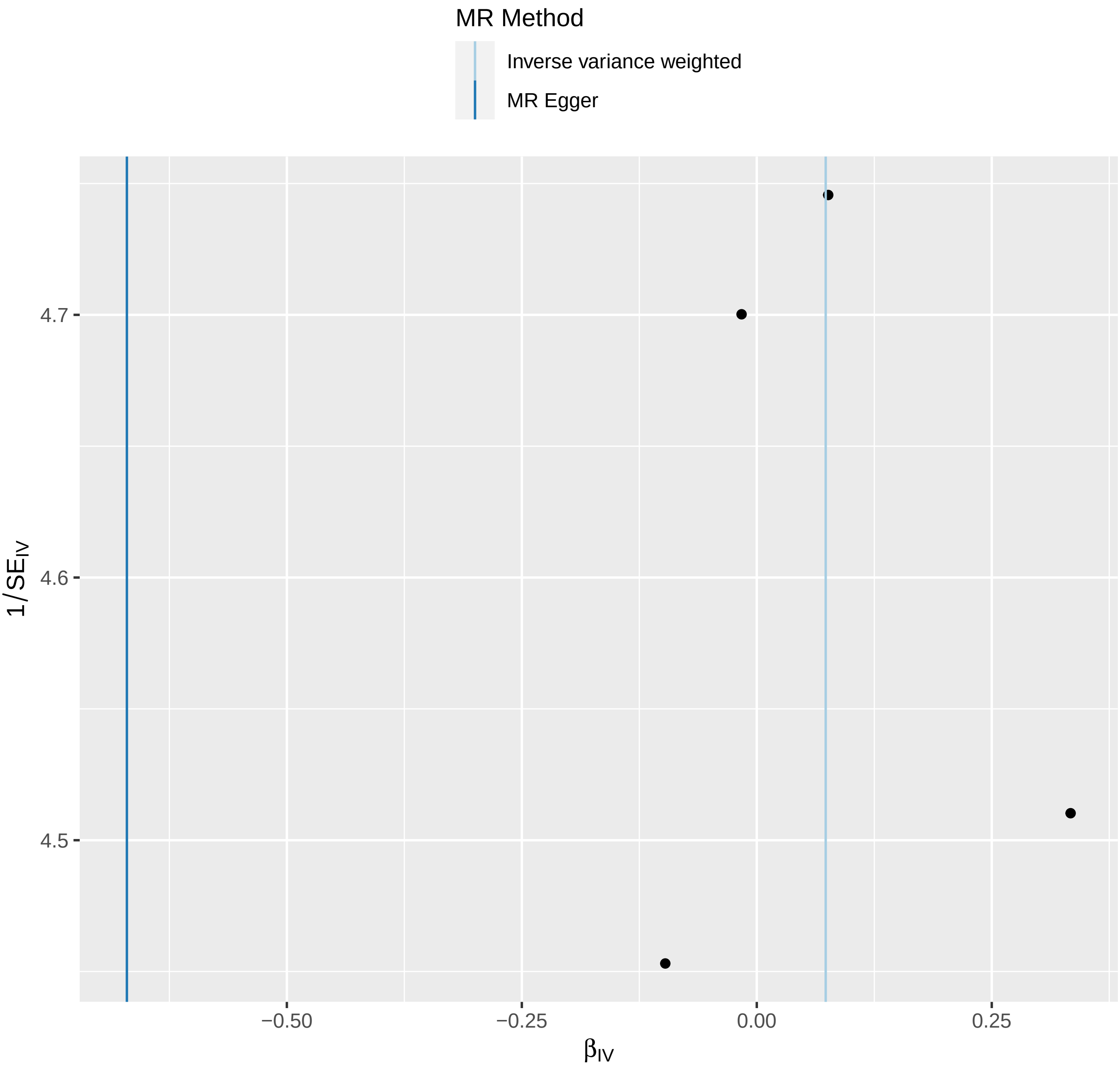

Batch 2−5380 : Gut microbiota abundance (genus Parasutterella id.2892) on Serpin A12
with a threshold of 1e−05

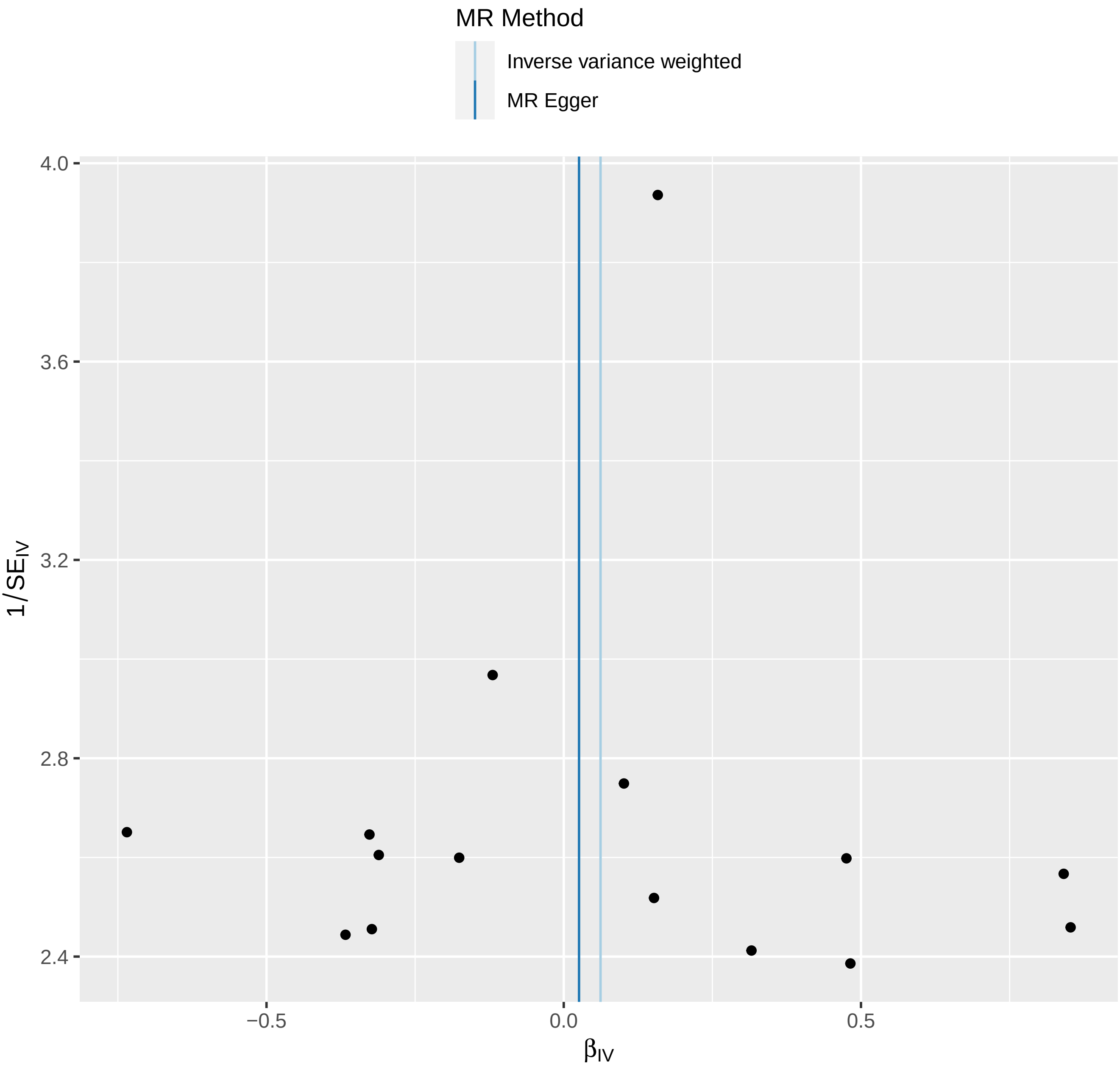

Batch 2−5381 : Gut microbiota abundance (genus Catenibacterium id.2153) on Kallistatin
with a threshold of 1e−05

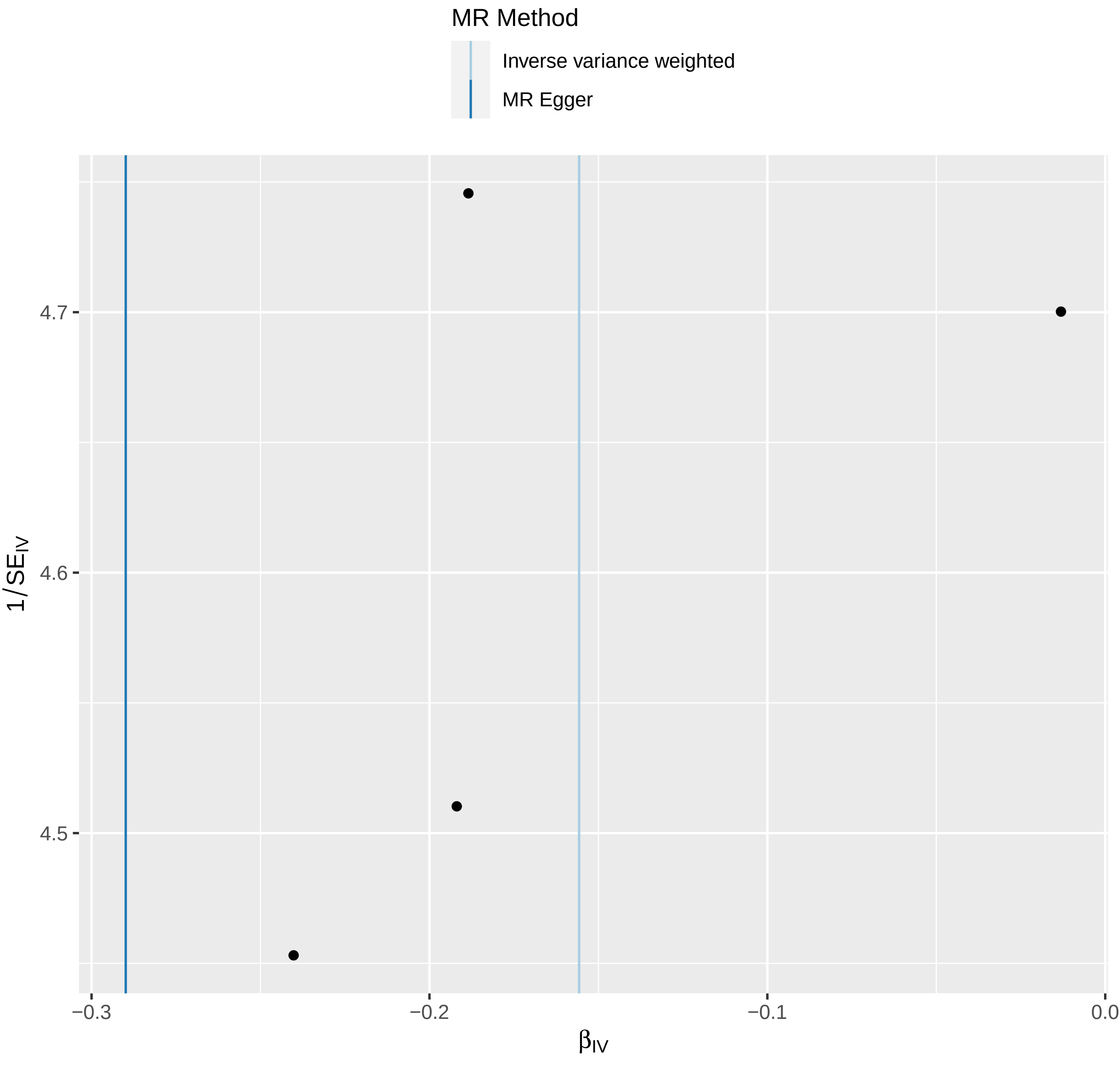

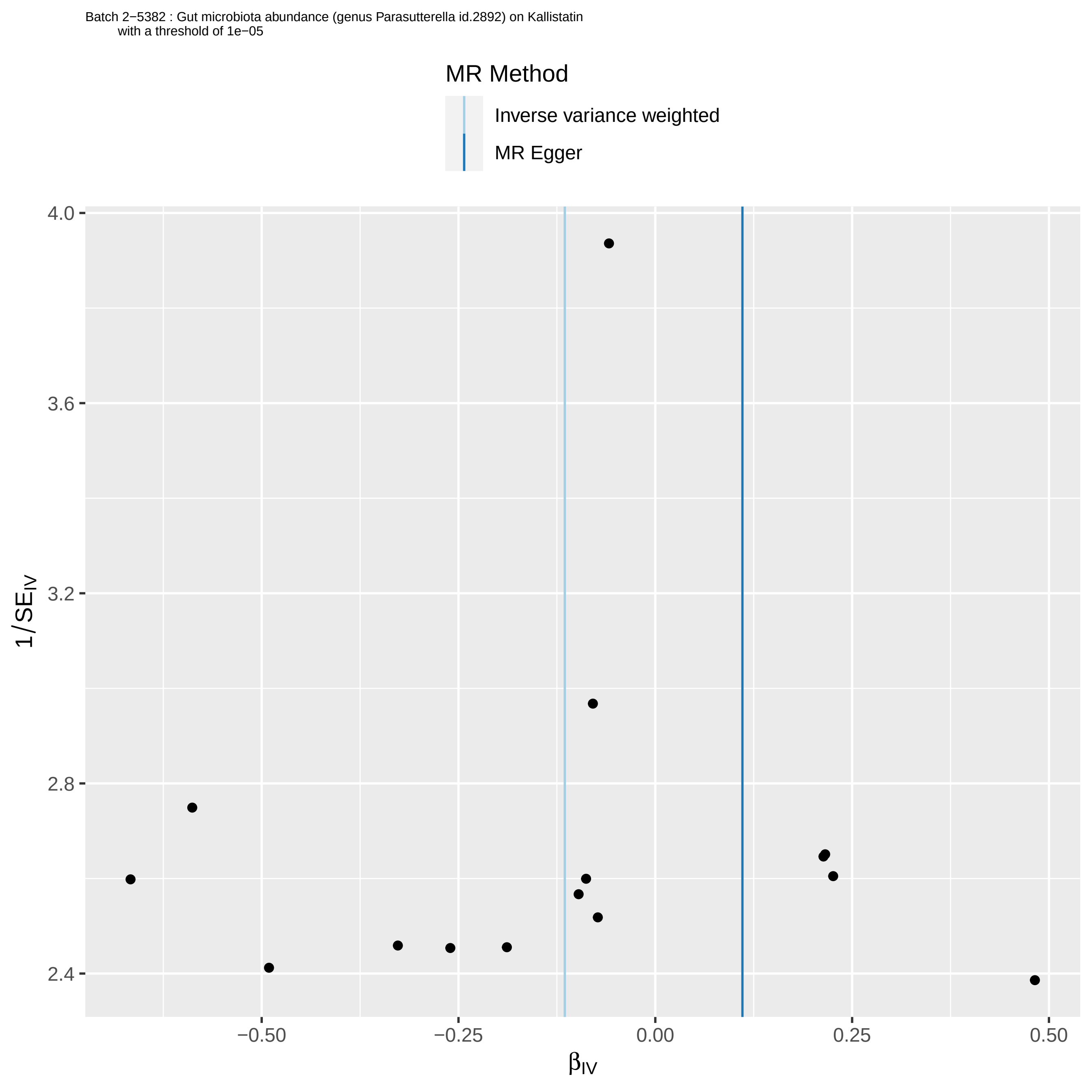
Batch 2−5382 : Gut microbiota abundance (genus Parasutterella id.2892) on Kallistatin
with a threshold of 1e−05
MR Method
Inverse variance weighted
MR Egger
4.0
3.6
3.2
2.8
2.4
1/SE_IV
−0.50
−0.25
0.00
0.25
0.50
β_IV

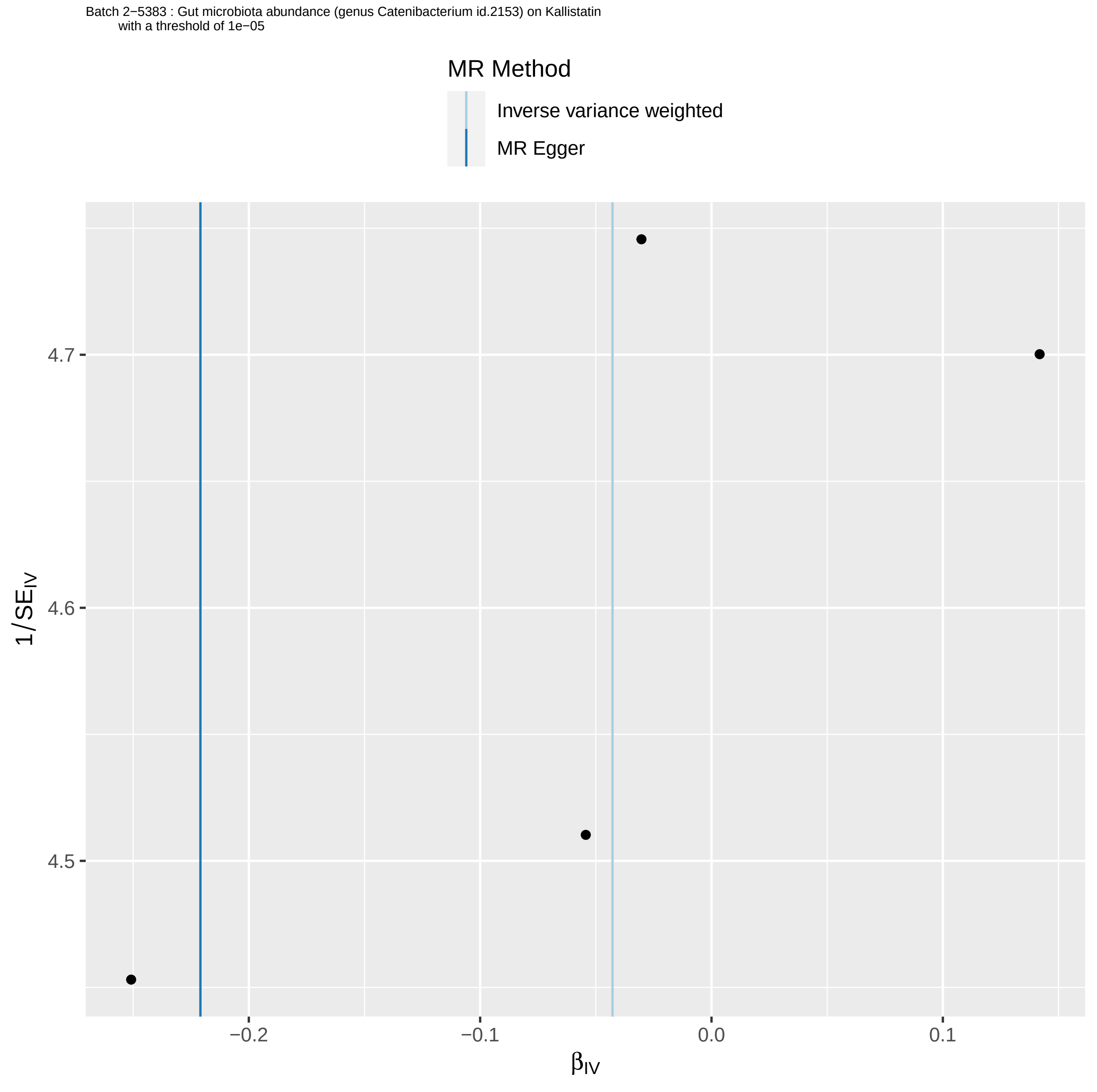
Batch 2−5383 : Gut microbiota abundance (genus Catenibacterium id.2153) on Kallistatin
with a threshold of 1e−05
MR Method
Inverse variance weighted
MR Egger
4.7
4.6
4.5
1/SE$_{IV}$
−0.2
−0.1
0.0
0.1
β$_{IV}$

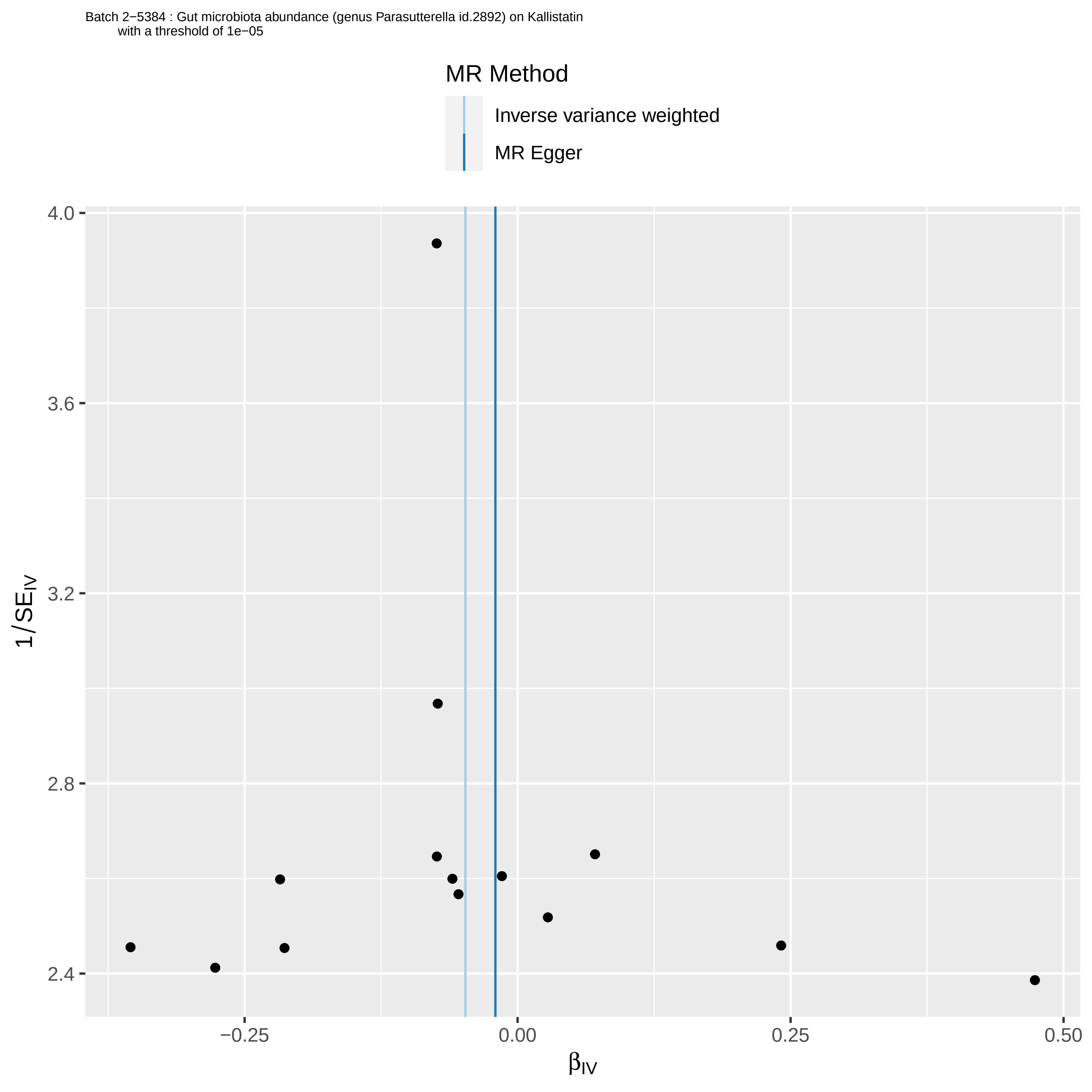
Batch 2−5384 : Gut microbiota abundance (genus Parasutterella id.2892) on Kallistatin
with a threshold of 1e−05
MR Method
Inverse variance weighted
MR Egger
4.0
3.6
3.2
2.8
2.4
1/SE_IV
−0.25
0.00
0.25
0.50
β_IV

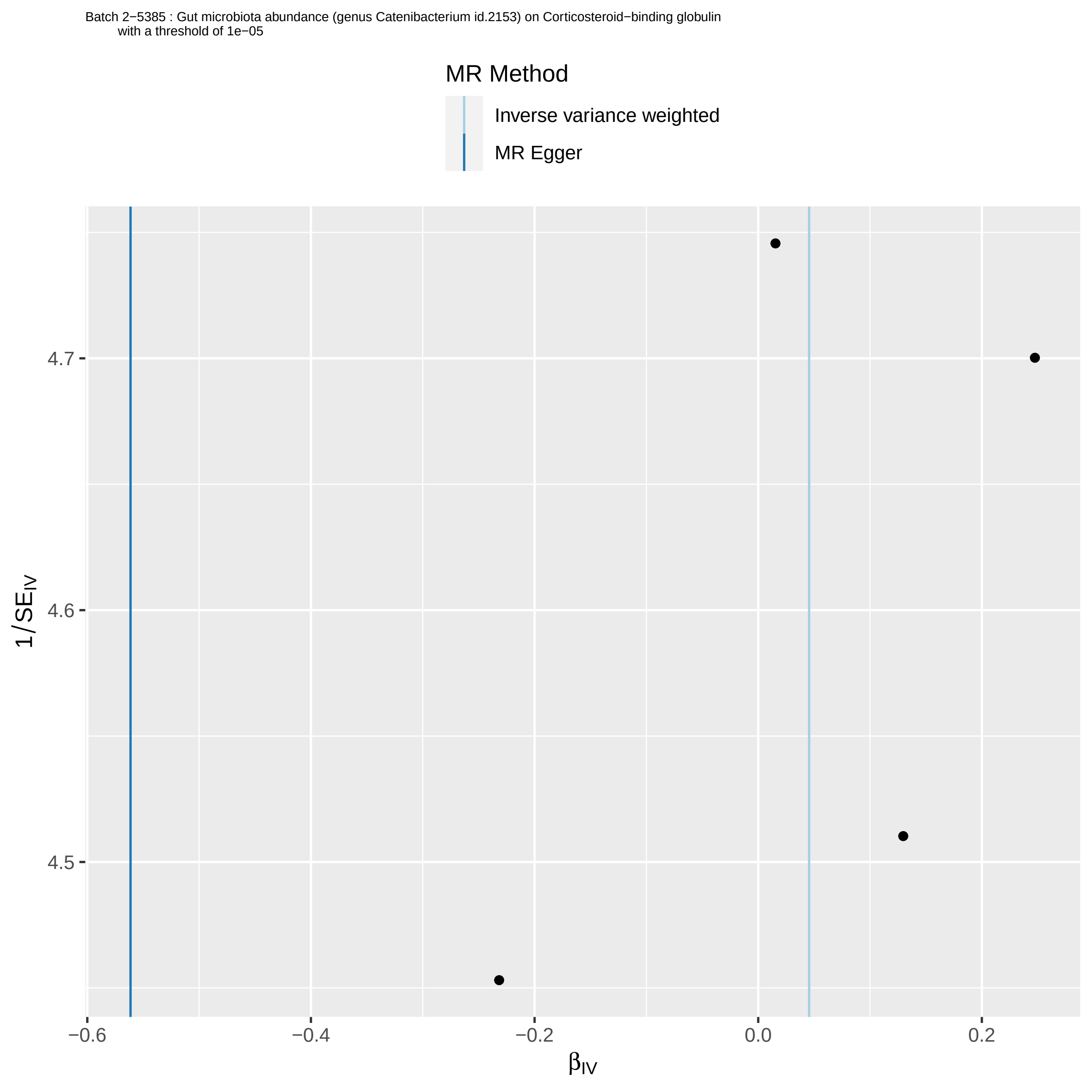

Batch 2−5385 : Gut microbiota abundance (genus Catenibacterium id.2153) on Corticosteroid−binding globulin
with a threshold of 1e−05
MR Method
Inverse variance weighted
MR Egger
4.7
4.6
4.5
1/SE_IV
−0.6
−0.4
−0.2
0.0
0.2
β_IV

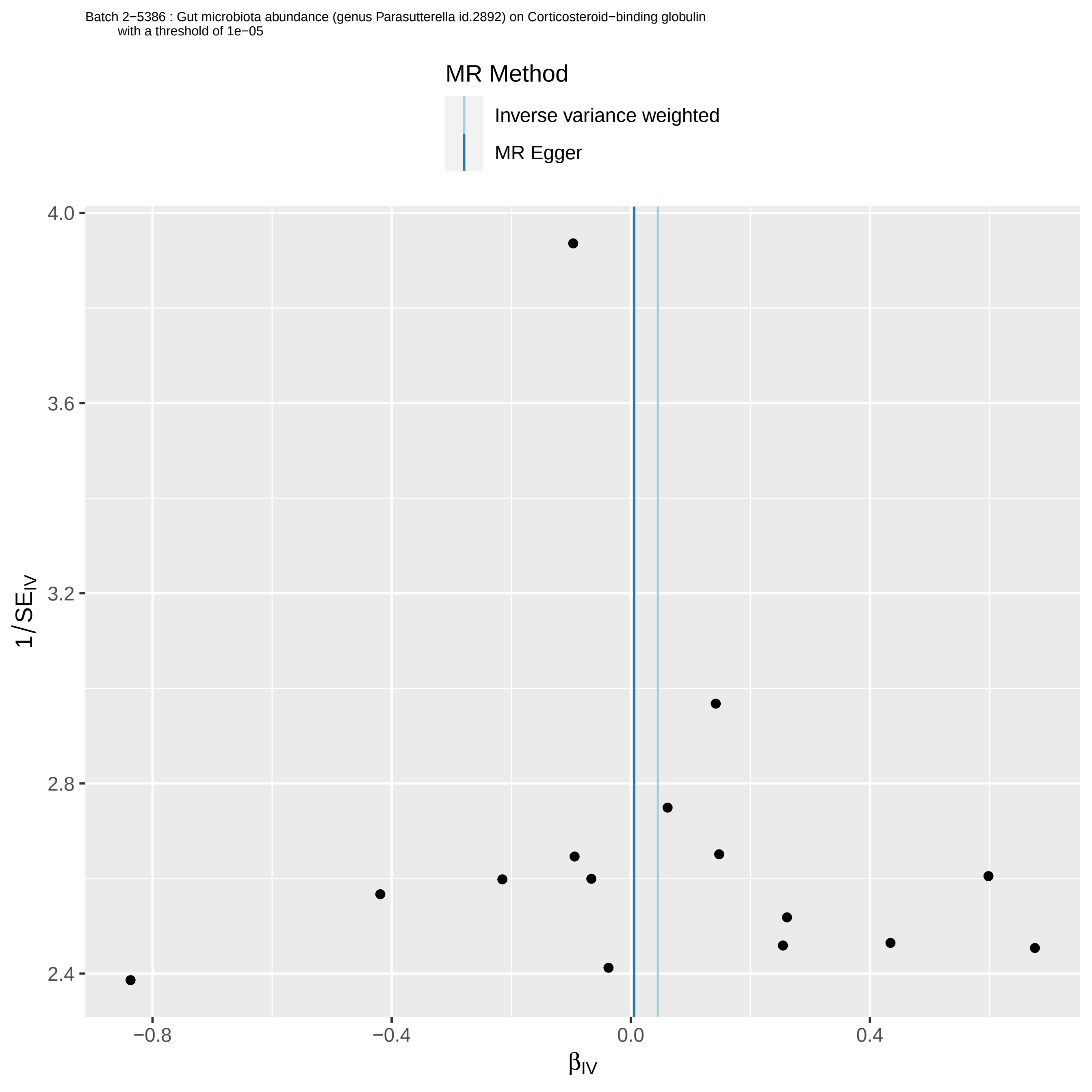
Batch 2−5386 : Gut microbiota abundance (genus Parasutterella id.2892) on Corticosteroid−binding globulin
with a threshold of 1e−05
MR Method
Inverse variance weighted
MR Egger
4.0
3.6
3.2
2.8
2.4
1/SE_IV
−0.8
−0.4
0.0
0.4
β_IV

Batch 2−5387 : Gut microbiota abundance (genus Catenibacterium id.2153) on Serpin A9
with a threshold of 1e−05

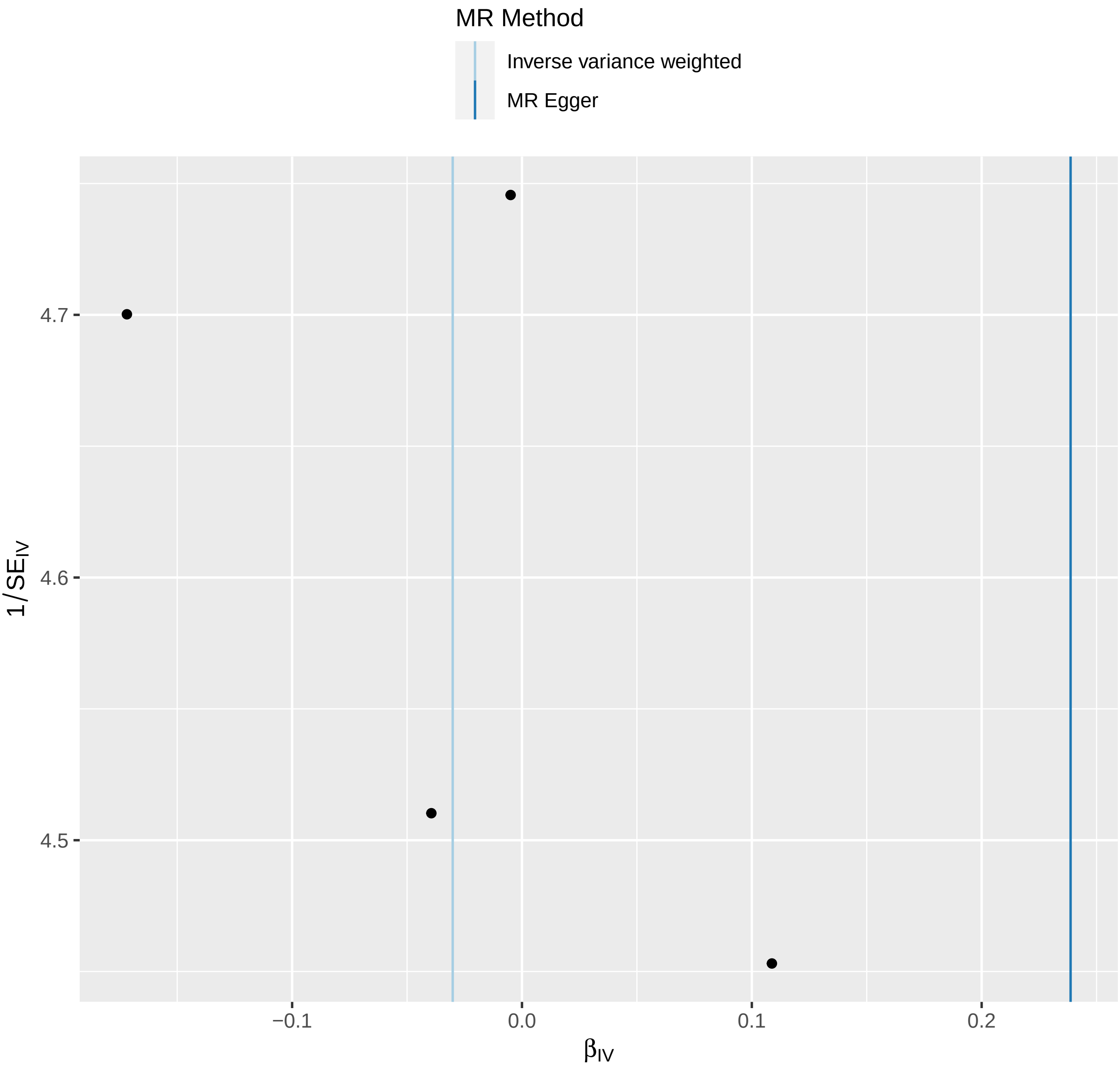

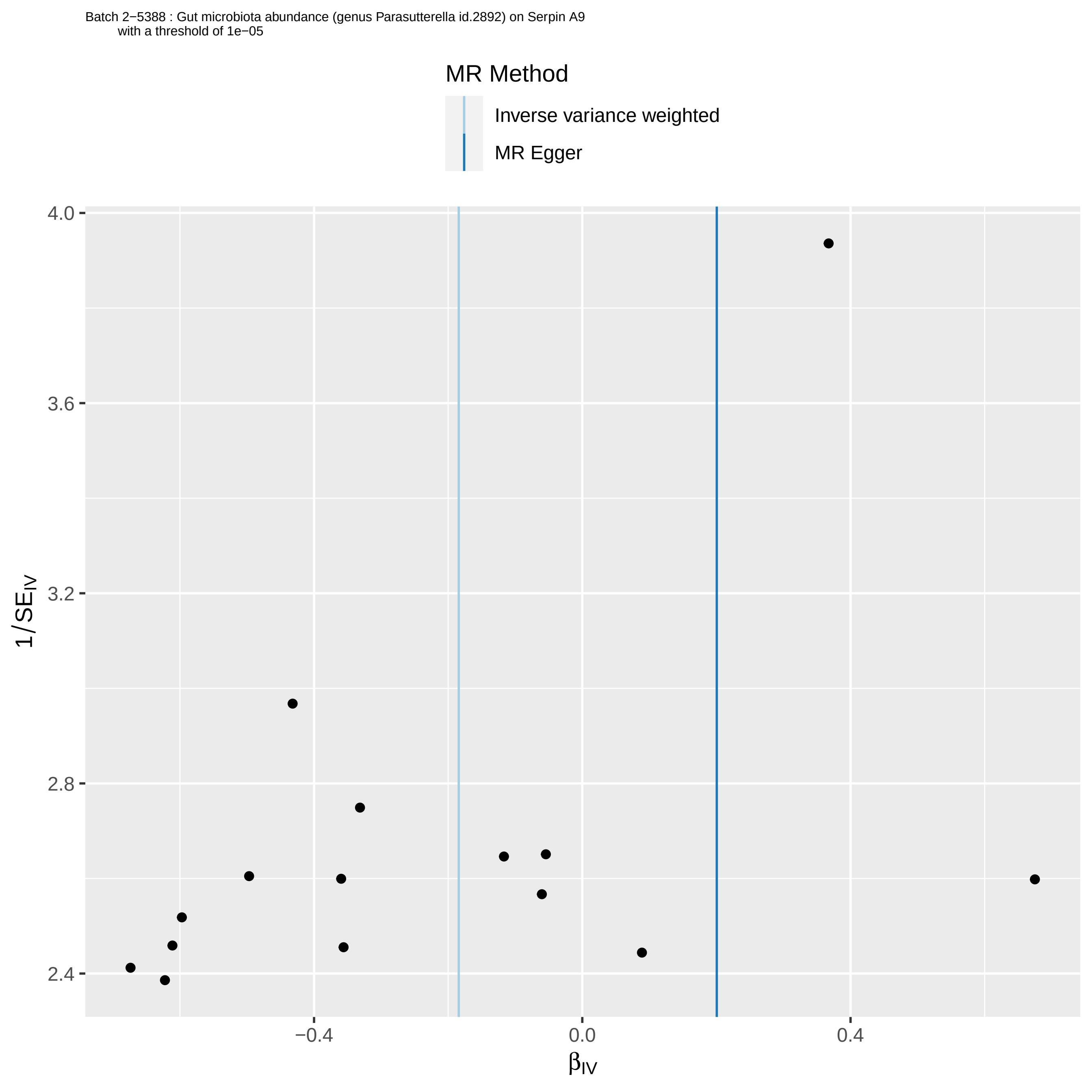

Batch 2−5388 : Gut microbiota abundance (genus Parasutterella id.2892) on Serpin A9
with a threshold of 1e−05
MR Method
Inverse variance weighted
MR Egger
4.0
3.6
3.2
2.8
2.4
1/SE$_{IV}$
−0.4
0.0
0.4
β$_{IV}$

Batch 2−5389 : Gut microbiota abundance (genus Catenibacterium id.2153) on Antithrombin−III
with a threshold of 1e−05

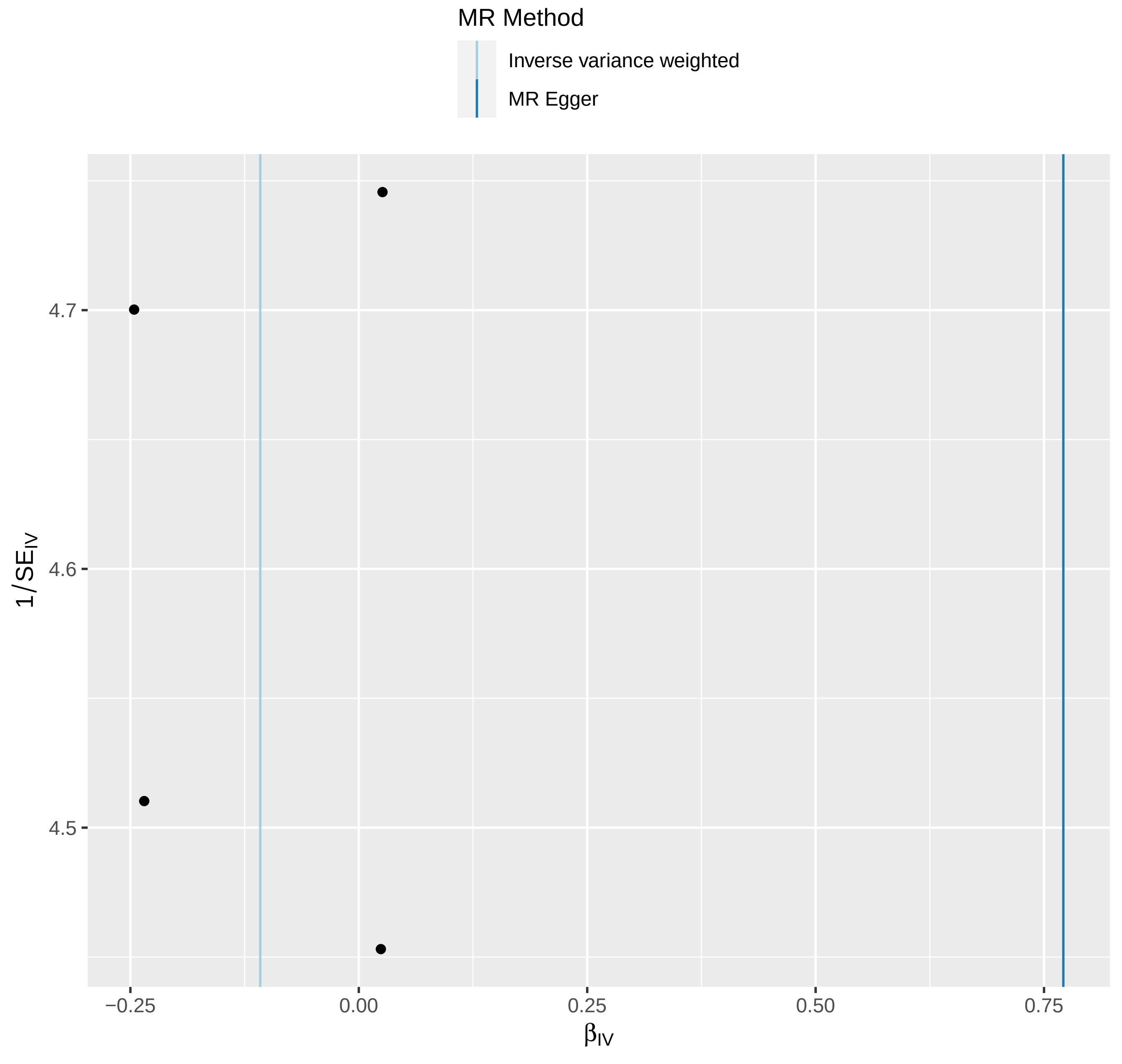

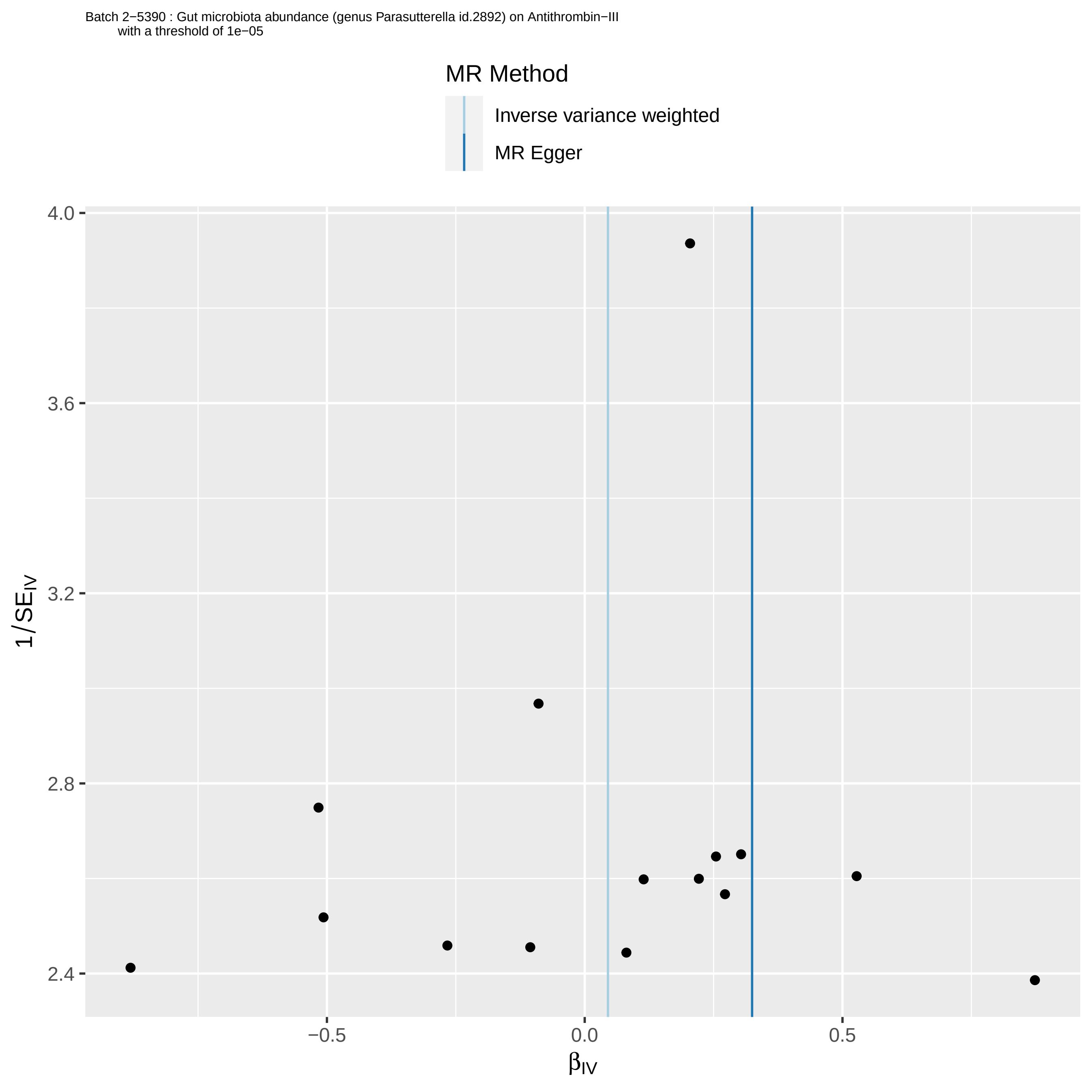

Batch 2−5390 : Gut microbiota abundance (genus Parasutterella id.2892) on Antithrombin−III
with a threshold of 1e−05
MR Method
Inverse variance weighted
MR Egger
4.0
3.6
3.2
2.8
2.4
1/SE_IV
−0.5
0.0
0.5
β_IV

Batch 2−5391 : Gut microbiota abundance (genus Catenibacterium id.2153) on Plasminogen activator inhibitor 1
with a threshold of 1e−05

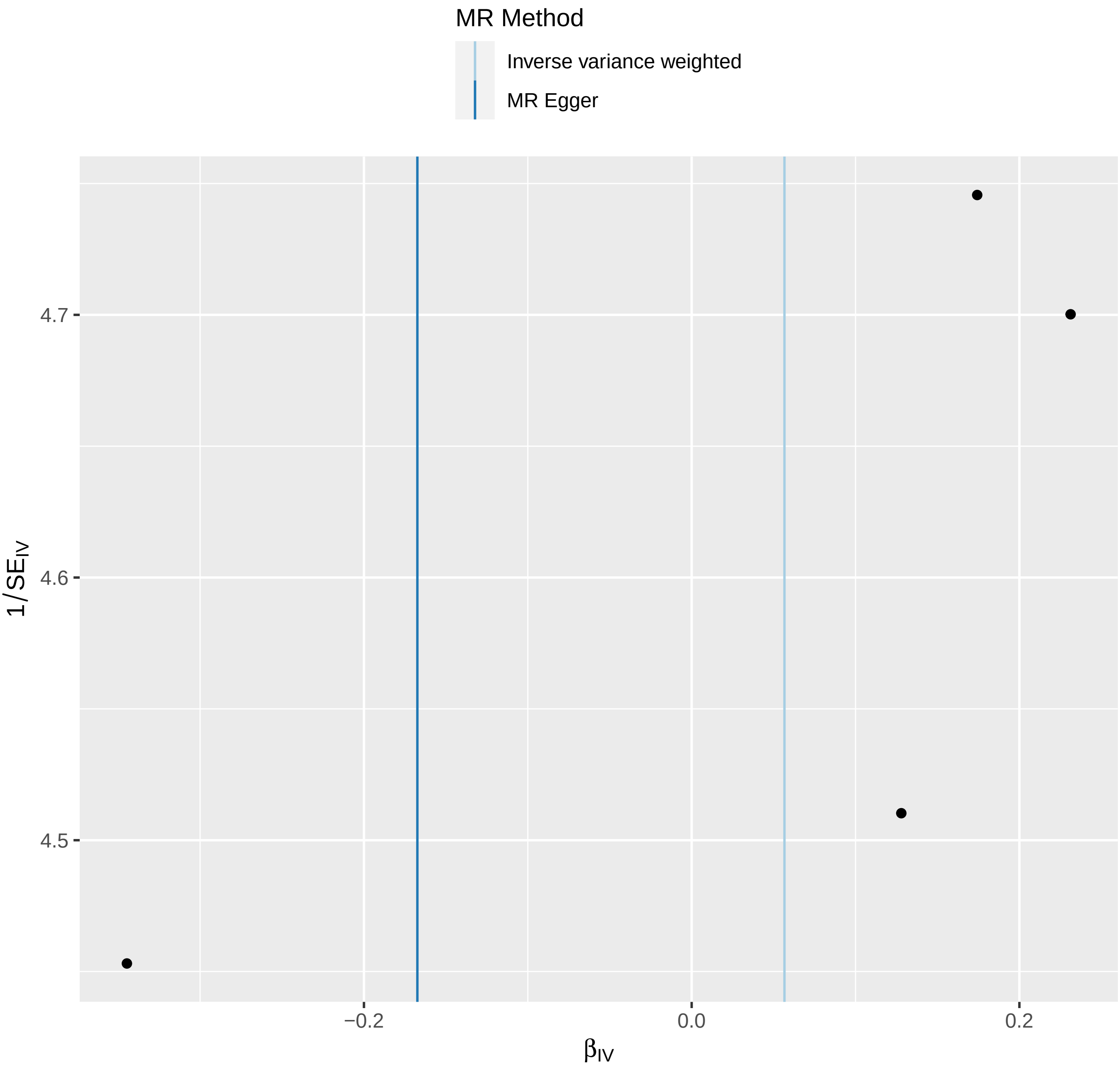

Batch 2−5392 : Gut microbiota abundance (genus Parasutterella id.2892) on Plasminogen activator inhibitor 1 with a threshold of 1e−05

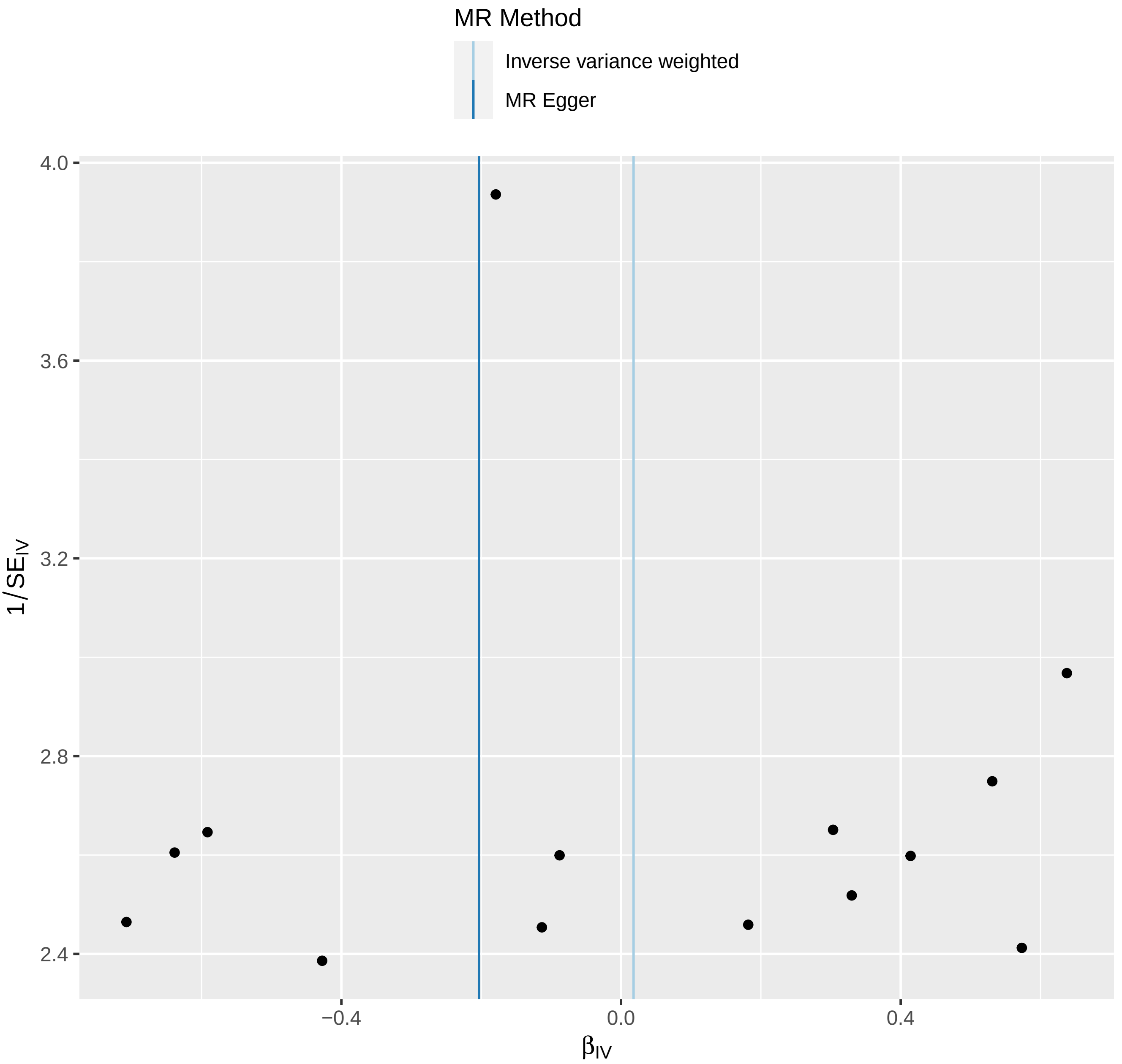

Batch 2−5393 : Gut microbiota abundance (genus Catenibacterium id.2153) on Glia−derived nexin
with a threshold of 1e−05

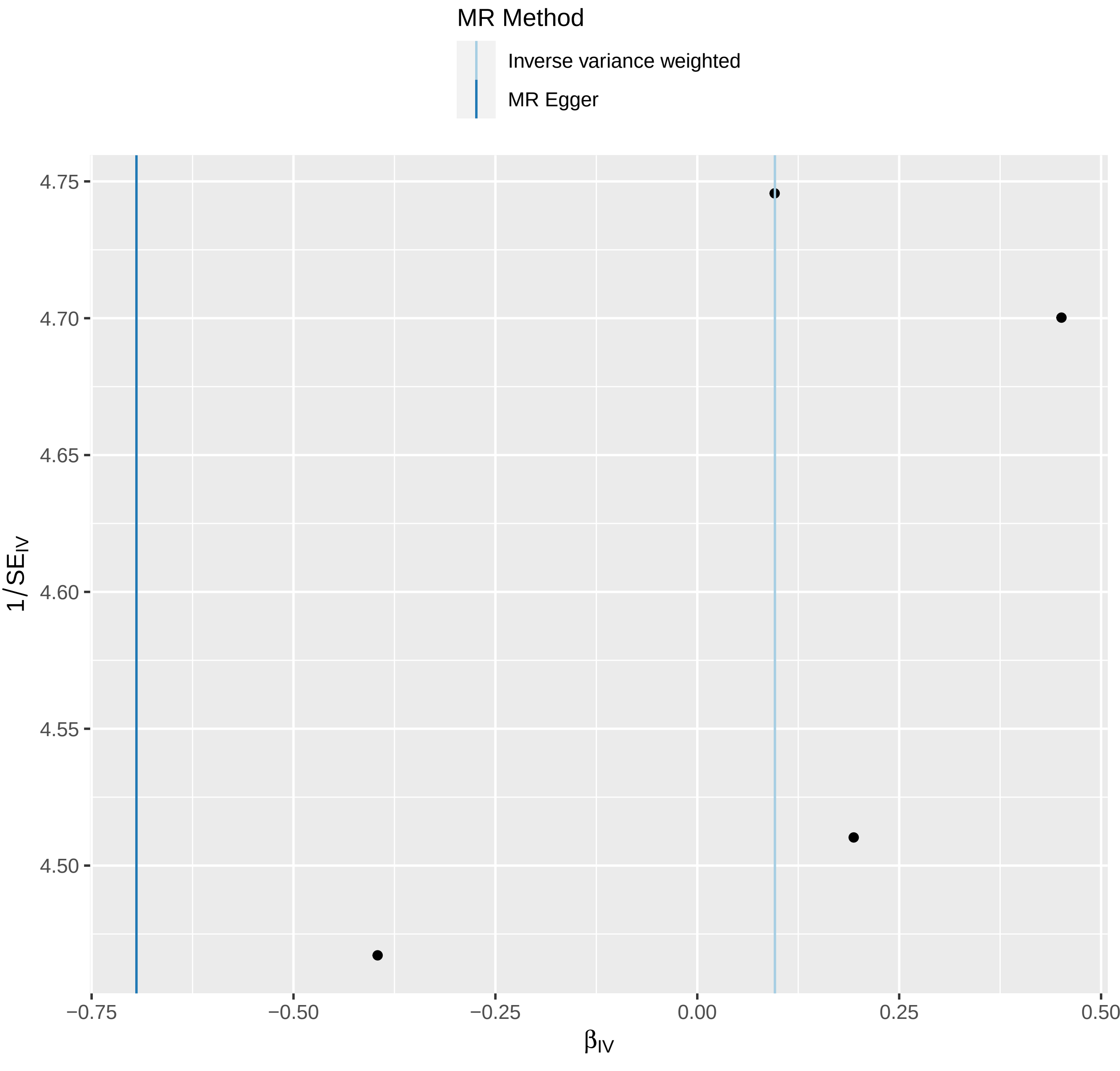

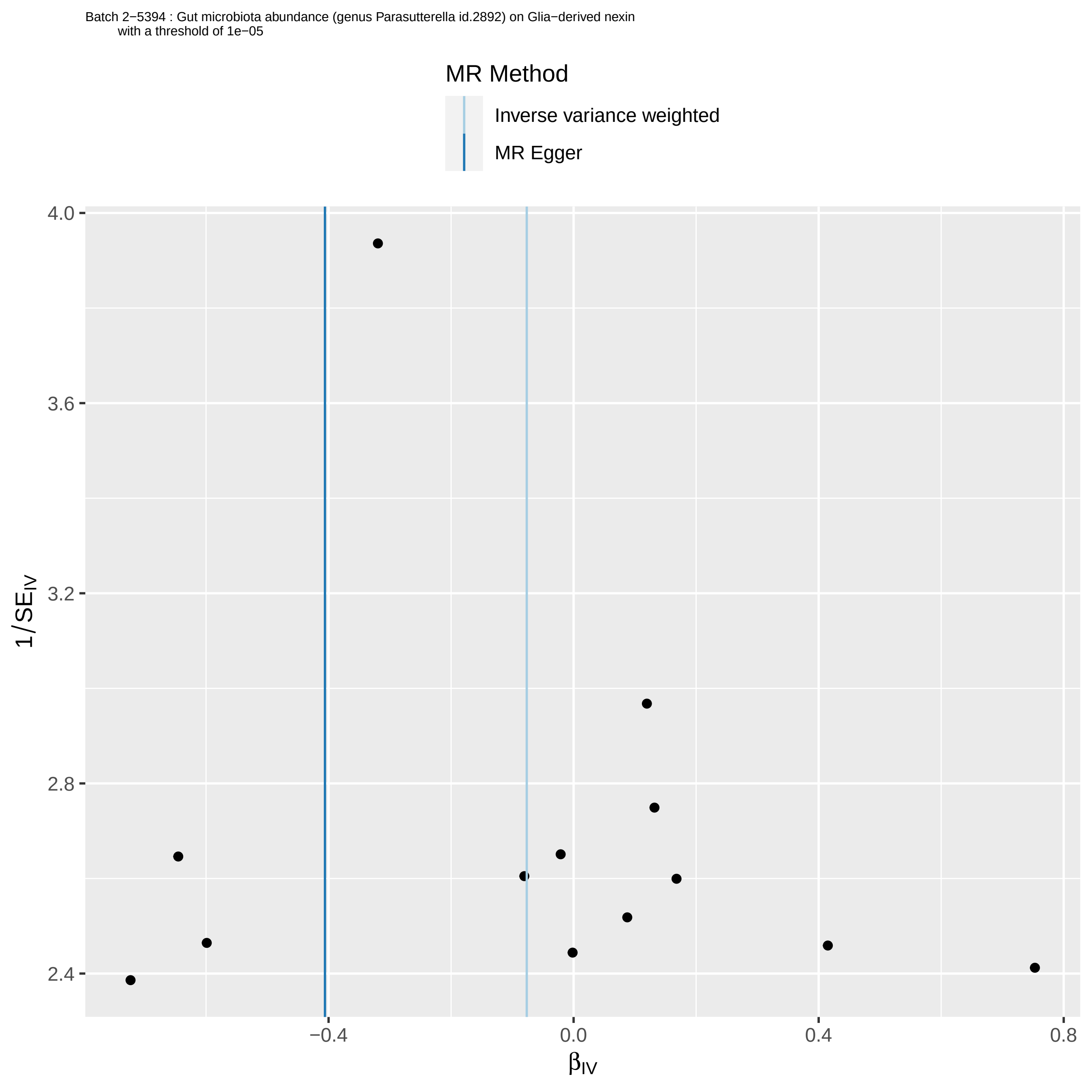
Batch 2−5394 : Gut microbiota abundance (genus Parasutterella id.2892) on Glia−derived nexin
with a threshold of 1e−05
MR Method
Inverse variance weighted
MR Egger
4.0
3.6
3.2
2.8
2.4
1/SE_IV
−0.4
0.0
0.4
0.8
β_IV

Batch 2−5395 : Gut microbiota abundance (genus Catenibacterium id.2153) on Pigment epithelium−derived factor
with a threshold of 1e−05

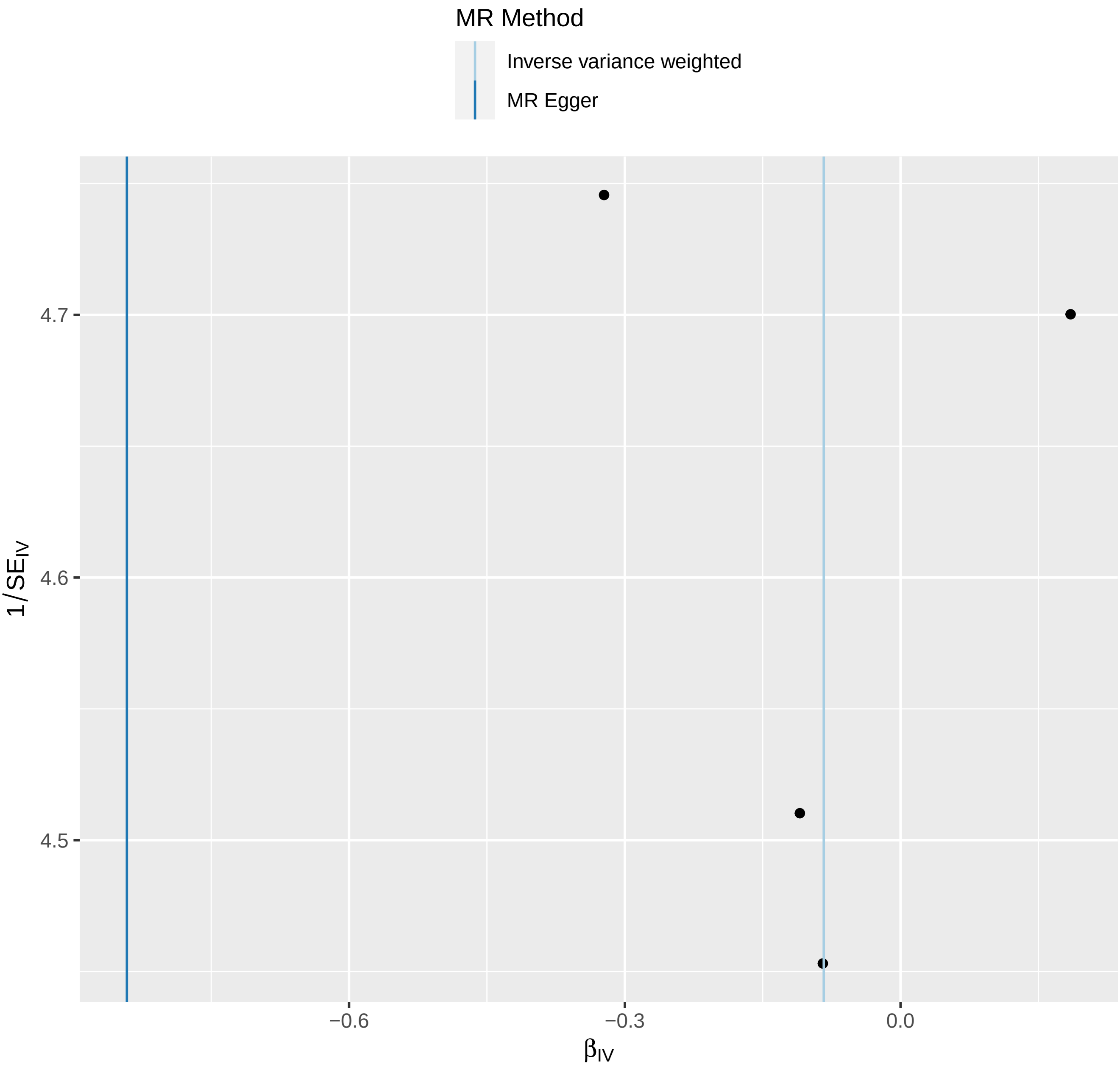

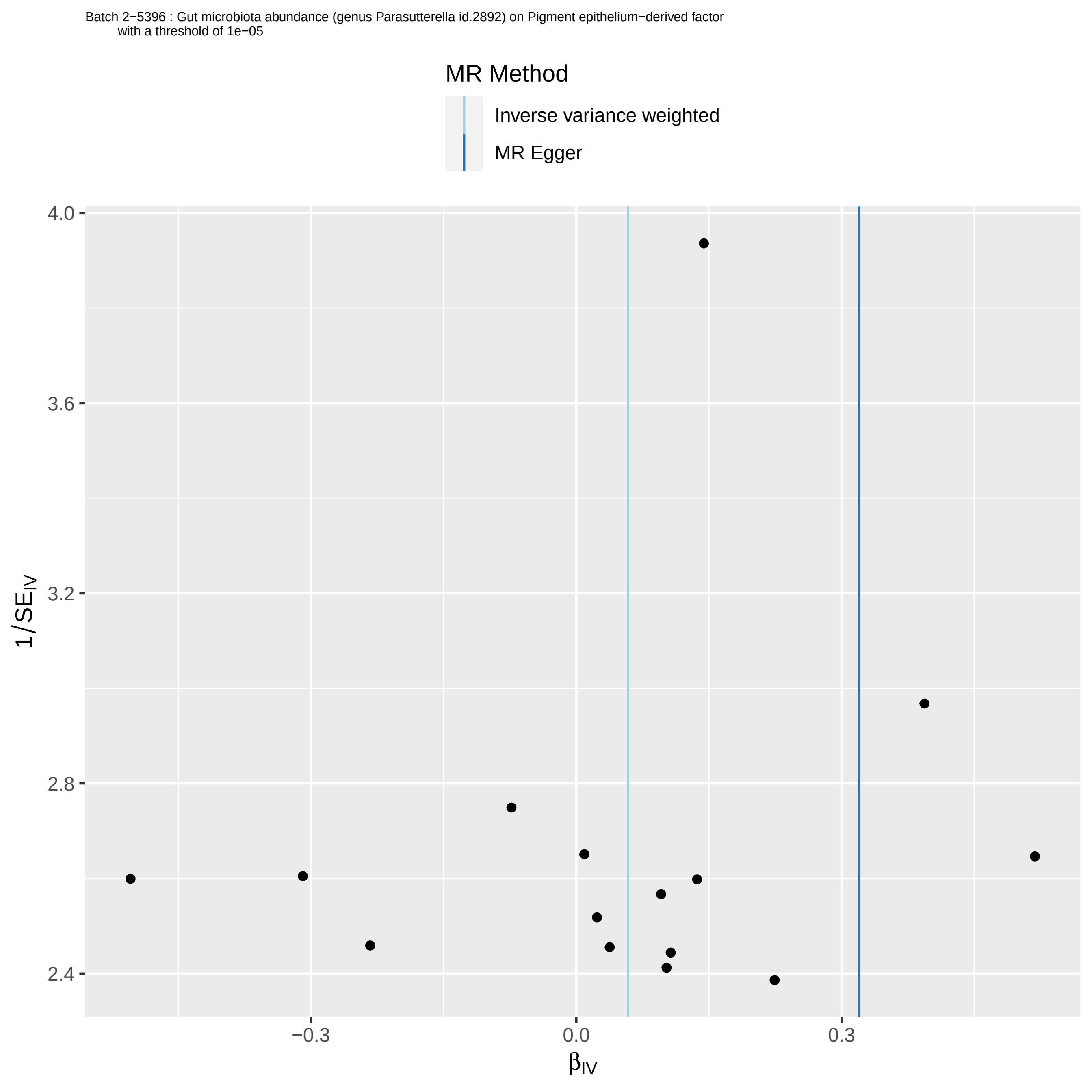

Batch 2−5396 : Gut microbiota abundance (genus Parasutterella id.2892) on Pigment epithelium−derived factor
with a threshold of 1e−05
MR Method
Inverse variance weighted
MR Egger
4.0
3.6
3.2
2.8
2.4
1/SE_IV
−0.3
0.0
0.3
β_IV

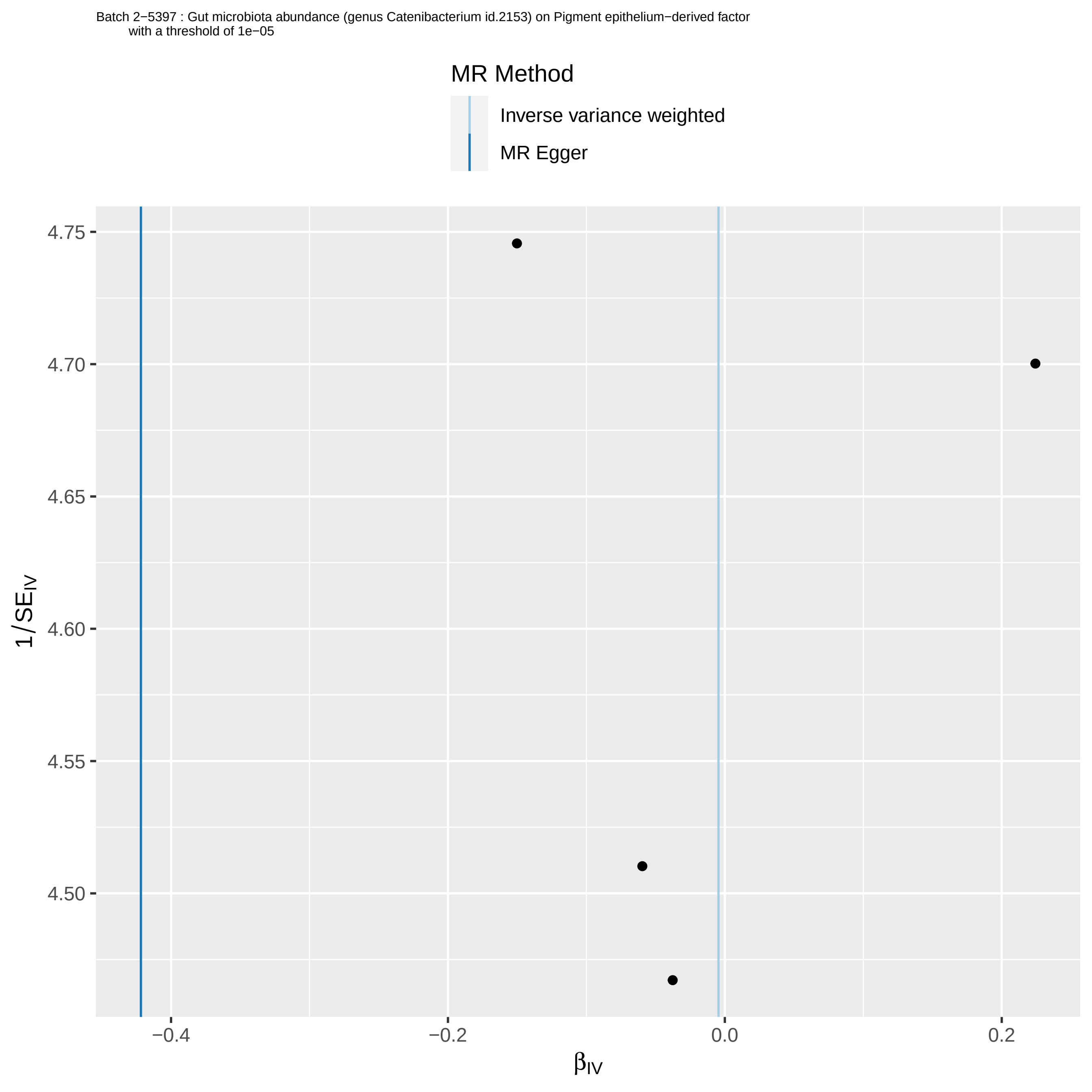

Batch 2−5397 : Gut microbiota abundance (genus Catenibacterium id.2153) on Pigment epithelium−derived factor
with a threshold of 1e−05
MR Method
Inverse variance weighted
MR Egger
4.75
4.70
4.65
4.60
4.55
4.50
1/SE_IV
−0.4
−0.2
0.0
0.2
β_IV

Batch 2−5398 : Gut microbiota abundance (genus Parasutterella id.2892) on Pigment epithelium−derived factor
with a threshold of 1e−05

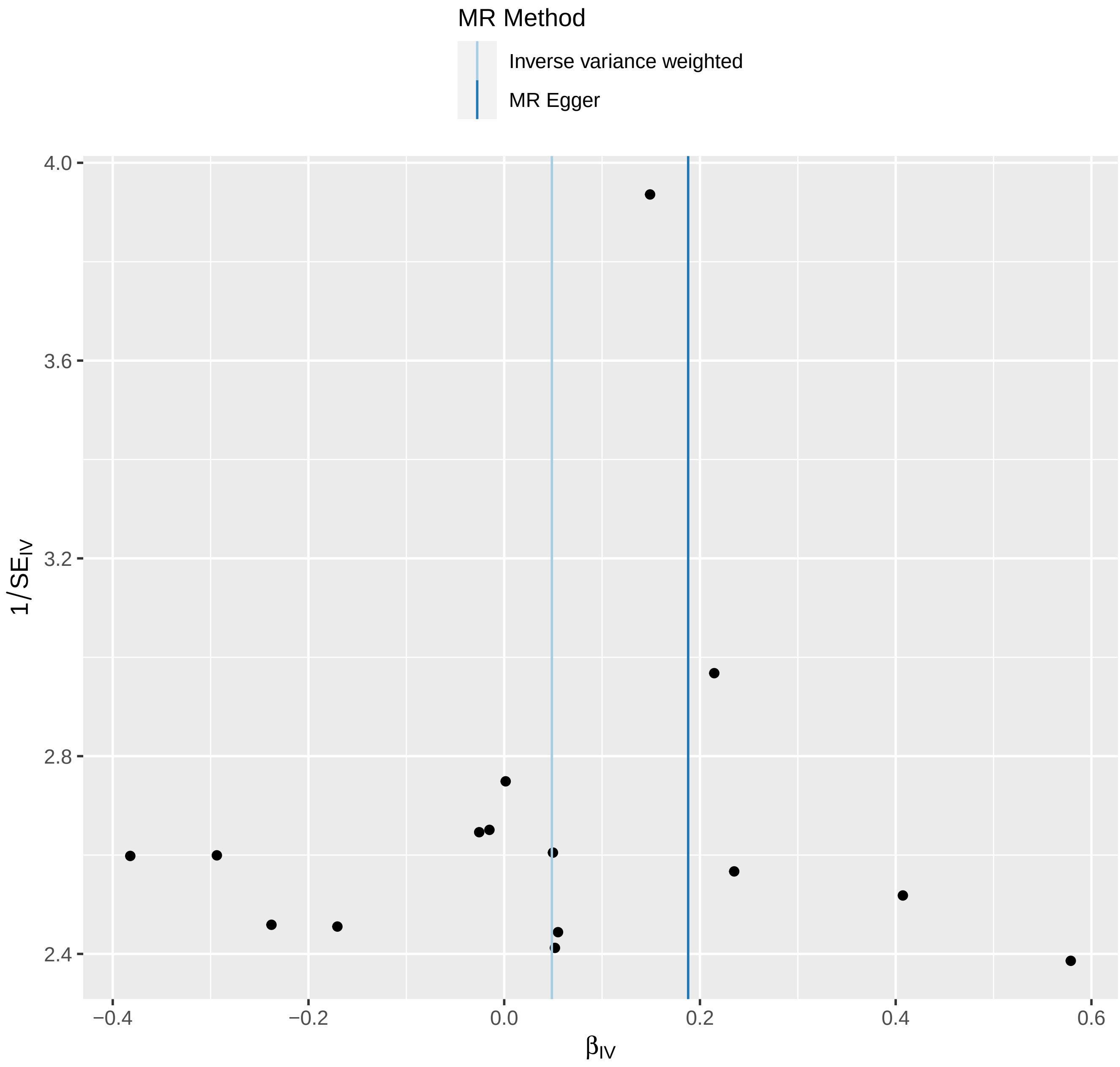

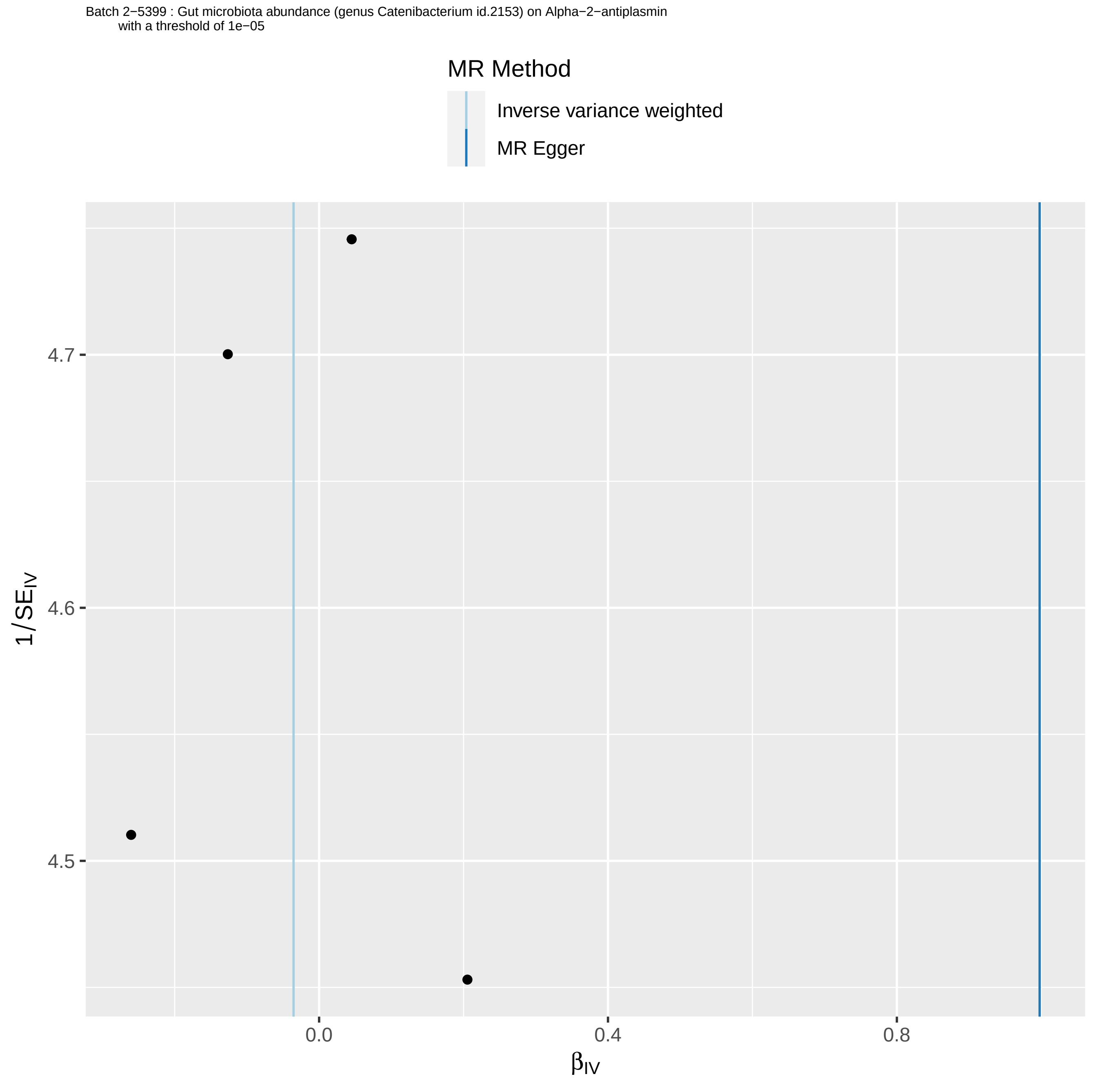
Batch 2−5399 : Gut microbiota abundance (genus Catenibacterium id.2153) on Alpha−2−antiplasmin
with a threshold of 1e−05
MR Method
Inverse variance weighted
MR Egger
4.7
4.6
4.5
1/SE_IV
0.0
0.4
0.8
β_IV

Batch 2−5400 : Gut microbiota abundance (genus Parasutterella id.2892) on Alpha−2−antiplasmin
with a threshold of 1e−05

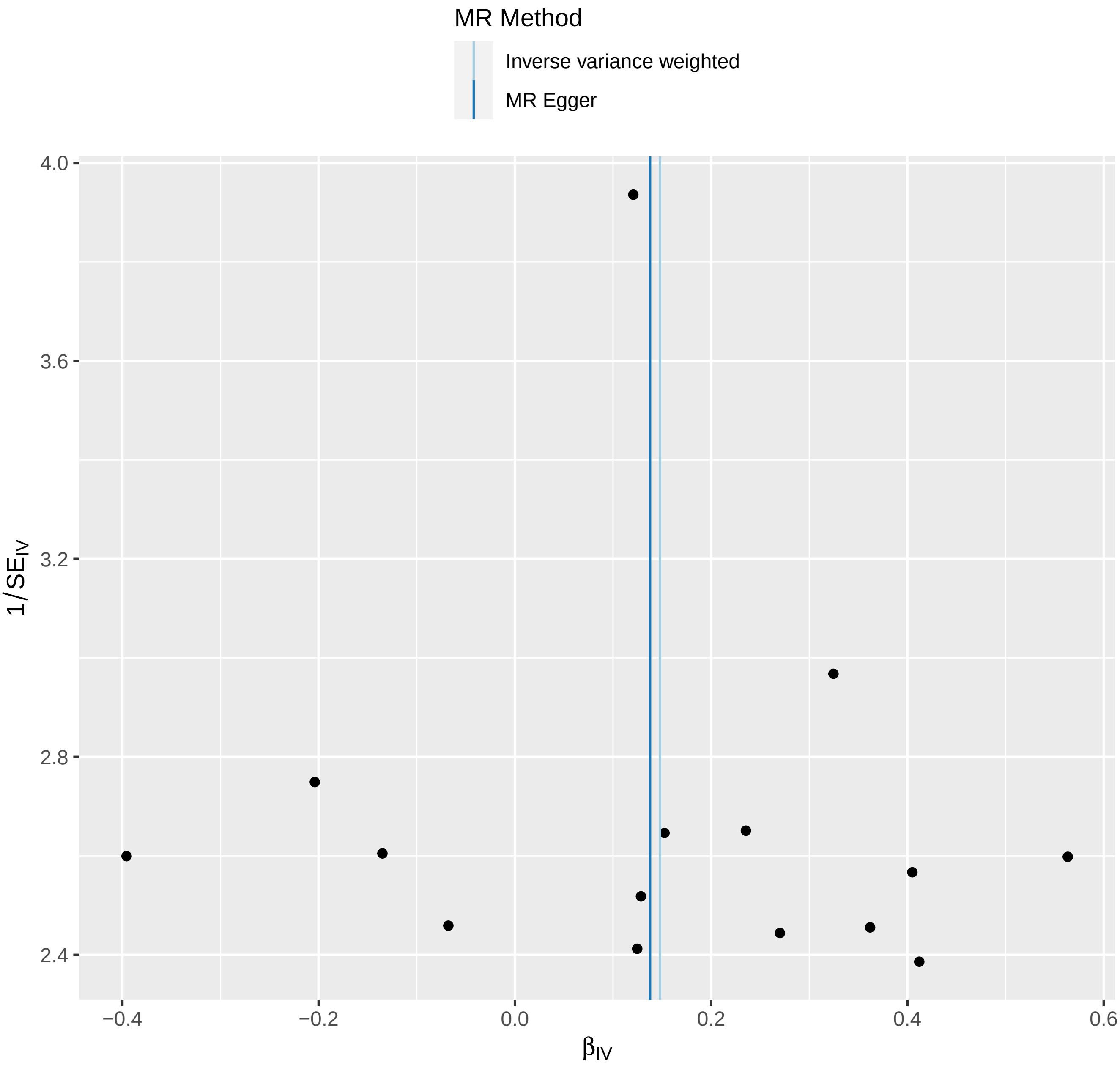

Batch 2−5401 : Gut microbiota abundance (genus Catenibacterium id.2153) on Plasma protease C1 inhibitor
with a threshold of 1e−05

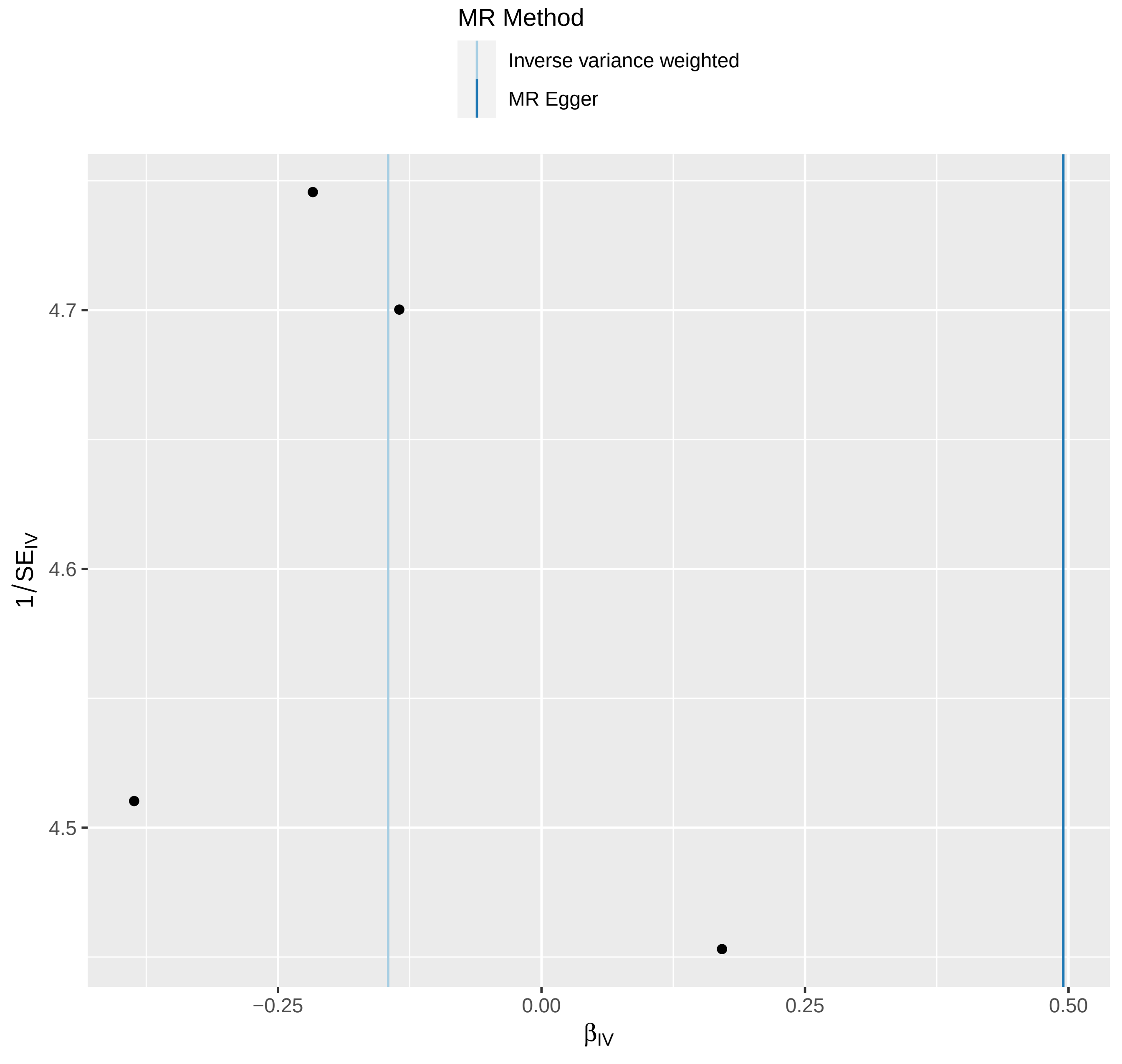

Batch 2−5402 : Gut microbiota abundance (genus Parasutterella id.2892) on Plasma protease C1 inhibitor
with a threshold of 1e−05

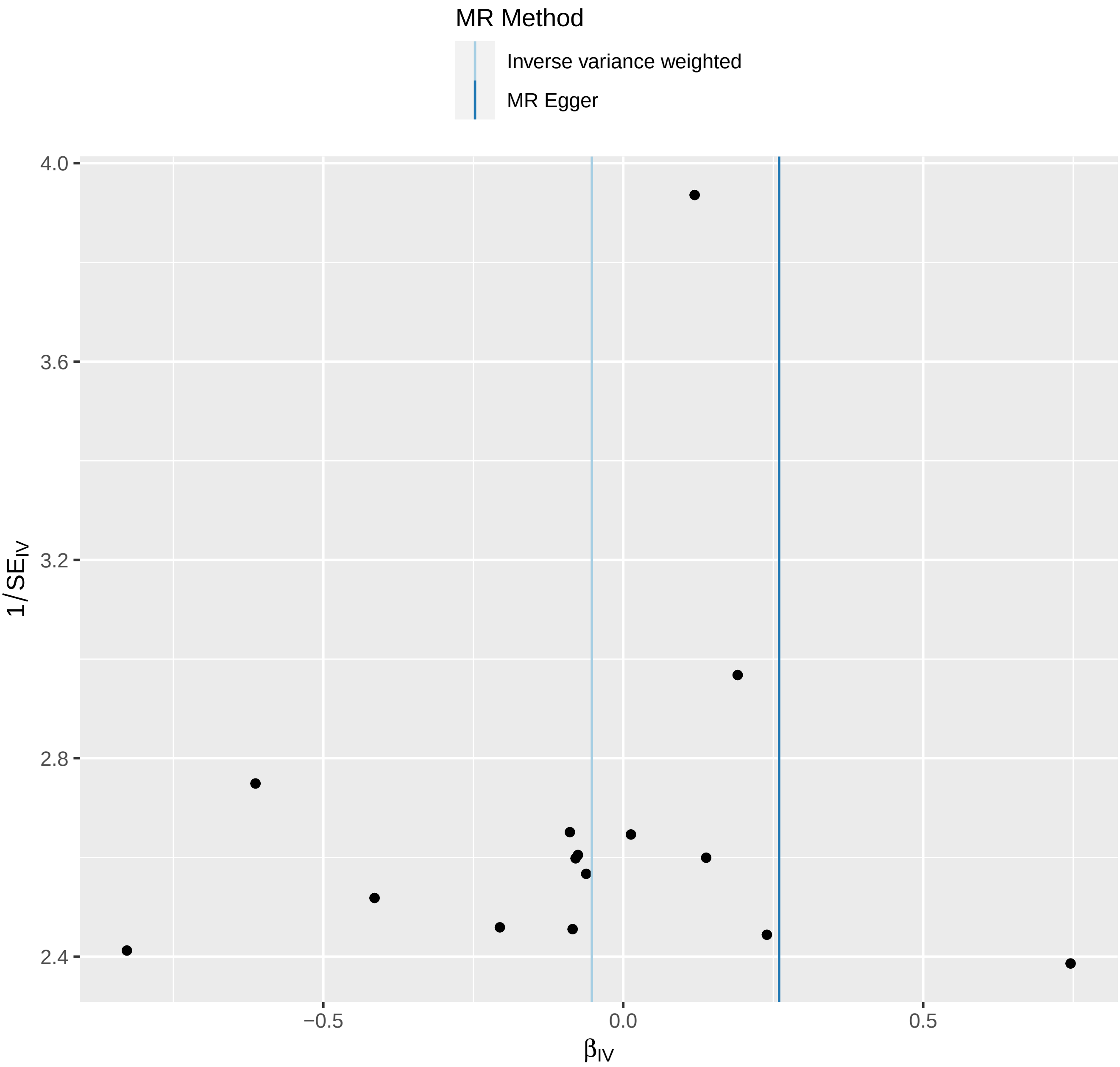

Batch 2−5403 : Gut microbiota abundance (genus Catenibacterium id.2153) on Serpin I2
with a threshold of 1e−05

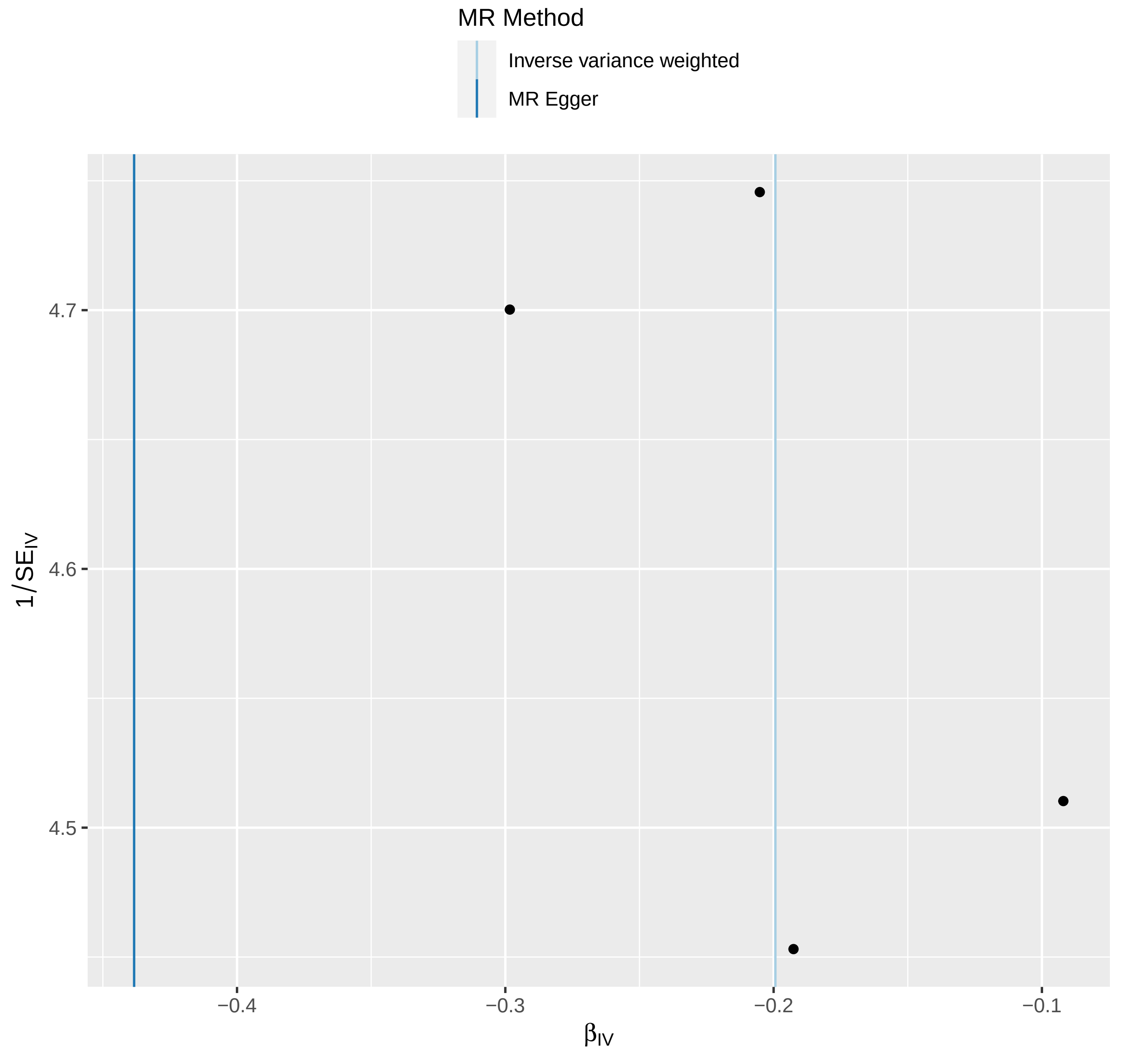

Batch 2−5404 : Gut microbiota abundance (genus Parasutterella id.2892) on Serpin I2
with a threshold of 1e−05

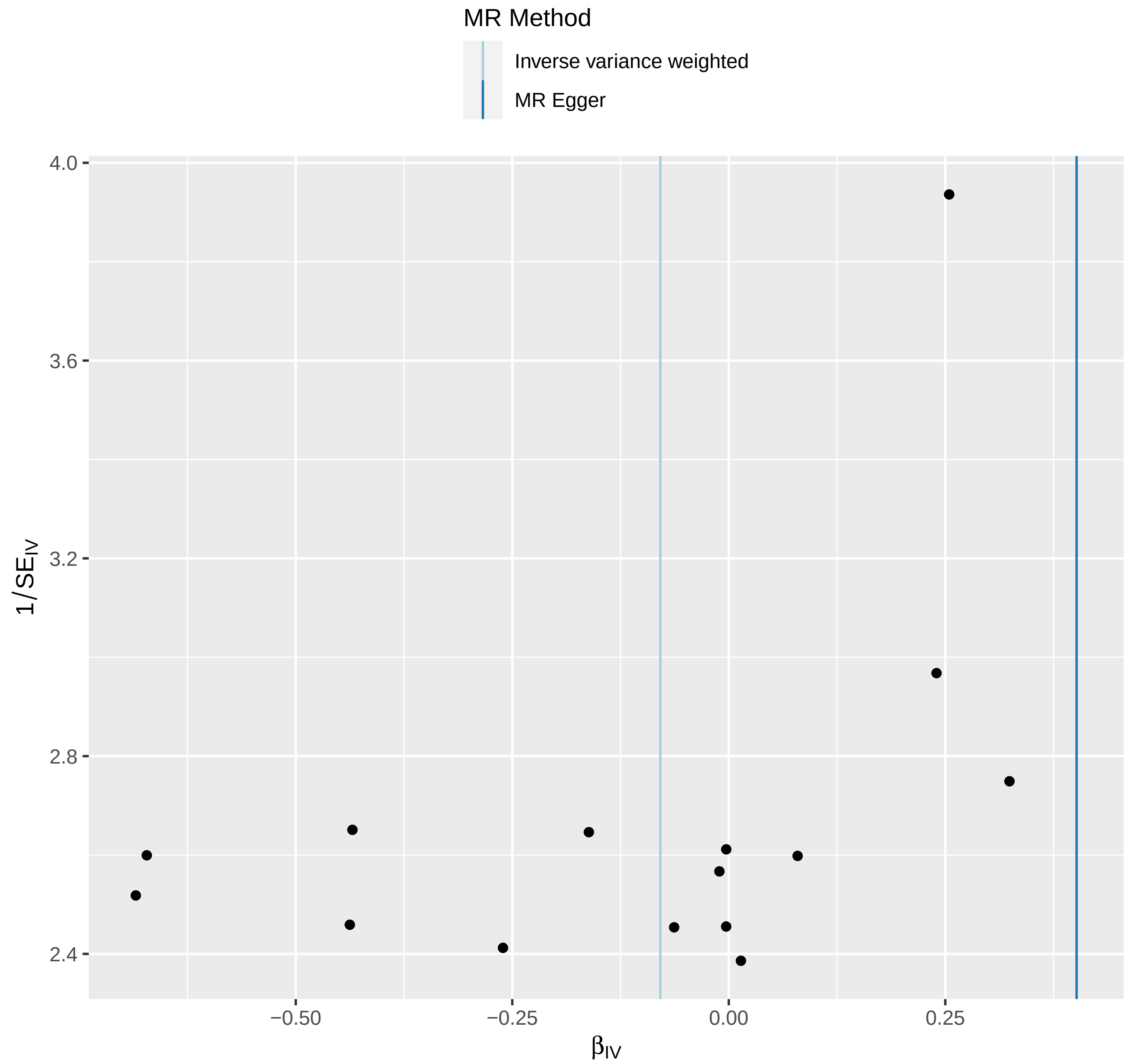

Batch 2−5405 : Gut microbiota abundance (genus Catenibacterium id.2153) on Histone−lysine N−methyltransferase SETD2
with a threshold of 1e−05

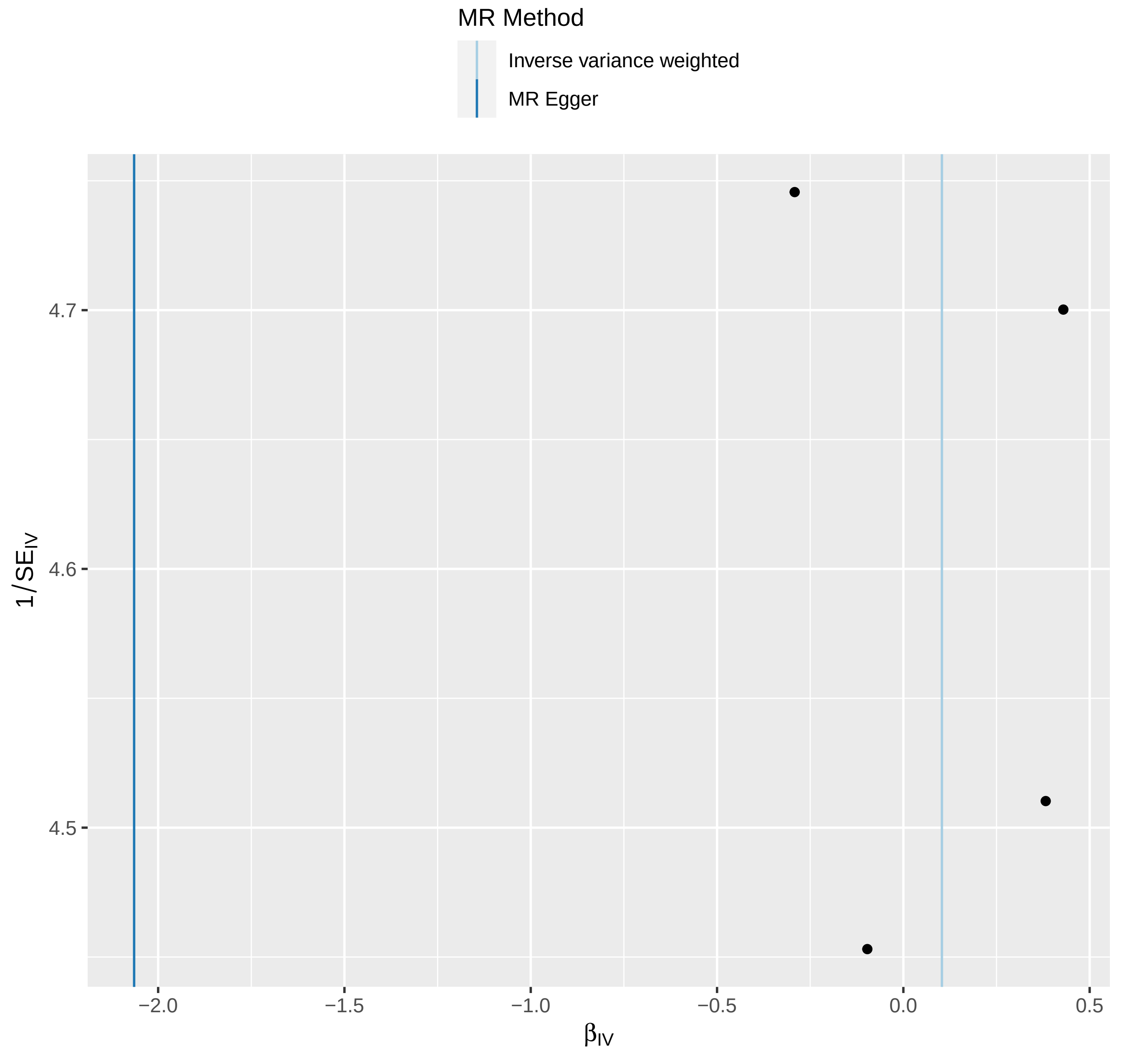

Batch 2−5406 : Gut microbiota abundance (genus Parasutterella id.2892) on Histone−lysine N−methyltransferase SETD2
with a threshold of 1e−05

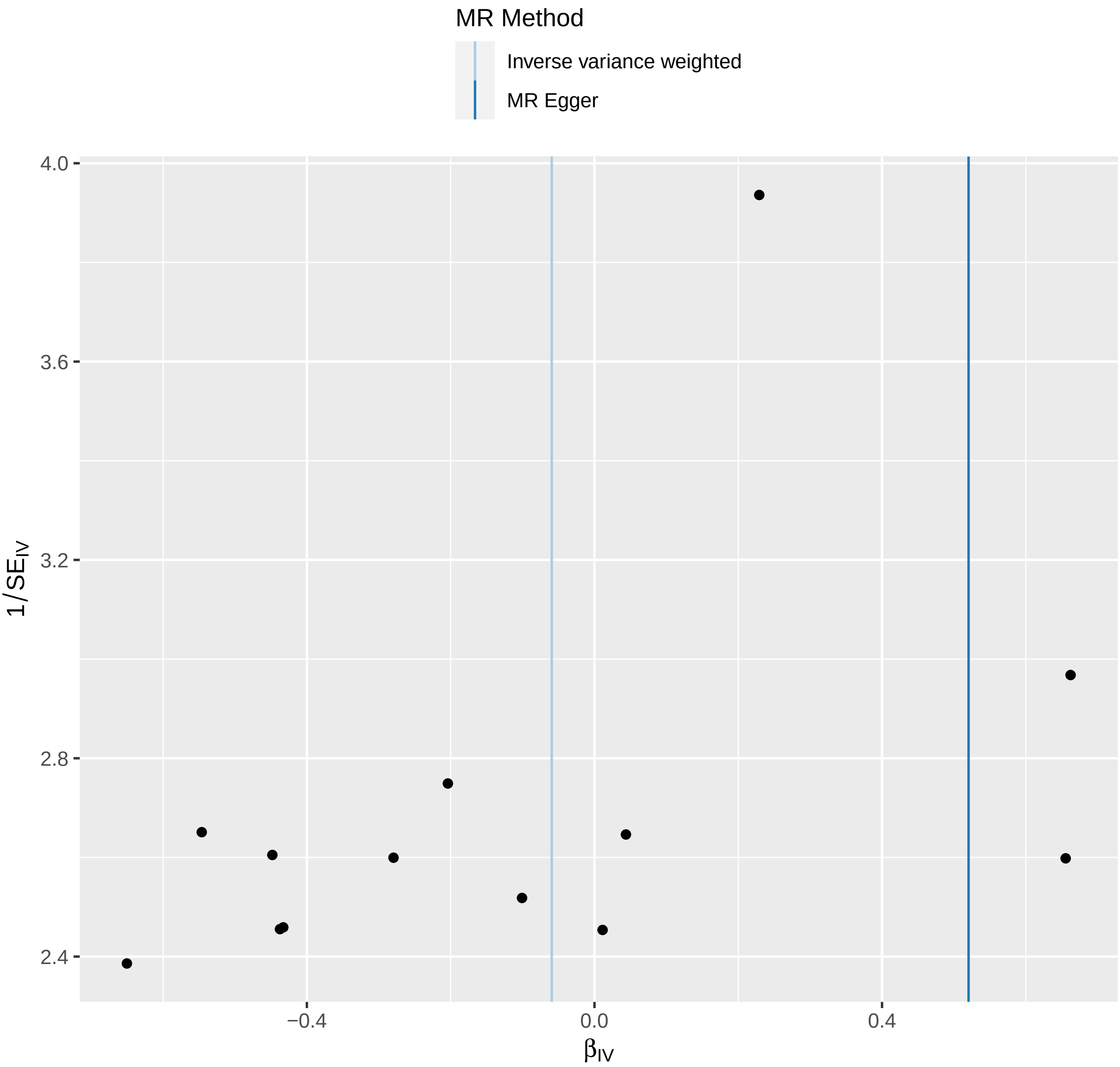

Batch 2−5407 : Gut microbiota abundance (genus Catenibacterium id.2153) on Histone−lysine N−methyltransferase SETMAR
with a threshold of 1e−05

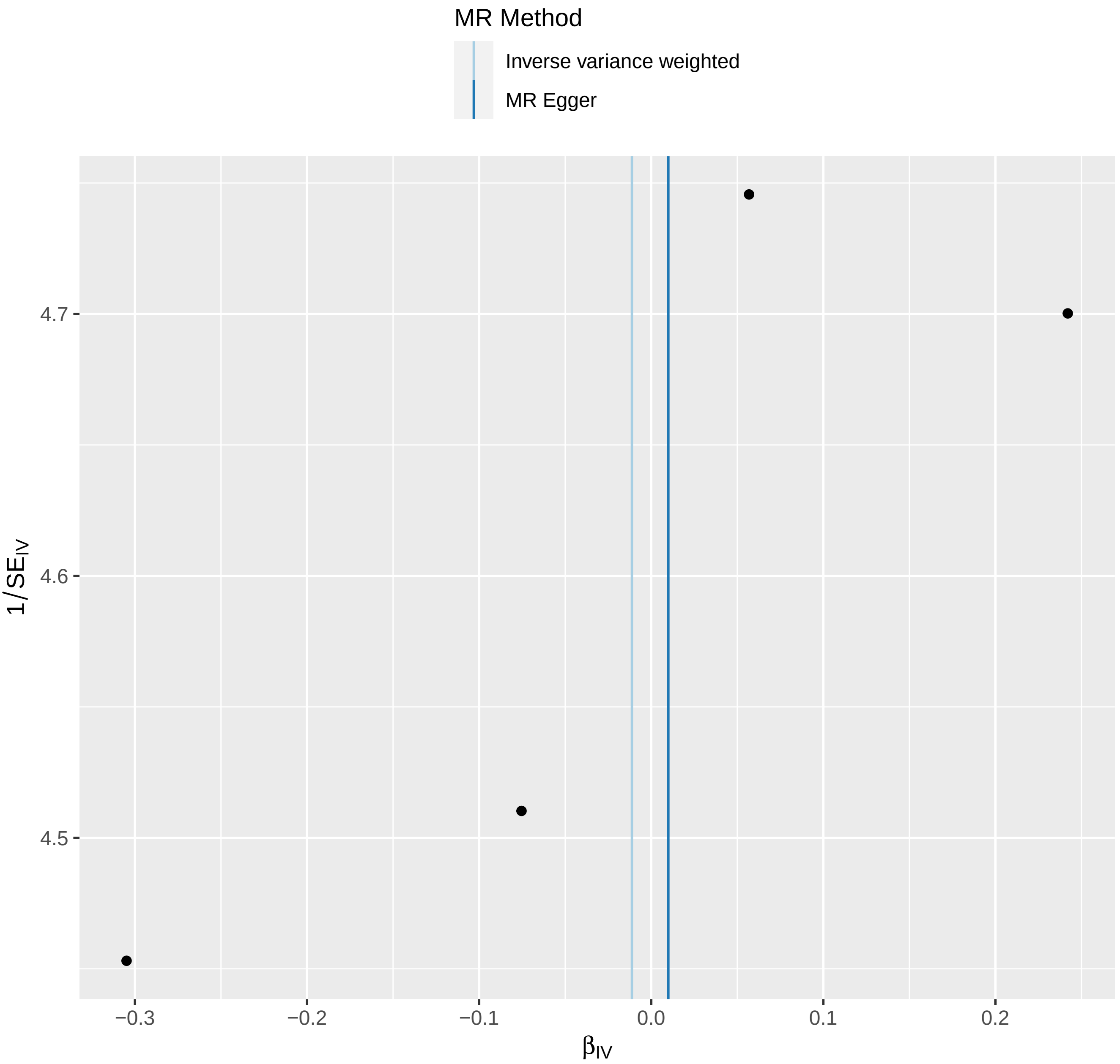

Batch 2−5408 : Gut microbiota abundance (genus Parasutterella id.2892) on Histone−lysine N−methyltransferase SETMAR
with a threshold of 1e−05

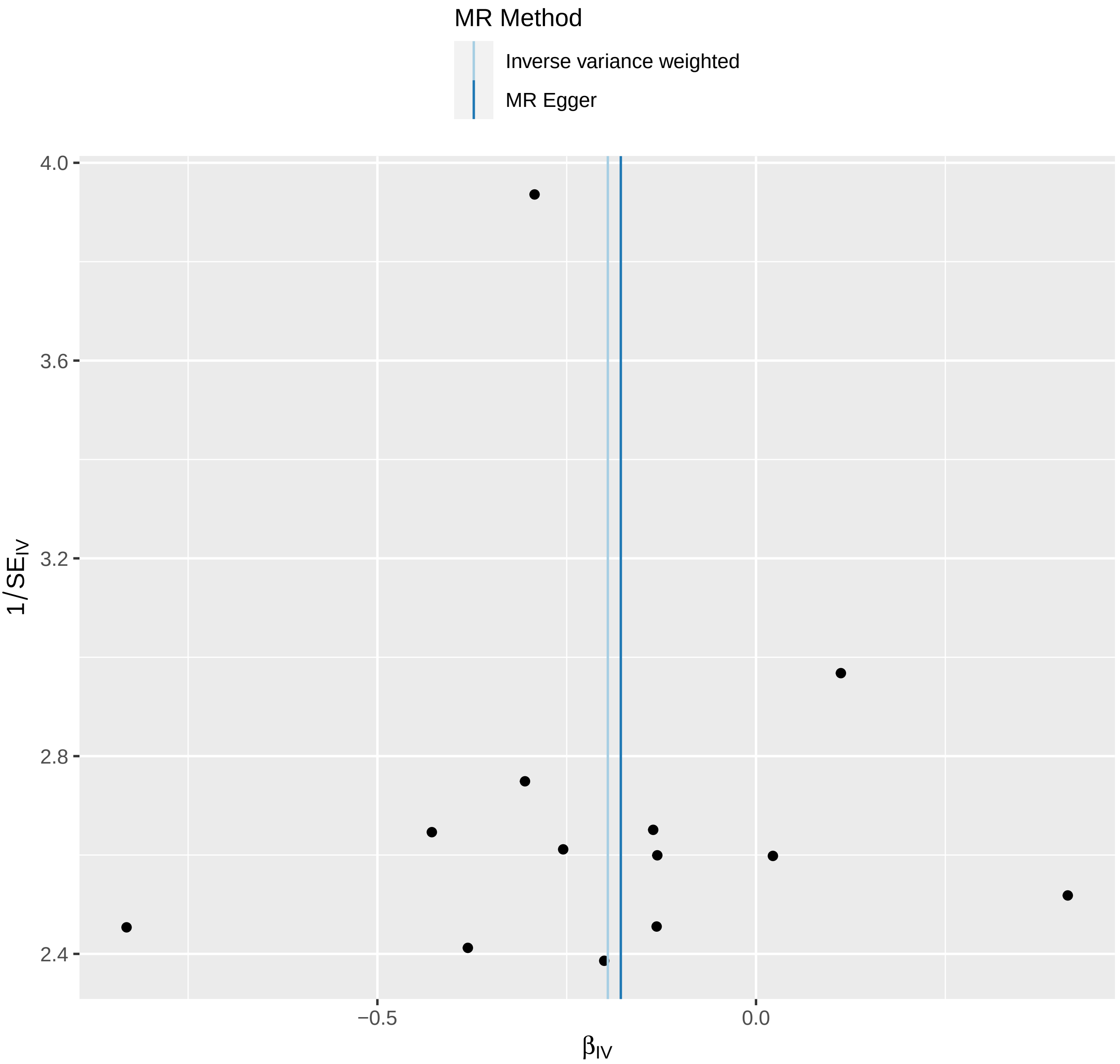

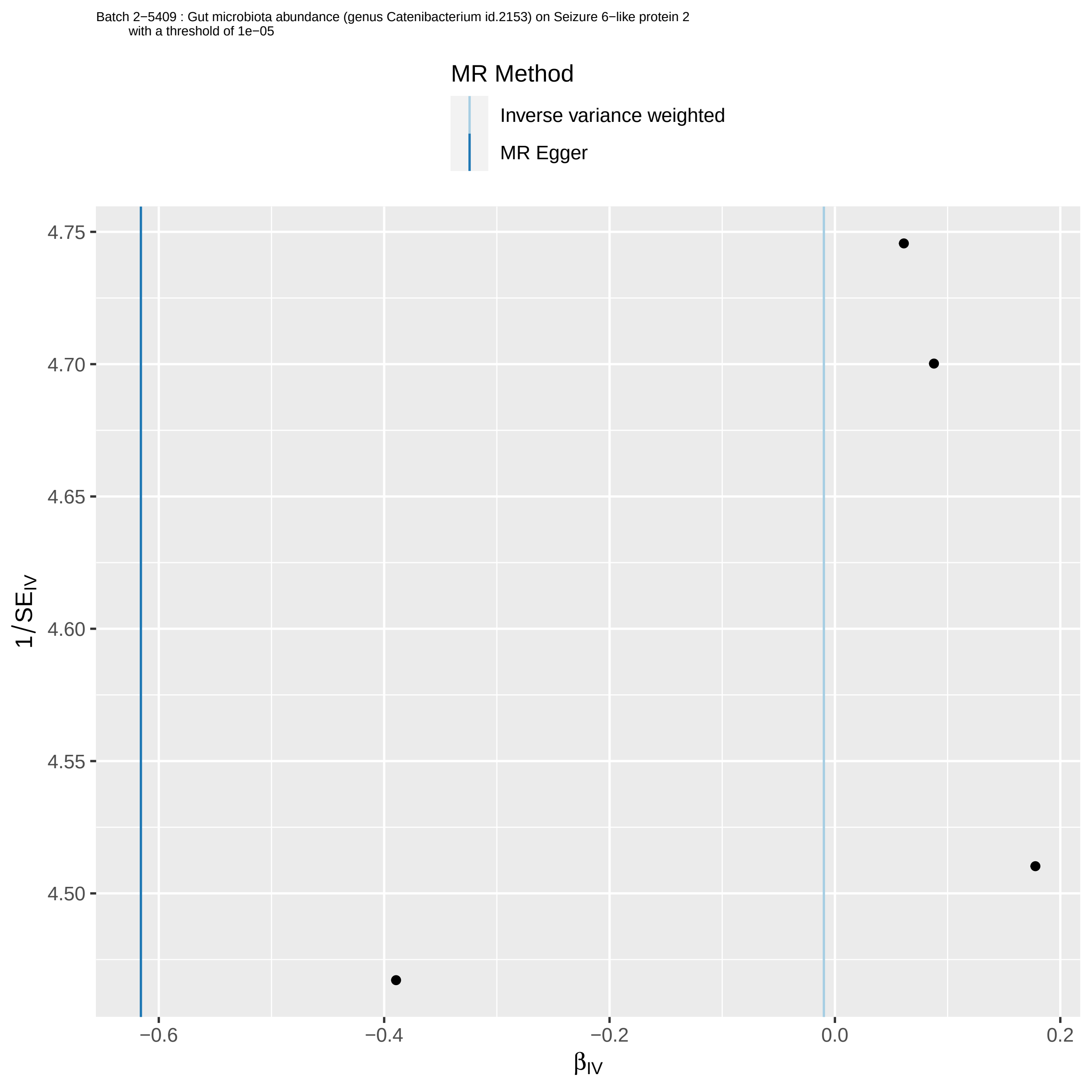
Batch 2−5409 : Gut microbiota abundance (genus Catenibacterium id.2153) on Seizure 6−like protein 2
with a threshold of 1e−05
MR Method
Inverse variance weighted
MR Egger
4.75
4.70
4.65
4.60
4.55
4.50
1/SE_IV
−0.6
−0.4
−0.2
0.0
0.2
β_IV

Batch 2−5410 : Gut microbiota abundance (genus Parasutterella id.2892) on Seizure 6−like protein 2
with a threshold of 1e−05

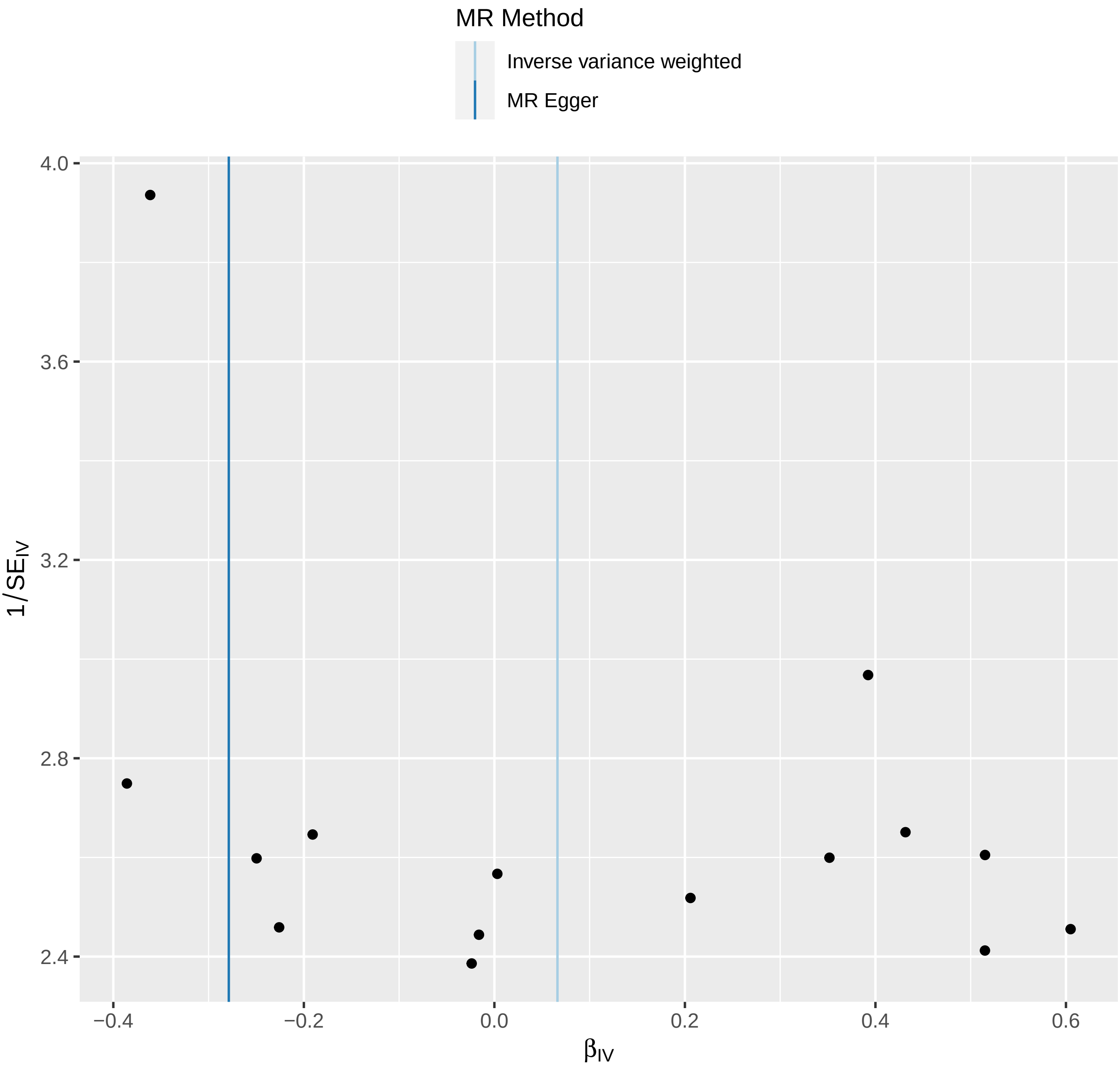

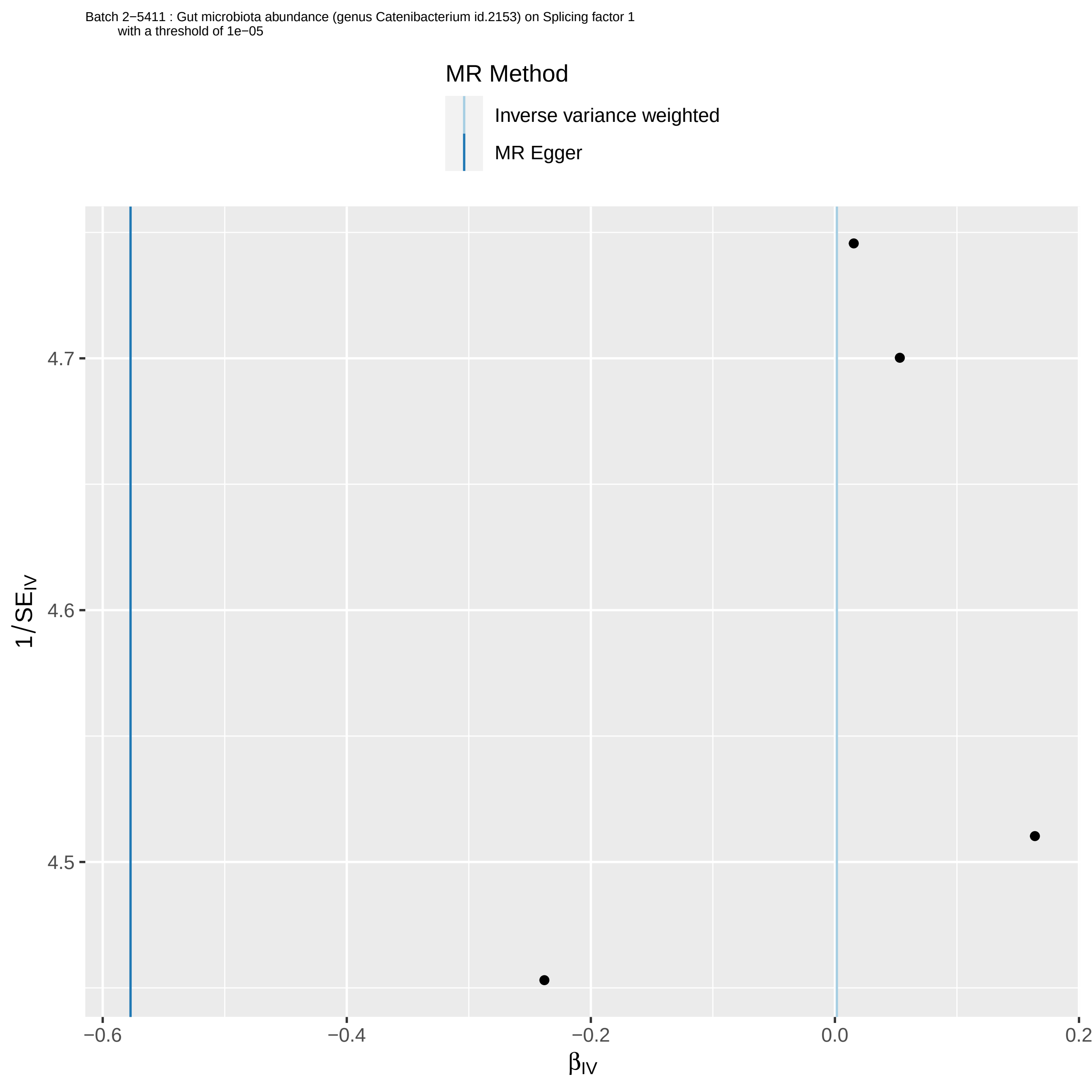
Batch 2−5411 : Gut microbiota abundance (genus Catenibacterium id.2153) on Splicing factor 1
with a threshold of 1e−05
MR Method
Inverse variance weighted
MR Egger
4.7
4.6
4.5
1/SE_IV
−0.6
−0.4
−0.2
0.0
0.2
β_IV

Batch 2−5412 : Gut microbiota abundance (genus Parasutterella id.2892) on Splicing factor 1
with a threshold of 1e−05

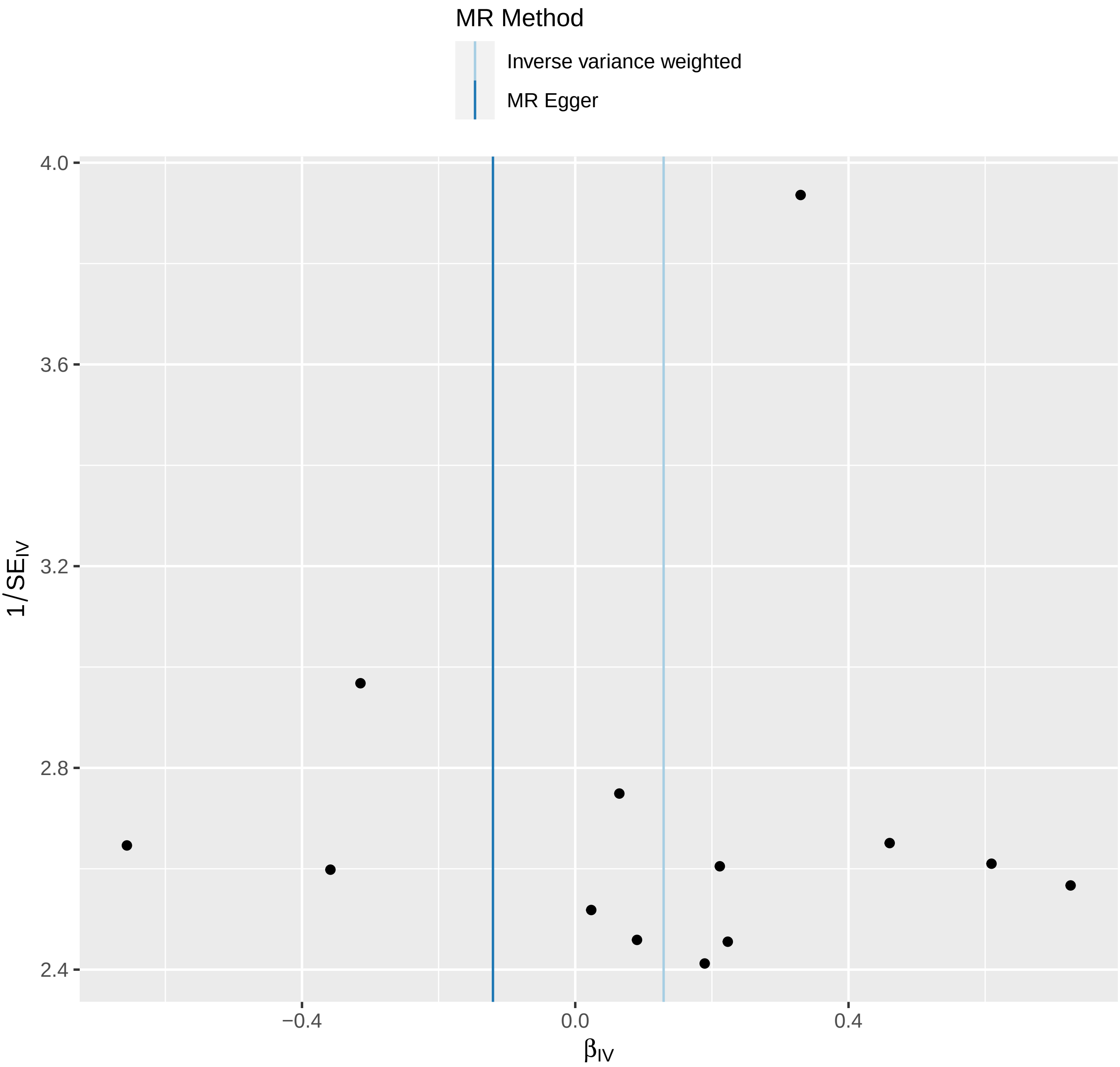

Batch 2−5413 : Gut microbiota abundance (genus Catenibacterium id.2153) on Splicing factor 3B subunit 4
with a threshold of 1e−05

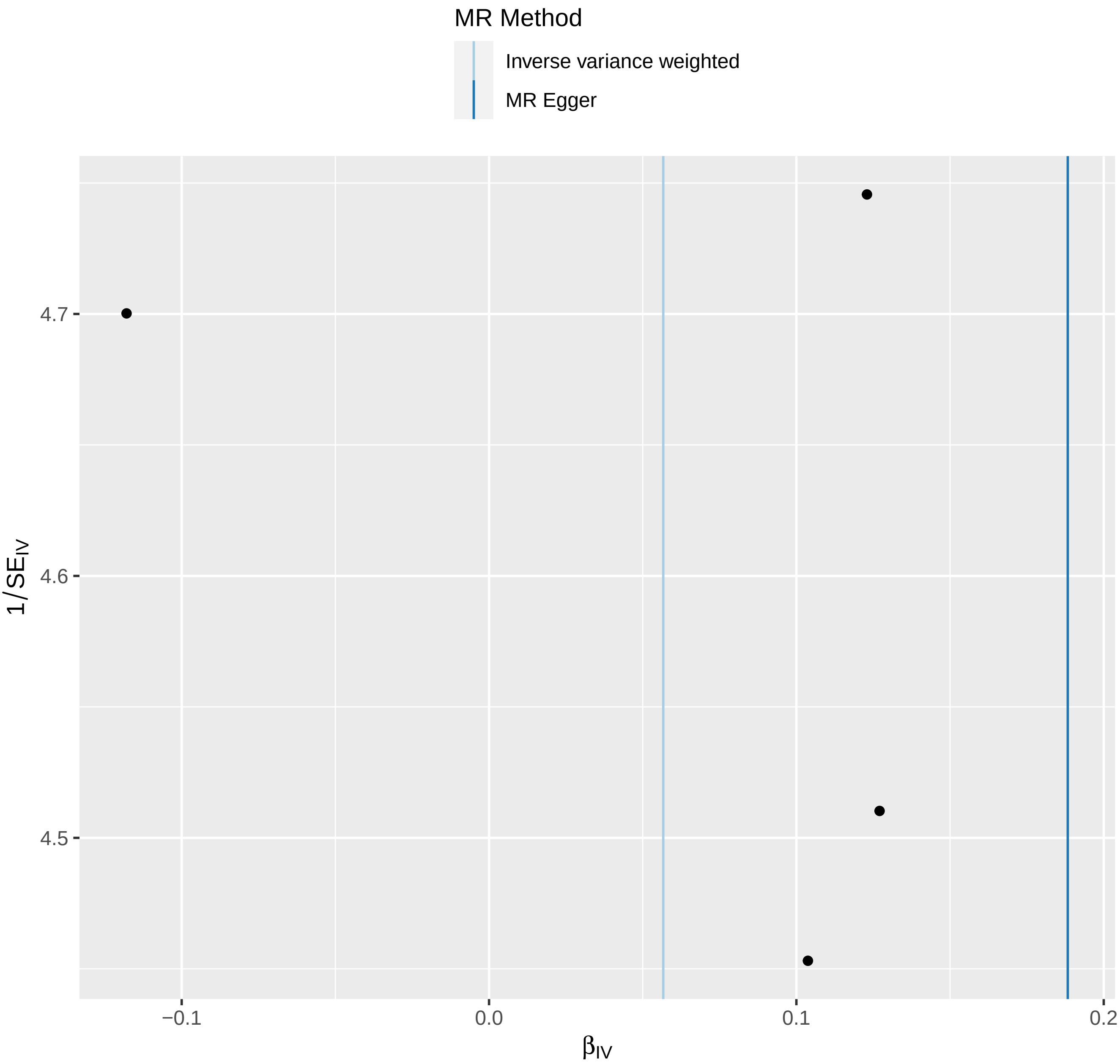

Batch 2−5414 : Gut microbiota abundance (genus Parasutterella id.2892) on Splicing factor 3B subunit 4
with a threshold of 1e−05

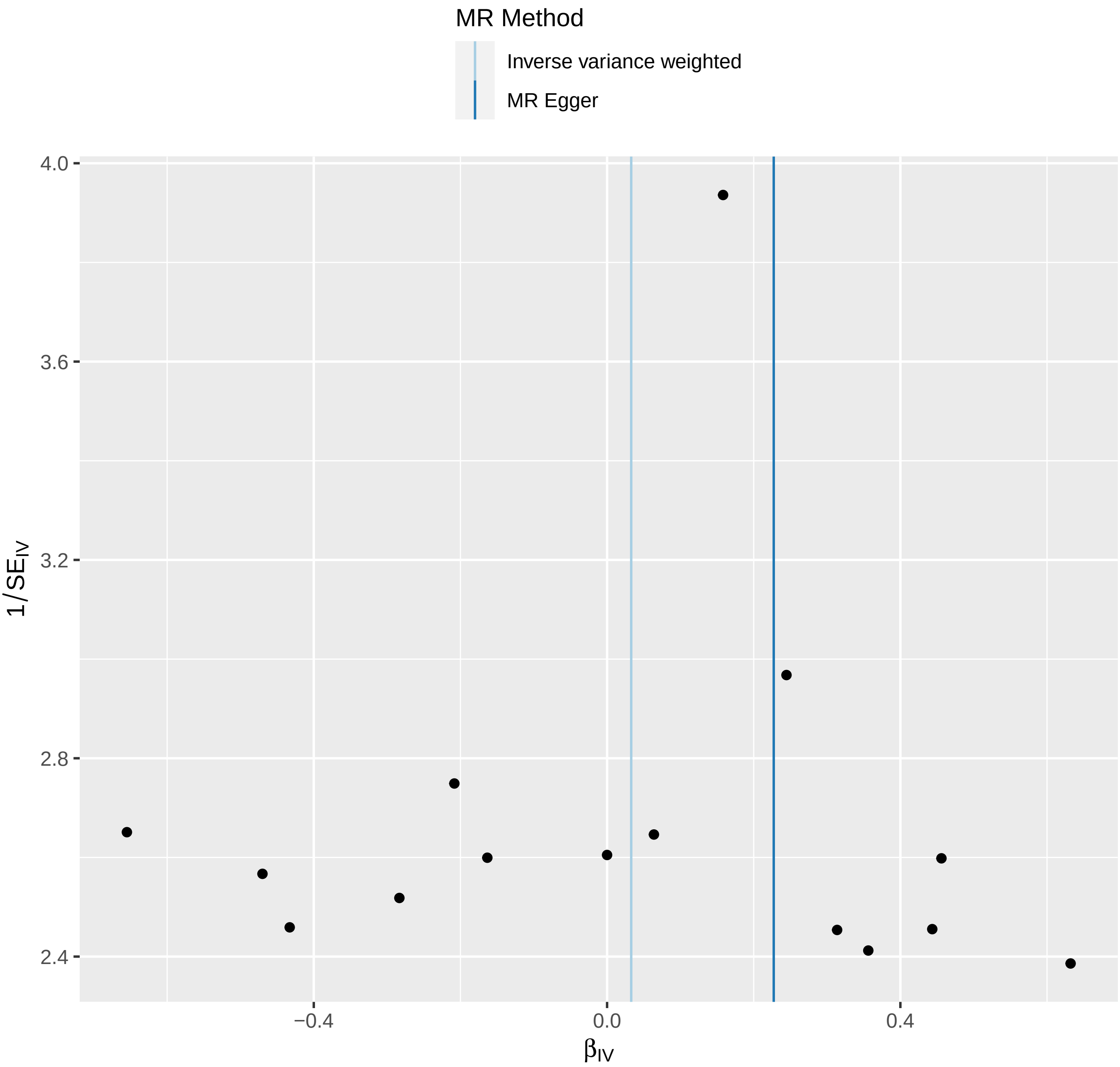

Batch 2−5415 : Gut microbiota abundance (genus Catenibacterium id.2153) on 14−3−3 protein sigma
with a threshold of 1e−05

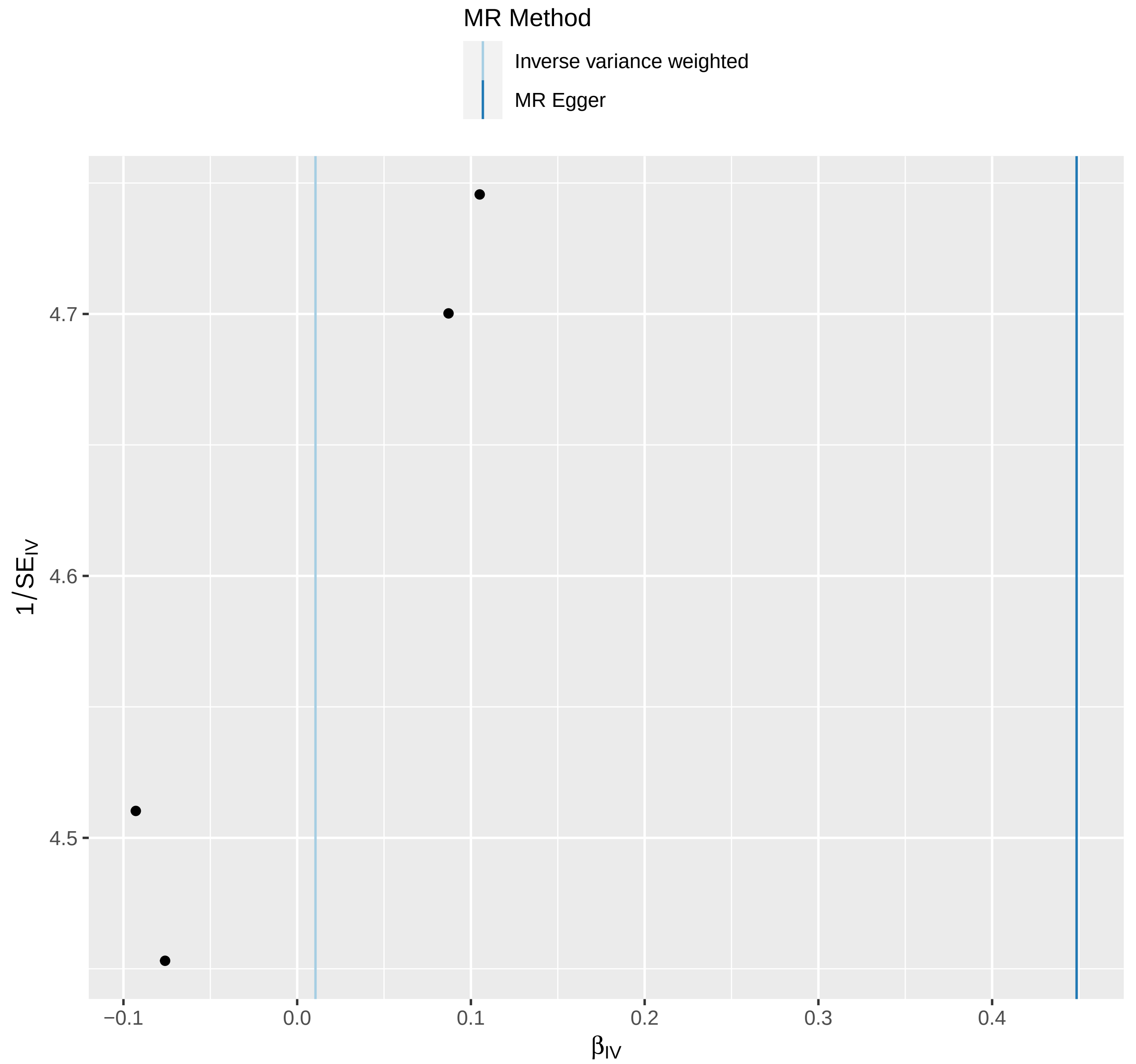

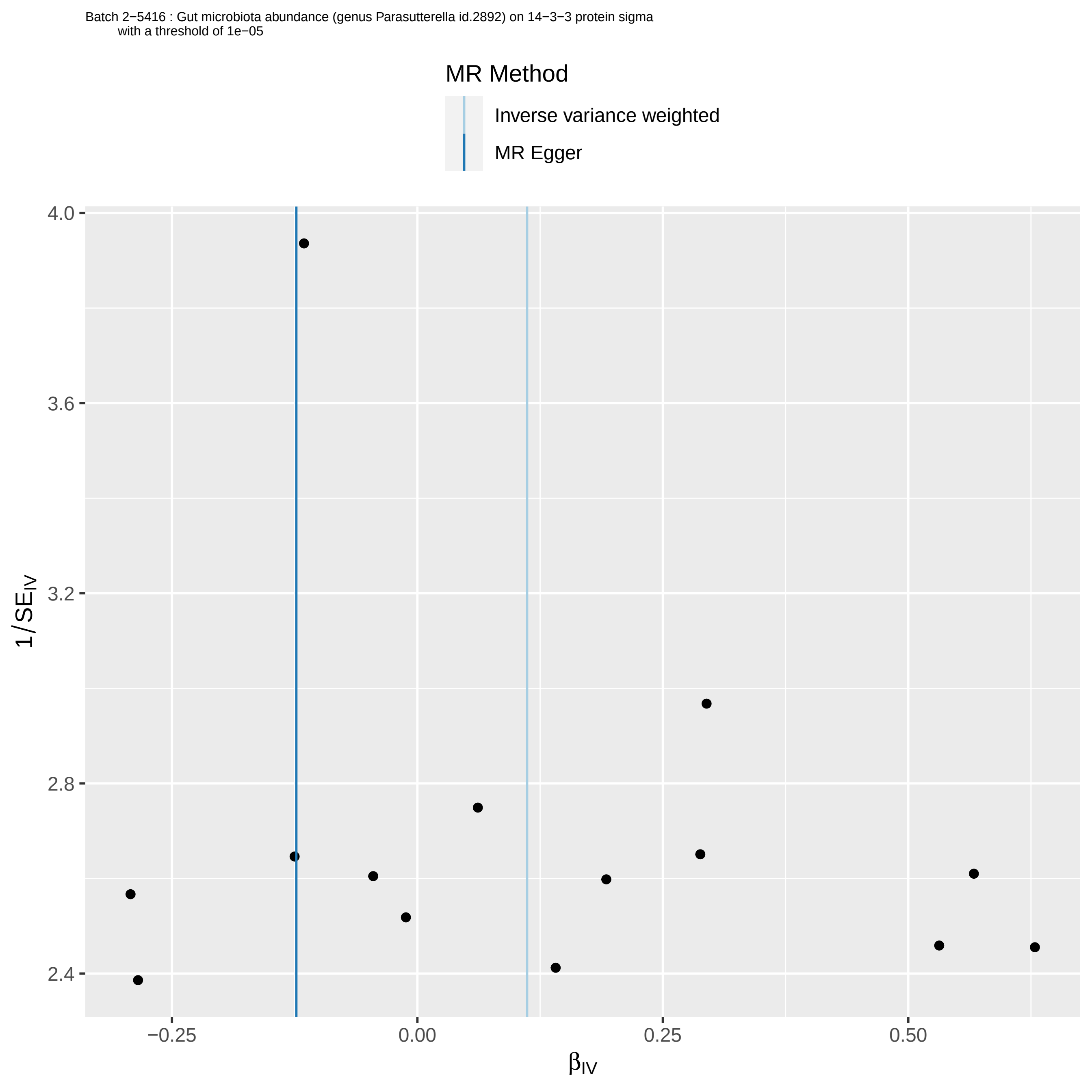

Batch 2−5416 : Gut microbiota abundance (genus Parasutterella id.2892) on 14−3−3 protein sigma
with a threshold of 1e−05
MR Method
Inverse variance weighted
MR Egger
4.0
3.6
3.2
2.8
2.4
1/SE_IV
−0.25
0.00
0.25
0.50
β_IV

Batch 2−5417 : Gut microbiota abundance (genus Catenibacterium id.2153) on Secreted frizzled−related protein 1
with a threshold of 1e−05

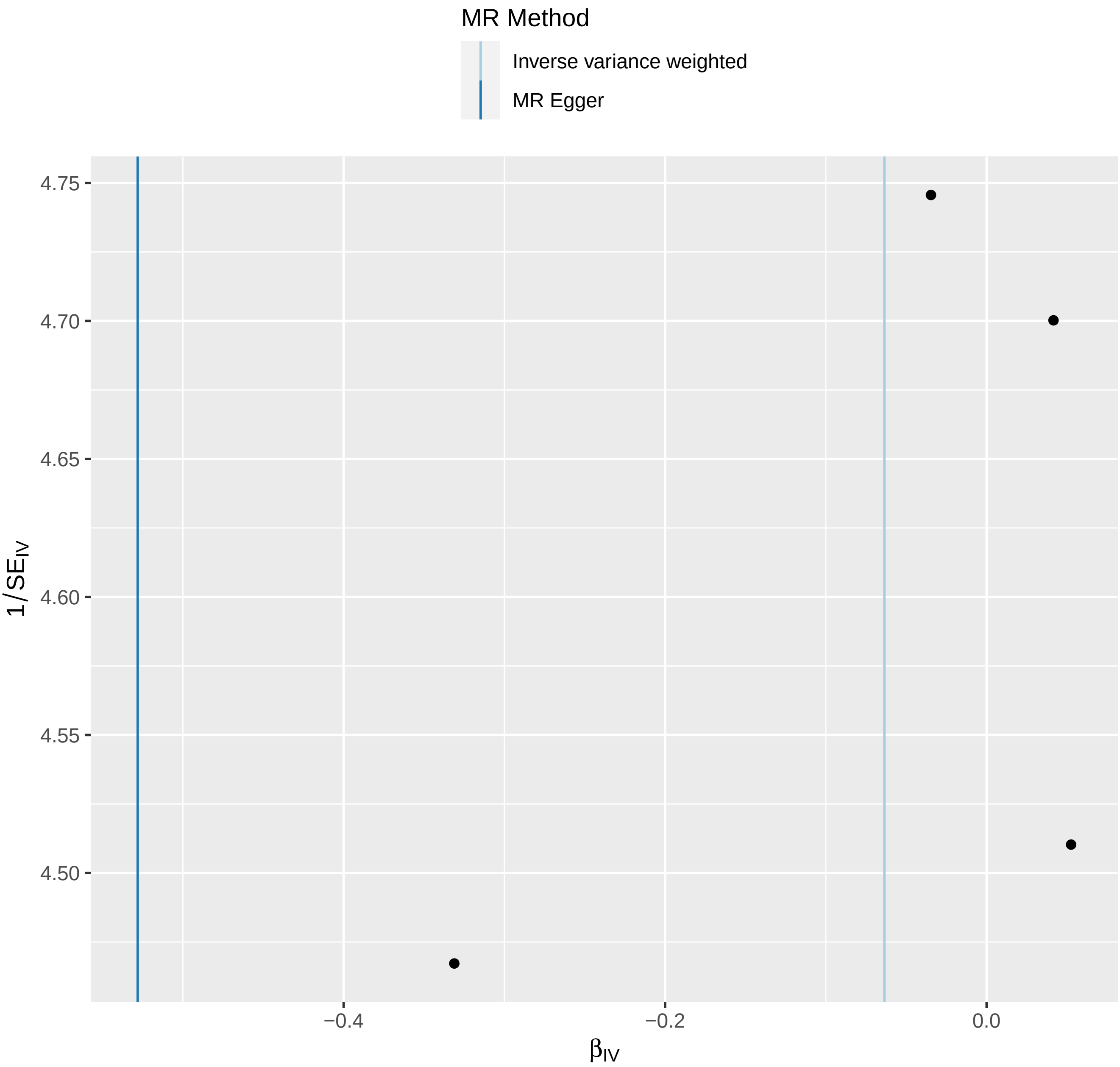

Batch 2−5418 : Gut microbiota abundance (genus Parasutterella id.2892) on Secreted frizzled−related protein 1
with a threshold of 1e−05

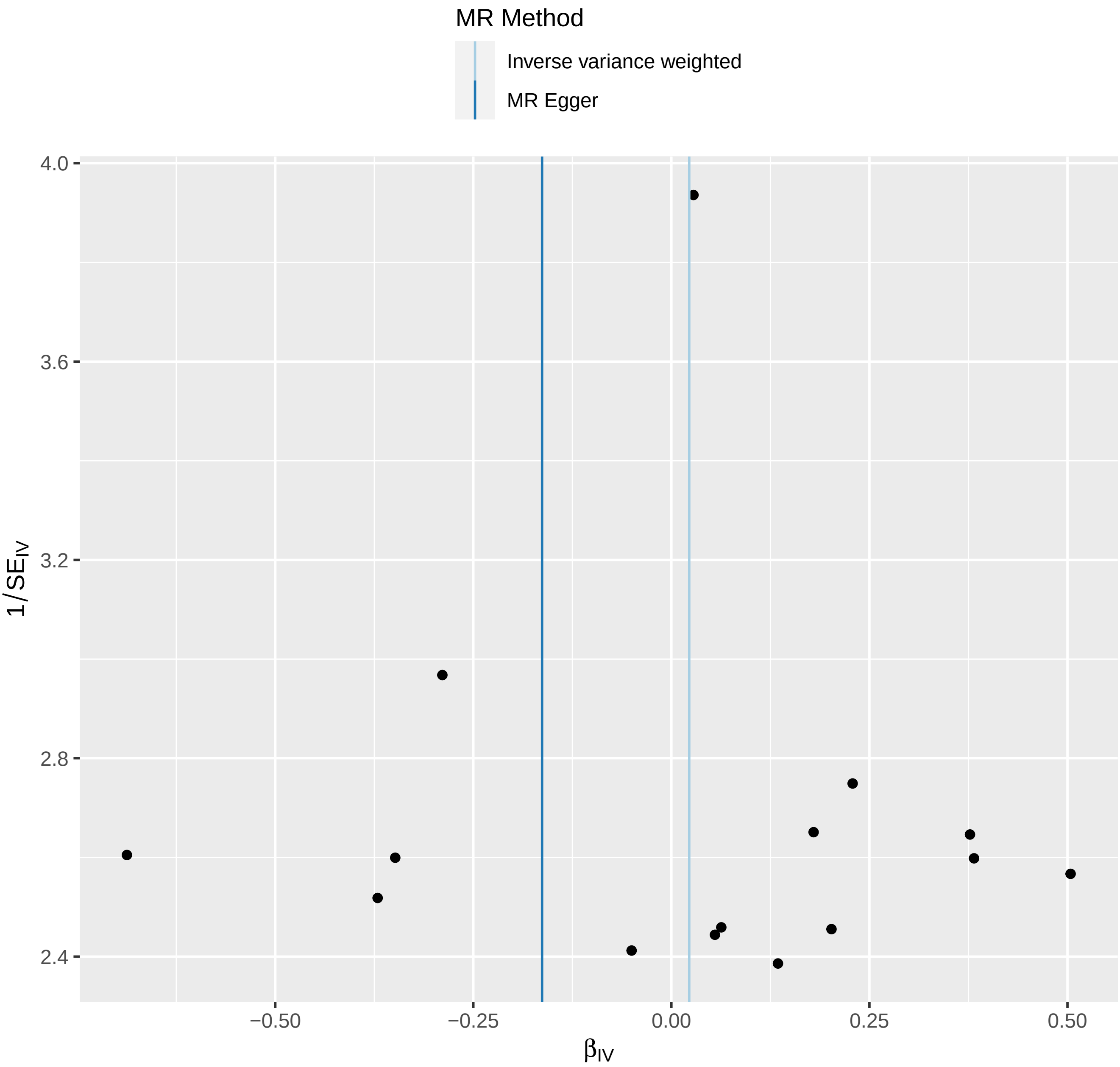

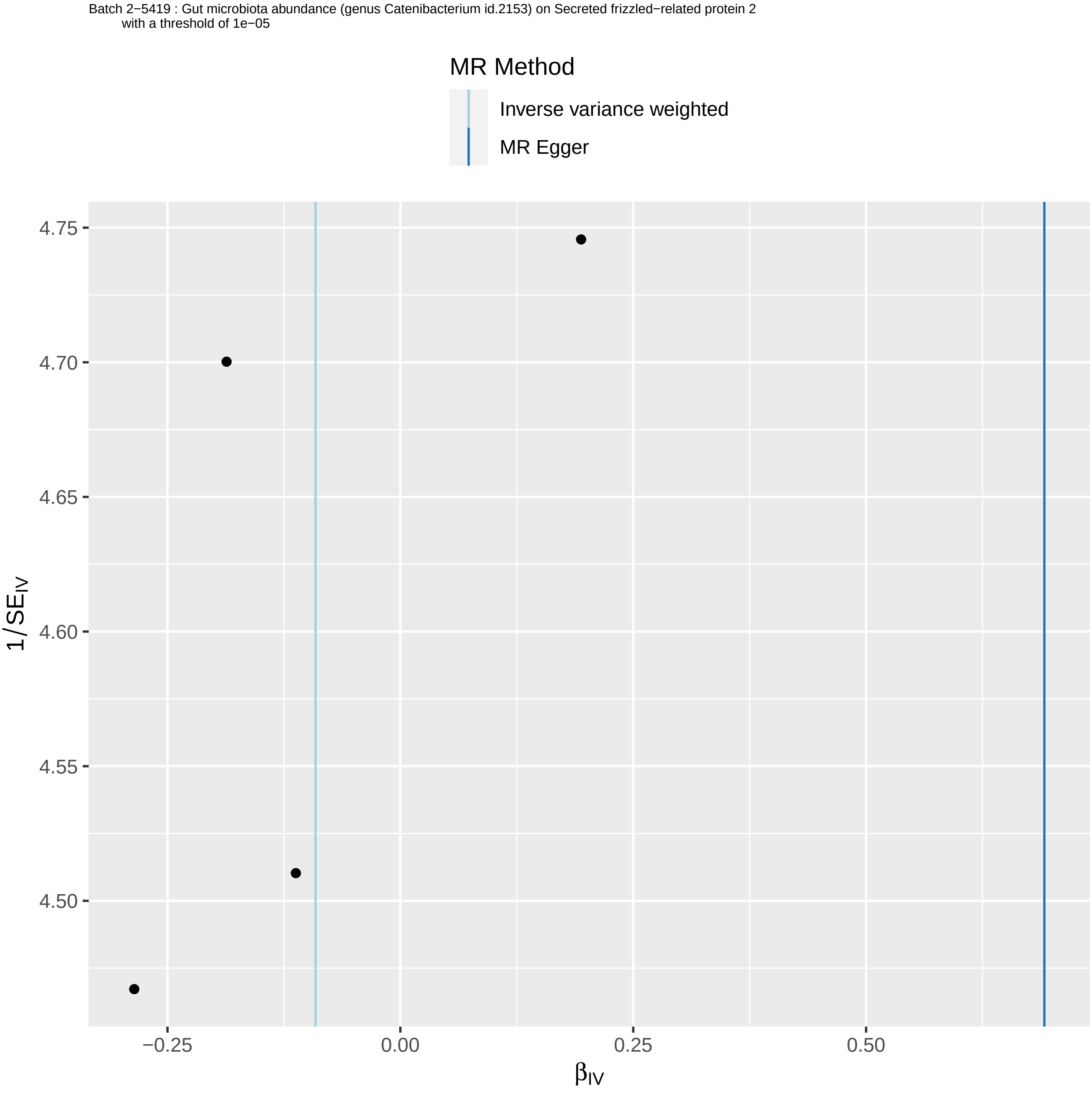
Batch 2−5419 : Gut microbiota abundance (genus Catenibacterium id.2153) on Secreted frizzled−related protein 2
with a threshold of 1e−05
MR Method
Inverse variance weighted
MR Egger
4.75
4.70
4.65
4.60
4.55
4.50
1/SE_IV
−0.25
0.00
0.25
0.50
β_IV

Batch 2−5420 : Gut microbiota abundance (genus Parasutterella id.2892) on Secreted frizzled−related protein 2 with a threshold of 1e−05

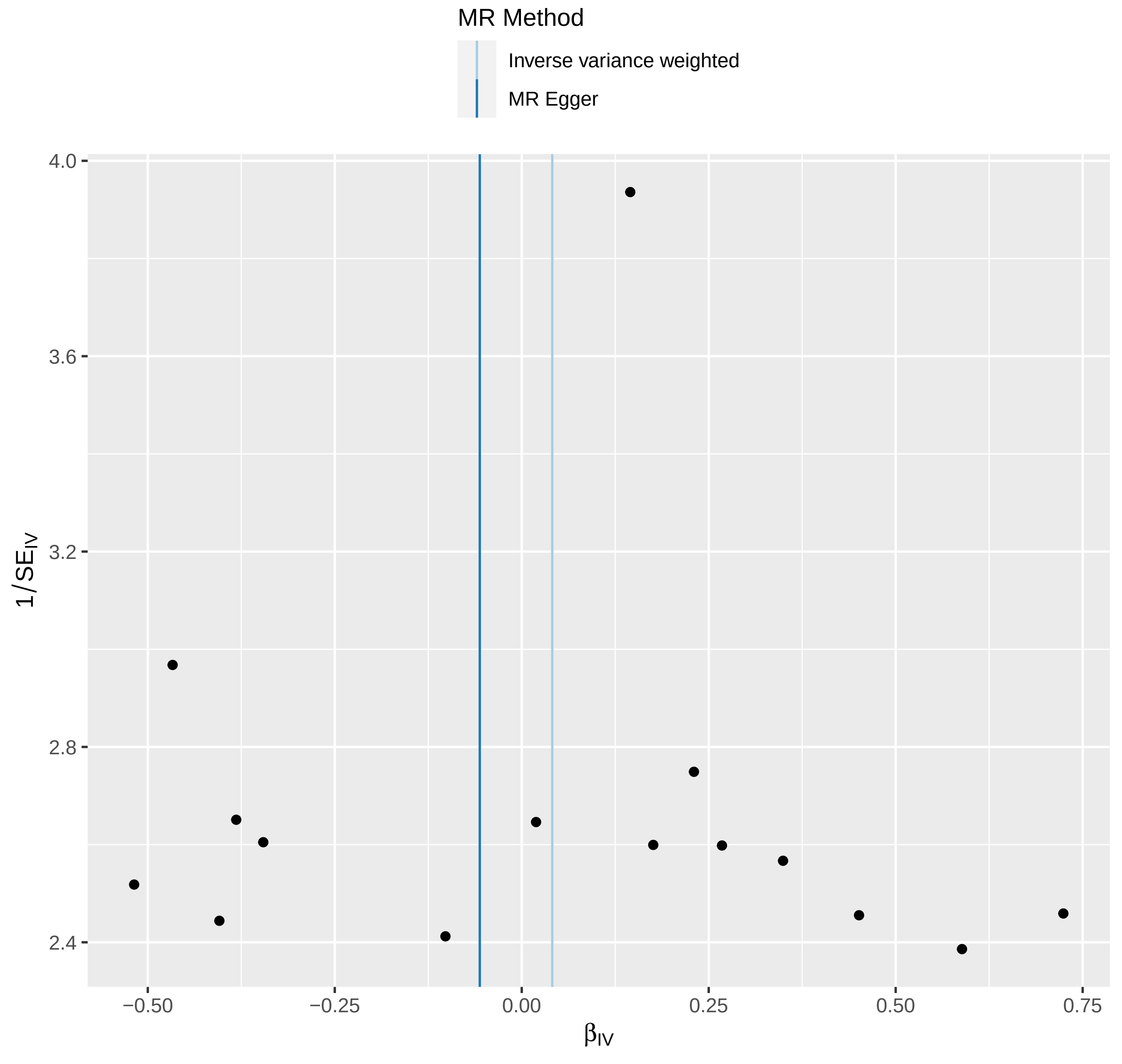

Batch 2−5421 : Gut microbiota abundance (genus Catenibacterium id.2153) on Pulmonary surfactant−associated protein C
with a threshold of 1e−05

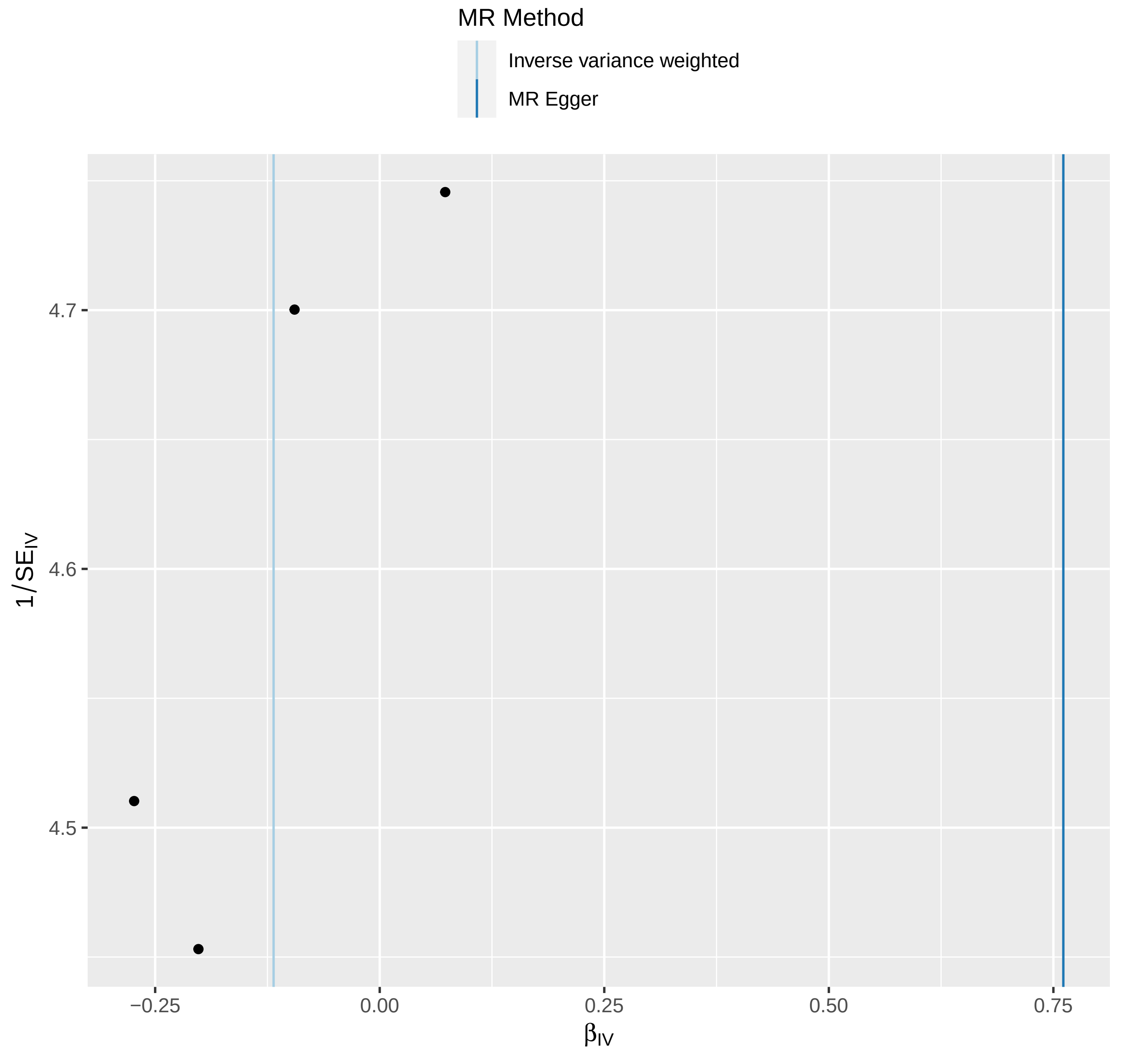

Batch 2−5422 : Gut microbiota abundance (genus Parasutterella id.2892) on Pulmonary surfactant−associated protein C
with a threshold of 1e−05

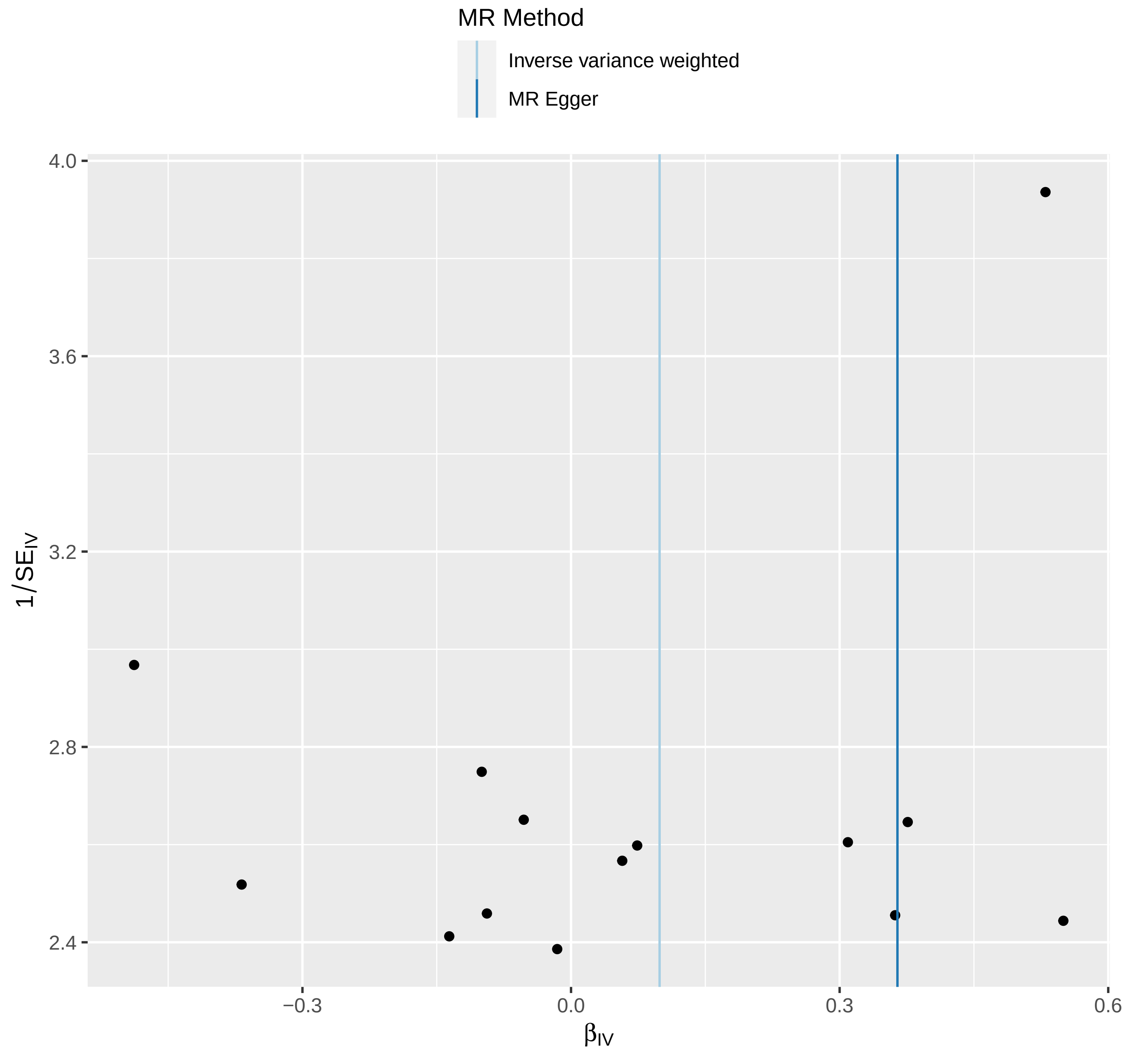

Batch 2−5423 : Gut microbiota abundance (genus Catenibacterium id.2153) on Pulmonary surfactant−associated protein D with a threshold of 1e−05

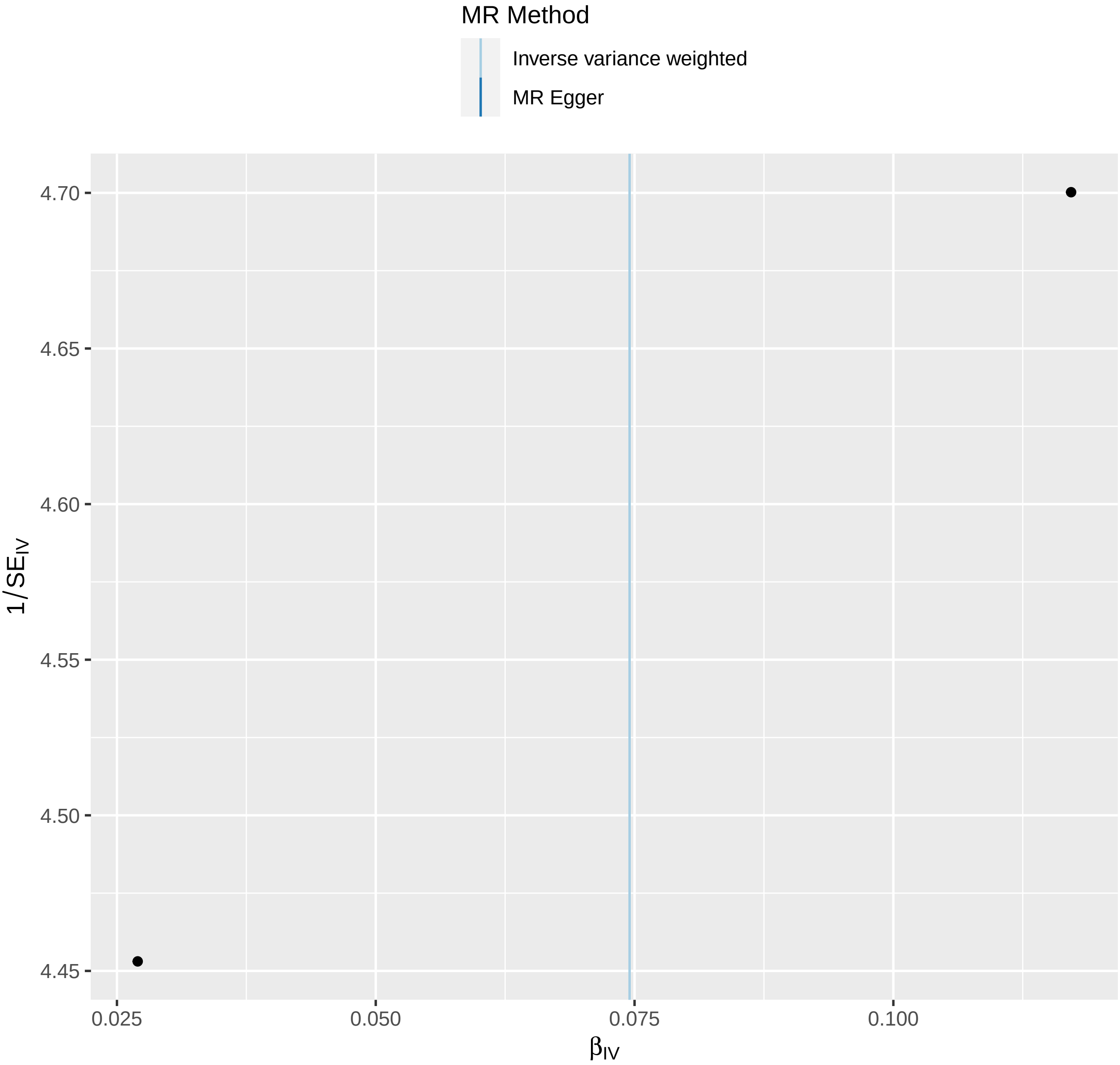

Batch 2−5424 : Gut microbiota abundance (genus Parasutterella id.2892) on Pulmonary surfactant−associated protein D
with a threshold of 1e−05

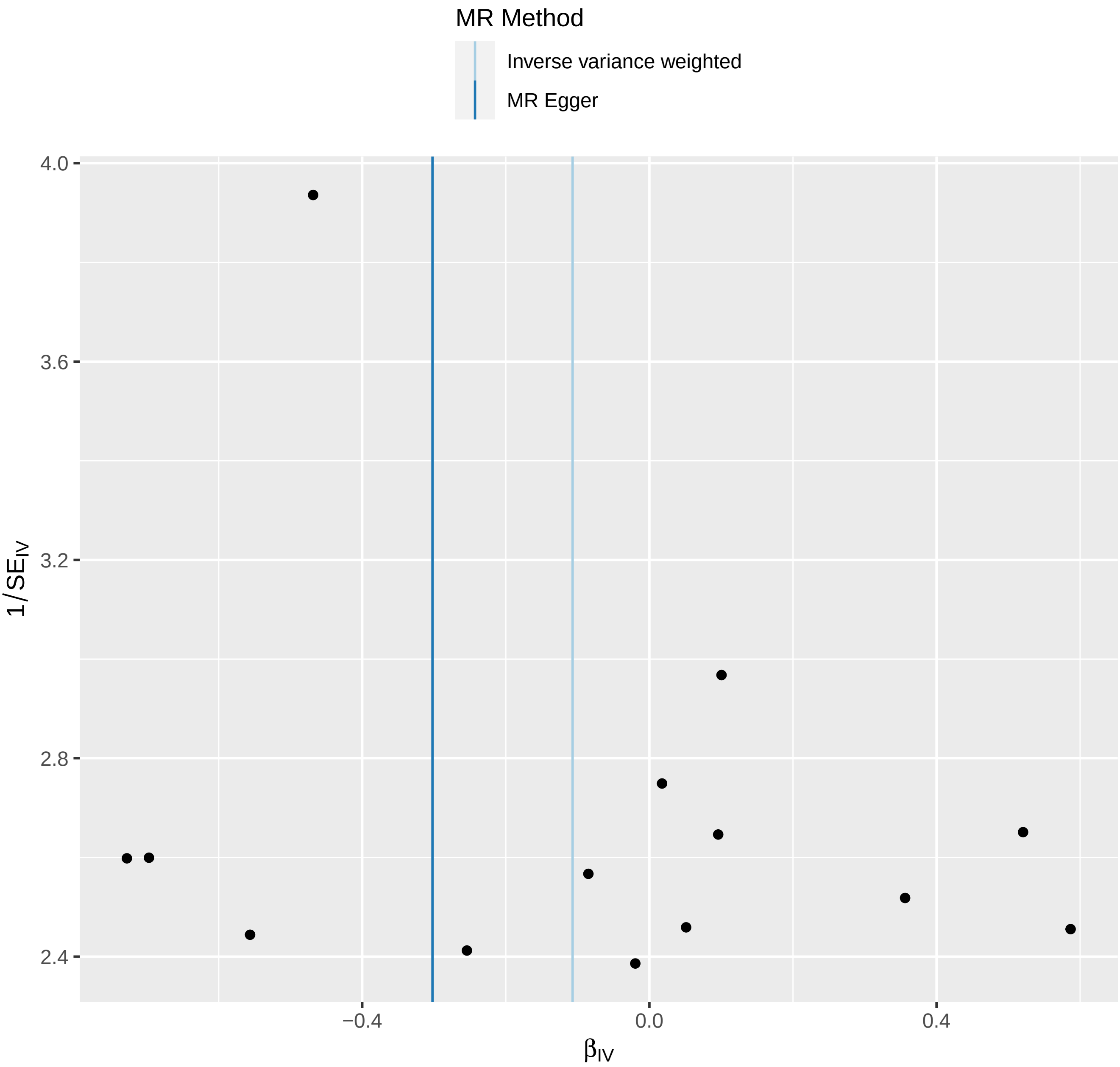

Batch 2−5425 : Gut microbiota abundance (genus Catenibacterium id.2153) on Beta−sarcoglycan
with a threshold of 1e−05

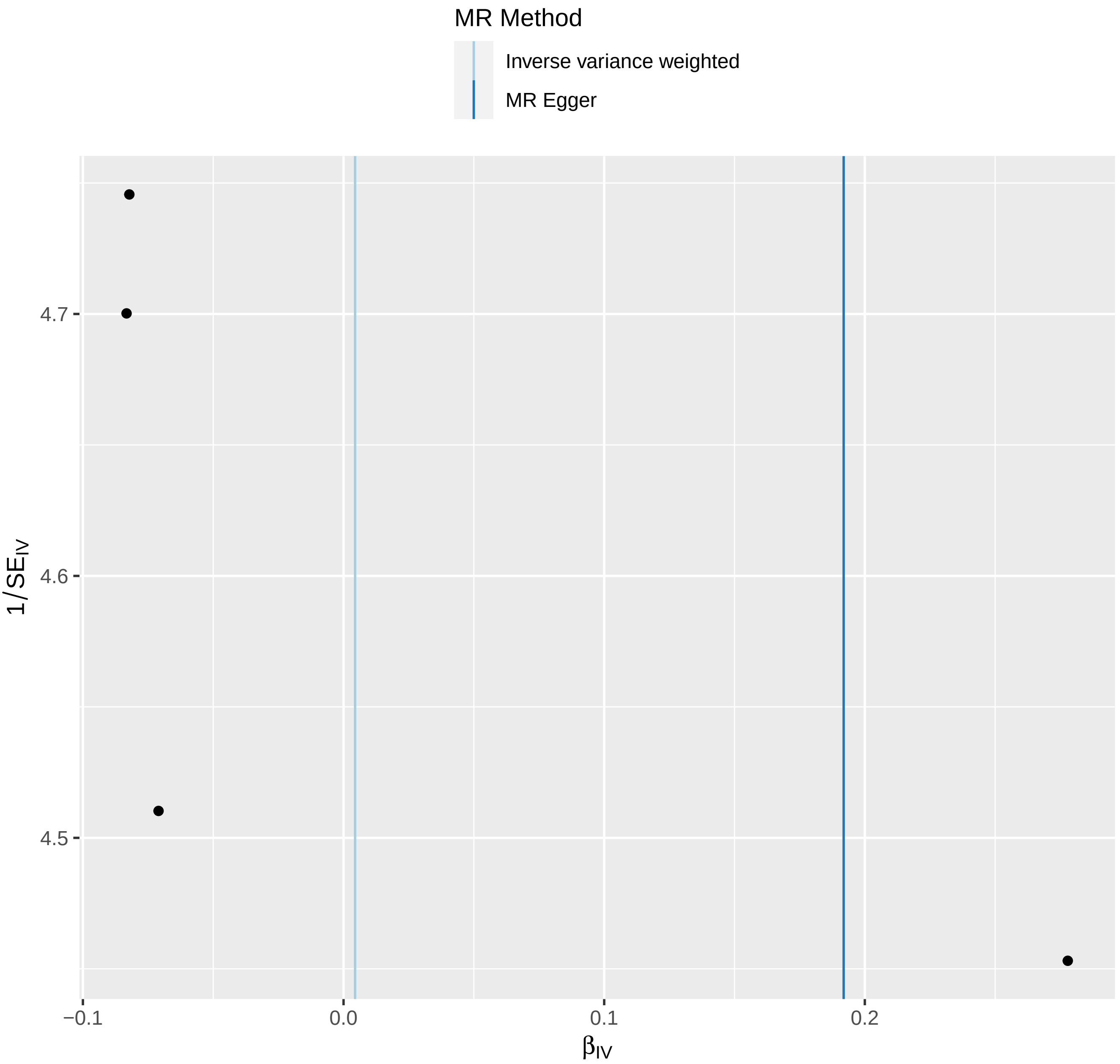

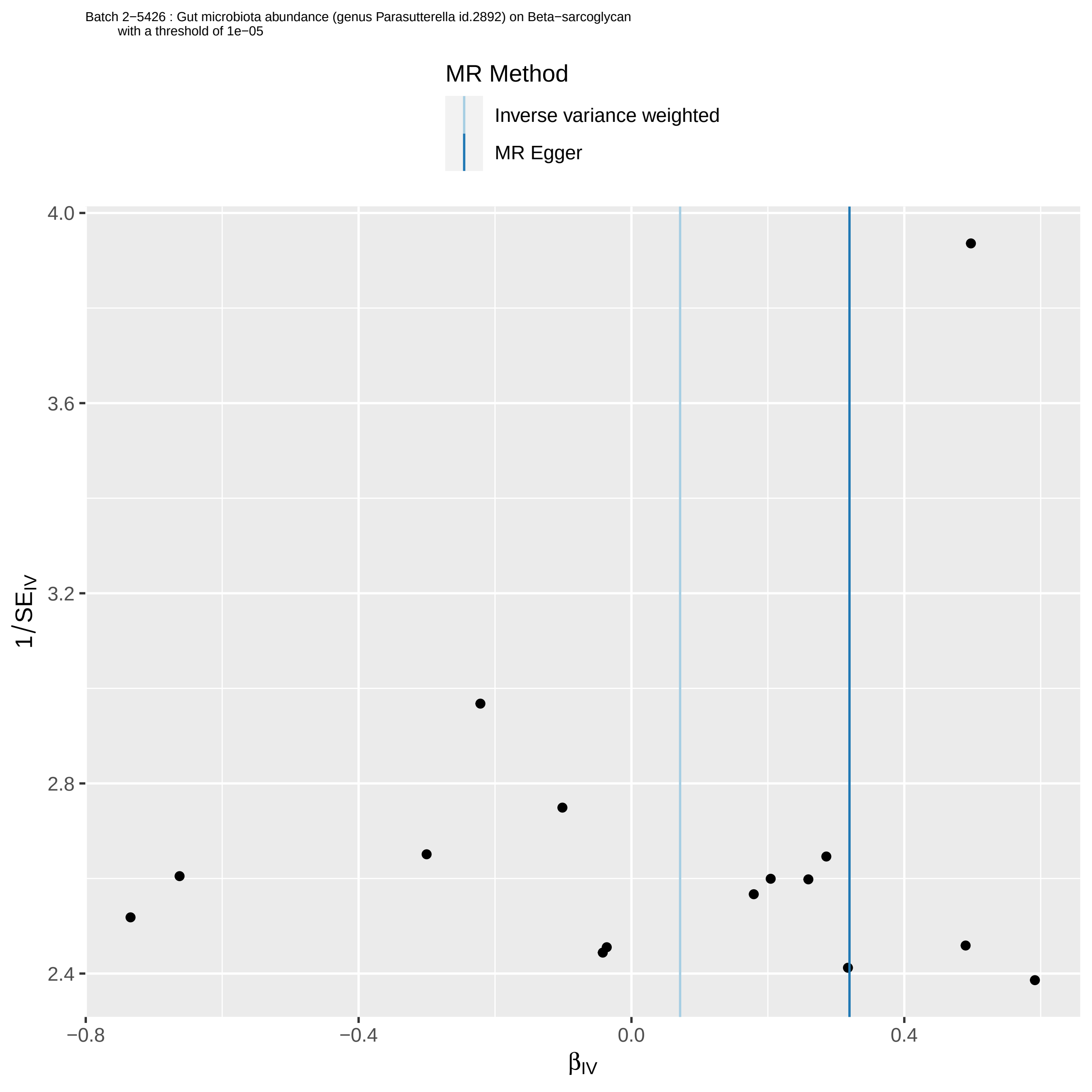
Batch 2−5426 : Gut microbiota abundance (genus Parasutterella id.2892) on Beta−sarcoglycan
with a threshold of 1e−05
MR Method
Inverse variance weighted
MR Egger
4.0
3.6
3.2
2.8
2.4
1/SE_IV
−0.8
−0.4
0.0
0.4
β_IV

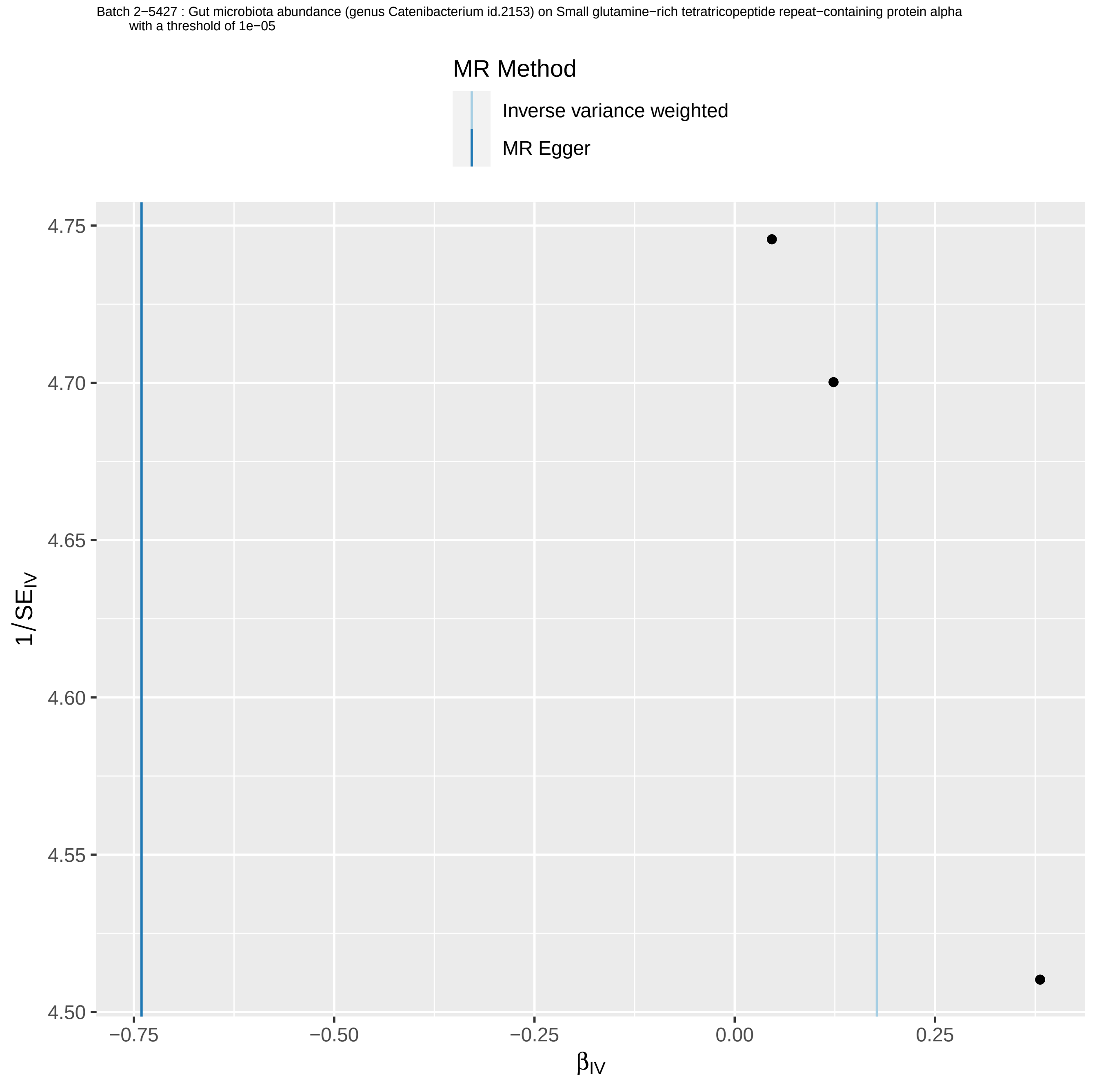

Batch 2−5427 : Gut microbiota abundance (genus Catenibacterium id.2153) on Small glutamine−rich tetratricopeptide repeat−containing protein alpha with a threshold of 1e−05
MR Method
Inverse variance weighted
MR Egger
4.75
4.70
4.65
4.60
4.55
4.50
1/SE_IV
−0.75
−0.50
−0.25
0.00
0.25
β_IV

Batch 2−5428 : Gut microbiota abundance (genus Parasutterella id.2892) on Small glutamine−rich tetratricopeptide repeat−containing protein alpha with a threshold of 1e−05

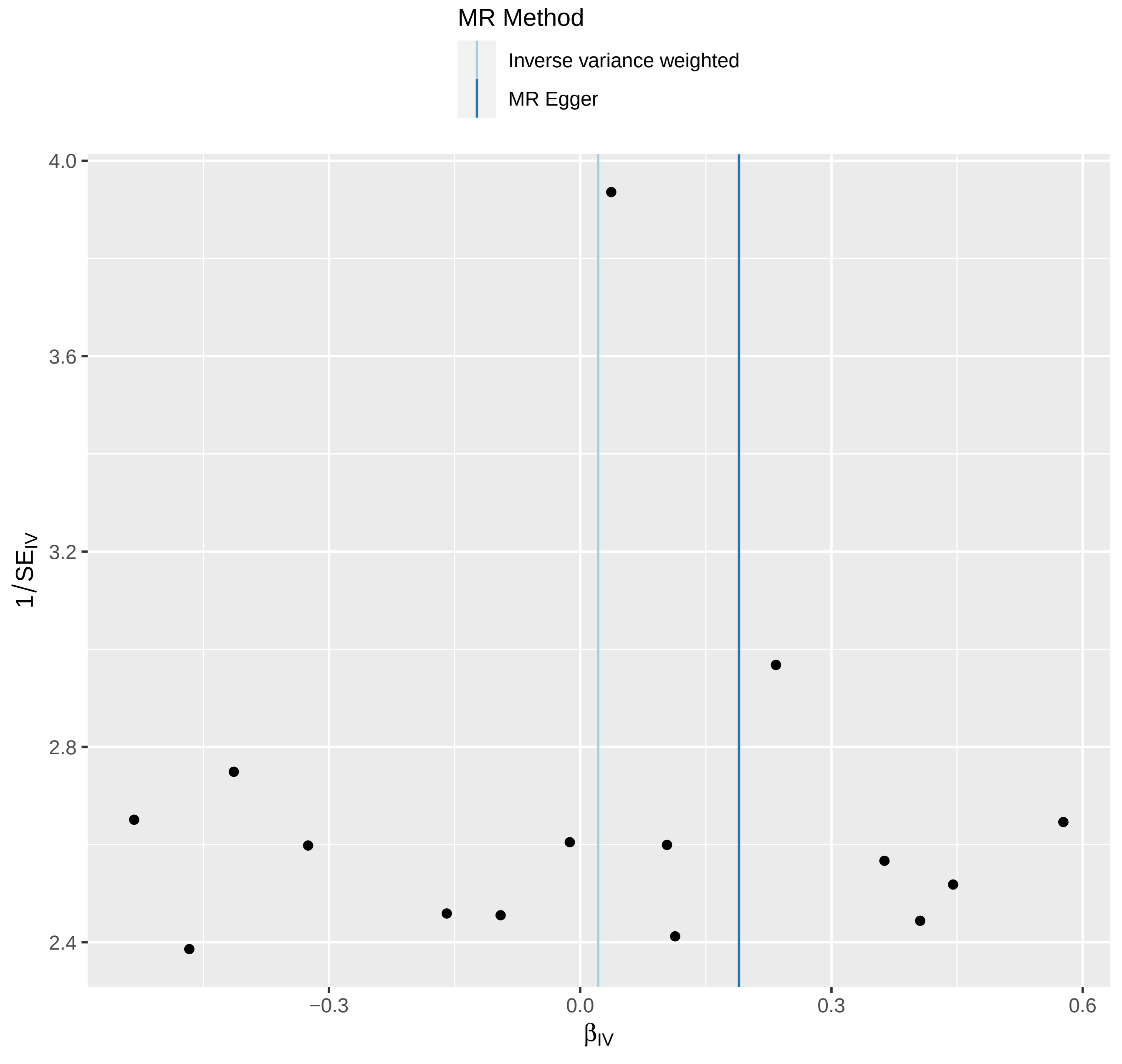

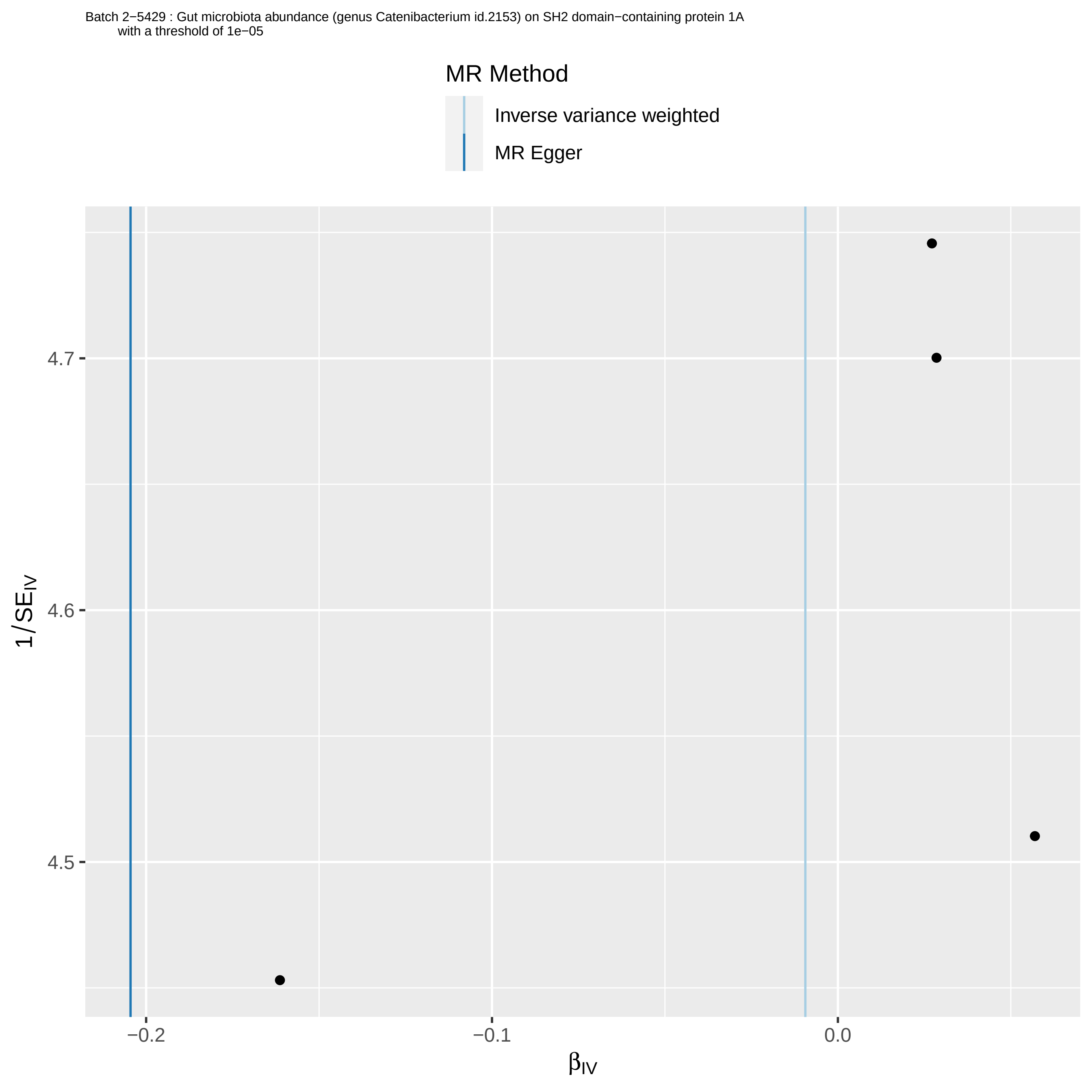
Batch 2−5429 : Gut microbiota abundance (genus Catenibacterium id.2153) on SH2 domain−containing protein 1A
with a threshold of 1e−05
MR Method
Inverse variance weighted
MR Egger
4.7
4.6
4.5
1/SE_IV
−0.2
−0.1
0.0
β_IV

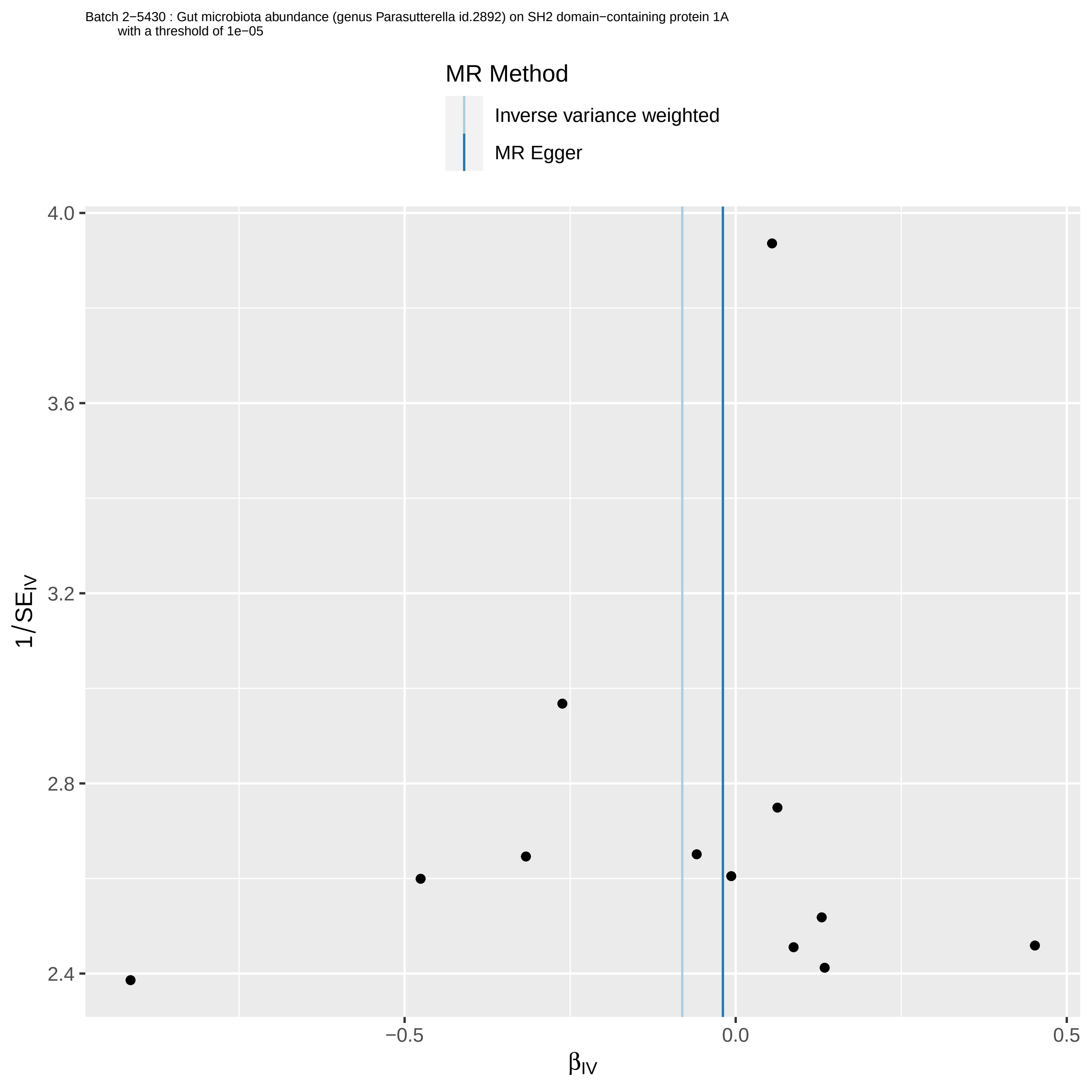

Batch 2−5430 : Gut microbiota abundance (genus Parasutterella id.2892) on SH2 domain−containing protein 1A
with a threshold of 1e−05
MR Method
Inverse variance weighted
MR Egger
4.0
3.6
3.2
2.8
2.4
$1/SE_{IV}$
−0.5
0.0
0.5
β_{IV}

Batch 2−5431 : Gut microbiota abundance (genus Catenibacterium id.2153) on SH3 domain−binding protein 2
with a threshold of 1e−05

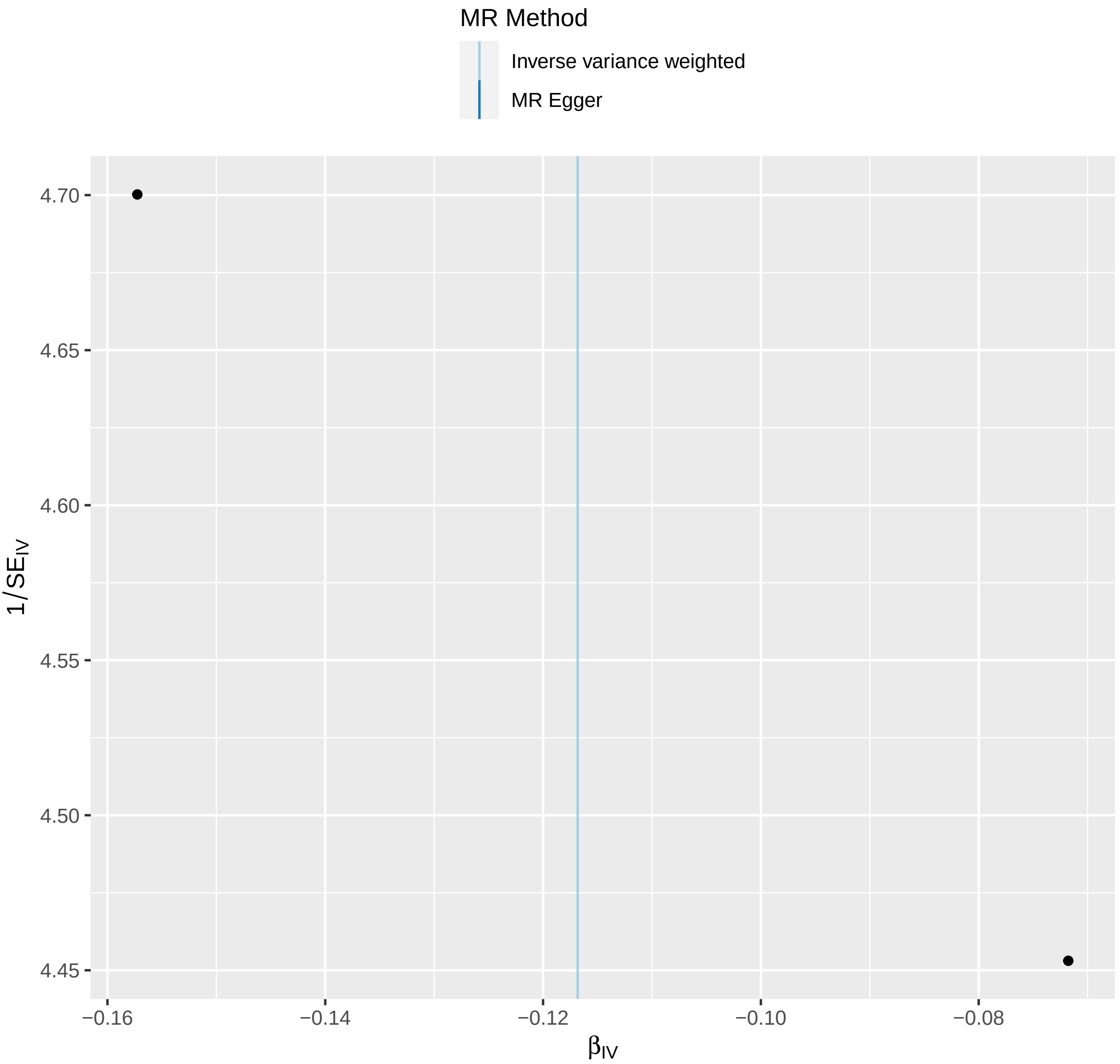

Batch 2−5432 : Gut microbiota abundance (genus Parasutterella id.2892) on SH3 domain−binding protein 2
with a threshold of 1e−05

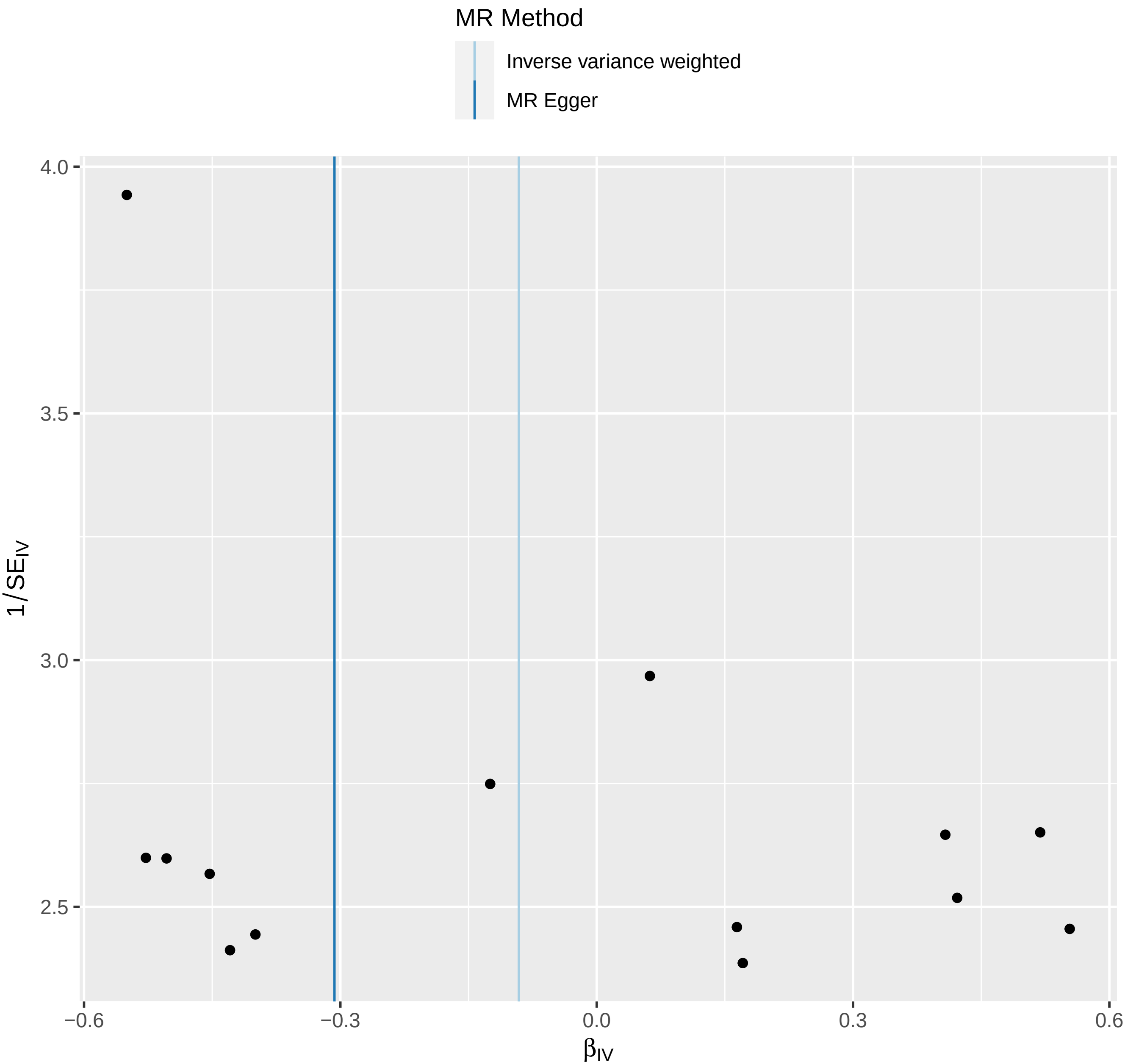

Batch 2−5433 : Gut microbiota abundance (genus Catenibacterium id.2153) on SHC−transforming protein 1
with a threshold of 1e−05

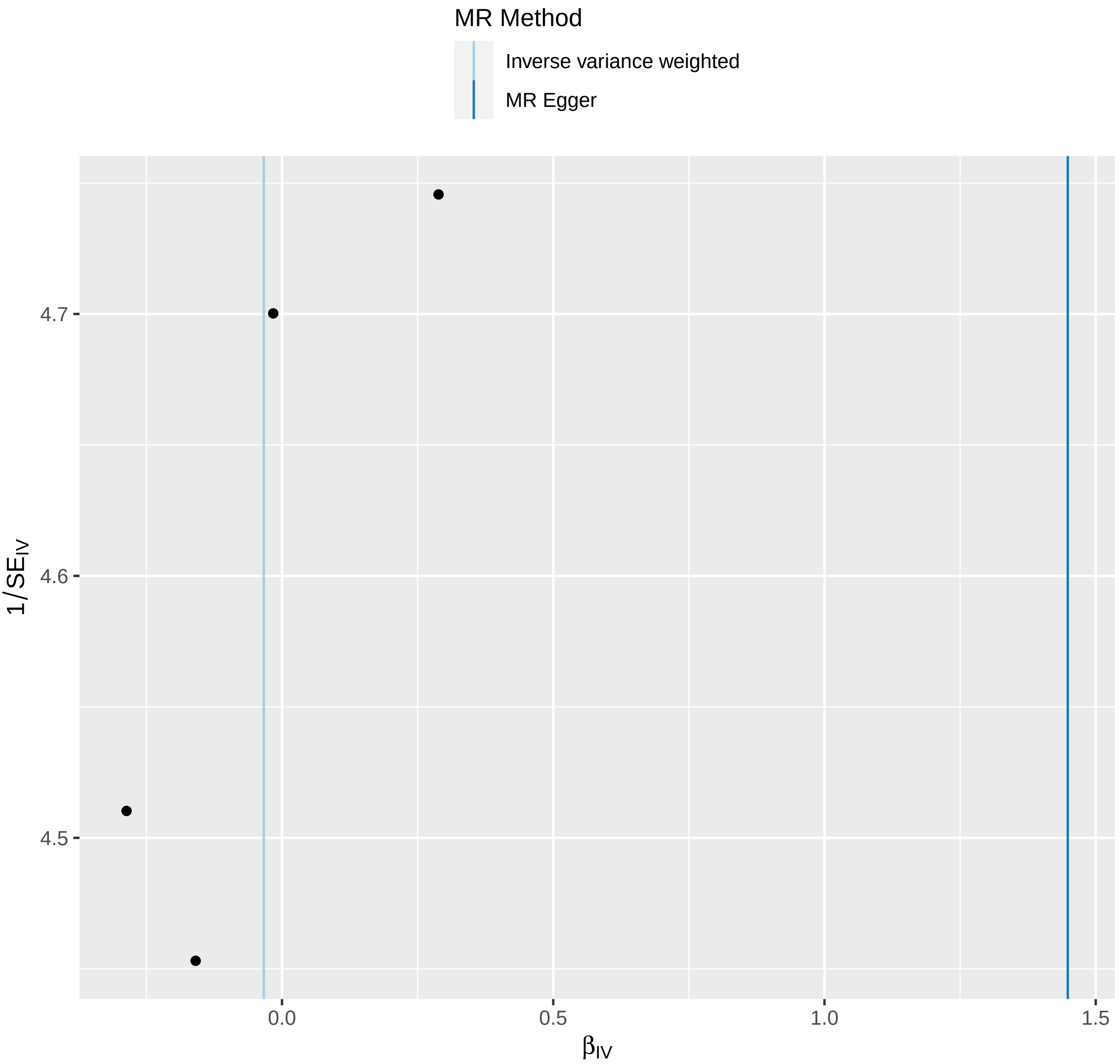

Batch 2−5434 : Gut microbiota abundance (genus Parasutterella id.2892) on SHC−transforming protein 1
with a threshold of 1e−05

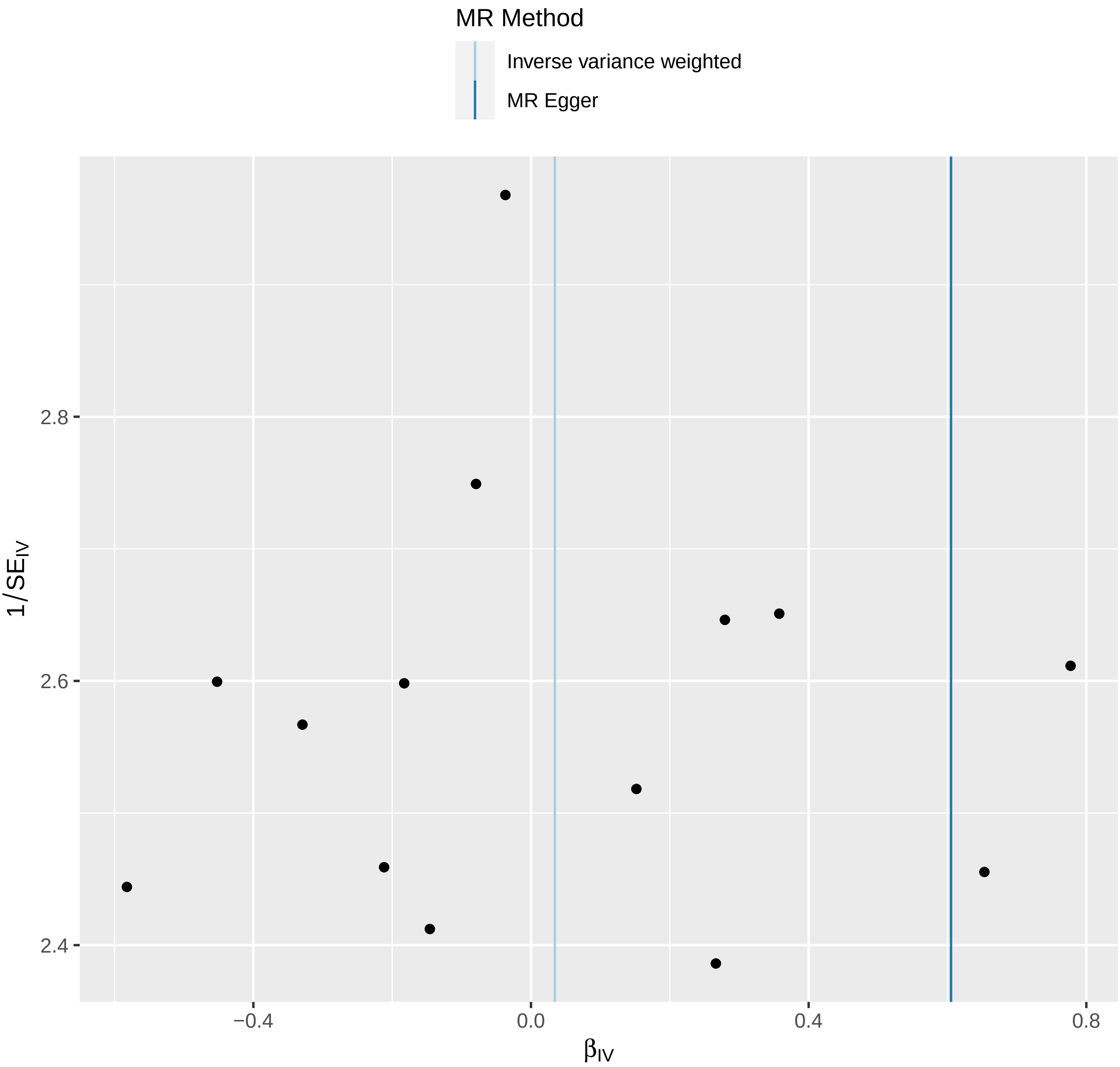

Batch 2−5435 : Gut microbiota abundance (genus Catenibacterium id.2153) on SHC−transforming protein 2
with a threshold of 1e−05

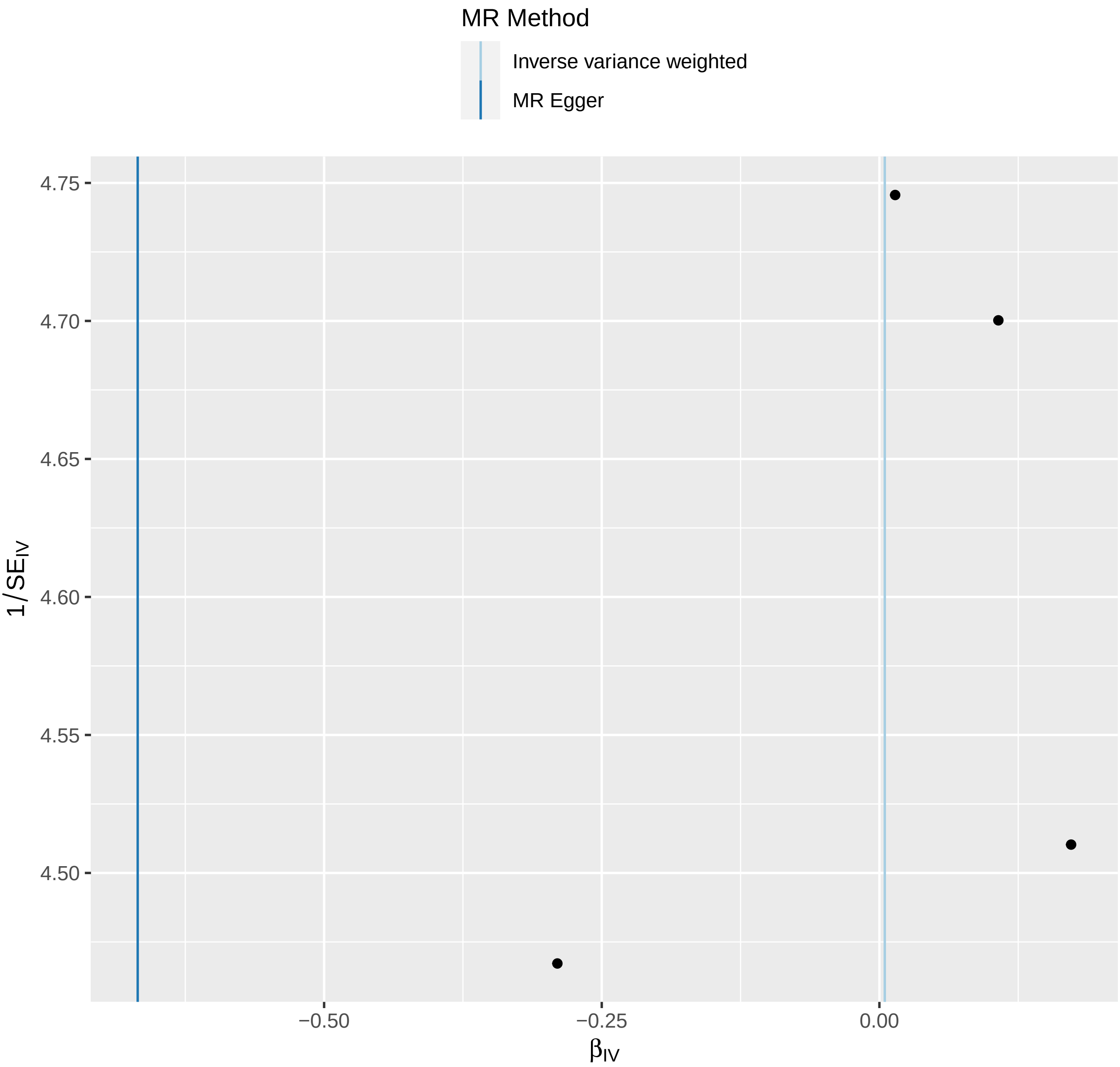

Batch 2−5436 : Gut microbiota abundance (genus Parasutterella id.2892) on SHC−transforming protein 2
with a threshold of 1e−05

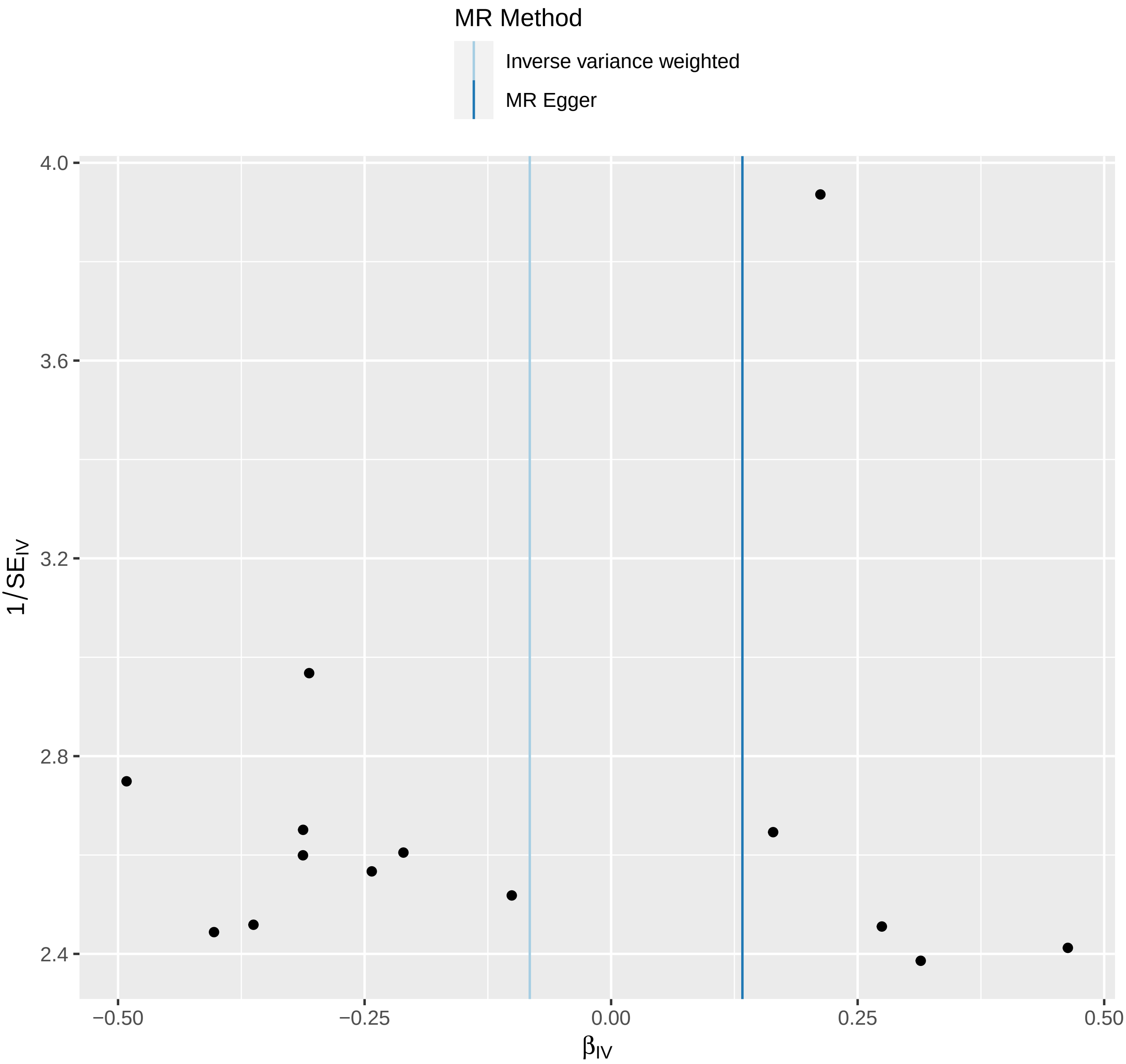

Batch 2−5437 : Gut microbiota abundance (genus Catenibacterium id.2153) on SHC−transforming protein 4
with a threshold of 1e−05

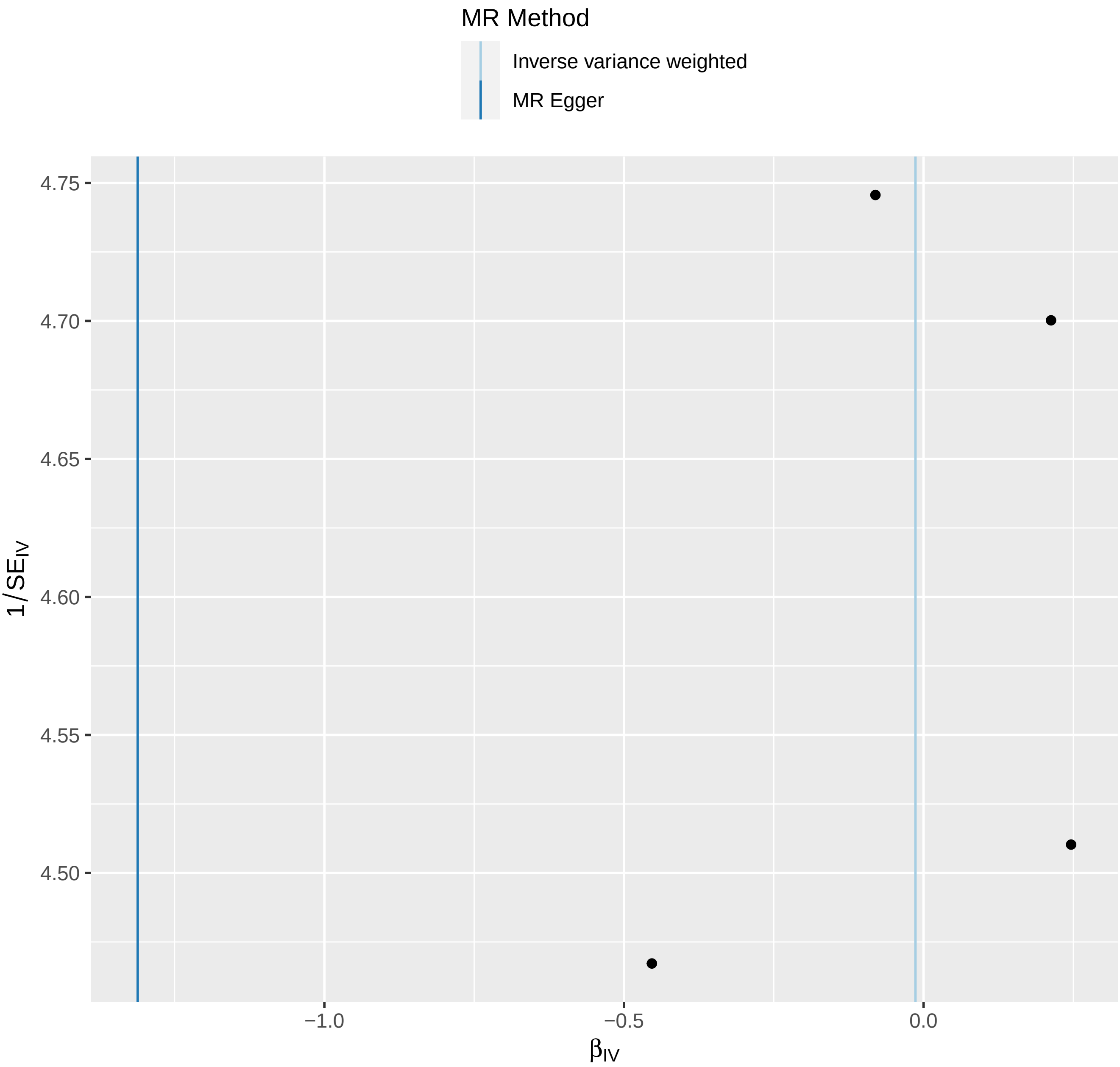

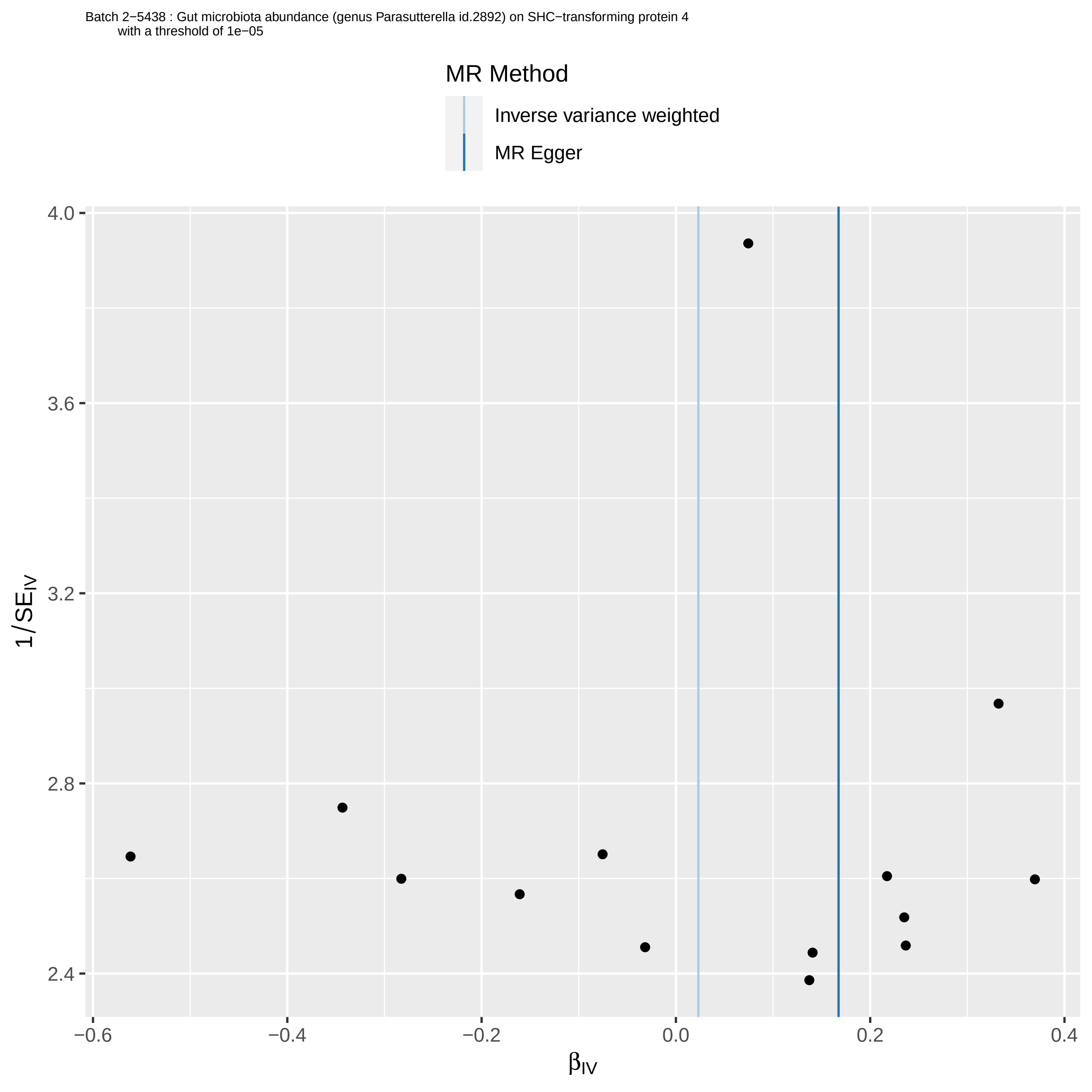
Batch 2−5438 : Gut microbiota abundance (genus Parasutterella id.2892) on SHC−transforming protein 4
with a threshold of 1e−05
MR Method
Inverse variance weighted
MR Egger
4.0
3.6
3.2
2.8
2.4
1/SE_IV
−0.6
−0.4
−0.2
0.0
0.2
0.4
β_IV

Batch 2−5439 : Gut microbiota abundance (genus Catenibacterium id.2153) on Protein shisa−3 homolog
with a threshold of 1e−05

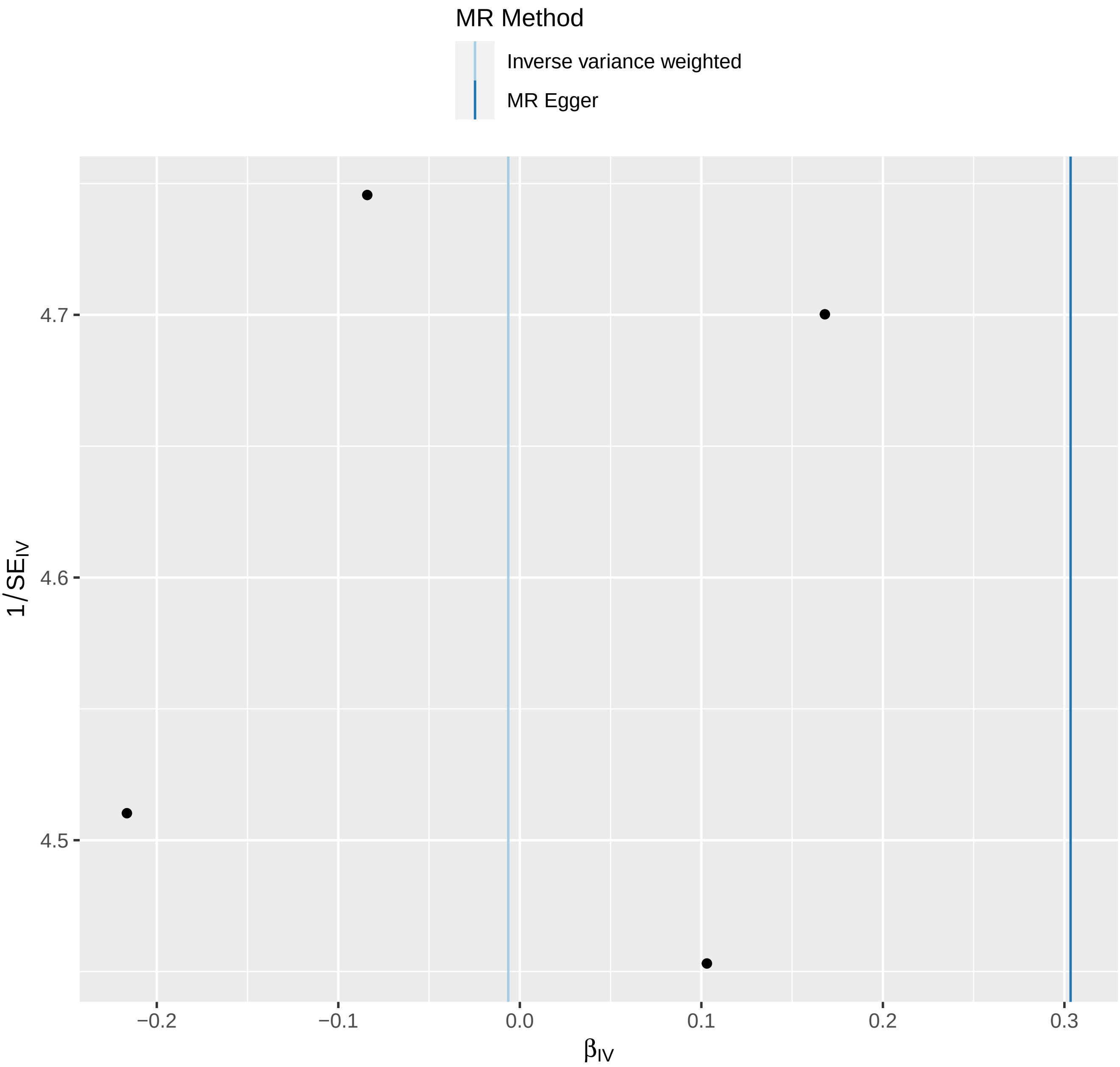

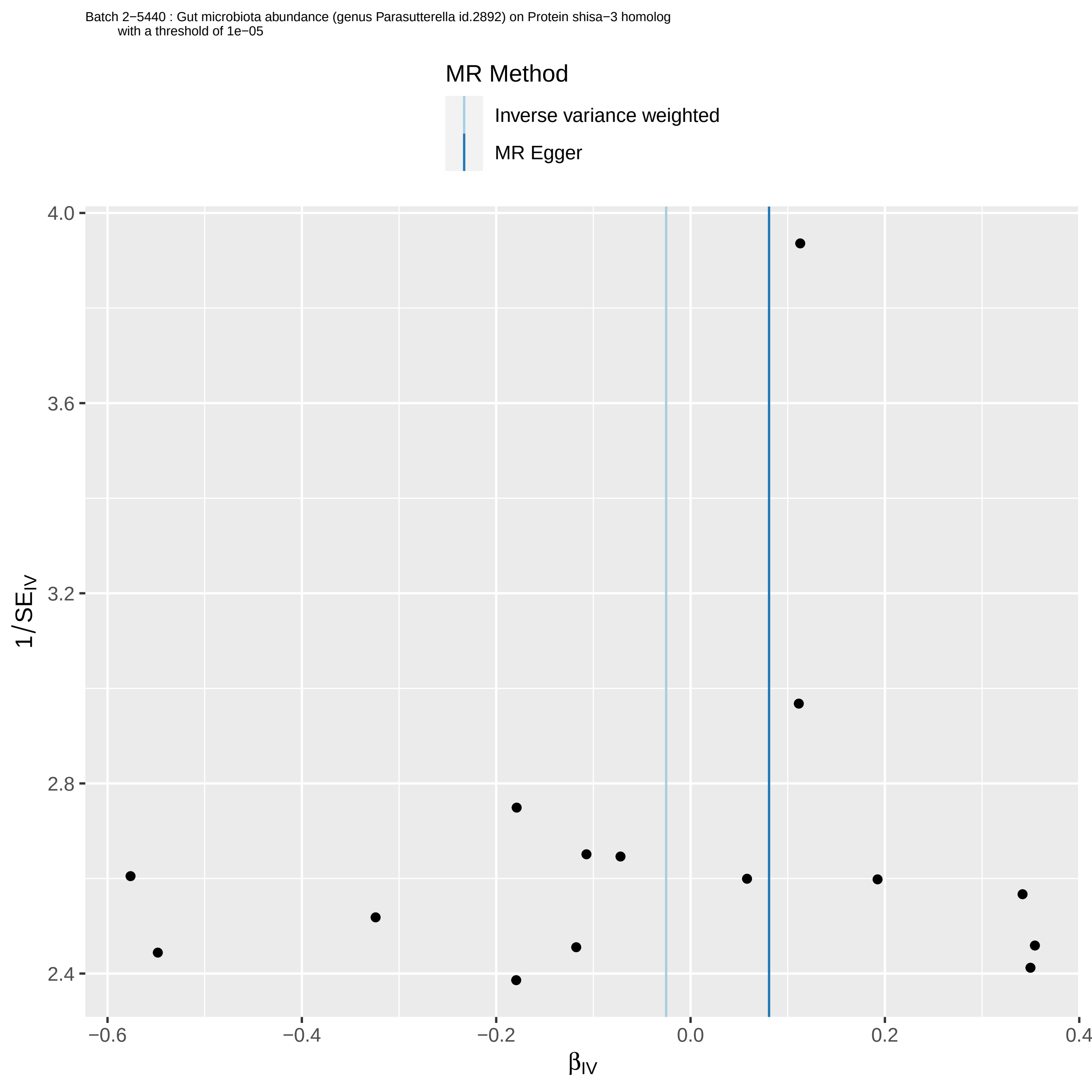
Batch 2−5440 : Gut microbiota abundance (genus Parasutterella id.2892) on Protein shisa−3 homolog
with a threshold of 1e−05
MR Method
Inverse variance weighted
MR Egger
4.0
3.6
3.2
2.8
2.4
1/SE_IV
−0.6
−0.4
−0.2
0.0
0.2
0.4
β_IV

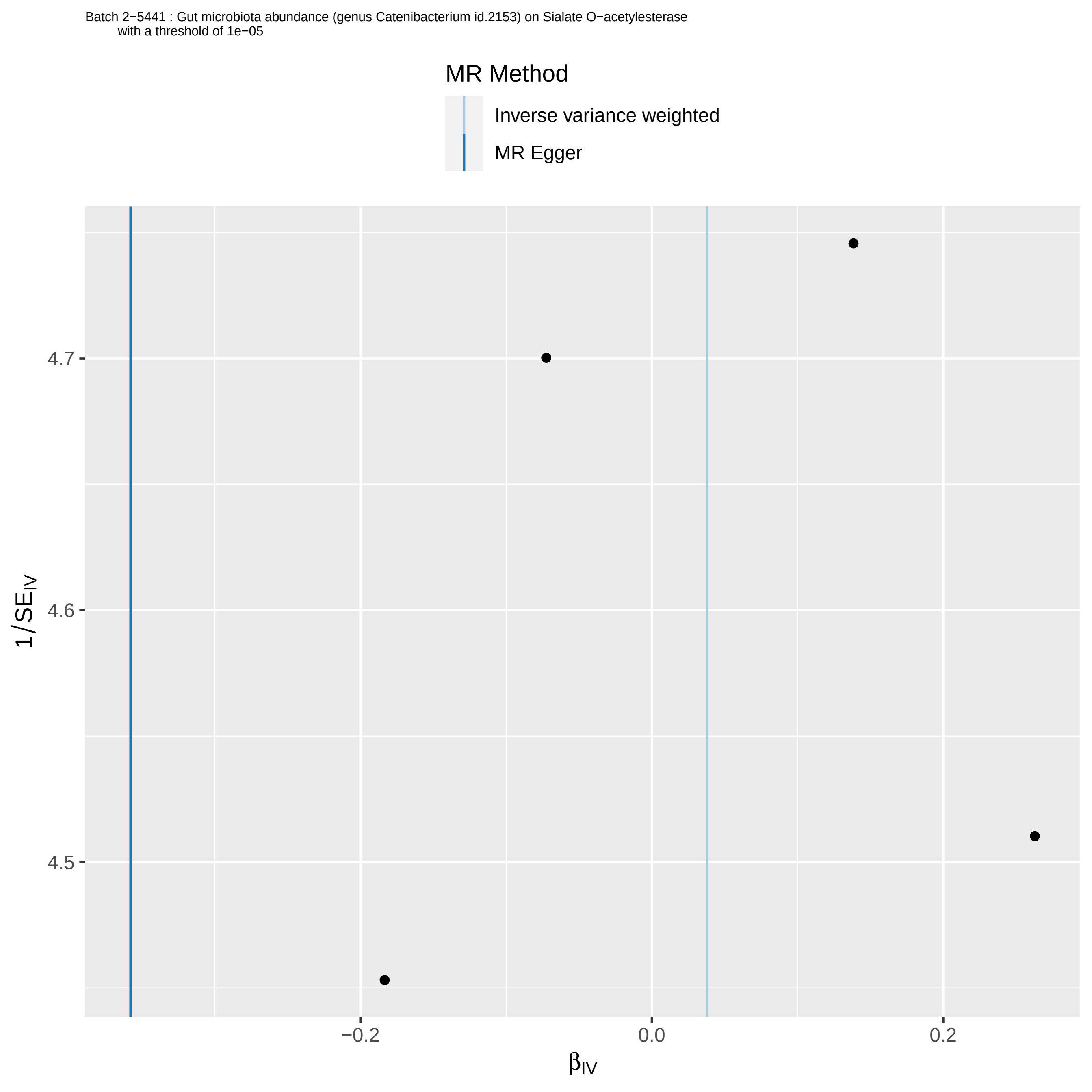
Batch 2−5441 : Gut microbiota abundance (genus Catenibacterium id.2153) on Sialate O−acetylesterase
with a threshold of 1e−05
MR Method
Inverse variance weighted
MR Egger
4.7
4.6
4.5
1/SE_IV
−0.2
0.0
0.2
β_IV

Batch 2−5442 : Gut microbiota abundance (genus Parasutterella id.2892) on Sialate O−acetylesterase
with a threshold of 1e−05

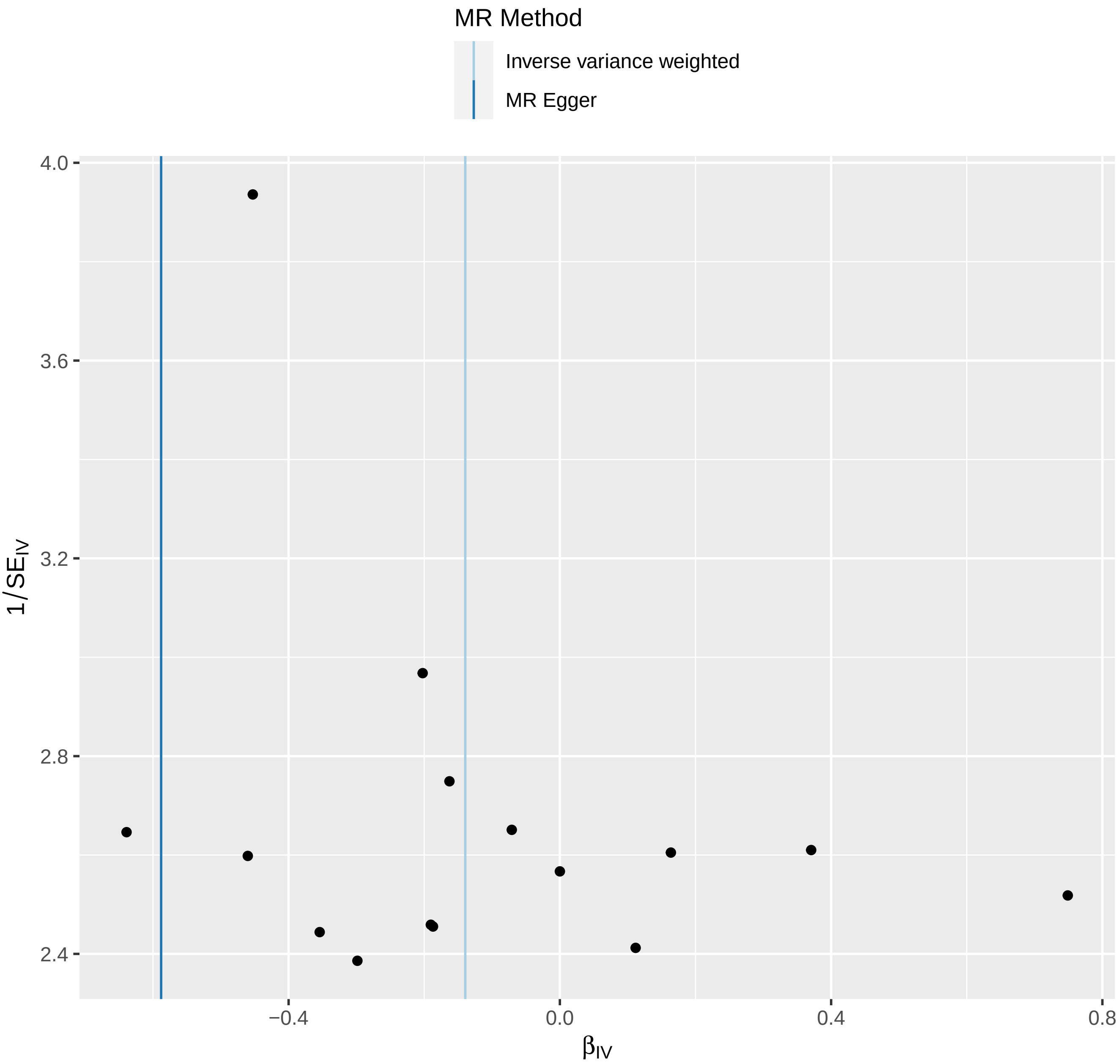

Batch 2−5443 : Gut microbiota abundance (genus Catenibacterium id.2153) on Single Ig IL−1−related receptor with a threshold of 1e−05

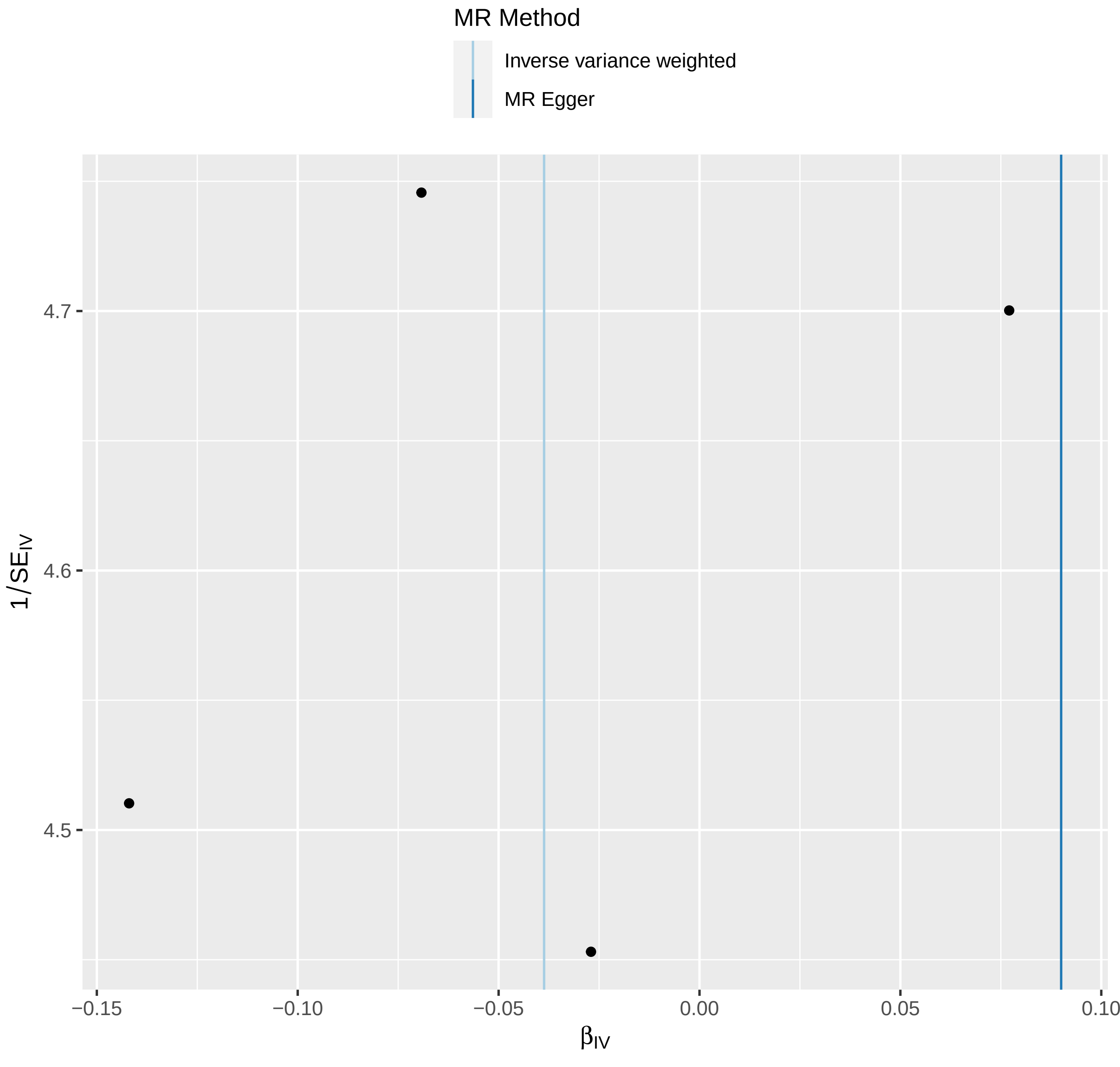

Batch 2−5444 : Gut microbiota abundance (genus Parasutterella id.2892) on Single Ig IL−1−related receptor
with a threshold of 1e−05

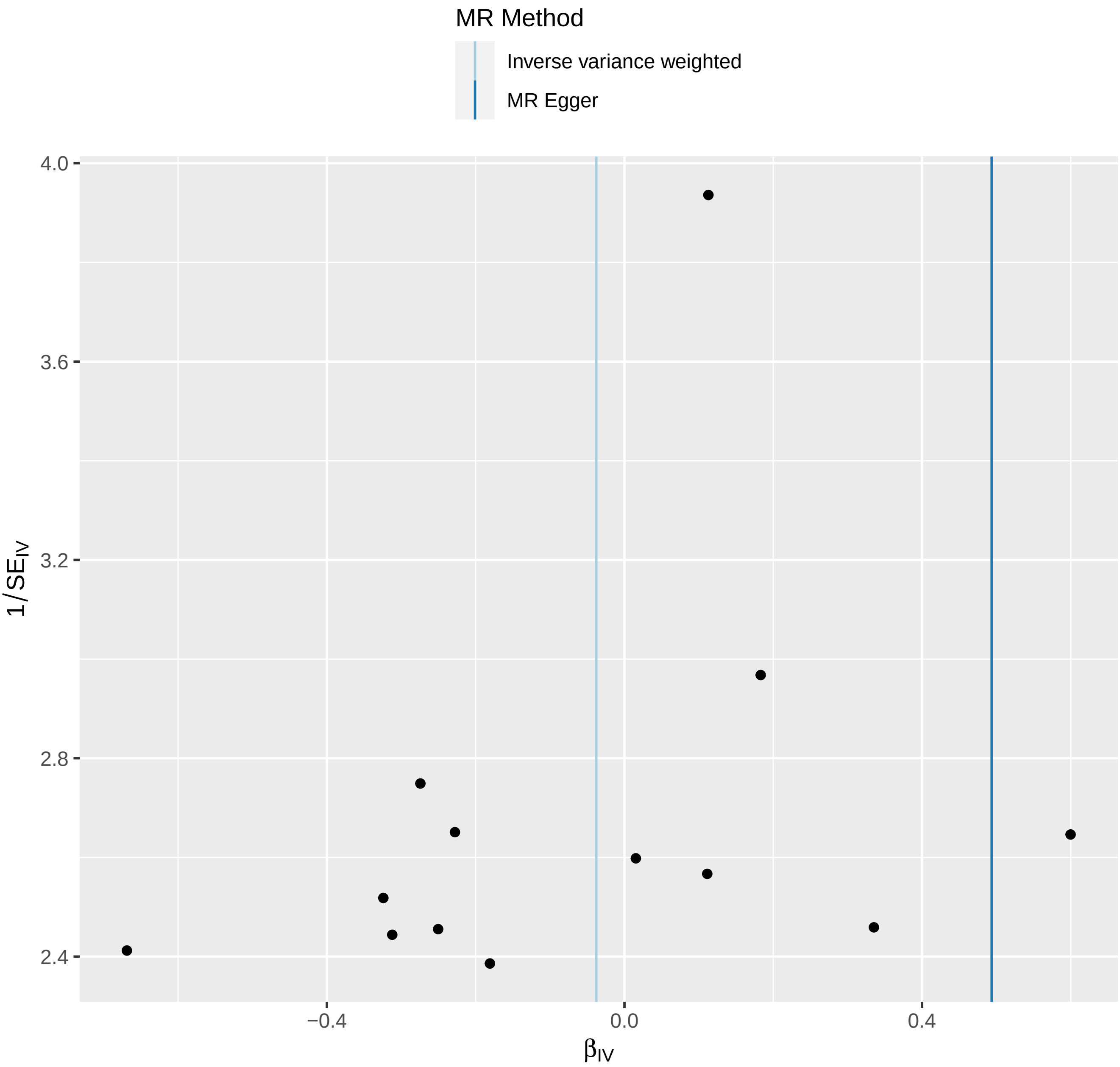

Batch 2−5445 : Gut microbiota abundance (genus Catenibacterium id.2153) on Sialic acid−binding Ig−like lectin 10
with a threshold of 1e−05

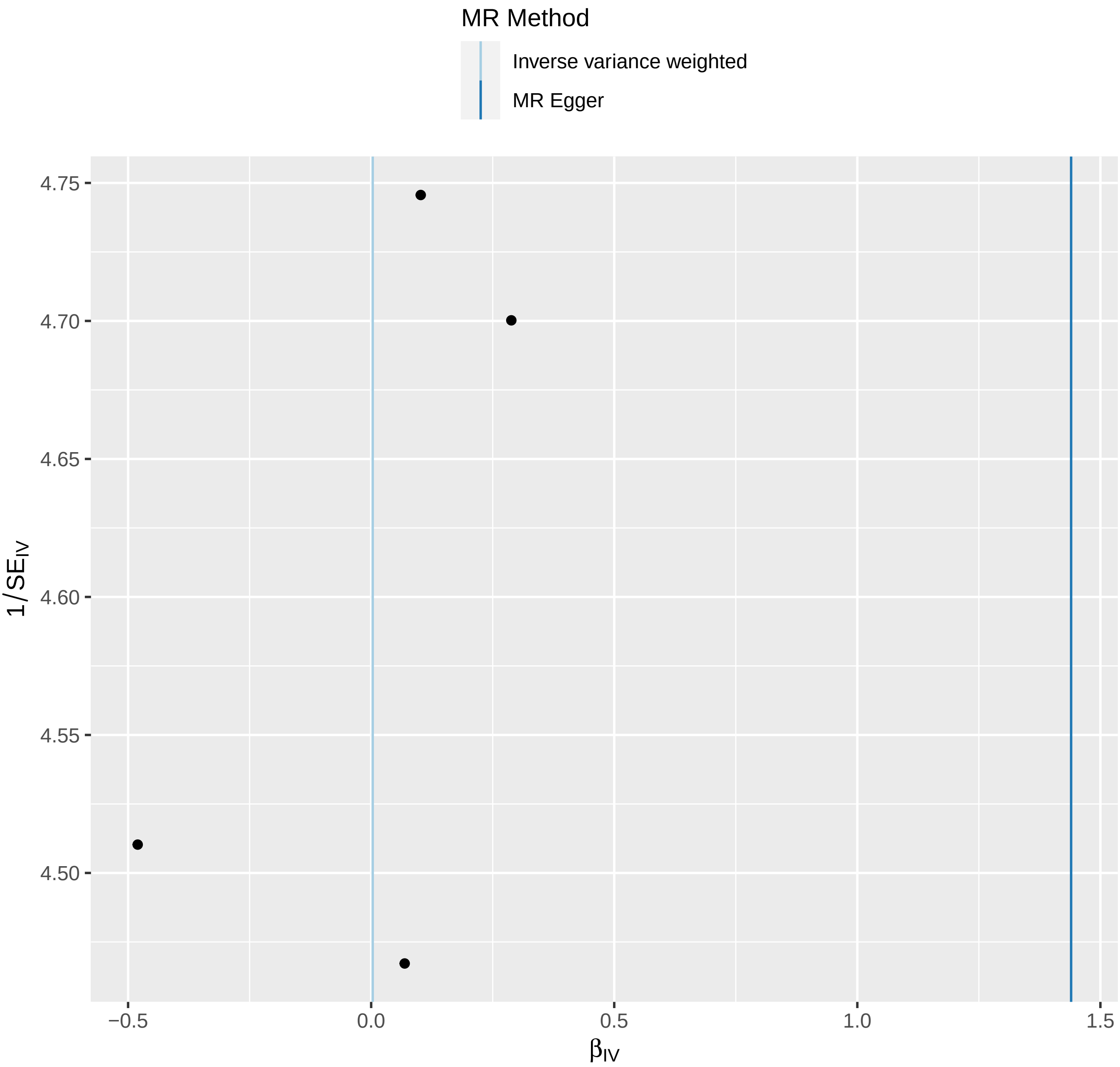

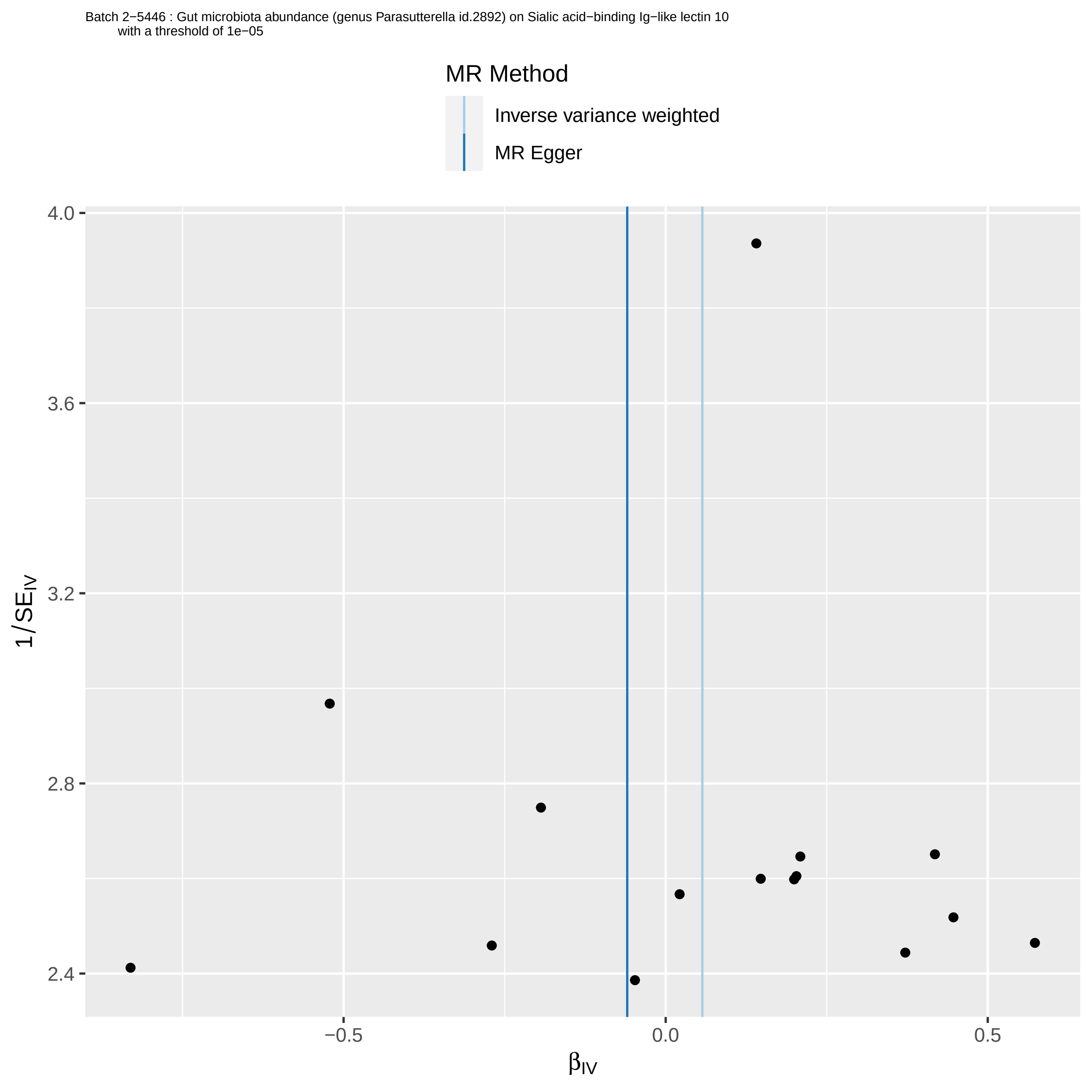

Batch 2−5446 : Gut microbiota abundance (genus Parasutterella id.2892) on Sialic acid−binding Ig−like lectin 10
with a threshold of 1e−05
MR Method
Inverse variance weighted
MR Egger
4.0
3.6
3.2
2.8
2.4
1/SE_IV
−0.5
0.0
0.5
β_IV

Batch 2−5447 : Gut microbiota abundance (genus Catenibacterium id.2153) on Sialic acid−binding Ig−like lectin 11
with a threshold of 1e−05

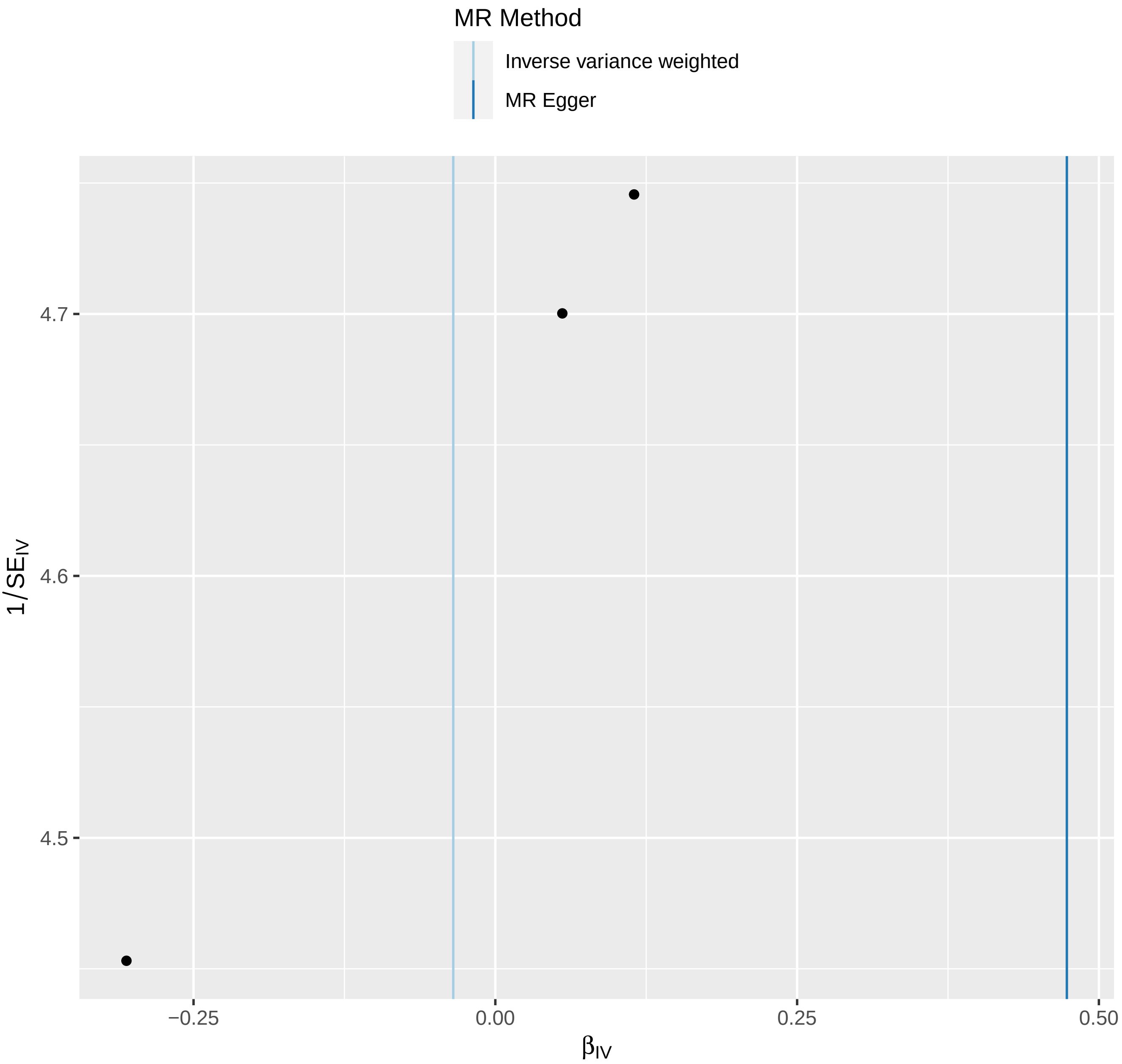

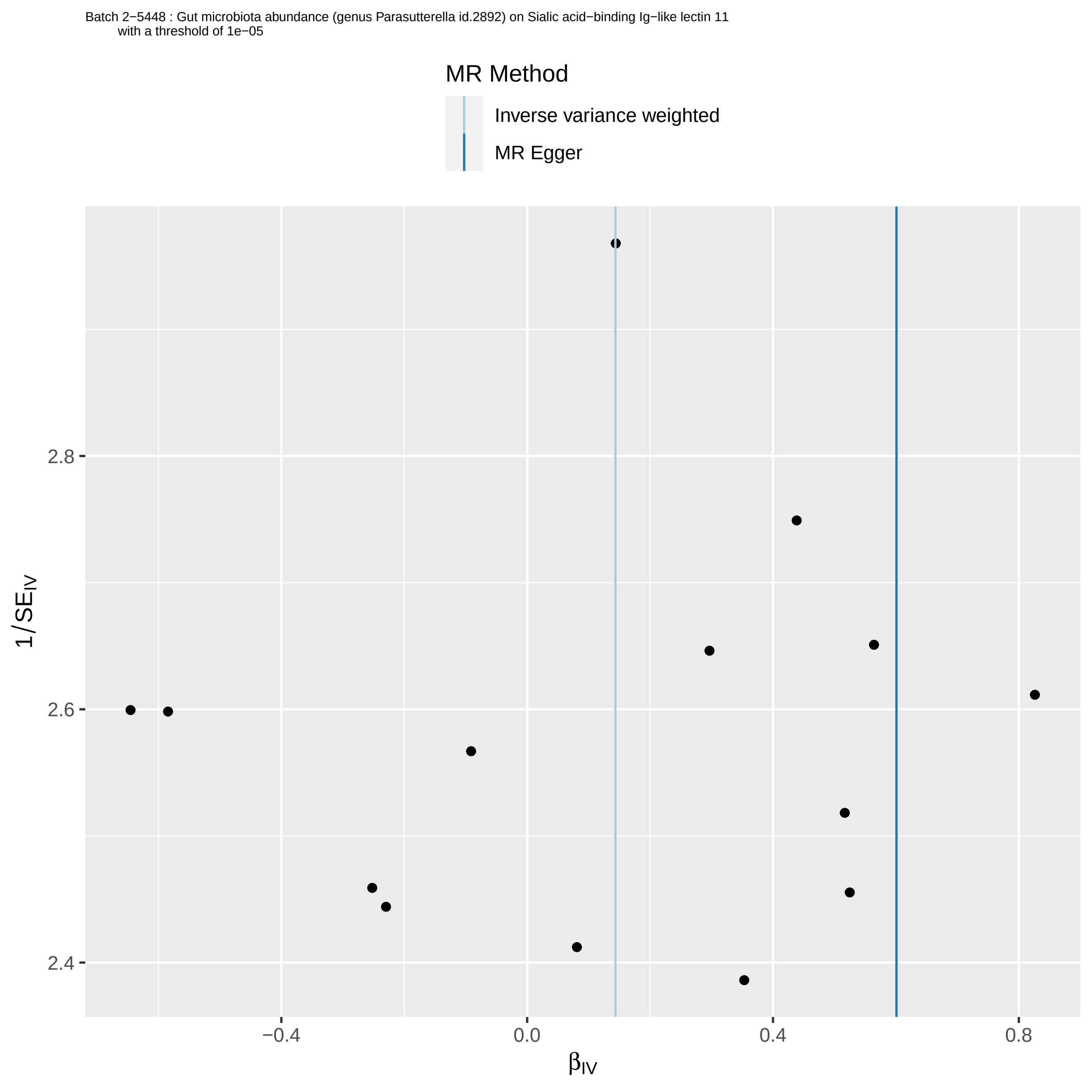
Batch 2−5448 : Gut microbiota abundance (genus Parasutterella id.2892) on Sialic acid−binding Ig−like lectin 11
with a threshold of 1e−05
MR Method
Inverse variance weighted
MR Egger
2.8
2.6
2.4
1/SE_IV
−0.4
0.0
0.4
0.8
β_IV

Batch 2−5449 : Gut microbiota abundance (genus Catenibacterium id.2153) on Sialic acid−binding Ig−like lectin 12
with a threshold of 1e−05

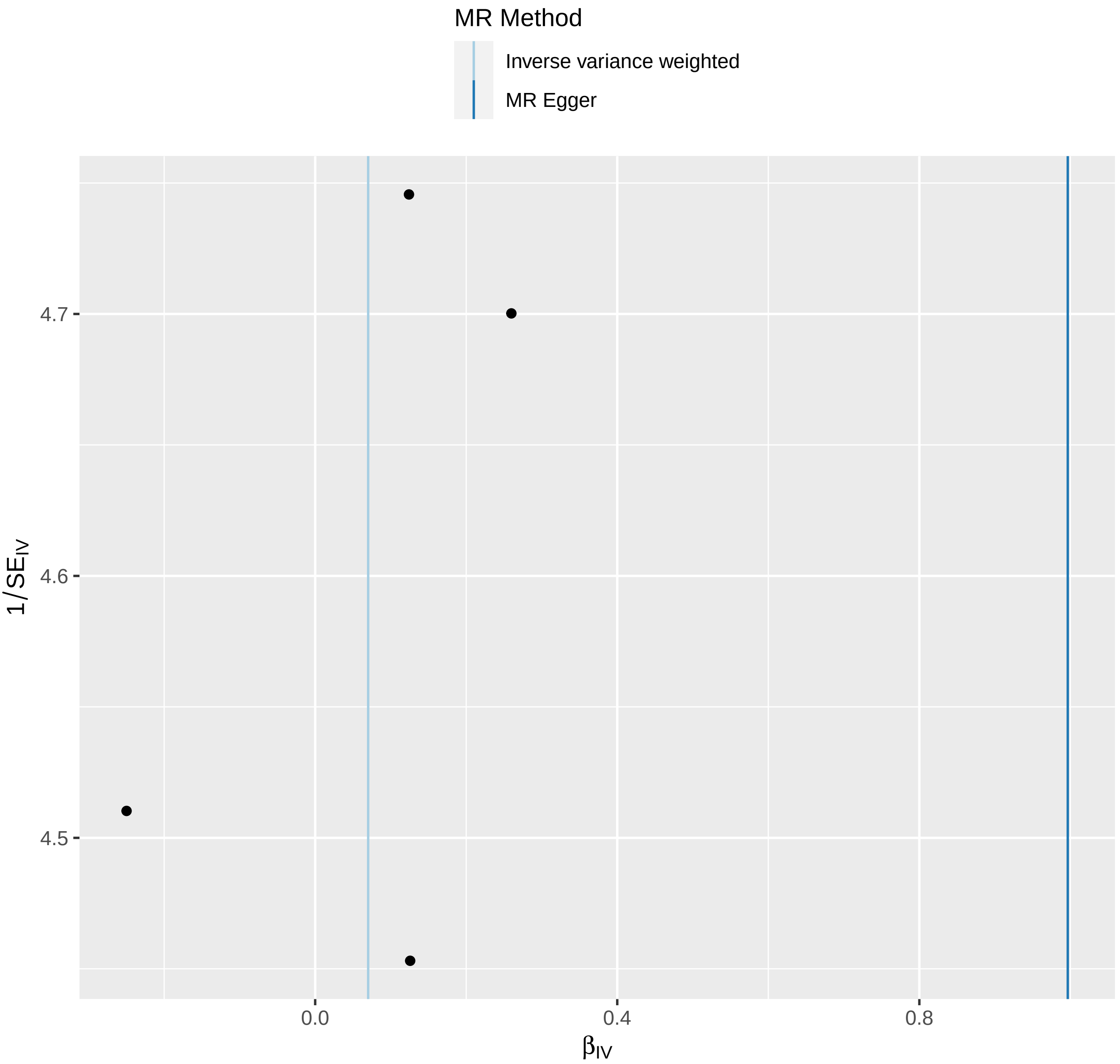

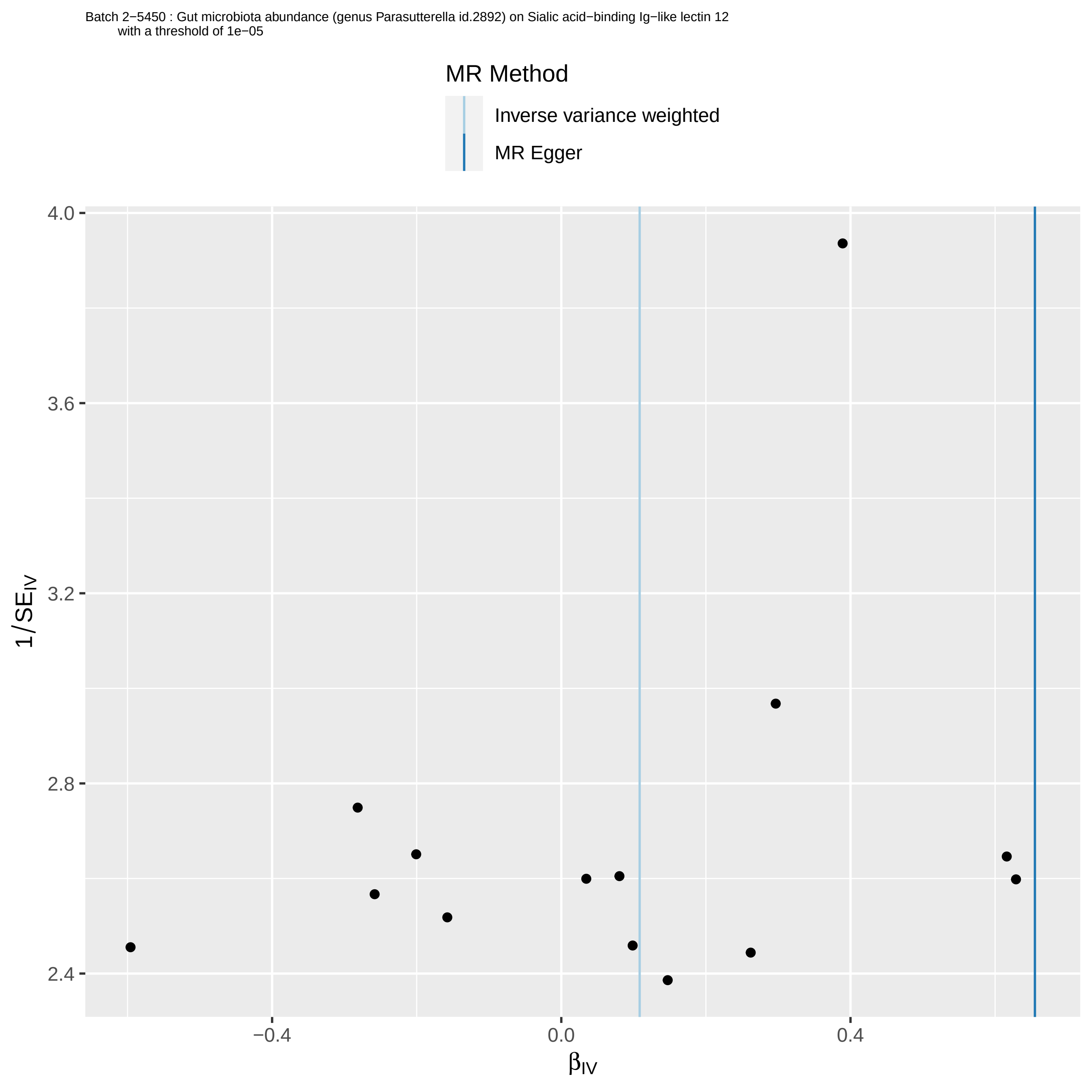

Batch 2−5450 : Gut microbiota abundance (genus Parasutterella id.2892) on Sialic acid−binding Ig−like lectin 12
with a threshold of 1e−05
MR Method
Inverse variance weighted
MR Egger
4.0
3.6
3.2
2.8
2.4
1/SE$_{IV}$
−0.4
0.0
0.4
β$_{IV}$

Batch 2−5451 : Gut microbiota abundance (genus Catenibacterium id.2153) on Sialic acid−binding Ig−like lectin 12
with a threshold of 1e−05

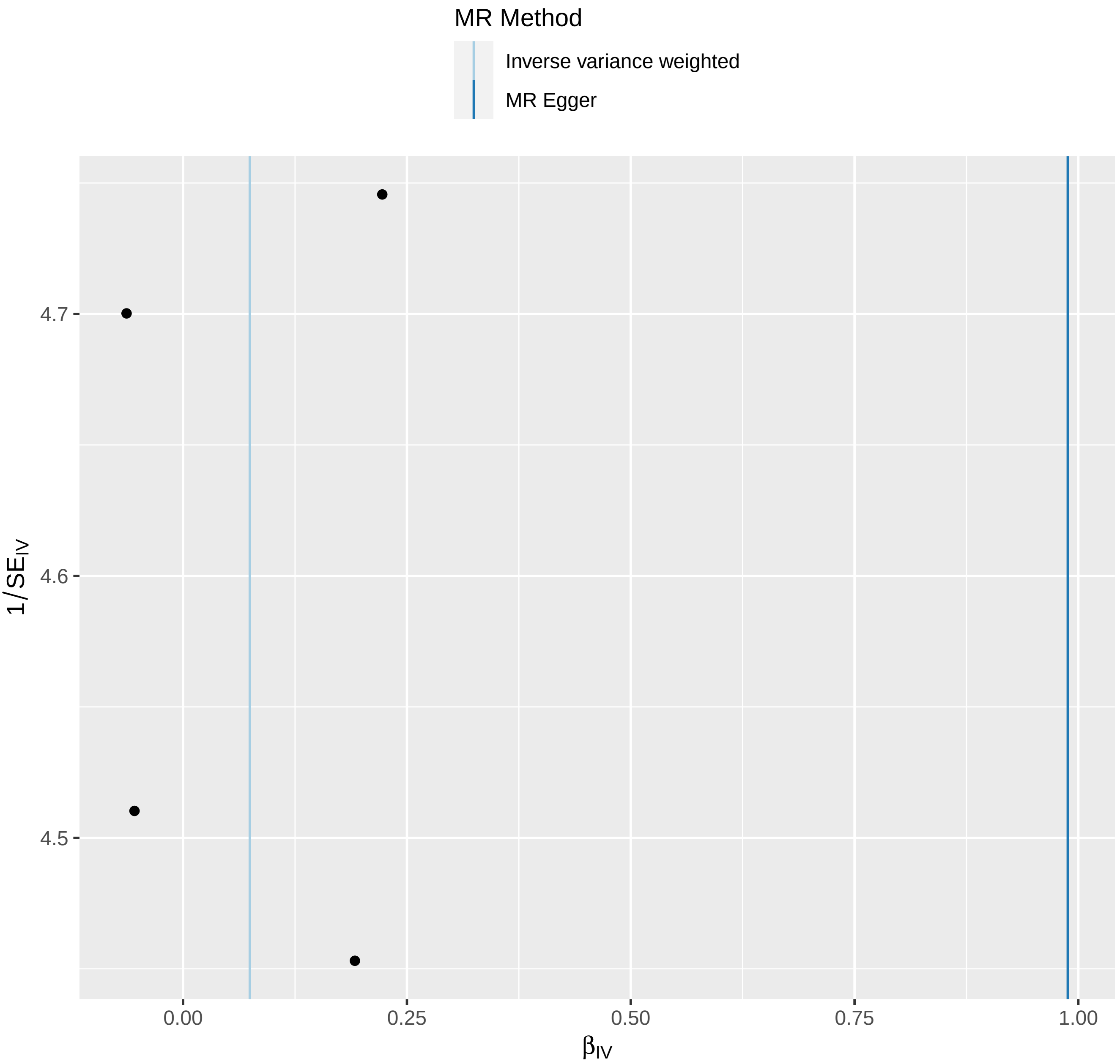

Batch 2−5452 : Gut microbiota abundance (genus Parasutterella id.2892) on Sialic acid−binding Ig−like lectin 12 with a threshold of 1e−05

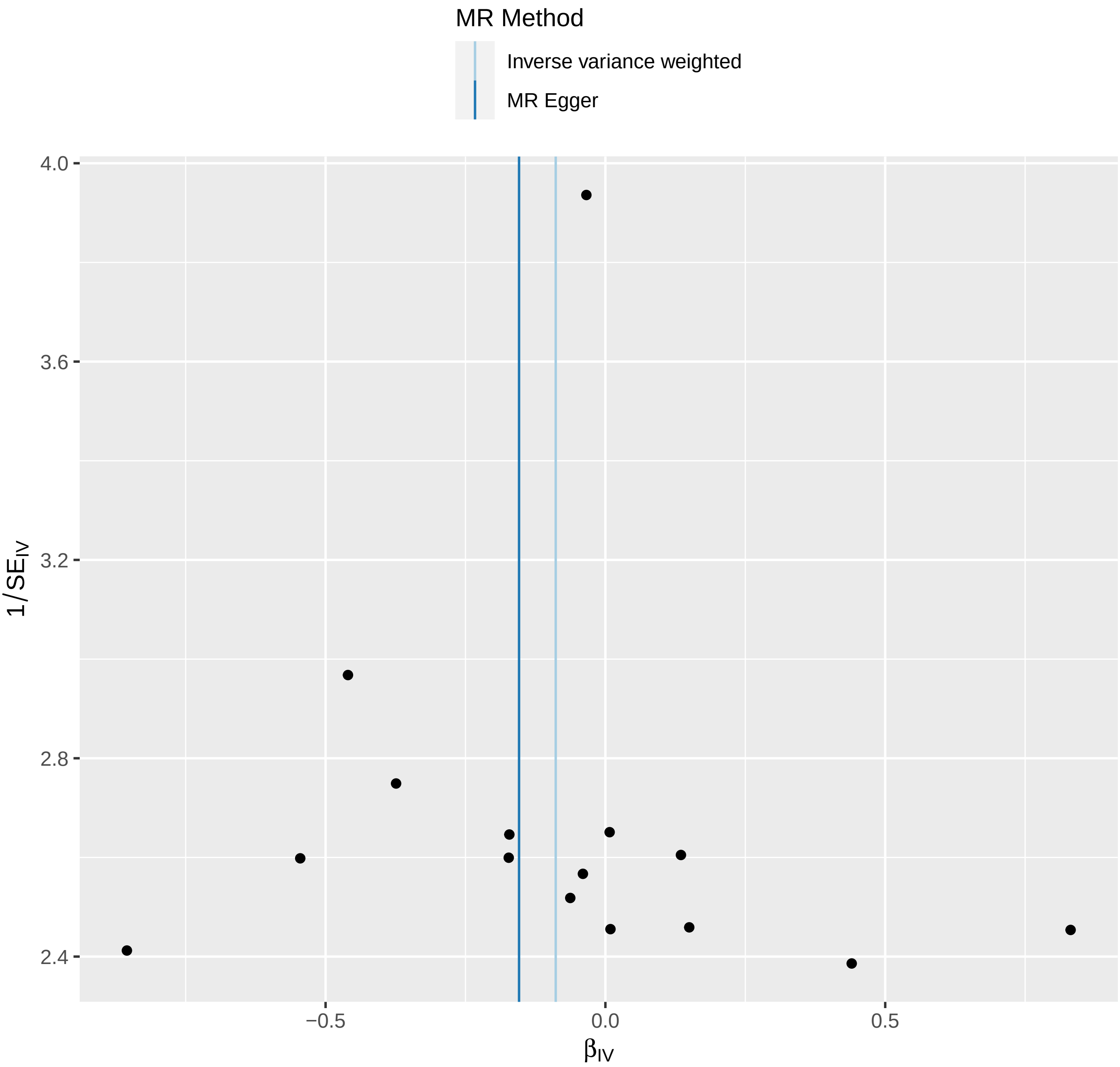

Batch 2−5453 : Gut microbiota abundance (genus Catenibacterium id.2153) on Sialic acid−binding Ig−like lectin 14
with a threshold of 1e−05

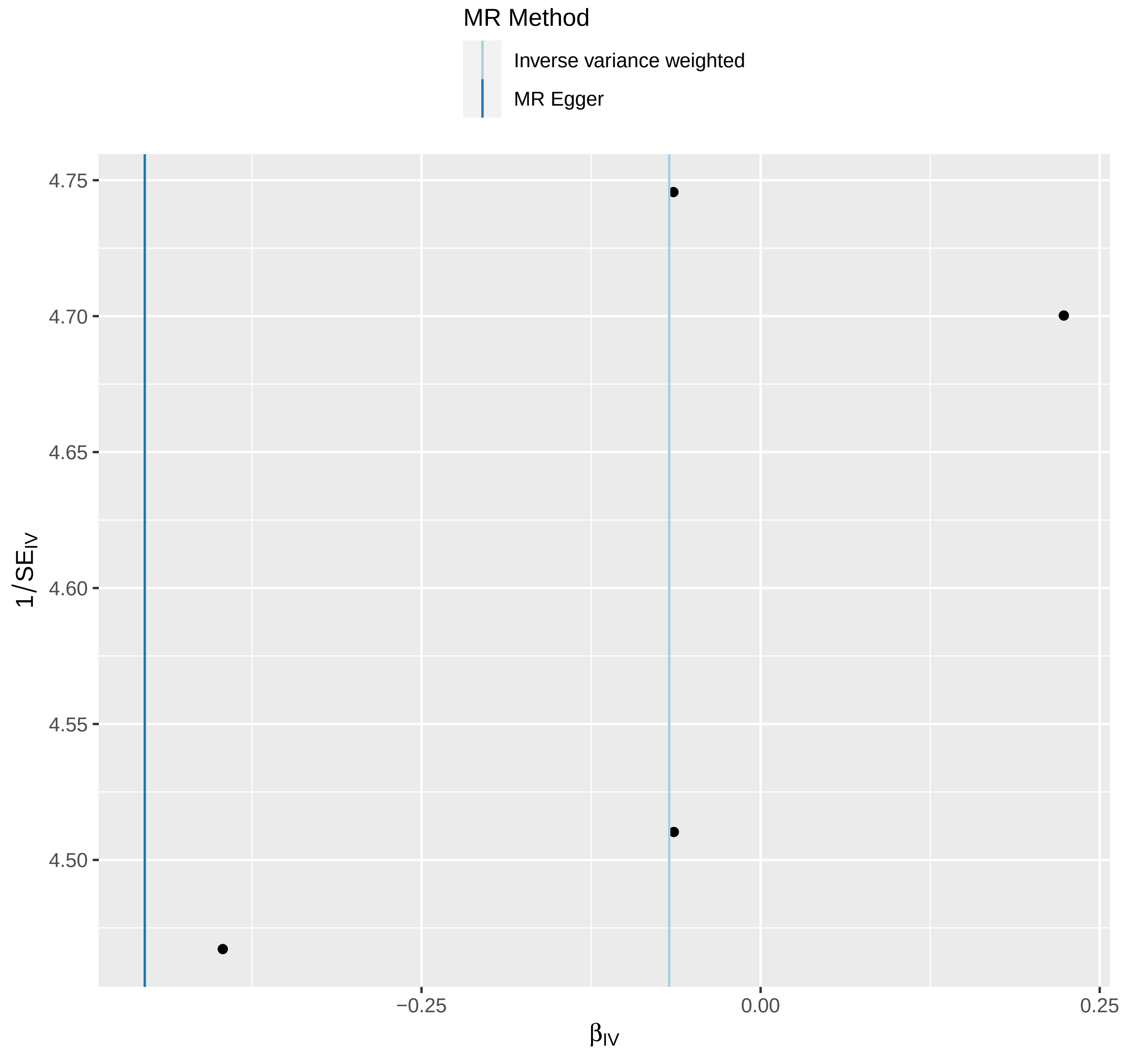

Batch 2−5454 : Gut microbiota abundance (genus Parasutterella id.2892) on Sialic acid−binding Ig−like lectin 14
with a threshold of 1e−05

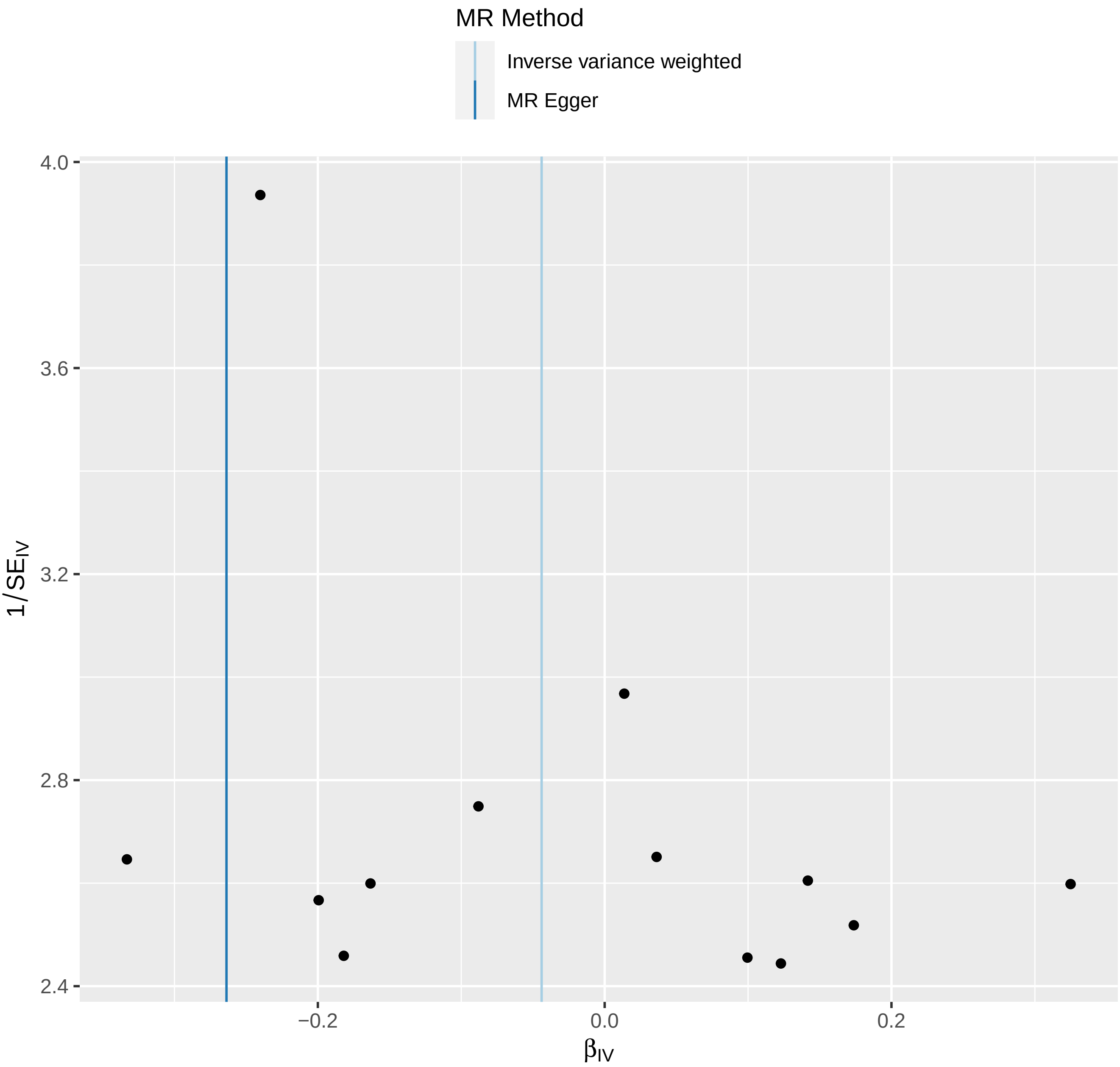

Batch 2−5455 : Gut microbiota abundance (genus Catenibacterium id.2153) on Sialic acid−binding Ig−like lectin 14
with a threshold of 1e−05

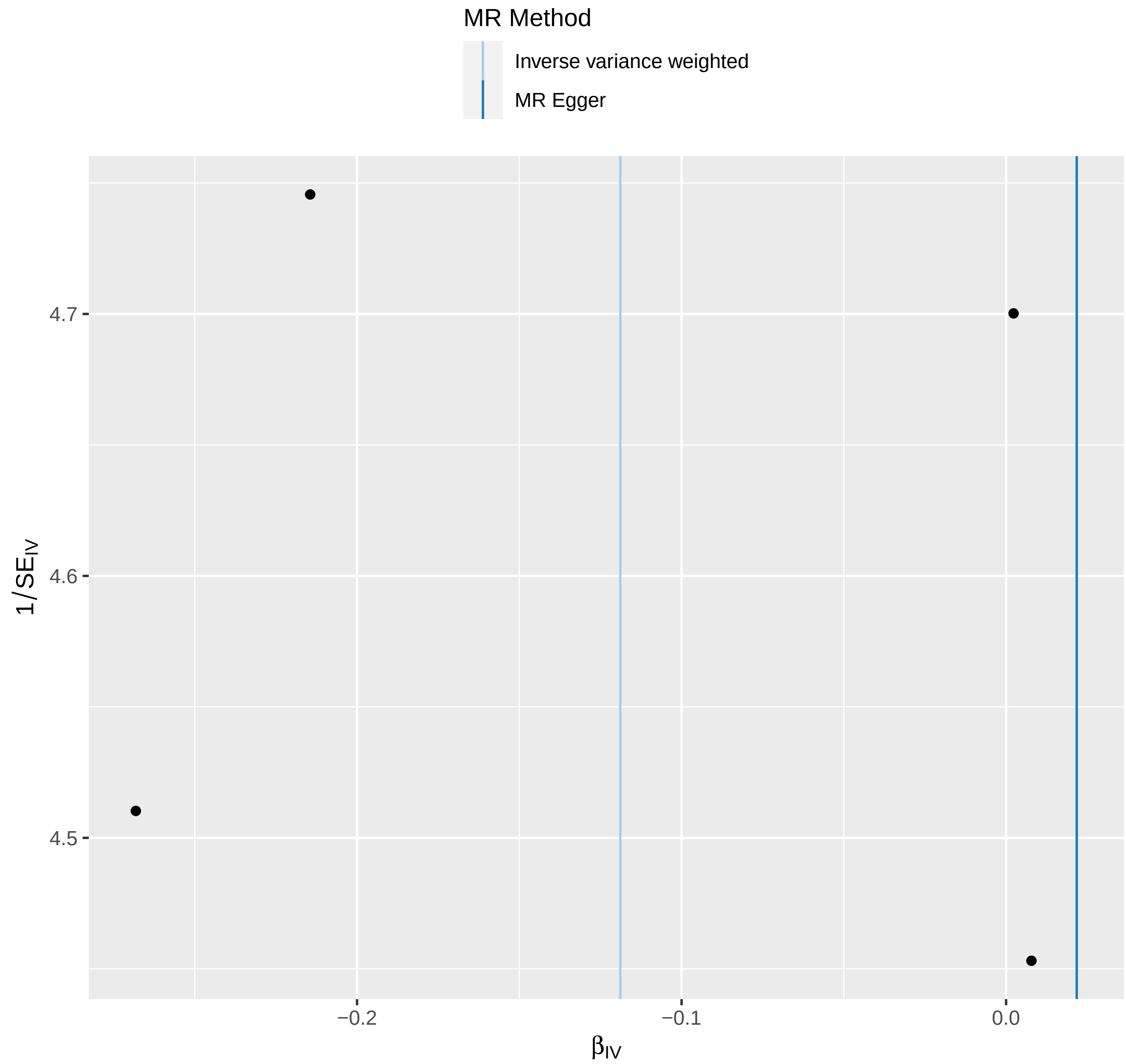

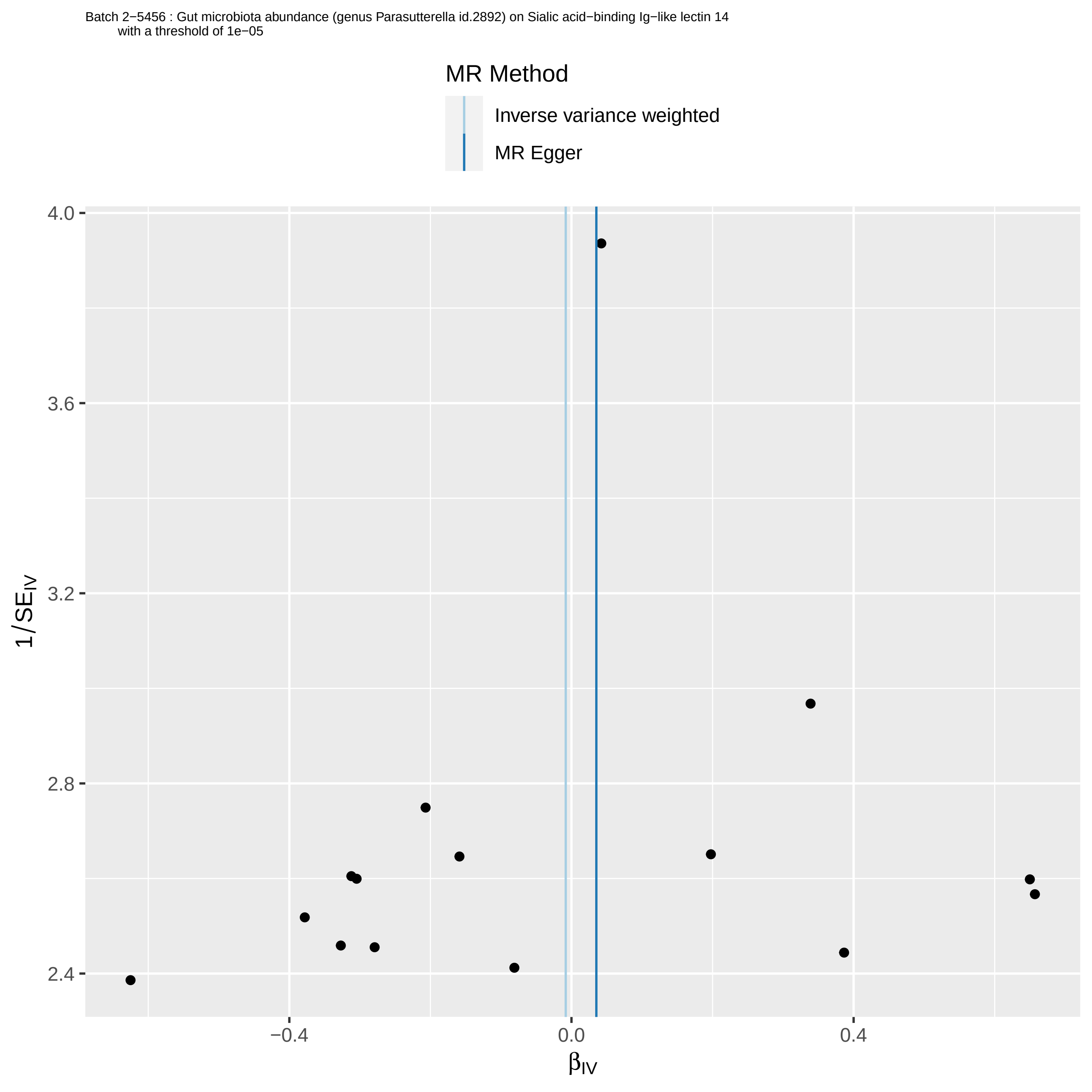

Batch 2−5456 : Gut microbiota abundance (genus Parasutterella id.2892) on Sialic acid−binding Ig−like lectin 14
with a threshold of 1e−05
MR Method
Inverse variance weighted
MR Egger
4.0
3.6
3.2
2.8
2.4
$1/SE_{IV}$
−0.4
0.0
0.4
β_{IV}

Batch 2−5457 : Gut microbiota abundance (genus Catenibacterium id.2153) on Sialic acid−binding Ig−like lectin 7
with a threshold of 1e−05

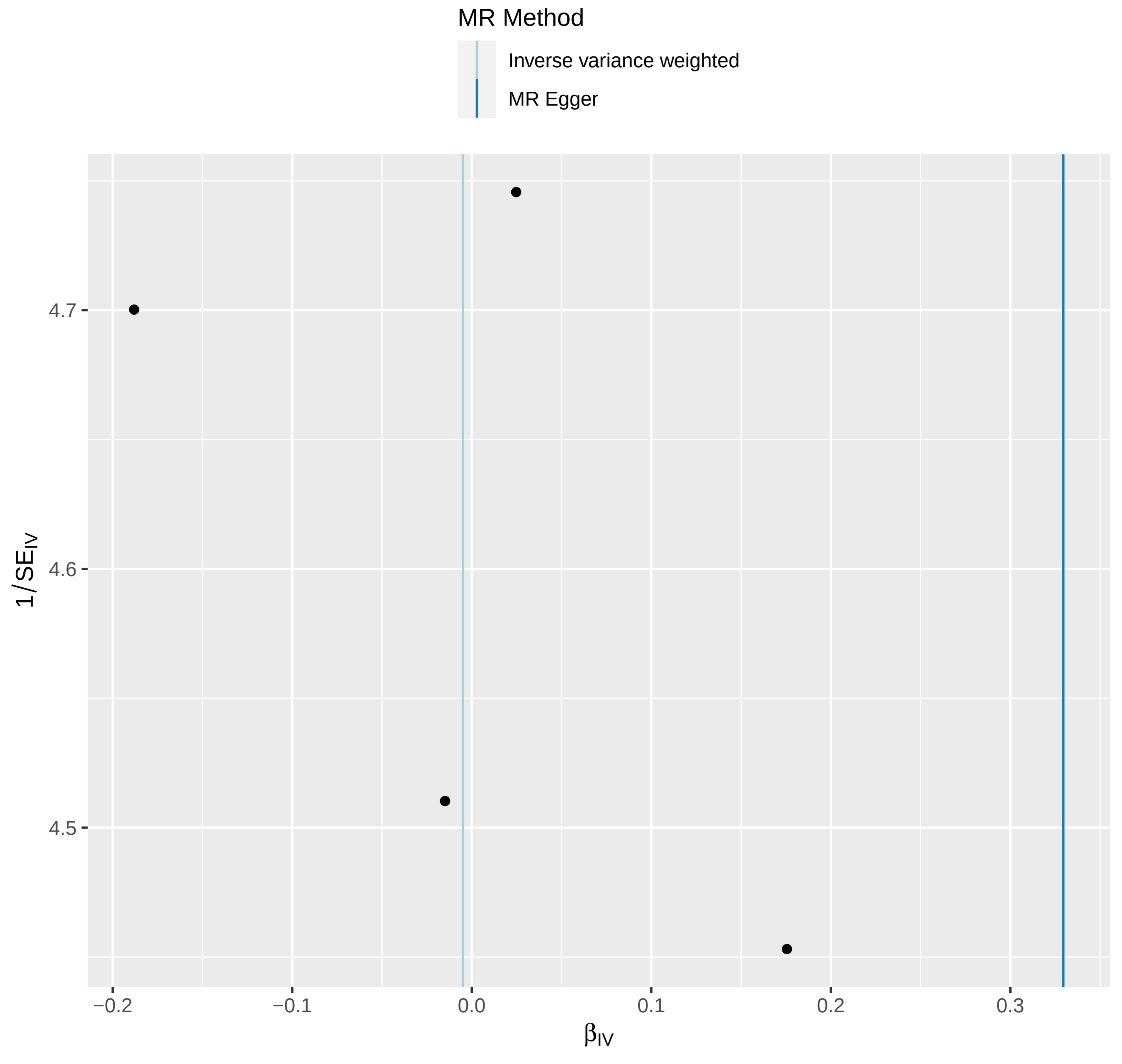

Batch 2−5458 : Gut microbiota abundance (genus Parasutterella id.2892) on Sialic acid−binding Ig−like lectin 7
with a threshold of 1e−05

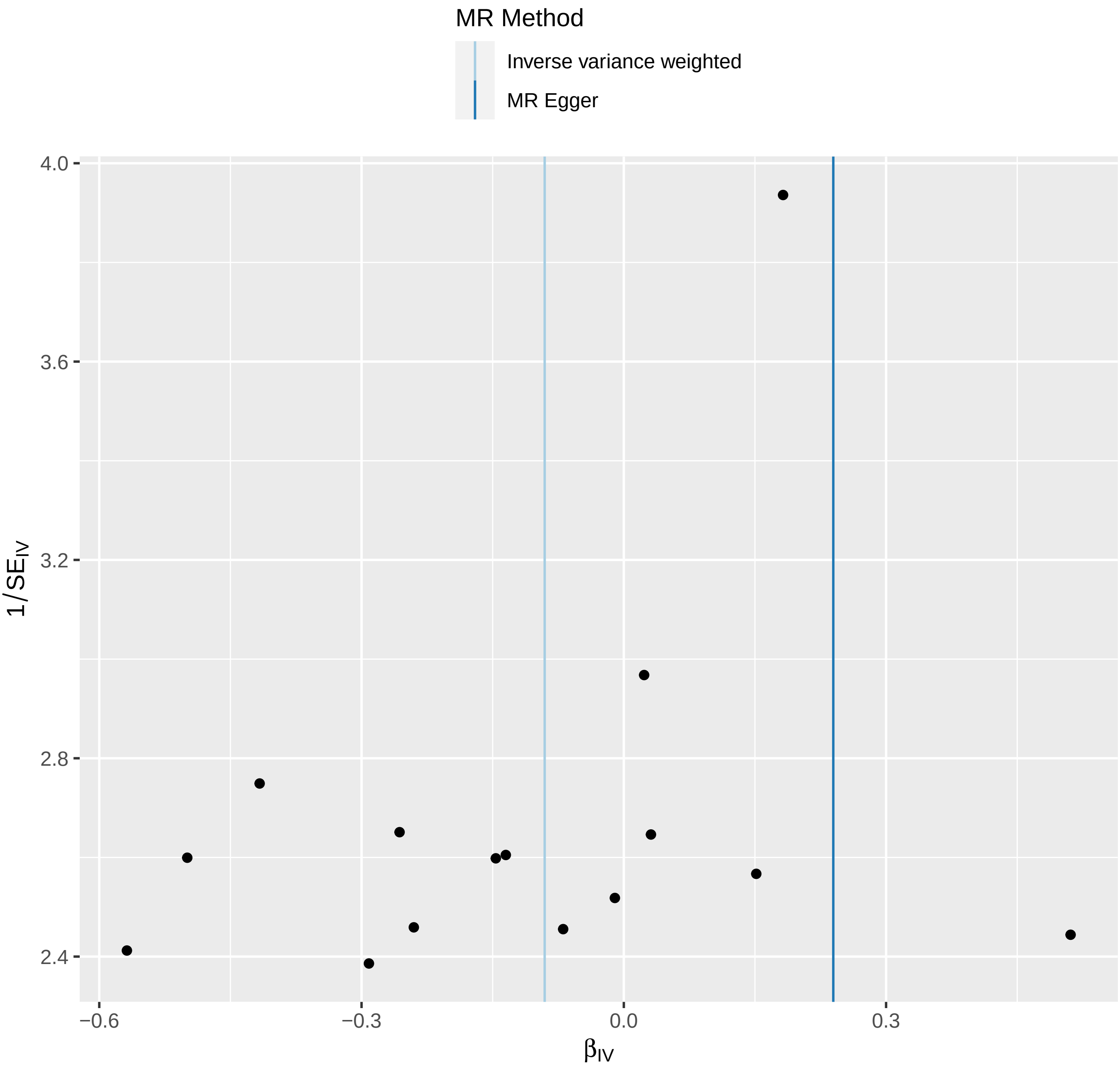

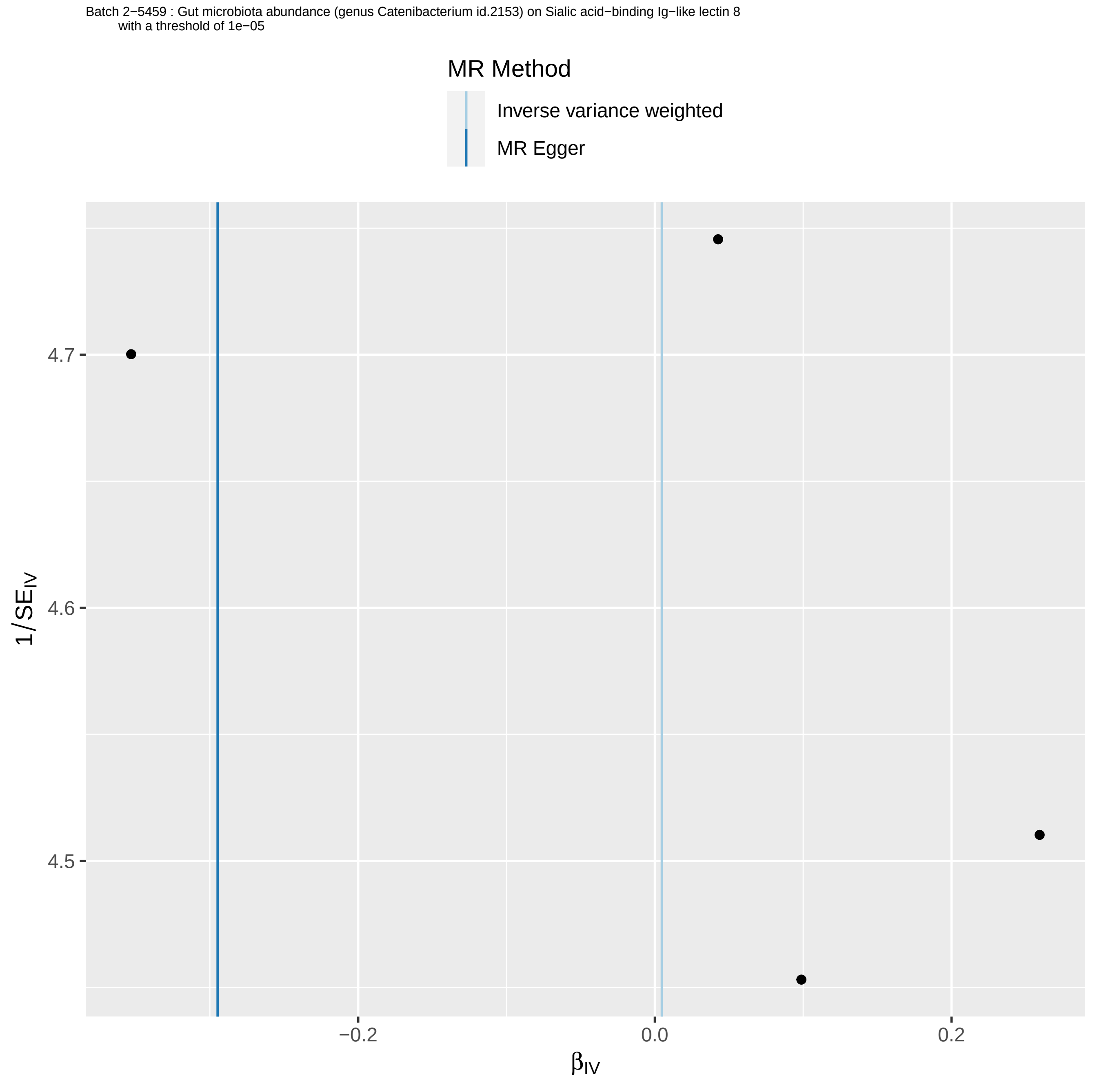

Batch 2−5459 : Gut microbiota abundance (genus Catenibacterium id.2153) on Sialic acid−binding Ig−like lectin 8
with a threshold of 1e−05
MR Method
Inverse variance weighted
MR Egger
4.7
4.6
4.5
1/SE_IV
−0.2
0.0
0.2
β_IV

Batch 2−5460 : Gut microbiota abundance (genus Parasutterella id.2892) on Sialic acid−binding Ig−like lectin 8
with a threshold of 1e−05

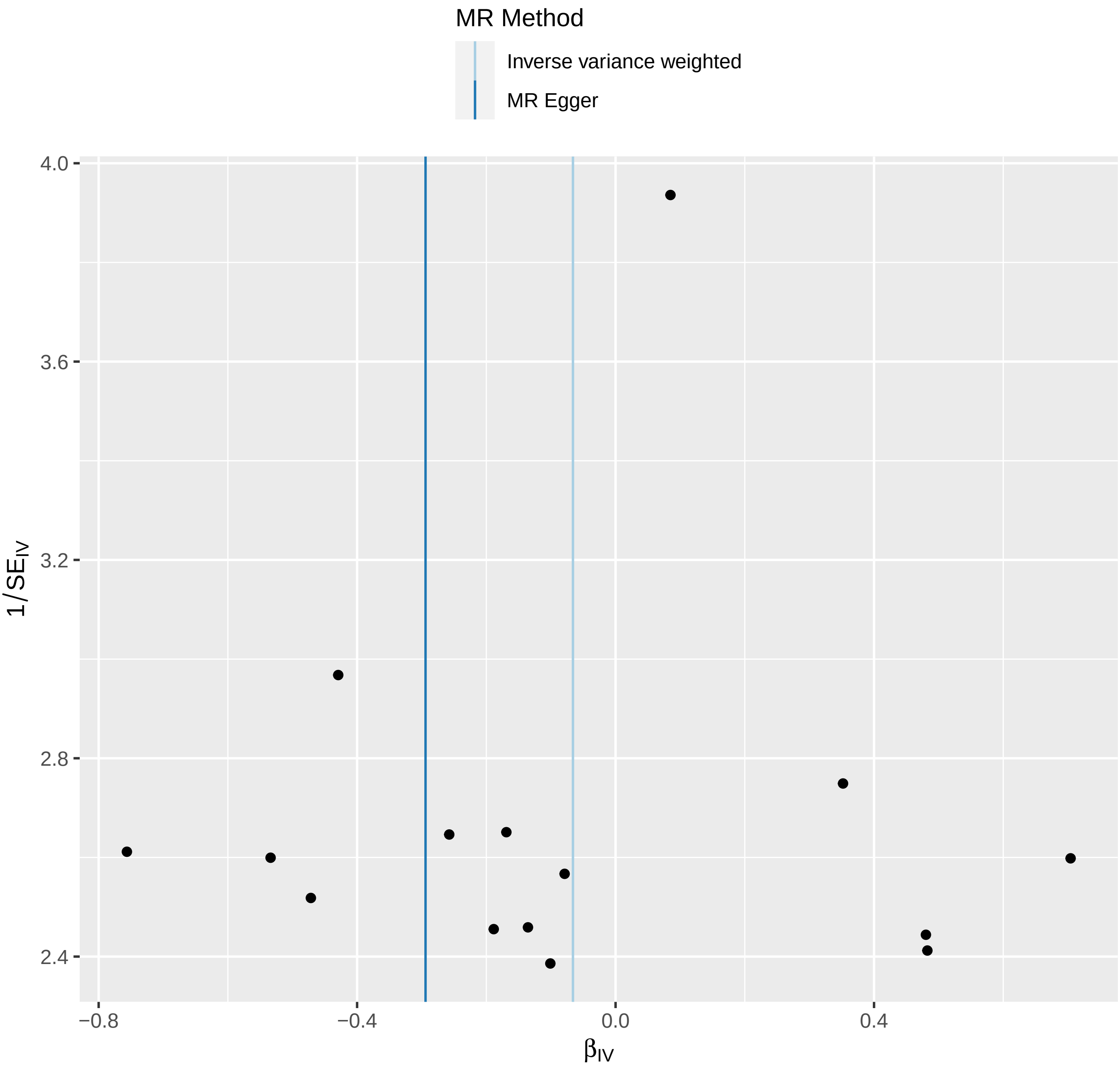

Batch 2−5461 : Gut microbiota abundance (genus Catenibacterium id.2153) on Sialic acid−binding Ig−like lectin 9
with a threshold of 1e−05

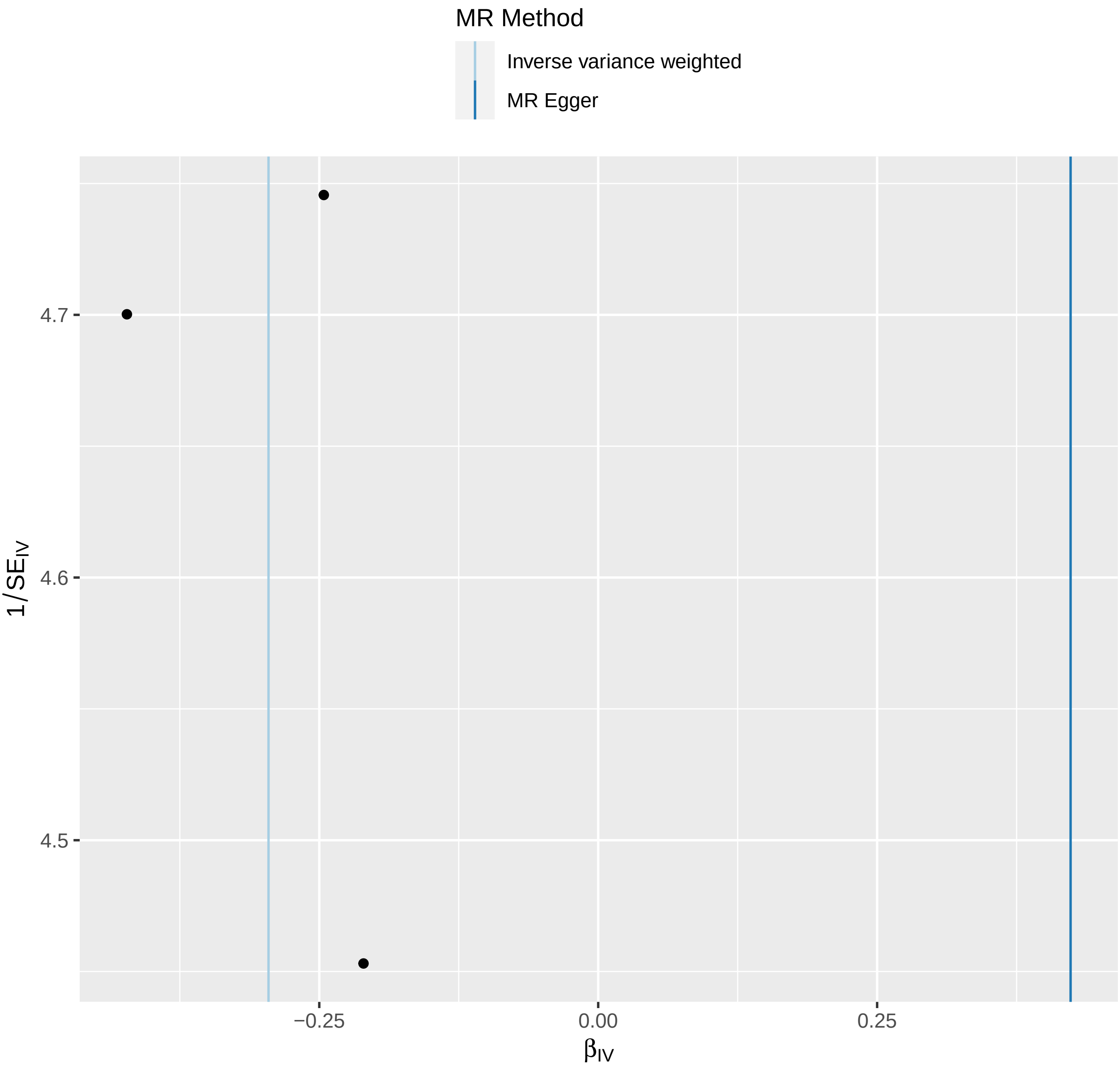

Batch 2−5462 : Gut microbiota abundance (genus Parasutterella id.2892) on Sialic acid−binding Ig−like lectin 9
with a threshold of 1e−05

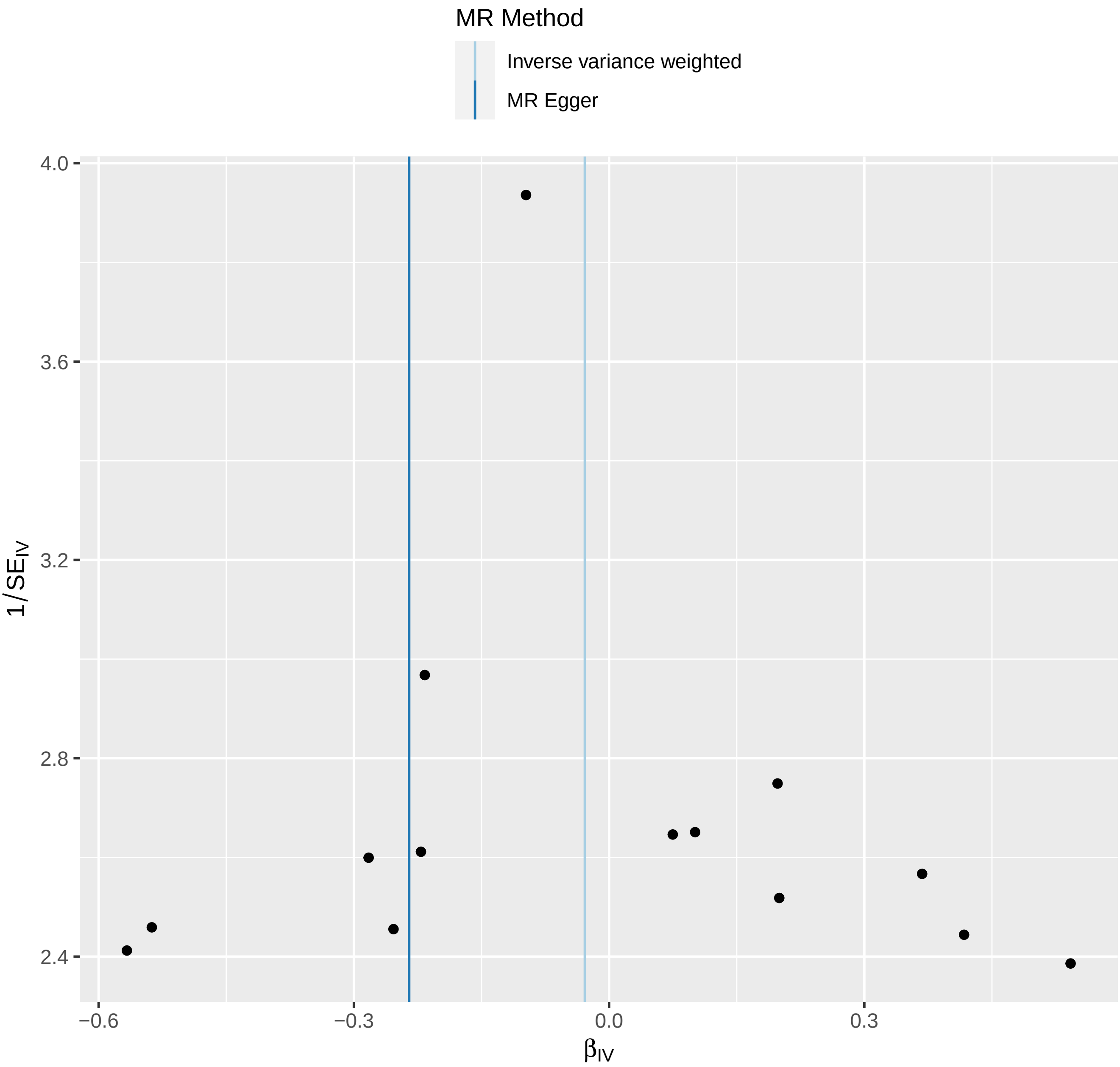

Batch 2−5463 : Gut microbiota abundance (genus Catenibacterium id.2153) on Tyrosine−protein phosphatase non−receptor type substrate 1 with a threshold of 1e−05

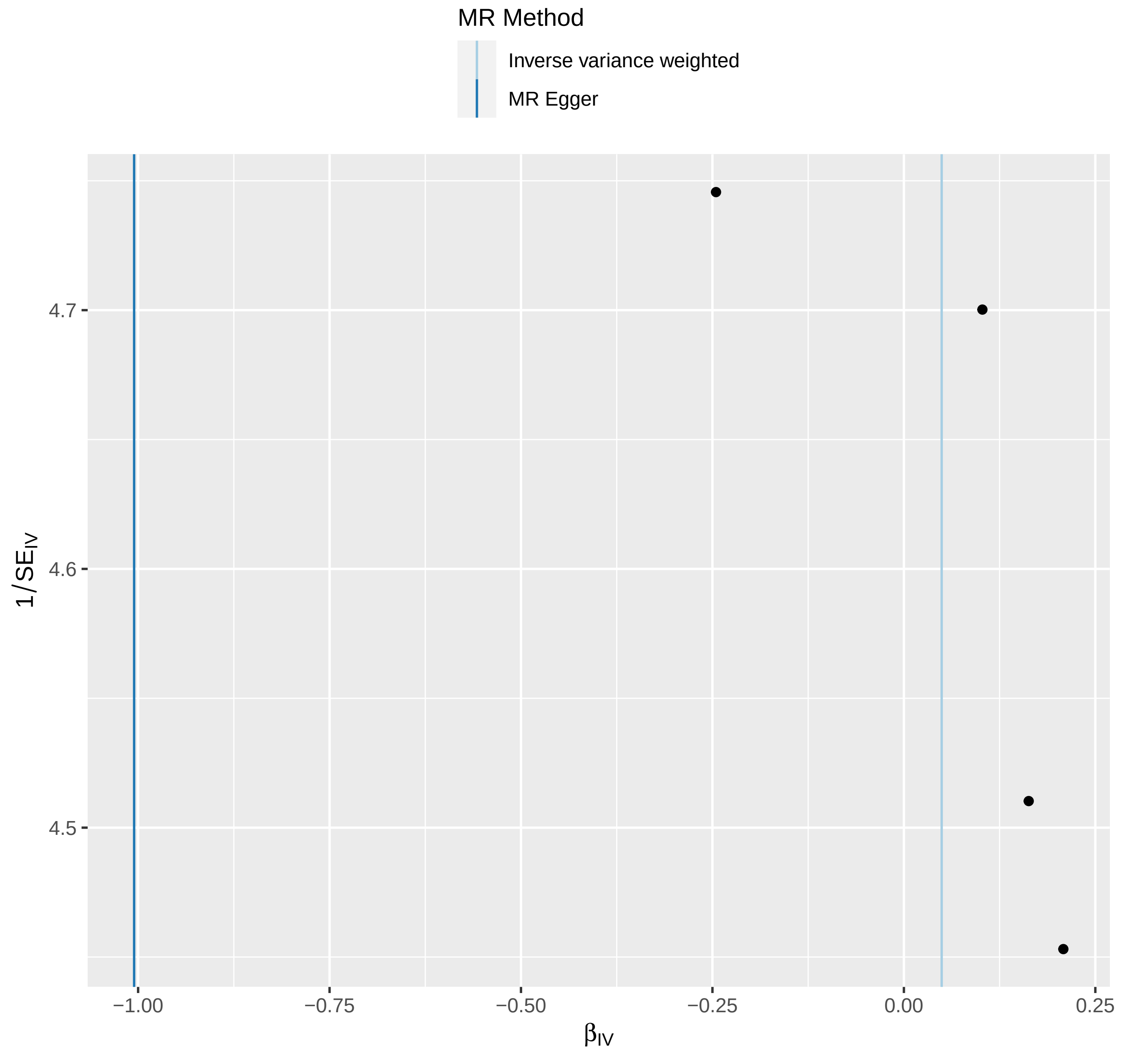

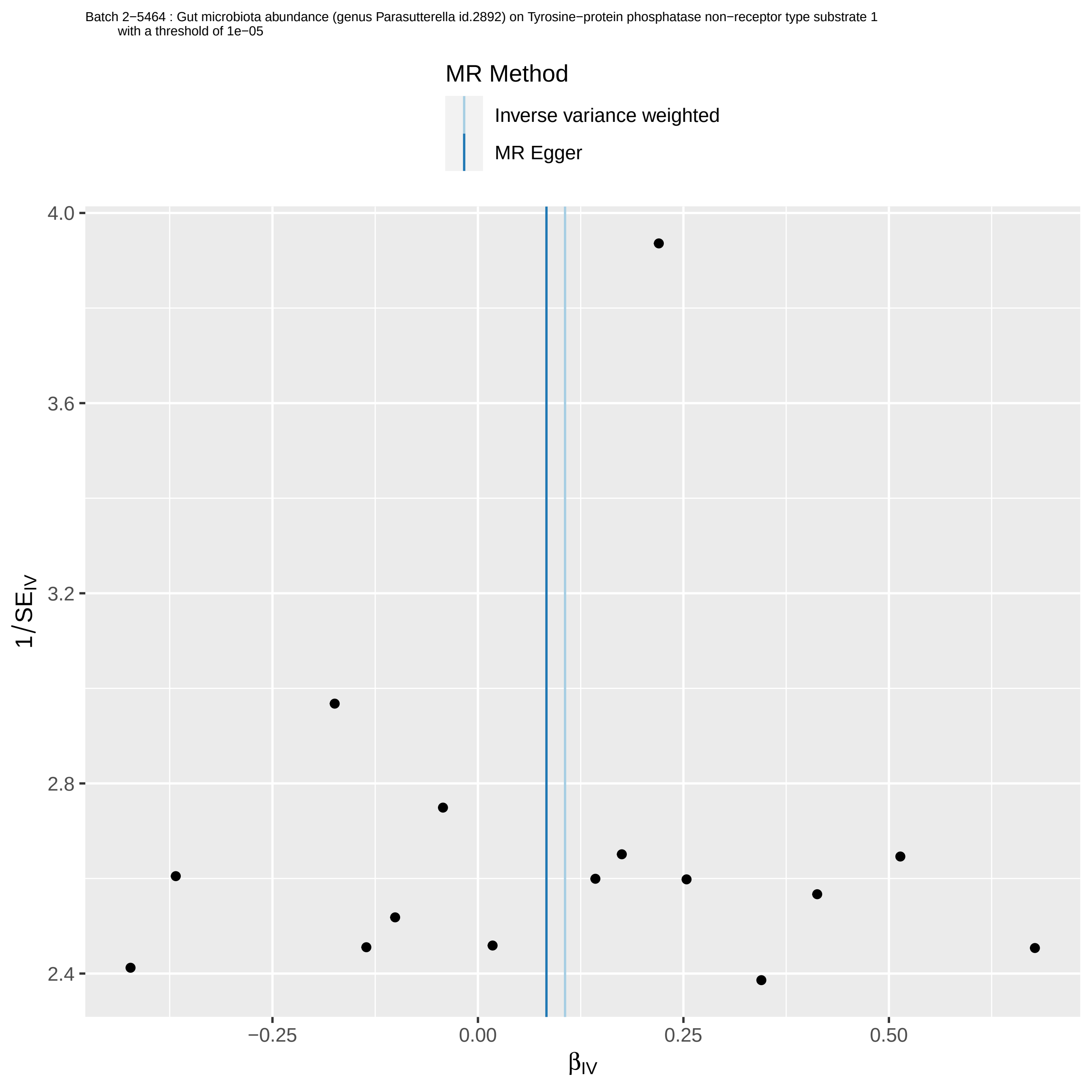

Batch 2−5464 : Gut microbiota abundance (genus Parasutterella id.2892) on Tyrosine−protein phosphatase non−receptor type substrate 1 with a threshold of 1e−05
MR Method
Inverse variance weighted
MR Egger
4.0
3.6
3.2
2.8
2.4
$1/SE_{IV}$
−0.25
0.00
0.25
0.50
β_{IV}

Batch 2−5465 : Gut microbiota abundance (genus Catenibacterium id.2153) on Signal−regulatory protein beta−1
with a threshold of 1e−05

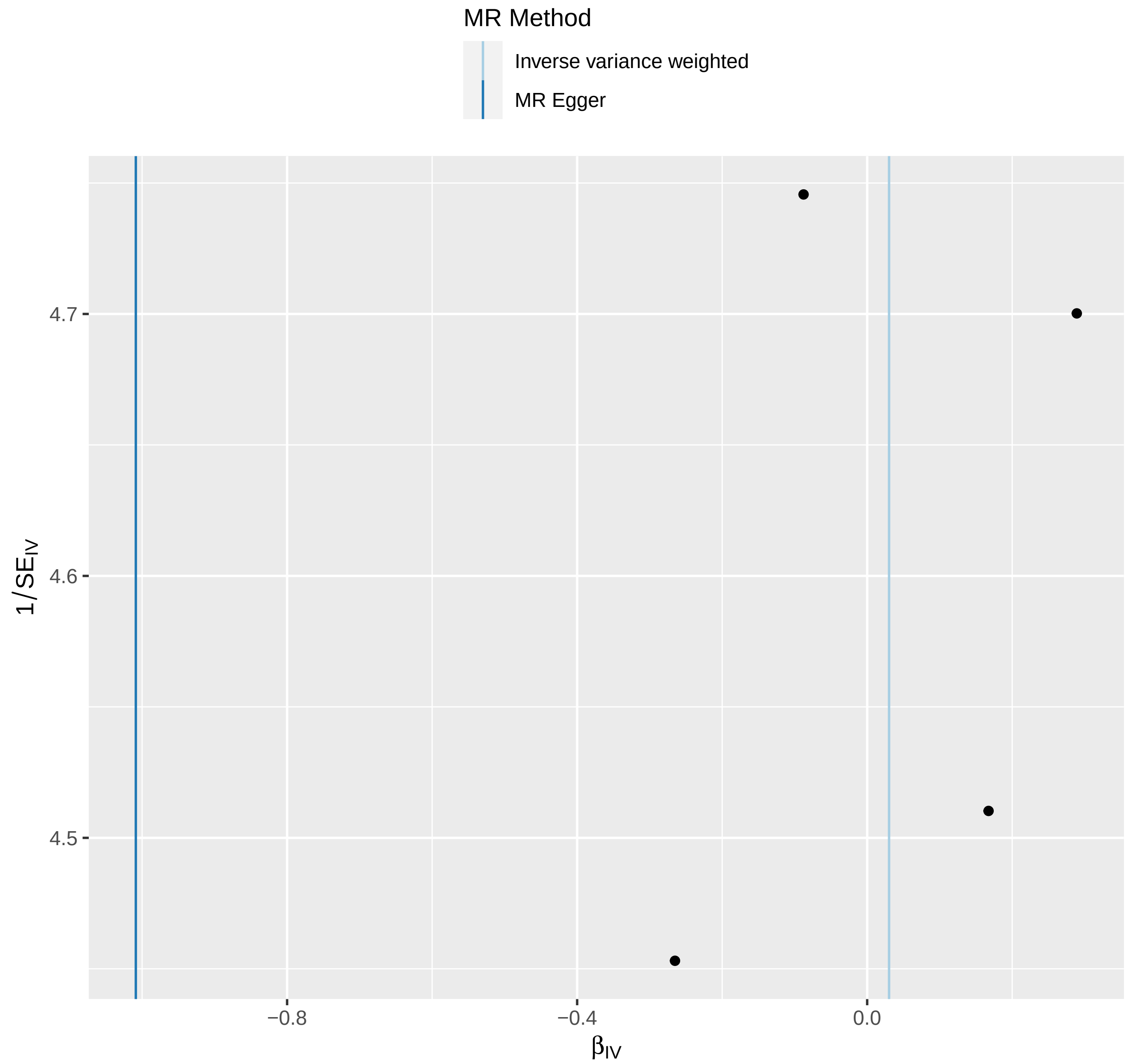

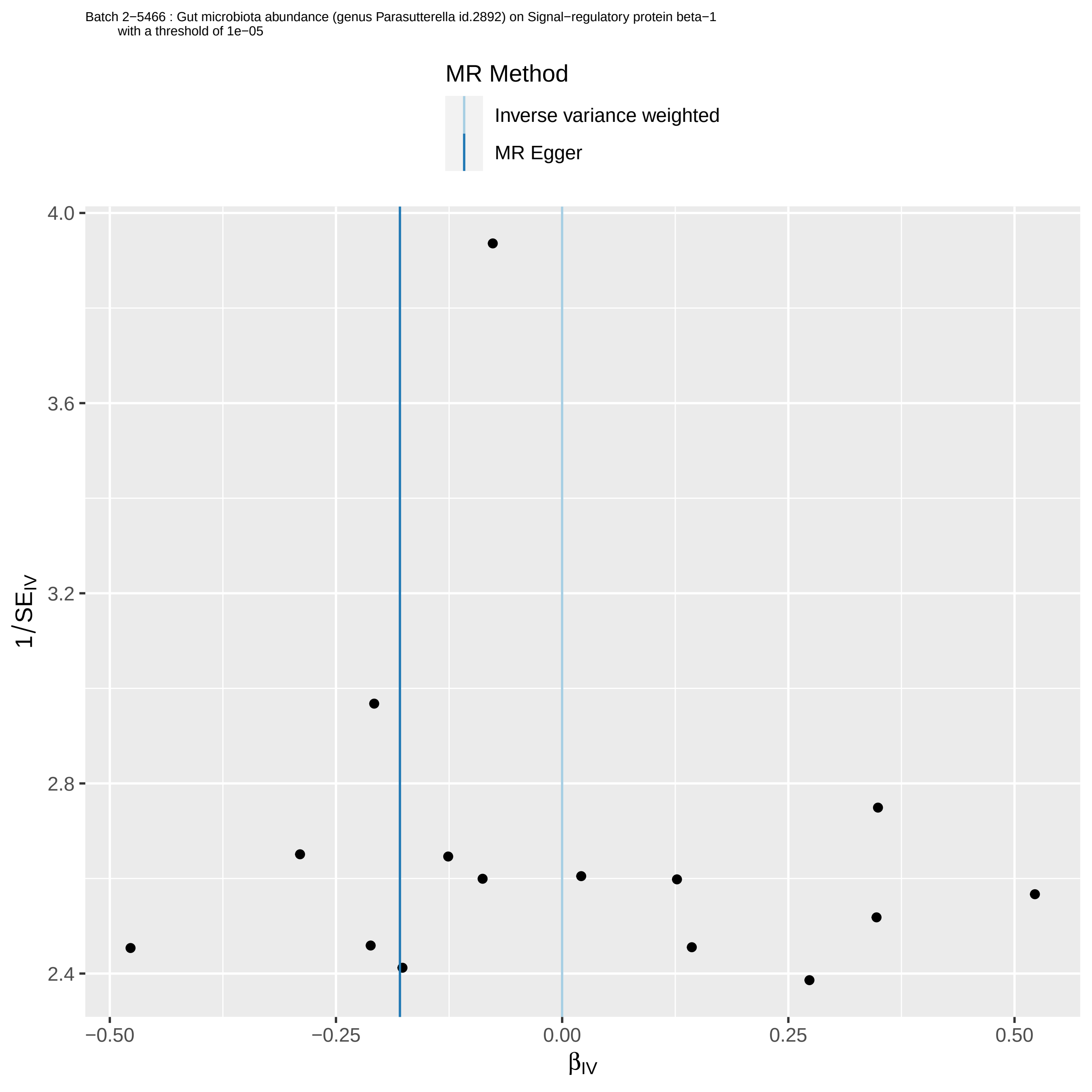
Batch 2−5466 : Gut microbiota abundance (genus Parasutterella id.2892) on Signal−regulatory protein beta−1
with a threshold of 1e−05
MR Method
Inverse variance weighted
MR Egger
4.0
3.6
3.2
2.8
2.4
1/SE$_{IV}$
−0.50
−0.25
0.00
0.25
0.50
β_{IV}

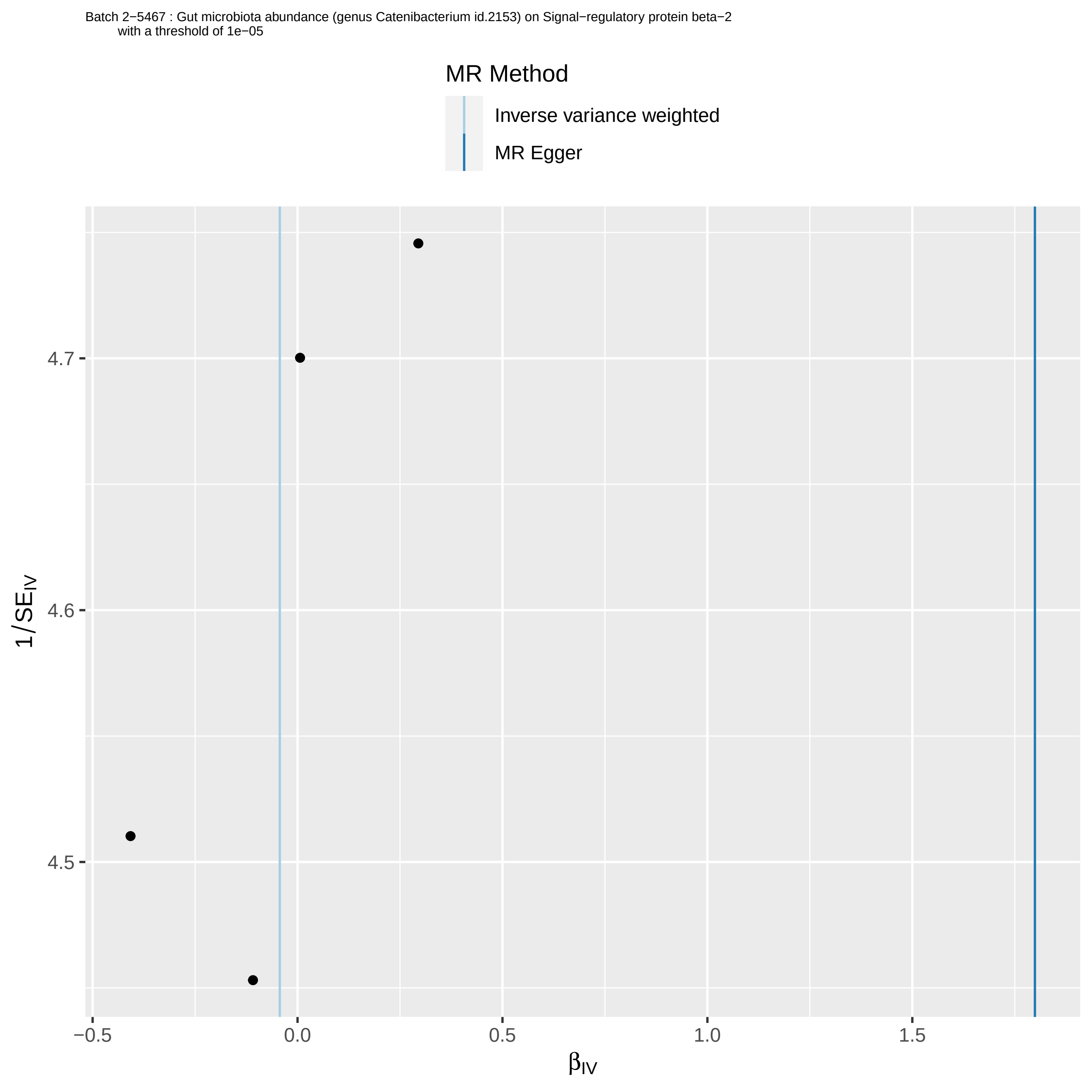

Batch 2−5467 : Gut microbiota abundance (genus Catenibacterium id.2153) on Signal−regulatory protein beta−2
with a threshold of 1e−05
MR Method
Inverse variance weighted
MR Egger
4.7
4.6
4.5
1/SE_IV
−0.5
0.0
0.5
1.0
1.5
β_IV

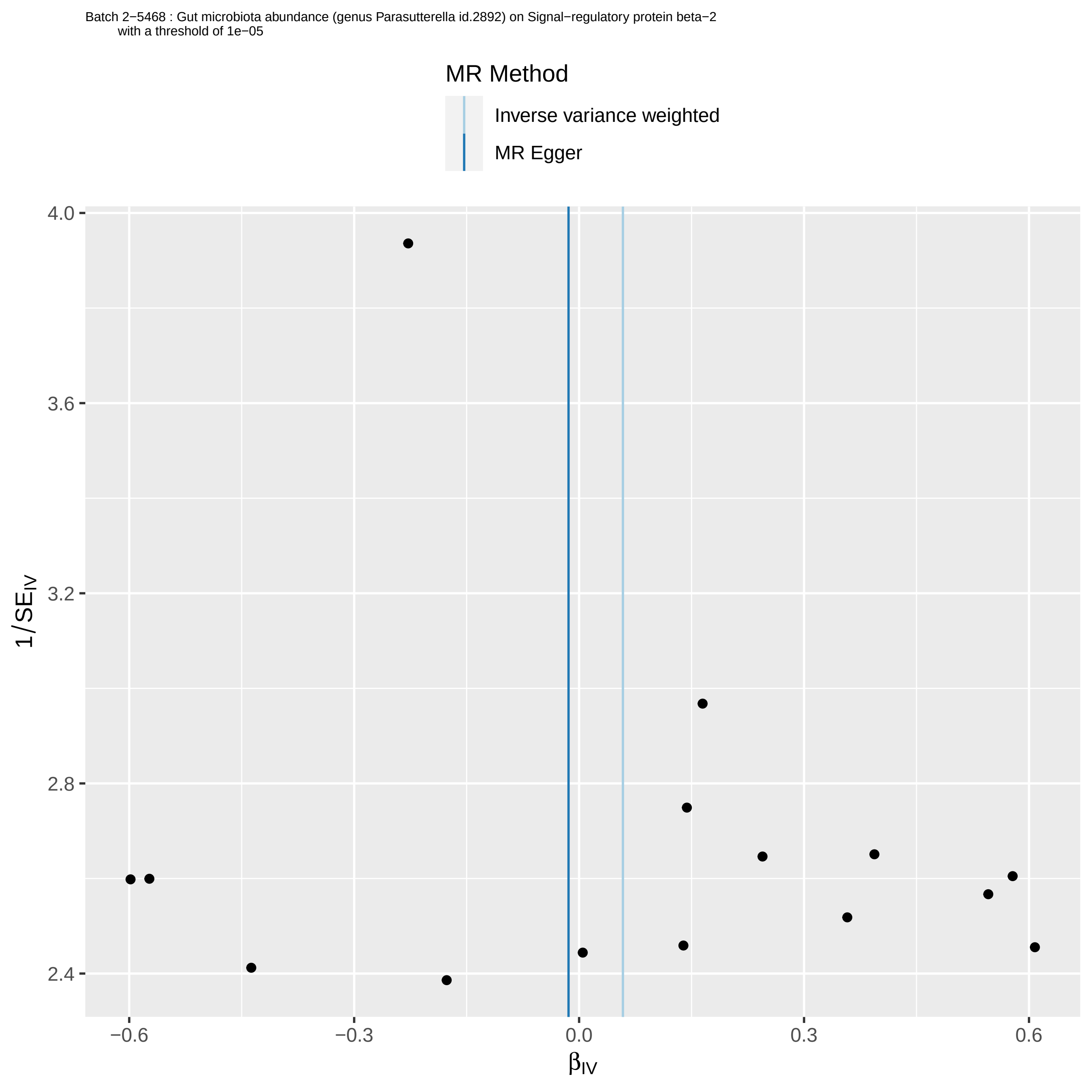
Batch 2−5468 : Gut microbiota abundance (genus Parasutterella id.2892) on Signal−regulatory protein beta−2
with a threshold of 1e−05
MR Method
Inverse variance weighted
MR Egger
4.0
3.6
3.2
2.8
2.4
1/SE$_{IV}$
−0.6
−0.3
0.0
0.3
0.6
β$_{IV}$

Batch 2−5469 : Gut microbiota abundance (genus Catenibacterium id.2153) on Signal−regulatory protein gamma
with a threshold of 1e−05

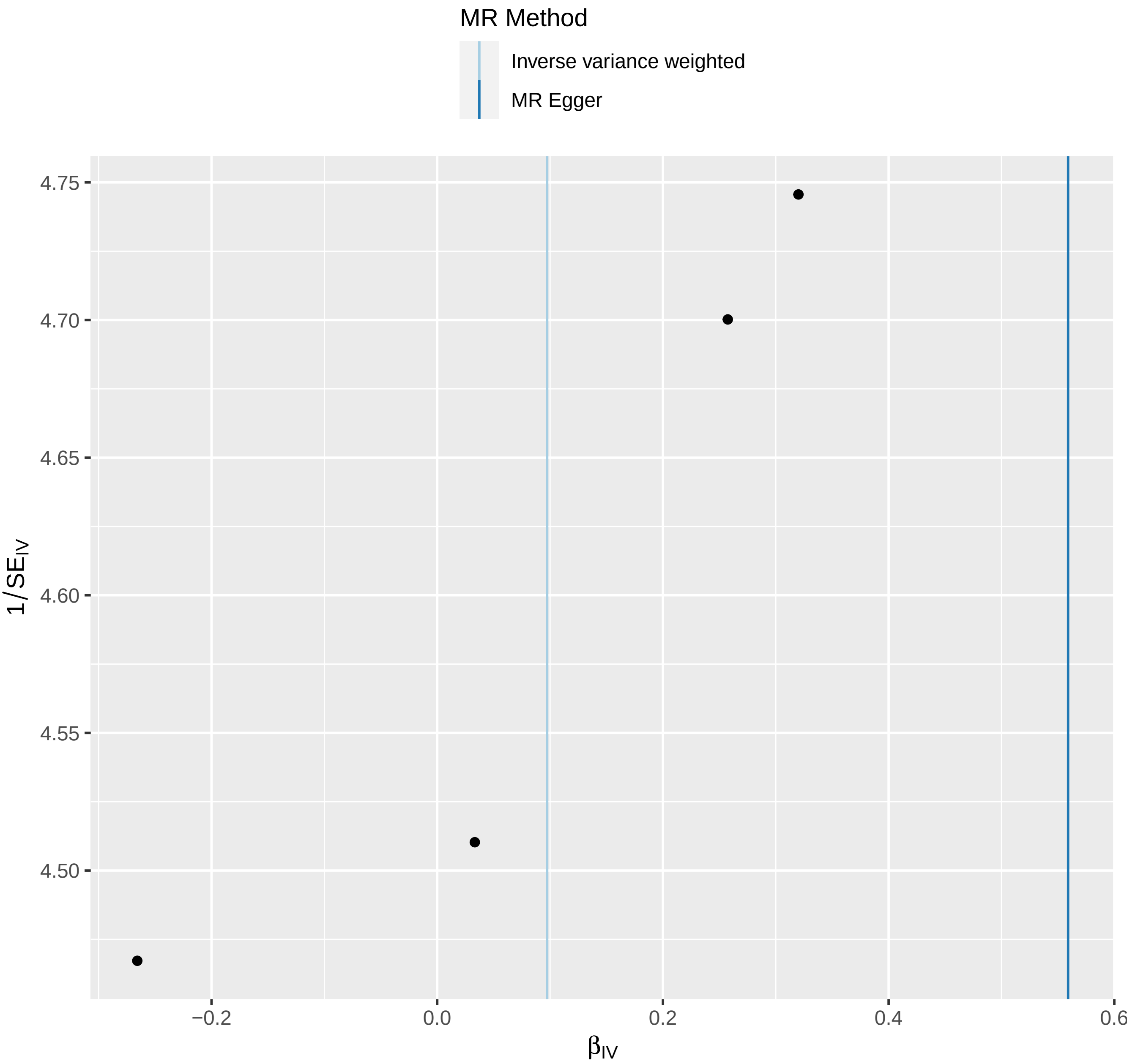

Batch 2−5470 : Gut microbiota abundance (genus Parasutterella id.2892) on Signal−regulatory protein gamma
with a threshold of 1e−05

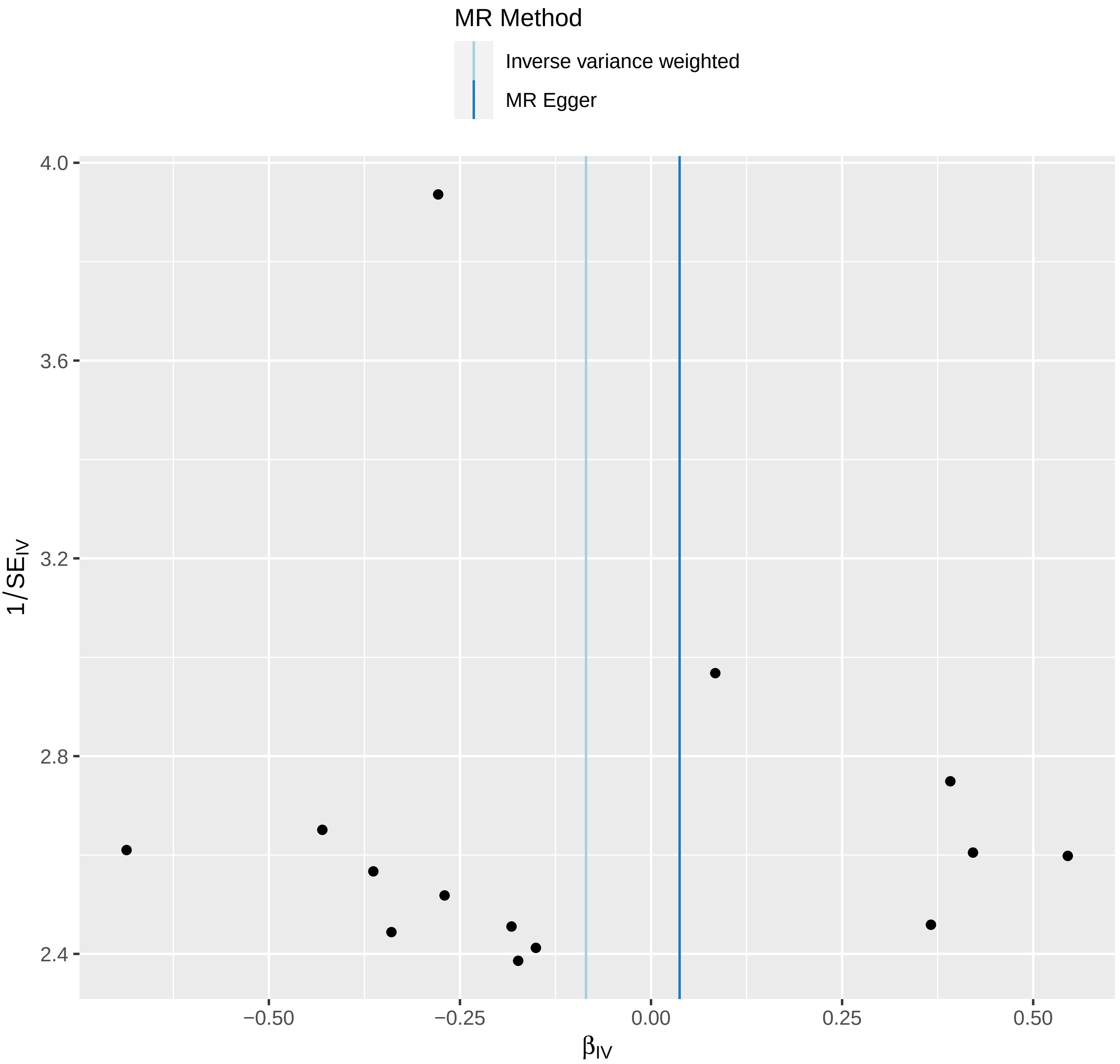

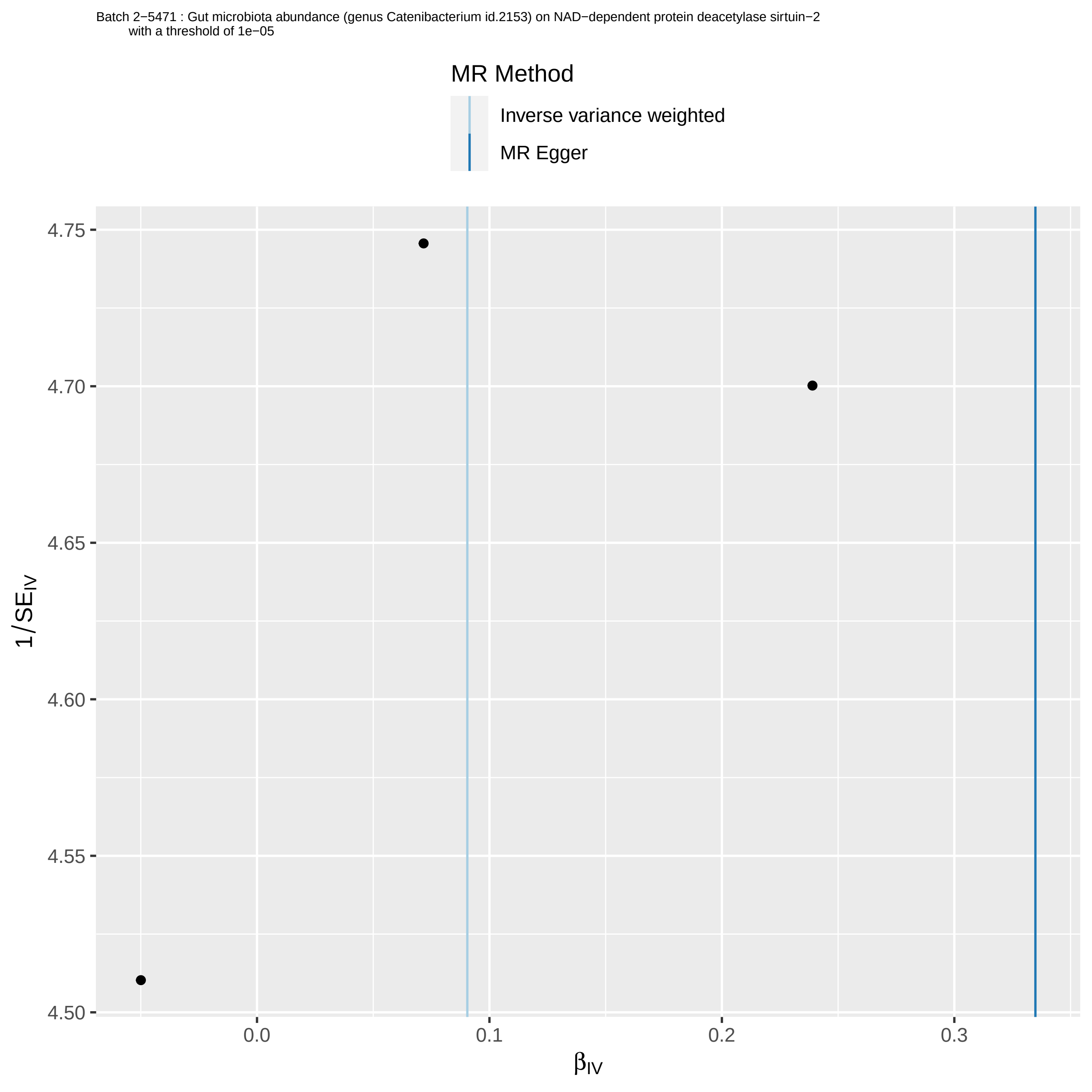

Batch 2−5471 : Gut microbiota abundance (genus Catenibacterium id.2153) on NAD−dependent protein deacetylase sirtuin−2
with a threshold of 1e−05
MR Method
Inverse variance weighted
MR Egger
4.75
4.70
4.65
4.60
4.55
4.50
1/SE_IV
0.0
0.1
0.2
0.3
β_IV

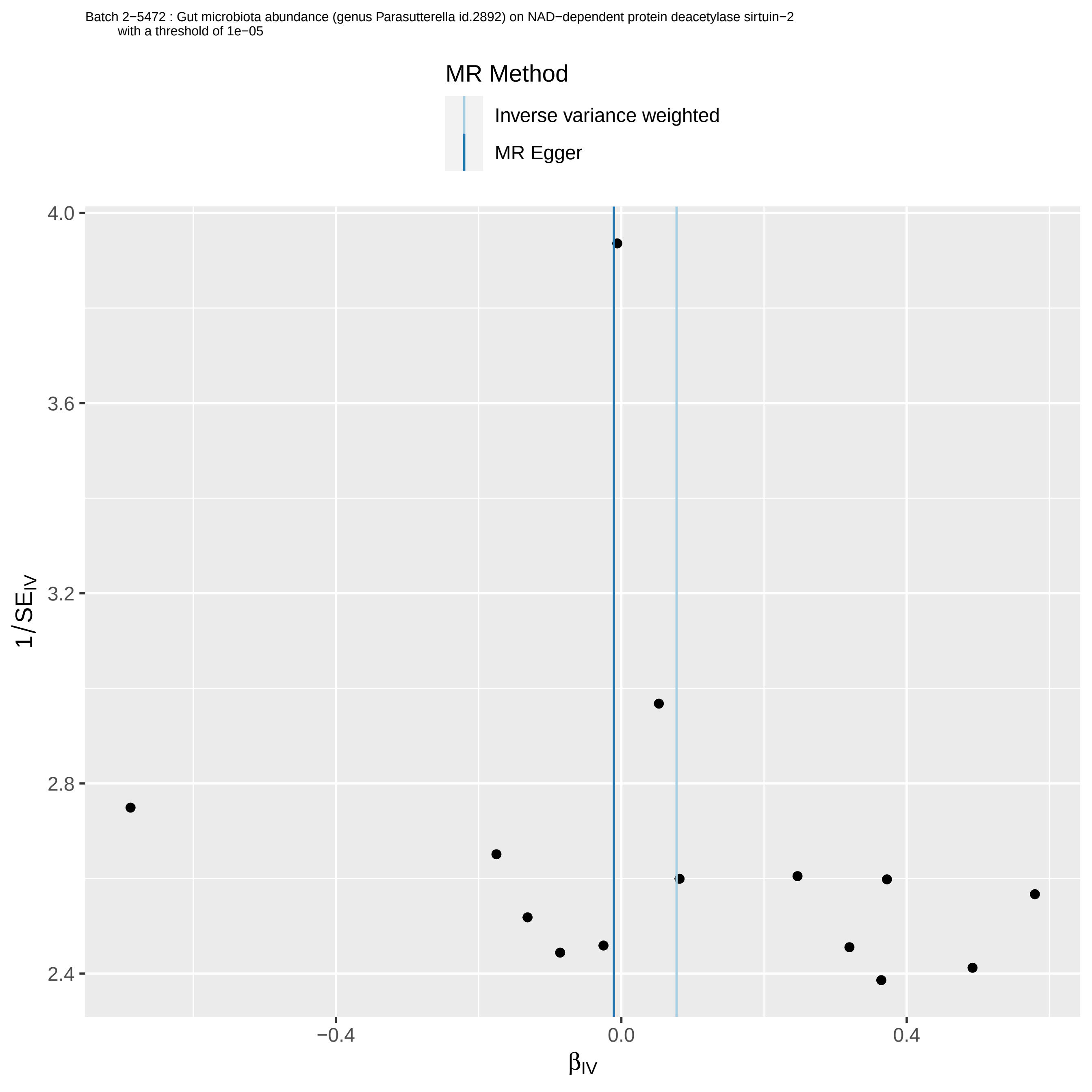
Batch 2−5472 : Gut microbiota abundance (genus Parasutterella id.2892) on NAD−dependent protein deacetylase sirtuin−2
with a threshold of 1e−05
MR Method
Inverse variance weighted
MR Egger
4.0
3.6
3.2
2.8
2.4
1/SE_IV
−0.4
0.0
0.4
β_IV

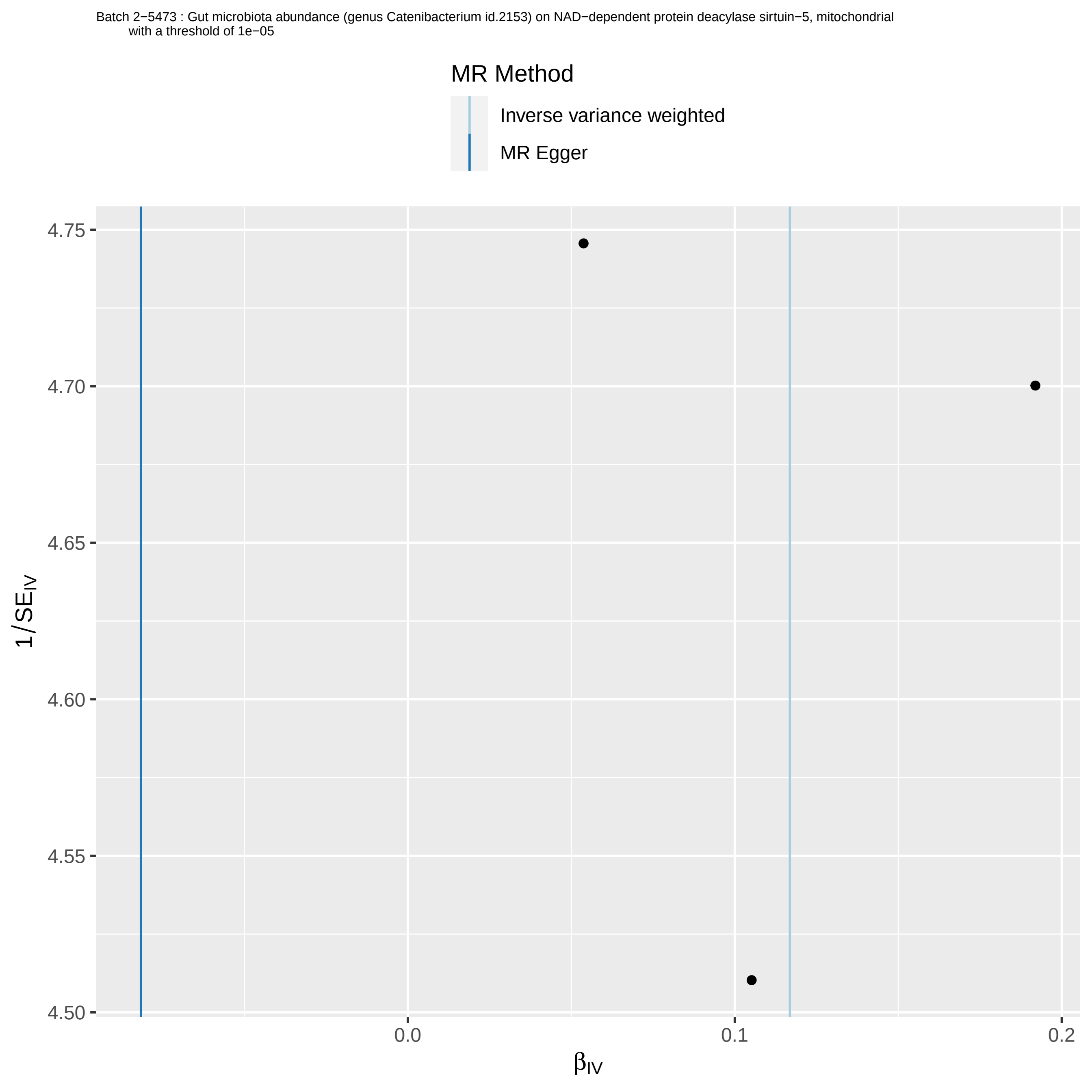

Batch 2−5473 : Gut microbiota abundance (genus Catenibacterium id.2153) on NAD−dependent protein deacylase sirtuin−5, mitochondrial
with a threshold of 1e−05
MR Method
Inverse variance weighted
MR Egger
4.75
4.70
4.65
4.60
4.55
4.50
1/SE_IV
0.0
0.1
0.2
β_IV

Batch 2−5474 : Gut microbiota abundance (genus Parasutterella id.2892) on NAD−dependent protein deacylase sirtuin−5, mitochondrial
with a threshold of 1e−05

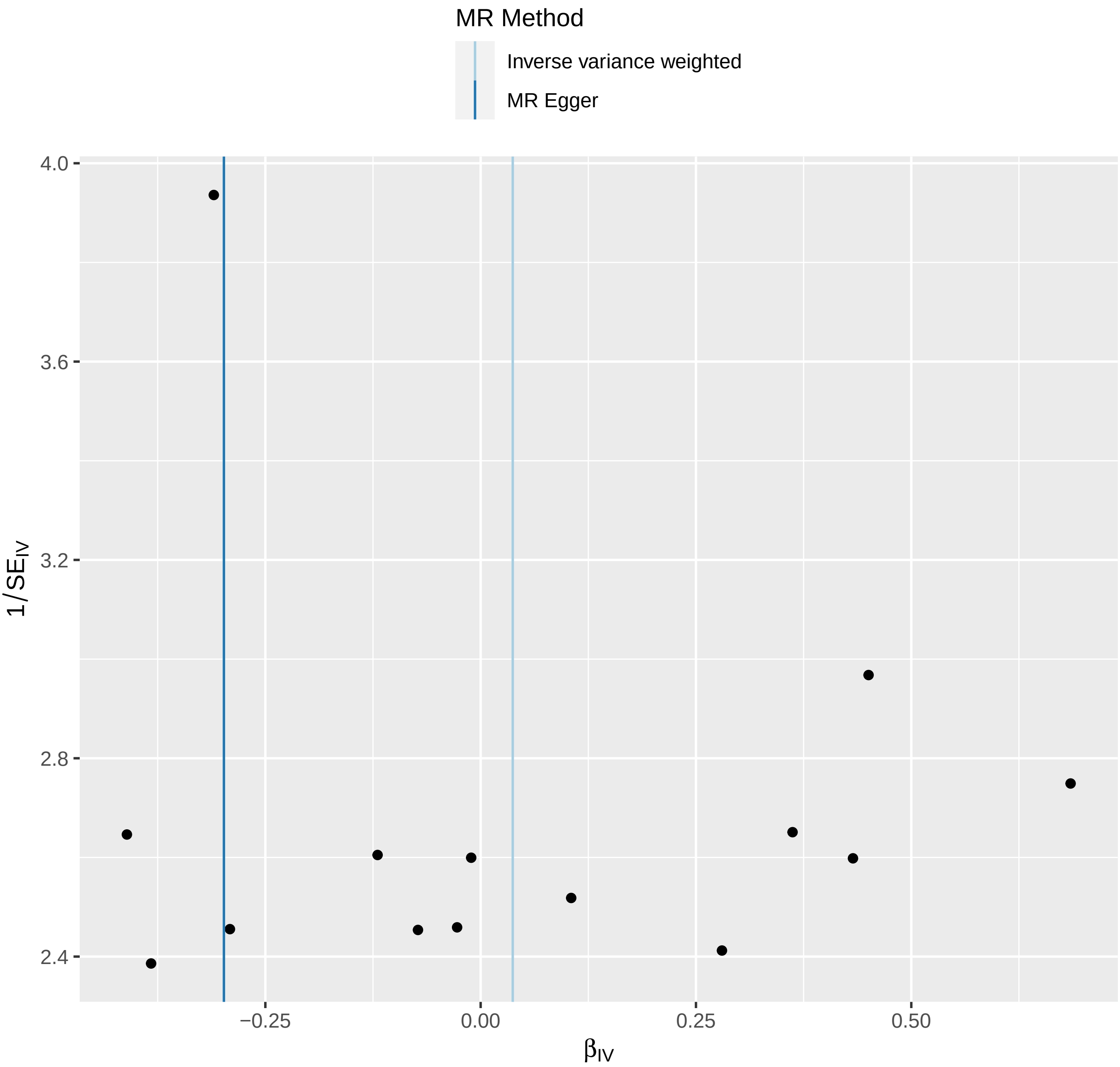

Batch 2−5475 : Gut microbiota abundance (genus Catenibacterium id.2153) on Signaling threshold−regulating transmembrane adapter 1 with a threshold of 1e−05

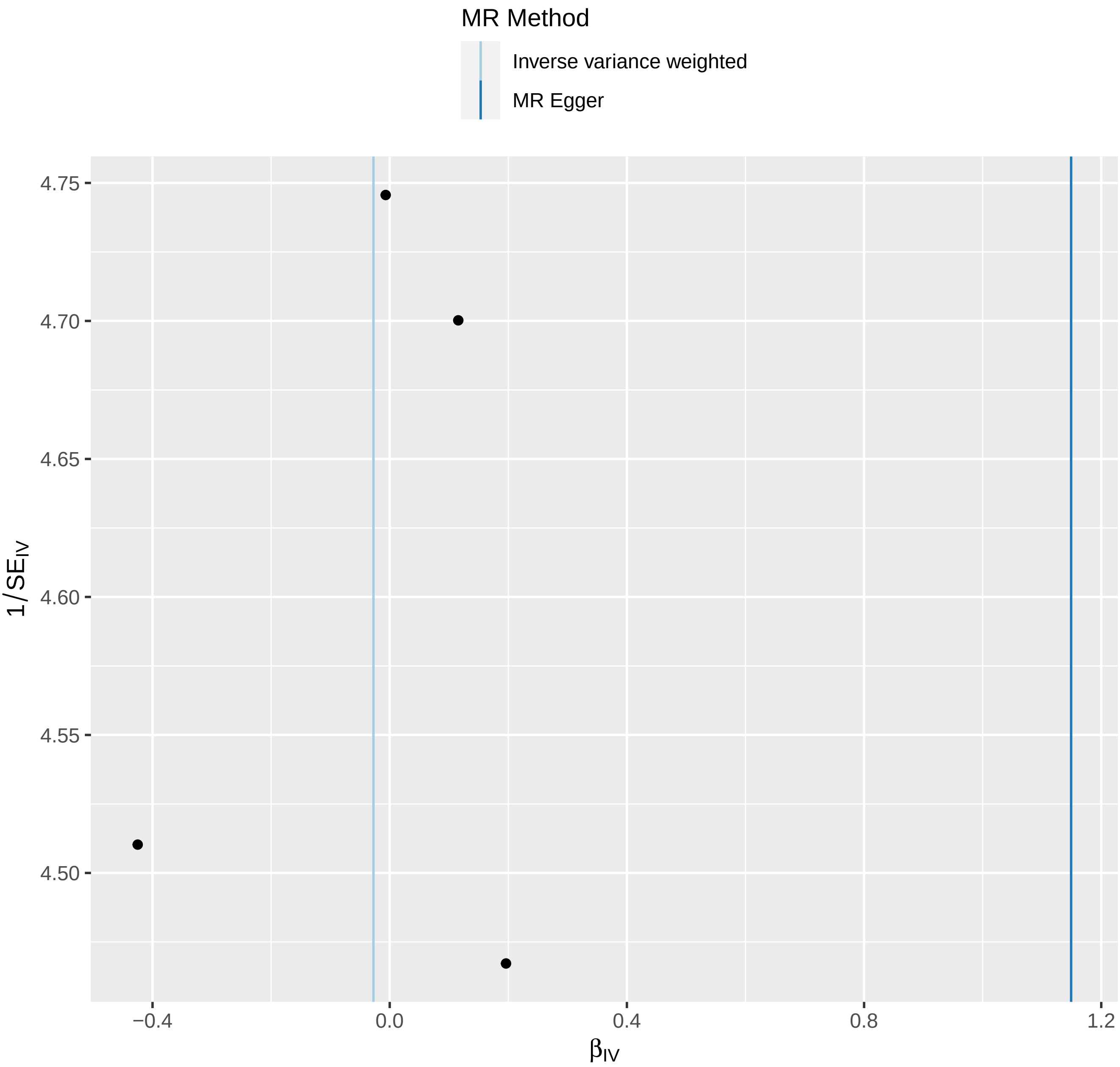

Batch 2−5476 : Gut microbiota abundance (genus Parasutterella id.2892) on Signaling threshold−regulating transmembrane adapter 1
with a threshold of 1e−05

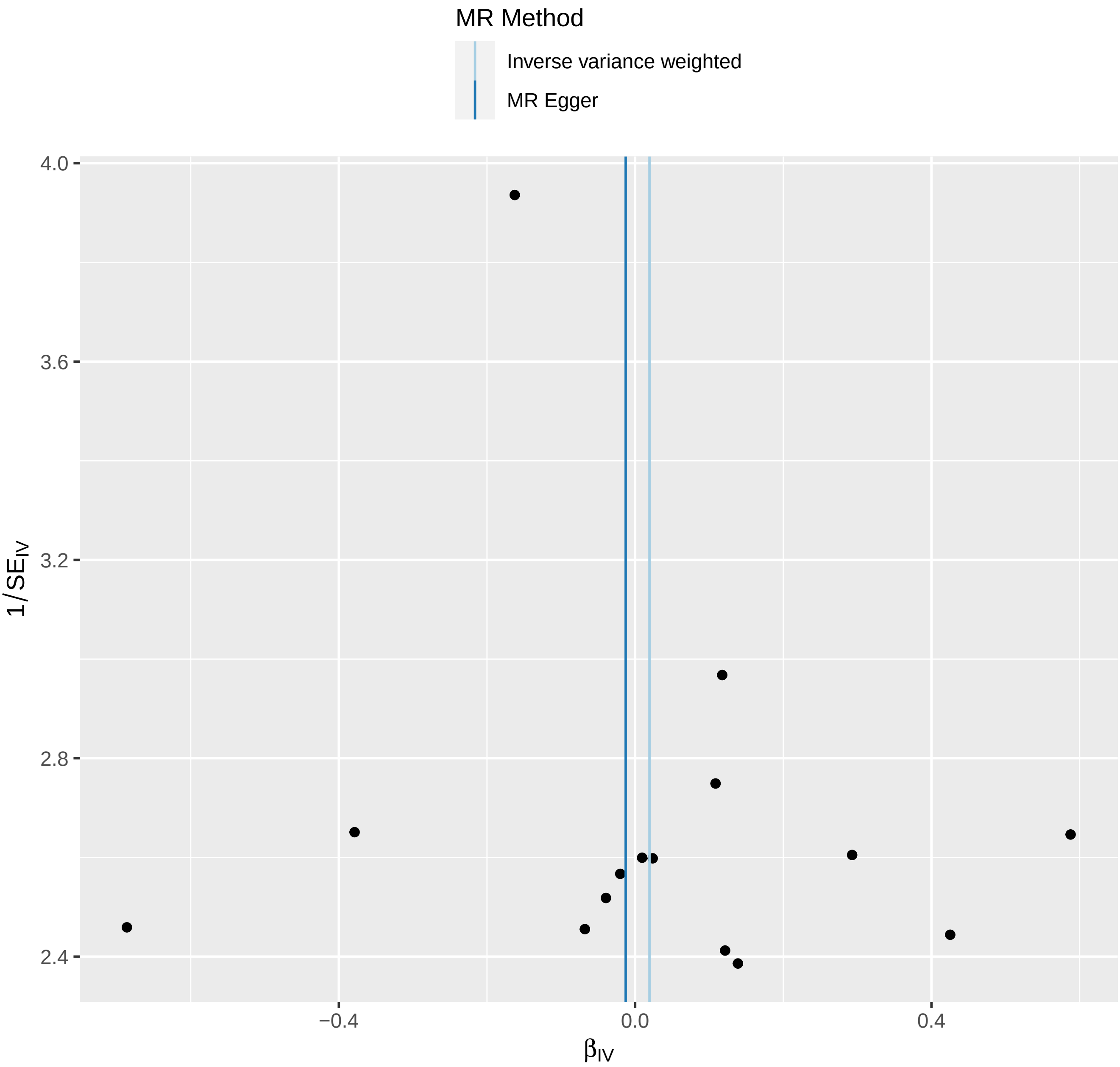

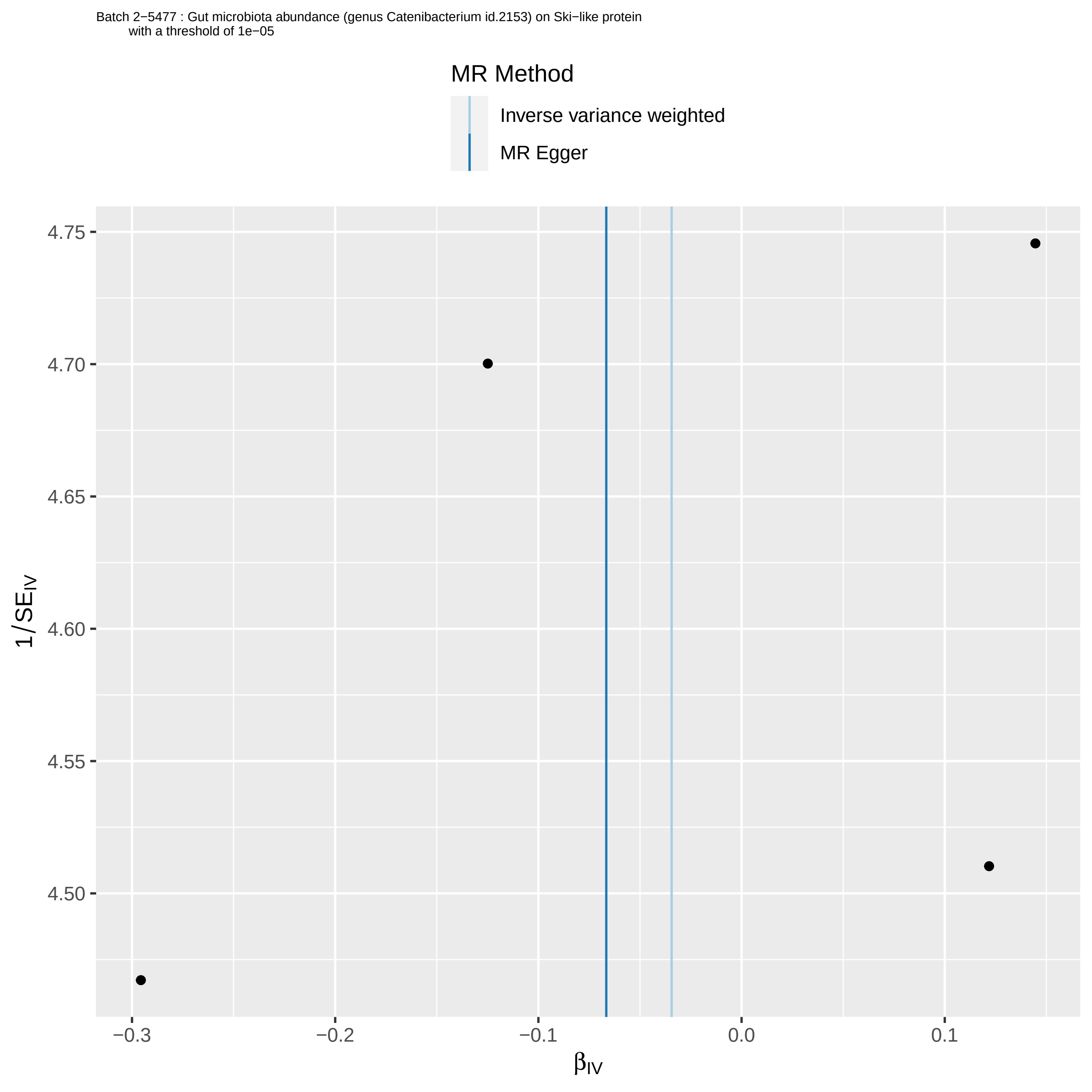

Batch 2−5477 : Gut microbiota abundance (genus Catenibacterium id.2153) on Ski−like protein
with a threshold of 1e−05
MR Method
Inverse variance weighted
MR Egger
4.75
4.70
4.65
4.60
4.55
4.50
1/SE_IV
−0.3
−0.2
−0.1
0.0
0.1
β_IV

Batch 2−5478 : Gut microbiota abundance (genus Parasutterella id.2892) on Ski−like protein
with a threshold of 1e−05

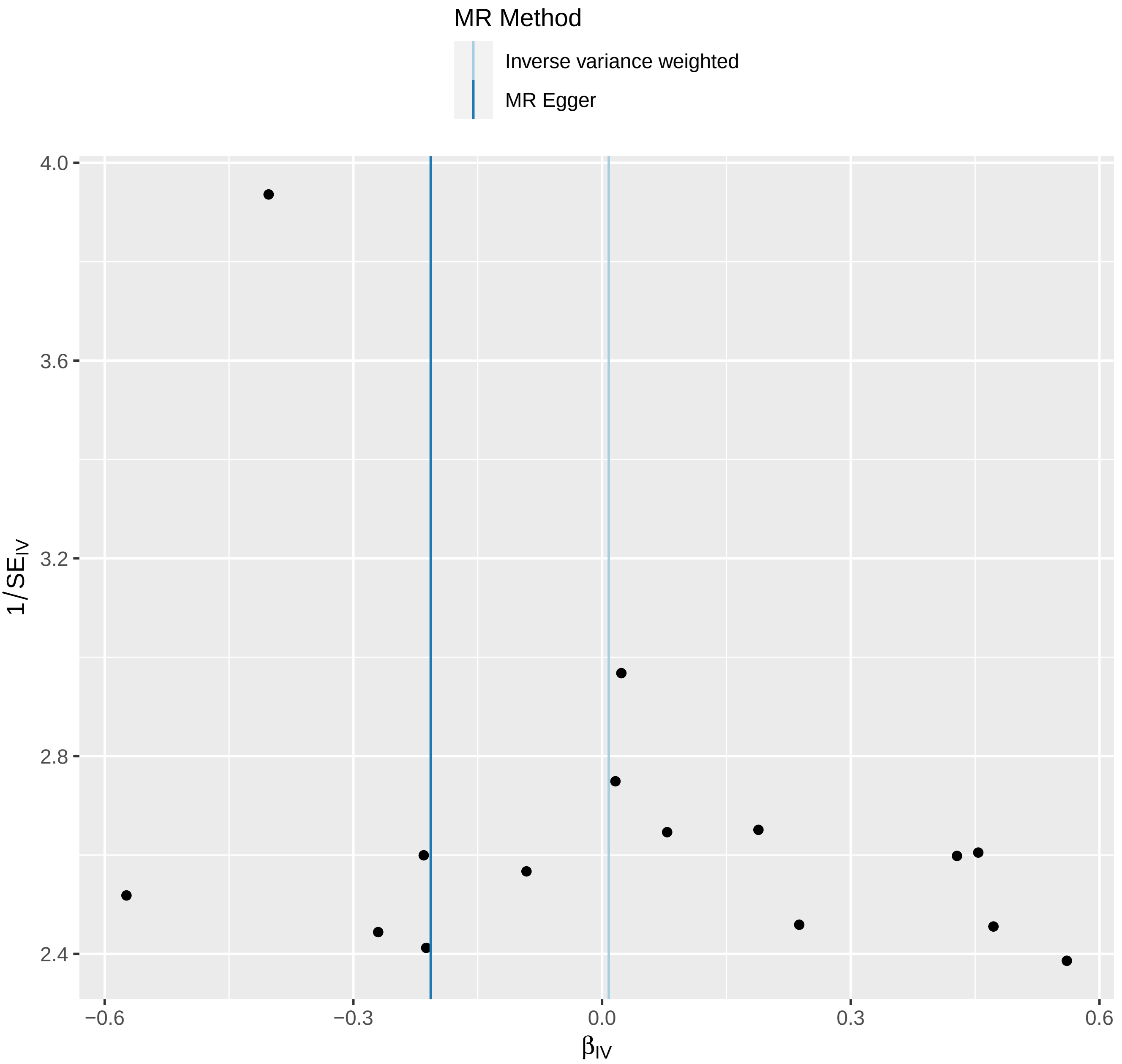

Batch 2−5479 : Gut microbiota abundance (genus Catenibacterium id.2153) on S−phase kinase−associated protein 1
with a threshold of 1e−05

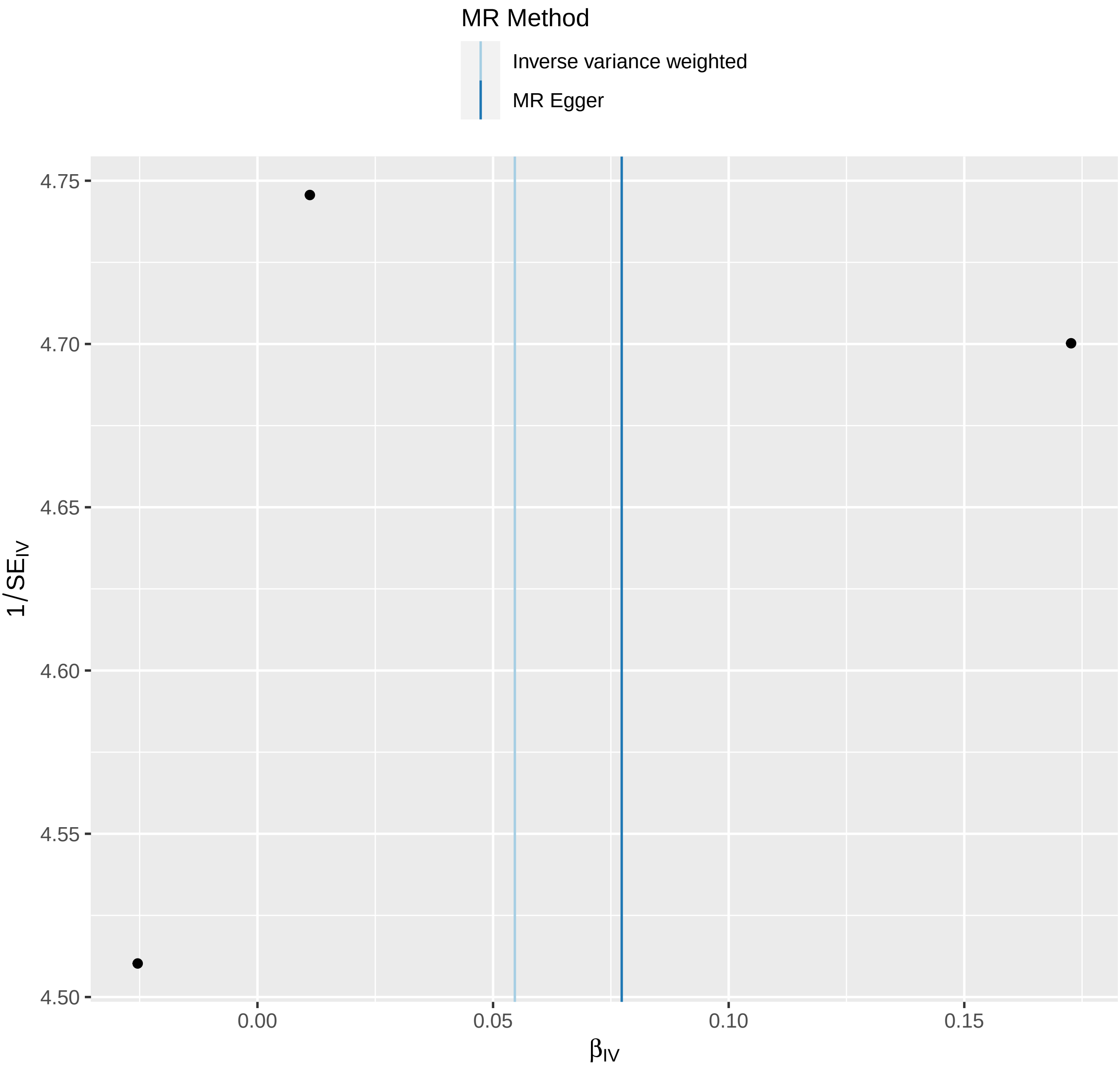

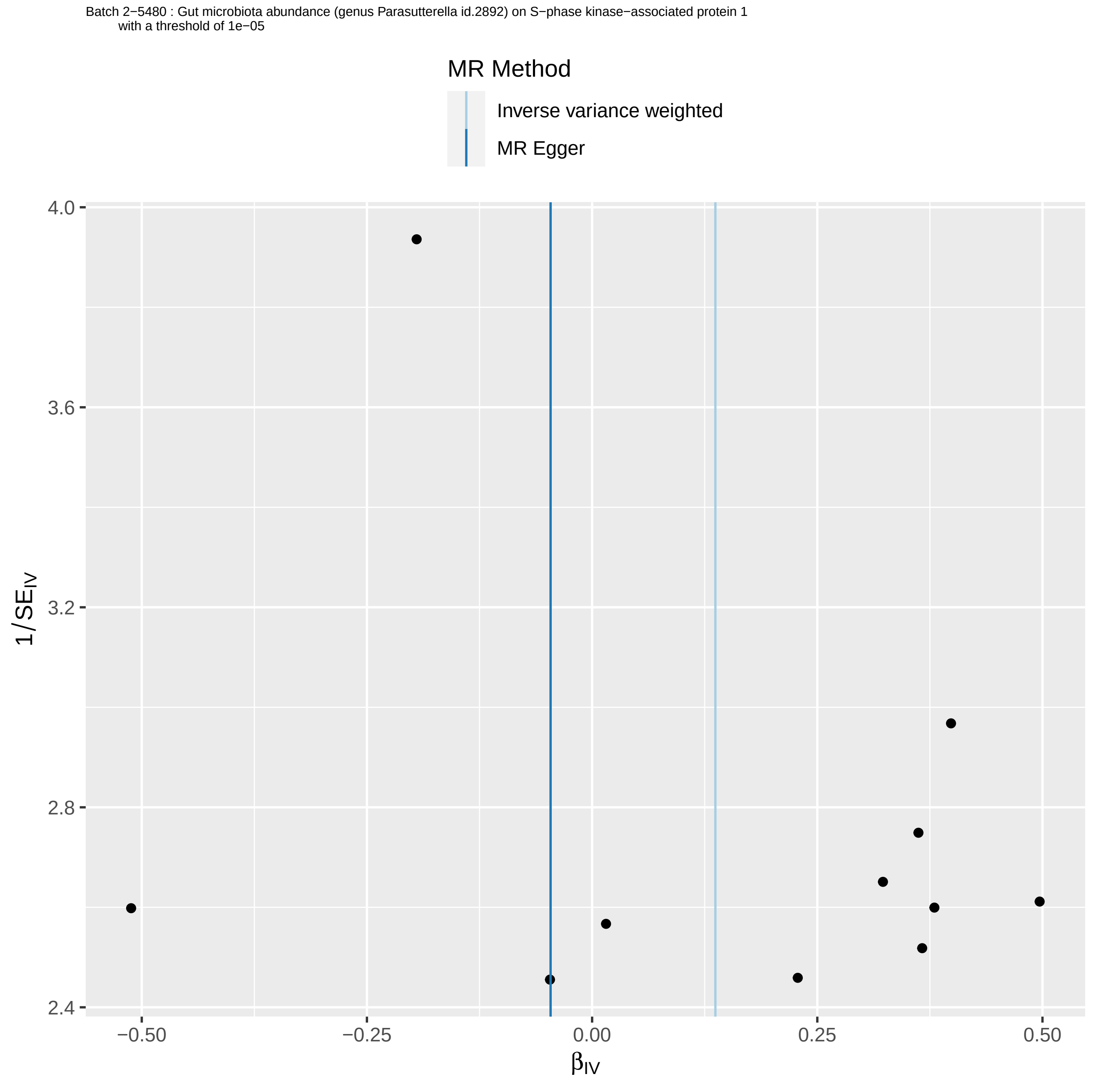
Batch 2−5480 : Gut microbiota abundance (genus Parasutterella id.2892) on S−phase kinase−associated protein 1
with a threshold of 1e−05
MR Method
Inverse variance weighted
MR Egger
4.0
3.6
3.2
2.8
2.4
1/SE_IV
−0.50
−0.25
0.00
0.25
0.50
β_IV

Batch 2−5481 : Gut microbiota abundance (genus Catenibacterium id.2153) on Signaling lymphocytic activation molecule
with a threshold of 1e−05

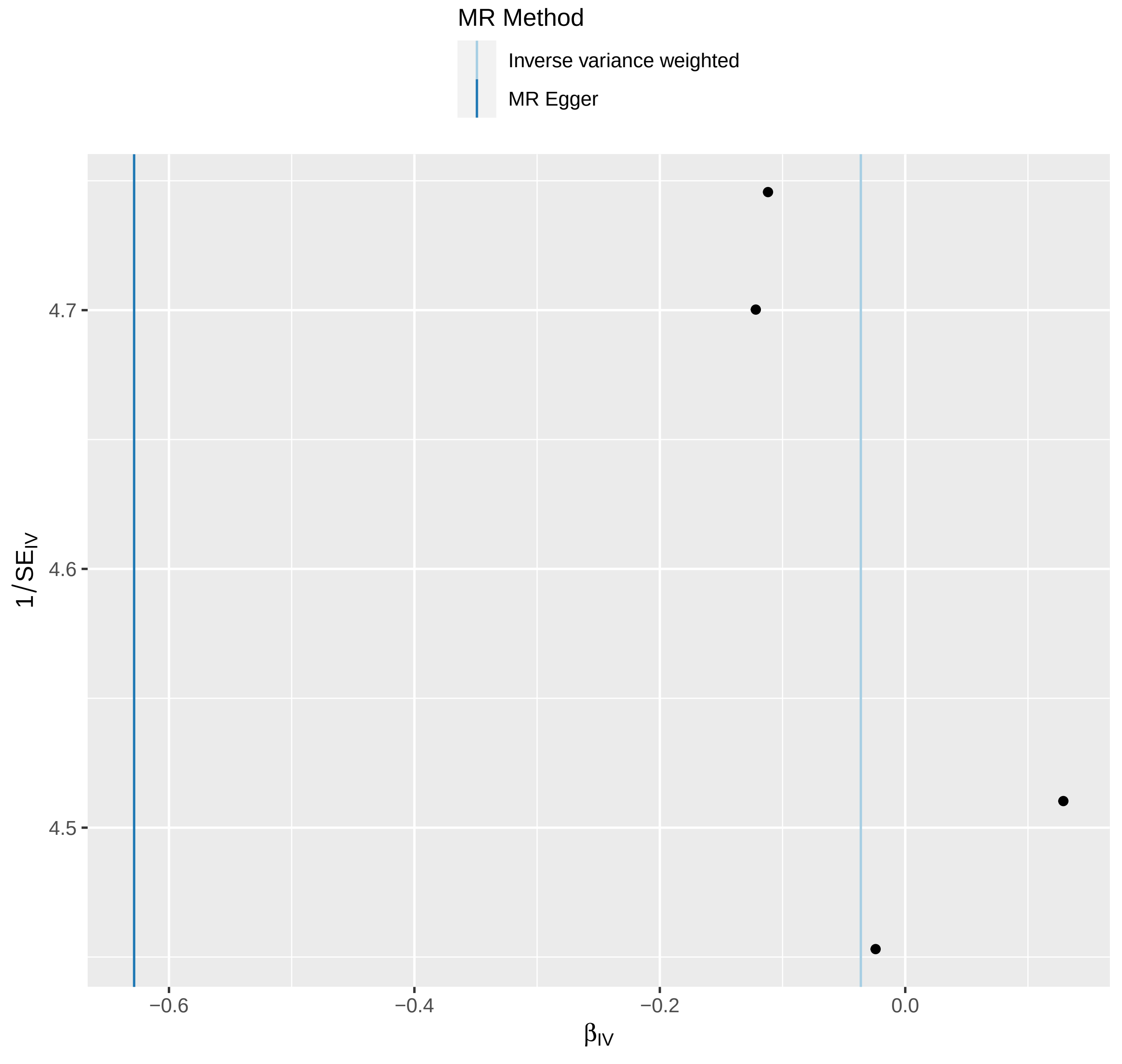

Batch 2−5482 : Gut microbiota abundance (genus Parasutterella id.2892) on Signaling lymphocytic activation molecule
with a threshold of 1e−05

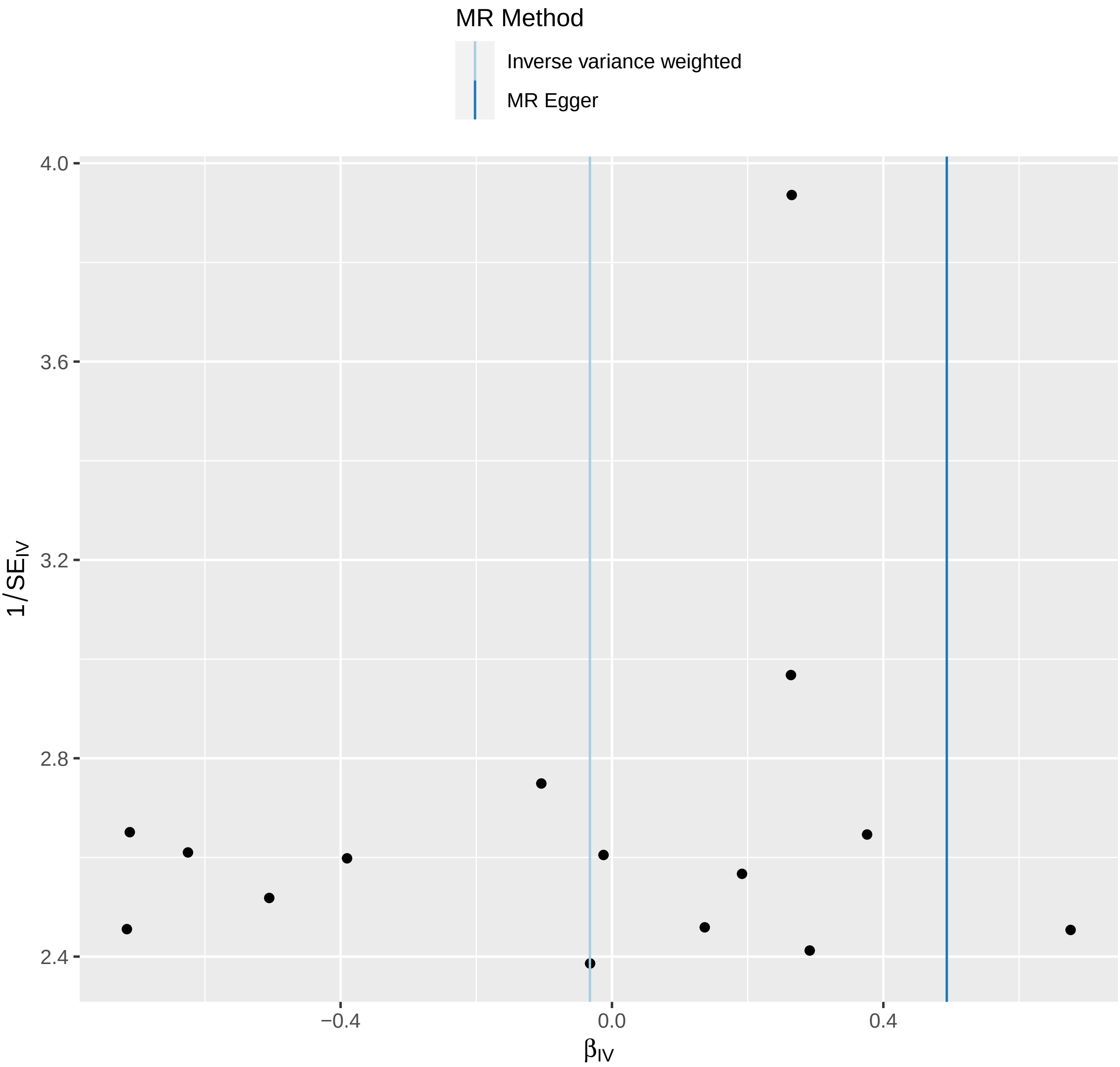

Batch 2−5483 : Gut microbiota abundance (genus Catenibacterium id.2153) on SLAM family member 6
with a threshold of 1e−05

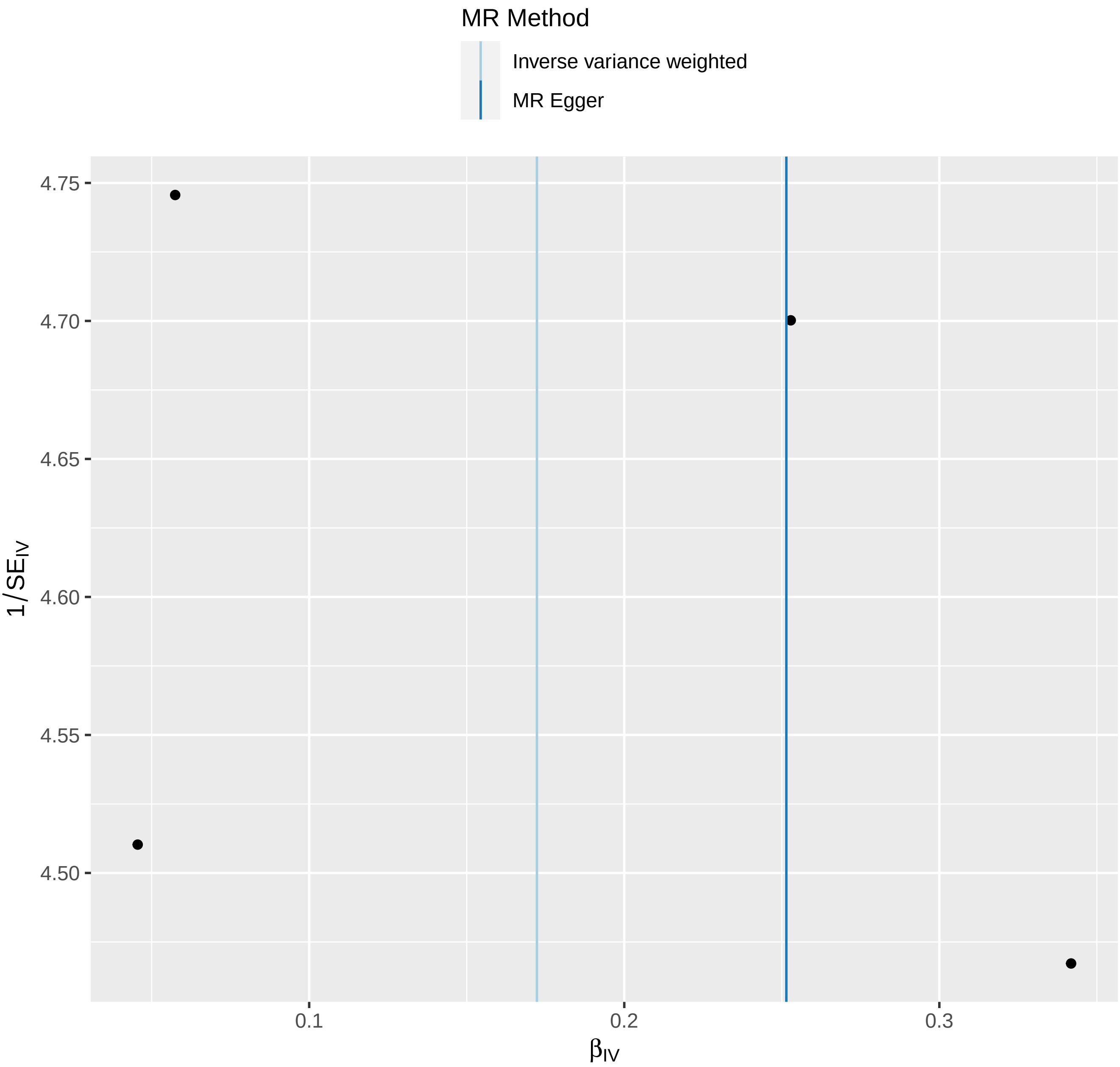

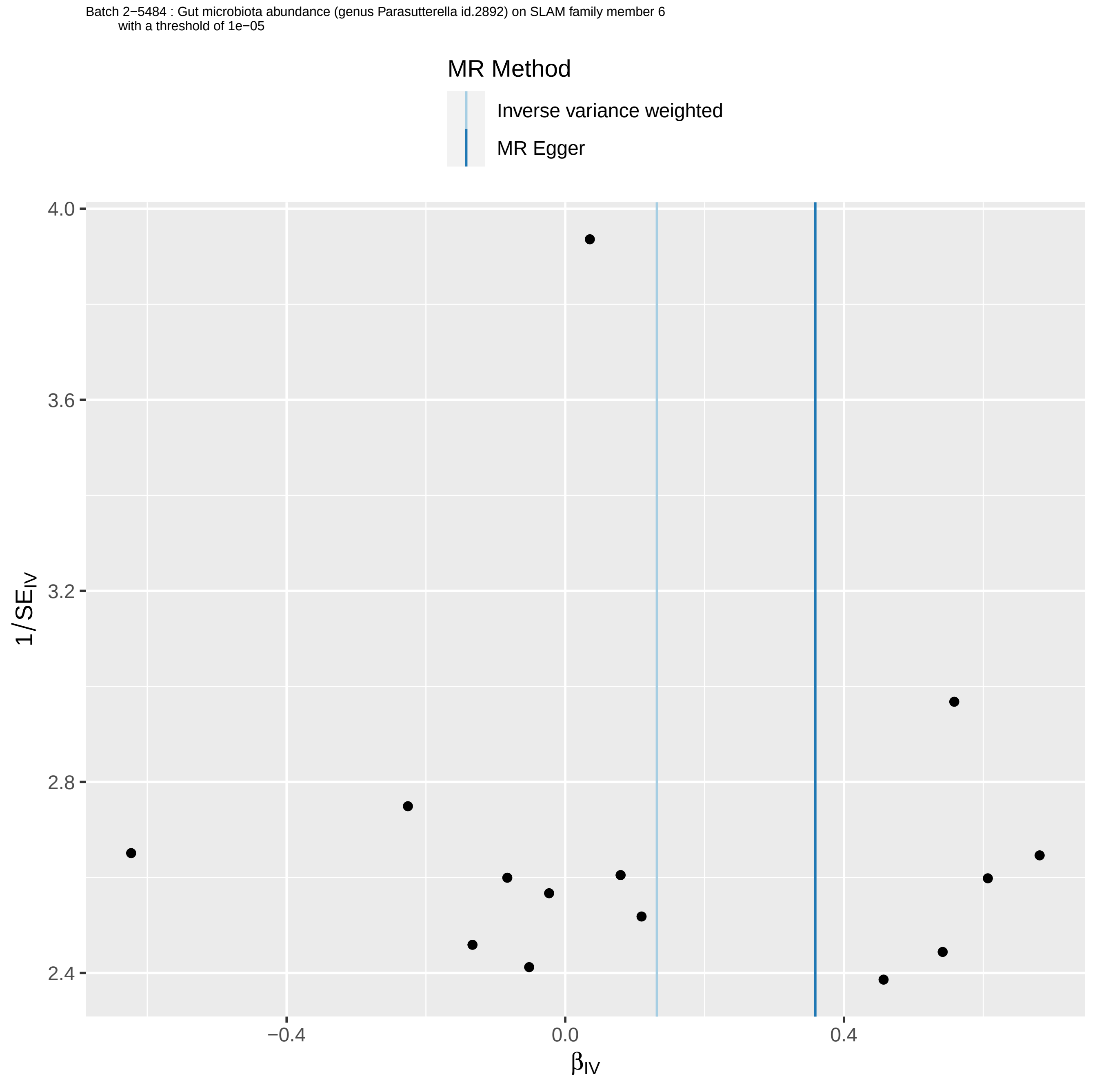
Batch 2−5484 : Gut microbiota abundance (genus Parasutterella id.2892) on SLAM family member 6
with a threshold of 1e−05
MR Method
Inverse variance weighted
MR Egger
4.0
3.6
3.2
2.8
2.4
1/SE_IV
−0.4
0.0
0.4
β_IV

Batch 2−5485 : Gut microbiota abundance (genus Catenibacterium id.2153) on SLAM family member 7
with a threshold of 1e−05

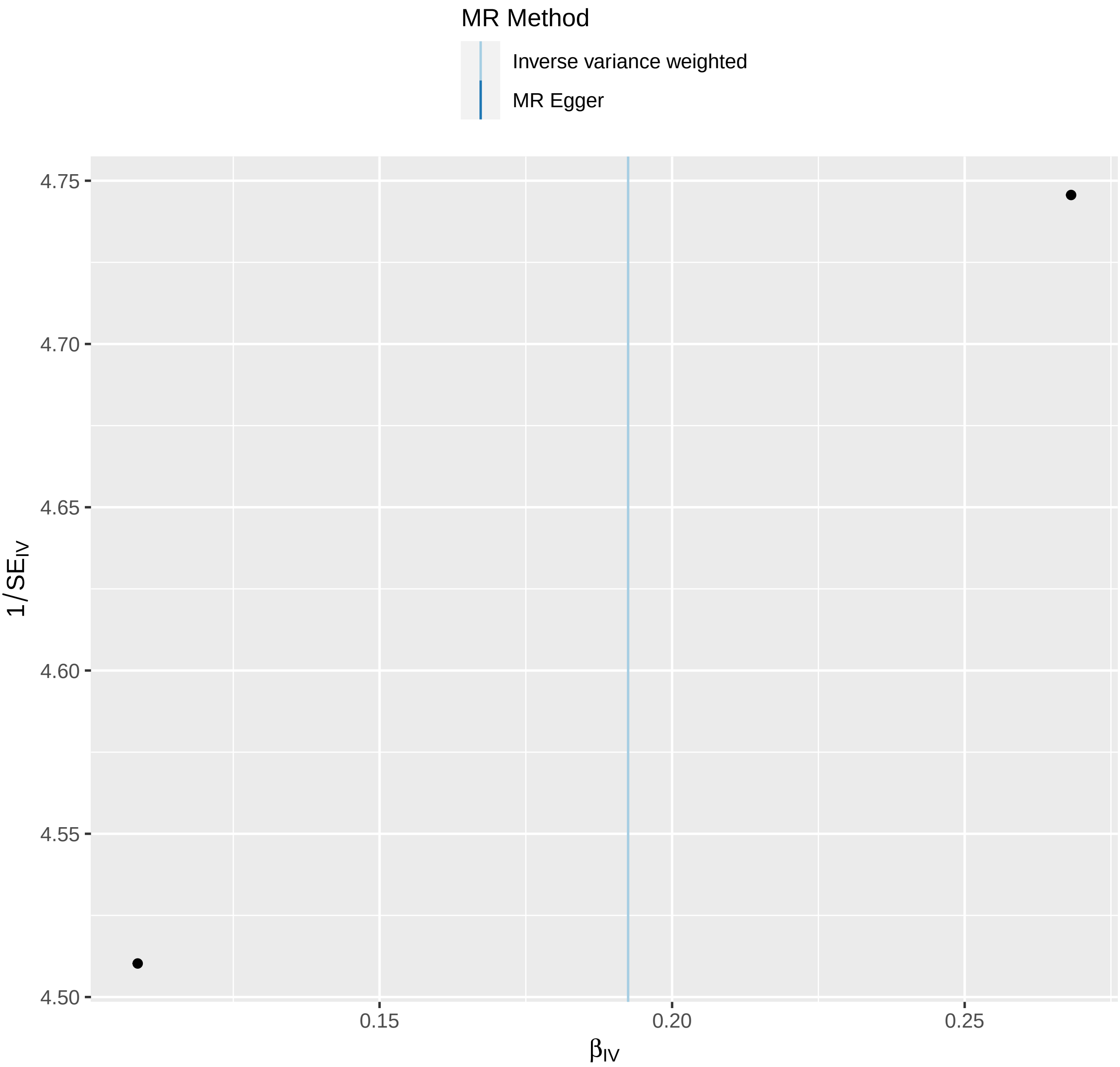

Batch 2−5486 : Gut microbiota abundance (genus Parasutterella id.2892) on SLAM family member 7
with a threshold of 1e−05

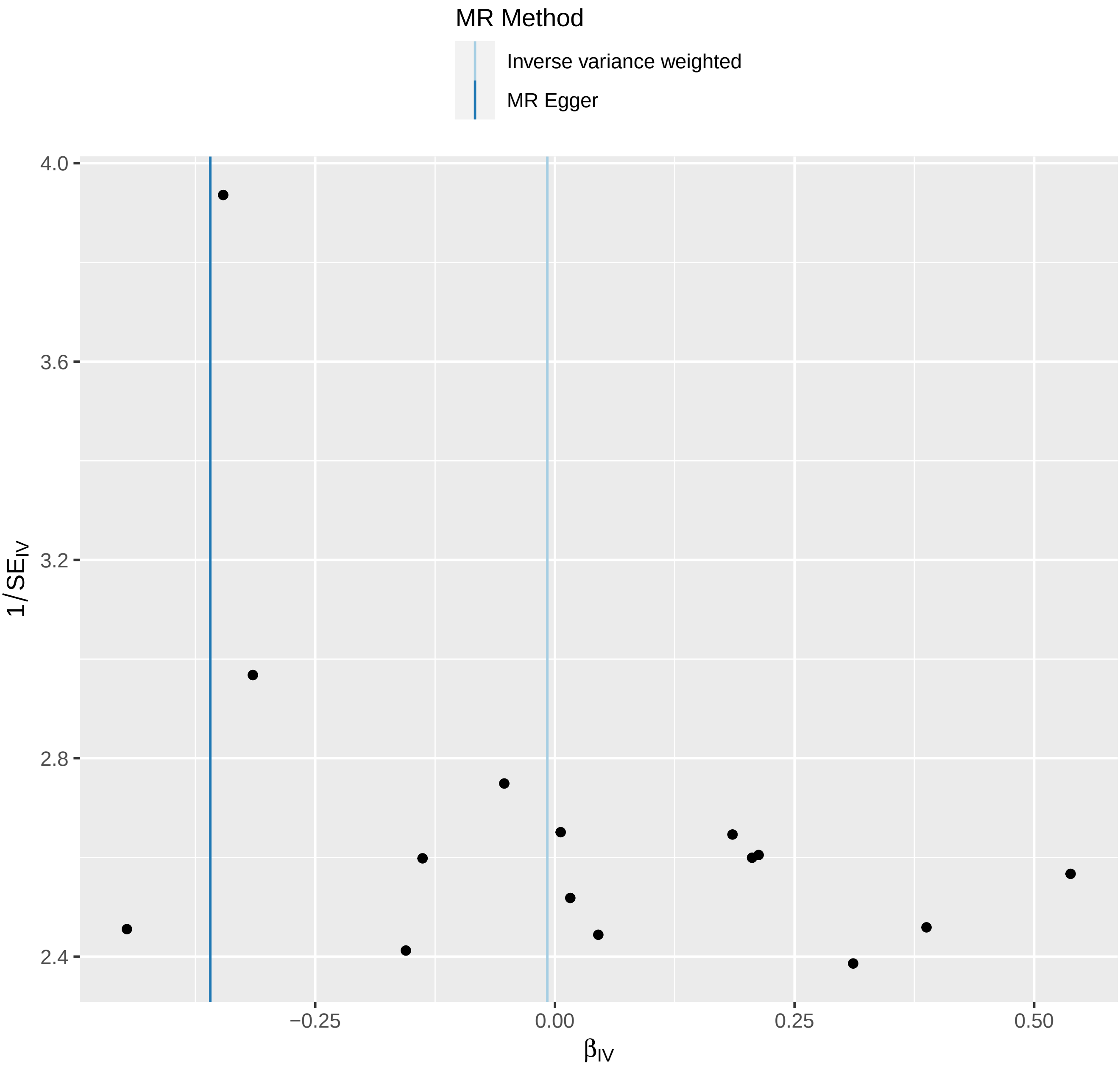

Batch 2−5487 : Gut microbiota abundance (genus Catenibacterium id.2153) on SLAM family member 7
with a threshold of 1e−05

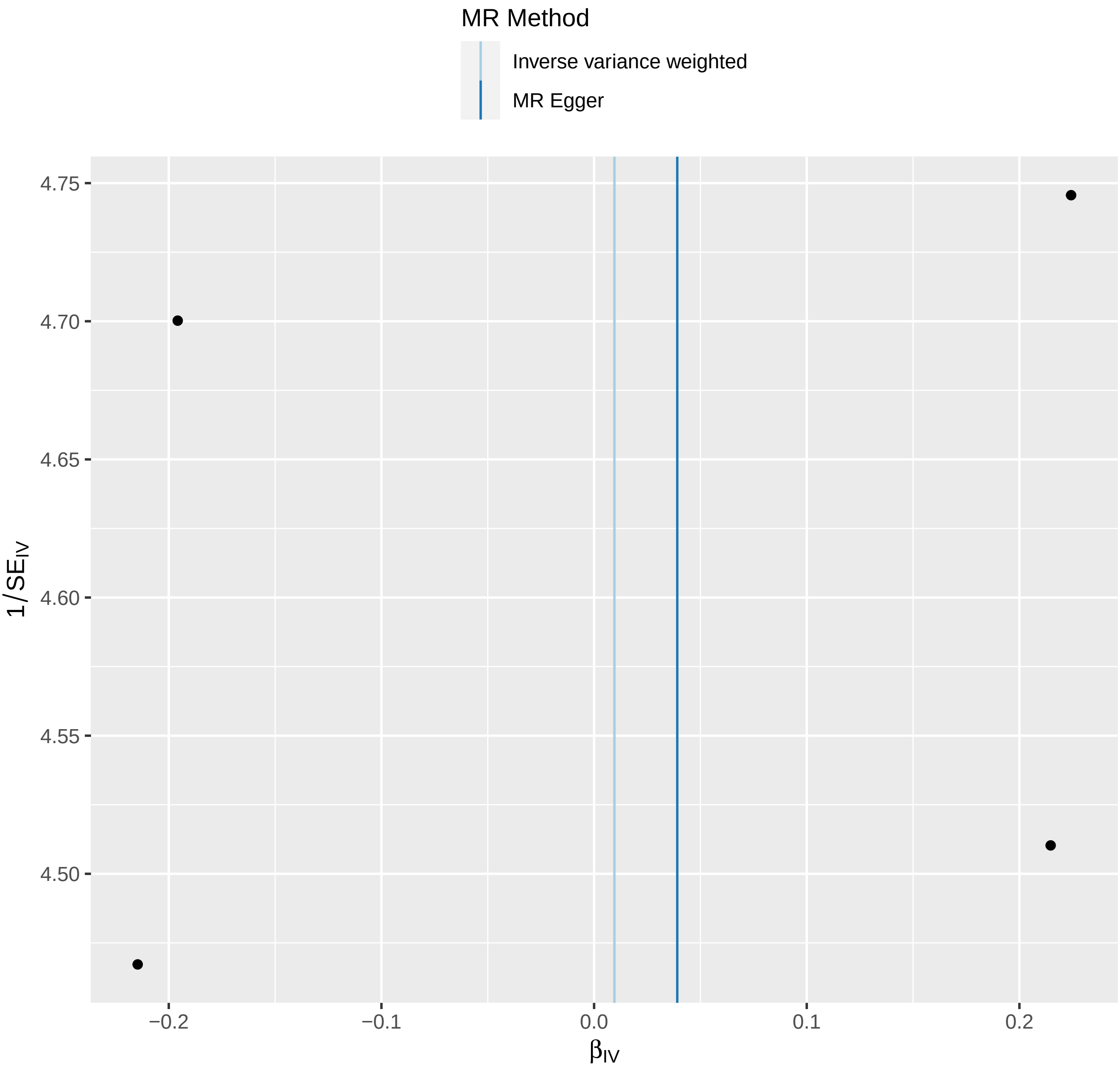

Batch 2−5488 : Gut microbiota abundance (genus Parasutterella id.2892) on SLAM family member 7
with a threshold of 1e−05

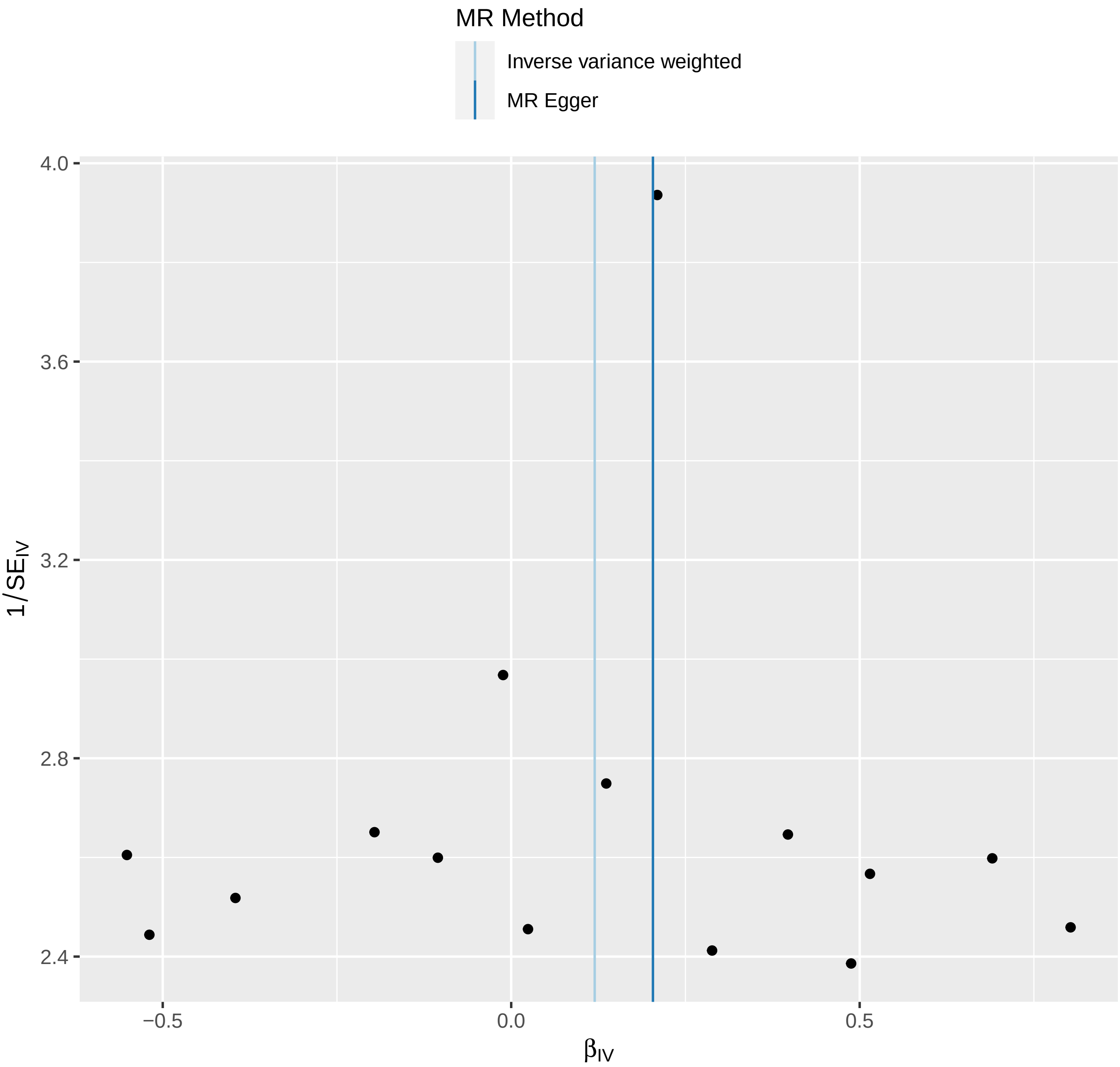

Batch 2−5489 : Gut microbiota abundance (genus Catenibacterium id.2153) on SLAM family member 8
with a threshold of 1e−05

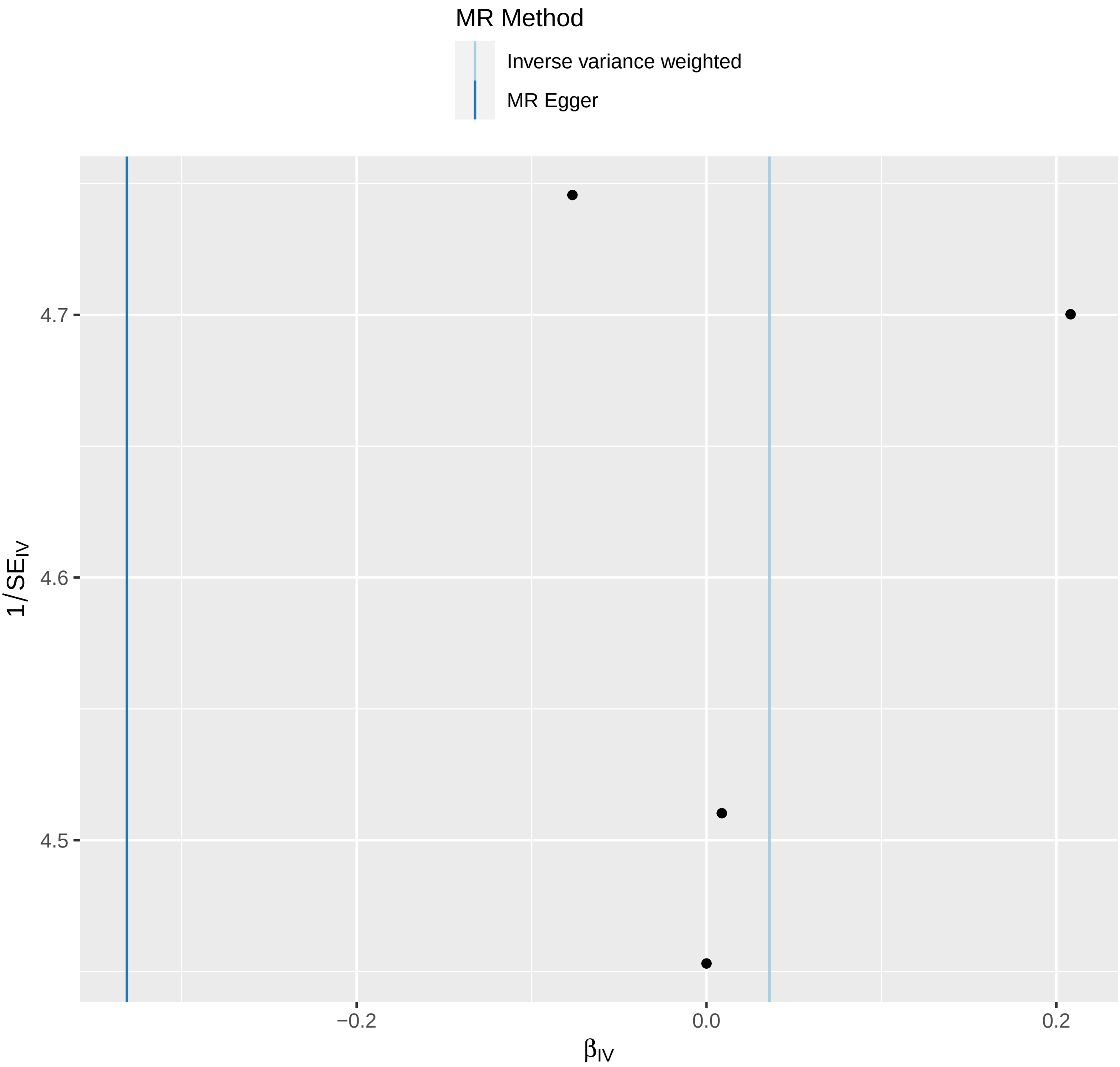

Batch 2−5490 : Gut microbiota abundance (genus Parasutterella id.2892) on SLAM family member 8
with a threshold of 1e−05

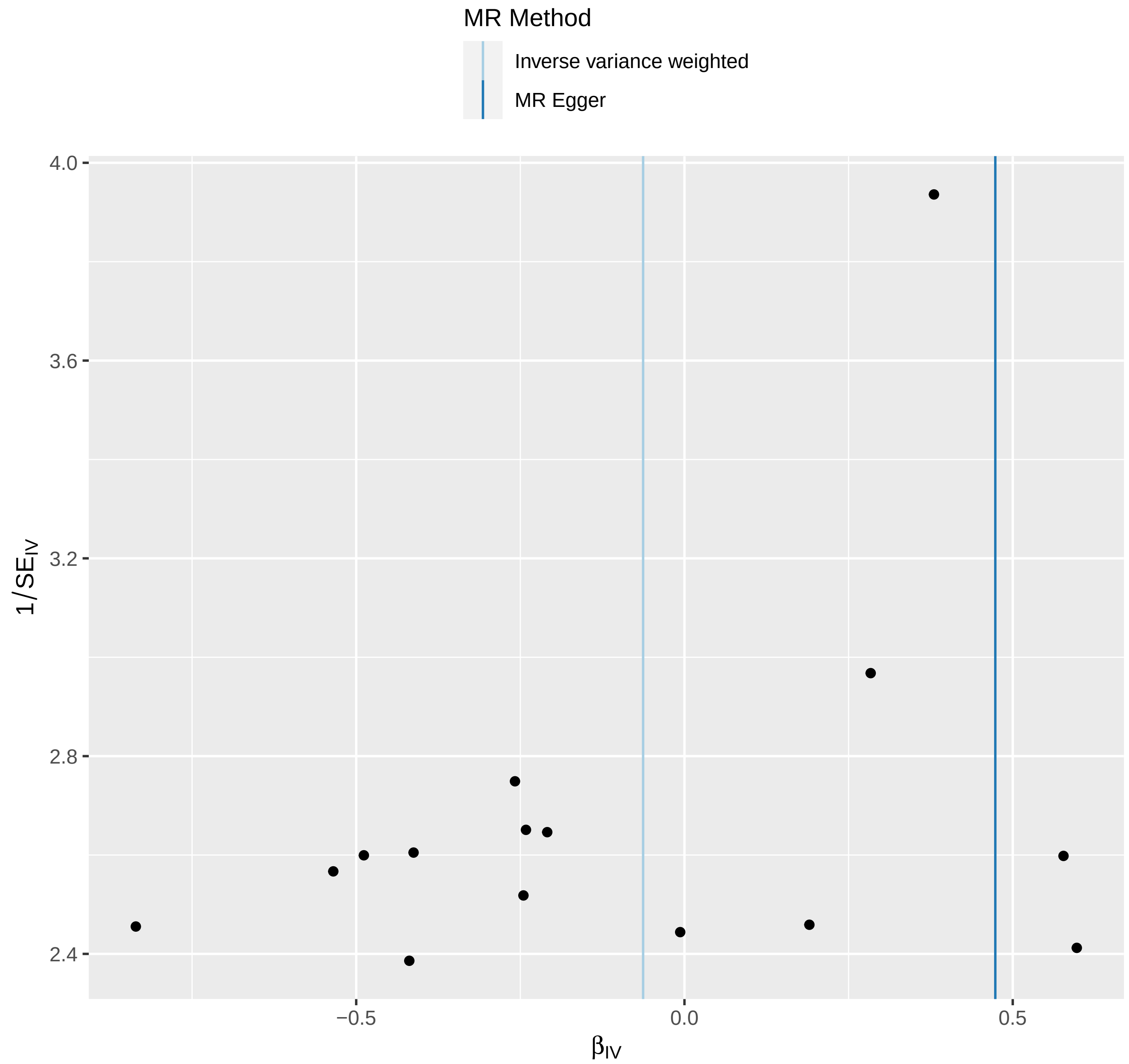

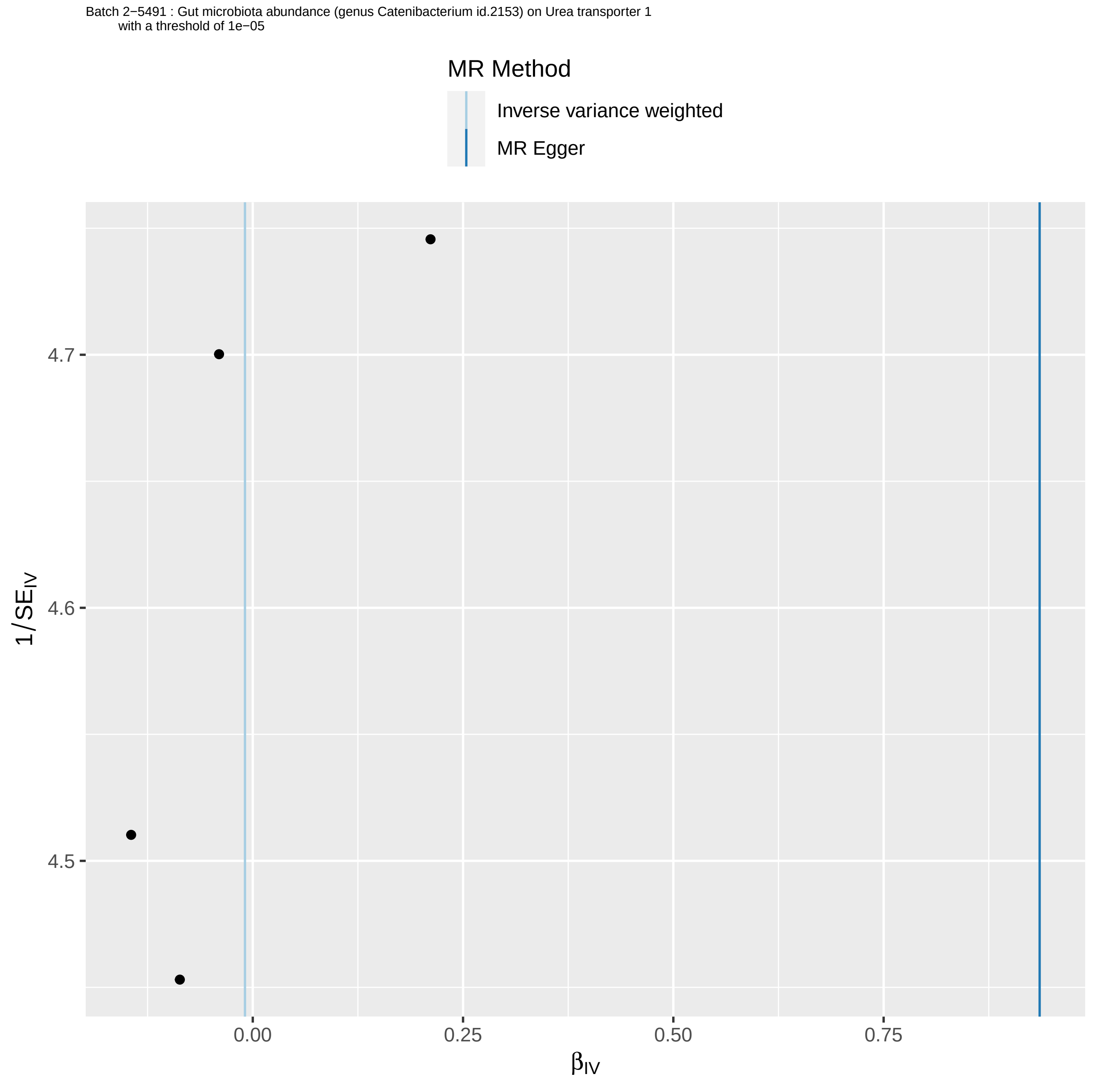

Batch 2−5491 : Gut microbiota abundance (genus Catenibacterium id.2153) on Urea transporter 1
with a threshold of 1e−05
MR Method
Inverse variance weighted
MR Egger
4.7
4.6
4.5
0.00
0.25
0.50
0.75
1/SE_IV
β_IV

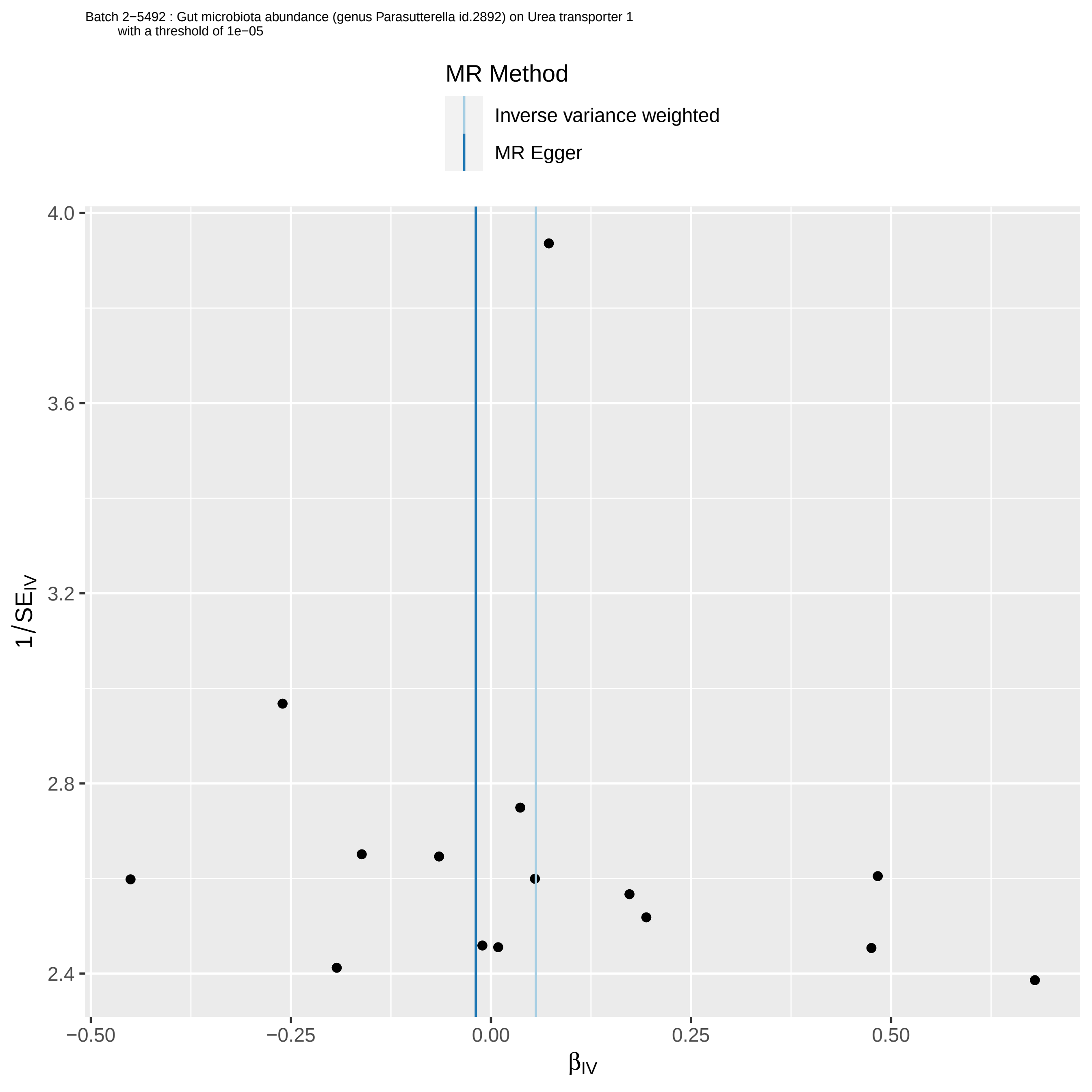

Batch 2−5492 : Gut microbiota abundance (genus Parasutterella id.2892) on Urea transporter 1
with a threshold of 1e−05
MR Method
Inverse variance weighted
MR Egger
4.0
3.6
3.2
2.8
2.4
1/SE_IV
−0.50
−0.25
0.00
0.25
0.50
β_IV

Batch 2−5493 : Gut microbiota abundance (genus Catenibacterium id.2153) on Urea transporter 2
with a threshold of 1e−05

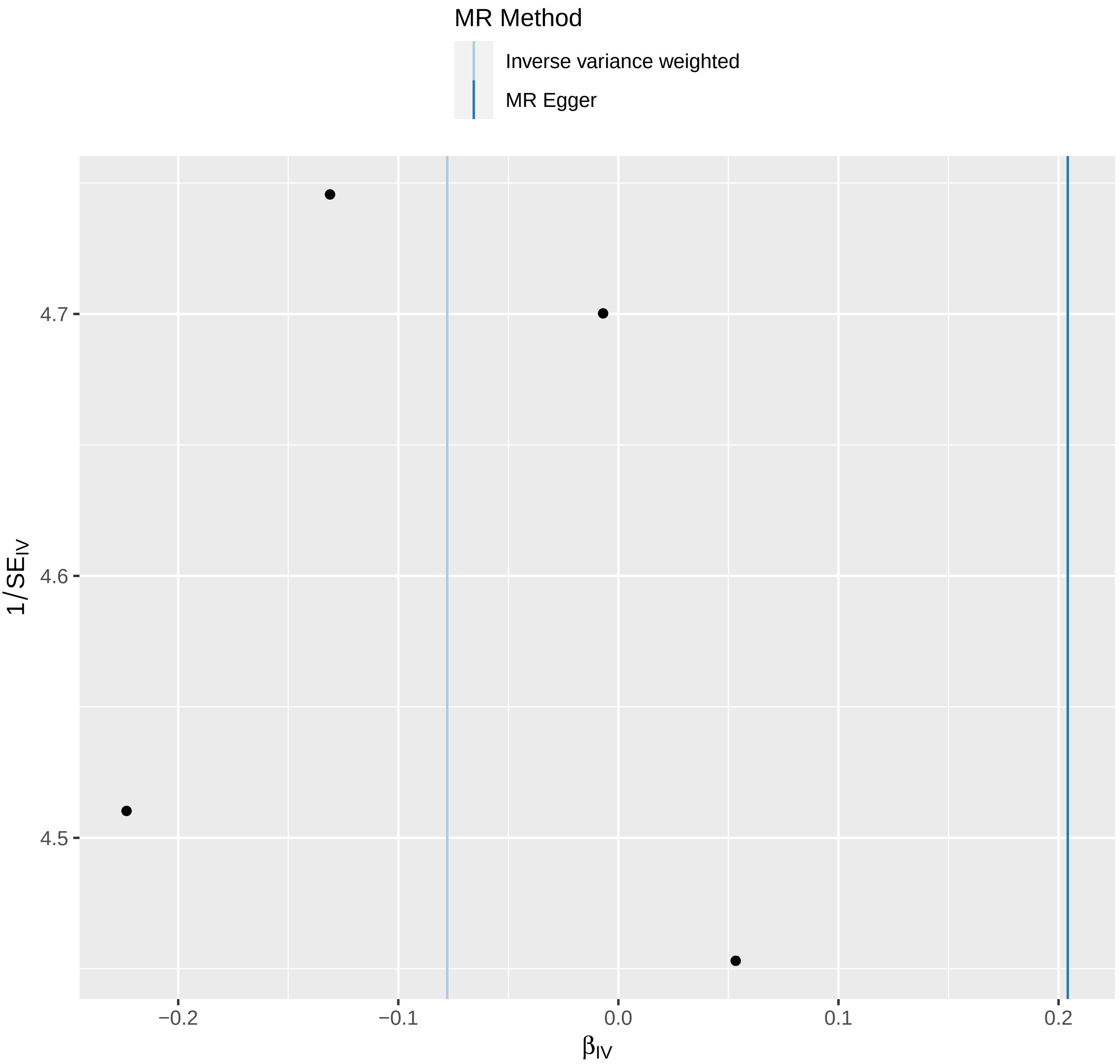

Batch 2−5494 : Gut microbiota abundance (genus Parasutterella id.2892) on Urea transporter 2
with a threshold of 1e−05

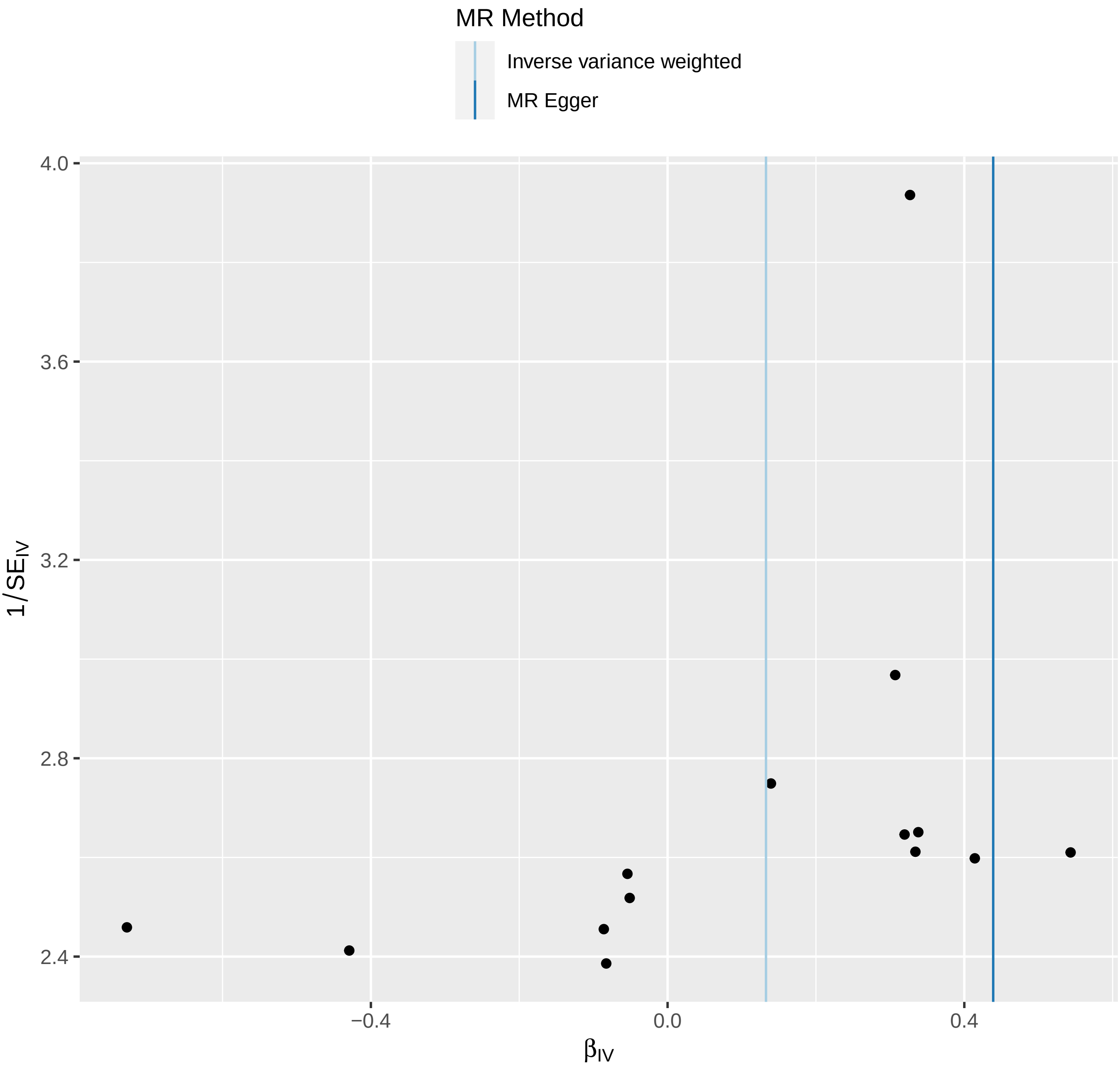

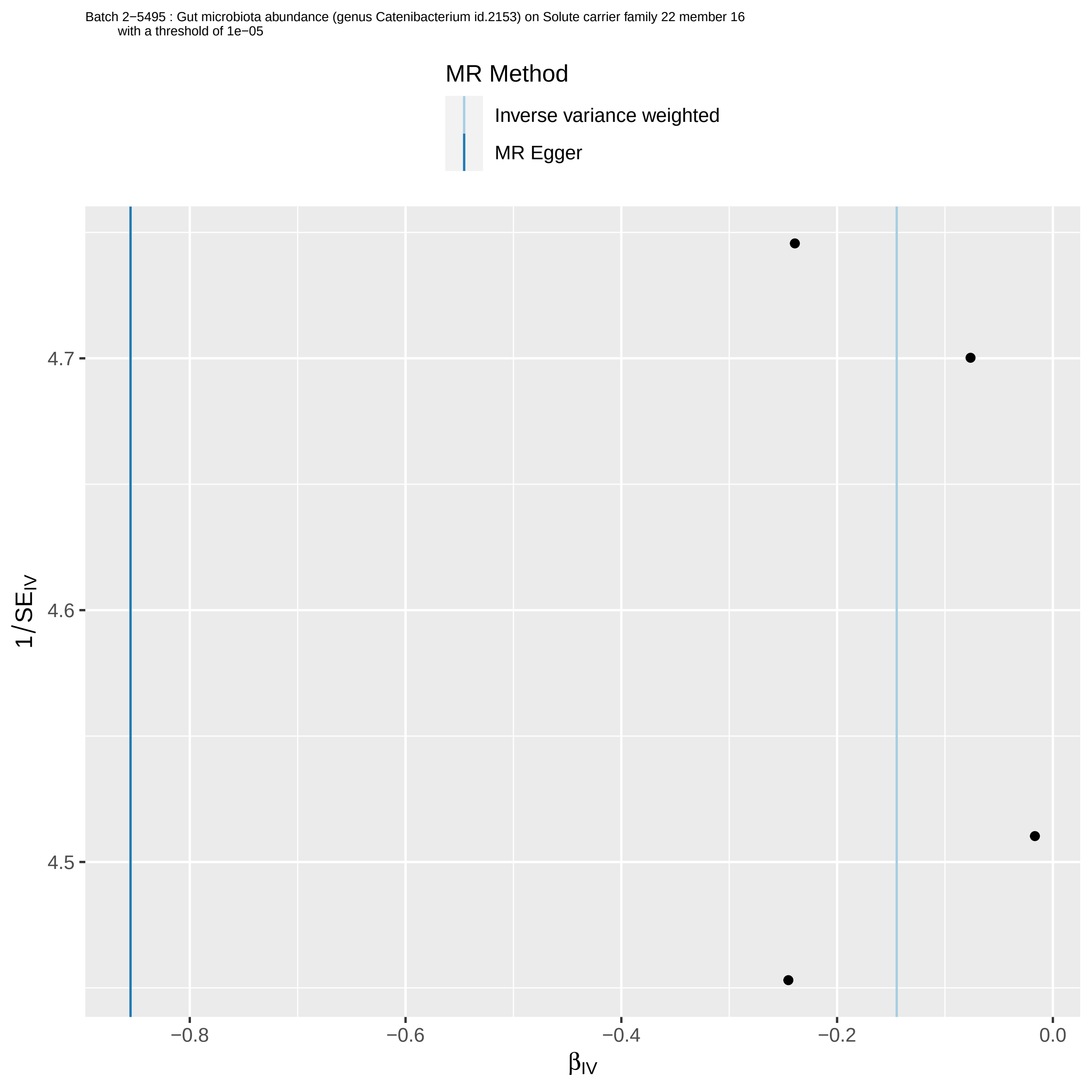

Batch 2−5495 : Gut microbiota abundance (genus Catenibacterium id.2153) on Solute carrier family 22 member 16
with a threshold of 1e−05
MR Method
Inverse variance weighted
MR Egger
4.7
4.6
4.5
1/SE_IV
−0.8
−0.6
−0.4
−0.2
0.0
β_IV

Batch 2−5496 : Gut microbiota abundance (genus Parasutterella id.2892) on Solute carrier family 22 member 16
with a threshold of 1e−05

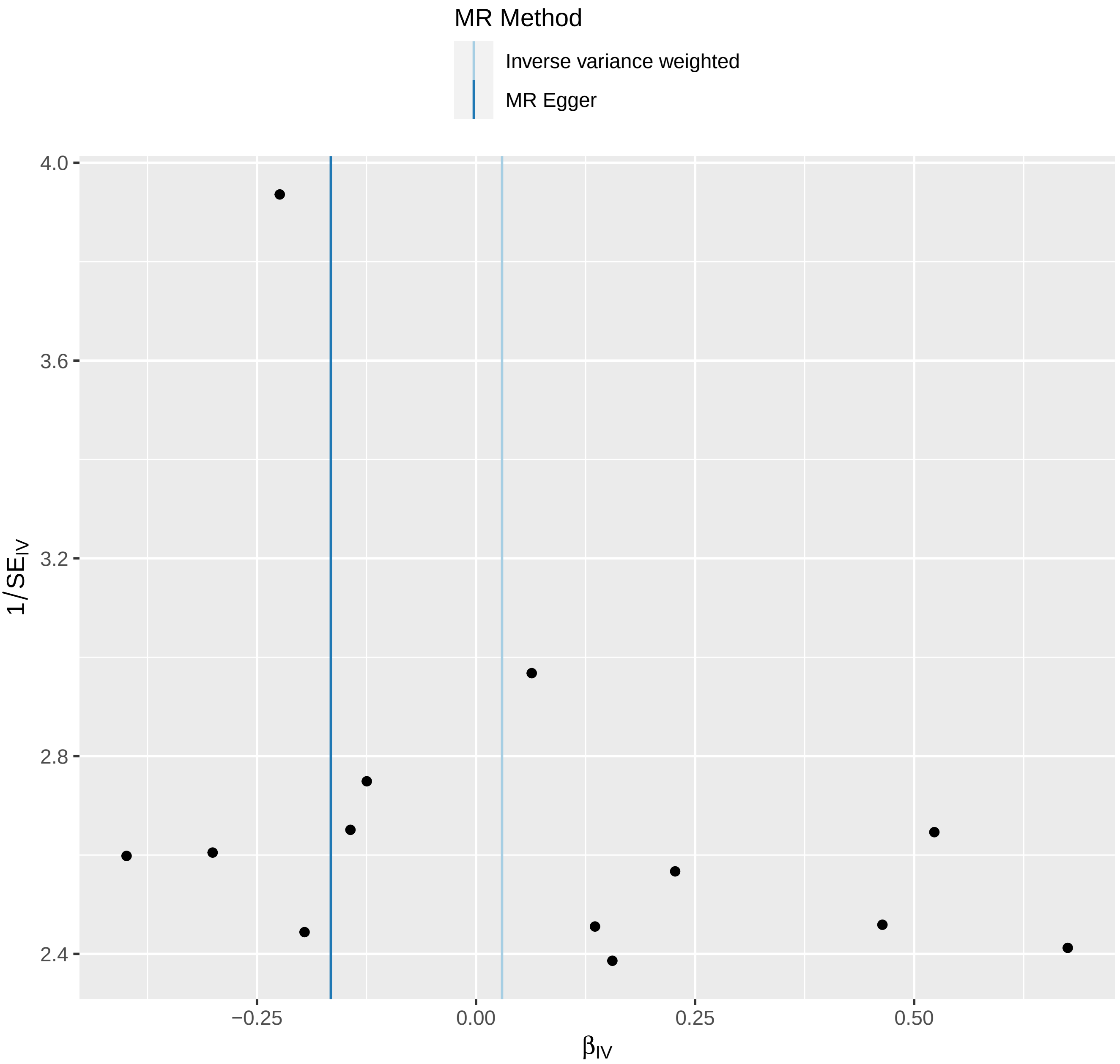

Batch 2−5497 : Gut microbiota abundance (genus Catenibacterium id.2153) on Mitochondrial glutamate carrier 2
with a threshold of 1e−05

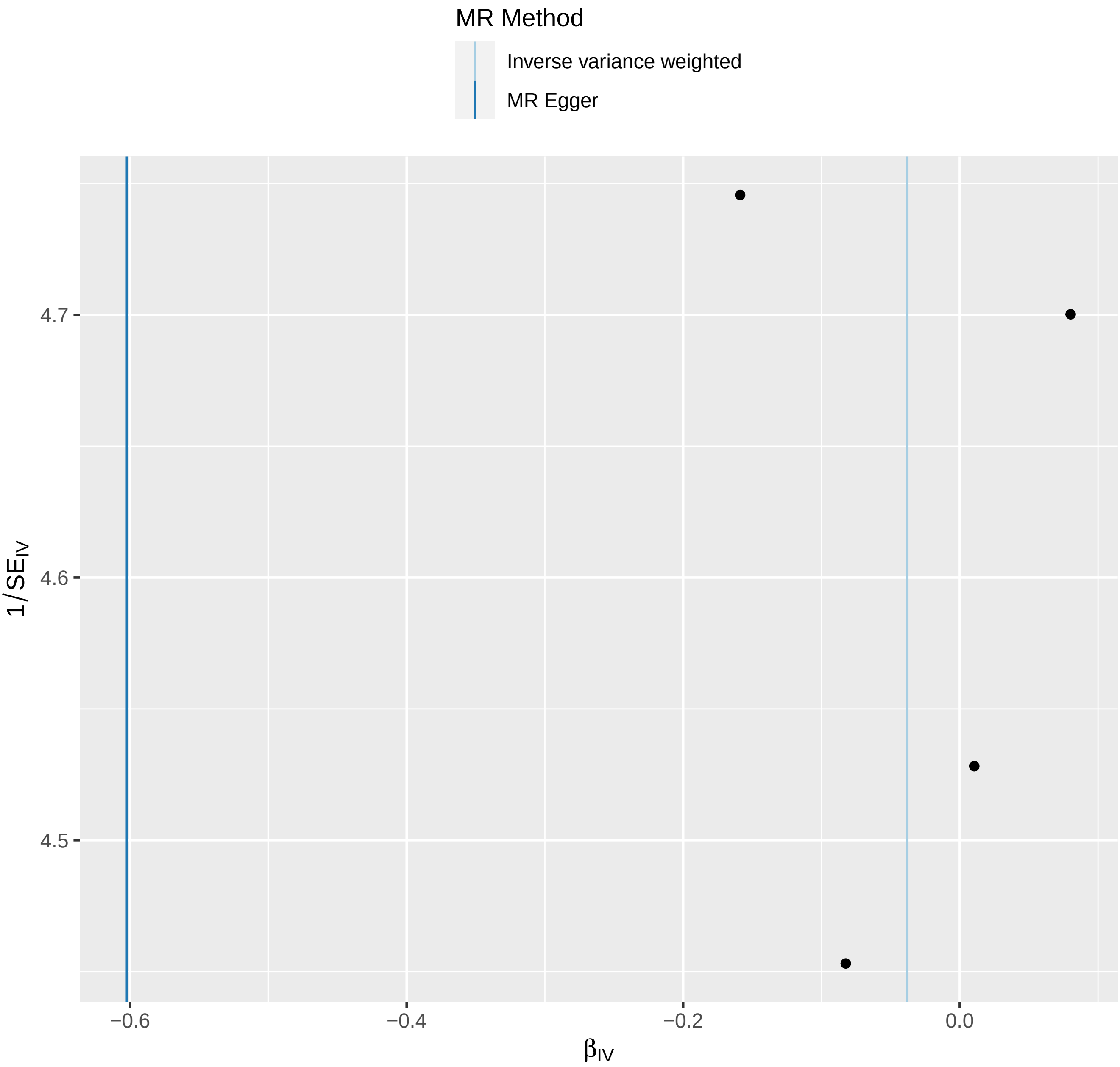

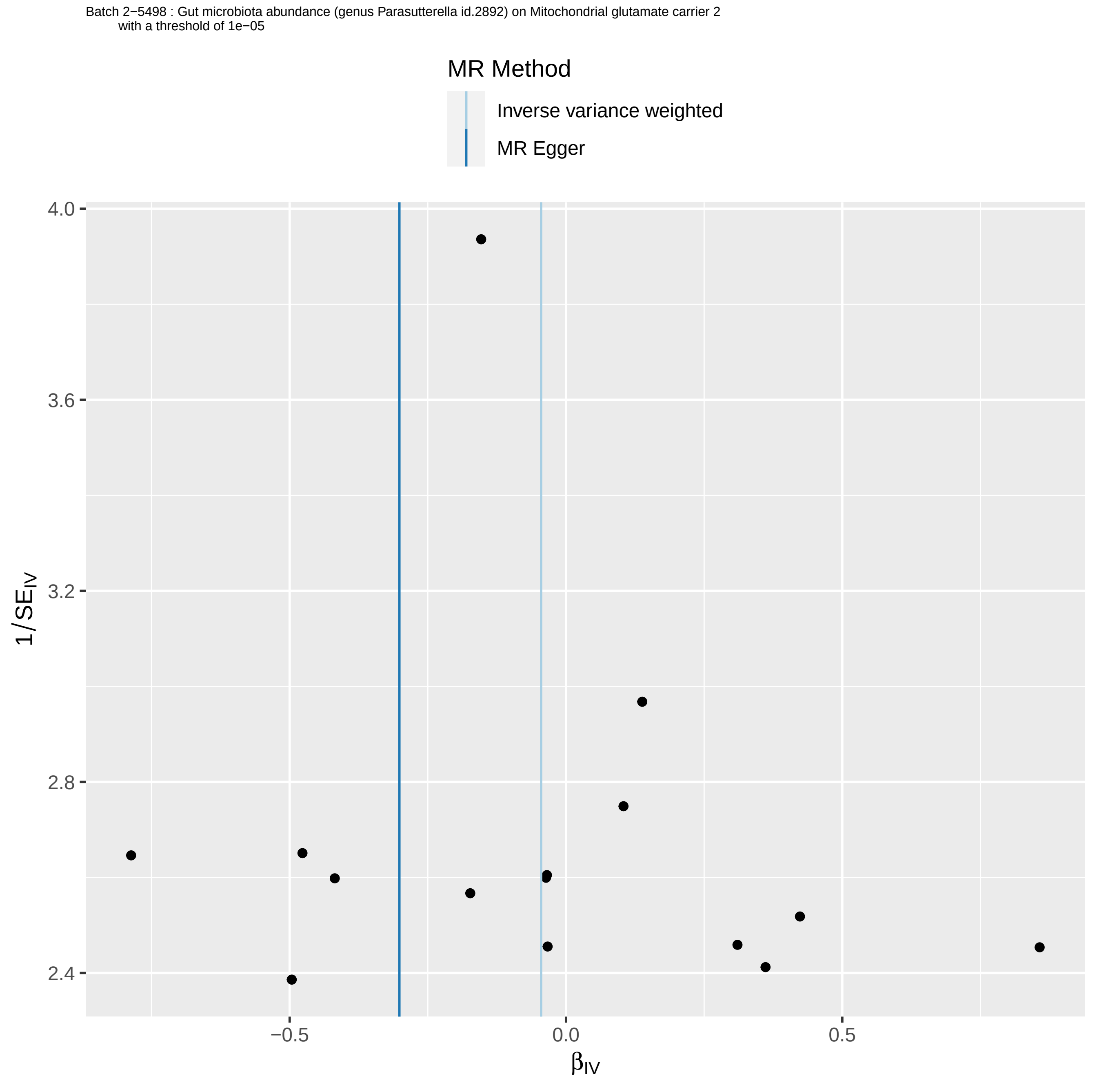

Batch 2−5498 : Gut microbiota abundance (genus Parasutterella id.2892) on Mitochondrial glutamate carrier 2
with a threshold of 1e−05
MR Method
Inverse variance weighted
MR Egger
4.0
3.6
3.2
2.8
2.4
1/SE$_{IV}$
−0.5
0.0
0.5
β$_{IV}$

Batch 2−5499 : Gut microbiota abundance (genus Catenibacterium id.2153) on Sodium−independent sulfate anion transporter with a threshold of 1e−05

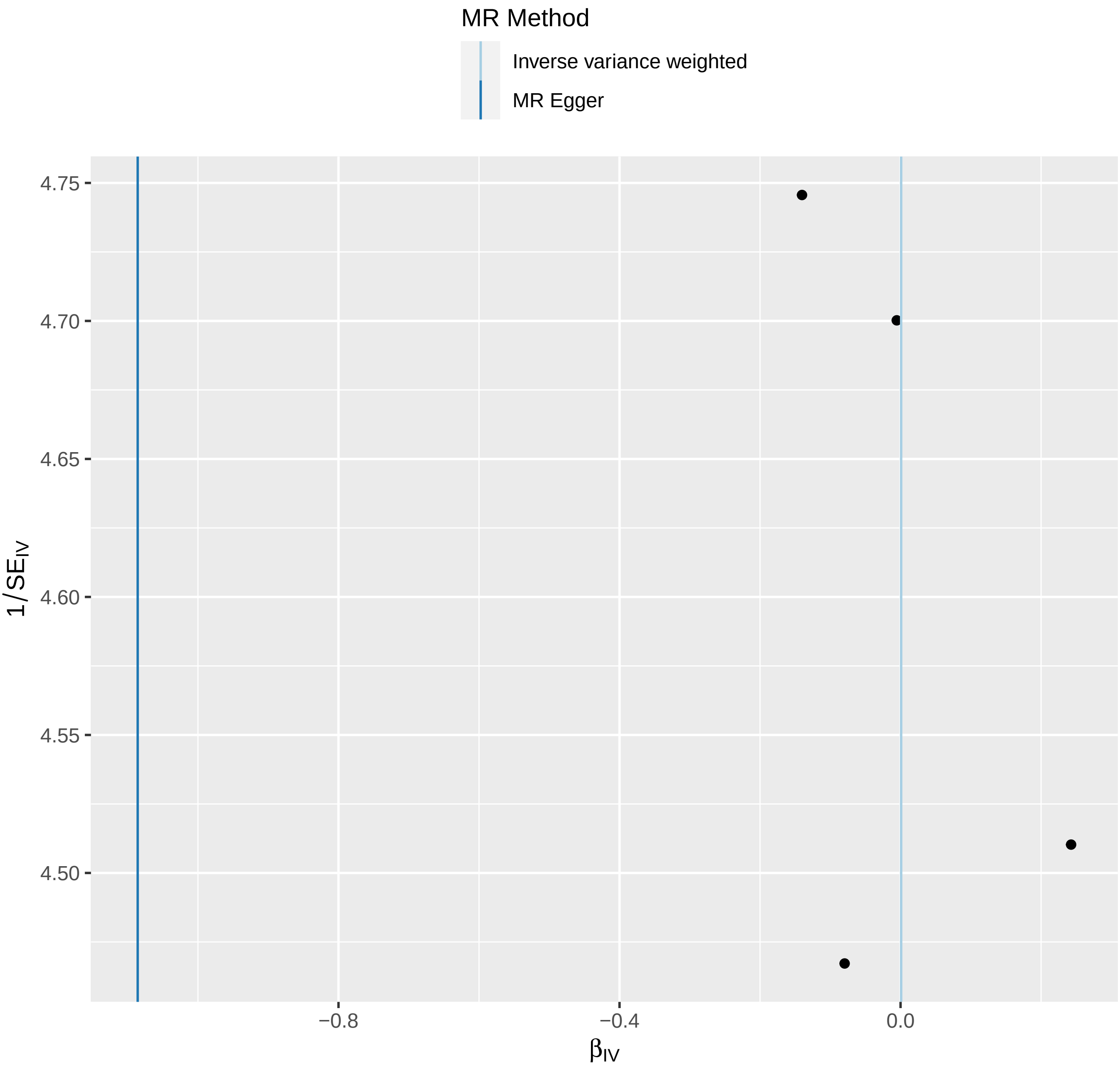

Batch 2−5500 : Gut microbiota abundance (genus Parasutterella id.2892) on Sodium−independent sulfate anion transporter
with a threshold of 1e−05

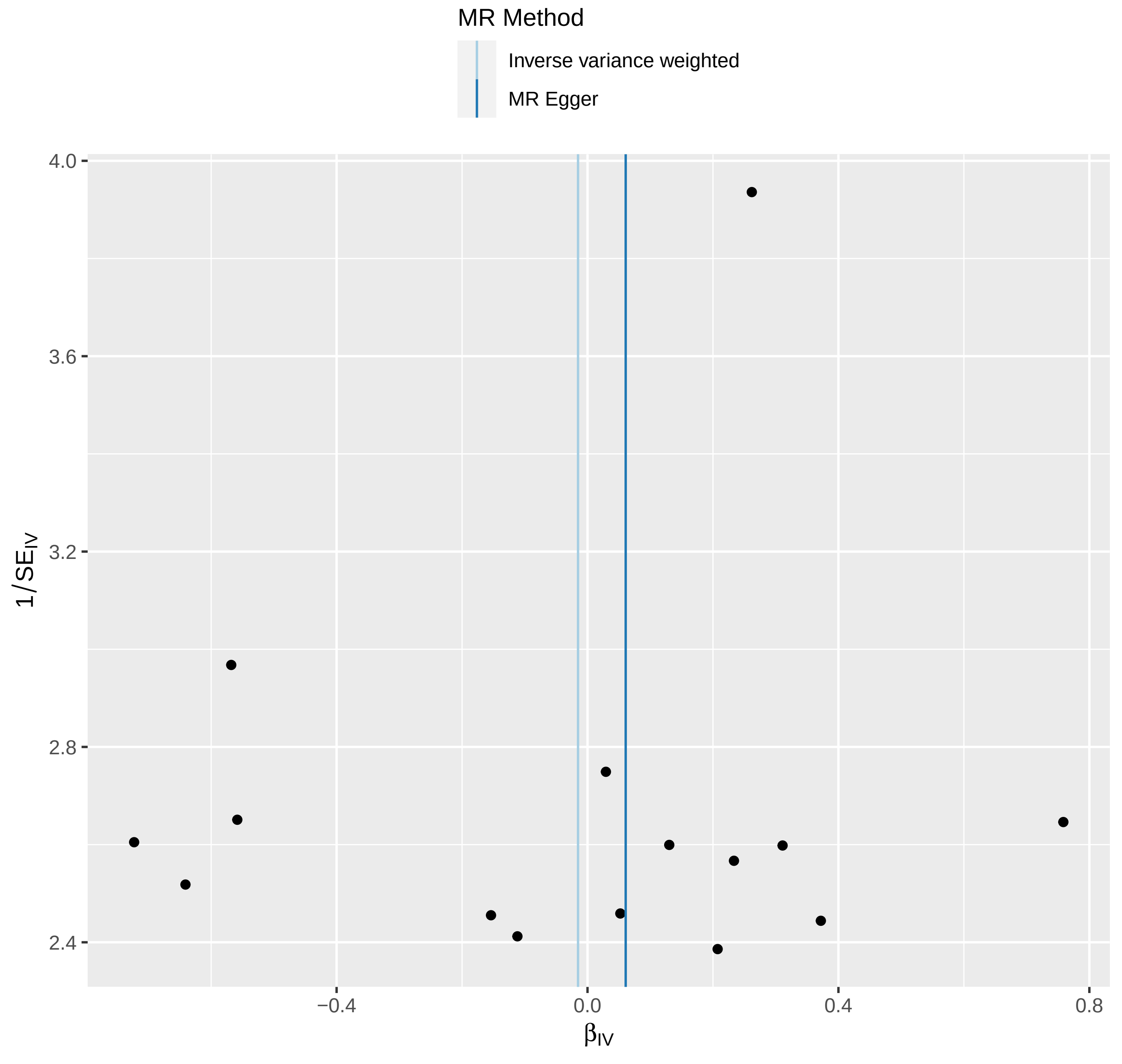

Batch 2−5501 : Gut microbiota abundance (genus Catenibacterium id.2153) on Prestin
with a threshold of 1e−05

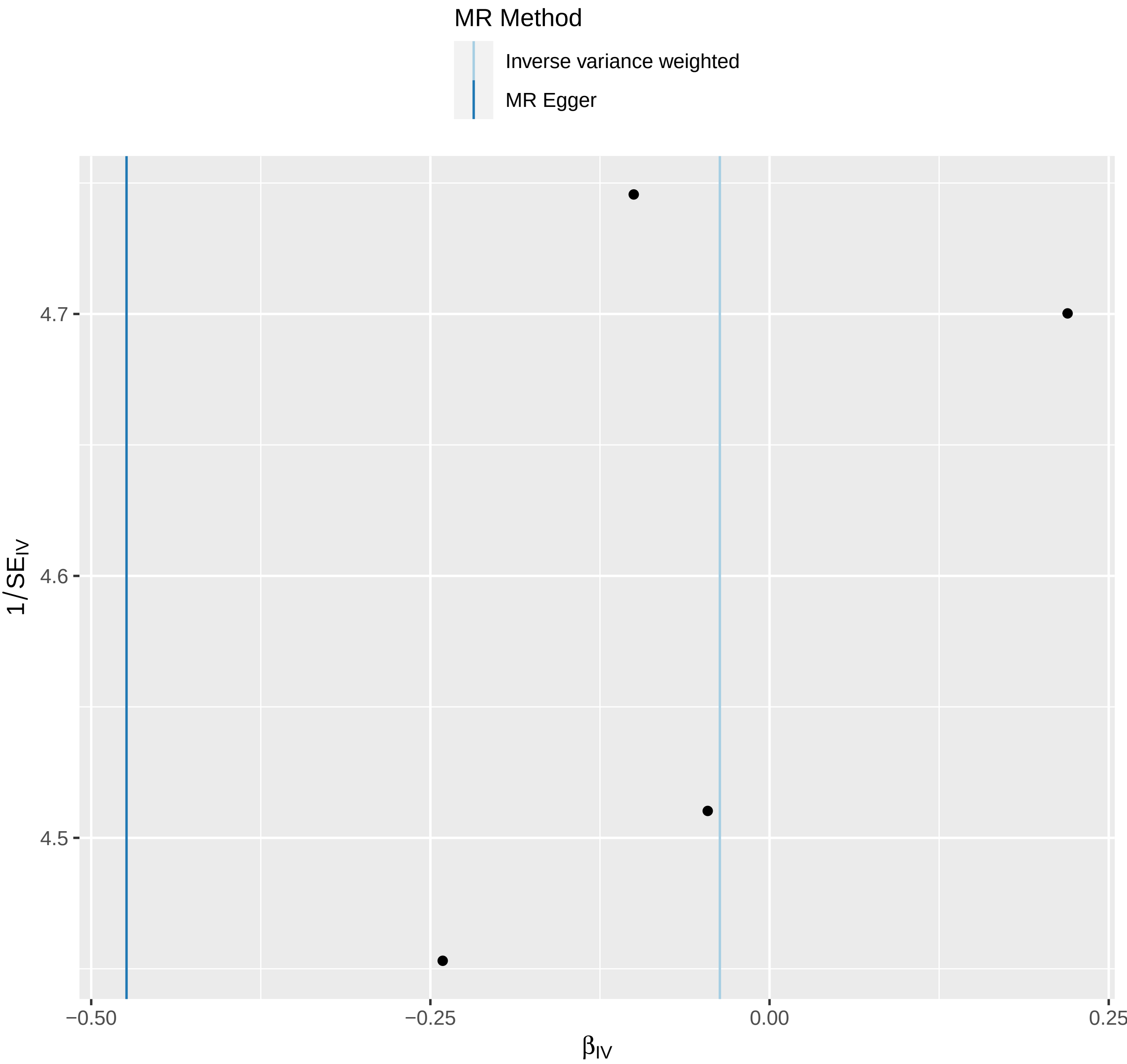

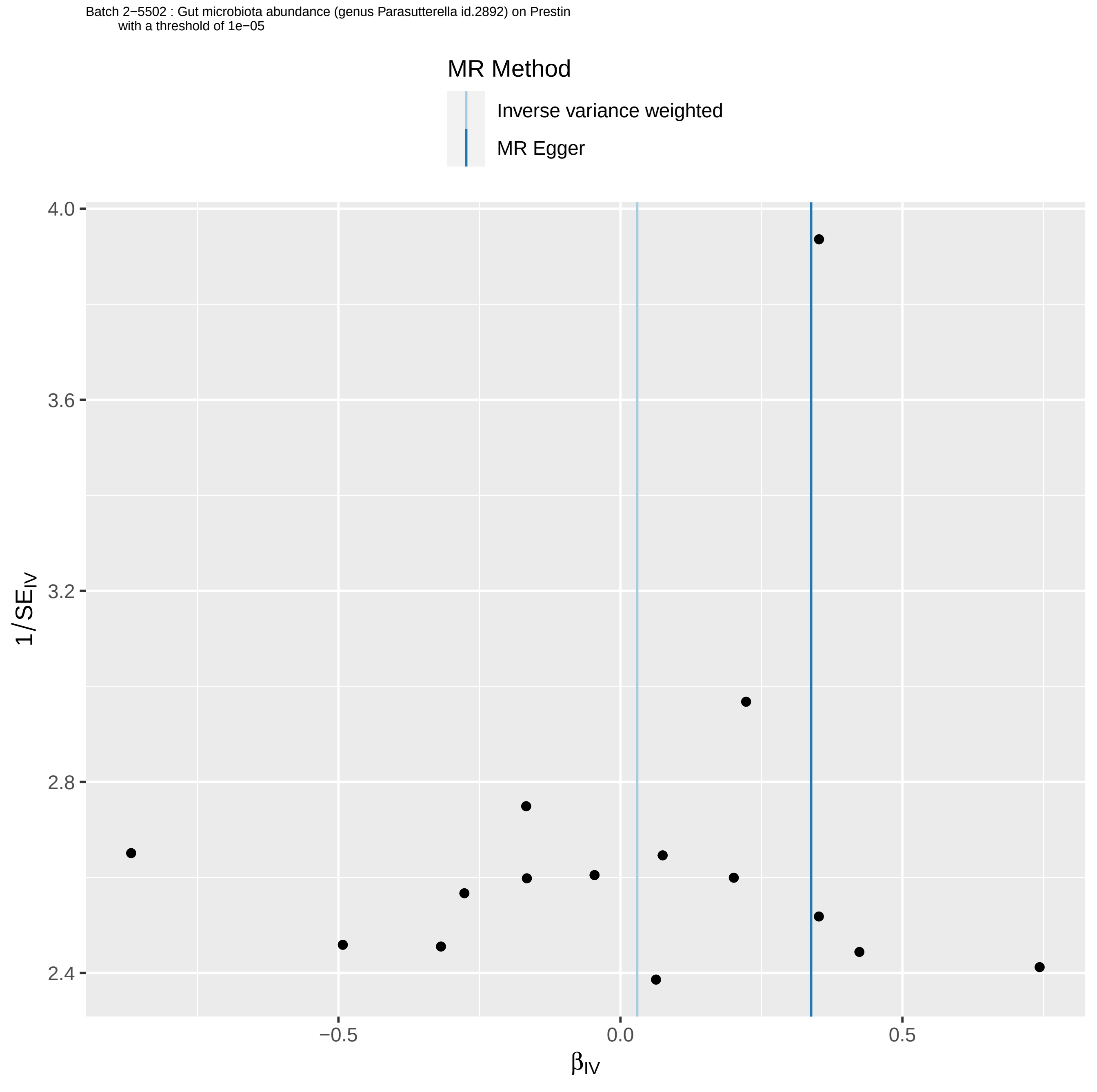

Batch 2−5502 : Gut microbiota abundance (genus Parasutterella id.2892) on Prestin
with a threshold of 1e−05
MR Method
Inverse variance weighted
MR Egger
4.0
3.6
3.2
2.8
2.4
1/SE_IV
−0.5
0.0
0.5
β_IV

Batch 2−5503 : Gut microbiota abundance (genus Catenibacterium id.2153) on Very long−chain acyl−CoA synthetase
with a threshold of 1e−05

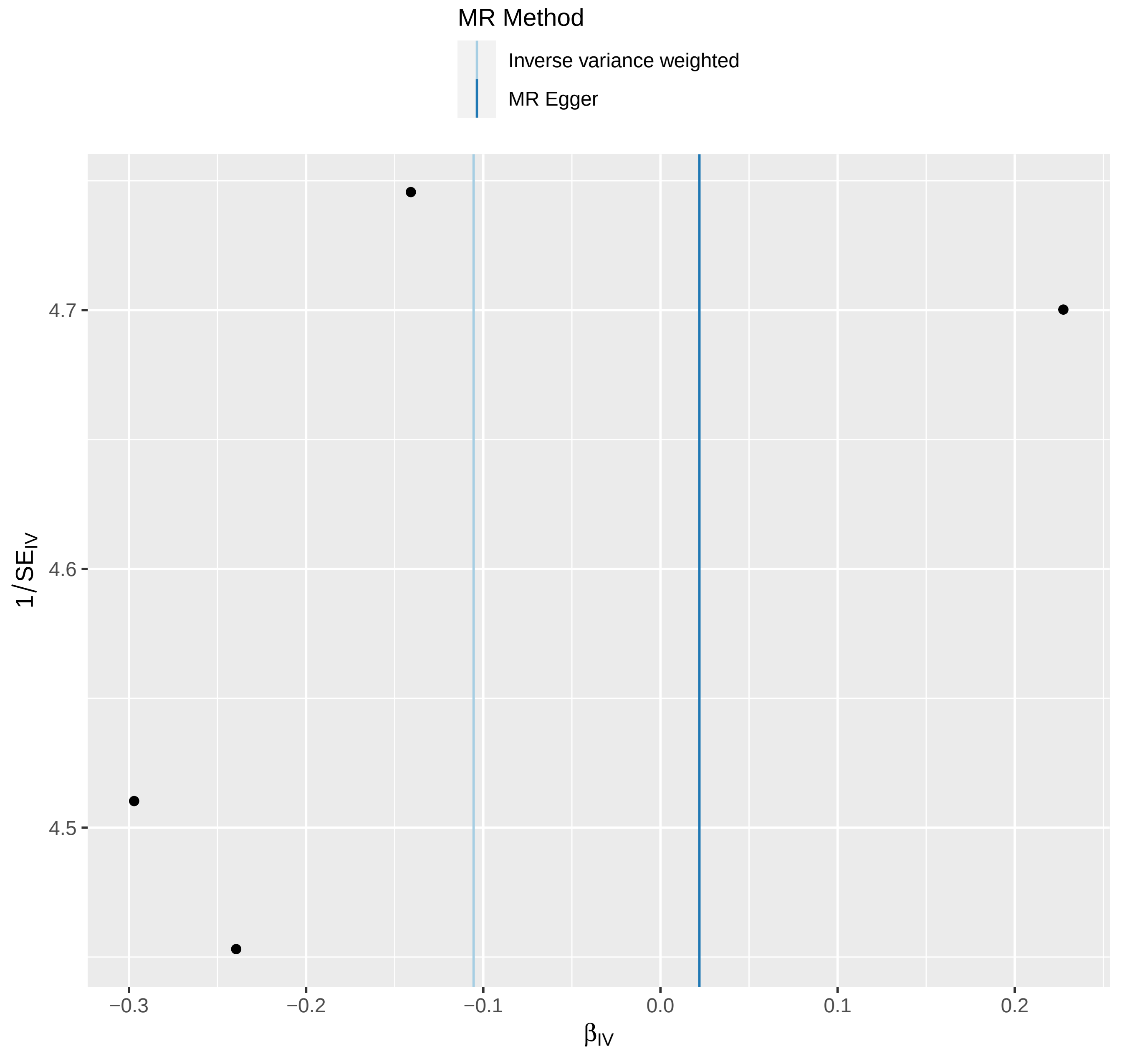

Batch 2−5504 : Gut microbiota abundance (genus Parasutterella id.2892) on Very long−chain acyl−CoA synthetase
with a threshold of 1e−05

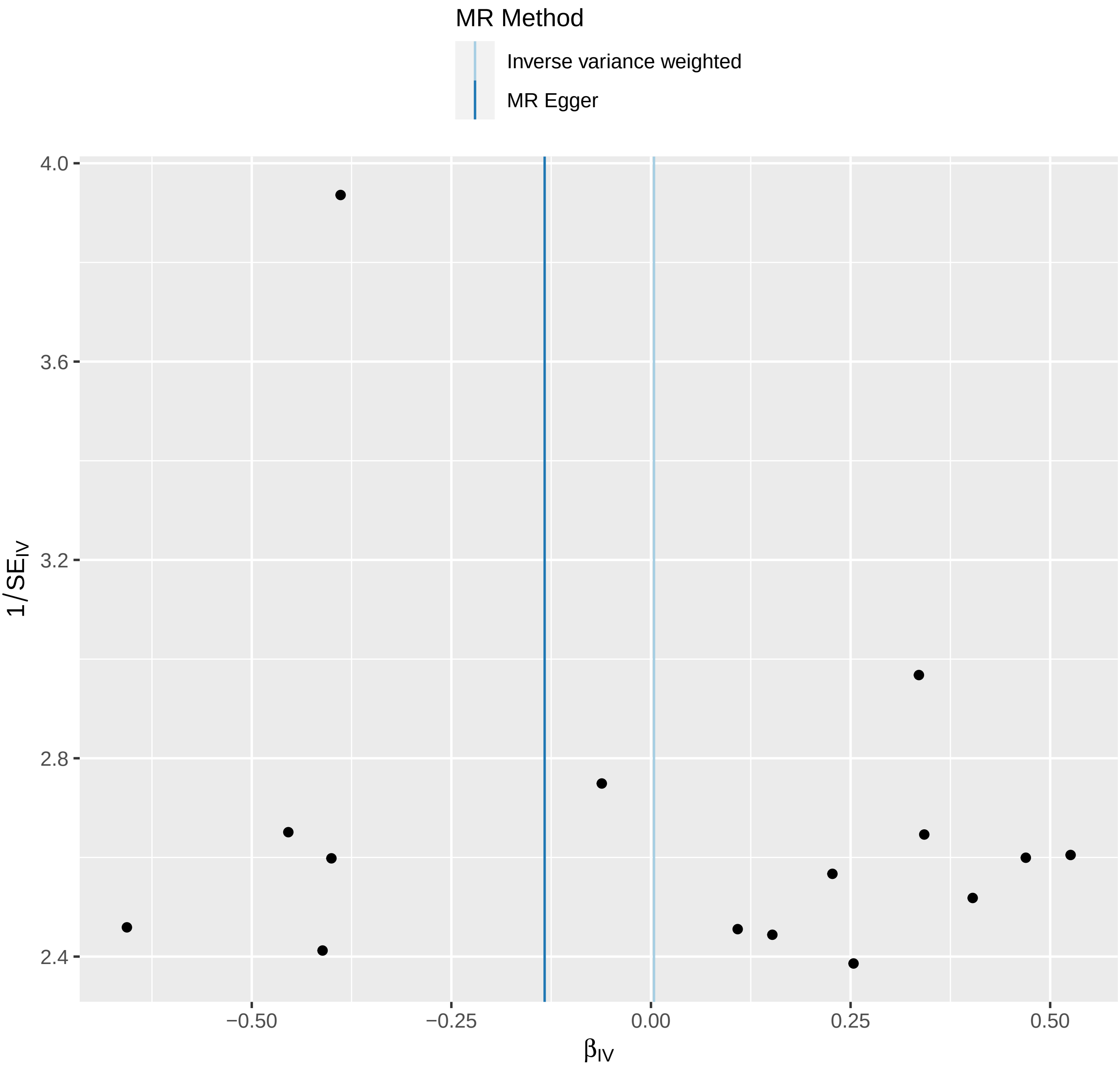

Batch 2−5505 : Gut microbiota abundance (genus Catenibacterium id.2153) on Zinc transporter 3
with a threshold of 1e−05

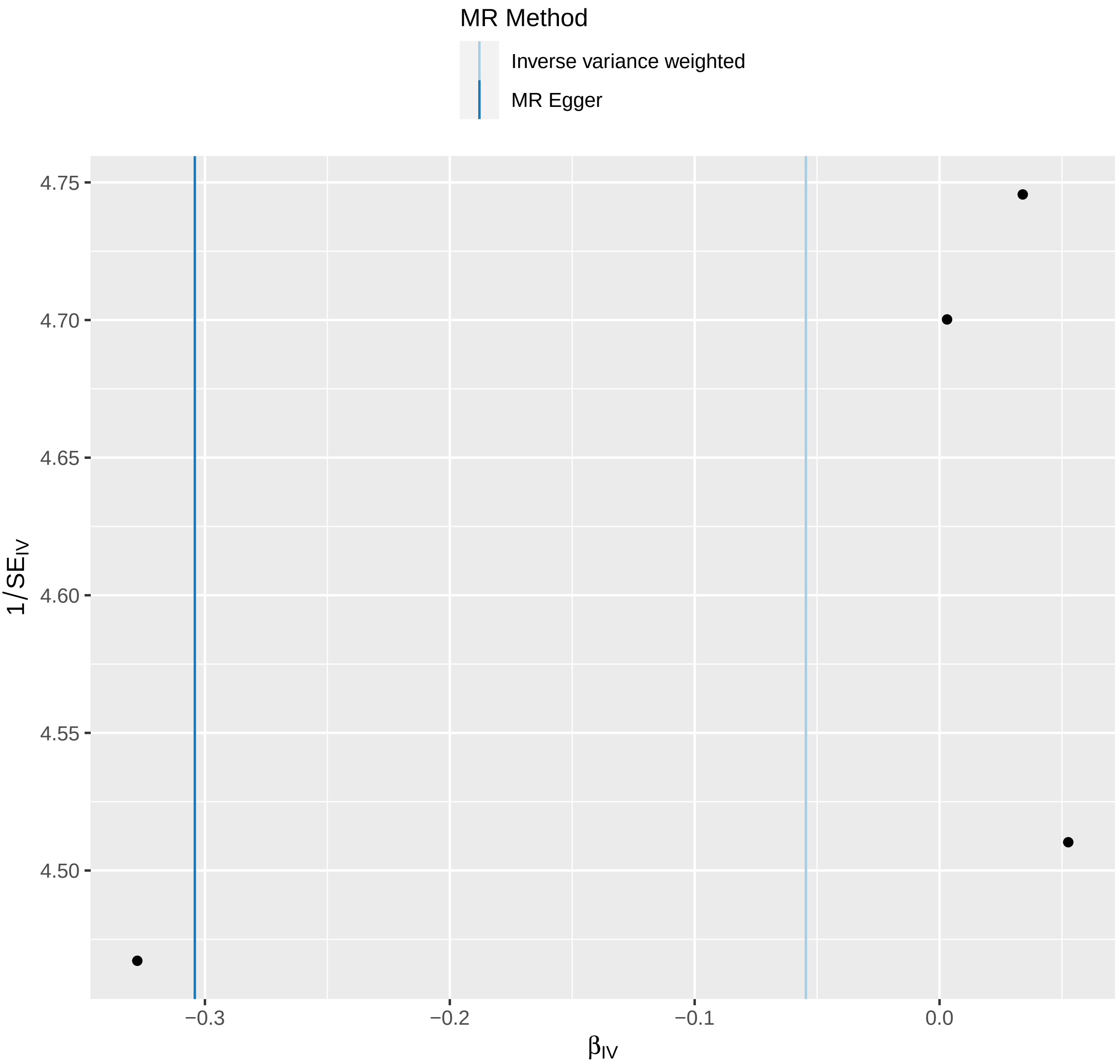

Batch 2−5506 : Gut microbiota abundance (genus Parasutterella id.2892) on Zinc transporter 3
with a threshold of 1e−05

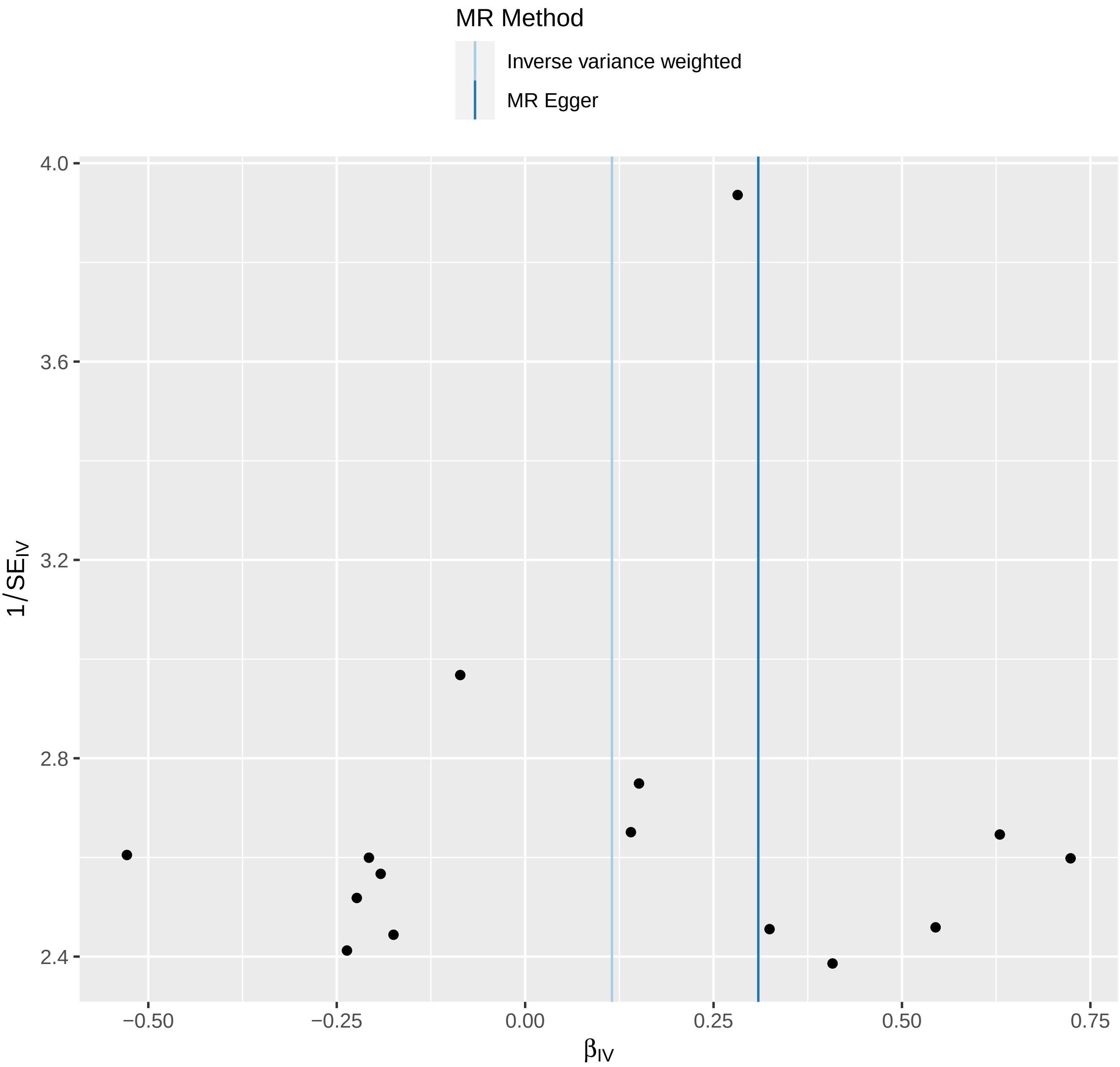

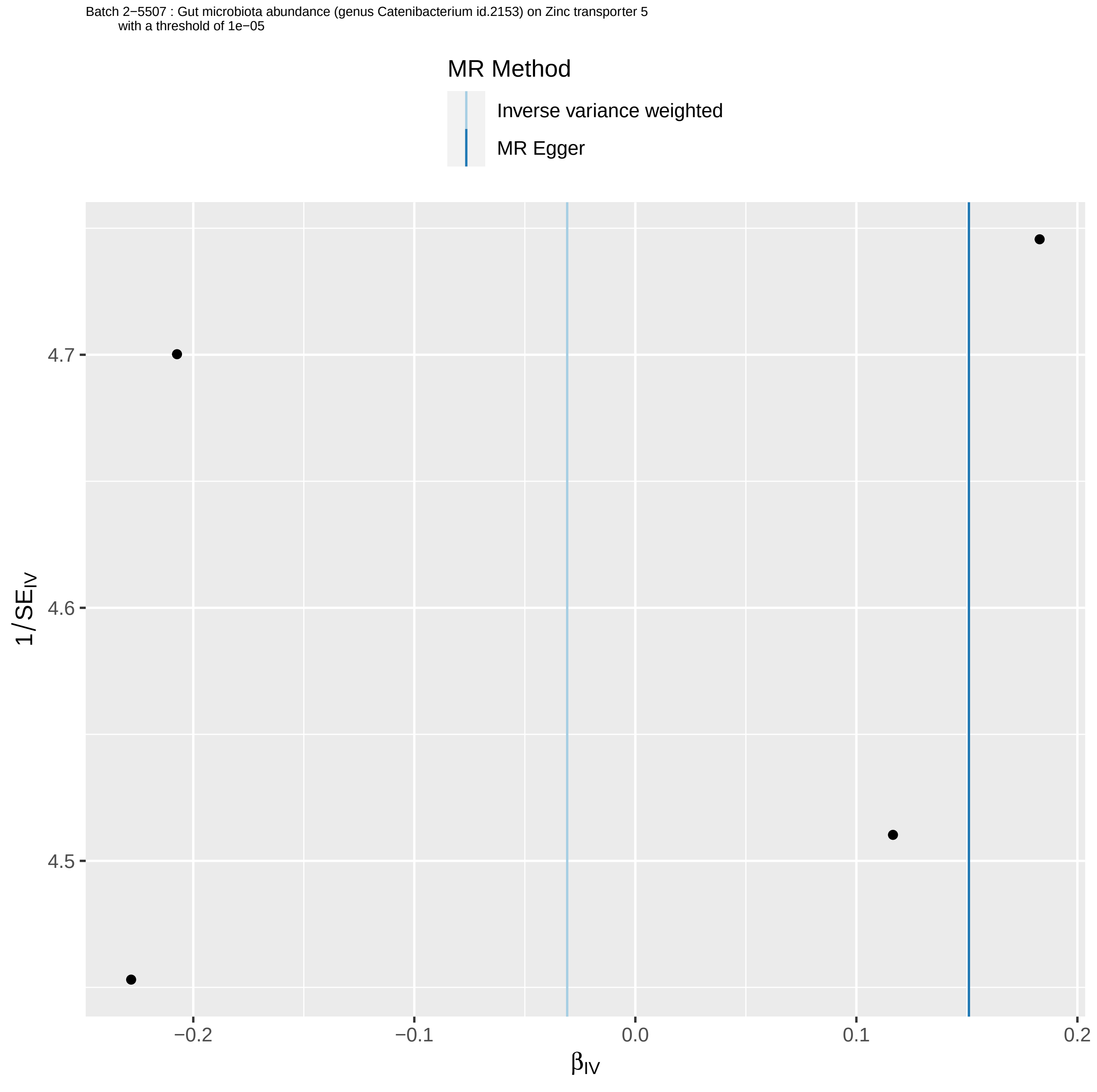

Batch 2−5507 : Gut microbiota abundance (genus Catenibacterium id.2153) on Zinc transporter 5
with a threshold of 1e−05
MR Method
Inverse variance weighted
MR Egger
4.7
4.6
4.5
1/SE_IV
−0.2
−0.1
0.0
0.1
0.2
β_IV

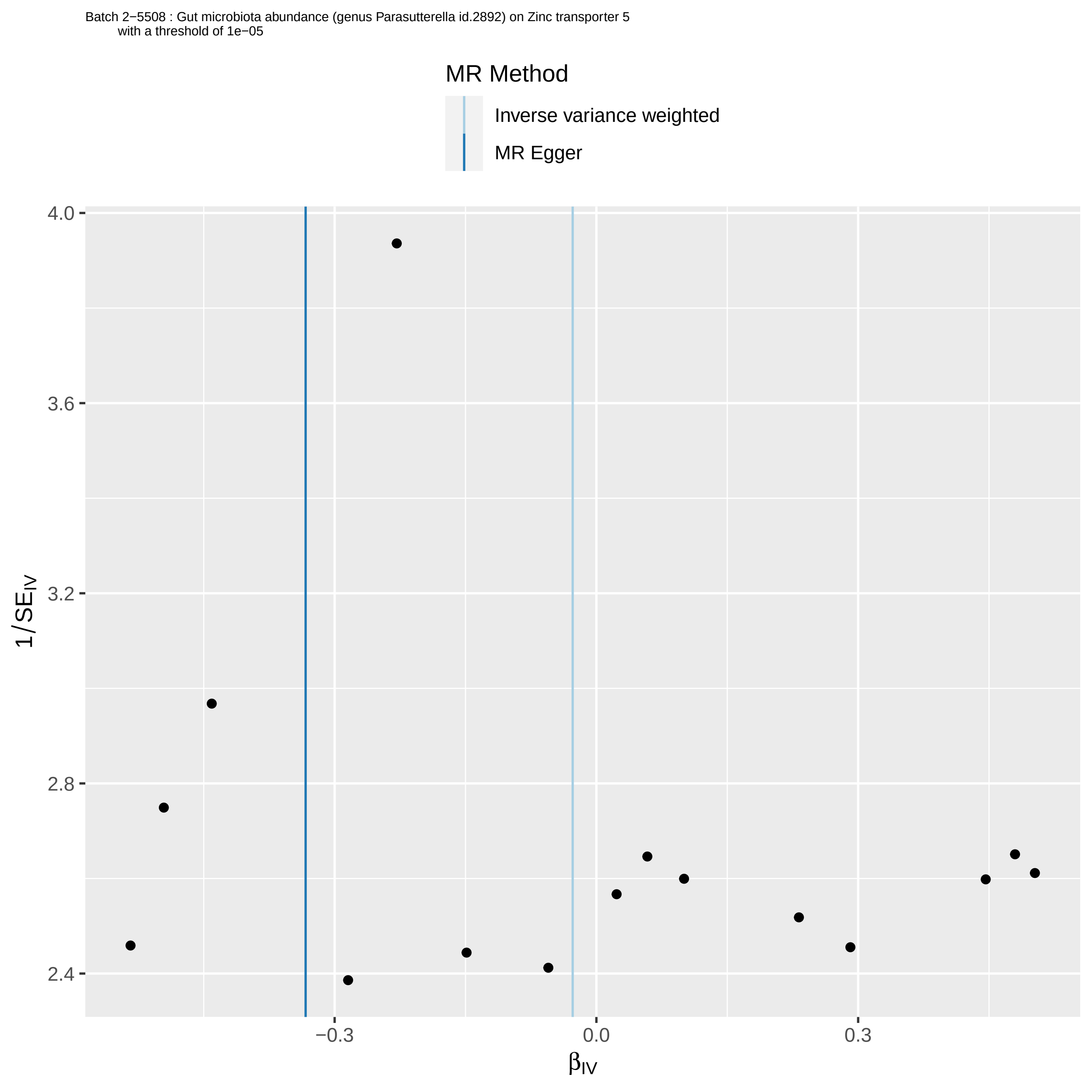
Batch 2−5508 : Gut microbiota abundance (genus Parasutterella id.2892) on Zinc transporter 5
with a threshold of 1e−05
MR Method
Inverse variance weighted
MR Egger
4.0
3.6
3.2
2.8
2.4
1/SE_IV
−0.3
0.0
0.3
β_IV

Batch 2−5509 : Gut microbiota abundance (genus Catenibacterium id.2153) on Adenosine 3'−phospho 5'−phosphosulfate transporter 2
with a threshold of 1e−05

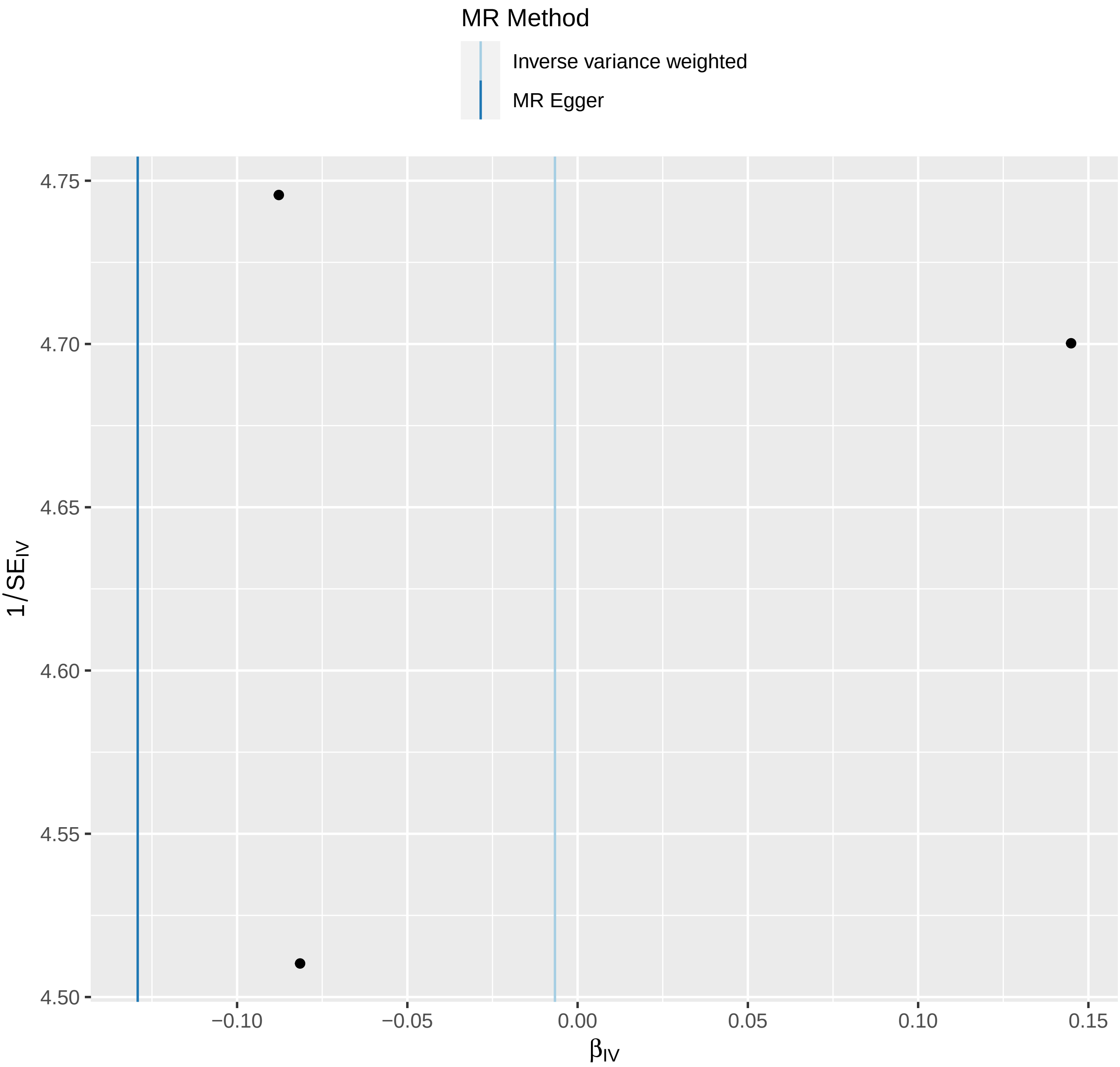

Batch 2−5510 : Gut microbiota abundance (genus Parasutterella id.2892) on Adenosine 3'−phospho 5'−phosphosulfate transporter 2
with a threshold of 1e−05

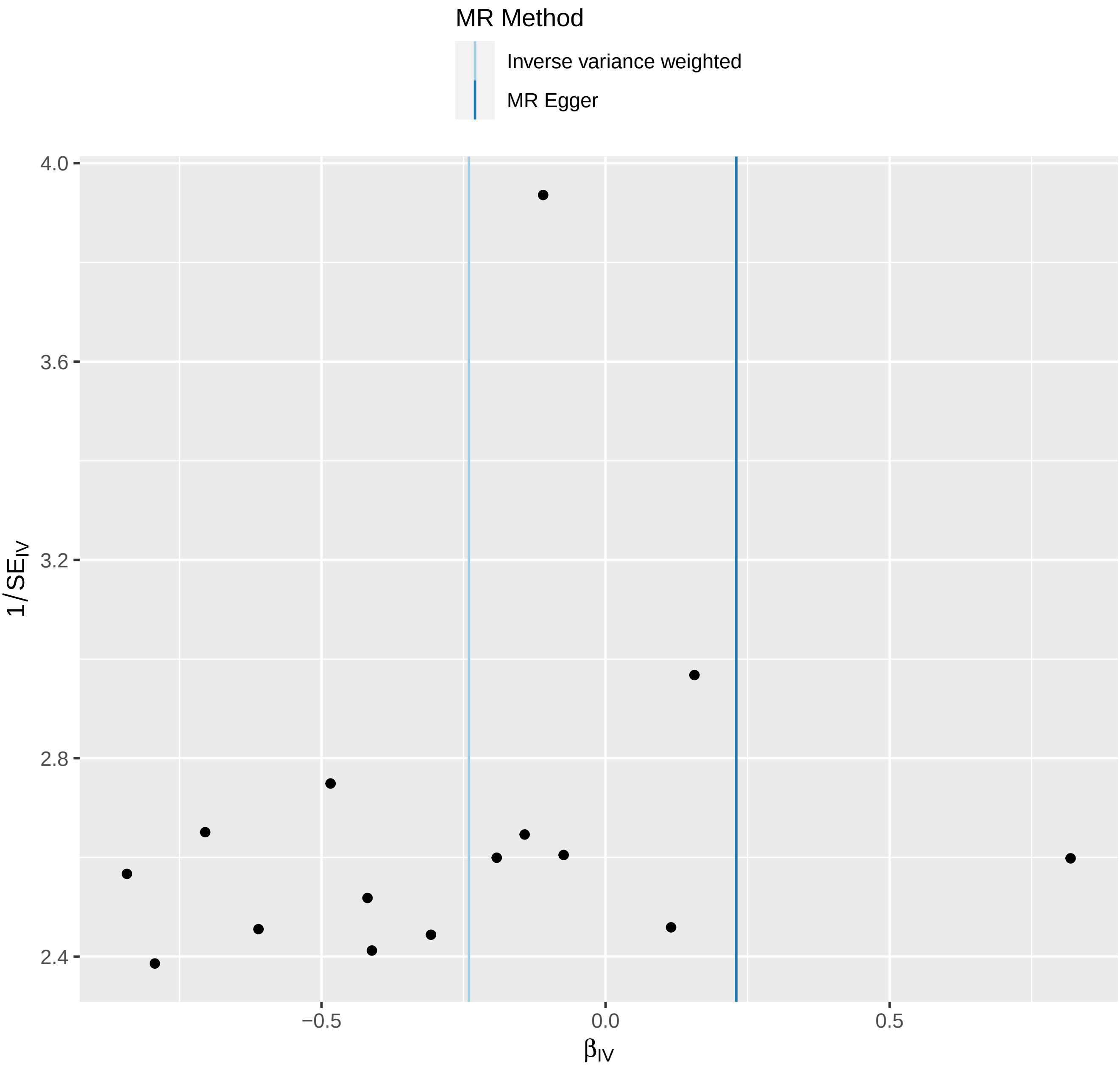

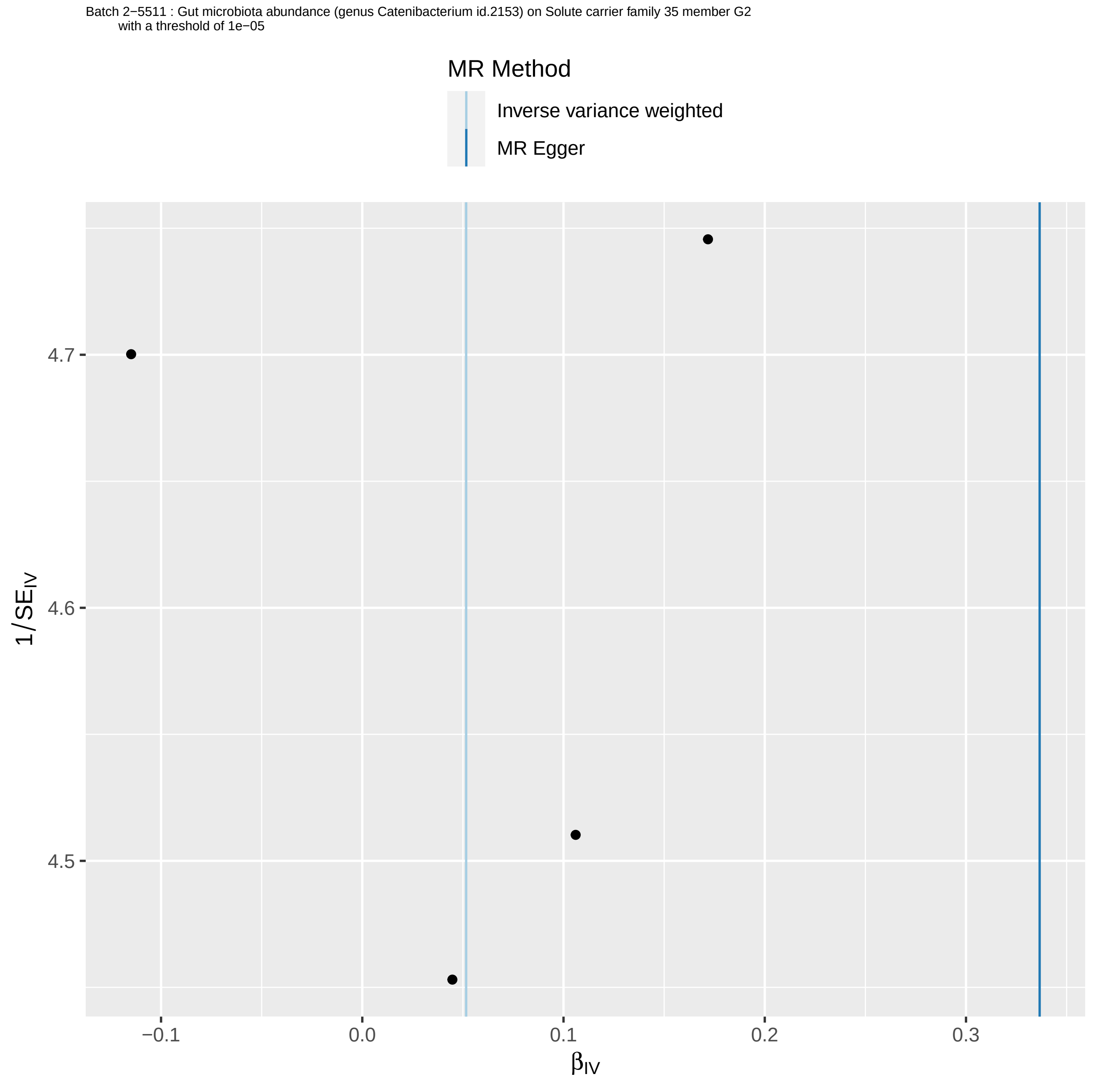

Batch 2−5511 : Gut microbiota abundance (genus Catenibacterium id.2153) on Solute carrier family 35 member G2
with a threshold of 1e−05
MR Method
Inverse variance weighted
MR Egger
4.7
4.6
4.5
1/SE_IV
−0.1
0.0
0.1
0.2
0.3
β_IV

Batch 2−5512 : Gut microbiota abundance (genus Parasutterella id.2892) on Solute carrier family 35 member G2
with a threshold of 1e−05

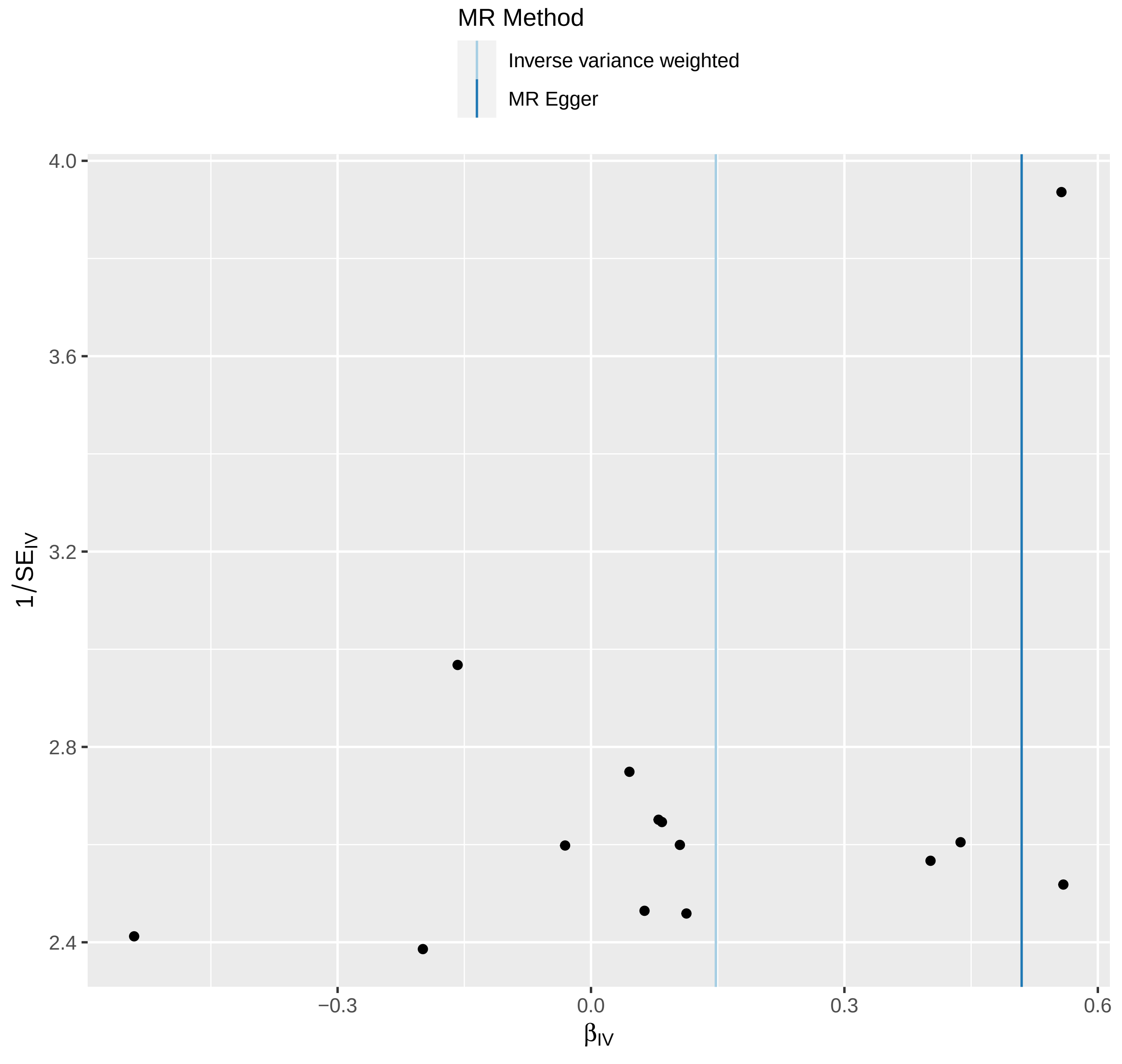

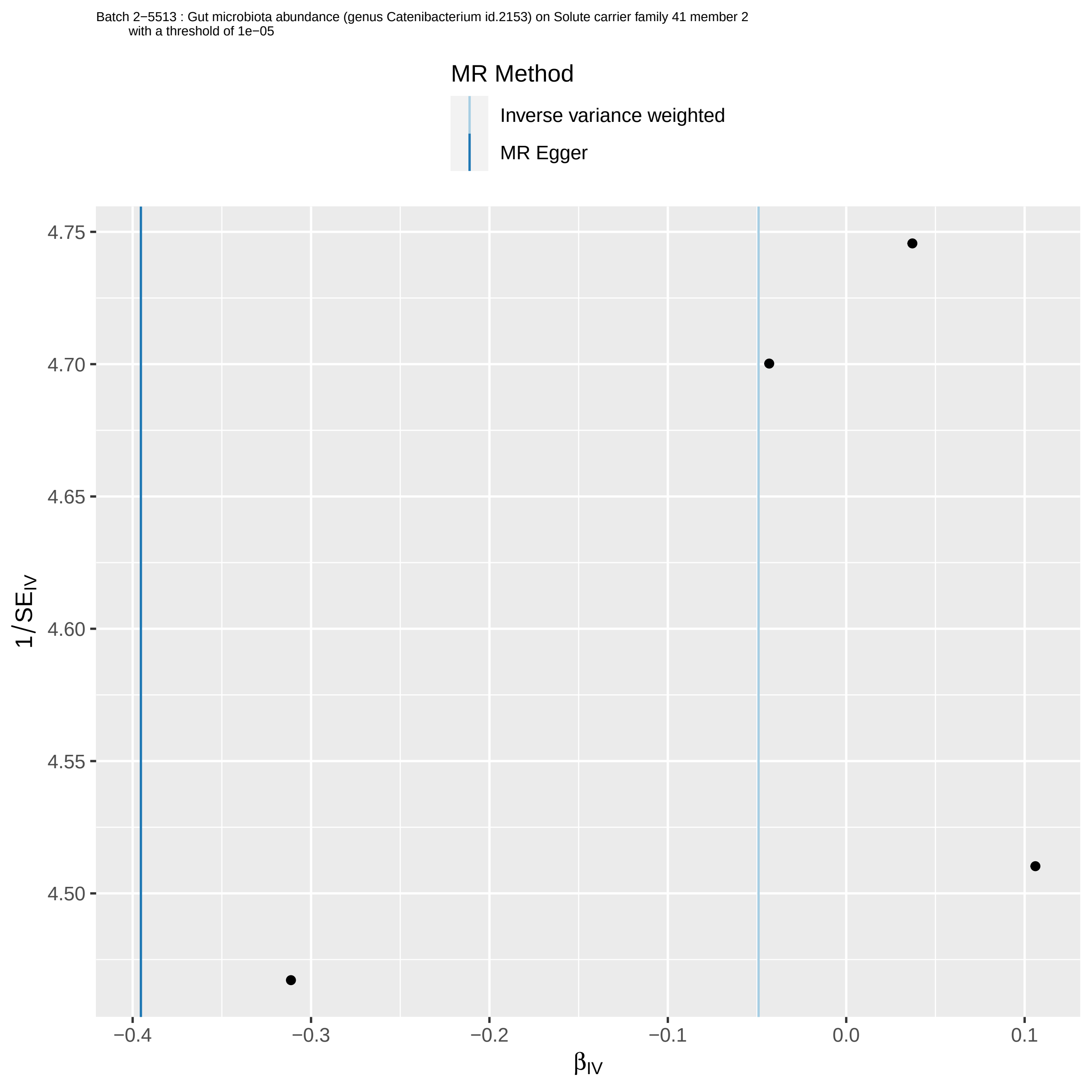

Batch 2−5513 : Gut microbiota abundance (genus Catenibacterium id.2153) on Solute carrier family 41 member 2
with a threshold of 1e−05
MR Method
Inverse variance weighted
MR Egger
4.75
4.70
4.65
4.60
4.55
4.50
1/SEIV
−0.4
−0.3
−0.2
−0.1
0.0
0.1
βIV

Batch 2−5514 : Gut microbiota abundance (genus Parasutterella id.2892) on Solute carrier family 41 member 2
with a threshold of 1e−05

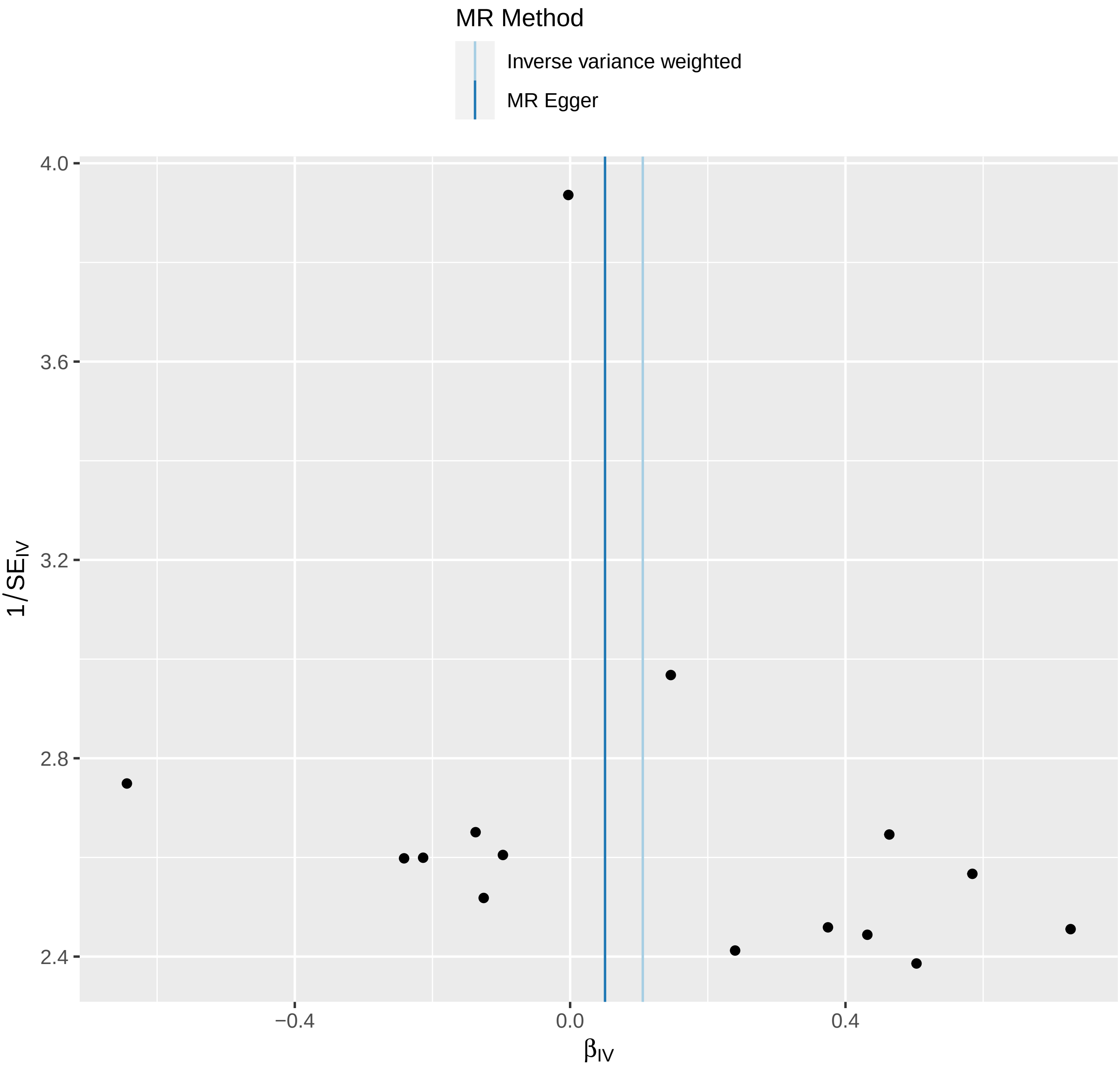

Batch 2−5515 : Gut microbiota abundance (genus Catenibacterium id.2153) on Electroneutral sodium bicarbonate exchanger 1 with a threshold of 1e−05

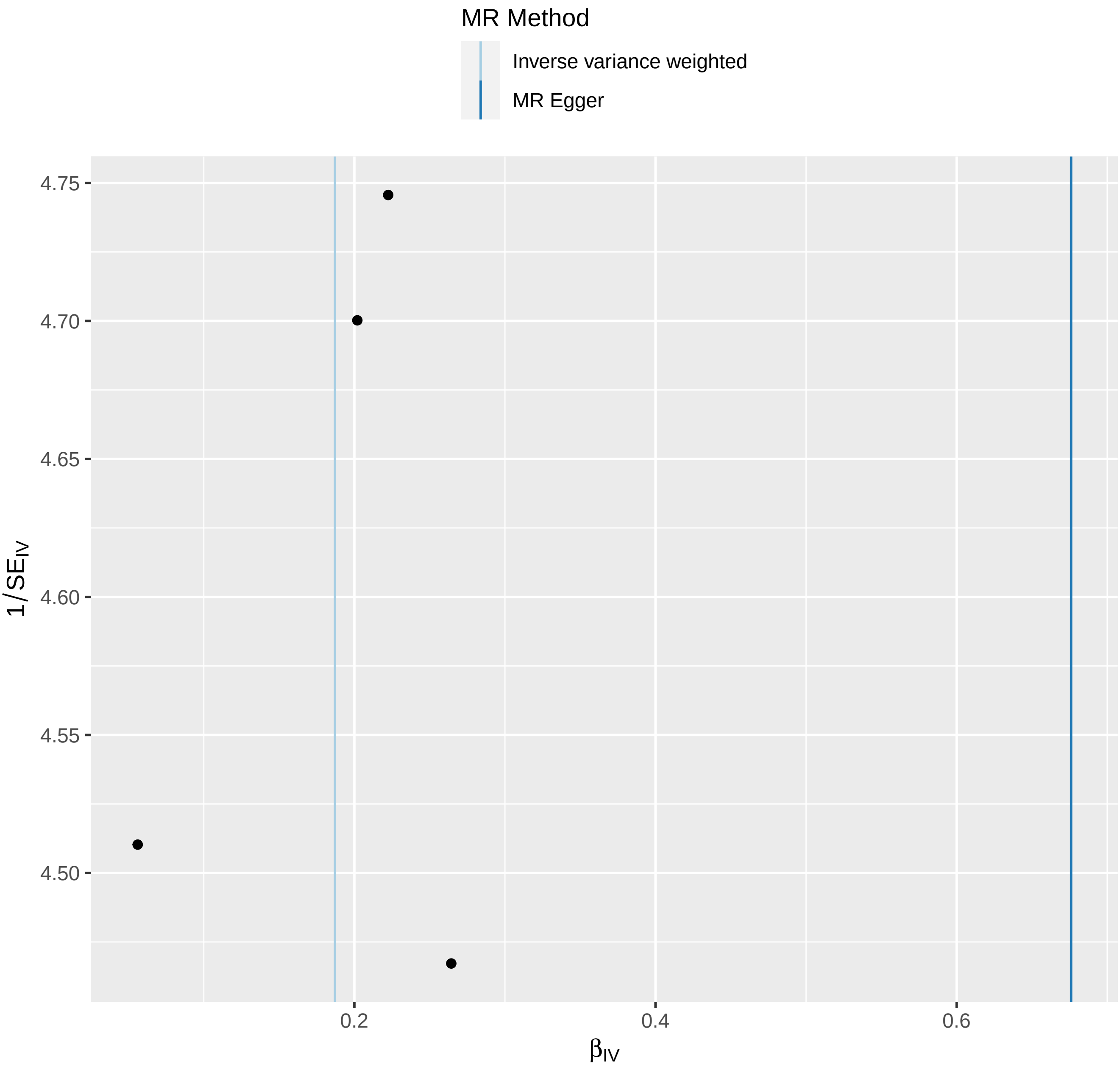

Batch 2−5516 : Gut microbiota abundance (genus Parasutterella id.2892) on Electroneutral sodium bicarbonate exchanger 1 with a threshold of 1e−05

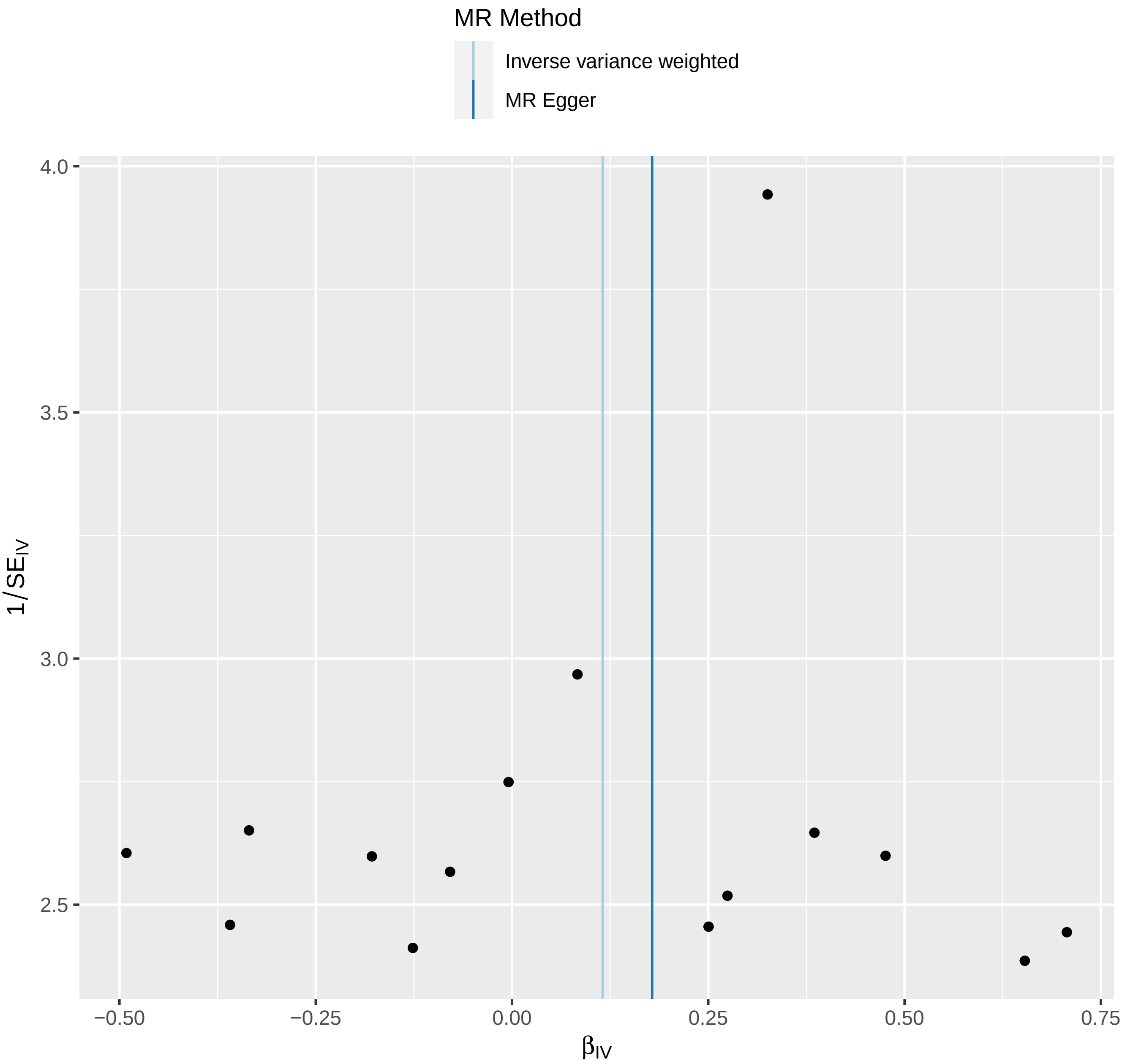

Batch 2−5517 : Gut microbiota abundance (genus Catenibacterium id.2153) on Sodium/iodide cotransporter
with a threshold of 1e−05

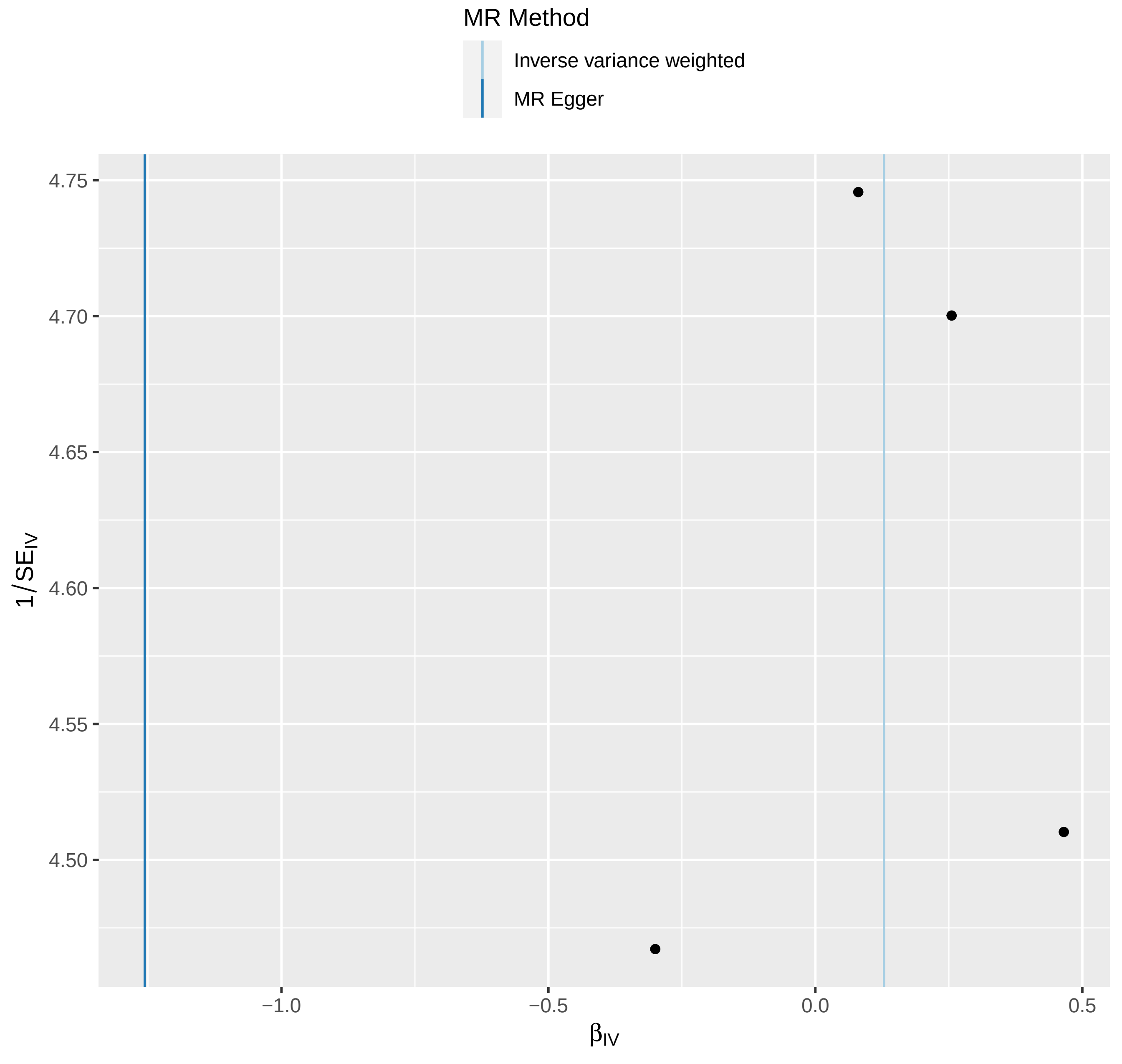

Batch 2−5518 : Gut microbiota abundance (genus Parasutterella id.2892) on Sodium/iodide cotransporter
with a threshold of 1e−05

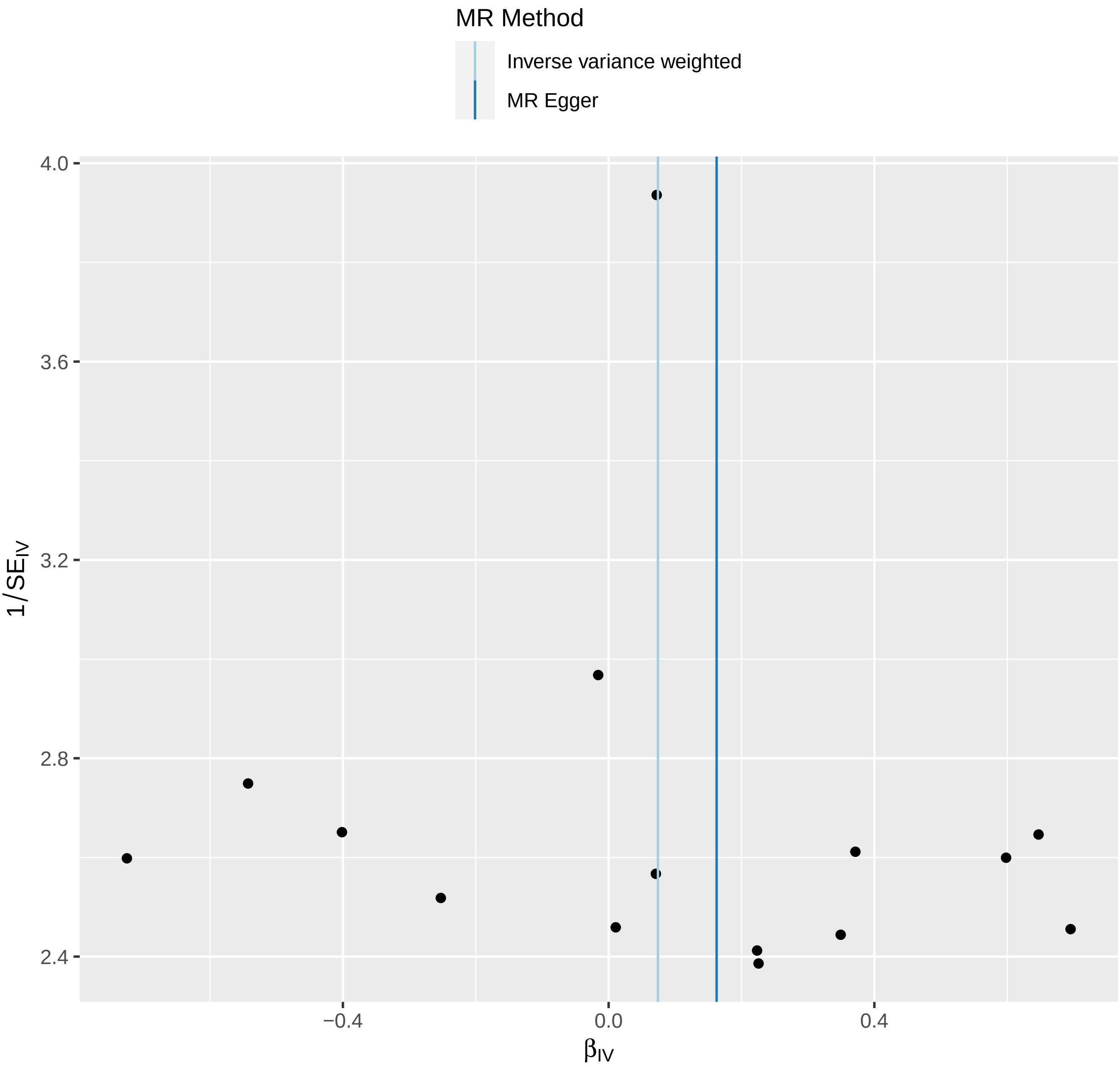

Batch 2−5519 : Gut microbiota abundance (genus Catenibacterium id.2153) on Sodium−coupled monocarboxylate transporter 1 with a threshold of 1e−05

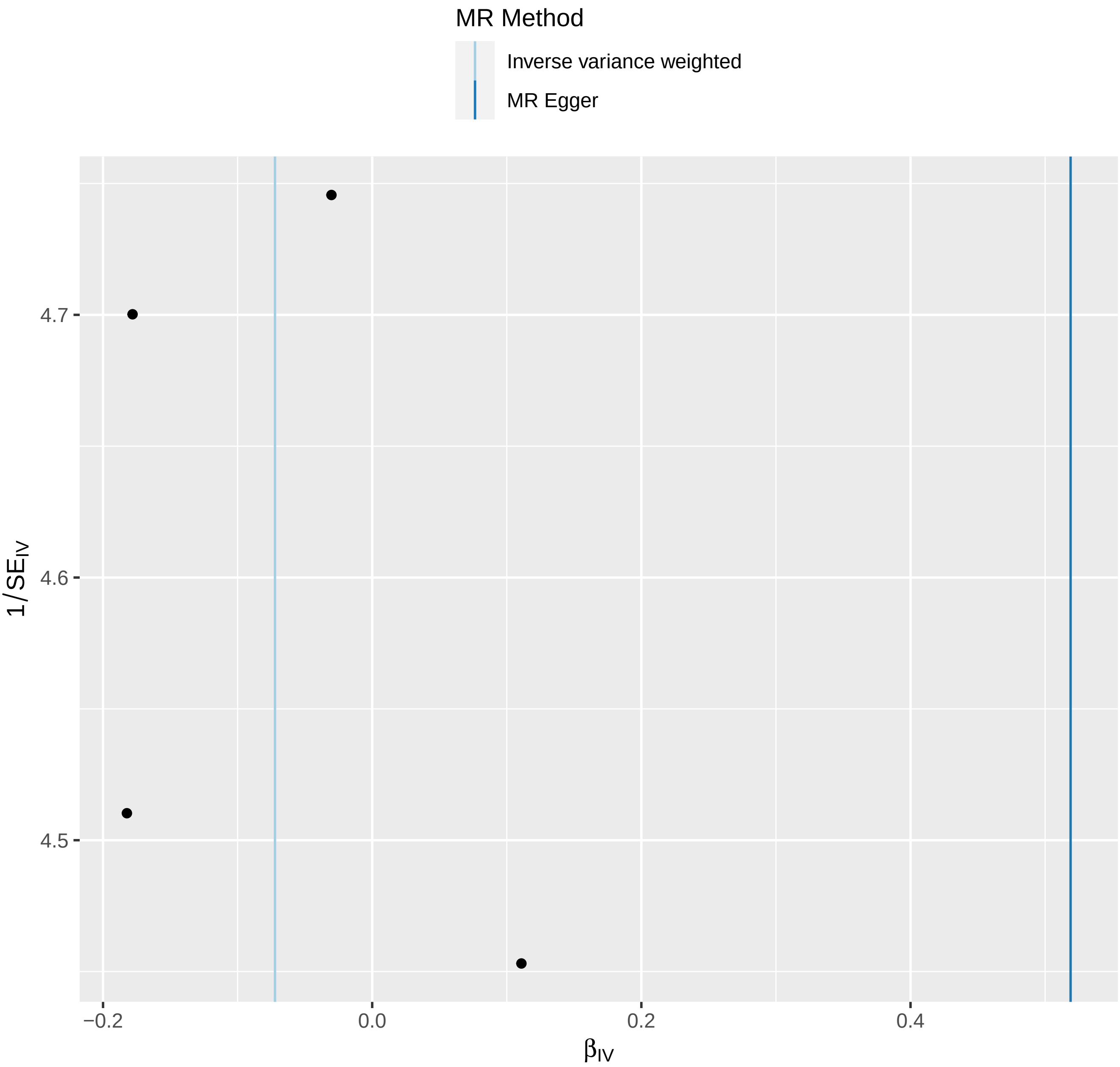

Batch 2−5520 : Gut microbiota abundance (genus Parasutterella id.2892) on Sodium−coupled monocarboxylate transporter 1
with a threshold of 1e−05

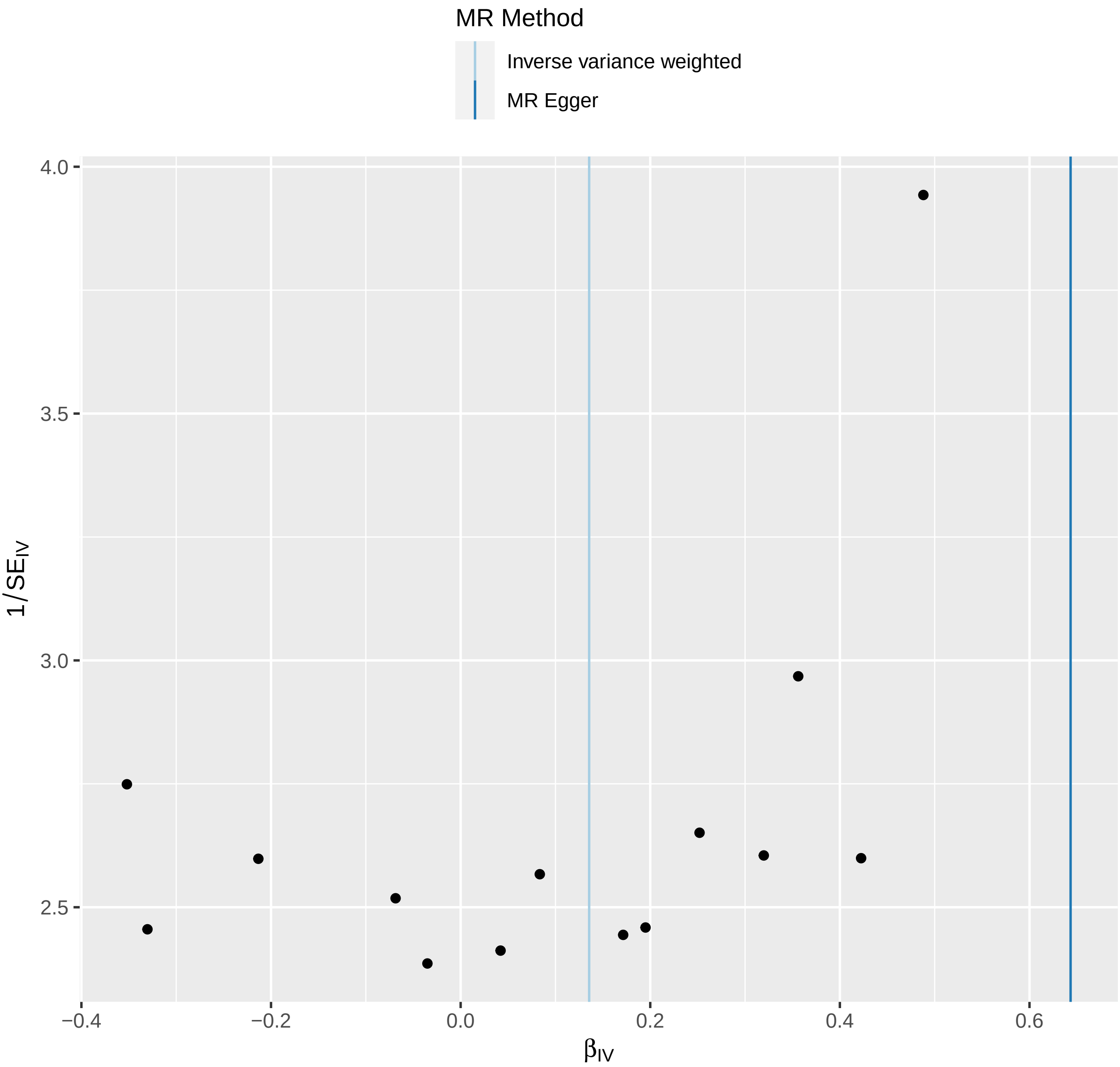

Batch 2−5521 : Gut microbiota abundance (genus Catenibacterium id.2153) on Sodium− and chloride−dependent neutral and basic amino acid transporter B(0+) with a threshold of 1e−05

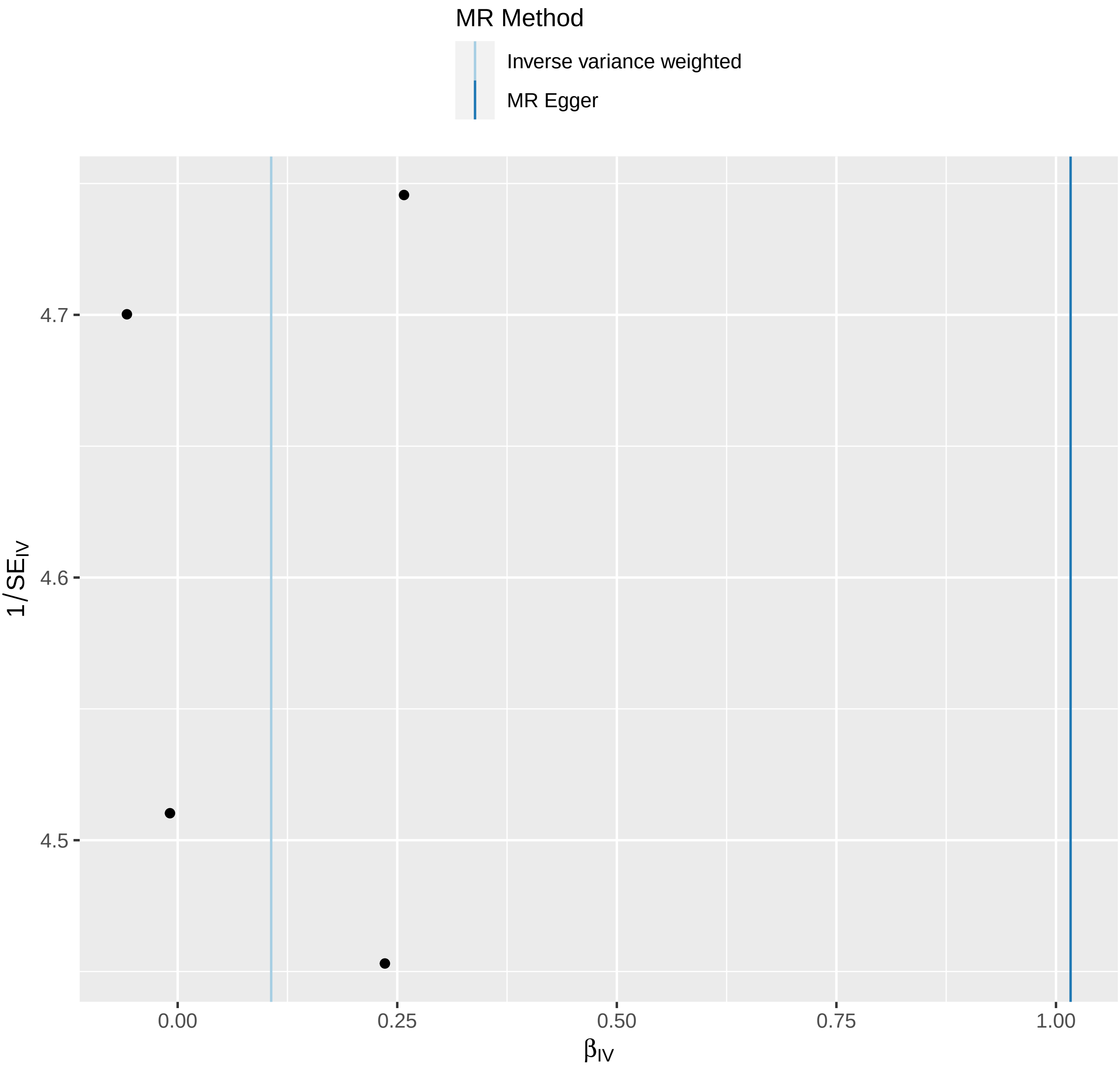

Batch 2−5522 : Gut microbiota abundance (genus Parasutterella id.2892) on Sodium− and chloride−dependent neutral and basic amino acid transporter B(0+)
with a threshold of 1e−05

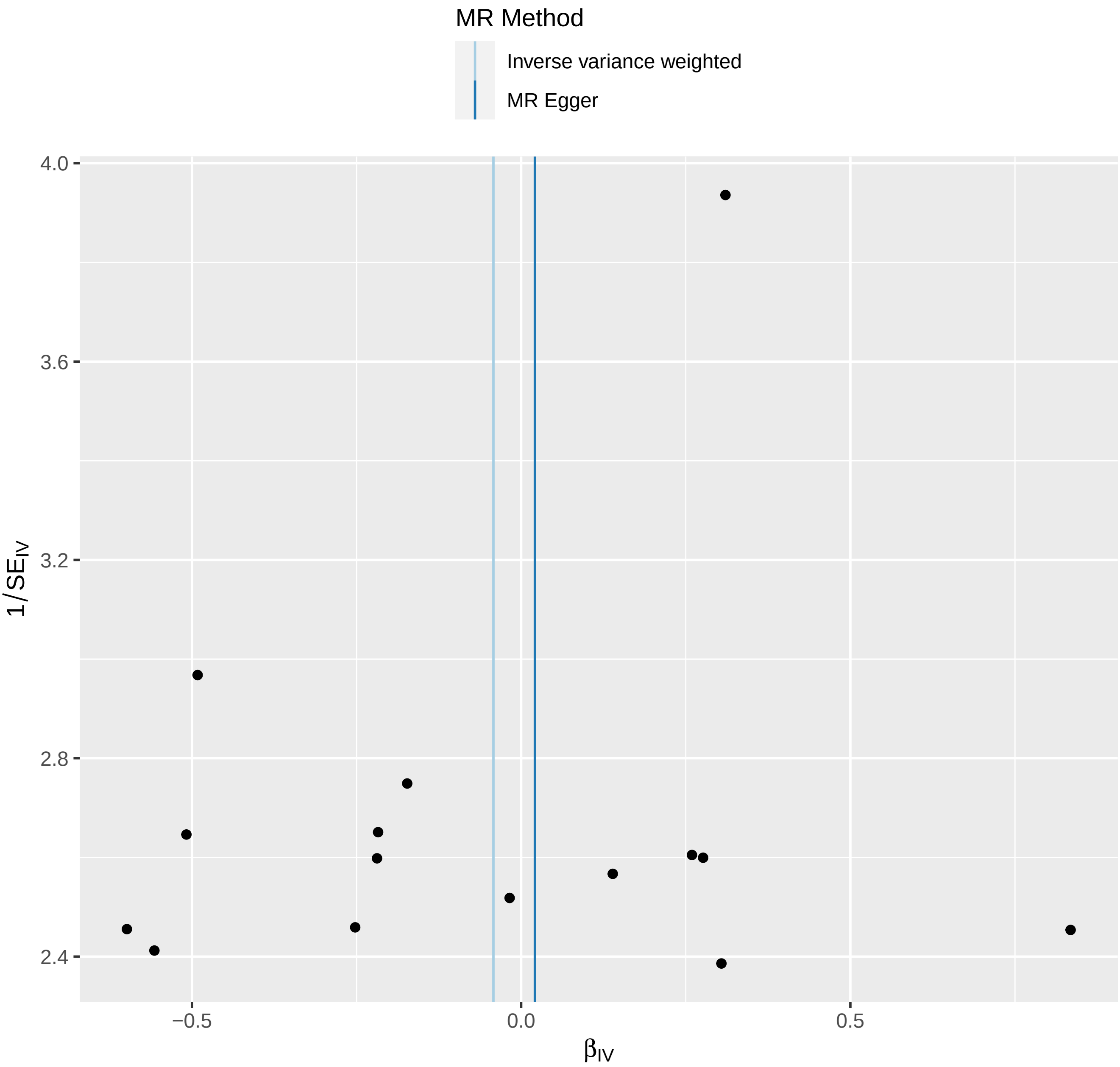

Batch 2−5523 : Gut microbiota abundance (genus Catenibacterium id.2153) on Orphan sodium− and chloride−dependent neurotransmitter transporter NTT5
with a threshold of 1e−05

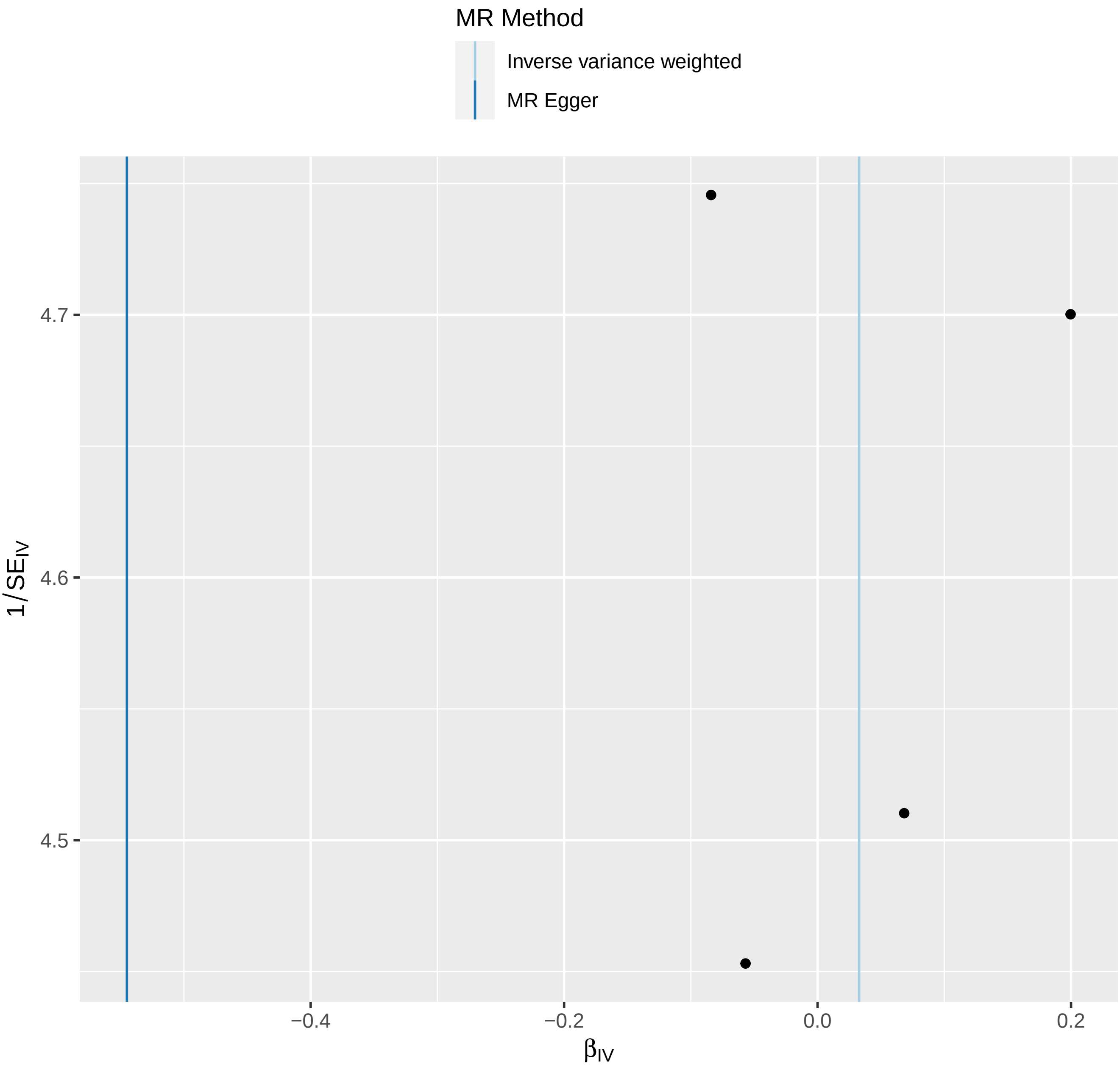

Batch 2−5524 : Gut microbiota abundance (genus Parasutterella id.2892) on Orphan sodium− and chloride−dependent neurotransmitter transporter NTT5 with a threshold of 1e−05

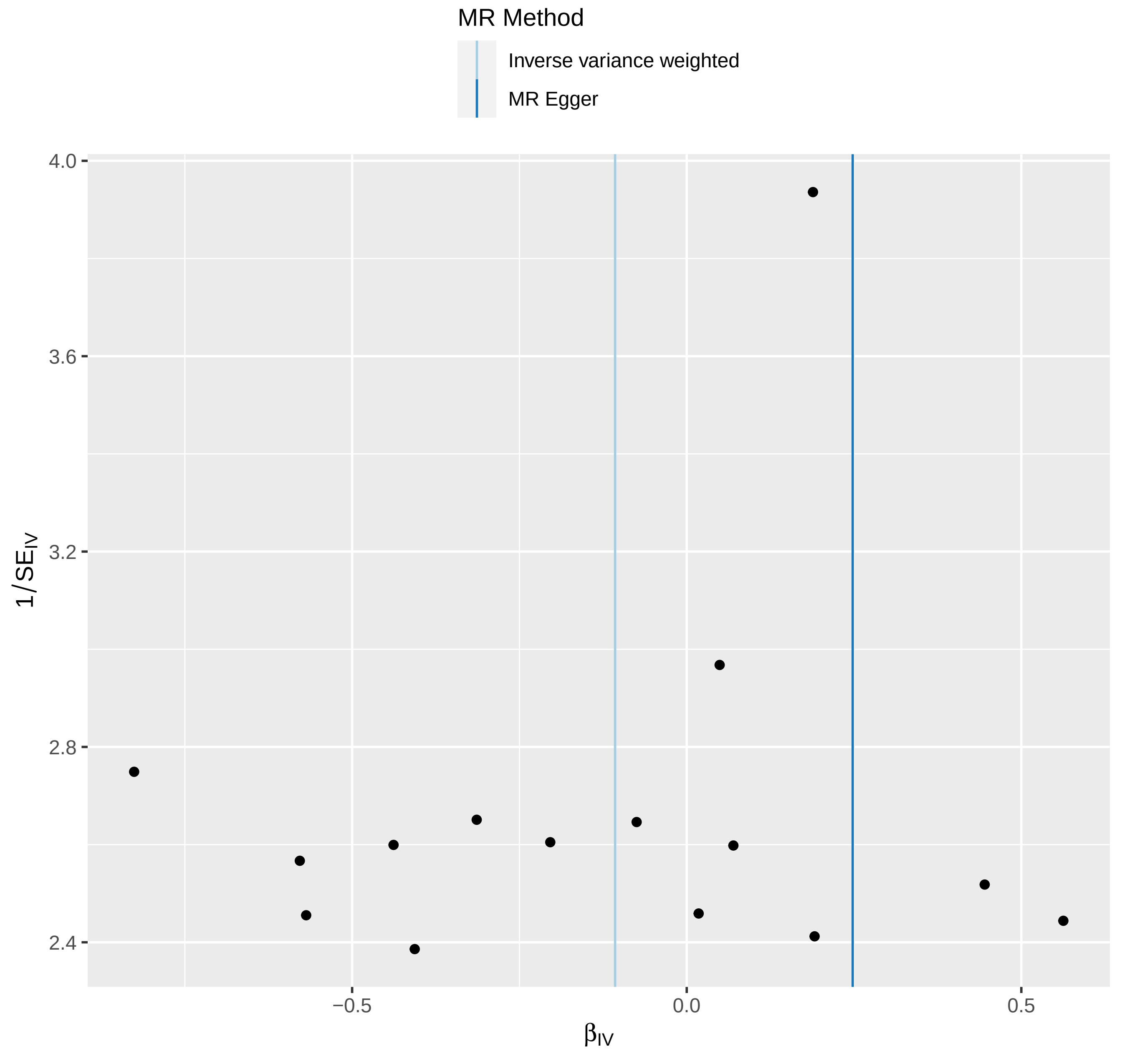

Batch 2−5525 : Gut microbiota abundance (genus Catenibacterium id.2153) on Sodium− and chloride−dependent glycine transporter 1 with a threshold of 1e−05

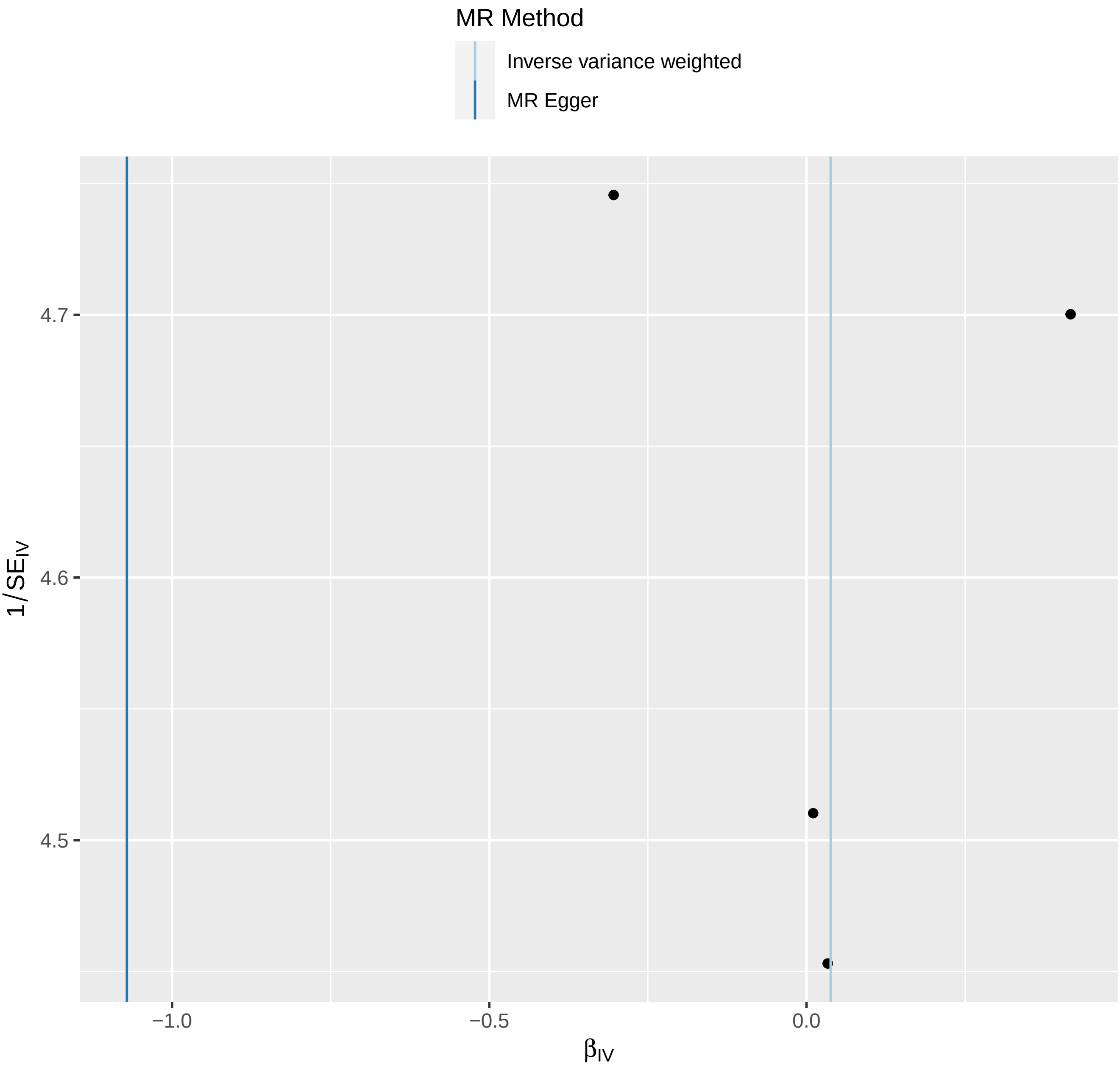

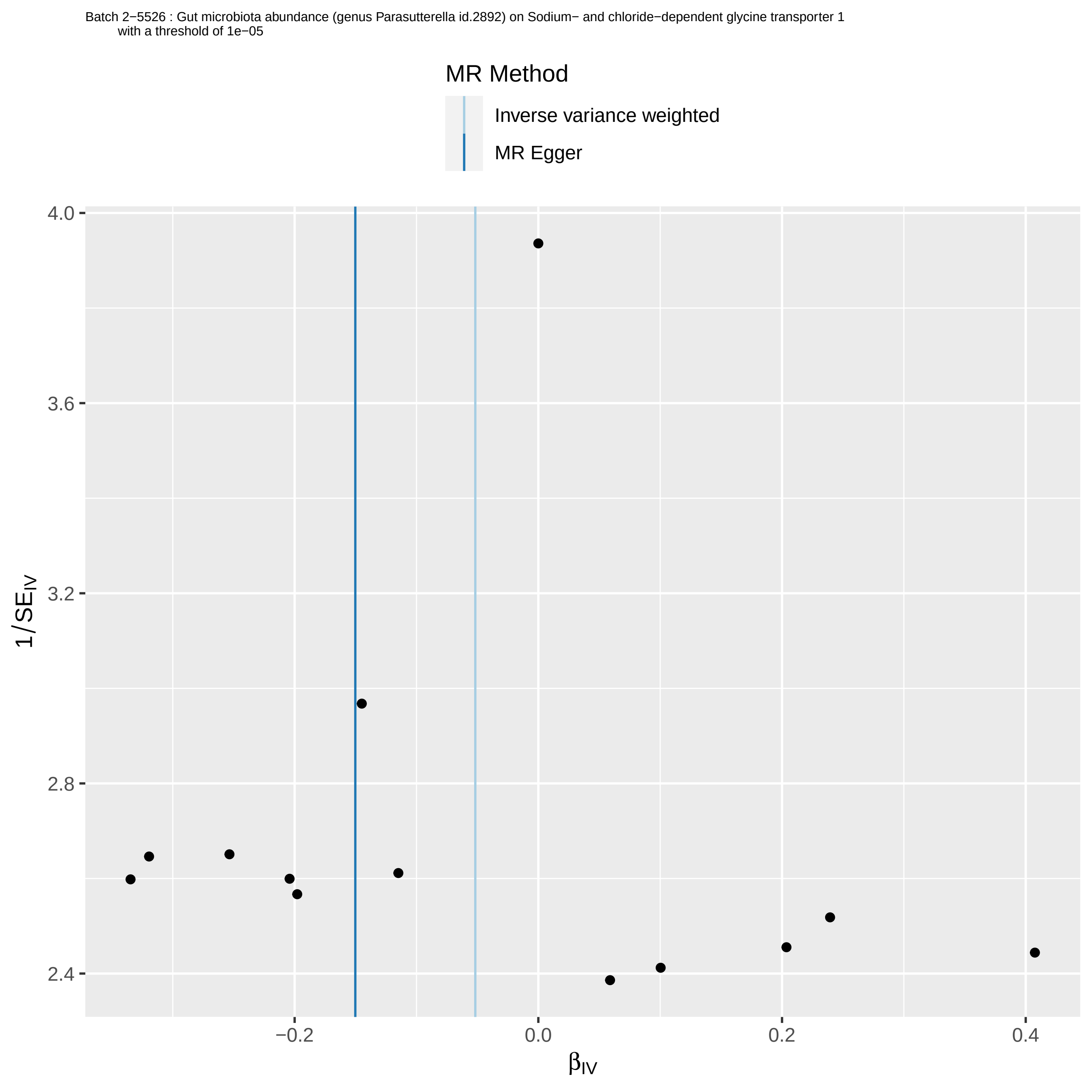

Batch 2−5526 : Gut microbiota abundance (genus Parasutterella id.2892) on Sodium− and chloride−dependent glycine transporter 1
with a threshold of 1e−05
MR Method
Inverse variance weighted
MR Egger
4.0
3.6
3.2
2.8
2.4
1/SE_IV
−0.2
0.0
0.2
0.4
β_IV

Batch 2−5527 : Gut microbiota abundance (genus Catenibacterium id.2153) on Mitochondrial sodium/hydrogen exchanger 9B2
with a threshold of 1e−05

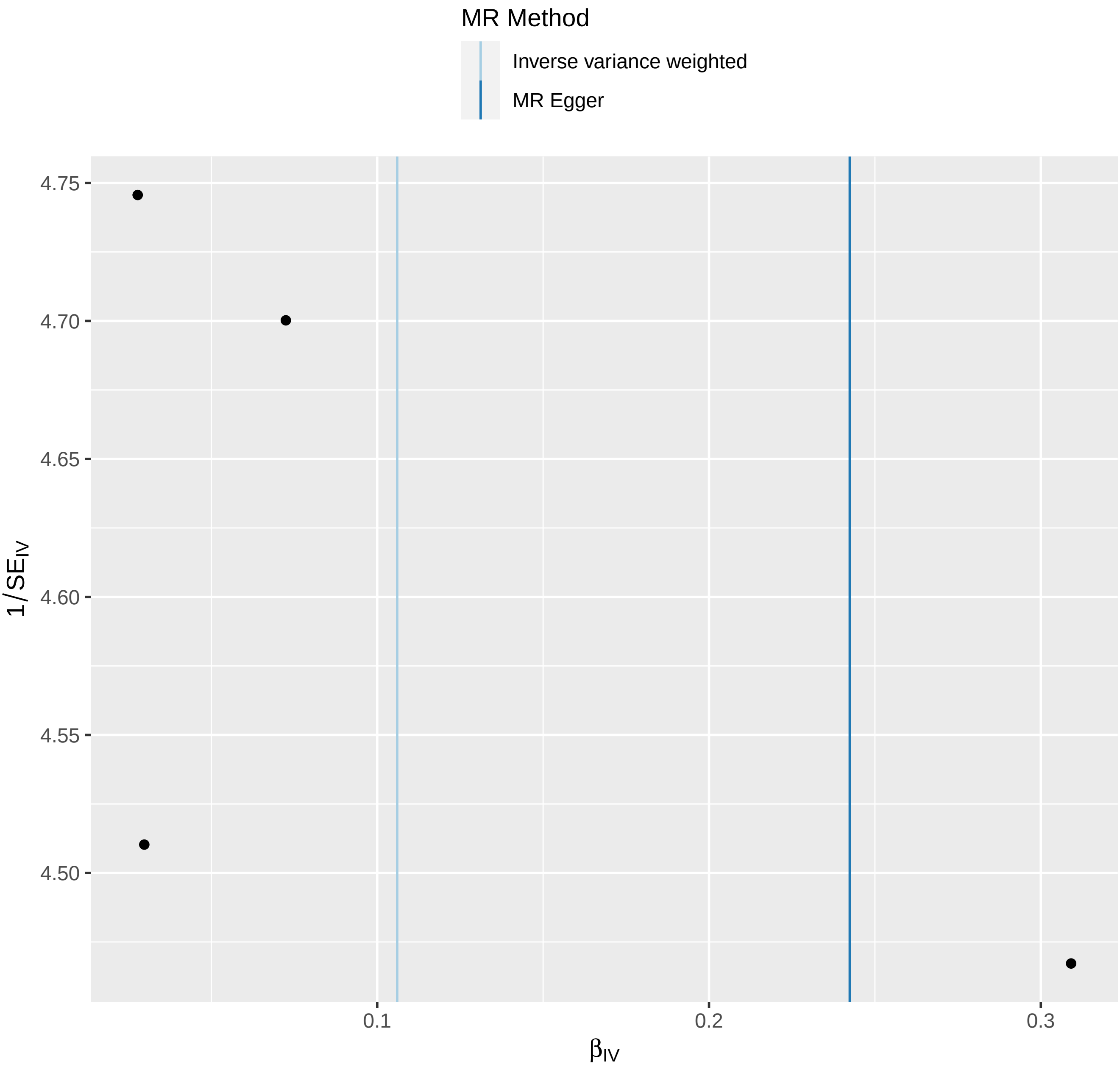

Batch 2−5528 : Gut microbiota abundance (genus Parasutterella id.2892) on Mitochondrial sodium/hydrogen exchanger 9B2
with a threshold of 1e−05

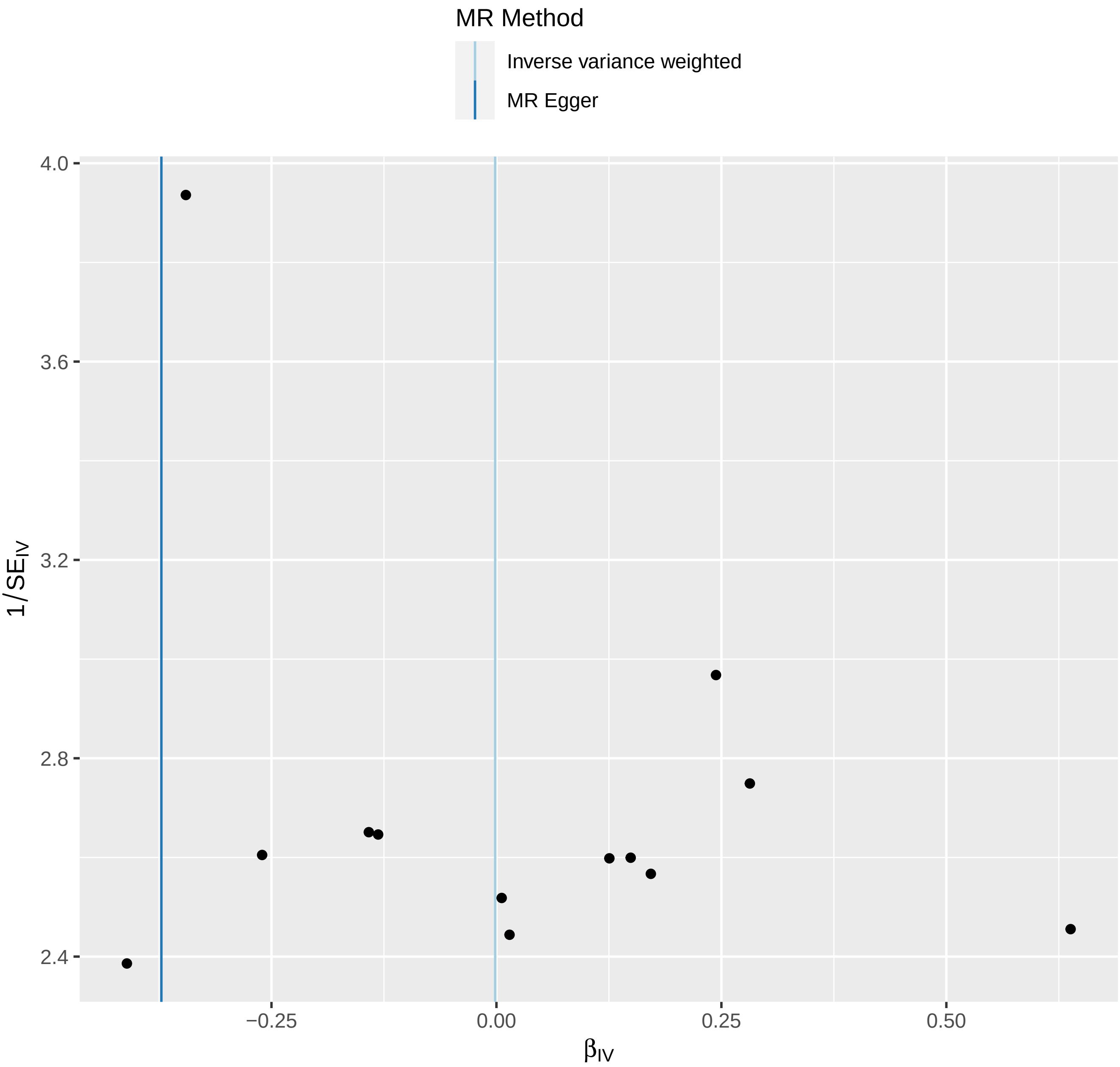

Batch 2−5529 : Gut microbiota abundance (genus Catenibacterium id.2153) on Solute carrier organic anion transporter family member 5A1 with a threshold of 1e−05

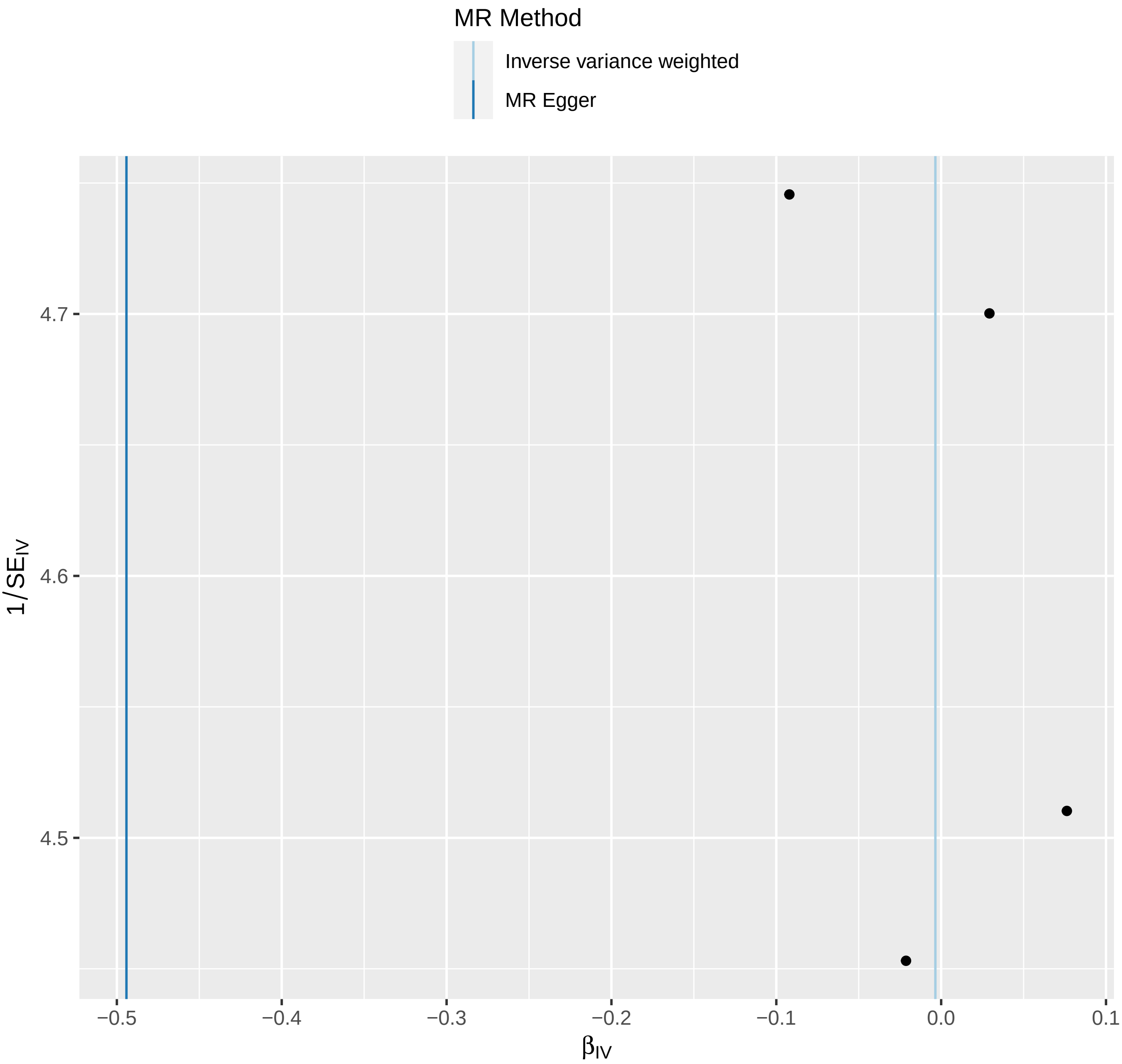

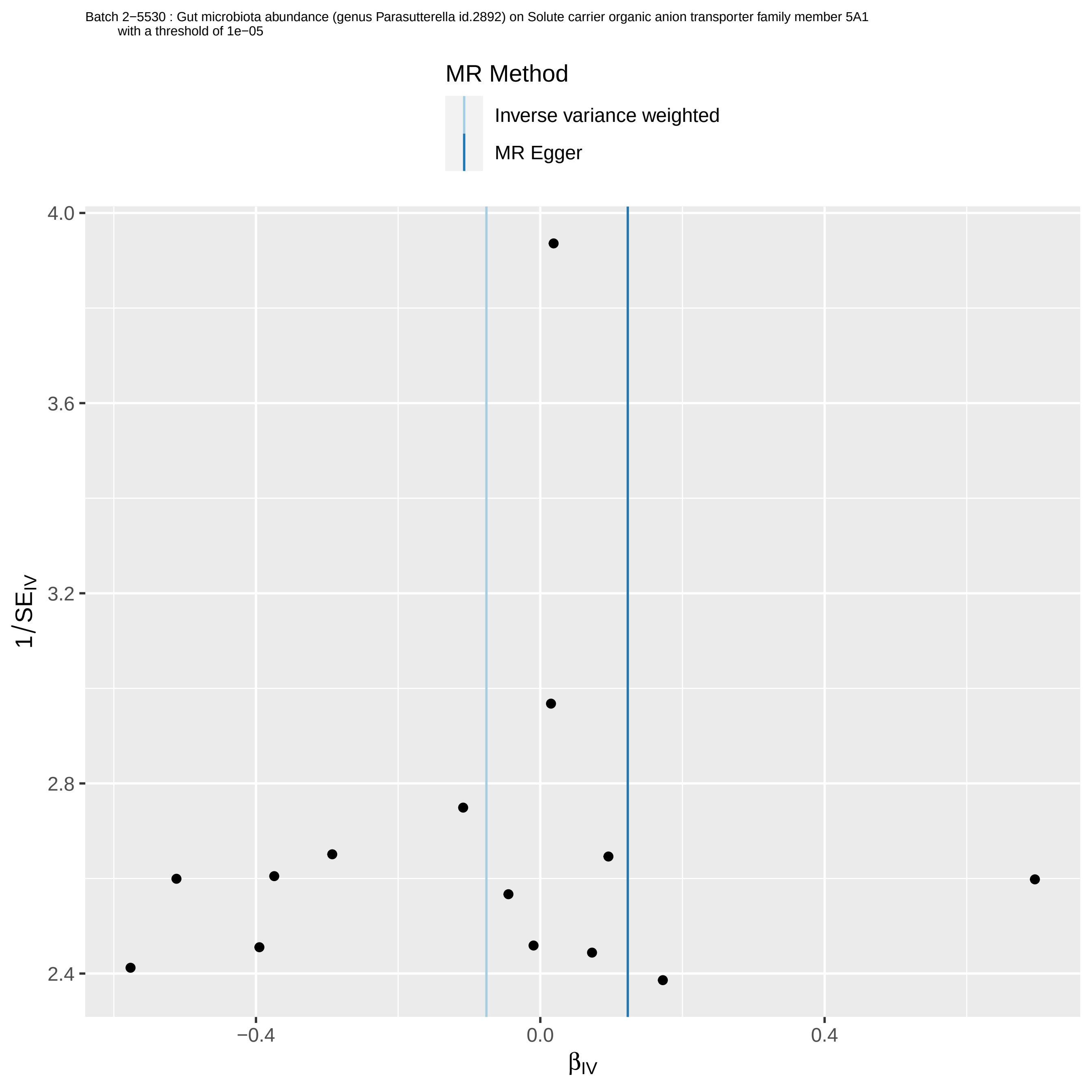

Batch 2−5530 : Gut microbiota abundance (genus Parasutterella id.2892) on Solute carrier organic anion transporter family member 5A1
with a threshold of 1e−05
MR Method
Inverse variance weighted
MR Egger
4.0
3.6
3.2
2.8
2.4
1/SE$_{IV}$
−0.4
0.0
0.4
β_{IV}

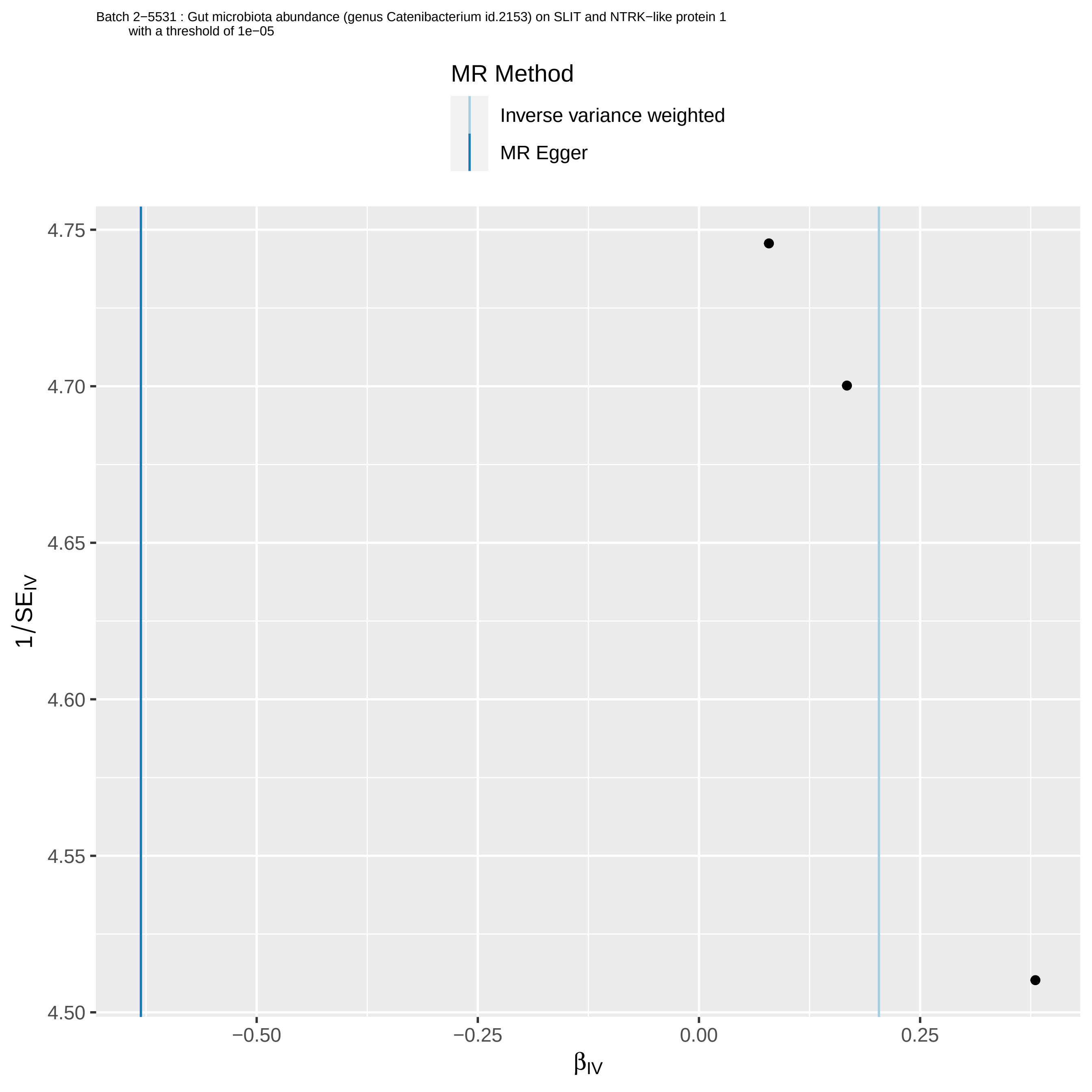
Batch 2−5531 : Gut microbiota abundance (genus Catenibacterium id.2153) on SLIT and NTRK−like protein 1
with a threshold of 1e−05
MR Method
Inverse variance weighted
MR Egger
4.75
4.70
4.65
4.60
4.55
4.50
1/SE_IV
−0.50
−0.25
0.00
0.25
β_IV

Batch 2−5532 : Gut microbiota abundance (genus Parasutterella id.2892) on SLIT and NTRK−like protein 1
with a threshold of 1e−05

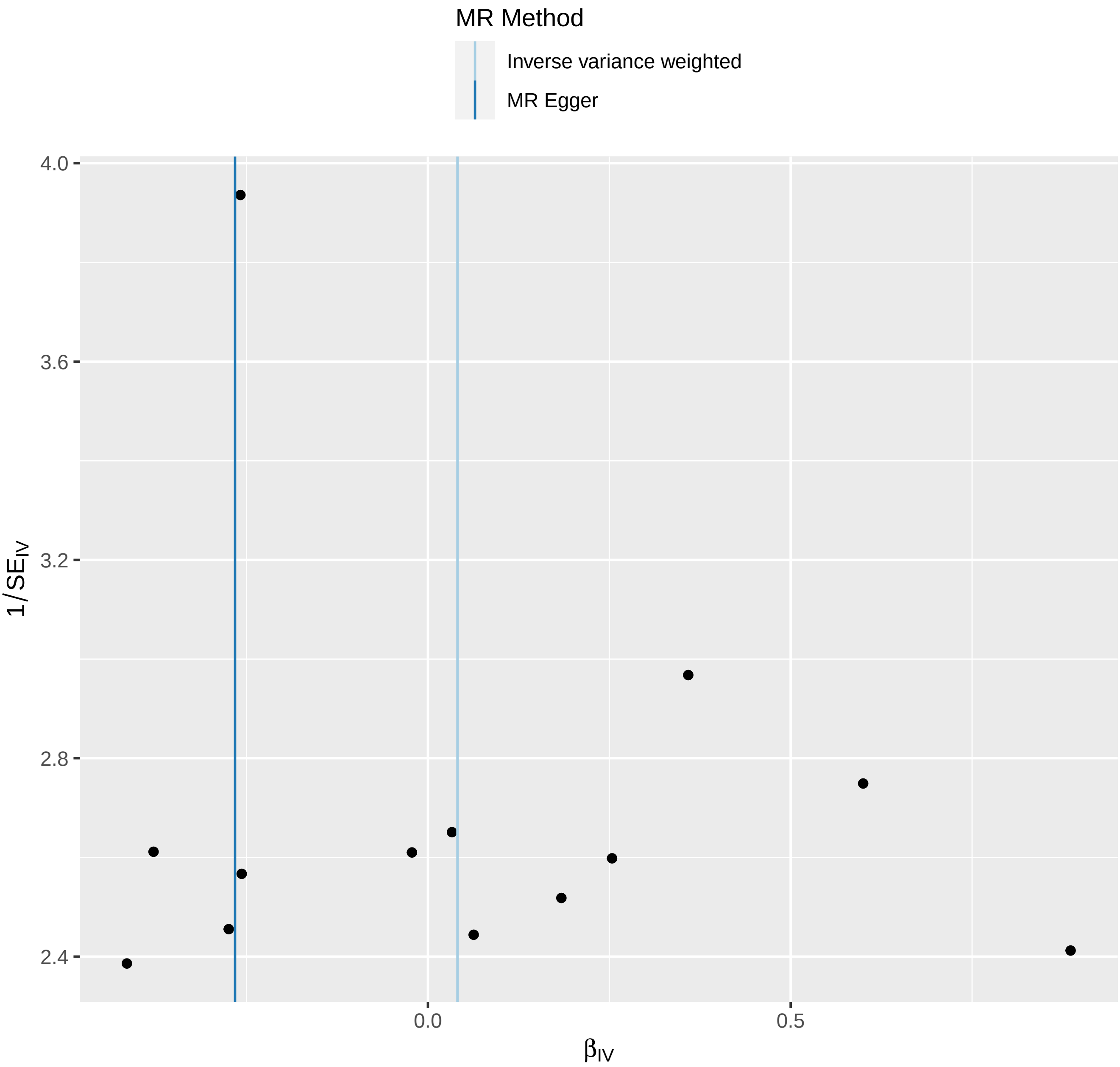

Batch 2−5533 : Gut microbiota abundance (genus Catenibacterium id.2153) on SLIT and NTRK−like protein 3
with a threshold of 1e−05

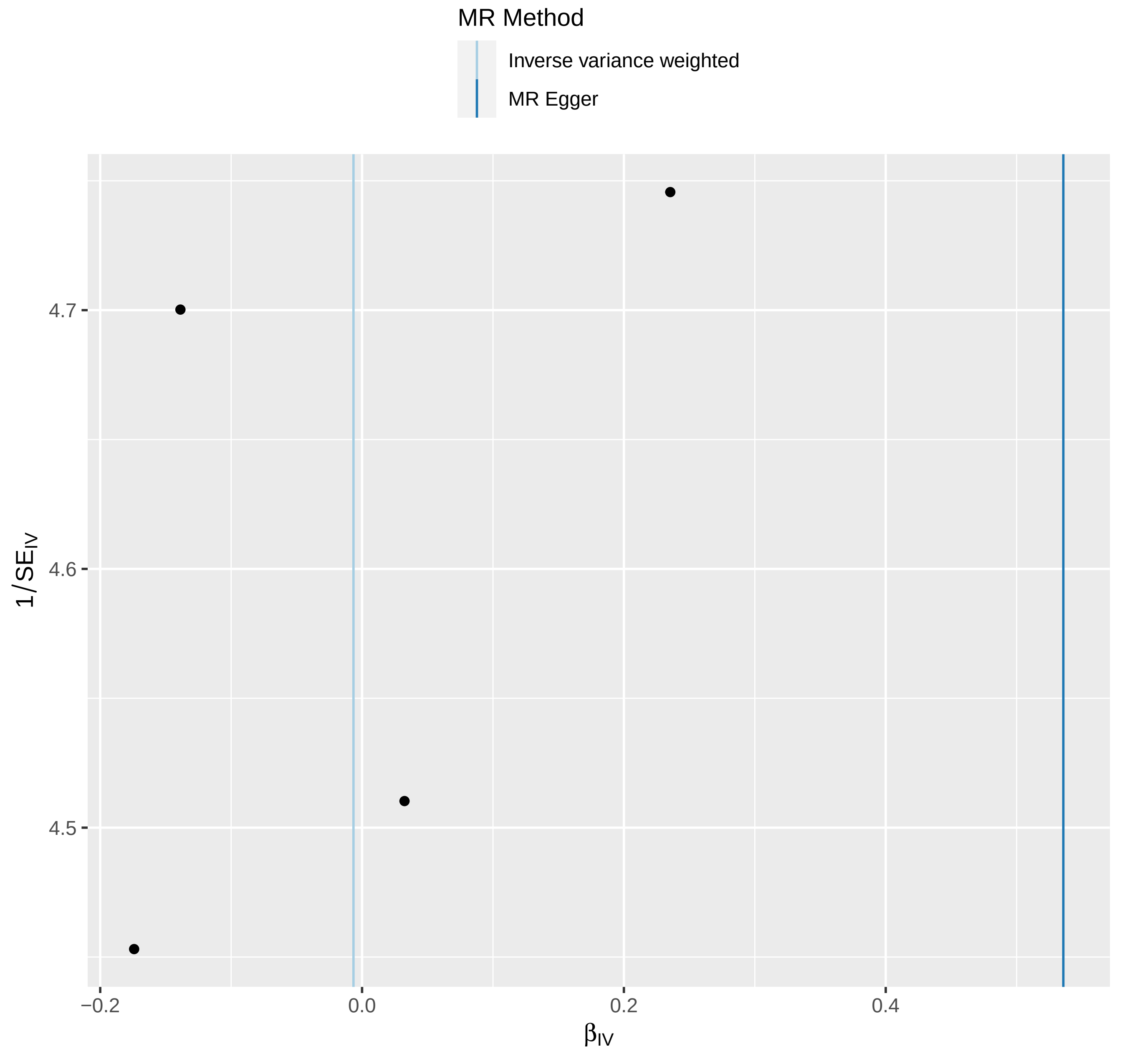

Batch 2−5534 : Gut microbiota abundance (genus Parasutterella id.2892) on SLIT and NTRK−like protein 3
with a threshold of 1e−05

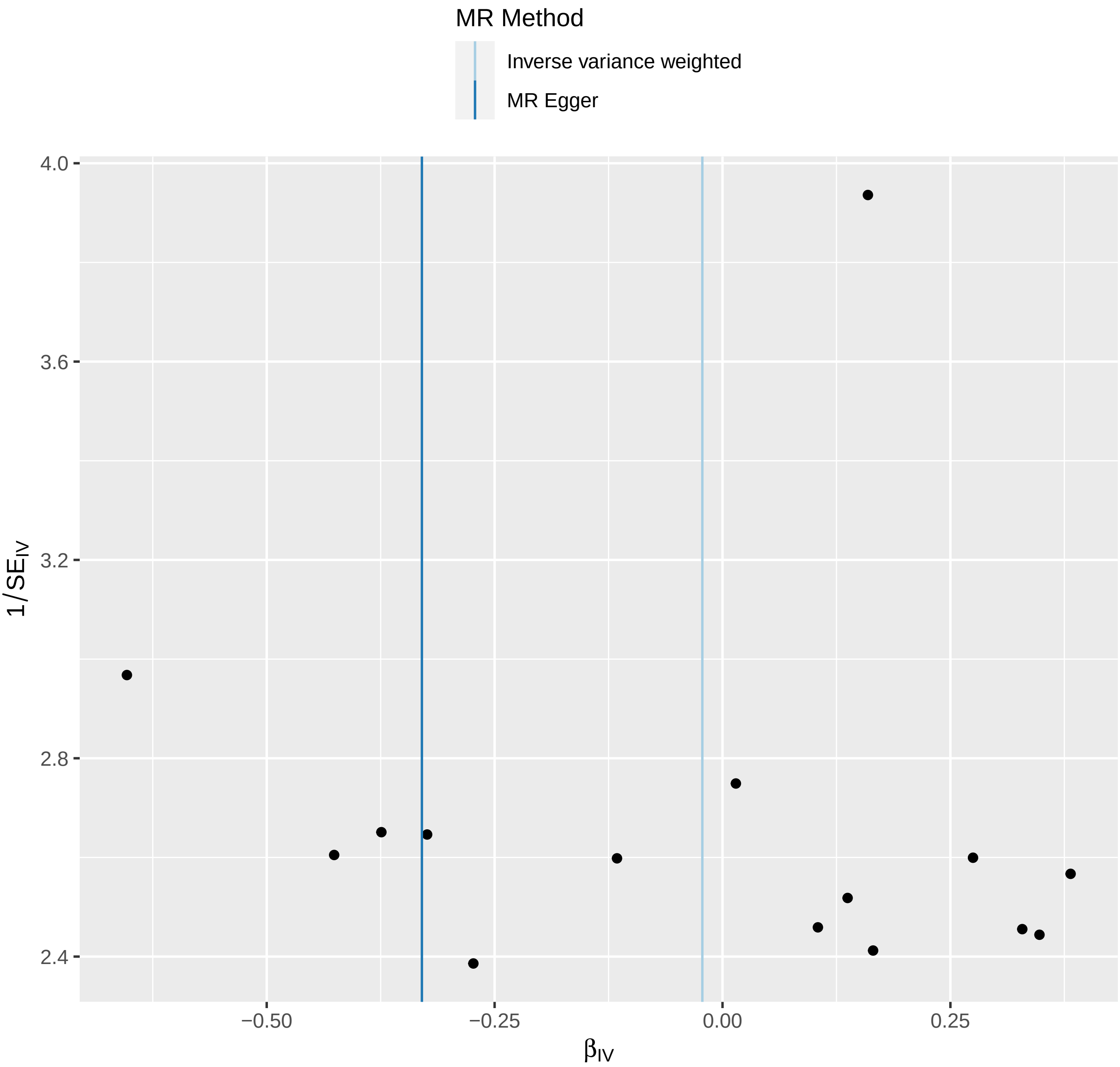

Batch 2−5535 : Gut microbiota abundance (genus Catenibacterium id.2153) on SLIT and NTRK−like protein 4
with a threshold of 1e−05

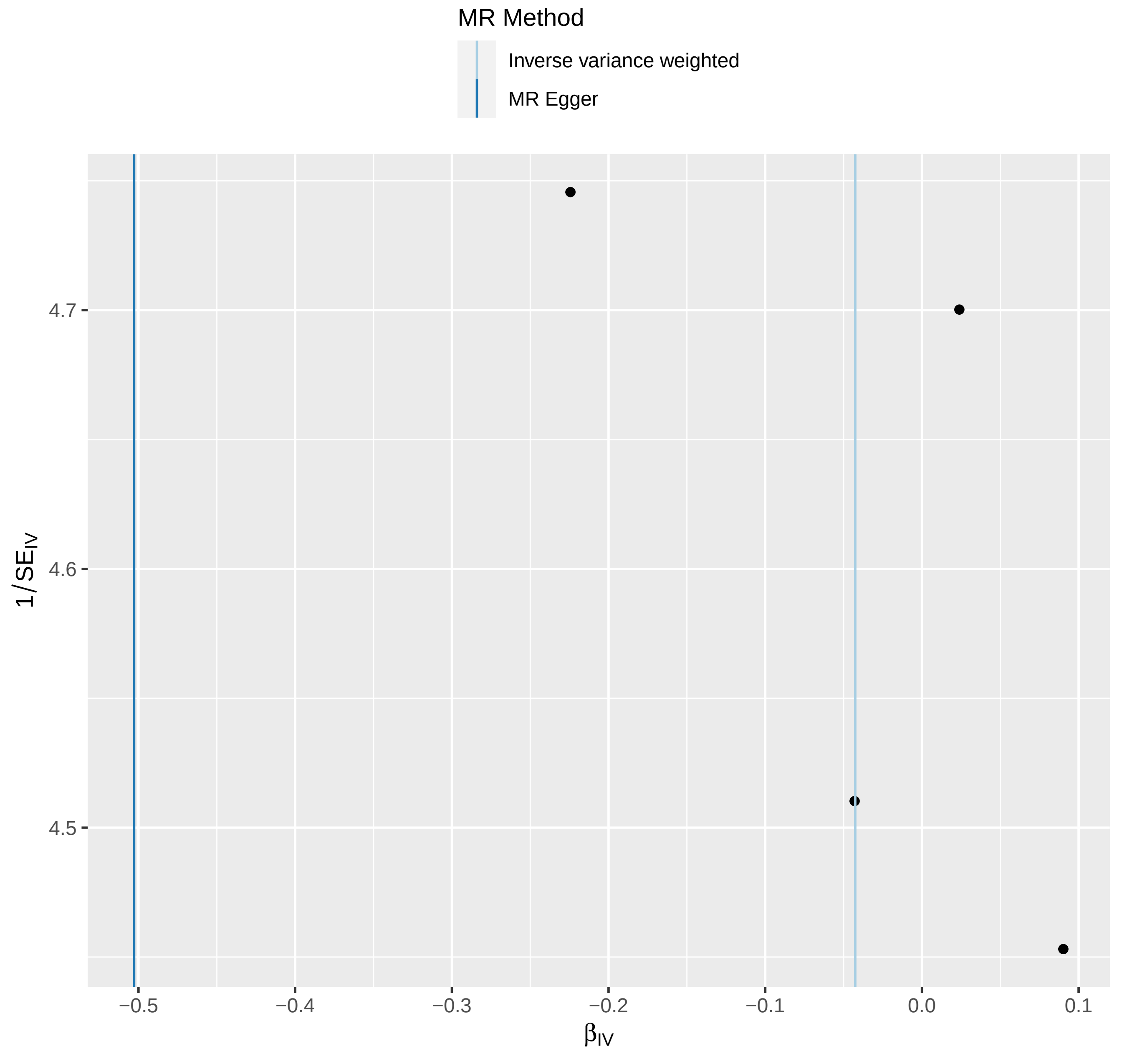

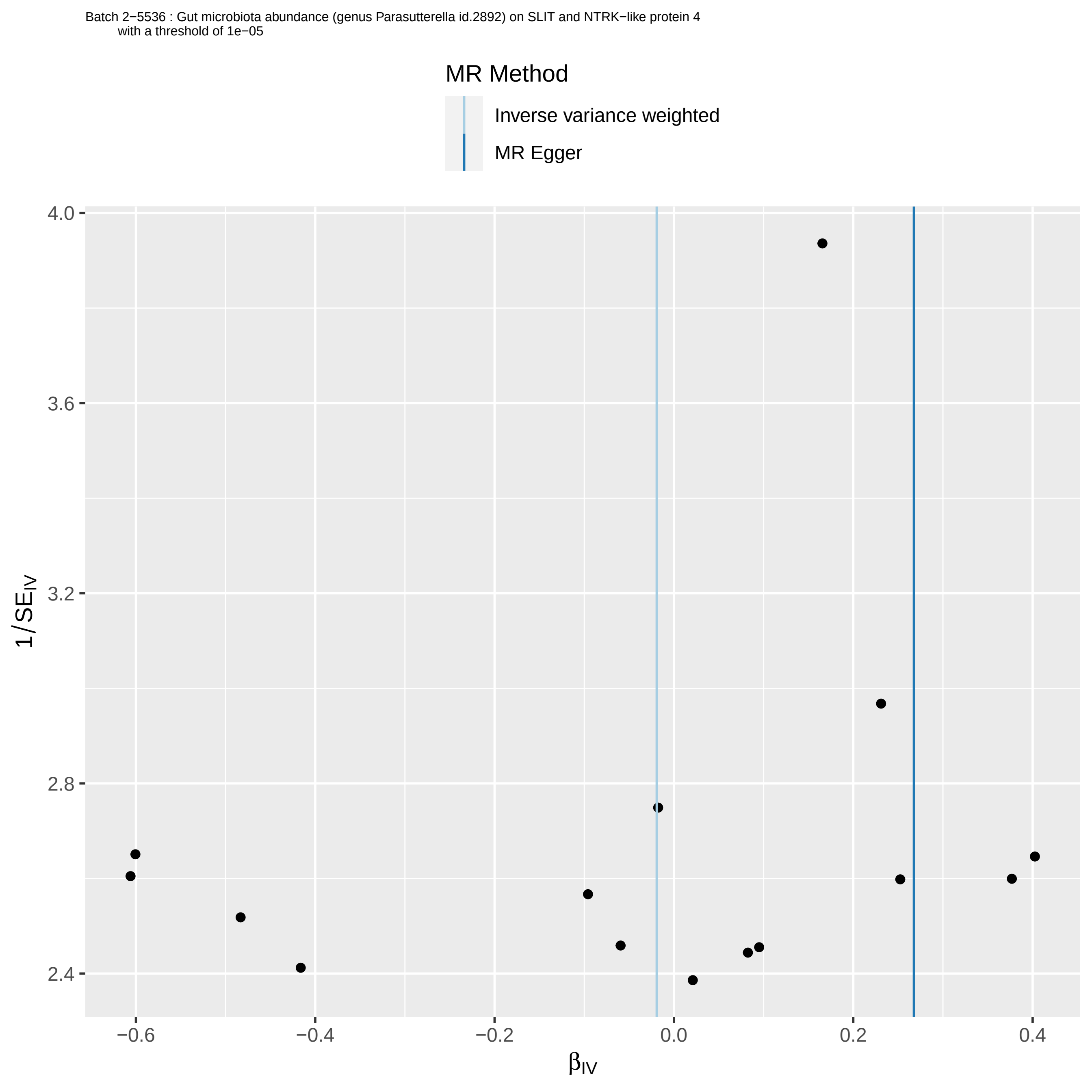

Batch 2−5536 : Gut microbiota abundance (genus Parasutterella id.2892) on SLIT and NTRK−like protein 4
with a threshold of 1e−05
MR Method
Inverse variance weighted
MR Egger
4.0
3.6
3.2
2.8
2.4
1/SE_IV
−0.6
−0.4
−0.2
0.0
0.2
0.4
β_IV

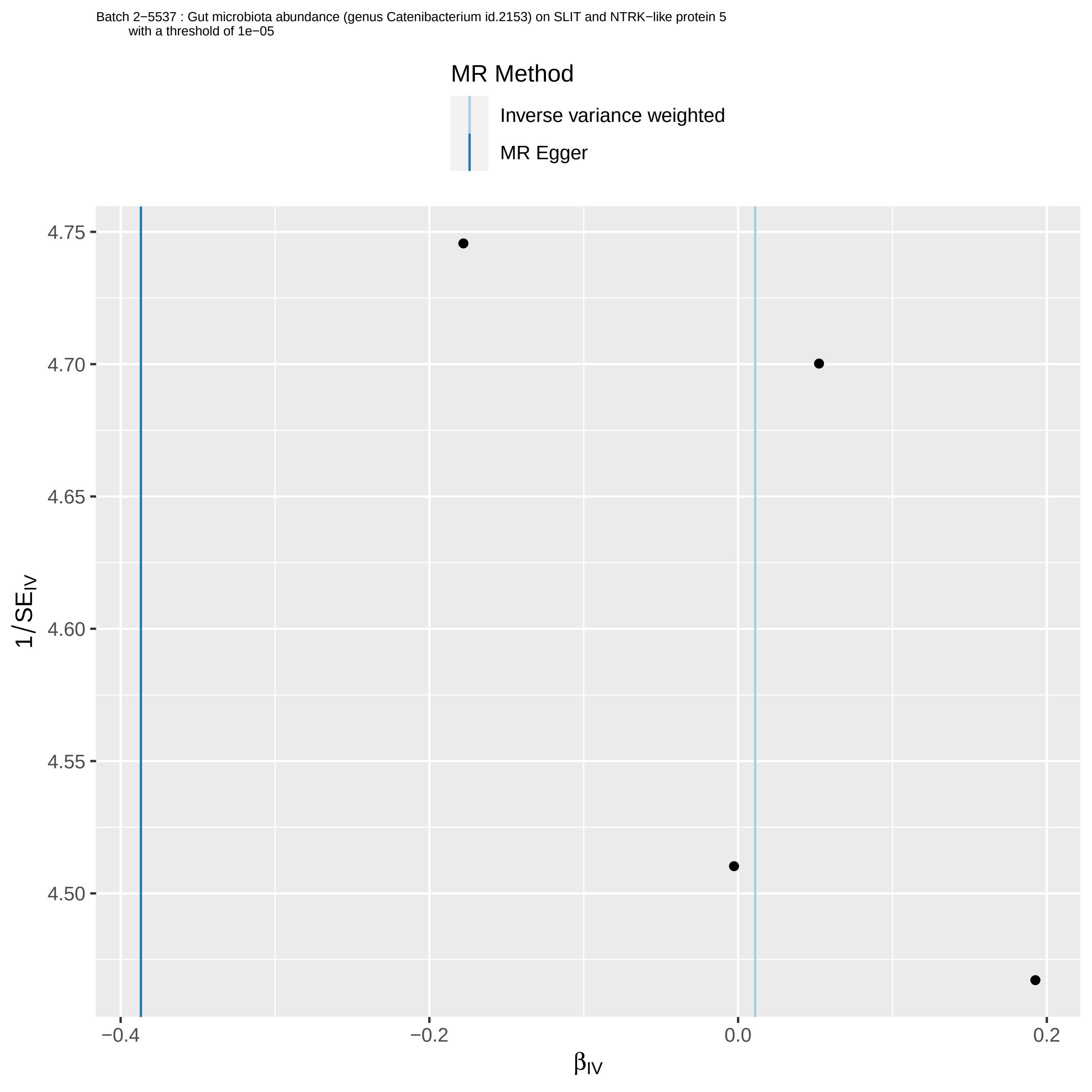
Batch 2−5537 : Gut microbiota abundance (genus Catenibacterium id.2153) on SLIT and NTRK−like protein 5
with a threshold of 1e−05
MR Method
Inverse variance weighted
MR Egger
4.75
4.70
4.65
4.60
4.55
4.50
1/SE_IV
−0.4
−0.2
0.0
0.2
β_IV

Batch 2−5538 : Gut microbiota abundance (genus Parasutterella id.2892) on SLIT and NTRK−like protein 5
with a threshold of 1e−05

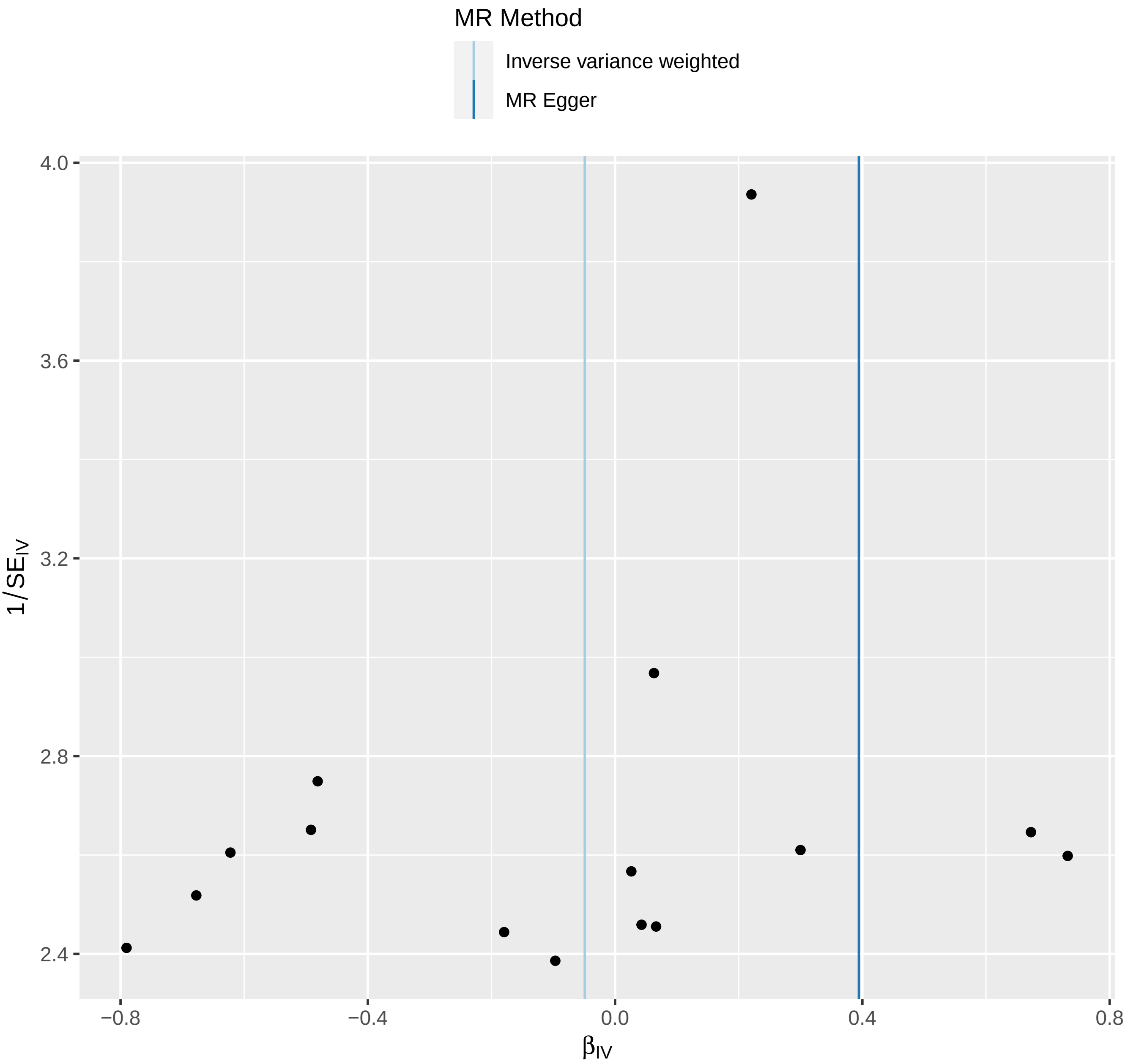

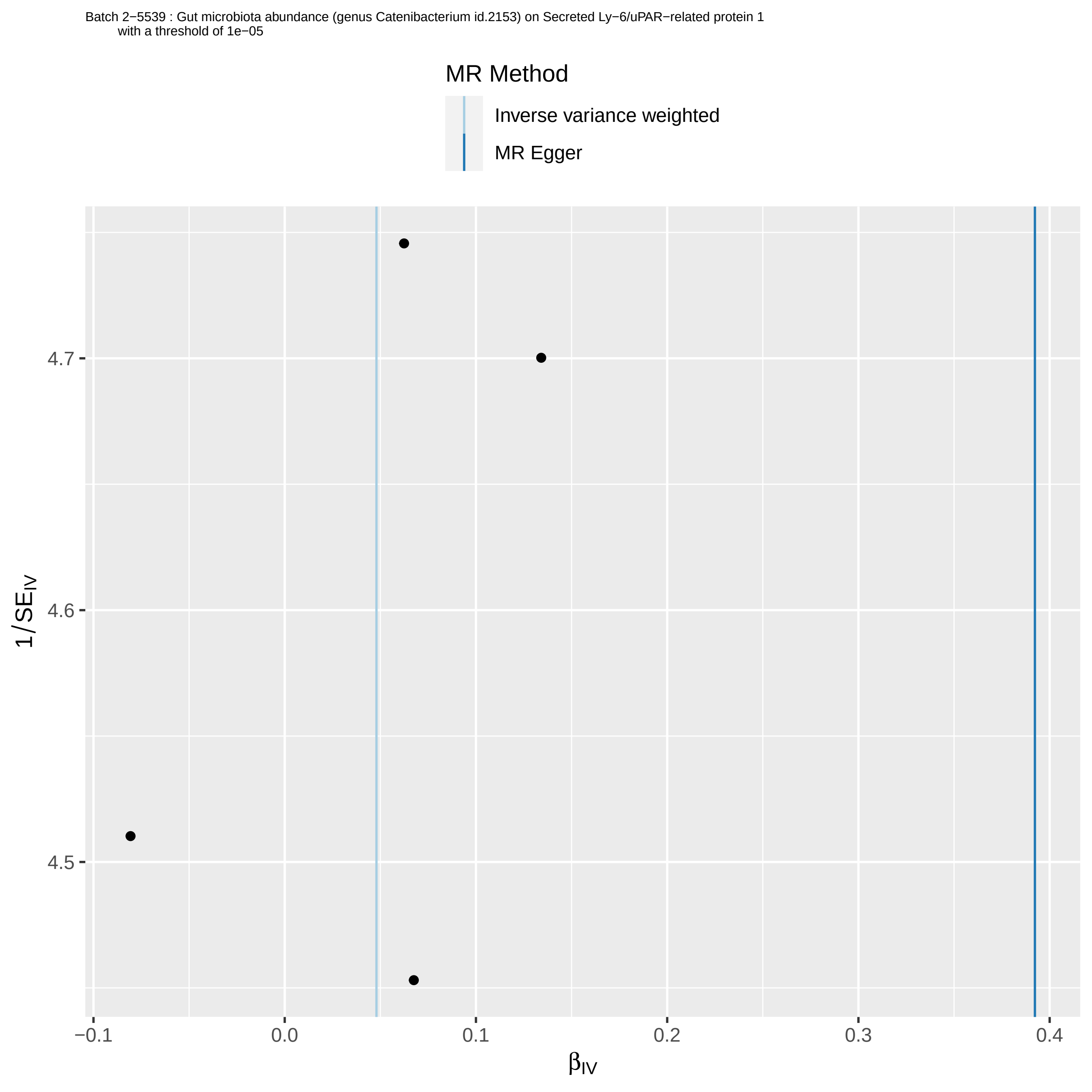
Batch 2−5539 : Gut microbiota abundance (genus Catenibacterium id.2153) on Secreted Ly−6/uPAR−related protein 1
with a threshold of 1e−05
MR Method
Inverse variance weighted
MR Egger
4.7
4.6
4.5
$1/SE_{IV}$
−0.1
0.0
0.1
0.2
0.3
0.4
β_{IV}

Batch 2−5540 : Gut microbiota abundance (genus Parasutterella id.2892) on Secreted Ly−6/uPAR−related protein 1
with a threshold of 1e−05

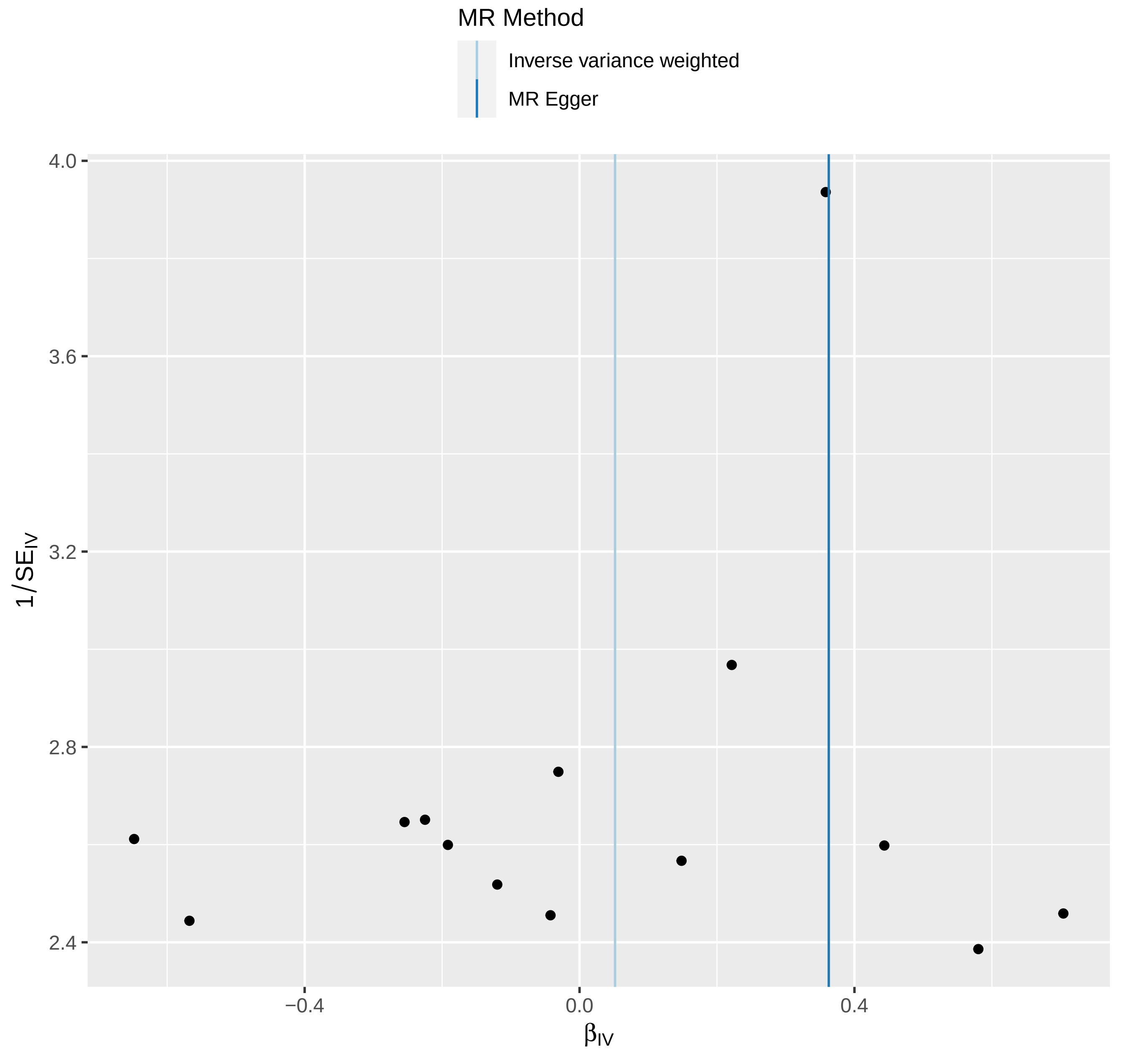

Batch 2−5541 : Gut microbiota abundance (genus Catenibacterium id.2153) on Mothers against decapentaplegic homolog 2 with a threshold of 1e−05

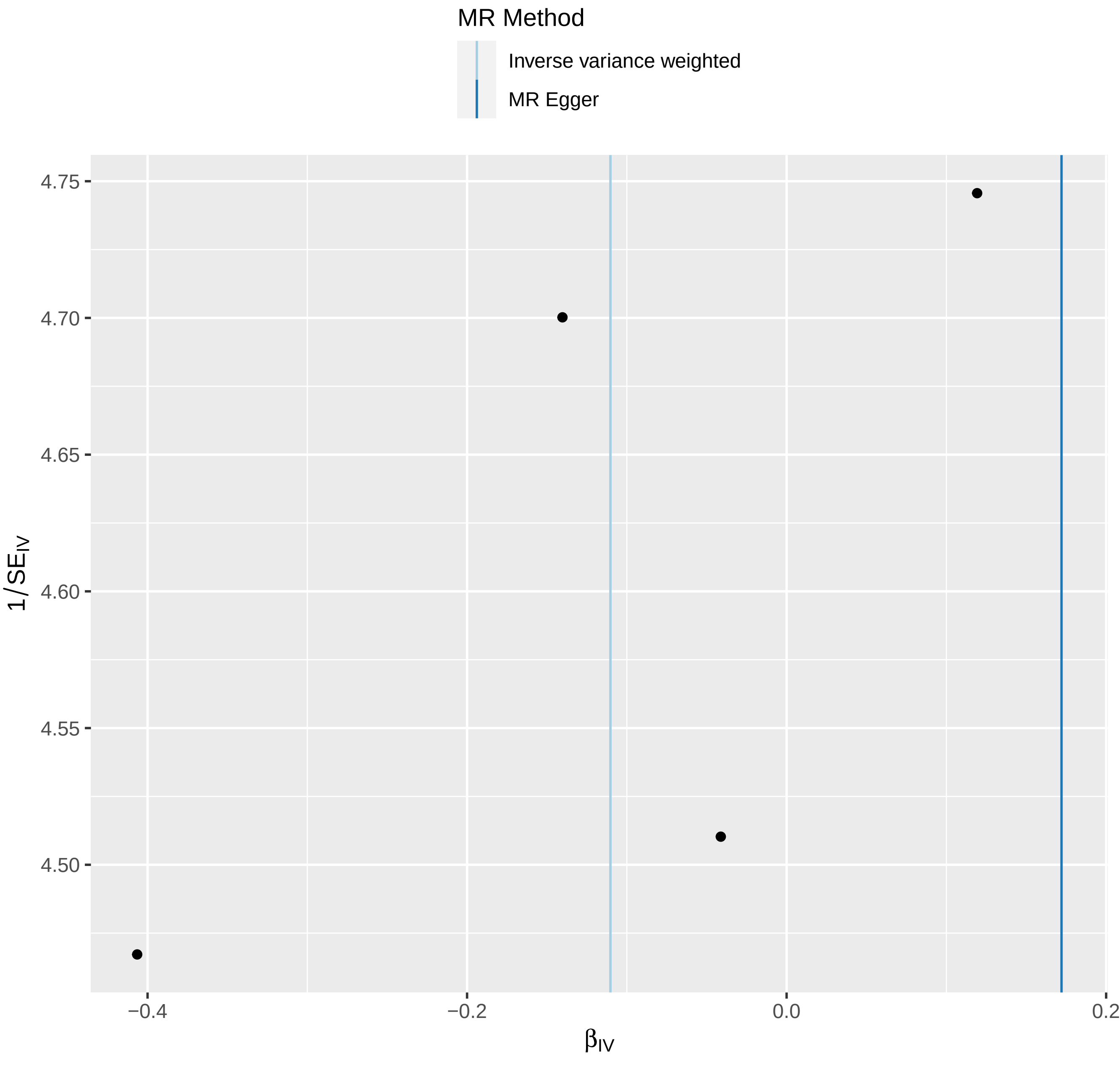

Batch 2−5542 : Gut microbiota abundance (genus Parasutterella id.2892) on Mothers against decapentaplegic homolog 2
with a threshold of 1e−05

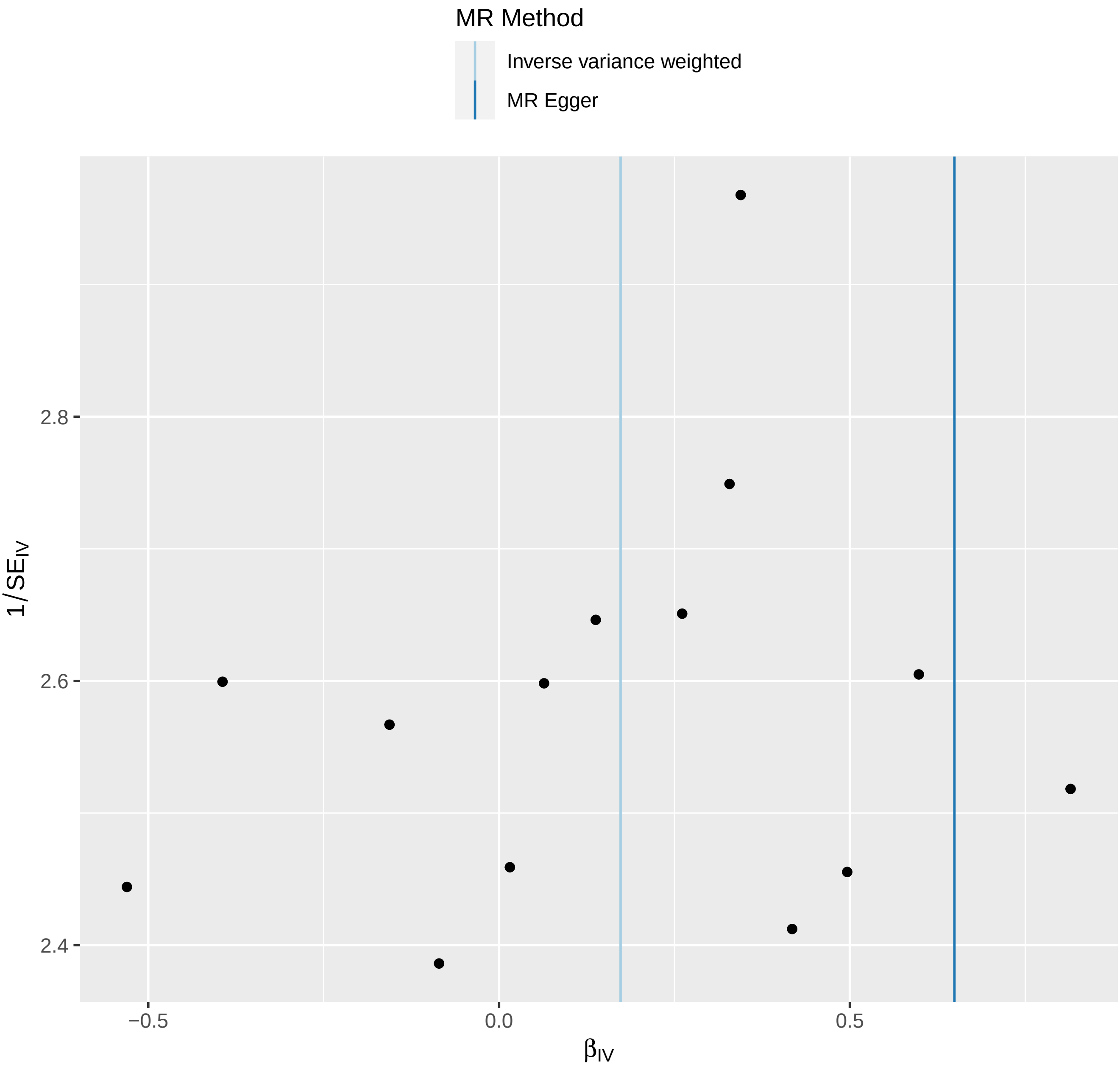

Batch 2−5543 : Gut microbiota abundance (genus Catenibacterium id.2153) on Mothers against decapentaplegic homolog 4
with a threshold of 1e−05

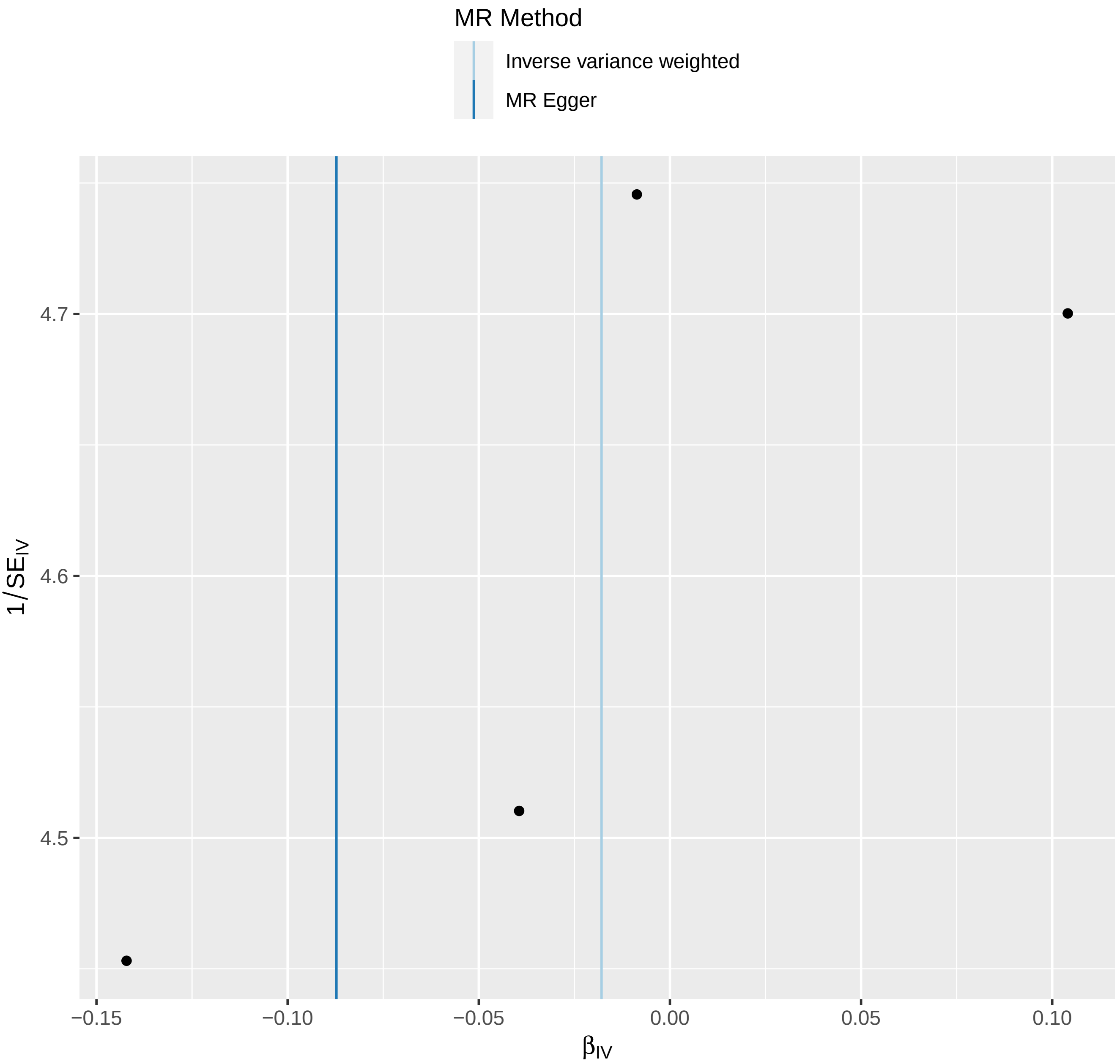

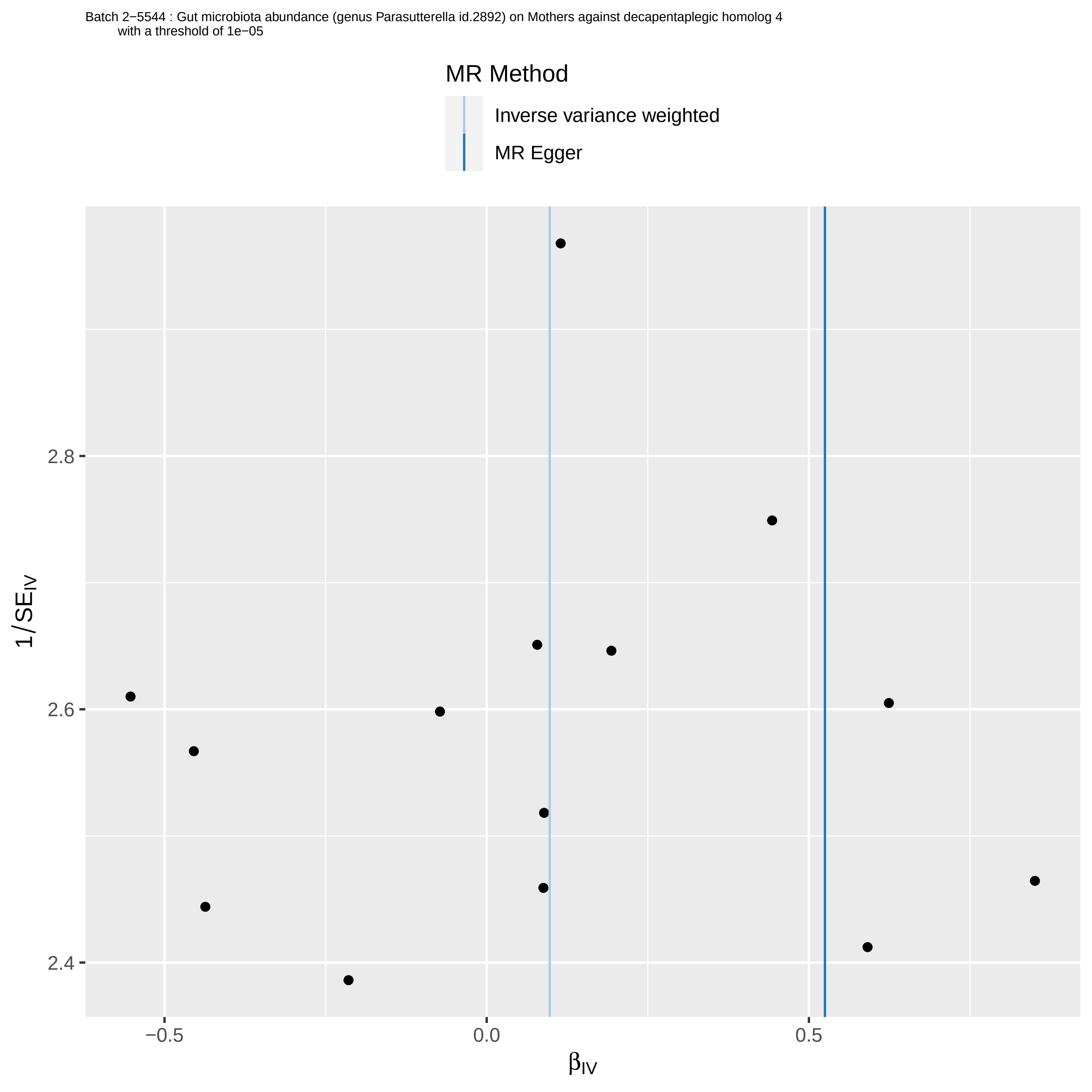
Batch 2−5544 : Gut microbiota abundance (genus Parasutterella id.2892) on Mothers against decapentaplegic homolog 4
with a threshold of 1e−05
MR Method
Inverse variance weighted
MR Egger
1/SE_IV
2.8
2.6
2.4
−0.5
0.0
0.5
β_IV

Batch 2−5545 : Gut microbiota abundance (genus Catenibacterium id.2153) on Stromal membrane−associated protein 1
with a threshold of 1e−05

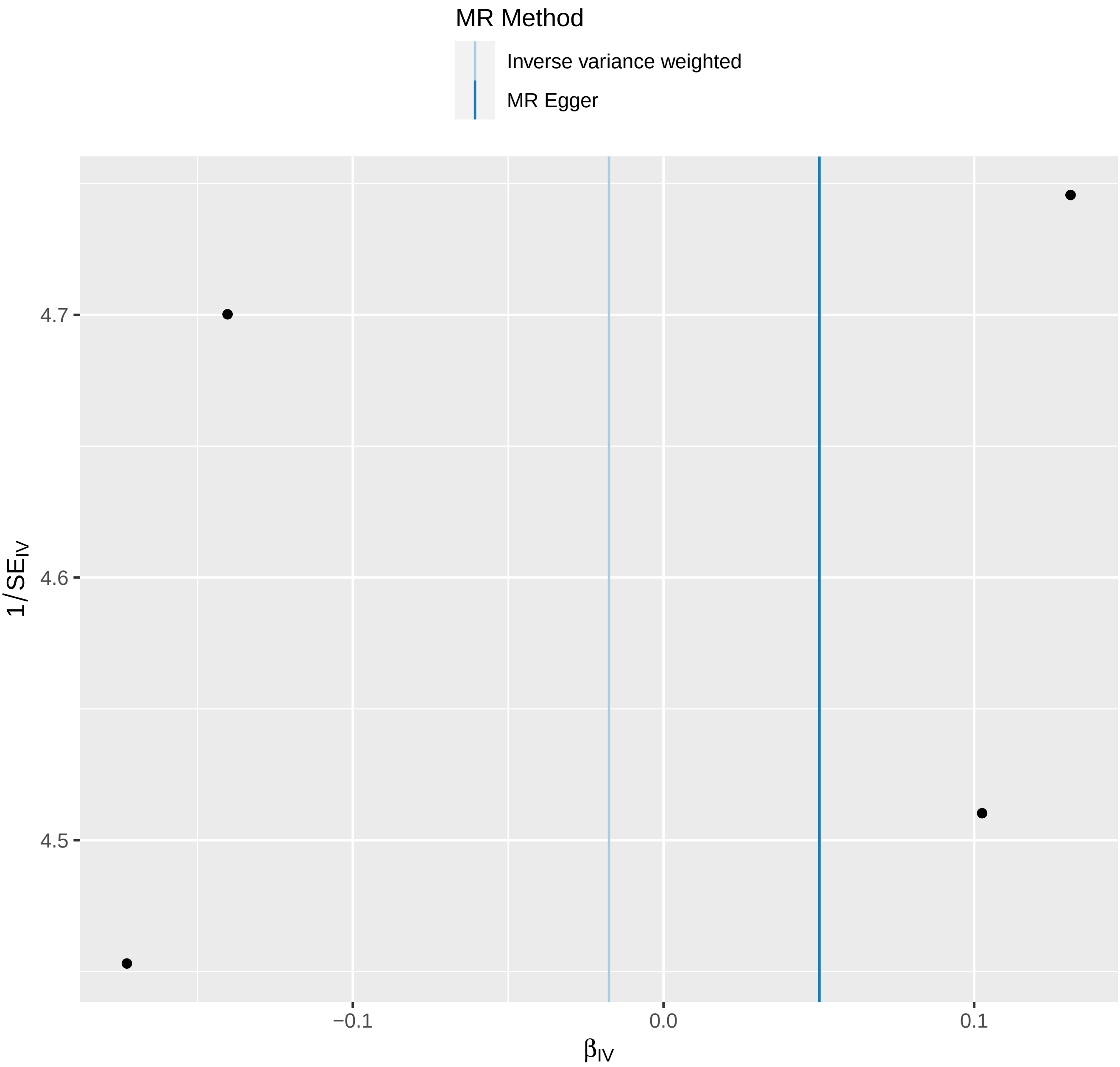

Batch 2−5546 : Gut microbiota abundance (genus Parasutterella id.2892) on Stromal membrane−associated protein 1
with a threshold of 1e−05

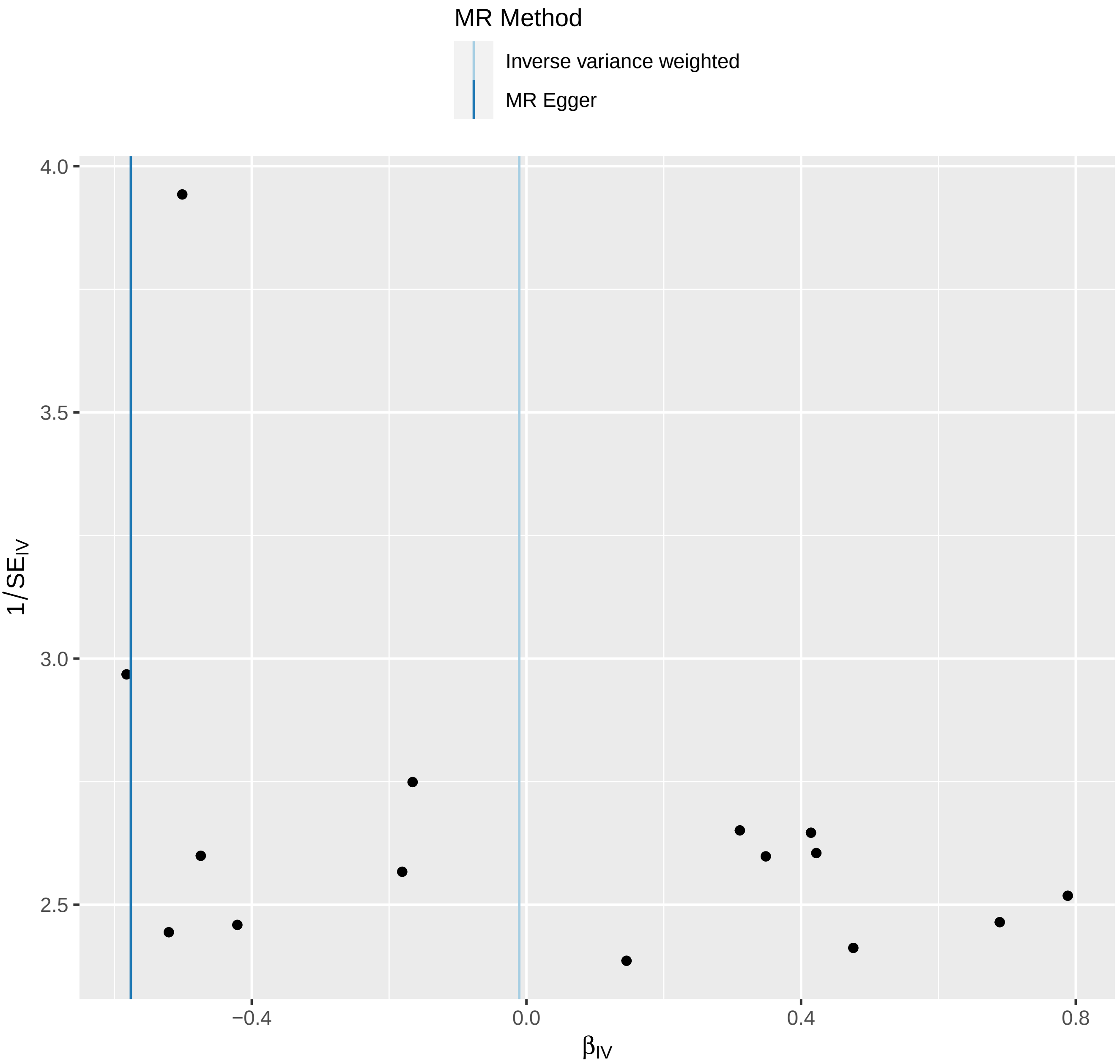

Batch 2−5547 : Gut microbiota abundance (genus Catenibacterium id.2153) on Structural maintenance of chromosomes protein 3 with a threshold of 1e−05

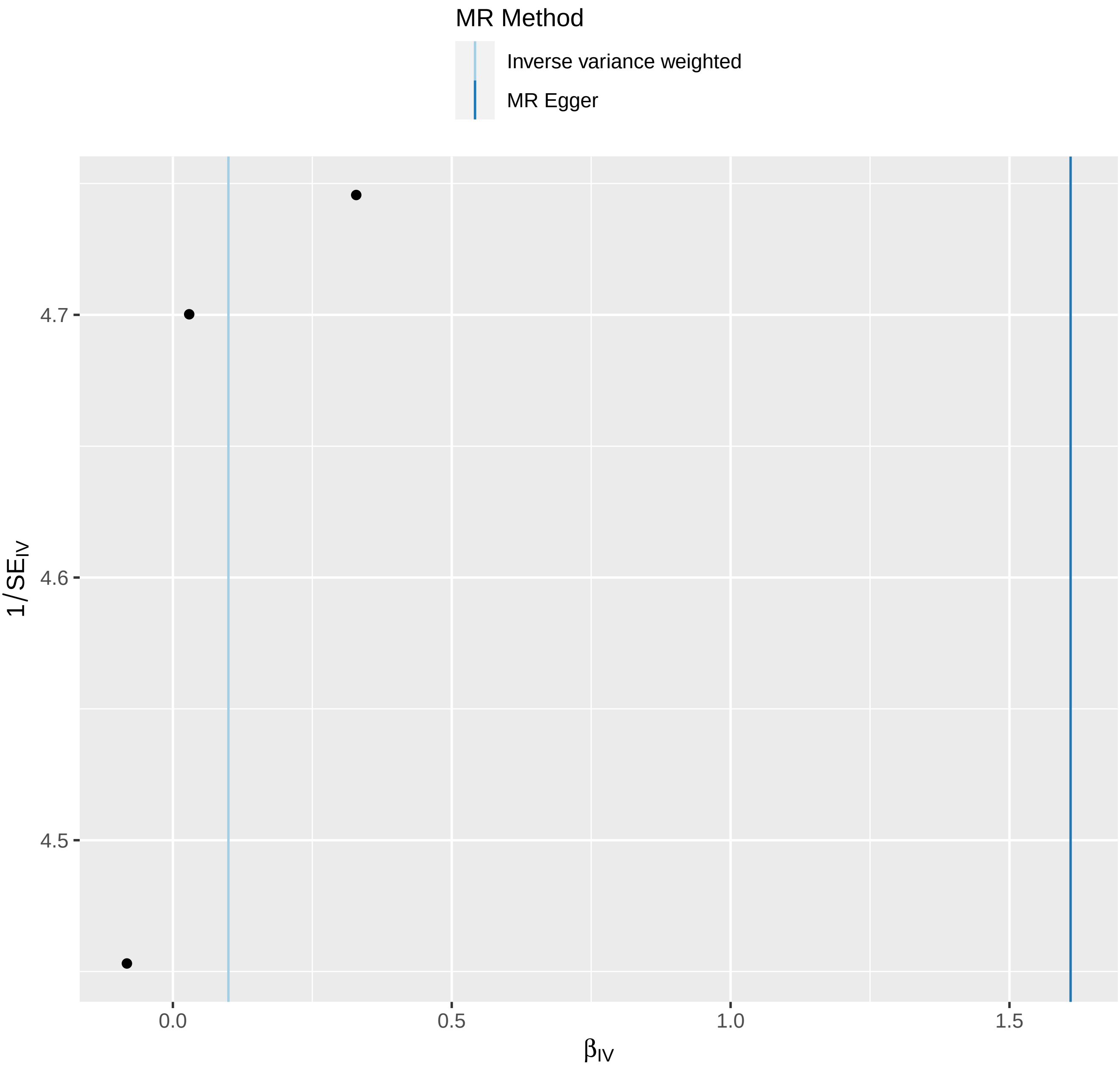

Batch 2−5548 : Gut microbiota abundance (genus Parasutterella id.2892) on Structural maintenance of chromosomes protein 3
with a threshold of 1e−05

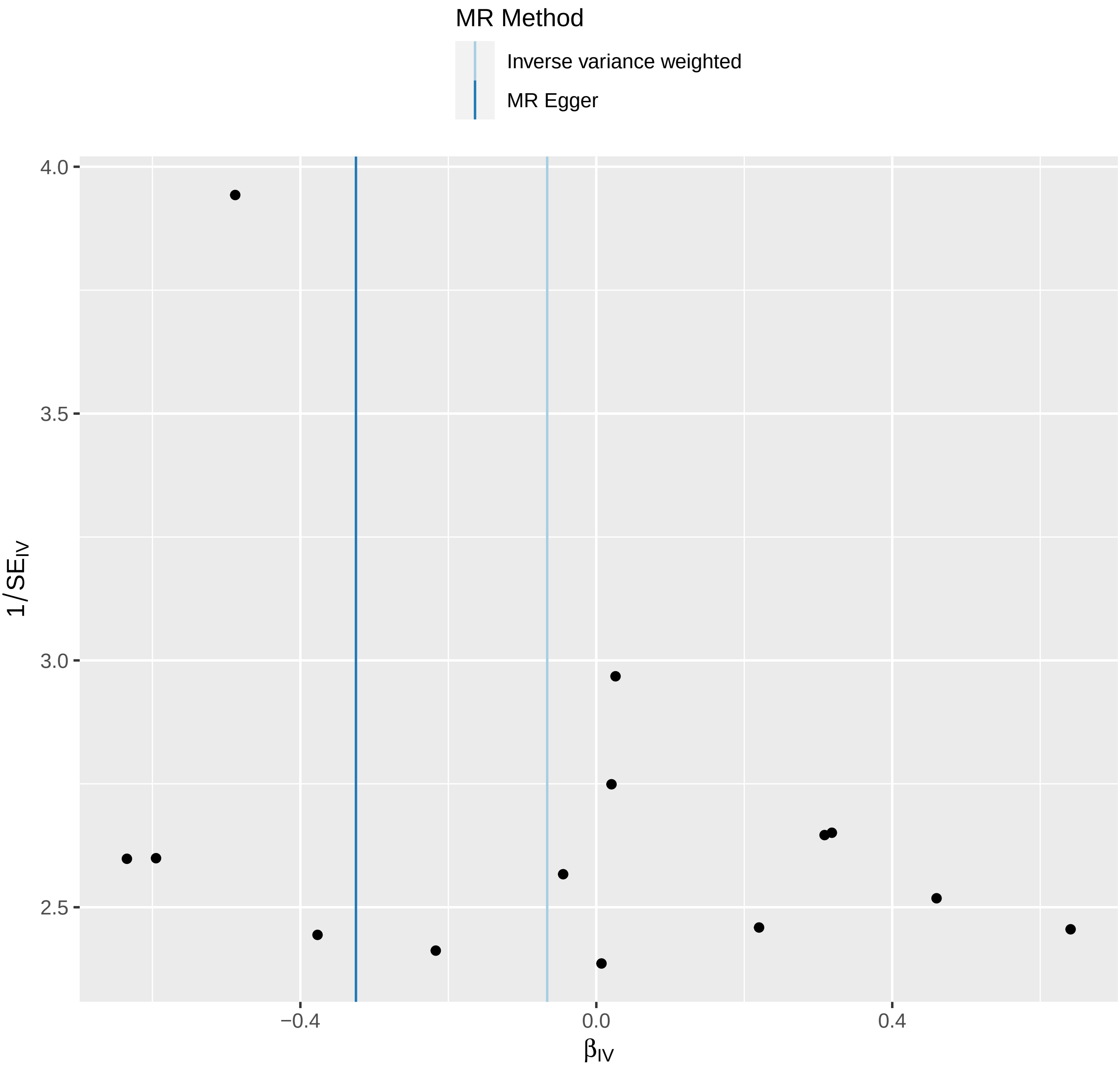

Batch 2−5549 : Gut microbiota abundance (genus Catenibacterium id.2153) on Single−pass membrane and coiled−coil domain−containing protein 2 with a threshold of 1e−05

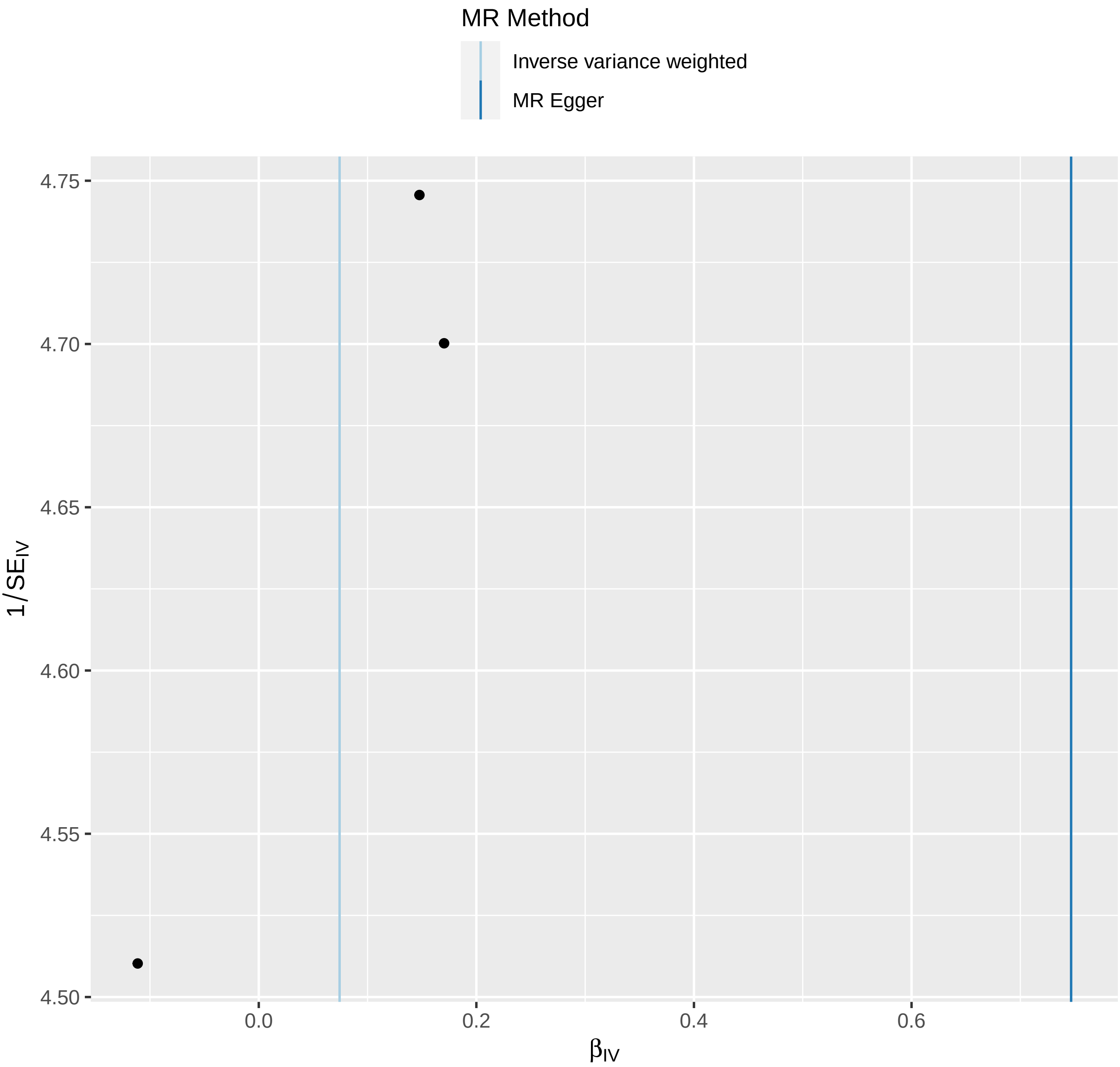

Batch 2−5550 : Gut microbiota abundance (genus Parasutterella id.2892) on Single−pass membrane and coiled−coil domain−containing protein 2 with a threshold of 1e−05

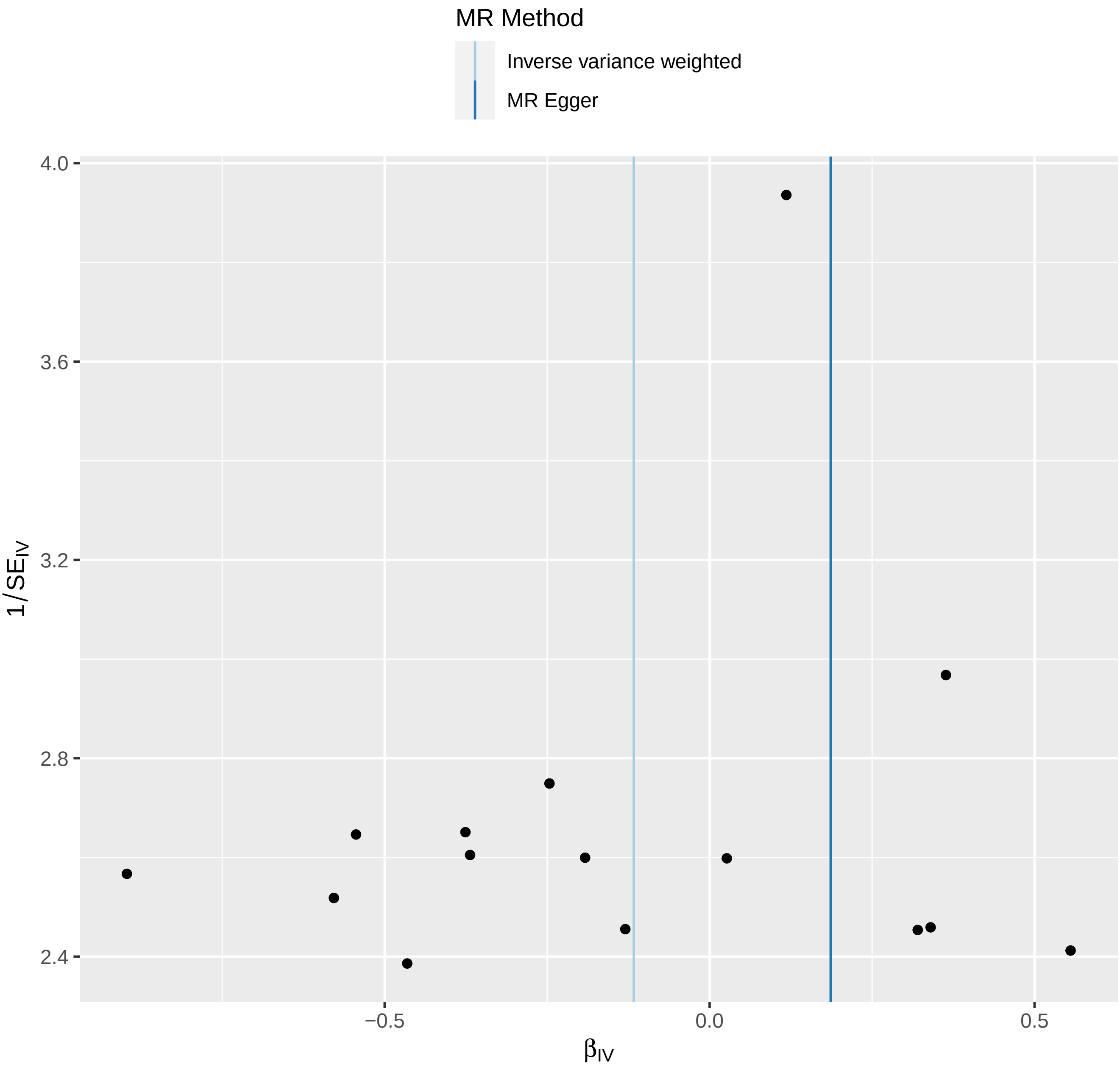

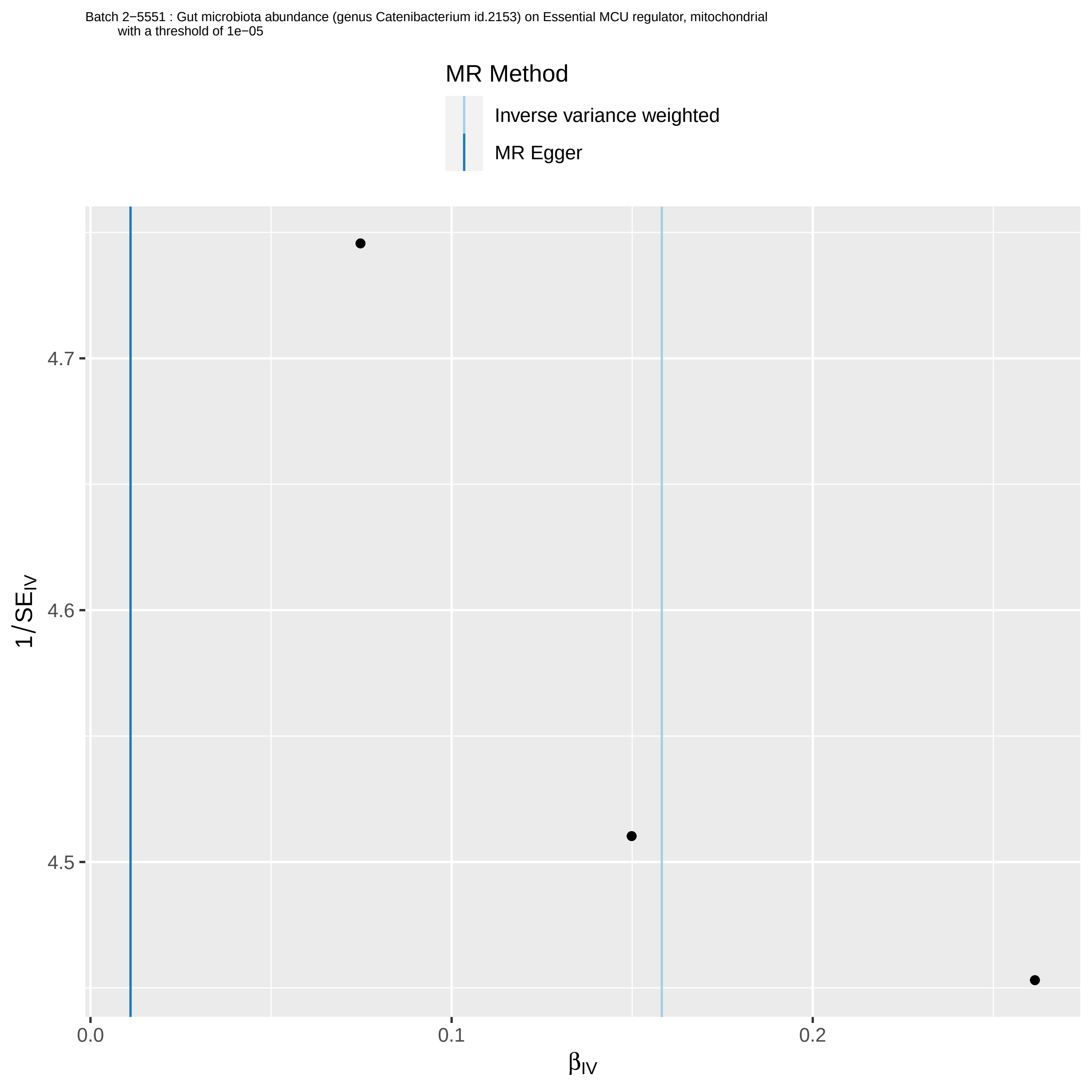
Batch 2−5551 : Gut microbiota abundance (genus Catenibacterium id.2153) on Essential MCU regulator, mitochondrial
with a threshold of 1e−05
MR Method
Inverse variance weighted
MR Egger
4.7
4.6
4.5
1/SE_IV
0.0
0.1
0.2
β_IV

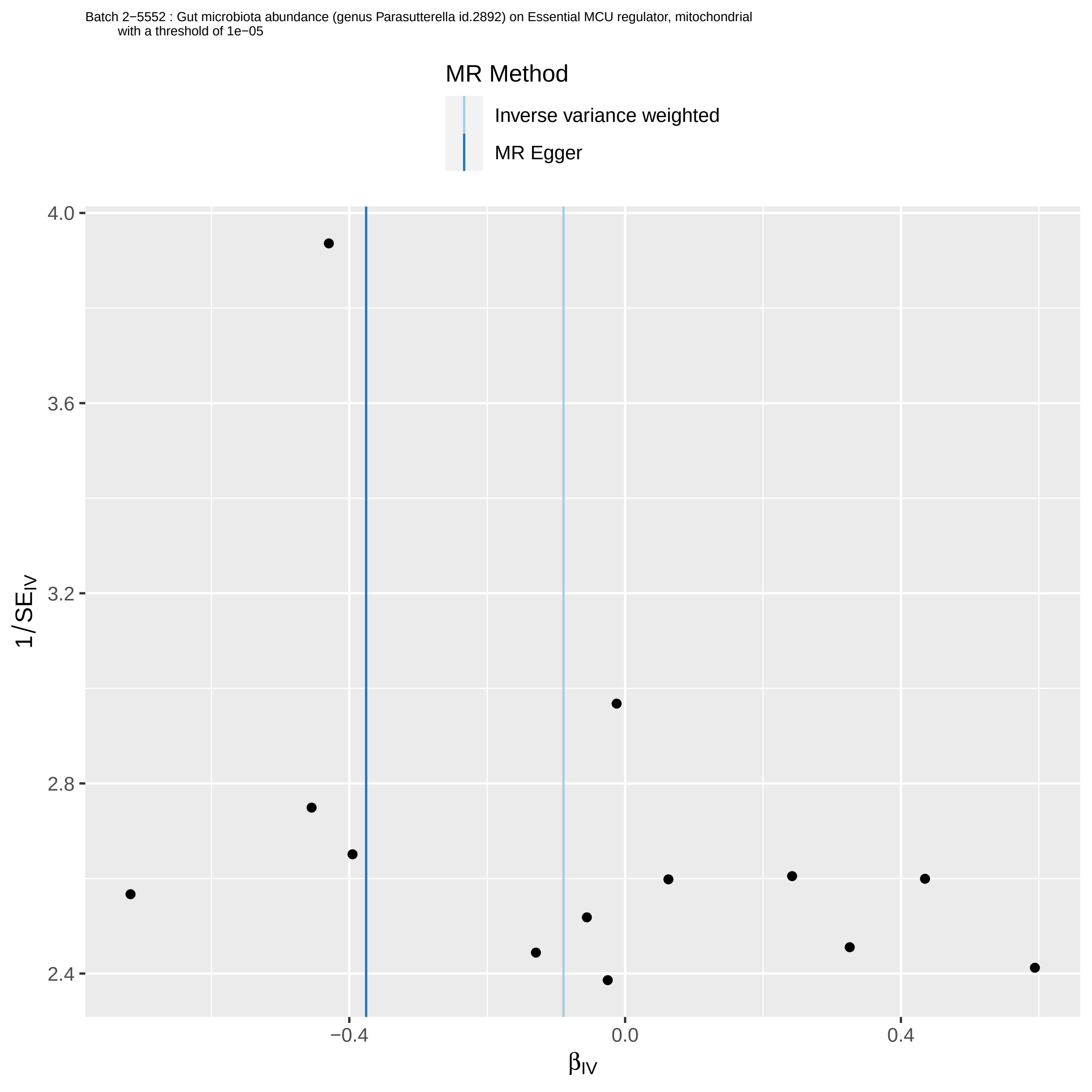
Batch 2−5552 : Gut microbiota abundance (genus Parasutterella id.2892) on Essential MCU regulator, mitochondrial
with a threshold of 1e−05
MR Method
Inverse variance weighted
MR Egger
4.0
3.6
3.2
2.8
2.4
1/SE_IV
−0.4
0.0
0.4
β_IV

Batch 2−5553 : Gut microbiota abundance (genus Catenibacterium id.2153) on Small integral membrane protein 10
with a threshold of 1e−05

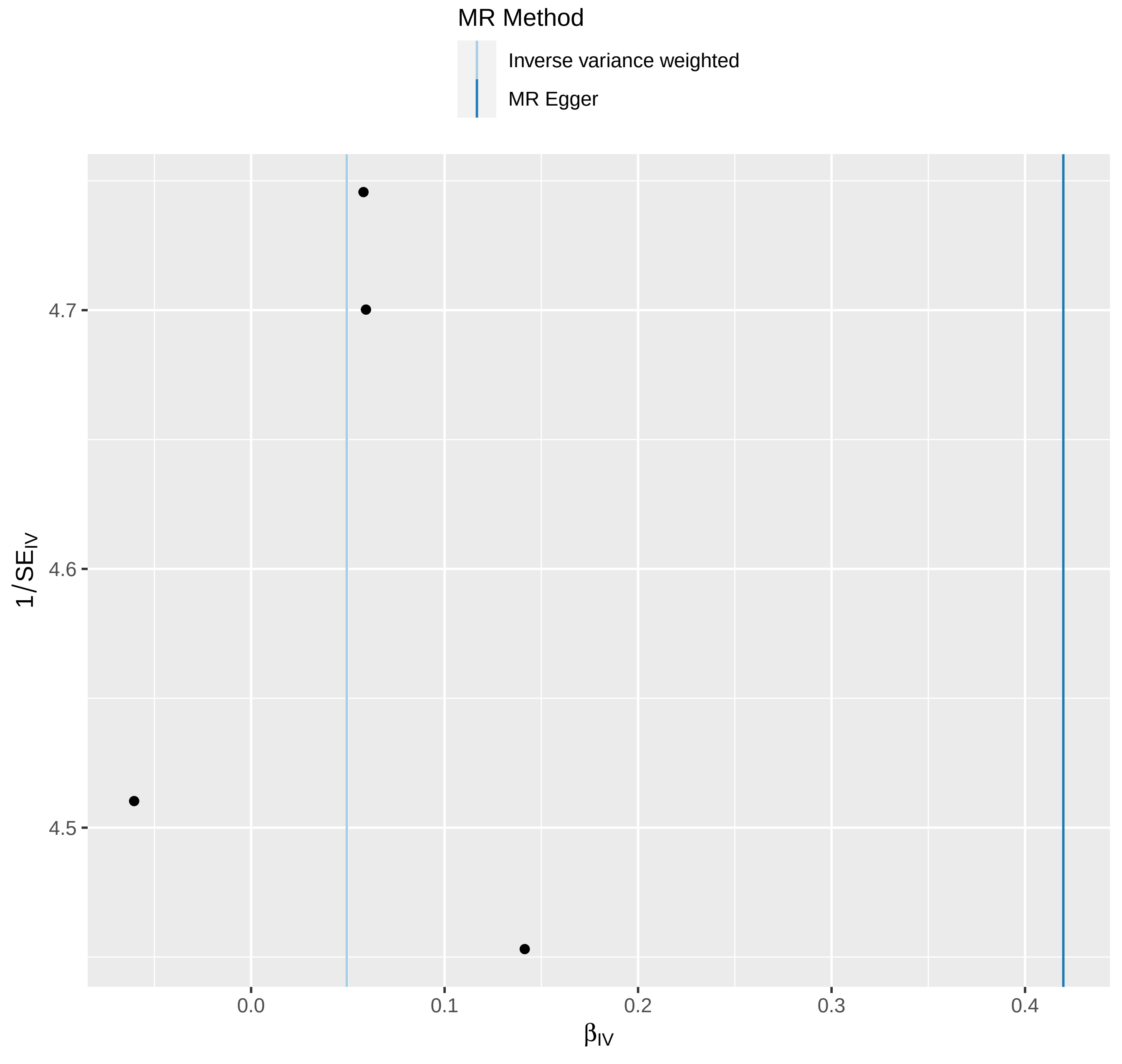

Batch 2−5554 : Gut microbiota abundance (genus Parasutterella id.2892) on Small integral membrane protein 10
with a threshold of 1e−05

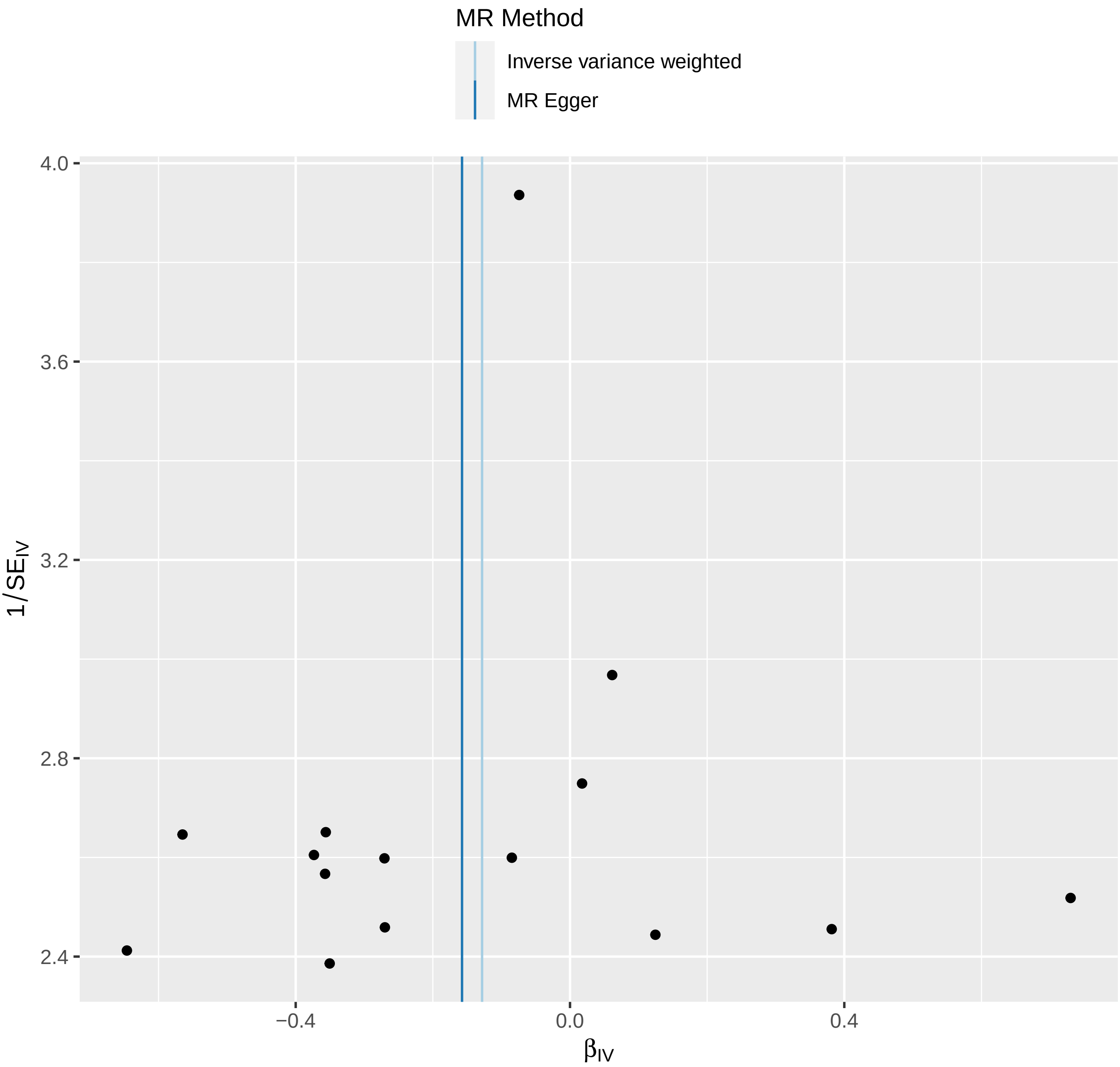

Batch 2−5555 : Gut microbiota abundance (genus Catenibacterium id.2153) on Small integral membrane protein 9
with a threshold of 1e−05

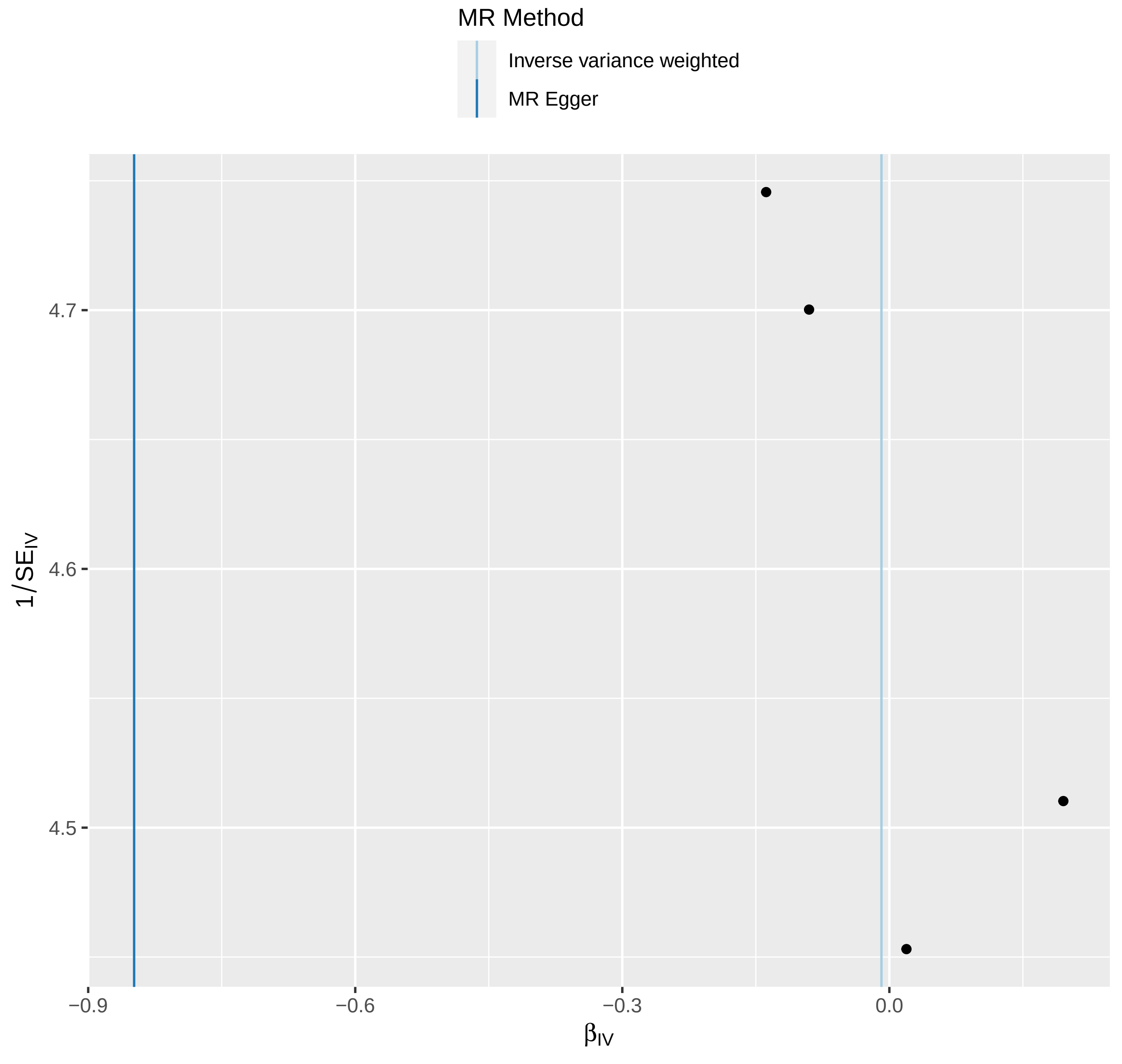

Batch 2−5556 : Gut microbiota abundance (genus Parasutterella id.2892) on Small integral membrane protein 9
with a threshold of 1e−05

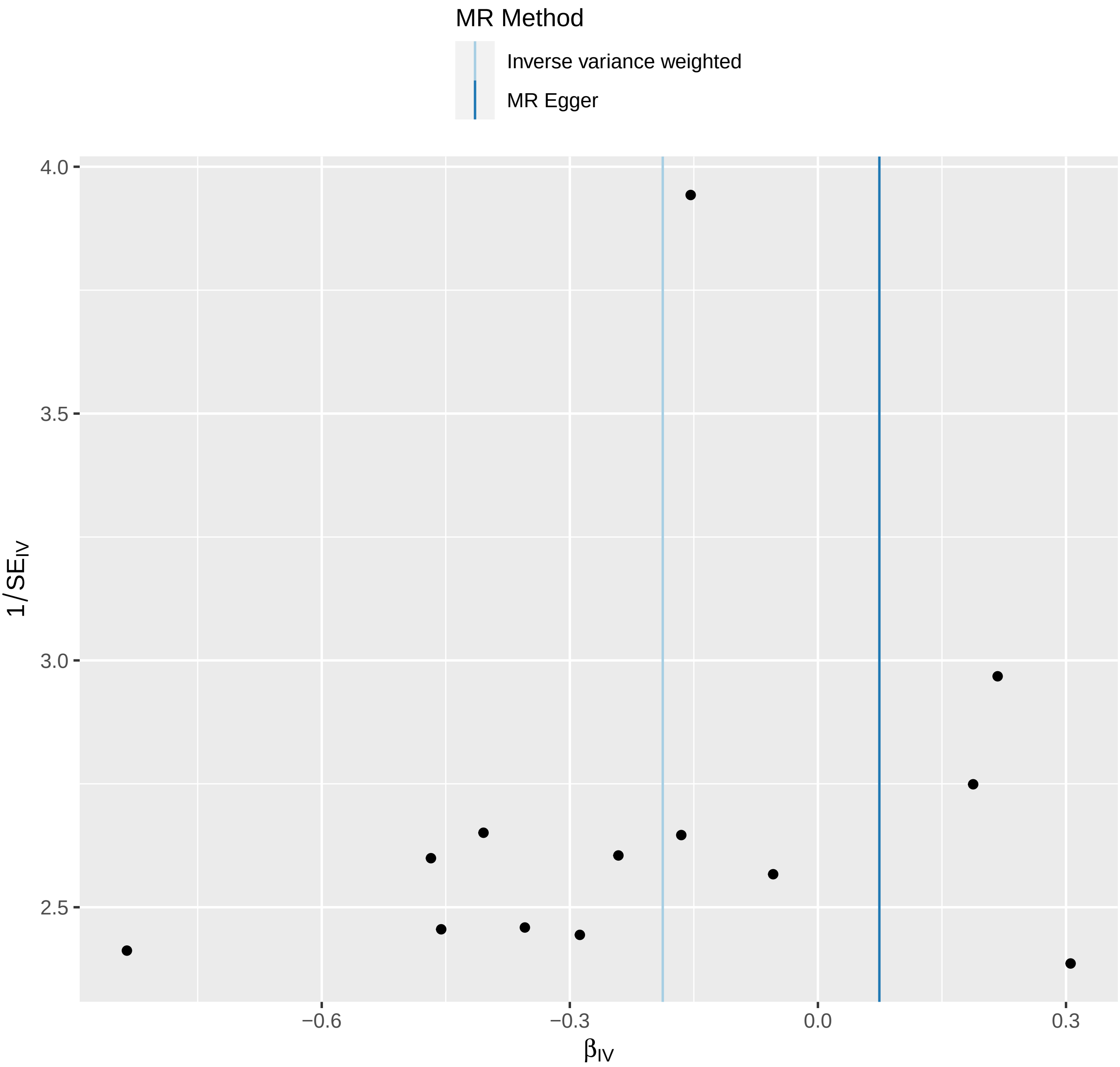

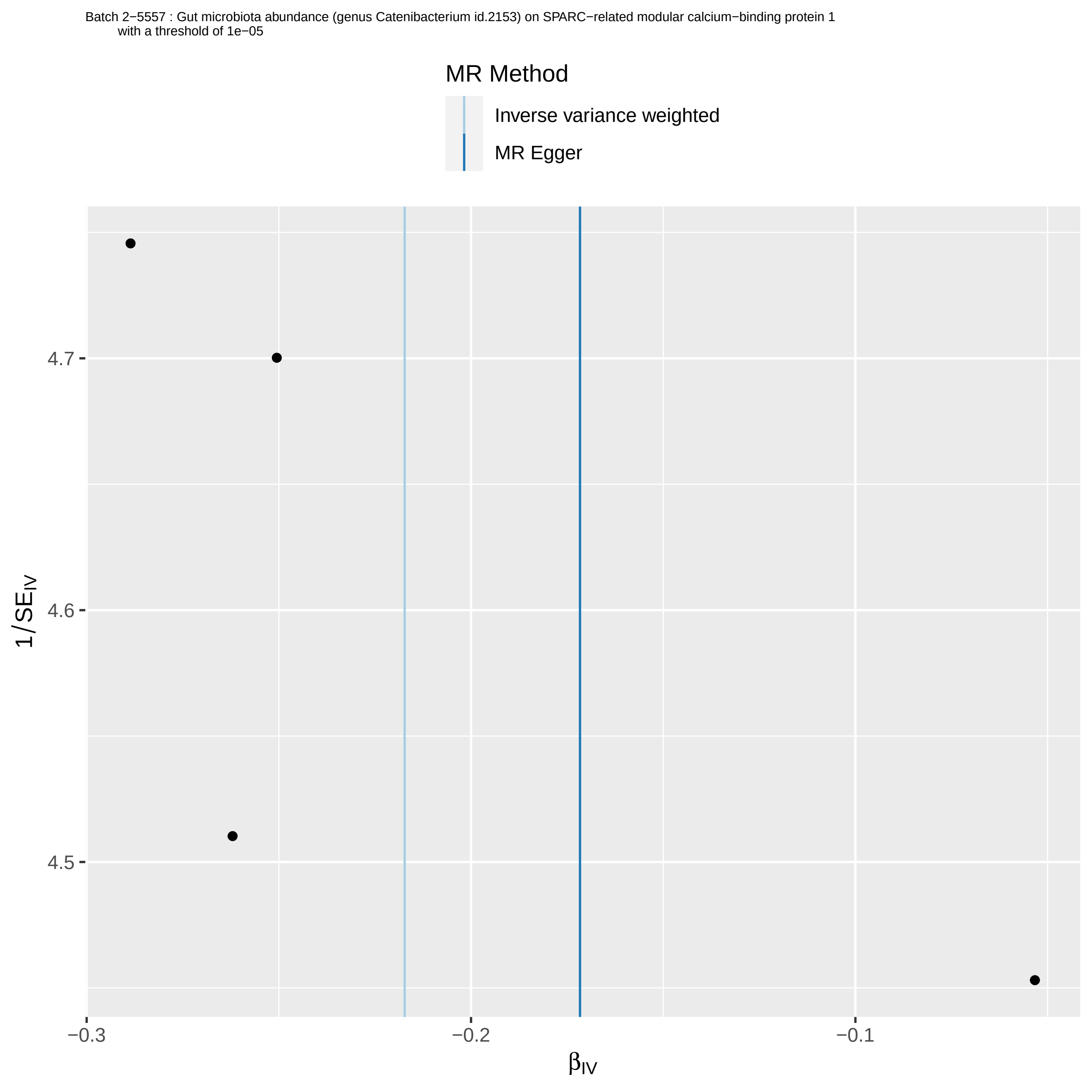
Batch 2−5557 : Gut microbiota abundance (genus Catenibacterium id.2153) on SPARC−related modular calcium−binding protein 1
with a threshold of 1e−05
MR Method
Inverse variance weighted
MR Egger
4.7
4.6
4.5
1/SE_IV
−0.3
−0.2
−0.1
β_IV

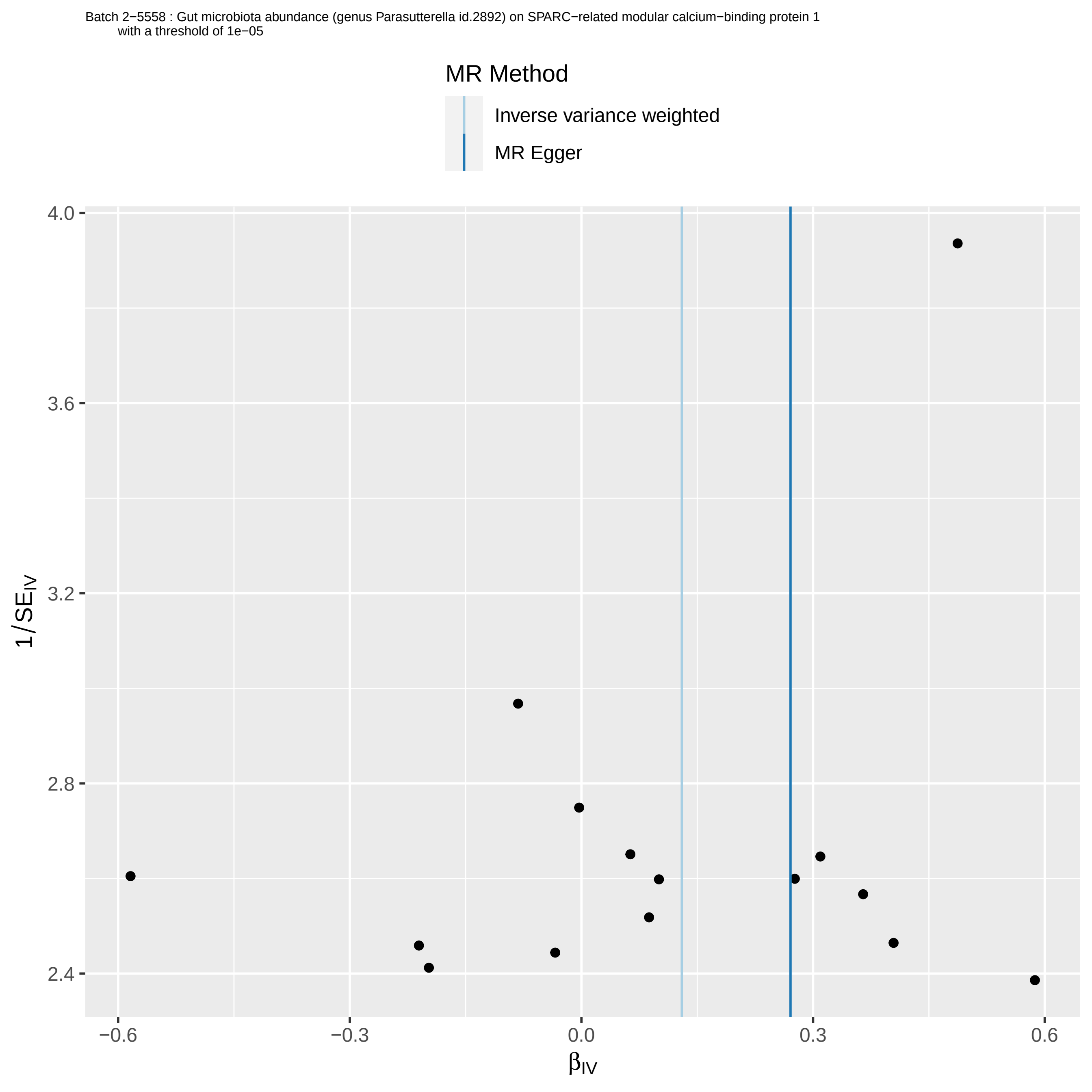

Batch 2−5558 : Gut microbiota abundance (genus Parasutterella id.2892) on SPARC−related modular calcium−binding protein 1
with a threshold of 1e−05
MR Method
Inverse variance weighted
MR Egger
4.0
3.6
3.2
2.8
2.4
1/SE$_{IV}$
−0.6
−0.3
0.0
0.3
0.6
β$_{IV}$

Batch 2−5559 : Gut microbiota abundance (genus Catenibacterium id.2153) on Acid sphingomyelinase−like phosphodiesterase 3a
with a threshold of 1e−05

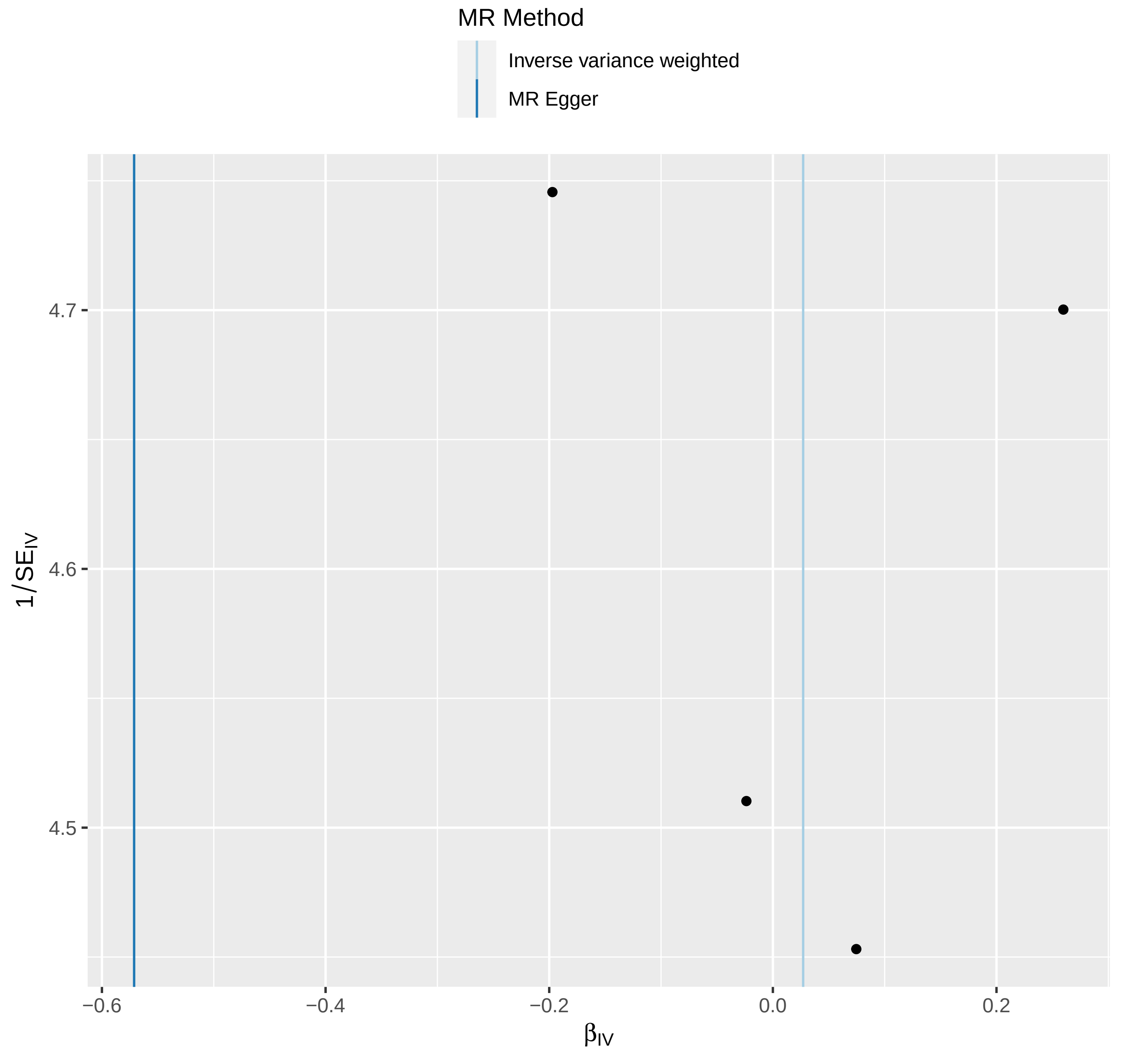

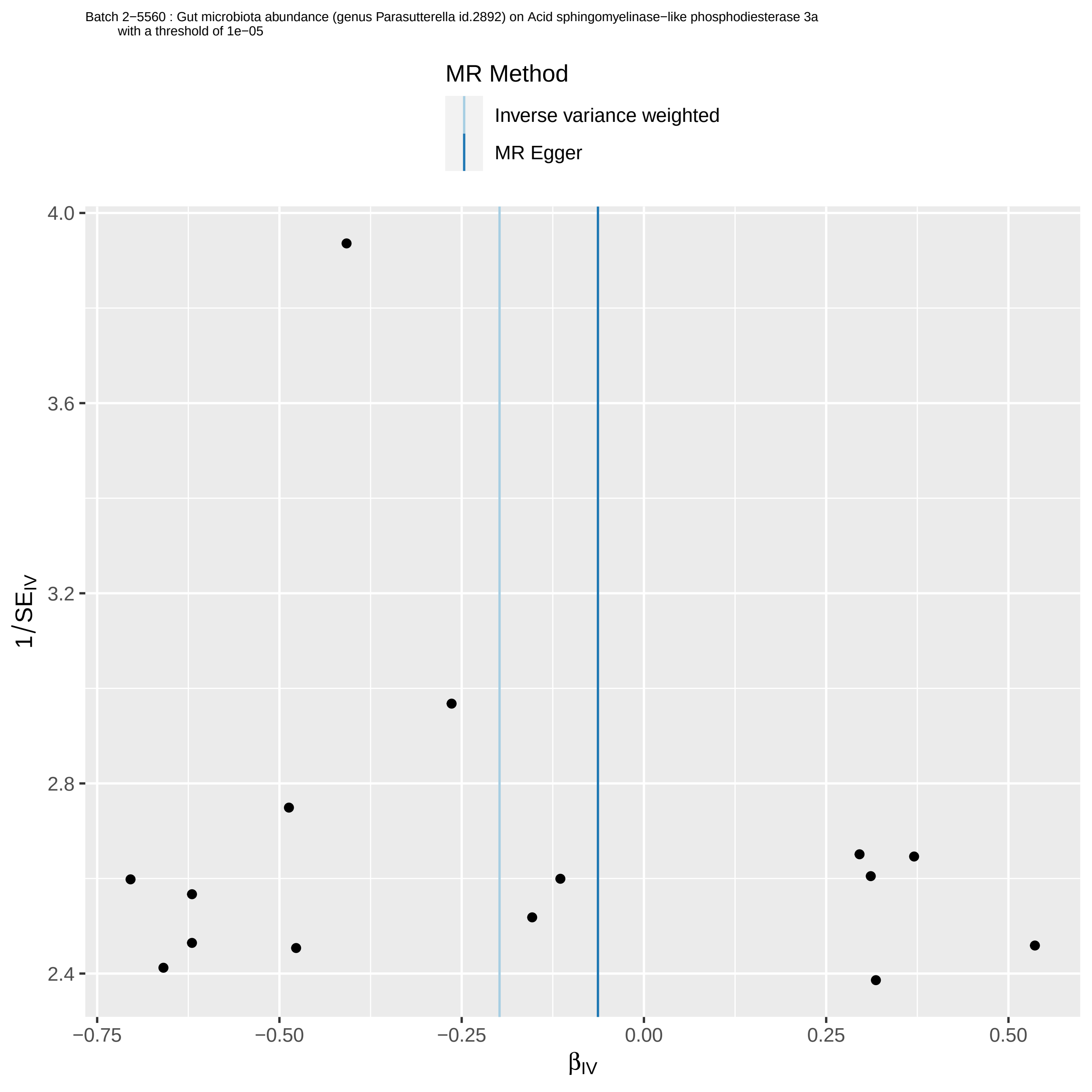

Batch 2−5560 : Gut microbiota abundance (genus Parasutterella id.2892) on Acid sphingomyelinase−like phosphodiesterase 3a
with a threshold of 1e−05
MR Method
Inverse variance weighted
MR Egger
4.0
3.6
3.2
2.8
2.4
1/SE$_{IV}$
−0.75
−0.50
−0.25
0.00
0.25
0.50
β$_{IV}$

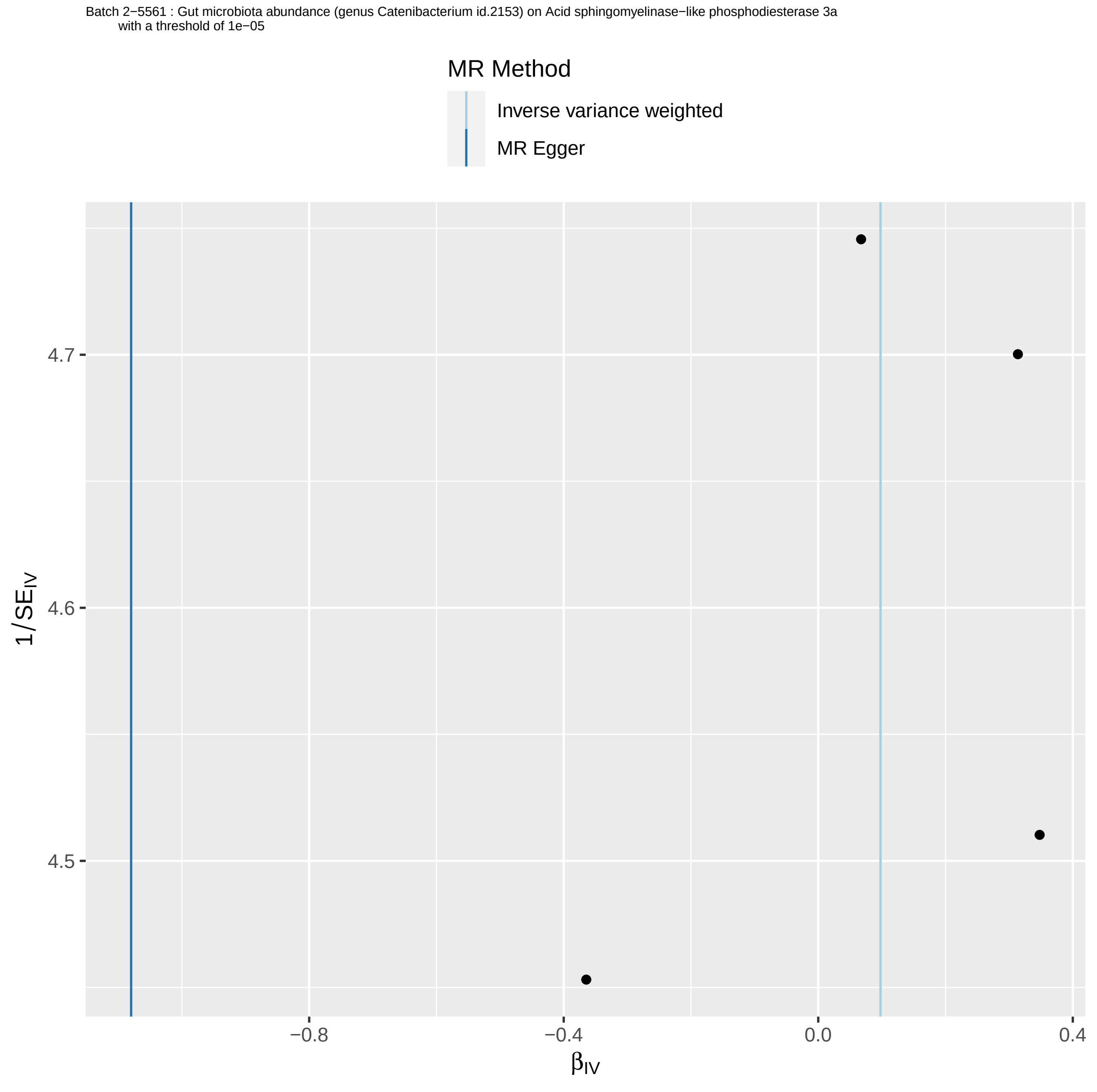

Batch 2−5561 : Gut microbiota abundance (genus Catenibacterium id.2153) on Acid sphingomyelinase−like phosphodiesterase 3a with a threshold of 1e−05
MR Method
Inverse variance weighted
MR Egger
4.7
4.6
4.5
1/SE$_{IV}$
−0.8
−0.4
0.0
0.4
β_{IV}

Batch 2−5562 : Gut microbiota abundance (genus Parasutterella id.2892) on Acid sphingomyelinase−like phosphodiesterase 3a
with a threshold of 1e−05

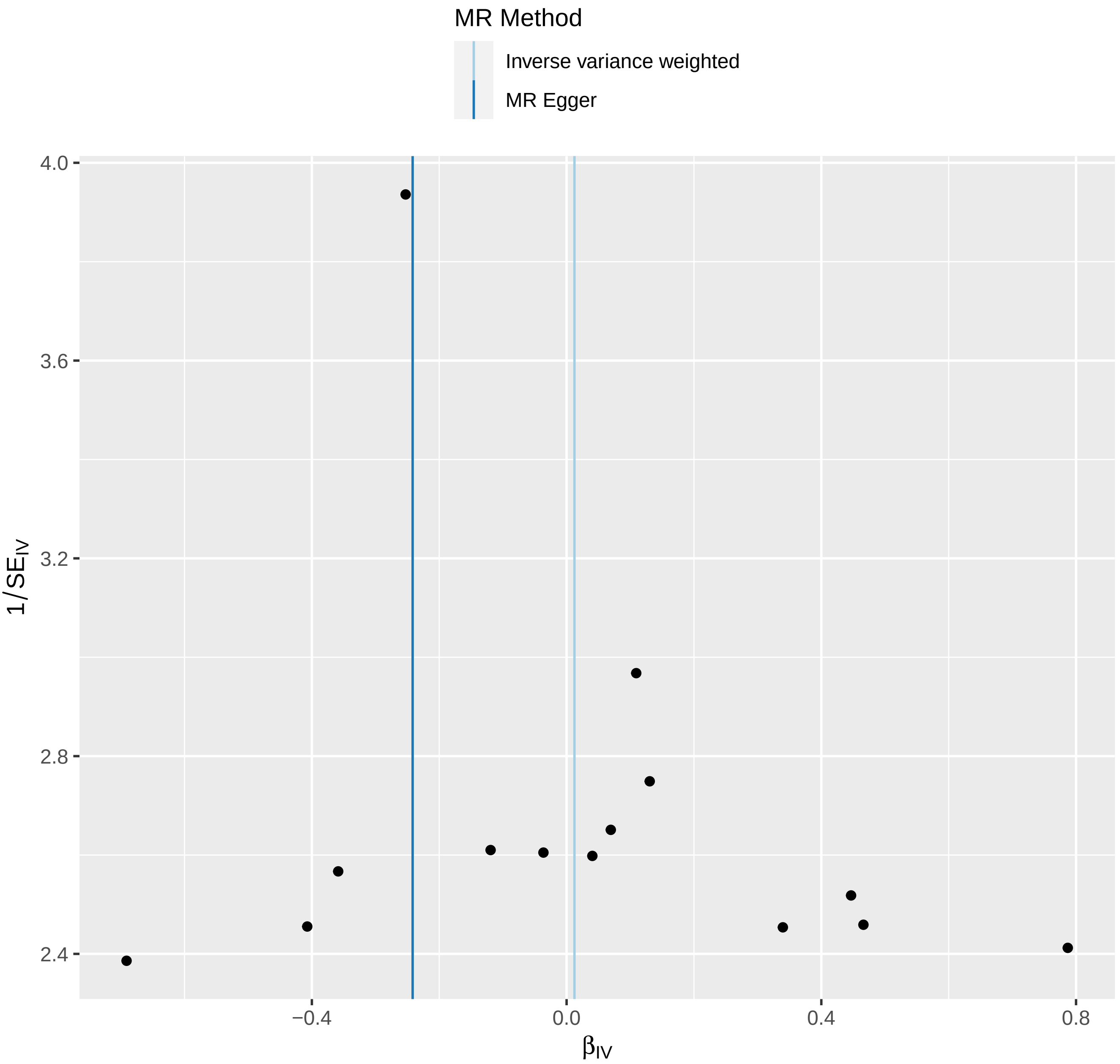

Batch 2−5563 : Gut microbiota abundance (genus Catenibacterium id.2153) on Submaxillary gland androgen−regulated protein 3A
with a threshold of 1e−05

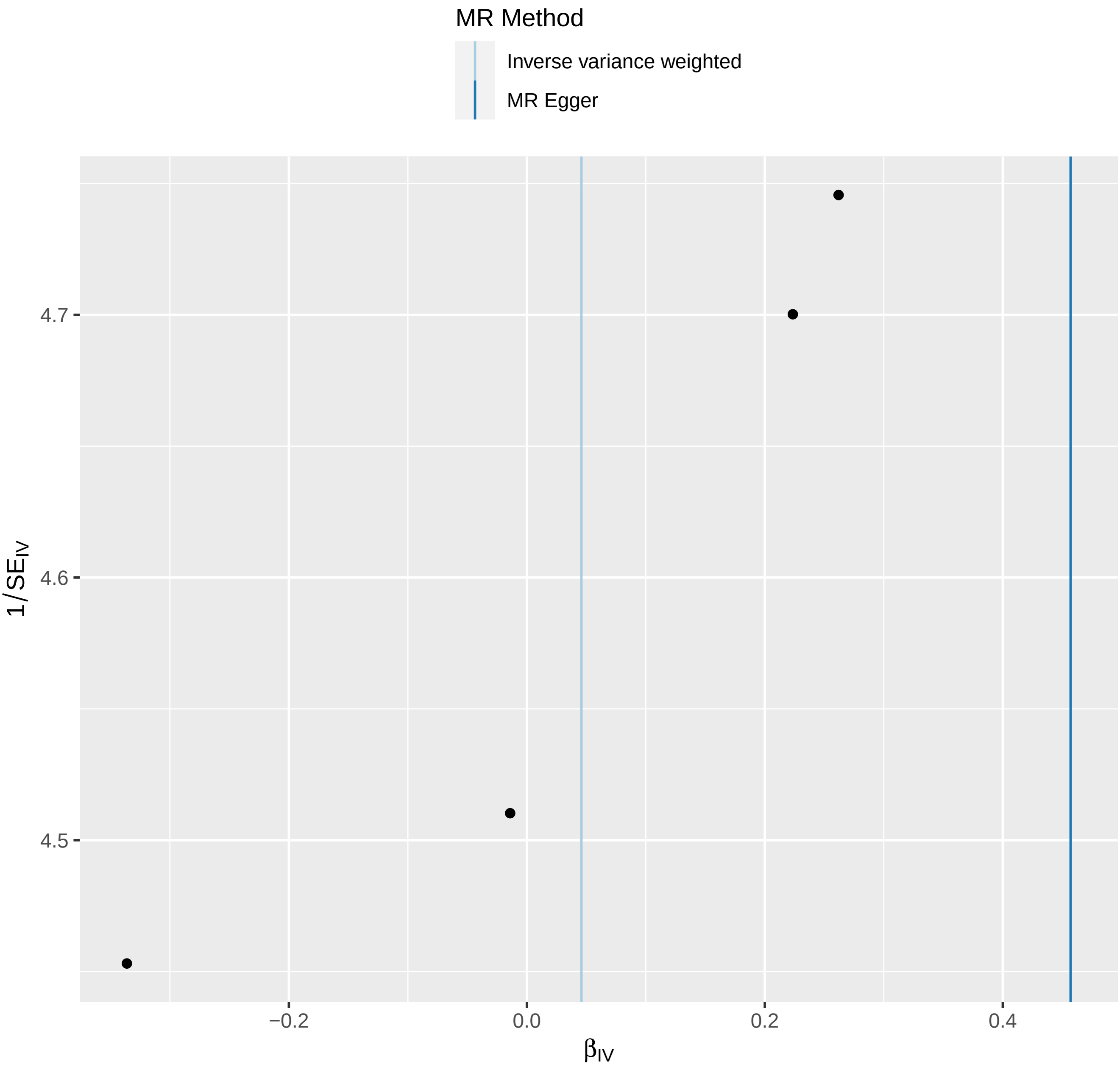

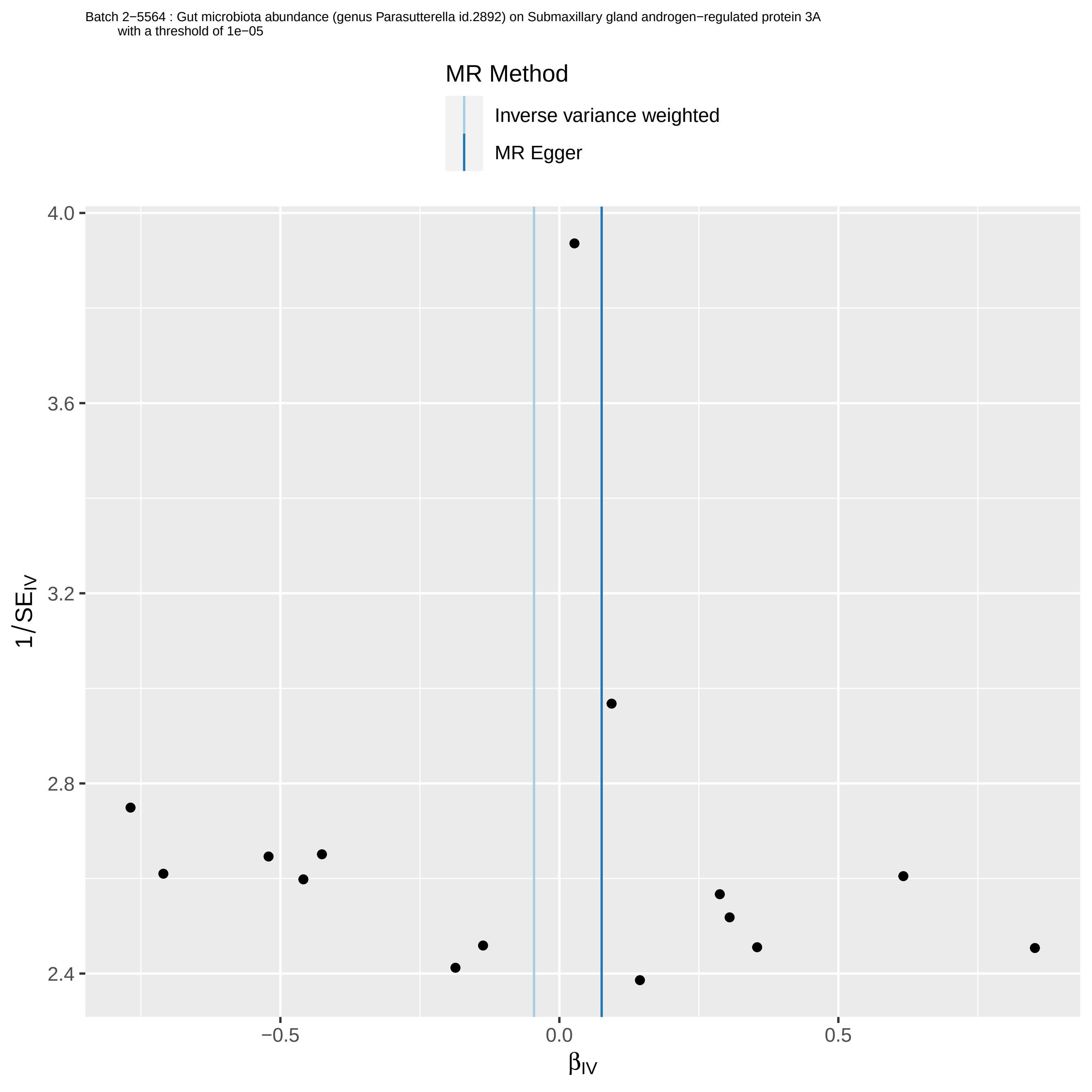
Batch 2−5564 : Gut microbiota abundance (genus Parasutterella id.2892) on Submaxillary gland androgen−regulated protein 3A
with a threshold of 1e−05
MR Method
Inverse variance weighted
MR Egger
4.0
3.6
3.2
2.8
2.4
1/SE_IV
−0.5
0.0
0.5
β_IV

Batch 2−5565 : Gut microbiota abundance (genus Catenibacterium id.2153) on Submaxillary gland androgen−regulated protein 3B
with a threshold of 1e−05

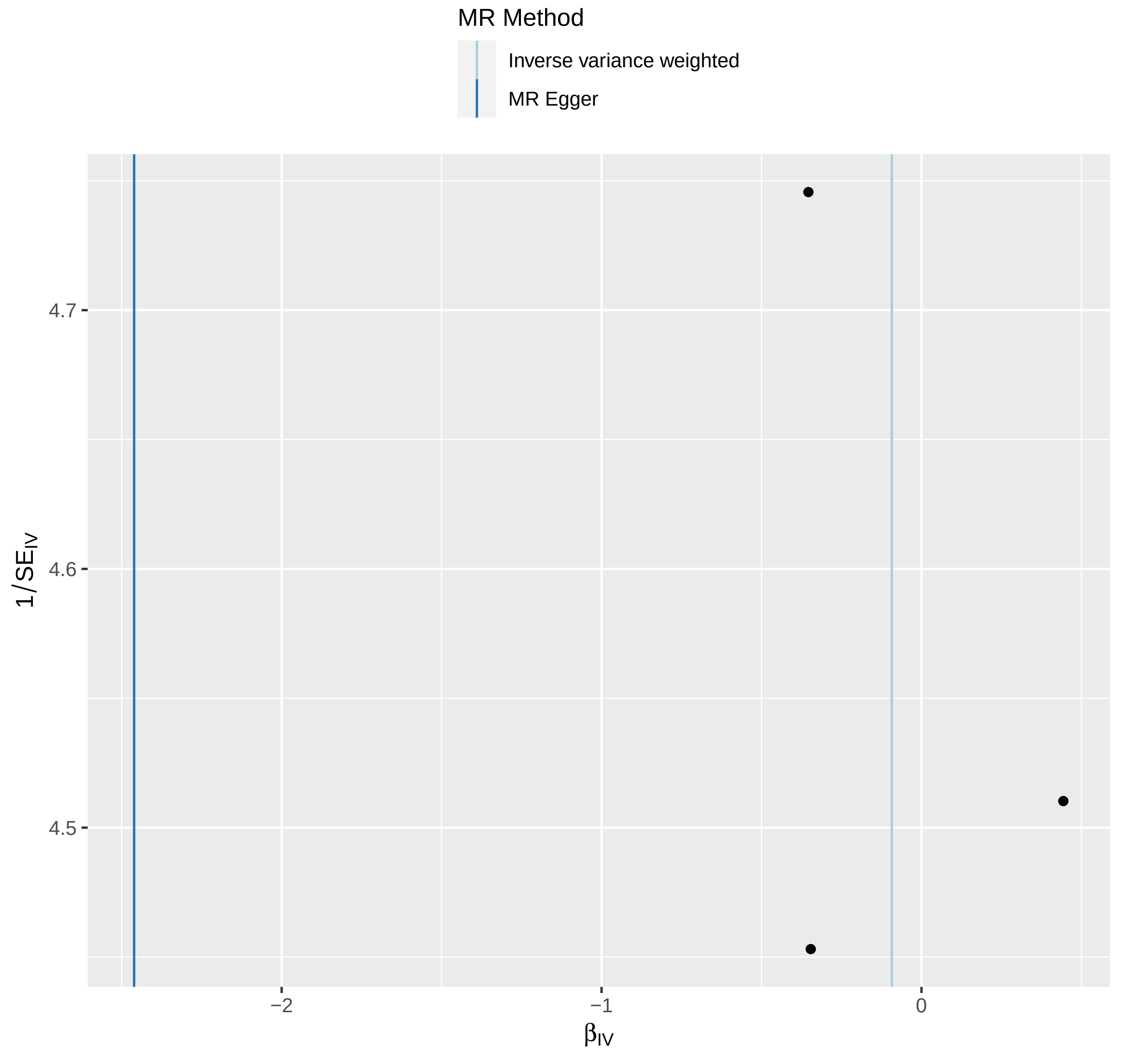

Batch 2−5566 : Gut microbiota abundance (genus Parasutterella id.2892) on Submaxillary gland androgen−regulated protein 3B
with a threshold of 1e−05

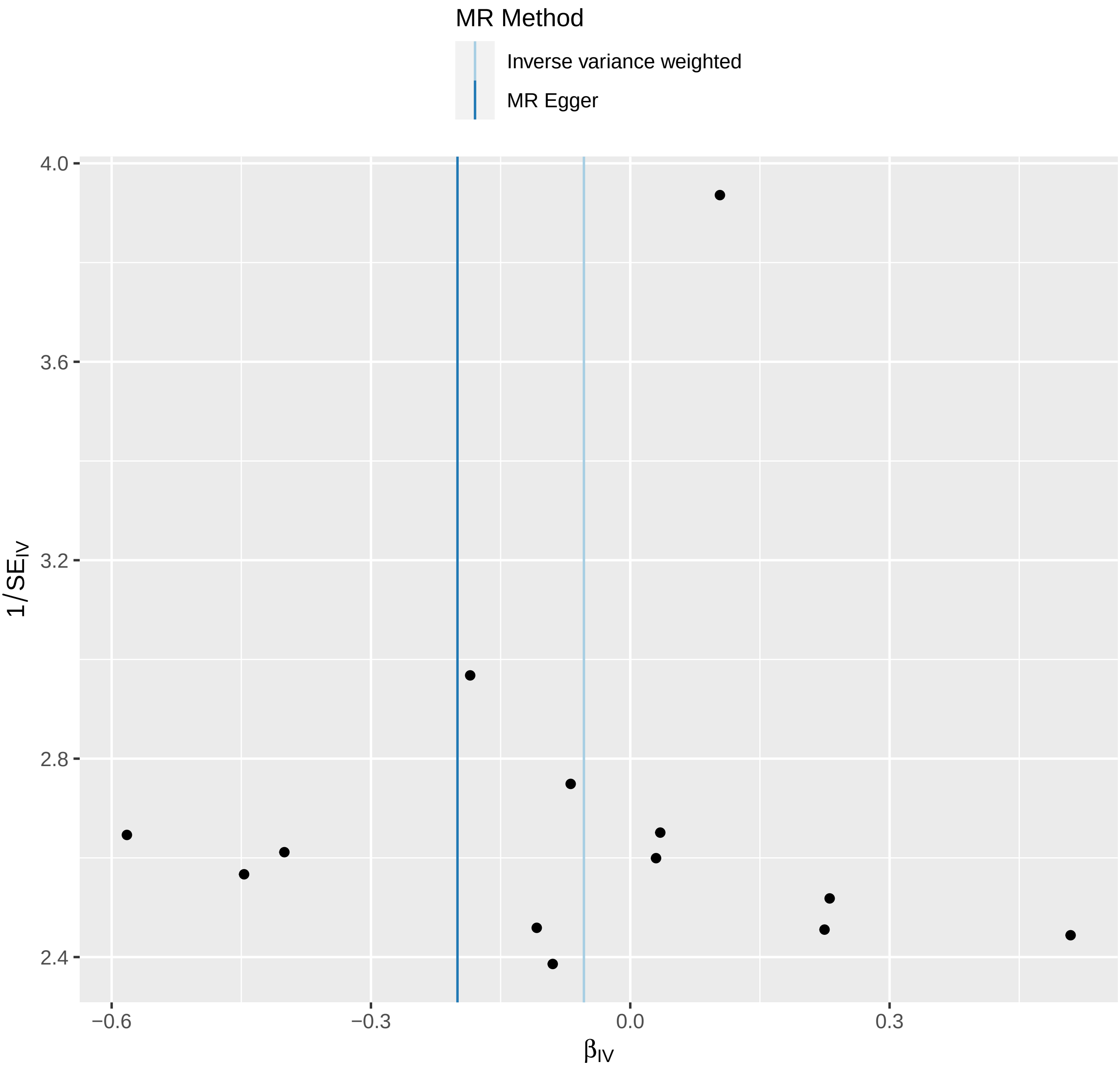

Batch 2−5567 : Gut microbiota abundance (genus Catenibacterium id.2153) on N−lysine methyltransferase SMYD2
with a threshold of 1e−05

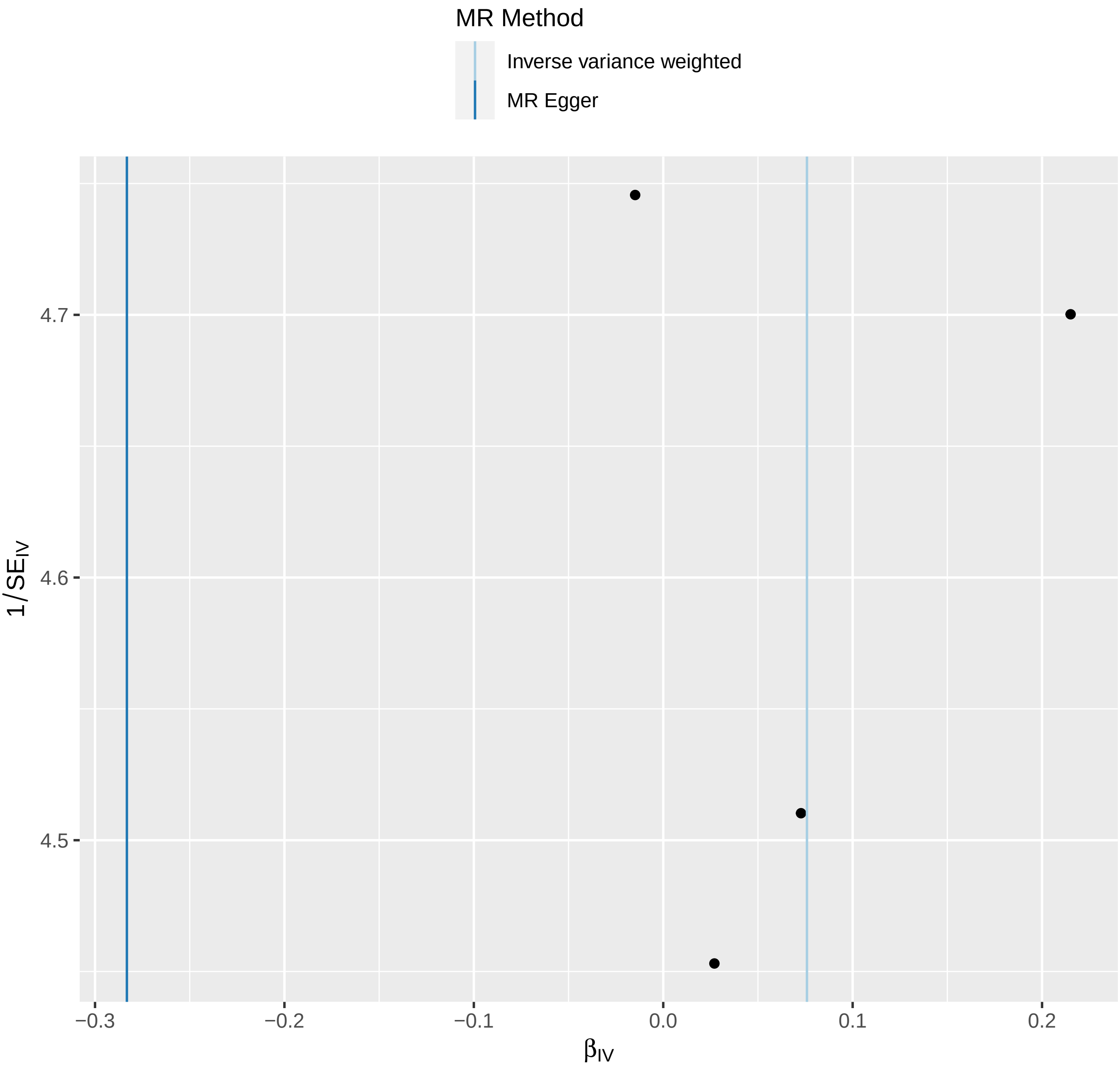

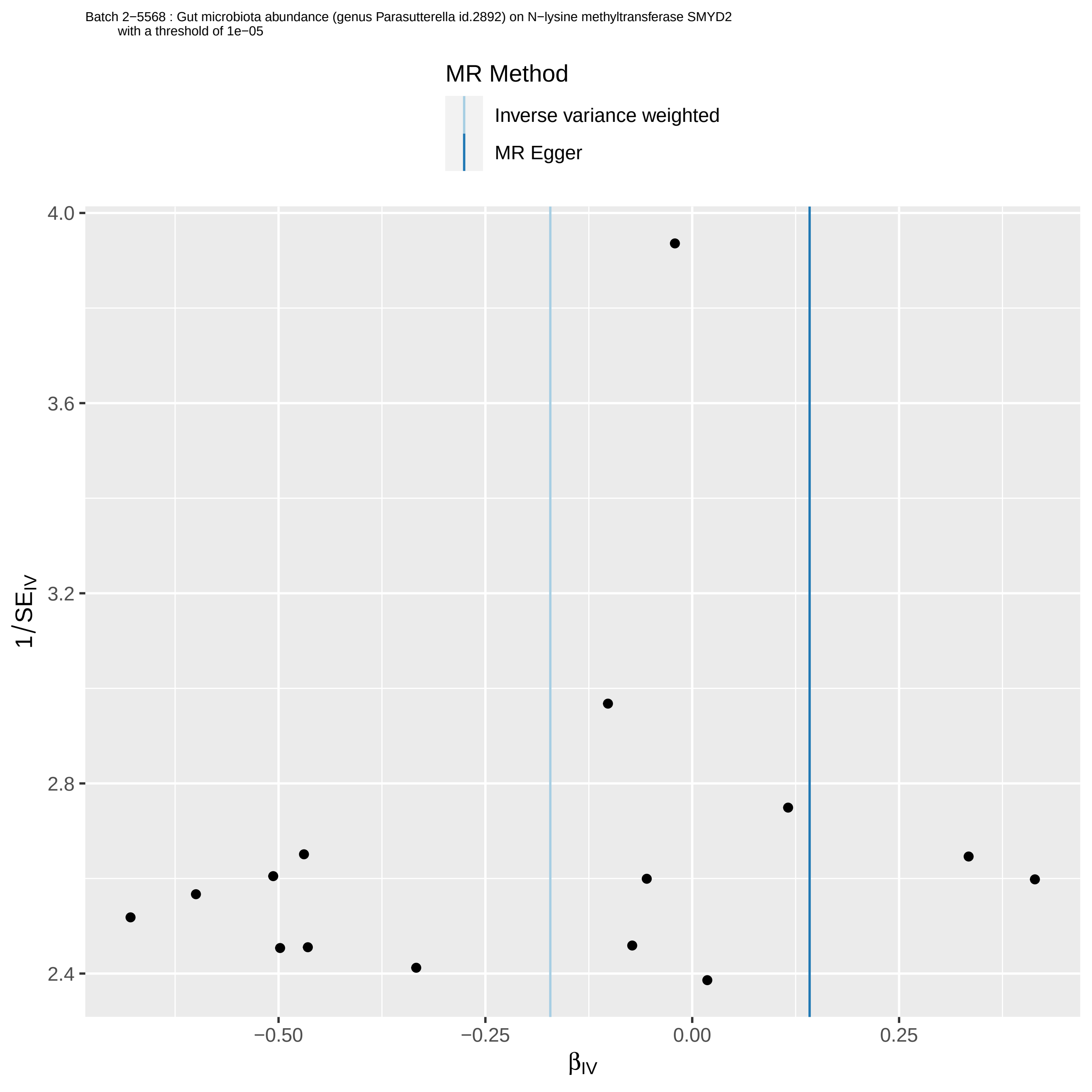

Batch 2−5568 : Gut microbiota abundance (genus Parasutterella id.2892) on N−lysine methyltransferase SMYD2
with a threshold of 1e−05
MR Method
Inverse variance weighted
MR Egger
4.0
3.6
3.2
2.8
2.4
1/SE$_{IV}$
−0.50
−0.25
0.00
0.25
β$_{IV}$

Batch 2−5569 : Gut microbiota abundance (genus Catenibacterium id.2153) on Zinc finger protein SNAI2
with a threshold of 1e−05

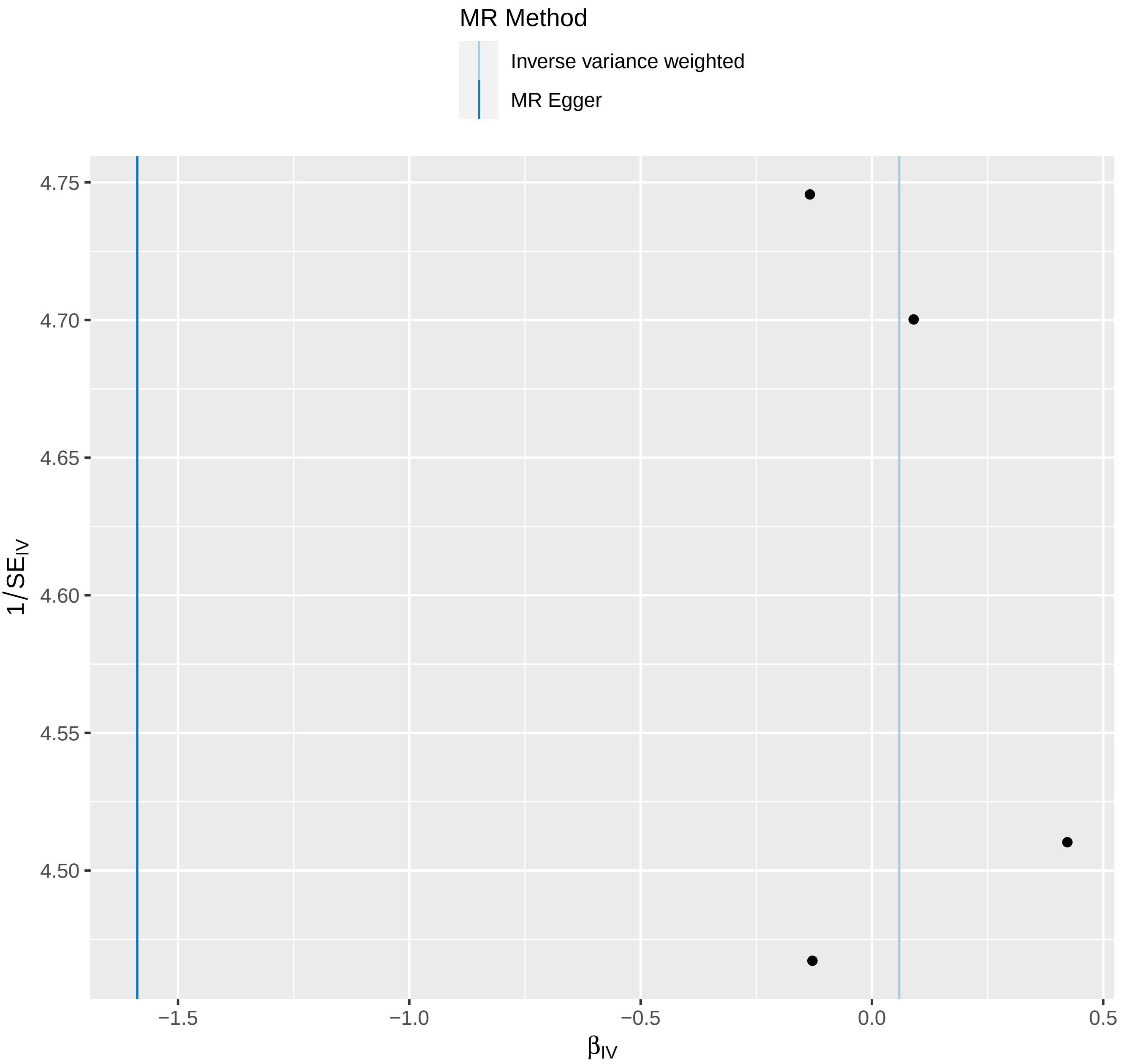

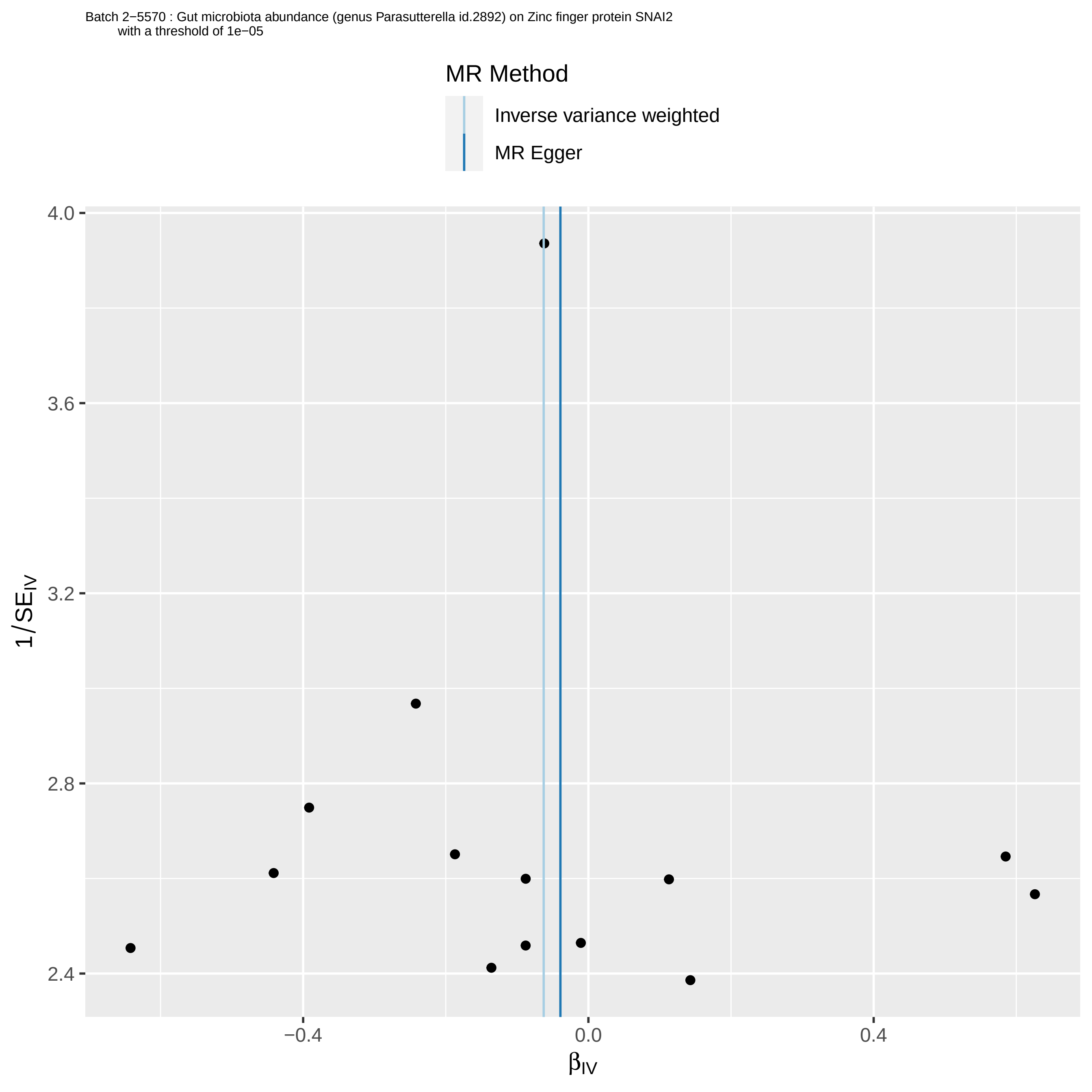
Batch 2−5570 : Gut microbiota abundance (genus Parasutterella id.2892) on Zinc finger protein SNAI2
with a threshold of 1e−05
MR Method
Inverse variance weighted
MR Egger
4.0
3.6
3.2
2.8
2.4
1/SE_IV
−0.4
0.0
0.4
β_IV

Batch 2−5571 : Gut microbiota abundance (genus Catenibacterium id.2153) on Synaptosomal−associated protein 25
with a threshold of 1e−05

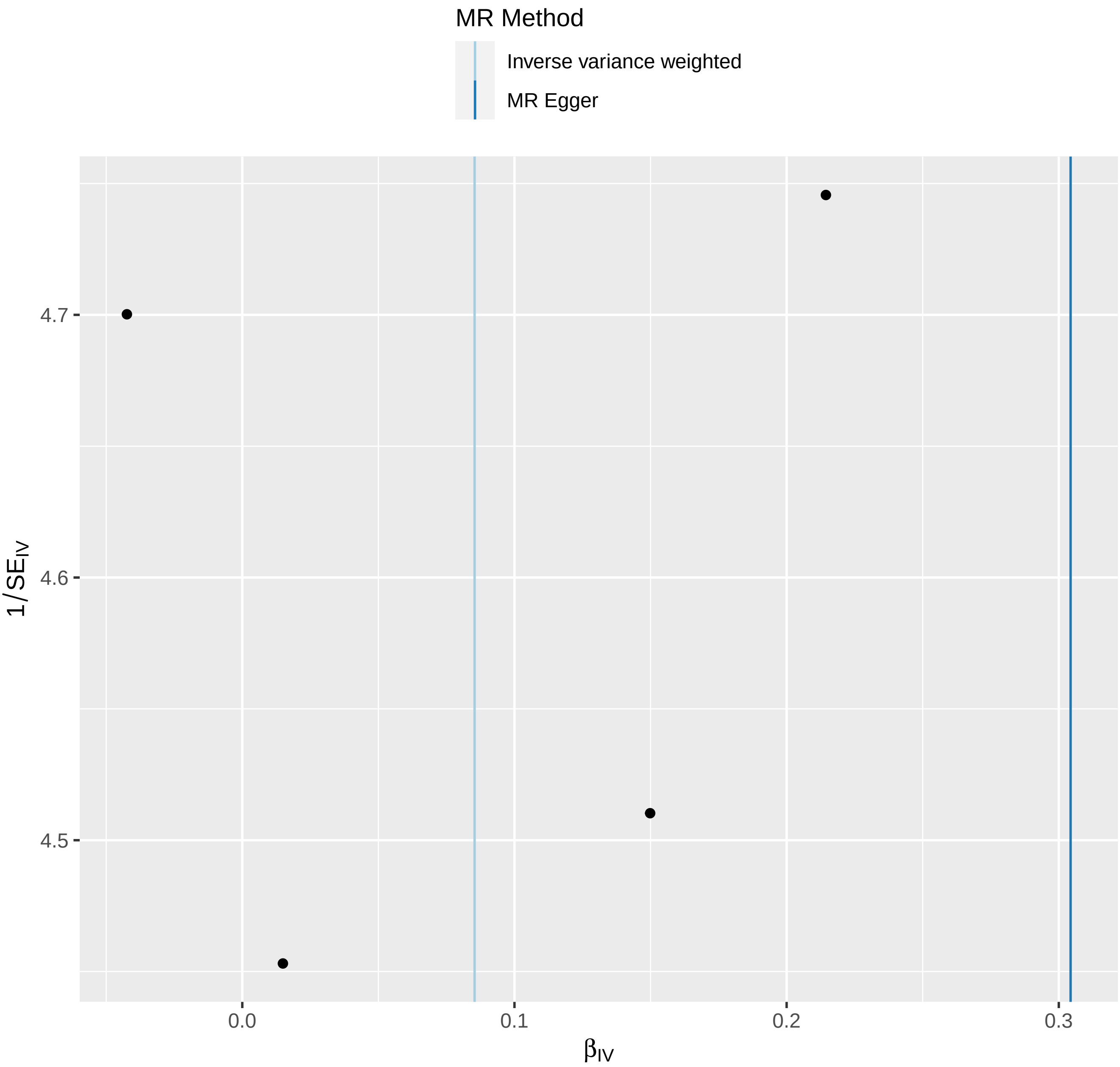

Batch 2−5572 : Gut microbiota abundance (genus Parasutterella id.2892) on Synaptosomal−associated protein 25
with a threshold of 1e−05

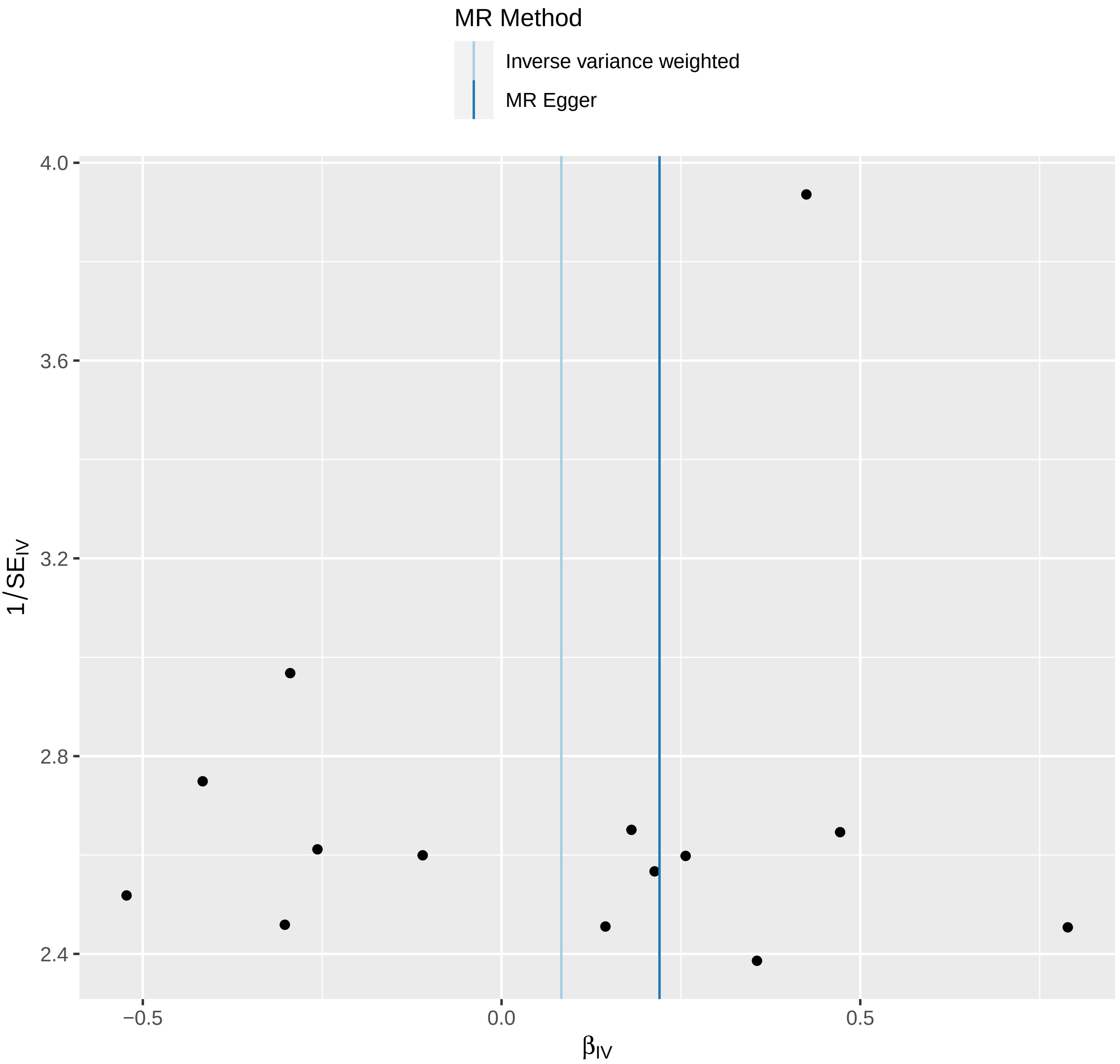

Batch 2−5573 : Gut microbiota abundance (genus Catenibacterium id.2153) on Synaptosomal−associated protein 29
with a threshold of 1e−05

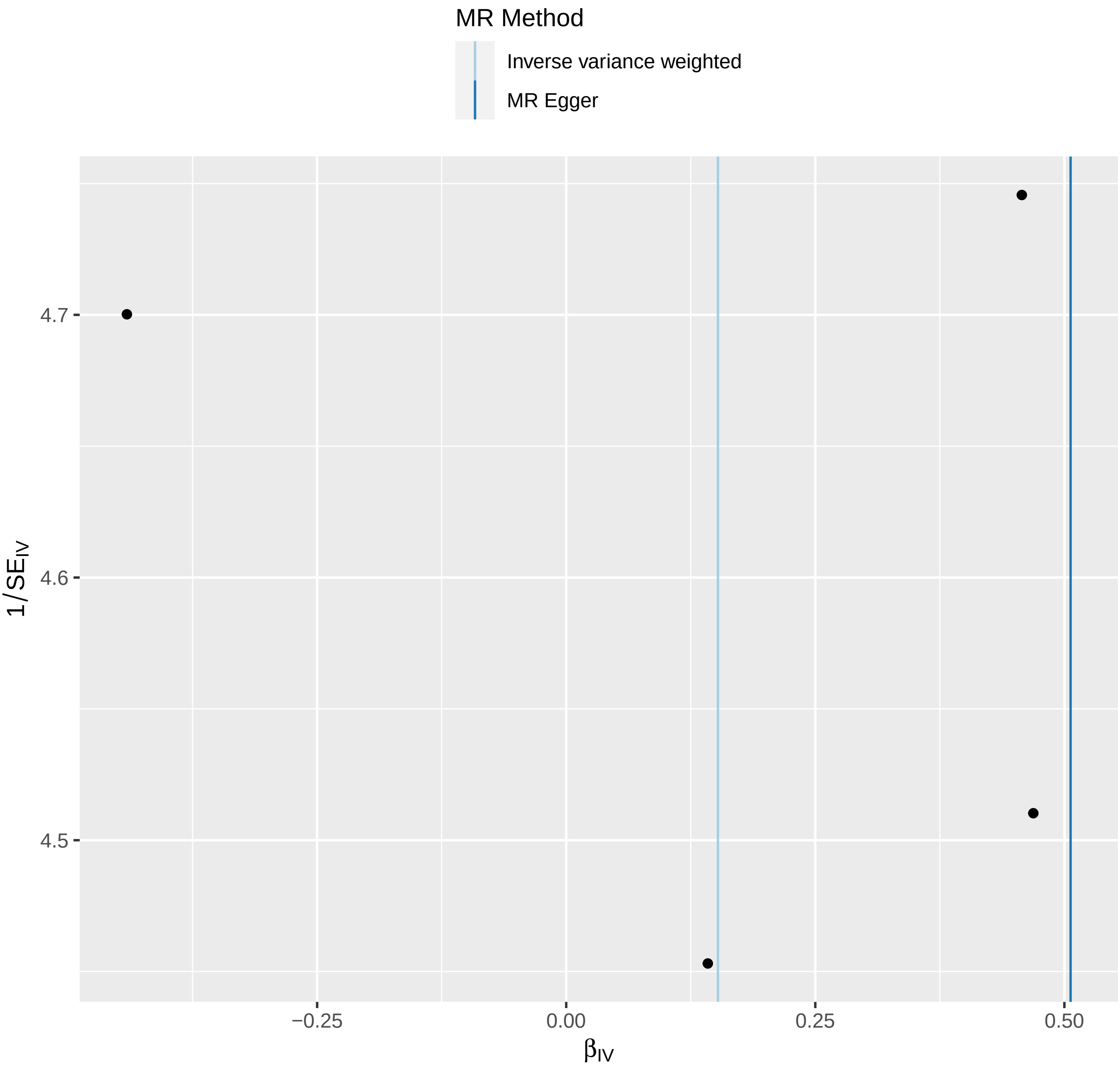

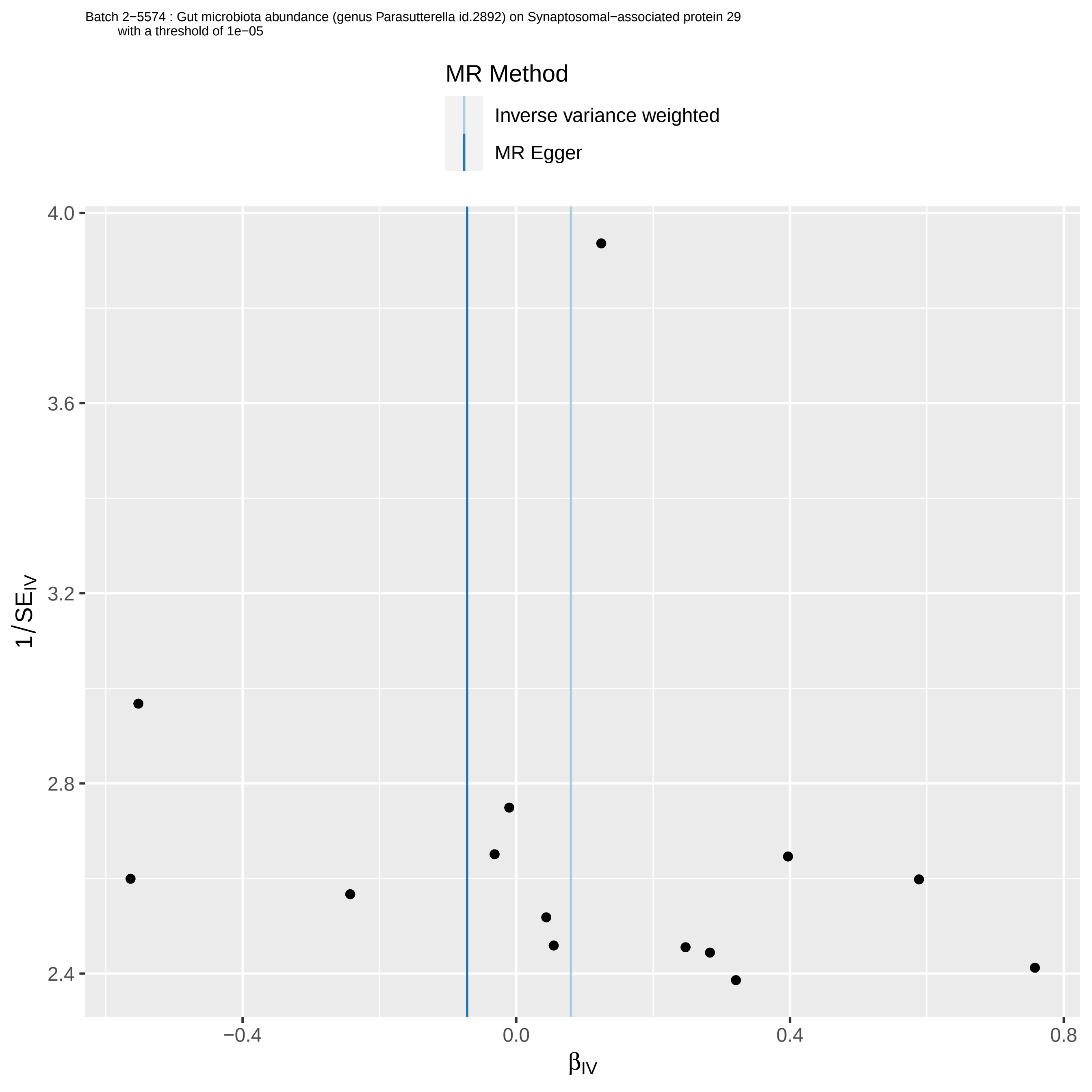
Batch 2−5574 : Gut microbiota abundance (genus Parasutterella id.2892) on Synaptosomal−associated protein 29
with a threshold of 1e−05
MR Method
Inverse variance weighted
MR Egger
4.0
3.6
3.2
2.8
2.4
1/SE_IV
−0.4
0.0
0.4
0.8
β_IV

Batch 2−5575 : Gut microbiota abundance (genus Catenibacterium id.2153) on SNARE−associated protein Snapin
with a threshold of 1e−05

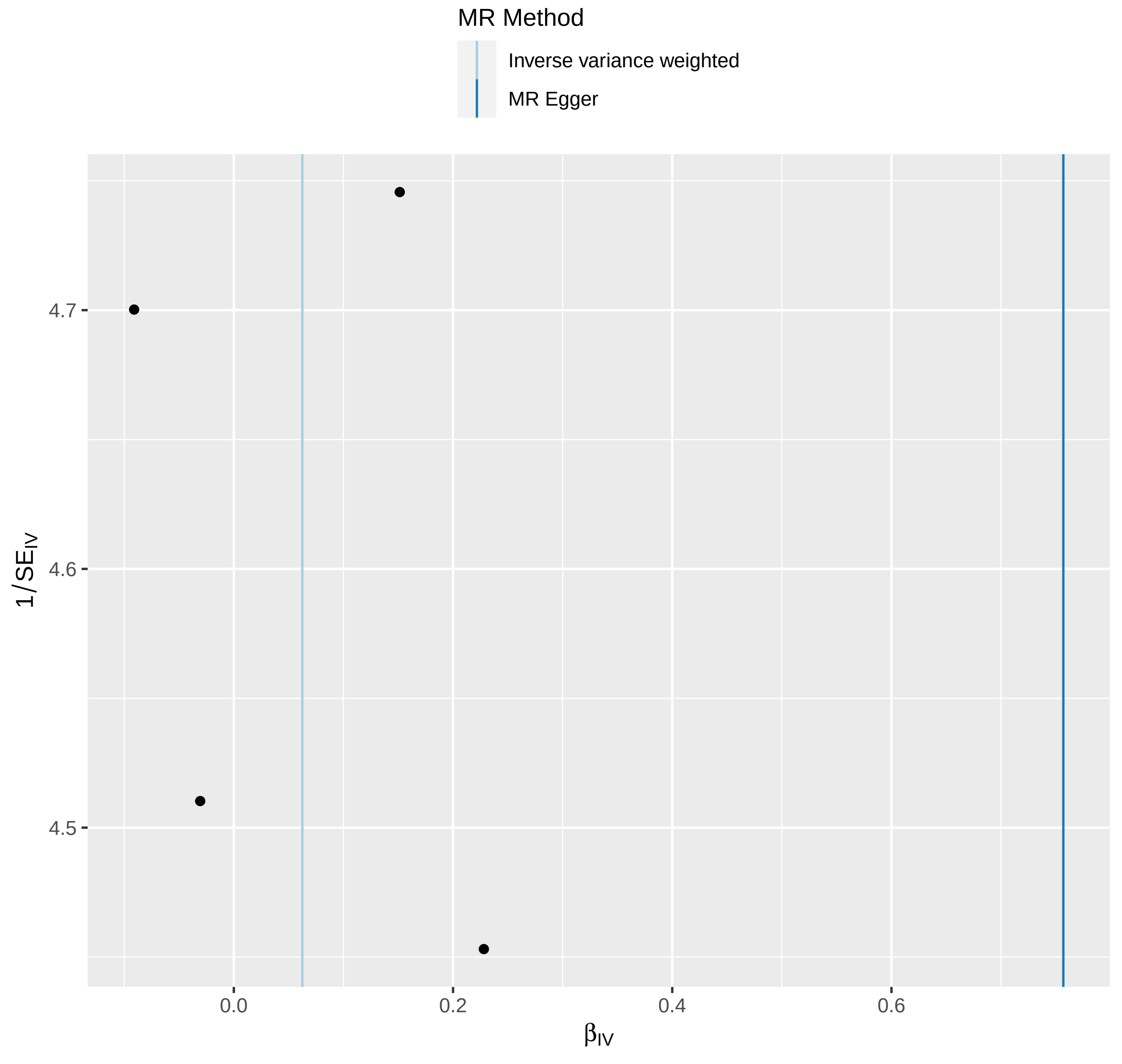

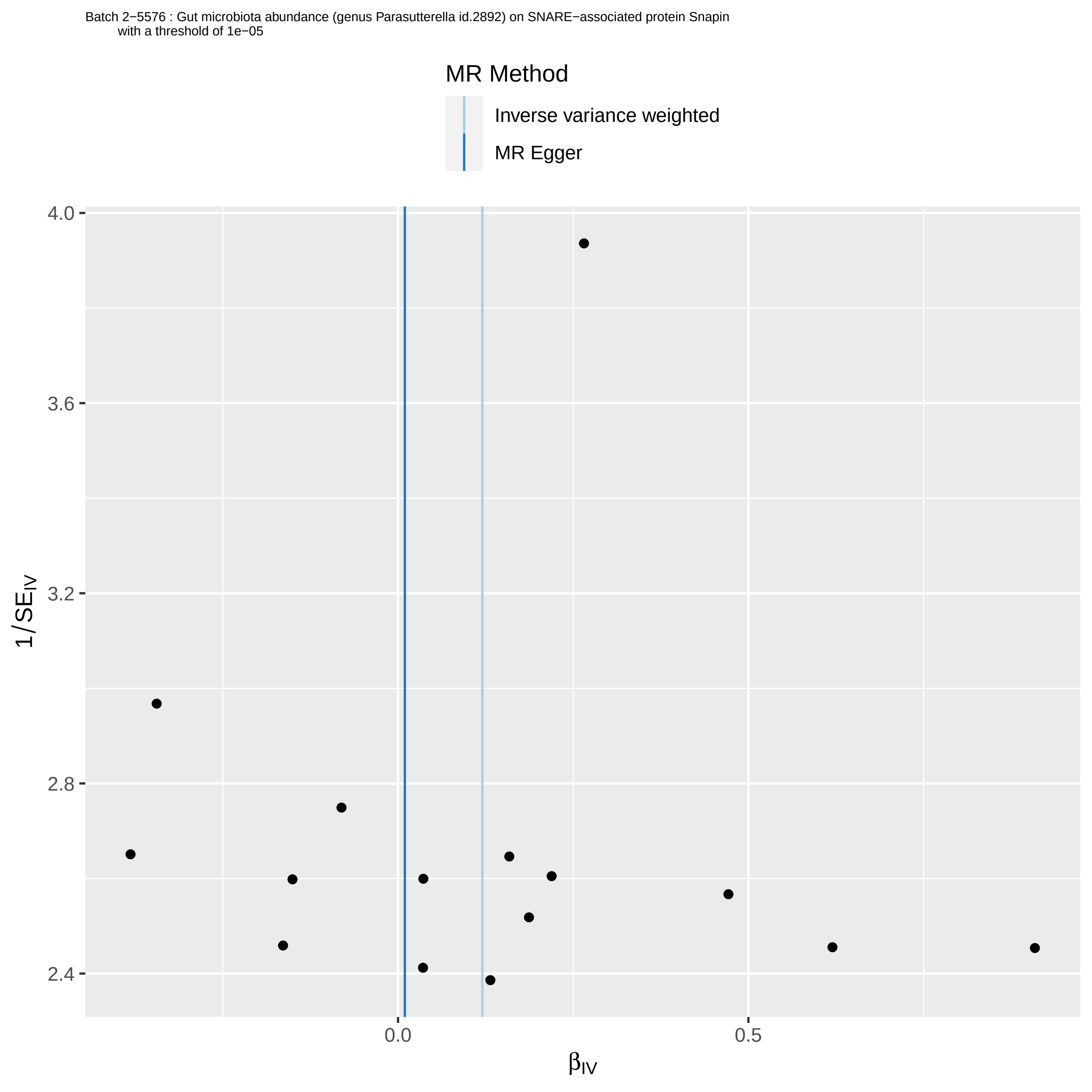

Batch 2−5576 : Gut microbiota abundance (genus Parasutterella id.2892) on SNARE−associated protein Snapin
with a threshold of 1e−05
MR Method
Inverse variance weighted
MR Egger
4.0
3.6
3.2
2.8
2.4
1/SE_IV
0.0
0.5
β_IV

Batch 2−5577 : Gut microbiota abundance (genus Catenibacterium id.2153) on Alpha−synuclein
with a threshold of 1e−05

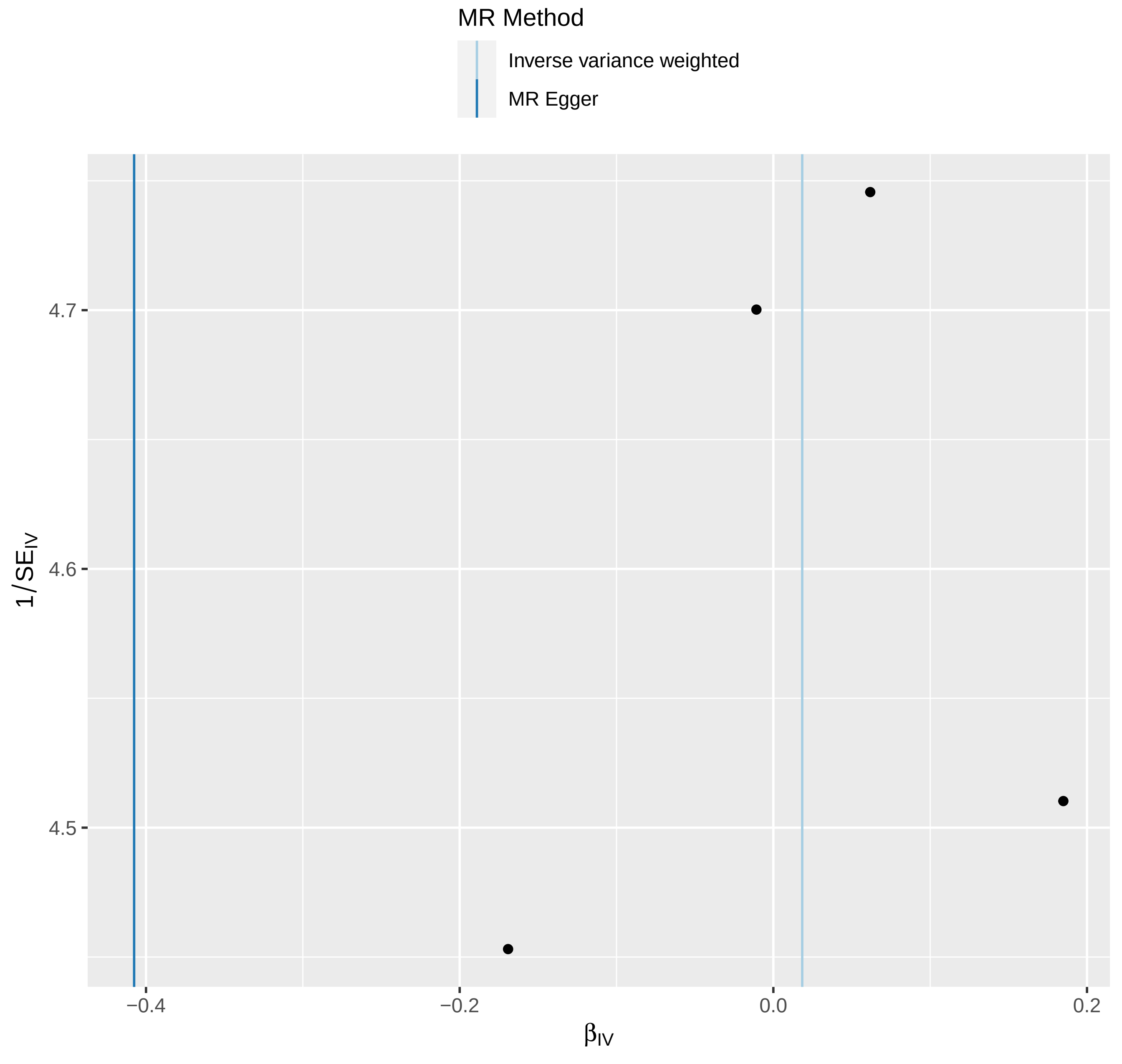

Batch 2−5578 : Gut microbiota abundance (genus Parasutterella id.2892) on Alpha−synuclein
with a threshold of 1e−05

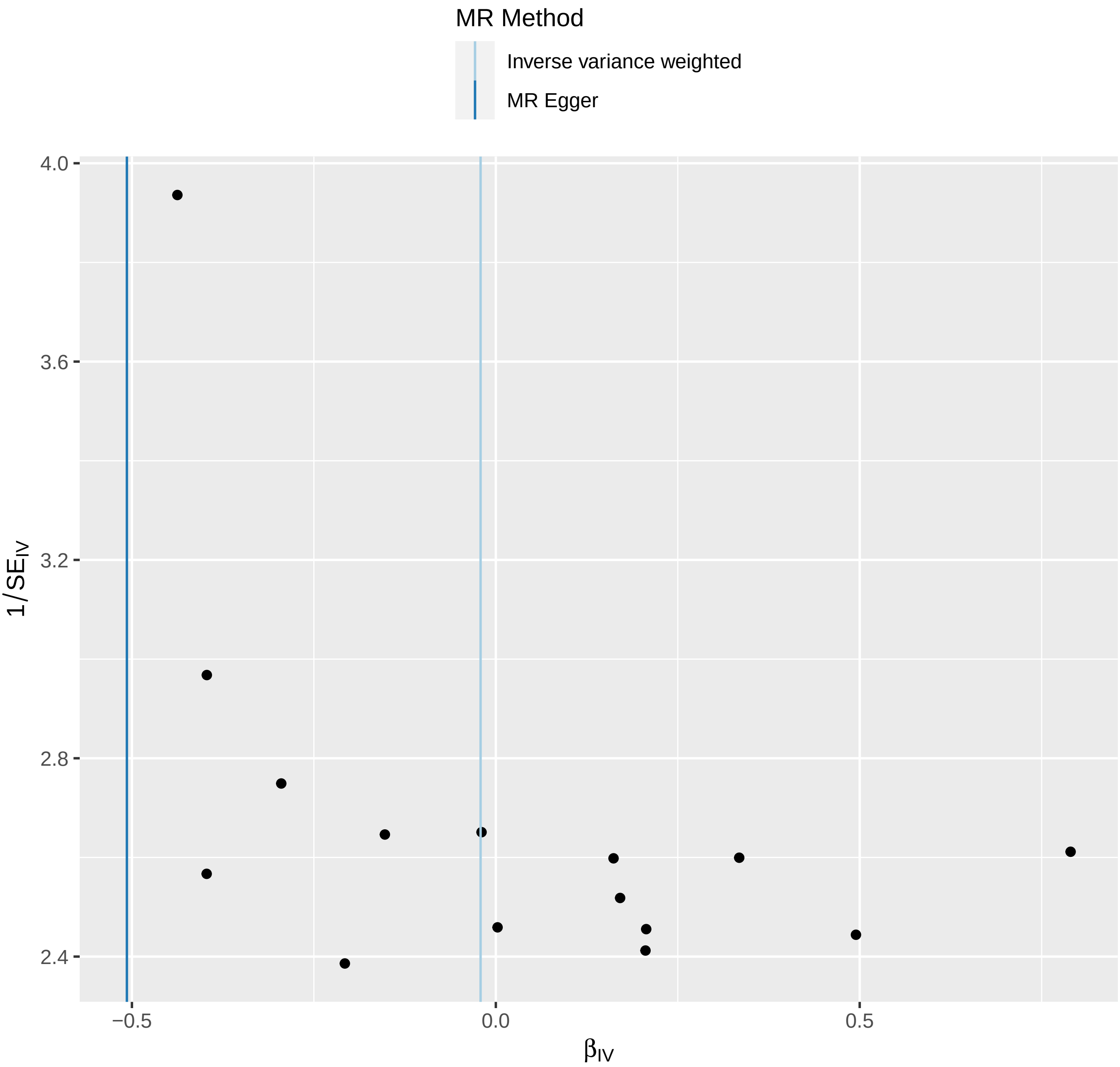

Batch 2−5579 : Gut microbiota abundance (genus Catenibacterium id.2153) on Alpha−synuclein
with a threshold of 1e−05

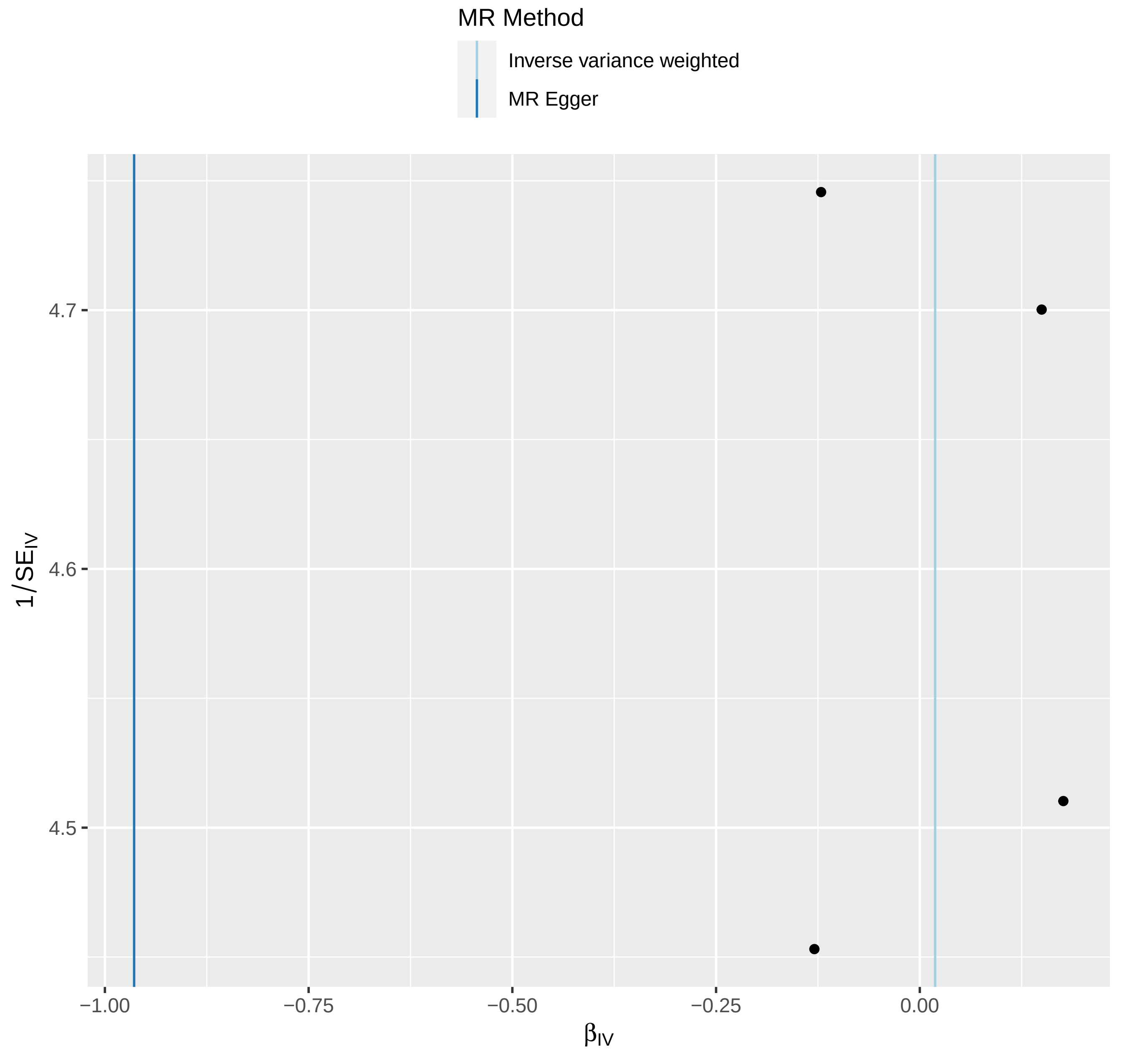

Batch 2−5580 : Gut microbiota abundance (genus Parasutterella id.2892) on Alpha−synuclein
with a threshold of 1e−05

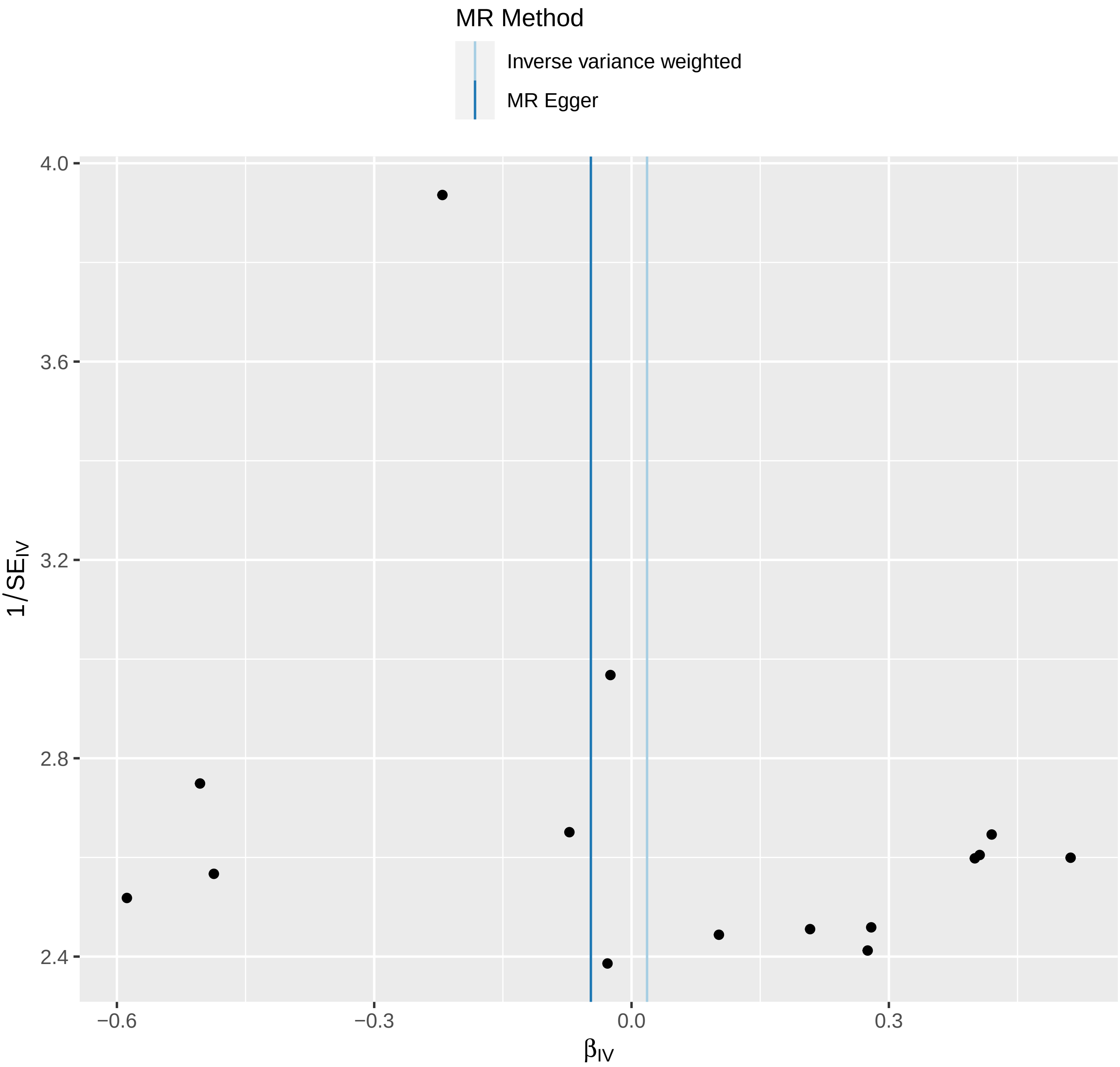

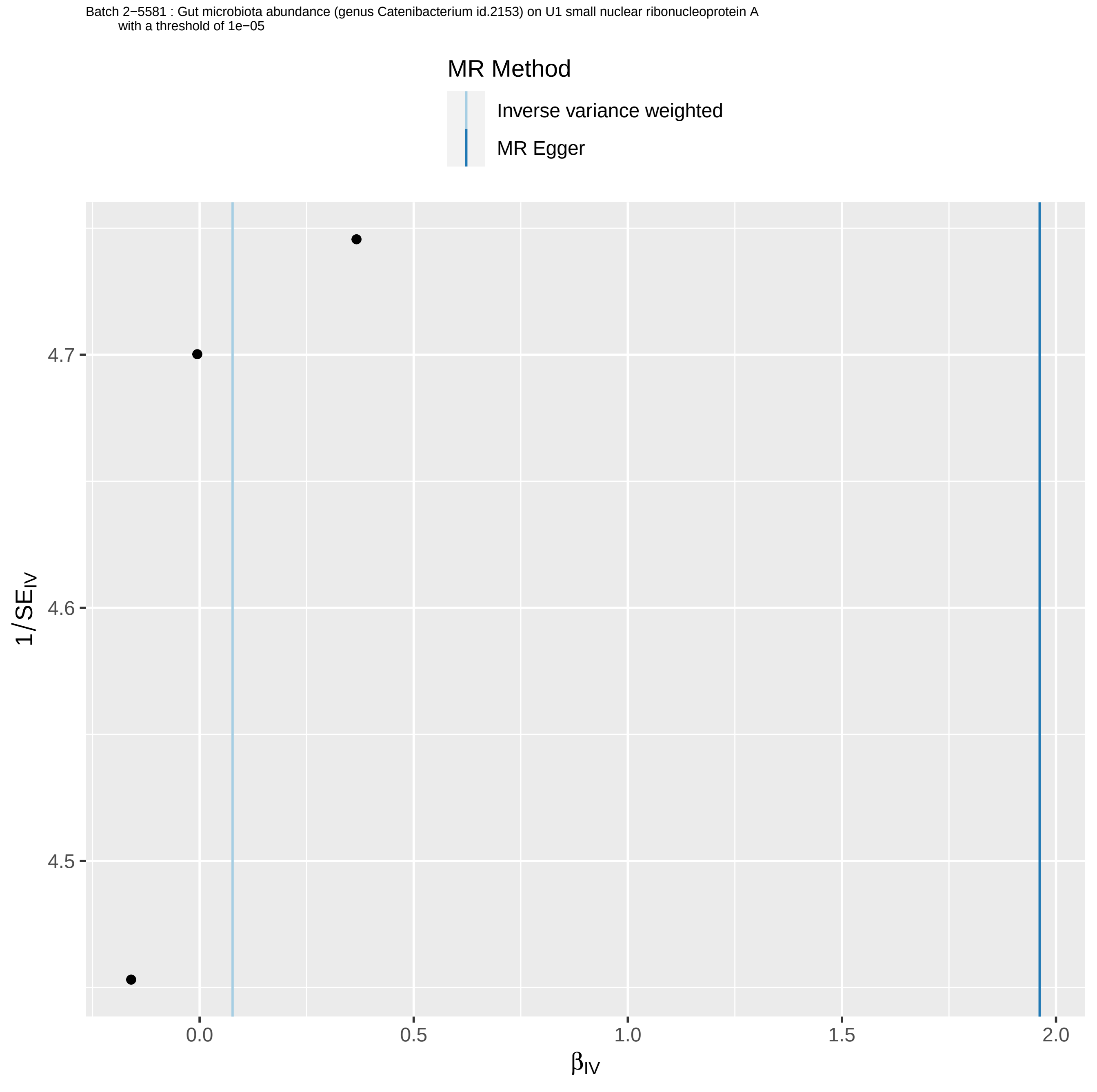
Batch 2−5581 : Gut microbiota abundance (genus Catenibacterium id.2153) on U1 small nuclear ribonucleoprotein A
with a threshold of 1e−05
MR Method
Inverse variance weighted
MR Egger
4.7
4.6
4.5
1/SE_IV
0.0
0.5
1.0
1.5
2.0
β_IV

Batch 2−5582 : Gut microbiota abundance (genus Parasutterella id.2892) on U1 small nuclear ribonucleoprotein A
with a threshold of 1e−05

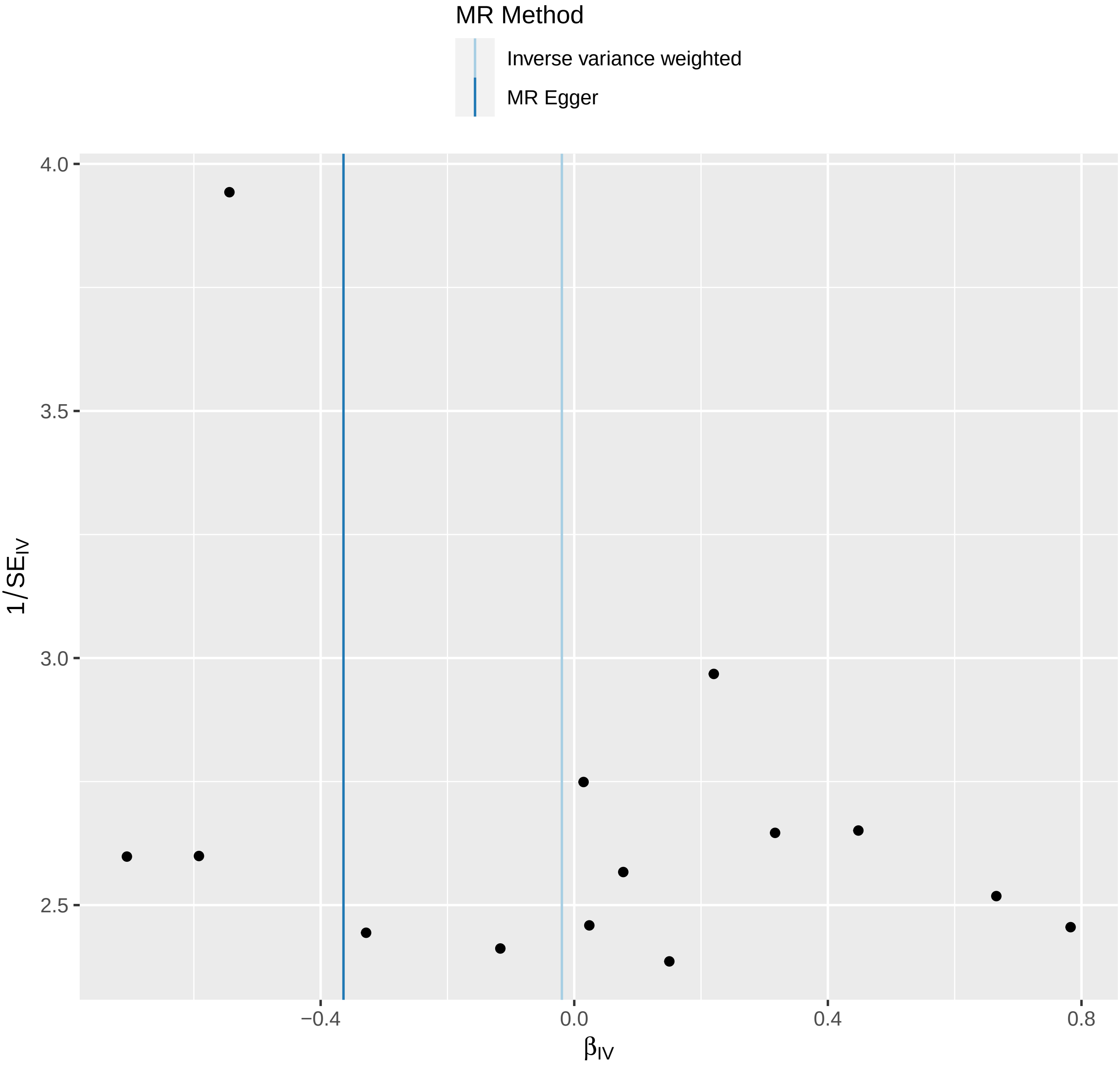

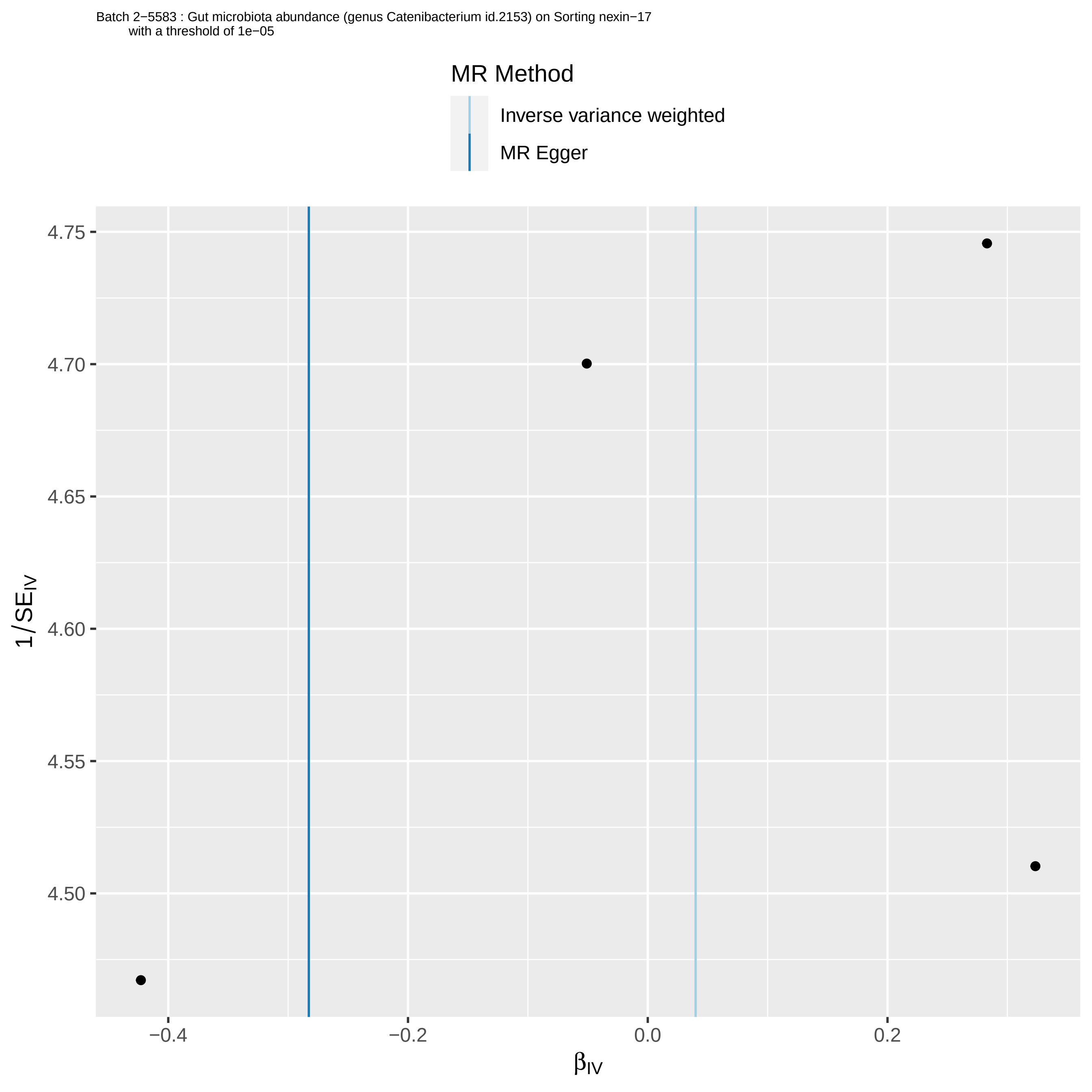
Batch 2−5583 : Gut microbiota abundance (genus Catenibacterium id.2153) on Sorting nexin−17
with a threshold of 1e−05
MR Method
Inverse variance weighted
MR Egger
4.75
4.70
4.65
4.60
4.55
4.50
1/SE_IV
−0.4
−0.2
0.0
0.2
β_IV

Batch 2−5584 : Gut microbiota abundance (genus Parasutterella id.2892) on Sorting nexin−17
with a threshold of 1e−05

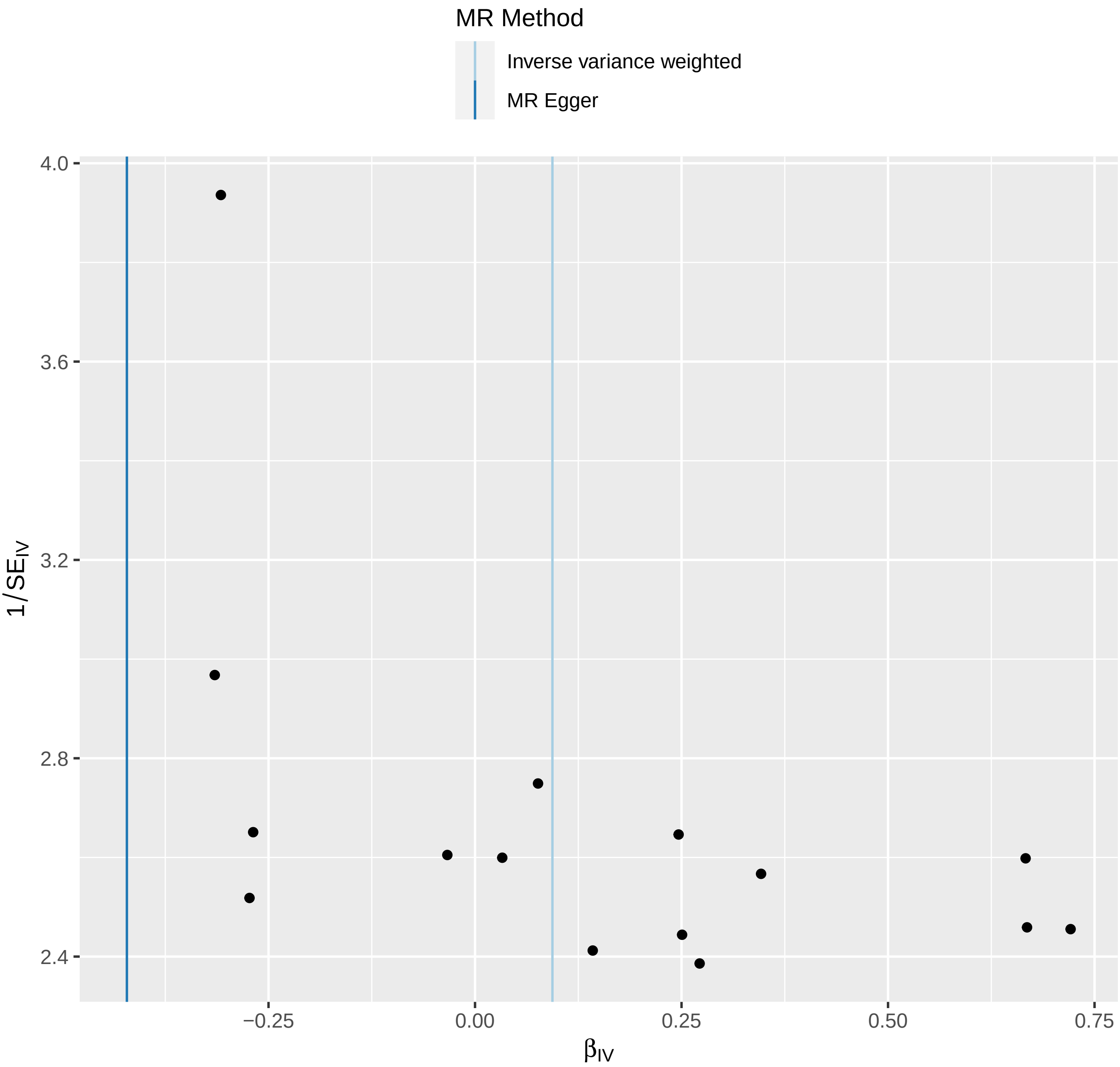

Batch 2−5585 : Gut microbiota abundance (genus Catenibacterium id.2153) on Sorting nexin−1
with a threshold of 1e−05

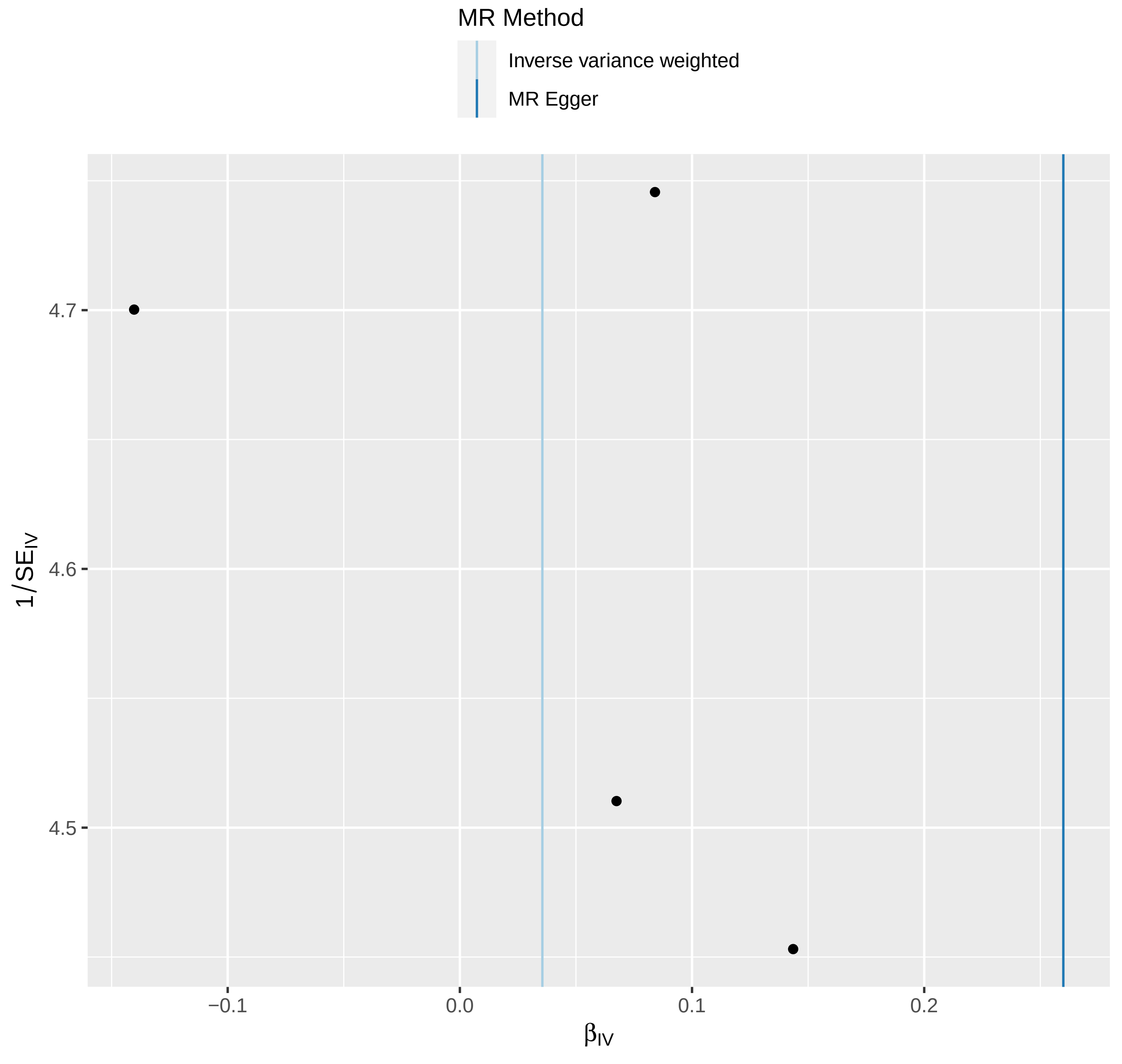

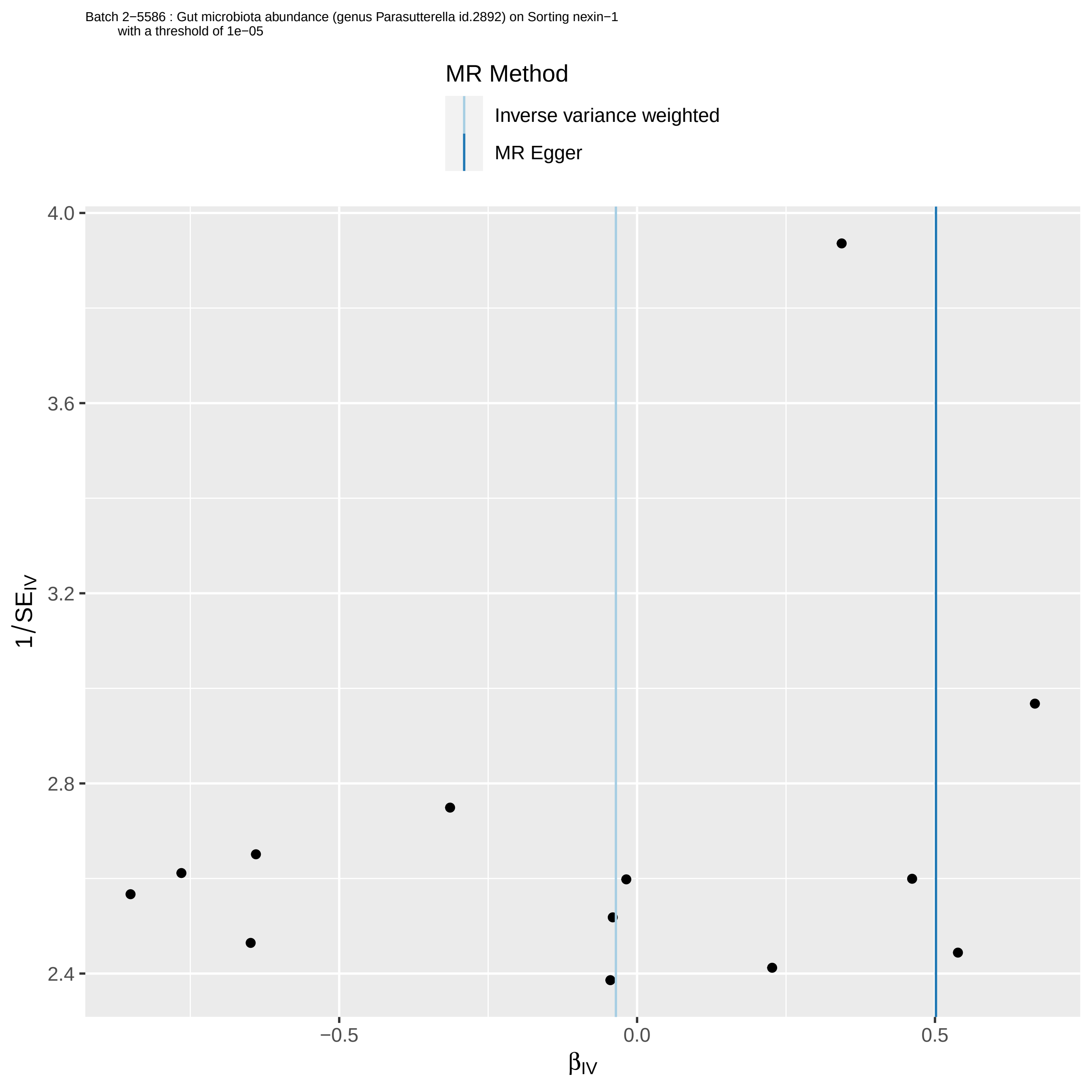

Batch 2−5586 : Gut microbiota abundance (genus Parasutterella id.2892) on Sorting nexin−1
with a threshold of 1e−05
MR Method
Inverse variance weighted
MR Egger
4.0
3.6
3.2
2.8
2.4
1/SE_IV
−0.5
0.0
0.5
β_IV

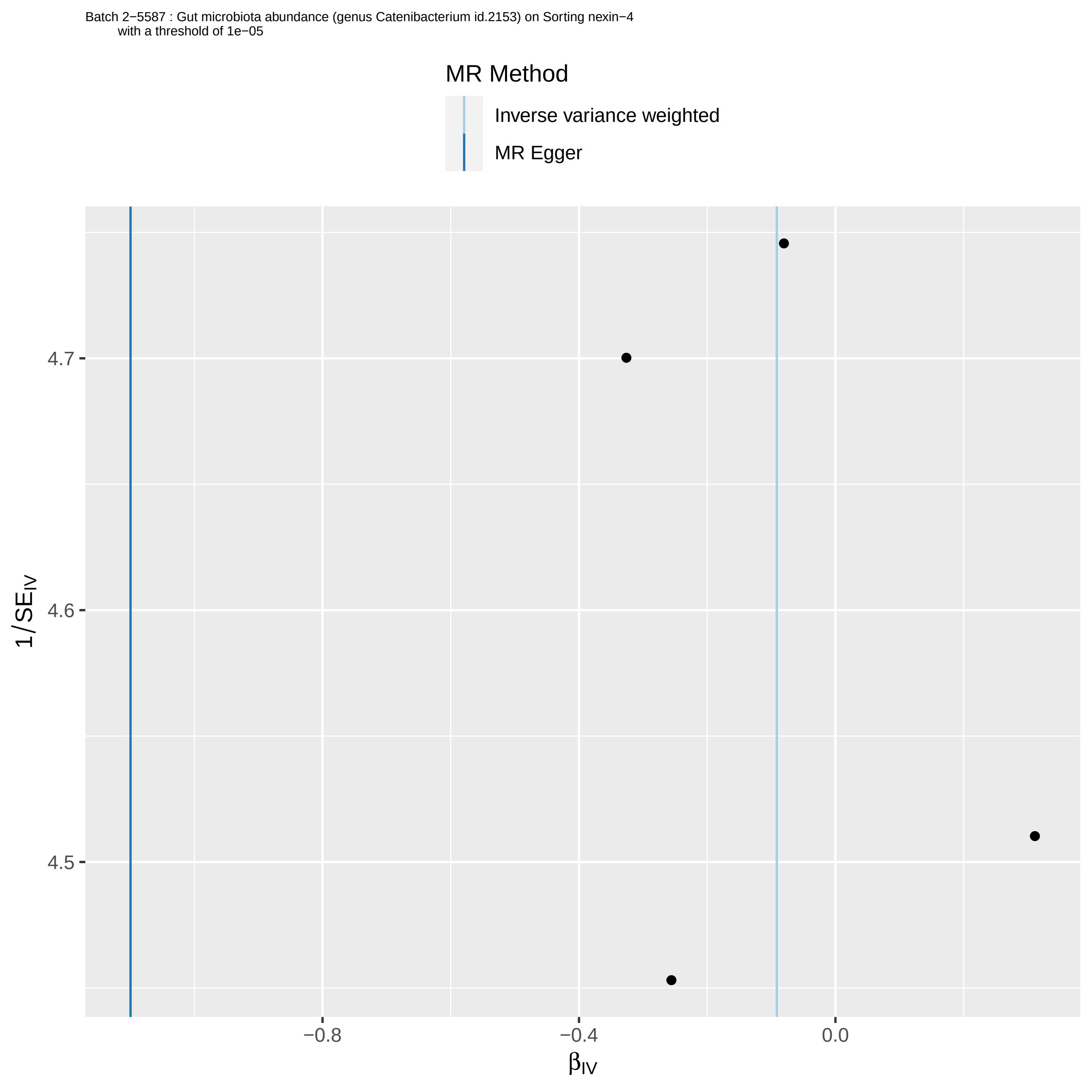

Batch 2−5587 : Gut microbiota abundance (genus Catenibacterium id.2153) on Sorting nexin−4
with a threshold of 1e−05
MR Method
Inverse variance weighted
MR Egger
4.7
4.6
4.5
1/SE_IV
−0.8
−0.4
0.0
β_IV

Batch 2−5588 : Gut microbiota abundance (genus Parasutterella id.2892) on Sorting nexin−4
with a threshold of 1e−05

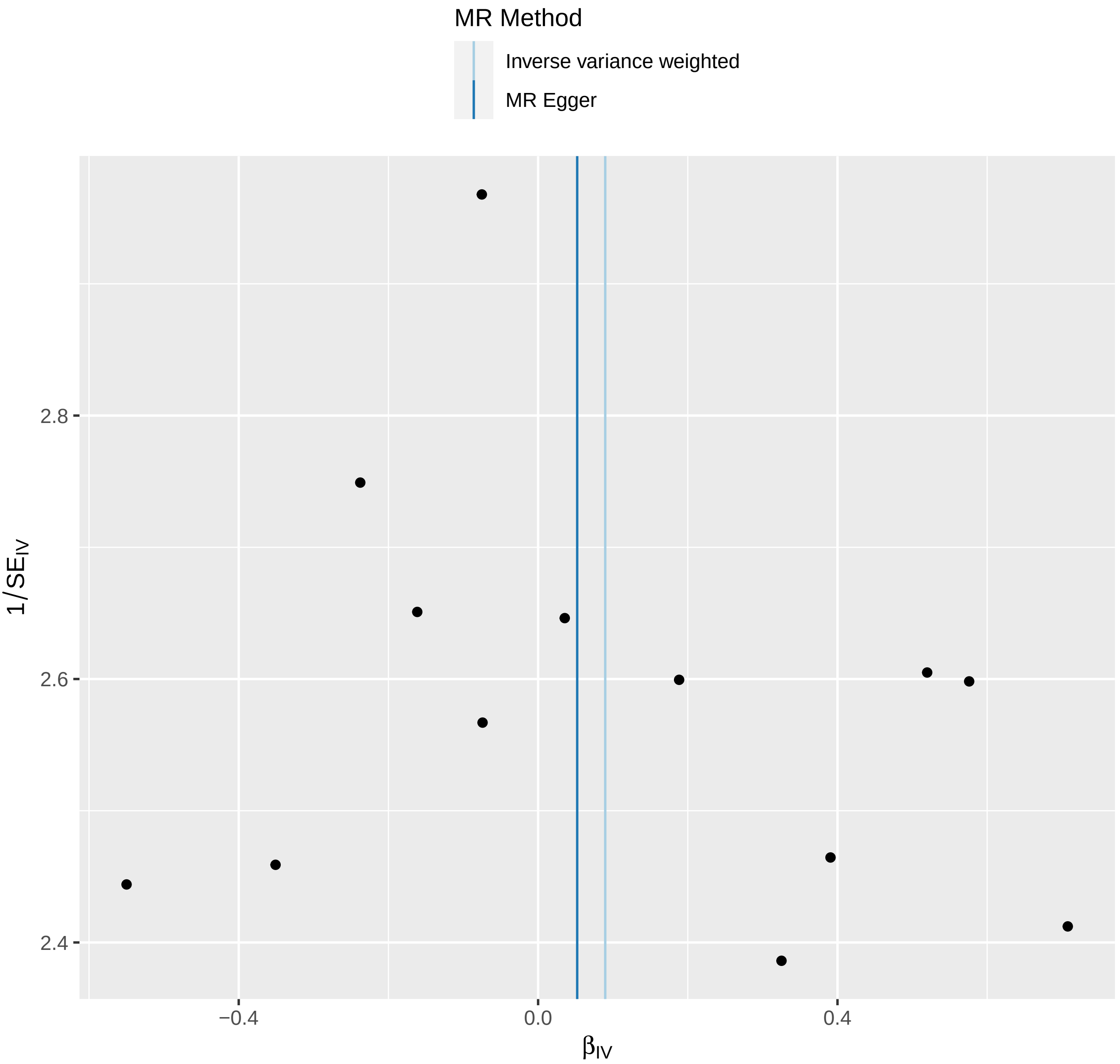

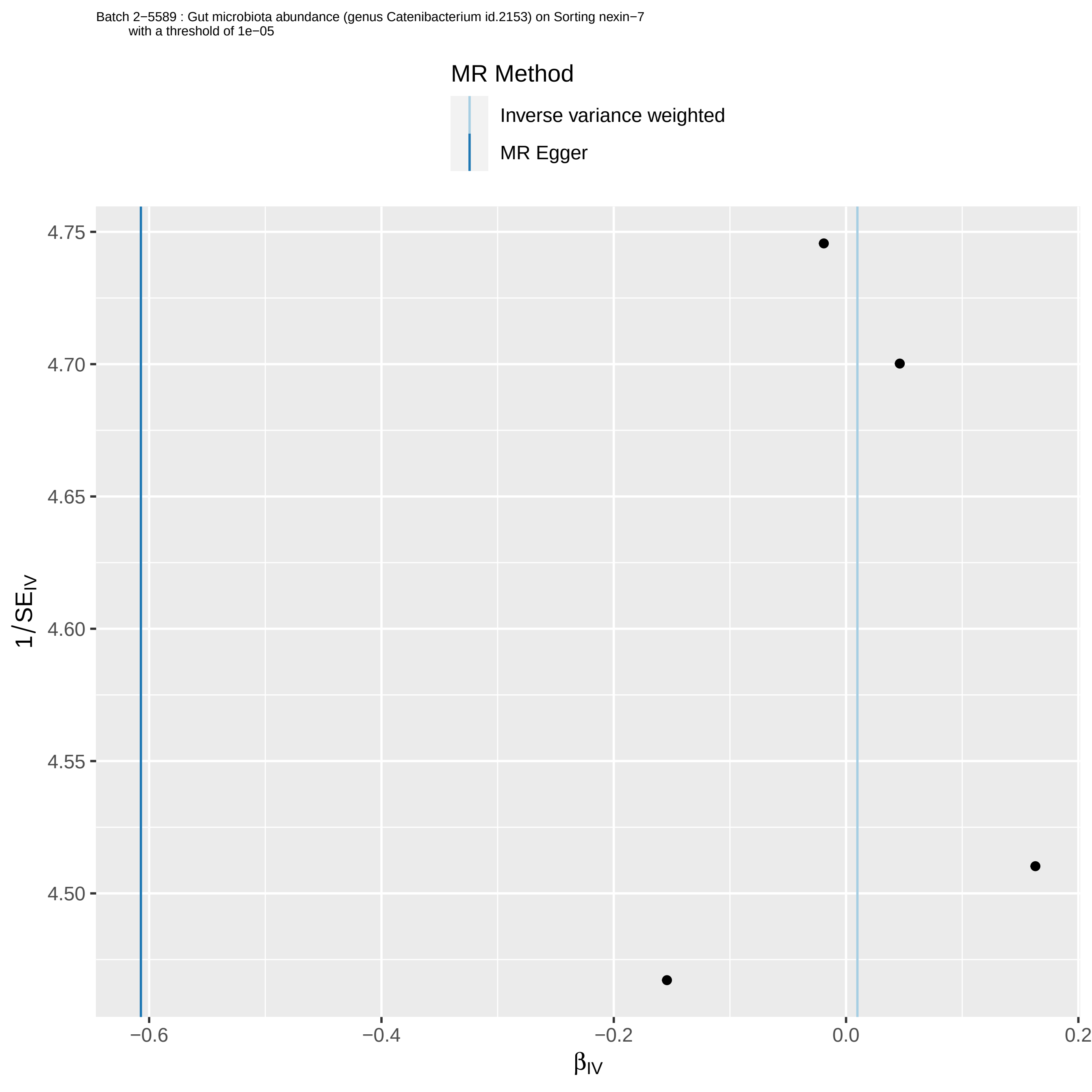
Batch 2−5589 : Gut microbiota abundance (genus Catenibacterium id.2153) on Sorting nexin−7
with a threshold of 1e−05
MR Method
Inverse variance weighted
MR Egger
4.75
4.70
4.65
4.60
4.55
4.50
1/SE_IV
−0.6
−0.4
−0.2
0.0
0.2
β_IV

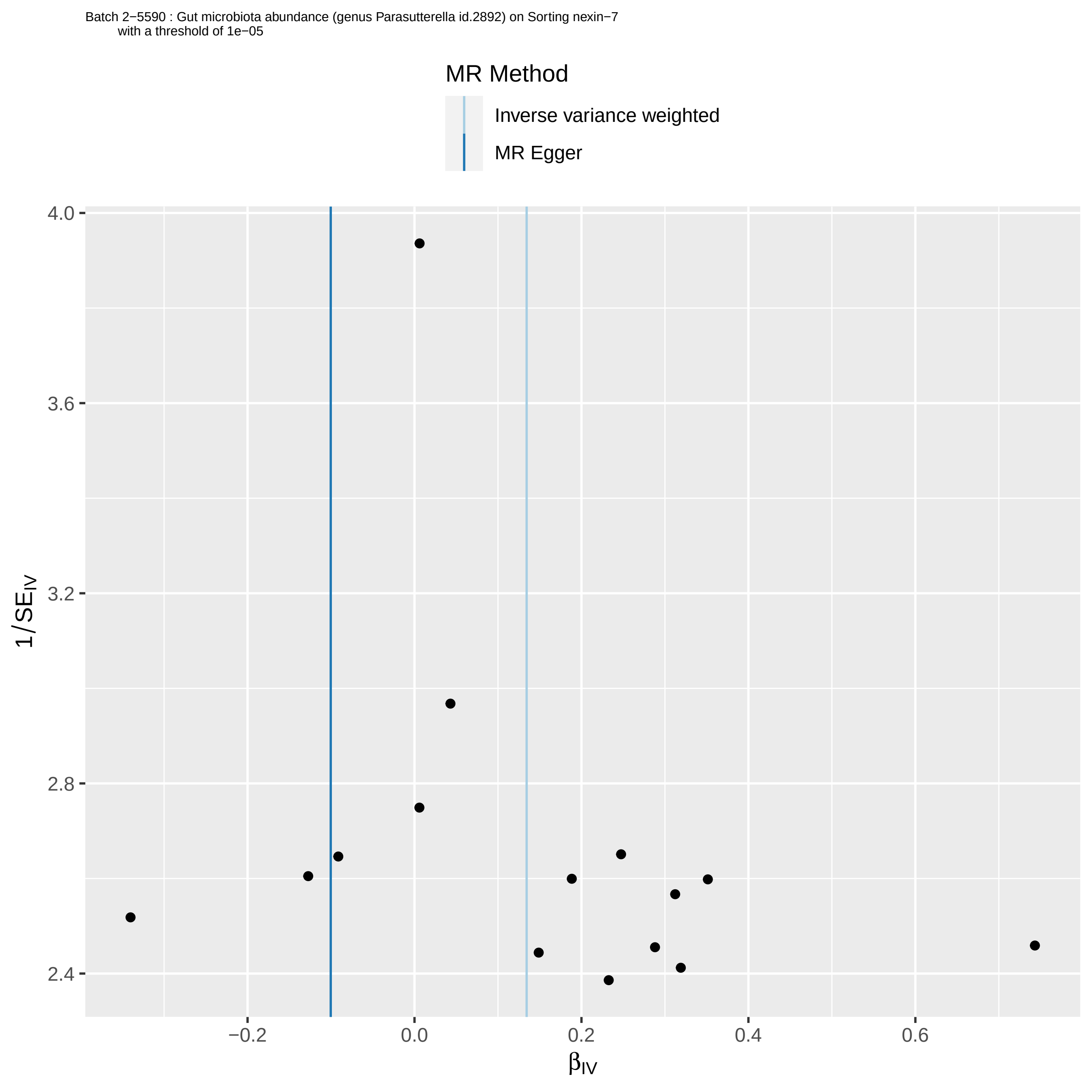

Batch 2−5590 : Gut microbiota abundance (genus Parasutterella id.2892) on Sorting nexin−7
with a threshold of 1e−05
MR Method
Inverse variance weighted
MR Egger
4.0
3.6
3.2
2.8
2.4
1/SE_IV
−0.2
0.0
0.2
0.4
0.6
β_IV

Batch 2−5591 : Gut microbiota abundance (genus Catenibacterium id.2153) on Sorting nexin−8
with a threshold of 1e−05

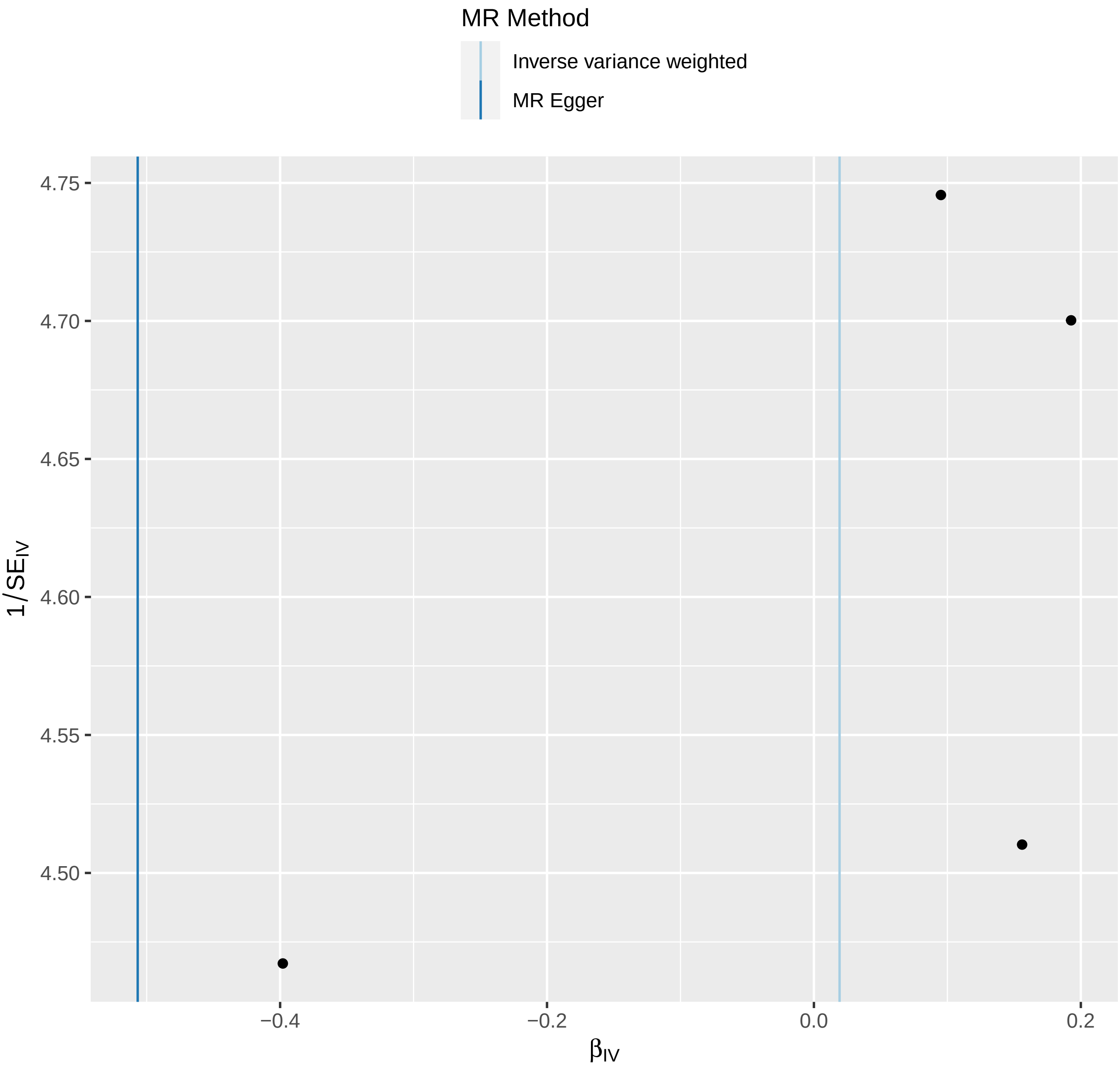

Batch 2−5592 : Gut microbiota abundance (genus Parasutterella id.2892) on Sorting nexin−8
with a threshold of 1e−05

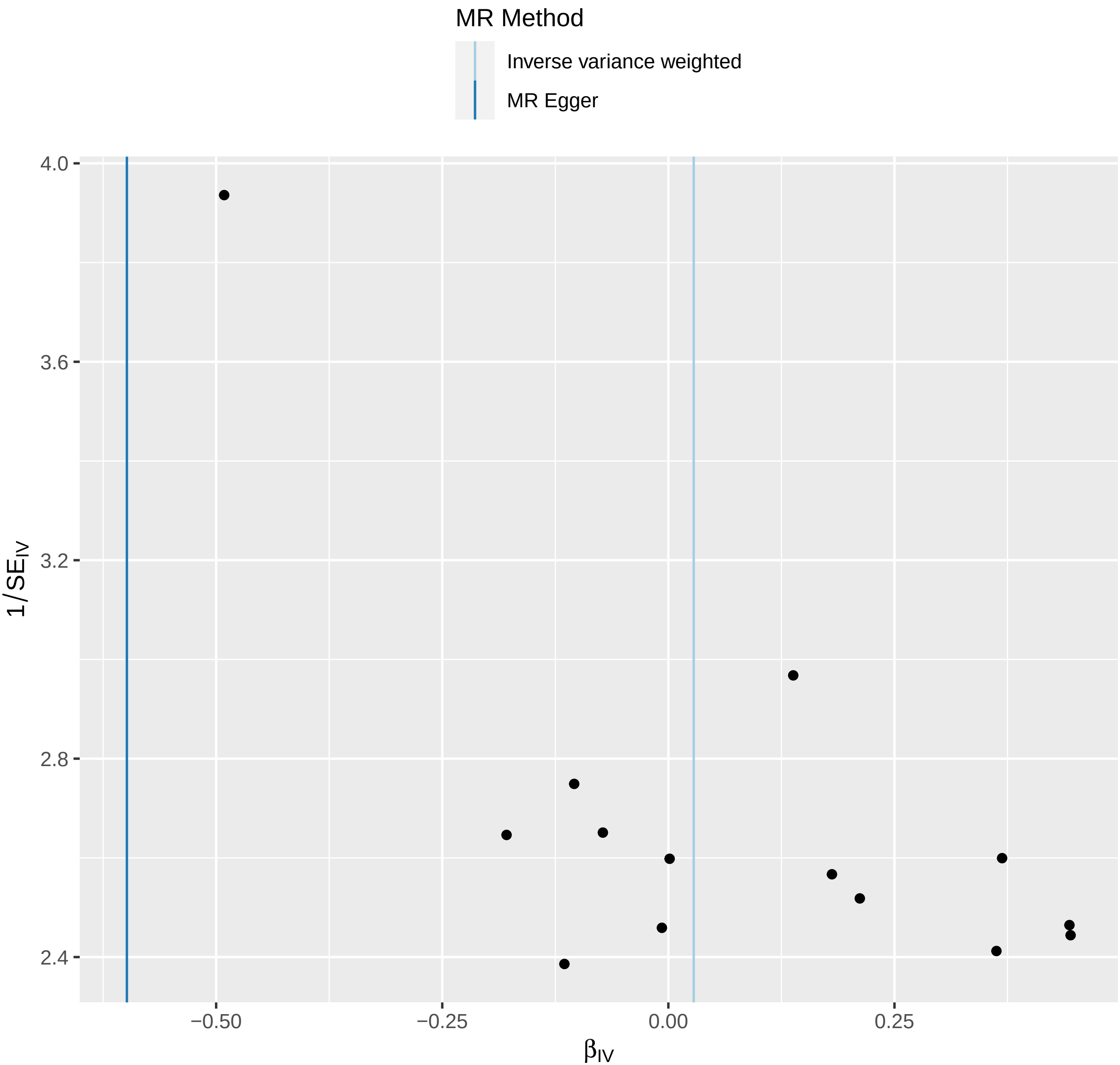

Batch 2−5593 : Gut microbiota abundance (genus Catenibacterium id.2153) on Suppressor of cytokine signaling 3
with a threshold of 1e−05

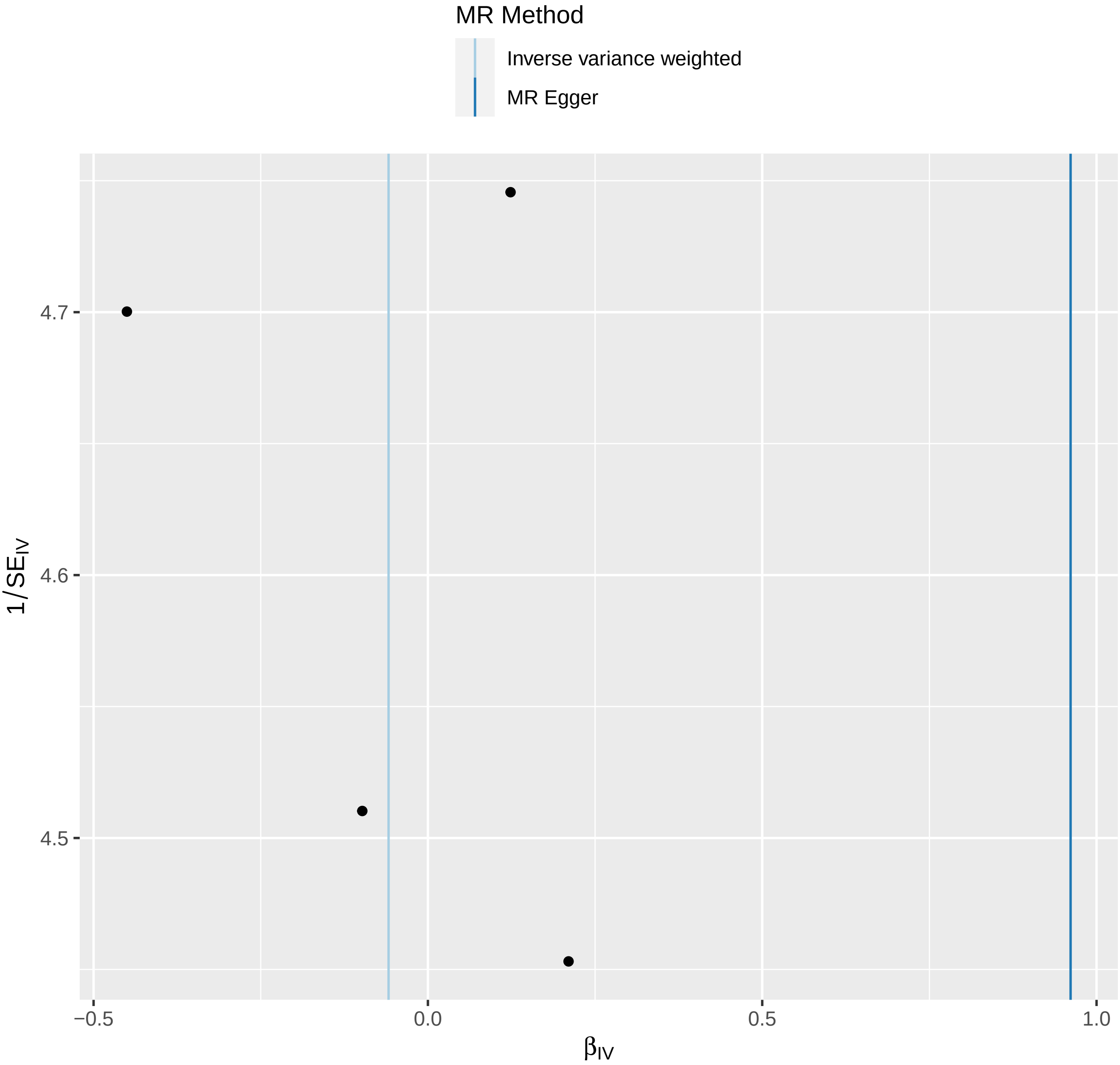

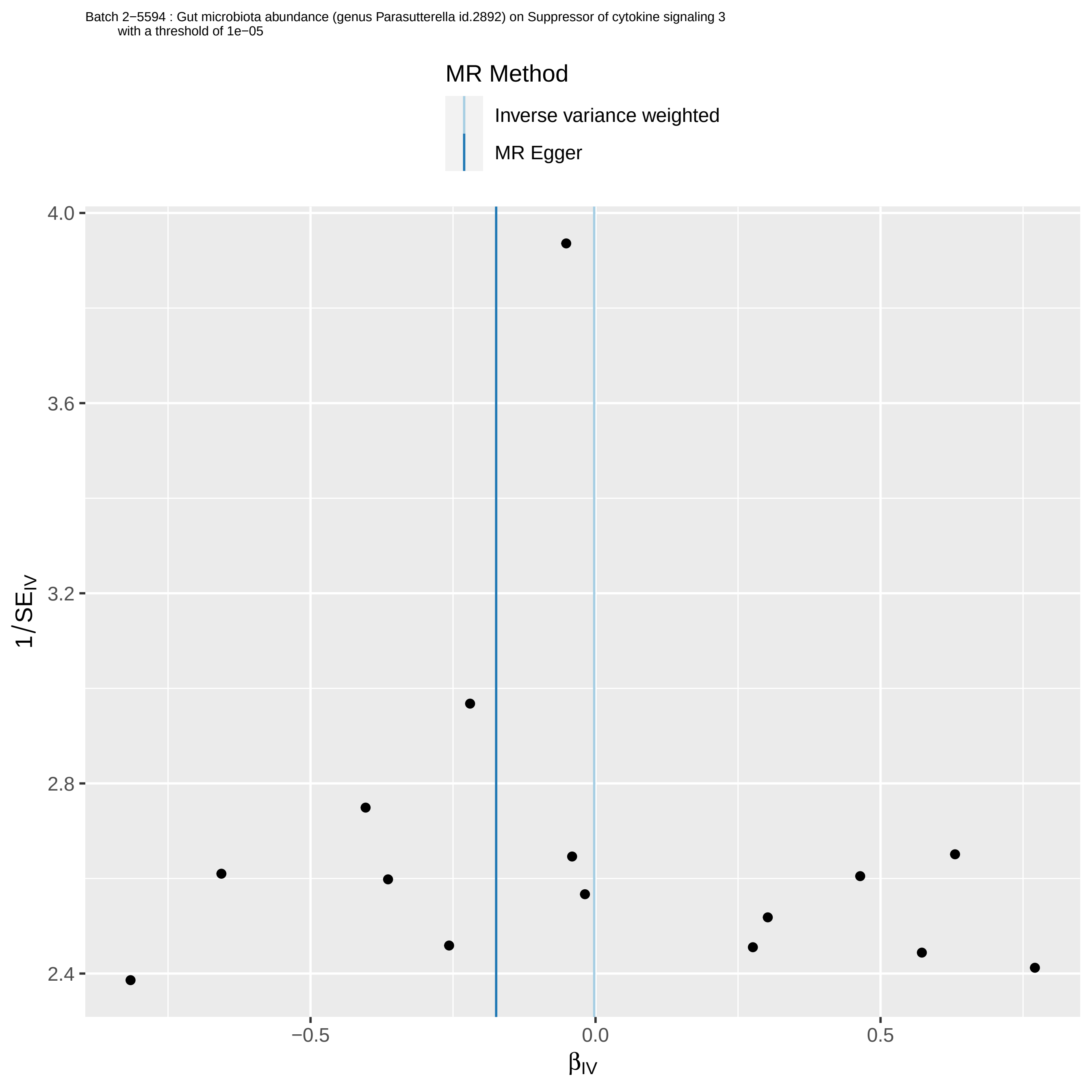

Batch 2−5594 : Gut microbiota abundance (genus Parasutterella id.2892) on Suppressor of cytokine signaling 3
with a threshold of 1e−05
MR Method
Inverse variance weighted
MR Egger
4.0
3.6
3.2
2.8
2.4
1/SE$_{IV}$
−0.5
0.0
0.5
β_{IV}

Batch 2−5595 : Gut microbiota abundance (genus Catenibacterium id.2153) on Suppressor of cytokine signaling 7 with a threshold of 1e−05

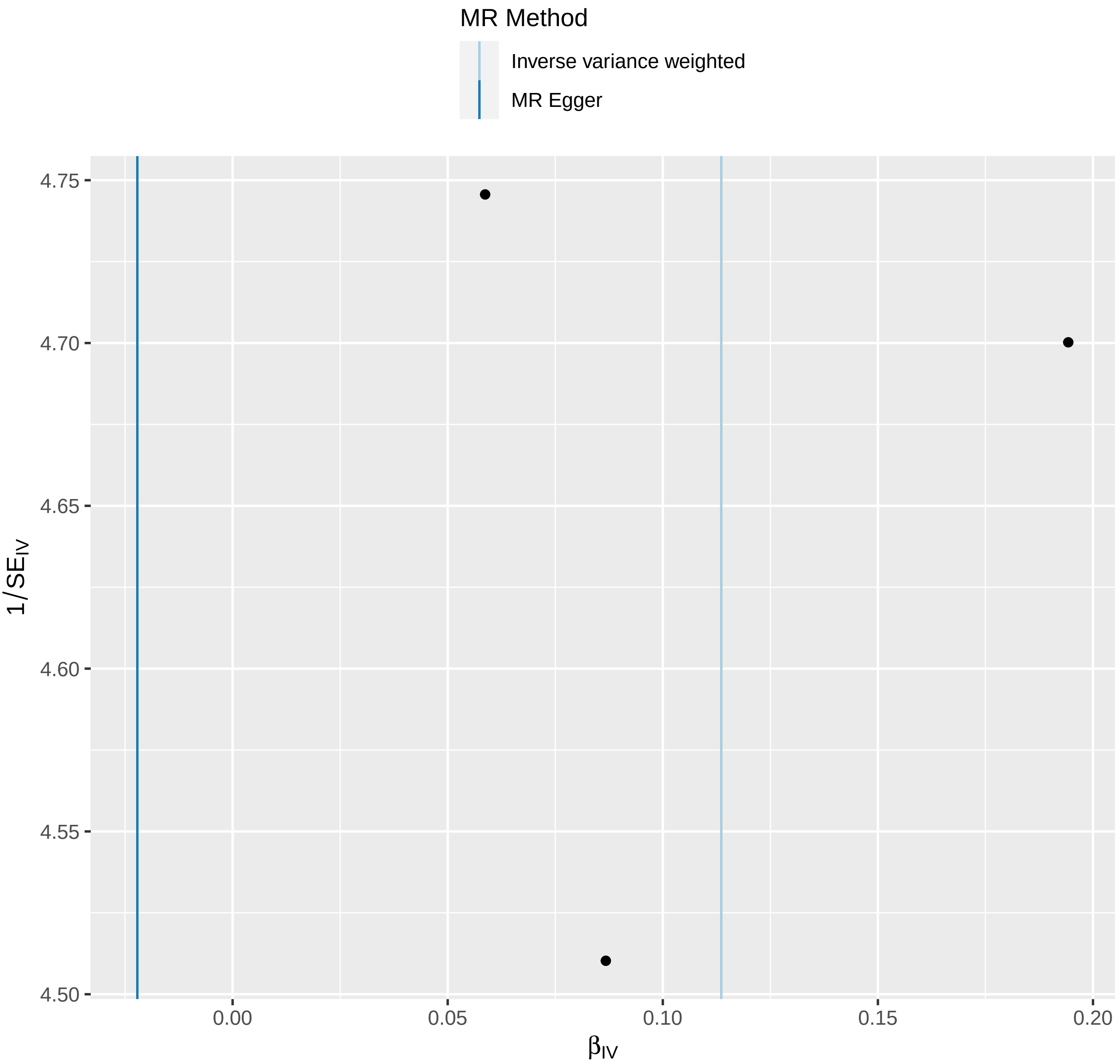

Batch 2−5596 : Gut microbiota abundance (genus Parasutterella id.2892) on Suppressor of cytokine signaling 7
with a threshold of 1e−05

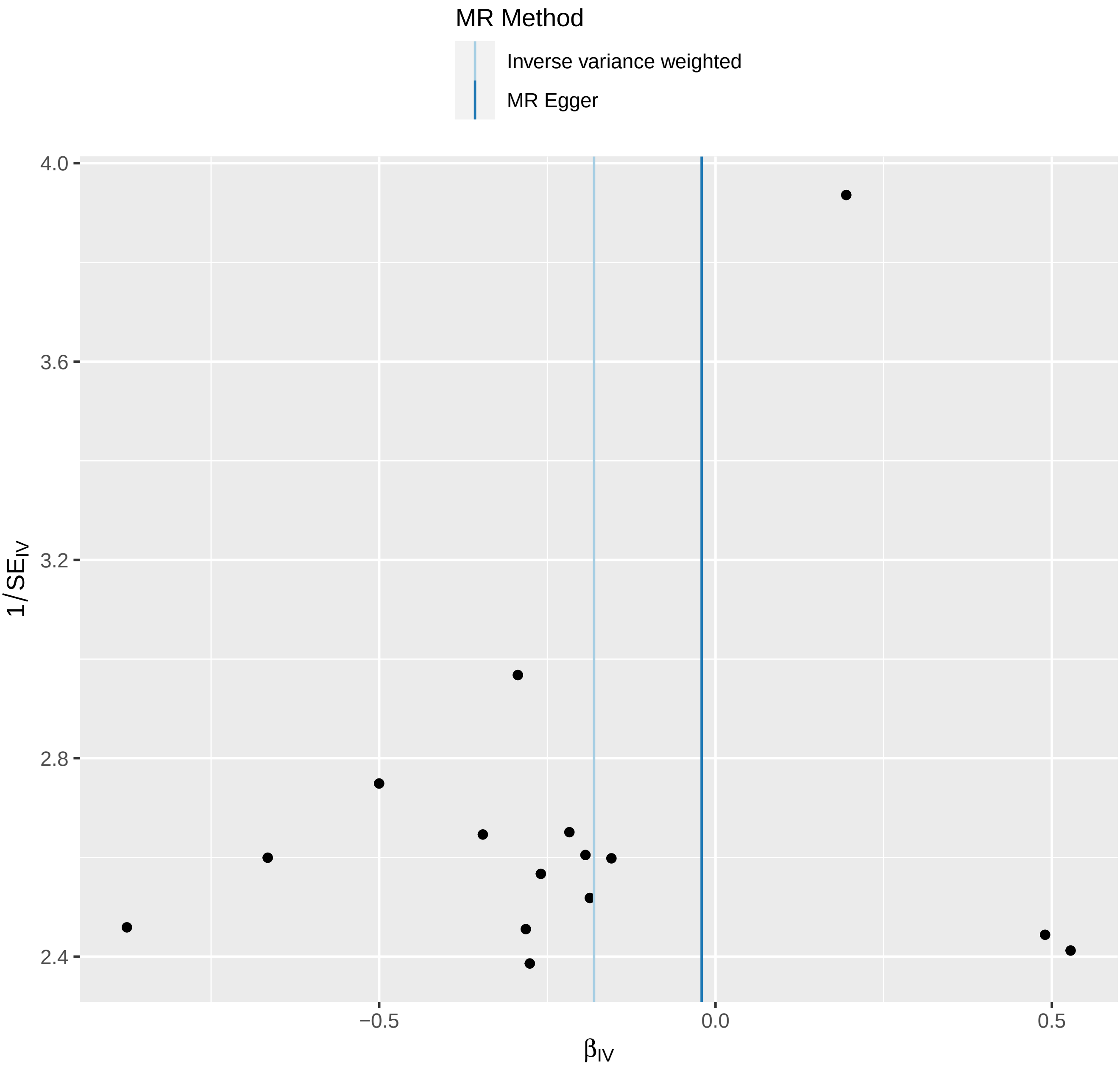

Batch 2−5597 : Gut microbiota abundance (genus Catenibacterium id.2153) on Superoxide dismutase [Mn], mitochondrial
with a threshold of 1e−05

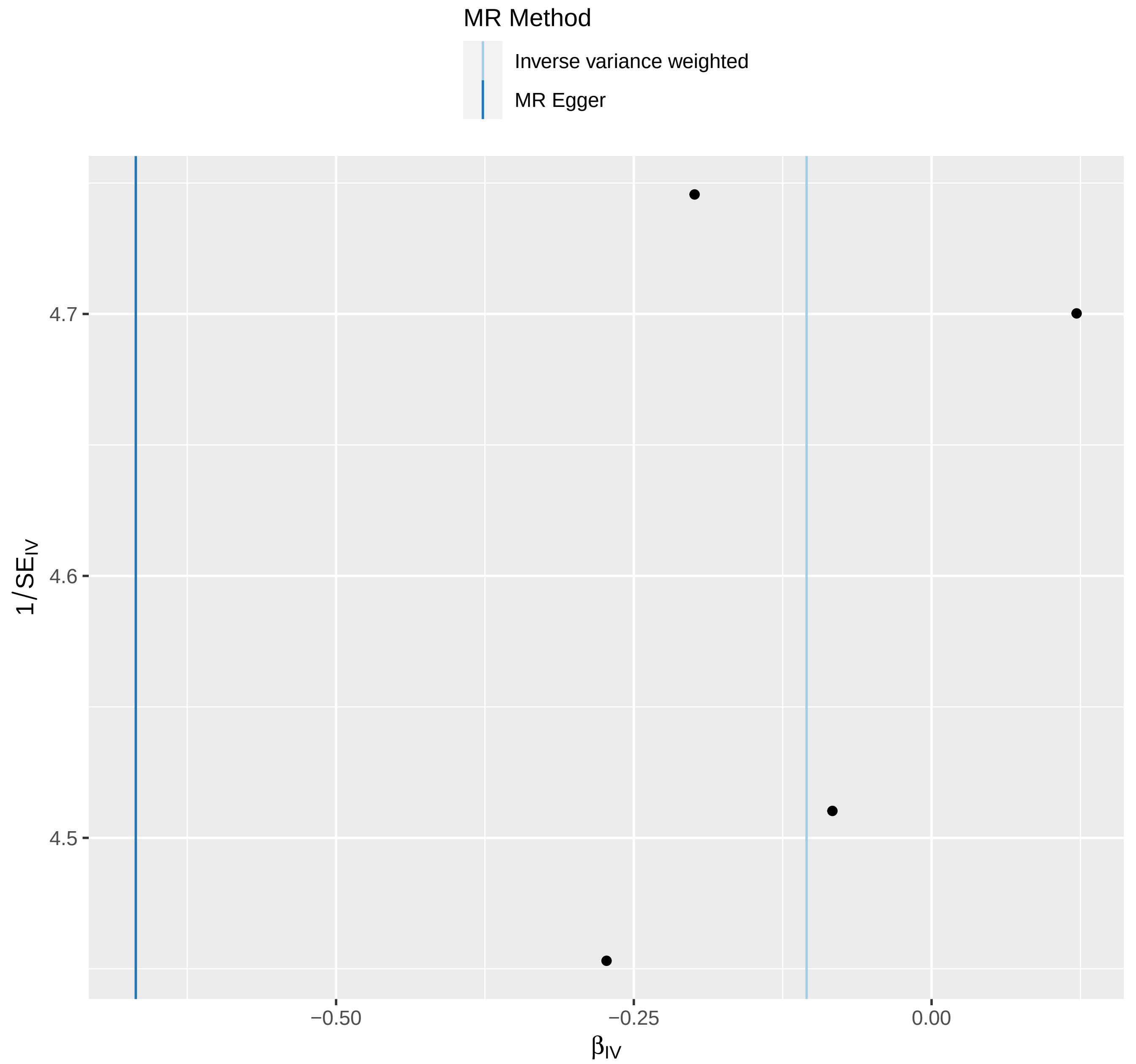

Batch 2−5598 : Gut microbiota abundance (genus Parasutterella id.2892) on Superoxide dismutase [Mn], mitochondrial
with a threshold of 1e−05

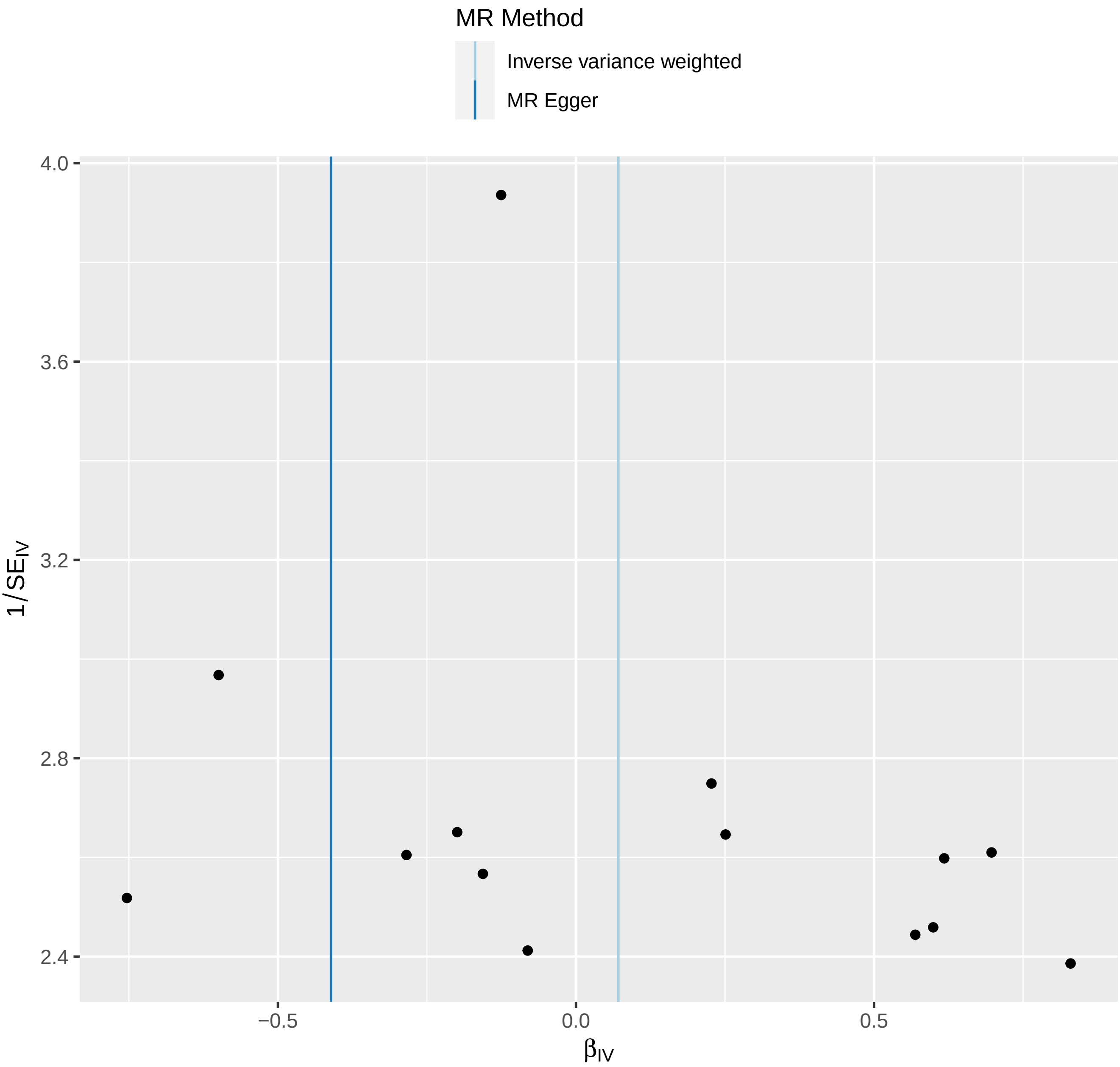

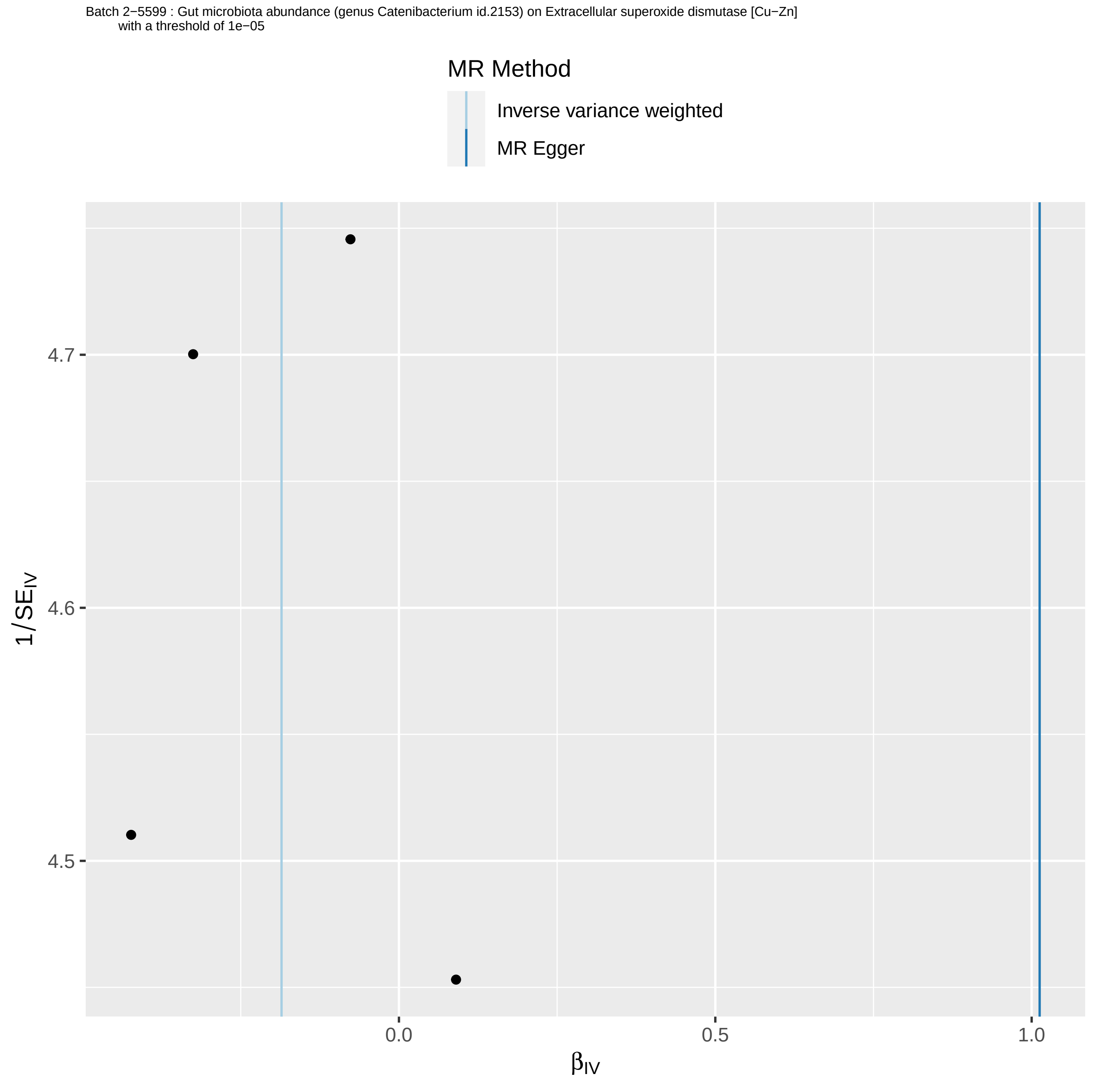
Batch 2−5599 : Gut microbiota abundance (genus Catenibacterium id.2153) on Extracellular superoxide dismutase [Cu−Zn] with a threshold of 1e−05
MR Method
Inverse variance weighted
MR Egger
4.7
4.6
4.5
1/SE$_{IV}$
0.0
0.5
1.0
β$_{IV}$

Batch 2−5600 : Gut microbiota abundance (genus Parasutterella id.2892) on Extracellular superoxide dismutase [Cu−Zn]
with a threshold of 1e−05

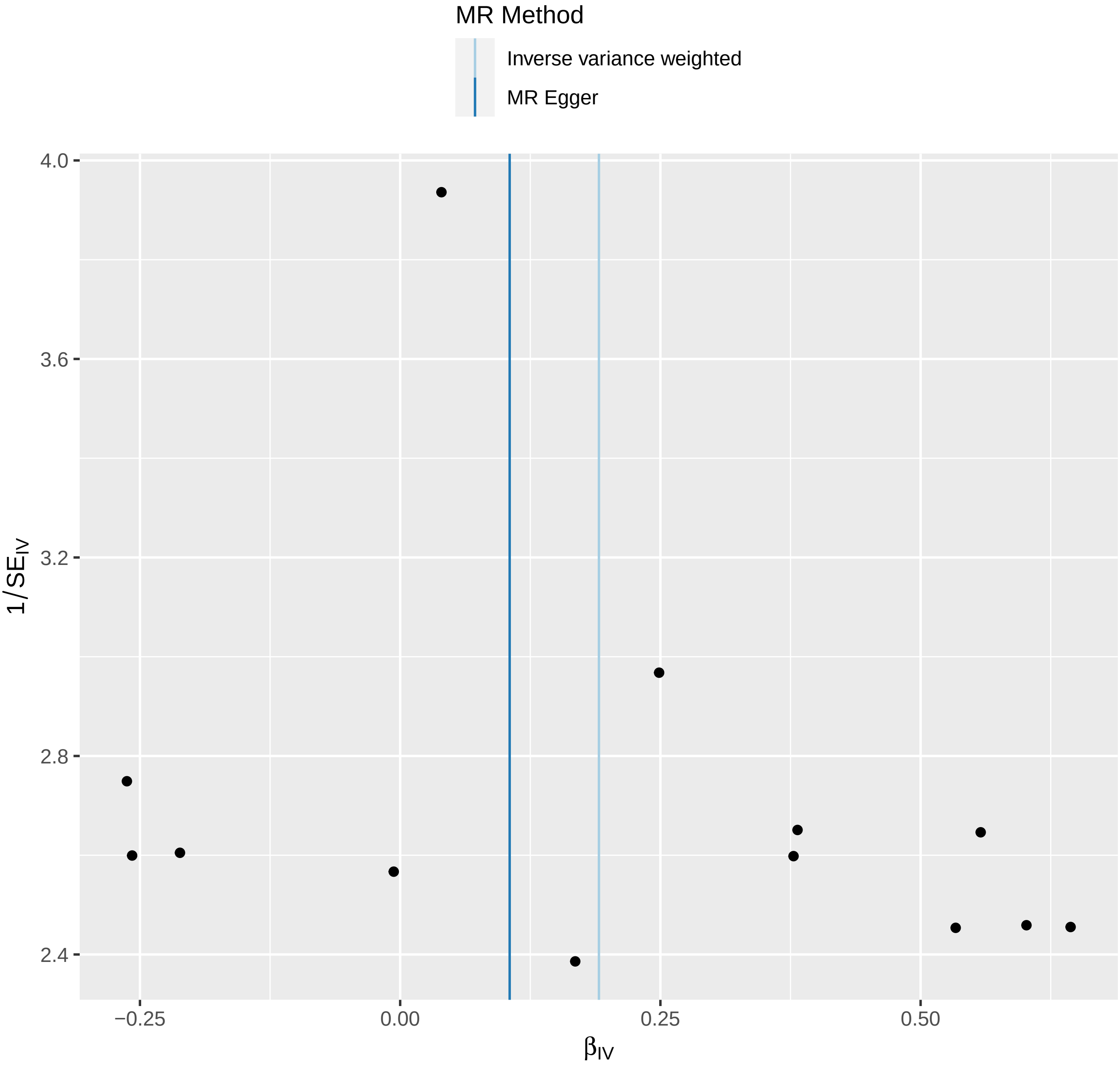

Batch 2−5601 : Gut microbiota abundance (genus Catenibacterium id.2153) on Vinexin b
with a threshold of 1e−05

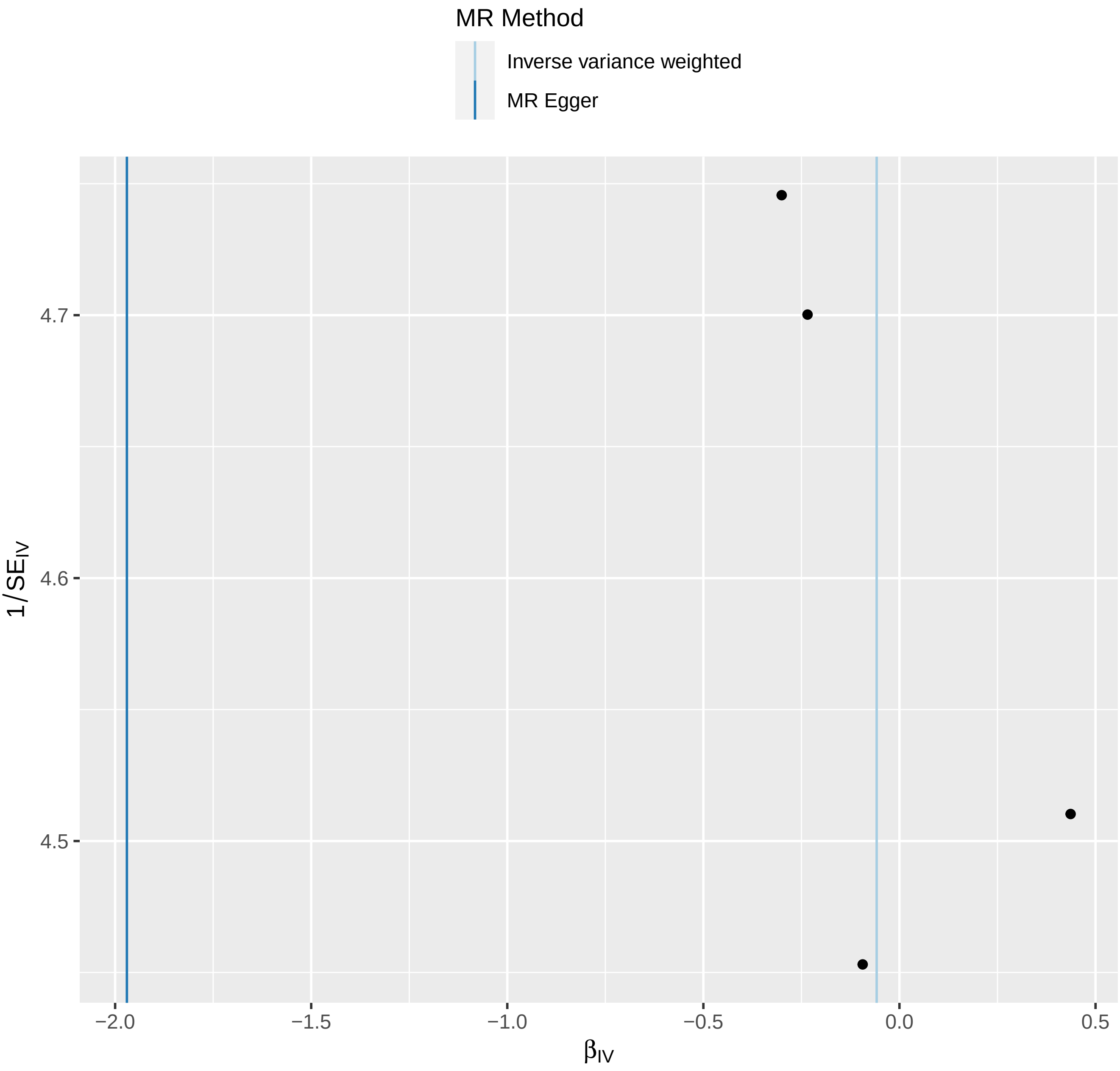

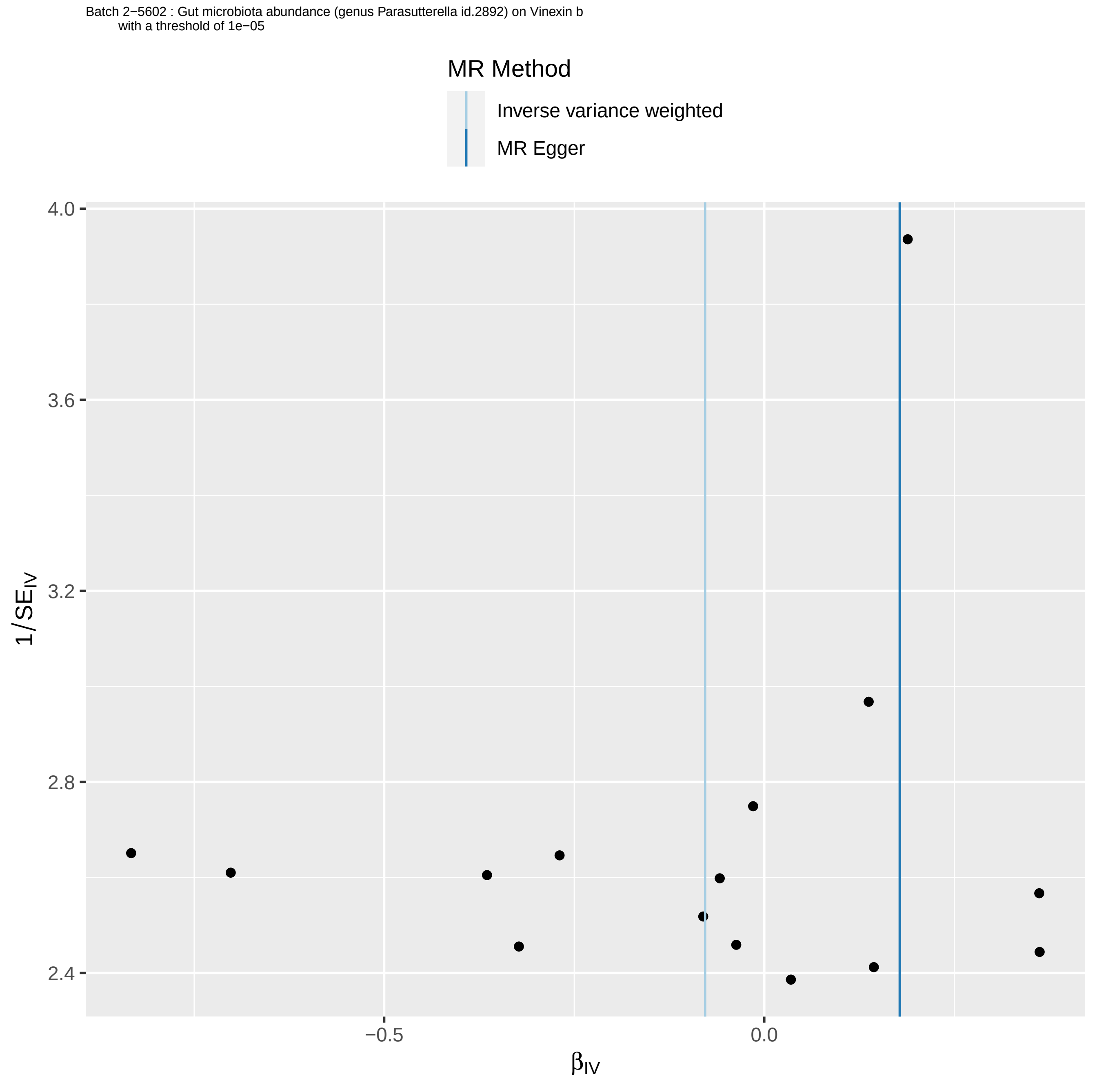
Batch 2−5602 : Gut microbiota abundance (genus Parasutterella id.2892) on Vinexin b
with a threshold of 1e−05
MR Method
Inverse variance weighted
MR Egger
4.0
3.6
3.2
2.8
2.4
1/SE_IV
−0.5
0.0
β_IV

Batch 2−5603 : Gut microbiota abundance (genus Catenibacterium id.2153) on Sortilin
with a threshold of 1e−05

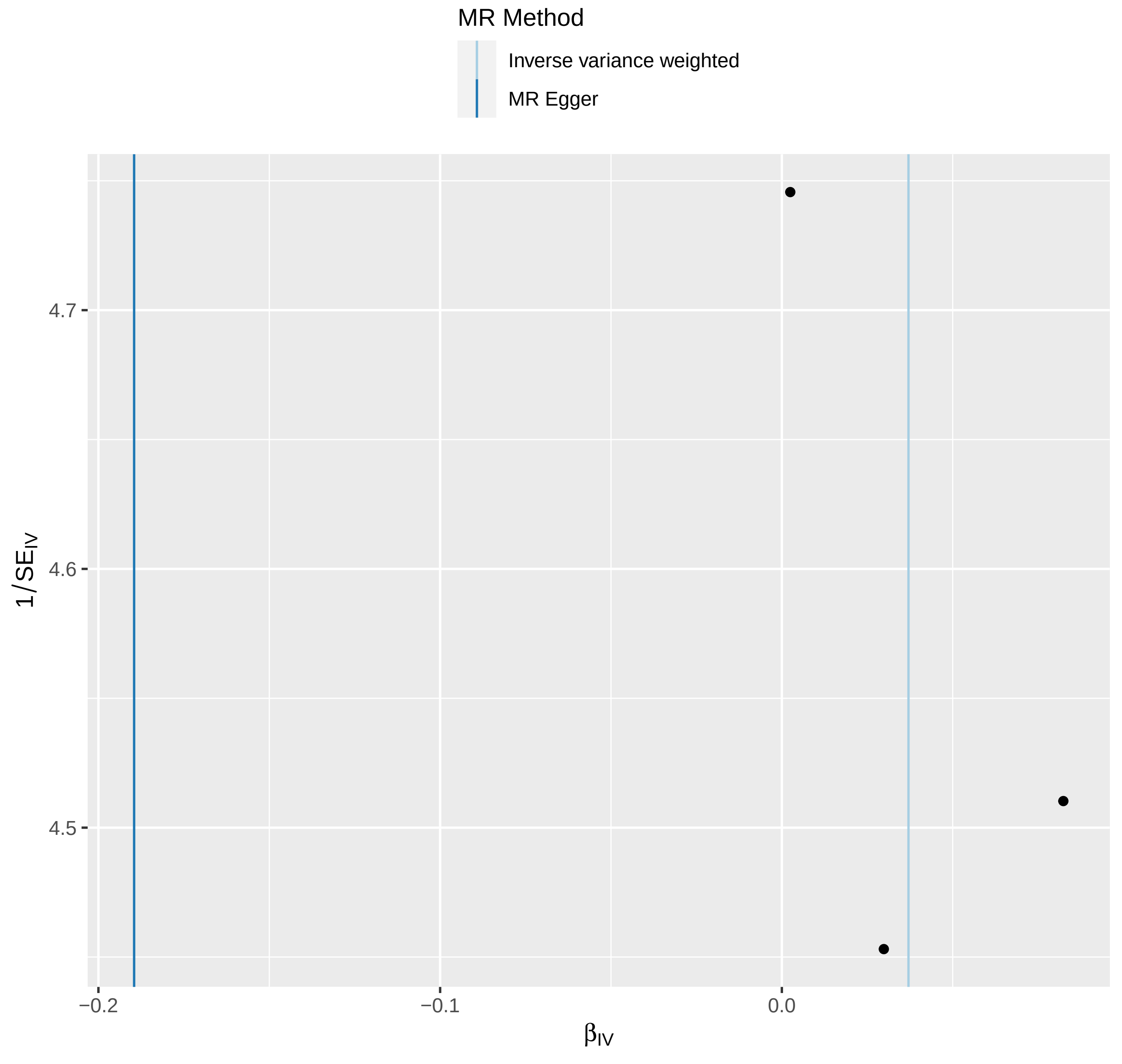

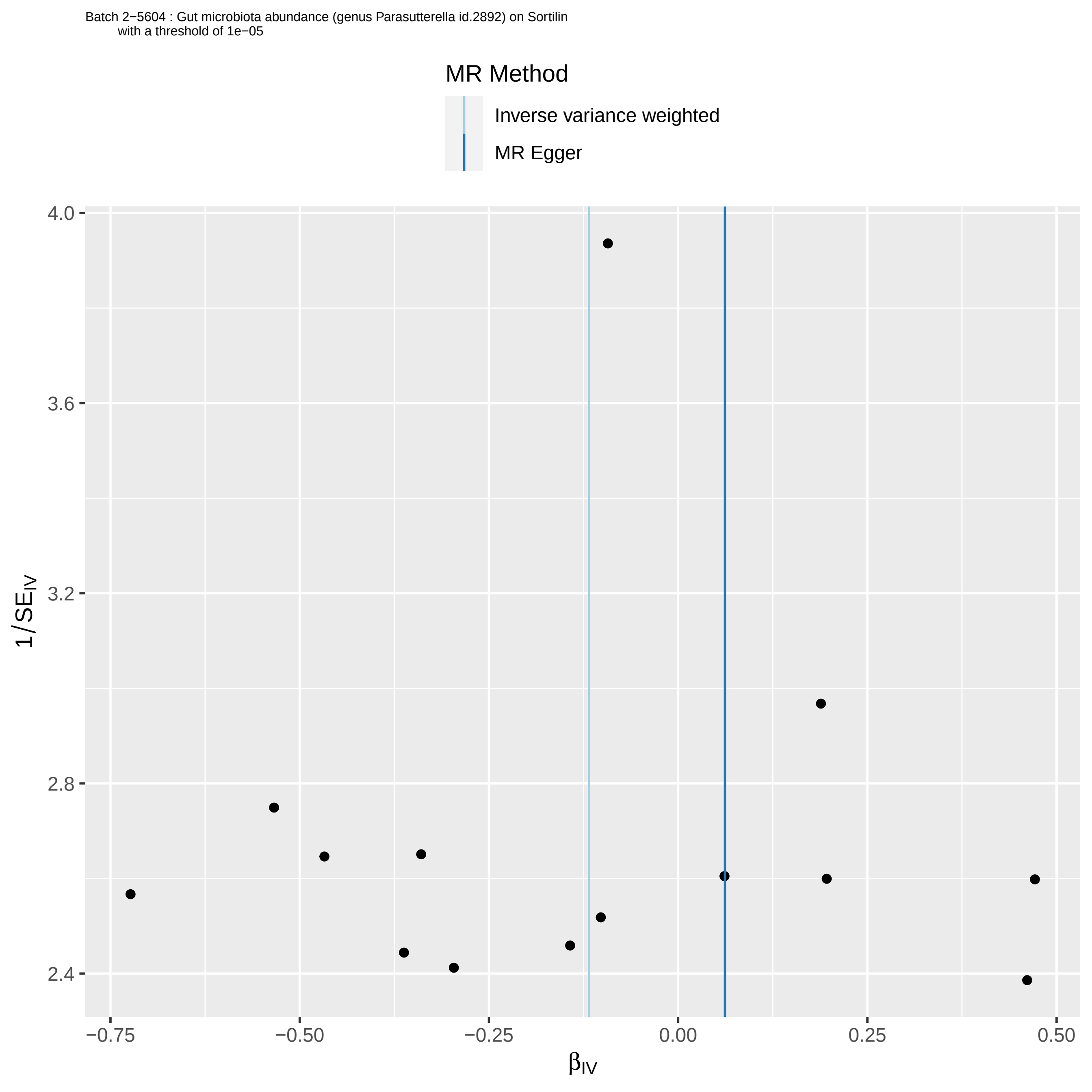
Batch 2−5604 : Gut microbiota abundance (genus Parasutterella id.2892) on Sortilin
with a threshold of 1e−05
MR Method
Inverse variance weighted
MR Egger
4.0
3.6
3.2
2.8
2.4
1/SE_IV
−0.75
−0.50
−0.25
0.00
0.25
0.50
β_IV

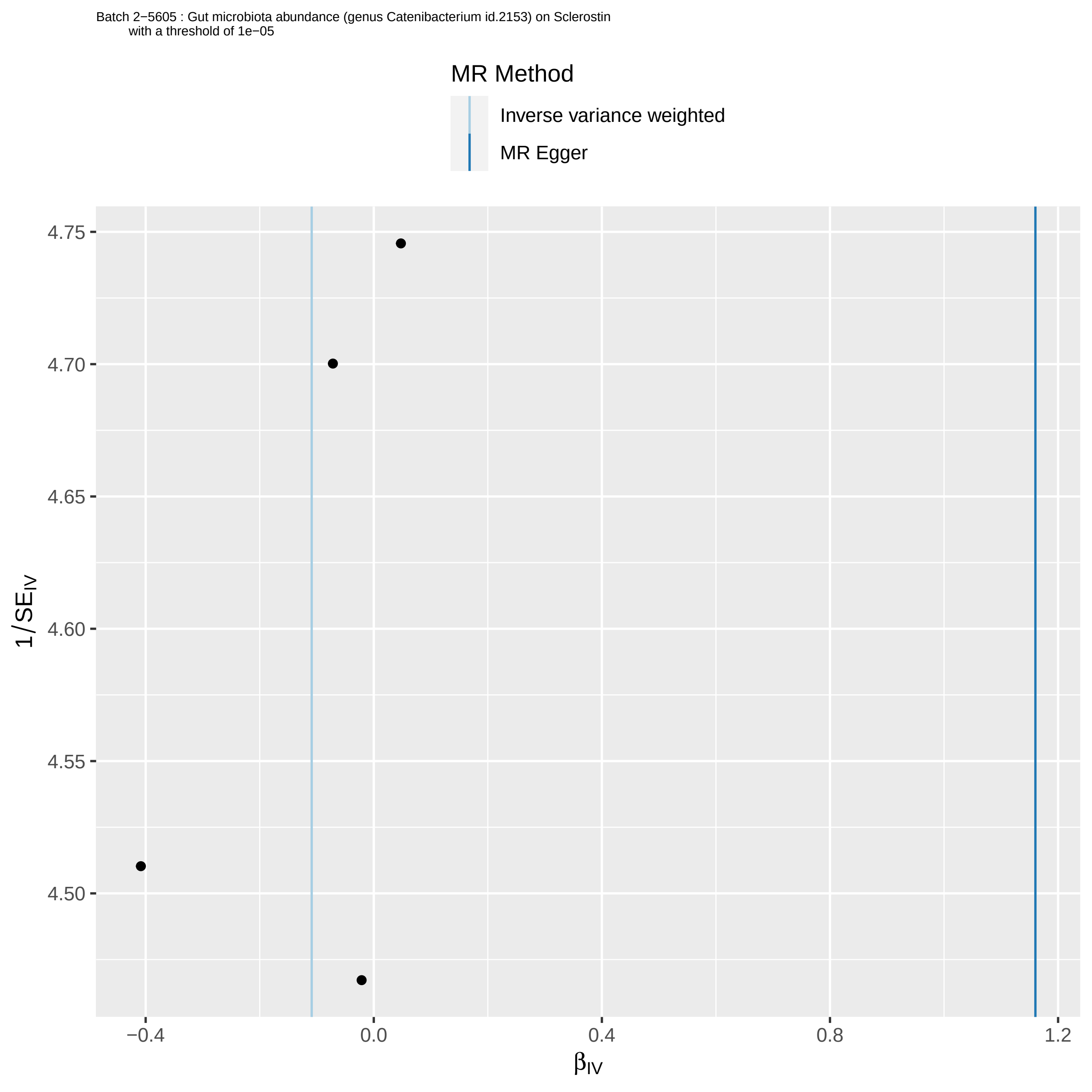
Batch 2−5605 : Gut microbiota abundance (genus Catenibacterium id.2153) on Sclerostin
with a threshold of 1e−05
MR Method
Inverse variance weighted
MR Egger
4.75
4.70
4.65
4.60
4.55
4.50
1/SE_IV
−0.4
0.0
0.4
0.8
1.2
β_IV

Batch 2−5606 : Gut microbiota abundance (genus Parasutterella id.2892) on Sclerostin
with a threshold of 1e−05

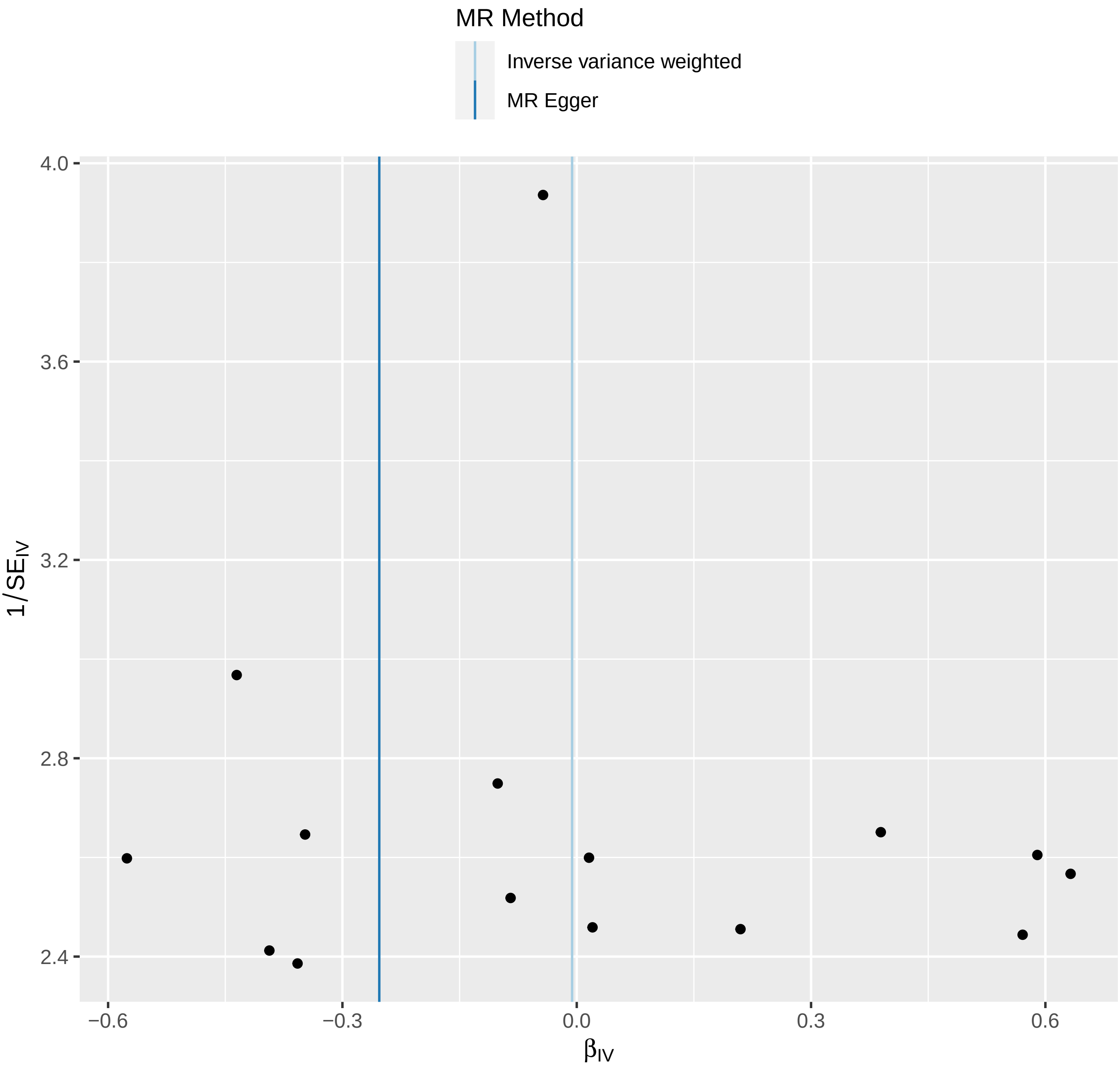

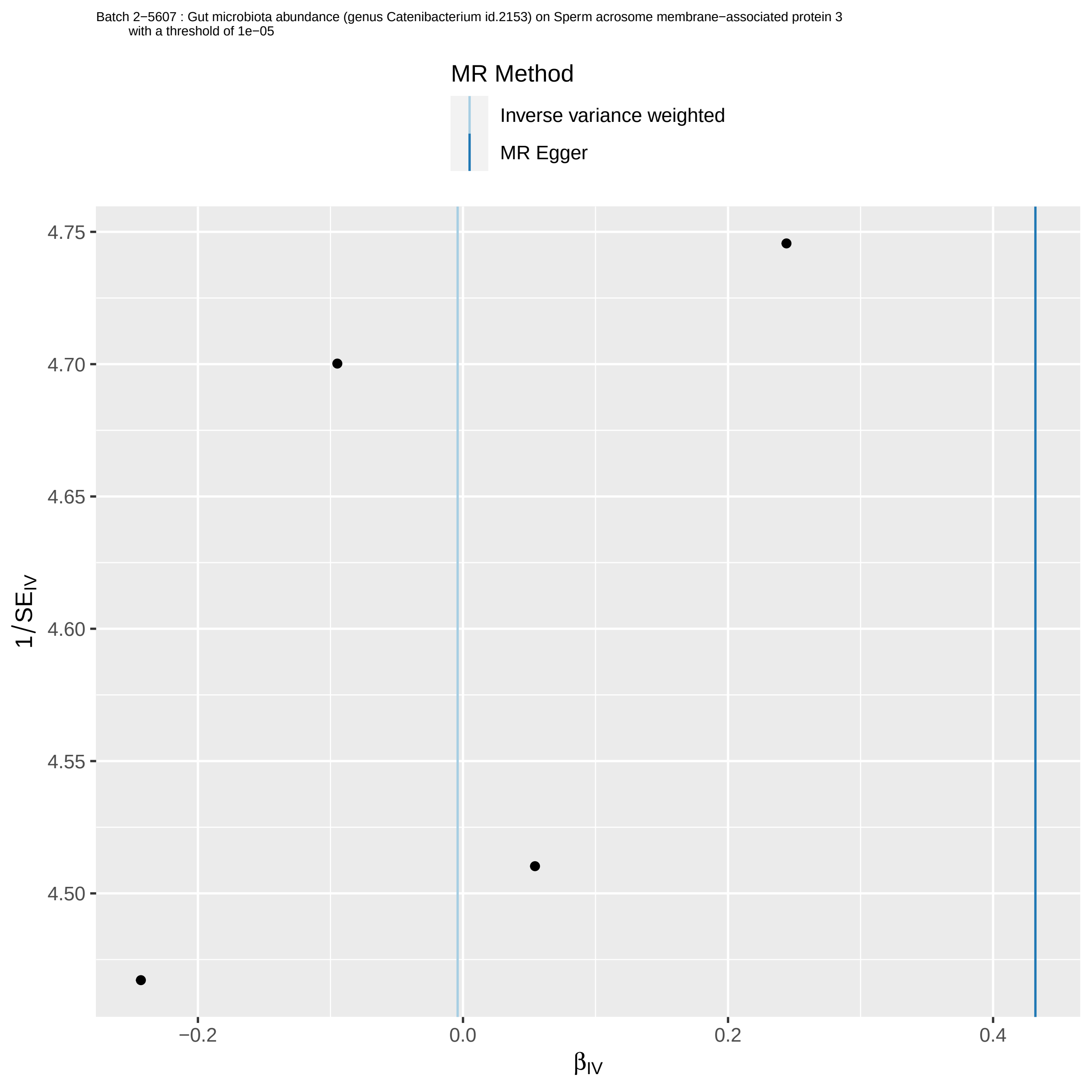

Batch 2−5607 : Gut microbiota abundance (genus Catenibacterium id.2153) on Sperm acrosome membrane−associated protein 3
with a threshold of 1e−05
MR Method
Inverse variance weighted
MR Egger
4.75
4.70
4.65
4.60
4.55
4.50
1/SE_IV
−0.2
0.0
0.2
0.4
β_IV

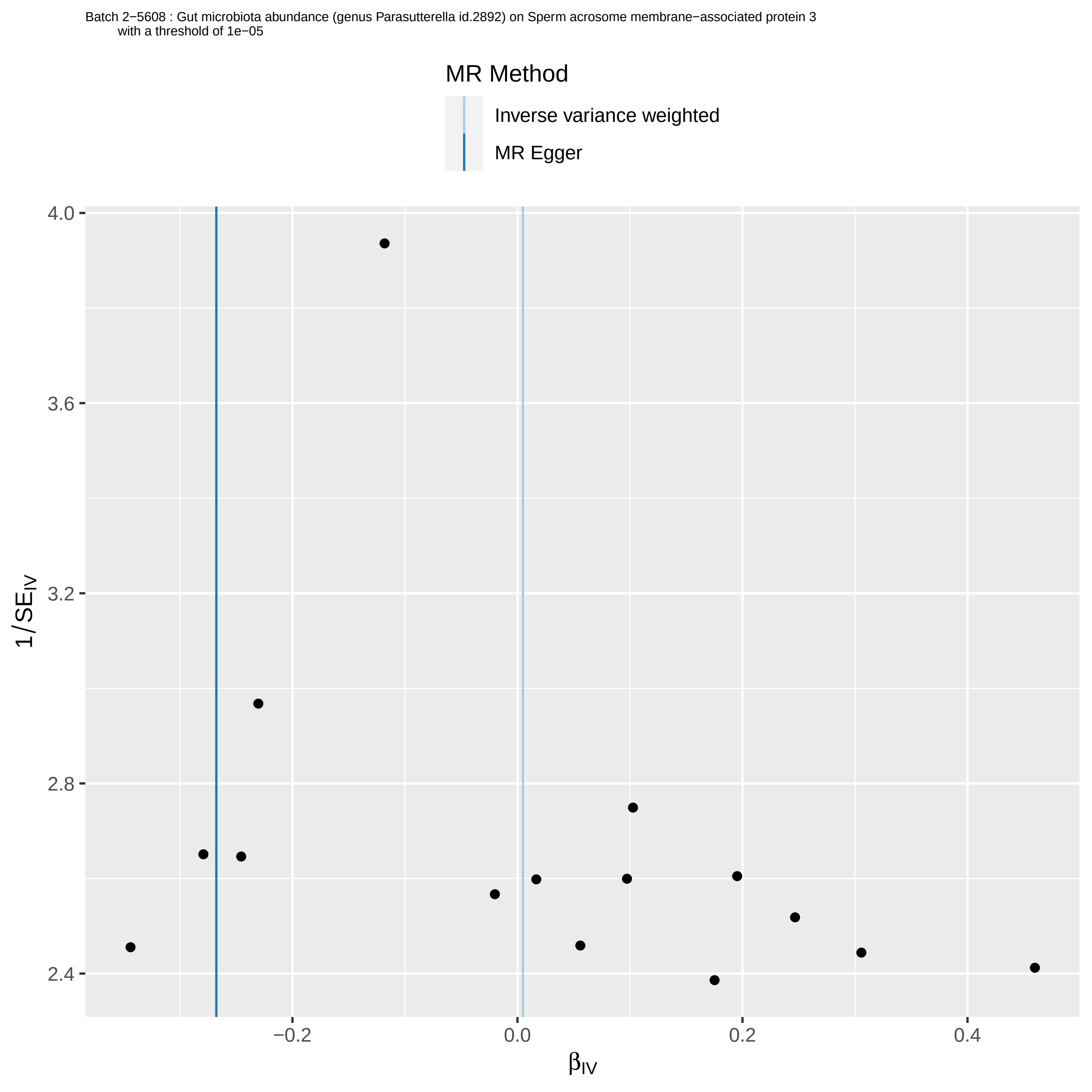

Batch 2−5608 : Gut microbiota abundance (genus Parasutterella id.2892) on Sperm acrosome membrane−associated protein 3
with a threshold of 1e−05
MR Method
Inverse variance weighted
MR Egger
4.0
3.6
3.2
2.8
2.4
1/SE$_{IV}$
−0.2
0.0
0.2
0.4
β$_{IV}$

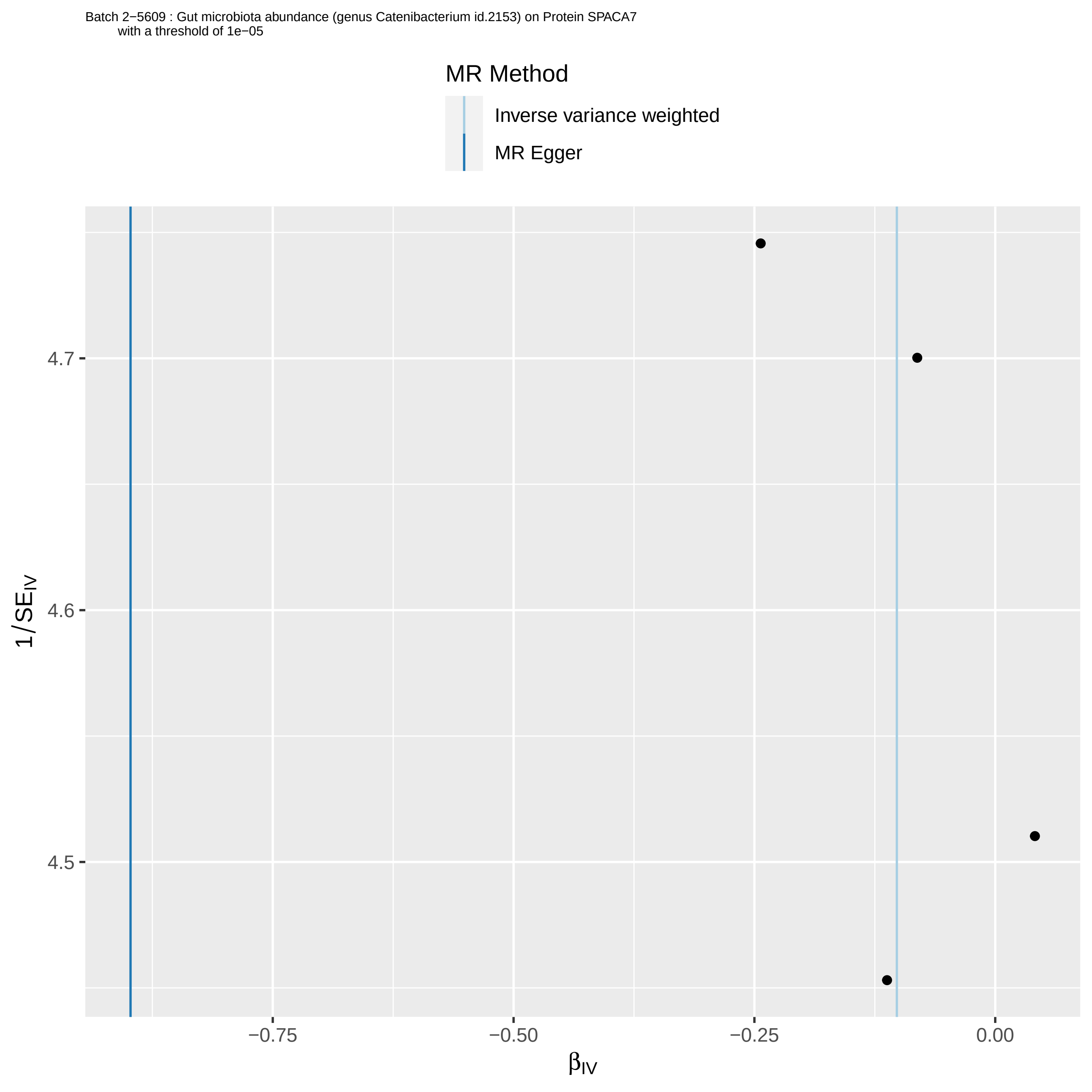
Batch 2−5609 : Gut microbiota abundance (genus Catenibacterium id.2153) on Protein SPACA7
with a threshold of 1e−05
MR Method
Inverse variance weighted
MR Egger
4.7
4.6
4.5
1/SE_IV
−0.75
−0.50
−0.25
0.00
β_IV

Batch 2−5610 : Gut microbiota abundance (genus Parasutterella id.2892) on Protein SPACA7
with a threshold of 1e−05

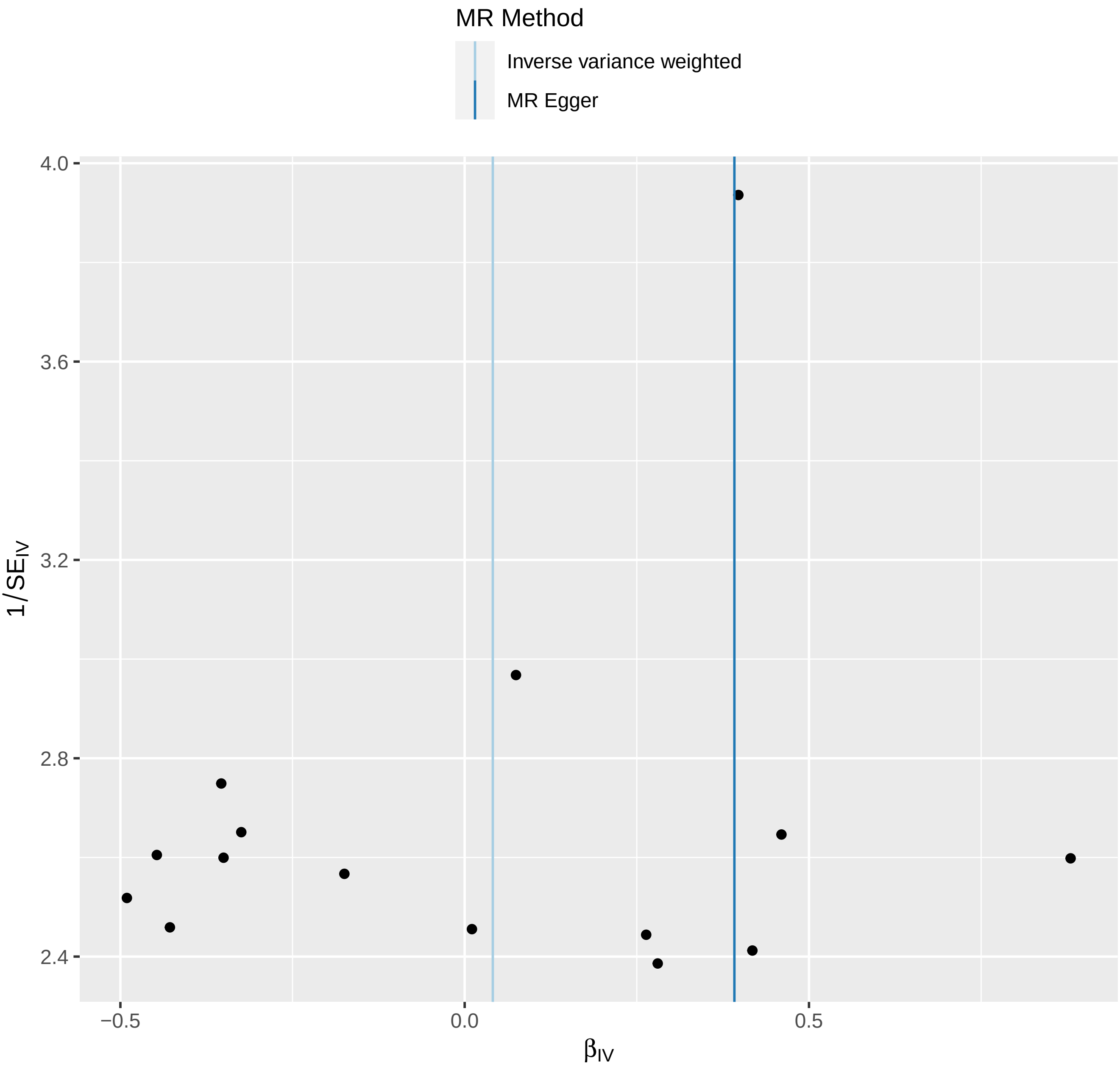

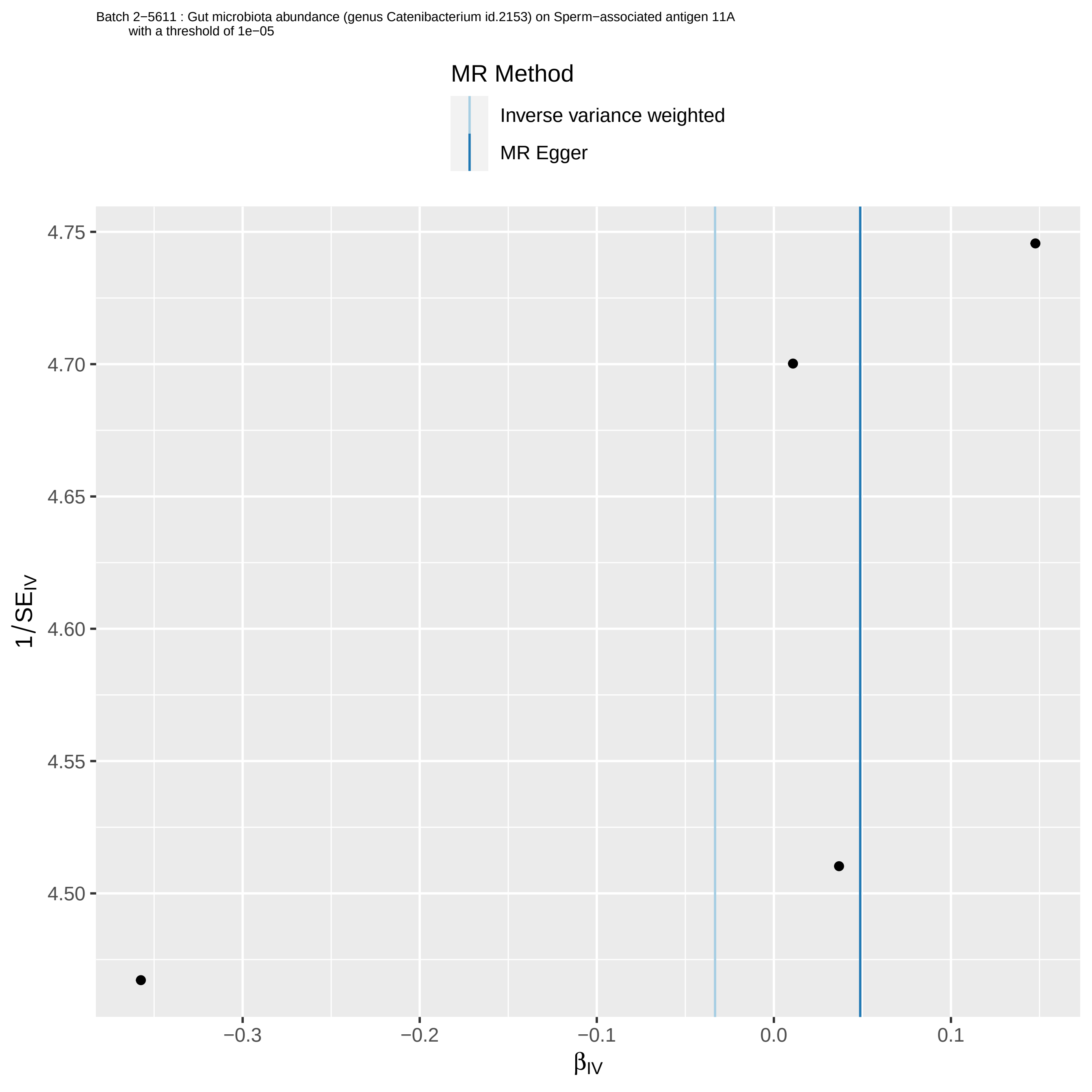

Batch 2−5611 : Gut microbiota abundance (genus Catenibacterium id.2153) on Sperm−associated antigen 11A
with a threshold of 1e−05
MR Method
Inverse variance weighted
MR Egger
4.75
4.70
4.65
4.60
4.55
4.50
1/SE_IV
−0.3
−0.2
−0.1
0.0
0.1
β_IV

Batch 2−5612 : Gut microbiota abundance (genus Parasutterella id.2892) on Sperm−associated antigen 11A
with a threshold of 1e−05

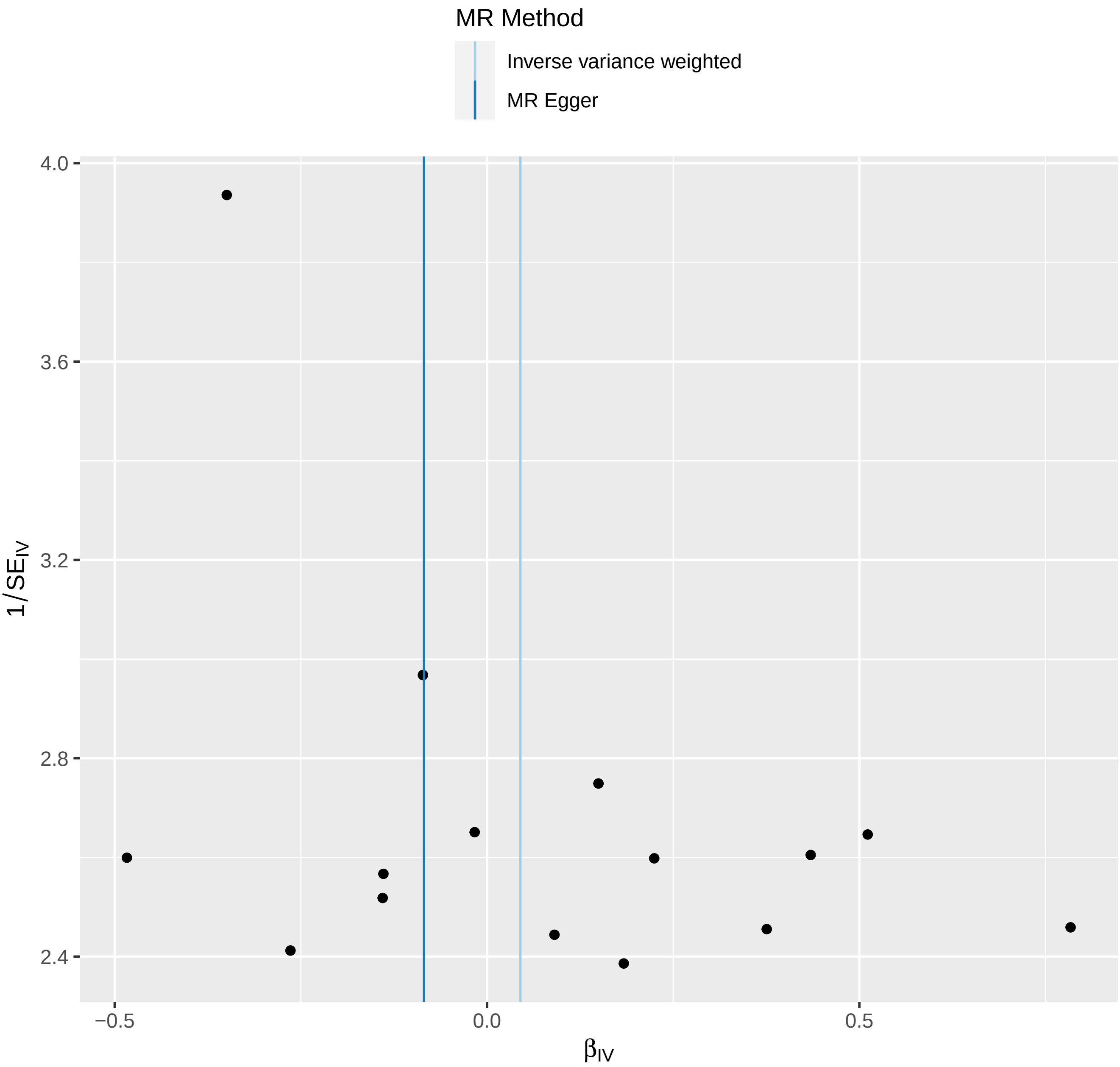

Batch 2−5613 : Gut microbiota abundance (genus Catenibacterium id.2153) on Sperm−associated antigen 11B
with a threshold of 1e−05

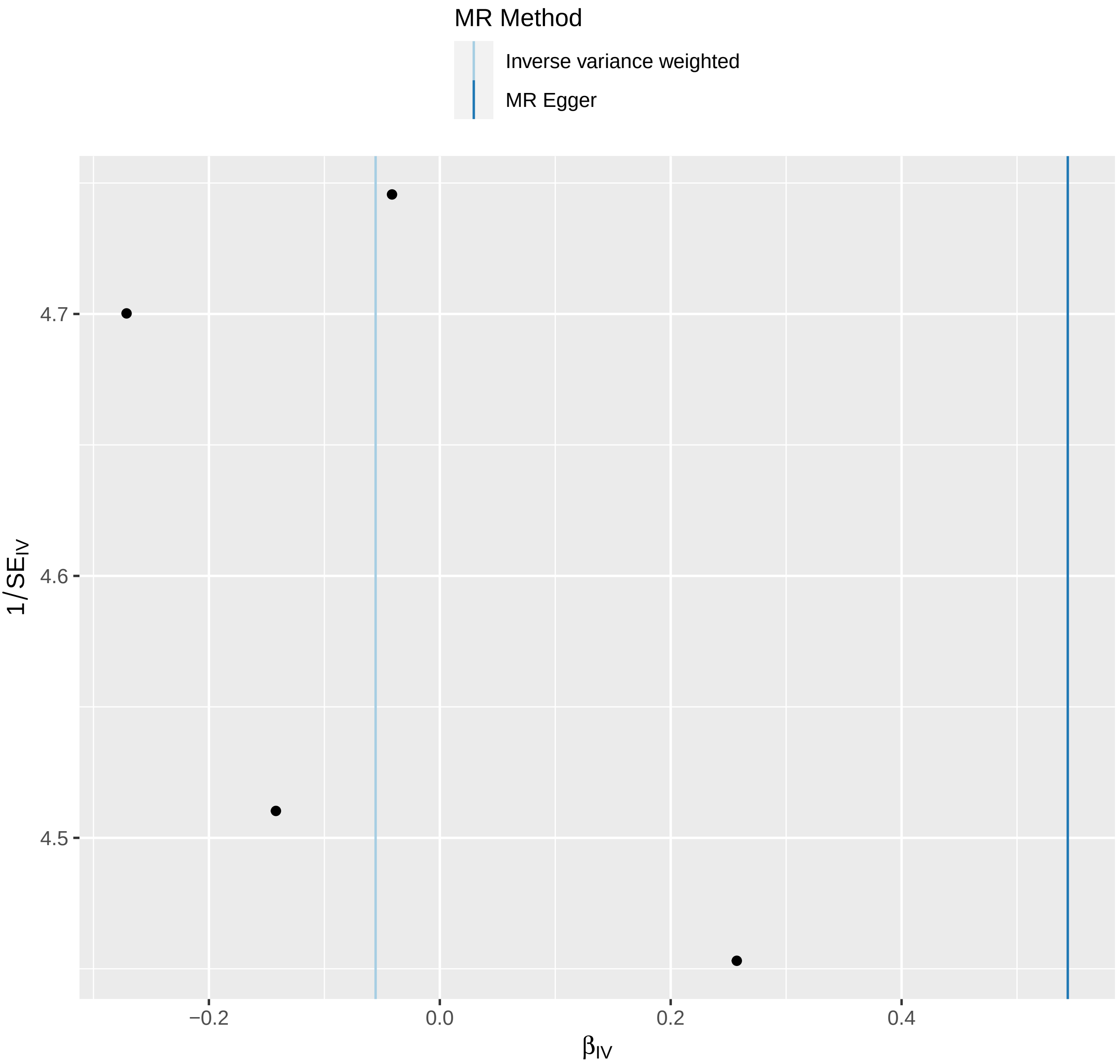

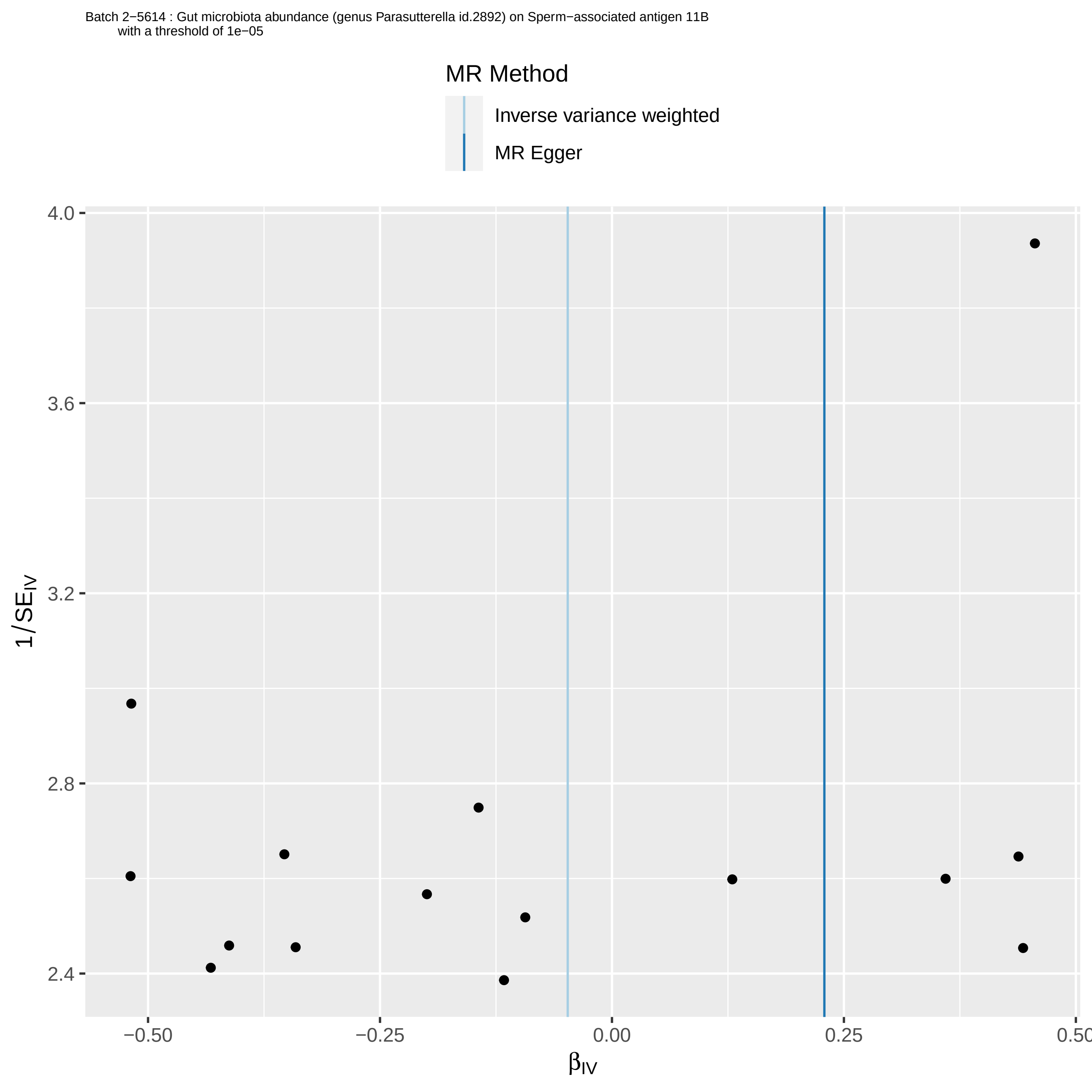

Batch 2−5614 : Gut microbiota abundance (genus Parasutterella id.2892) on Sperm−associated antigen 11B
with a threshold of 1e−05
MR Method
Inverse variance weighted
MR Egger
4.0
3.6
3.2
2.8
2.4
1/SE_IV
−0.50
−0.25
0.00
0.25
0.50
β_IV

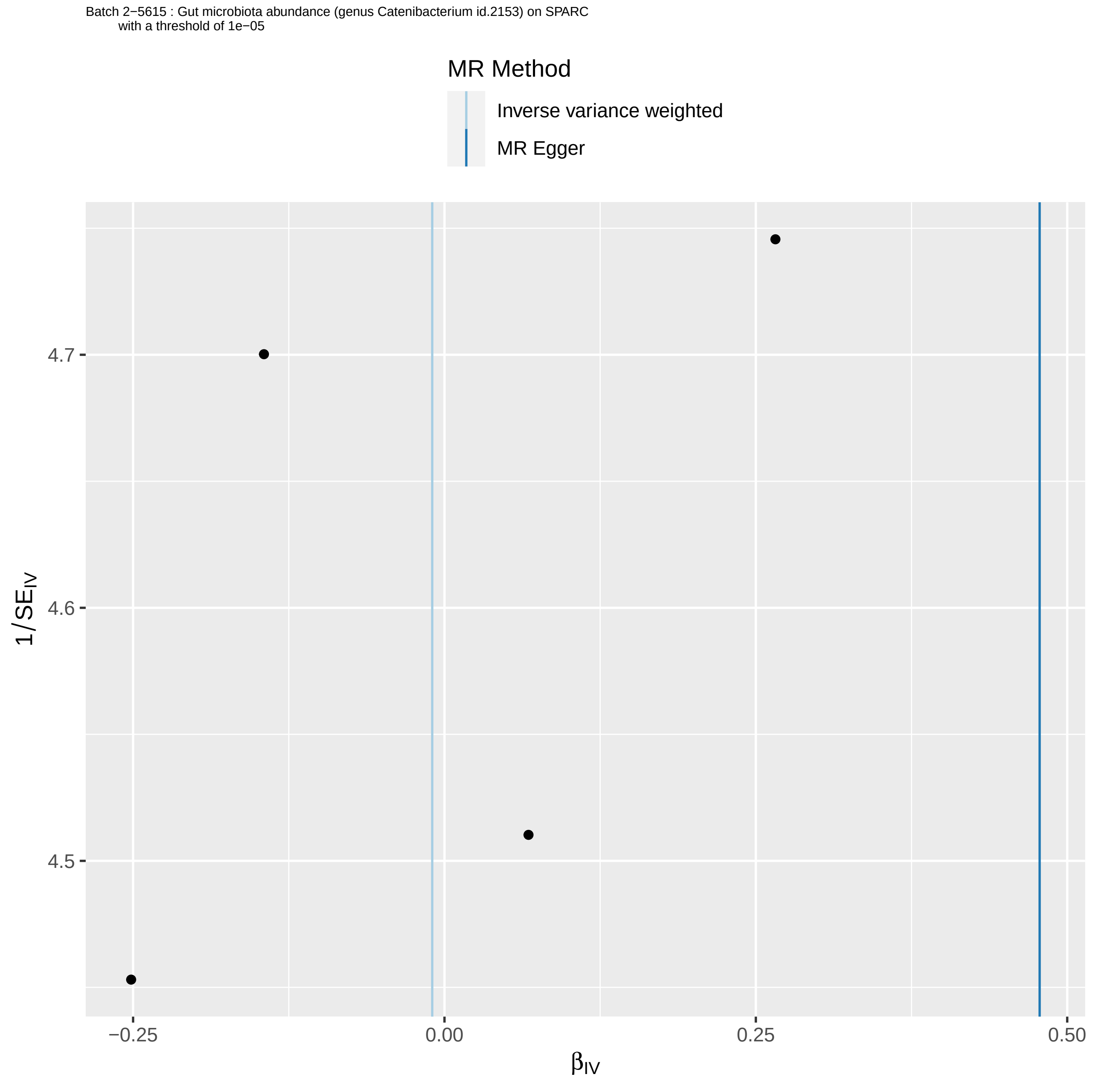

Batch 2−5615 : Gut microbiota abundance (genus Catenibacterium id.2153) on SPARC
with a threshold of 1e−05
MR Method
Inverse variance weighted
MR Egger
4.7
4.6
4.5
1/SE_IV
−0.25
0.00
0.25
0.50
β_IV

Batch 2−5616 : Gut microbiota abundance (genus Parasutterella id.2892) on SPARC
with a threshold of 1e−05

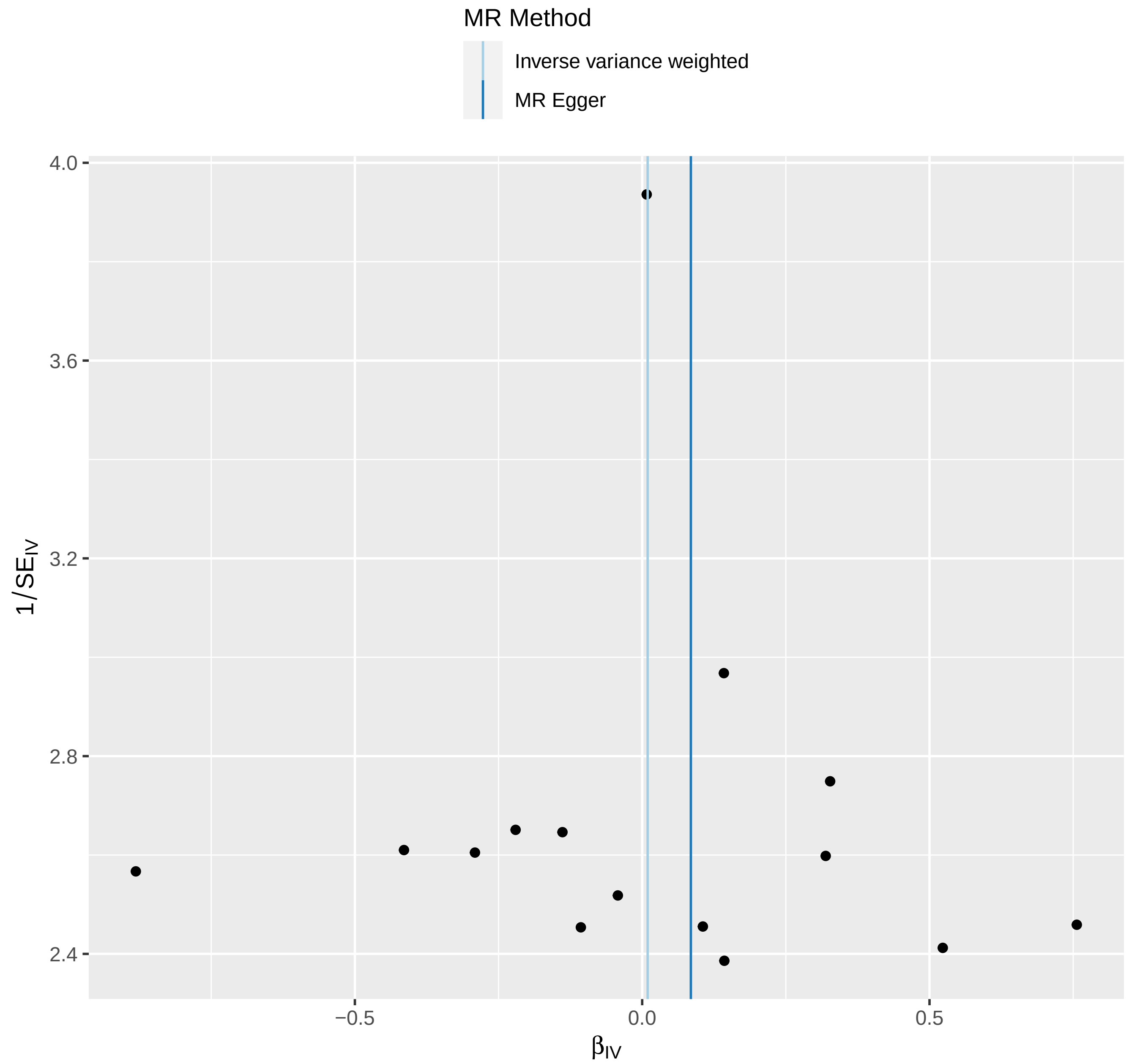

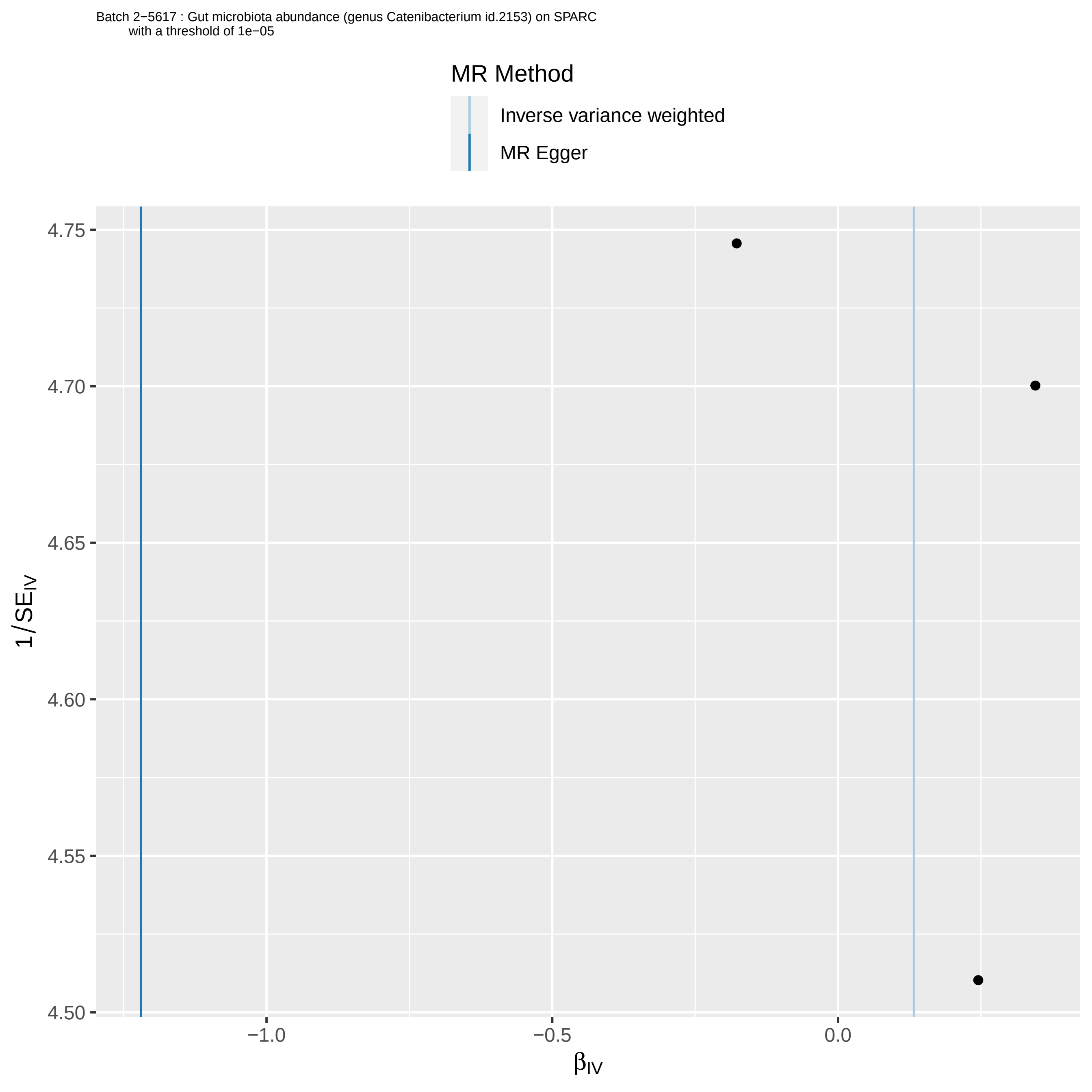
Batch 2−5617 : Gut microbiota abundance (genus Catenibacterium id.2153) on SPARC
with a threshold of 1e−05
MR Method
Inverse variance weighted
MR Egger
4.75
4.70
4.65
4.60
4.55
4.50
1/SE_IV
−1.0
−0.5
0.0
β_IV

Batch 2−5618 : Gut microbiota abundance (genus Parasutterella id.2892) on SPARC
with a threshold of 1e−05

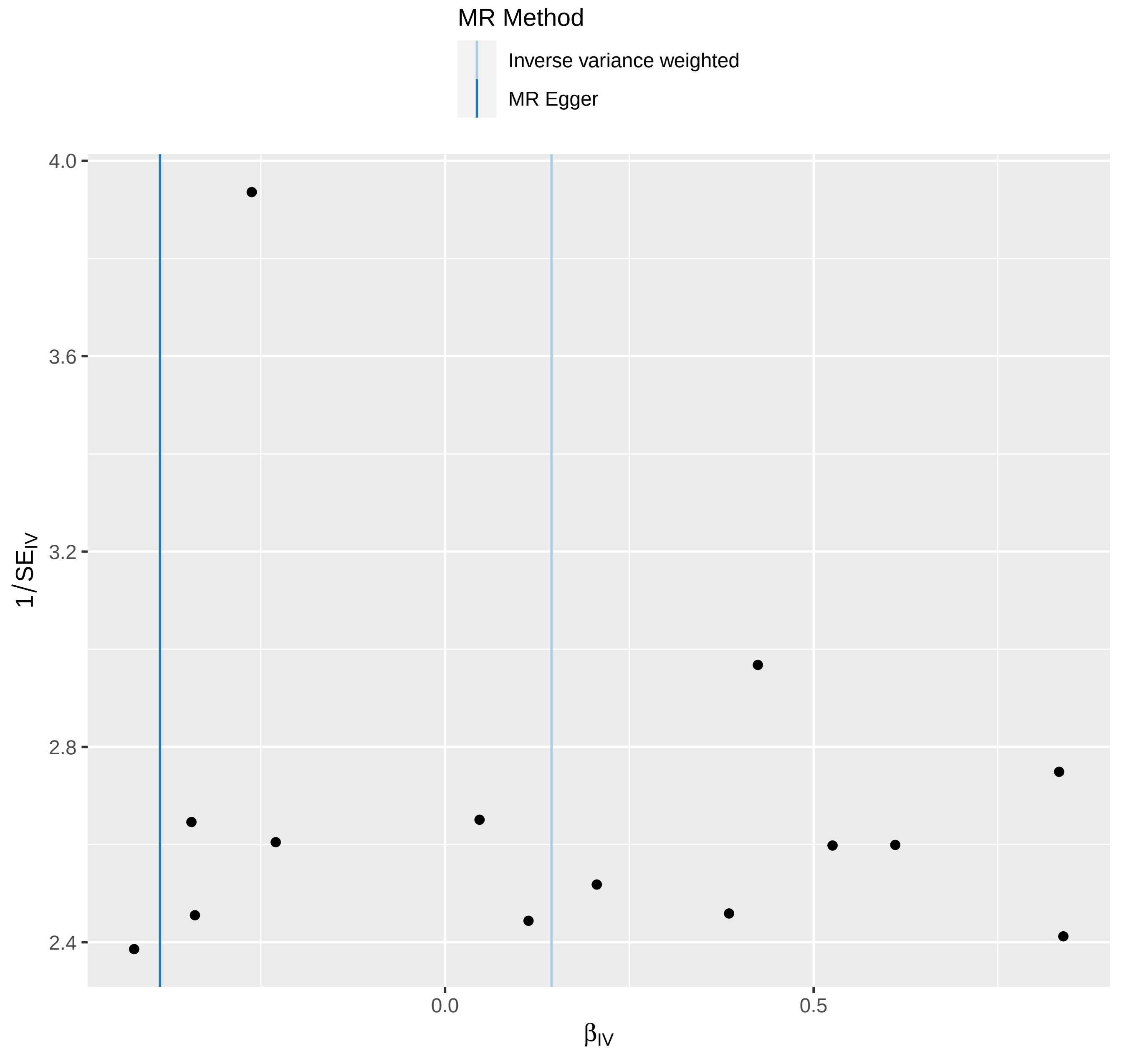

Batch 2−5619 : Gut microbiota abundance (genus Catenibacterium id.2153) on SPARC−like protein 1
with a threshold of 1e−05

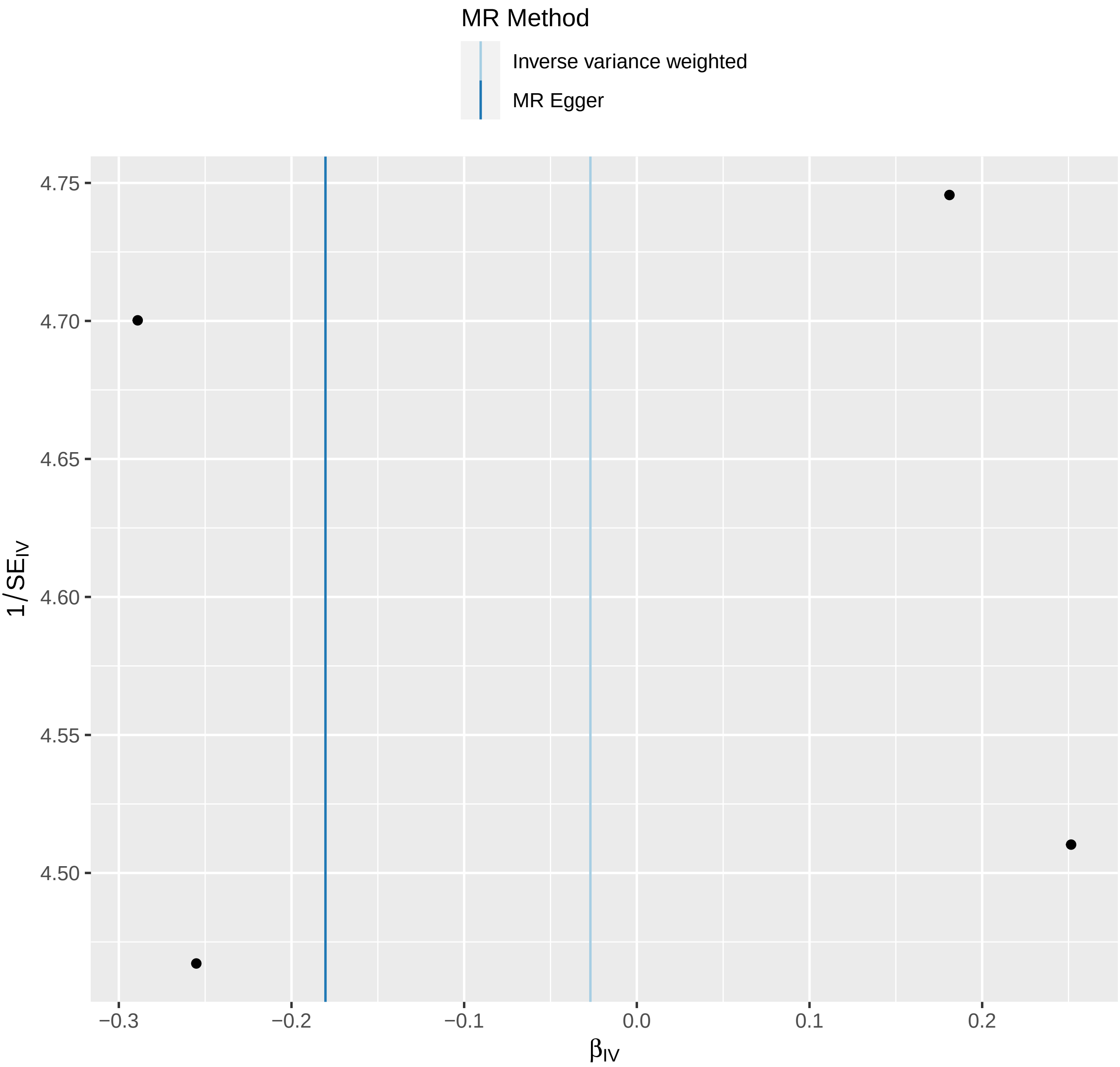

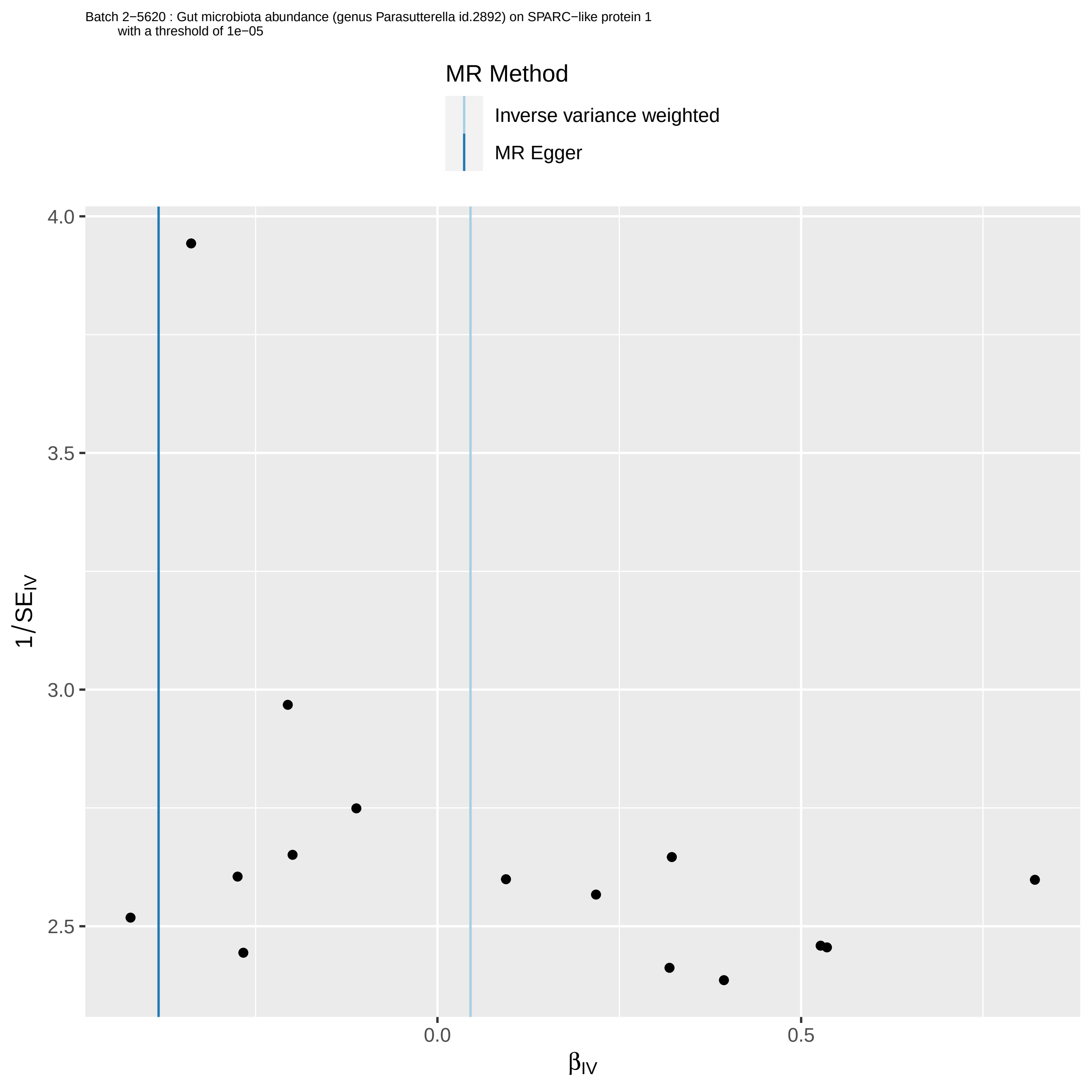
Batch 2−5620 : Gut microbiota abundance (genus Parasutterella id.2892) on SPARC−like protein 1
with a threshold of 1e−05
MR Method
Inverse variance weighted
MR Egger
4.0
3.5
3.0
2.5
1/SE_IV
0.0
0.5
β_IV

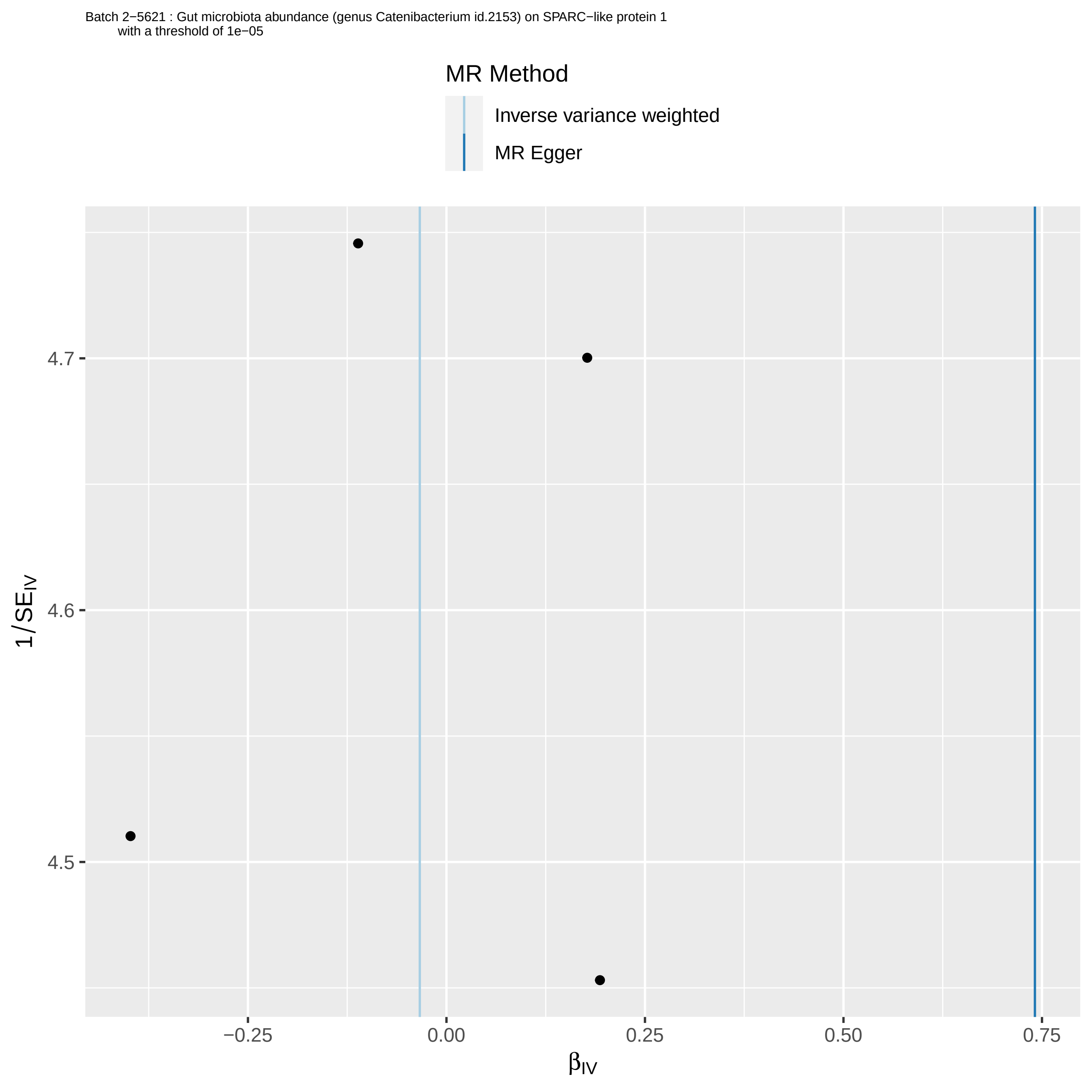

Batch 2−5621 : Gut microbiota abundance (genus Catenibacterium id.2153) on SPARC−like protein 1
with a threshold of 1e−05
MR Method
Inverse variance weighted
MR Egger
4.7
4.6
4.5
1/SE_IV
−0.25
0.00
0.25
0.50
0.75
β_IV

Batch 2−5622 : Gut microbiota abundance (genus Parasutterella id.2892) on SPARC−like protein 1
with a threshold of 1e−05

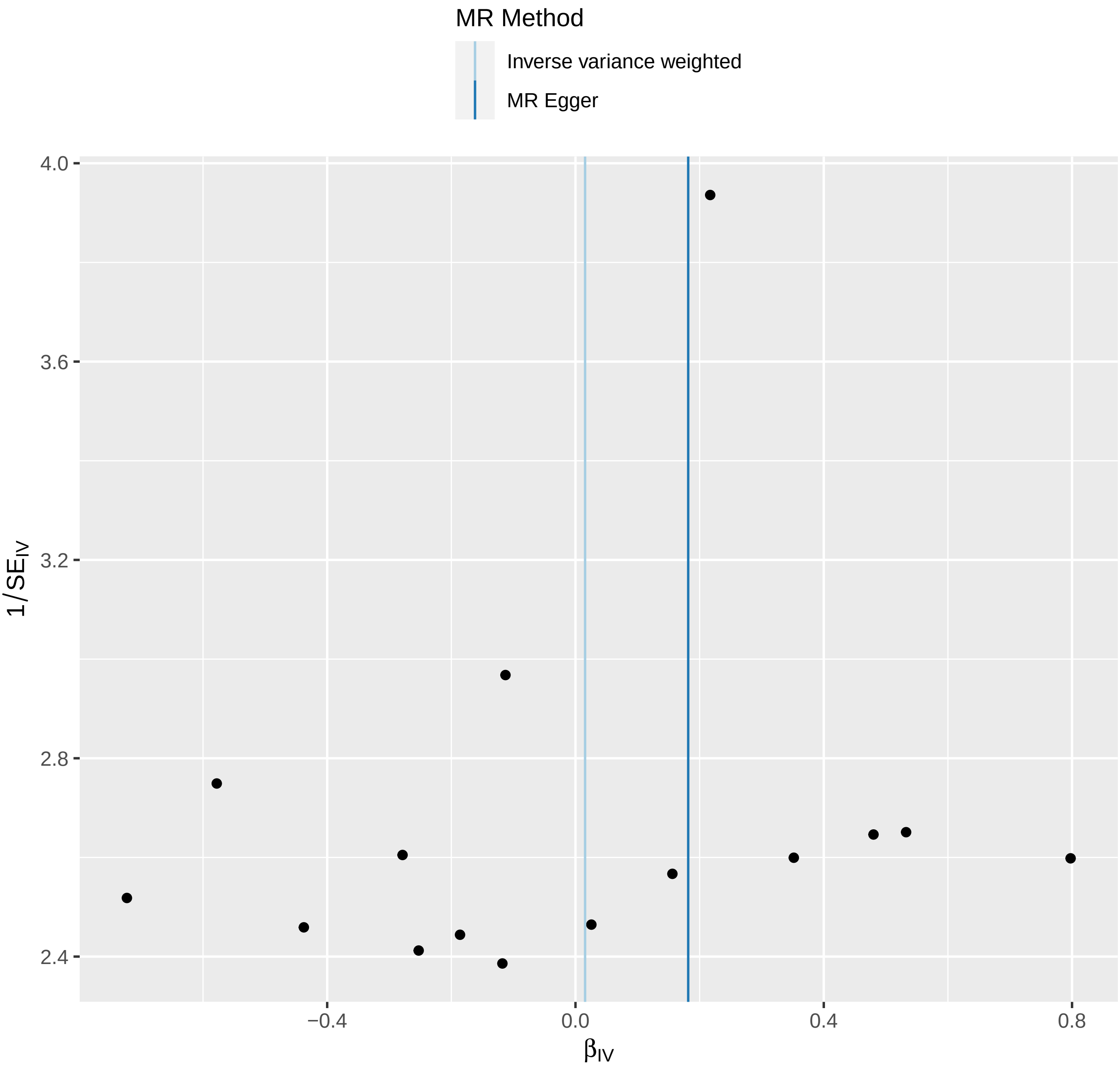

Batch 2−5623 : Gut microbiota abundance (genus Catenibacterium id.2153) on Spastin
with a threshold of 1e−05

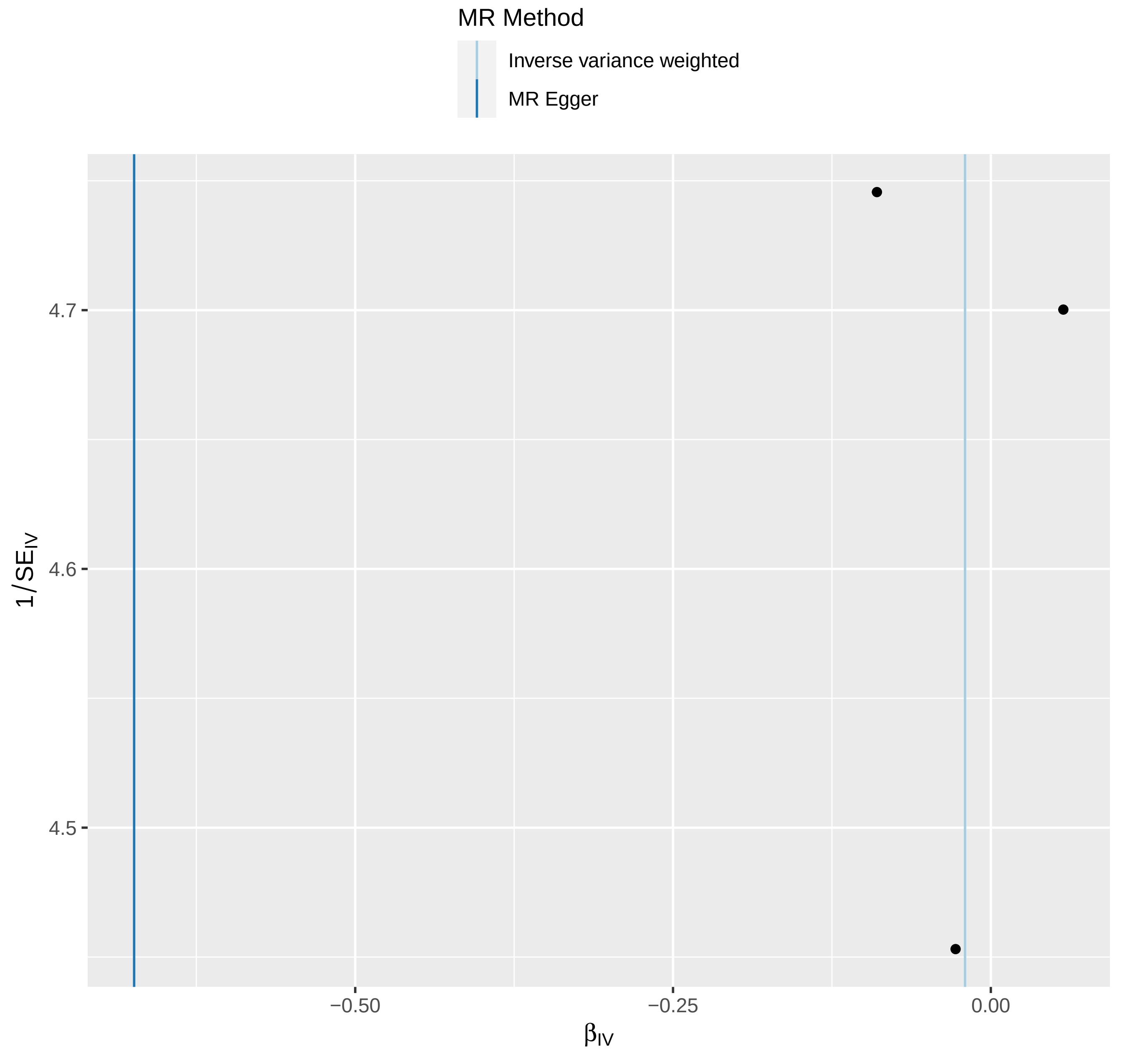

Batch 2−5624 : Gut microbiota abundance (genus Parasutterella id.2892) on Spastin
with a threshold of 1e−05

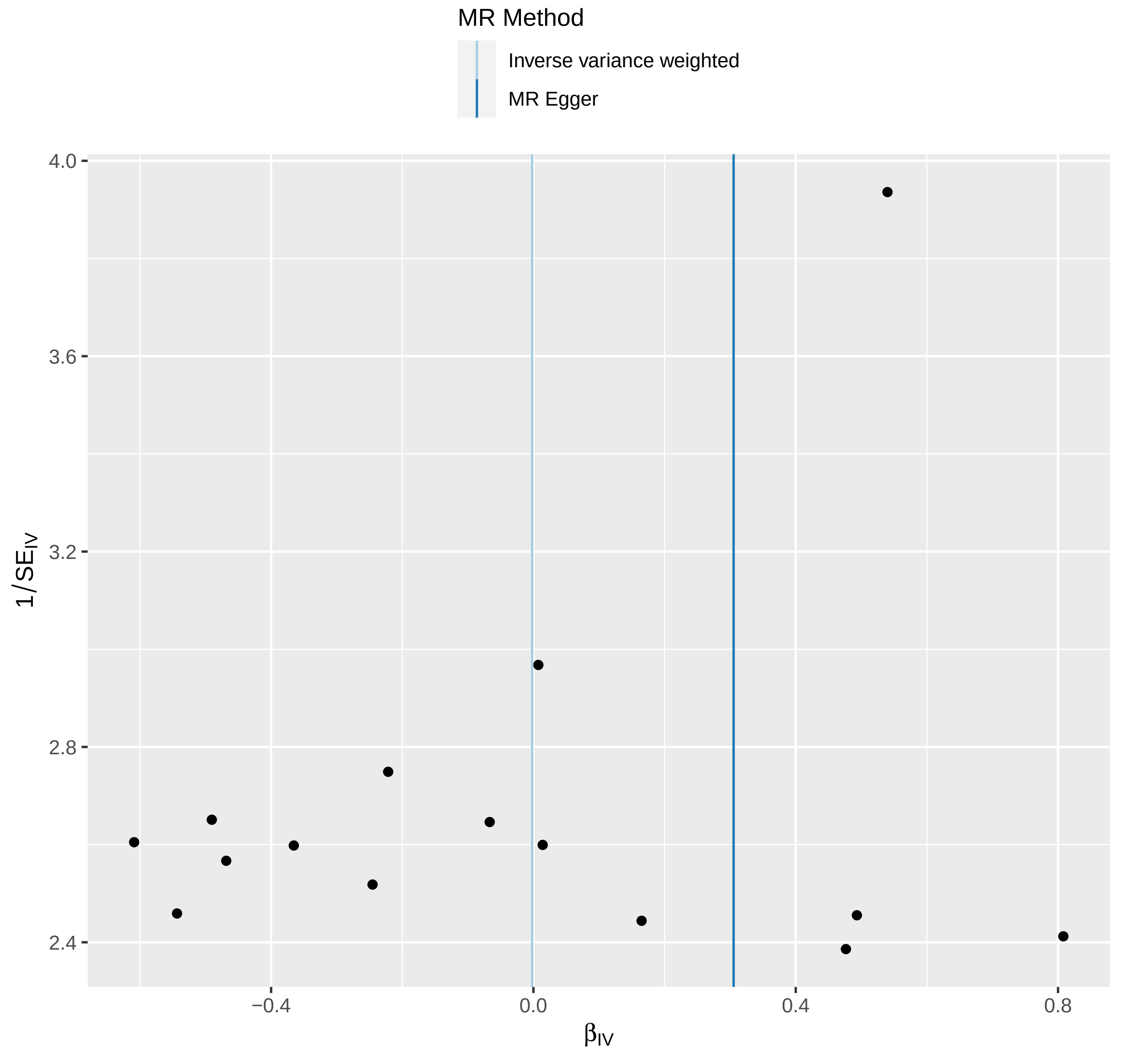

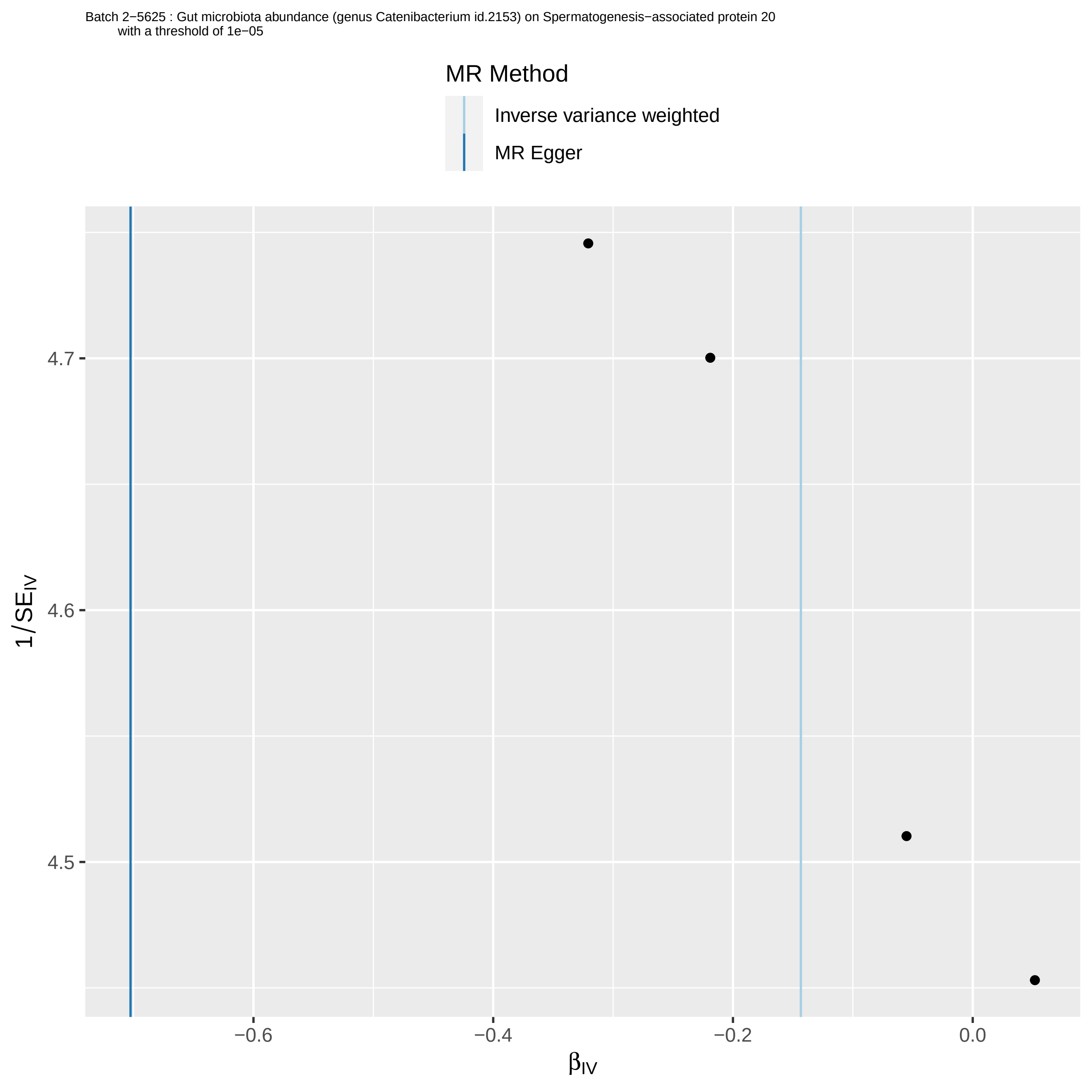
Batch 2−5625 : Gut microbiota abundance (genus Catenibacterium id.2153) on Spermatogenesis−associated protein 20
with a threshold of 1e−05
MR Method
Inverse variance weighted
MR Egger
4.7
4.6
4.5
1/SE_IV
−0.6
−0.4
−0.2
0.0
β_IV

Batch 2−5626 : Gut microbiota abundance (genus Parasutterella id.2892) on Spermatogenesis−associated protein 20 with a threshold of 1e−05

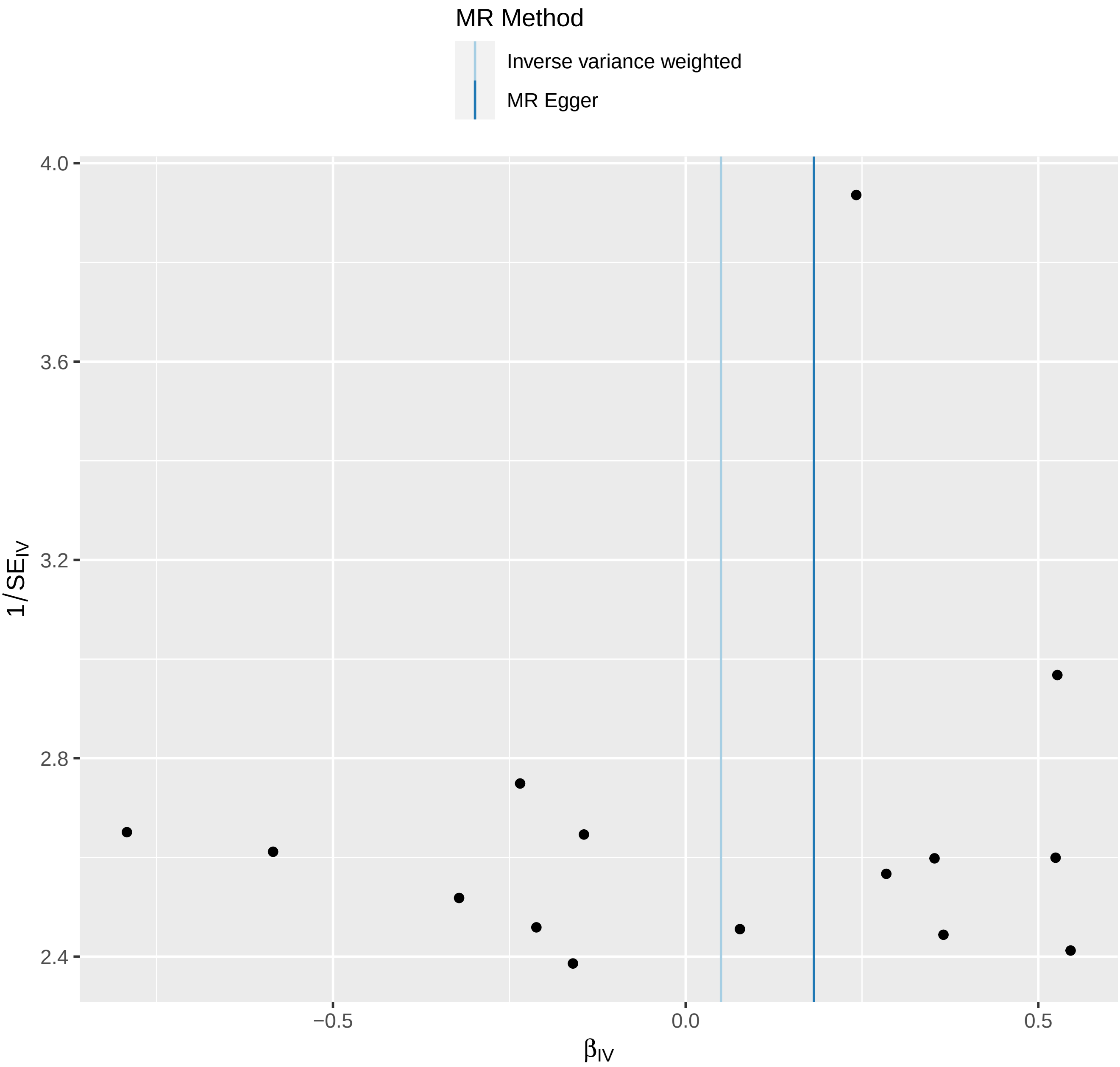

Batch 2−5627 : Gut microbiota abundance (genus Catenibacterium id.2153) on Spermatogenesis−associated protein 9 with a threshold of 1e−05

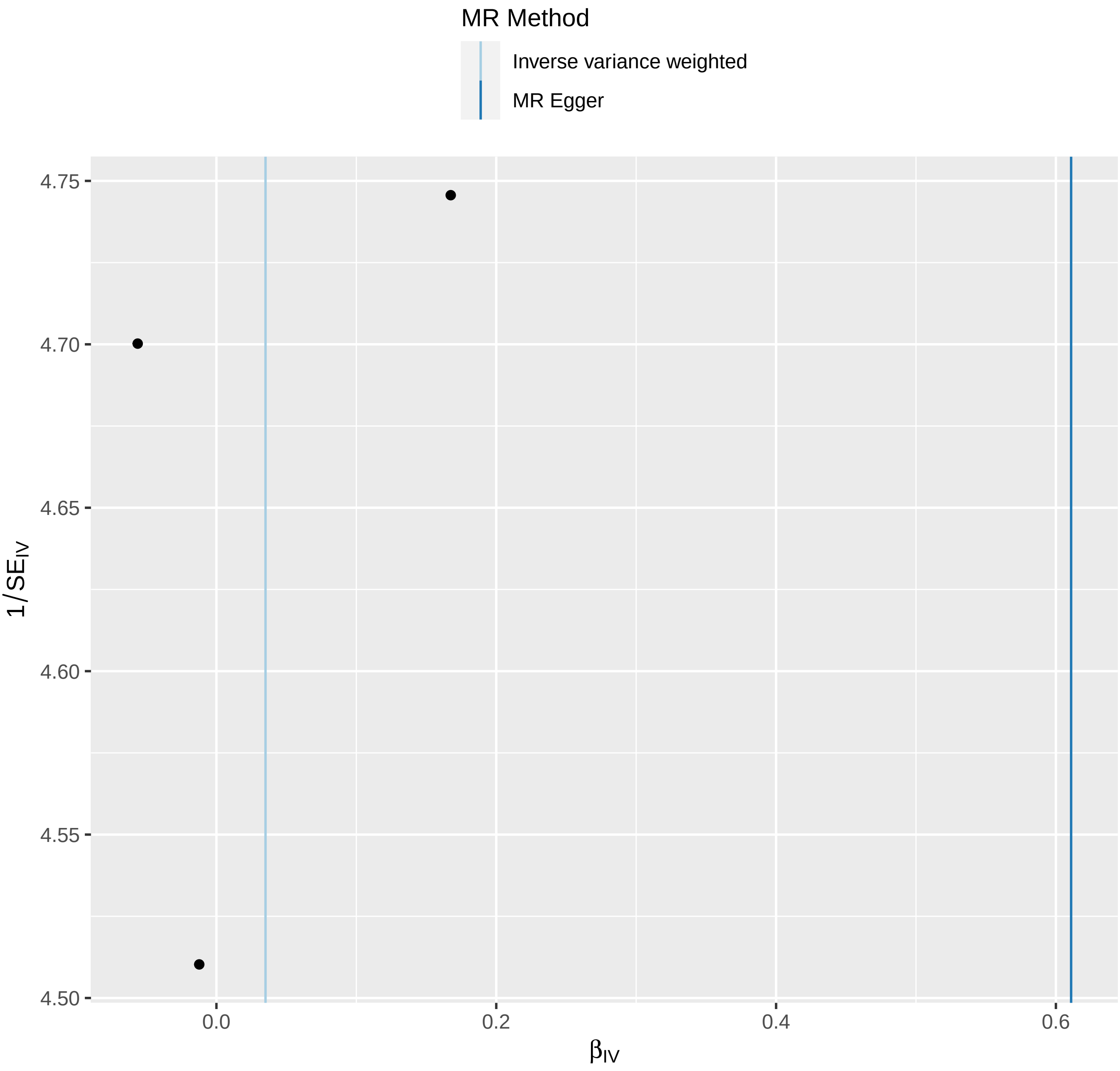

Batch 2−5628 : Gut microbiota abundance (genus Parasutterella id.2892) on Spermatogenesis−associated protein 9
with a threshold of 1e−05

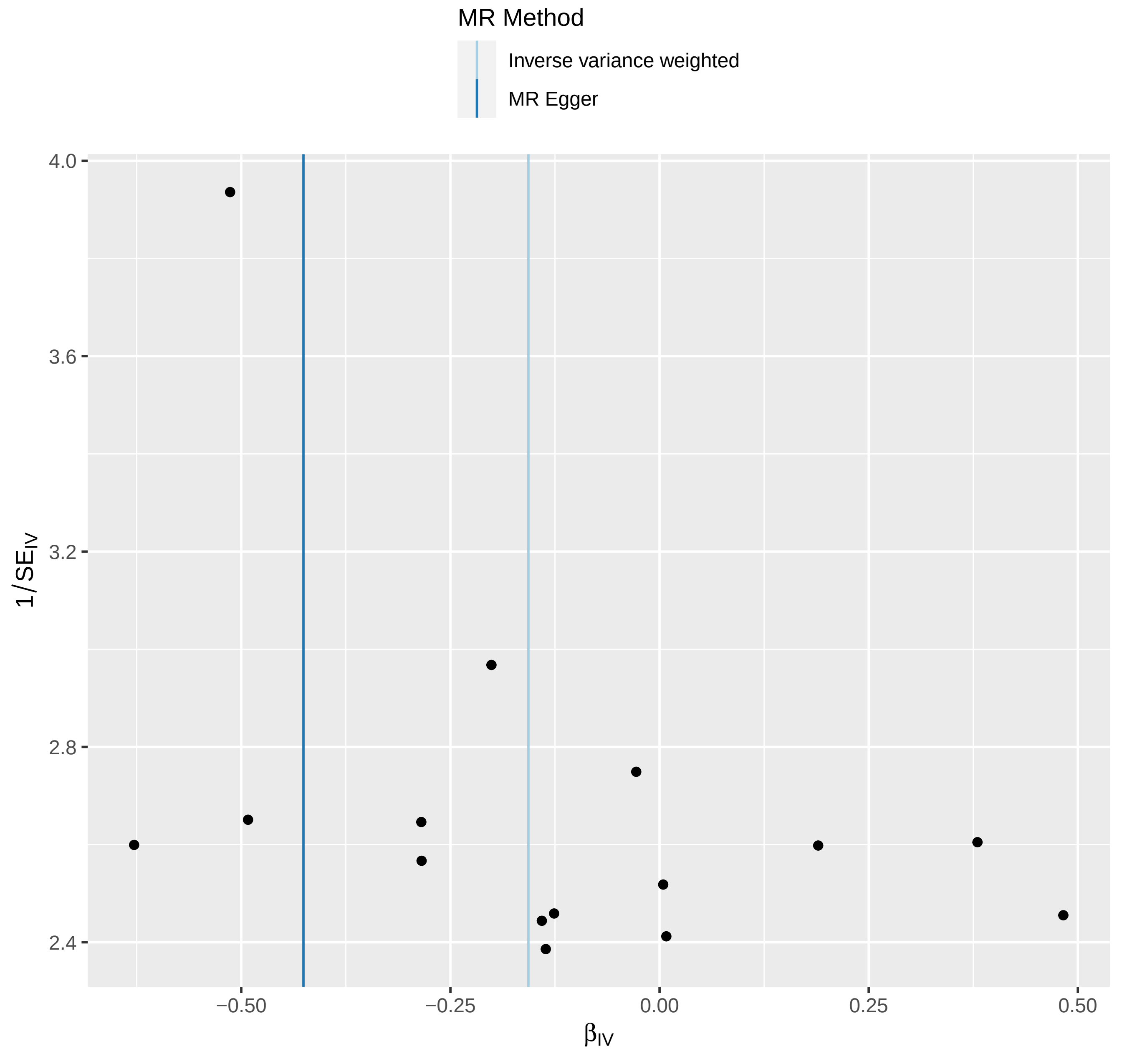

Batch 2−5629 : Gut microbiota abundance (genus Catenibacterium id.2153) on Maspardin
with a threshold of 1e−05

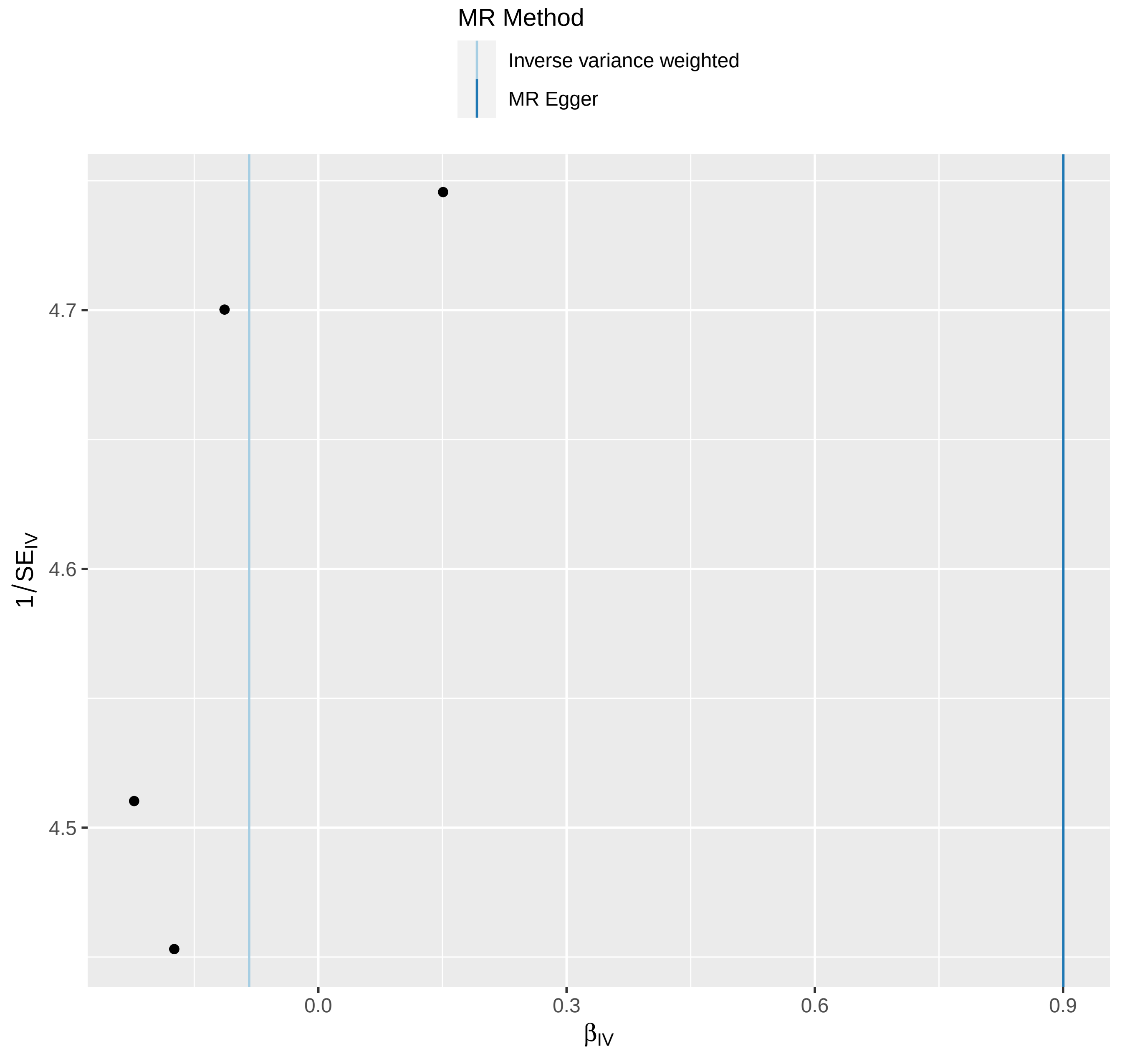

Batch 2−5630 : Gut microbiota abundance (genus Parasutterella id.2892) on Maspardin
with a threshold of 1e−05

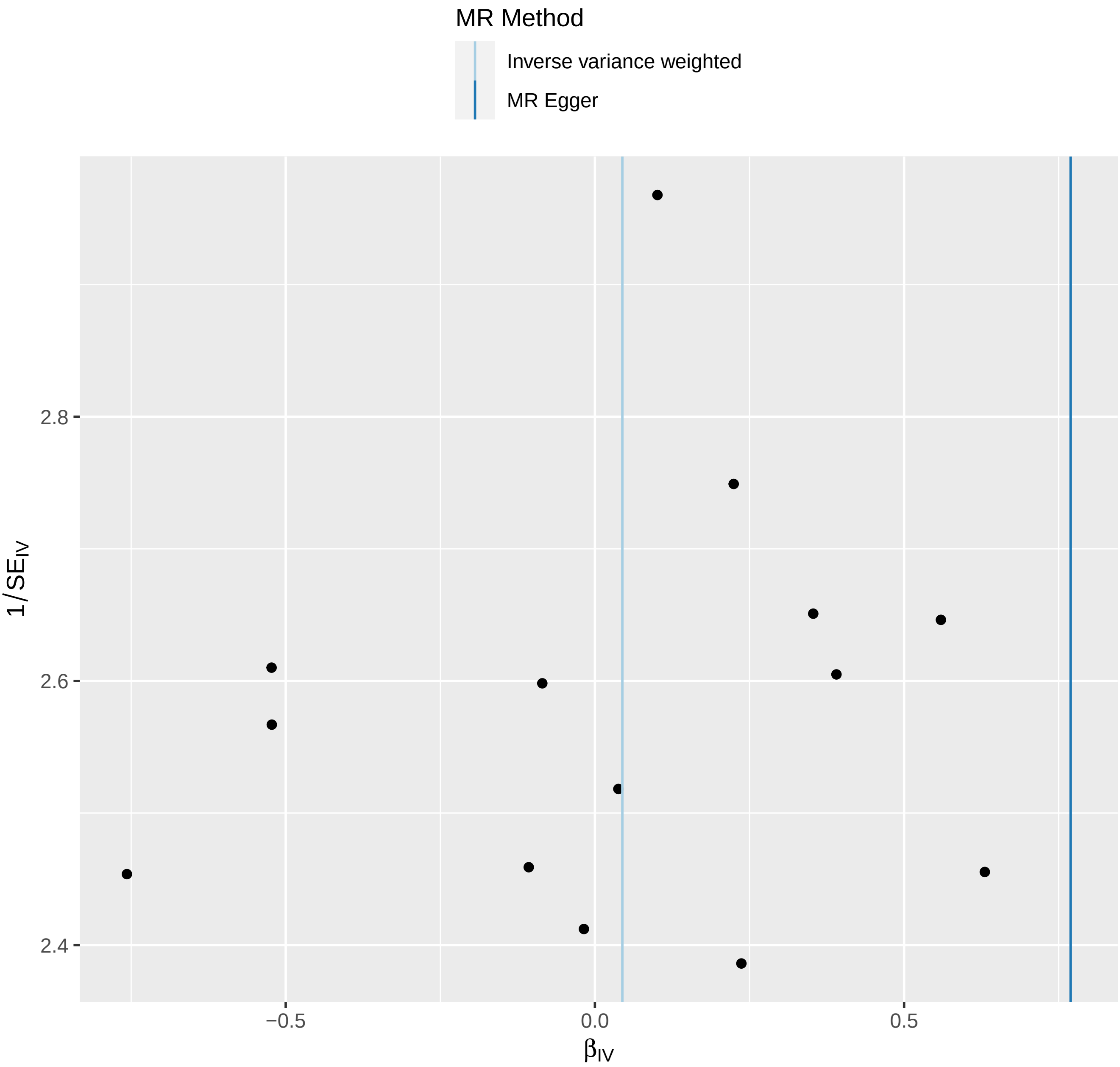

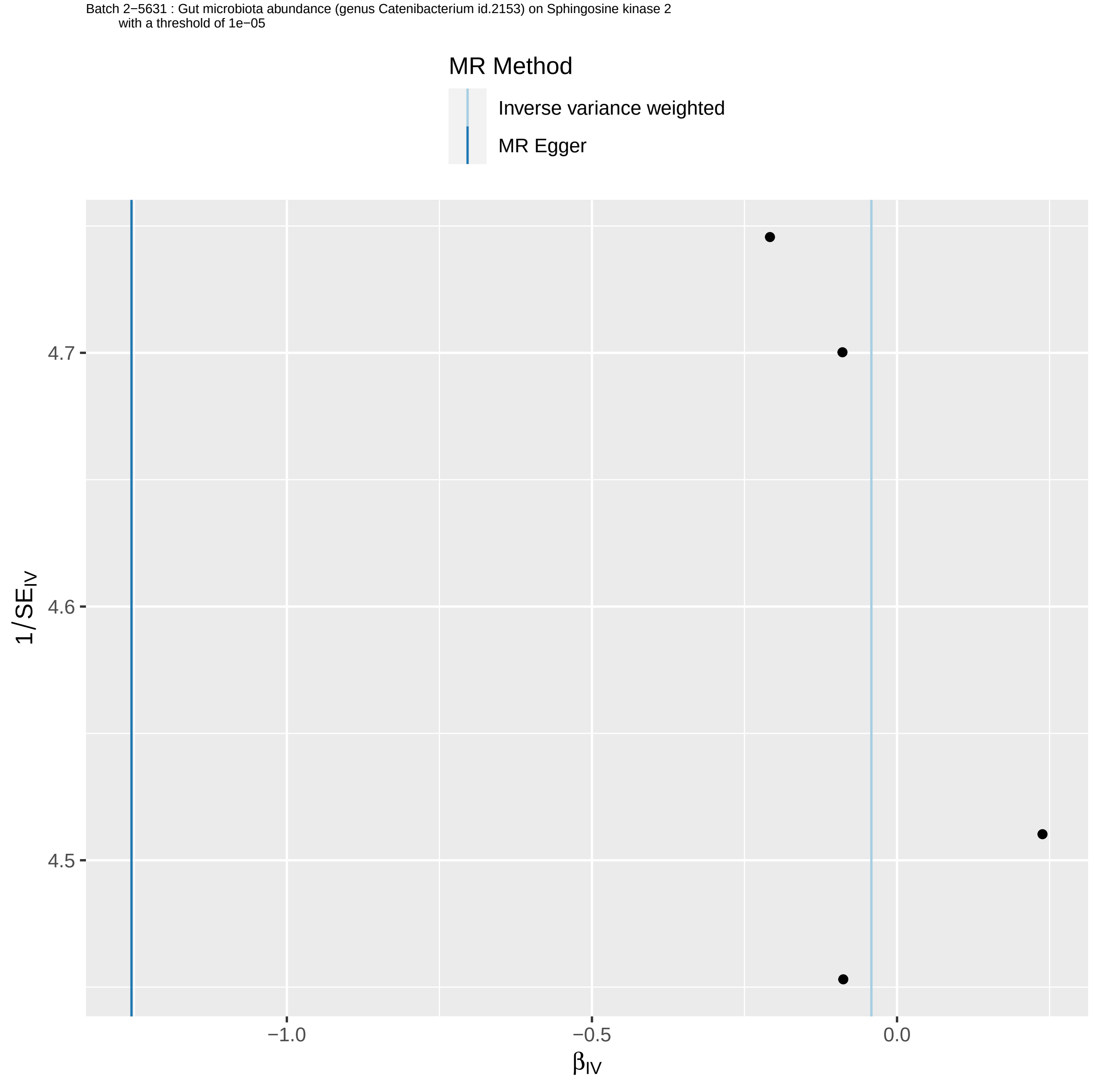

Batch 2−5631 : Gut microbiota abundance (genus Catenibacterium id.2153) on Sphingosine kinase 2
with a threshold of 1e−05
MR Method
Inverse variance weighted
MR Egger
4.7
4.6
4.5
1/SE_IV
−1.0
−0.5
0.0
β_IV

Batch 2−5632 : Gut microbiota abundance (genus Parasutterella id.2892) on Sphingosine kinase 2
with a threshold of 1e−05

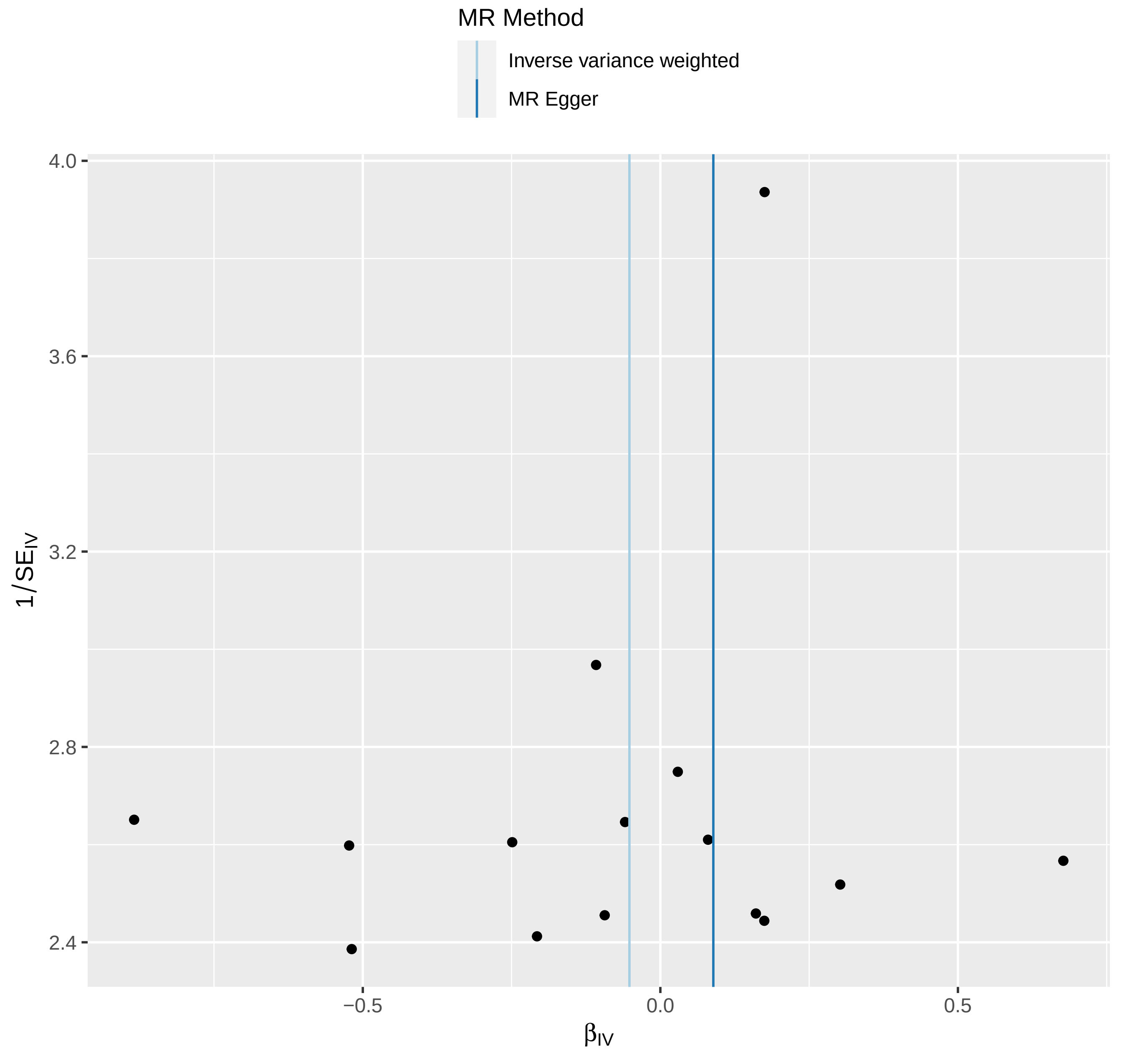

Batch 2−5633 : Gut microbiota abundance (genus Catenibacterium id.2153) on Serine protease inhibitor Kazal−type 13 with a threshold of 1e−05

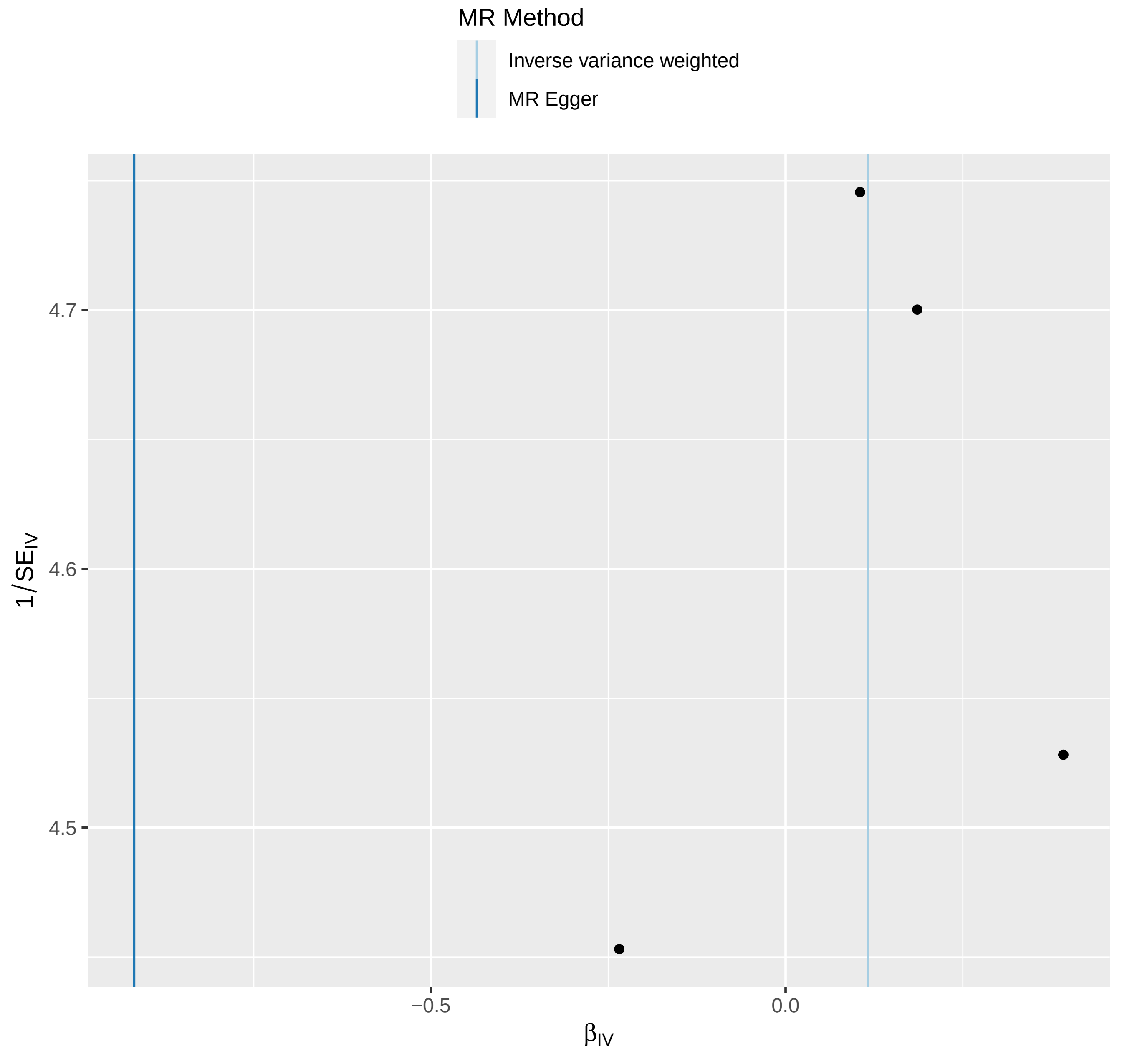

Batch 2−5634 : Gut microbiota abundance (genus Parasutterella id.2892) on Serine protease inhibitor Kazal−type 13
with a threshold of 1e−05

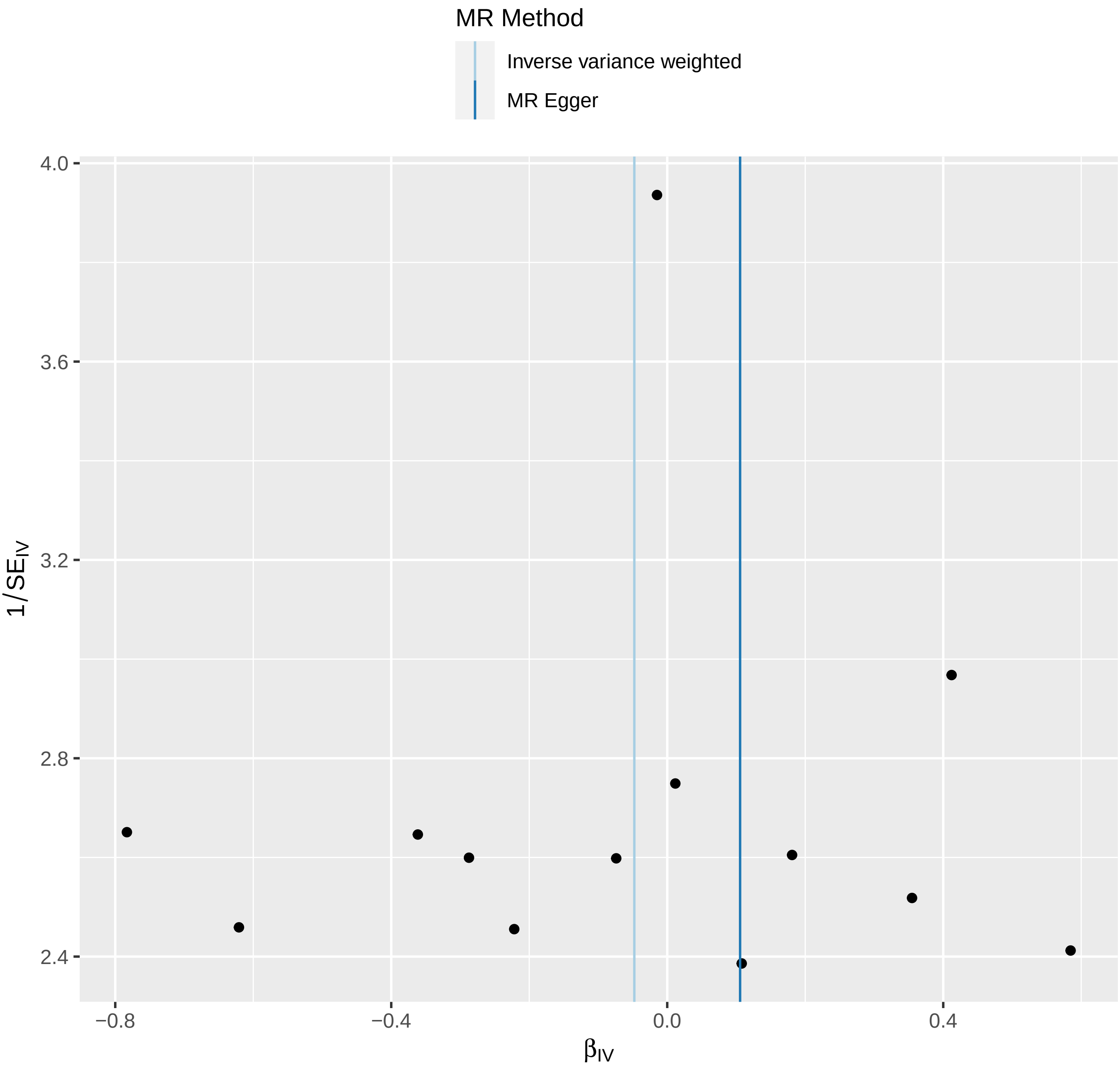

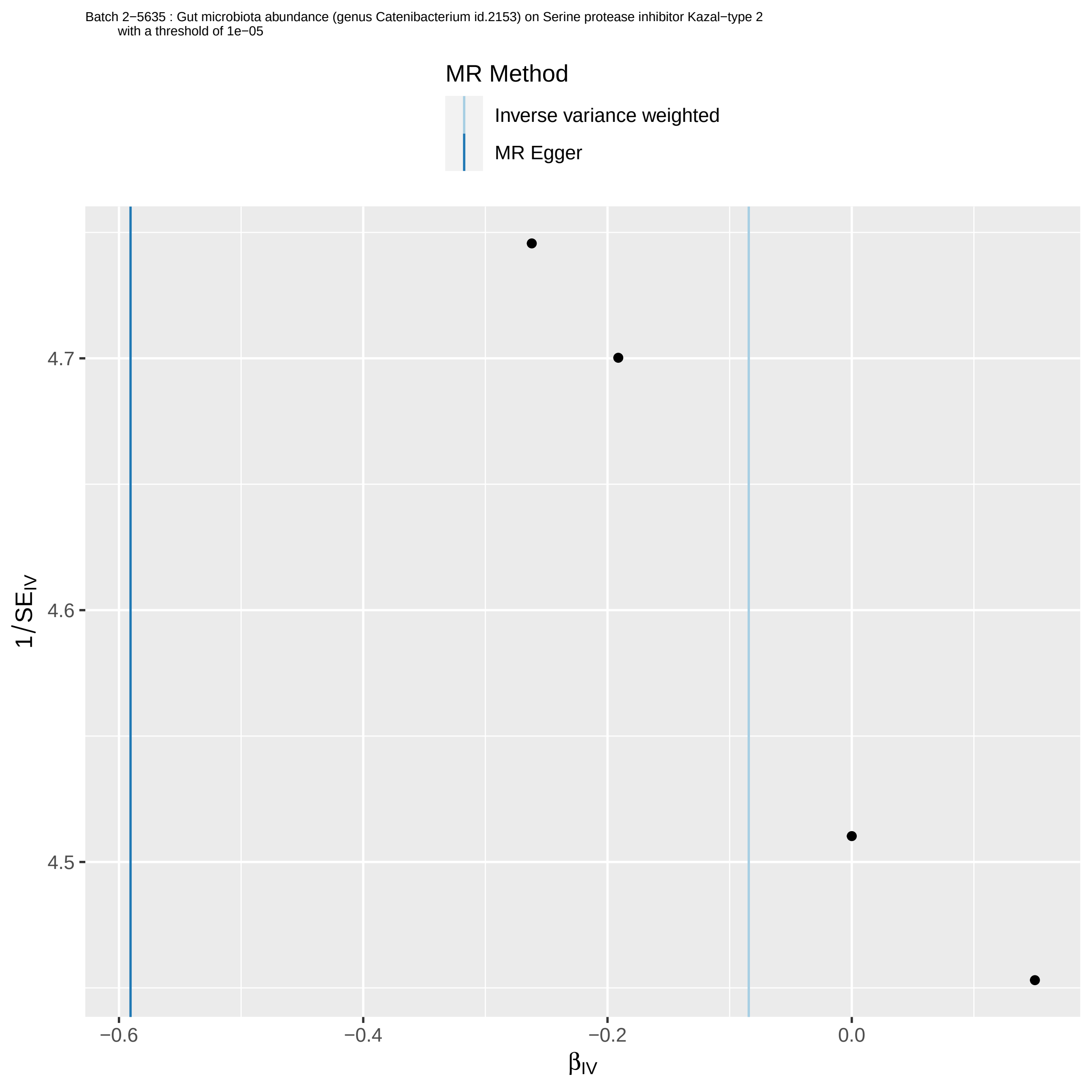

Batch 2−5635 : Gut microbiota abundance (genus Catenibacterium id.2153) on Serine protease inhibitor Kazal−type 2
with a threshold of 1e−05
MR Method
Inverse variance weighted
MR Egger
4.7
4.6
4.5
1/SE_IV
−0.6
−0.4
−0.2
0.0
β_IV

Batch 2−5636 : Gut microbiota abundance (genus Parasutterella id.2892) on Serine protease inhibitor Kazal−type 2
with a threshold of 1e−05

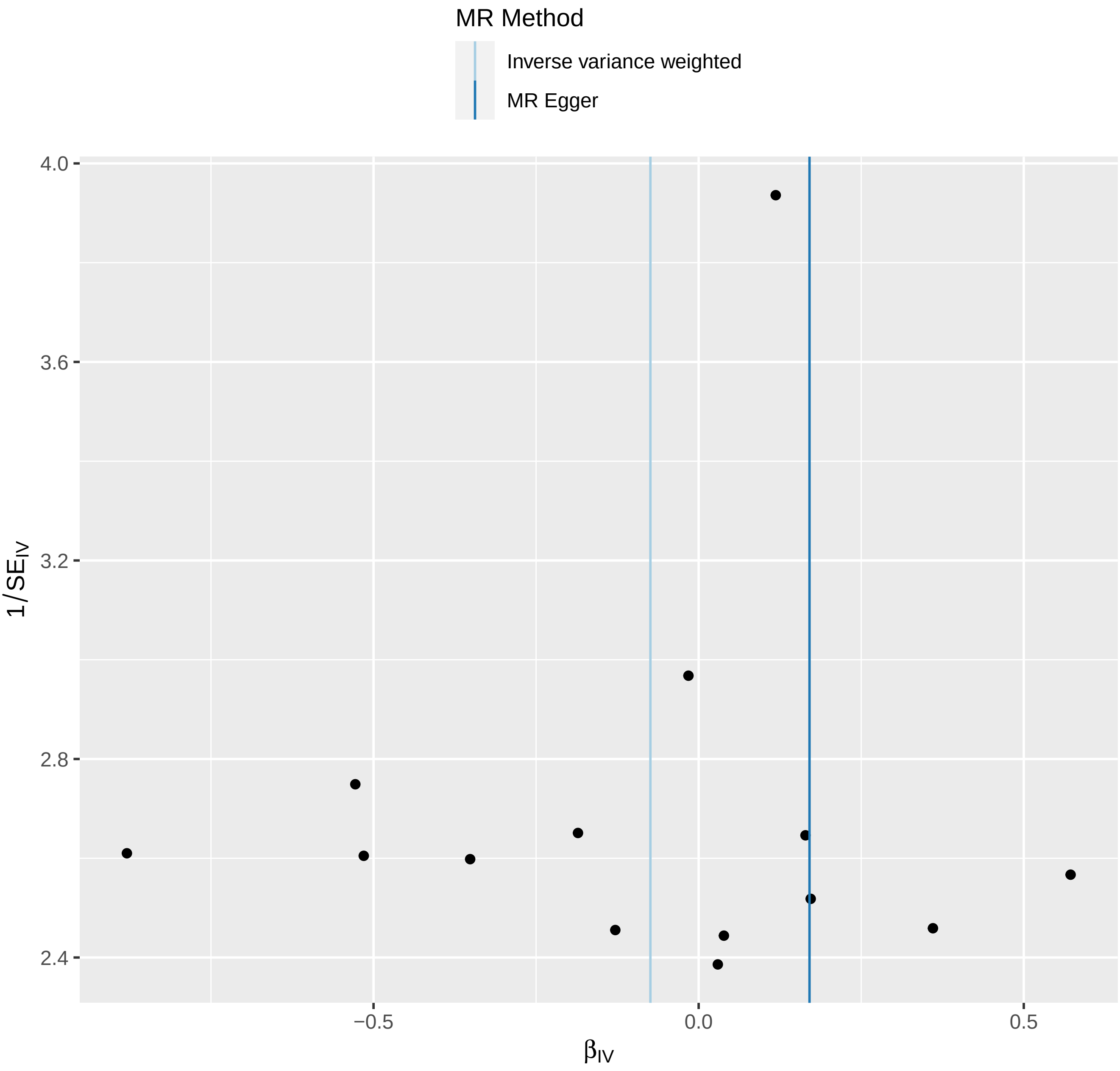

Batch 2−5637 : Gut microbiota abundance (genus Catenibacterium id.2153) on Serine protease inhibitor Kazal−type 5
with a threshold of 1e−05

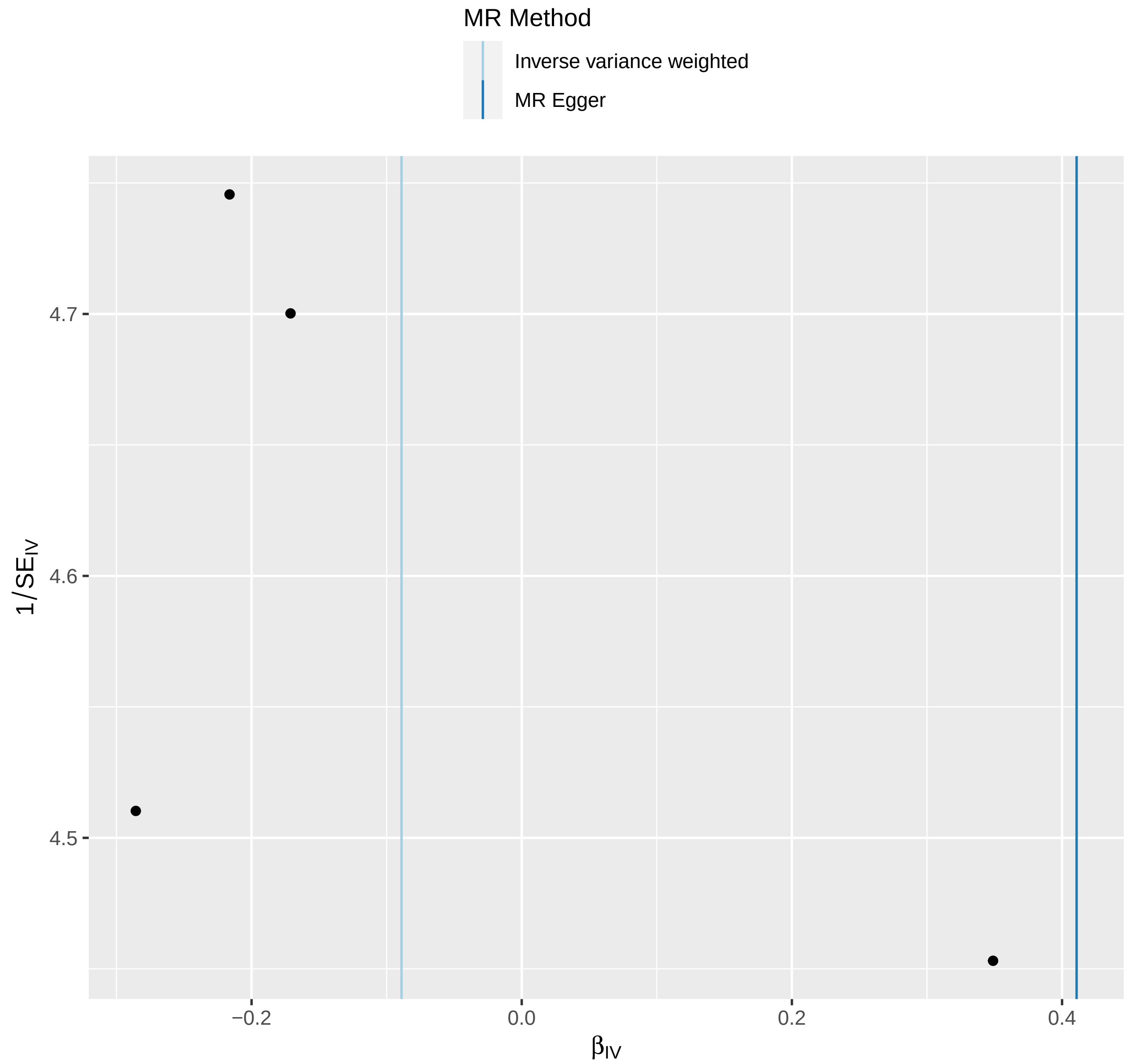

Batch 2−5638 : Gut microbiota abundance (genus Parasutterella id.2892) on Serine protease inhibitor Kazal−type 5
with a threshold of 1e−05

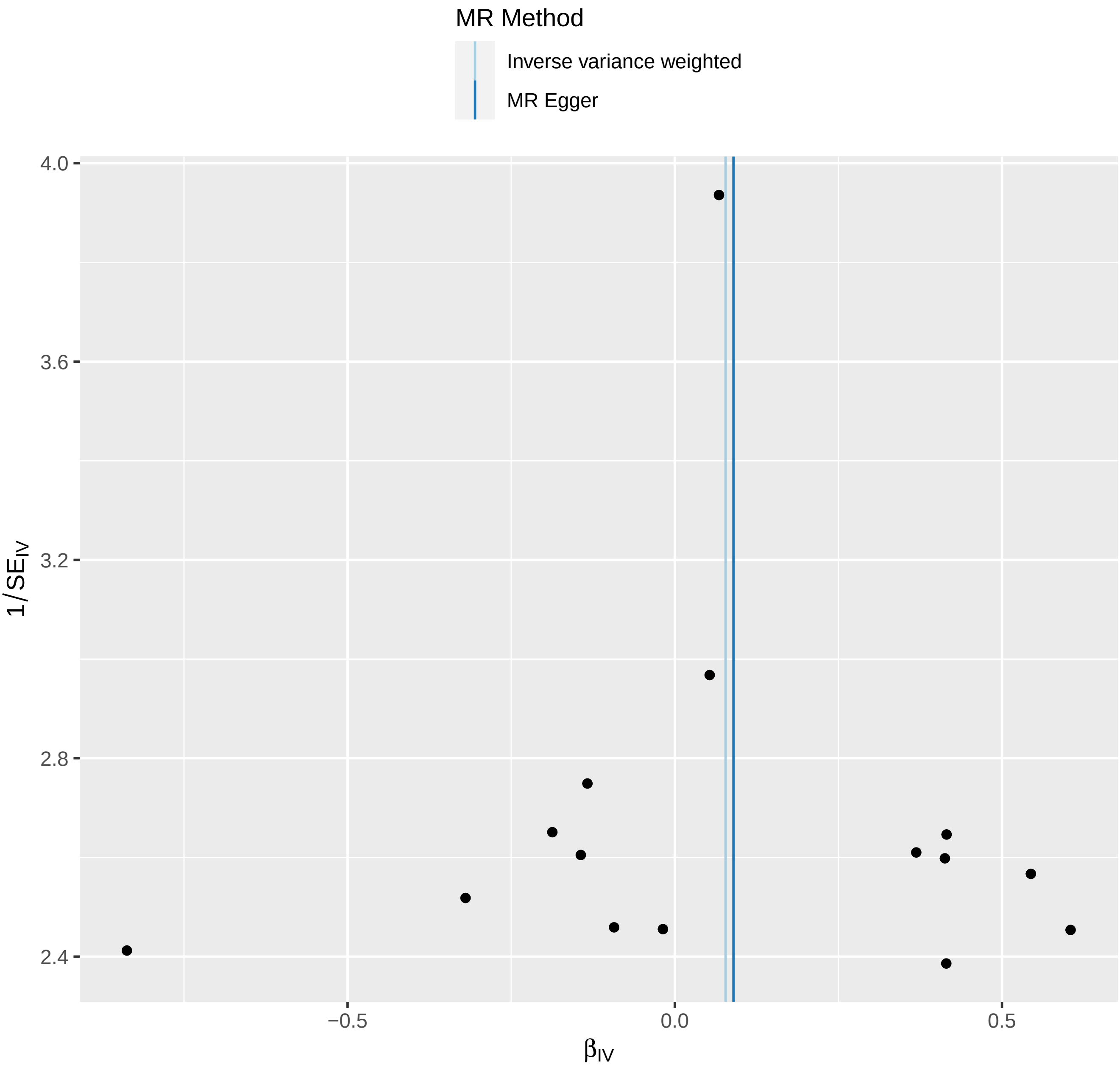

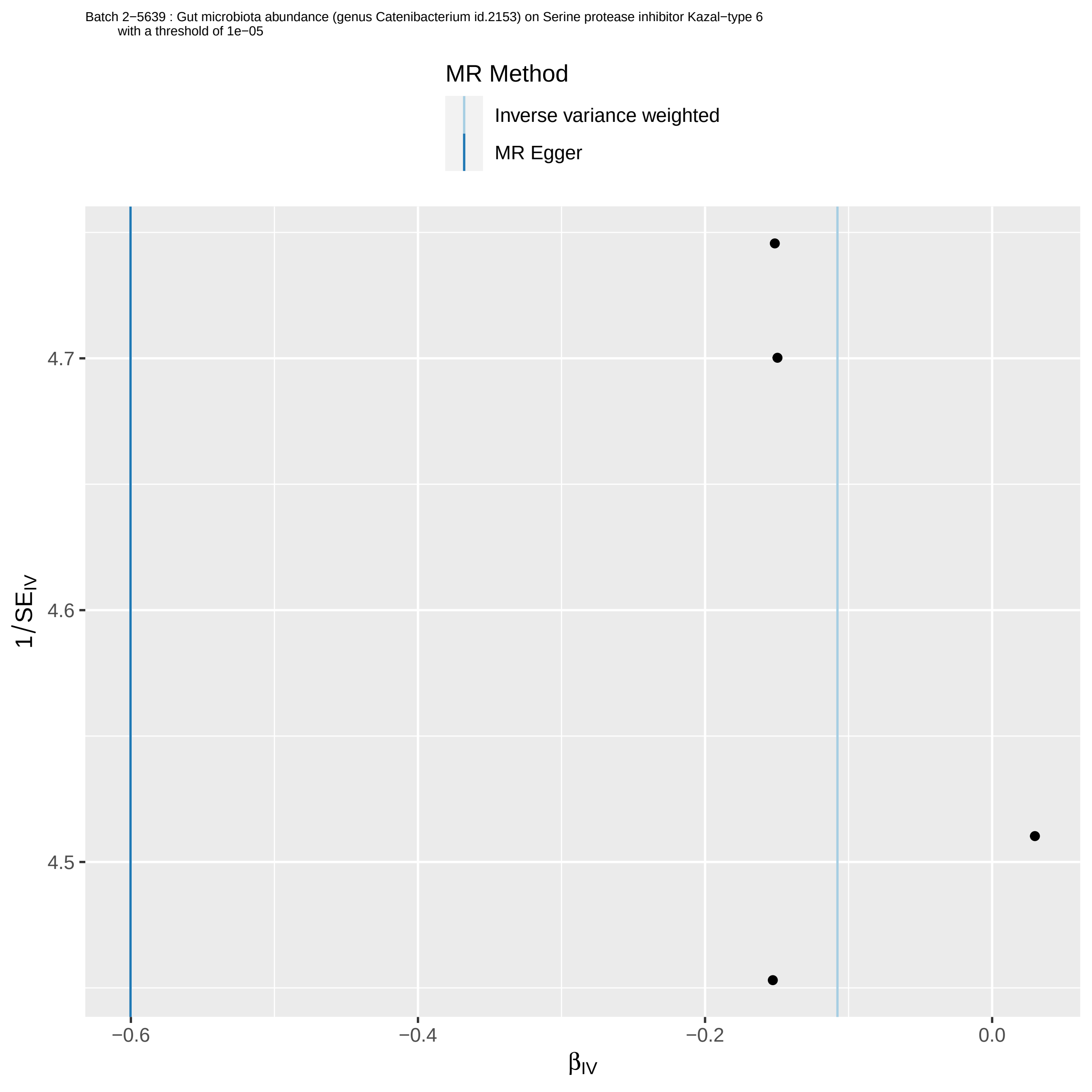
Batch 2−5639 : Gut microbiota abundance (genus Catenibacterium id.2153) on Serine protease inhibitor Kazal−type 6
with a threshold of 1e−05
MR Method
Inverse variance weighted
MR Egger
4.7
4.6
4.5
1/SE_IV
−0.6
−0.4
−0.2
0.0
β_IV

Batch 2−5640 : Gut microbiota abundance (genus Parasutterella id.2892) on Serine protease inhibitor Kazal−type 6
with a threshold of 1e−05

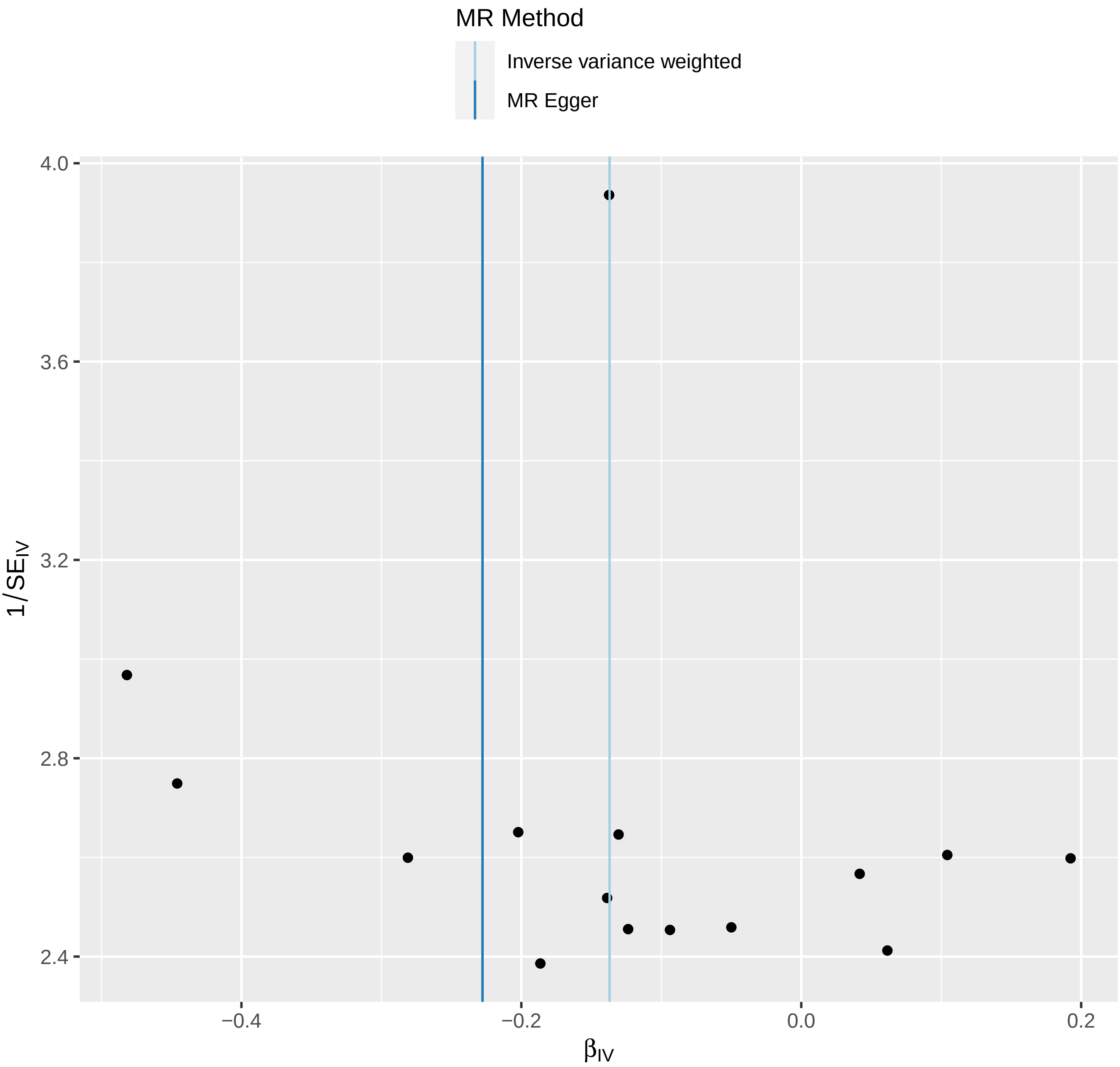

Batch 2−5641 : Gut microbiota abundance (genus Catenibacterium id.2153) on Serine protease inhibitor Kazal−type 8
with a threshold of 1e−05

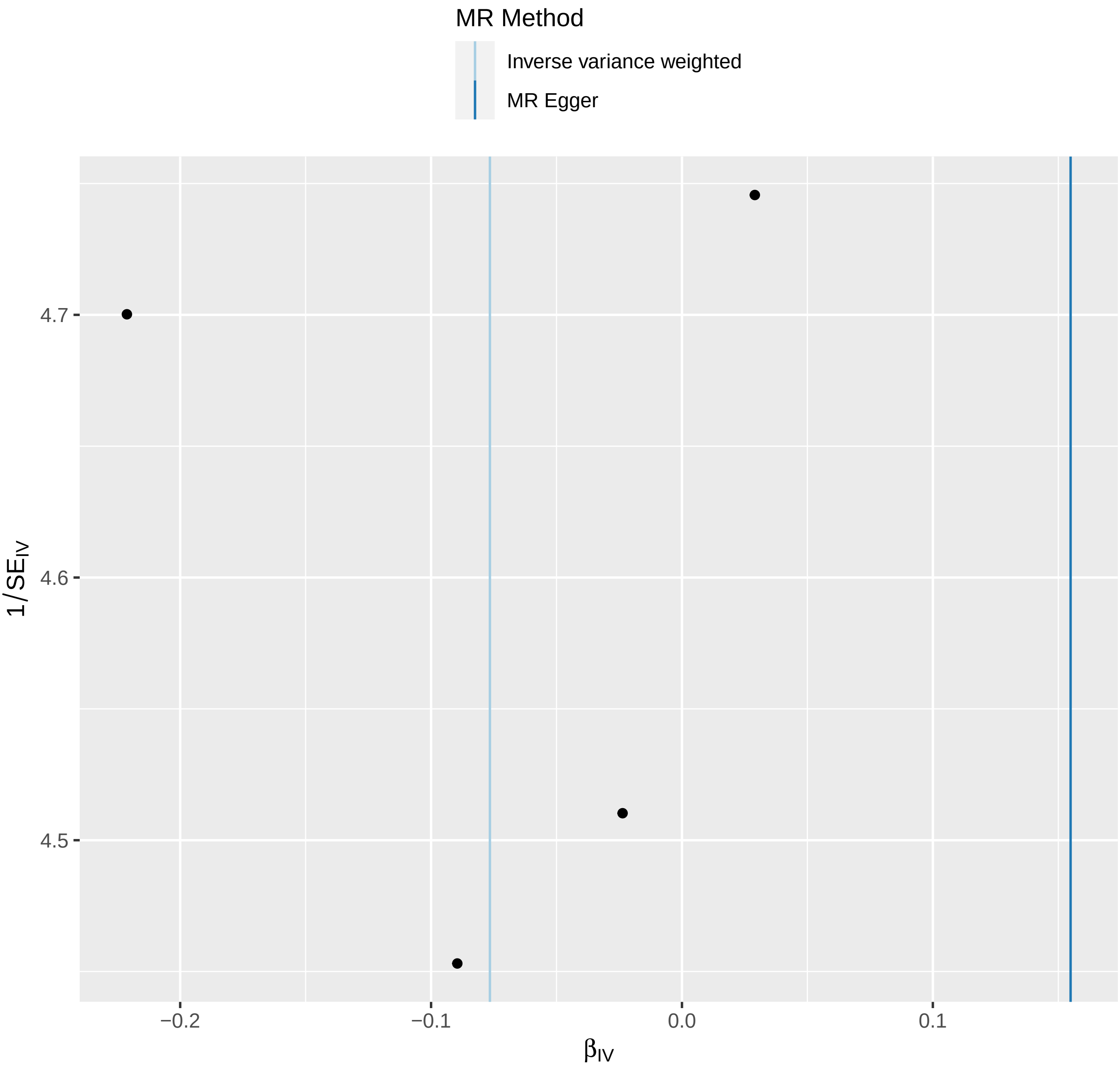

Batch 2−5642 : Gut microbiota abundance (genus Parasutterella id.2892) on Serine protease inhibitor Kazal−type 8
with a threshold of 1e−05

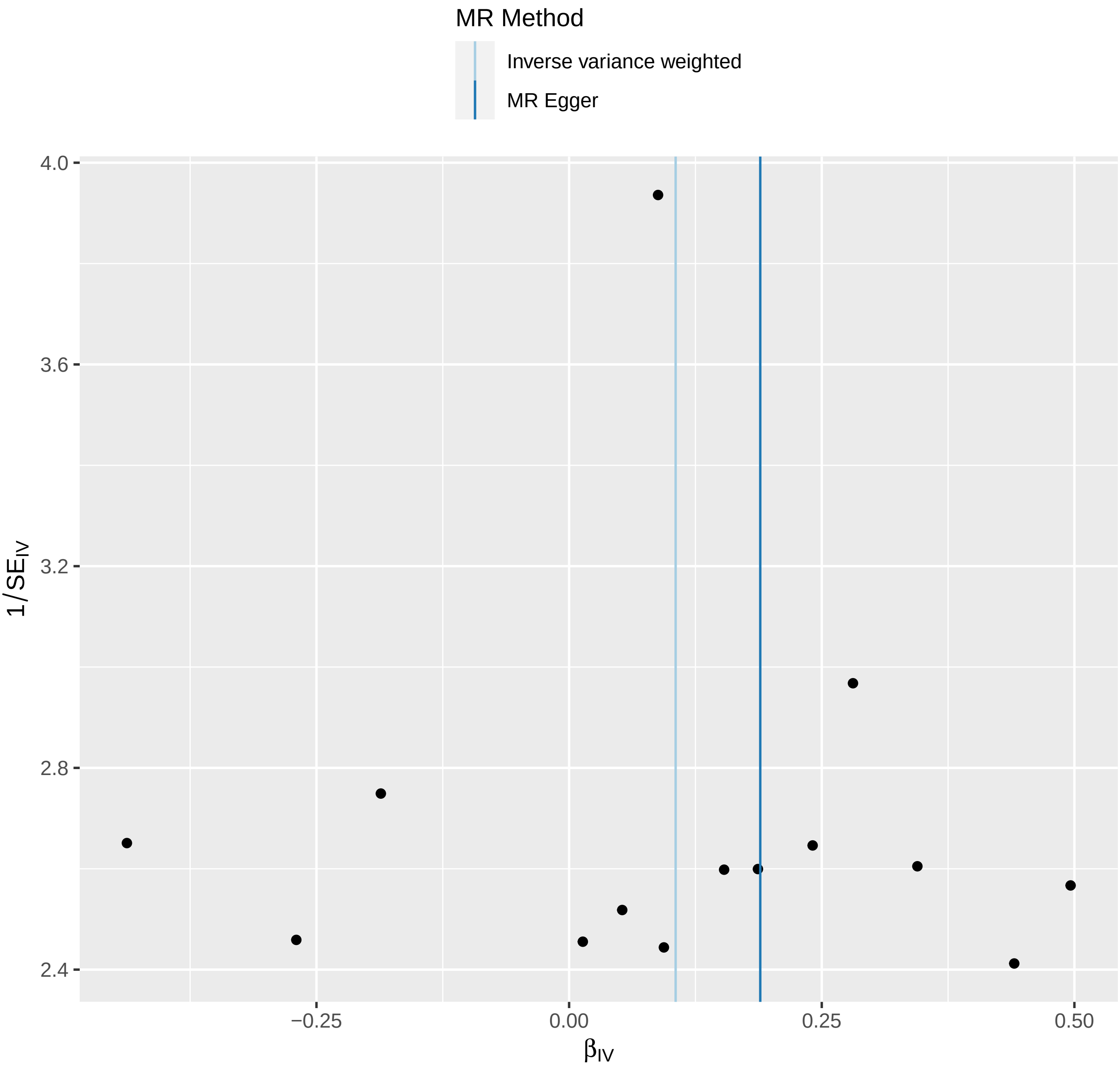

Batch 2−5643 : Gut microbiota abundance (genus Catenibacterium id.2153) on Serine protease inhibitor Kazal−type 9
with a threshold of 1e−05

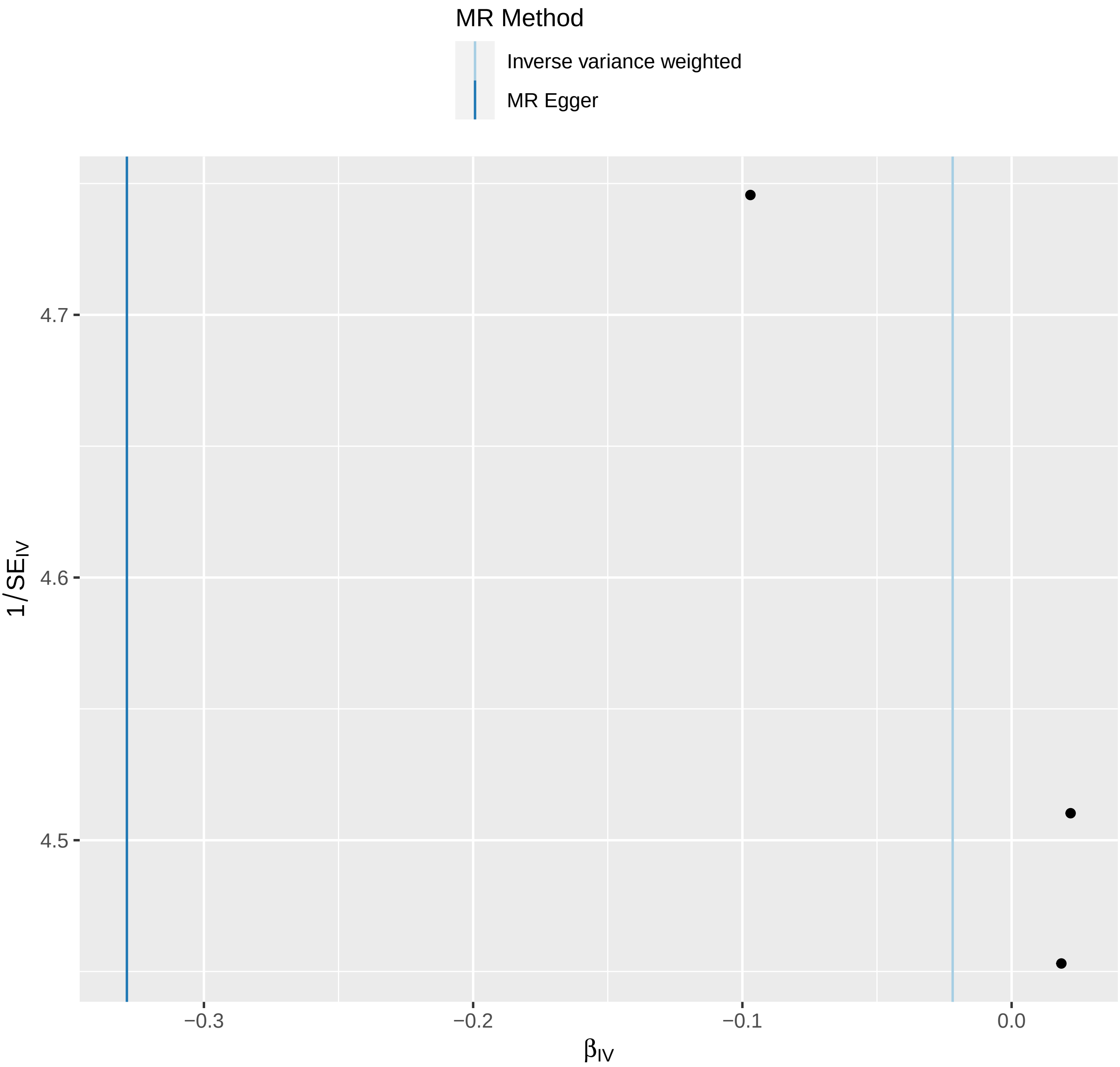

Batch 2−5644 : Gut microbiota abundance (genus Parasutterella id.2892) on Serine protease inhibitor Kazal−type 9
with a threshold of 1e−05

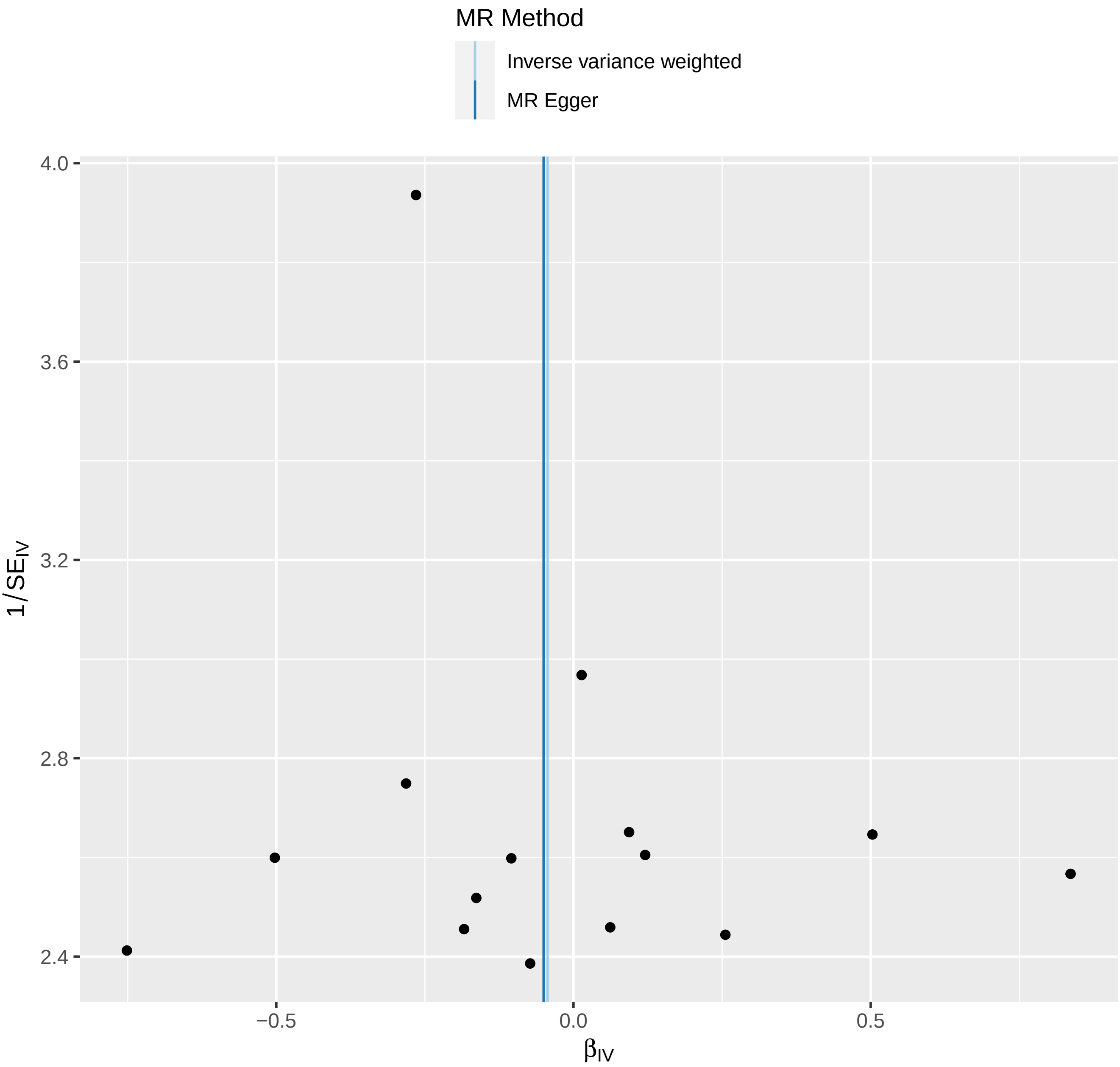

Batch 2−5645 : Gut microbiota abundance (genus Catenibacterium id.2153) on Kunitz−type protease inhibitor 1
with a threshold of 1e−05

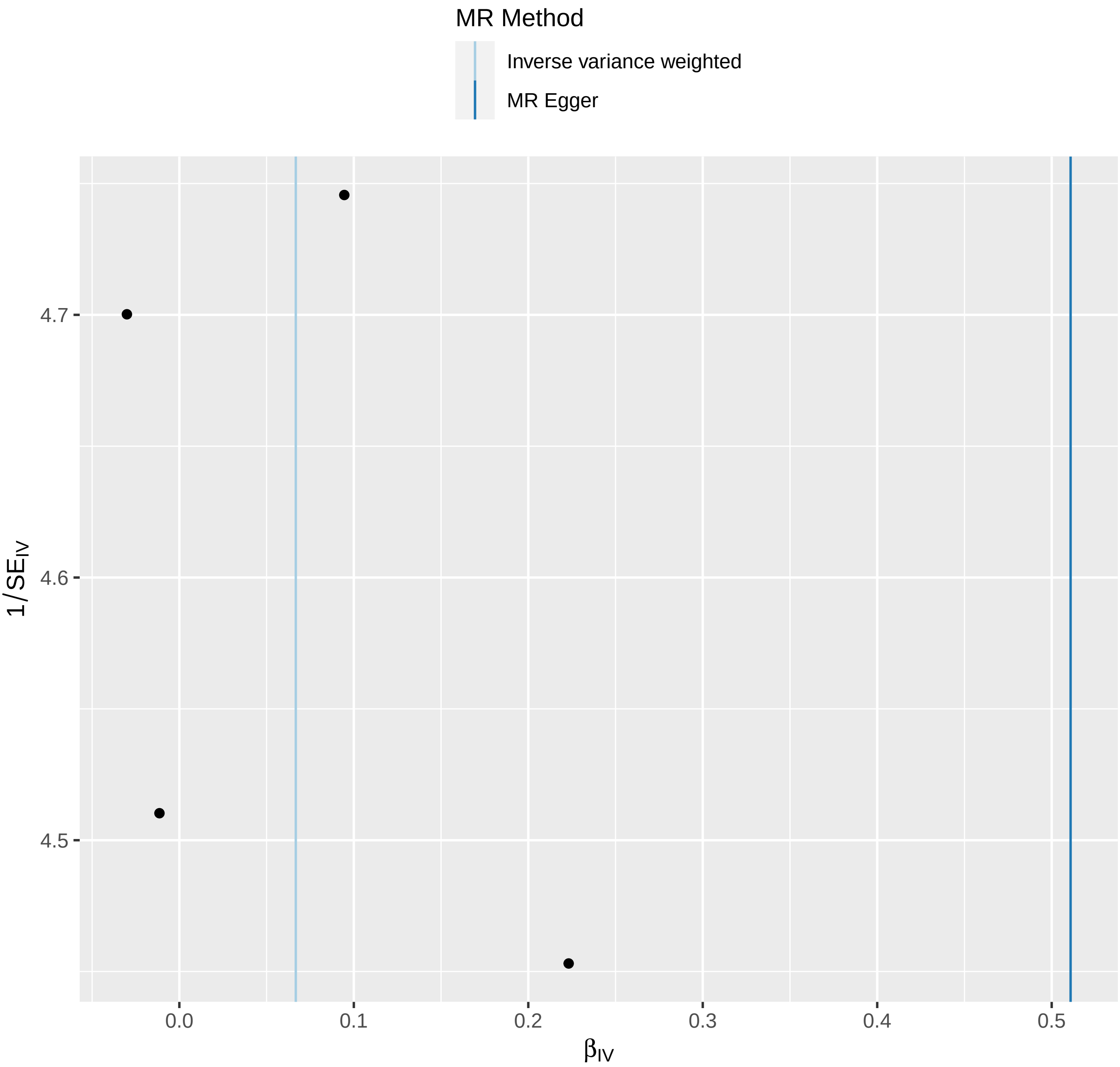

Batch 2−5646 : Gut microbiota abundance (genus Parasutterella id.2892) on Kunitz−type protease inhibitor 1
with a threshold of 1e−05

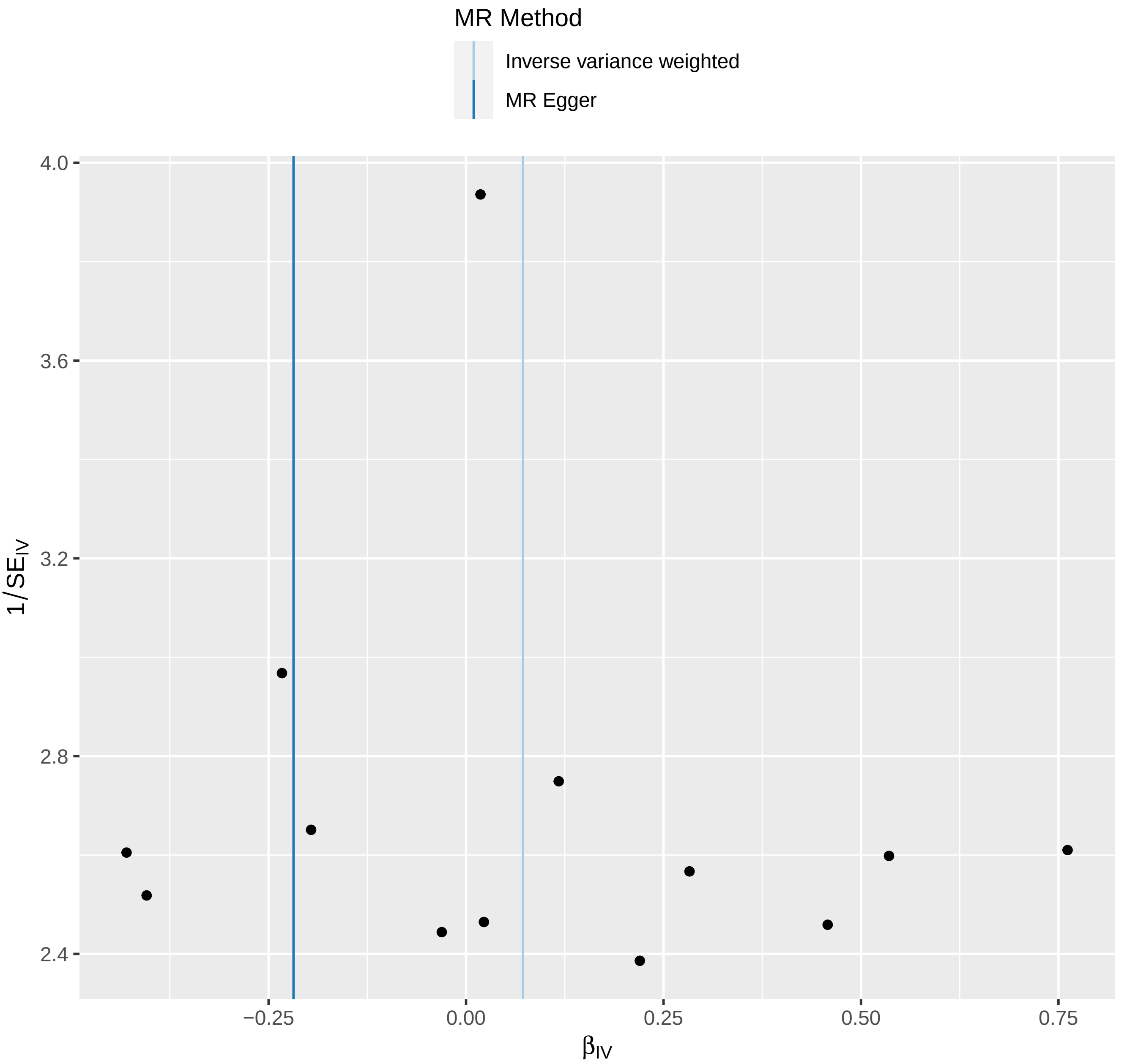

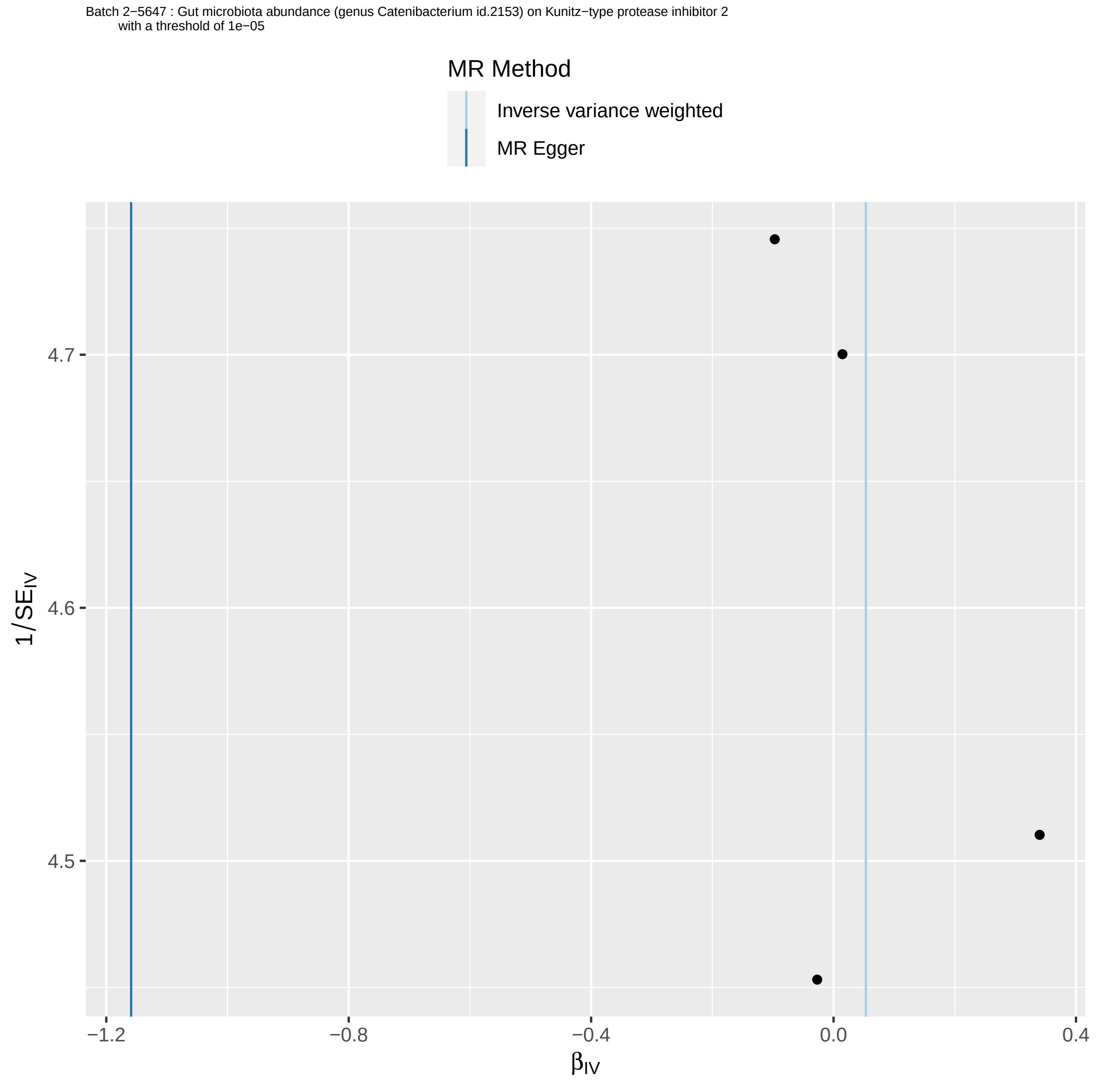
Batch 2−5647 : Gut microbiota abundance (genus Catenibacterium id.2153) on Kunitz−type protease inhibitor 2
with a threshold of 1e−05
MR Method
Inverse variance weighted
MR Egger
4.7
4.6
4.5
$1/SE_{IV}$
−1.2
−0.8
−0.4
0.0
0.4
β_{IV}

Batch 2−5648 : Gut microbiota abundance (genus Parasutterella id.2892) on Kunitz−type protease inhibitor 2
with a threshold of 1e−05

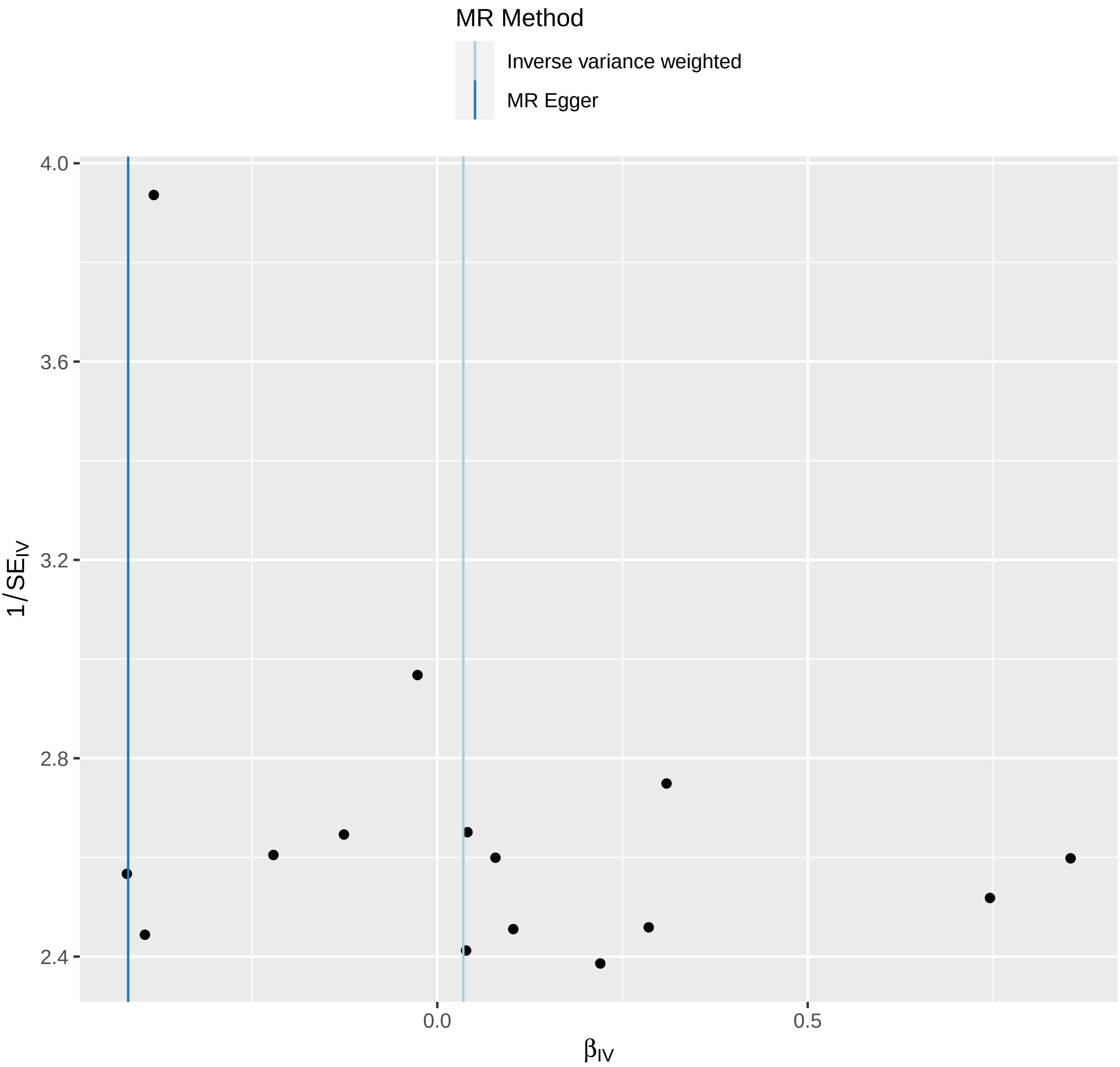

Batch 2−5649 : Gut microbiota abundance (genus Catenibacterium id.2153) on Kunitz−type protease inhibitor 3
with a threshold of 1e−05

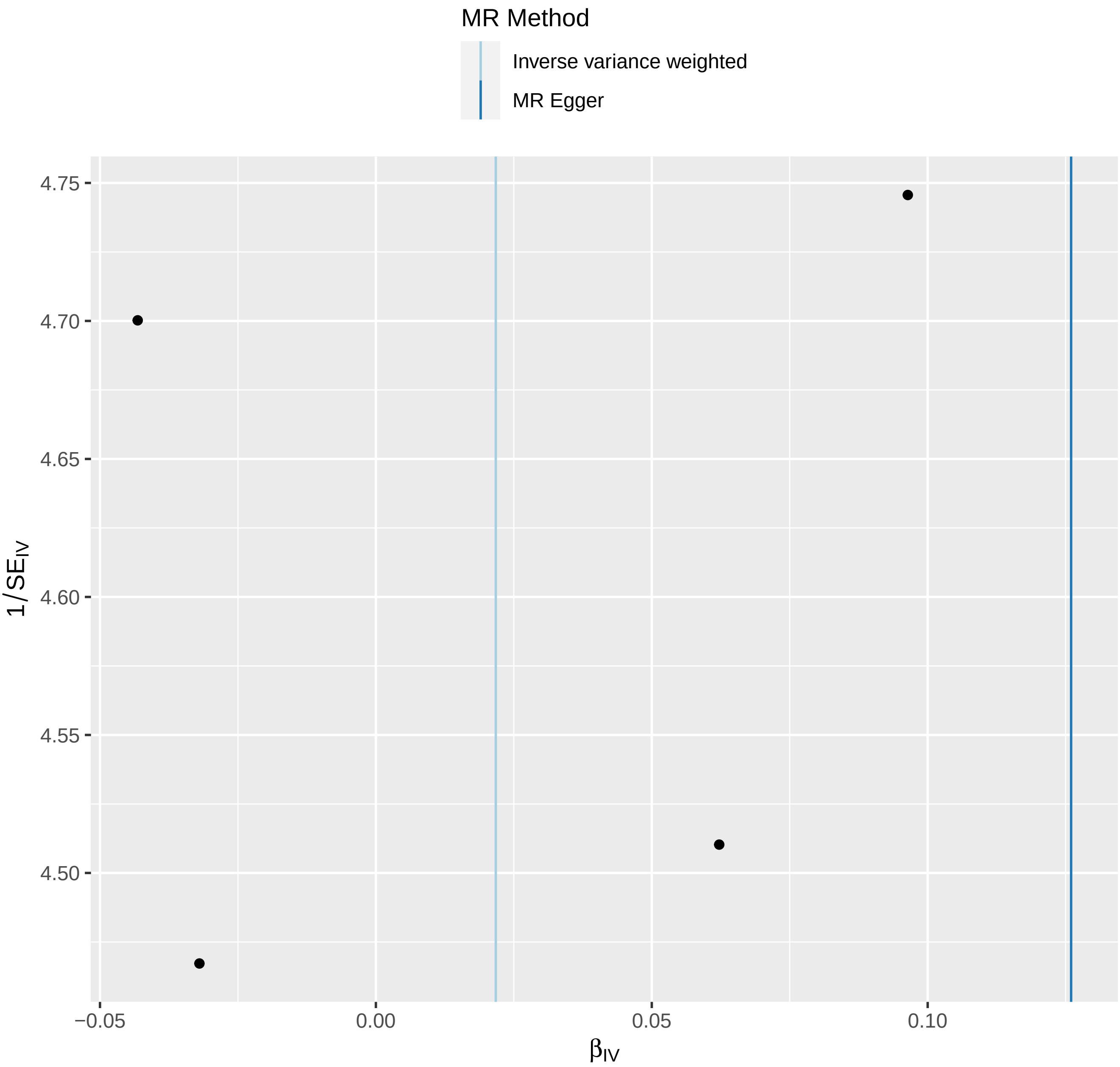

Batch 2−5650 : Gut microbiota abundance (genus Parasutterella id.2892) on Kunitz−type protease inhibitor 3
with a threshold of 1e−05

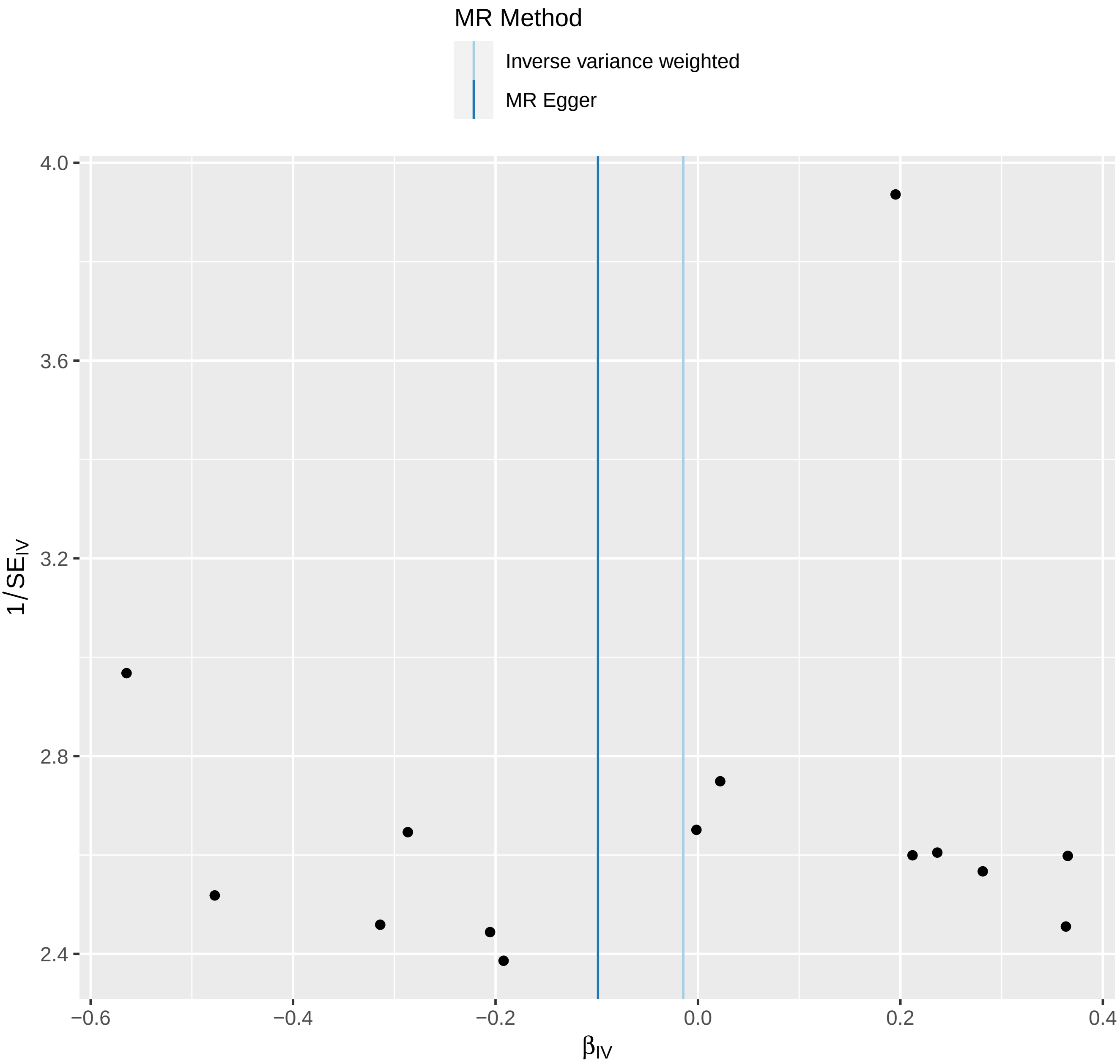

Batch 2−5651 : Gut microbiota abundance (genus Catenibacterium id.2153) on Testican−1
with a threshold of 1e−05

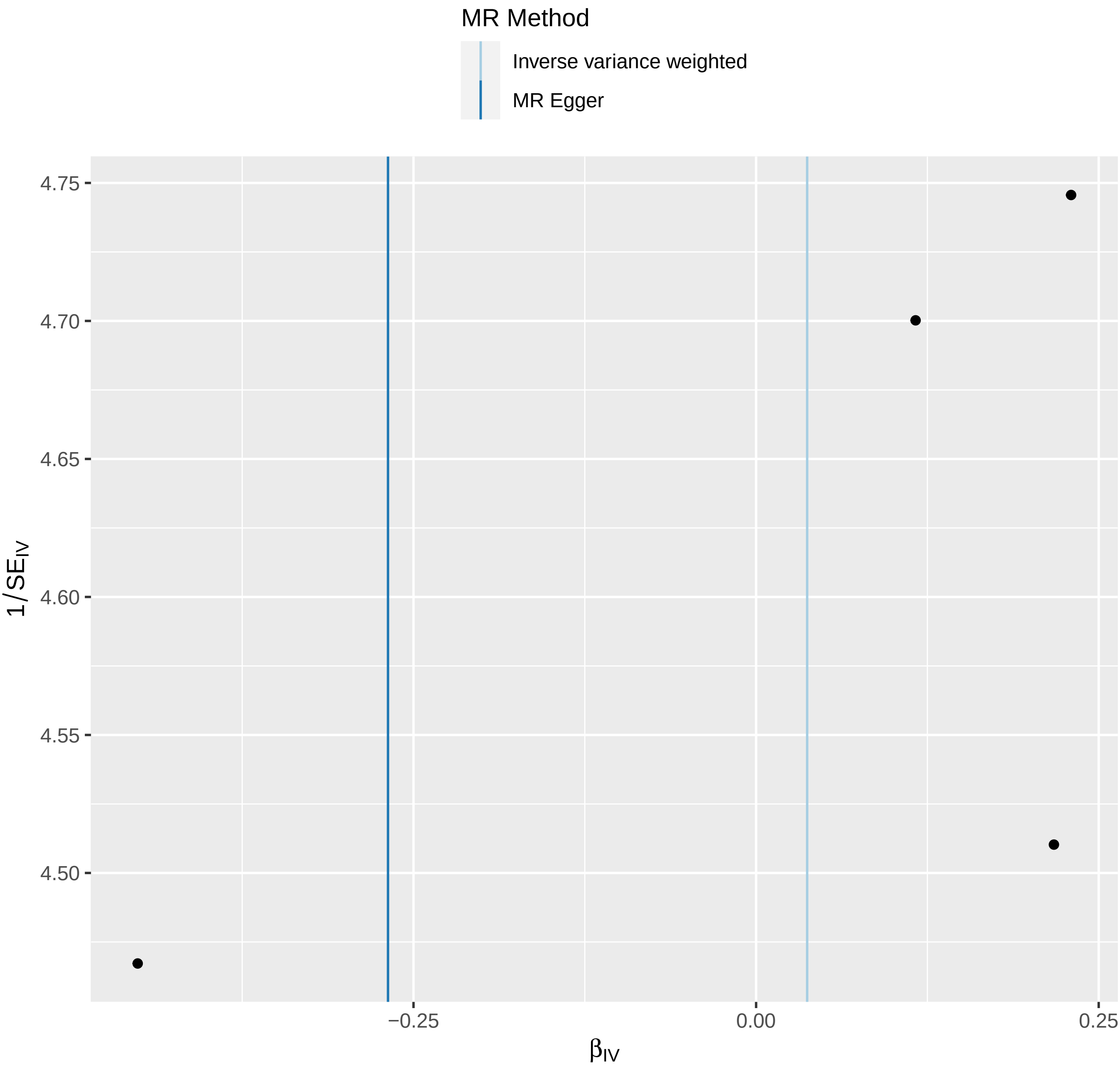

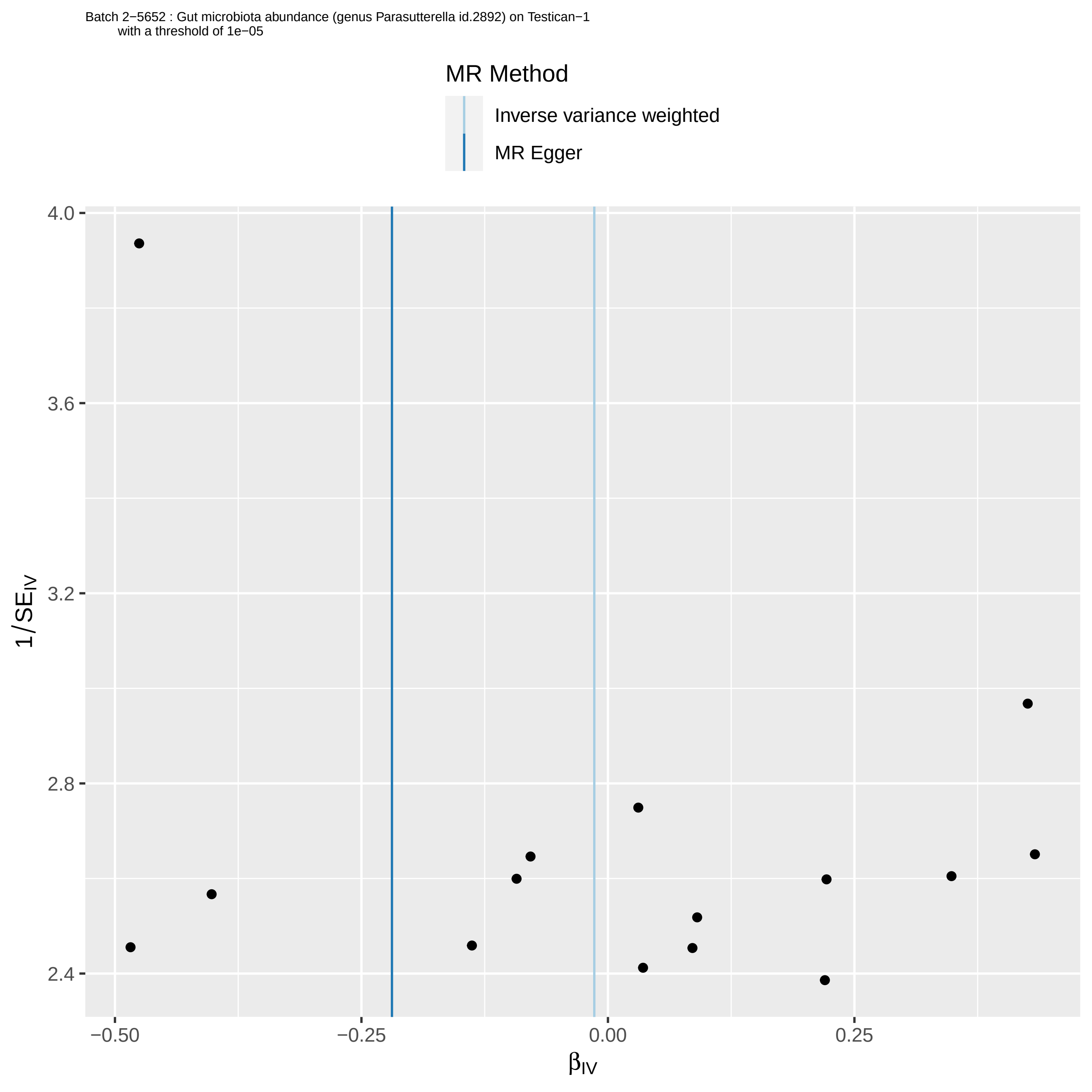
Batch 2−5652 : Gut microbiota abundance (genus Parasutterella id.2892) on Testican−1
with a threshold of 1e−05
MR Method
Inverse variance weighted
MR Egger
4.0
3.6
3.2
2.8
2.4
1/SE_IV
−0.50
−0.25
0.00
0.25
β_IV

Batch 2−5653 : Gut microbiota abundance (genus Catenibacterium id.2153) on Testican−2
with a threshold of 1e−05

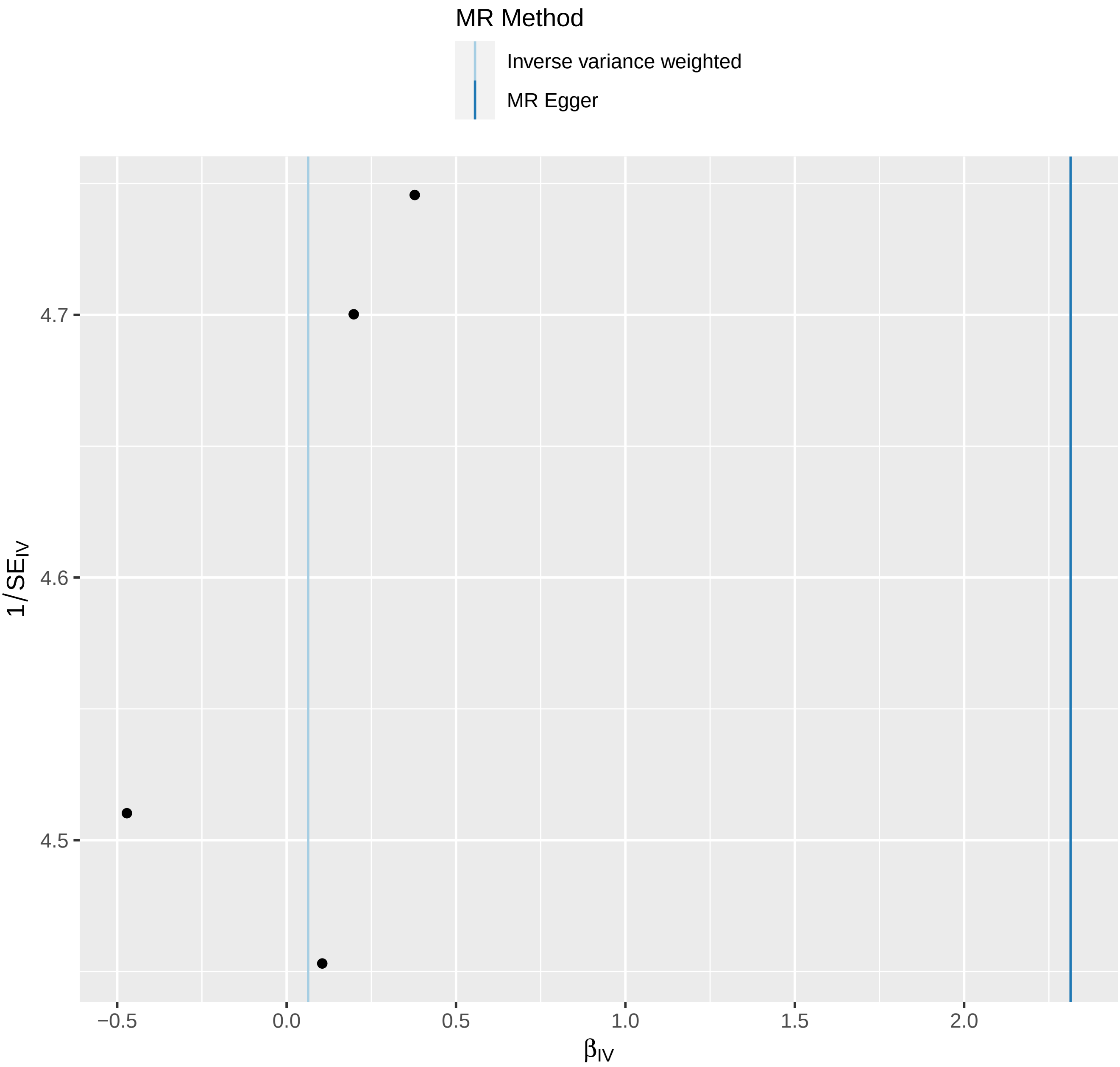

Batch 2−5654 : Gut microbiota abundance (genus Parasutterella id.2892) on Testican−2
with a threshold of 1e−05

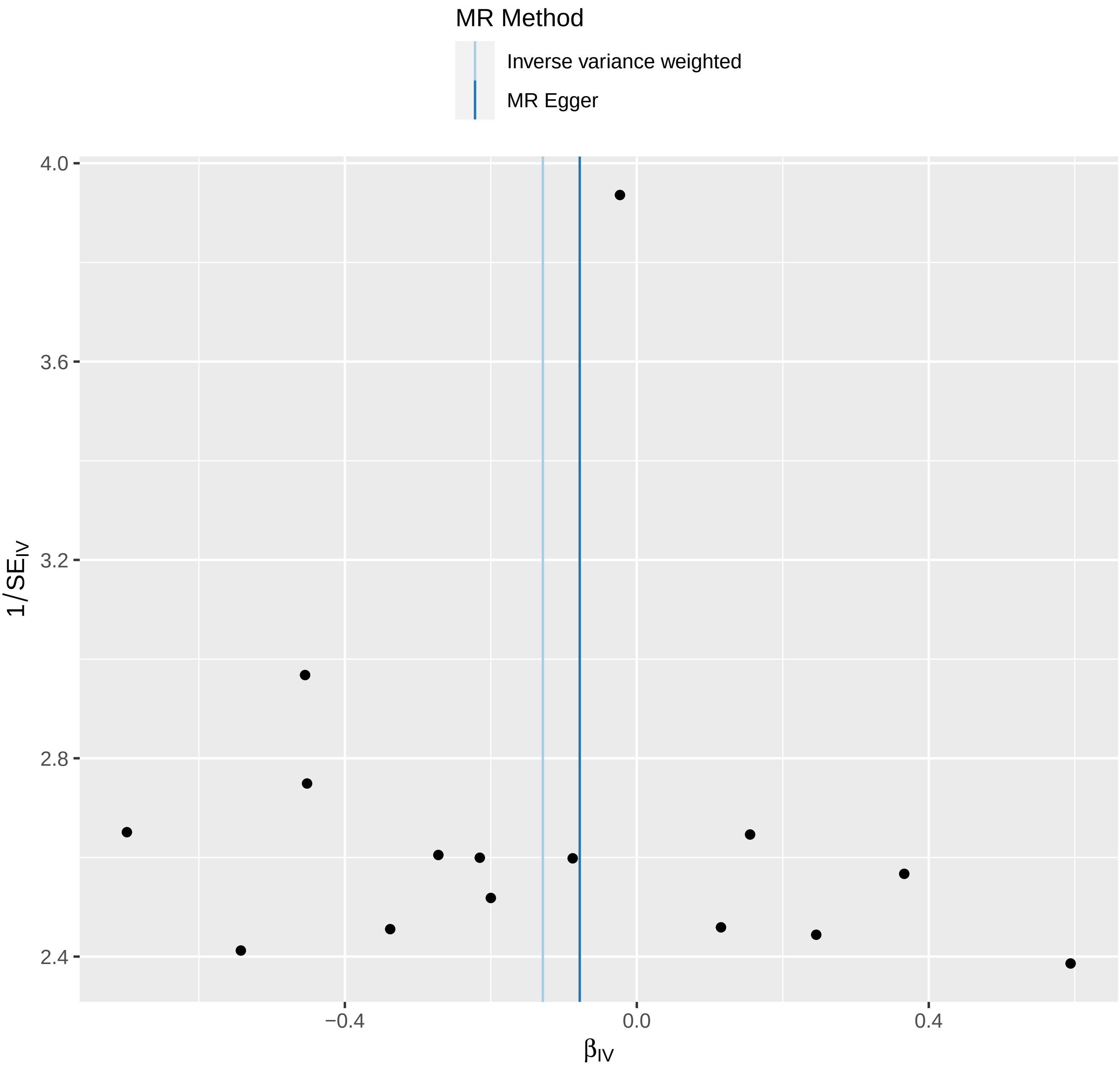

Batch 2−5655 : Gut microbiota abundance (genus Catenibacterium id.2153) on Testican−3
with a threshold of 1e−05

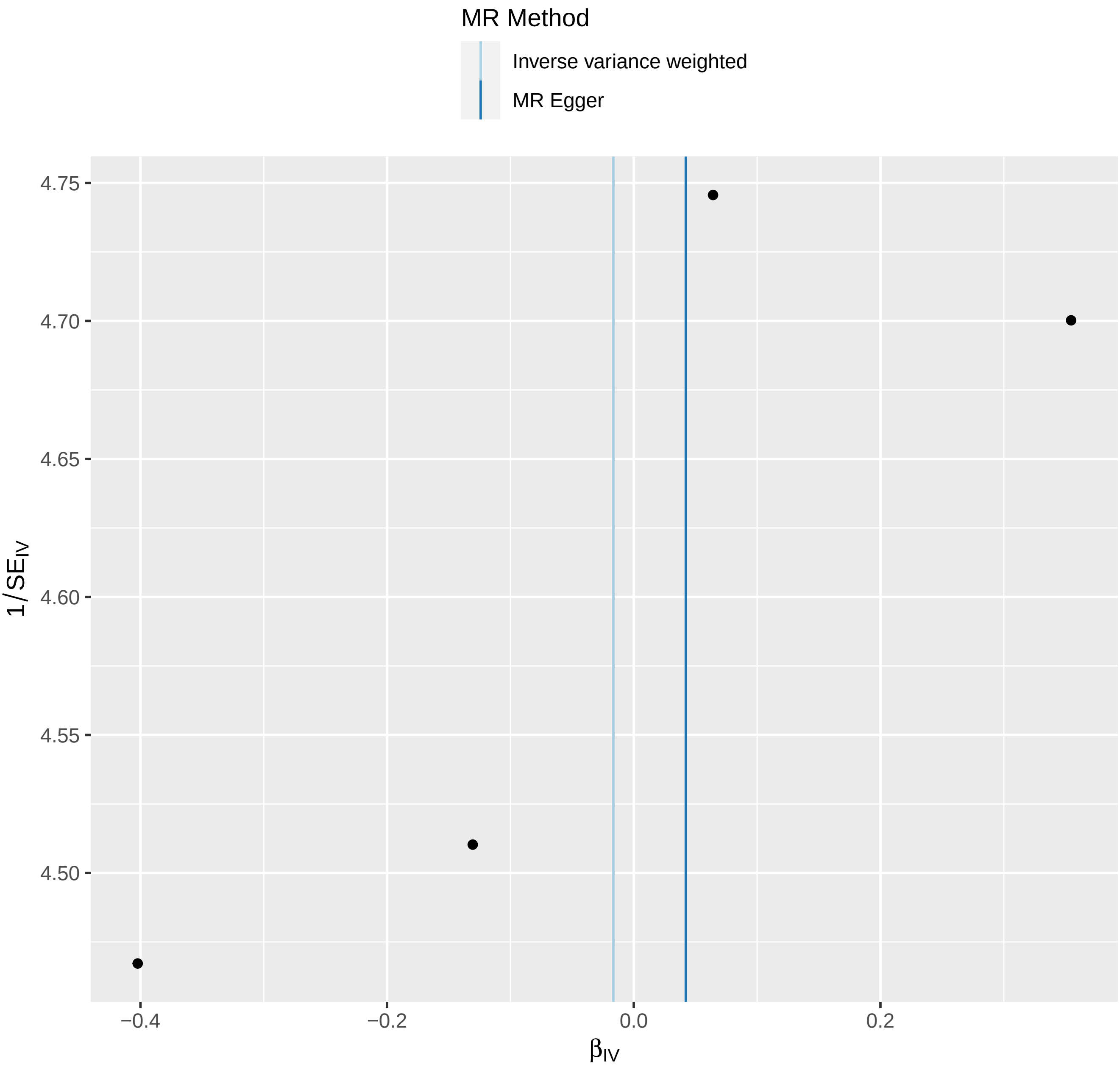

Batch 2−5656 : Gut microbiota abundance (genus Parasutterella id.2892) on Testican−3
with a threshold of 1e−05

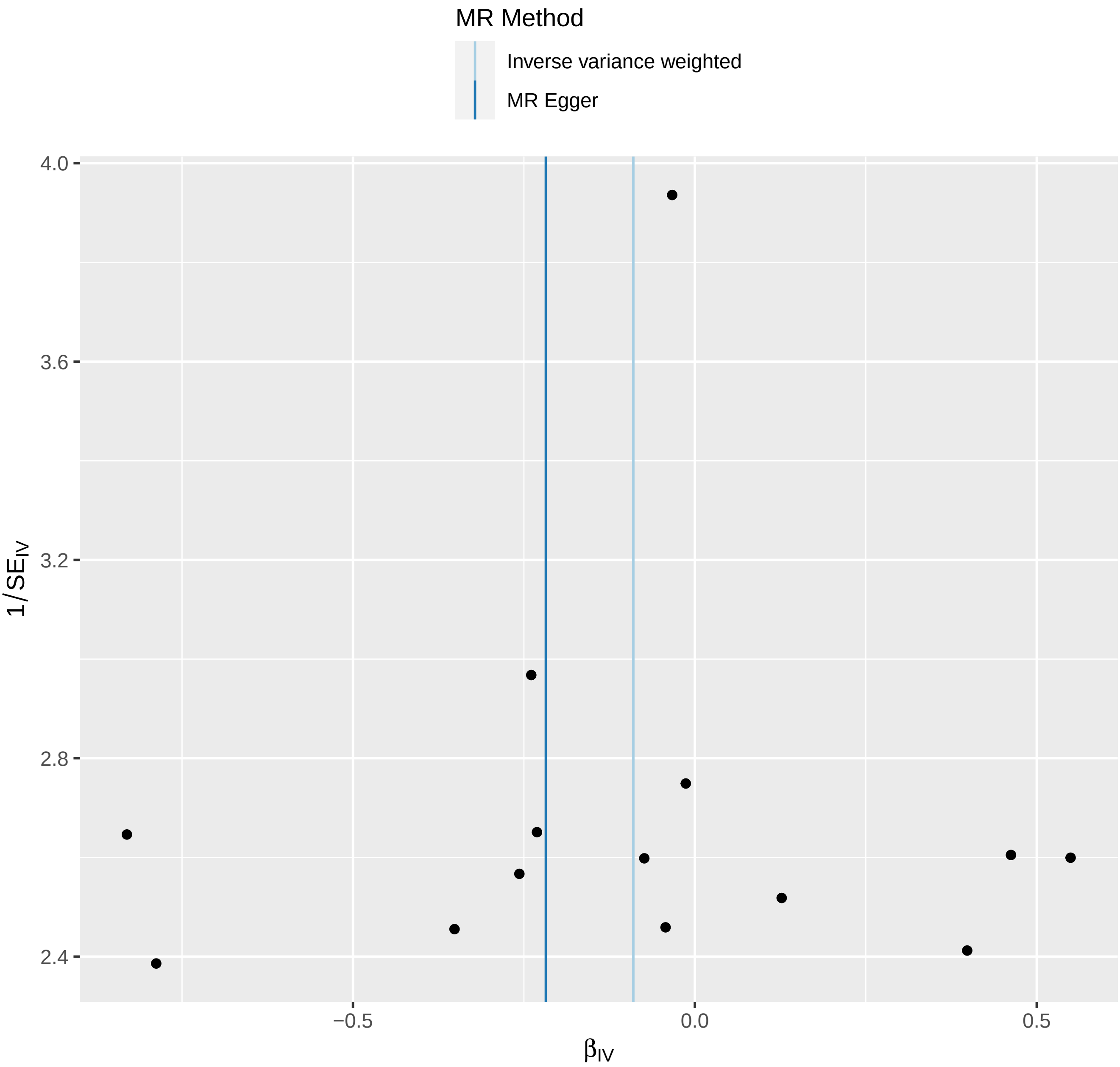

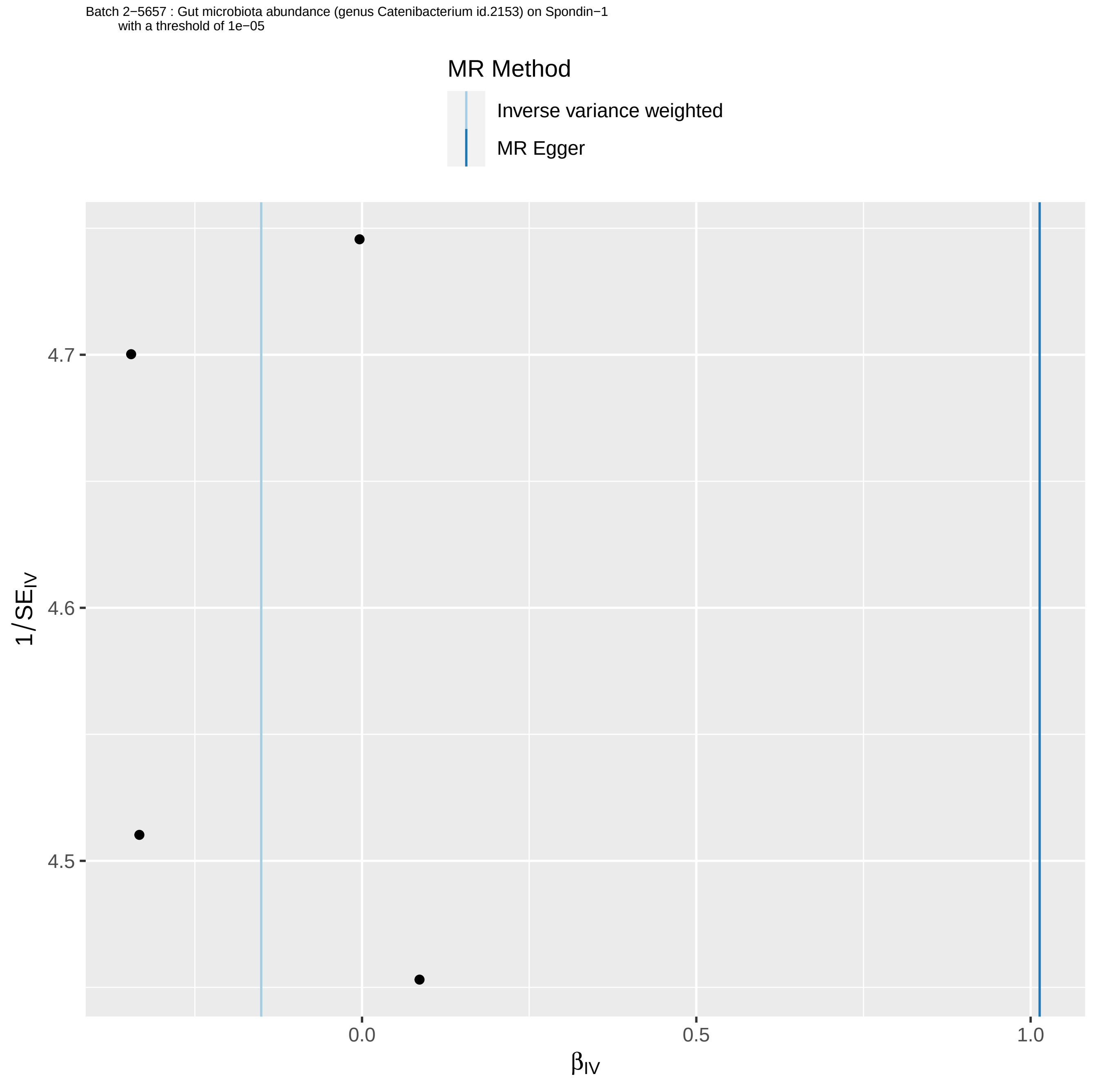

Batch 2−5657 : Gut microbiota abundance (genus Catenibacterium id.2153) on Spondin−1
with a threshold of 1e−05
MR Method
Inverse variance weighted
MR Egger
4.7
4.6
4.5
1/SE_IV
0.0
0.5
1.0
β_IV

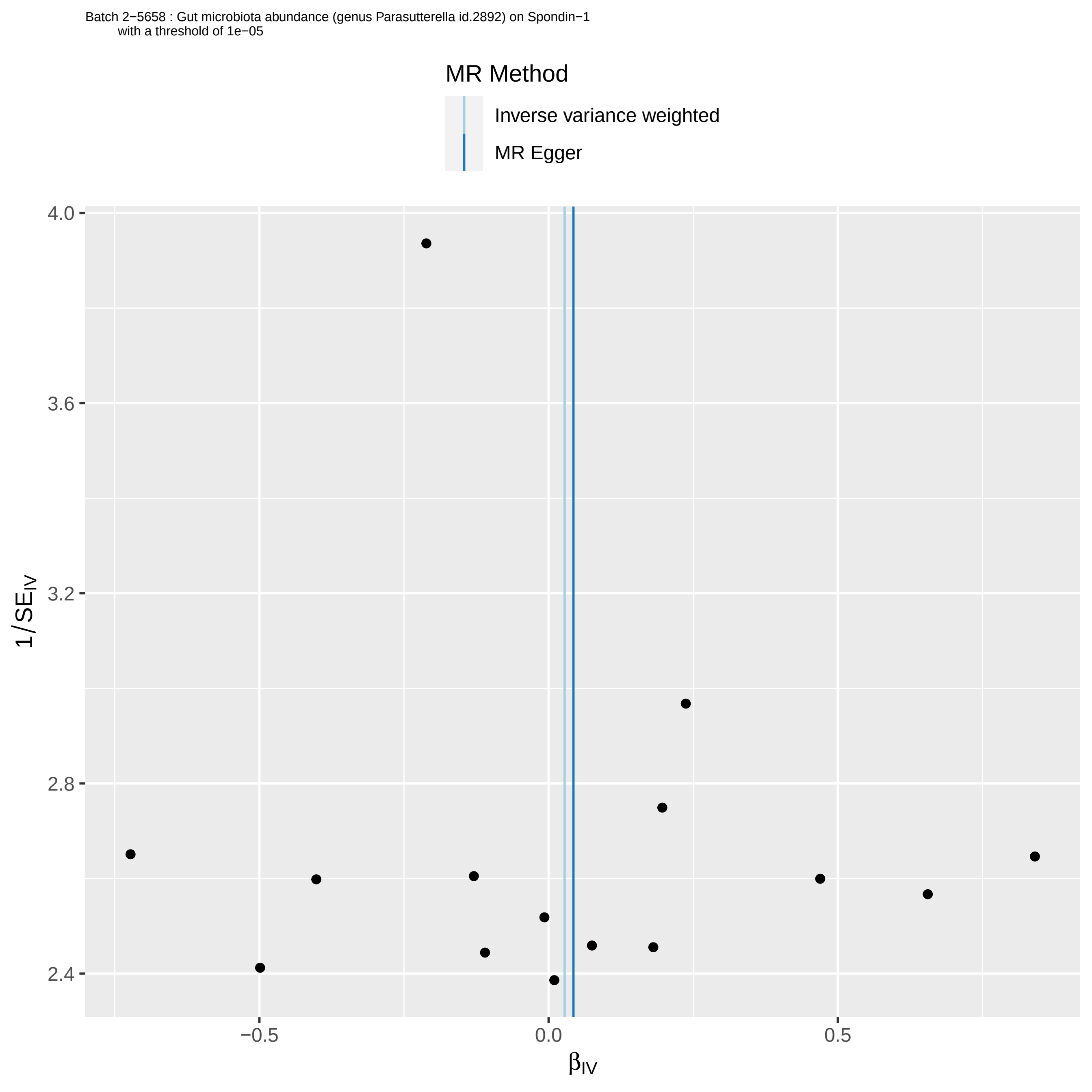

Batch 2−5658 : Gut microbiota abundance (genus Parasutterella id.2892) on Spondin−1
with a threshold of 1e−05
MR Method
Inverse variance weighted
MR Egger
4.0
3.6
3.2
2.8
2.4
1/SE_IV
−0.5
0.0
0.5
β_IV

Batch 2−5659 : Gut microbiota abundance (genus Catenibacterium id.2153) on Speckle−type POZ protein
with a threshold of 1e−05

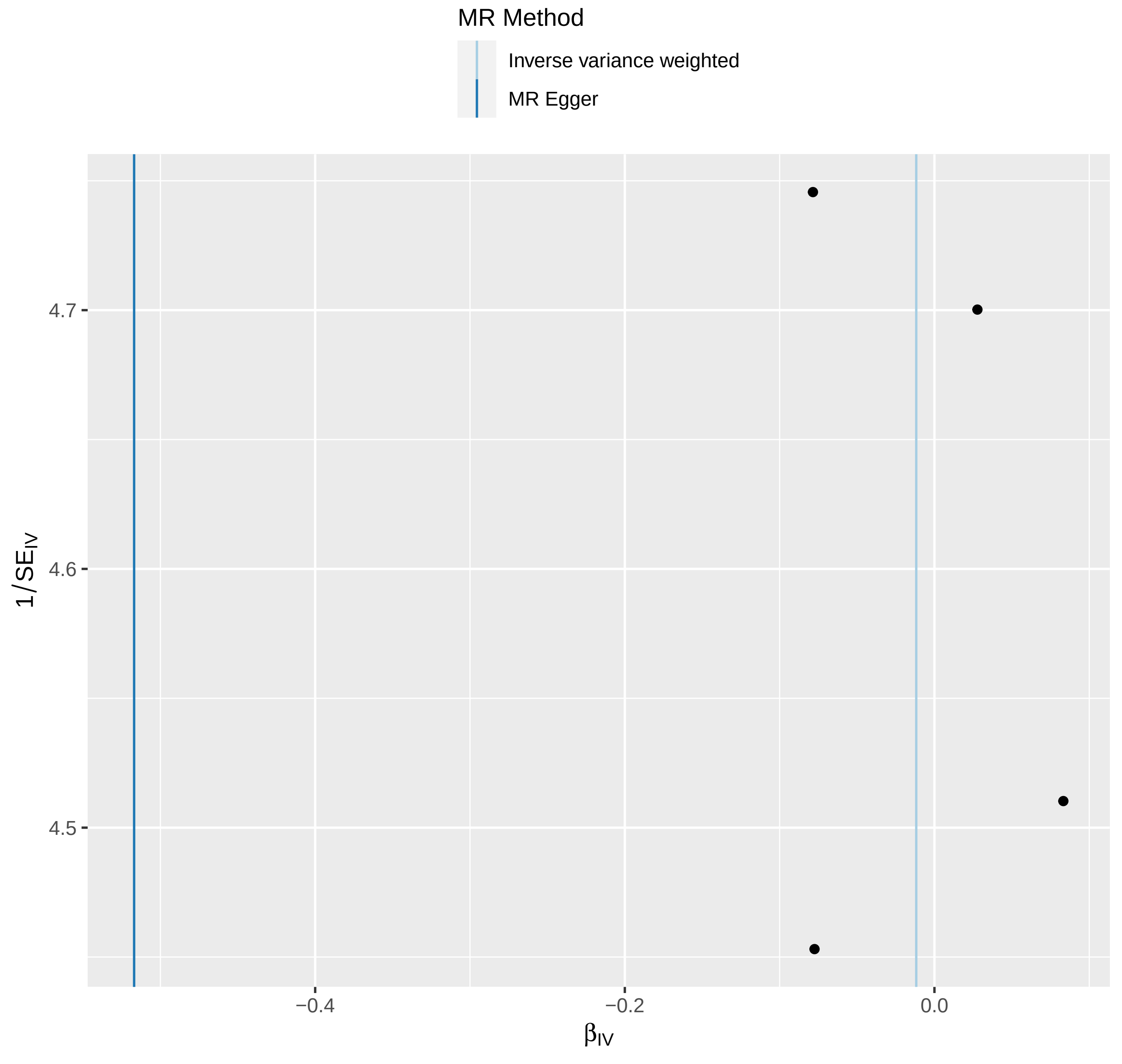

Batch 2−5660 : Gut microbiota abundance (genus Parasutterella id.2892) on Speckle−type POZ protein
with a threshold of 1e−05

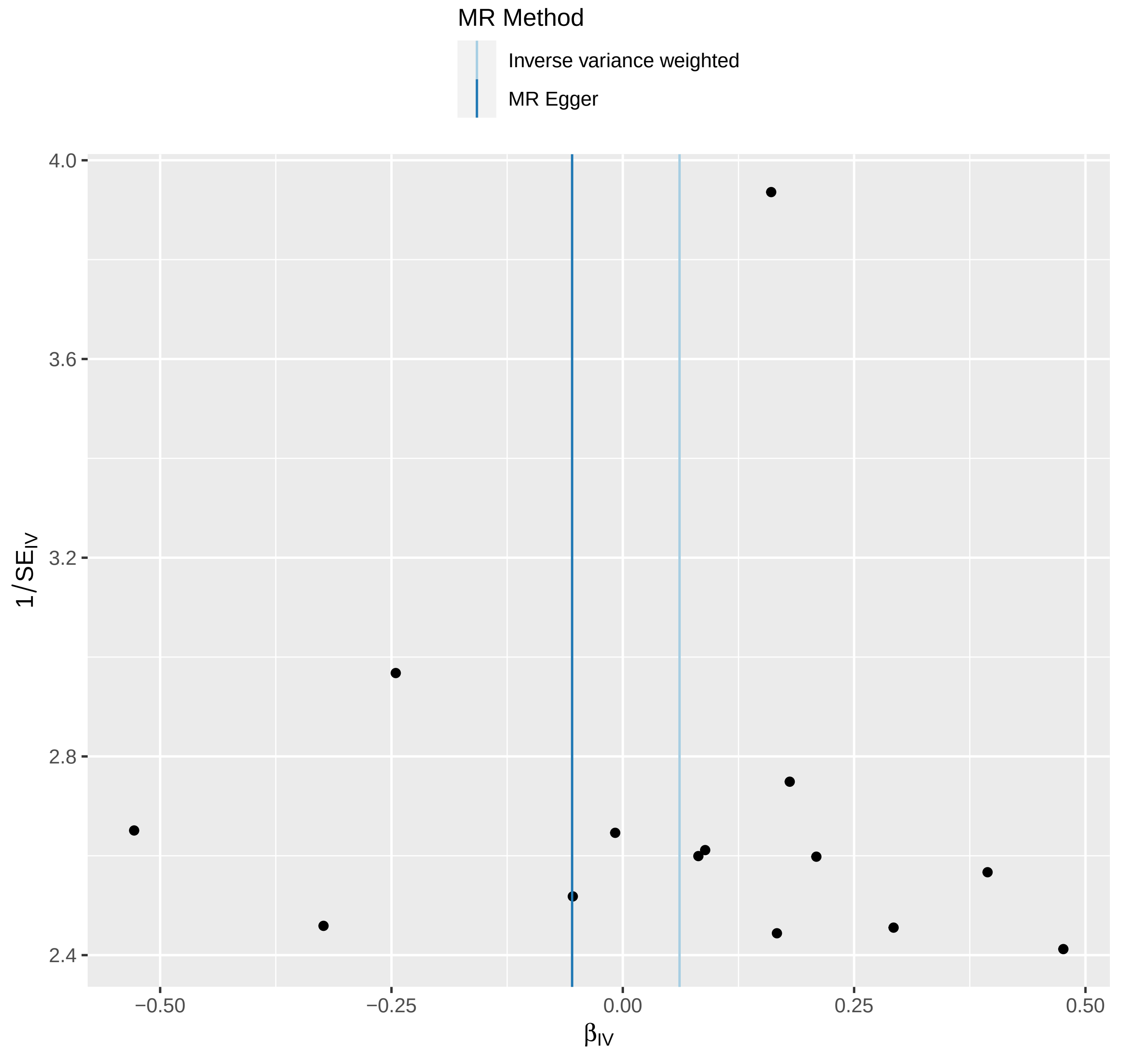

Batch 2−5661 : Gut microbiota abundance (genus Catenibacterium id.2153) on Sepiapterin reductase
with a threshold of 1e−05

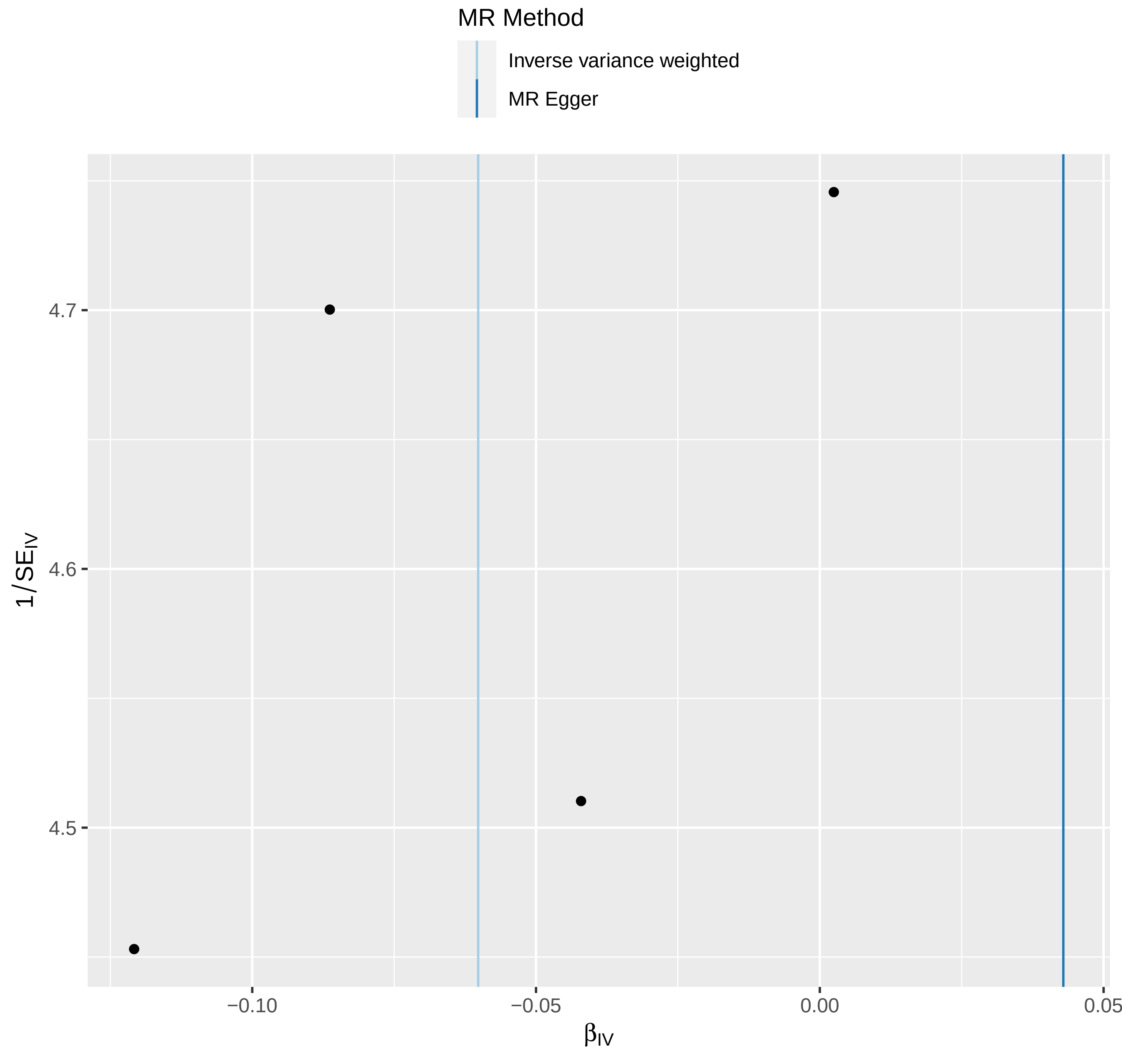

Batch 2−5662 : Gut microbiota abundance (genus Parasutterella id.2892) on Sepiapterin reductase
with a threshold of 1e−05

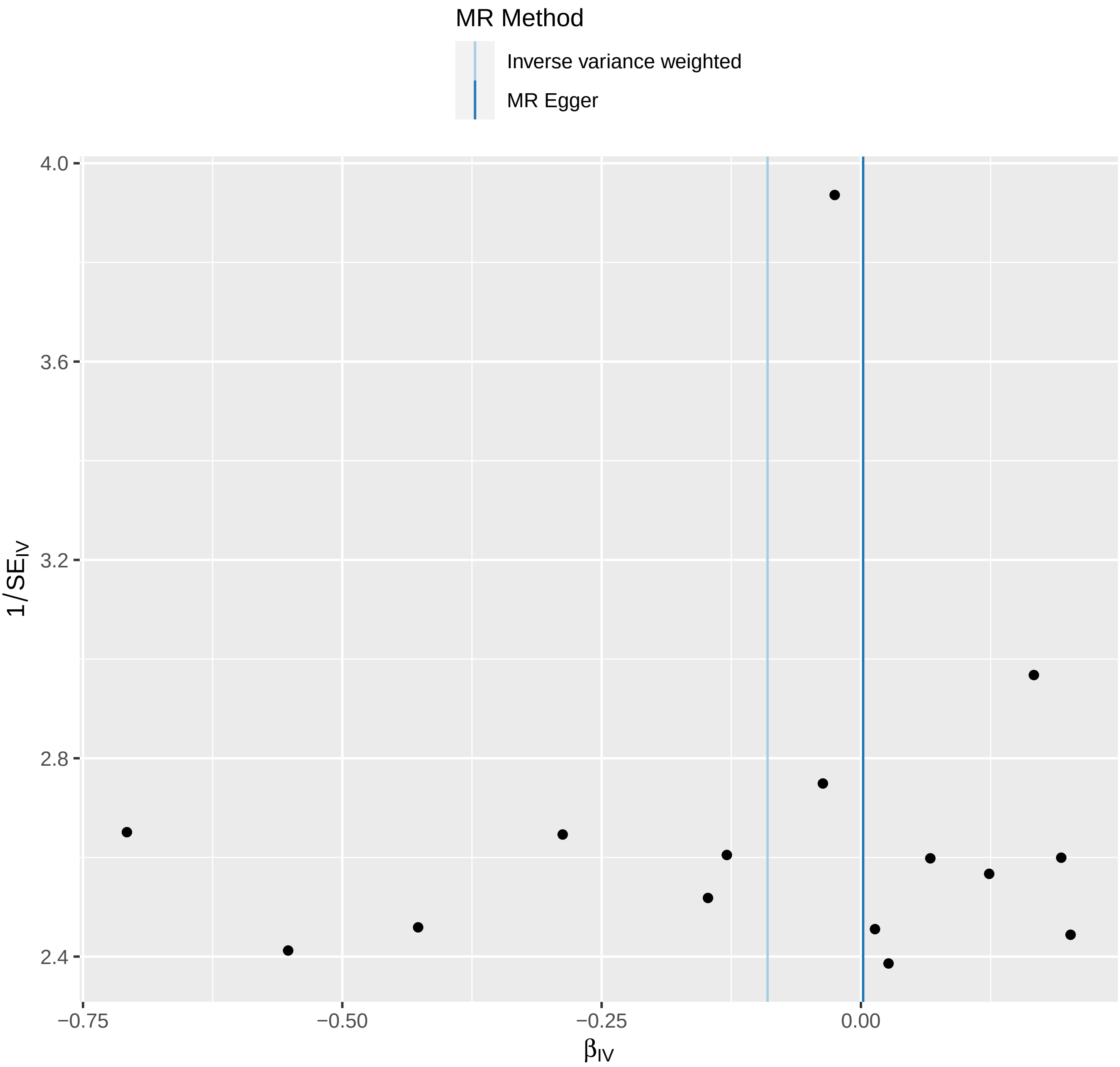

Batch 2−5663 : Gut microbiota abundance (genus Catenibacterium id.2153) on Sprouty−related, EVH1 domain−containing protein 1
with a threshold of 1e−05

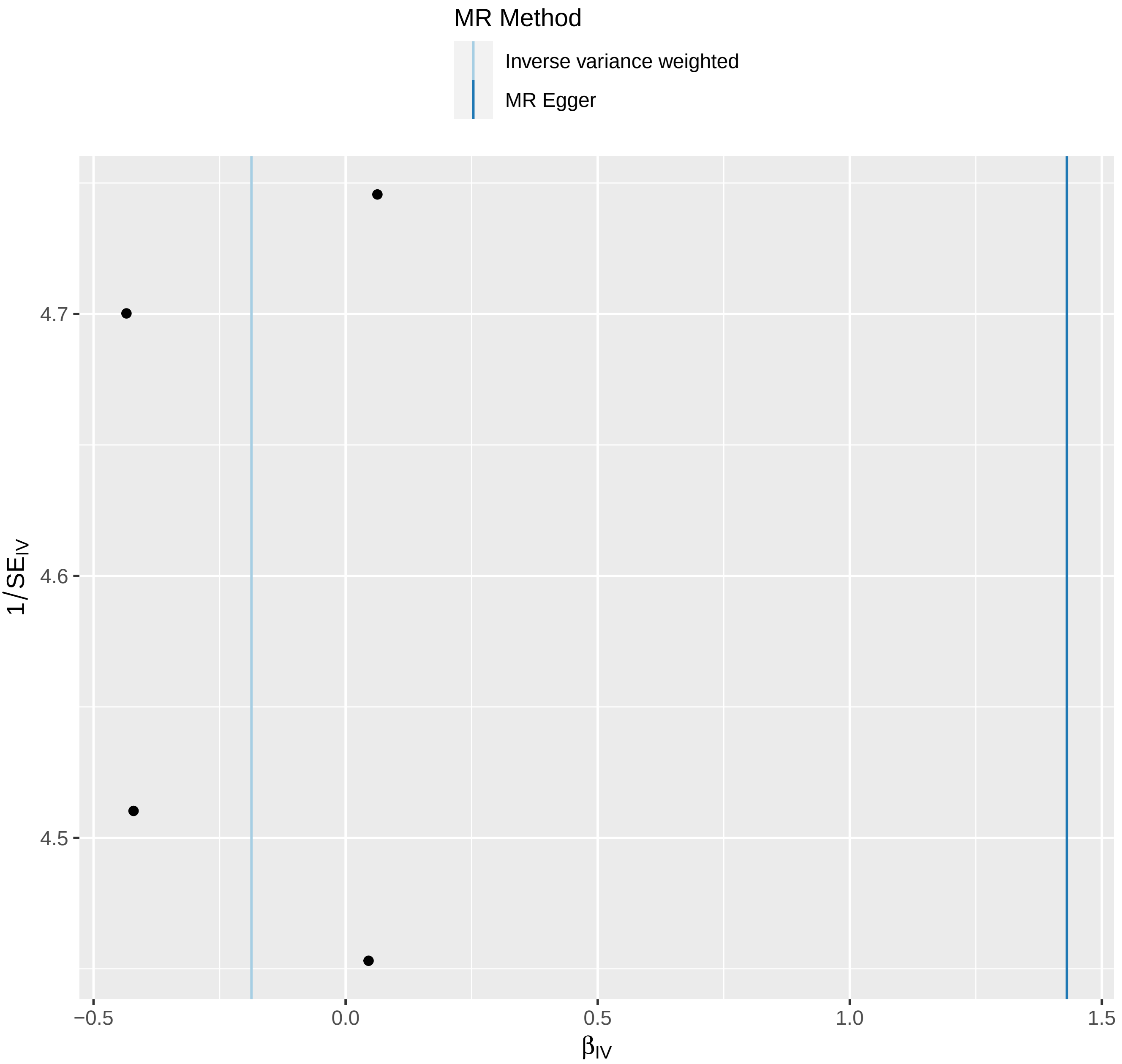

Batch 2−5664 : Gut microbiota abundance (genus Parasutterella id.2892) on Sprouty−related, EVH1 domain−containing protein 1 with a threshold of 1e−05

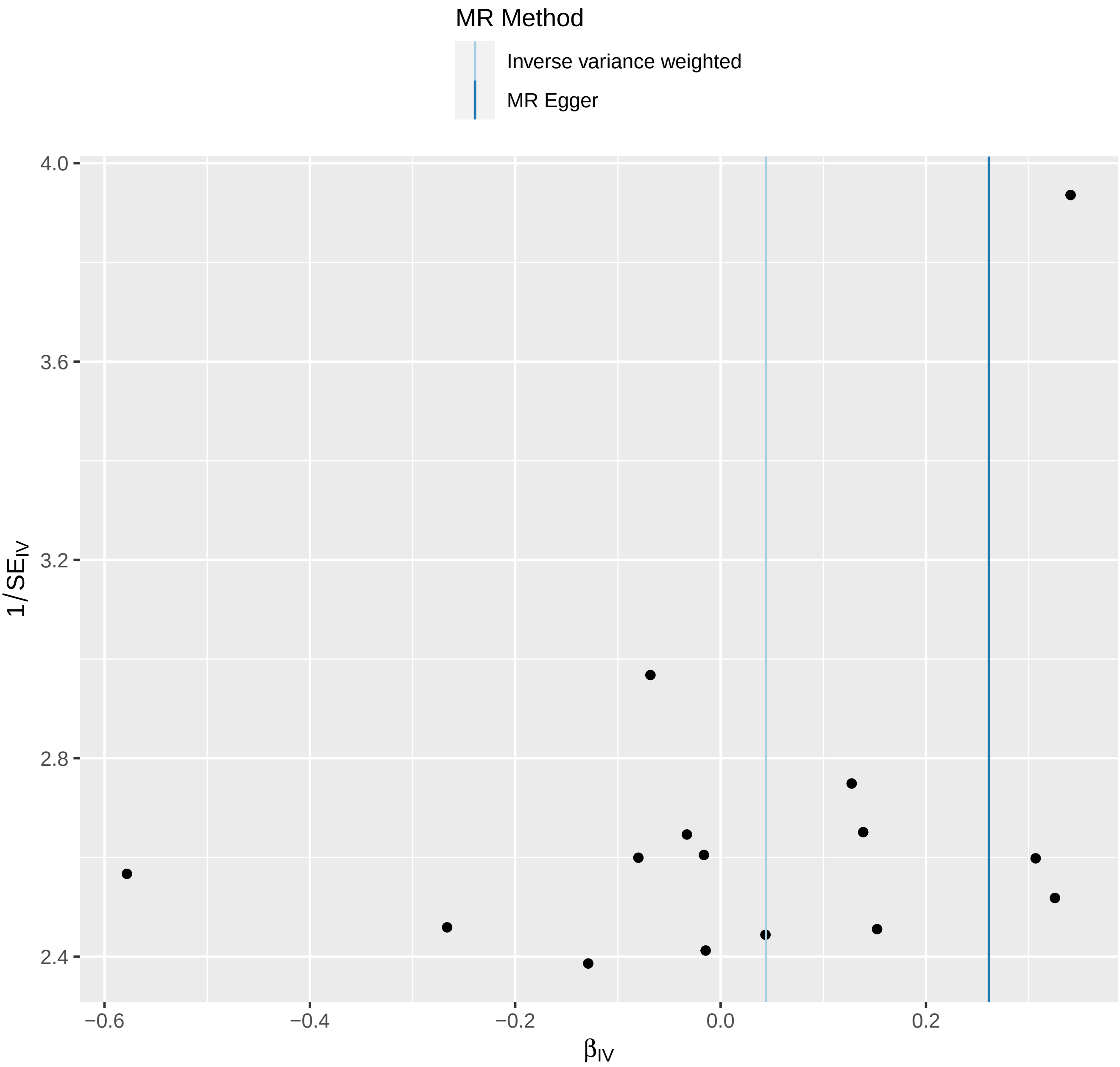

Batch 2−5665 : Gut microbiota abundance (genus Catenibacterium id.2153) on Protein sprouty homolog 1
with a threshold of 1e−05

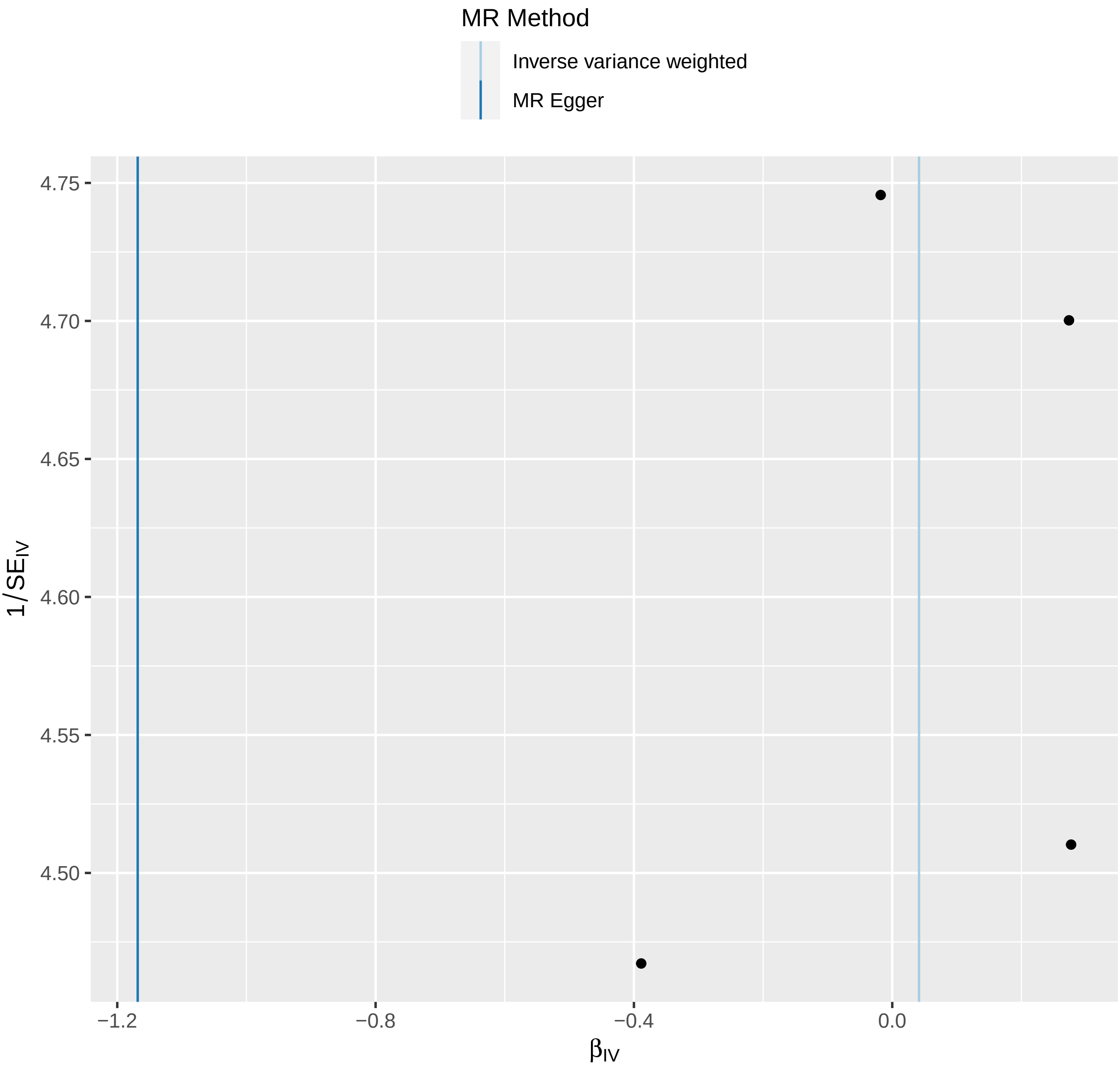

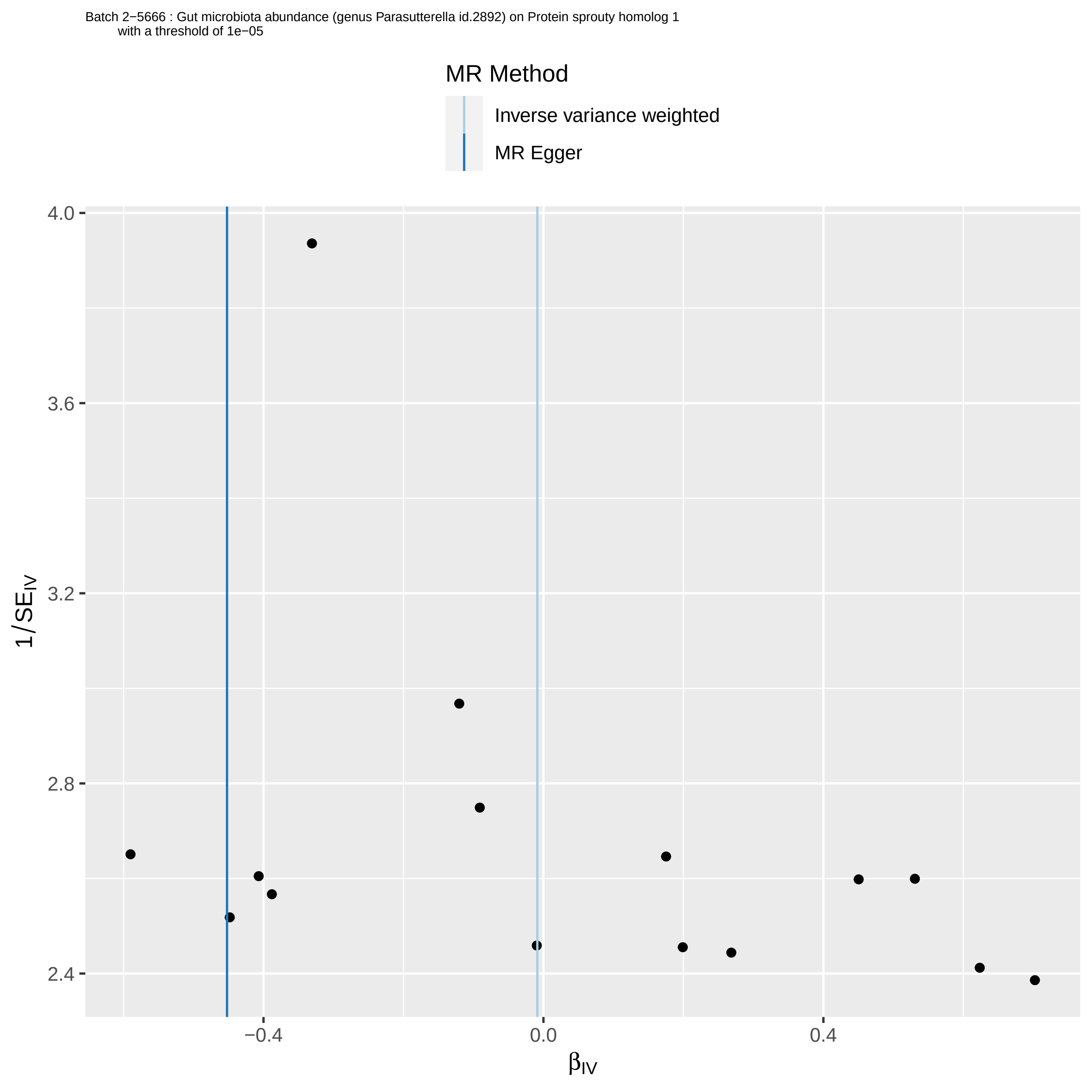

Batch 2−5666 : Gut microbiota abundance (genus Parasutterella id.2892) on Protein sprouty homolog 1
with a threshold of 1e−05
MR Method
Inverse variance weighted
MR Egger
4.0
3.6
3.2
2.8
2.4
1/SE_IV
−0.4
0.0
0.4
β_IV

Batch 2−5667 : Gut microbiota abundance (genus Catenibacterium id.2153) on SPRY domain−containing SOCS box protein 1
with a threshold of 1e−05

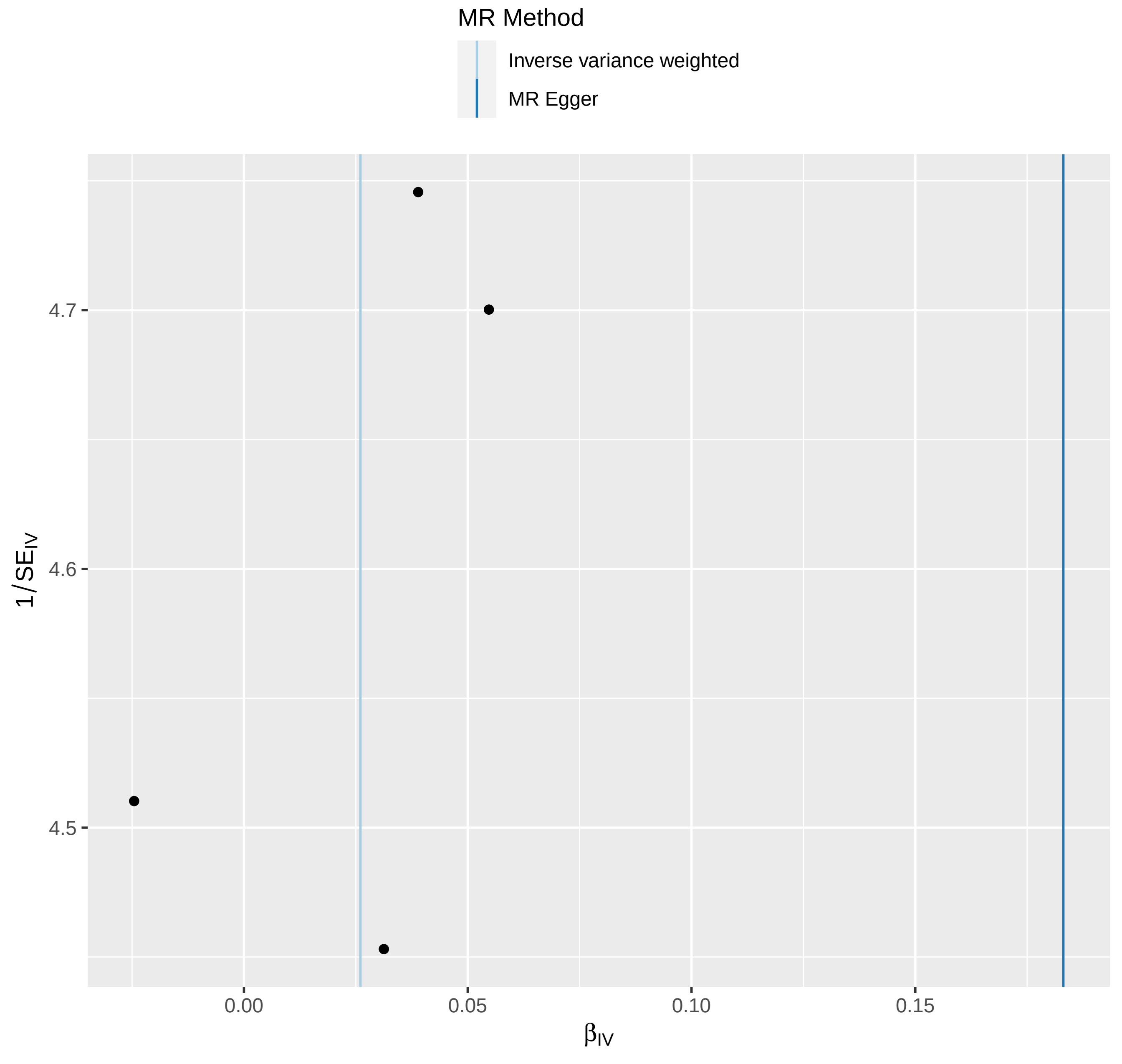

Batch 2−5668 : Gut microbiota abundance (genus Parasutterella id.2892) on SPRY domain−containing SOCS box protein 1
with a threshold of 1e−05

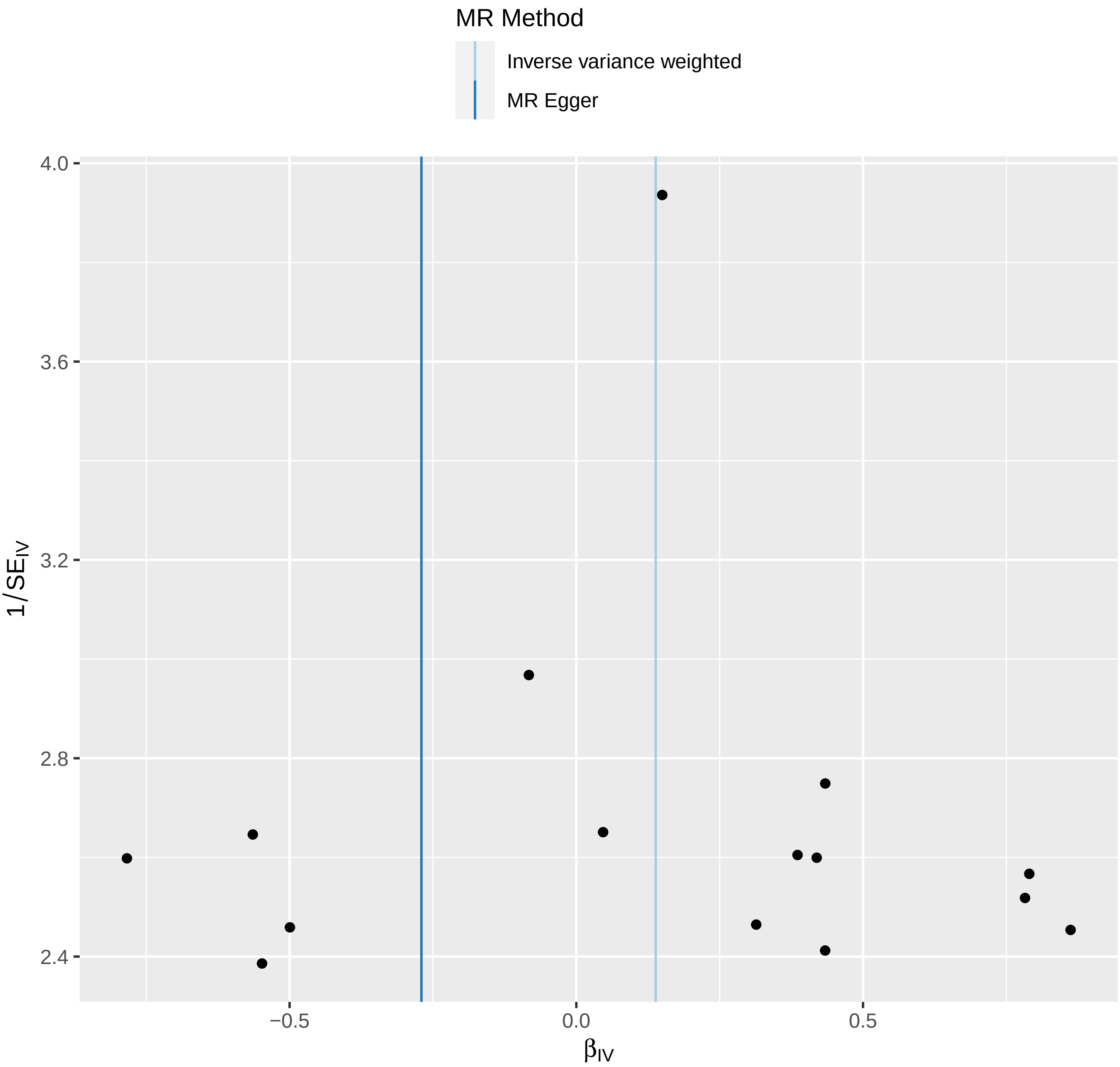

Batch 2−5669 : Gut microbiota abundance (genus Catenibacterium id.2153) on Spectrin alpha chain, non−erythrocytic 1
with a threshold of 1e−05

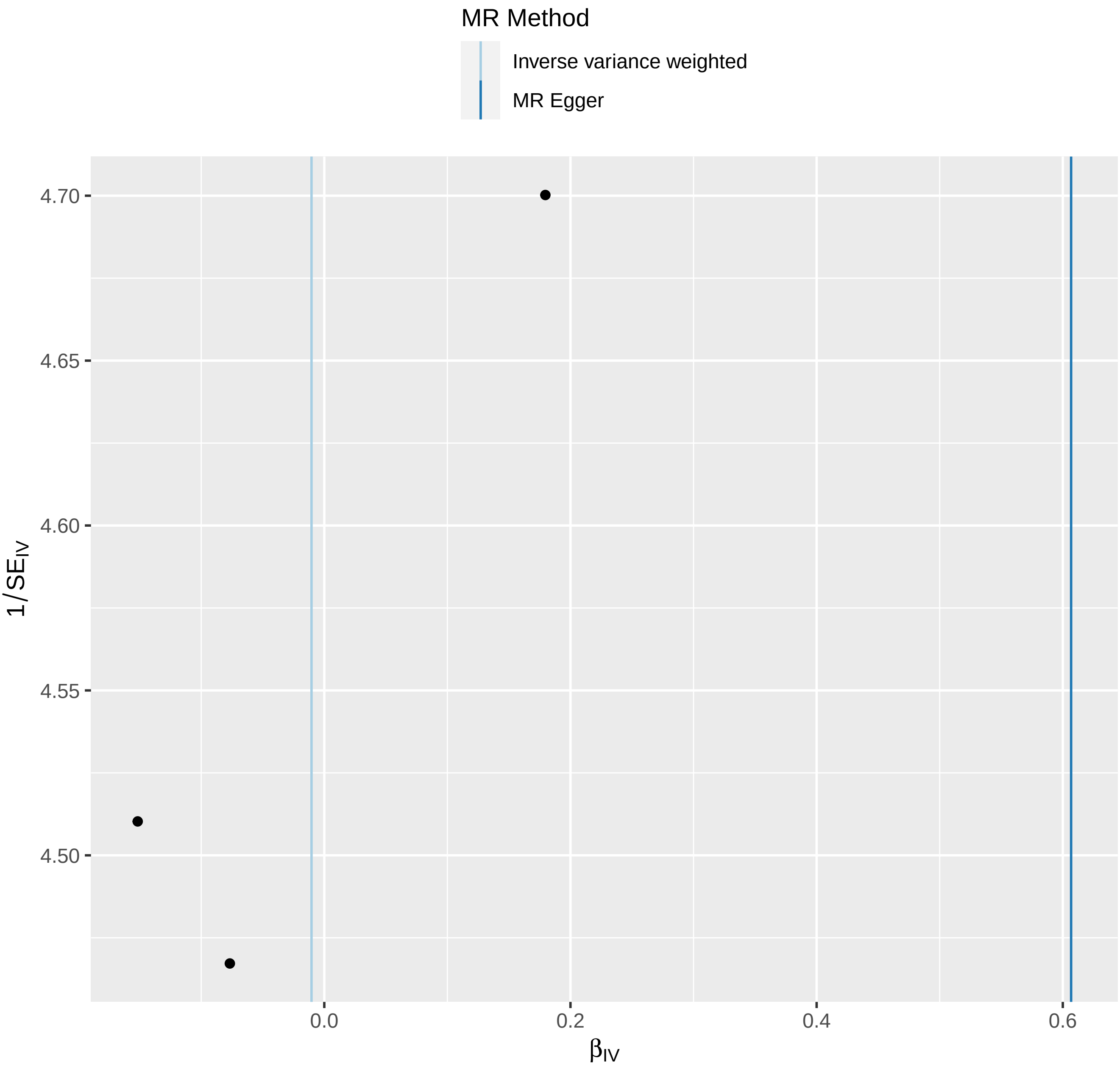

Batch 2−5670 : Gut microbiota abundance (genus Parasutterella id.2892) on Spectrin alpha chain, non−erythrocytic 1
with a threshold of 1e−05

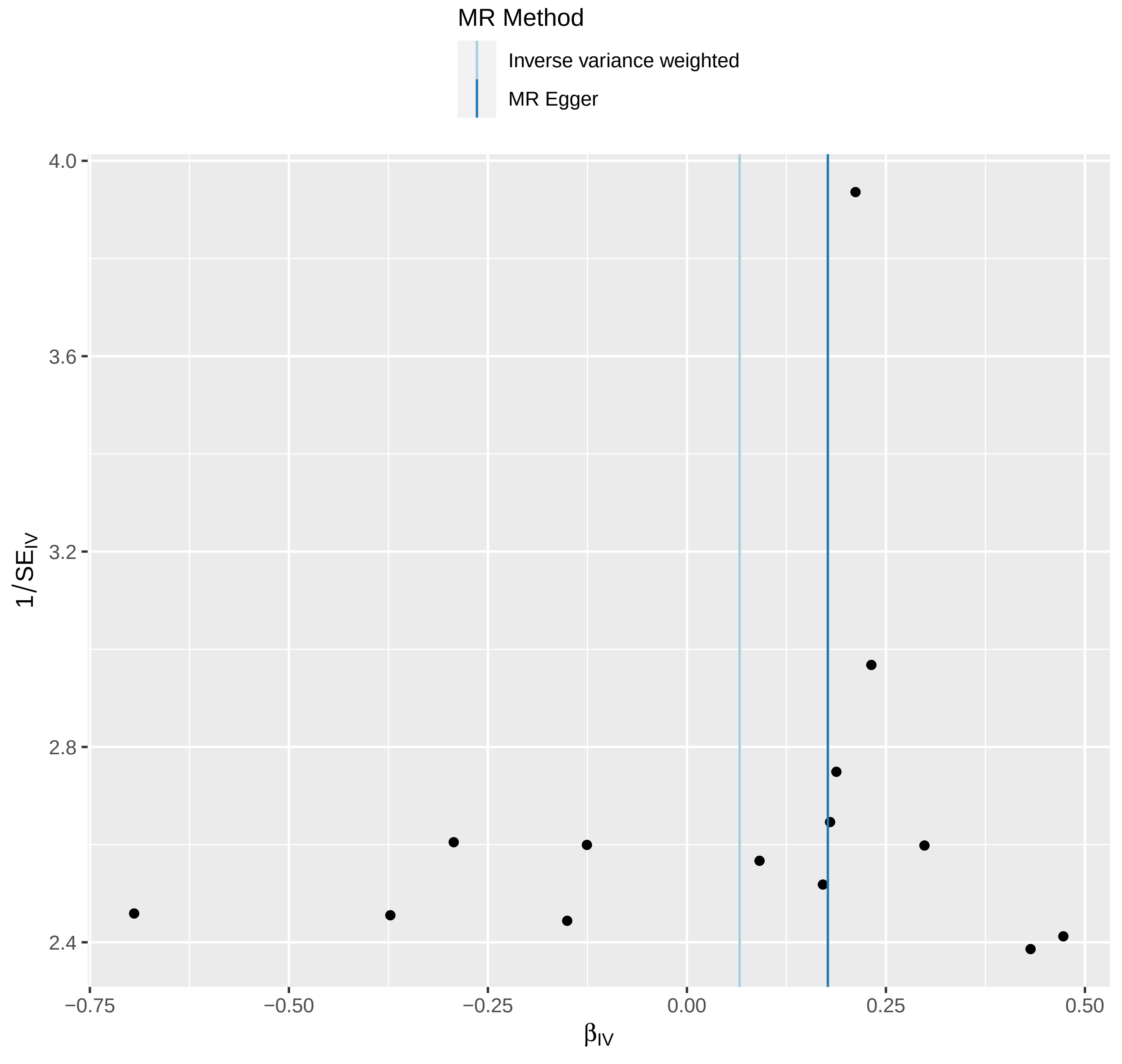

Batch 2−5671 : Gut microbiota abundance (genus Catenibacterium id.2153) on Serine palmitoyltransferase 1
with a threshold of 1e−05

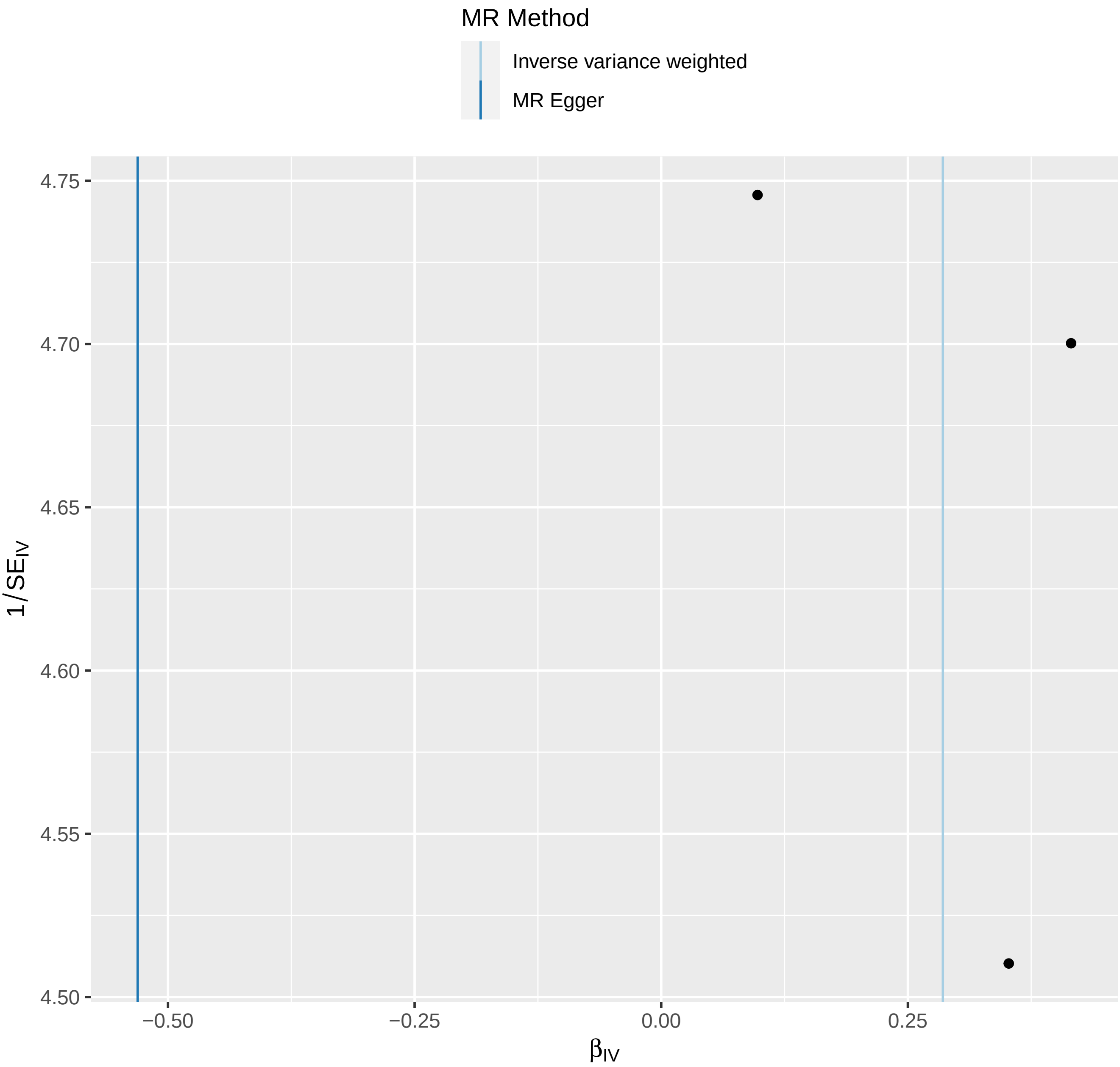

Batch 2−5672 : Gut microbiota abundance (genus Parasutterella id.2892) on Serine palmitoyltransferase 1
with a threshold of 1e−05

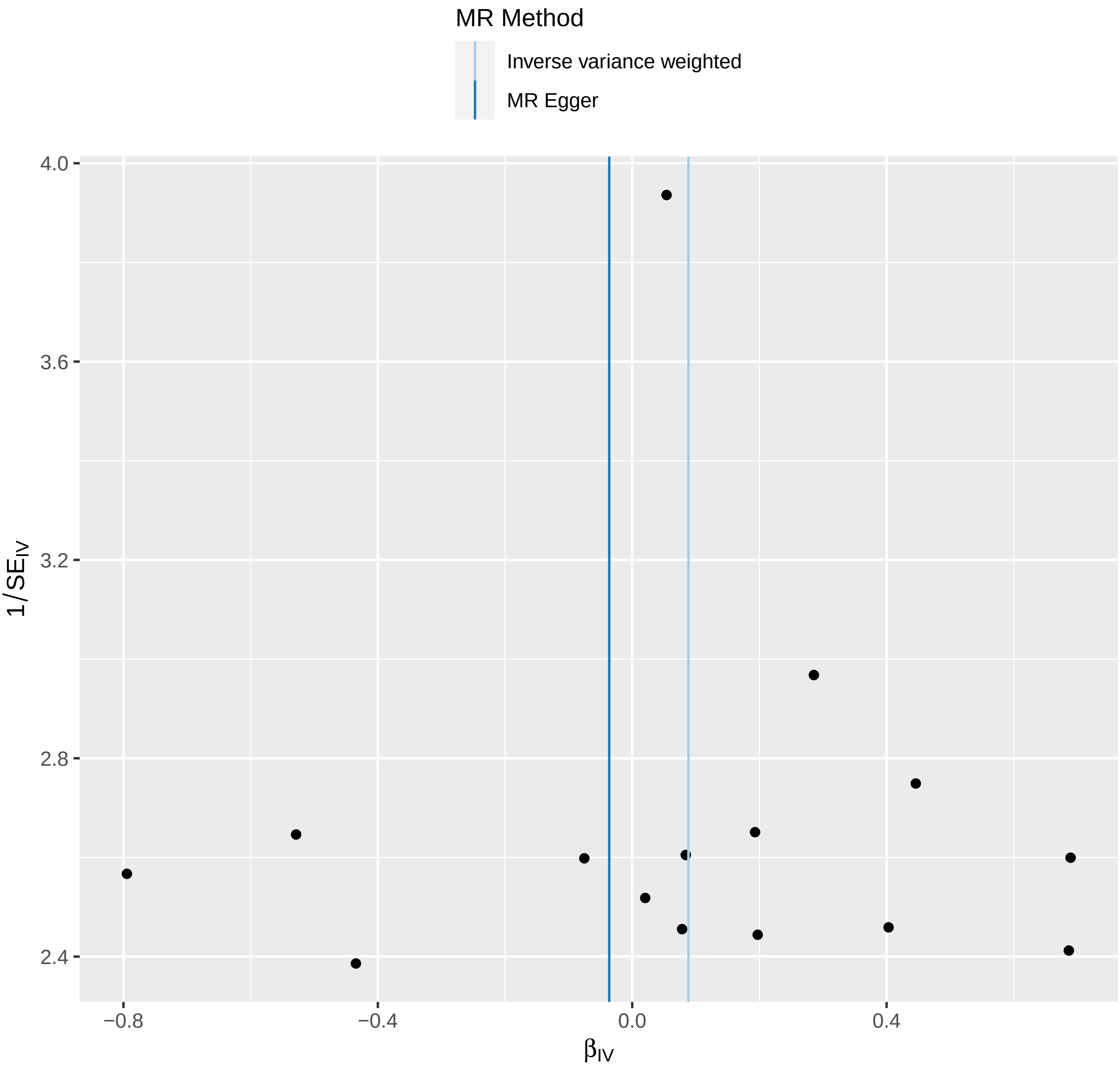

Batch 2−5673 : Gut microbiota abundance (genus Catenibacterium id.2153) on Serine palmitoyltransferase 2
with a threshold of 1e−05

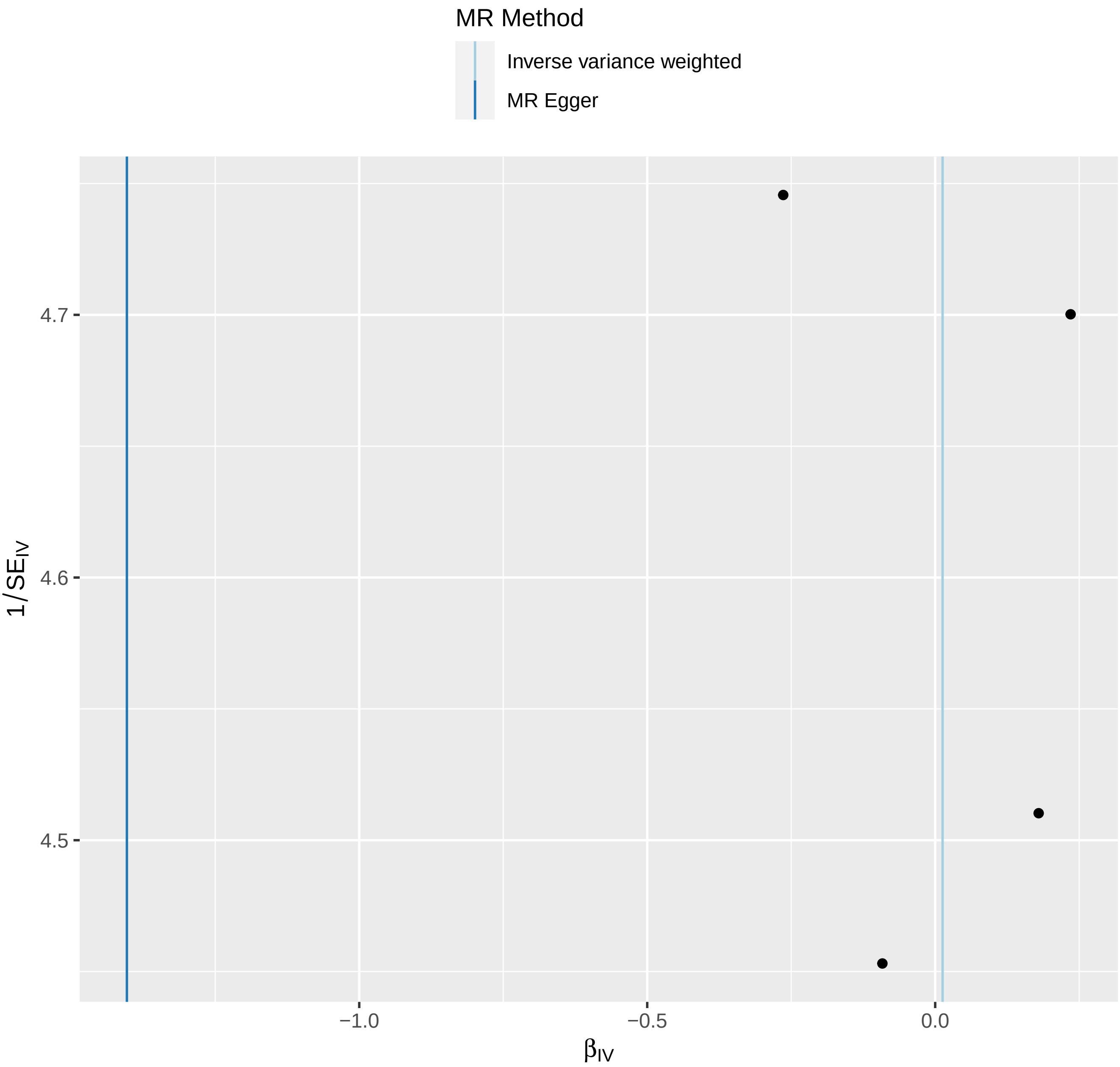

Batch 2−5674 : Gut microbiota abundance (genus Parasutterella id.2892) on Serine palmitoyltransferase 2
with a threshold of 1e−05

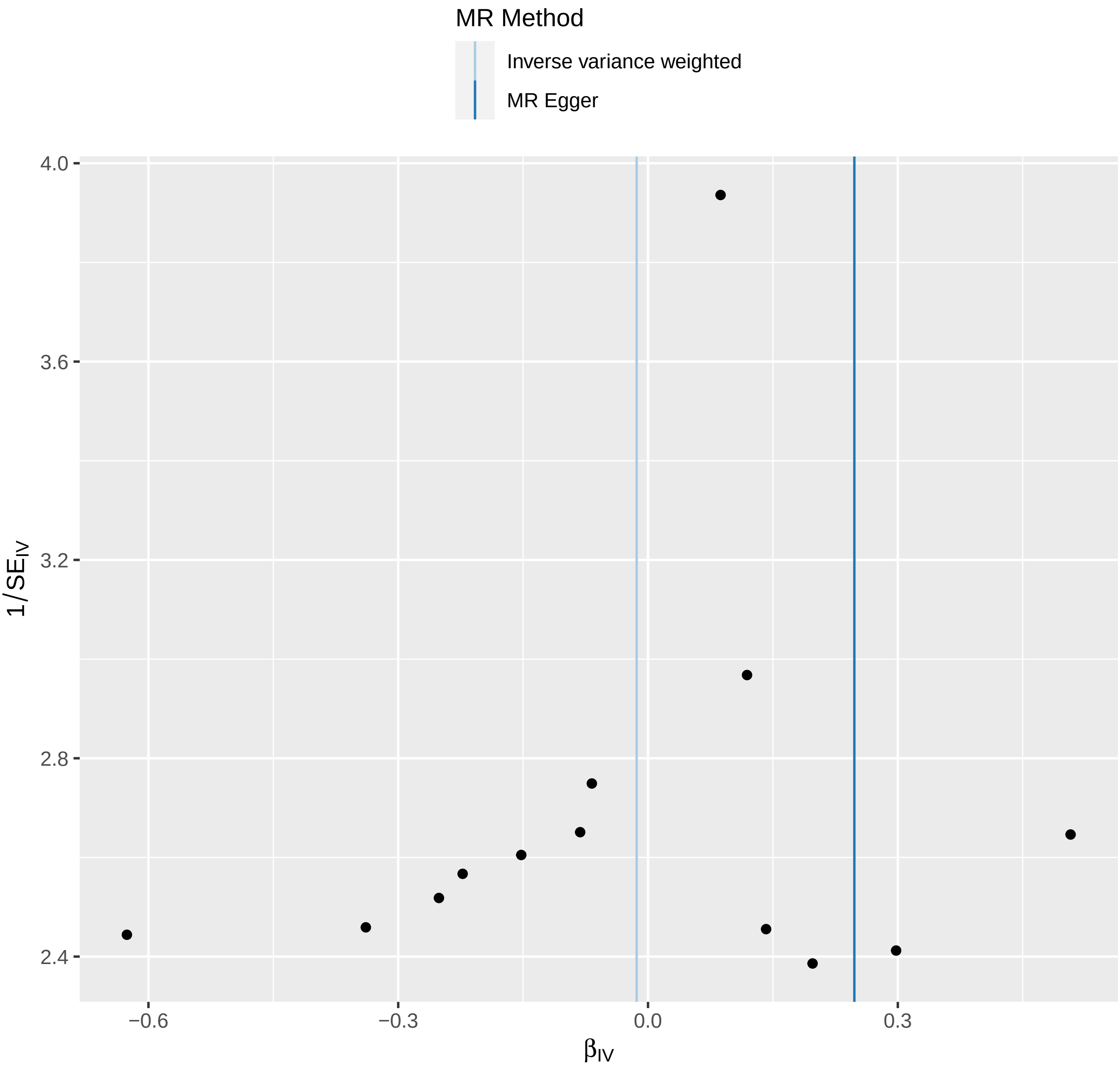

Batch 2−5675 : Gut microbiota abundance (genus Catenibacterium id.2153) on Serine/arginine−rich splicing factor 6
with a threshold of 1e−05

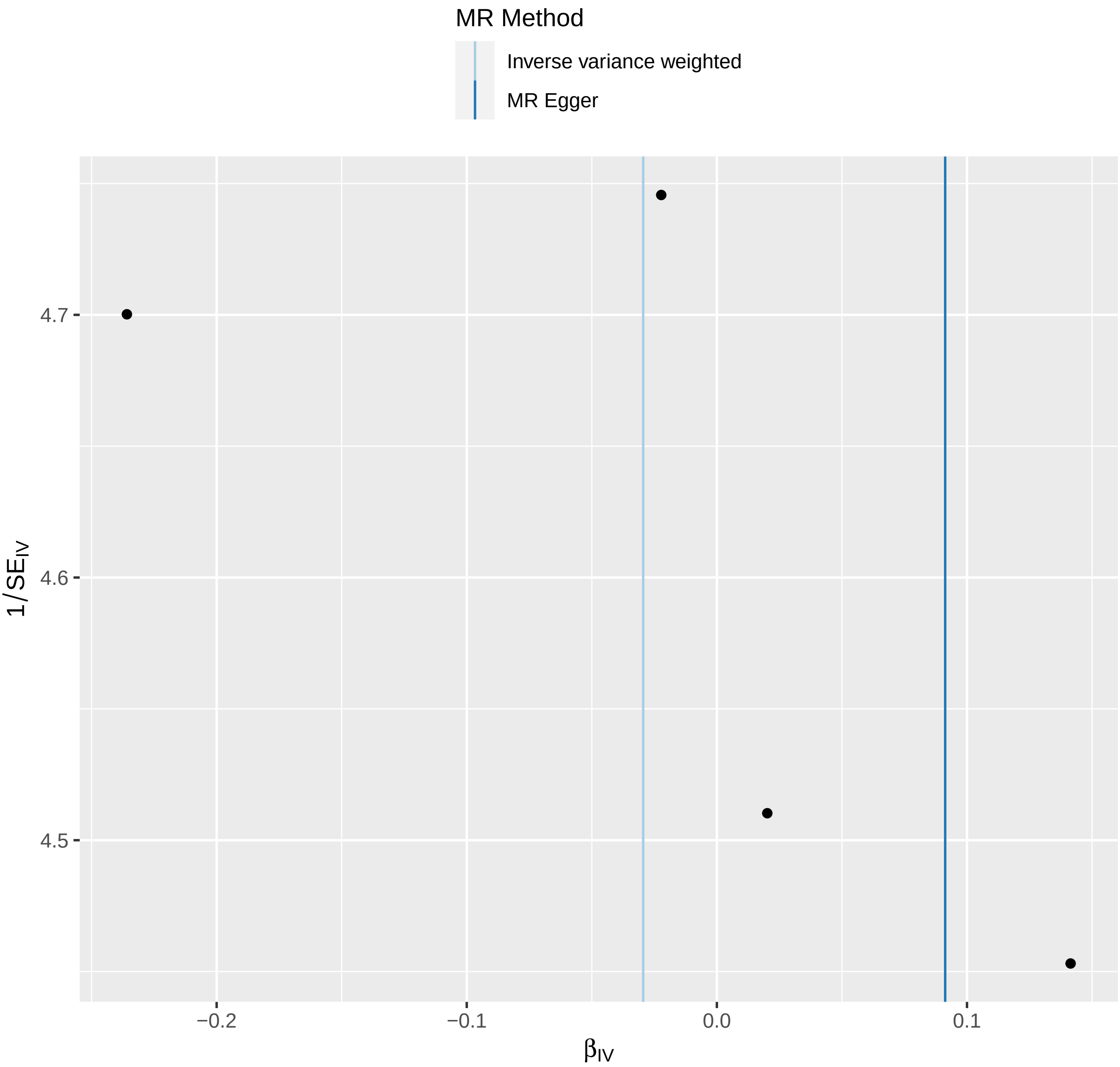

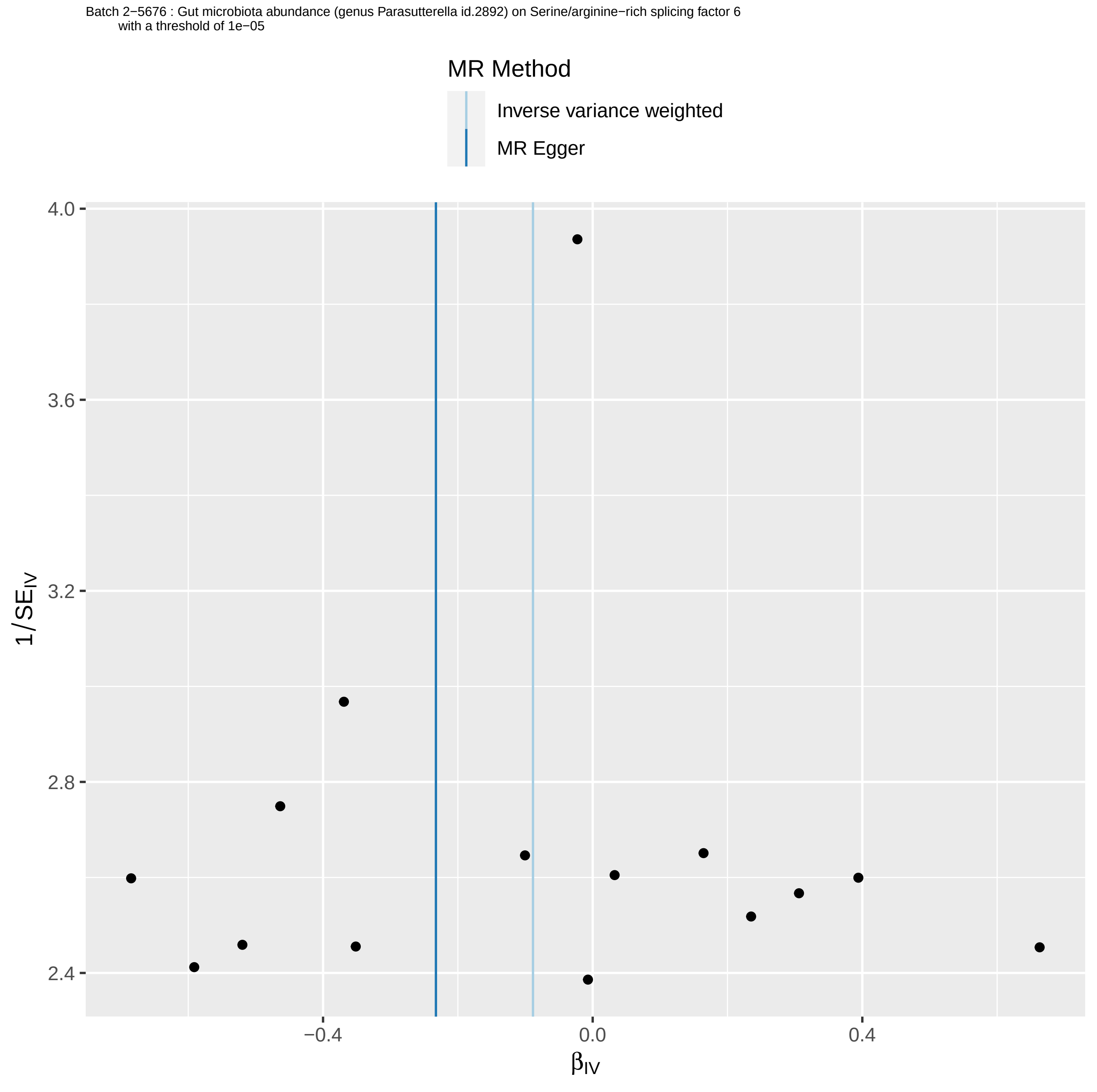

Batch 2−5676 : Gut microbiota abundance (genus Parasutterella id.2892) on Serine/arginine−rich splicing factor 6
with a threshold of 1e−05
MR Method
Inverse variance weighted
MR Egger
4.0
3.6
3.2
2.8
2.4
1/SE_IV
−0.4
0.0
0.4
β_IV

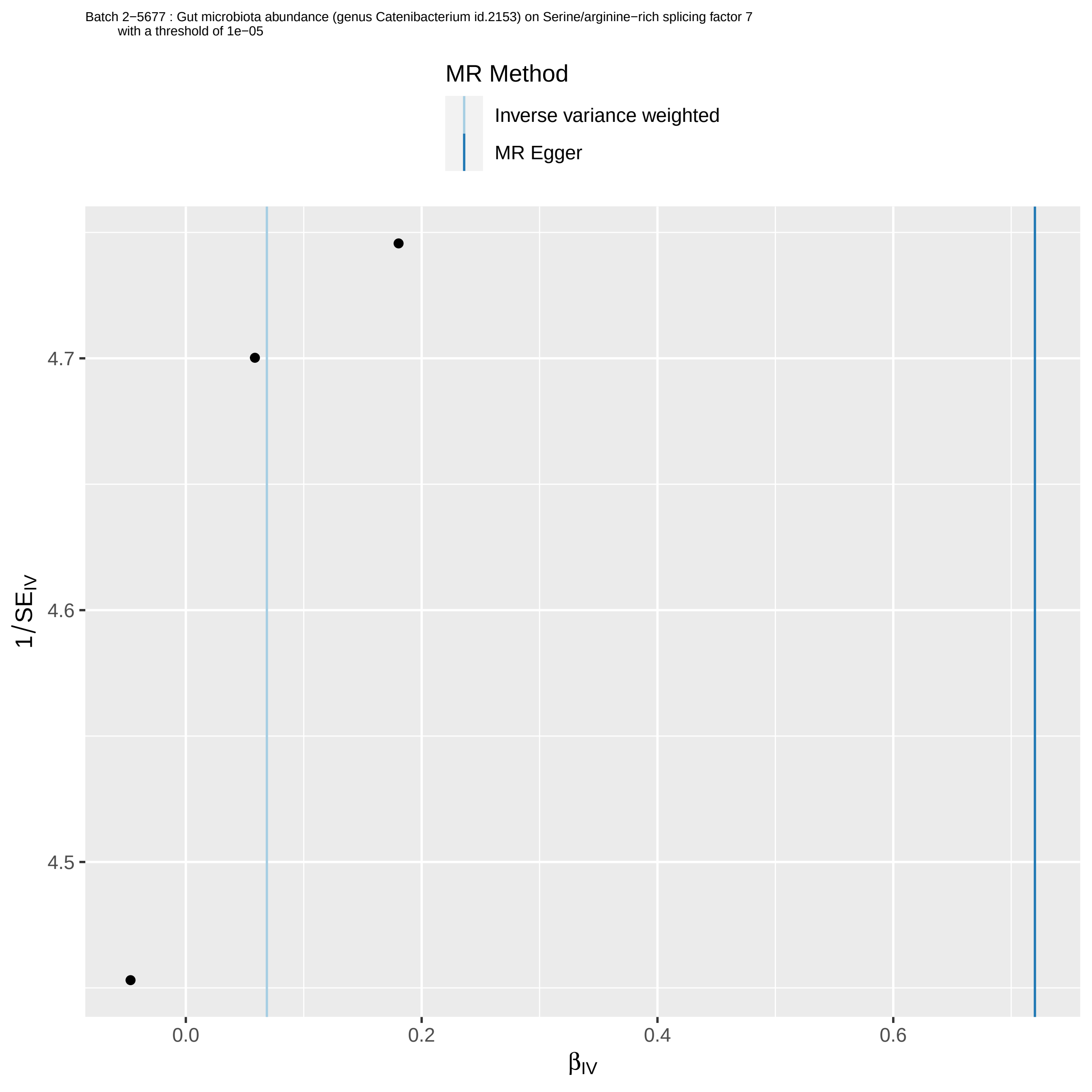
Batch 2−5677 : Gut microbiota abundance (genus Catenibacterium id.2153) on Serine/arginine−rich splicing factor 7
with a threshold of 1e−05
MR Method
Inverse variance weighted
MR Egger
4.7
4.6
4.5
1/SE_IV
0.0
0.2
0.4
0.6
β_IV

Batch 2−5678 : Gut microbiota abundance (genus Parasutterella id.2892) on Serine/arginine−rich splicing factor 7
with a threshold of 1e−05

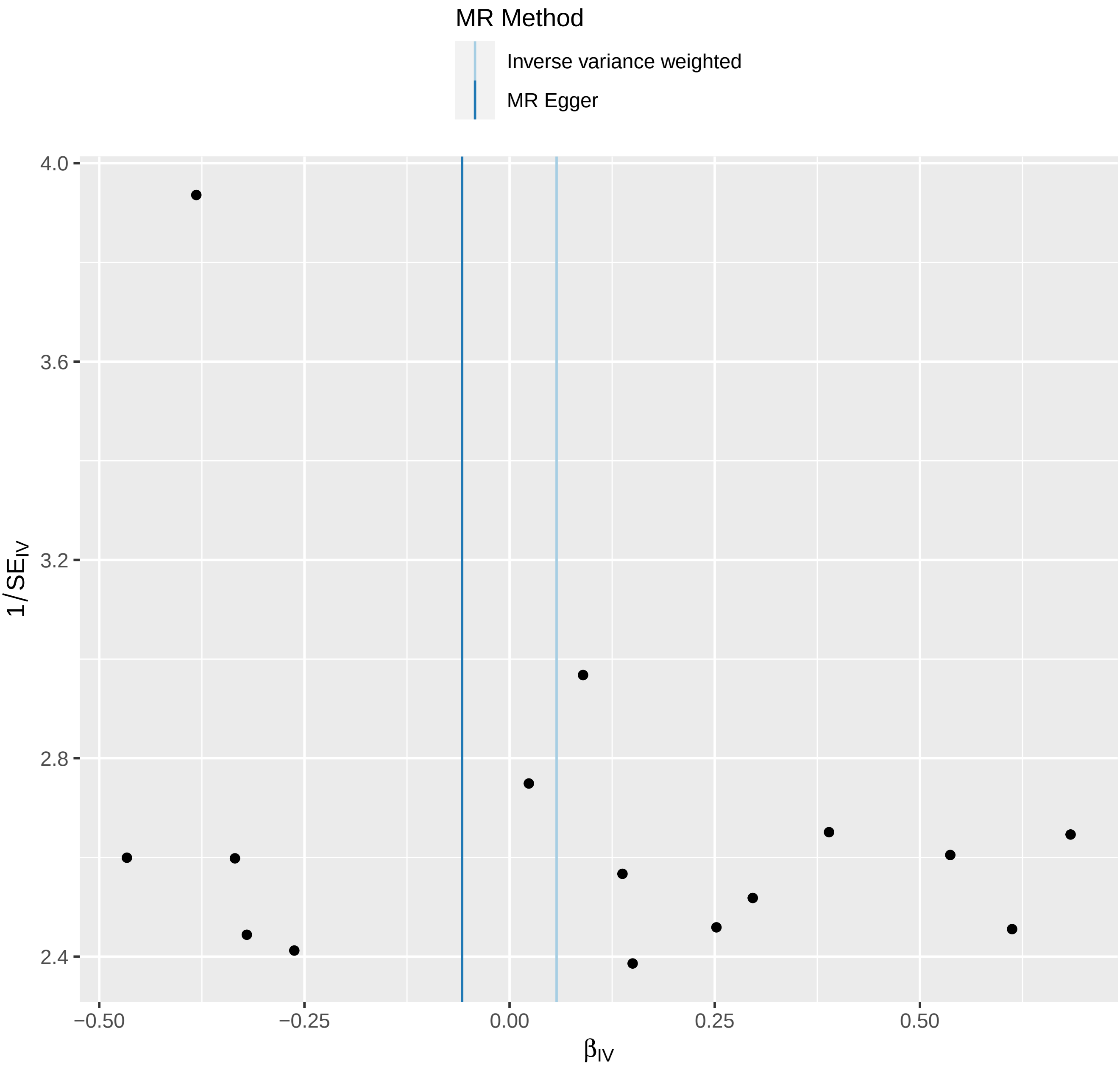

Batch 2−5679 : Gut microbiota abundance (genus Catenibacterium id.2153) on Sulfiredoxin−1
with a threshold of 1e−05

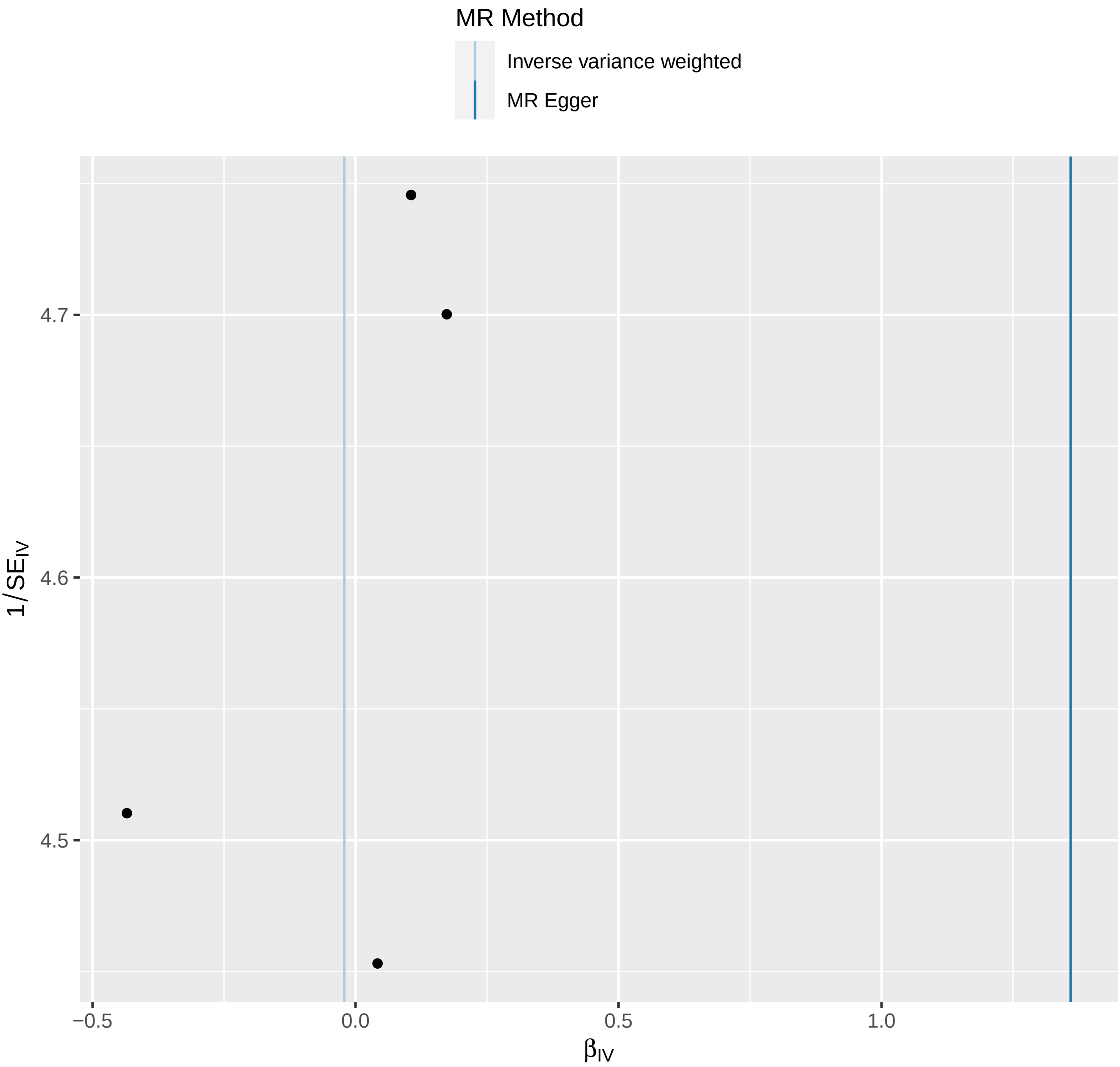

Batch 2−5680 : Gut microbiota abundance (genus Parasutterella id.2892) on Sulfiredoxin−1
with a threshold of 1e−05

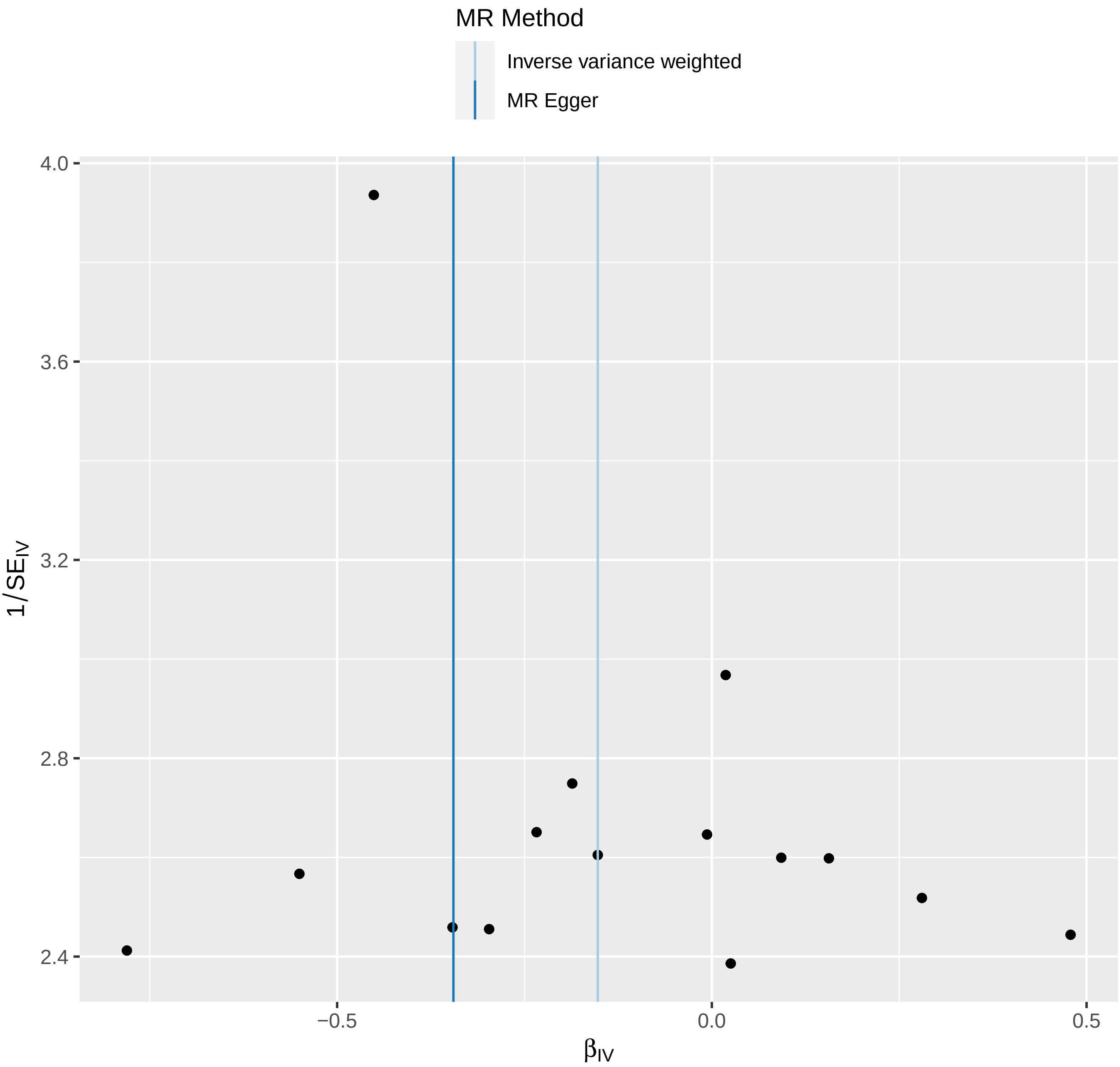

Batch 2−5681 : Gut microbiota abundance (genus Catenibacterium id.2153) on Lupus La protein
with a threshold of 1e−05

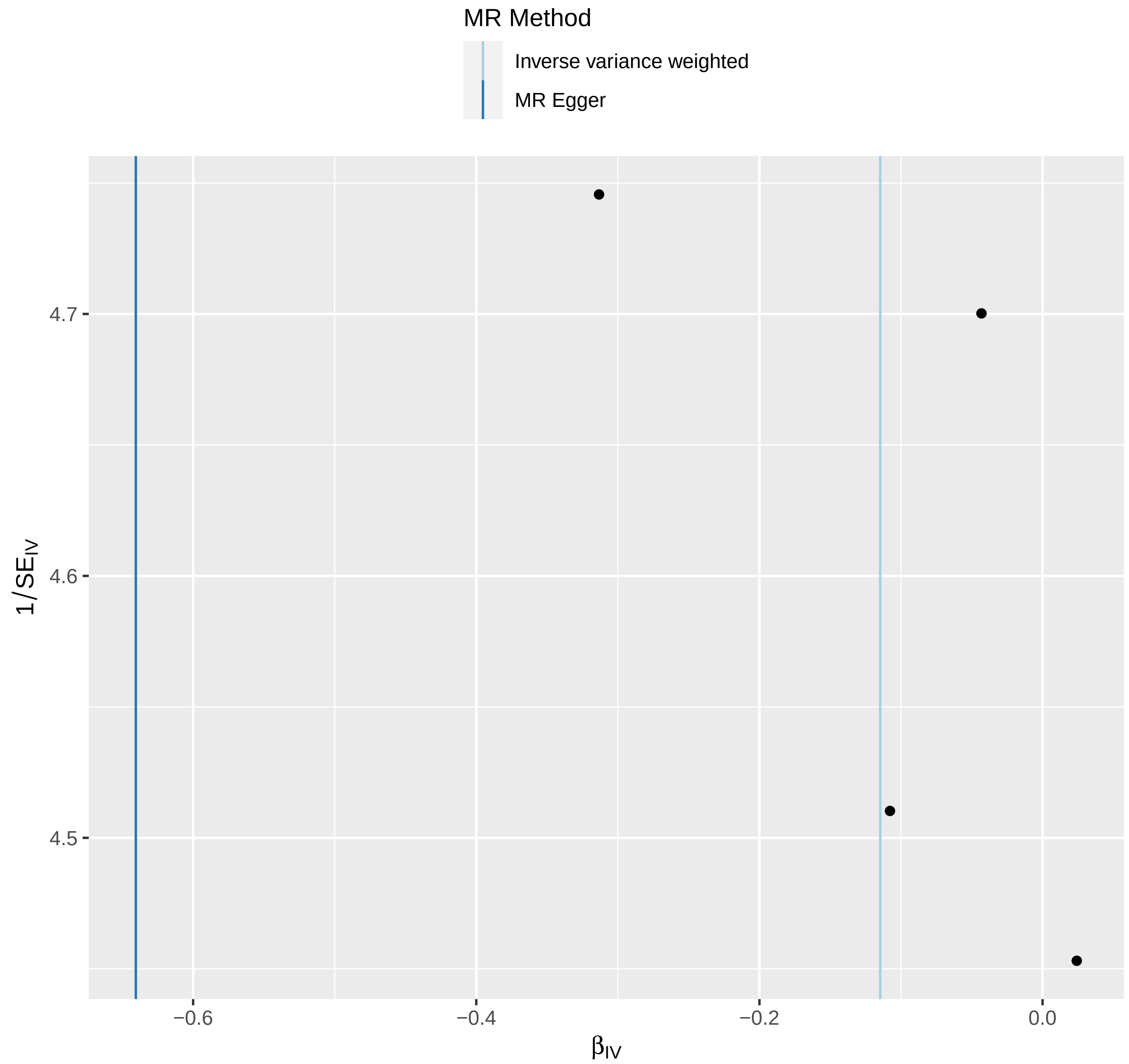

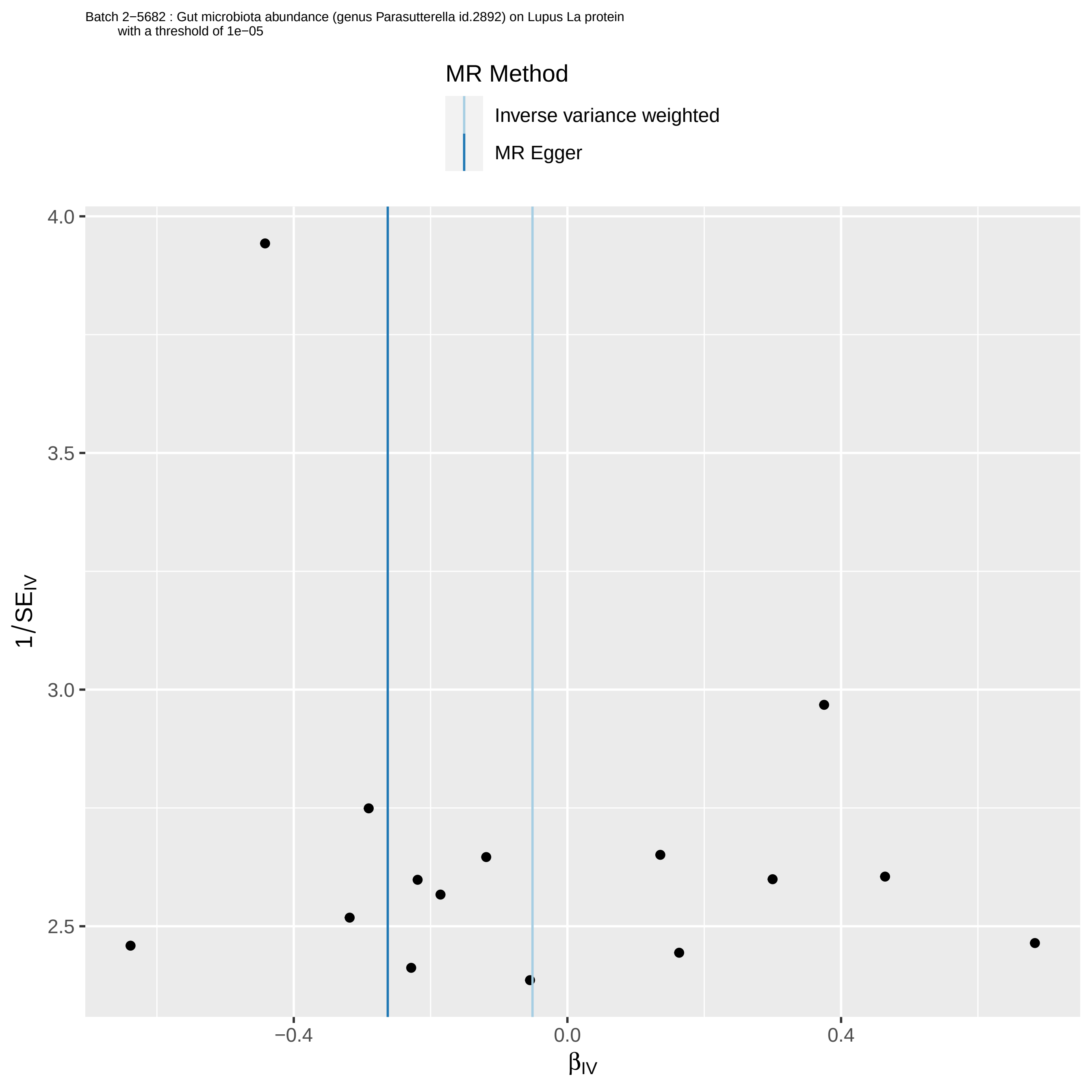
Batch 2−5682 : Gut microbiota abundance (genus Parasutterella id.2892) on Lupus La protein
with a threshold of 1e−05
MR Method
Inverse variance weighted
MR Egger
4.0
3.5
3.0
2.5
1/SE_IV
−0.4
0.0
0.4
β_IV

Batch 2−5683 : Gut microbiota abundance (genus Catenibacterium id.2153) on Lupus La protein
with a threshold of 1e−05

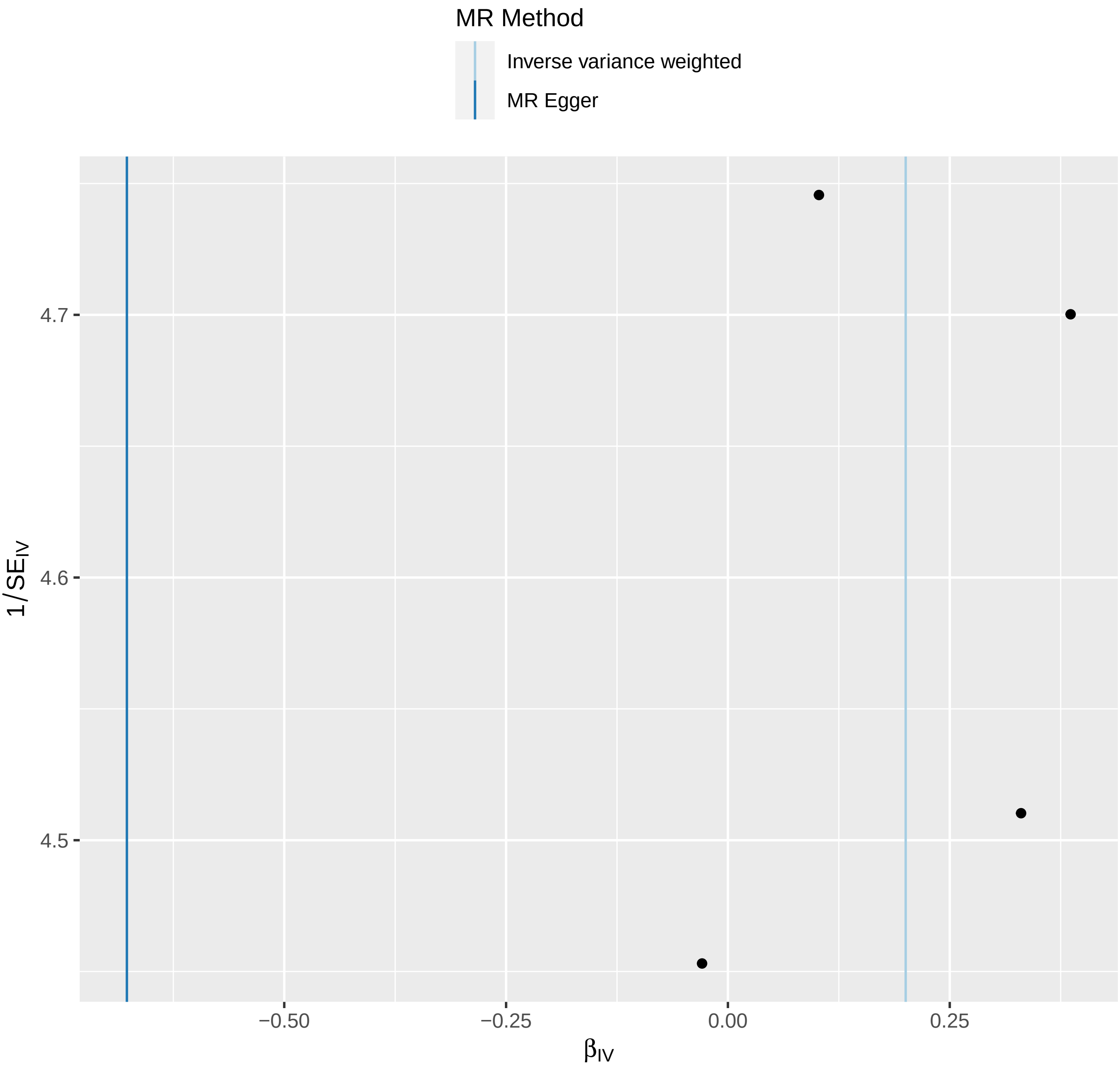

Batch 2−5684 : Gut microbiota abundance (genus Parasutterella id.2892) on Lupus La protein
with a threshold of 1e−05

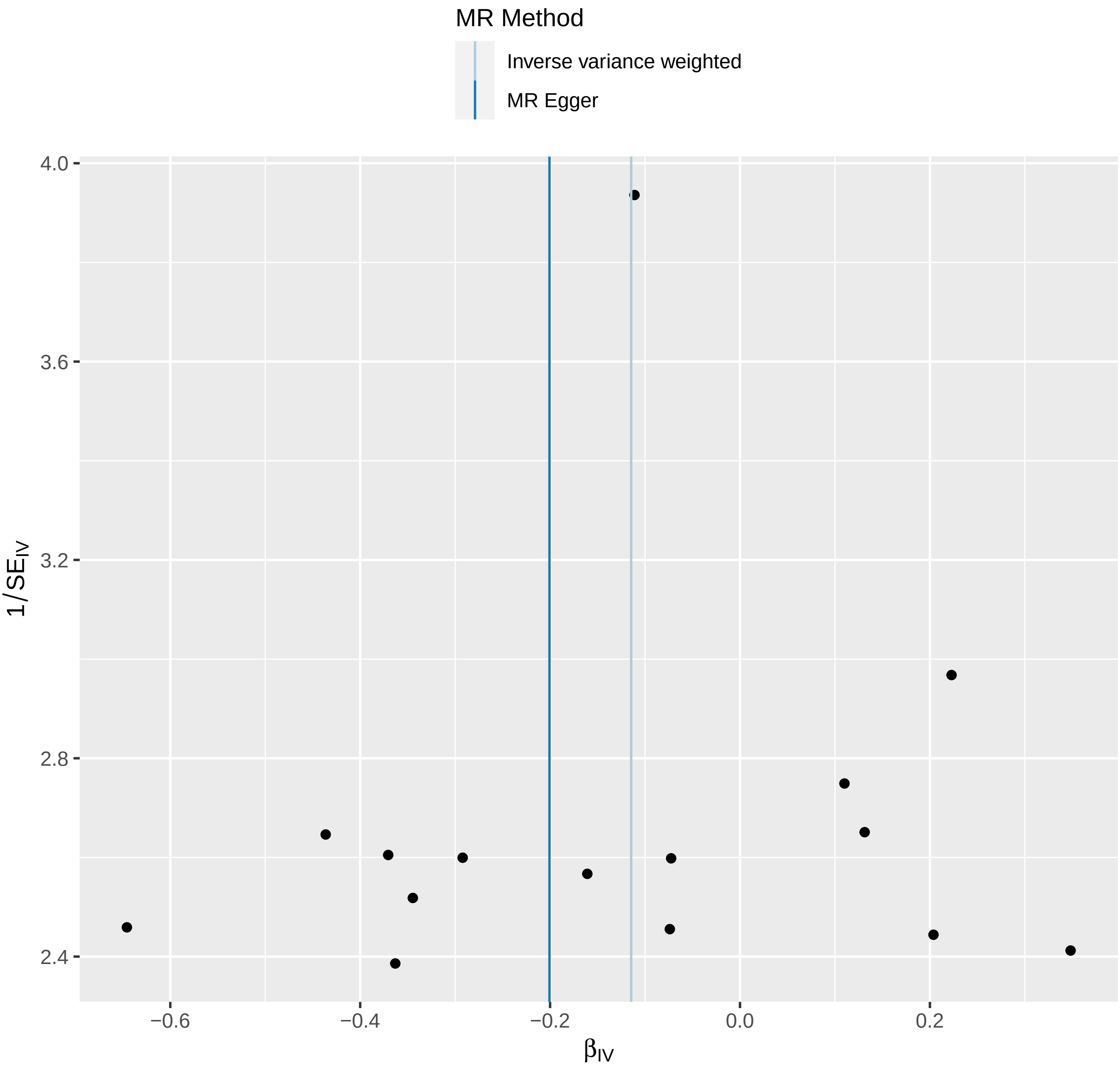

Batch 2−5685 : Gut microbiota abundance (genus Catenibacterium id.2153) on Serine−rich single−pass membrane protein 1
with a threshold of 1e−05

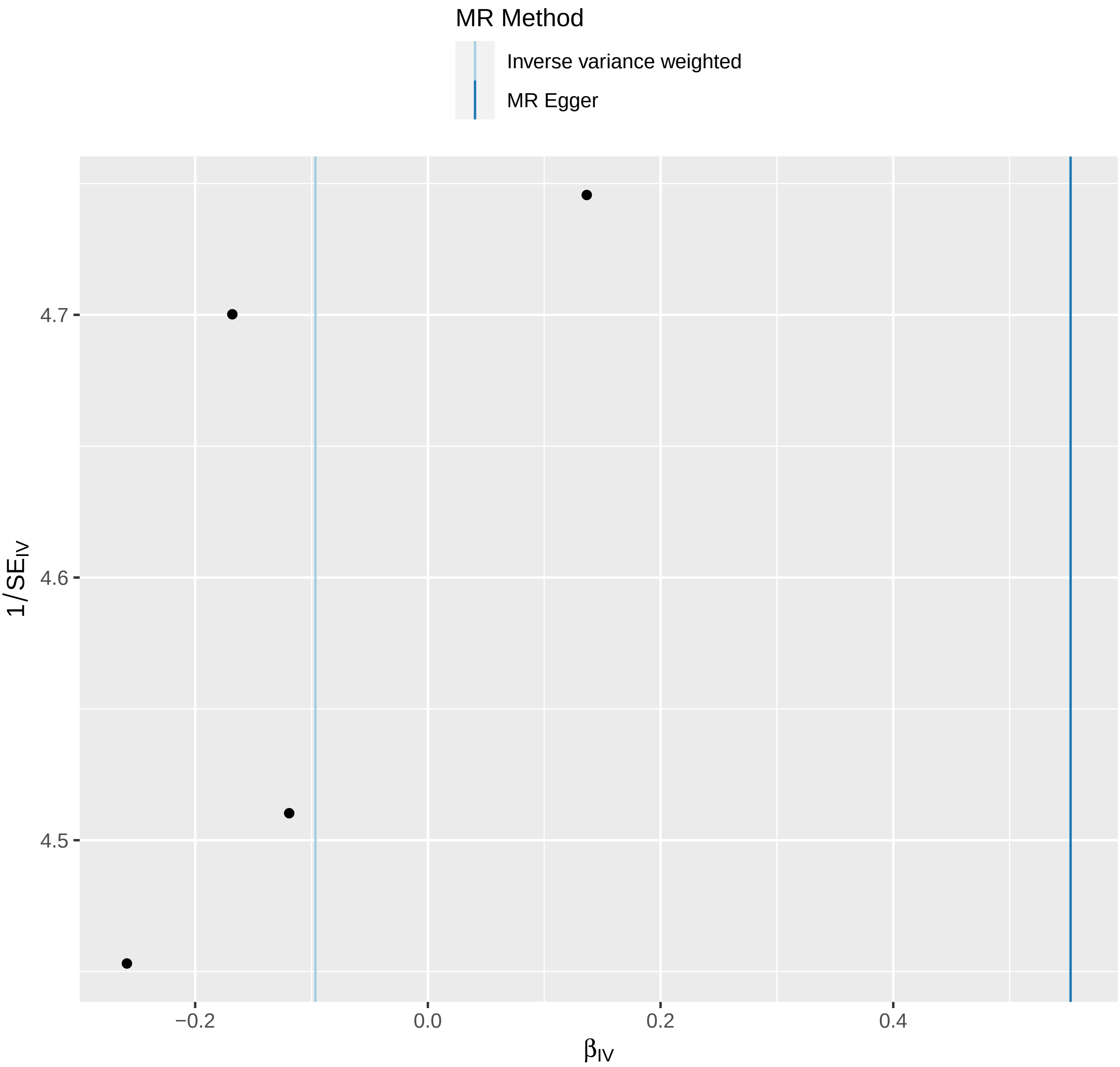

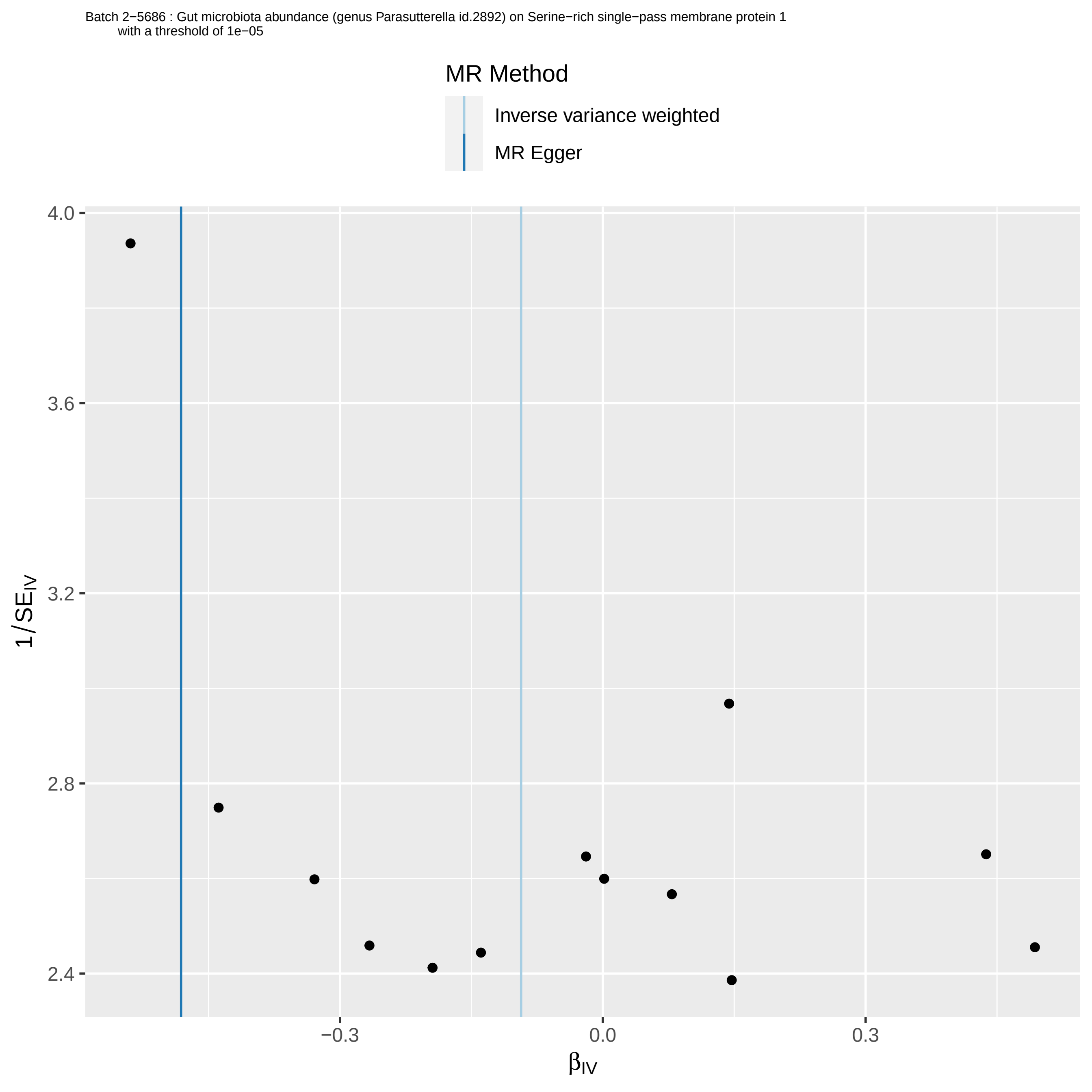

Batch 2−5686 : Gut microbiota abundance (genus Parasutterella id.2892) on Serine−rich single−pass membrane protein 1
with a threshold of 1e−05
MR Method
Inverse variance weighted
MR Egger
4.0
3.6
3.2
2.8
2.4
1/SE_IV
−0.3
0.0
0.3
β_IV

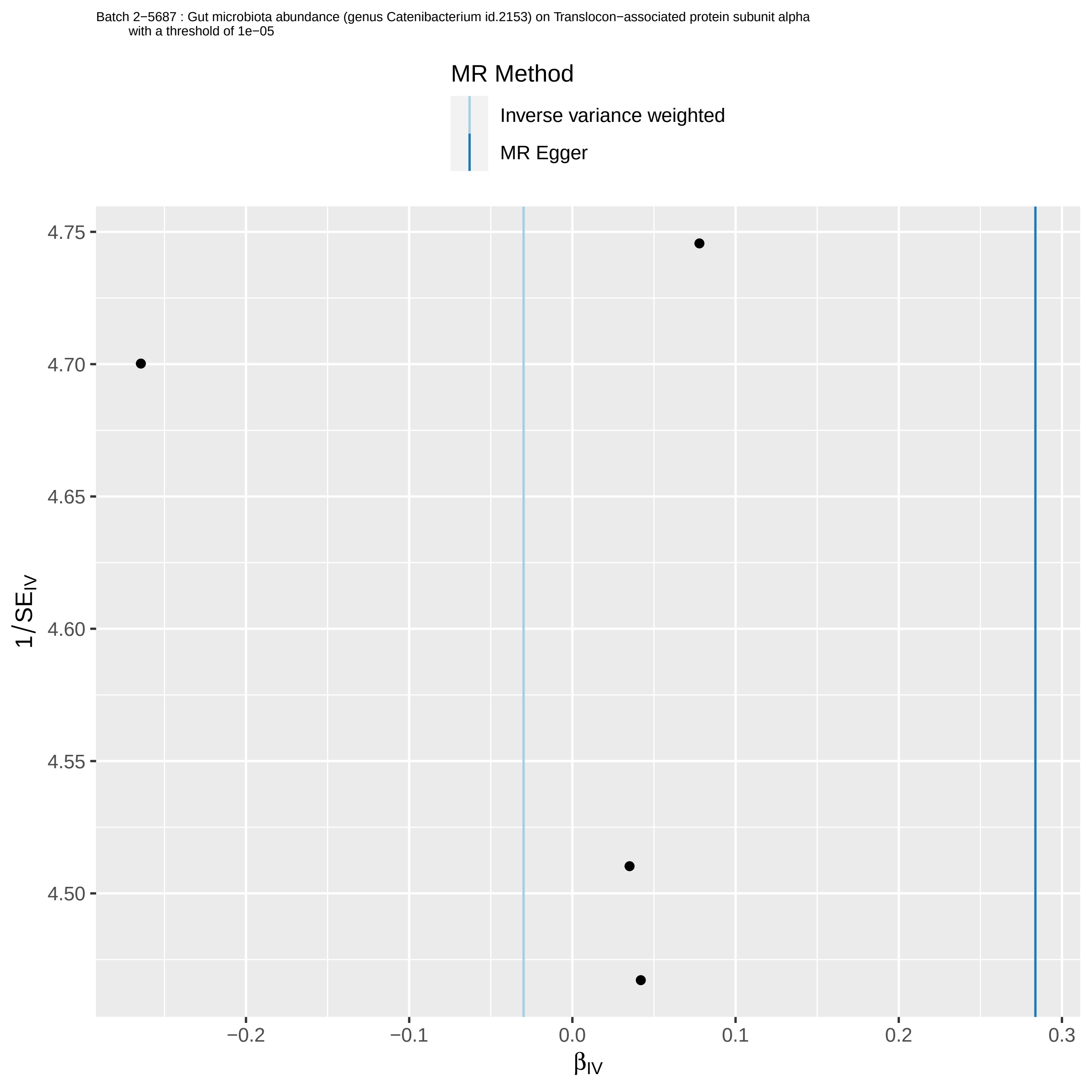

Batch 2−5687 : Gut microbiota abundance (genus Catenibacterium id.2153) on Translocon−associated protein subunit alpha
with a threshold of 1e−05
MR Method
Inverse variance weighted
MR Egger
4.75
4.70
4.65
4.60
4.55
4.50
1/SE$_{IV}$
−0.2
−0.1
0.0
0.1
0.2
0.3
β$_{IV}$

Batch 2−5688 : Gut microbiota abundance (genus Parasutterella id.2892) on Translocon−associated protein subunit alpha
with a threshold of 1e−05

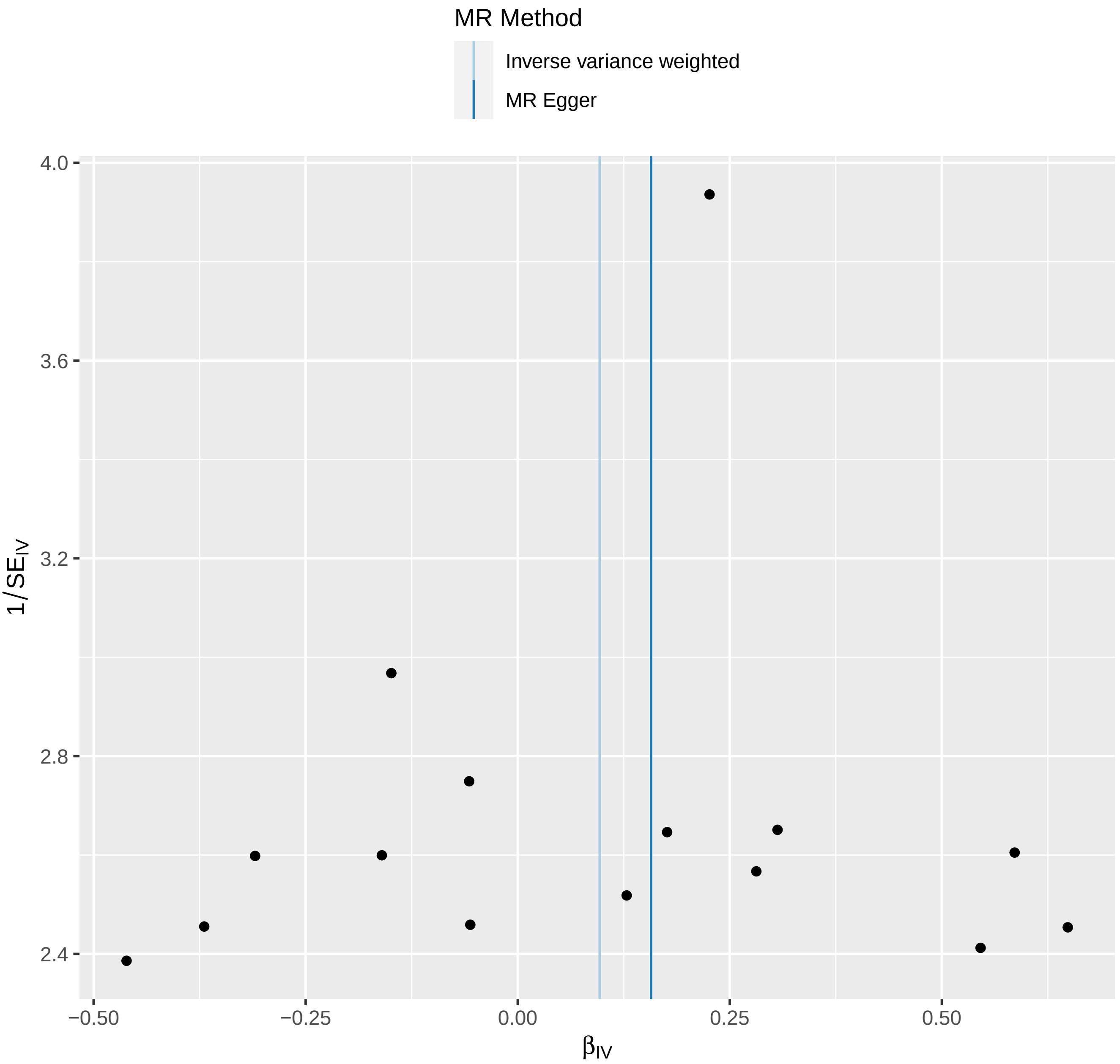

Batch 2−5689 : Gut microbiota abundance (genus Catenibacterium id.2153) on FACT complex subunit SSRP1
with a threshold of 1e−05

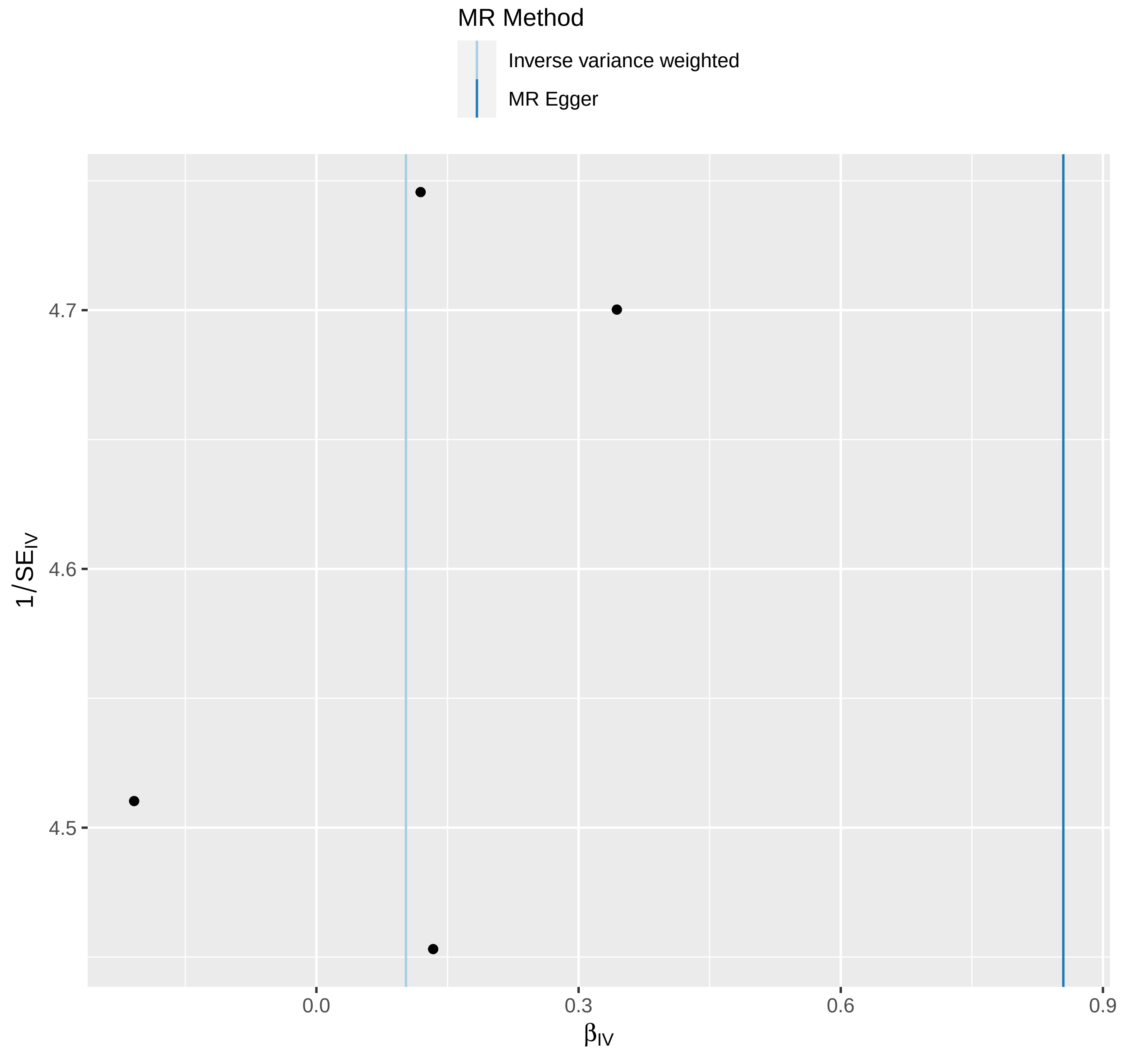

Batch 2−5690 : Gut microbiota abundance (genus Parasutterella id.2892) on FACT complex subunit SSRP1
with a threshold of 1e−05

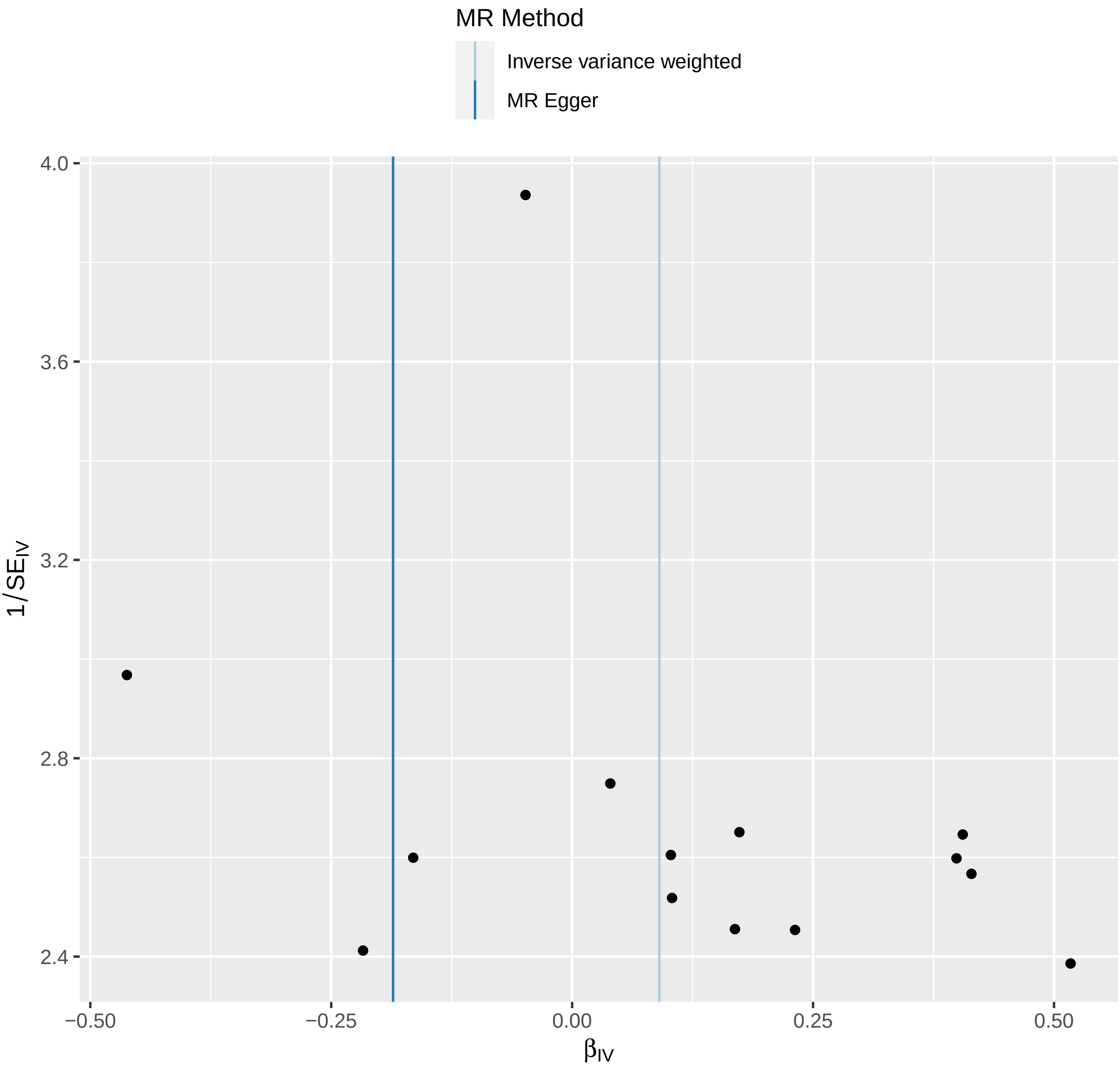

Batch 2−5691 : Gut microbiota abundance (genus Catenibacterium id.2153) on Somatostatin−28
with a threshold of 1e−05

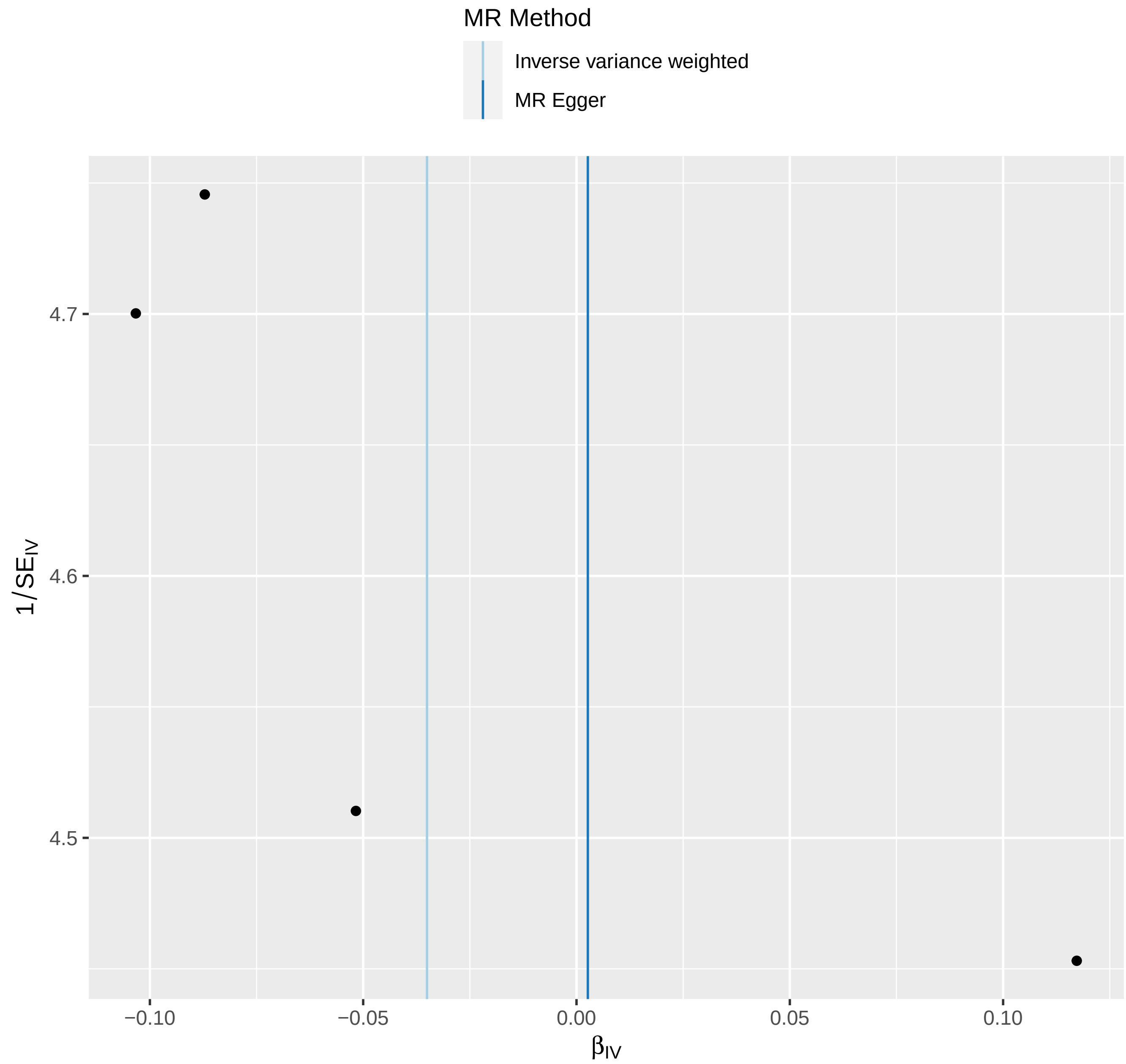

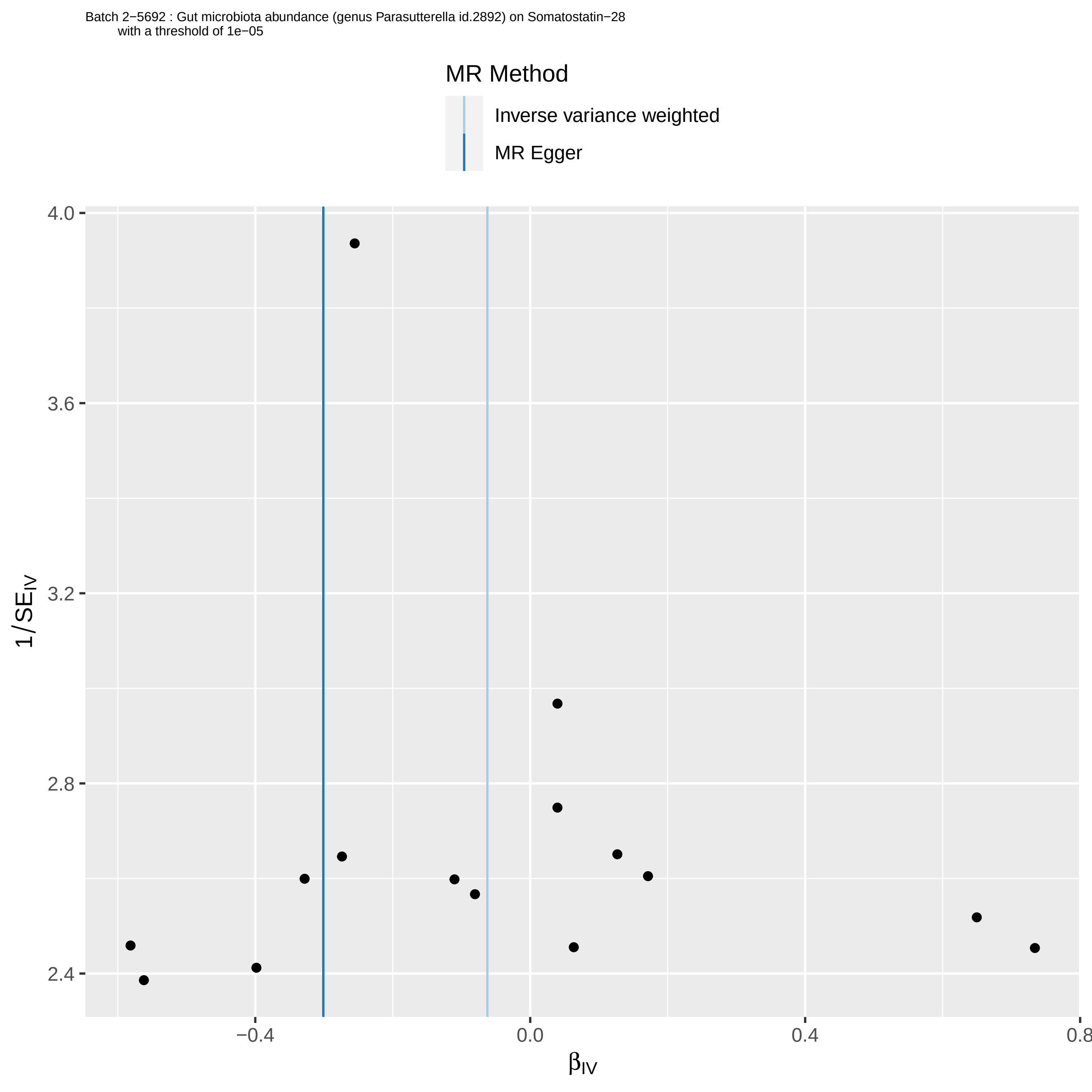

Batch 2−5692 : Gut microbiota abundance (genus Parasutterella id.2892) on Somatostatin−28
with a threshold of 1e−05
MR Method
Inverse variance weighted
MR Egger
4.0
3.6
3.2
2.8
2.4
1/SE_IV
−0.4
0.0
0.4
0.8
β_IV

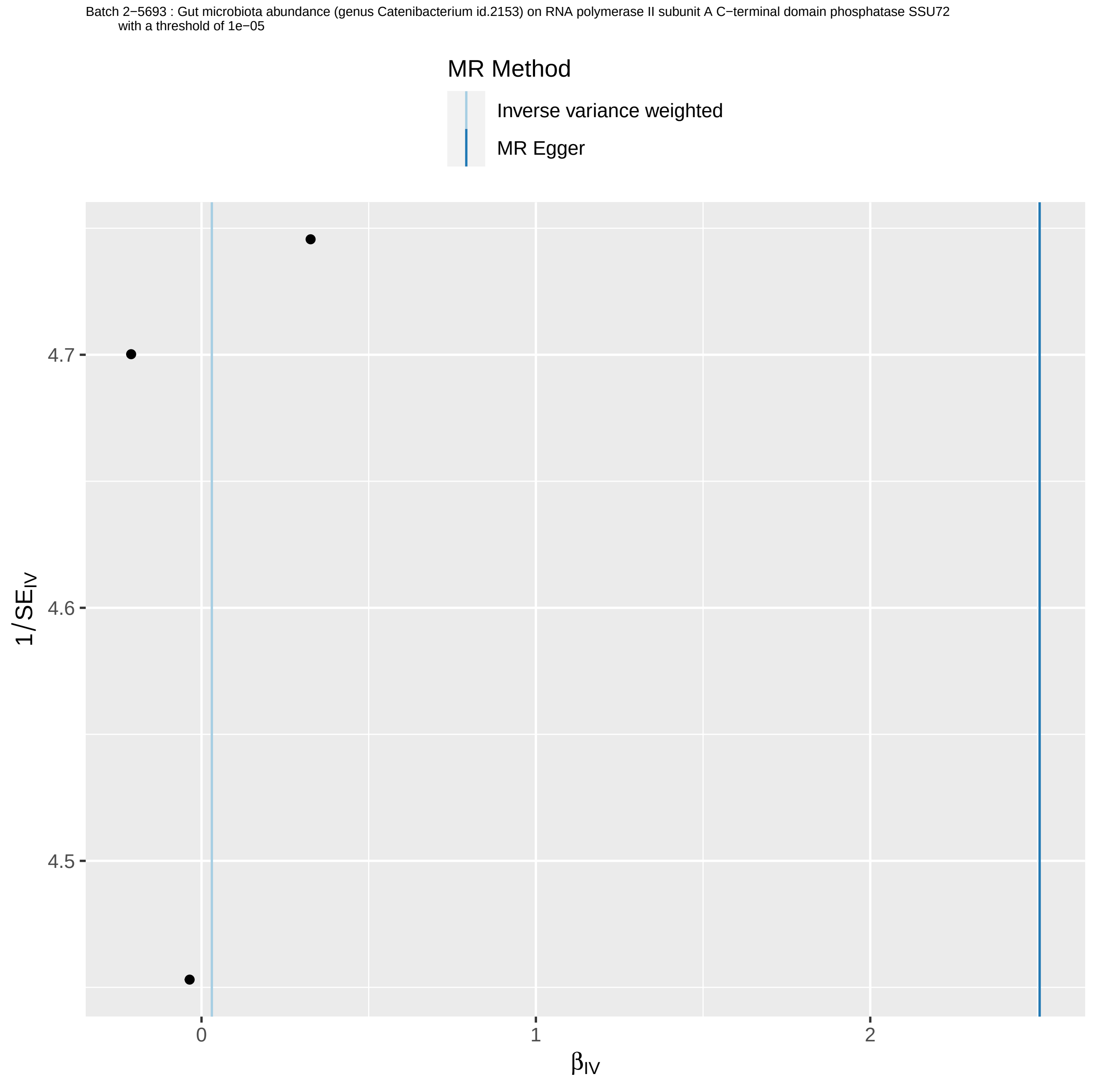

Batch 2−5693 : Gut microbiota abundance (genus Catenibacterium id.2153) on RNA polymerase II subunit A C−terminal domain phosphatase SSU72
with a threshold of 1e−05
MR Method
Inverse variance weighted
MR Egger
4.7
4.6
4.5
1/SE_IV
0
1
2
β_IV

Batch 2−5694 : Gut microbiota abundance (genus Parasutterella id.2892) on RNA polymerase II subunit A C−terminal domain phosphatase SSU72
with a threshold of 1e−05

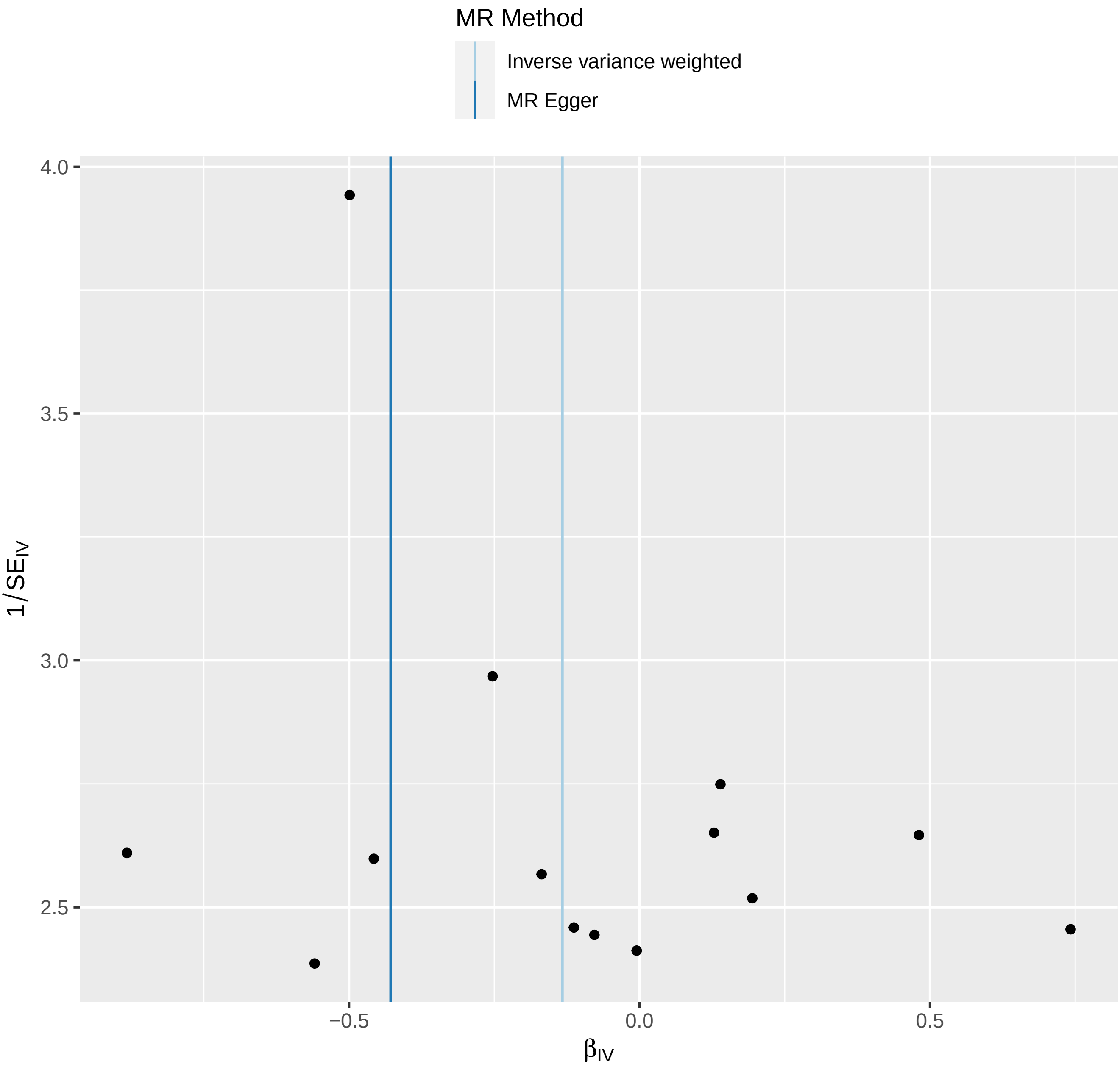

Batch 2−5695 : Gut microbiota abundance (genus Catenibacterium id.2153) on CMP−N−acetylneuraminate−beta−galactosamide−alpha−2,3−sialyltransferase 1 with a threshold of 1e−05

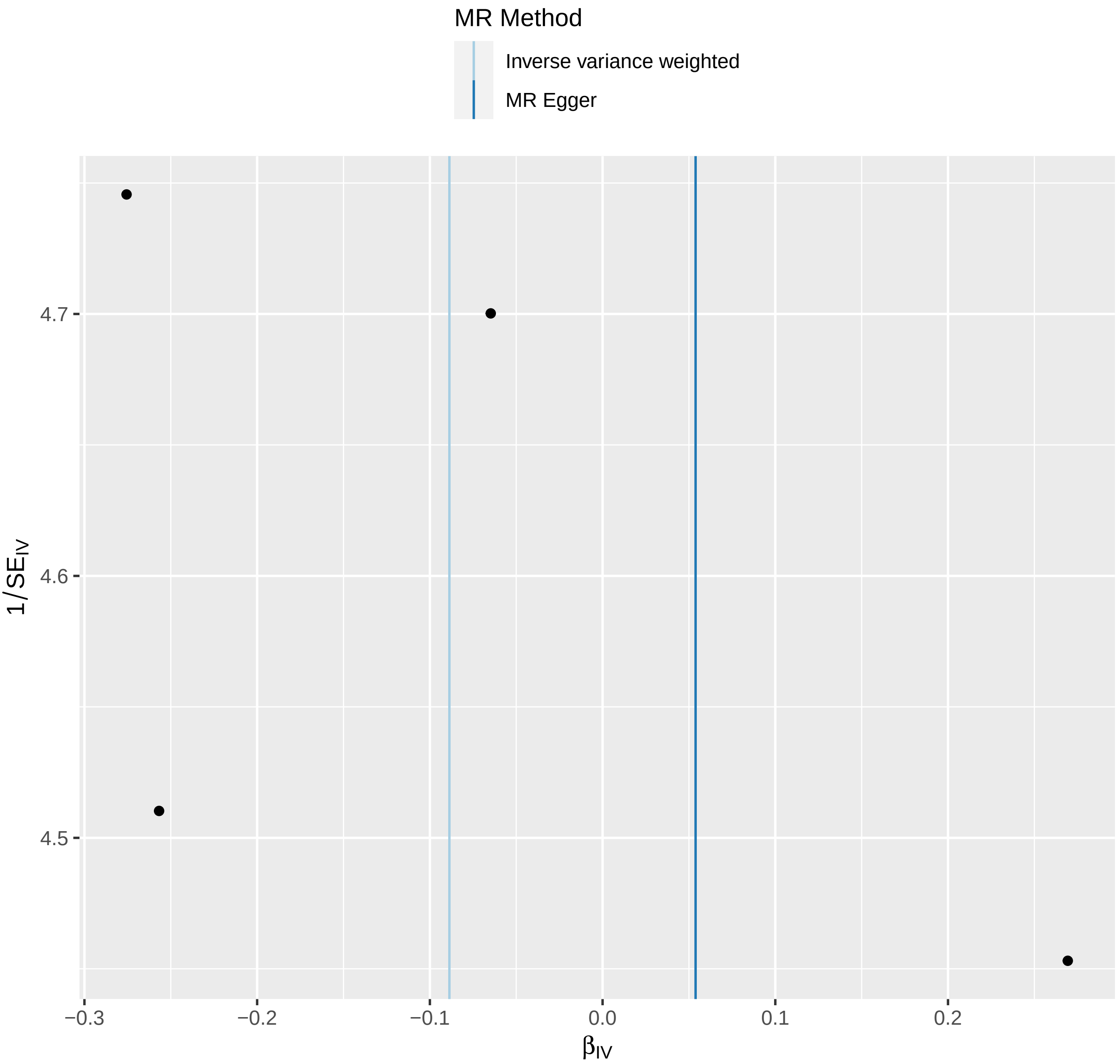

Batch 2−5696 : Gut microbiota abundance (genus Parasutterella id.2892) on CMP−N−acetylneuraminate−beta−galactosamide−alpha−2,3−sialyltransferase 1 with a threshold of 1e−05

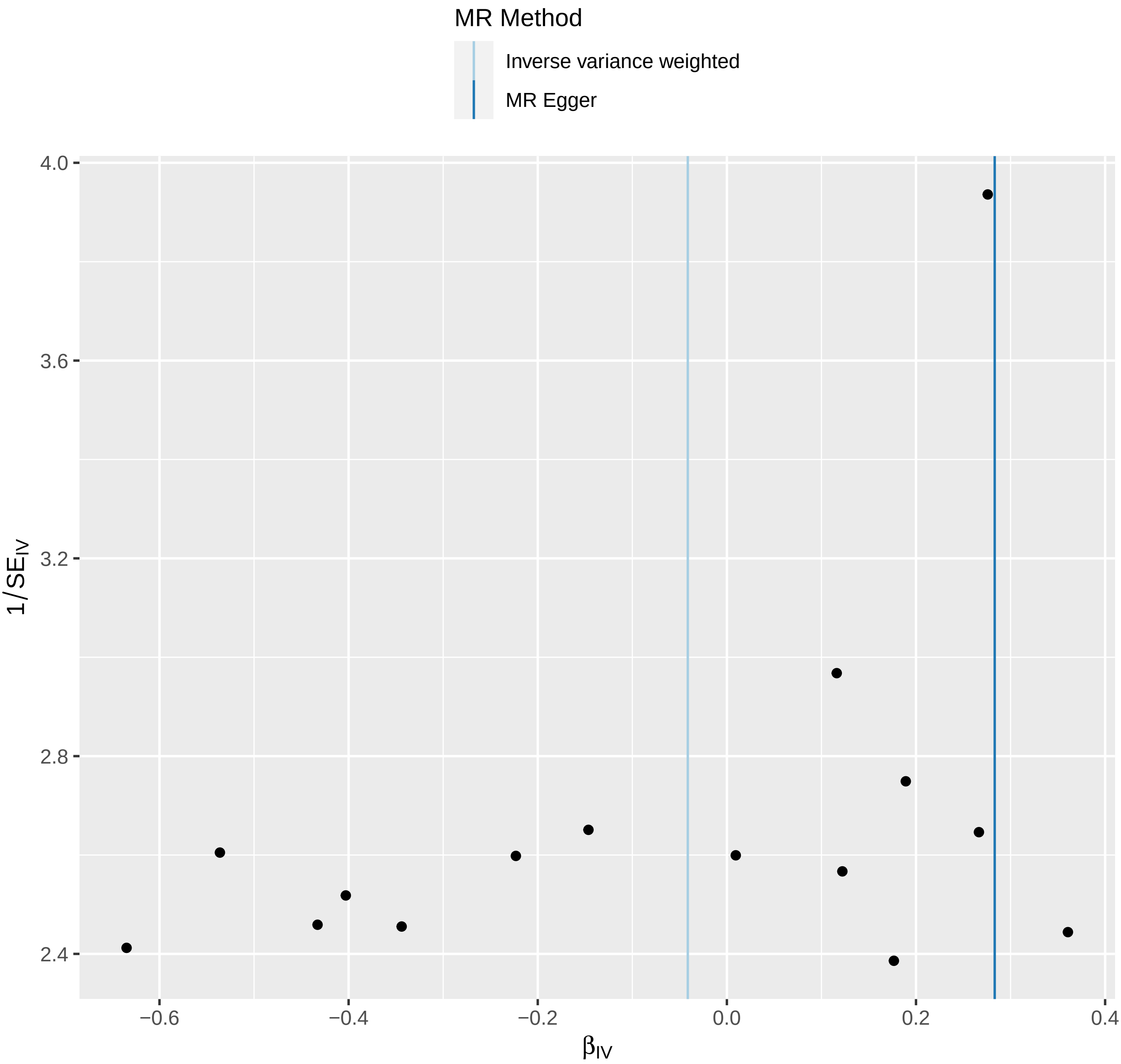

Batch 2−5697 : Gut microbiota abundance (genus Catenibacterium id.2153) on CMP−N−acetylneuraminate−beta−galactosamide−alpha−2,3−sialyltransferase 2 with a threshold of 1e−05

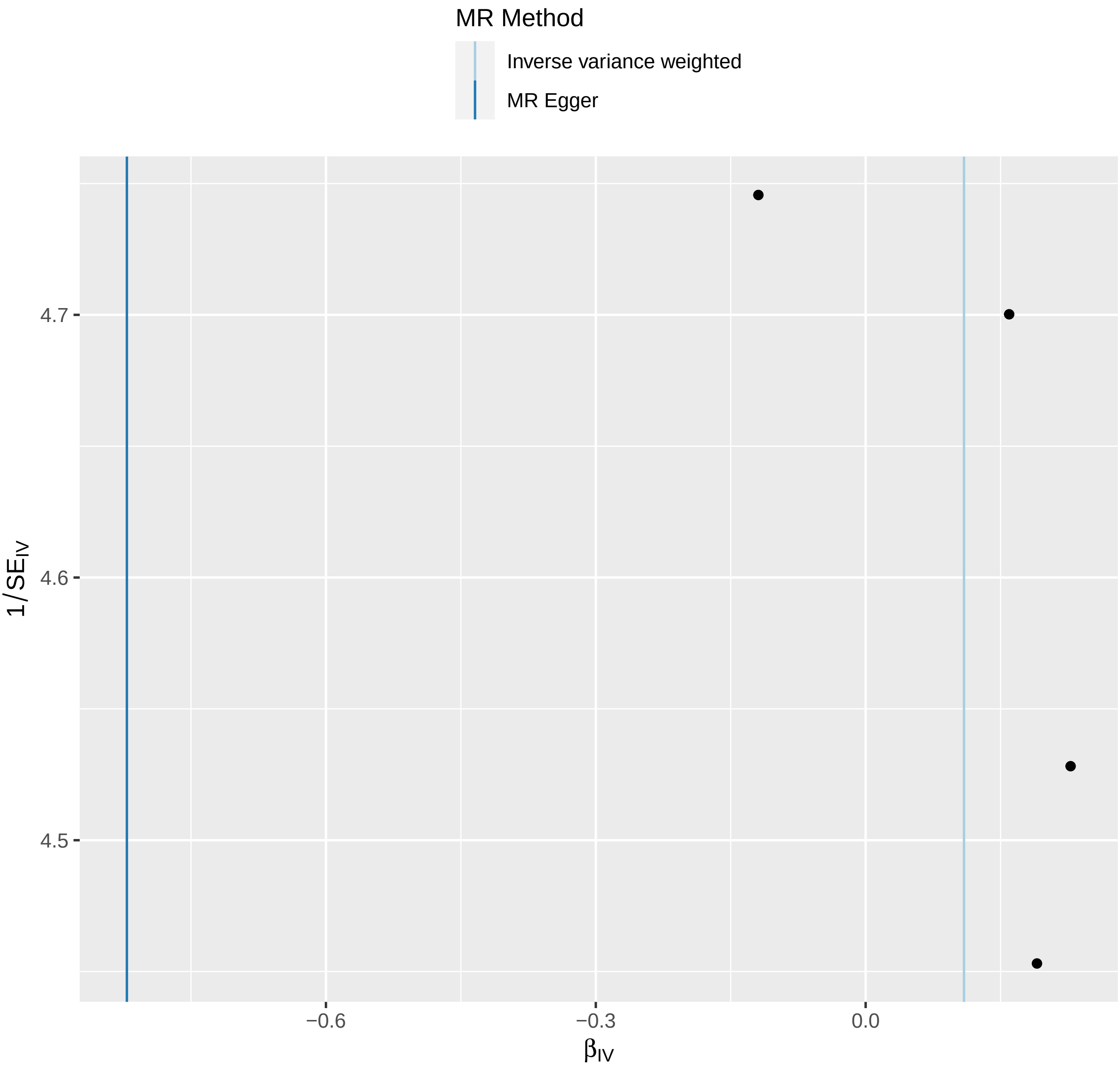

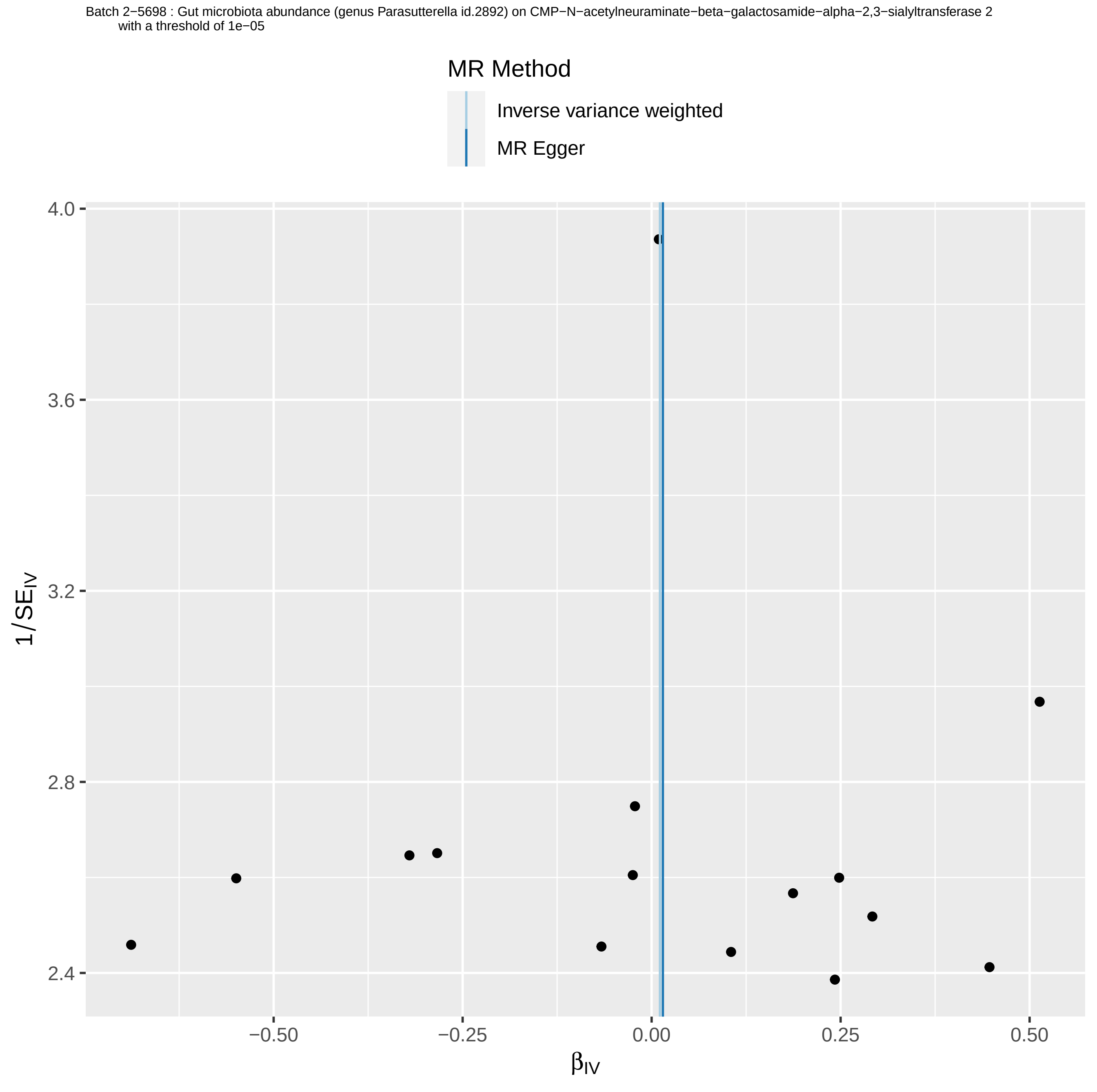
Batch 2−5698 : Gut microbiota abundance (genus Parasutterella id.2892) on CMP−N−acetylneuraminate−beta−galactosamide−alpha−2,3−sialyltransferase 2
with a threshold of 1e−05
MR Method
Inverse variance weighted
MR Egger
4.0
3.6
3.2
2.8
2.4
1/SE_IV
−0.50
−0.25
0.00
0.25
0.50
β_IV

Batch 2−5699 : Gut microbiota abundance (genus Catenibacterium id.2153) on Type 2 lactosamine alpha−2,3−sialyltransferase
with a threshold of 1e−05

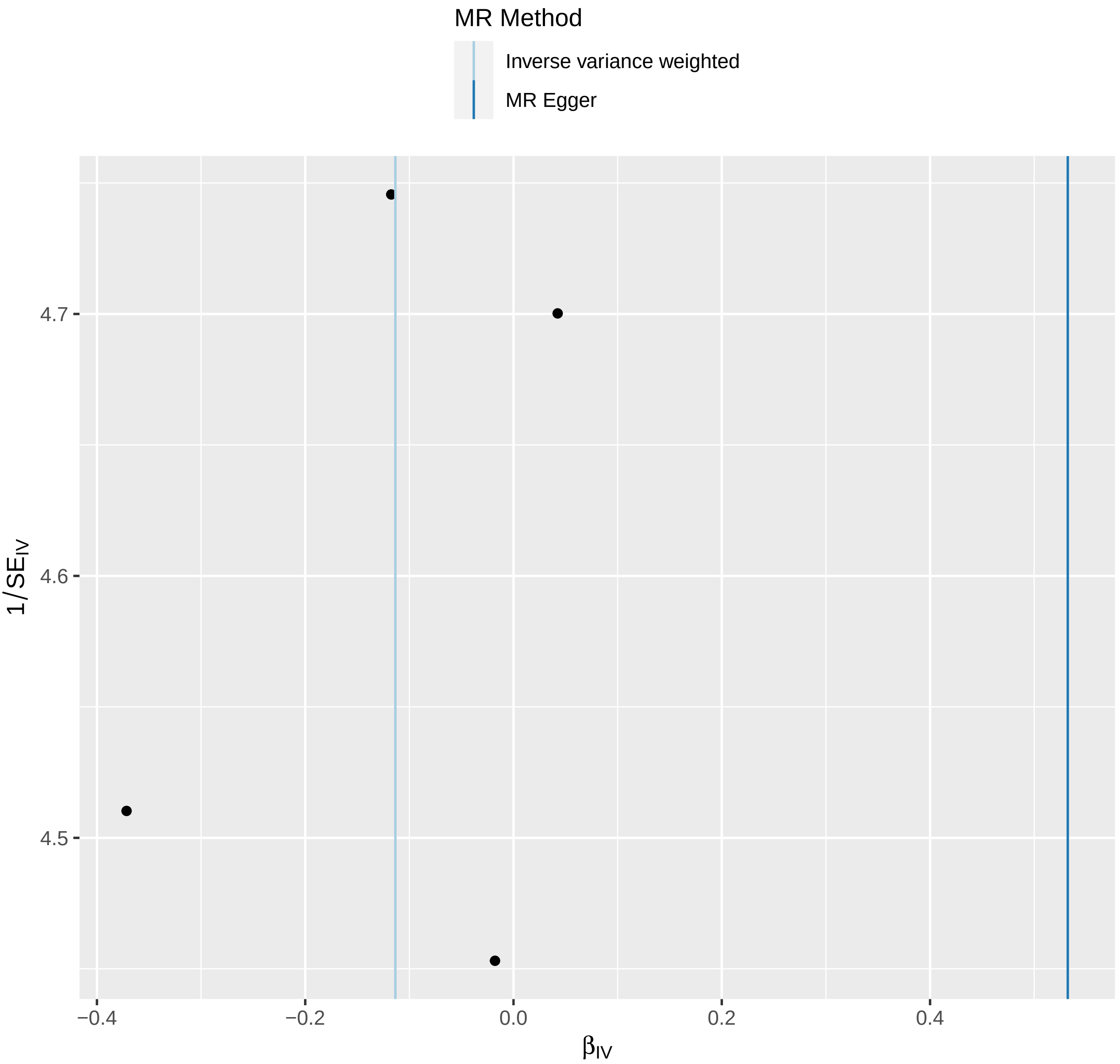

Batch 2−5700 : Gut microbiota abundance (genus Parasutterella id.2892) on Type 2 lactosamine alpha−2,3−sialyltransferase
with a threshold of 1e−05

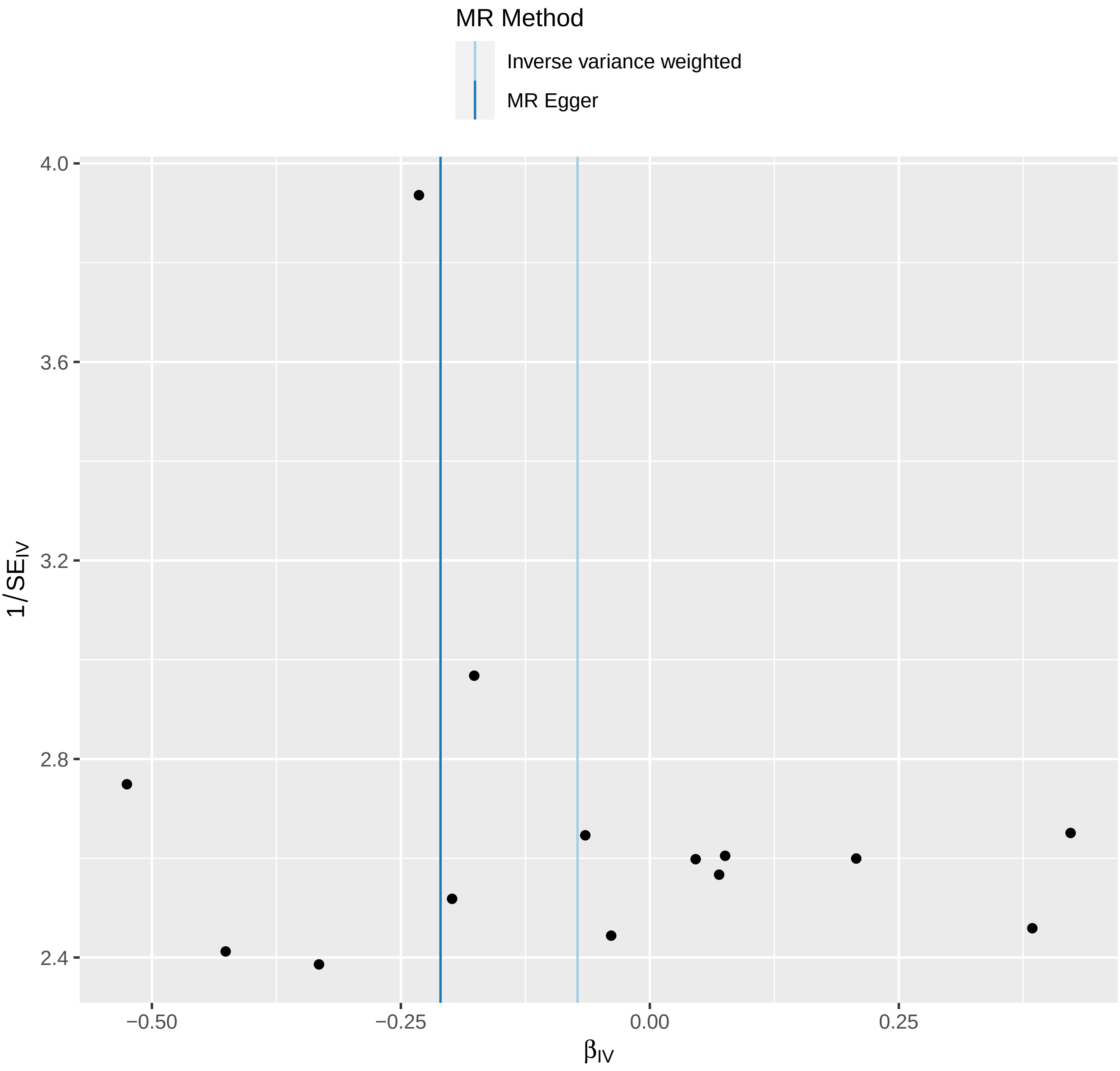

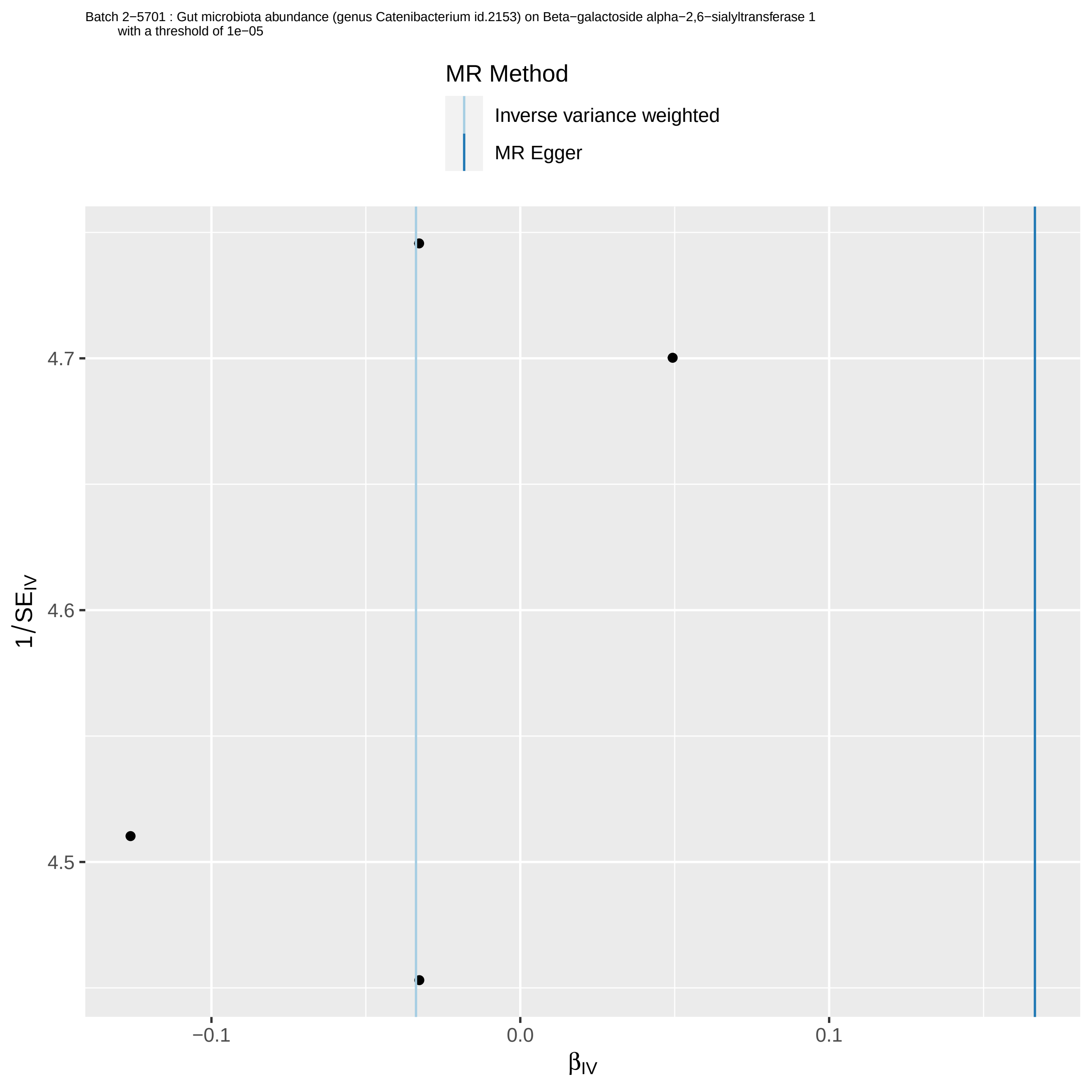

Batch 2−5701 : Gut microbiota abundance (genus Catenibacterium id.2153) on Beta−galactoside alpha−2,6−sialyltransferase 1
with a threshold of 1e−05
MR Method
Inverse variance weighted
MR Egger
4.7
4.6
4.5
1/SE_IV
−0.1
0.0
0.1
β_IV

Batch 2−5702 : Gut microbiota abundance (genus Parasutterella id.2892) on Beta−galactoside alpha−2,6−sialyltransferase 1
with a threshold of 1e−05

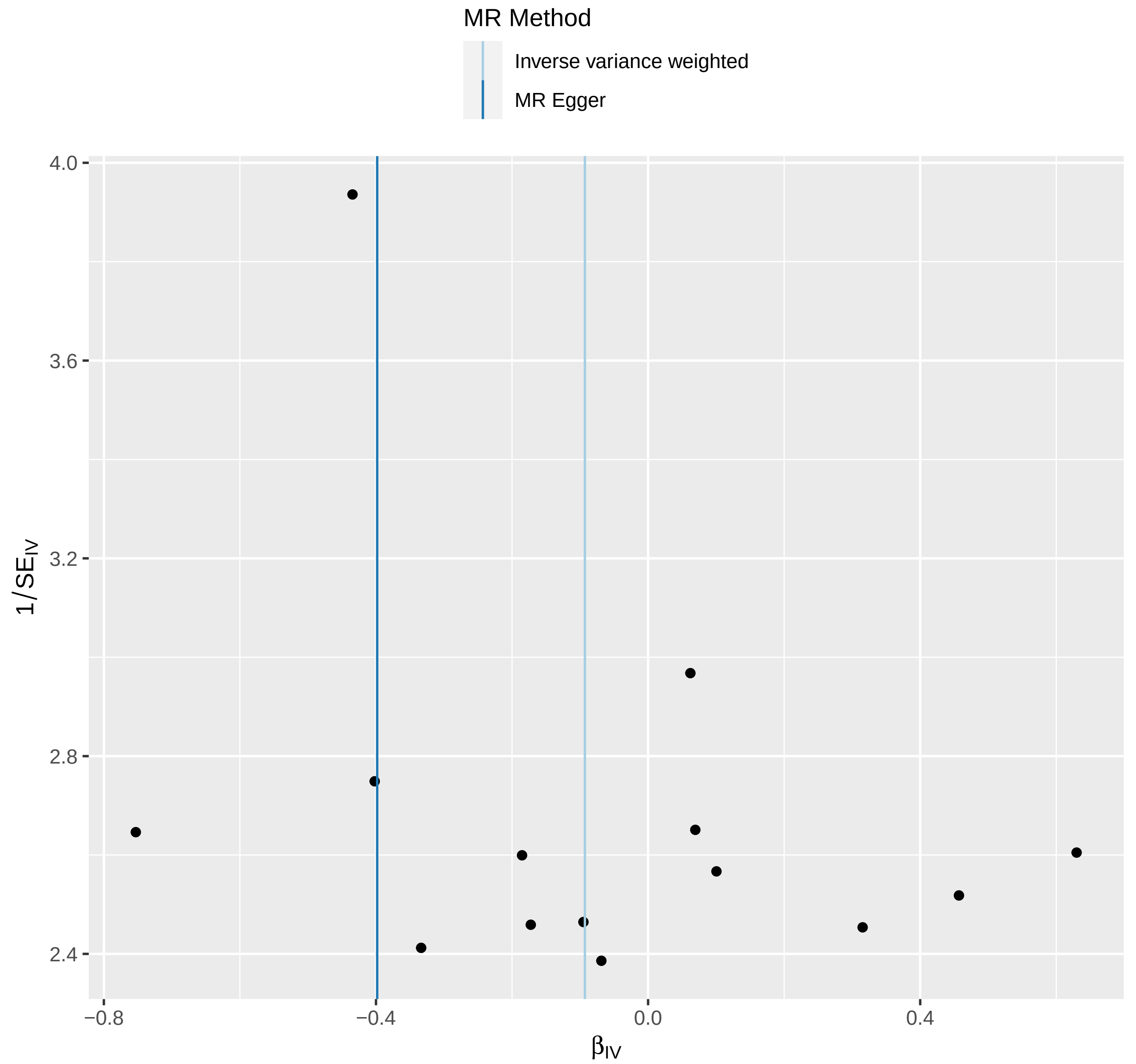

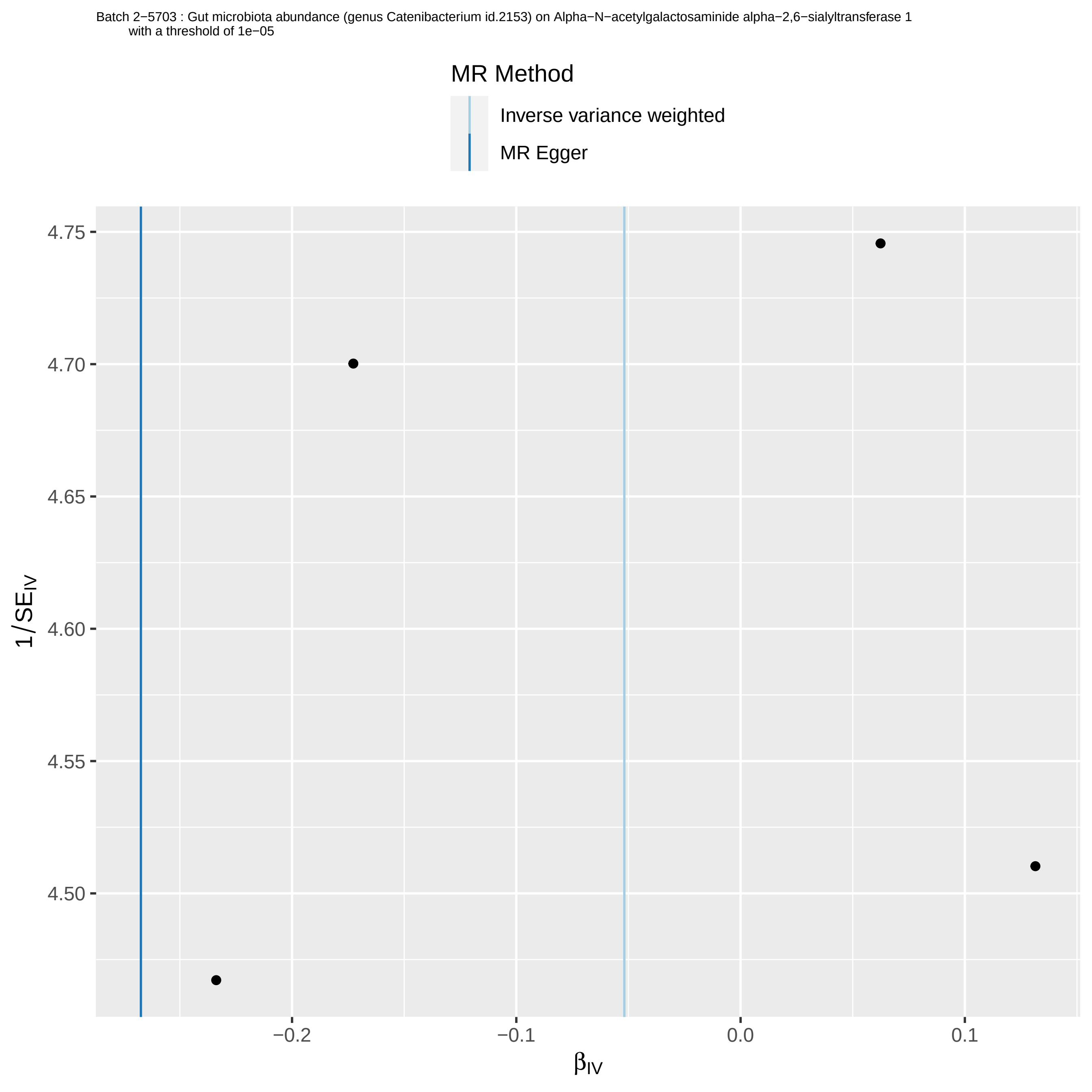

Batch 2−5703 : Gut microbiota abundance (genus Catenibacterium id.2153) on Alpha−N−acetylgalactosaminide alpha−2,6−sialyltransferase 1
with a threshold of 1e−05
MR Method
Inverse variance weighted
MR Egger
4.75
4.70
4.65
4.60
4.55
4.50
1/SE_IV
−0.2
−0.1
0.0
0.1
β_IV

Batch 2−5704 : Gut microbiota abundance (genus Parasutterella id.2892) on Alpha−N−acetylgalactosaminide alpha−2,6−sialyltransferase 1
with a threshold of 1e−05

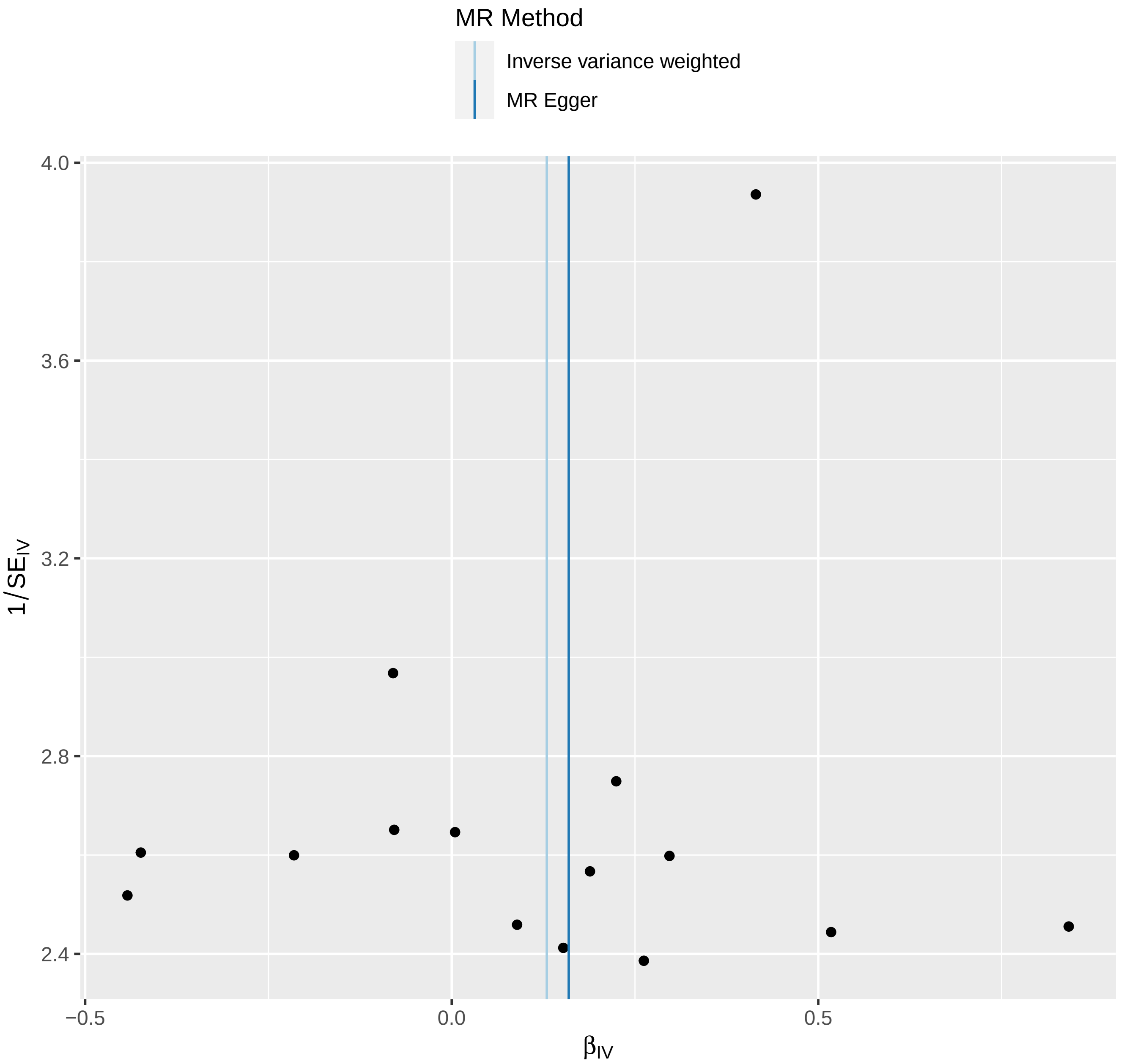

Batch 2−5705 : Gut microbiota abundance (genus Catenibacterium id.2153) on Alpha−N−acetylgalactosaminide alpha−2,6−sialyltransferase 2
with a threshold of 1e−05

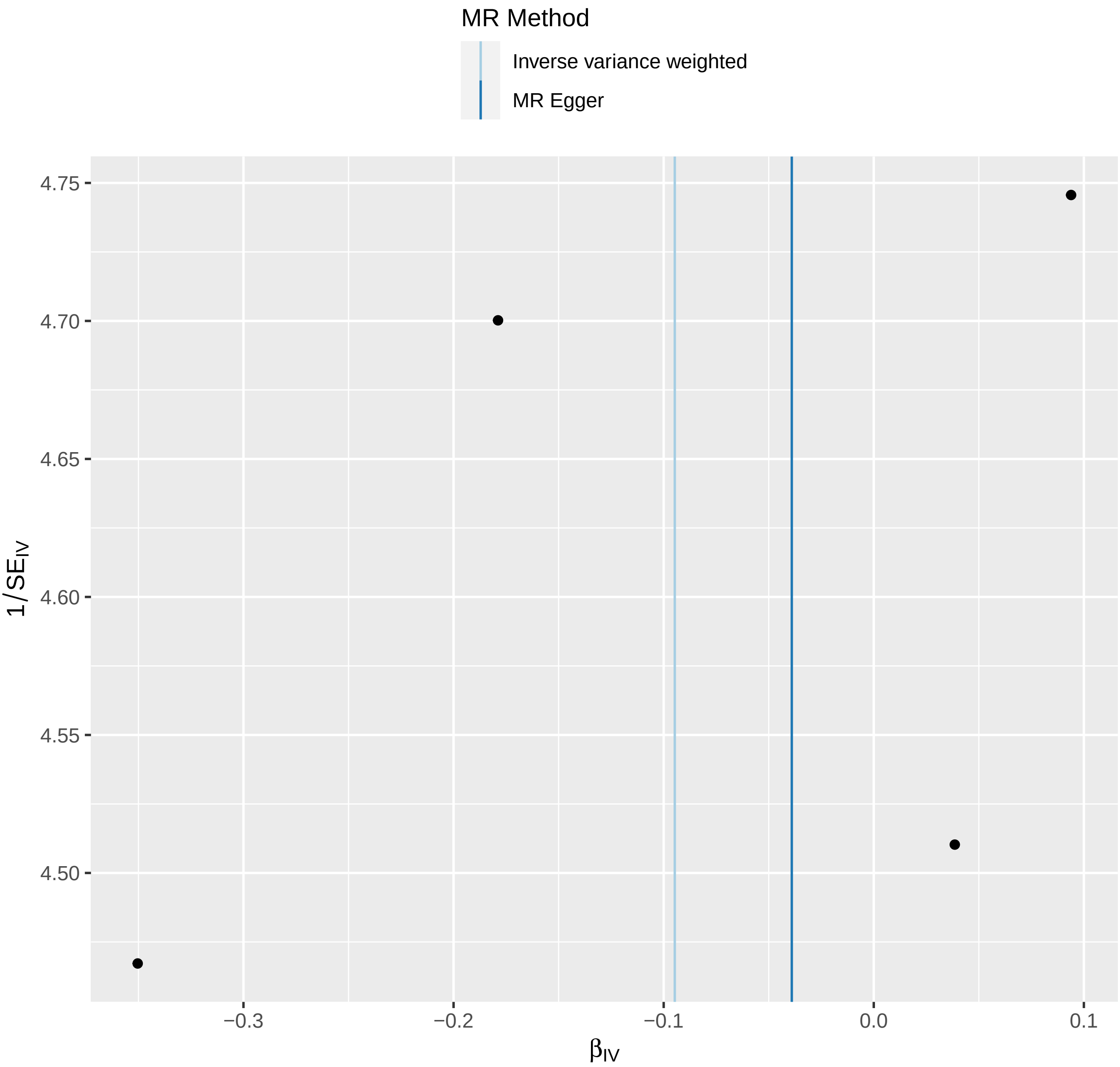

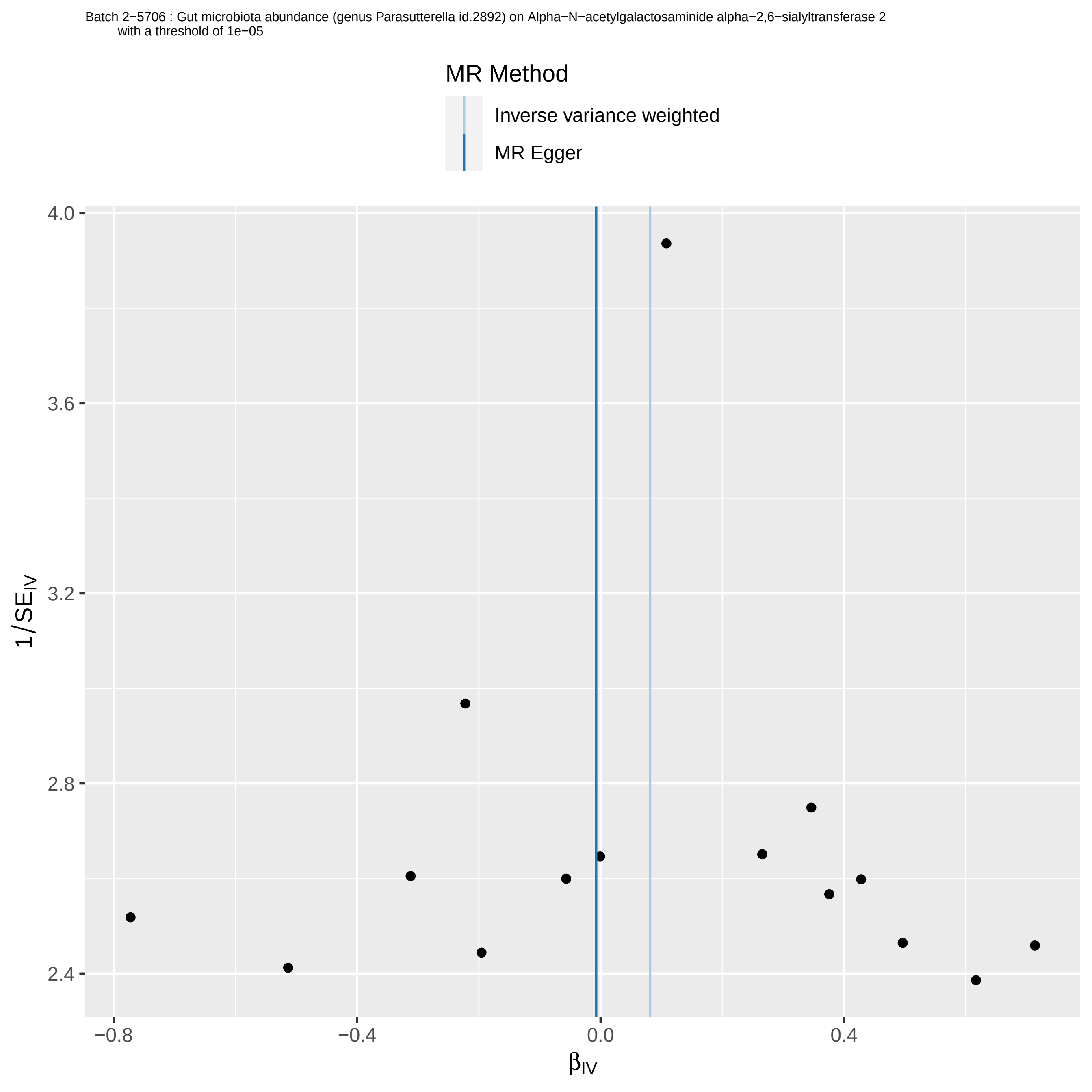

Batch 2−5706 : Gut microbiota abundance (genus Parasutterella id.2892) on Alpha−N−acetylgalactosaminide alpha−2,6−sialyltransferase 2
with a threshold of 1e−05
MR Method
Inverse variance weighted
MR Egger
4.0
3.6
3.2
2.8
2.4
1/SE$_{IV}$
−0.8
−0.4
0.0
0.4
β$_{IV}$

Batch 2−5707 : Gut microbiota abundance (genus Catenibacterium id.2153) on Alpha−N−acetylgalactosaminide alpha−2,6−sialyltransferase 3 with a threshold of 1e−05

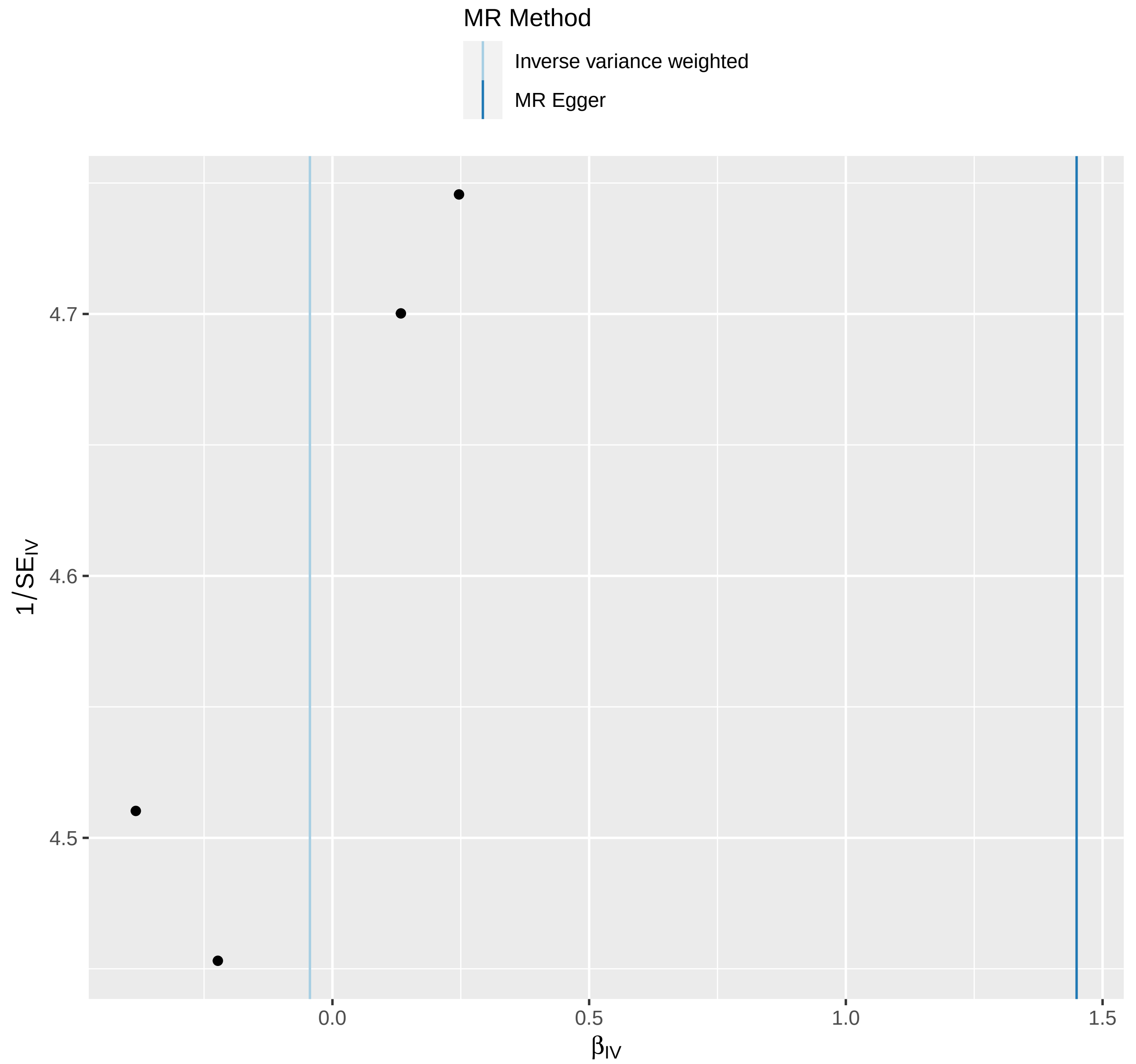

Batch 2−5708 : Gut microbiota abundance (genus Parasutterella id.2892) on Alpha−N−acetylgalactosaminide alpha−2,6−sialyltransferase 3
with a threshold of 1e−05

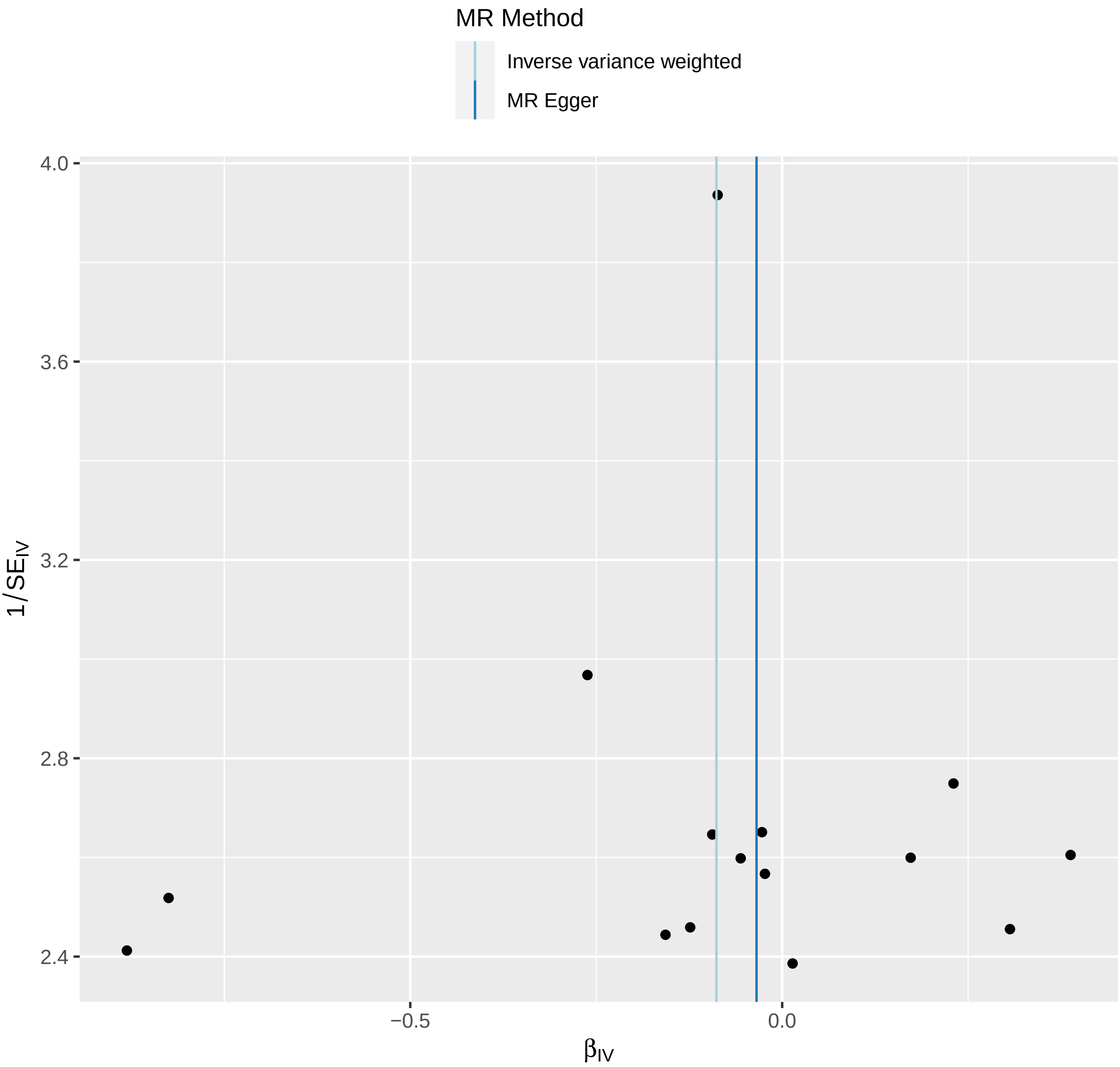

Batch 2−5709 : Gut microbiota abundance (genus Catenibacterium id.2153) on Alpha−N−acetylgalactosaminide alpha−2,6−sialyltransferase 3 with a threshold of 1e−05

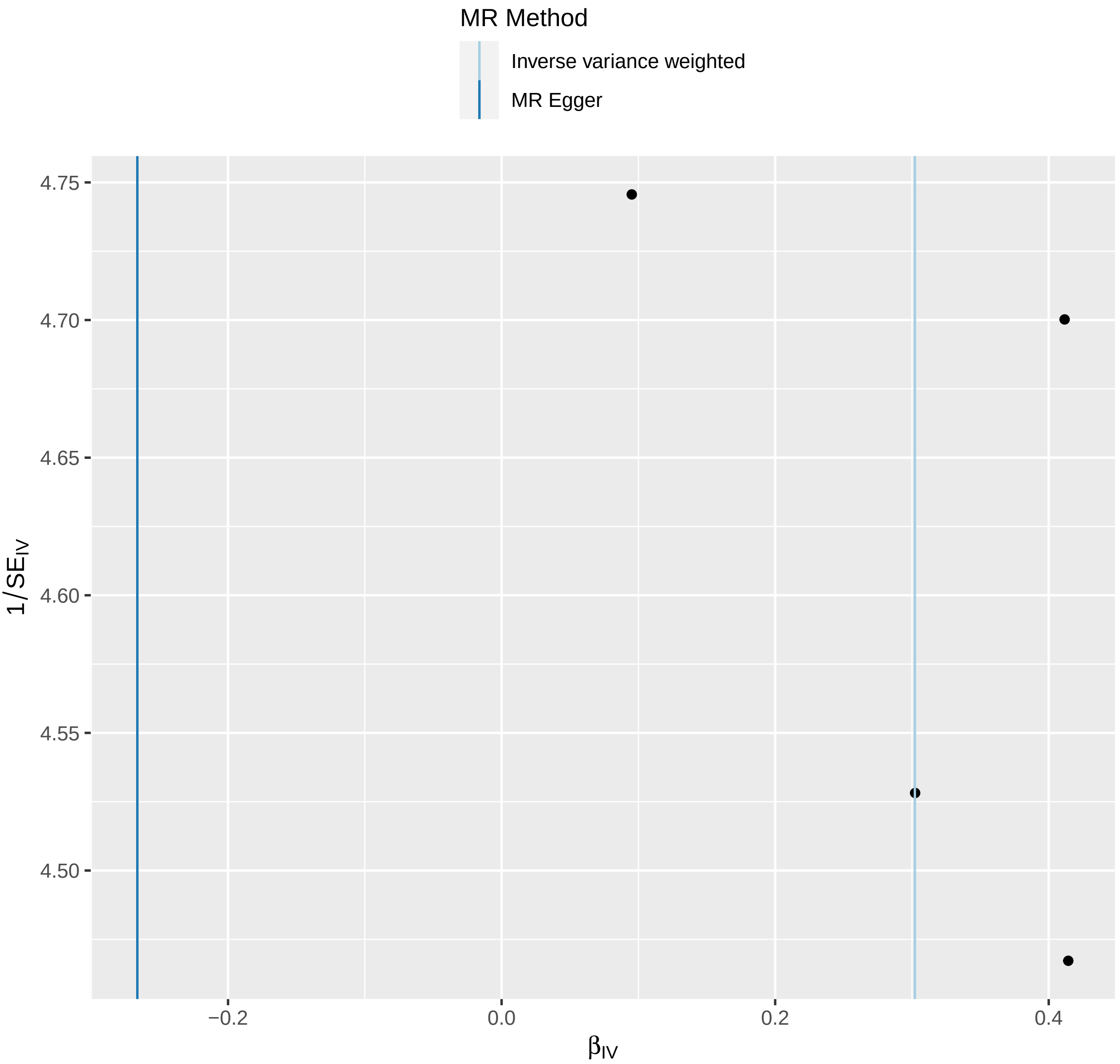

Batch 2−5710 : Gut microbiota abundance (genus Parasutterella id.2892) on Alpha−N−acetylgalactosaminide alpha−2,6−sialyltransferase 3
with a threshold of 1e−05

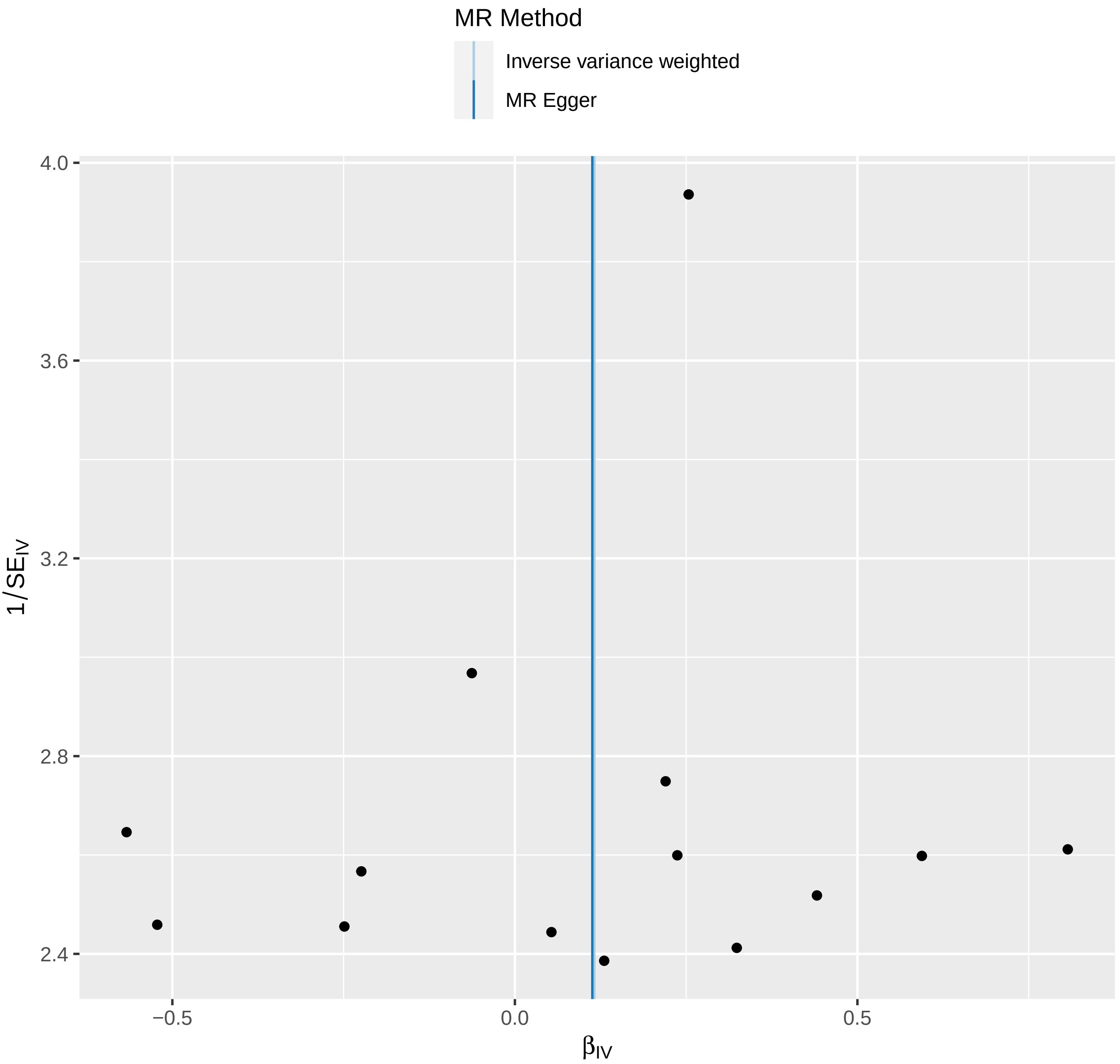

Batch 2−5711 : Gut microbiota abundance (genus Catenibacterium id.2153) on Alpha−N−acetylgalactosaminide alpha−2,6−sialyltransferase 5 with a threshold of 1e−05

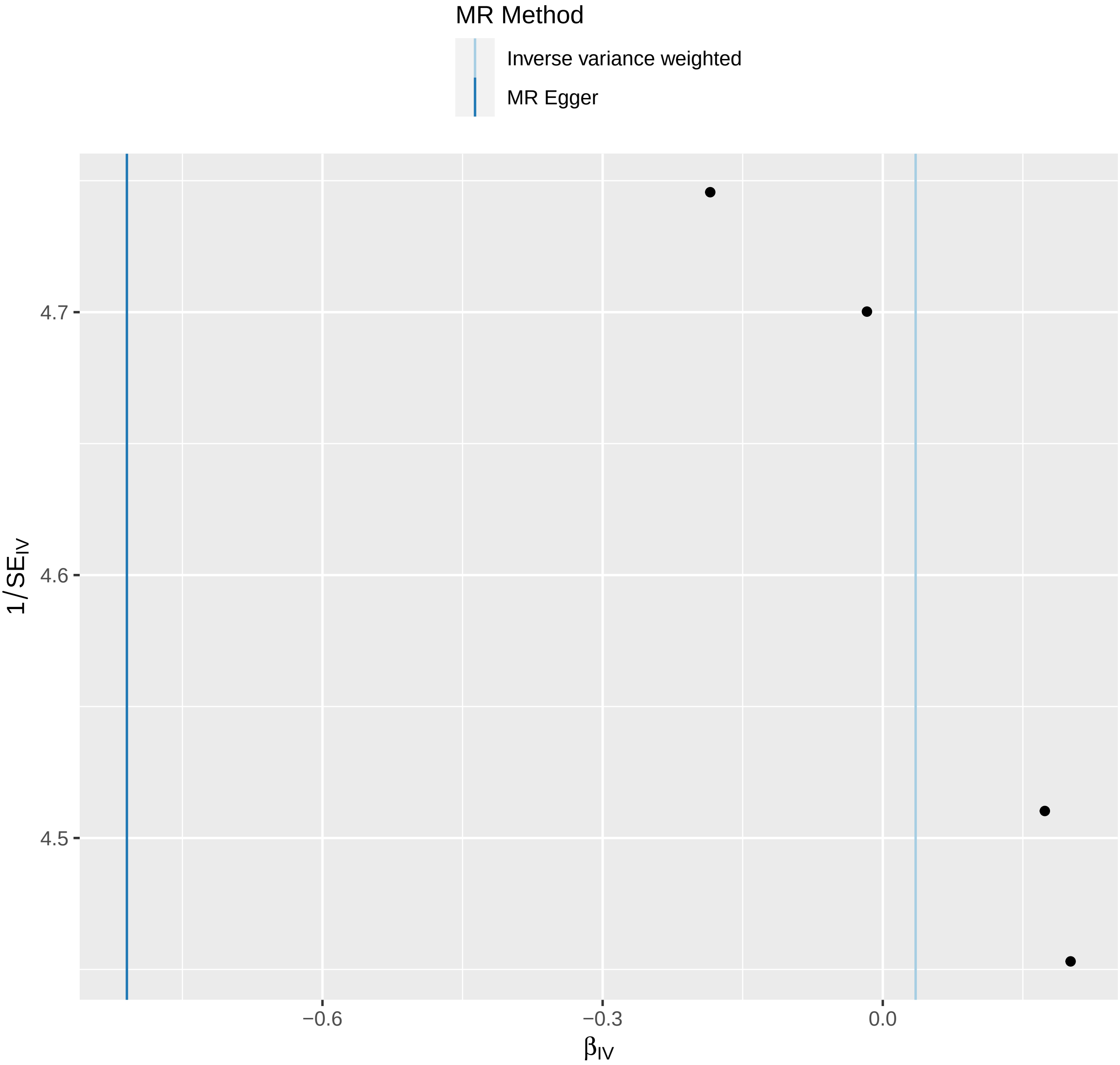

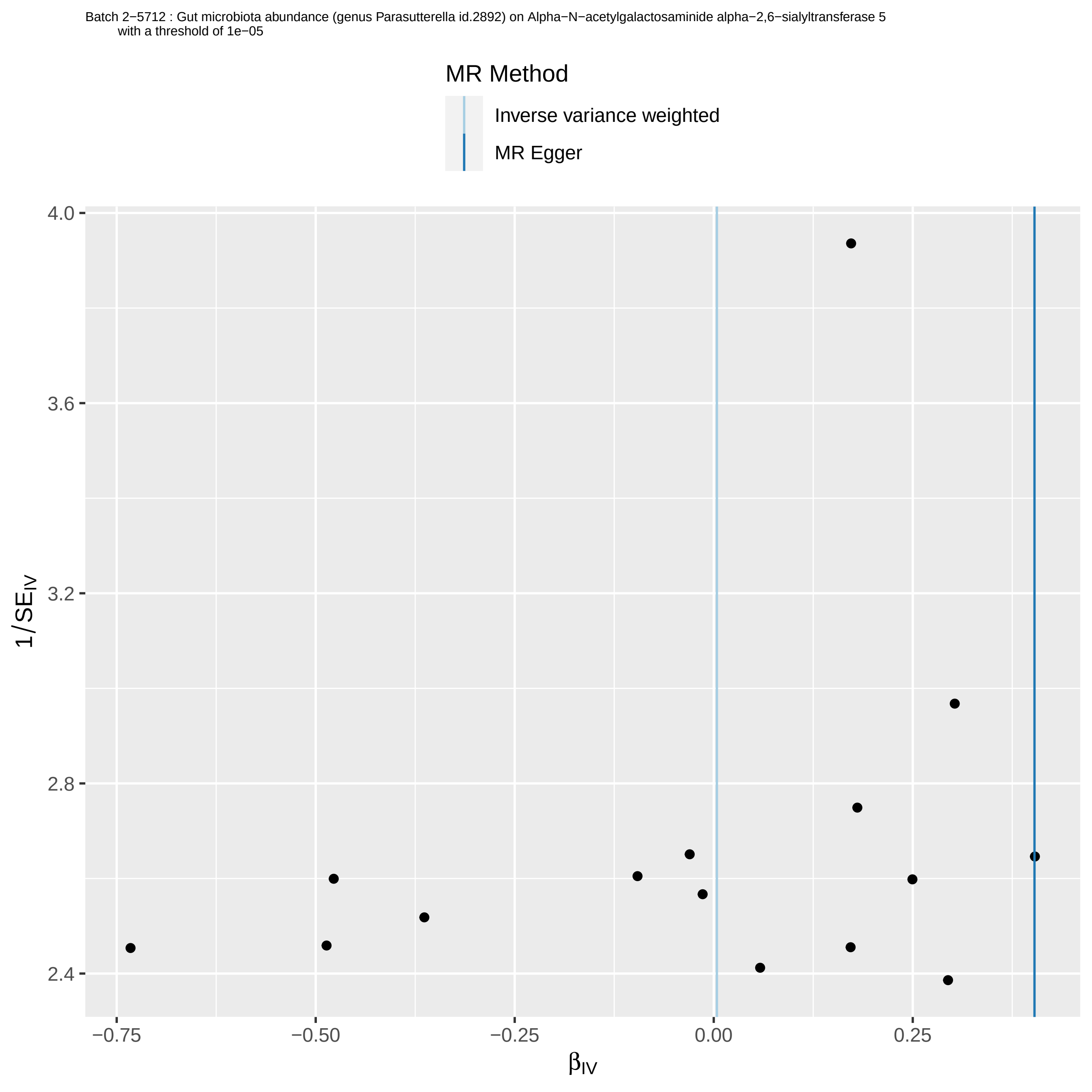
Batch 2−5712 : Gut microbiota abundance (genus Parasutterella id.2892) on Alpha−N−acetylgalactosaminide alpha−2,6−sialyltransferase 5
with a threshold of 1e−05
MR Method
Inverse variance weighted
MR Egger
4.0
3.6
3.2
2.8
2.4
1/SE_IV
−0.75
−0.50
−0.25
0.00
0.25
β_IV

Batch 2−5713 : Gut microbiota abundance (genus Catenibacterium id.2153) on Alpha−N−acetylgalactosaminide alpha−2,6−sialyltransferase 6 with a threshold of 1e−05

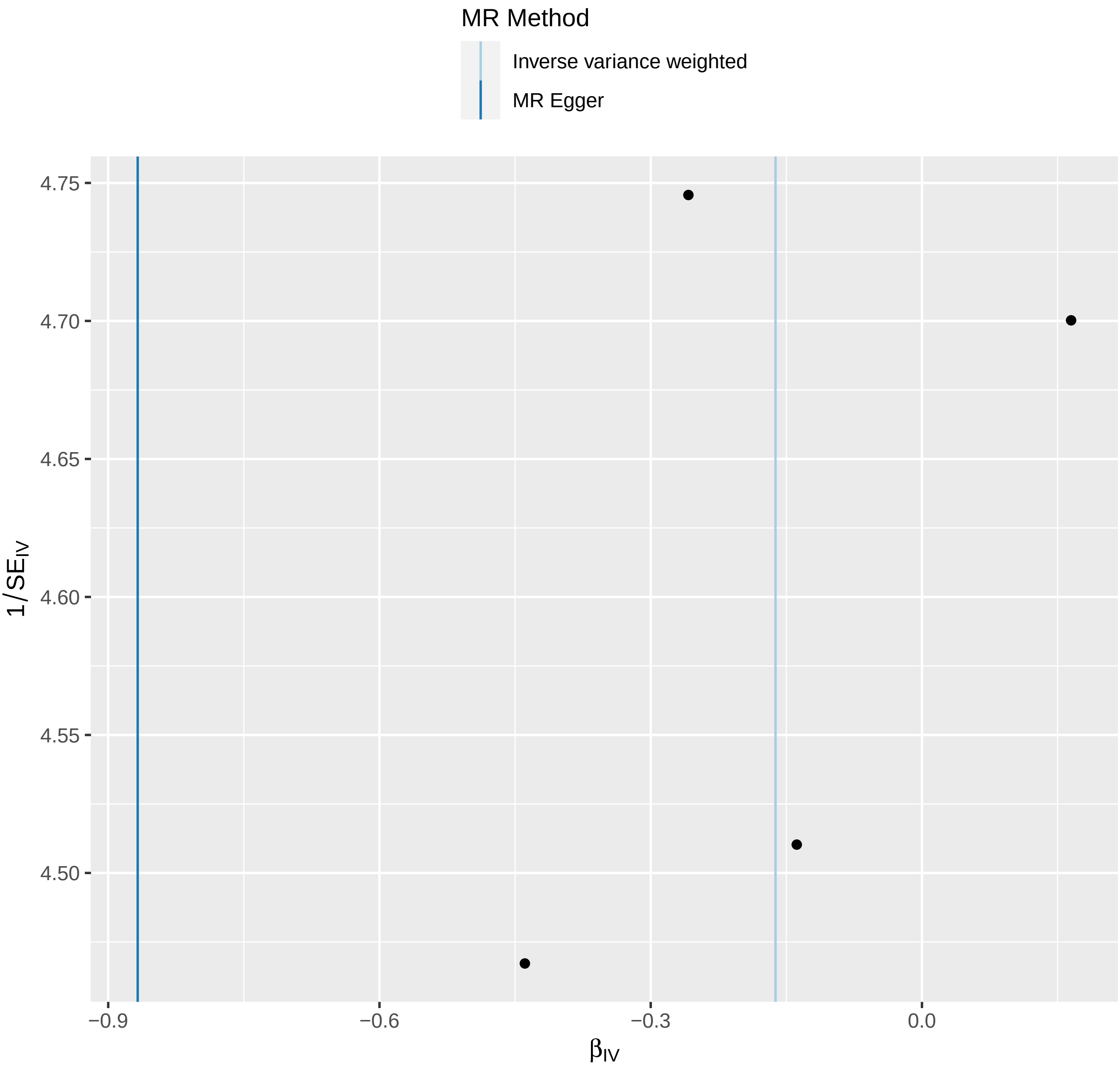

Batch 2−5714 : Gut microbiota abundance (genus Parasutterella id.2892) on Alpha−N−acetylgalactosaminide alpha−2,6−sialyltransferase 6
with a threshold of 1e−05

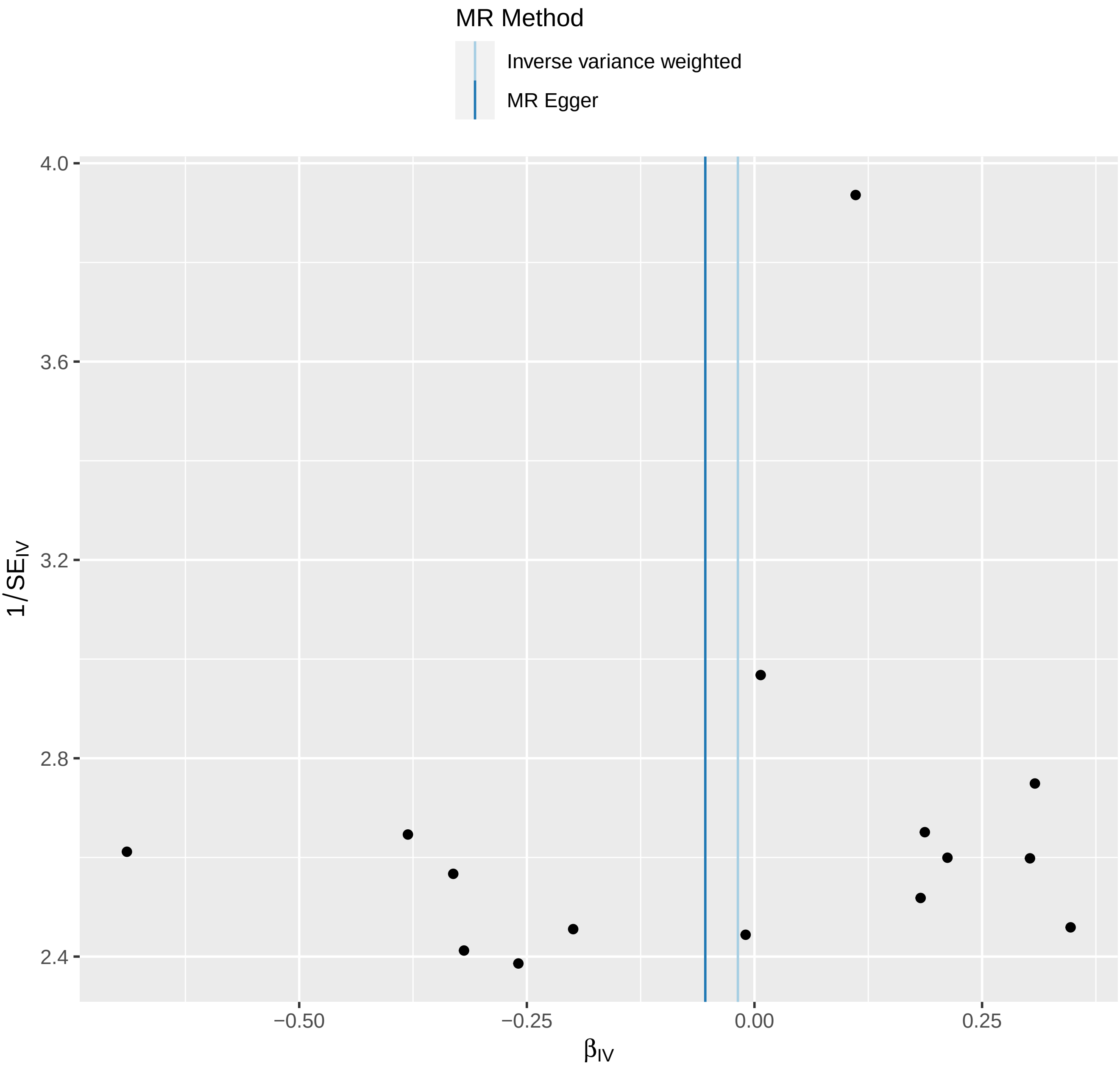

Batch 2−5715 : Gut microbiota abundance (genus Catenibacterium id.2153) on Alpha−2,8−sialyltransferase 8B
with a threshold of 1e−05

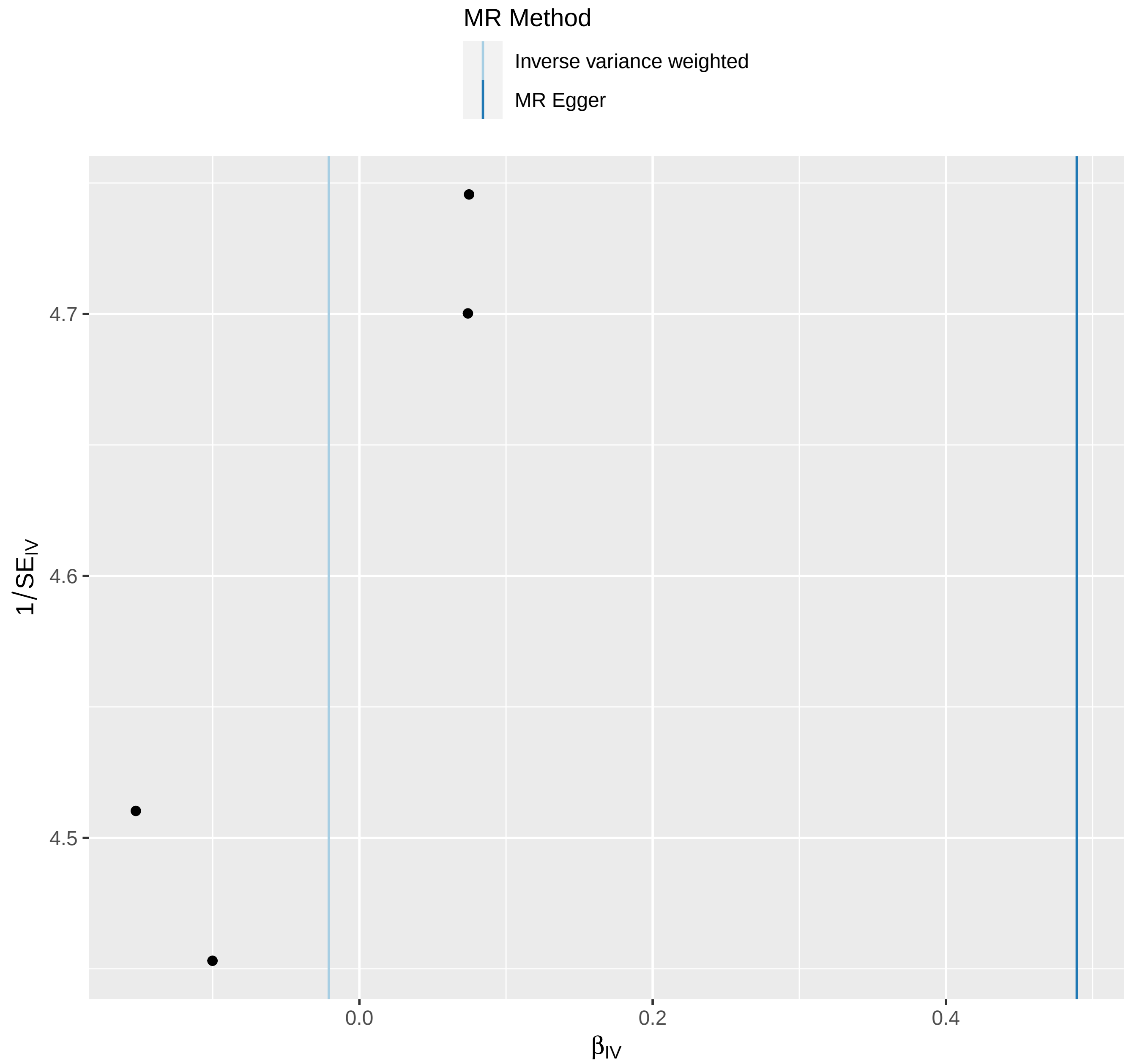

Batch 2−5716 : Gut microbiota abundance (genus Parasutterella id.2892) on Alpha−2,8−sialyltransferase 8B
with a threshold of 1e−05

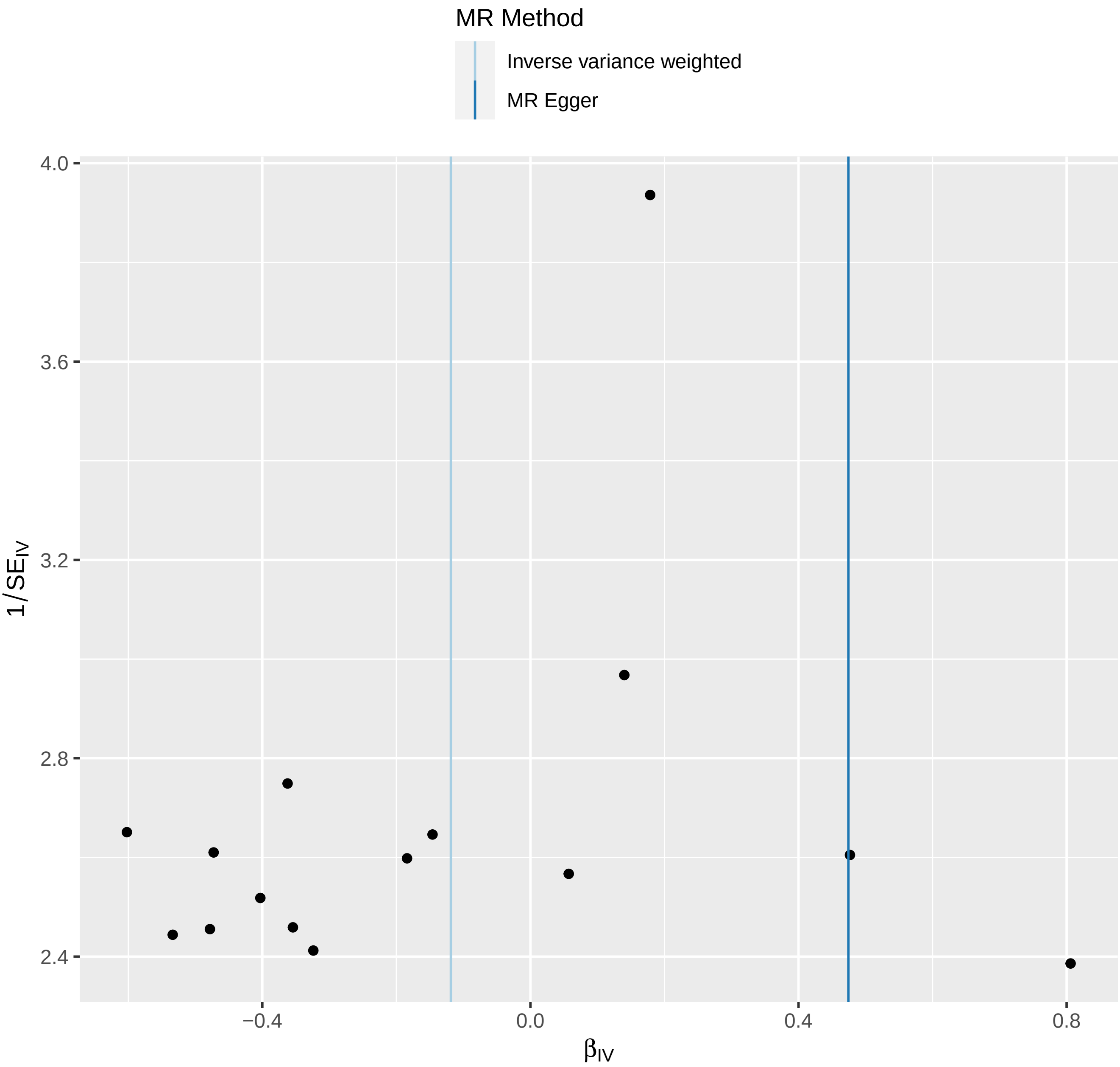

Batch 2−5717 : Gut microbiota abundance (genus Catenibacterium id.2153) on Sia−alpha−2,3−Gal−beta−1,4−GlcNAc−R:alpha 2,8−sialyltransferase with a threshold of 1e−05

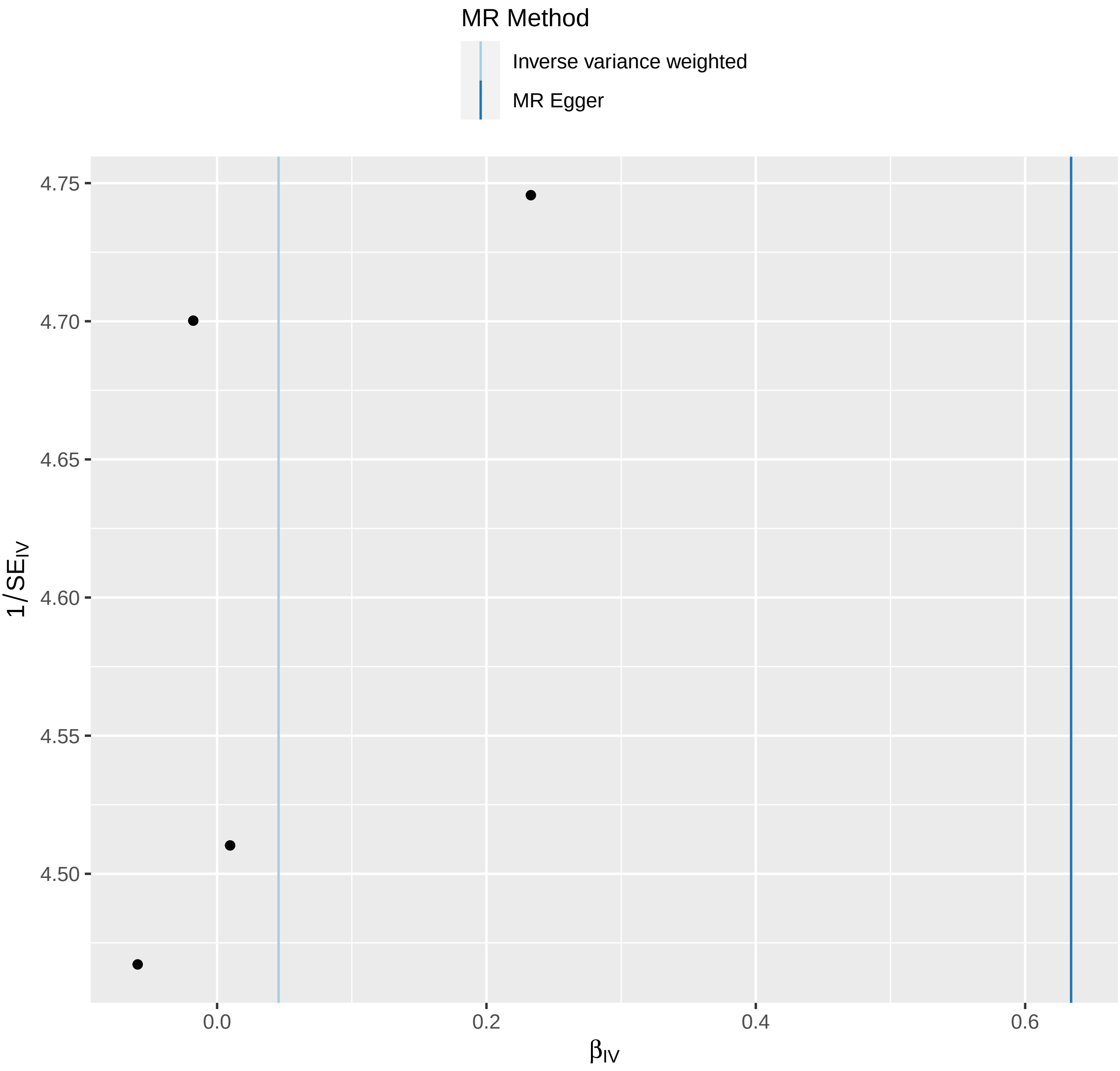

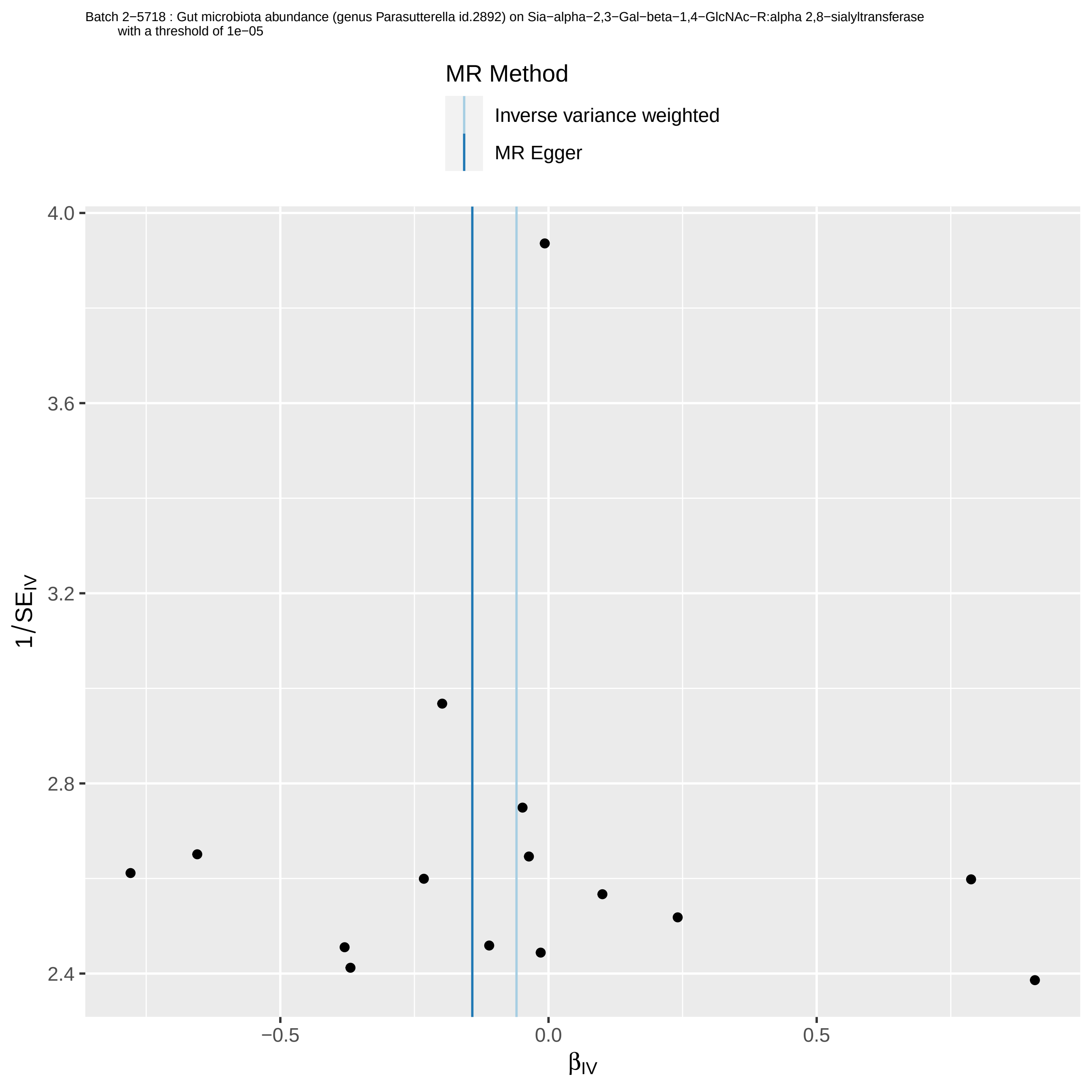

Batch 2−5718 : Gut microbiota abundance (genus Parasutterella id.2892) on Sia−alpha−2,3−Gal−beta−1,4−GlcNAc−R:alpha 2,8−sialyltransferase
with a threshold of 1e−05
MR Method
Inverse variance weighted
MR Egger
4.0
3.6
3.2
2.8
2.4
1/SE_IV
−0.5
0.0
0.5
β_IV

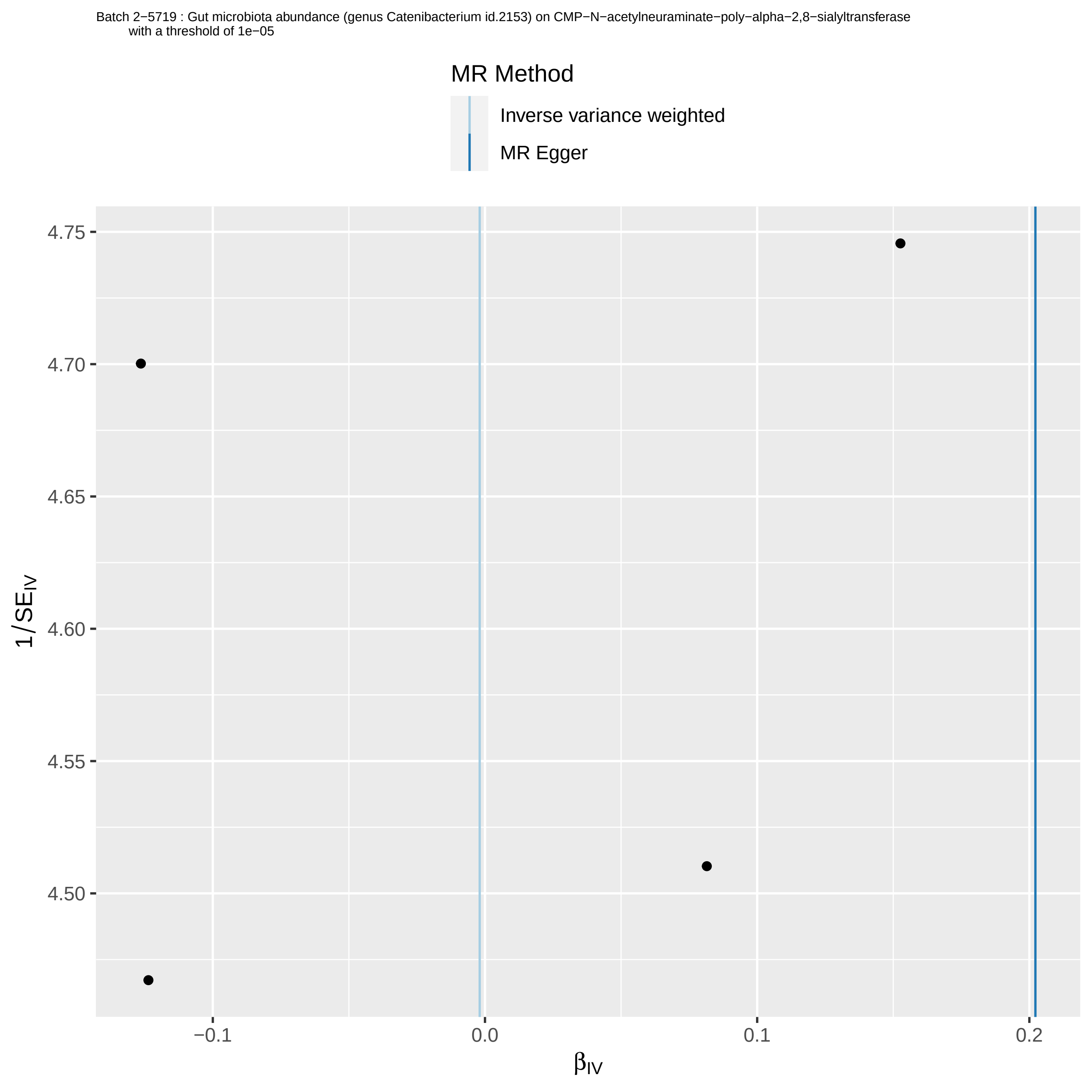
Batch 2−5719 : Gut microbiota abundance (genus Catenibacterium id.2153) on CMP−N−acetylneuraminate−poly−alpha−2,8−sialyltransferase with a threshold of 1e−05
MR Method
Inverse variance weighted
MR Egger
4.75
4.70
4.65
4.60
4.55
4.50
1/SE_IV
−0.1
0.0
0.1
0.2
β_IV

Batch 2−5720 : Gut microbiota abundance (genus Parasutterella id.2892) on CMP−N−acetylneuraminate−poly−alpha−2,8−sialyltransferase with a threshold of 1e−05

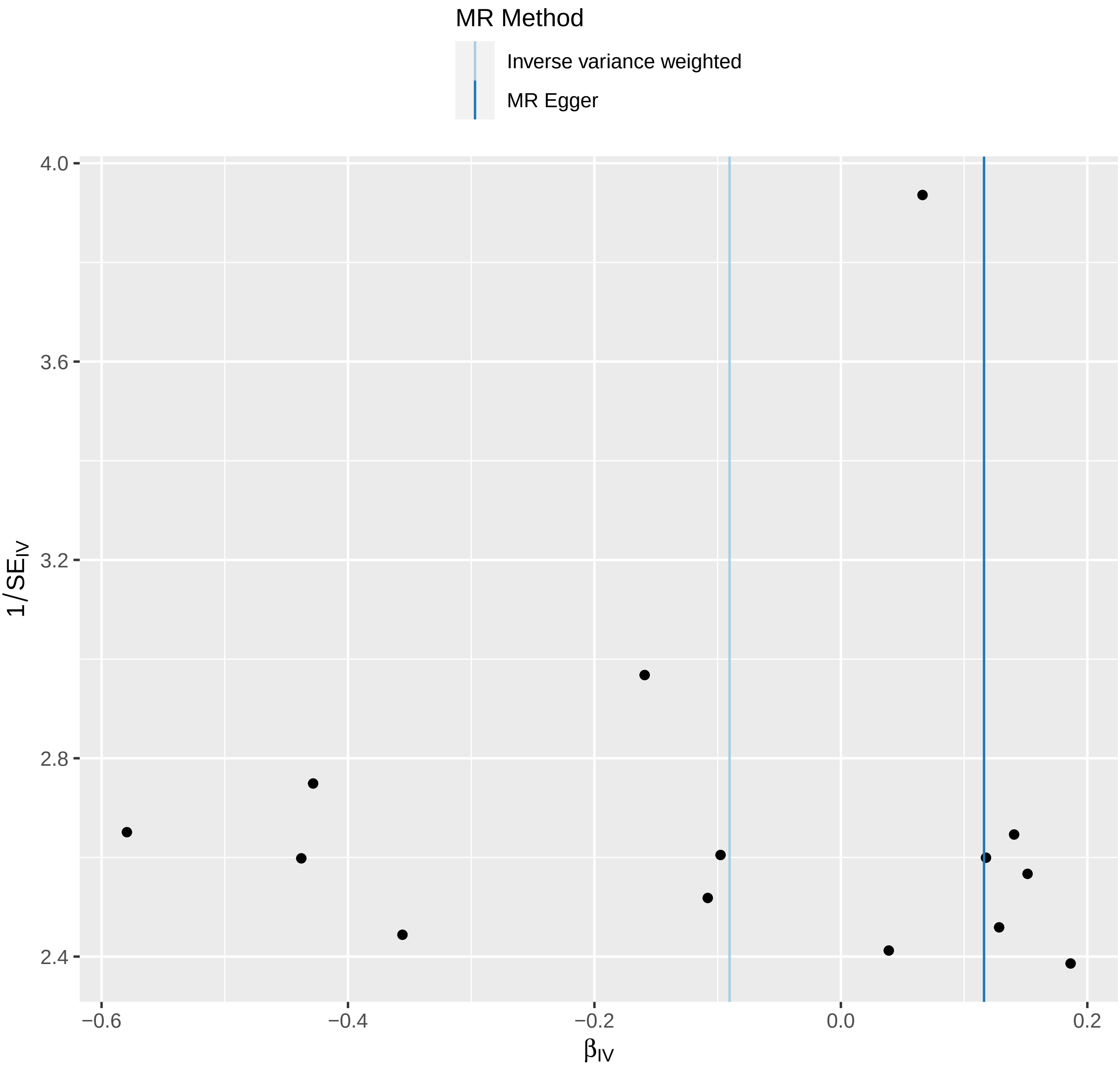

Batch 2−5721 : Gut microbiota abundance (genus Catenibacterium id.2153) on Alpha−2,8−sialyltransferase 8F
with a threshold of 1e−05

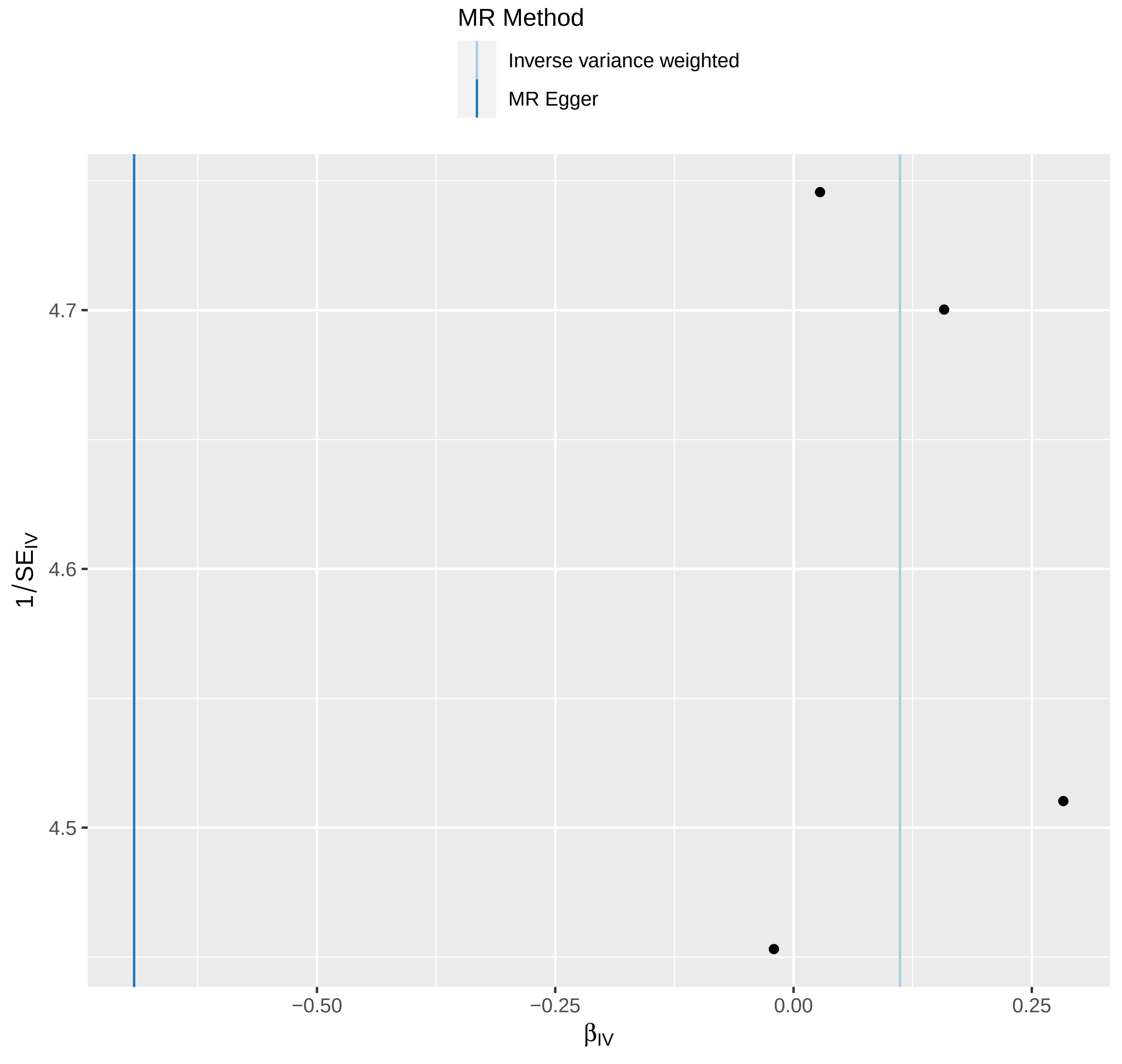

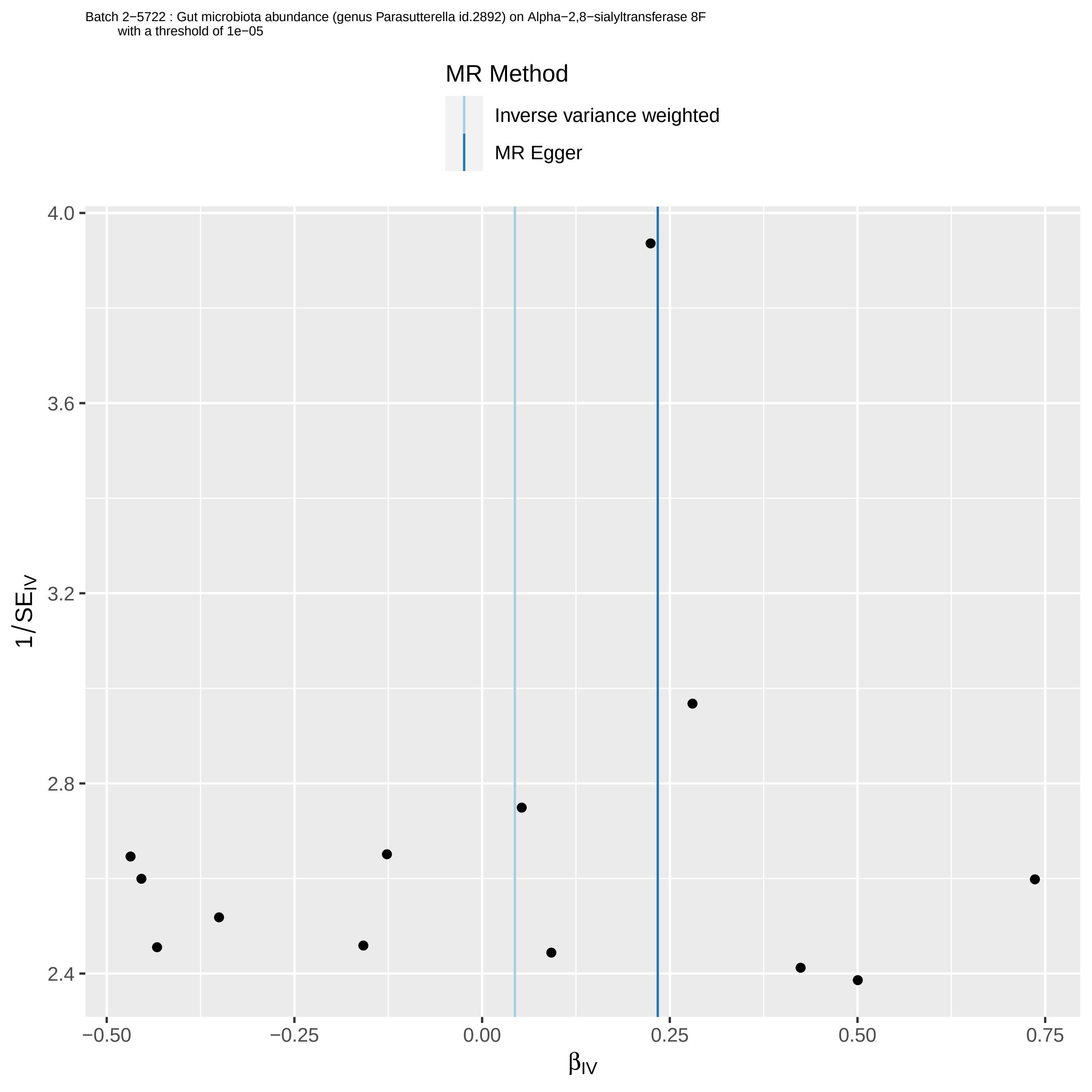
Batch 2−5722 : Gut microbiota abundance (genus Parasutterella id.2892) on Alpha−2,8−sialyltransferase 8F
with a threshold of 1e−05
MR Method
Inverse variance weighted
MR Egger
4.0
3.6
3.2
2.8
2.4
1/SE_IV
−0.50
−0.25
0.00
0.25
0.50
0.75
β_IV

Batch 2−5723 : Gut microbiota abundance (genus Catenibacterium id.2153) on Stabilin−1
with a threshold of 1e−05

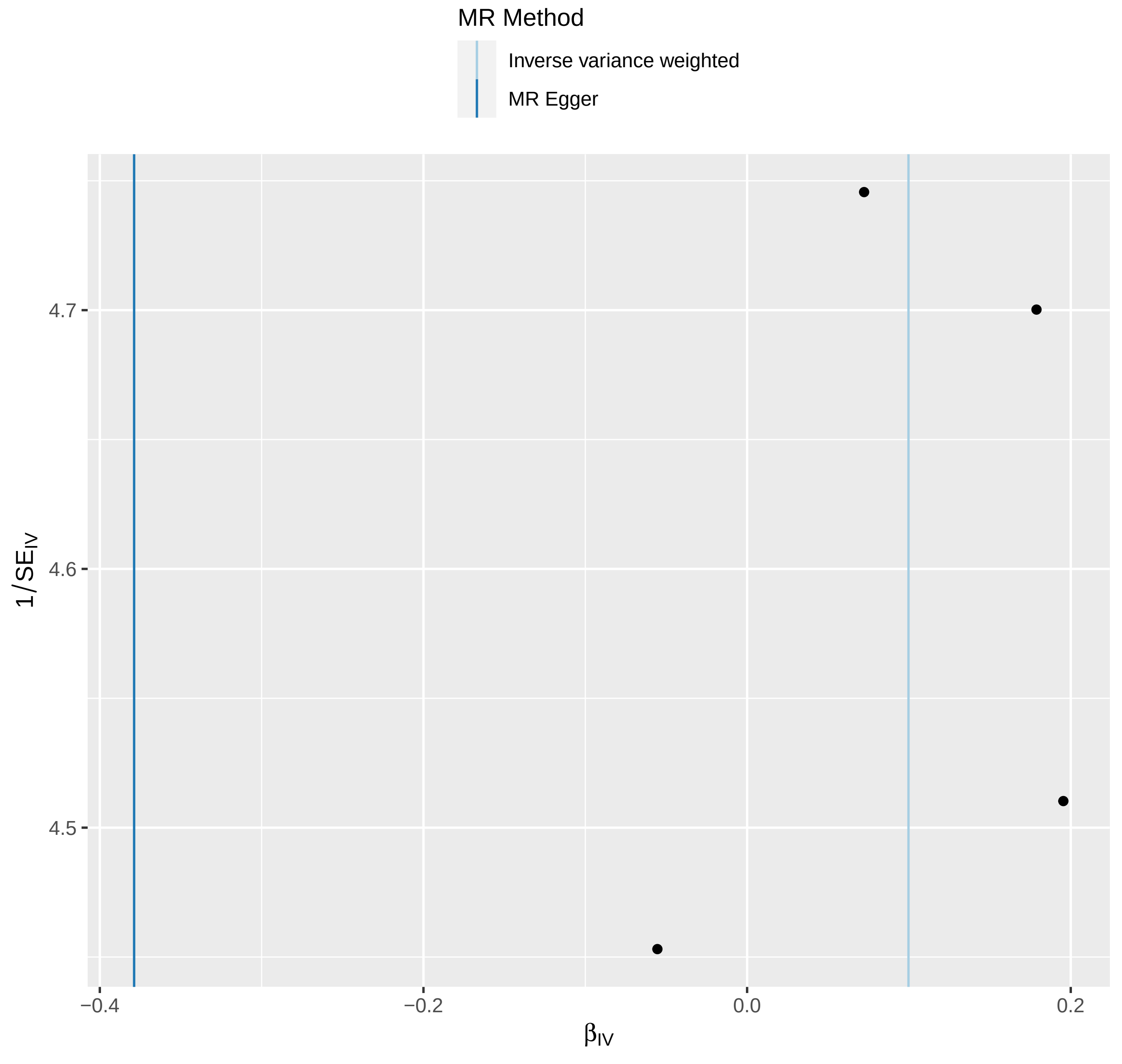

Batch 2−5724 : Gut microbiota abundance (genus Parasutterella id.2892) on Stabilin−1
with a threshold of 1e−05

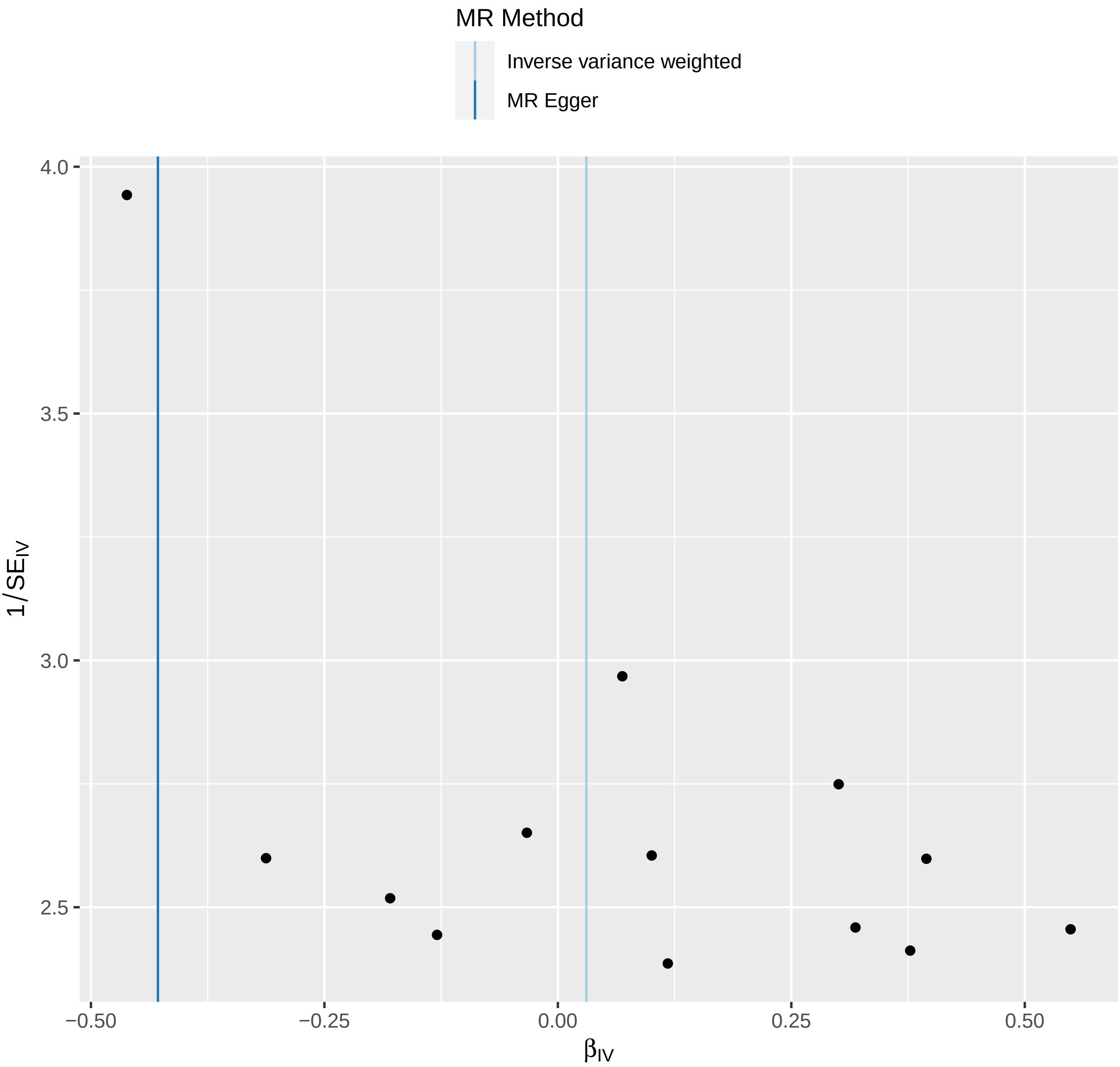

Batch 2−5725 : Gut microbiota abundance (genus Catenibacterium id.2153) on Stabilin−2
with a threshold of 1e−05

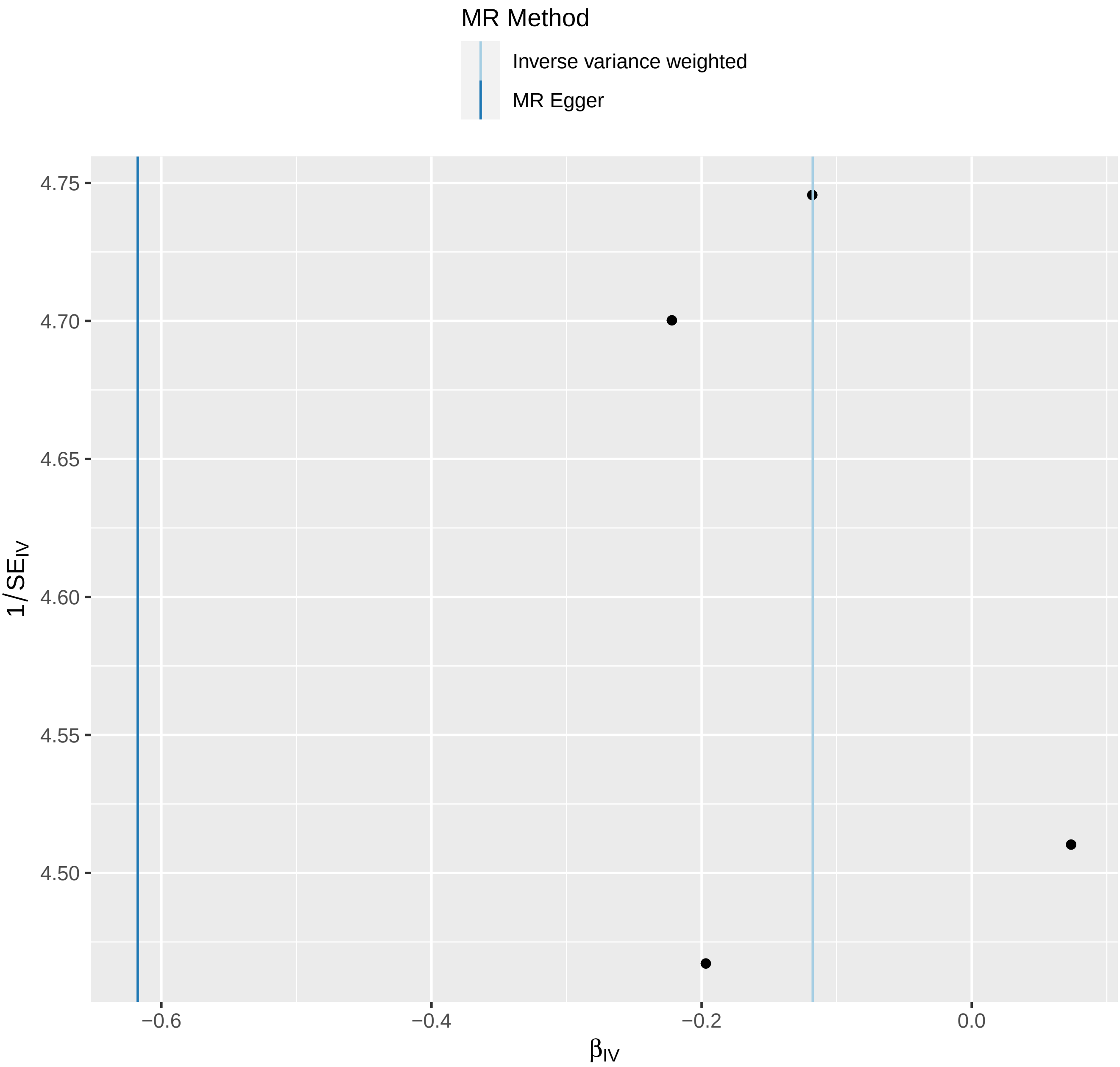

Batch 2−5726 : Gut microbiota abundance (genus Parasutterella id.2892) on Stabilin−2
with a threshold of 1e−05

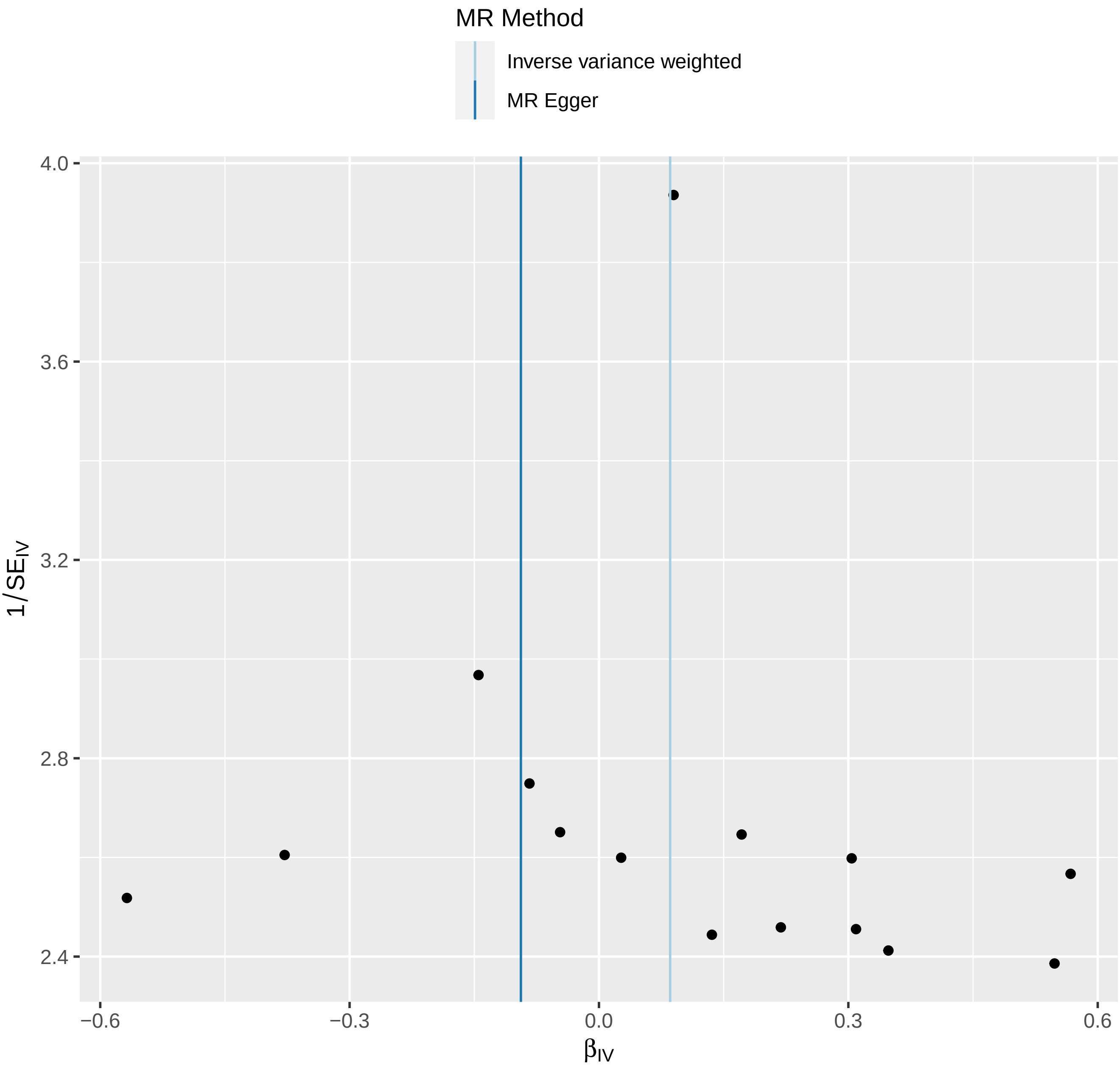

Batch 2−5727 : Gut microbiota abundance (genus Catenibacterium id.2153) on AMSH−like protease
with a threshold of 1e−05

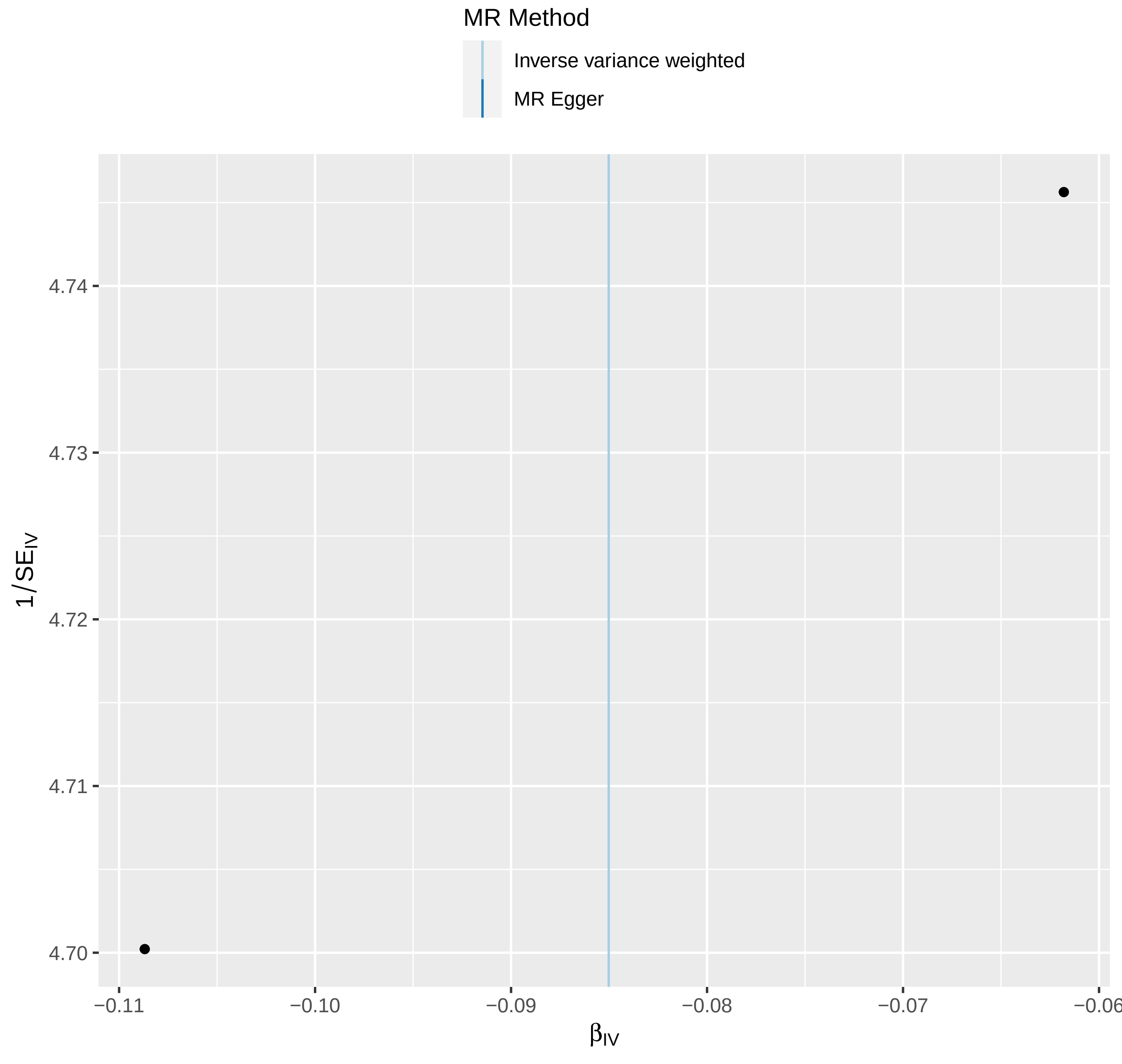

Batch 2−5728 : Gut microbiota abundance (genus Parasutterella id.2892) on AMSH−like protease
with a threshold of 1e−05

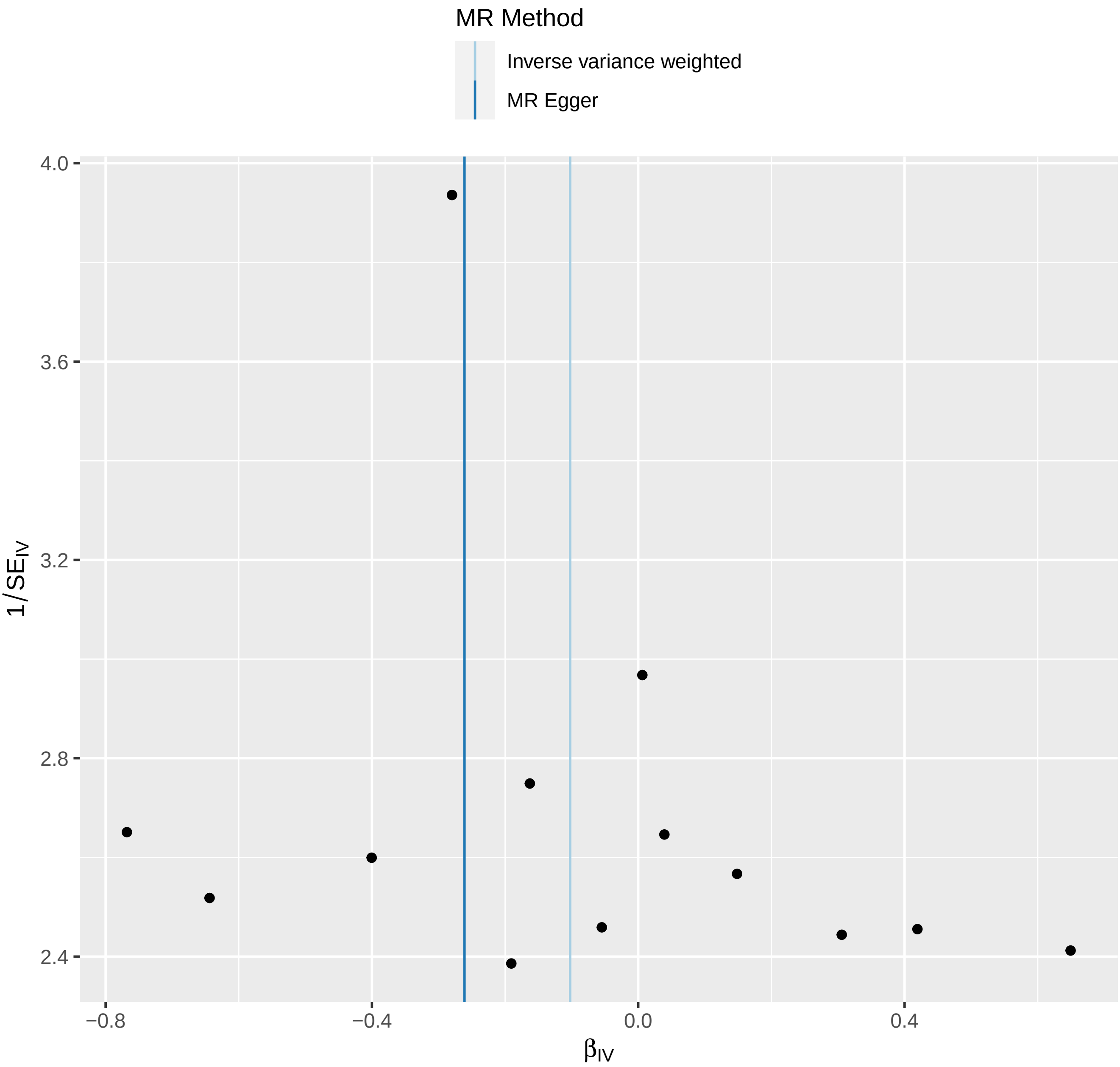

Batch 2−5729 : Gut microbiota abundance (genus Catenibacterium id.2153) on Signal−transducing adaptor protein 1 with a threshold of 1e−05

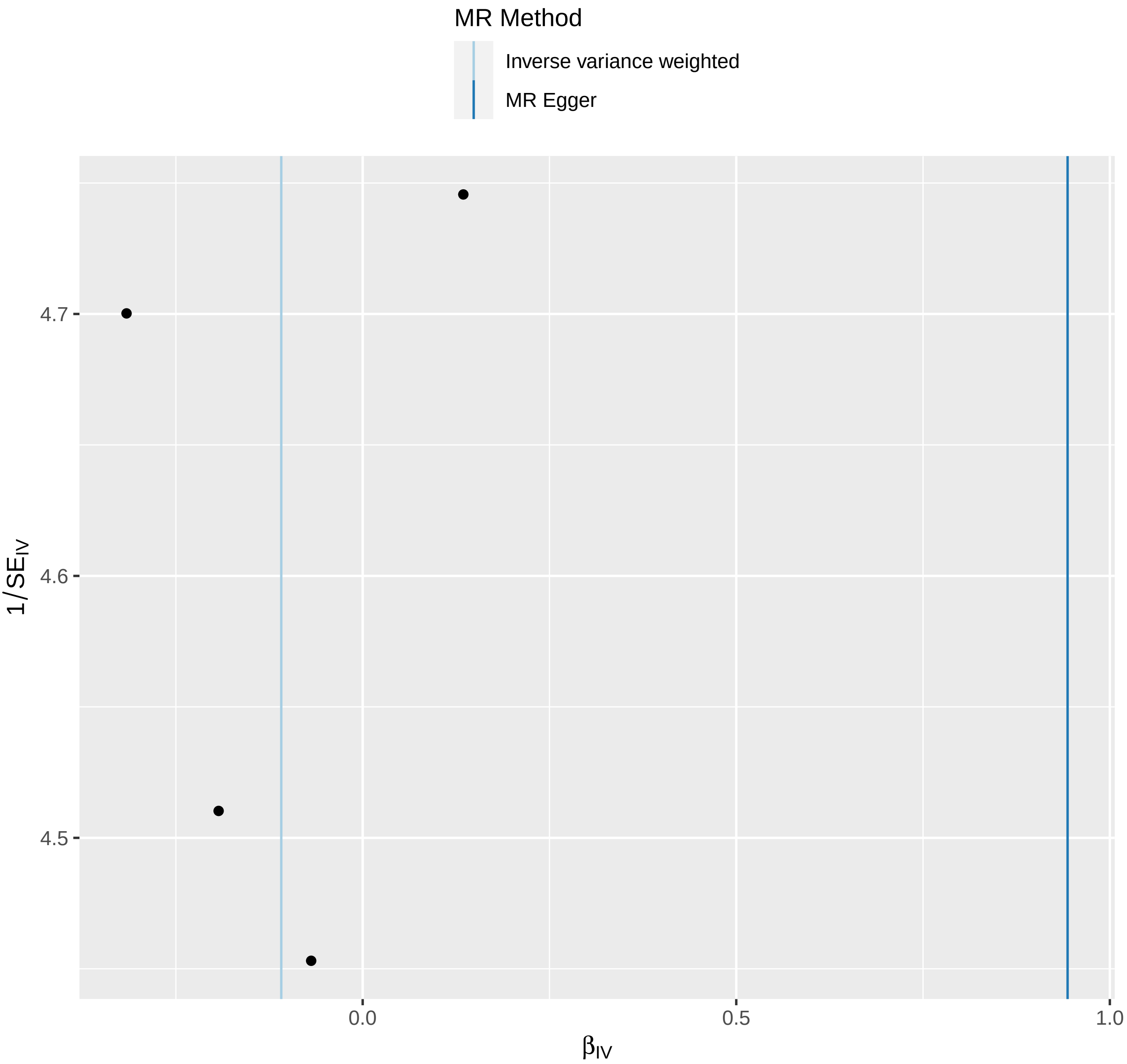

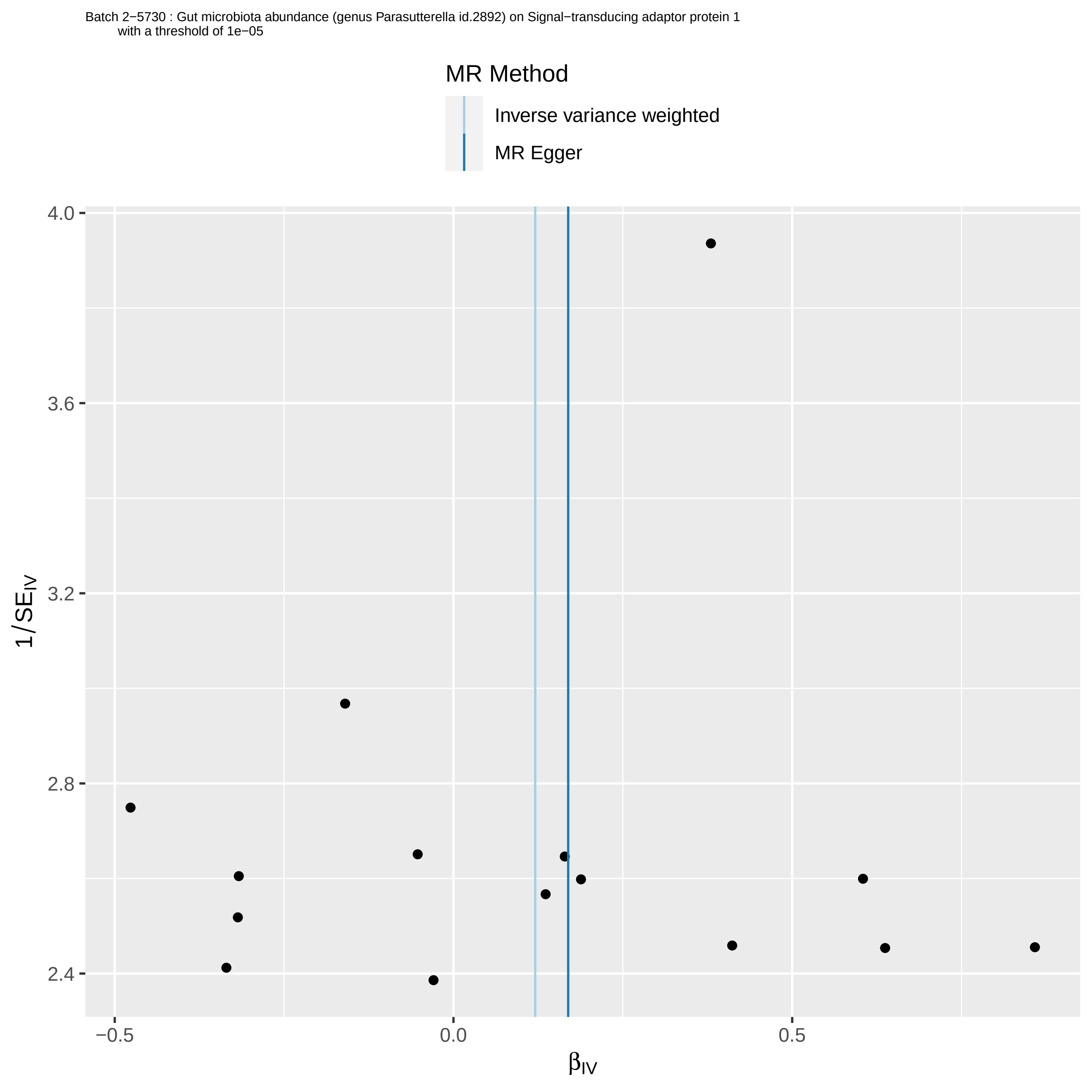
Batch 2−5730 : Gut microbiota abundance (genus Parasutterella id.2892) on Signal−transducing adaptor protein 1
with a threshold of 1e−05
MR Method
Inverse variance weighted
MR Egger
4.0
3.6
3.2
2.8
2.4
1/SE_IV
−0.5
0.0
0.5
β_IV

Batch 2−5731 : Gut microbiota abundance (genus Catenibacterium id.2153) on Steroidogenic acute regulatory protein, mitochondrial
with a threshold of 1e−05

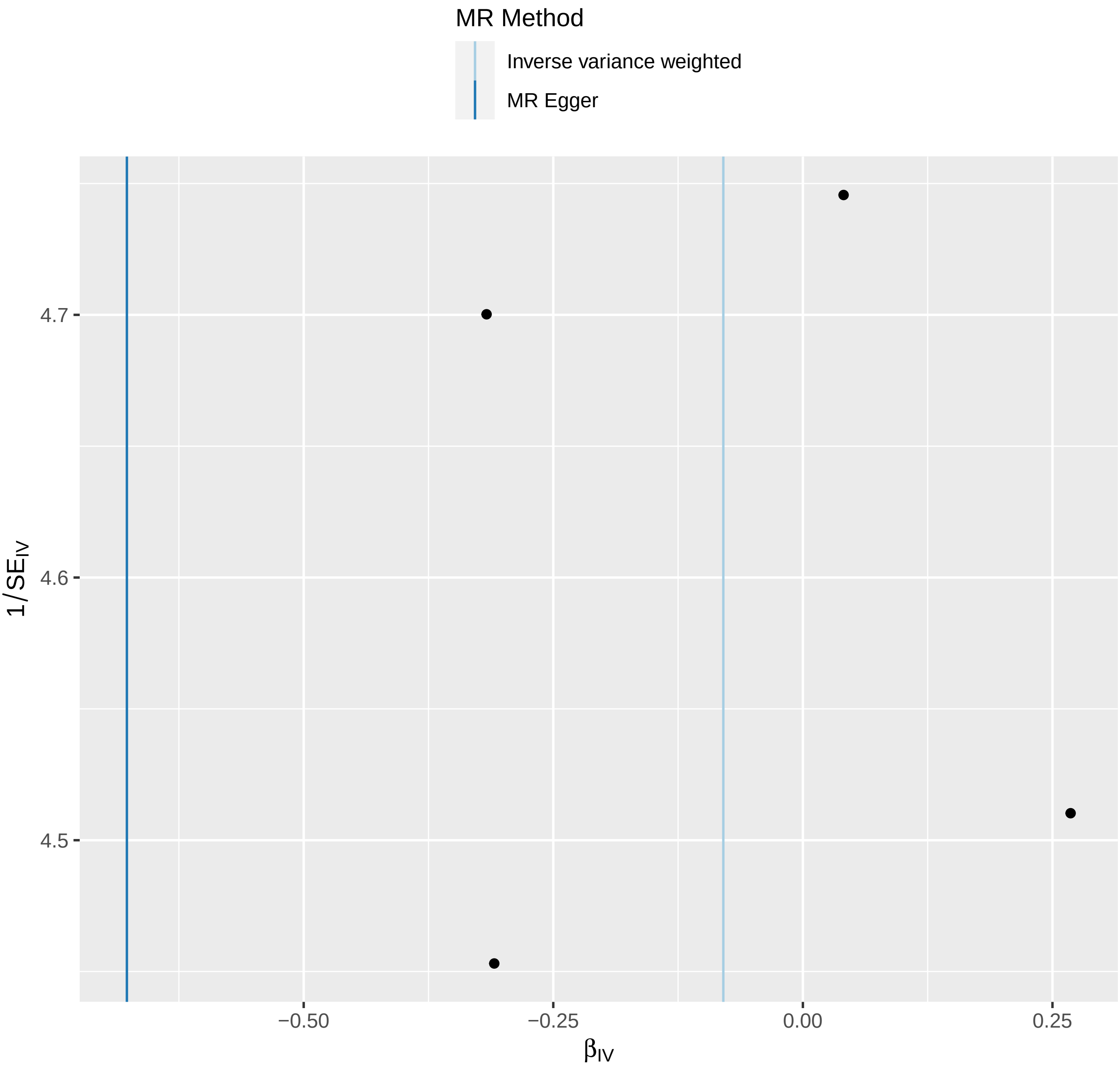

Batch 2−5732 : Gut microbiota abundance (genus Parasutterella id.2892) on Steroidogenic acute regulatory protein, mitochondrial
with a threshold of 1e−05

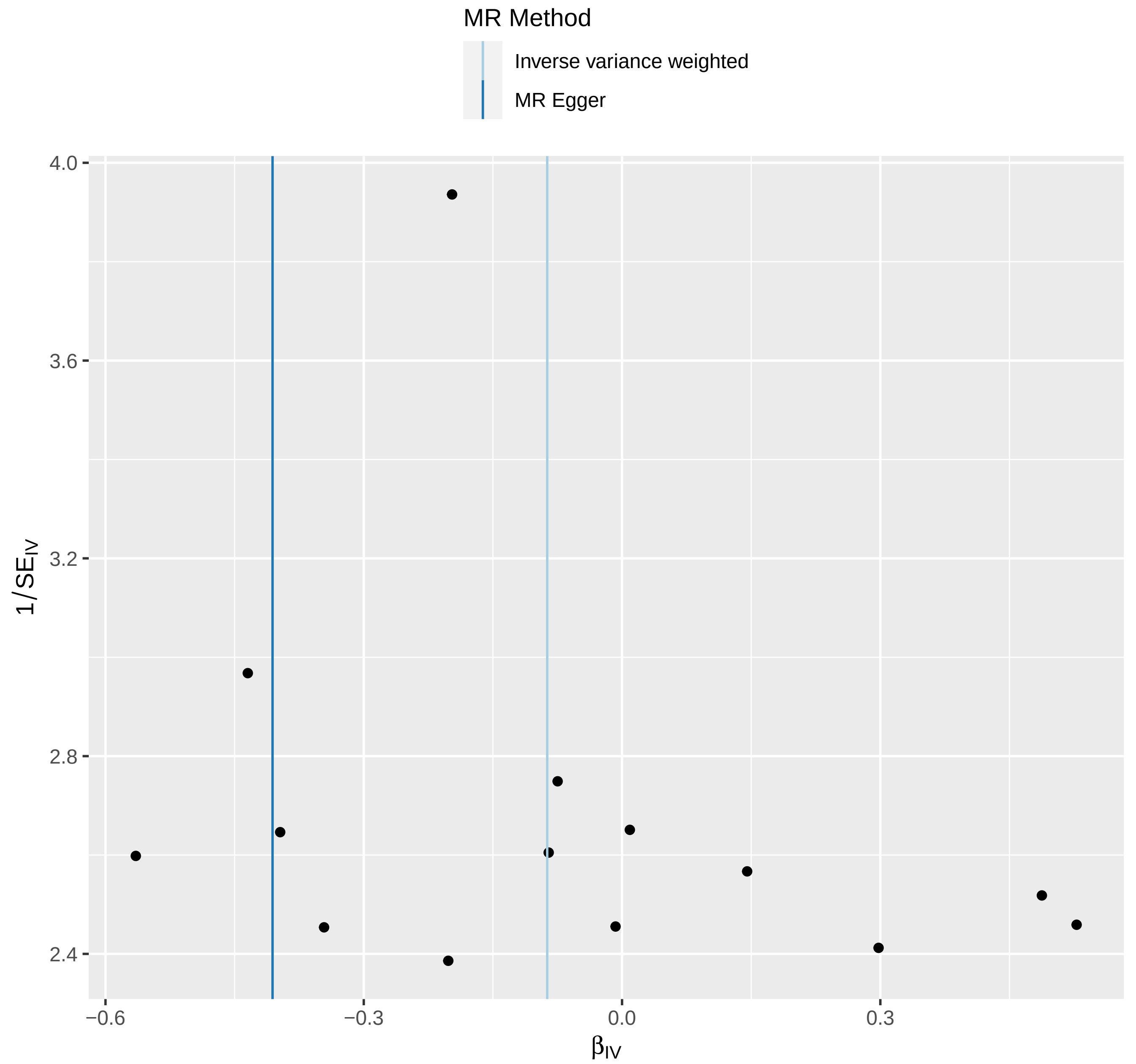

Batch 2−5733 : Gut microbiota abundance (genus Catenibacterium id.2153) on Signal transducer and activator of transcription 1−alpha/beta with a threshold of 1e−05

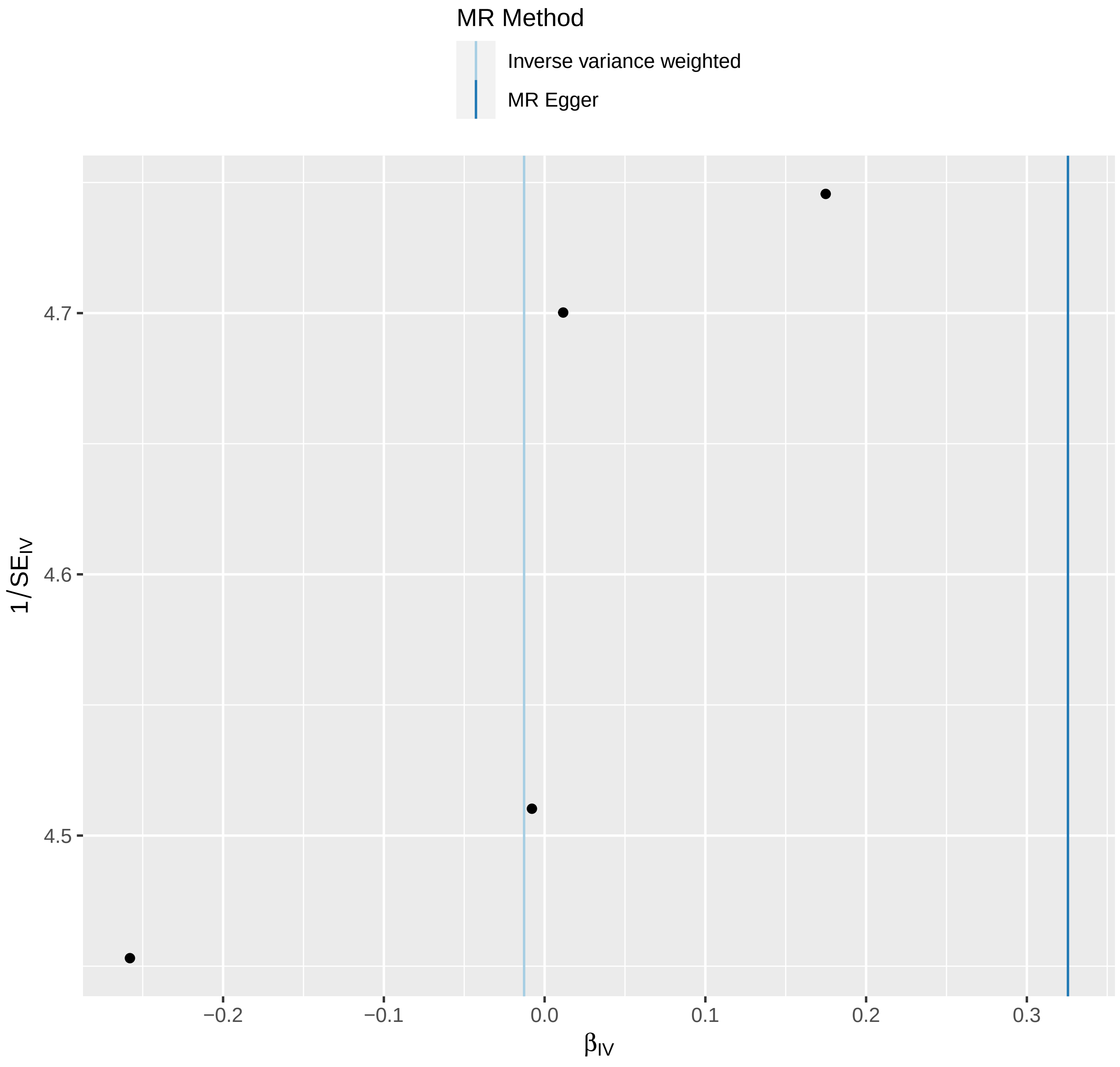

Batch 2−5734 : Gut microbiota abundance (genus Parasutterella id.2892) on Signal transducer and activator of transcription 1−alpha/beta
with a threshold of 1e−05

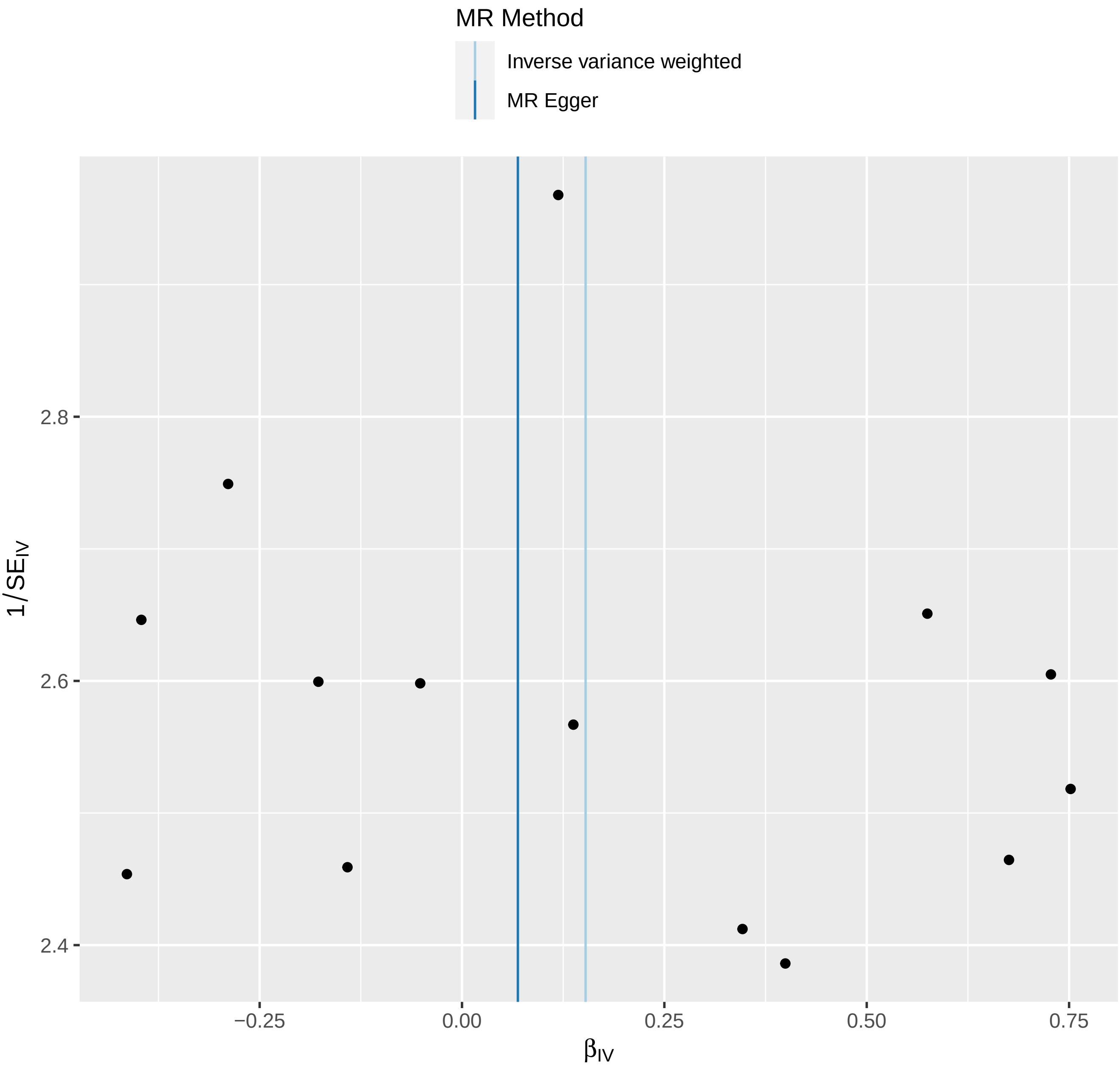

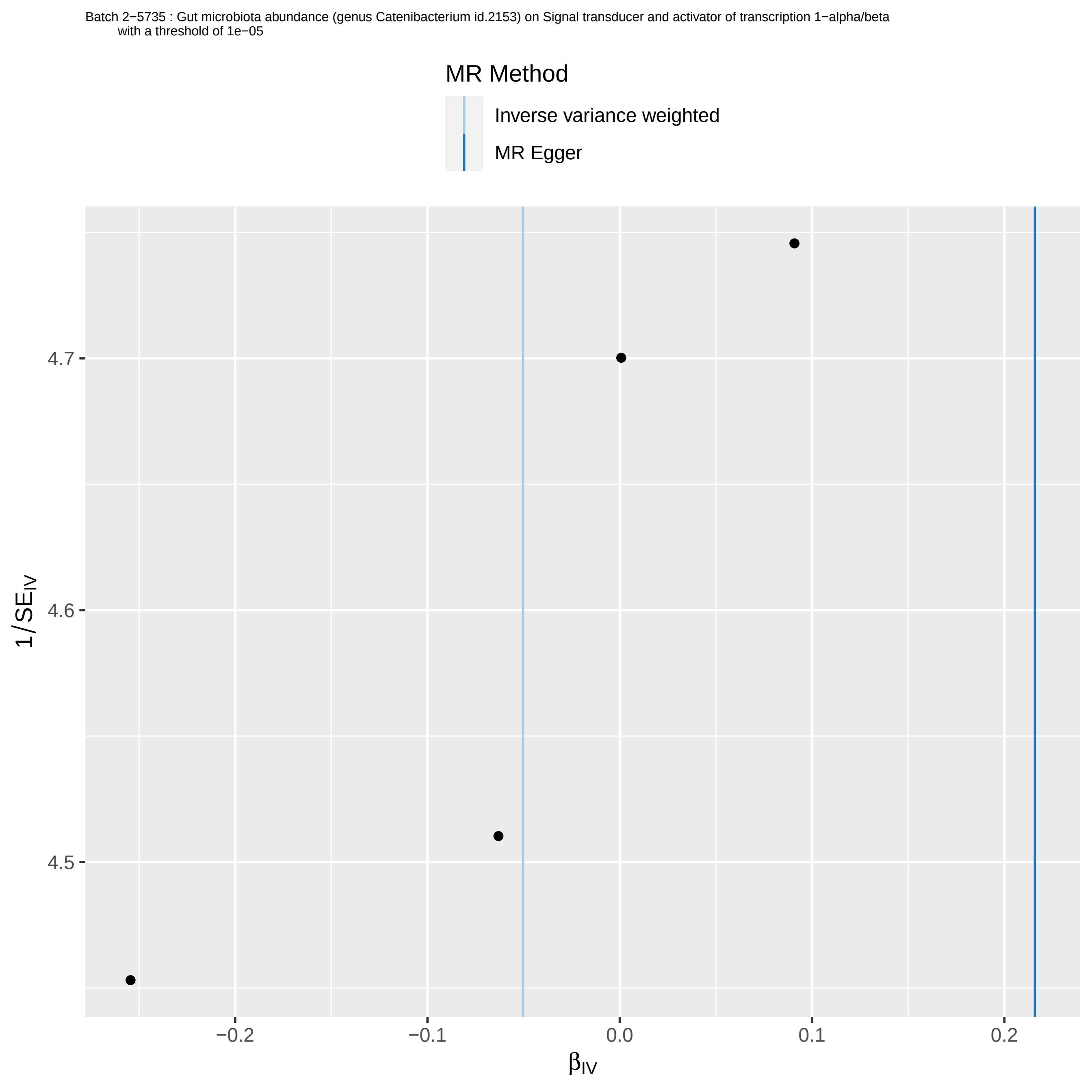

Batch 2−5735 : Gut microbiota abundance (genus Catenibacterium id.2153) on Signal transducer and activator of transcription 1−alpha/beta
with a threshold of 1e−05
MR Method
Inverse variance weighted
MR Egger
4.7
4.6
4.5
1/SE_IV
−0.2
−0.1
0.0
0.1
0.2
β_IV

Batch 2−5736 : Gut microbiota abundance (genus Parasutterella id.2892) on Signal transducer and activator of transcription 1−alpha/beta with a threshold of 1e−05

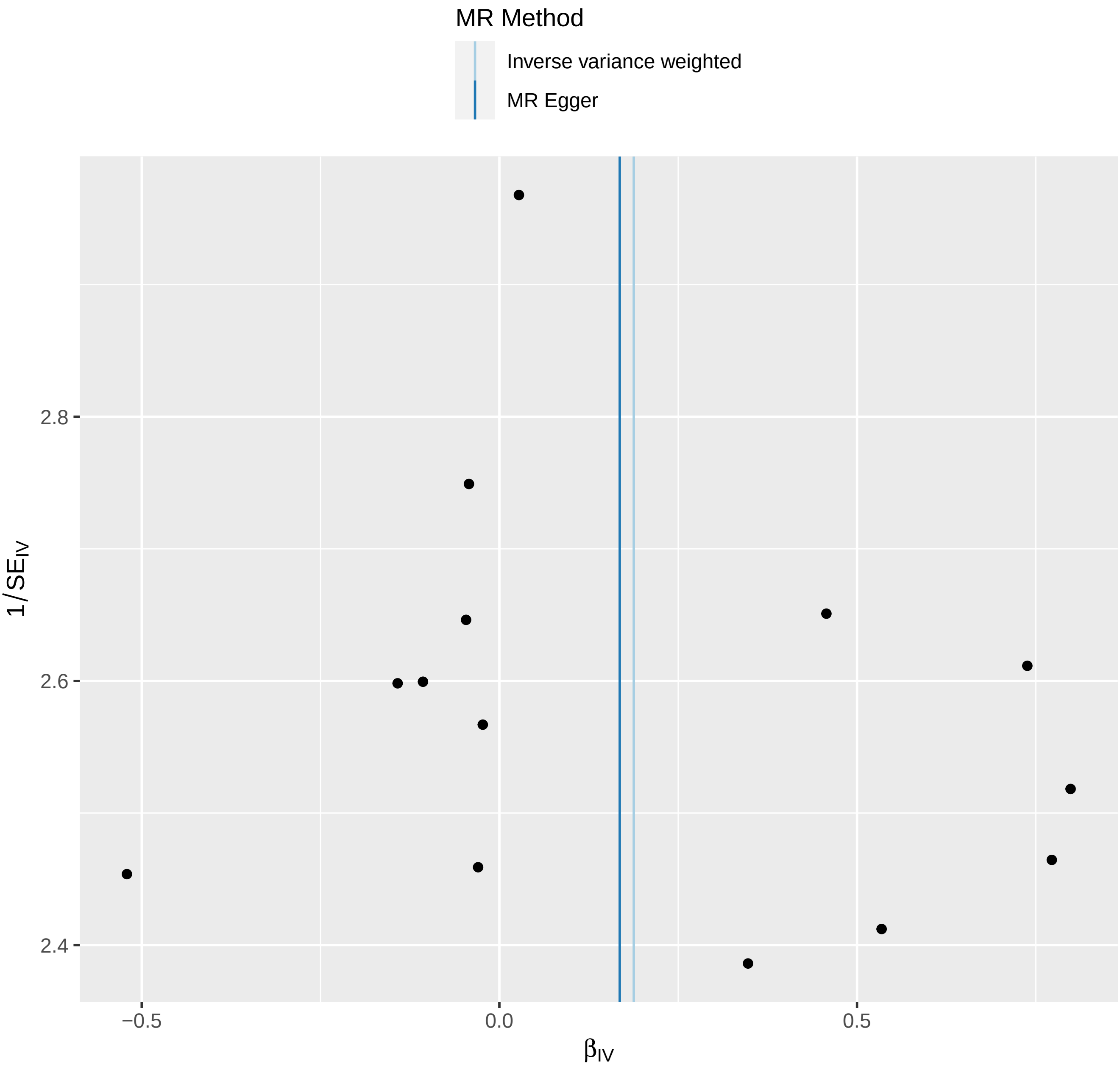

Batch 2−5737 : Gut microbiota abundance (genus Catenibacterium id.2153) on Signal transducer and activator of transcription 3 with a threshold of 1e−05

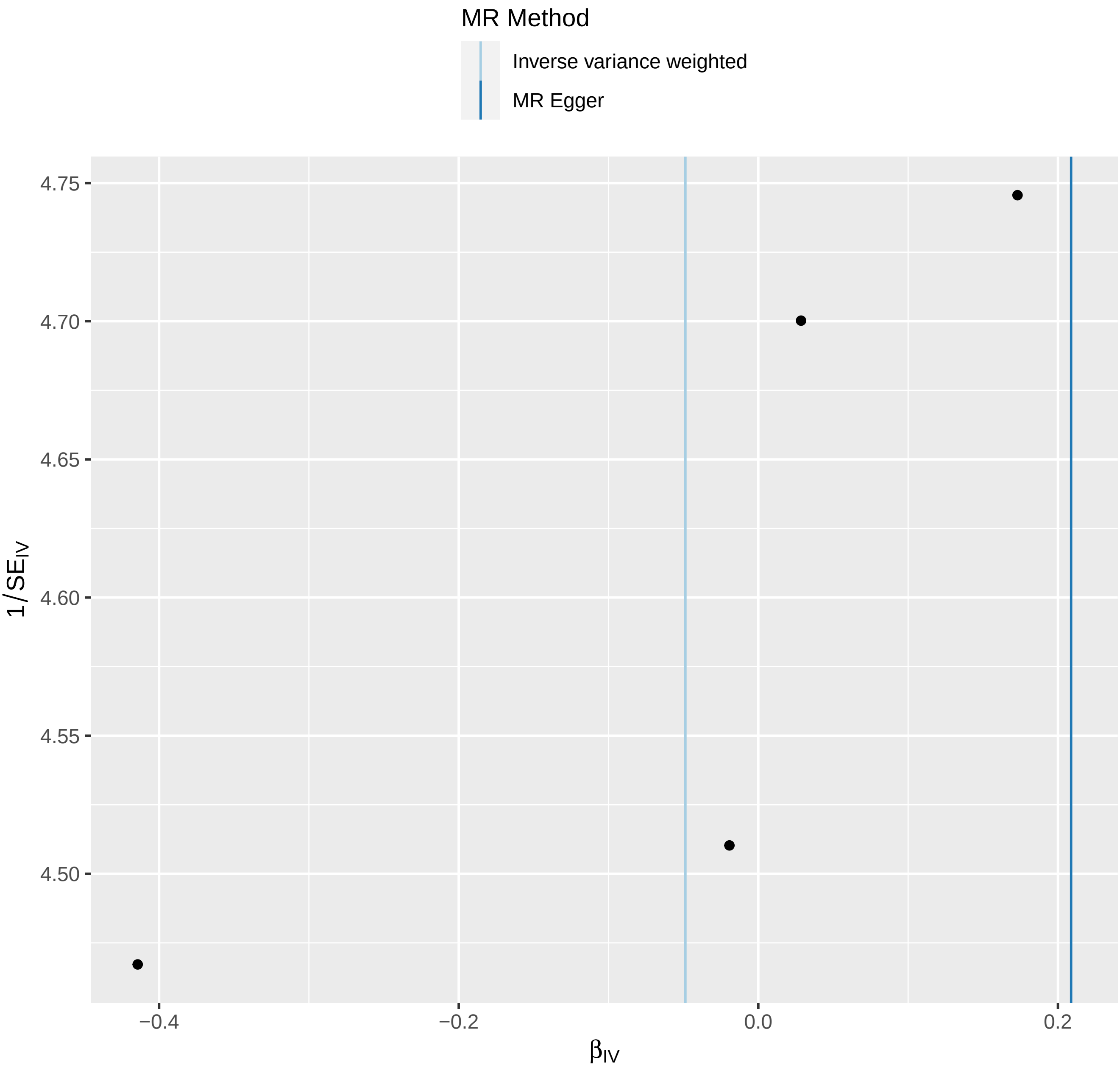

Batch 2−5738 : Gut microbiota abundance (genus Parasutterella id.2892) on Signal transducer and activator of transcription 3
with a threshold of 1e−05

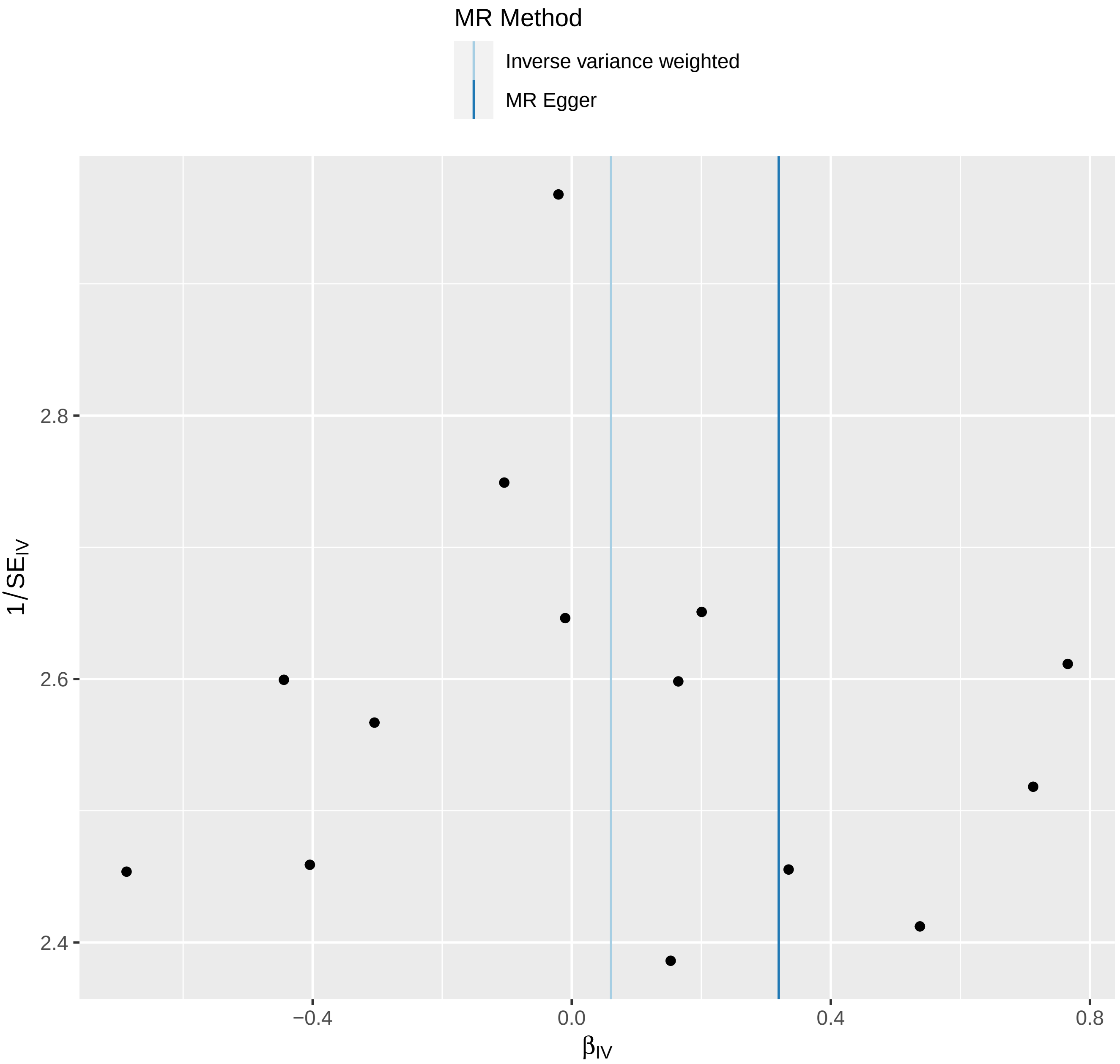

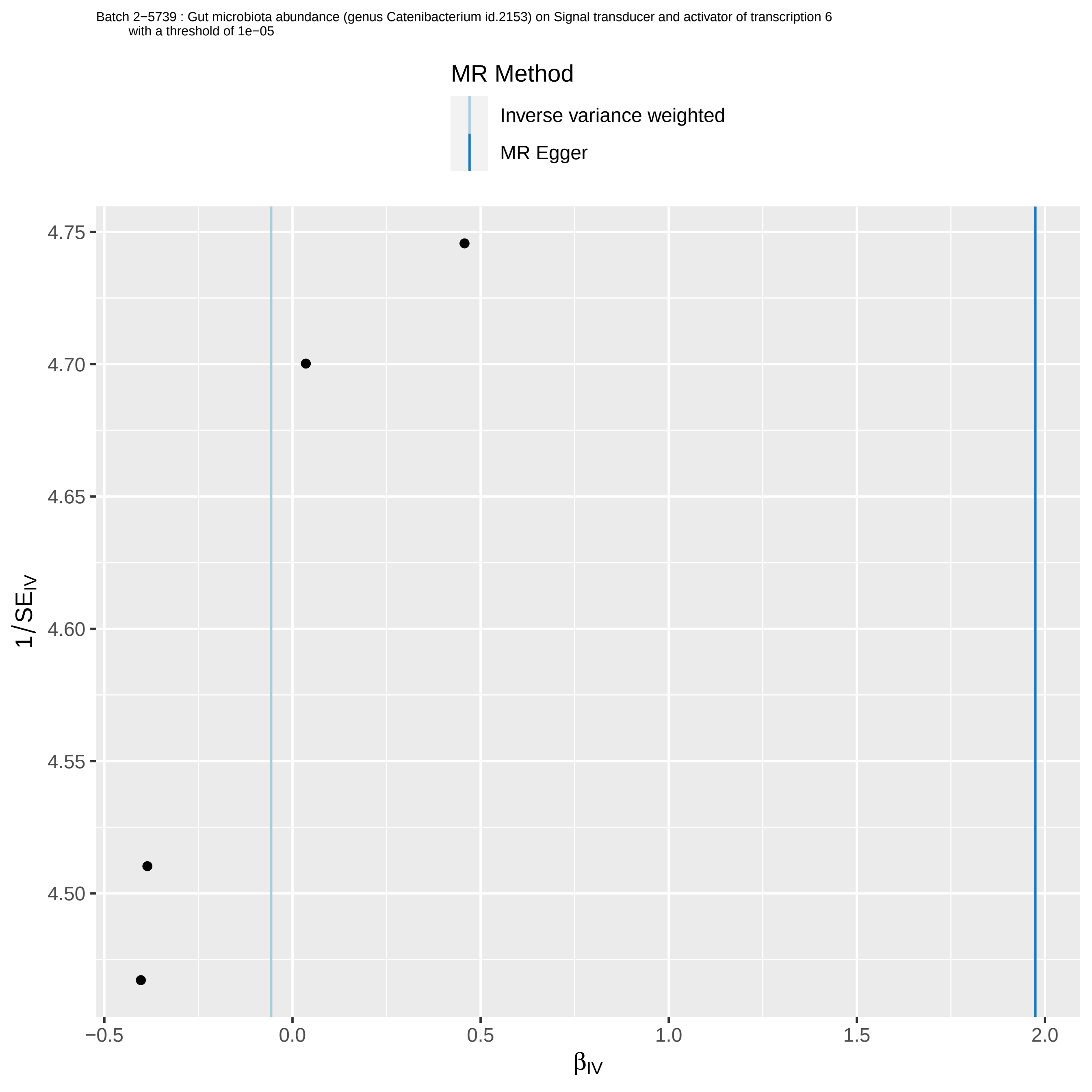
Batch 2−5739 : Gut microbiota abundance (genus Catenibacterium id.2153) on Signal transducer and activator of transcription 6
with a threshold of 1e−05
MR Method
Inverse variance weighted
MR Egger
4.75
4.70
4.65
4.60
4.55
4.50
1/SE_IV
−0.5
0.0
0.5
1.0
1.5
2.0
β_IV

Batch 2−5740 : Gut microbiota abundance (genus Parasutterella id.2892) on Signal transducer and activator of transcription 6
with a threshold of 1e−05

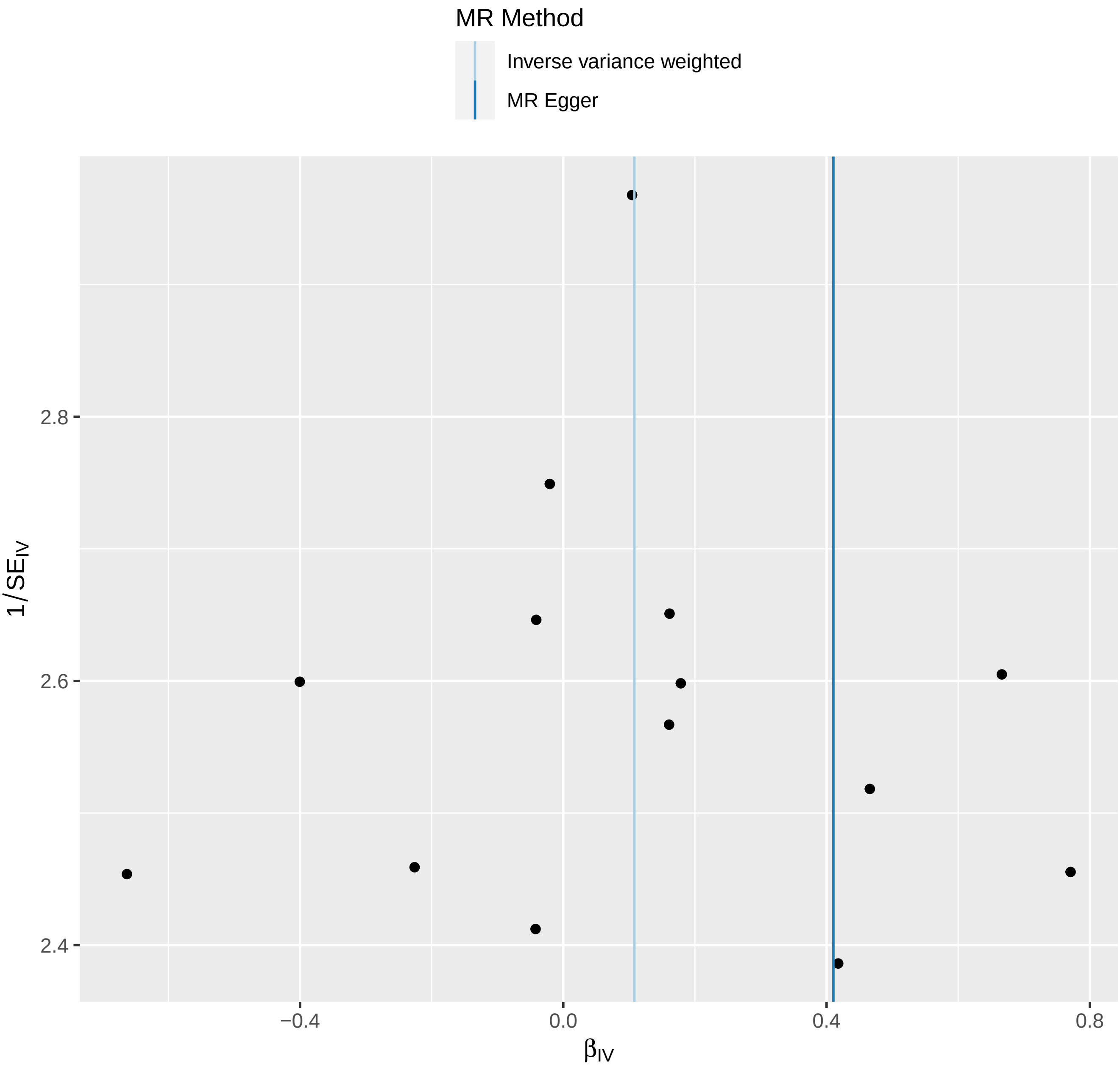

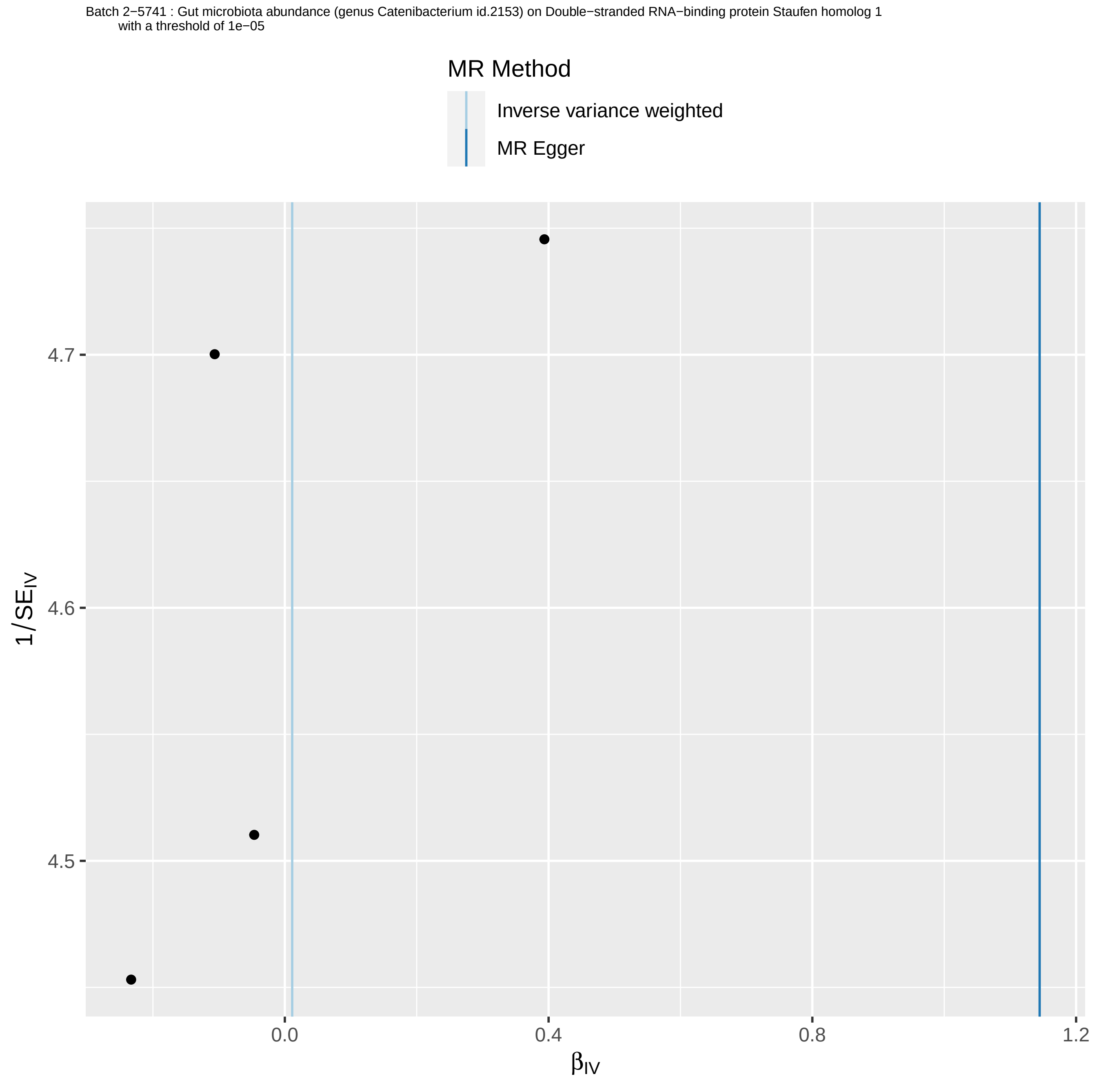

Batch 2−5741 : Gut microbiota abundance (genus Catenibacterium id.2153) on Double−stranded RNA−binding protein Staufen homolog 1
with a threshold of 1e−05
MR Method
Inverse variance weighted
MR Egger
4.7
4.6
4.5
$1/SE_{IV}$
0.0
0.4
0.8
1.2
β_{IV}

Batch 2−5742 : Gut microbiota abundance (genus Parasutterella id.2892) on Double−stranded RNA−binding protein Staufen homolog 1 with a threshold of 1e−05

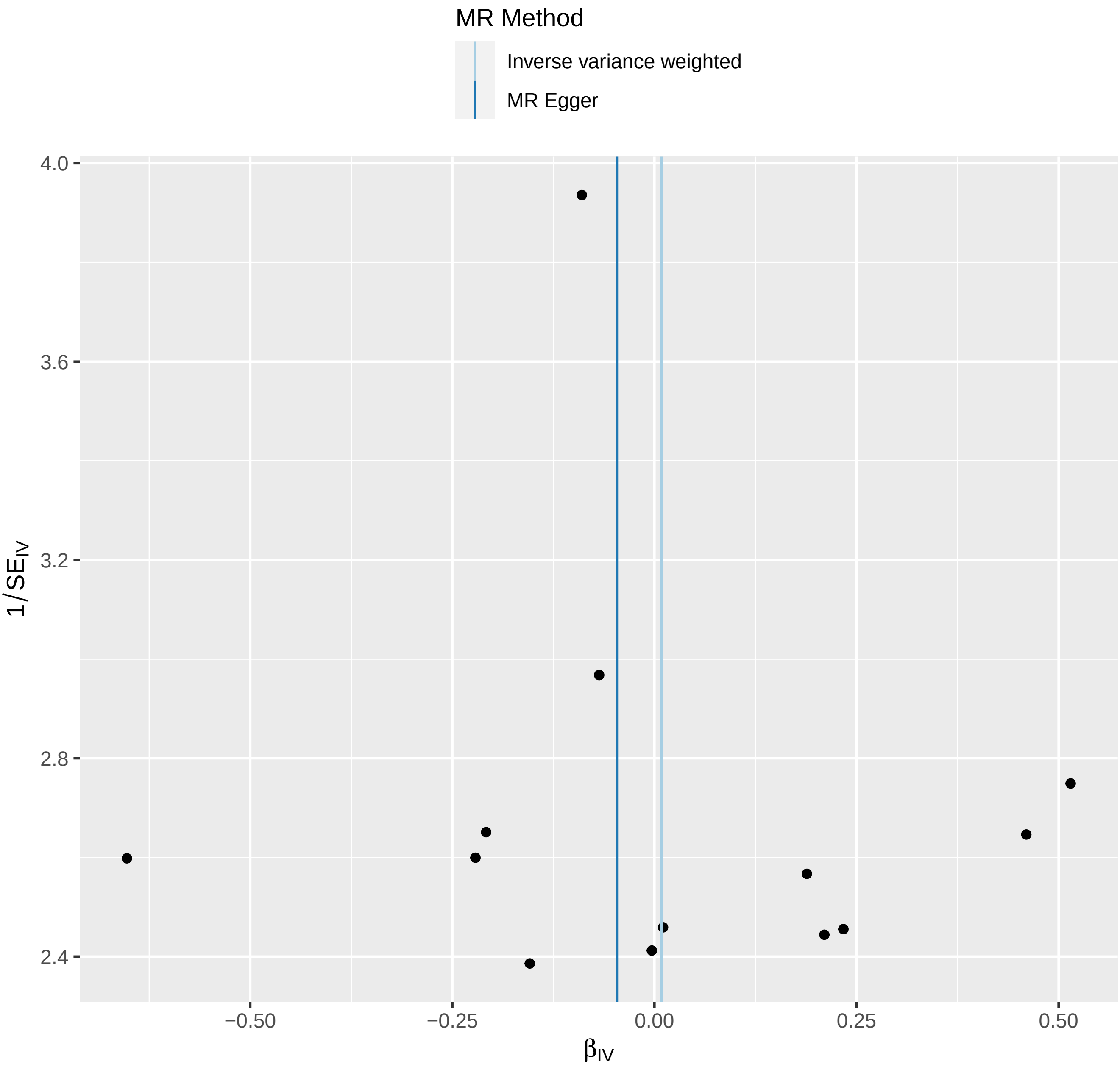

Batch 2−5743 : Gut microbiota abundance (genus Catenibacterium id.2153) on Double−stranded RNA−binding protein Staufen homolog 2 with a threshold of 1e−05

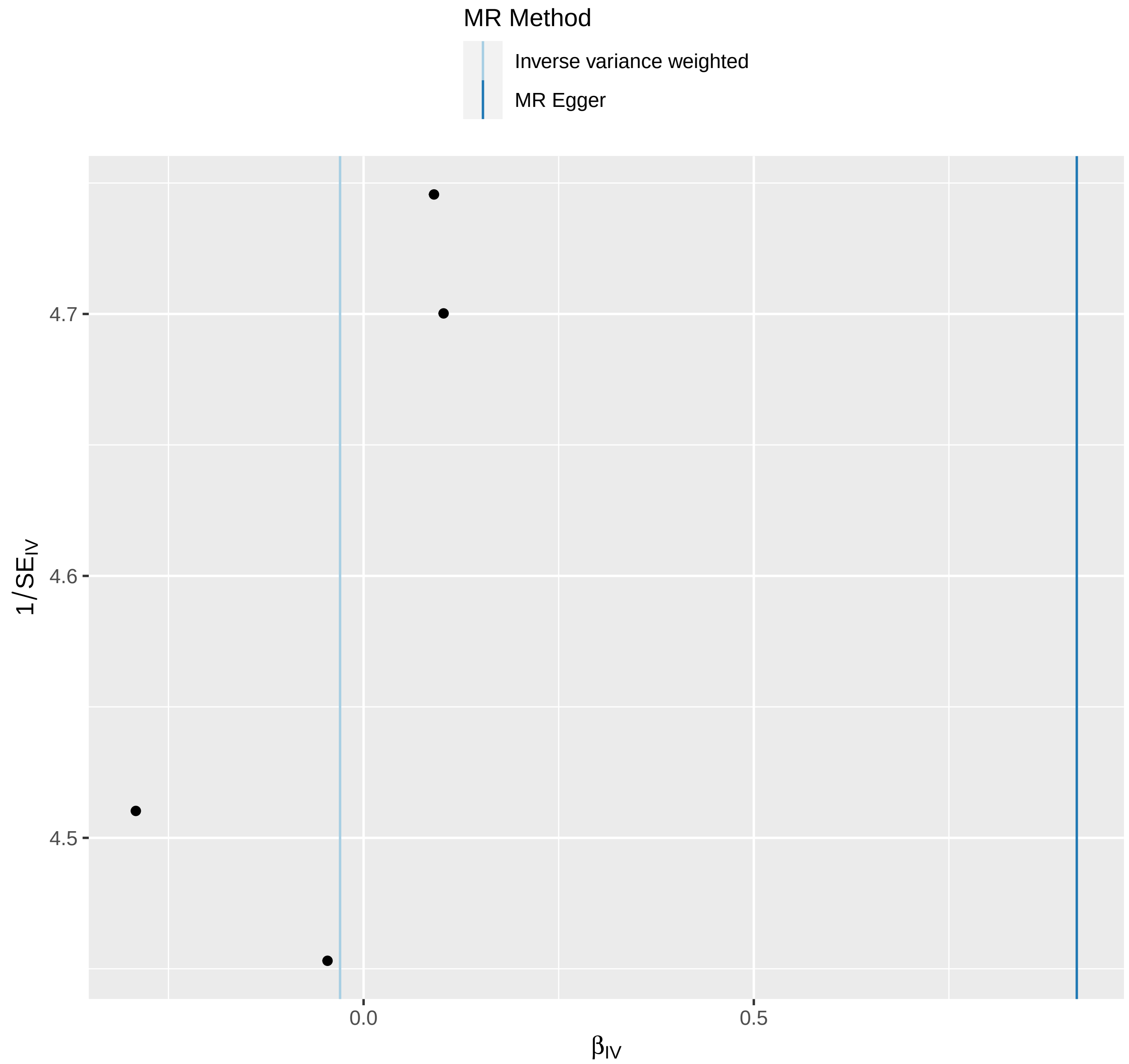

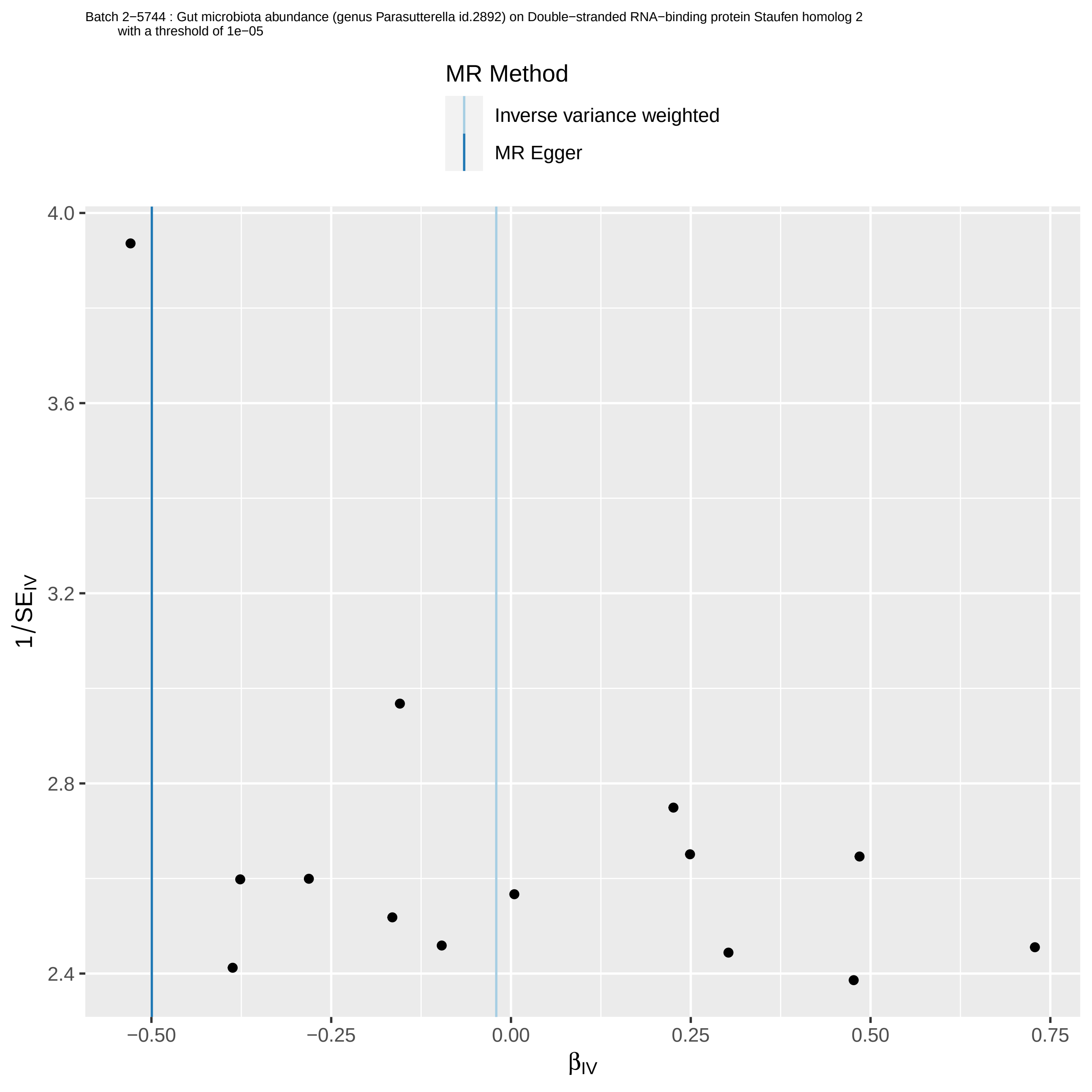

Batch 2−5744 : Gut microbiota abundance (genus Parasutterella id.2892) on Double−stranded RNA−binding protein Staufen homolog 2
with a threshold of 1e−05
MR Method
Inverse variance weighted
MR Egger
4.0
3.6
3.2
2.8
2.4
$1/SE_{IV}$
−0.50
−0.25
0.00
0.25
0.50
0.75
β_{IV}

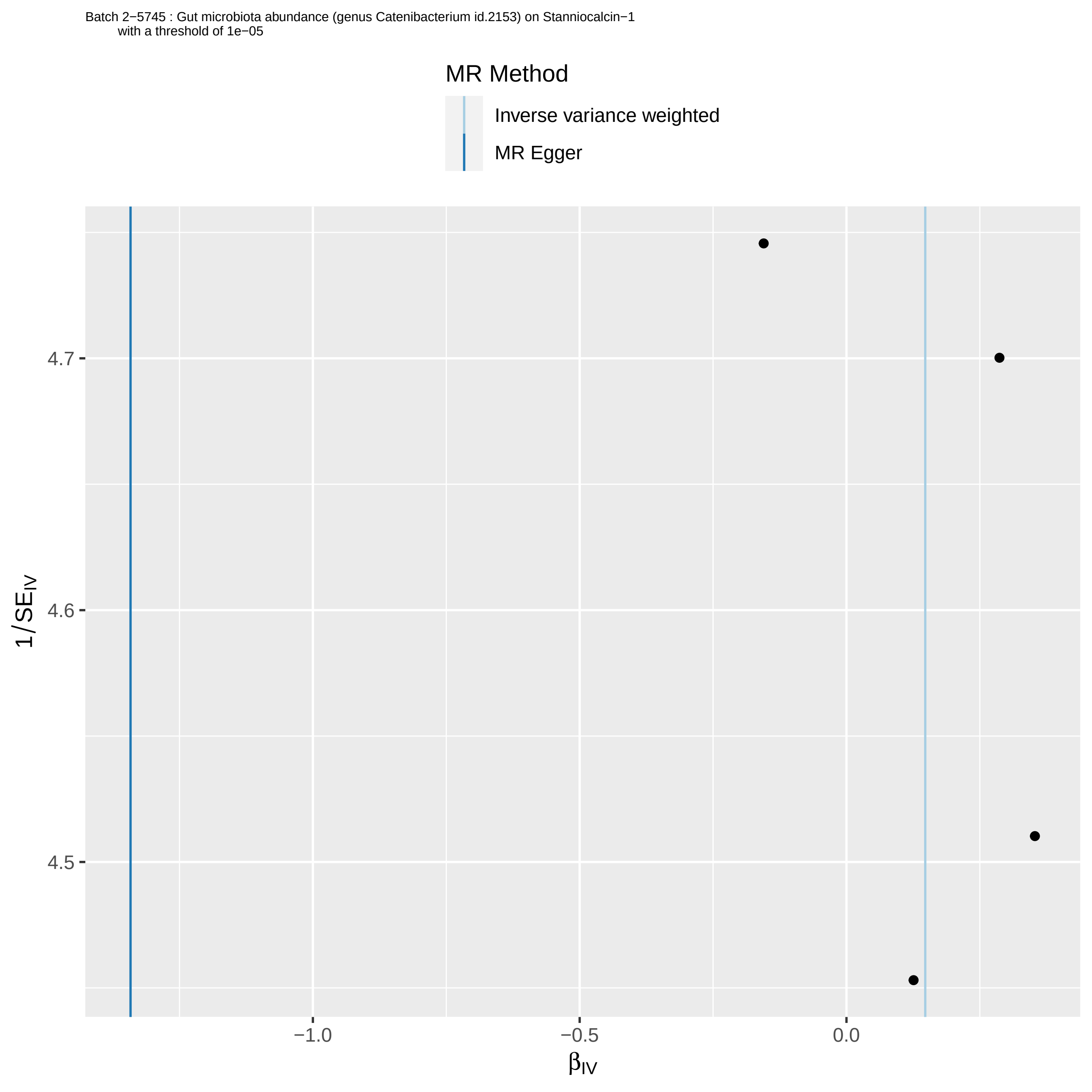

Batch 2−5745 : Gut microbiota abundance (genus Catenibacterium id.2153) on Stanniocalcin−1
with a threshold of 1e−05
MR Method
Inverse variance weighted
MR Egger
4.7
4.6
4.5
1/SE$_{IV}$
−1.0
−0.5
0.0
β$_{IV}$

Batch 2−5746 : Gut microbiota abundance (genus Parasutterella id.2892) on Stanniocalcin−1
with a threshold of 1e−05

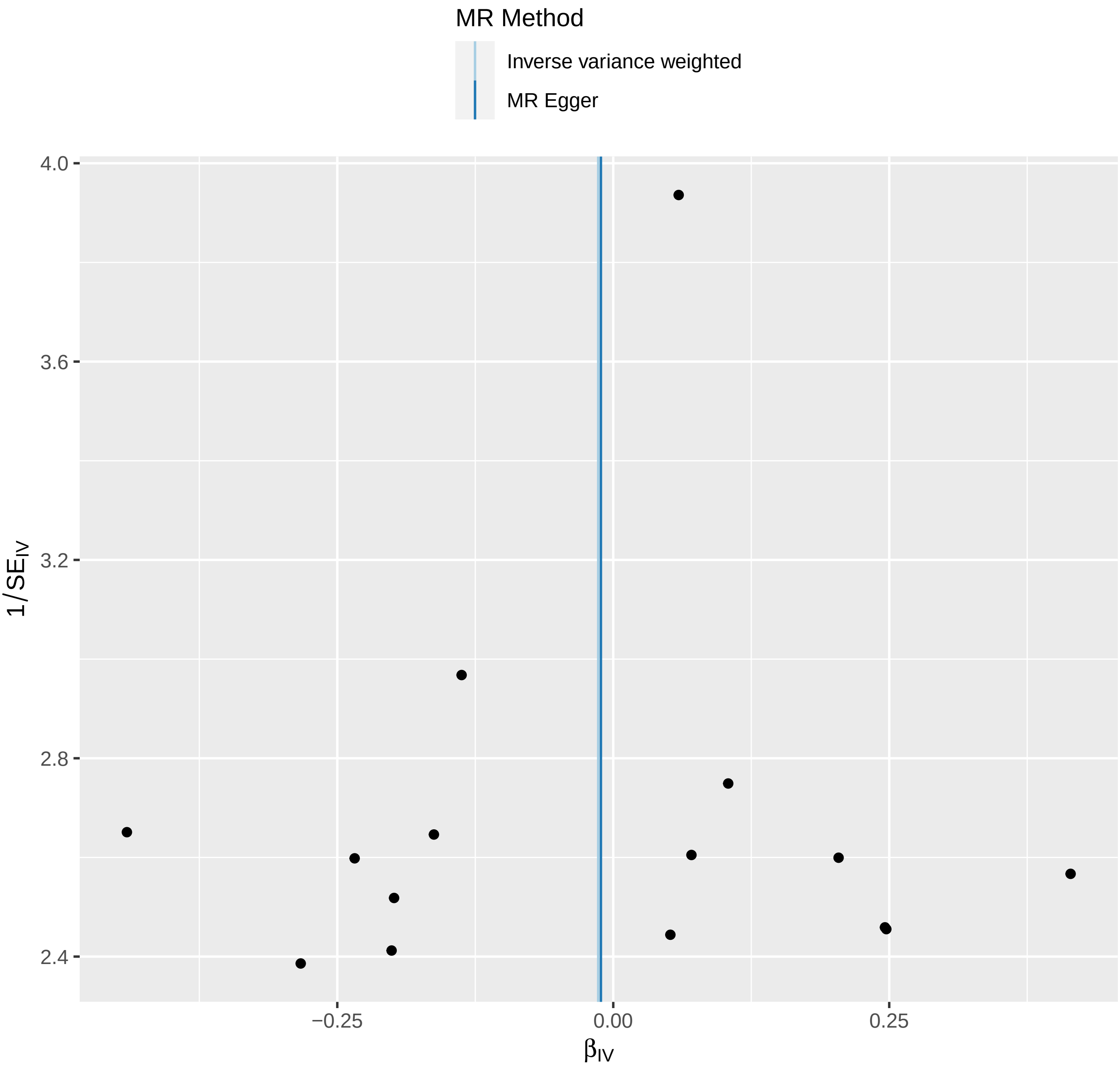

Batch 2−5747 : Gut microbiota abundance (genus Catenibacterium id.2153) on Stanniocalcin−2
with a threshold of 1e−05

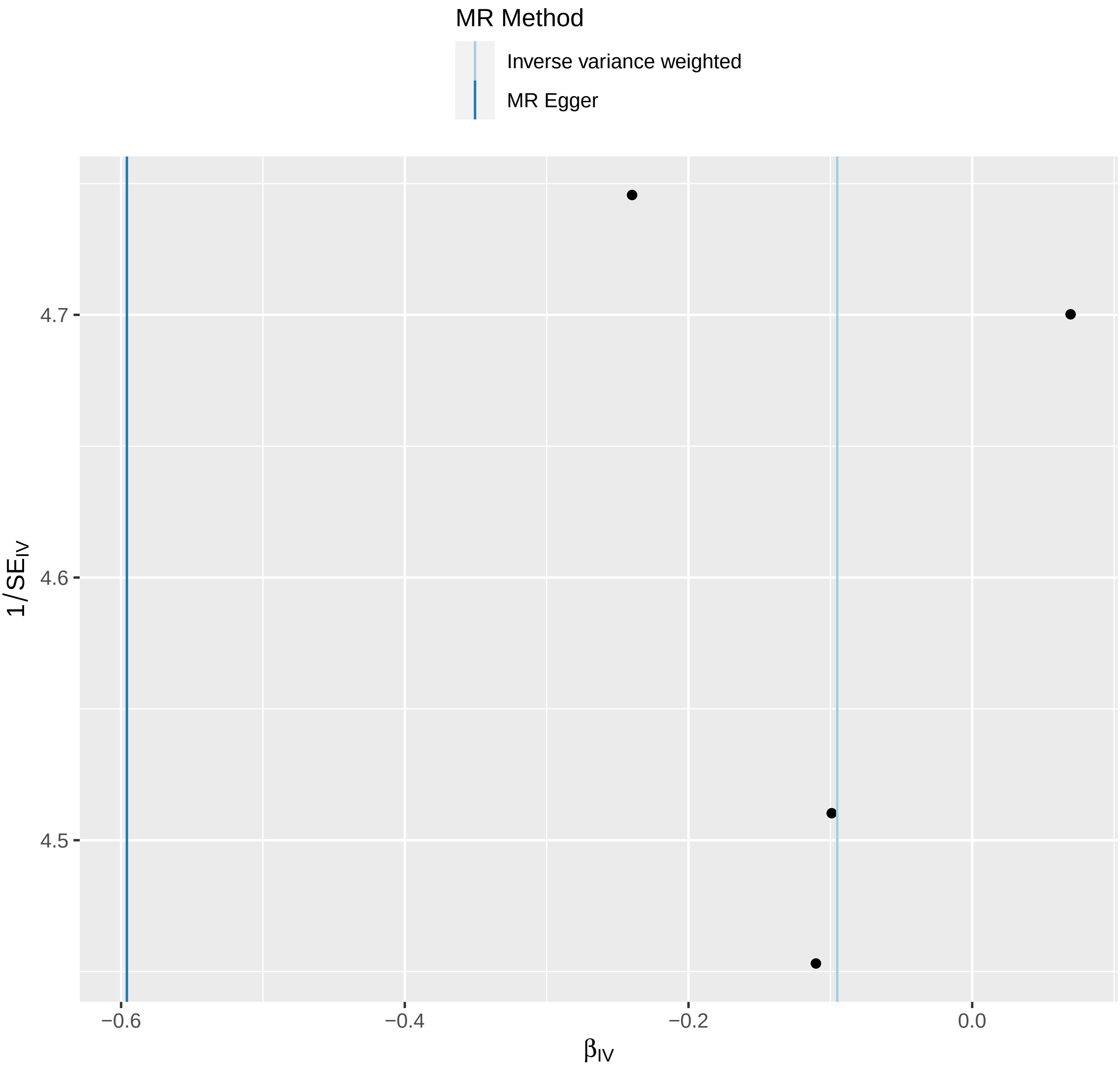

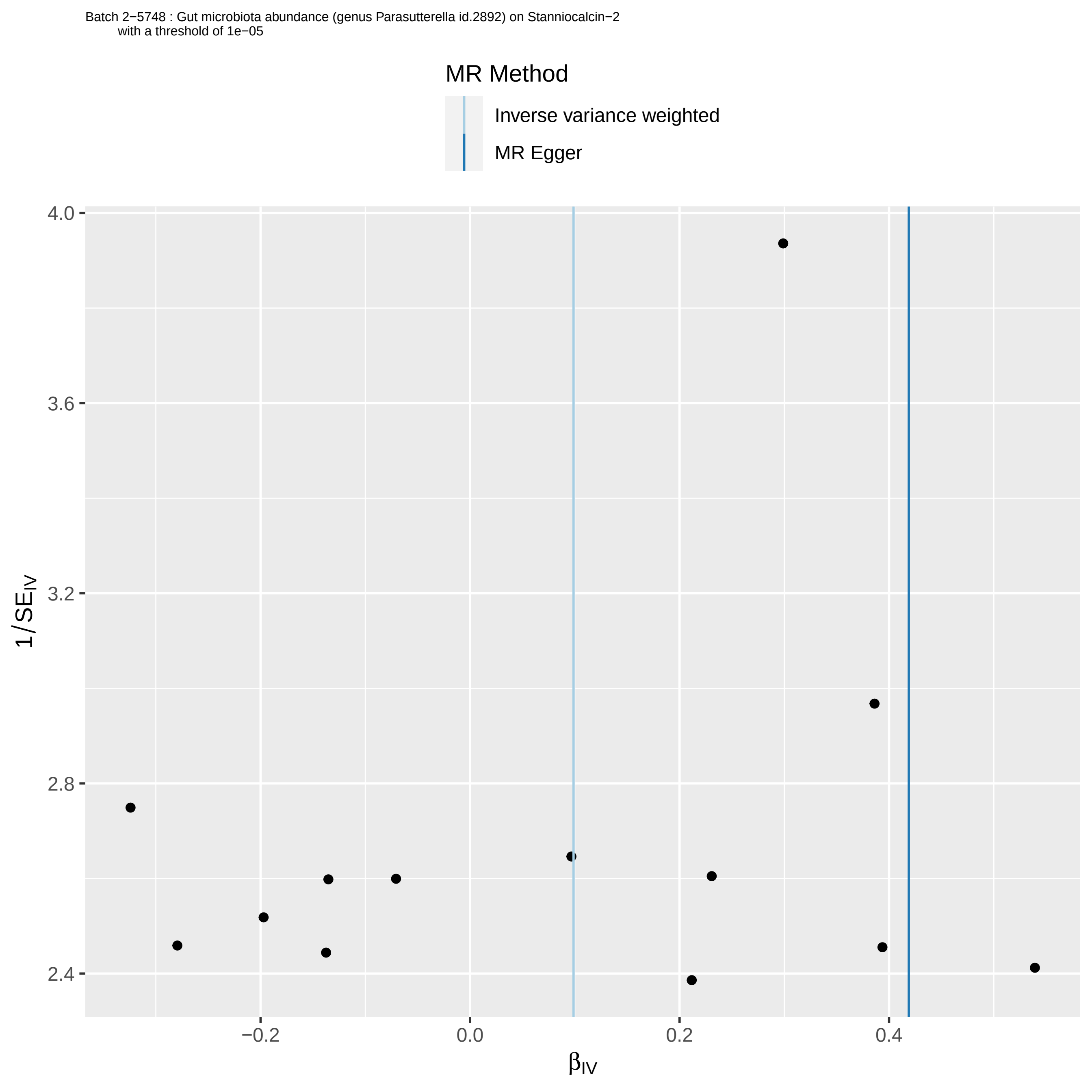

Batch 2−5748 : Gut microbiota abundance (genus Parasutterella id.2892) on Stanniocalcin−2
with a threshold of 1e−05
MR Method
Inverse variance weighted
MR Egger
4.0
3.6
3.2
2.8
2.4
$1/SE_{IV}$
−0.2
0.0
0.2
0.4
β_{IV}

Batch 2−5749 : Gut microbiota abundance (genus Catenibacterium id.2153) on Stromal interaction molecule 1 with a threshold of 1e−05

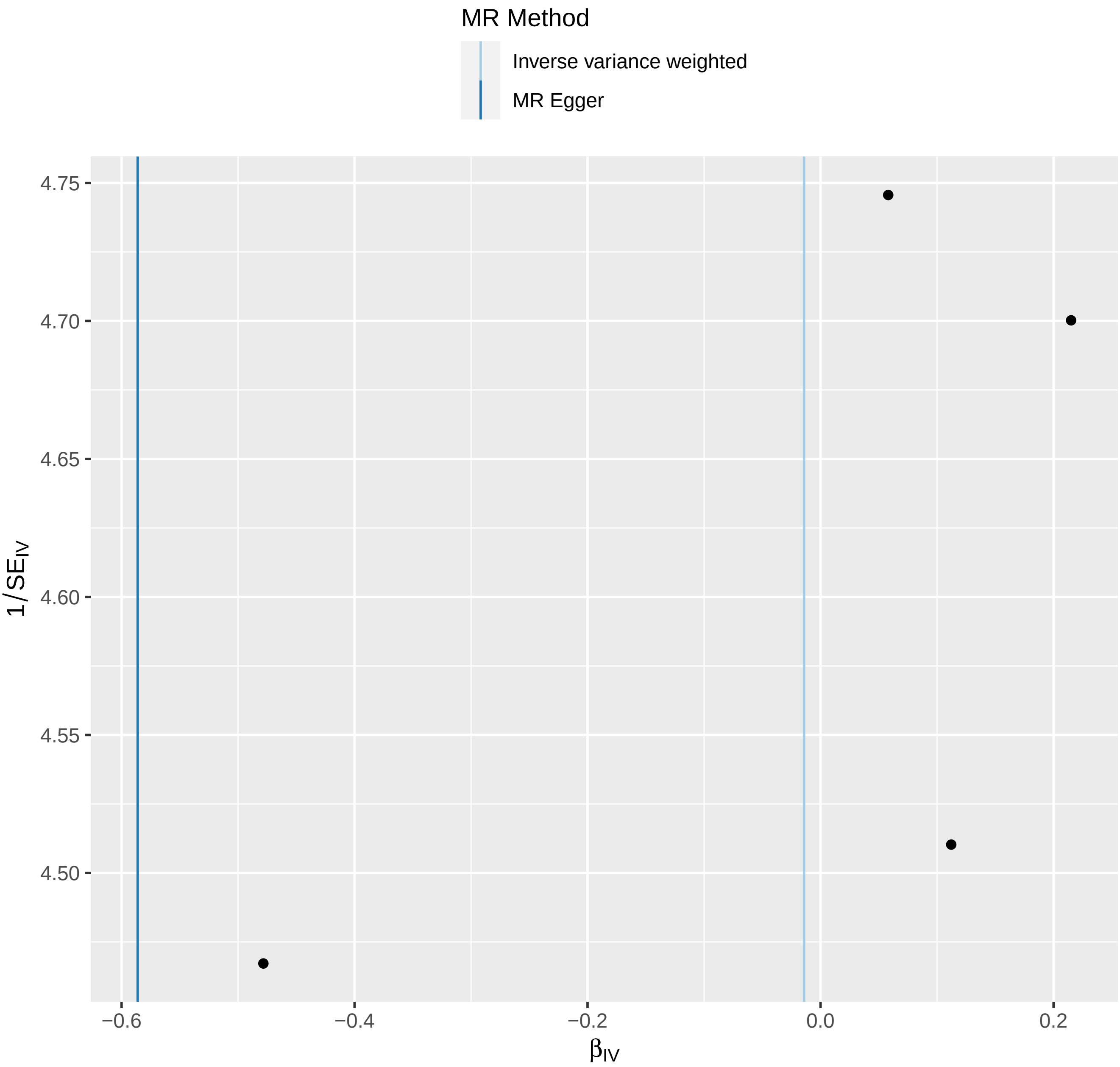

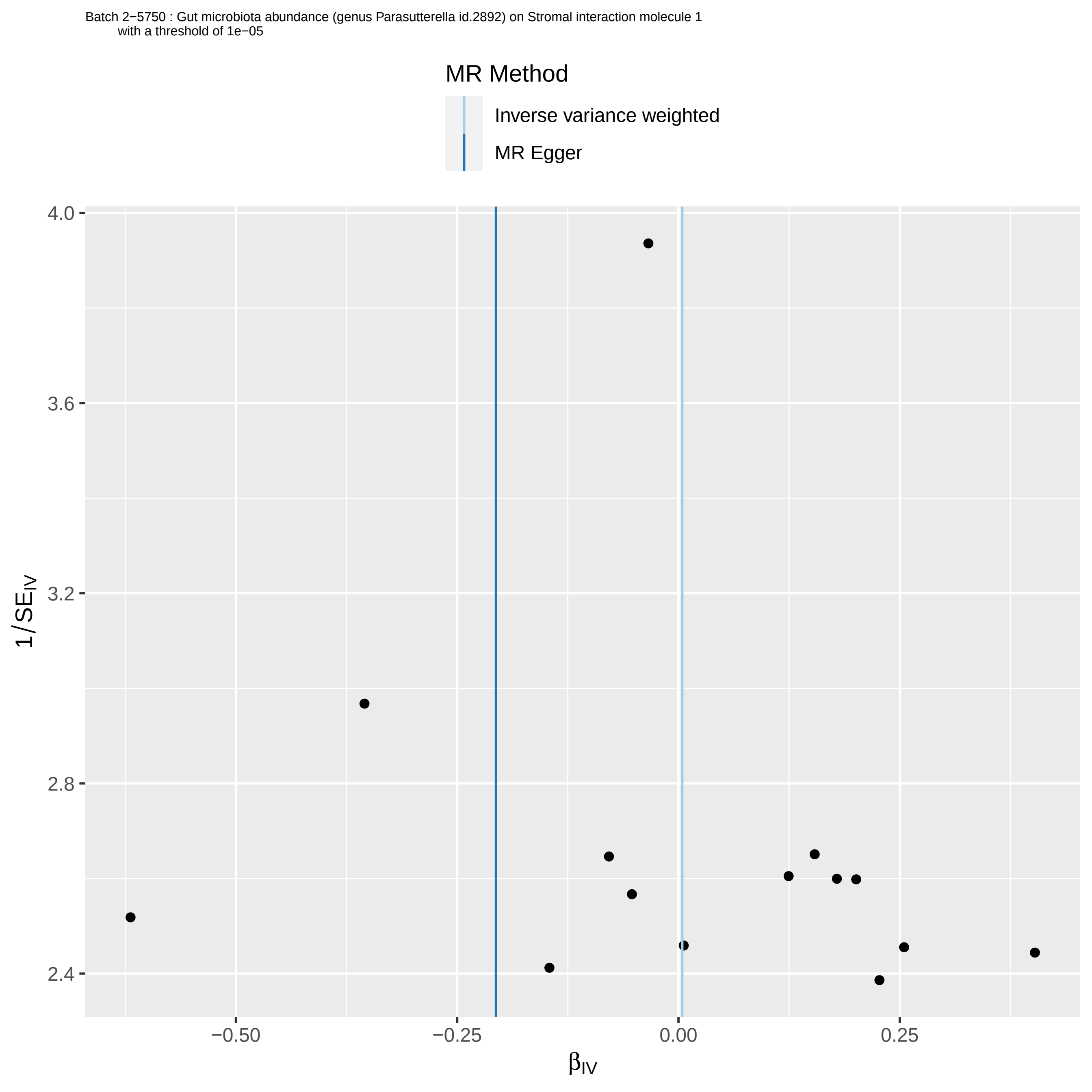

Batch 2−5750 : Gut microbiota abundance (genus Parasutterella id.2892) on Stromal interaction molecule 1
with a threshold of 1e−05
MR Method
Inverse variance weighted
MR Egger
4.0
3.6
3.2
2.8
2.4
1/SE$_{IV}$
−0.50
−0.25
0.00
0.25
β$_{IV}$

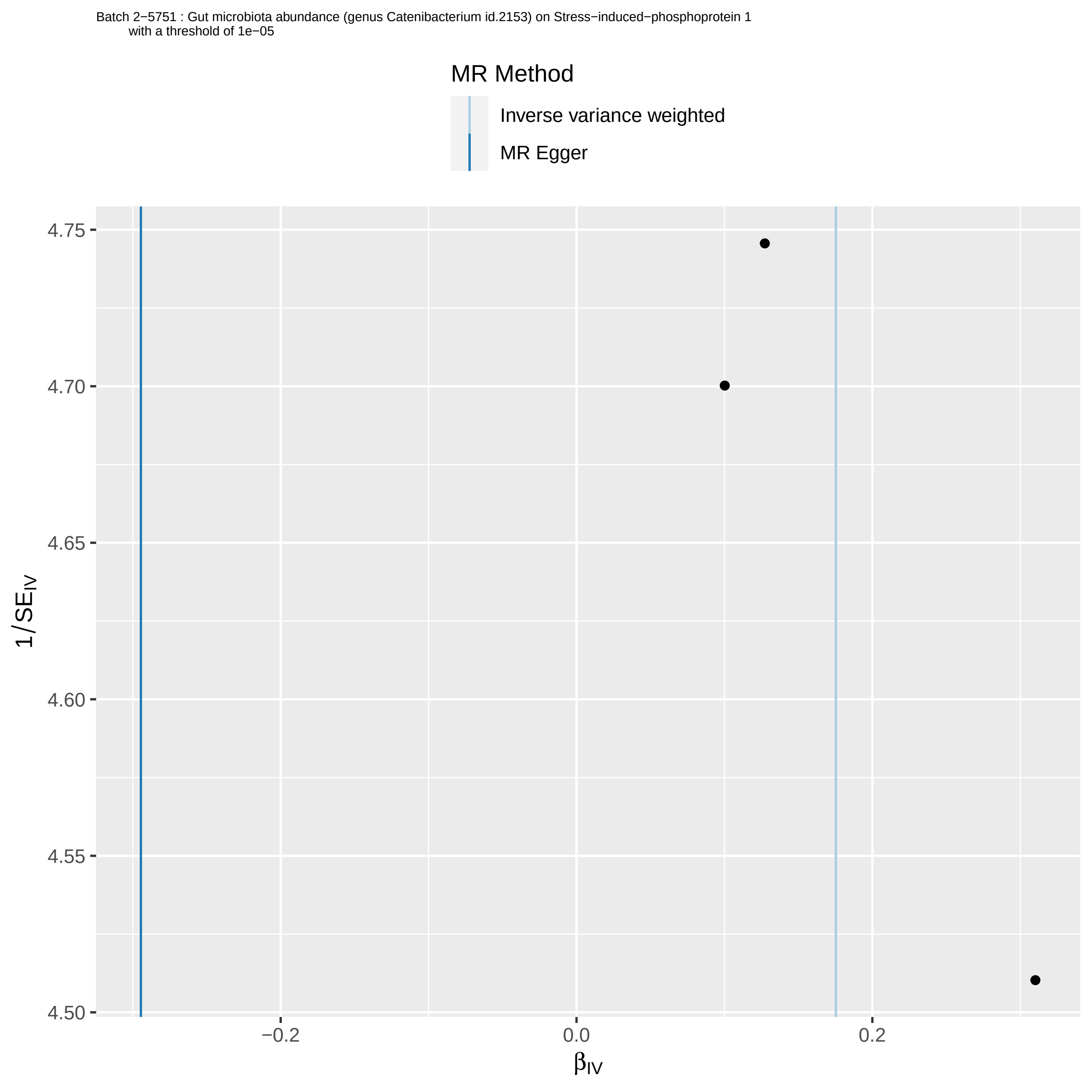
Batch 2−5751 : Gut microbiota abundance (genus Catenibacterium id.2153) on Stress−induced−phosphoprotein 1
with a threshold of 1e−05
MR Method
Inverse variance weighted
MR Egger
4.75
4.70
4.65
4.60
4.55
4.50
1/SE_IV
−0.2
0.0
0.2
β_IV

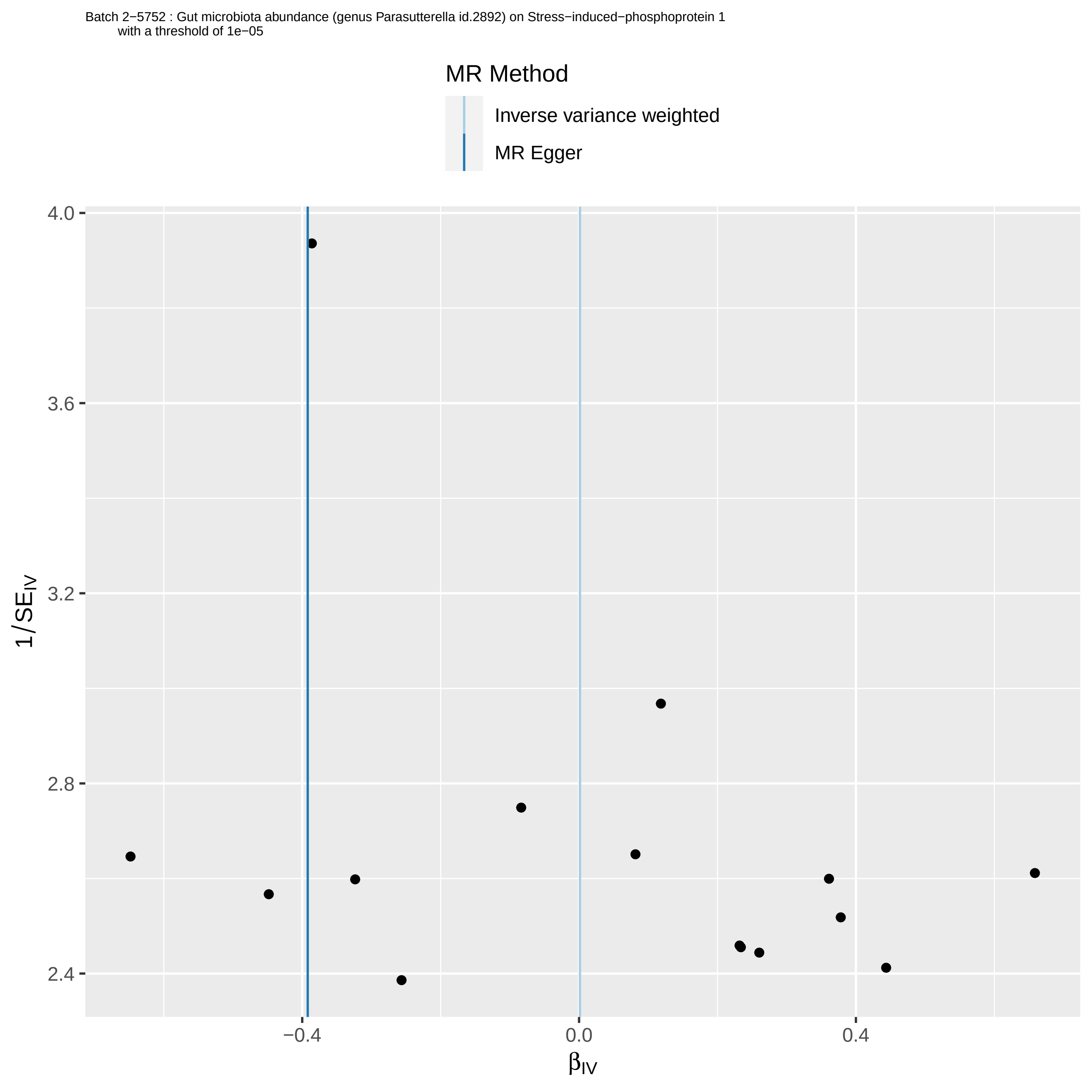
Batch 2−5752 : Gut microbiota abundance (genus Parasutterella id.2892) on Stress−induced−phosphoprotein 1
with a threshold of 1e−05
MR Method
Inverse variance weighted
MR Egger
4.0
3.6
3.2
2.8
2.4
1/SE$_{IV}$
−0.4
0.0
0.4
β$_{IV}$

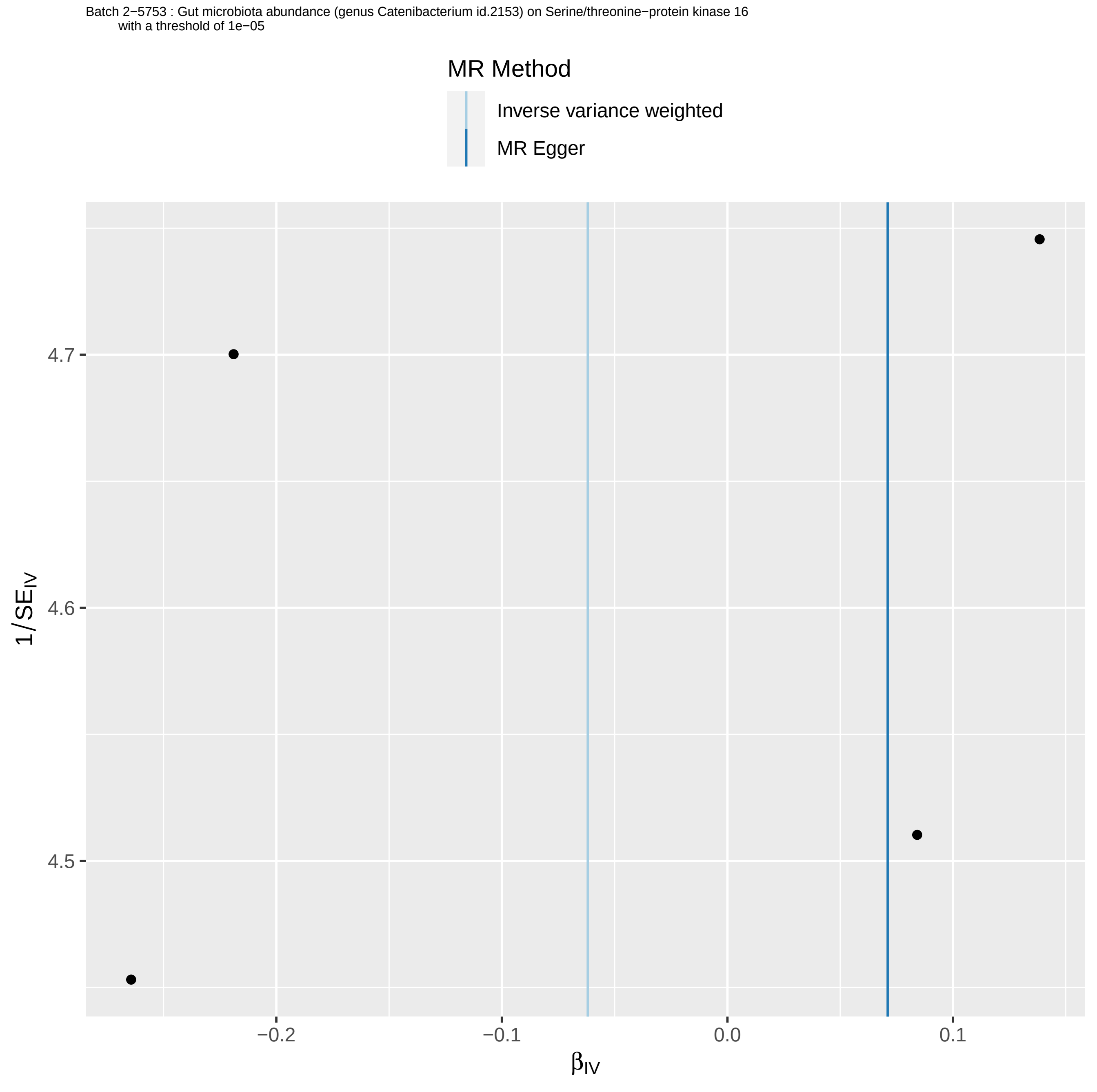
Batch 2−5753 : Gut microbiota abundance (genus Catenibacterium id.2153) on Serine/threonine−protein kinase 16
with a threshold of 1e−05
MR Method
Inverse variance weighted
MR Egger
4.7
4.6
4.5
1/SE_IV
−0.2
−0.1
0.0
0.1
β_IV

Batch 2−5754 : Gut microbiota abundance (genus Parasutterella id.2892) on Serine/threonine−protein kinase 16
with a threshold of 1e−05

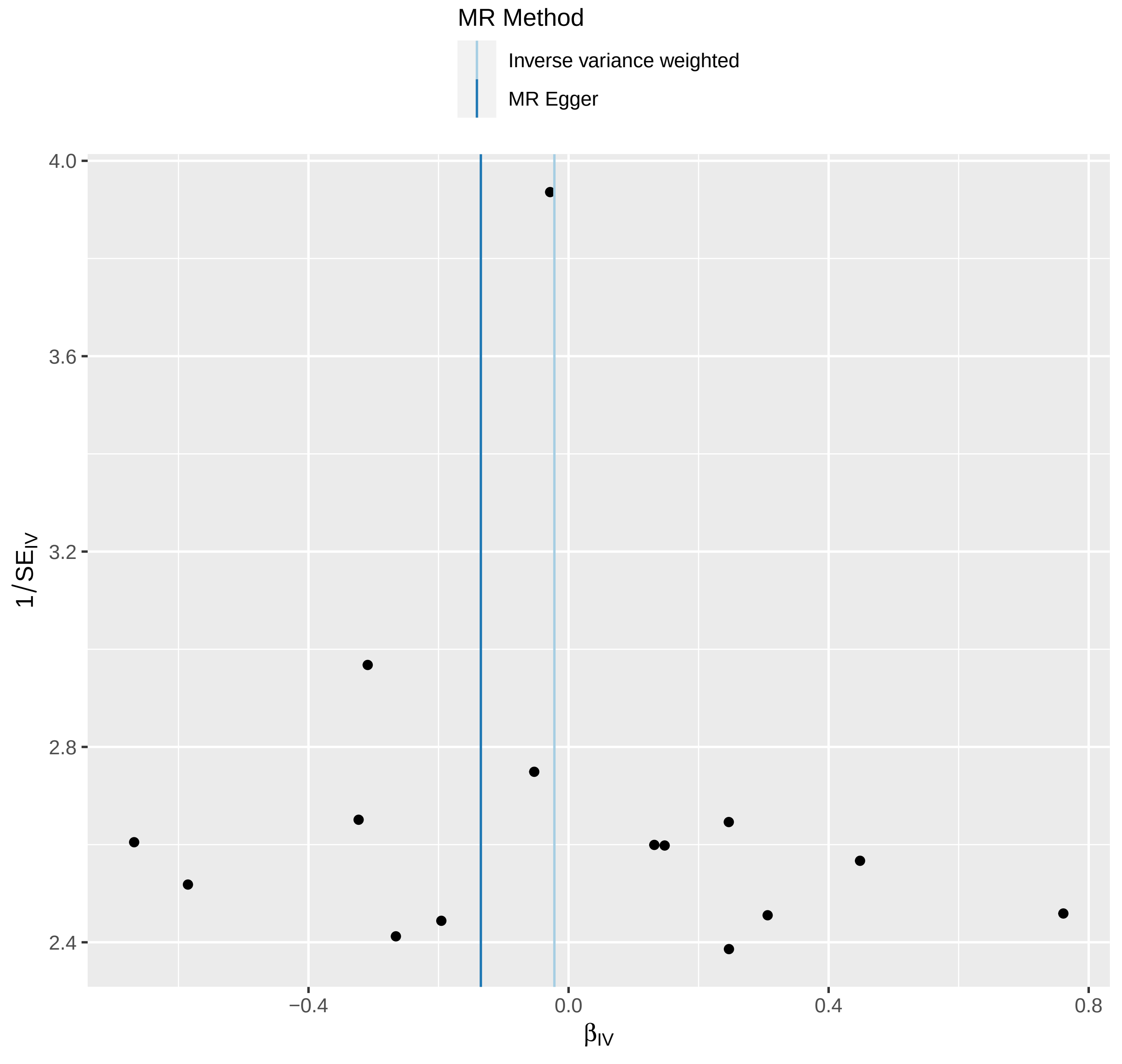

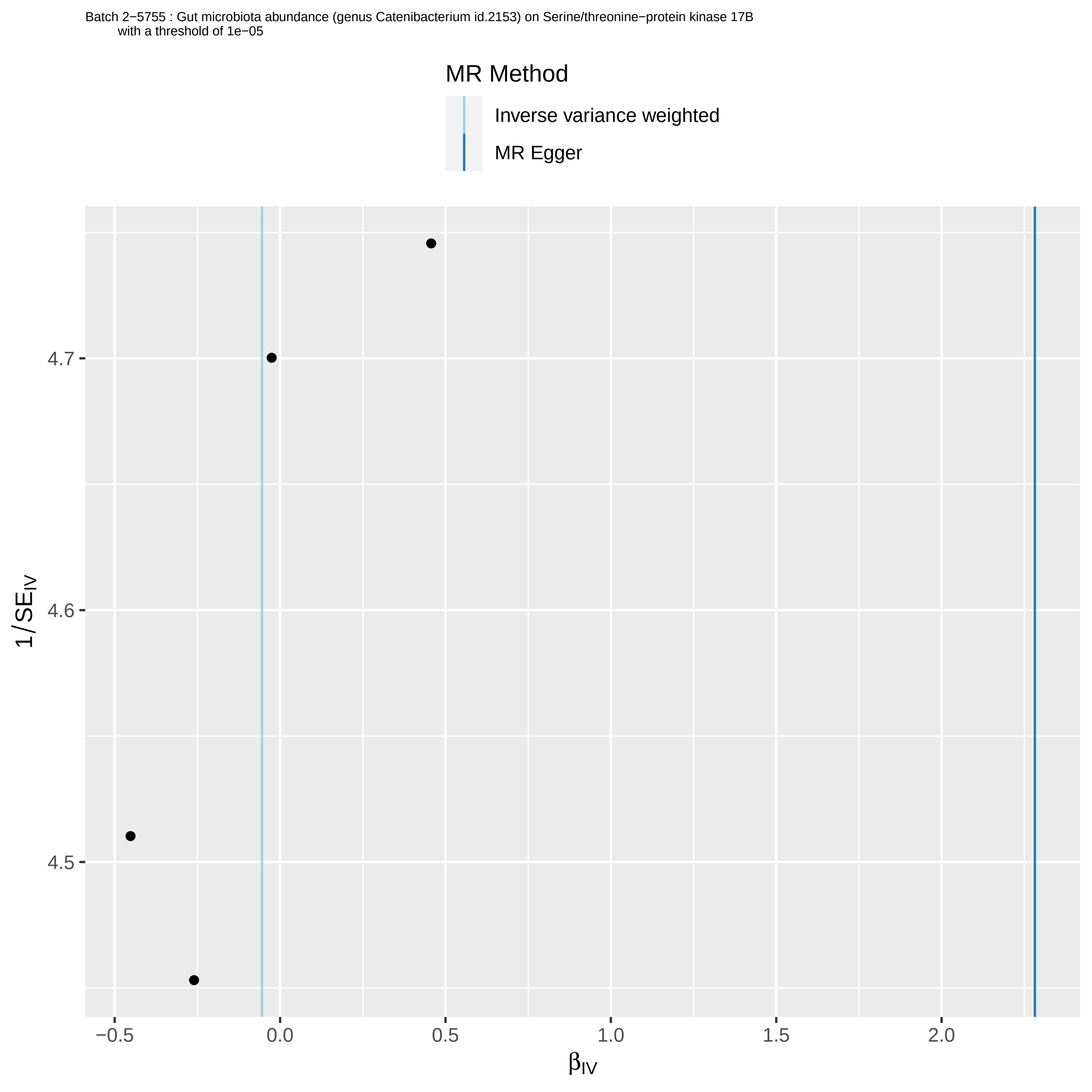

Batch 2−5755 : Gut microbiota abundance (genus Catenibacterium id.2153) on Serine/threonine−protein kinase 17B
with a threshold of 1e−05
MR Method
Inverse variance weighted
MR Egger
4.7
4.6
4.5
1/SE_IV
−0.5
0.0
0.5
1.0
1.5
2.0
β_IV

Batch 2−5756 : Gut microbiota abundance (genus Parasutterella id.2892) on Serine/threonine−protein kinase 17B
with a threshold of 1e−05

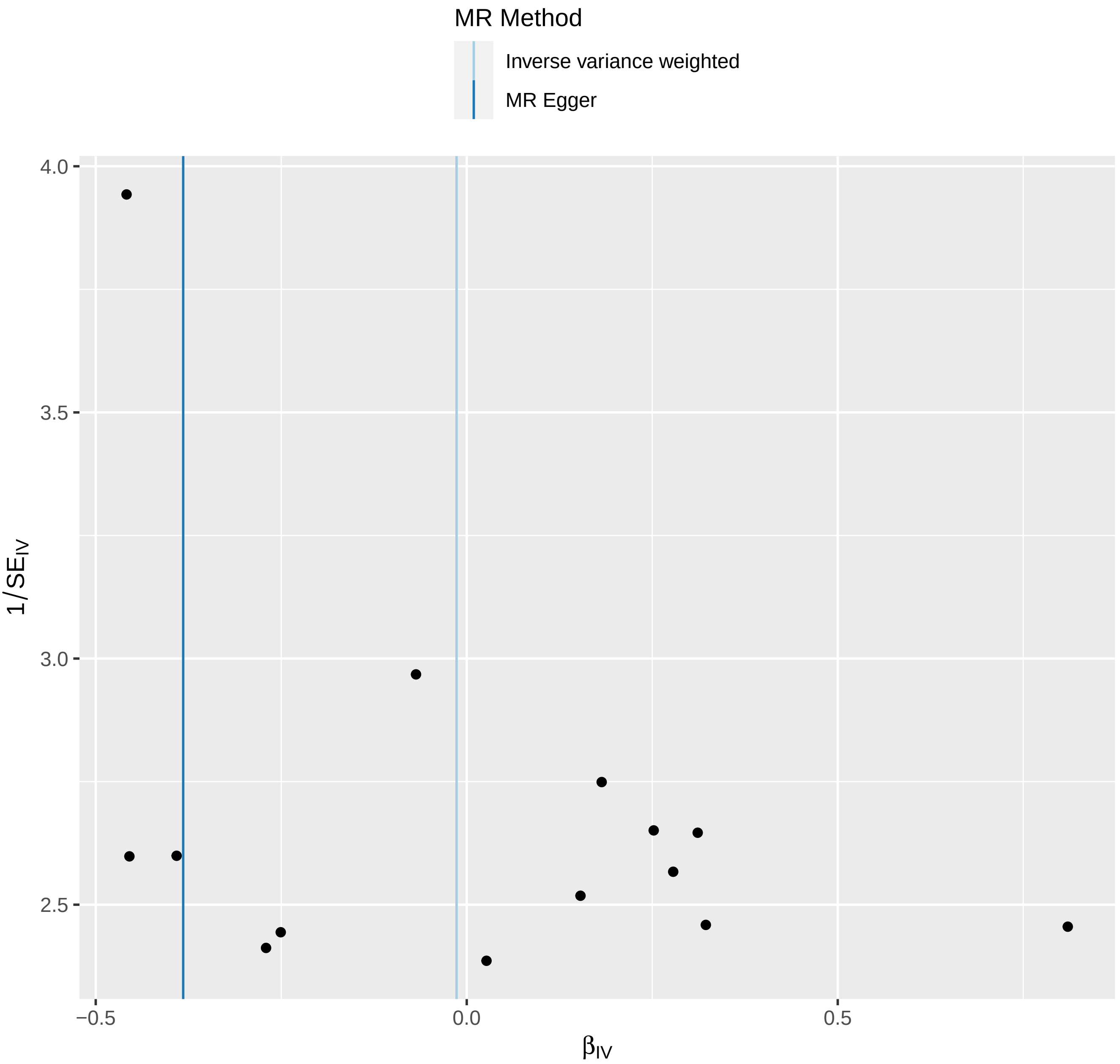

Batch 2−5757 : Gut microbiota abundance (genus Catenibacterium id.2153) on Stathmin−2
with a threshold of 1e−05

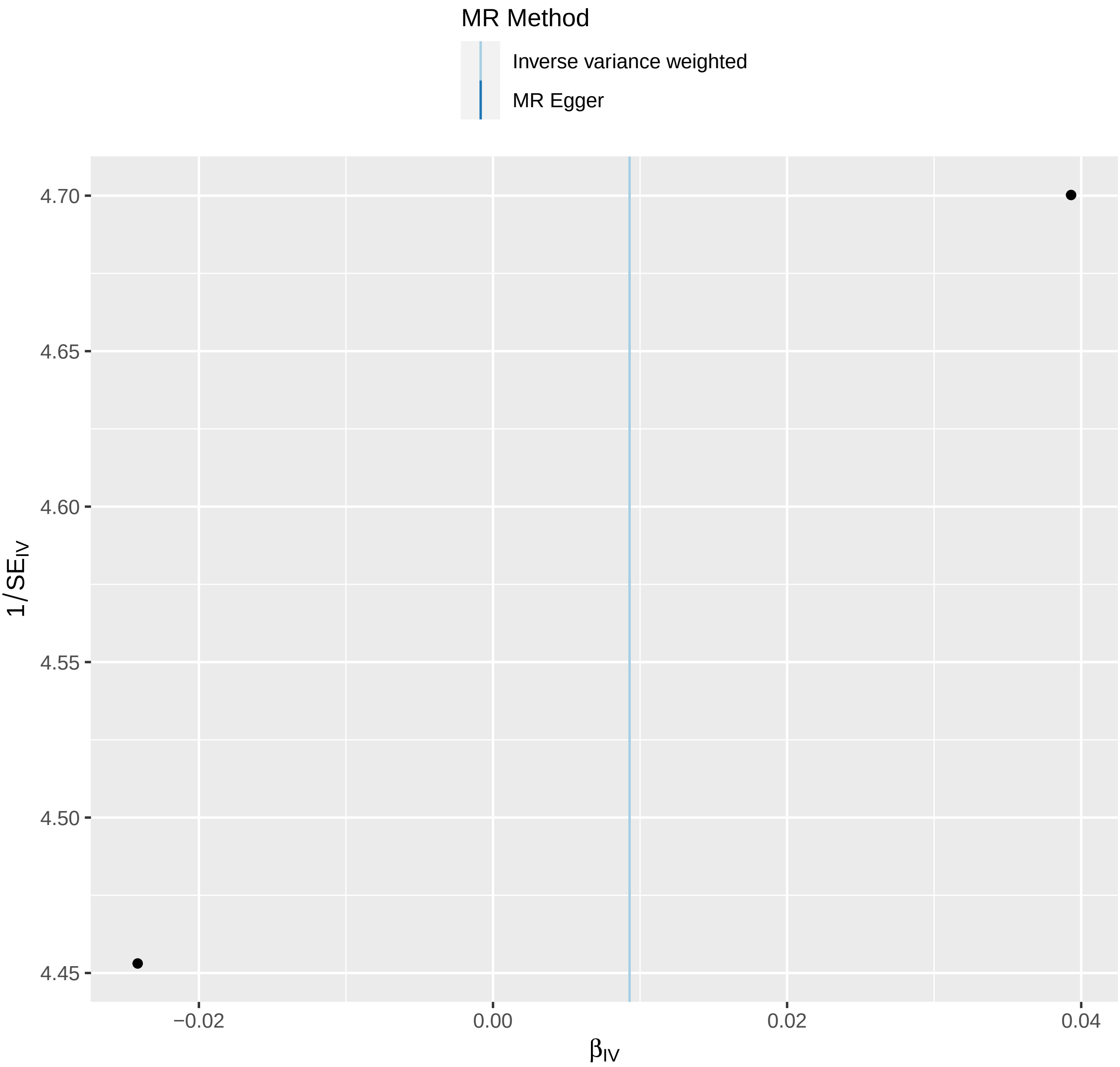

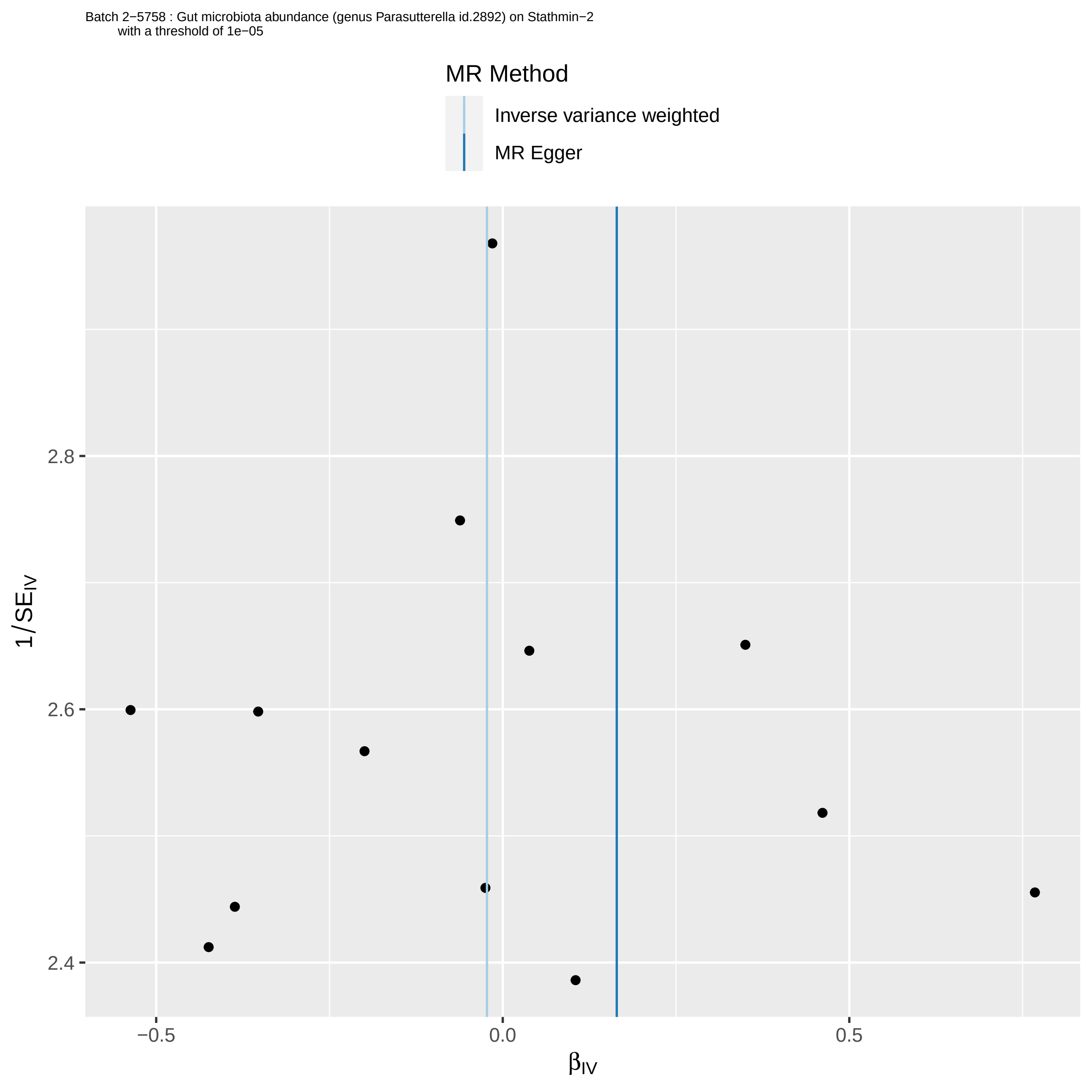

Batch 2−5758 : Gut microbiota abundance (genus Parasutterella id.2892) on Stathmin−2
with a threshold of 1e−05
MR Method
Inverse variance weighted
MR Egger
2.8
2.6
2.4
1/SE_IV
−0.5
0.0
0.5
β_IV

Batch 2−5759 : Gut microbiota abundance (genus Catenibacterium id.2153) on Stathmin−3
with a threshold of 1e−05

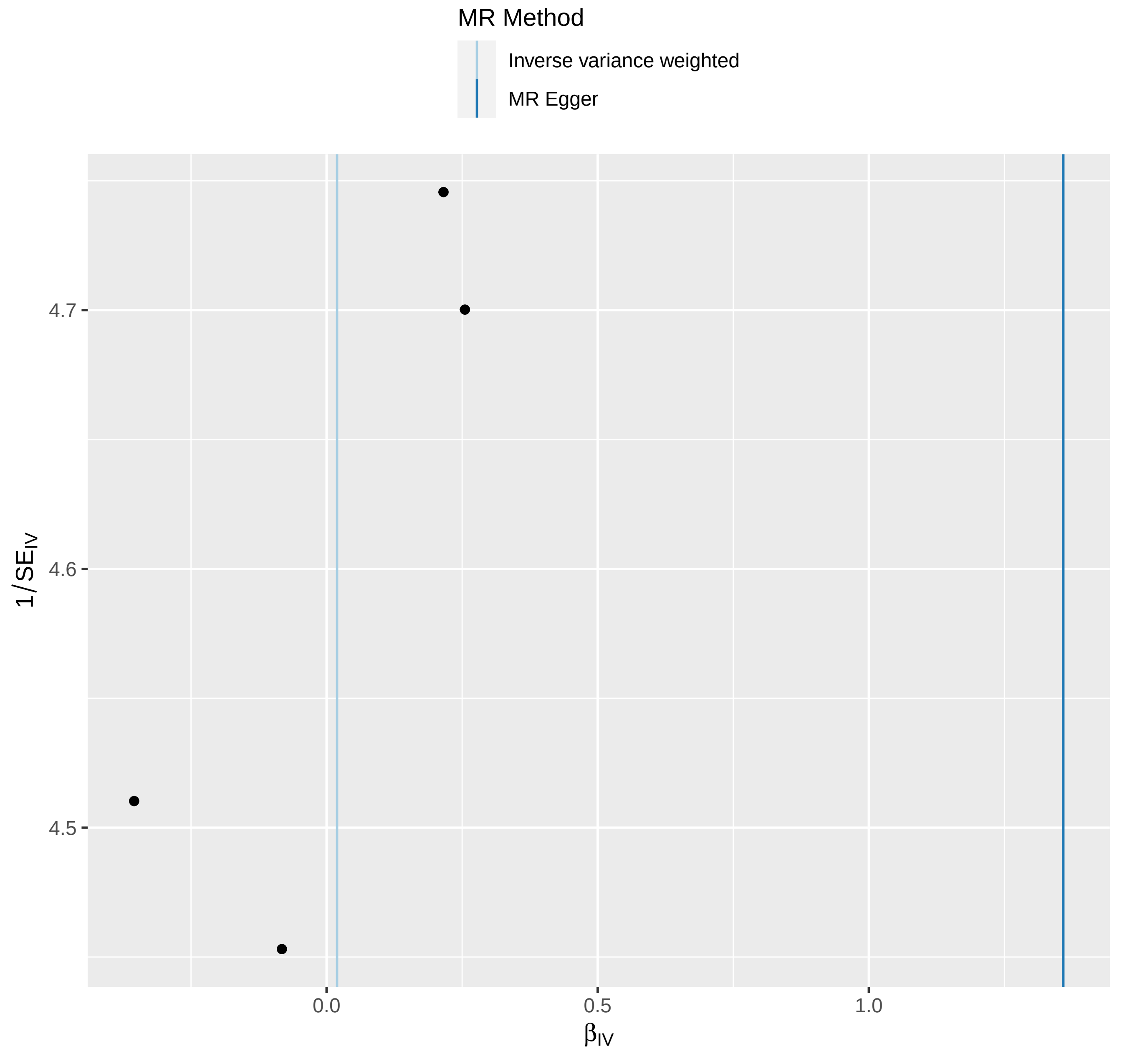

Batch 2−5760 : Gut microbiota abundance (genus Parasutterella id.2892) on Stathmin−3
with a threshold of 1e−05

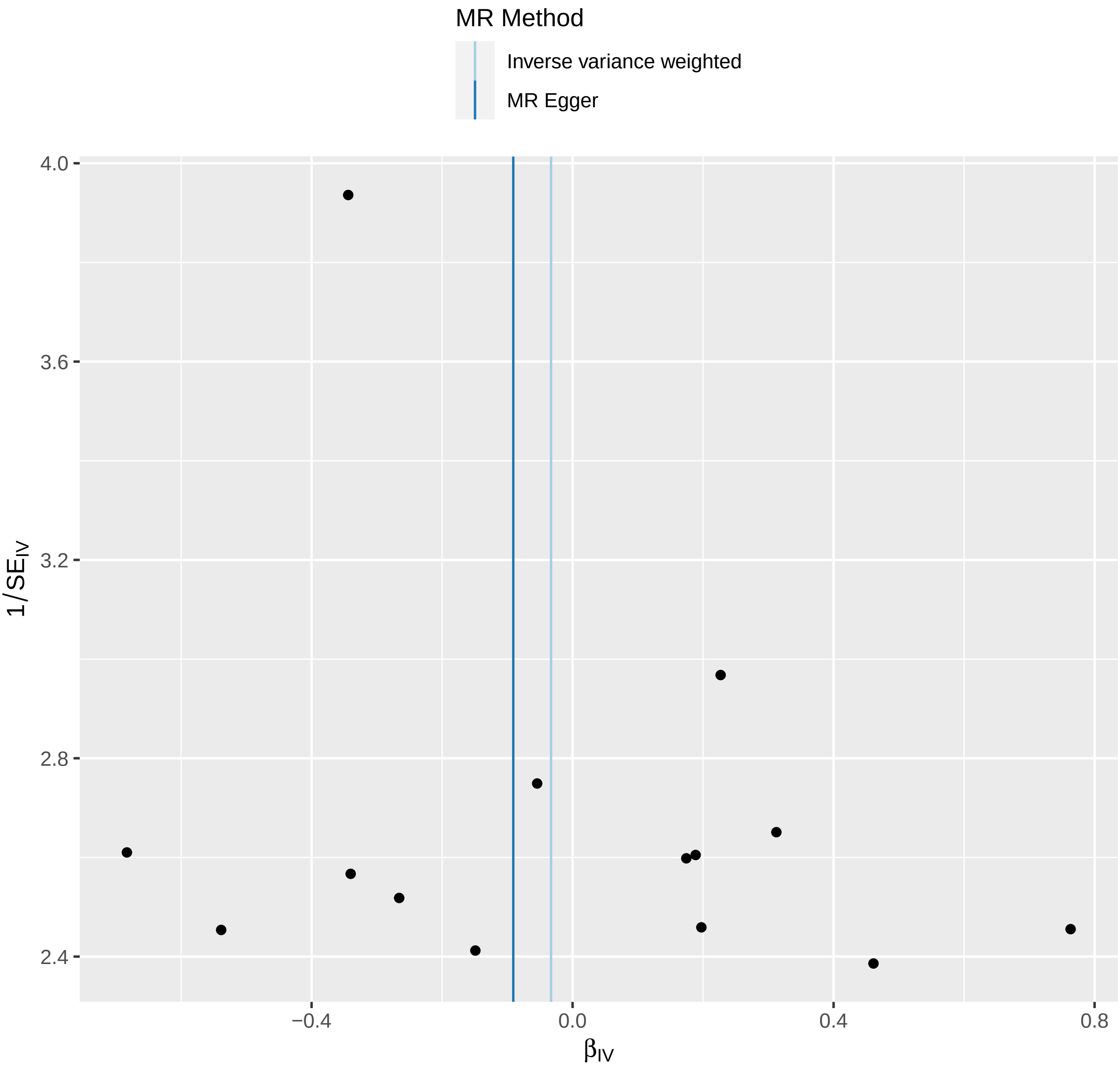

Batch 2−5761 : Gut microbiota abundance (genus Catenibacterium id.2153) on Stathmin−4
with a threshold of 1e−05

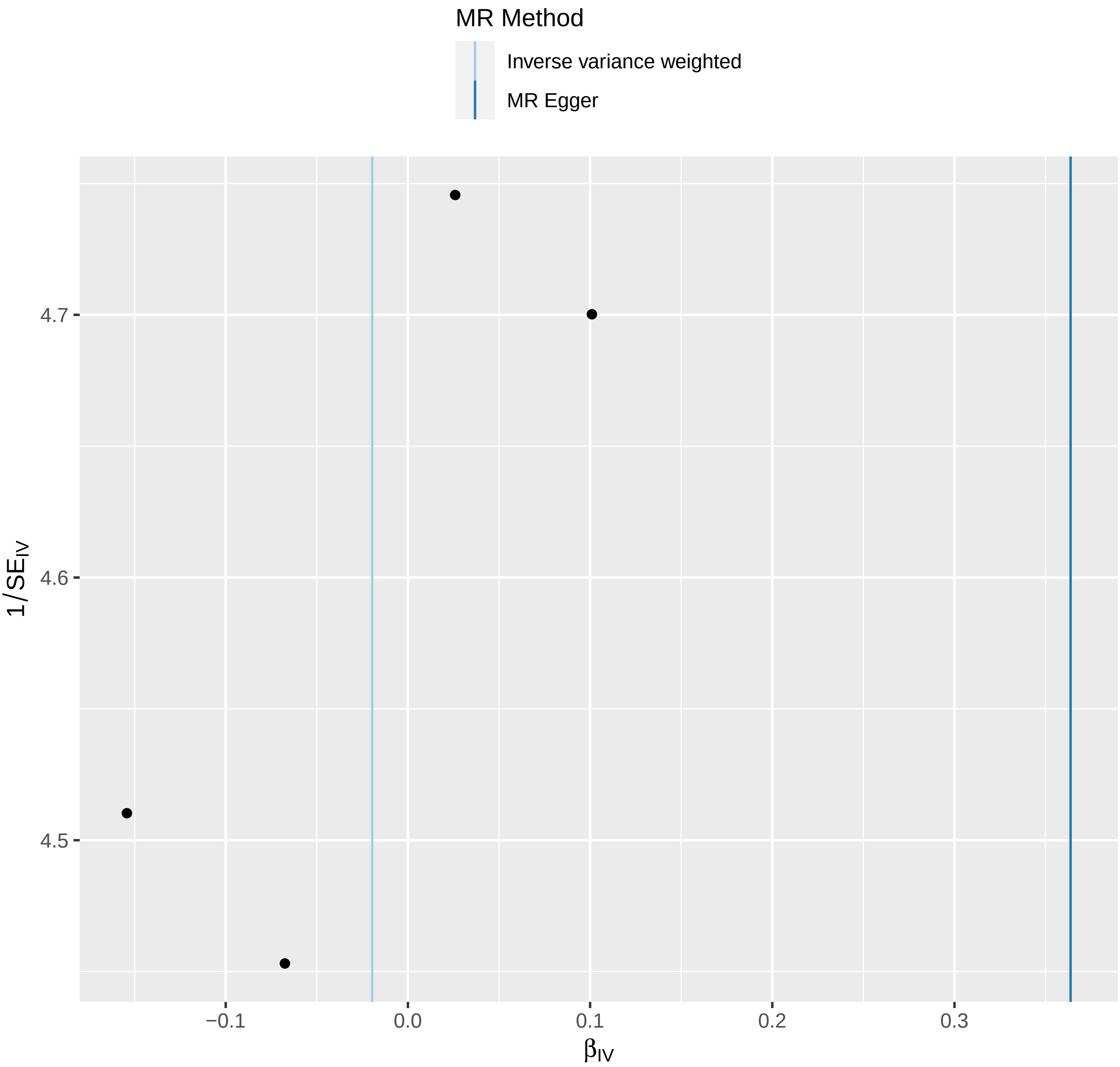

Batch 2−5762 : Gut microbiota abundance (genus Parasutterella id.2892) on Stathmin−4
with a threshold of 1e−05

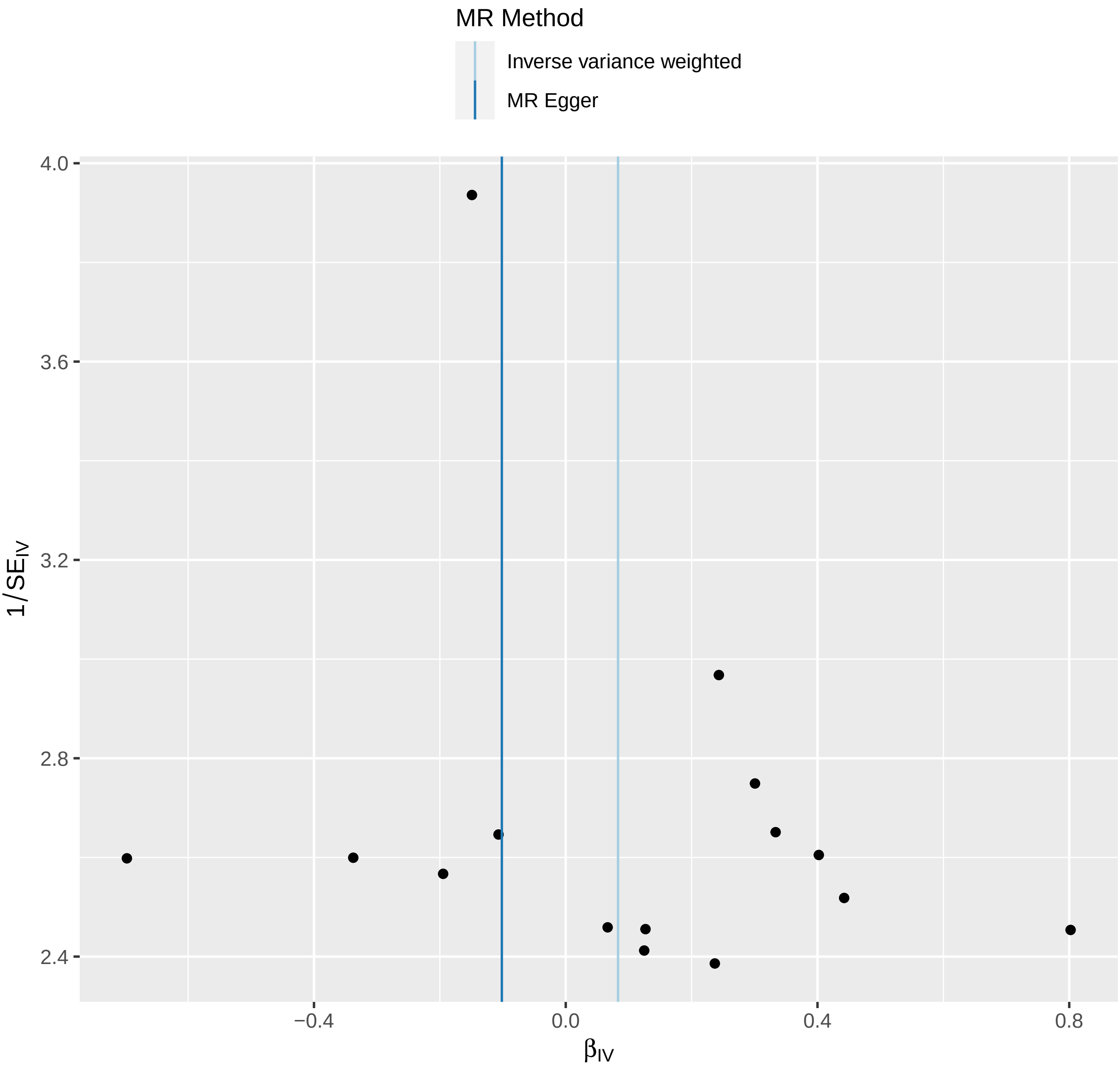

Batch 2−5763 : Gut microbiota abundance (genus Catenibacterium id.2153) on Erythrocyte band 7 integral membrane protein with a threshold of 1e−05

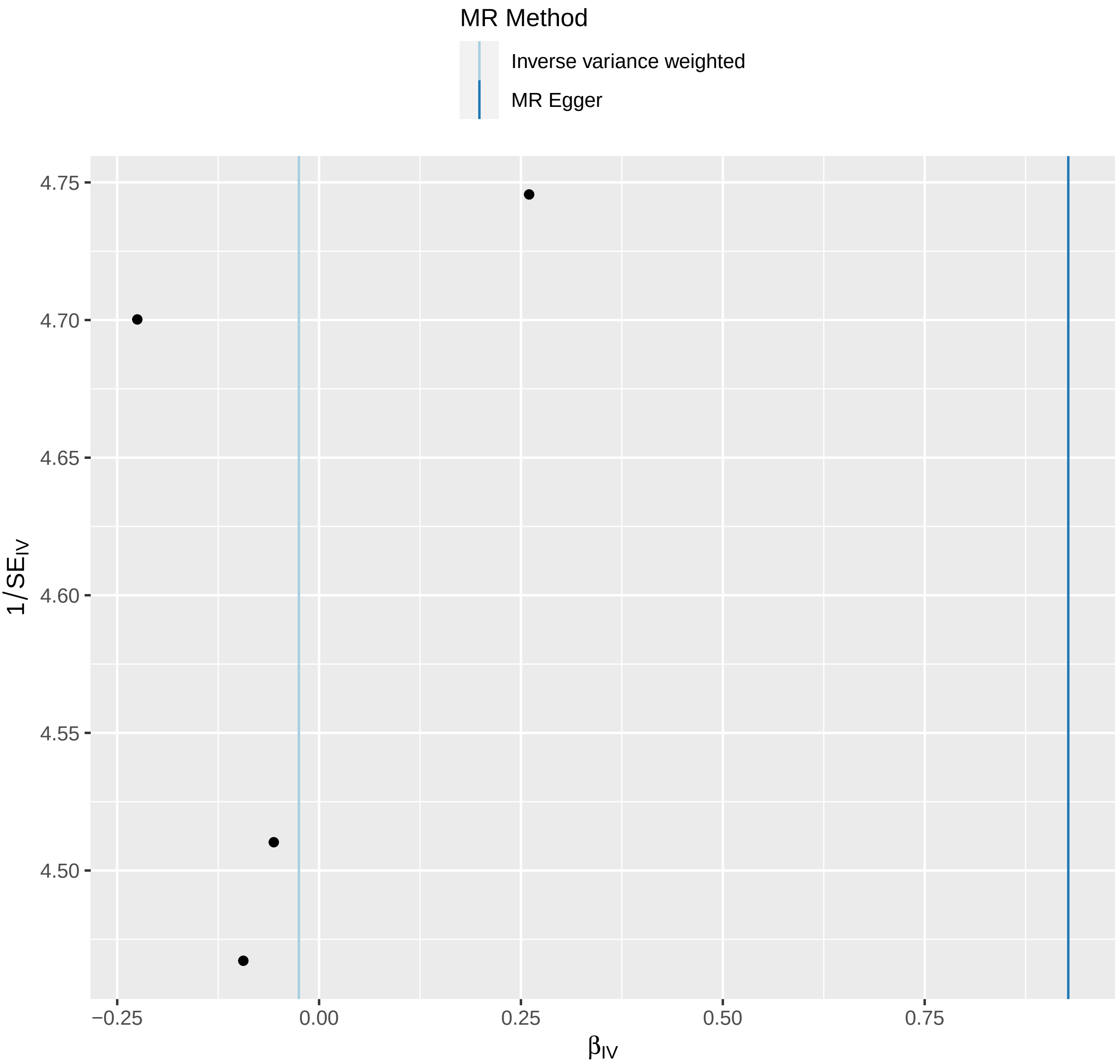

Batch 2−5764 : Gut microbiota abundance (genus Parasutterella id.2892) on Erythrocyte band 7 integral membrane protein
with a threshold of 1e−05

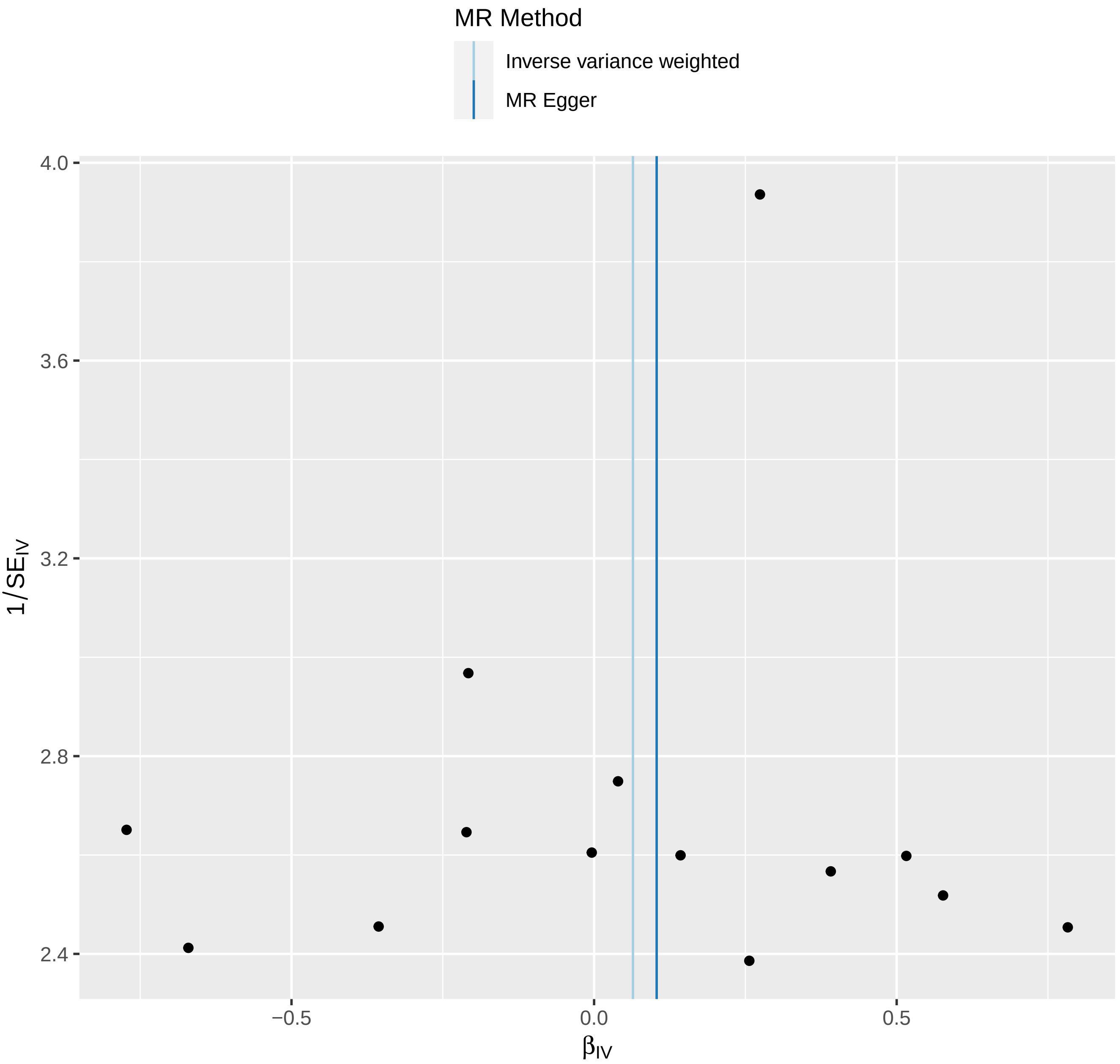

Batch 2−5765 : Gut microbiota abundance (genus Catenibacterium id.2153) on Syntaxin−10
with a threshold of 1e−05

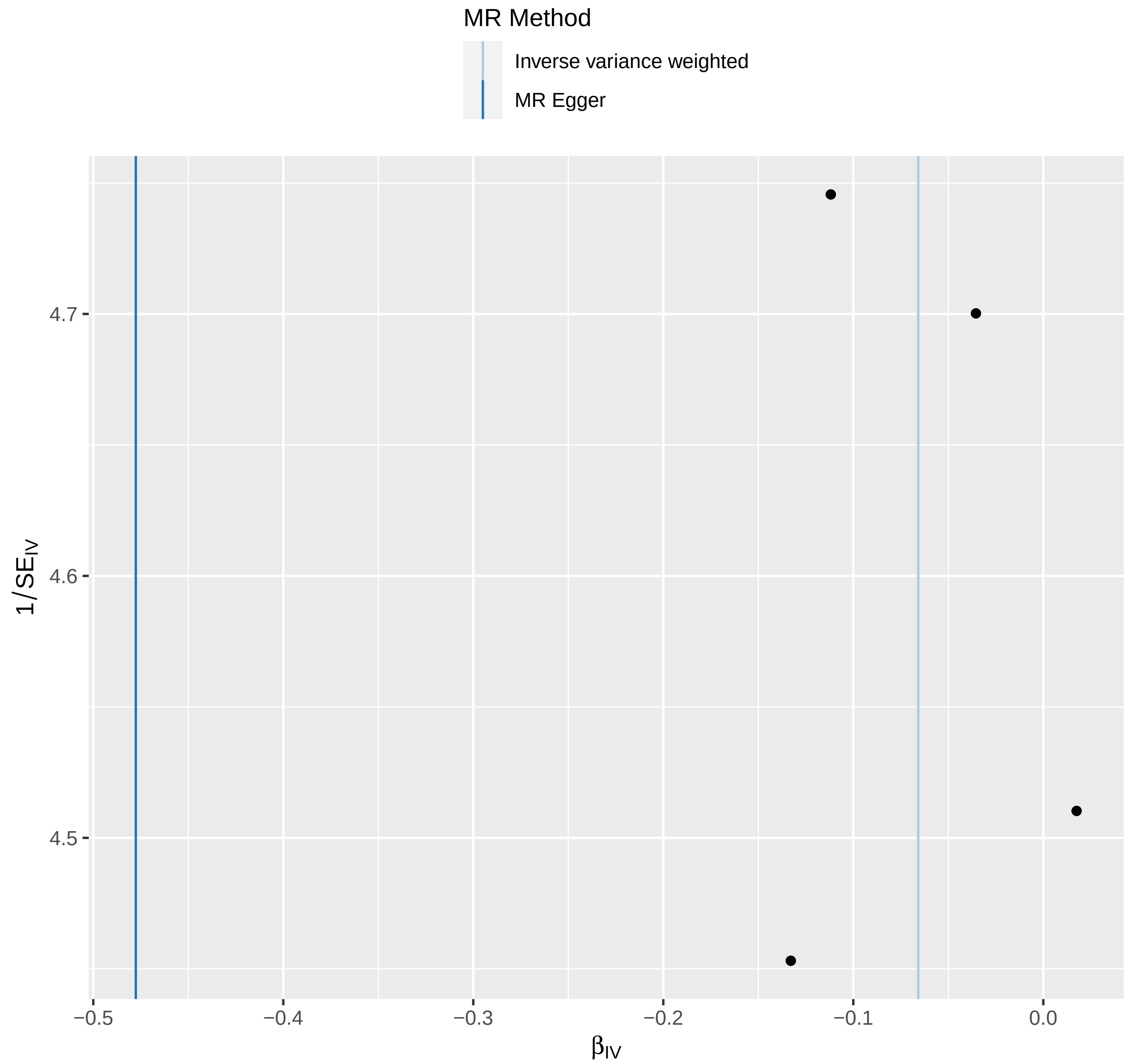

Batch 2−5766 : Gut microbiota abundance (genus Parasutterella id.2892) on Syntaxin−10
with a threshold of 1e−05

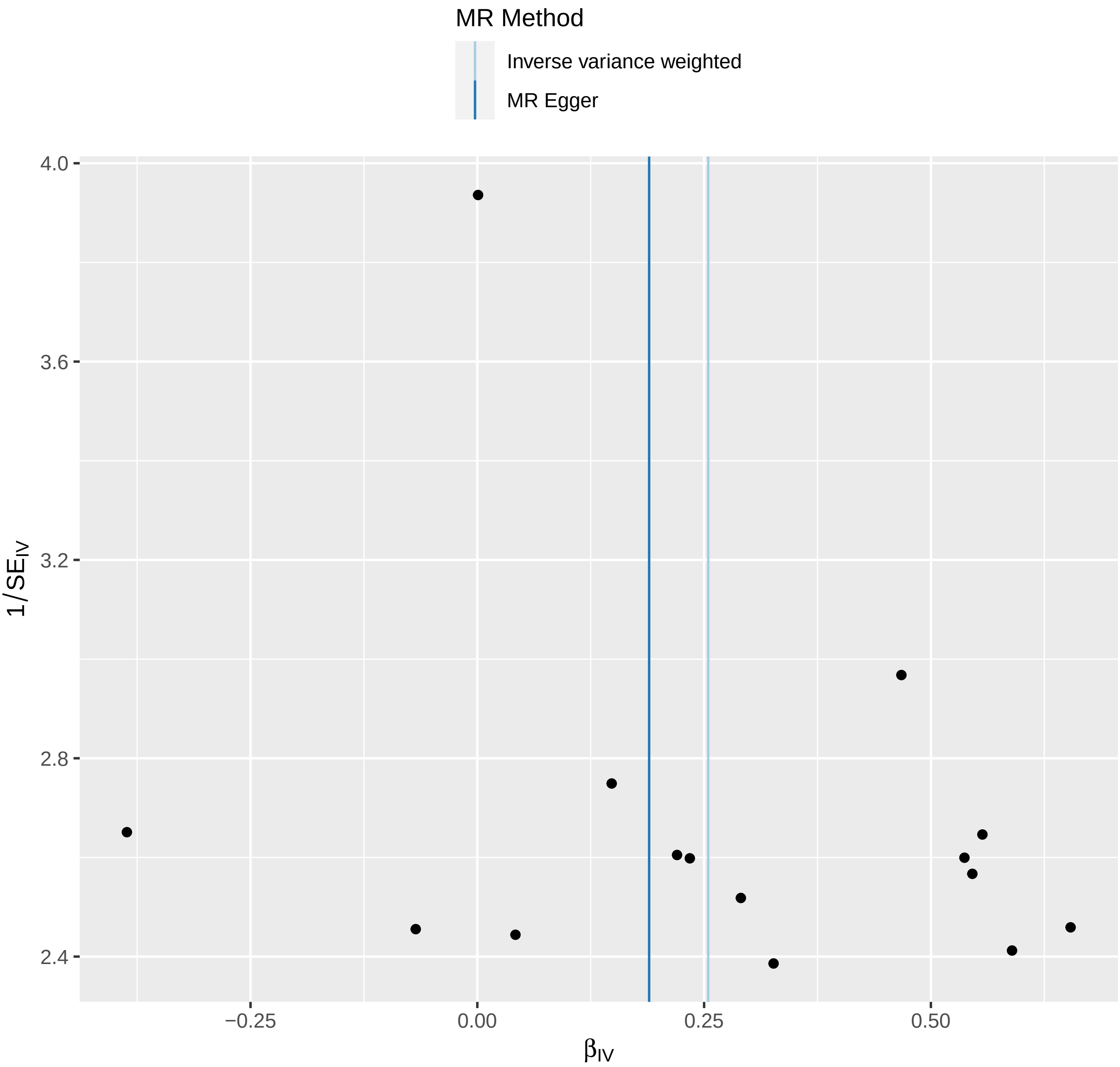

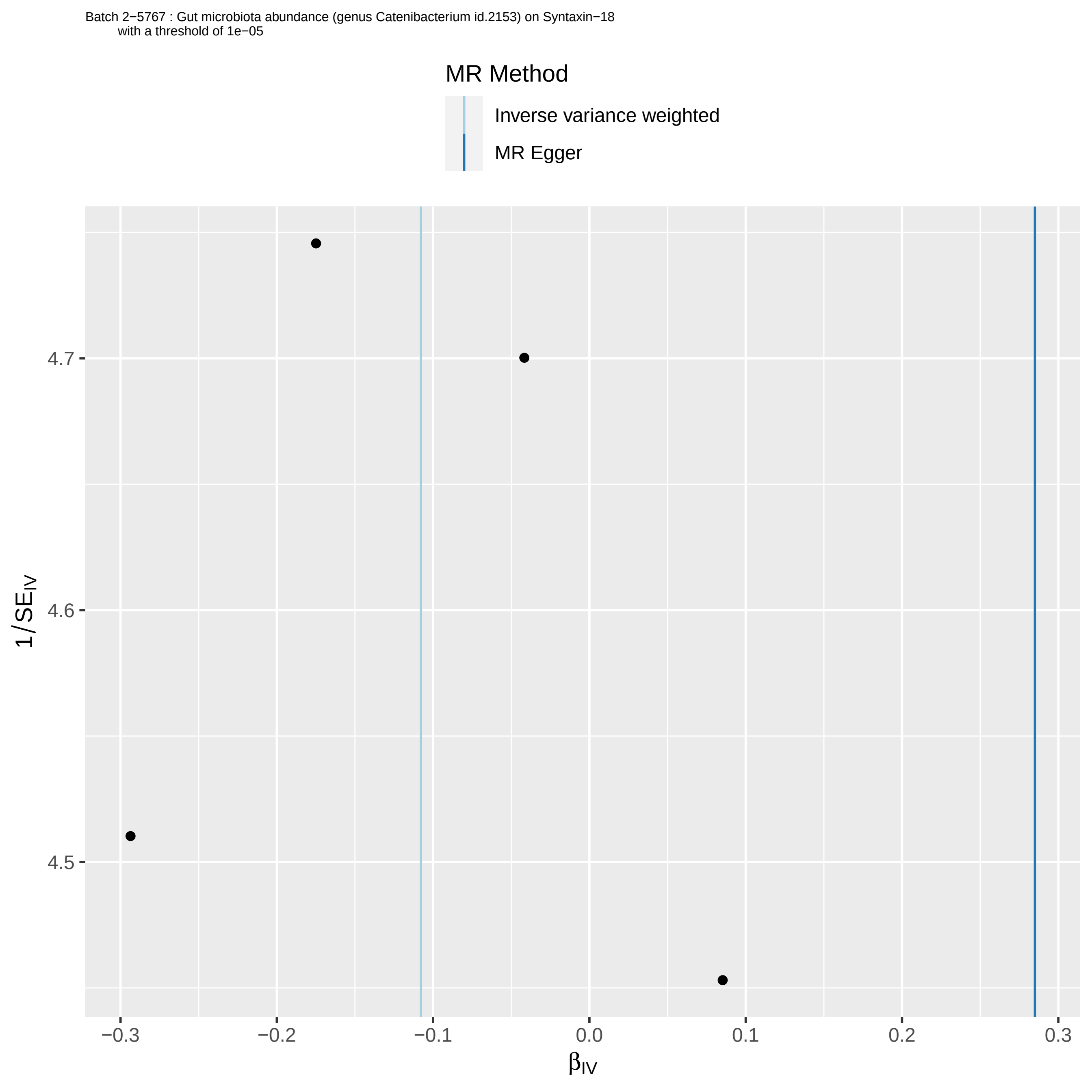
Batch 2−5767 : Gut microbiota abundance (genus Catenibacterium id.2153) on Syntaxin−18
with a threshold of 1e−05
MR Method
Inverse variance weighted
MR Egger
4.7
4.6
4.5
1/SE_IV
−0.3
−0.2
−0.1
0.0
0.1
0.2
0.3
β_IV

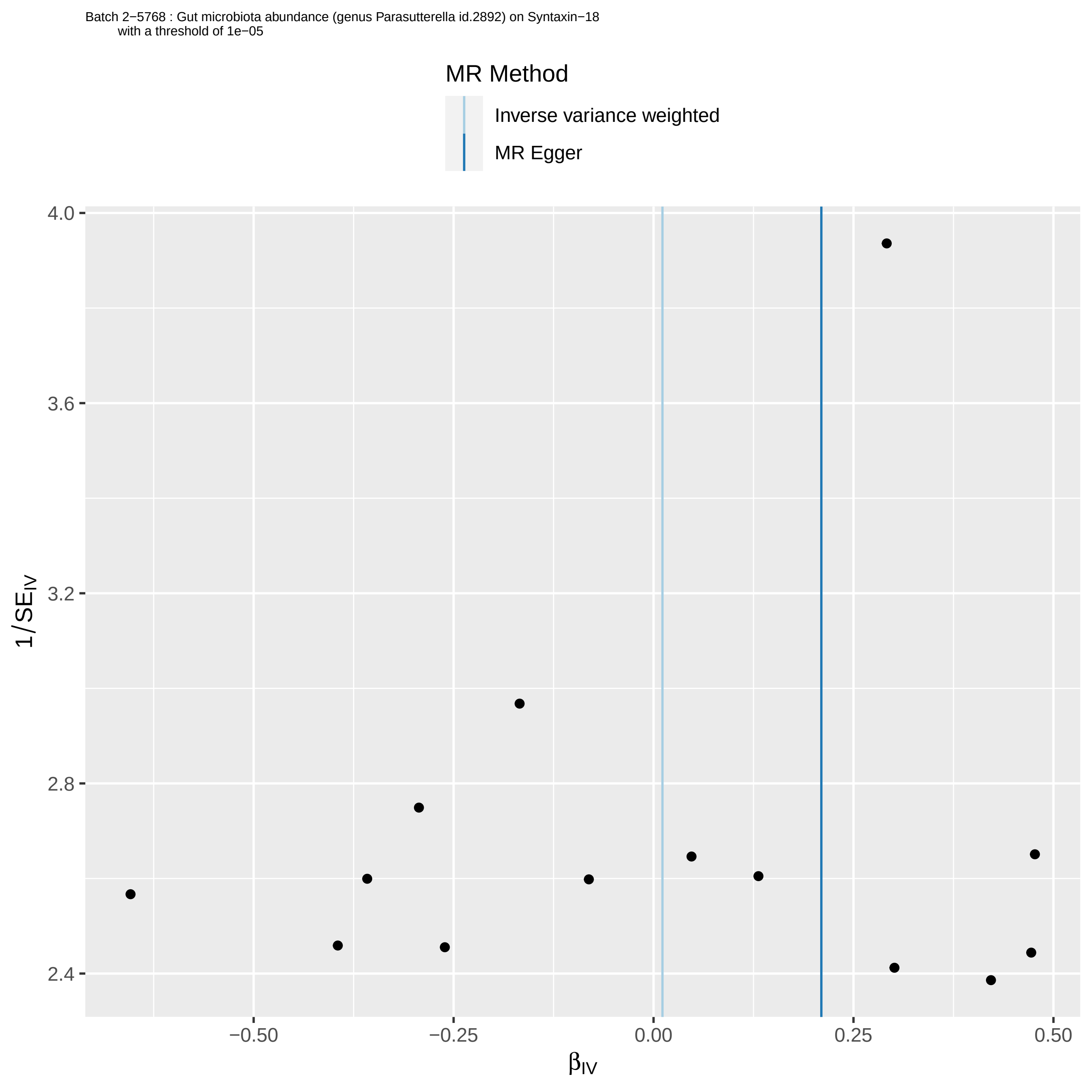

Batch 2−5768 : Gut microbiota abundance (genus Parasutterella id.2892) on Syntaxin−18
with a threshold of 1e−05
MR Method
Inverse variance weighted
MR Egger
4.0
3.6
3.2
2.8
2.4
1/SE_IV
−0.50
−0.25
0.00
0.25
0.50
β_IV

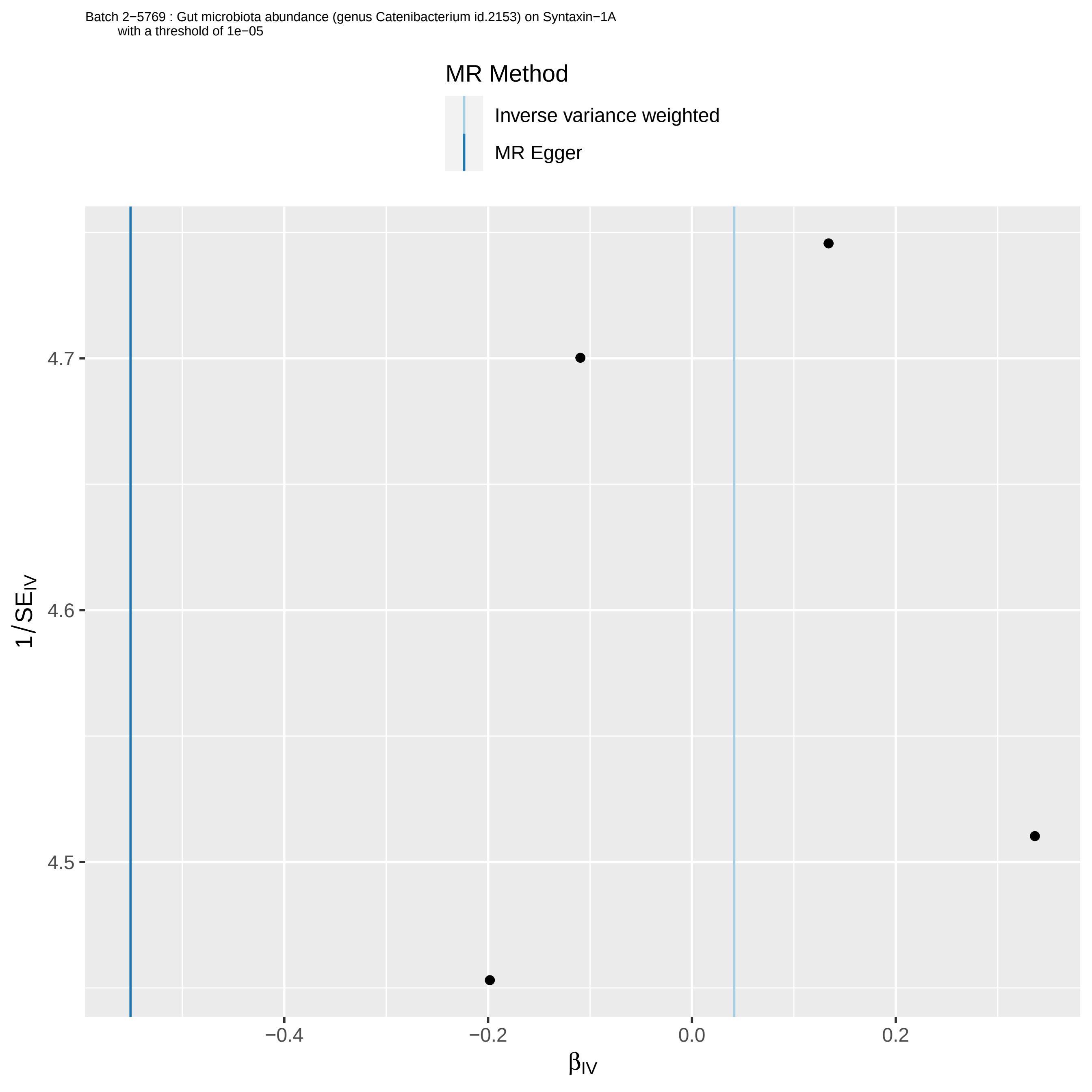
Batch 2−5769 : Gut microbiota abundance (genus Catenibacterium id.2153) on Syntaxin−1A
with a threshold of 1e−05
MR Method
Inverse variance weighted
MR Egger
4.7
4.6
4.5
1/SE$_{IV}$
−0.4
−0.2
0.0
0.2
β_{IV}

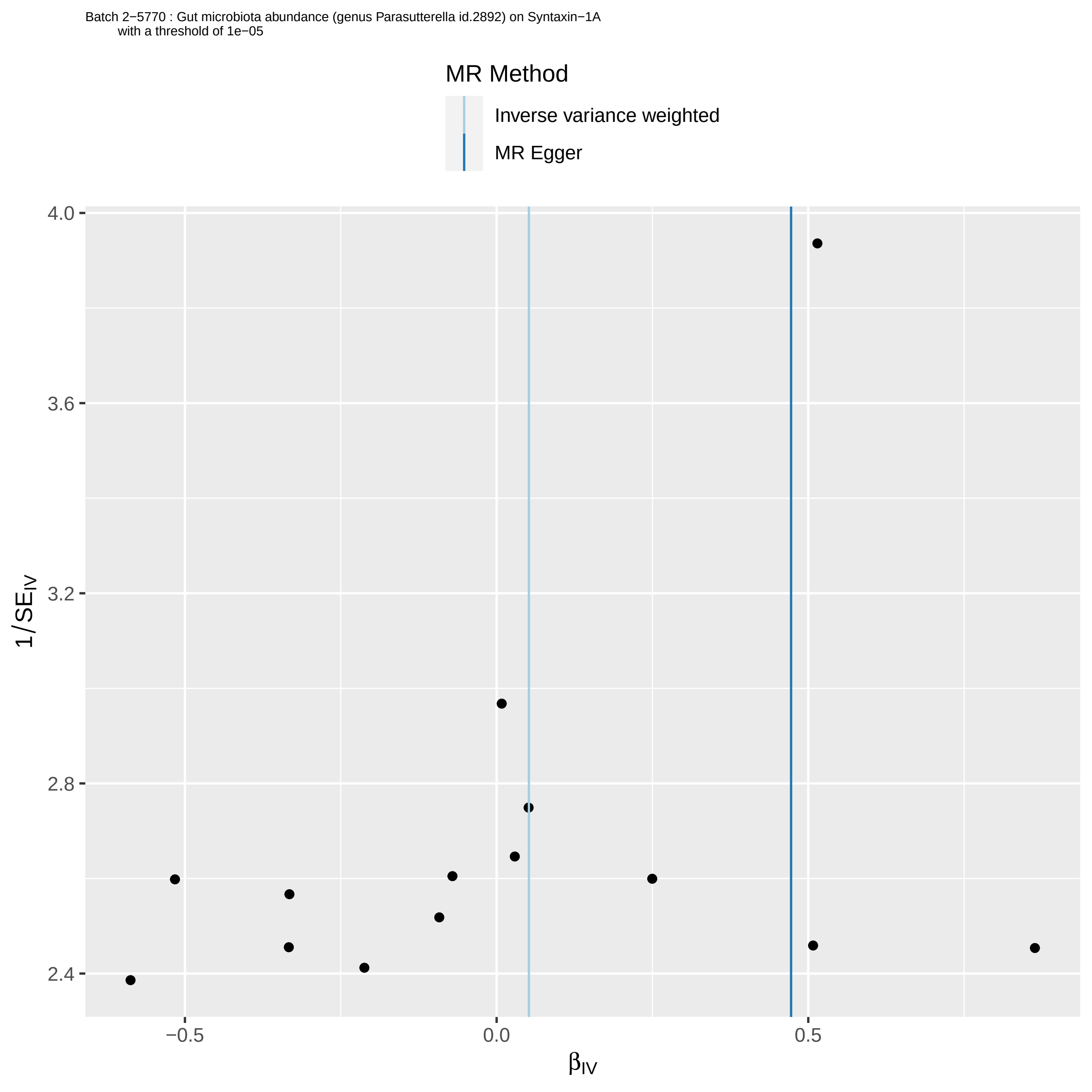
Batch 2−5770 : Gut microbiota abundance (genus Parasutterella id.2892) on Syntaxin−1A
with a threshold of 1e−05
MR Method
Inverse variance weighted
MR Egger
4.0
3.6
3.2
2.8
2.4
1/SE_IV
−0.5
0.0
0.5
β_IV

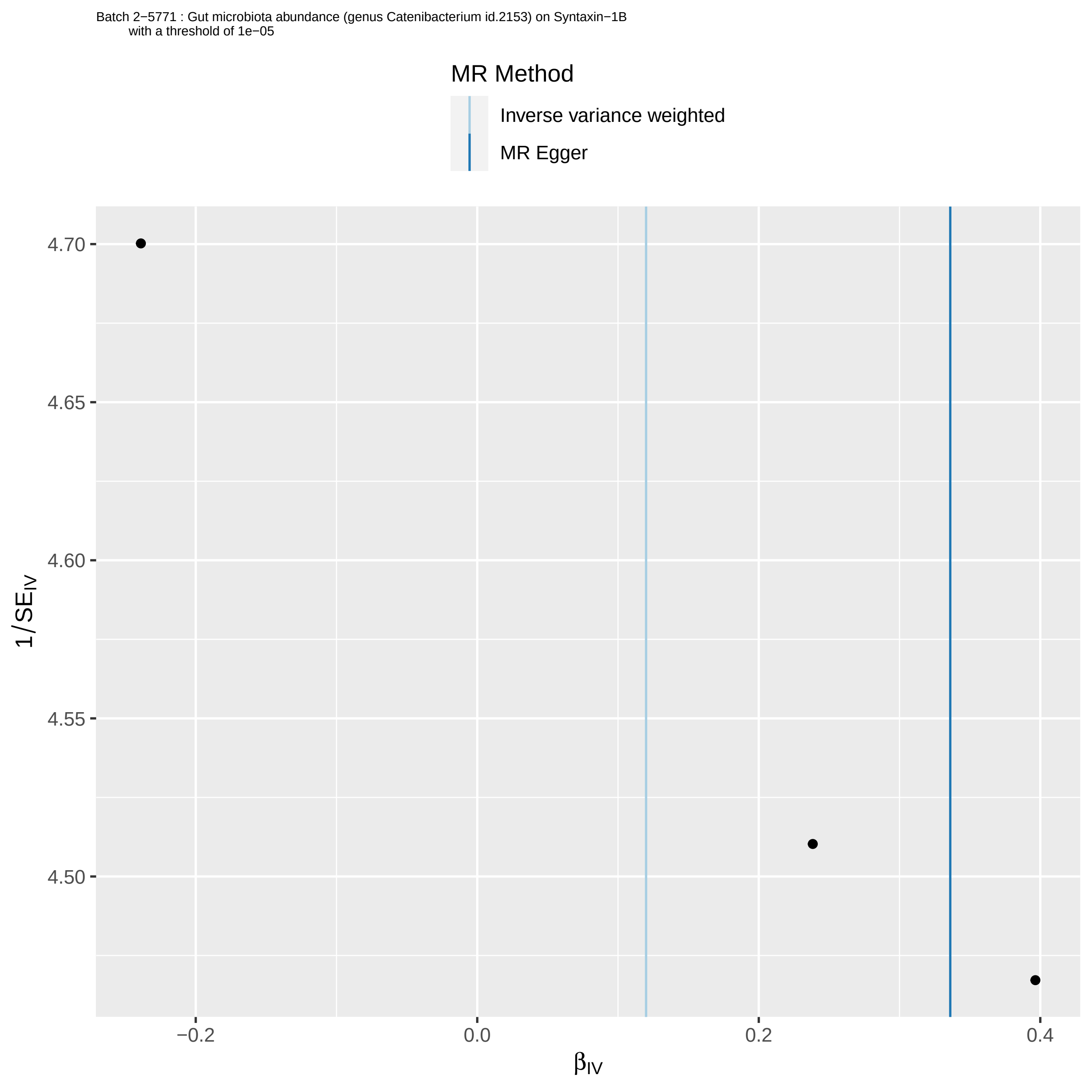

Batch 2−5771 : Gut microbiota abundance (genus Catenibacterium id.2153) on Syntaxin−1B
with a threshold of 1e−05
MR Method
Inverse variance weighted
MR Egger
4.70
4.65
4.60
4.55
4.50
1/SE_IV
−0.2
0.0
0.2
0.4
β_IV

Batch 2−5772 : Gut microbiota abundance (genus Parasutterella id.2892) on Syntaxin−1B
with a threshold of 1e−05

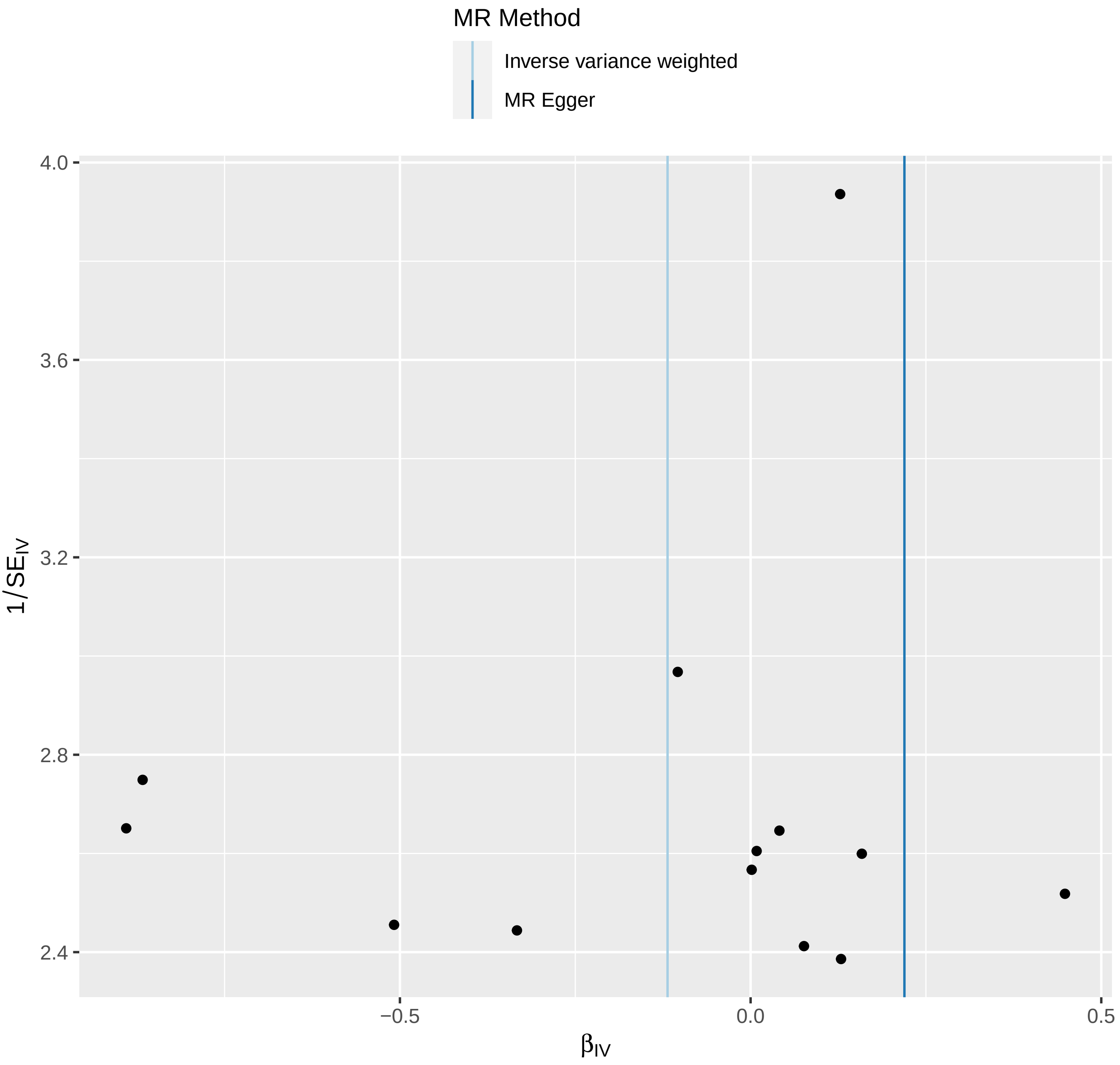

Batch 2−5773 : Gut microbiota abundance (genus Catenibacterium id.2153) on Syntaxin−2 with a threshold of 1e−05

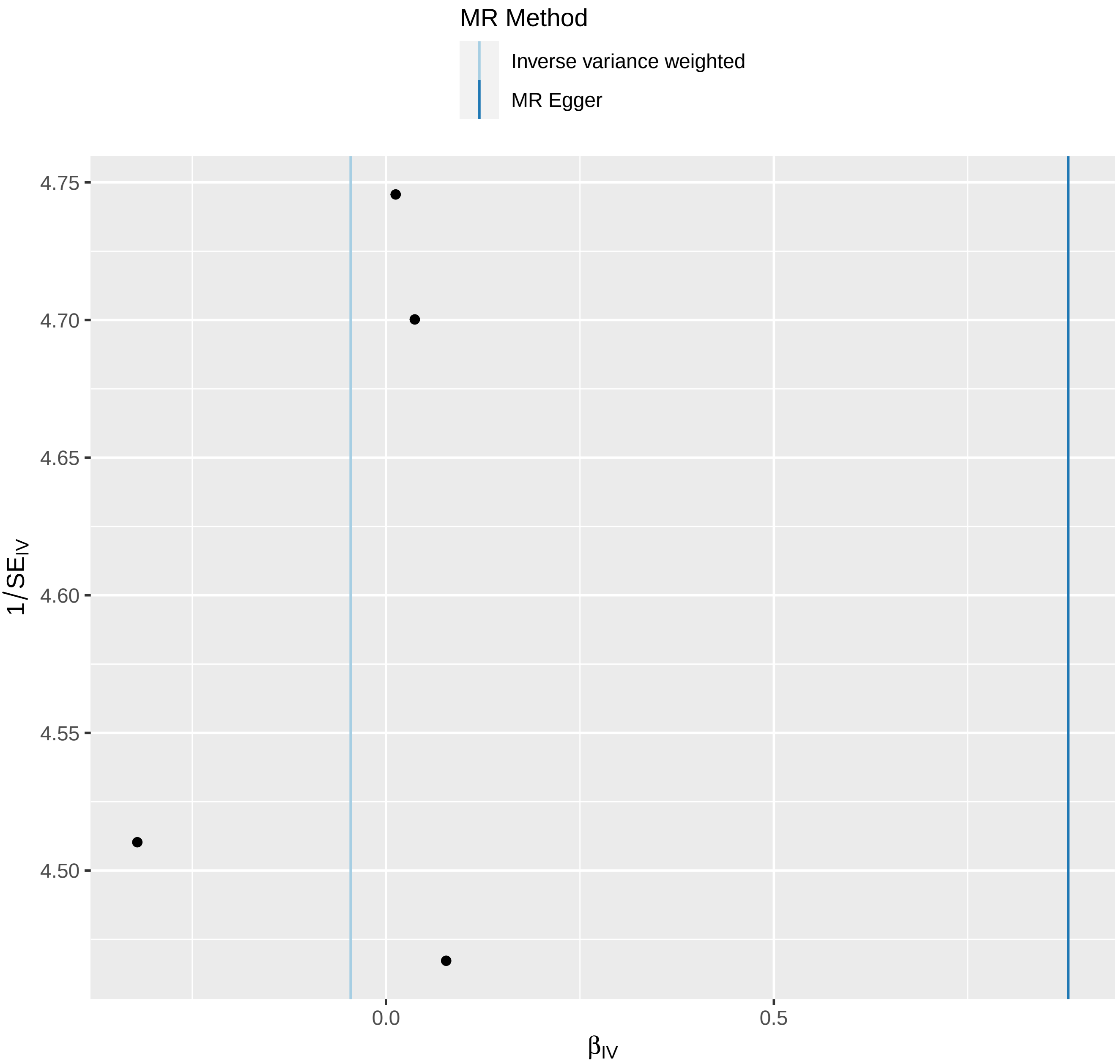

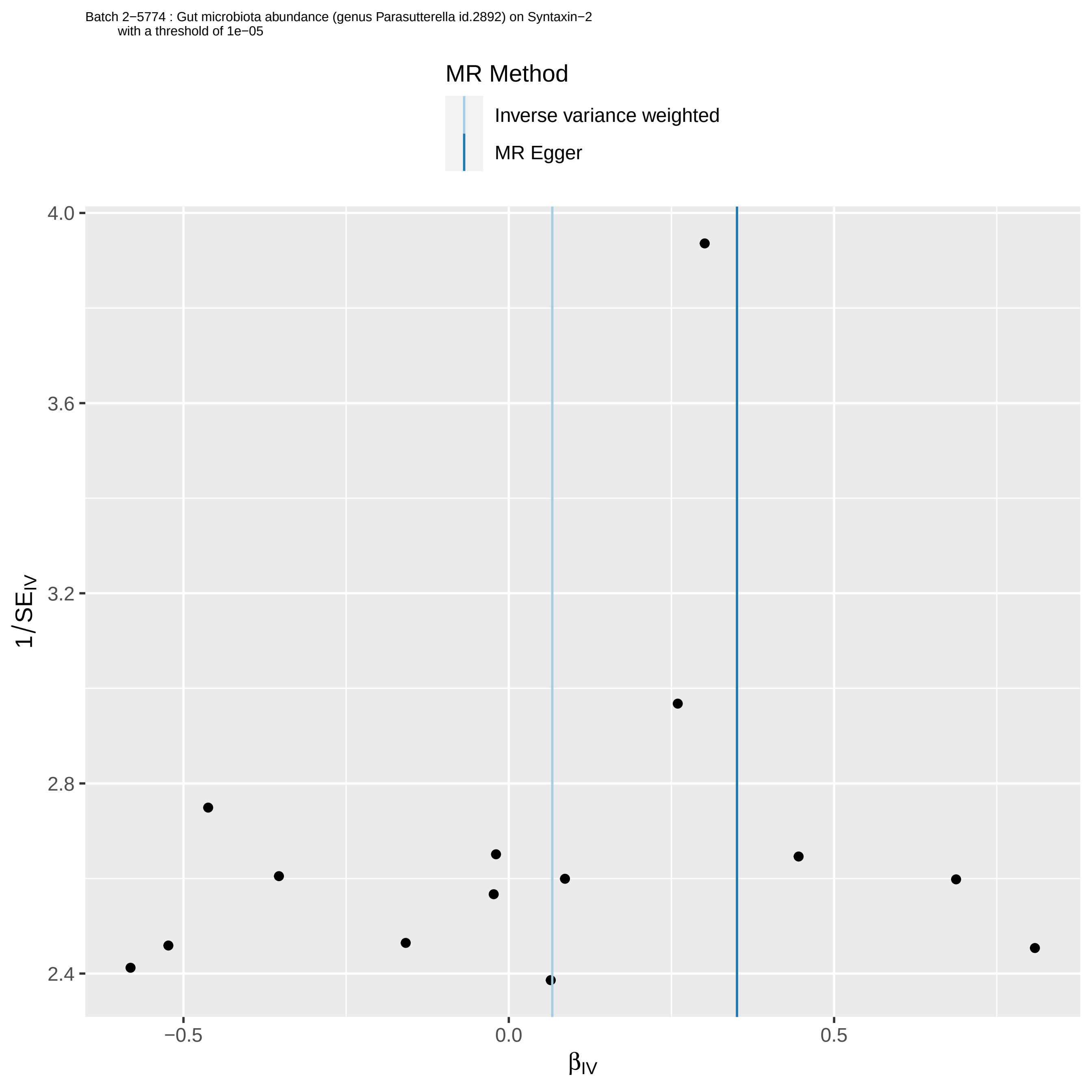

Batch 2−5774 : Gut microbiota abundance (genus Parasutterella id.2892) on Syntaxin−2
with a threshold of 1e−05
MR Method
Inverse variance weighted
MR Egger
4.0
3.6
3.2
2.8
2.4
1/SE_IV
−0.5
0.0
0.5
β_IV

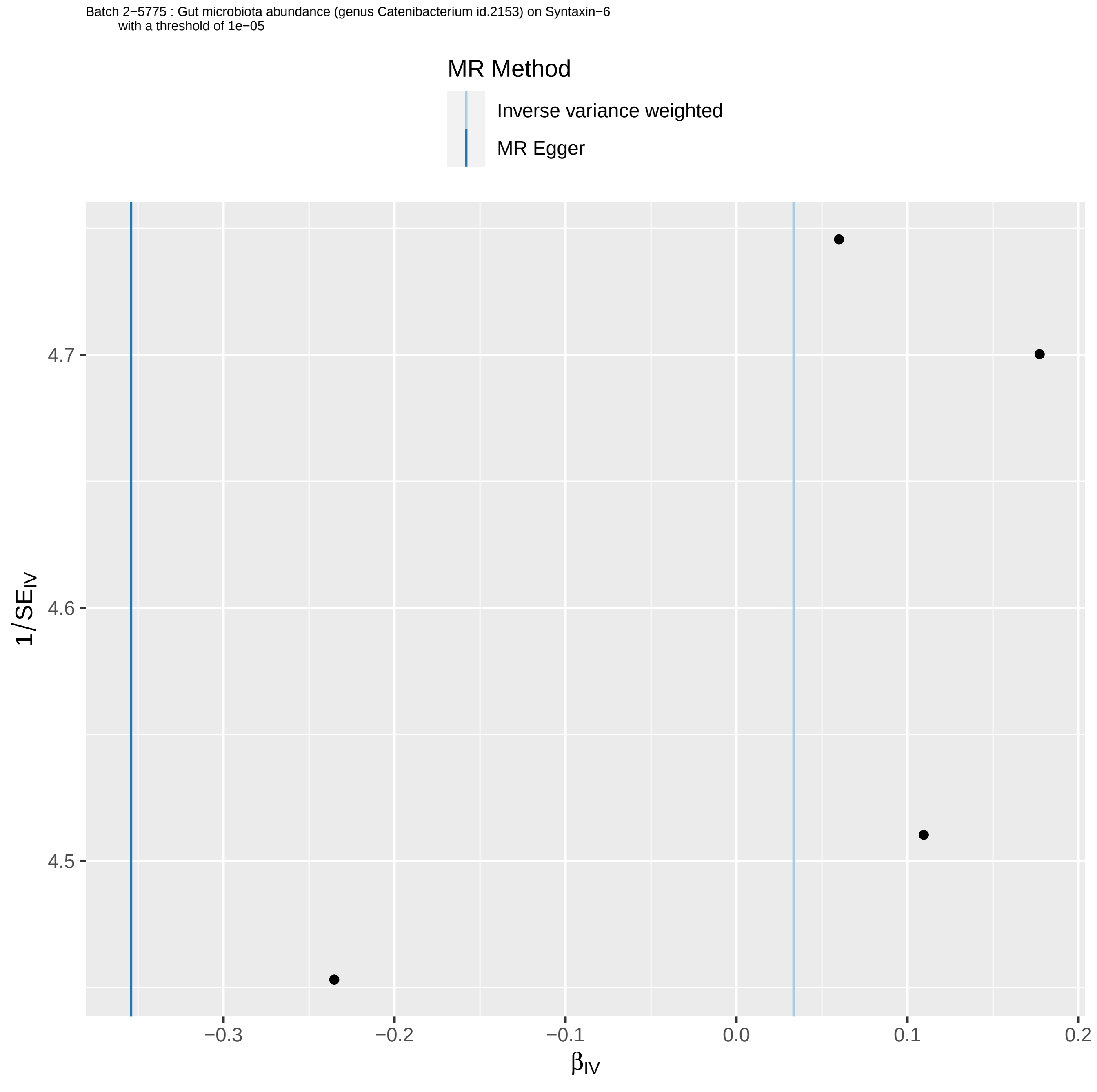
Batch 2−5775 : Gut microbiota abundance (genus Catenibacterium id.2153) on Syntaxin−6
with a threshold of 1e−05
MR Method
Inverse variance weighted
MR Egger
4.7
4.6
4.5
1/SE$_{IV}$
−0.3
−0.2
−0.1
0.0
0.1
0.2
β$_{IV}$

Batch 2−5776 : Gut microbiota abundance (genus Parasutterella id.2892) on Syntaxin−6
with a threshold of 1e−05

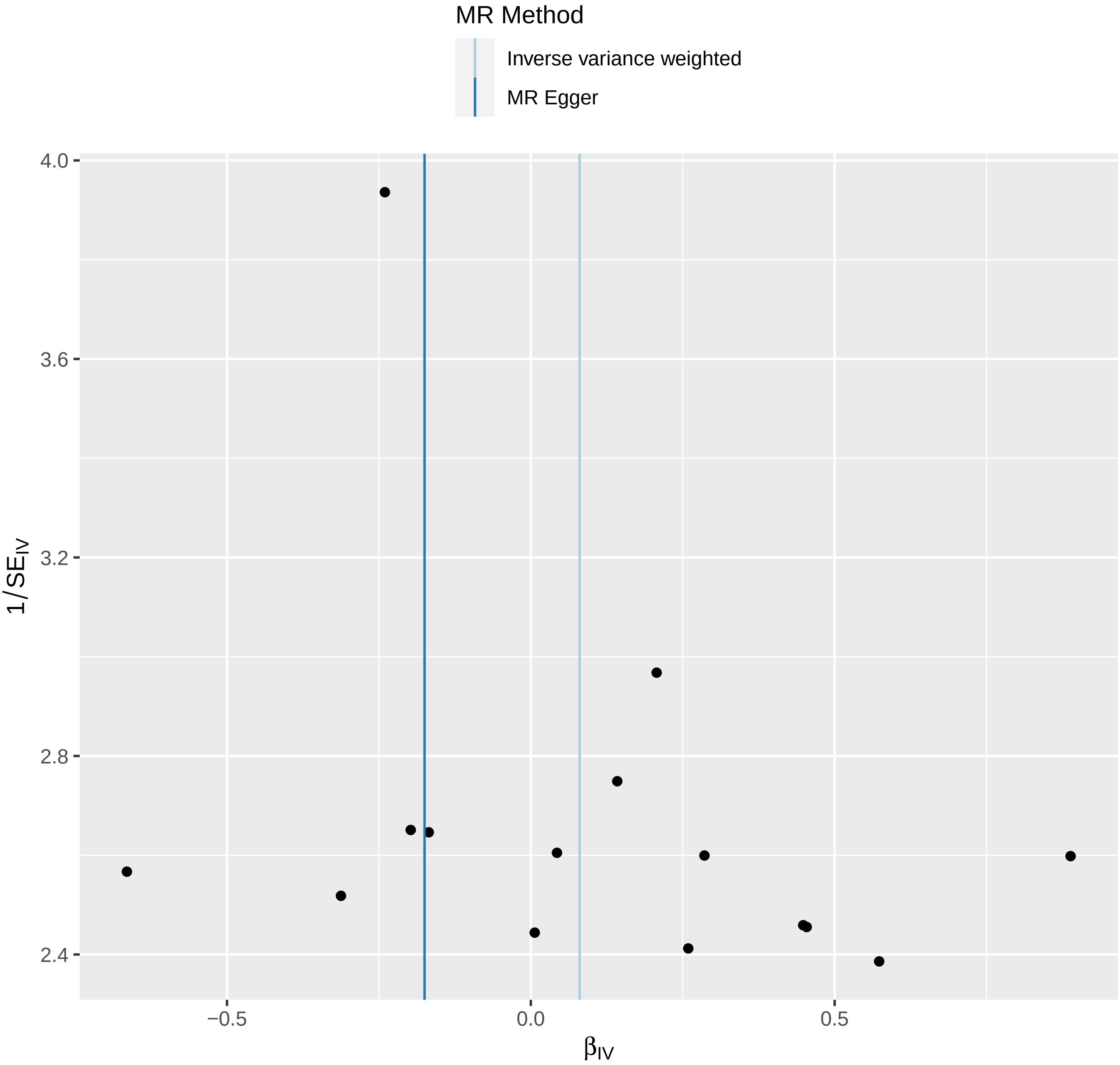

Batch 2−5777 : Gut microbiota abundance (genus Catenibacterium id.2153) on Syntaxin−7
with a threshold of 1e−05

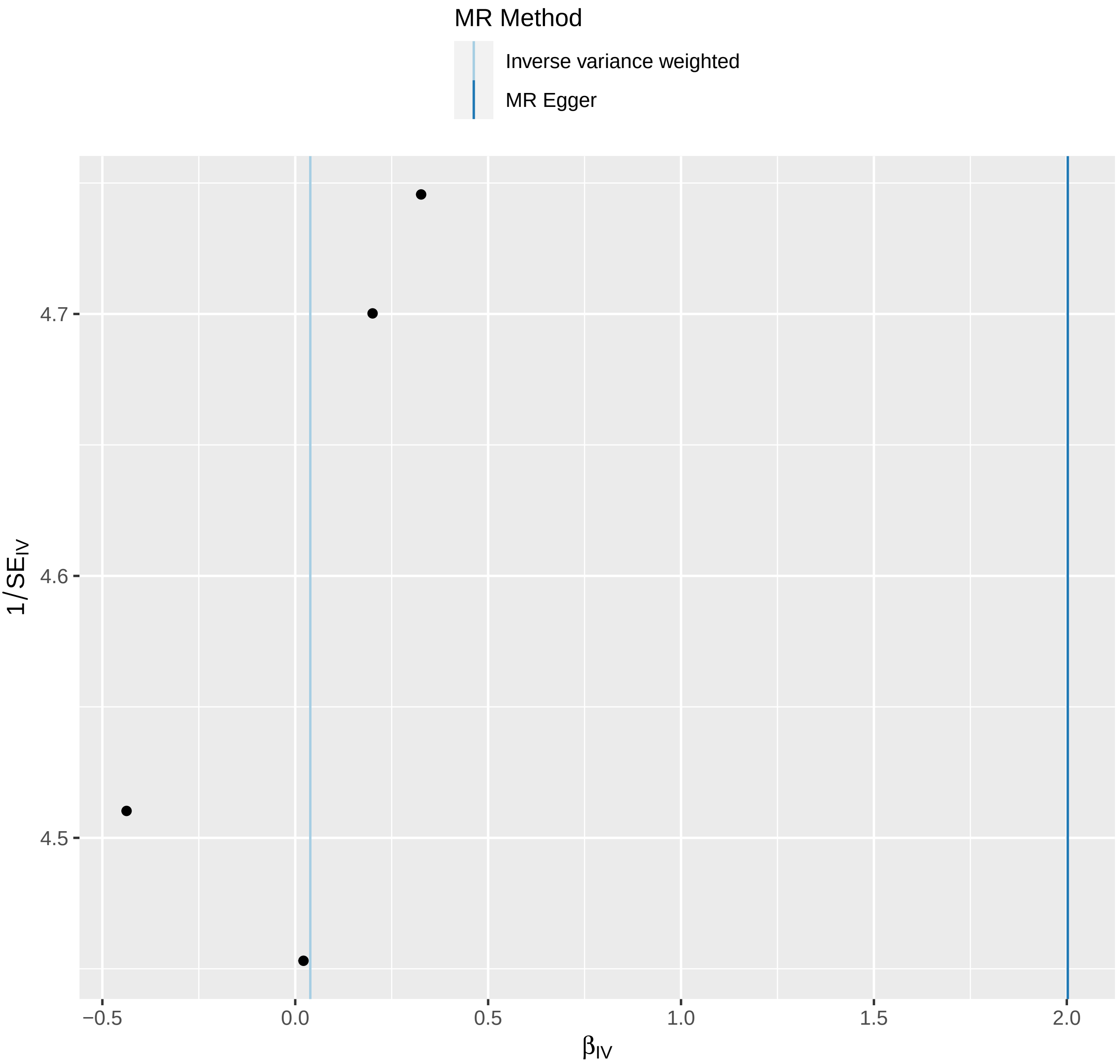

Batch 2−5778 : Gut microbiota abundance (genus Parasutterella id.2892) on Syntaxin−7
with a threshold of 1e−05

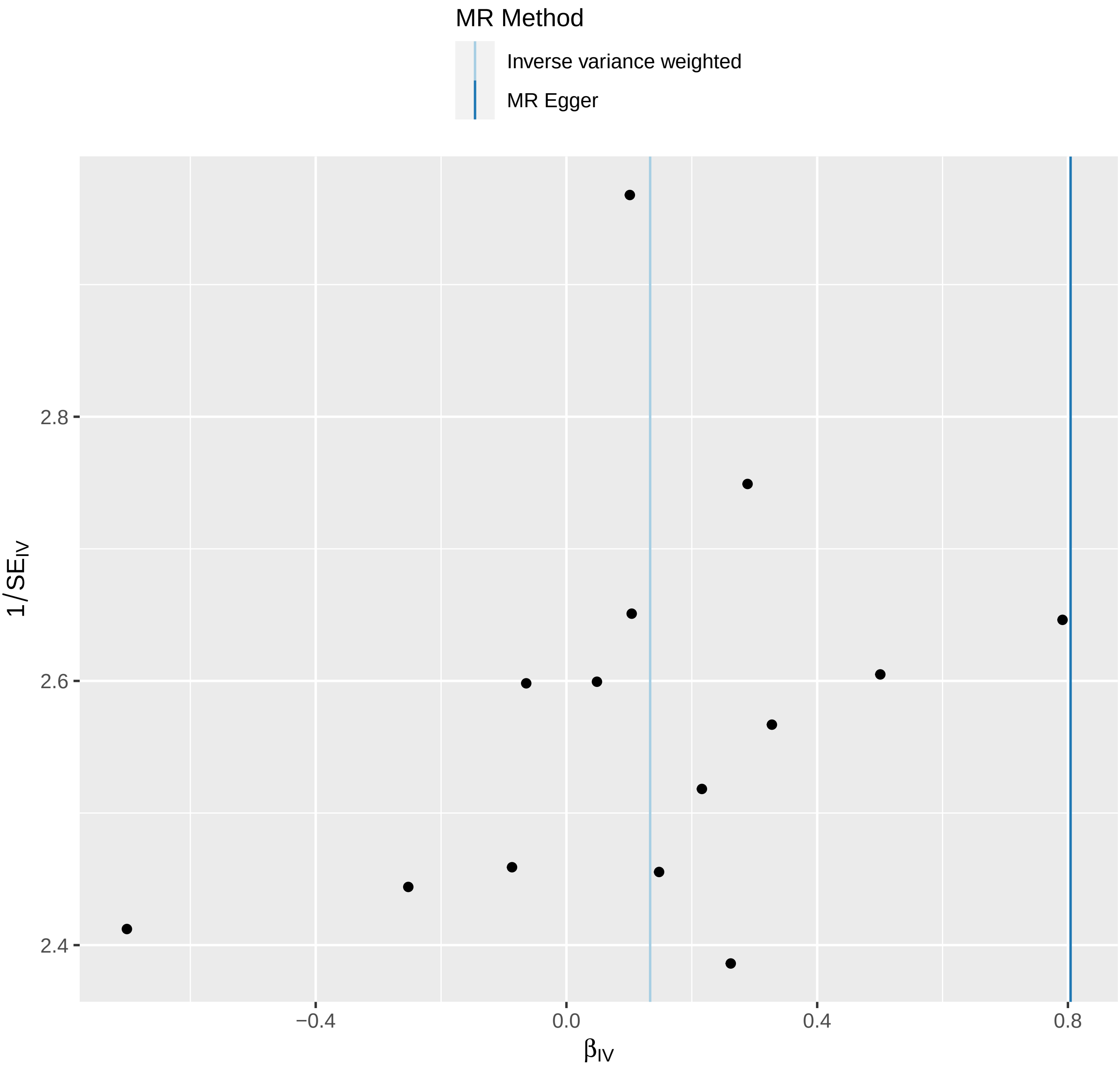

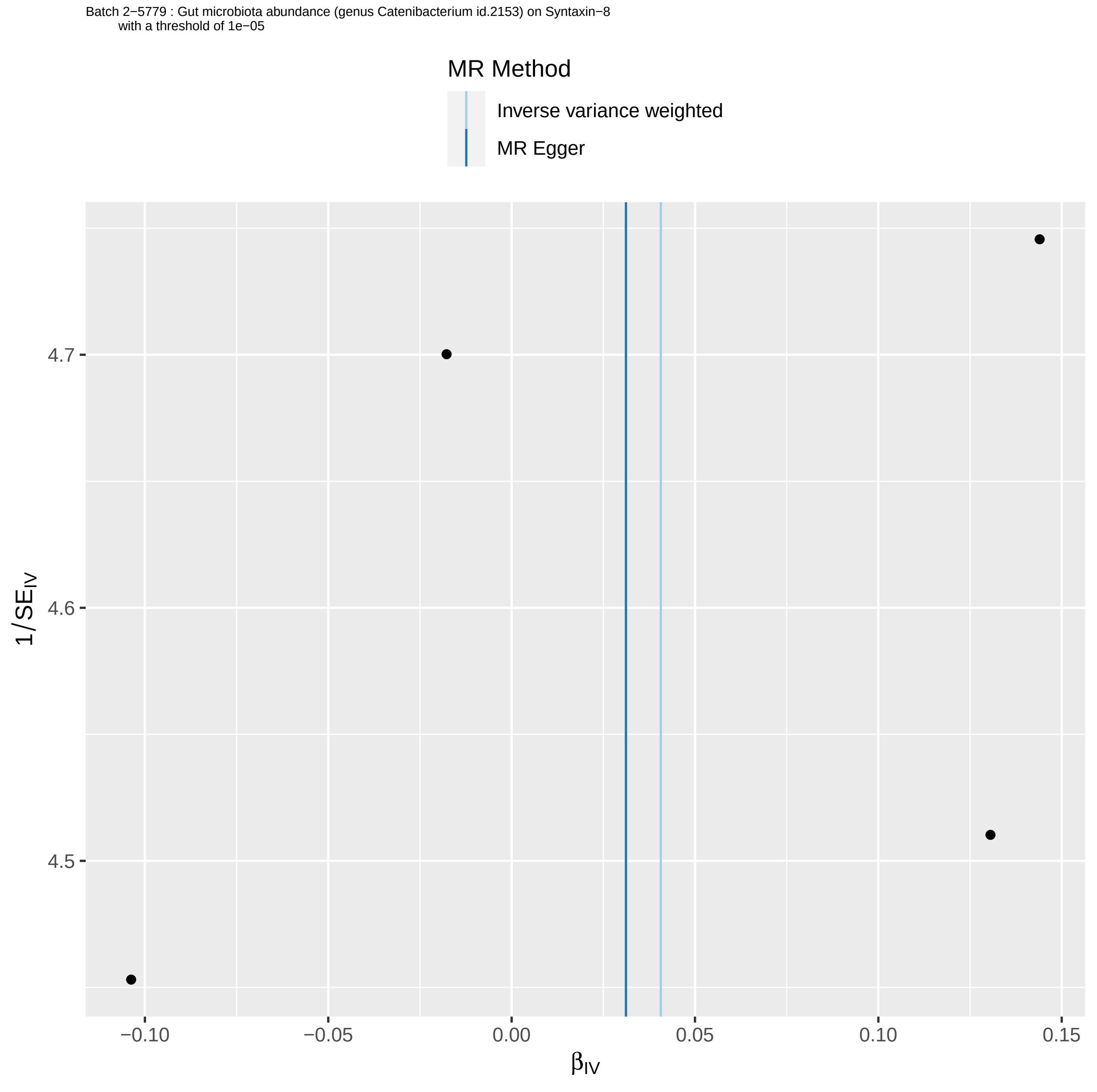
Batch 2−5779 : Gut microbiota abundance (genus Catenibacterium id.2153) on Syntaxin−8
with a threshold of 1e−05
MR Method
Inverse variance weighted
MR Egger
4.7
4.6
4.5
1/SE_IV
−0.10
−0.05
0.00
0.05
0.10
0.15
β_IV

Batch 2−5780 : Gut microbiota abundance (genus Parasutterella id.2892) on Syntaxin−8
with a threshold of 1e−05

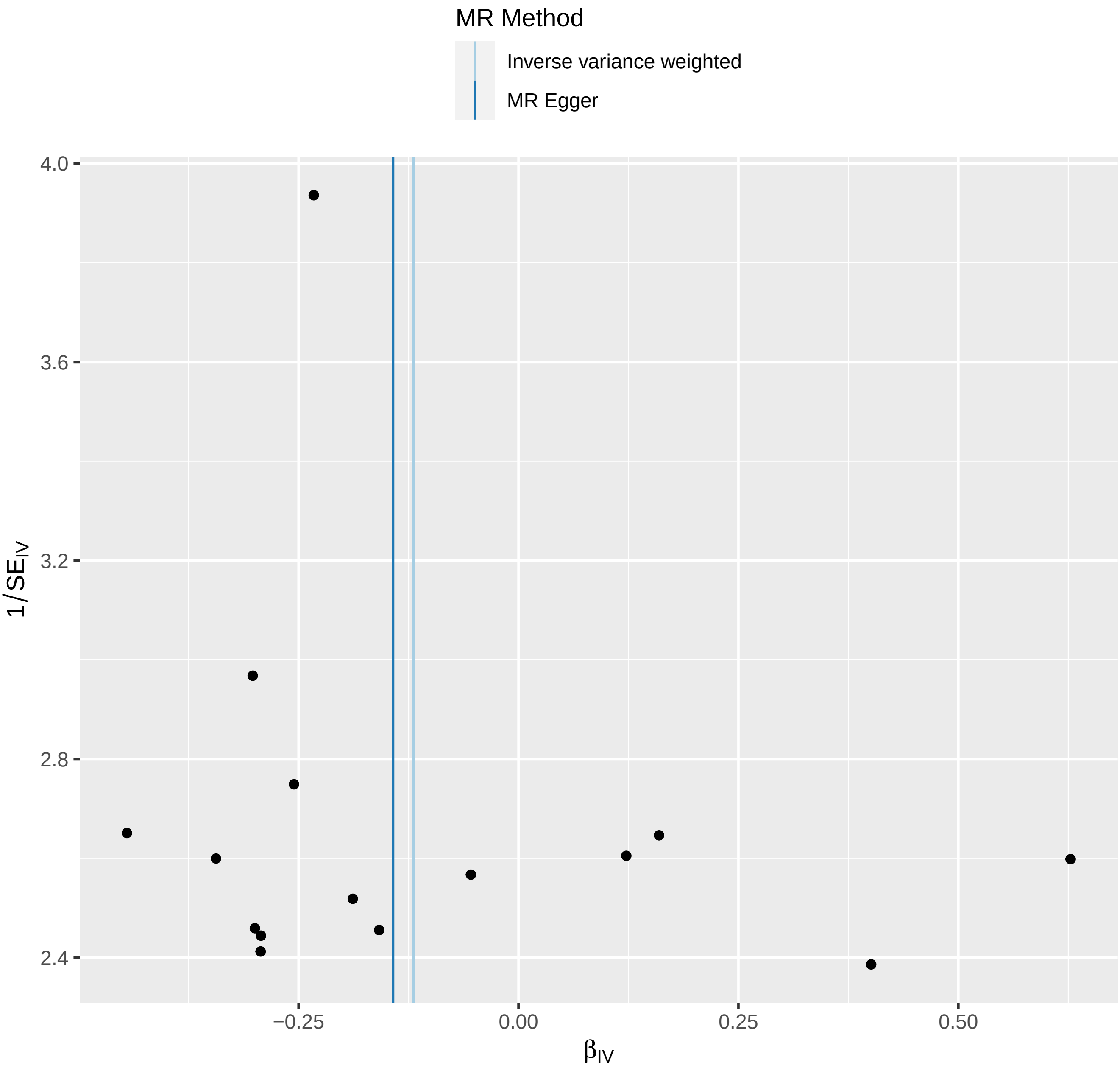

Batch 2−5781 : Gut microbiota abundance (genus Catenibacterium id.2153) on Extracellular sulfatase Sulf−2
with a threshold of 1e−05

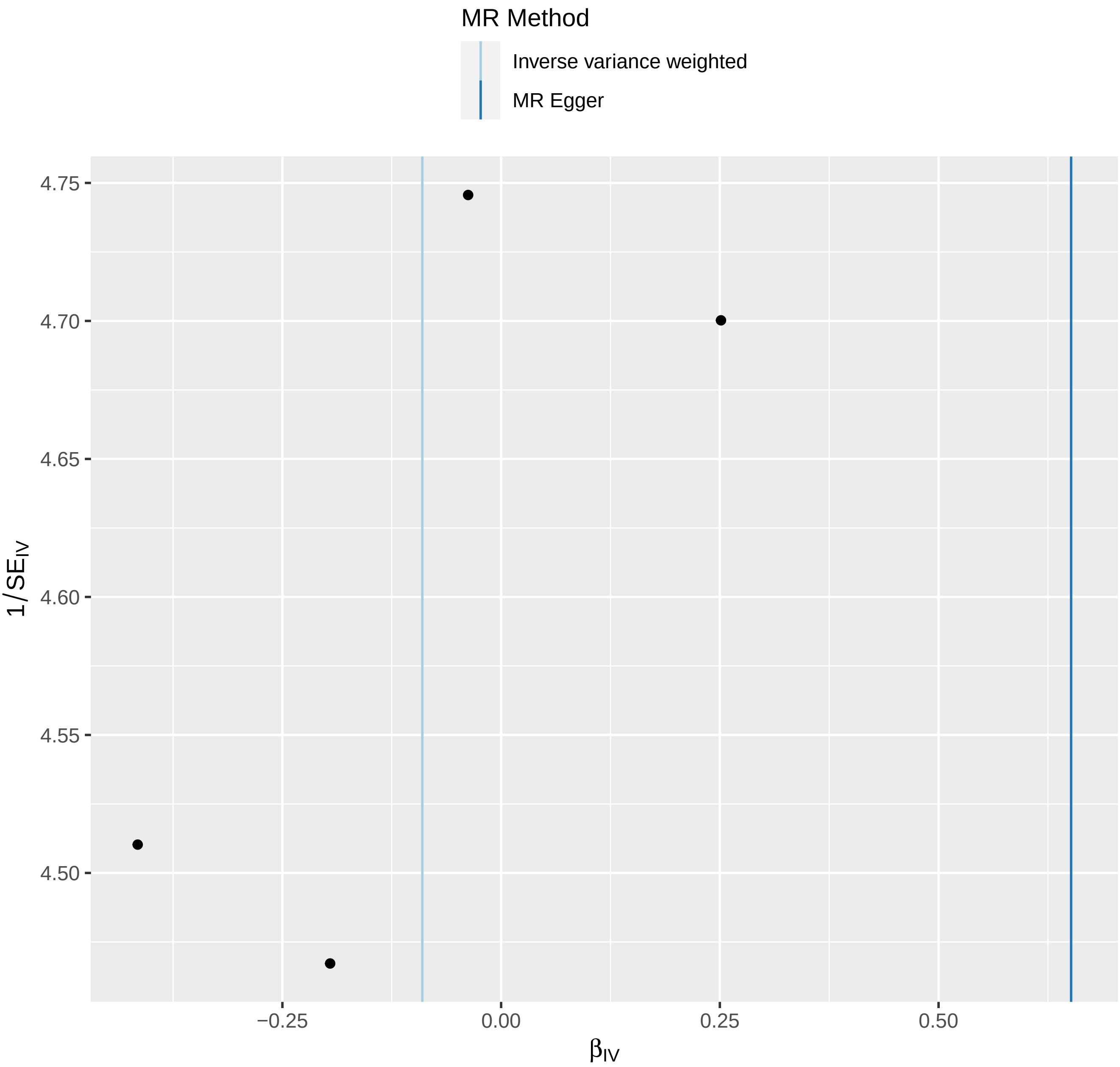

Batch 2−5782 : Gut microbiota abundance (genus Parasutterella id.2892) on Extracellular sulfatase Sulf−2
with a threshold of 1e−05

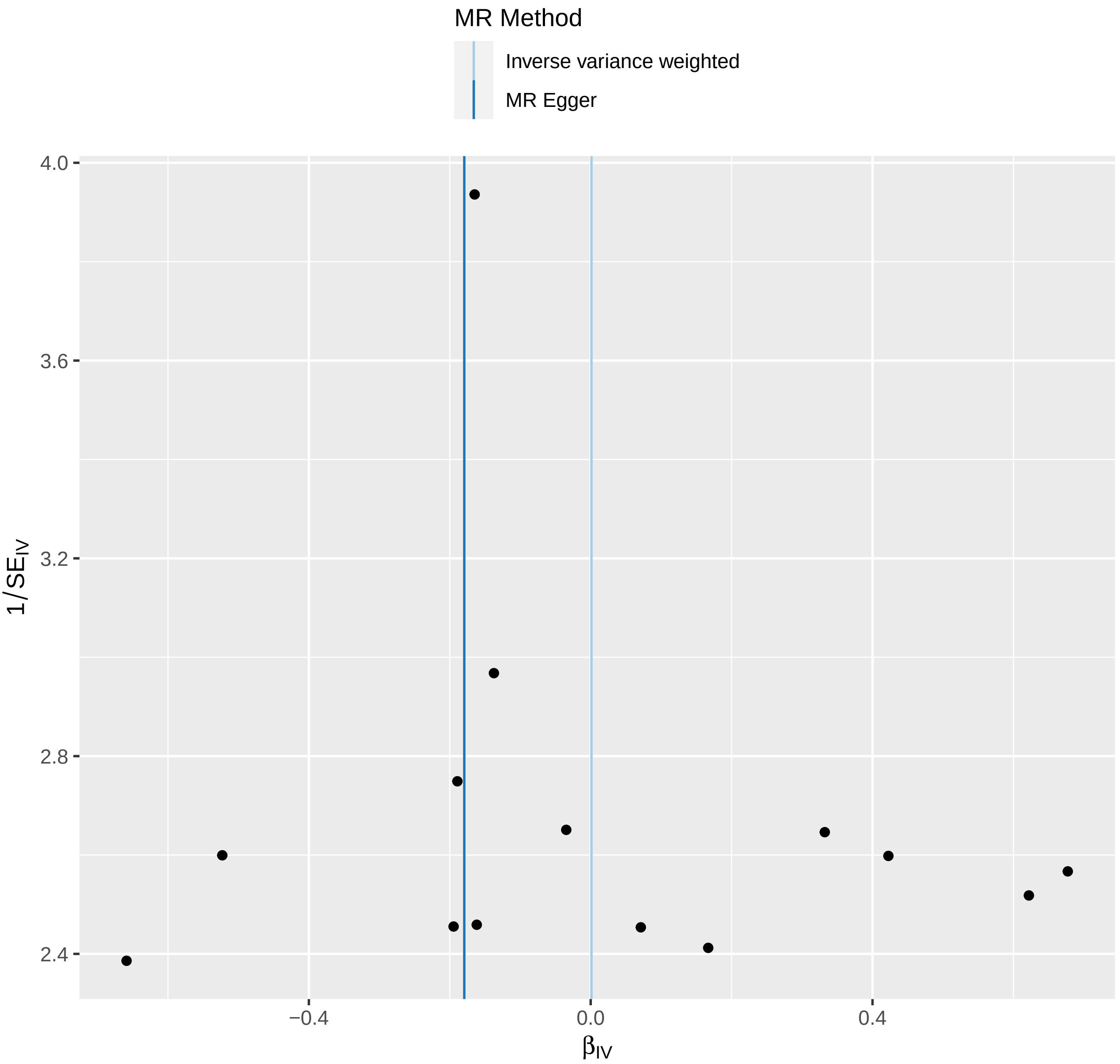

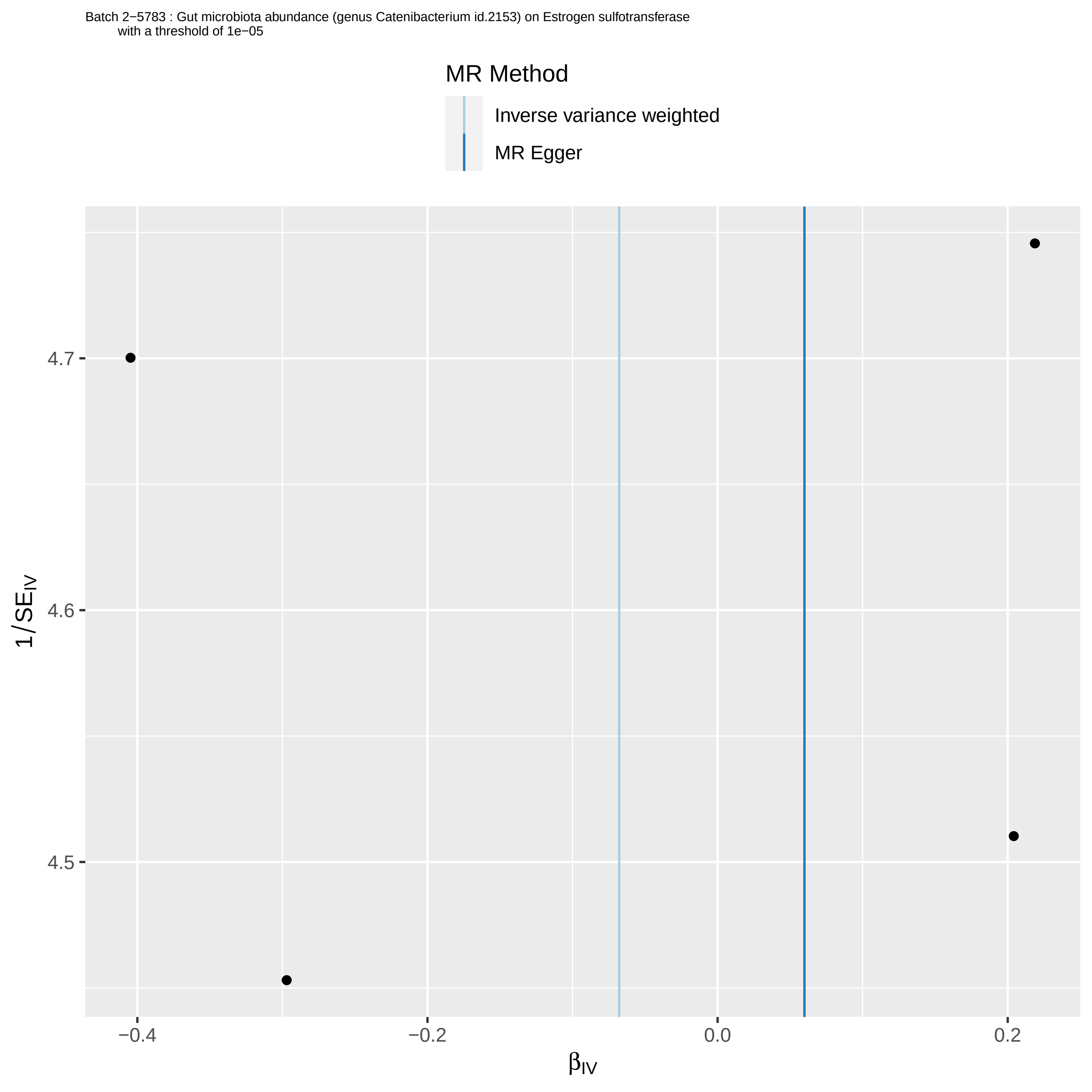

Batch 2−5783 : Gut microbiota abundance (genus Catenibacterium id.2153) on Estrogen sulfotransferase
with a threshold of 1e−05
MR Method
Inverse variance weighted
MR Egger
4.7
4.6
4.5
1/SE_IV
−0.4
−0.2
0.0
0.2
β_IV

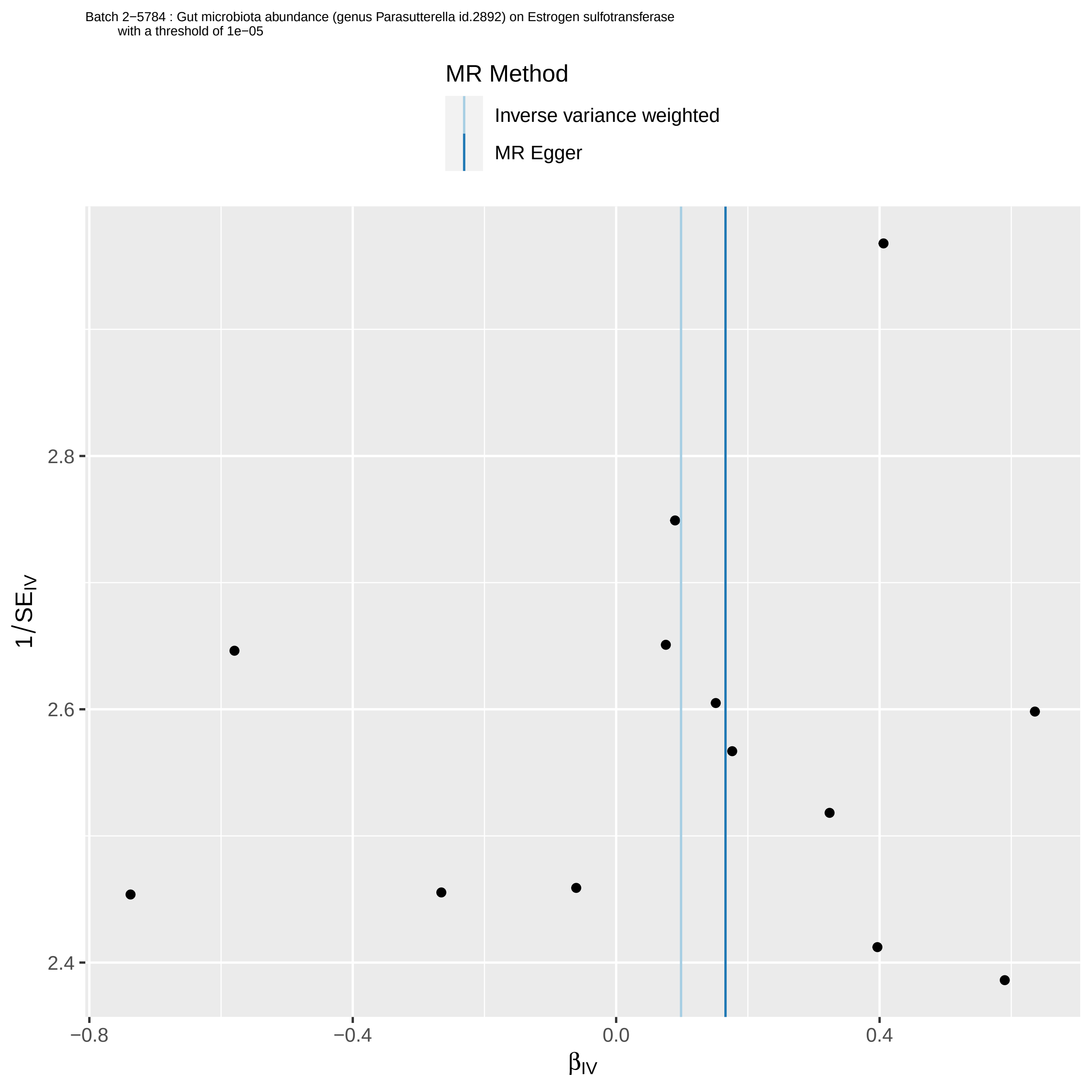
Batch 2−5784 : Gut microbiota abundance (genus Parasutterella id.2892) on Estrogen sulfotransferase
with a threshold of 1e−05
MR Method
Inverse variance weighted
MR Egger
2.8
2.6
2.4
1/SE_IV
−0.8
−0.4
0.0
0.4
β_IV

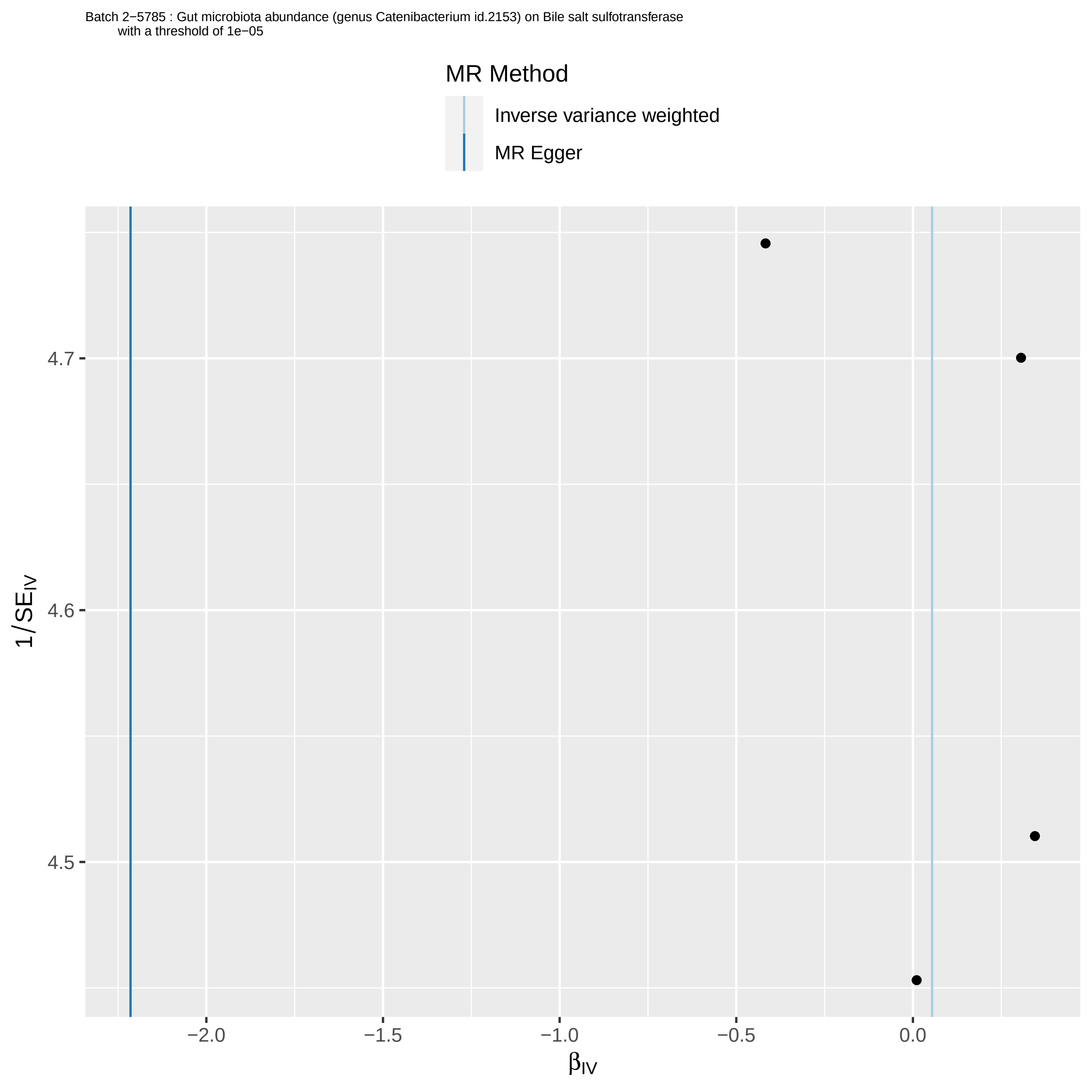

Batch 2−5785 : Gut microbiota abundance (genus Catenibacterium id.2153) on Bile salt sulfotransferase
with a threshold of 1e−05
MR Method
Inverse variance weighted
MR Egger
4.7
4.6
4.5
1/SE_IV
−2.0
−1.5
−1.0
−0.5
0.0
β_IV

Batch 2−5786 : Gut microbiota abundance (genus Parasutterella id.2892) on Bile salt sulfotransferase
with a threshold of 1e−05

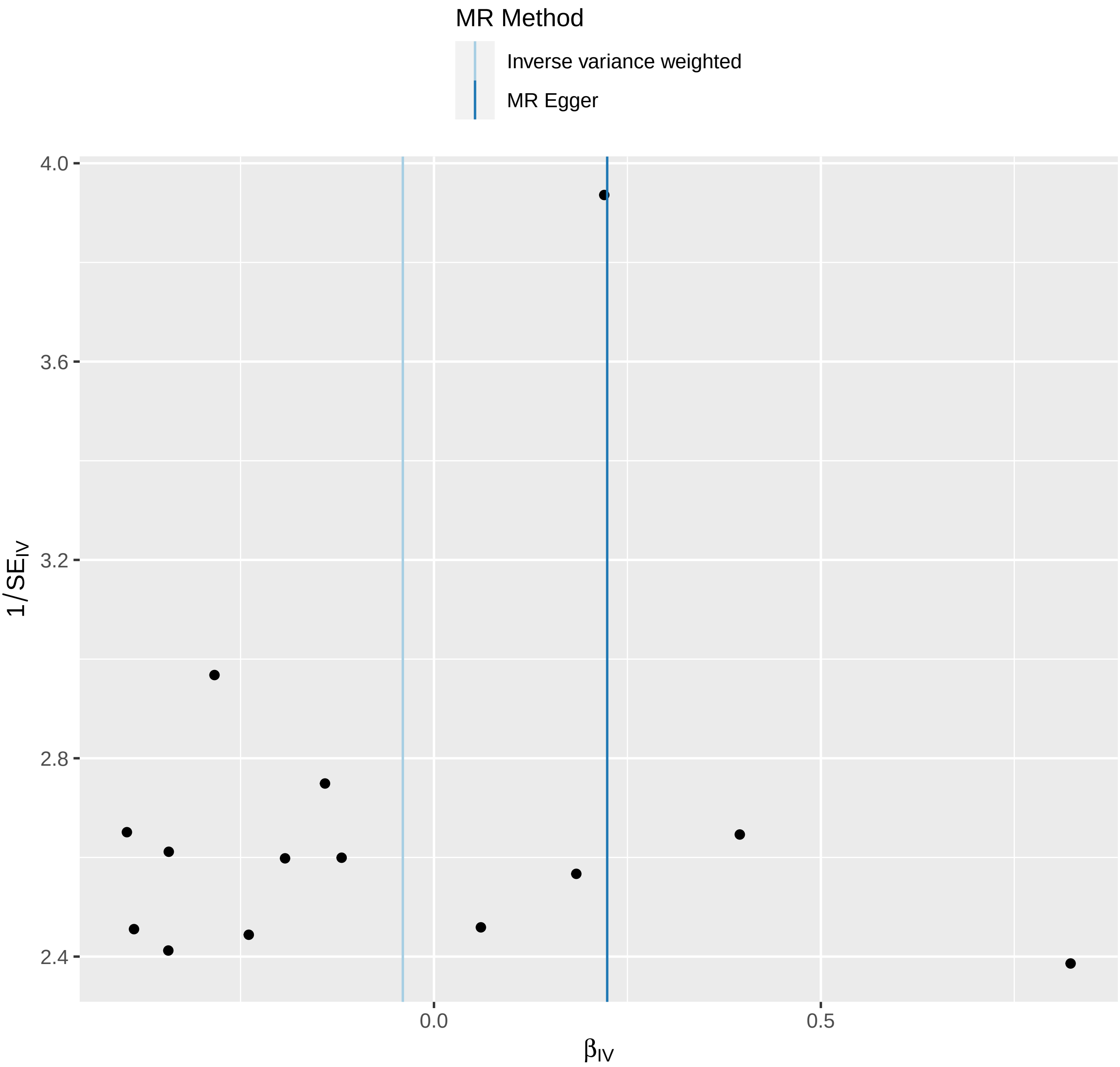

Batch 2−5787 : Gut microbiota abundance (genus Catenibacterium id.2153) on Sulfotransferase 4A1
with a threshold of 1e−05

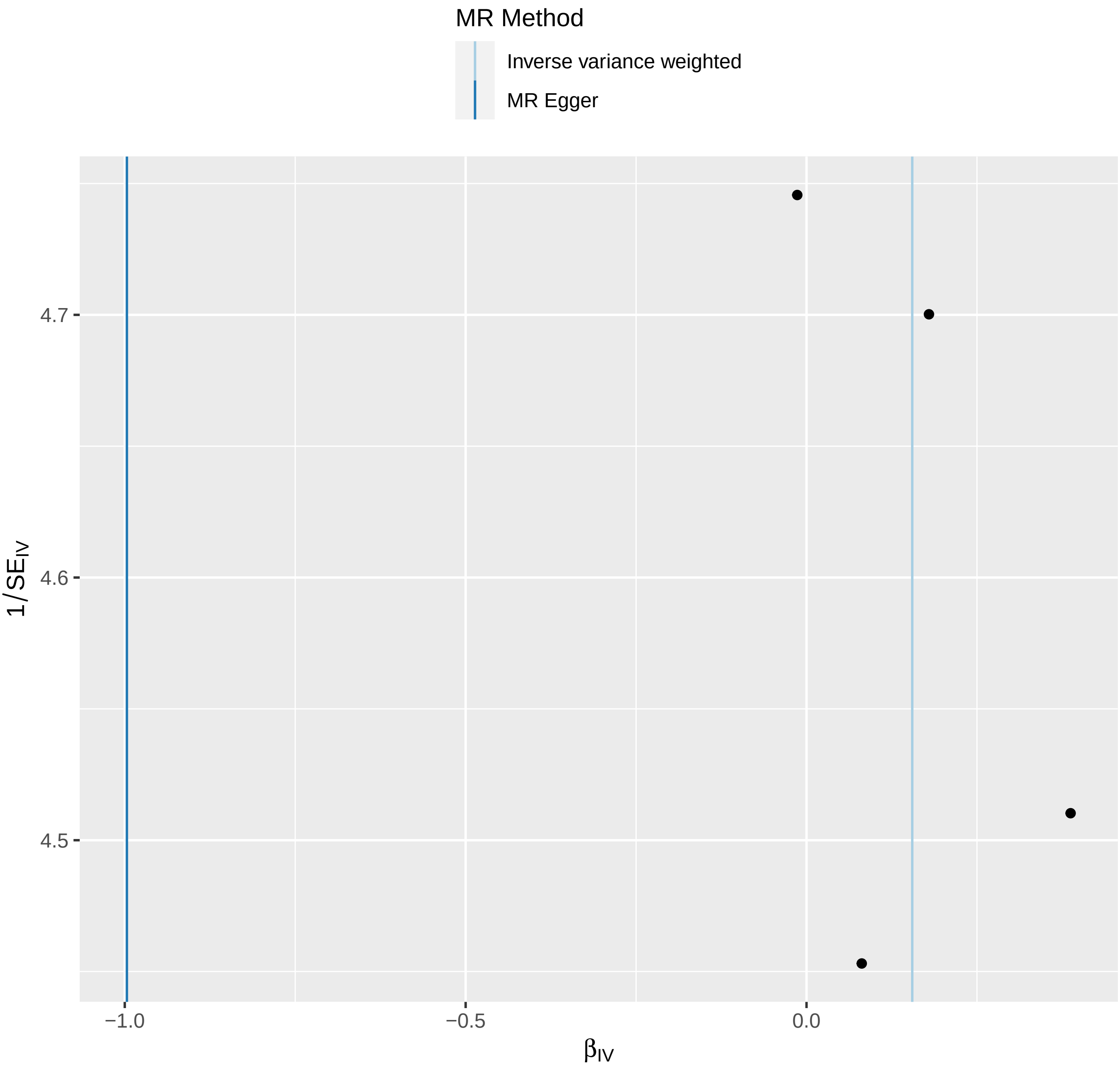

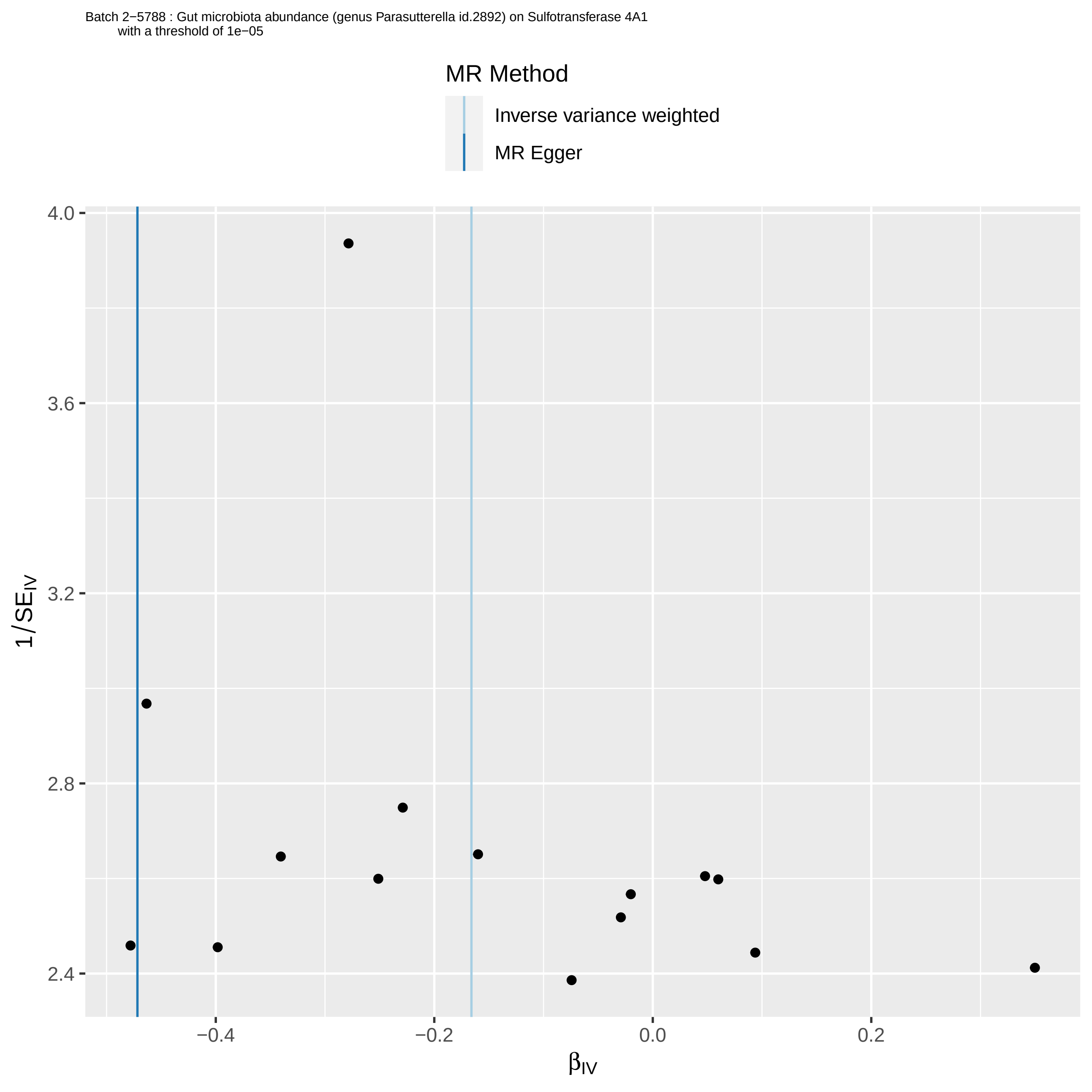

Batch 2−5788 : Gut microbiota abundance (genus Parasutterella id.2892) on Sulfotransferase 4A1
with a threshold of 1e−05
MR Method
Inverse variance weighted
MR Egger
4.0
3.6
3.2
2.8
2.4
1/SE_IV
−0.4
−0.2
0.0
0.2
β_IV

Batch 2−5789 : Gut microbiota abundance (genus Catenibacterium id.2153) on Sulfatase−modifying factor 1
with a threshold of 1e−05

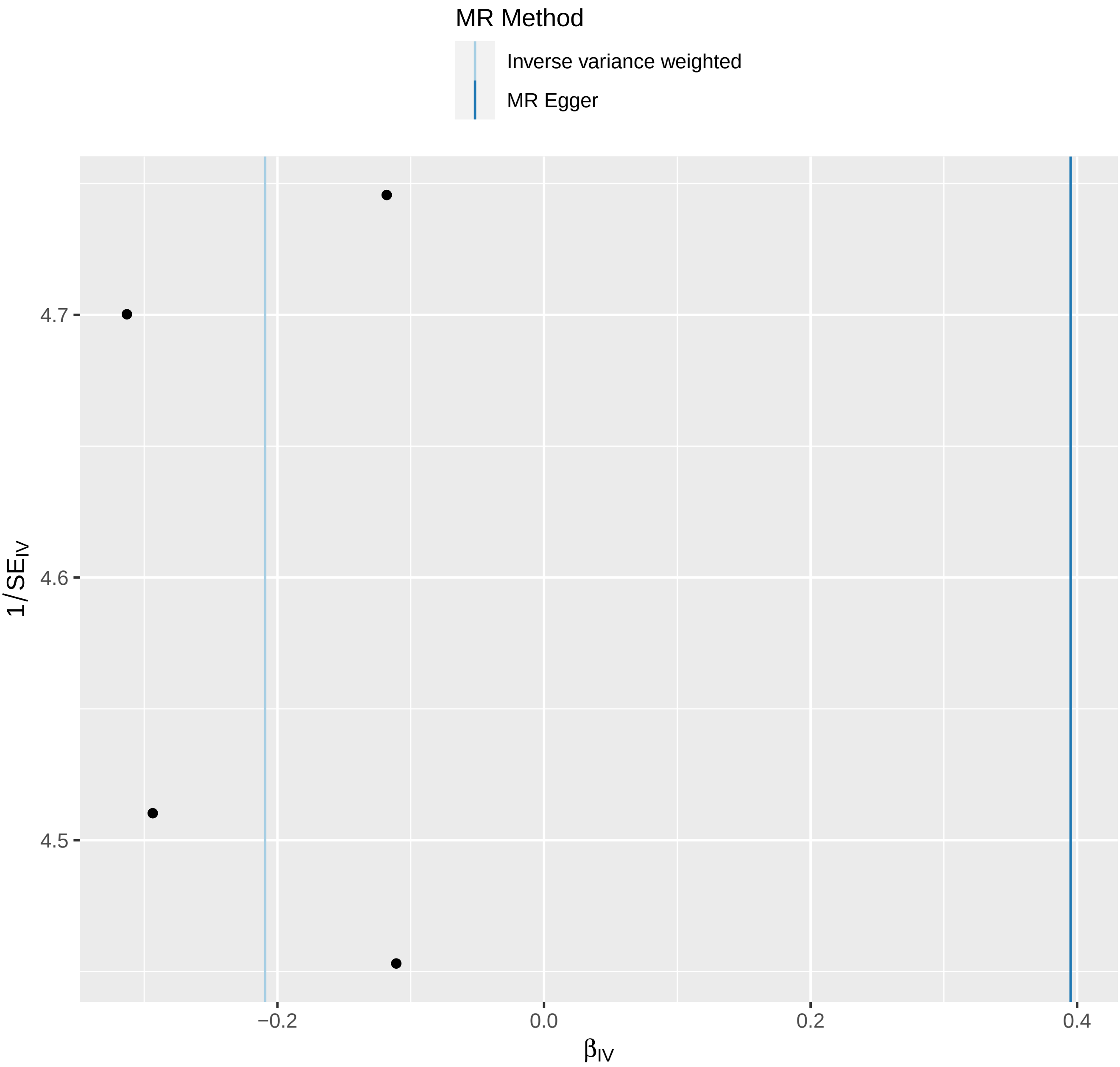

Batch 2−5790 : Gut microbiota abundance (genus Parasutterella id.2892) on Sulfatase−modifying factor 1
with a threshold of 1e−05

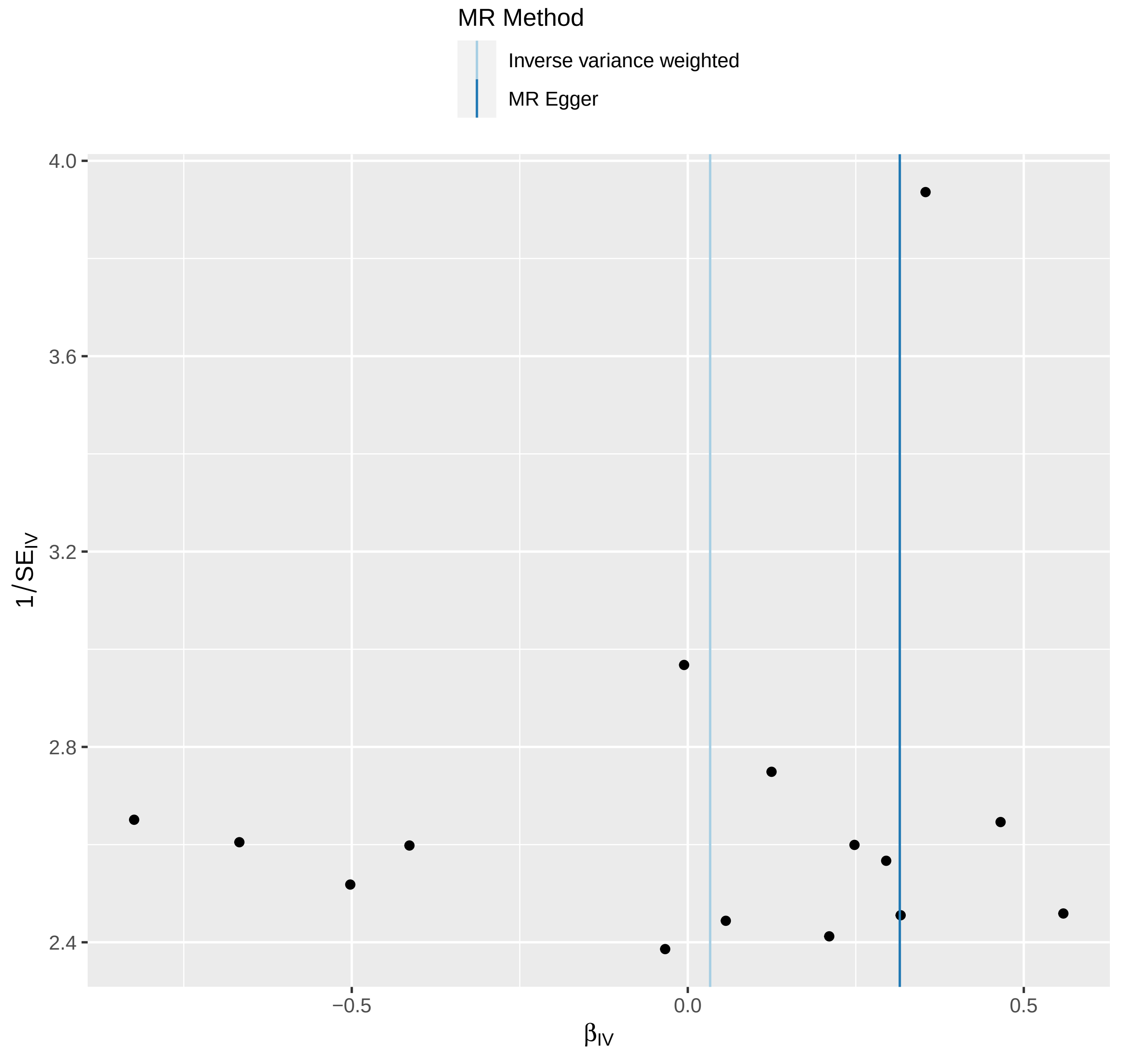

Batch 2−5791 : Gut microbiota abundance (genus Catenibacterium id.2153) on Small ubiquitin−related modifier 3
with a threshold of 1e−05

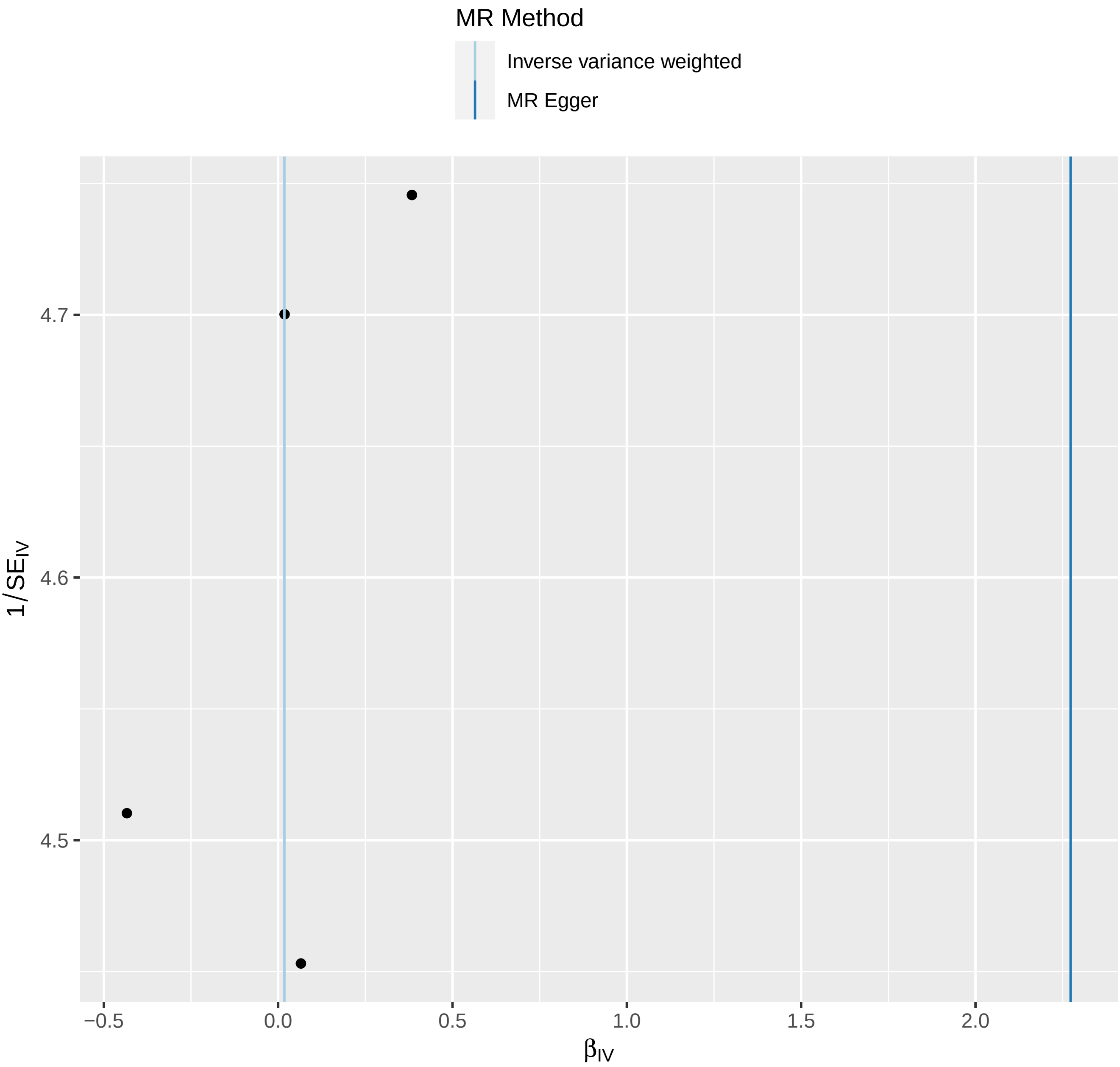

Batch 2−5792 : Gut microbiota abundance (genus Parasutterella id.2892) on Small ubiquitin−related modifier 3 with a threshold of 1e−05

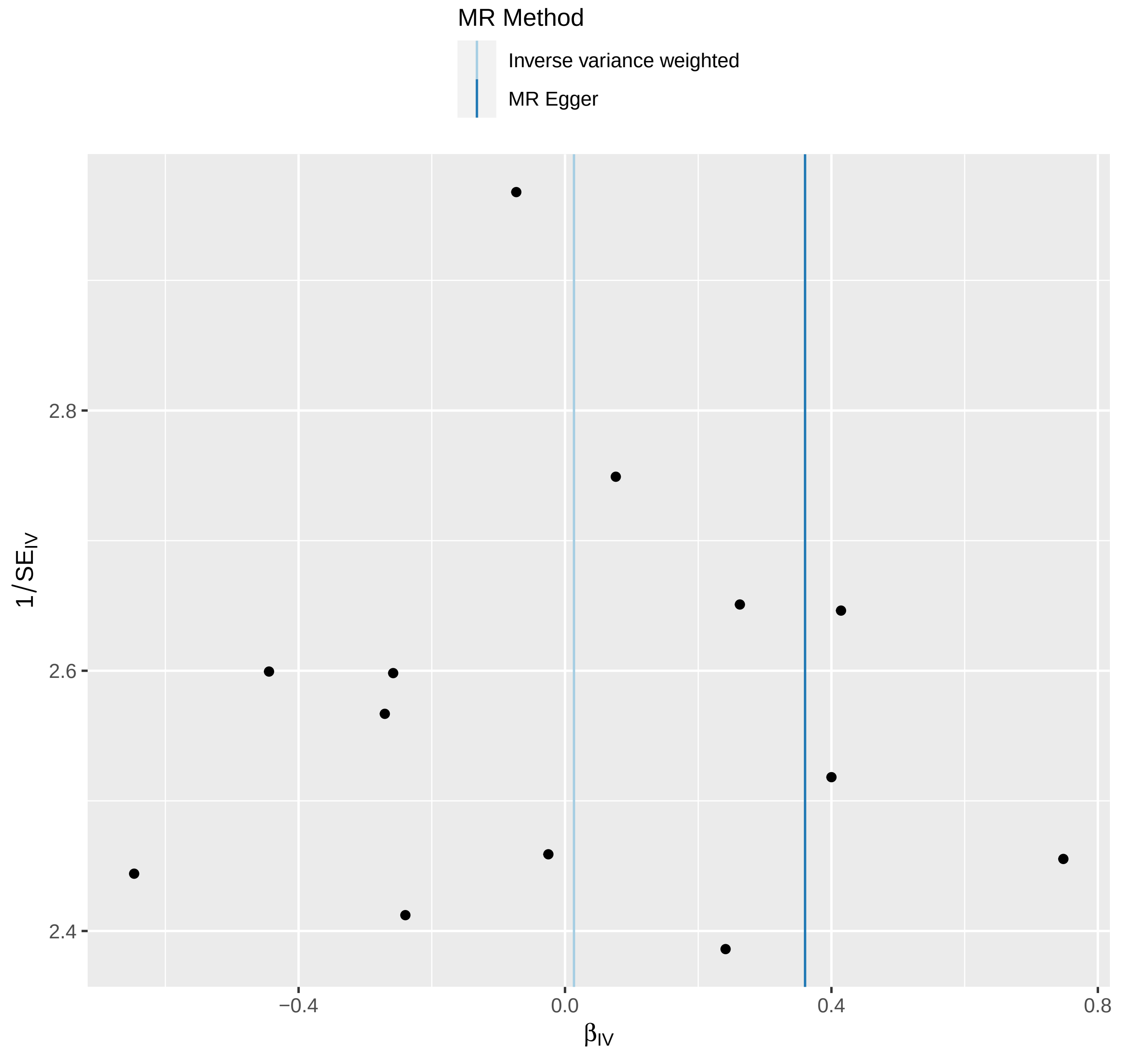

Batch 2−5793 : Gut microbiota abundance (genus Catenibacterium id.2153) on Small ubiquitin−related modifier 3
with a threshold of 1e−05

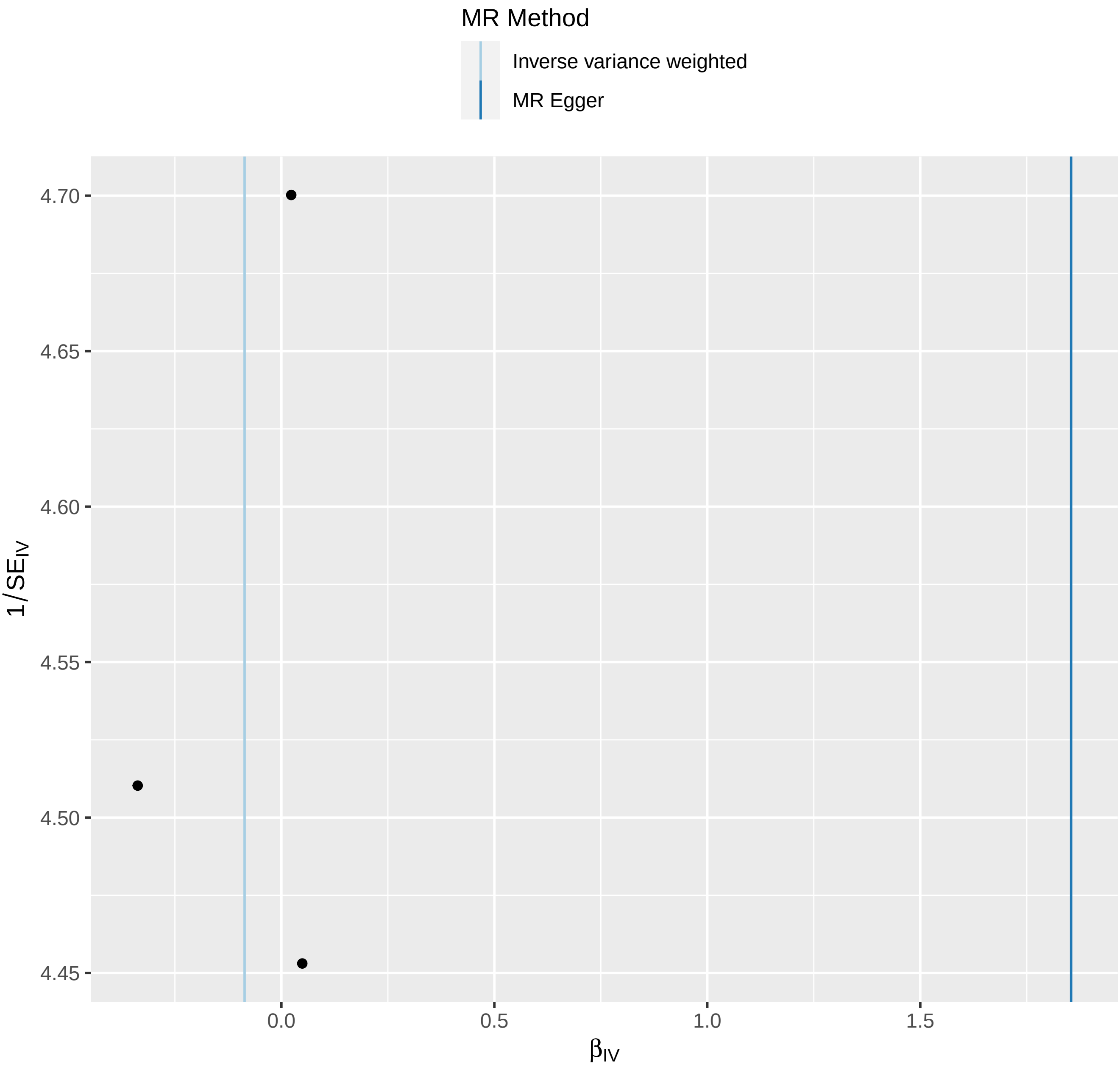

Batch 2−5794 : Gut microbiota abundance (genus Parasutterella id.2892) on Small ubiquitin−related modifier 3
with a threshold of 1e−05

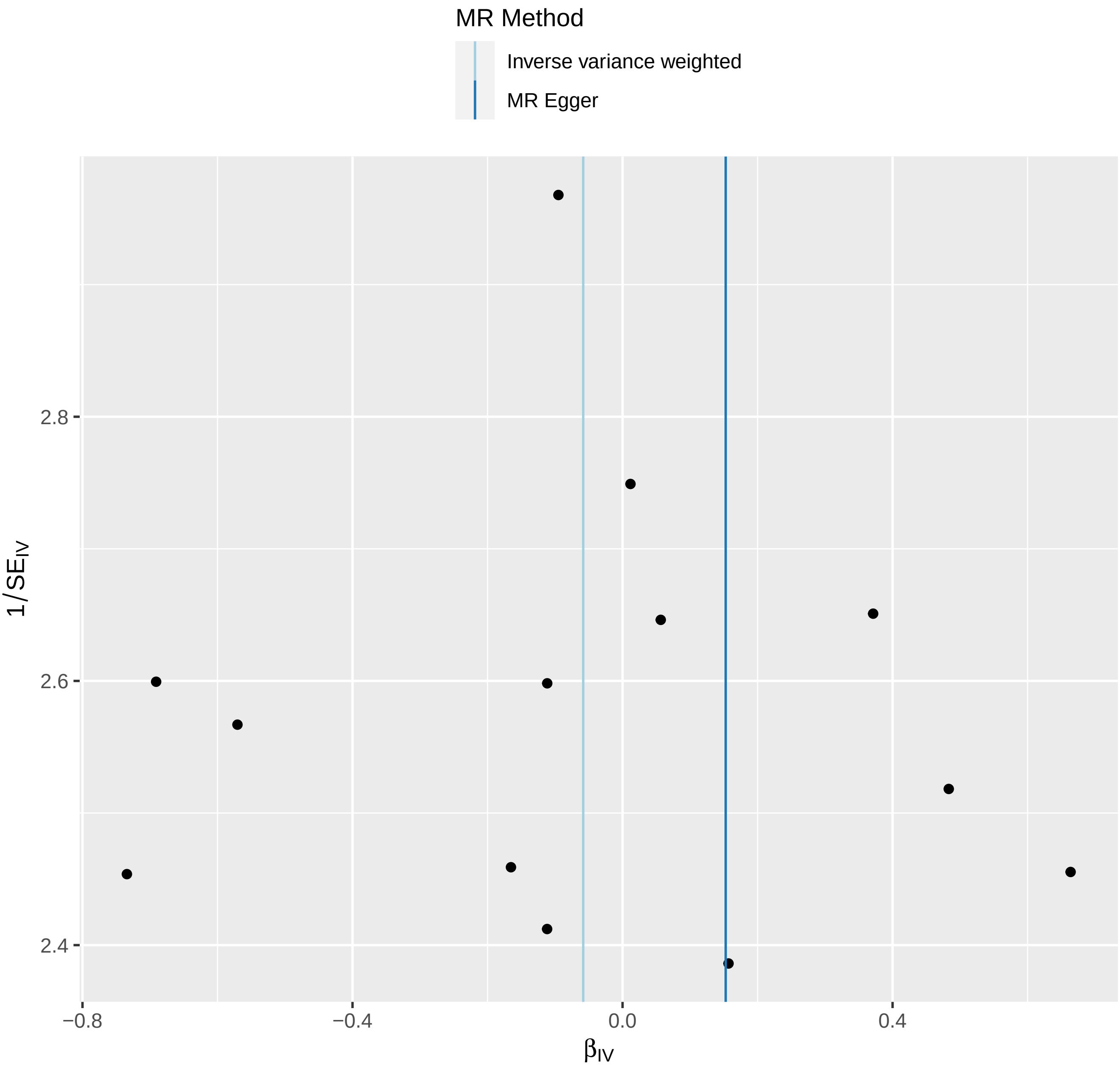

Batch 2−5795 : Gut microbiota abundance (genus Catenibacterium id.2153) on SUN domain−containing protein 3
with a threshold of 1e−05

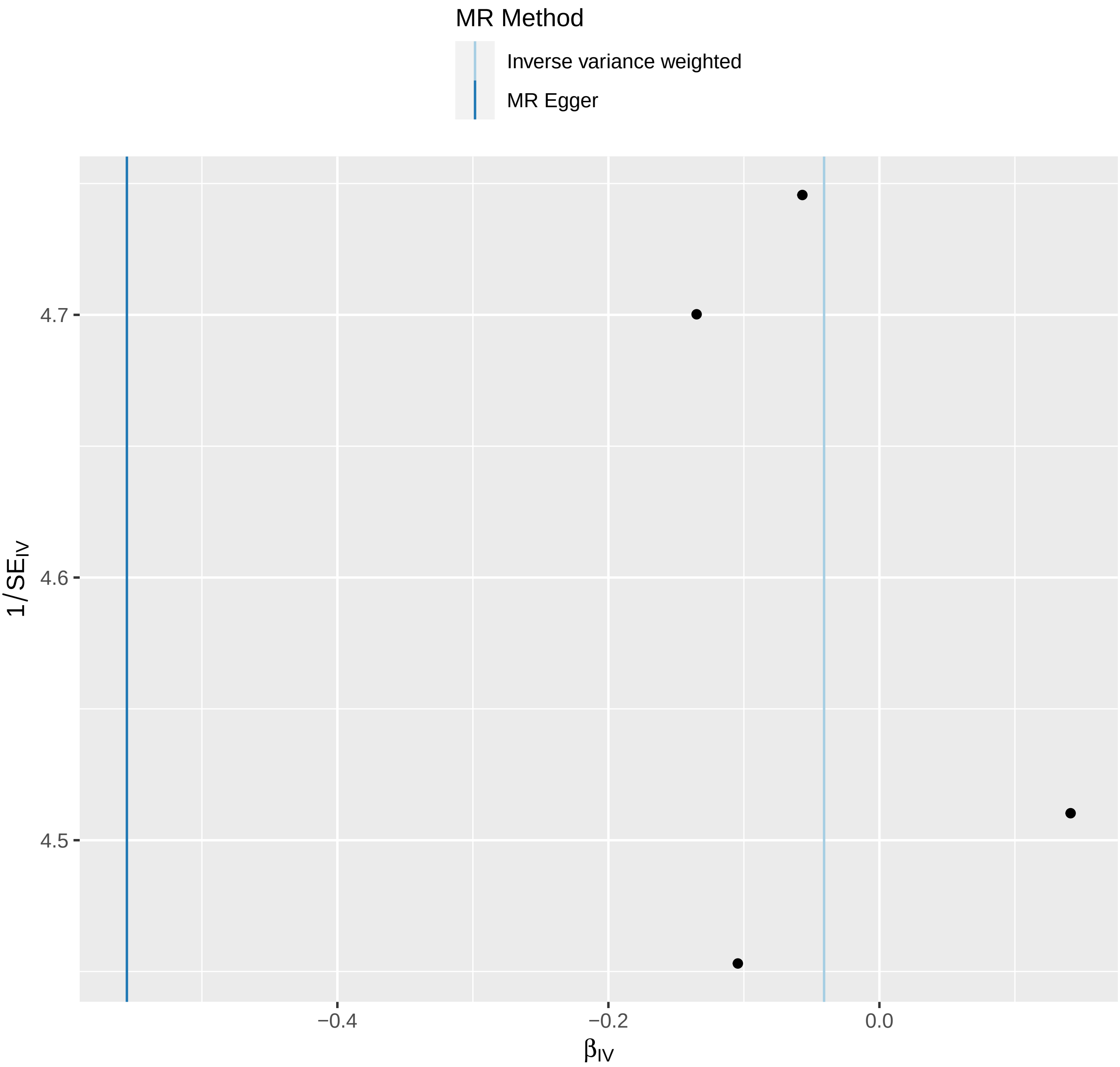

Batch 2−5796 : Gut microbiota abundance (genus Parasutterella id.2892) on SUN domain−containing protein 3
with a threshold of 1e−05

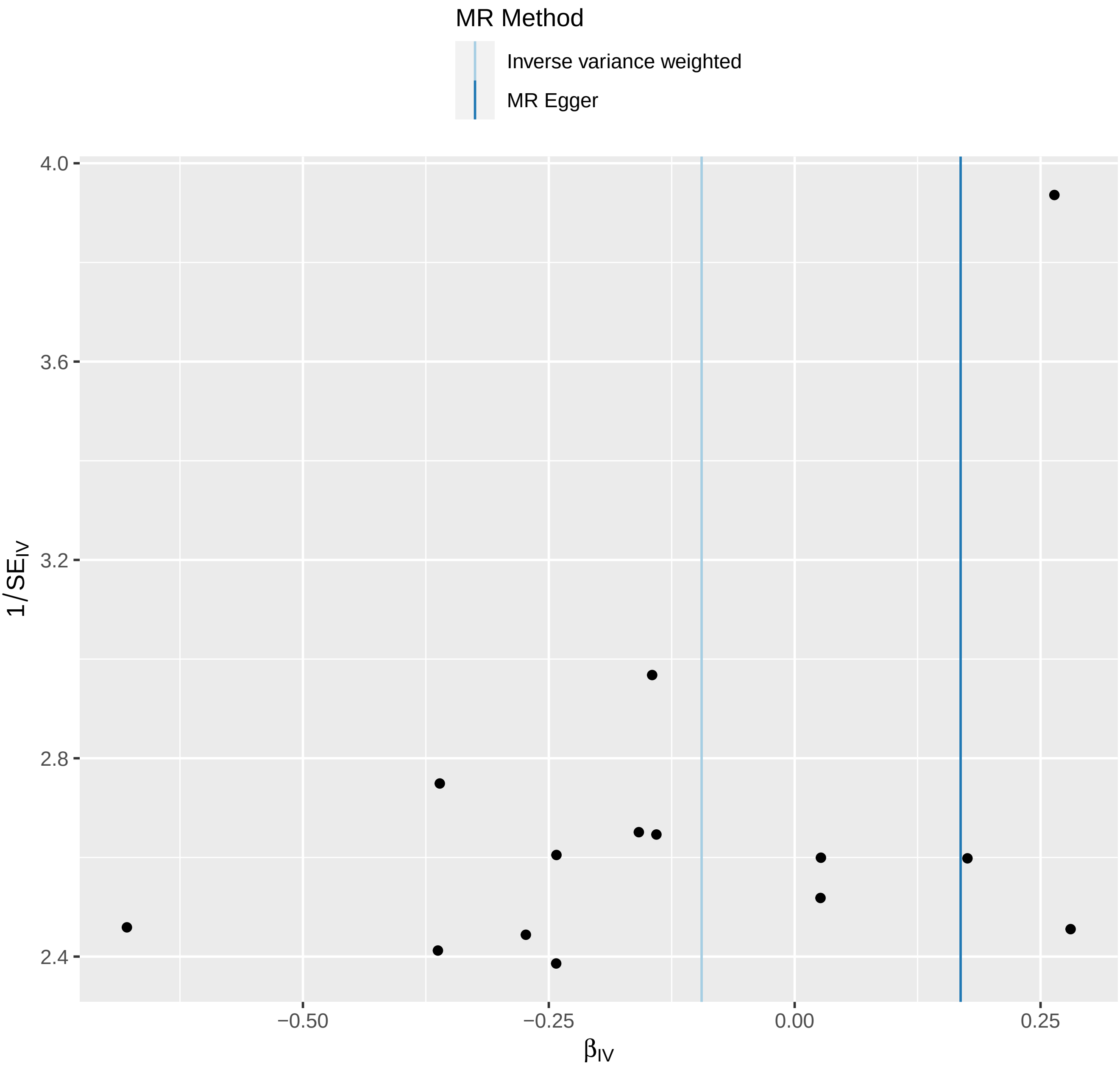

Batch 2−5797 : Gut microbiota abundance (genus Catenibacterium id.2153) on SUN domain−containing protein 5
with a threshold of 1e−05

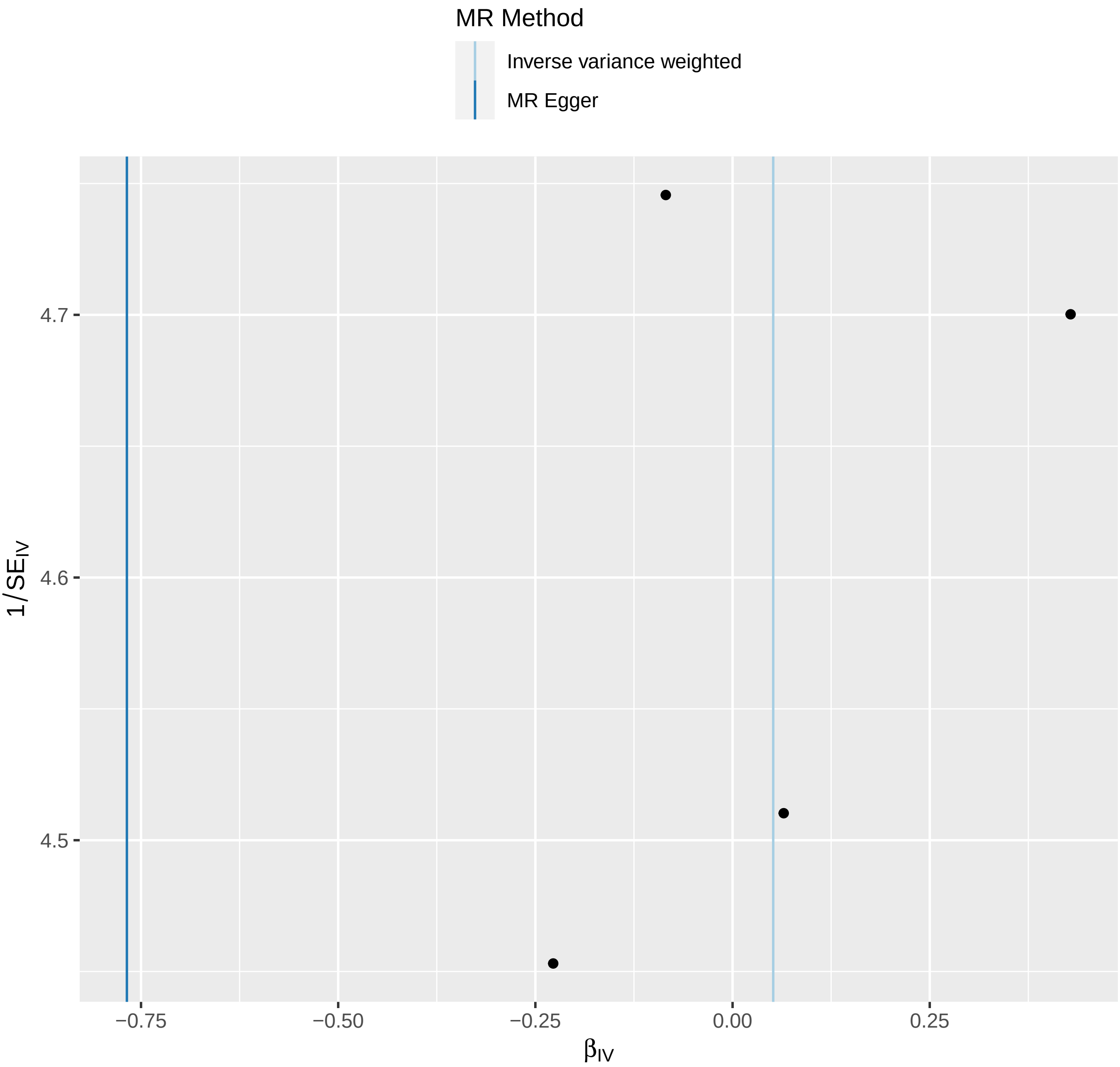

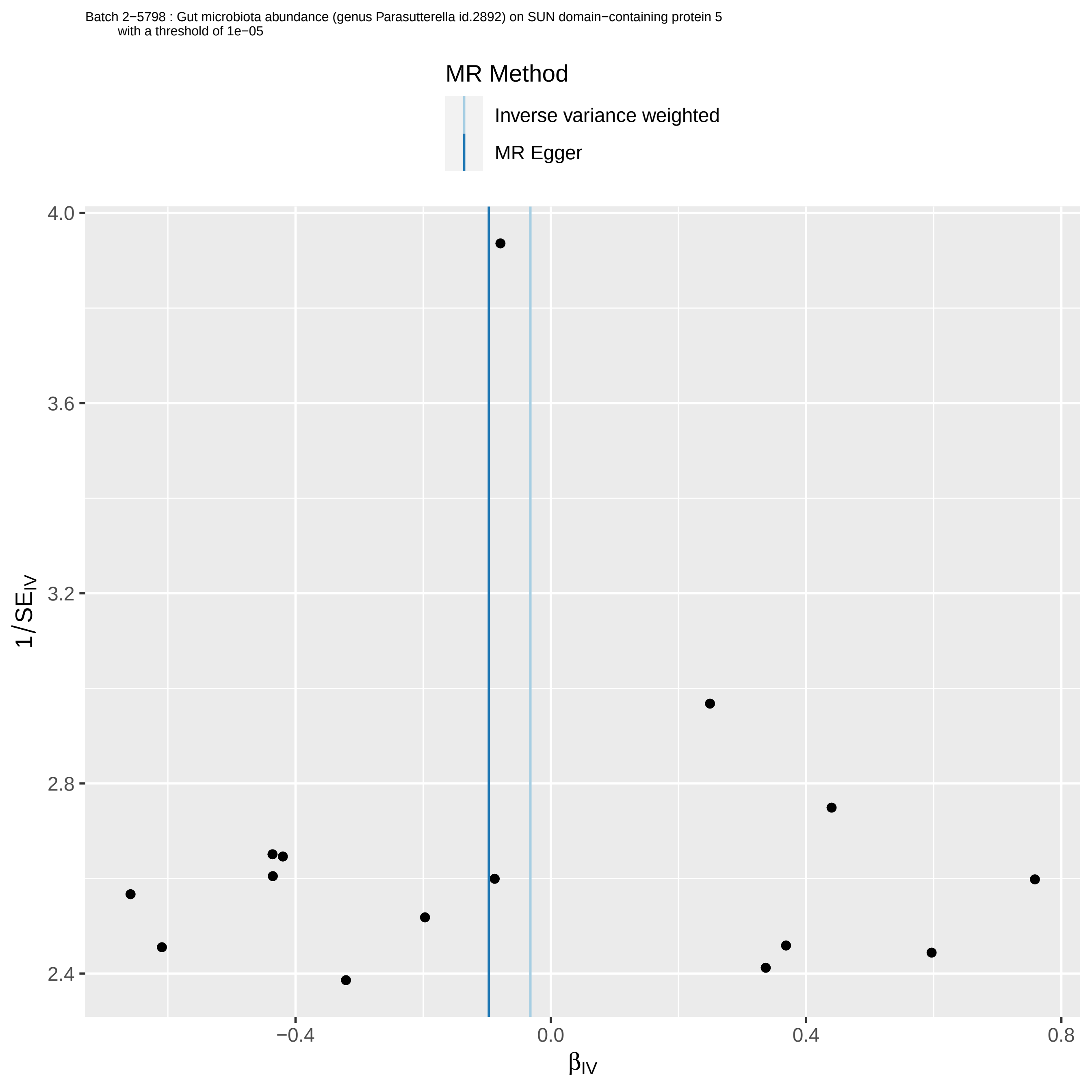

Batch 2−5798 : Gut microbiota abundance (genus Parasutterella id.2892) on SUN domain−containing protein 5
with a threshold of 1e−05
MR Method
Inverse variance weighted
MR Egger
4.0
3.6
3.2
2.8
2.4
1/SE_IV
−0.4
0.0
0.4
0.8
β_IV

Batch 2−5799 : Gut microbiota abundance (genus Catenibacterium id.2153) on Surfeit locus protein 1
with a threshold of 1e−05

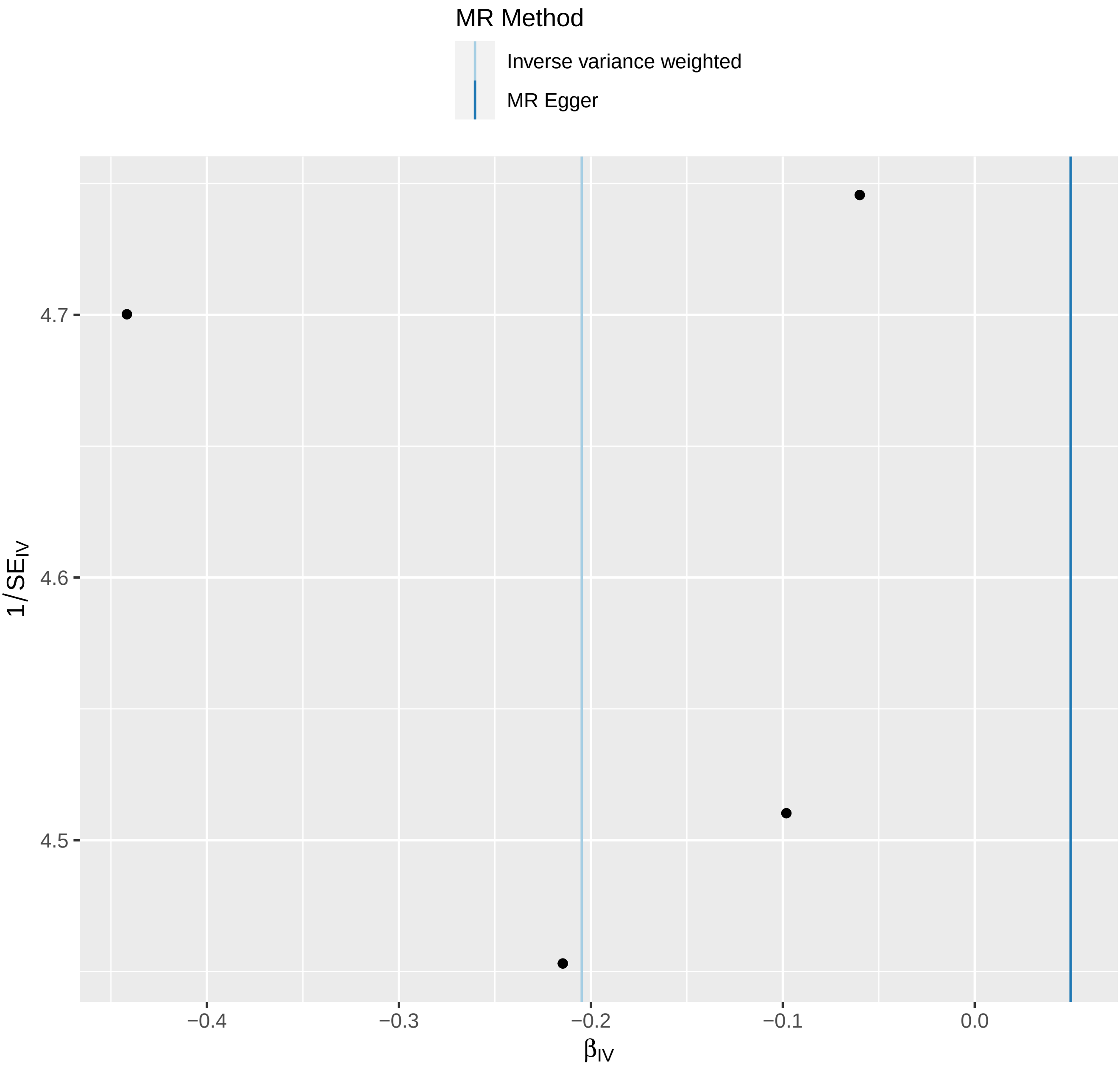

Batch 2−5800 : Gut microbiota abundance (genus Parasutterella id.2892) on Surfeit locus protein 1
with a threshold of 1e−05

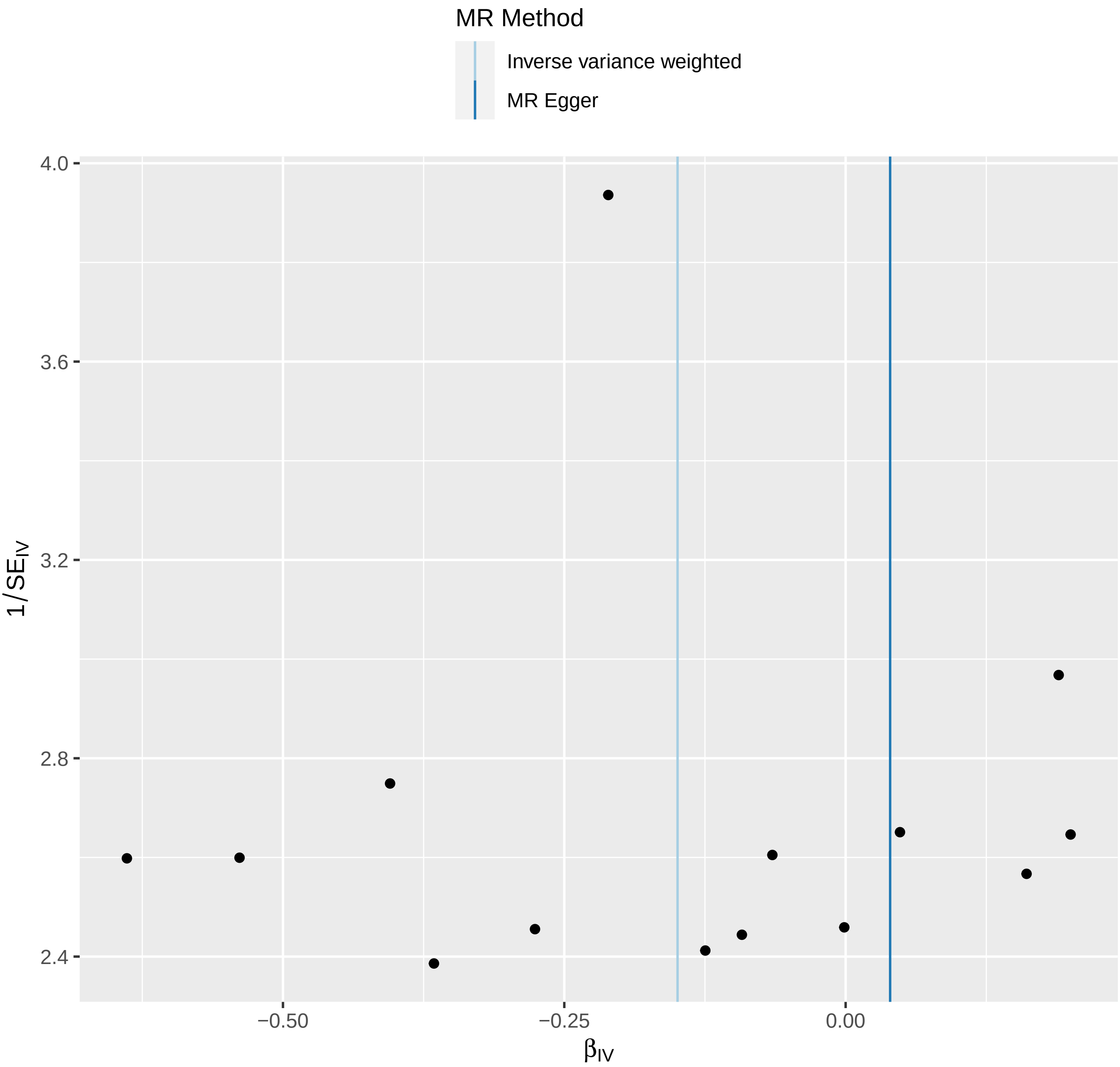

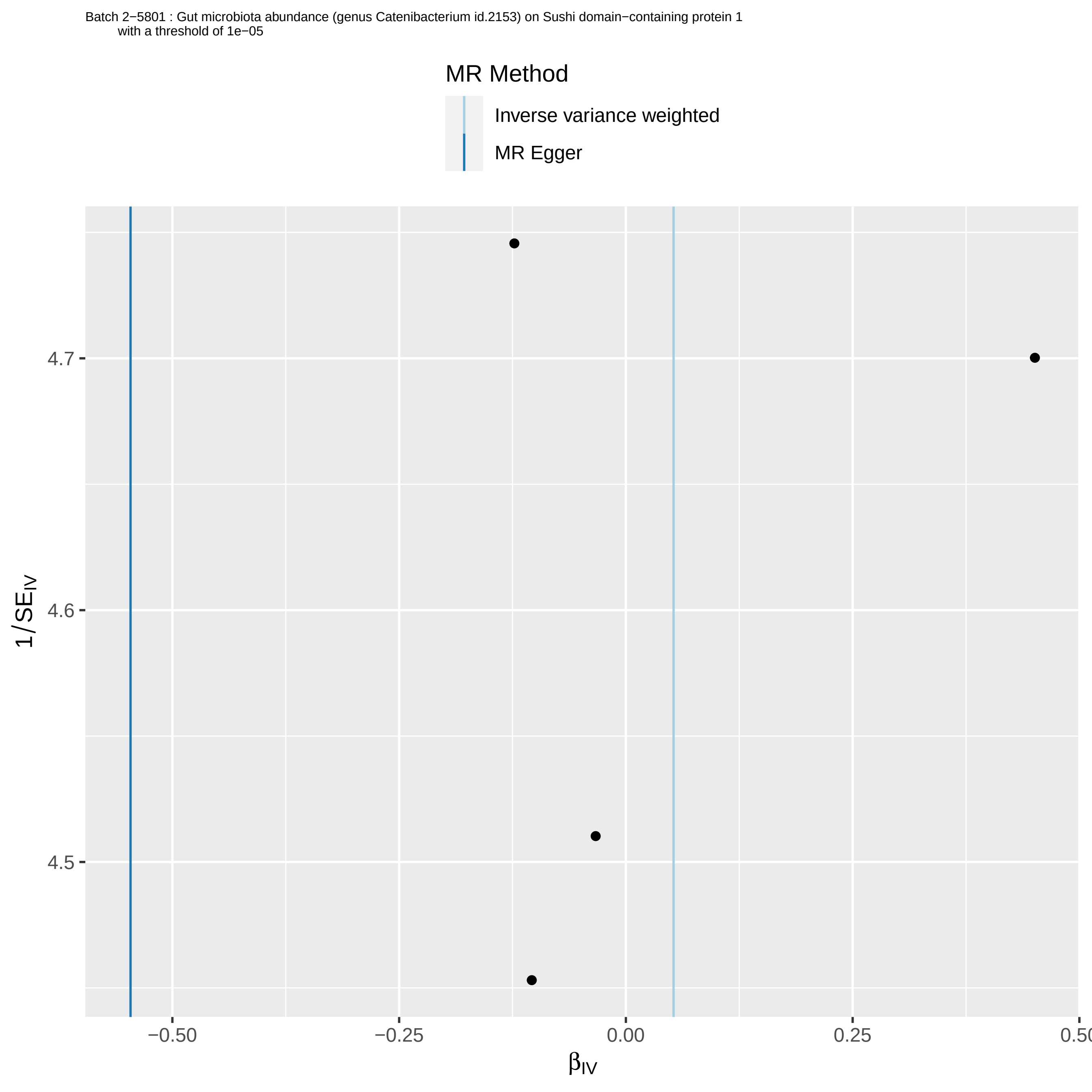

Batch 2−5801 : Gut microbiota abundance (genus Catenibacterium id.2153) on Sushi domain−containing protein 1
with a threshold of 1e−05
MR Method
Inverse variance weighted
MR Egger
4.7
4.6
4.5
1/SE_IV
−0.50
−0.25
0.00
0.25
0.50
β_IV

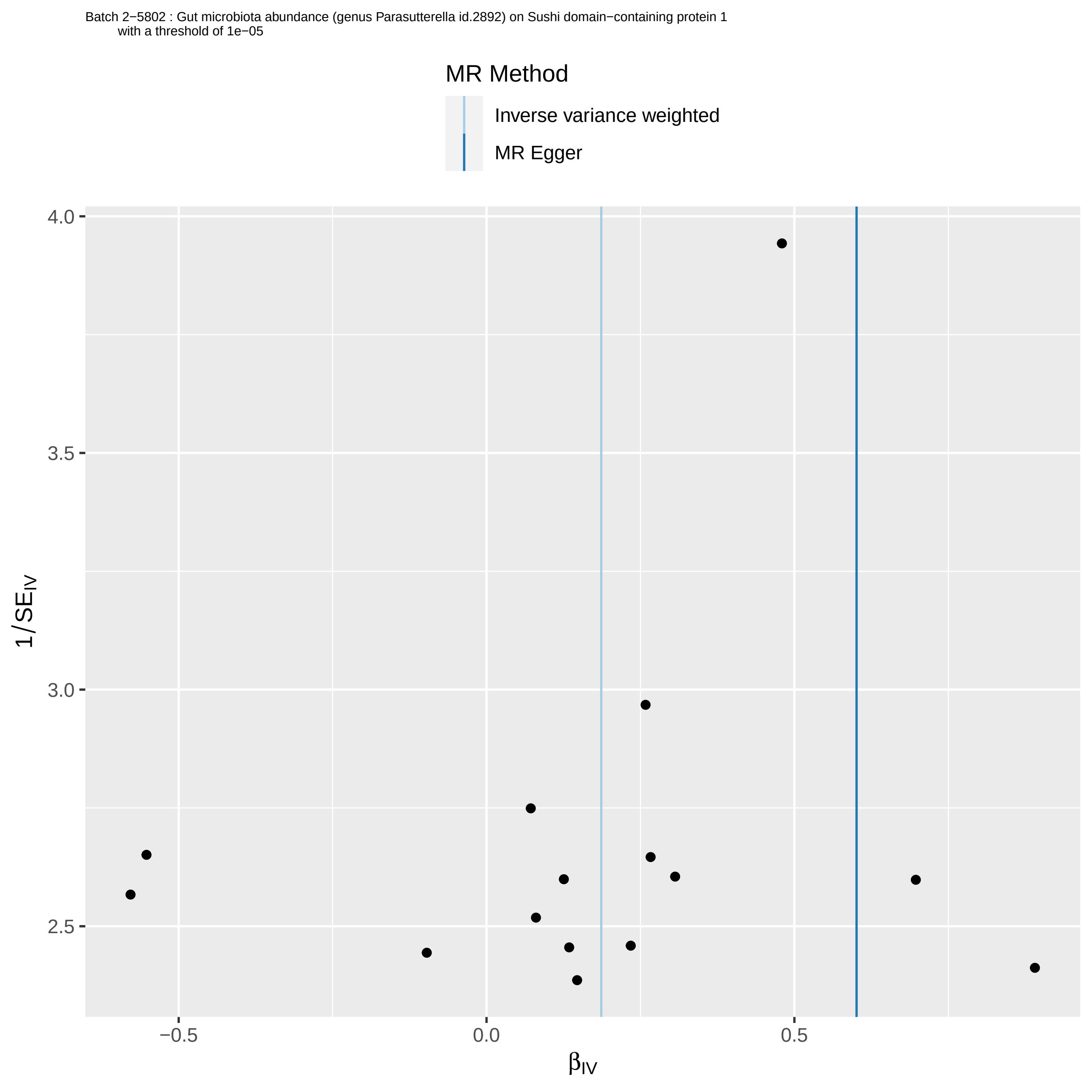
Batch 2−5802 : Gut microbiota abundance (genus Parasutterella id.2892) on Sushi domain−containing protein 1
with a threshold of 1e−05
MR Method
Inverse variance weighted
MR Egger
4.0
3.5
3.0
2.5
1/SE_IV
−0.5
0.0
0.5
β_IV

Batch 2−5803 : Gut microbiota abundance (genus Catenibacterium id.2153) on Sushi domain−containing protein 2
with a threshold of 1e−05

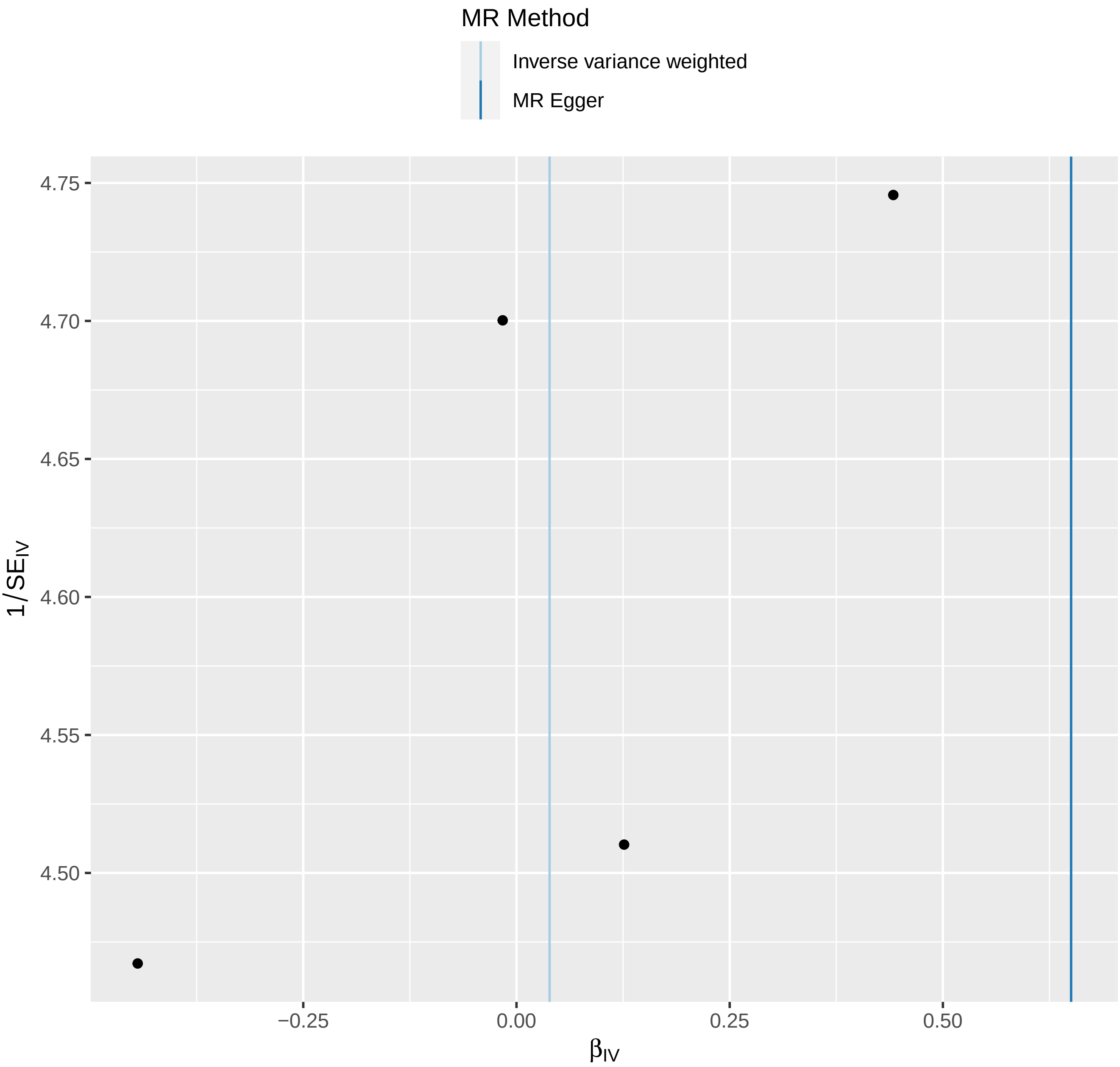

Batch 2−5804 : Gut microbiota abundance (genus Parasutterella id.2892) on Sushi domain−containing protein 2
with a threshold of 1e−05

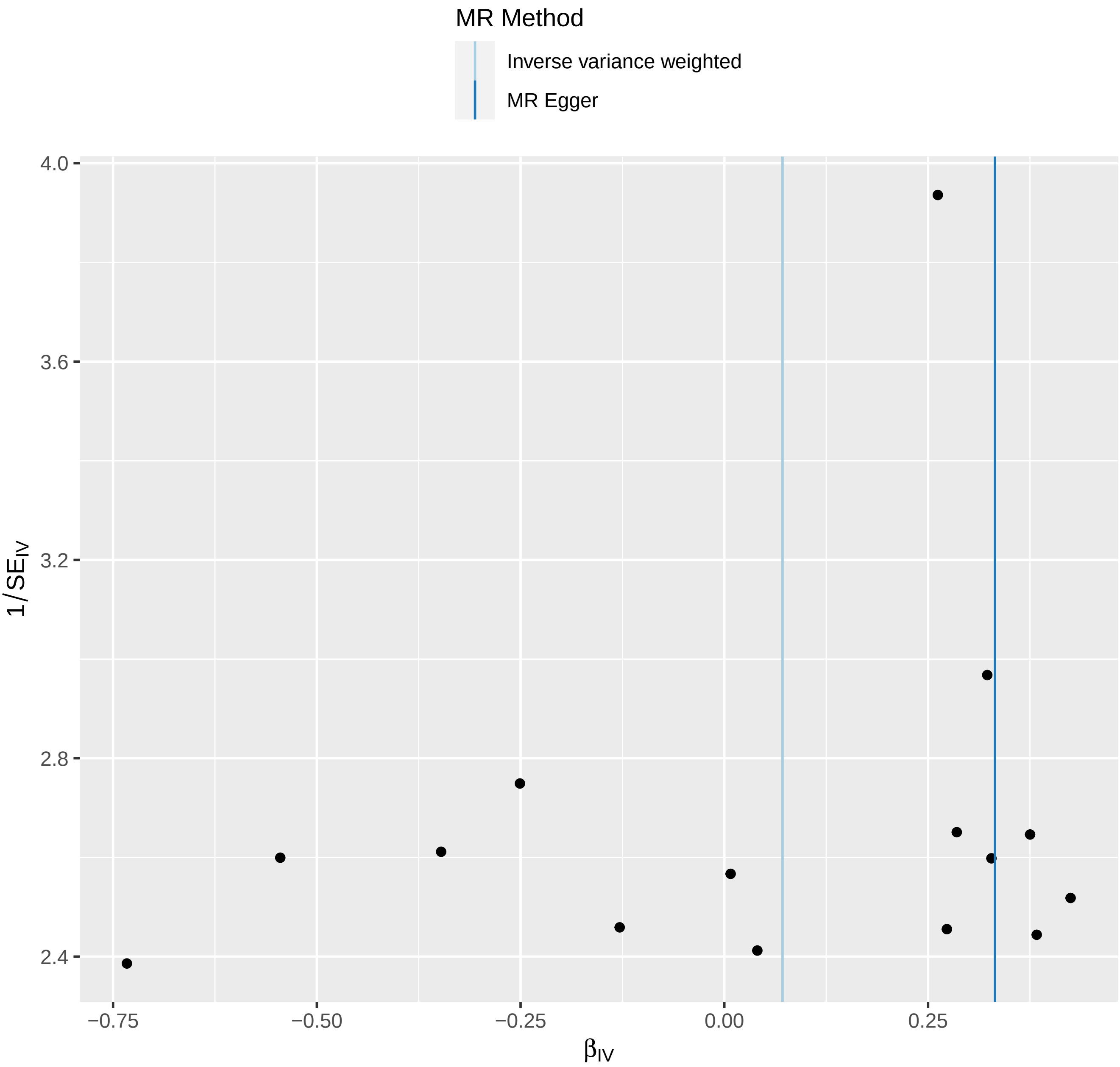

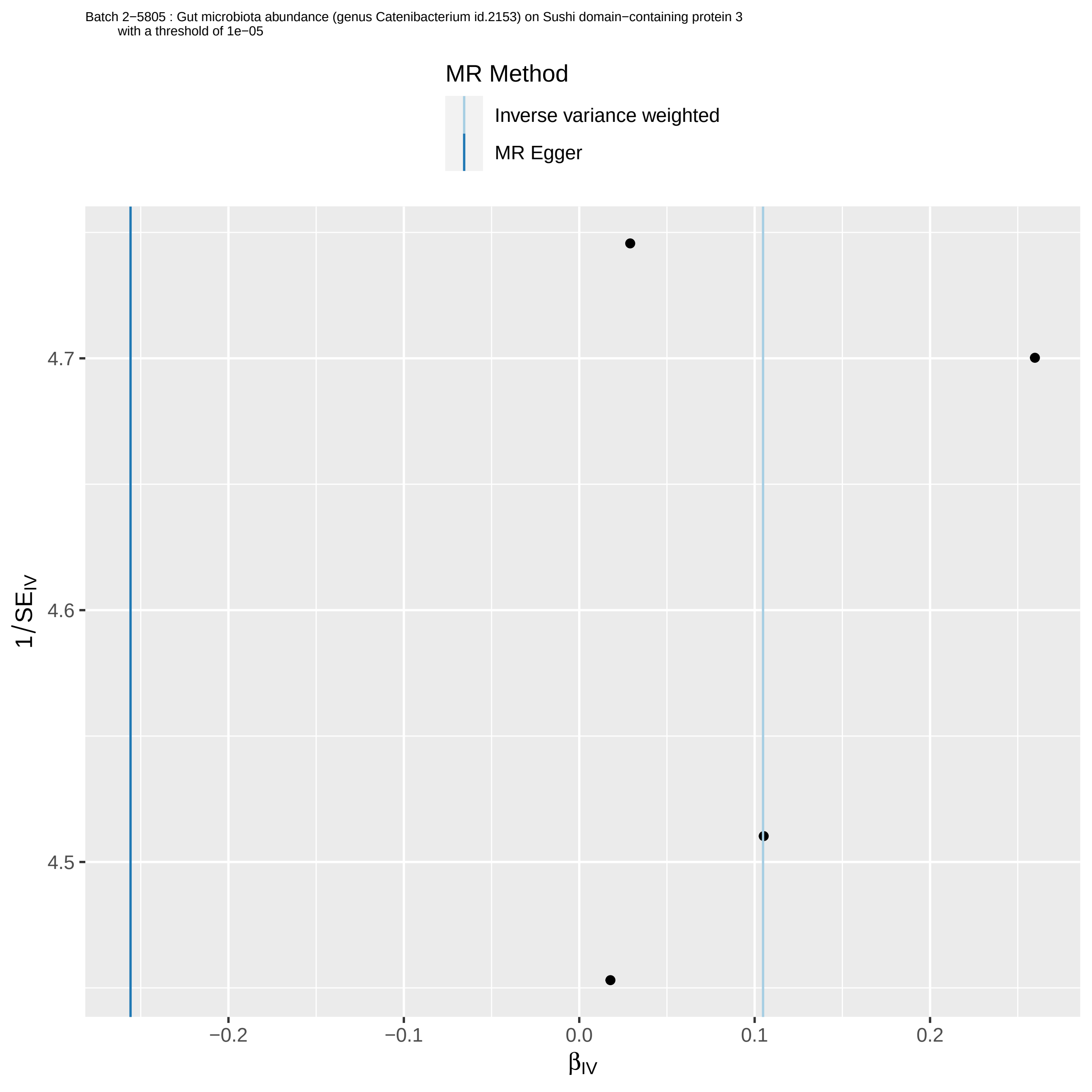
Batch 2−5805 : Gut microbiota abundance (genus Catenibacterium id.2153) on Sushi domain−containing protein 3
with a threshold of 1e−05
MR Method
Inverse variance weighted
MR Egger
4.7
4.6
4.5
1/SE$_{IV}$
−0.2
−0.1
0.0
0.1
0.2
β$_{IV}$

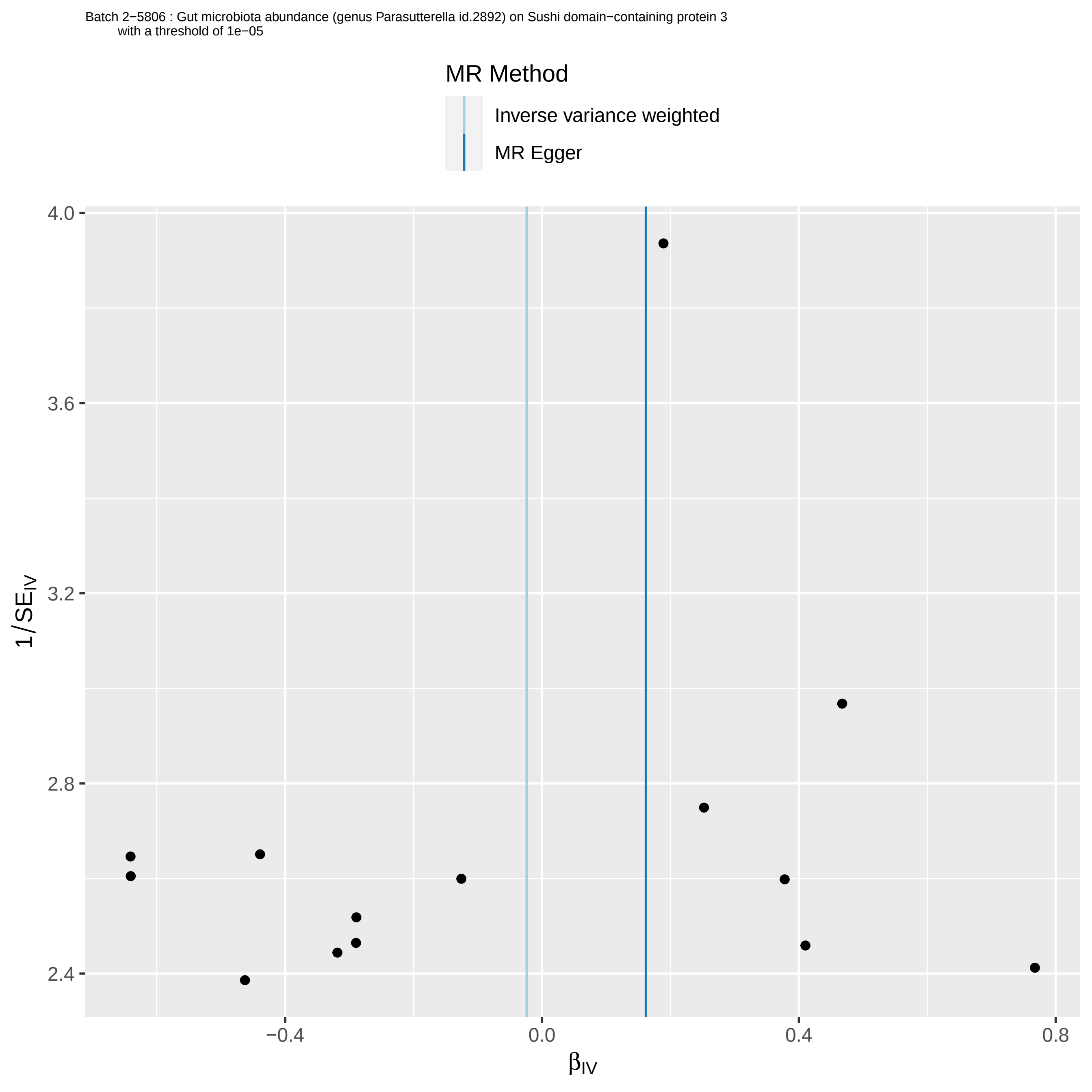
Batch 2−5806 : Gut microbiota abundance (genus Parasutterella id.2892) on Sushi domain−containing protein 3
with a threshold of 1e−05
MR Method
Inverse variance weighted
MR Egger
1/SE_IV
4.0
3.6
3.2
2.8
2.4
−0.4
0.0
0.4
0.8
β_IV

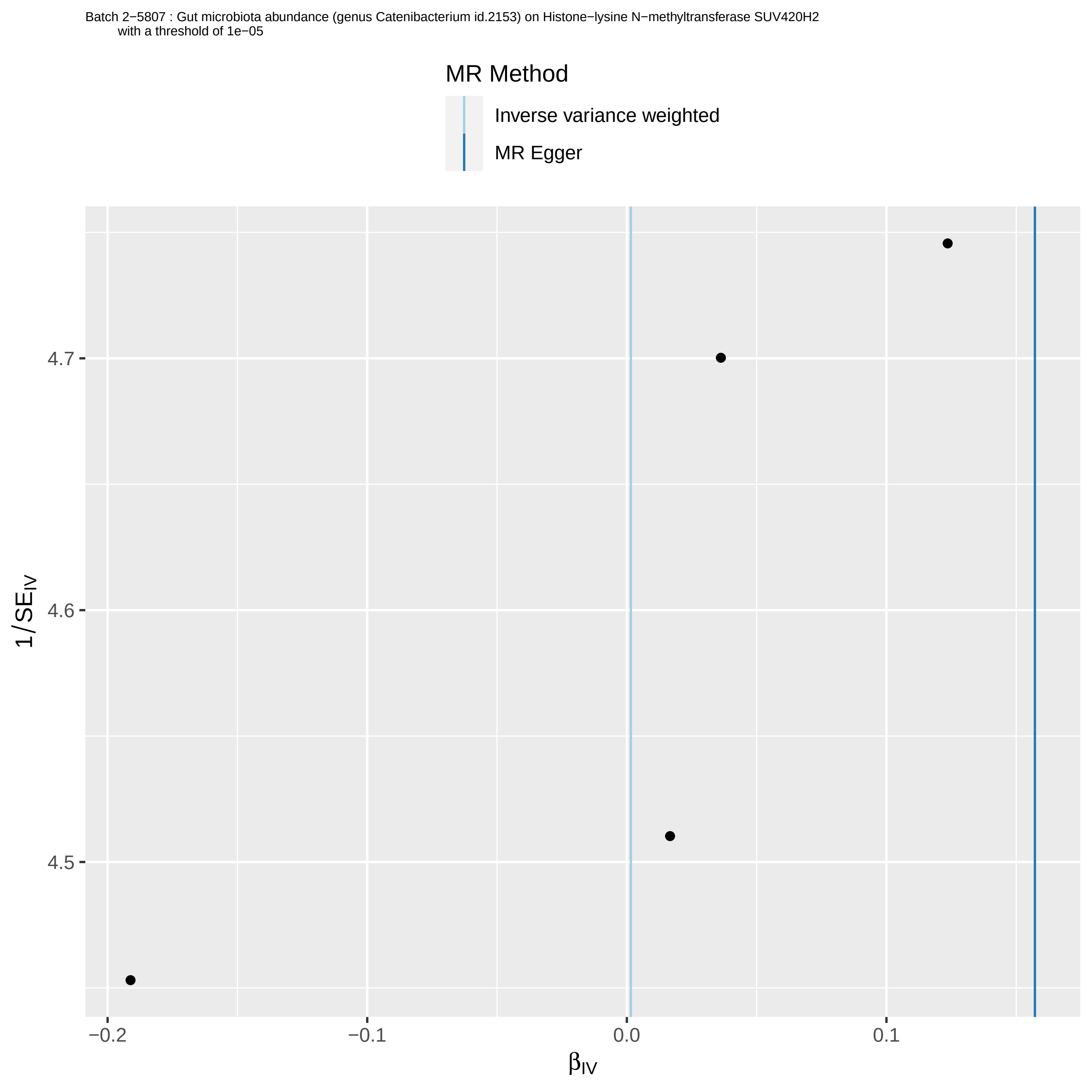
Batch 2−5807 : Gut microbiota abundance (genus Catenibacterium id.2153) on Histone−lysine N−methyltransferase SUV420H2
with a threshold of 1e−05
MR Method
Inverse variance weighted
MR Egger
4.7
4.6
4.5
1/SE$_{IV}$
−0.2
−0.1
0.0
0.1
β$_{IV}$

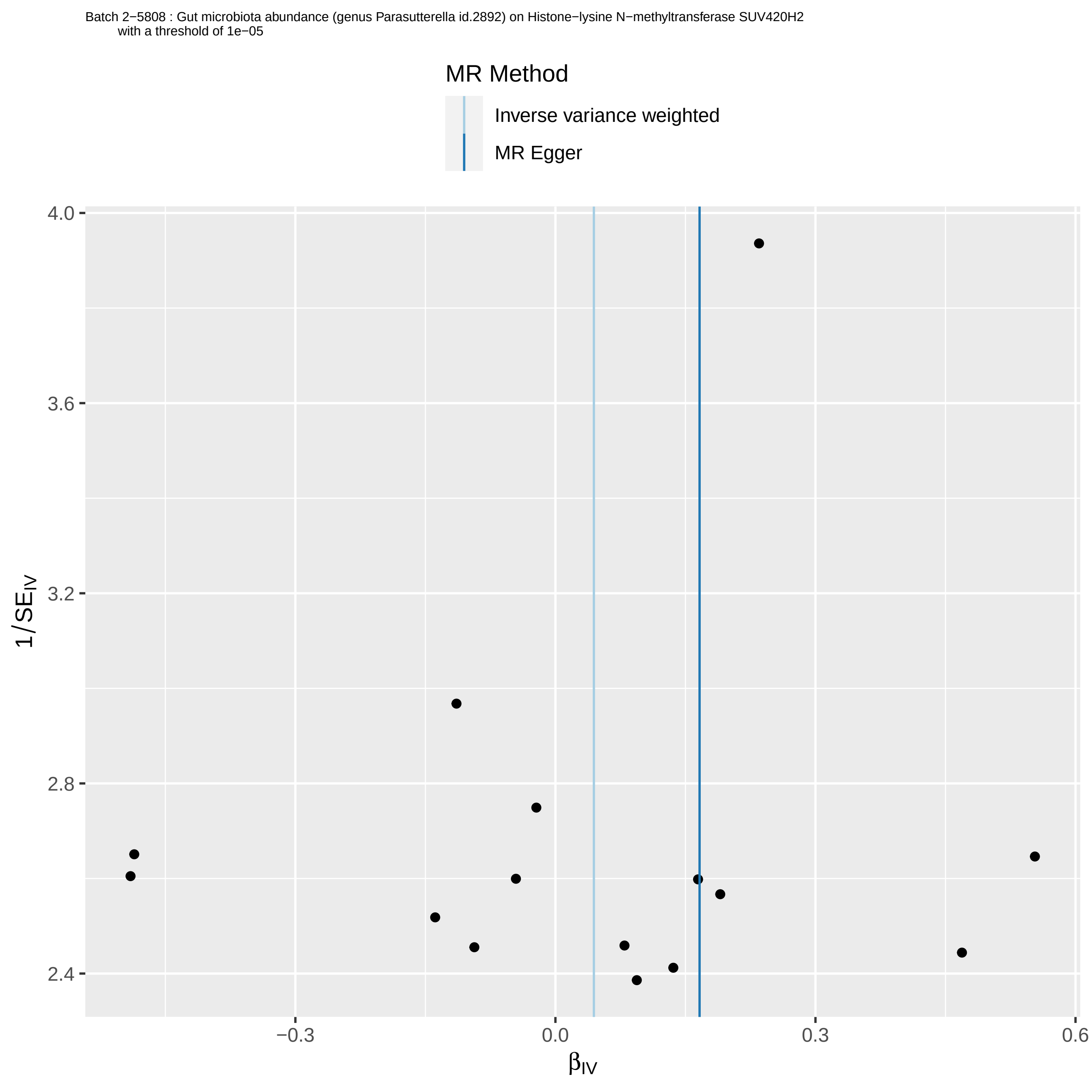

Batch 2−5808 : Gut microbiota abundance (genus Parasutterella id.2892) on Histone−lysine N−methyltransferase SUV420H2
with a threshold of 1e−05
MR Method
Inverse variance weighted
MR Egger
4.0
3.6
3.2
2.8
2.4
1/SE$_{IV}$
−0.3
0.0
0.3
0.6
β$_{IV}$

Batch 2−5809 : Gut microbiota abundance (genus Catenibacterium id.2153) on Synaptic vesicle glycoprotein 2A
with a threshold of 1e−05

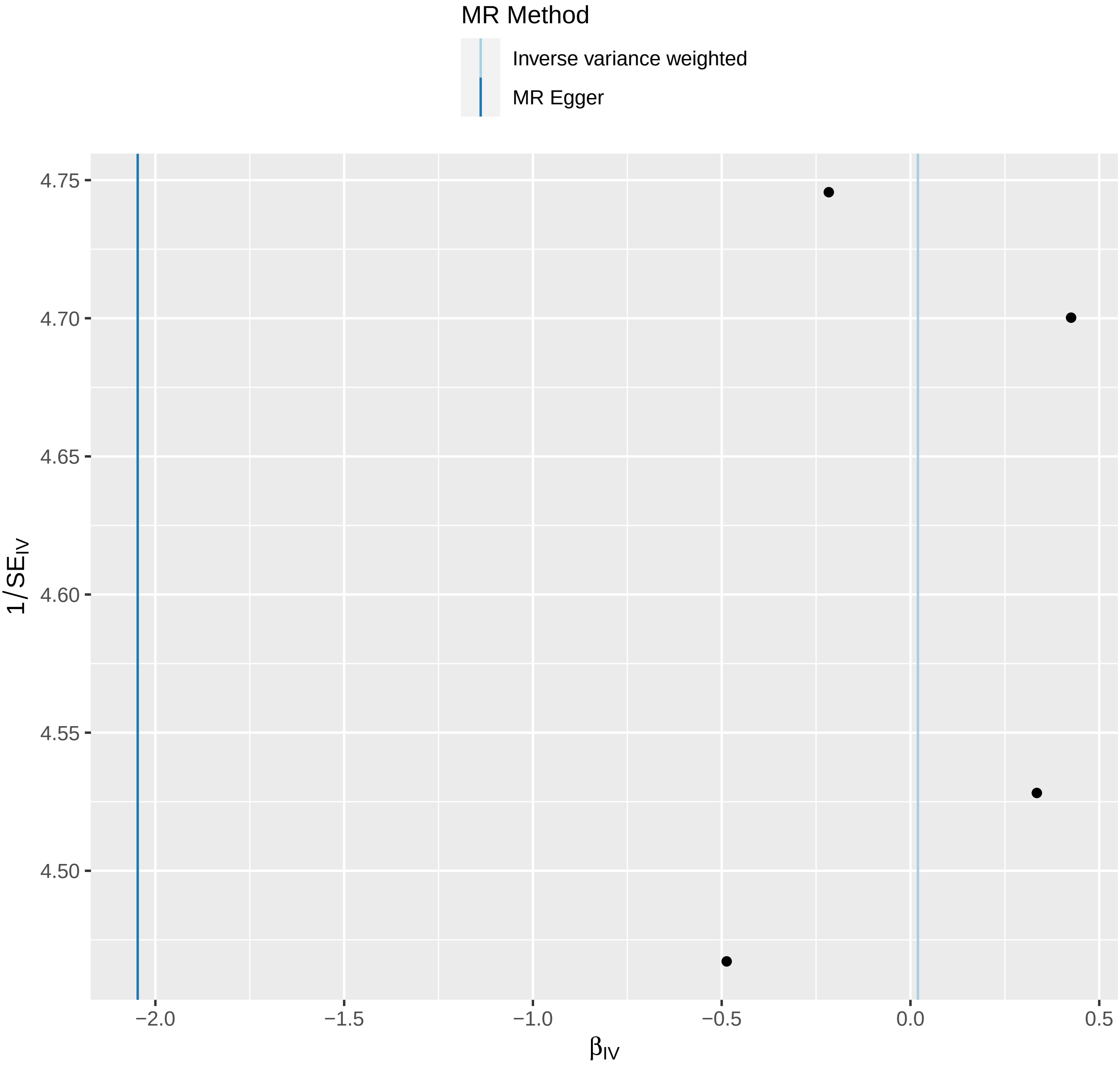

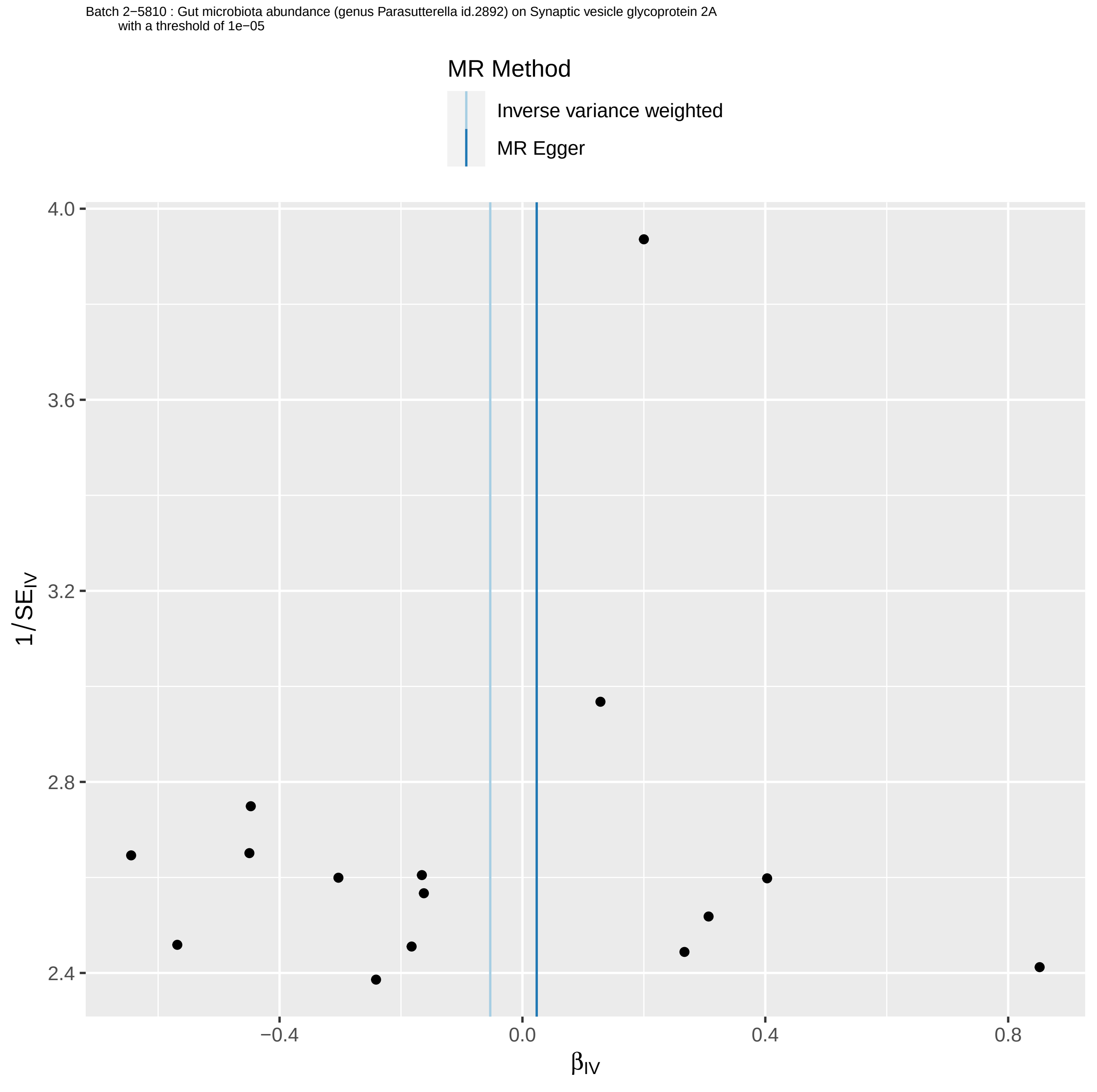
Batch 2−5810 : Gut microbiota abundance (genus Parasutterella id.2892) on Synaptic vesicle glycoprotein 2A
with a threshold of 1e−05
MR Method
Inverse variance weighted
MR Egger
4.0
3.6
3.2
2.8
2.4
1/SE$_{IV}$
−0.4
0.0
0.4
0.8
β$_{IV}$

Batch 2−5811 : Gut microbiota abundance (genus Catenibacterium id.2153) on Sushi, von Willebrand factor type A, EGF and pentraxin domain−containing protein 1 with a threshold of 1e−05

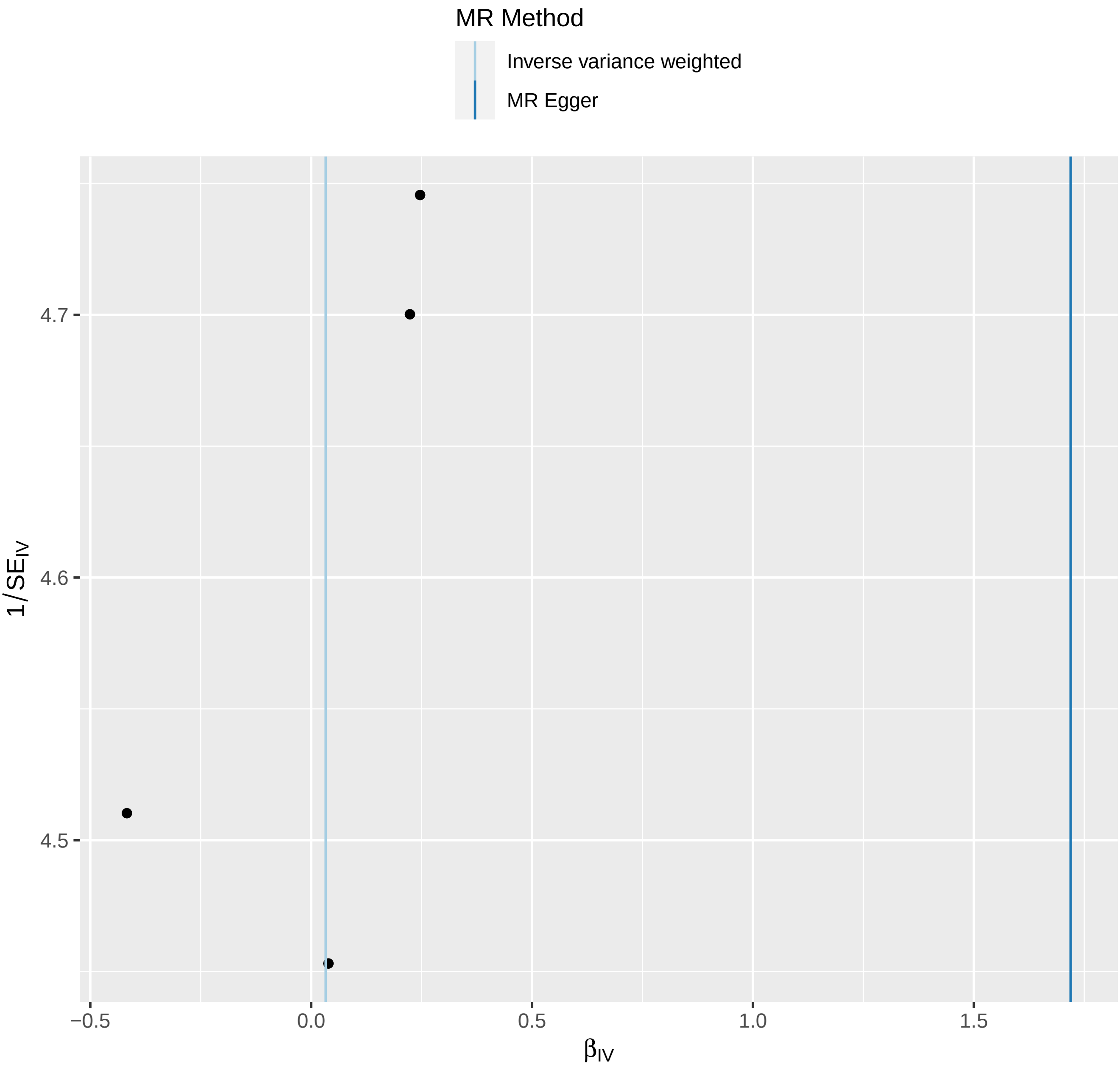

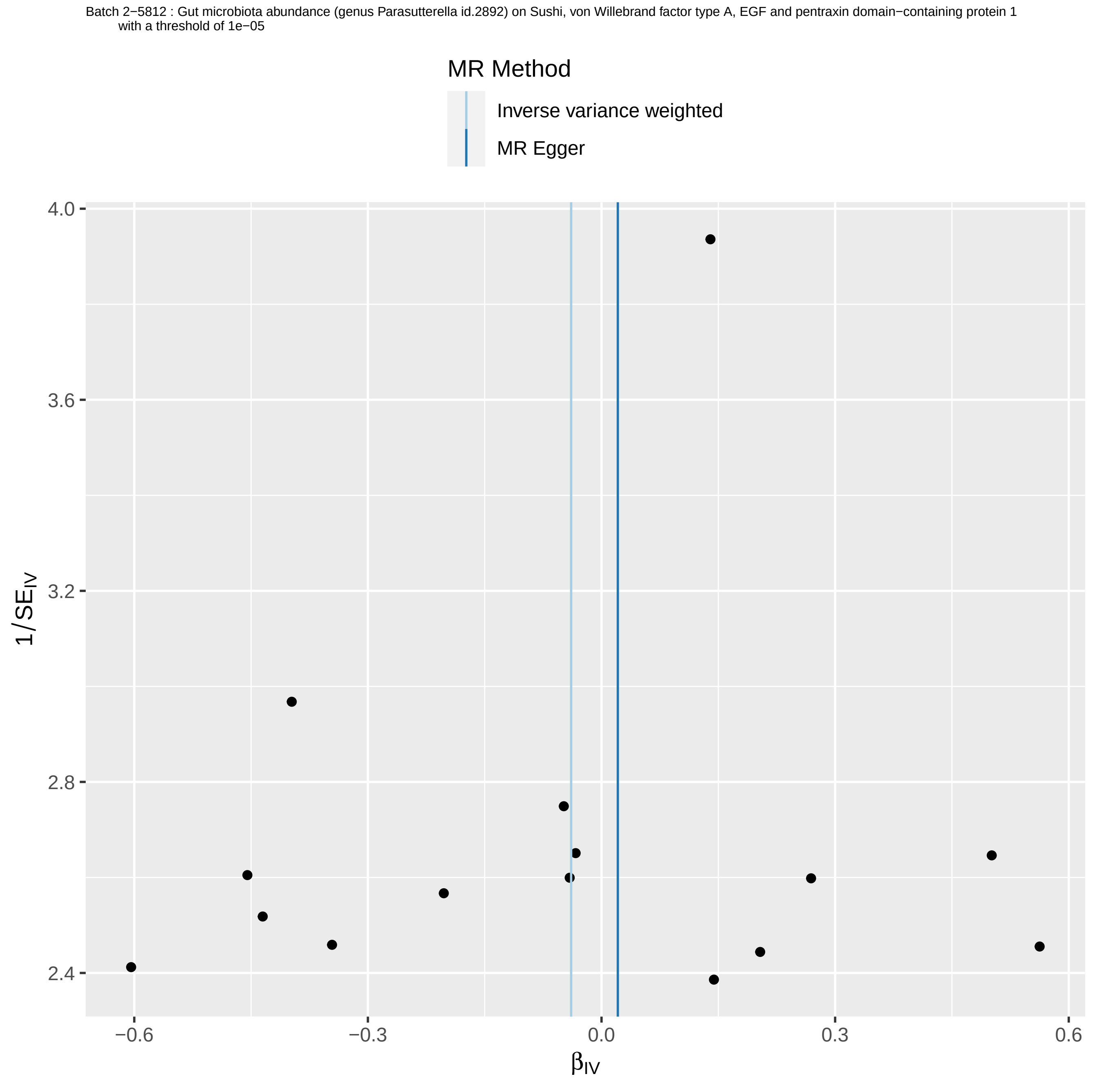

Batch 2−5812 : Gut microbiota abundance (genus Parasutterella id.2892) on Sushi, von Willebrand factor type A, EGF and pentraxin domain−containing protein 1
with a threshold of 1e−05
MR Method
Inverse variance weighted
MR Egger
4.0
3.6
3.2
2.8
2.4
1/SE_IV
−0.6
−0.3
0.0
0.3
0.6
β_IV

Batch 2−5813 : Gut microbiota abundance (genus Catenibacterium id.2153) on Sushi, von Willebrand factor type A, EGF and pentraxin domain−containing protein 1 with a threshold of 1e−05

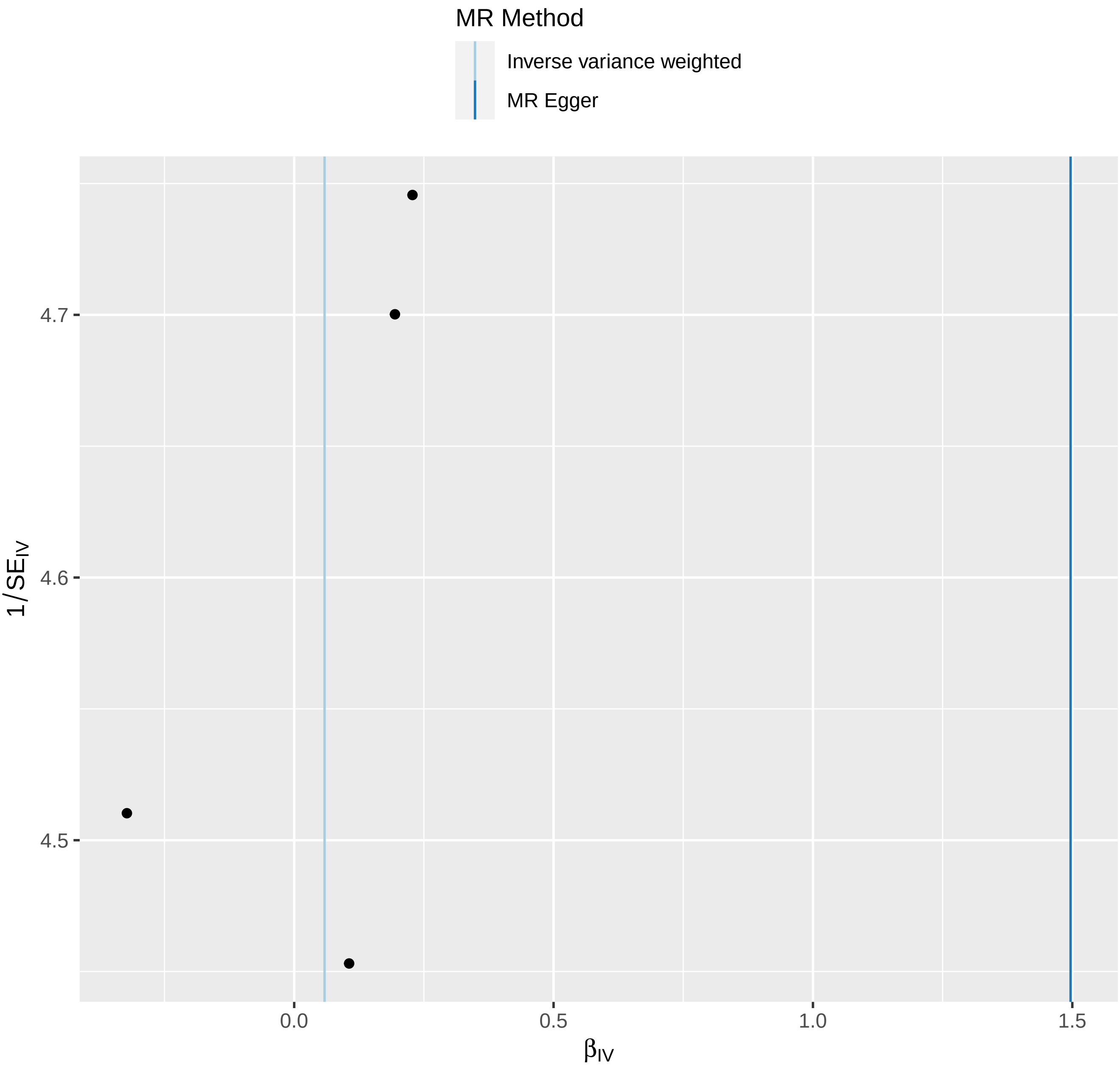

Batch 2−5814 : Gut microbiota abundance (genus Parasutterella id.2892) on Sushi, von Willebrand factor type A, EGF and pentraxin domain−containing protein 1 with a threshold of 1e−05

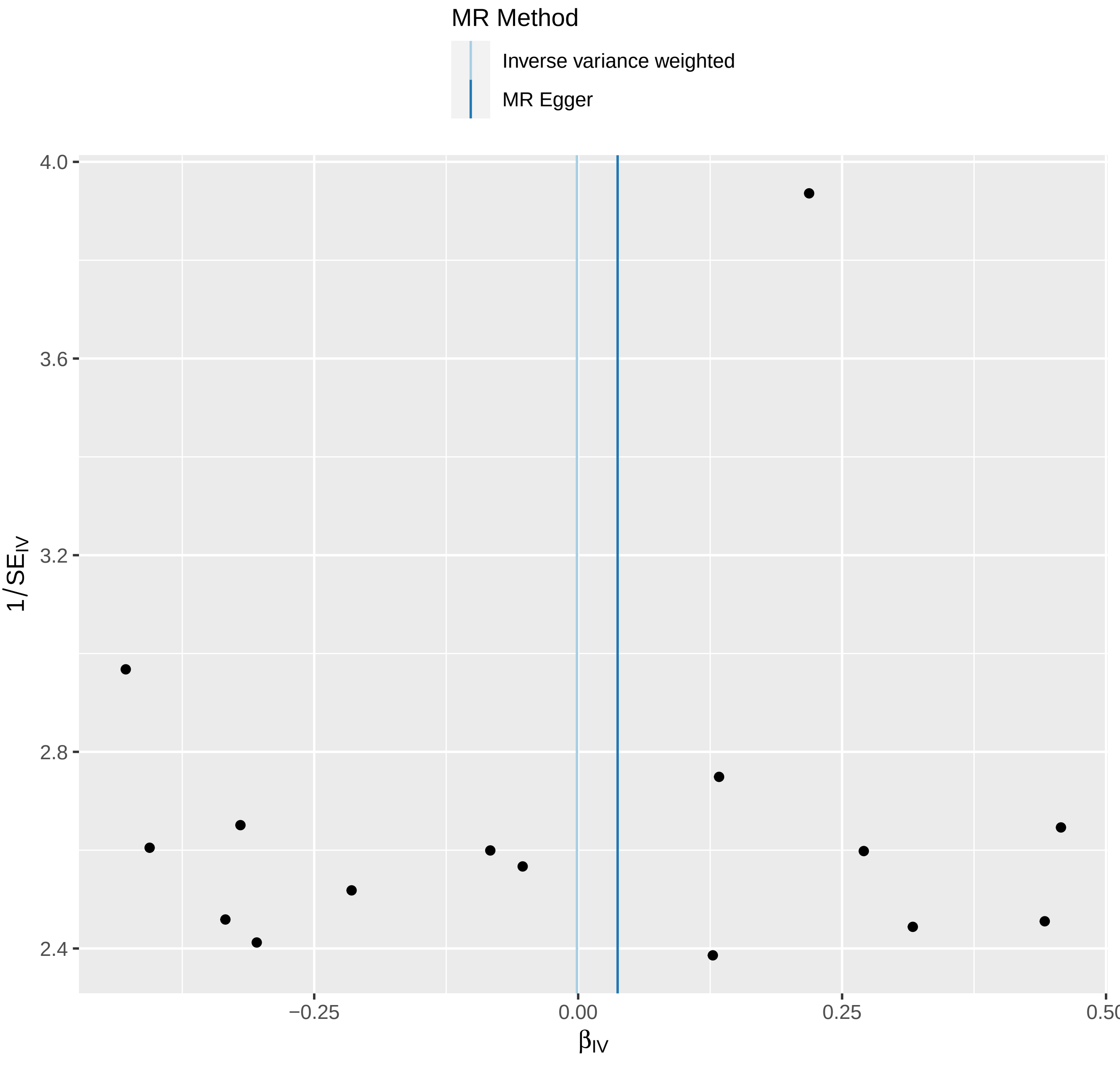

Batch 2−5815 : Gut microbiota abundance (genus Catenibacterium id.2153) on Switch−associated protein 70 with a threshold of 1e−05

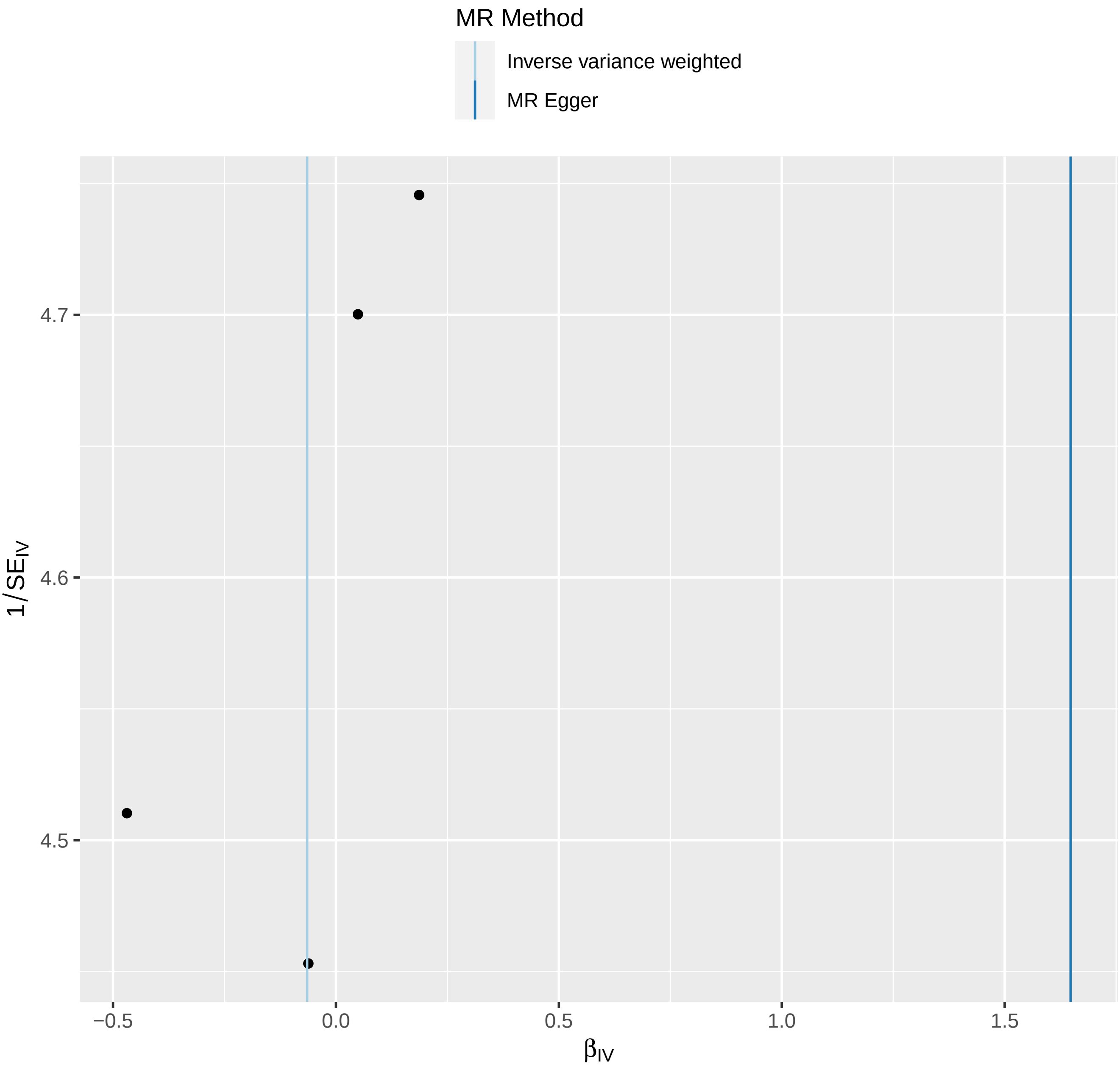

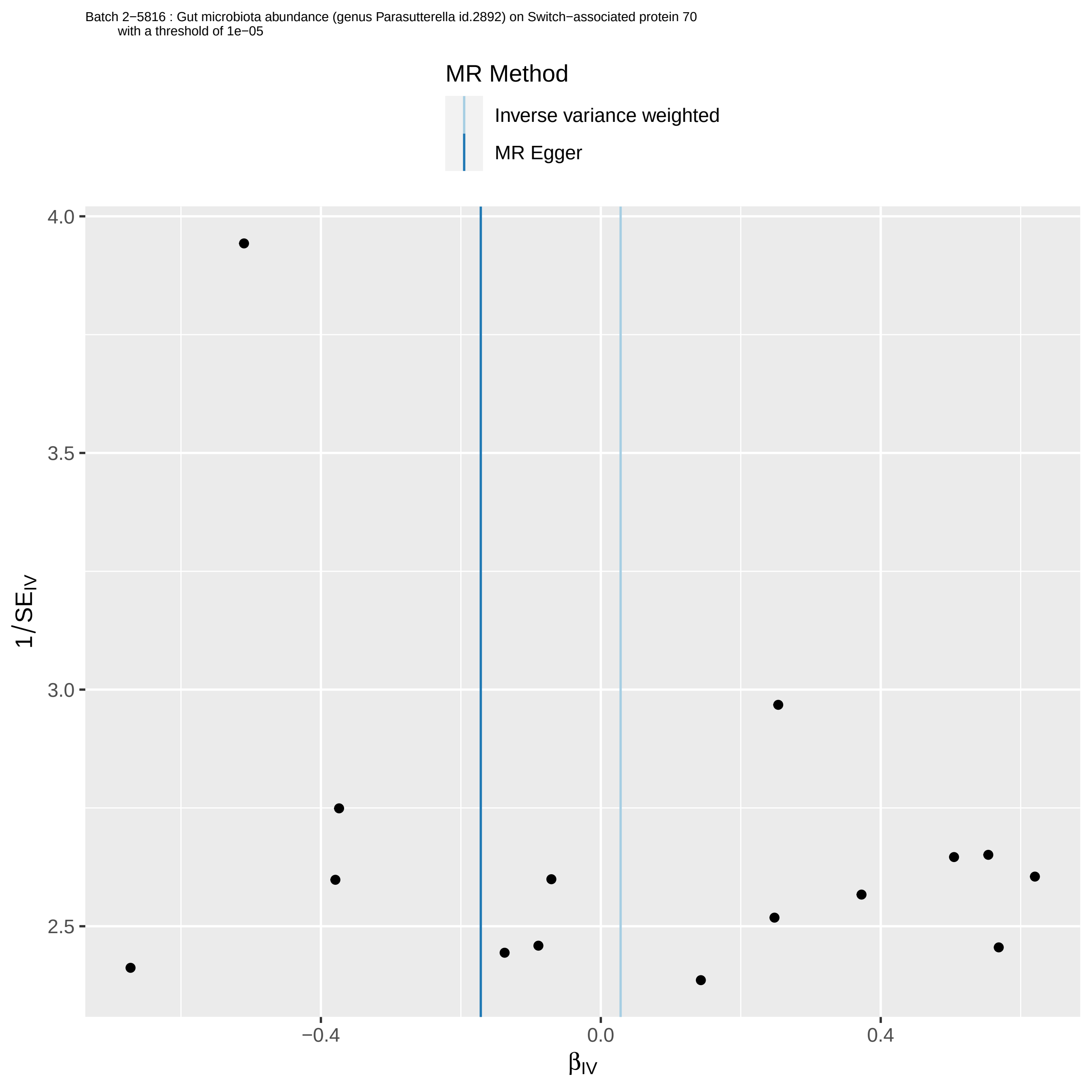

Batch 2−5816 : Gut microbiota abundance (genus Parasutterella id.2892) on Switch−associated protein 70
with a threshold of 1e−05
MR Method
Inverse variance weighted
MR Egger
4.0
3.5
3.0
2.5
1/SE_IV
−0.4
0.0
0.4
β_IV

Batch 2−5817 : Gut microbiota abundance (genus Catenibacterium id.2153) on Heterogeneous nuclear ribonucleoprotein Q
with a threshold of 1e−05

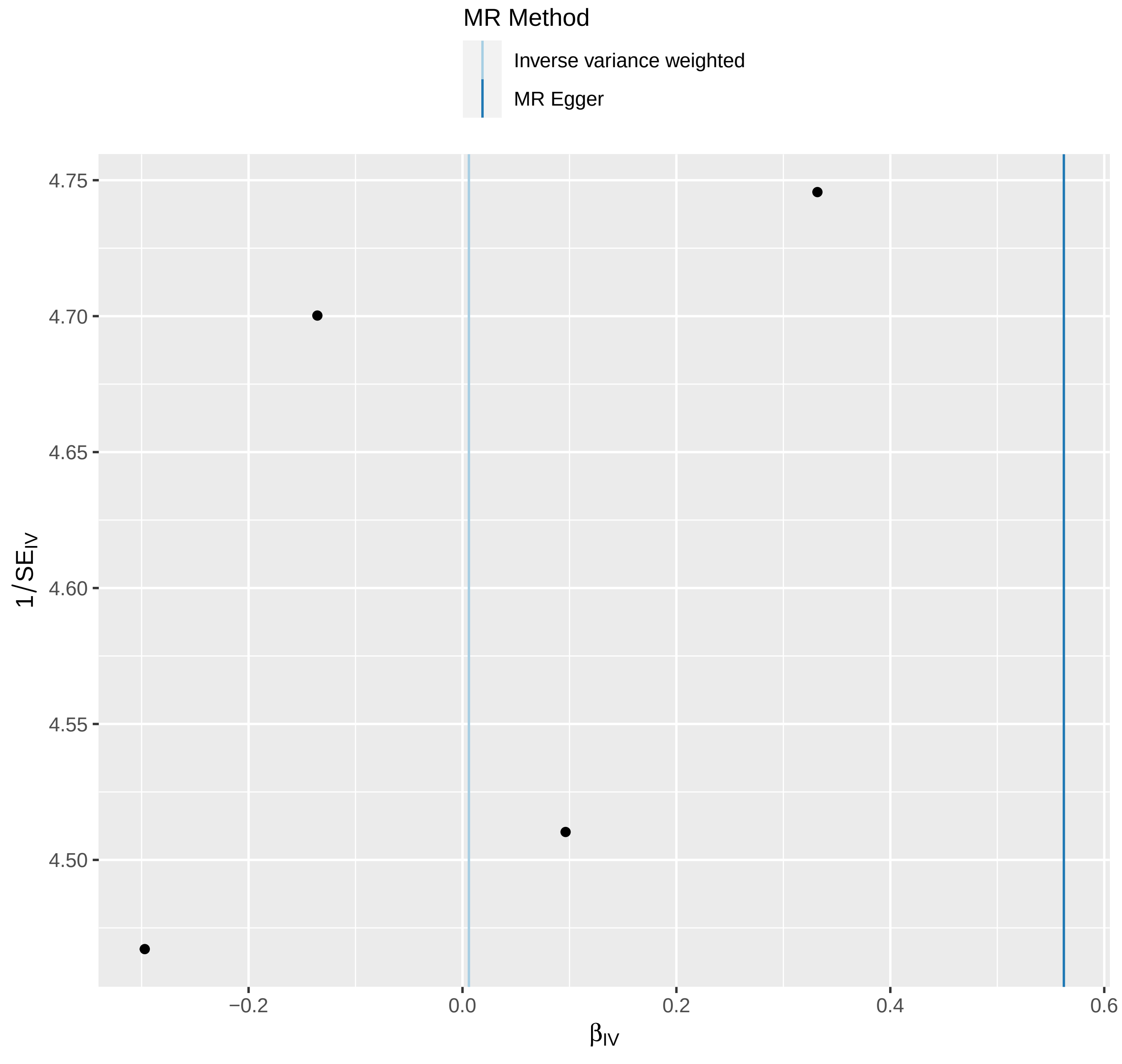

Batch 2−5818 : Gut microbiota abundance (genus Parasutterella id.2892) on Heterogeneous nuclear ribonucleoprotein Q
with a threshold of 1e−05

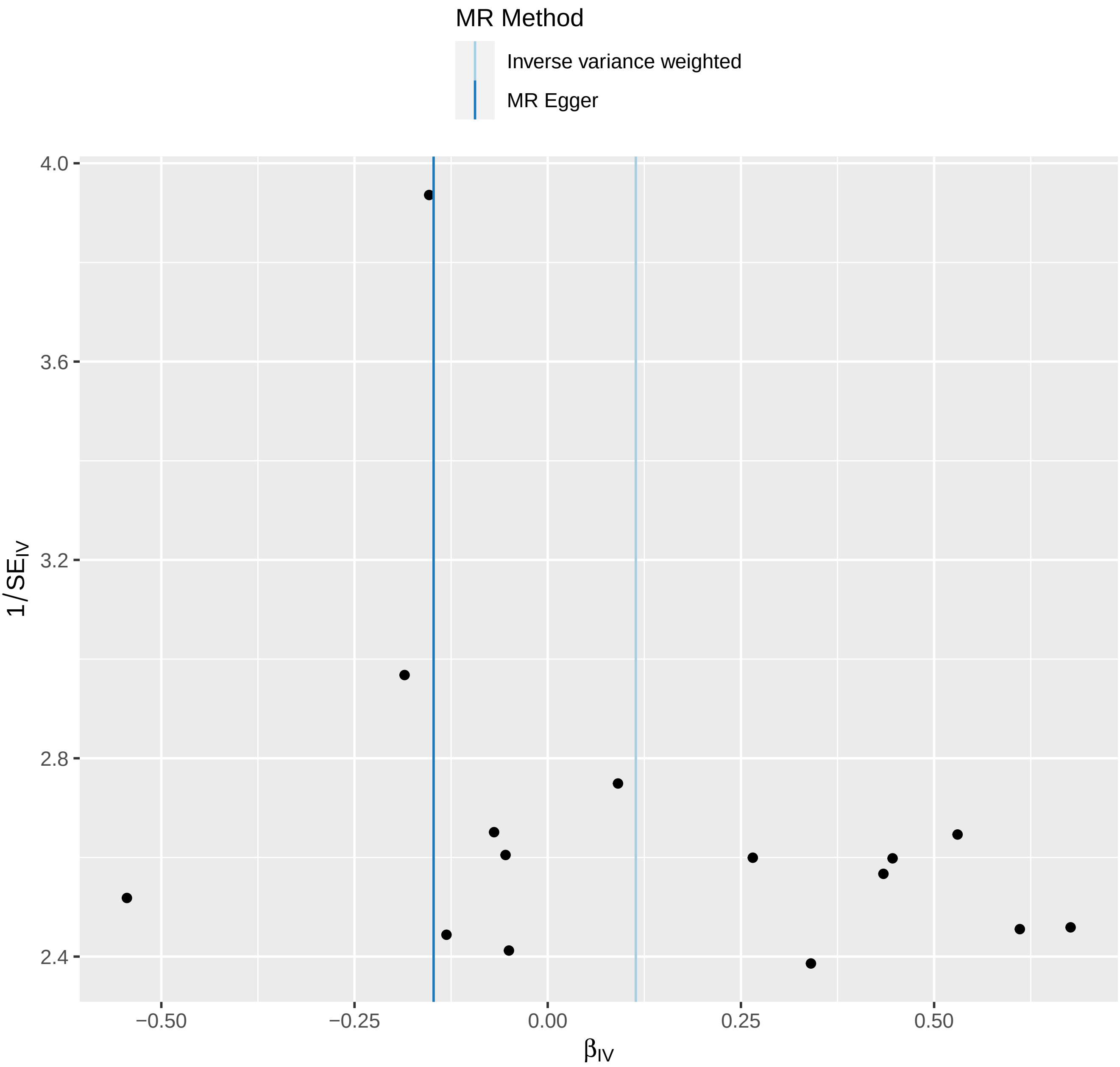

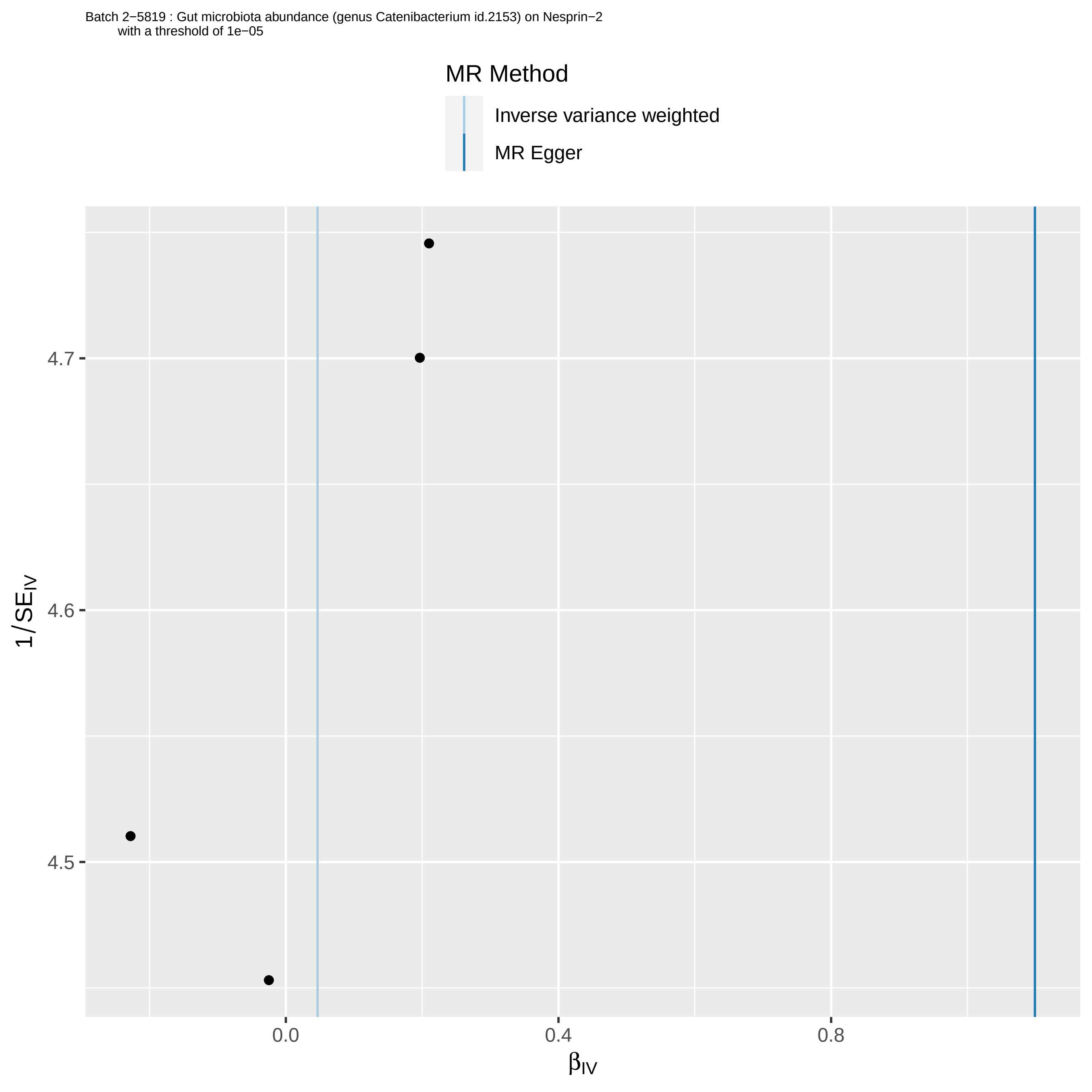
Batch 2−5819 : Gut microbiota abundance (genus Catenibacterium id.2153) on Nesprin−2
with a threshold of 1e−05
MR Method
Inverse variance weighted
MR Egger
4.7
4.6
4.5
1/SE$_{IV}$
0.0
0.4
0.8
β$_{IV}$

Batch 2−5820 : Gut microbiota abundance (genus Parasutterella id.2892) on Nesprin−2
with a threshold of 1e−05

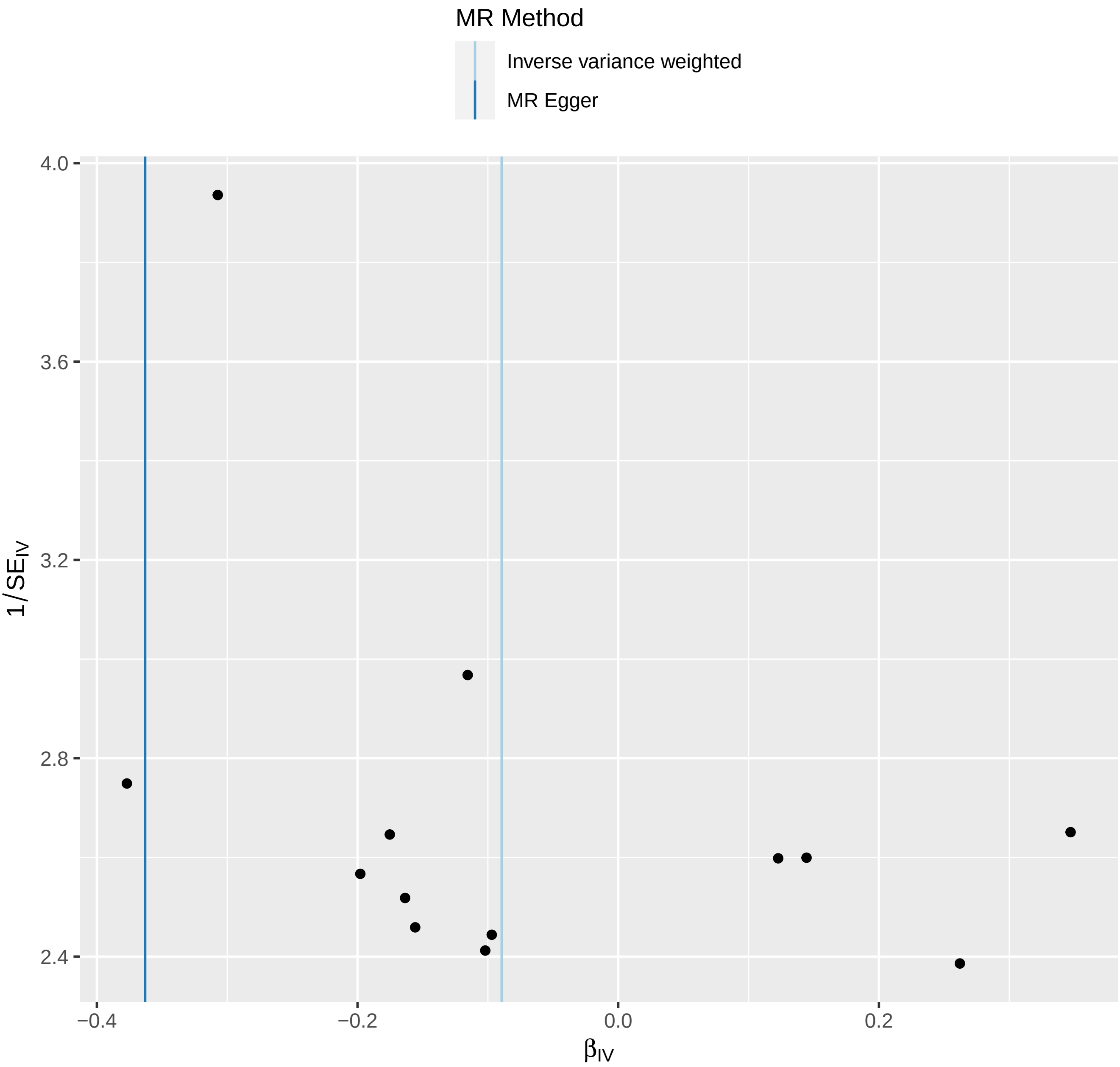

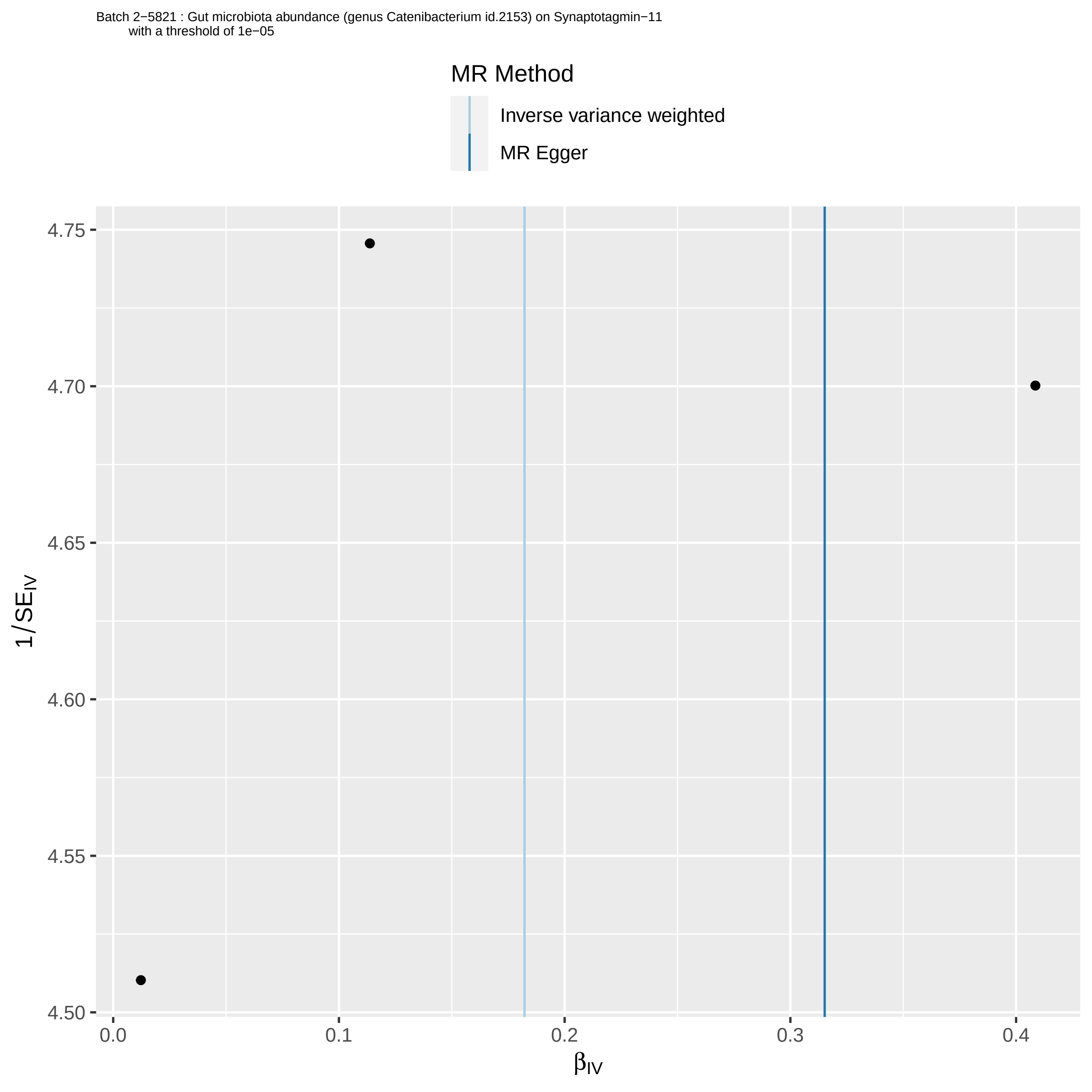

Batch 2−5821 : Gut microbiota abundance (genus Catenibacterium id.2153) on Synaptotagmin−11
with a threshold of 1e−05
MR Method
Inverse variance weighted
MR Egger
4.75
4.70
4.65
4.60
4.55
4.50
1/SE_IV
0.0
0.1
0.2
0.3
0.4
β_IV

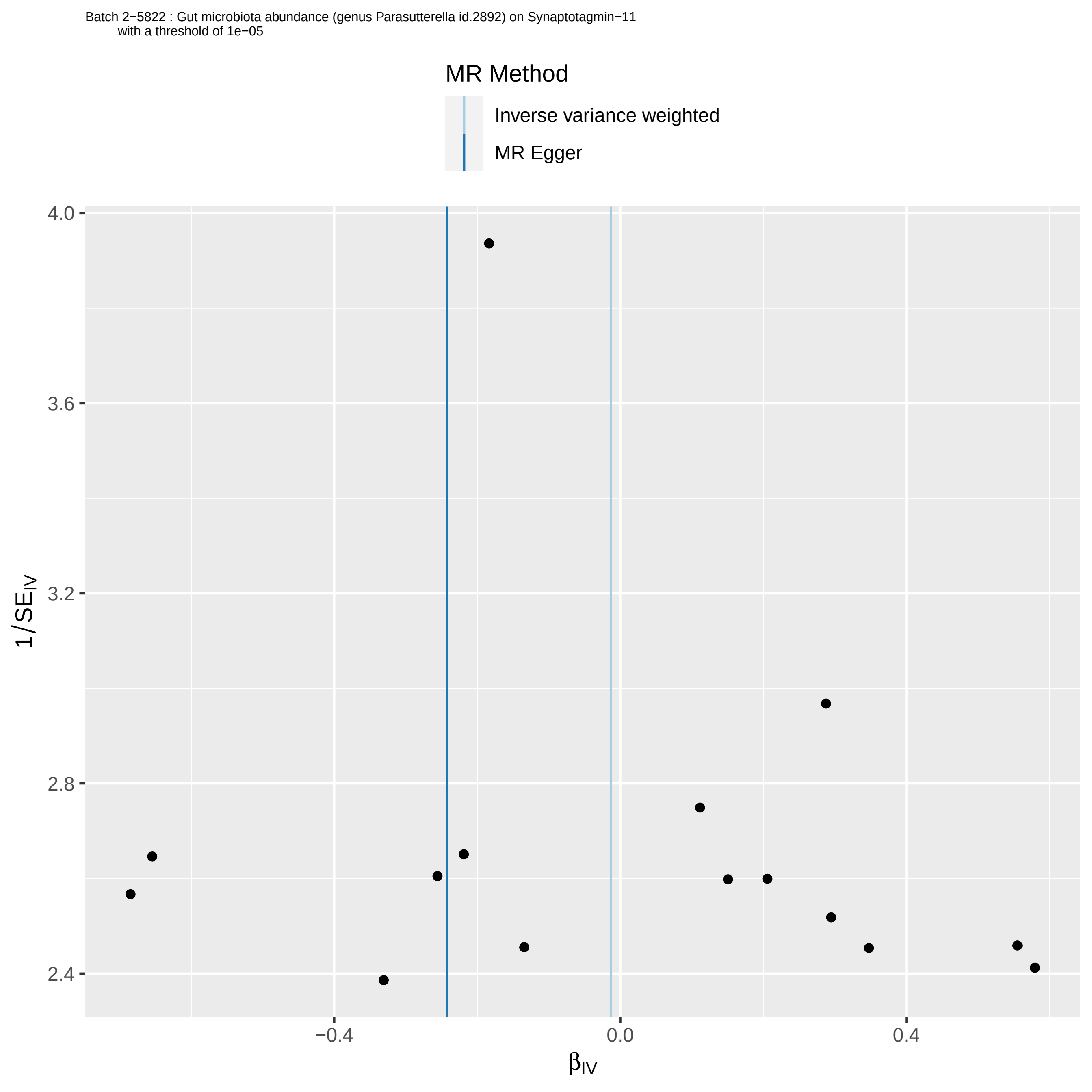

Batch 2−5822 : Gut microbiota abundance (genus Parasutterella id.2892) on Synaptotagmin−11
with a threshold of 1e−05
MR Method
Inverse variance weighted
MR Egger
4.0
3.6
3.2
2.8
2.4
1/SE_IV
−0.4
0.0
0.4
β_IV

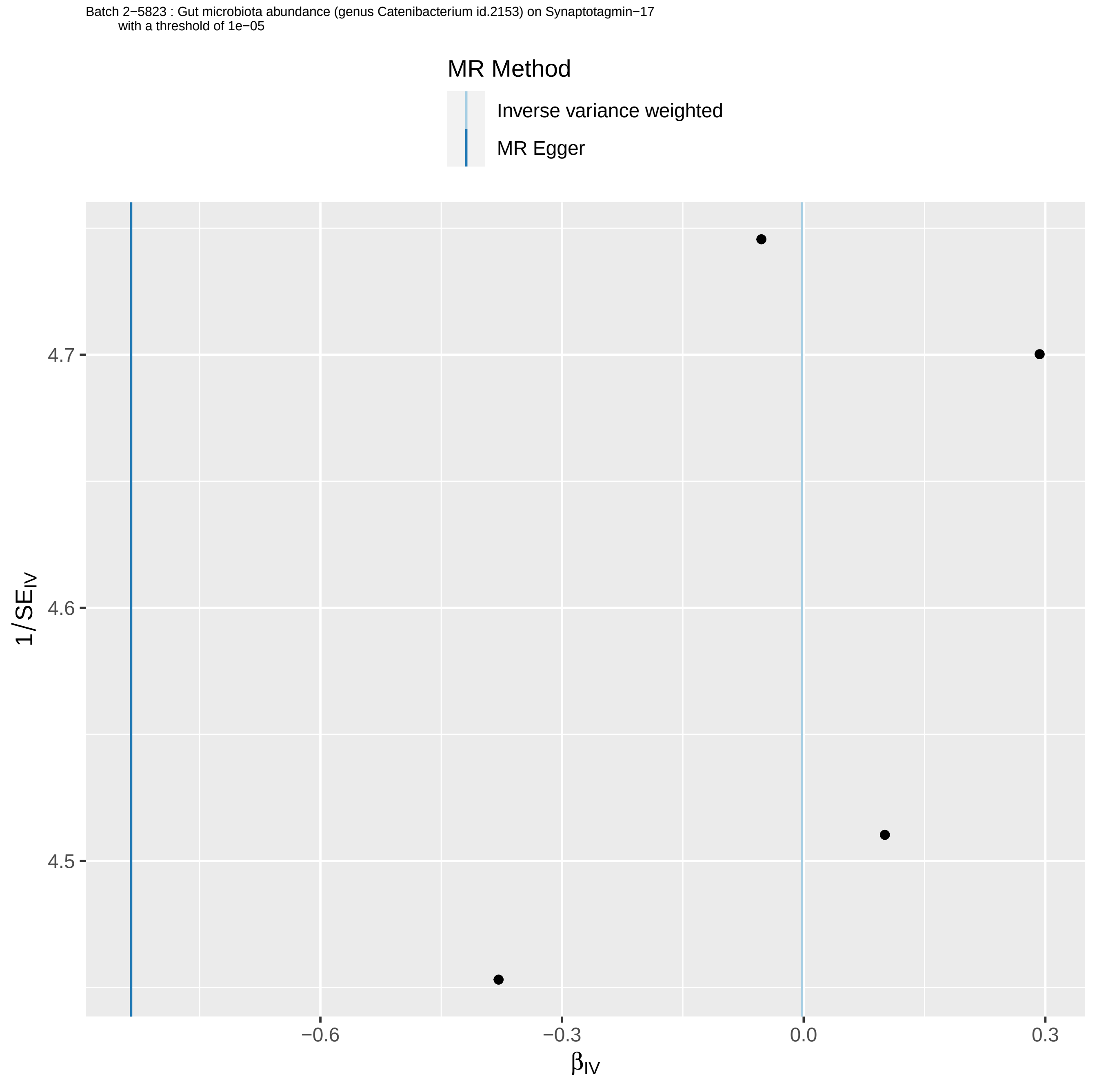
Batch 2−5823 : Gut microbiota abundance (genus Catenibacterium id.2153) on Synaptotagmin−17
with a threshold of 1e−05
MR Method
Inverse variance weighted
MR Egger
4.7
4.6
4.5
$1/SE_{IV}$
−0.6
−0.3
0.0
0.3
β_{IV}

Batch 2−5824 : Gut microbiota abundance (genus Parasutterella id.2892) on Synaptotagmin−17
with a threshold of 1e−05

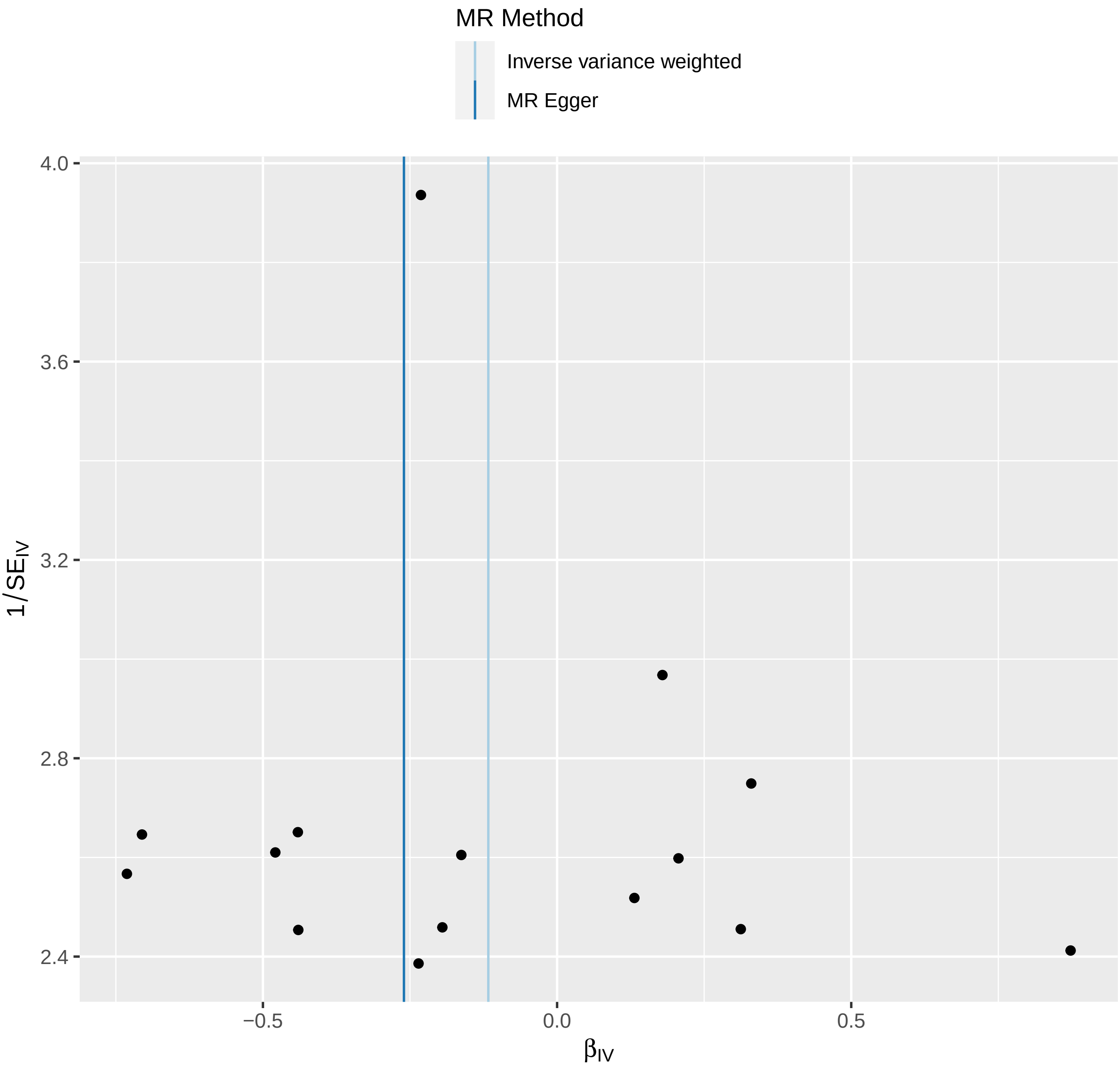

Batch 2−5825 : Gut microbiota abundance (genus Catenibacterium id.2153) on Synaptotagmin−2
with a threshold of 1e−05

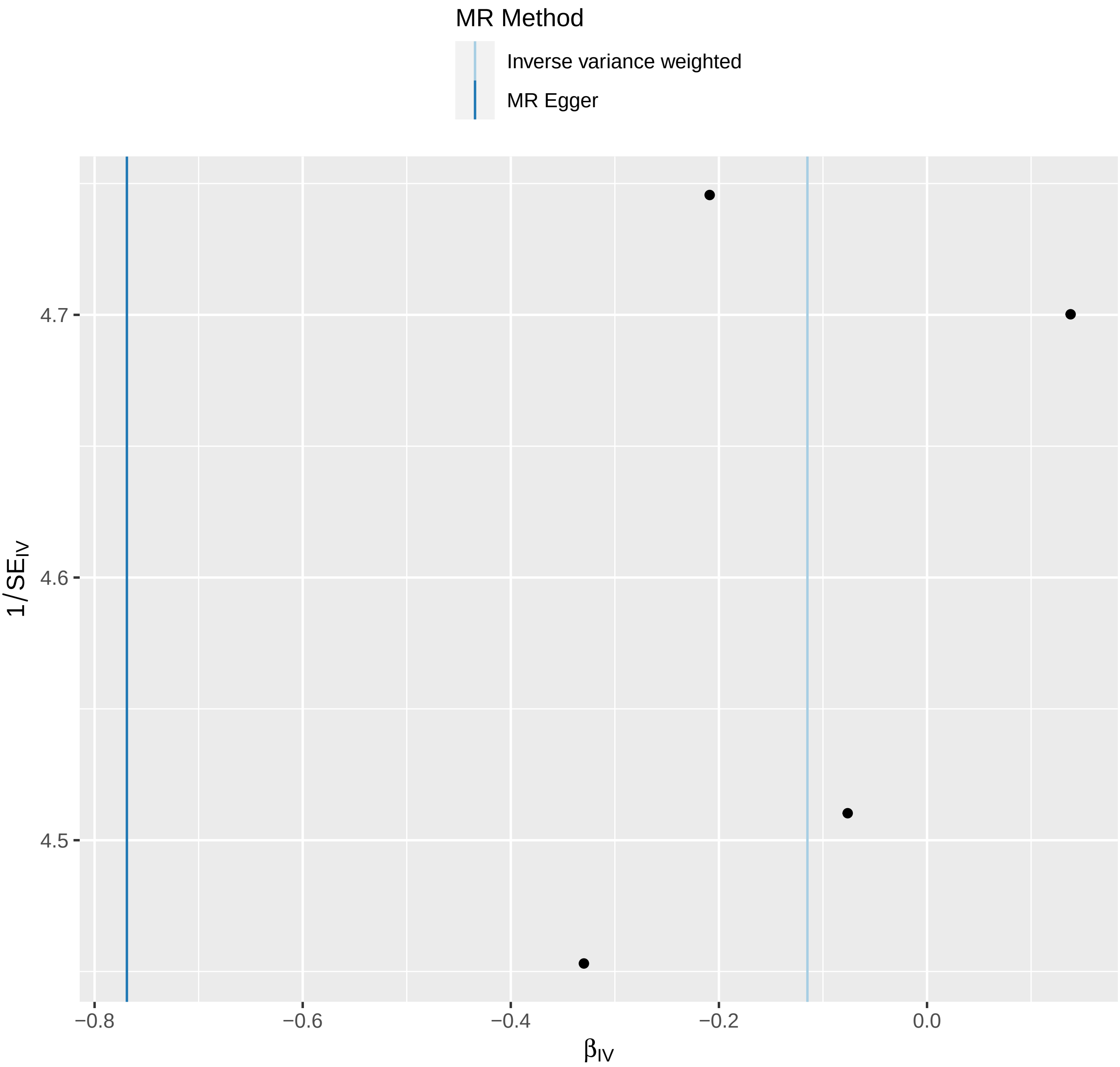

Batch 2−5826 : Gut microbiota abundance (genus Parasutterella id.2892) on Synaptotagmin−2
with a threshold of 1e−05

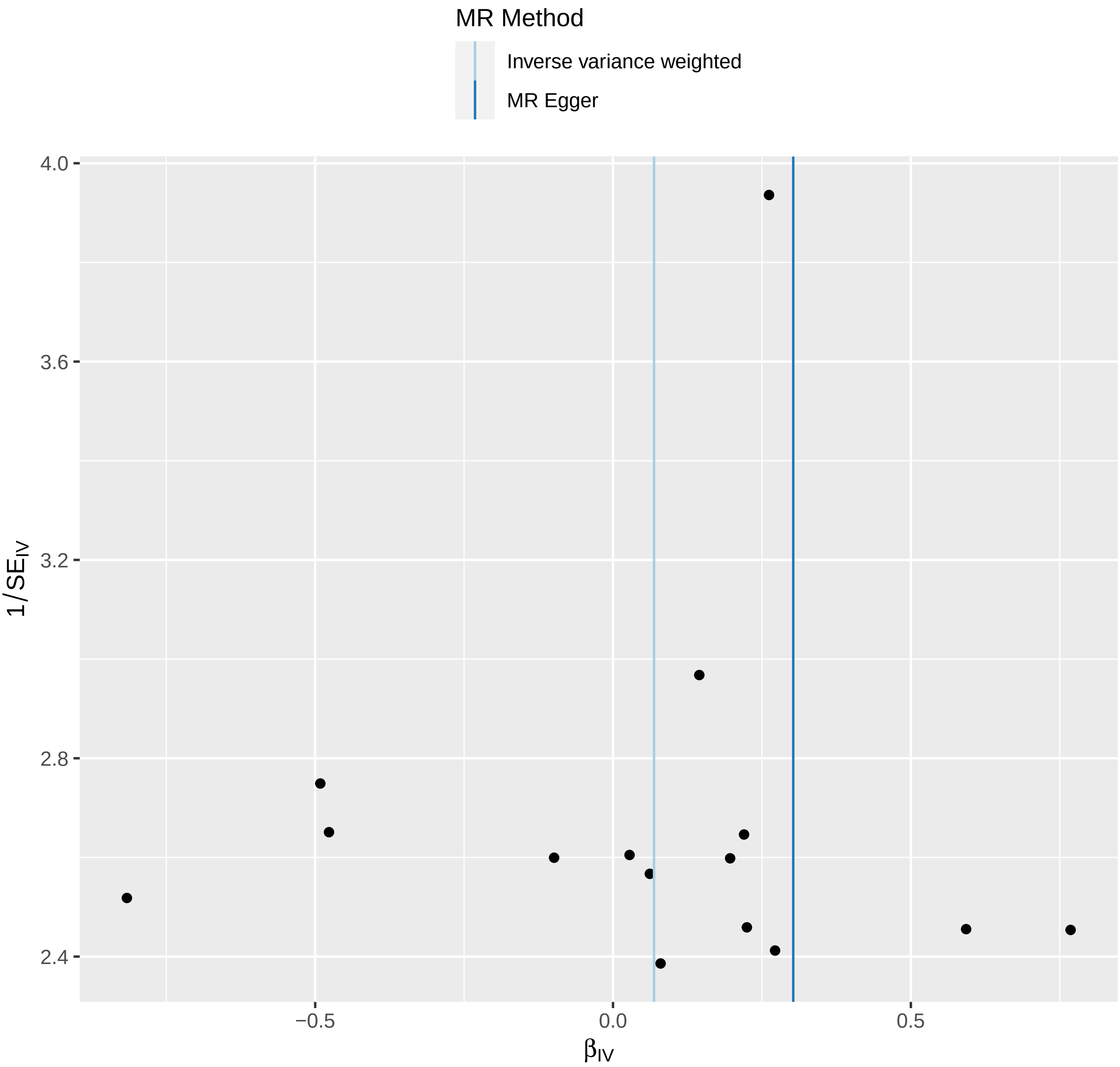

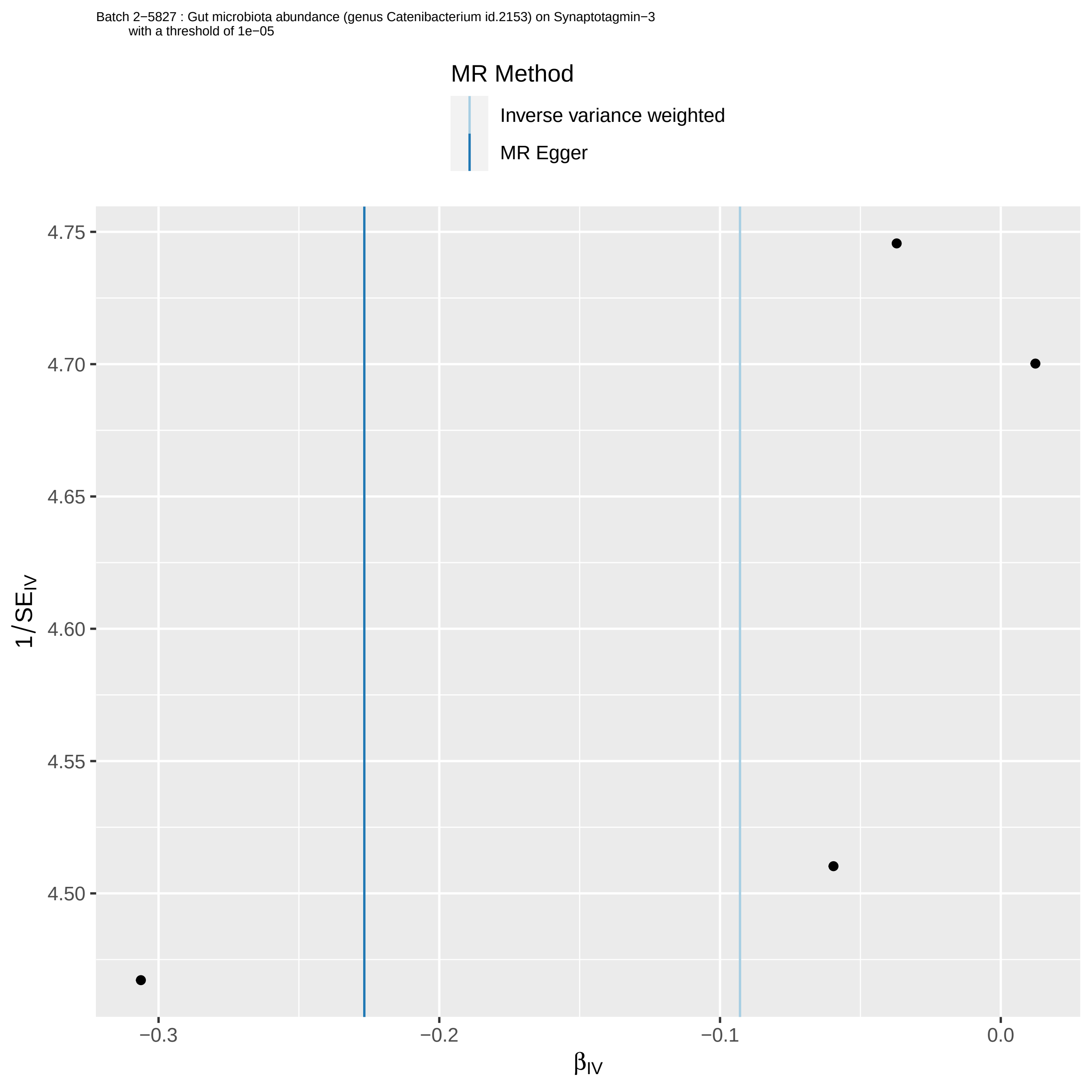

Batch 2−5827 : Gut microbiota abundance (genus Catenibacterium id.2153) on Synaptotagmin−3
with a threshold of 1e−05
MR Method
Inverse variance weighted
MR Egger
4.75
4.70
4.65
4.60
4.55
4.50
1/SE_IV
−0.3
−0.2
−0.1
0.0
β_IV

Batch 2−5828 : Gut microbiota abundance (genus Parasutterella id.2892) on Synaptotagmin−3
with a threshold of 1e−05

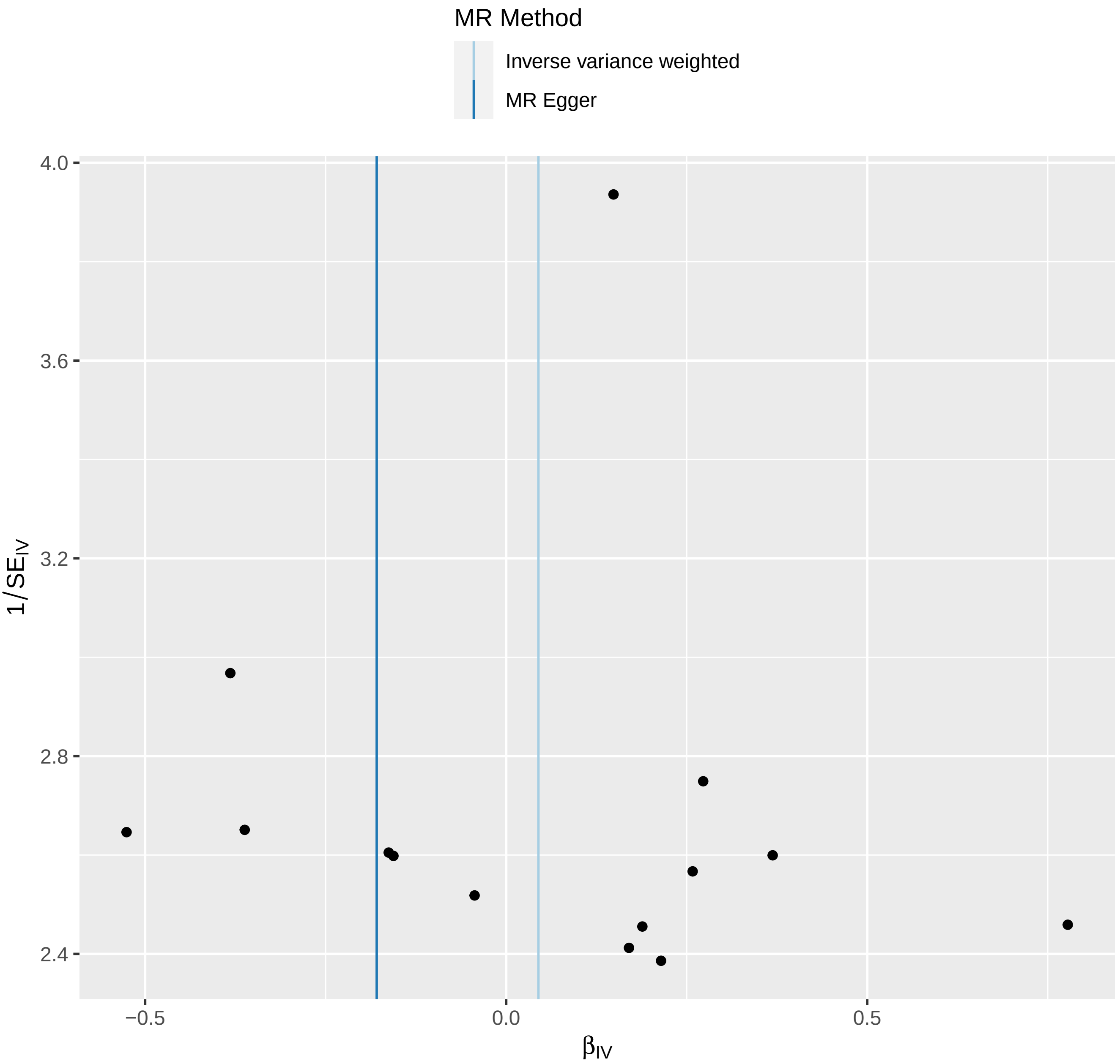

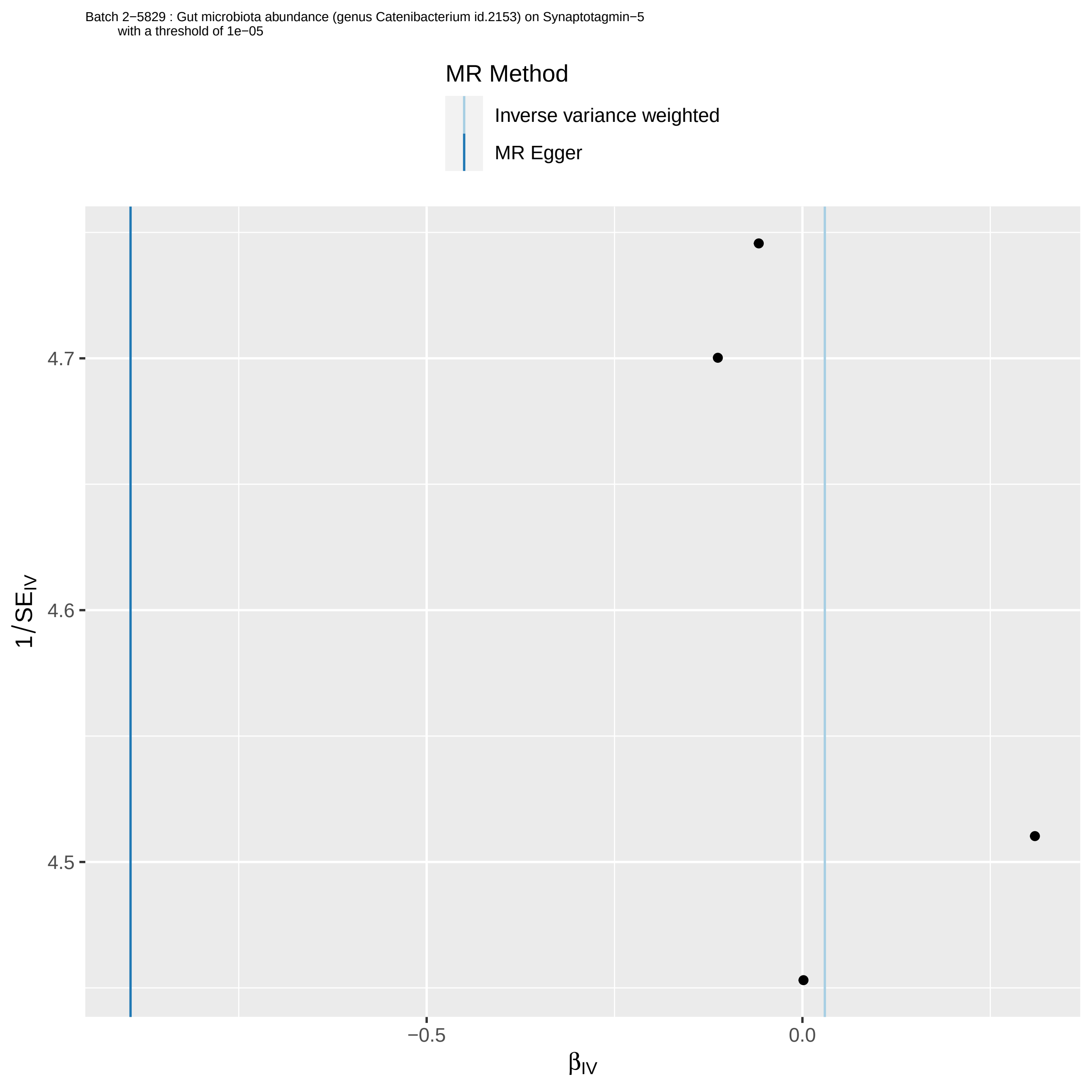
Batch 2−5829 : Gut microbiota abundance (genus Catenibacterium id.2153) on Synaptotagmin−5
with a threshold of 1e−05
MR Method
Inverse variance weighted
MR Egger
4.7
4.6
4.5
1/SE_IV
−0.5
0.0
β_IV

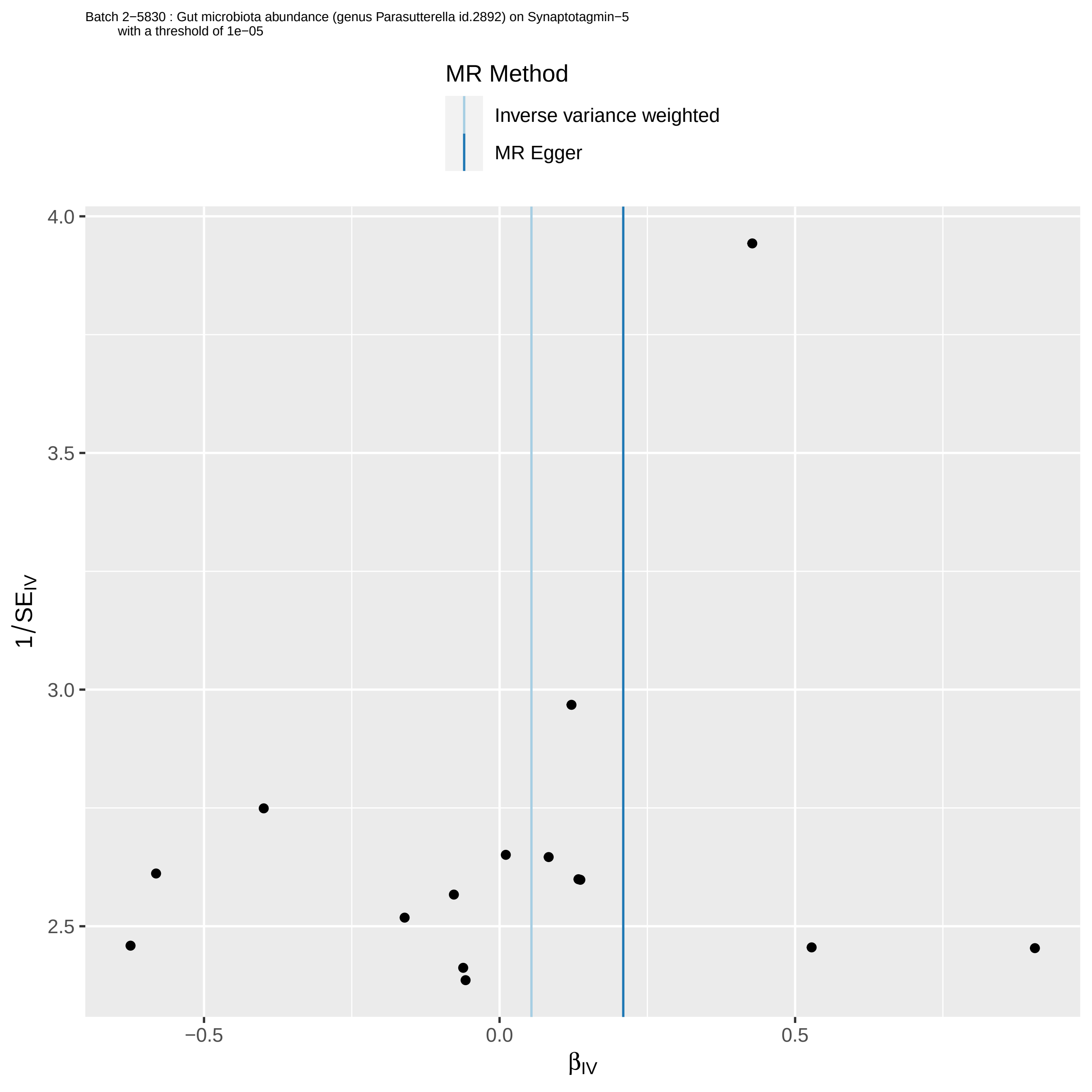

Batch 2−5830 : Gut microbiota abundance (genus Parasutterella id.2892) on Synaptotagmin−5
with a threshold of 1e−05
MR Method
Inverse variance weighted
MR Egger
4.0
3.5
3.0
2.5
1/SE_IV
−0.5
0.0
0.5
β_IV

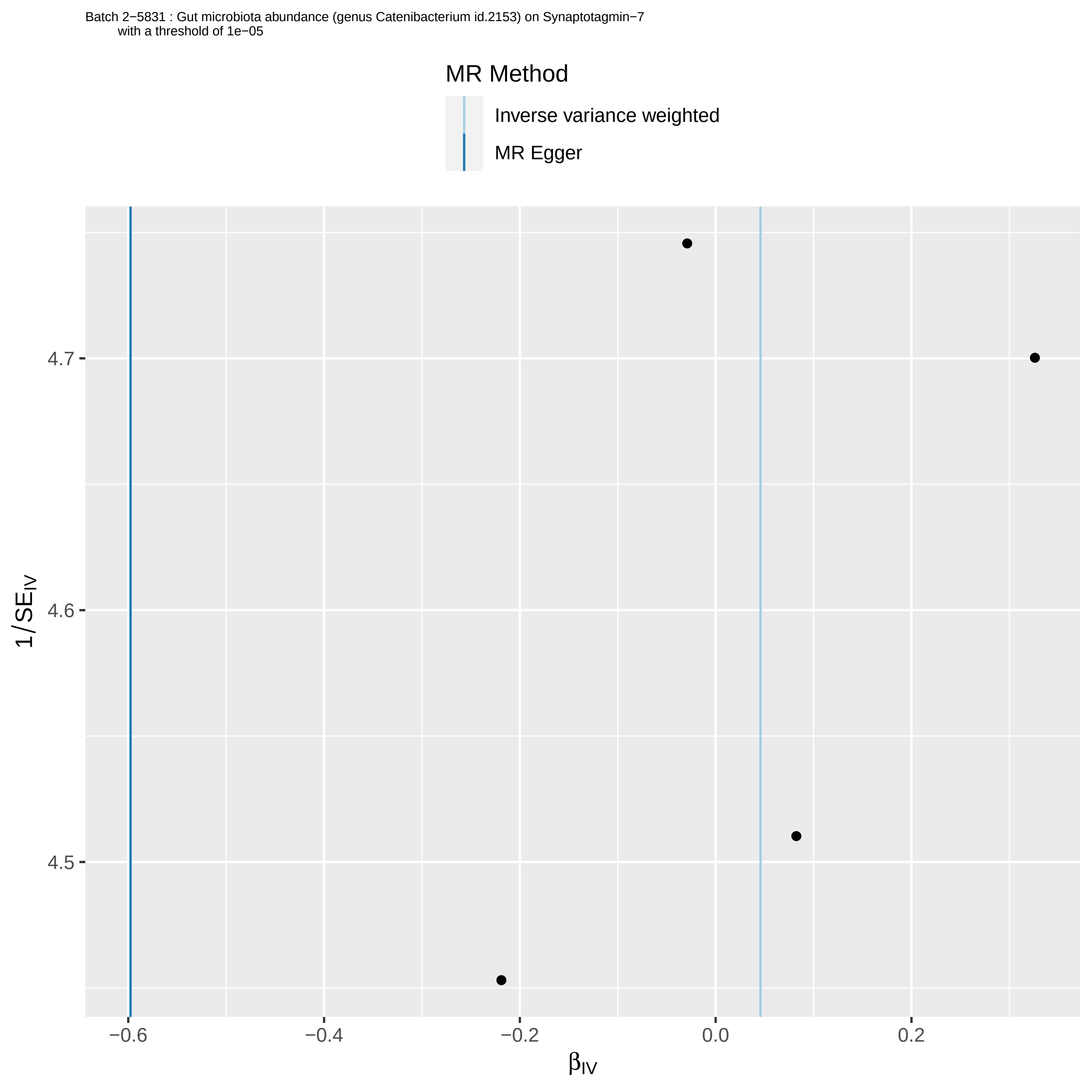

Batch 2−5831 : Gut microbiota abundance (genus Catenibacterium id.2153) on Synaptotagmin−7
with a threshold of 1e−05
MR Method
Inverse variance weighted
MR Egger
4.7
4.6
4.5
1/SE_IV
−0.6
−0.4
−0.2
0.0
0.2
β_IV

Batch 2−5832 : Gut microbiota abundance (genus Parasutterella id.2892) on Synaptotagmin−7
with a threshold of 1e−05

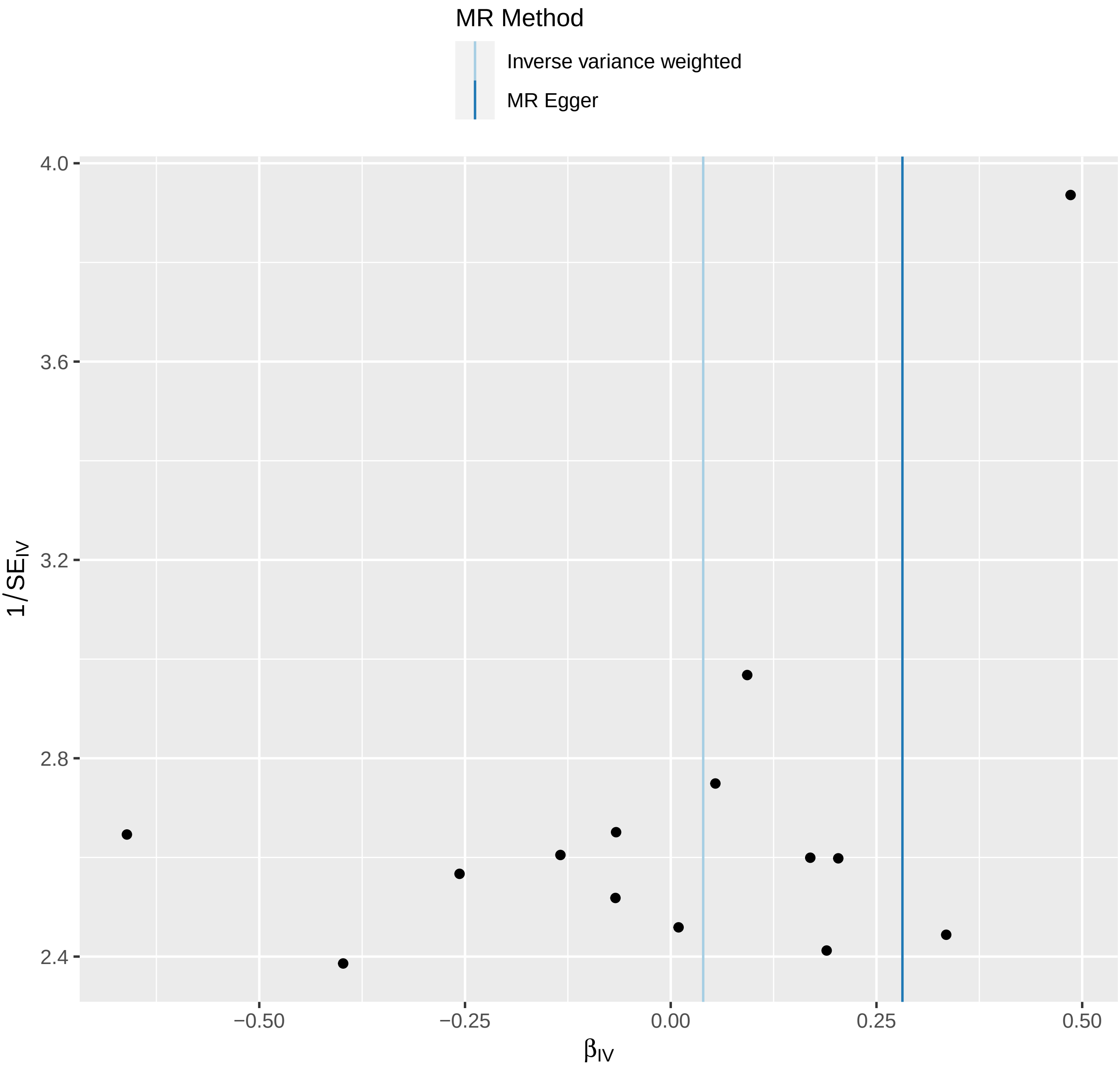

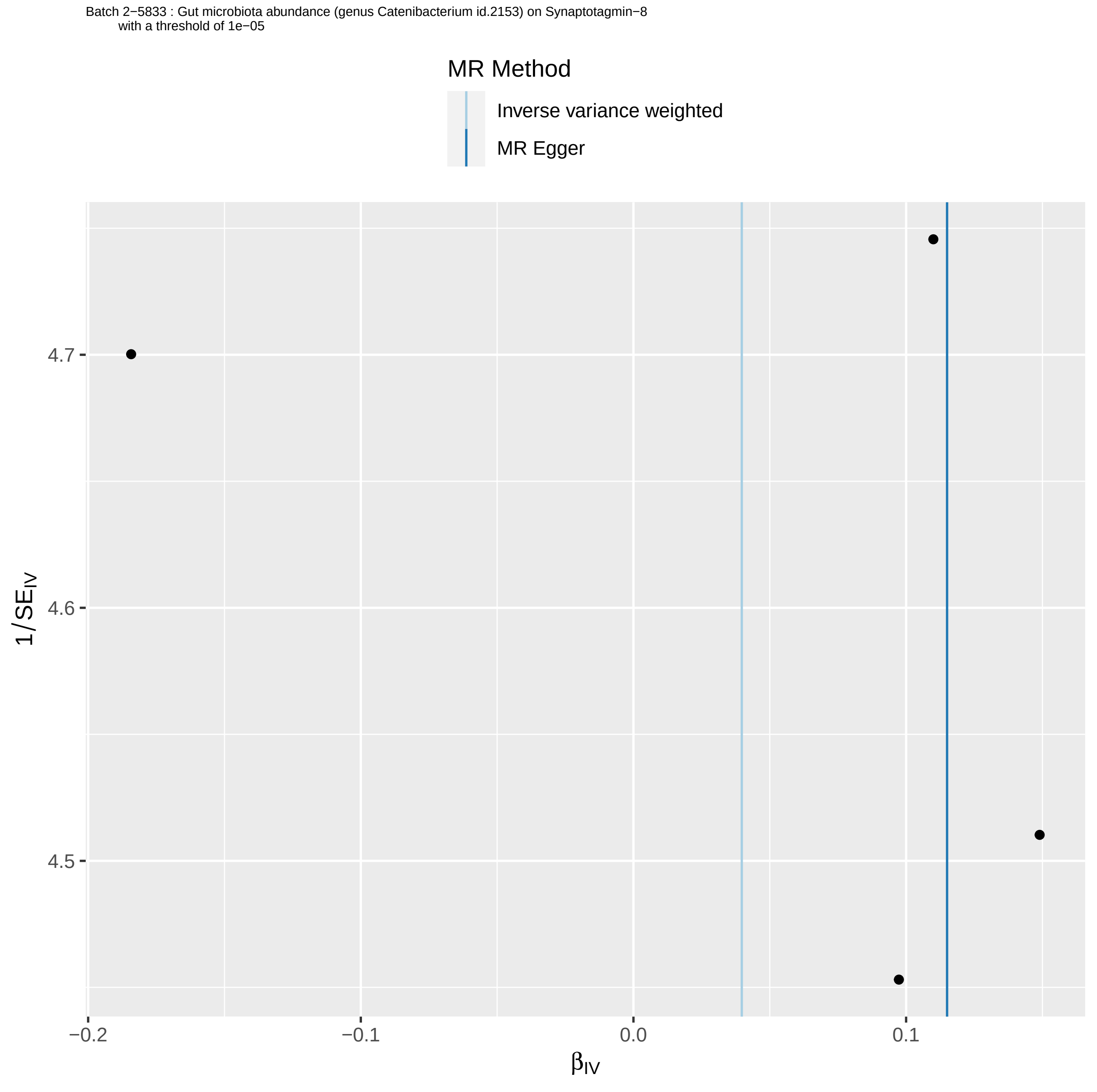
Batch 2−5833 : Gut microbiota abundance (genus Catenibacterium id.2153) on Synaptotagmin−8
with a threshold of 1e−05
MR Method
Inverse variance weighted
MR Egger
4.7
4.6
4.5
1/SE$_{IV}$
−0.2
−0.1
0.0
0.1
β_{IV}

Batch 2−5834 : Gut microbiota abundance (genus Parasutterella id.2892) on Synaptotagmin−8
with a threshold of 1e−05

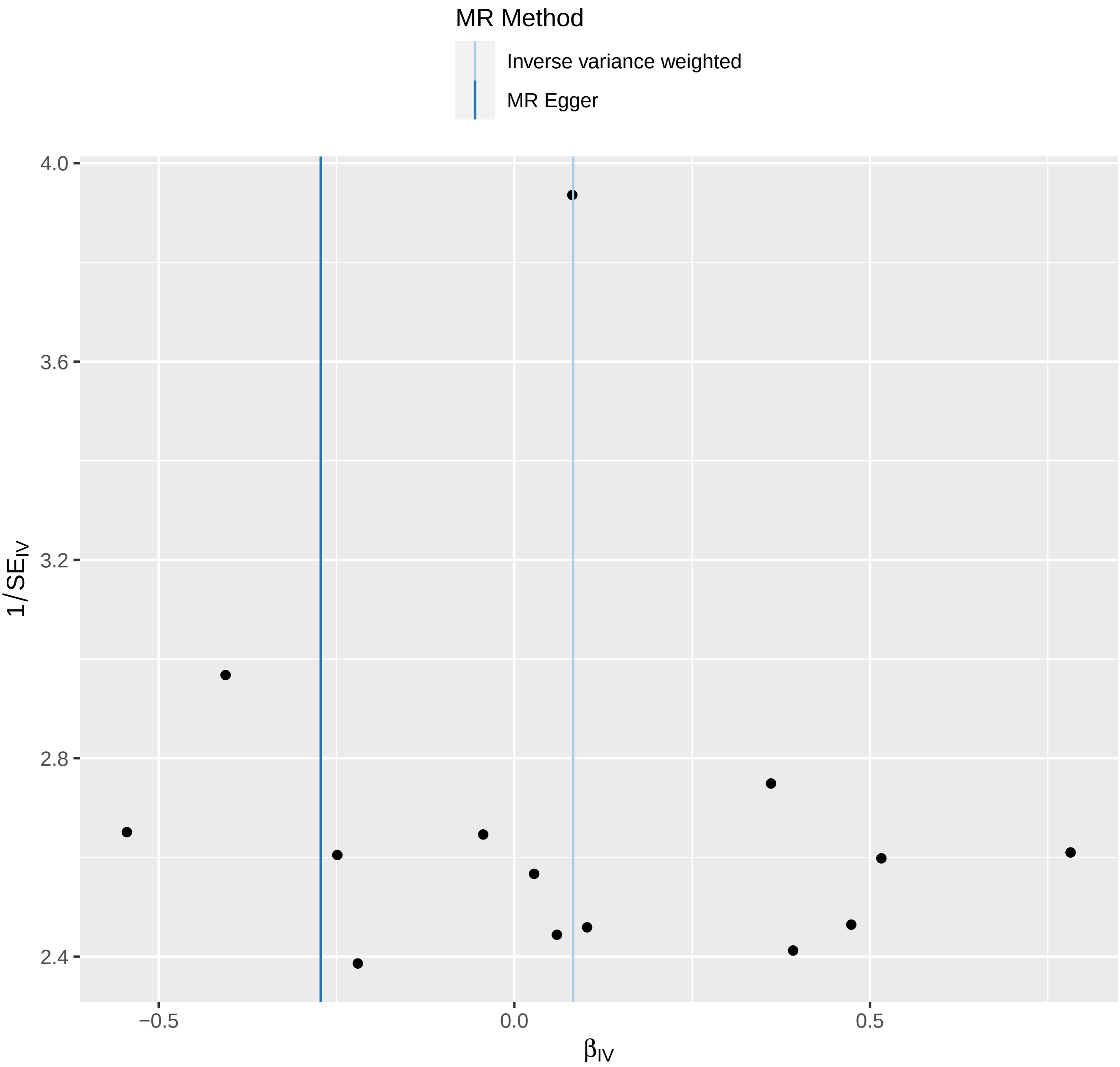

Batch 2−5835 : Gut microbiota abundance (genus Catenibacterium id.2153) on Synaptotagmin−like protein 1
with a threshold of 1e−05

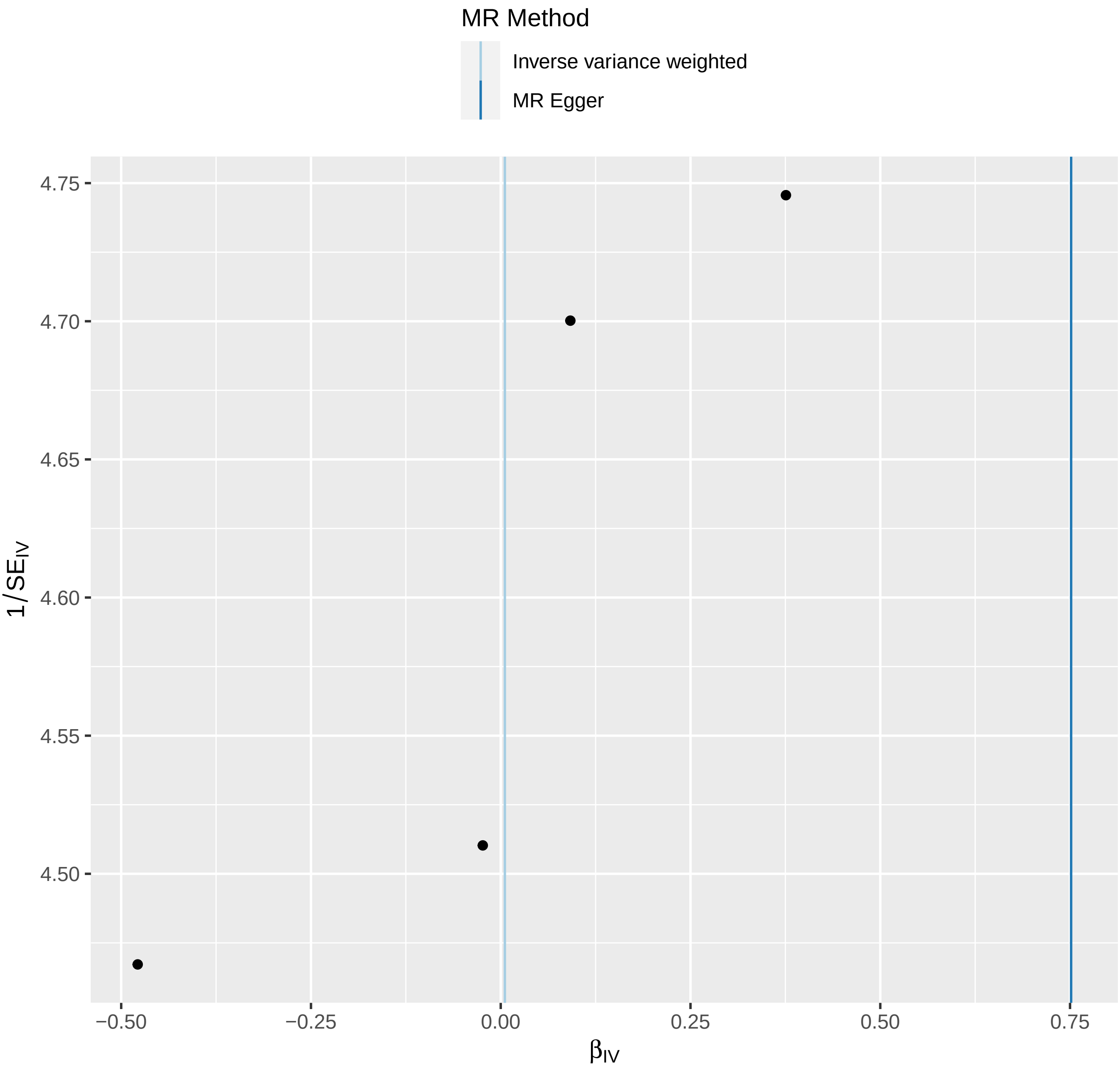

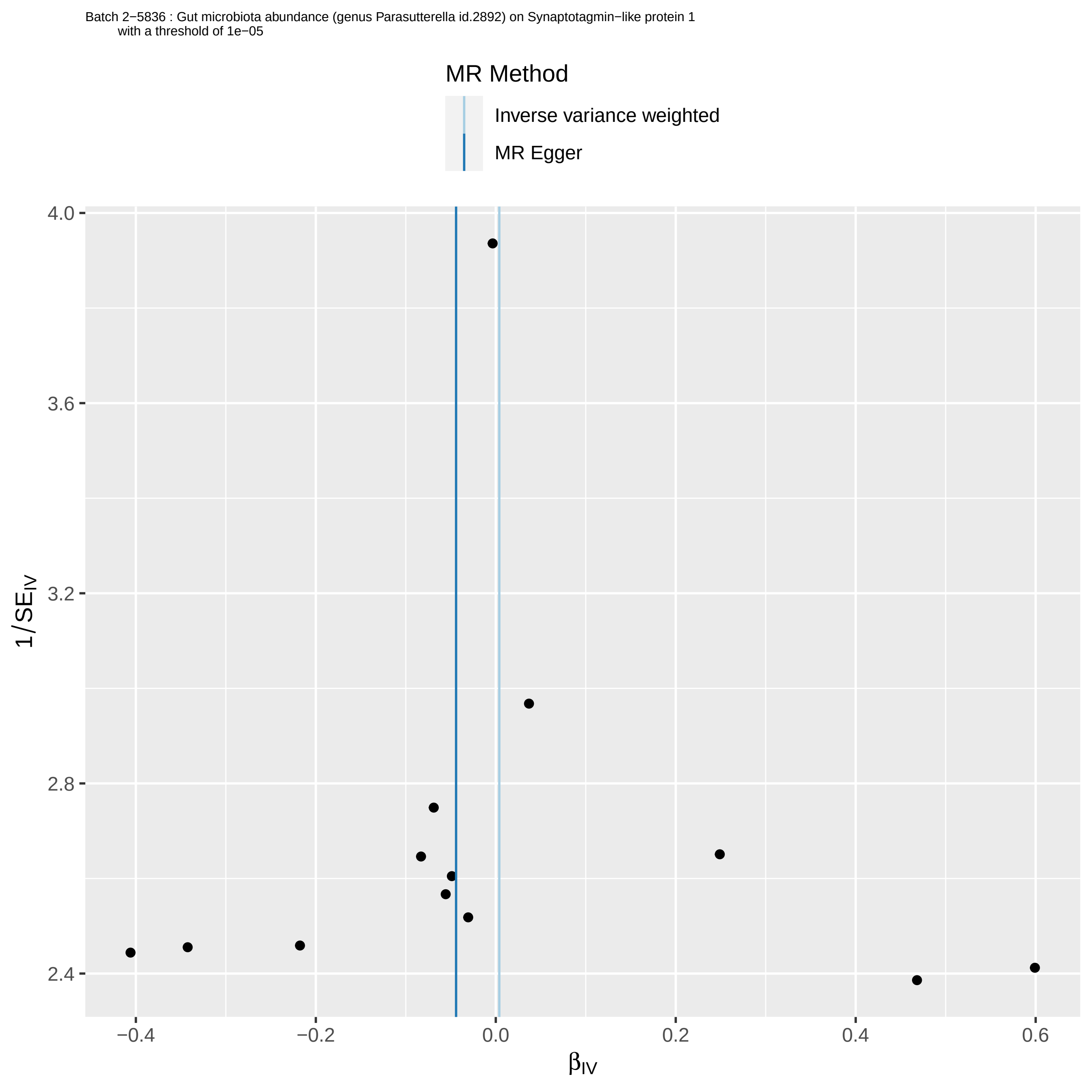

Batch 2−5836 : Gut microbiota abundance (genus Parasutterella id.2892) on Synaptotagmin−like protein 1
with a threshold of 1e−05
MR Method
Inverse variance weighted
MR Egger
4.0
3.6
3.2
2.8
2.4
1/SE_IV
−0.4
−0.2
0.0
0.2
0.4
0.6
β_IV

Batch 2−5837 : Gut microbiota abundance (genus Catenibacterium id.2153) on Synaptotagmin−like protein 4
with a threshold of 1e−05

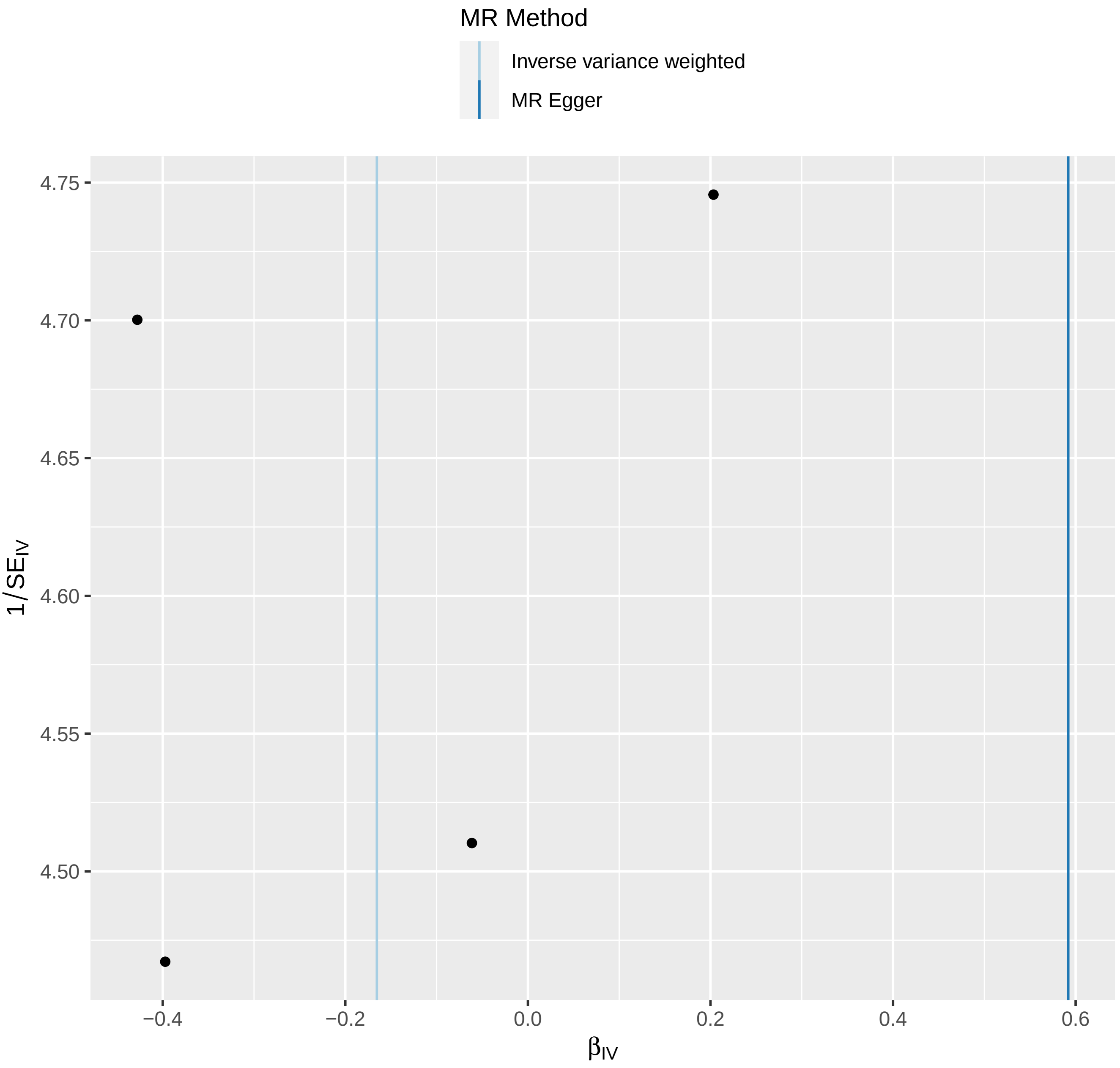

Batch 2−5838 : Gut microbiota abundance (genus Parasutterella id.2892) on Synaptotagmin−like protein 4
with a threshold of 1e−05

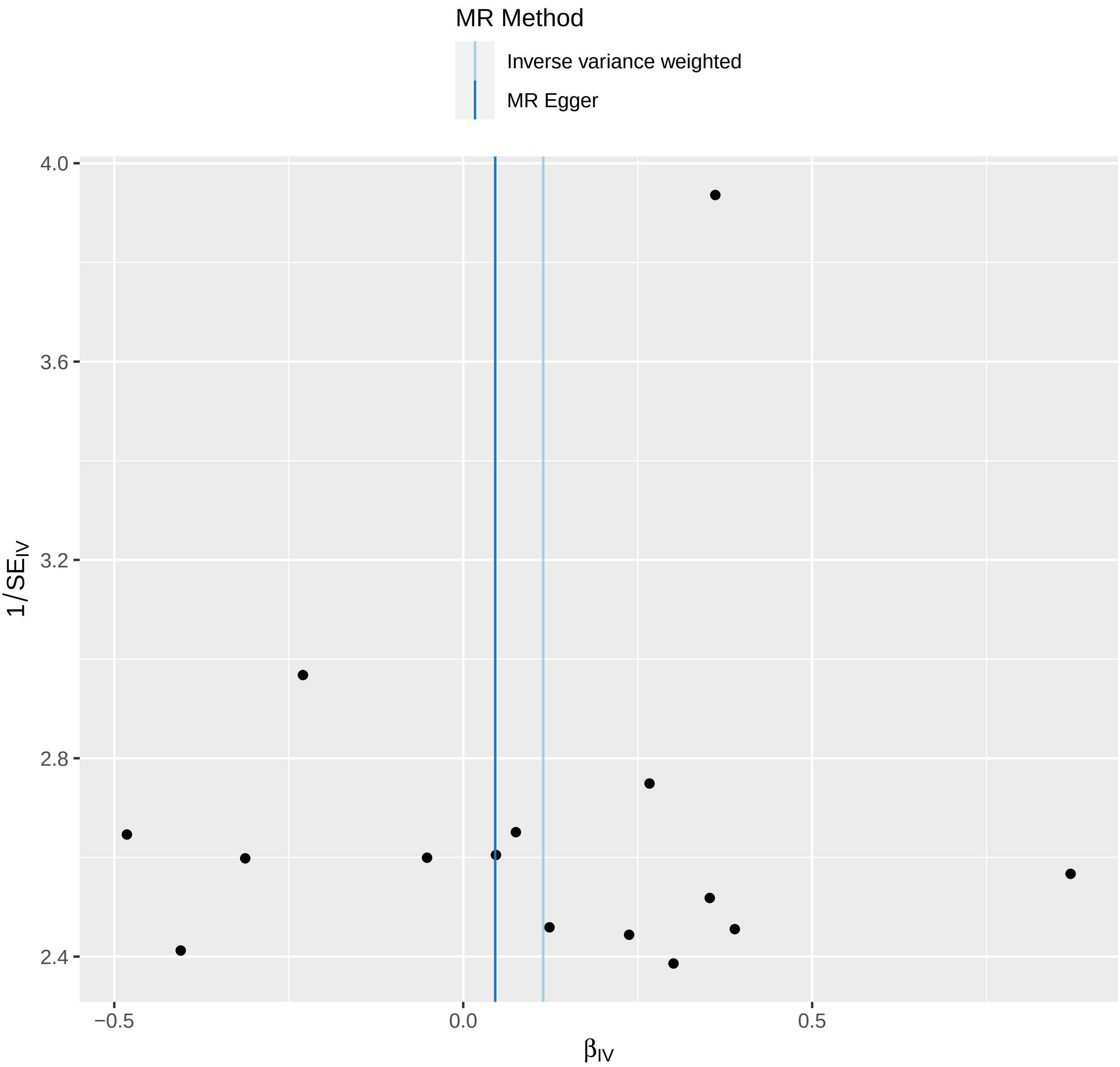

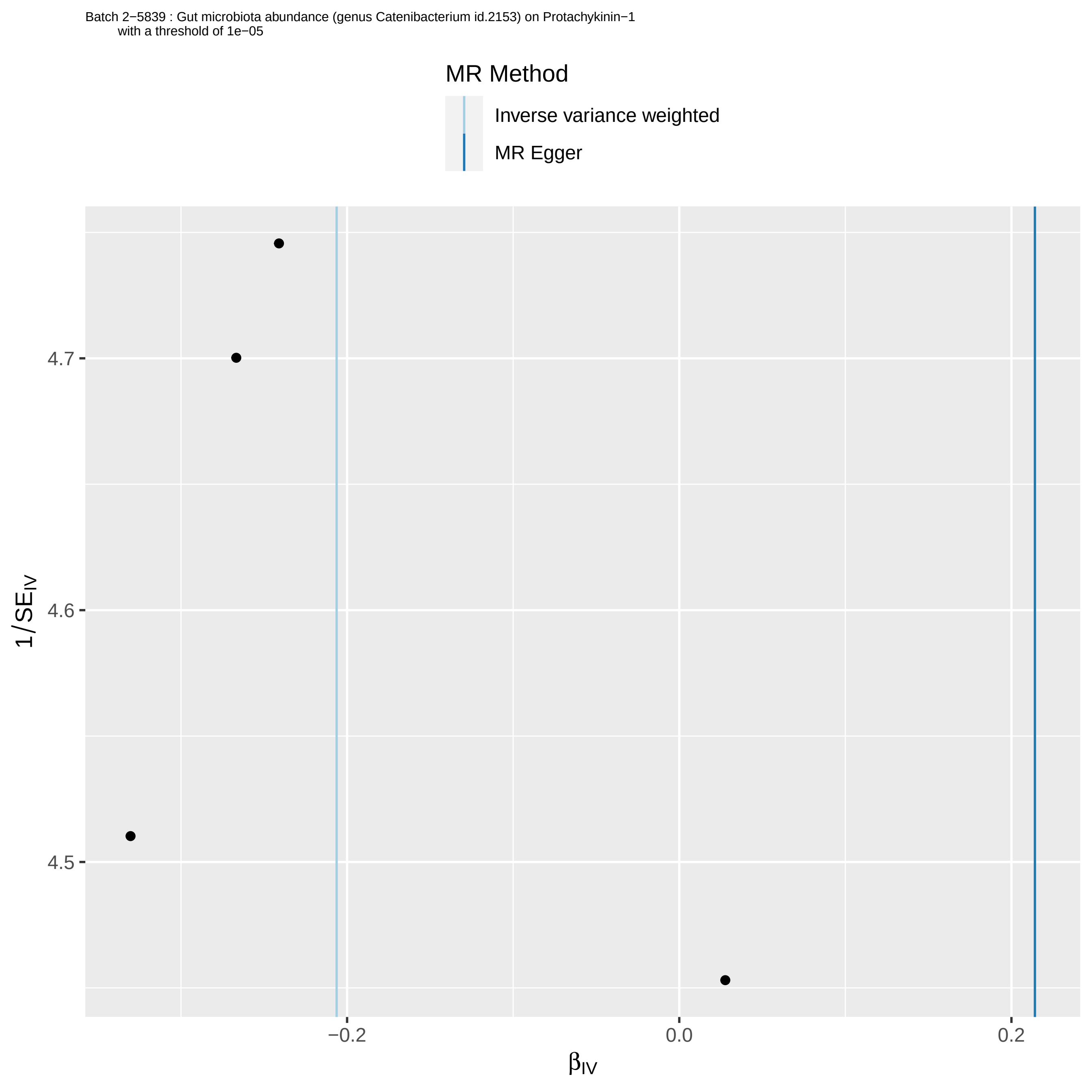

Batch 2−5839 : Gut microbiota abundance (genus Catenibacterium id.2153) on Protachykinin−1
with a threshold of 1e−05
MR Method
Inverse variance weighted
MR Egger
4.7
4.6
4.5
1/SE_IV
−0.2
0.0
0.2
β_IV

Batch 2−5840 : Gut microbiota abundance (genus Parasutterella id.2892) on Protachykinin−1
with a threshold of 1e−05

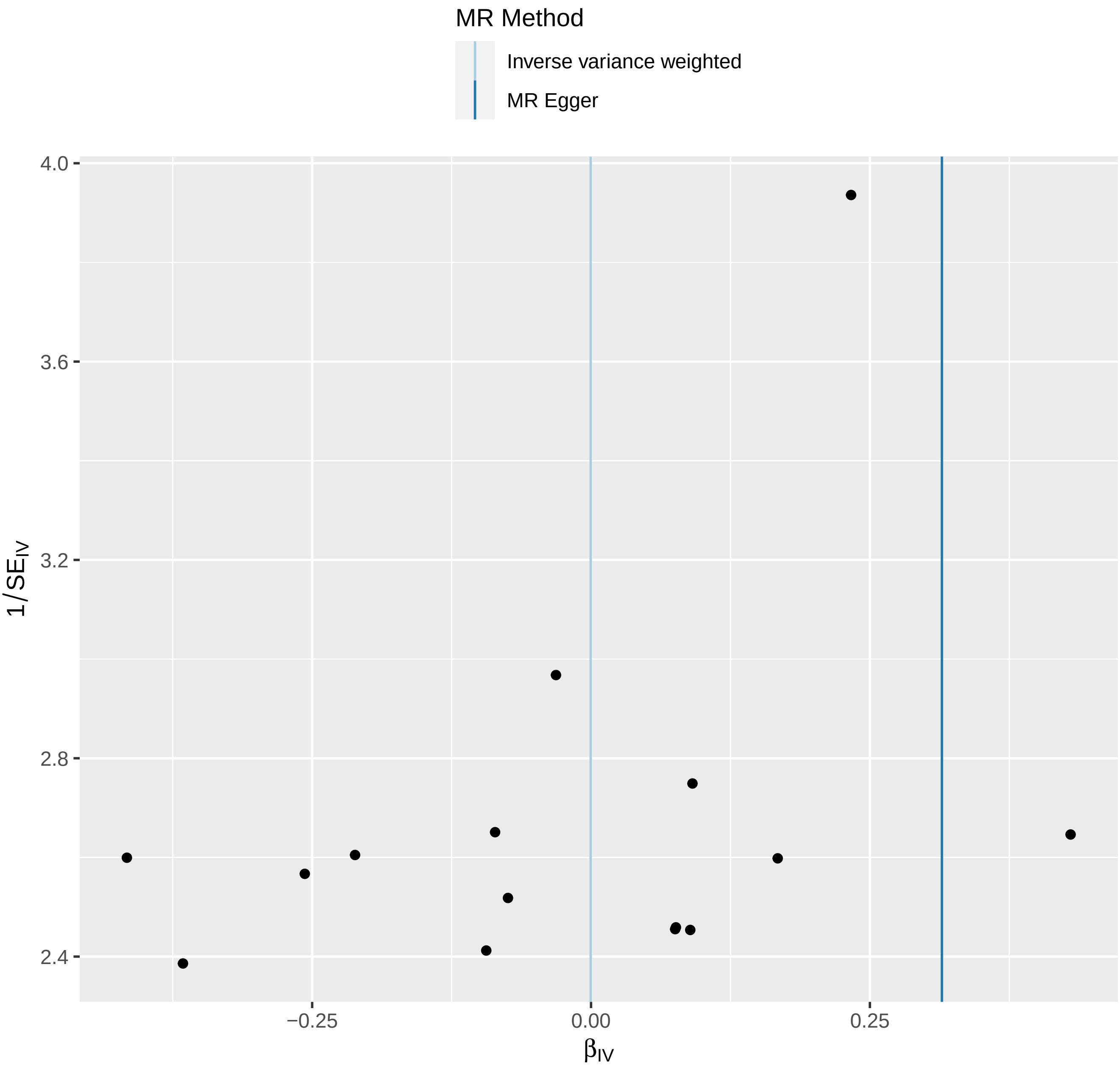

Batch 2−5841 : Gut microbiota abundance (genus Catenibacterium id.2153) on Tachykinin−3
with a threshold of 1e−05

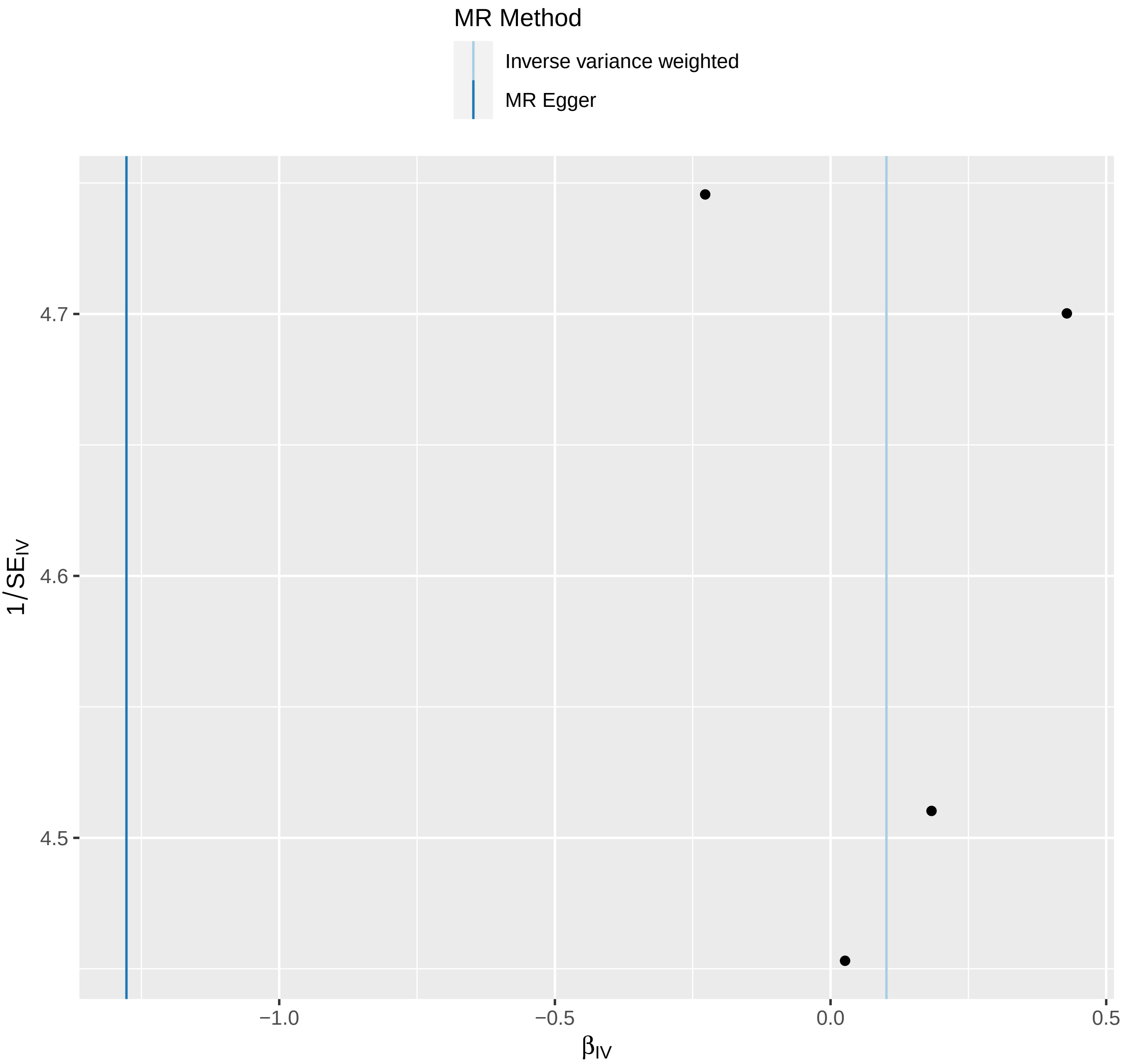

Batch 2−5842 : Gut microbiota abundance (genus Parasutterella id.2892) on Tachykinin−3
with a threshold of 1e−05

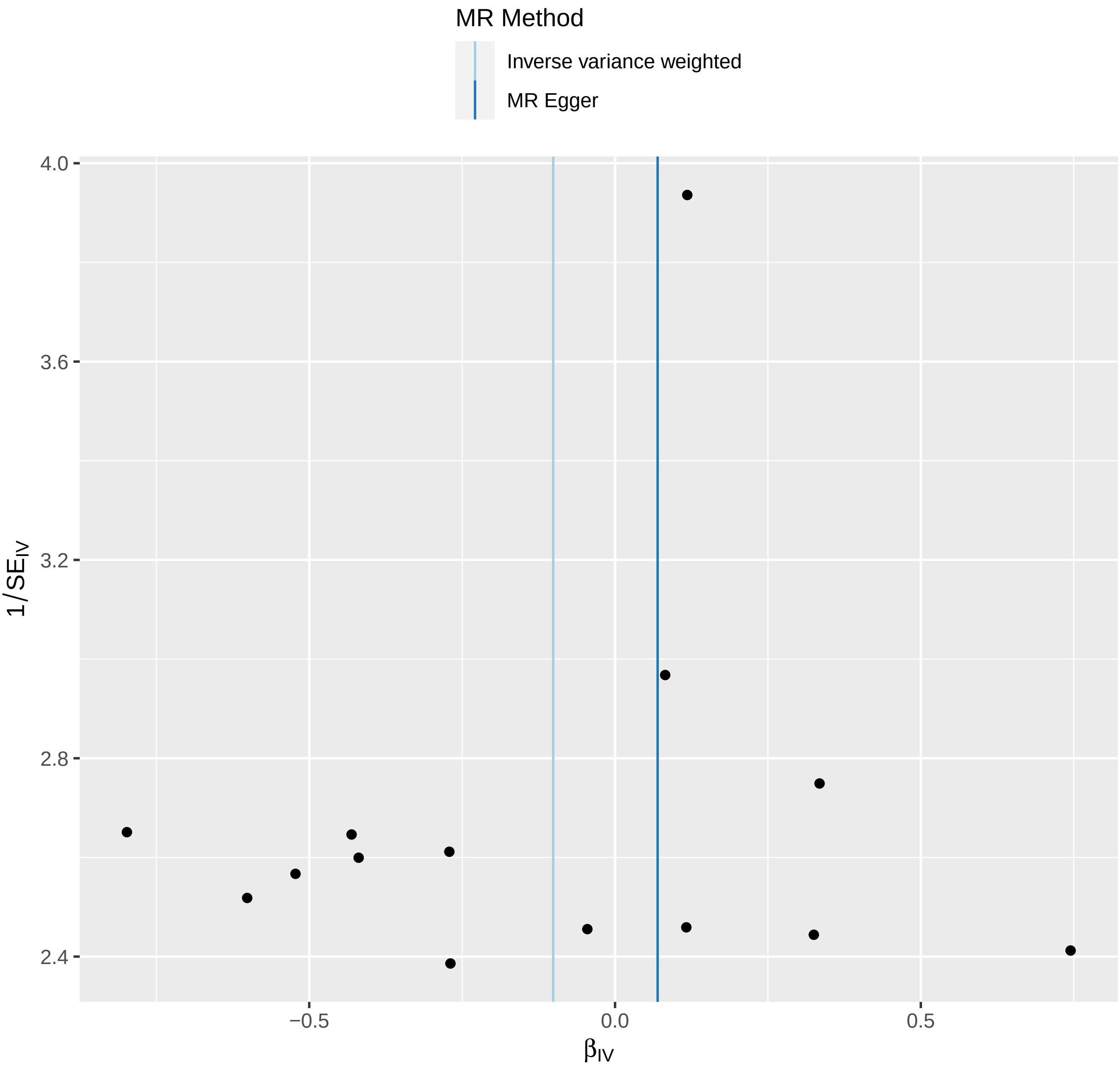

Batch 2−5843 : Gut microbiota abundance (genus Catenibacterium id.2153) on Tachykinin−4
with a threshold of 1e−05

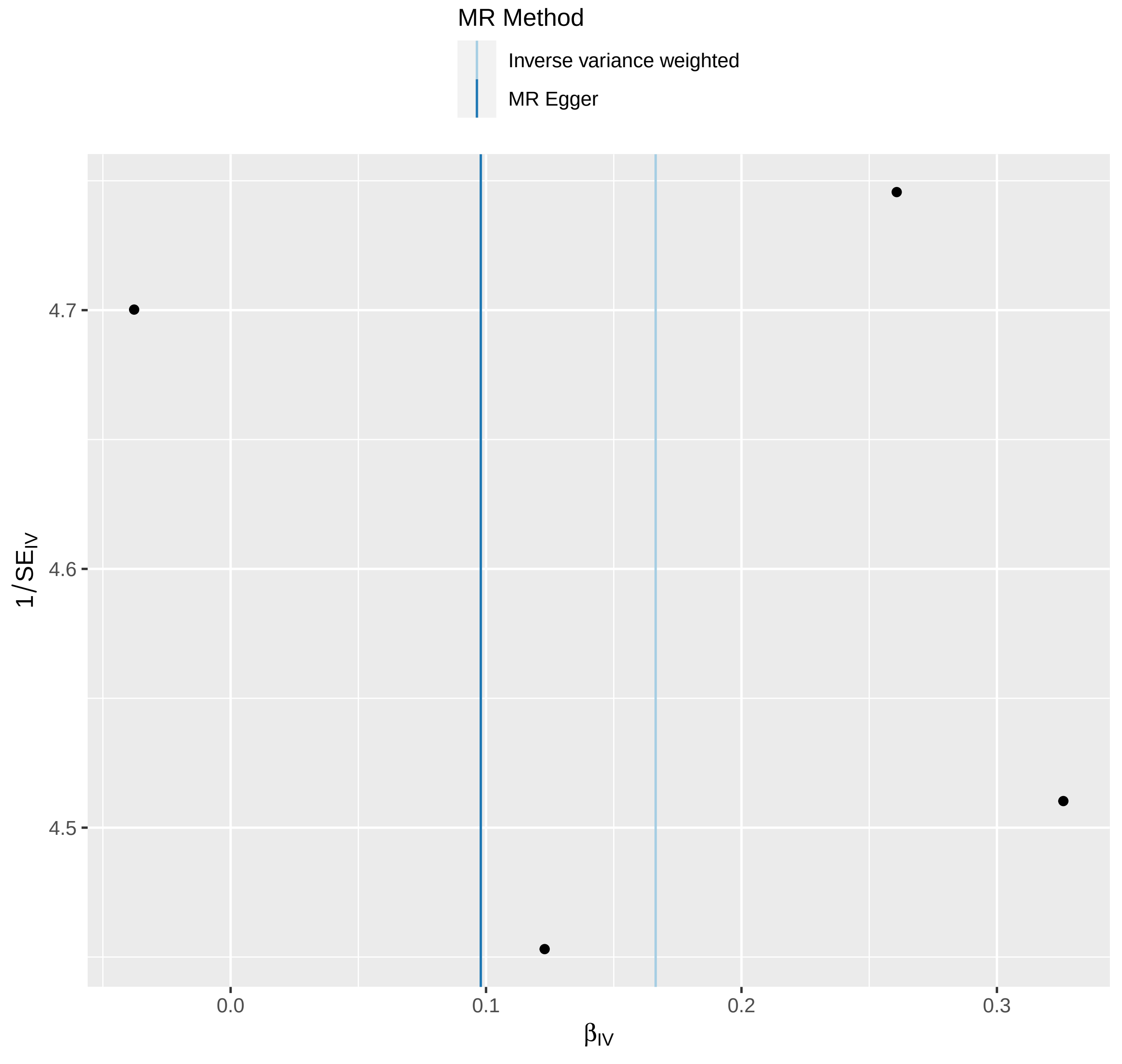

Batch 2−5844 : Gut microbiota abundance (genus Parasutterella id.2892) on Tachykinin−4
with a threshold of 1e−05

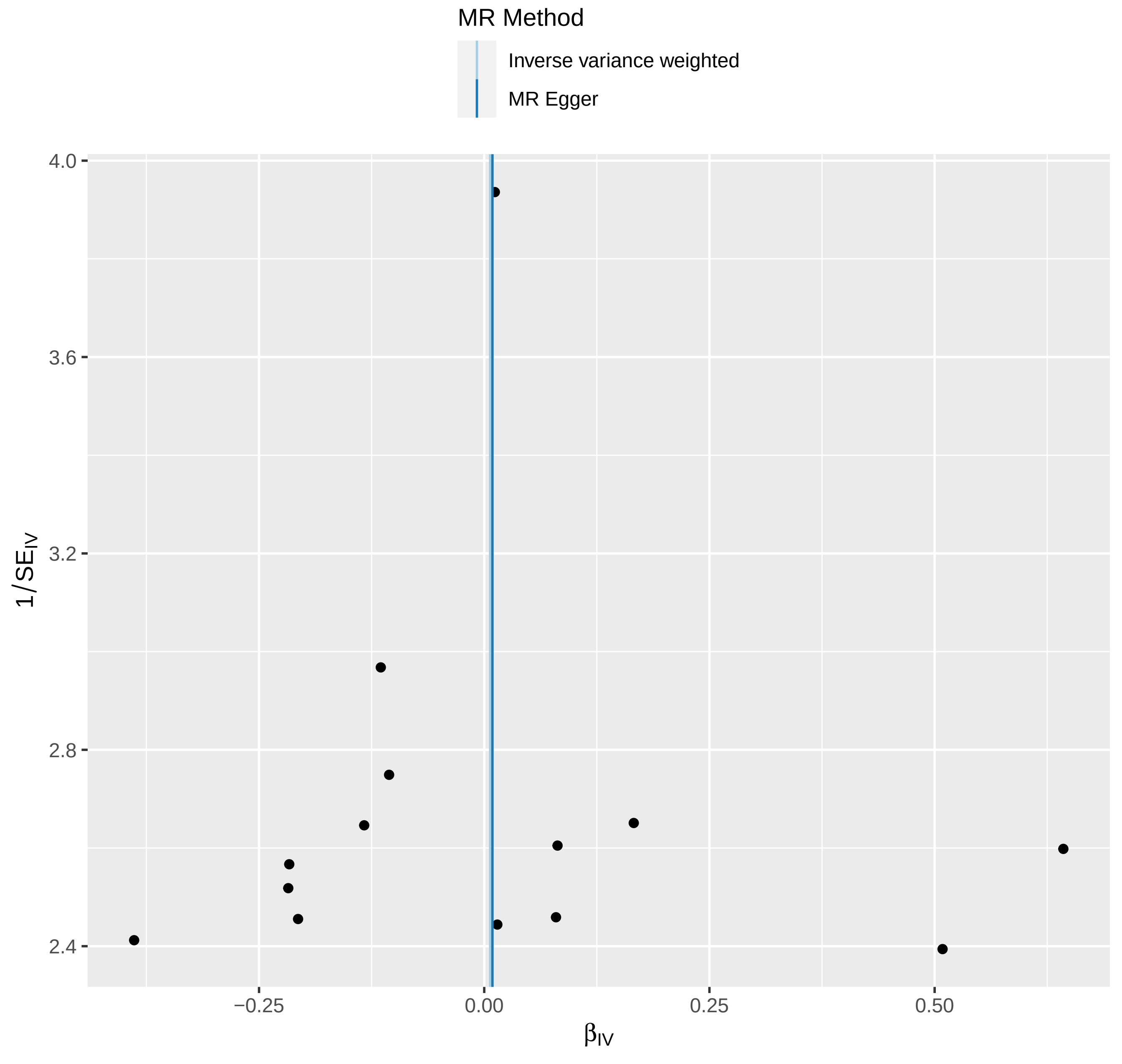

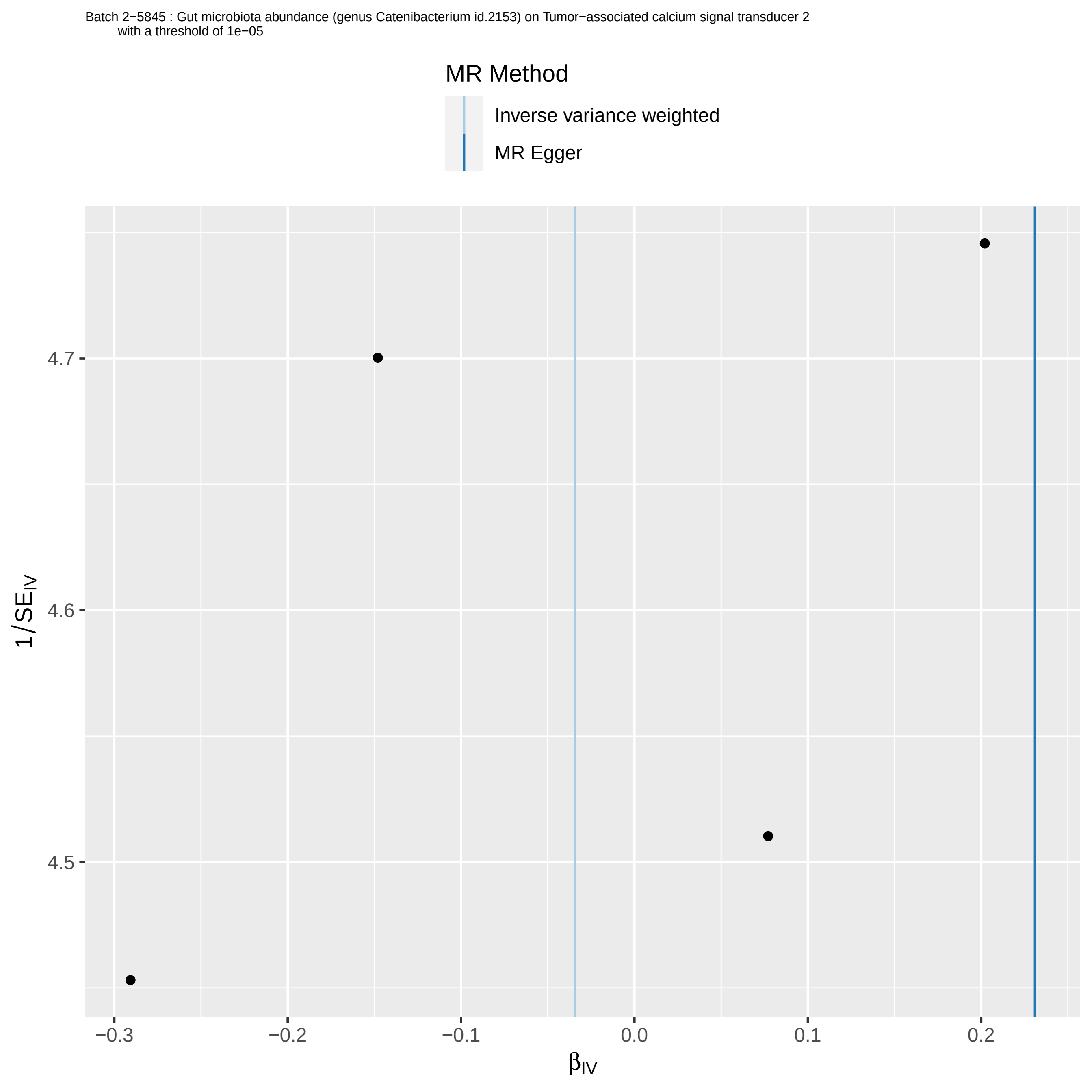

Batch 2−5845 : Gut microbiota abundance (genus Catenibacterium id.2153) on Tumor−associated calcium signal transducer 2
with a threshold of 1e−05
MR Method
Inverse variance weighted
MR Egger
4.7
4.6
4.5
1/SE_IV
−0.3
−0.2
−0.1
0.0
0.1
0.2
β_IV

Batch 2−5846 : Gut microbiota abundance (genus Parasutterella id.2892) on Tumor−associated calcium signal transducer 2
with a threshold of 1e−05

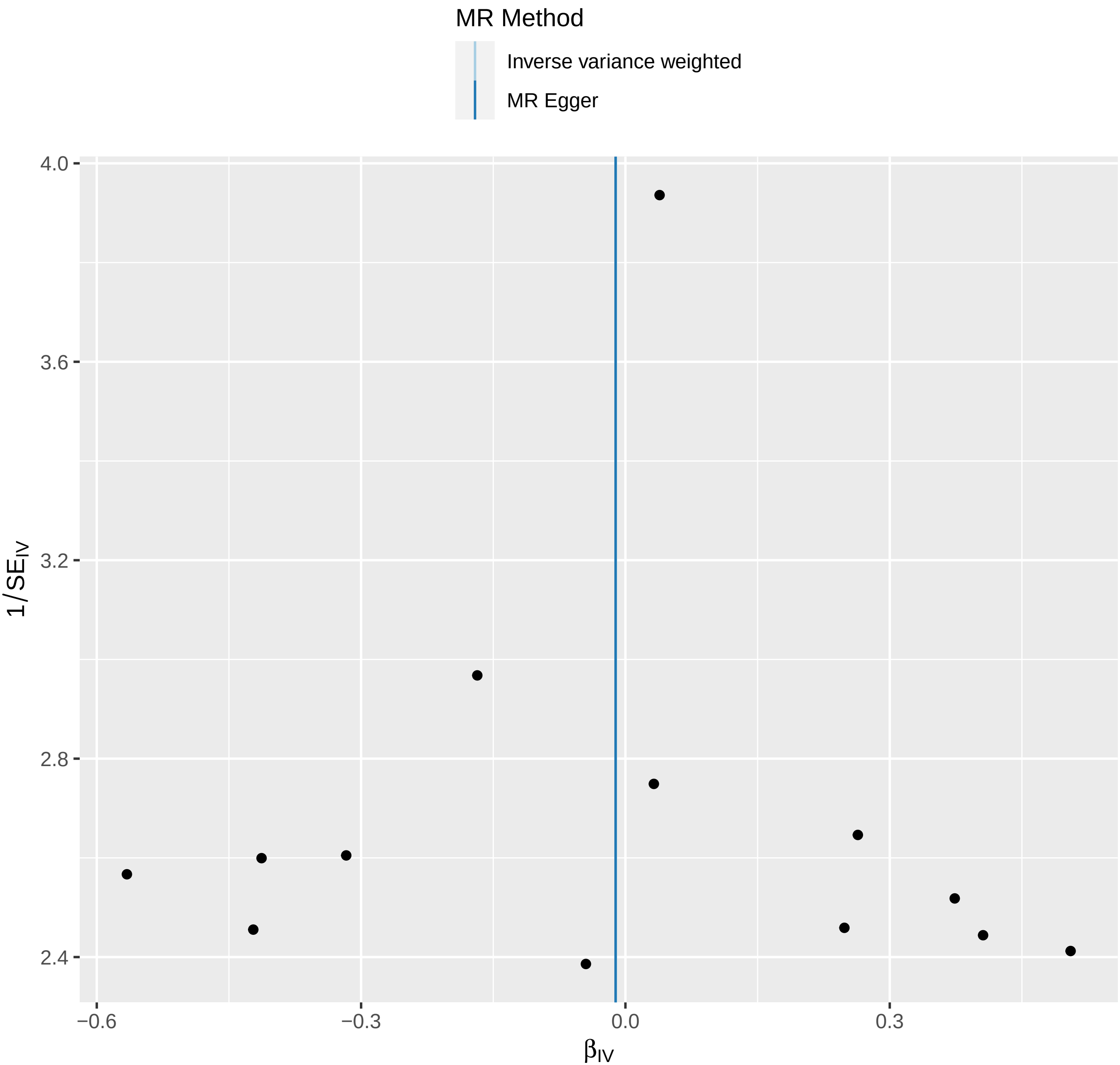

Batch 2−5847 : Gut microbiota abundance (genus Catenibacterium id.2153) on Tumor−associated calcium signal transducer 2
with a threshold of 1e−05

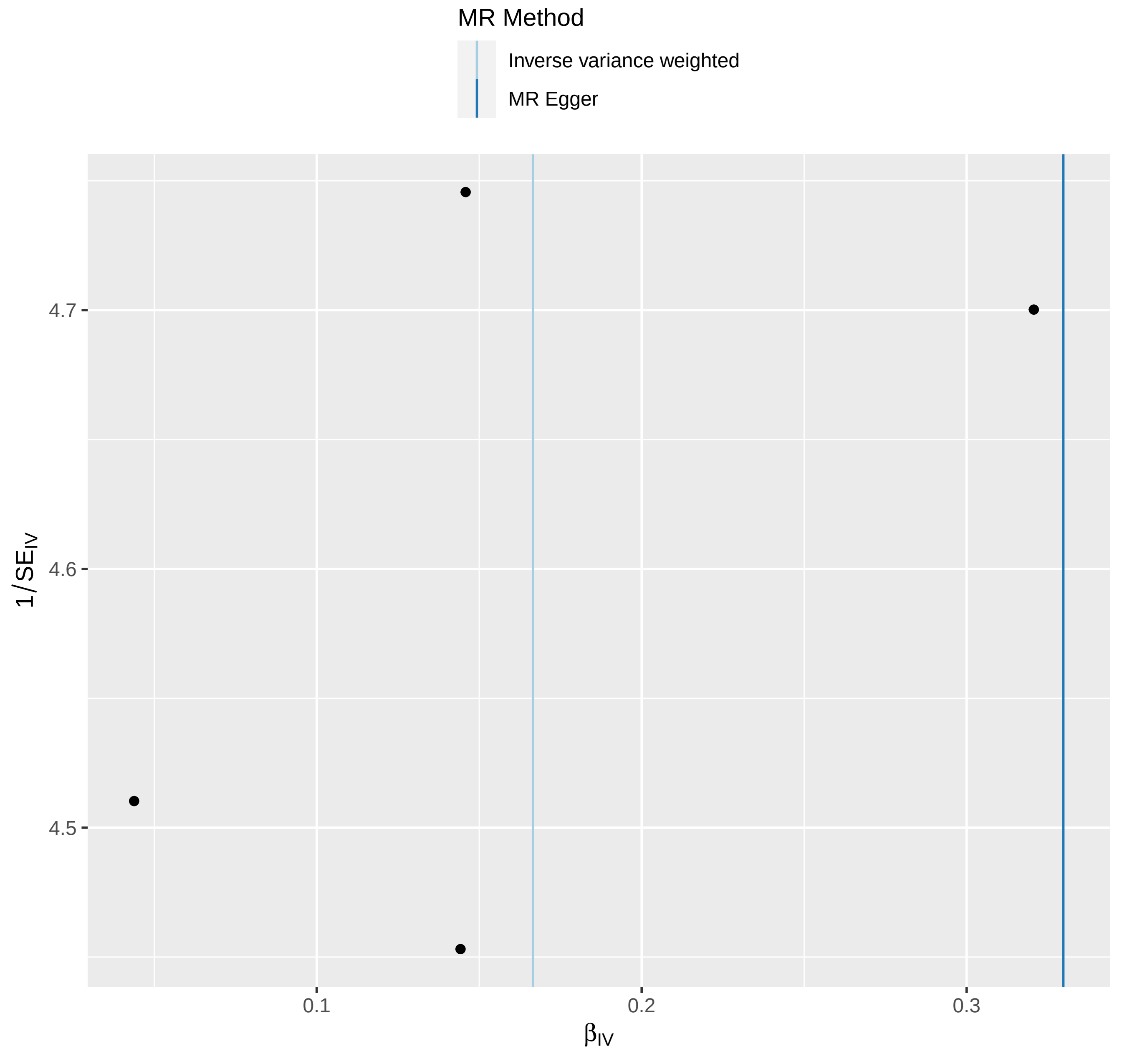

Batch 2−5848 : Gut microbiota abundance (genus Parasutterella id.2892) on Tumor−associated calcium signal transducer 2
with a threshold of 1e−05

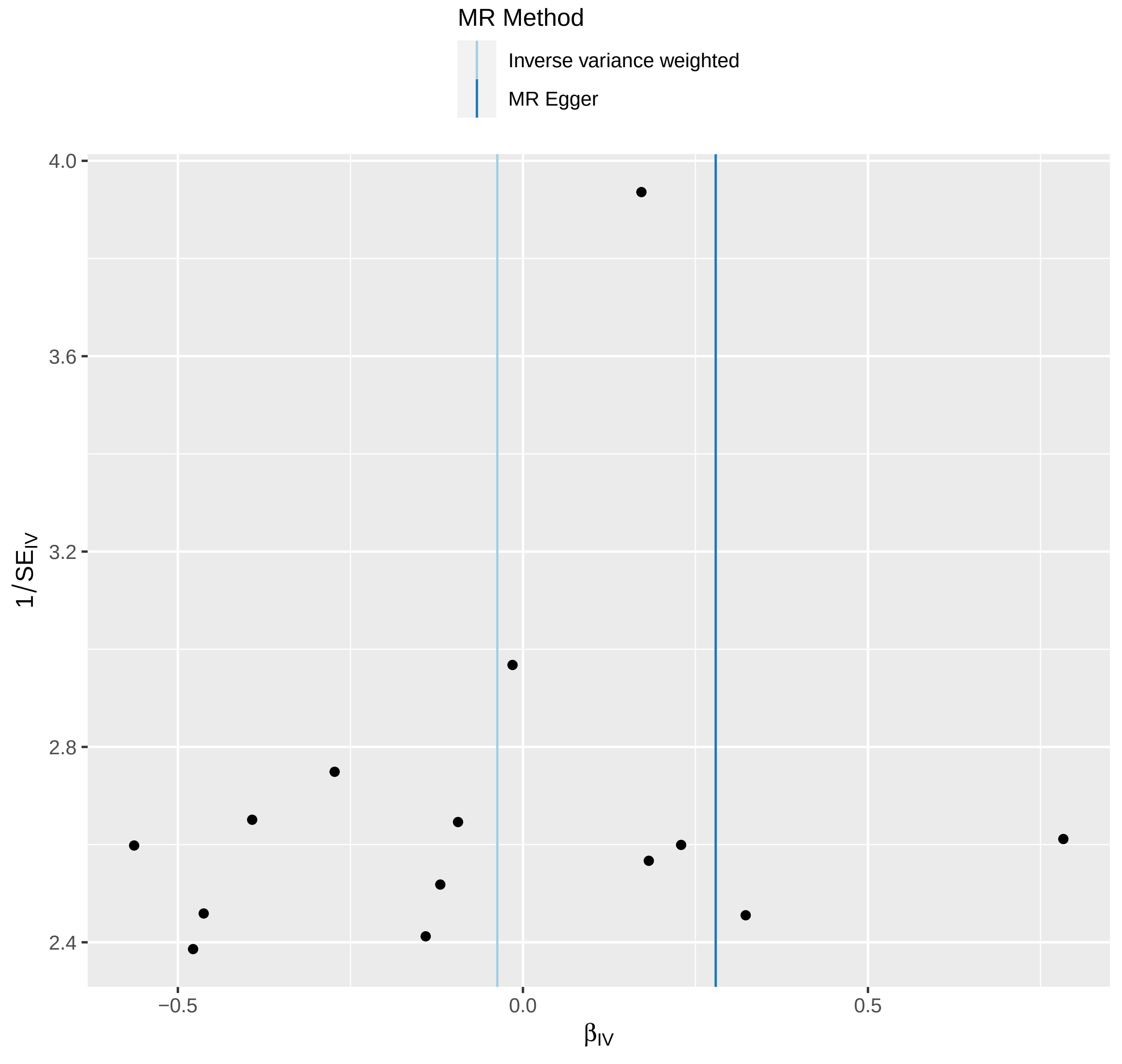

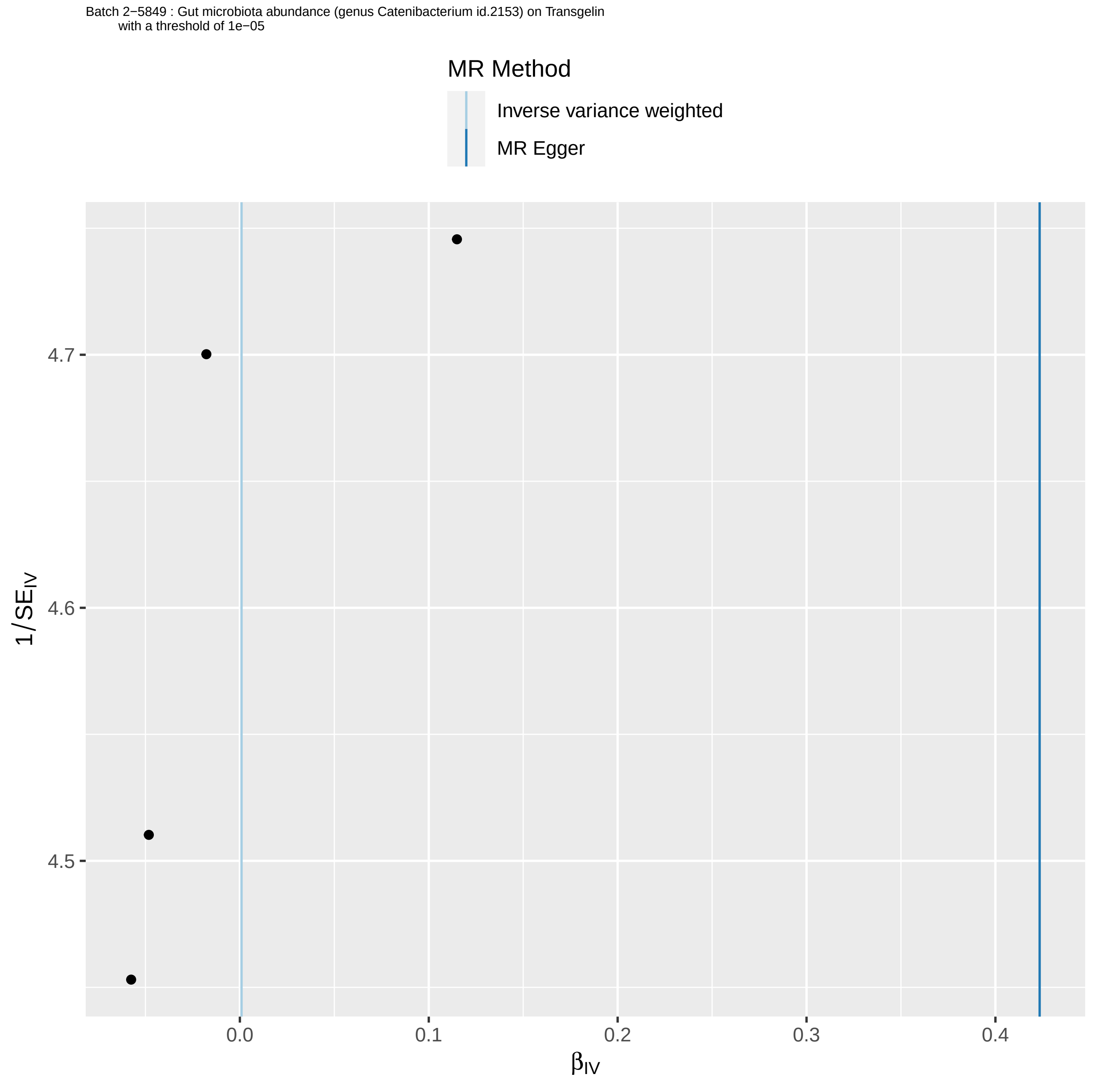
Batch 2−5849 : Gut microbiota abundance (genus Catenibacterium id.2153) on Transgelin
with a threshold of 1e−05
MR Method
Inverse variance weighted
MR Egger
4.7
4.6
4.5
1/SE_IV
0.0
0.1
0.2
0.3
0.4
β_IV

Batch 2−5850 : Gut microbiota abundance (genus Parasutterella id.2892) on Transgelin
with a threshold of 1e−05

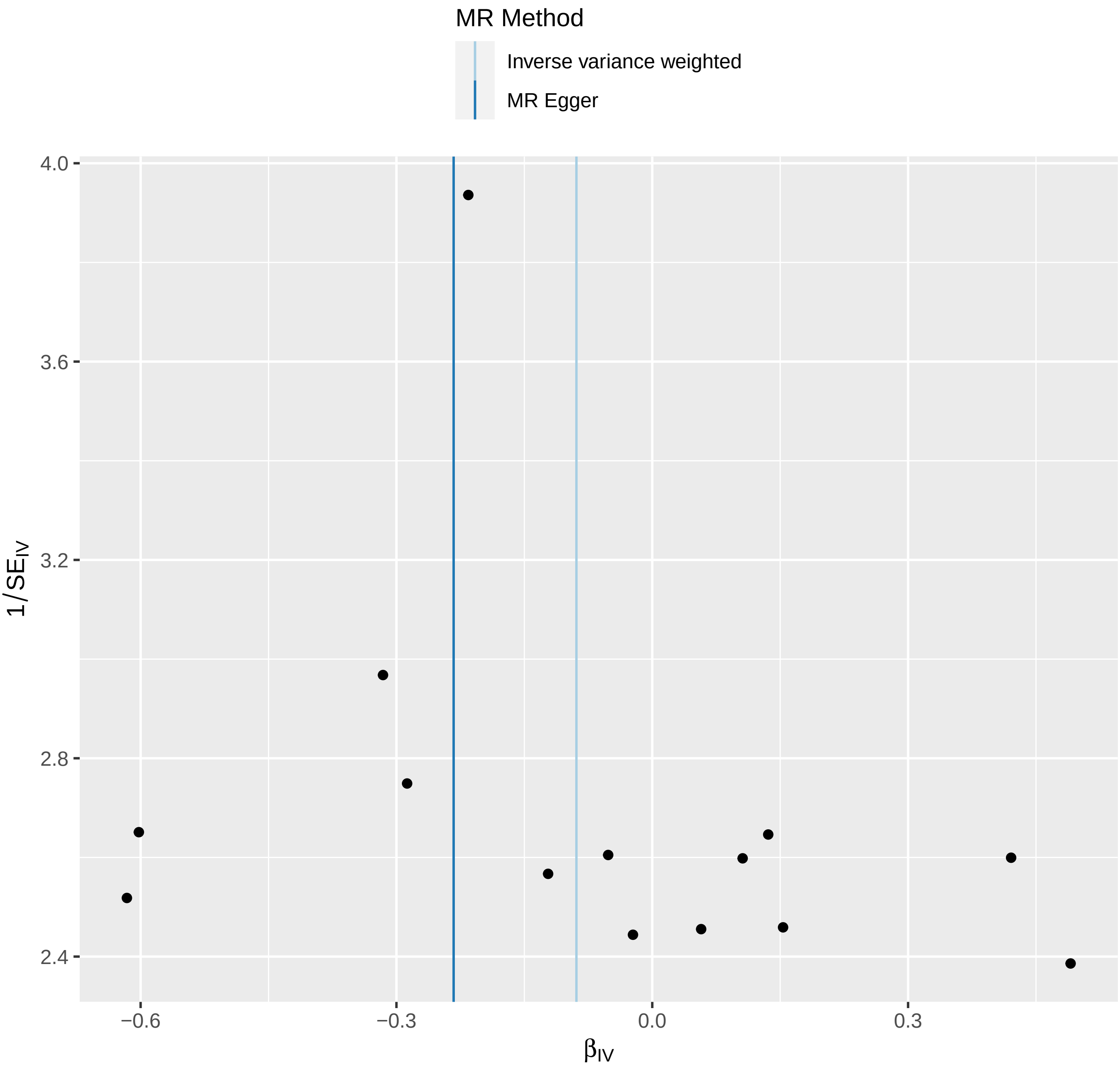

Batch 2−5851 : Gut microbiota abundance (genus Catenibacterium id.2153) on Tapasin
with a threshold of 1e−05

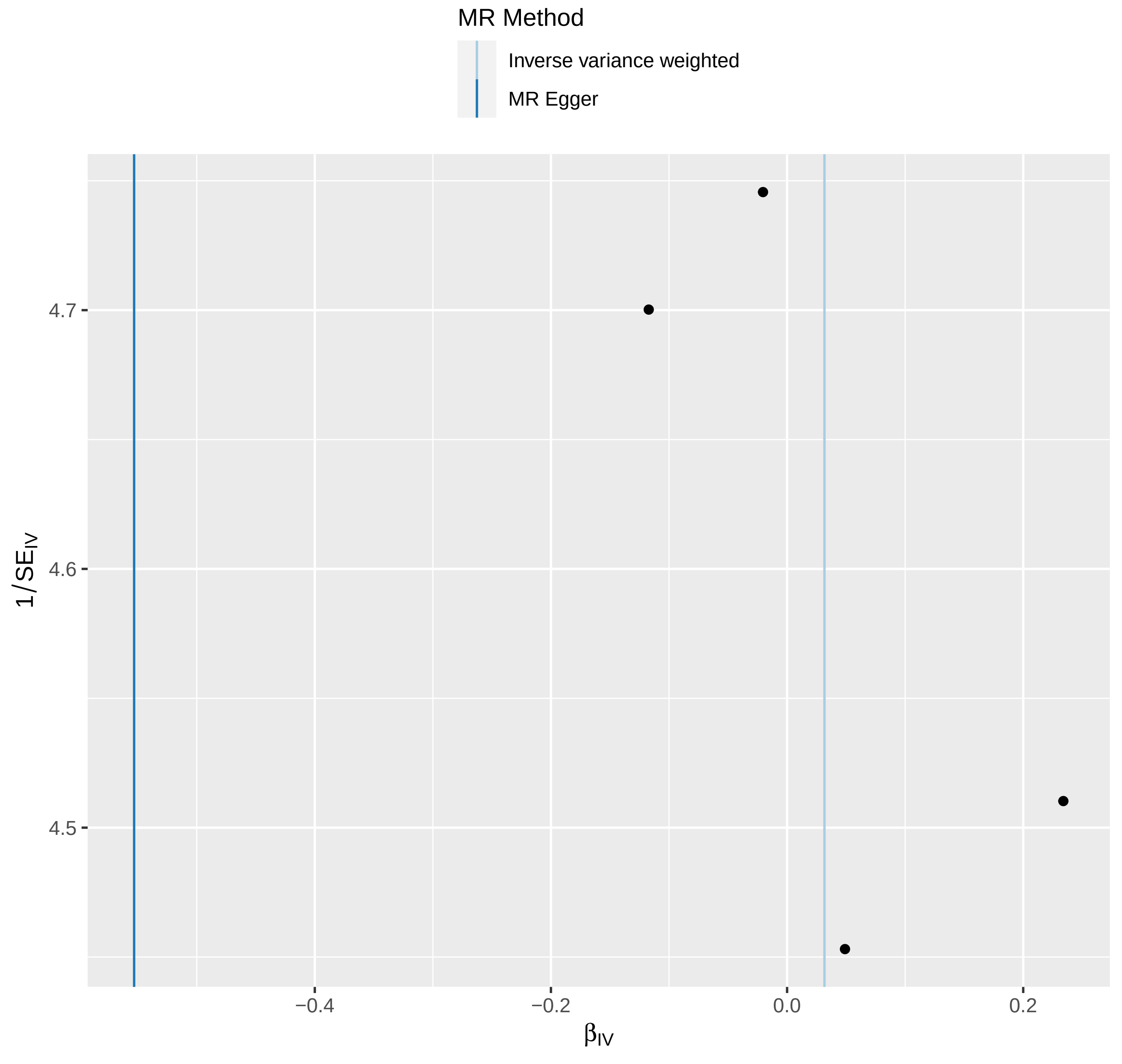

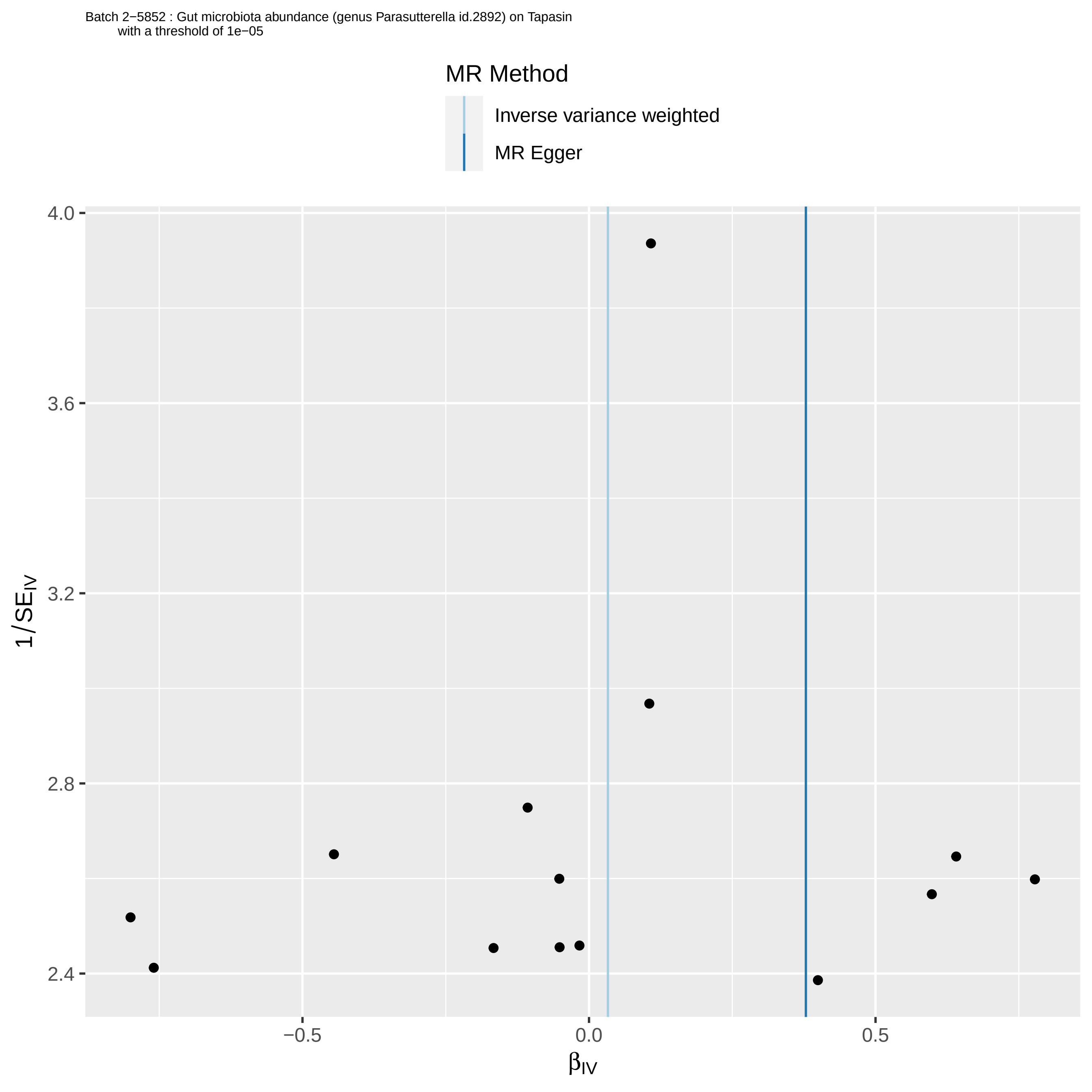

Batch 2−5852 : Gut microbiota abundance (genus Parasutterella id.2892) on Tapasin
with a threshold of 1e−05
MR Method
Inverse variance weighted
MR Egger
4.0
3.6
3.2
2.8
2.4
1/SE_IV
−0.5
0.0
0.5
β_IV

Batch 2−5853 : Gut microbiota abundance (genus Catenibacterium id.2153) on Tapasin−related protein
with a threshold of 1e−05

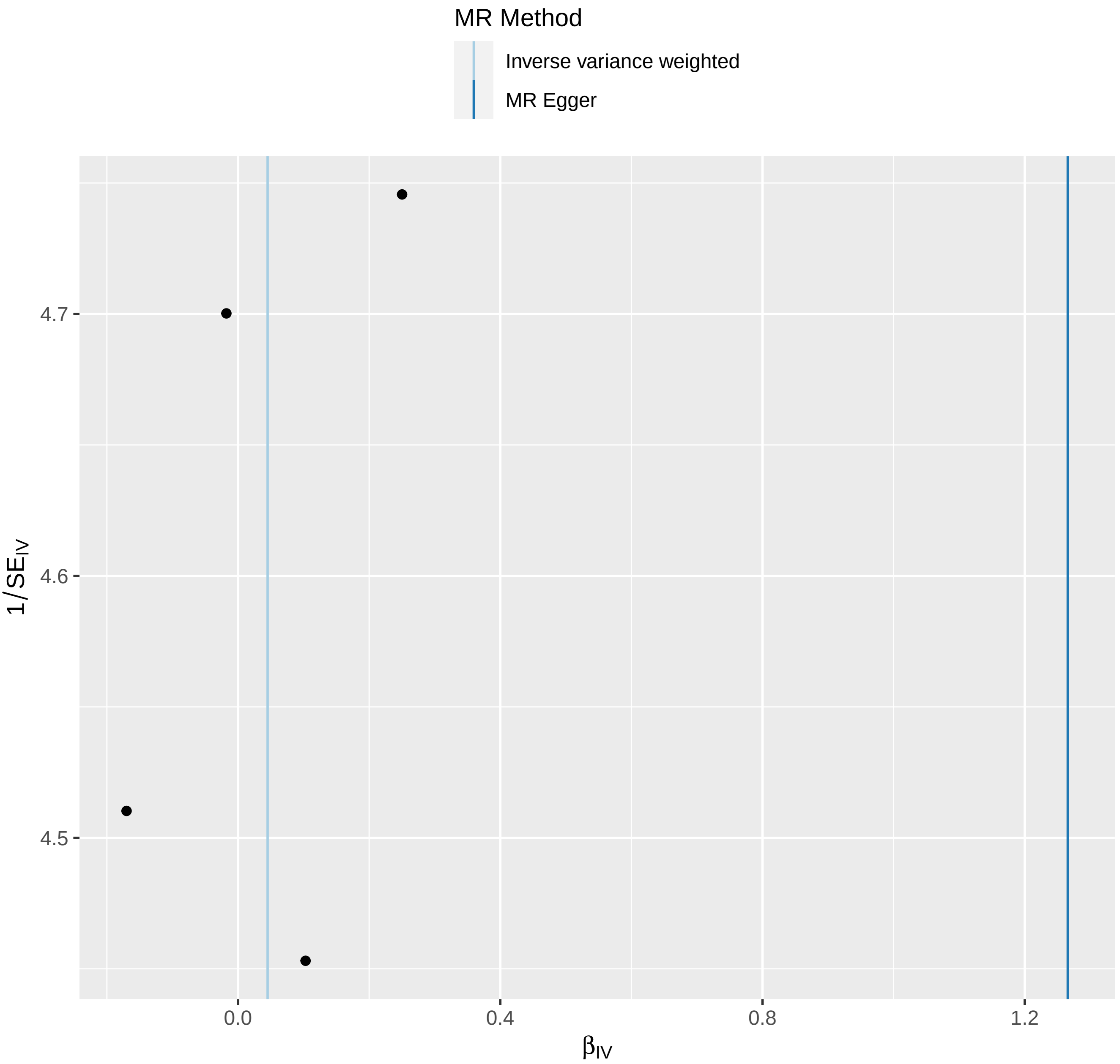

Batch 2−5854 : Gut microbiota abundance (genus Parasutterella id.2892) on Tapasin−related protein
with a threshold of 1e−05

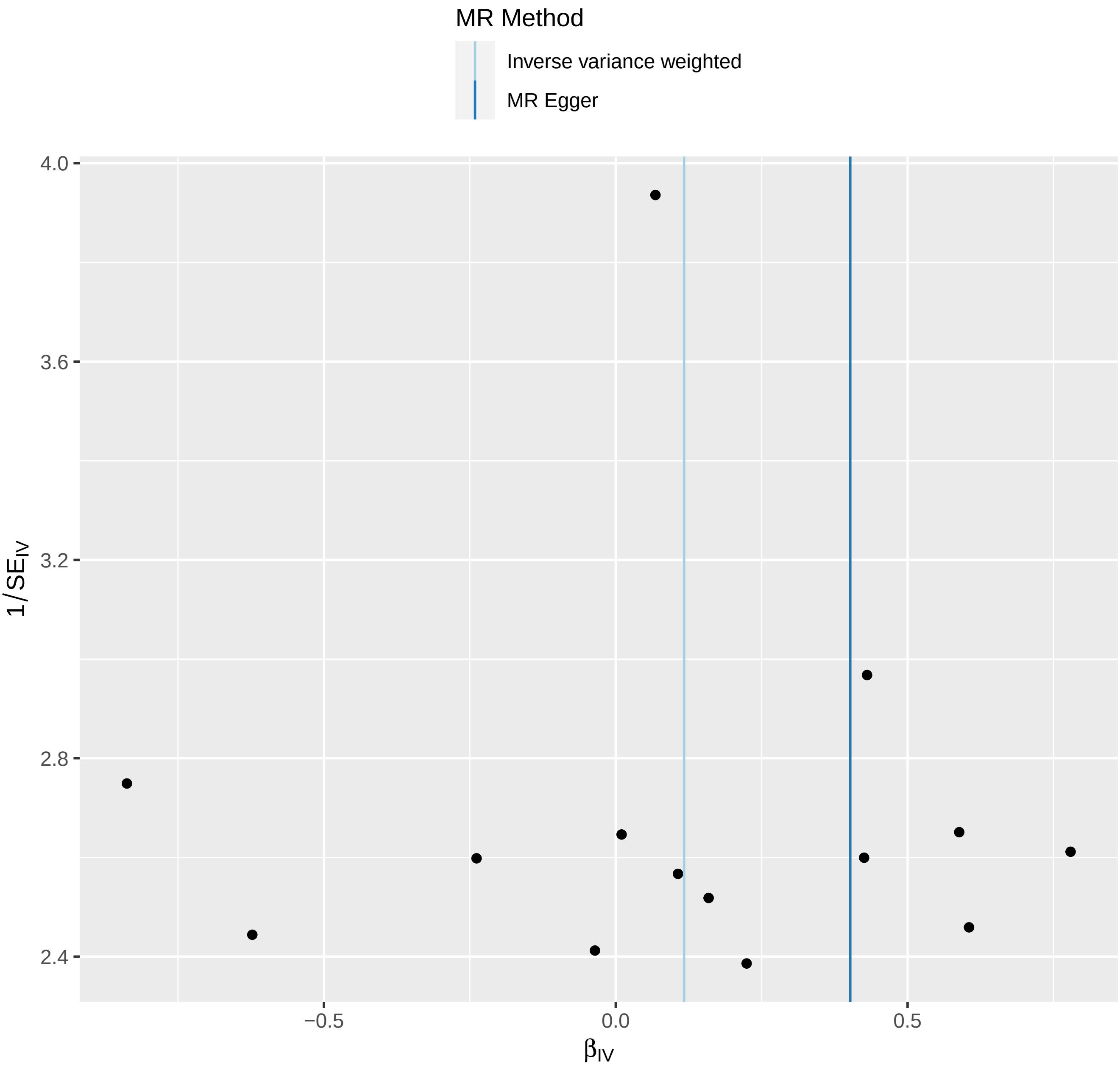

Batch 2−5855 : Gut microbiota abundance (genus Catenibacterium id.2153) on Tax1−binding protein 1
with a threshold of 1e−05

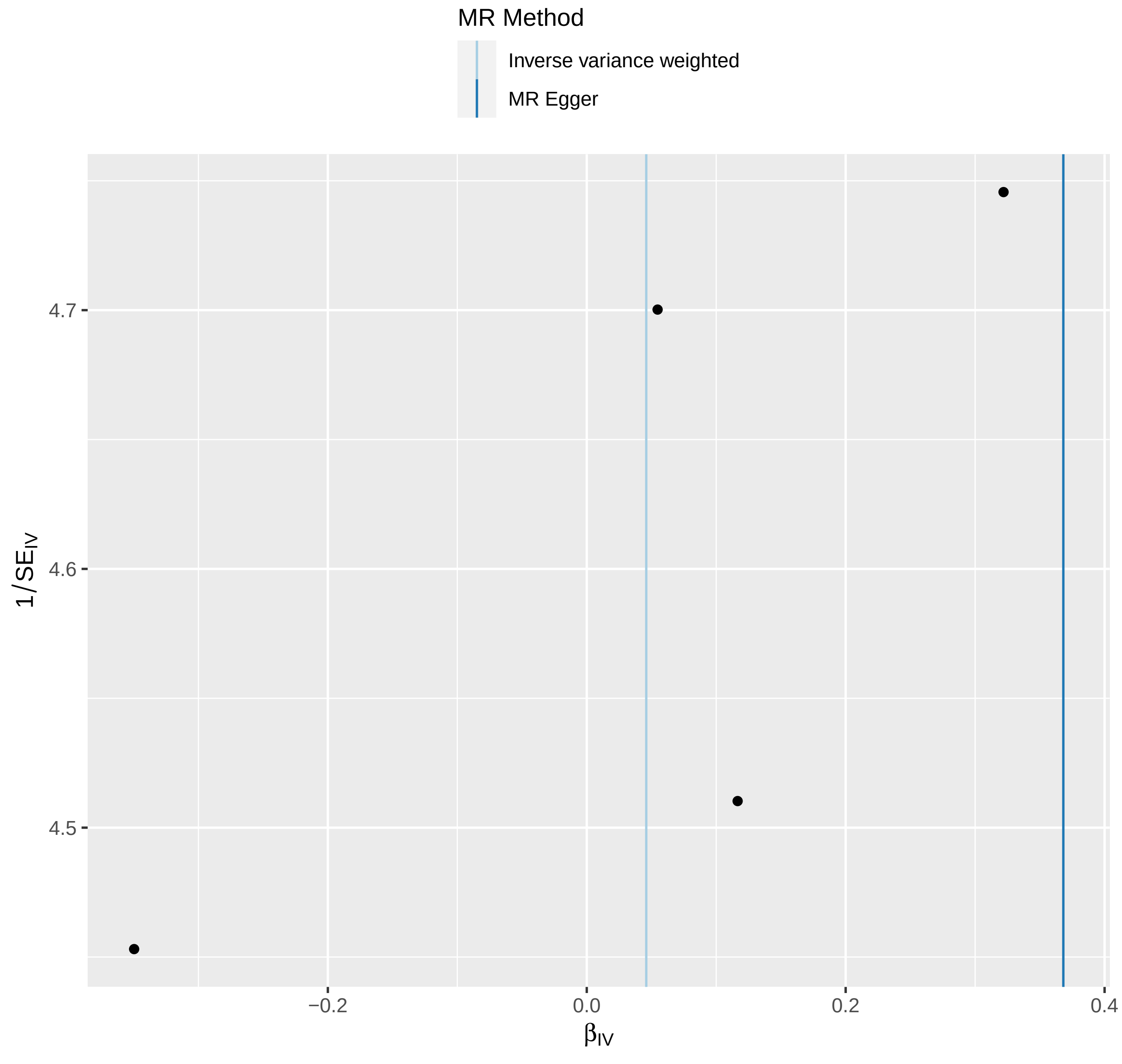

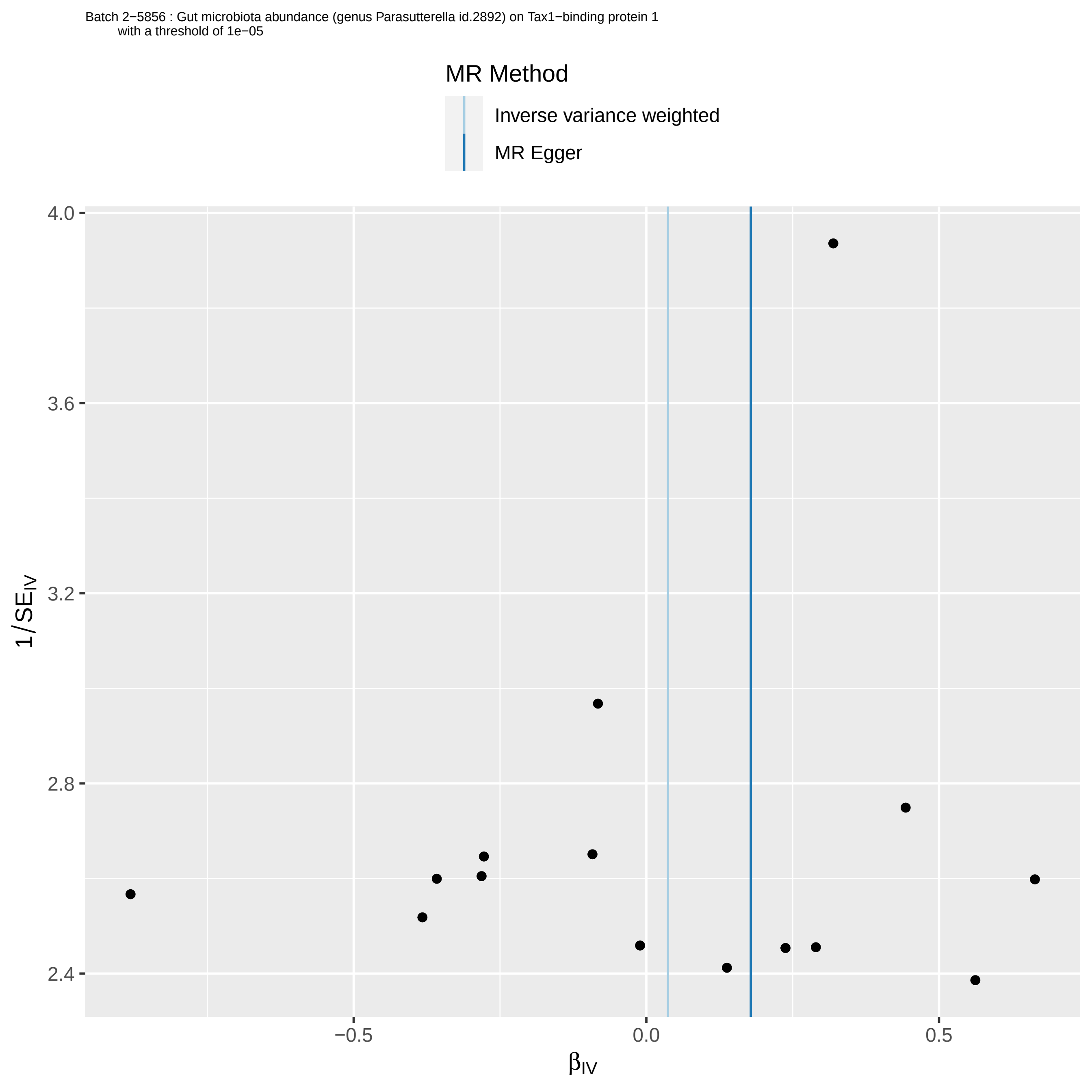
Batch 2−5856 : Gut microbiota abundance (genus Parasutterella id.2892) on Tax1−binding protein 1
with a threshold of 1e−05
MR Method
Inverse variance weighted
MR Egger
4.0
3.6
3.2
2.8
2.4
$1/SE_{IV}$
−0.5
0.0
0.5
β_{IV}

Batch 2−5857 : Gut microbiota abundance (genus Catenibacterium id.2153) on Tax1−binding protein 3
with a threshold of 1e−05

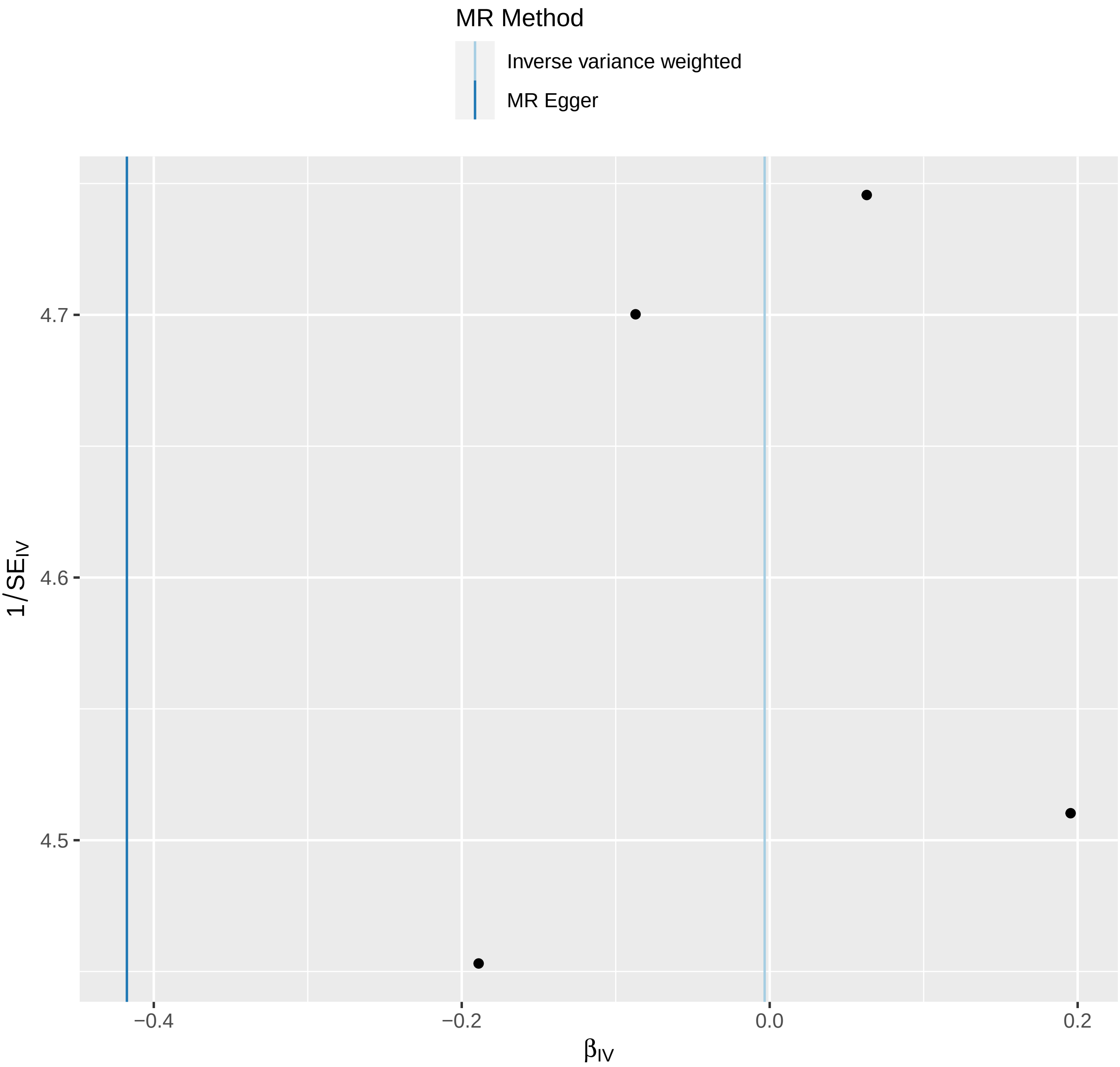

Batch 2−5858 : Gut microbiota abundance (genus Parasutterella id.2892) on Tax1−binding protein 3
with a threshold of 1e−05

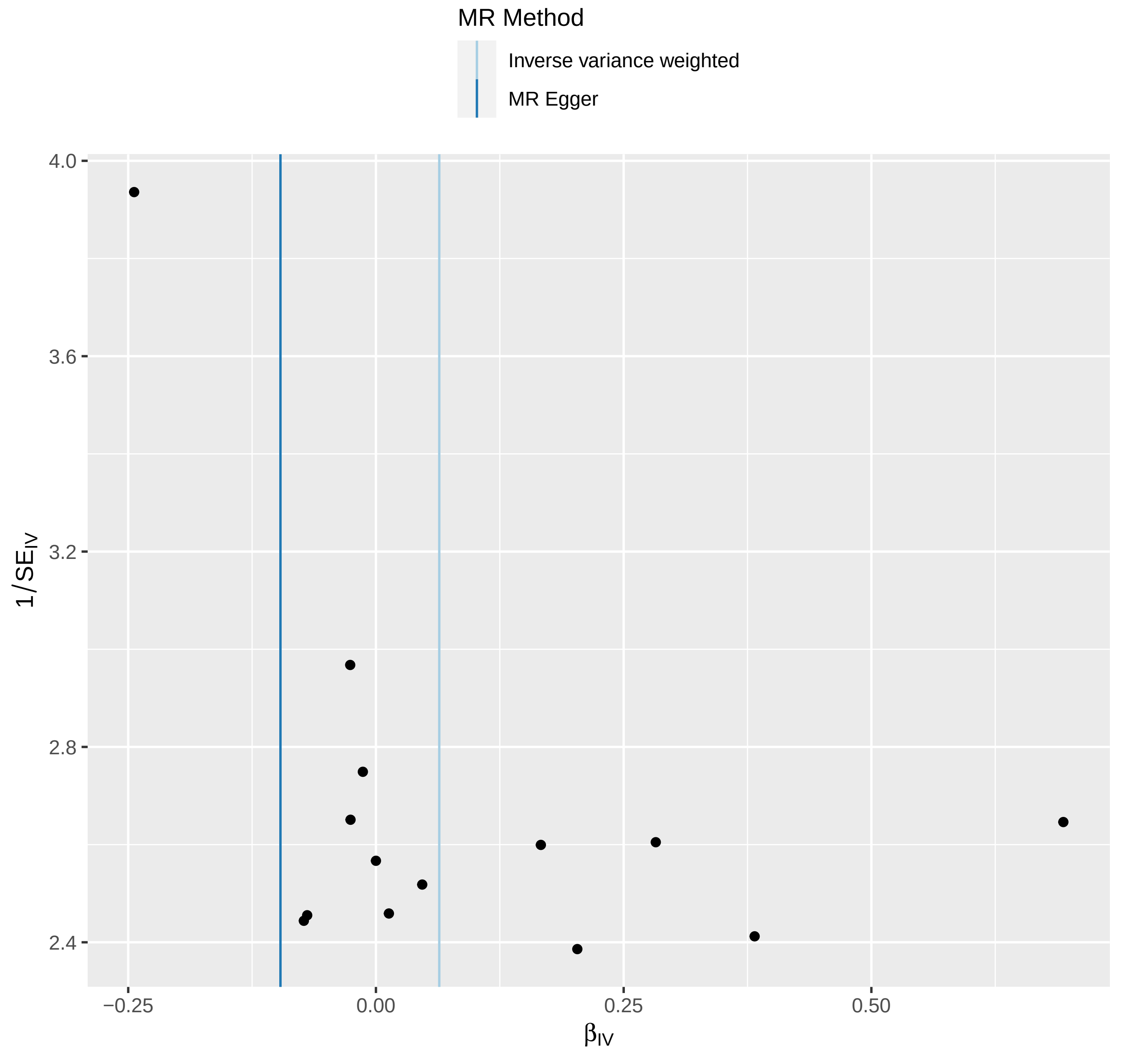

Batch 2−5859 : Gut microbiota abundance (genus Catenibacterium id.2153) on Tubulin−specific chaperone A with a threshold of 1e−05

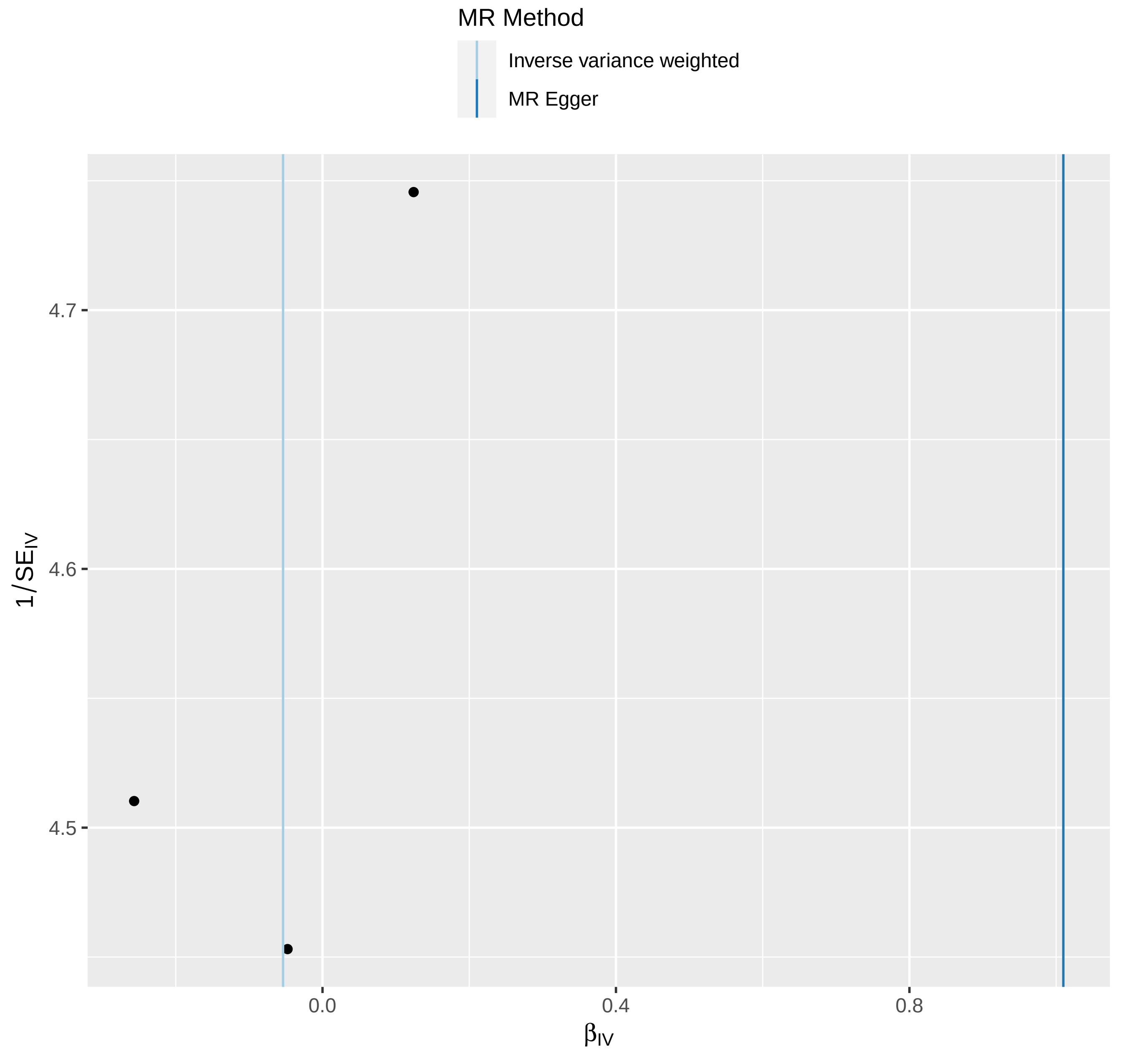

Batch 2−5860 : Gut microbiota abundance (genus Parasutterella id.2892) on Tubulin−specific chaperone A
with a threshold of 1e−05

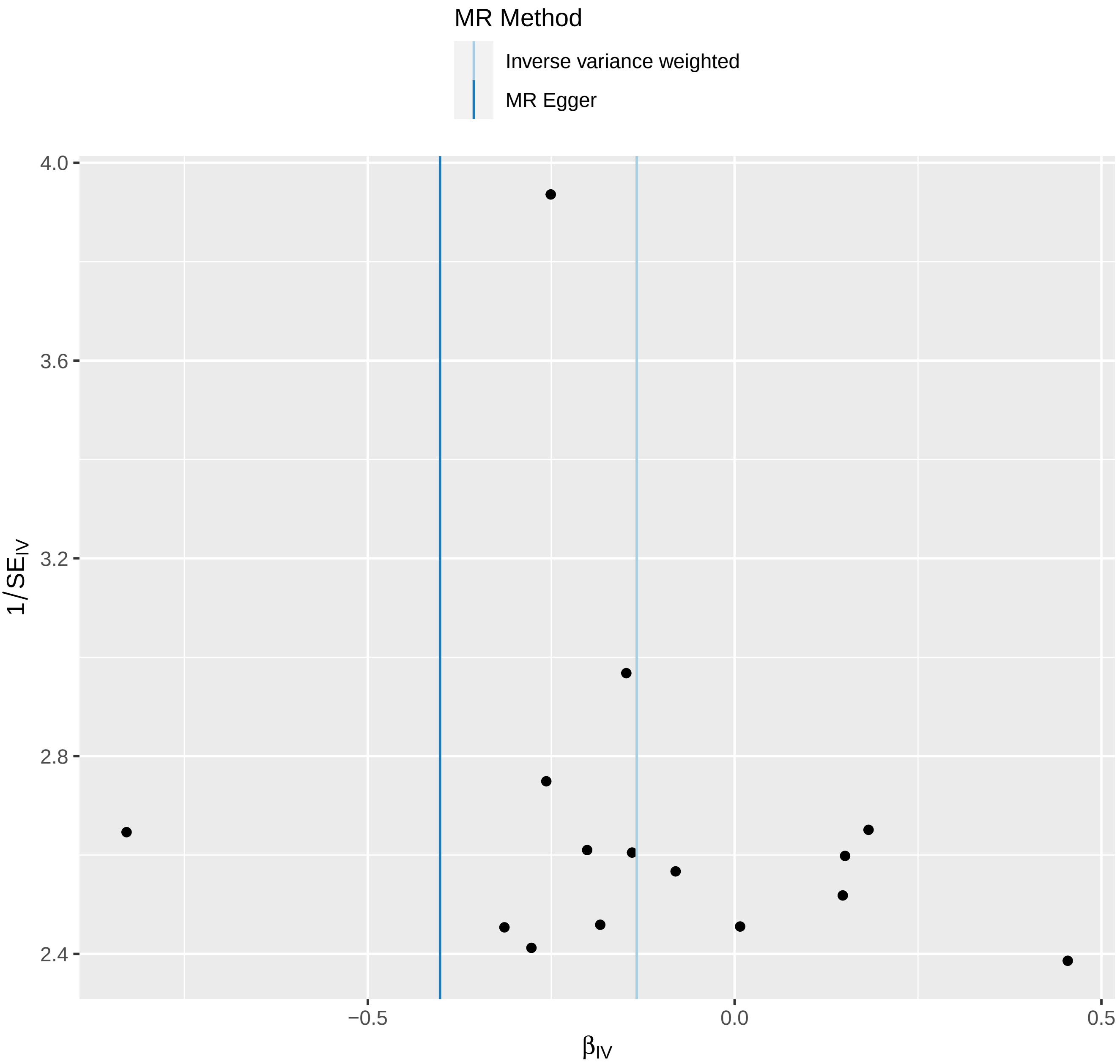

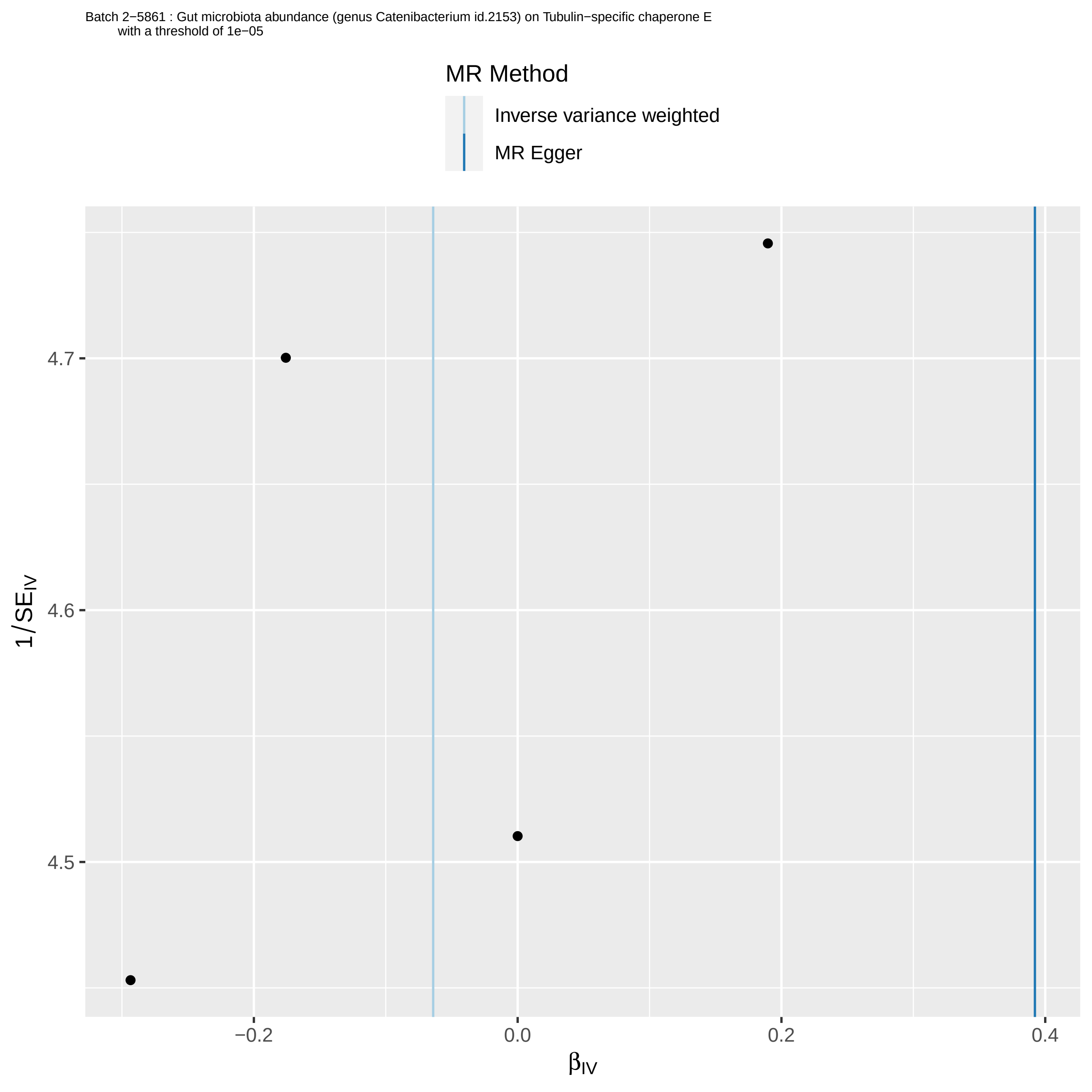

Batch 2−5861 : Gut microbiota abundance (genus Catenibacterium id.2153) on Tubulin−specific chaperone E
with a threshold of 1e−05
MR Method
Inverse variance weighted
MR Egger
4.7
4.6
4.5
1/SE_IV
−0.2
0.0
0.2
0.4
β_IV

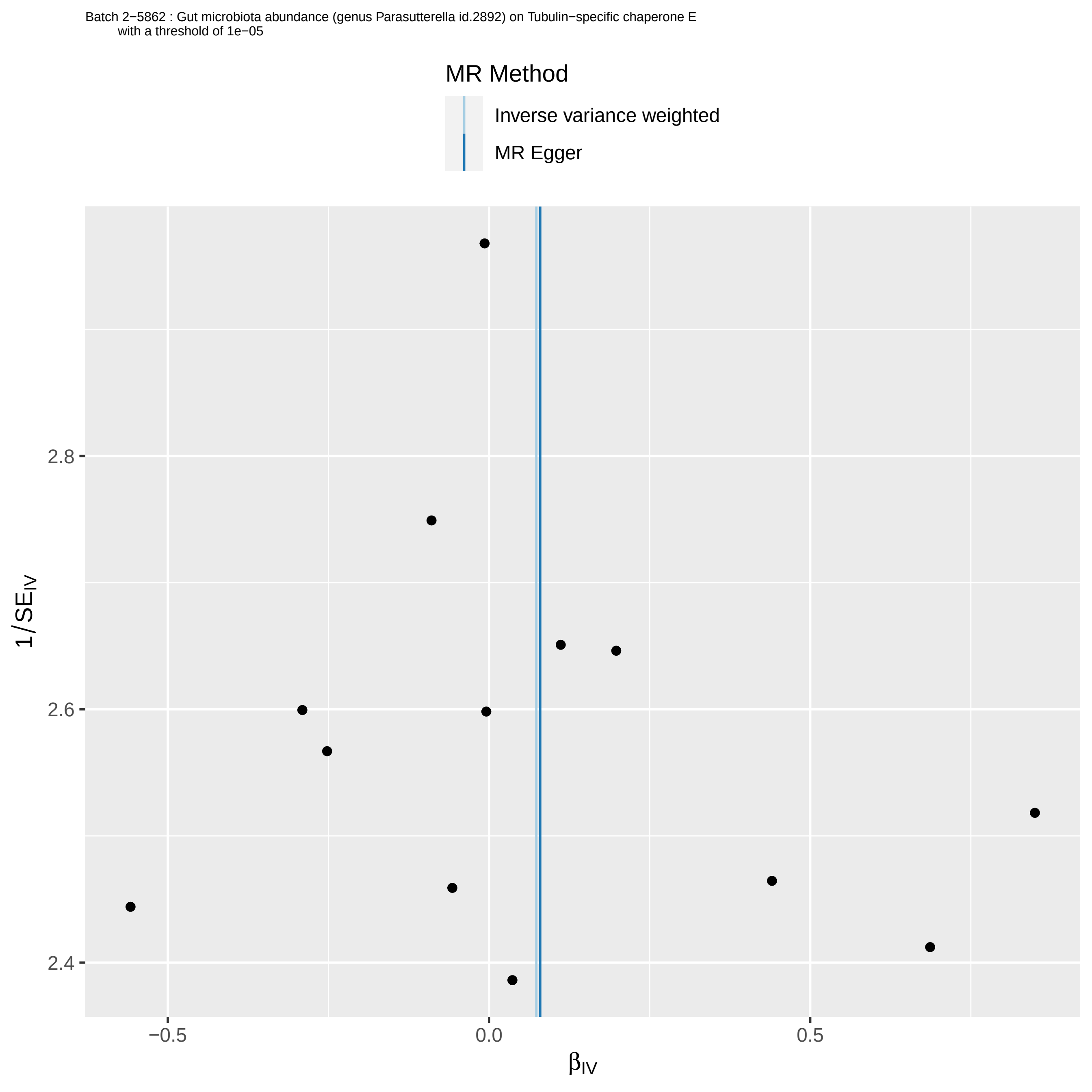
Batch 2−5862 : Gut microbiota abundance (genus Parasutterella id.2892) on Tubulin−specific chaperone E
with a threshold of 1e−05
MR Method
Inverse variance weighted
MR Egger
2.8
2.6
2.4
1/SE_IV
−0.5
0.0
0.5
β_IV

Batch 2−5863 : Gut microbiota abundance (genus Catenibacterium id.2153) on Serine/threonine−protein kinase TBK1
with a threshold of 1e−05

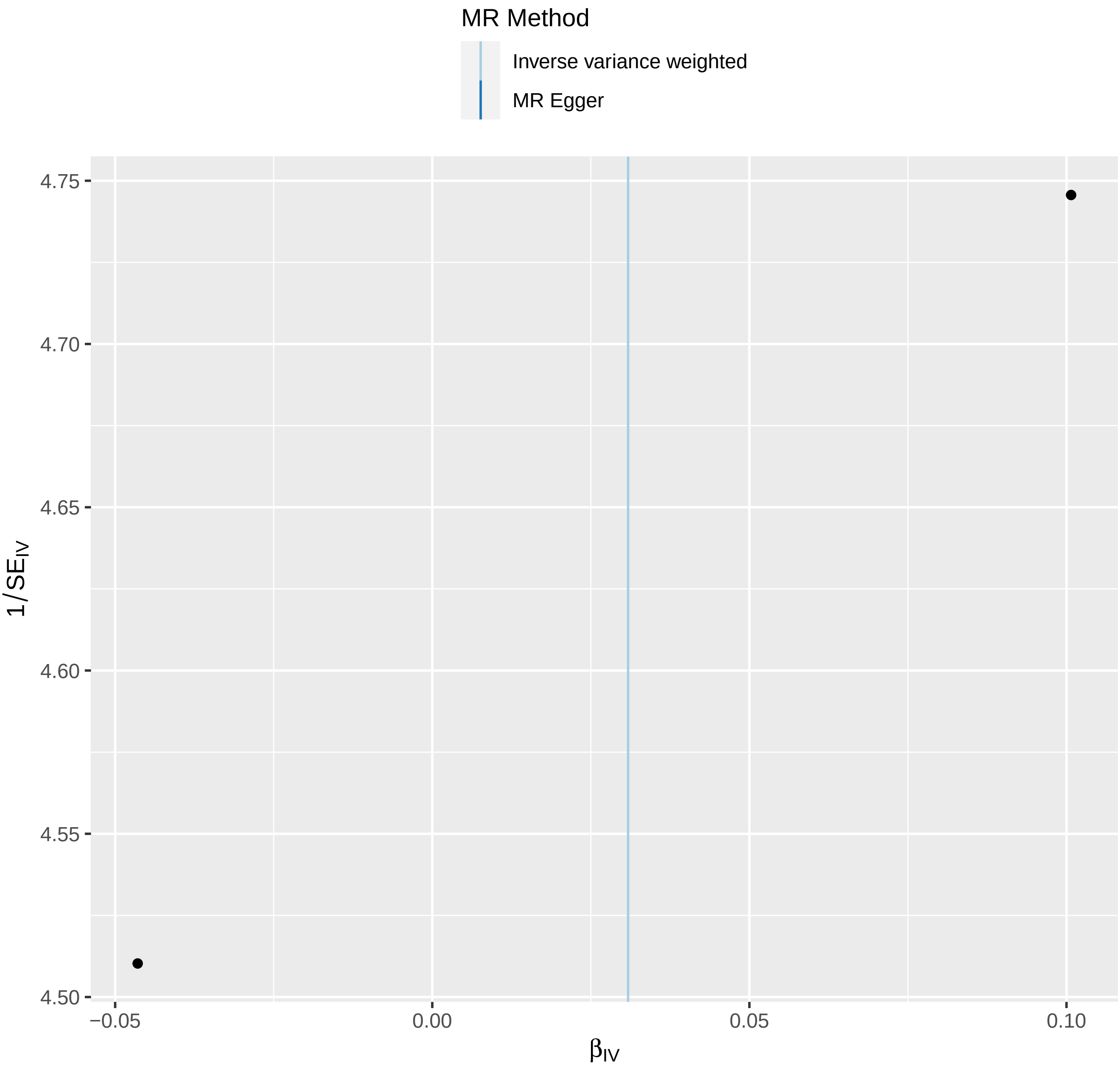

Batch 2−5864 : Gut microbiota abundance (genus Parasutterella id.2892) on Serine/threonine−protein kinase TBK1
with a threshold of 1e−05

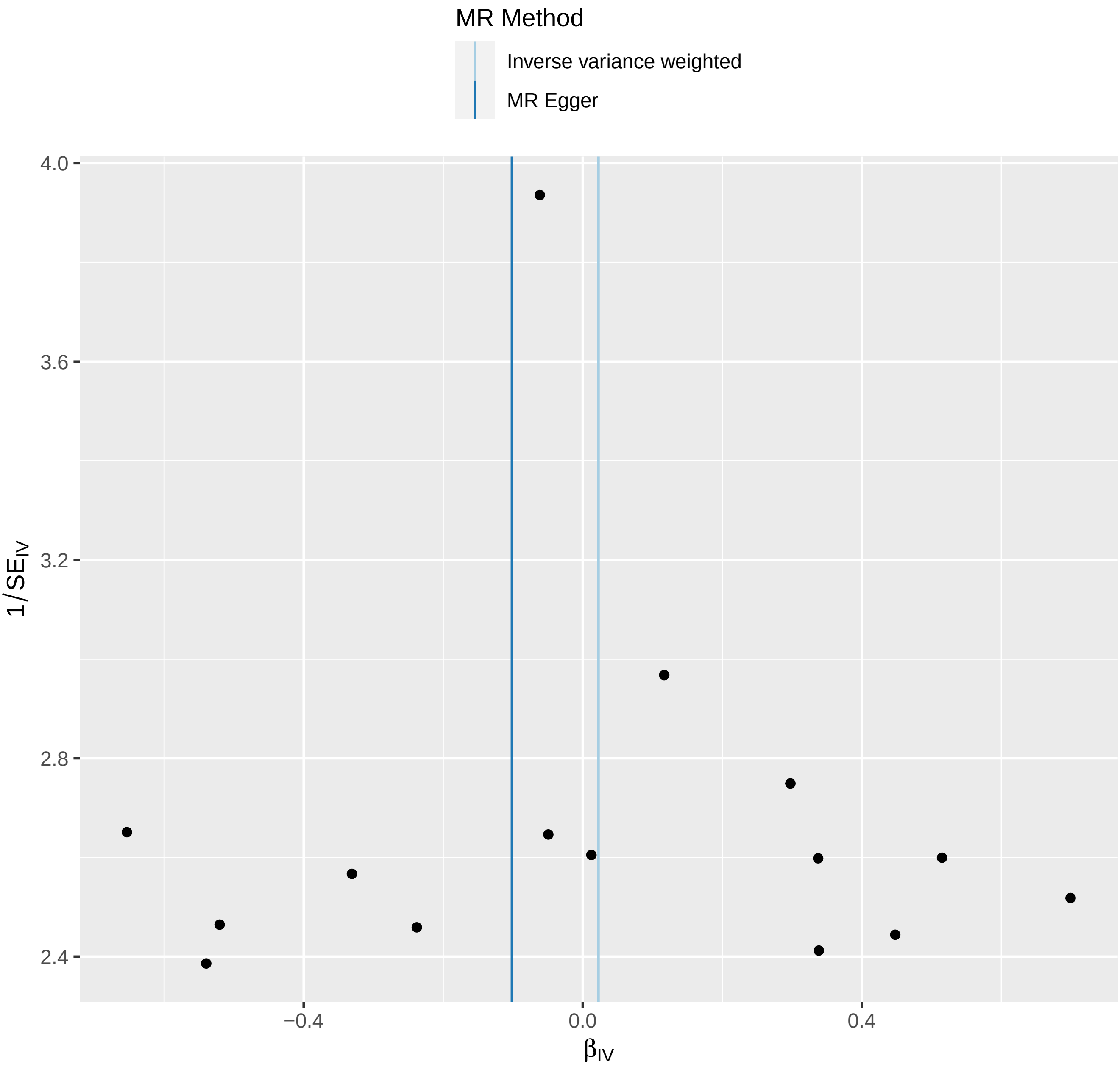

Batch 2−5865 : Gut microbiota abundance (genus Catenibacterium id.2153) on TATA−box−binding protein
with a threshold of 1e−05

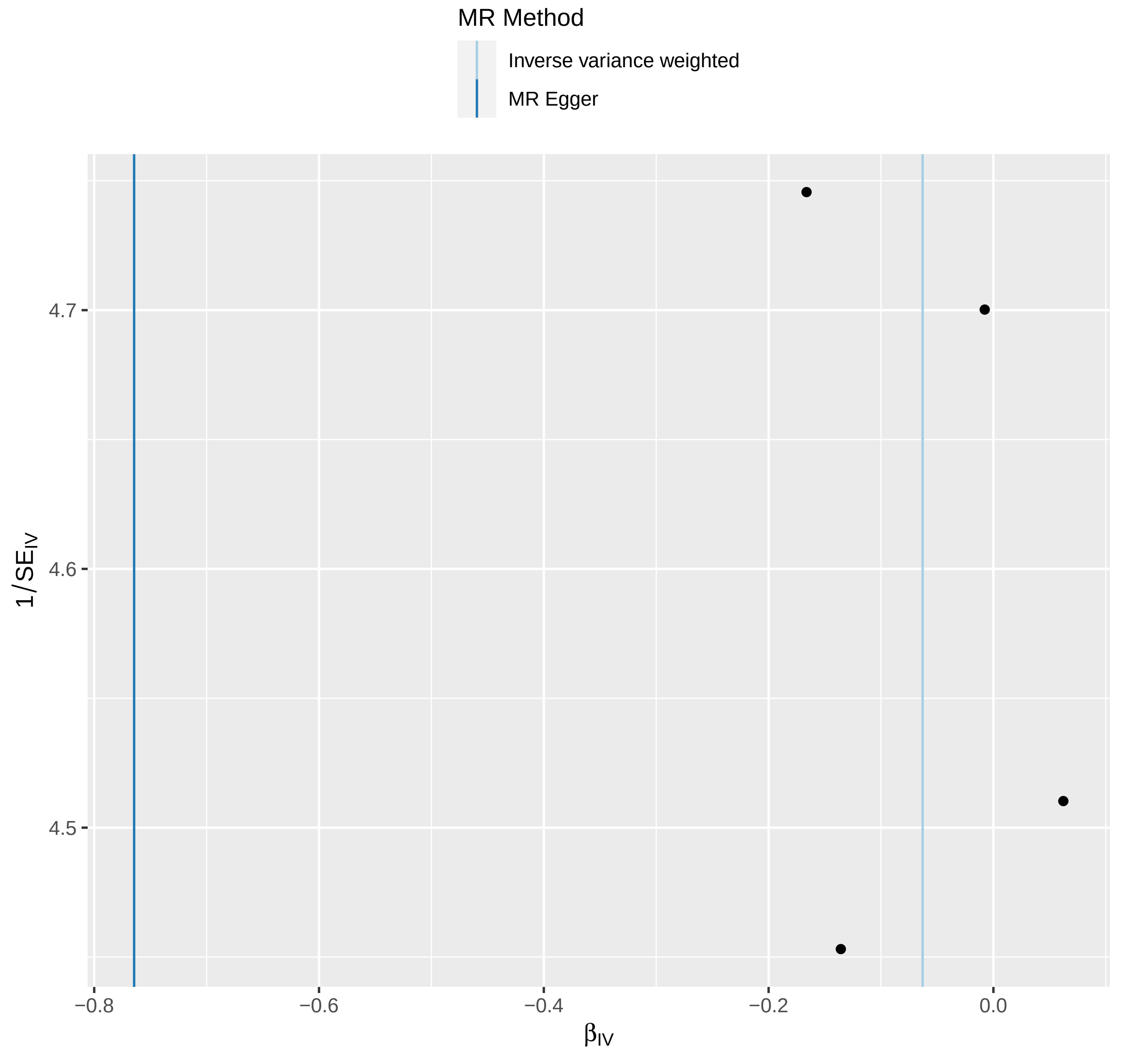

Batch 2−5866 : Gut microbiota abundance (genus Parasutterella id.2892) on TATA−box−binding protein
with a threshold of 1e−05

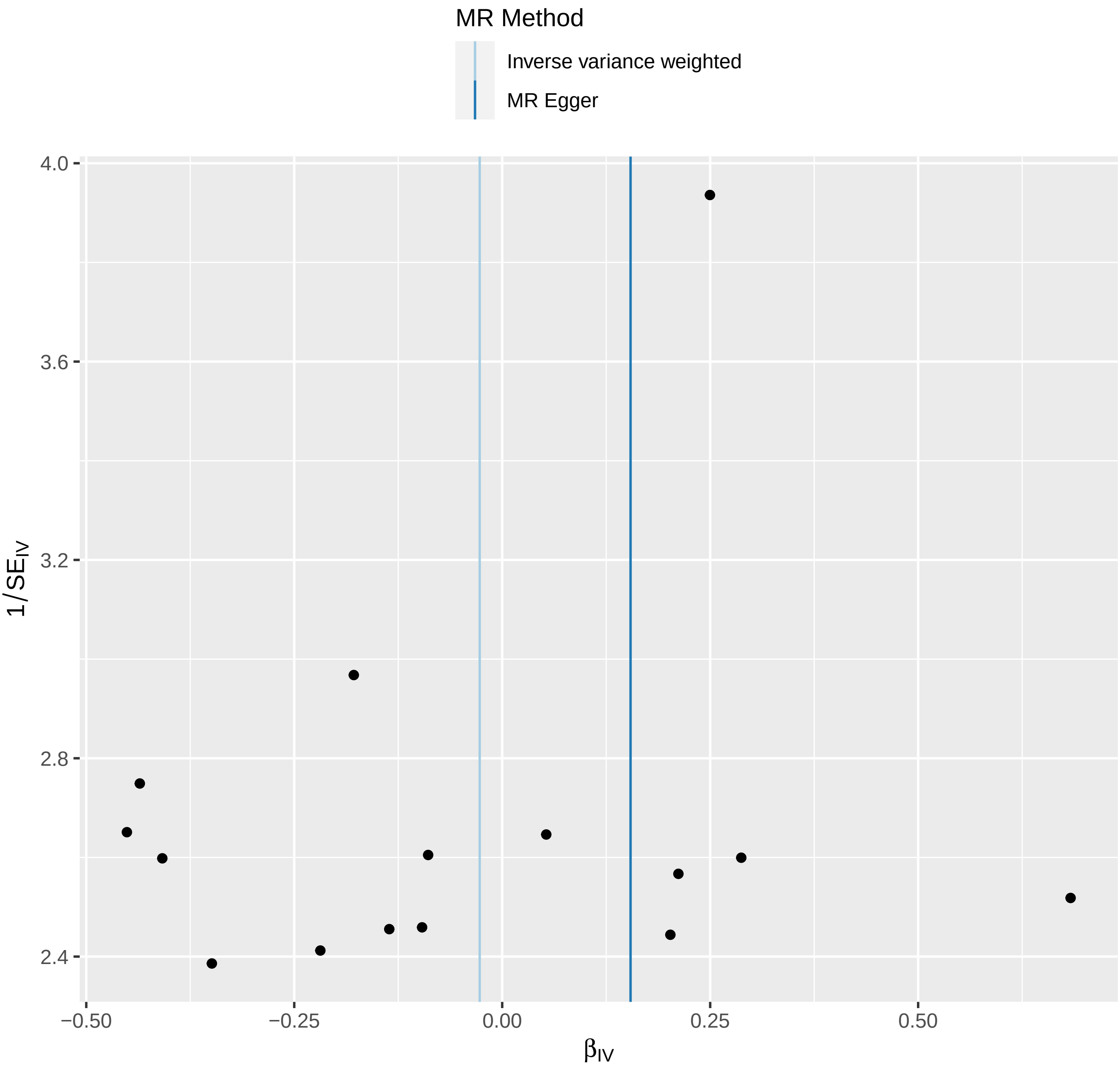

Batch 2−5867 : Gut microbiota abundance (genus Catenibacterium id.2153) on T−box transcription factor TBX22
with a threshold of 1e−05

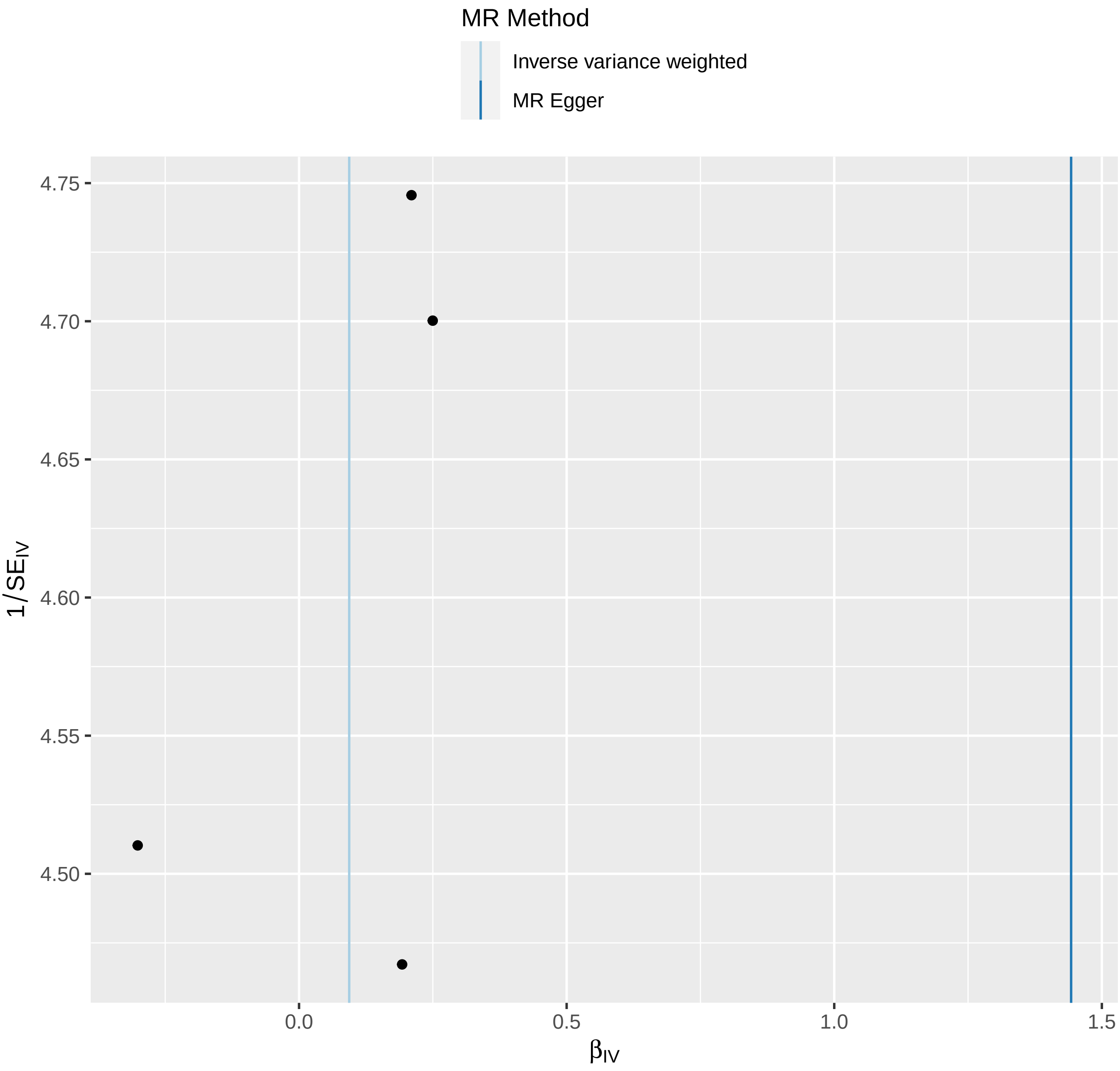

Batch 2−5868 : Gut microbiota abundance (genus Parasutterella id.2892) on T−box transcription factor TBX22 with a threshold of 1e−05

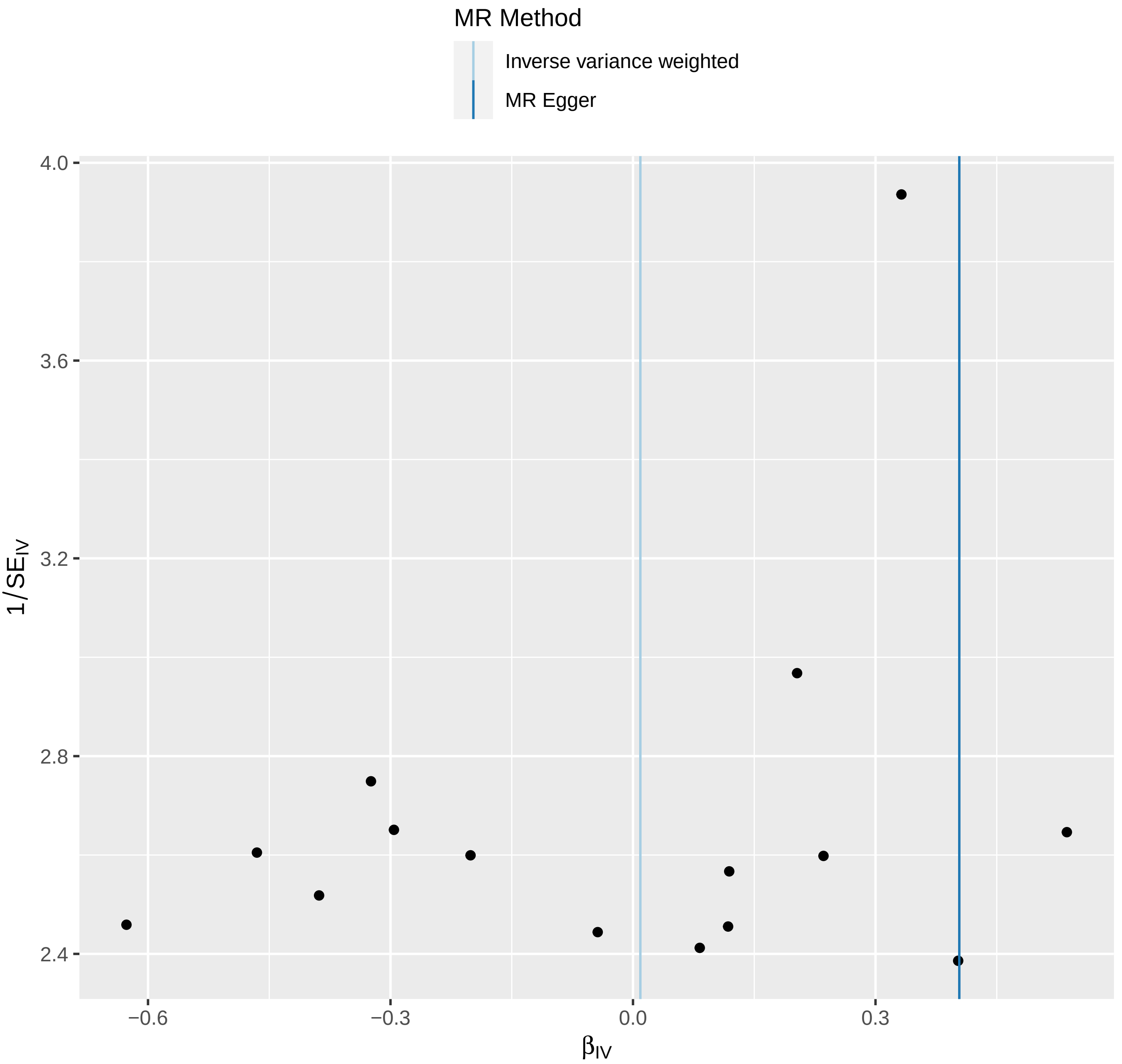

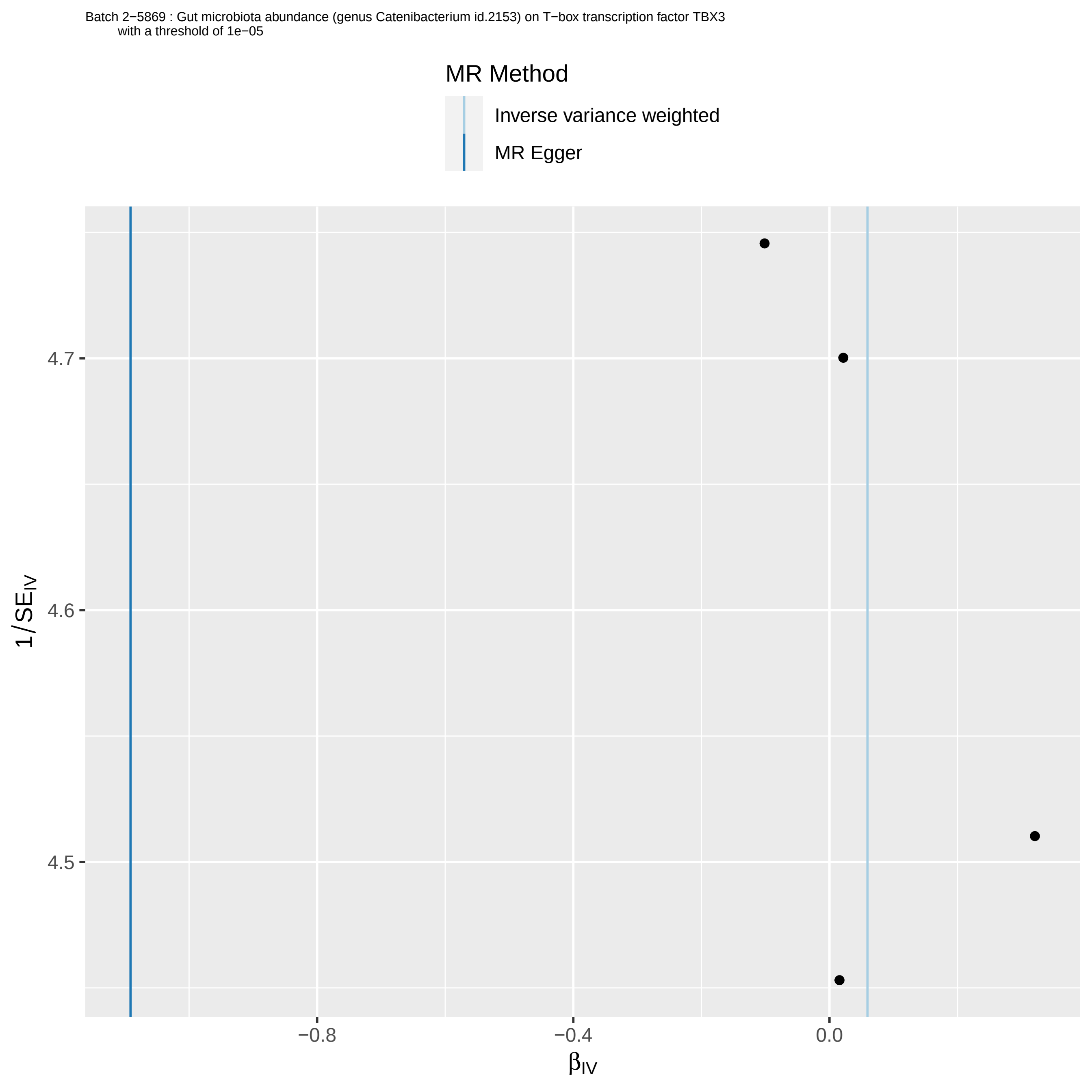
Batch 2−5869 : Gut microbiota abundance (genus Catenibacterium id.2153) on T−box transcription factor TBX3
with a threshold of 1e−05
MR Method
Inverse variance weighted
MR Egger
4.7
4.6
4.5
1/SE_IV
−0.8
−0.4
0.0
β_IV

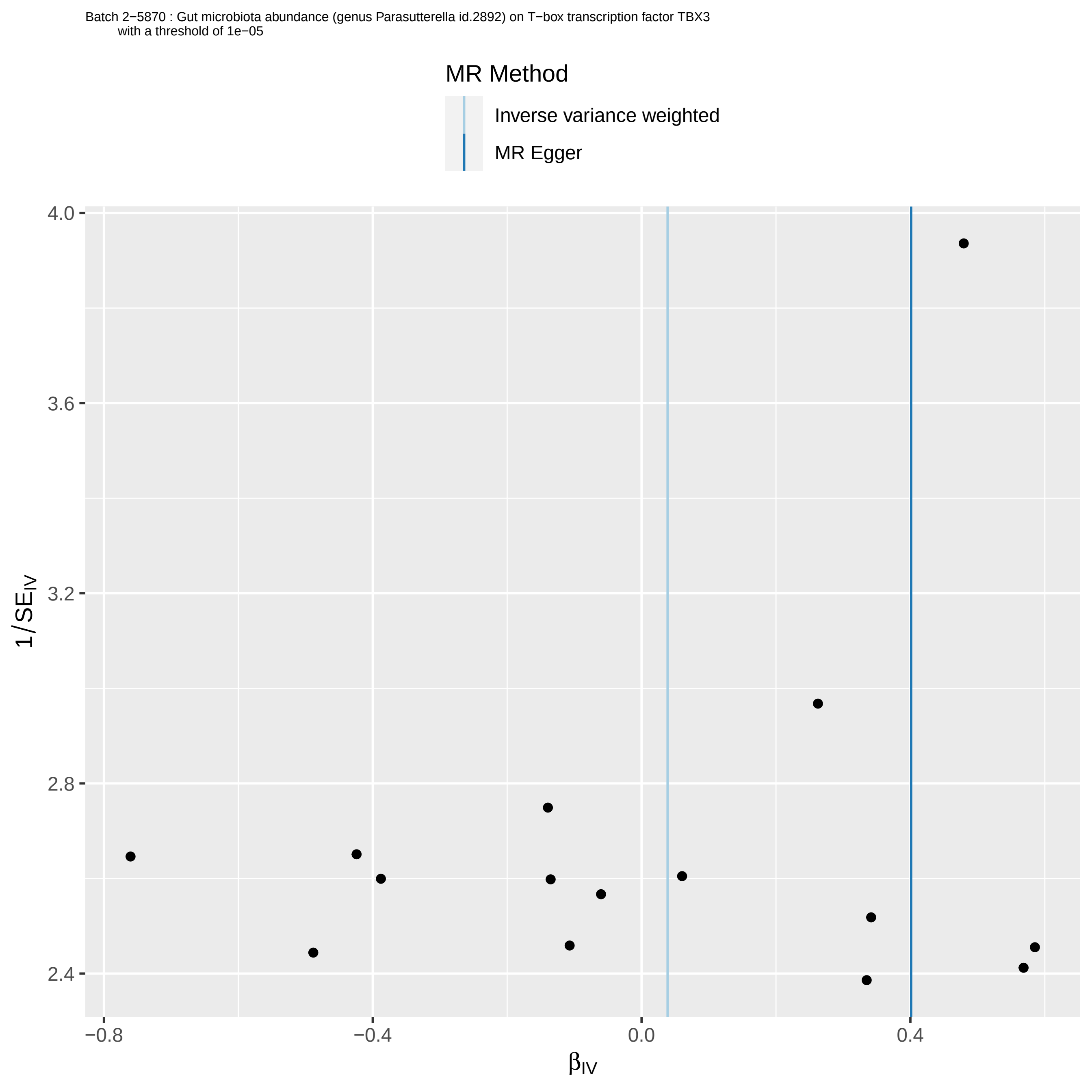
Batch 2−5870 : Gut microbiota abundance (genus Parasutterella id.2892) on T−box transcription factor TBX3
with a threshold of 1e−05
MR Method
Inverse variance weighted
MR Egger
4.0
3.6
3.2
2.8
2.4
1/SE_IV
−0.8
−0.4
0.0
0.4
β_IV

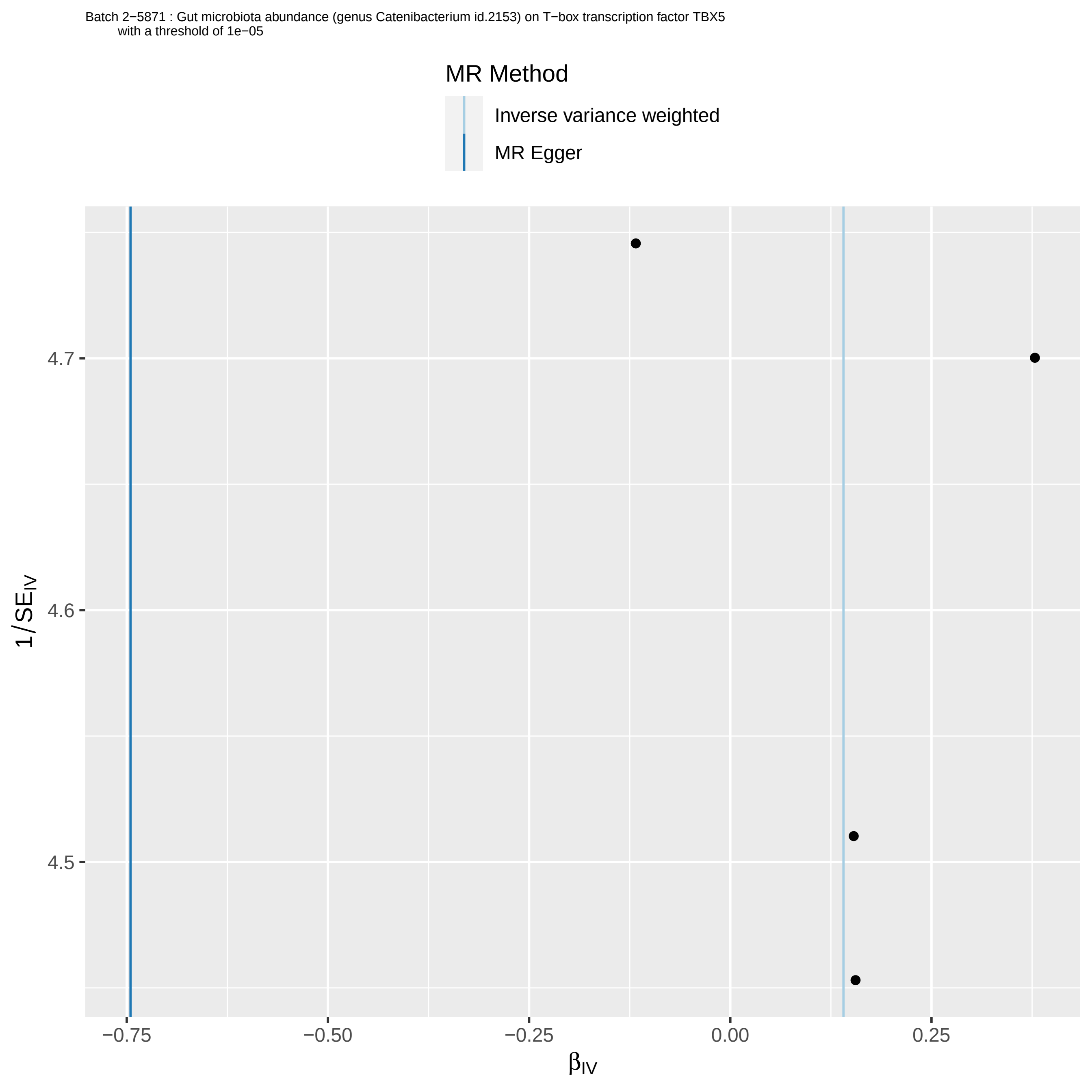

Batch 2−5871 : Gut microbiota abundance (genus Catenibacterium id.2153) on T−box transcription factor TBX5
with a threshold of 1e−05
MR Method
Inverse variance weighted
MR Egger
4.7
4.6
4.5
1/SE_IV
−0.75
−0.50
−0.25
0.00
0.25
β_IV

Batch 2−5872 : Gut microbiota abundance (genus Parasutterella id.2892) on T−box transcription factor TBX5
with a threshold of 1e−05

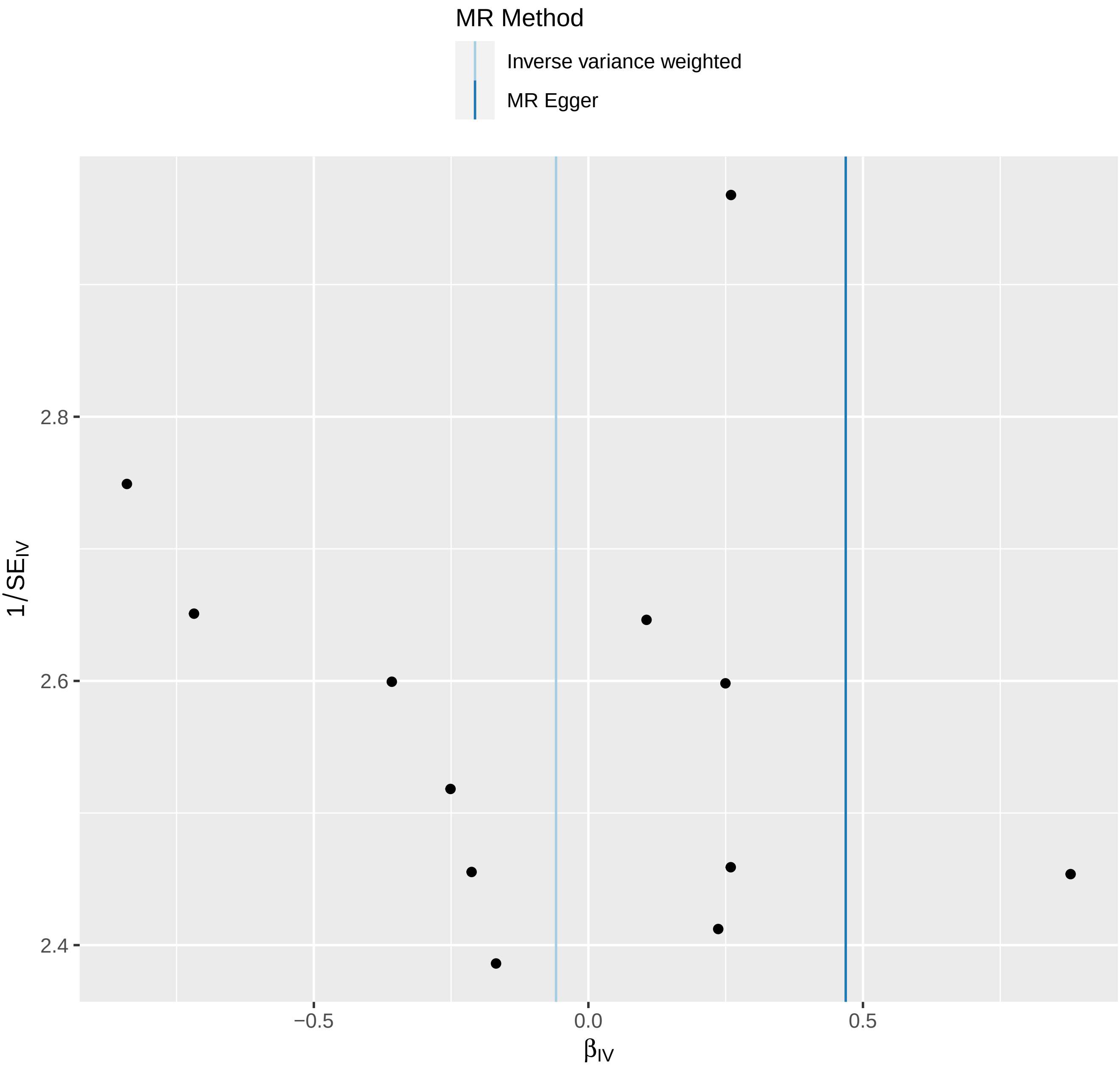

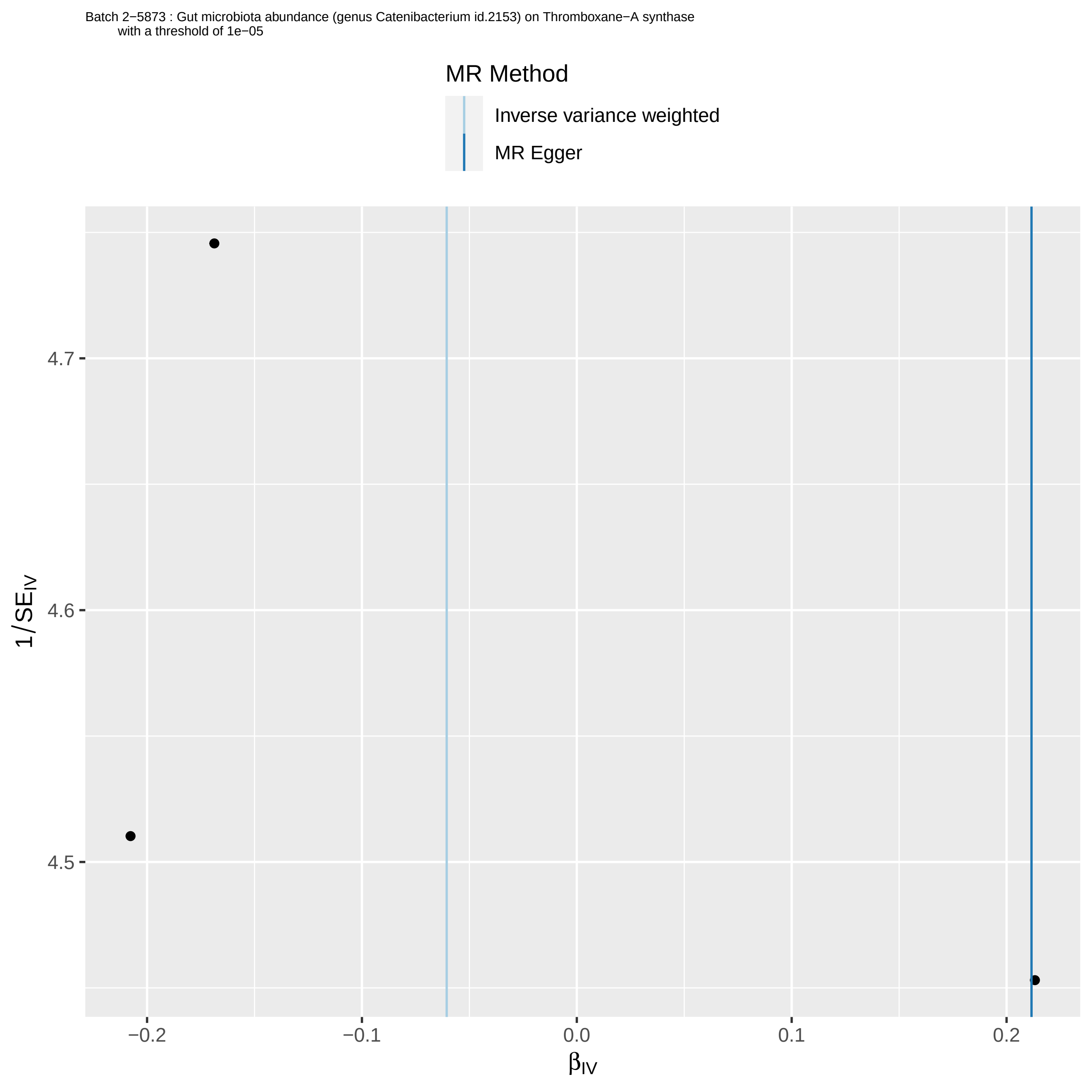

Batch 2−5873 : Gut microbiota abundance (genus Catenibacterium id.2153) on Thromboxane−A synthase
with a threshold of 1e−05
MR Method
Inverse variance weighted
MR Egger
4.7
4.6
4.5
1/SE_IV
−0.2
−0.1
0.0
0.1
0.2
β_IV

Batch 2−5874 : Gut microbiota abundance (genus Parasutterella id.2892) on Thromboxane−A synthase
with a threshold of 1e−05

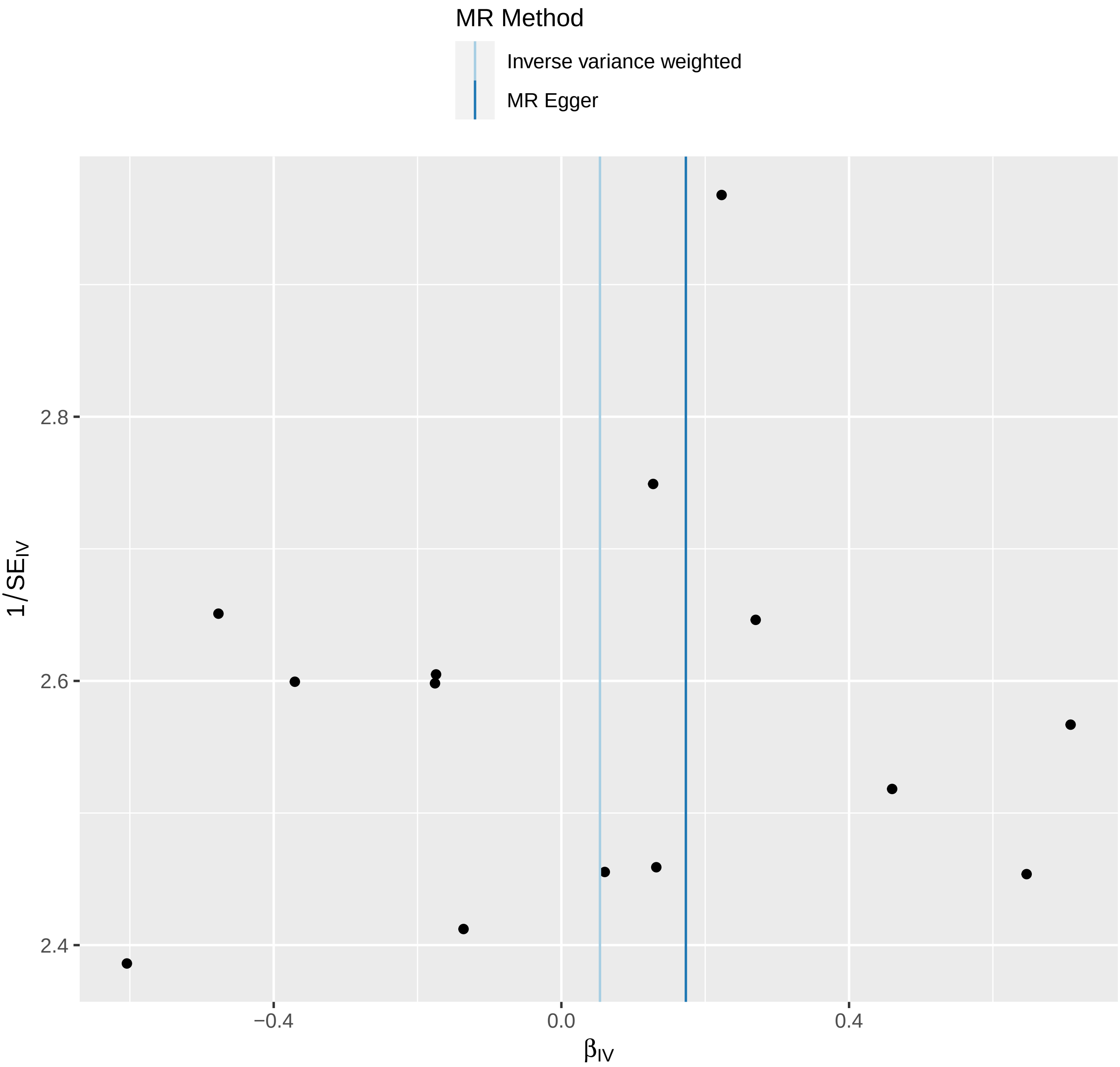

Batch 2−5875 : Gut microbiota abundance (genus Catenibacterium id.2153) on Transcobalamin−1
with a threshold of 1e−05

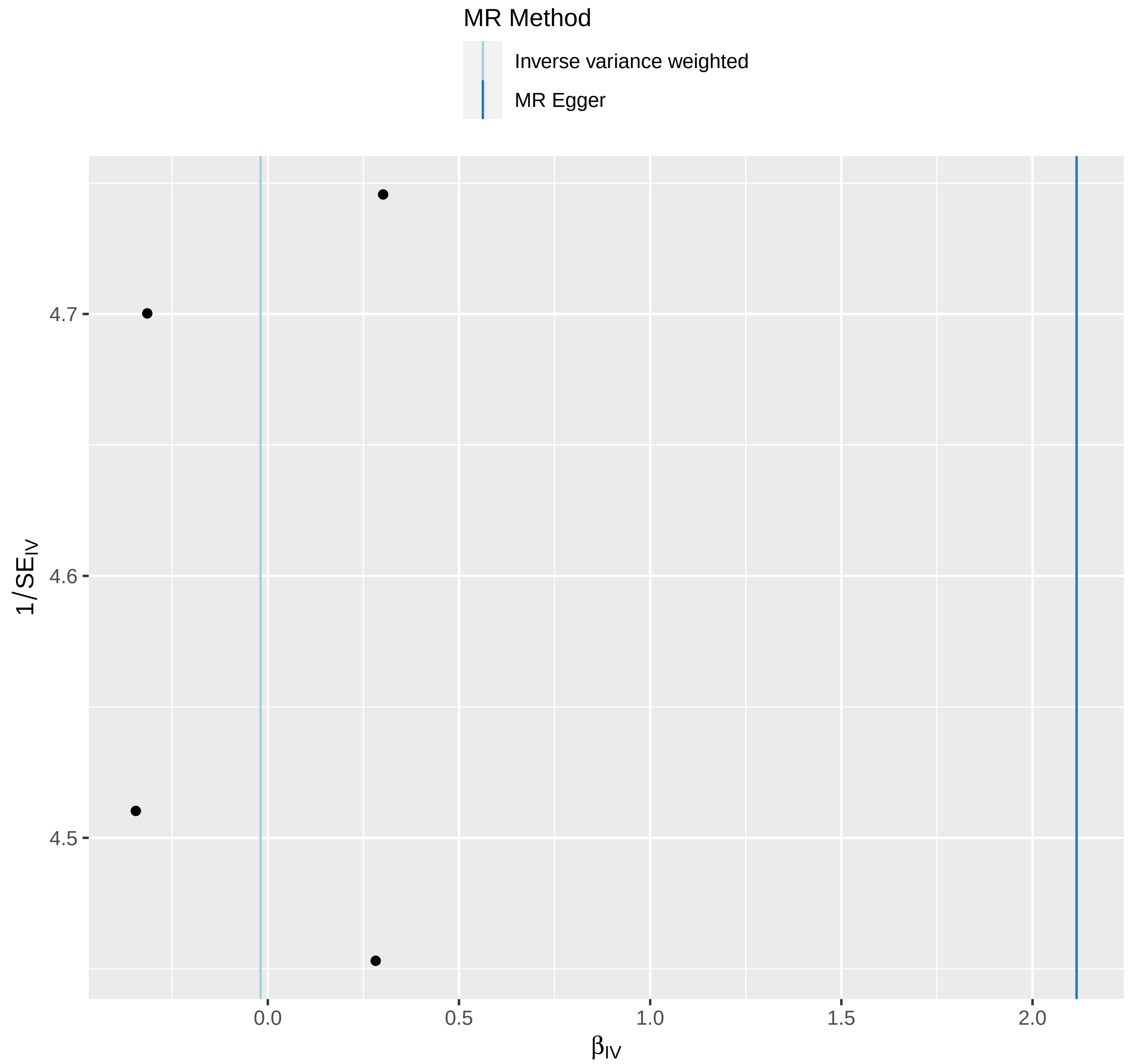

Batch 2−5876 : Gut microbiota abundance (genus Parasutterella id.2892) on Transcobalamin−1
with a threshold of 1e−05

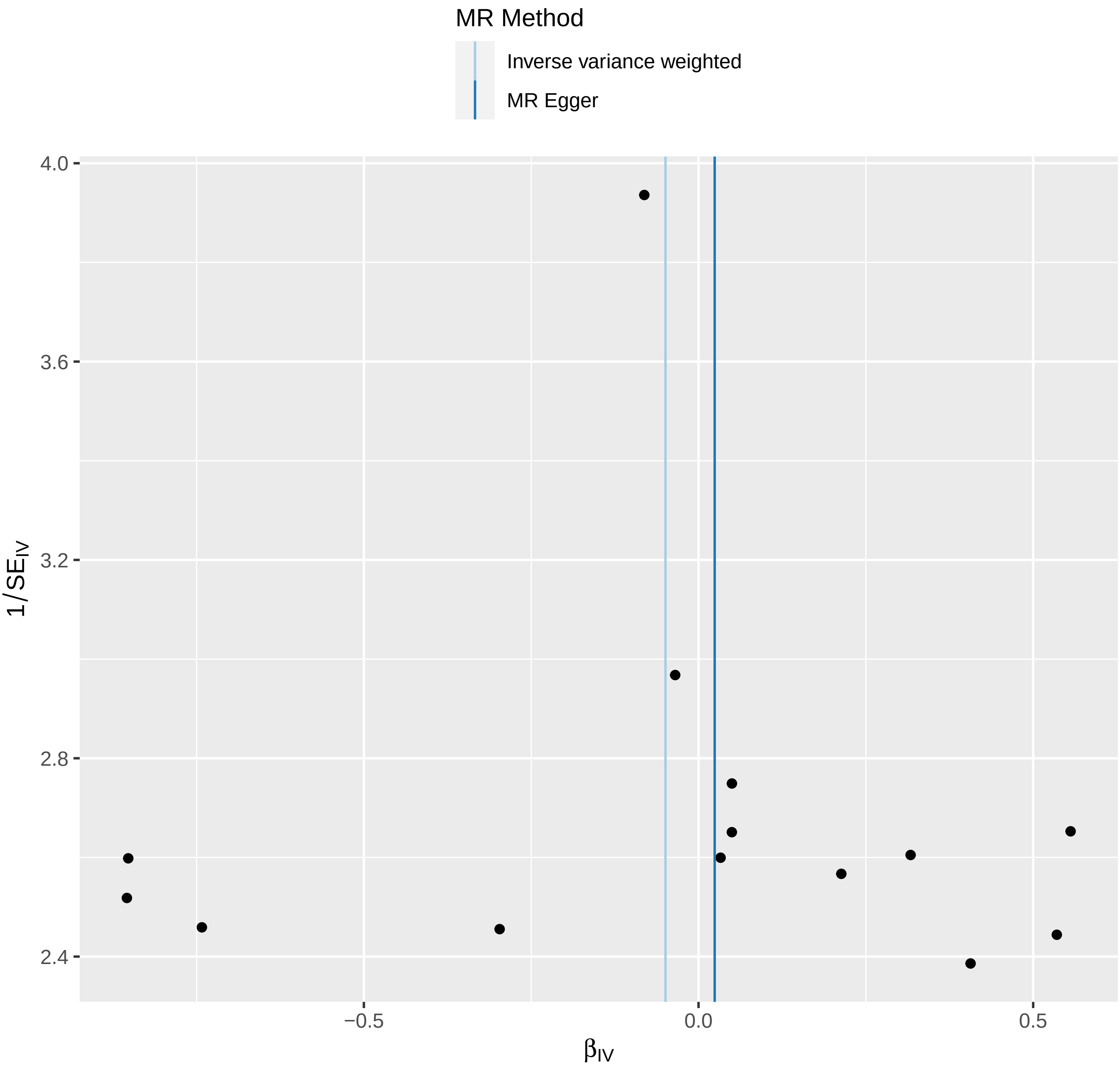

Batch 2−5877 : Gut microbiota abundance (genus Catenibacterium id.2153) on Holo−Transcobalamin−2
with a threshold of 1e−05

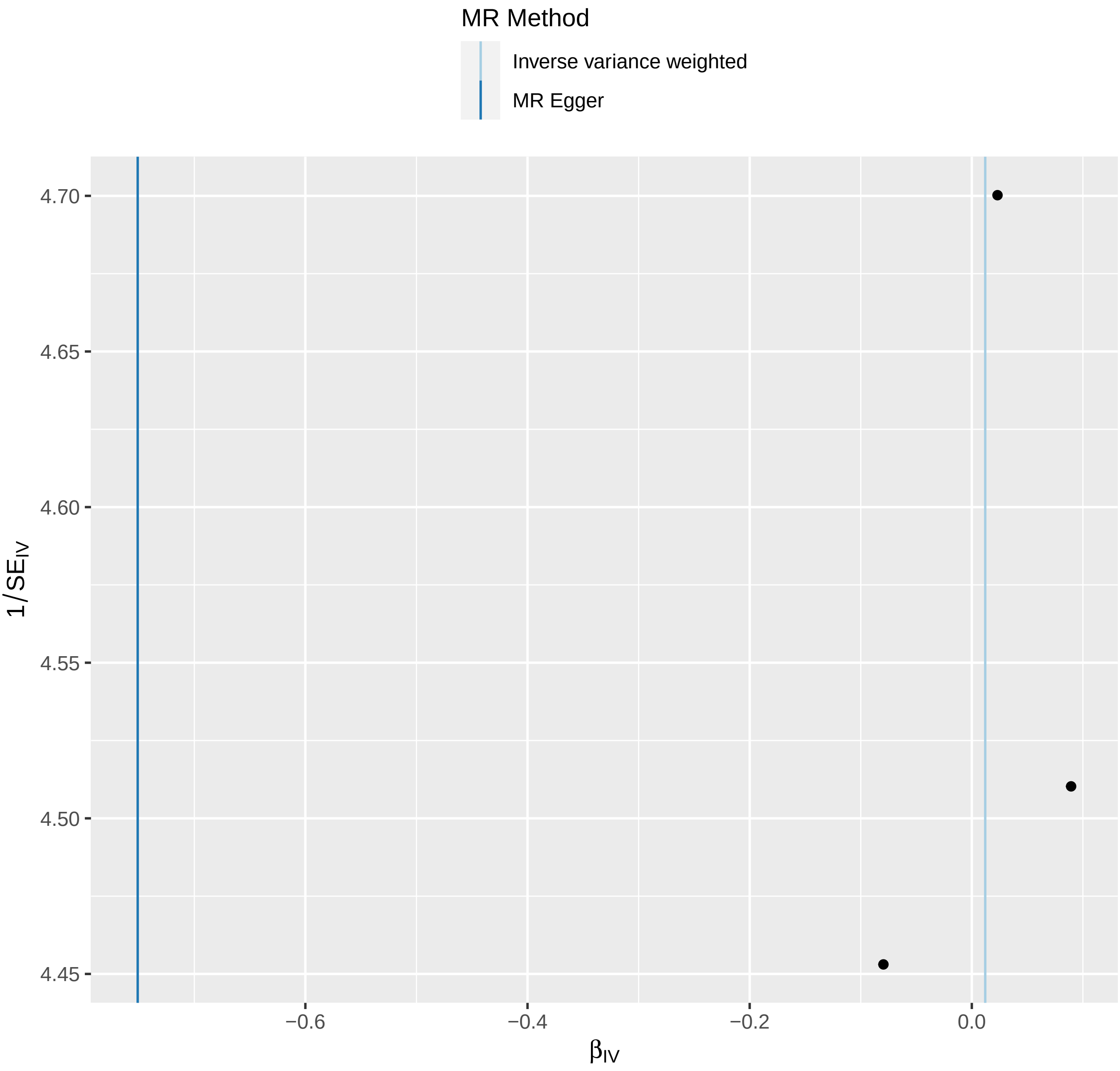

Batch 2−5878 : Gut microbiota abundance (genus Parasutterella id.2892) on Holo−Transcobalamin−2
with a threshold of 1e−05

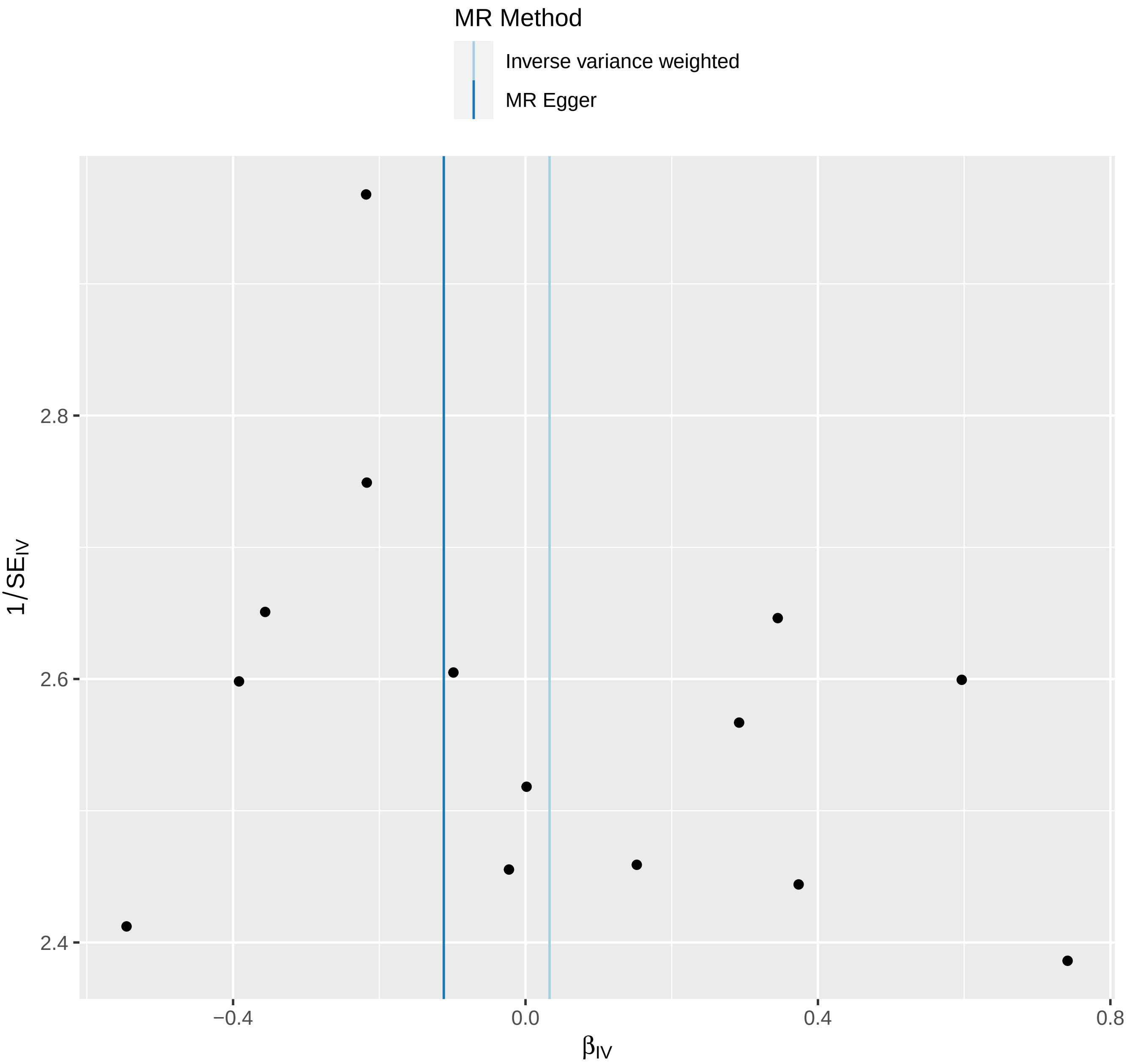

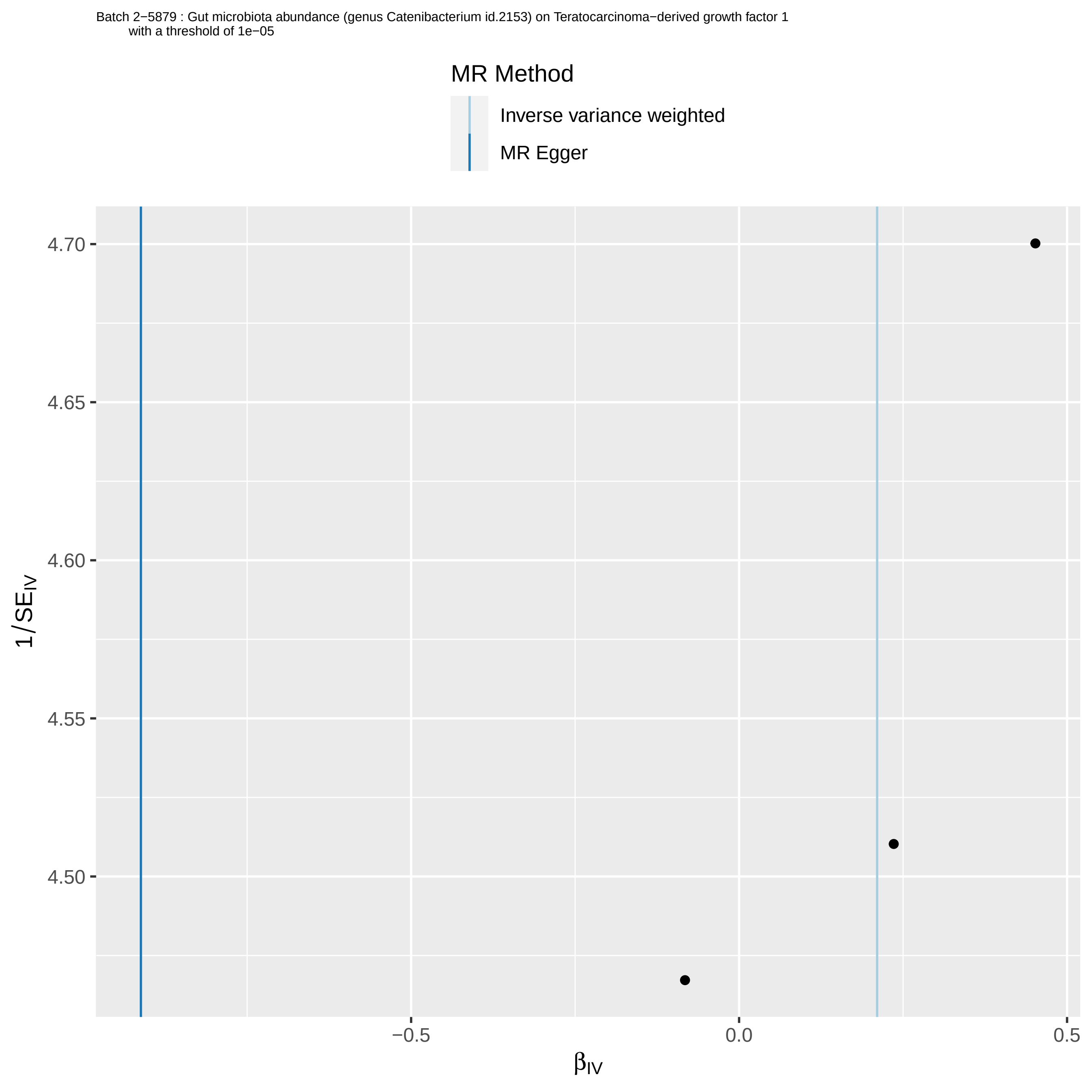
Batch 2−5879 : Gut microbiota abundance (genus Catenibacterium id.2153) on Teratocarcinoma−derived growth factor 1
with a threshold of 1e−05
MR Method
Inverse variance weighted
MR Egger
4.70
4.65
4.60
4.55
4.50
1/SE_IV
−0.5
0.0
0.5
β_IV

Batch 2−5880 : Gut microbiota abundance (genus Parasutterella id.2892) on Teratocarcinoma−derived growth factor 1
with a threshold of 1e−05

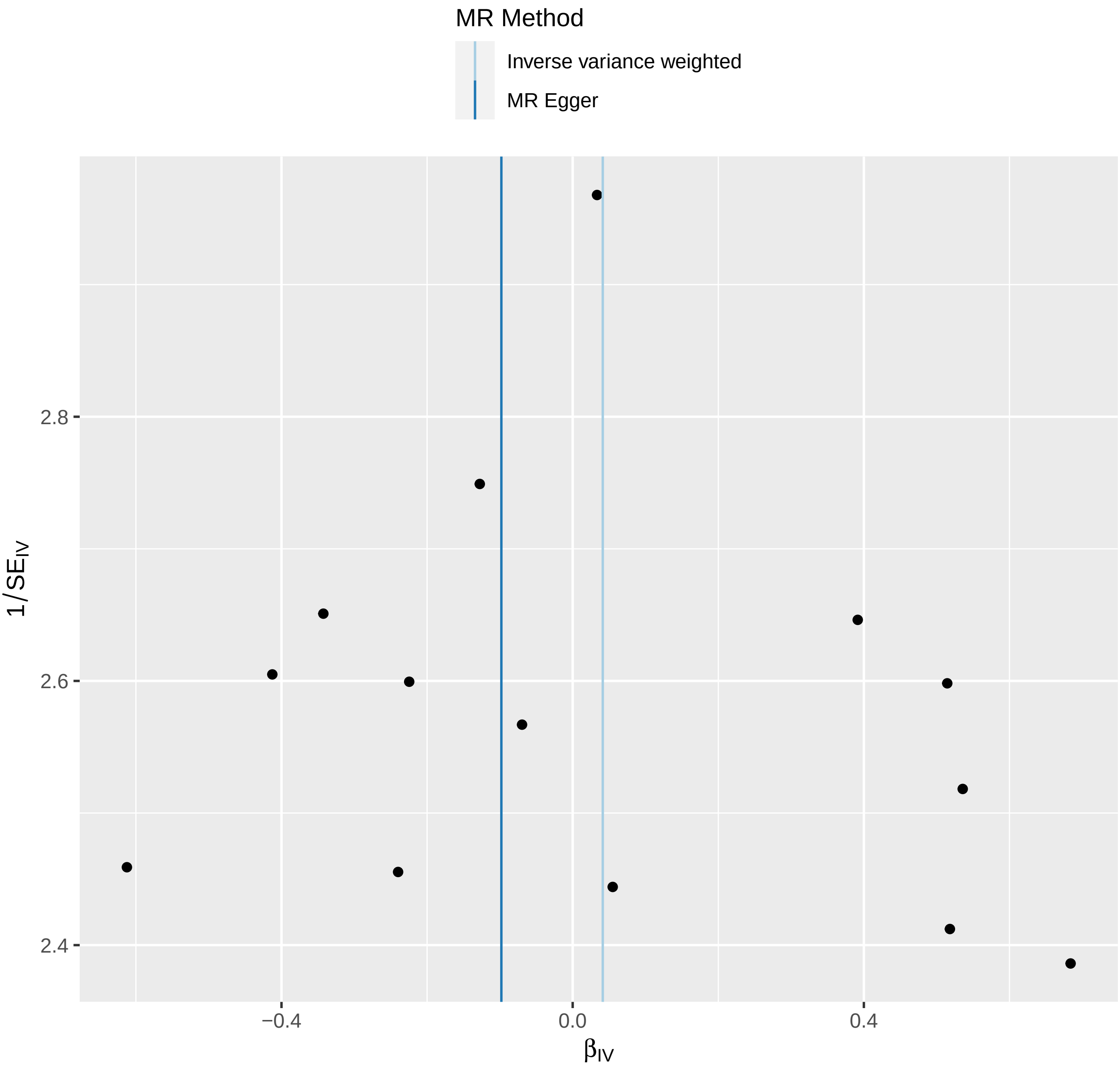

Batch 2−5881 : Gut microbiota abundance (genus Catenibacterium id.2153) on Tyrosyl−DNA phosphodiesterase 1
with a threshold of 1e−05

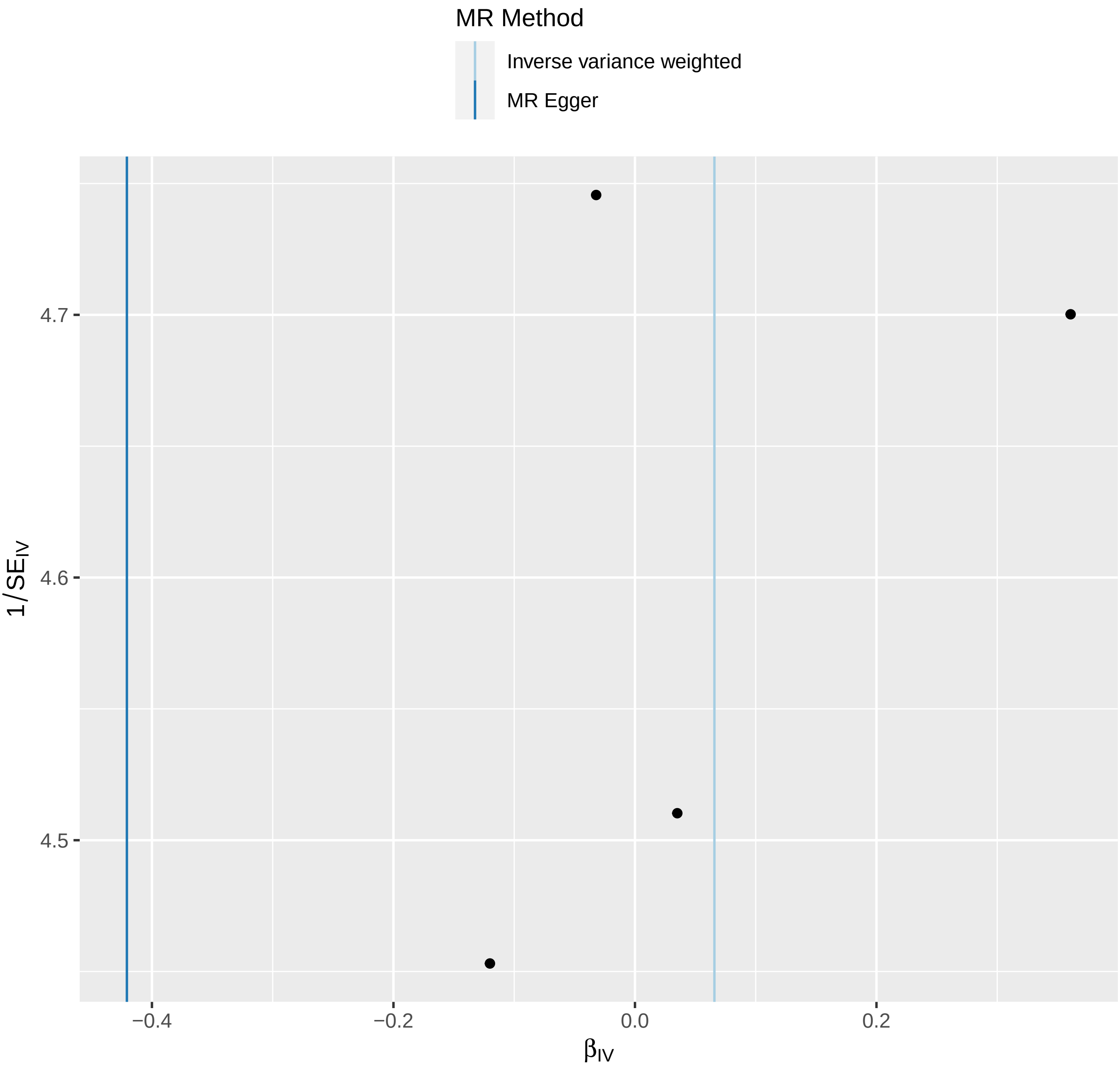

Batch 2−5882 : Gut microbiota abundance (genus Parasutterella id.2892) on Tyrosyl−DNA phosphodiesterase 1
with a threshold of 1e−05

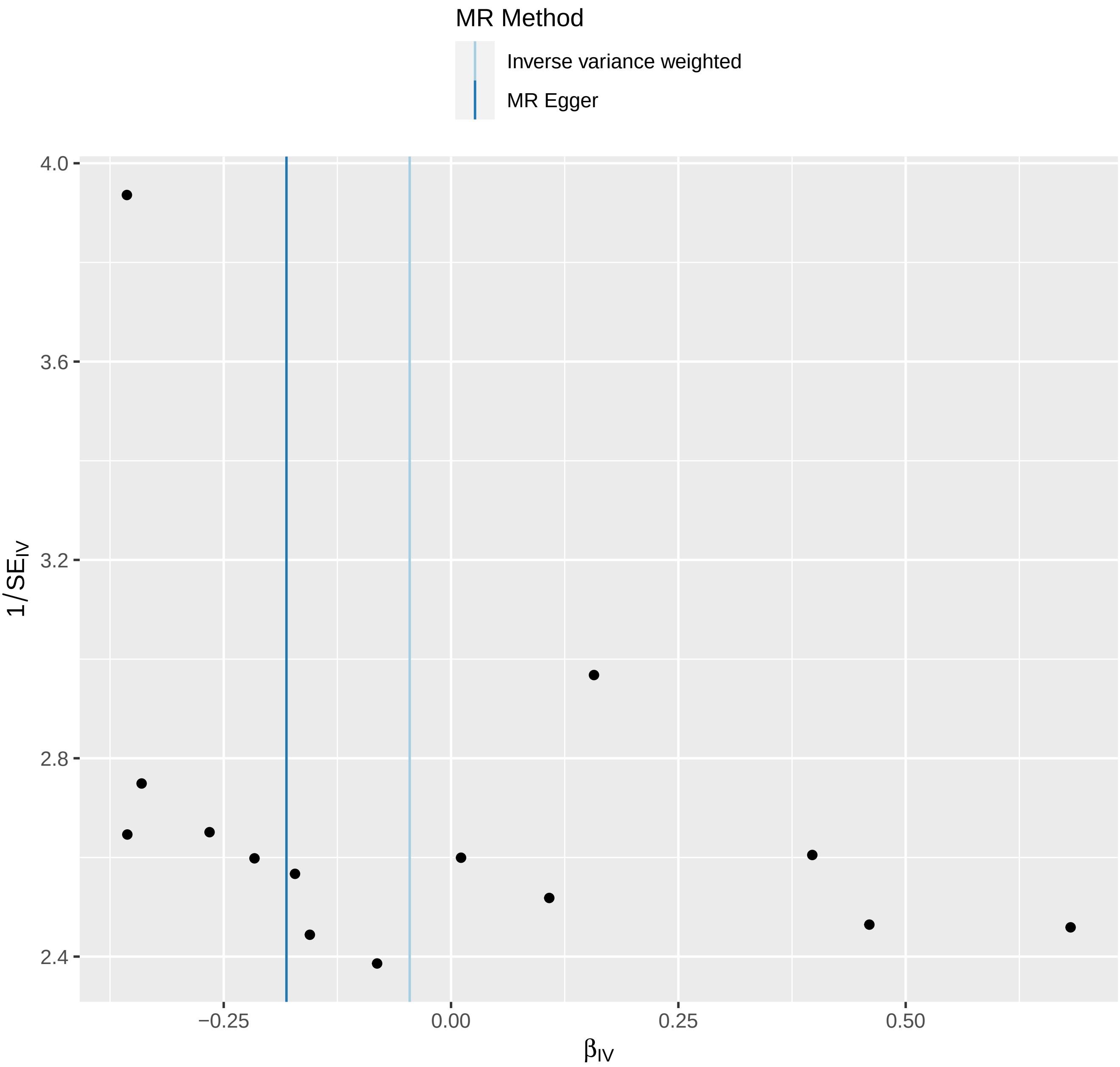

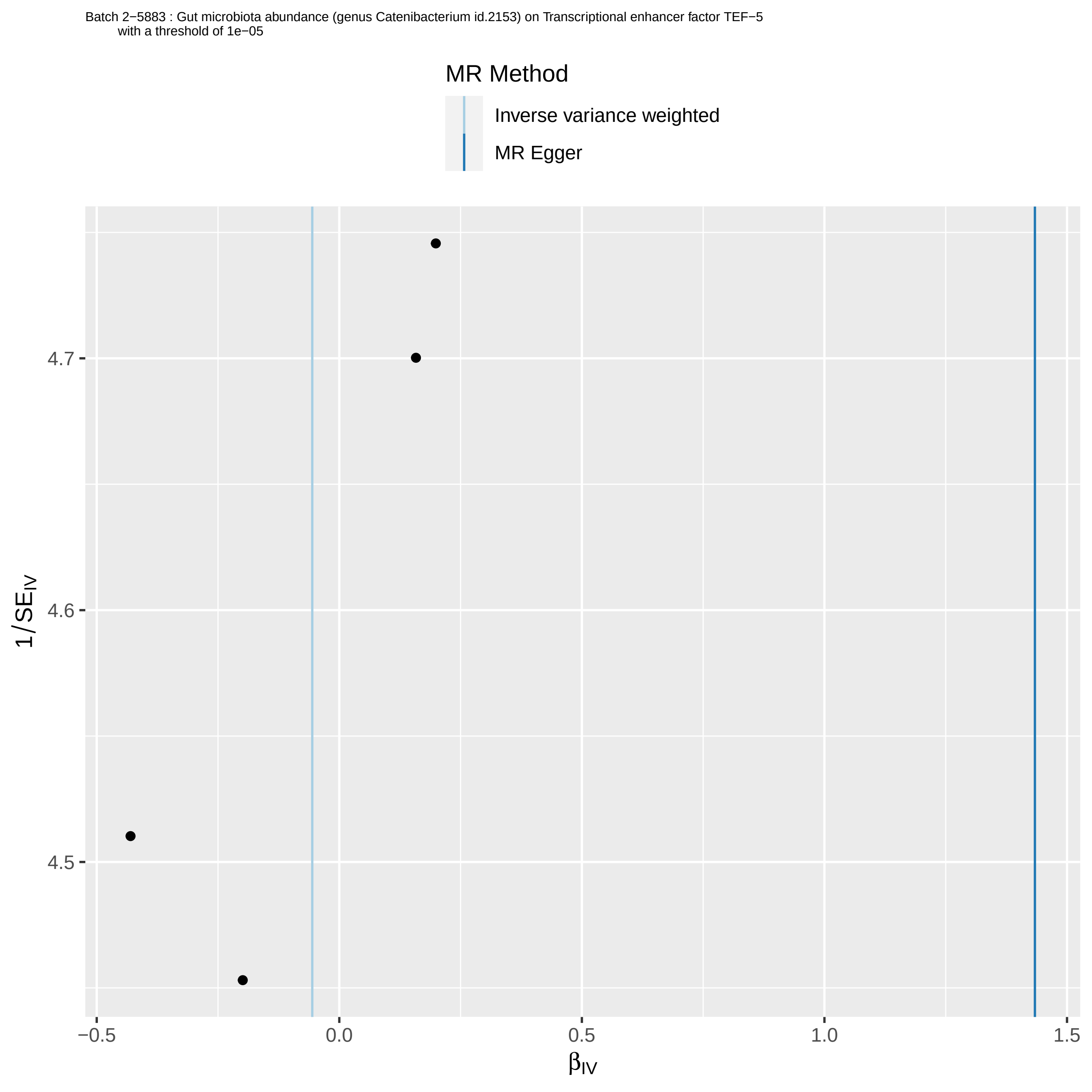

Batch 2−5883 : Gut microbiota abundance (genus Catenibacterium id.2153) on Transcriptional enhancer factor TEF−5
with a threshold of 1e−05
MR Method
Inverse variance weighted
MR Egger
4.7
4.6
4.5
$1/SE_{IV}$
−0.5
0.0
0.5
1.0
1.5
β_{IV}

Batch 2−5884 : Gut microbiota abundance (genus Parasutterella id.2892) on Transcriptional enhancer factor TEF−5
with a threshold of 1e−05

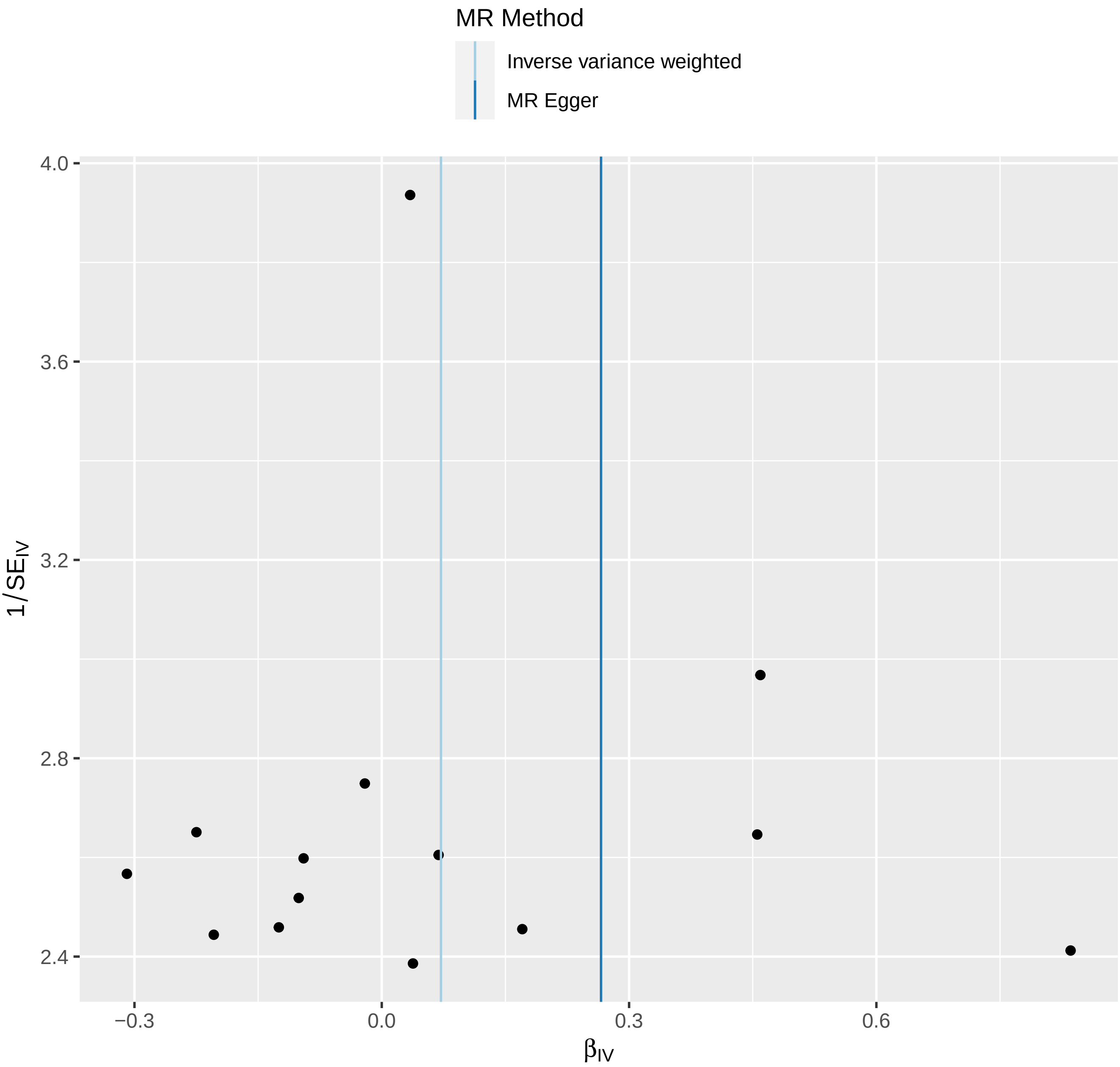

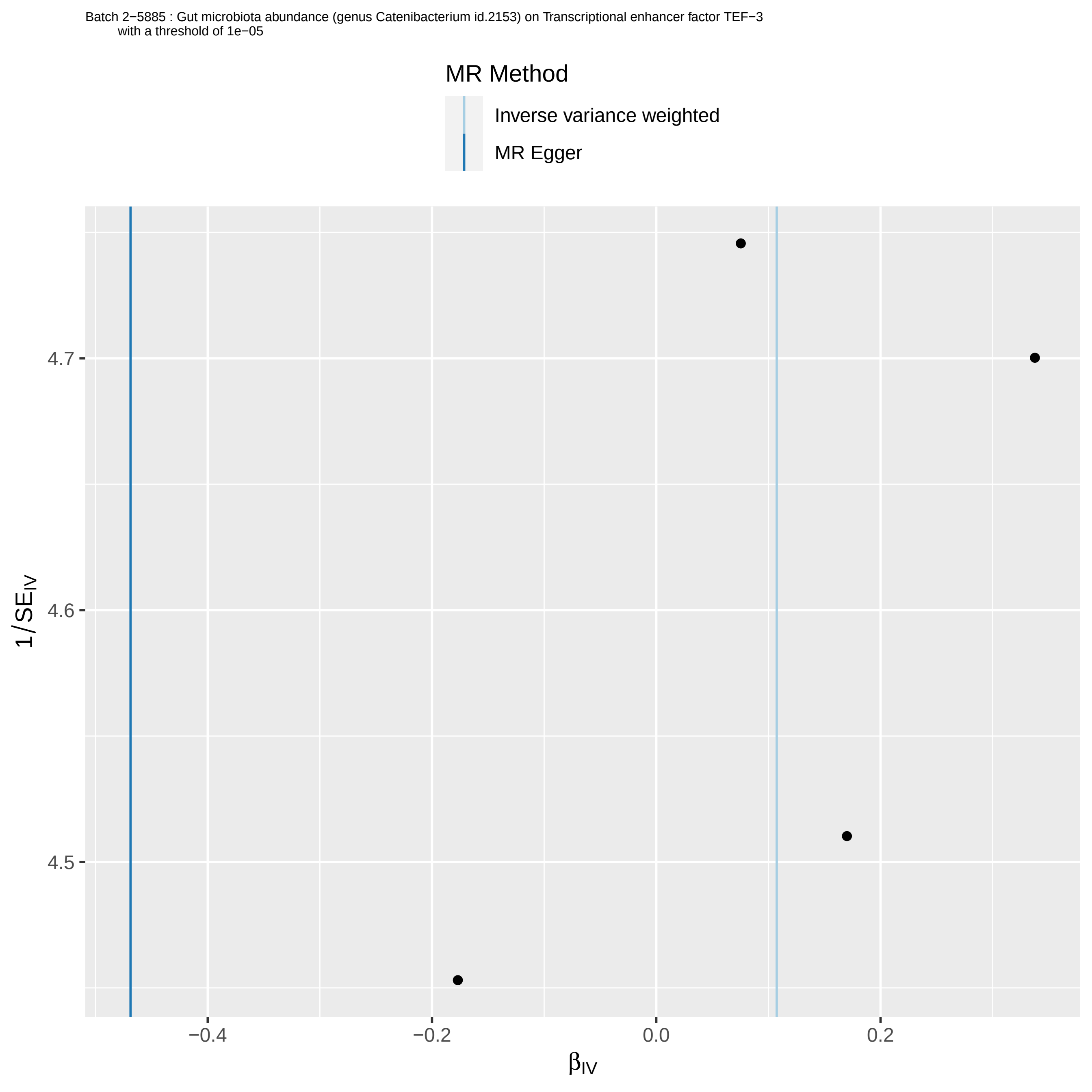

Batch 2−5885 : Gut microbiota abundance (genus Catenibacterium id.2153) on Transcriptional enhancer factor TEF−3
with a threshold of 1e−05
MR Method
Inverse variance weighted
MR Egger
4.7
4.6
4.5
1/SE_IV
−0.4
−0.2
0.0
0.2
β_IV

Batch 2−5886 : Gut microbiota abundance (genus Parasutterella id.2892) on Transcriptional enhancer factor TEF−3
with a threshold of 1e−05

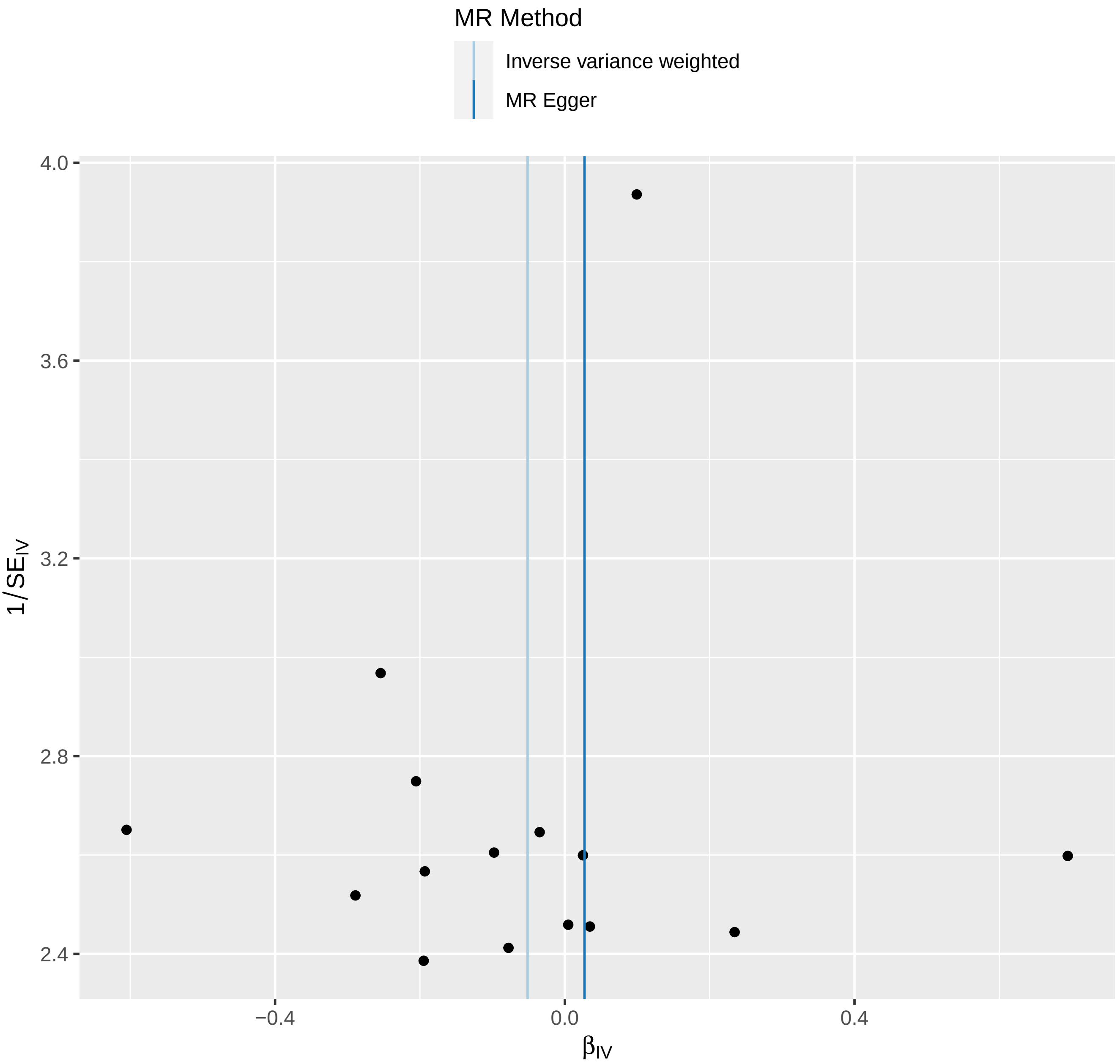

Batch 2−5887 : Gut microbiota abundance (genus Catenibacterium id.2153) on Angiopoietin−1 receptor, soluble
with a threshold of 1e−05

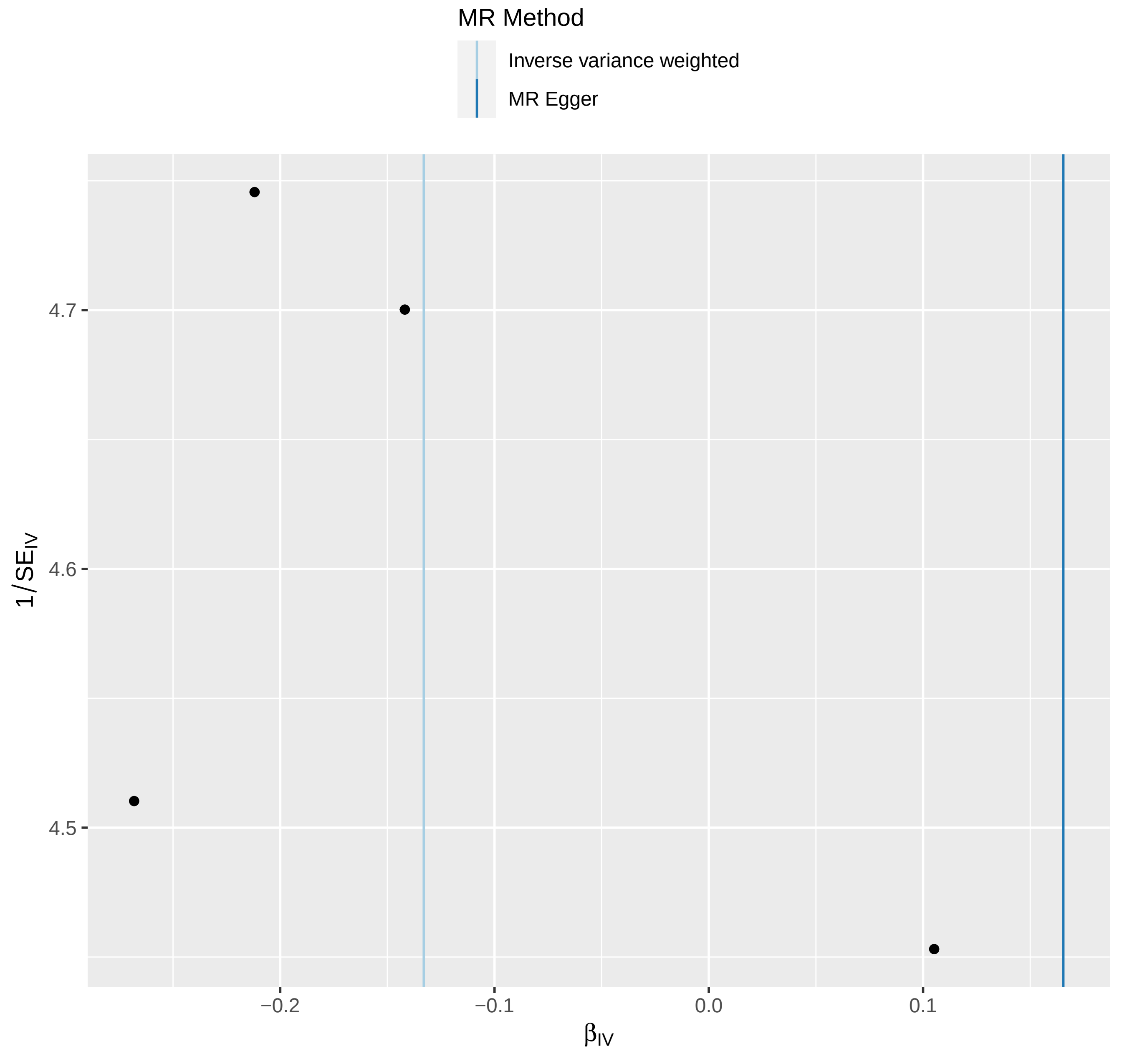

Batch 2−5888 : Gut microbiota abundance (genus Parasutterella id.2892) on Angiopoietin−1 receptor, soluble
with a threshold of 1e−05

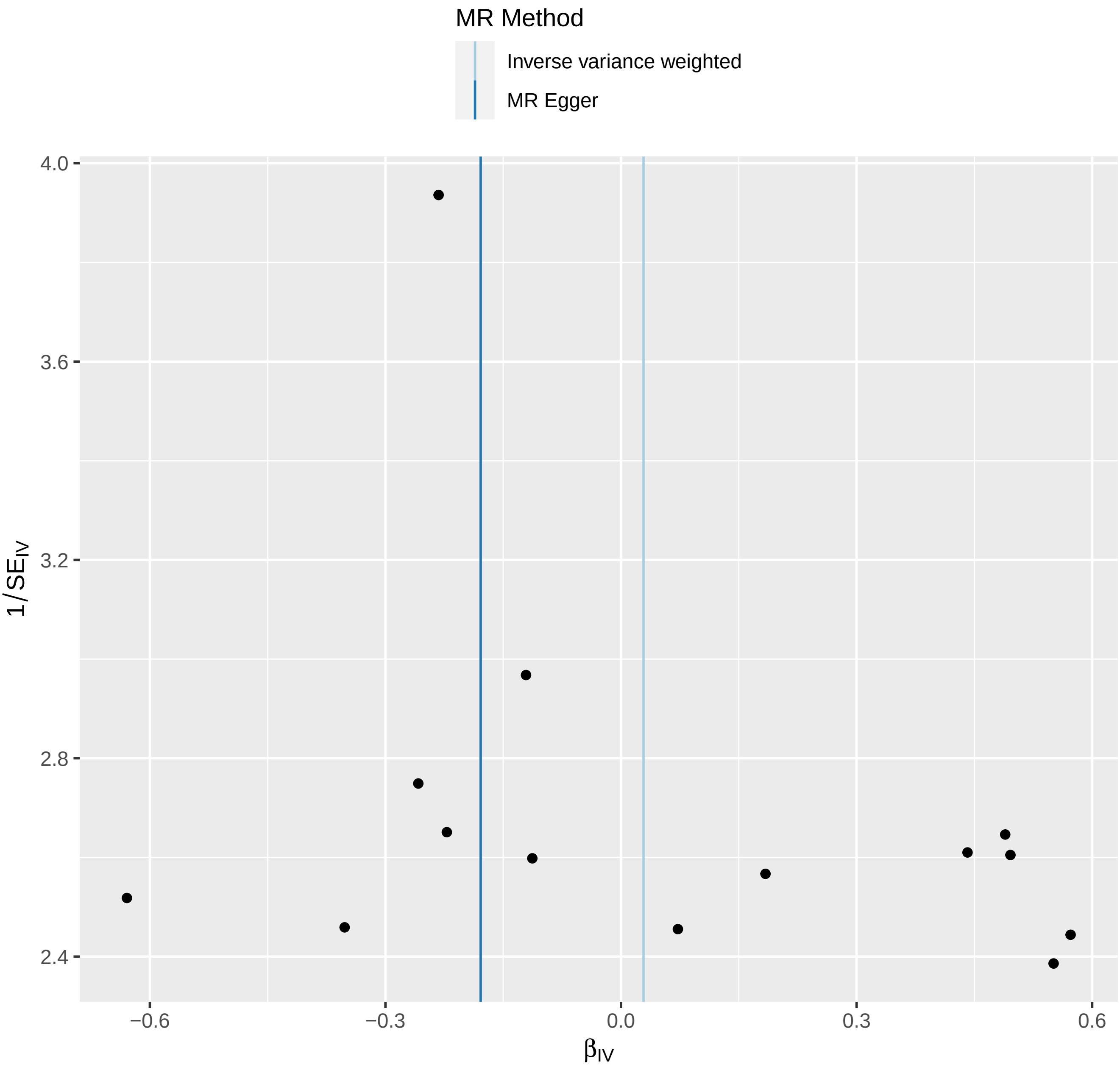

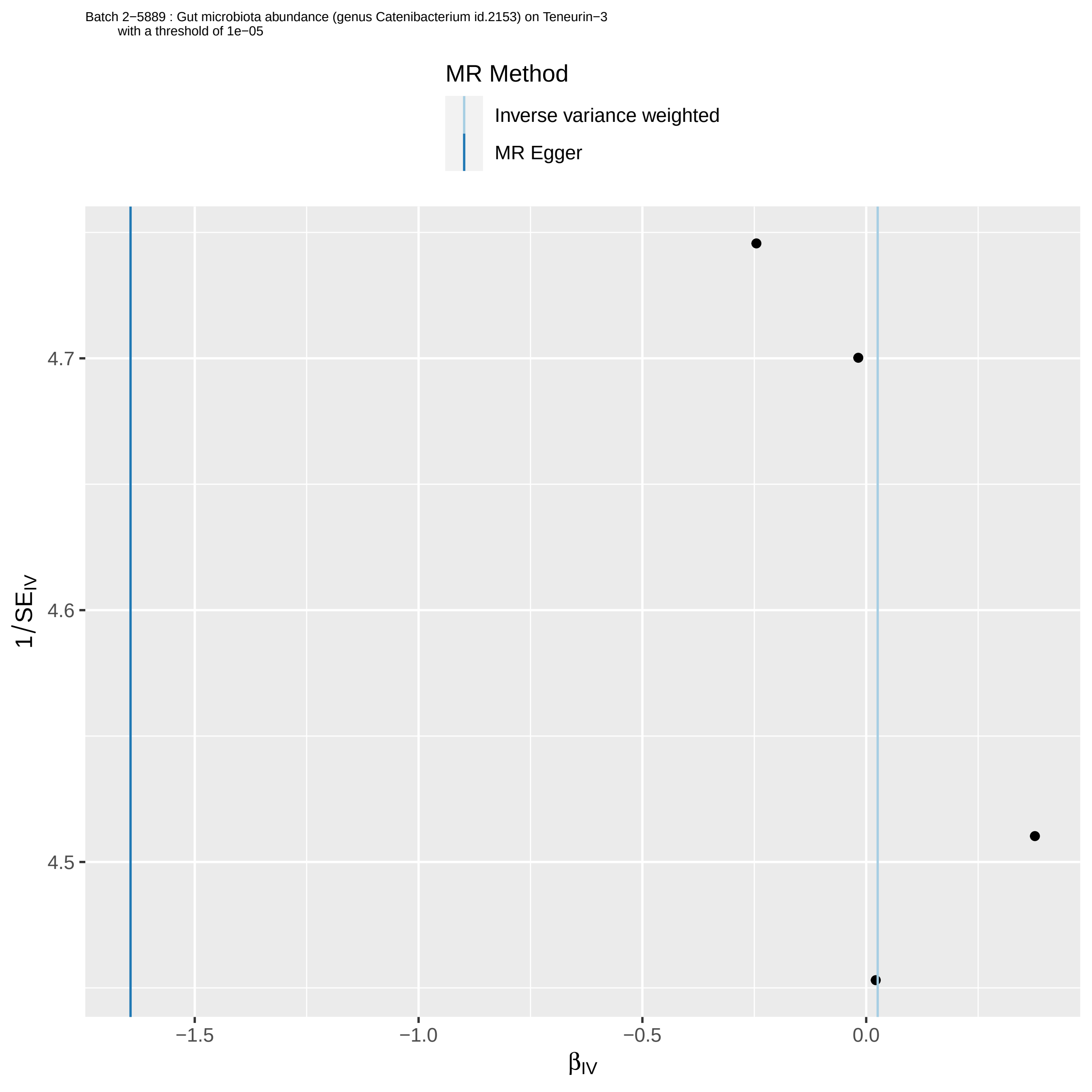

Batch 2−5889 : Gut microbiota abundance (genus Catenibacterium id.2153) on Teneurin−3
with a threshold of 1e−05
MR Method
Inverse variance weighted
MR Egger
4.7
4.6
4.5
1/SE$_{IV}$
−1.5
−1.0
−0.5
0.0
β$_{IV}$

Batch 2−5890 : Gut microbiota abundance (genus Parasutterella id.2892) on Teneurin−3
with a threshold of 1e−05

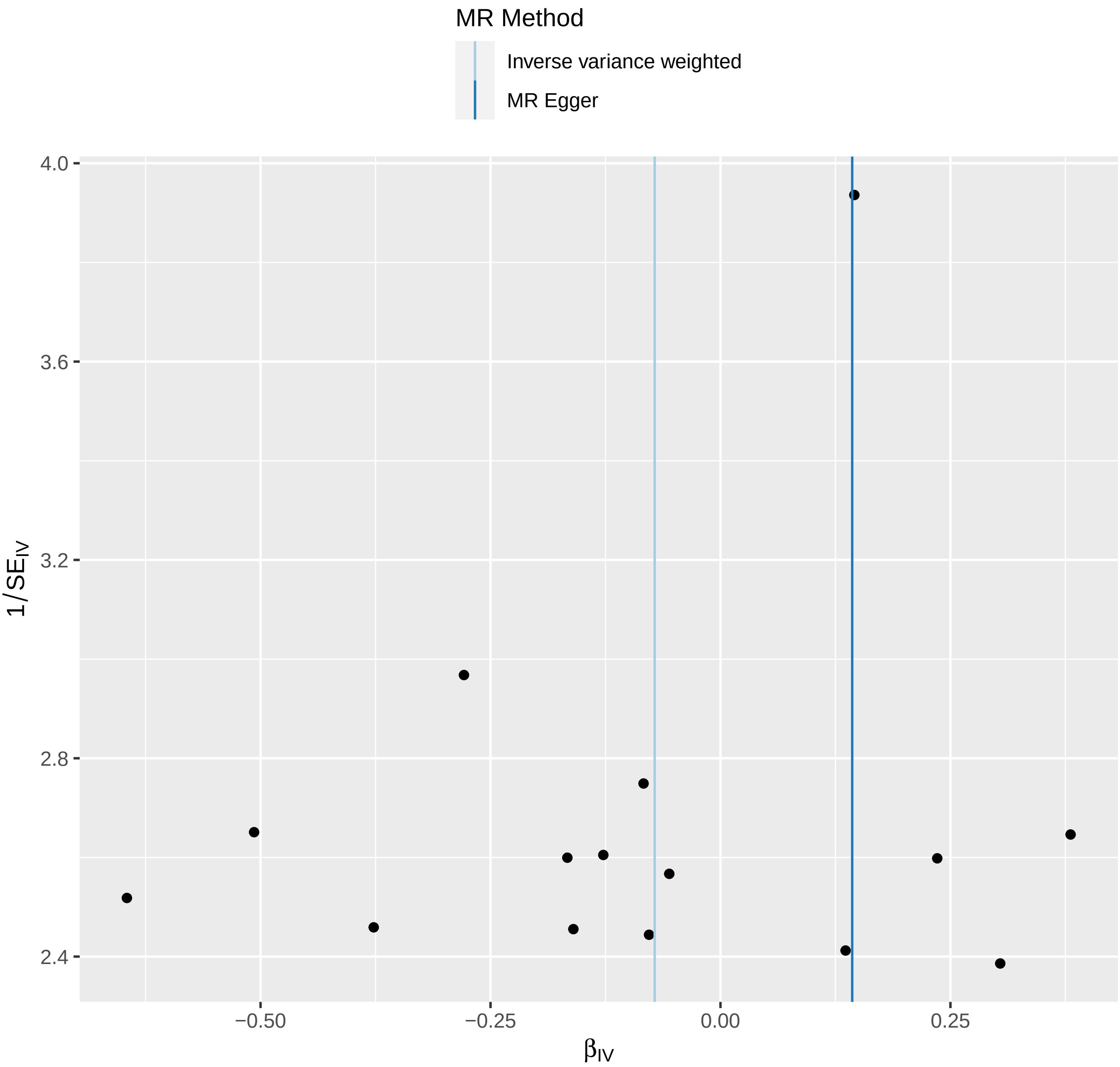

Batch 2−5891 : Gut microbiota abundance (genus Catenibacterium id.2153) on Teneurin−4
with a threshold of 1e−05

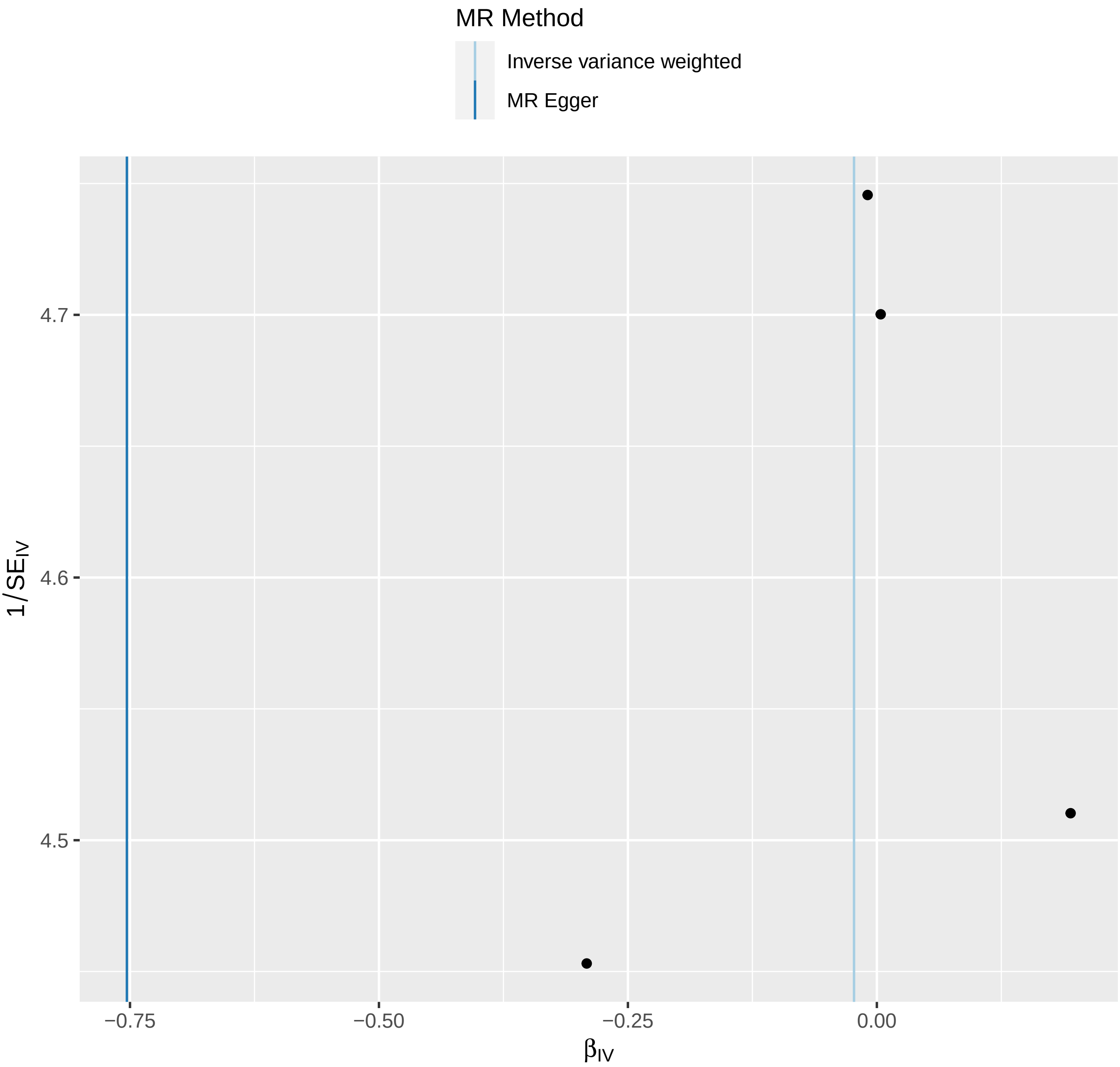

Batch 2−5892 : Gut microbiota abundance (genus Parasutterella id.2892) on Teneurin−4
with a threshold of 1e−05

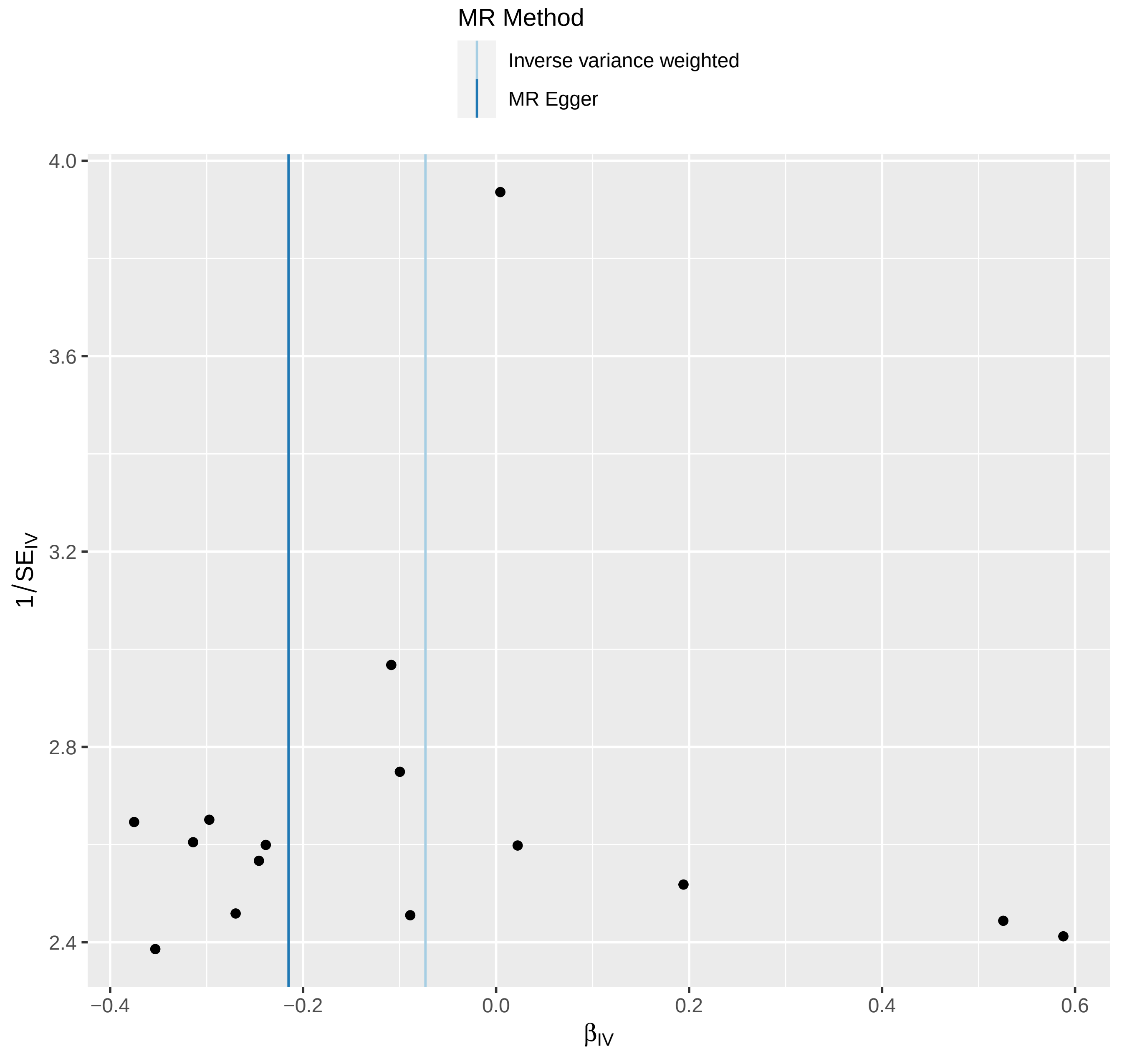

Batch 2−5893 : Gut microbiota abundance (genus Catenibacterium id.2153) on Calcineurin B homologous protein 3
with a threshold of 1e−05

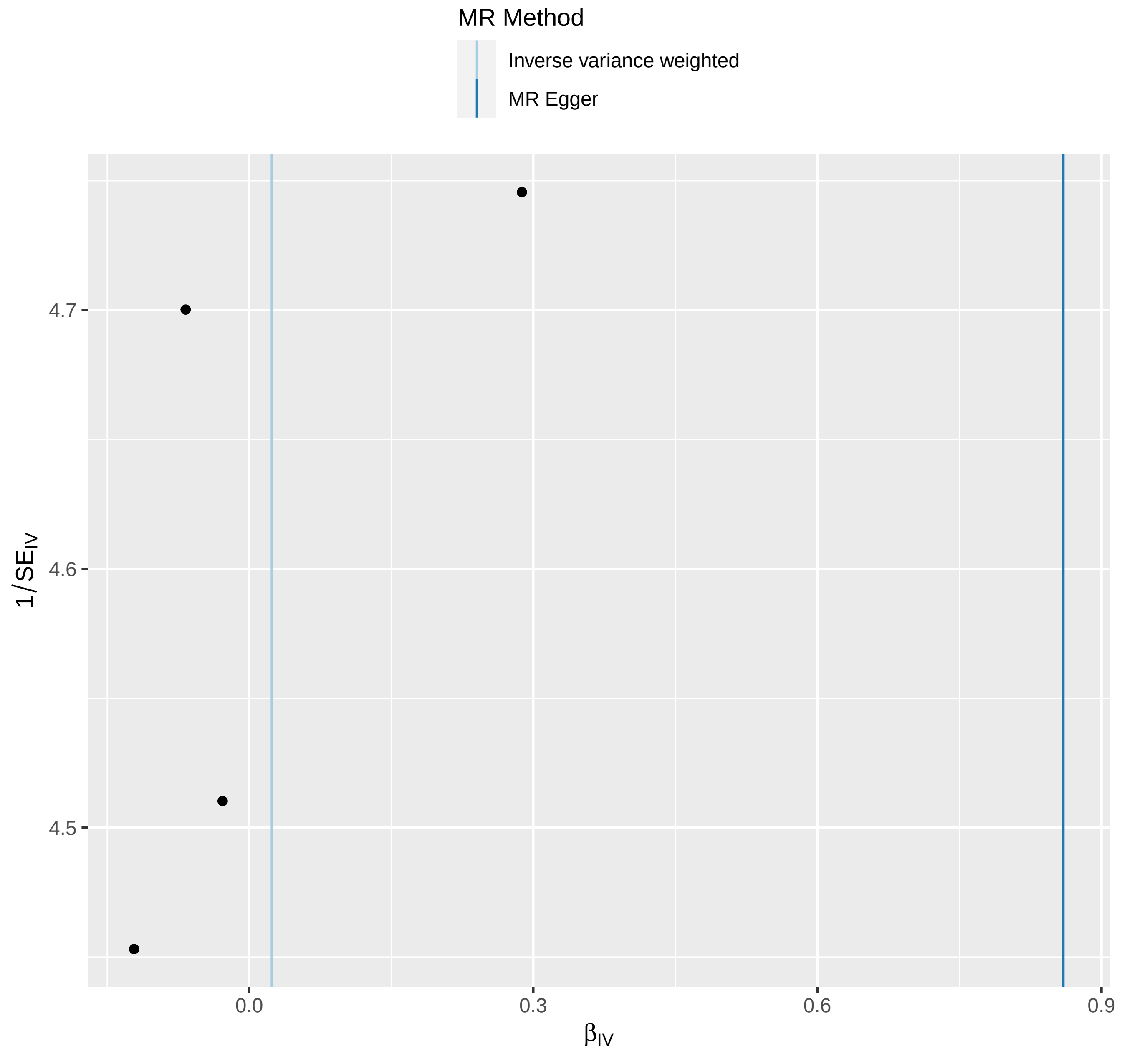

Batch 2−5894 : Gut microbiota abundance (genus Parasutterella id.2892) on Calcineurin B homologous protein 3
with a threshold of 1e−05

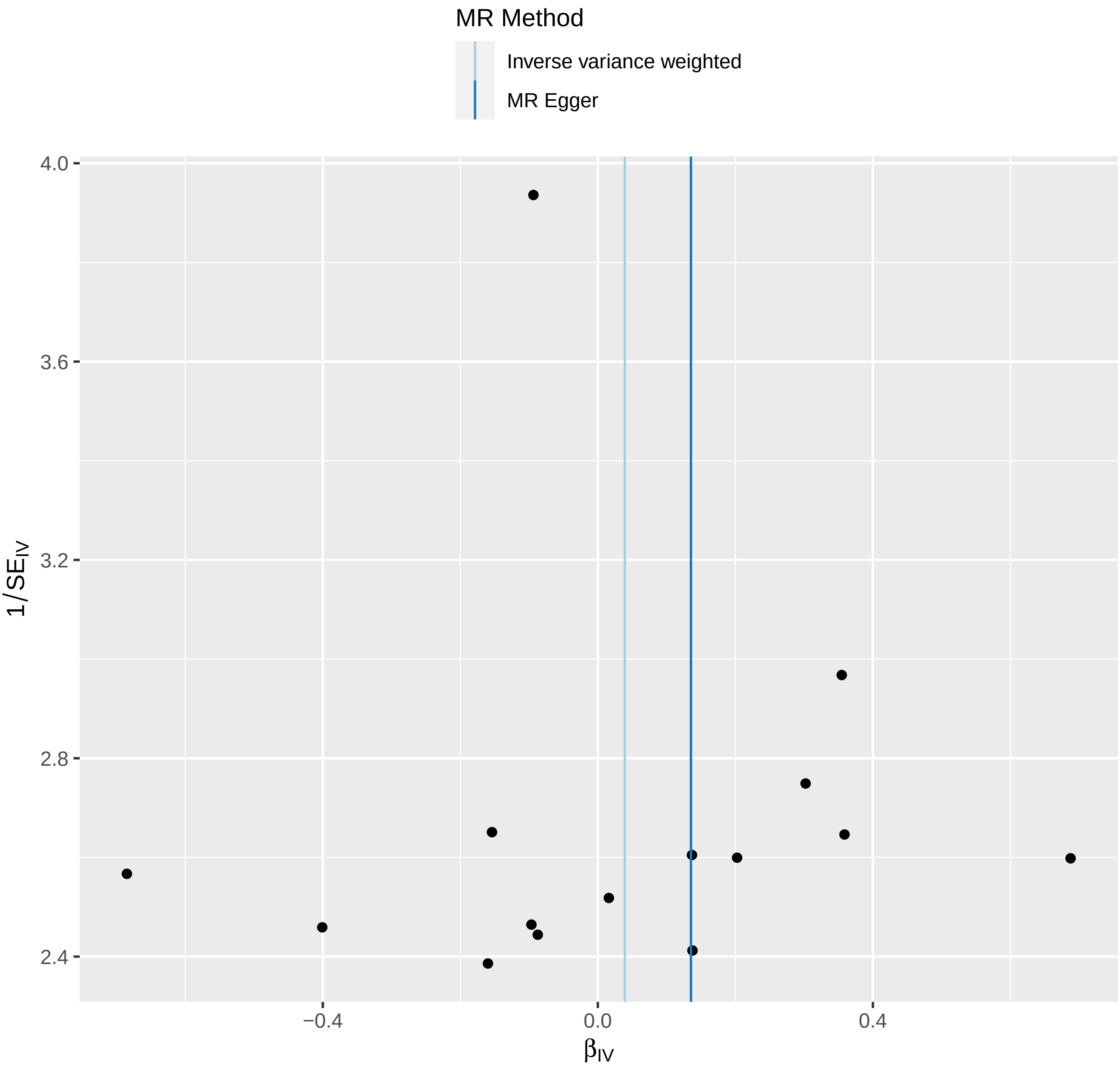

Batch 2−5895 : Gut microbiota abundance (genus Catenibacterium id.2153) on Testis−expressed sequence 29 protein
with a threshold of 1e−05

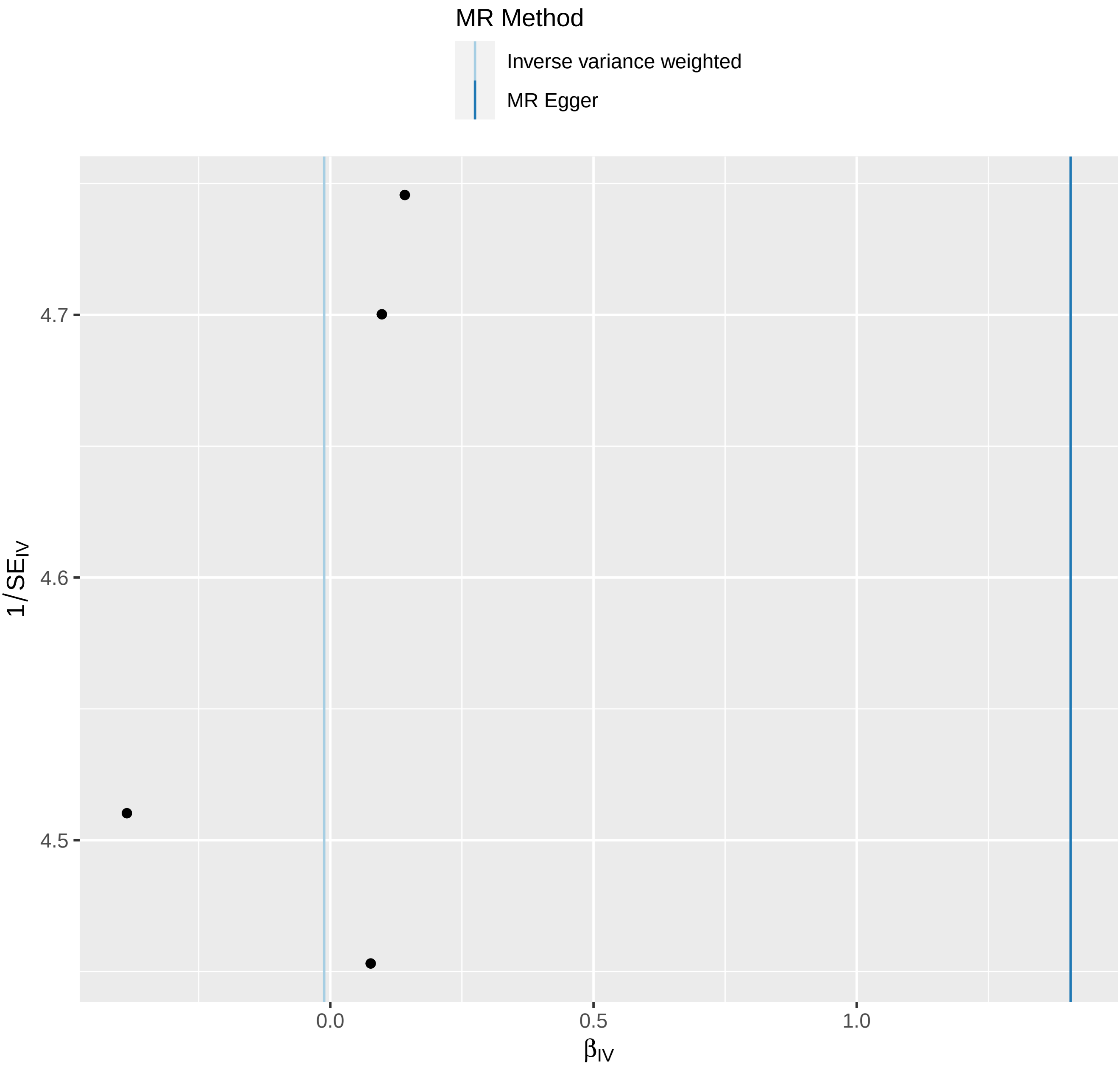

Batch 2−5896 : Gut microbiota abundance (genus Parasutterella id.2892) on Testis−expressed sequence 29 protein
with a threshold of 1e−05

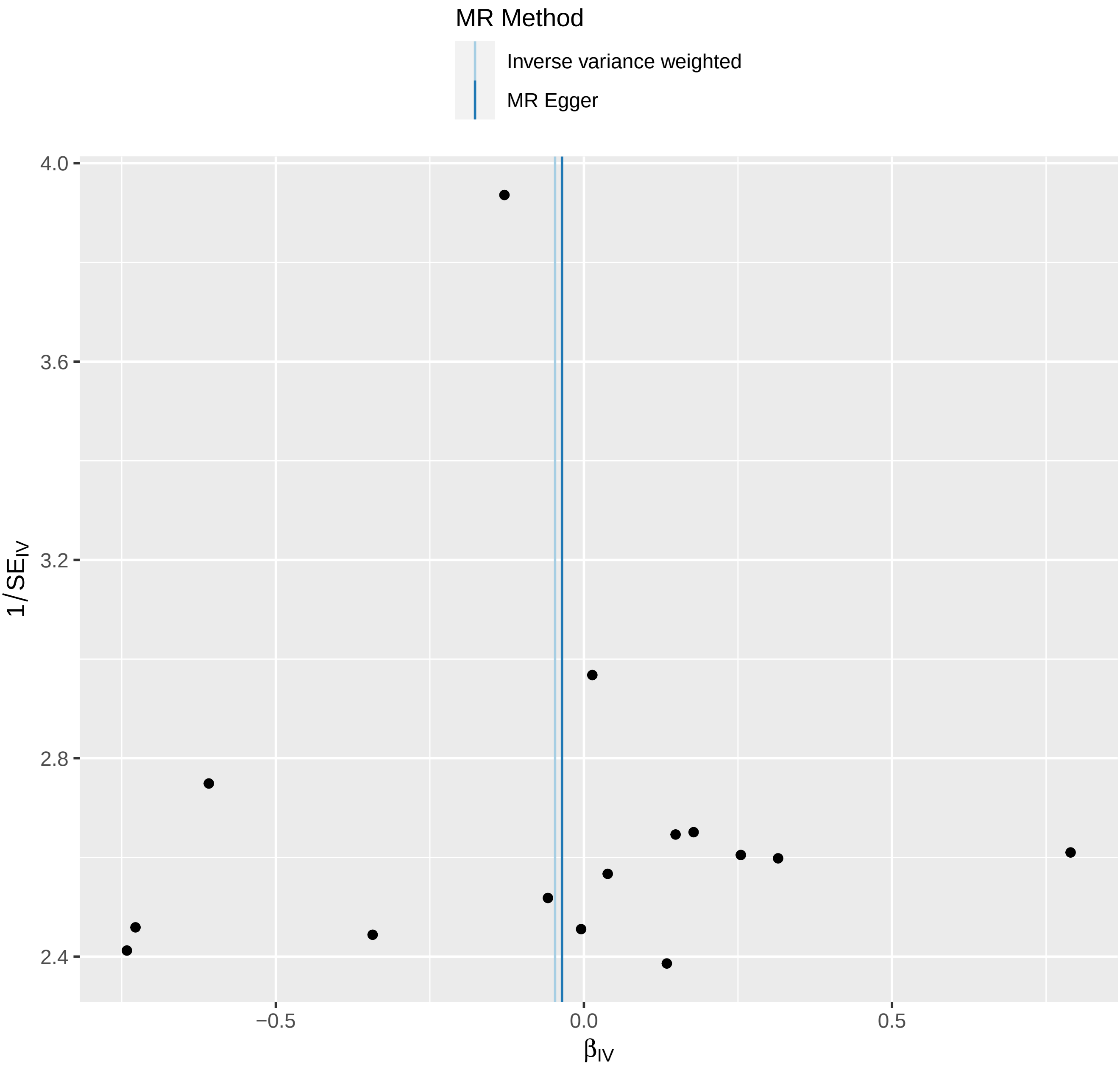

Batch 2−5897 : Gut microbiota abundance (genus Catenibacterium id.2153) on Testis−expressed sequence 29 protein
with a threshold of 1e−05

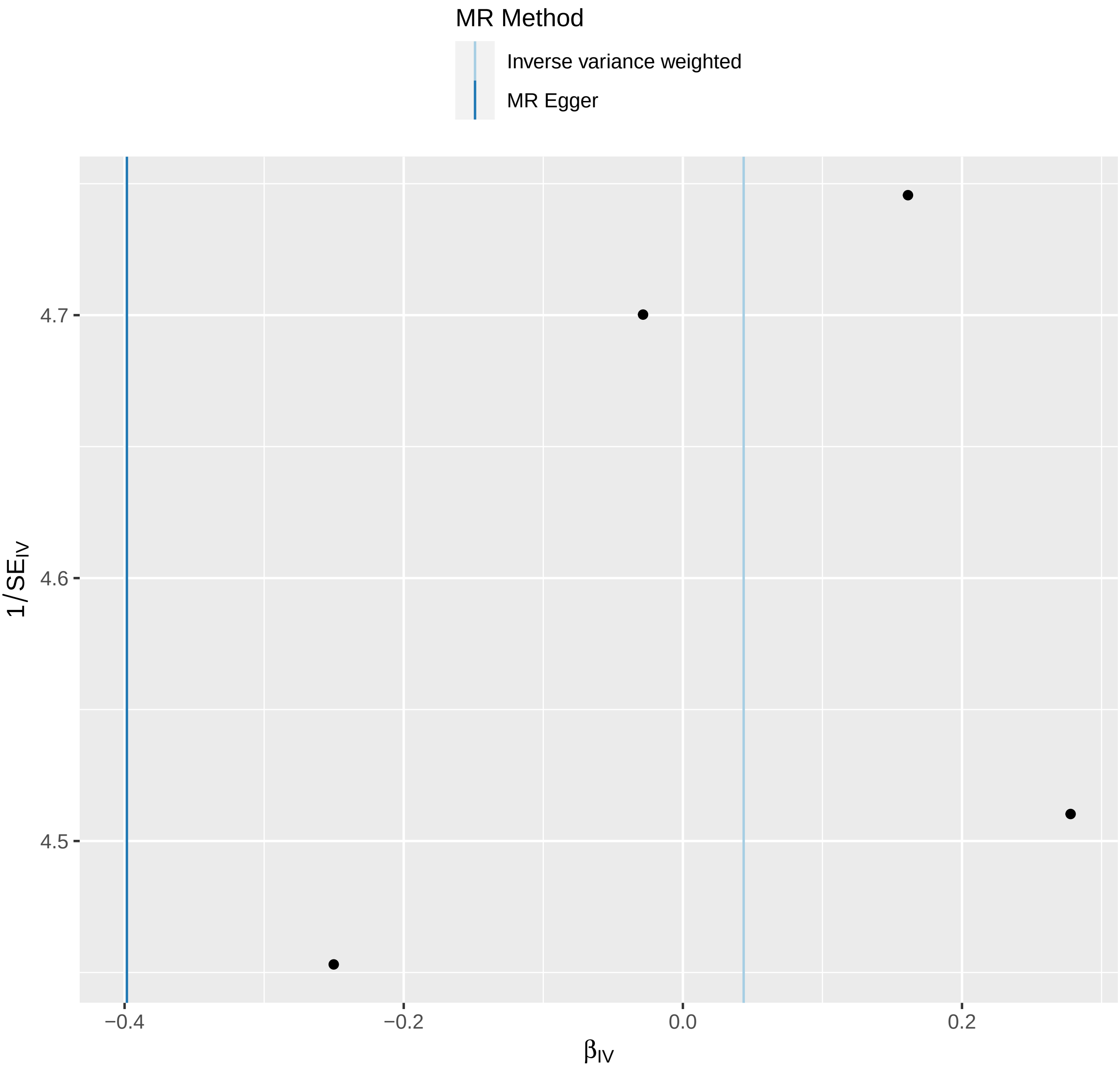

Batch 2−5898 : Gut microbiota abundance (genus Parasutterella id.2892) on Testis−expressed sequence 29 protein with a threshold of 1e−05

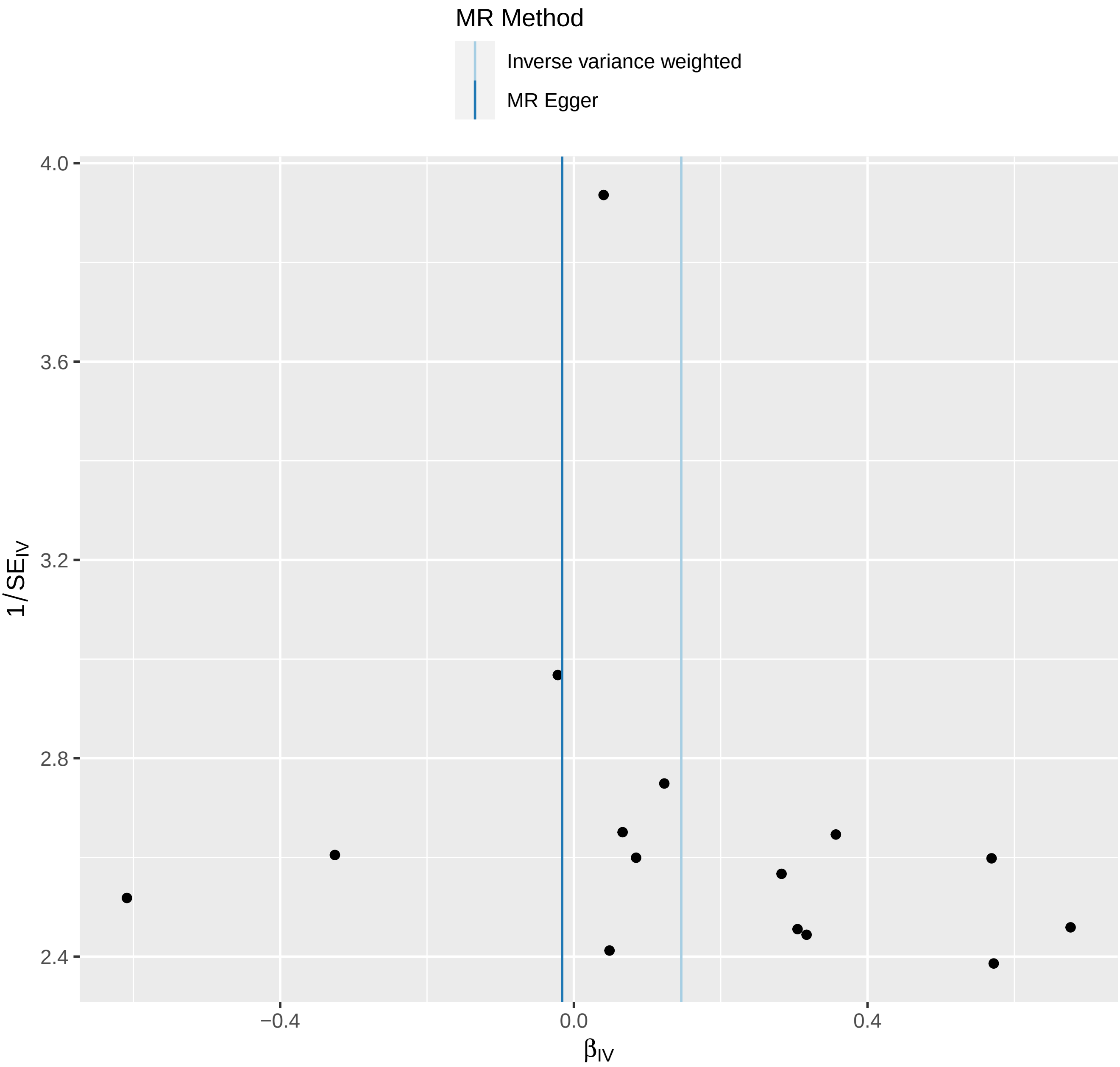

Batch 2−5899 : Gut microbiota abundance (genus Catenibacterium id.2153) on Serotransferrin
with a threshold of 1e−05

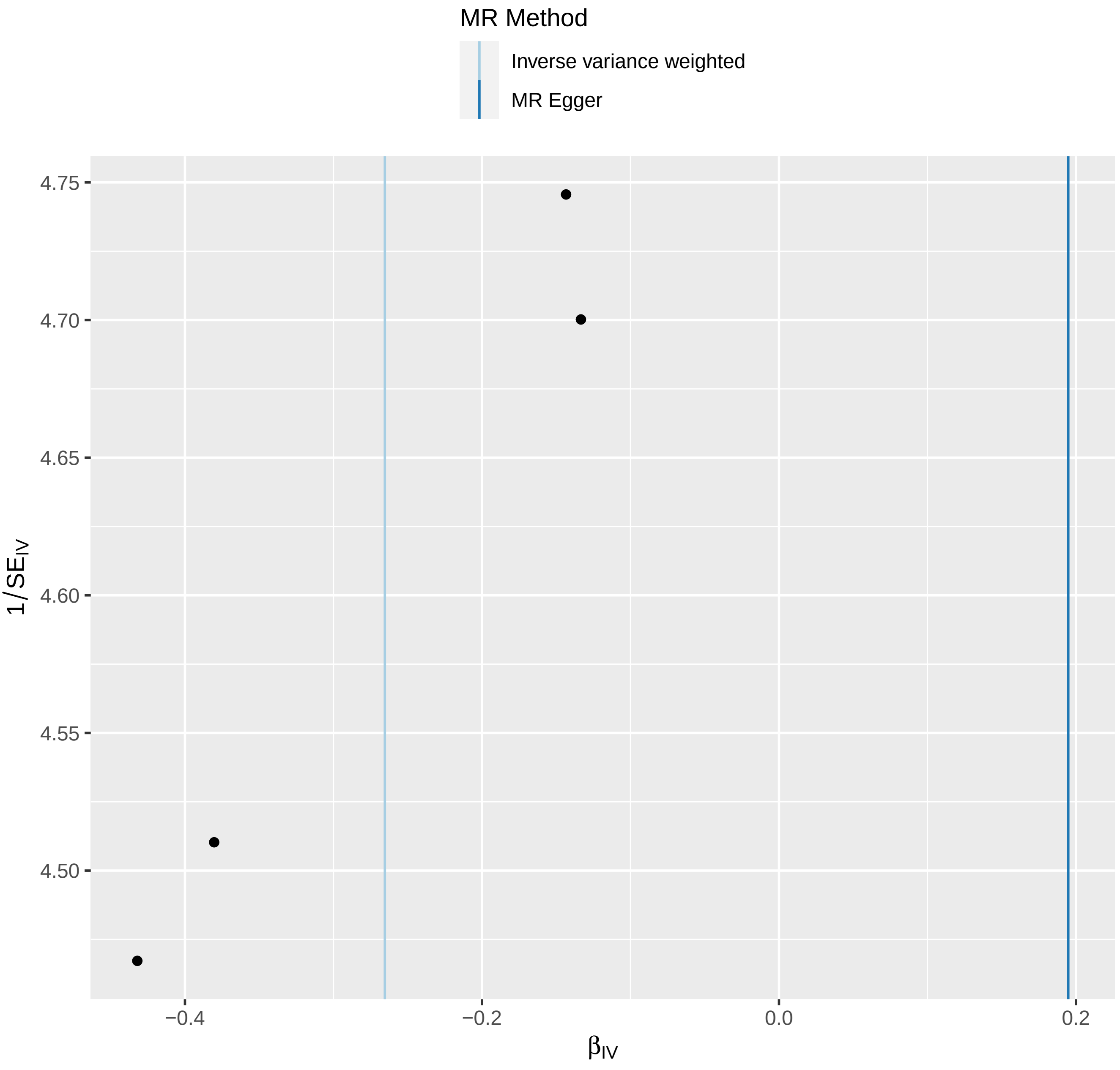

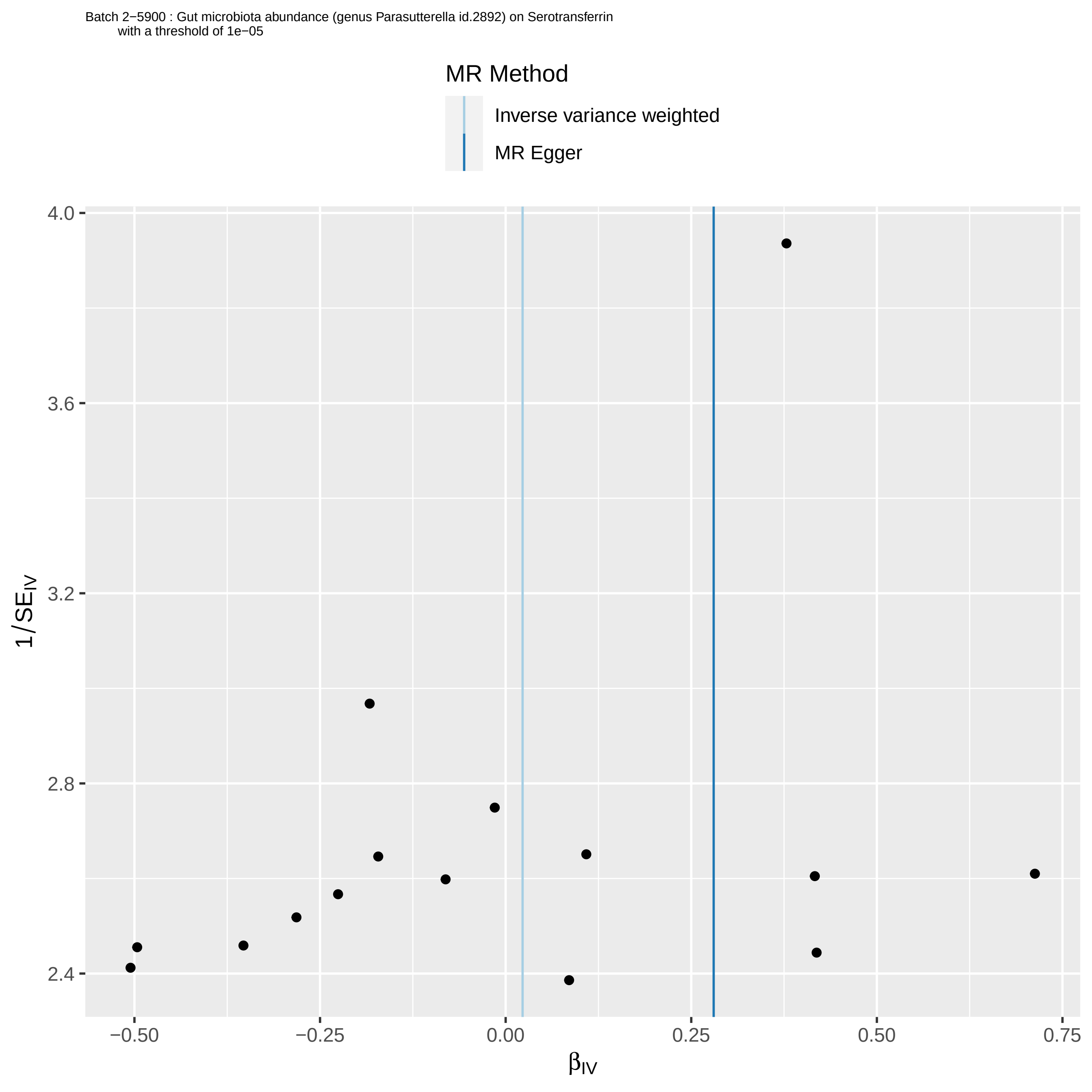
Batch 2−5900 : Gut microbiota abundance (genus Parasutterella id.2892) on Serotransferrin
with a threshold of 1e−05
MR Method
Inverse variance weighted
MR Egger
4.0
3.6
3.2
2.8
2.4
1/SE_IV
−0.50
−0.25
0.00
0.25
0.50
0.75
β_IV

Batch 2−5901 : Gut microbiota abundance (genus Catenibacterium id.2153) on Trefoil factor 1
with a threshold of 1e−05

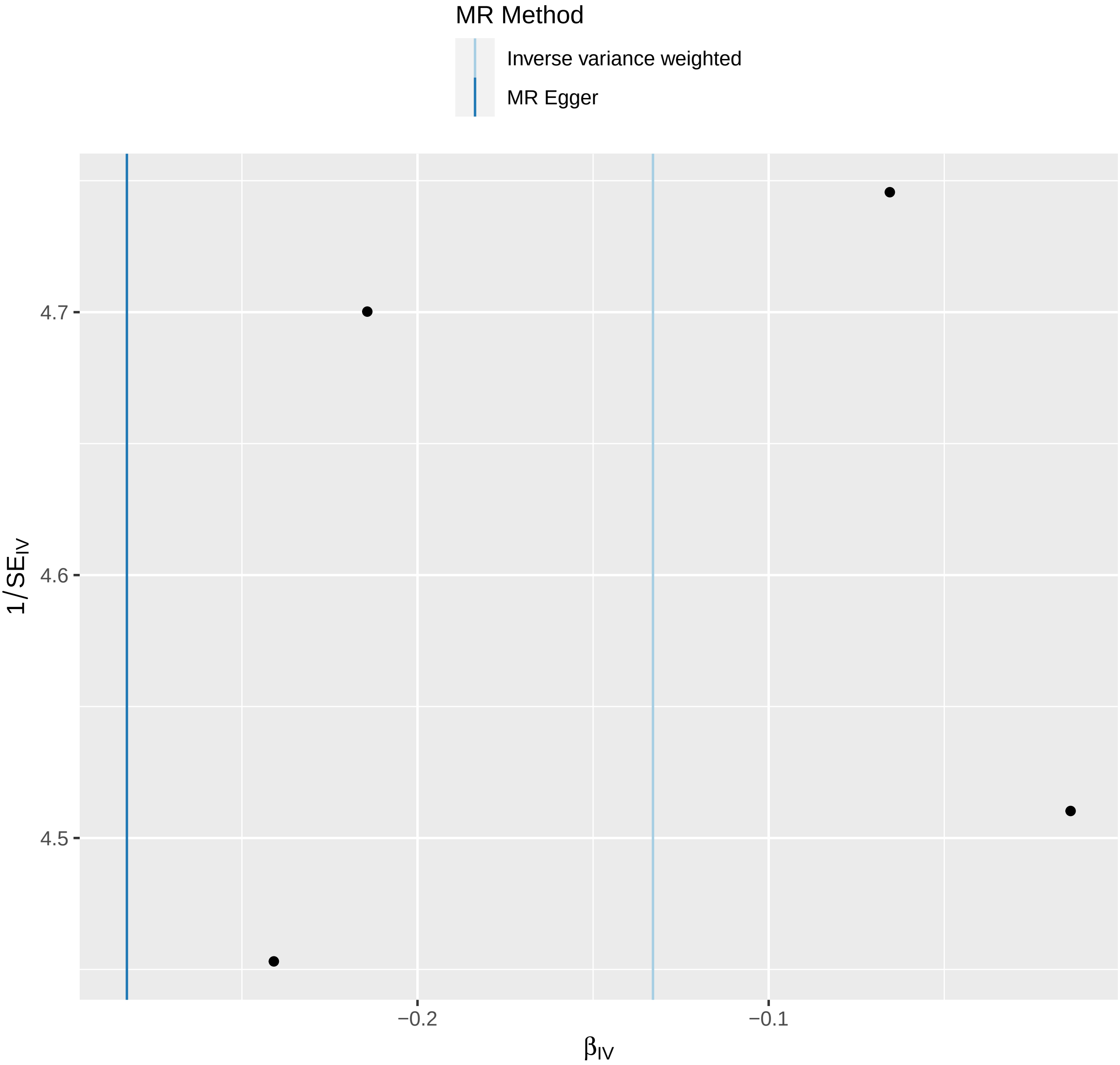

Batch 2−5902 : Gut microbiota abundance (genus Parasutterella id.2892) on Trefoil factor 1
with a threshold of 1e−05

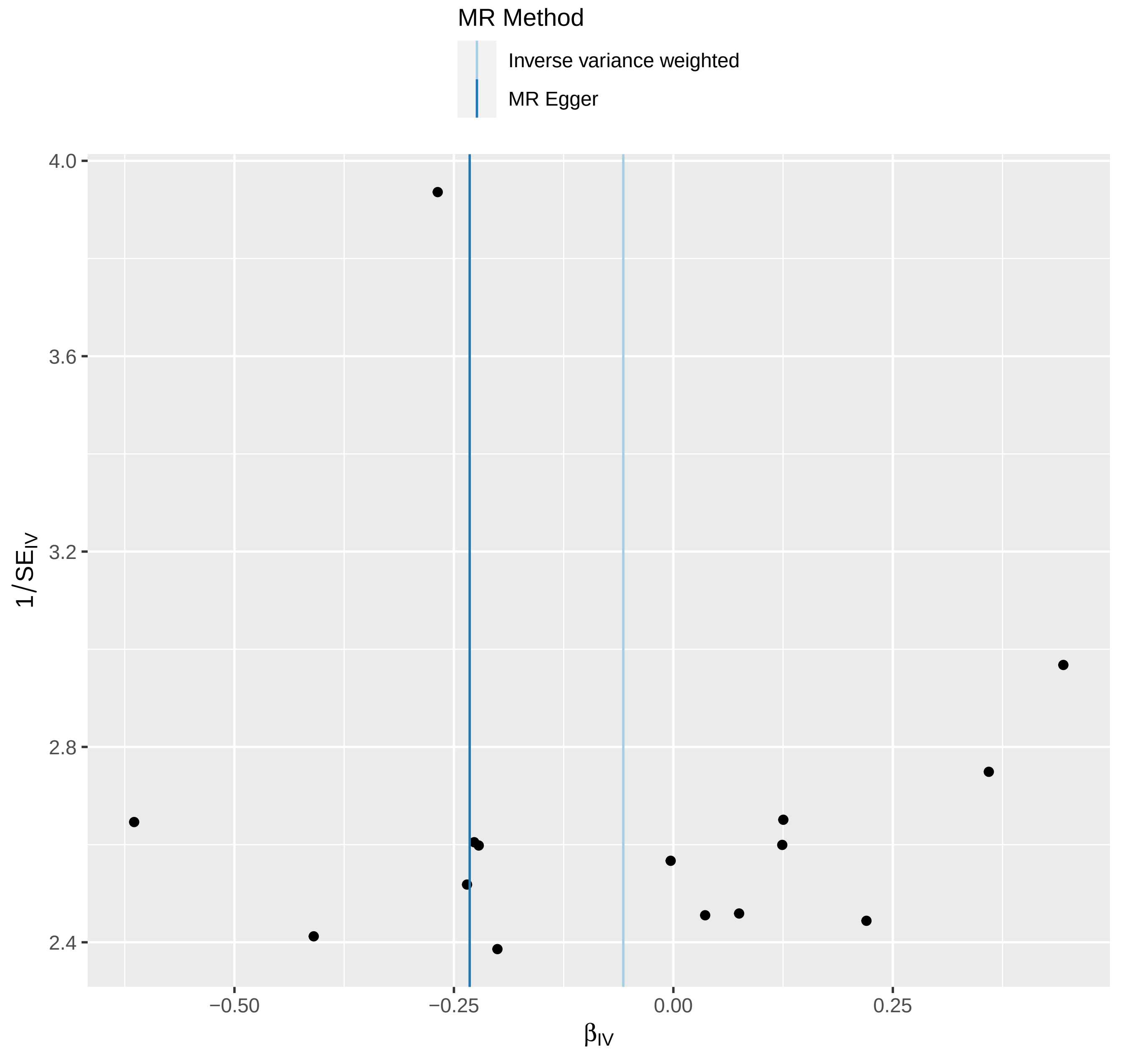

Batch 2−5903 : Gut microbiota abundance (genus Catenibacterium id.2153) on Trefoil factor 2
with a threshold of 1e−05

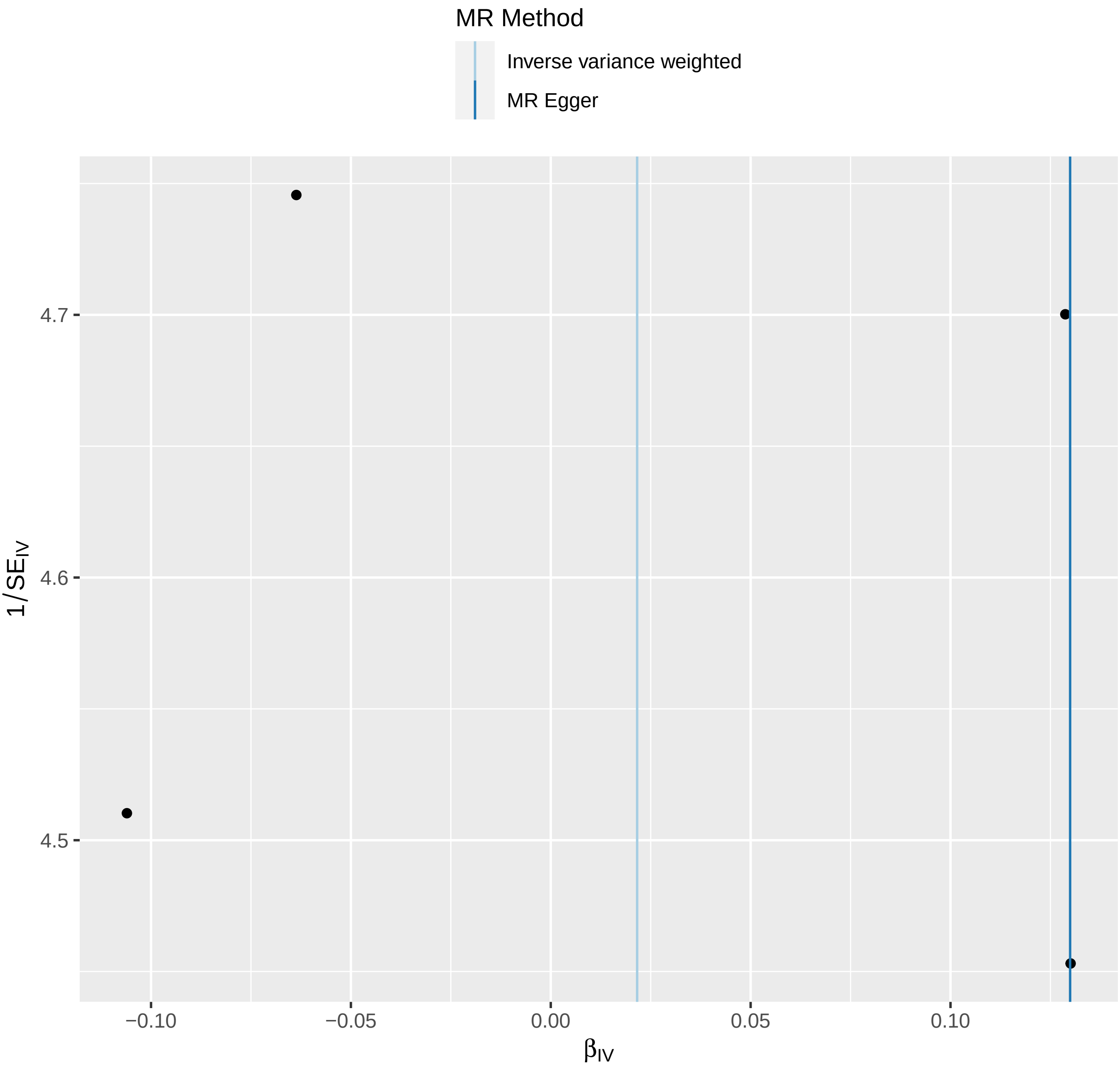

Batch 2−5904 : Gut microbiota abundance (genus Parasutterella id.2892) on Trefoil factor 2
with a threshold of 1e−05

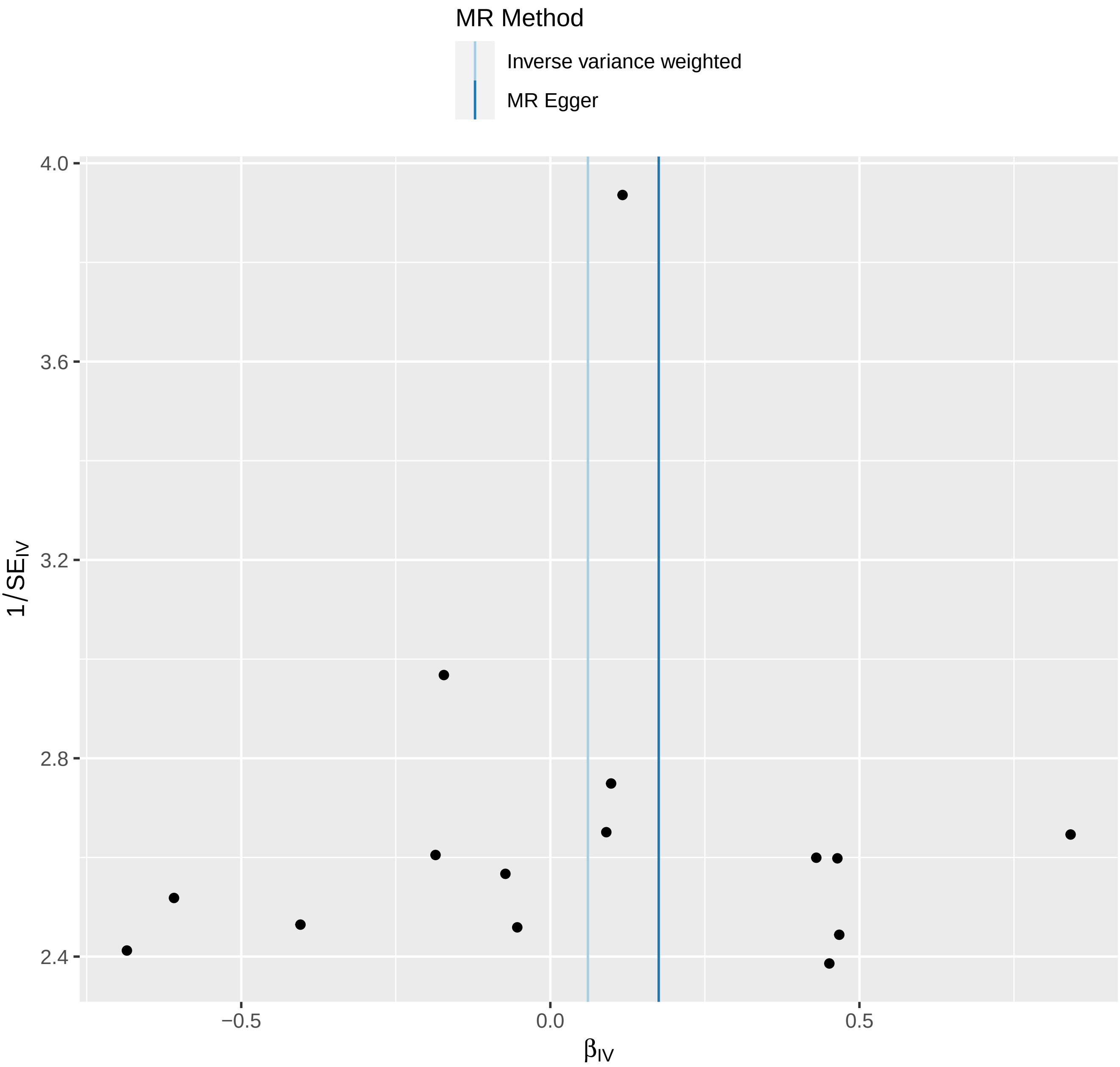

Batch 2−5905 : Gut microbiota abundance (genus Catenibacterium id.2153) on Trefoil factor 3
with a threshold of 1e−05

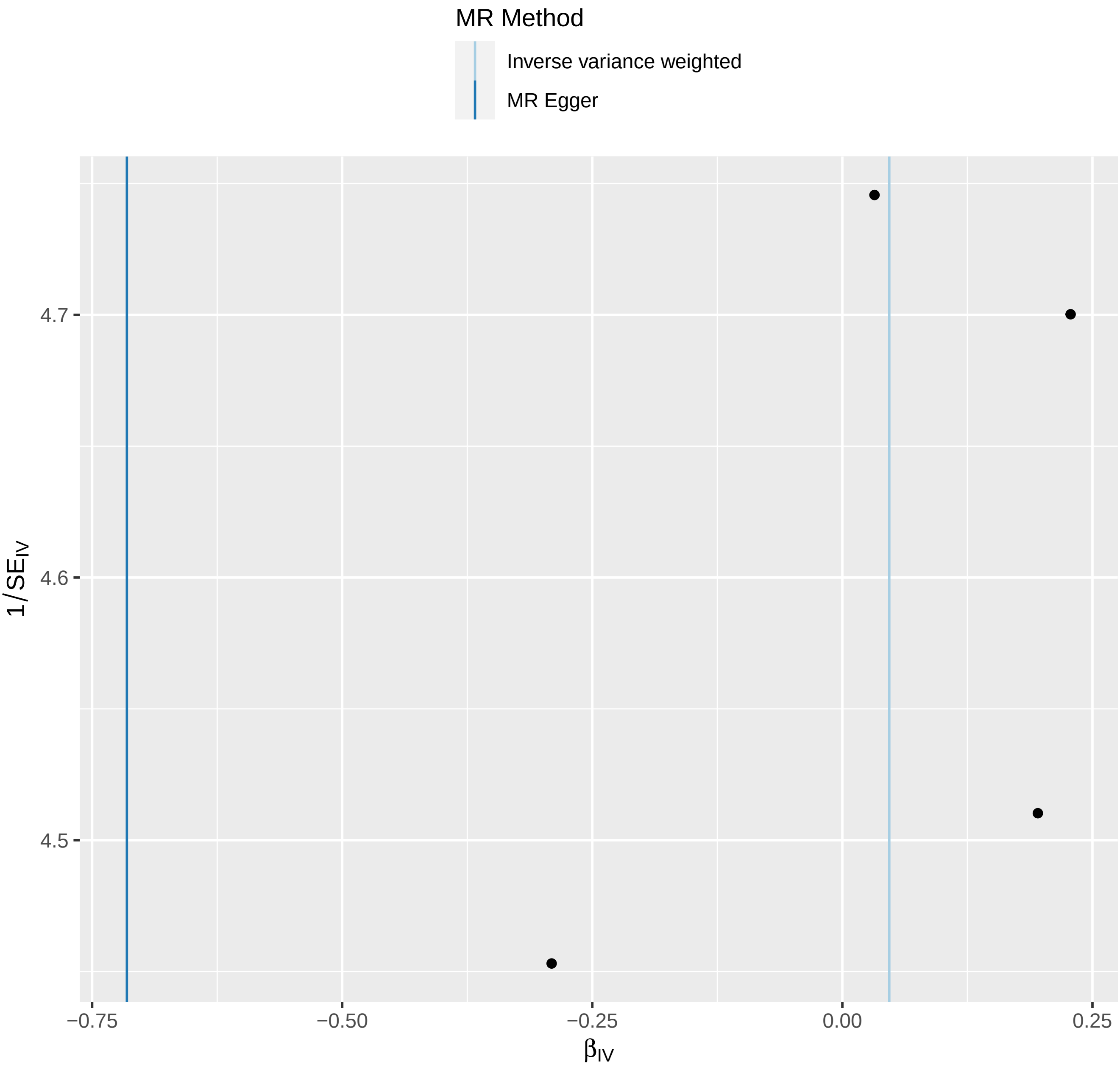

Batch 2−5906 : Gut microbiota abundance (genus Parasutterella id.2892) on Trefoil factor 3
with a threshold of 1e−05

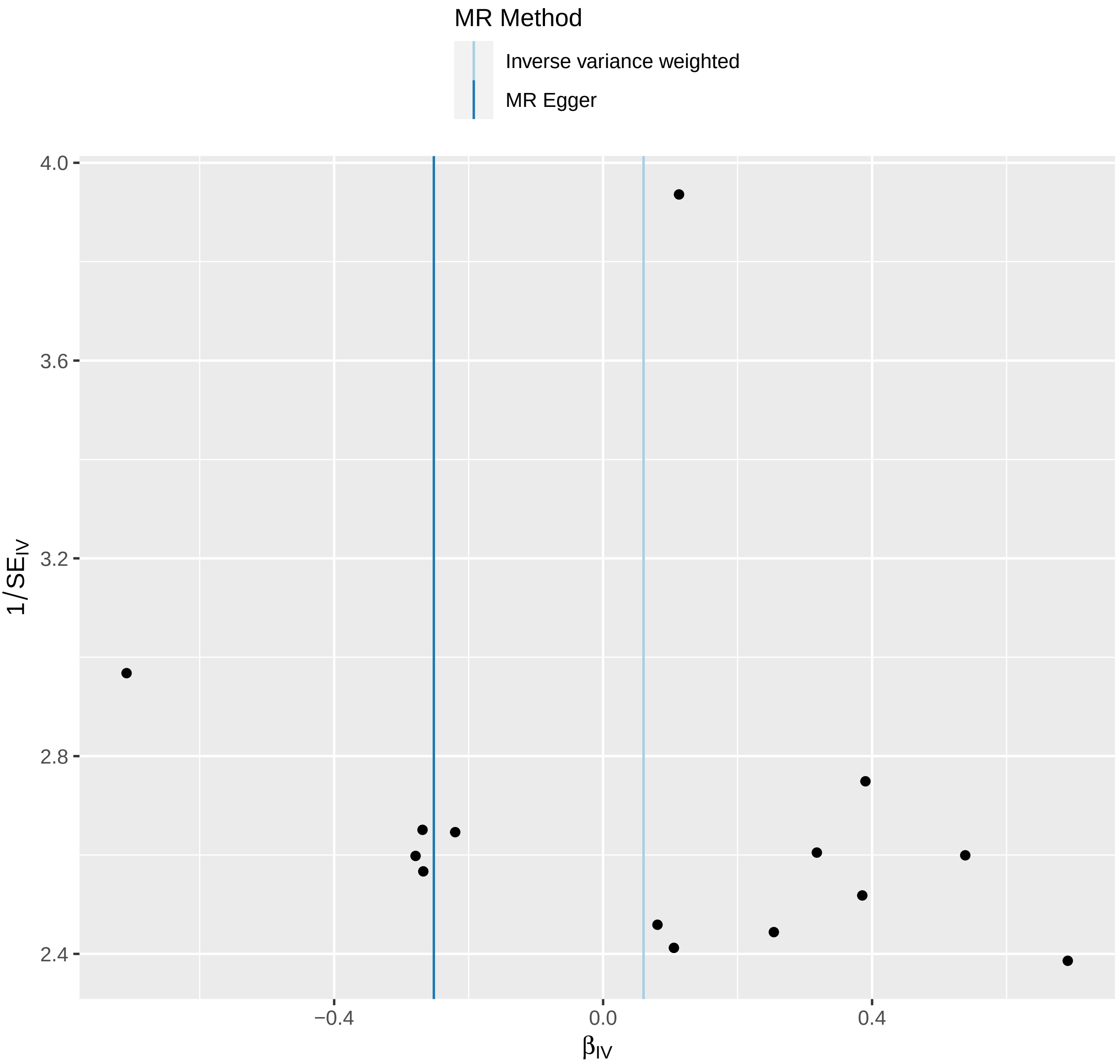

Batch 2−5907 : Gut microbiota abundance (genus Catenibacterium id.2153) on Trefoil factor 3
with a threshold of 1e−05

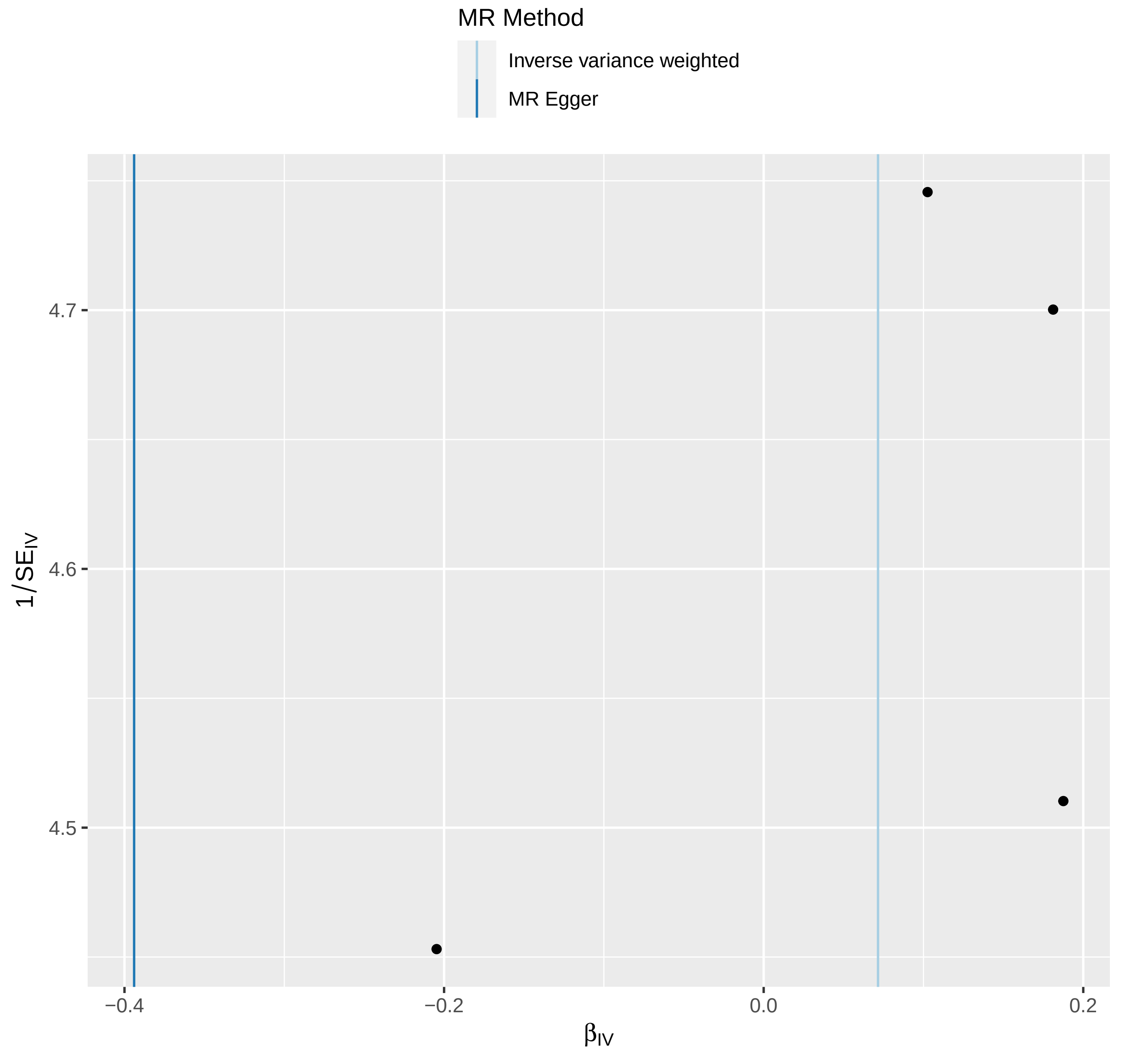

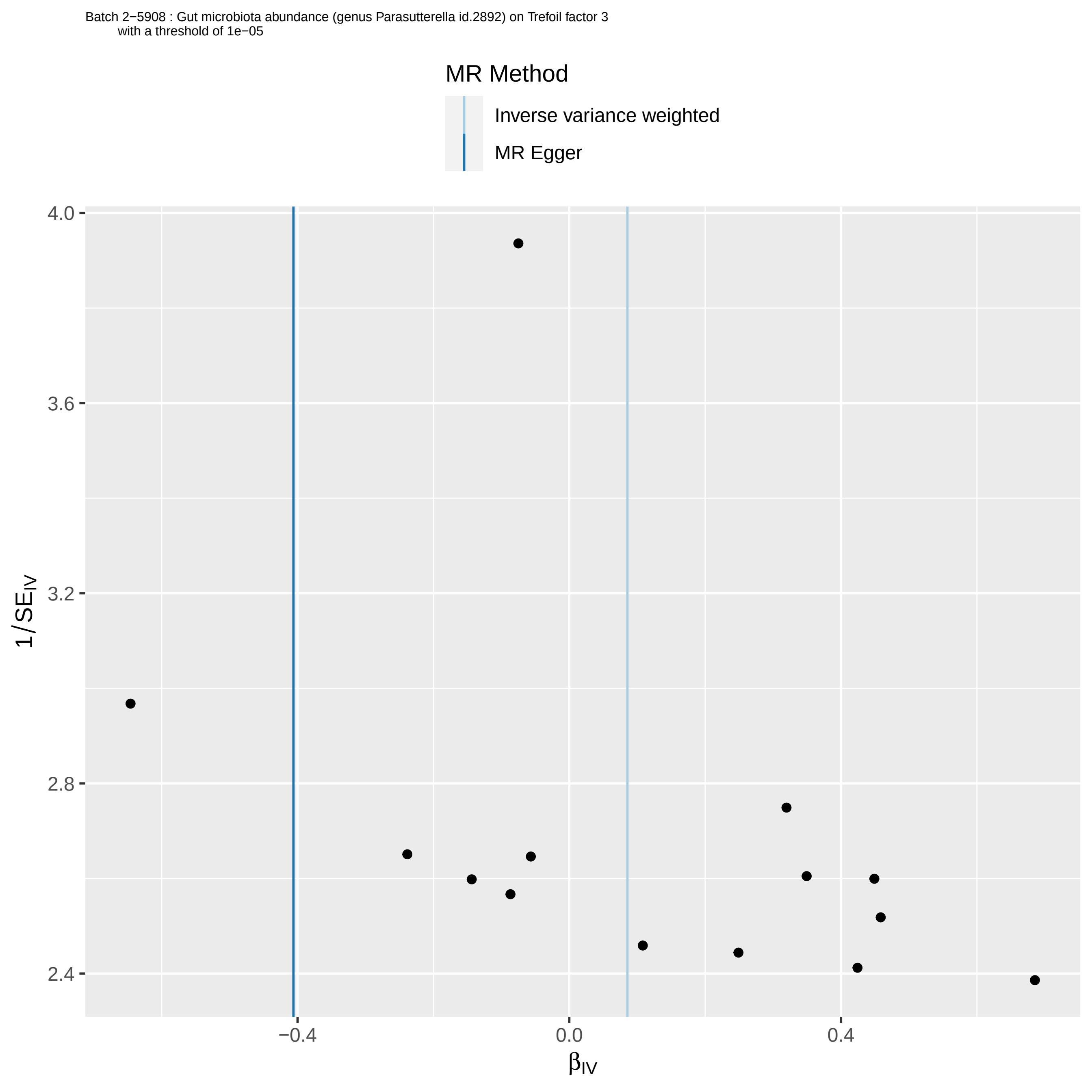

Batch 2−5908 : Gut microbiota abundance (genus Parasutterella id.2892) on Trefoil factor 3
with a threshold of 1e−05
MR Method
Inverse variance weighted
MR Egger
4.0
3.6
3.2
2.8
2.4
1/SE_IV
−0.4
0.0
0.4
β_IV

Batch 2−5909 : Gut microbiota abundance (genus Catenibacterium id.2153) on Tuftelin−interacting protein 11
with a threshold of 1e−05

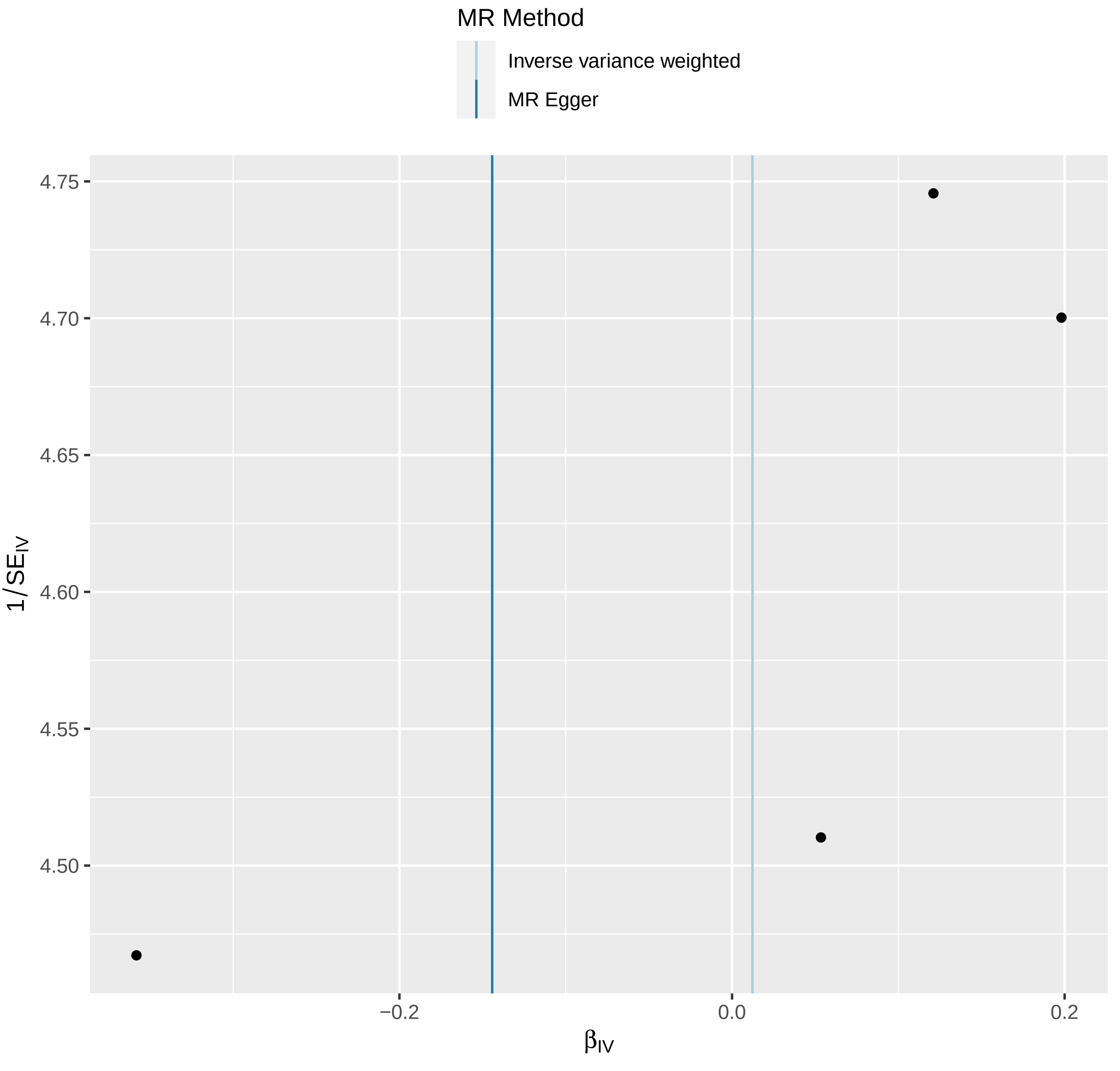

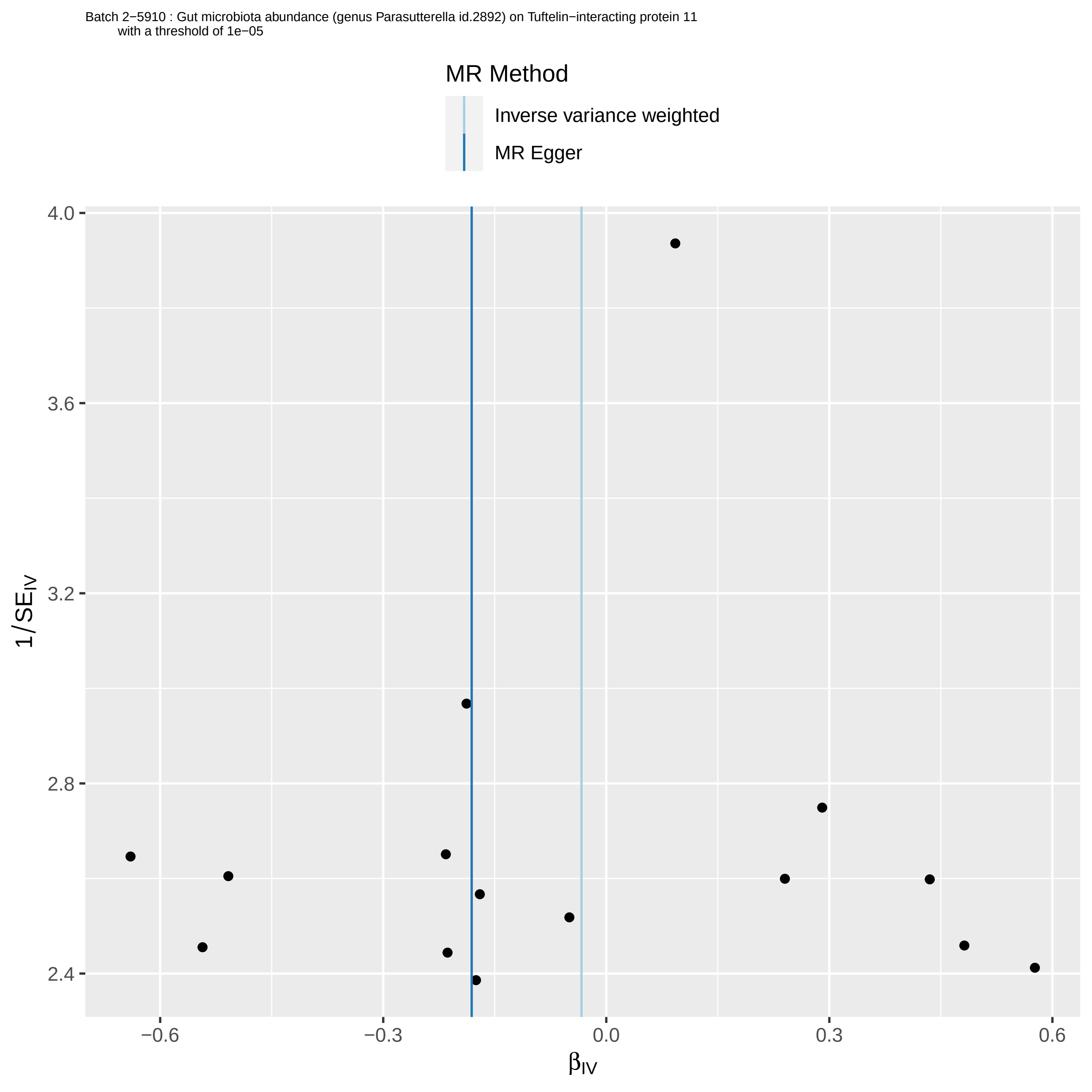
Batch 2−5910 : Gut microbiota abundance (genus Parasutterella id.2892) on Tuftelin−interacting protein 11
with a threshold of 1e−05
MR Method
Inverse variance weighted
MR Egger
1/SE_IV
4.0
3.6
3.2
2.8
2.4
−0.6
−0.3
0.0
0.3
0.6
β_IV

Batch 2−5911 : Gut microbiota abundance (genus Catenibacterium id.2153) on Tissue factor pathway inhibitor 2
with a threshold of 1e−05

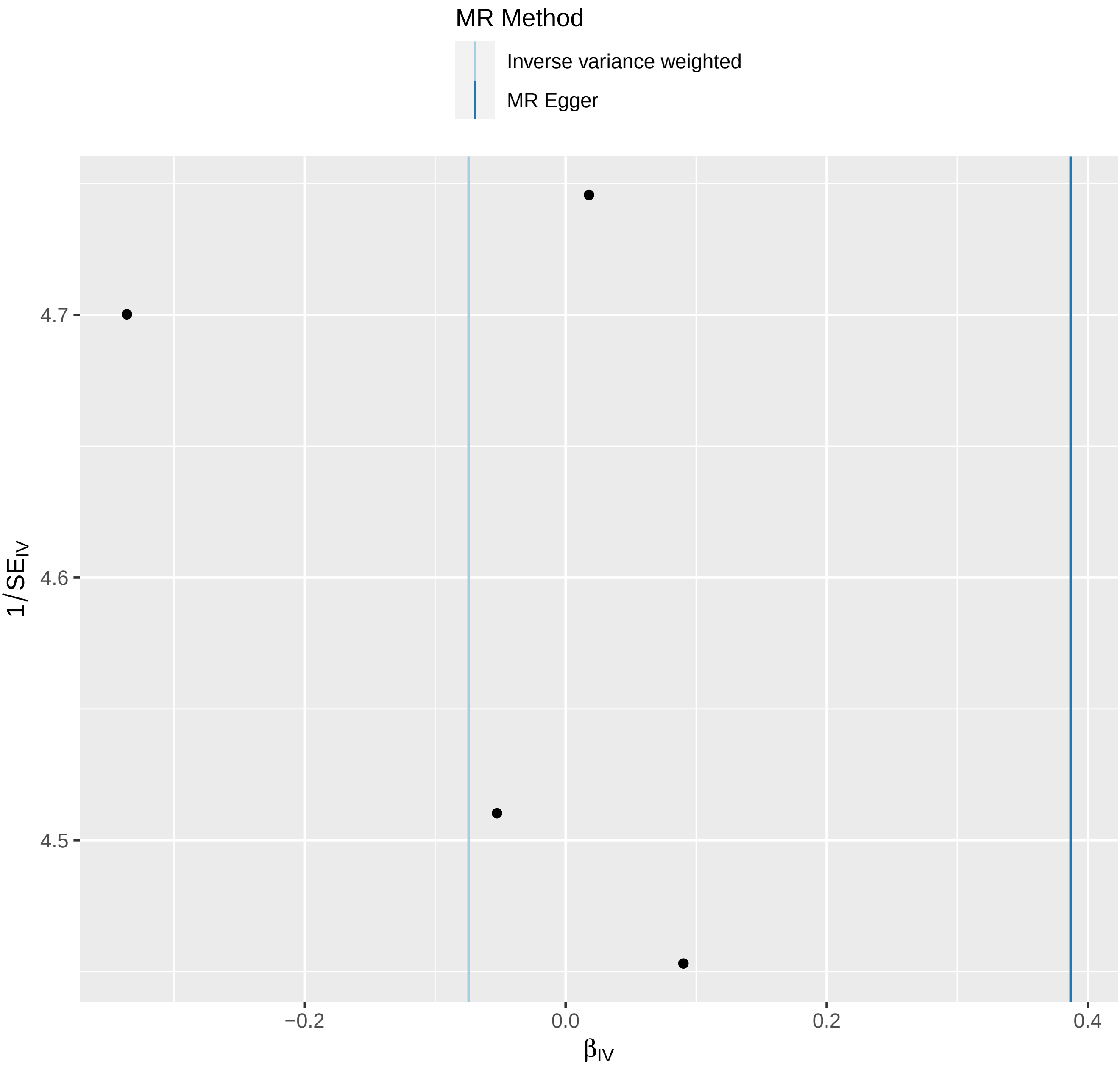

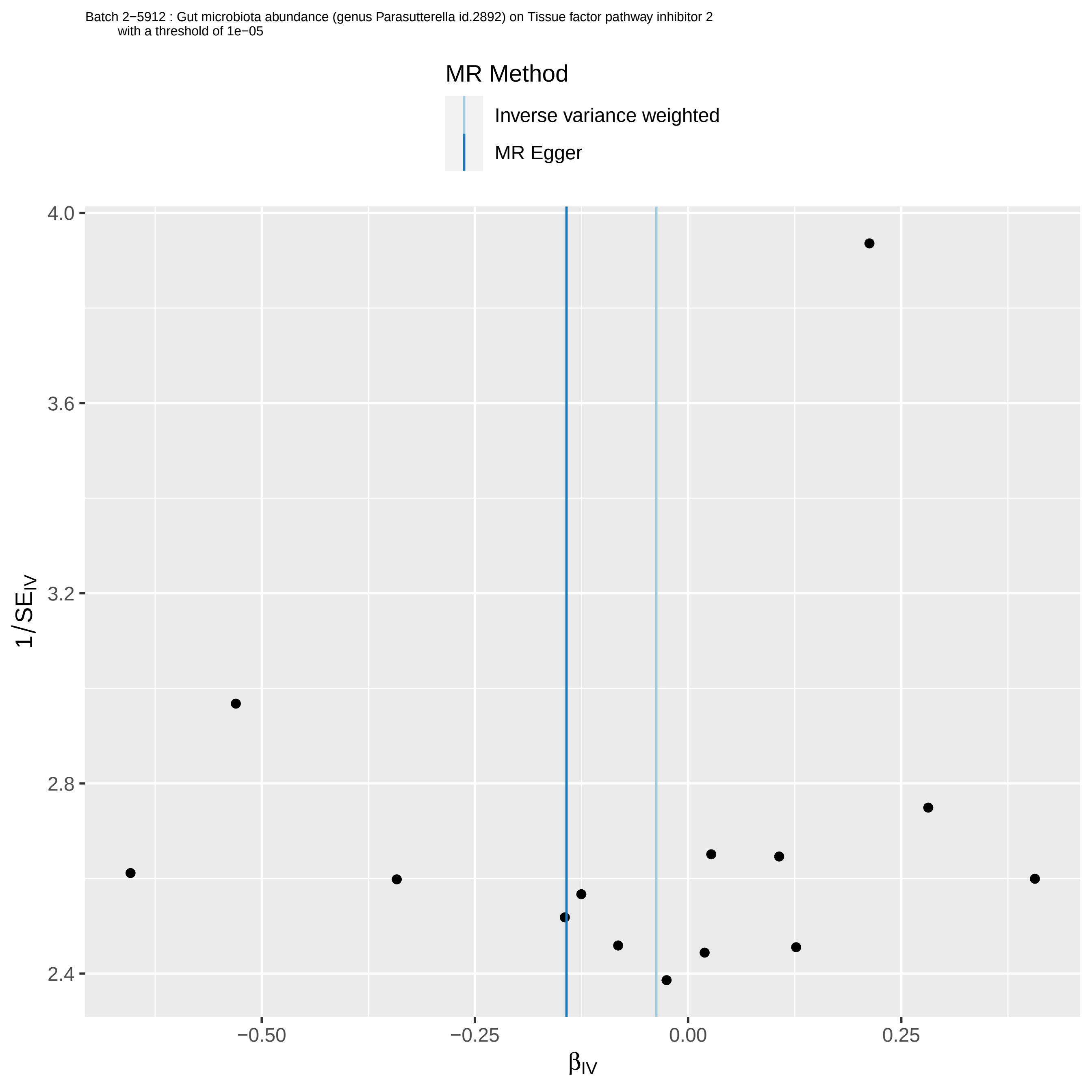

Batch 2−5912 : Gut microbiota abundance (genus Parasutterella id.2892) on Tissue factor pathway inhibitor 2
with a threshold of 1e−05
MR Method
Inverse variance weighted
MR Egger
4.0
3.6
3.2
2.8
2.4
1/SE_IV
−0.50
−0.25
0.00
0.25
β_IV

Batch 2−5913 : Gut microbiota abundance (genus Catenibacterium id.2153) on Tissue factor pathway inhibitor
with a threshold of 1e−05

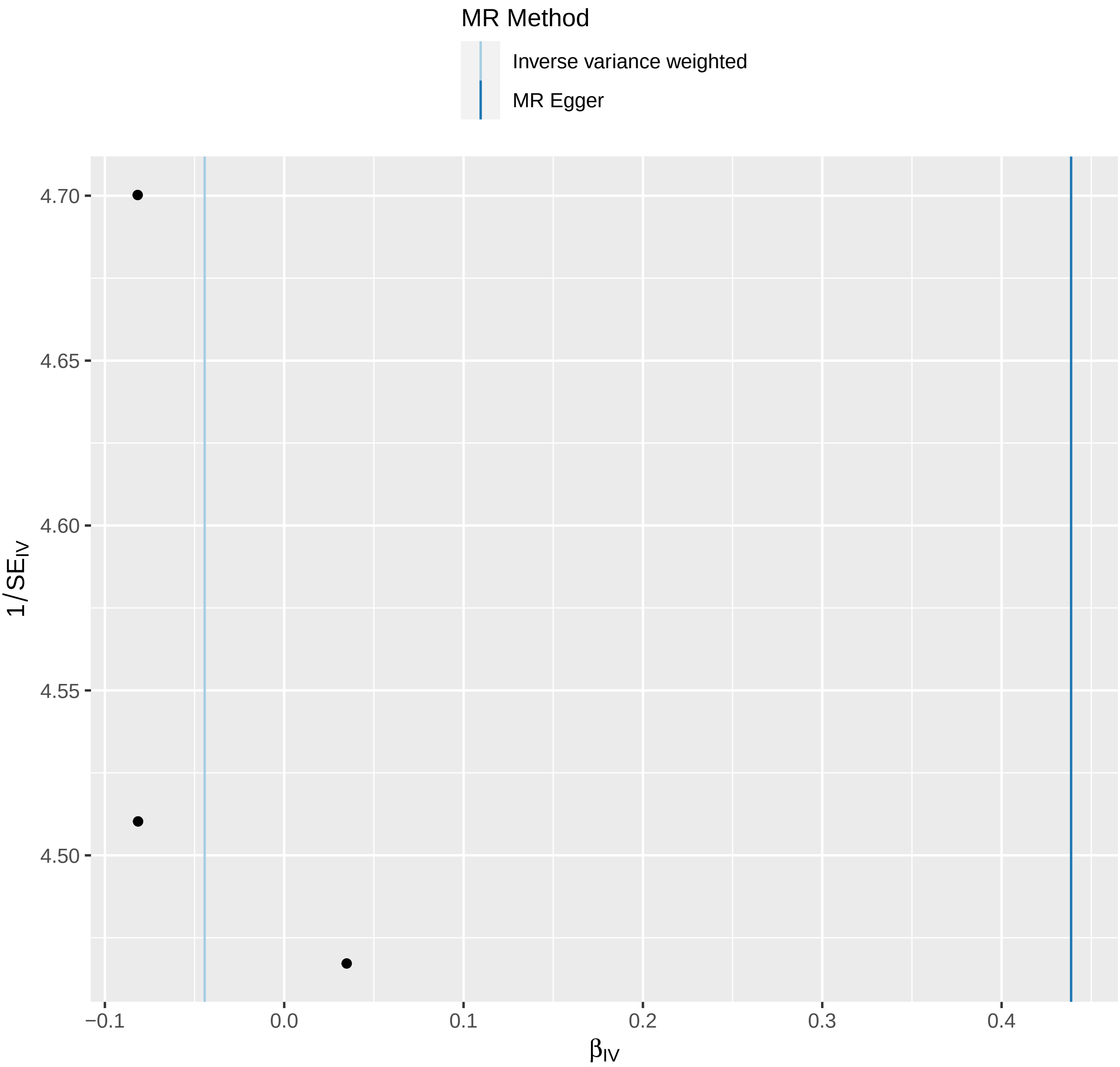

Batch 2−5914 : Gut microbiota abundance (genus Parasutterella id.2892) on Tissue factor pathway inhibitor with a threshold of 1e−05

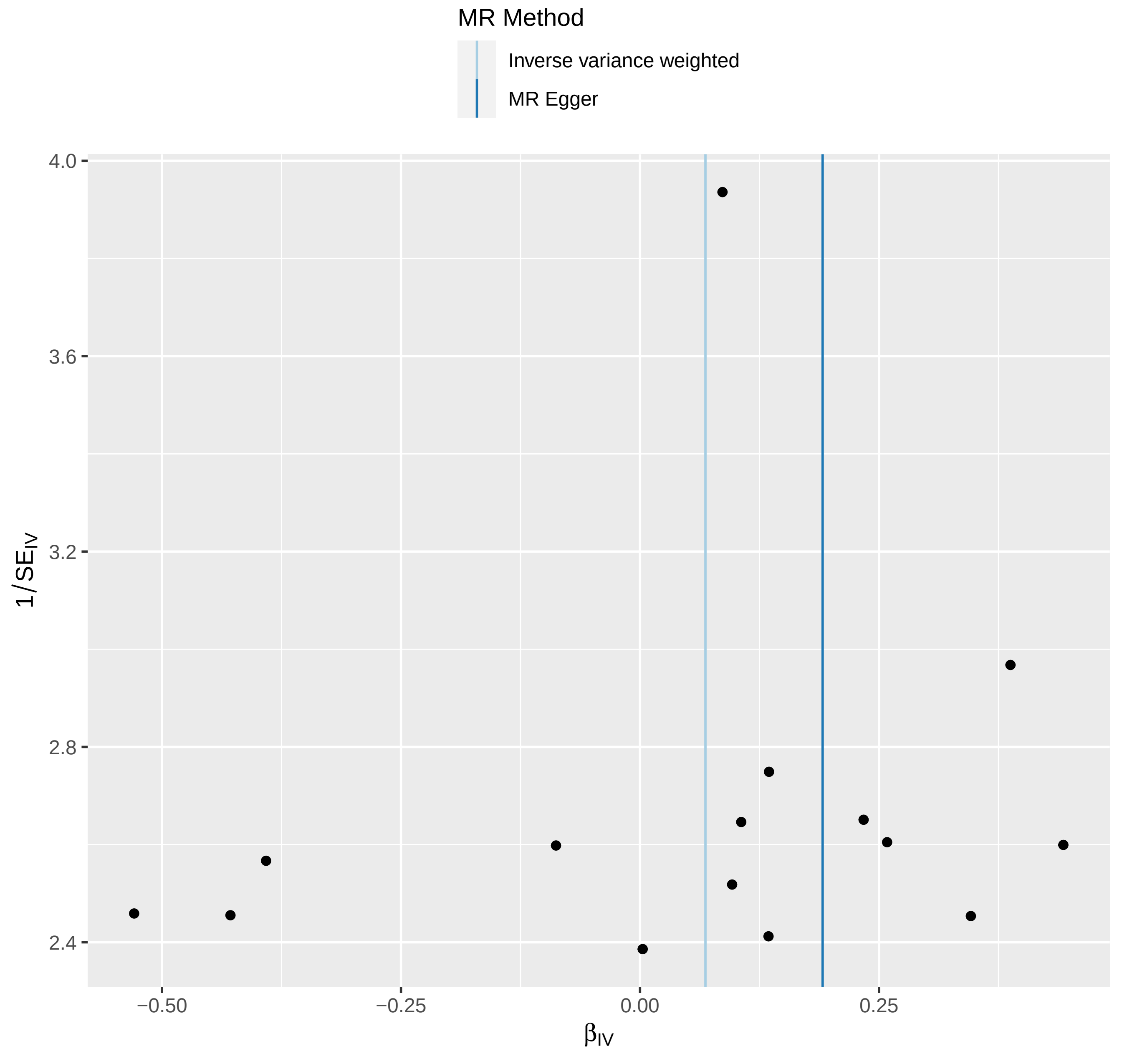

Batch 2−5915 : Gut microbiota abundance (genus Catenibacterium id.2153) on Transferrin receptor protein 1
with a threshold of 1e−05

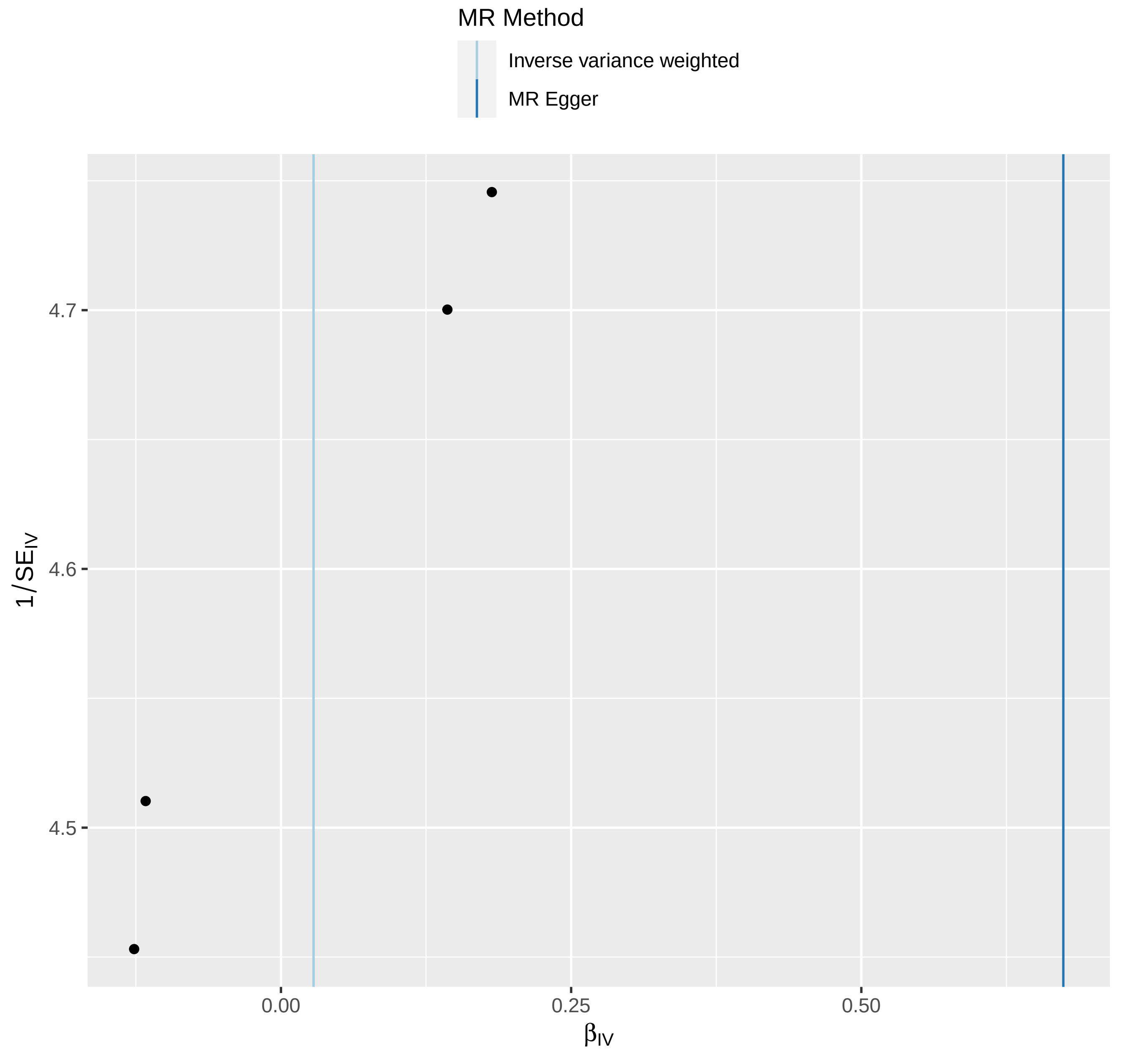

Batch 2−5916 : Gut microbiota abundance (genus Parasutterella id.2892) on Transferrin receptor protein 1
with a threshold of 1e−05

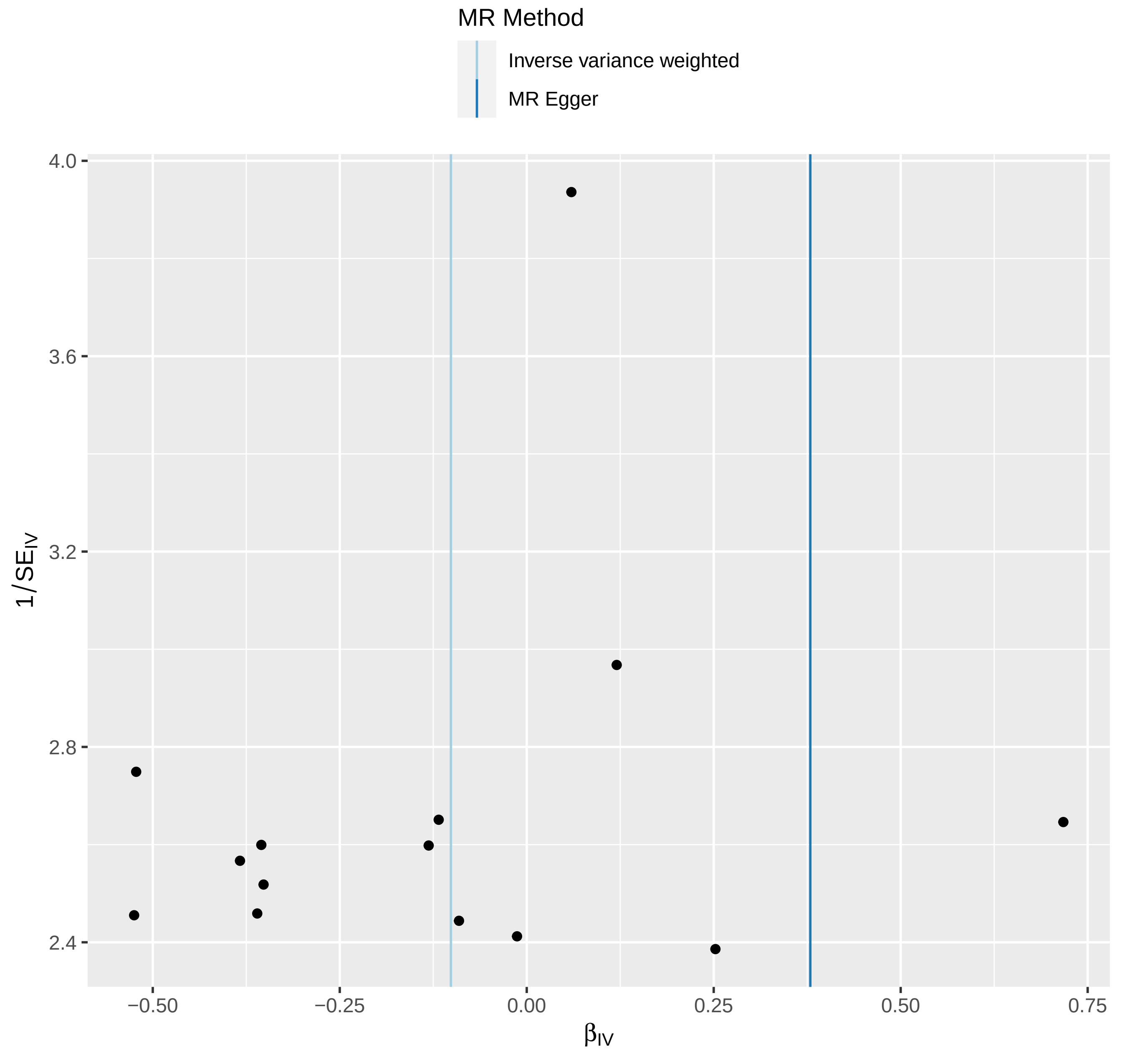

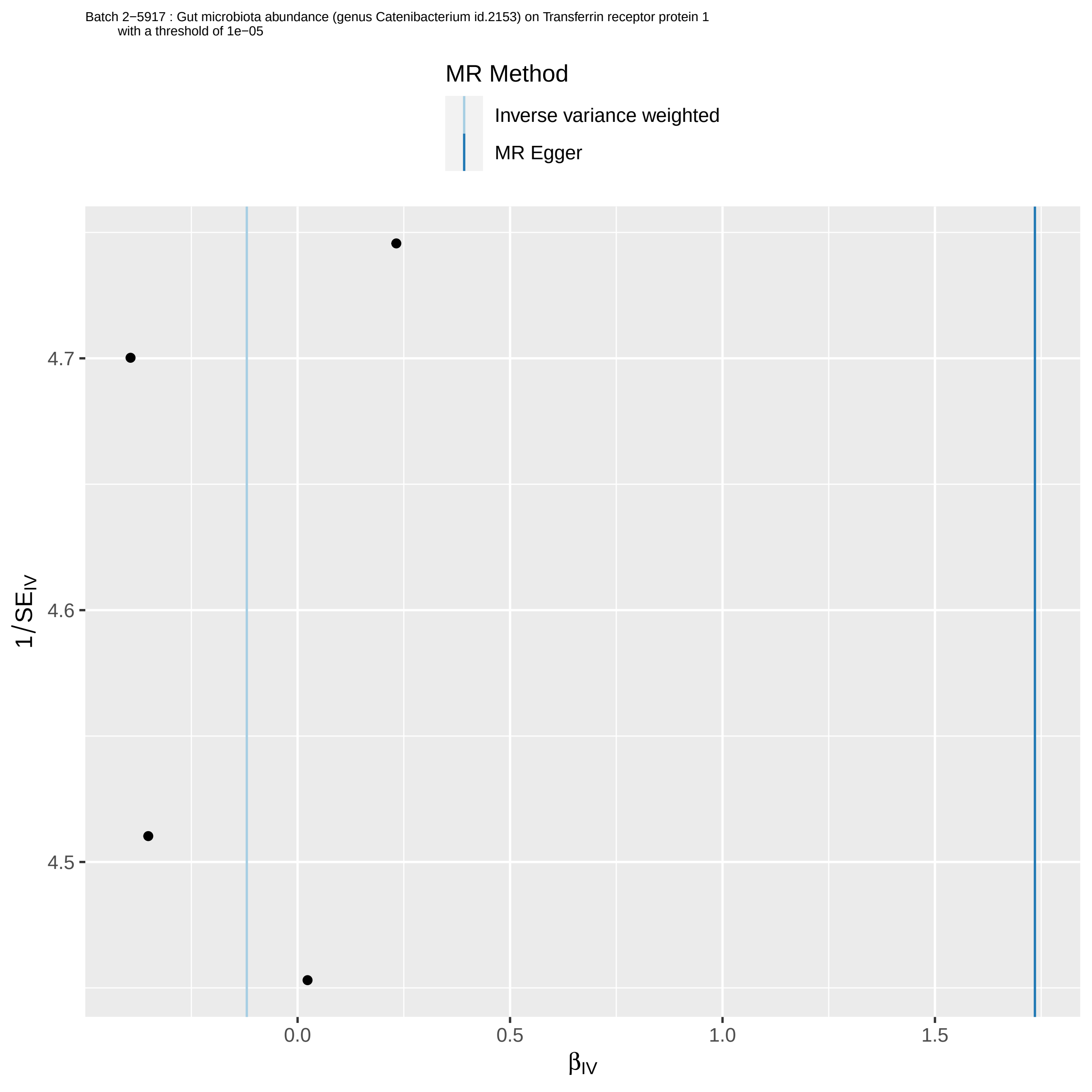

Batch 2−5917 : Gut microbiota abundance (genus Catenibacterium id.2153) on Transferrin receptor protein 1
with a threshold of 1e−05
MR Method
Inverse variance weighted
MR Egger
4.7
4.6
4.5
1/SE_IV
0.0
0.5
1.0
1.5
β_IV

Batch 2−5918 : Gut microbiota abundance (genus Parasutterella id.2892) on Transferrin receptor protein 1
with a threshold of 1e−05

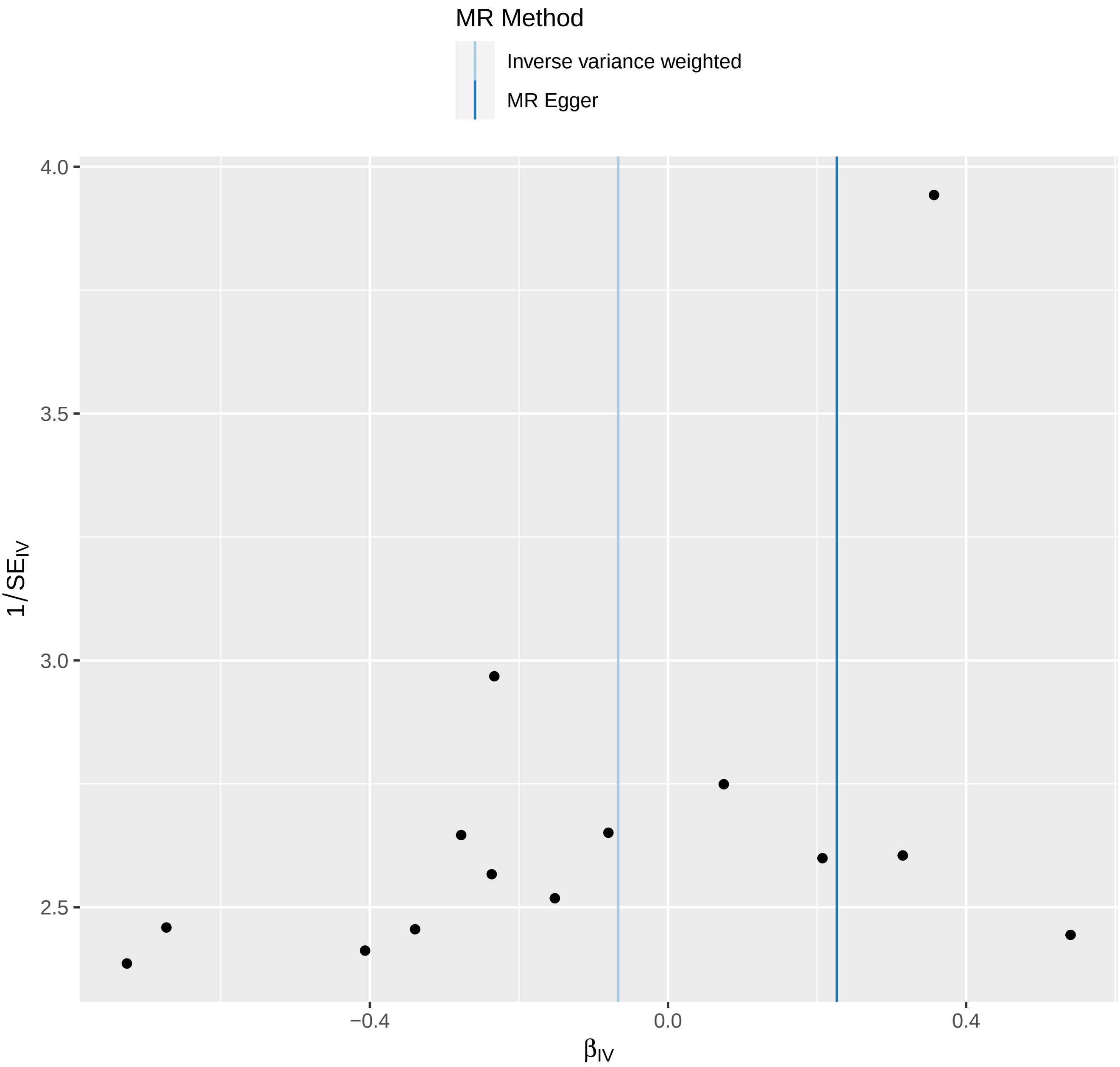

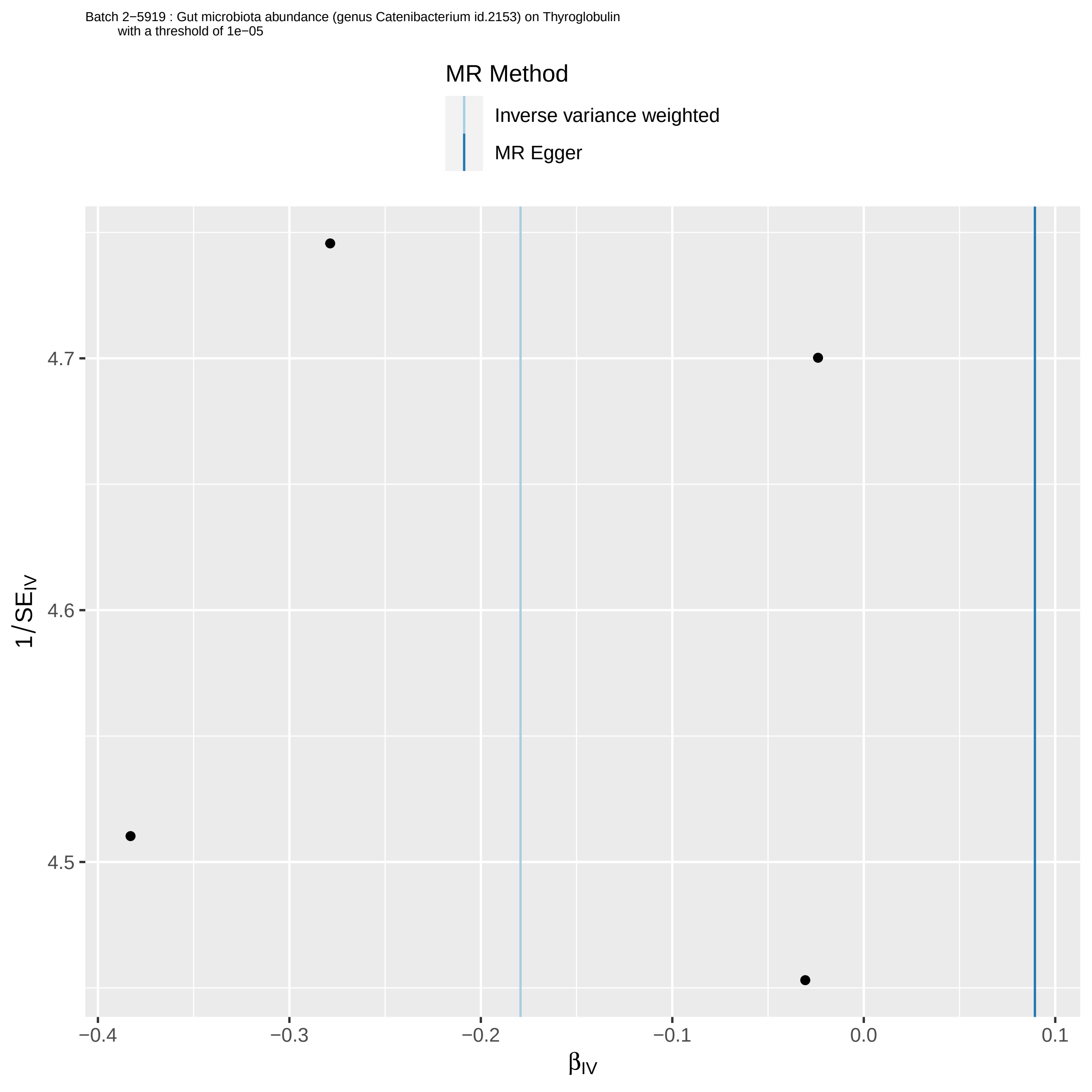

Batch 2−5919 : Gut microbiota abundance (genus Catenibacterium id.2153) on Thyroglobulin
with a threshold of 1e−05
MR Method
Inverse variance weighted
MR Egger
4.7
4.6
4.5
1/SE_IV
−0.4
−0.3
−0.2
−0.1
0.0
0.1
β_IV

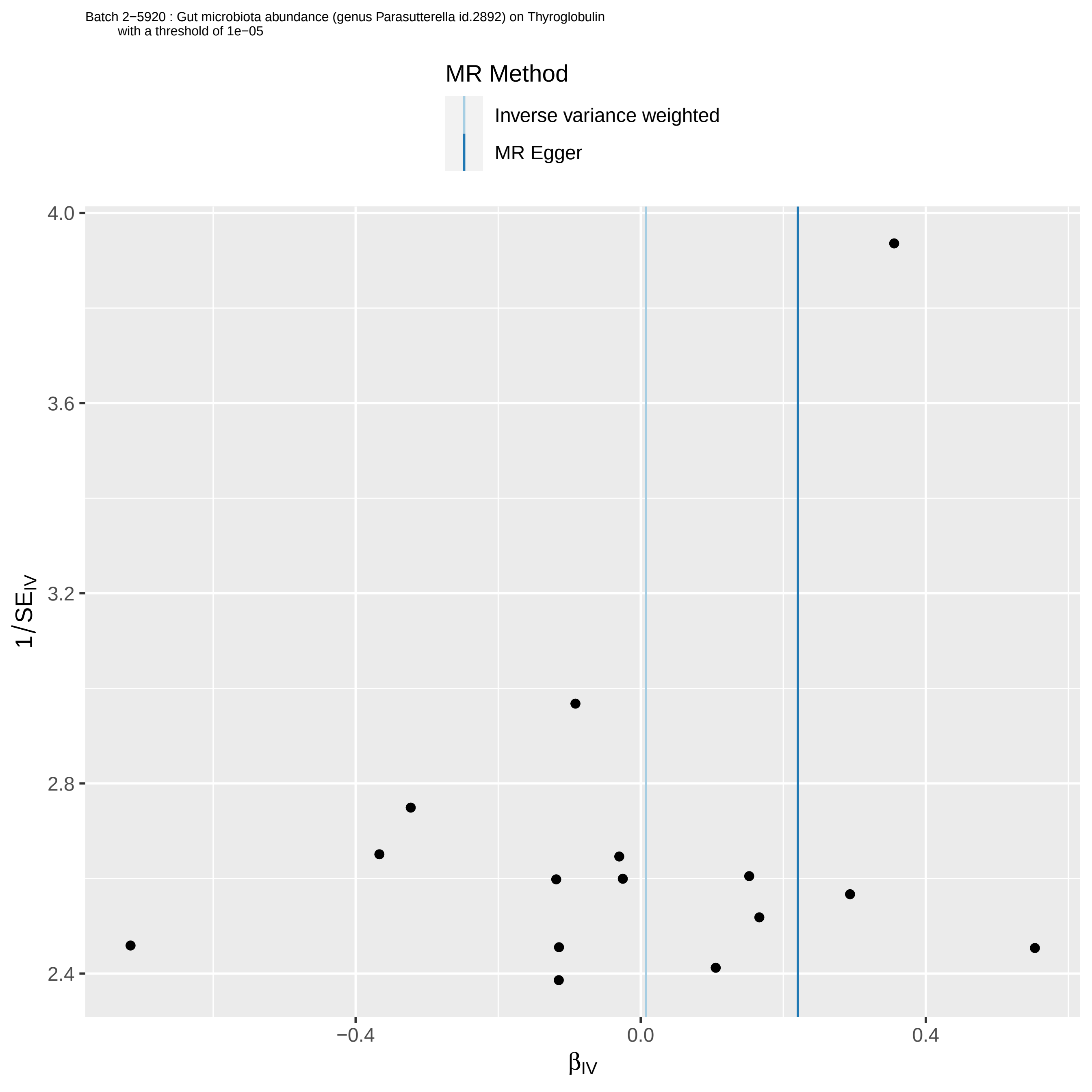

Batch 2−5920 : Gut microbiota abundance (genus Parasutterella id.2892) on Thyroglobulin
with a threshold of 1e−05
MR Method
Inverse variance weighted
MR Egger
4.0
3.6
3.2
2.8
2.4
1/SE_IV
−0.4
0.0
0.4
β_IV

Batch 2−5921 : Gut microbiota abundance (genus Catenibacterium id.2153) on Transforming growth factor alpha
with a threshold of 1e−05

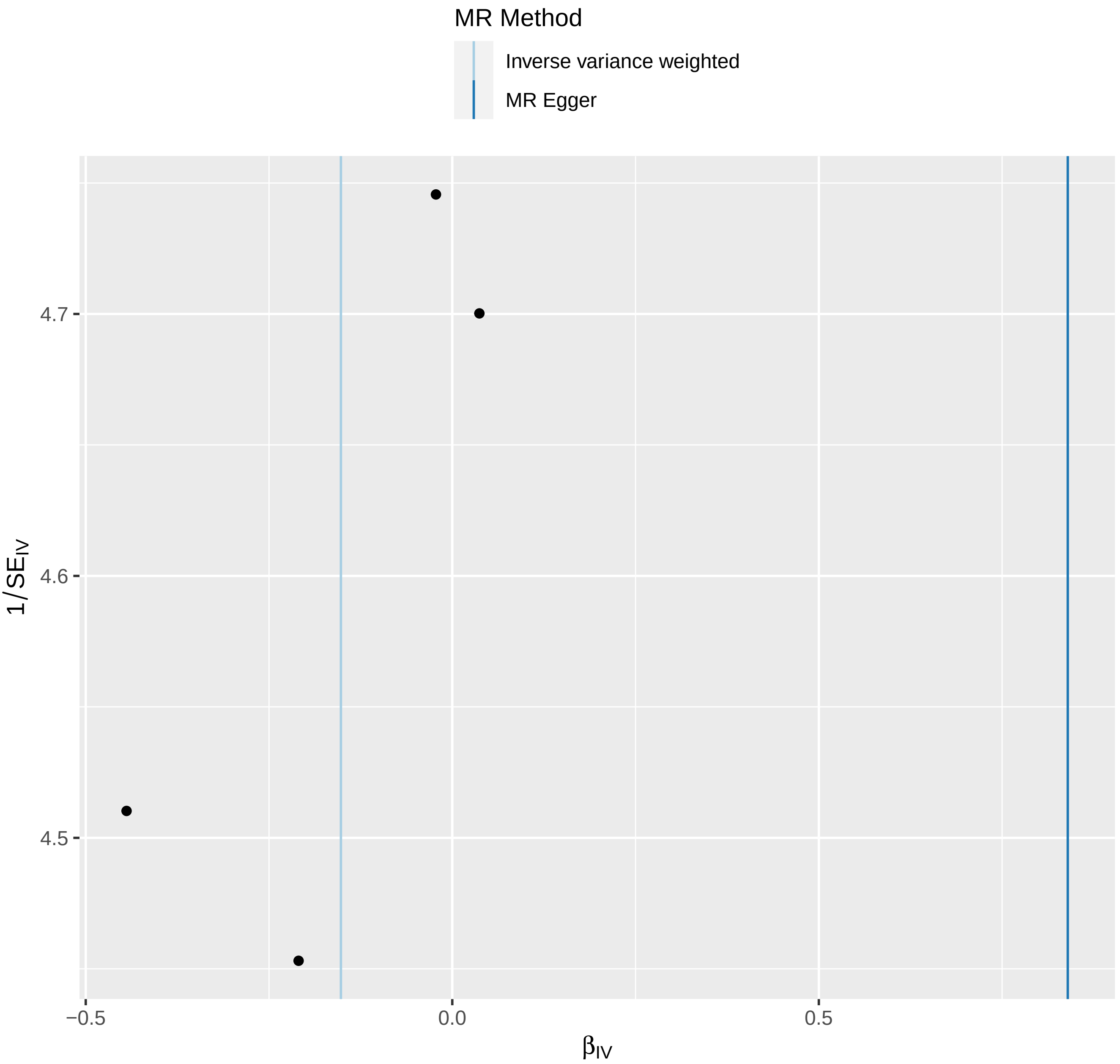

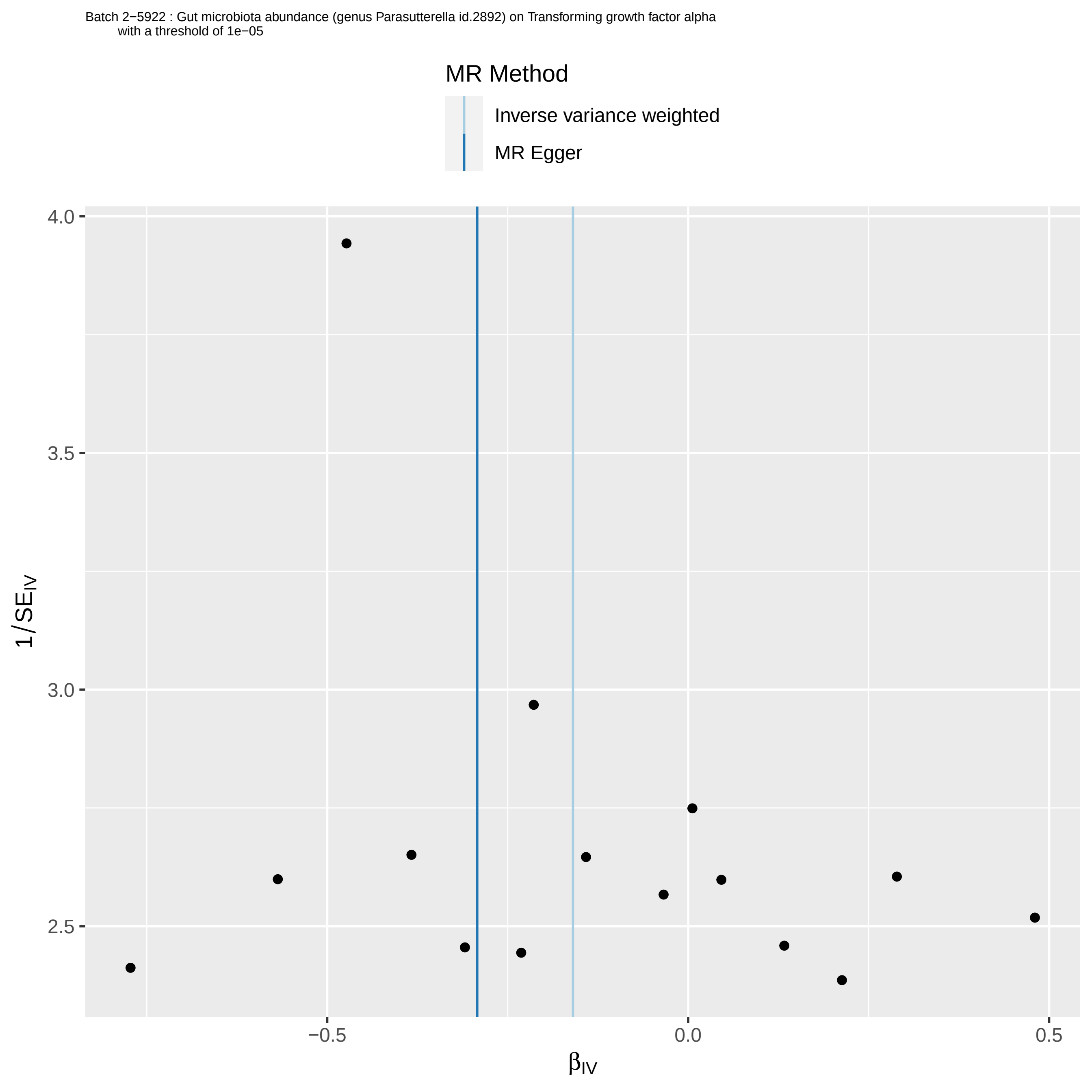

Batch 2−5922 : Gut microbiota abundance (genus Parasutterella id.2892) on Transforming growth factor alpha with a threshold of 1e−05
MR Method
Inverse variance weighted
MR Egger
4.0
3.5
3.0
2.5
1/SE_IV
−0.5
0.0
0.5
β_IV

Batch 2−5923 : Gut microbiota abundance (genus Catenibacterium id.2153) on Transforming growth factor beta−1
with a threshold of 1e−05

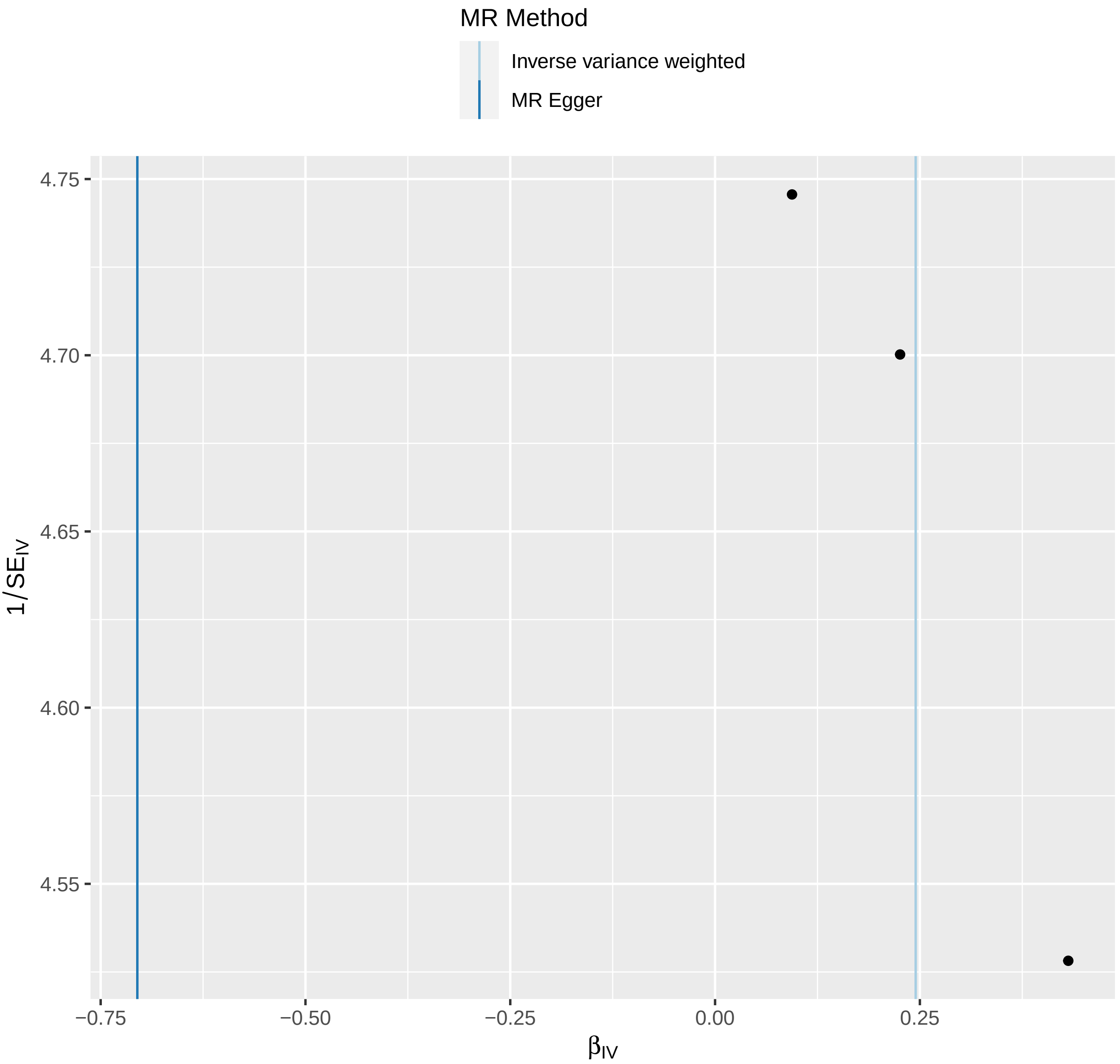

Batch 2−5924 : Gut microbiota abundance (genus Parasutterella id.2892) on Transforming growth factor beta−1
with a threshold of 1e−05

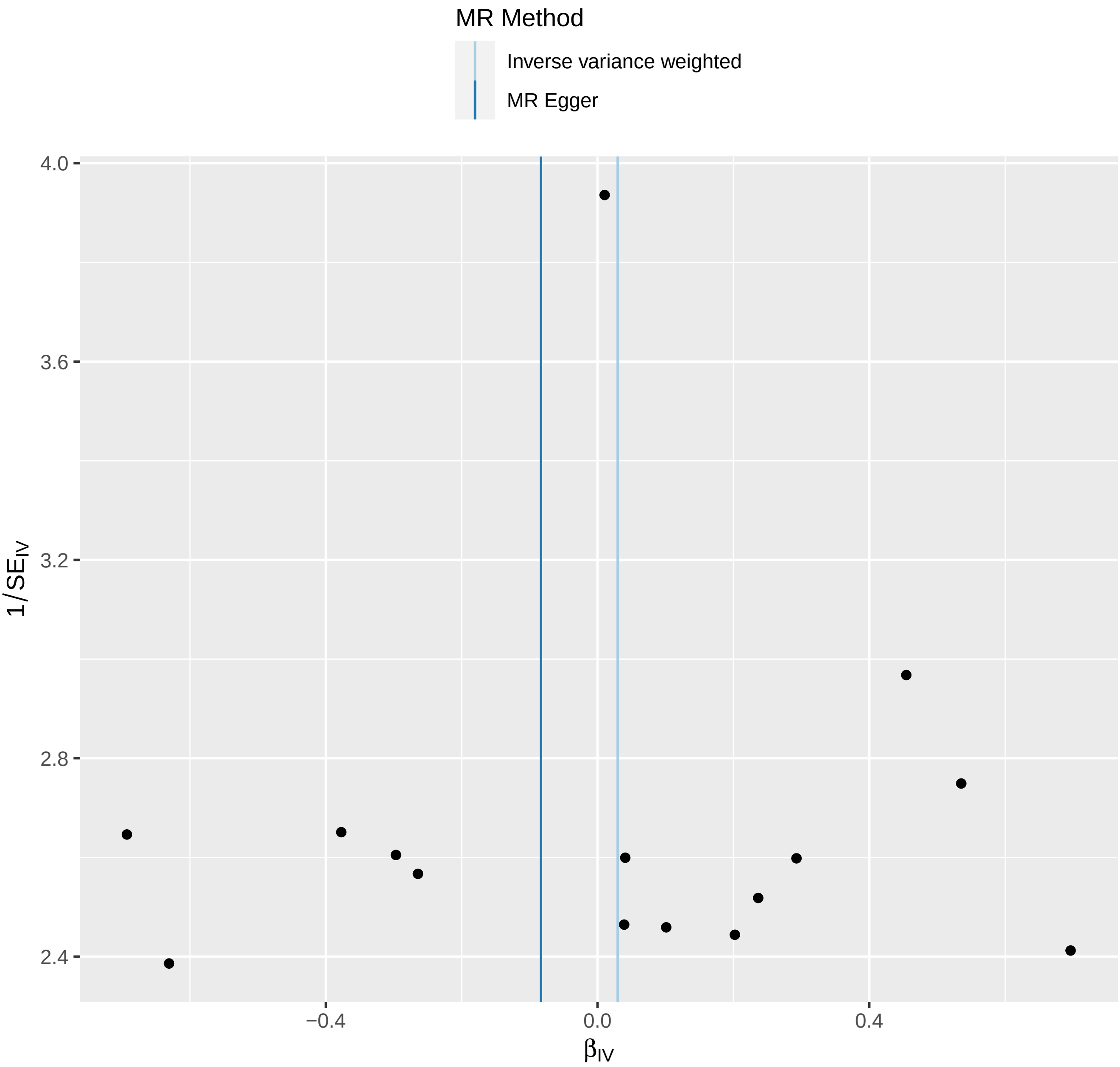

Batch 2−5925 : Gut microbiota abundance (genus Catenibacterium id.2153) on Transforming growth factor beta−1−induced transcript 1 protein with a threshold of 1e−05

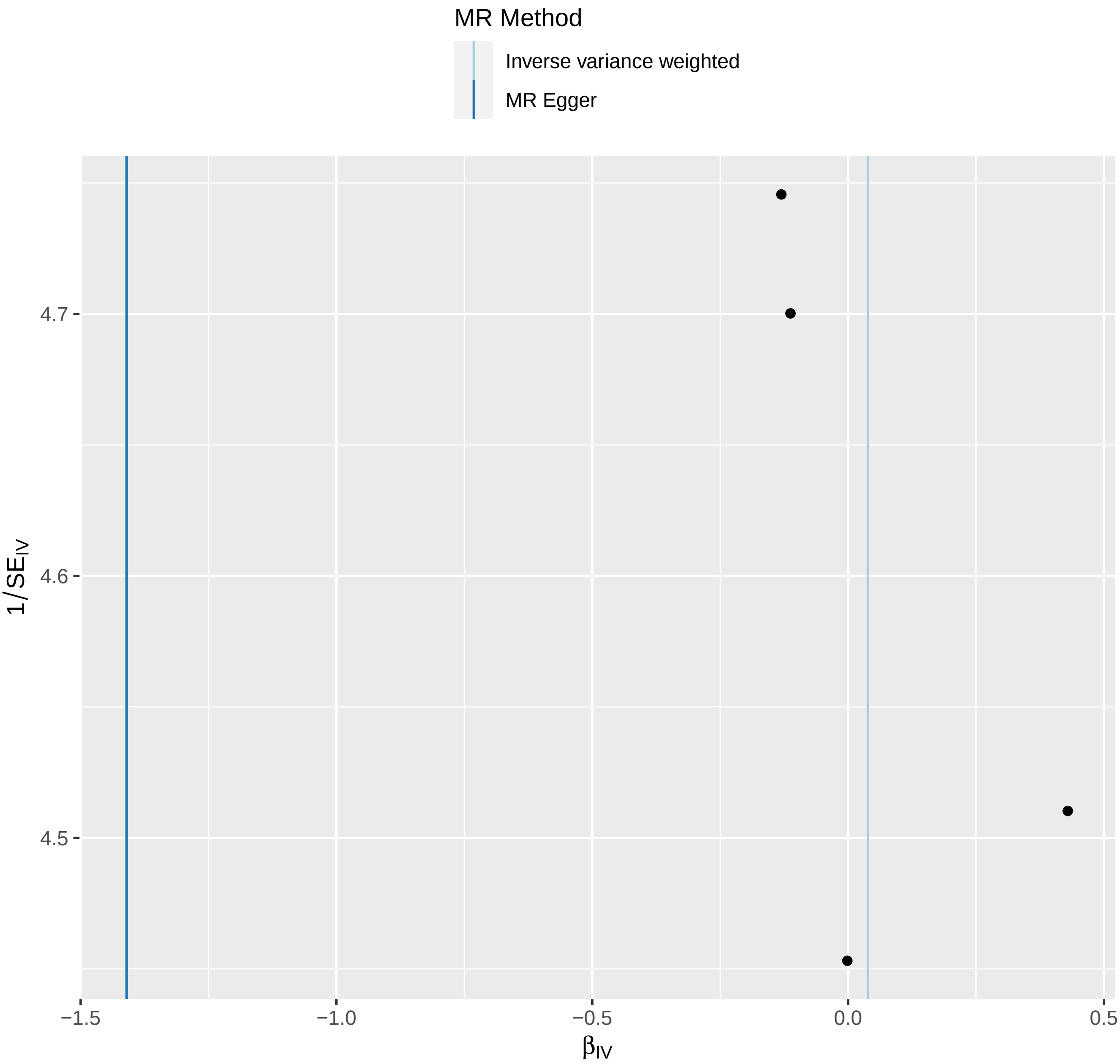

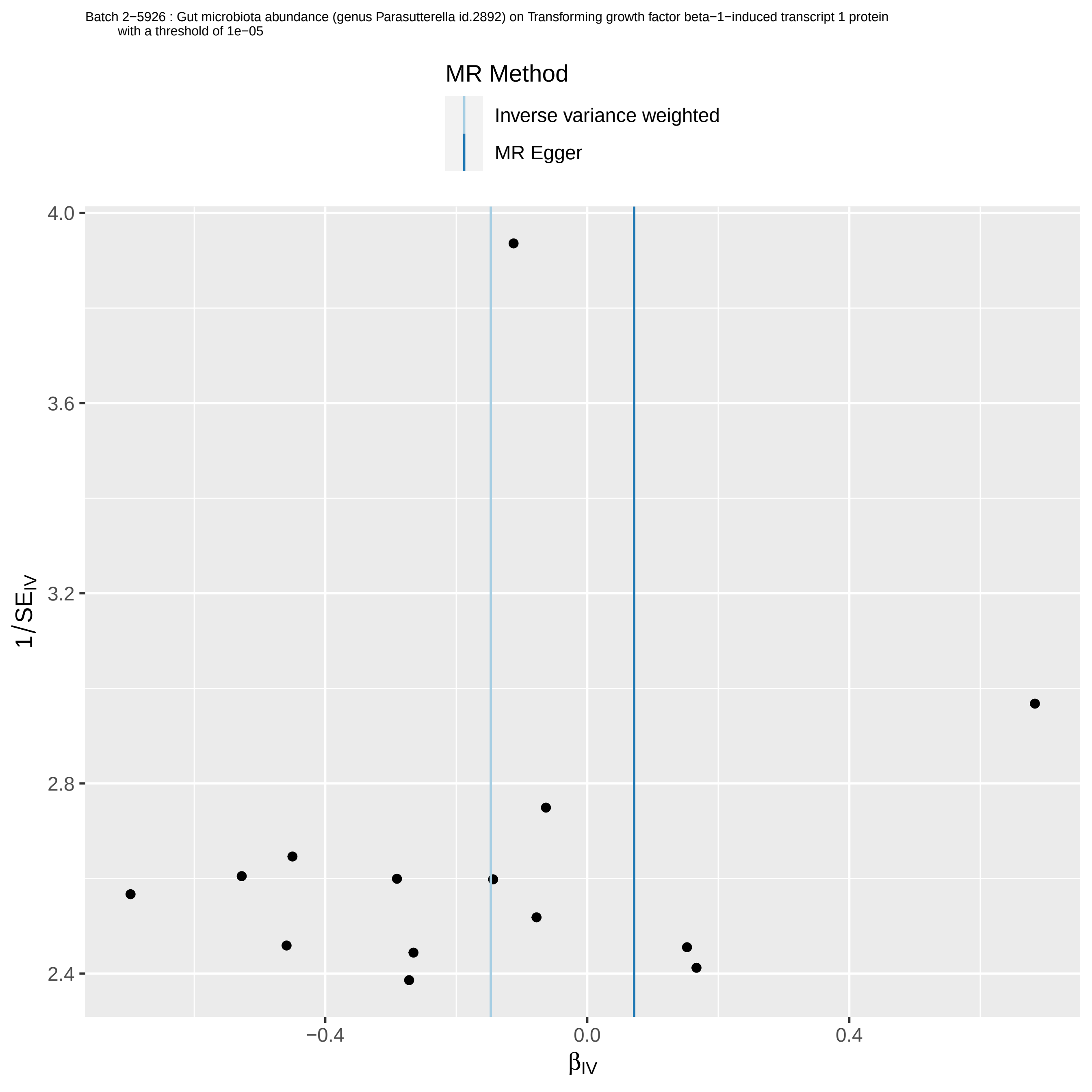

Batch 2−5926 : Gut microbiota abundance (genus Parasutterella id.2892) on Transforming growth factor beta−1−induced transcript 1 protein
with a threshold of 1e−05
MR Method
Inverse variance weighted
MR Egger
4.0
3.6
3.2
2.8
2.4
1/SE_IV
−0.4
0.0
0.4
β_IV

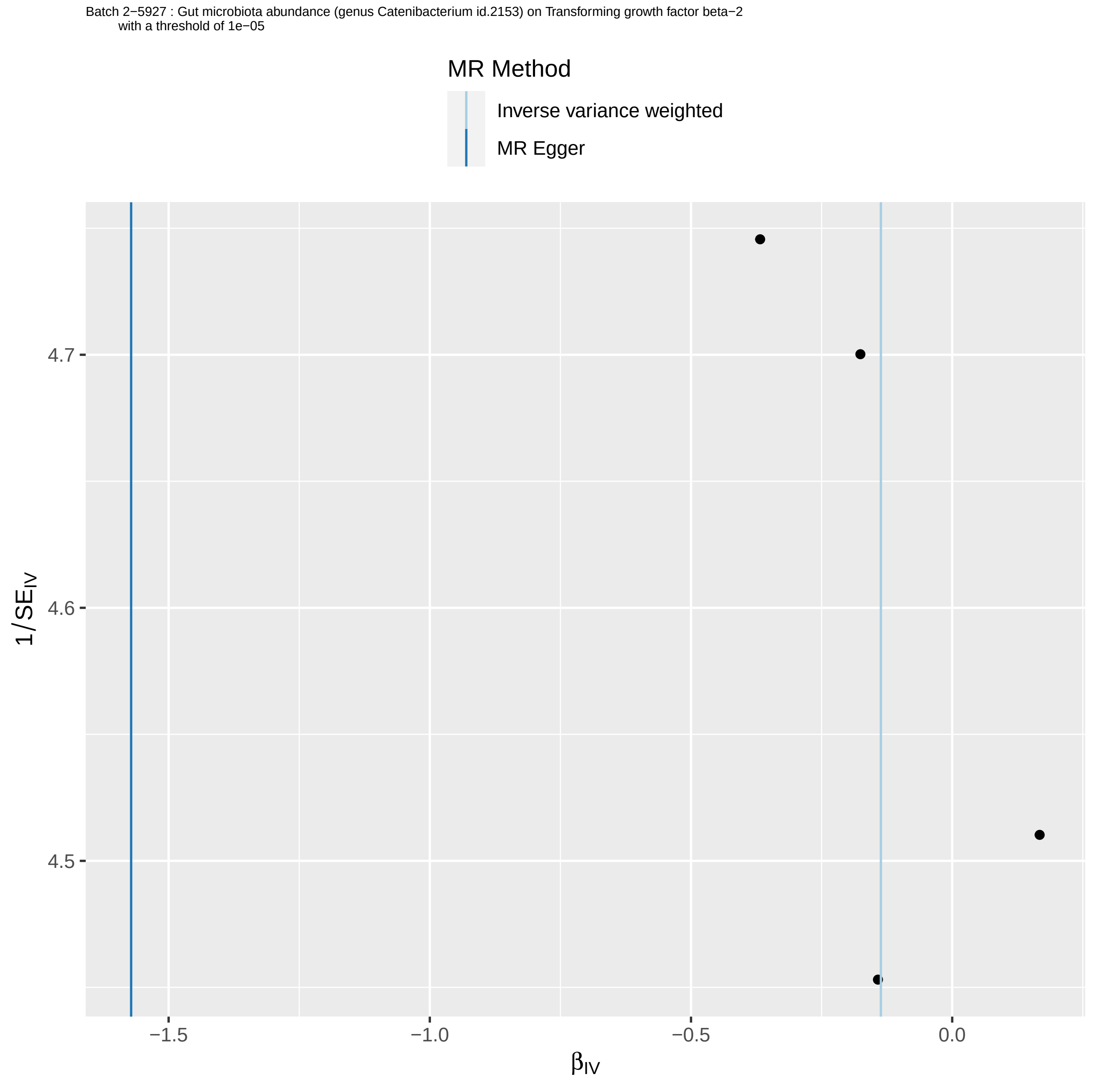

Batch 2−5927 : Gut microbiota abundance (genus Catenibacterium id.2153) on Transforming growth factor beta−2
with a threshold of 1e−05
MR Method
Inverse variance weighted
MR Egger
4.7
4.6
4.5
$1/SE_{IV}$
−1.5
−1.0
−0.5
0.0
β_{IV}

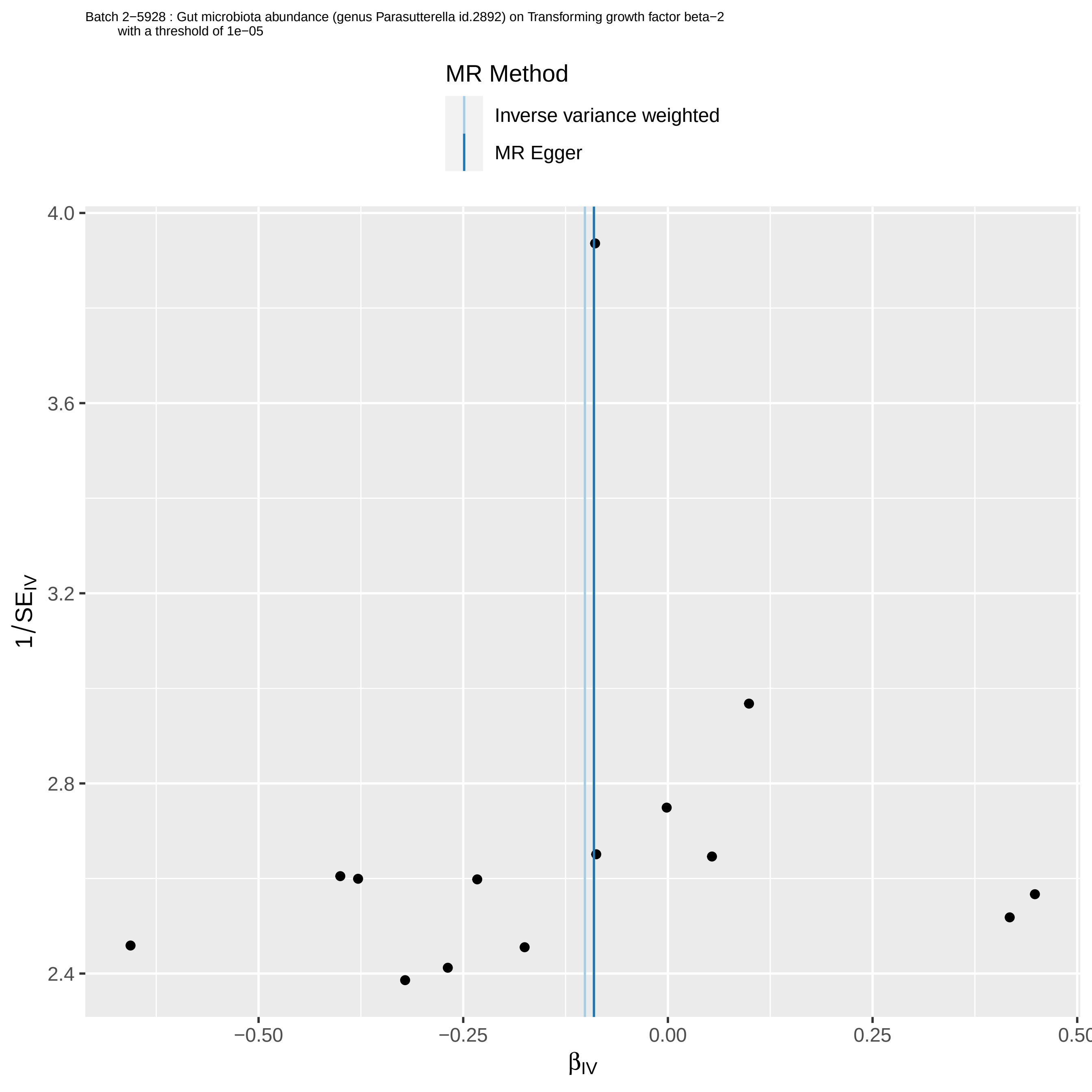
Batch 2−5928 : Gut microbiota abundance (genus Parasutterella id.2892) on Transforming growth factor beta−2
with a threshold of 1e−05
MR Method
Inverse variance weighted
MR Egger
4.0
3.6
3.2
2.8
2.4
1/SE_IV
−0.50
−0.25
0.00
0.25
0.50
β_IV

Batch 2−5929 : Gut microbiota abundance (genus Catenibacterium id.2153) on Transforming growth factor beta−3
with a threshold of 1e−05

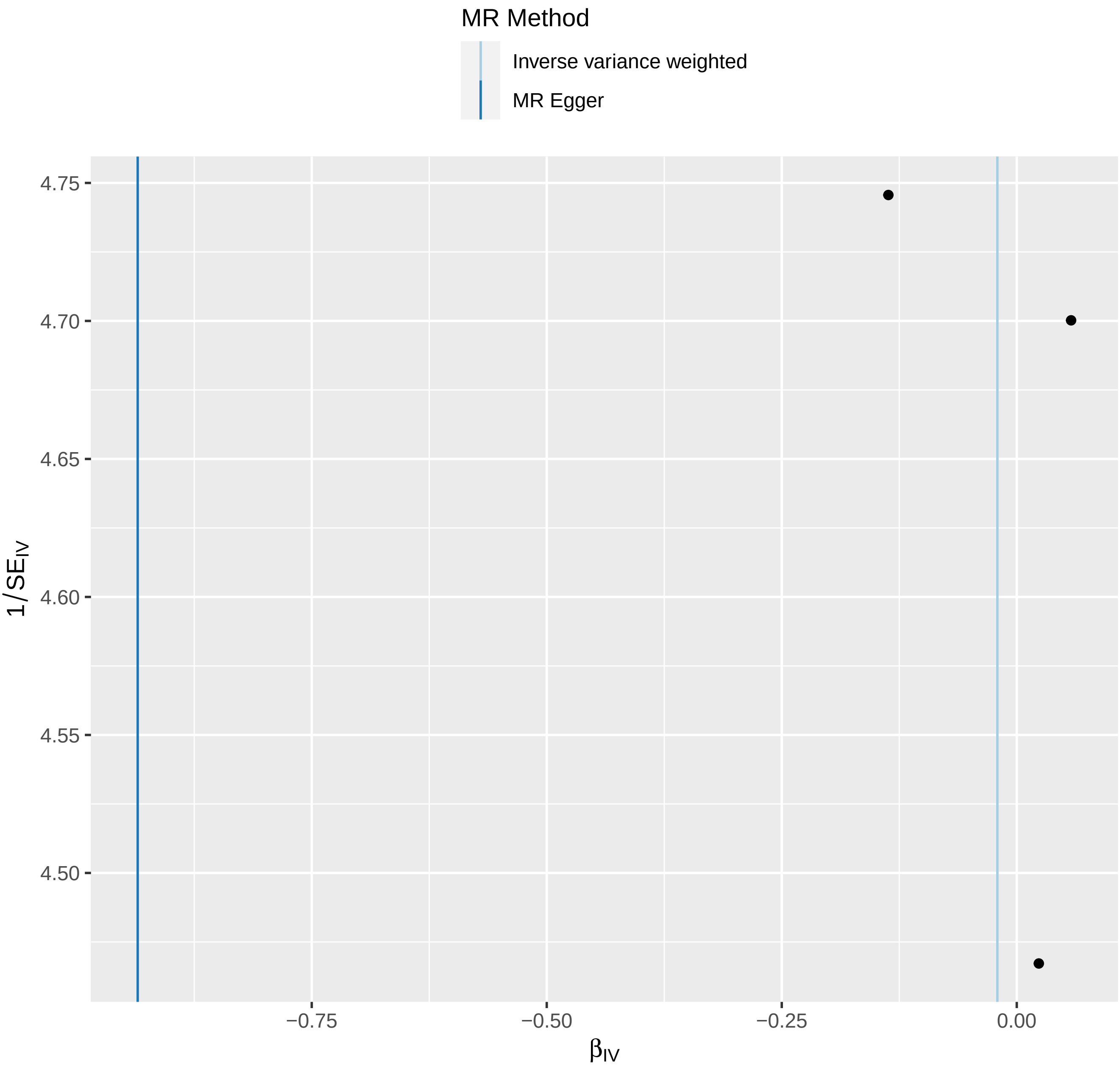

Batch 2−5930 : Gut microbiota abundance (genus Parasutterella id.2892) on Transforming growth factor beta−3
with a threshold of 1e−05

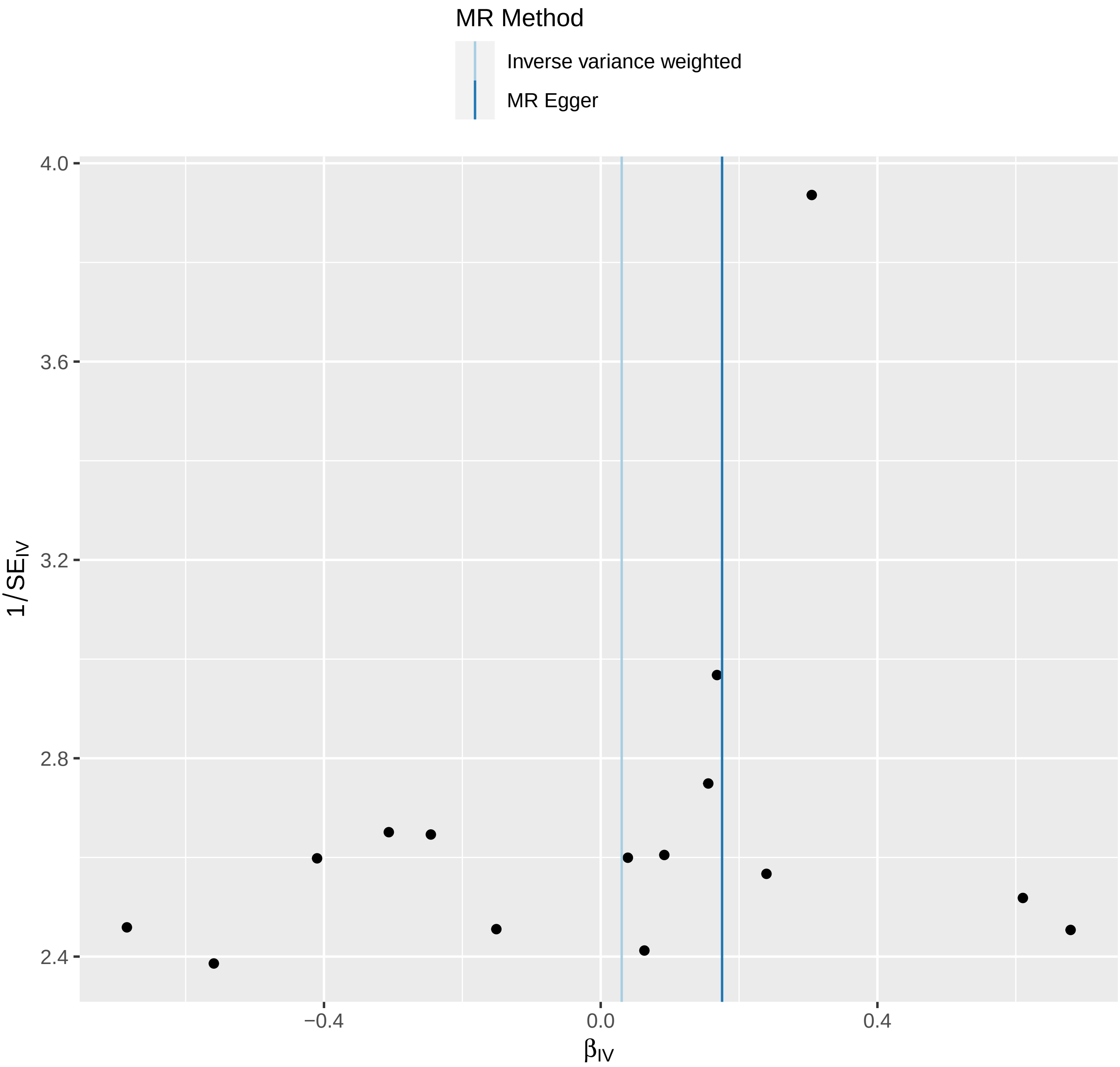

Batch 2−5931 : Gut microbiota abundance (genus Catenibacterium id.2153) on Transforming growth factor−beta−induced protein ig−h3
with a threshold of 1e−05

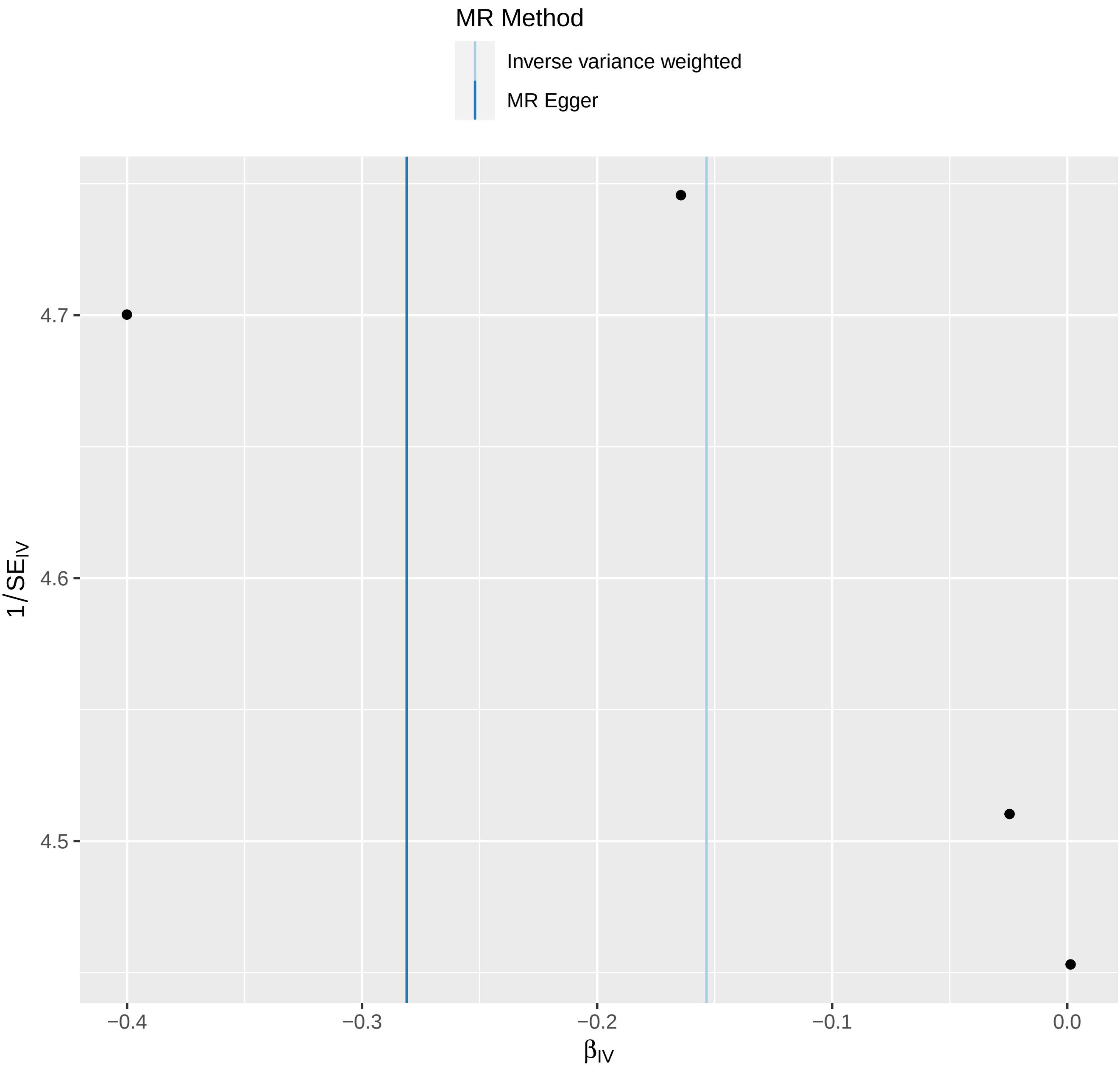

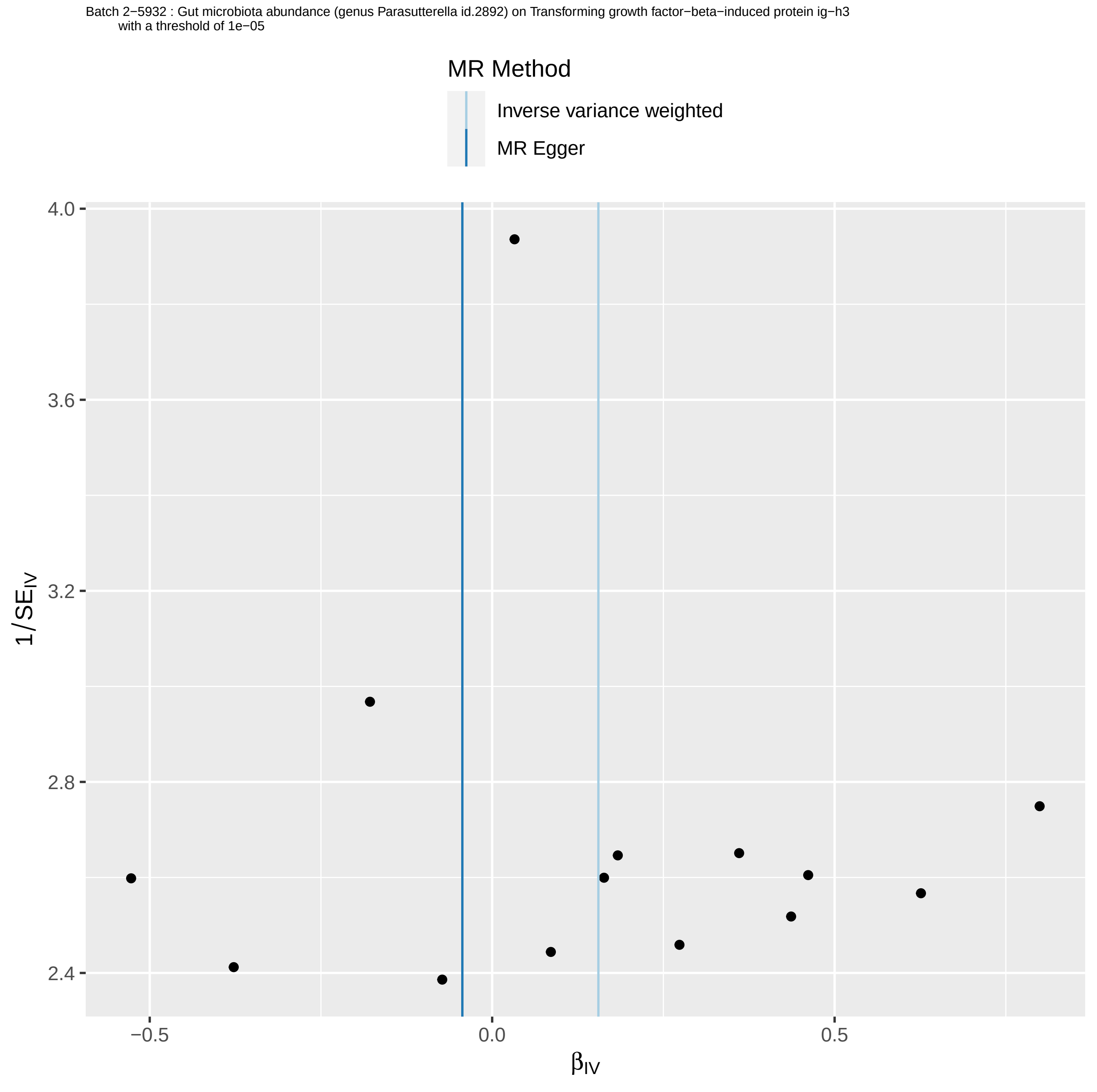
Batch 2−5932 : Gut microbiota abundance (genus Parasutterella id.2892) on Transforming growth factor−beta−induced protein ig−h3
with a threshold of 1e−05
MR Method
Inverse variance weighted
MR Egger
4.0
3.6
3.2
2.8
2.4
1/SE_IV
−0.5
0.0
0.5
β_IV

Batch 2−5933 : Gut microbiota abundance (genus Catenibacterium id.2153) on TGF−beta receptor type−2
with a threshold of 1e−05

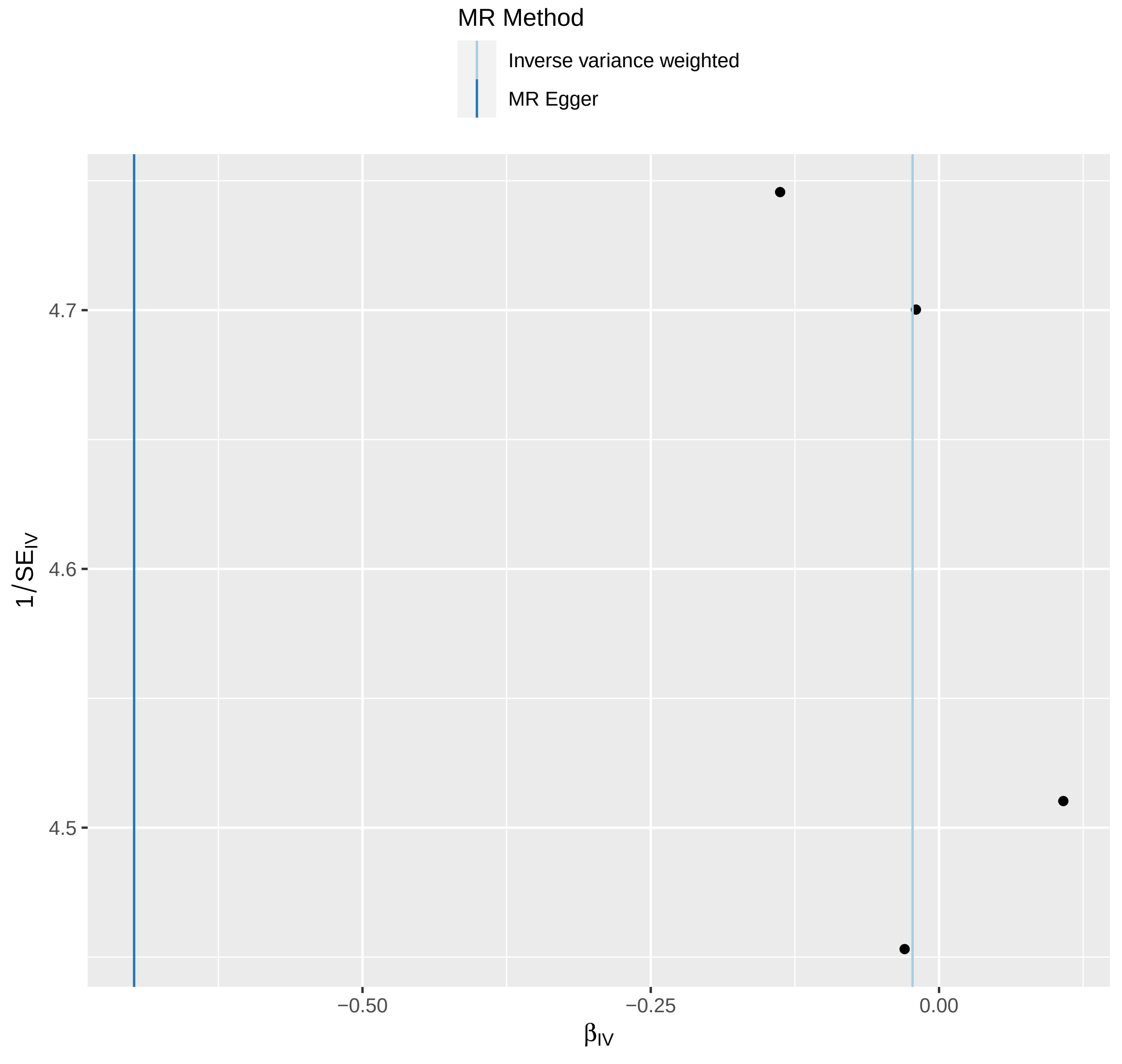

Batch 2−5934 : Gut microbiota abundance (genus Parasutterella id.2892) on TGF−beta receptor type−2
with a threshold of 1e−05

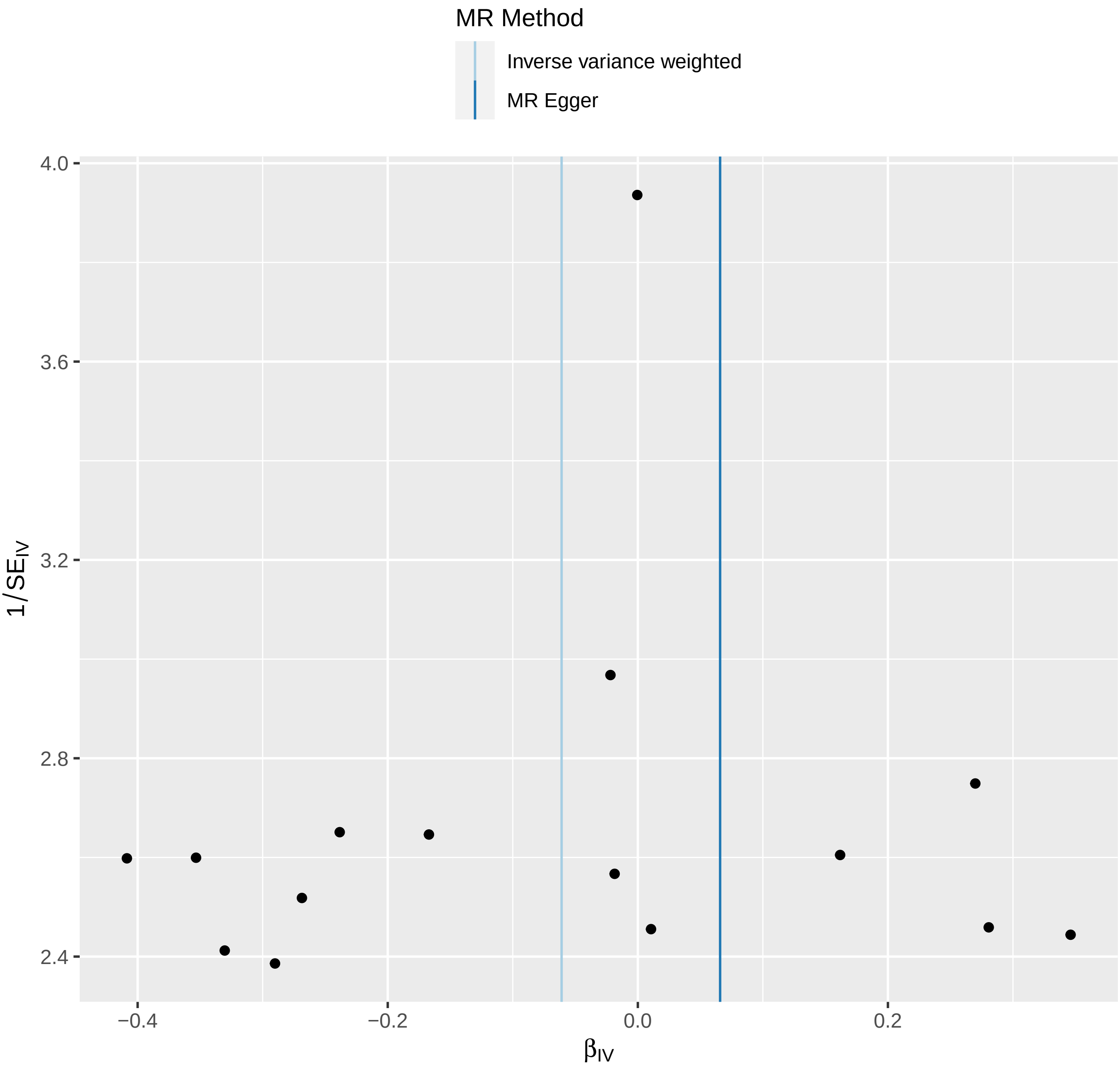

Batch 2−5935 : Gut microbiota abundance (genus Catenibacterium id.2153) on Transforming growth factor beta receptor type 3
with a threshold of 1e−05

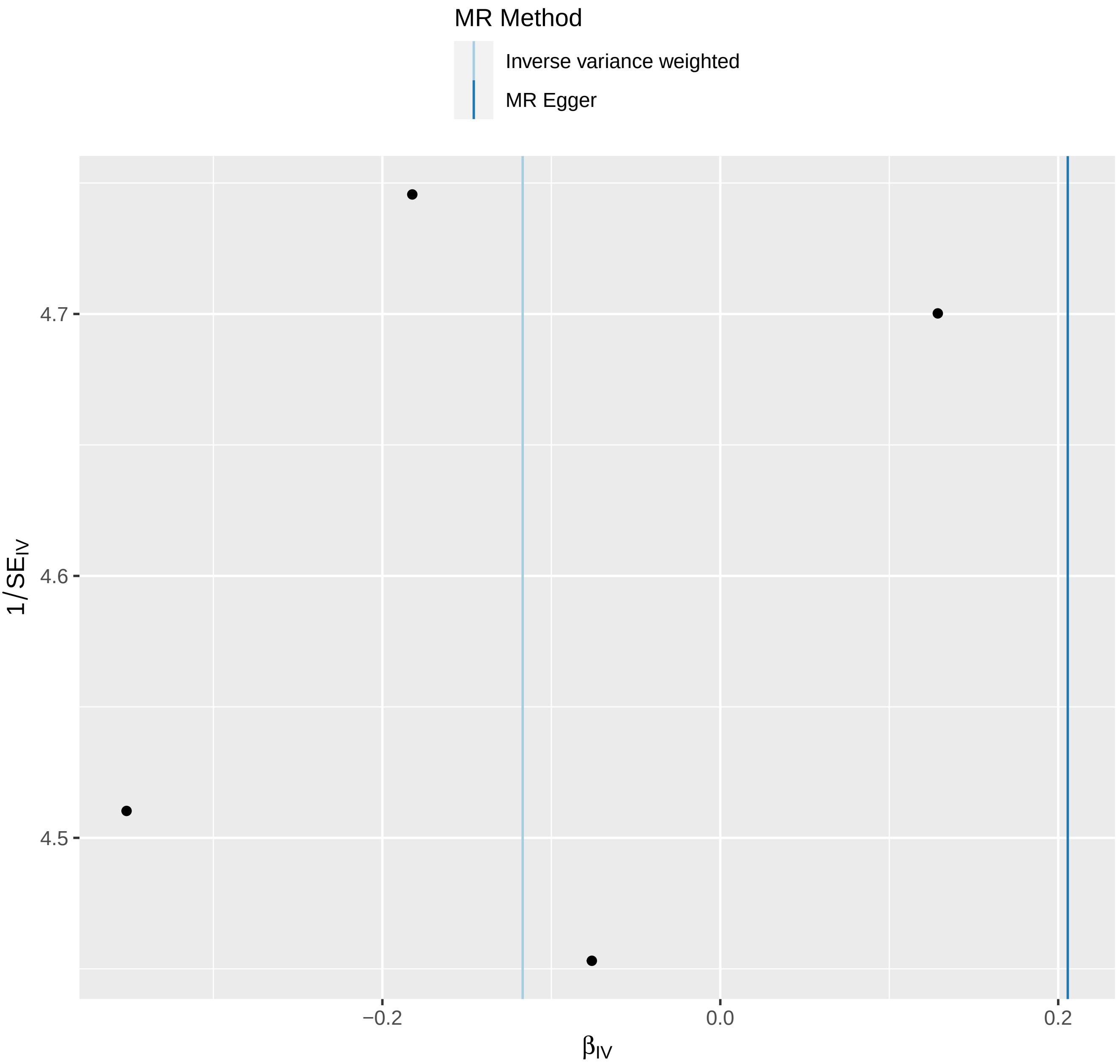

Batch 2−5936 : Gut microbiota abundance (genus Parasutterella id.2892) on Transforming growth factor beta receptor type 3
with a threshold of 1e−05

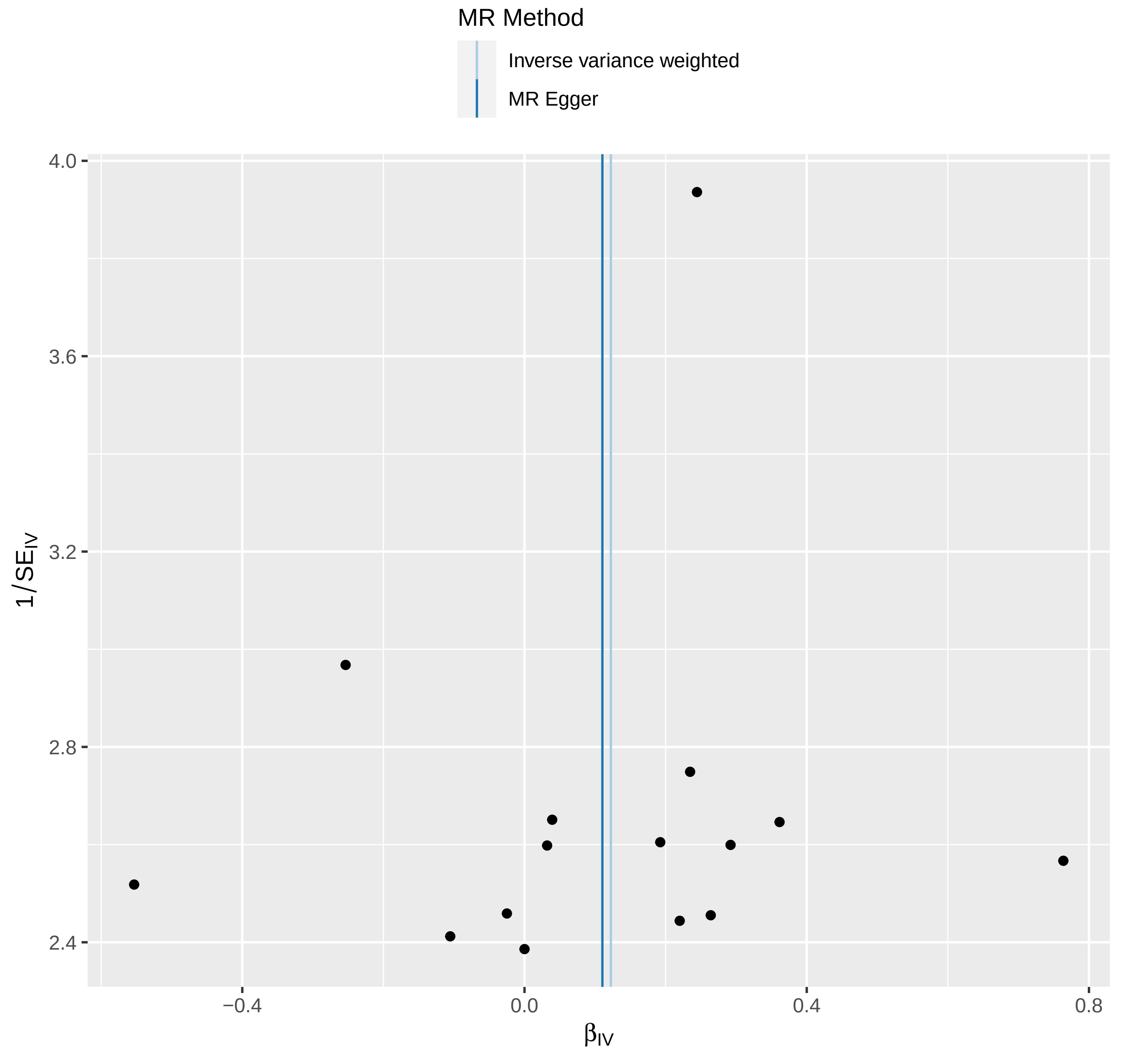

Batch 2−5937 : Gut microbiota abundance (genus Catenibacterium id.2153) on Homeobox protein TGIF2
with a threshold of 1e−05

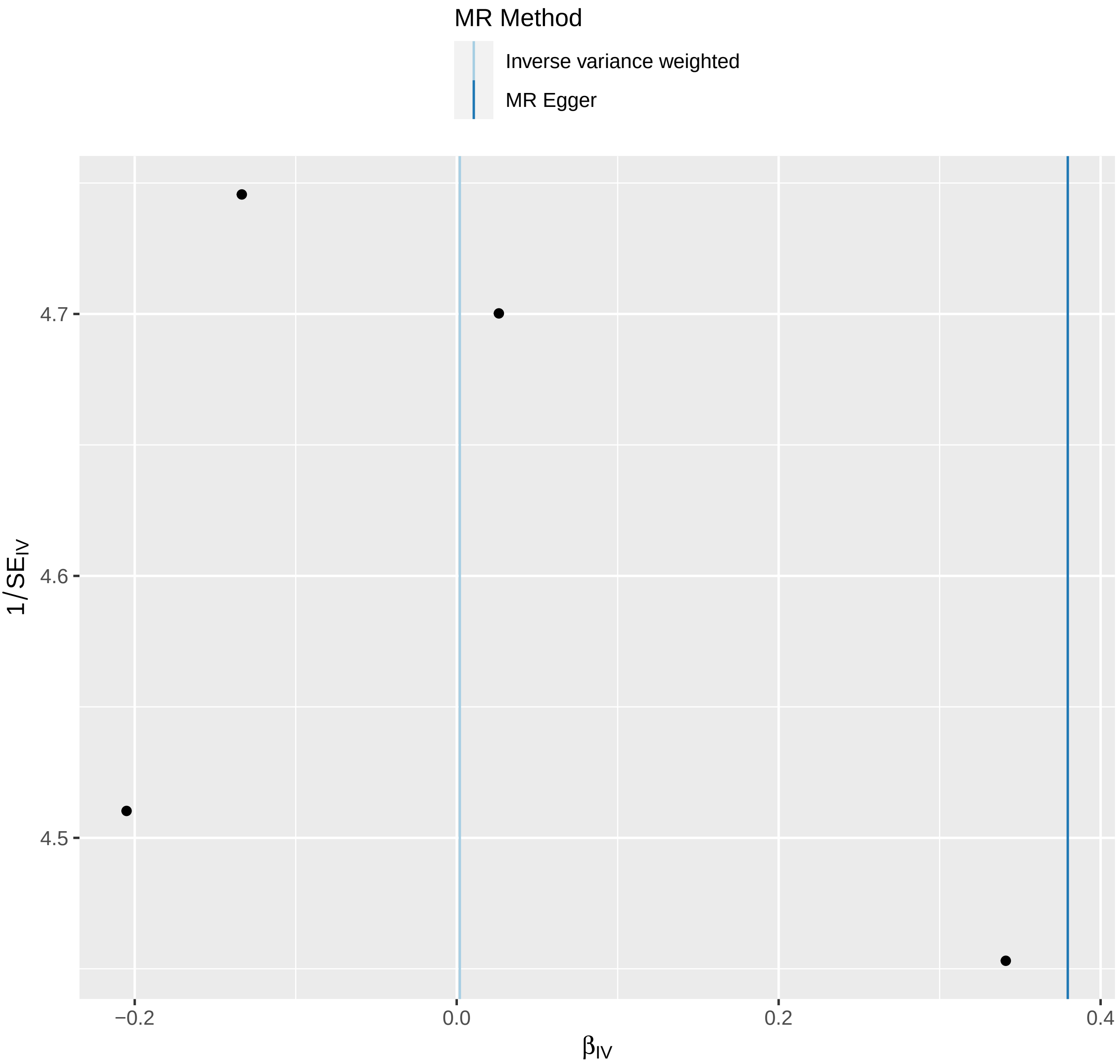

Batch 2−5938 : Gut microbiota abundance (genus Parasutterella id.2892) on Homeobox protein TGIF2
with a threshold of 1e−05

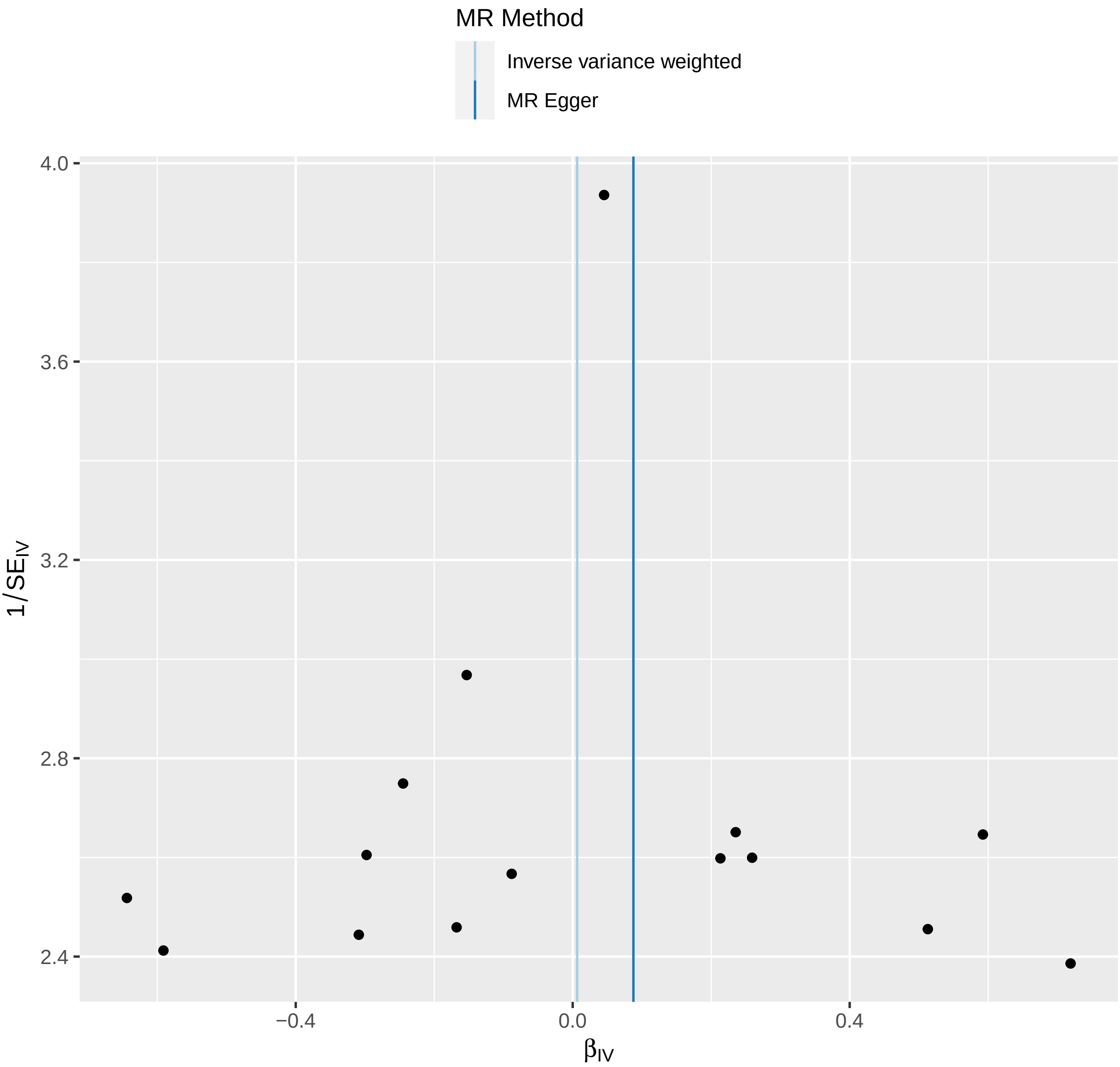

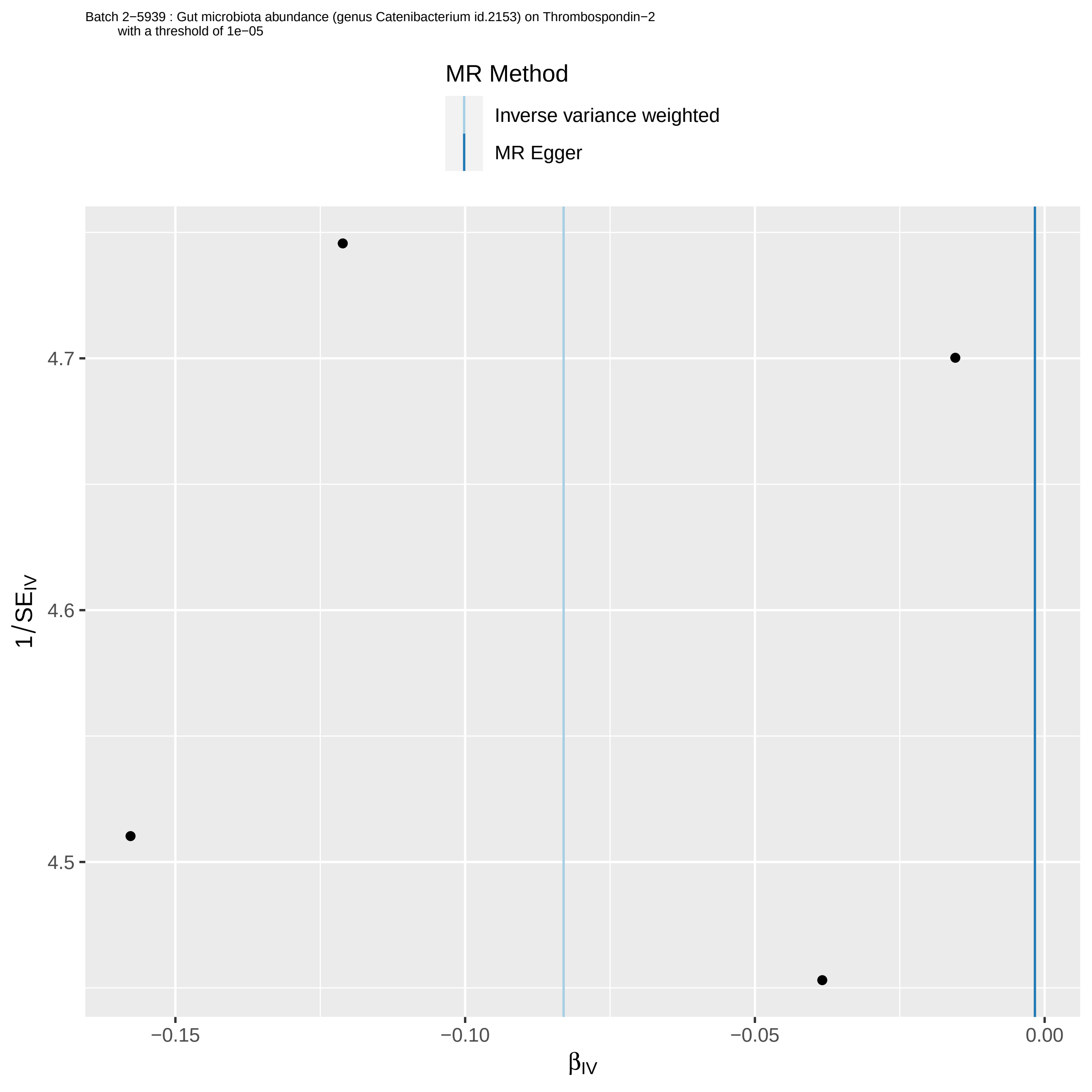
Batch 2−5939 : Gut microbiota abundance (genus Catenibacterium id.2153) on Thrombospondin−2
with a threshold of 1e−05
MR Method
Inverse variance weighted
MR Egger
4.7
4.6
4.5
1/SE_IV
−0.15
−0.10
−0.05
0.00
β_IV

Batch 2−5940 : Gut microbiota abundance (genus Parasutterella id.2892) on Thrombospondin−2
with a threshold of 1e−05

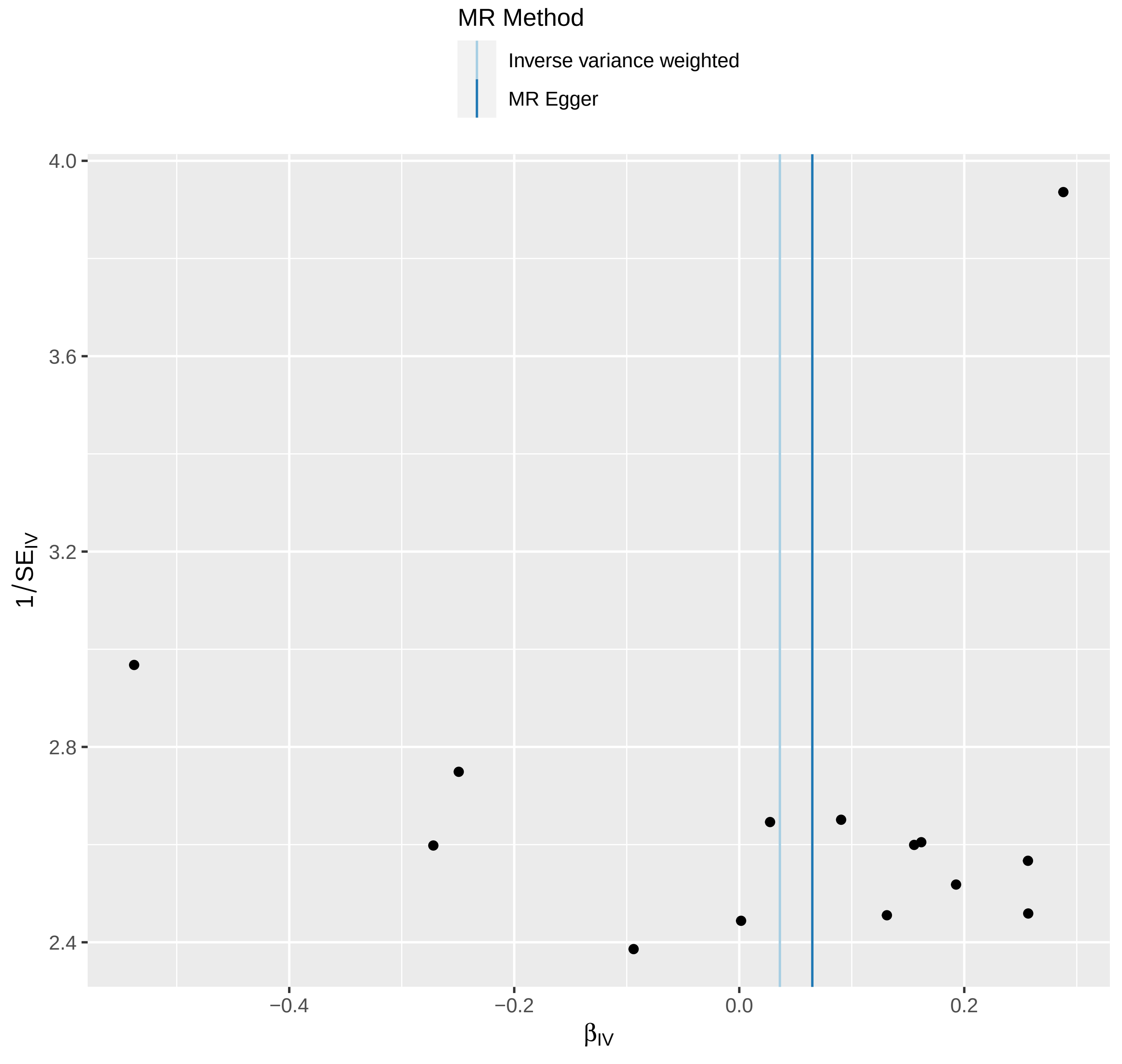

Batch 2−5941 : Gut microbiota abundance (genus Catenibacterium id.2153) on Thrombospondin−3
with a threshold of 1e−05

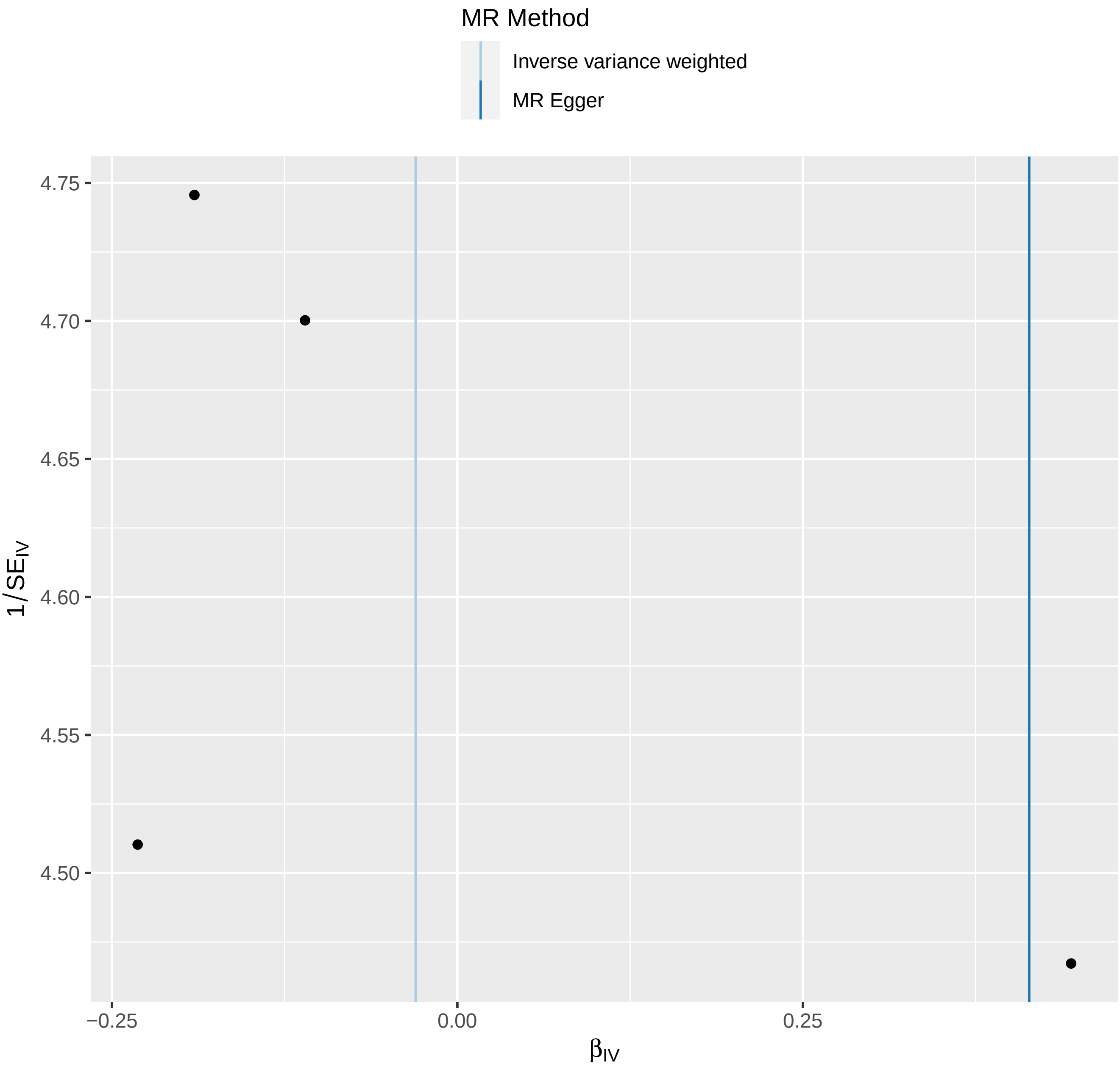

Batch 2−5942 : Gut microbiota abundance (genus Parasutterella id.2892) on Thrombospondin−3
with a threshold of 1e−05

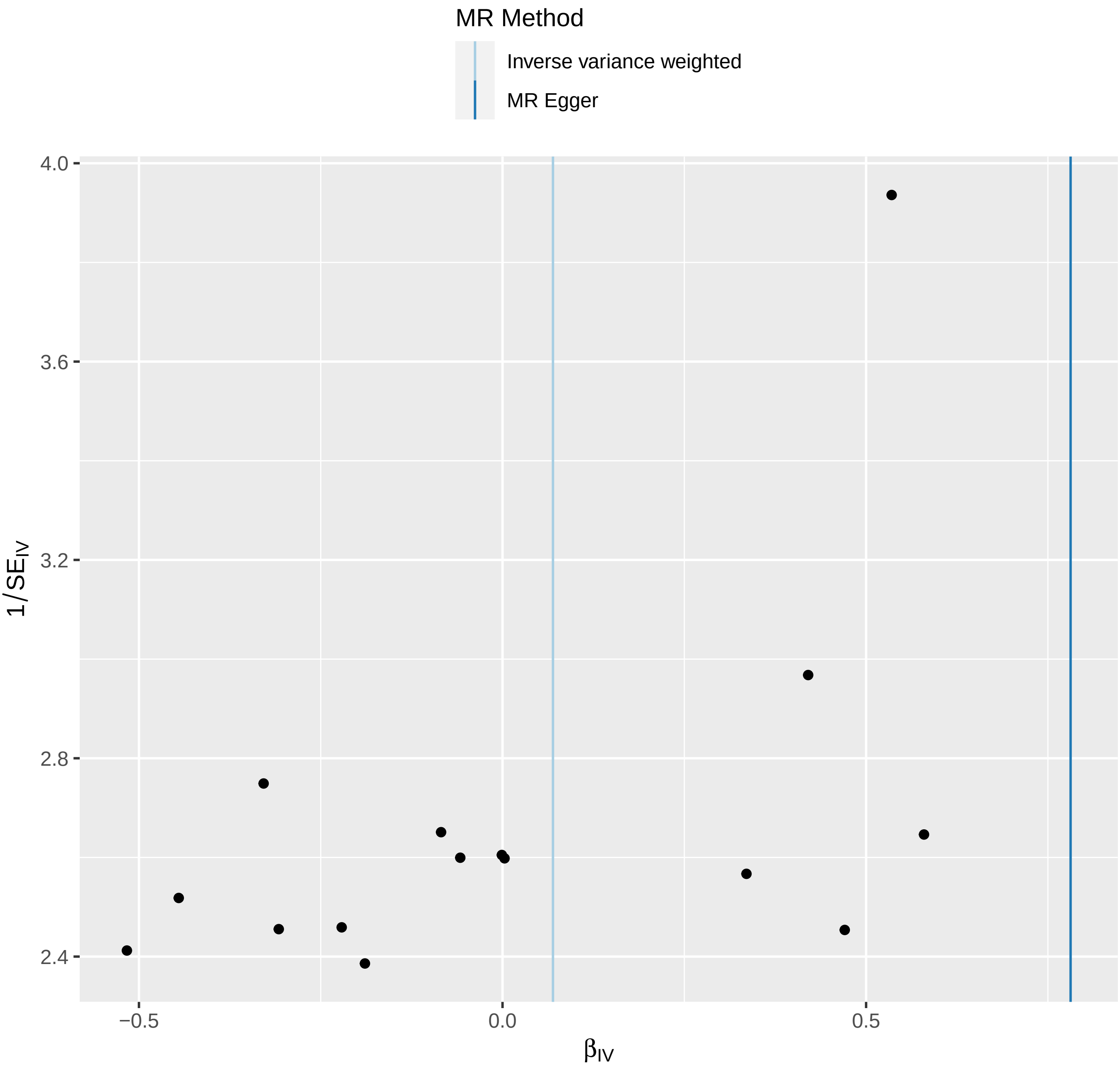

Batch 2−5943 : Gut microbiota abundance (genus Catenibacterium id.2153) on Thrombopoietin
with a threshold of 1e−05

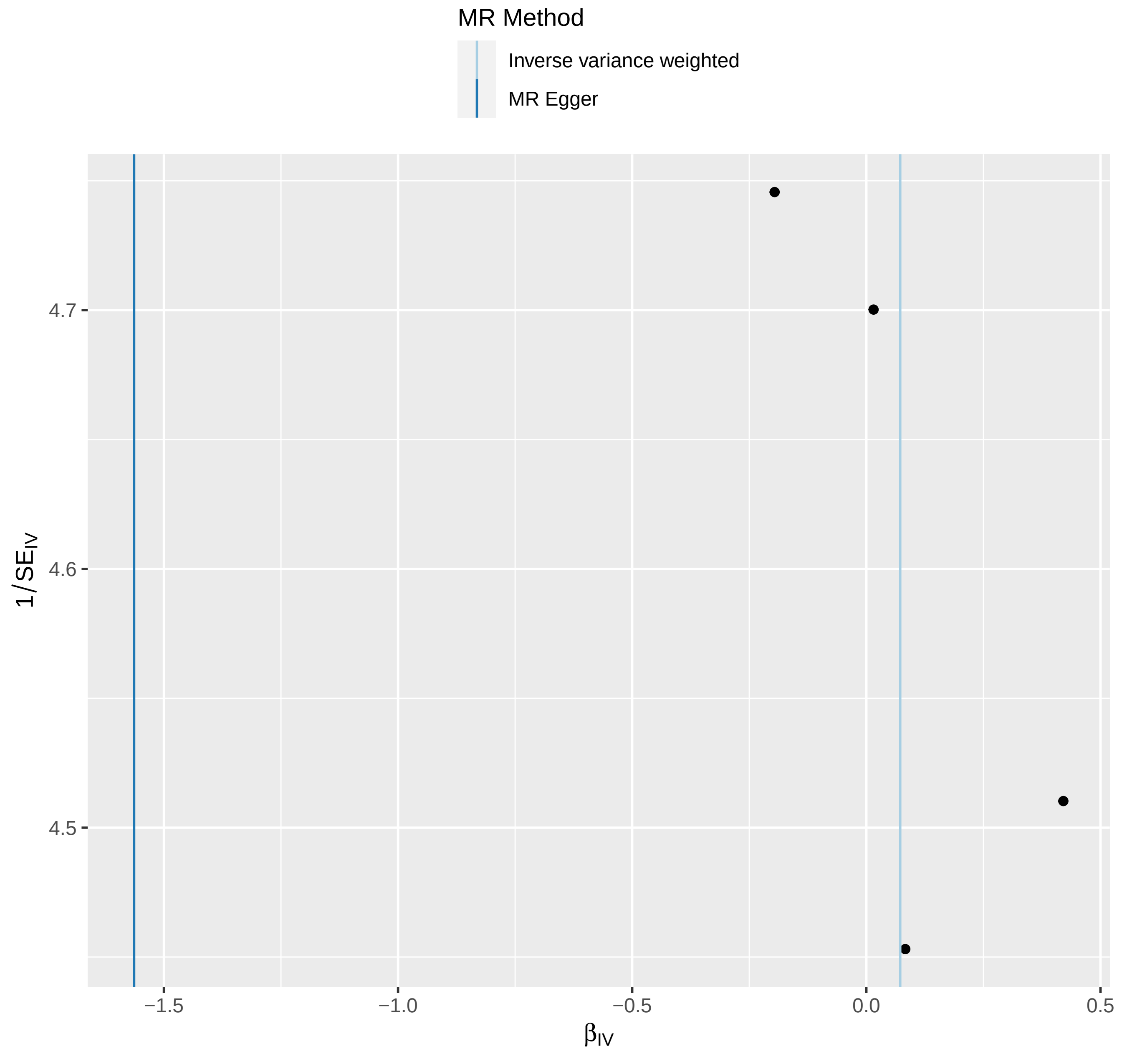

Batch 2−5944 : Gut microbiota abundance (genus Parasutterella id.2892) on Thrombopoietin
with a threshold of 1e−05

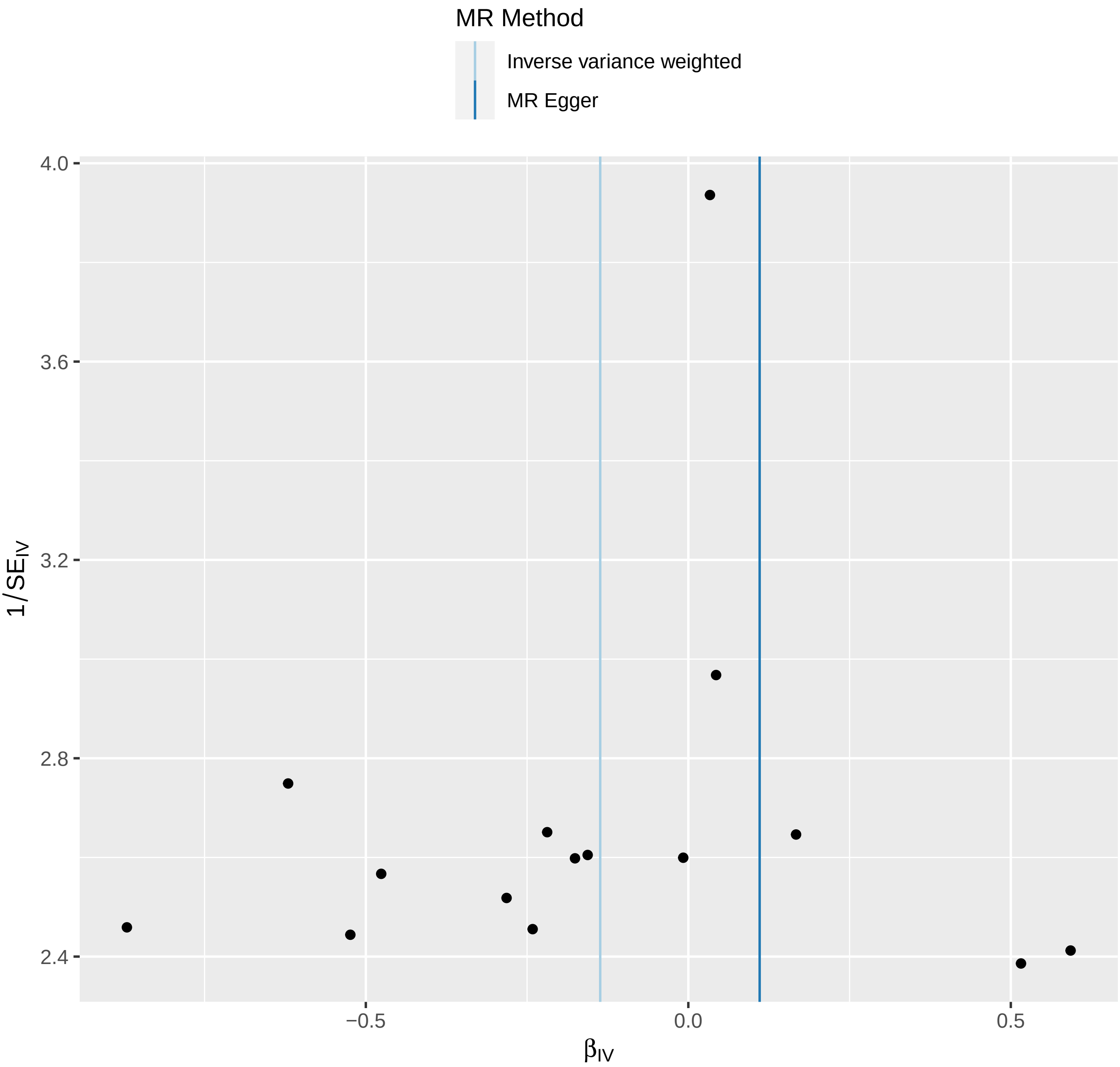

Batch 2−5945 : Gut microbiota abundance (genus Catenibacterium id.2153) on Thrombopoietin
with a threshold of 1e−05

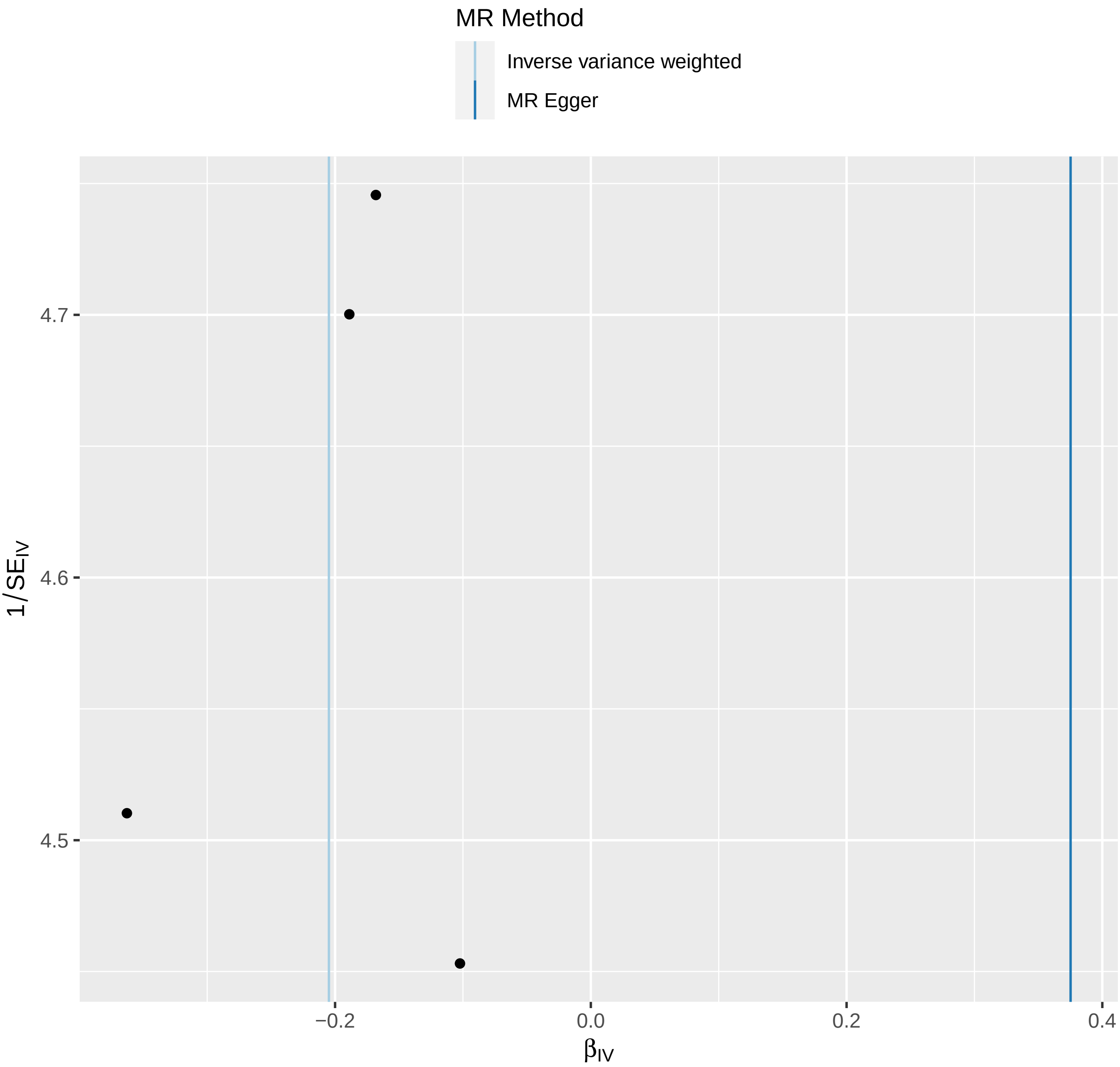

Batch 2−5946 : Gut microbiota abundance (genus Parasutterella id.2892) on Thrombopoietin
with a threshold of 1e−05

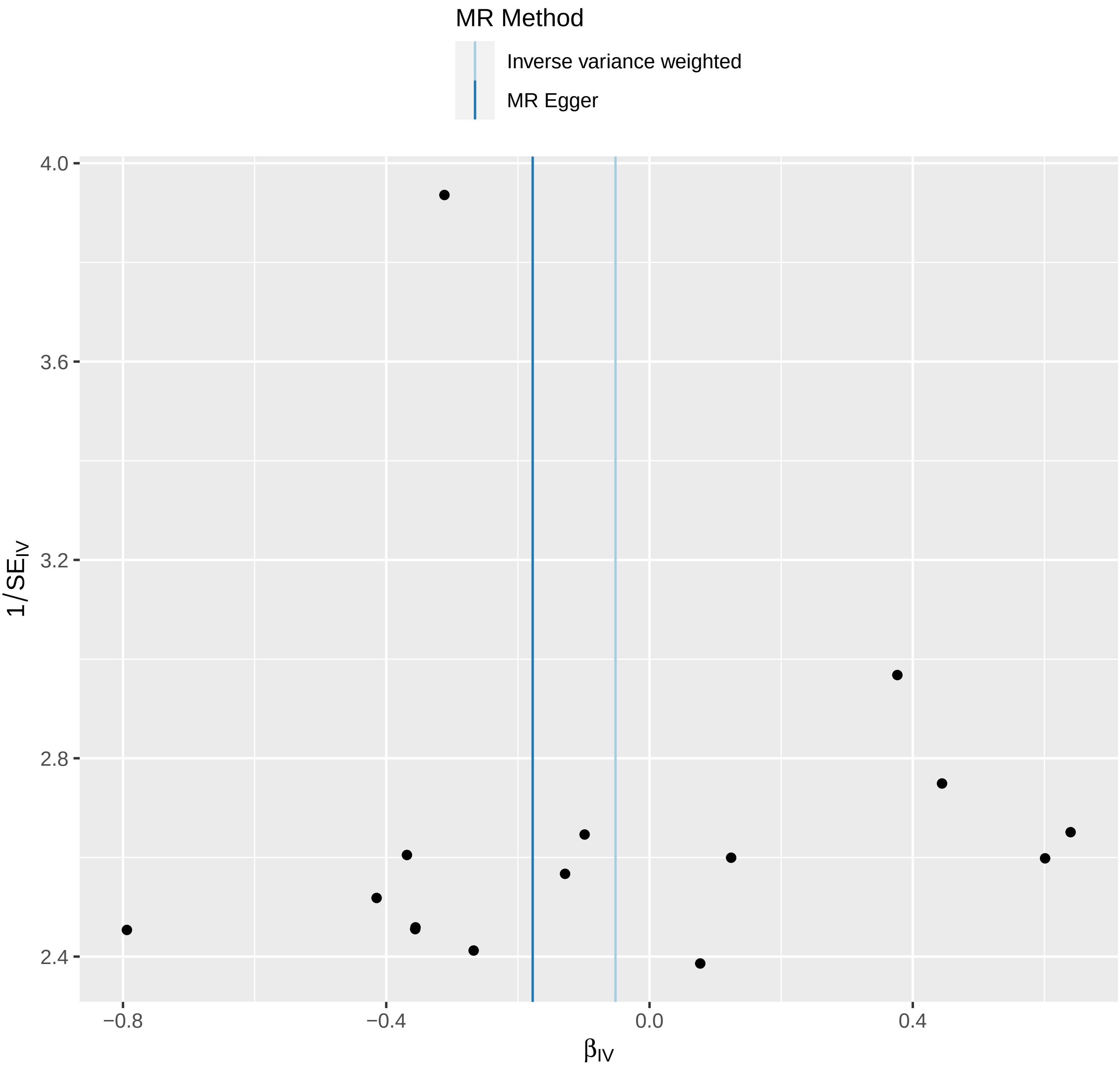

Batch 2−5947 : Gut microbiota abundance (genus Catenibacterium id.2153) on Thyroid hormone receptor alpha
with a threshold of 1e−05

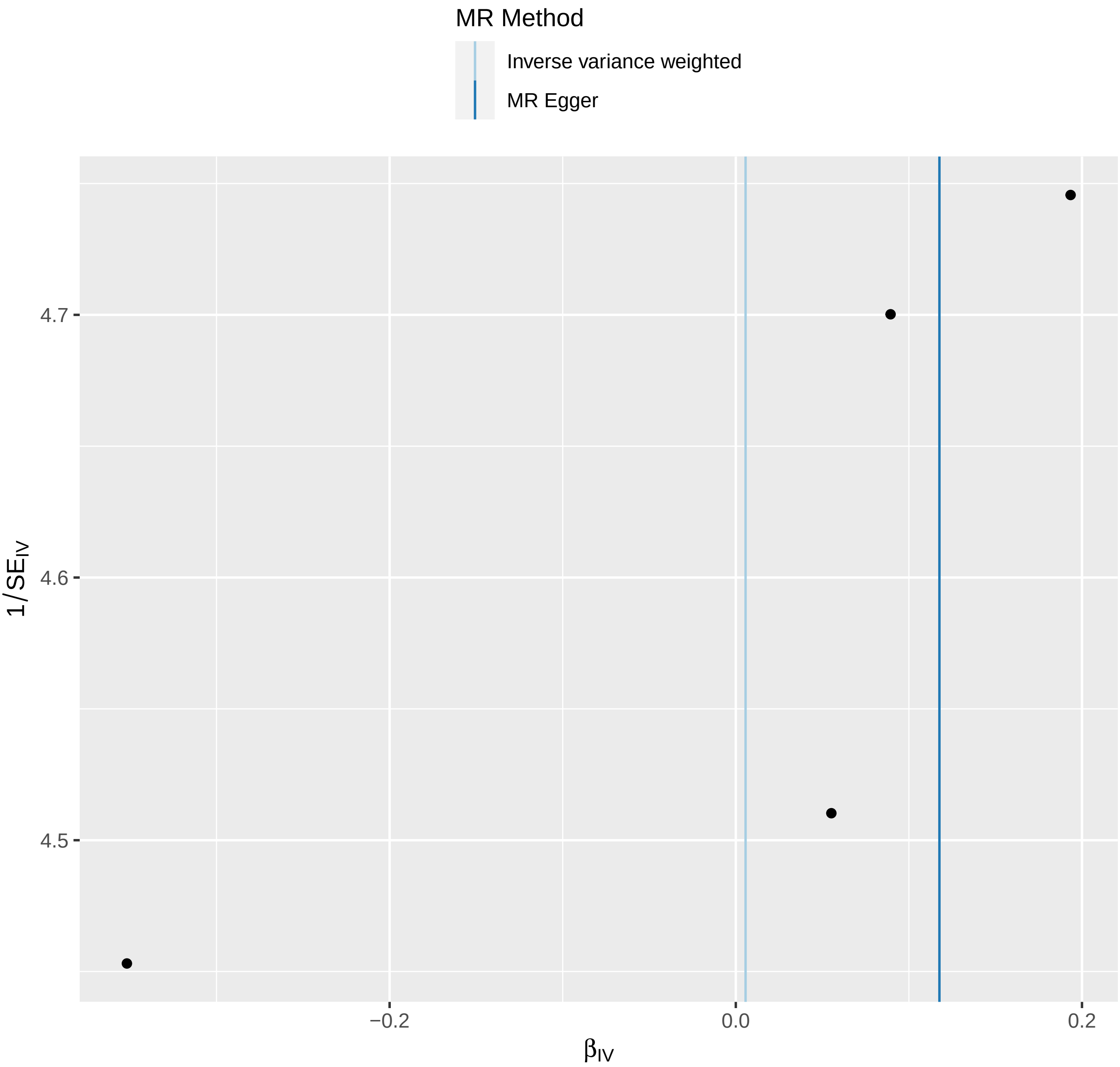

Batch 2−5948 : Gut microbiota abundance (genus Parasutterella id.2892) on Thyroid hormone receptor alpha
with a threshold of 1e−05

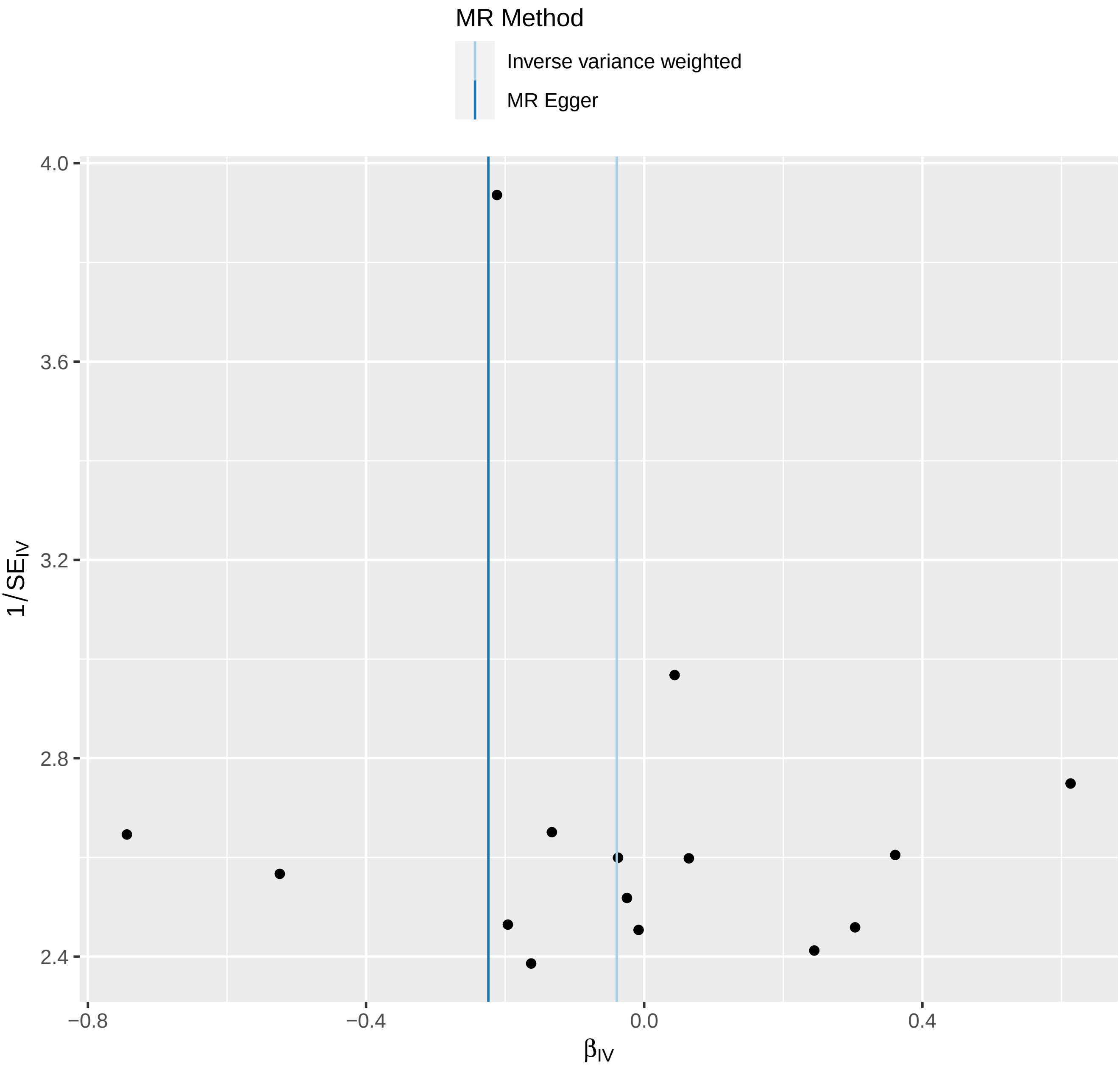

Batch 2−5949 : Gut microbiota abundance (genus Catenibacterium id.2153) on Thrombospondin type−1 domain−containing protein 1 with a threshold of 1e−05

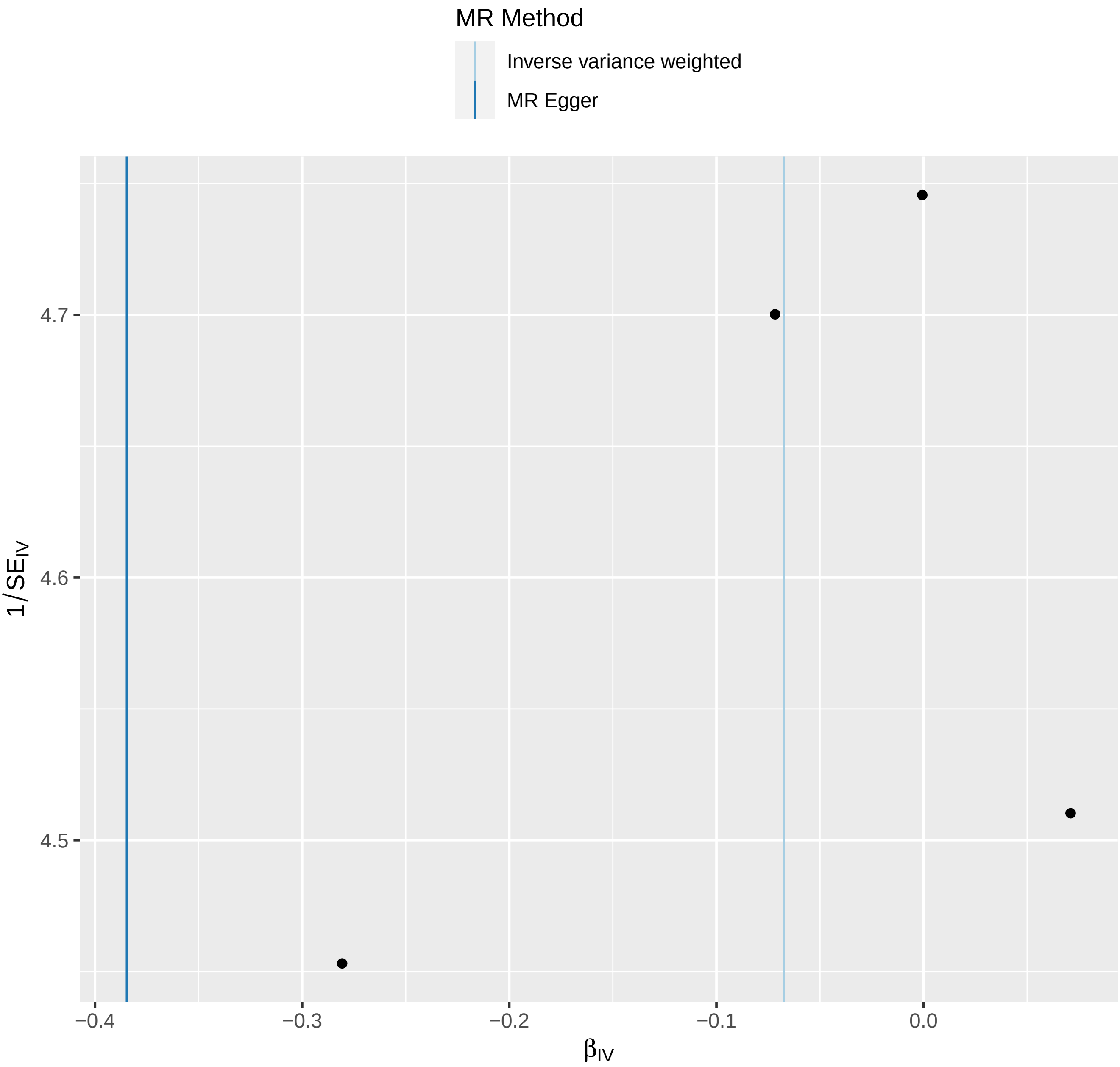

Batch 2−5950 : Gut microbiota abundance (genus Parasutterella id.2892) on Thrombospondin type−1 domain−containing protein 1 with a threshold of 1e−05

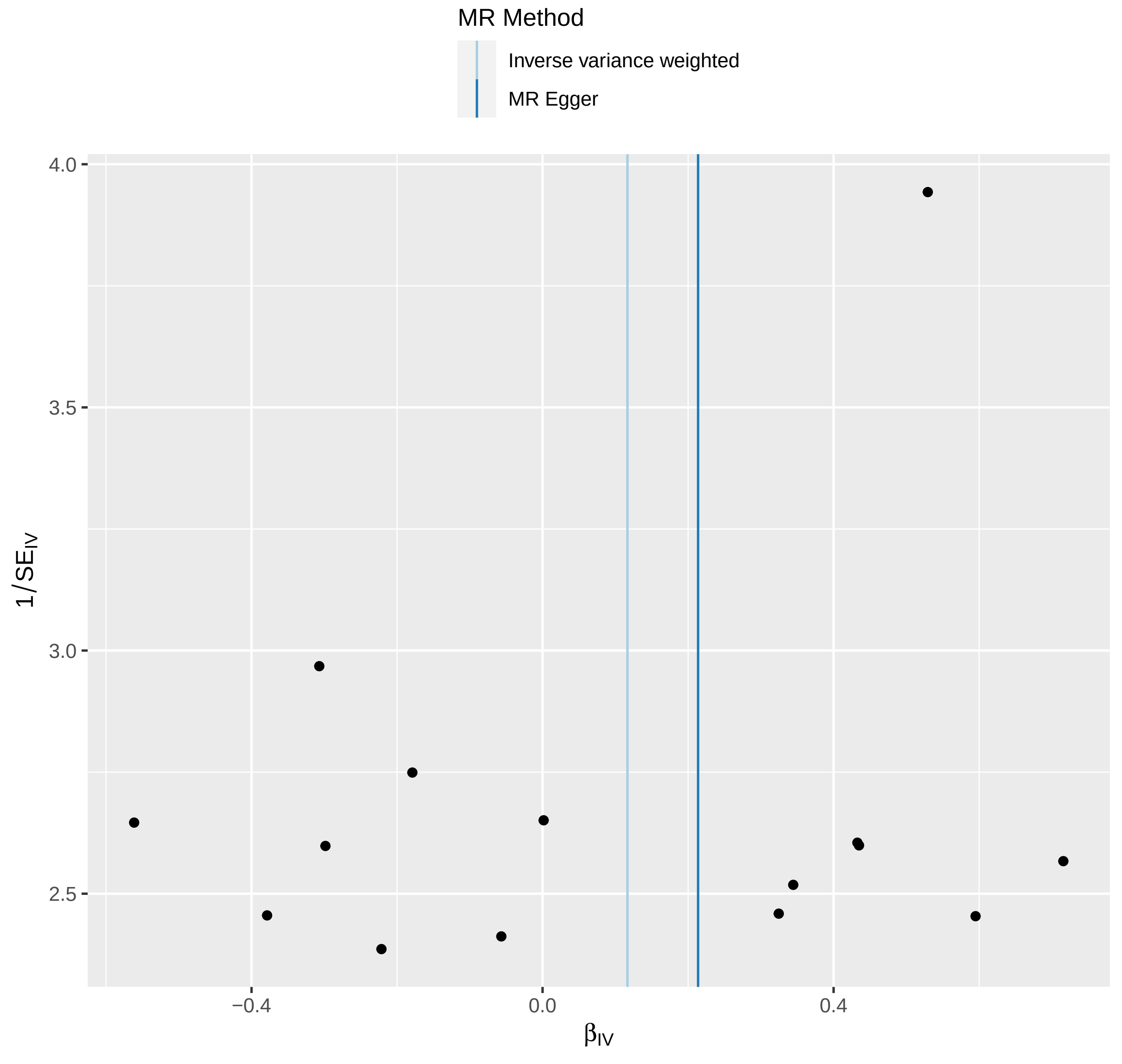

Batch 2−5951 : Gut microbiota abundance (genus Catenibacterium id.2153) on Thrombospondin type−1 domain−containing protein 7A
with a threshold of 1e−05

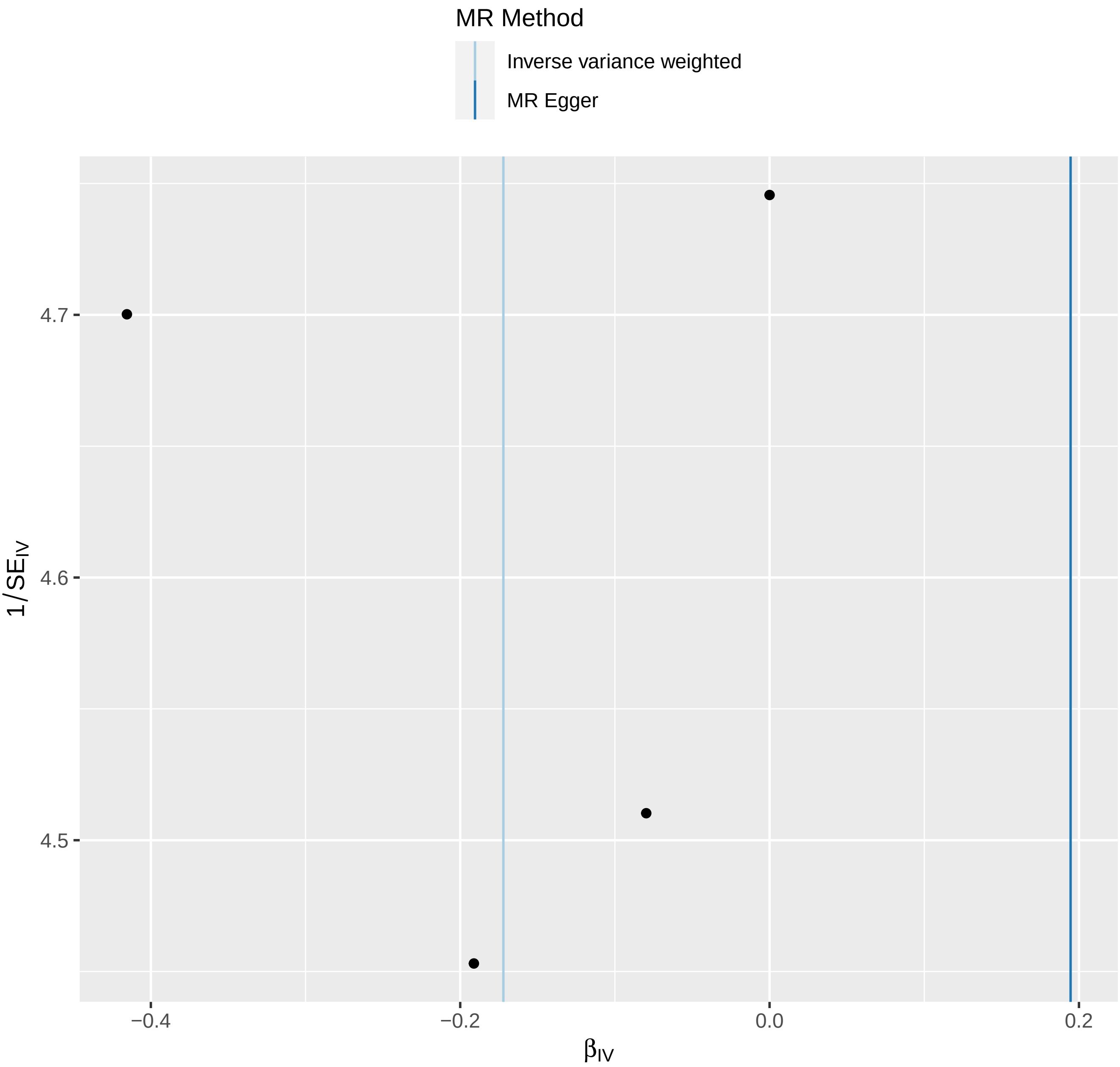

Batch 2−5952 : Gut microbiota abundance (genus Parasutterella id.2892) on Thrombospondin type−1 domain−containing protein 7A
with a threshold of 1e−05

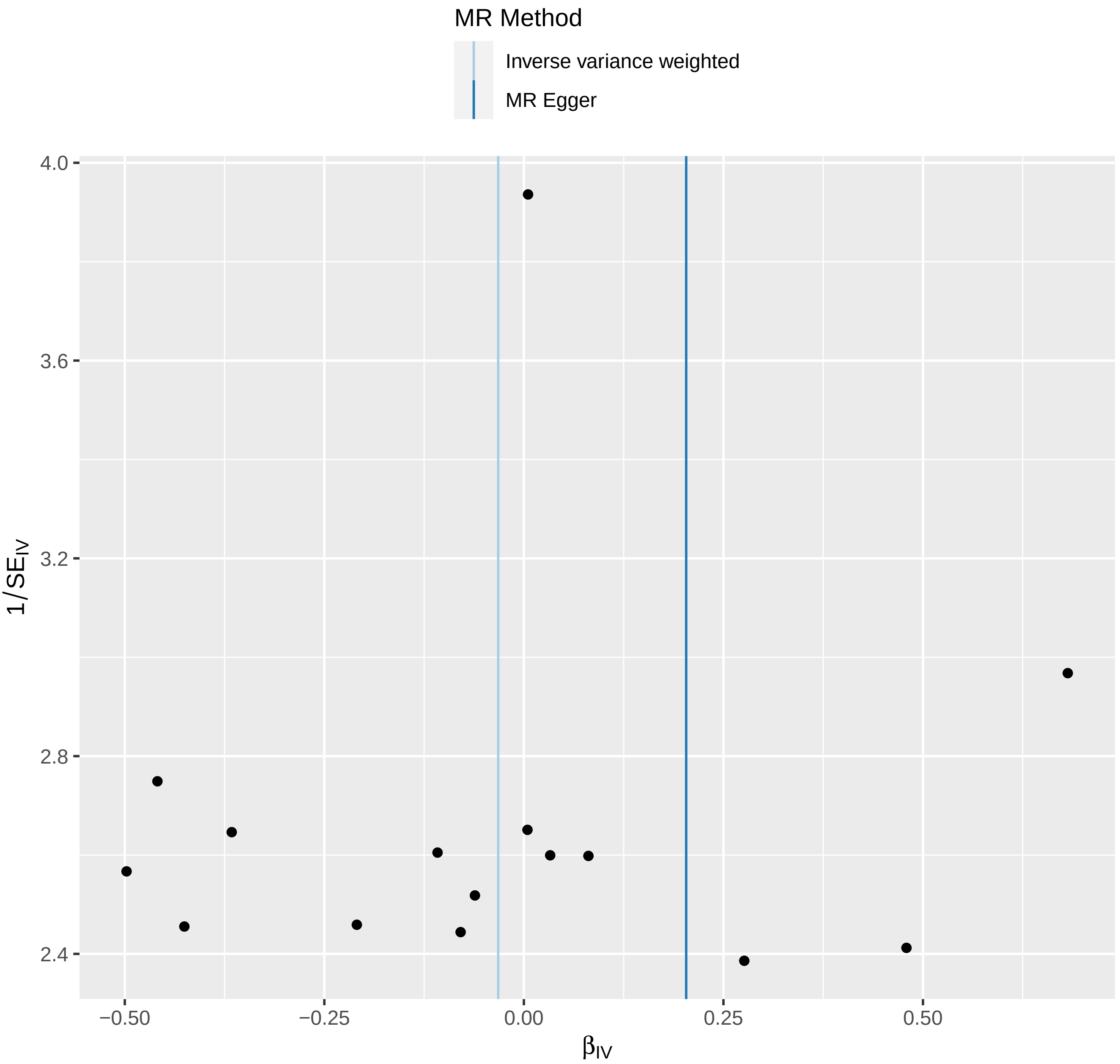

Batch 2−5953 : Gut microbiota abundance (genus Catenibacterium id.2153) on Thymocyte nuclear protein 1
with a threshold of 1e−05

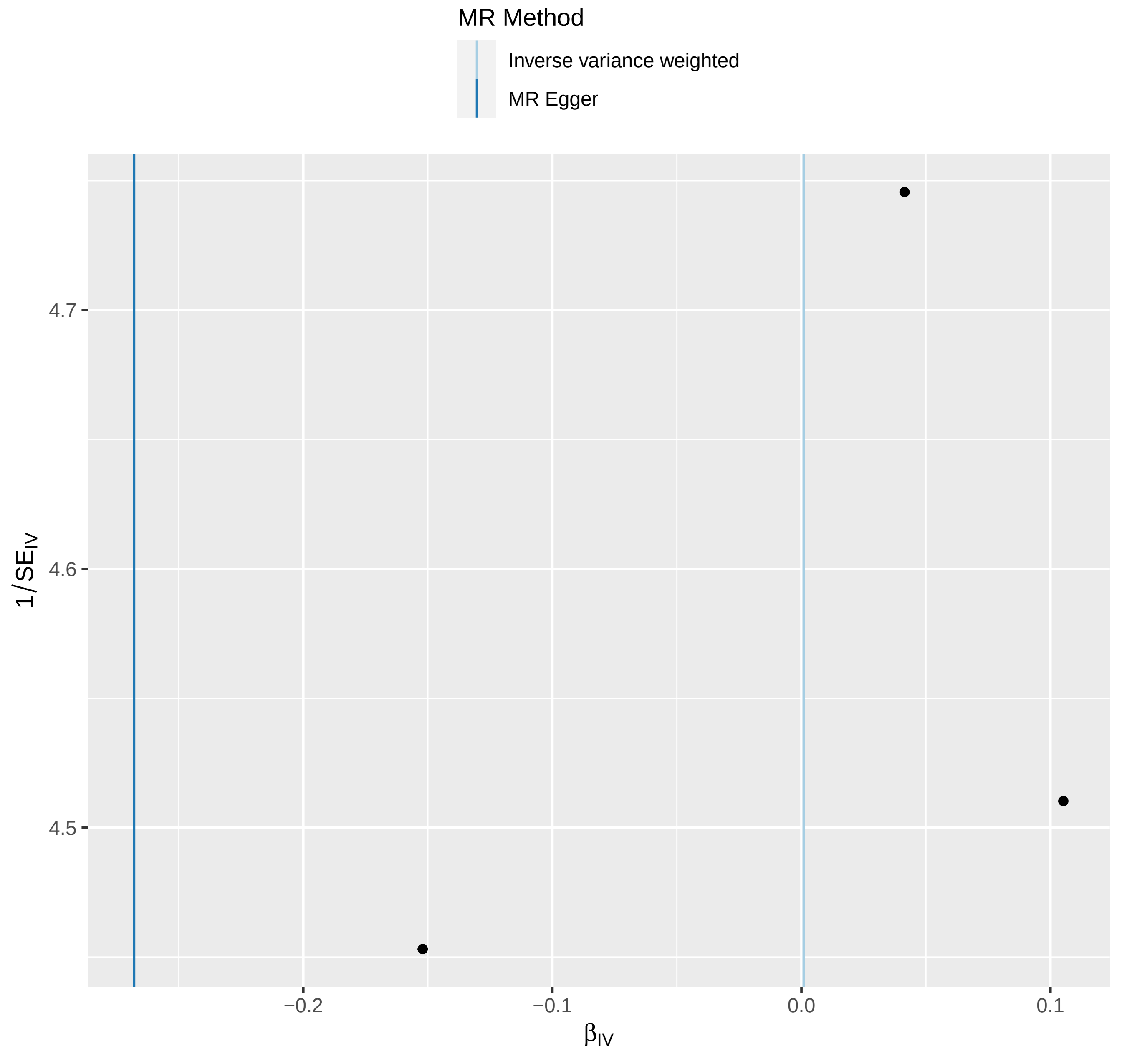

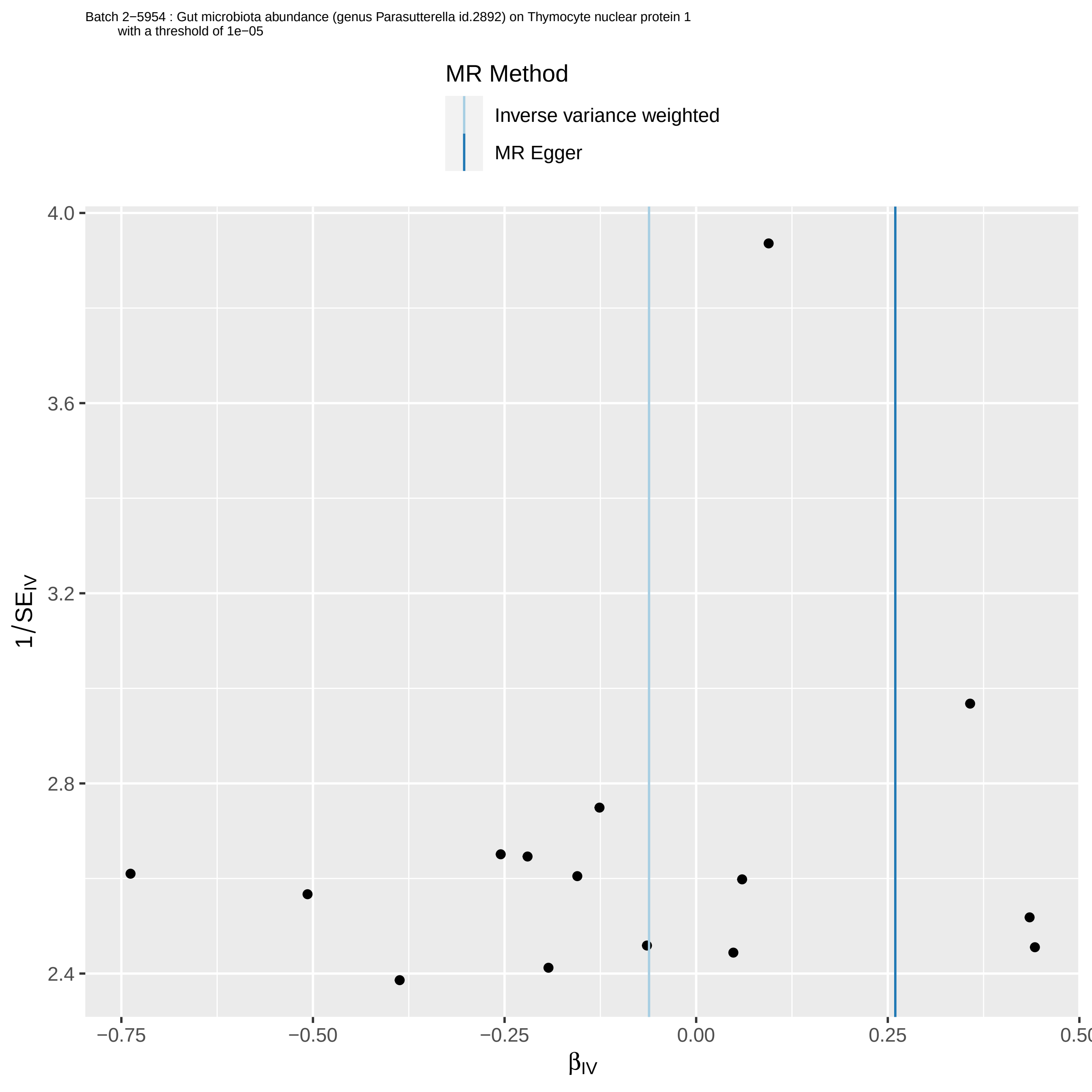
Batch 2−5954 : Gut microbiota abundance (genus Parasutterella id.2892) on Thymocyte nuclear protein 1
with a threshold of 1e−05
MR Method
Inverse variance weighted
MR Egger
4.0
3.6
3.2
2.8
2.4
1/SE_IV
−0.75
−0.50
−0.25
0.00
0.25
0.50
β_IV

Batch 2−5955 : Gut microbiota abundance (genus Catenibacterium id.2153) on Tyrosine−protein kinase receptor Tie−1, soluble with a threshold of 1e−05

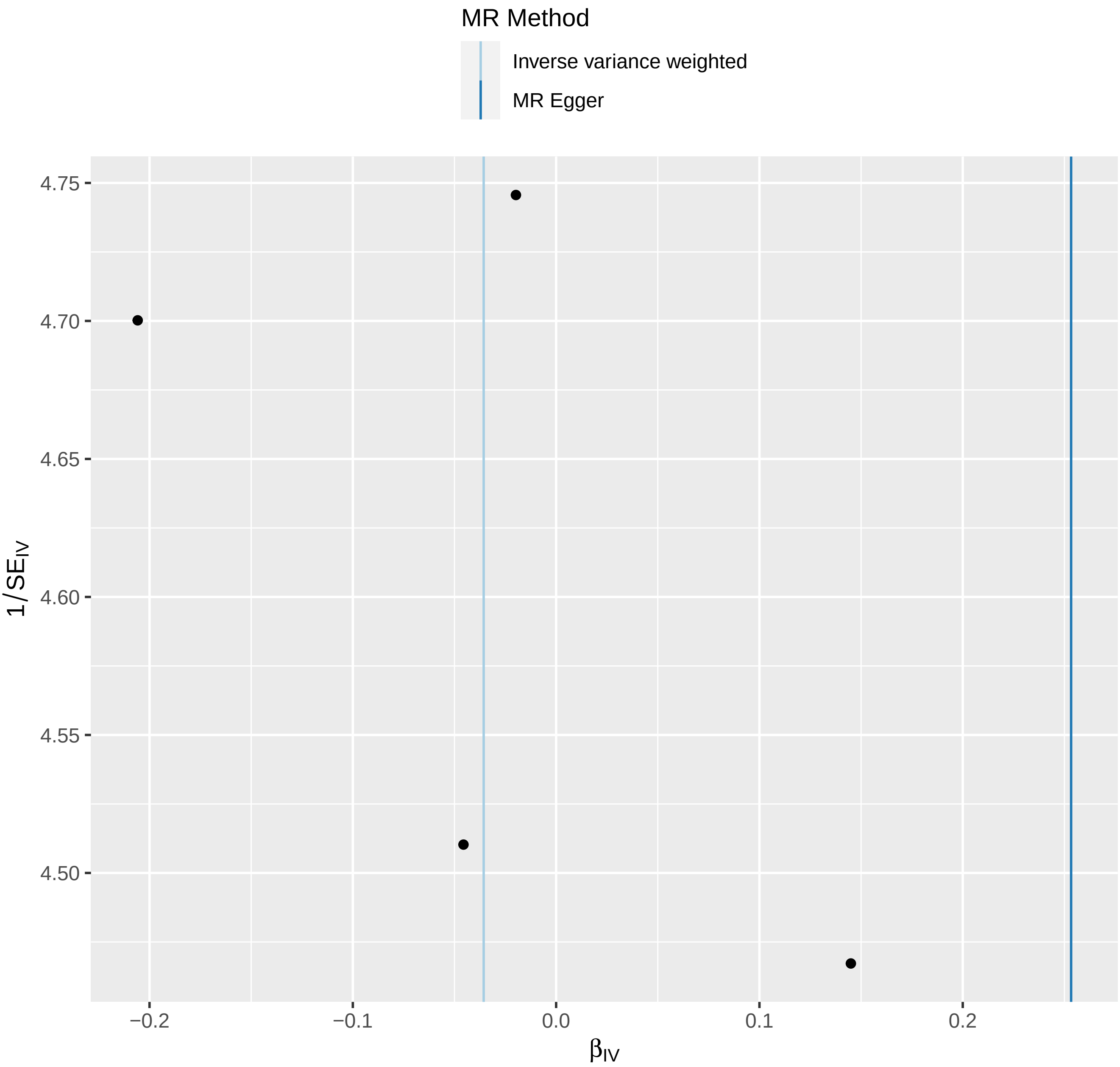

Batch 2−5956 : Gut microbiota abundance (genus Parasutterella id.2892) on Tyrosine−protein kinase receptor Tie−1, soluble
with a threshold of 1e−05

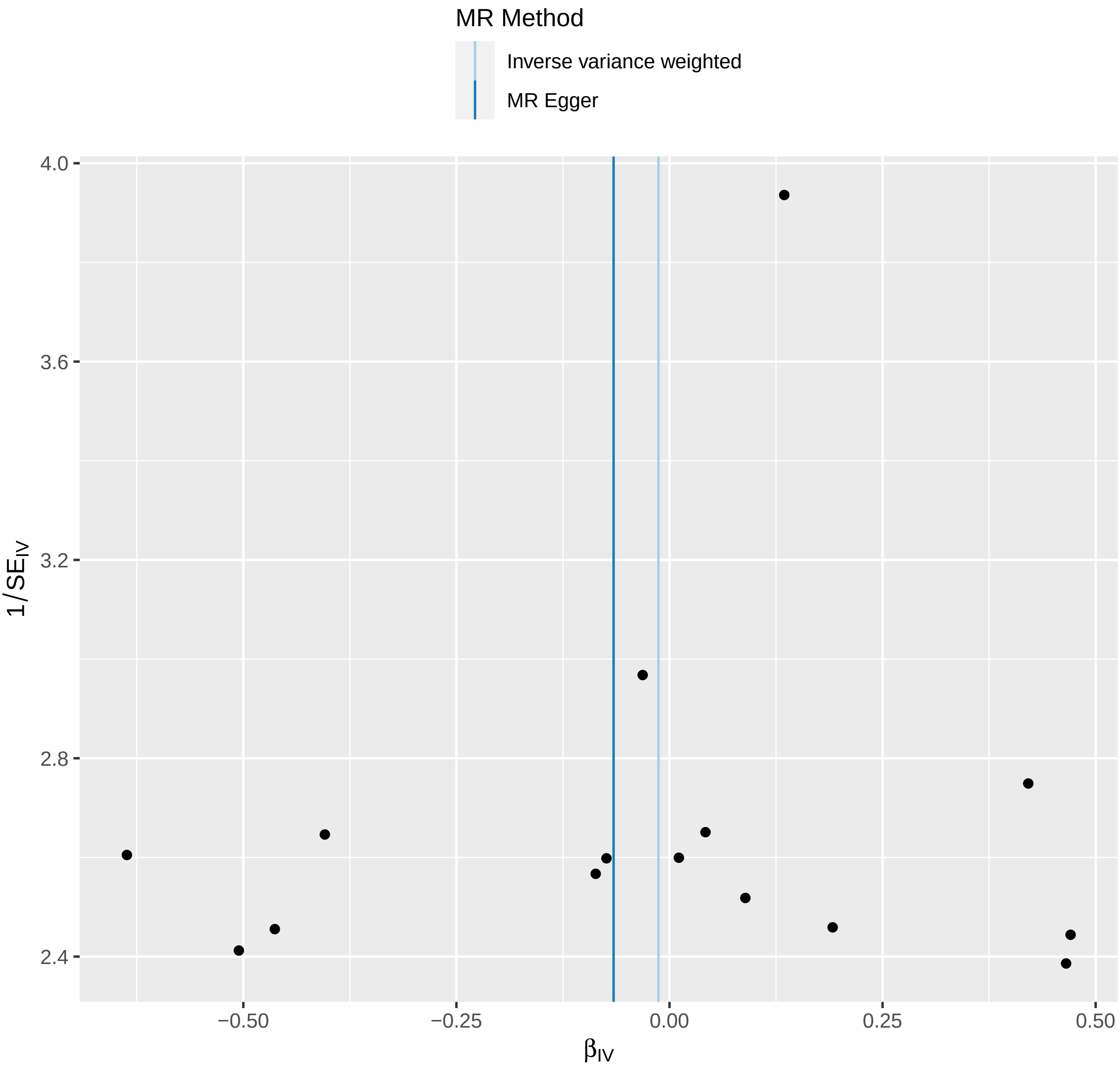

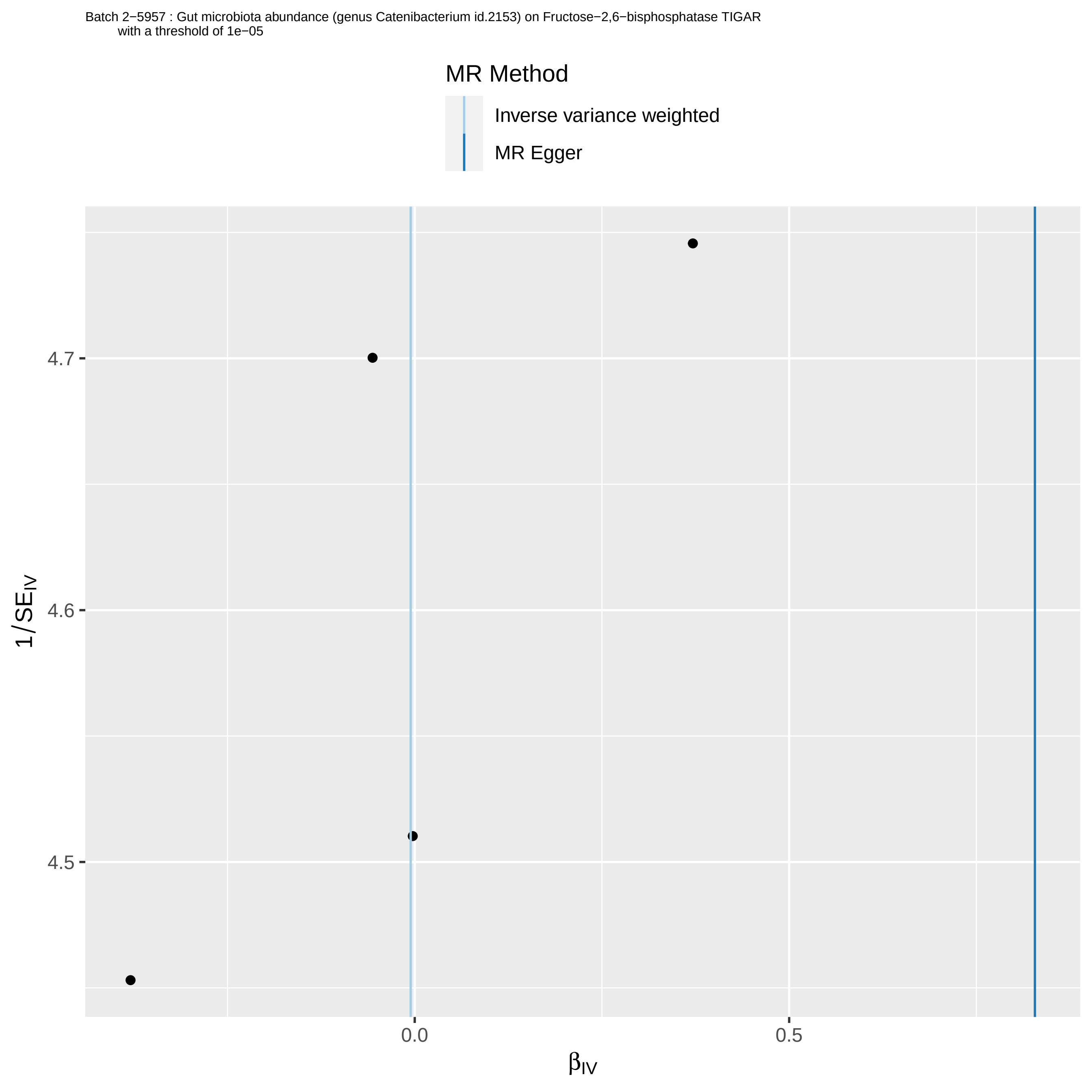

Batch 2−5957 : Gut microbiota abundance (genus Catenibacterium id.2153) on Fructose−2,6−bisphosphatase TIGAR
with a threshold of 1e−05
MR Method
Inverse variance weighted
MR Egger
4.7
4.6
4.5
0.0
0.5
1/SE_IV
β_IV

Batch 2−5958 : Gut microbiota abundance (genus Parasutterella id.2892) on Fructose−2,6−bisphosphatase TIGAR
with a threshold of 1e−05

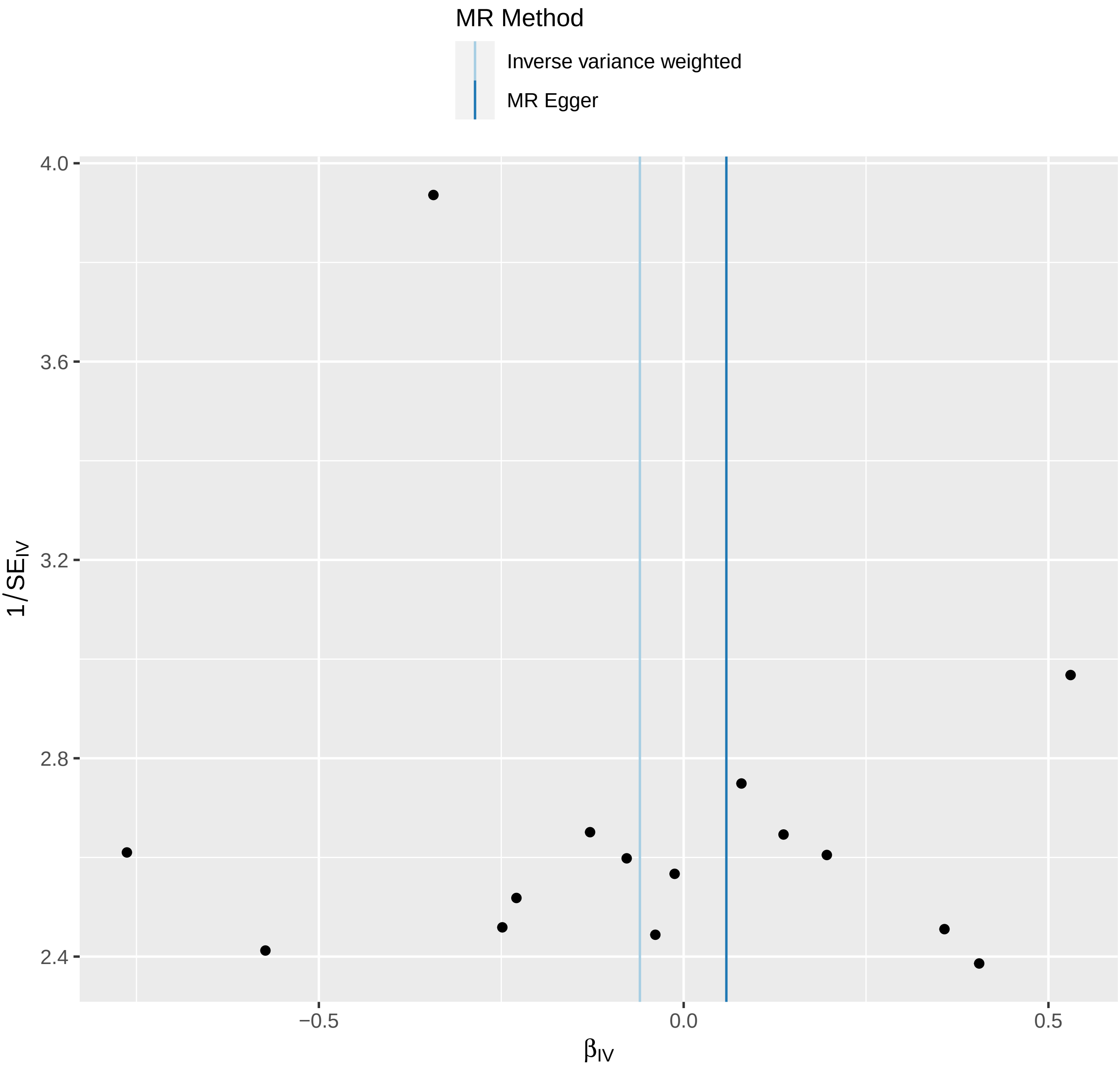

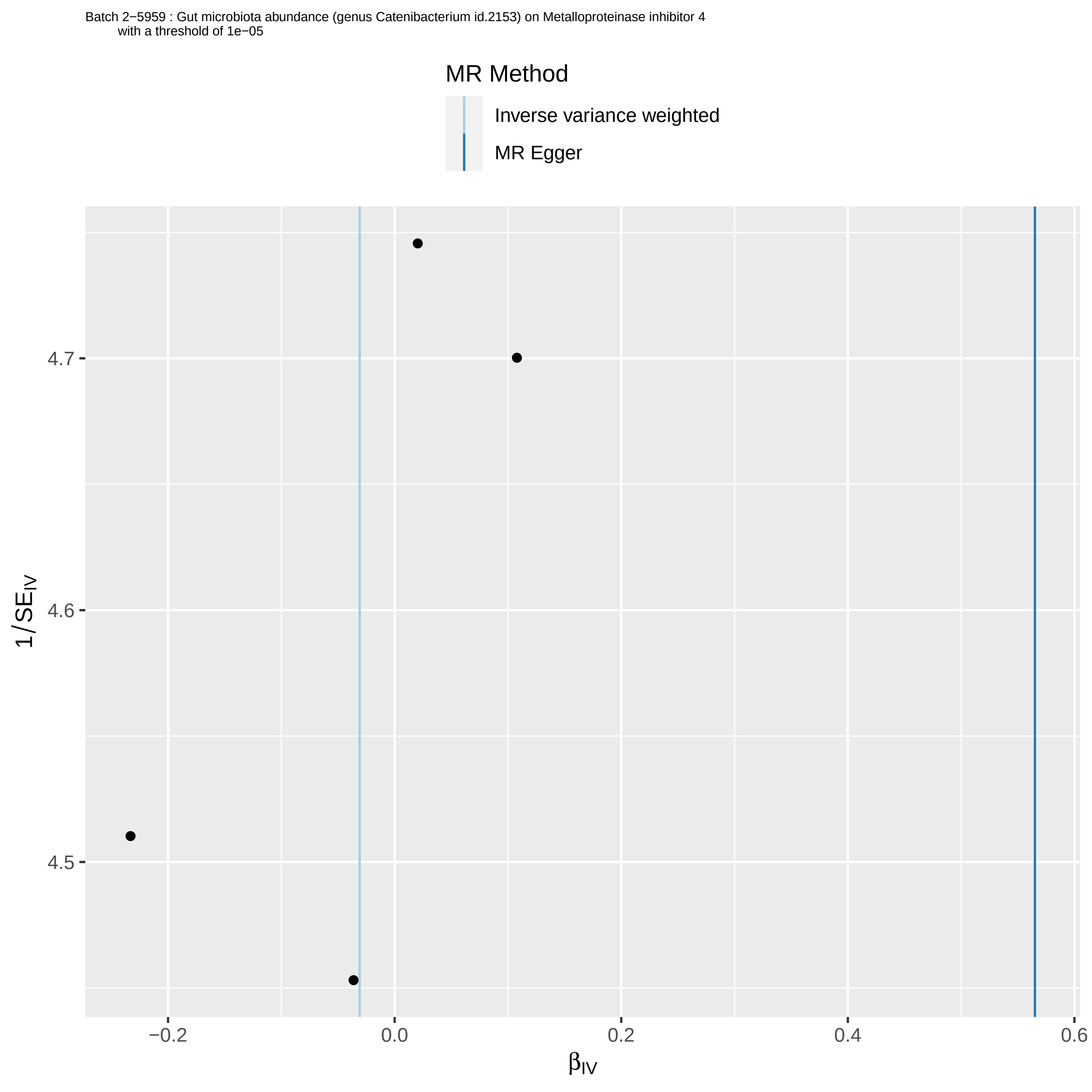
Batch 2−5959 : Gut microbiota abundance (genus Catenibacterium id.2153) on Metalloproteinase inhibitor 4
with a threshold of 1e−05
MR Method
Inverse variance weighted
MR Egger
4.7
4.6
4.5
1/SE$_{IV}$
−0.2
0.0
0.2
0.4
0.6
β$_{IV}$

Batch 2−5960 : Gut microbiota abundance (genus Parasutterella id.2892) on Metalloproteinase inhibitor 4
with a threshold of 1e−05

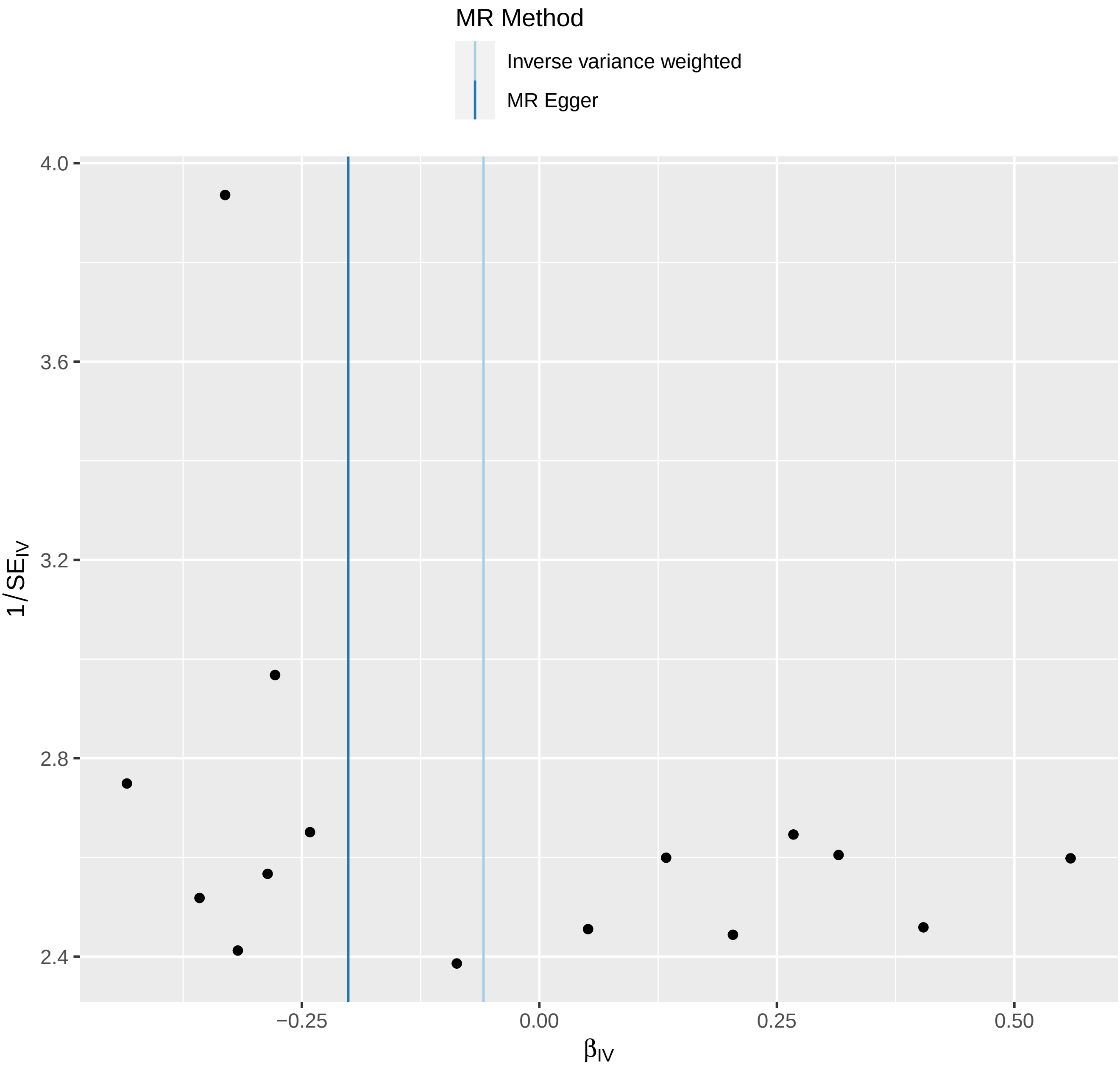

Batch 2−5961 : Gut microbiota abundance (genus Catenibacterium id.2153) on Tubulointerstitial nephritis antigen−like with a threshold of 1e−05

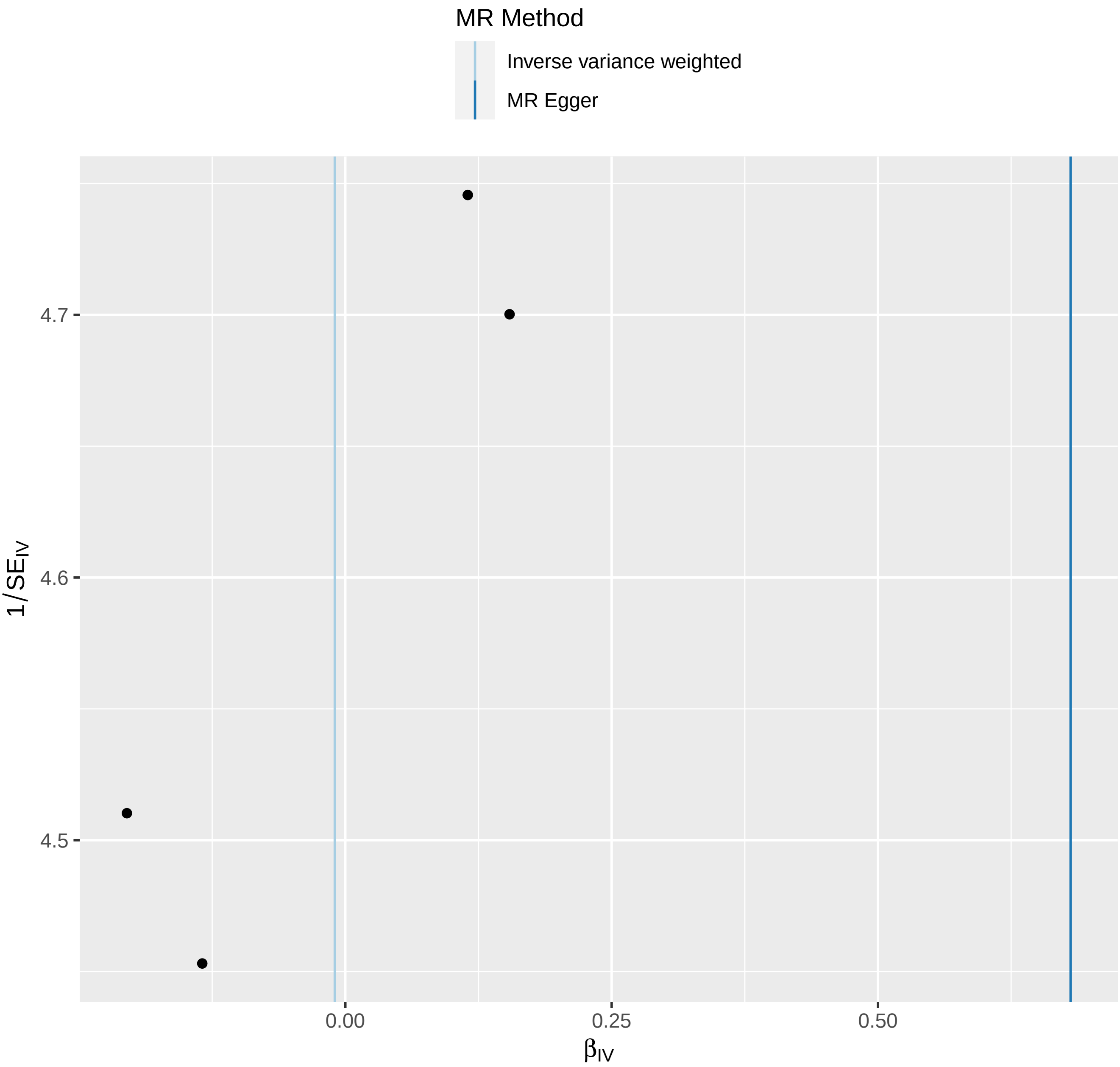

Batch 2−5962 : Gut microbiota abundance (genus Parasutterella id.2892) on Tubulointerstitial nephritis antigen−like
with a threshold of 1e−05

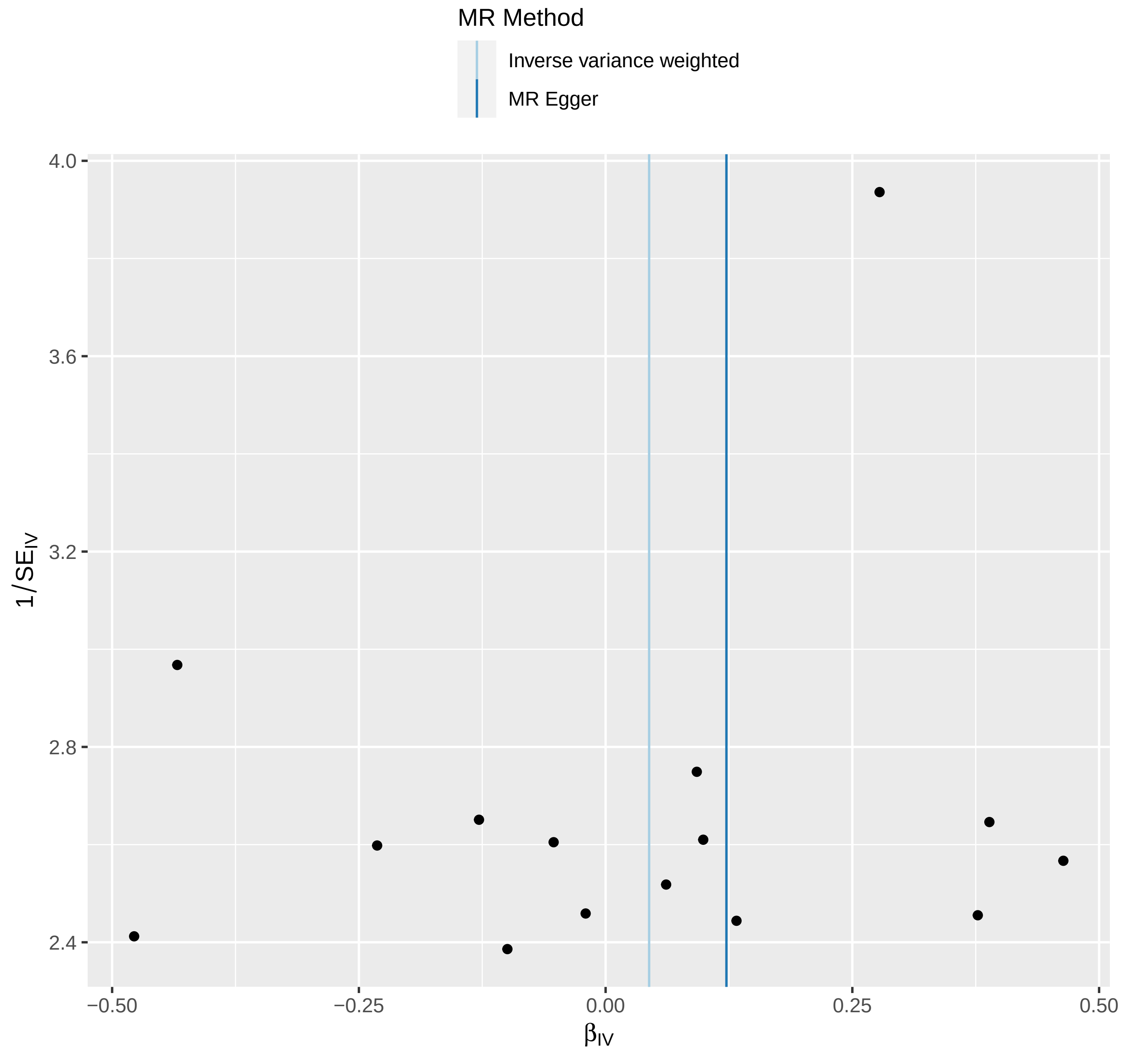

Batch 2−5963 : Gut microbiota abundance (genus Catenibacterium id.2153) on Toll/interleukin−1 receptor domain−containing adapter protein with a threshold of 1e−05

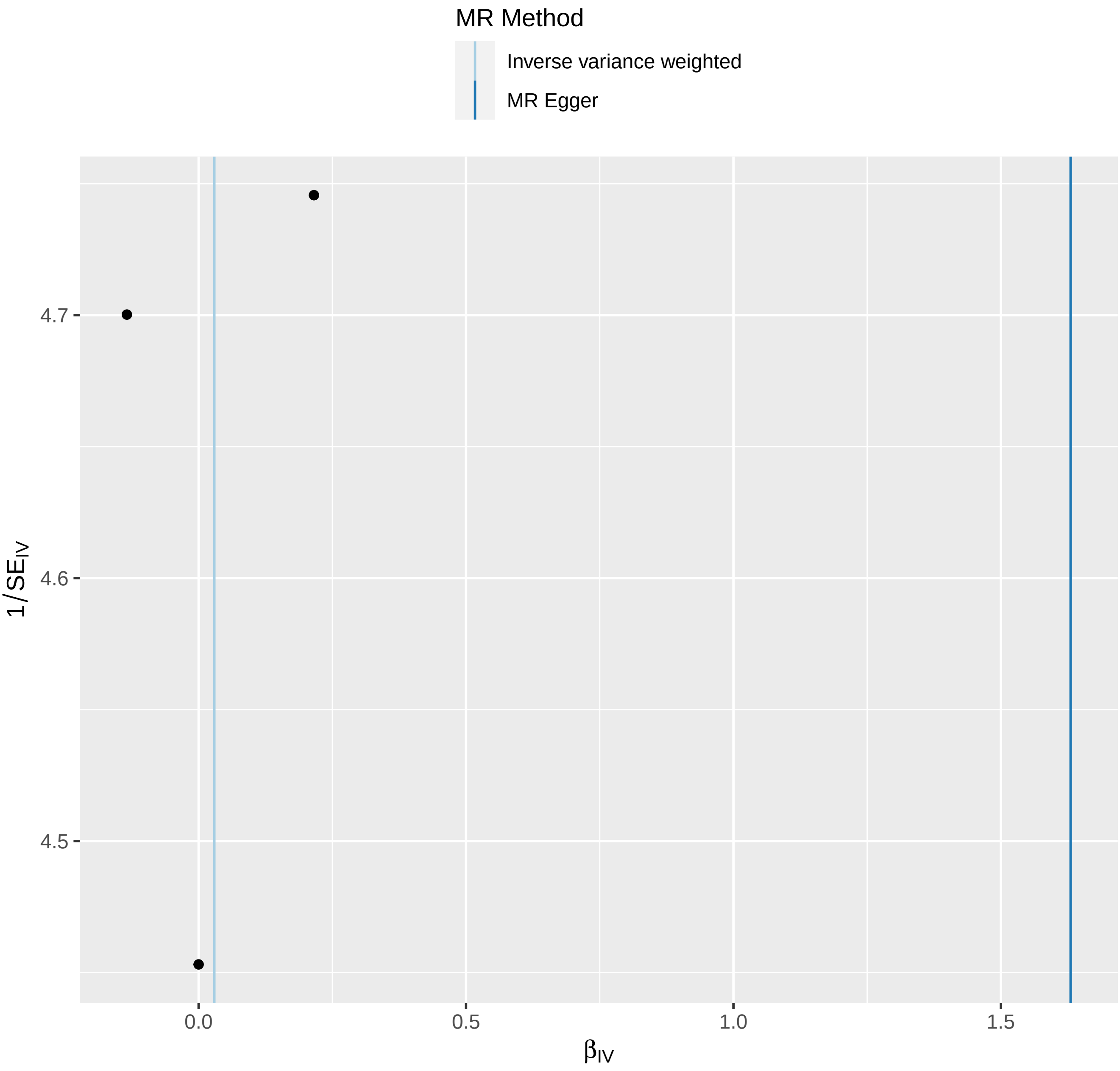

Batch 2−5964 : Gut microbiota abundance (genus Parasutterella id.2892) on Toll/interleukin−1 receptor domain−containing adapter protein with a threshold of 1e−05

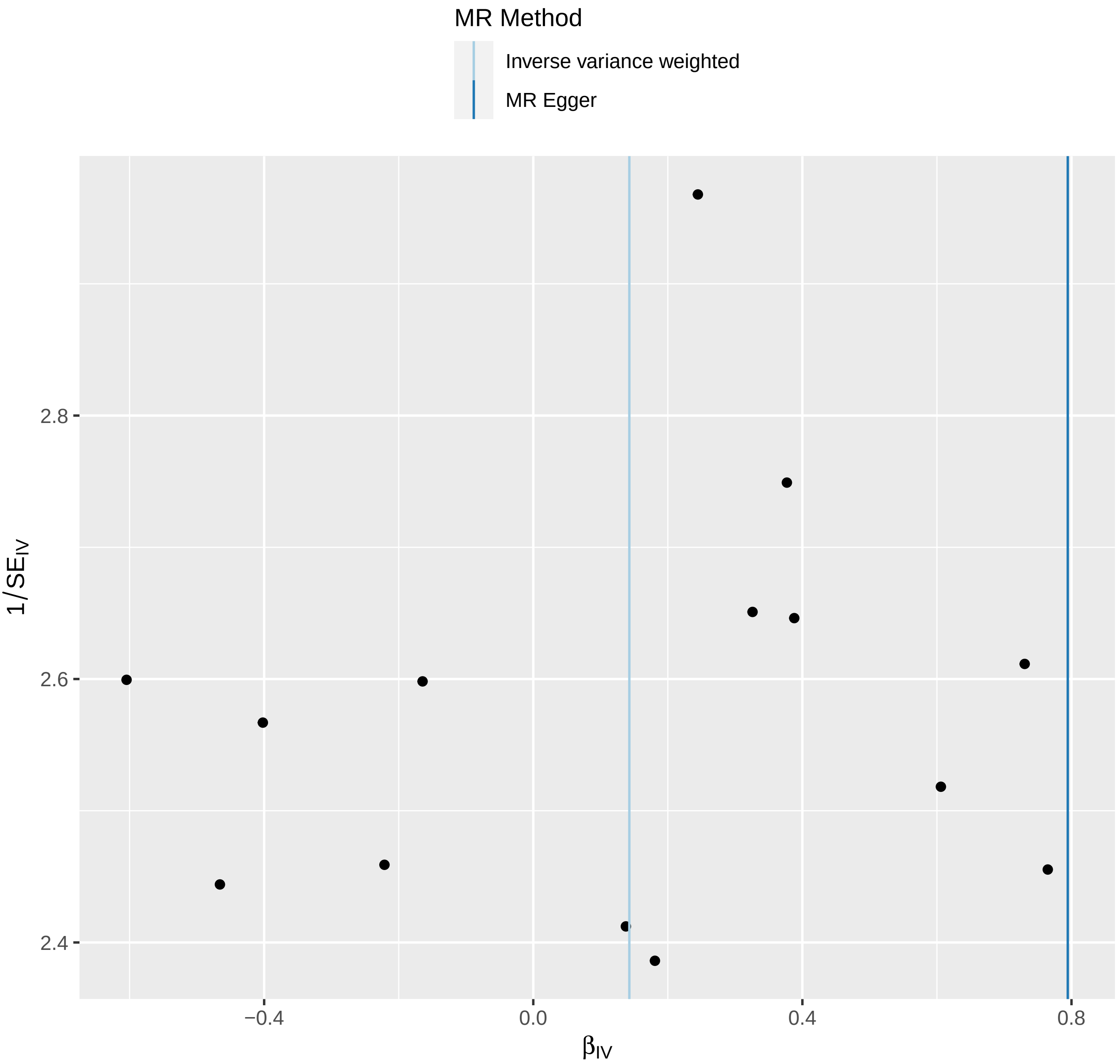

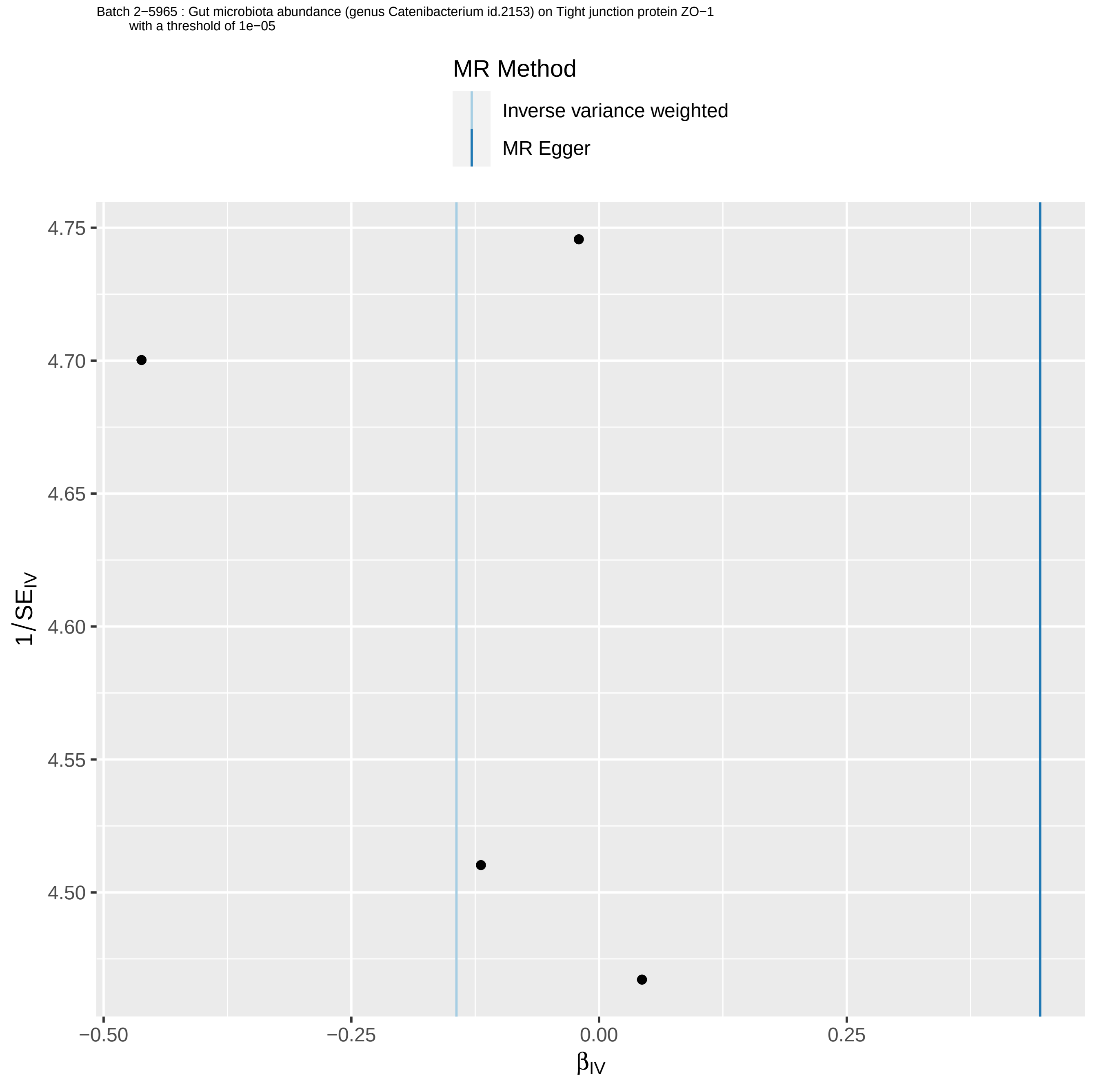

Batch 2−5965 : Gut microbiota abundance (genus Catenibacterium id.2153) on Tight junction protein ZO−1
with a threshold of 1e−05
MR Method
Inverse variance weighted
MR Egger
4.75
4.70
4.65
4.60
4.55
4.50
1/SE$_{IV}$
−0.50
−0.25
0.00
0.25
β_{IV}

Batch 2−5966 : Gut microbiota abundance (genus Parasutterella id.2892) on Tight junction protein ZO−1
with a threshold of 1e−05

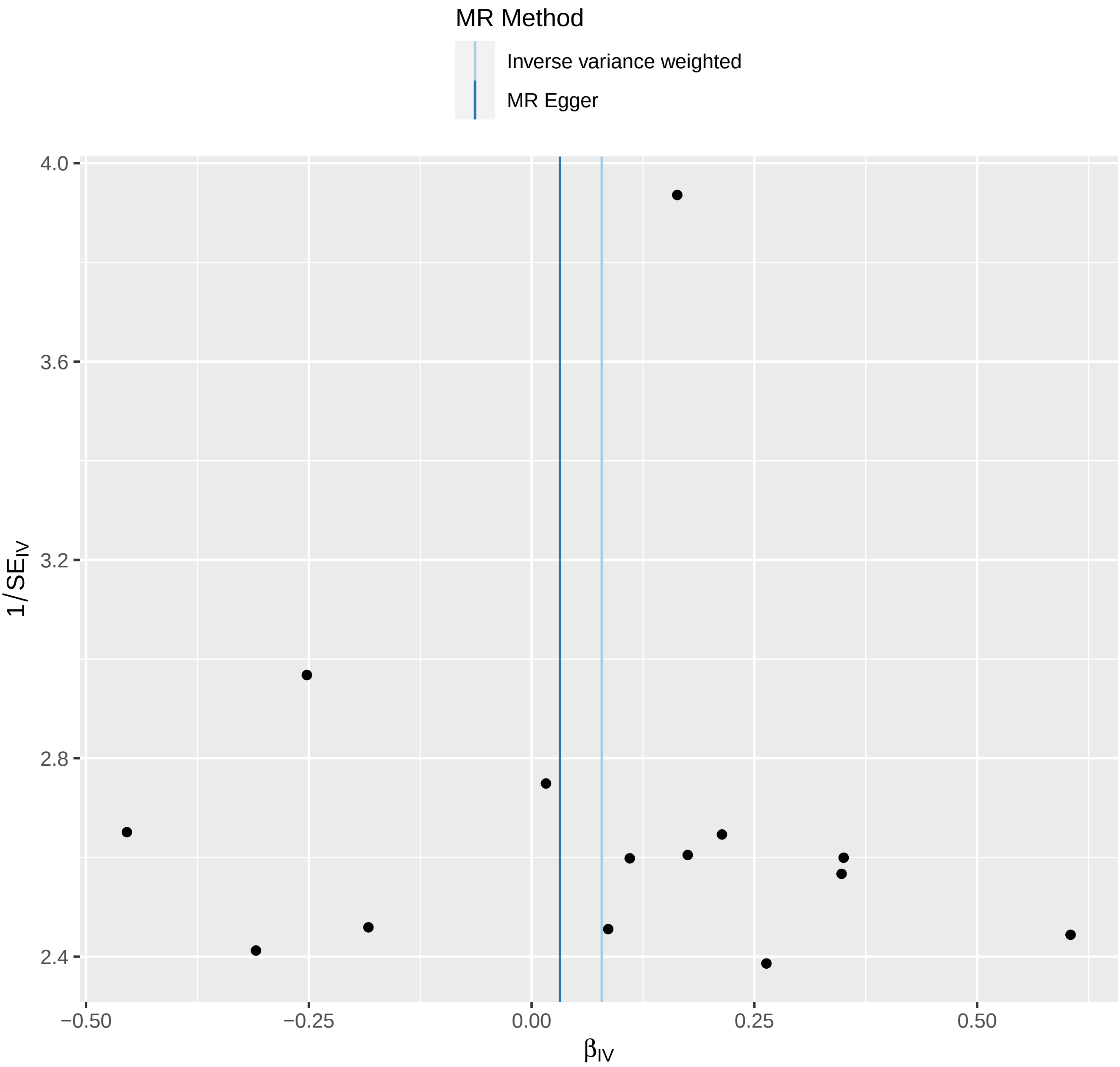

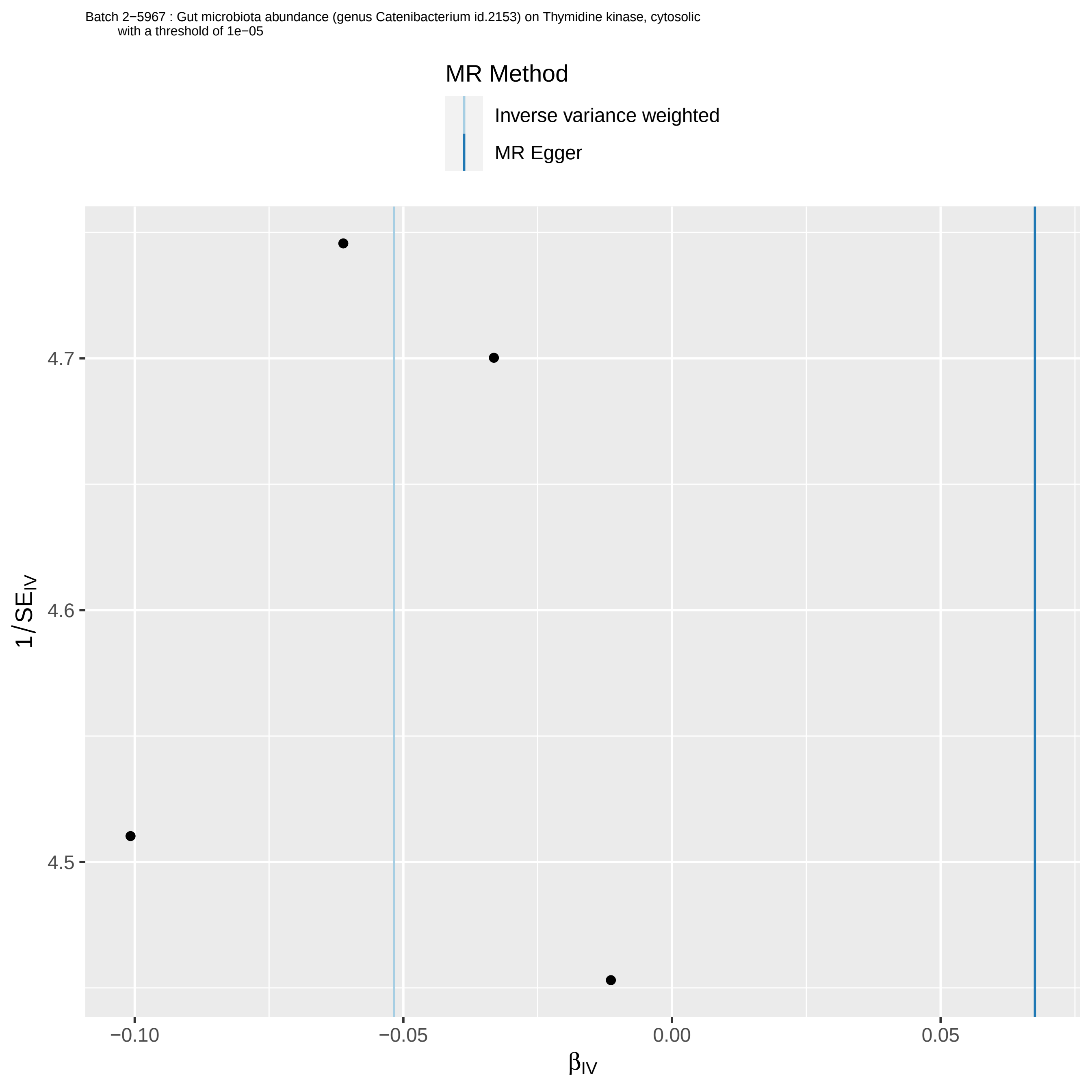
Batch 2−5967 : Gut microbiota abundance (genus Catenibacterium id.2153) on Thymidine kinase, cytosolic
with a threshold of 1e−05
MR Method
Inverse variance weighted
MR Egger
4.7
4.6
4.5
1/SE_IV
−0.10
−0.05
0.00
0.05
β_IV

Batch 2−5968 : Gut microbiota abundance (genus Parasutterella id.2892) on Thymidine kinase, cytosolic
with a threshold of 1e−05

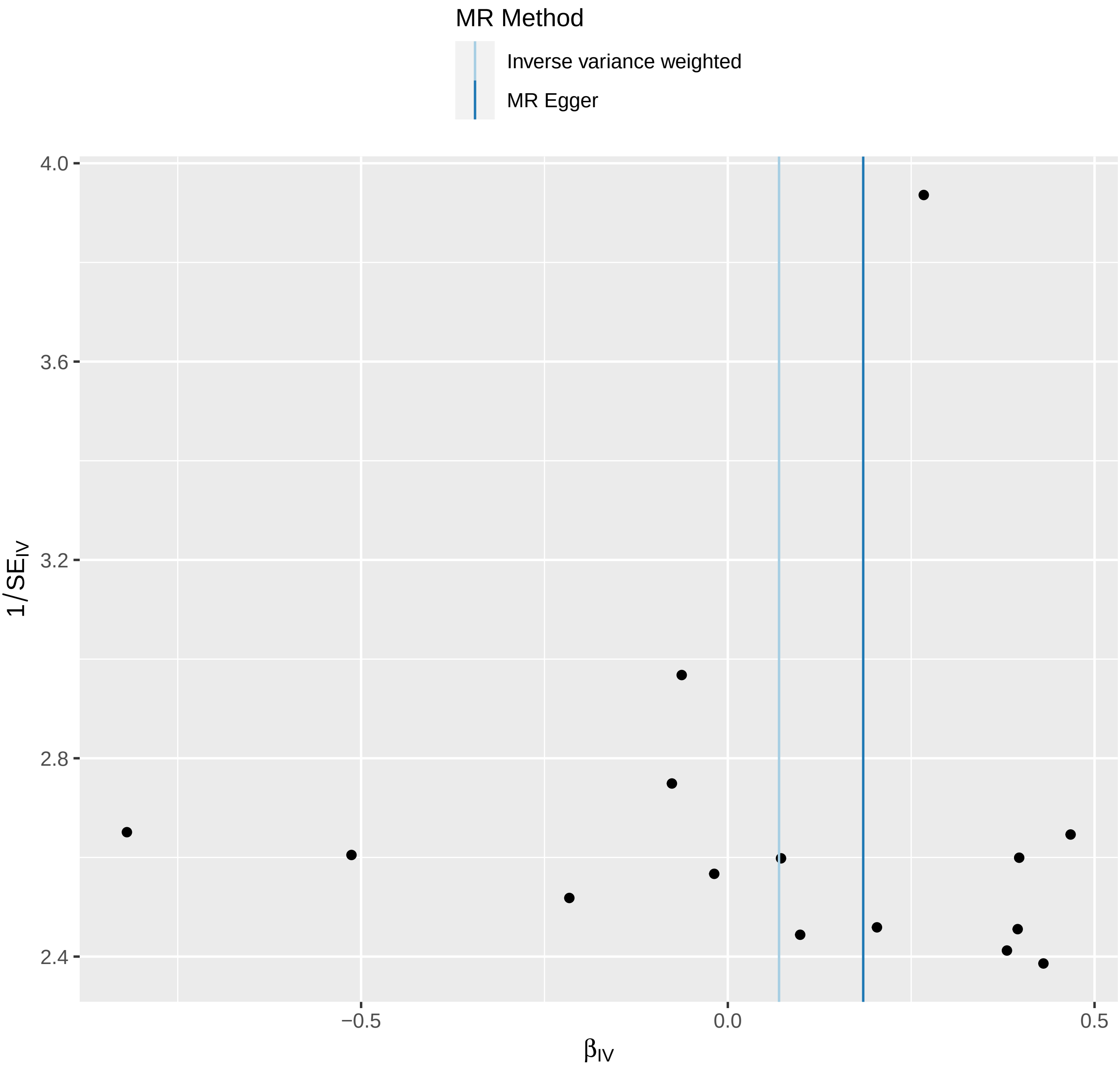

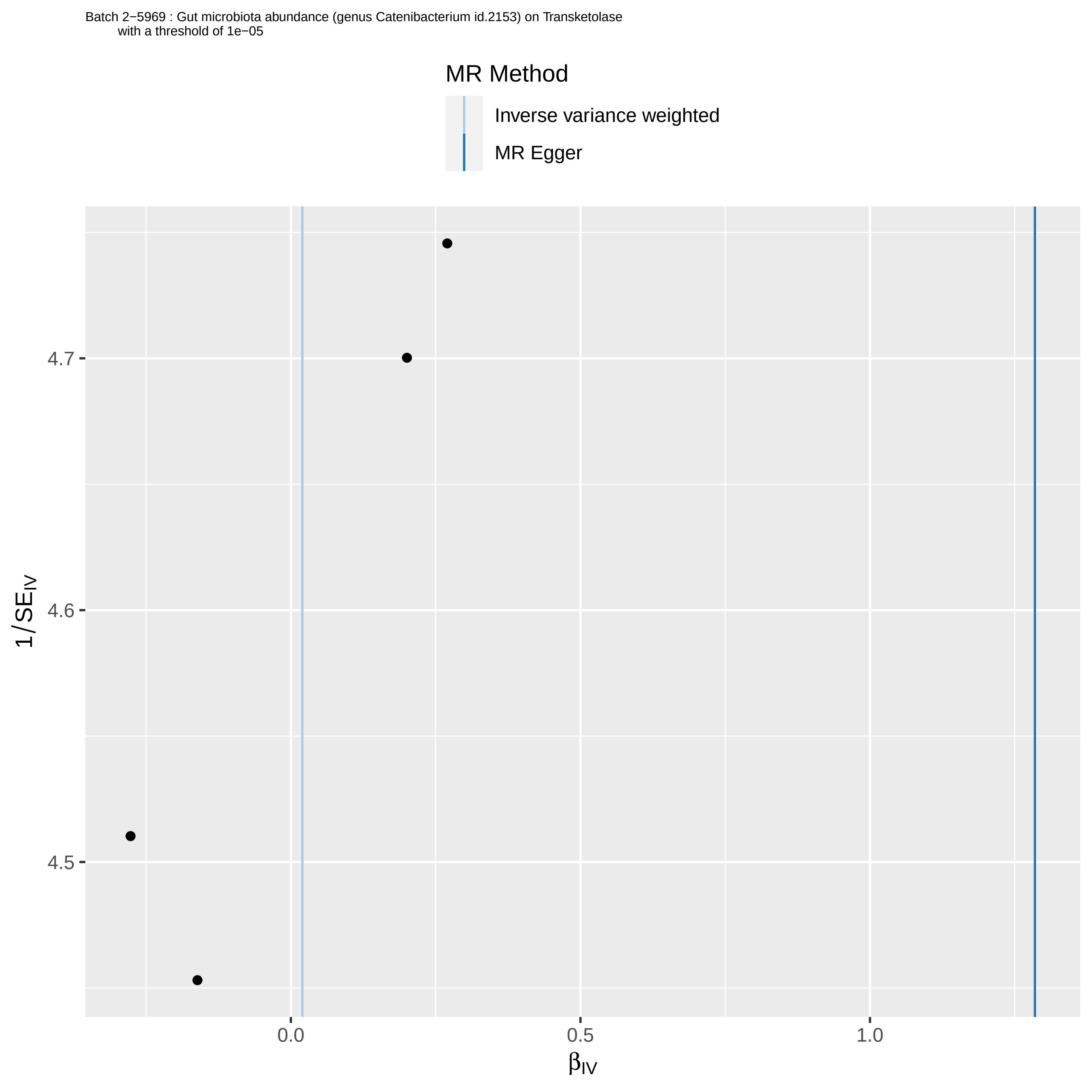

Batch 2−5969 : Gut microbiota abundance (genus Catenibacterium id.2153) on Transketolase
with a threshold of 1e−05
MR Method
Inverse variance weighted
MR Egger
4.7
4.6
4.5
1/SE$_{IV}$
0.0
0.5
1.0
β$_{IV}$

Batch 2−5970 : Gut microbiota abundance (genus Parasutterella id.2892) on Transketolase
with a threshold of 1e−05

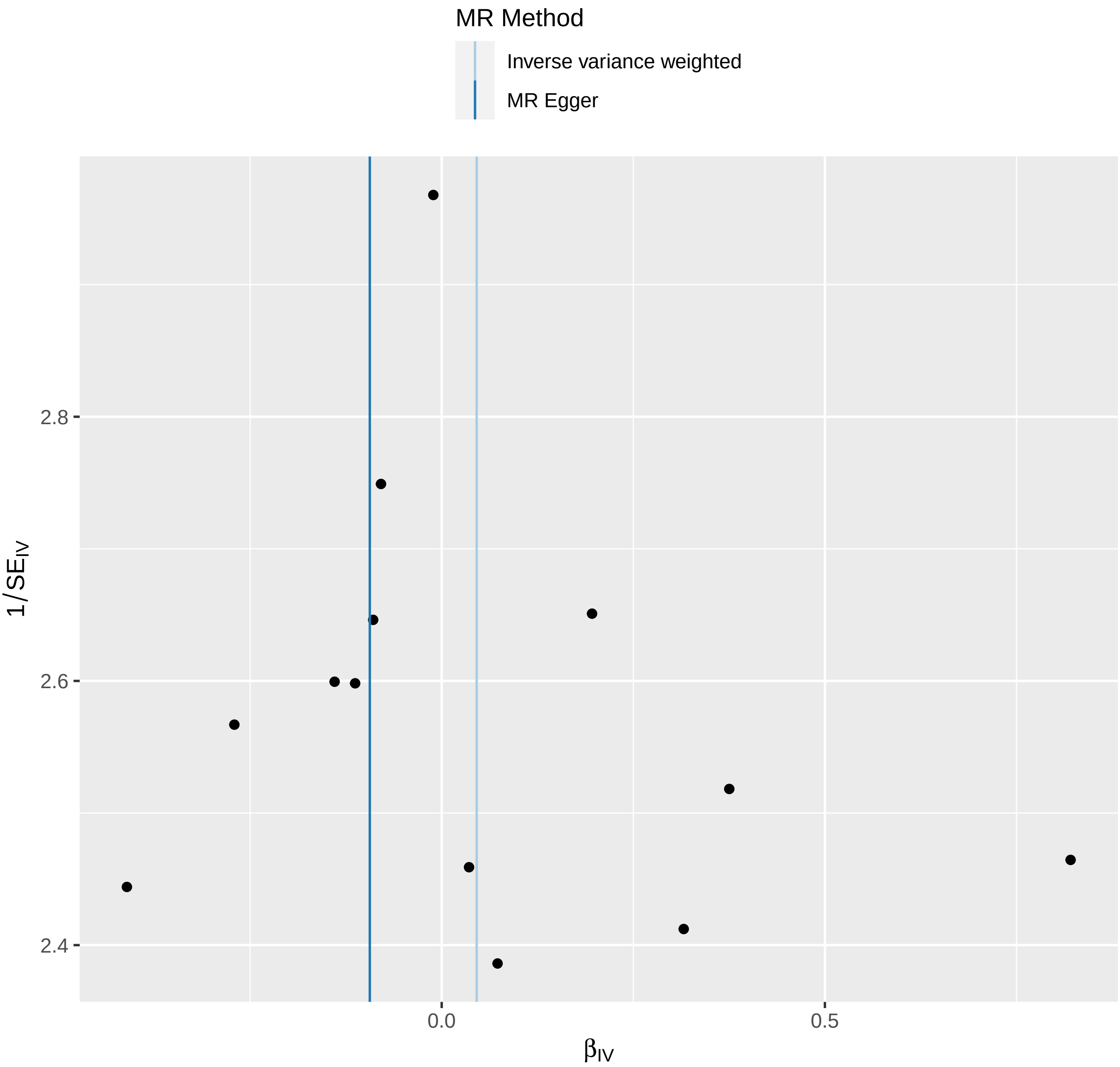

Batch 2−5971 : Gut microbiota abundance (genus Catenibacterium id.2153) on Tolloid−like protein 1
with a threshold of 1e−05

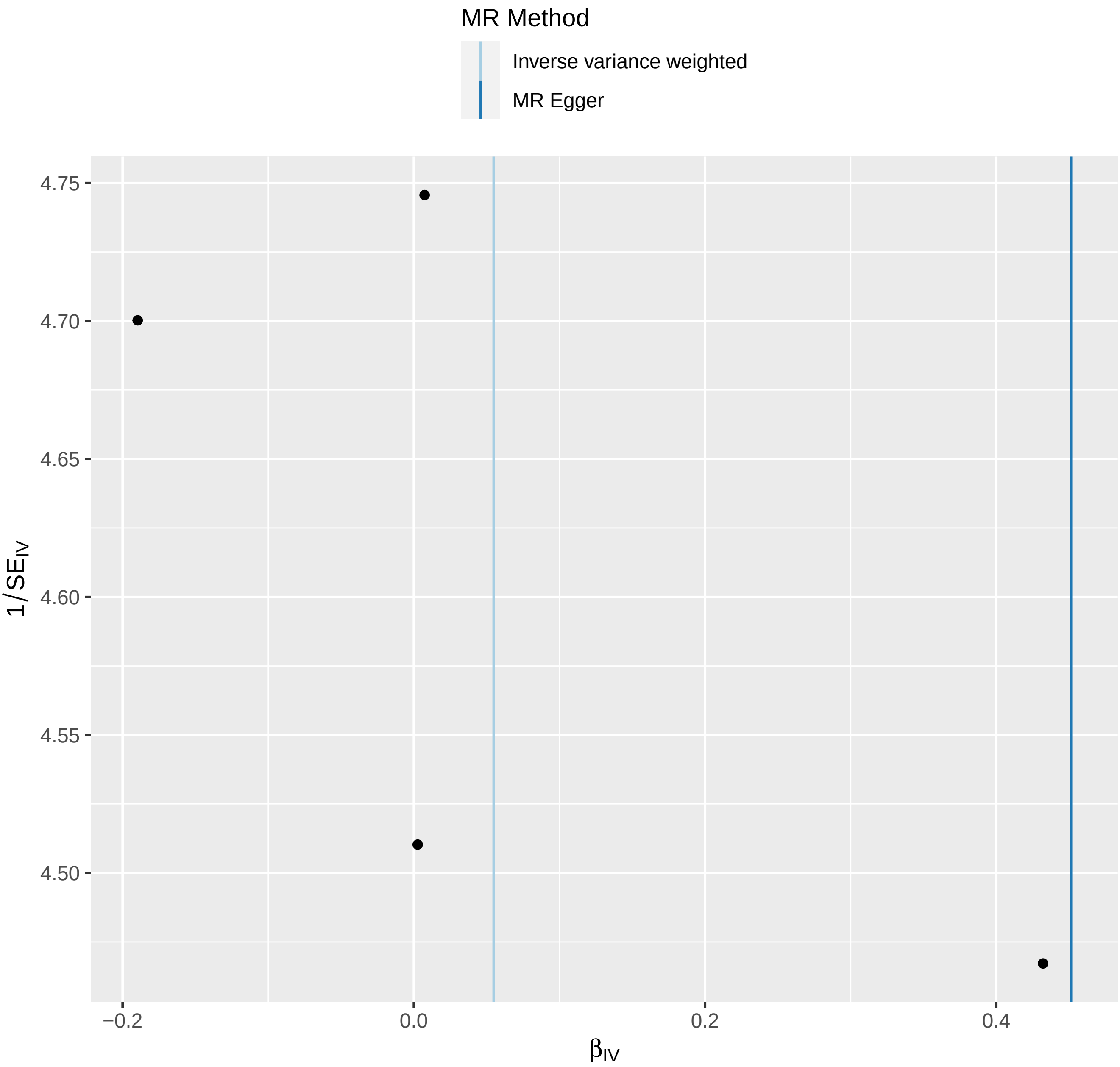

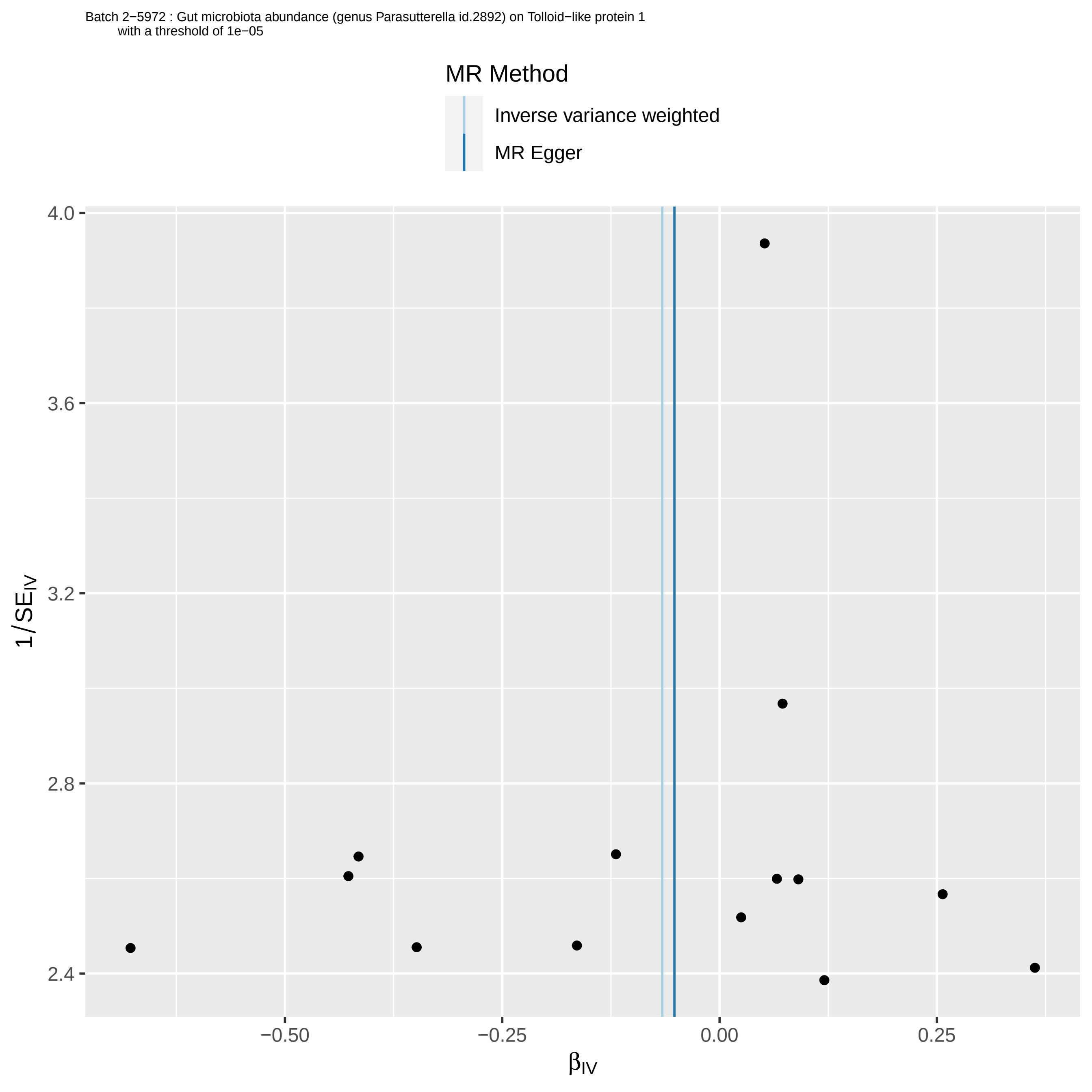
Batch 2−5972 : Gut microbiota abundance (genus Parasutterella id.2892) on Tolloid−like protein 1
with a threshold of 1e−05
MR Method
Inverse variance weighted
MR Egger
4.0
3.6
3.2
2.8
2.4
1/SE_IV
−0.50
−0.25
0.00
0.25
β_IV

Batch 2−5973 : Gut microbiota abundance (genus Catenibacterium id.2153) on Talin−2
with a threshold of 1e−05

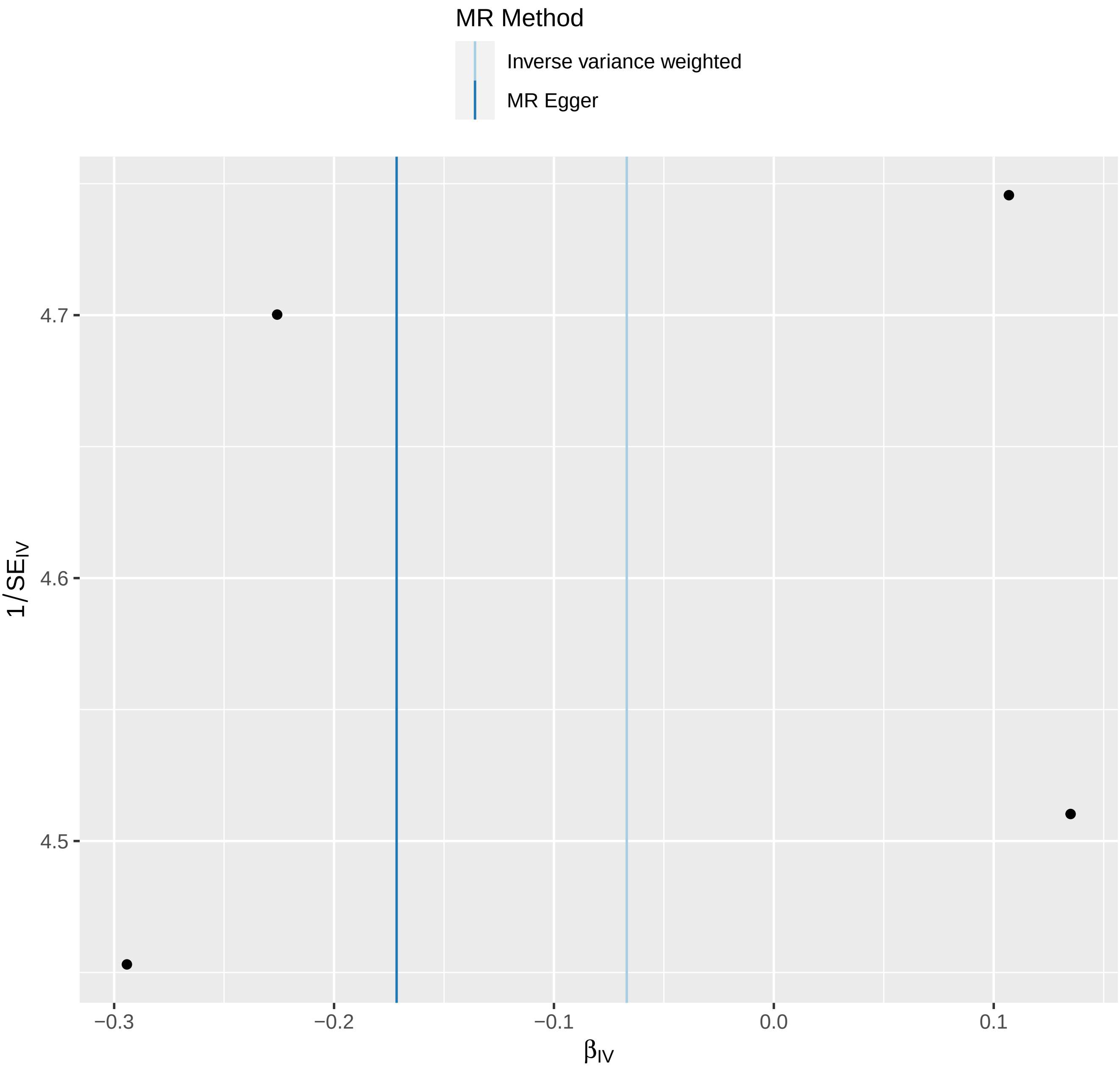

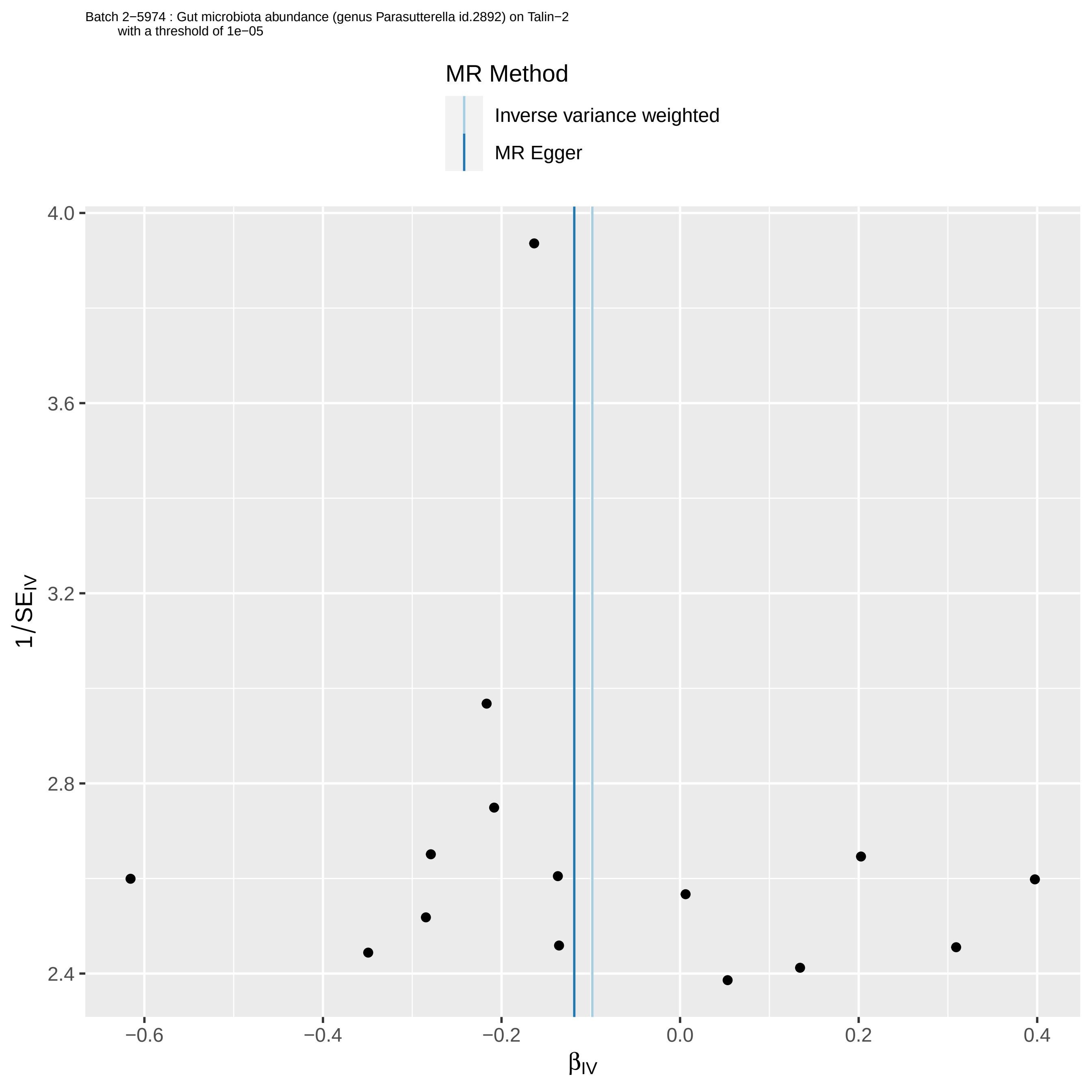

Batch 2−5974 : Gut microbiota abundance (genus Parasutterella id.2892) on Talin−2
with a threshold of 1e−05
MR Method
Inverse variance weighted
MR Egger
4.0
3.6
3.2
2.8
2.4
1/SE_IV
−0.6
−0.4
−0.2
0.0
0.2
0.4
β_IV

Batch 2−5975 : Gut microbiota abundance (genus Catenibacterium id.2153) on Toll−like receptor 1
with a threshold of 1e−05

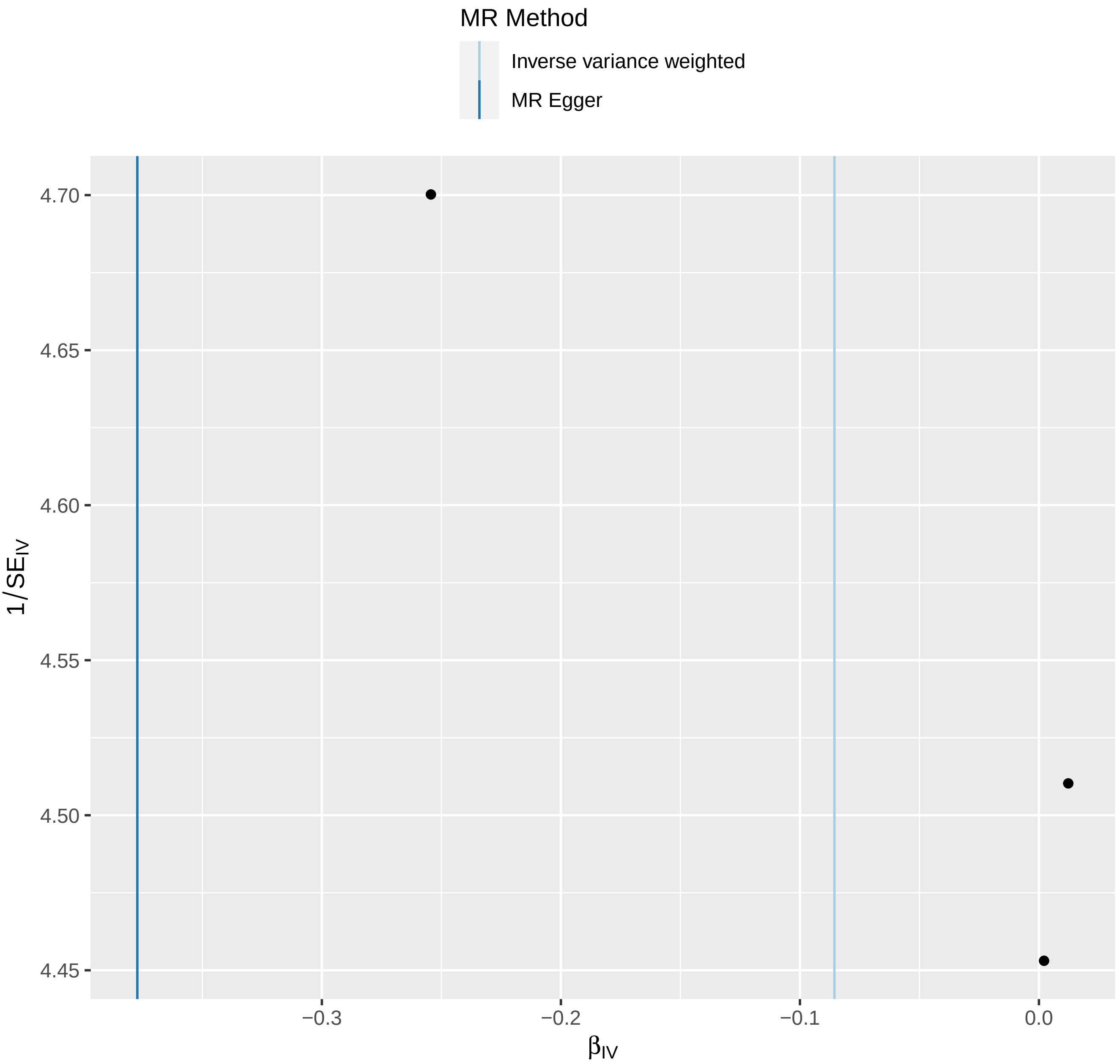

Batch 2−5976 : Gut microbiota abundance (genus Parasutterella id.2892) on Toll−like receptor 1
with a threshold of 1e−05

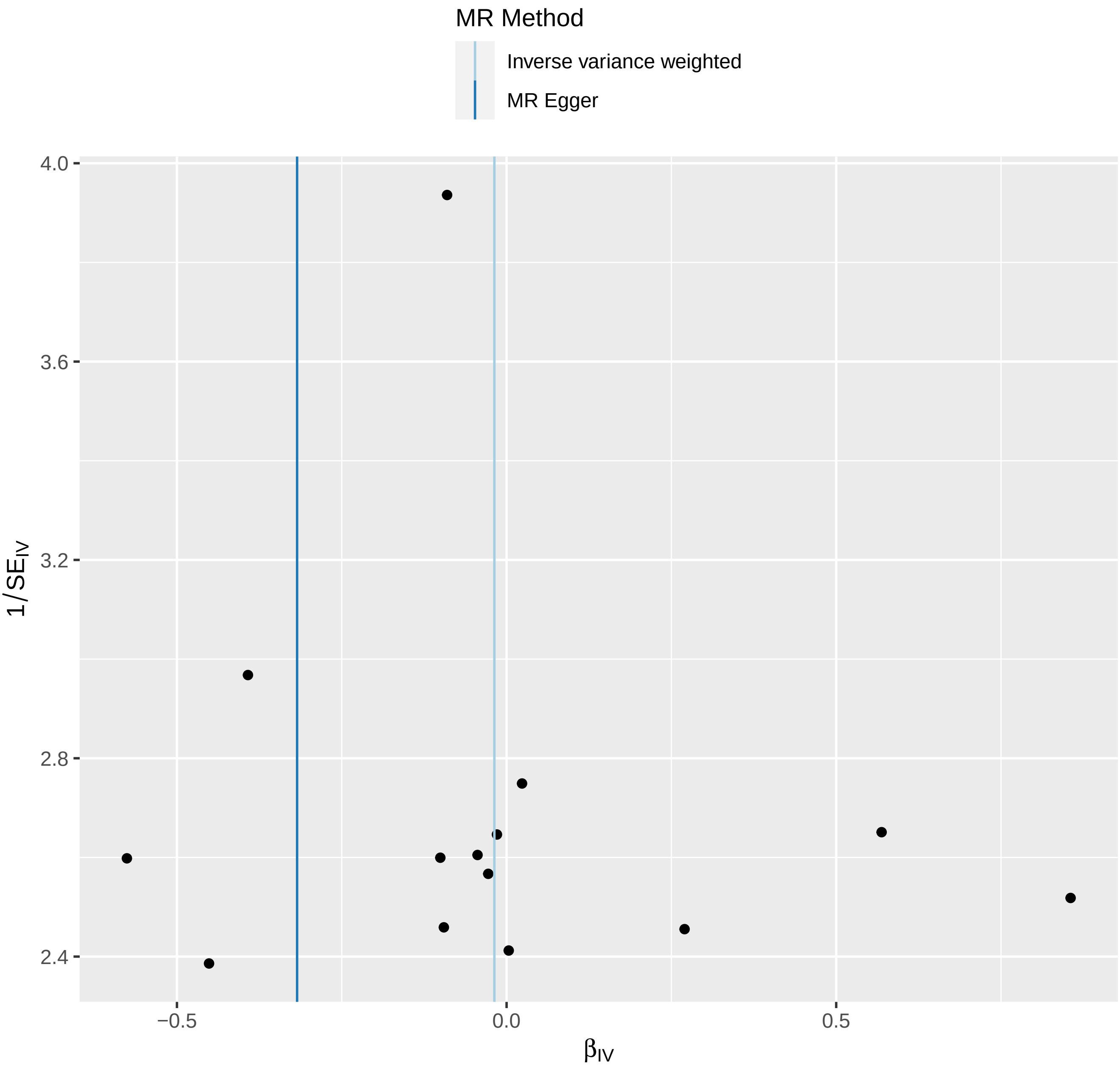

Batch 2−5977 : Gut microbiota abundance (genus Catenibacterium id.2153) on Toll−like receptor 2
with a threshold of 1e−05

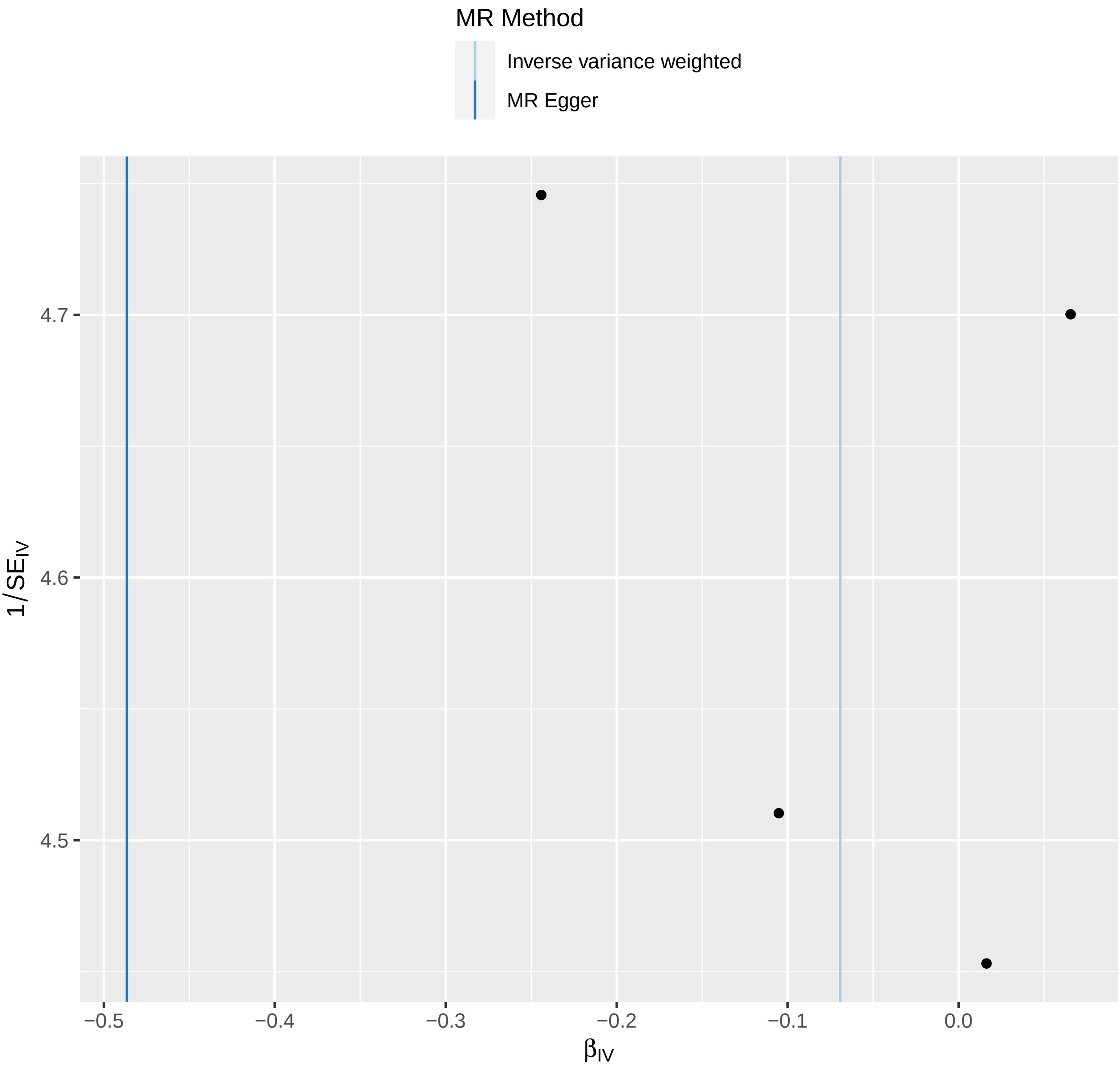

Batch 2−5978 : Gut microbiota abundance (genus Parasutterella id.2892) on Toll−like receptor 2
with a threshold of 1e−05

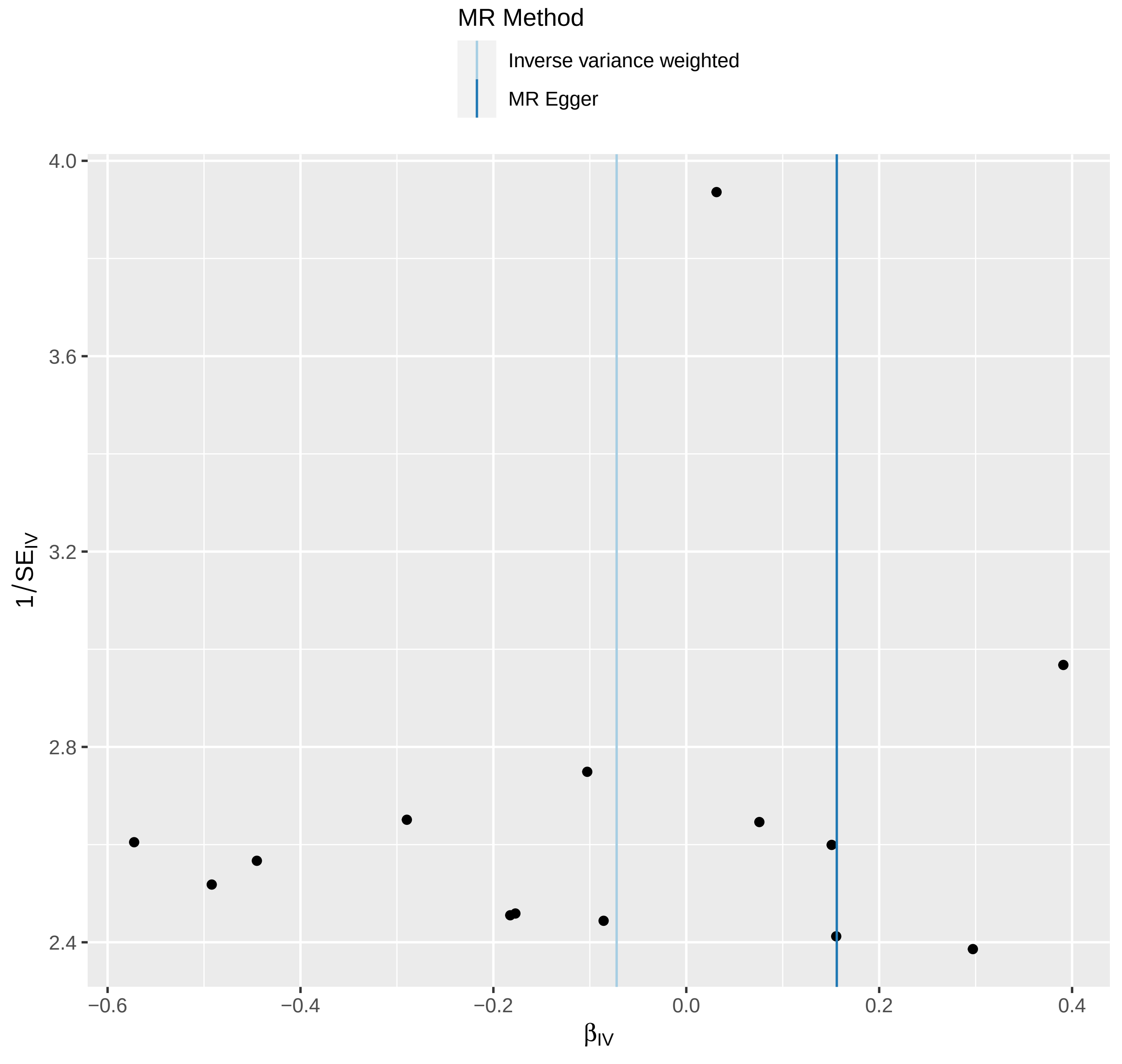

Batch 2−5979 : Gut microbiota abundance (genus Catenibacterium id.2153) on Toll−like receptor 4 with a threshold of 1e−05

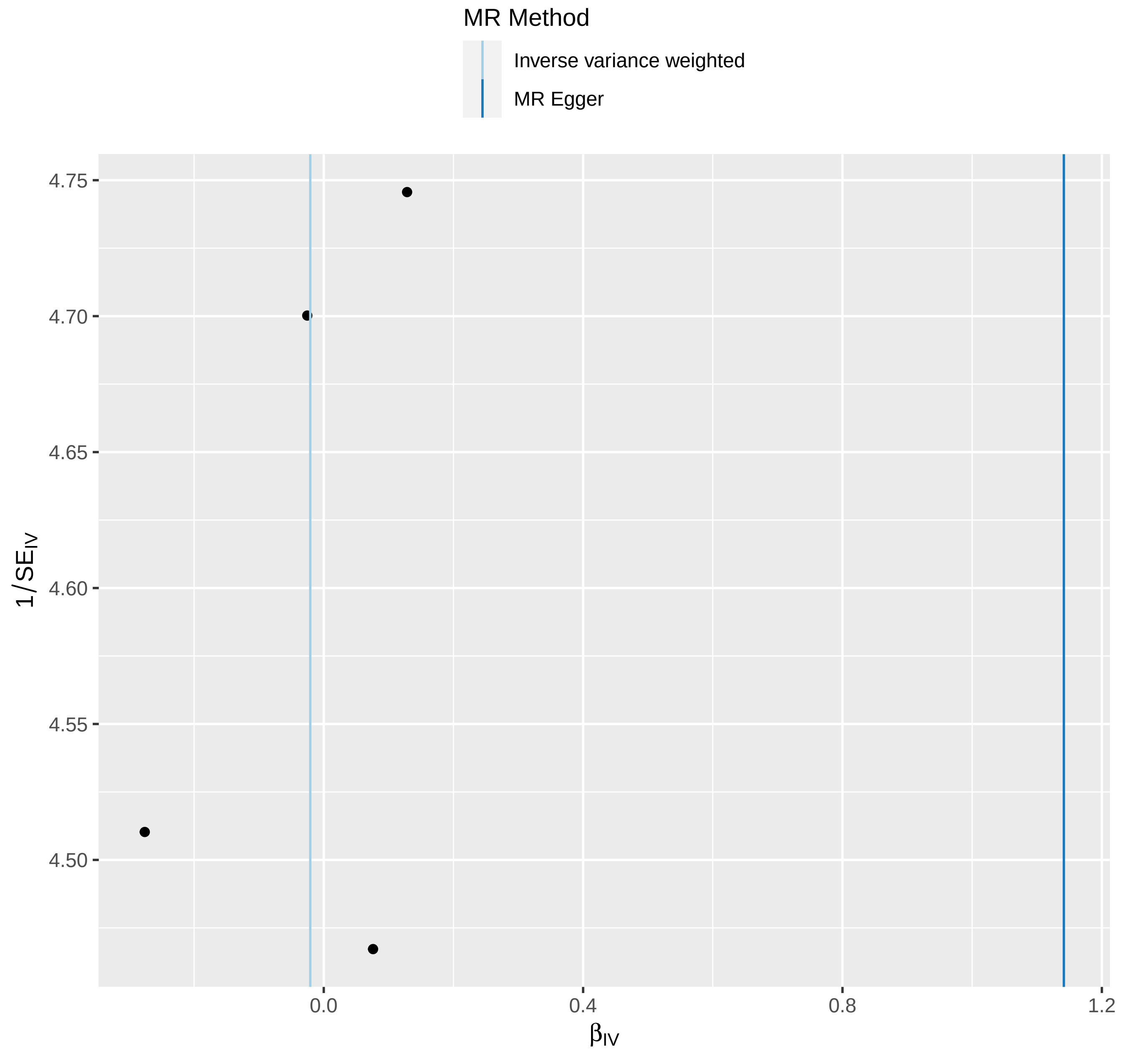

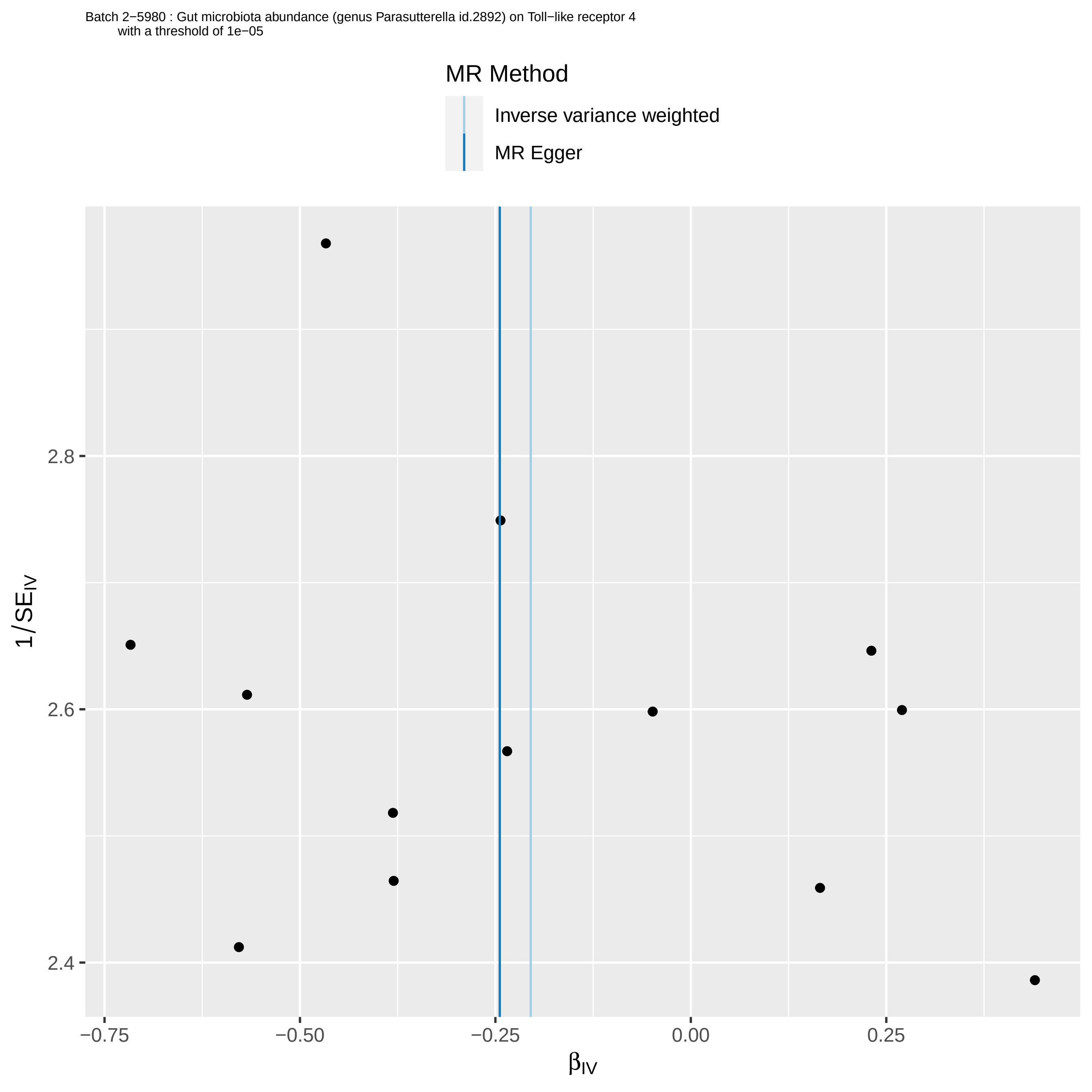
Batch 2−5980 : Gut microbiota abundance (genus Parasutterella id.2892) on Toll−like receptor 4
with a threshold of 1e−05
MR Method
Inverse variance weighted
MR Egger
2.8
2.6
2.4
1/SE_IV
−0.75
−0.50
−0.25
0.00
0.25
β_IV

Batch 2−5981 : Gut microbiota abundance (genus Catenibacterium id.2153) on Toll−like receptor 4:Lymphocyte antigen 96 complex with a threshold of 1e−05

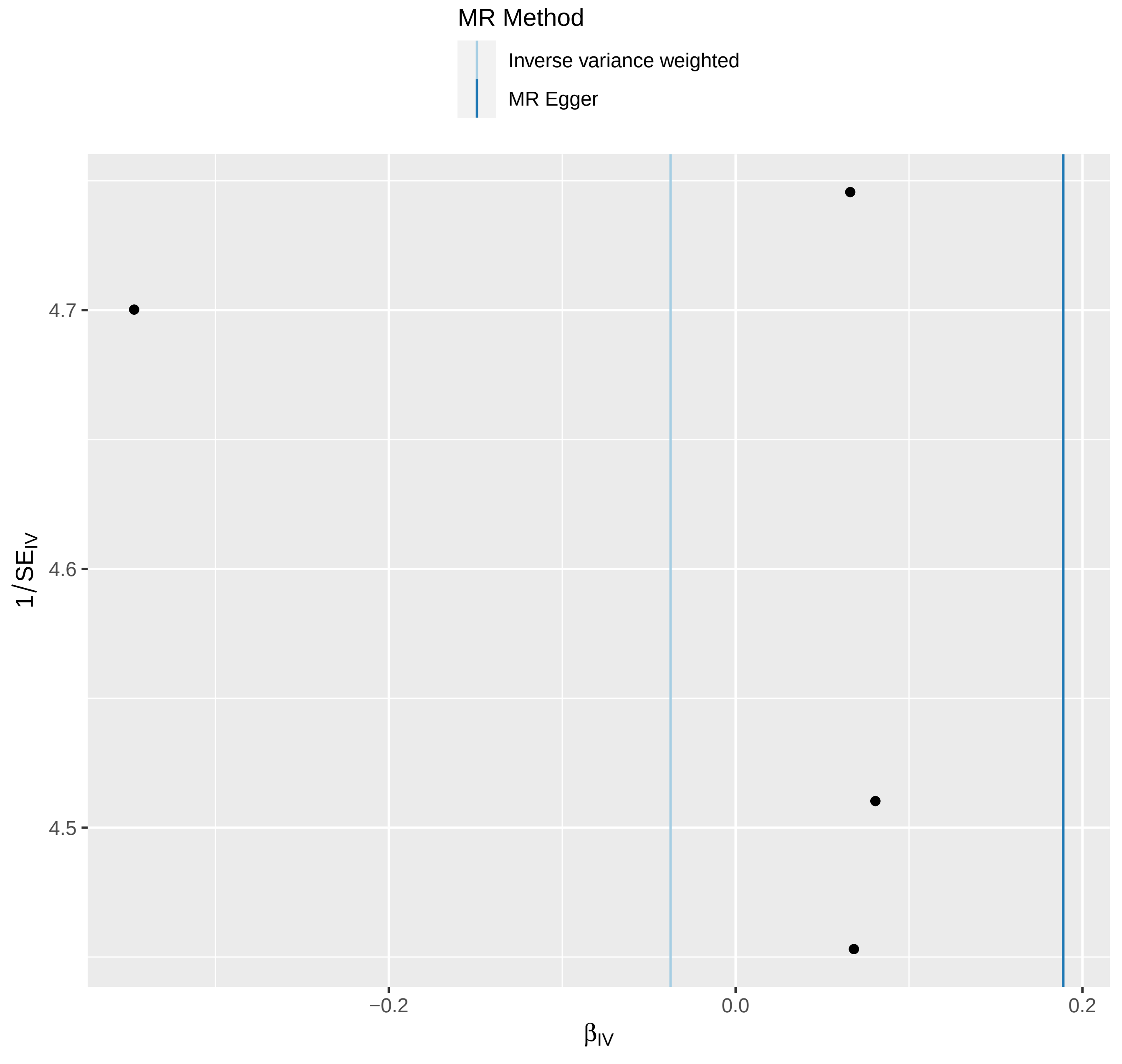

Batch 2−5982 : Gut microbiota abundance (genus Parasutterella id.2892) on Toll−like receptor 4:Lymphocyte antigen 96 complex with a threshold of 1e−05

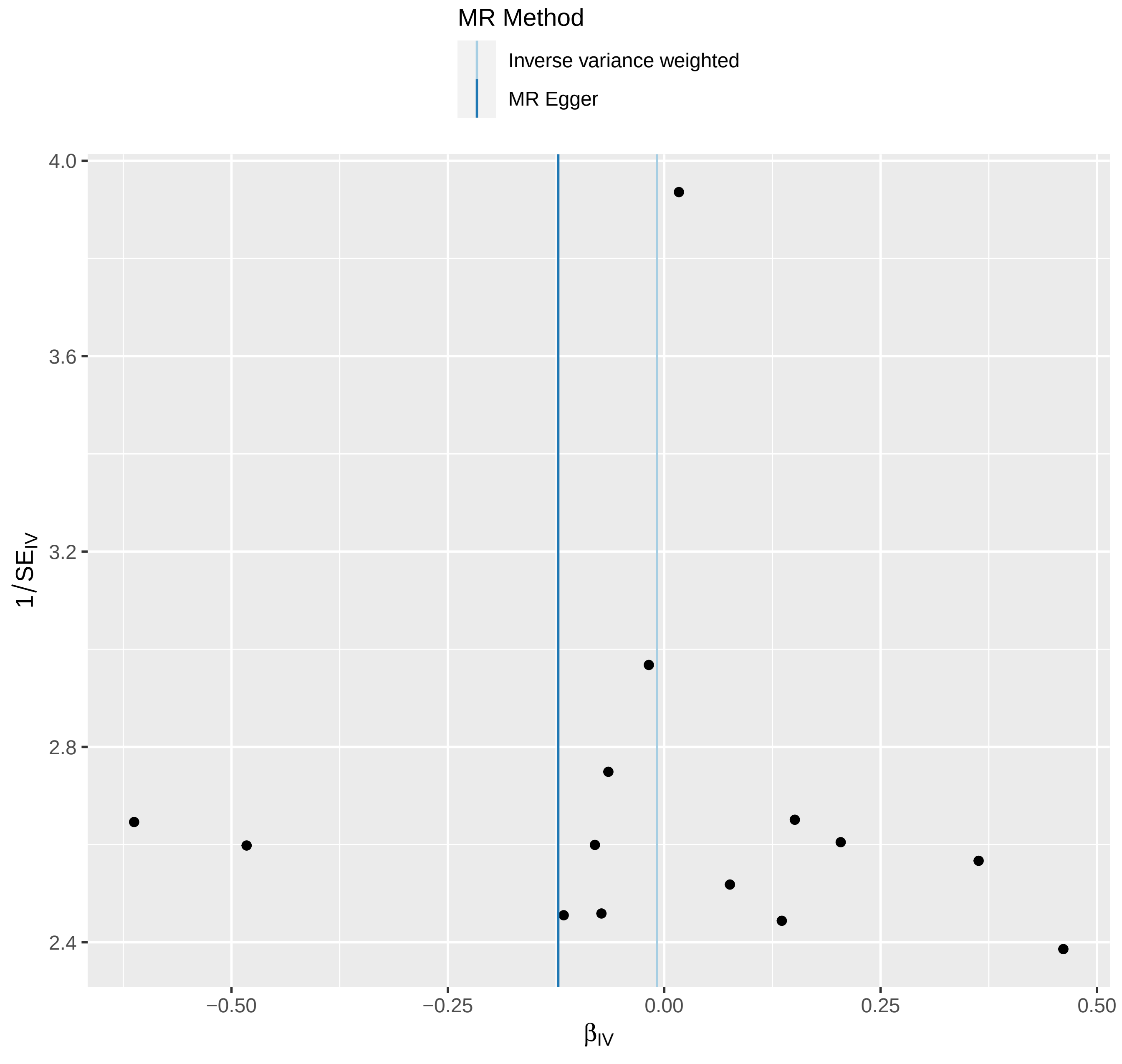

Batch 2−5983 : Gut microbiota abundance (genus Catenibacterium id.2153) on Transmembrane and coiled−coil domain−containing protein 5A with a threshold of 1e−05

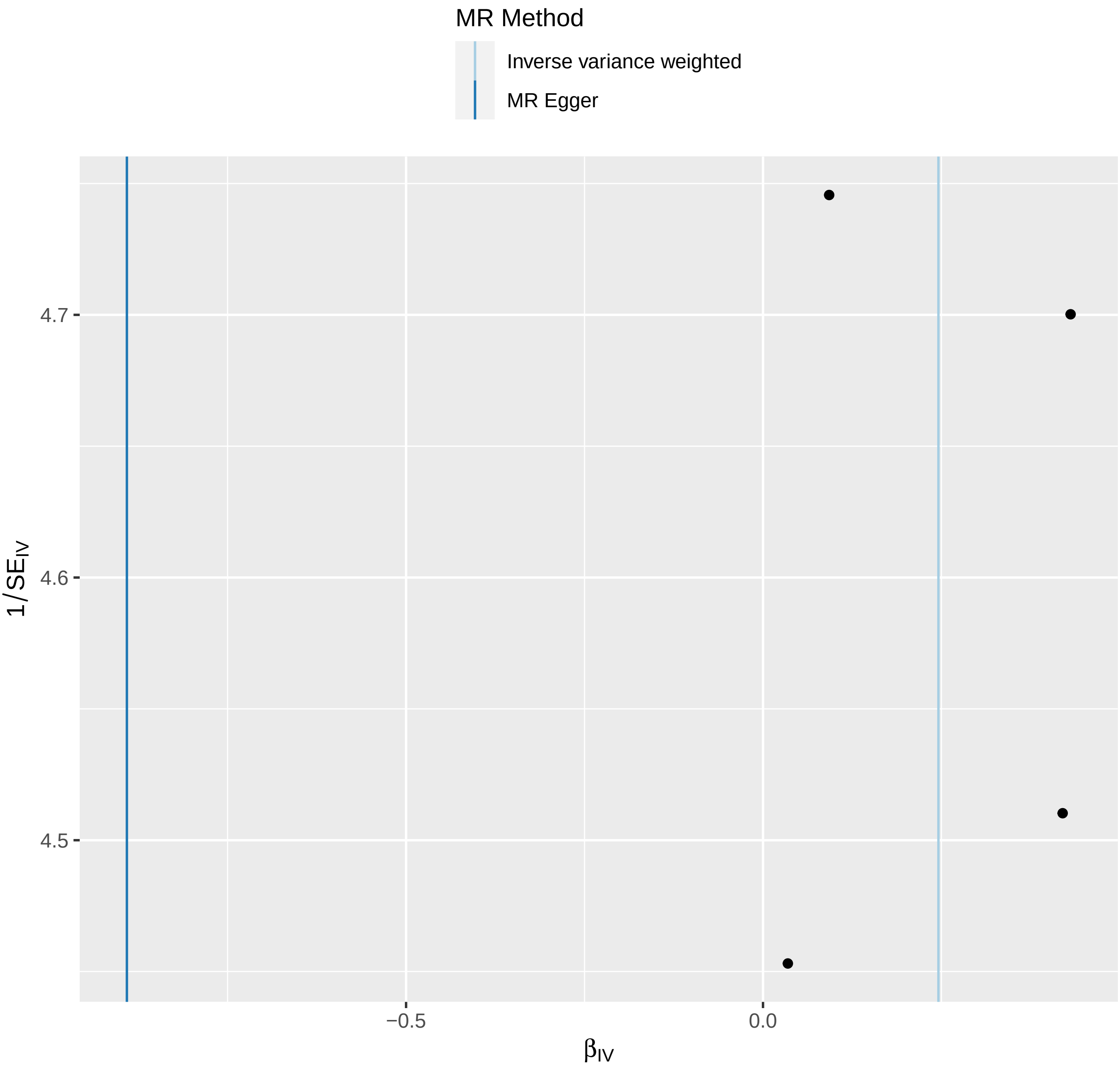

Batch 2−5984 : Gut microbiota abundance (genus Parasutterella id.2892) on Transmembrane and coiled−coil domain−containing protein 5A
with a threshold of 1e−05

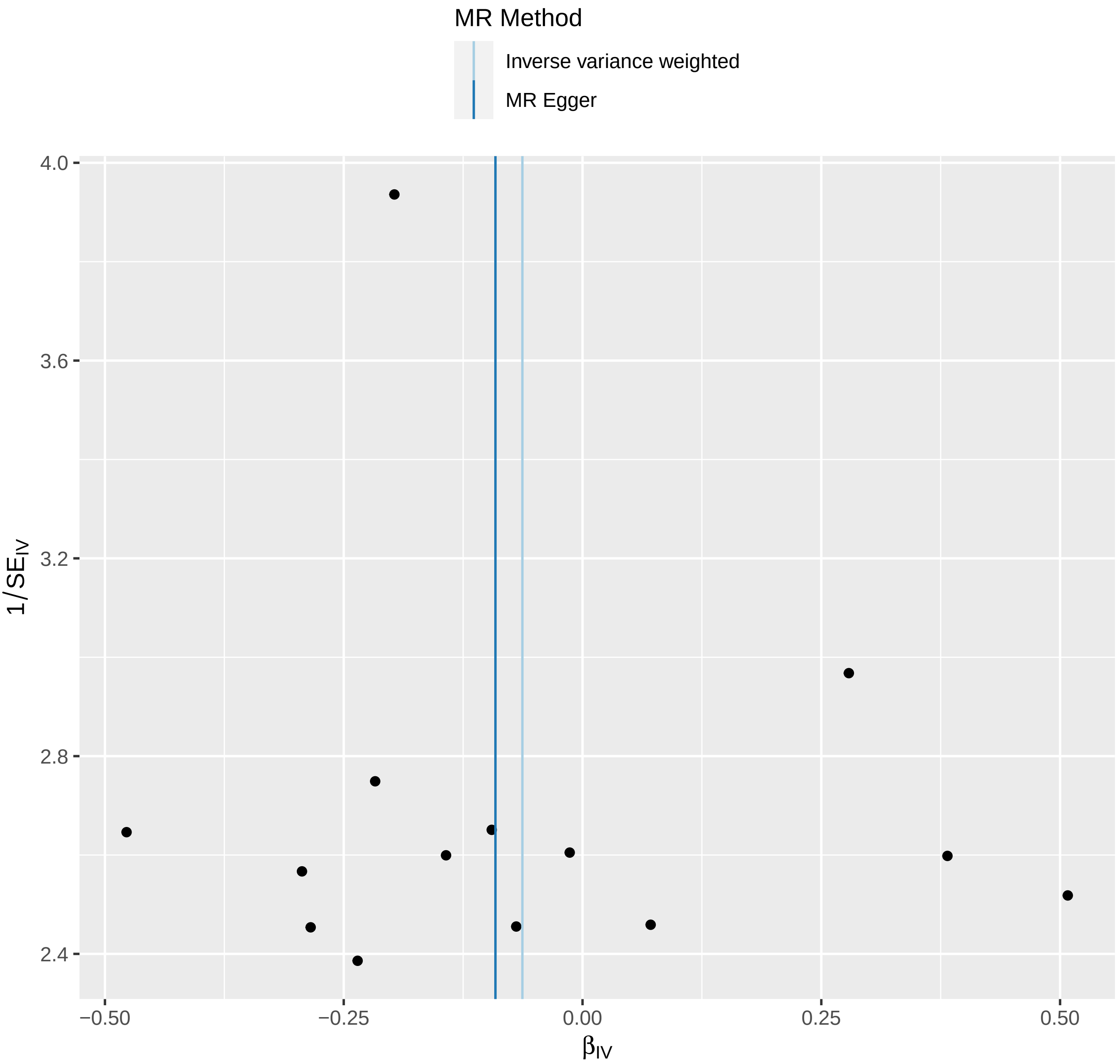

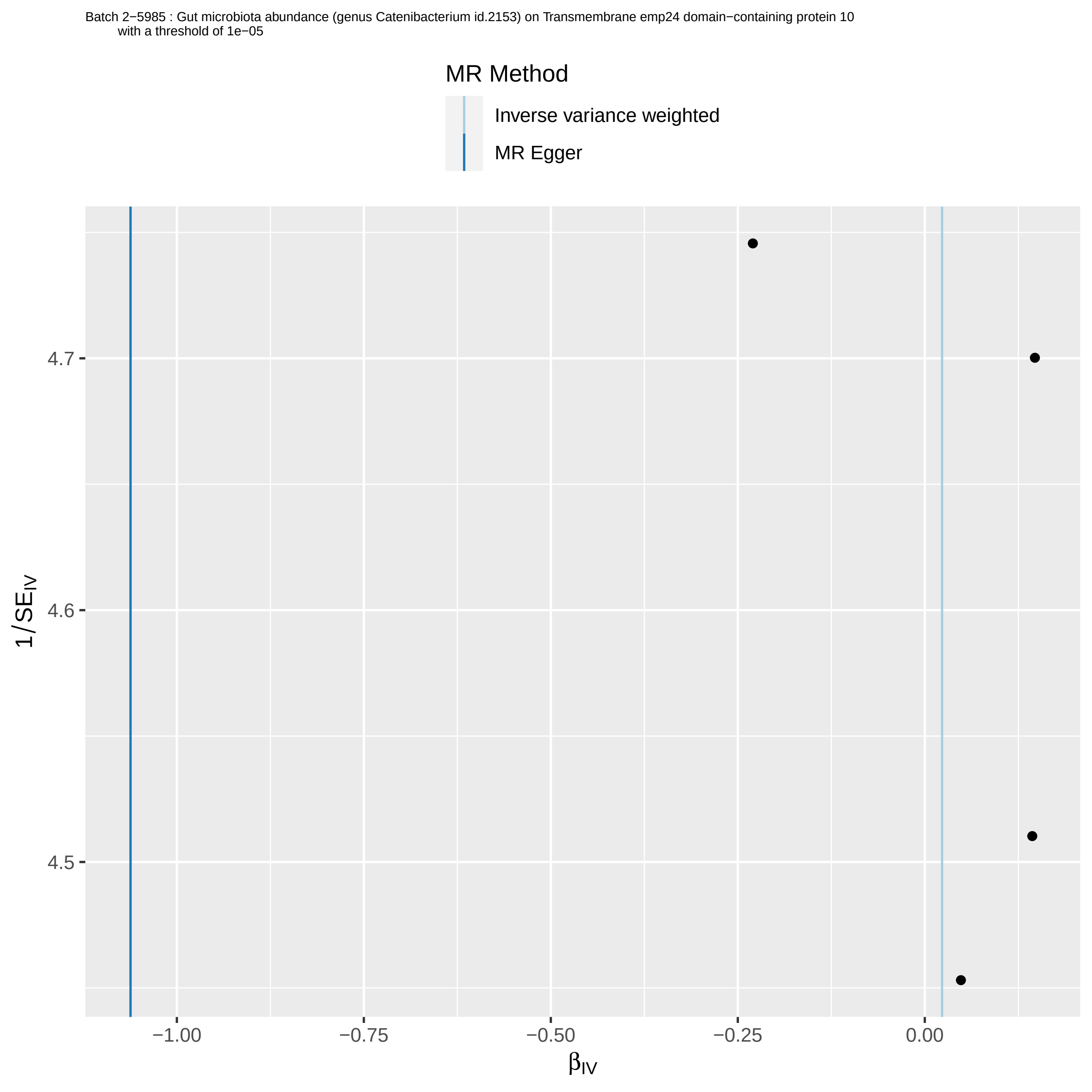

Batch 2−5985 : Gut microbiota abundance (genus Catenibacterium id.2153) on Transmembrane emp24 domain−containing protein 10
with a threshold of 1e−05
MR Method
Inverse variance weighted
MR Egger
4.7
4.6
4.5
1/SE_IV
−1.00
−0.75
−0.50
−0.25
0.00
β_IV

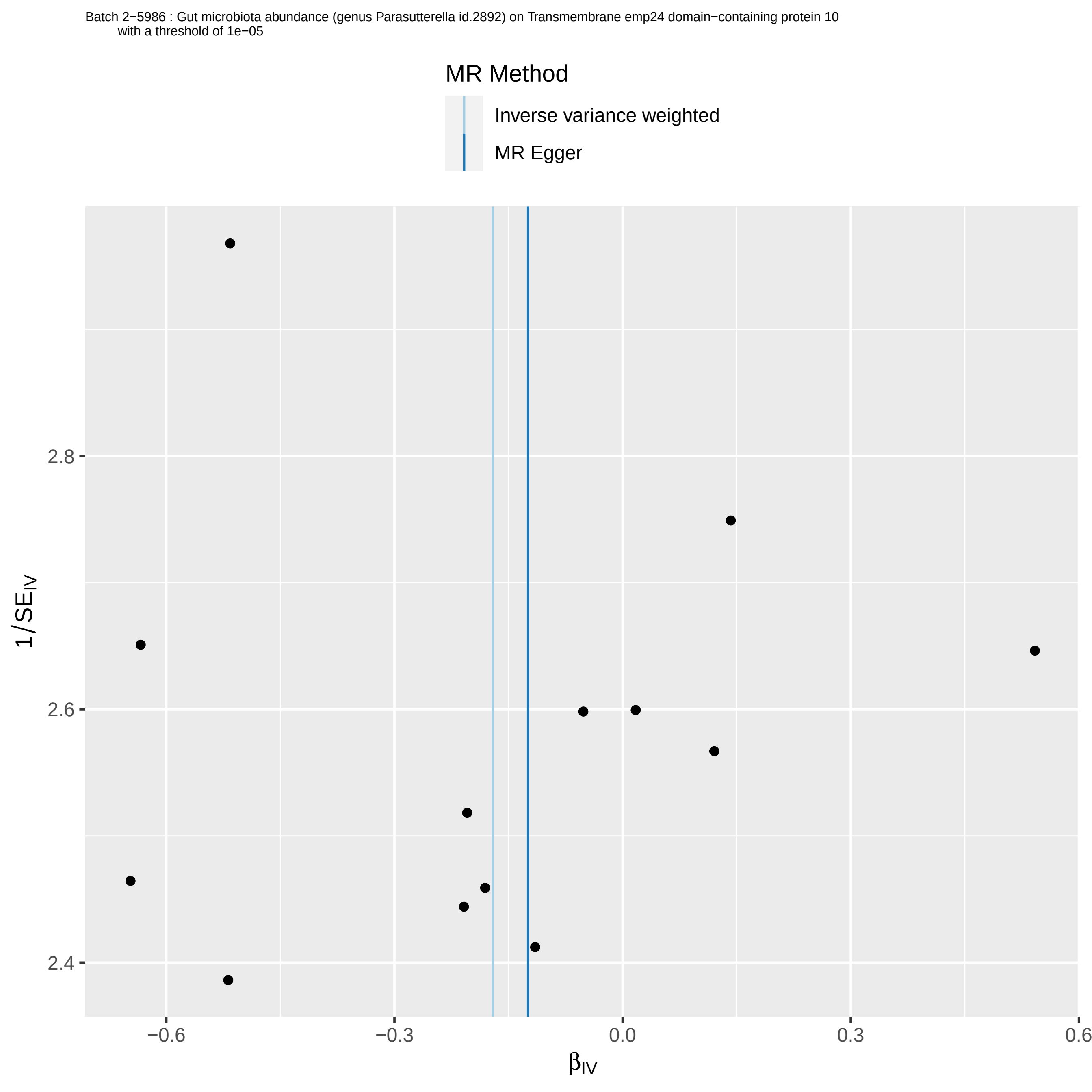

Batch 2−5986 : Gut microbiota abundance (genus Parasutterella id.2892) on Transmembrane emp24 domain−containing protein 10
with a threshold of 1e−05
MR Method
Inverse variance weighted
MR Egger
2.8
2.6
2.4
1/SE_IV
−0.6
−0.3
0.0
0.3
0.6
β_IV

Batch 2−5987 : Gut microbiota abundance (genus Catenibacterium id.2153) on Transmembrane emp24 domain−containing protein 2 with a threshold of 1e−05

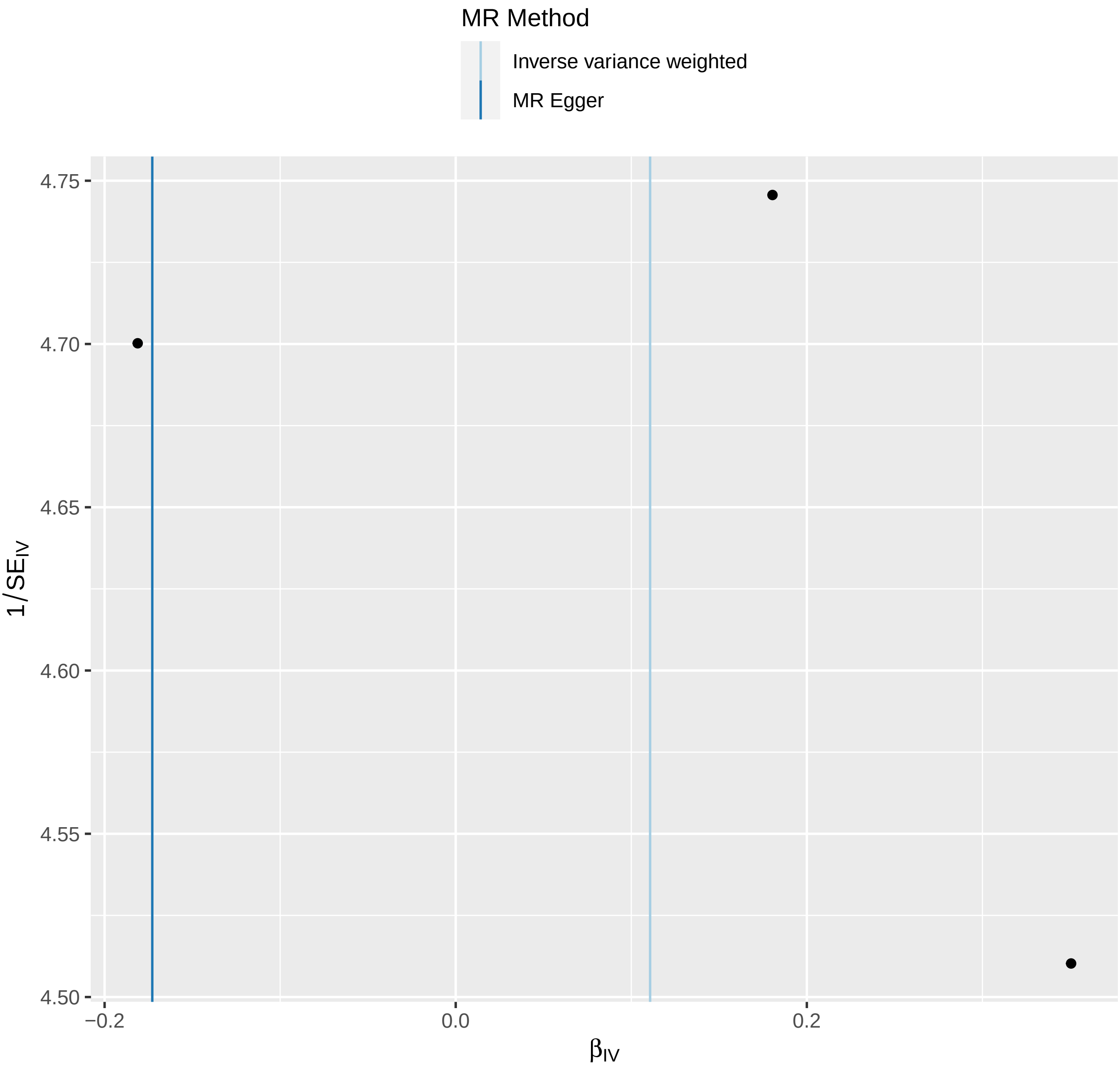

Batch 2−5988 : Gut microbiota abundance (genus Parasutterella id.2892) on Transmembrane emp24 domain−containing protein 2 with a threshold of 1e−05

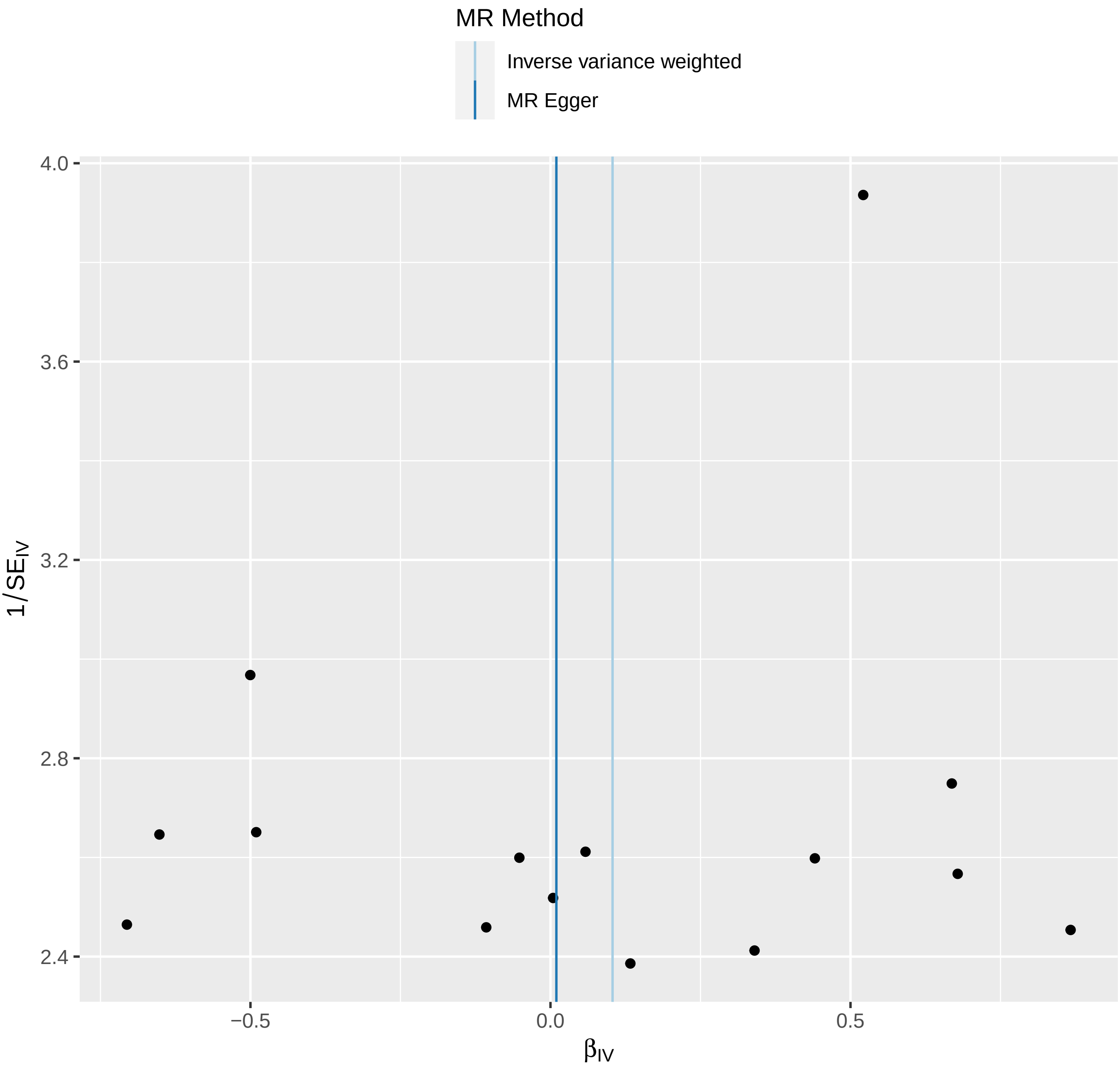

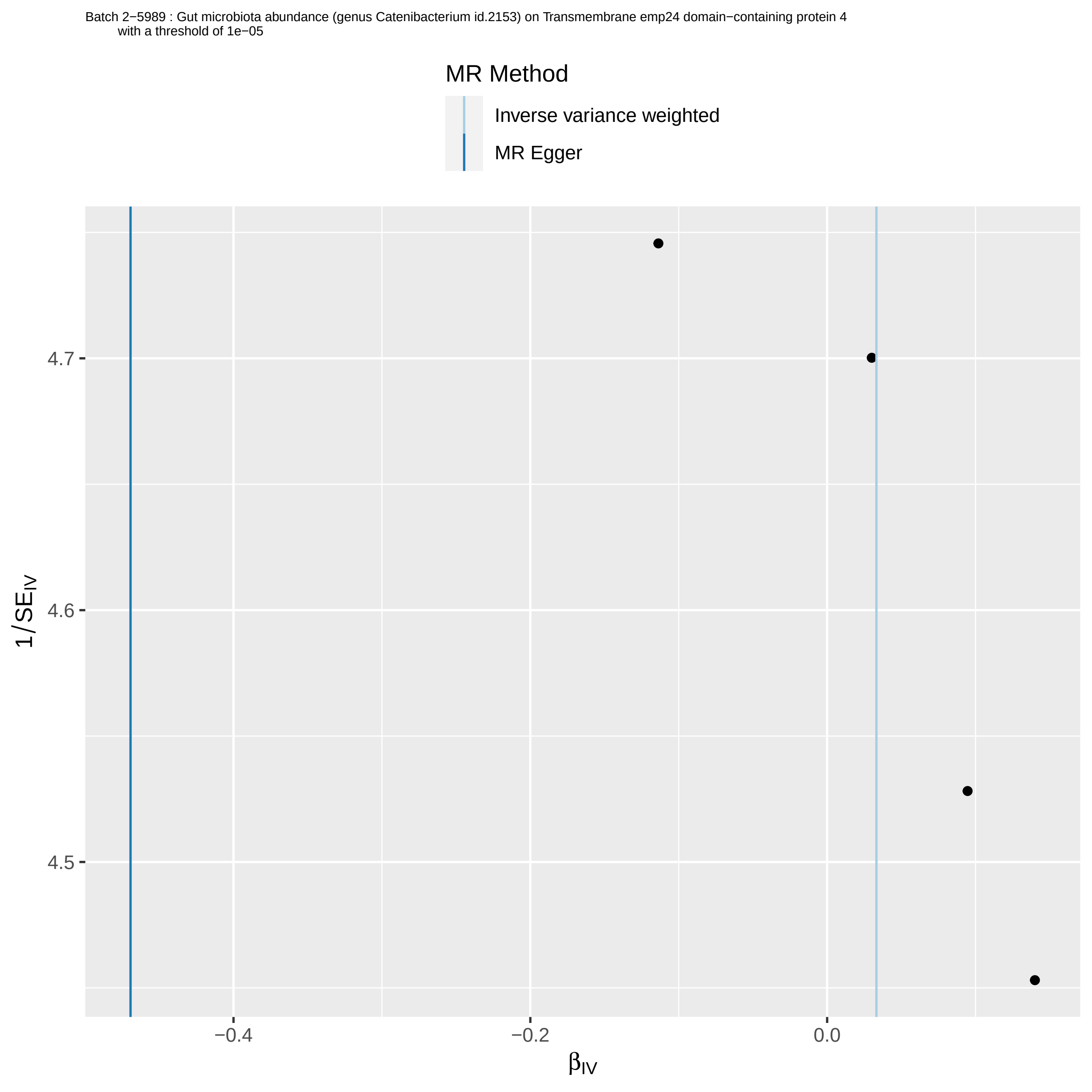

Batch 2−5989 : Gut microbiota abundance (genus Catenibacterium id.2153) on Transmembrane emp24 domain−containing protein 4
with a threshold of 1e−05
MR Method
Inverse variance weighted
MR Egger
4.7
4.6
4.5
1/SE$_{IV}$
−0.4
−0.2
0.0
β$_{IV}$

Batch 2−5990 : Gut microbiota abundance (genus Parasutterella id.2892) on Transmembrane emp24 domain−containing protein 4 with a threshold of 1e−05

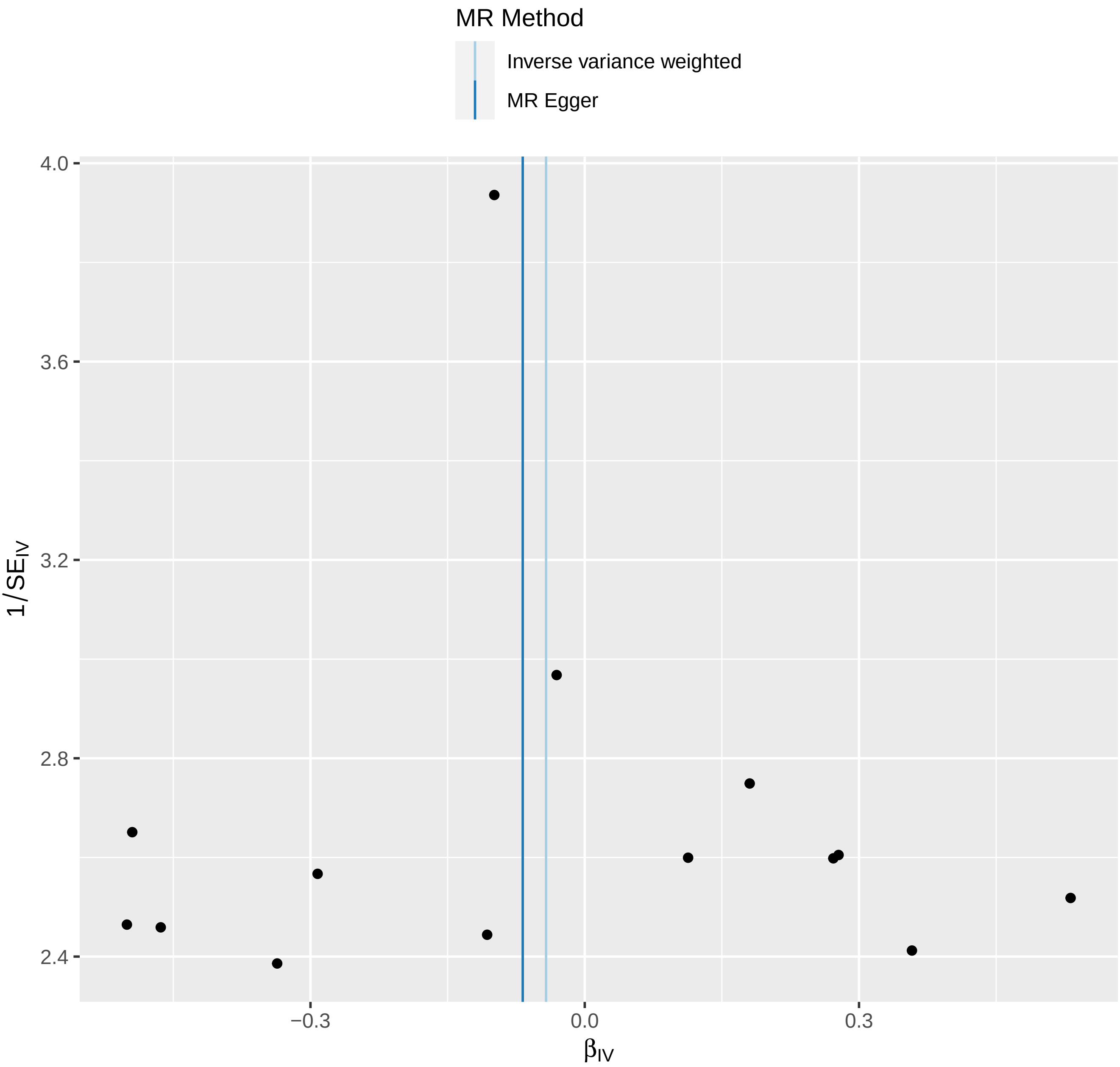

Batch 2−5991 : Gut microbiota abundance (genus Catenibacterium id.2153) on Transmembrane protein 119
with a threshold of 1e−05

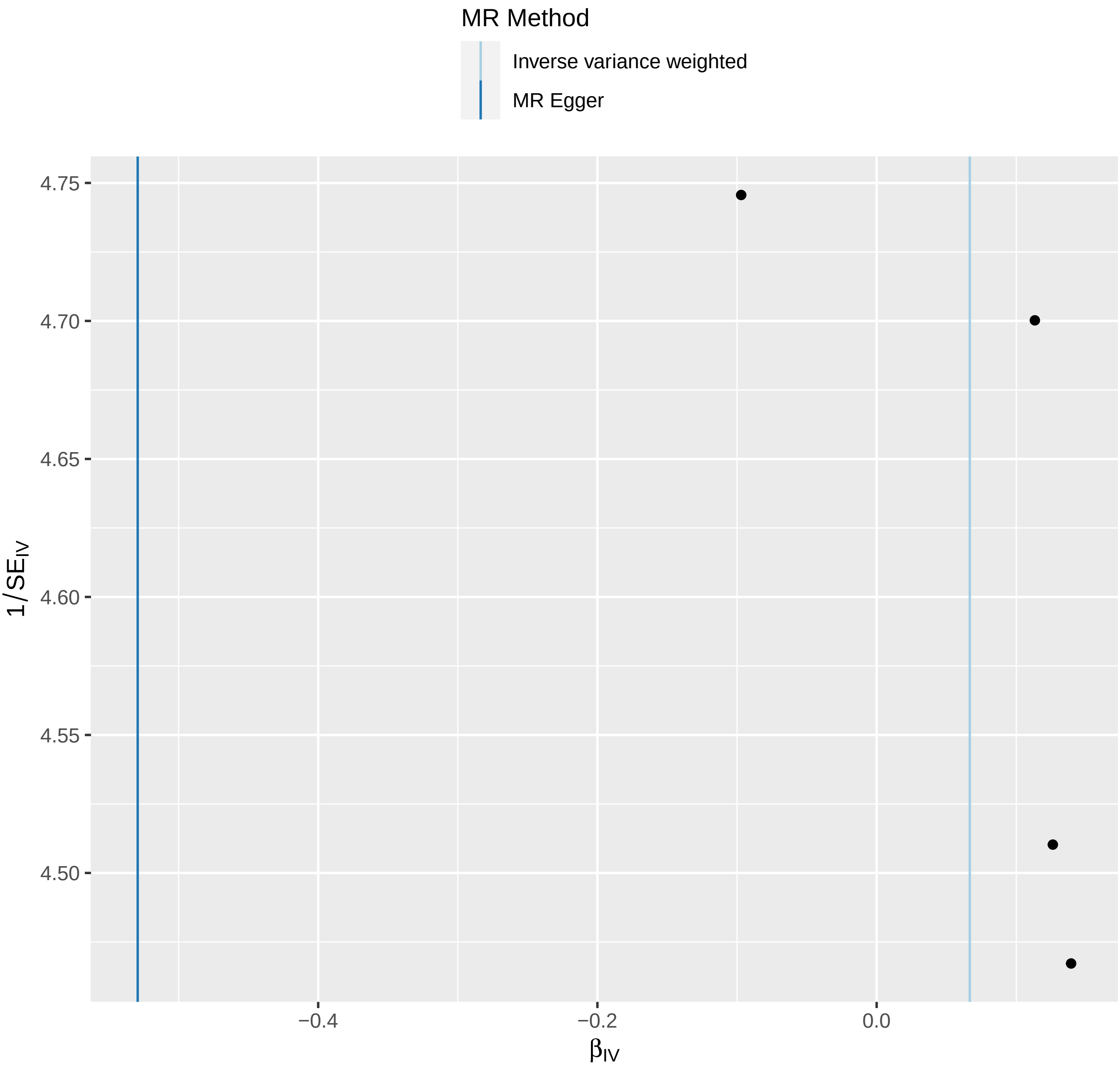

Batch 2−5992 : Gut microbiota abundance (genus Parasutterella id.2892) on Transmembrane protein 119
with a threshold of 1e−05

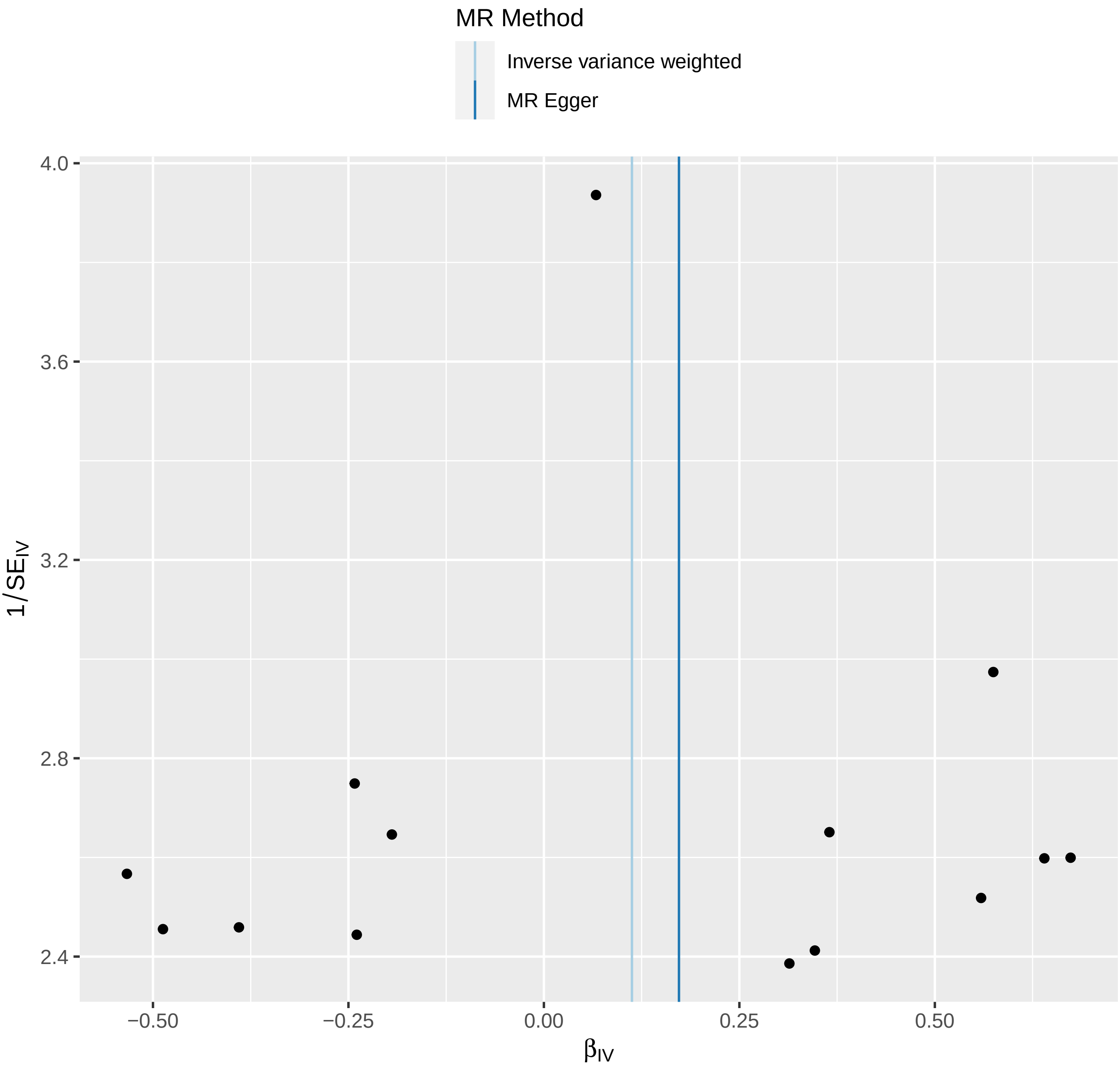

Batch 2−5993 : Gut microbiota abundance (genus Catenibacterium id.2153) on Transmembrane protein 132A
with a threshold of 1e−05

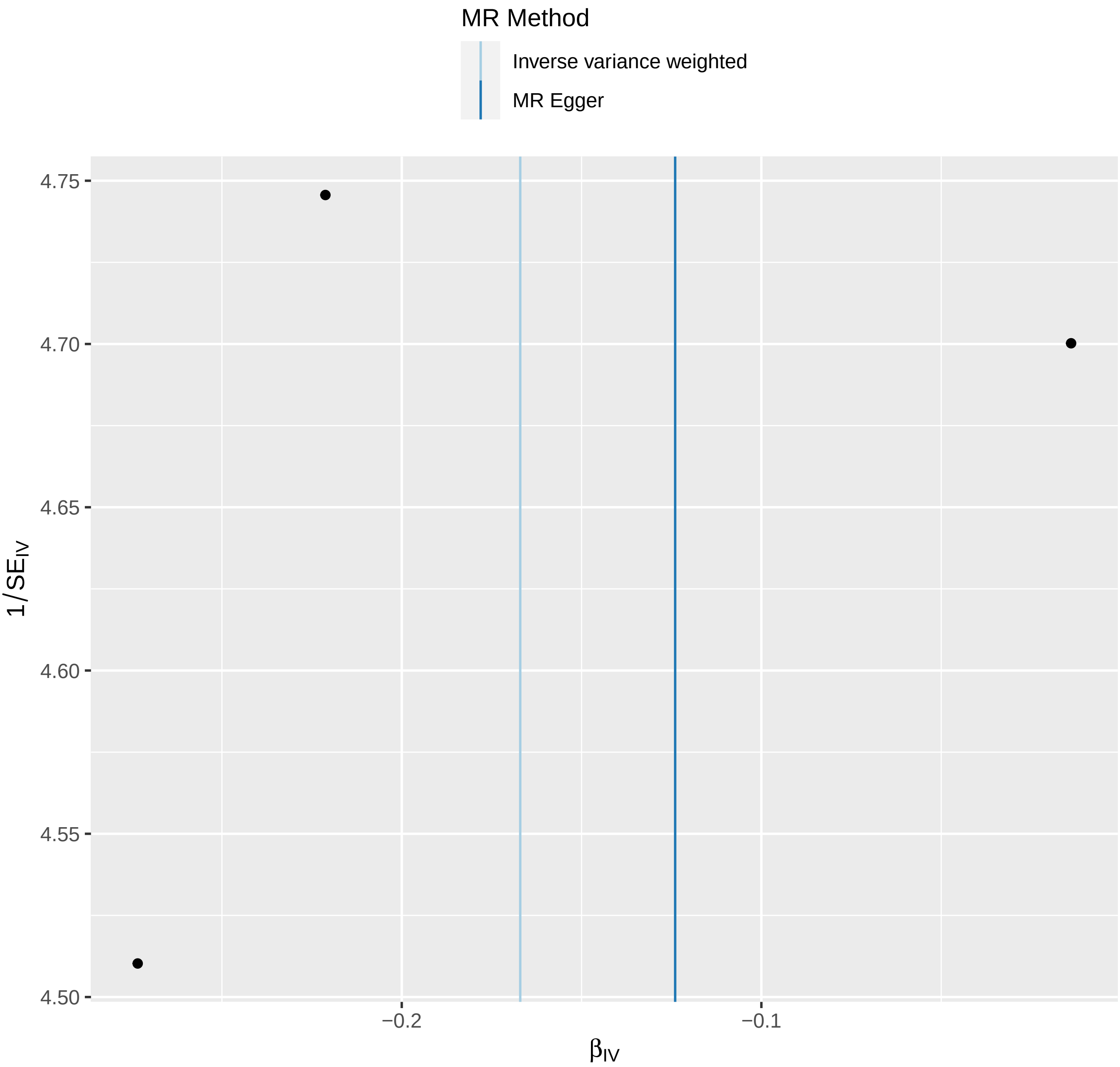

Batch 2−5994 : Gut microbiota abundance (genus Parasutterella id.2892) on Transmembrane protein 132A
with a threshold of 1e−05

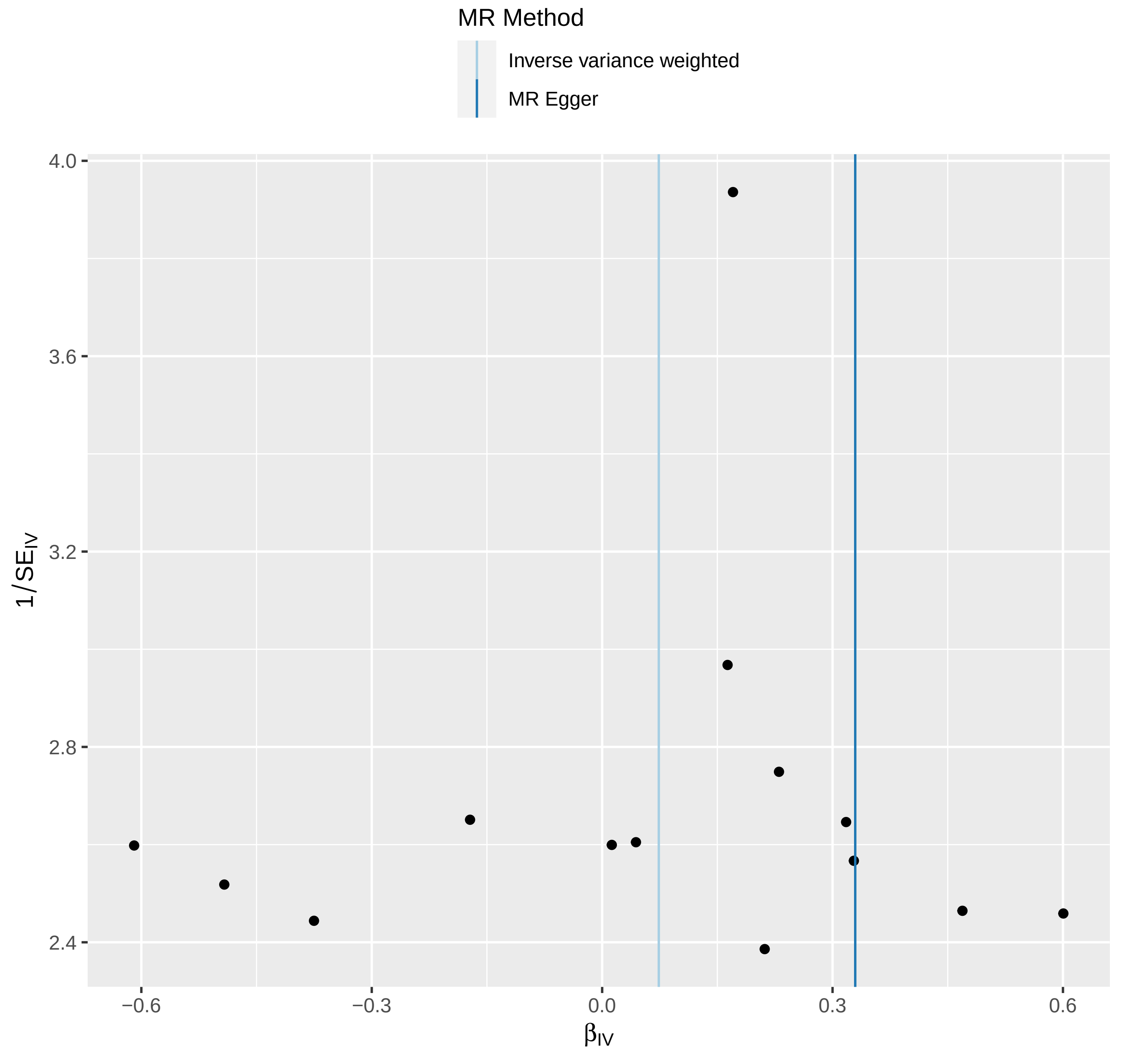

Batch 2−5995 : Gut microbiota abundance (genus Catenibacterium id.2153) on Transmembrane protein 132B
with a threshold of 1e−05

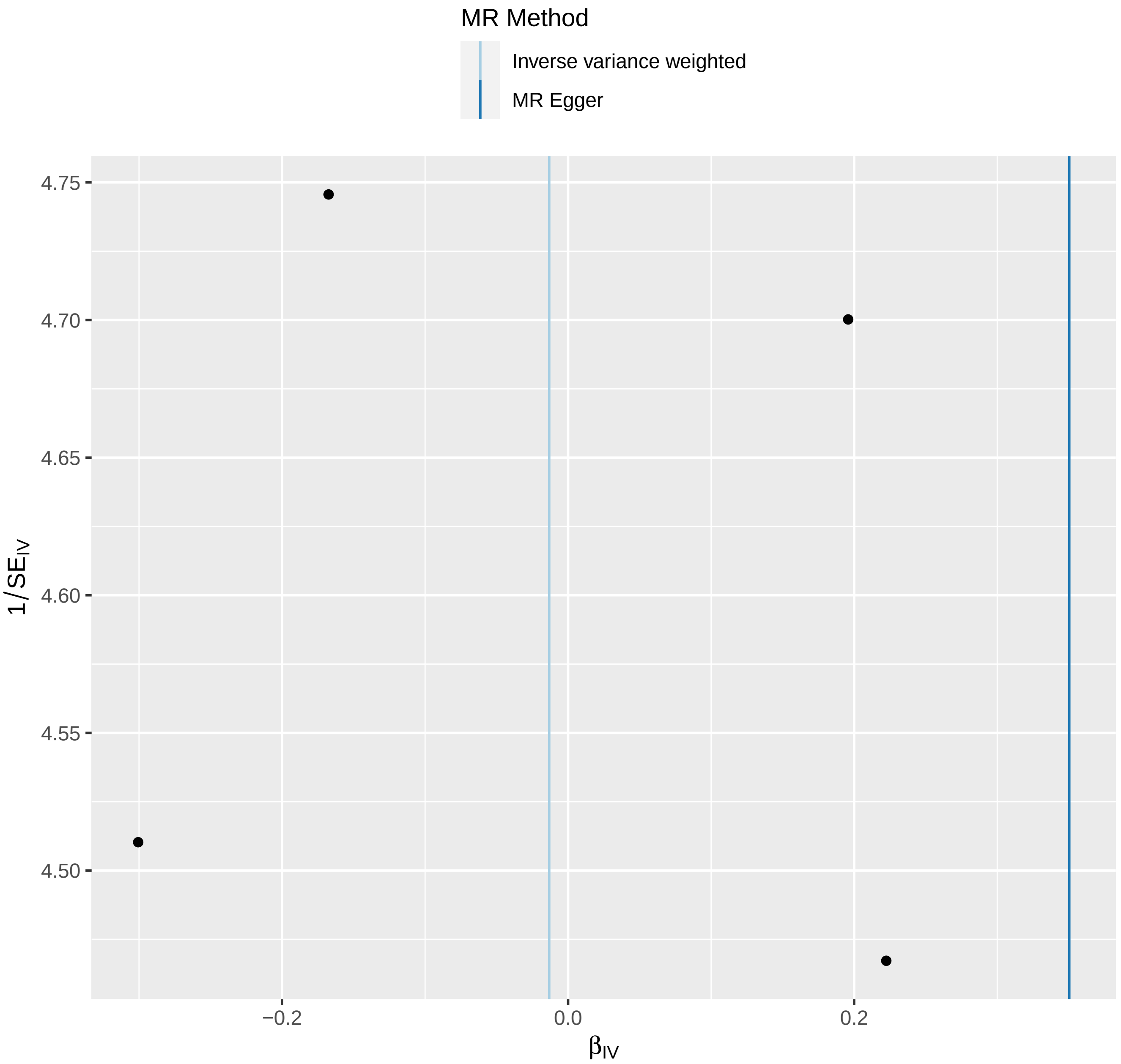

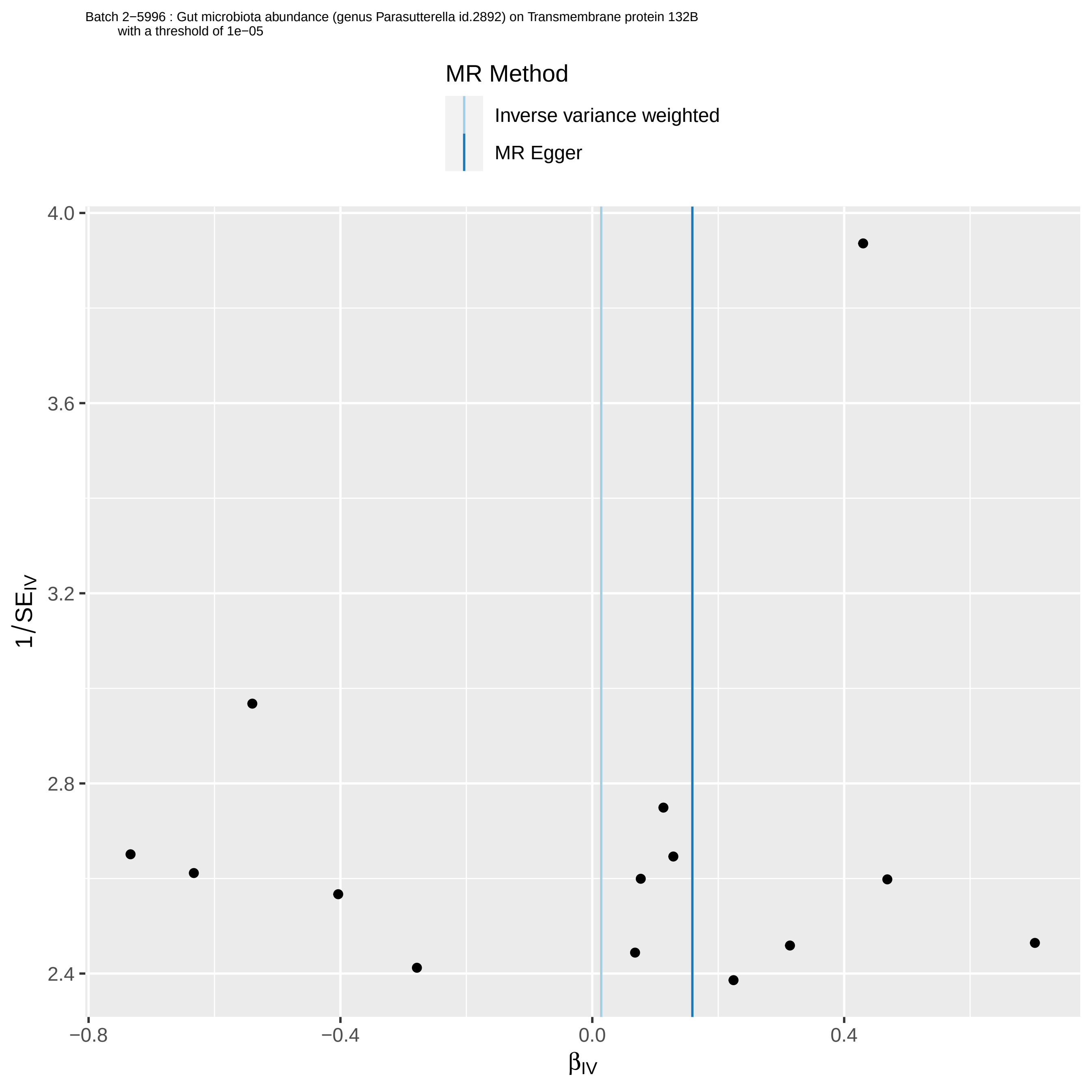

Batch 2−5996 : Gut microbiota abundance (genus Parasutterella id.2892) on Transmembrane protein 132B
with a threshold of 1e−05
MR Method
Inverse variance weighted
MR Egger
1/SE_IV
4.0
3.6
3.2
2.8
2.4
β_IV
−0.8
−0.4
0.0
0.4

Batch 2−5997 : Gut microbiota abundance (genus Catenibacterium id.2153) on Transmembrane protein 132C
with a threshold of 1e−05

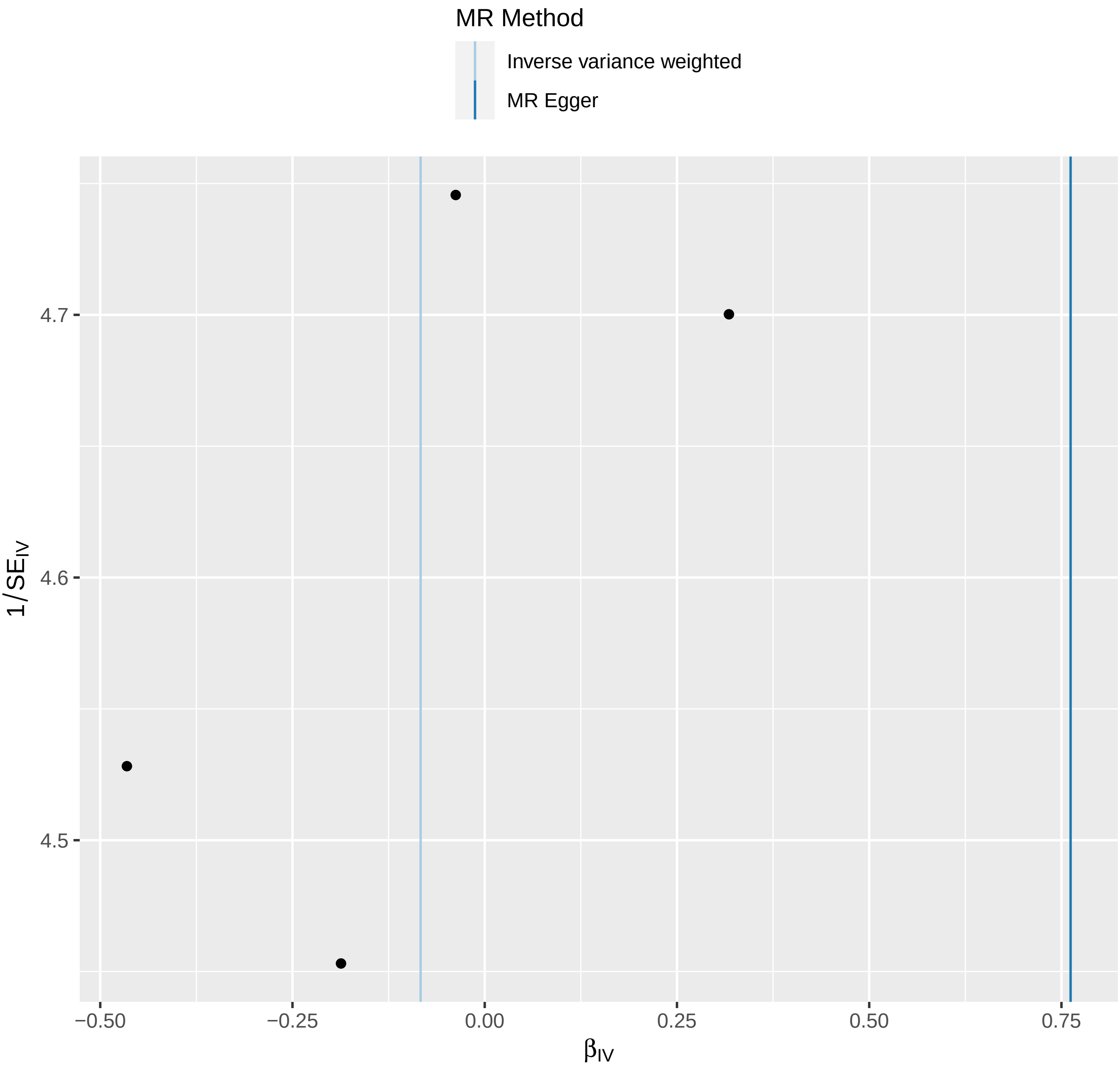

Batch 2−5998 : Gut microbiota abundance (genus Parasutterella id.2892) on Transmembrane protein 132C
with a threshold of 1e−05

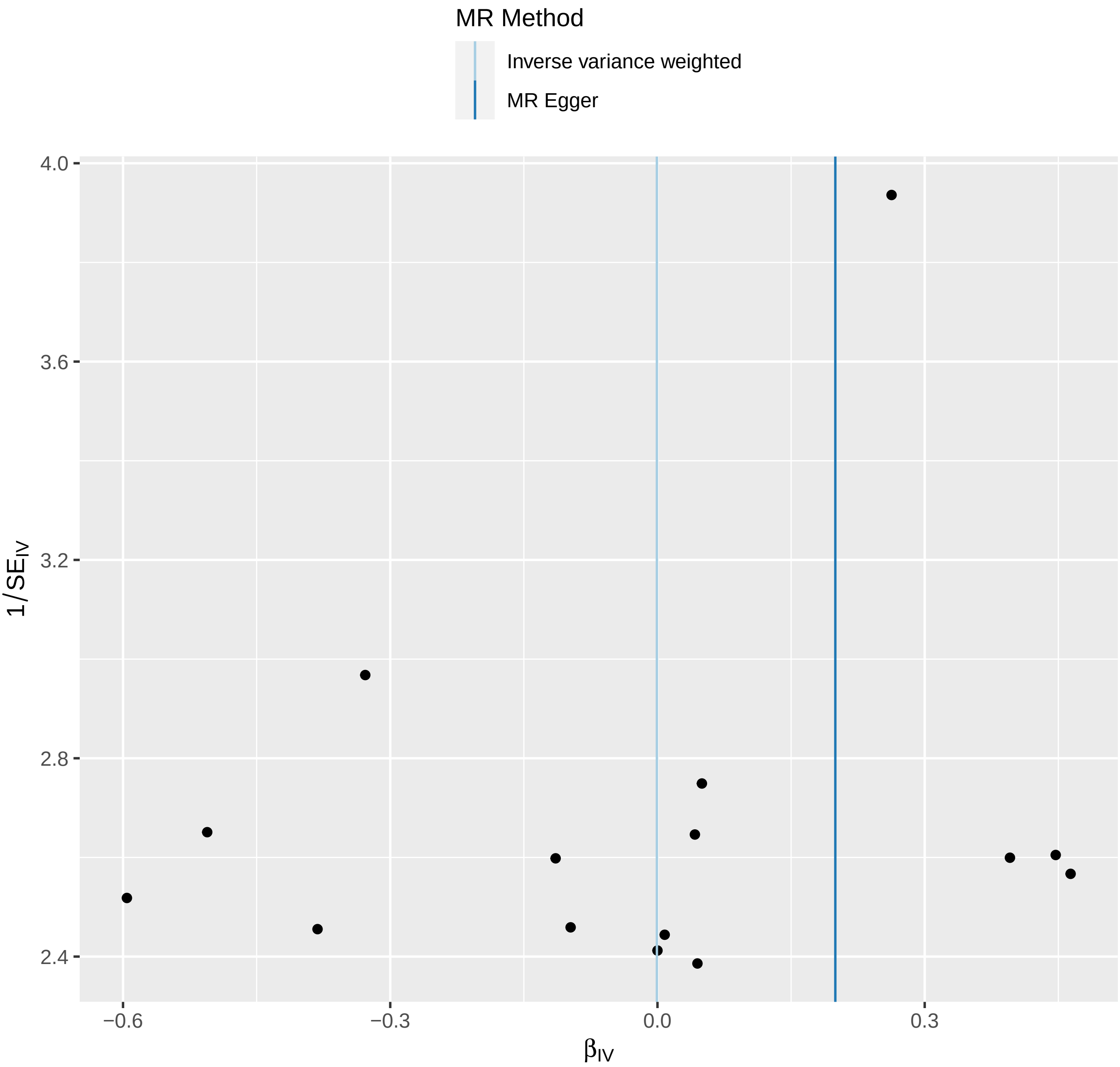

Batch 2−5999 : Gut microbiota abundance (genus Catenibacterium id.2153) on Transmembrane protein 132C
with a threshold of 1e−05

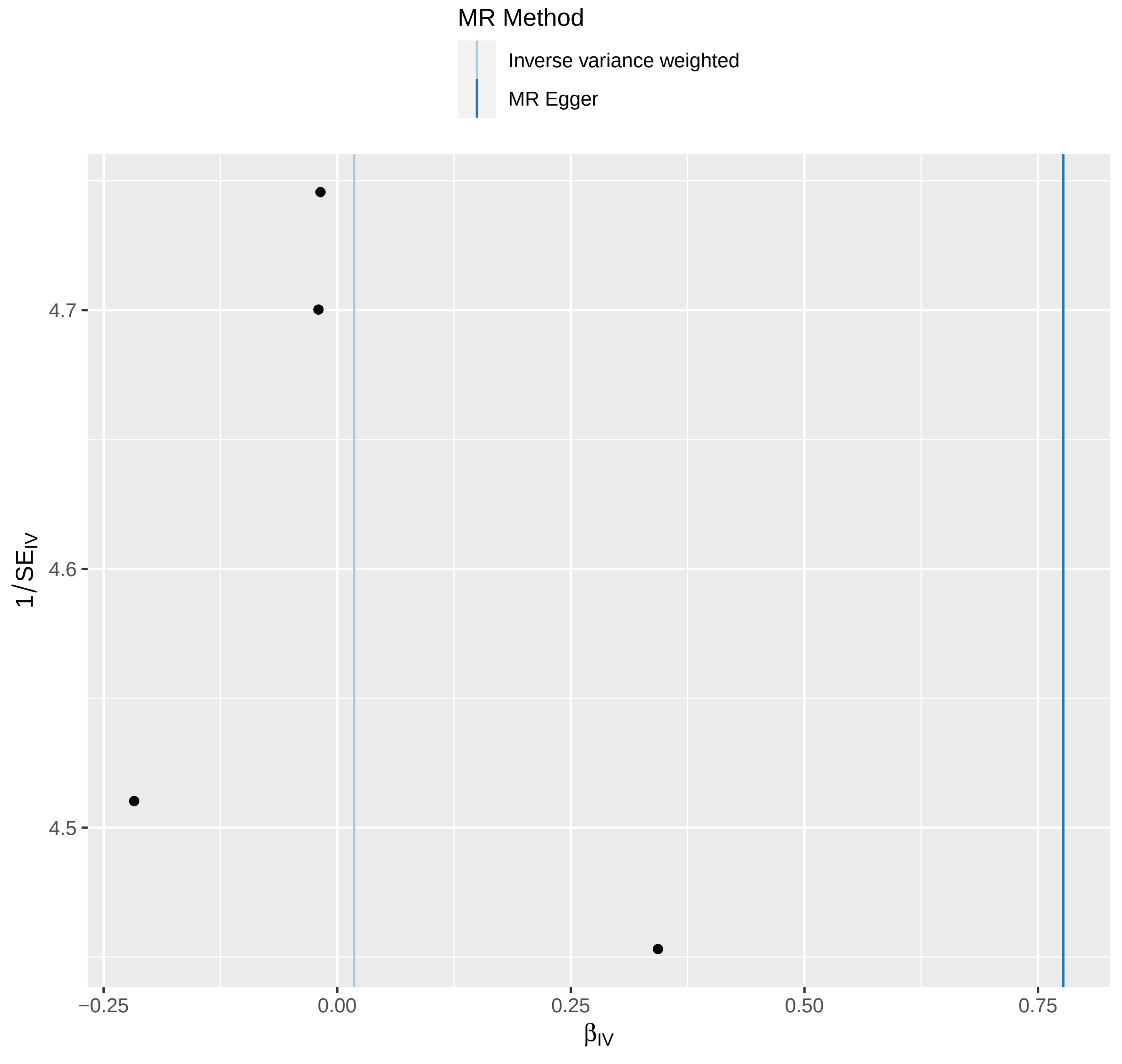

Batch 2−6000 : Gut microbiota abundance (genus Parasutterella id.2892) on Transmembrane protein 132C
with a threshold of 1e−05

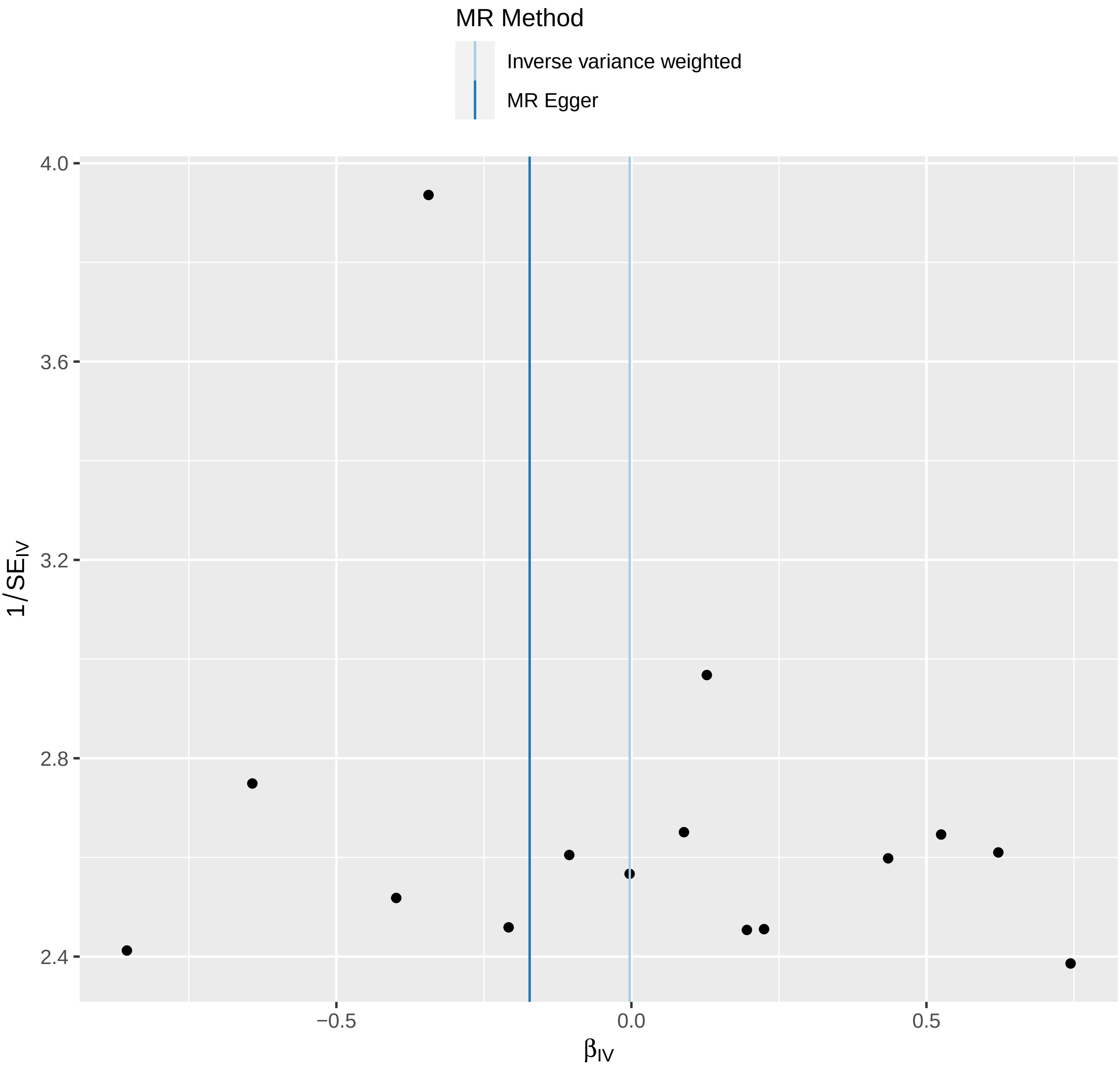

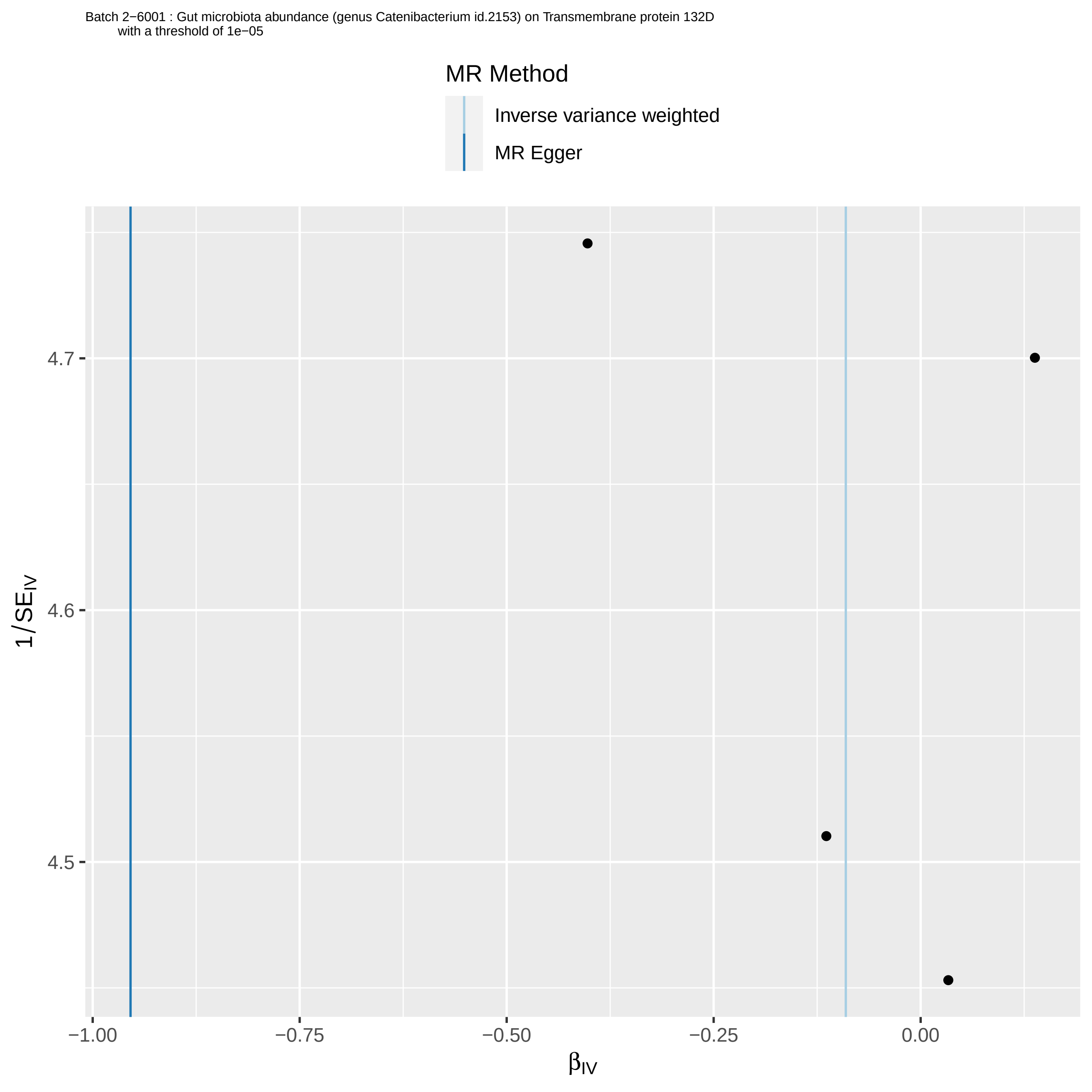
Batch 2−6001 : Gut microbiota abundance (genus Catenibacterium id.2153) on Transmembrane protein 132D
with a threshold of 1e−05
MR Method
Inverse variance weighted
MR Egger
4.7
4.6
4.5
1/SE_IV
−1.00
−0.75
−0.50
−0.25
0.00
β_IV

Batch 2−6002 : Gut microbiota abundance (genus Parasutterella id.2892) on Transmembrane protein 132D
with a threshold of 1e−05

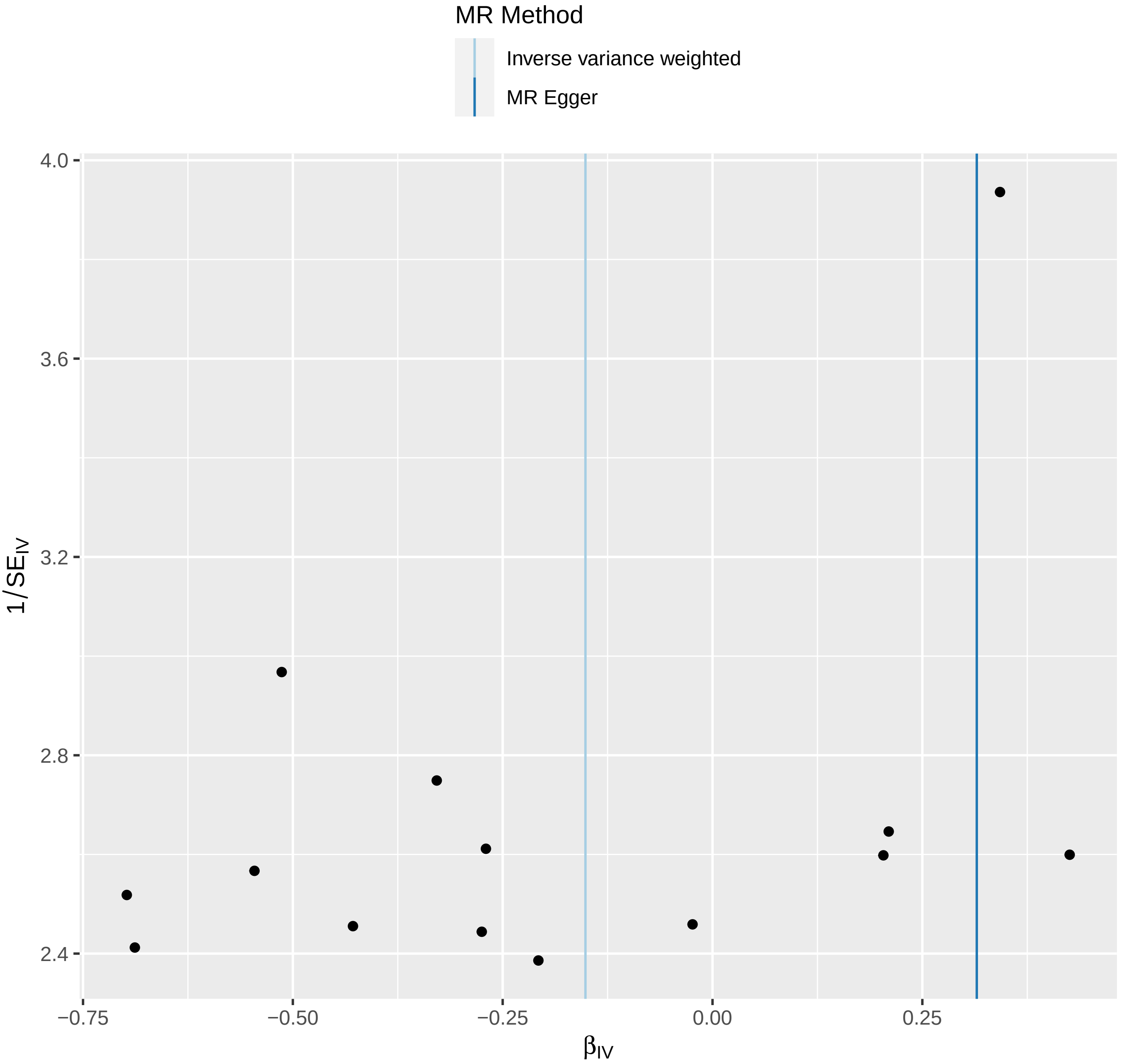

Batch 2−6003 : Gut microbiota abundance (genus Catenibacterium id.2153) on Protein kish−A
with a threshold of 1e−05

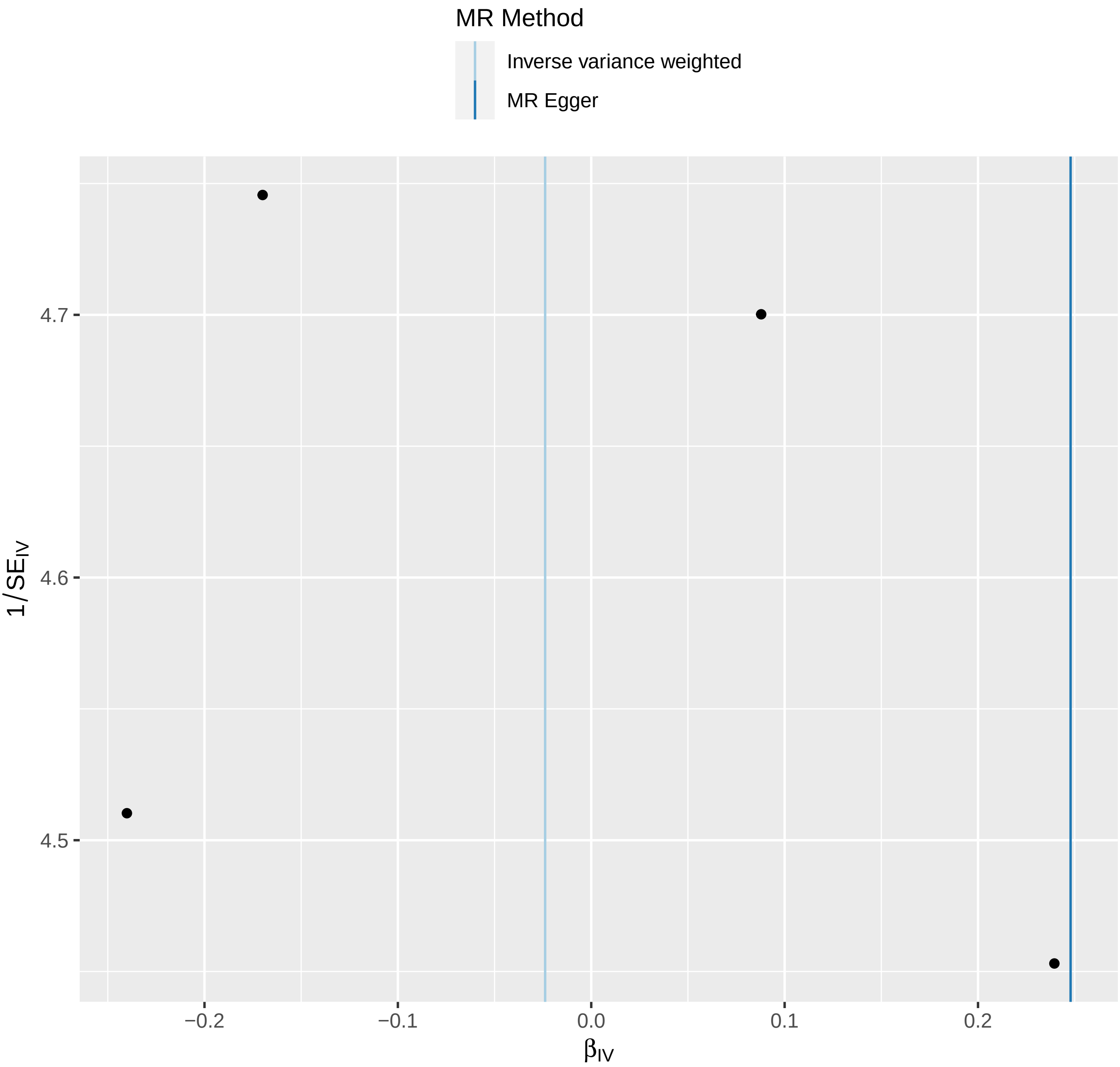

Batch 2−6004 : Gut microbiota abundance (genus Parasutterella id.2892) on Protein kish−A
with a threshold of 1e−05

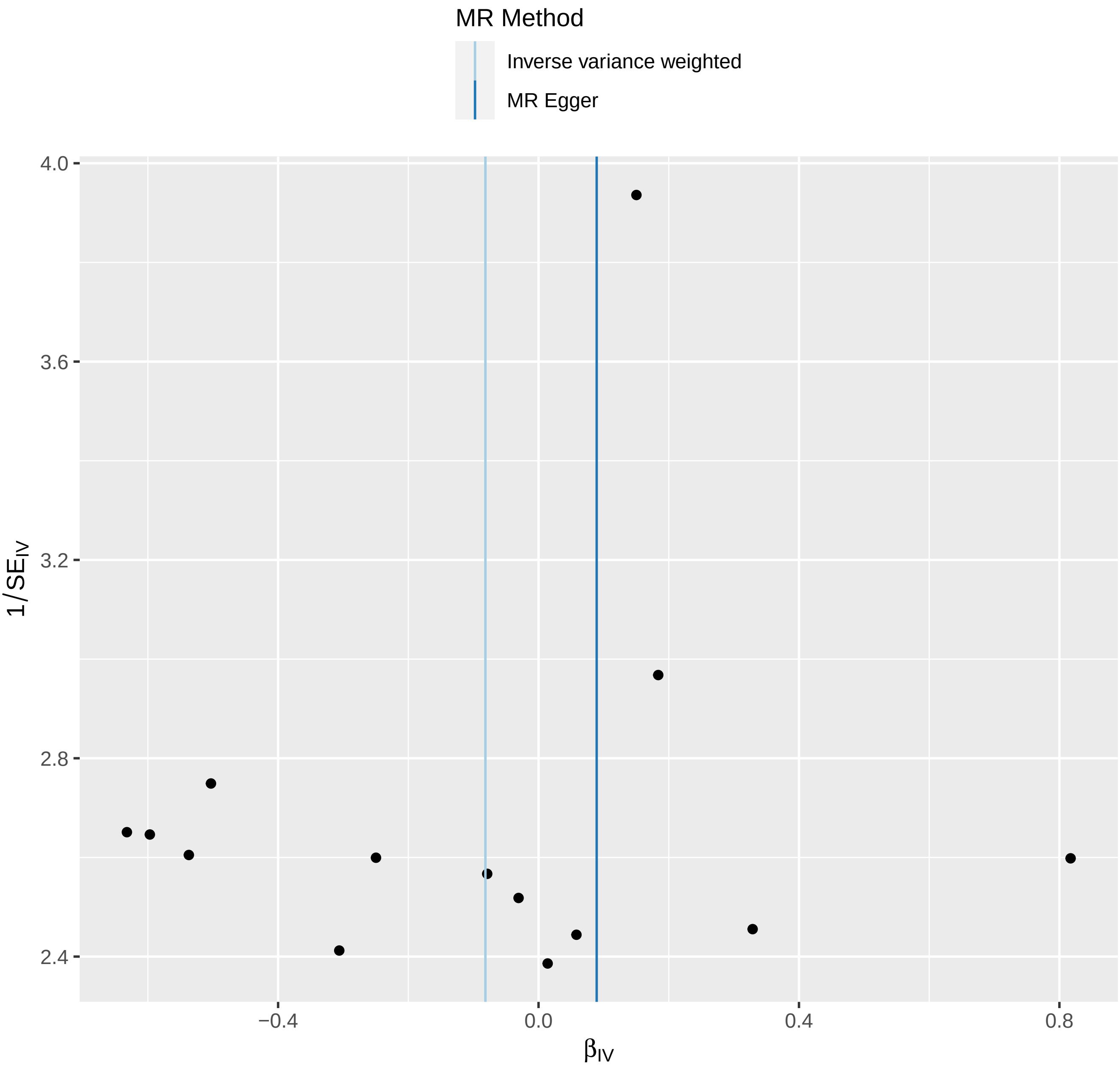

Batch 2−6005 : Gut microbiota abundance (genus Catenibacterium id.2153) on Protein kish−B
with a threshold of 1e−05

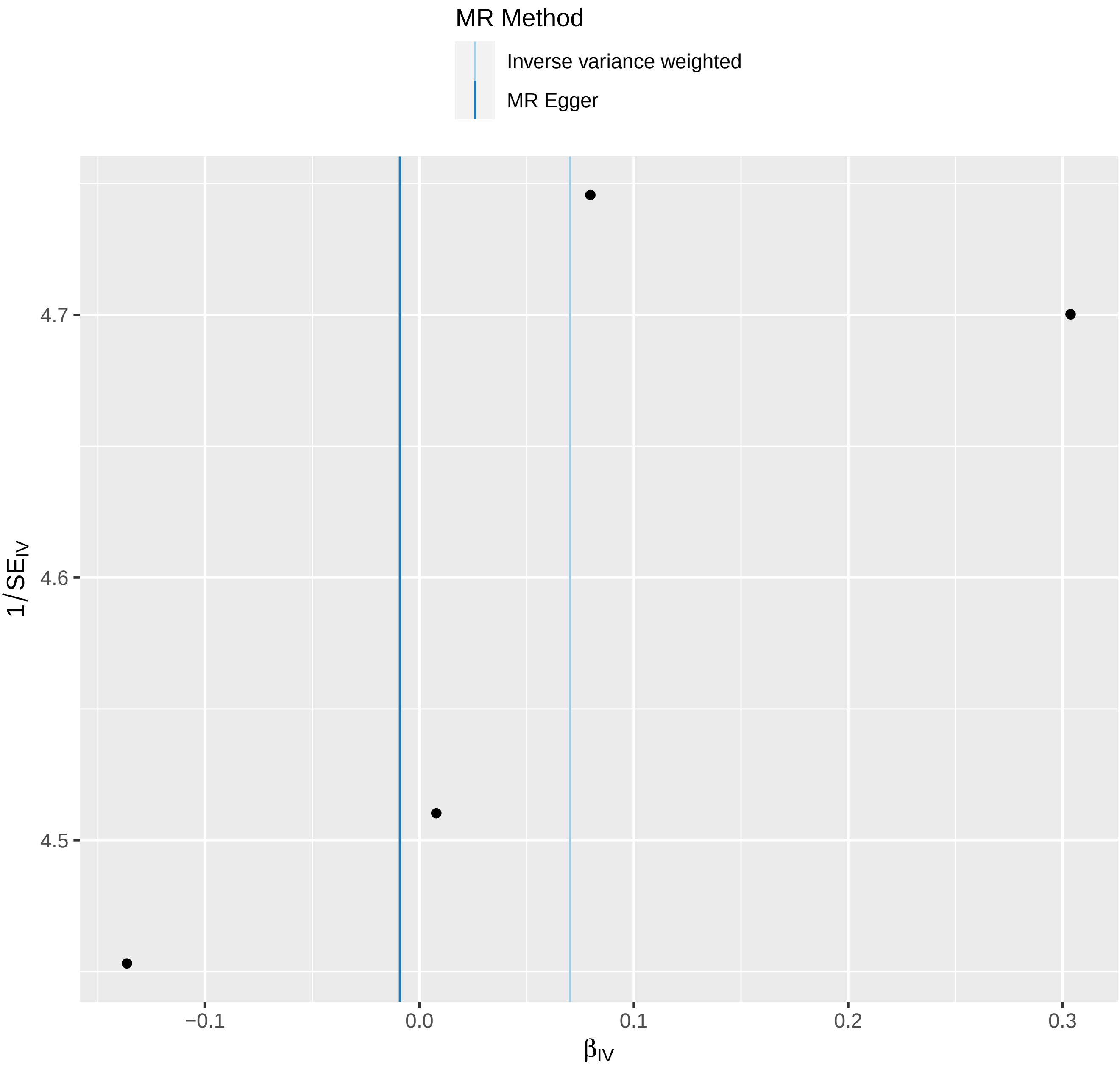

Batch 2−6006 : Gut microbiota abundance (genus Parasutterella id.2892) on Protein kish−B
with a threshold of 1e−05

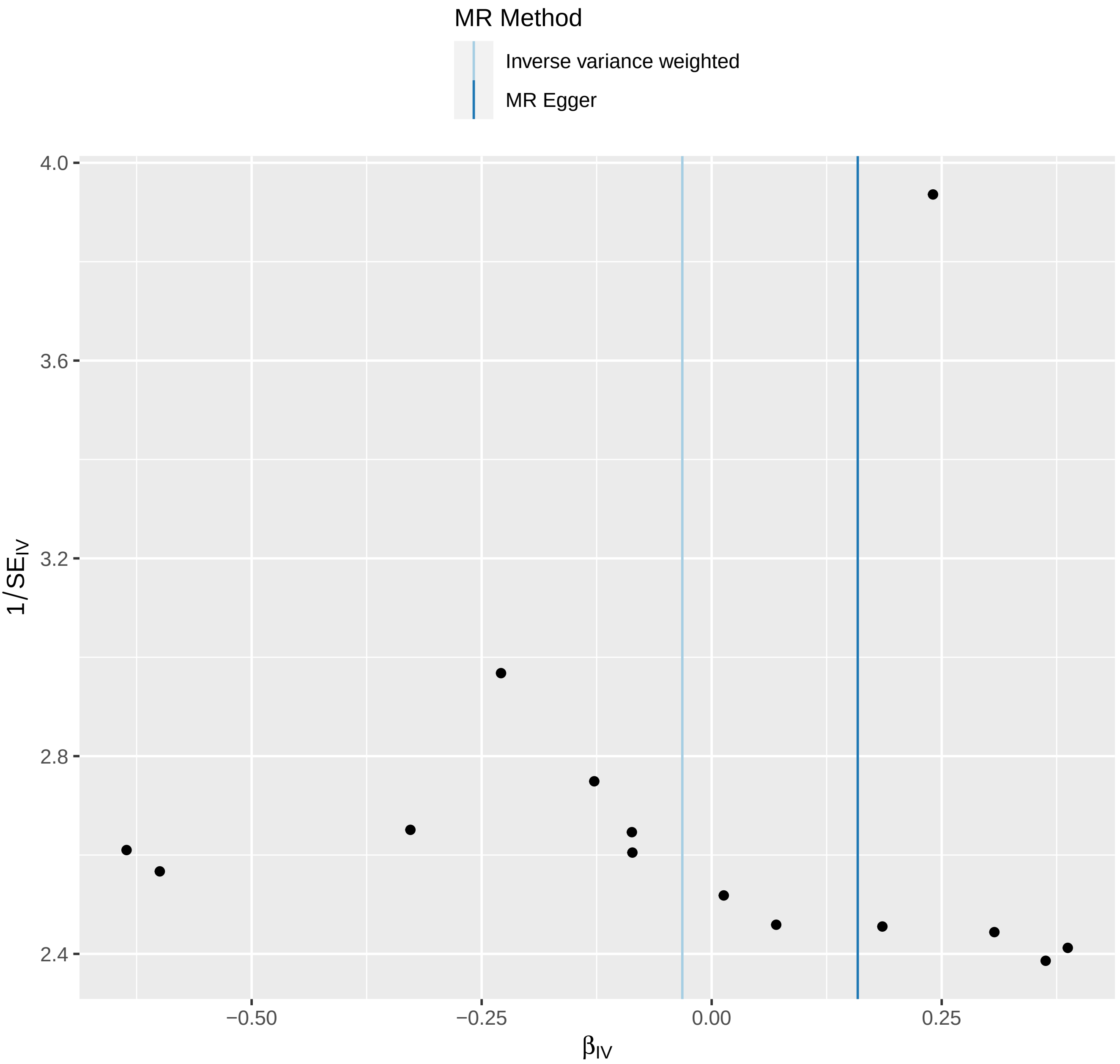

Batch 2−6007 : Gut microbiota abundance (genus Catenibacterium id.2153) on Transmembrane protein 190
with a threshold of 1e−05

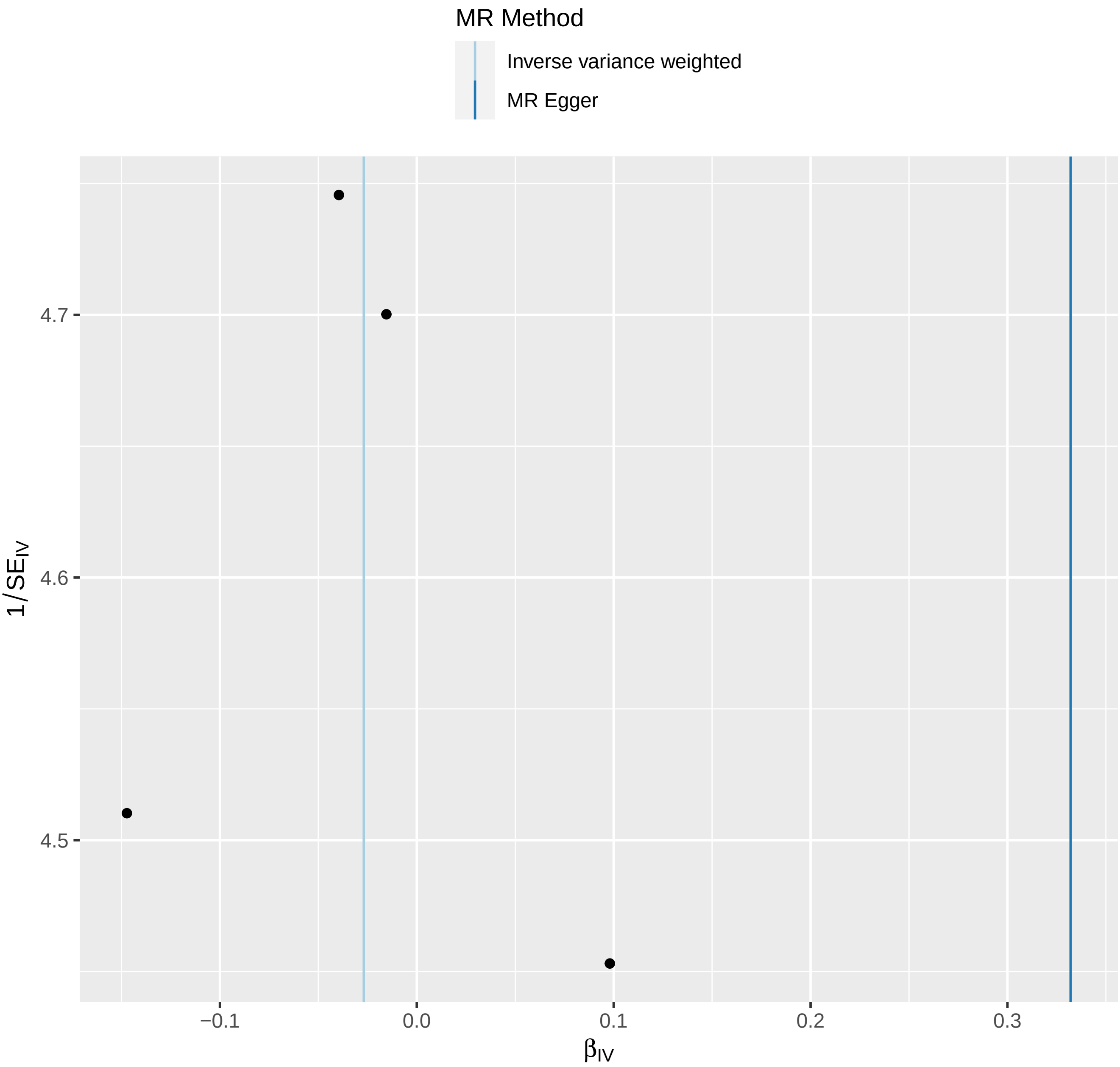

Batch 2−6008 : Gut microbiota abundance (genus Parasutterella id.2892) on Transmembrane protein 190
with a threshold of 1e−05

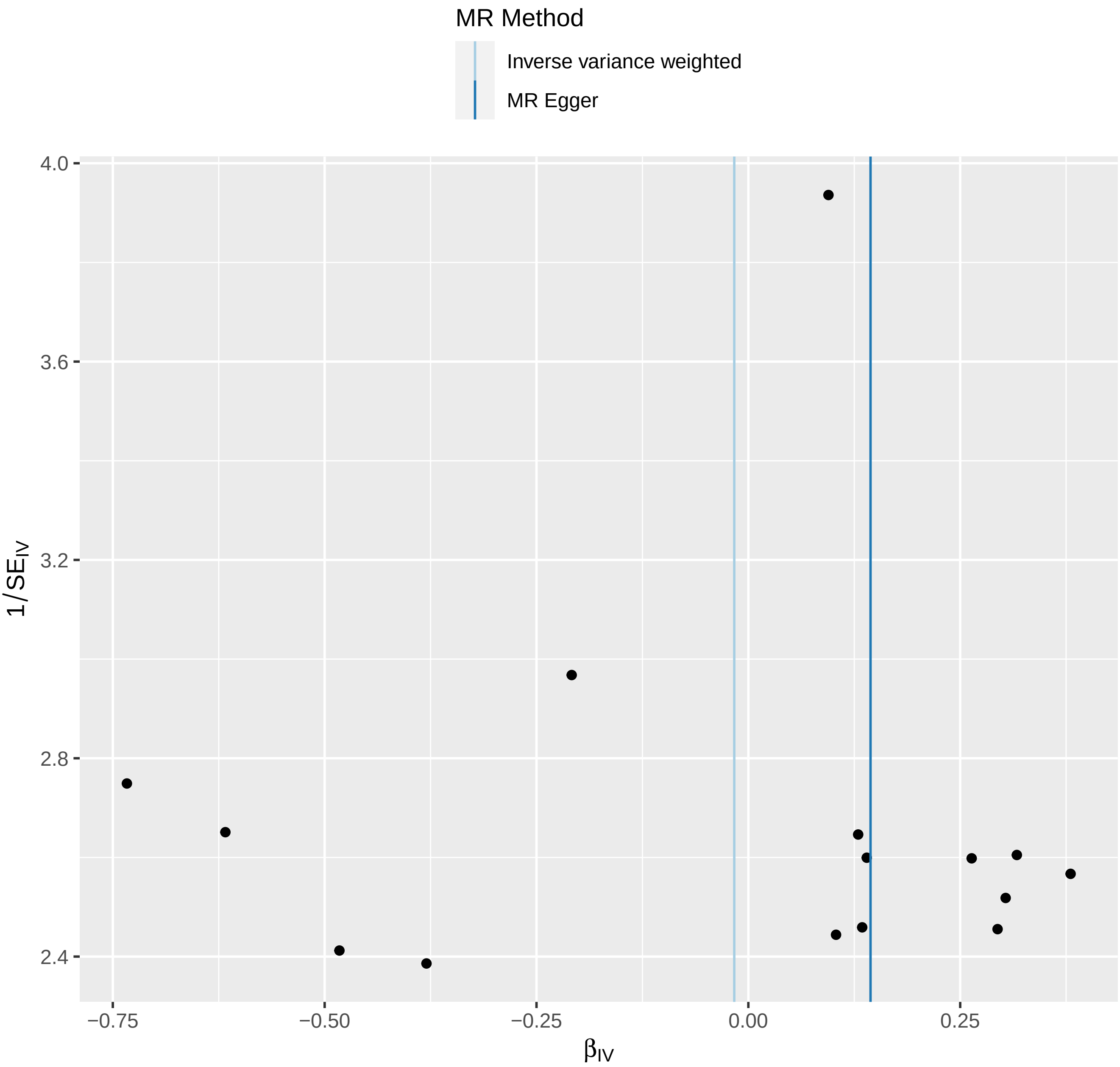

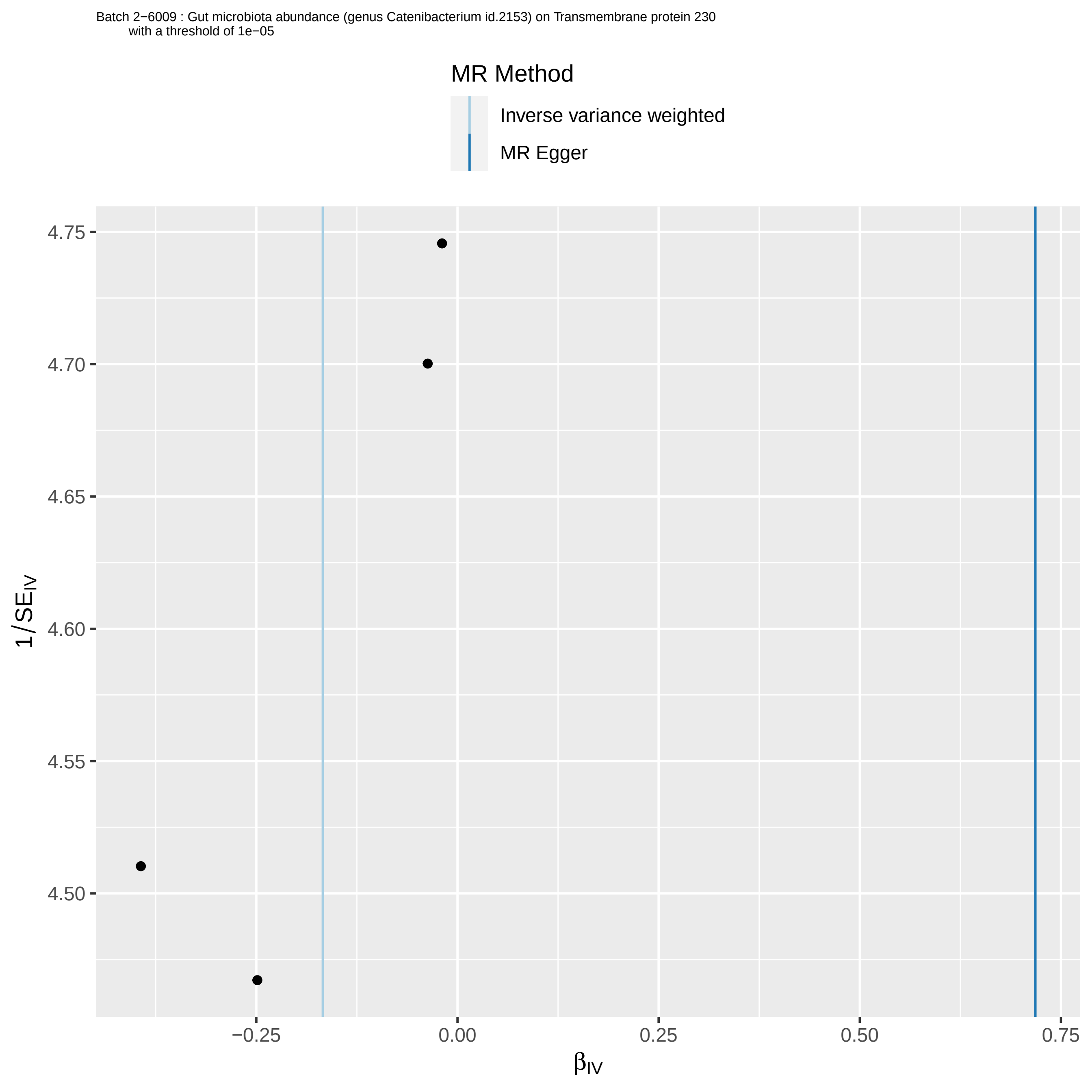
Batch 2−6009 : Gut microbiota abundance (genus Catenibacterium id.2153) on Transmembrane protein 230
with a threshold of 1e−05
MR Method
Inverse variance weighted
MR Egger
4.75
4.70
4.65
4.60
4.55
4.50
1/SEIV
−0.25
0.00
0.25
0.50
0.75
βIV

Batch 2−6010 : Gut microbiota abundance (genus Parasutterella id.2892) on Transmembrane protein 230
with a threshold of 1e−05

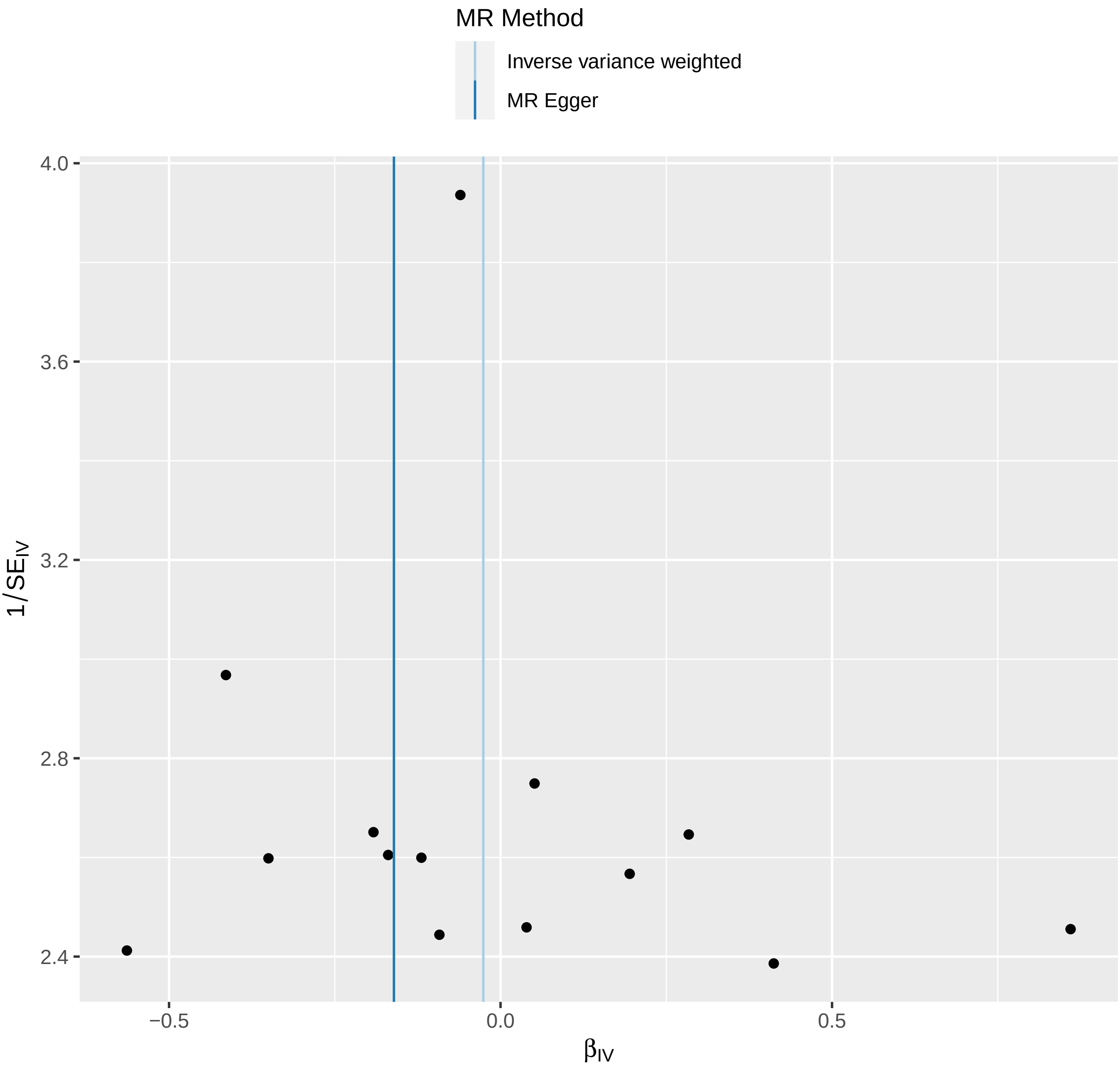

Batch 2−6011 : Gut microbiota abundance (genus Catenibacterium id.2153) on Transmembrane protein 234
with a threshold of 1e−05

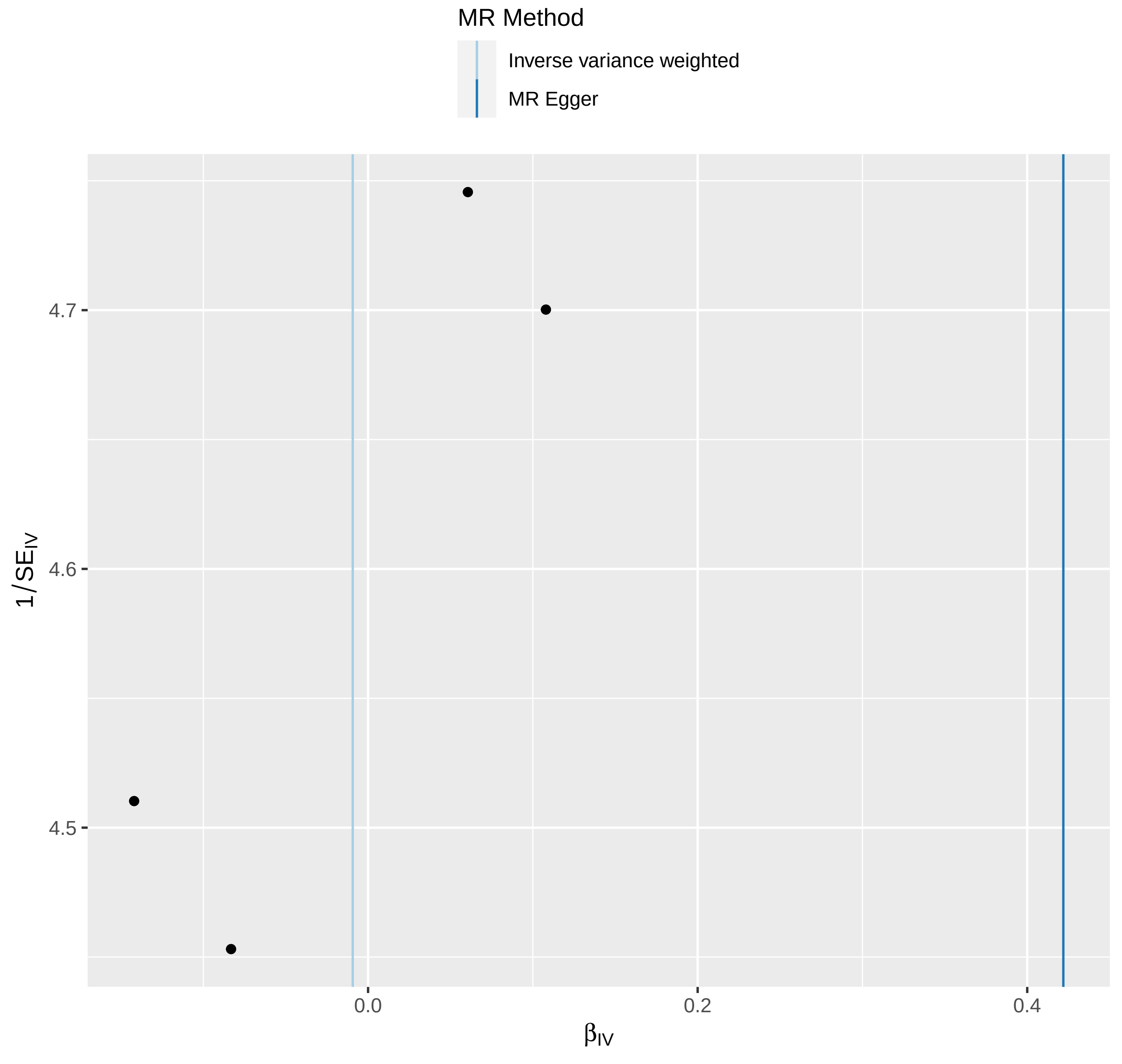

Batch 2−6012 : Gut microbiota abundance (genus Parasutterella id.2892) on Transmembrane protein 234
with a threshold of 1e−05

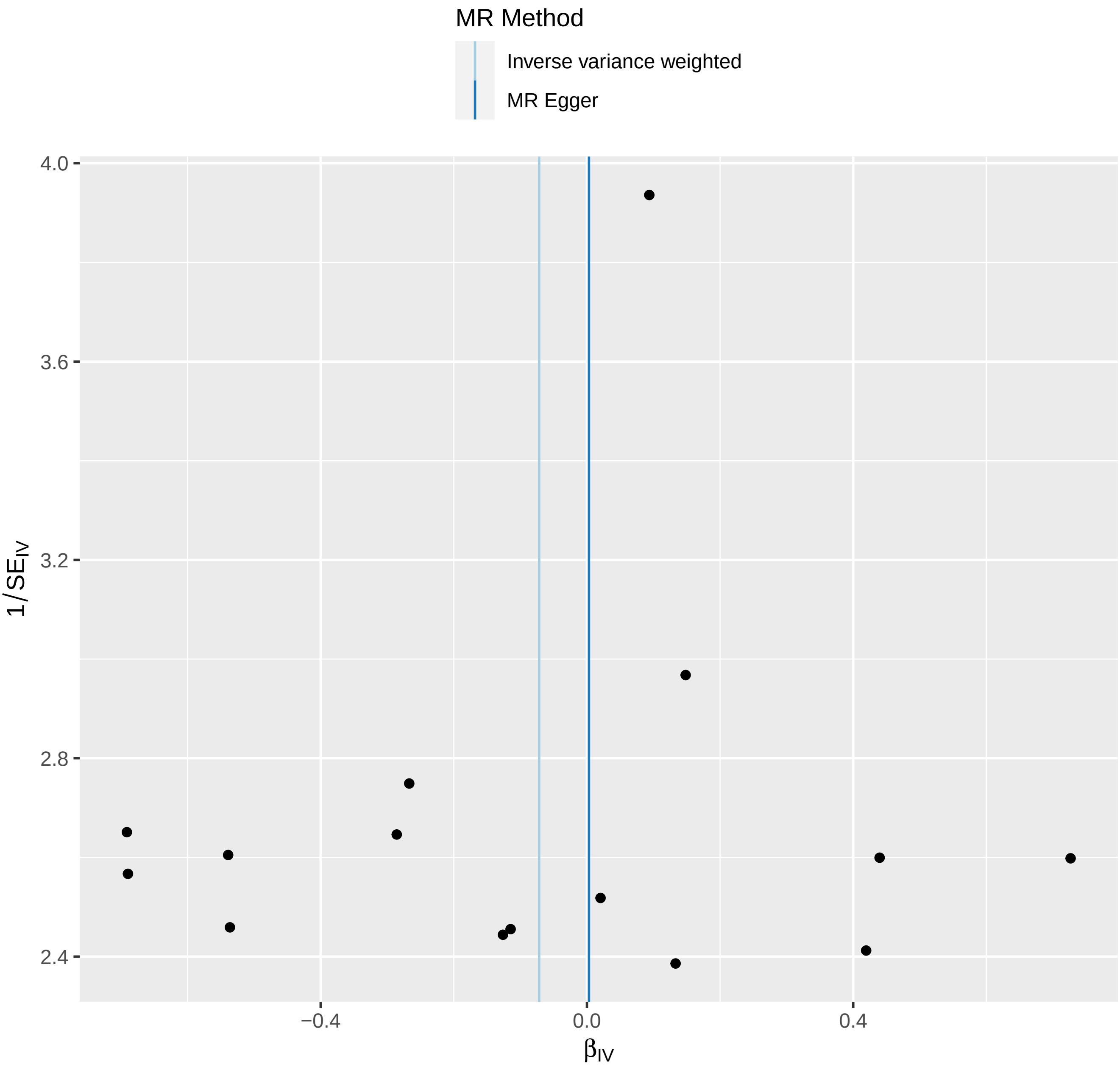

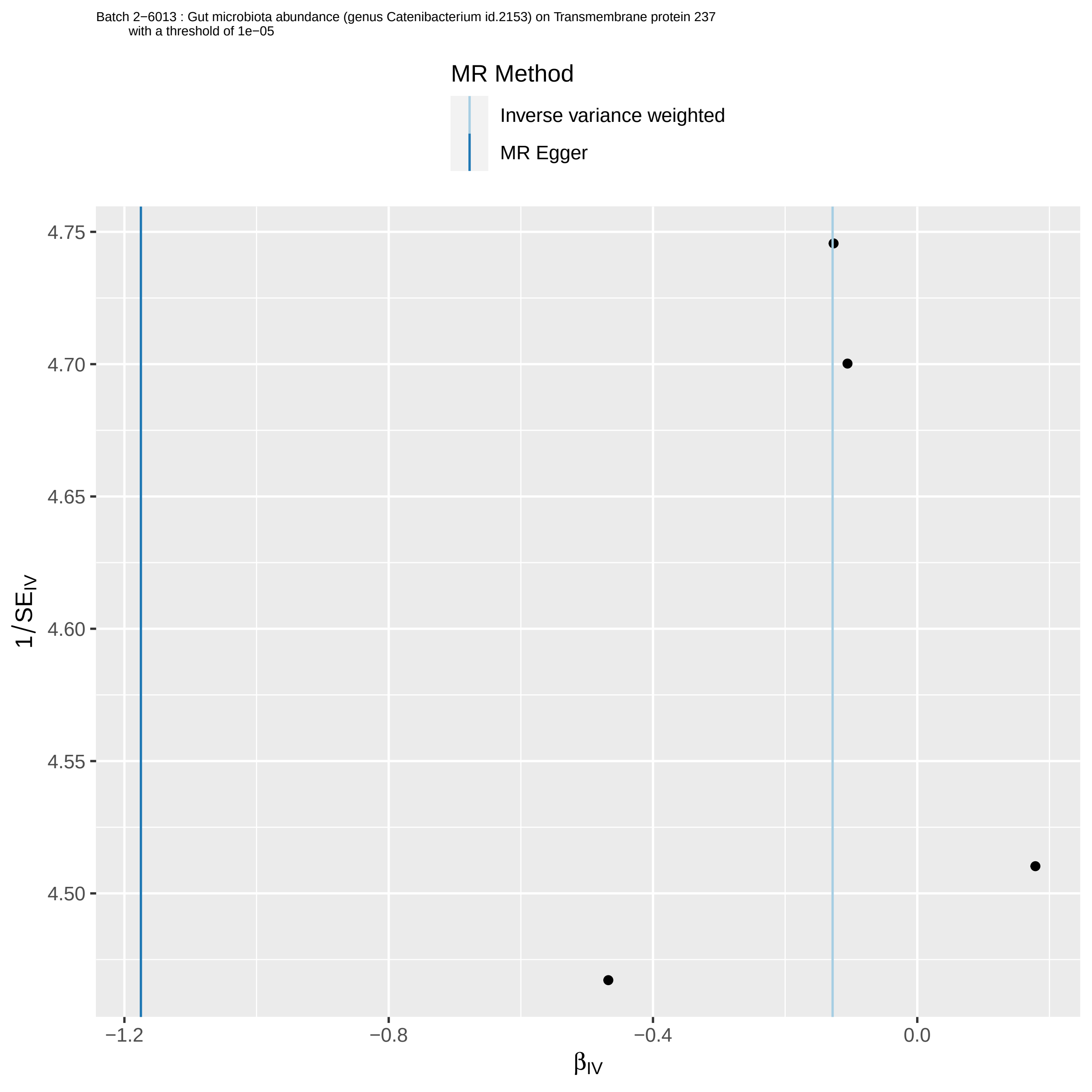
Batch 2−6013 : Gut microbiota abundance (genus Catenibacterium id.2153) on Transmembrane protein 237
with a threshold of 1e−05
MR Method
Inverse variance weighted
MR Egger
4.75
4.70
4.65
4.60
4.55
4.50
1/SE_IV
−1.2
−0.8
−0.4
0.0
β_IV

Batch 2−6014 : Gut microbiota abundance (genus Parasutterella id.2892) on Transmembrane protein 237
with a threshold of 1e−05

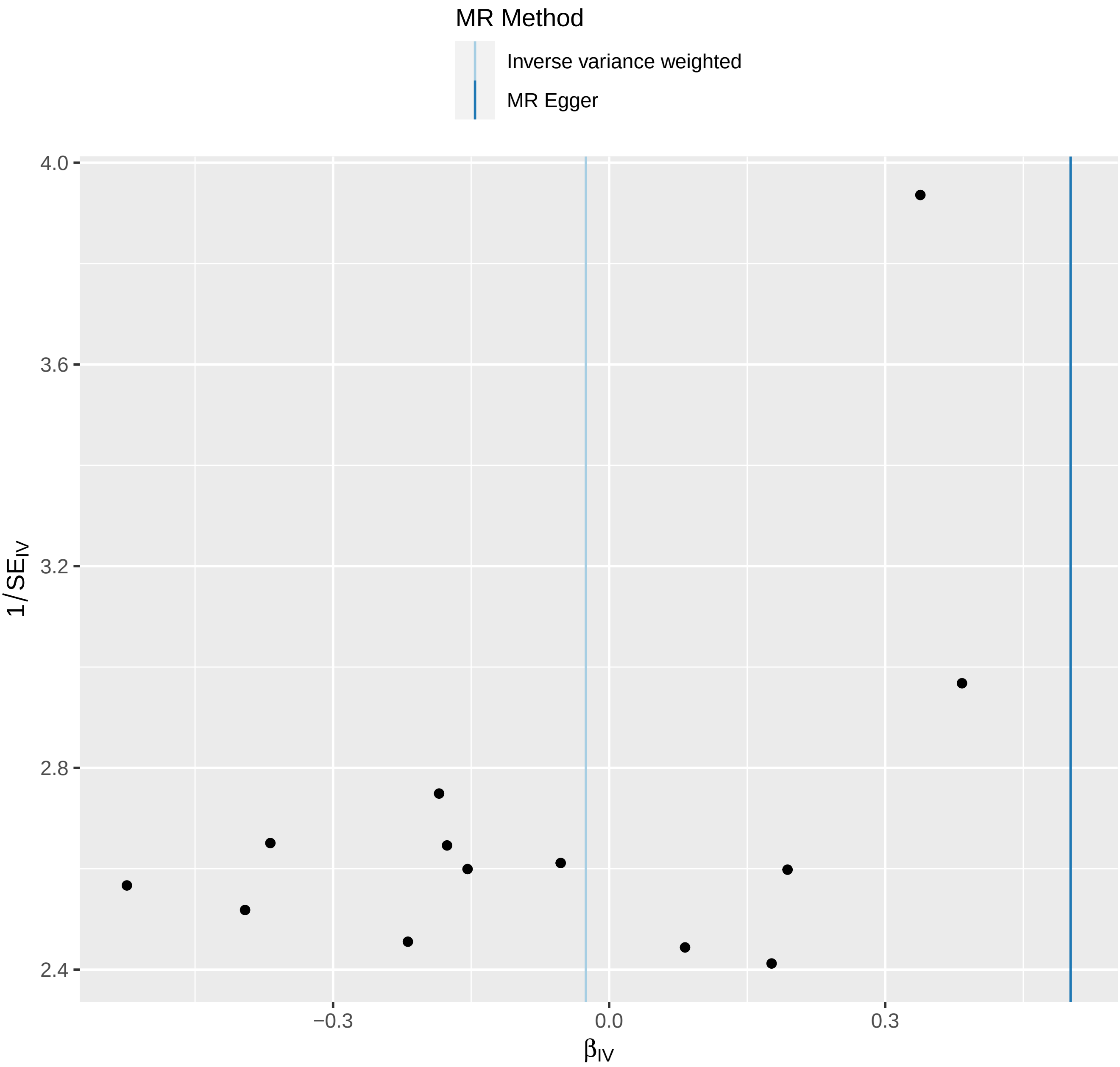

Batch 2−6015 : Gut microbiota abundance (genus Catenibacterium id.2153) on Collectrin
with a threshold of 1e−05

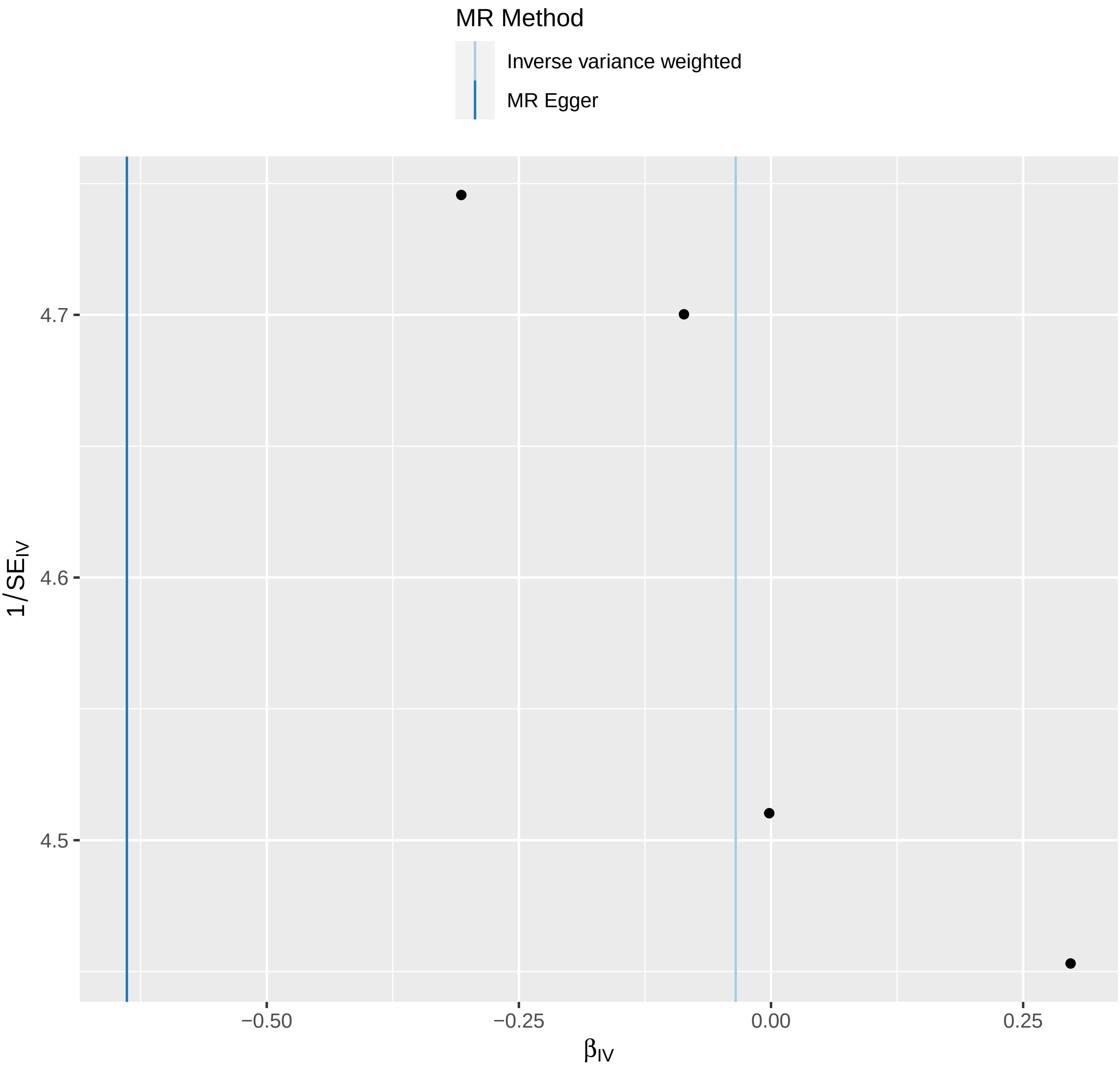

Batch 2−6016 : Gut microbiota abundance (genus Parasutterella id.2892) on Collectrin
with a threshold of 1e−05

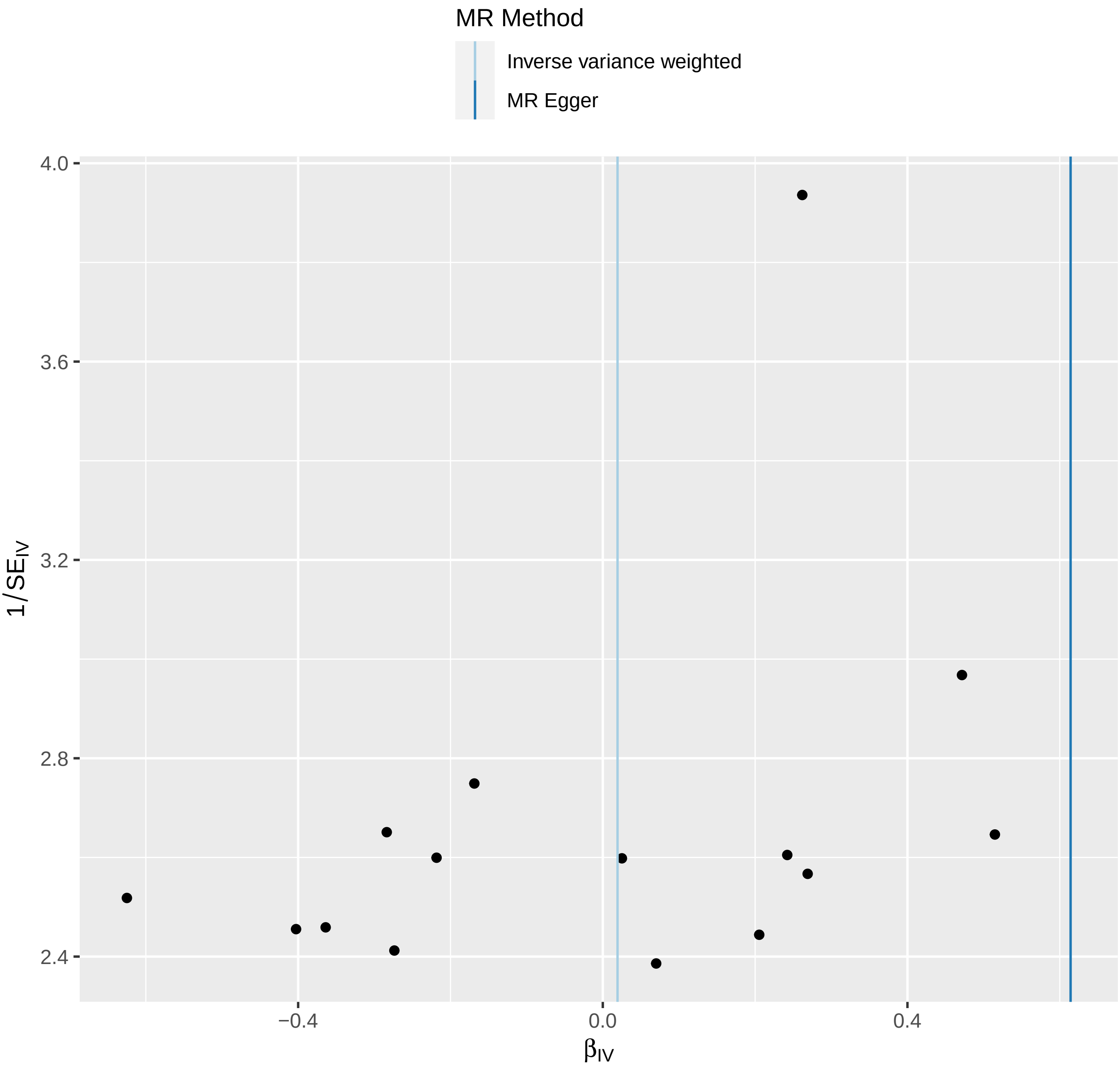

Batch 2−6017 : Gut microbiota abundance (genus Catenibacterium id.2153) on Transmembrane protein 2
with a threshold of 1e−05

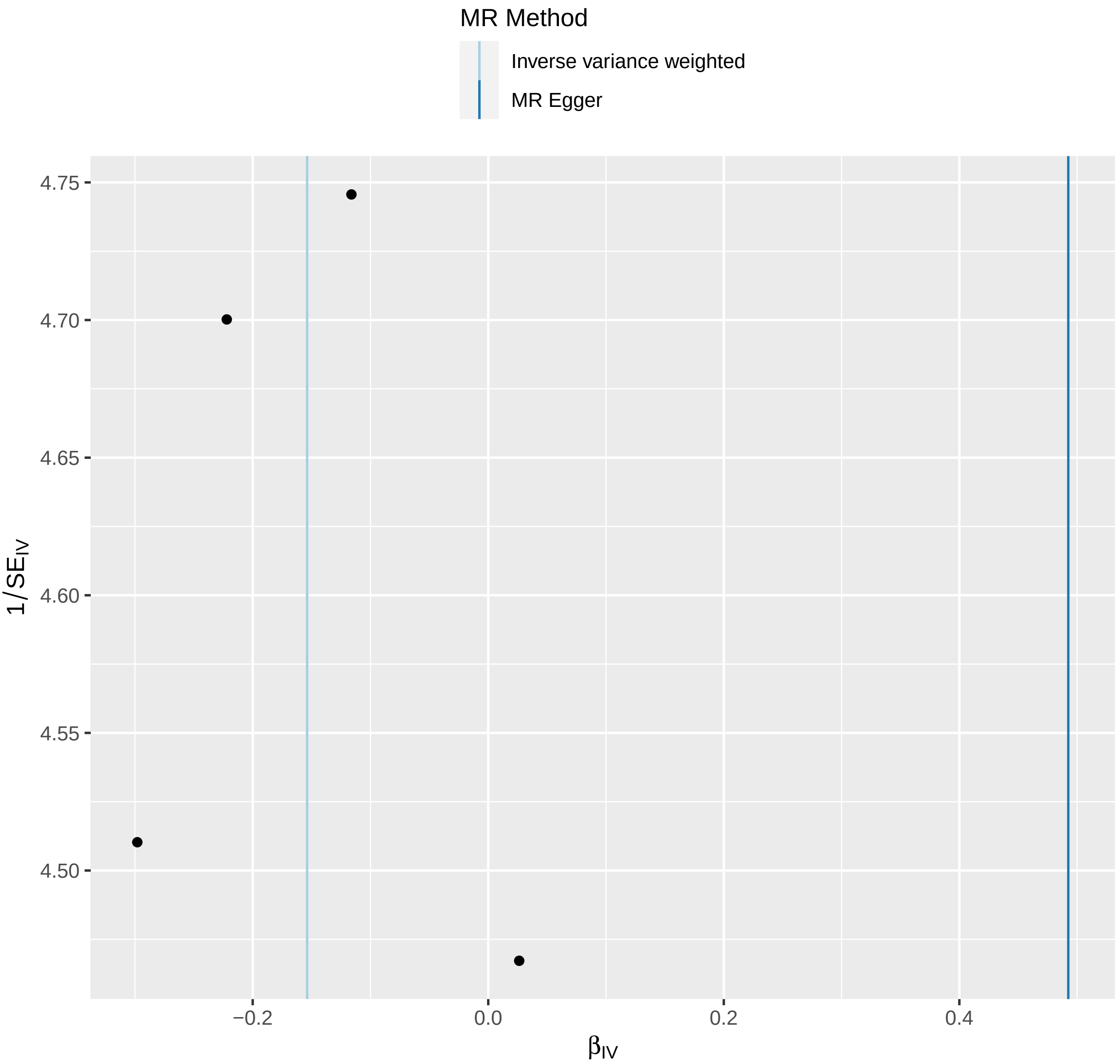

Batch 2−6018 : Gut microbiota abundance (genus Parasutterella id.2892) on Transmembrane protein 2
with a threshold of 1e−05

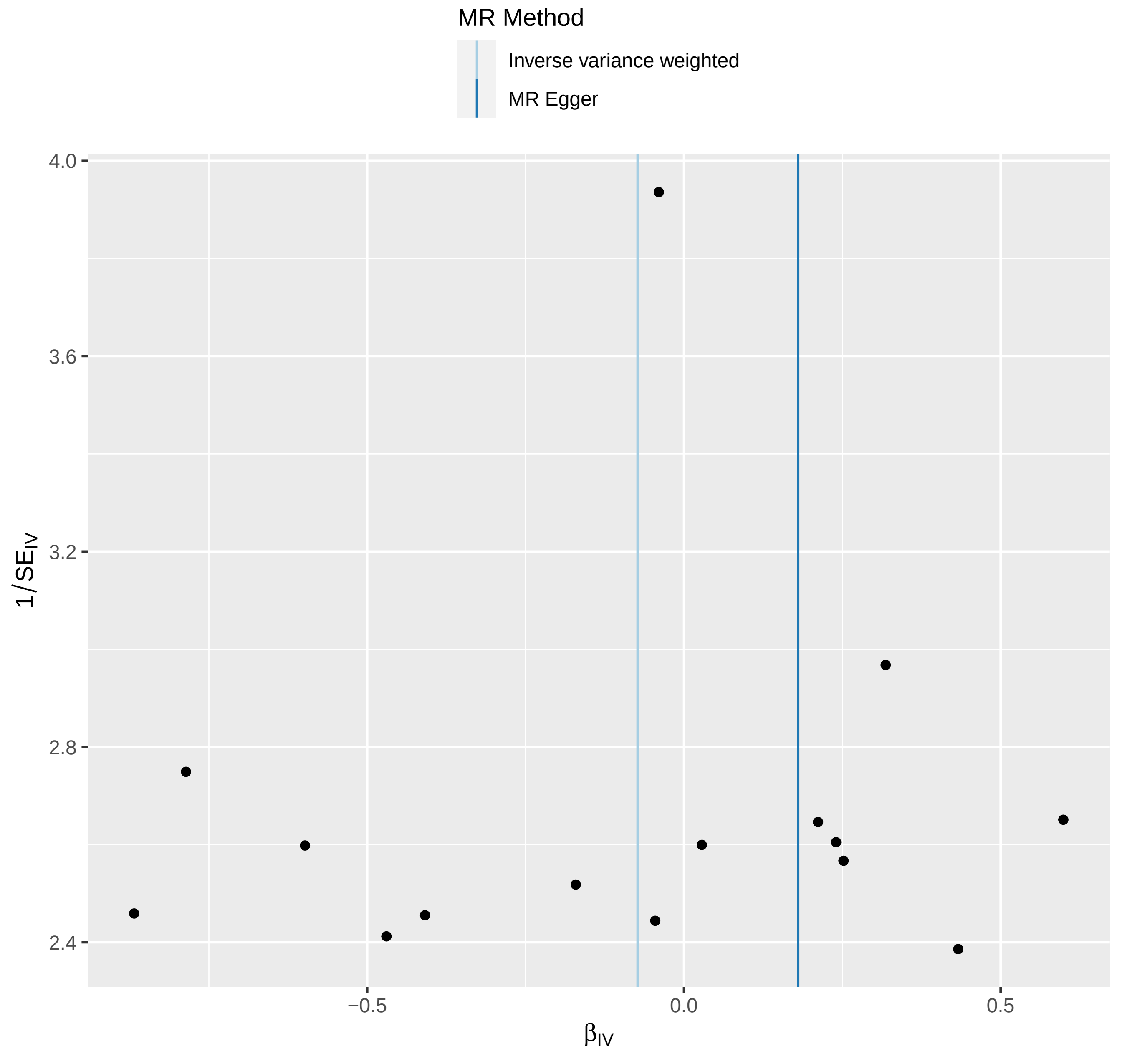

Batch 2−6019 : Gut microbiota abundance (genus Catenibacterium id.2153) on Transmembrane protein 40
with a threshold of 1e−05

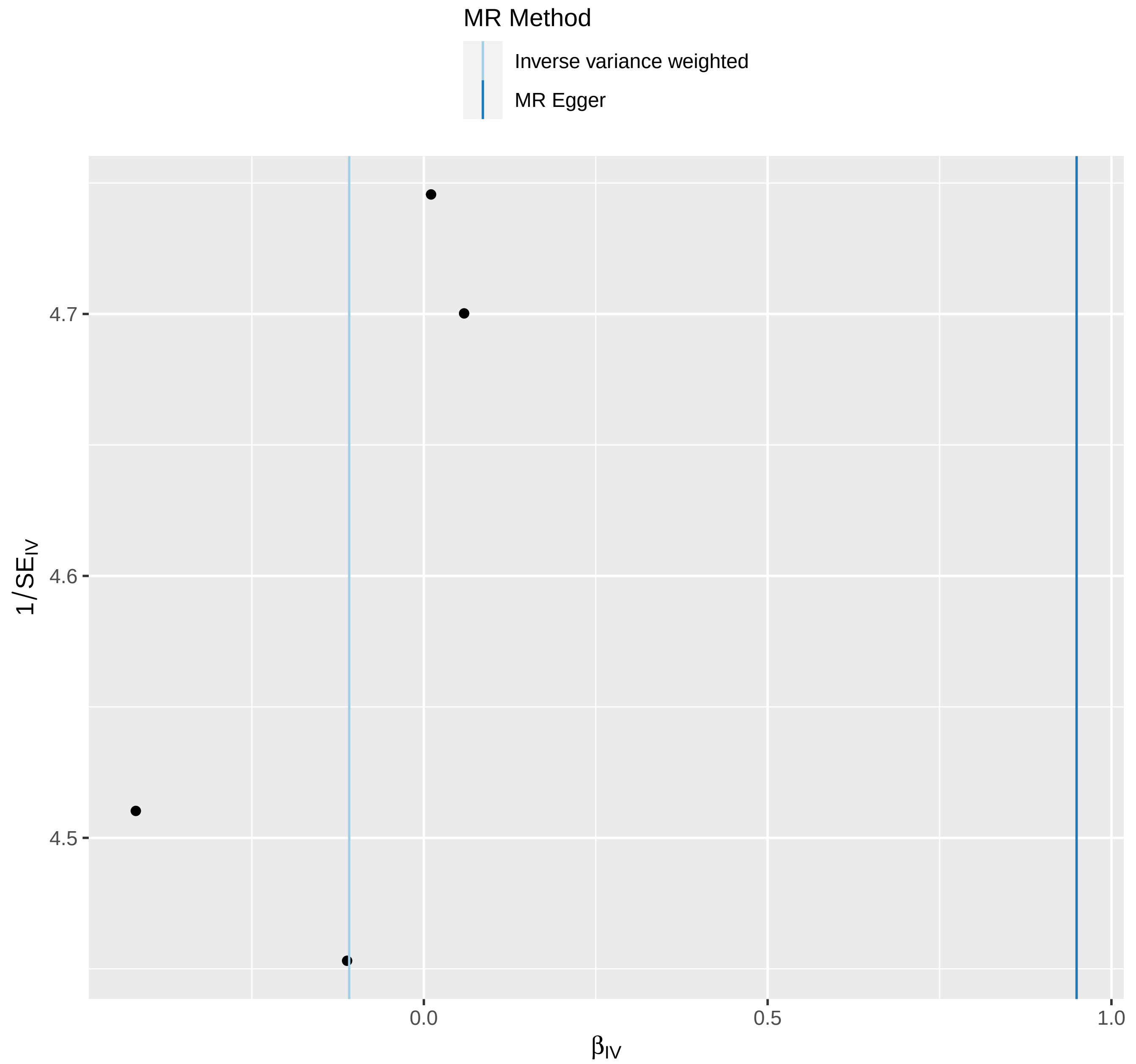

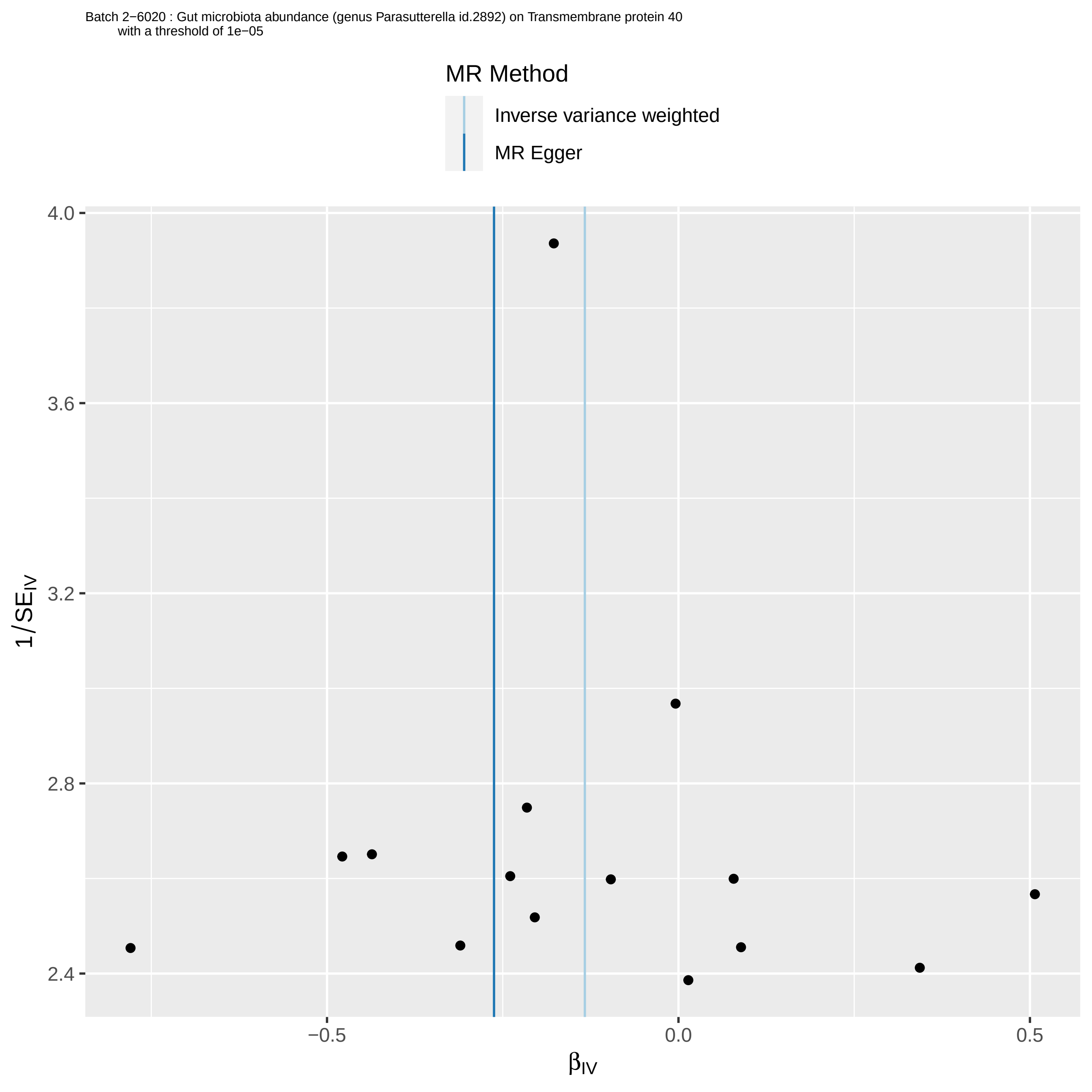

Batch 2−6020 : Gut microbiota abundance (genus Parasutterella id.2892) on Transmembrane protein 40
with a threshold of 1e−05
MR Method
Inverse variance weighted
MR Egger
4.0
3.6
3.2
2.8
2.4
1/SE_IV
−0.5
0.0
0.5
β_IV

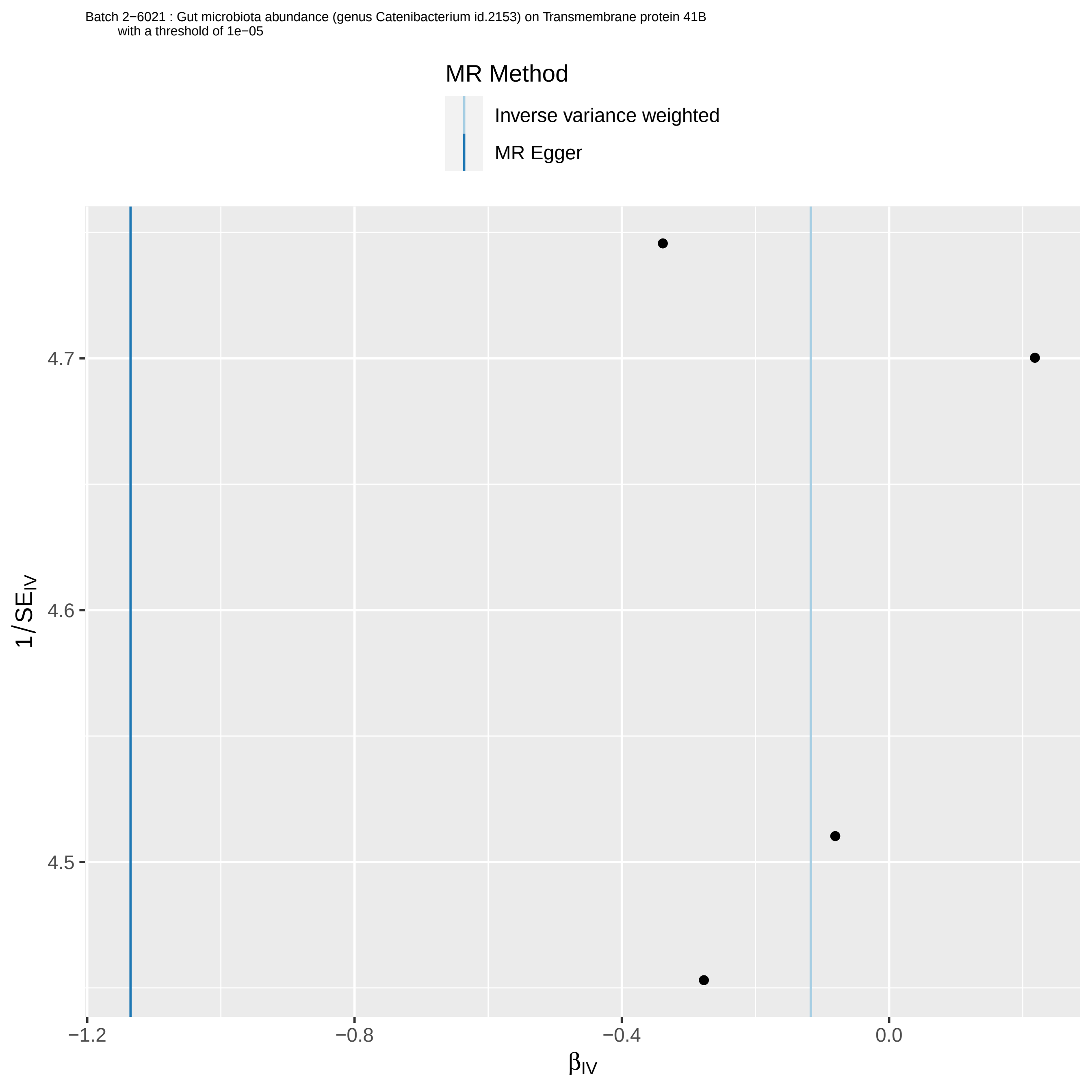

Batch 2−6021 : Gut microbiota abundance (genus Catenibacterium id.2153) on Transmembrane protein 41B
with a threshold of 1e−05
MR Method
Inverse variance weighted
MR Egger
4.7
4.6
4.5
1/SE_IV
−1.2
−0.8
−0.4
0.0
β_IV

Batch 2−6022 : Gut microbiota abundance (genus Parasutterella id.2892) on Transmembrane protein 41B
with a threshold of 1e−05

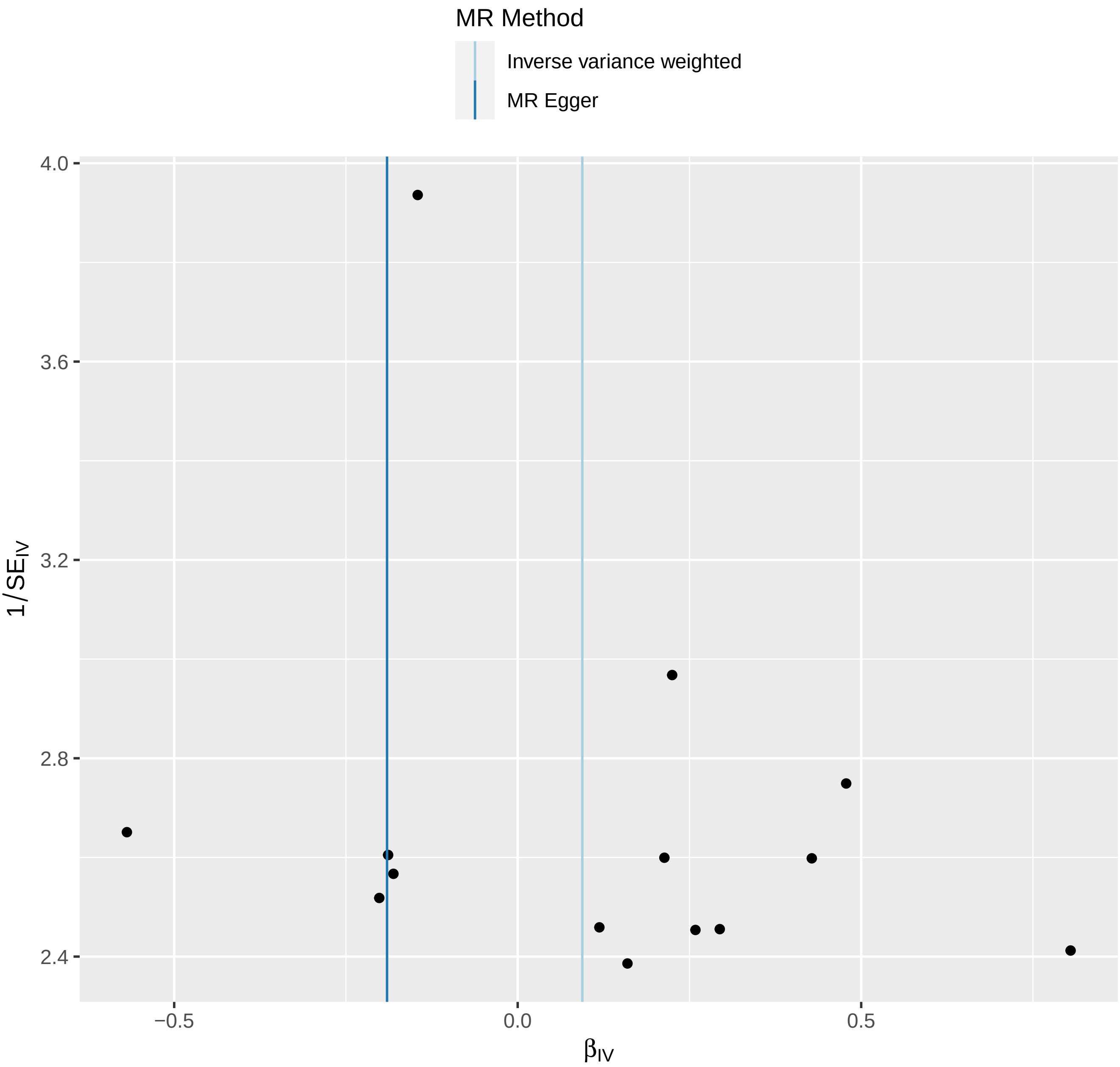

Batch 2−6023 : Gut microbiota abundance (genus Catenibacterium id.2153) on Transmembrane protein 52
with a threshold of 1e−05

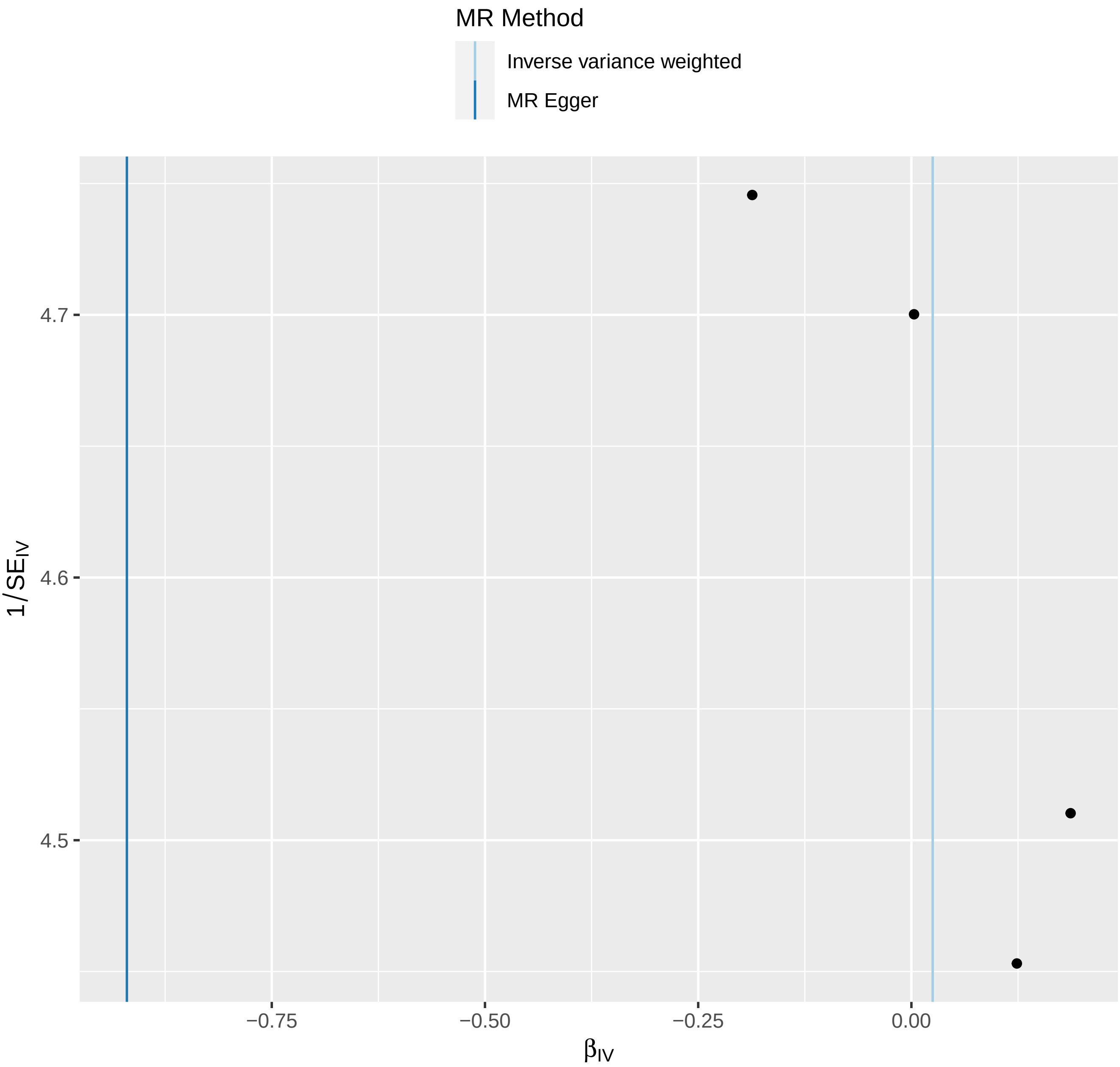

Batch 2−6024 : Gut microbiota abundance (genus Parasutterella id.2892) on Transmembrane protein 52
with a threshold of 1e−05

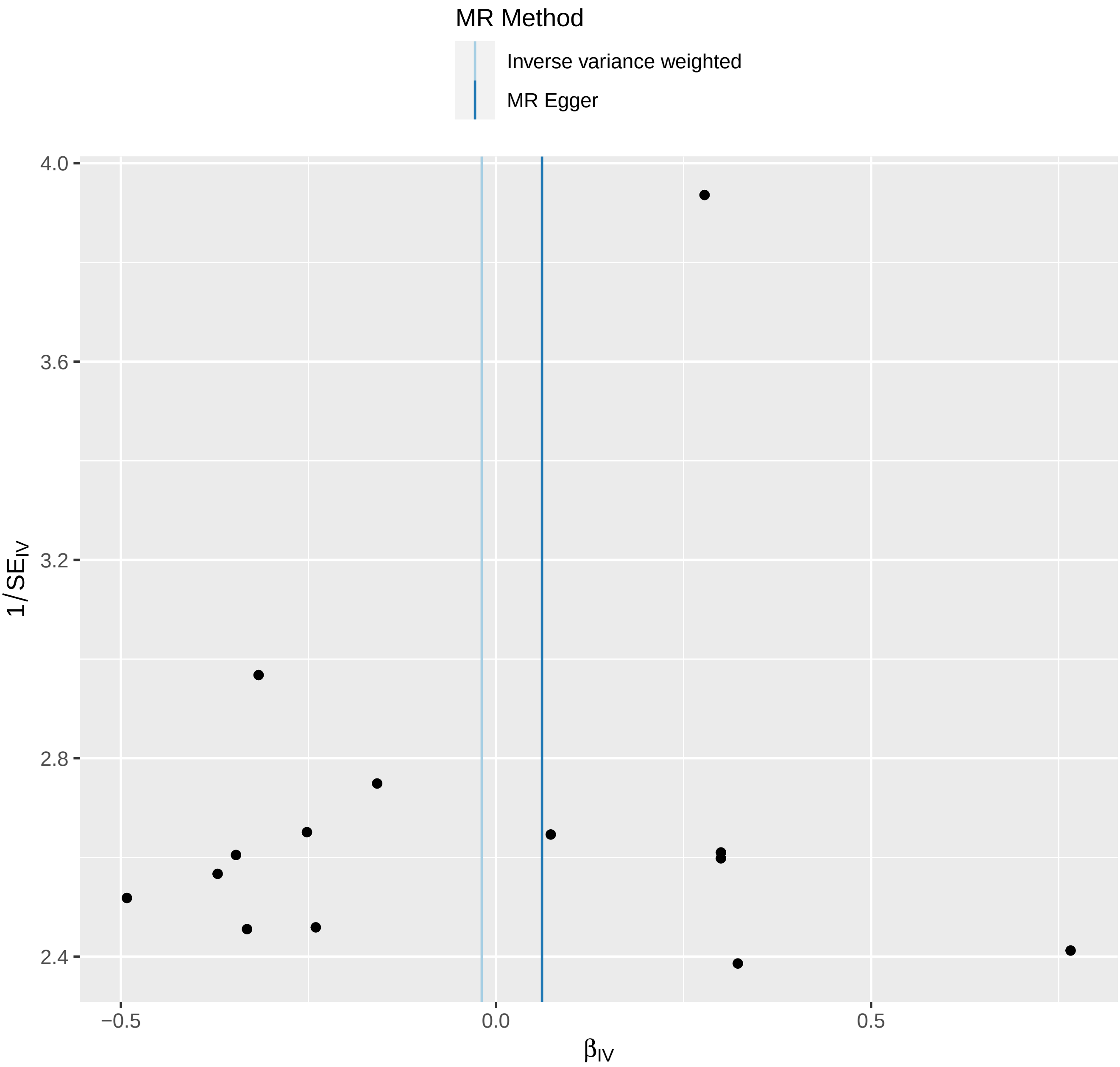

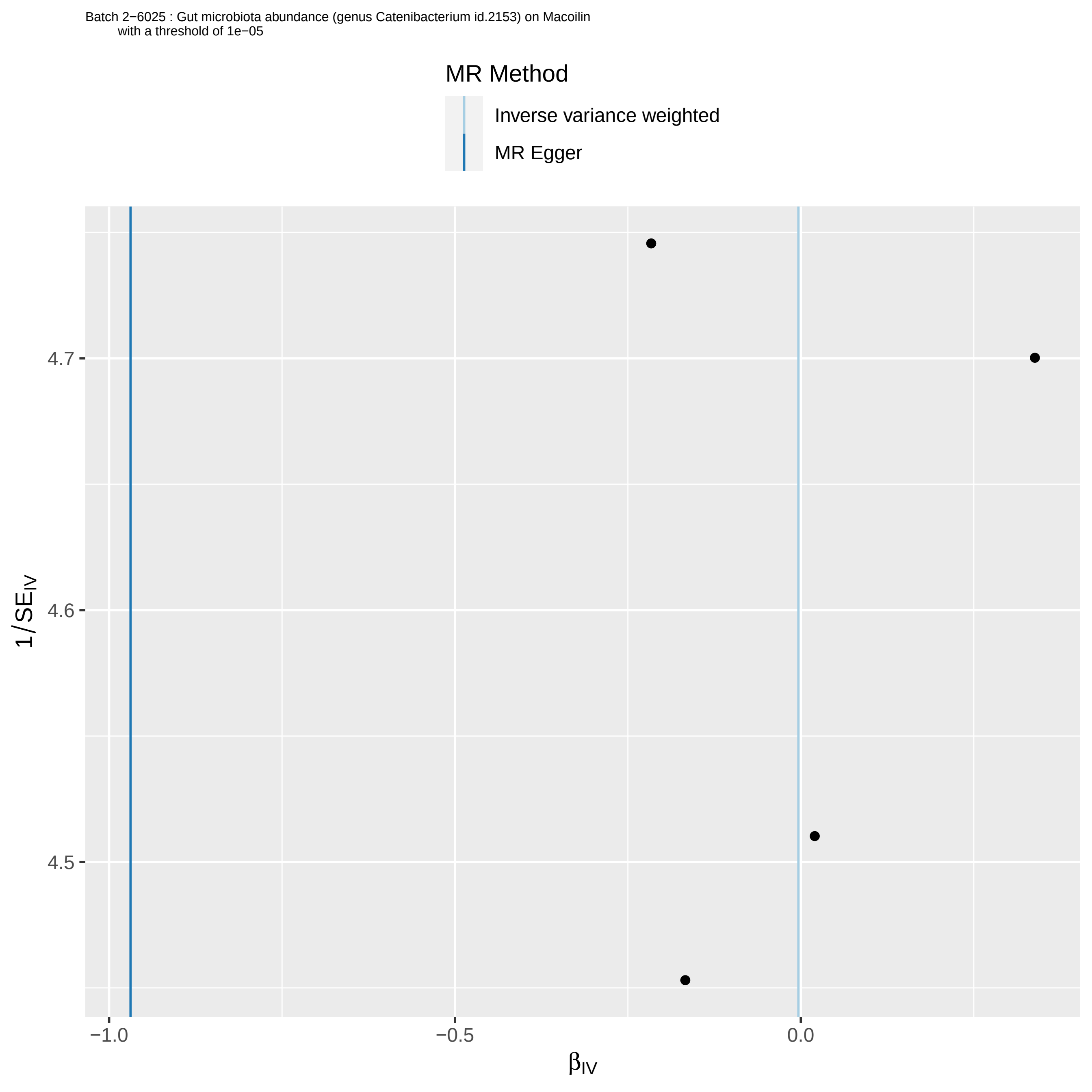

Batch 2−6025 : Gut microbiota abundance (genus Catenibacterium id.2153) on Macoilin
with a threshold of 1e−05
MR Method
Inverse variance weighted
MR Egger
4.7
4.6
4.5
1/SE_IV
−1.0
−0.5
0.0
β_IV

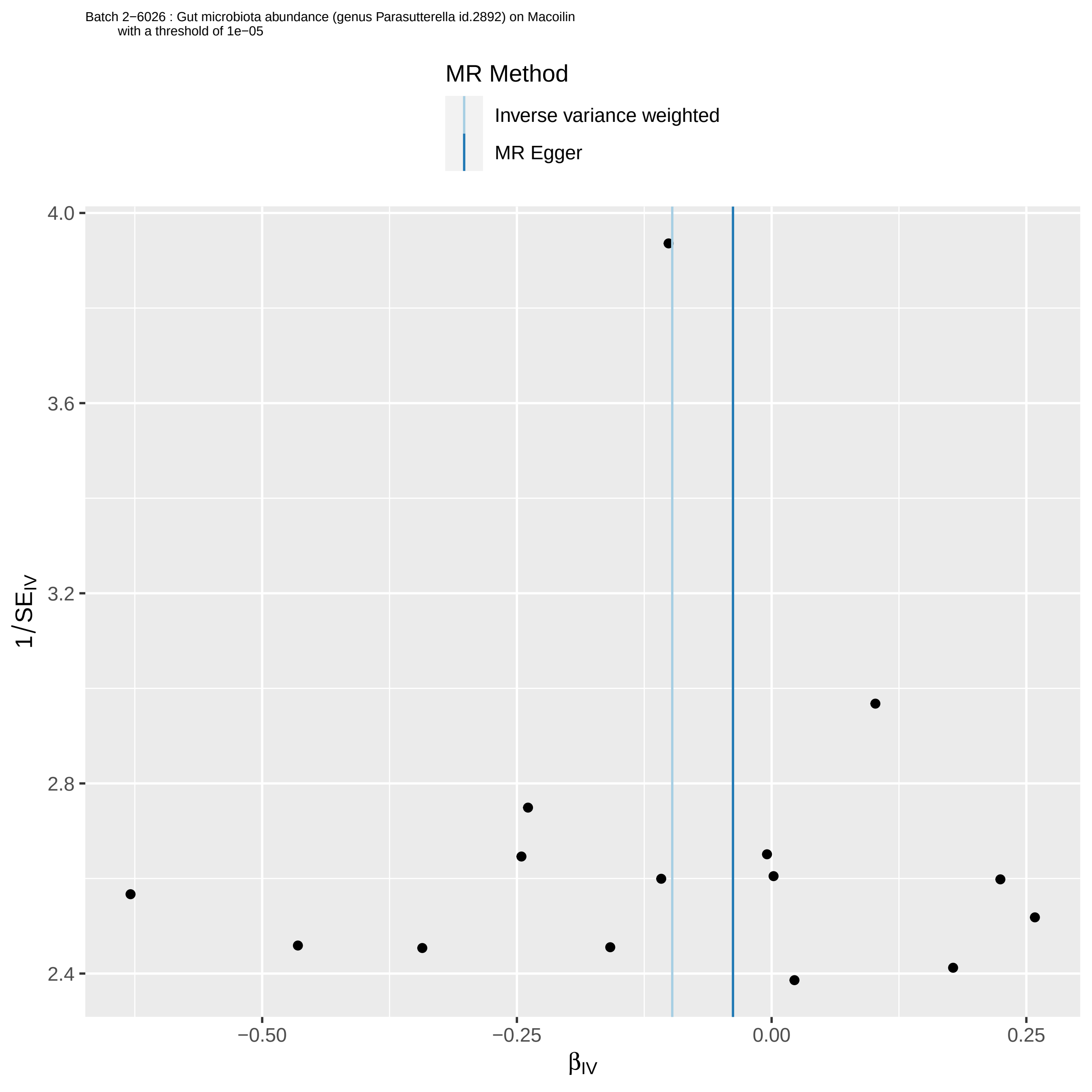

Batch 2−6026 : Gut microbiota abundance (genus Parasutterella id.2892) on Macoilin
with a threshold of 1e−05
MR Method
Inverse variance weighted
MR Egger
4.0
3.6
3.2
2.8
2.4
1/SE_IV
−0.50
−0.25
0.00
0.25
β_IV

Batch 2−6027 : Gut microbiota abundance (genus Catenibacterium id.2153) on Transmembrane protein 59−like with a threshold of 1e−05

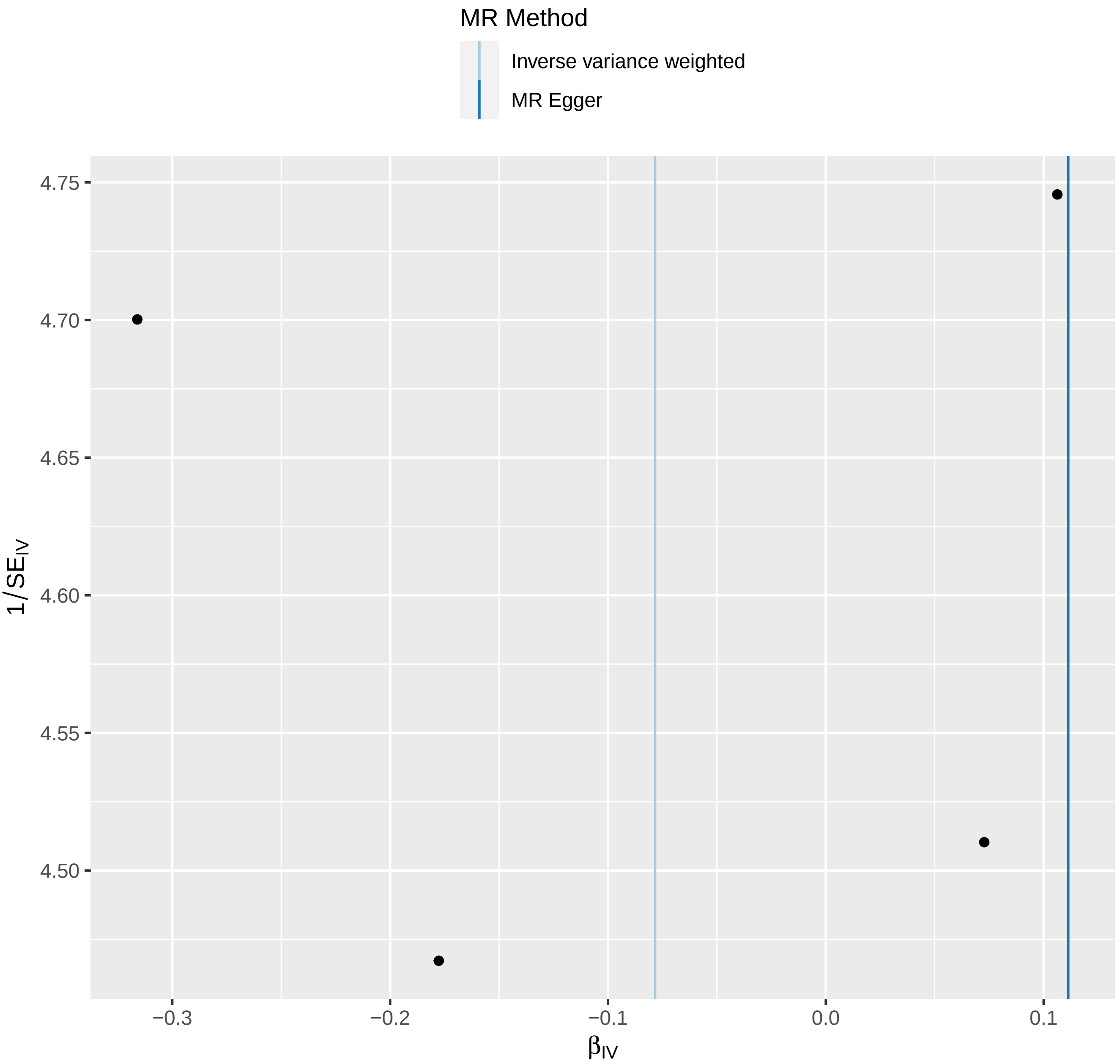

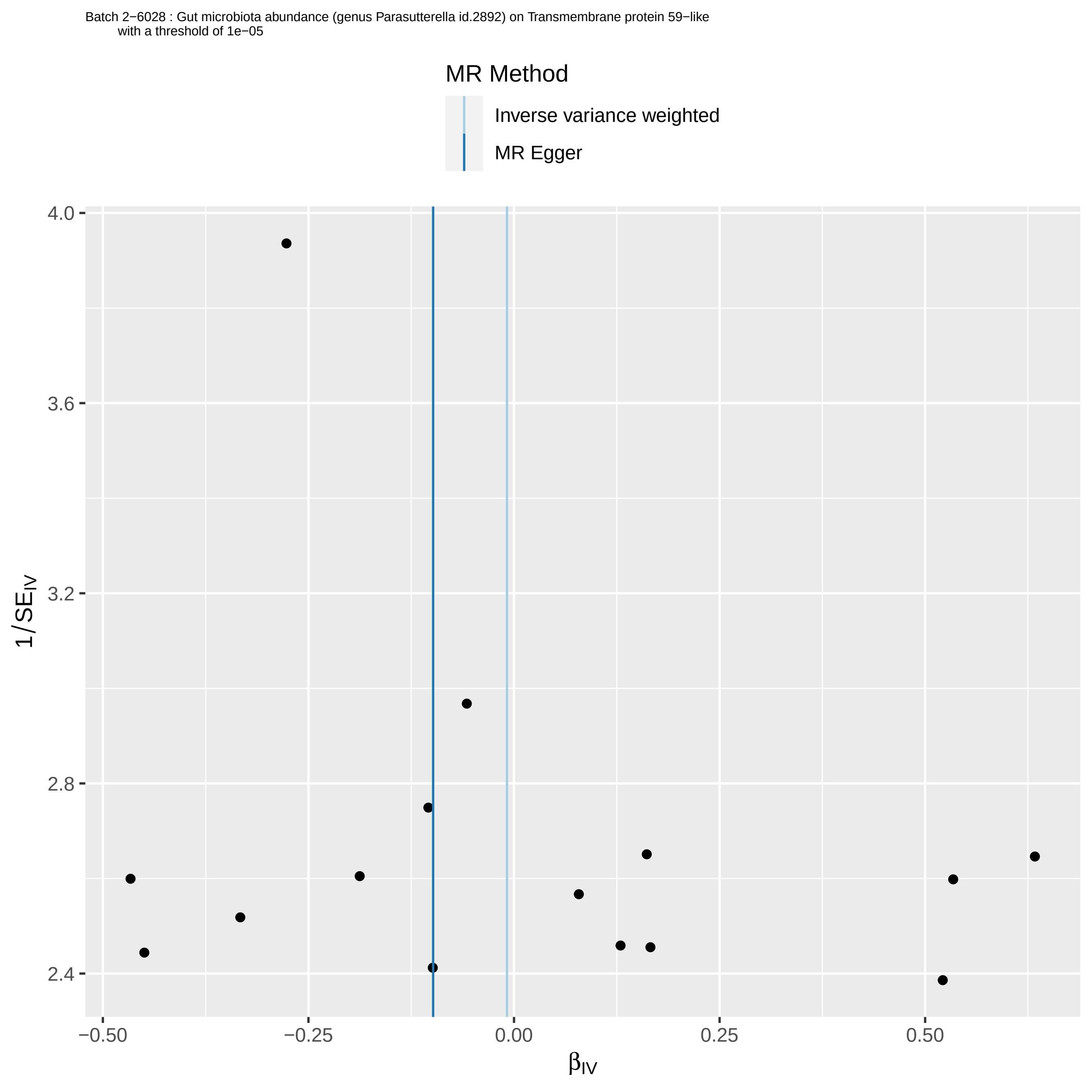

Batch 2−6028 : Gut microbiota abundance (genus Parasutterella id.2892) on Transmembrane protein 59−like
with a threshold of 1e−05
MR Method
Inverse variance weighted
MR Egger
4.0
3.6
3.2
2.8
2.4
1/SE_IV
−0.50
−0.25
0.00
0.25
0.50
β_IV

Batch 2−6029 : Gut microbiota abundance (genus Catenibacterium id.2153) on Transmembrane protein 70, mitochondrial with a threshold of 1e−05

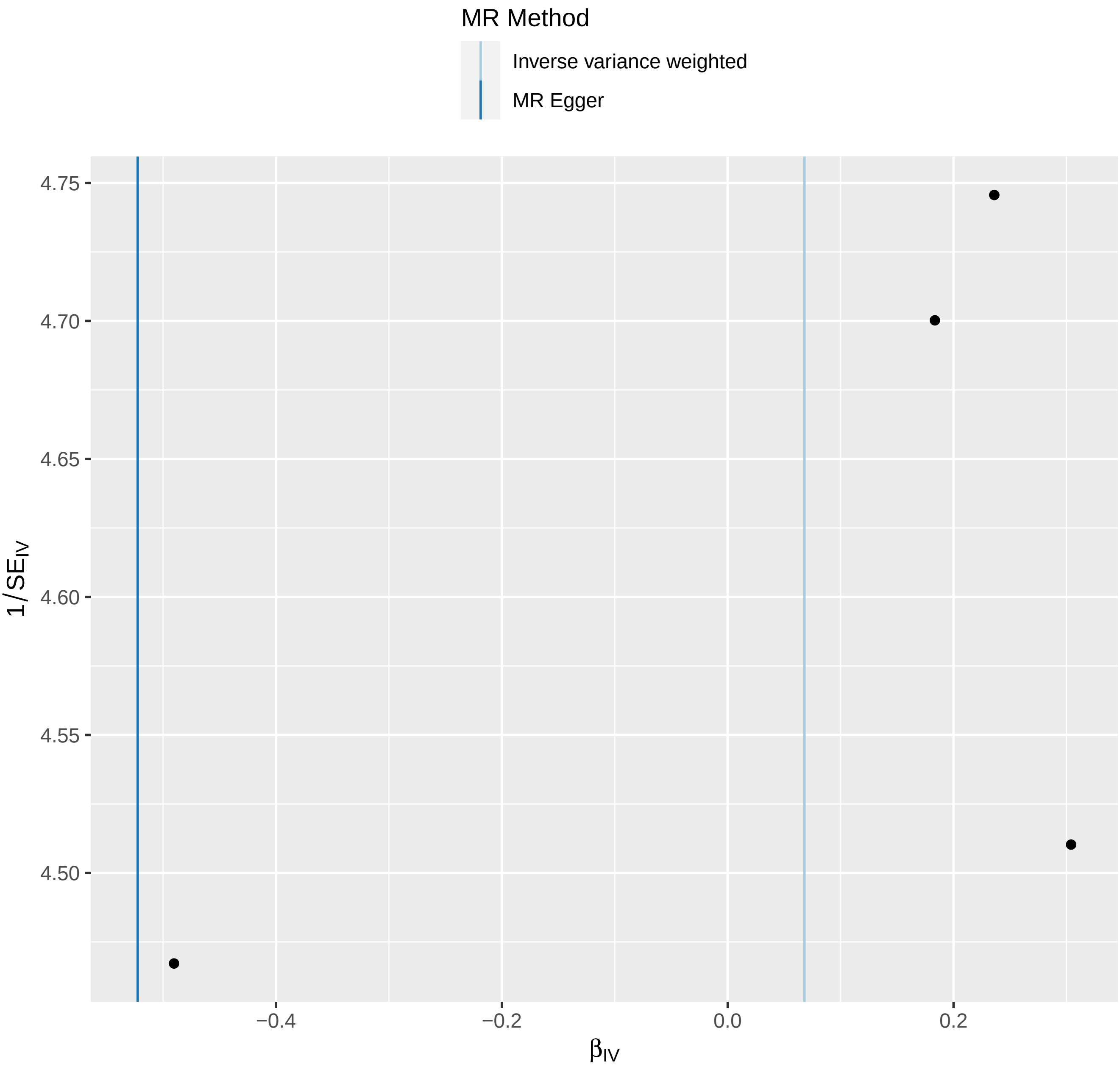

Batch 2−6030 : Gut microbiota abundance (genus Parasutterella id.2892) on Transmembrane protein 70, mitochondrial
with a threshold of 1e−05

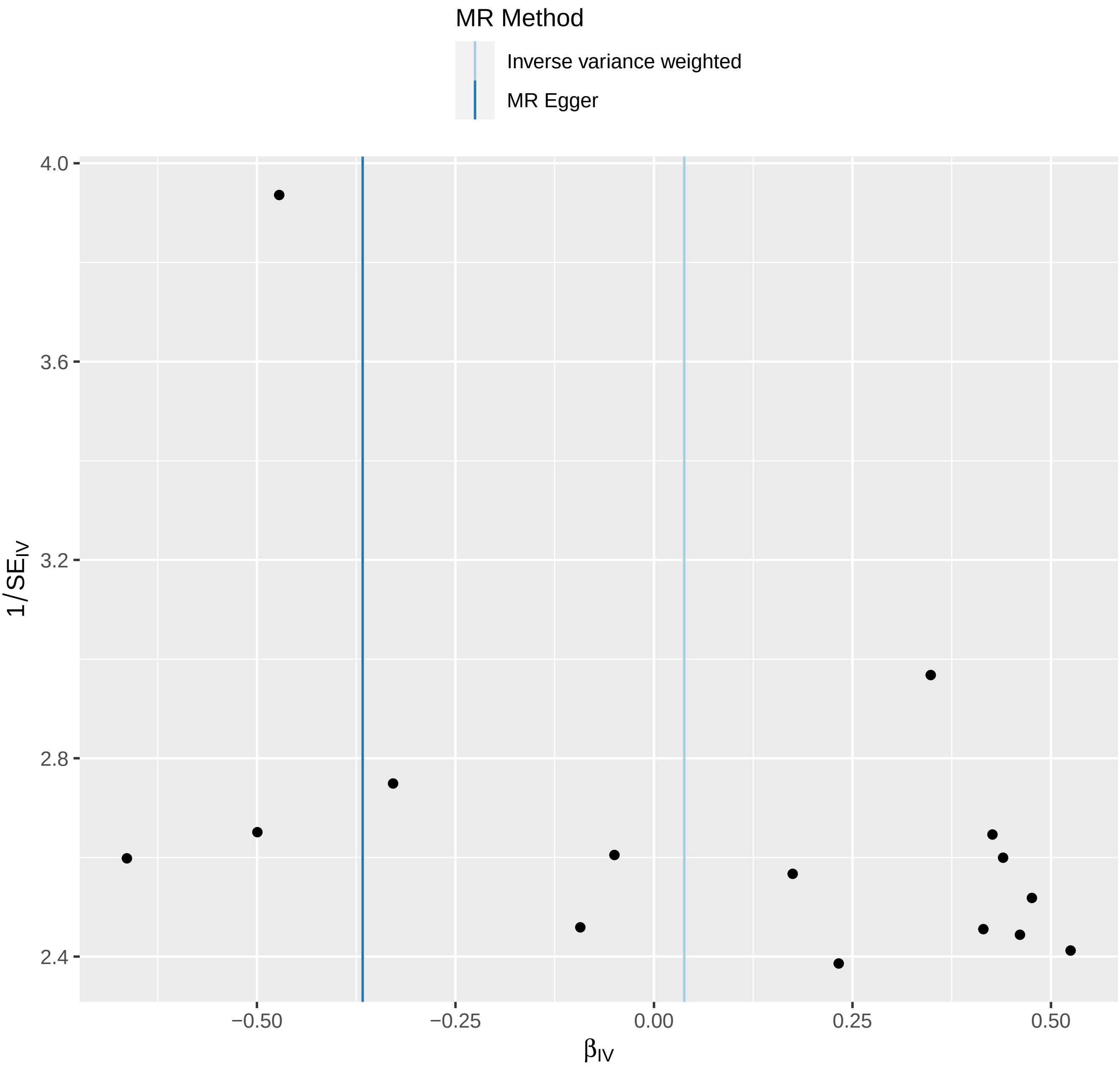

Batch 2−6031 : Gut microbiota abundance (genus Catenibacterium id.2153) on Transmembrane protein 87B
with a threshold of 1e−05

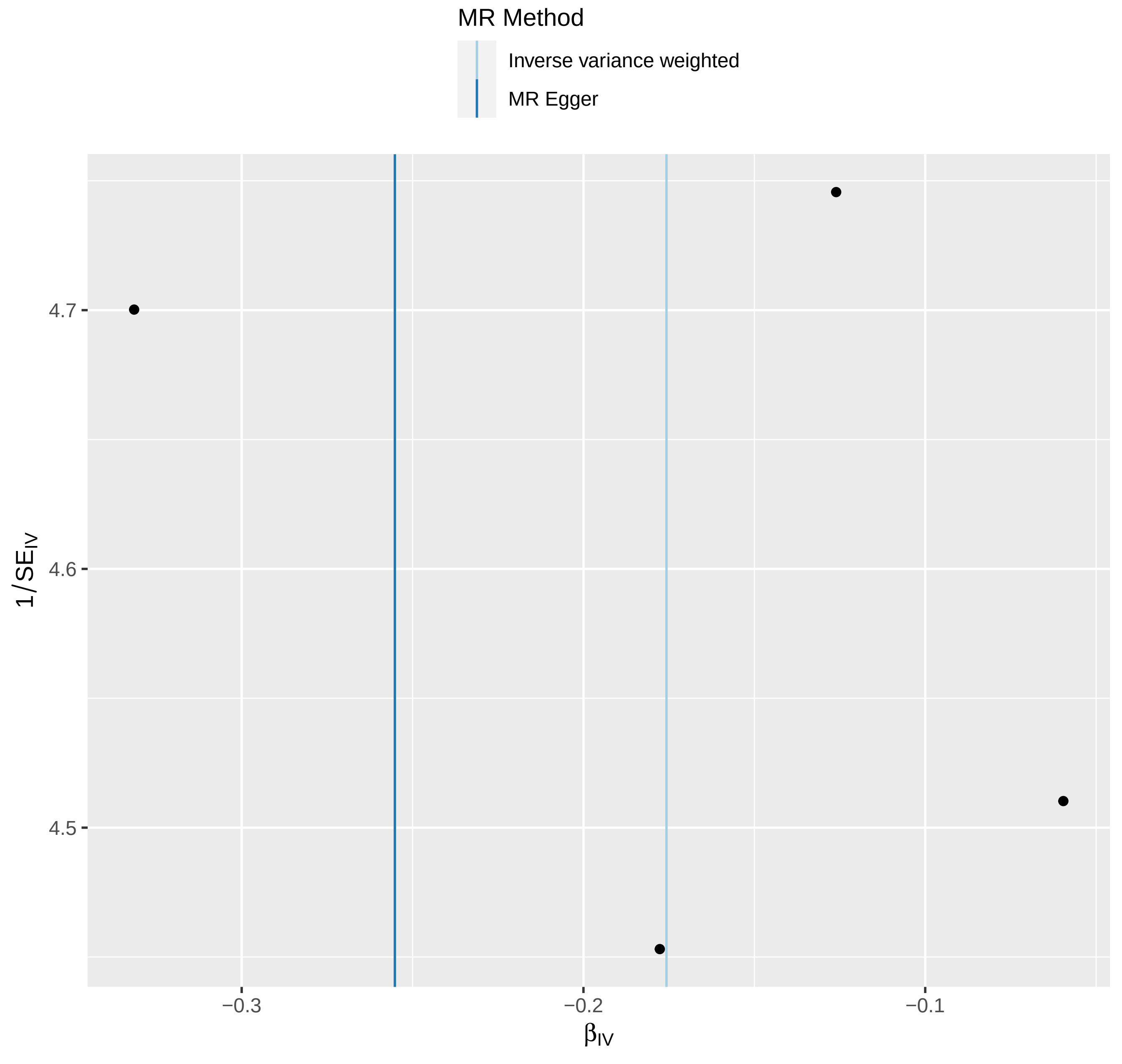

Batch 2−6032 : Gut microbiota abundance (genus Parasutterella id.2892) on Transmembrane protein 87B
with a threshold of 1e−05

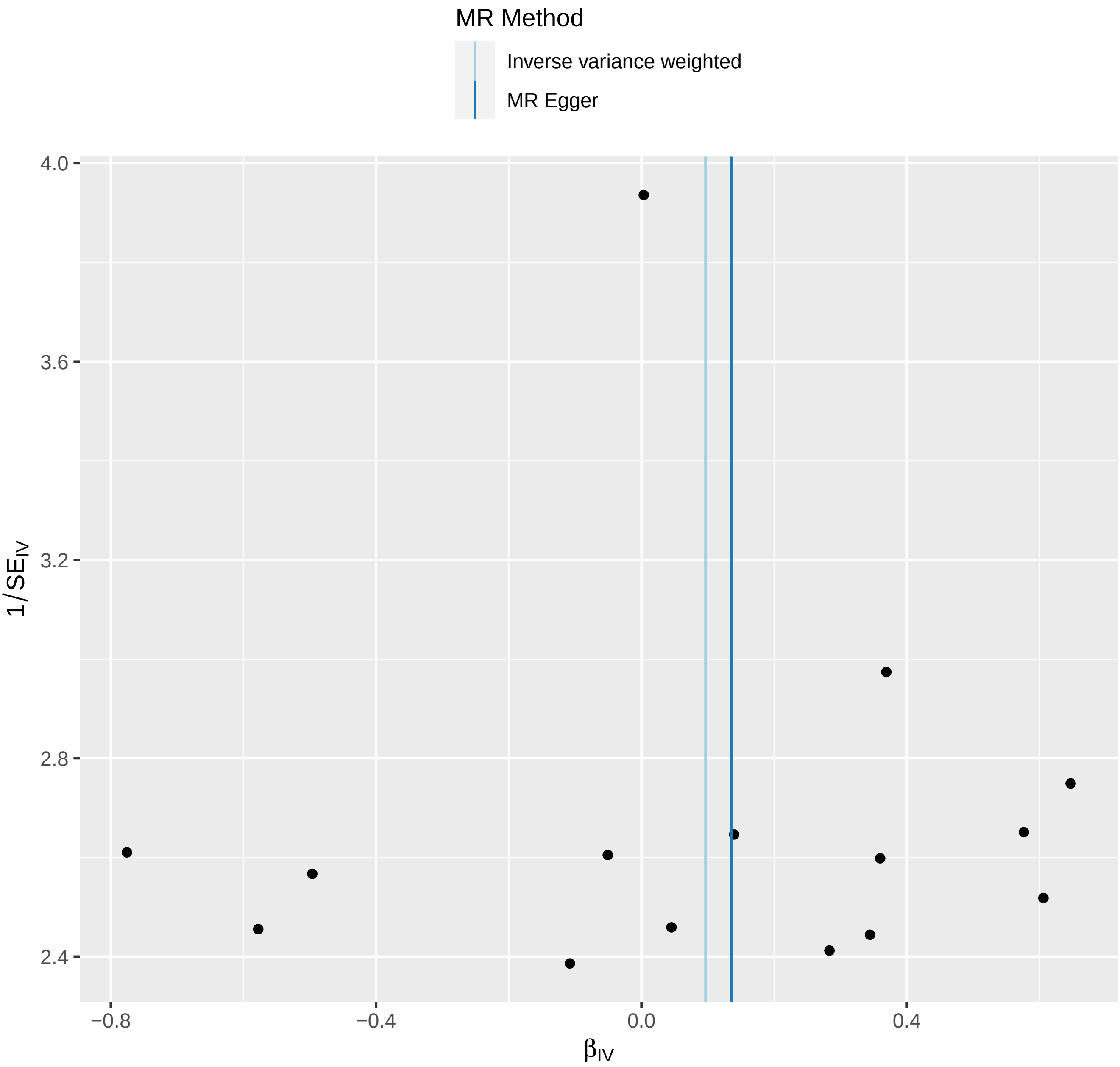

Batch 2−6033 : Gut microbiota abundance (genus Catenibacterium id.2153) on Transmembrane protein 8B
with a threshold of 1e−05

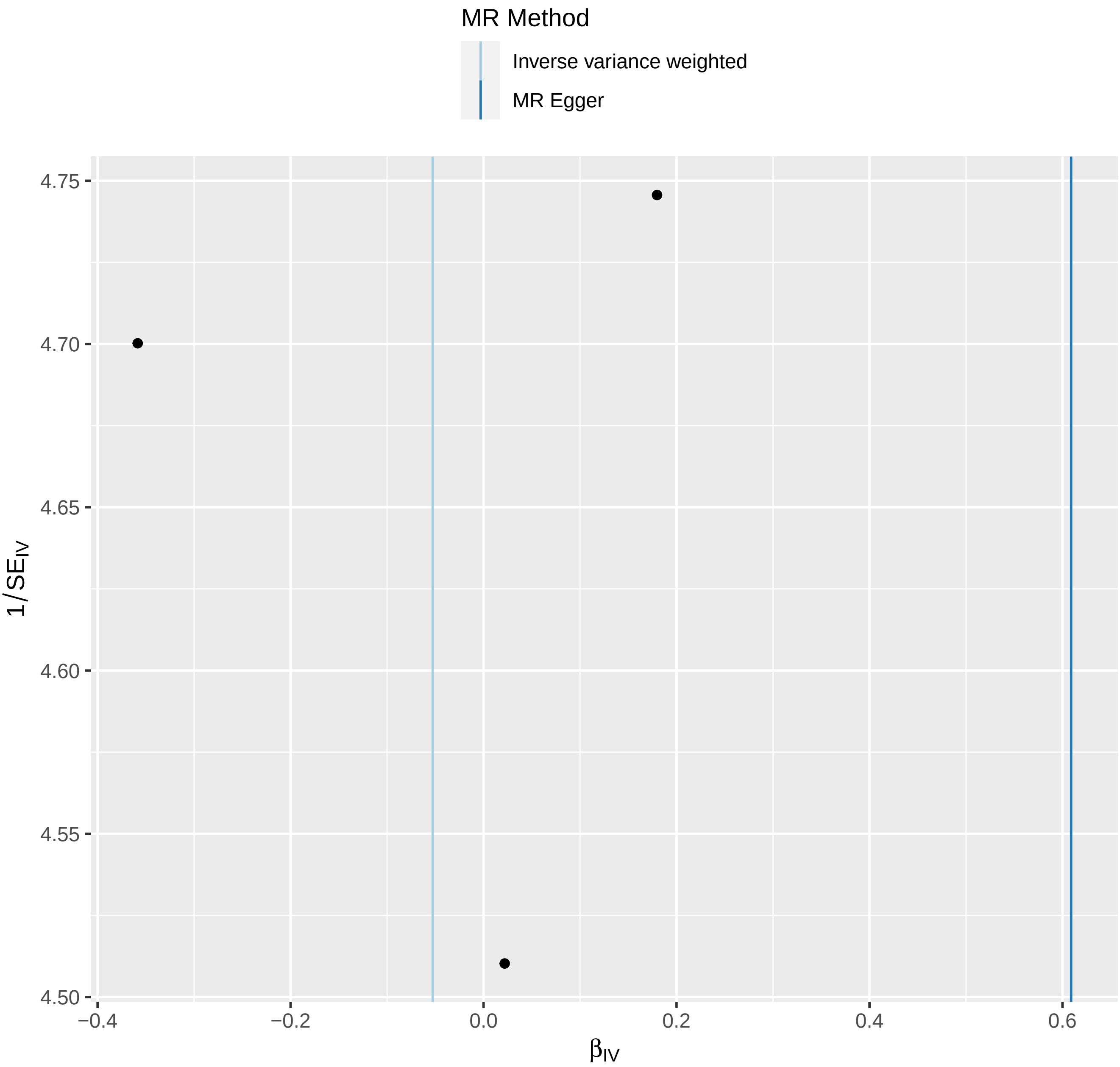

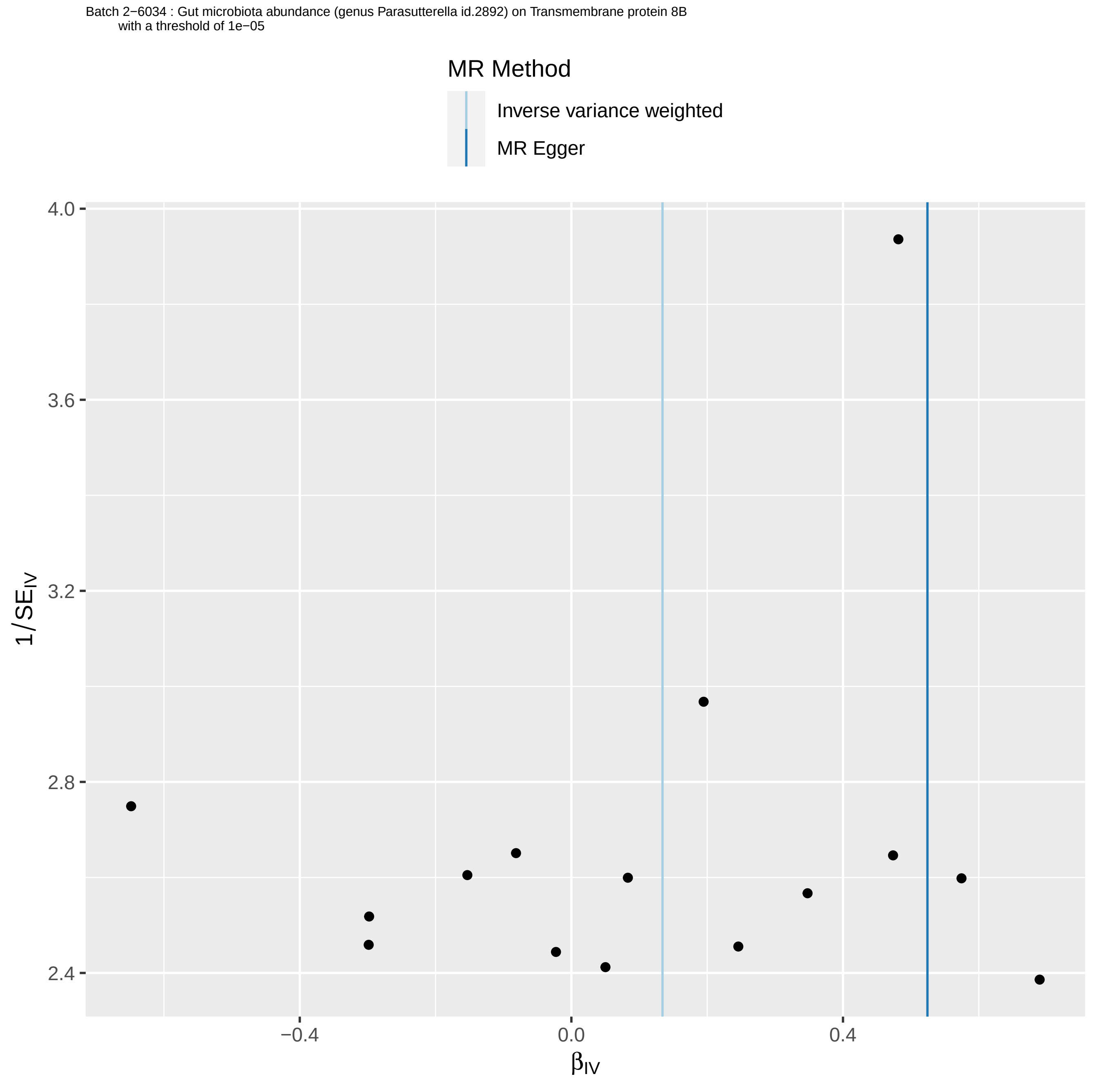
Batch 2−6034 : Gut microbiota abundance (genus Parasutterella id.2892) on Transmembrane protein 8B
with a threshold of 1e−05
MR Method
Inverse variance weighted
MR Egger
4.0
3.6
3.2
2.8
2.4
1/SE_IV
−0.4
0.0
0.4
β_IV

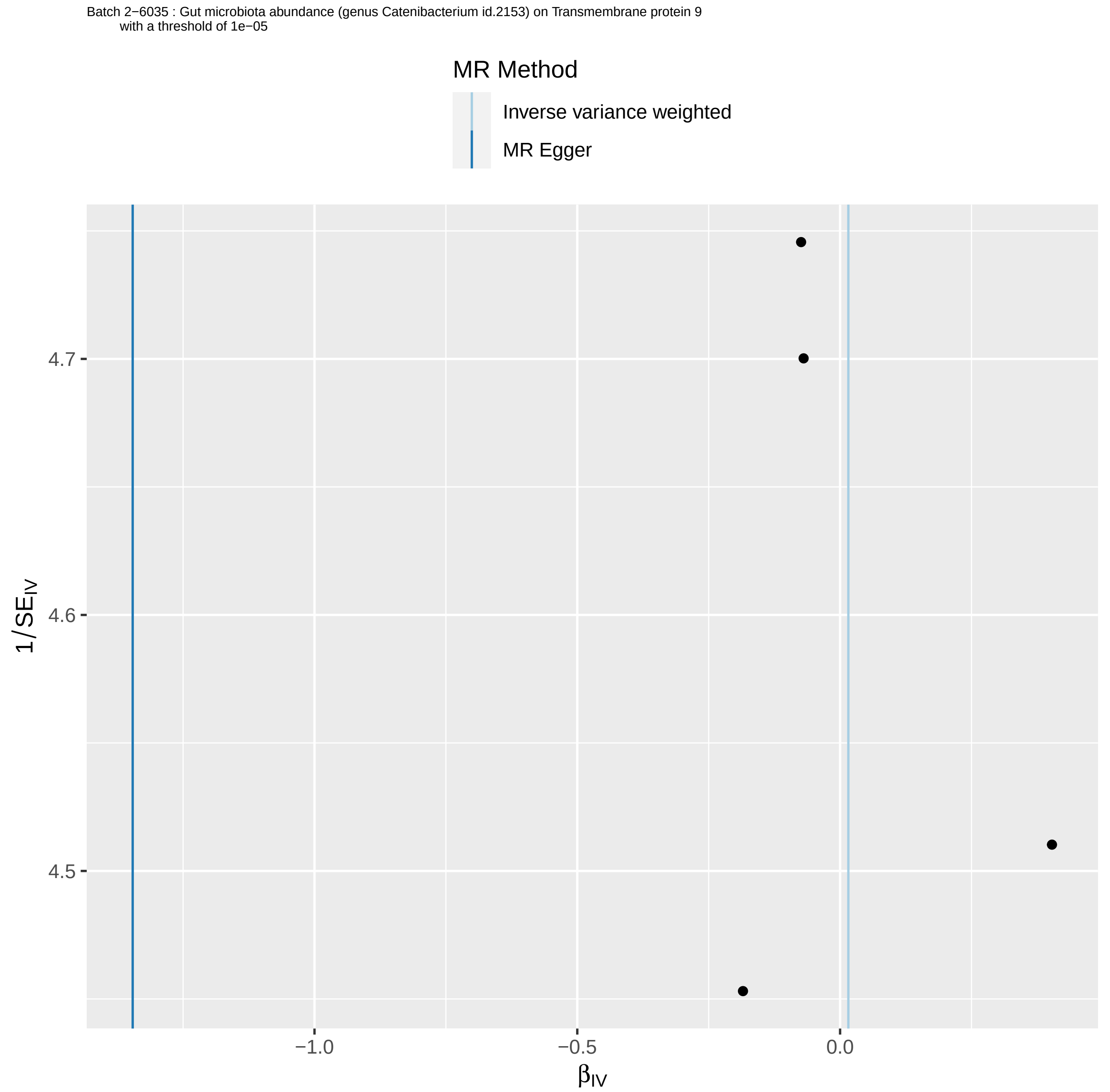
Batch 2−6035 : Gut microbiota abundance (genus Catenibacterium id.2153) on Transmembrane protein 9
with a threshold of 1e−05
MR Method
Inverse variance weighted
MR Egger
4.7
4.6
4.5
1/SE$_{IV}$
−1.0
−0.5
0.0
β$_{IV}$

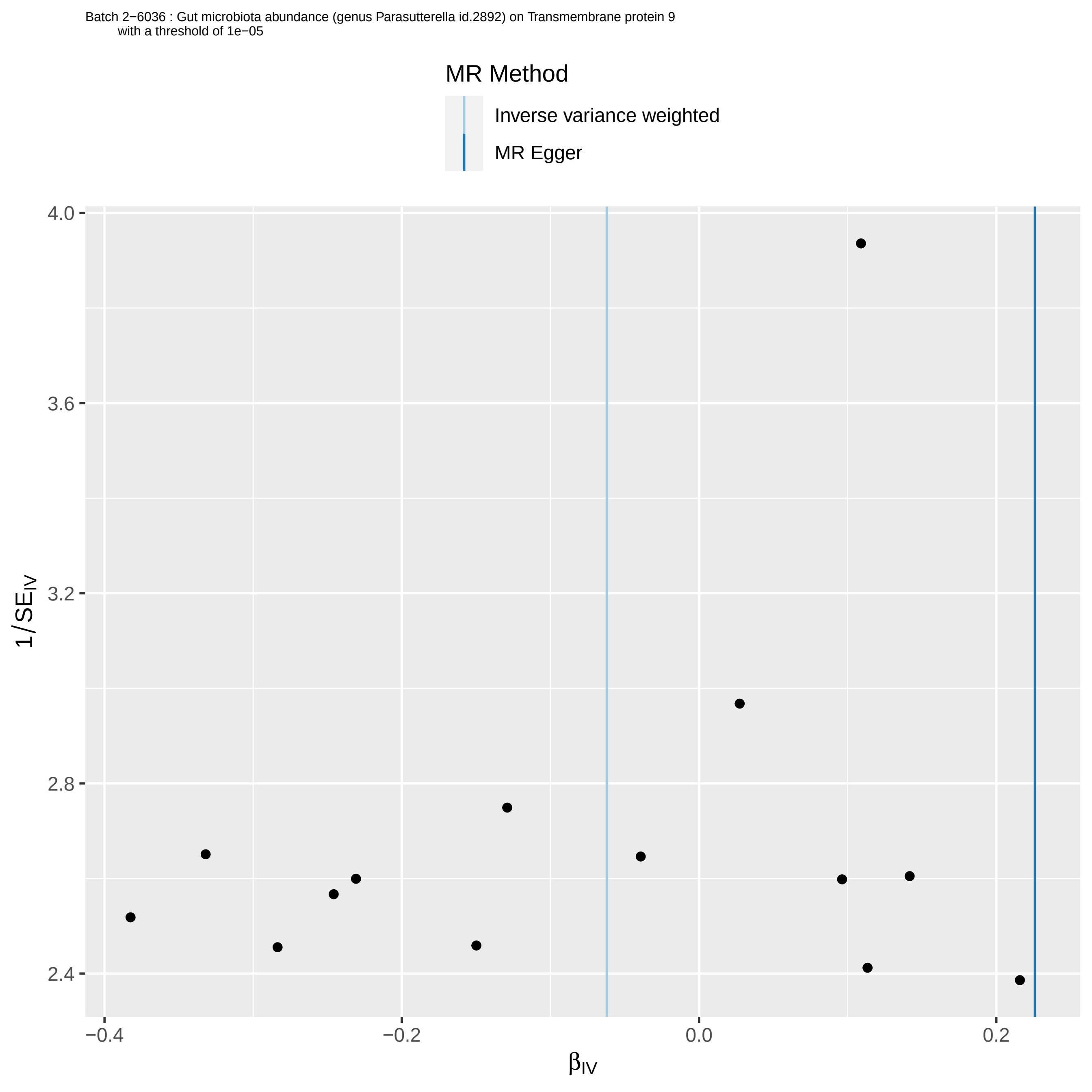

Batch 2−6036 : Gut microbiota abundance (genus Parasutterella id.2892) on Transmembrane protein 9
with a threshold of 1e−05
MR Method
Inverse variance weighted
MR Egger
4.0
3.6
3.2
2.8
2.4
1/SE_IV
−0.4
−0.2
0.0
0.2
β_IV

Batch 2−6037 : Gut microbiota abundance (genus Catenibacterium id.2153) on Transmembrane inner ear expressed protein with a threshold of 1e−05

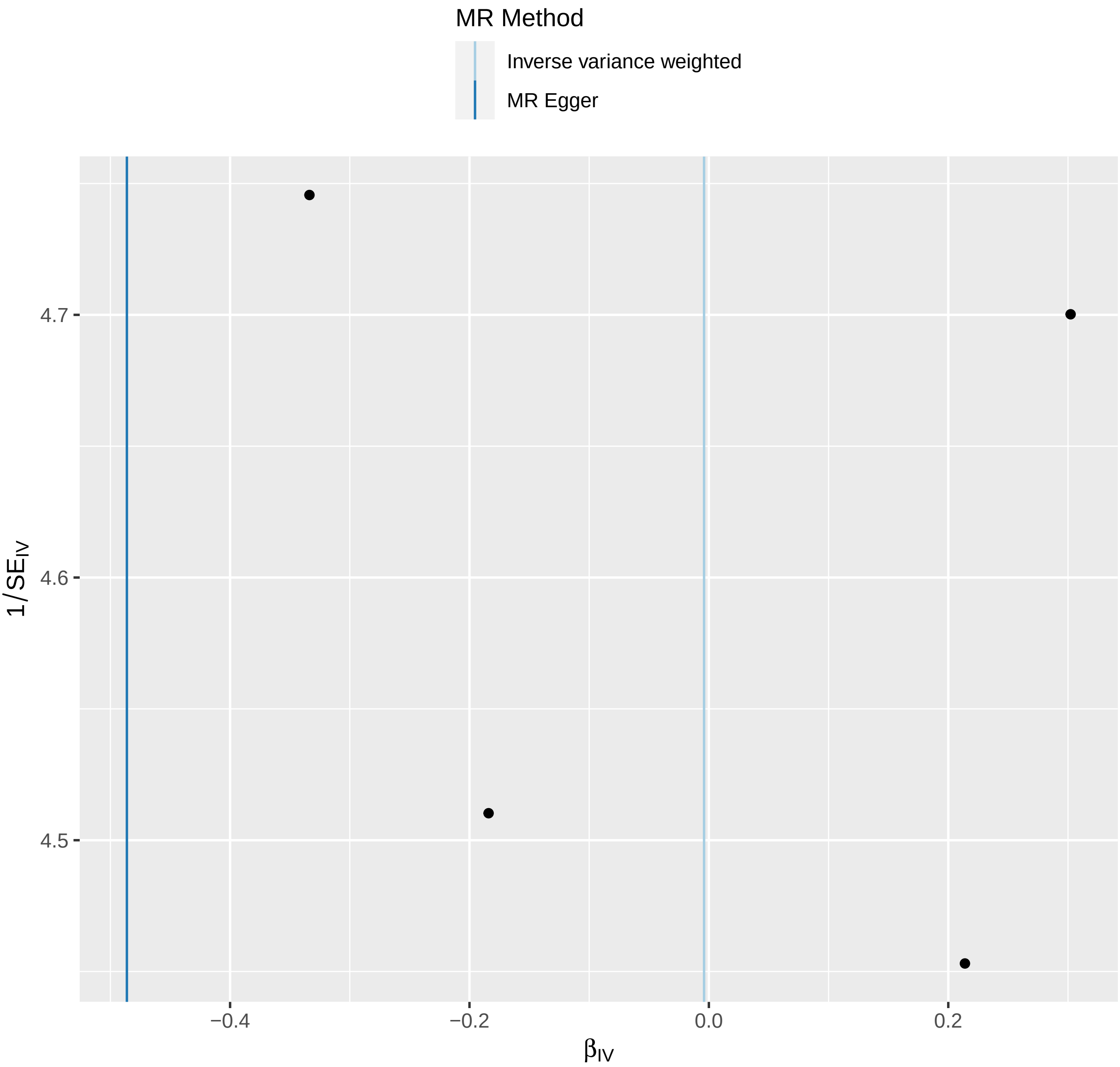

Batch 2−6038 : Gut microbiota abundance (genus Parasutterella id.2892) on Transmembrane inner ear expressed protein with a threshold of 1e−05

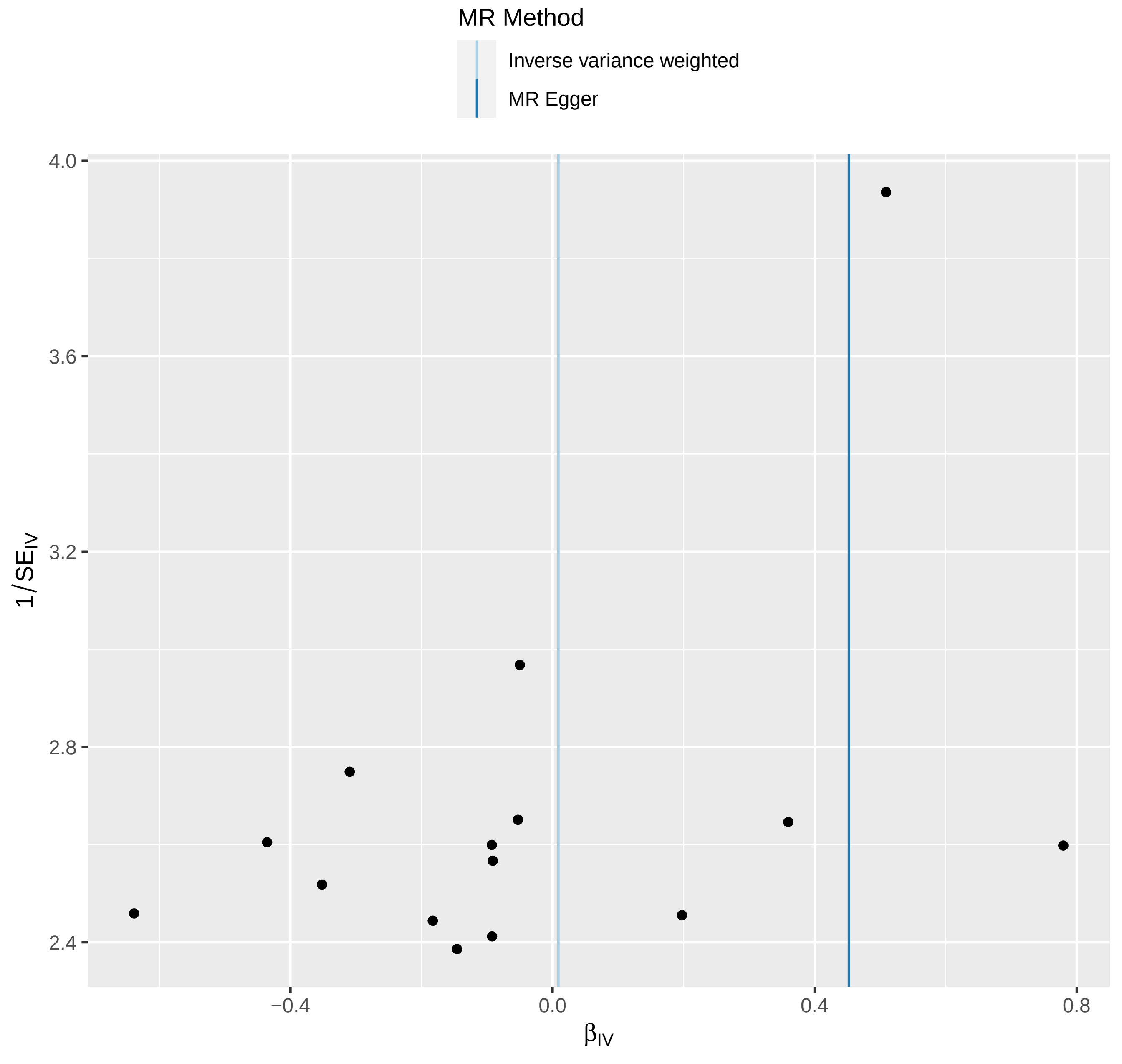

Batch 2−6039 : Gut microbiota abundance (genus Catenibacterium id.2153) on Tropomodulin−1
with a threshold of 1e−05

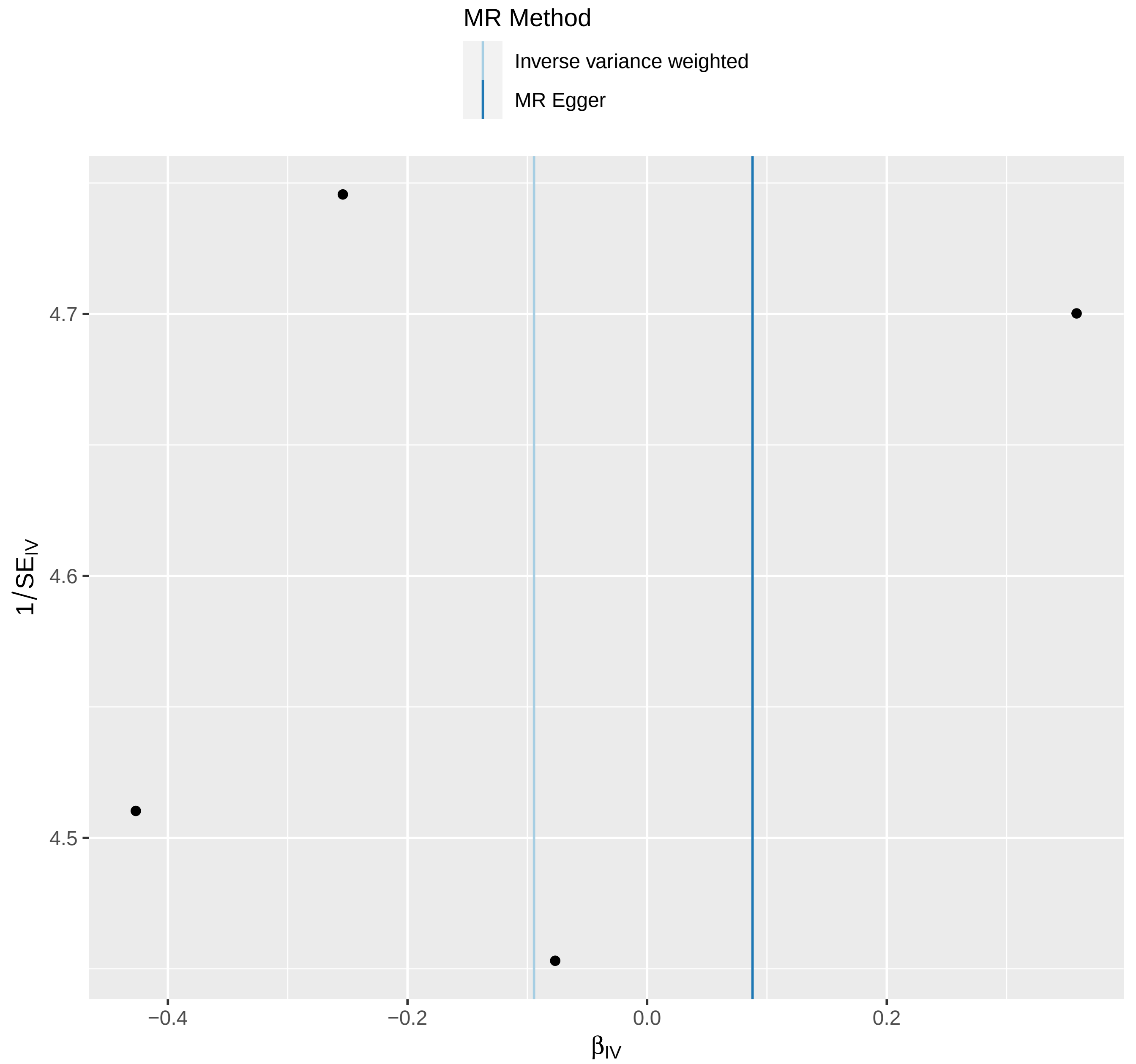

Batch 2−6040 : Gut microbiota abundance (genus Parasutterella id.2892) on Tropomodulin−1 with a threshold of 1e−05

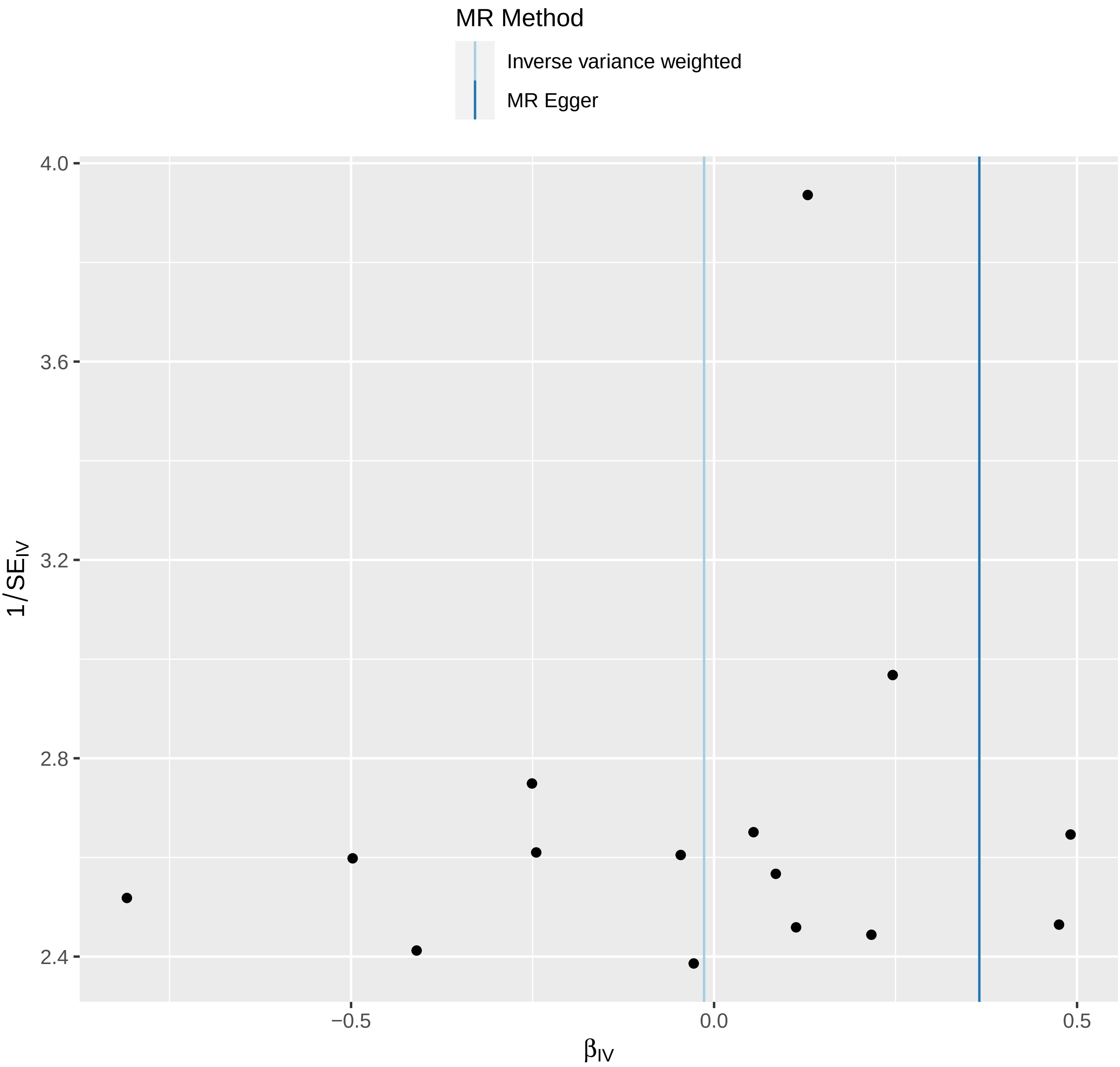

Batch 2−6041 : Gut microbiota abundance (genus Catenibacterium id.2153) on Lamina−associated polypeptide 2, isoforms beta/gamma with a threshold of 1e−05

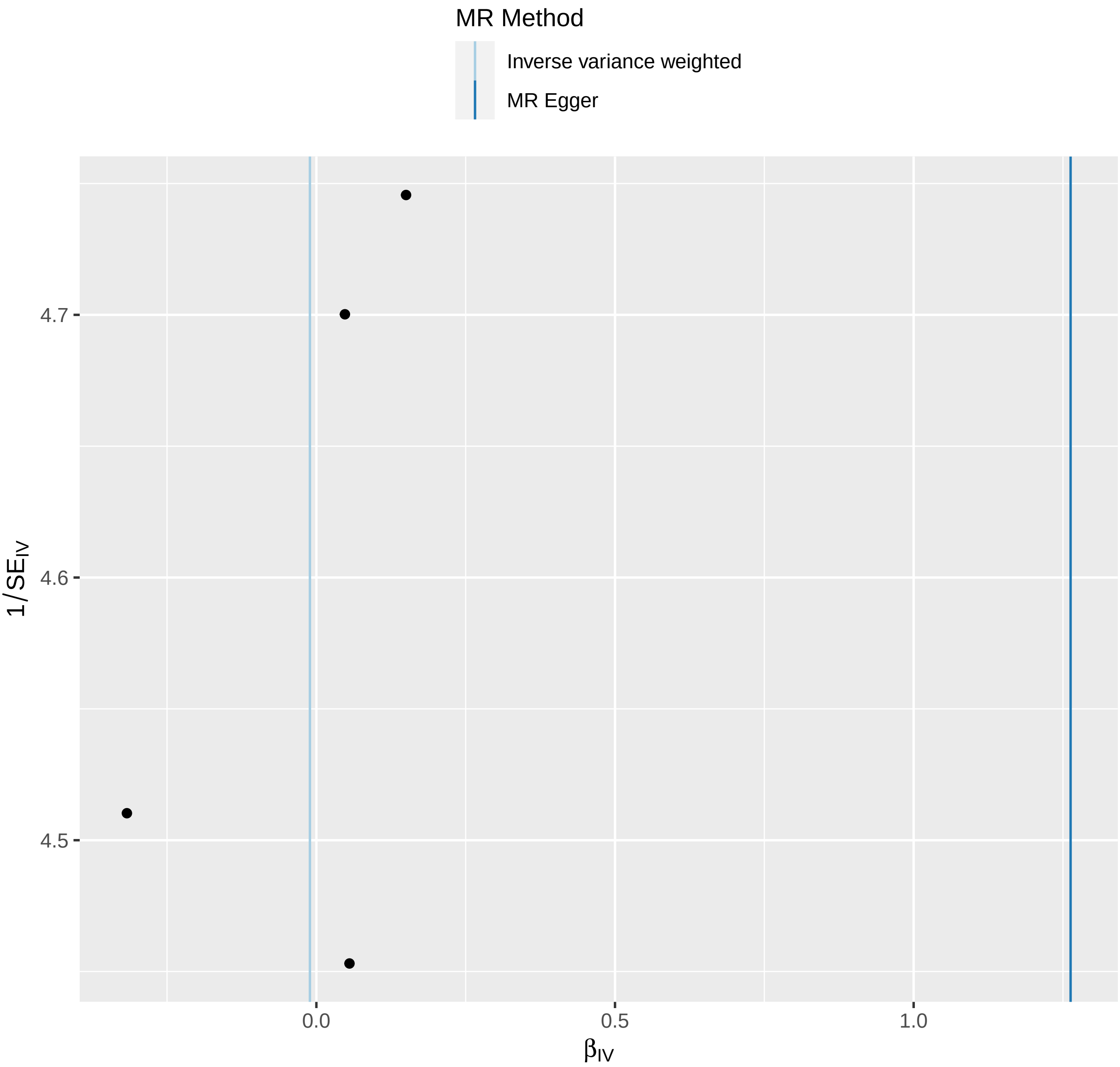

Batch 2−6042 : Gut microbiota abundance (genus Parasutterella id.2892) on Lamina−associated polypeptide 2, isoforms beta/gamma with a threshold of 1e−05

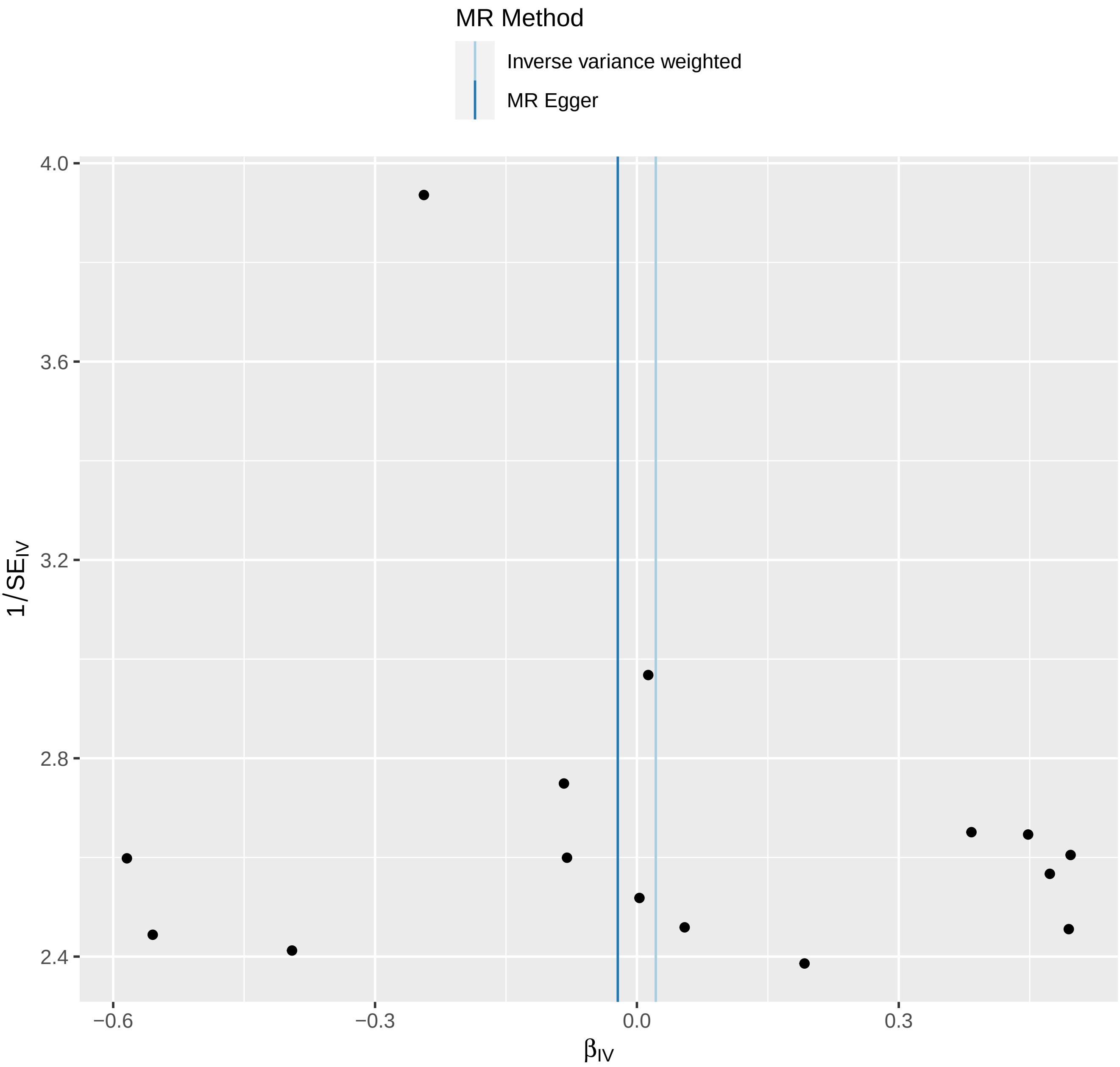

Batch 2−6043 : Gut microbiota abundance (genus Catenibacterium id.2153) on Transmembrane protease serine 11A
with a threshold of 1e−05

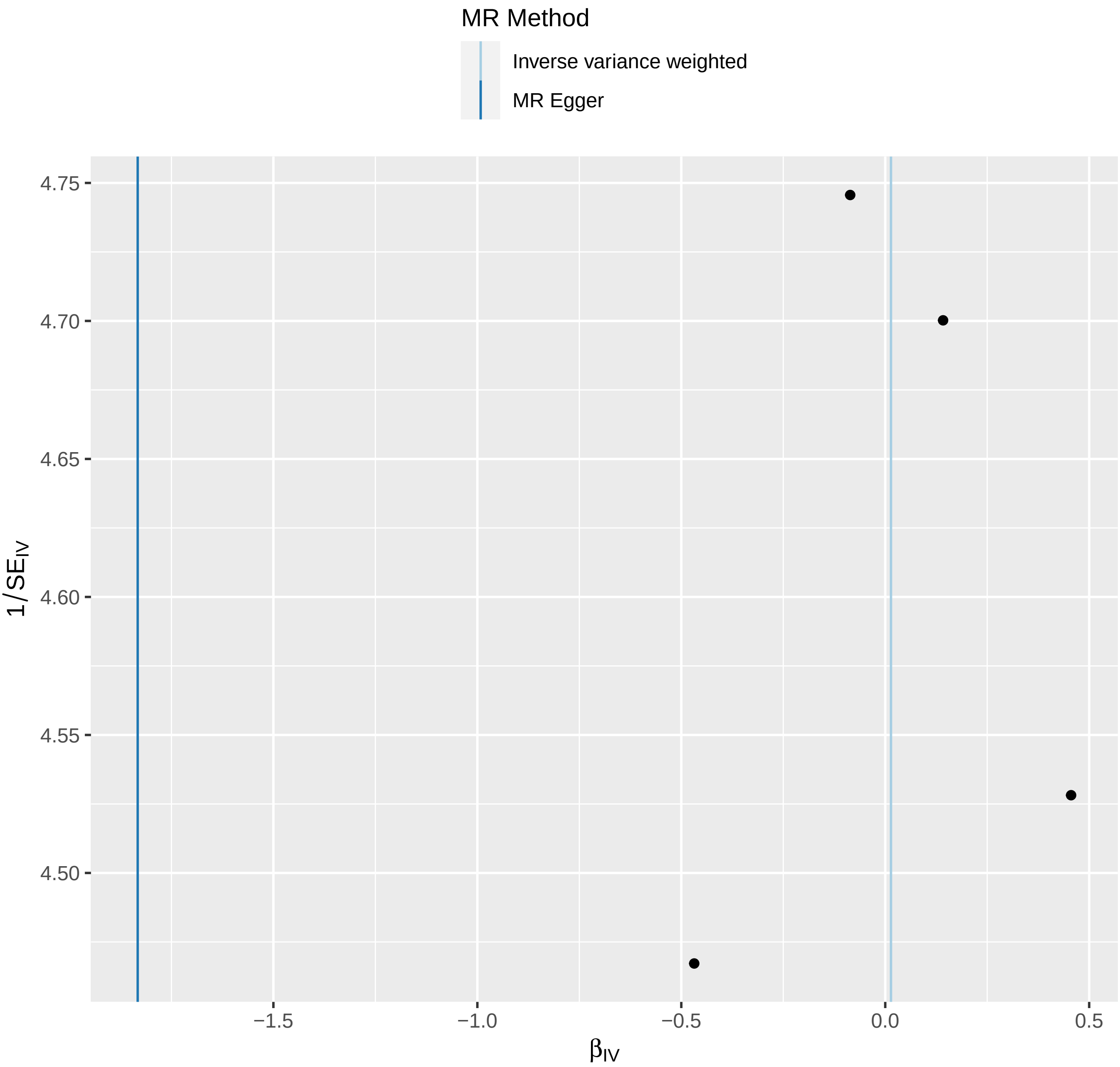

Batch 2−6044 : Gut microbiota abundance (genus Parasutterella id.2892) on Transmembrane protease serine 11A
with a threshold of 1e−05

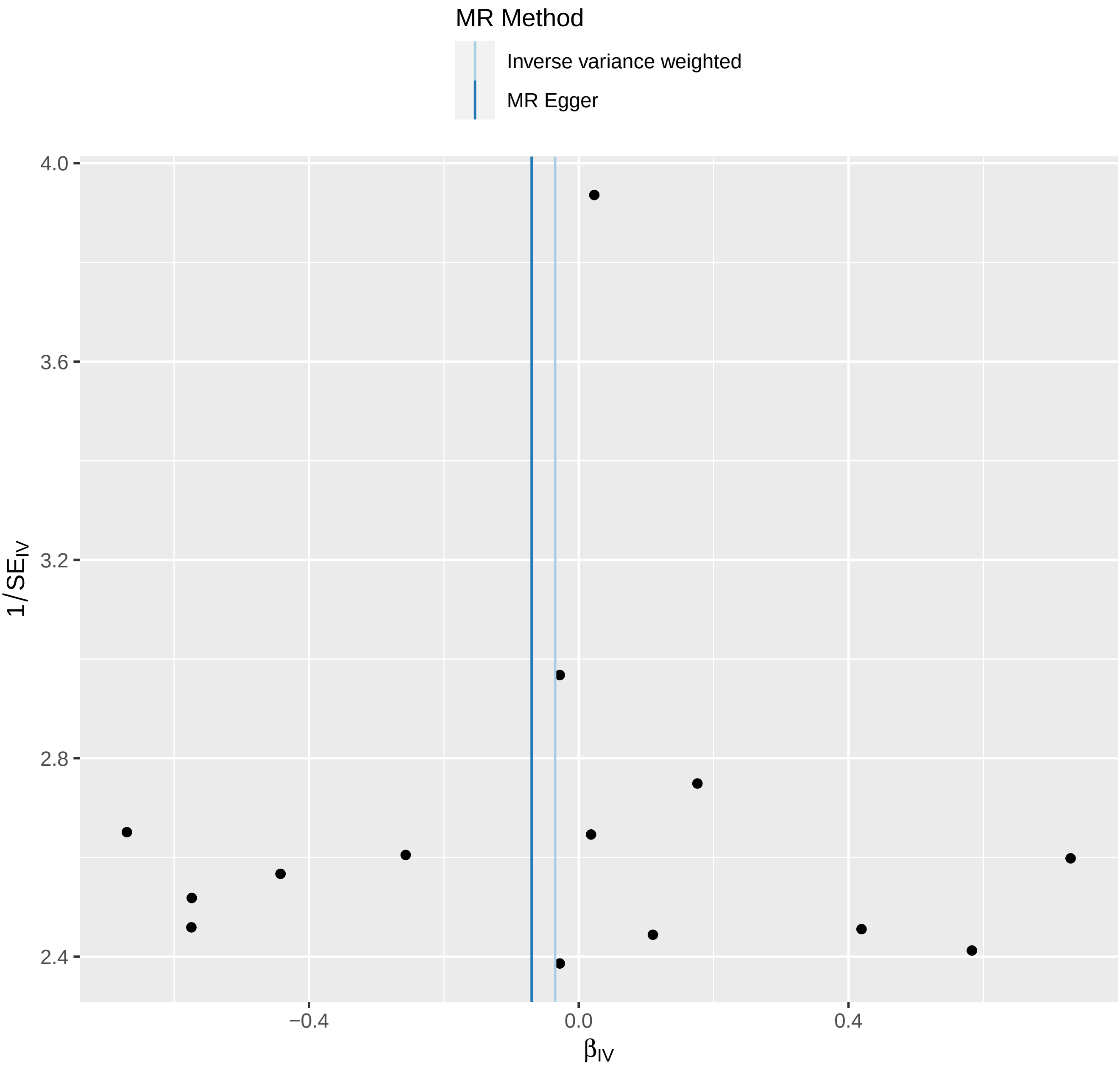

Batch 2−6045 : Gut microbiota abundance (genus Catenibacterium id.2153) on Transmembrane protease serine 11B with a threshold of 1e−05

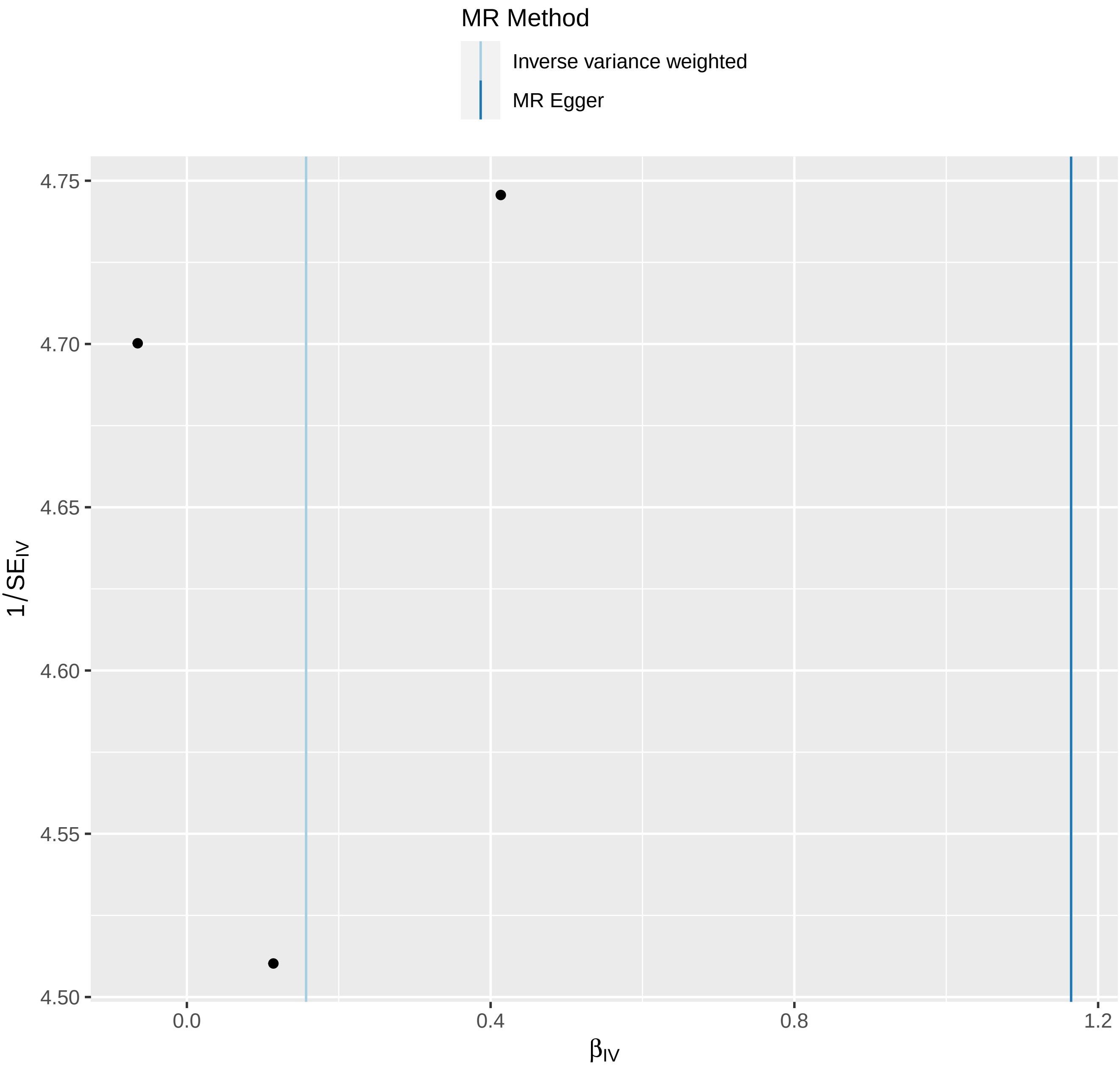

Batch 2−6046 : Gut microbiota abundance (genus Parasutterella id.2892) on Transmembrane protease serine 11B
with a threshold of 1e−05

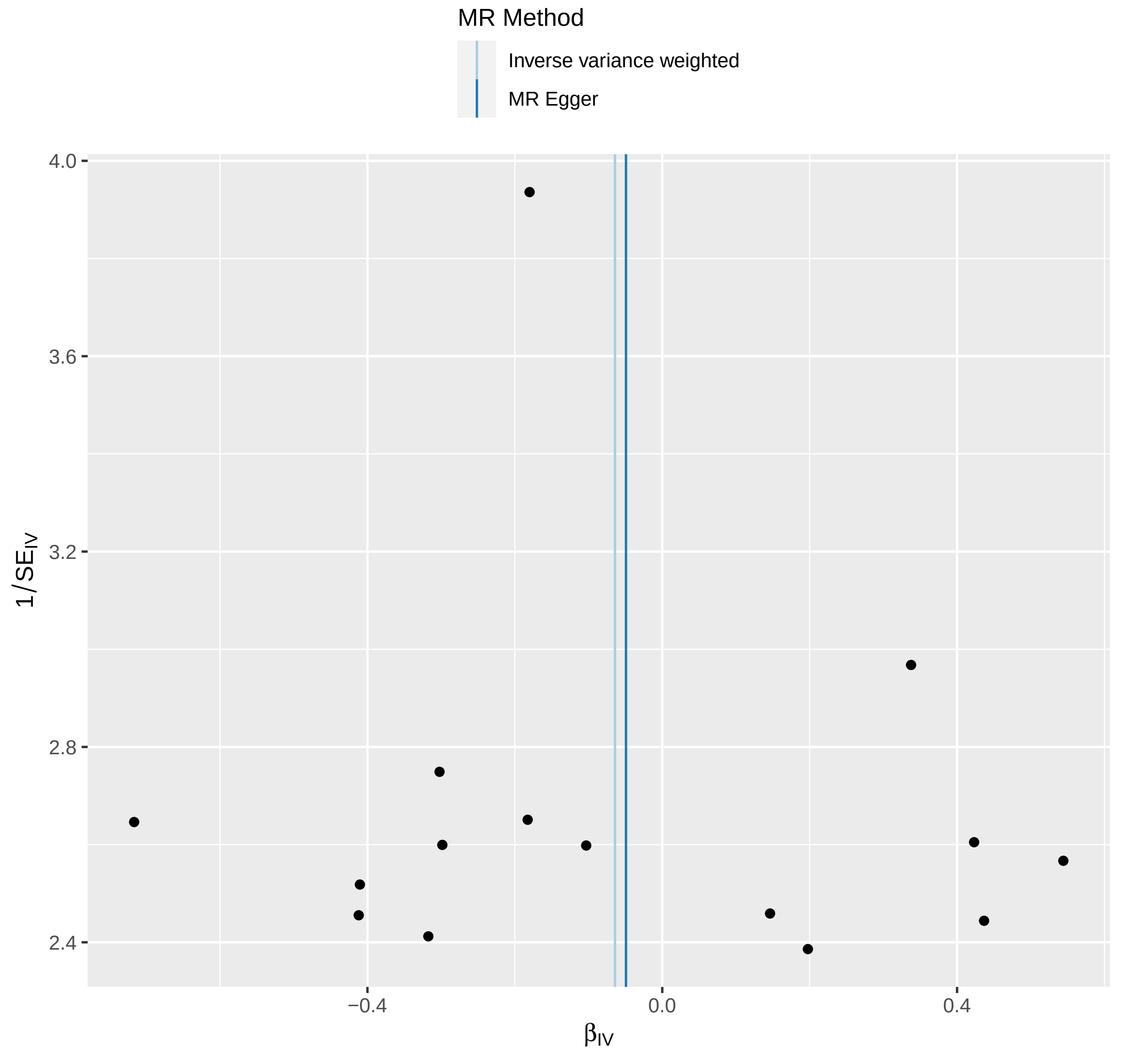

Batch 2−6047 : Gut microbiota abundance (genus Catenibacterium id.2153) on Transmembrane protease serine 11D
with a threshold of 1e−05

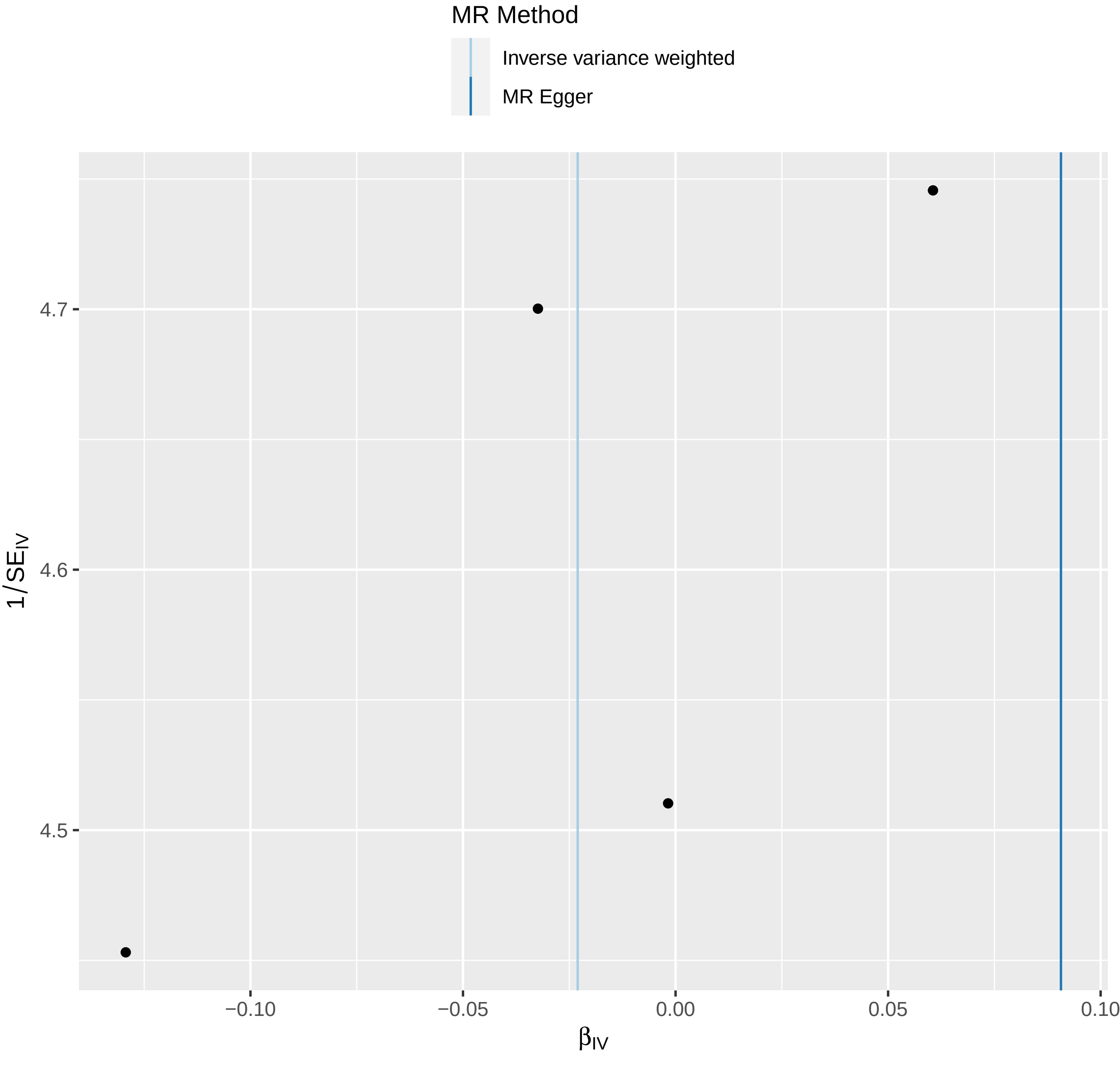

Batch 2−6048 : Gut microbiota abundance (genus Parasutterella id.2892) on Transmembrane protease serine 11D
with a threshold of 1e−05

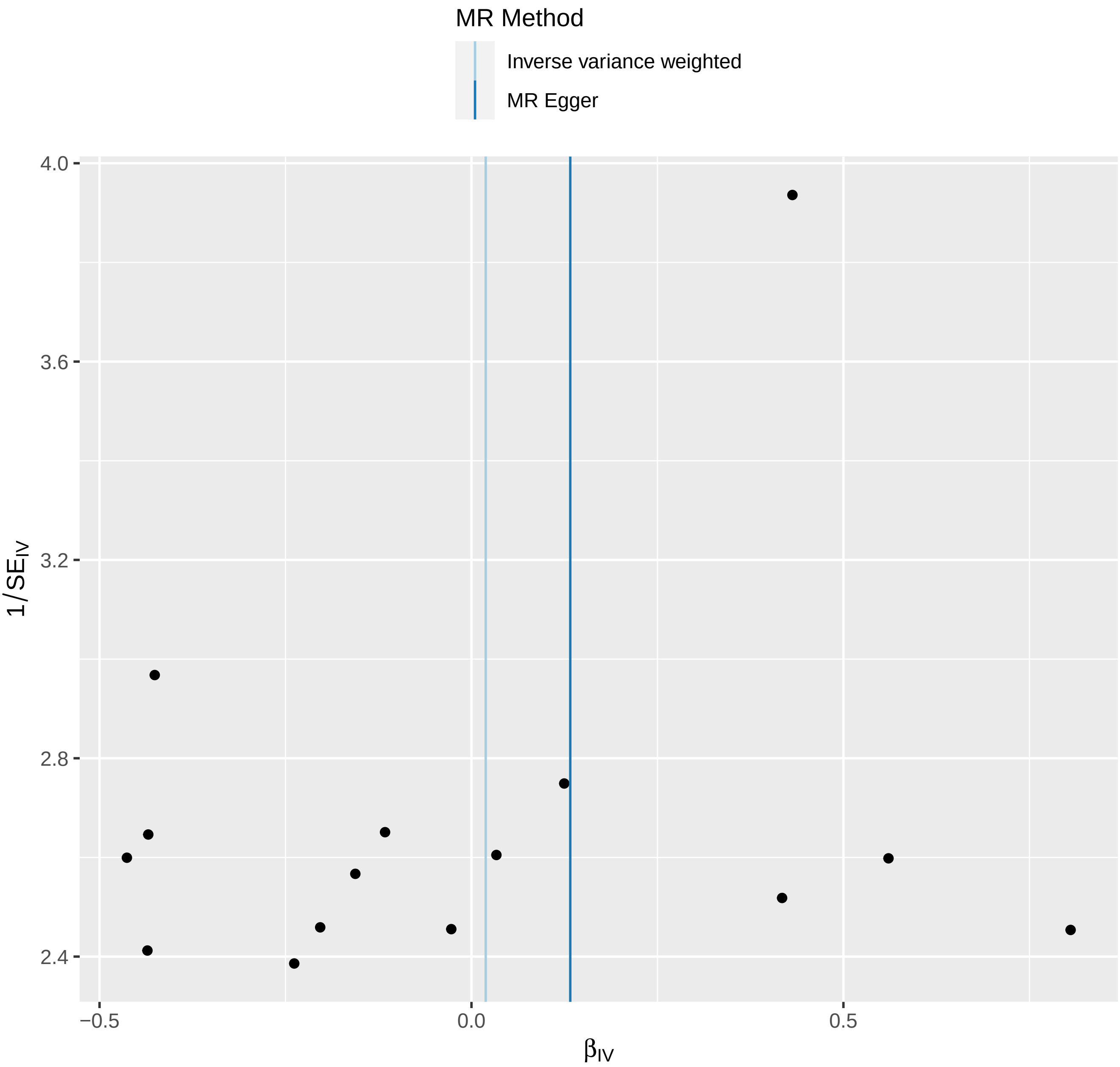

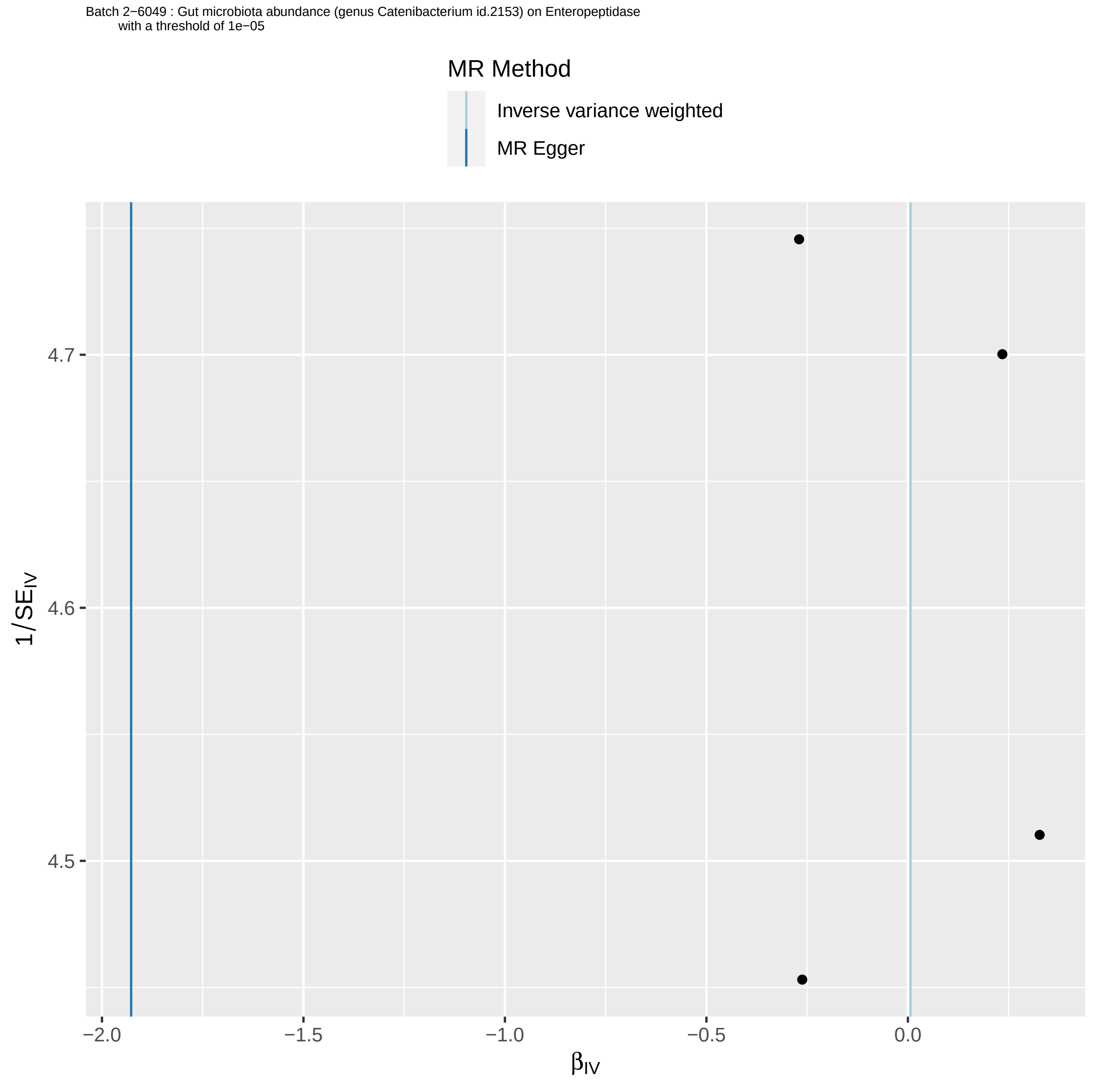
Batch 2−6049 : Gut microbiota abundance (genus Catenibacterium id.2153) on Enteropeptidase
with a threshold of 1e−05
MR Method
Inverse variance weighted
MR Egger
4.7
4.6
4.5
1/SE_IV
−2.0
−1.5
−1.0
−0.5
0.0
β_IV

Batch 2−6050 : Gut microbiota abundance (genus Parasutterella id.2892) on Enteropeptidase
with a threshold of 1e−05

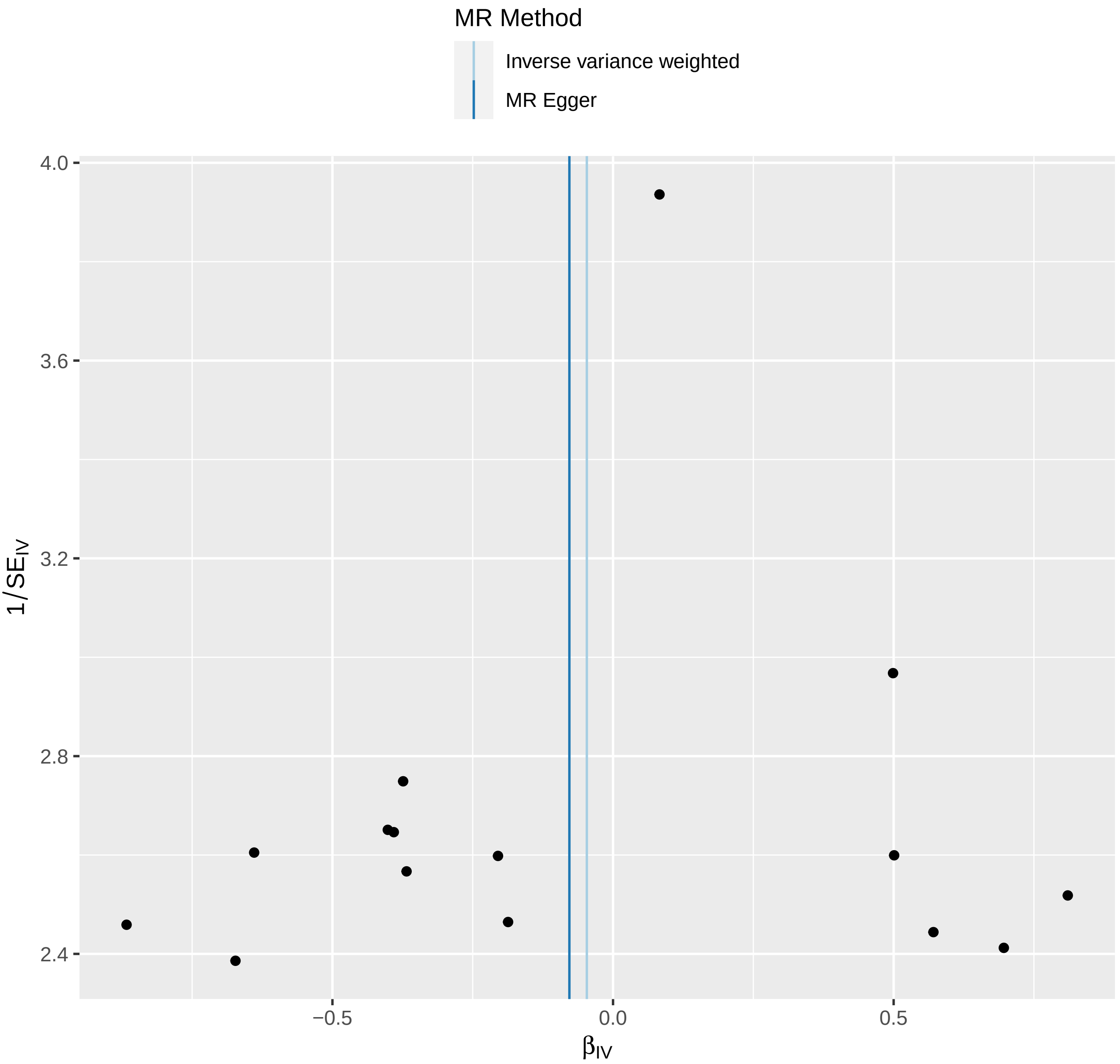

Batch 2−6051 : Gut microbiota abundance (genus Catenibacterium id.2153) on Transmembrane protease serine 5
with a threshold of 1e−05

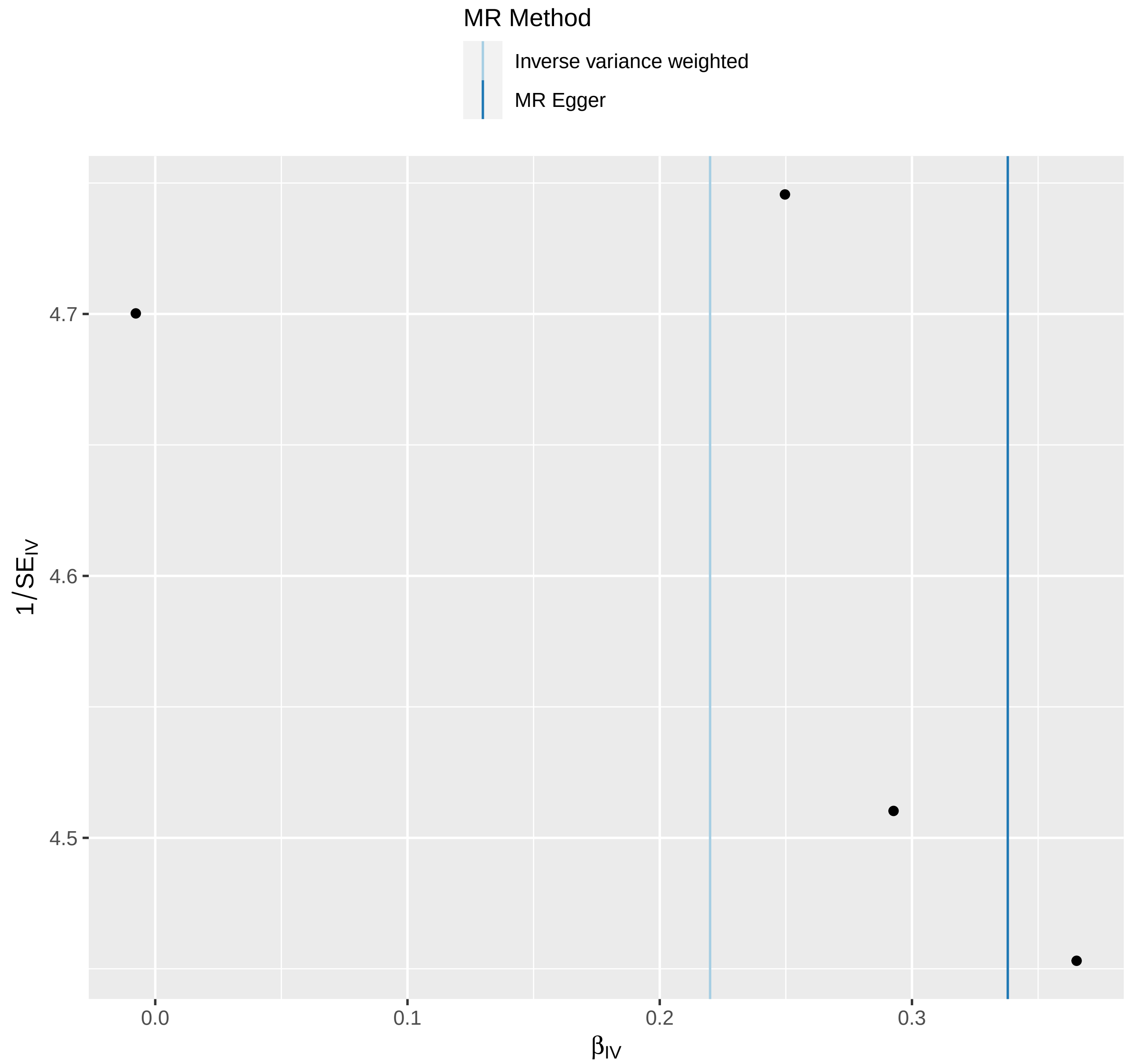

Batch 2−6052 : Gut microbiota abundance (genus Parasutterella id.2892) on Transmembrane protease serine 5
with a threshold of 1e−05

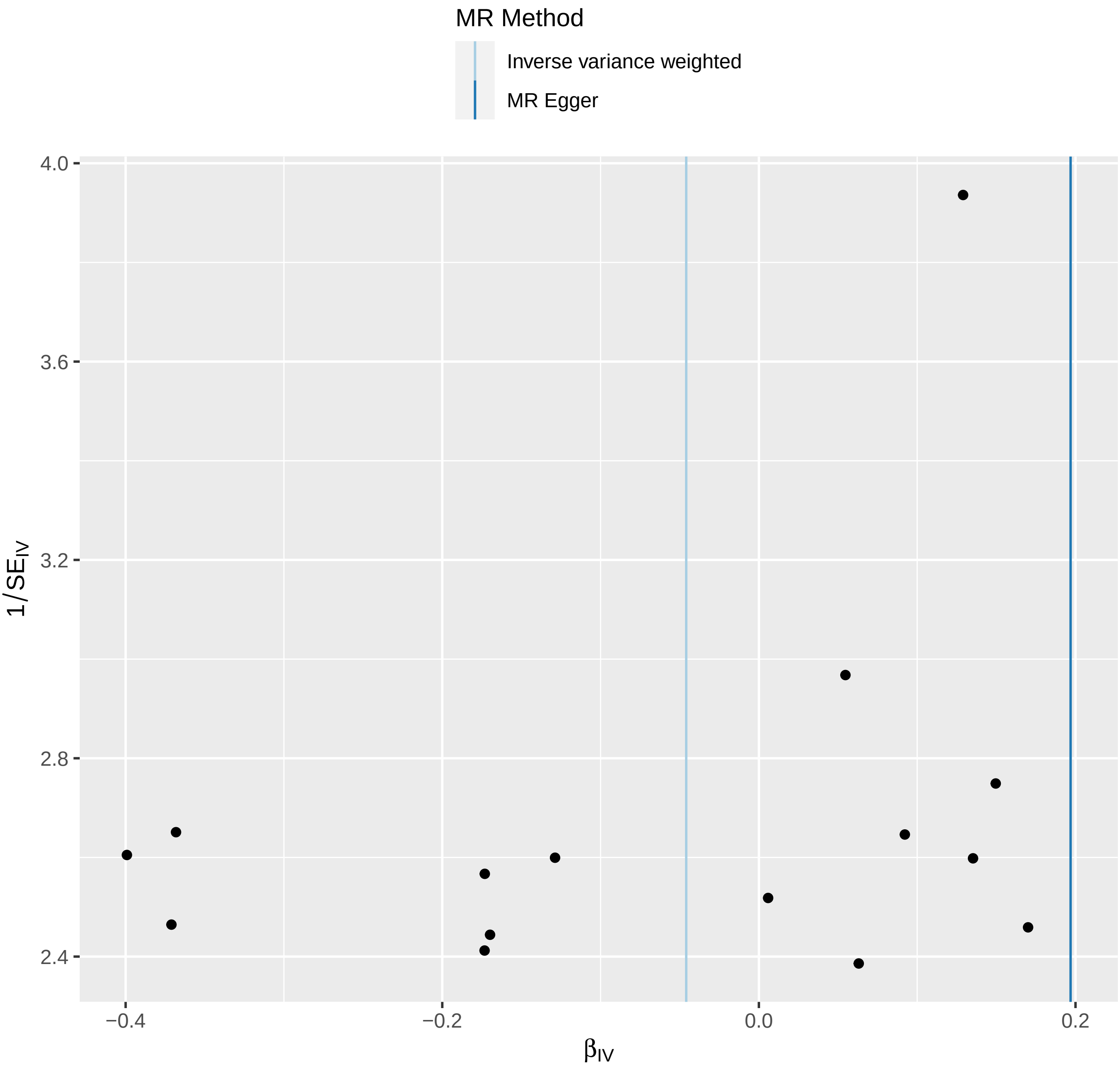

Batch 2−6053 : Gut microbiota abundance (genus Catenibacterium id.2153) on Transmembrane and ubiquitin−like domain−containing protein 2 with a threshold of 1e−05

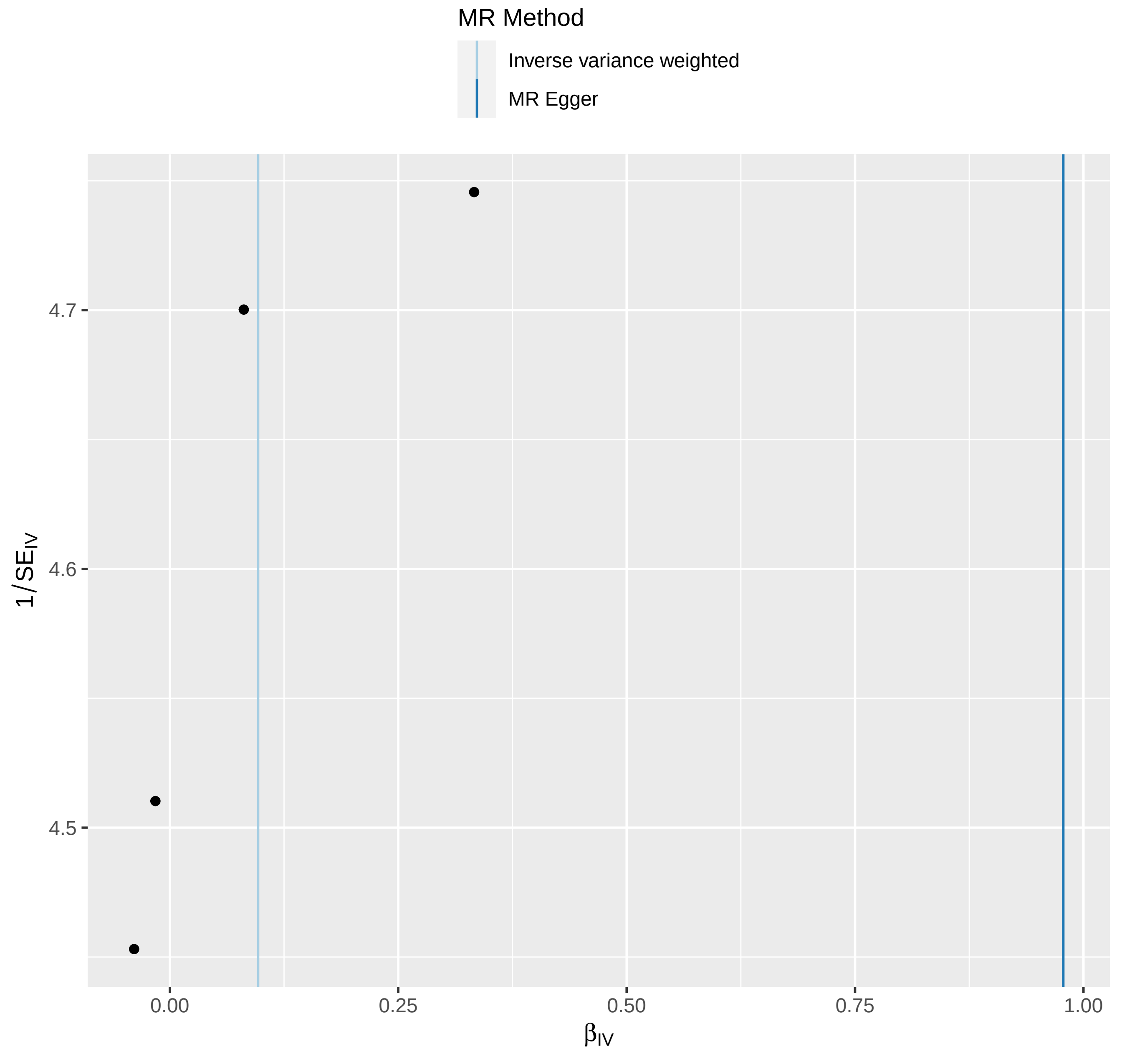

Batch 2−6054 : Gut microbiota abundance (genus Parasutterella id.2892) on Transmembrane and ubiquitin−like domain−containing protein 2 with a threshold of 1e−05

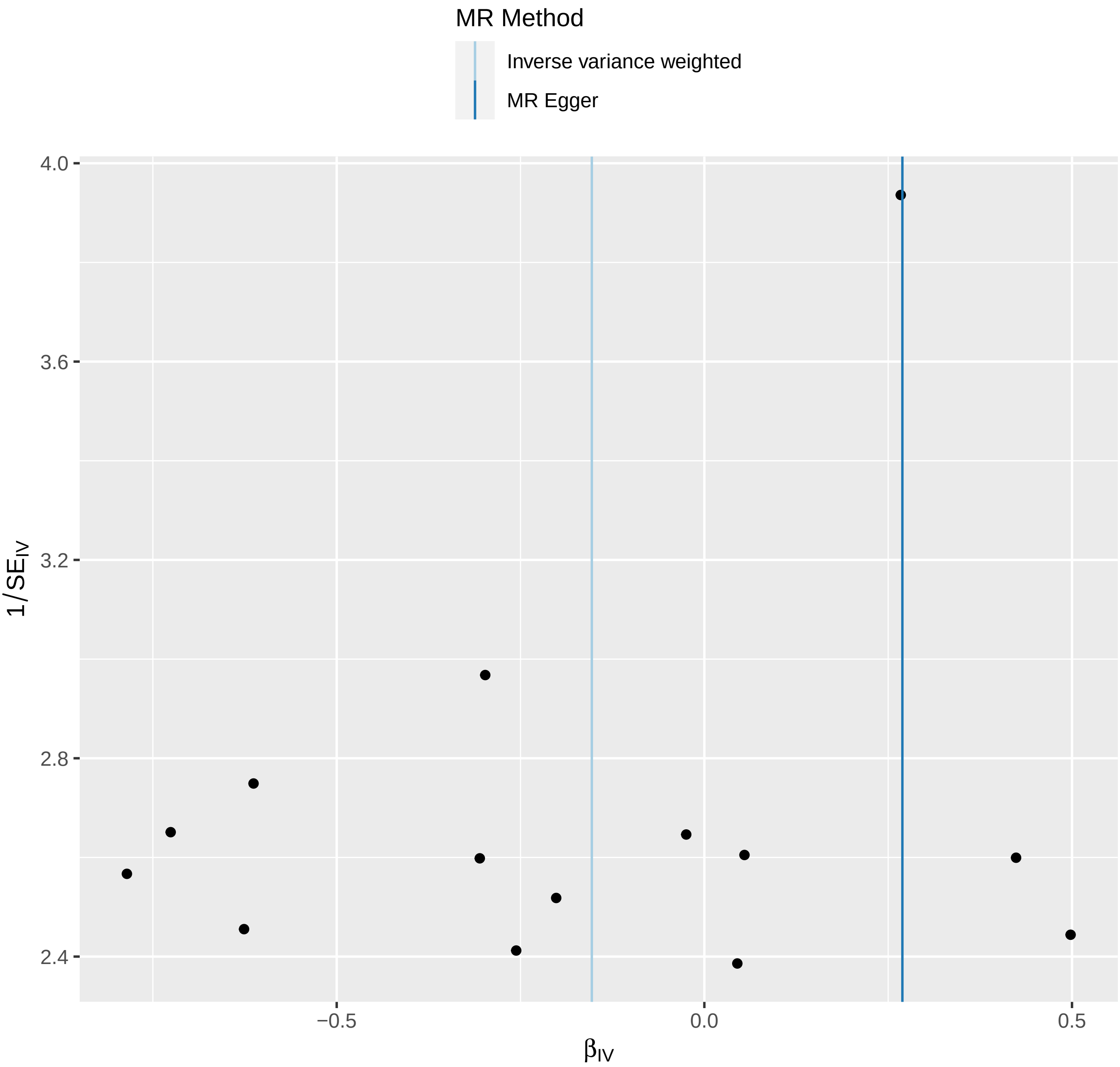

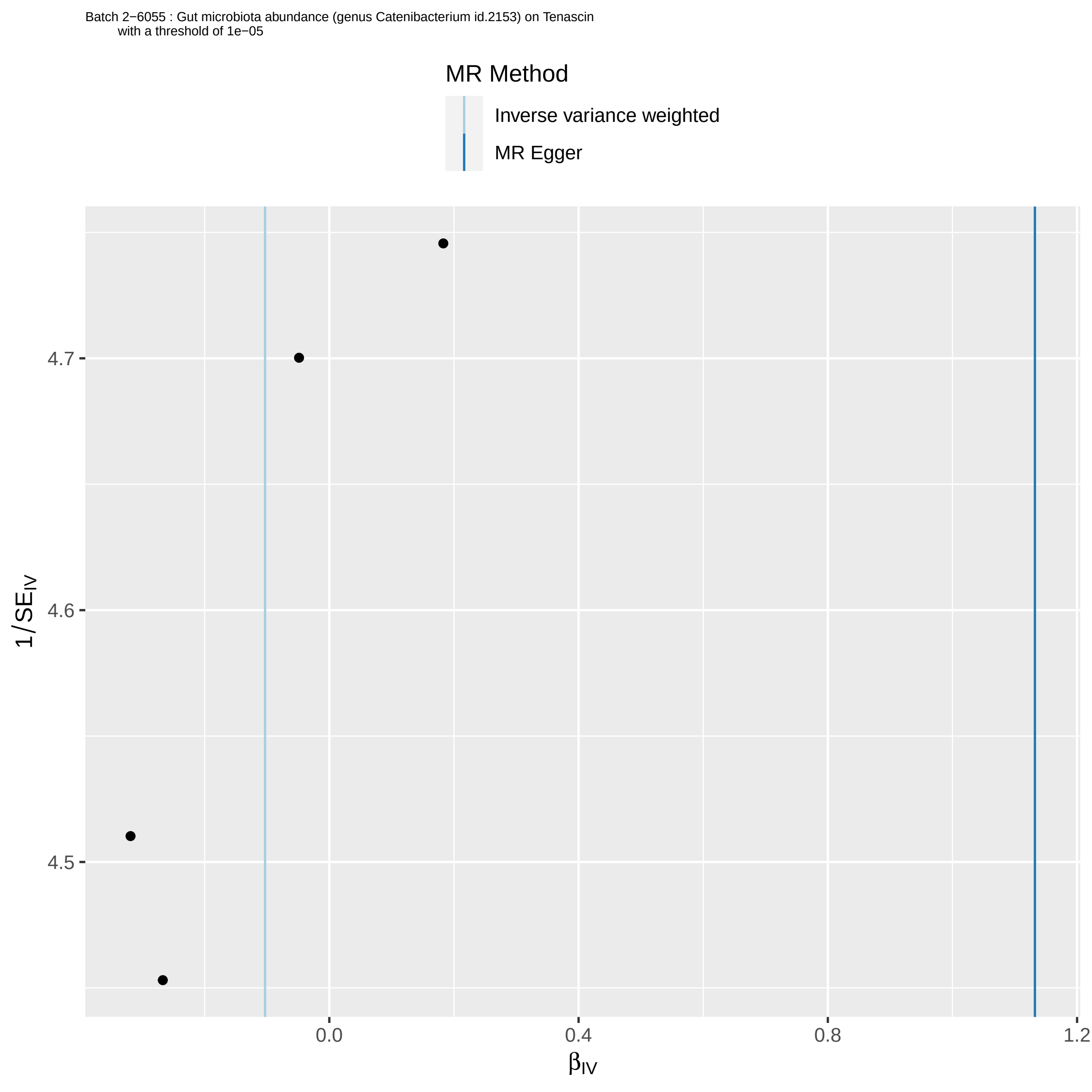
Batch 2−6055 : Gut microbiota abundance (genus Catenibacterium id.2153) on Tenascin
with a threshold of 1e−05
MR Method
Inverse variance weighted
MR Egger
4.7
4.6
4.5
1/SE_IV
0.0
0.4
0.8
1.2
β_IV

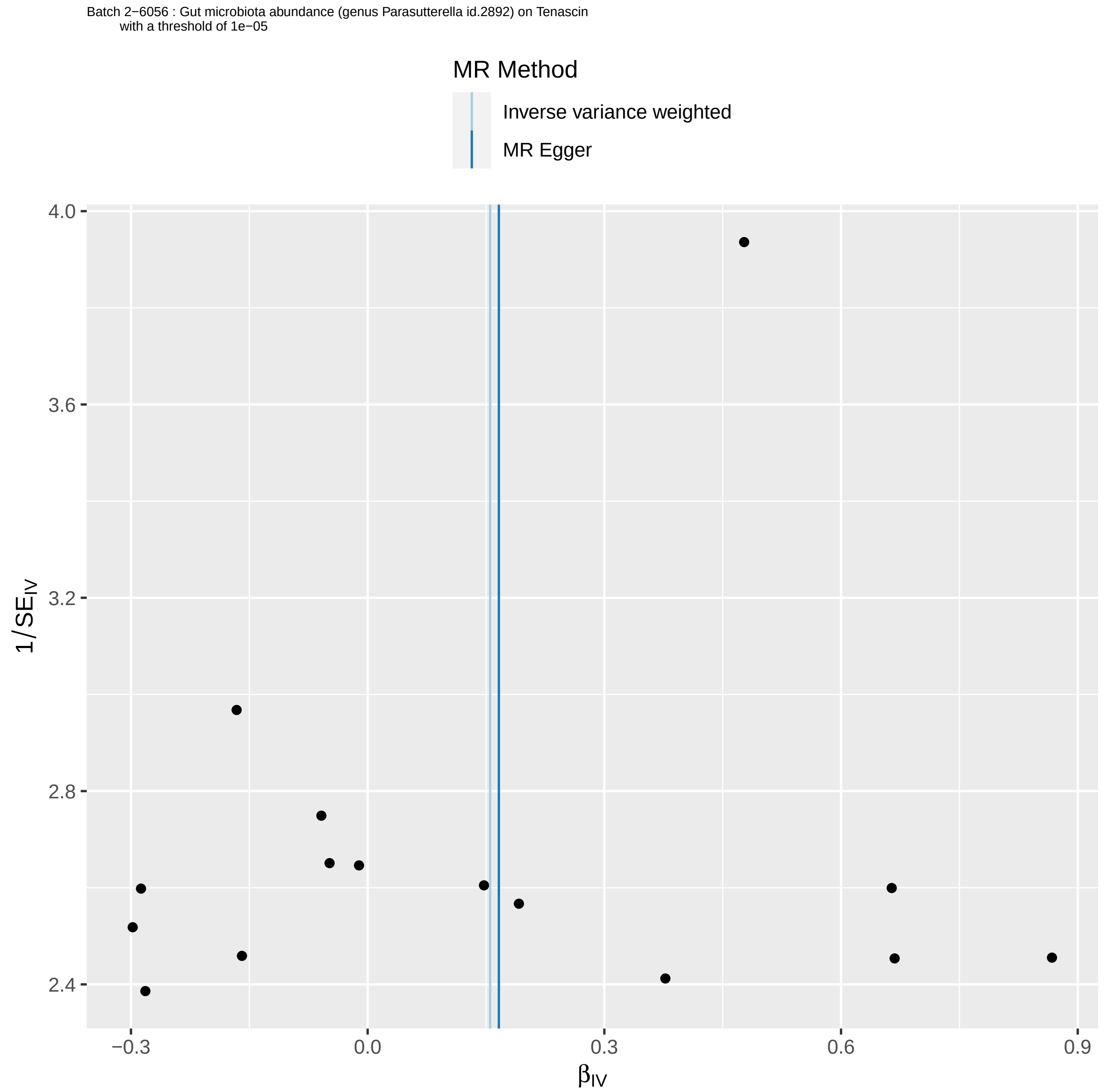

Batch 2−6056 : Gut microbiota abundance (genus Parasutterella id.2892) on Tenascin
with a threshold of 1e−05
MR Method
Inverse variance weighted
MR Egger
4.0
3.6
3.2
2.8
2.4
1/SE$_{IV}$
−0.3
0.0
0.3
0.6
0.9
β$_{IV}$

Batch 2−6057 : Gut microbiota abundance (genus Catenibacterium id.2153) on Tumor necrosis factor
with a threshold of 1e−05

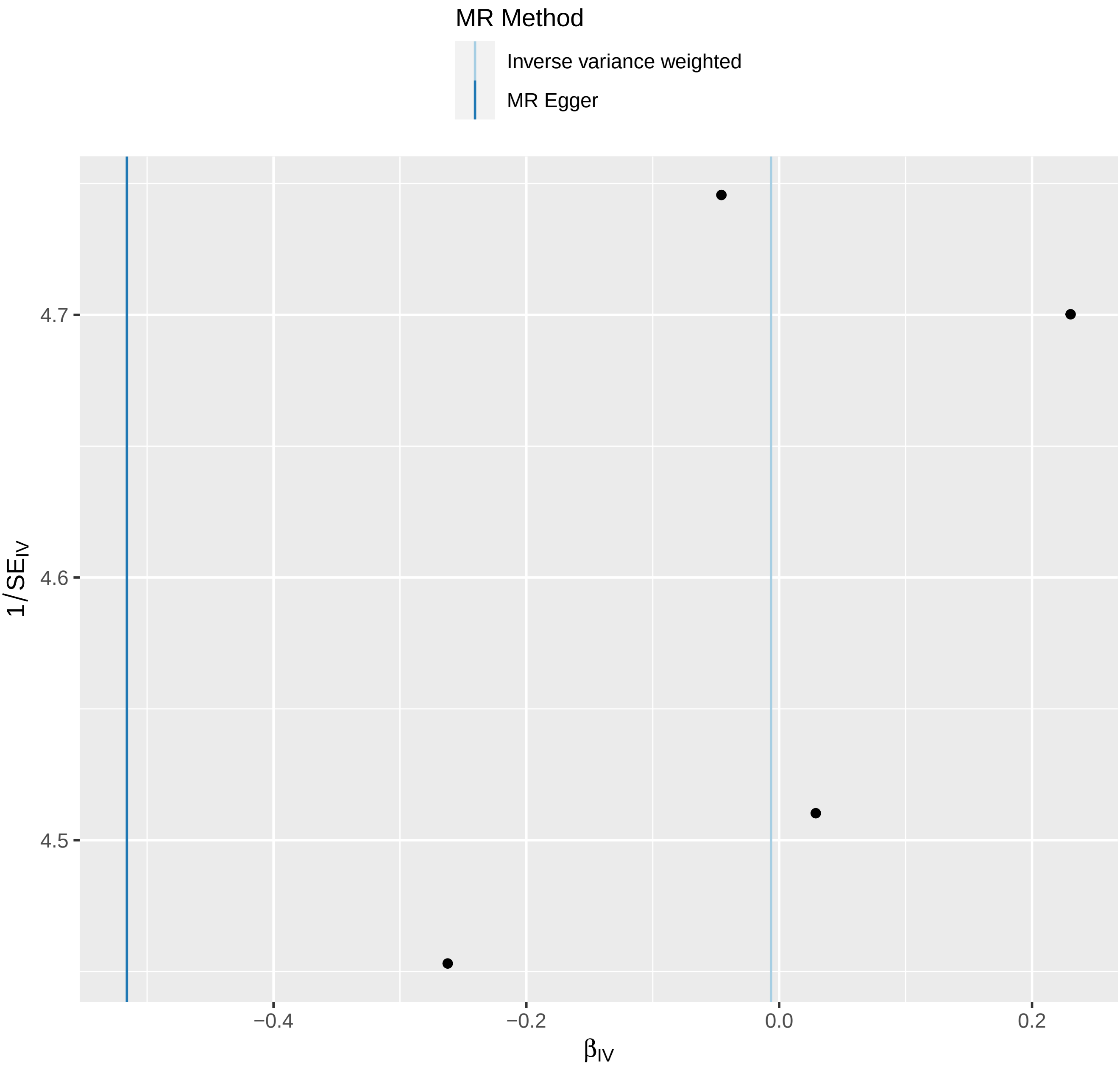

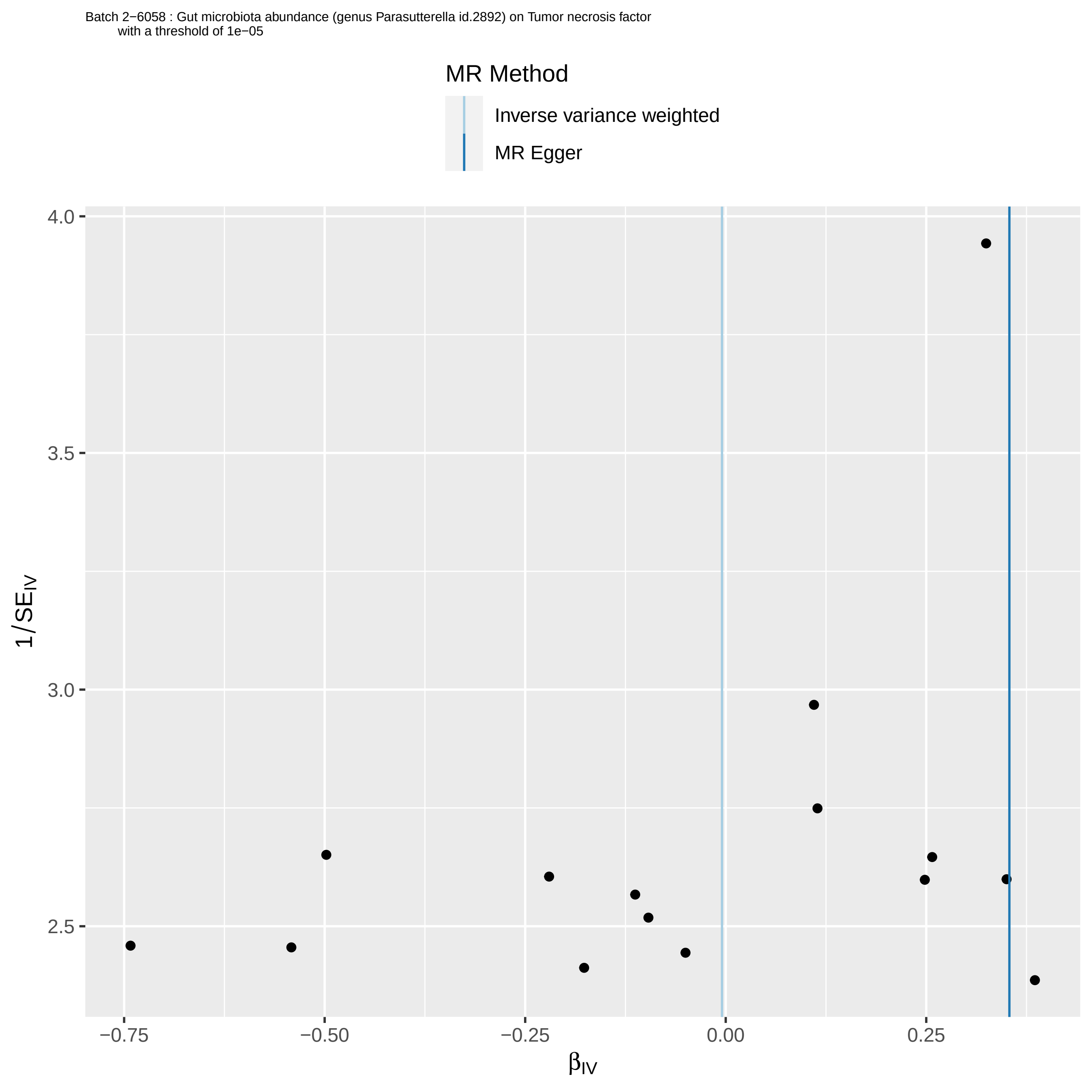
Batch 2−6058 : Gut microbiota abundance (genus Parasutterella id.2892) on Tumor necrosis factor
with a threshold of 1e−05
MR Method
Inverse variance weighted
MR Egger
4.0
3.5
3.0
2.5
1/SE_IV
−0.75
−0.50
−0.25
0.00
0.25
β_IV

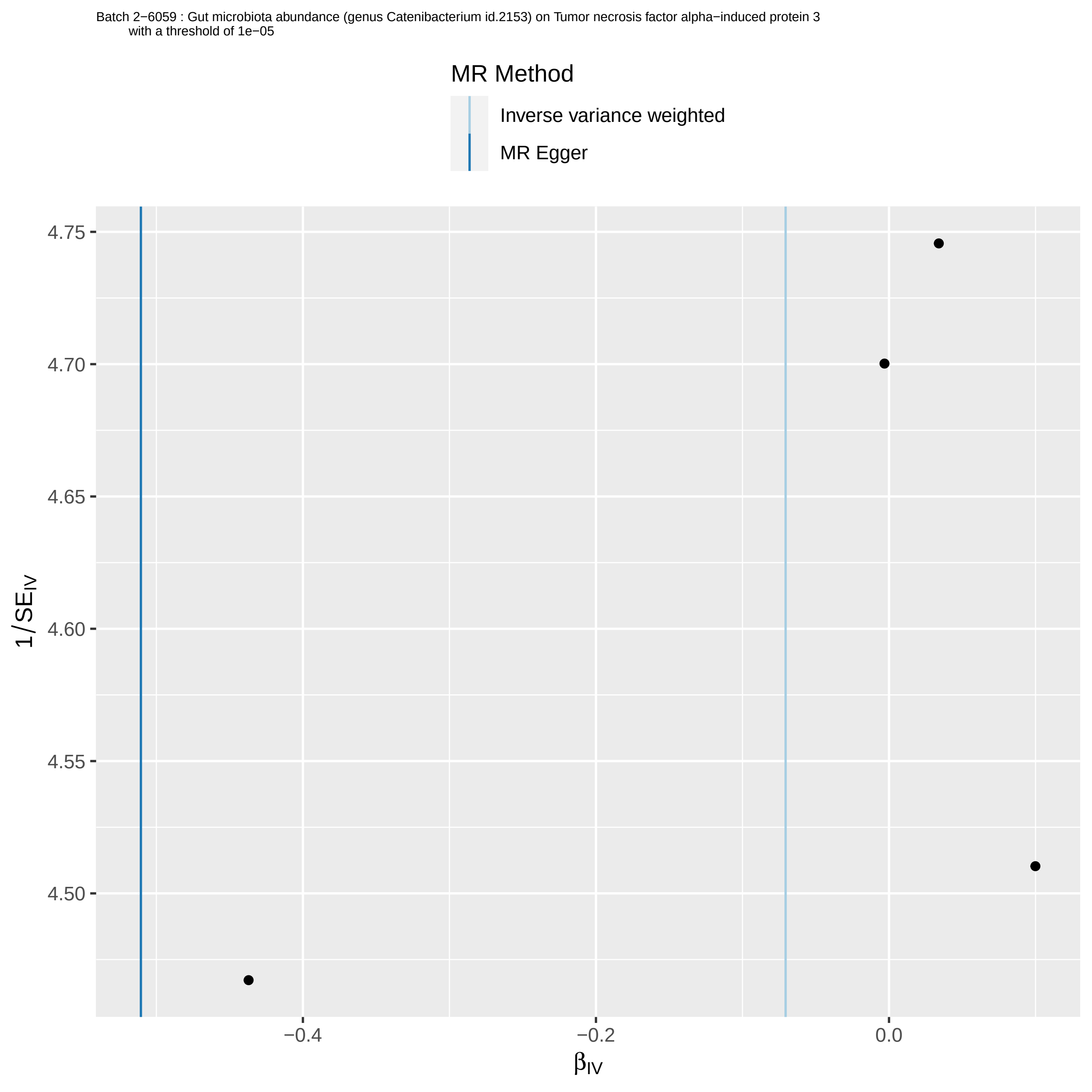

Batch 2−6059 : Gut microbiota abundance (genus Catenibacterium id.2153) on Tumor necrosis factor alpha−induced protein 3
with a threshold of 1e−05
MR Method
Inverse variance weighted
MR Egger
4.75
4.70
4.65
4.60
4.55
4.50
1/SE_IV
−0.4
−0.2
0.0
β_IV

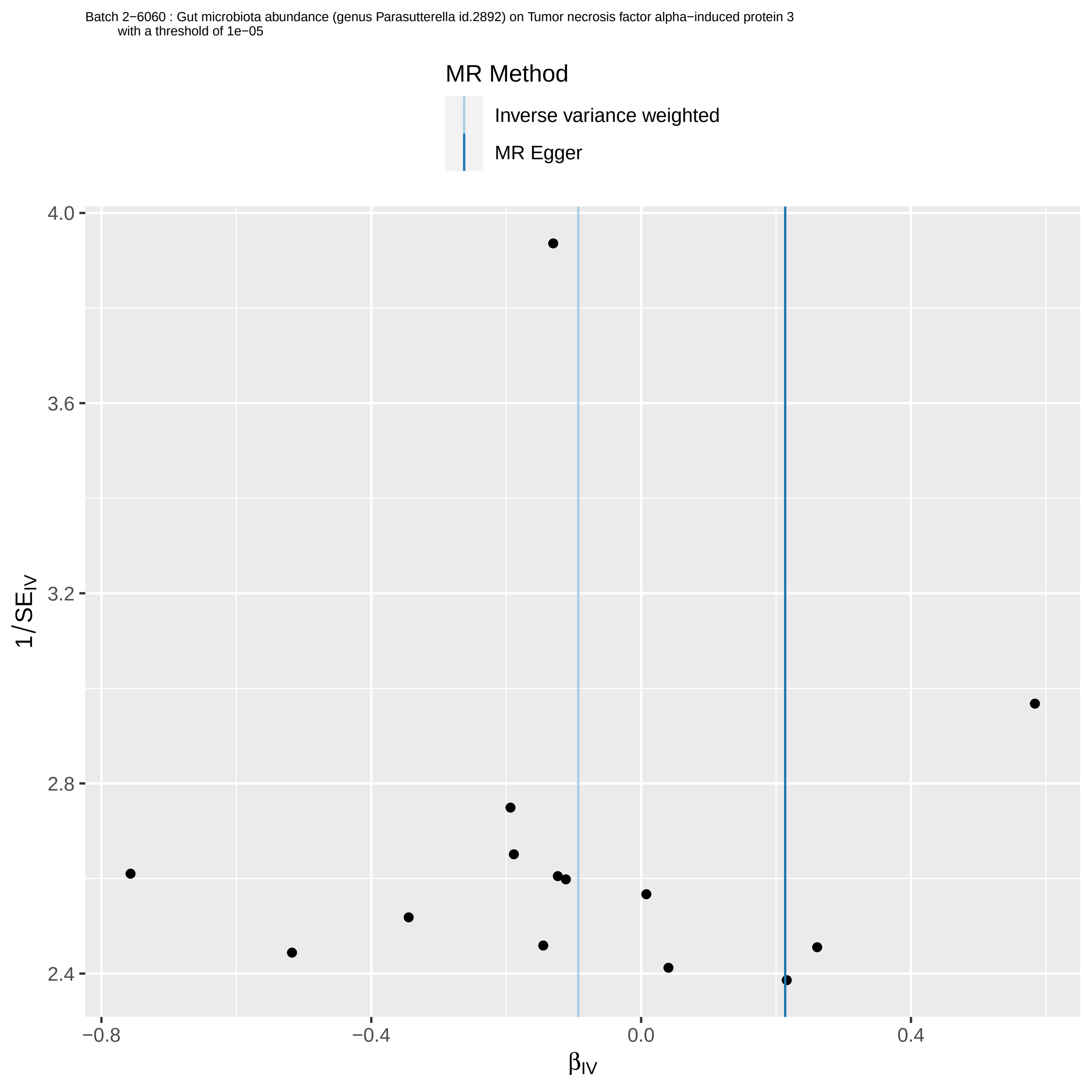
Batch 2−6060 : Gut microbiota abundance (genus Parasutterella id.2892) on Tumor necrosis factor alpha−induced protein 3
with a threshold of 1e−05
MR Method
Inverse variance weighted
MR Egger
4.0
3.6
3.2
2.8
2.4
1/SE_IV
−0.8
−0.4
0.0
0.4
β_IV

Batch 2−6061 : Gut microbiota abundance (genus Catenibacterium id.2153) on Tumor necrosis factor−inducible gene 6 protein with a threshold of 1e−05

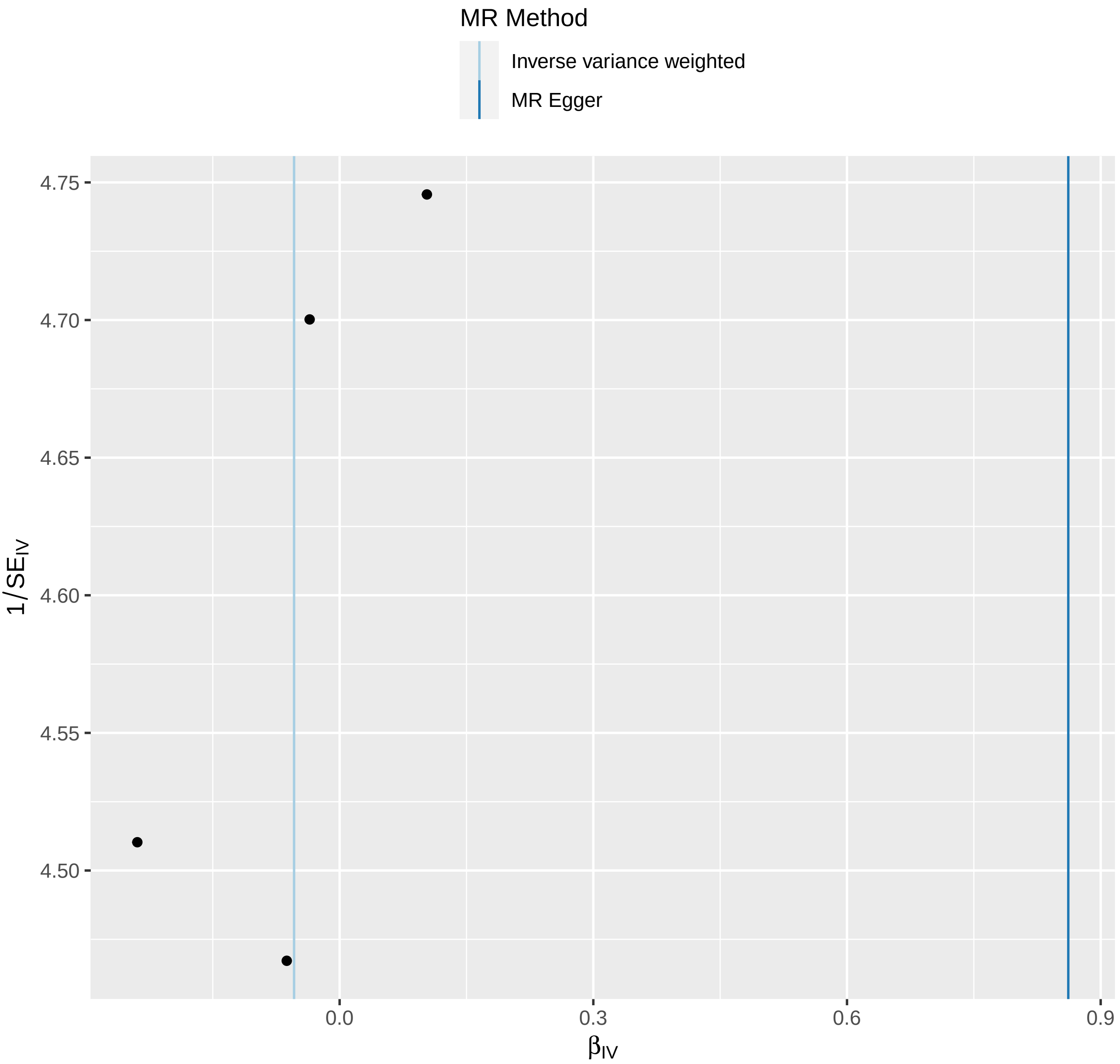

Batch 2−6062 : Gut microbiota abundance (genus Parasutterella id.2892) on Tumor necrosis factor−inducible gene 6 protein
with a threshold of 1e−05

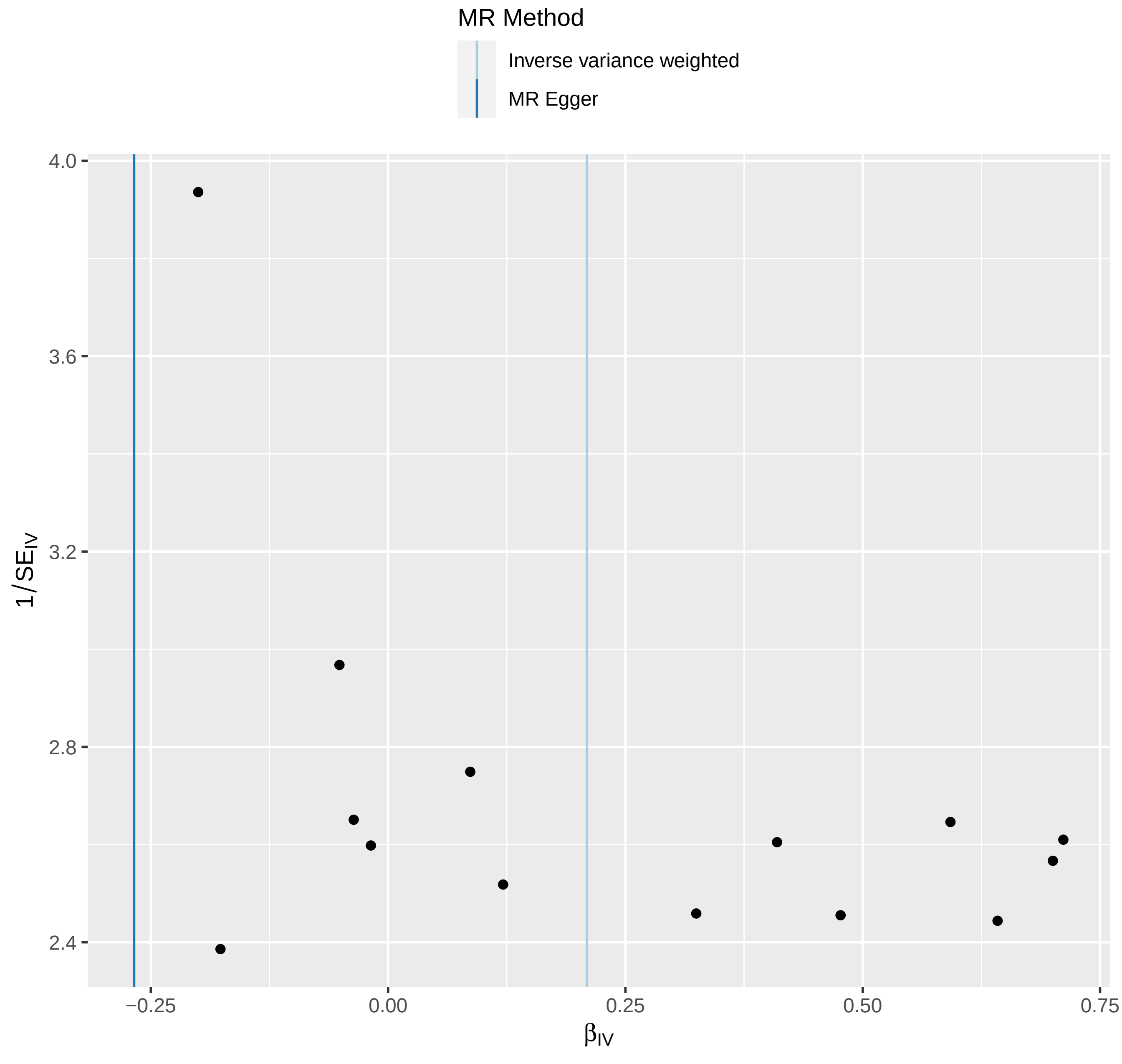

Batch 2−6063 : Gut microbiota abundance (genus Catenibacterium id.2153) on Tumor necrosis factor receptor superfamily member 10A
with a threshold of 1e−05

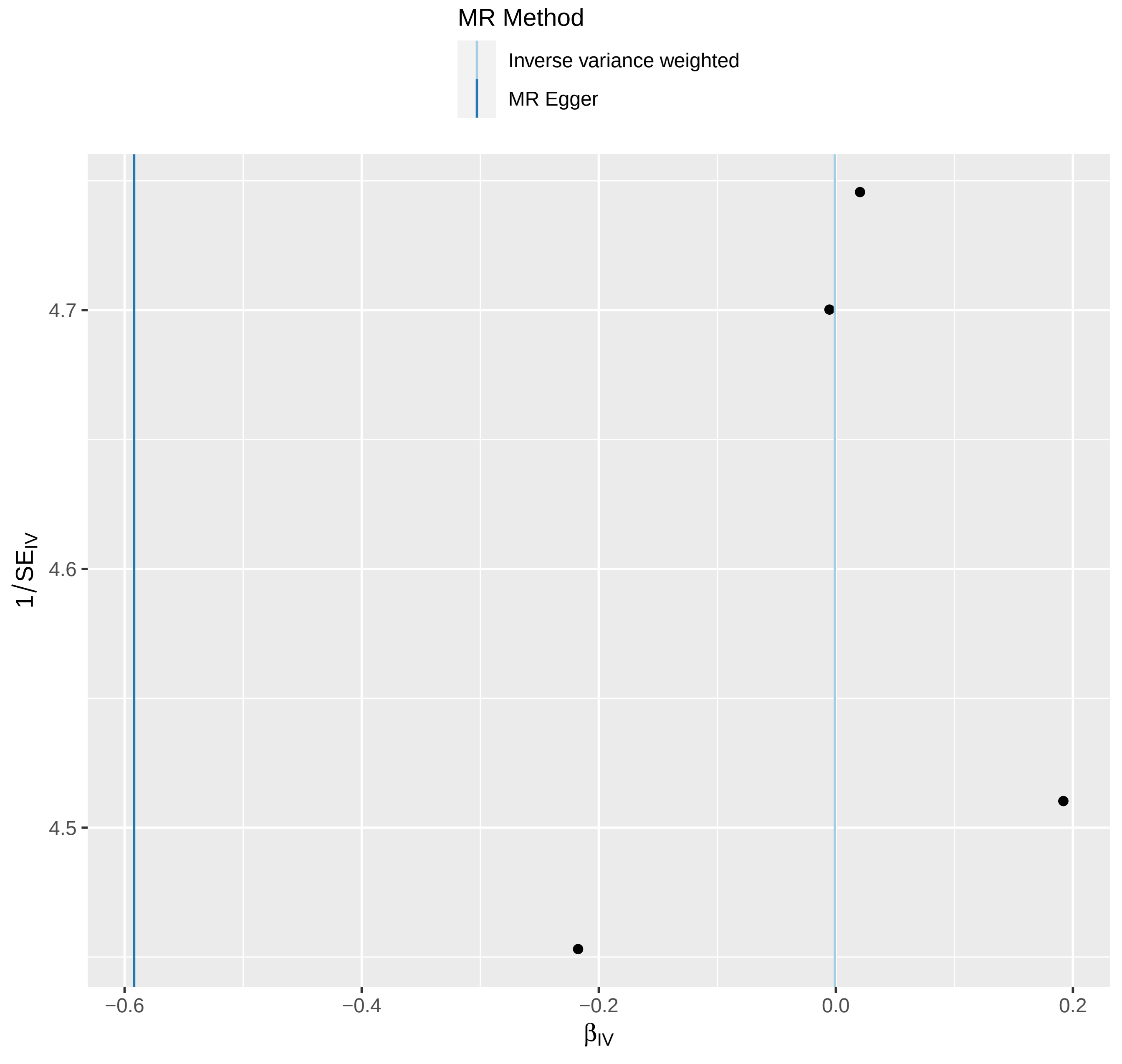

Batch 2−6064 : Gut microbiota abundance (genus Parasutterella id.2892) on Tumor necrosis factor receptor superfamily member 10A
with a threshold of 1e−05

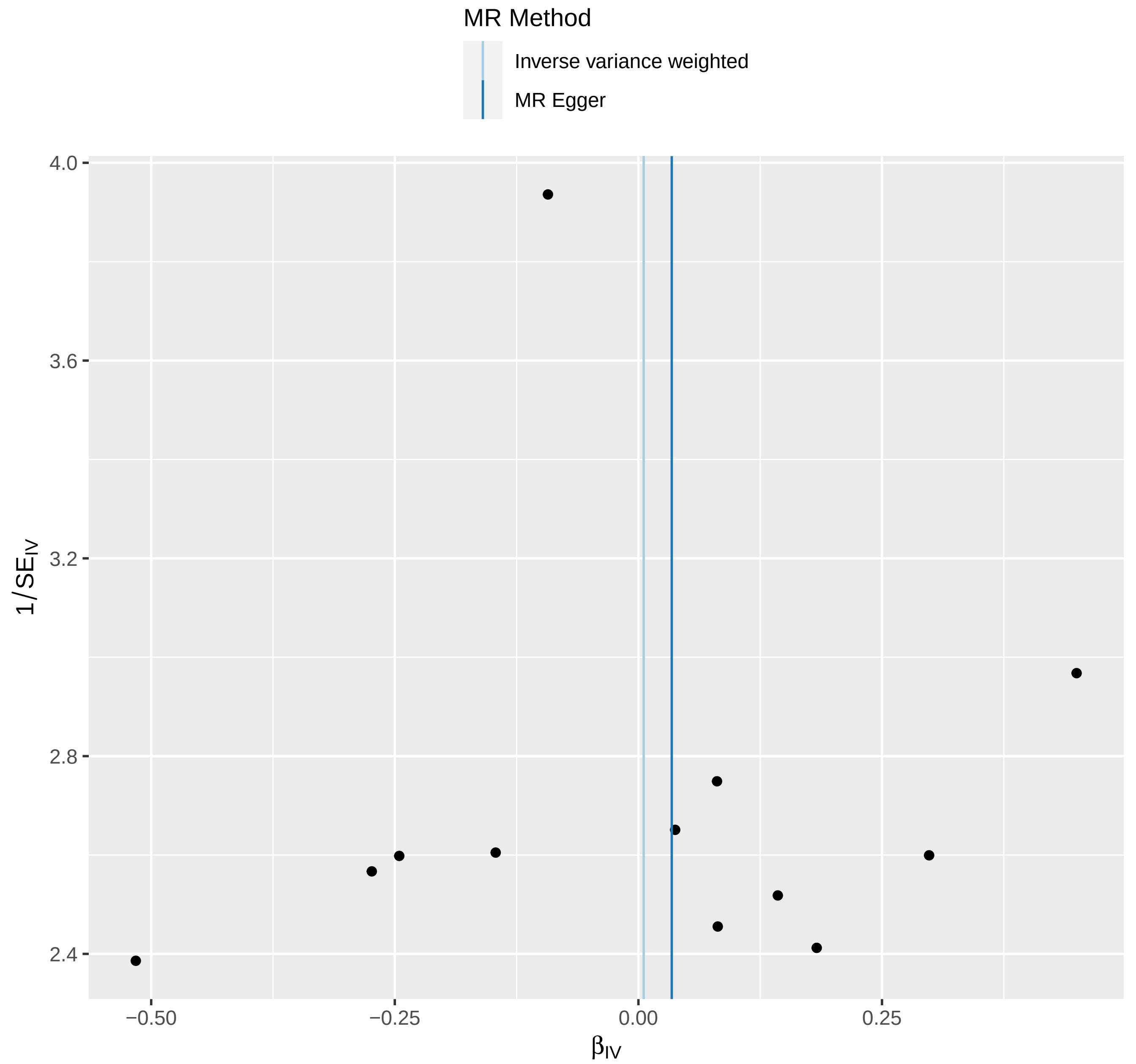

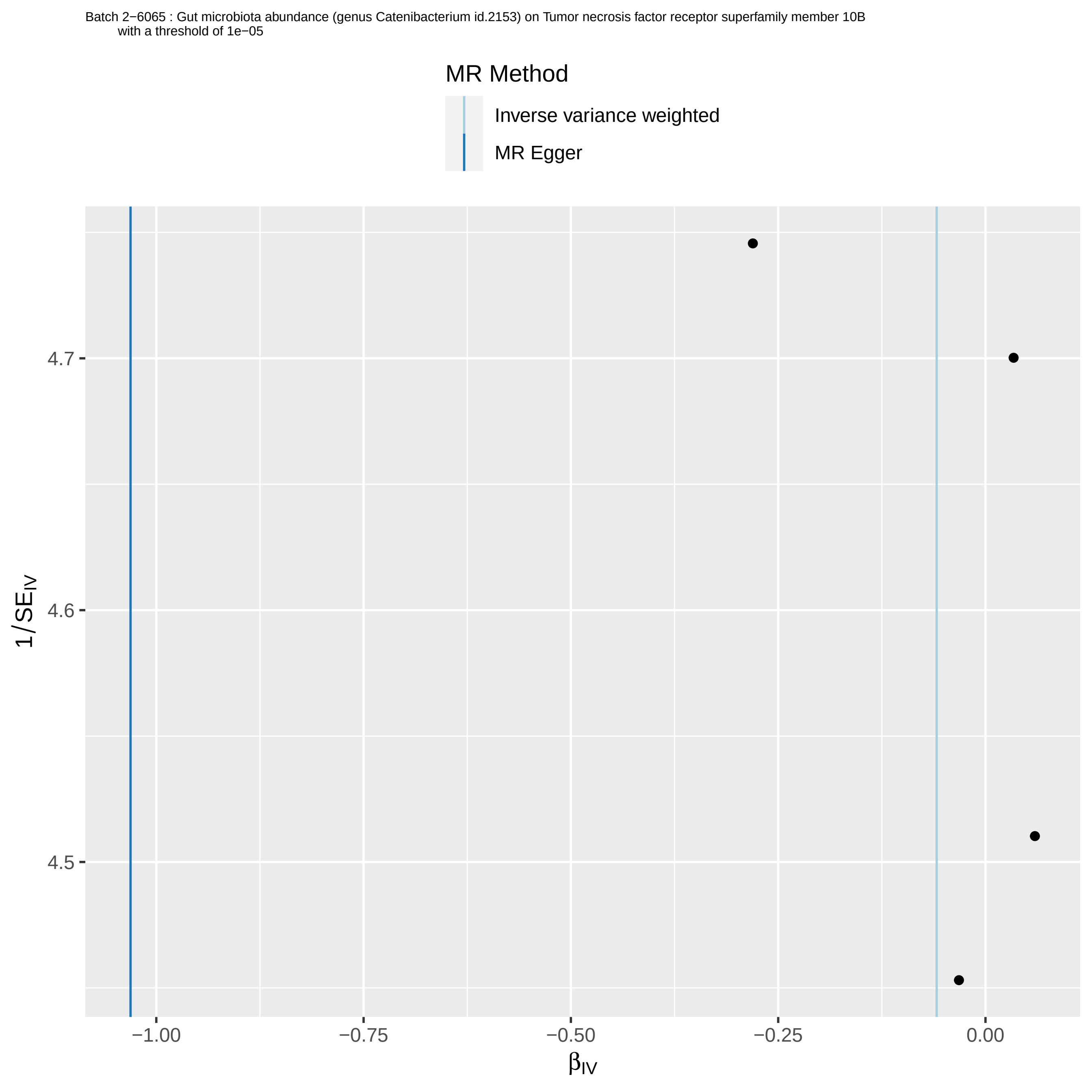
Batch 2−6065 : Gut microbiota abundance (genus Catenibacterium id.2153) on Tumor necrosis factor receptor superfamily member 10B
with a threshold of 1e−05
MR Method
Inverse variance weighted
MR Egger
4.7
4.6
4.5
1/SE_IV
−1.00
−0.75
−0.50
−0.25
0.00
β_IV

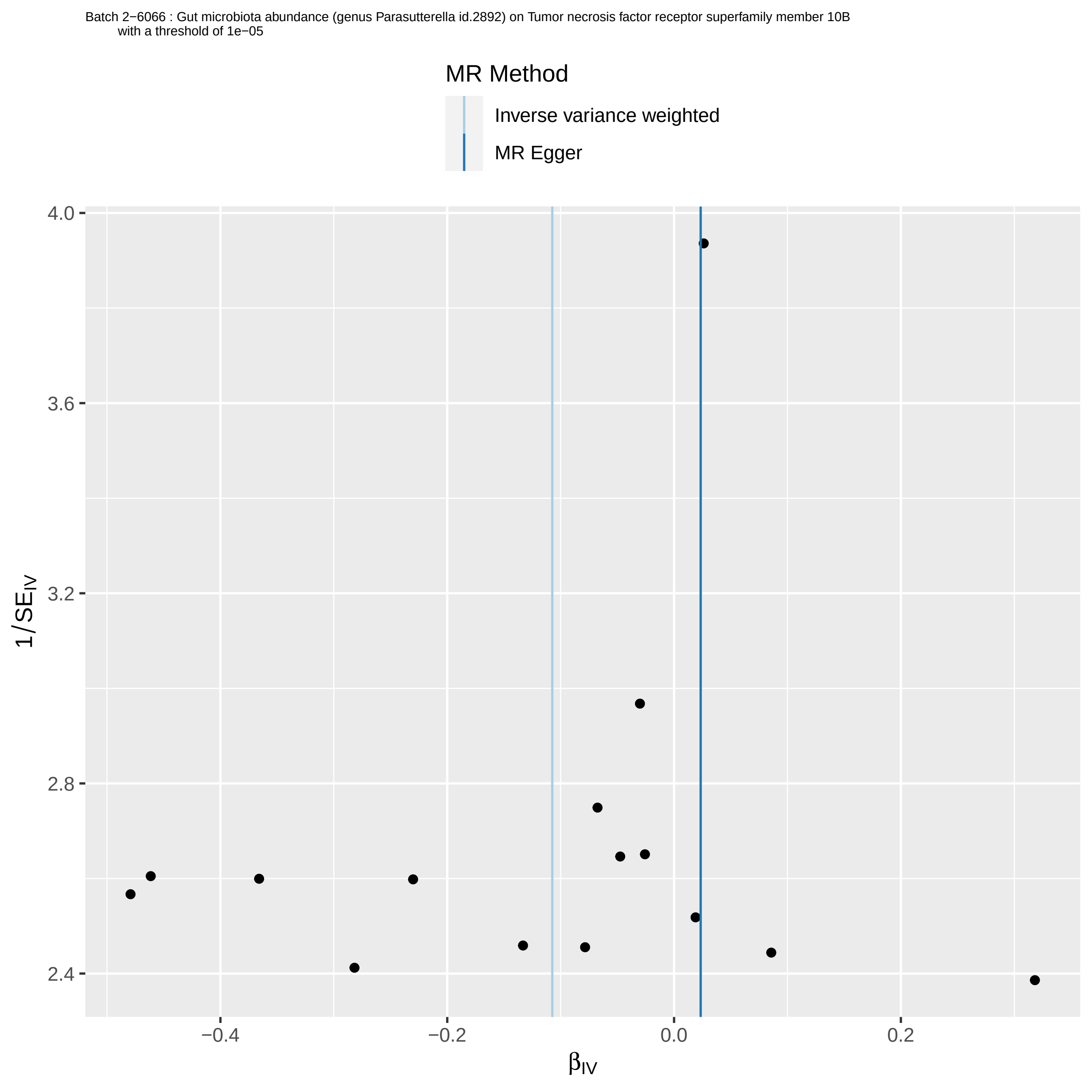

Batch 2−6066 : Gut microbiota abundance (genus Parasutterella id.2892) on Tumor necrosis factor receptor superfamily member 10B
with a threshold of 1e−05
MR Method
Inverse variance weighted
MR Egger
4.0
3.6
3.2
2.8
2.4
1/SE_IV
−0.4
−0.2
0.0
0.2
β_IV

Batch 2−6067 : Gut microbiota abundance (genus Catenibacterium id.2153) on Tumor necrosis factor receptor superfamily member 10D with a threshold of 1e−05

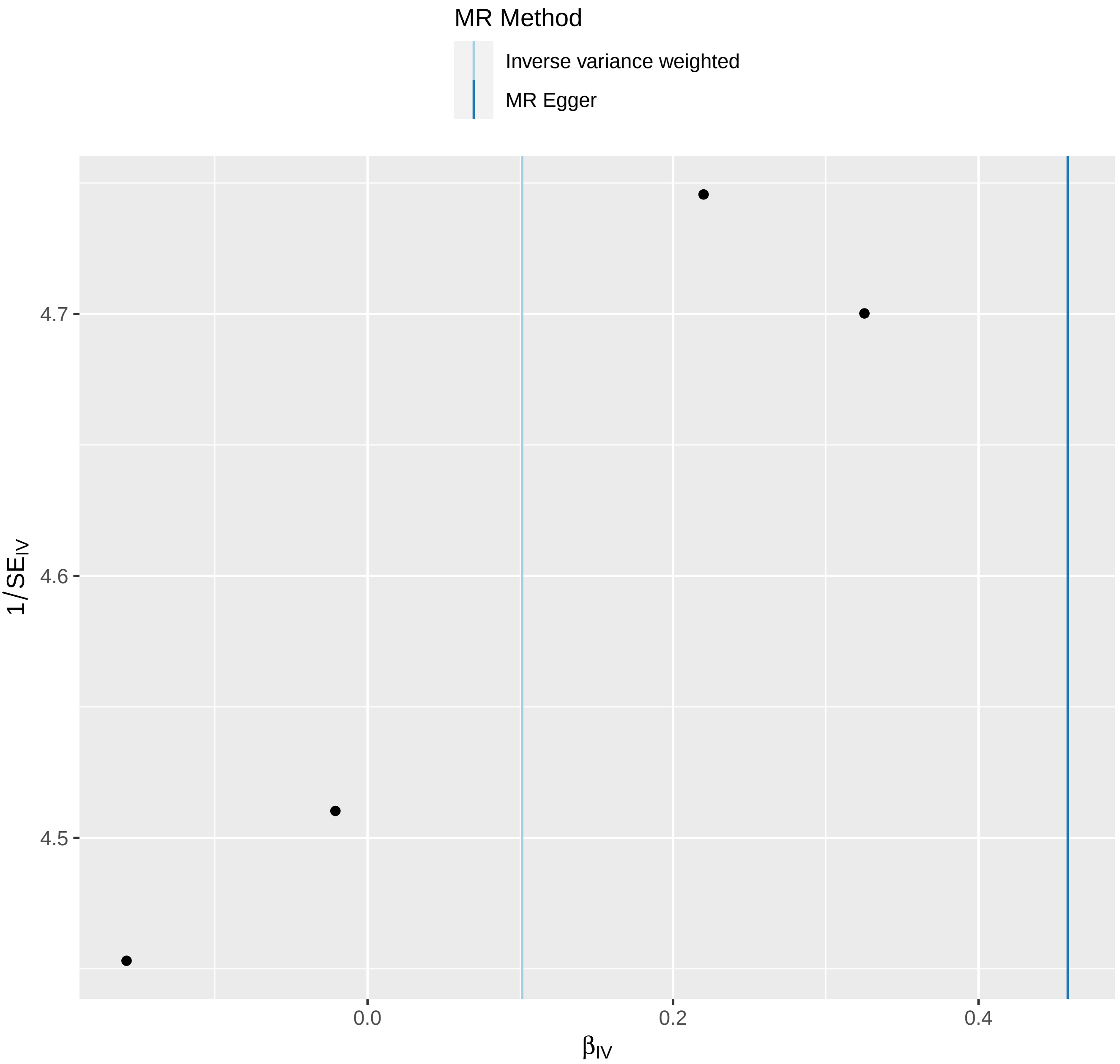

Batch 2−6068 : Gut microbiota abundance (genus Parasutterella id.2892) on Tumor necrosis factor receptor superfamily member 10D
with a threshold of 1e−05

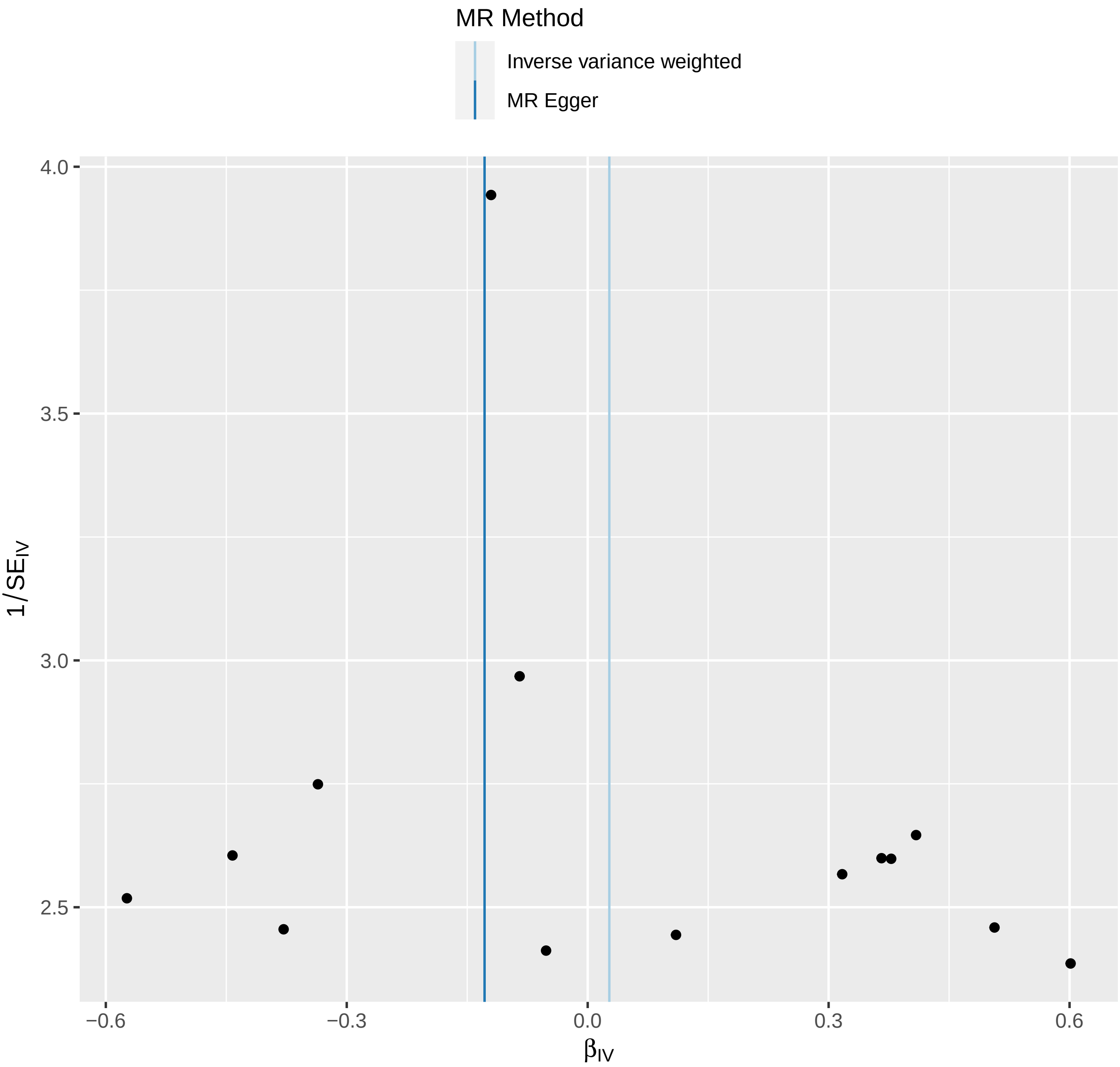

Batch 2−6069 : Gut microbiota abundance (genus Catenibacterium id.2153) on Tumor necrosis factor receptor superfamily member 11A
with a threshold of 1e−05

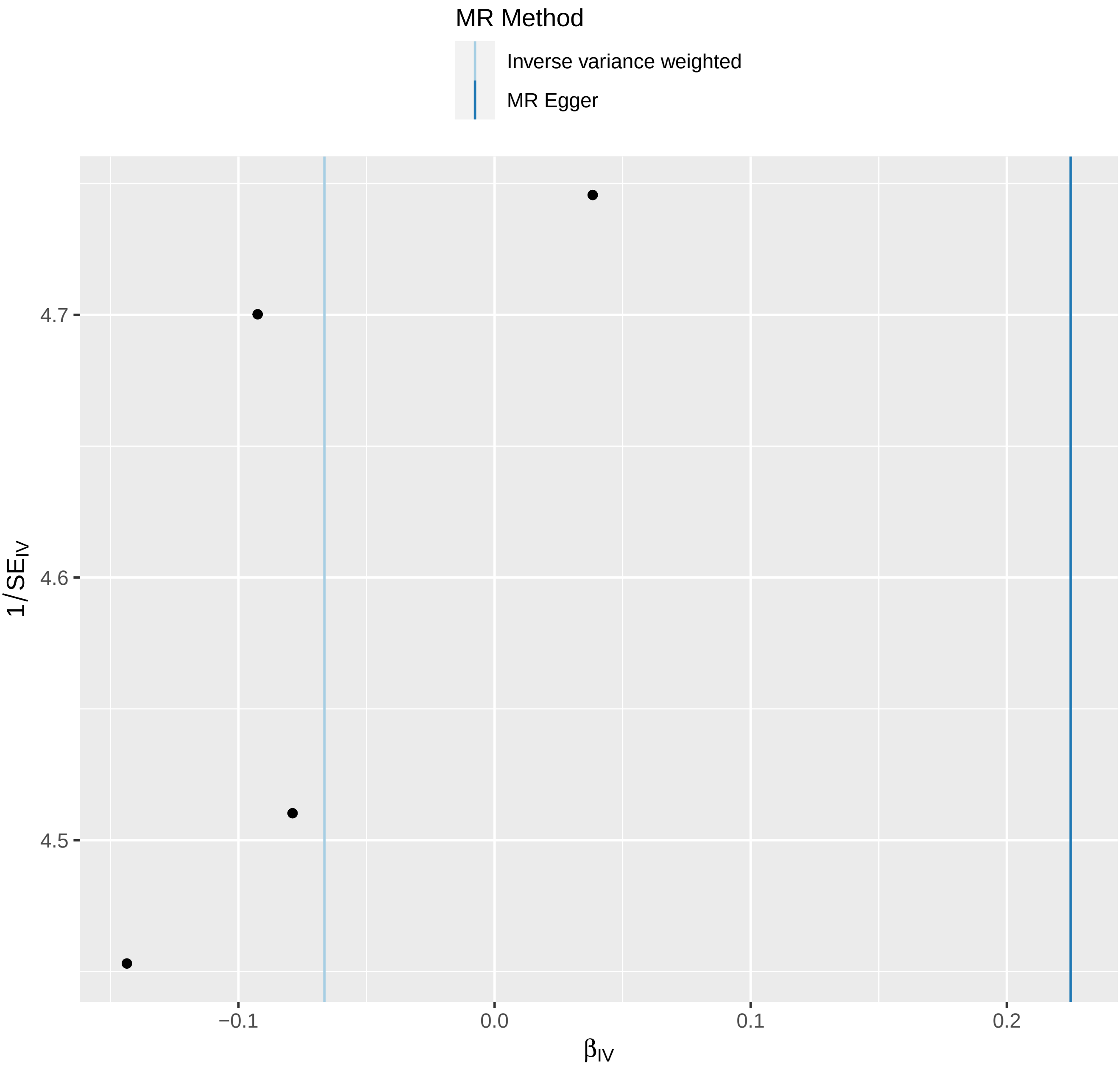

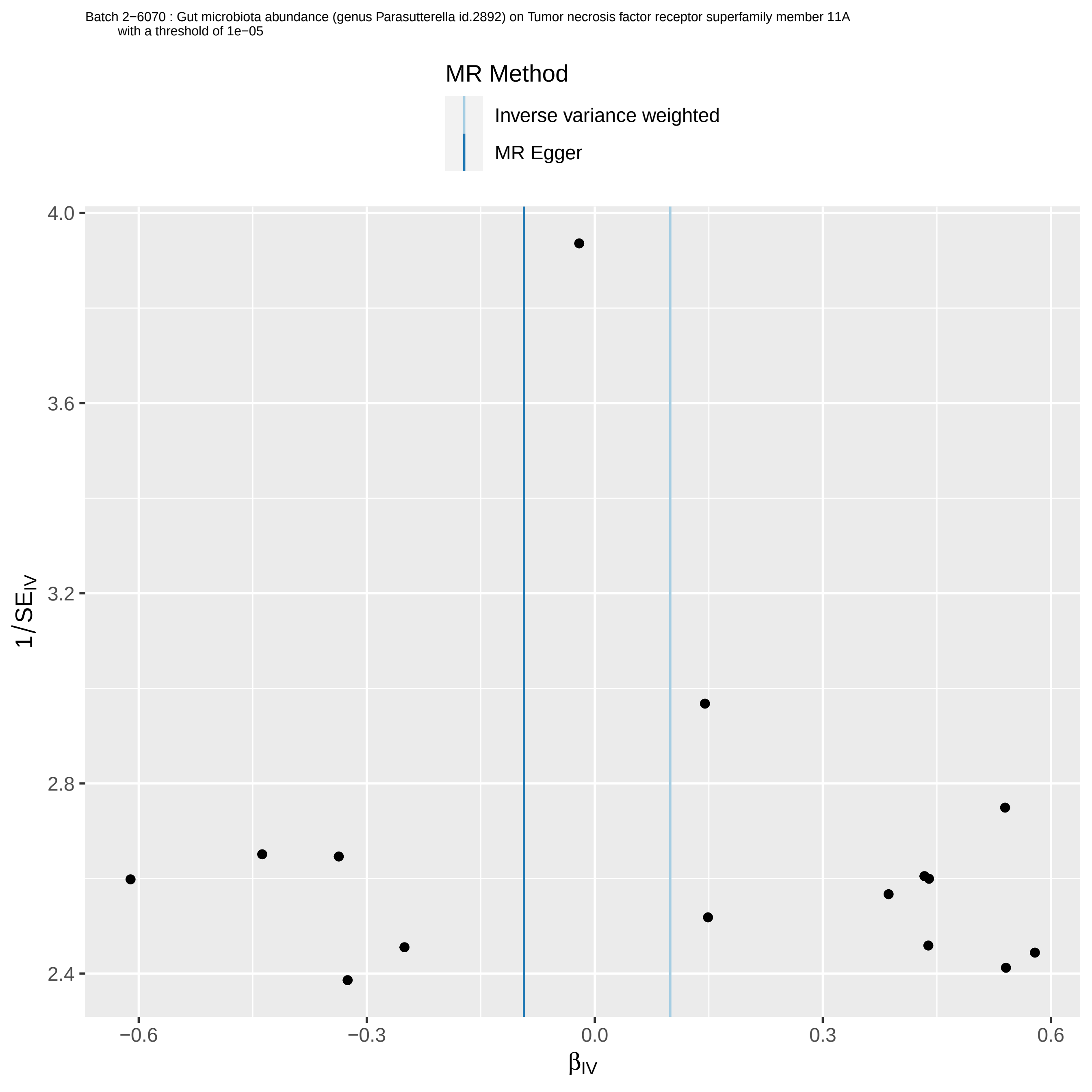

Batch 2−6070 : Gut microbiota abundance (genus Parasutterella id.2892) on Tumor necrosis factor receptor superfamily member 11A
with a threshold of 1e−05
MR Method
Inverse variance weighted
MR Egger
4.0
3.6
3.2
2.8
2.4
1/SE_IV
−0.6
−0.3
0.0
0.3
0.6
β_IV

Batch 2−6071 : Gut microbiota abundance (genus Catenibacterium id.2153) on Tumor necrosis factor receptor superfamily member 11A
with a threshold of 1e−05

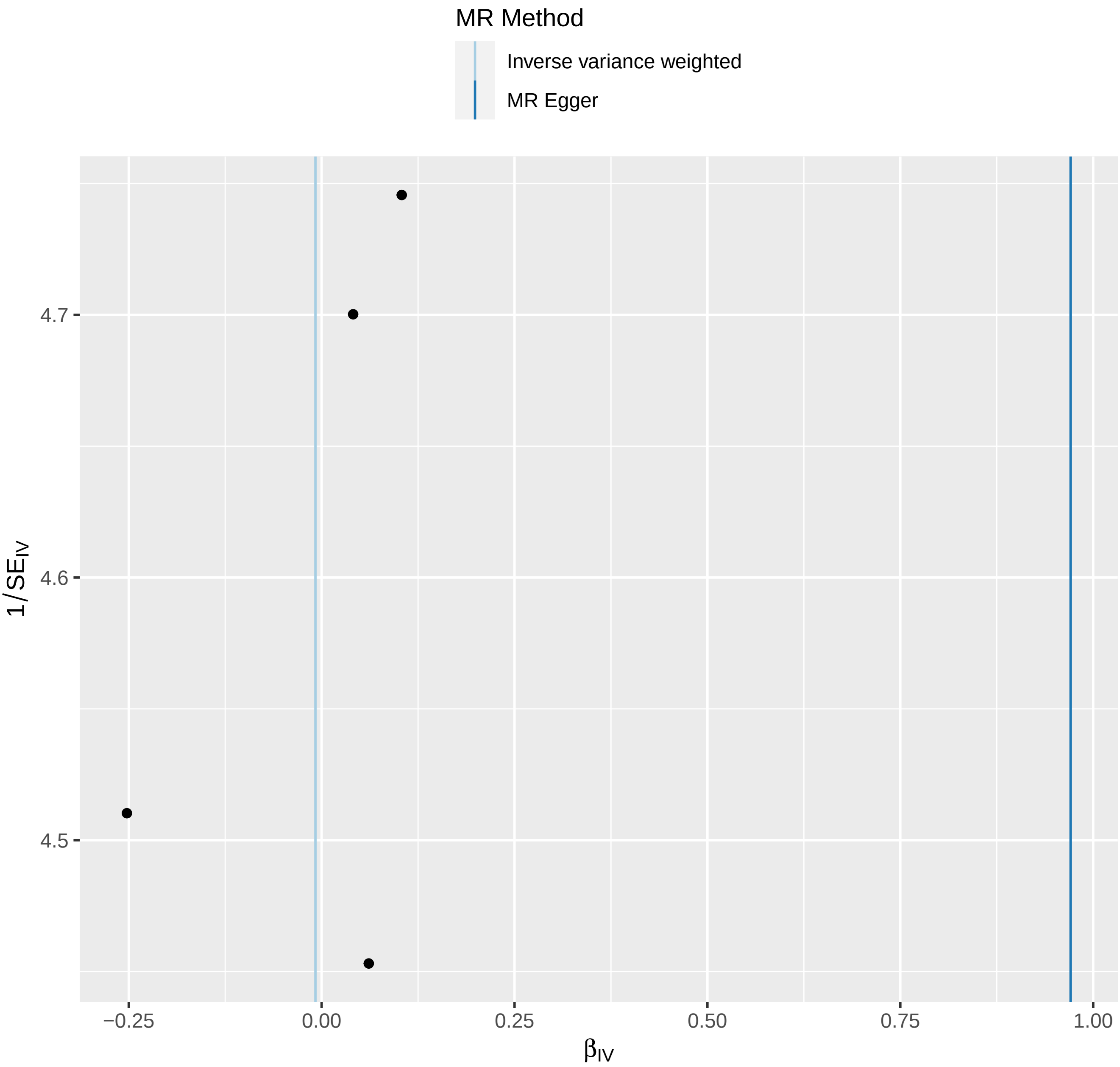

Batch 2−6072 : Gut microbiota abundance (genus Parasutterella id.2892) on Tumor necrosis factor receptor superfamily member 11A
with a threshold of 1e−05

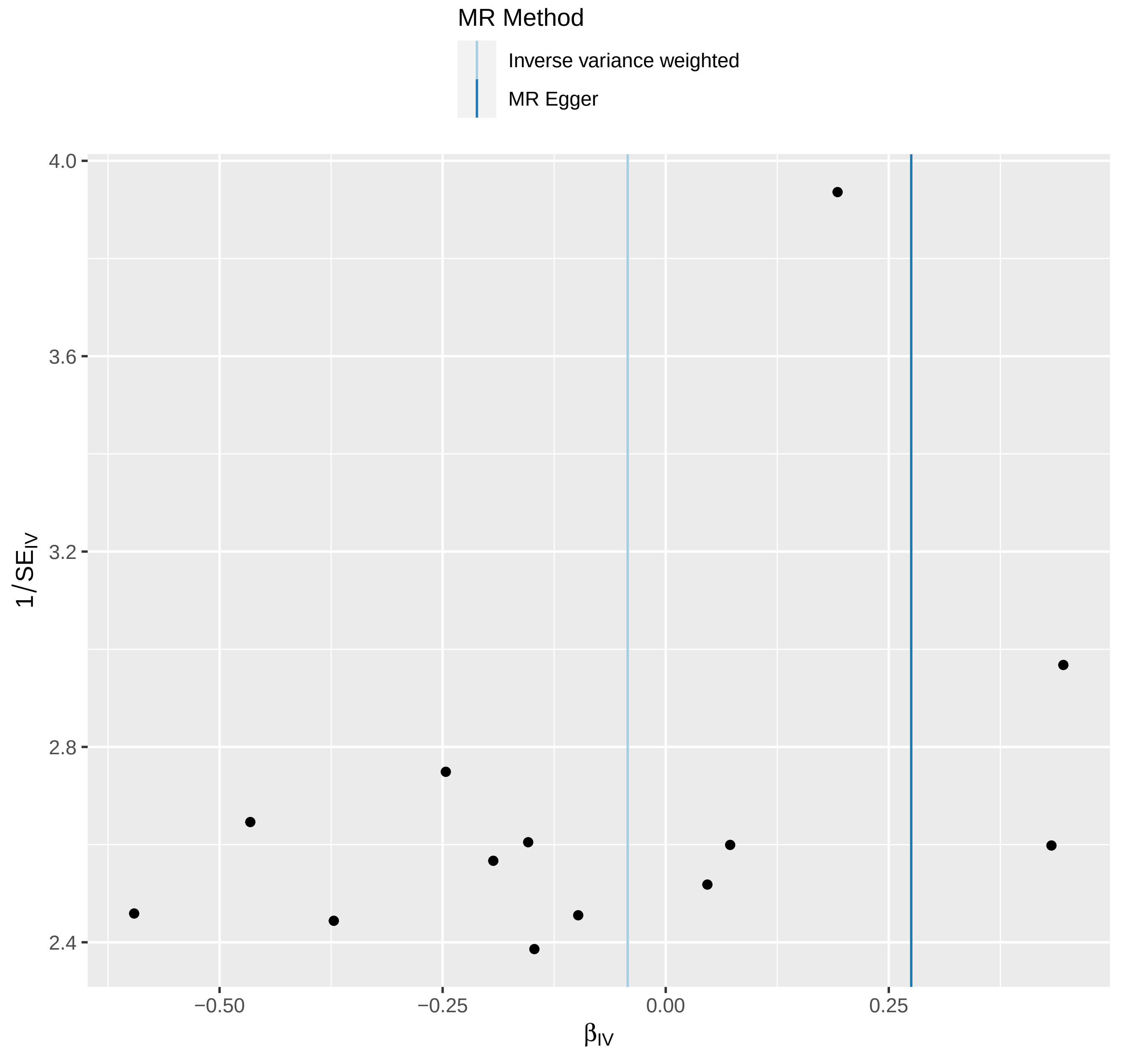

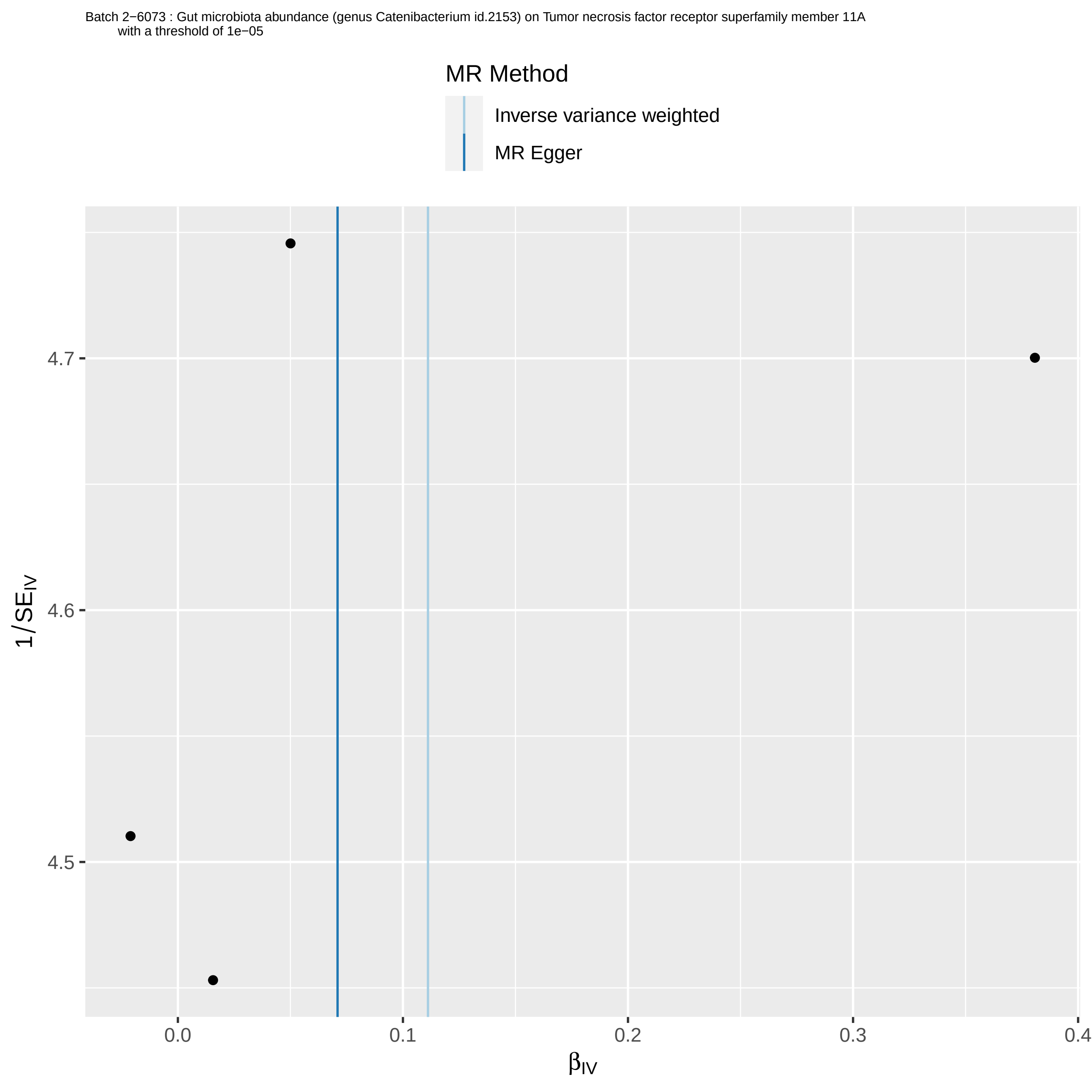

Batch 2−6073 : Gut microbiota abundance (genus Catenibacterium id.2153) on Tumor necrosis factor receptor superfamily member 11A
with a threshold of 1e−05
MR Method
Inverse variance weighted
MR Egger
4.7
4.6
4.5
1/SE_IV
0.0
0.1
0.2
0.3
0.4
β_IV

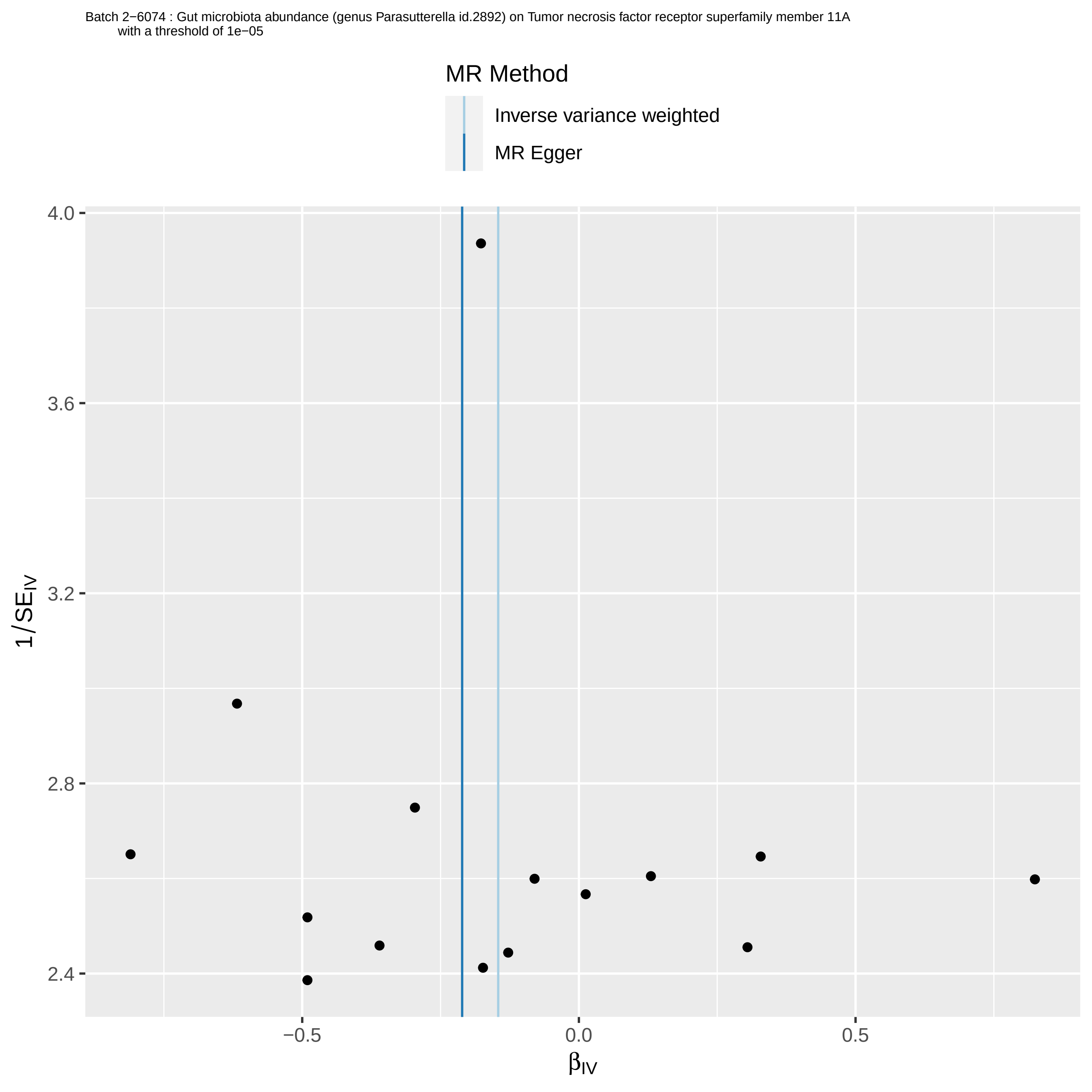
Batch 2−6074 : Gut microbiota abundance (genus Parasutterella id.2892) on Tumor necrosis factor receptor superfamily member 11A
with a threshold of 1e−05
MR Method
Inverse variance weighted
MR Egger
4.0
3.6
3.2
2.8
2.4
1/SE_IV
−0.5
0.0
0.5
β_IV

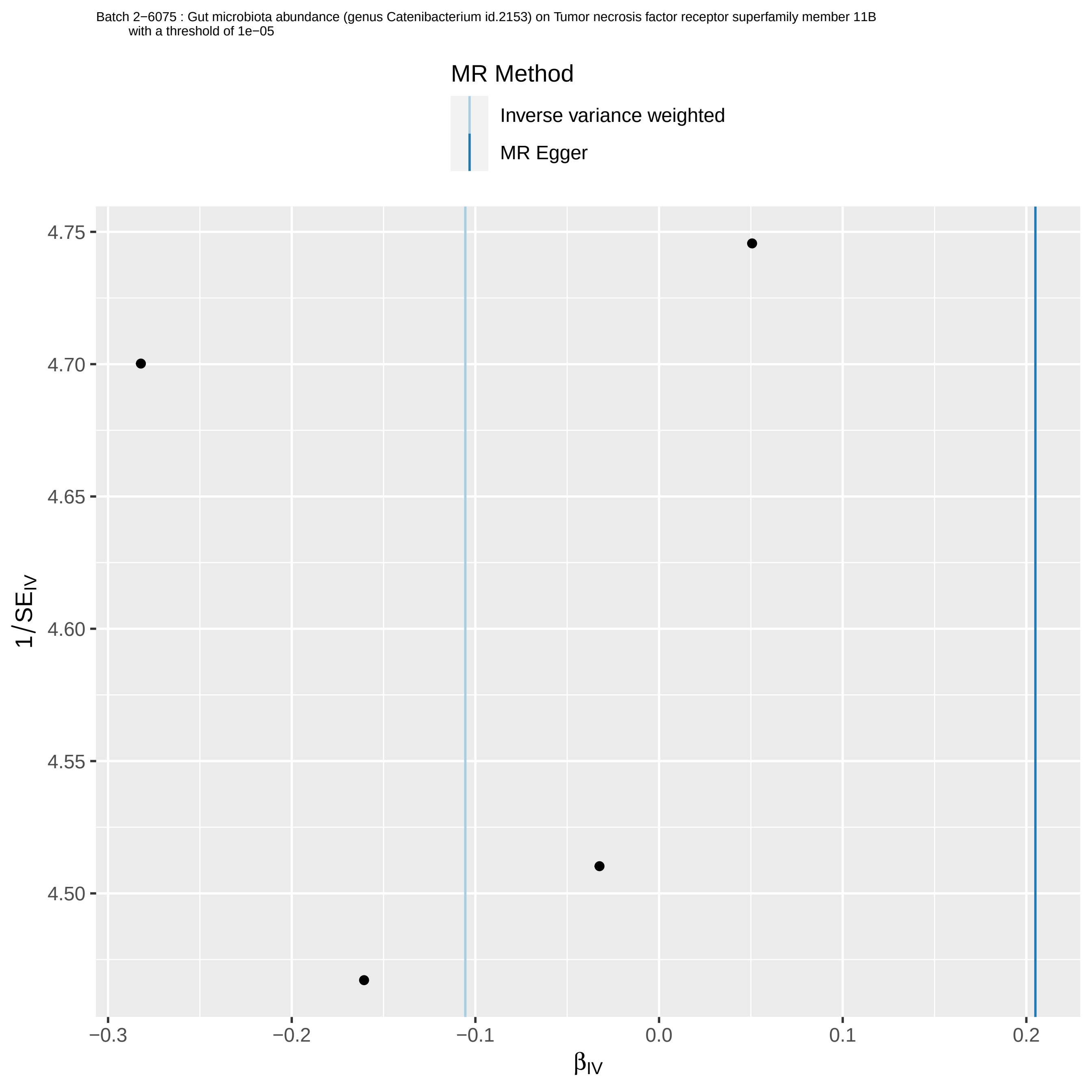

Batch 2−6075 : Gut microbiota abundance (genus Catenibacterium id.2153) on Tumor necrosis factor receptor superfamily member 11B
with a threshold of 1e−05
MR Method
Inverse variance weighted
MR Egger
4.75
4.70
4.65
4.60
4.55
4.50
1/SE_IV
−0.3
−0.2
−0.1
0.0
0.1
0.2
β_IV

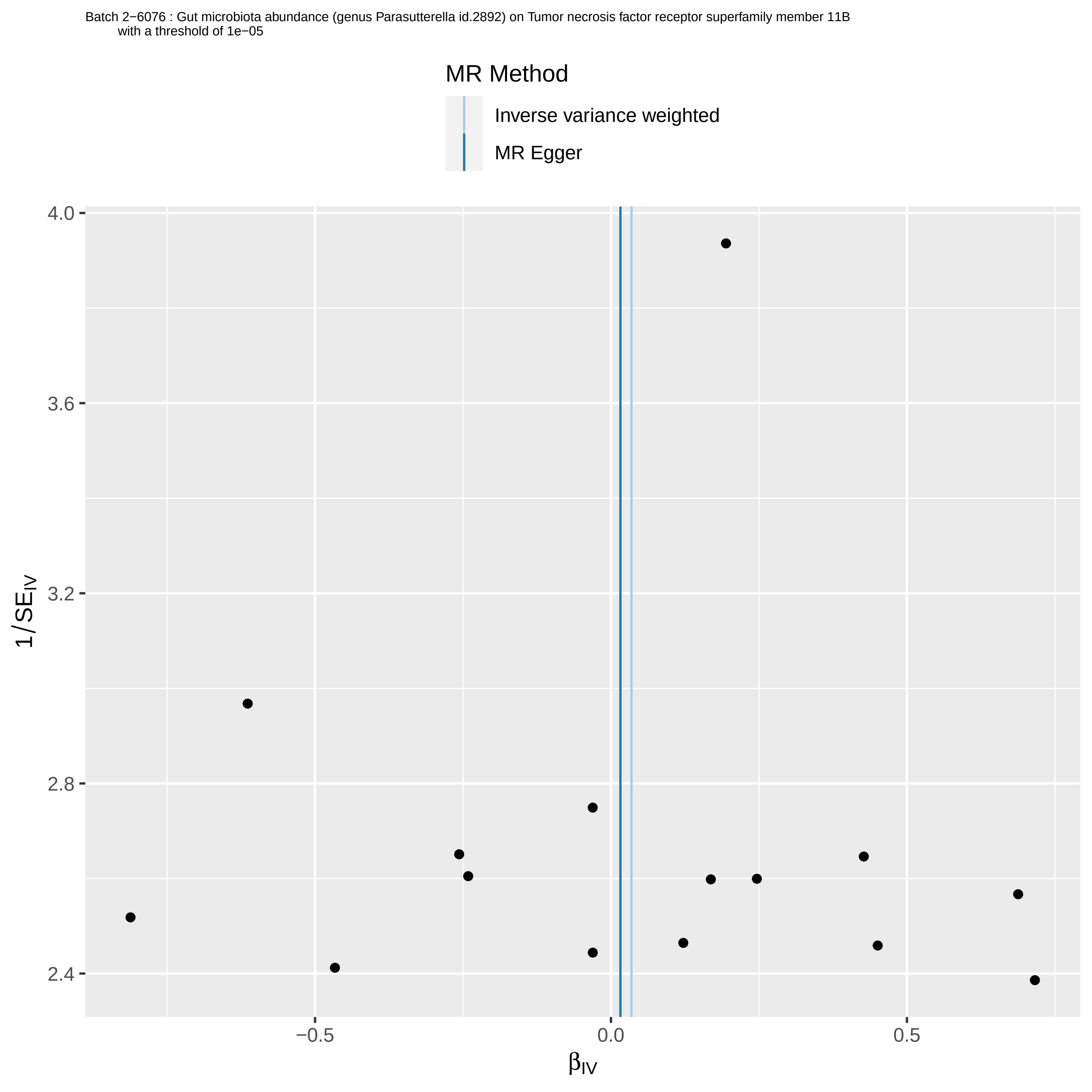

Batch 2−6076 : Gut microbiota abundance (genus Parasutterella id.2892) on Tumor necrosis factor receptor superfamily member 11B
with a threshold of 1e−05
MR Method
Inverse variance weighted
MR Egger
4.0
3.6
3.2
2.8
2.4
1/SE_IV
−0.5
0.0
0.5
β_IV

Batch 2−6077 : Gut microbiota abundance (genus Catenibacterium id.2153) on Tumor necrosis factor receptor superfamily member 11B
with a threshold of 1e−05

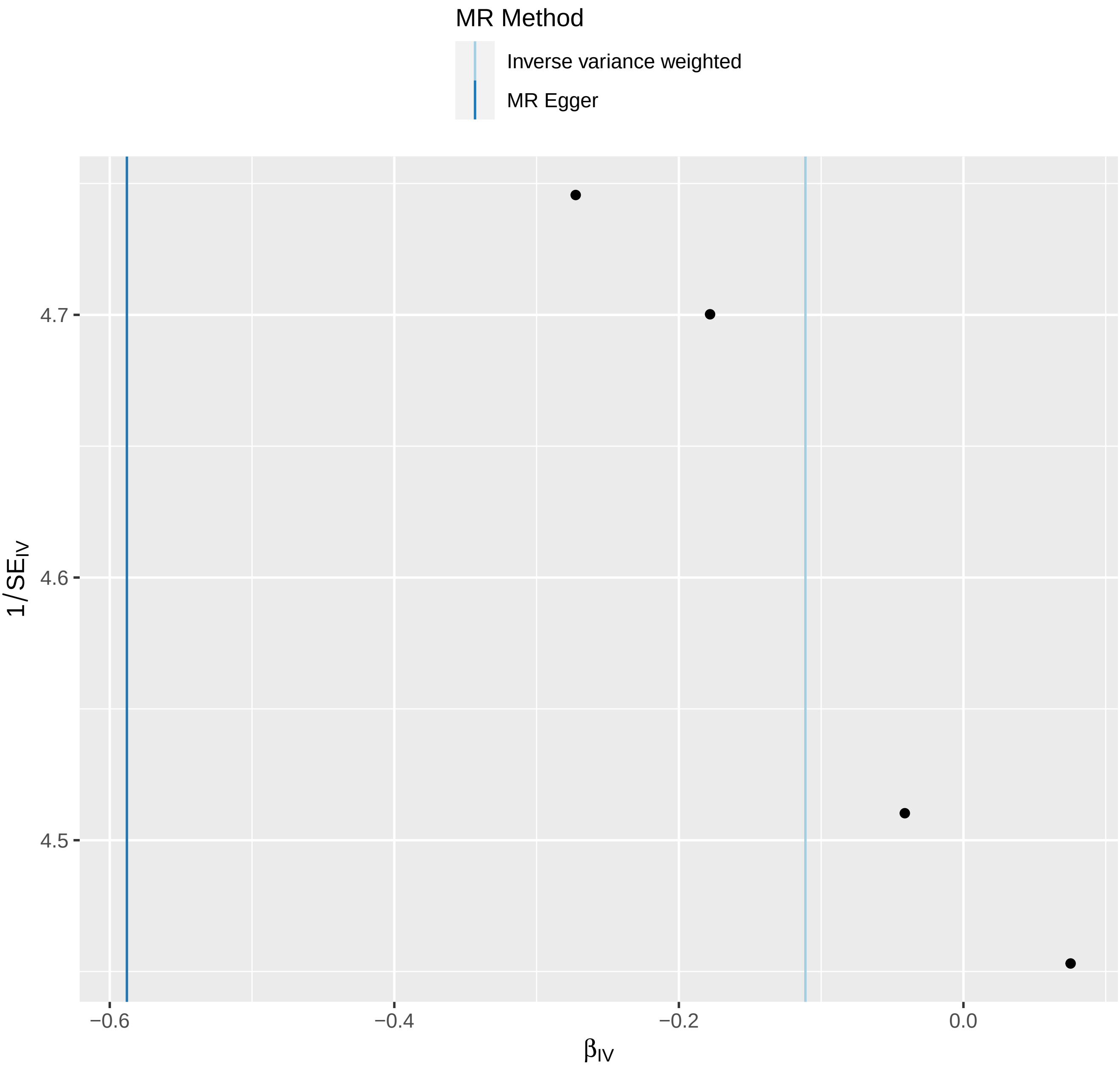

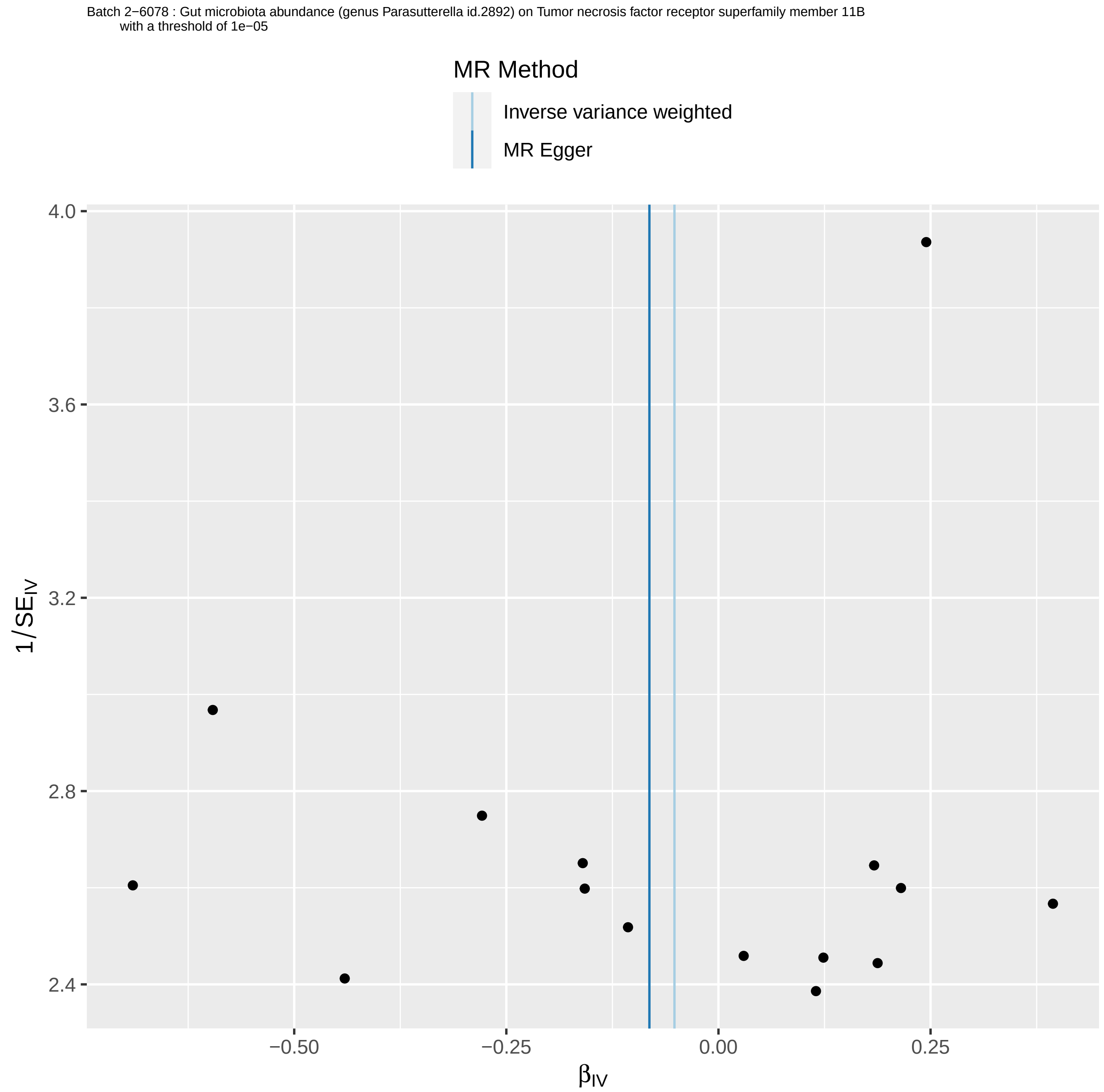
Batch 2−6078 : Gut microbiota abundance (genus Parasutterella id.2892) on Tumor necrosis factor receptor superfamily member 11B
with a threshold of 1e−05
MR Method
Inverse variance weighted
MR Egger
4.0
3.6
3.2
2.8
2.4
1/SE_IV
−0.50
−0.25
0.00
0.25
β_IV

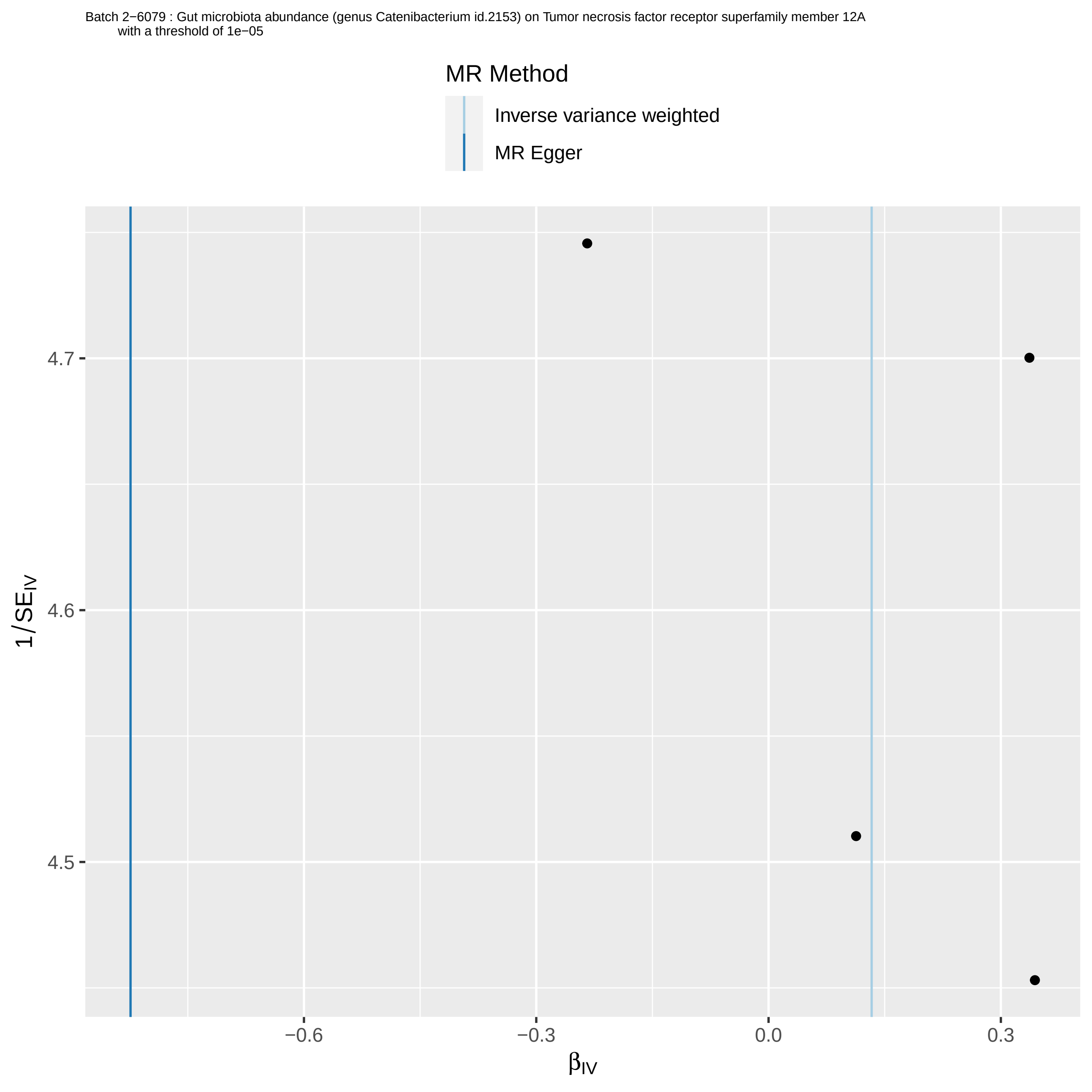

Batch 2−6079 : Gut microbiota abundance (genus Catenibacterium id.2153) on Tumor necrosis factor receptor superfamily member 12A
with a threshold of 1e−05
MR Method
Inverse variance weighted
MR Egger
4.7
4.6
4.5
1/SE_IV
−0.6
−0.3
0.0
0.3
β_IV

Batch 2−6080 : Gut microbiota abundance (genus Parasutterella id.2892) on Tumor necrosis factor receptor superfamily member 12A with a threshold of 1e−05

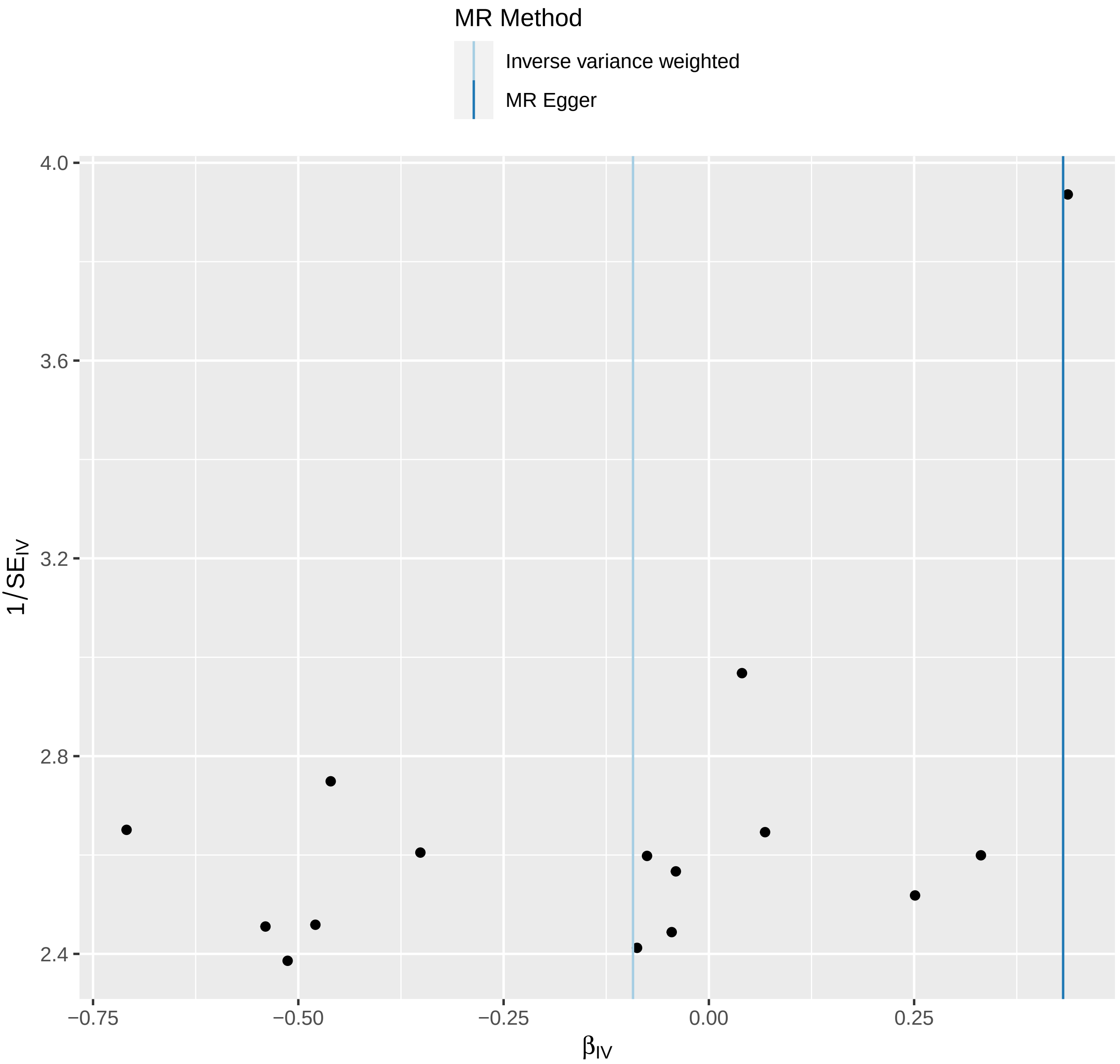

Batch 2−6081 : Gut microbiota abundance (genus Catenibacterium id.2153) on Tumor necrosis factor receptor superfamily member 13B with a threshold of 1e−05

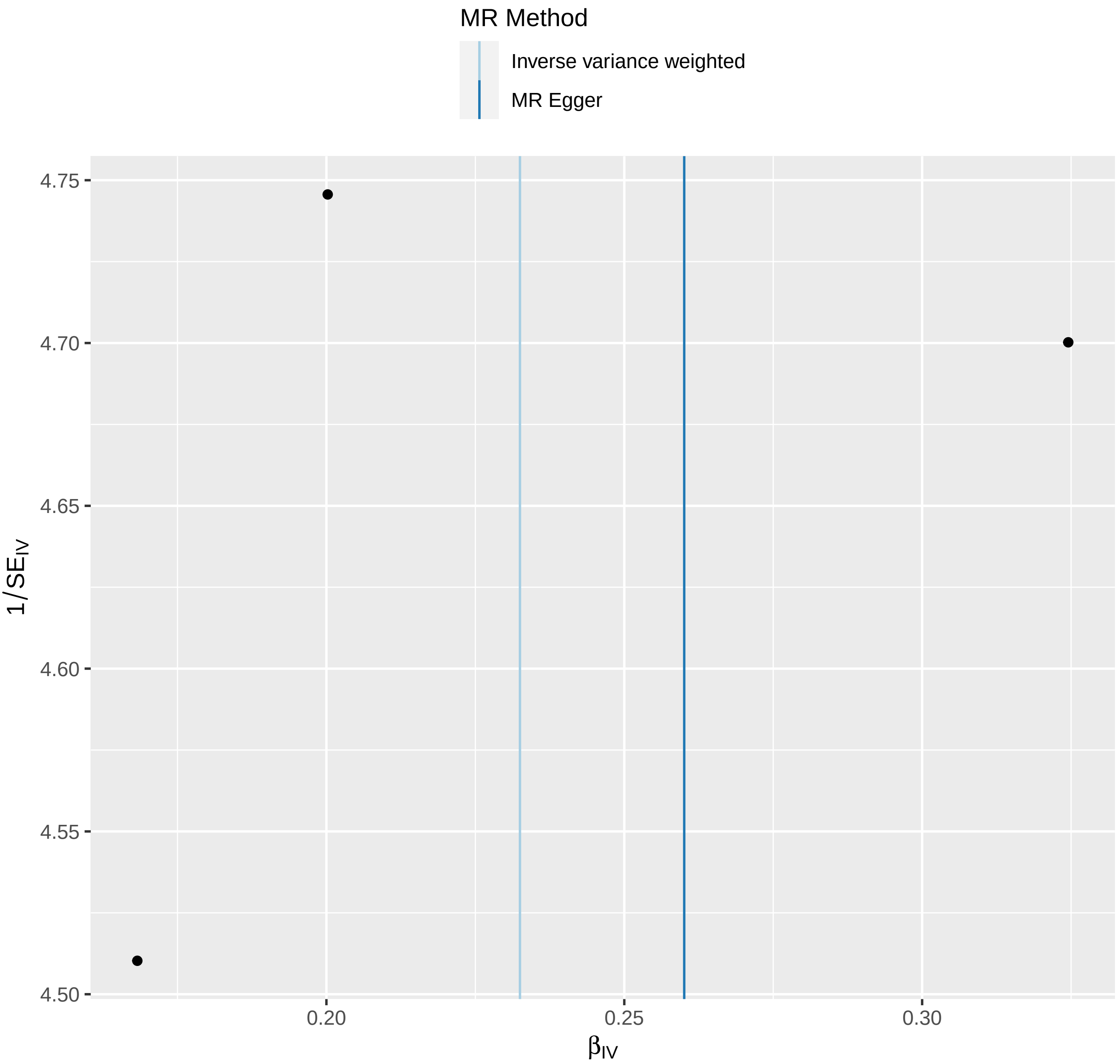

Batch 2−6082 : Gut microbiota abundance (genus Parasutterella id.2892) on Tumor necrosis factor receptor superfamily member 13B
with a threshold of 1e−05

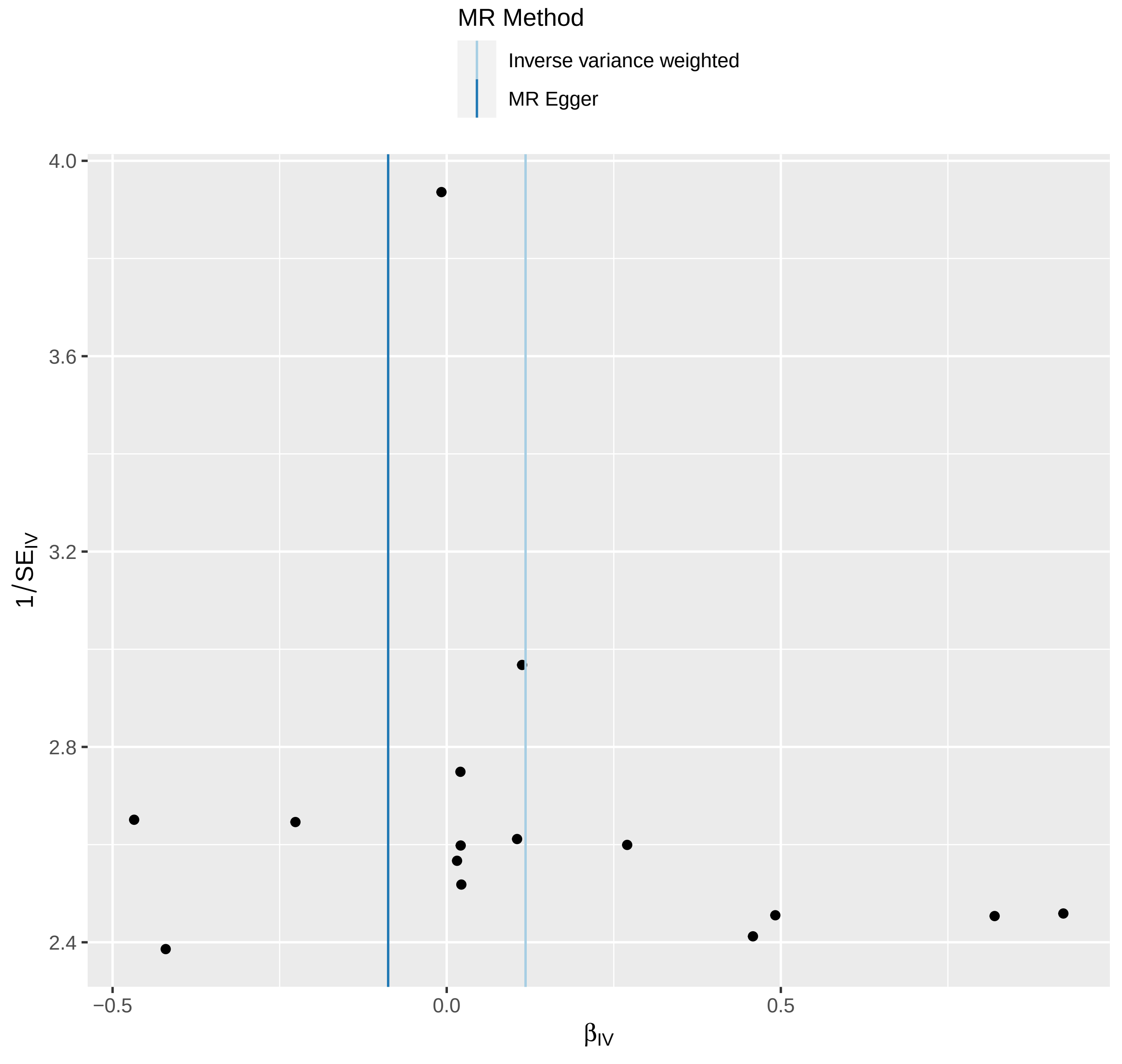

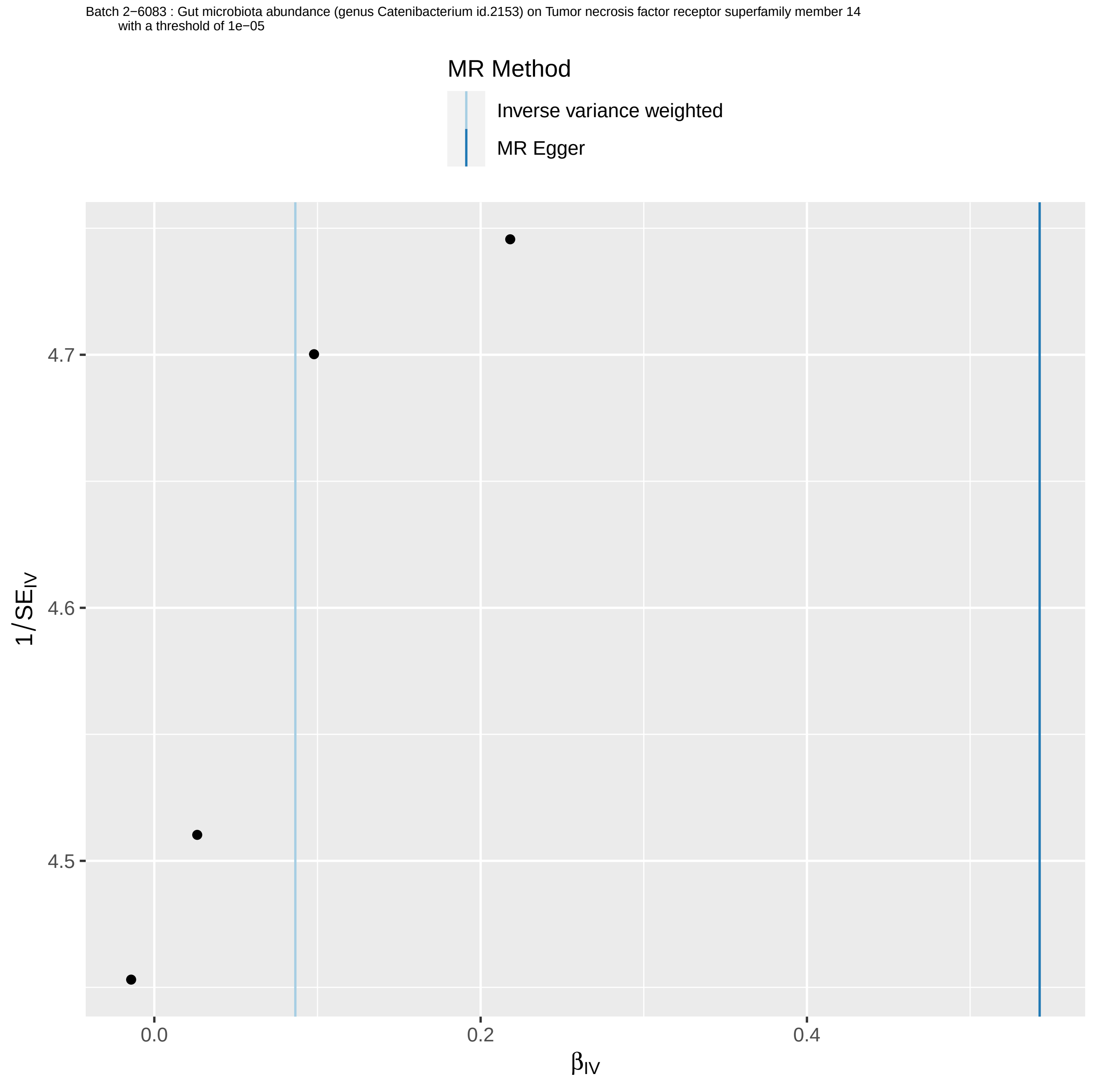

Batch 2−6083 : Gut microbiota abundance (genus Catenibacterium id.2153) on Tumor necrosis factor receptor superfamily member 14
with a threshold of 1e−05
MR Method
Inverse variance weighted
MR Egger
4.7
4.6
4.5
1/SE_IV
0.0
0.2
0.4
β_IV

Batch 2−6084 : Gut microbiota abundance (genus Parasutterella id.2892) on Tumor necrosis factor receptor superfamily member 14
with a threshold of 1e−05

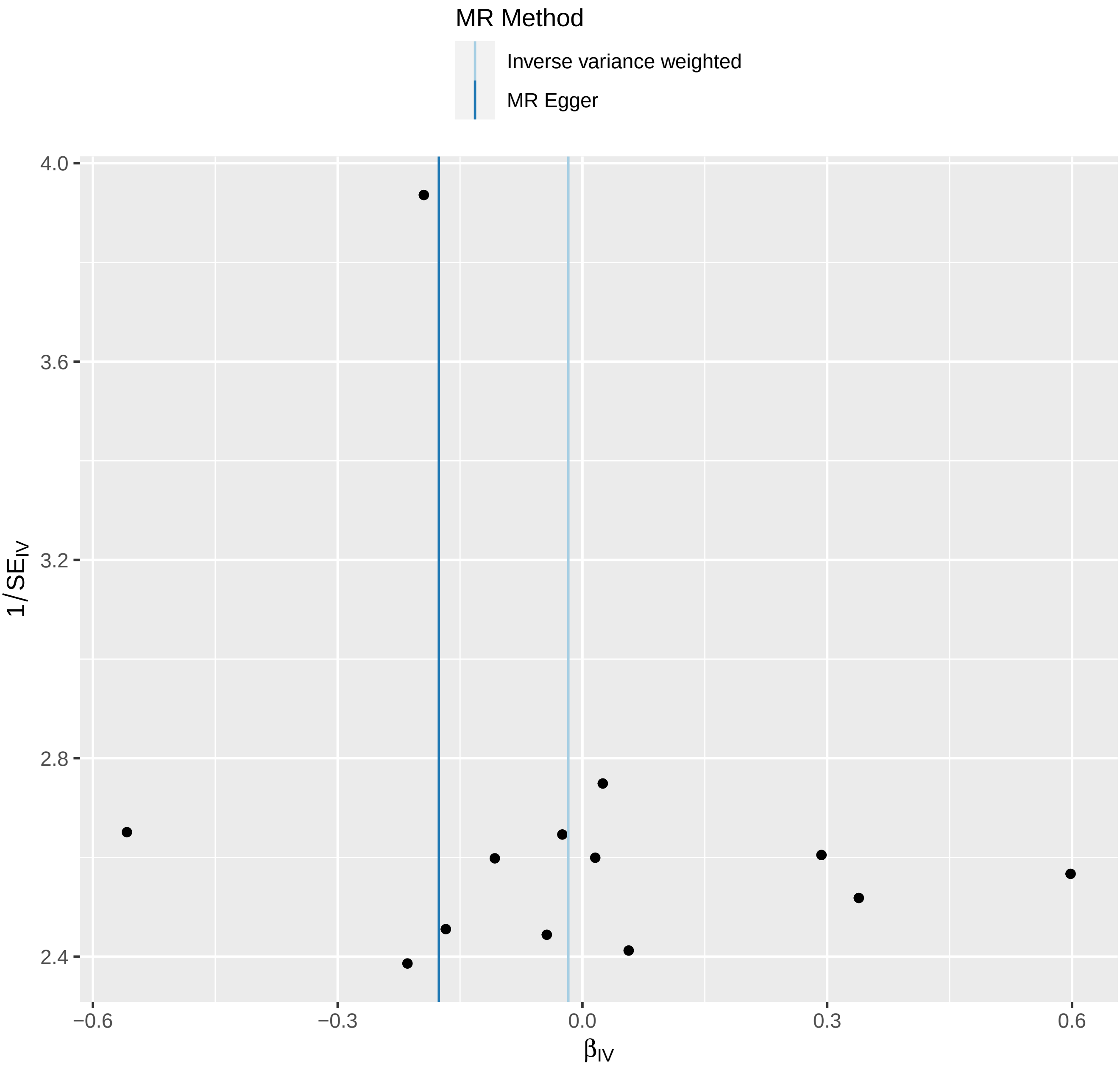

Batch 2−6085 : Gut microbiota abundance (genus Catenibacterium id.2153) on Tumor necrosis factor receptor superfamily member 17
with a threshold of 1e−05

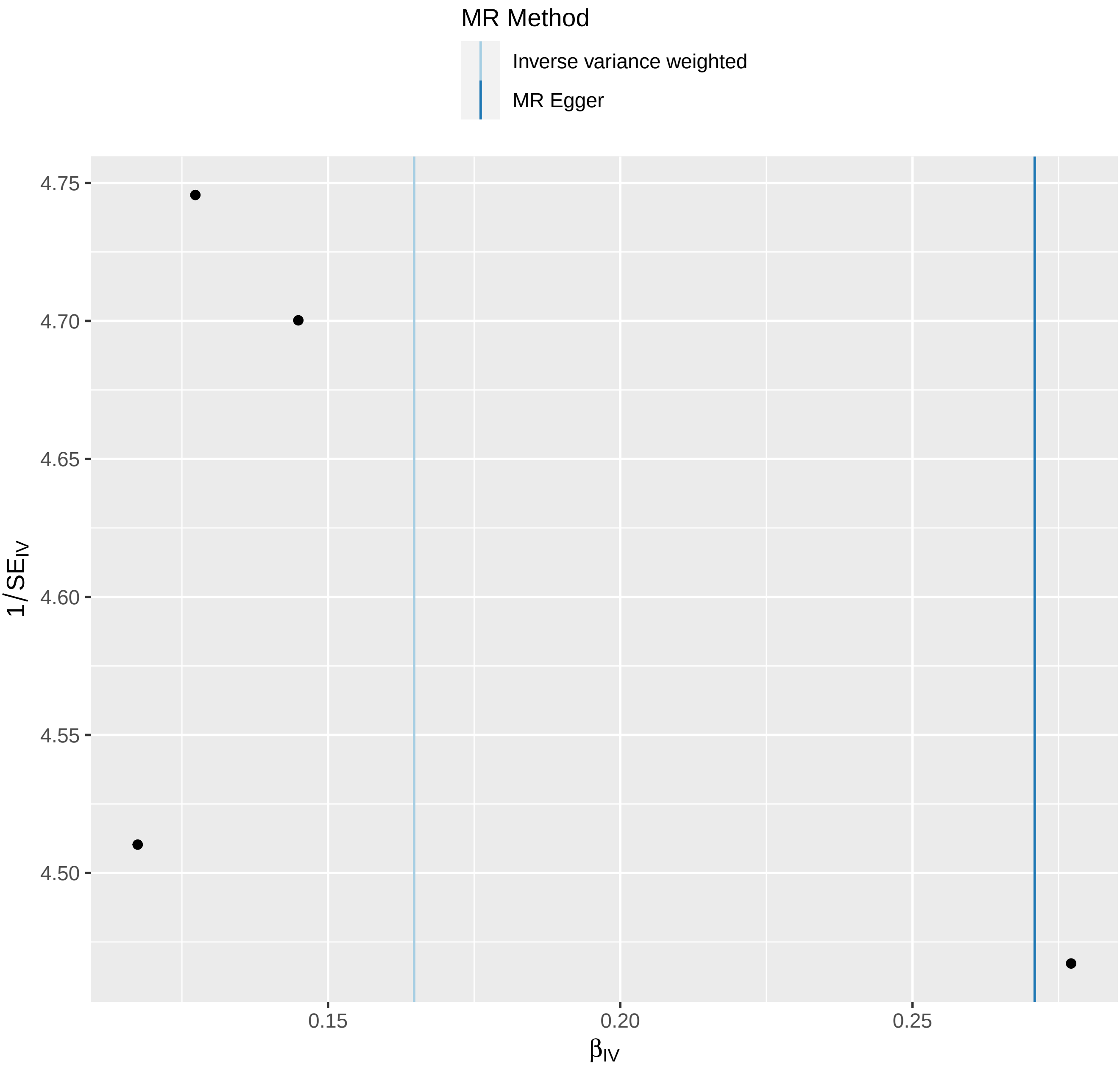

Batch 2−6086 : Gut microbiota abundance (genus Parasutterella id.2892) on Tumor necrosis factor receptor superfamily member 17
with a threshold of 1e−05

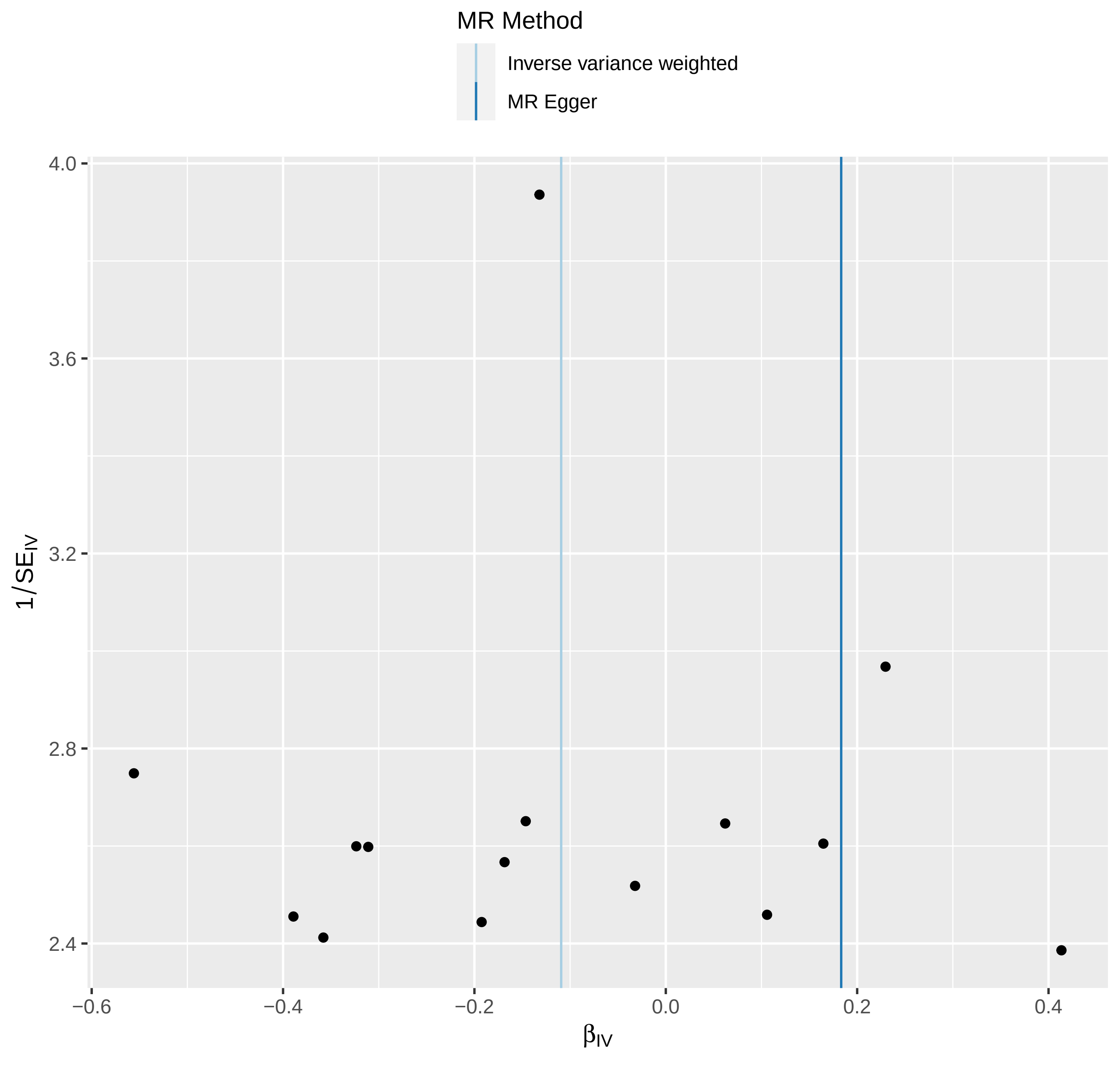

Batch 2−6087 : Gut microbiota abundance (genus Catenibacterium id.2153) on Tumor necrosis factor receptor superfamily member 18
with a threshold of 1e−05

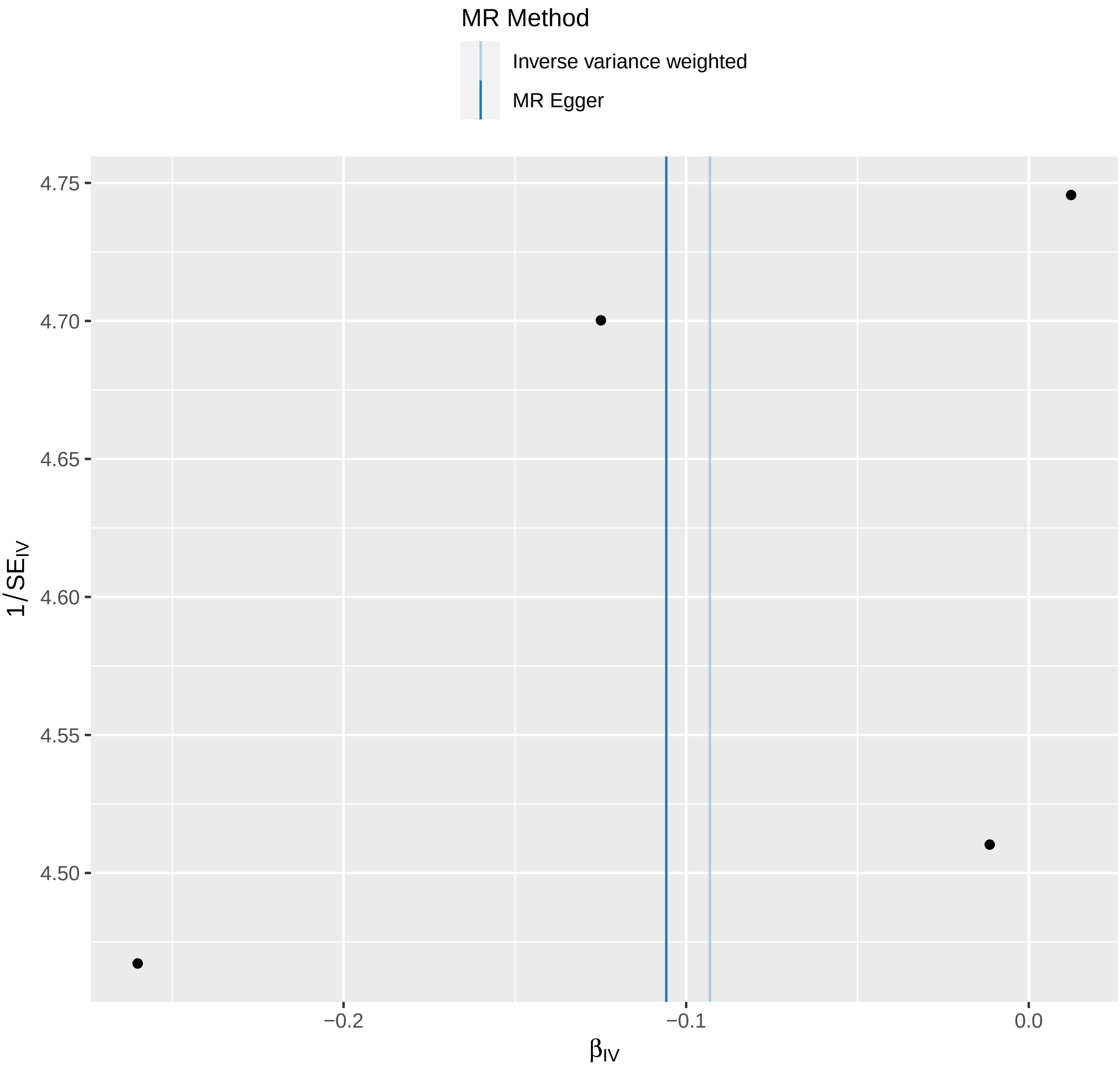

Batch 2−6088 : Gut microbiota abundance (genus Parasutterella id.2892) on Tumor necrosis factor receptor superfamily member 18
with a threshold of 1e−05

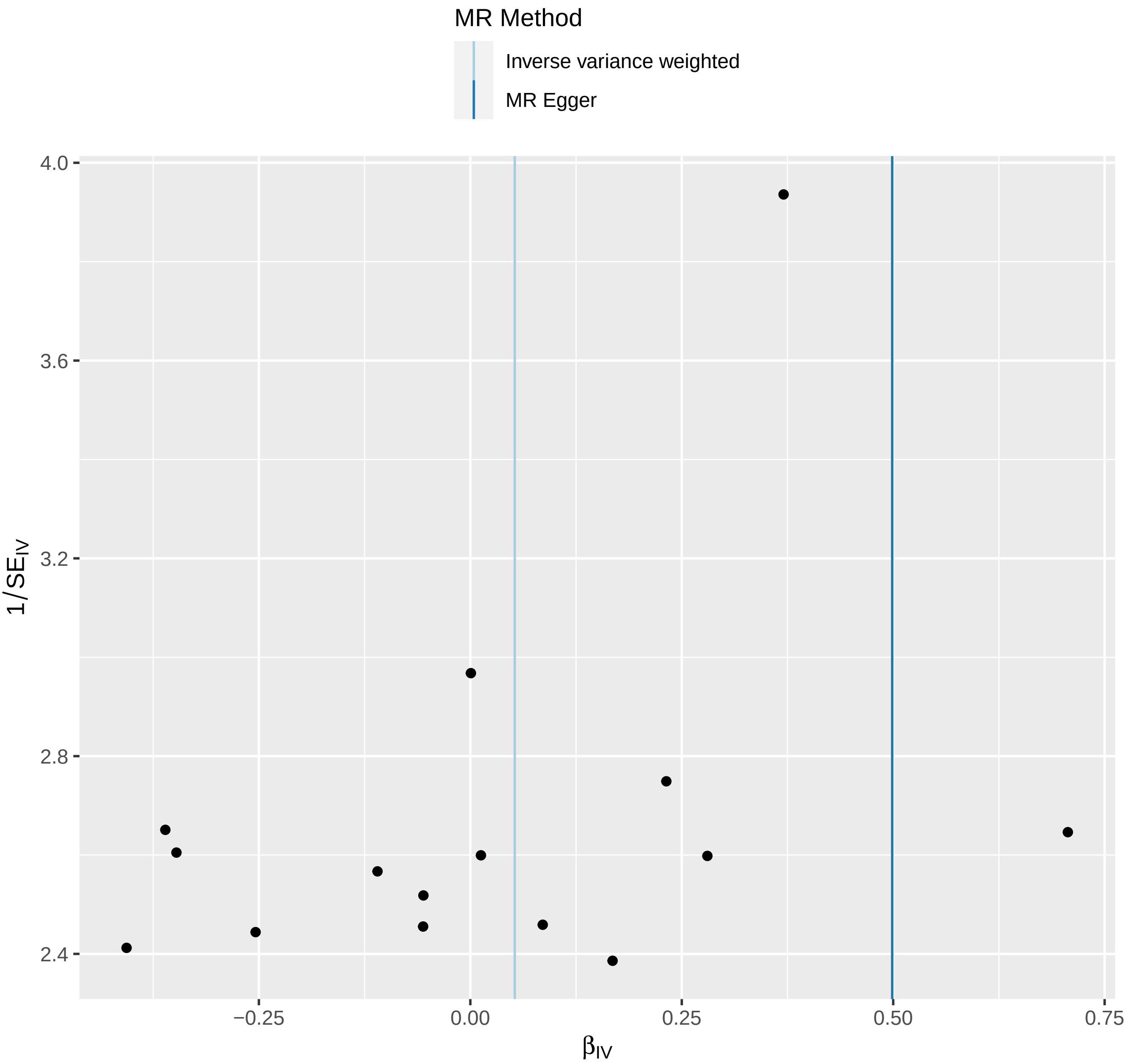

Batch 2−6089 : Gut microbiota abundance (genus Catenibacterium id.2153) on Tumor necrosis factor receptor superfamily member 19
with a threshold of 1e−05

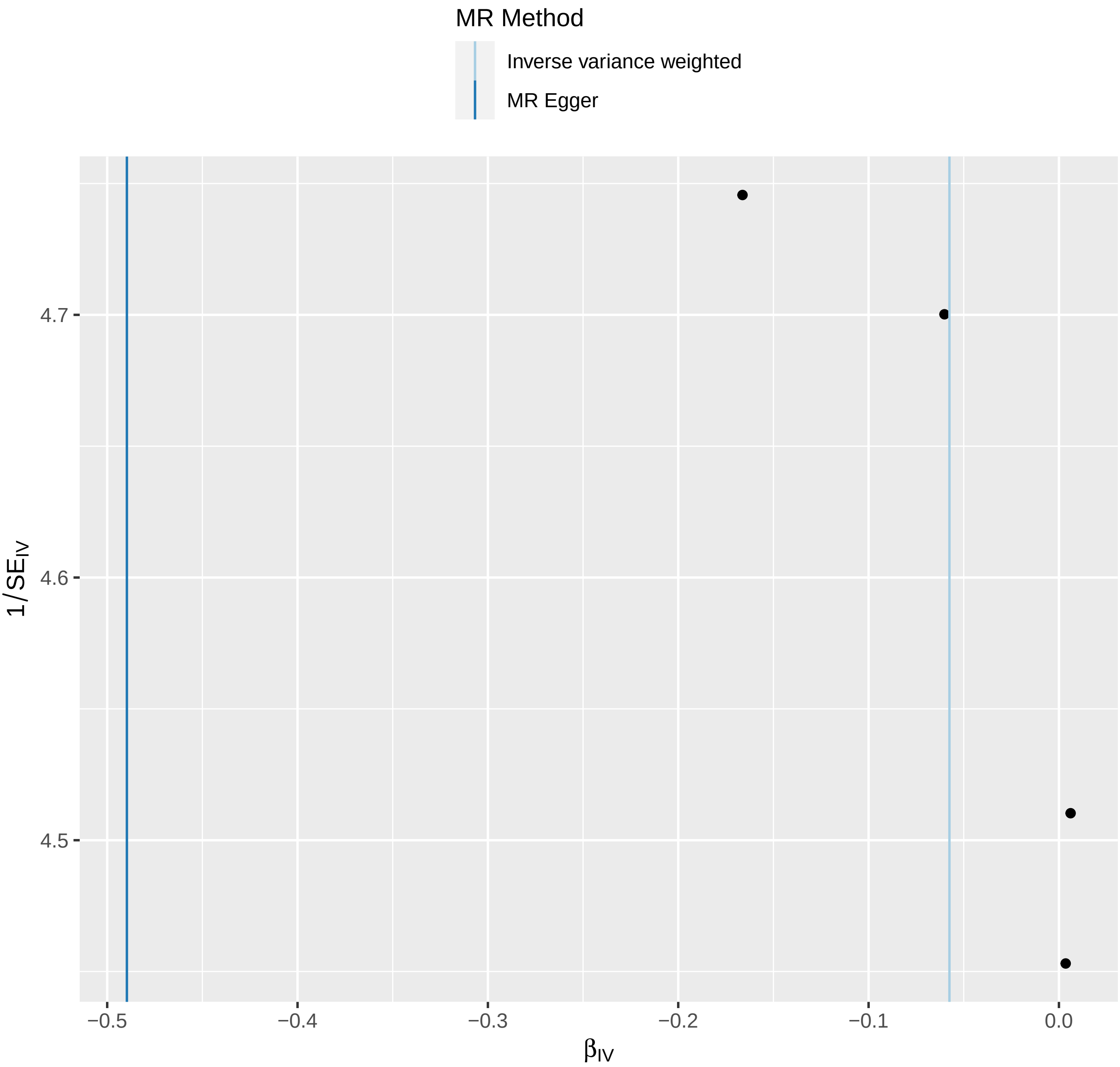

Batch 2−6090 : Gut microbiota abundance (genus Parasutterella id.2892) on Tumor necrosis factor receptor superfamily member 19 with a threshold of 1e−05

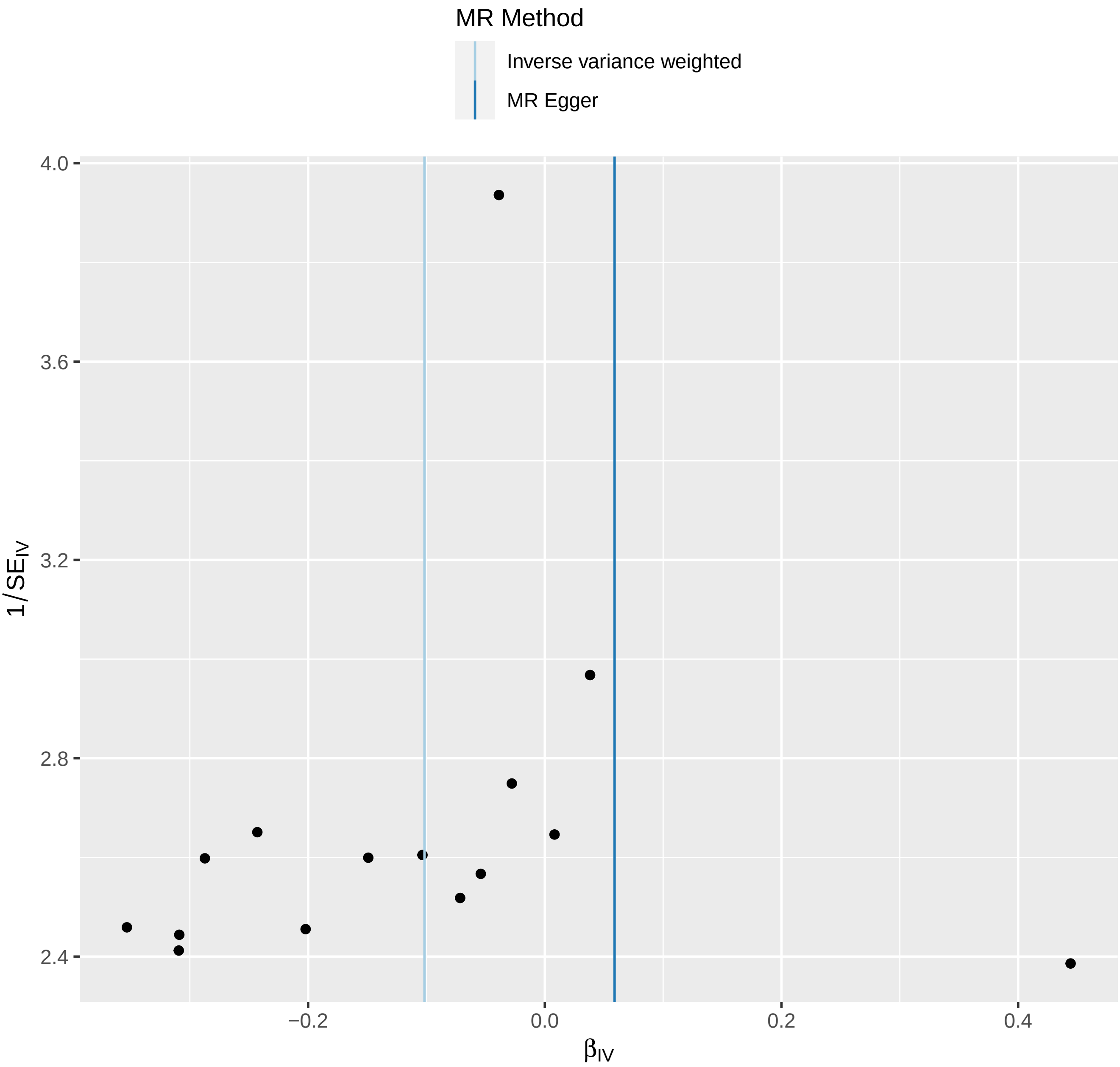

Batch 2−6091 : Gut microbiota abundance (genus Catenibacterium id.2153) on Tumor necrosis factor receptor superfamily member 1A with a threshold of 1e−05

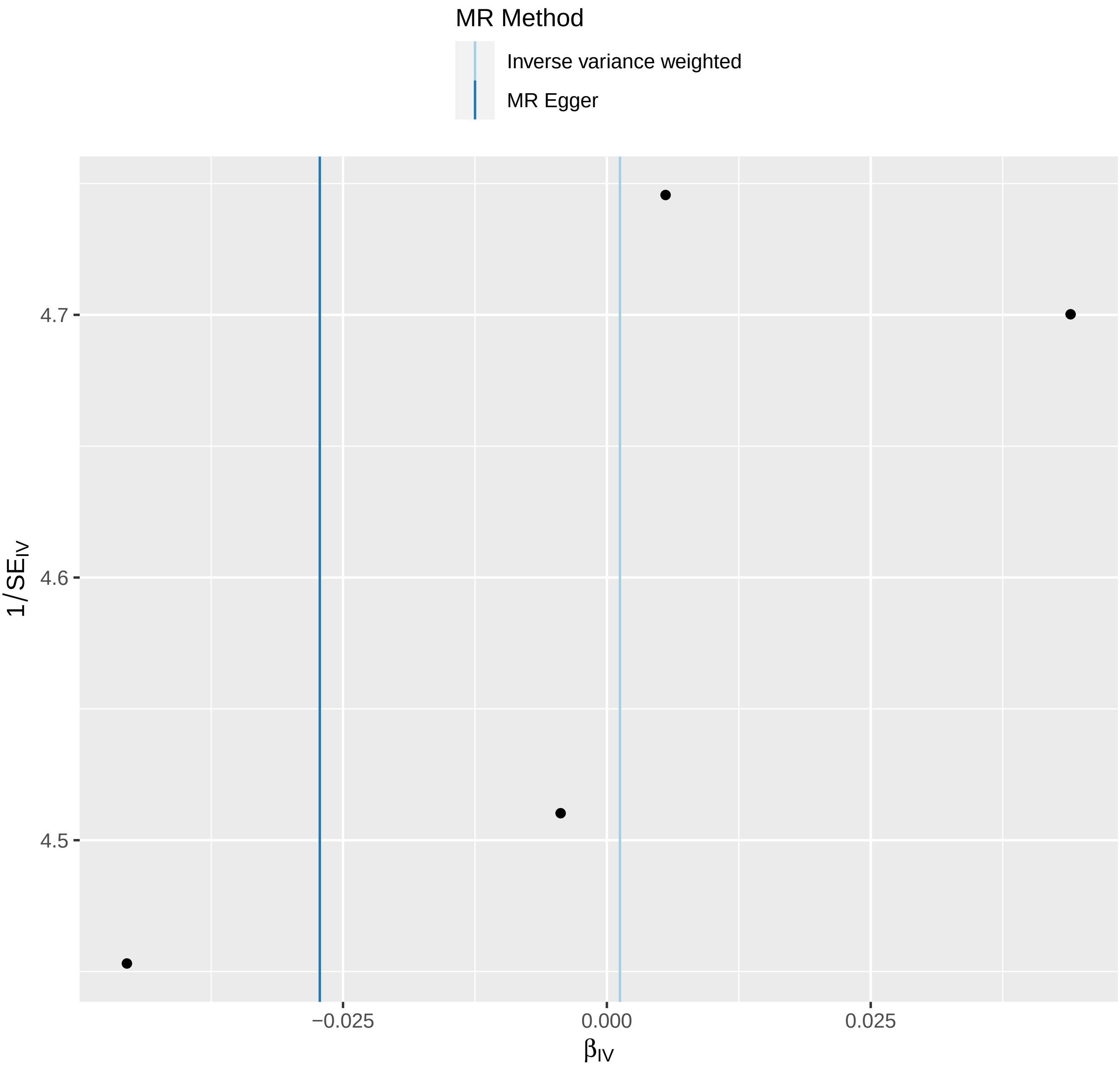

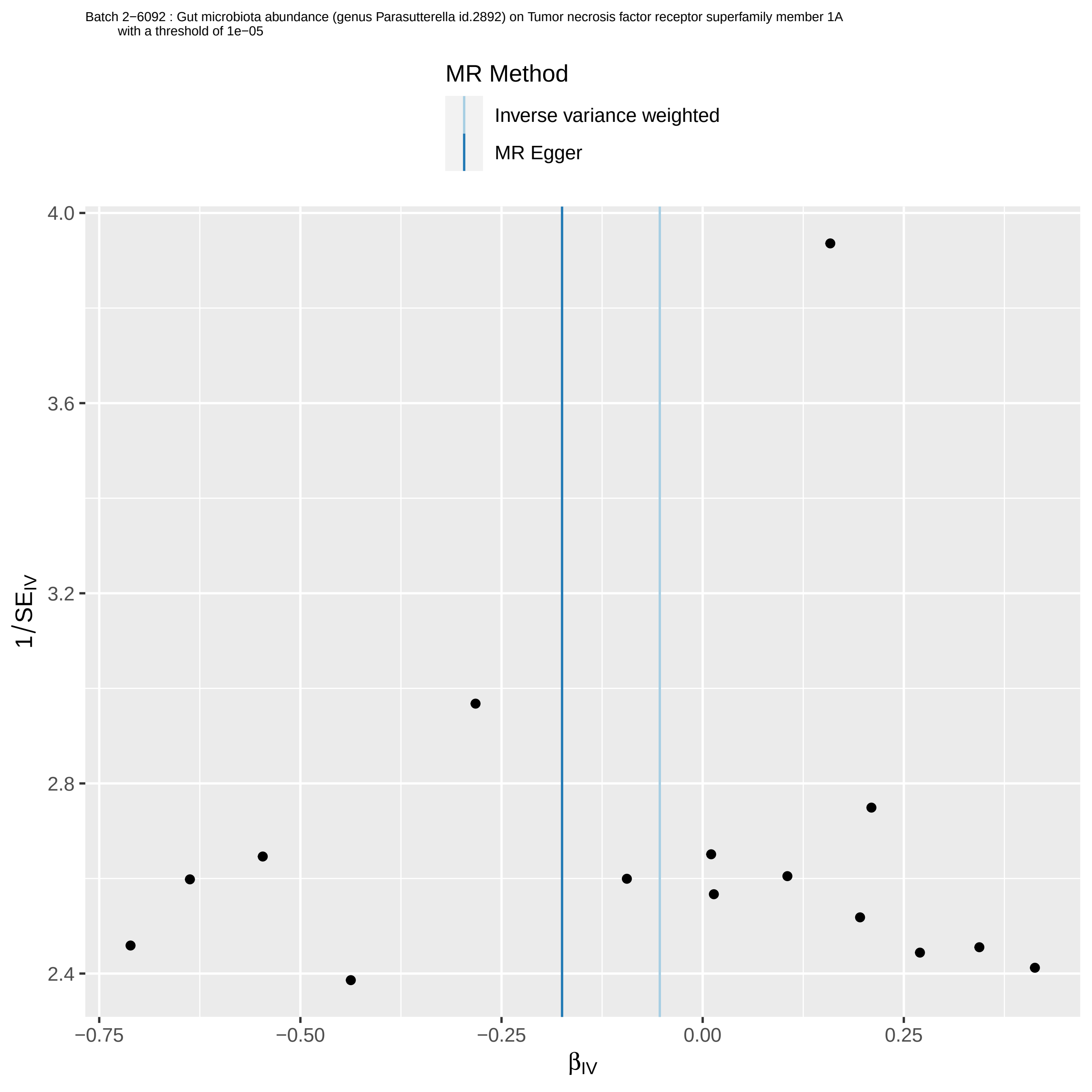

Batch 2−6092 : Gut microbiota abundance (genus Parasutterella id.2892) on Tumor necrosis factor receptor superfamily member 1A
with a threshold of 1e−05
MR Method
Inverse variance weighted
MR Egger
4.0
3.6
3.2
2.8
2.4
1/SE_IV
−0.75
−0.50
−0.25
0.00
0.25
β_IV

Batch 2−6093 : Gut microbiota abundance (genus Catenibacterium id.2153) on Tumor necrosis factor receptor superfamily member 1B
with a threshold of 1e−05

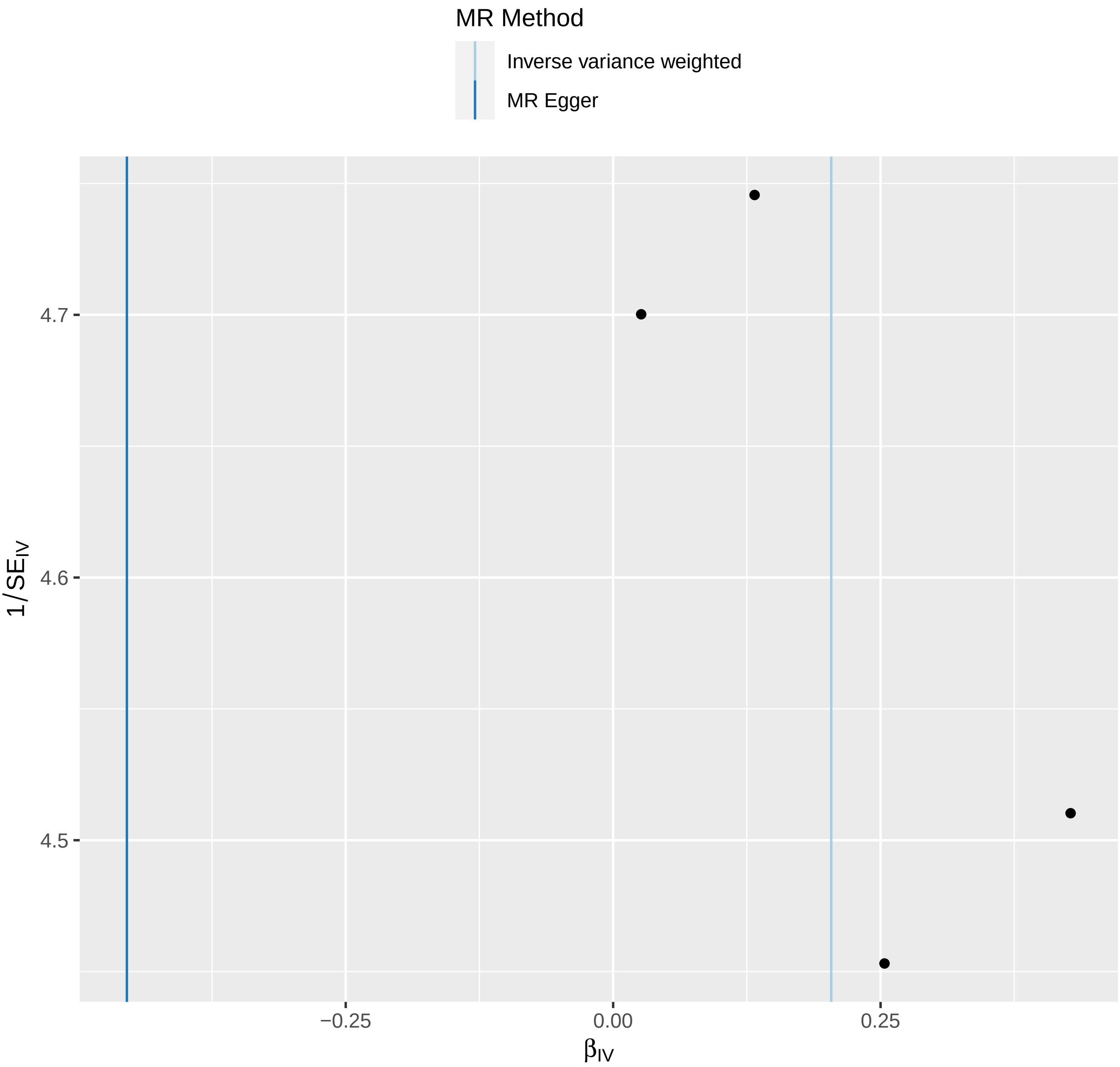

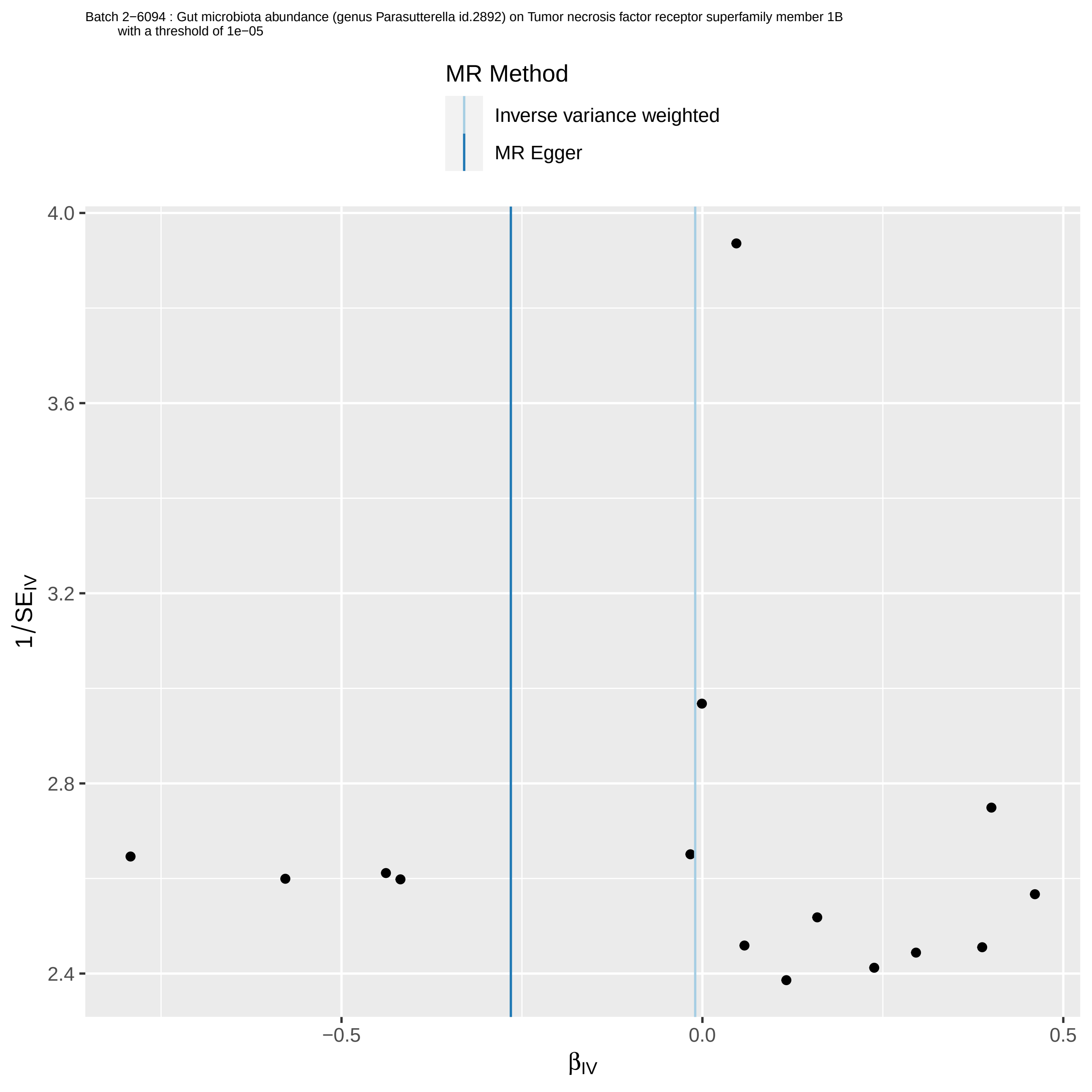

Batch 2−6094 : Gut microbiota abundance (genus Parasutterella id.2892) on Tumor necrosis factor receptor superfamily member 1B
with a threshold of 1e−05
MR Method
Inverse variance weighted
MR Egger
4.0
3.6
3.2
2.8
2.4
1/SE_IV
−0.5
0.0
0.5
β_IV

Batch 2−6095 : Gut microbiota abundance (genus Catenibacterium id.2153) on Tumor necrosis factor receptor superfamily member 1B
with a threshold of 1e−05

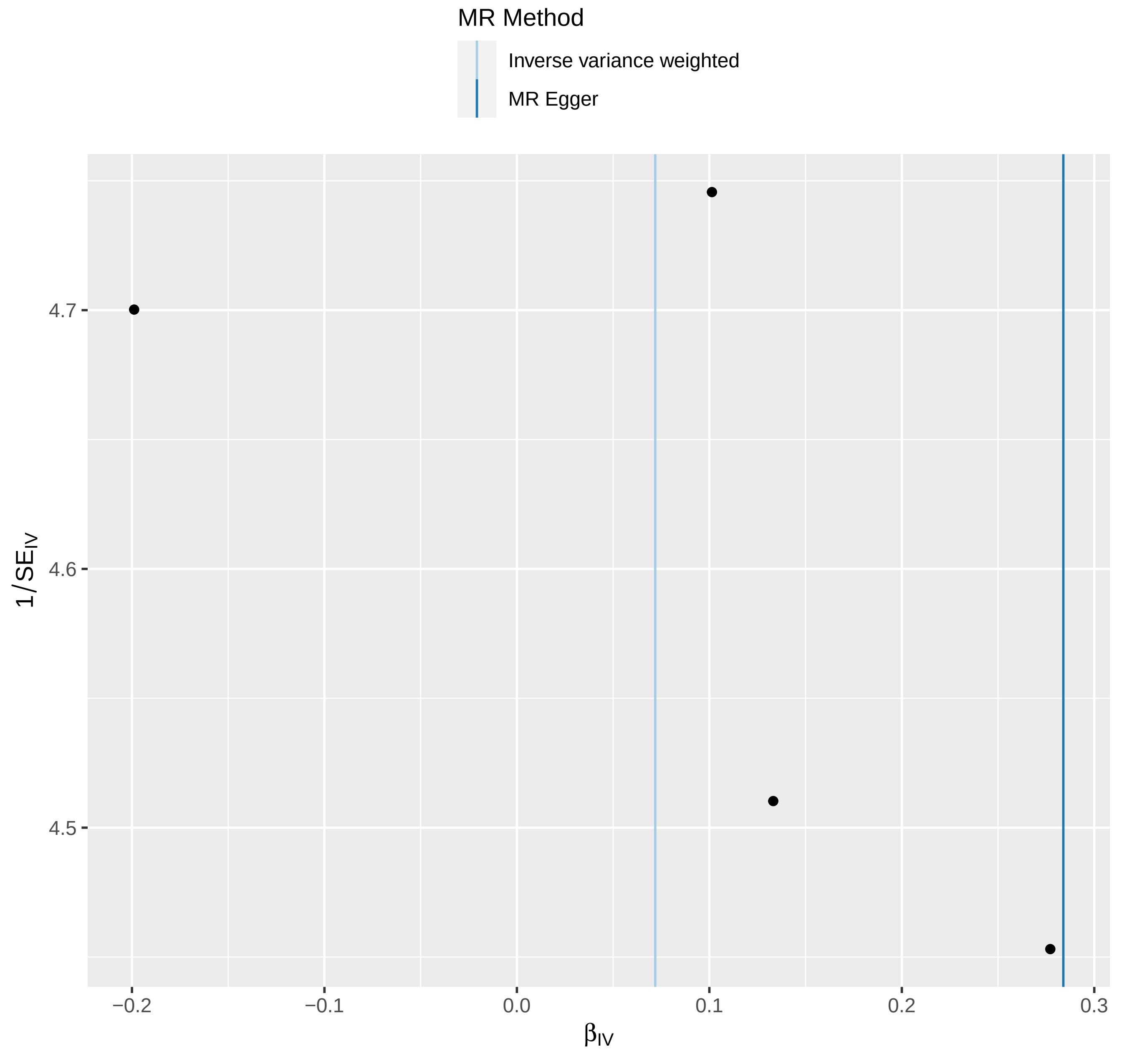

Batch 2−6096 : Gut microbiota abundance (genus Parasutterella id.2892) on Tumor necrosis factor receptor superfamily member 1B
with a threshold of 1e−05

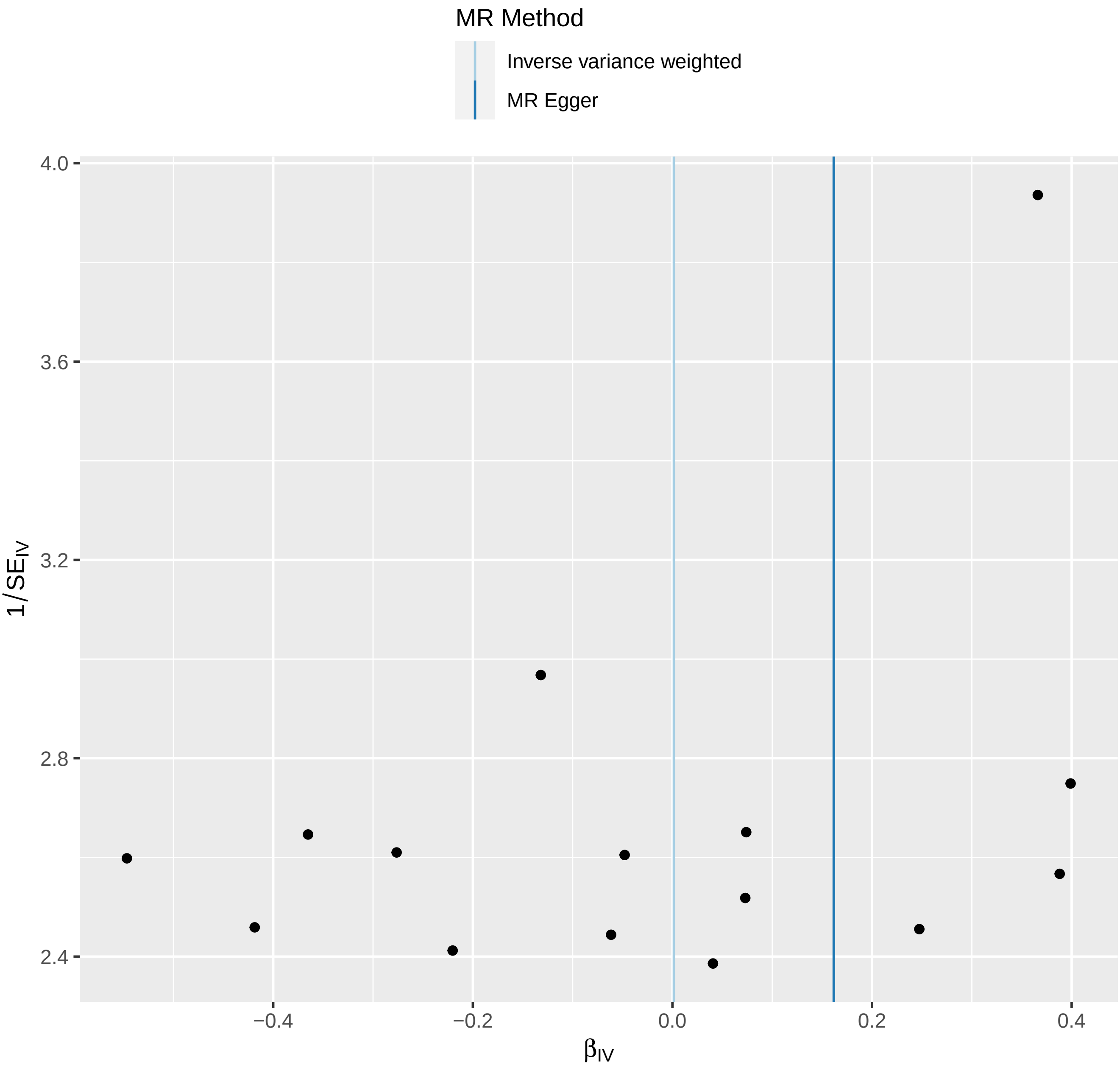

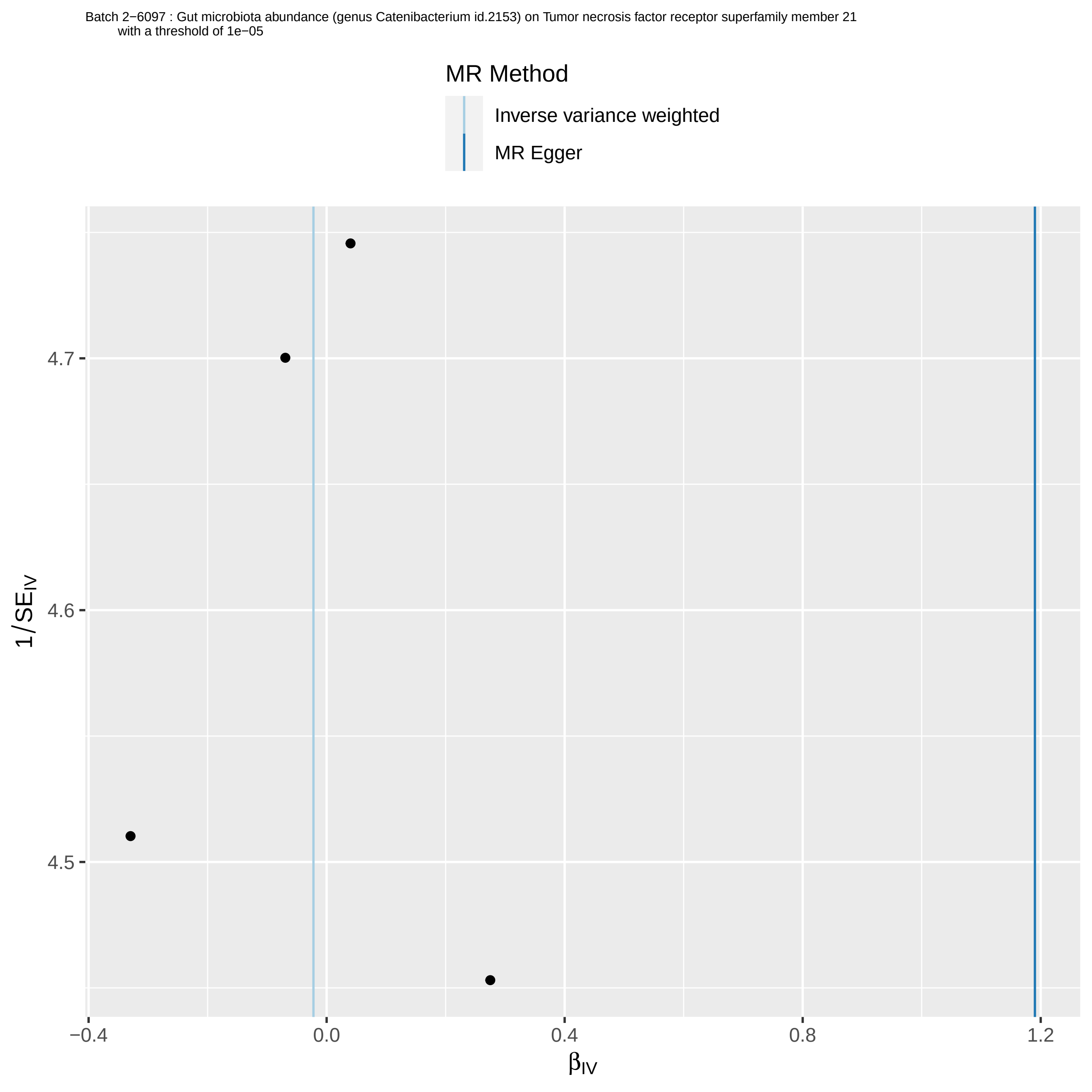

Batch 2−6097 : Gut microbiota abundance (genus Catenibacterium id.2153) on Tumor necrosis factor receptor superfamily member 21
with a threshold of 1e−05
MR Method
Inverse variance weighted
MR Egger
4.7
4.6
4.5
1/SE_IV
−0.4
0.0
0.4
0.8
1.2
β_IV

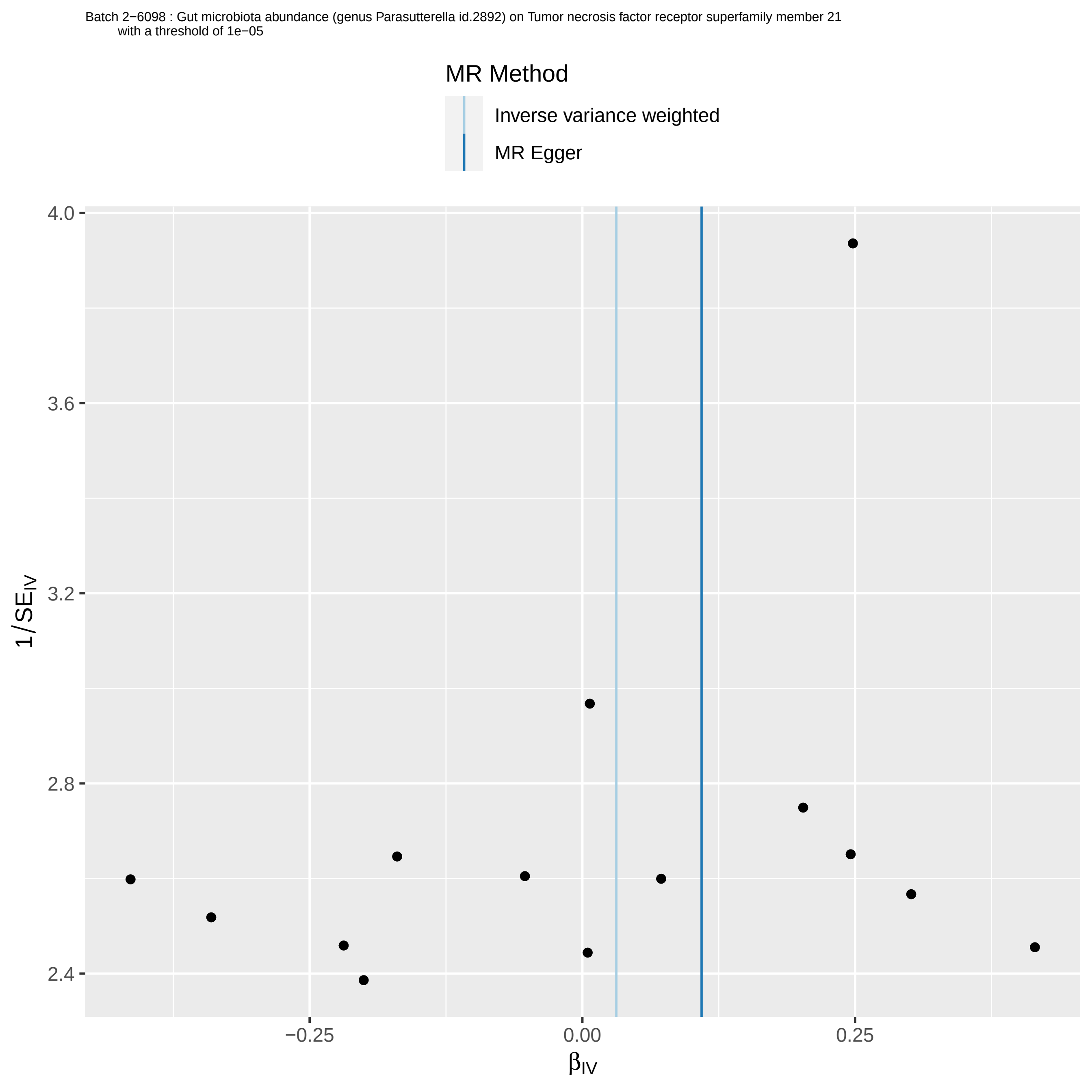
Batch 2−6098 : Gut microbiota abundance (genus Parasutterella id.2892) on Tumor necrosis factor receptor superfamily member 21
with a threshold of 1e−05
MR Method
Inverse variance weighted
MR Egger
4.0
3.6
3.2
2.8
2.4
$1/SE_{IV}$
−0.25
0.00
0.25
β_{IV}

Batch 2−6099 : Gut microbiota abundance (genus Catenibacterium id.2153) on Tumor necrosis factor receptor superfamily member 21 with a threshold of 1e−05

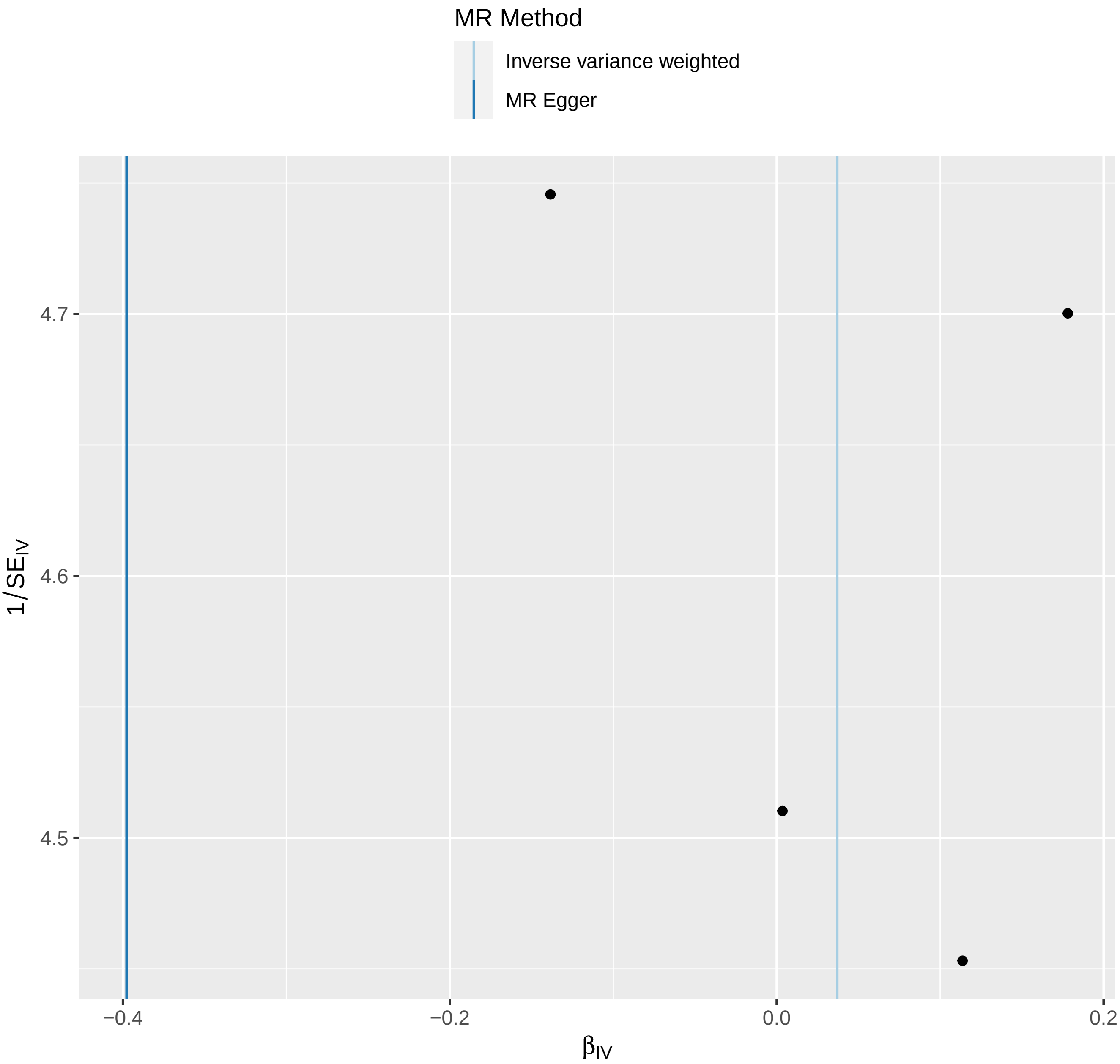

Batch 2−6100 : Gut microbiota abundance (genus Parasutterella id.2892) on Tumor necrosis factor receptor superfamily member 21
with a threshold of 1e−05

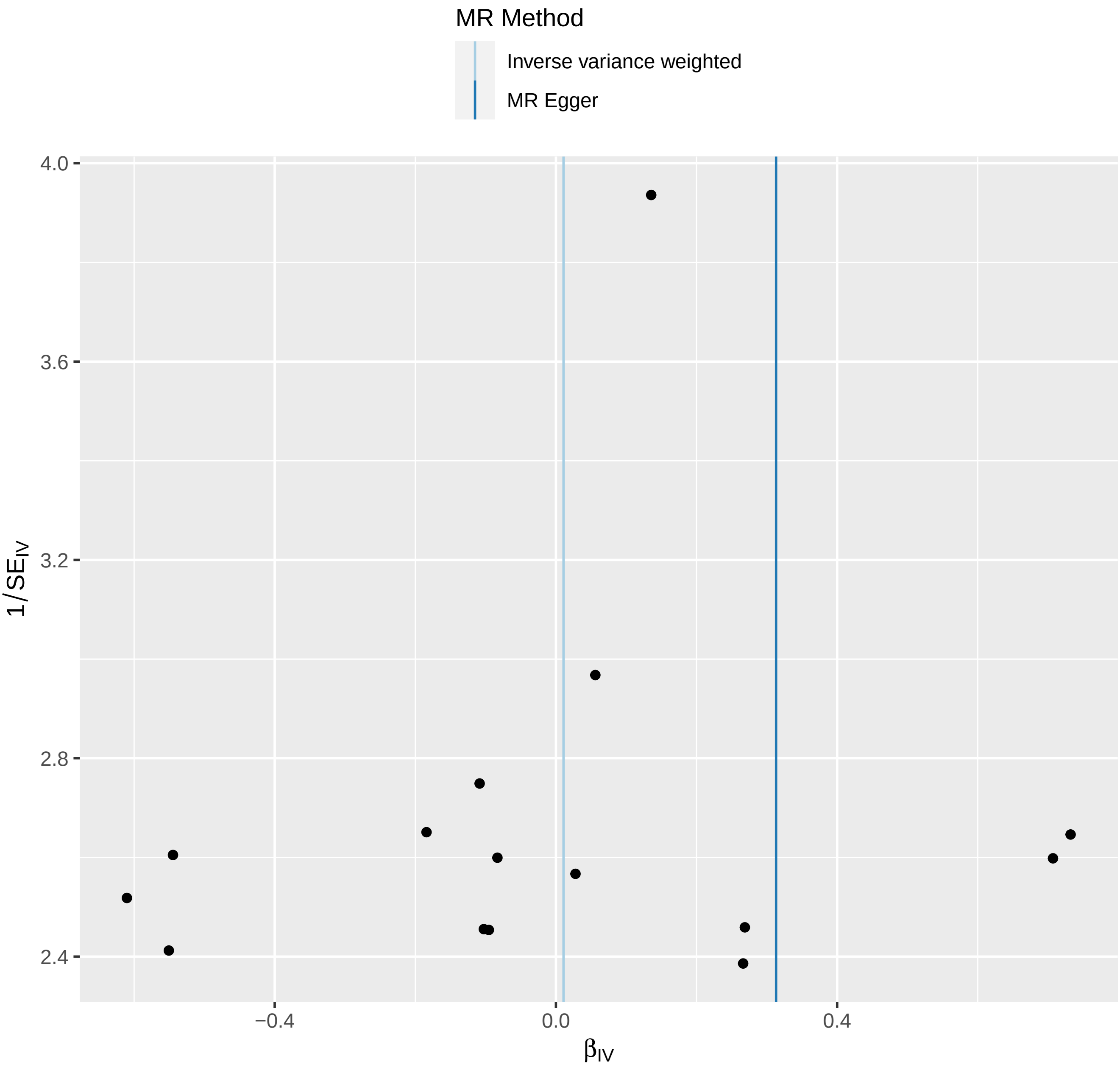

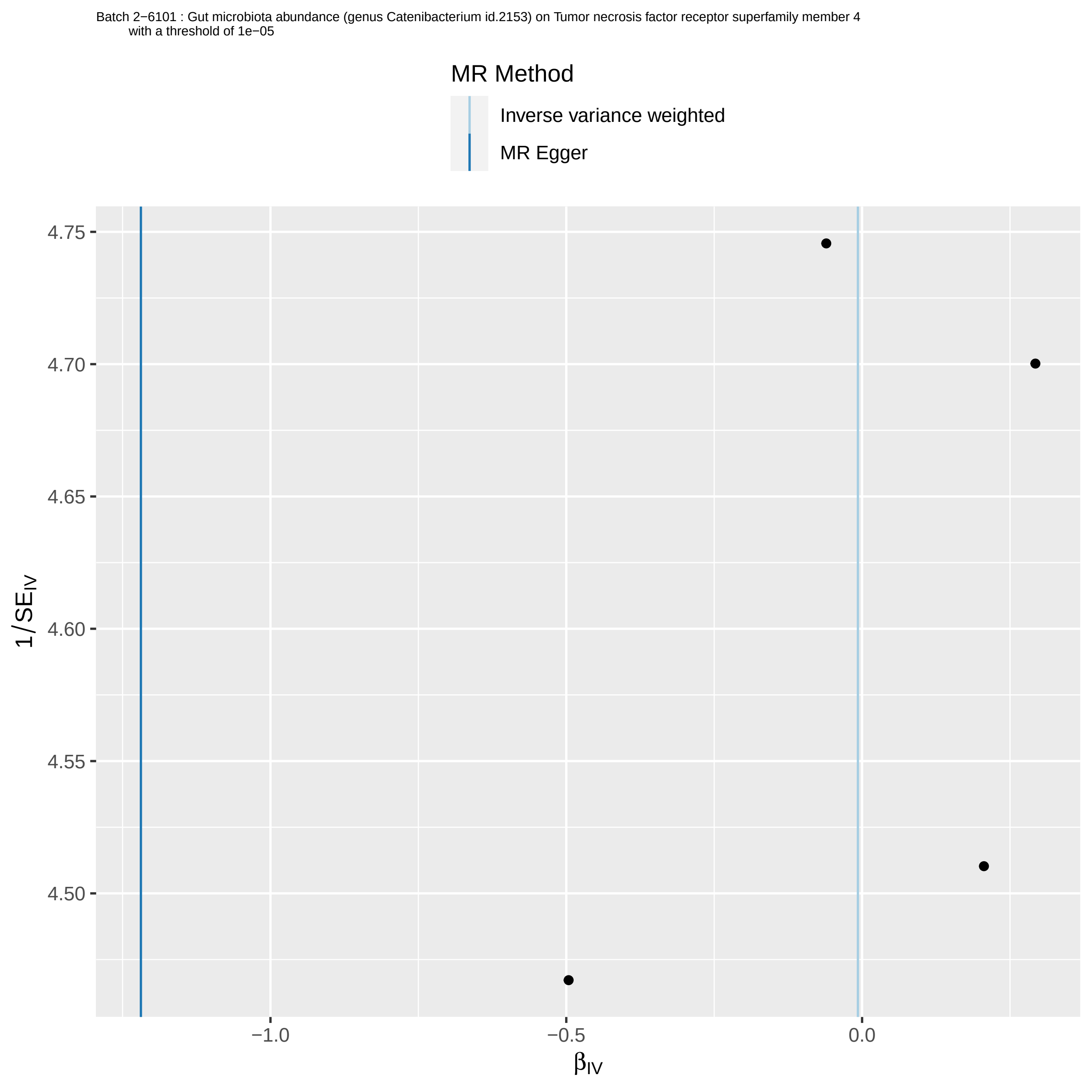
Batch 2−6101 : Gut microbiota abundance (genus Catenibacterium id.2153) on Tumor necrosis factor receptor superfamily member 4
with a threshold of 1e−05
MR Method
Inverse variance weighted
MR Egger
4.75
4.70
4.65
4.60
4.55
4.50
$1/SE_{IV}$
−1.0
−0.5
0.0
β_{IV}

Batch 2−6102 : Gut microbiota abundance (genus Parasutterella id.2892) on Tumor necrosis factor receptor superfamily member 4
with a threshold of 1e−05

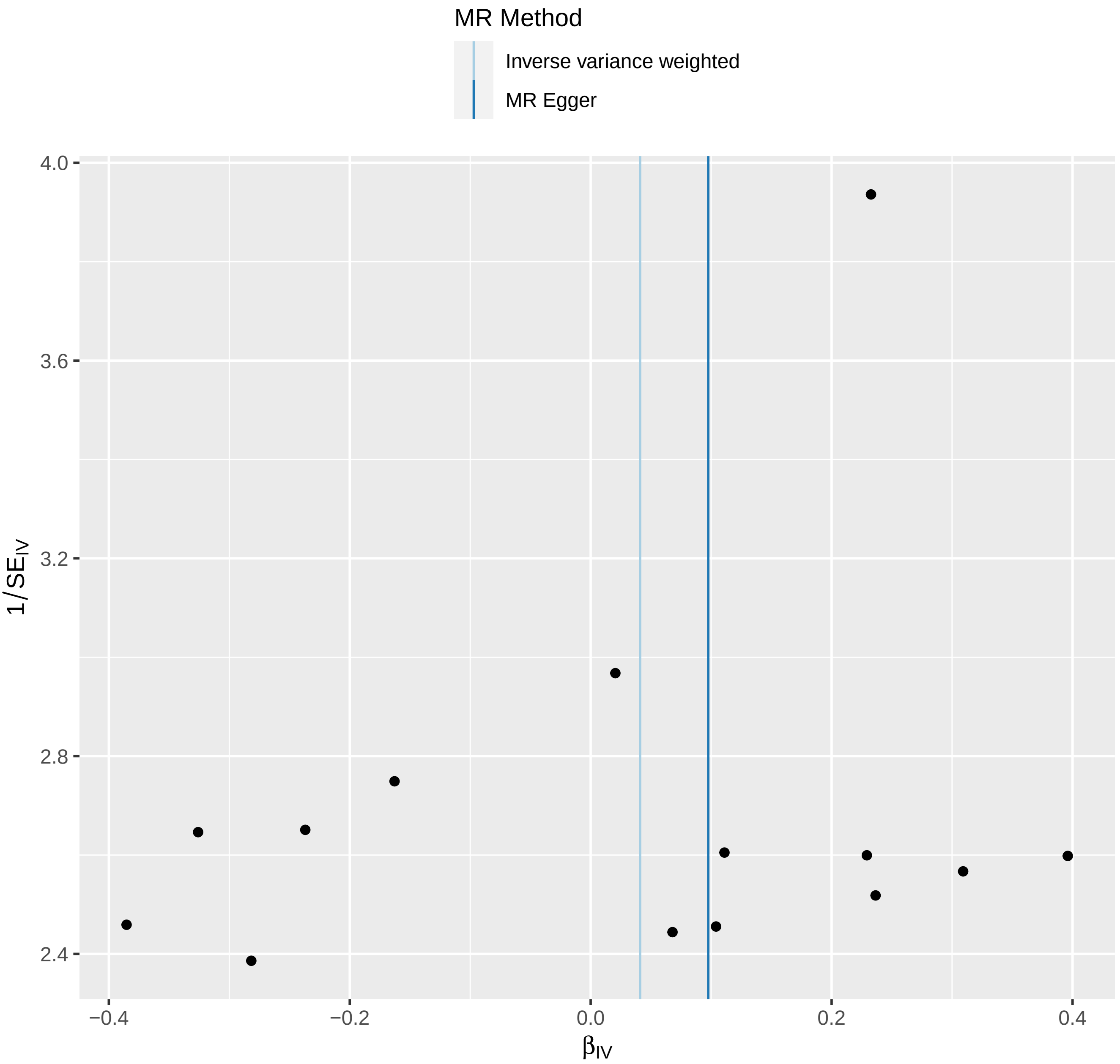

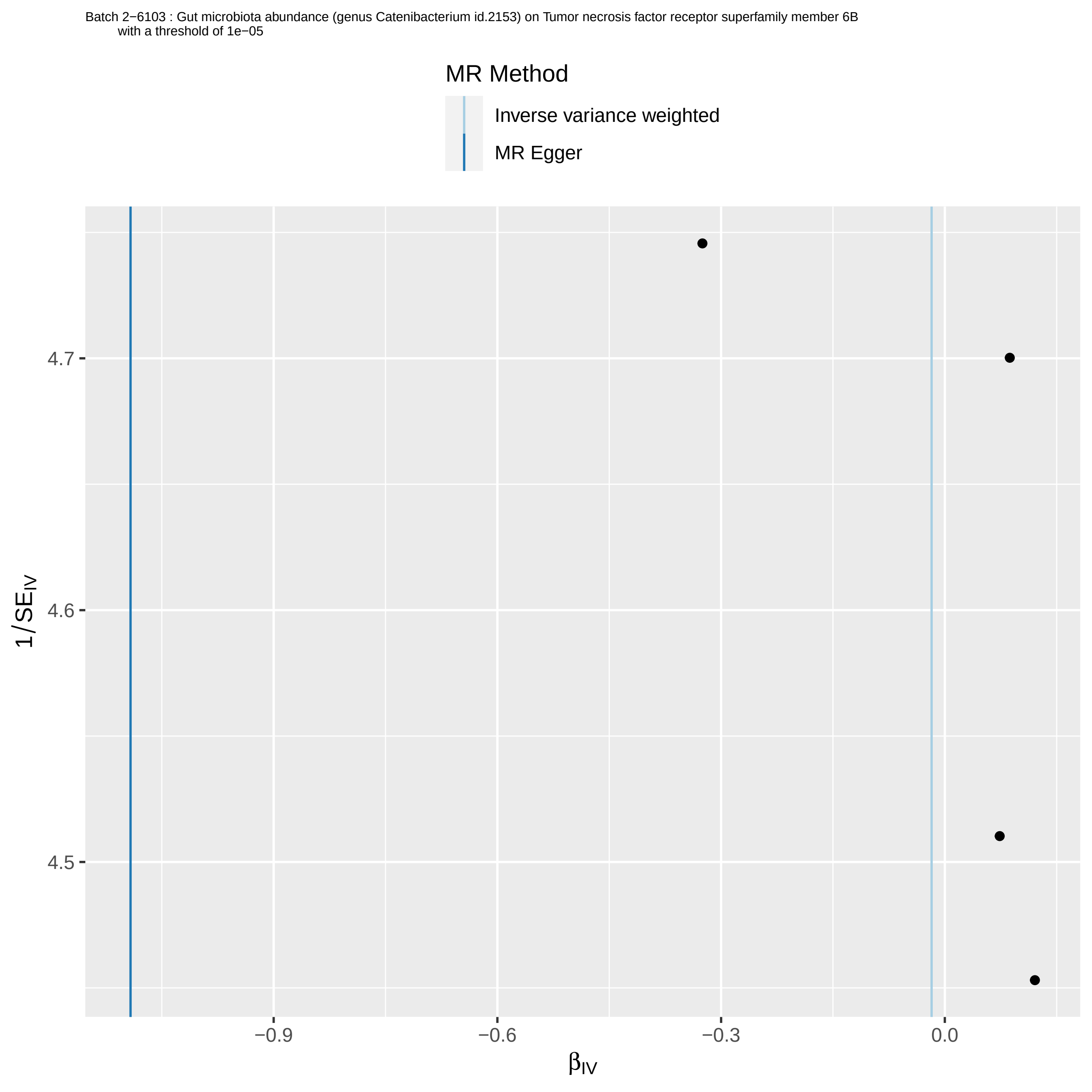
Batch 2−6103 : Gut microbiota abundance (genus Catenibacterium id.2153) on Tumor necrosis factor receptor superfamily member 6B
with a threshold of 1e−05
MR Method
Inverse variance weighted
MR Egger
4.7
4.6
4.5
1/SE_IV
−0.9
−0.6
−0.3
0.0
β_IV

Batch 2−6104 : Gut microbiota abundance (genus Parasutterella id.2892) on Tumor necrosis factor receptor superfamily member 6B
with a threshold of 1e−05

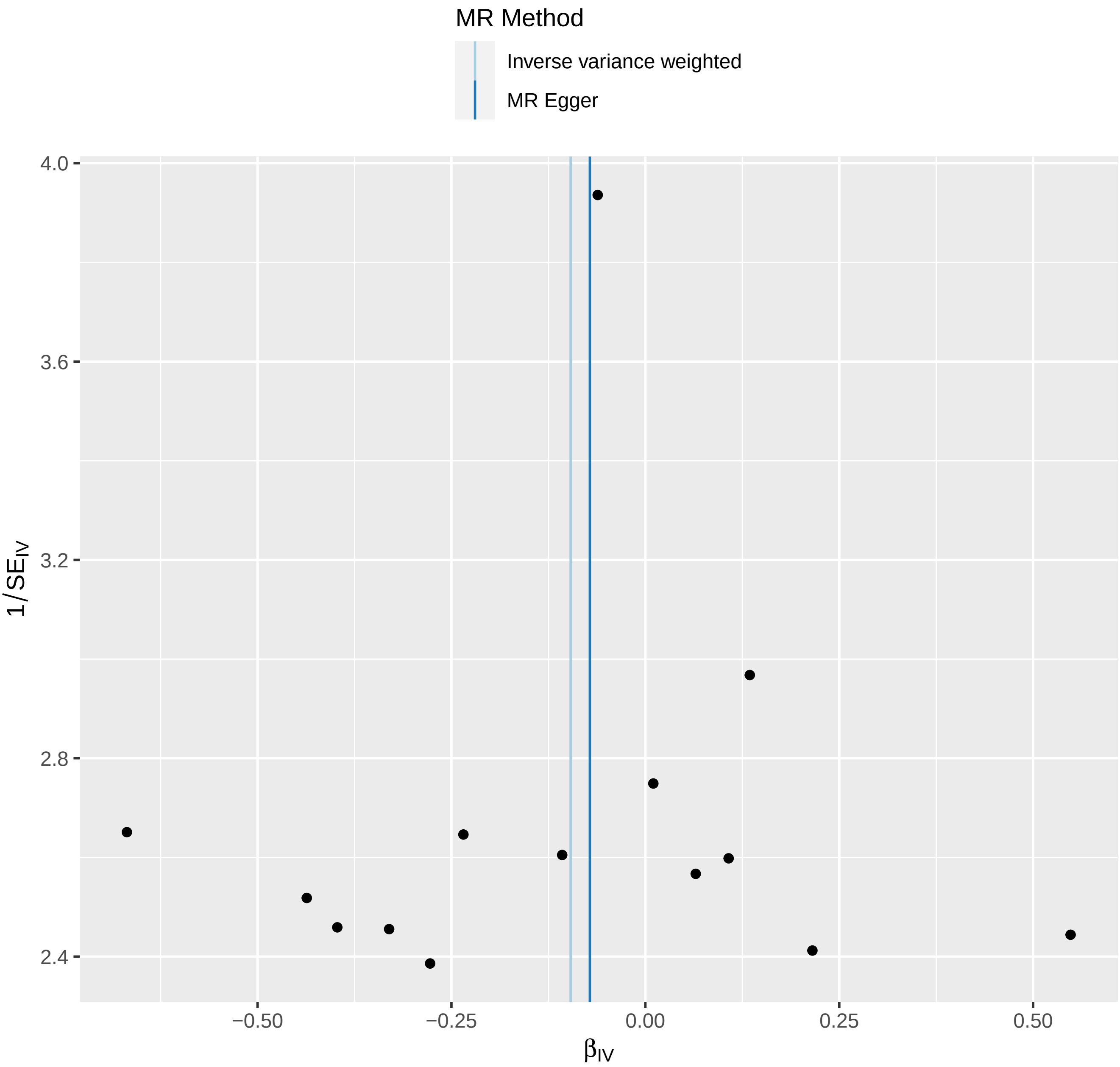

Batch 2−6105 : Gut microbiota abundance (genus Catenibacterium id.2153) on Tumor necrosis factor receptor superfamily member 8
with a threshold of 1e−05

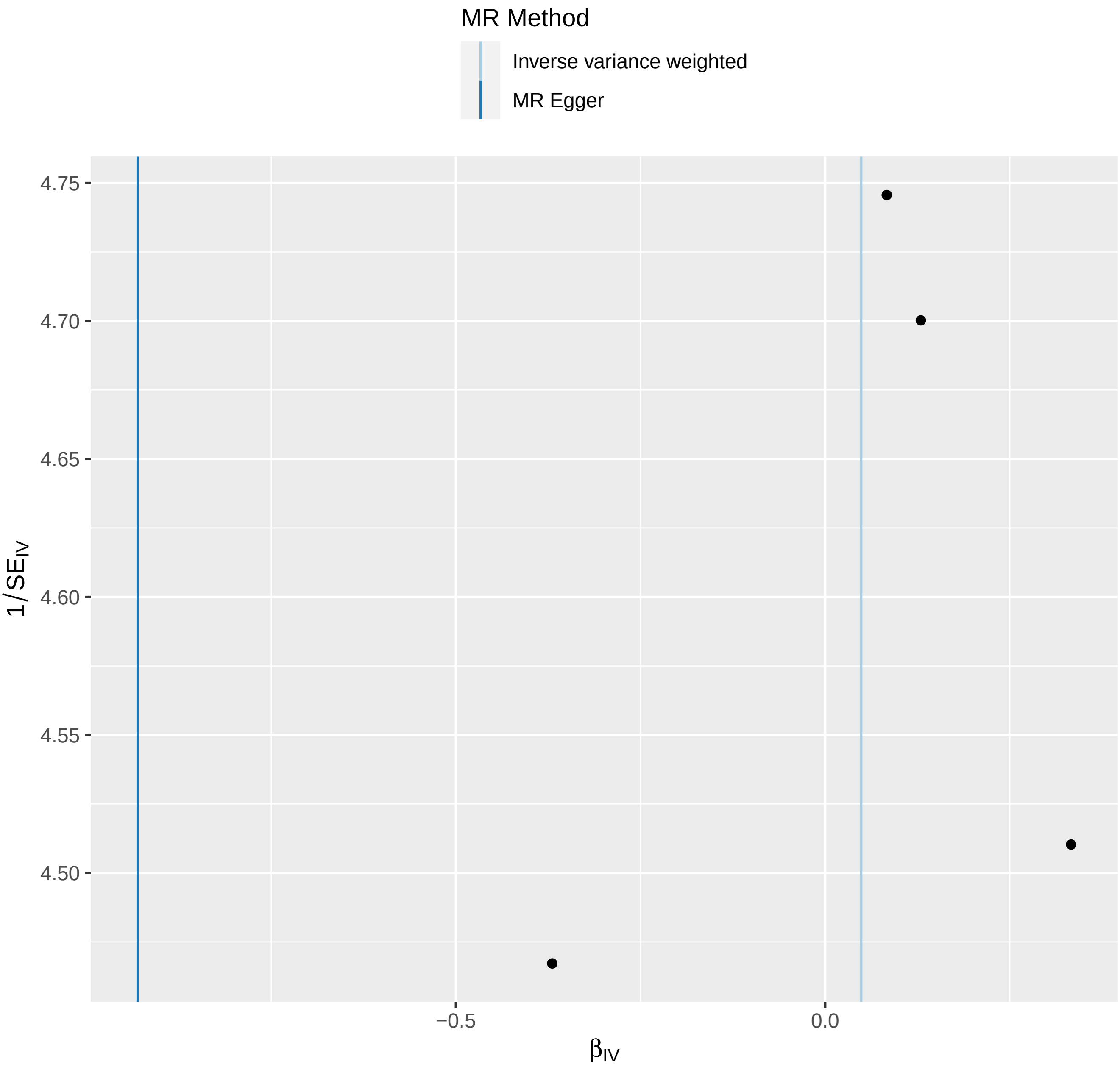

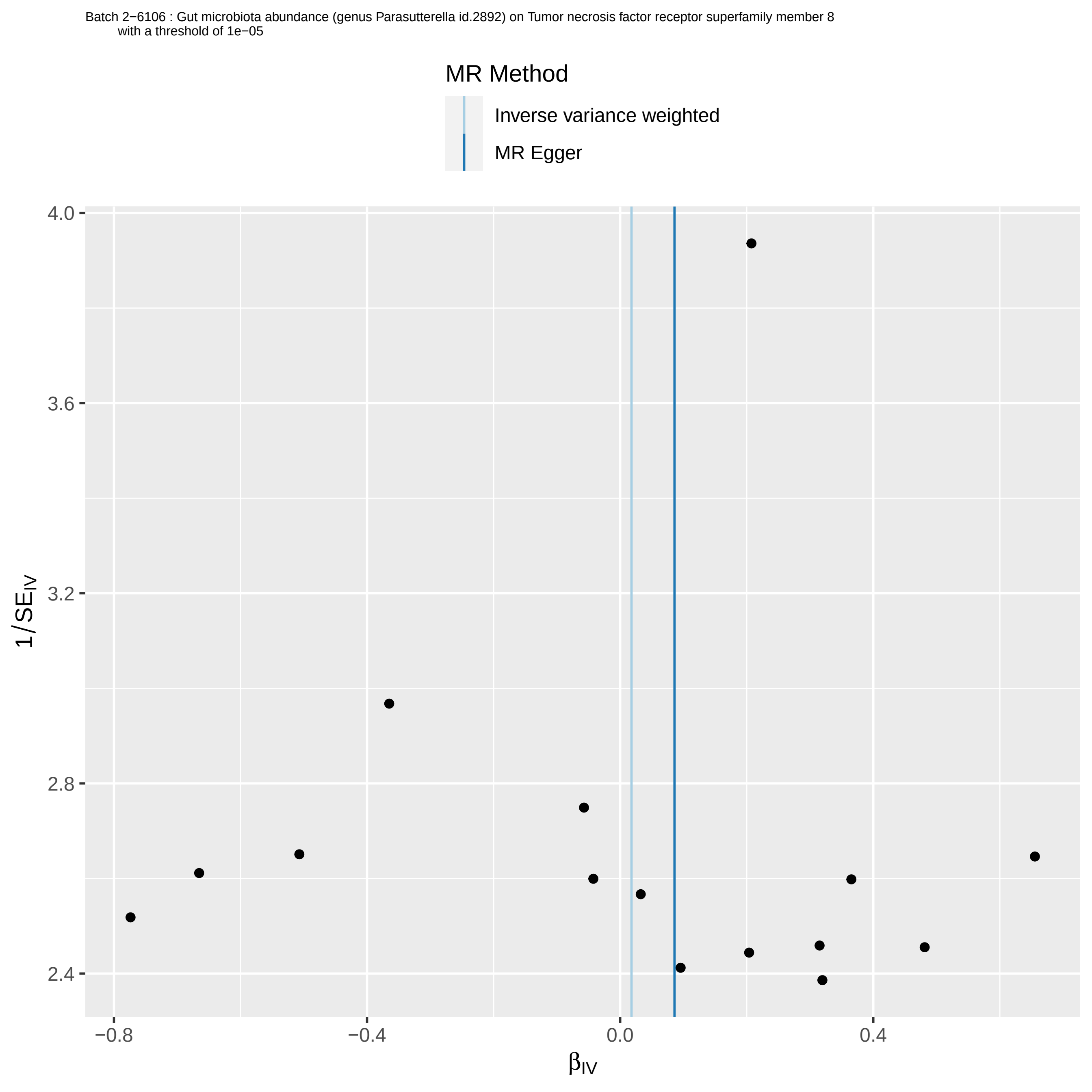

Batch 2−6106 : Gut microbiota abundance (genus Parasutterella id.2892) on Tumor necrosis factor receptor superfamily member 8
with a threshold of 1e−05
MR Method
Inverse variance weighted
MR Egger
4.0
3.6
3.2
2.8
2.4
1/SE_IV
−0.8
−0.4
0.0
0.4
β_IV

Batch 2−6107 : Gut microbiota abundance (genus Catenibacterium id.2153) on Tumor necrosis factor ligand superfamily member 11 with a threshold of 1e−05

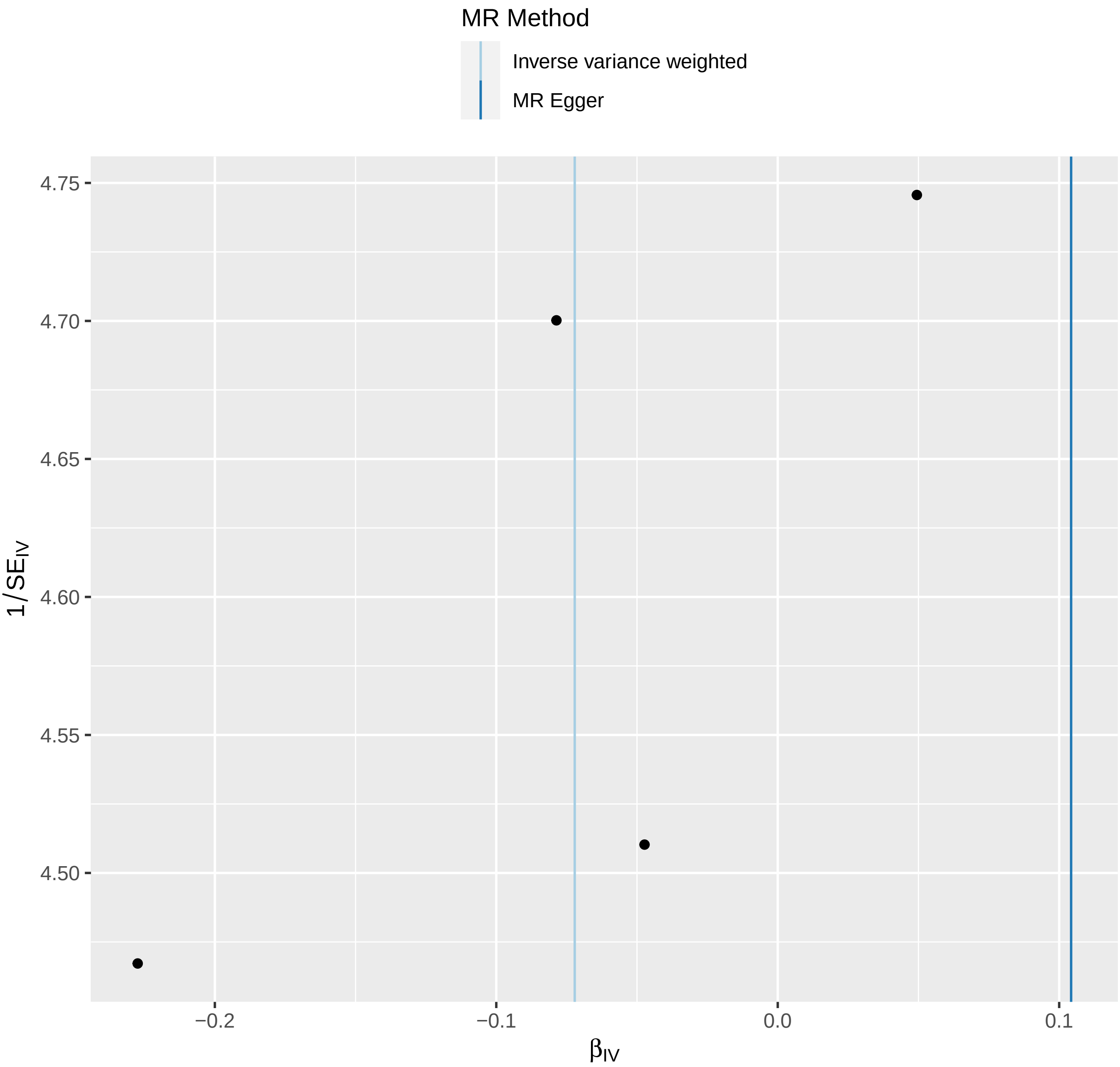

Batch 2−6108 : Gut microbiota abundance (genus Parasutterella id.2892) on Tumor necrosis factor ligand superfamily member 11
with a threshold of 1e−05

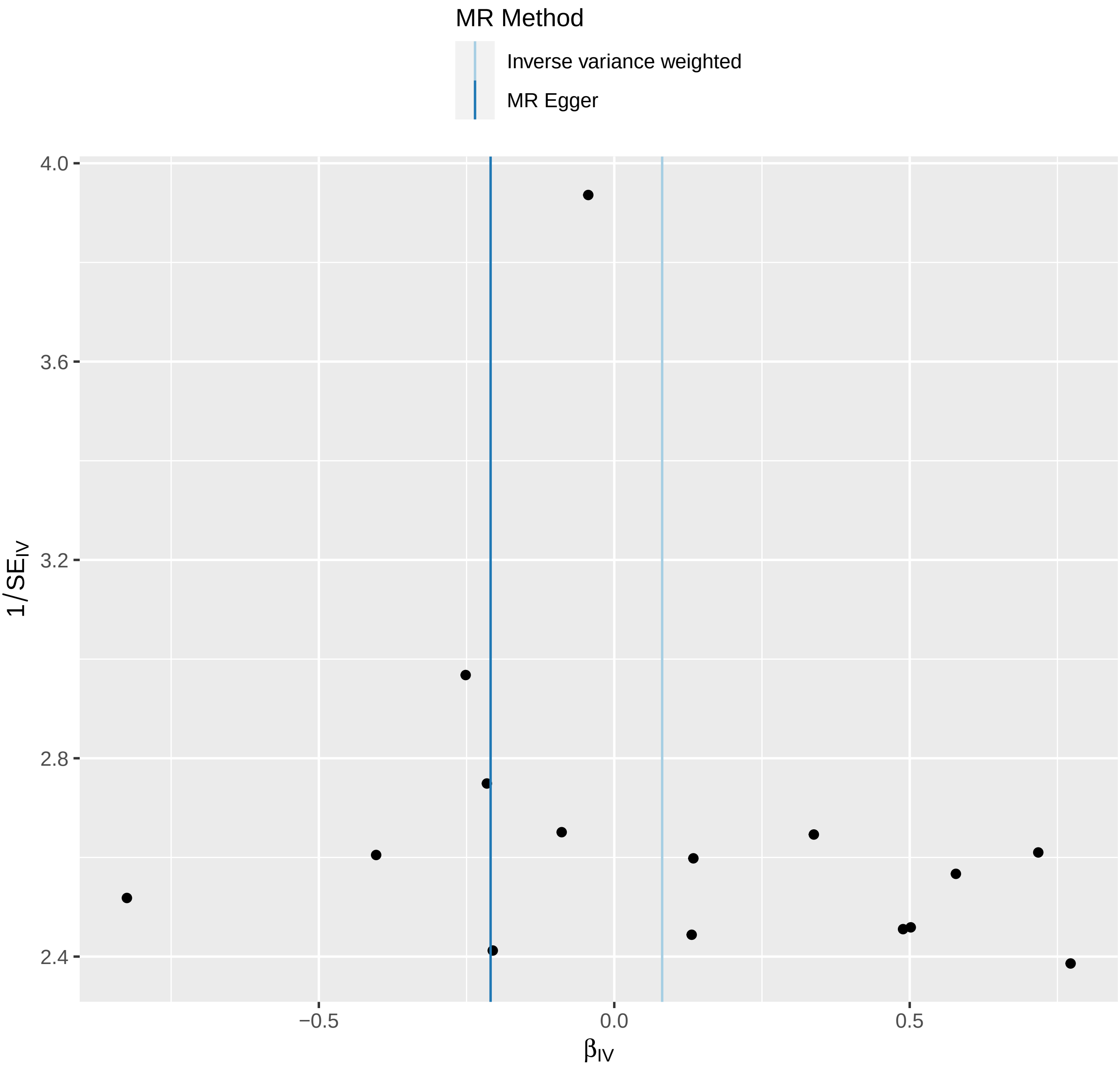

Batch 2−6109 : Gut microbiota abundance (genus Catenibacterium id.2153) on Tumor necrosis factor ligand superfamily member 12
with a threshold of 1e−05

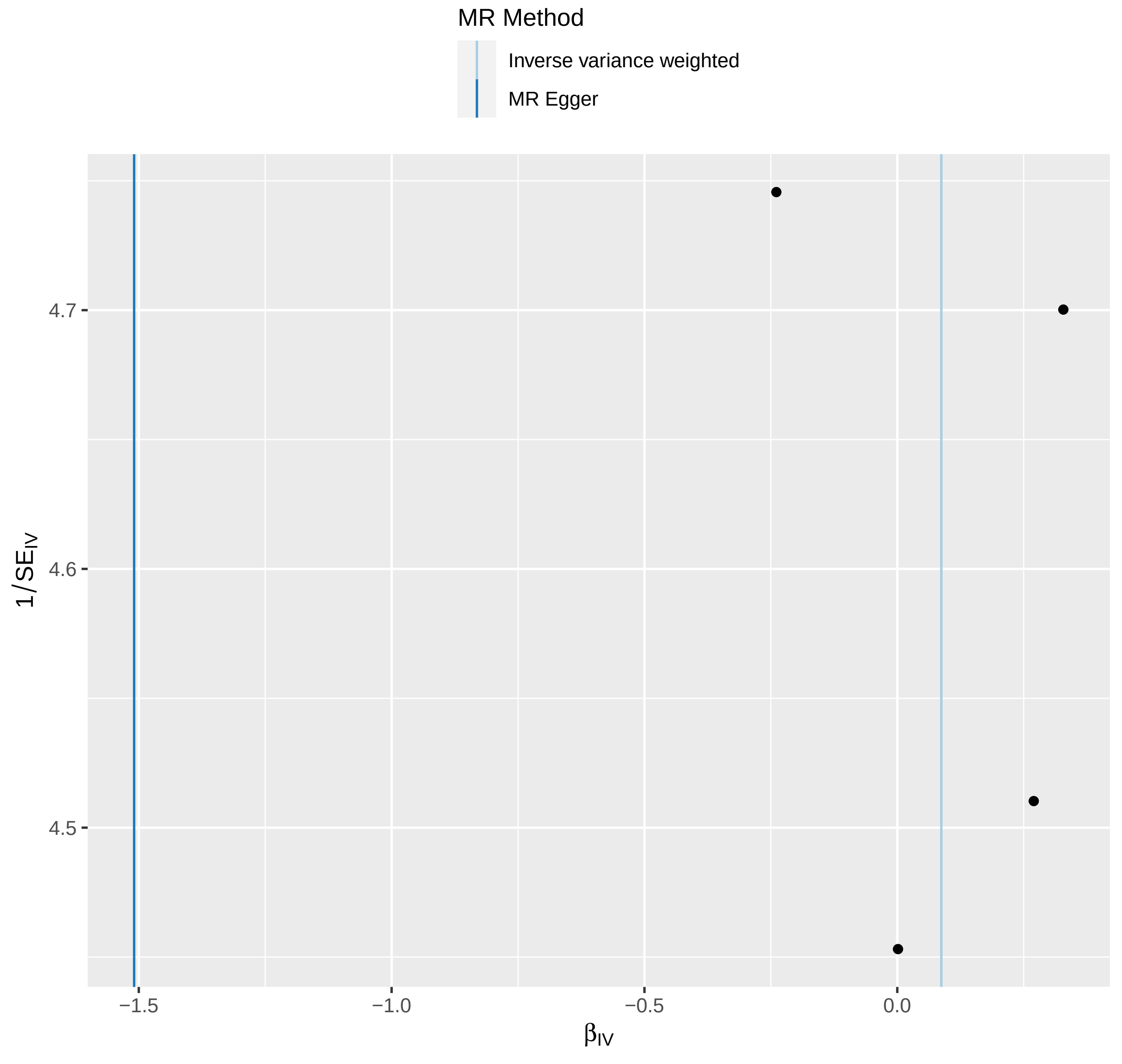

Batch 2−6110 : Gut microbiota abundance (genus Parasutterella id.2892) on Tumor necrosis factor ligand superfamily member 12
with a threshold of 1e−05

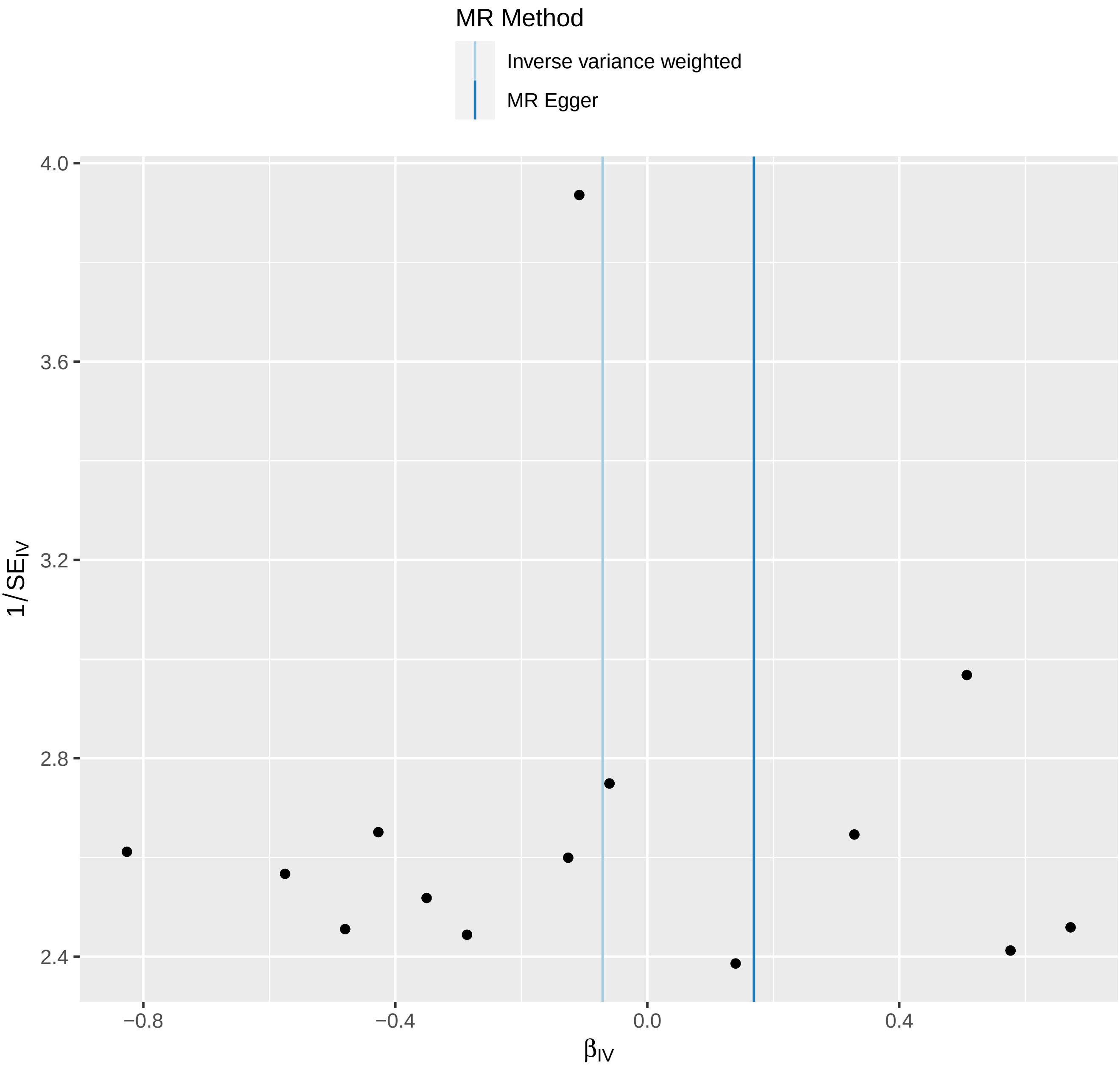

Batch 2−6111 : Gut microbiota abundance (genus Catenibacterium id.2153) on Tumor necrosis factor ligand superfamily member 13B
with a threshold of 1e−05

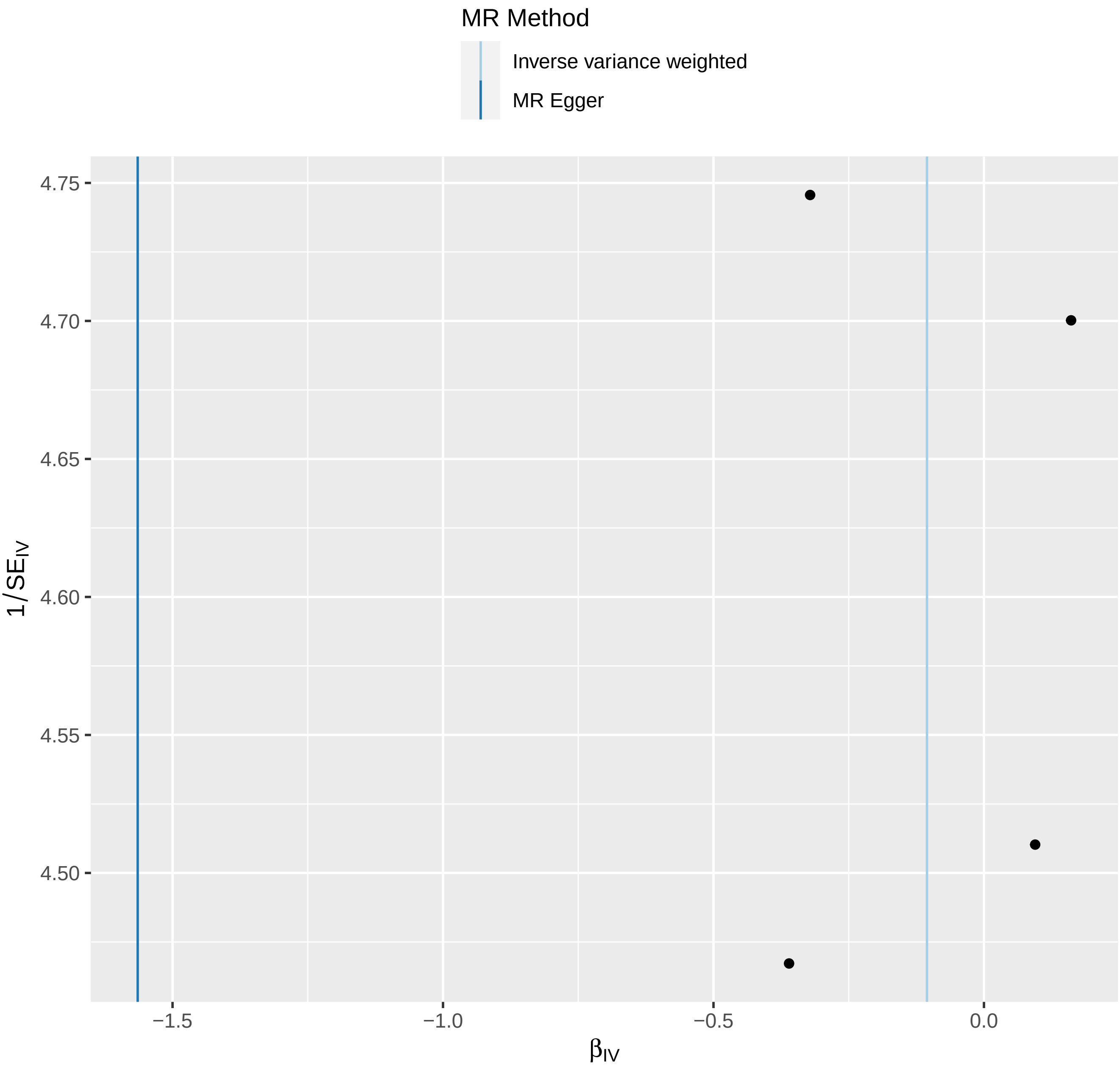

Batch 2−6112 : Gut microbiota abundance (genus Parasutterella id.2892) on Tumor necrosis factor ligand superfamily member 13B
with a threshold of 1e−05

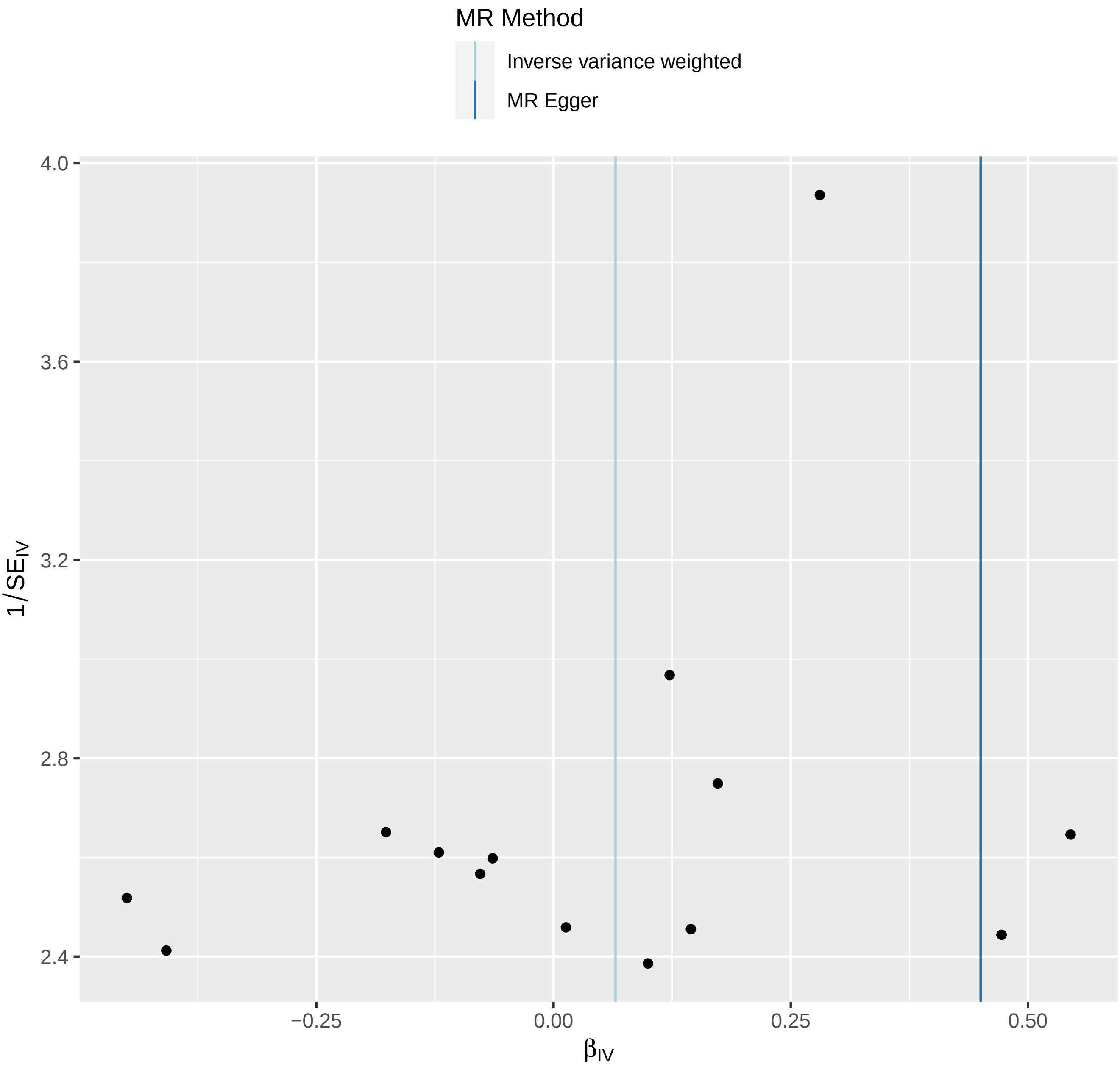

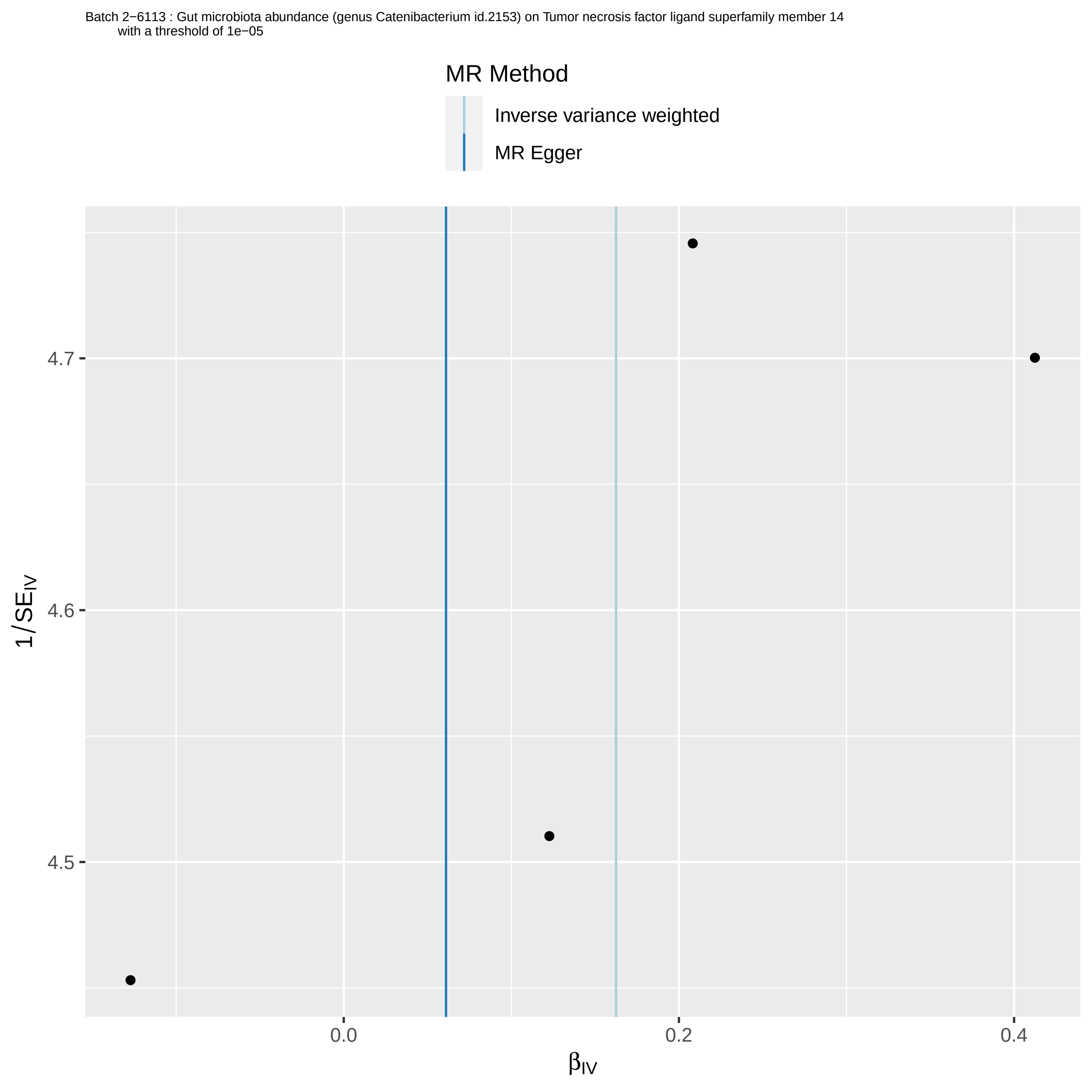

Batch 2−6113 : Gut microbiota abundance (genus Catenibacterium id.2153) on Tumor necrosis factor ligand superfamily member 14
with a threshold of 1e−05
MR Method
Inverse variance weighted
MR Egger
4.7
4.6
4.5
1/SE_IV
0.0
0.2
0.4
β_IV

Batch 2−6114 : Gut microbiota abundance (genus Parasutterella id.2892) on Tumor necrosis factor ligand superfamily member 14
with a threshold of 1e−05

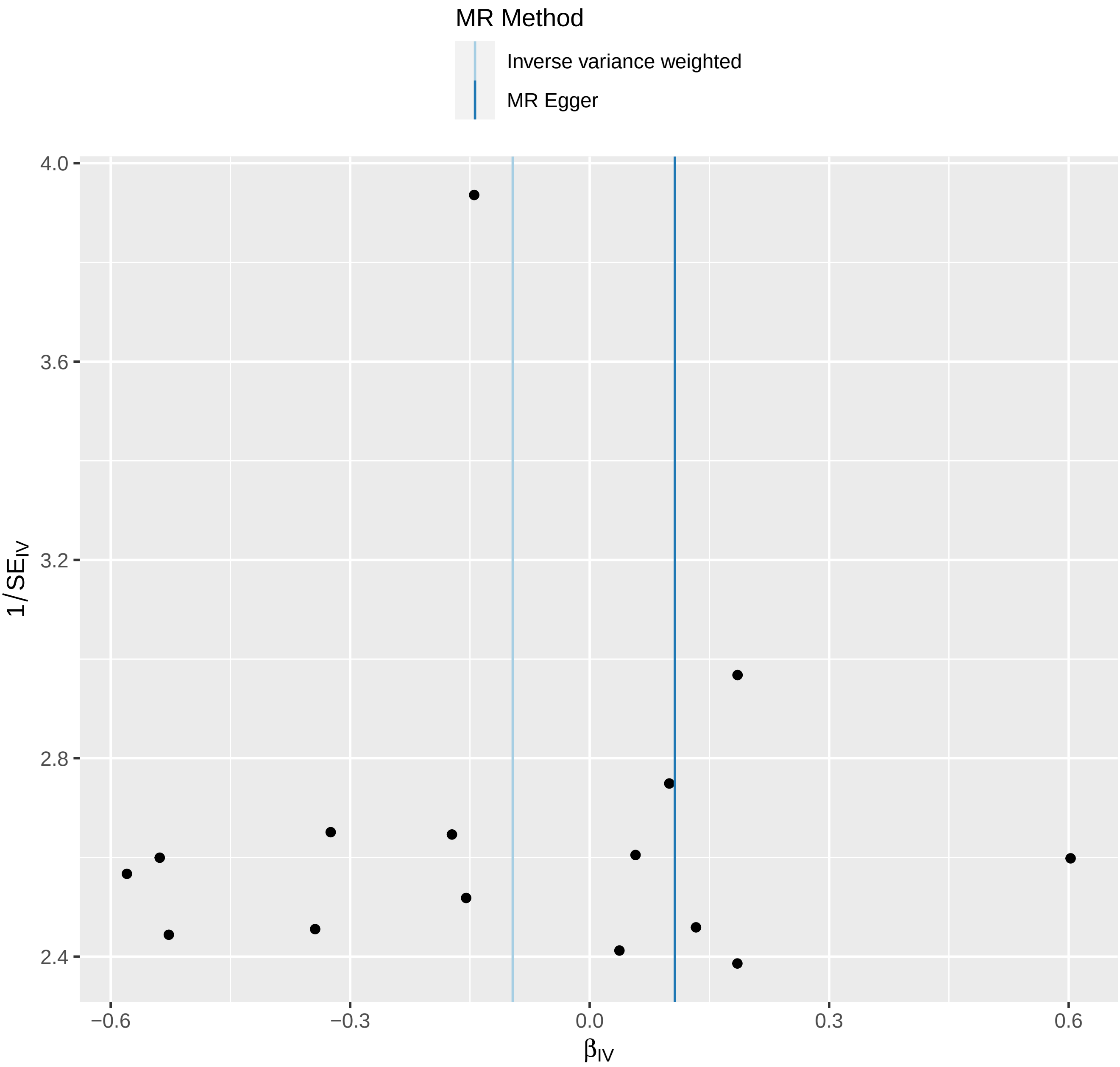

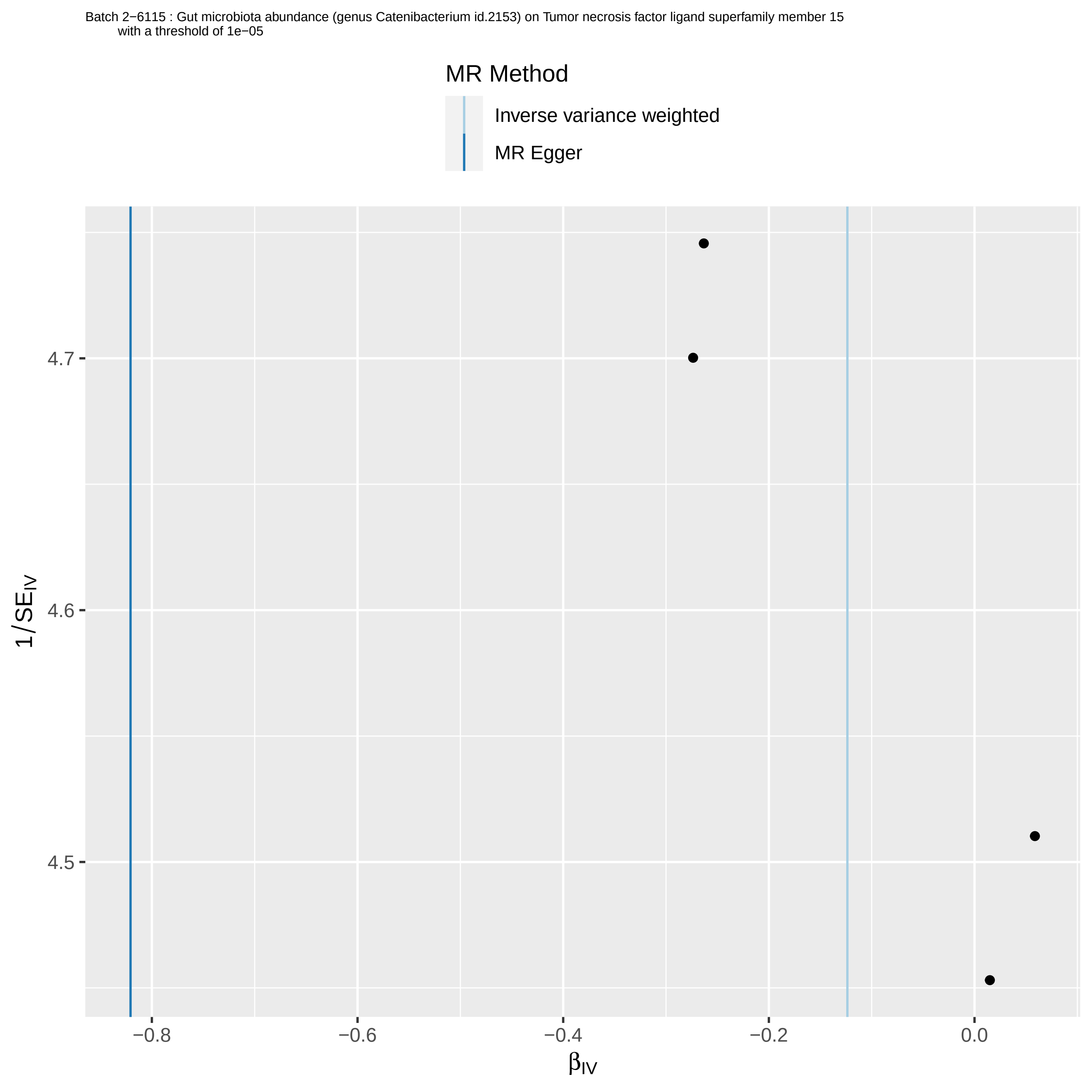
Batch 2−6115 : Gut microbiota abundance (genus Catenibacterium id.2153) on Tumor necrosis factor ligand superfamily member 15
with a threshold of 1e−05
MR Method
Inverse variance weighted
MR Egger
4.7
4.6
4.5
1/SE$_{IV}$
−0.8
−0.6
−0.4
−0.2
0.0
β_{IV}

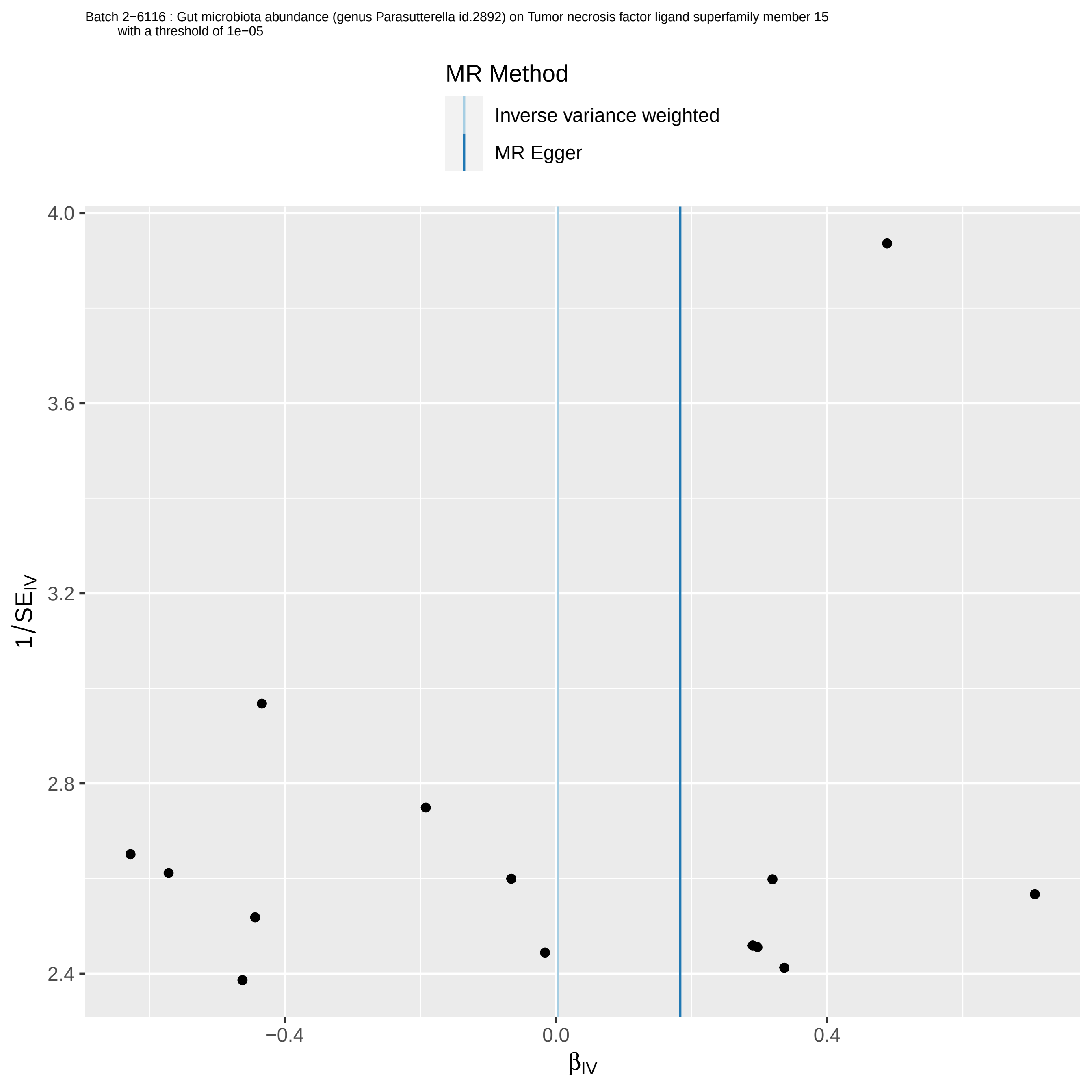

Batch 2−6116 : Gut microbiota abundance (genus Parasutterella id.2892) on Tumor necrosis factor ligand superfamily member 15
with a threshold of 1e−05
MR Method
Inverse variance weighted
MR Egger
4.0
3.6
3.2
2.8
2.4
1/SE_IV
−0.4
0.0
0.4
β_IV

Batch 2−6117 : Gut microbiota abundance (genus Catenibacterium id.2153) on Tumor necrosis factor ligand superfamily member 15
with a threshold of 1e−05

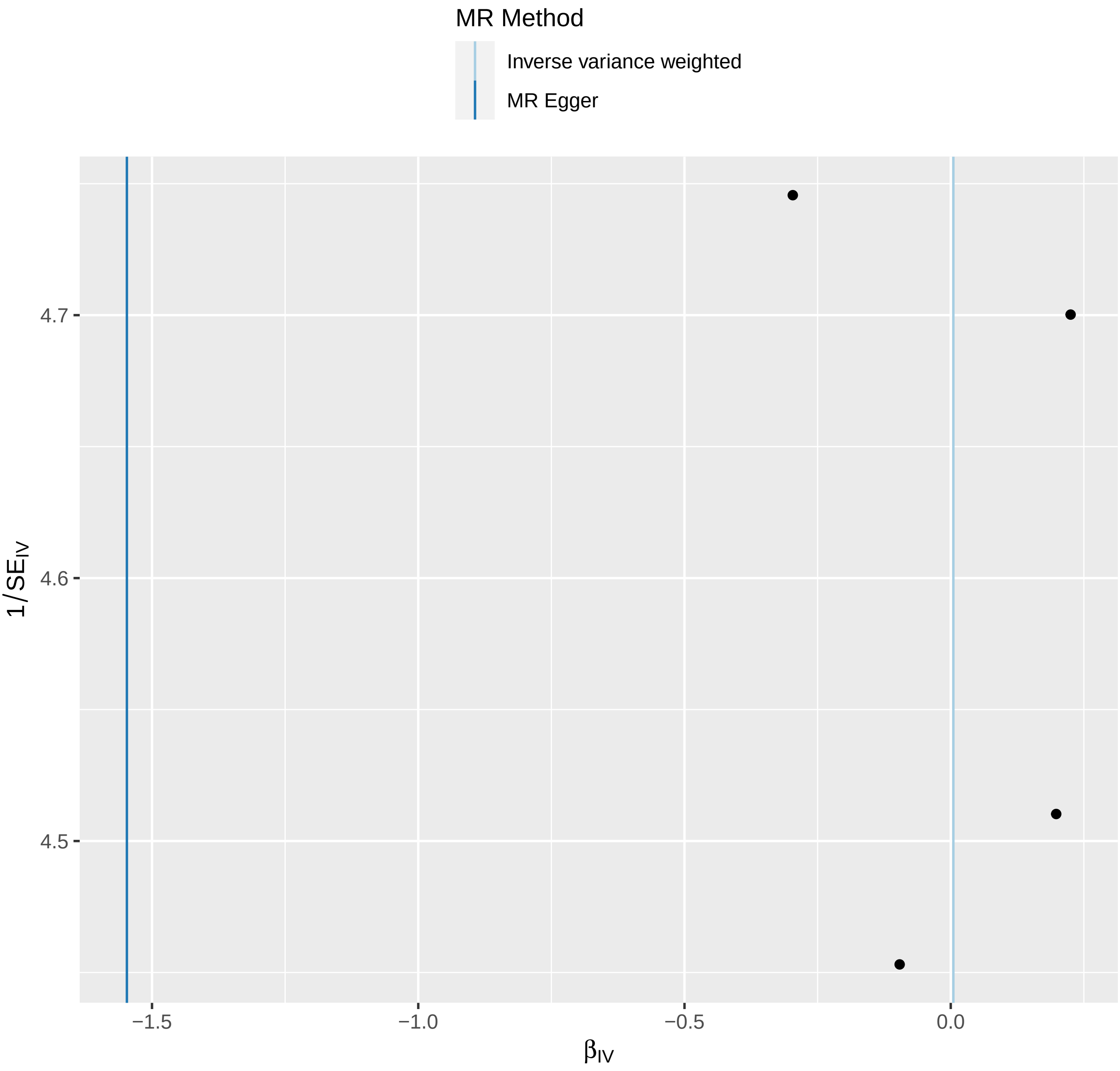

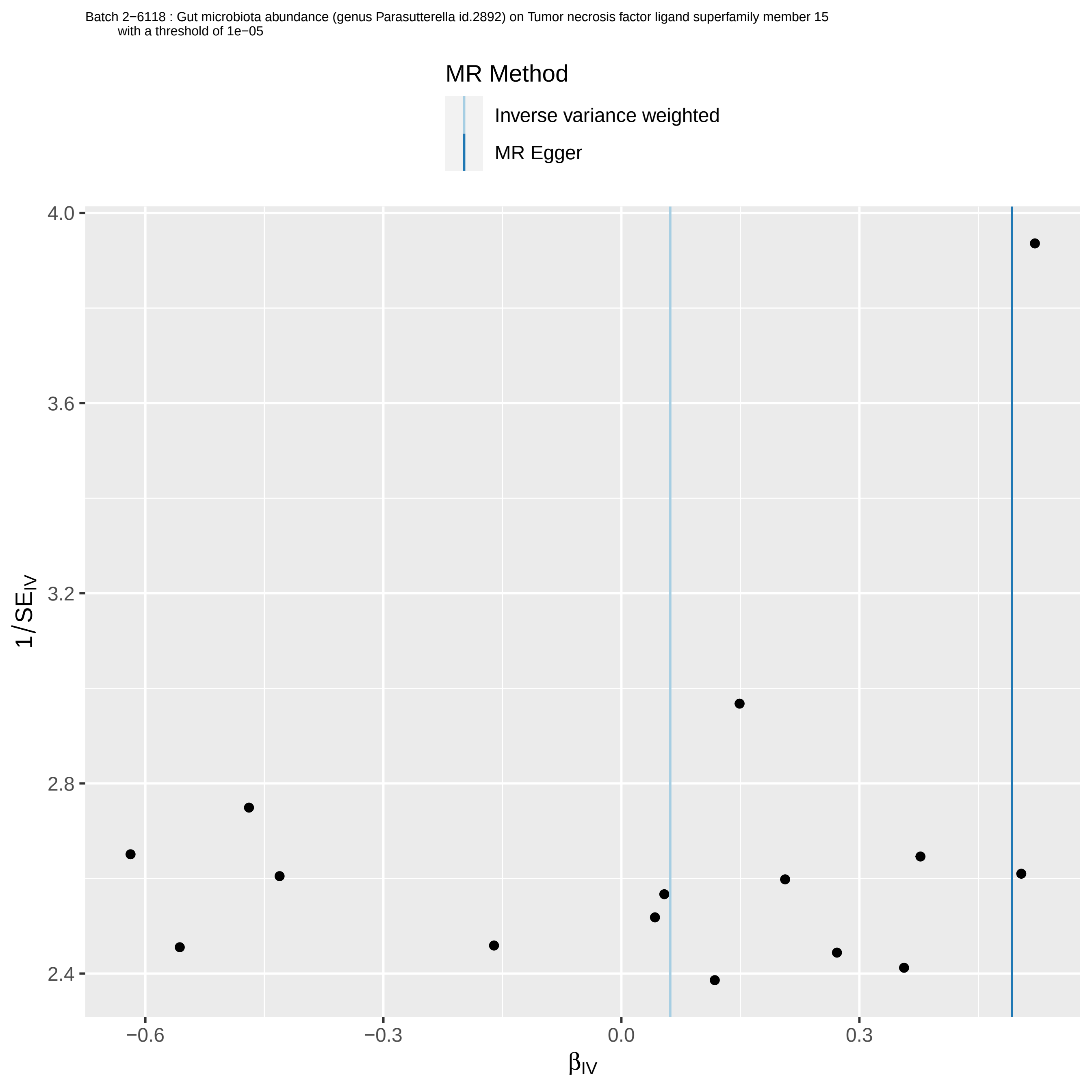
Batch 2−6118 : Gut microbiota abundance (genus Parasutterella id.2892) on Tumor necrosis factor ligand superfamily member 15
with a threshold of 1e−05
MR Method
Inverse variance weighted
MR Egger
4.0
3.6
3.2
2.8
2.4
1/SE_IV
−0.6
−0.3
0.0
0.3
β_IV

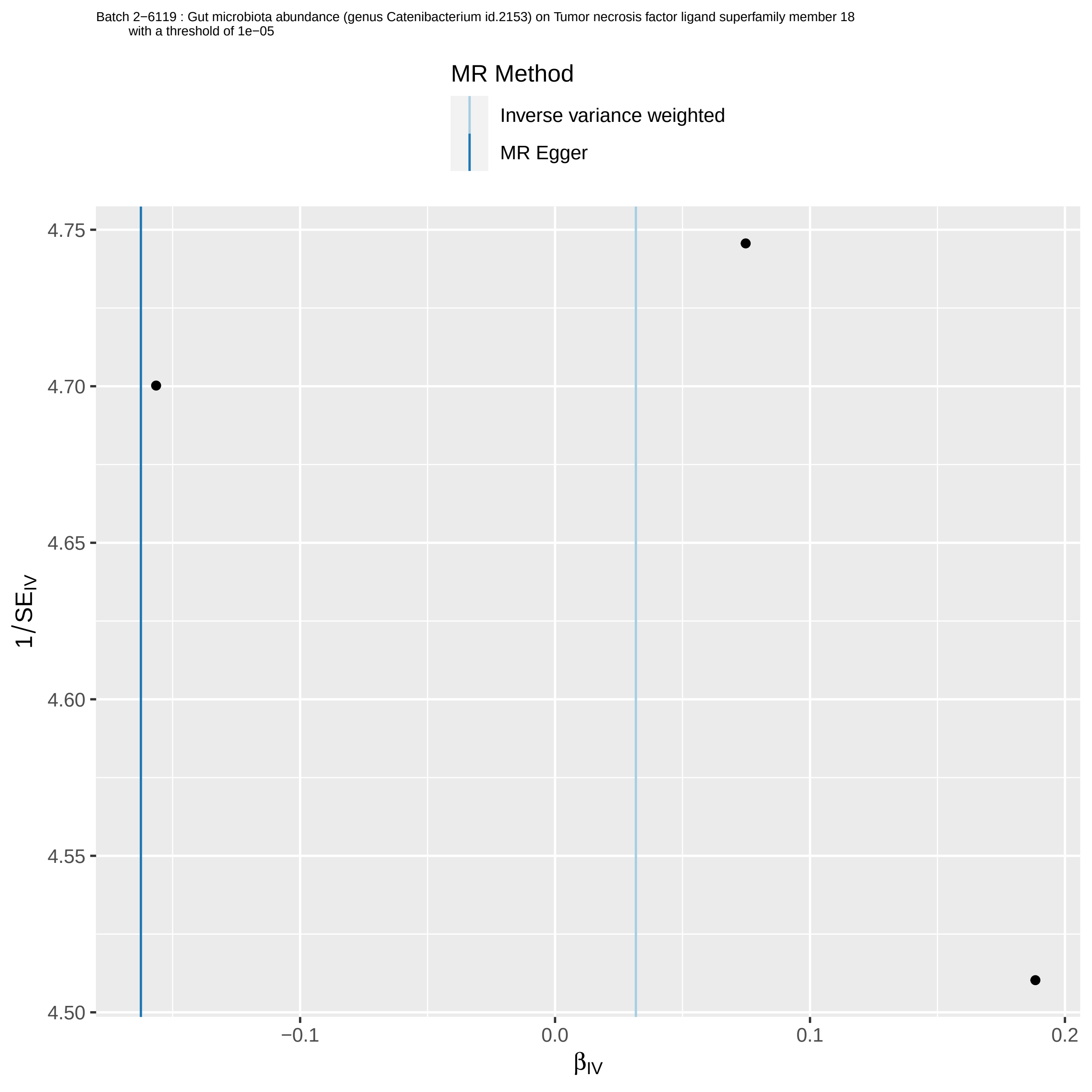

Batch 2−6119 : Gut microbiota abundance (genus Catenibacterium id.2153) on Tumor necrosis factor ligand superfamily member 18
with a threshold of 1e−05
MR Method
Inverse variance weighted
MR Egger
4.75
4.70
4.65
4.60
4.55
4.50
1/SE_IV
−0.1
0.0
0.1
0.2
β_IV

Batch 2−6120 : Gut microbiota abundance (genus Parasutterella id.2892) on Tumor necrosis factor ligand superfamily member 18
with a threshold of 1e−05

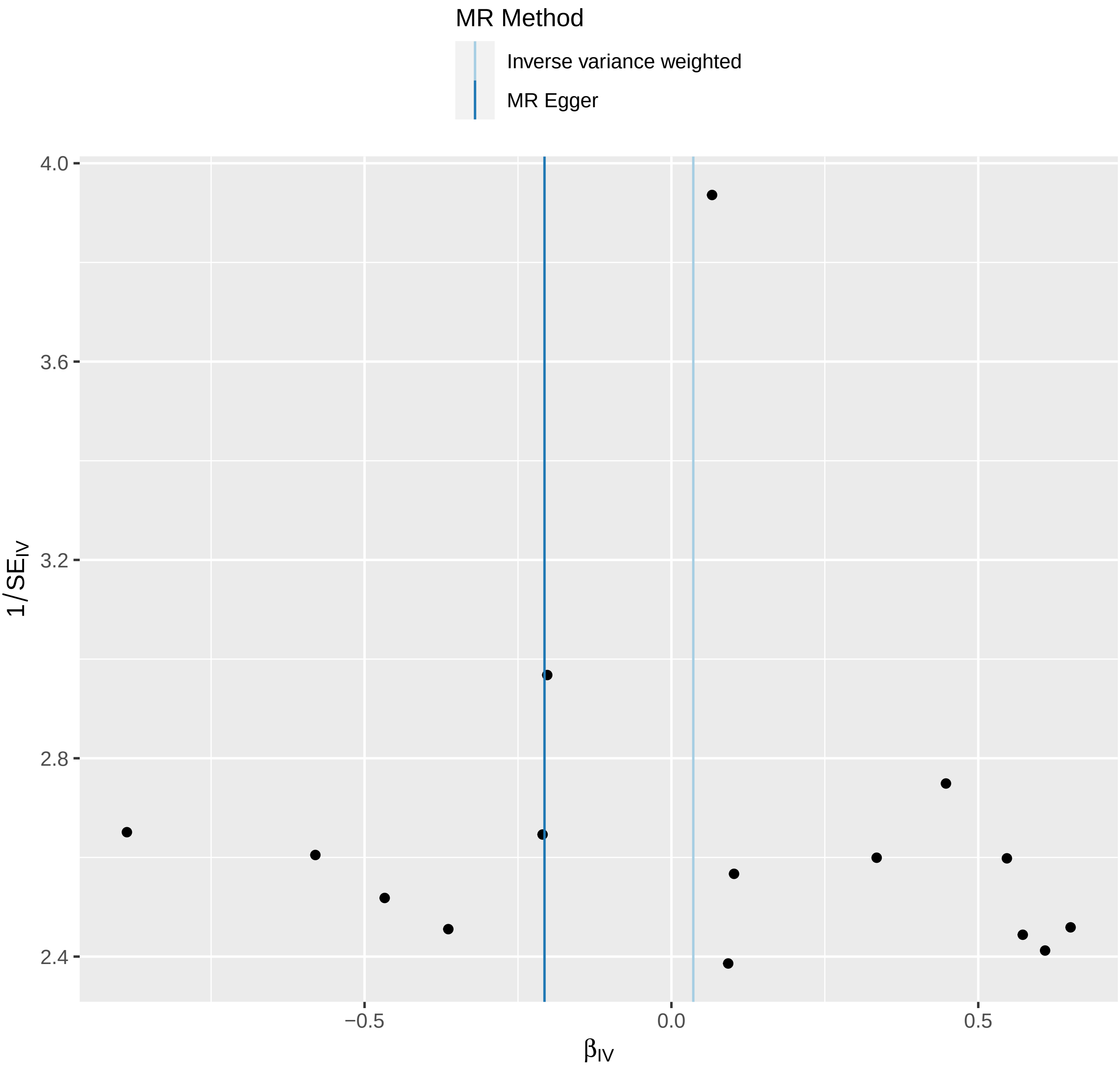

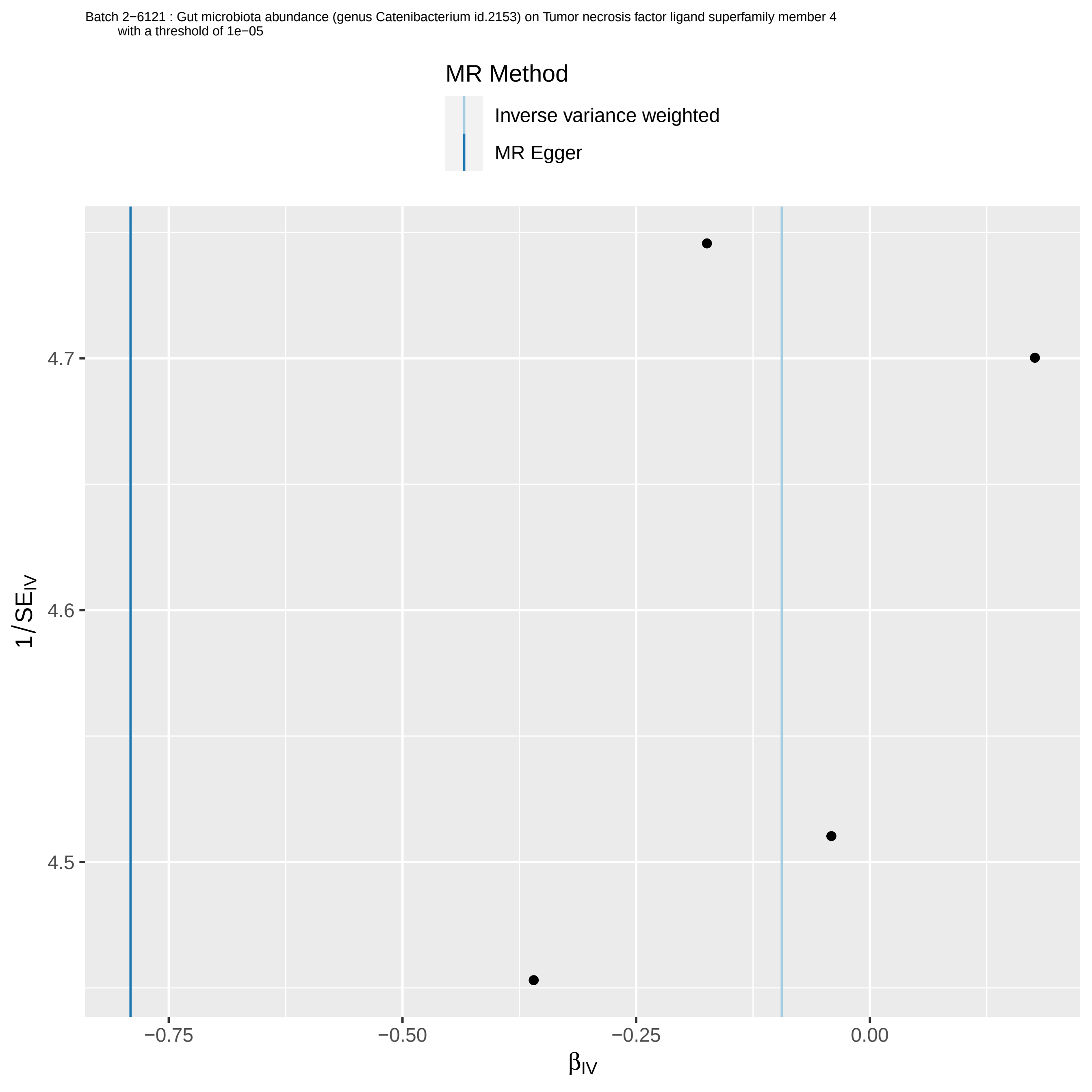
Batch 2−6121 : Gut microbiota abundance (genus Catenibacterium id.2153) on Tumor necrosis factor ligand superfamily member 4
with a threshold of 1e−05
MR Method
Inverse variance weighted
MR Egger
4.7
4.6
4.5
1/SE_IV
−0.75
−0.50
−0.25
0.00
β_IV

Batch 2−6122 : Gut microbiota abundance (genus Parasutterella id.2892) on Tumor necrosis factor ligand superfamily member 4
with a threshold of 1e−05

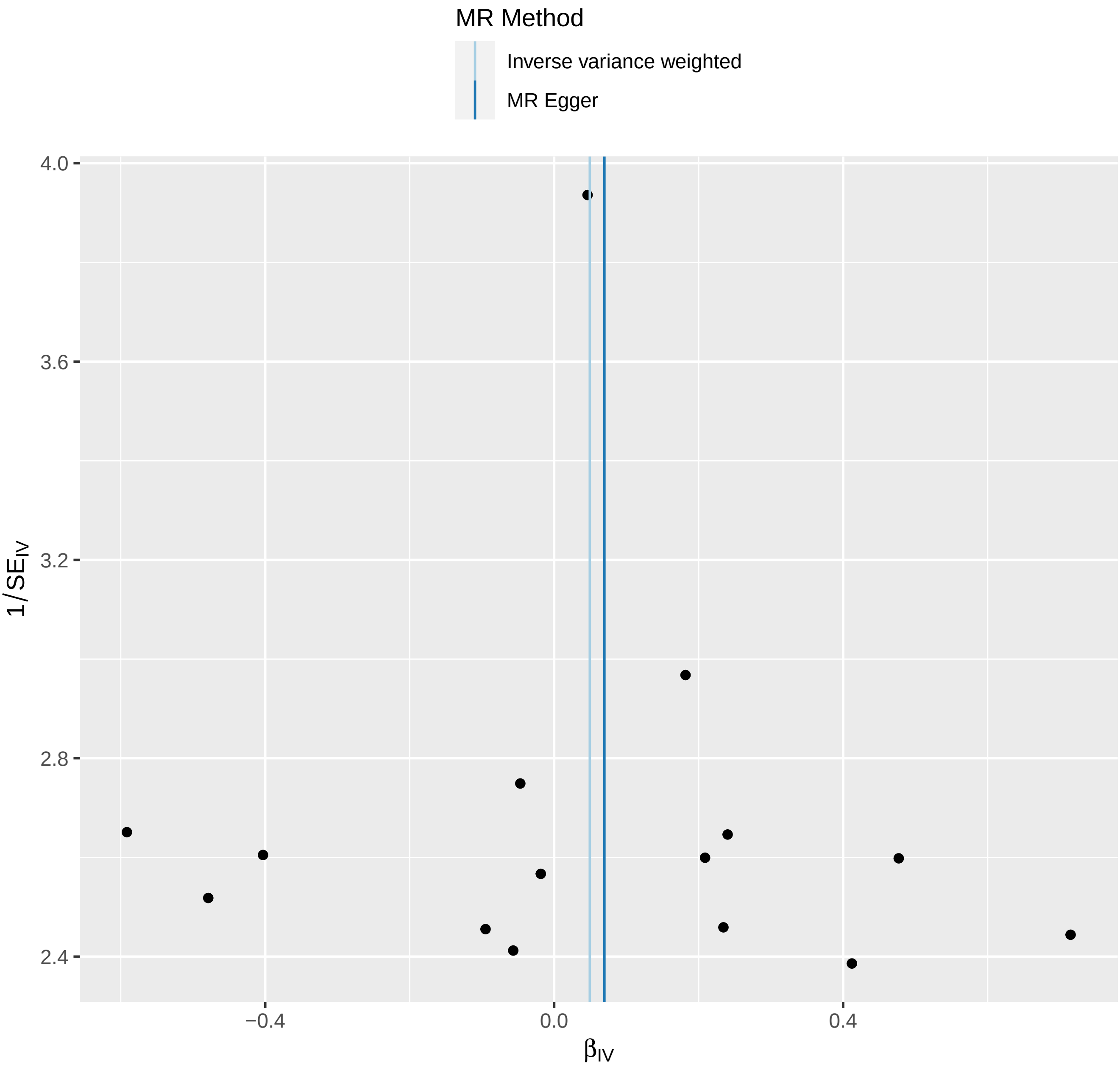

Batch 2−6123 : Gut microbiota abundance (genus Catenibacterium id.2153) on Tumor necrosis factor ligand superfamily member 4 with a threshold of 1e−05

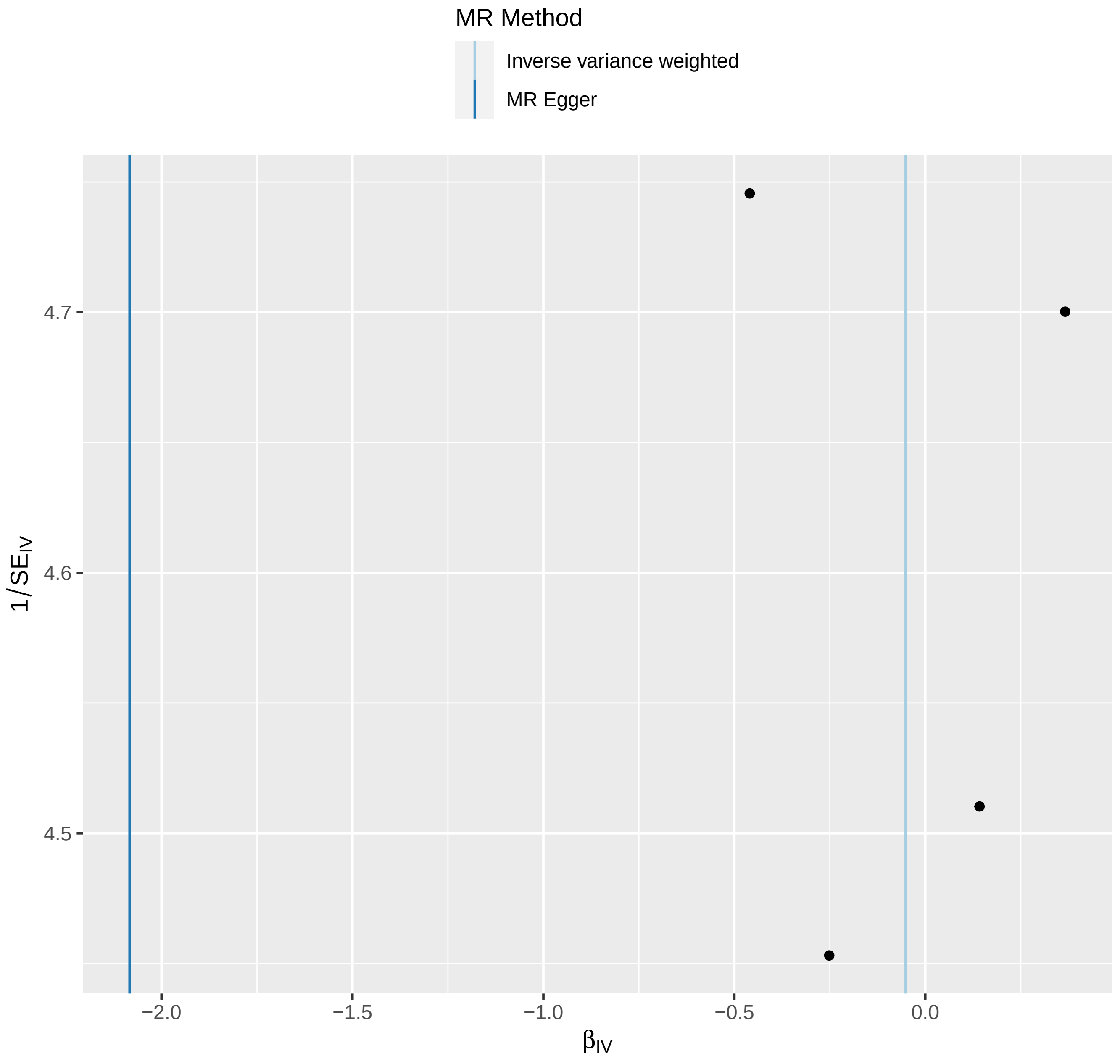

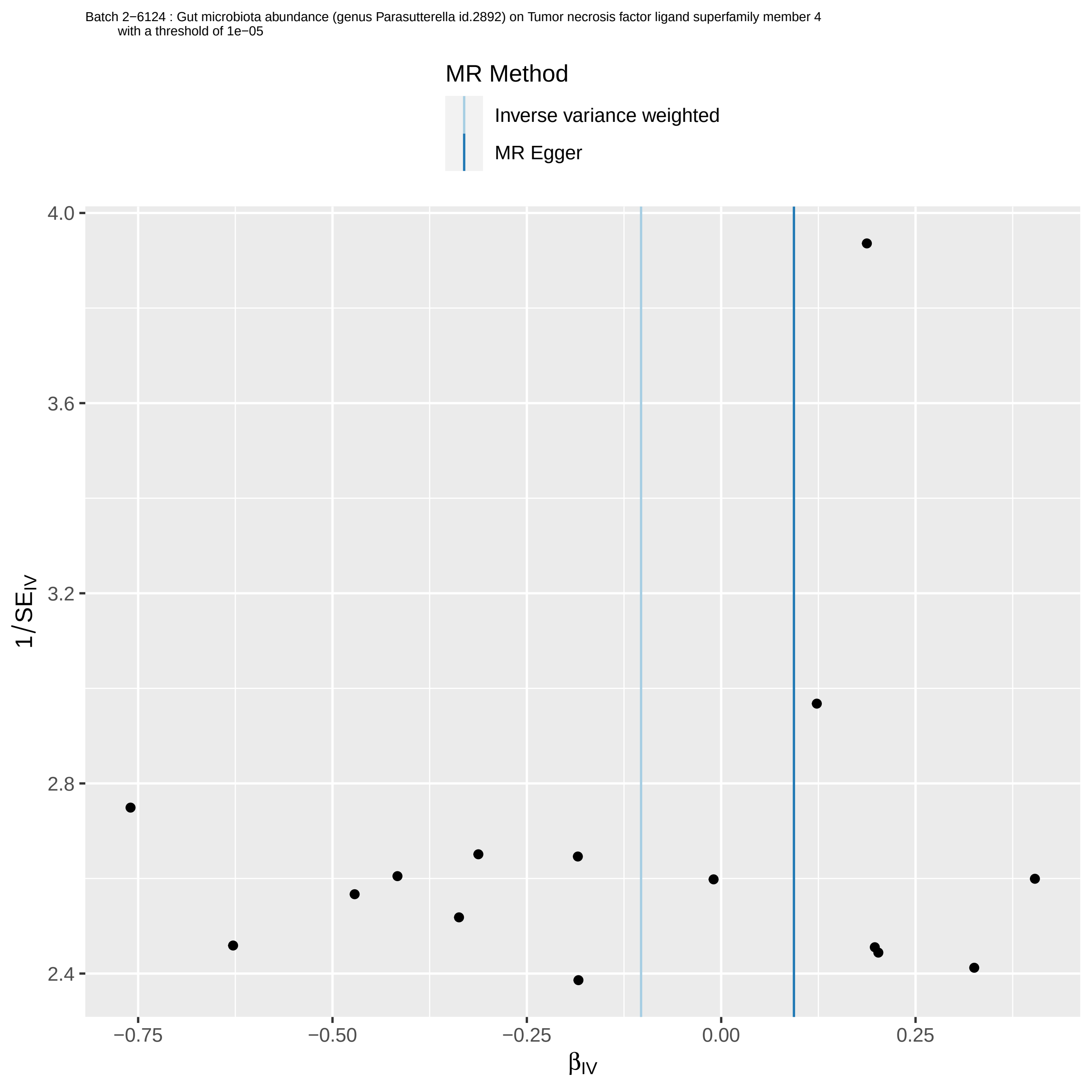
Batch 2−6124 : Gut microbiota abundance (genus Parasutterella id.2892) on Tumor necrosis factor ligand superfamily member 4
with a threshold of 1e−05
MR Method
Inverse variance weighted
MR Egger
4.0
3.6
3.2
2.8
2.4
1/SE$_{IV}$
−0.75
−0.50
−0.25
0.00
0.25
β_{IV}

Batch 2−6125 : Gut microbiota abundance (genus Catenibacterium id.2153) on Tumor necrosis factor ligand superfamily member 8 with a threshold of 1e−05

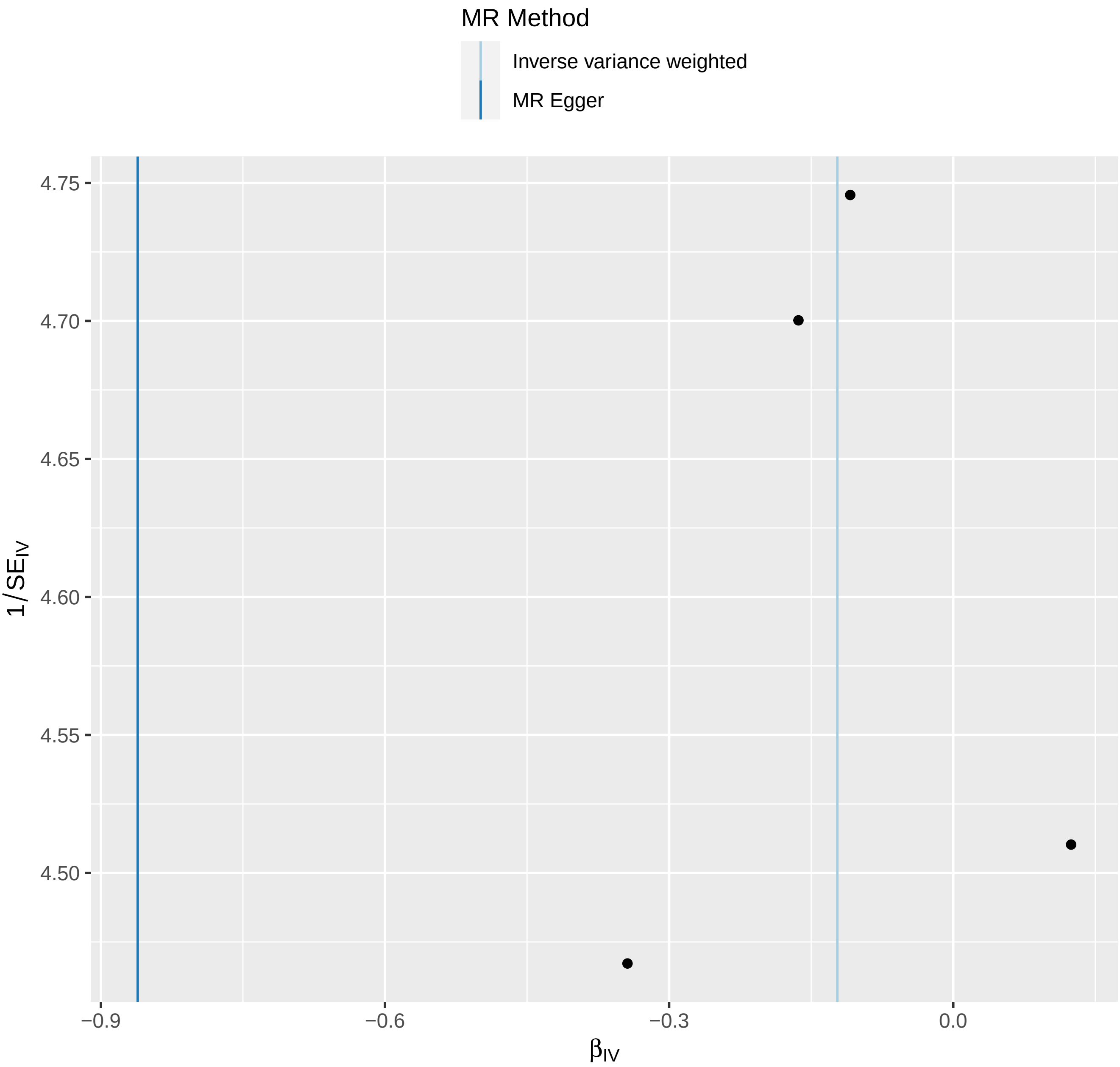

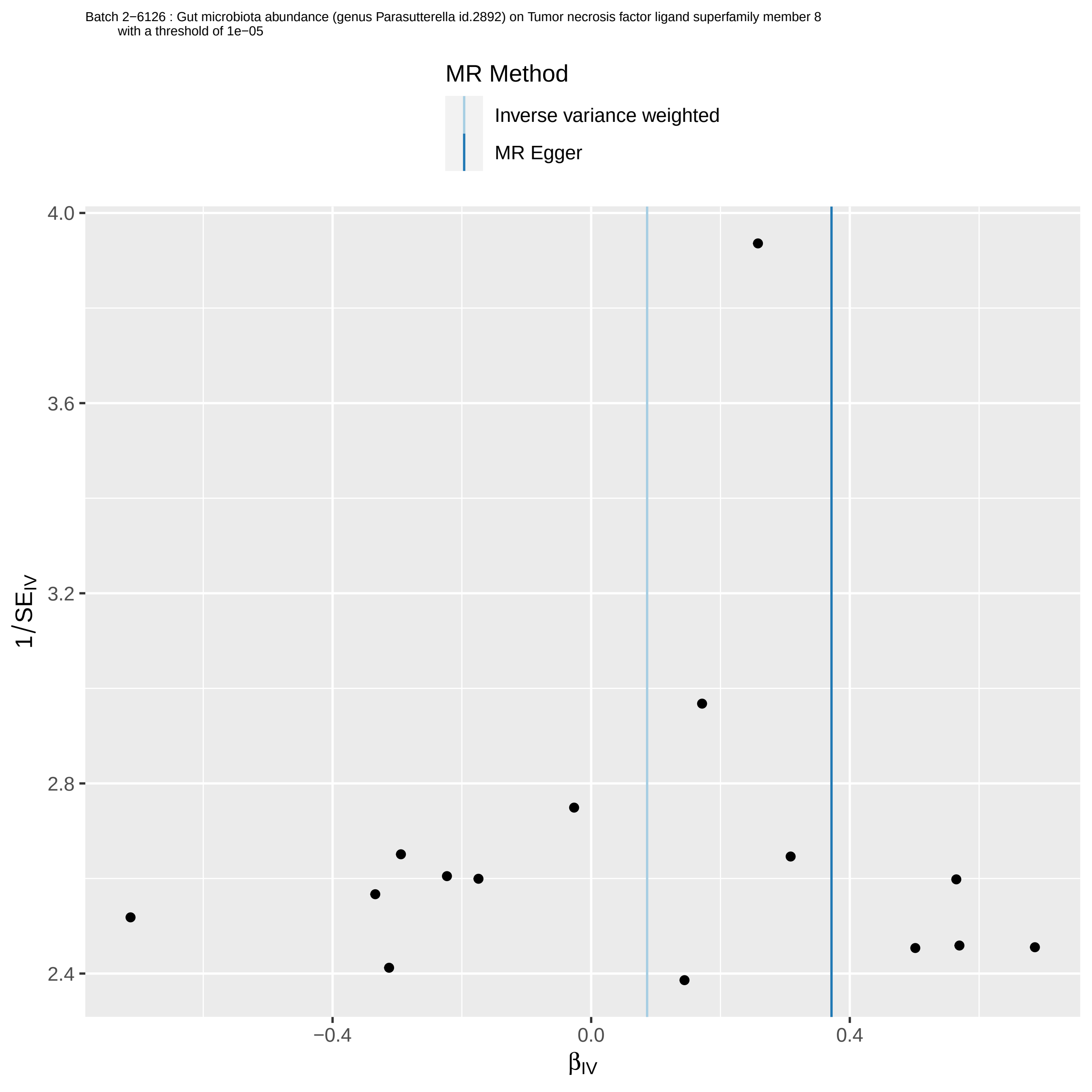

Batch 2−6126 : Gut microbiota abundance (genus Parasutterella id.2892) on Tumor necrosis factor ligand superfamily member 8
with a threshold of 1e−05
MR Method
Inverse variance weighted
MR Egger
4.0
3.6
3.2
2.8
2.4
1/SE_IV
−0.4
0.0
0.4
β_IV

Batch 2−6127 : Gut microbiota abundance (genus Catenibacterium id.2153) on Tumor necrosis factor ligand superfamily member 9
with a threshold of 1e−05

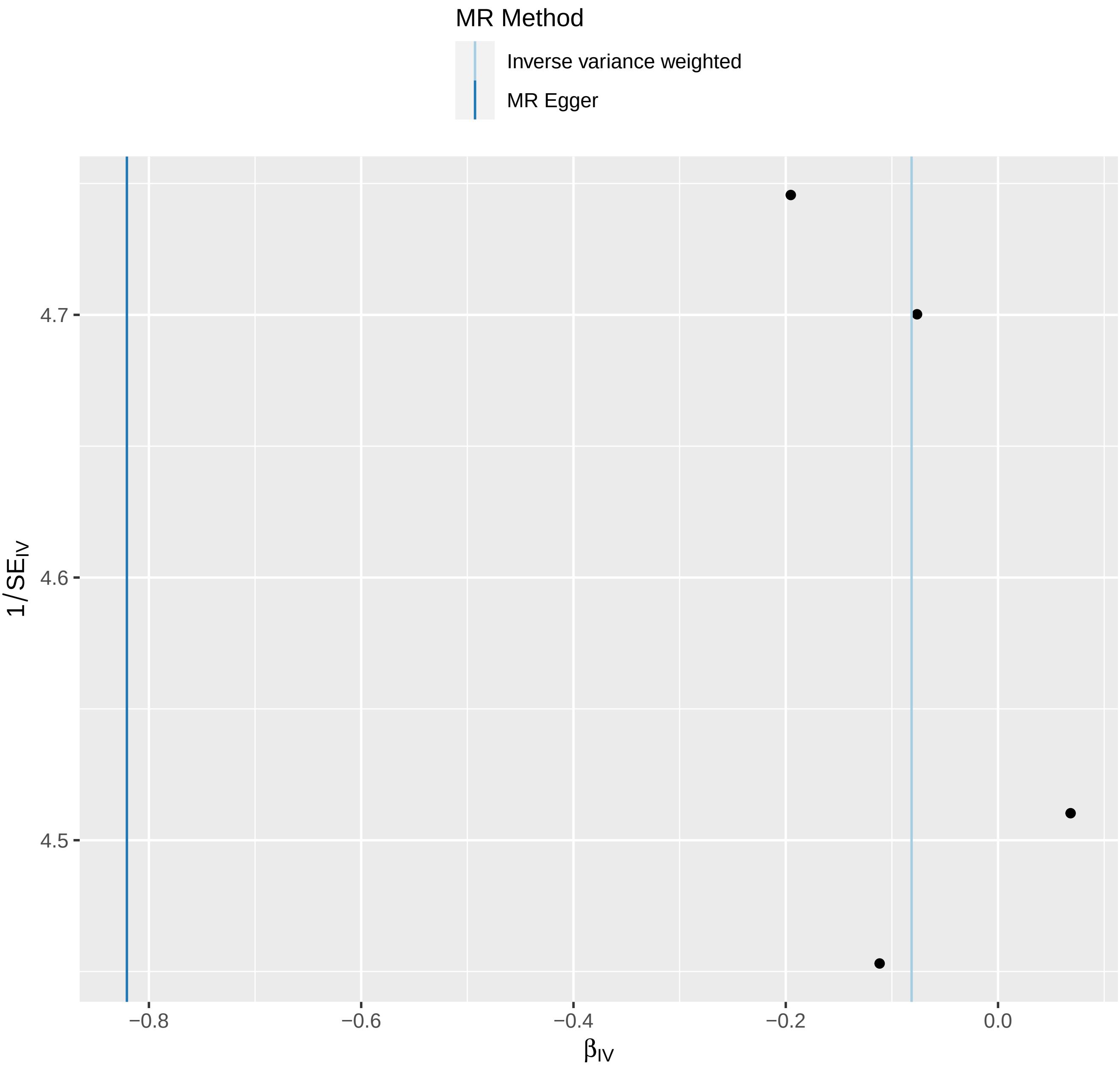

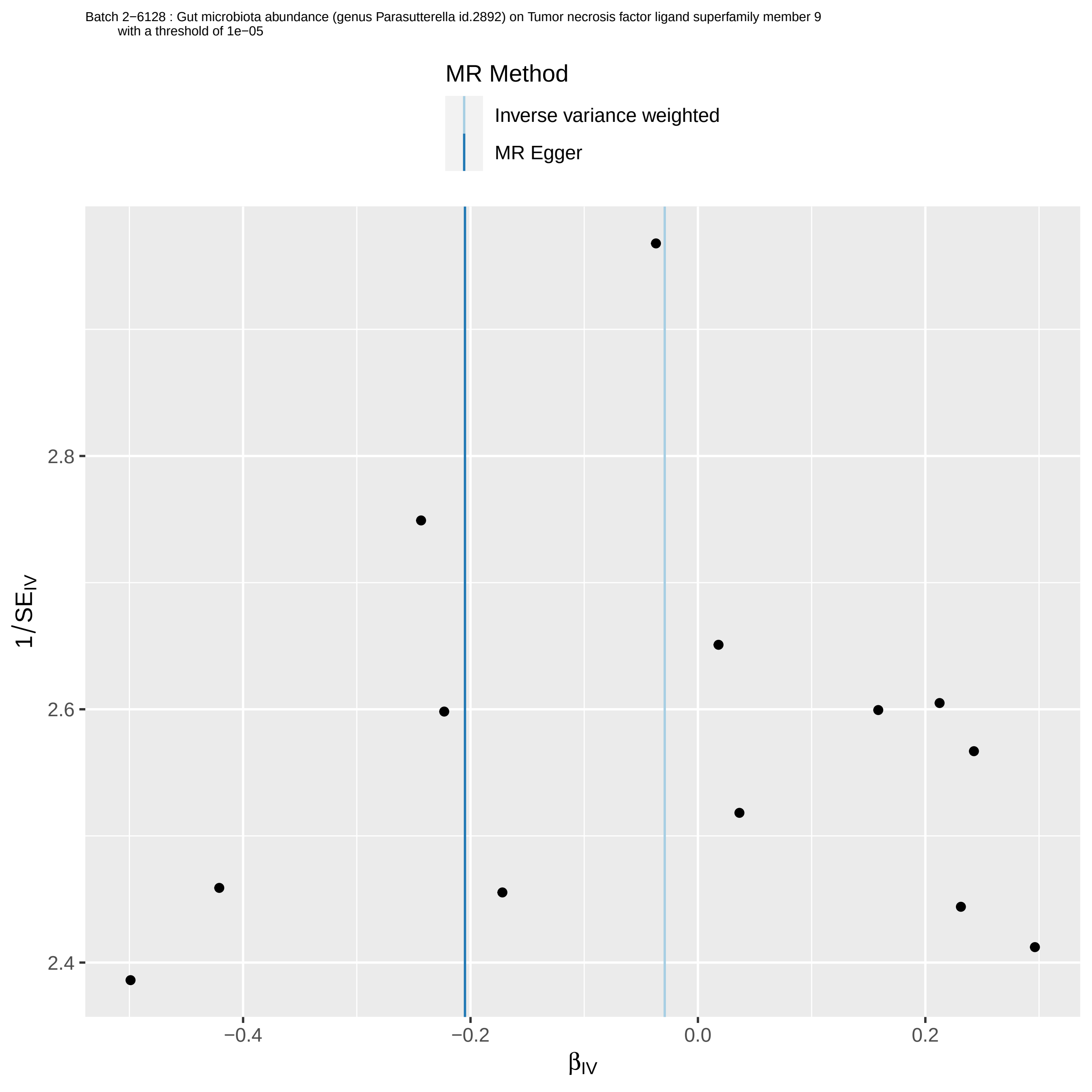

Batch 2−6128 : Gut microbiota abundance (genus Parasutterella id.2892) on Tumor necrosis factor ligand superfamily member 9
with a threshold of 1e−05
MR Method
Inverse variance weighted
MR Egger
2.8
2.6
2.4
1/SE_IV
−0.4
−0.2
0.0
0.2
β_IV

Batch 2−6129 : Gut microbiota abundance (genus Catenibacterium id.2153) on Tenomodulin
with a threshold of 1e−05

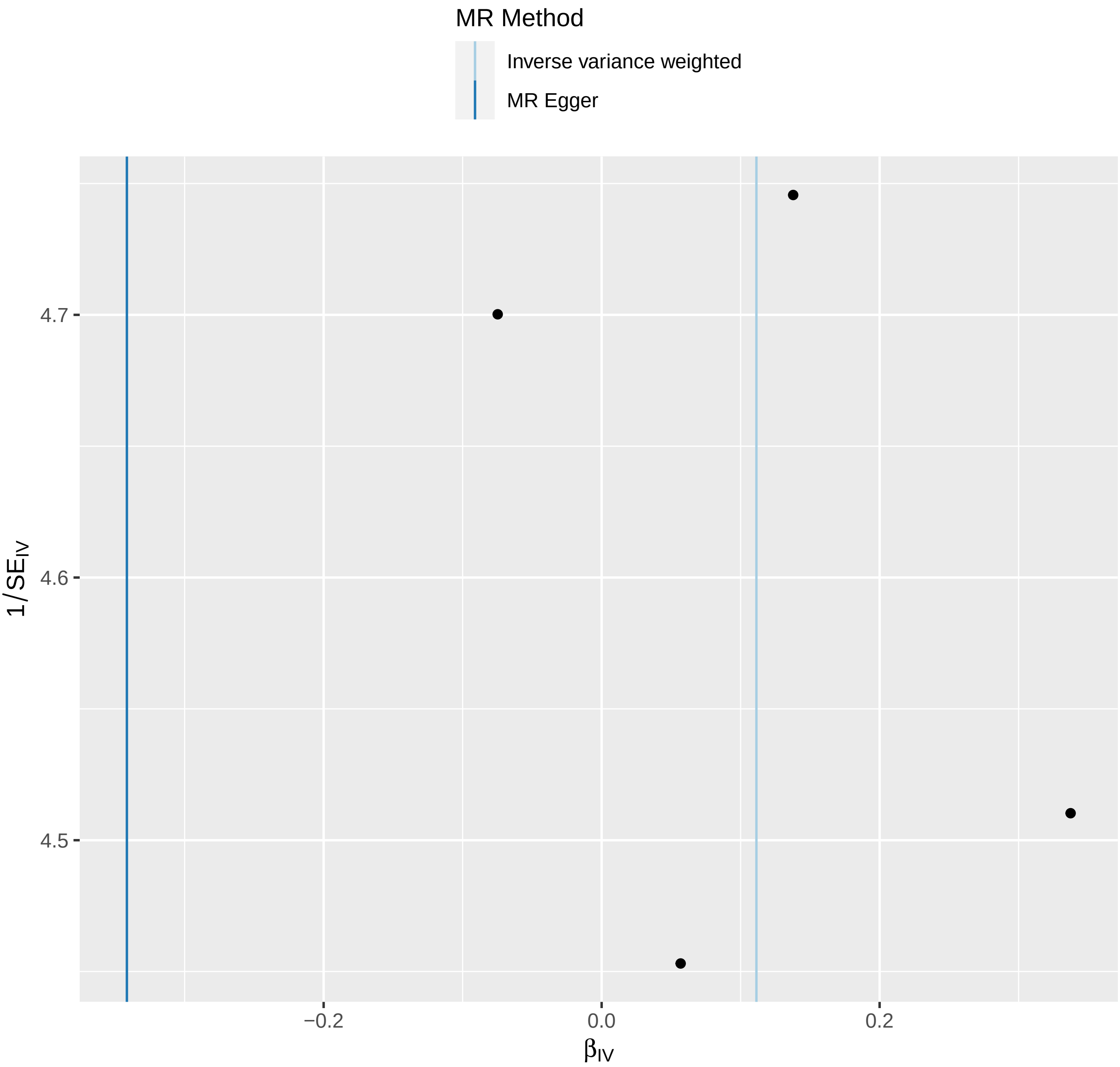

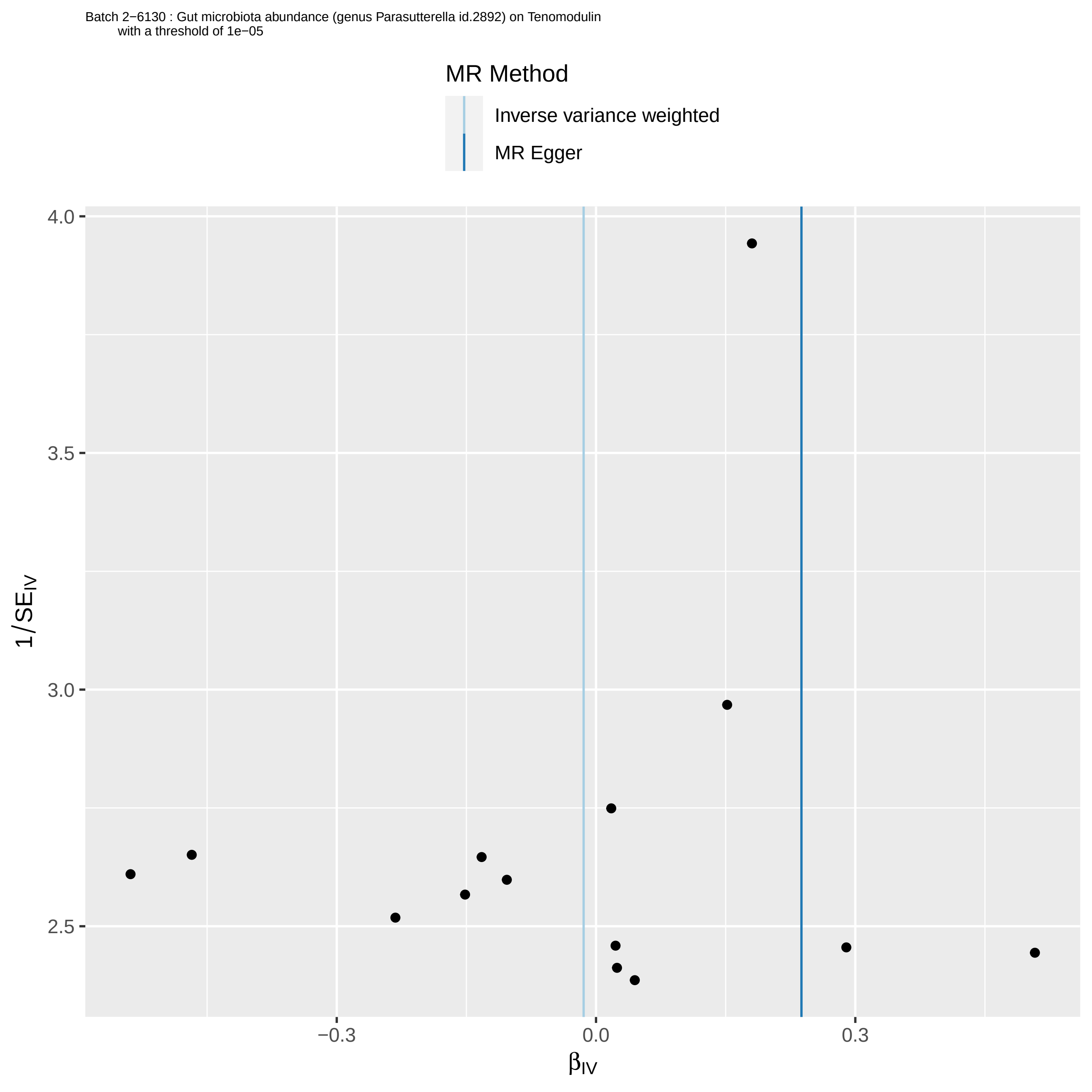
Batch 2−6130 : Gut microbiota abundance (genus Parasutterella id.2892) on Tenomodulin
with a threshold of 1e−05
MR Method
Inverse variance weighted
MR Egger
4.0
3.5
3.0
2.5
1/SE_IV
−0.3
0.0
0.3
β_IV

Batch 2−6131 : Gut microbiota abundance (genus Catenibacterium id.2153) on Troponin I, cardiac muscle
with a threshold of 1e−05

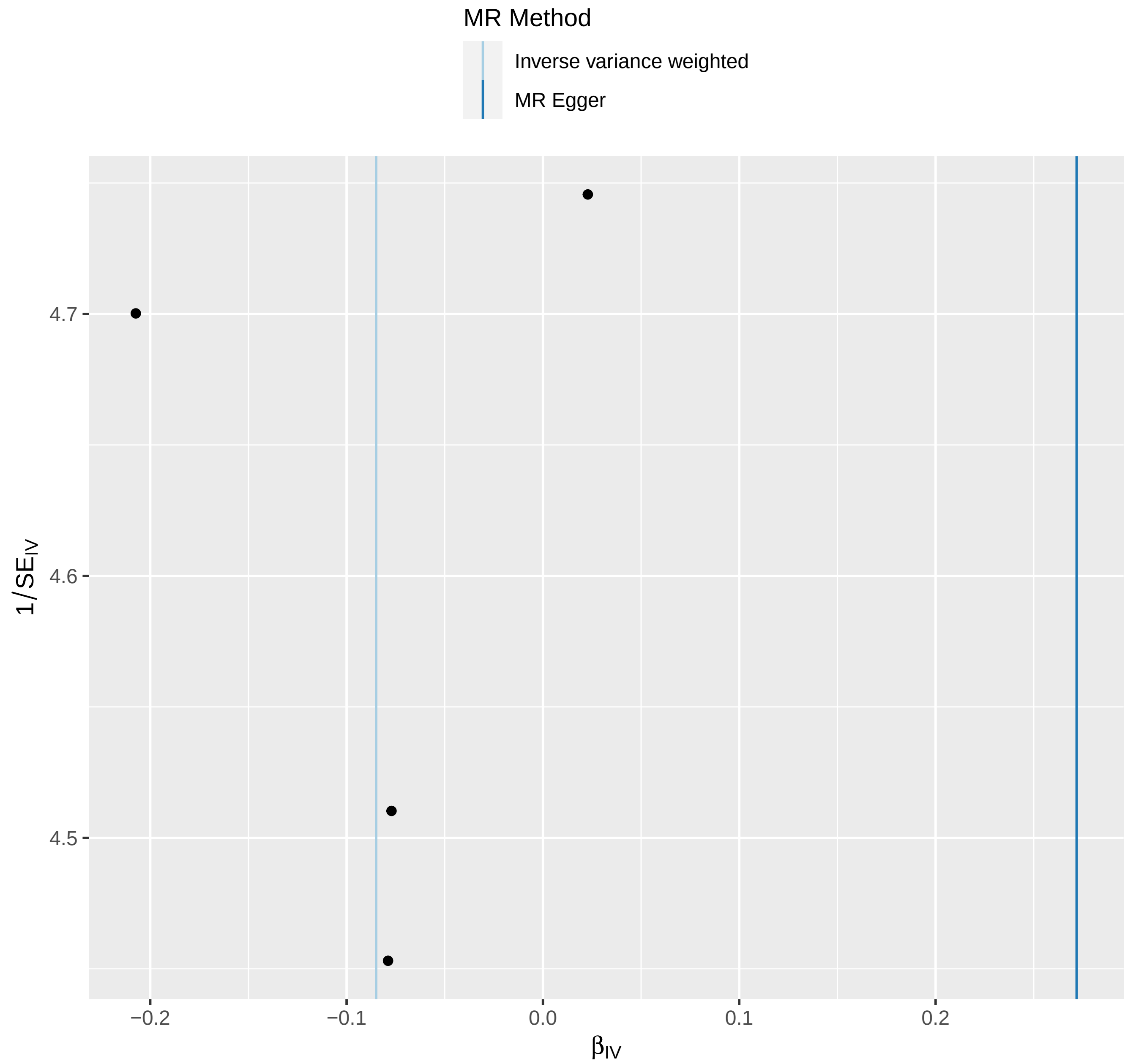

Batch 2−6132 : Gut microbiota abundance (genus Parasutterella id.2892) on Troponin I, cardiac muscle
with a threshold of 1e−05

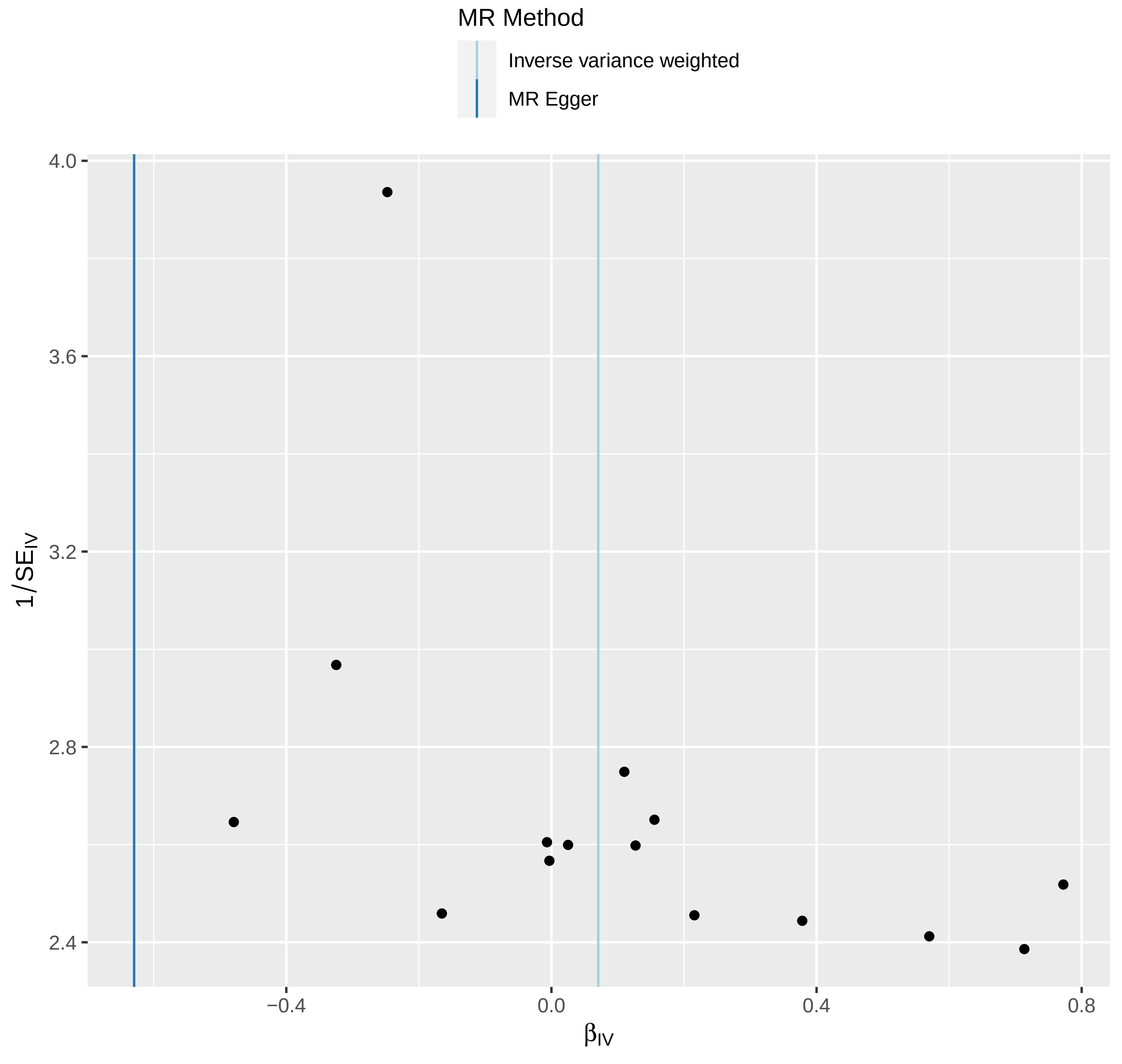

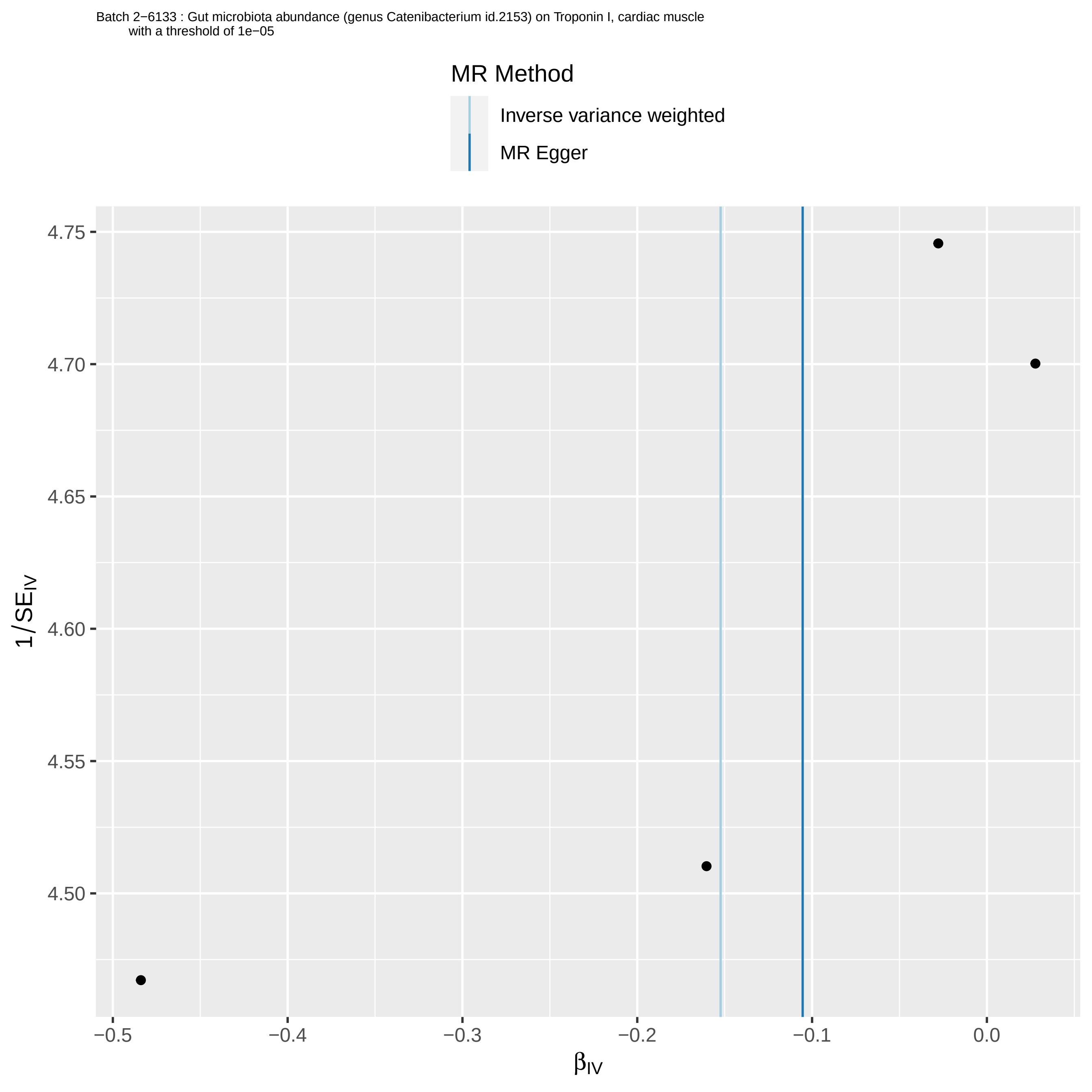
Batch 2−6133 : Gut microbiota abundance (genus Catenibacterium id.2153) on Troponin I, cardiac muscle
with a threshold of 1e−05
MR Method
Inverse variance weighted
MR Egger
4.75
4.70
4.65
4.60
4.55
4.50
1/SE_IV
−0.5
−0.4
−0.3
−0.2
−0.1
0.0
β_IV

Batch 2−6134 : Gut microbiota abundance (genus Parasutterella id.2892) on Troponin I, cardiac muscle
with a threshold of 1e−05

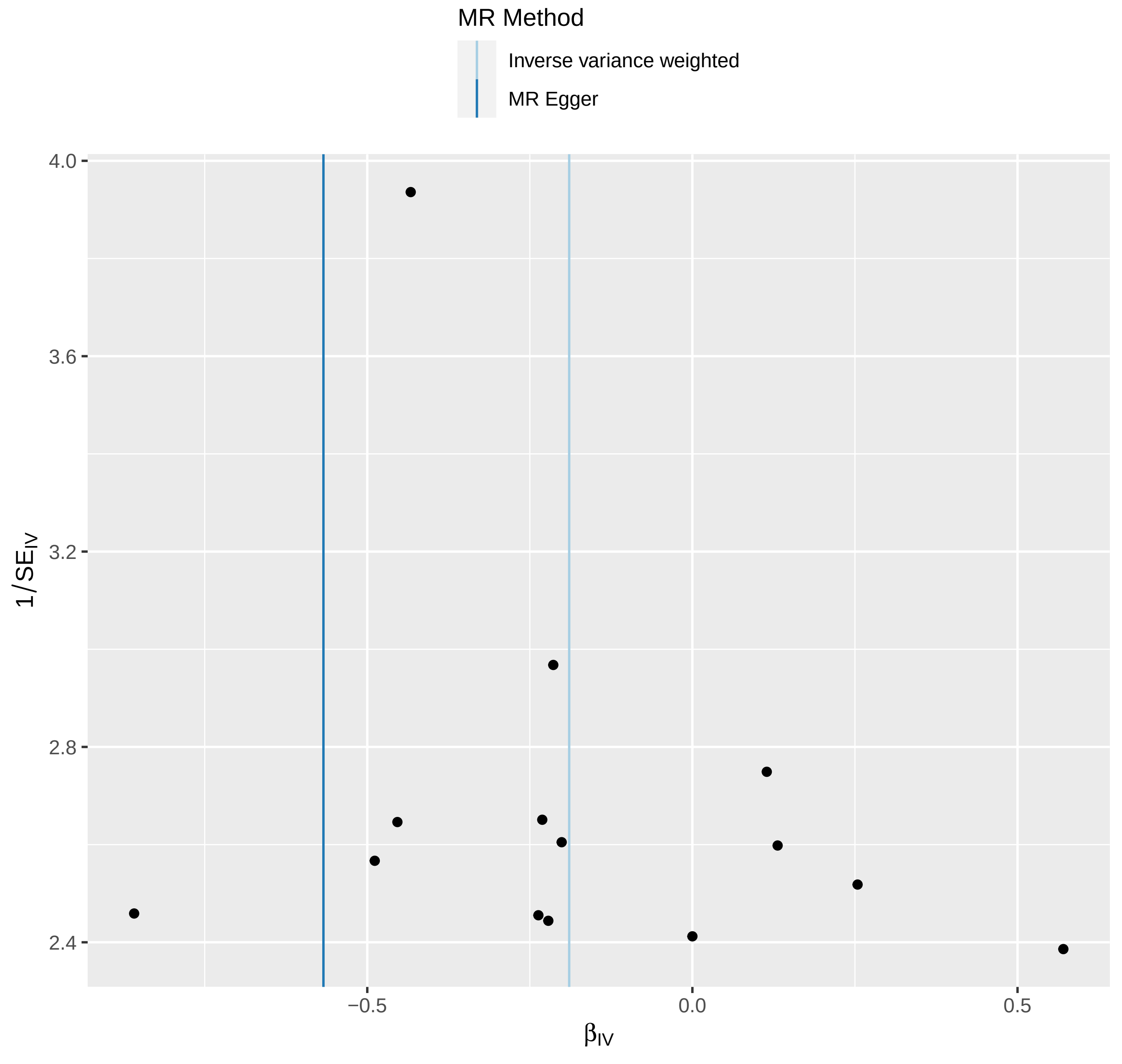

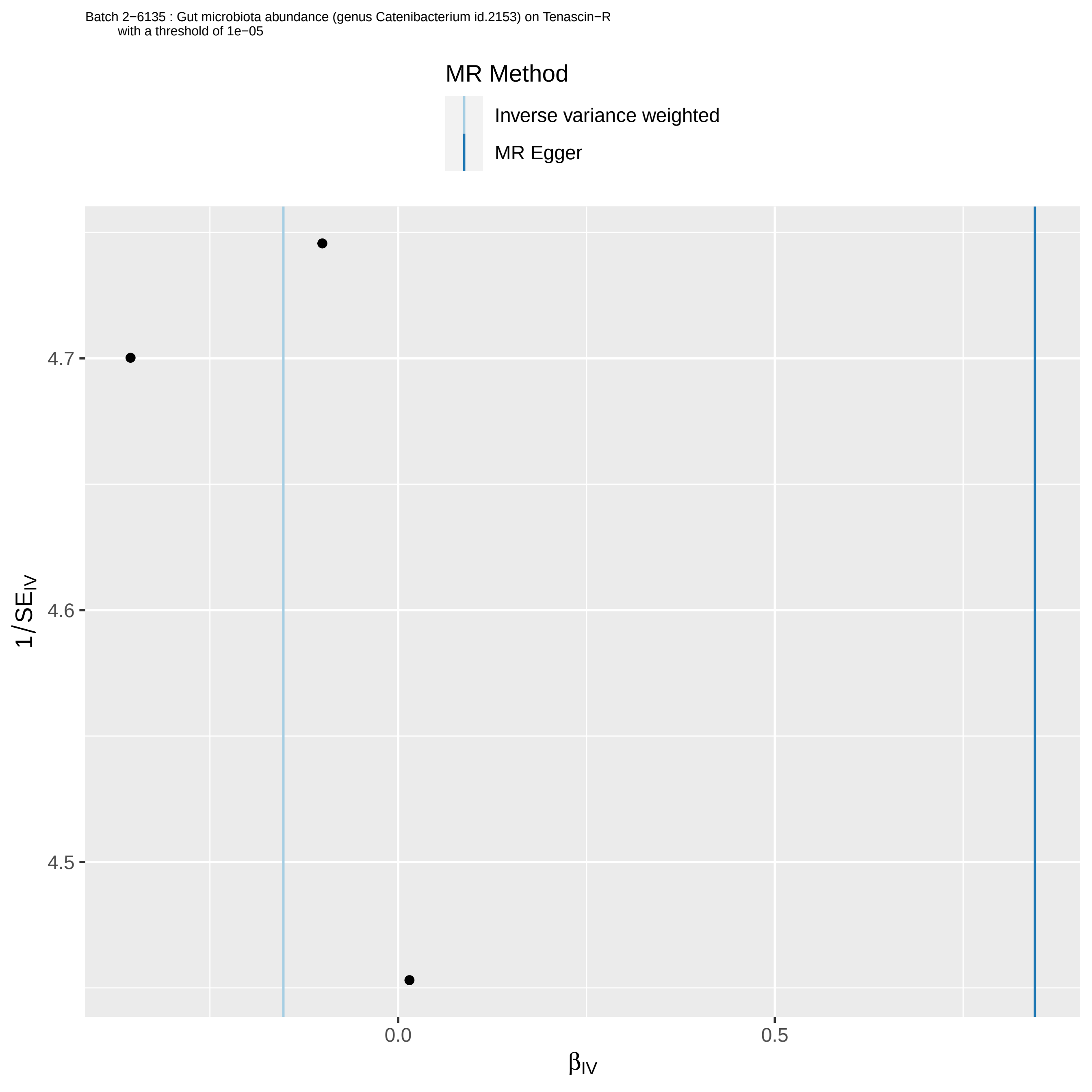
Batch 2−6135 : Gut microbiota abundance (genus Catenibacterium id.2153) on Tenascin−R
with a threshold of 1e−05
MR Method
Inverse variance weighted
MR Egger
4.7
4.6
4.5
1/SE_IV
0.0
0.5
β_IV

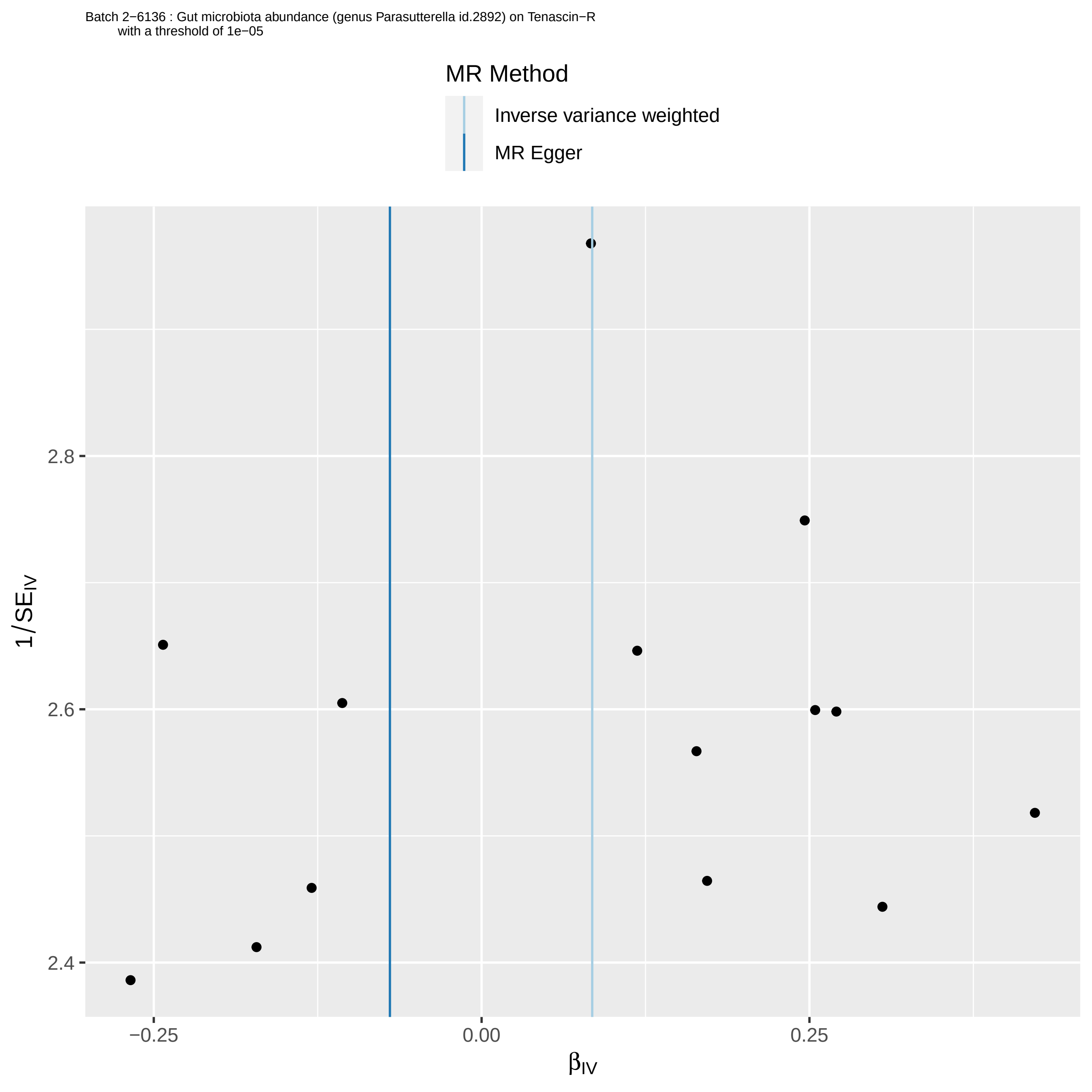
Batch 2−6136 : Gut microbiota abundance (genus Parasutterella id.2892) on Tenascin−R
with a threshold of 1e−05
MR Method
Inverse variance weighted
MR Egger
2.8
2.6
2.4
1/SE_IV
−0.25
0.00
0.25
β_IV

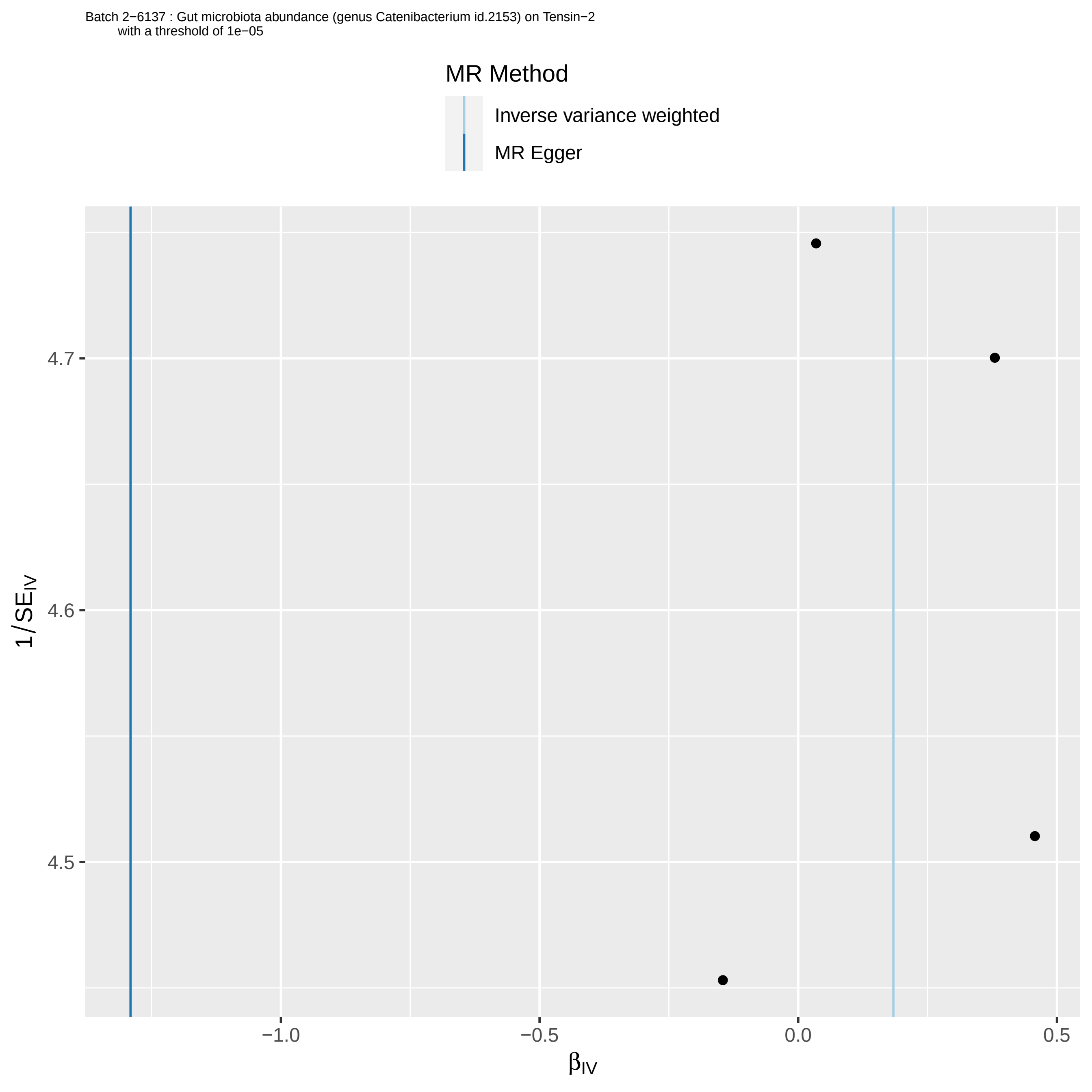

Batch 2−6137 : Gut microbiota abundance (genus Catenibacterium id.2153) on Tensin−2
with a threshold of 1e−05
MR Method
Inverse variance weighted
MR Egger
4.7
4.6
4.5
1/SE_IV
−1.0
−0.5
0.0
0.5
β_IV

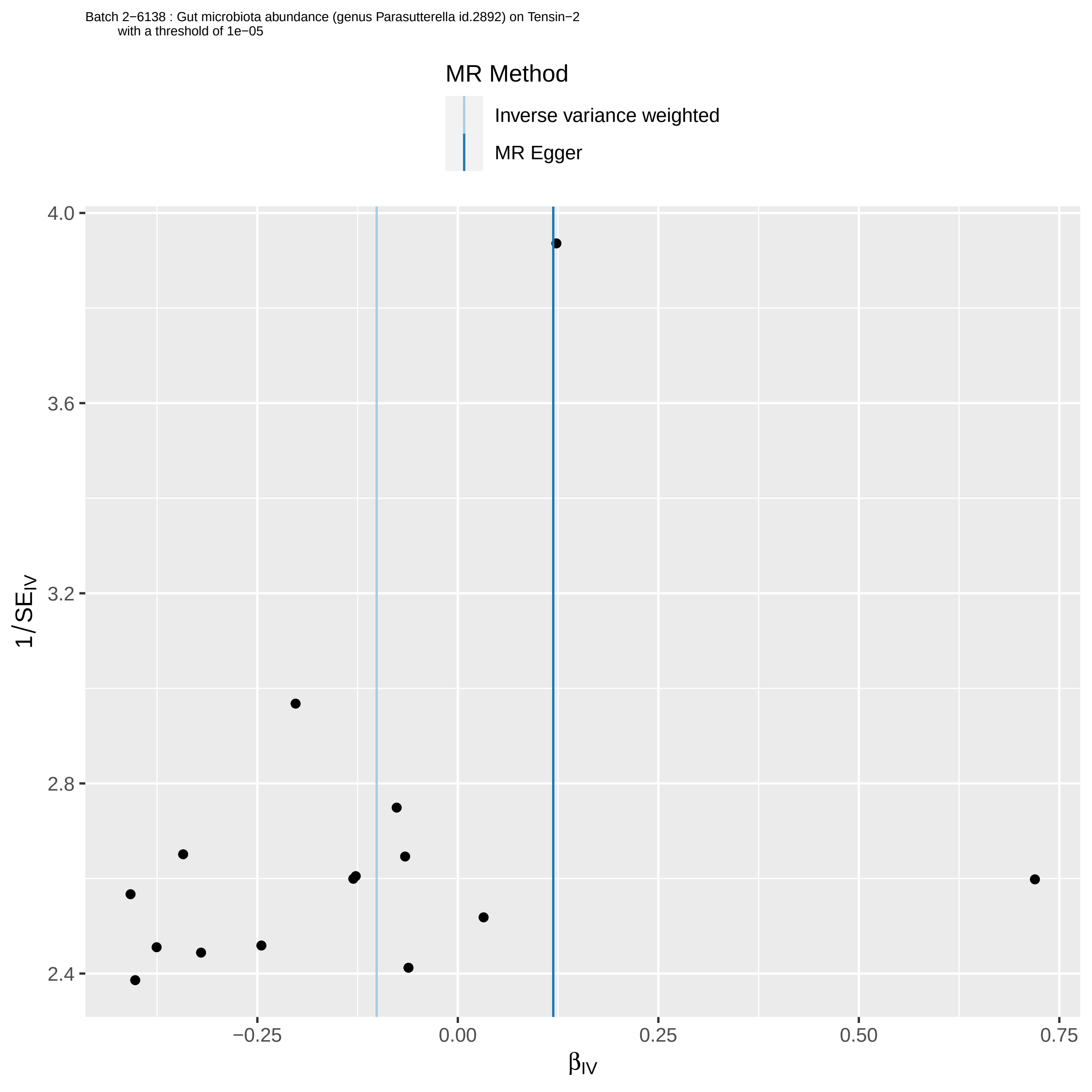
Batch 2−6138 : Gut microbiota abundance (genus Parasutterella id.2892) on Tensin−2
with a threshold of 1e−05
MR Method
Inverse variance weighted
MR Egger
4.0
3.6
3.2
2.8
2.4
1/SE_IV
−0.25
0.00
0.25
0.50
0.75
β_IV

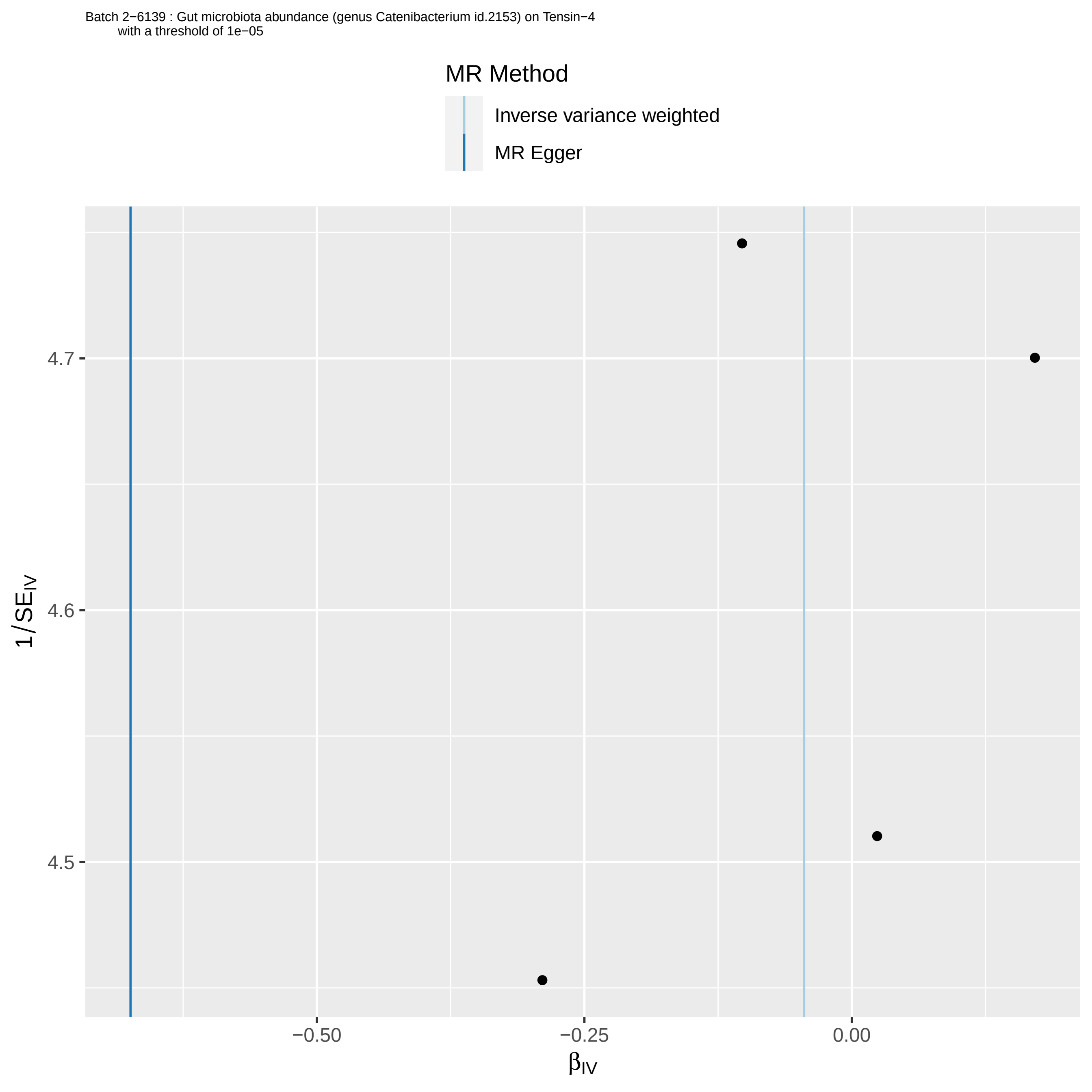
Batch 2−6139 : Gut microbiota abundance (genus Catenibacterium id.2153) on Tensin−4
with a threshold of 1e−05
MR Method
Inverse variance weighted
MR Egger
4.7
4.6
4.5
1/SE_IV
−0.50
−0.25
0.00
β_IV

Batch 2−6140 : Gut microbiota abundance (genus Parasutterella id.2892) on Tensin−4
with a threshold of 1e−05

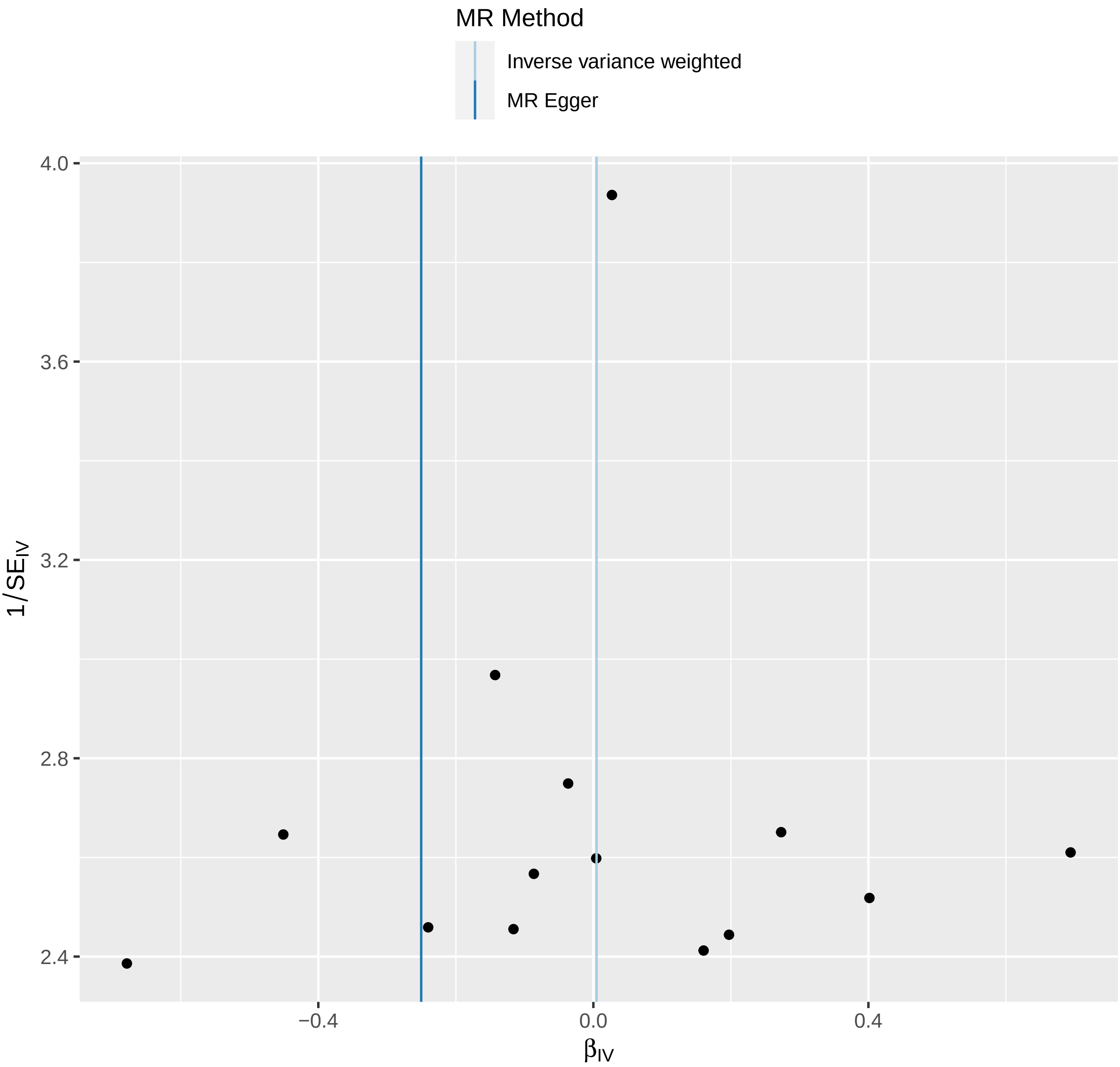

Batch 2−6141 : Gut microbiota abundance (genus Catenibacterium id.2153) on Tenascin−X
with a threshold of 1e−05

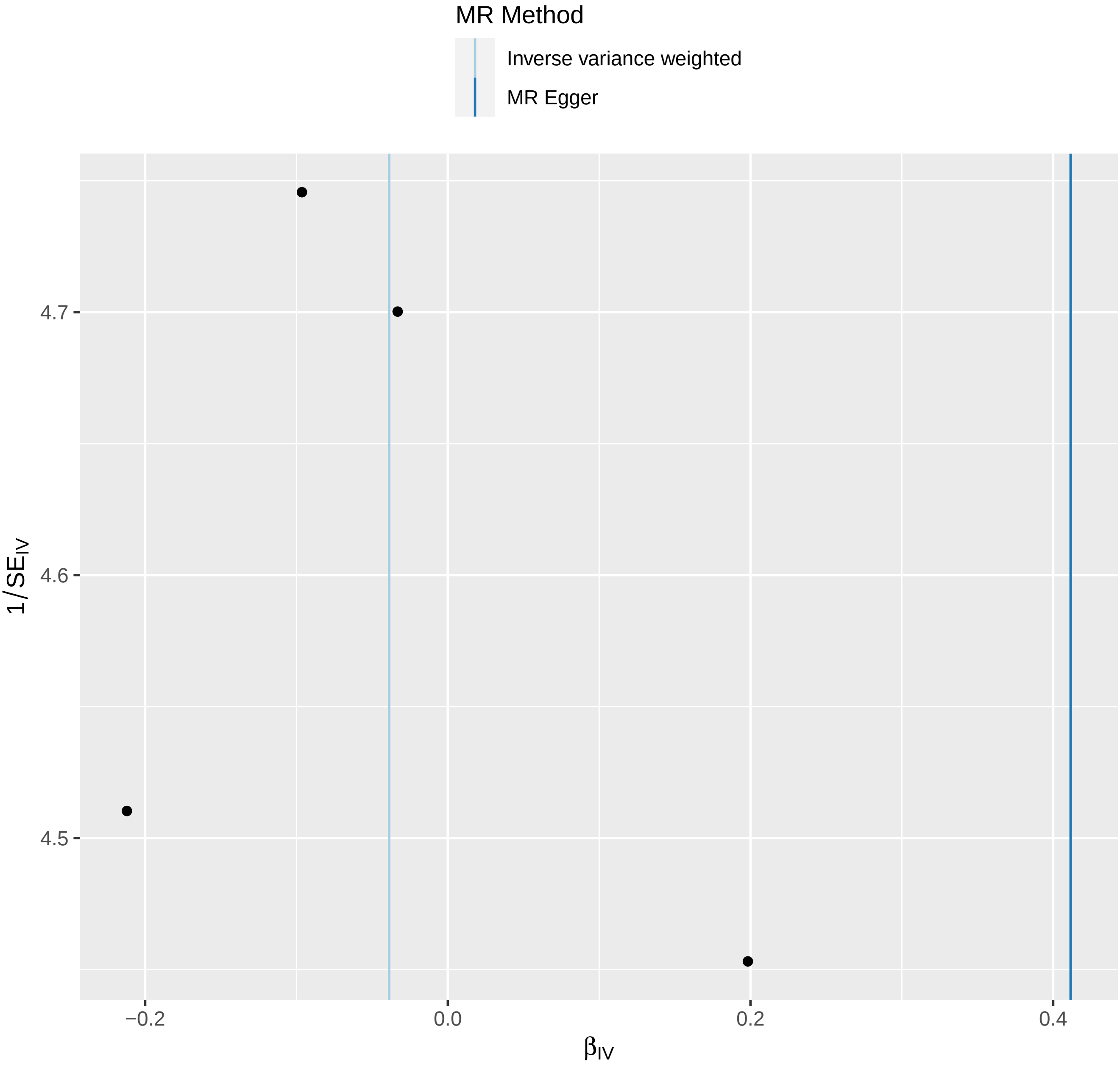

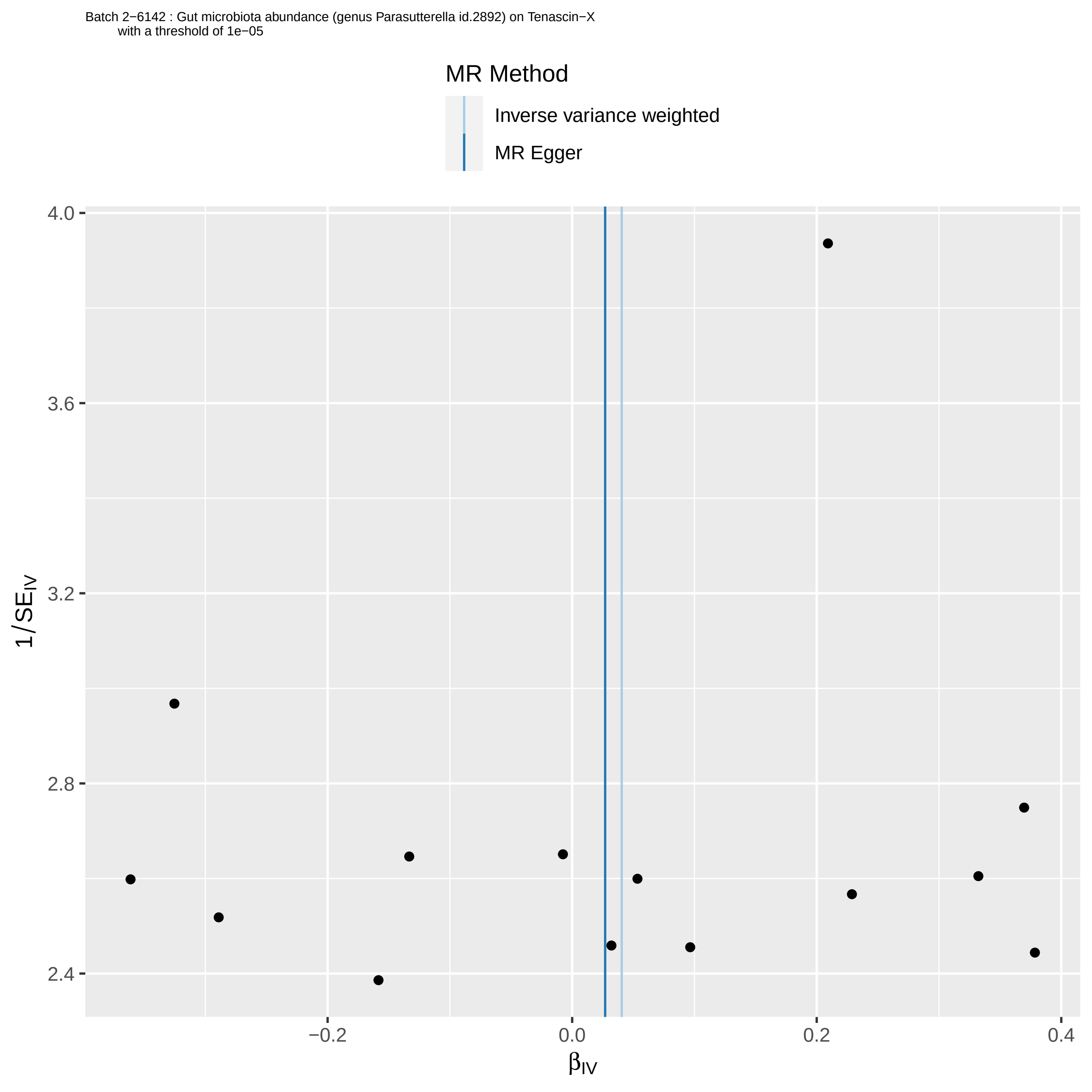
Batch 2−6142 : Gut microbiota abundance (genus Parasutterella id.2892) on Tenascin−X
with a threshold of 1e−05
MR Method
Inverse variance weighted
MR Egger
4.0
3.6
3.2
2.8
2.4
1/SE_IV
−0.2
0.0
0.2
0.4
β_IV

Batch 2−6143 : Gut microbiota abundance (genus Catenibacterium id.2153) on Toll−interacting protein
with a threshold of 1e−05

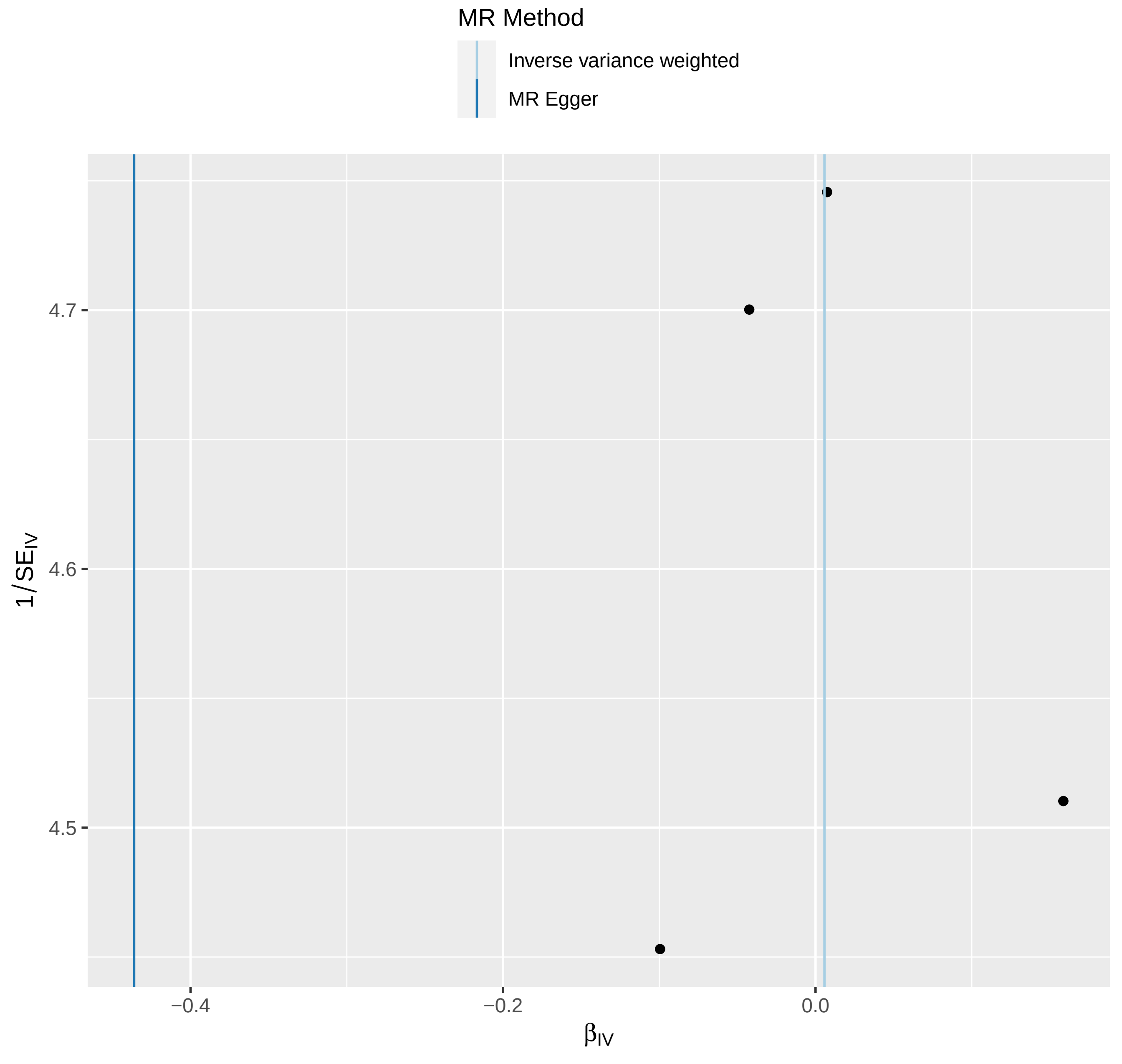

Batch 2−6144 : Gut microbiota abundance (genus Parasutterella id.2892) on Toll−interacting protein
with a threshold of 1e−05

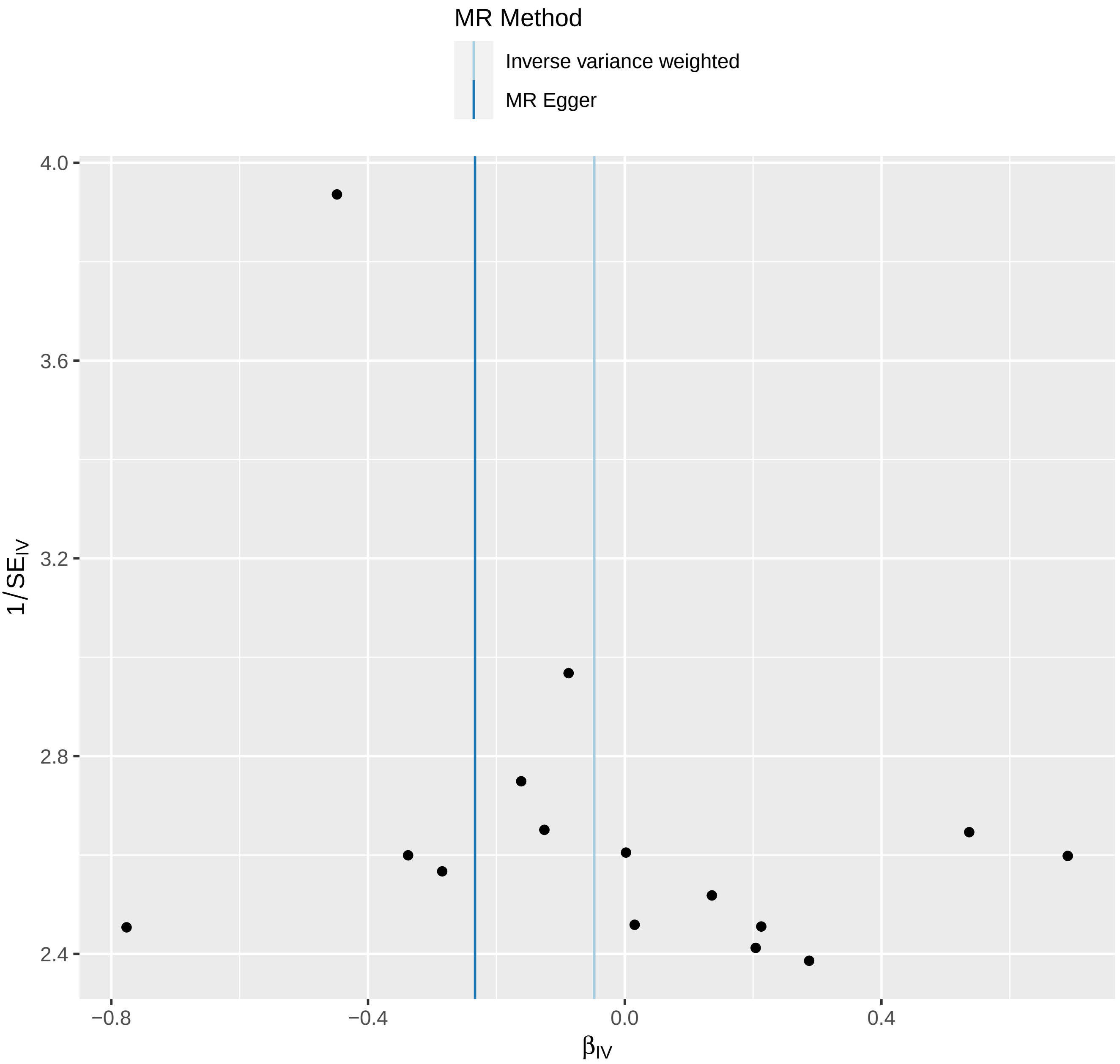

Batch 2−6145 : Gut microbiota abundance (genus Catenibacterium id.2153) on TOM1−like protein 1
with a threshold of 1e−05

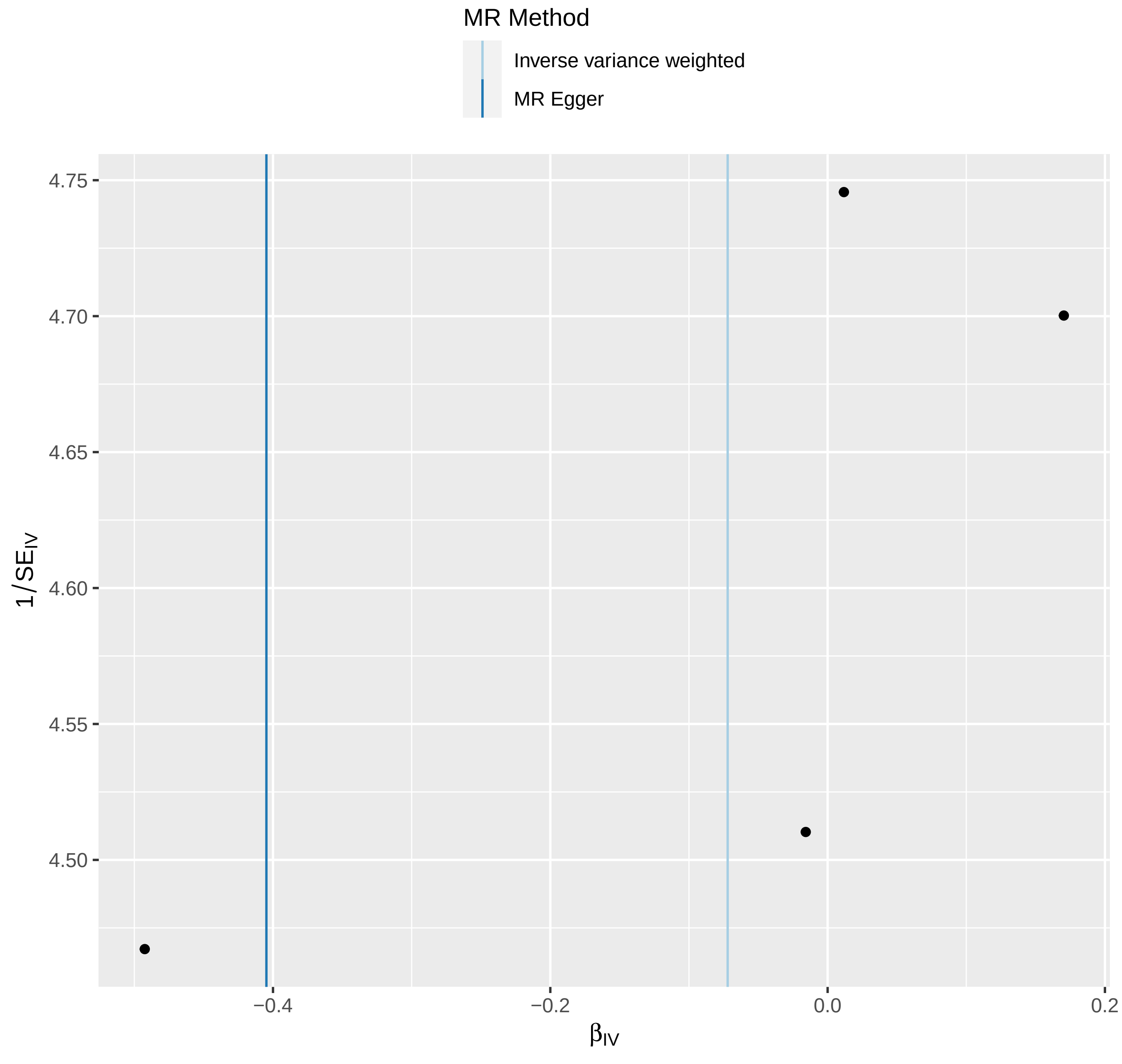

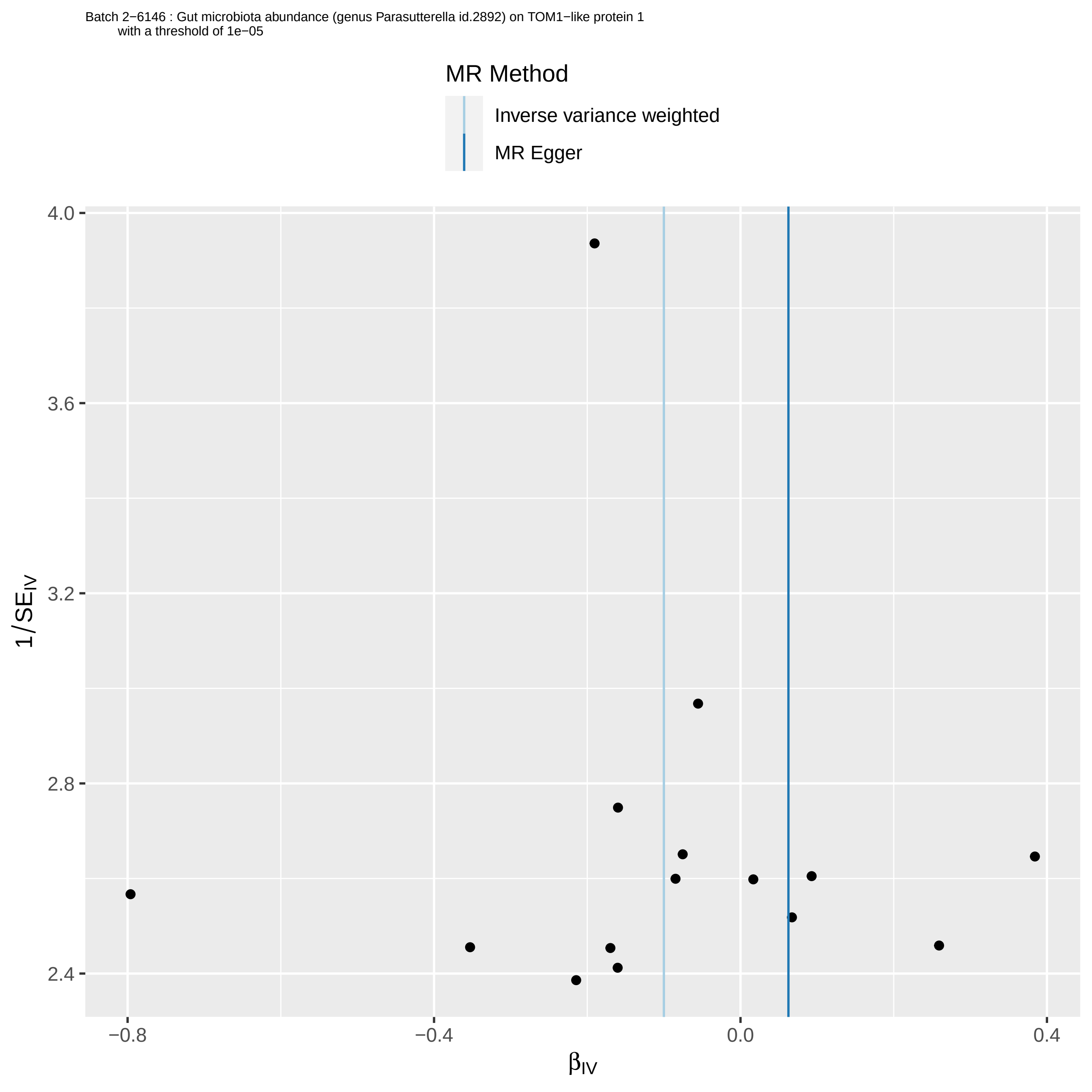
Batch 2−6146 : Gut microbiota abundance (genus Parasutterella id.2892) on TOM1−like protein 1
with a threshold of 1e−05
MR Method
Inverse variance weighted
MR Egger
4.0
3.6
3.2
2.8
2.4
1/SE_IV
−0.8
−0.4
0.0
0.4
β_IV

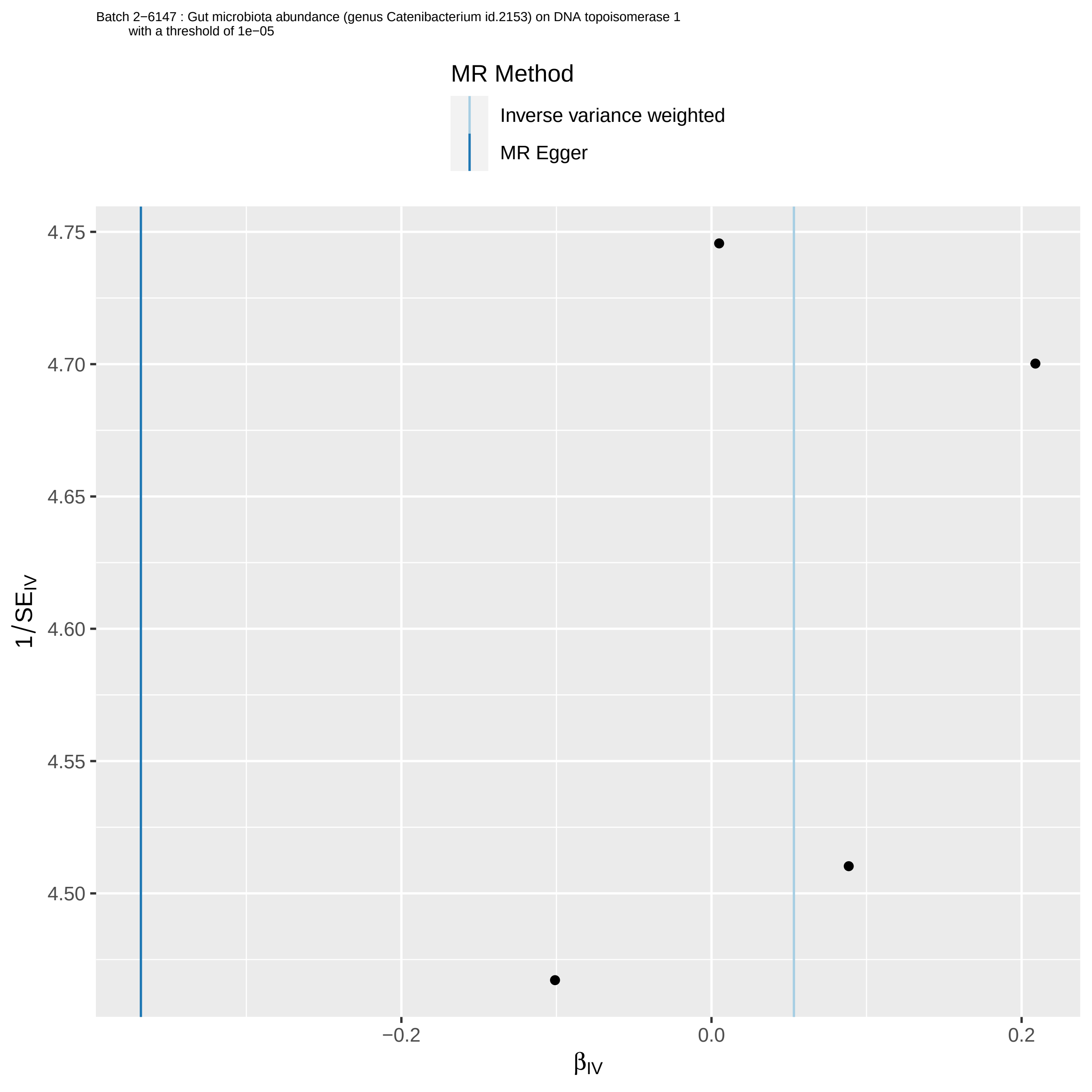
Batch 2−6147 : Gut microbiota abundance (genus Catenibacterium id.2153) on DNA topoisomerase 1
with a threshold of 1e−05
MR Method
Inverse variance weighted
MR Egger
4.75
4.70
4.65
4.60
4.55
4.50
−0.2
0.0
0.2
1/SE_IV
β_IV

Batch 2−6148 : Gut microbiota abundance (genus Parasutterella id.2892) on DNA topoisomerase 1
with a threshold of 1e−05

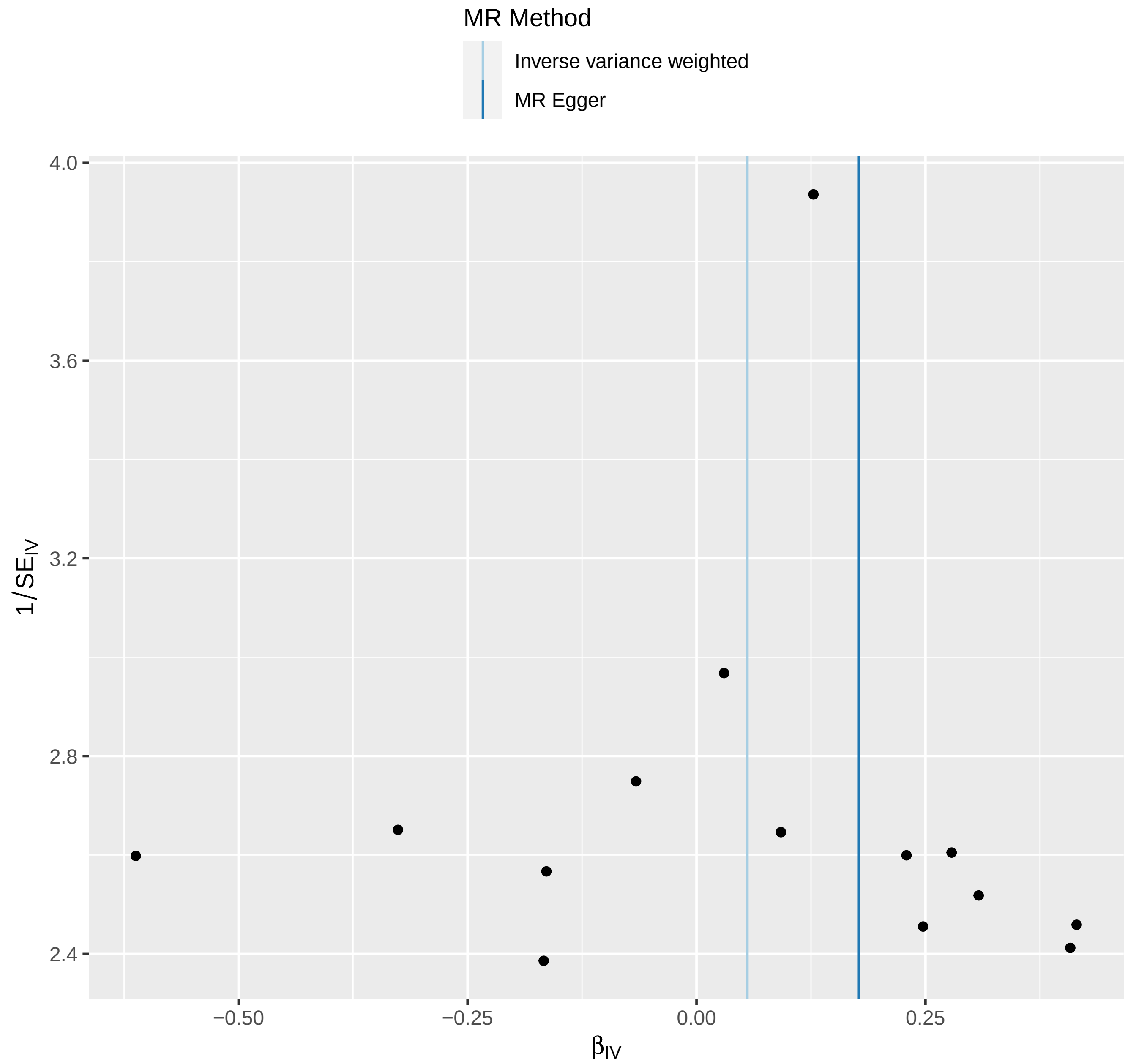

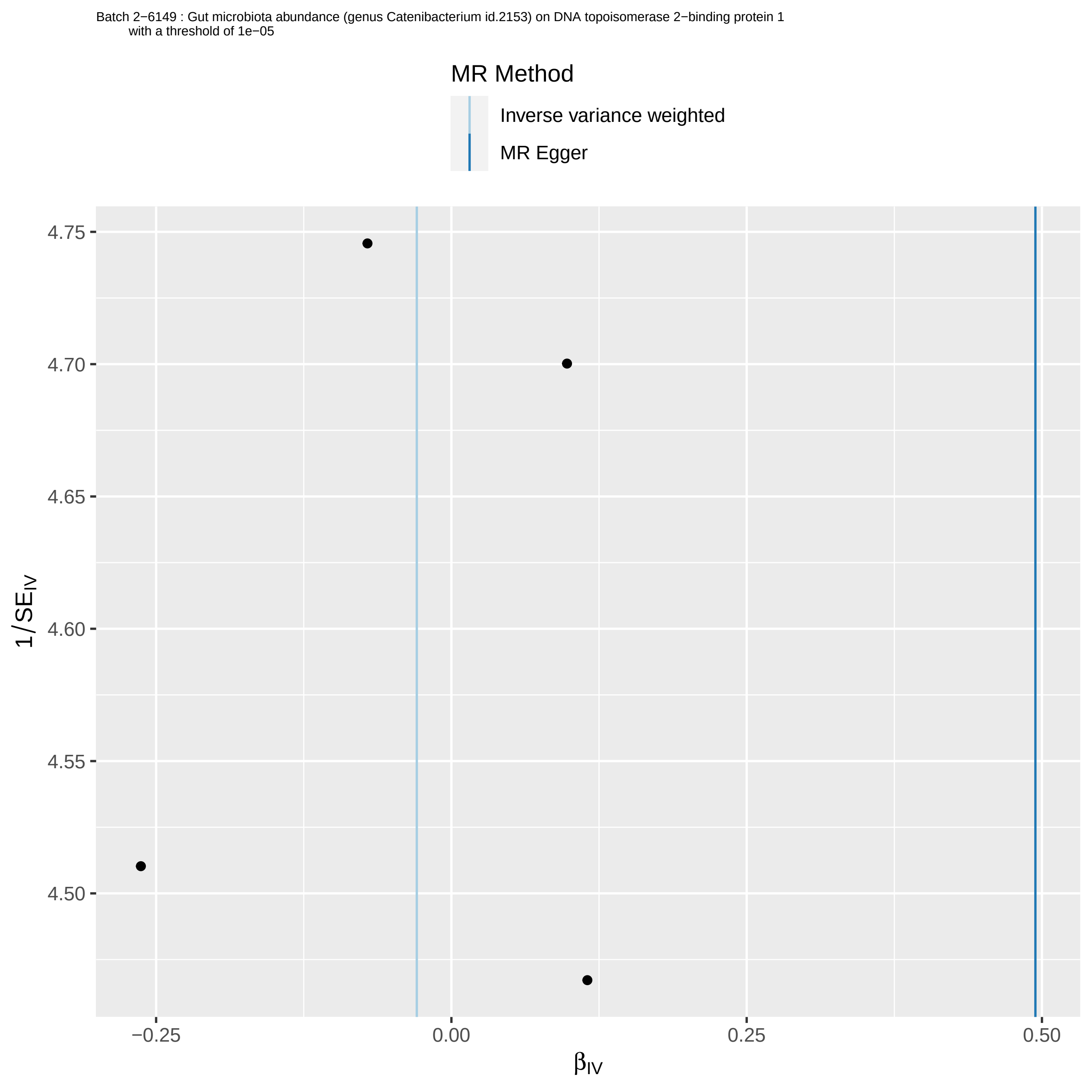

Batch 2−6149 : Gut microbiota abundance (genus Catenibacterium id.2153) on DNA topoisomerase 2−binding protein 1
with a threshold of 1e−05
MR Method
Inverse variance weighted
MR Egger
4.75
4.70
4.65
4.60
4.55
4.50
1/SEIV
−0.25
0.00
0.25
0.50
βIV

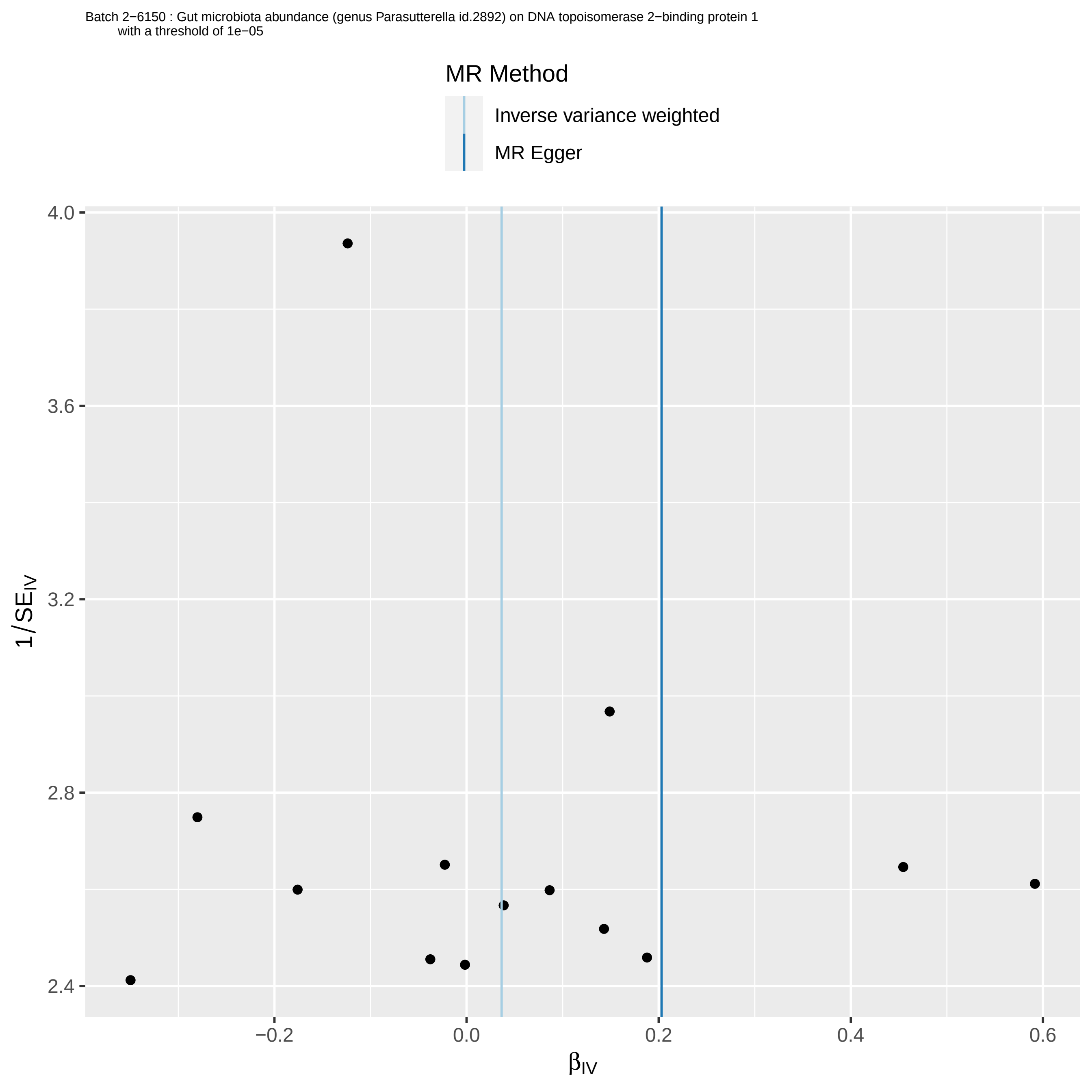

Batch 2−6150 : Gut microbiota abundance (genus Parasutterella id.2892) on DNA topoisomerase 2−binding protein 1
with a threshold of 1e−05
MR Method
Inverse variance weighted
MR Egger
4.0
3.6
3.2
2.8
2.4
1/SE_IV
−0.2
0.0
0.2
0.4
0.6
β_IV

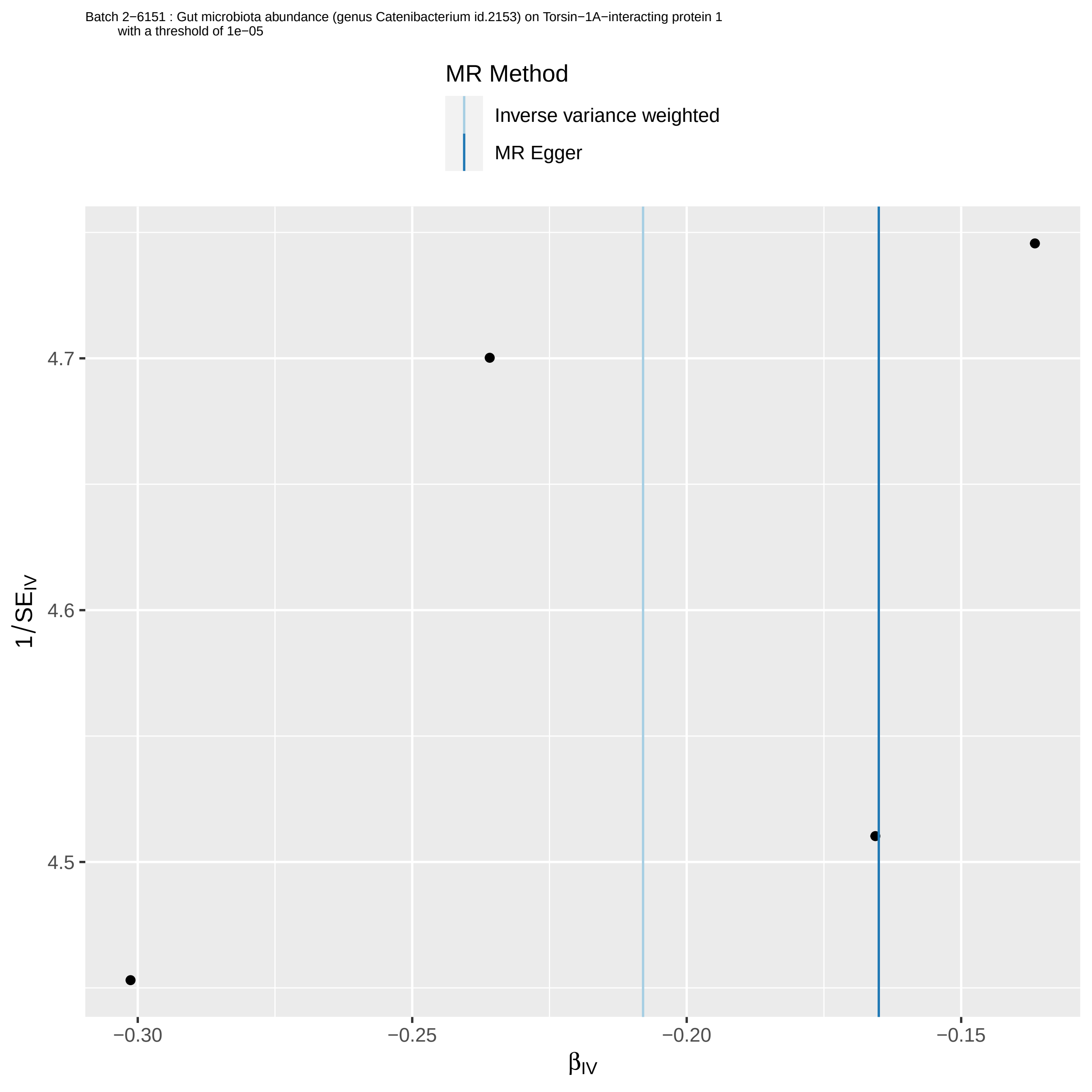
Batch 2−6151 : Gut microbiota abundance (genus Catenibacterium id.2153) on Torsin−1A−interacting protein 1
with a threshold of 1e−05
MR Method
Inverse variance weighted
MR Egger
4.7
4.6
4.5
1/SE_IV
−0.30
−0.25
−0.20
−0.15
β_IV

Batch 2−6152 : Gut microbiota abundance (genus Parasutterella id.2892) on Torsin−1A−interacting protein 1
with a threshold of 1e−05

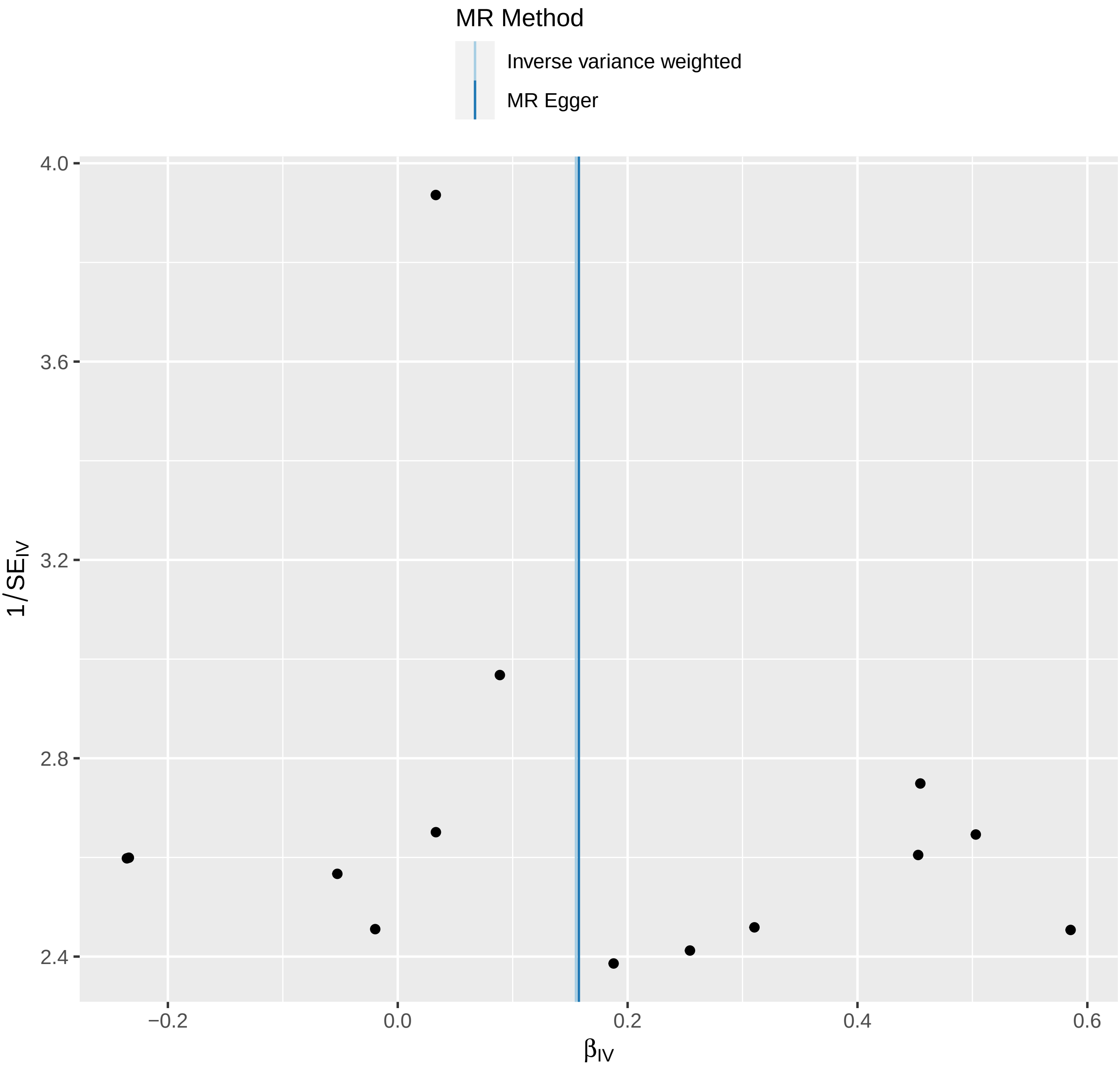

Batch 2−6153 : Gut microbiota abundance (genus Catenibacterium id.2153) on Torsin−1A−interacting protein 2 with a threshold of 1e−05

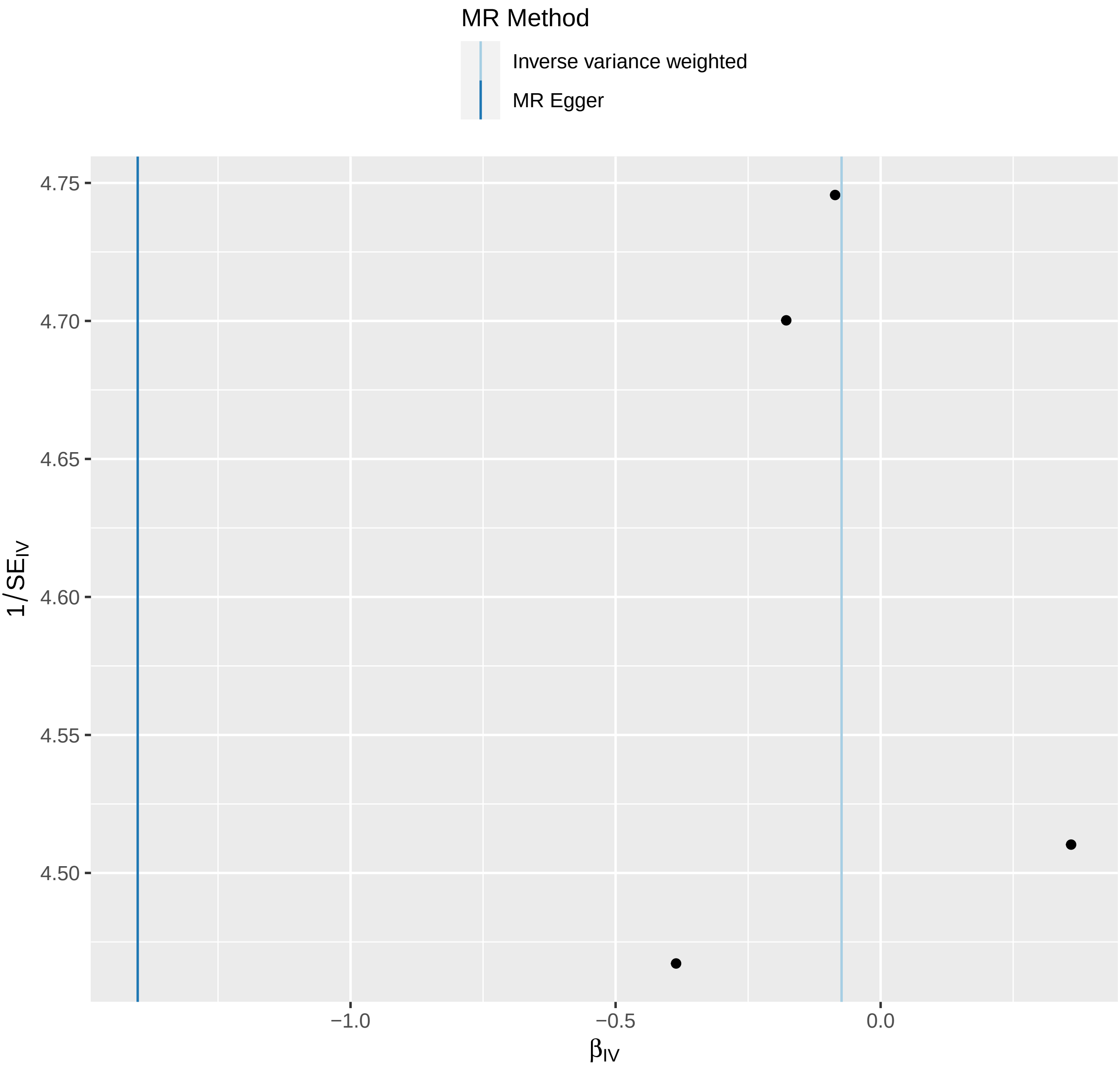

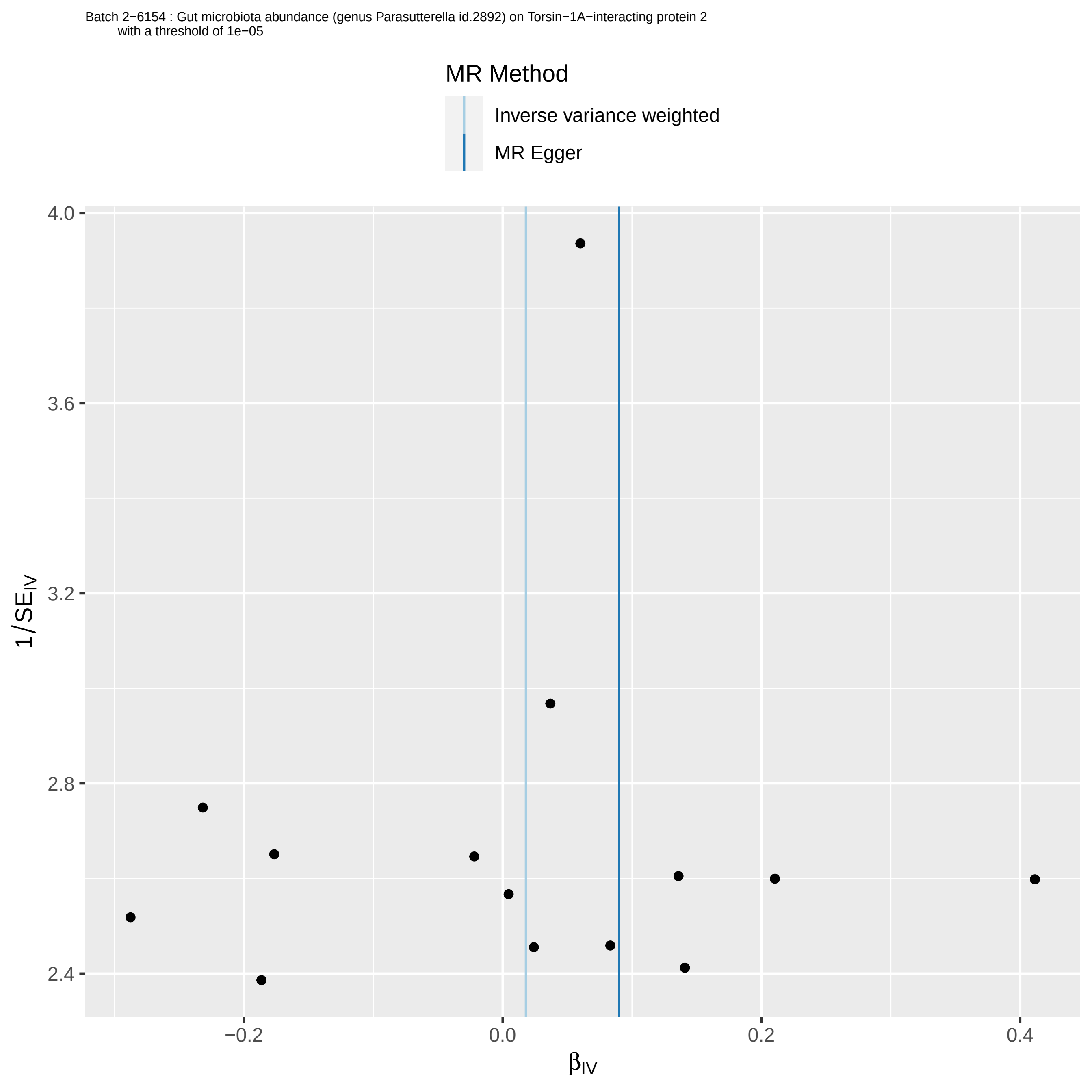
Batch 2−6154 : Gut microbiota abundance (genus Parasutterella id.2892) on Torsin−1A−interacting protein 2
with a threshold of 1e−05
MR Method
Inverse variance weighted
MR Egger
4.0
3.6
3.2
2.8
2.4
1/SE$_{IV}$
−0.2
0.0
0.2
0.4
β$_{IV}$

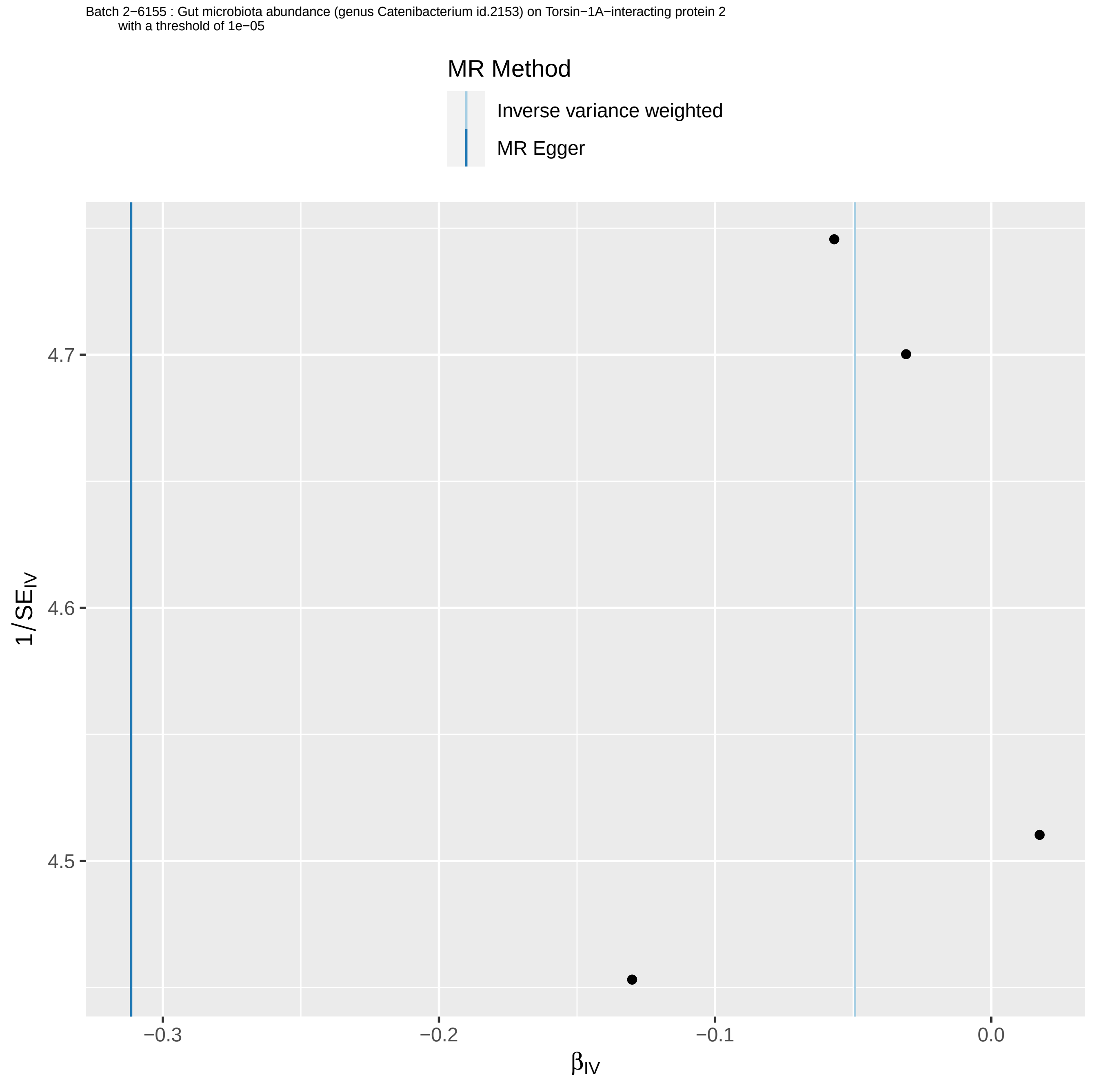

Batch 2−6155 : Gut microbiota abundance (genus Catenibacterium id.2153) on Torsin−1A−interacting protein 2
with a threshold of 1e−05
MR Method
Inverse variance weighted
MR Egger
4.7
4.6
4.5
$1/SE_{IV}$
−0.3
−0.2
−0.1
0.0
β_{IV}

Batch 2−6156 : Gut microbiota abundance (genus Parasutterella id.2892) on Torsin−1A−interacting protein 2
with a threshold of 1e−05

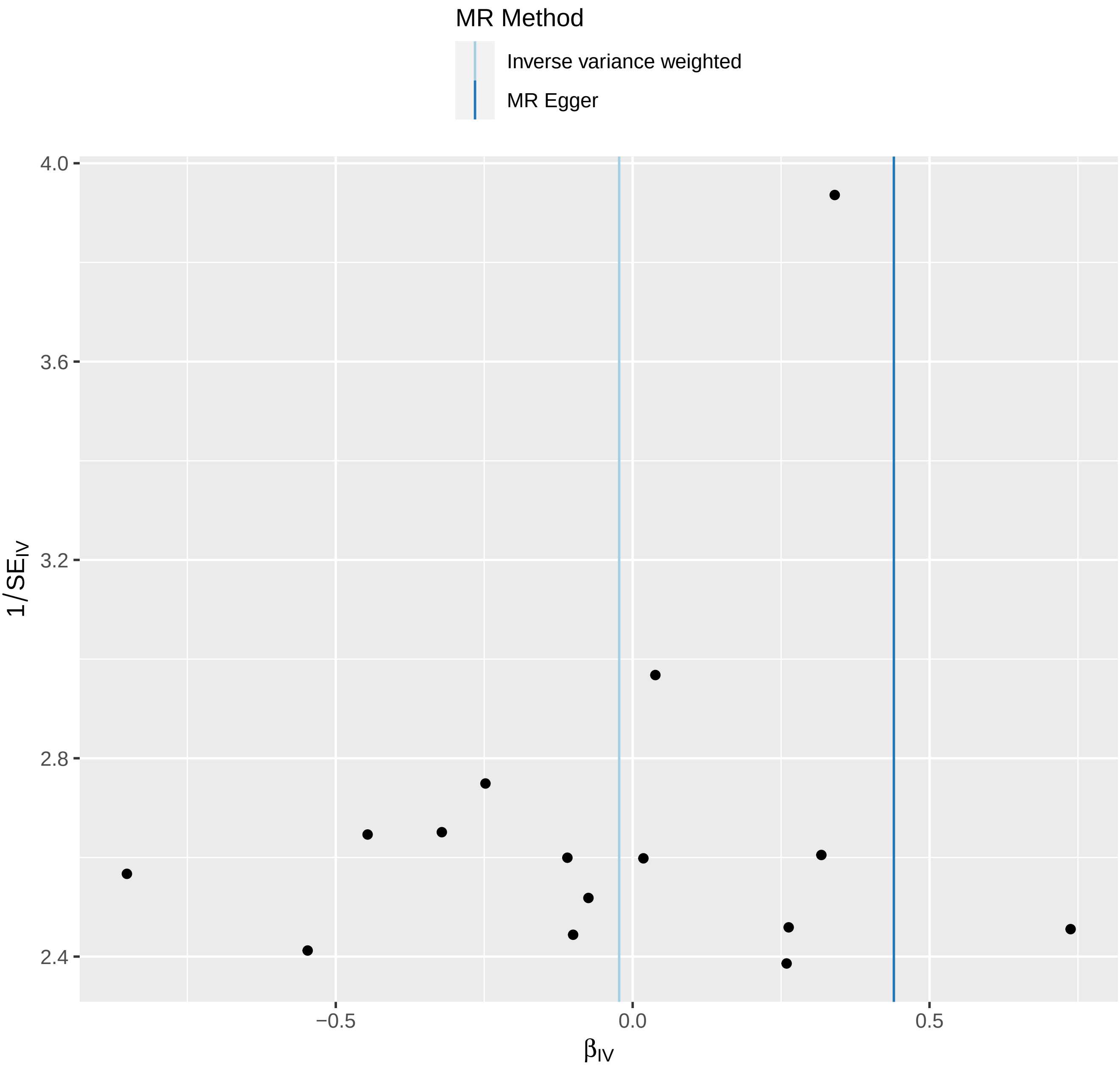

Batch 2−6157 : Gut microbiota abundance (genus Catenibacterium id.2153) on TOX high mobility group box family member 3
with a threshold of 1e−05

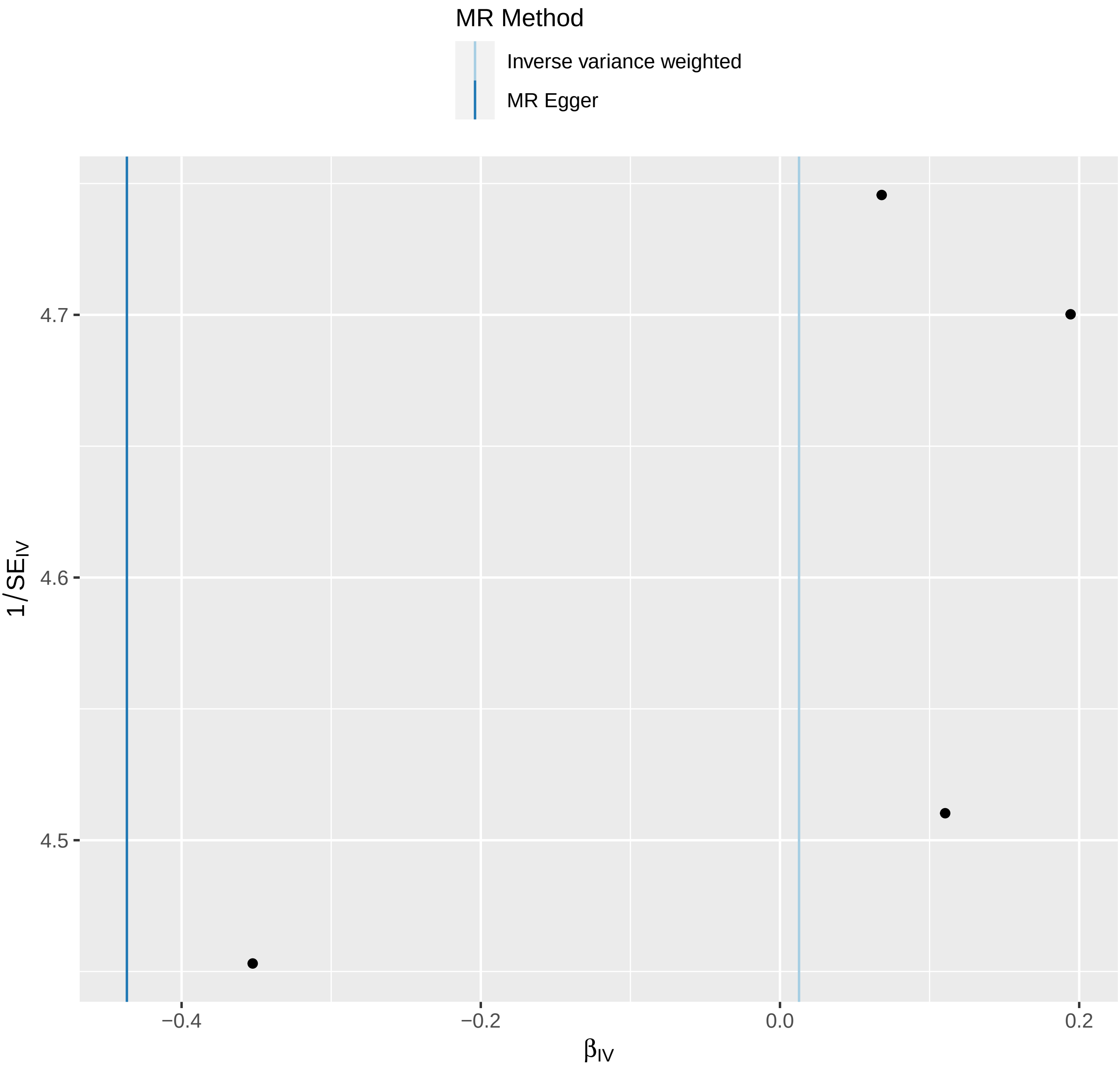

Batch 2−6158 : Gut microbiota abundance (genus Parasutterella id.2892) on TOX high mobility group box family member 3 with a threshold of 1e−05

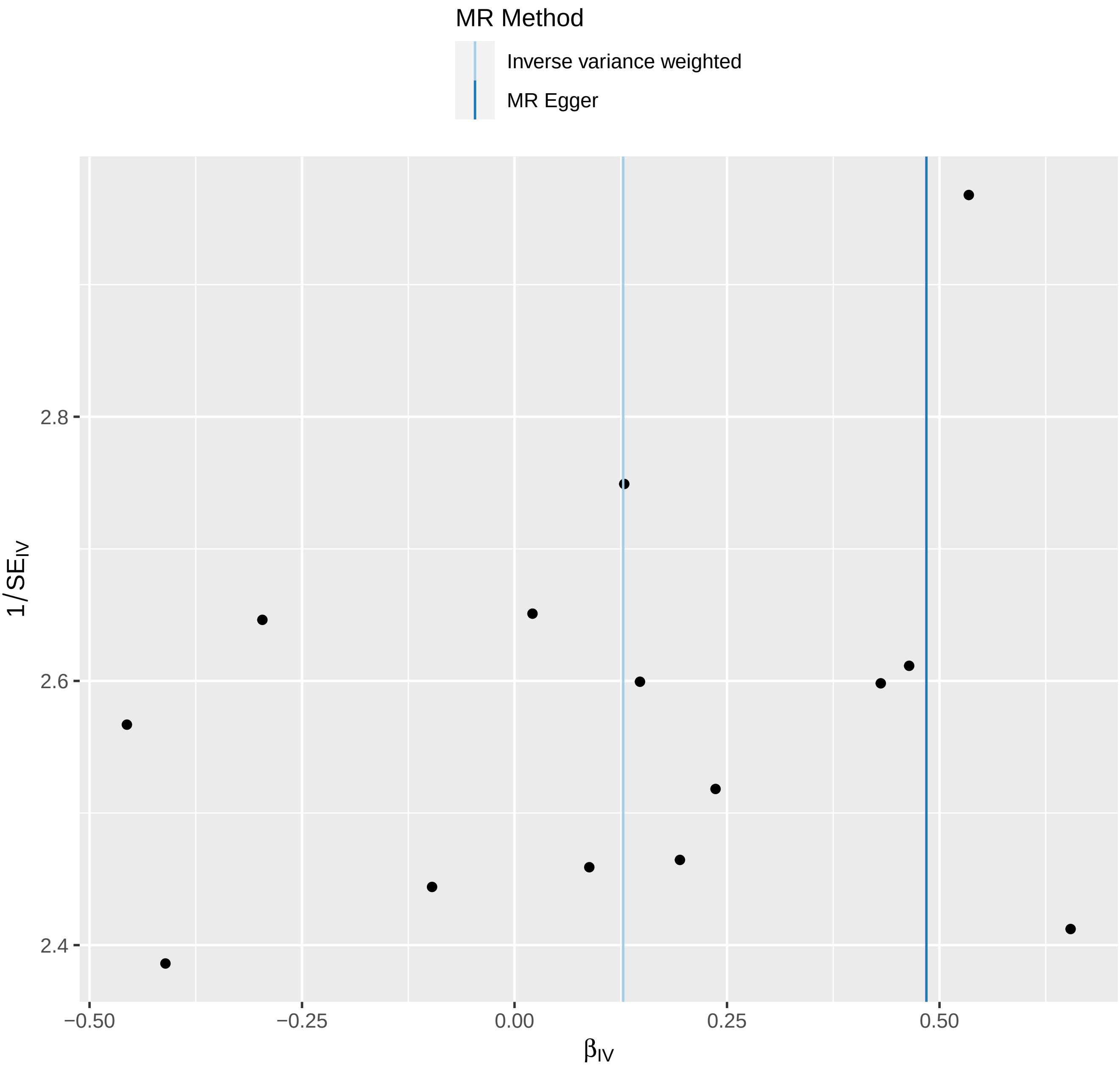

Batch 2−6159 : Gut microbiota abundance (genus Catenibacterium id.2153) on Cellular tumor antigen p53
with a threshold of 1e−05

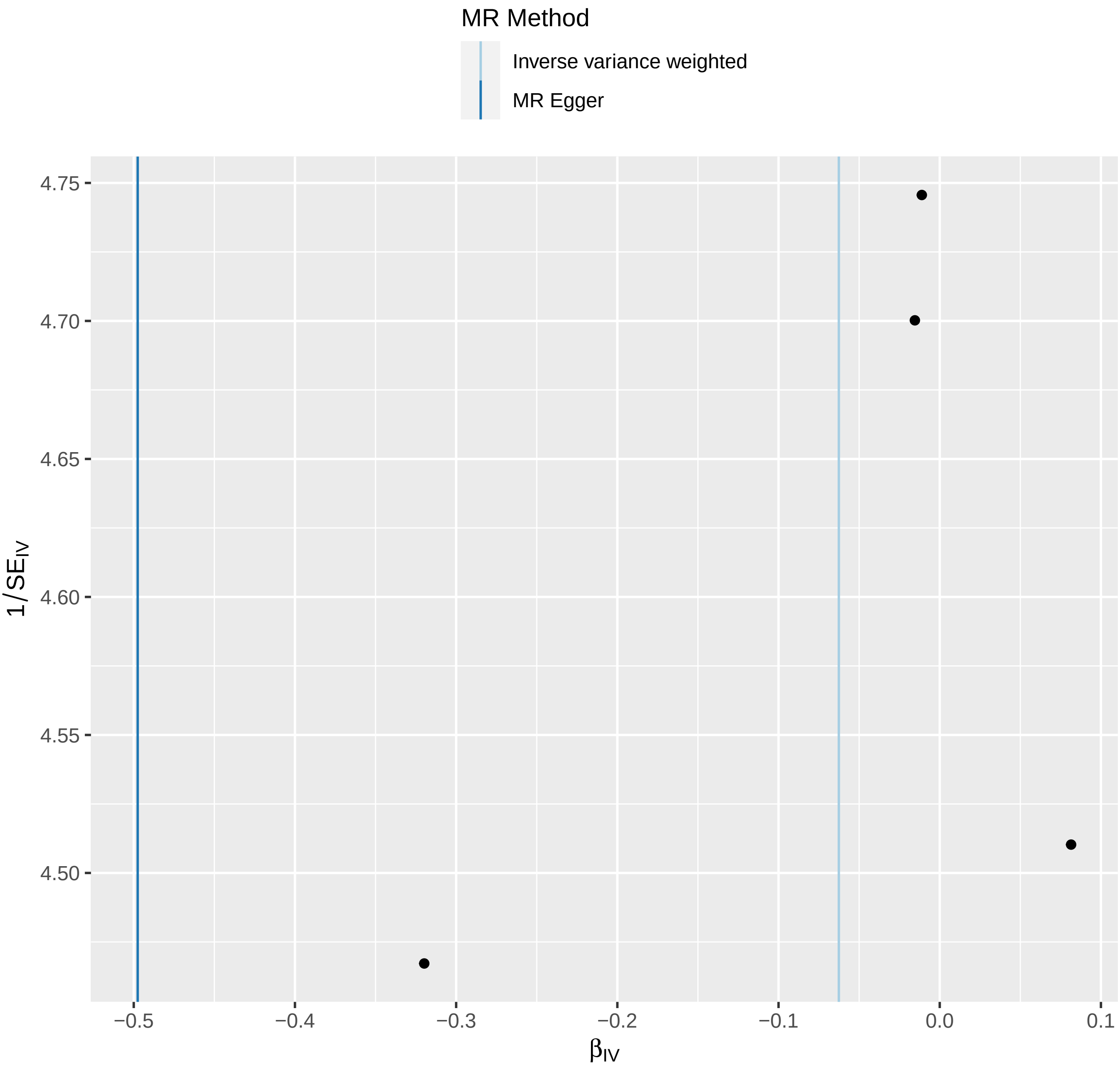

Batch 2−6160 : Gut microbiota abundance (genus Parasutterella id.2892) on Cellular tumor antigen p53
with a threshold of 1e−05

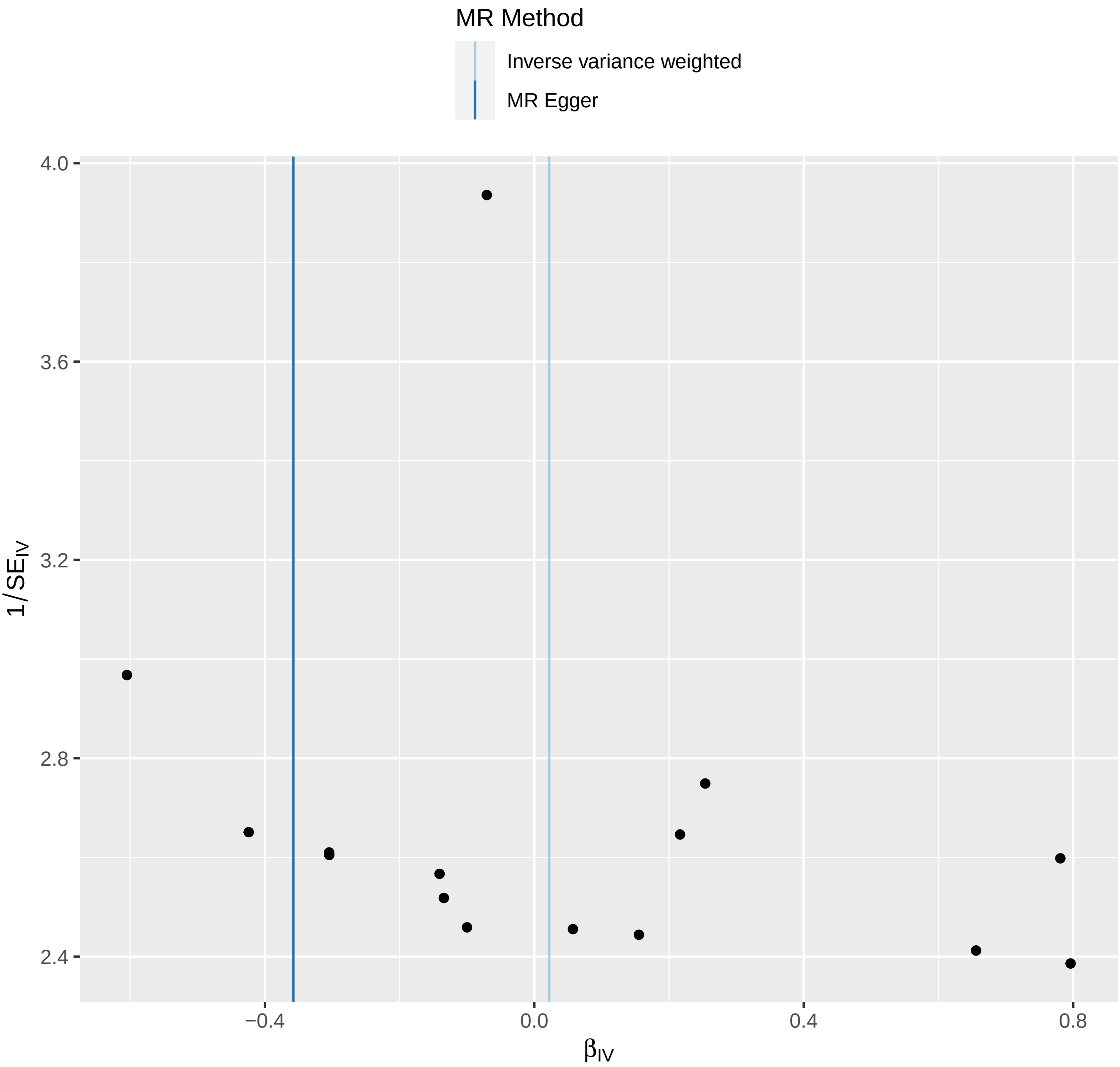

Batch 2−6161 : Gut microbiota abundance (genus Catenibacterium id.2153) on Tumor protein p53−inducible protein 11
with a threshold of 1e−05

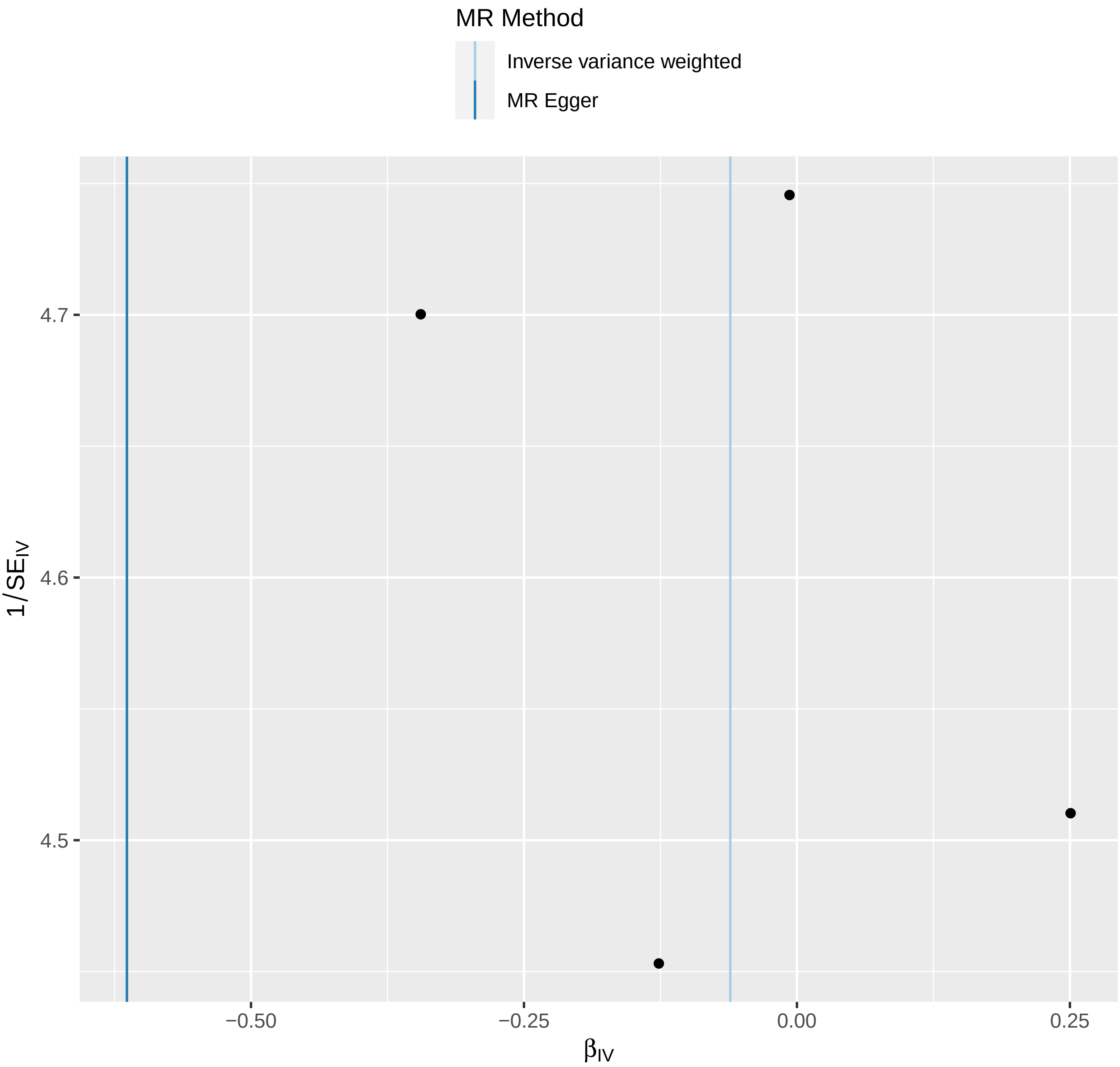

Batch 2−6162 : Gut microbiota abundance (genus Parasutterella id.2892) on Tumor protein p53−inducible protein 11
with a threshold of 1e−05

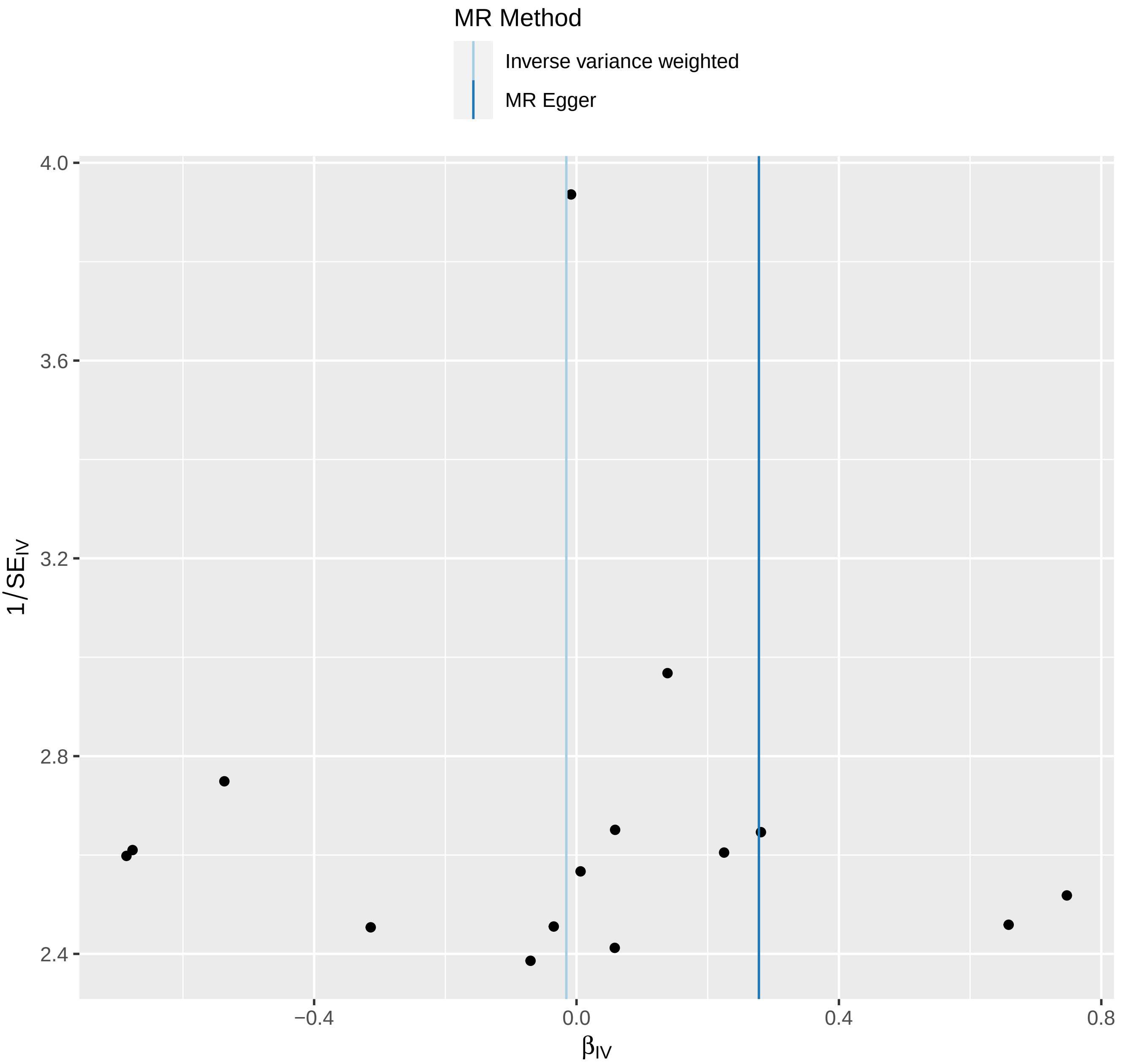

Batch 2−6163 : Gut microbiota abundance (genus Catenibacterium id.2153) on Tumor protein 63
with a threshold of 1e−05

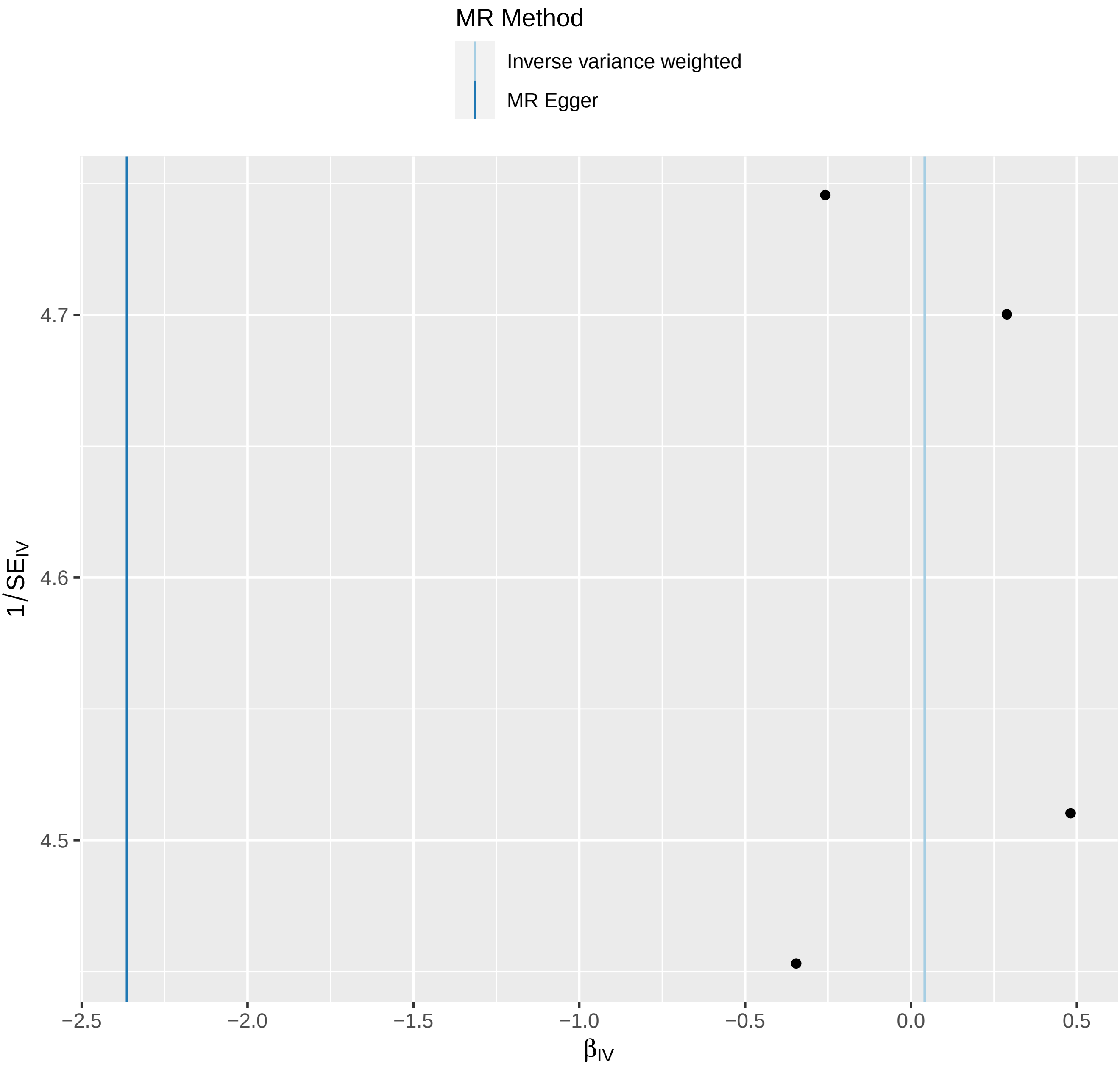

Batch 2−6164 : Gut microbiota abundance (genus Parasutterella id.2892) on Tumor protein 63
with a threshold of 1e−05

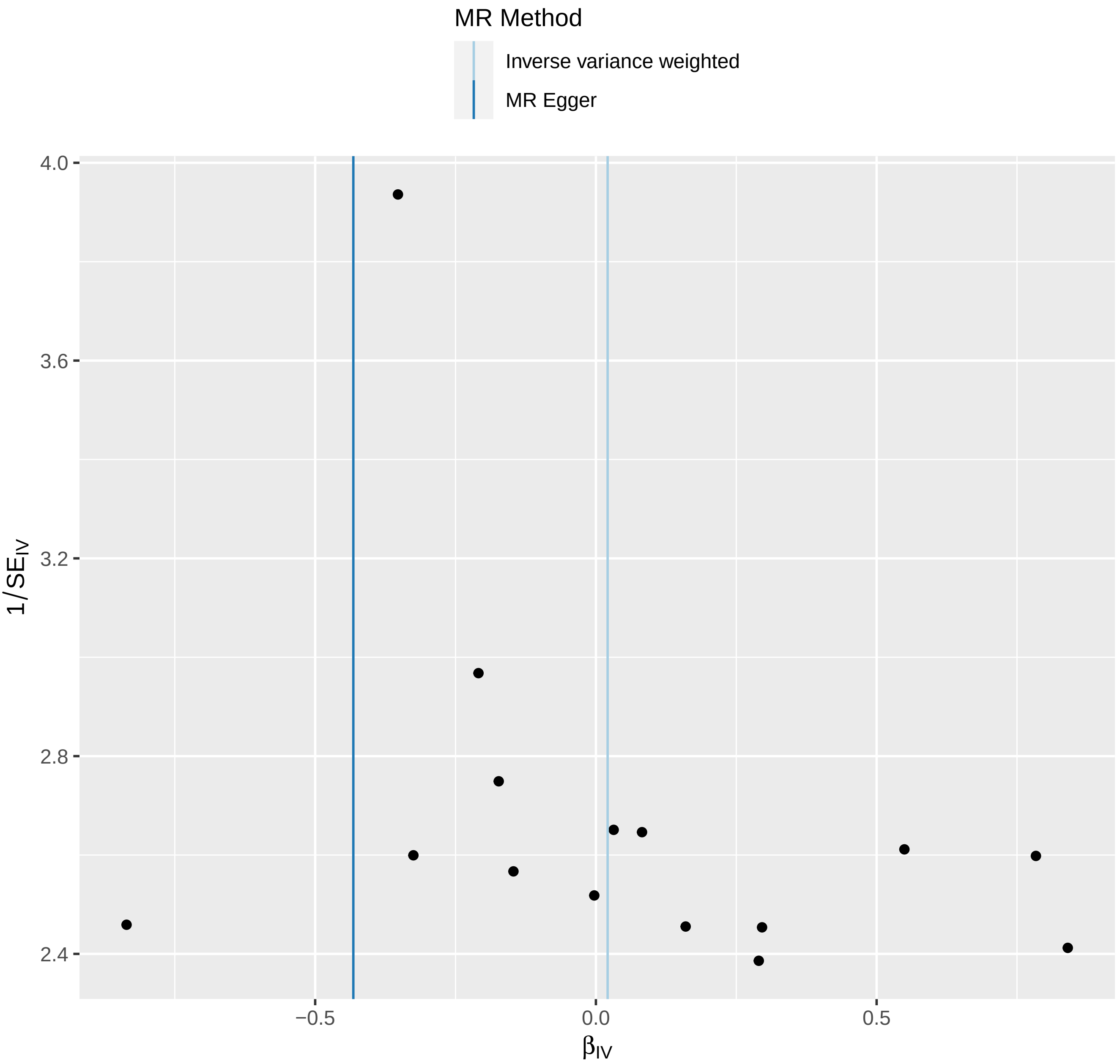

Batch 2−6165 : Gut microbiota abundance (genus Catenibacterium id.2153) on Triosephosphate isomerase
with a threshold of 1e−05

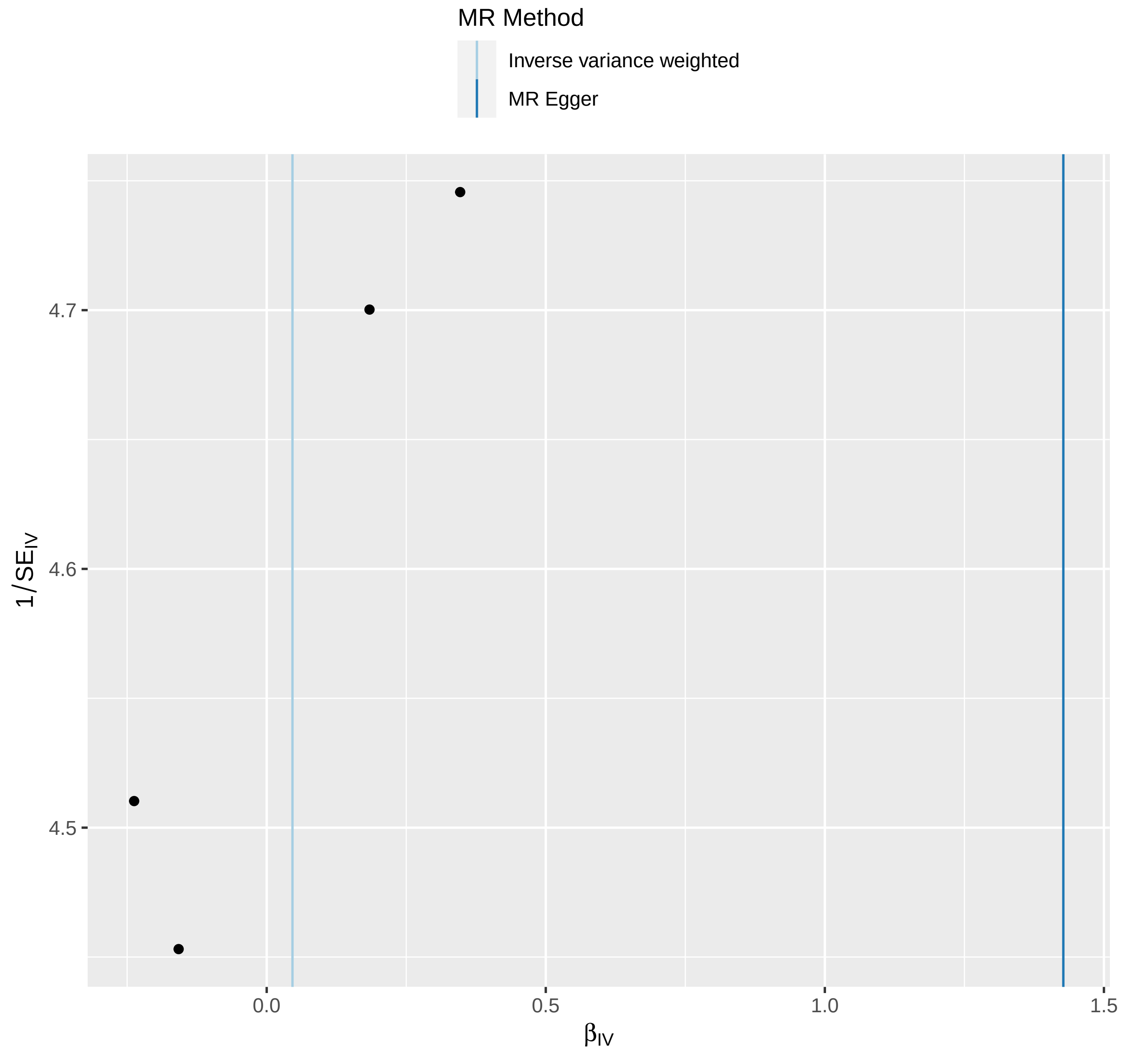

Batch 2−6166 : Gut microbiota abundance (genus Parasutterella id.2892) on Triosephosphate isomerase
with a threshold of 1e−05

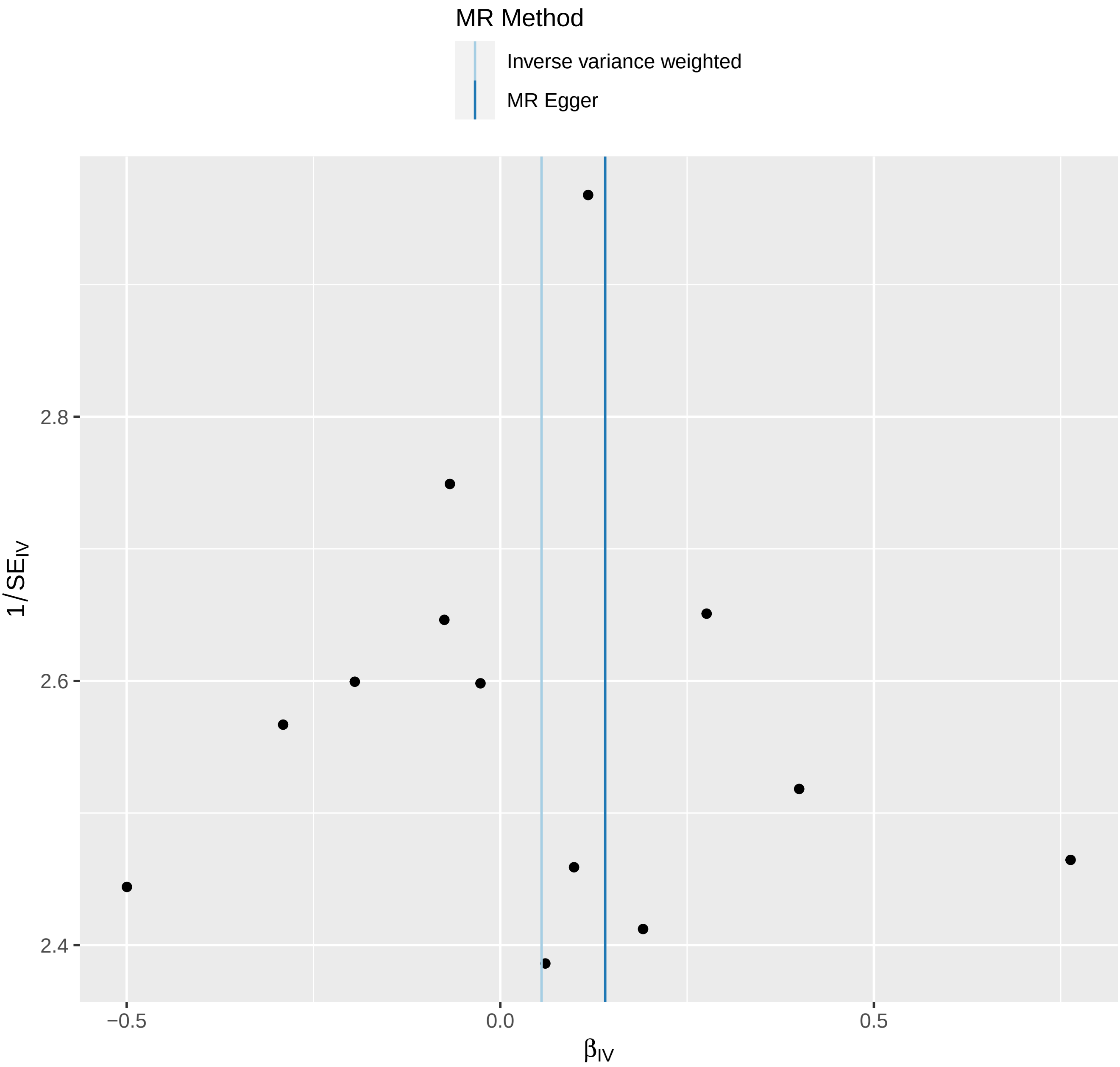

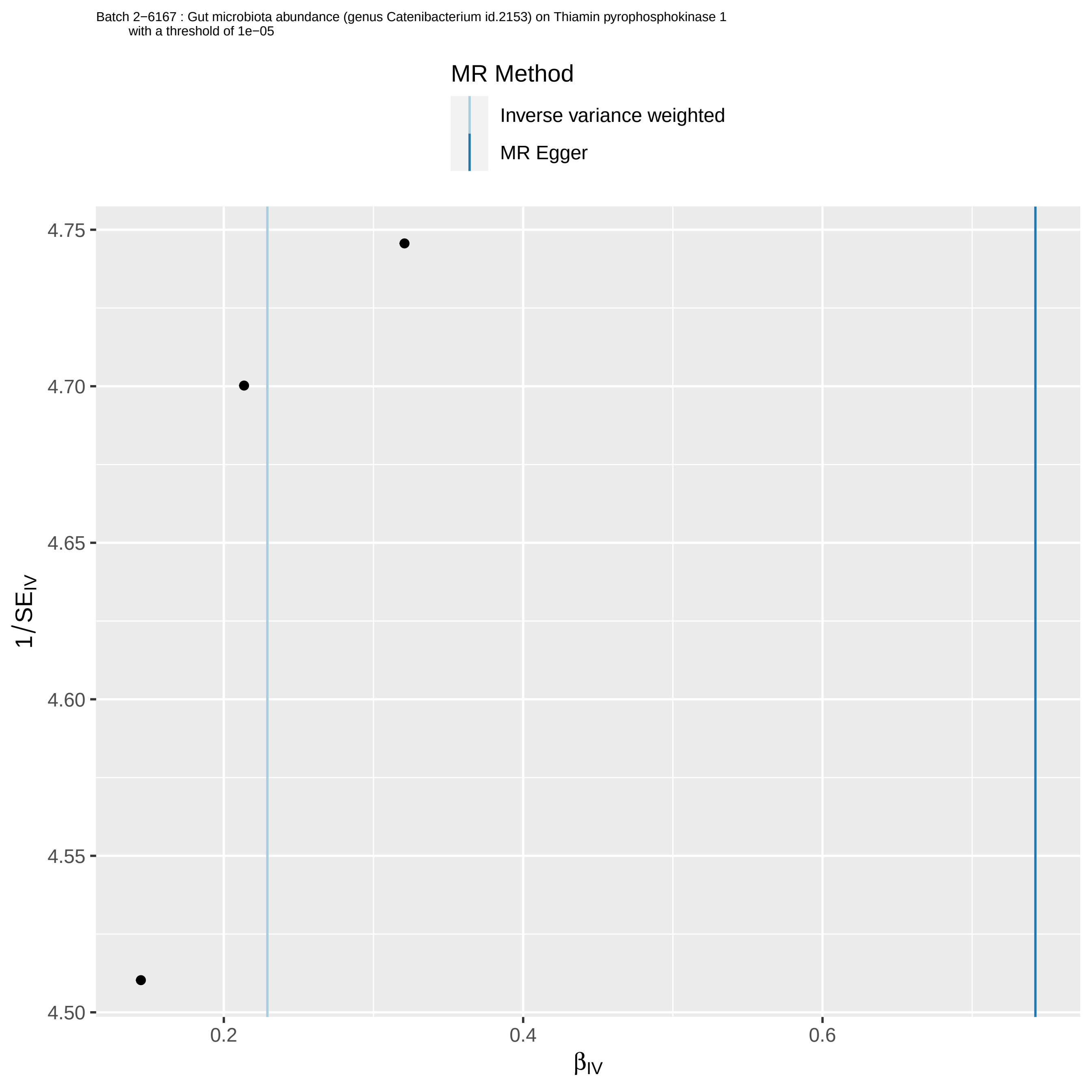
Batch 2−6167 : Gut microbiota abundance (genus Catenibacterium id.2153) on Thiamin pyrophosphokinase 1
with a threshold of 1e−05
MR Method
Inverse variance weighted
MR Egger
4.75
4.70
4.65
4.60
4.55
4.50
1/SE$_{IV}$
0.2
0.4
0.6
β$_{IV}$

Batch 2−6168 : Gut microbiota abundance (genus Parasutterella id.2892) on Thiamin pyrophosphokinase 1
with a threshold of 1e−05

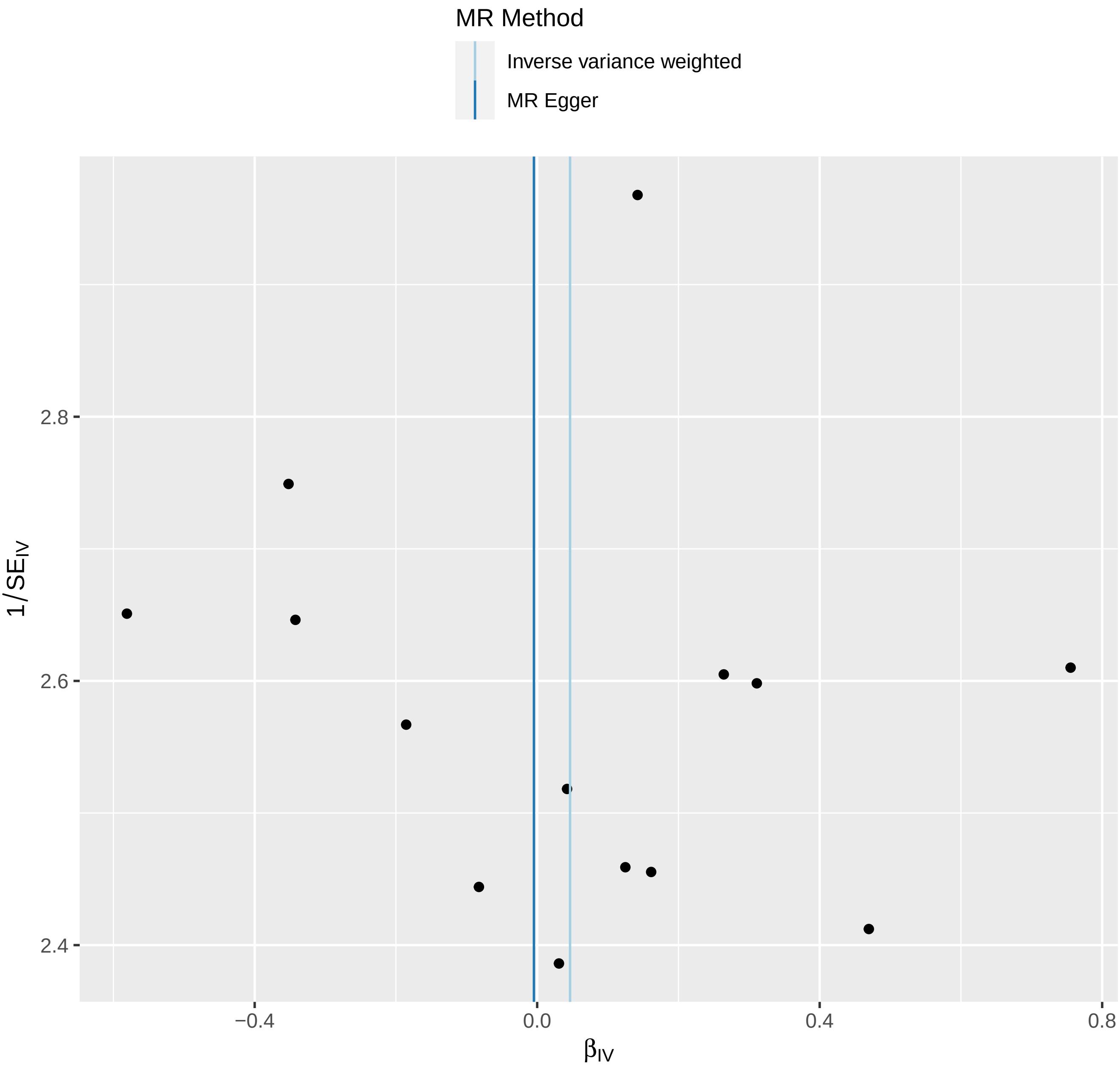

Batch 2−6169 : Gut microbiota abundance (genus Catenibacterium id.2153) on Tropomyosin alpha−1 chain
with a threshold of 1e−05

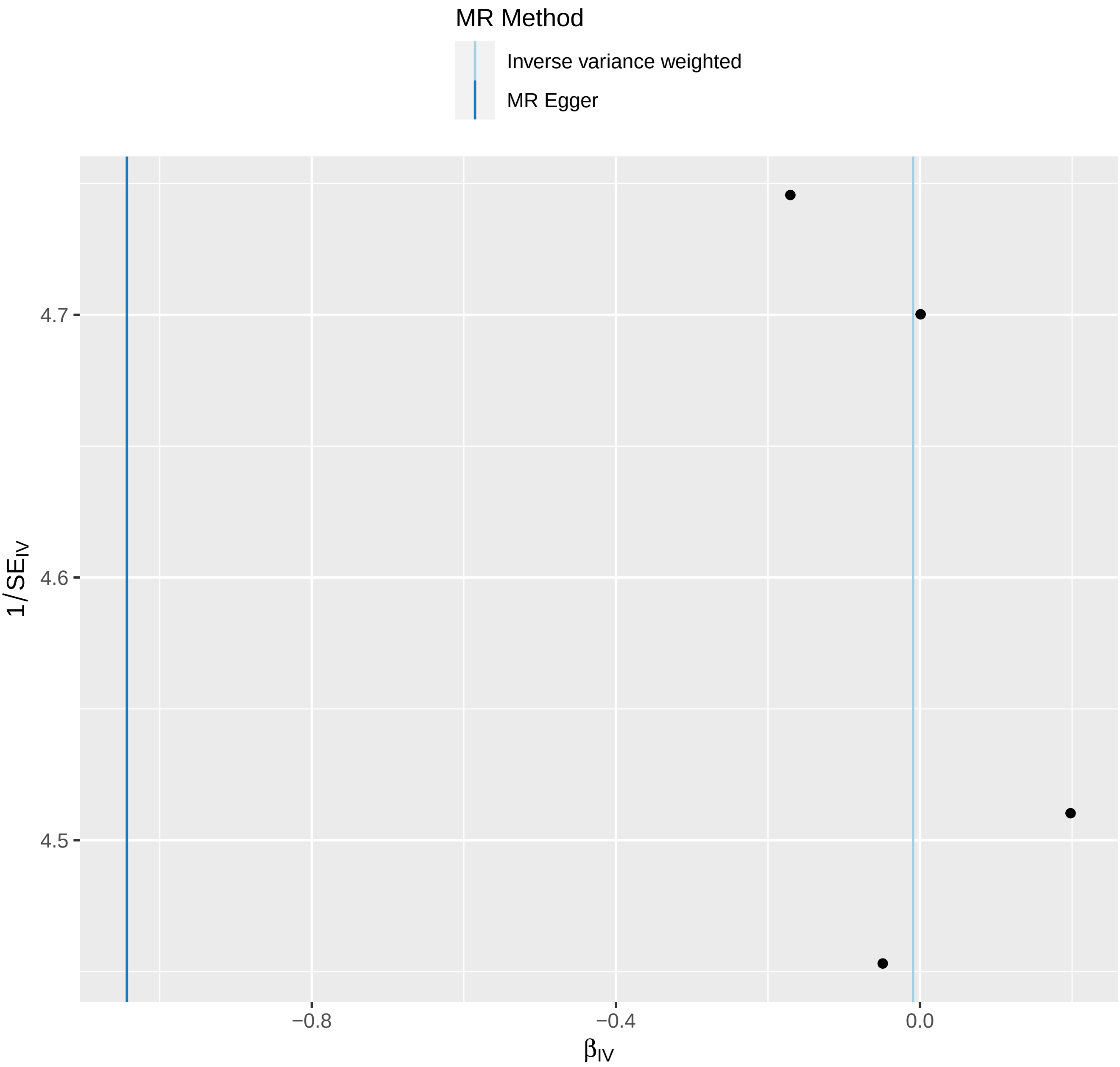

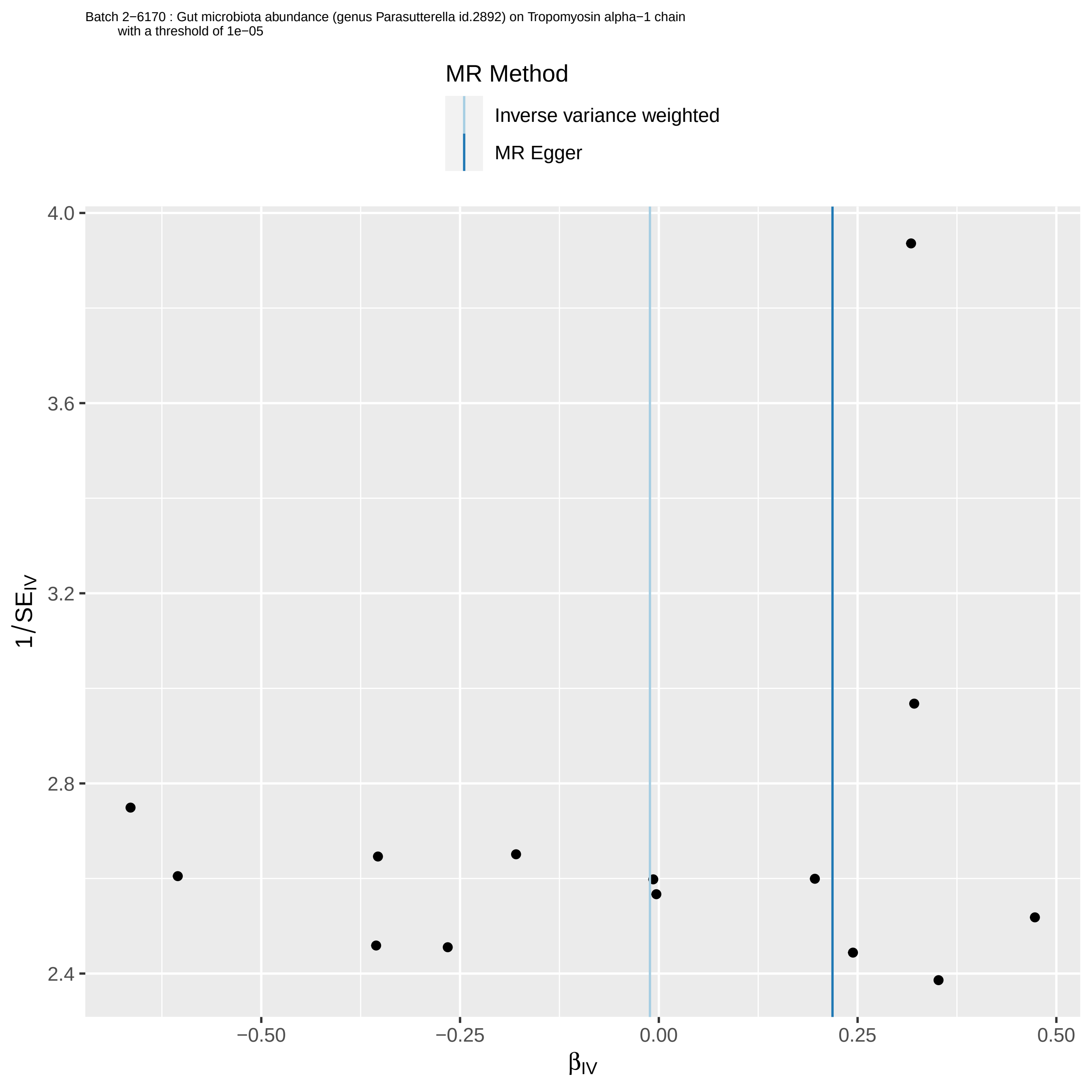
Batch 2−6170 : Gut microbiota abundance (genus Parasutterella id.2892) on Tropomyosin alpha−1 chain
with a threshold of 1e−05
MR Method
Inverse variance weighted
MR Egger
4.0
3.6
3.2
2.8
2.4
1/SE_IV
−0.50
−0.25
0.00
0.25
0.50
β_IV

Batch 2−6171 : Gut microbiota abundance (genus Catenibacterium id.2153) on Tropomyosin beta chain
with a threshold of 1e−05

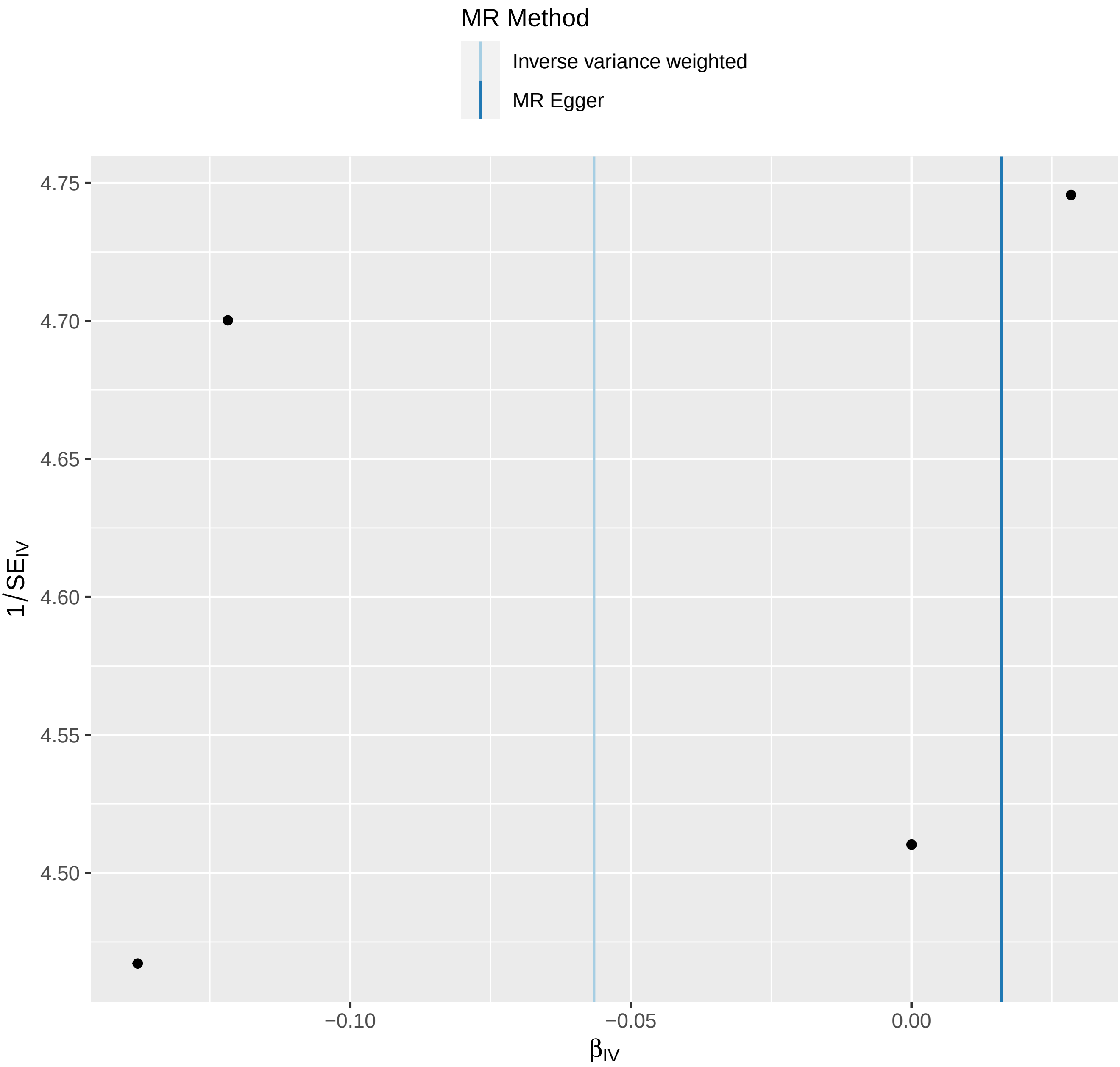

Batch 2−6172 : Gut microbiota abundance (genus Parasutterella id.2892) on Tropomyosin beta chain
with a threshold of 1e−05

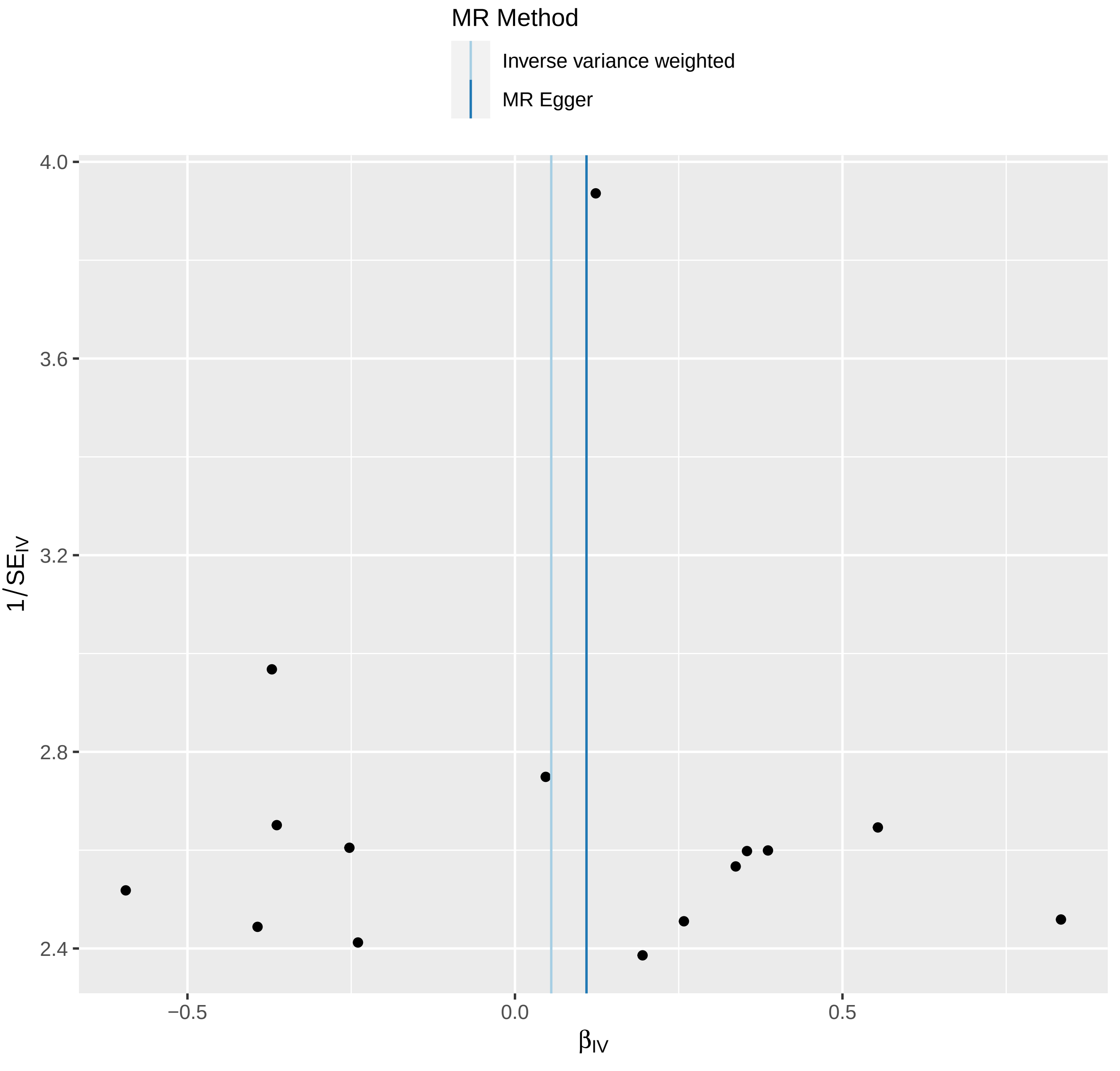

Batch 2−6173 : Gut microbiota abundance (genus Catenibacterium id.2153) on Tropomyosin alpha−3 chain
with a threshold of 1e−05

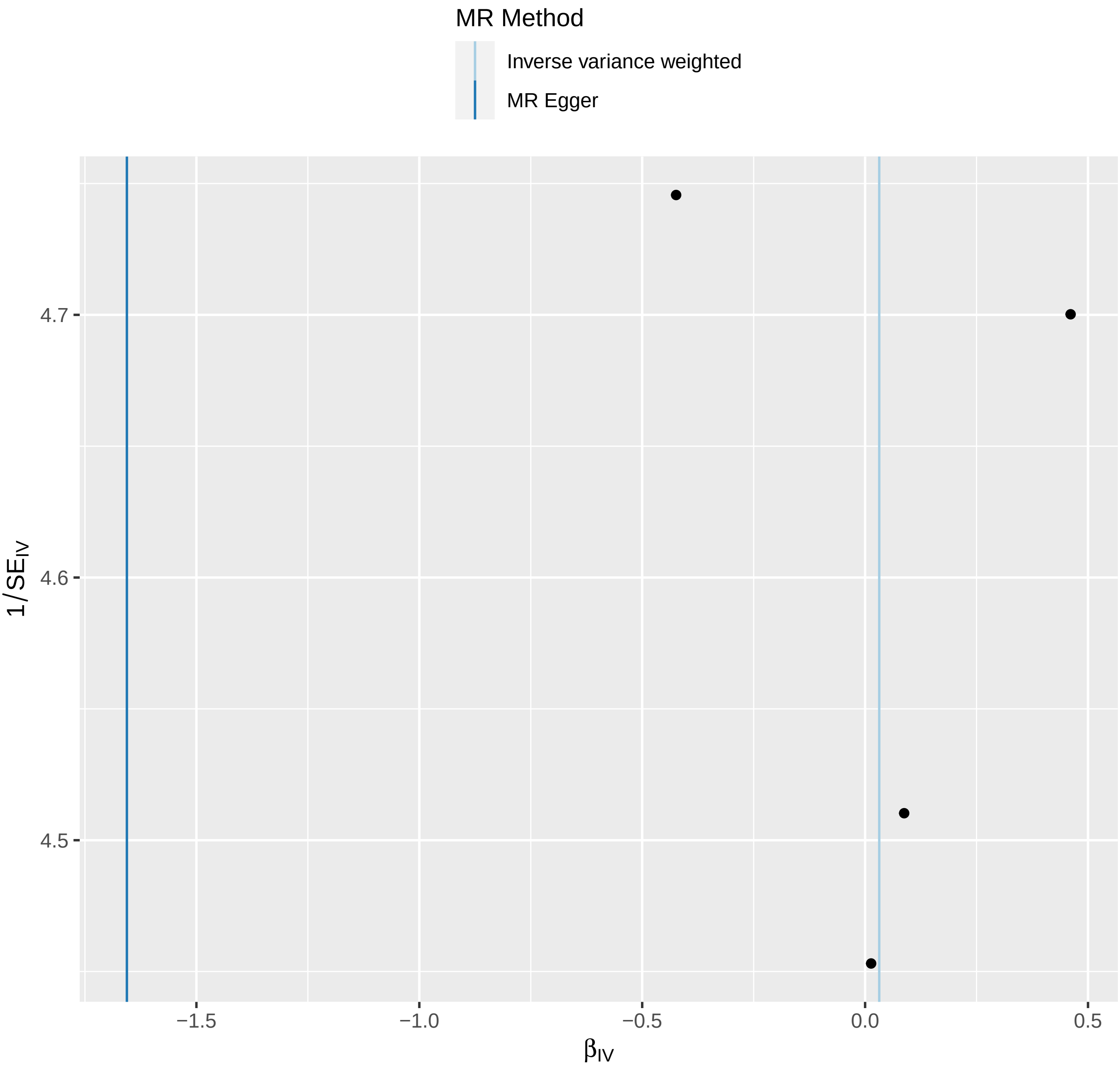

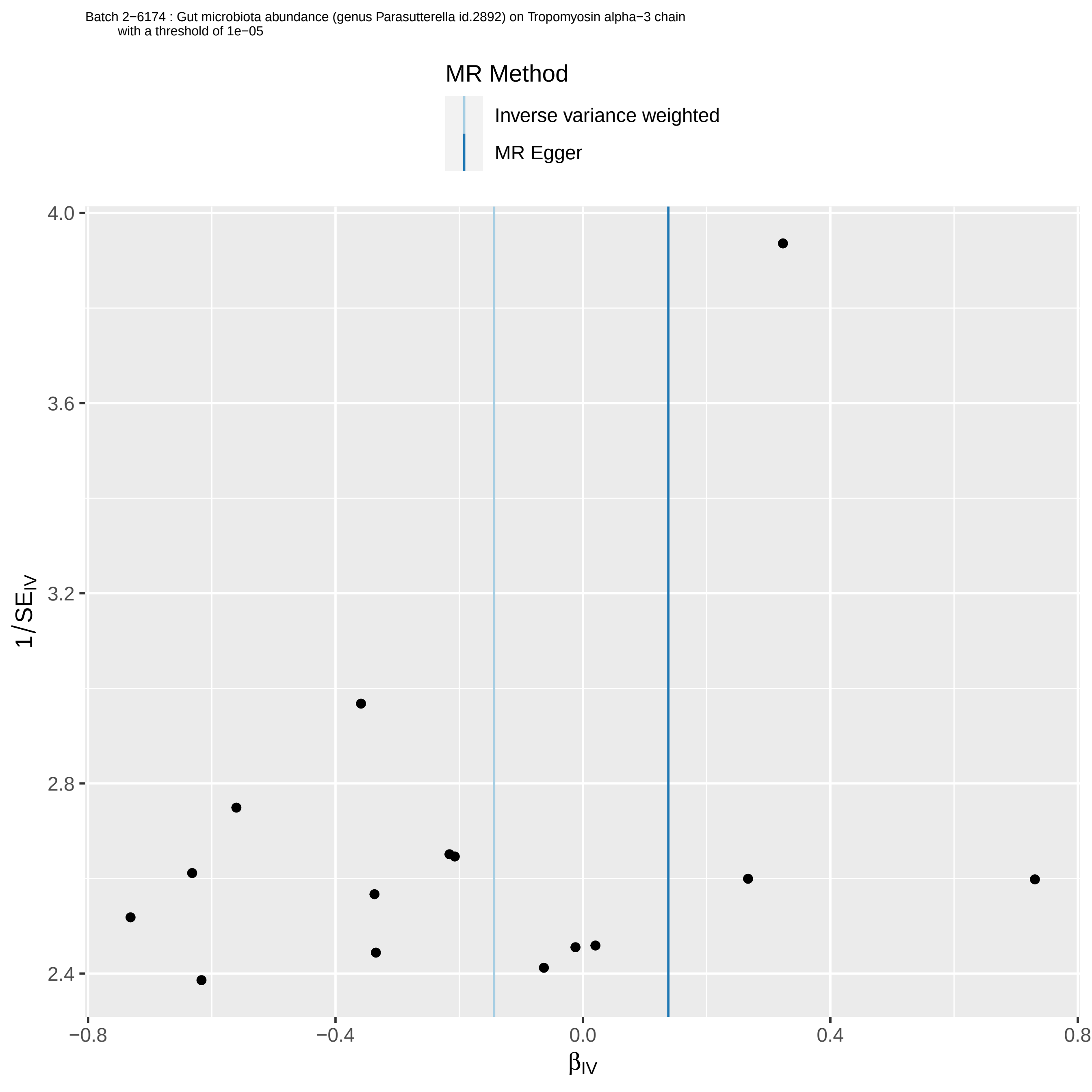

Batch 2−6174 : Gut microbiota abundance (genus Parasutterella id.2892) on Tropomyosin alpha−3 chain
with a threshold of 1e−05
MR Method
Inverse variance weighted
MR Egger
4.0
3.6
3.2
2.8
2.4
1/SE_IV
−0.8
−0.4
0.0
0.4
0.8
β_IV

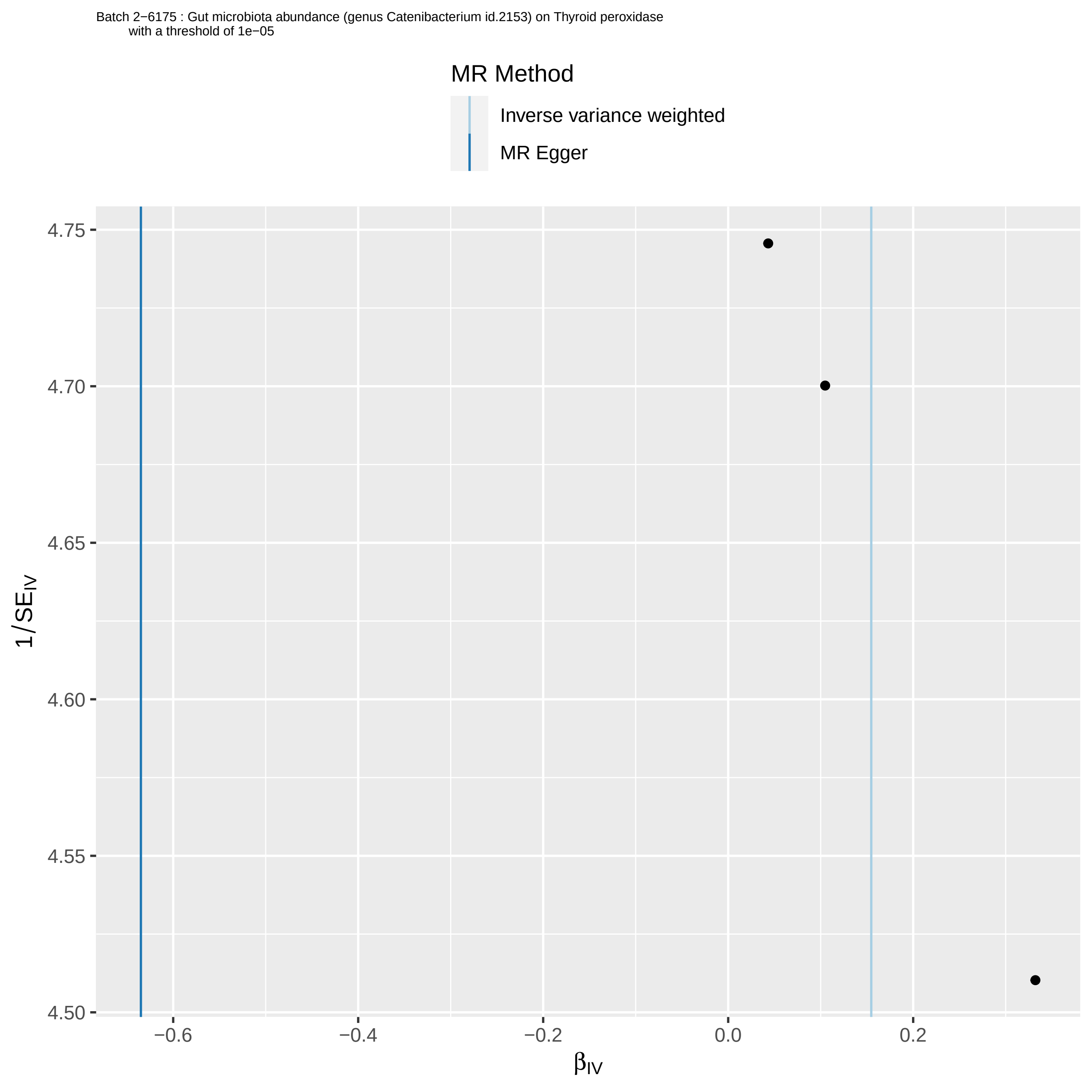

Batch 2−6175 : Gut microbiota abundance (genus Catenibacterium id.2153) on Thyroid peroxidase
with a threshold of 1e−05
MR Method
Inverse variance weighted
MR Egger
4.75
4.70
4.65
4.60
4.55
4.50
1/SE_IV
−0.6
−0.4
−0.2
0.0
0.2
β_IV

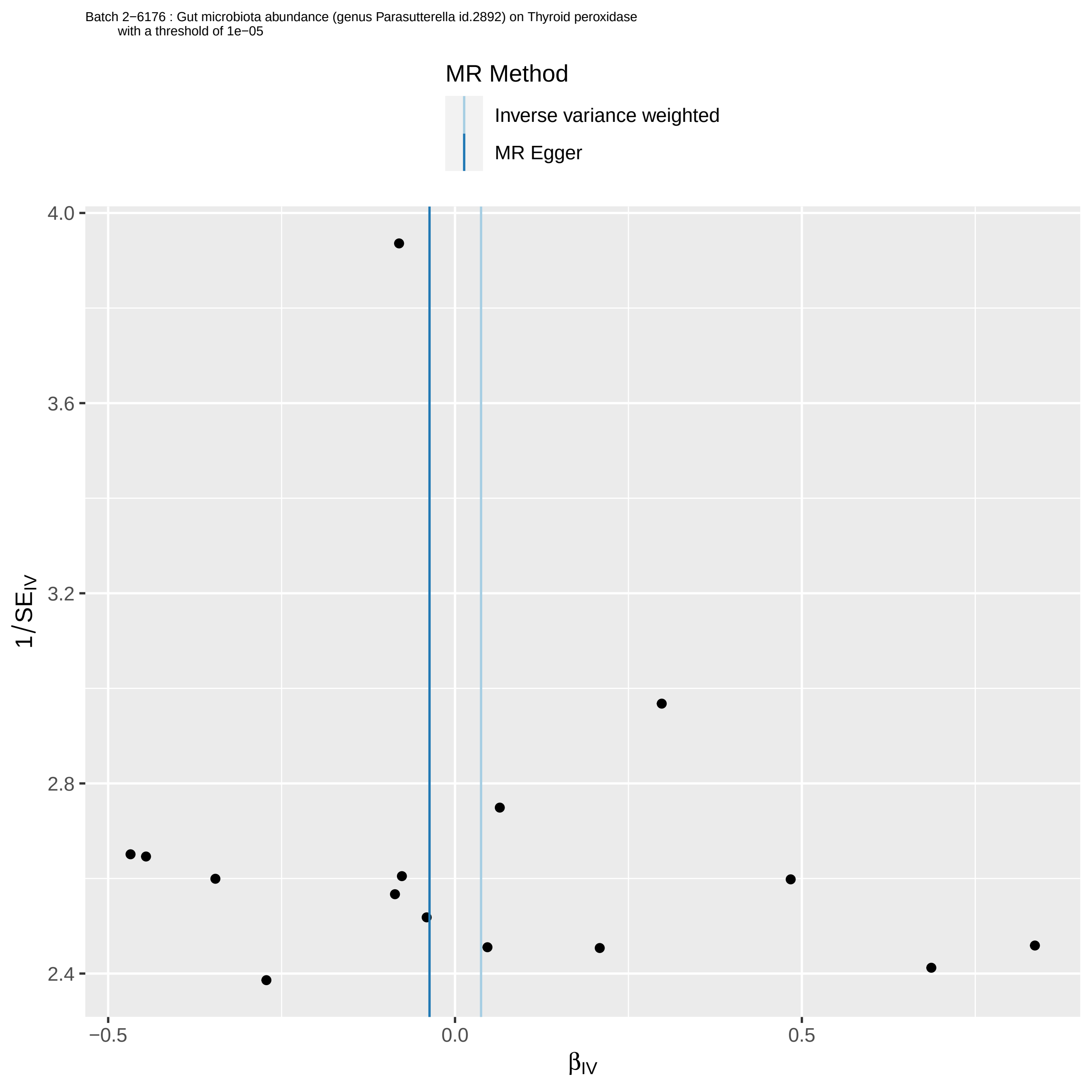

Batch 2−6176 : Gut microbiota abundance (genus Parasutterella id.2892) on Thyroid peroxidase
with a threshold of 1e−05
MR Method
Inverse variance weighted
MR Egger
4.0
3.6
3.2
2.8
2.4
1/SE$_{IV}$
−0.5
0.0
0.5
β$_{IV}$

Batch 2−6177 : Gut microbiota abundance (genus Catenibacterium id.2153) on Tubulin polymerization−promoting protein family member 2 with a threshold of 1e−05

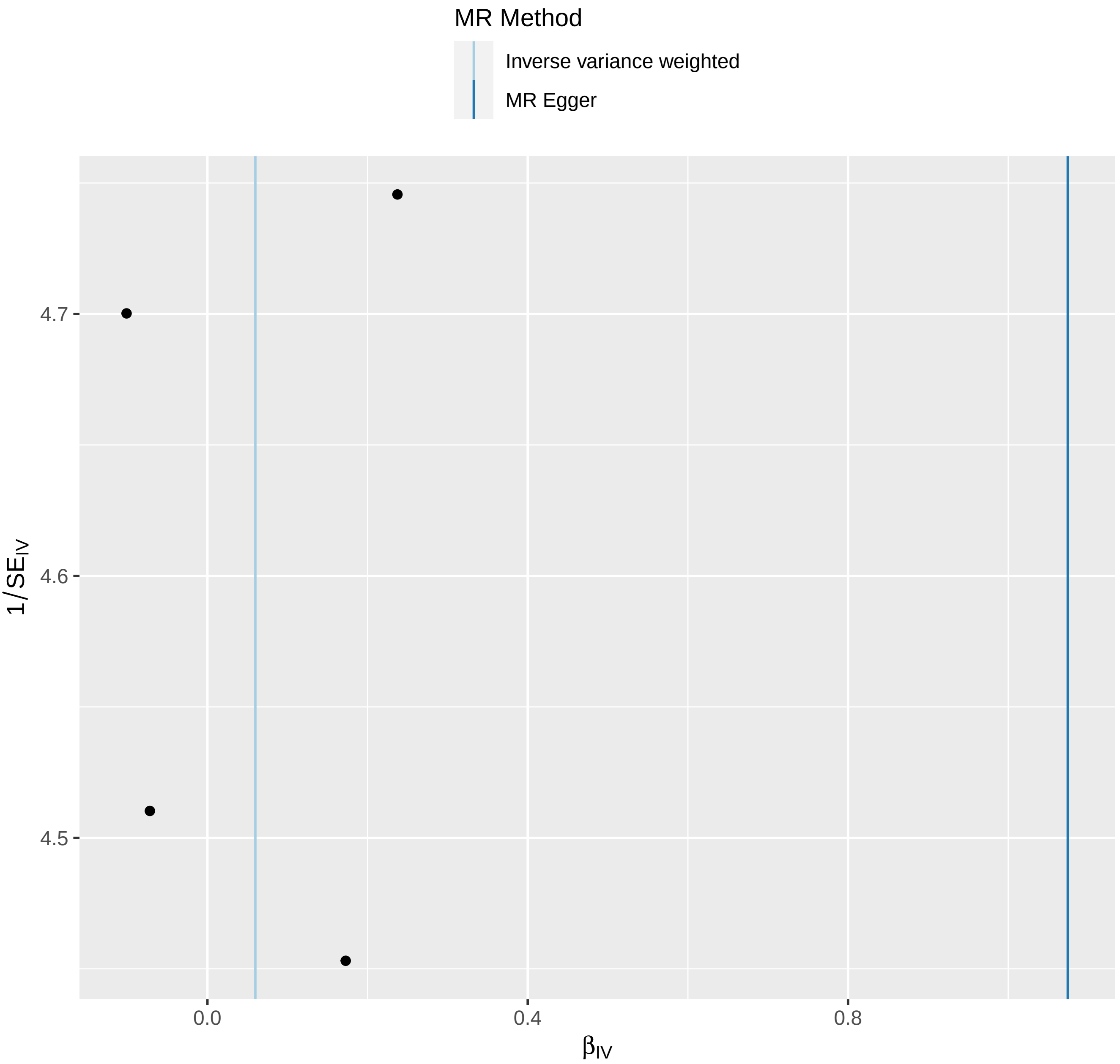

Batch 2−6178 : Gut microbiota abundance (genus Parasutterella id.2892) on Tubulin polymerization−promoting protein family member 2
with a threshold of 1e−05

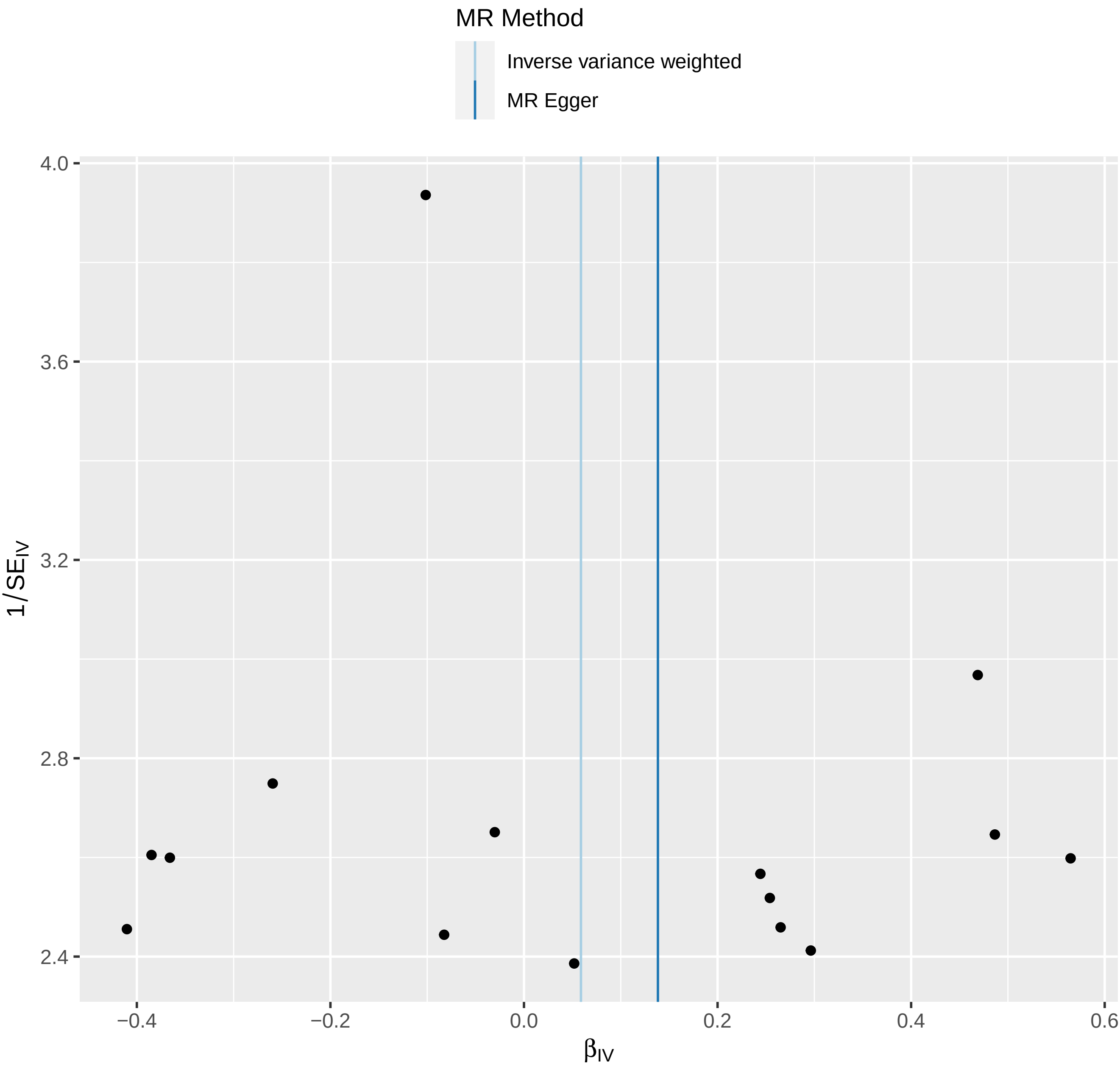

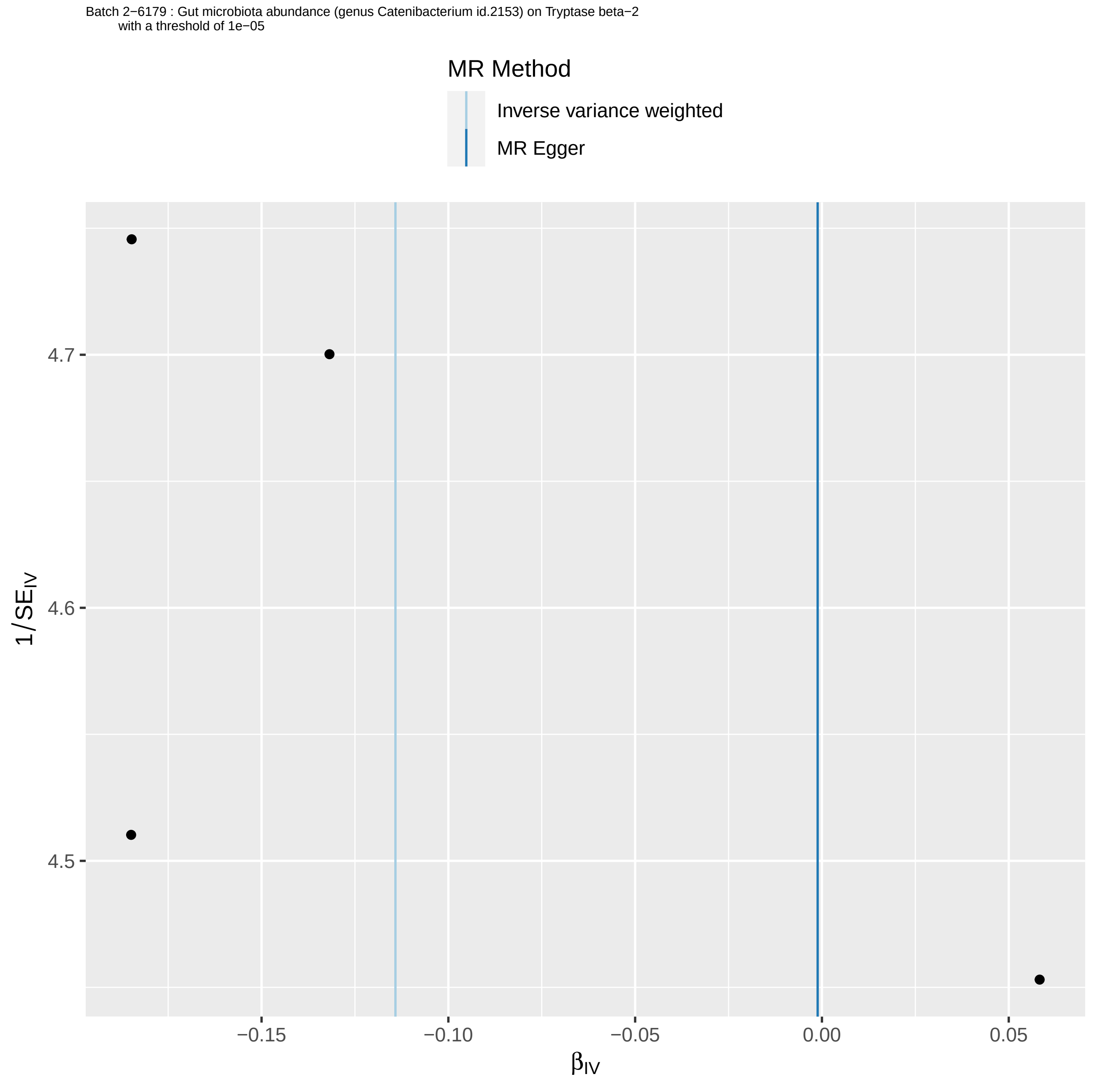
Batch 2−6179 : Gut microbiota abundance (genus Catenibacterium id.2153) on Tryptase beta−2
with a threshold of 1e−05
MR Method
Inverse variance weighted
MR Egger
4.7
4.6
4.5
1/SE$_{IV}$
−0.15
−0.10
−0.05
0.00
0.05
β$_{IV}$

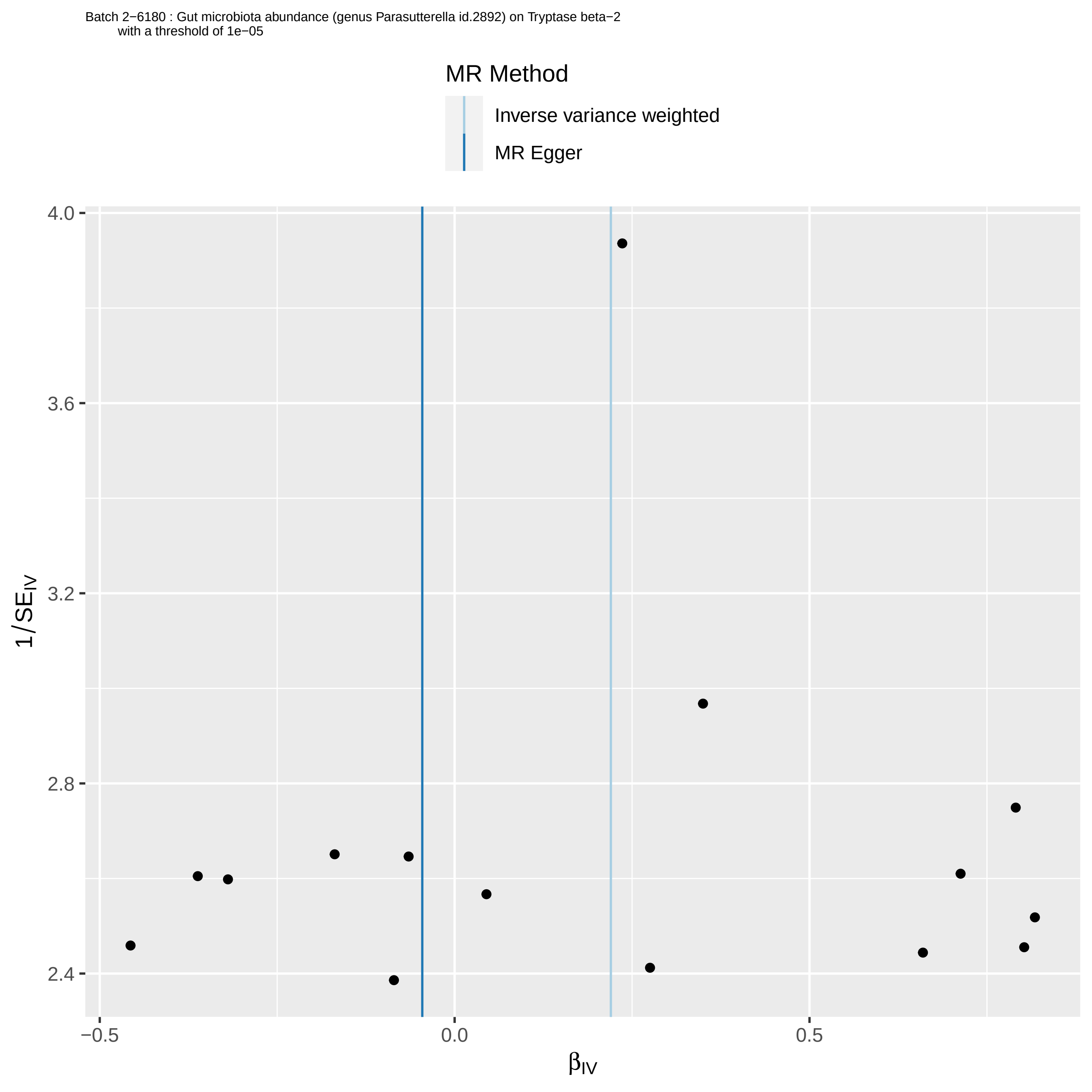

Batch 2−6180 : Gut microbiota abundance (genus Parasutterella id.2892) on Tryptase beta−2
with a threshold of 1e−05
MR Method
Inverse variance weighted
MR Egger
4.0
3.6
3.2
2.8
2.4
1/SE_IV
−0.5
0.0
0.5
β_IV

Batch 2−6181 : Gut microbiota abundance (genus Catenibacterium id.2153) on Tryptase gamma
with a threshold of 1e−05

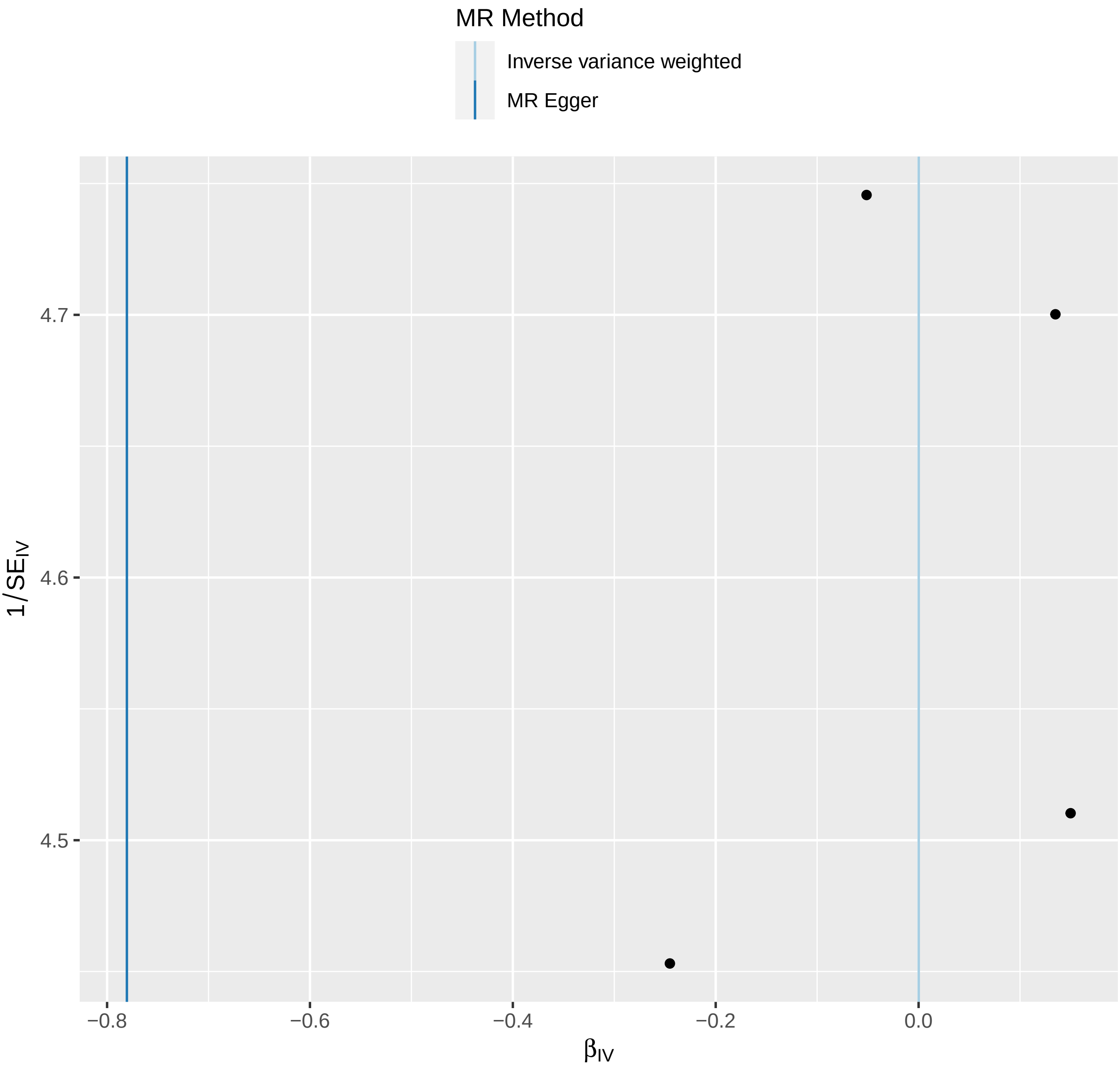

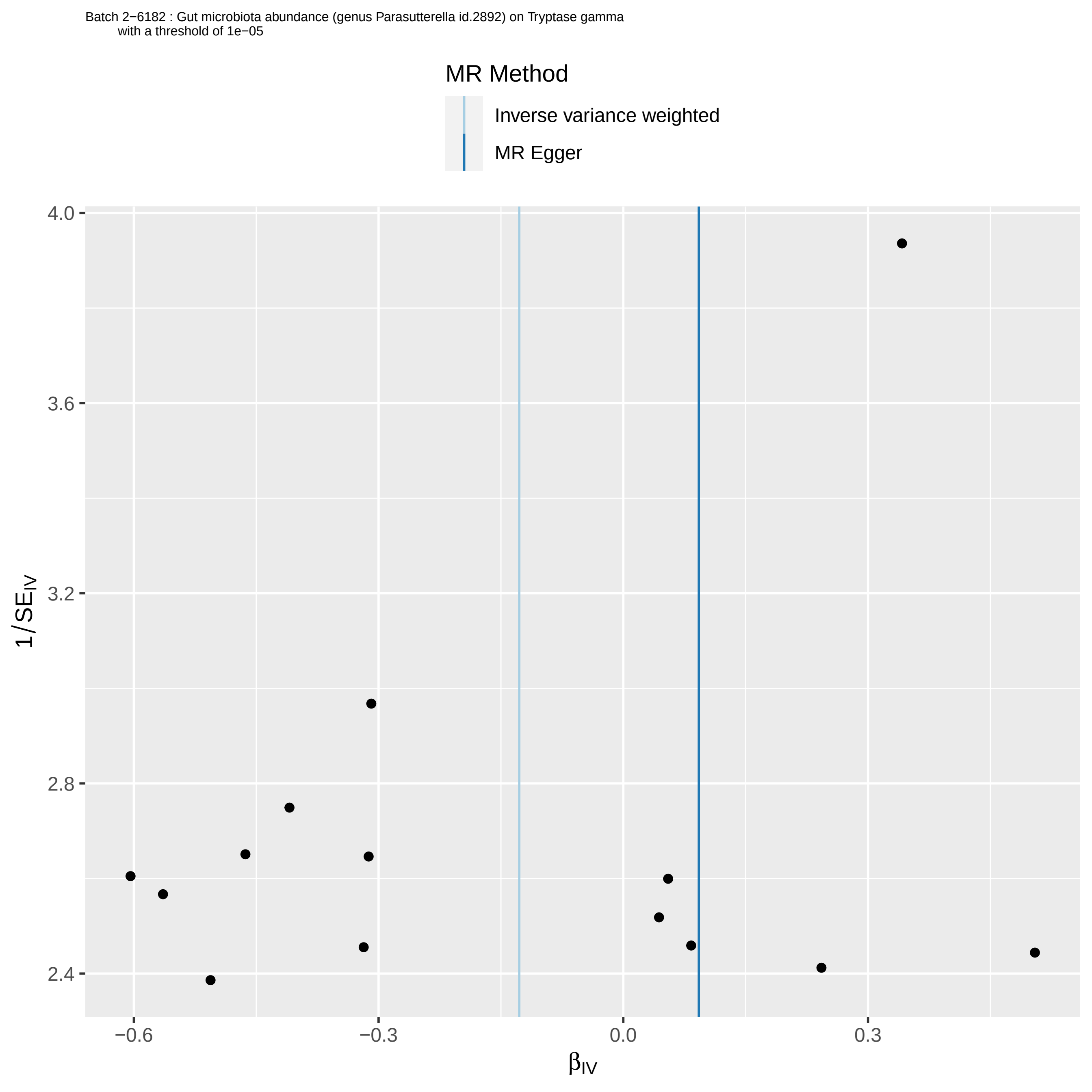
Batch 2−6182 : Gut microbiota abundance (genus Parasutterella id.2892) on Tryptase gamma
with a threshold of 1e−05
MR Method
Inverse variance weighted
MR Egger
4.0
3.6
3.2
2.8
2.4
1/SE_IV
−0.6
−0.3
0.0
0.3
β_IV

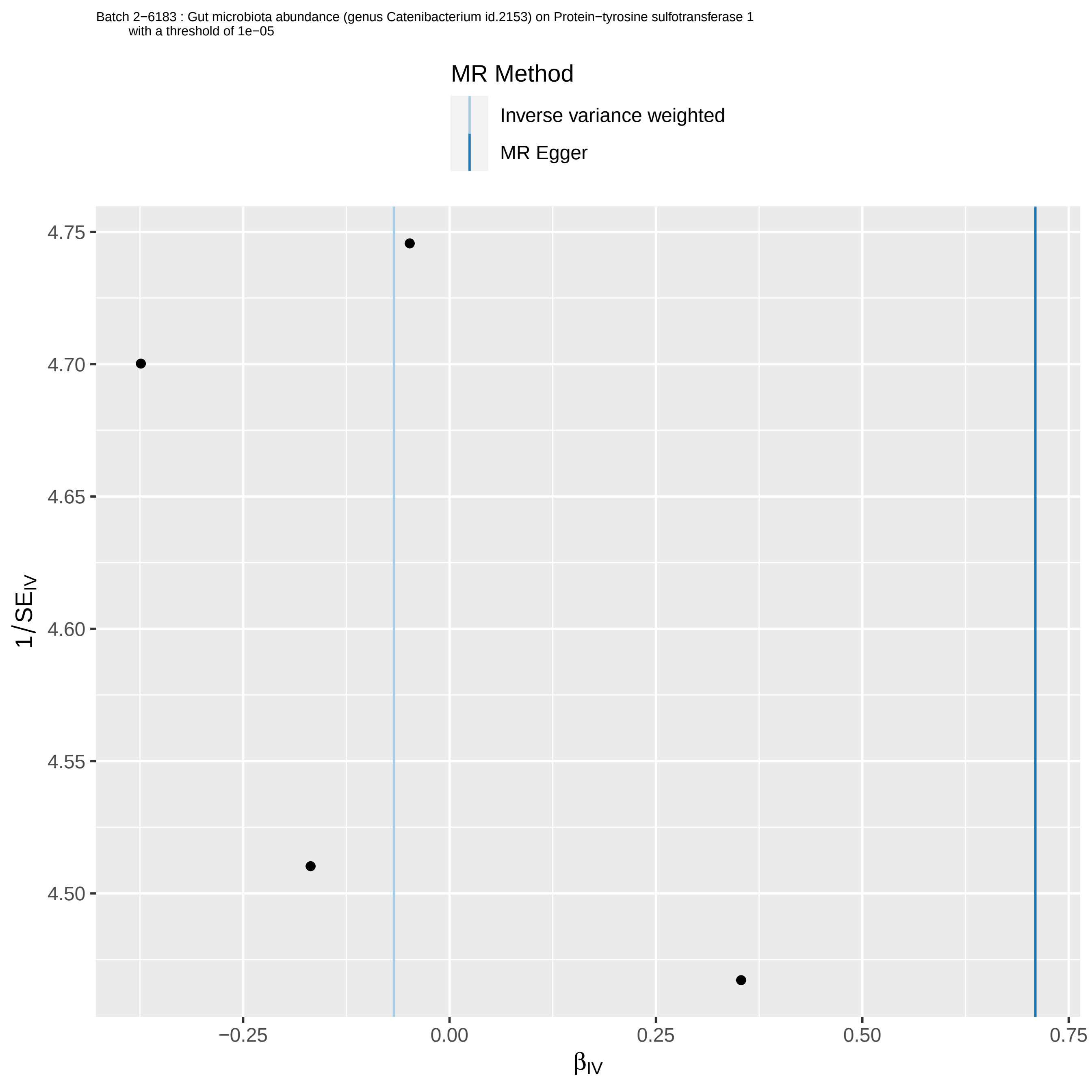
Batch 2−6183 : Gut microbiota abundance (genus Catenibacterium id.2153) on Protein−tyrosine sulfotransferase 1
with a threshold of 1e−05
MR Method
Inverse variance weighted
MR Egger
4.75
4.70
4.65
4.60
4.55
4.50
1/SE$_{IV}$
−0.25
0.00
0.25
0.50
0.75
β$_{IV}$

Batch 2−6184 : Gut microbiota abundance (genus Parasutterella id.2892) on Protein−tyrosine sulfotransferase 1
with a threshold of 1e−05

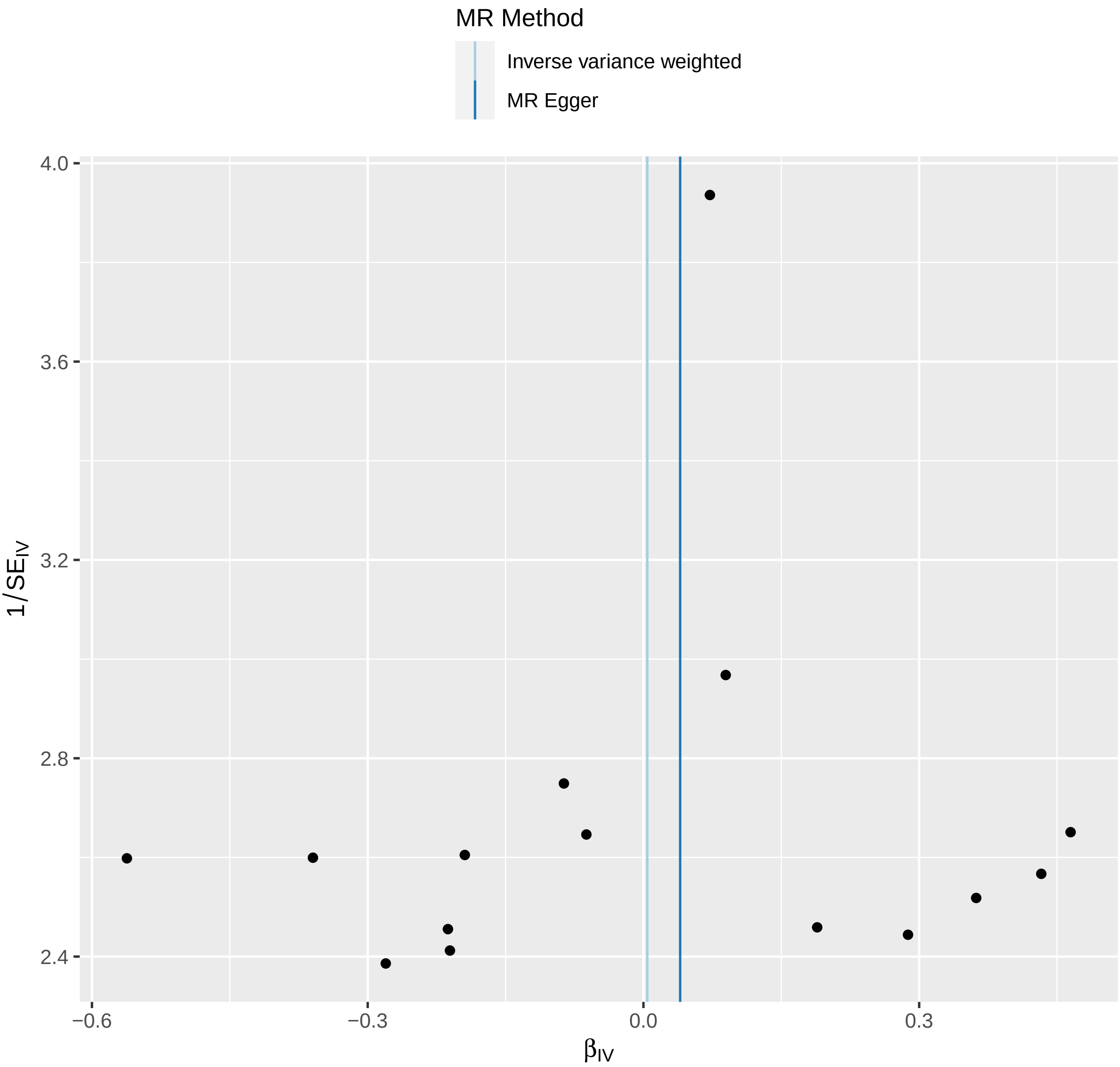

Batch 2−6185 : Gut microbiota abundance (genus Catenibacterium id.2153) on Protein−tyrosine sulfotransferase 2
with a threshold of 1e−05

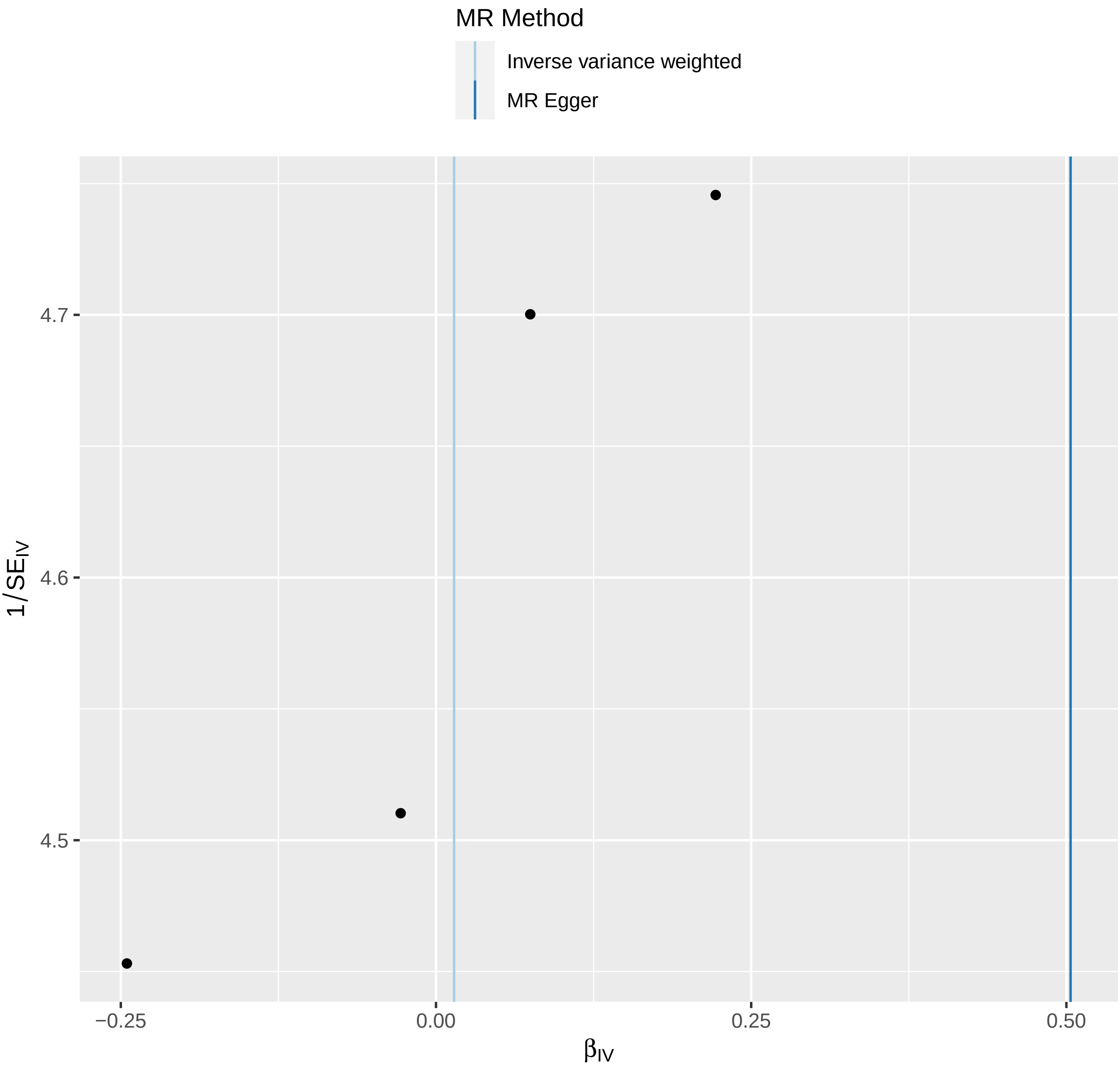

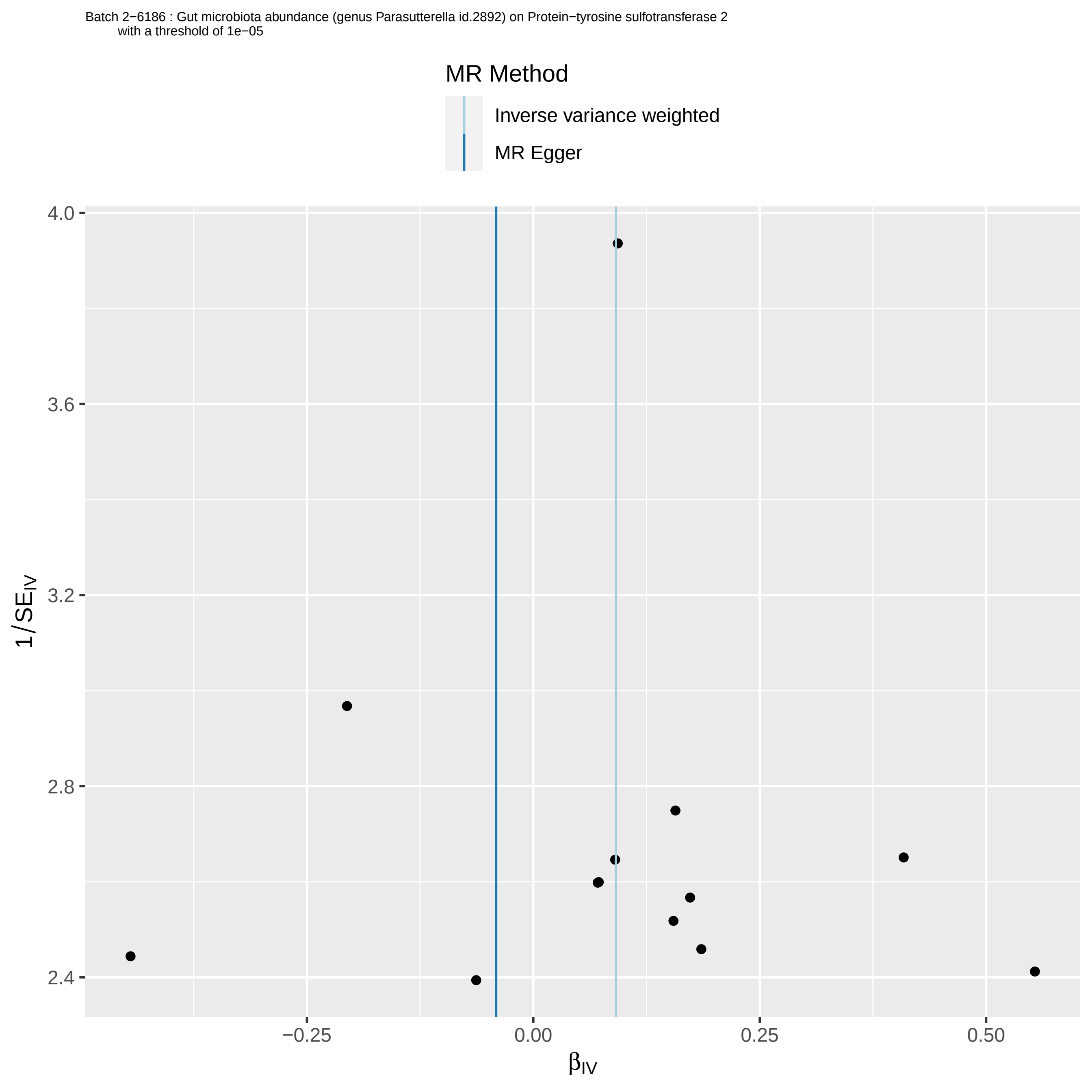

Batch 2−6186 : Gut microbiota abundance (genus Parasutterella id.2892) on Protein−tyrosine sulfotransferase 2
with a threshold of 1e−05
MR Method
Inverse variance weighted
MR Egger
4.0
3.6
3.2
2.8
2.4
1/SE_IV
−0.25
0.00
0.25
0.50
β_IV

Batch 2−6187 : Gut microbiota abundance (genus Catenibacterium id.2153) on Translationally−controlled tumor protein
with a threshold of 1e−05

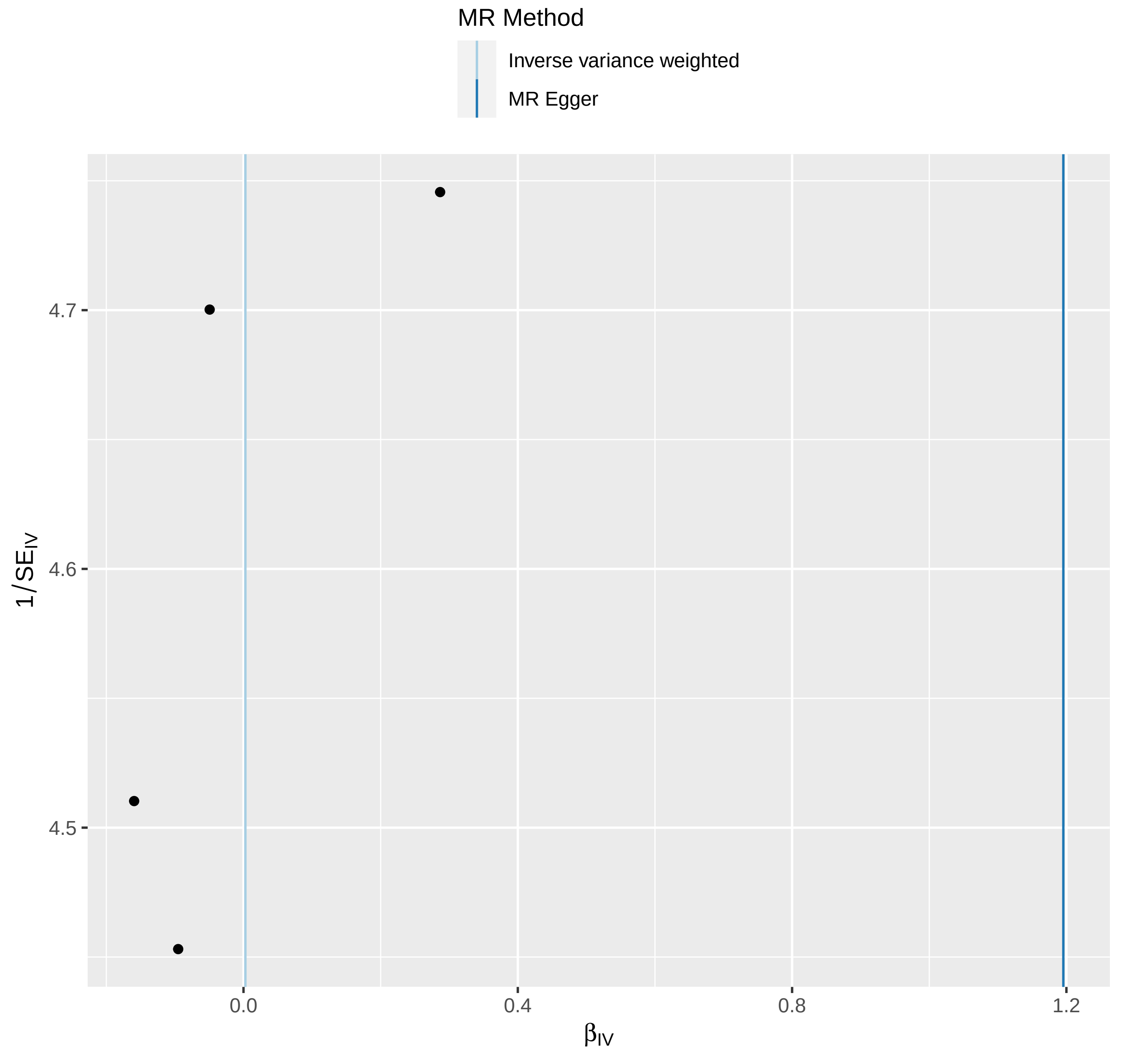

Batch 2−6188 : Gut microbiota abundance (genus Parasutterella id.2892) on Translationally−controlled tumor protein
with a threshold of 1e−05

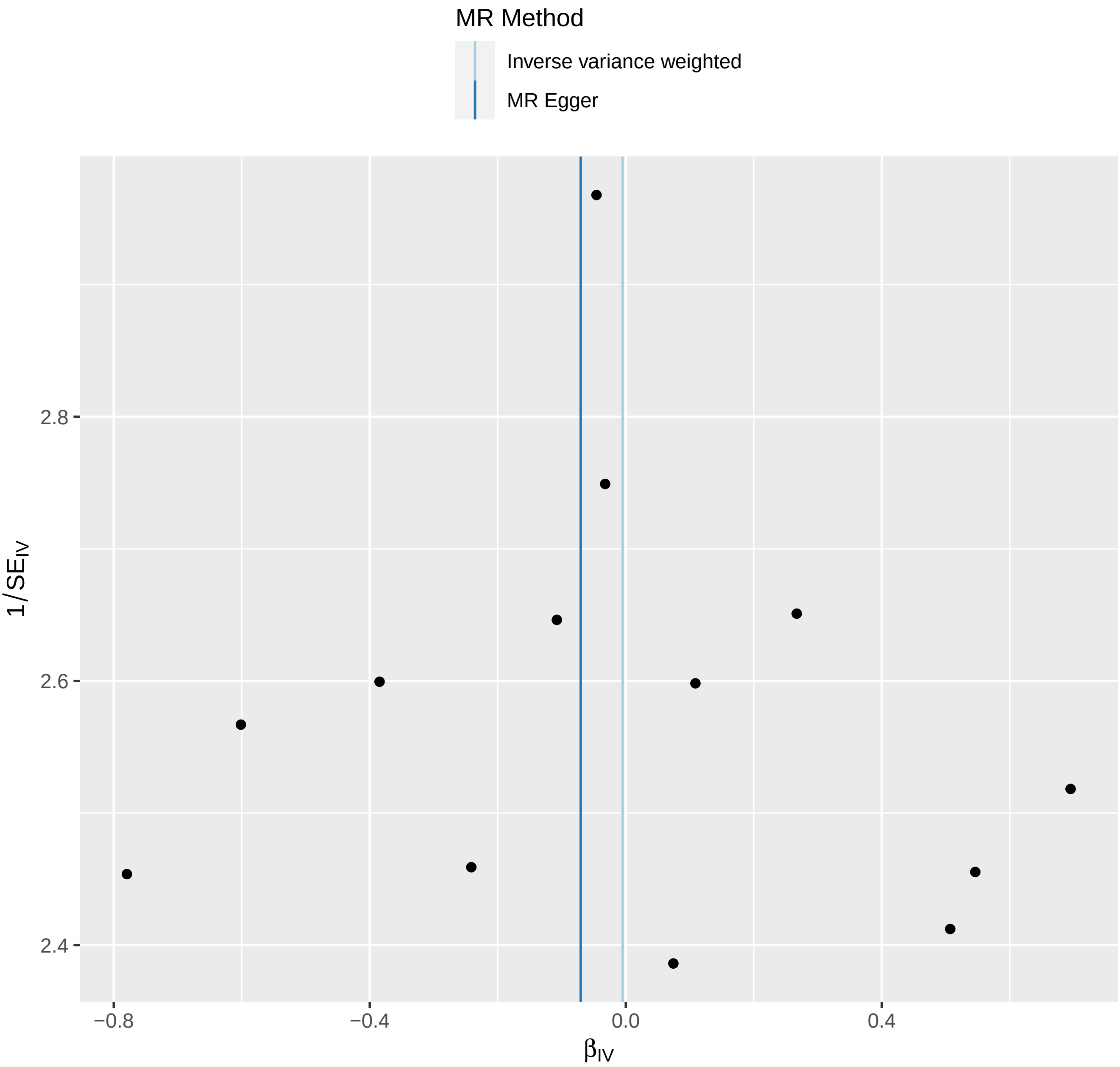

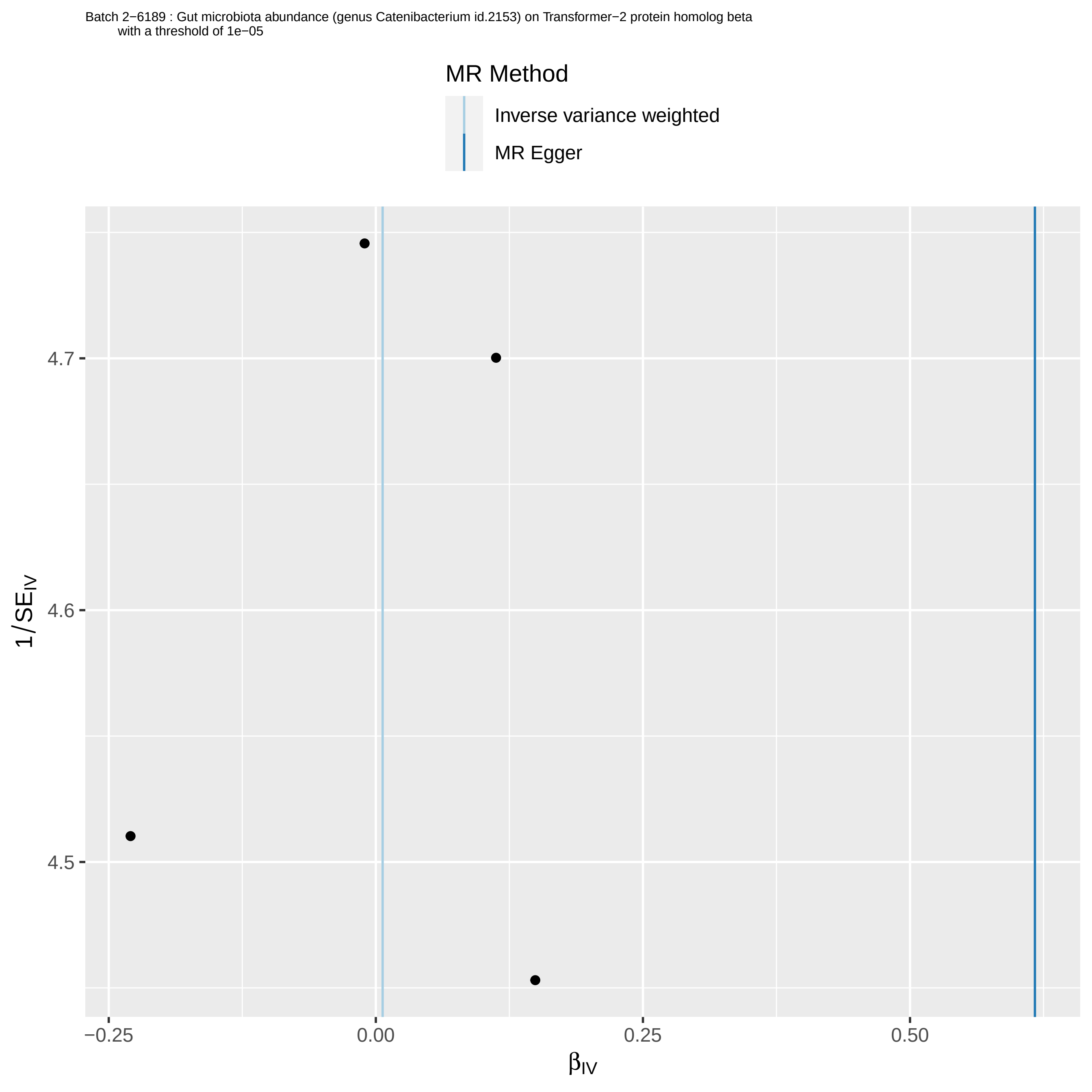

Batch 2−6189 : Gut microbiota abundance (genus Catenibacterium id.2153) on Transformer−2 protein homolog beta
with a threshold of 1e−05
MR Method
Inverse variance weighted
MR Egger
4.7
4.6
4.5
1/SE_IV
−0.25
0.00
0.25
0.50
β_IV

Batch 2−6190 : Gut microbiota abundance (genus Parasutterella id.2892) on Transformer−2 protein homolog beta
with a threshold of 1e−05

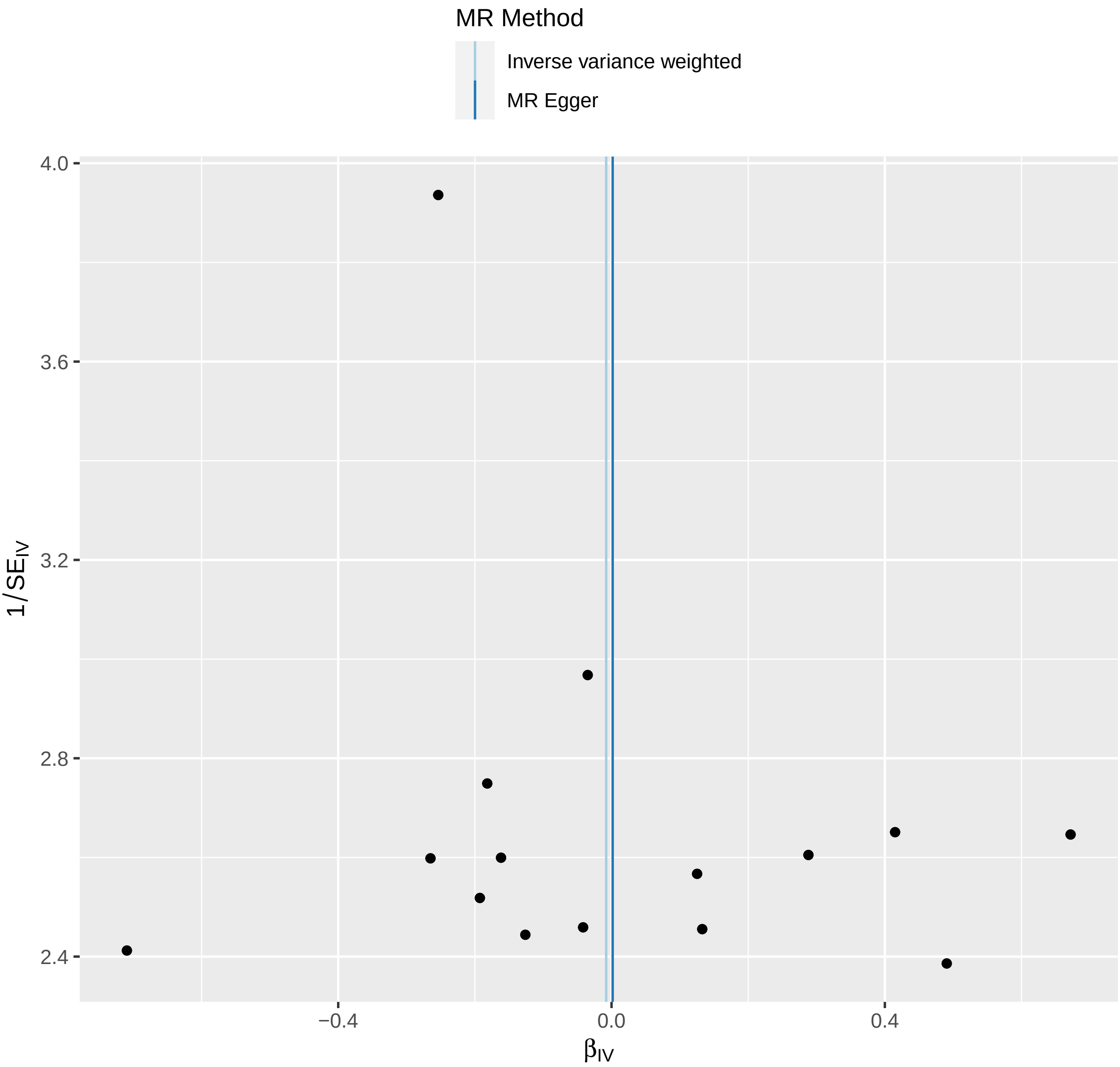

Batch 2−6191 : Gut microbiota abundance (genus Catenibacterium id.2153) on TNF receptor−associated factor 4
with a threshold of 1e−05

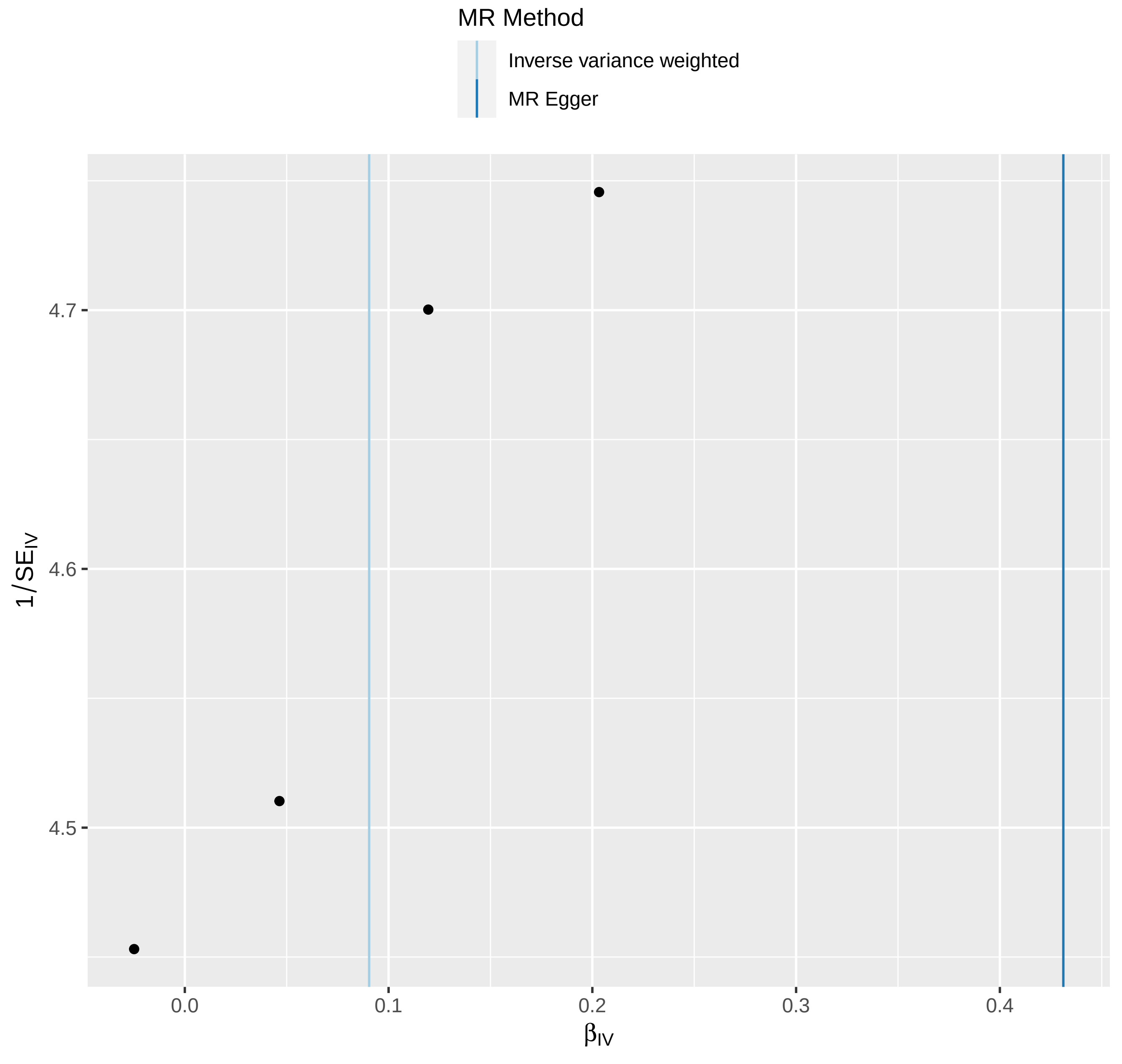

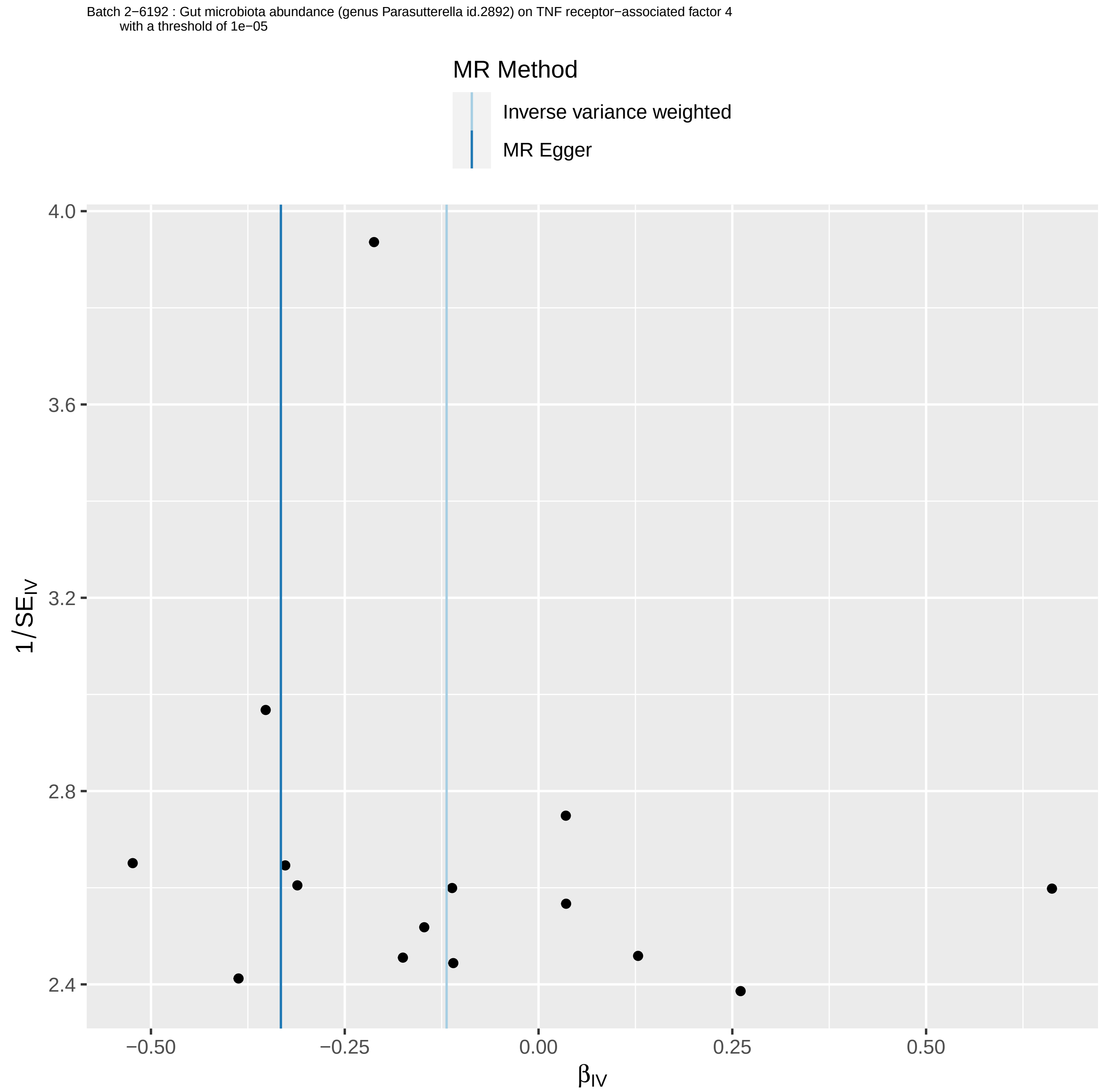
Batch 2−6192 : Gut microbiota abundance (genus Parasutterella id.2892) on TNF receptor−associated factor 4
with a threshold of 1e−05
MR Method
Inverse variance weighted
MR Egger
4.0
3.6
3.2
2.8
2.4
1/SE_IV
−0.50
−0.25
0.00
0.25
0.50
β_IV

Batch 2−6193 : Gut microbiota abundance (genus Catenibacterium id.2153) on T−cell receptor−associated transmembrane adapter 1 with a threshold of 1e−05

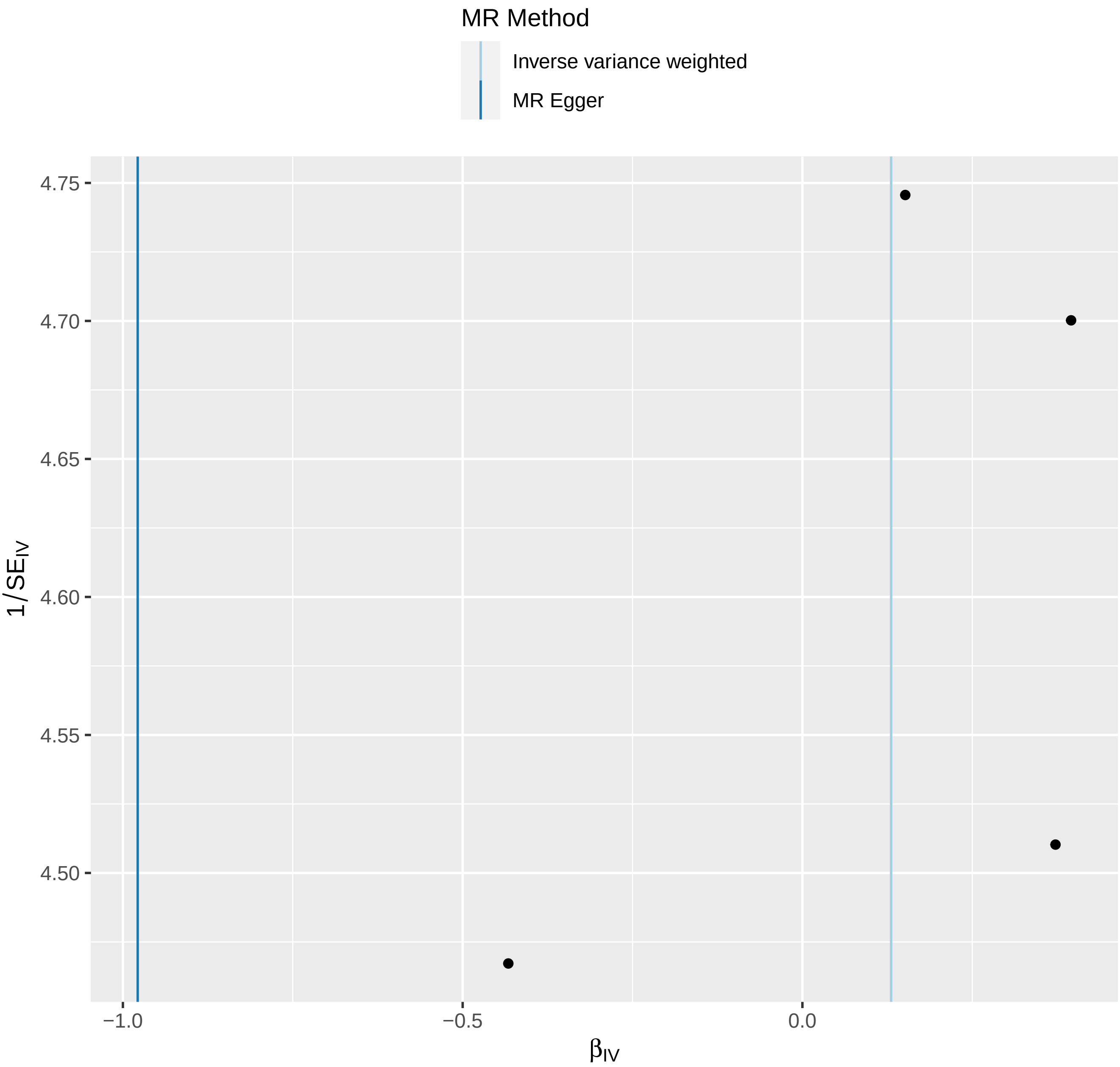

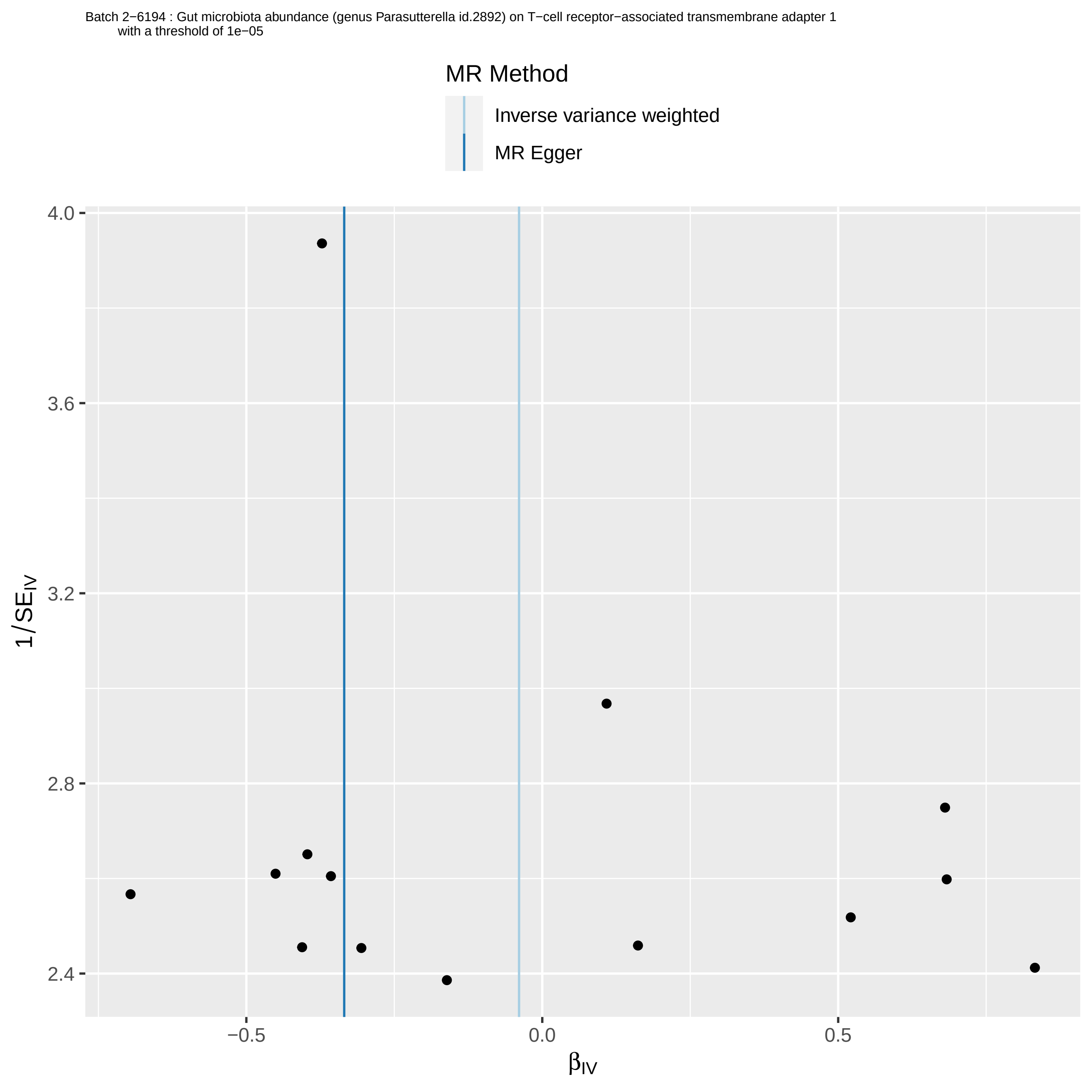
Batch 2−6194 : Gut microbiota abundance (genus Parasutterella id.2892) on T−cell receptor−associated transmembrane adapter 1
with a threshold of 1e−05
MR Method
Inverse variance weighted
MR Egger
4.0
3.6
3.2
2.8
2.4
1/SE_IV
−0.5
0.0
0.5
β_IV

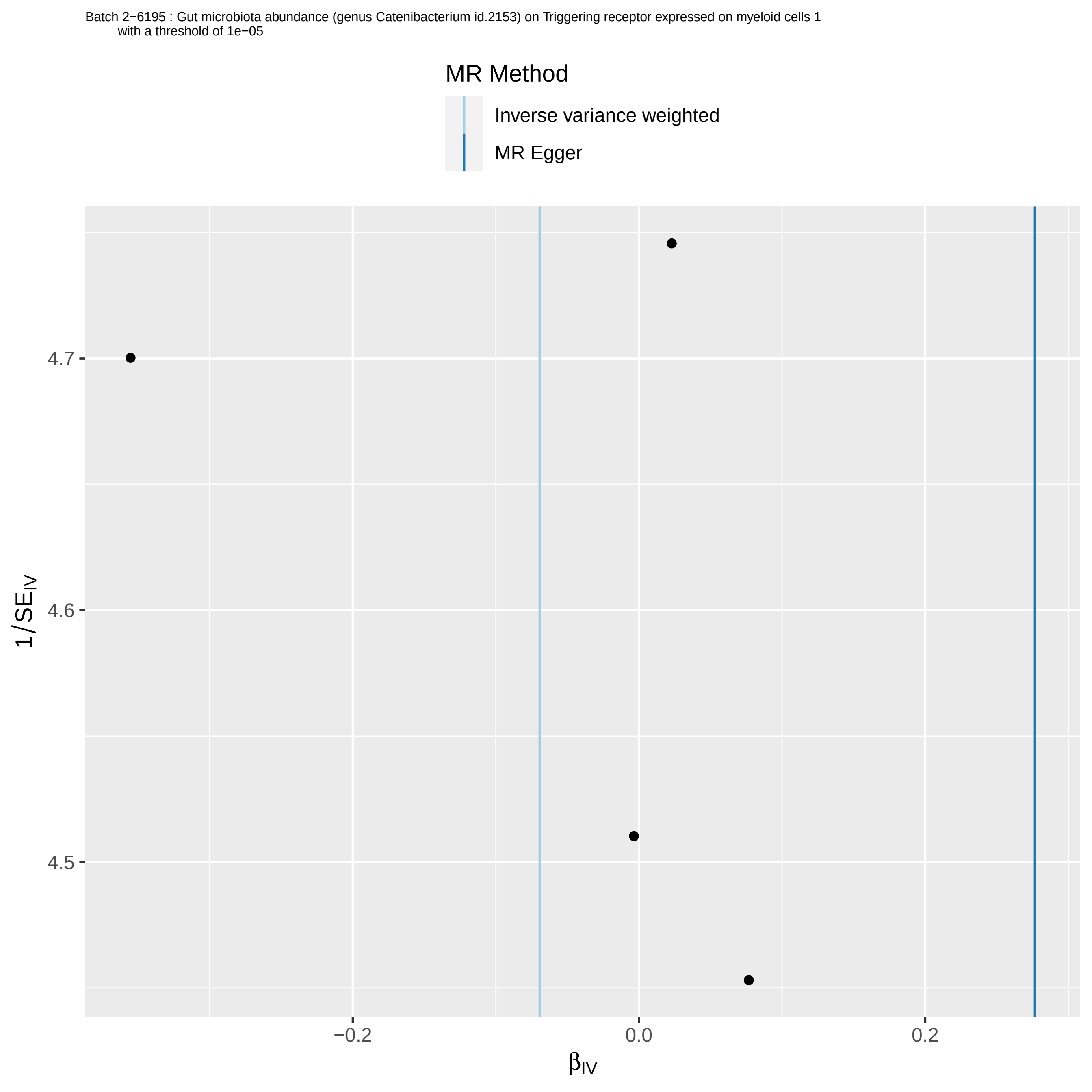
Batch 2−6195 : Gut microbiota abundance (genus Catenibacterium id.2153) on Triggering receptor expressed on myeloid cells 1
with a threshold of 1e−05
MR Method
Inverse variance weighted
MR Egger
4.7
4.6
4.5
$1/SE_{IV}$
−0.2
0.0
0.2
β_{IV}

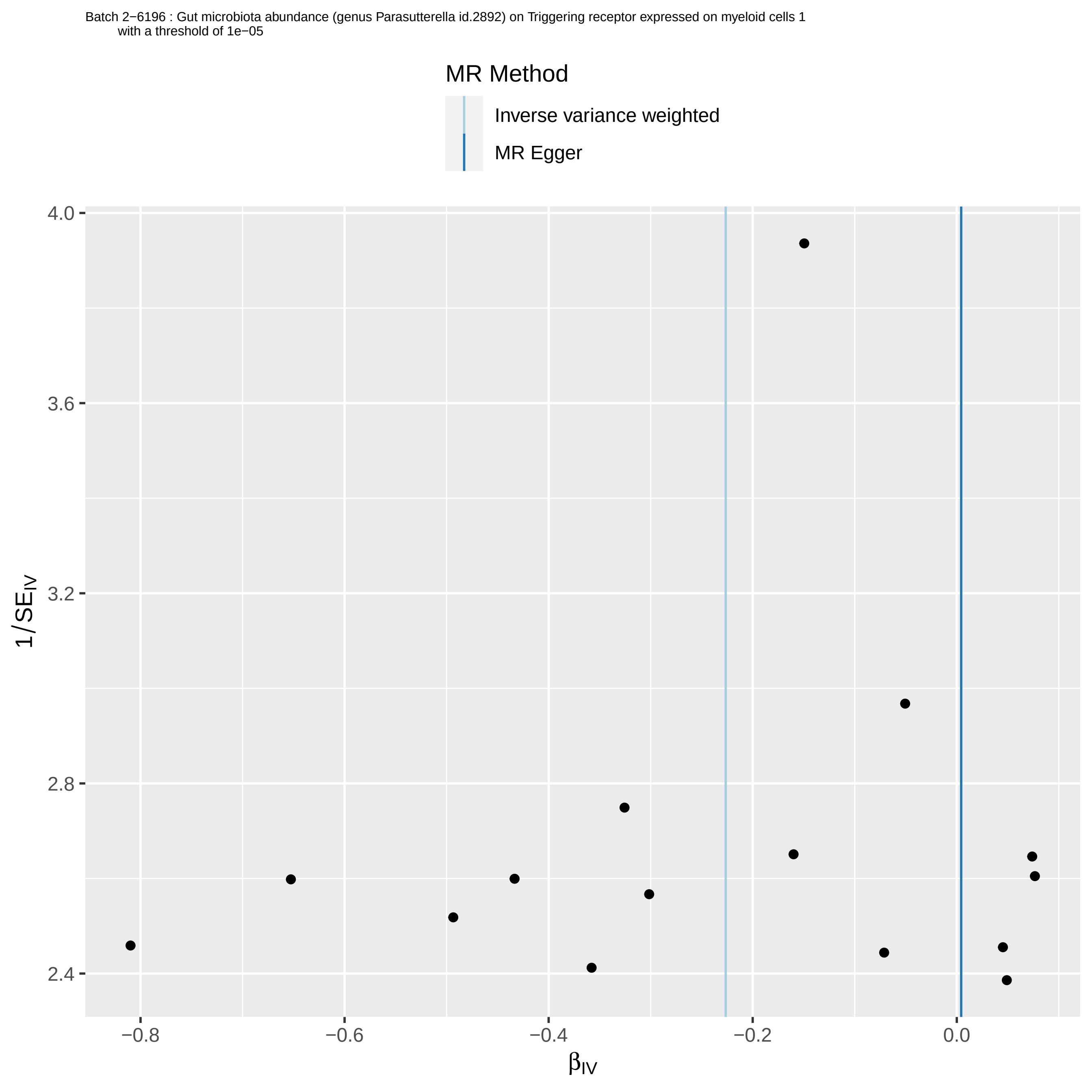
Batch 2−6196 : Gut microbiota abundance (genus Parasutterella id.2892) on Triggering receptor expressed on myeloid cells 1
with a threshold of 1e−05
MR Method
Inverse variance weighted
MR Egger
4.0
3.6
3.2
2.8
2.4
1/SE_IV
−0.8
−0.6
−0.4
−0.2
0.0
β_IV

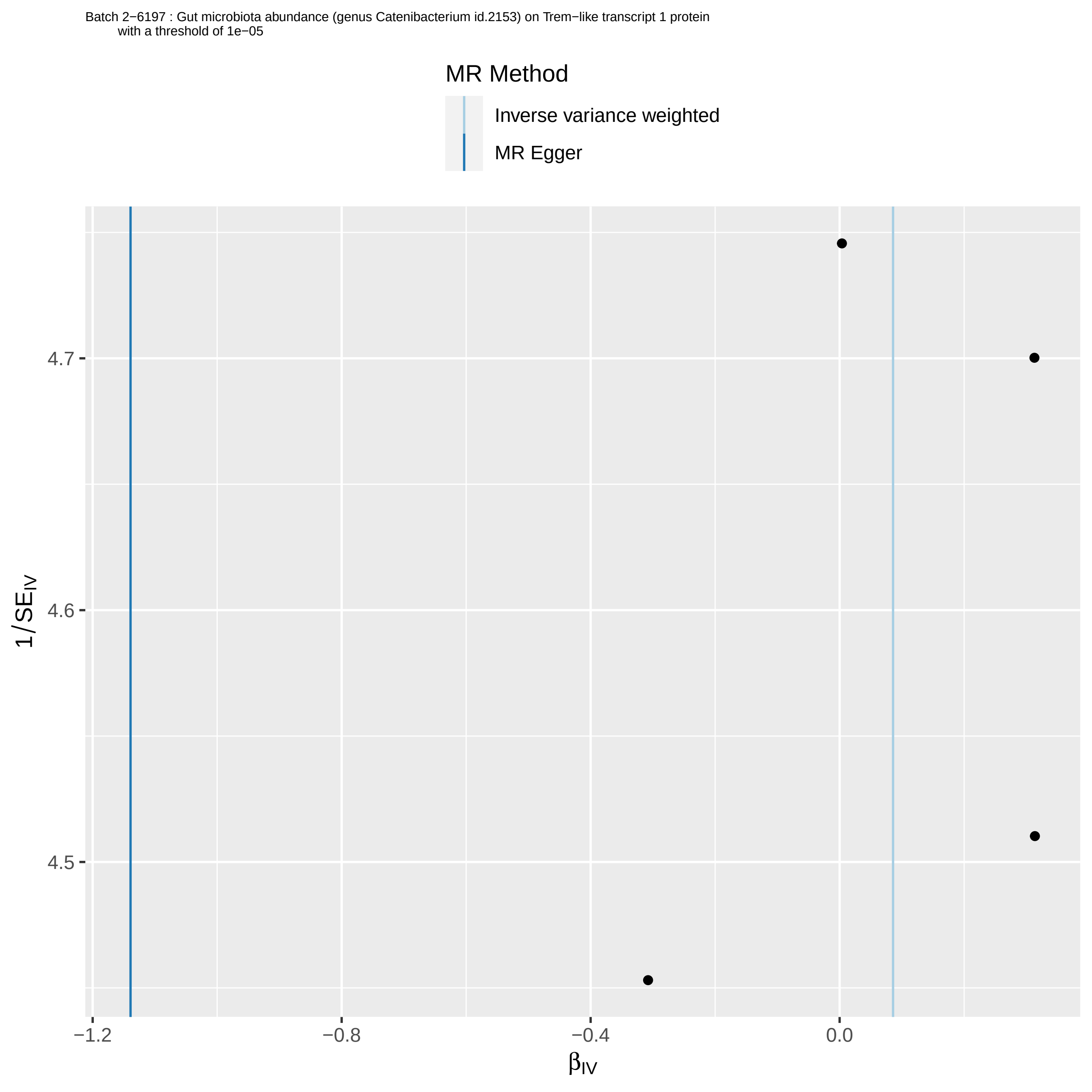

Batch 2−6197 : Gut microbiota abundance (genus Catenibacterium id.2153) on Trem−like transcript 1 protein
with a threshold of 1e−05
MR Method
Inverse variance weighted
MR Egger
4.7
4.6
4.5
$1/SE_{IV}$
−1.2
−0.8
−0.4
0.0
β_{IV}

Batch 2−6198 : Gut microbiota abundance (genus Parasutterella id.2892) on Trem−like transcript 1 protein
with a threshold of 1e−05

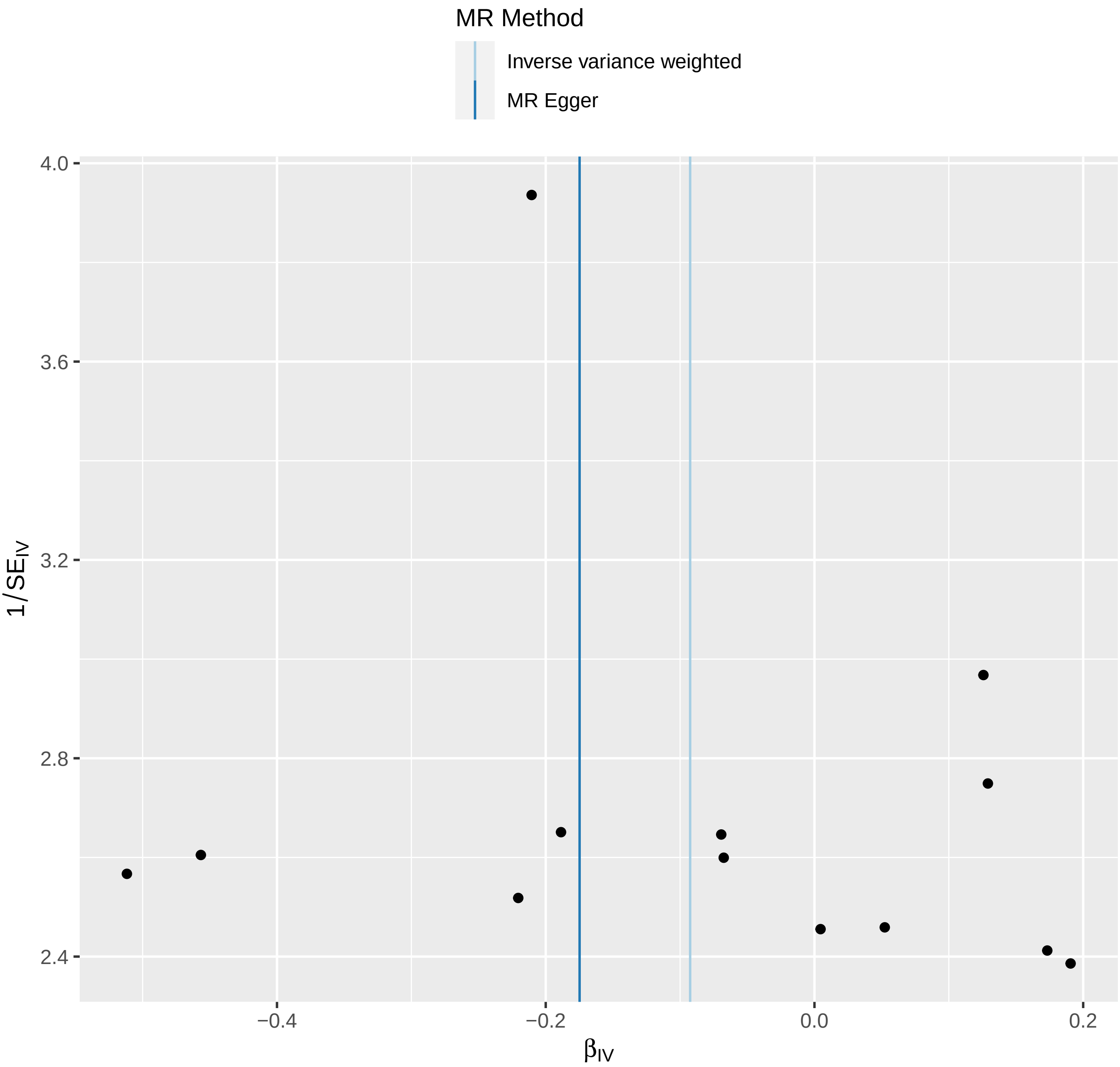

Batch 2−6199 : Gut microbiota abundance (genus Catenibacterium id.2153) on Trem−like transcript 2 protein
with a threshold of 1e−05

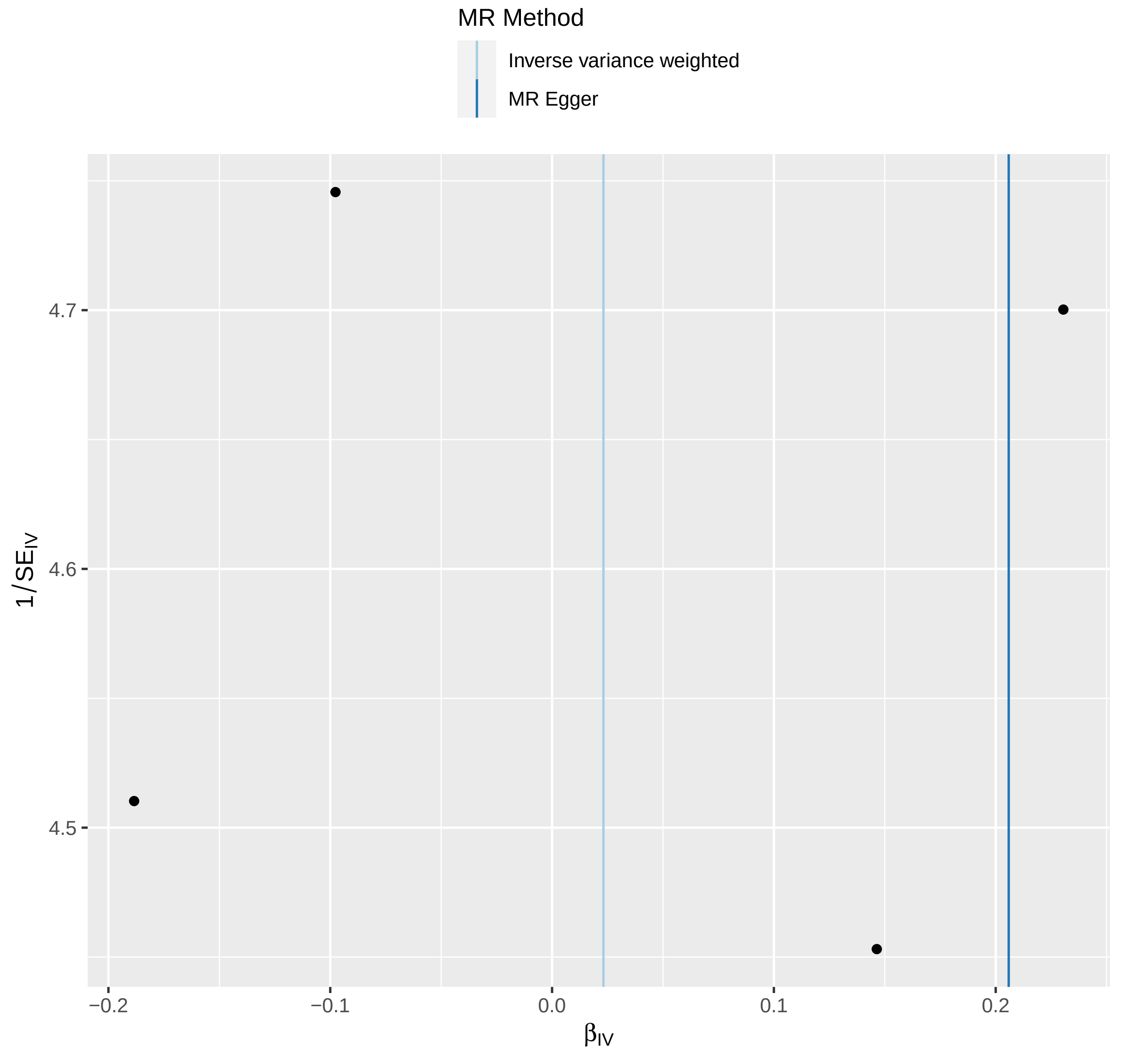

Batch 2−6200 : Gut microbiota abundance (genus Parasutterella id.2892) on Trem−like transcript 2 protein
with a threshold of 1e−05

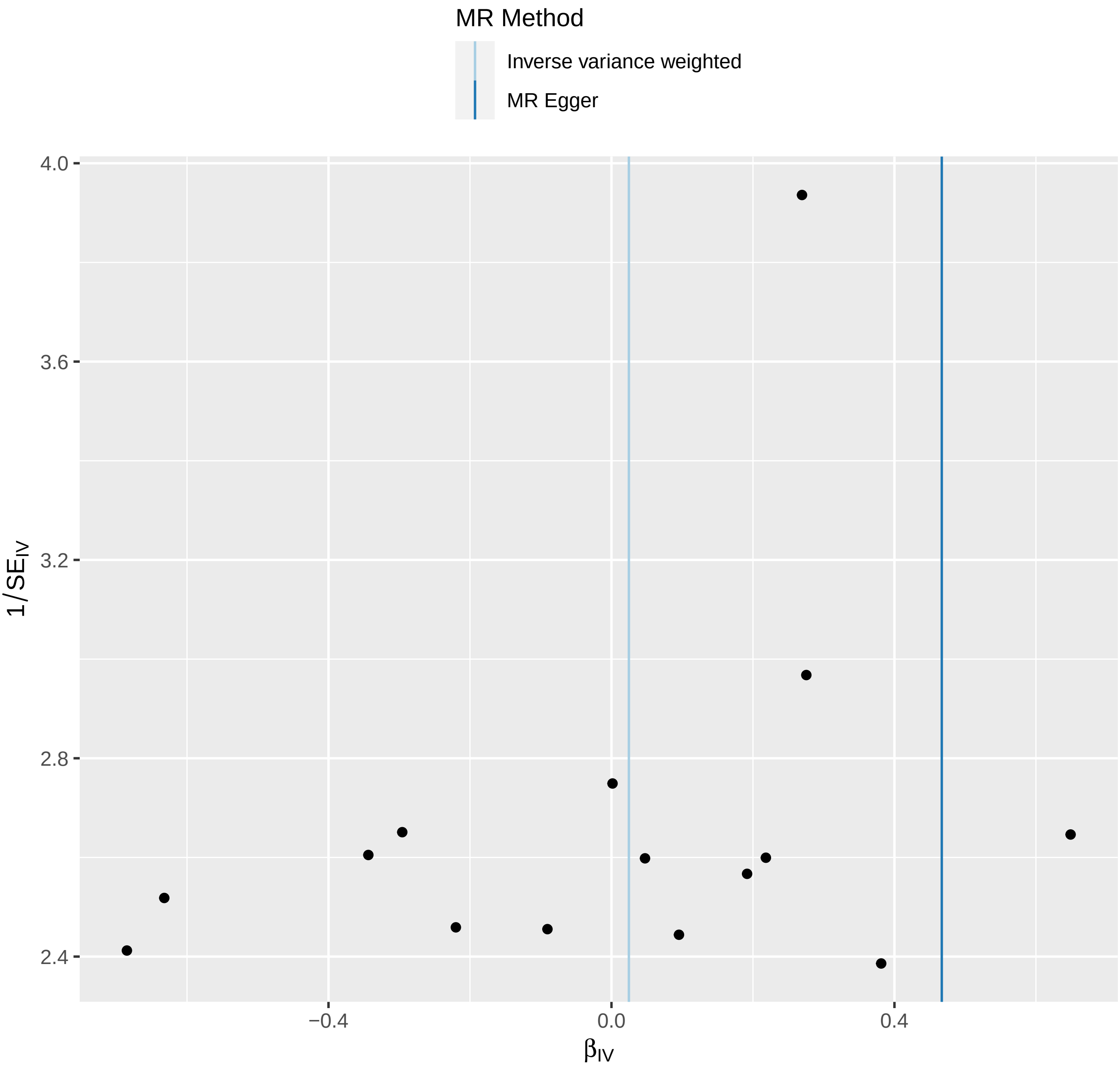

Batch 2−6201 : Gut microbiota abundance (genus Catenibacterium id.2153) on Trem−like transcript 4 protein
with a threshold of 1e−05

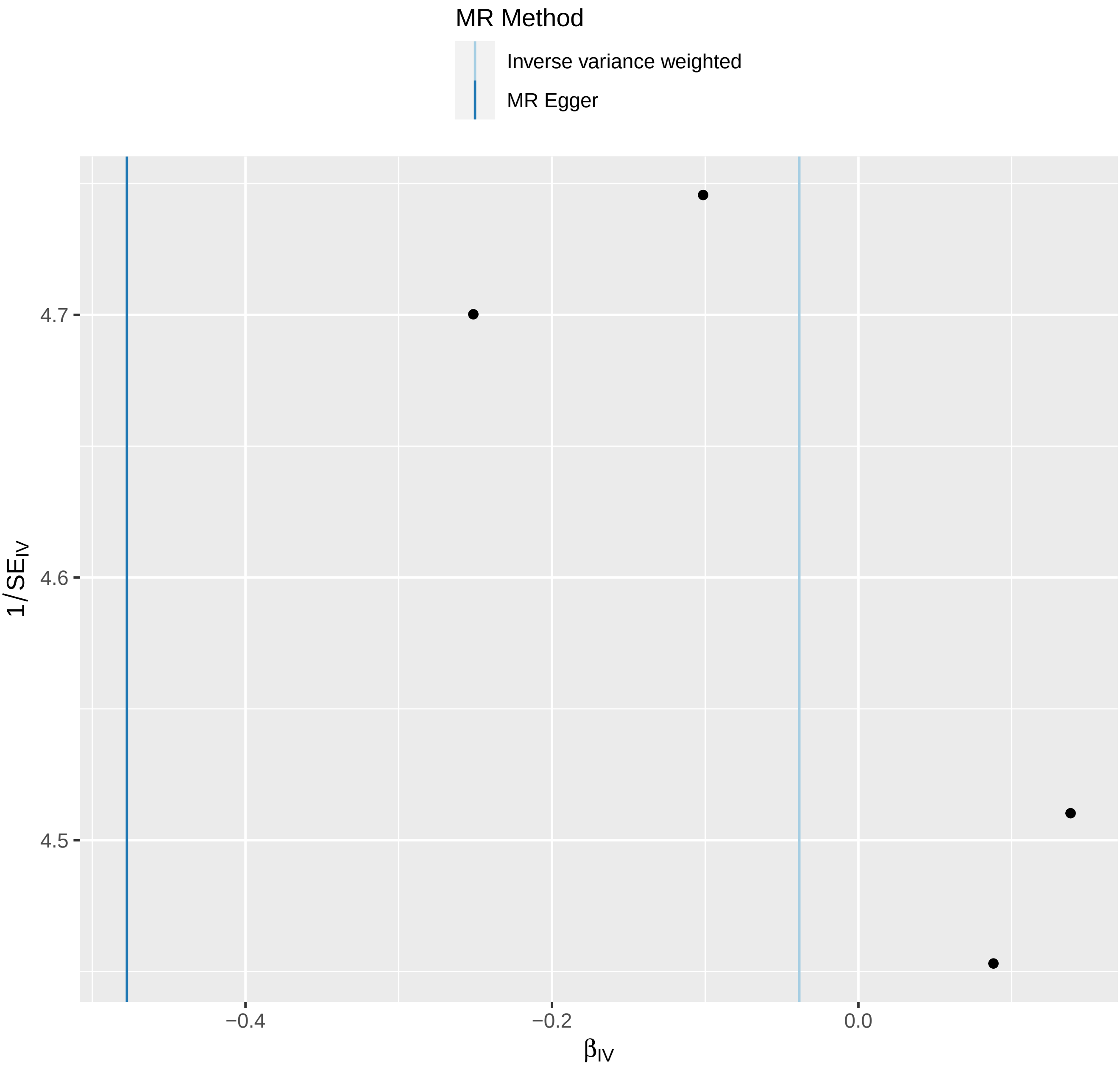

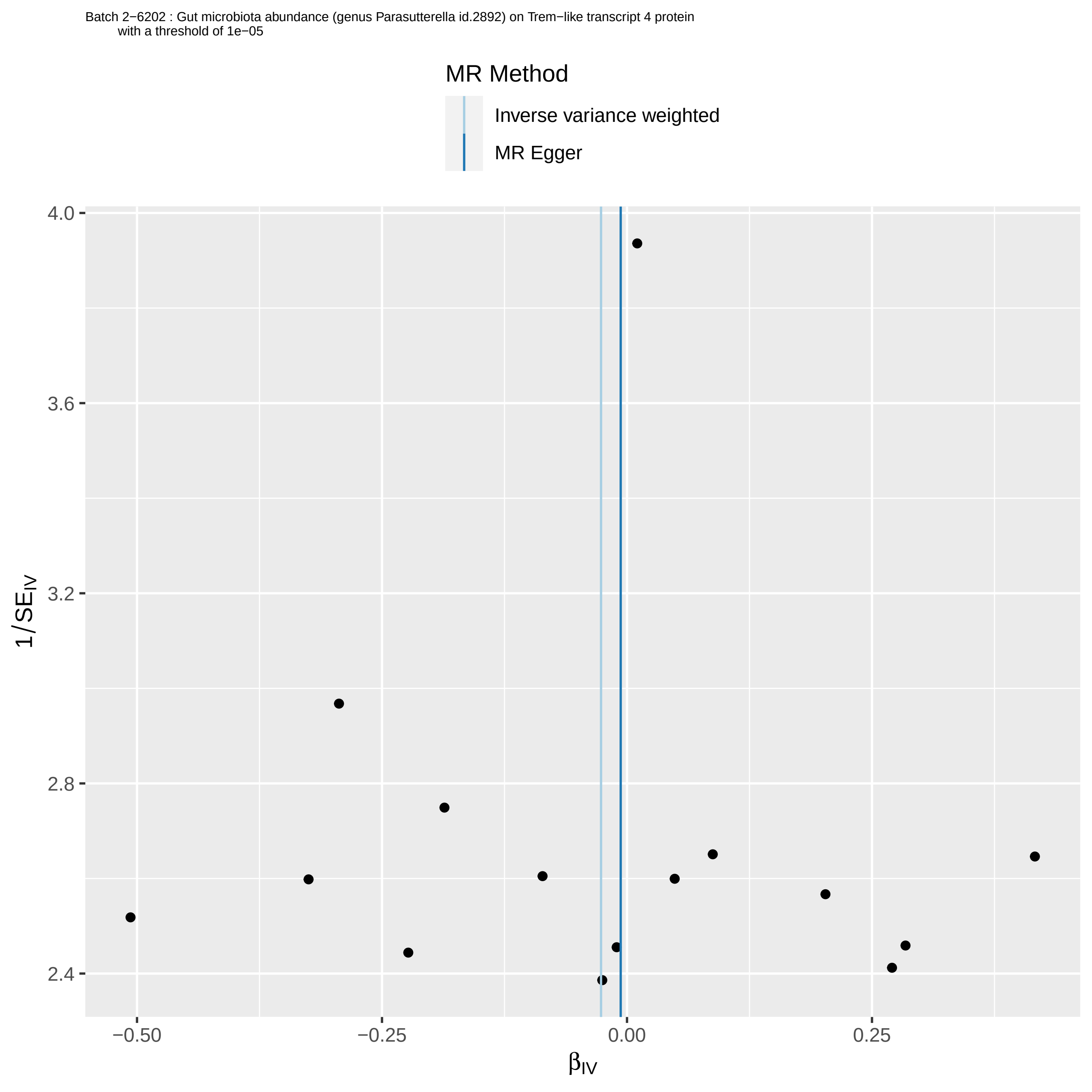
Batch 2−6202 : Gut microbiota abundance (genus Parasutterella id.2892) on Trem−like transcript 4 protein
with a threshold of 1e−05
MR Method
Inverse variance weighted
MR Egger
4.0
3.6
3.2
2.8
2.4
1/SE_IV
−0.50
−0.25
0.00
0.25
β_IV

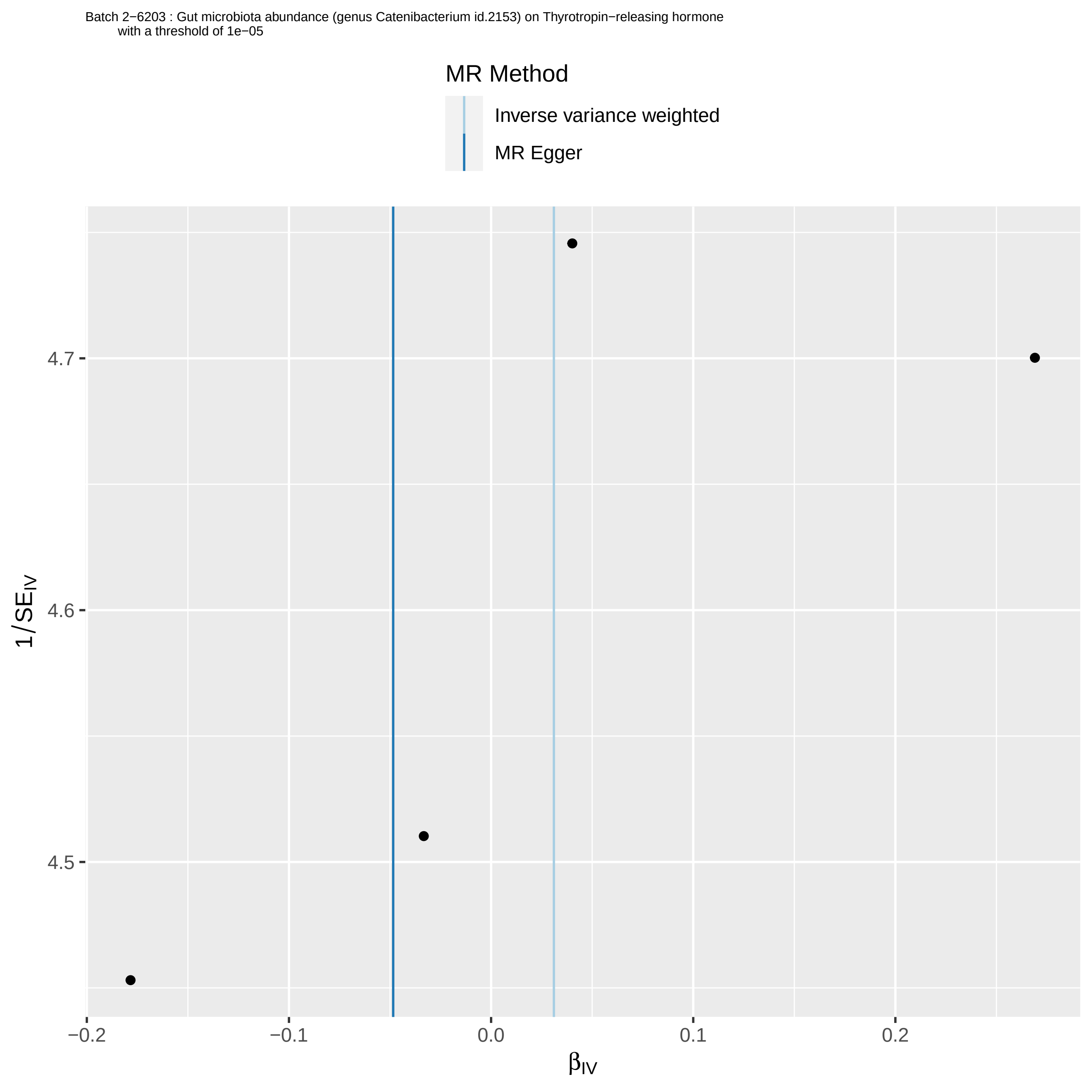

Batch 2−6203 : Gut microbiota abundance (genus Catenibacterium id.2153) on Thyrotropin−releasing hormone
with a threshold of 1e−05
MR Method
Inverse variance weighted
MR Egger
4.7
4.6
4.5
1/SE_IV
−0.2
−0.1
0.0
0.1
0.2
β_IV

Batch 2−6204 : Gut microbiota abundance (genus Parasutterella id.2892) on Thyrotropin−releasing hormone
with a threshold of 1e−05

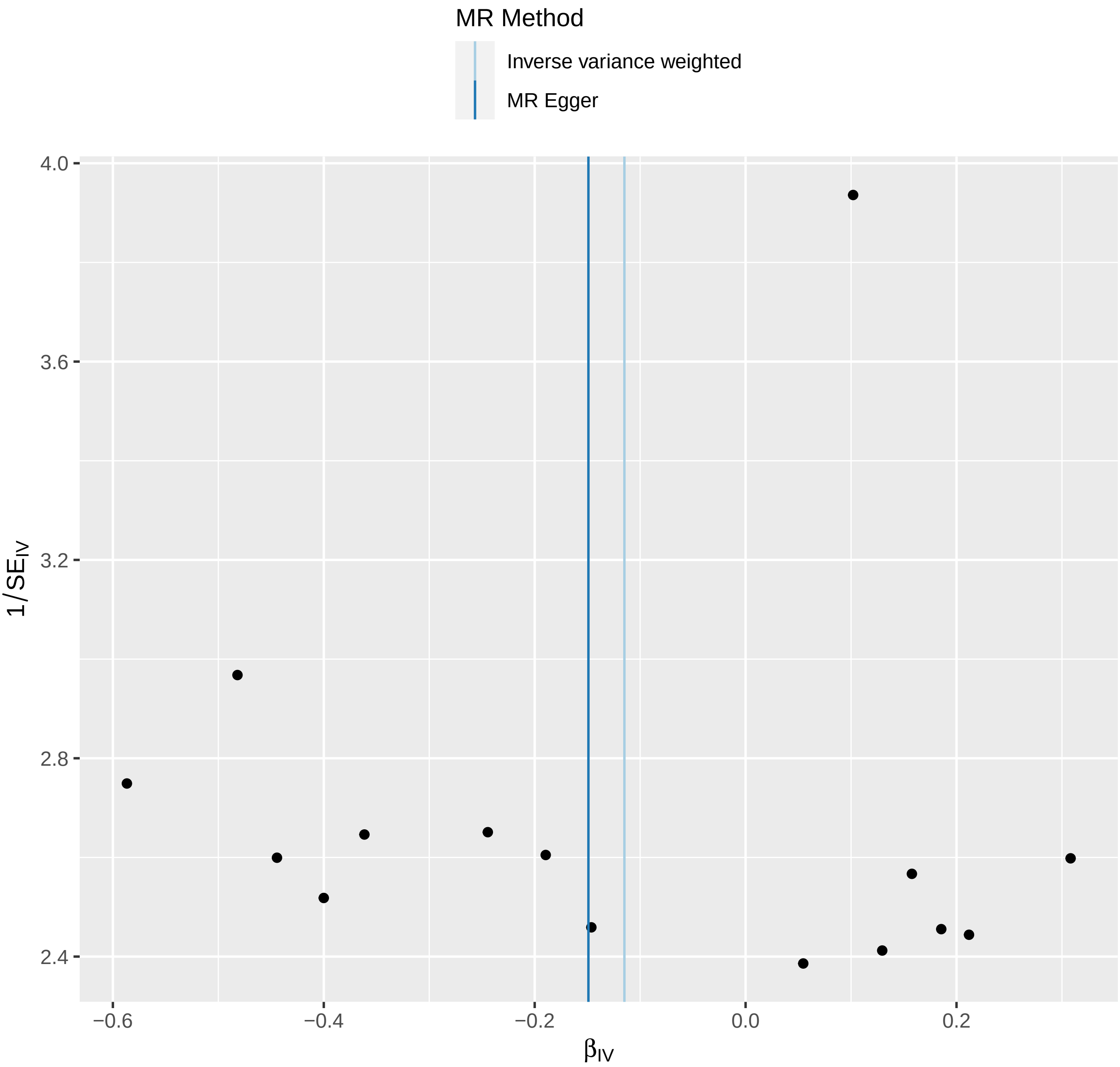

Batch 2−6205 : Gut microbiota abundance (genus Catenibacterium id.2153) on Tumor susceptibility gene 101 protein
with a threshold of 1e−05

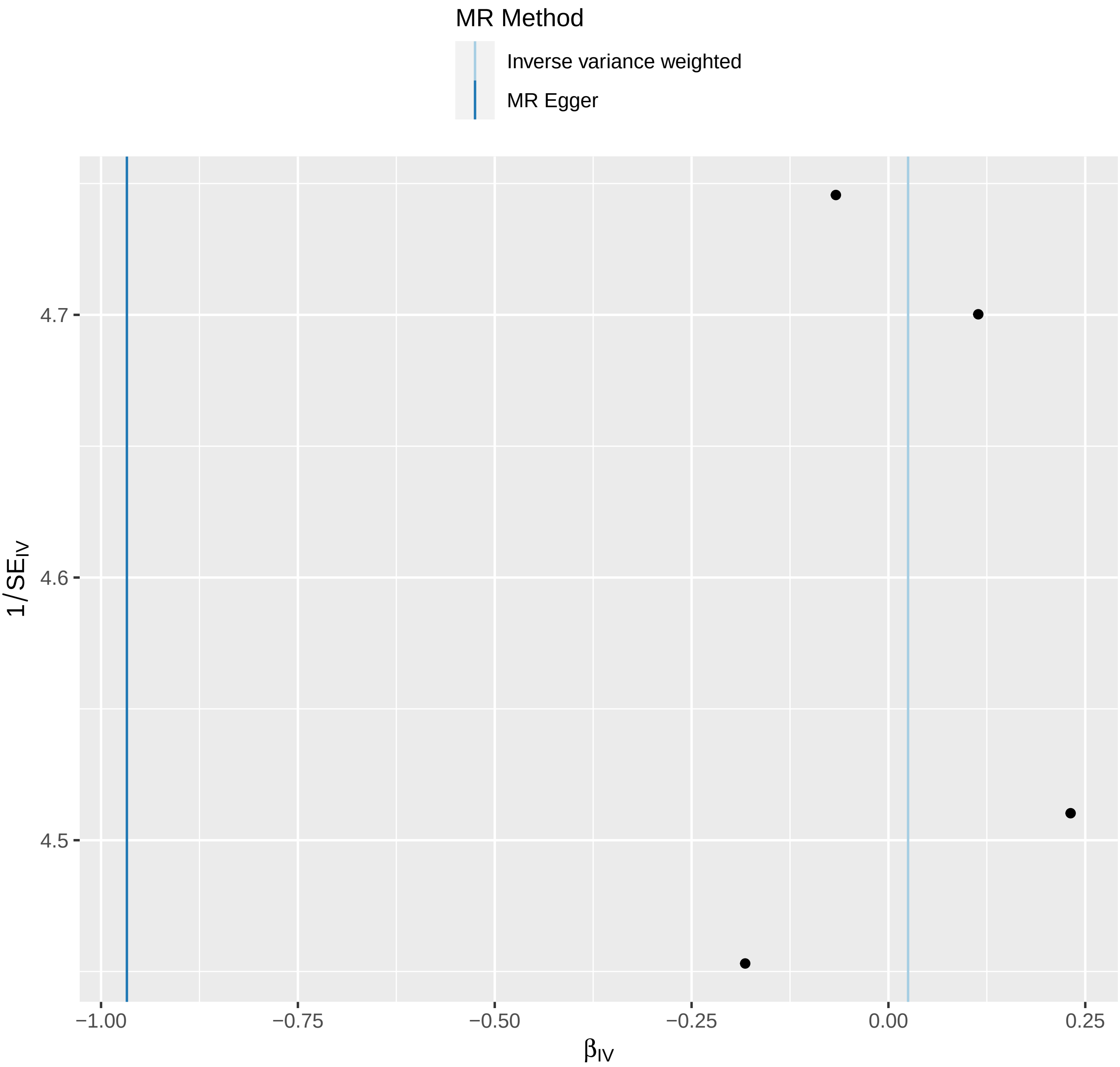

Batch 2−6206 : Gut microbiota abundance (genus Parasutterella id.2892) on Tumor susceptibility gene 101 protein
with a threshold of 1e−05

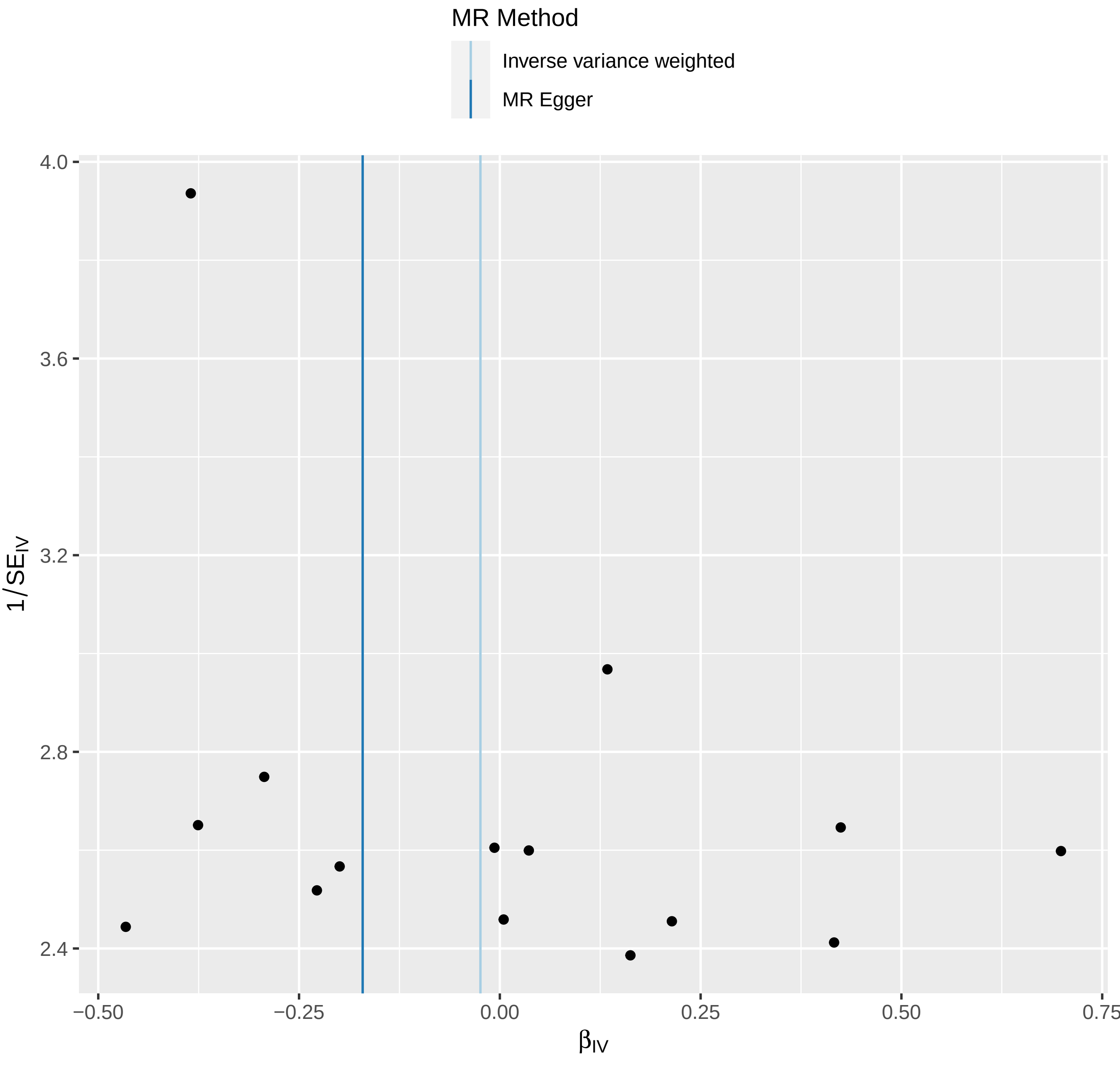

Batch 2−6207 : Gut microbiota abundance (genus Catenibacterium id.2153) on Thymic stromal lymphopoietin
with a threshold of 1e−05

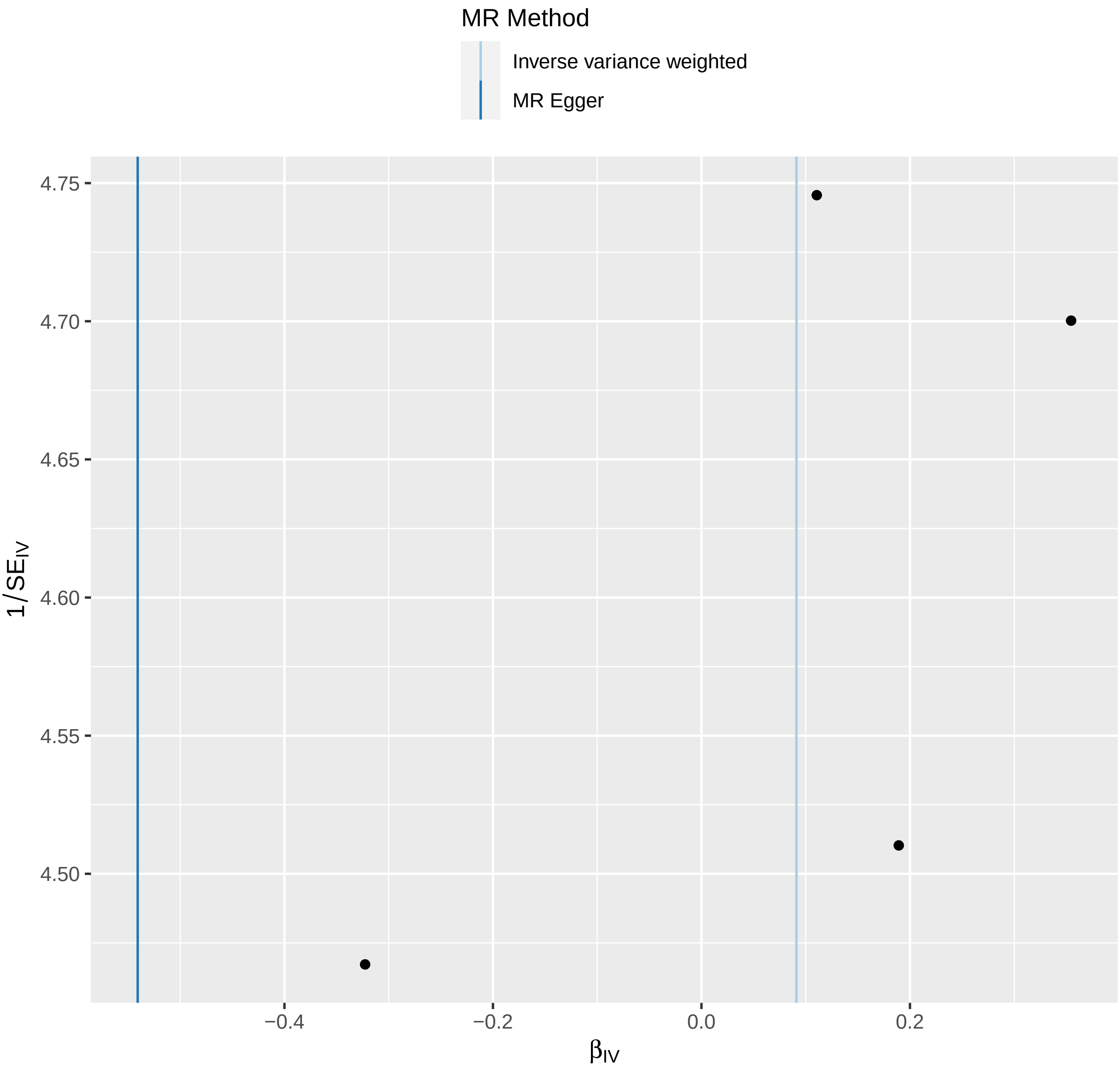

Batch 2−6208 : Gut microbiota abundance (genus Parasutterella id.2892) on Thymic stromal lymphopoietin
with a threshold of 1e−05

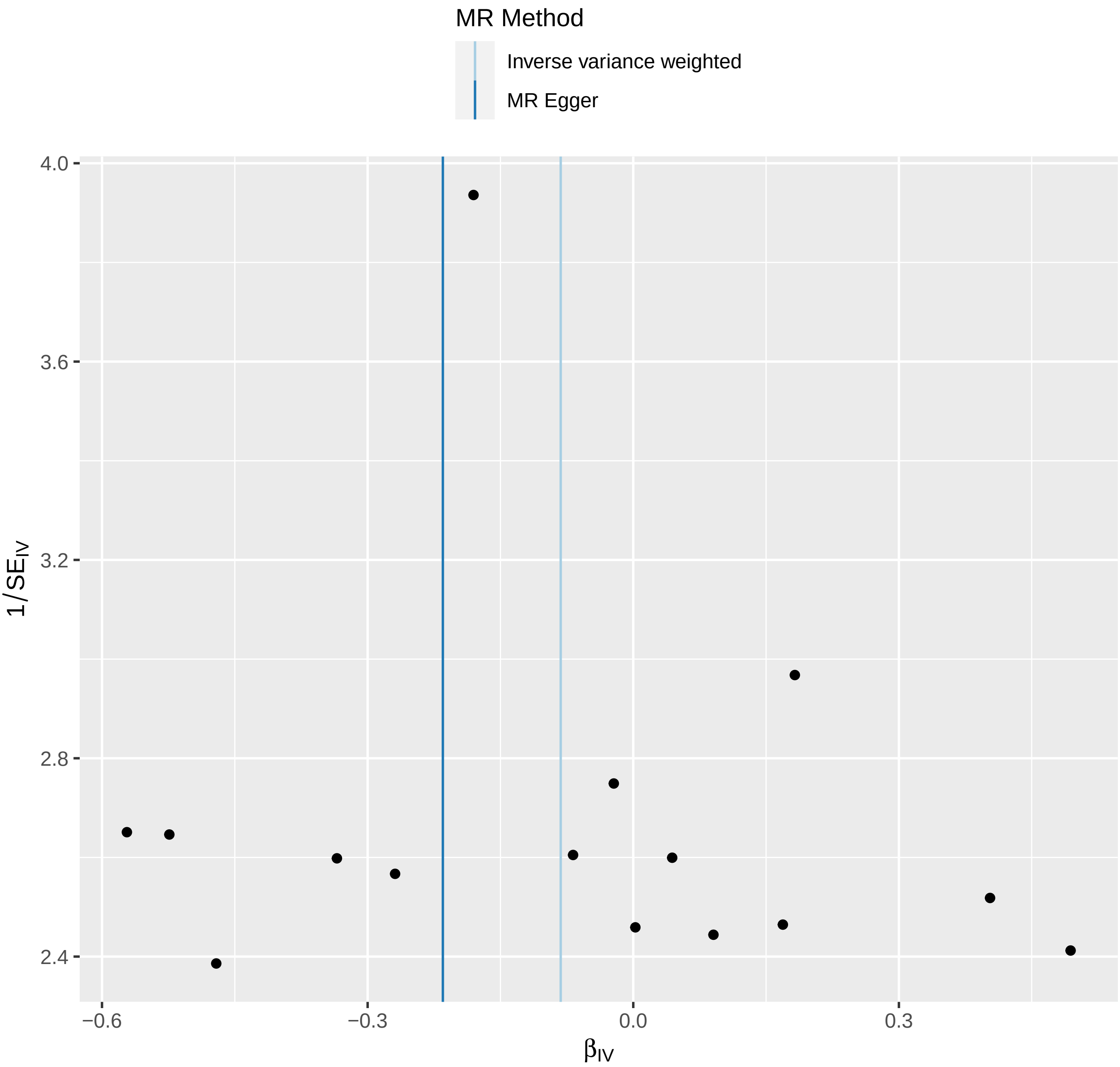

Batch 2−6209 : Gut microbiota abundance (genus Catenibacterium id.2153) on Translin
with a threshold of 1e−05

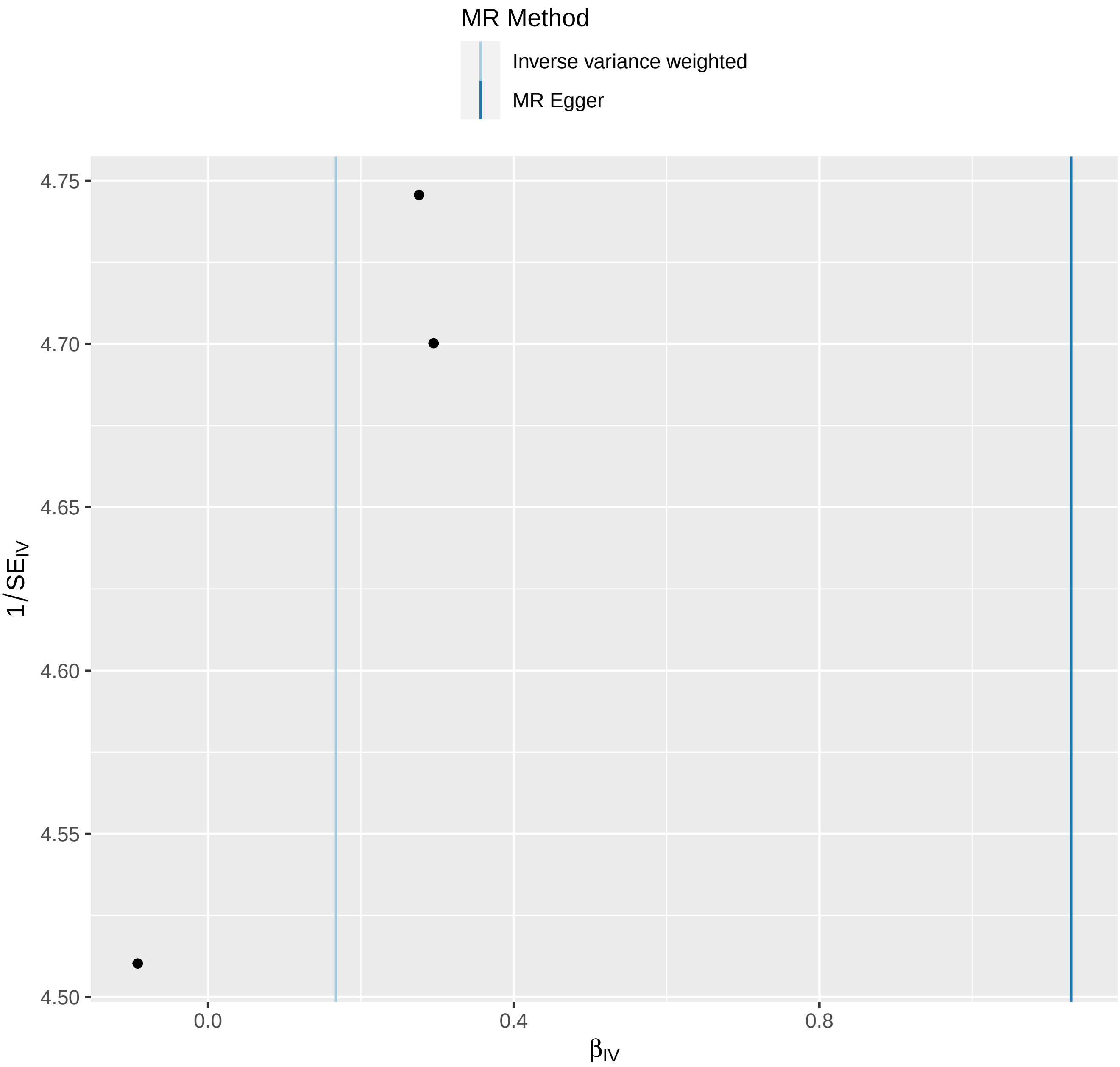

Batch 2−6210 : Gut microbiota abundance (genus Parasutterella id.2892) on Translin
with a threshold of 1e−05

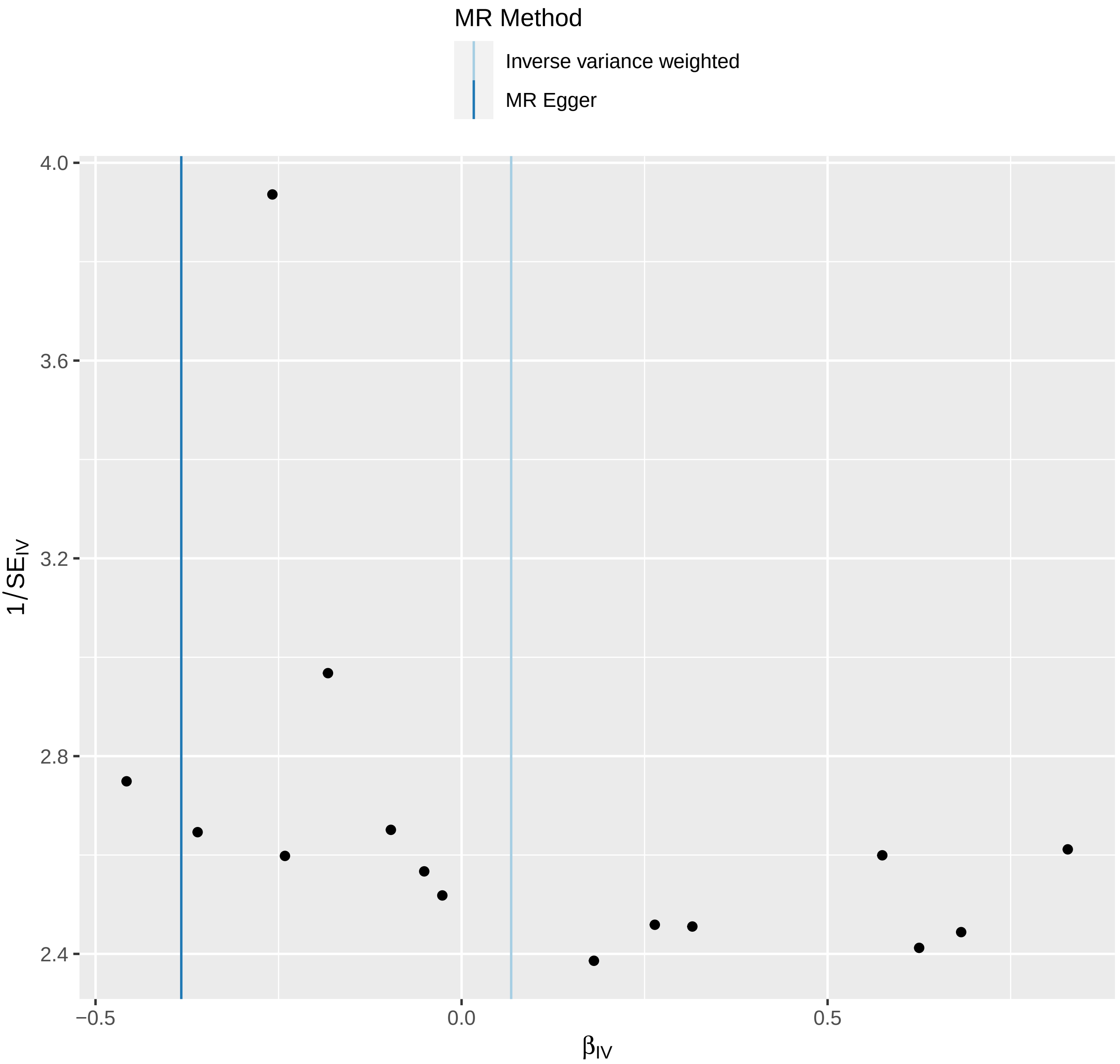

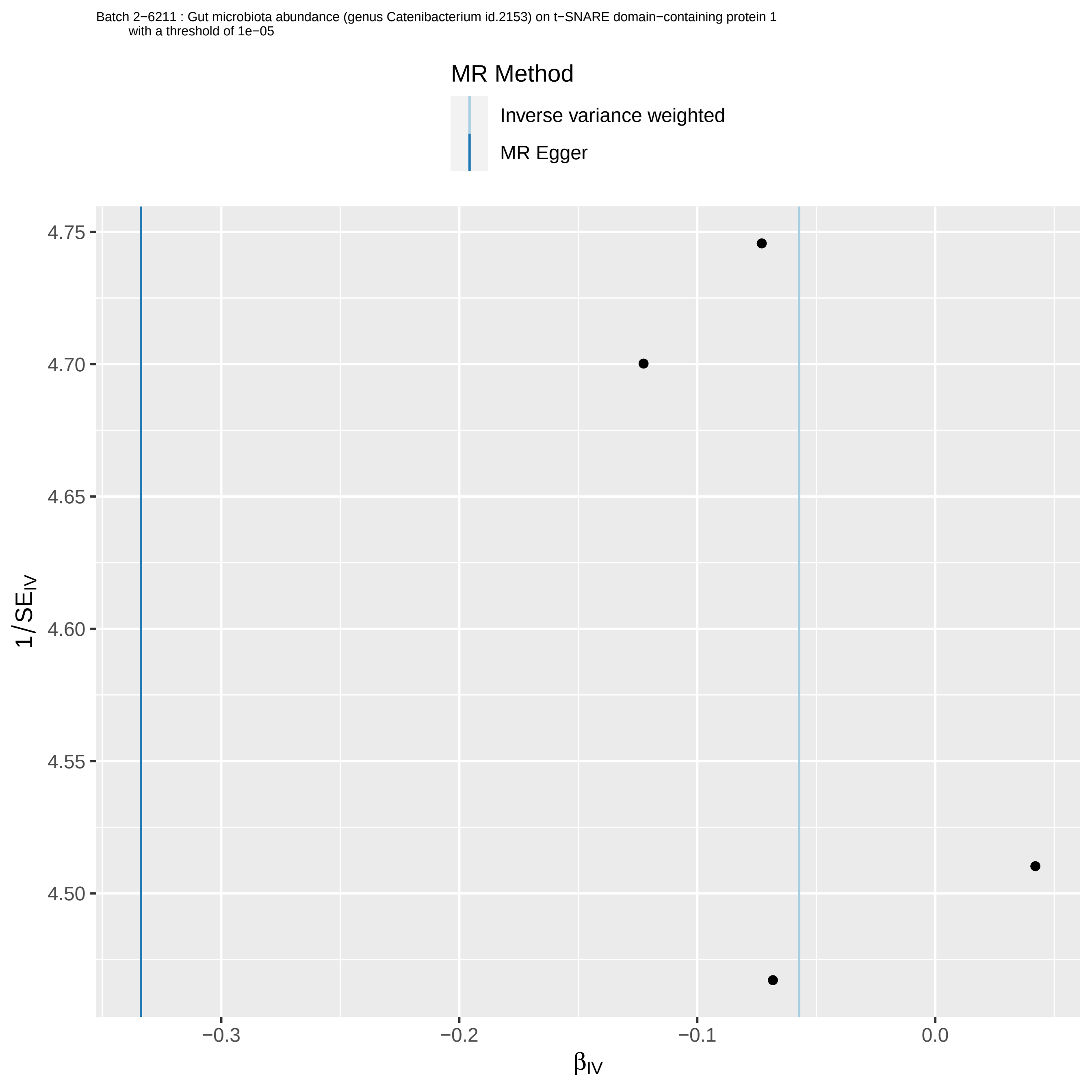

Batch 2−6211 : Gut microbiota abundance (genus Catenibacterium id.2153) on t−SNARE domain−containing protein 1
with a threshold of 1e−05
MR Method
Inverse variance weighted
MR Egger
4.75
4.70
4.65
4.60
4.55
4.50
1/SE_IV
−0.3
−0.2
−0.1
0.0
β_IV

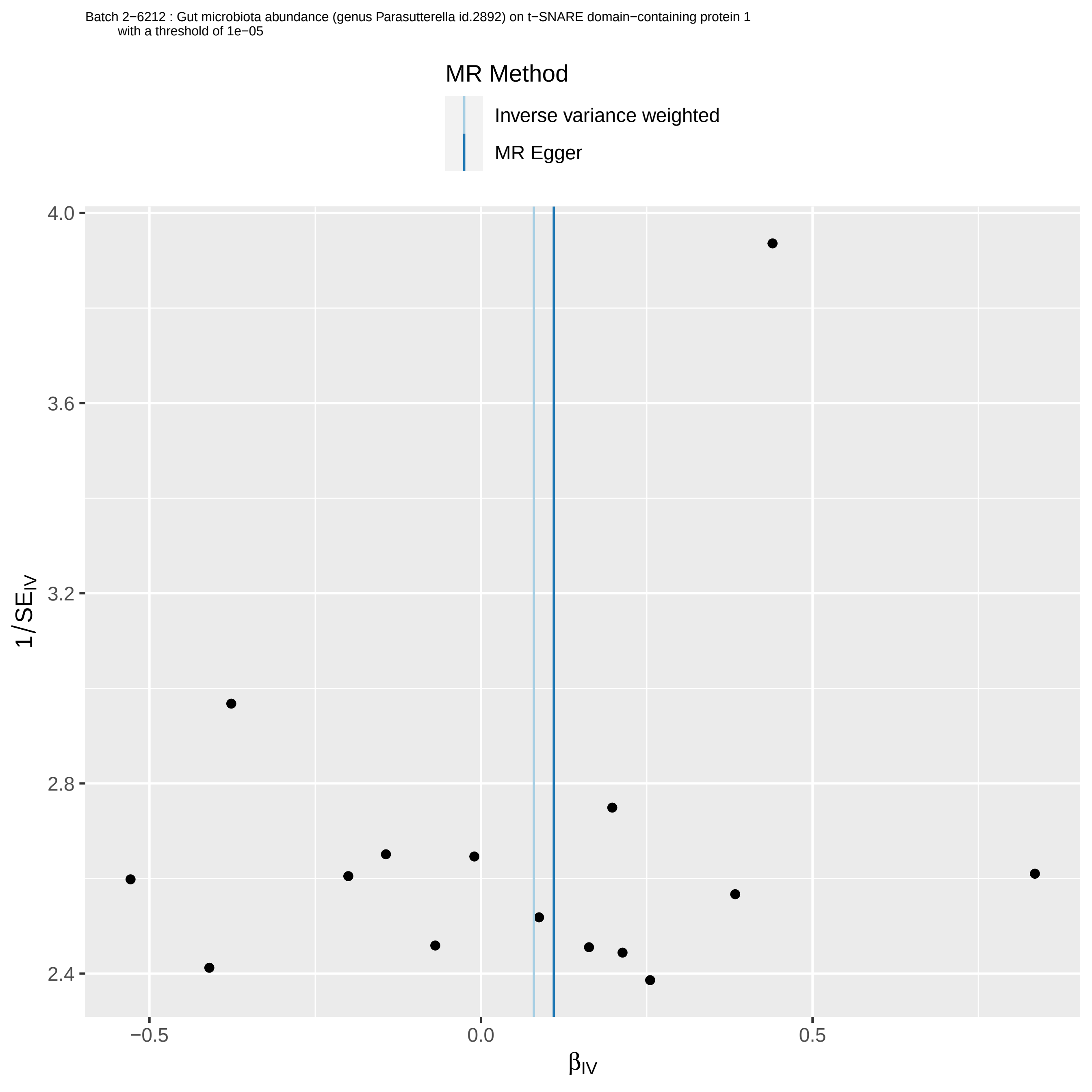

Batch 2−6212 : Gut microbiota abundance (genus Parasutterella id.2892) on t−SNARE domain−containing protein 1
with a threshold of 1e−05
MR Method
Inverse variance weighted
MR Egger
4.0
3.6
3.2
2.8
2.4
1/SE_IV
−0.5
0.0
0.5
β_IV

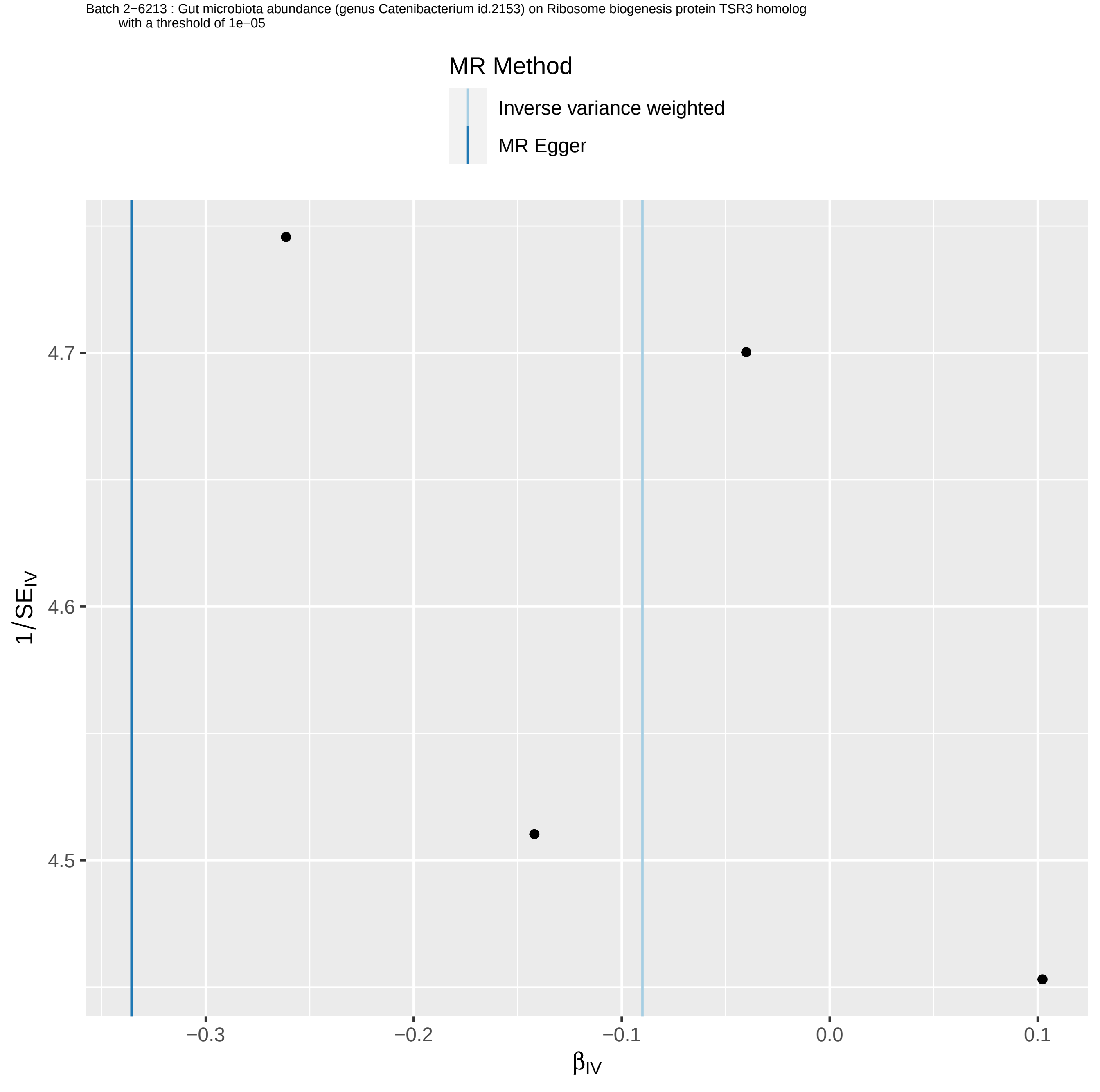

Batch 2−6213 : Gut microbiota abundance (genus Catenibacterium id.2153) on Ribosome biogenesis protein TSR3 homolog
with a threshold of 1e−05
MR Method
Inverse variance weighted
MR Egger
4.7
4.6
4.5
1/SE_IV
−0.3
−0.2
−0.1
0.0
0.1
β_IV

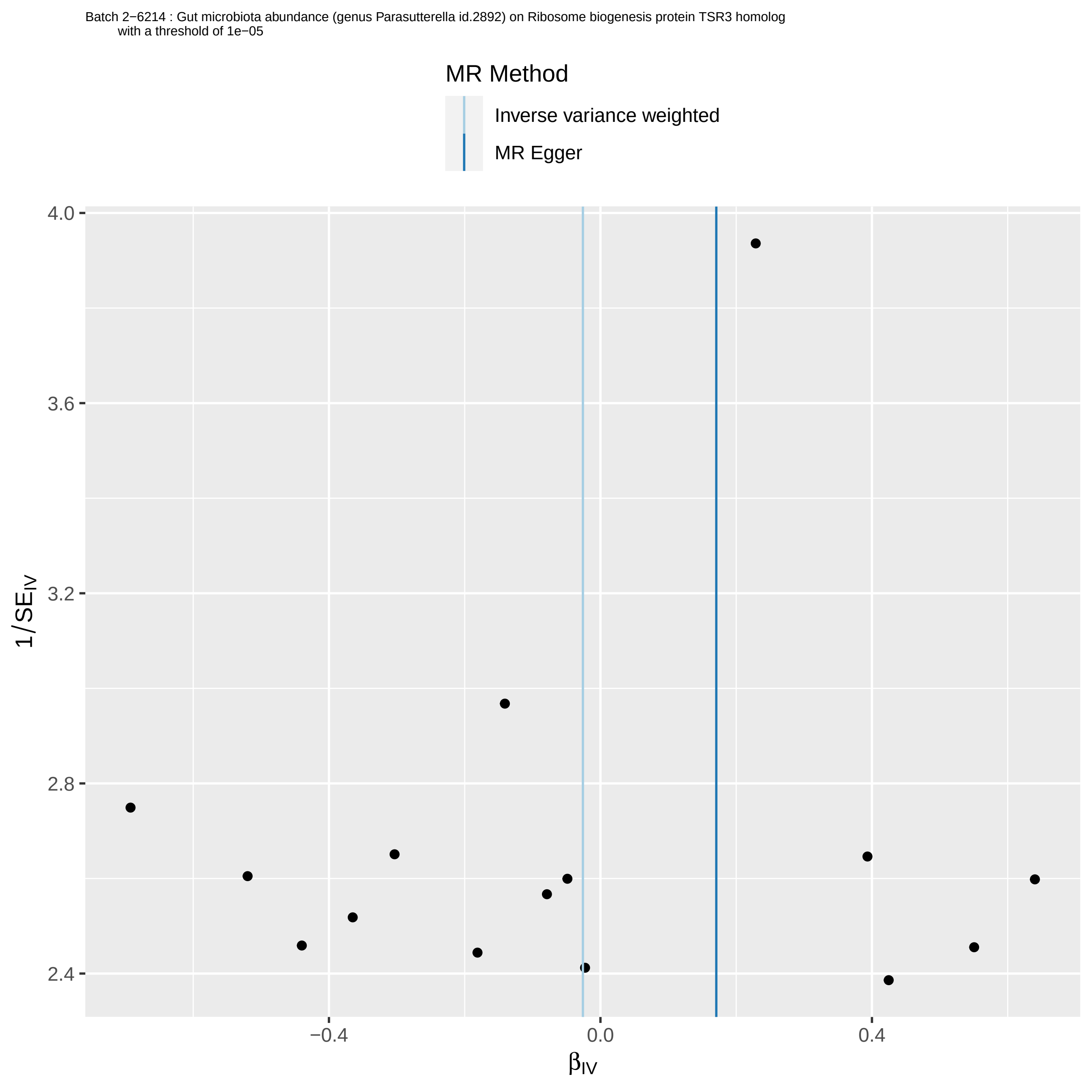
Batch 2−6214 : Gut microbiota abundance (genus Parasutterella id.2892) on Ribosome biogenesis protein TSR3 homolog
with a threshold of 1e−05
MR Method
Inverse variance weighted
MR Egger
4.0
3.6
3.2
2.8
2.4
1/SE_IV
−0.4
0.0
0.4
β_IV

Batch 2−6215 : Gut microbiota abundance (genus Catenibacterium id.2153) on Testis−specific serine/threonine−protein kinase 2 with a threshold of 1e−05

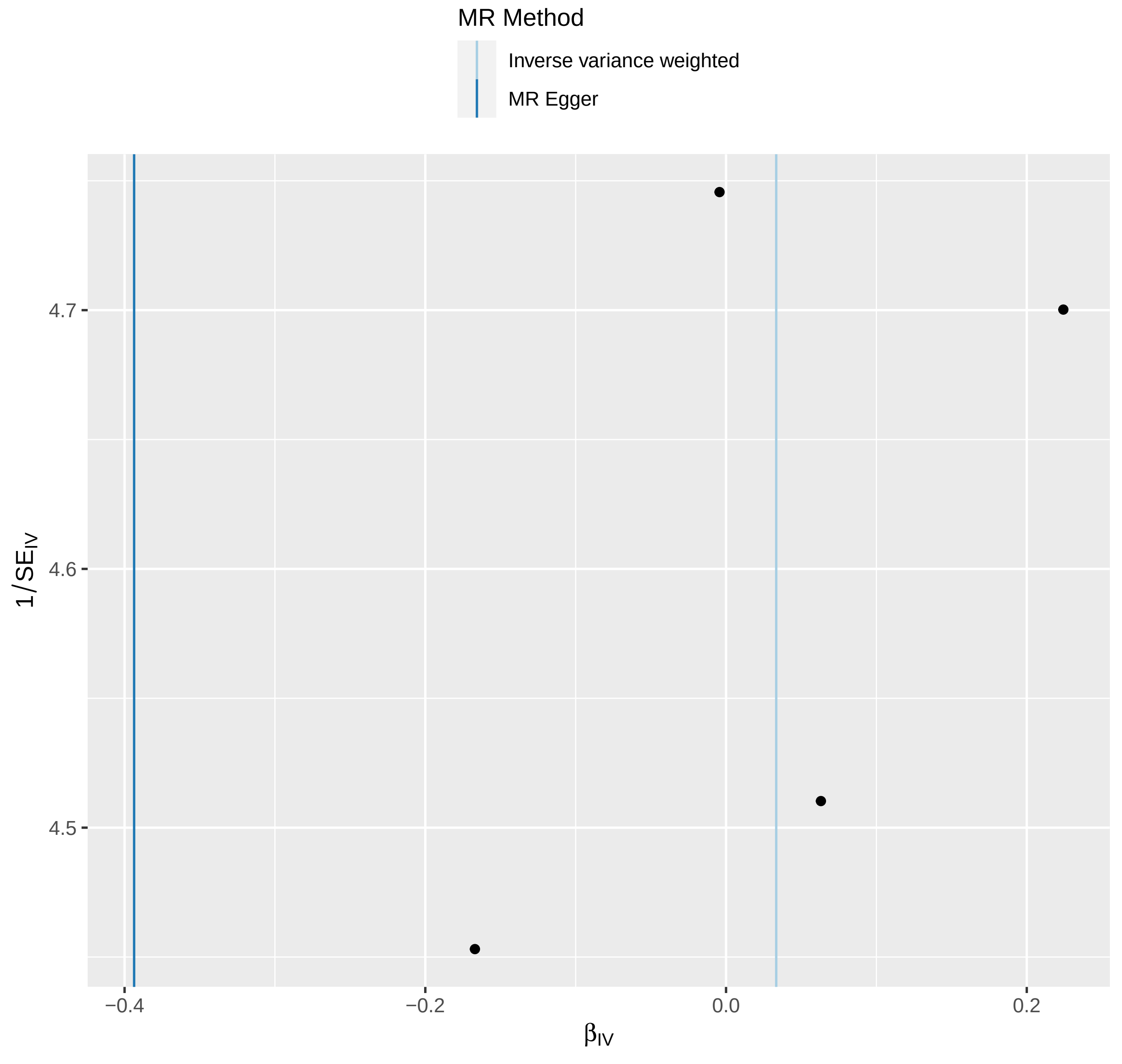

Batch 2−6216 : Gut microbiota abundance (genus Parasutterella id.2892) on Testis−specific serine/threonine−protein kinase 2
with a threshold of 1e−05

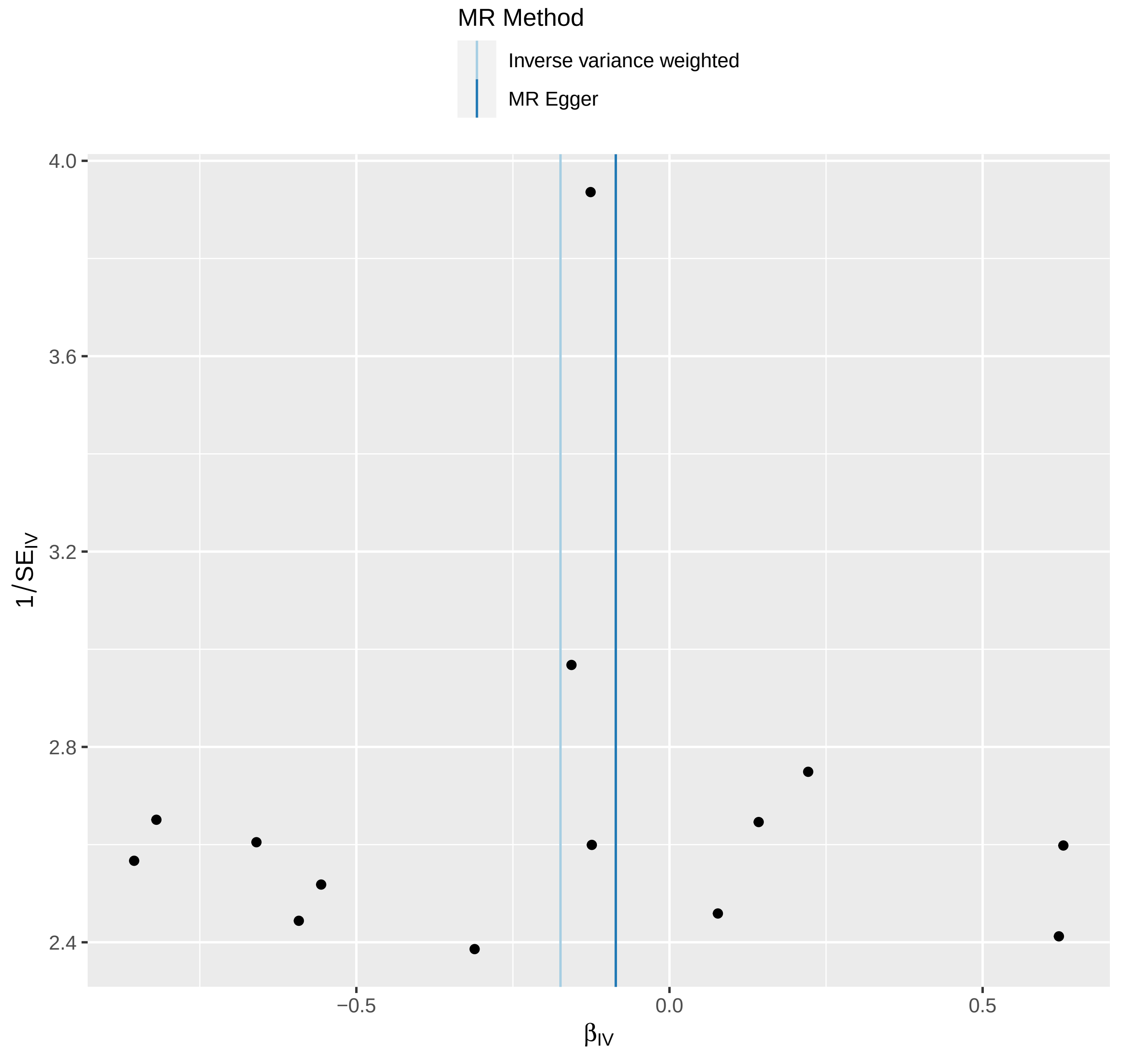

Batch 2−6217 : Gut microbiota abundance (genus Catenibacterium id.2153) on Thiosulfate sulfurtransferase
with a threshold of 1e−05

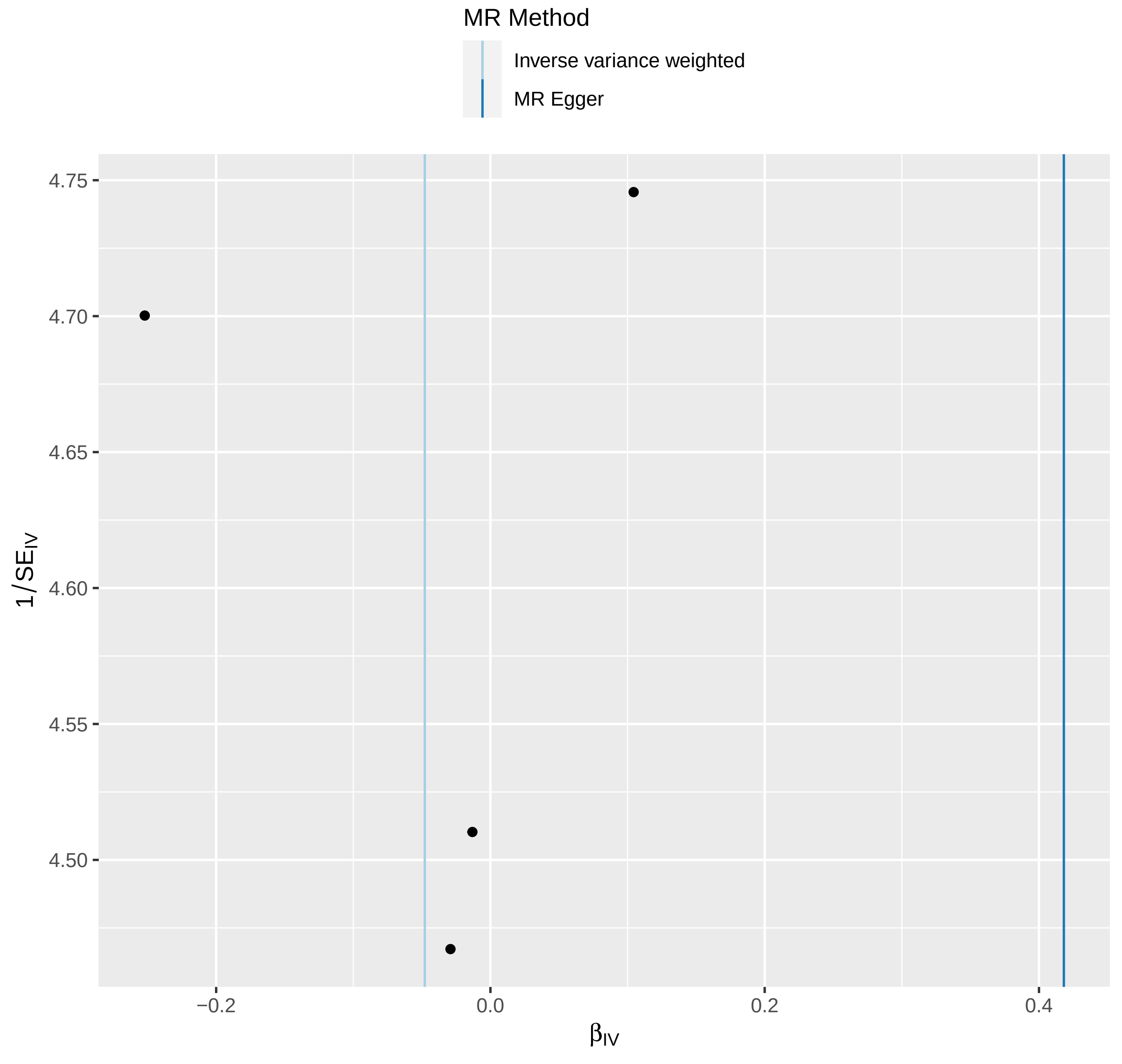

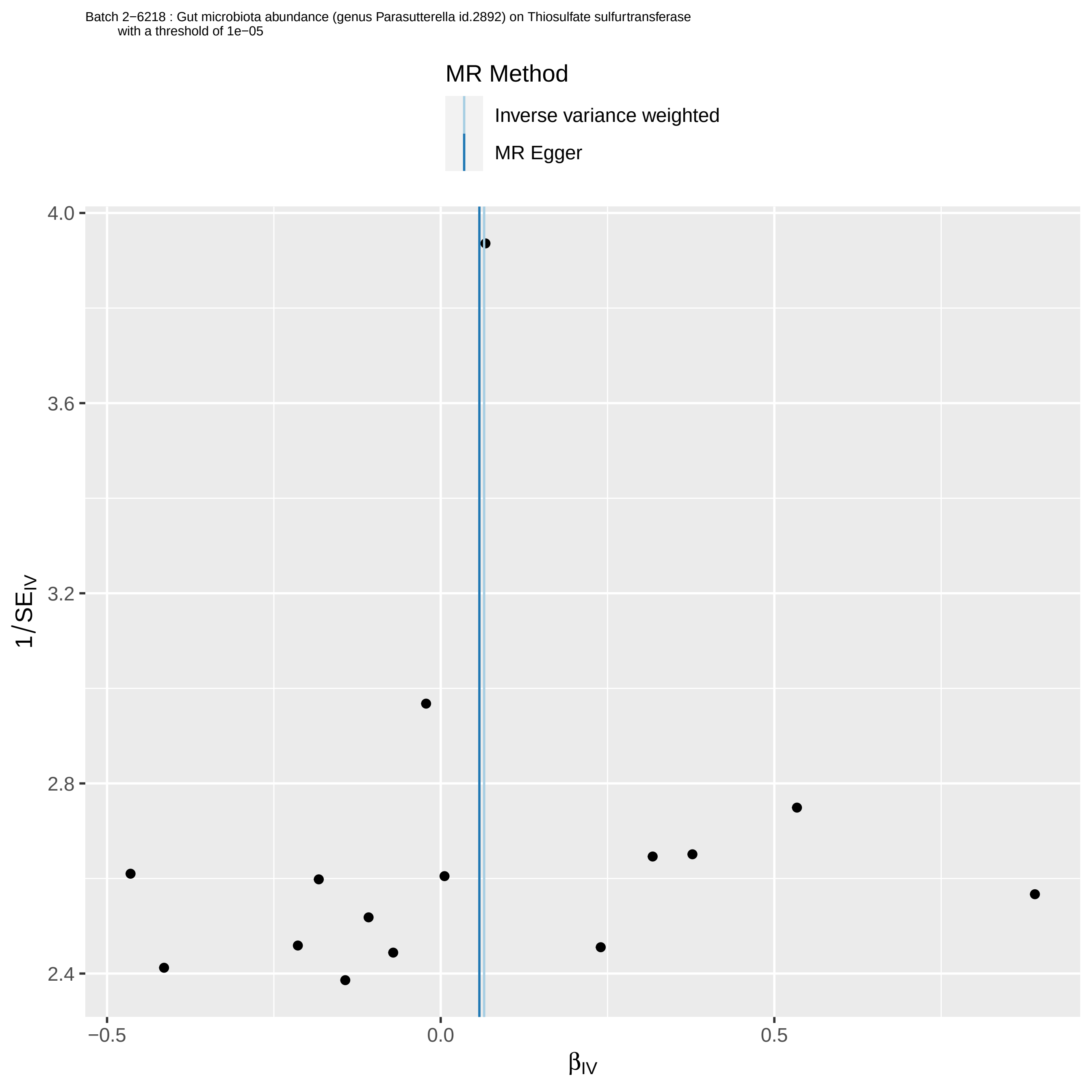
Batch 2−6218 : Gut microbiota abundance (genus Parasutterella id.2892) on Thiosulfate sulfurtransferase
with a threshold of 1e−05
MR Method
Inverse variance weighted
MR Egger
4.0
3.6
3.2
2.8
2.4
1/SE_IV
−0.5
0.0
0.5
β_IV

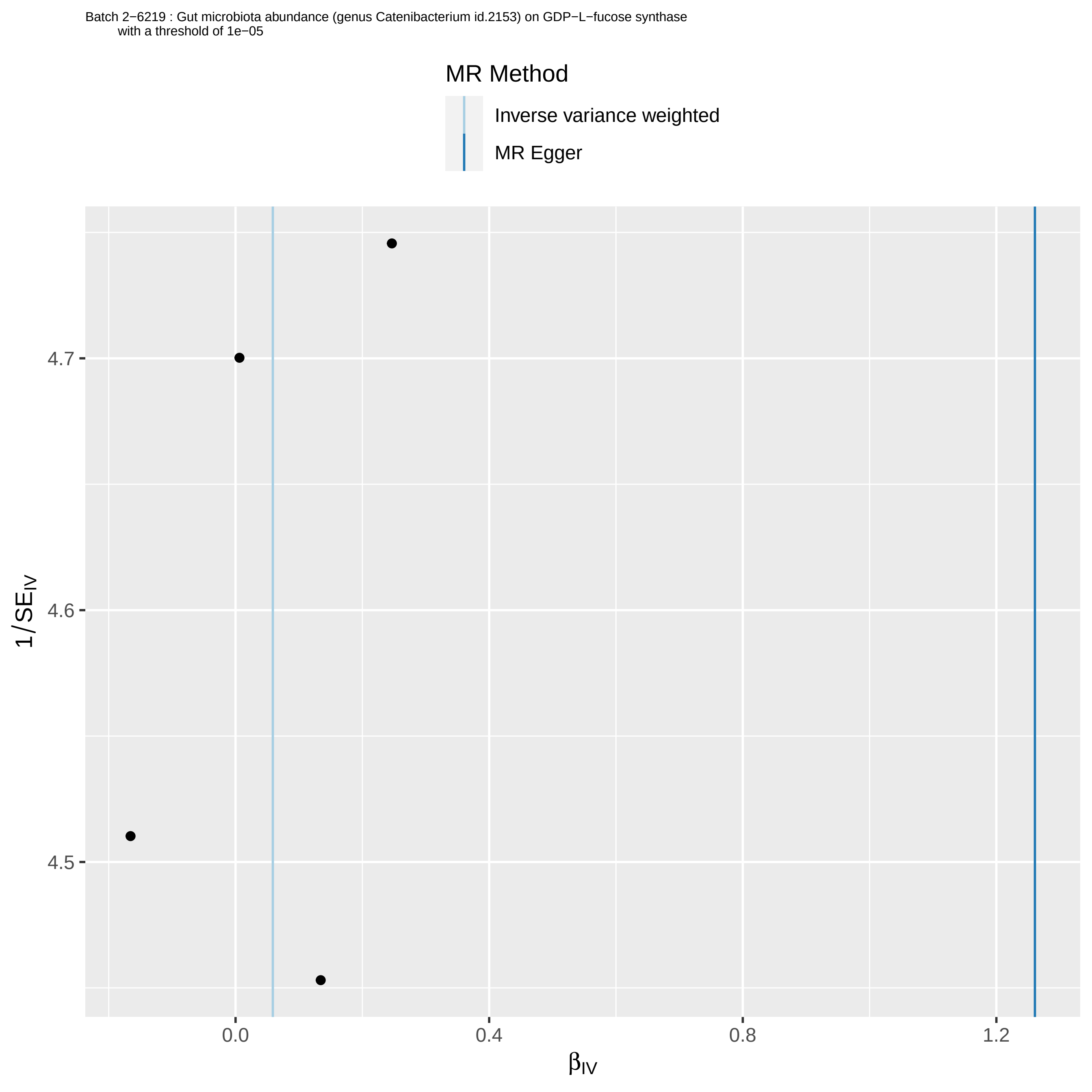
Batch 2−6219 : Gut microbiota abundance (genus Catenibacterium id.2153) on GDP−L−fucose synthase
with a threshold of 1e−05
MR Method
Inverse variance weighted
MR Egger
4.7
4.6
4.5
1/SE_IV
0.0
0.4
0.8
1.2
β_IV

Batch 2−6220 : Gut microbiota abundance (genus Parasutterella id.2892) on GDP−L−fucose synthase
with a threshold of 1e−05

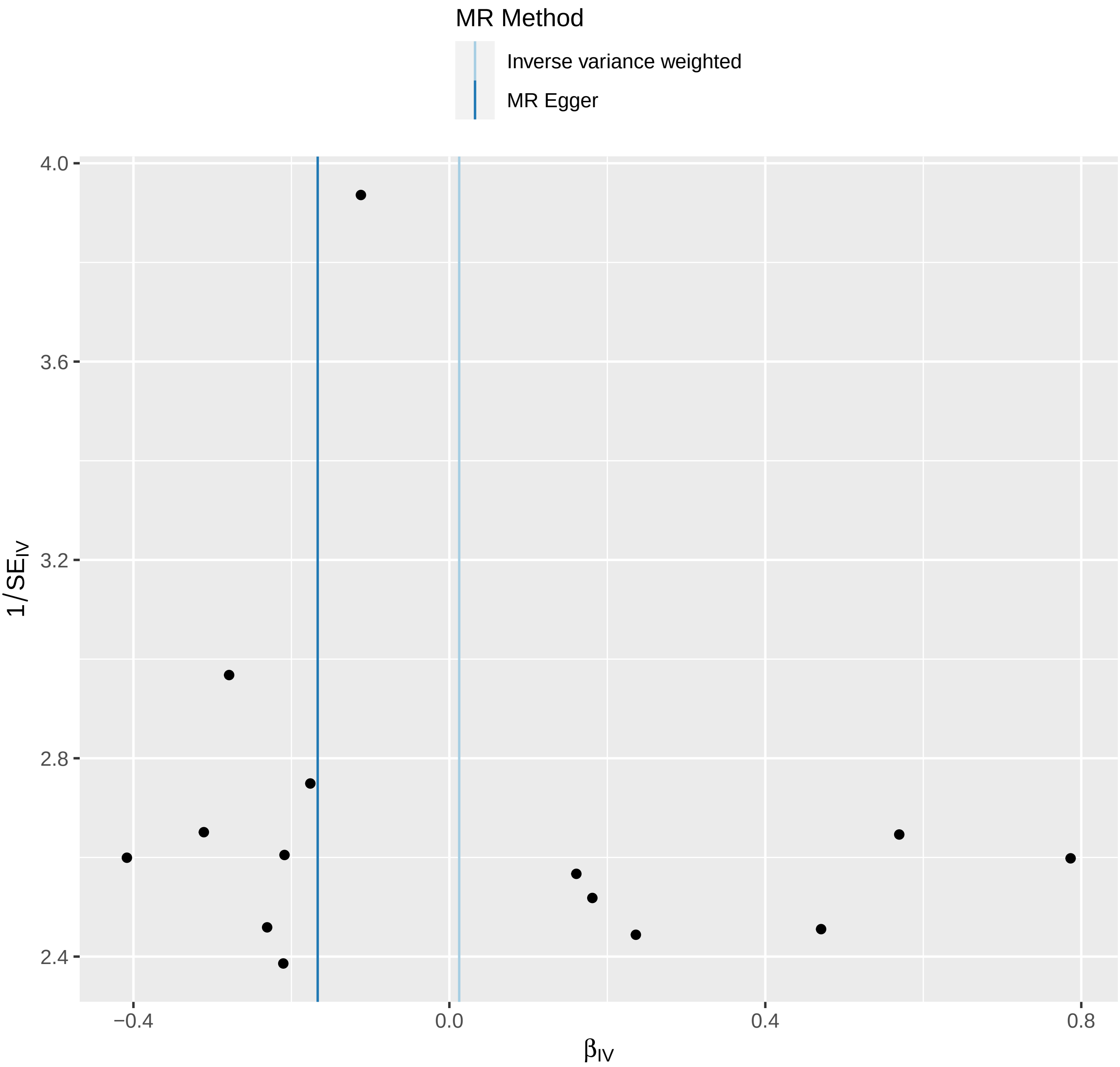

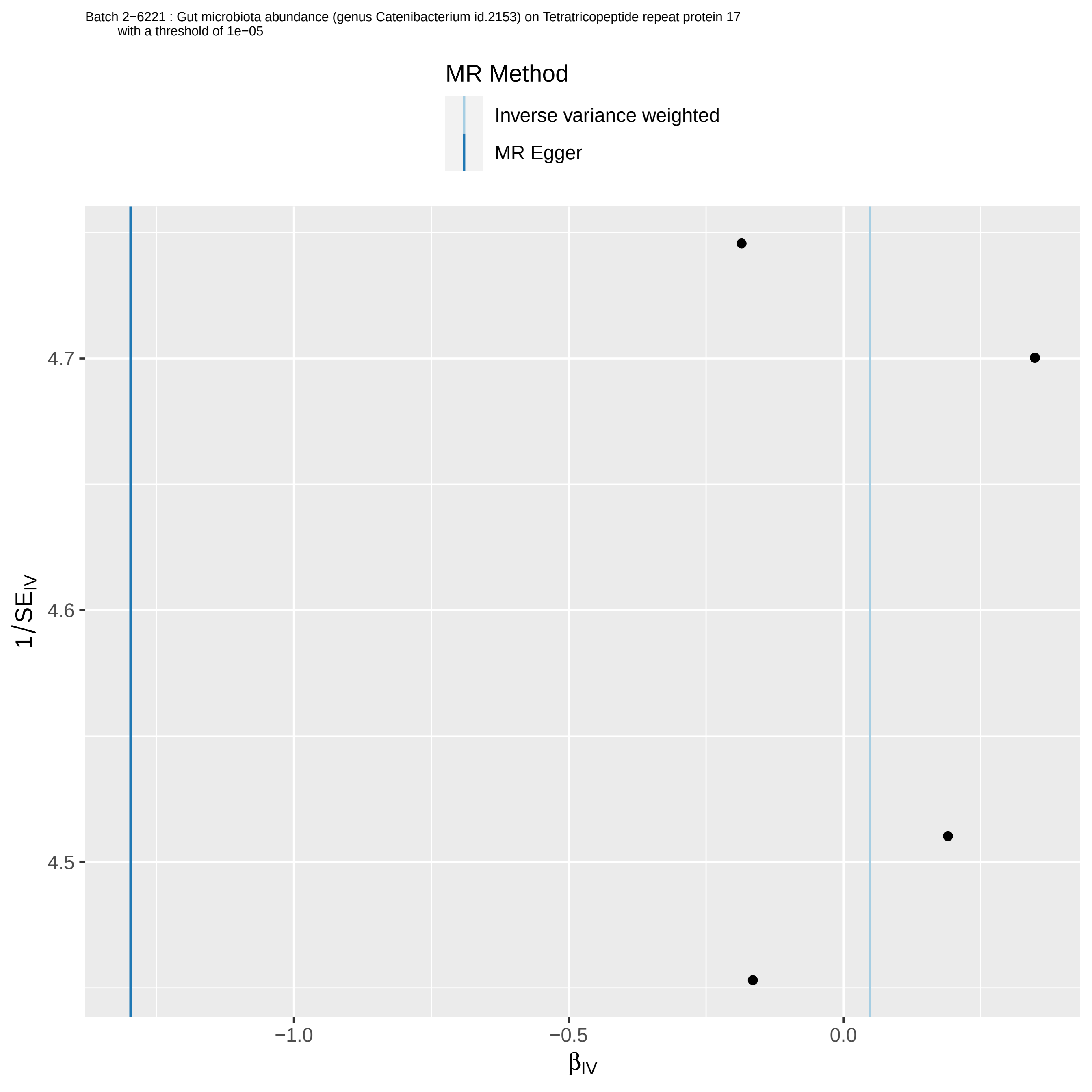

Batch 2−6221 : Gut microbiota abundance (genus Catenibacterium id.2153) on Tetratricopeptide repeat protein 17
with a threshold of 1e−05
MR Method
Inverse variance weighted
MR Egger
4.7
4.6
4.5
1/SE$_{IV}$
−1.0
−0.5
0.0
β$_{IV}$

Batch 2−6222 : Gut microbiota abundance (genus Parasutterella id.2892) on Tetratricopeptide repeat protein 17
with a threshold of 1e−05

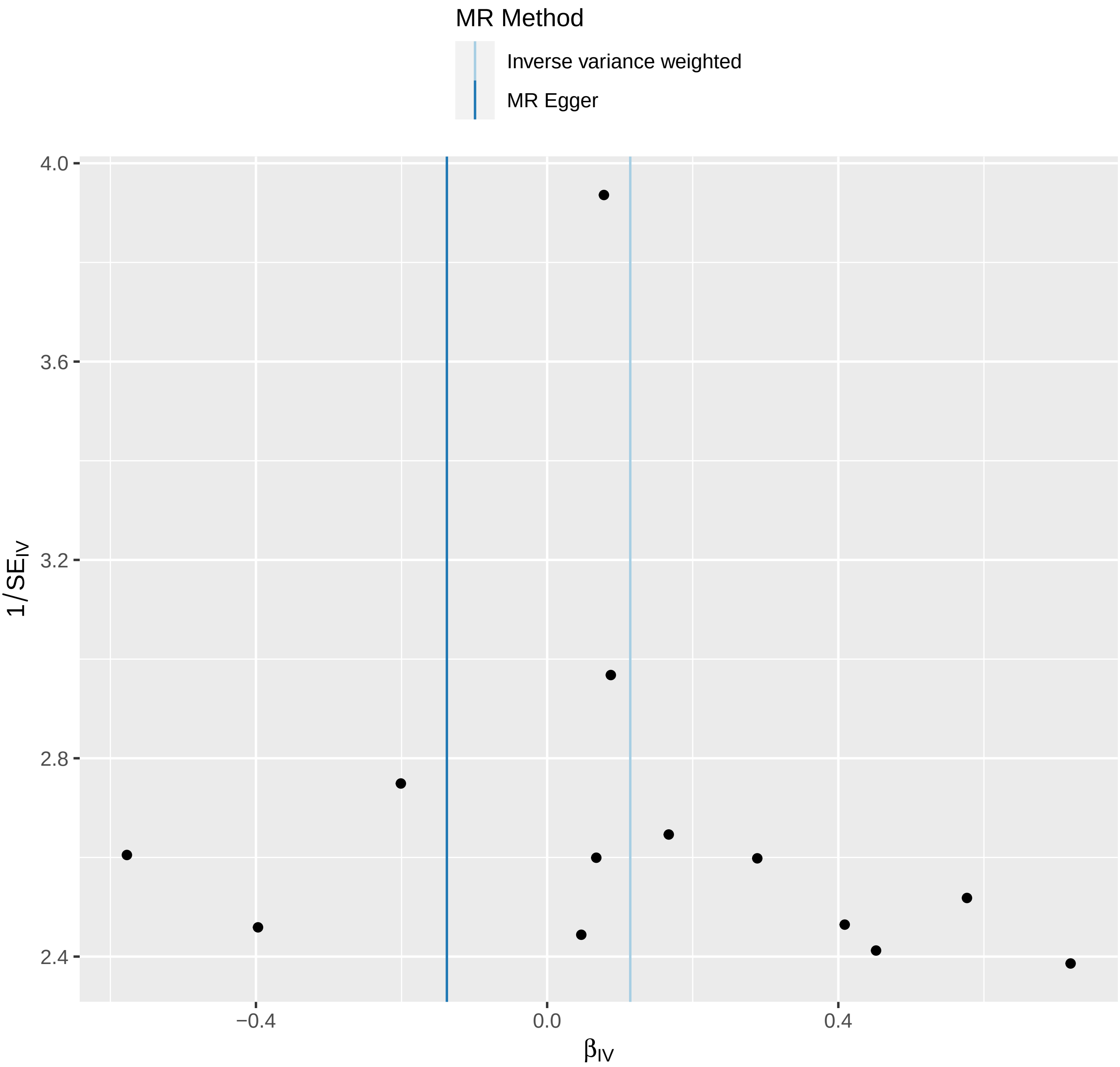

Batch 2−6223 : Gut microbiota abundance (genus Catenibacterium id.2153) on Tetratricopeptide repeat protein 17
with a threshold of 1e−05

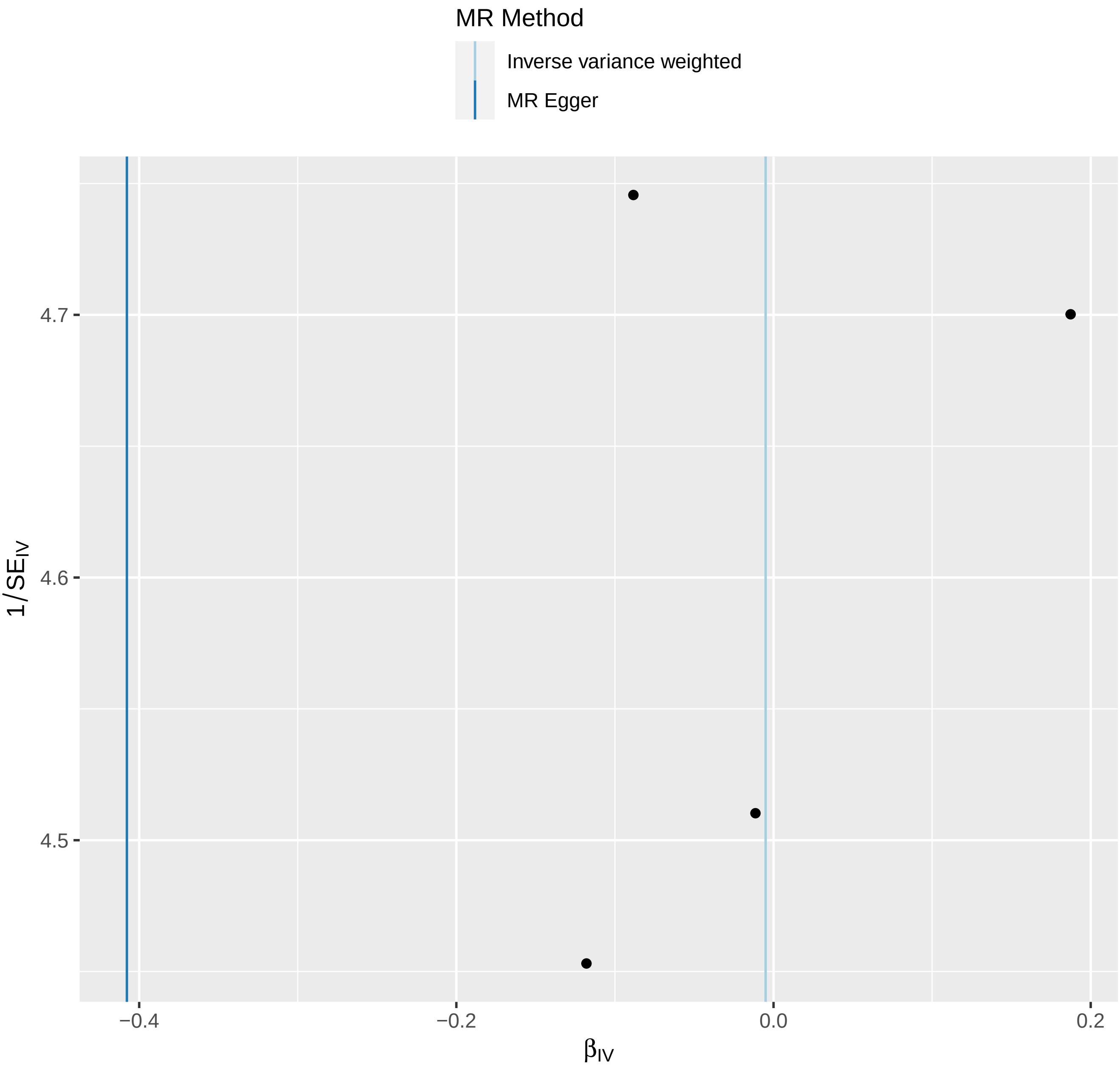

Batch 2−6224 : Gut microbiota abundance (genus Parasutterella id.2892) on Tetratricopeptide repeat protein 17
with a threshold of 1e−05

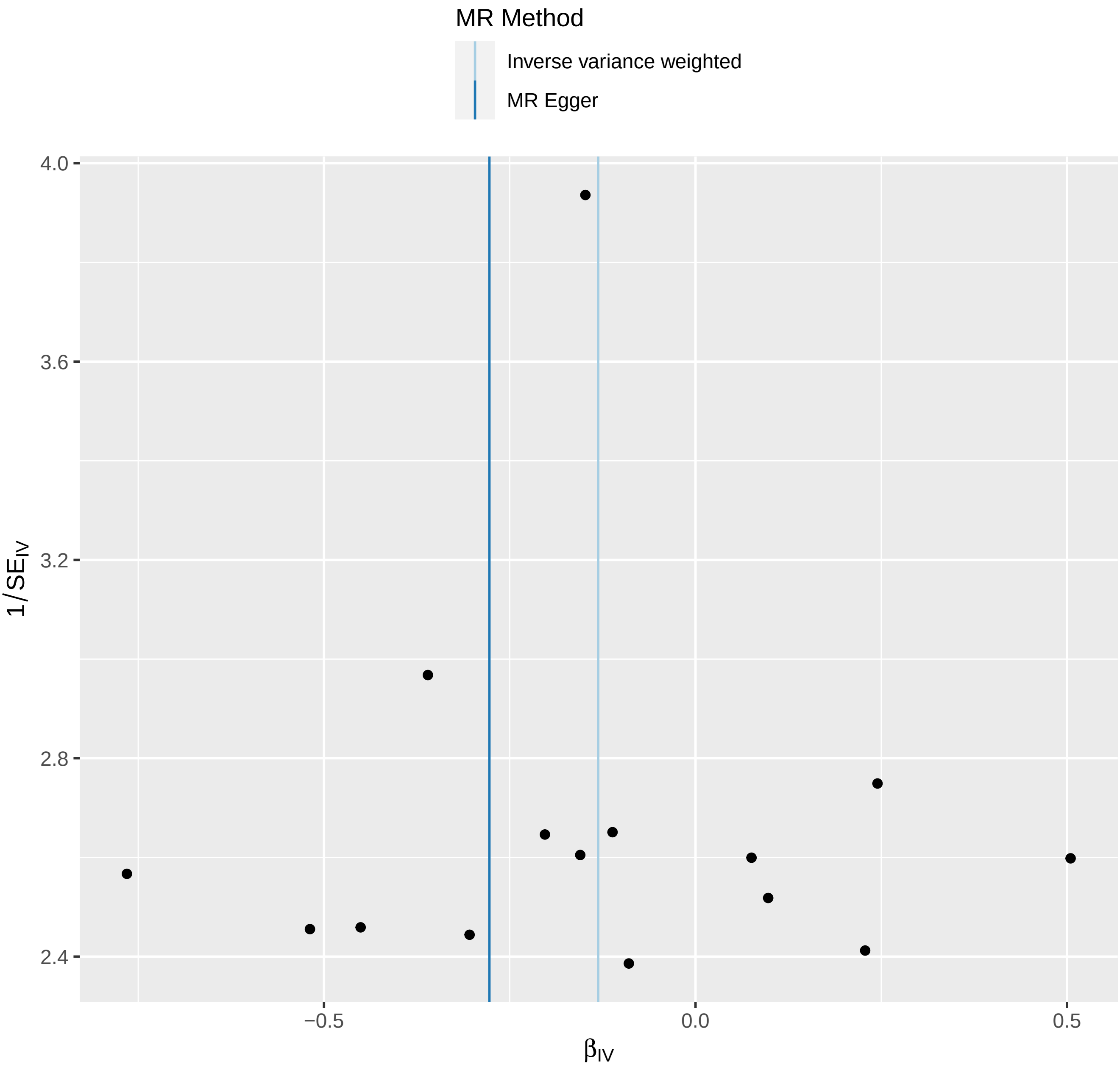

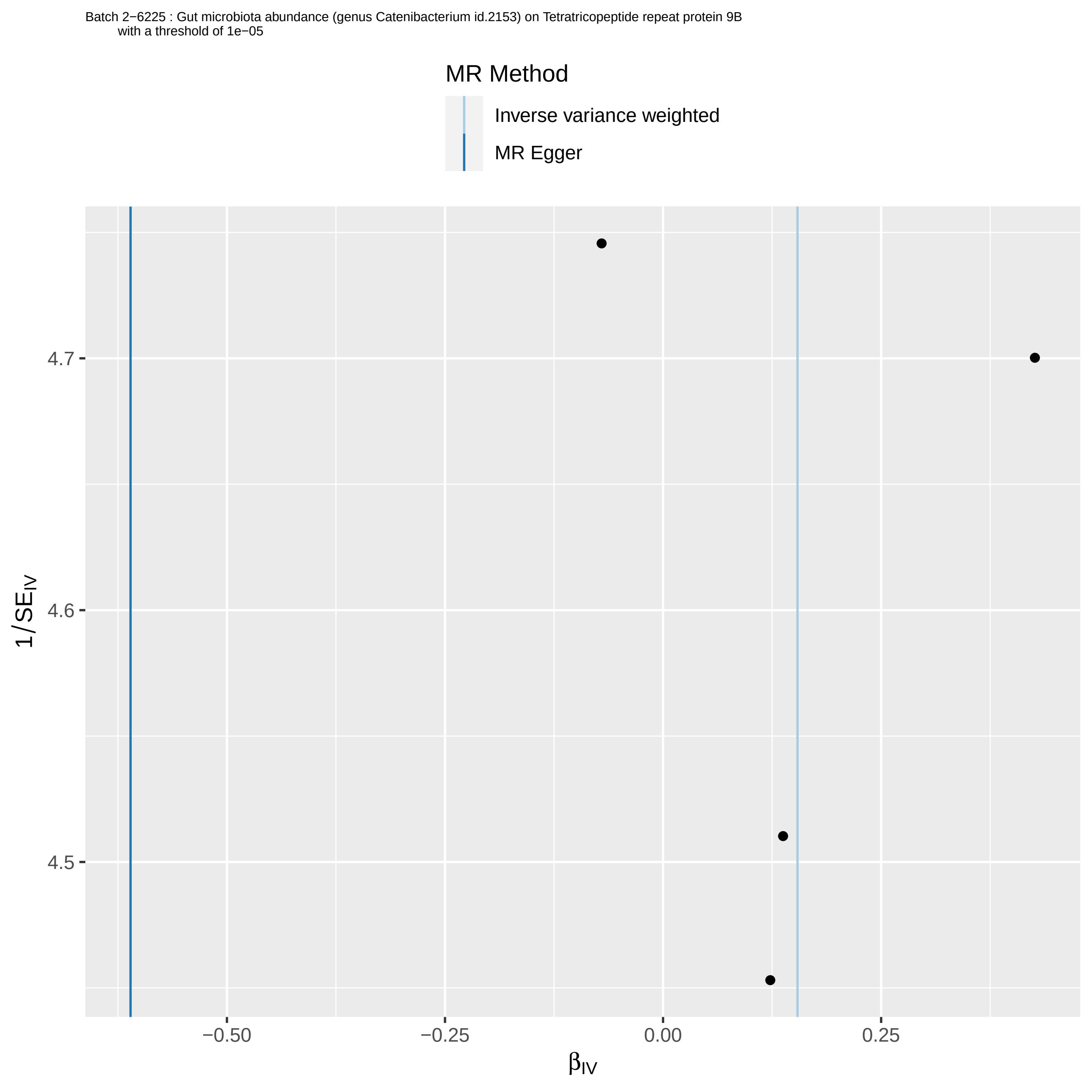

Batch 2−6225 : Gut microbiota abundance (genus Catenibacterium id.2153) on Tetratricopeptide repeat protein 9B
with a threshold of 1e−05
MR Method
Inverse variance weighted
MR Egger
4.7
4.6
4.5
1/SE_IV
−0.50
−0.25
0.00
0.25
β_IV

Batch 2−6226 : Gut microbiota abundance (genus Parasutterella id.2892) on Tetratricopeptide repeat protein 9B
with a threshold of 1e−05

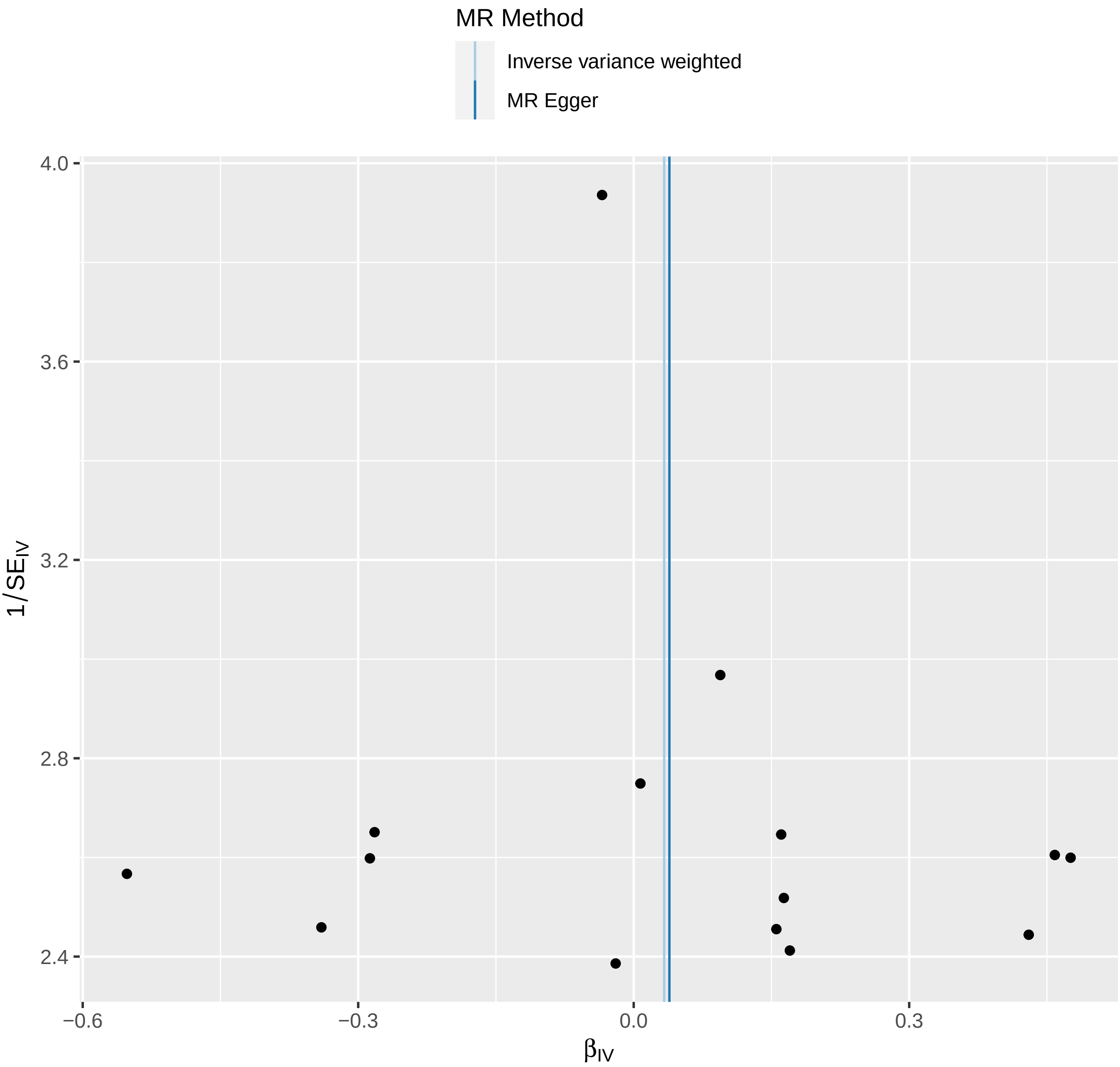

Batch 2−6227 : Gut microbiota abundance (genus Catenibacterium id.2153) on Tubulin−−tyrosine ligase
with a threshold of 1e−05

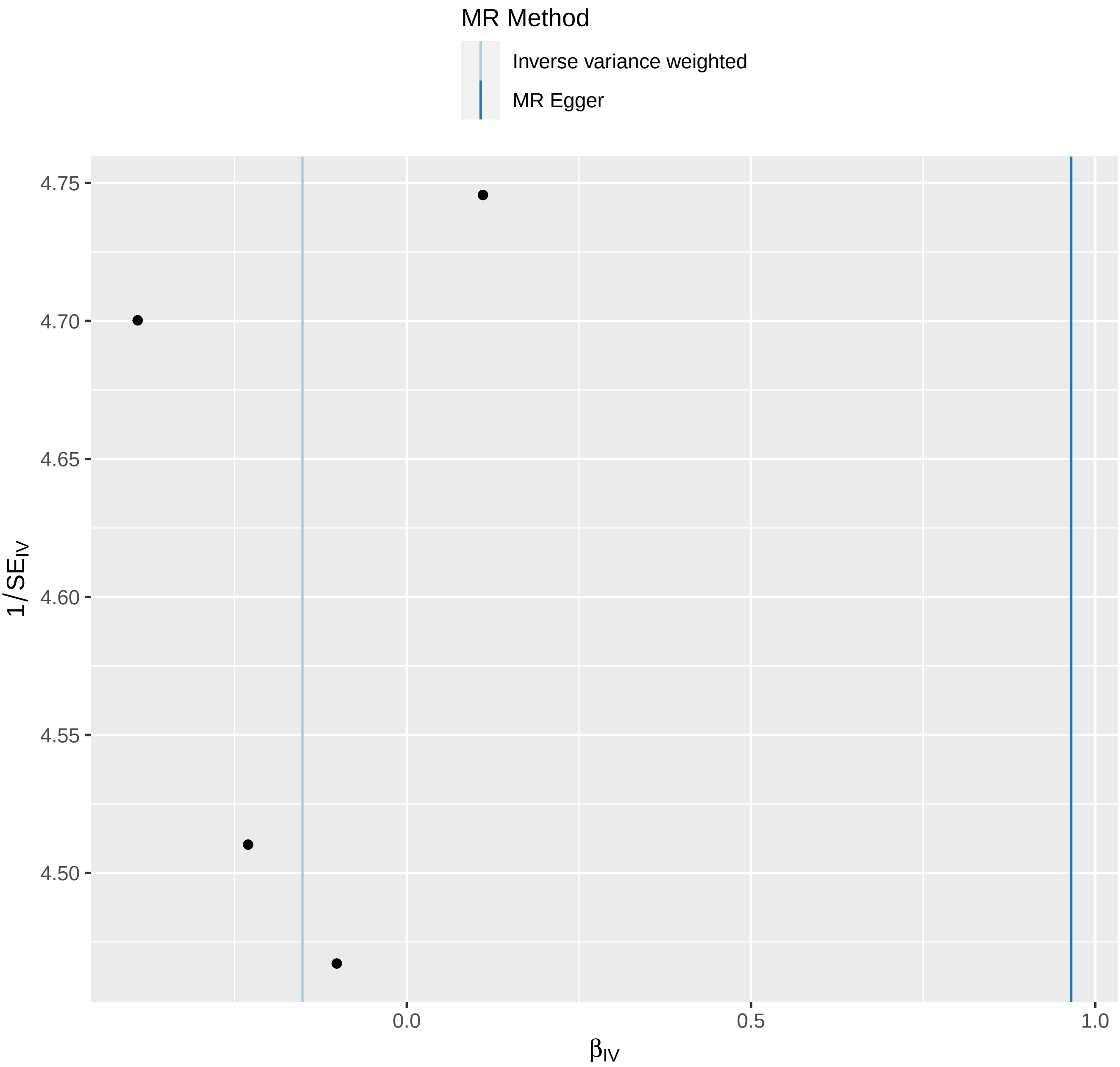

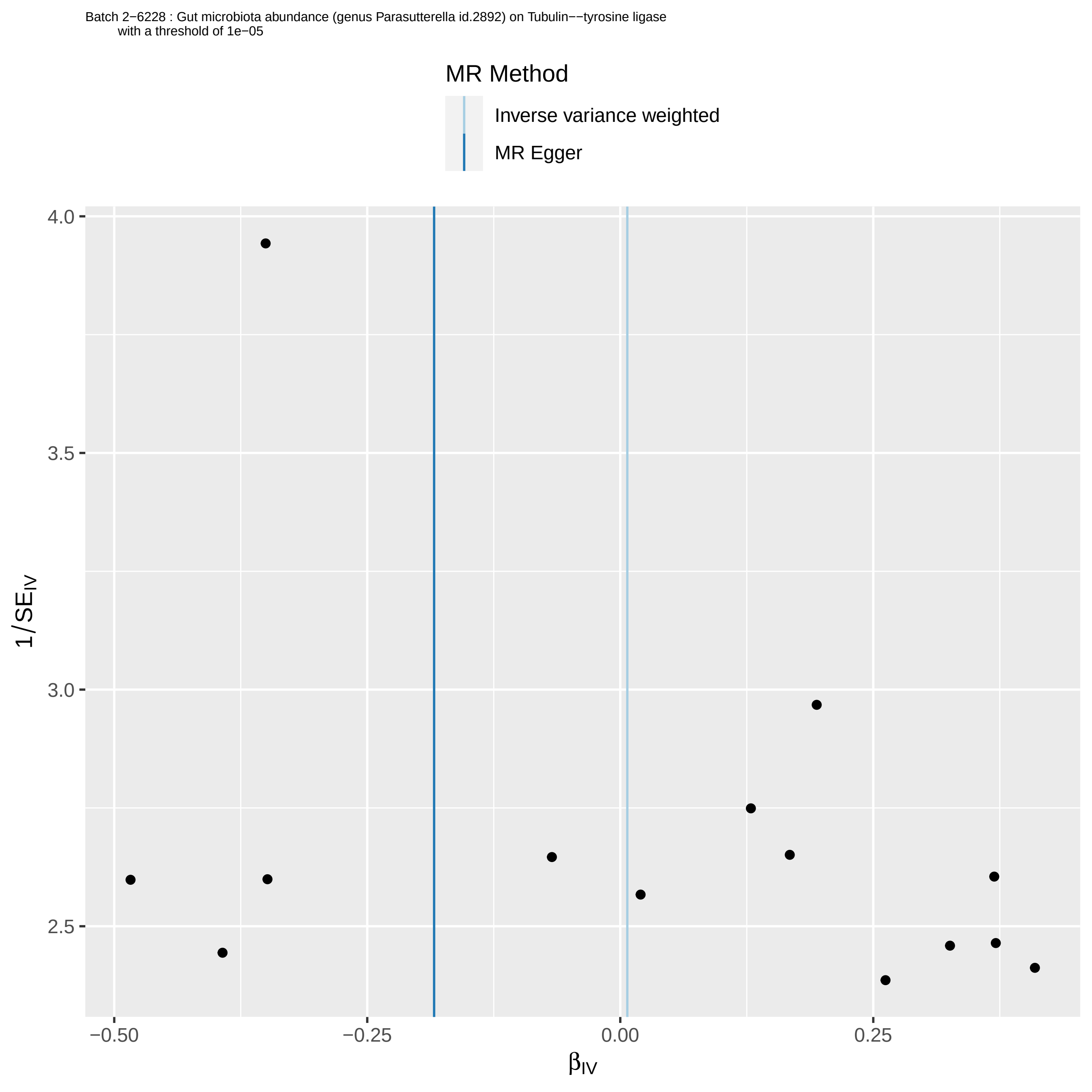

Batch 2−6228 : Gut microbiota abundance (genus Parasutterella id.2892) on Tubulin−−tyrosine ligase
with a threshold of 1e−05
MR Method
Inverse variance weighted
MR Egger
4.0
3.5
3.0
2.5
1/SE$_{IV}$
−0.50
−0.25
0.00
0.25
β$_{IV}$

Batch 2−6229 : Gut microbiota abundance (genus Catenibacterium id.2153) on TPA−induced transmembrane protein
with a threshold of 1e−05

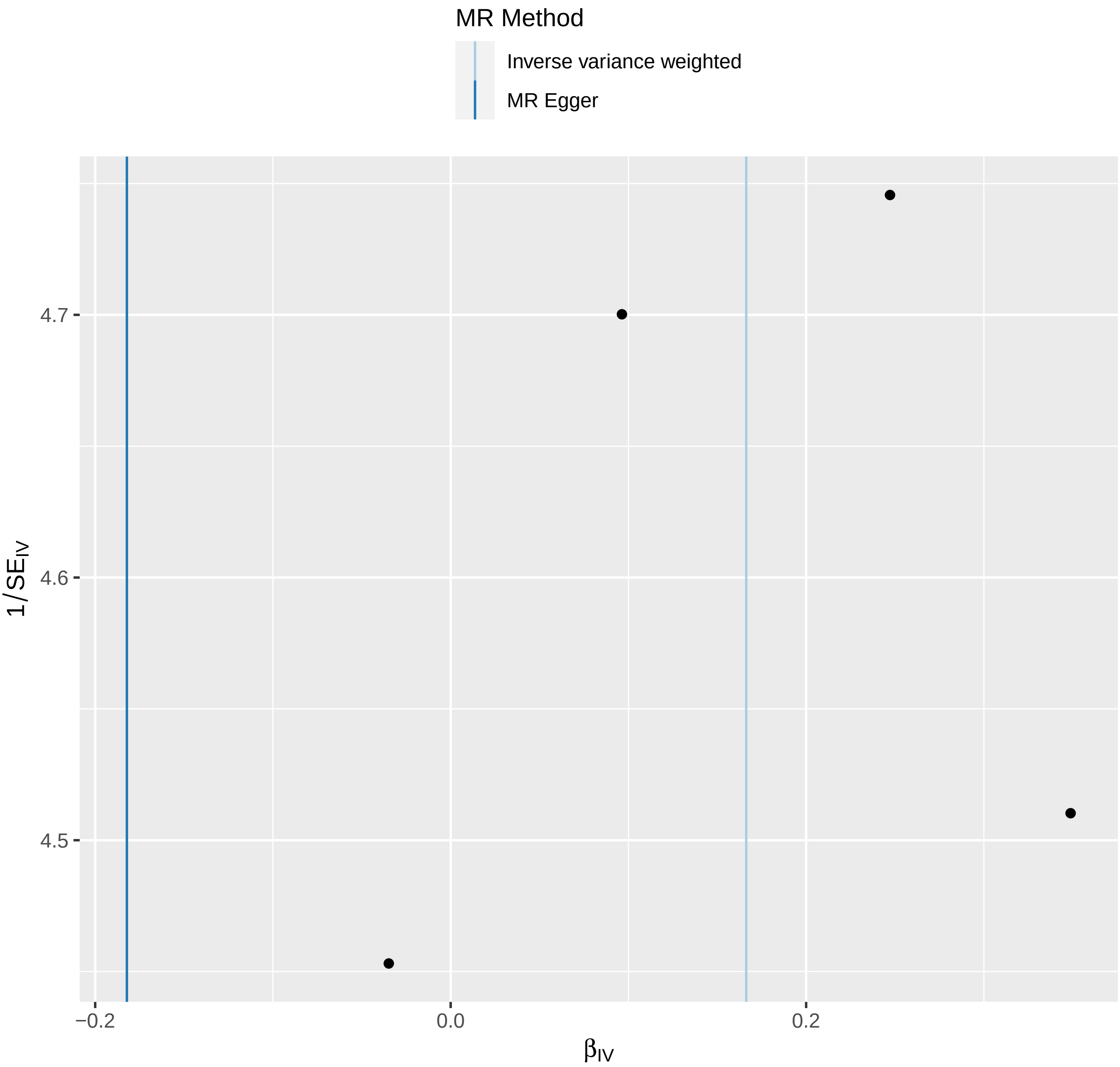

Batch 2−6230 : Gut microbiota abundance (genus Parasutterella id.2892) on TPA−induced transmembrane protein with a threshold of 1e−05

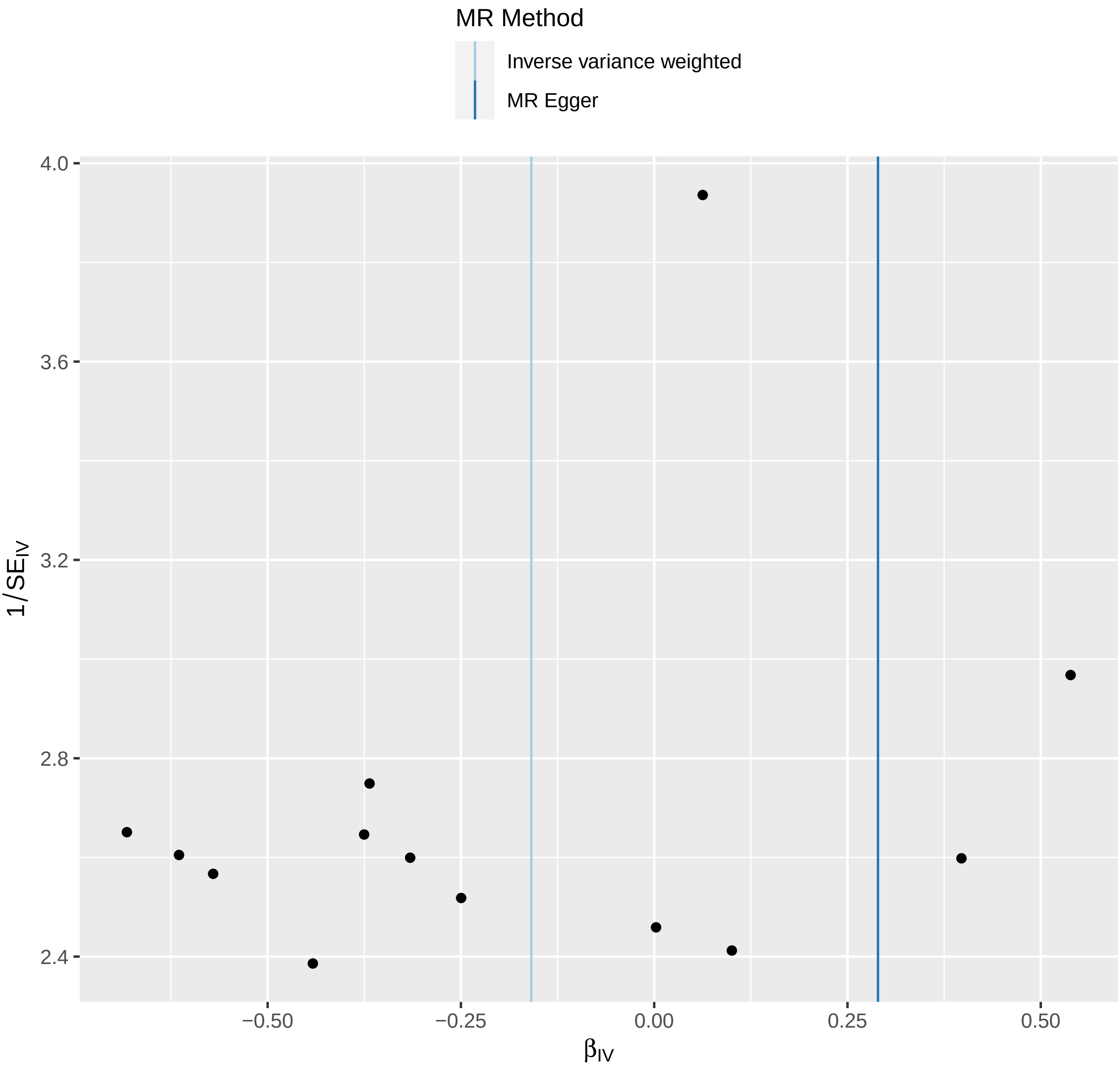

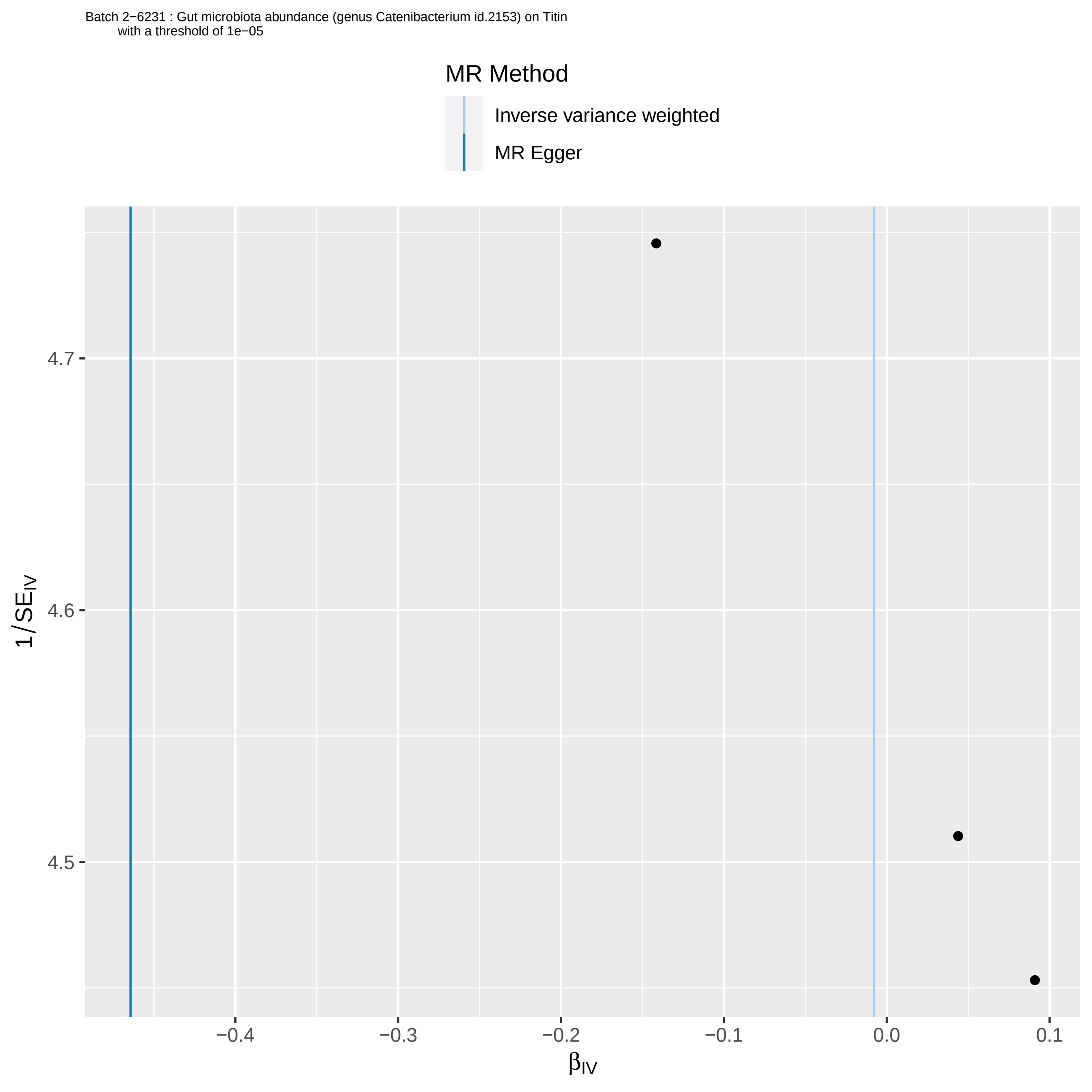
Batch 2−6231 : Gut microbiota abundance (genus Catenibacterium id.2153) on Titin
with a threshold of 1e−05
MR Method
Inverse variance weighted
MR Egger
4.7
4.6
4.5
1/SE_IV
−0.4
−0.3
−0.2
−0.1
0.0
0.1
β_IV

Batch 2−6232 : Gut microbiota abundance (genus Parasutterella id.2892) on Titin
with a threshold of 1e−05

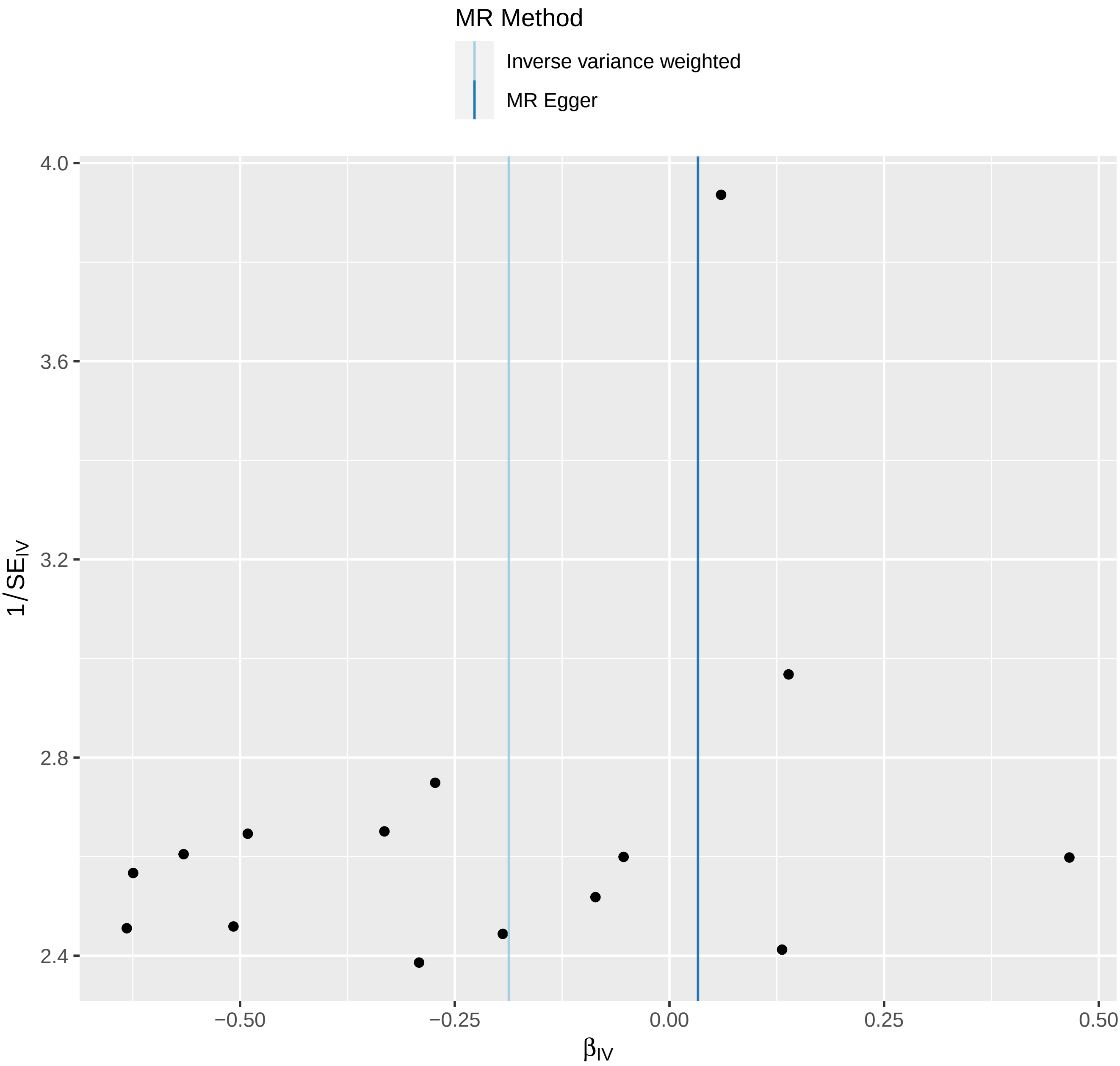

Batch 2−6233 : Gut microbiota abundance (genus Catenibacterium id.2153) on Tuftelin
with a threshold of 1e−05

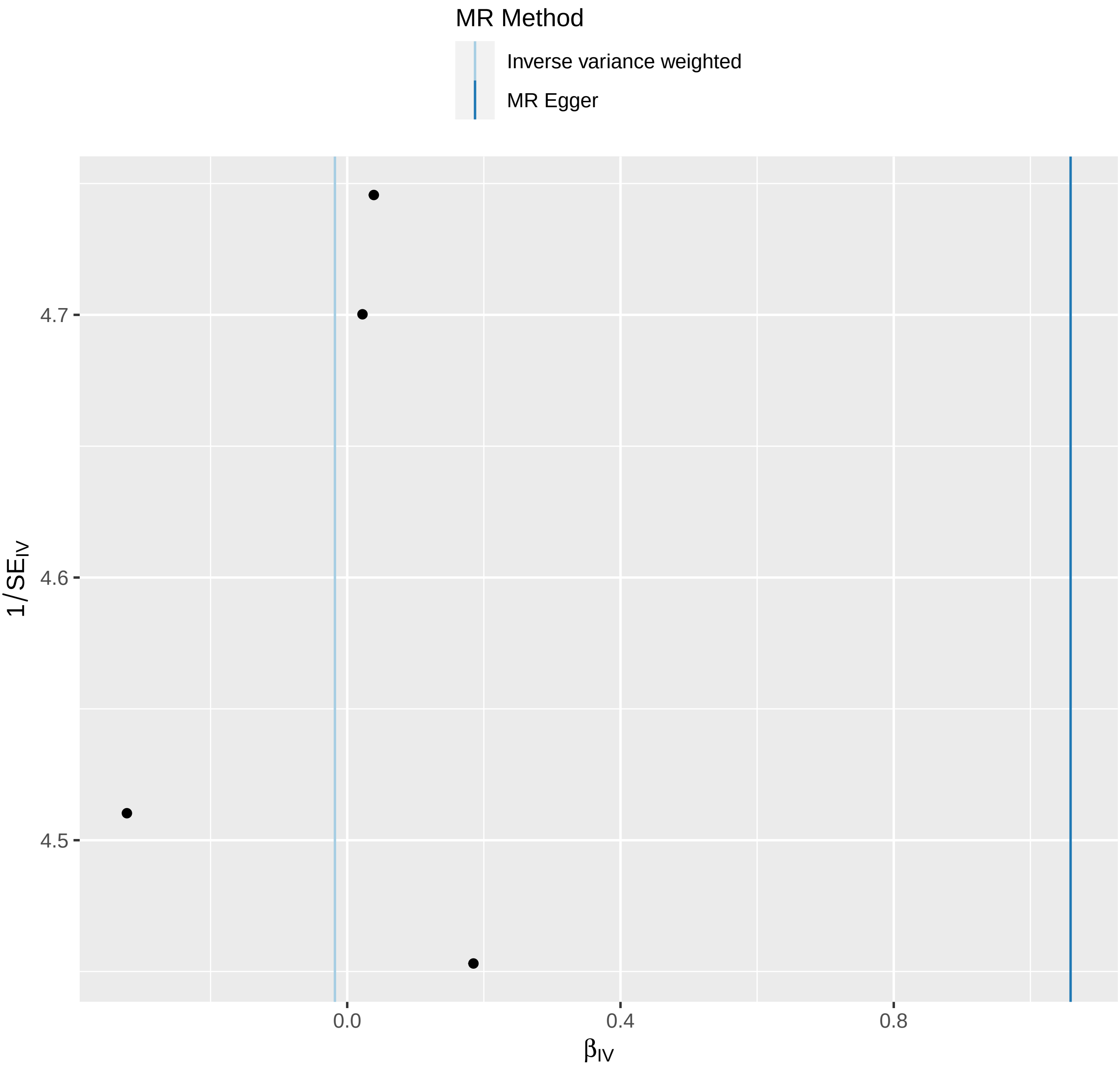

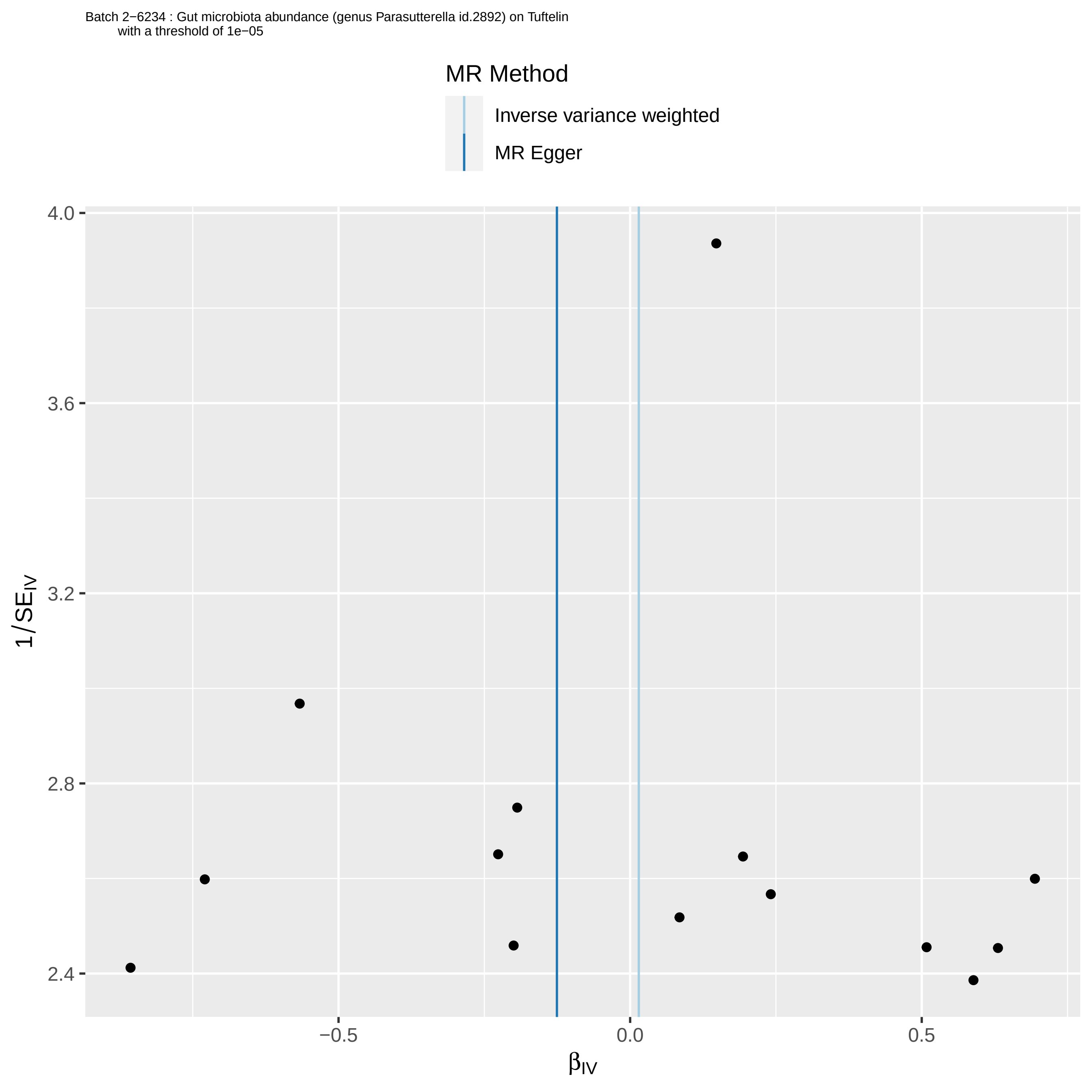

Batch 2−6234 : Gut microbiota abundance (genus Parasutterella id.2892) on Tuftelin
with a threshold of 1e−05
MR Method
Inverse variance weighted
MR Egger
4.0
3.6
3.2
2.8
2.4
1/SE_IV
−0.5
0.0
0.5
β_IV

Batch 2−6235 : Gut microbiota abundance (genus Catenibacterium id.2153) on Twinfilin−1
with a threshold of 1e−05

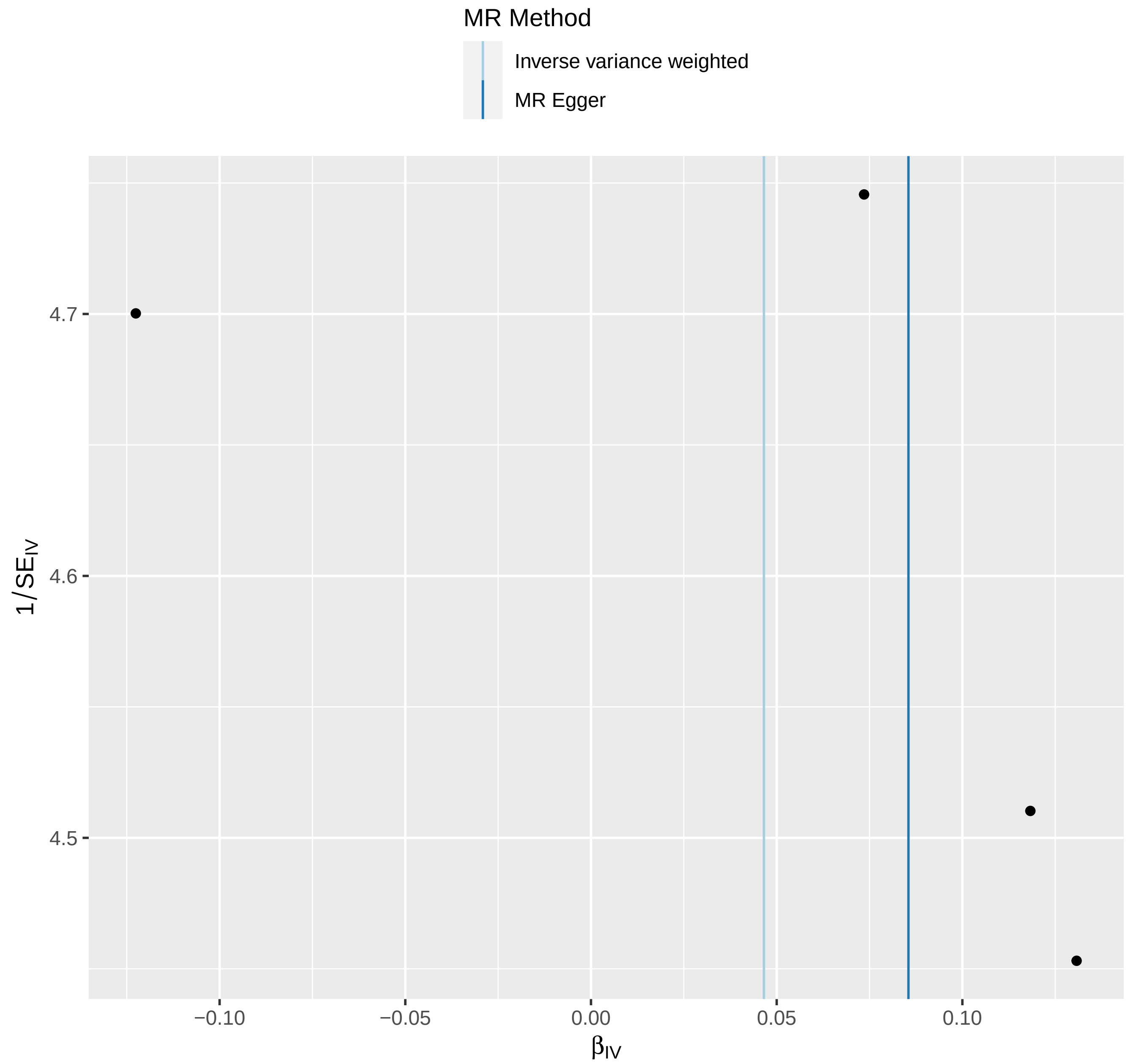

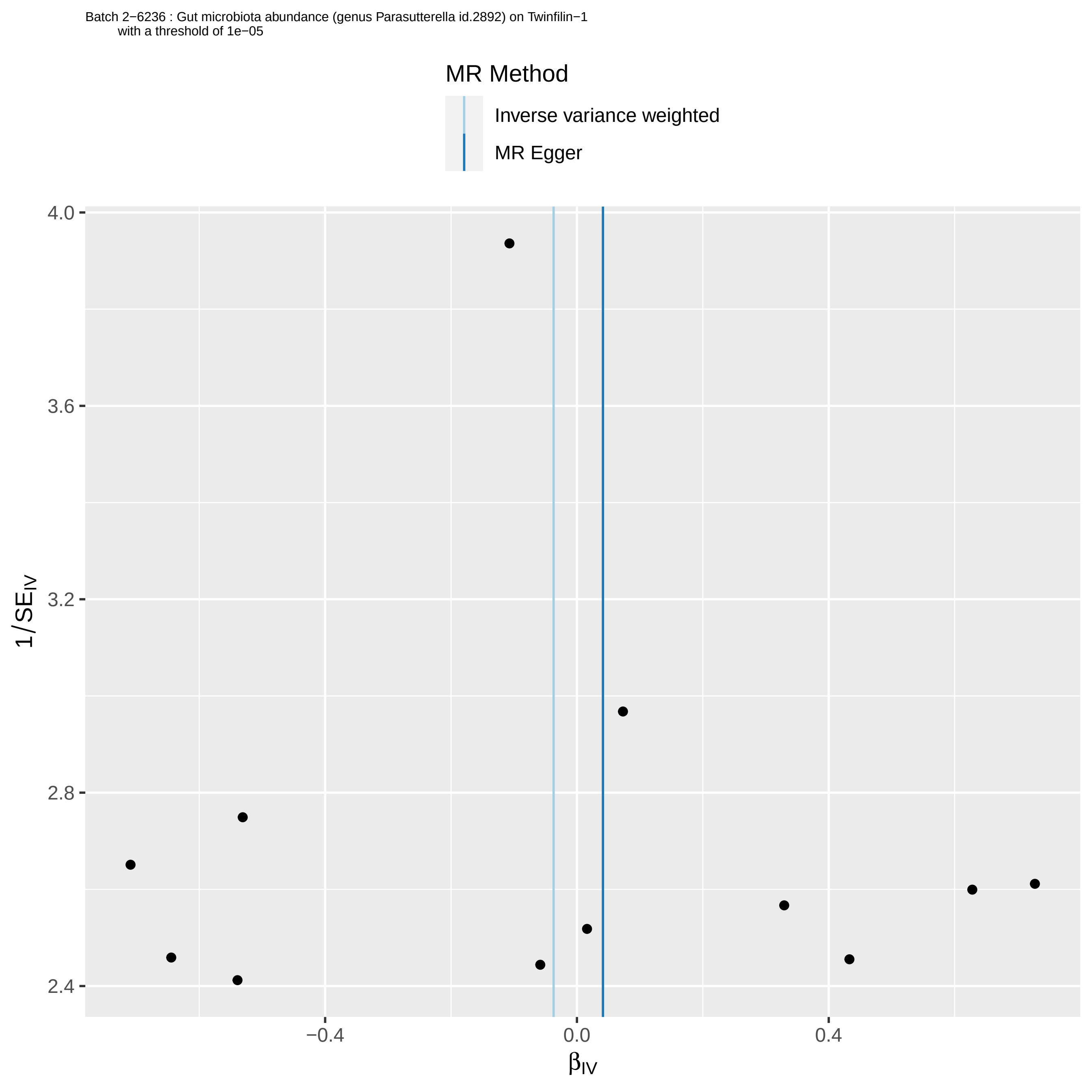

Batch 2−6236 : Gut microbiota abundance (genus Parasutterella id.2892) on Twinfilin−1
with a threshold of 1e−05
MR Method
Inverse variance weighted
MR Egger
4.0
3.6
3.2
2.8
2.4
1/SE_IV
−0.4
0.0
0.4
β_IV

Batch 2−6237 : Gut microbiota abundance (genus Catenibacterium id.2153) on Twisted gastrulation protein homolog 1
with a threshold of 1e−05

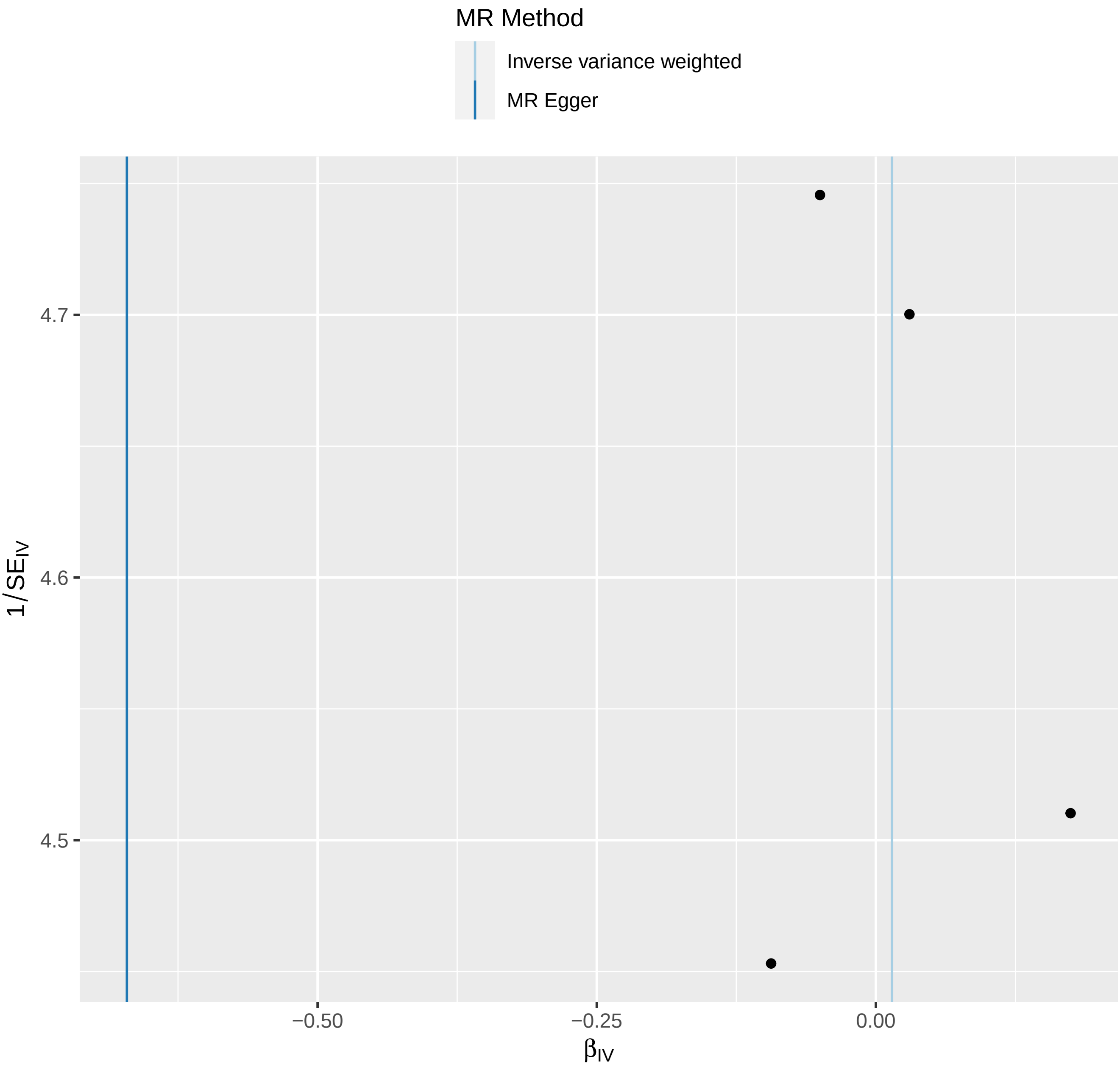

Batch 2−6238 : Gut microbiota abundance (genus Parasutterella id.2892) on Twisted gastrulation protein homolog 1
with a threshold of 1e−05

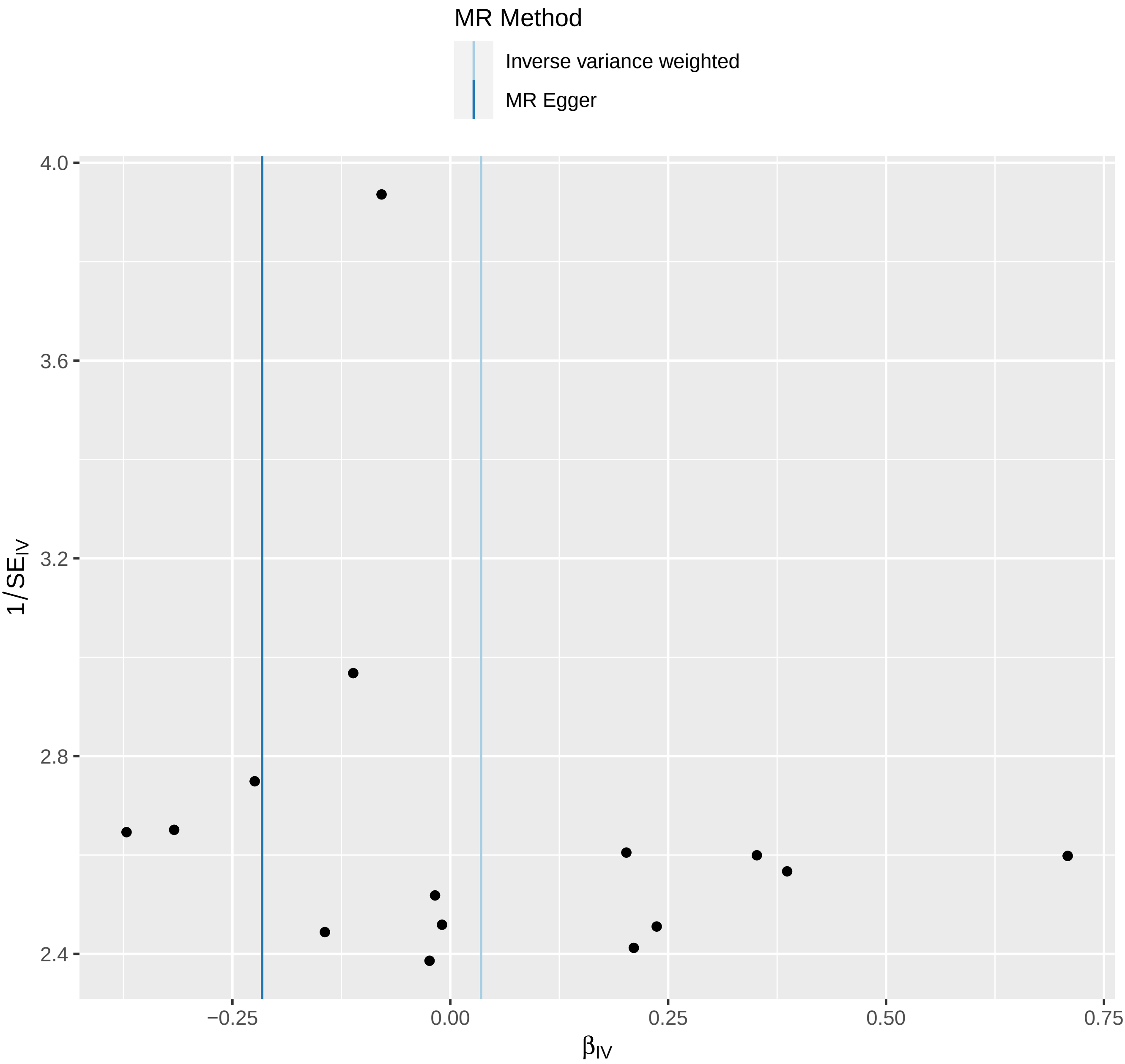

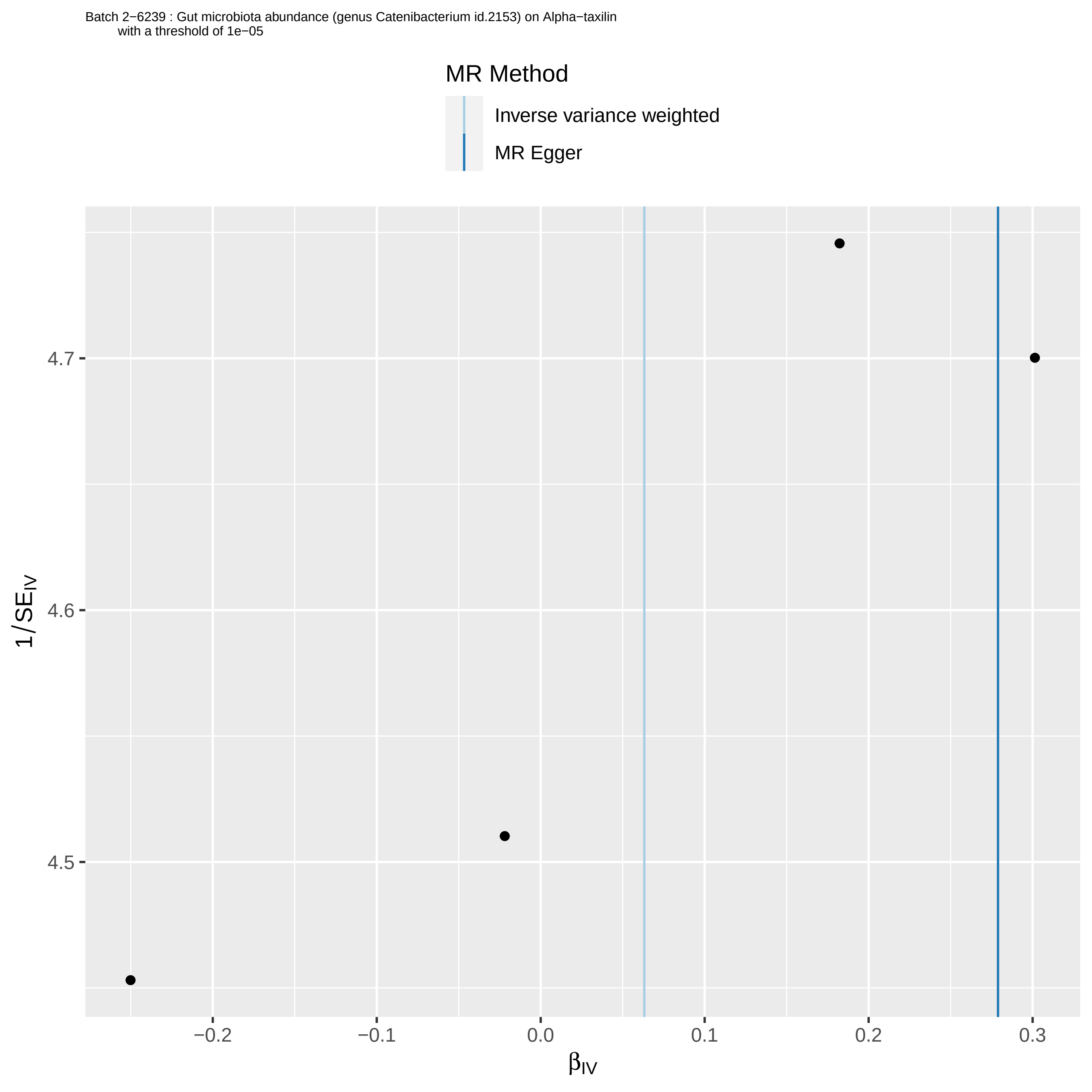

Batch 2−6239 : Gut microbiota abundance (genus Catenibacterium id.2153) on Alpha−taxilin
with a threshold of 1e−05
MR Method
Inverse variance weighted
MR Egger
4.7
4.6
4.5
1/SE_IV
−0.2
−0.1
0.0
0.1
0.2
0.3
β_IV

Batch 2−6240 : Gut microbiota abundance (genus Parasutterella id.2892) on Alpha−taxilin
with a threshold of 1e−05

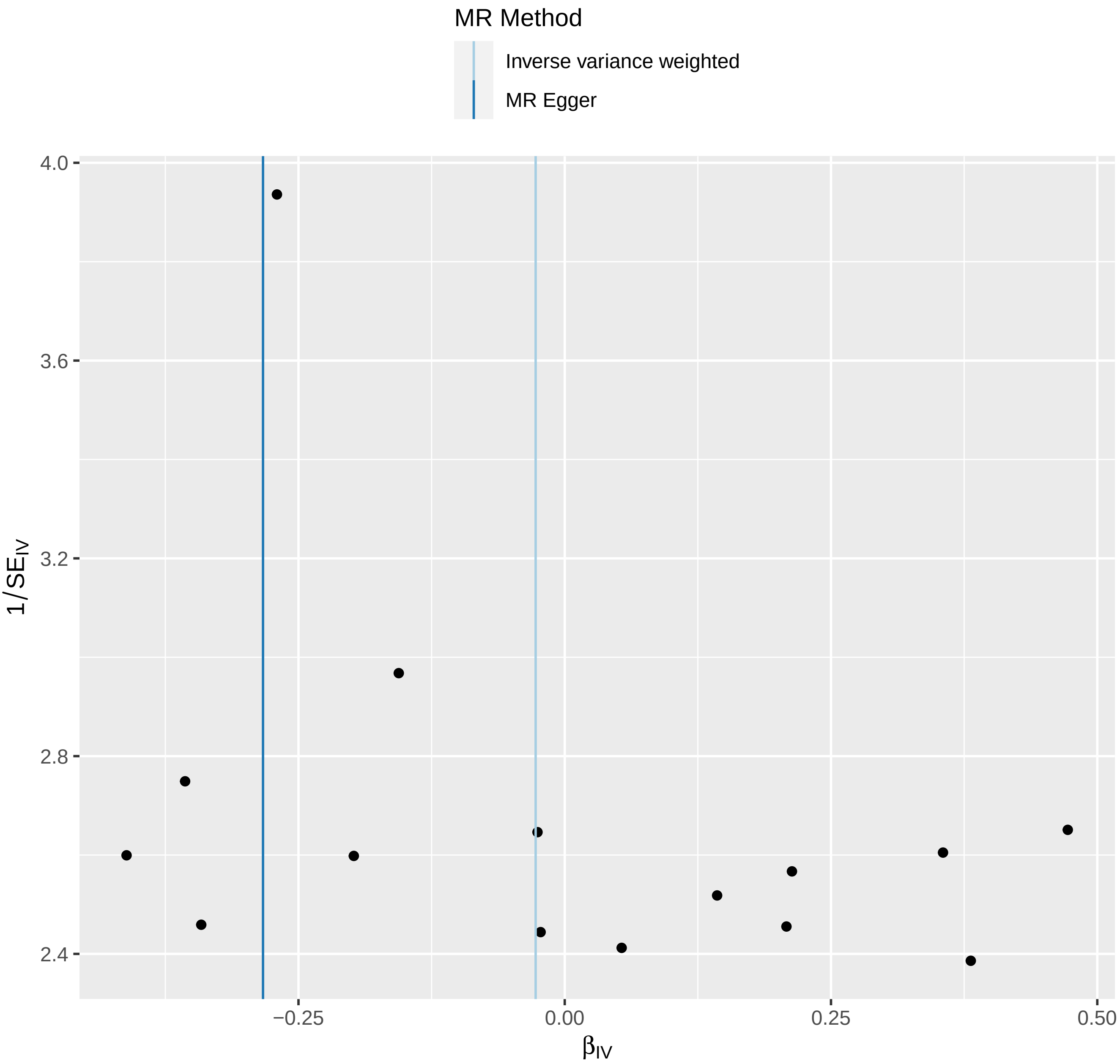

Batch 2−6241 : Gut microbiota abundance (genus Catenibacterium id.2153) on Thioredoxin
with a threshold of 1e−05

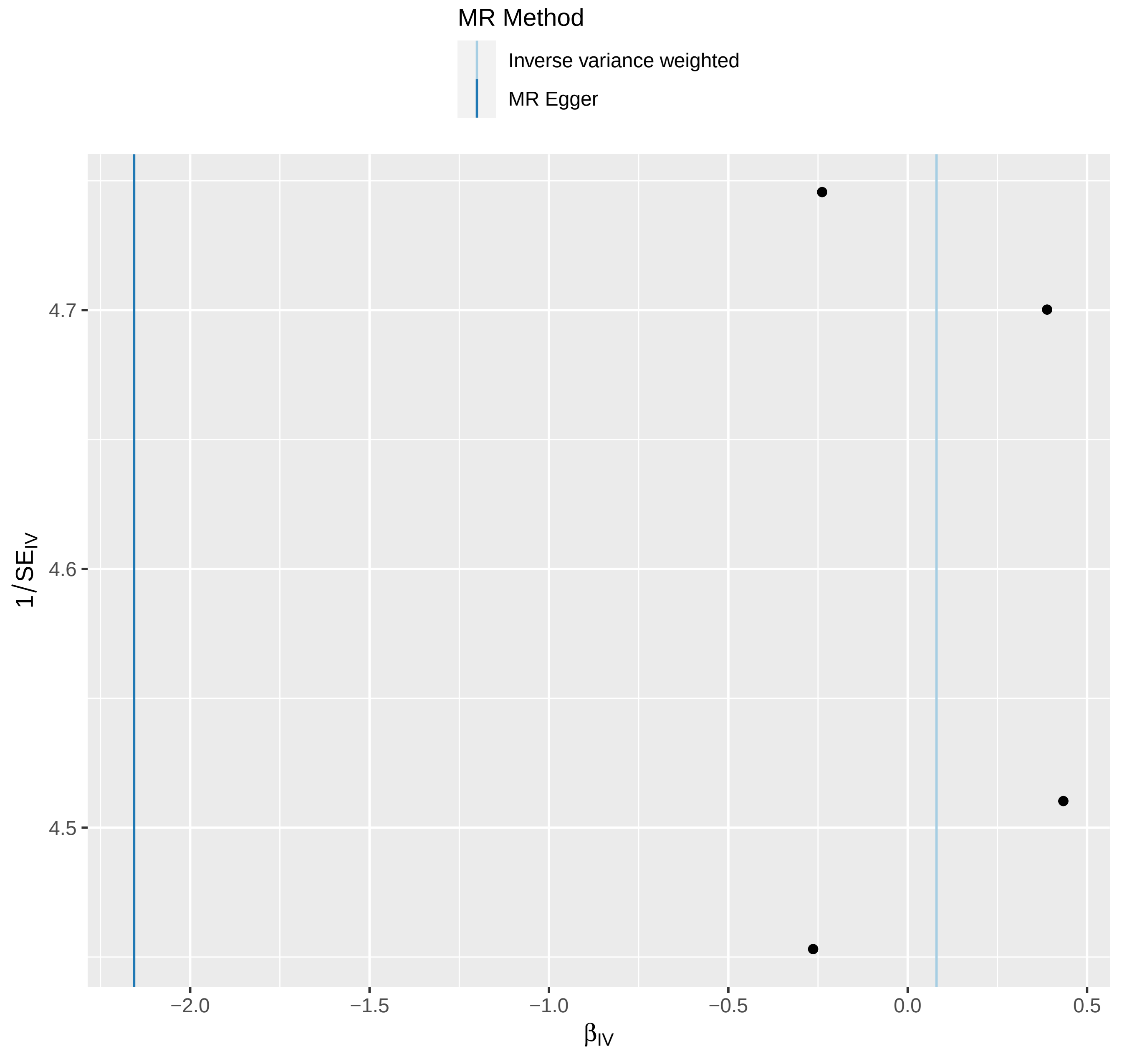

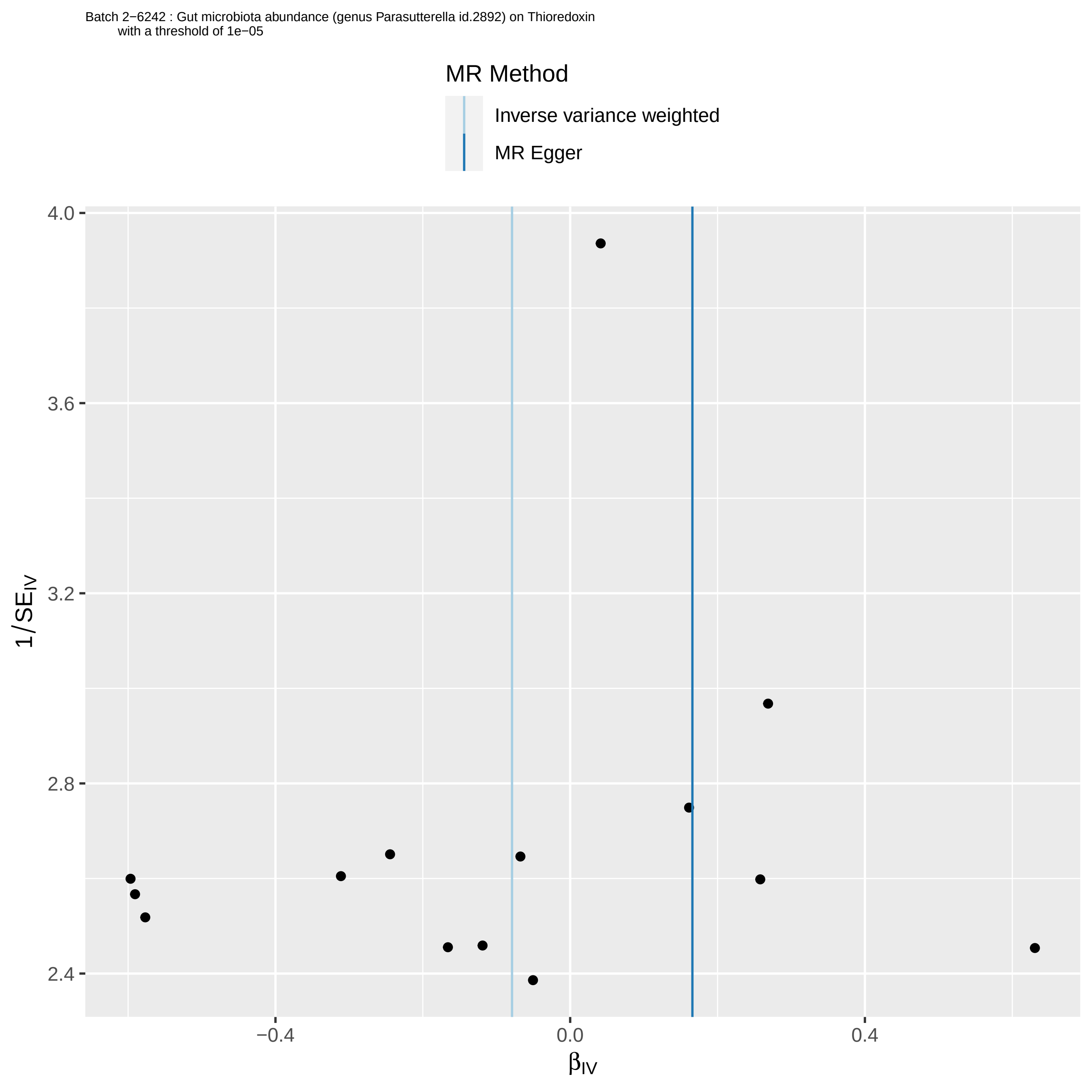

Batch 2−6242 : Gut microbiota abundance (genus Parasutterella id.2892) on Thioredoxin
with a threshold of 1e−05
MR Method
Inverse variance weighted
MR Egger
4.0
3.6
3.2
2.8
2.4
1/SE_IV
−0.4
0.0
0.4
β_IV

Batch 2−6243 : Gut microbiota abundance (genus Catenibacterium id.2153) on Thioredoxin domain−containing protein 11 with a threshold of 1e−05

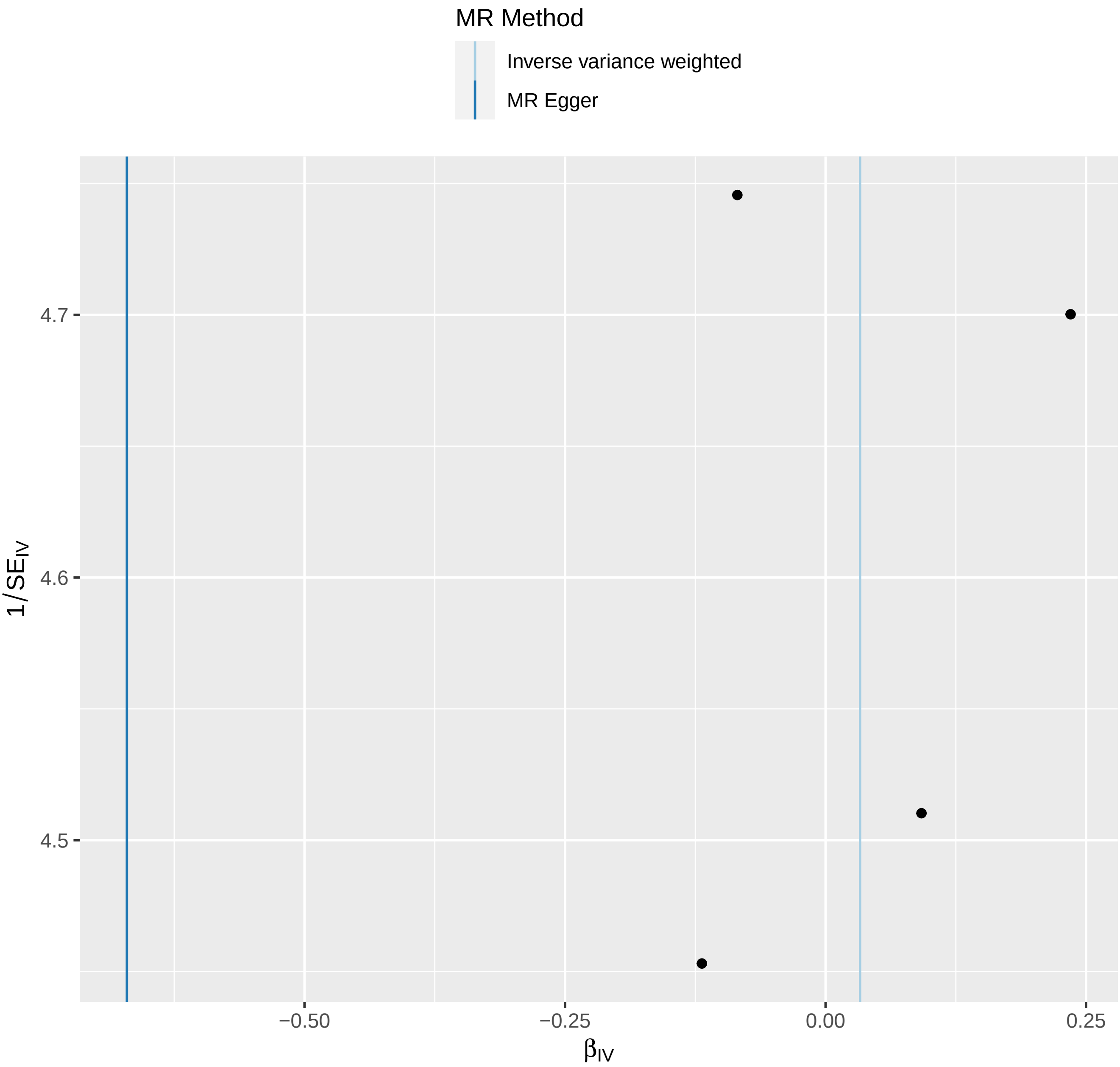

Batch 2−6244 : Gut microbiota abundance (genus Parasutterella id.2892) on Thioredoxin domain−containing protein 11
with a threshold of 1e−05

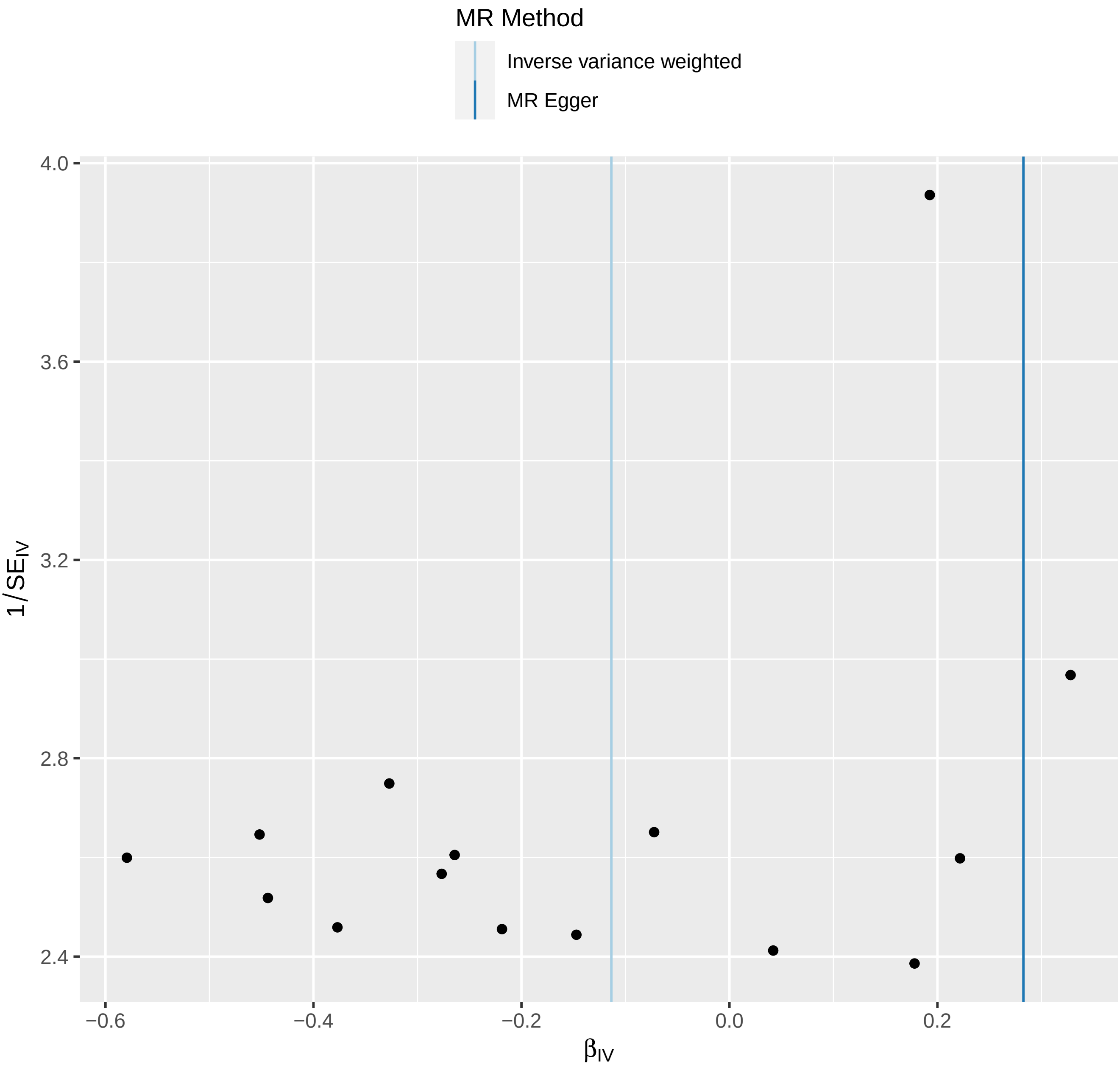

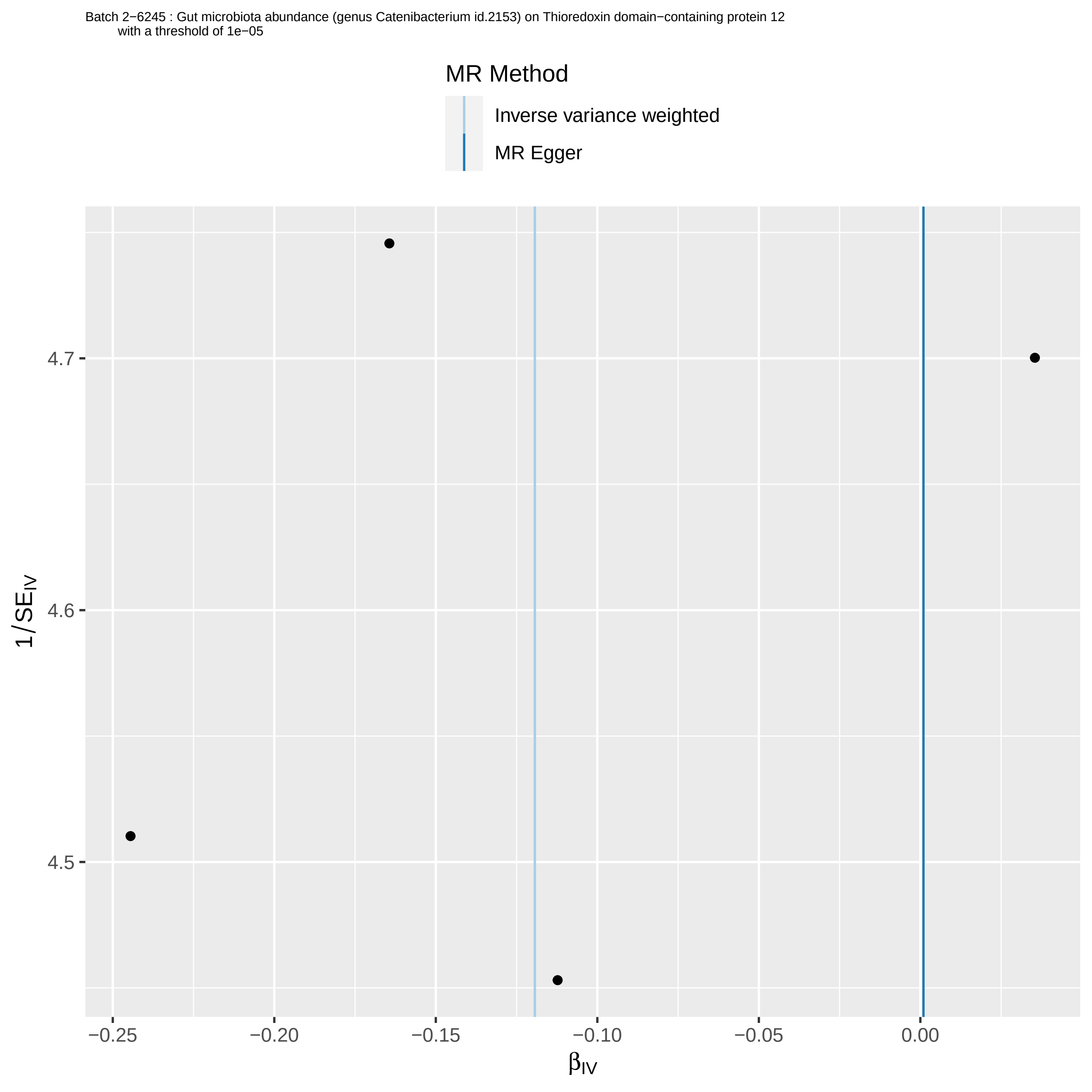

Batch 2−6245 : Gut microbiota abundance (genus Catenibacterium id.2153) on Thioredoxin domain−containing protein 12
with a threshold of 1e−05
MR Method
Inverse variance weighted
MR Egger
4.7
4.6
4.5
1/SE_IV
−0.25
−0.20
−0.15
−0.10
−0.05
0.00
β_IV

Batch 2−6246 : Gut microbiota abundance (genus Parasutterella id.2892) on Thioredoxin domain−containing protein 12
with a threshold of 1e−05

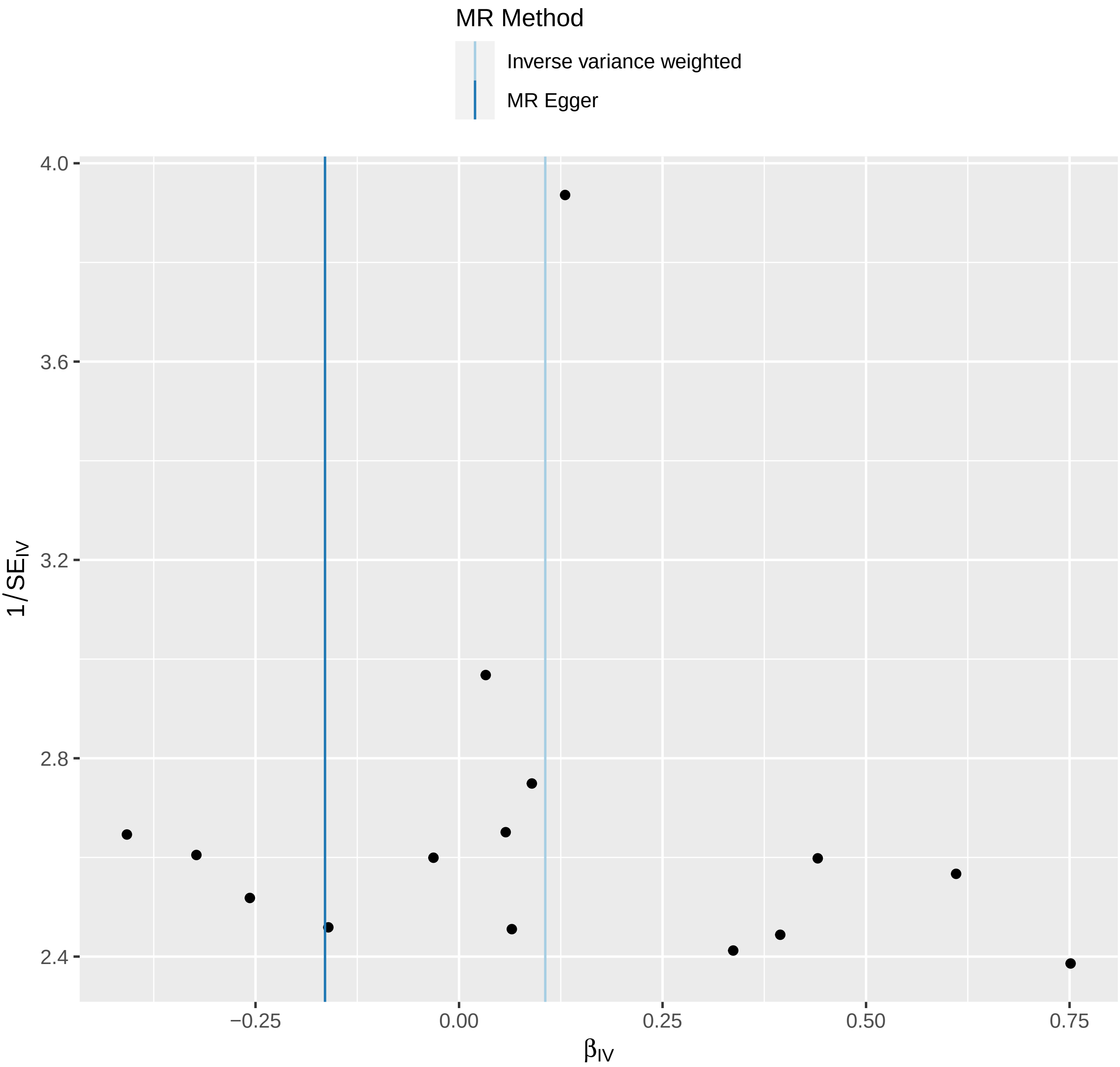

Batch 2−6247 : Gut microbiota abundance (genus Catenibacterium id.2153) on Thioredoxin domain−containing protein 5
with a threshold of 1e−05

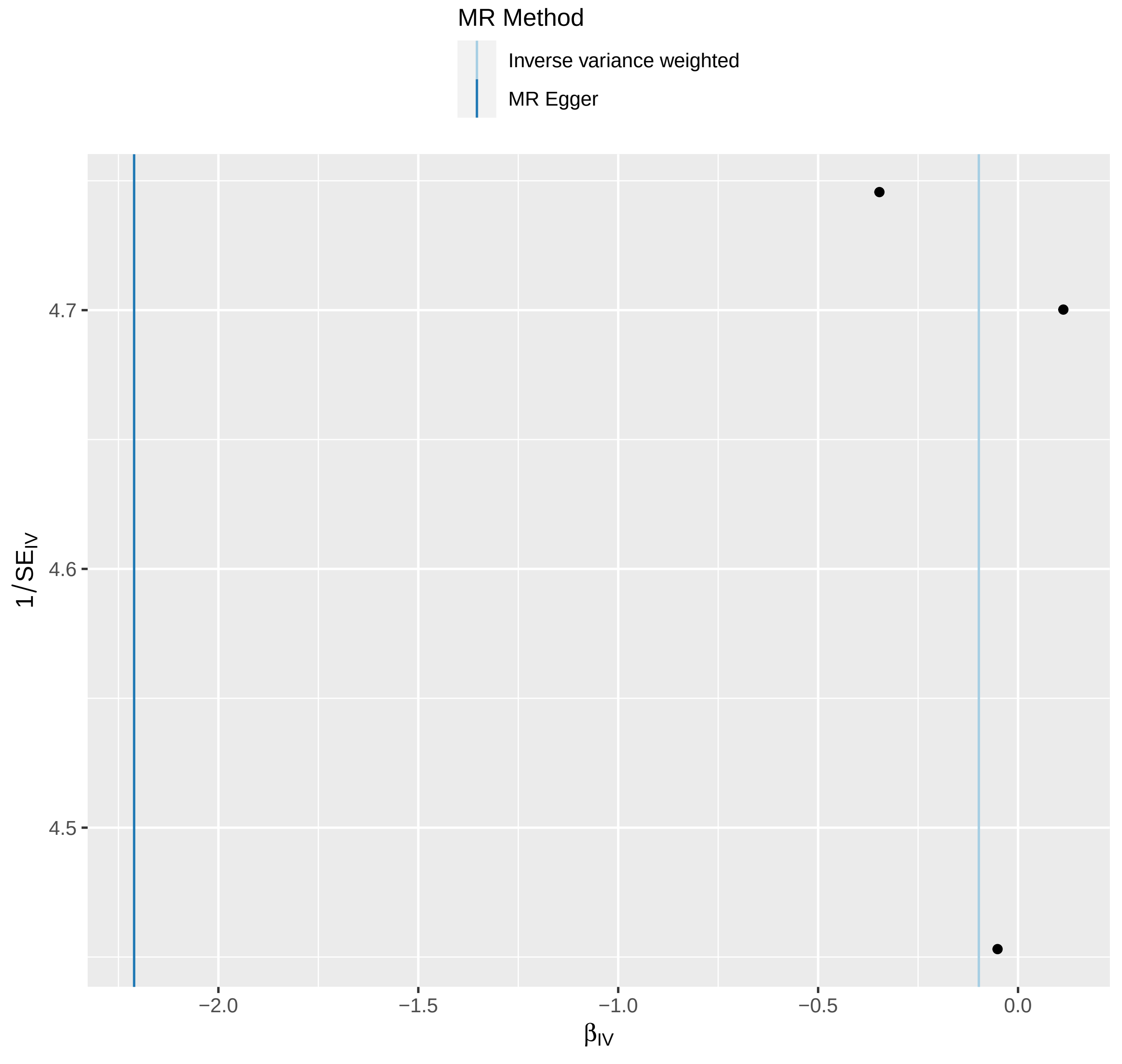

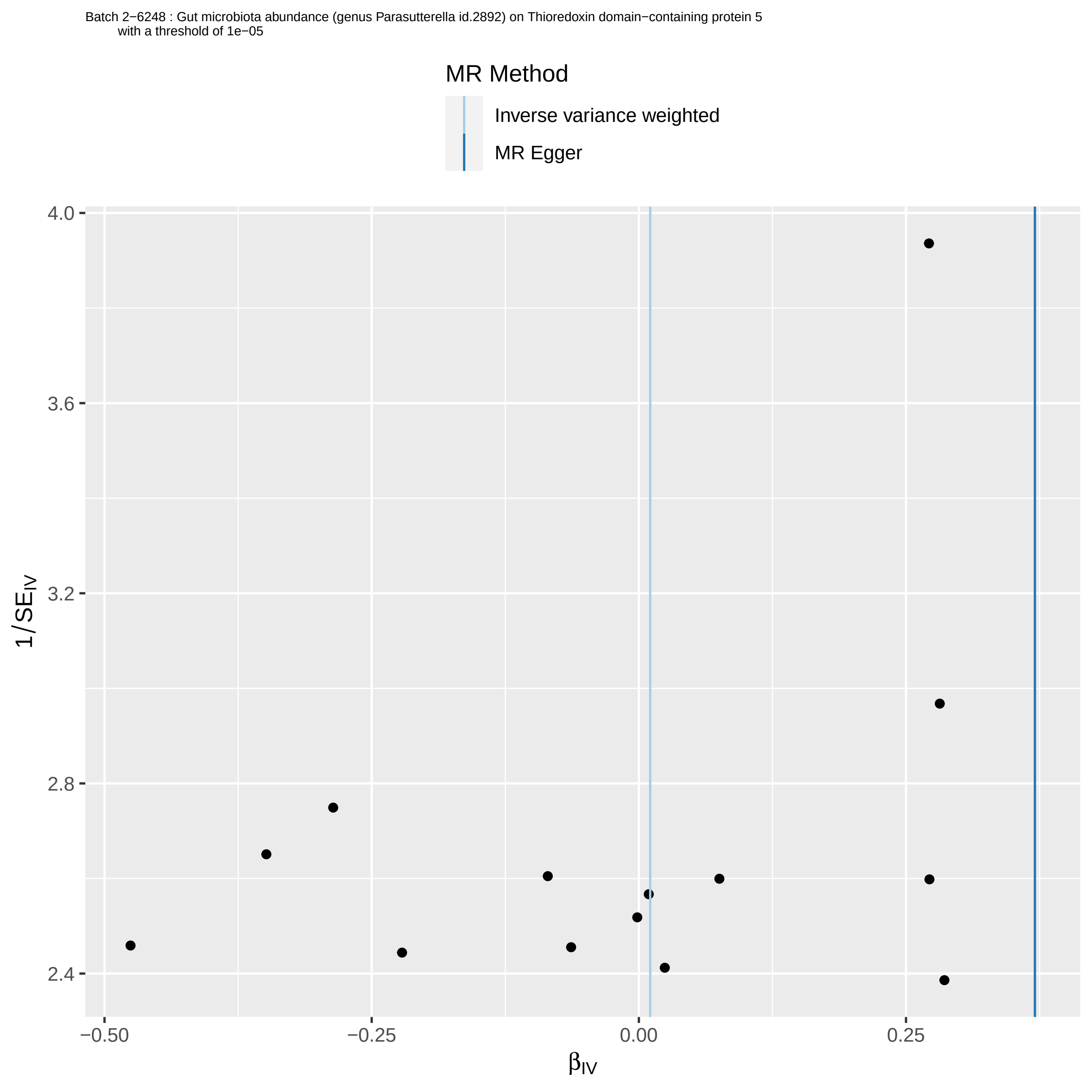

Batch 2−6248 : Gut microbiota abundance (genus Parasutterella id.2892) on Thioredoxin domain−containing protein 5
with a threshold of 1e−05
MR Method
Inverse variance weighted
MR Egger
4.0
3.6
3.2
2.8
2.4
1/SE_IV
−0.50
−0.25
0.00
0.25
β_IV

Batch 2−6249 : Gut microbiota abundance (genus Catenibacterium id.2153) on Thioredoxin−interacting protein with a threshold of 1e−05

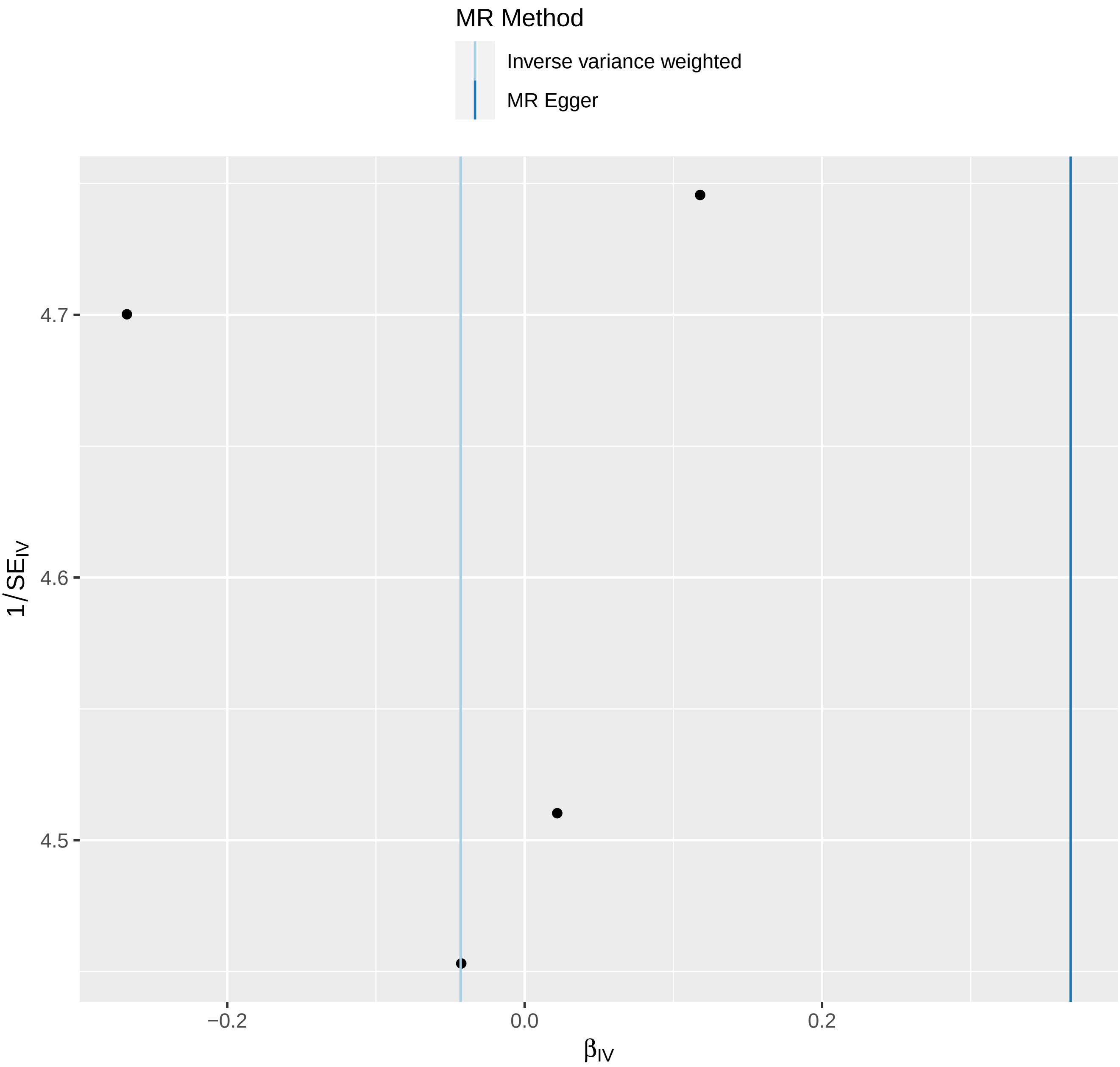

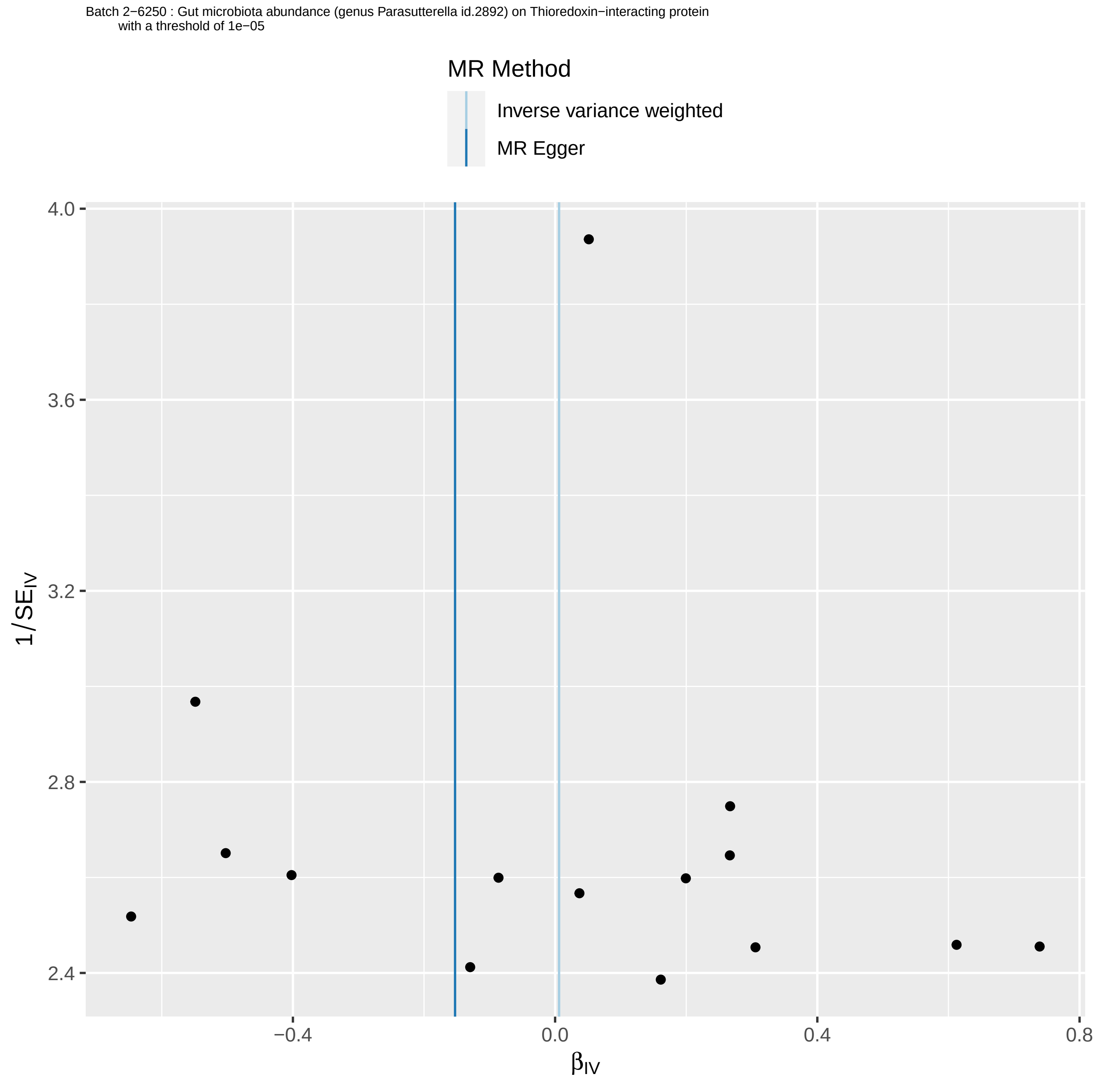
Batch 2−6250 : Gut microbiota abundance (genus Parasutterella id.2892) on Thioredoxin−interacting protein
with a threshold of 1e−05
MR Method
Inverse variance weighted
MR Egger
4.0
3.6
3.2
2.8
2.4
1/SE_IV
−0.4
0.0
0.4
0.8
β_IV

Batch 2−6251 : Gut microbiota abundance (genus Catenibacterium id.2153) on Thioredoxin reductase 1, cytoplasmic
with a threshold of 1e−05

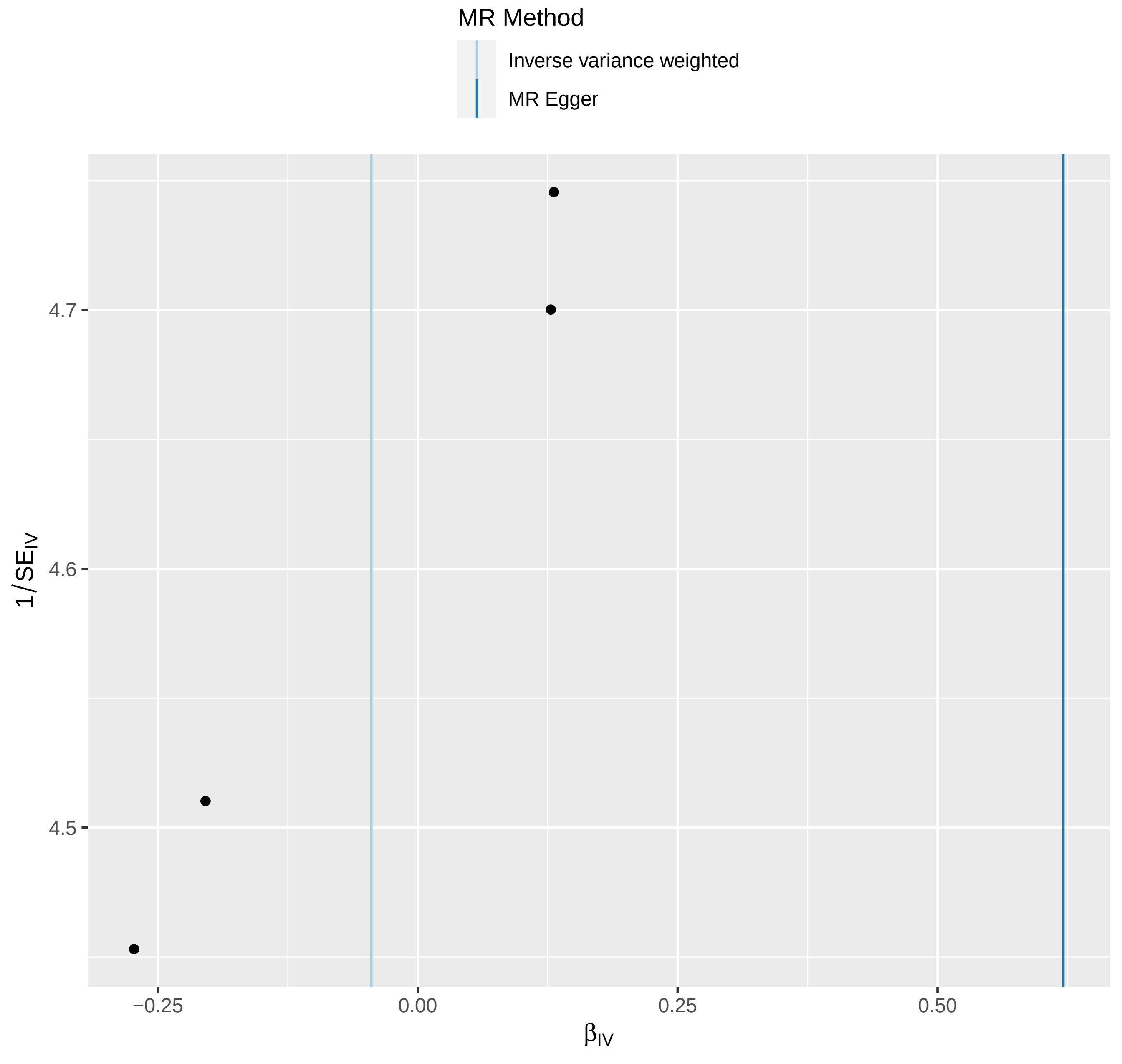

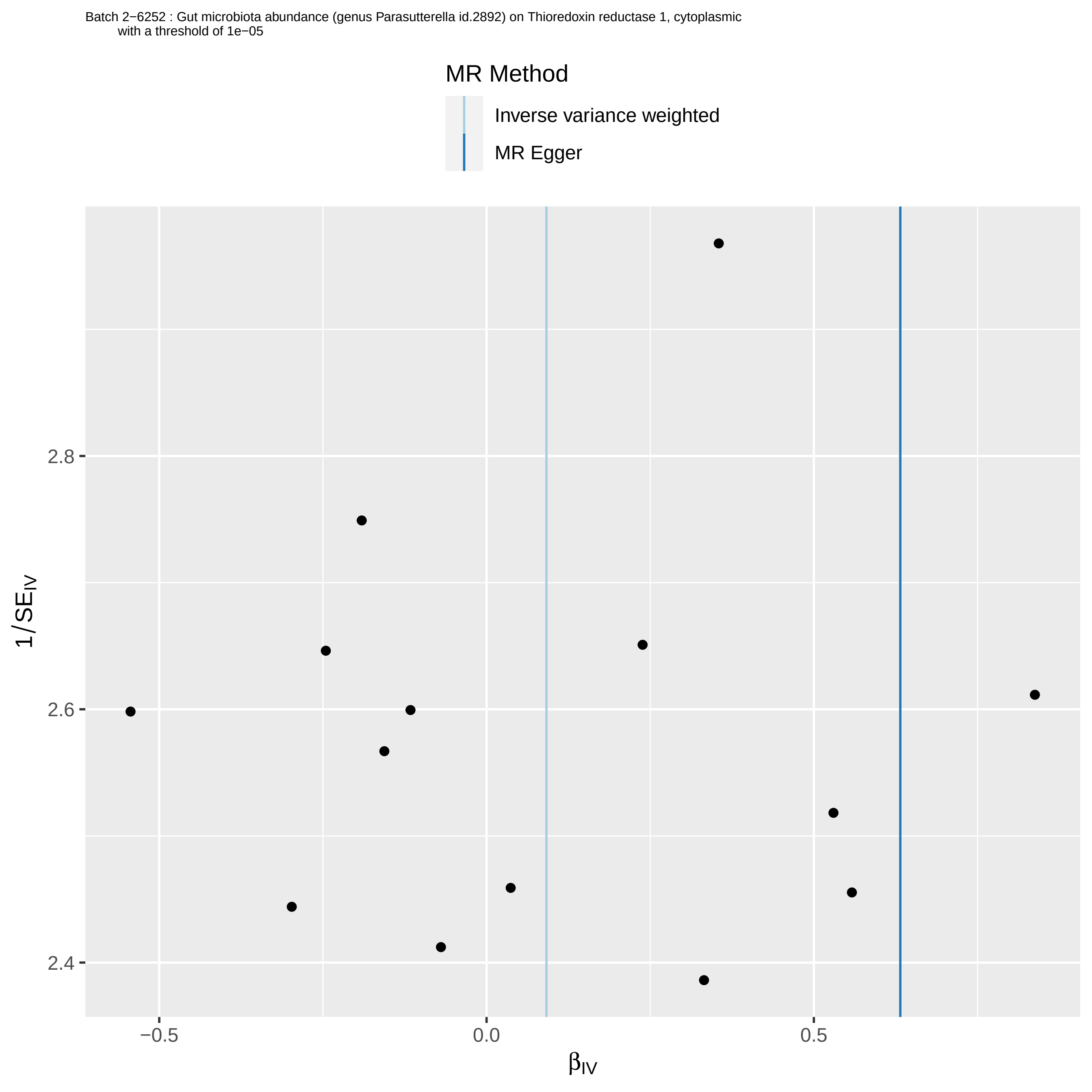

Batch 2−6252 : Gut microbiota abundance (genus Parasutterella id.2892) on Thioredoxin reductase 1, cytoplasmic
with a threshold of 1e−05
MR Method
Inverse variance weighted
MR Egger
2.8
2.6
2.4
1/SE_IV
−0.5
0.0
0.5
β_IV

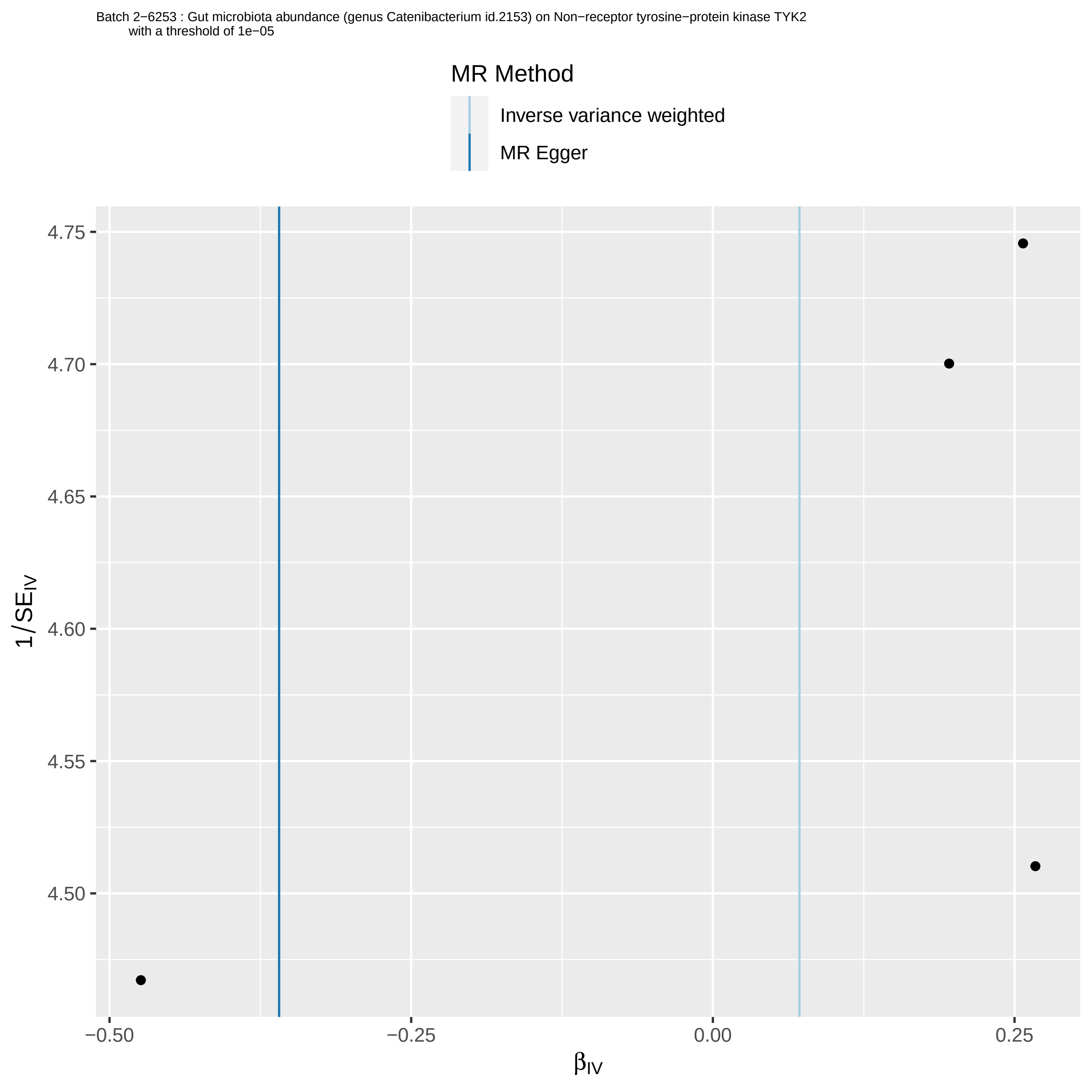
Batch 2−6253 : Gut microbiota abundance (genus Catenibacterium id.2153) on Non−receptor tyrosine−protein kinase TYK2
with a threshold of 1e−05
MR Method
Inverse variance weighted
MR Egger
4.75
4.70
4.65
4.60
4.55
4.50
$1/SE_{IV}$
−0.50
−0.25
0.00
0.25
β_{IV}

Batch 2−6254 : Gut microbiota abundance (genus Parasutterella id.2892) on Non−receptor tyrosine−protein kinase TYK2
with a threshold of 1e−05

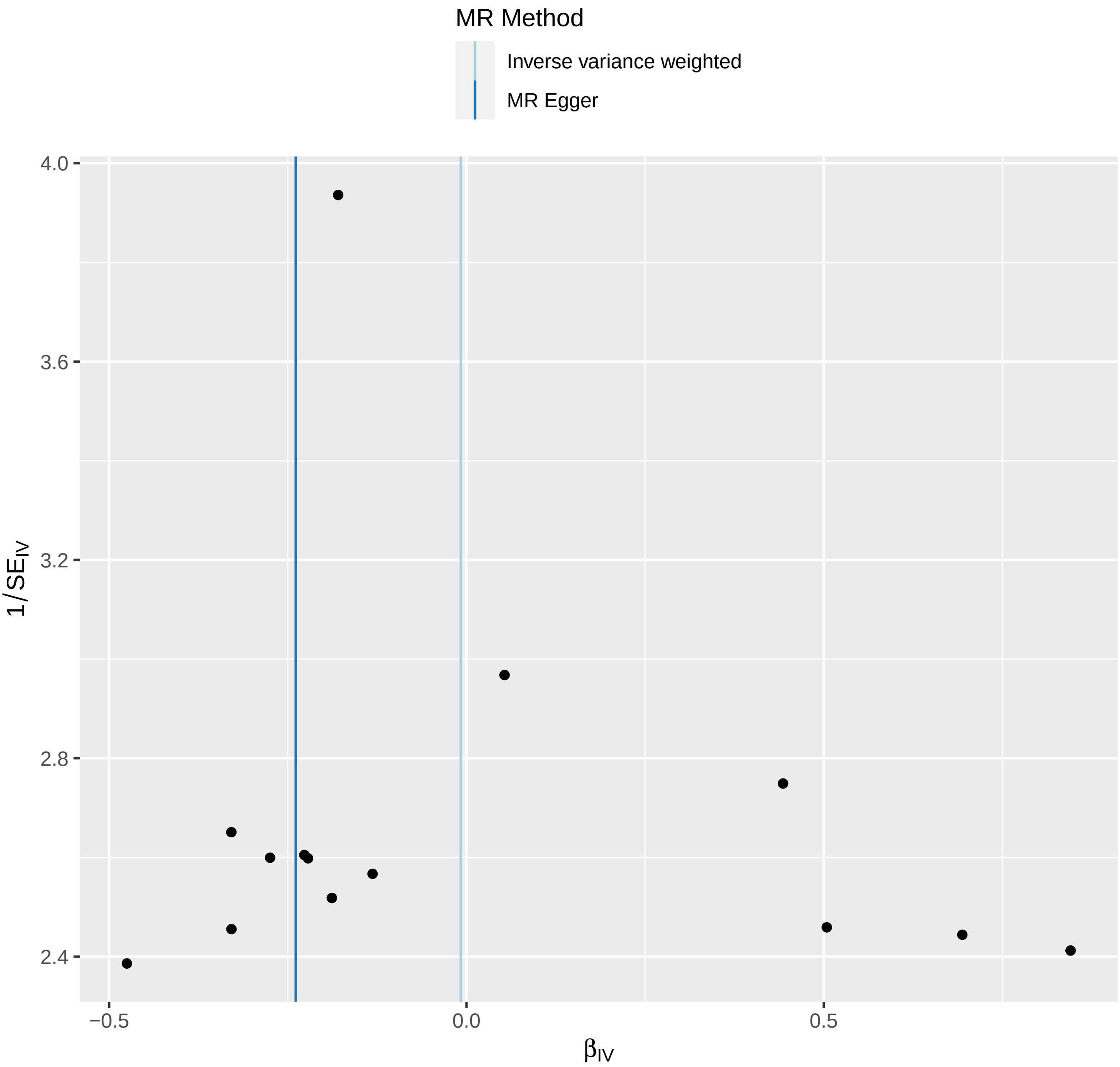

Batch 2−6255 : Gut microbiota abundance (genus Catenibacterium id.2153) on TYMS opposite strand protein
with a threshold of 1e−05

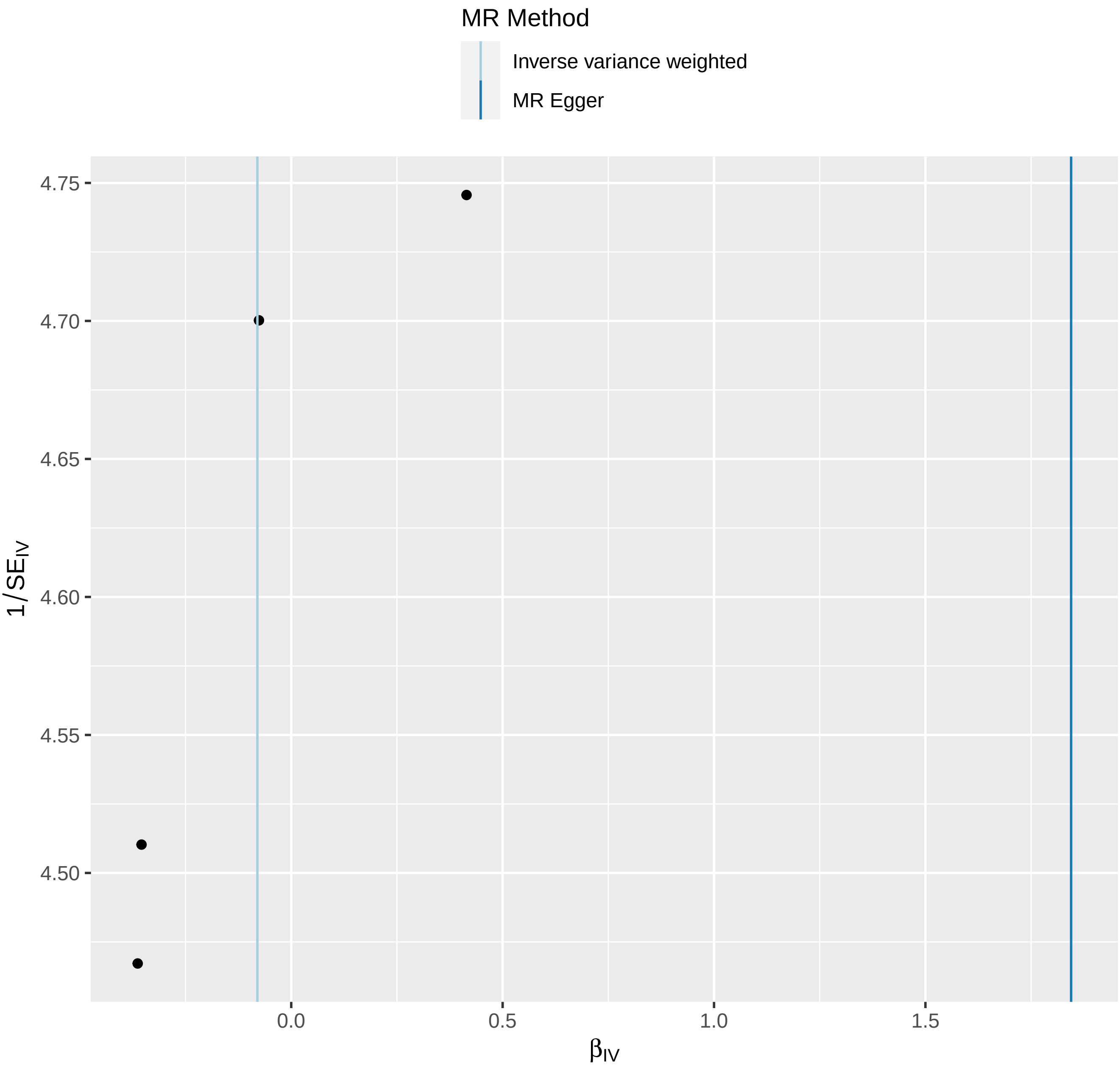

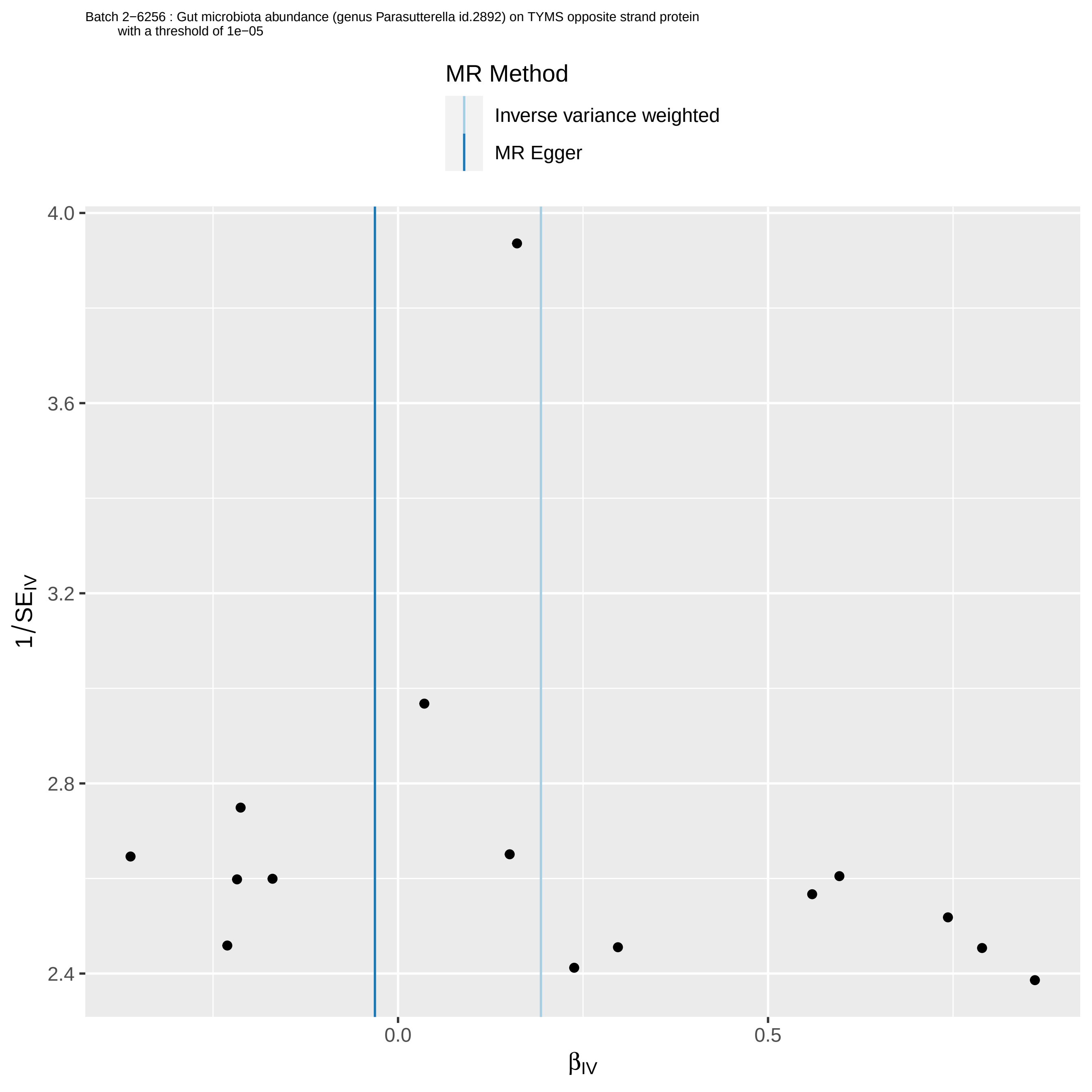

Batch 2−6256 : Gut microbiota abundance (genus Parasutterella id.2892) on TYMS opposite strand protein
with a threshold of 1e−05
MR Method
Inverse variance weighted
MR Egger
4.0
3.6
3.2
2.8
2.4
1/SE$_{IV}$
0.0
0.5
β_{IV}

Batch 2−6257 : Gut microbiota abundance (genus Catenibacterium id.2153) on Tyrosine−protein kinase receptor TYRO3
with a threshold of 1e−05

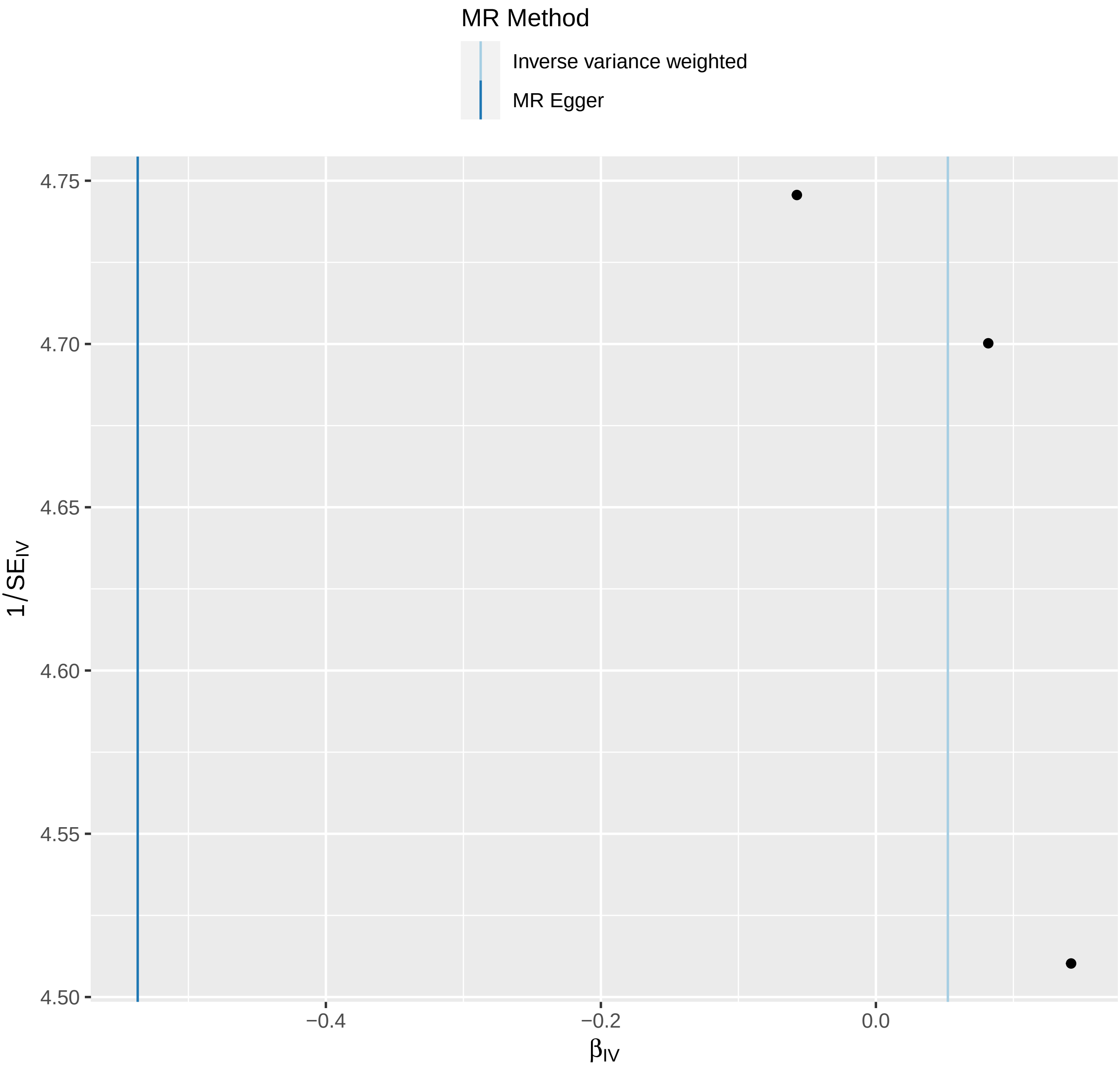

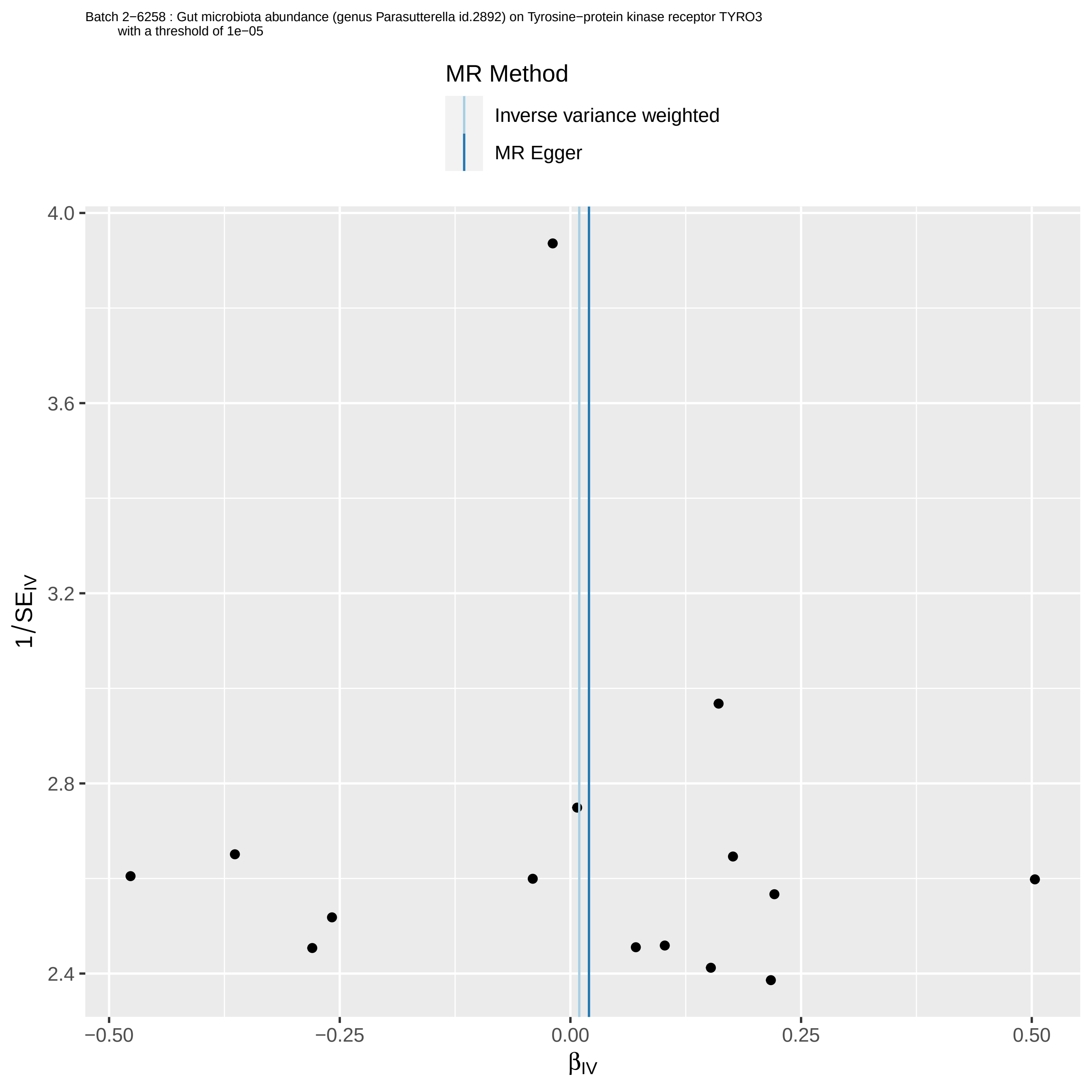
Batch 2−6258 : Gut microbiota abundance (genus Parasutterella id.2892) on Tyrosine−protein kinase receptor TYRO3
with a threshold of 1e−05
MR Method
Inverse variance weighted
MR Egger
4.0
3.6
3.2
2.8
2.4
1/SE_IV
−0.50
−0.25
0.00
0.25
0.50
β_IV

Batch 2−6259 : Gut microbiota abundance (genus Catenibacterium id.2153) on Splicing factor U2AF 65 kDa subunit
with a threshold of 1e−05

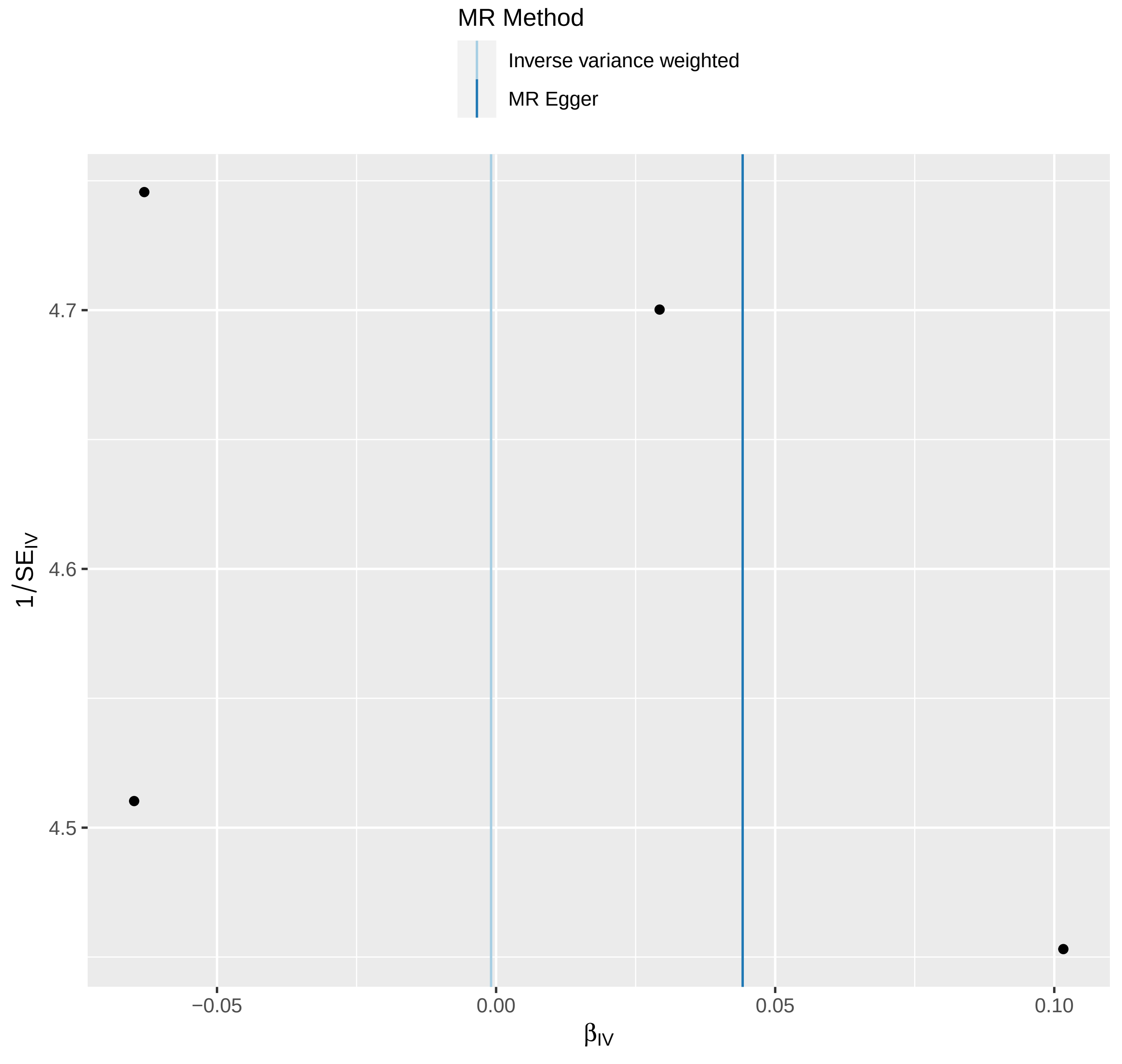

Batch 2−6260 : Gut microbiota abundance (genus Parasutterella id.2892) on Splicing factor U2AF 65 kDa subunit
with a threshold of 1e−05

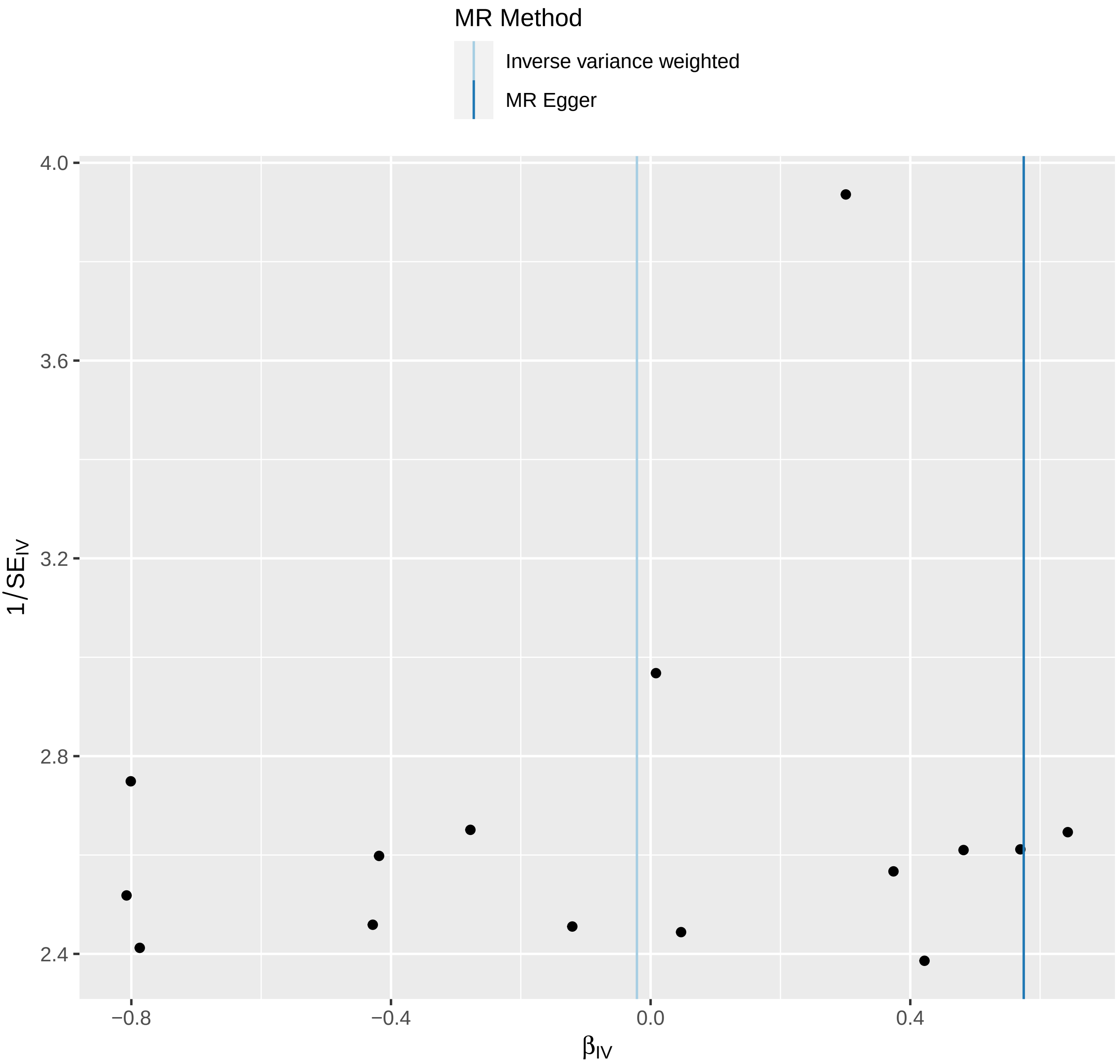

Batch 2−6261 : Gut microbiota abundance (genus Catenibacterium id.2153) on UDP−N−acetylhexosamine pyrophosphorylase
with a threshold of 1e−05

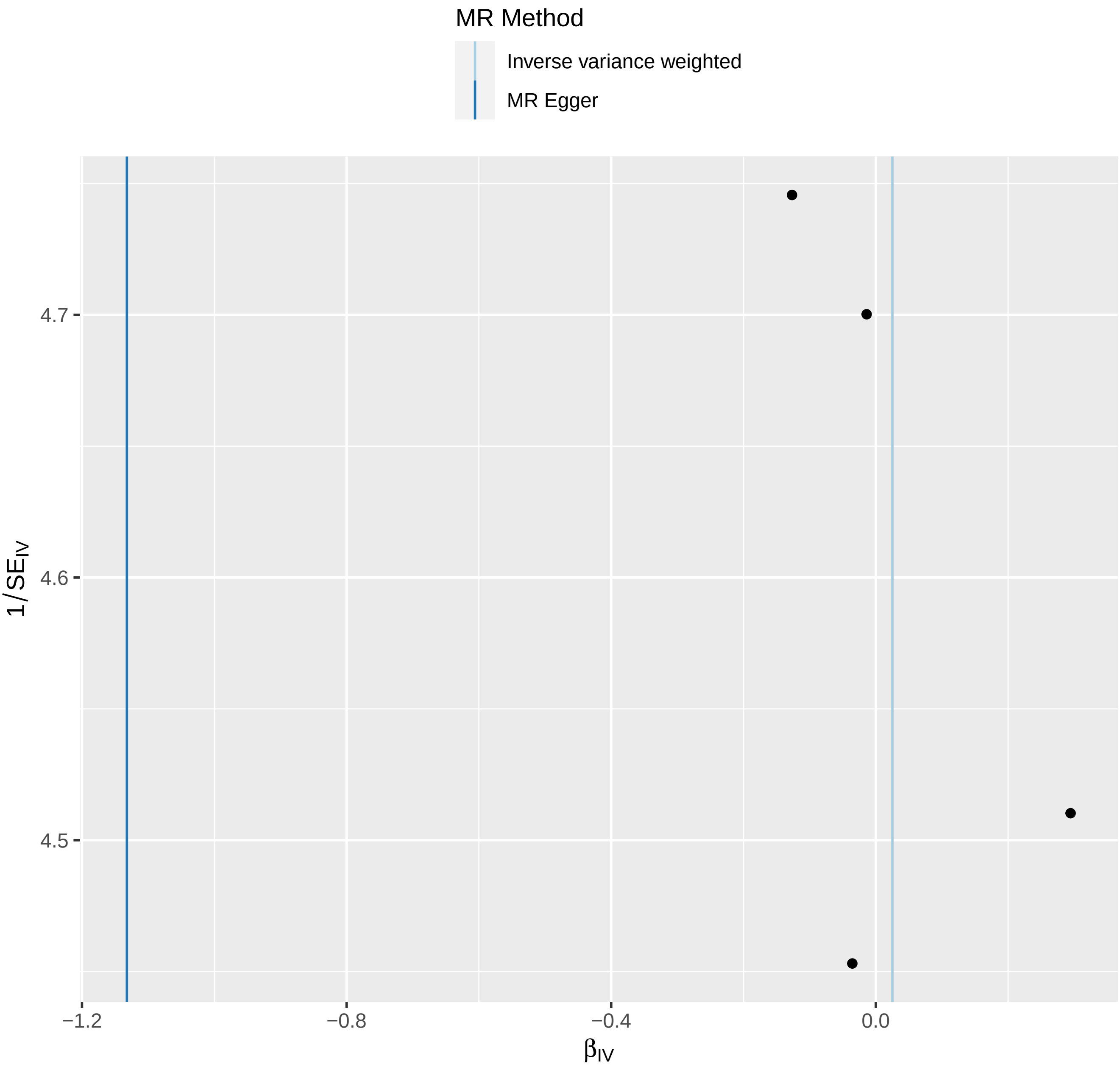

Batch 2−6262 : Gut microbiota abundance (genus Parasutterella id.2892) on UDP−N−acetylhexosamine pyrophosphorylase
with a threshold of 1e−05

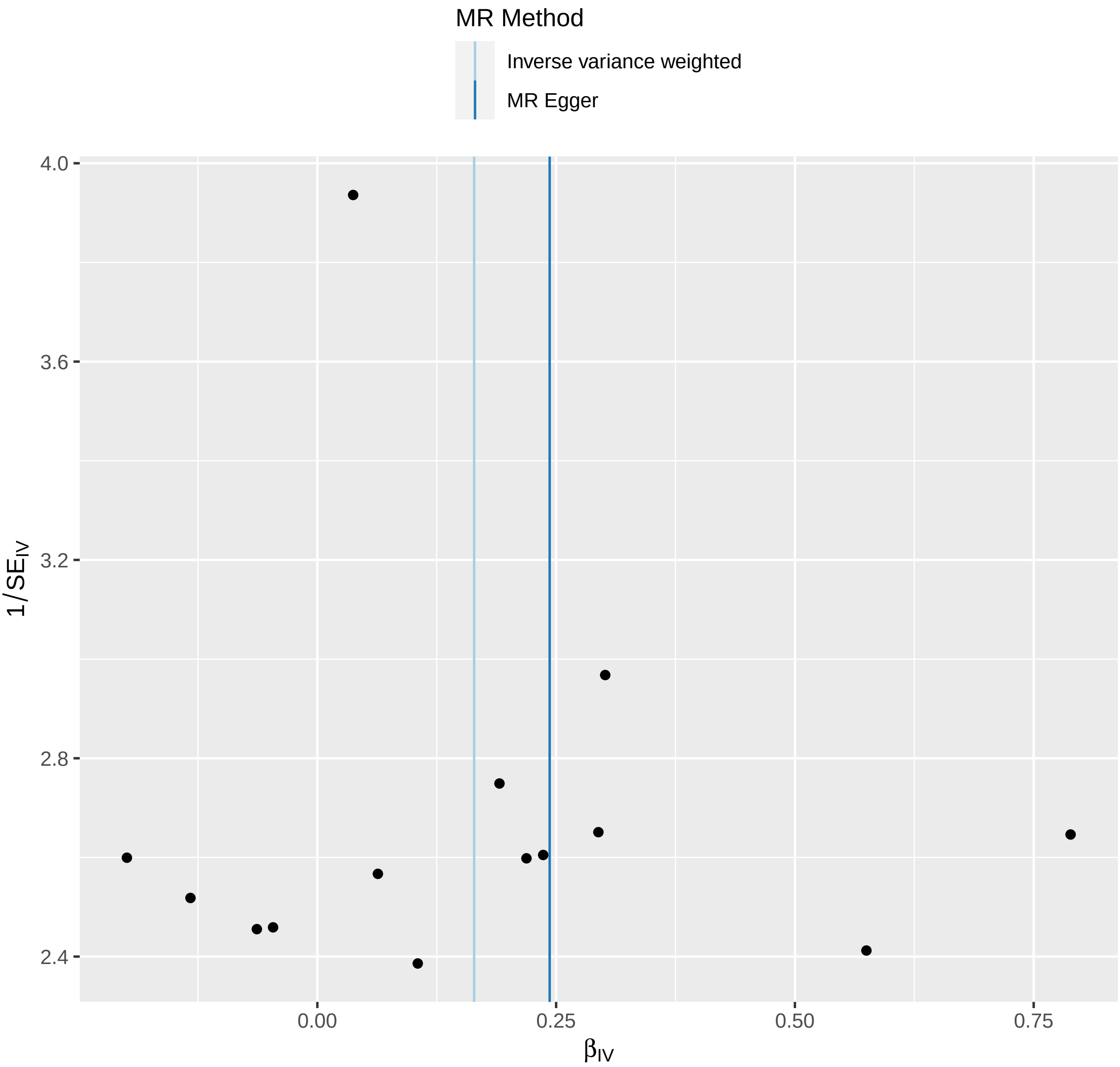

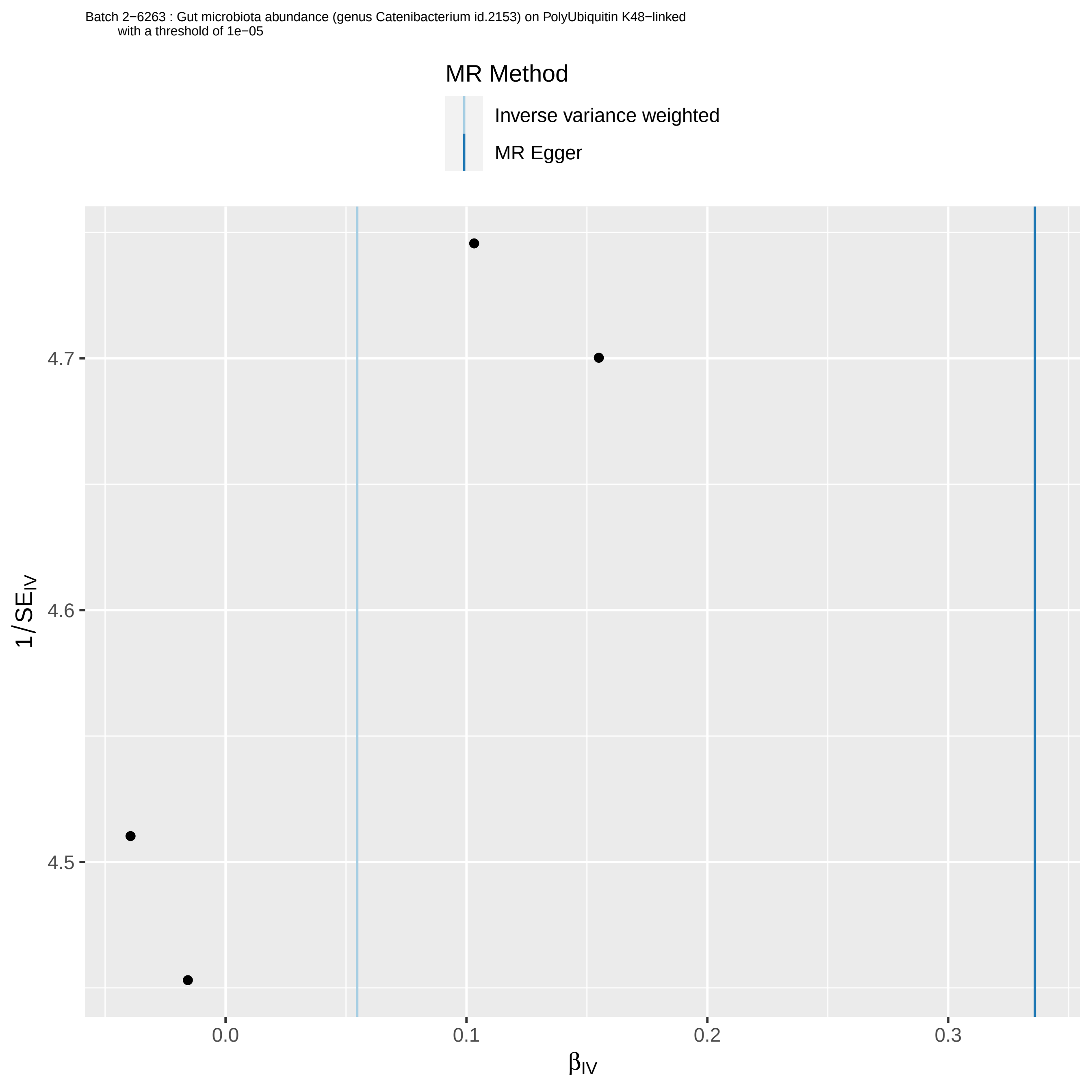
Batch 2−6263 : Gut microbiota abundance (genus Catenibacterium id.2153) on PolyUbiquitin K48−linked
with a threshold of 1e−05
MR Method
Inverse variance weighted
MR Egger
4.7
4.6
4.5
1/SE_IV
0.0
0.1
0.2
0.3
β_IV

Batch 2−6264 : Gut microbiota abundance (genus Parasutterella id.2892) on PolyUbiquitin K48−linked
with a threshold of 1e−05

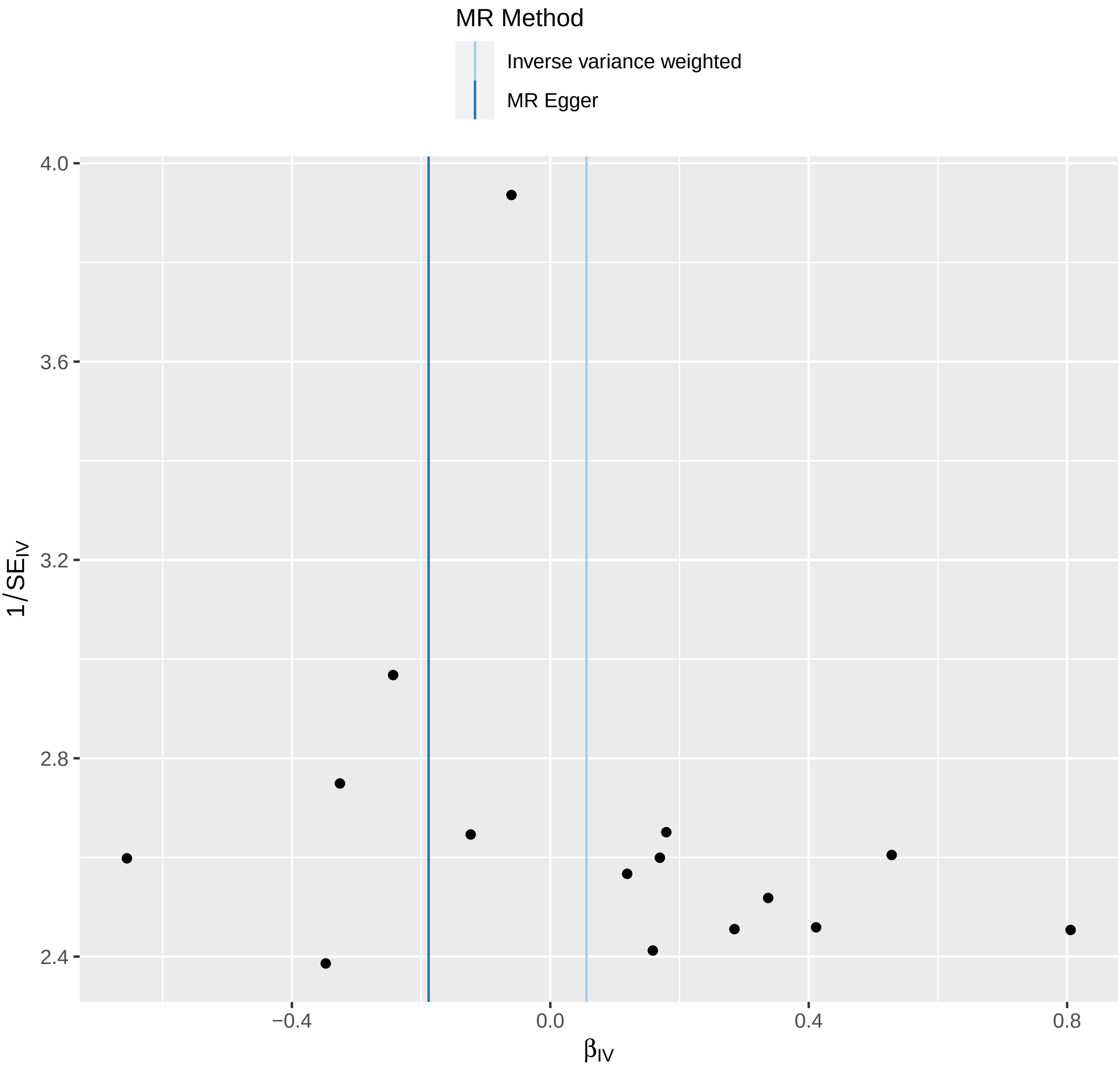

Batch 2−6265 : Gut microbiota abundance (genus Catenibacterium id.2153) on PolyUbiquitin K48−linked
with a threshold of 1e−05

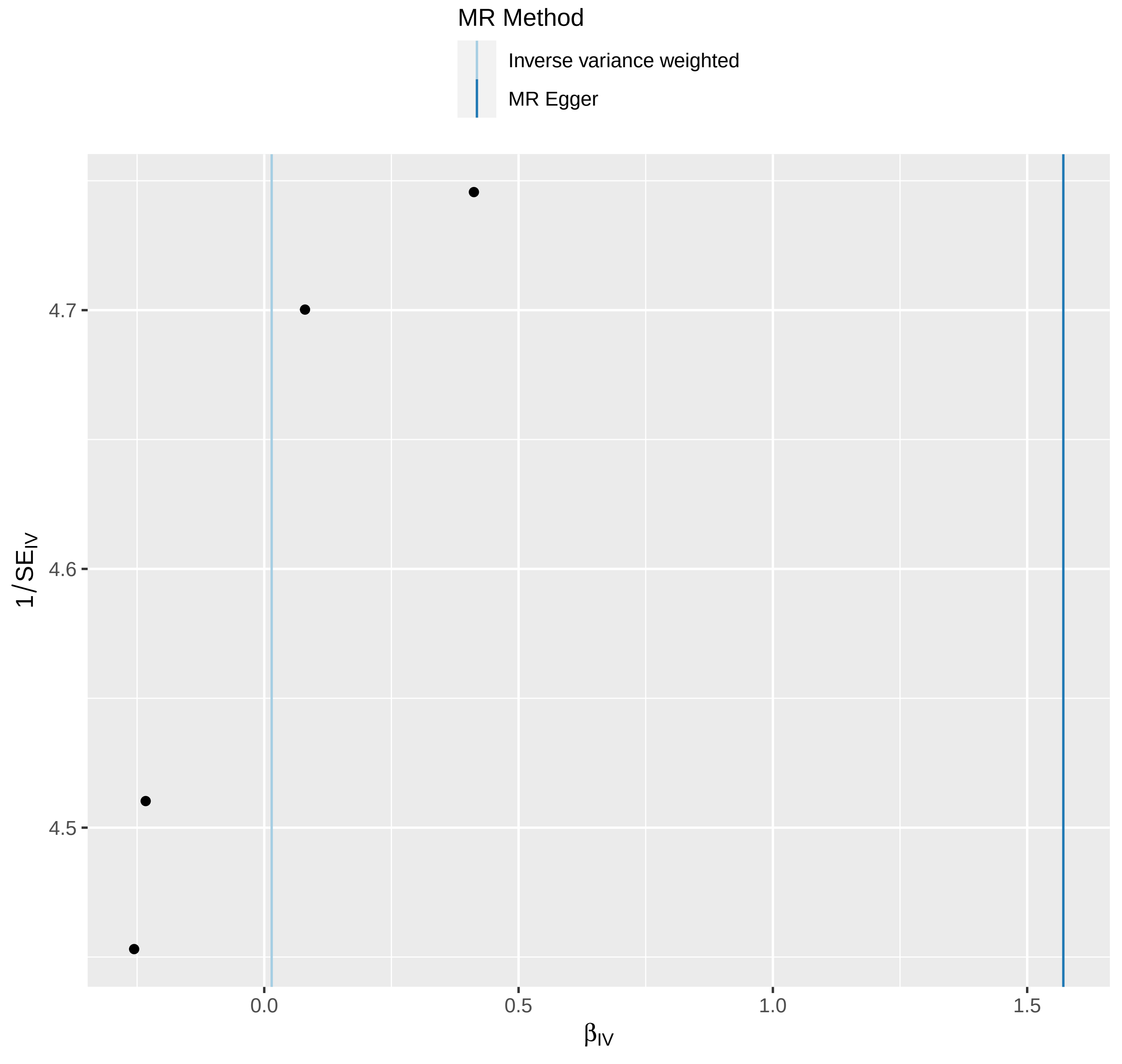

Batch 2−6266 : Gut microbiota abundance (genus Parasutterella id.2892) on PolyUbiquitin K48−linked
with a threshold of 1e−05

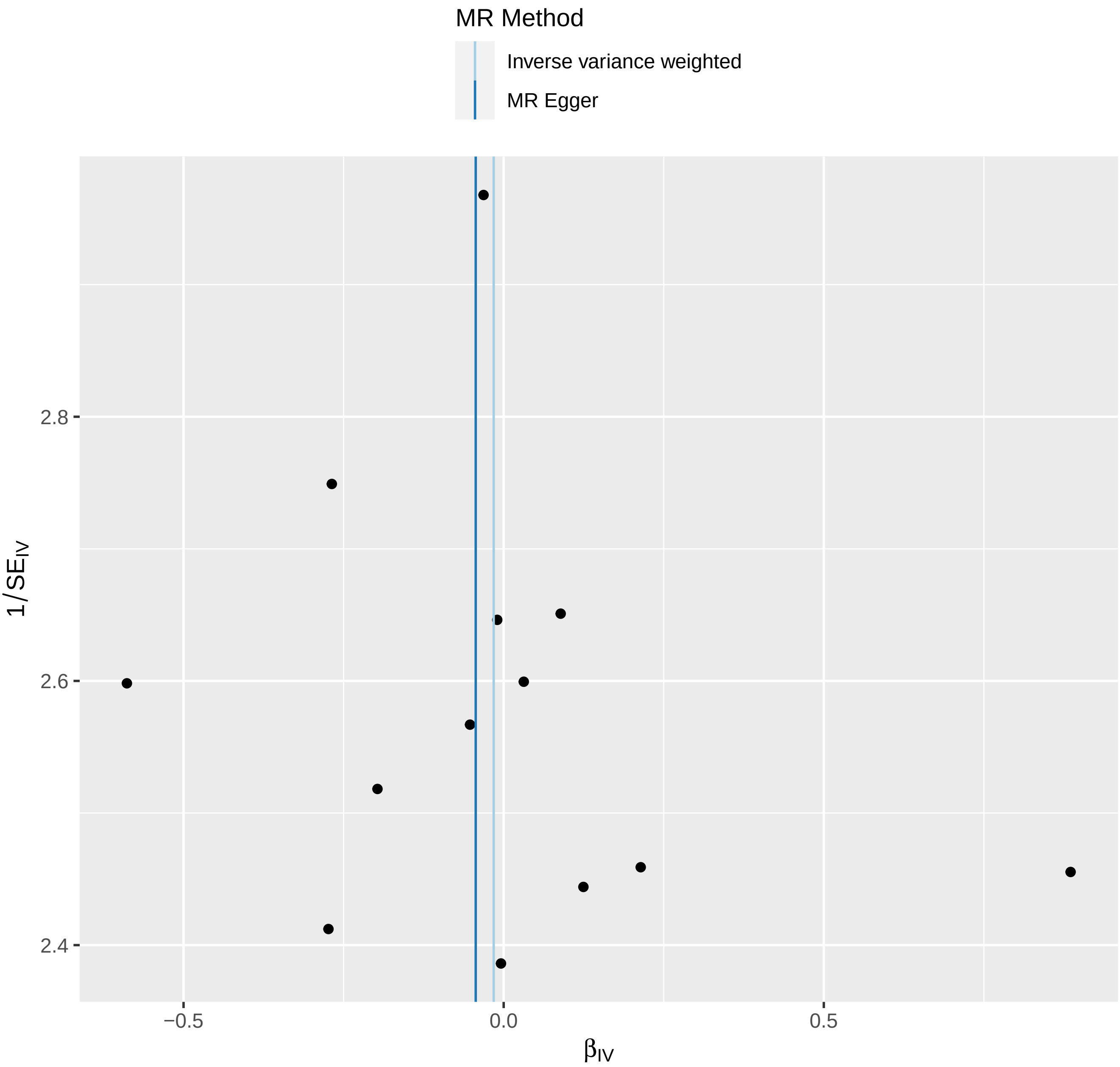

Batch 2−6267 : Gut microbiota abundance (genus Catenibacterium id.2153) on PolyUbiquitin K63−linked
with a threshold of 1e−05

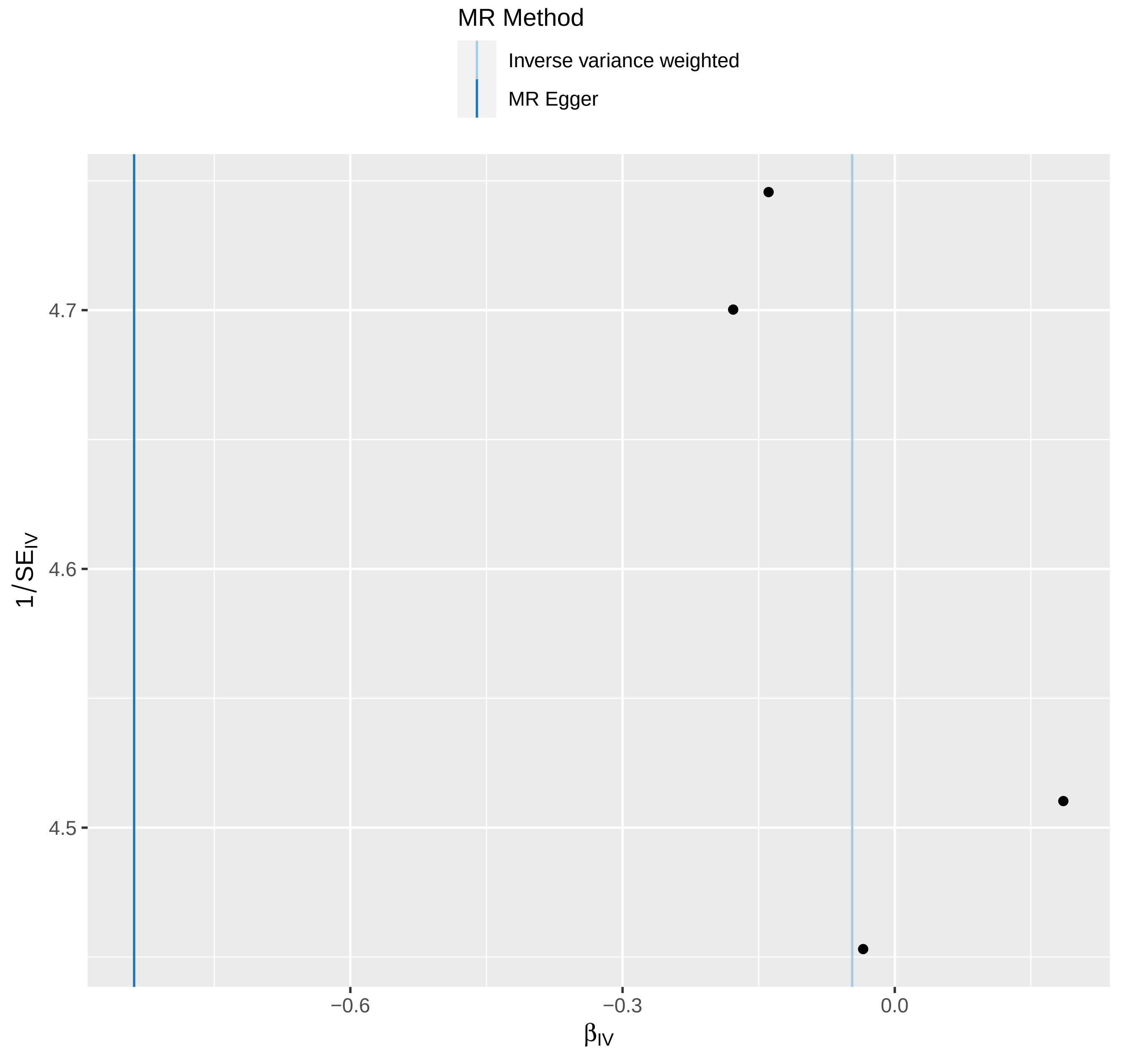

Batch 2−6268 : Gut microbiota abundance (genus Parasutterella id.2892) on PolyUbiquitin K63−linked
with a threshold of 1e−05

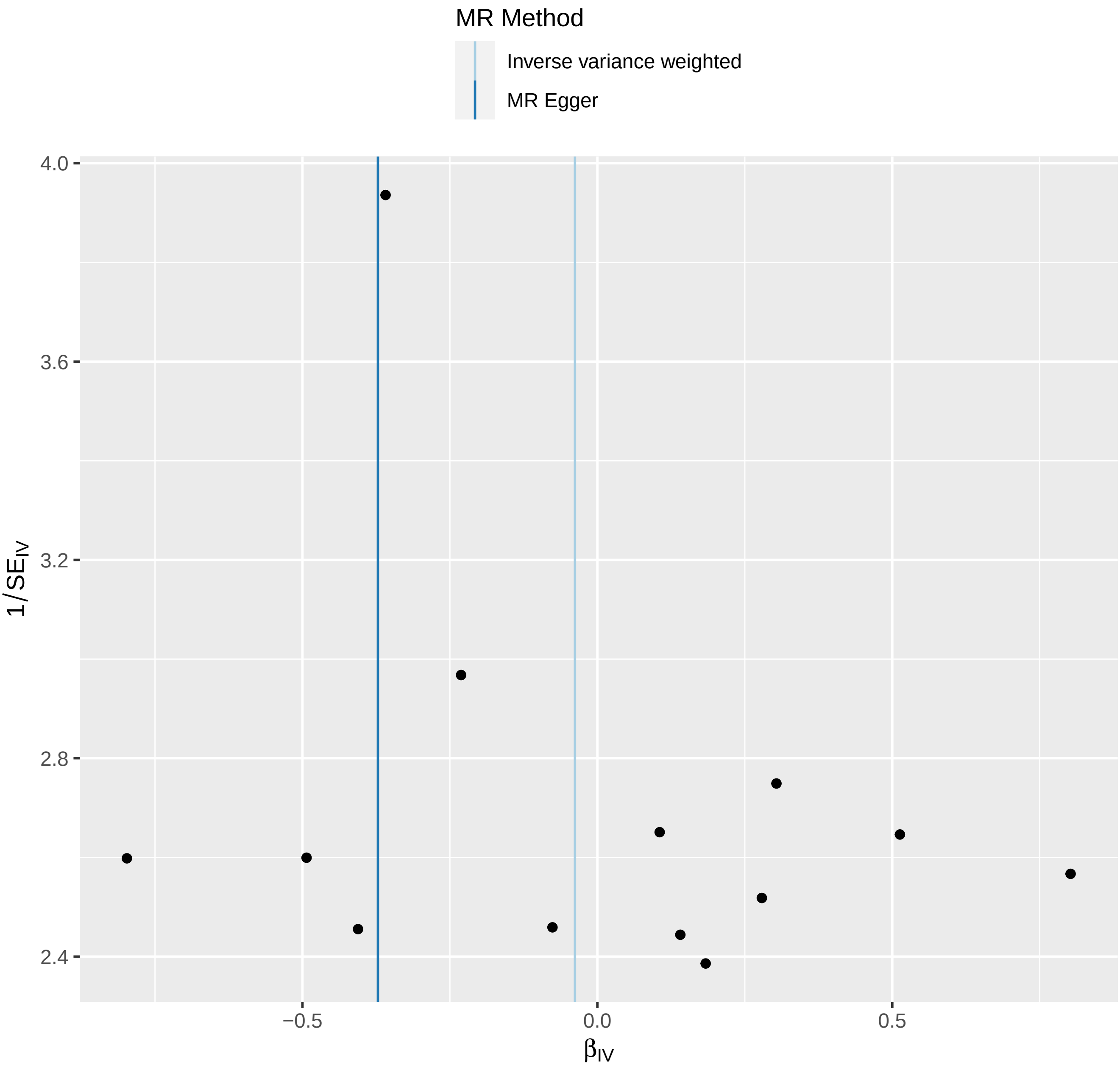

Batch 2−6269 : Gut microbiota abundance (genus Catenibacterium id.2153) on Ubiquitin−conjugating enzyme E2 B
with a threshold of 1e−05

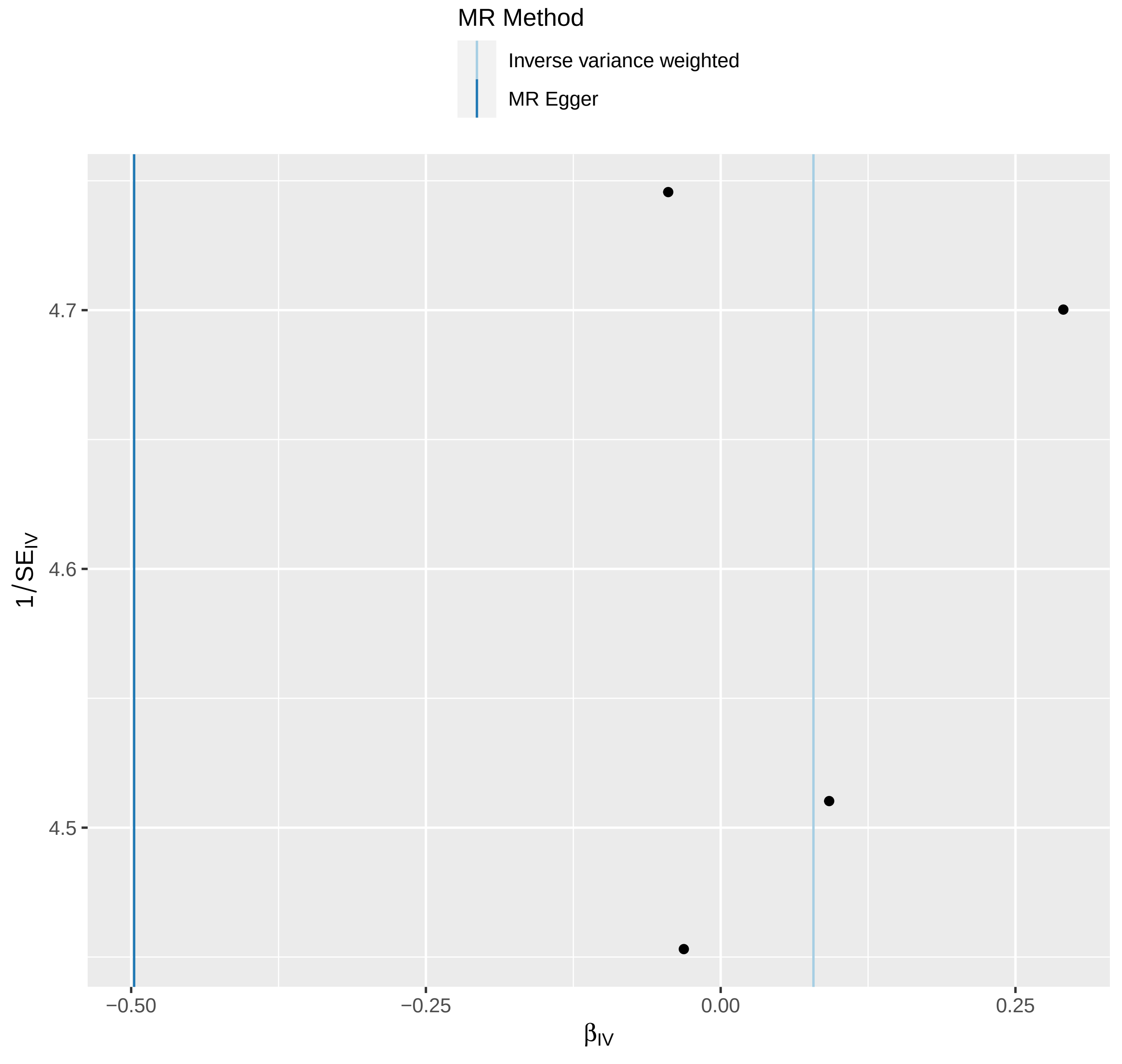

Batch 2−6270 : Gut microbiota abundance (genus Parasutterella id.2892) on Ubiquitin−conjugating enzyme E2 B
with a threshold of 1e−05

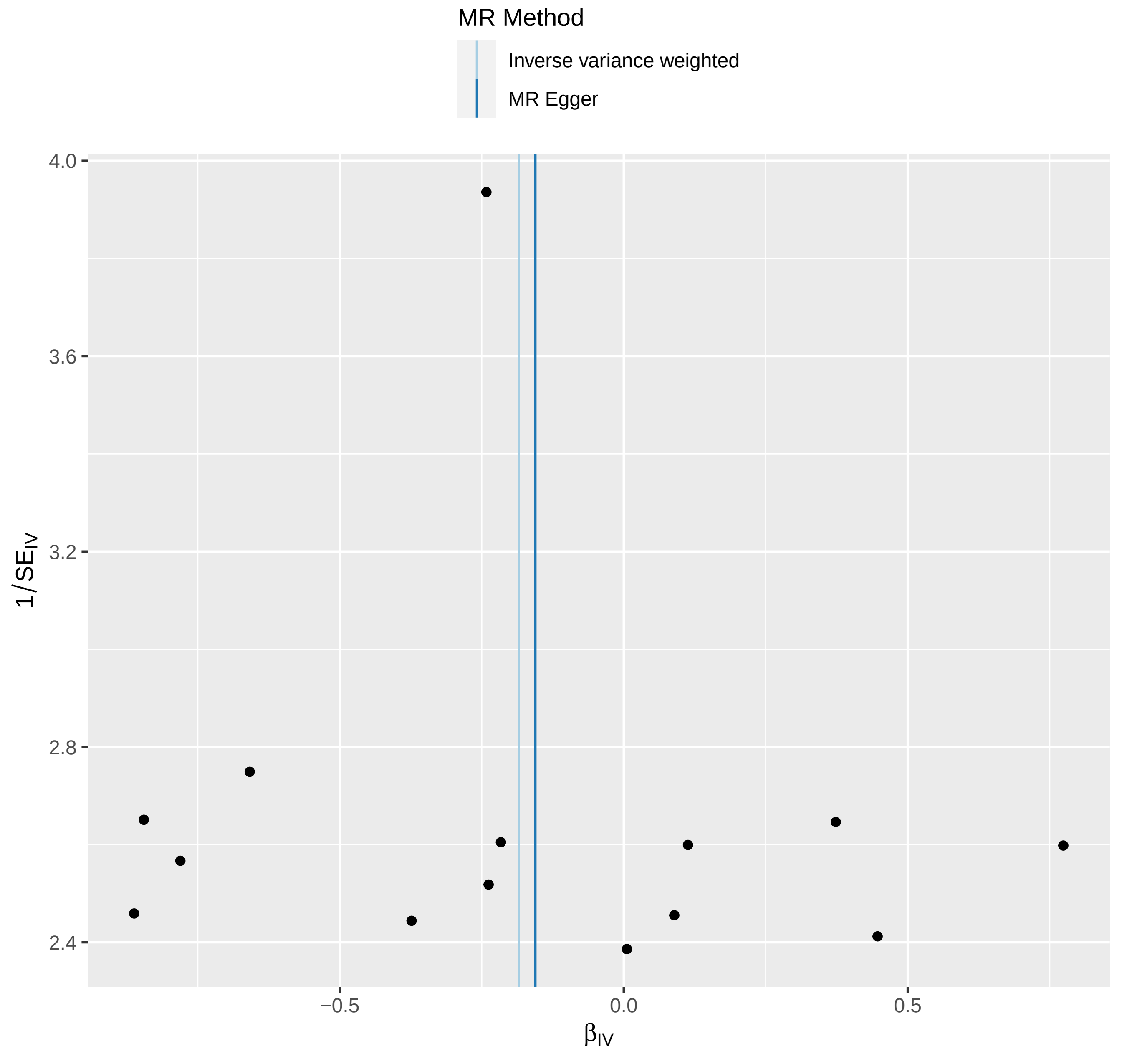

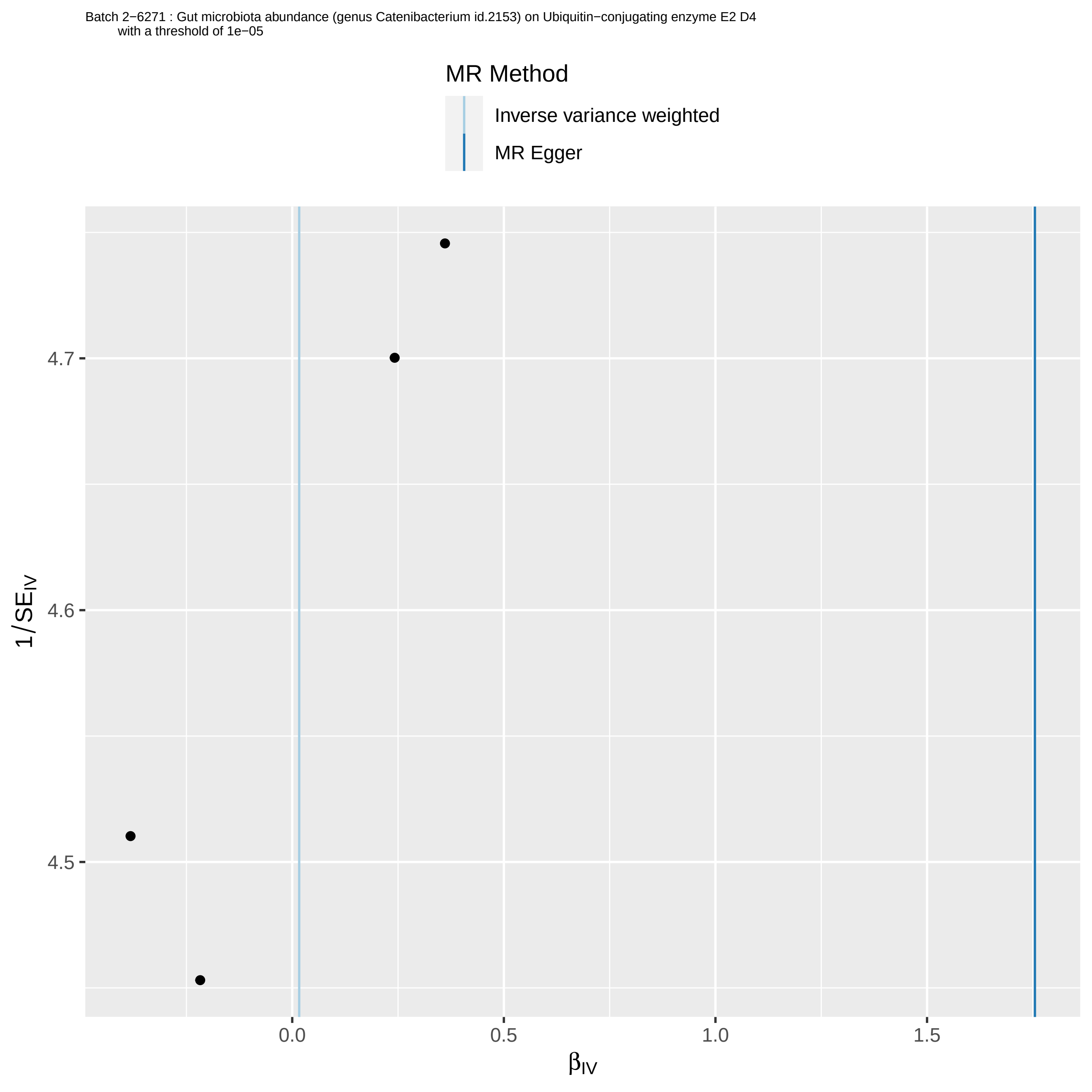

Batch 2−6271 : Gut microbiota abundance (genus Catenibacterium id.2153) on Ubiquitin−conjugating enzyme E2 D4
with a threshold of 1e−05
MR Method
Inverse variance weighted
MR Egger
4.7
4.6
4.5
1/SE_IV
0.0
0.5
1.0
1.5
β_IV

Batch 2−6272 : Gut microbiota abundance (genus Parasutterella id.2892) on Ubiquitin−conjugating enzyme E2 D4
with a threshold of 1e−05

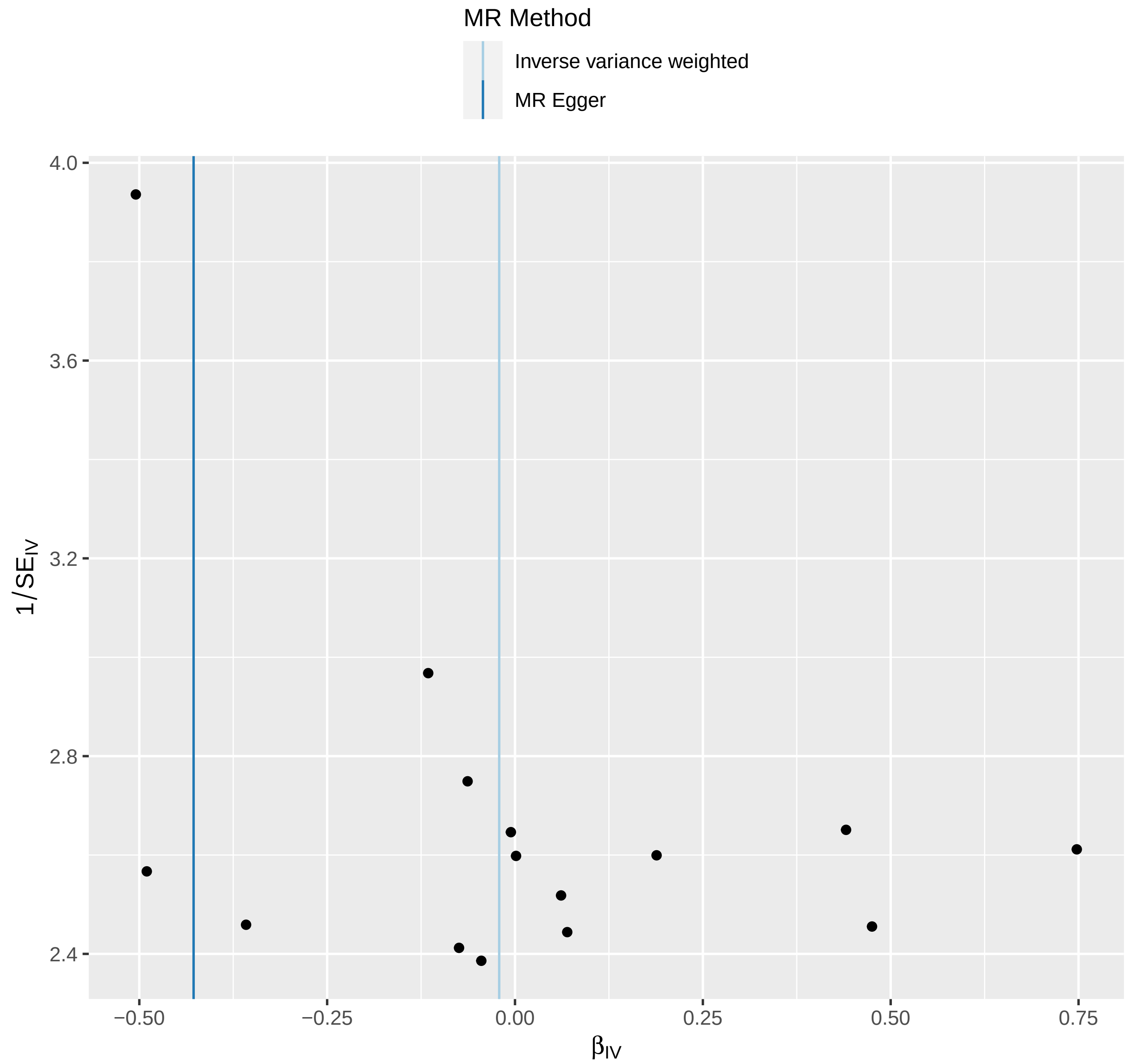

Batch 2−6273 : Gut microbiota abundance (genus Catenibacterium id.2153) on Ubiquitin−conjugating enzyme E2 G2
with a threshold of 1e−05

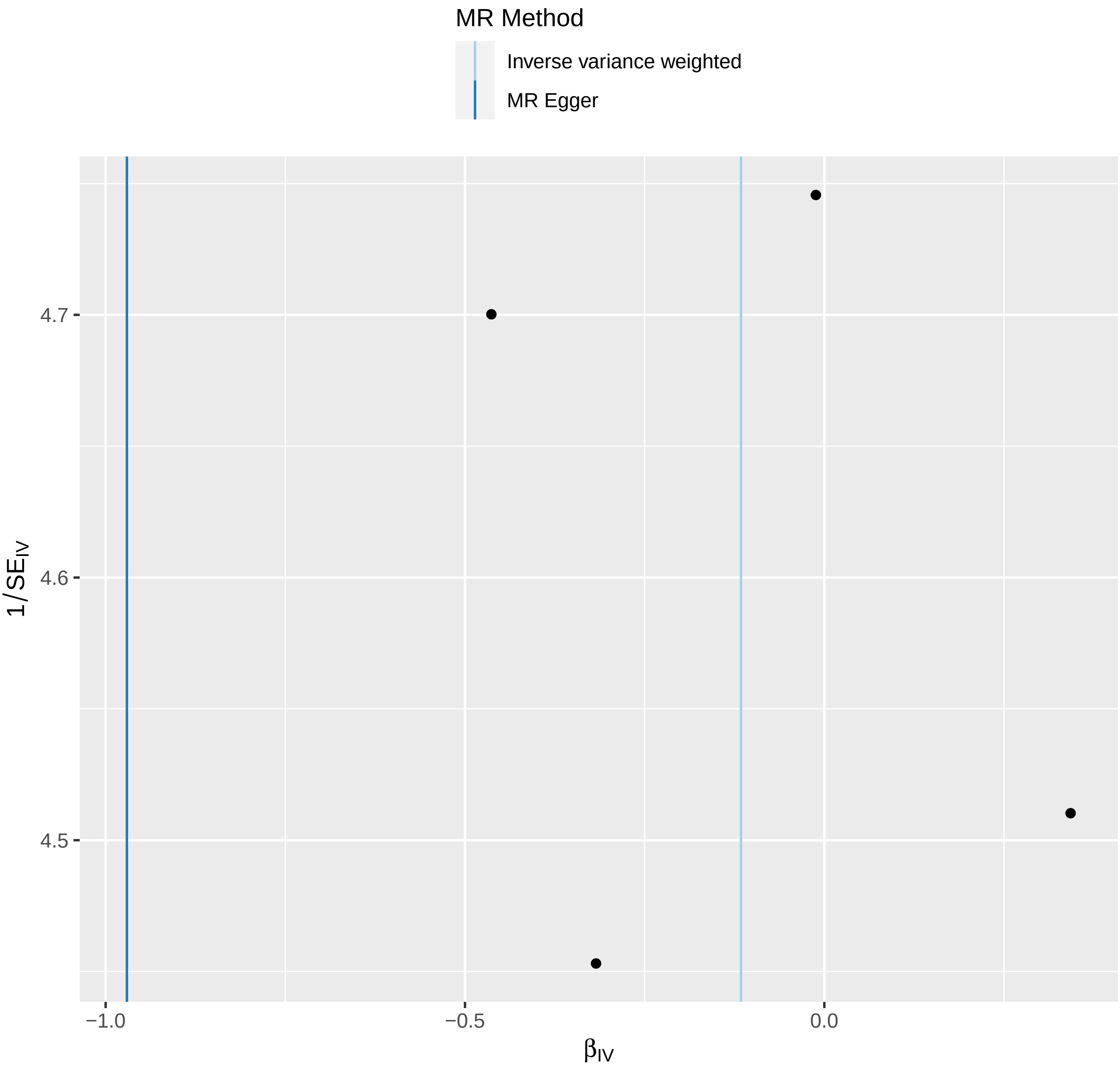

Batch 2−6274 : Gut microbiota abundance (genus Parasutterella id.2892) on Ubiquitin−conjugating enzyme E2 G2
with a threshold of 1e−05

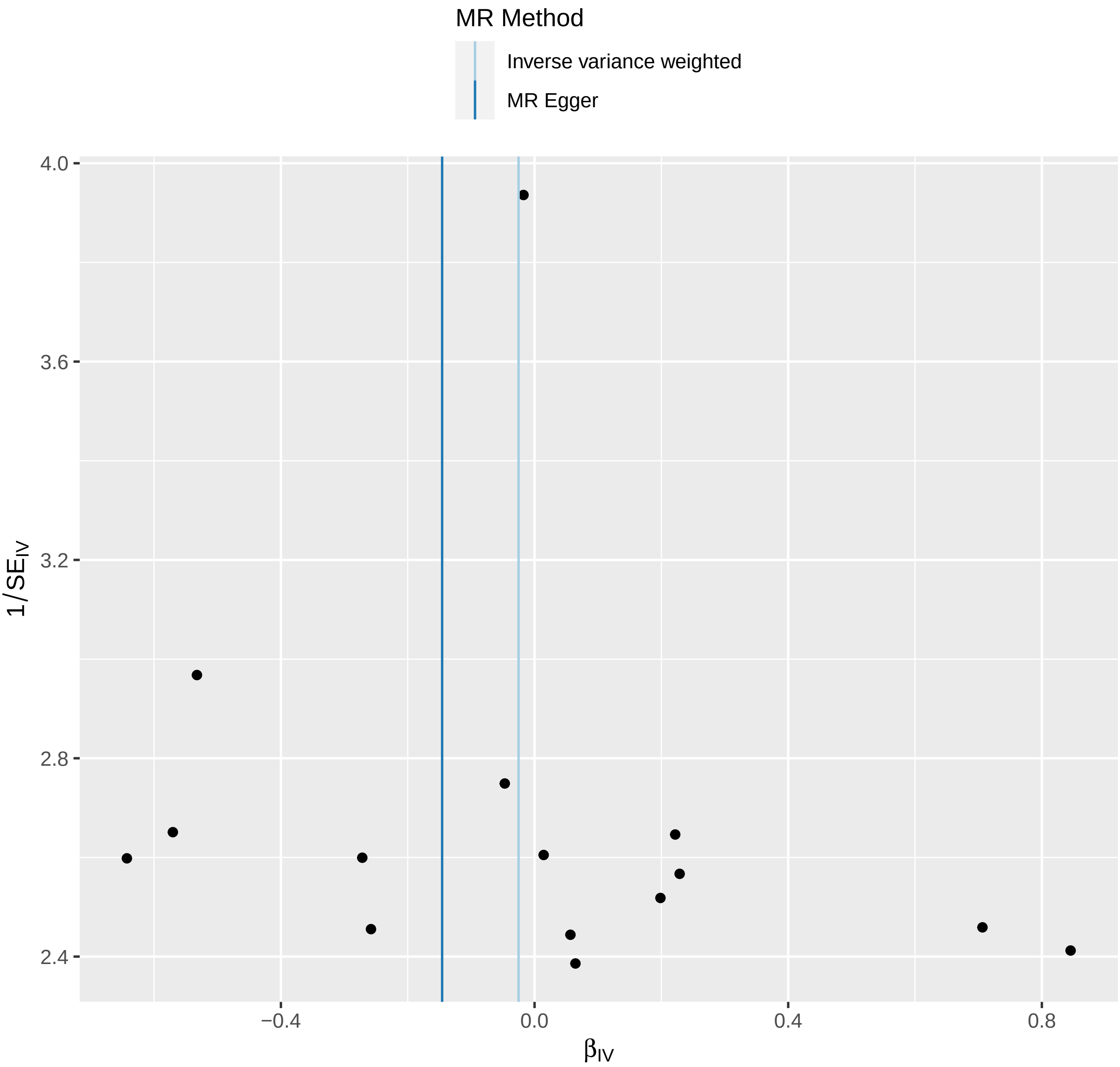

Batch 2−6275 : Gut microbiota abundance (genus Catenibacterium id.2153) on SUMO−conjugating enzyme UBC9
with a threshold of 1e−05

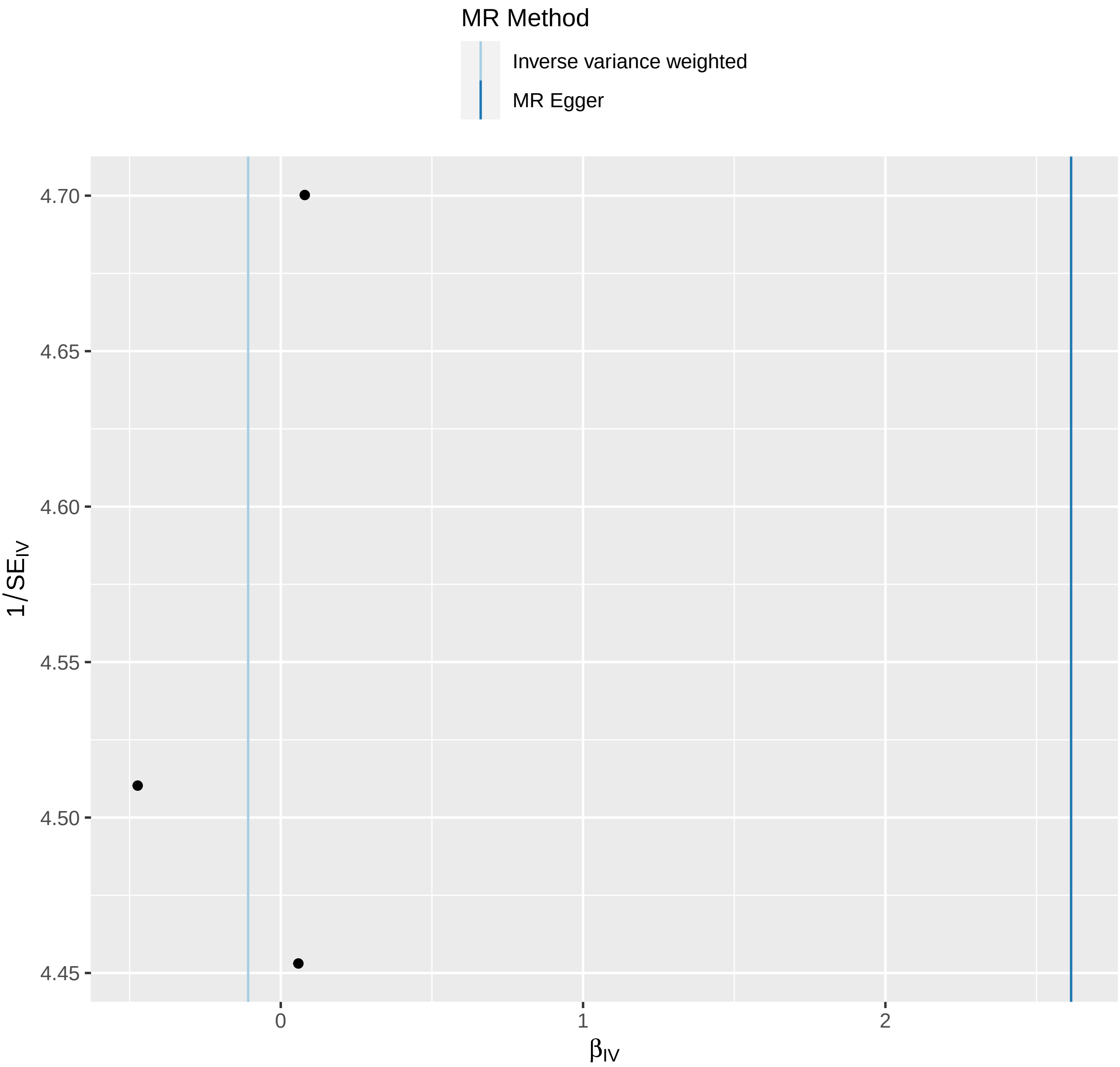

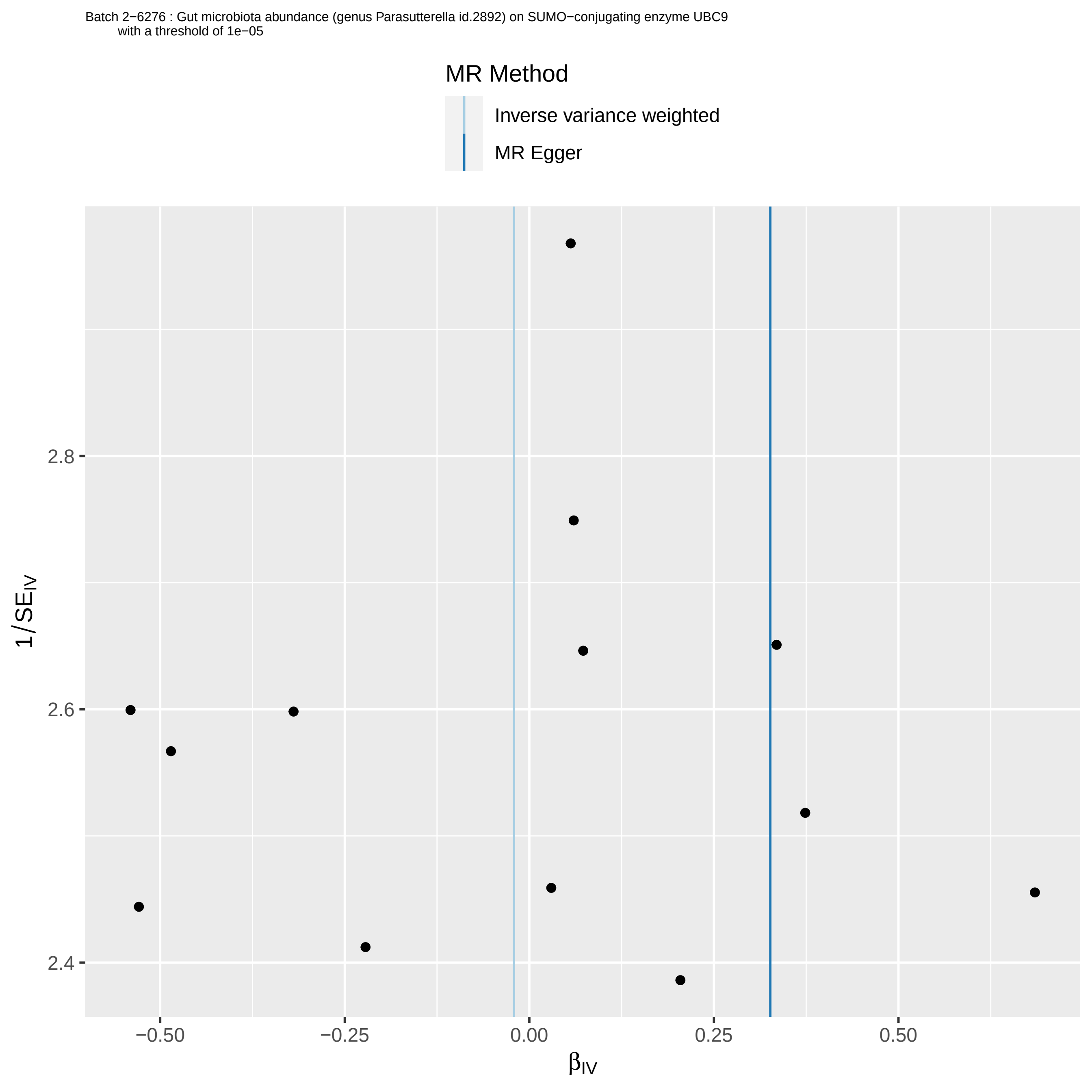
Batch 2−6276 : Gut microbiota abundance (genus Parasutterella id.2892) on SUMO−conjugating enzyme UBC9
with a threshold of 1e−05
MR Method
Inverse variance weighted
MR Egger
2.8
2.6
2.4
1/SE_IV
−0.50
−0.25
0.00
0.25
0.50
β_IV

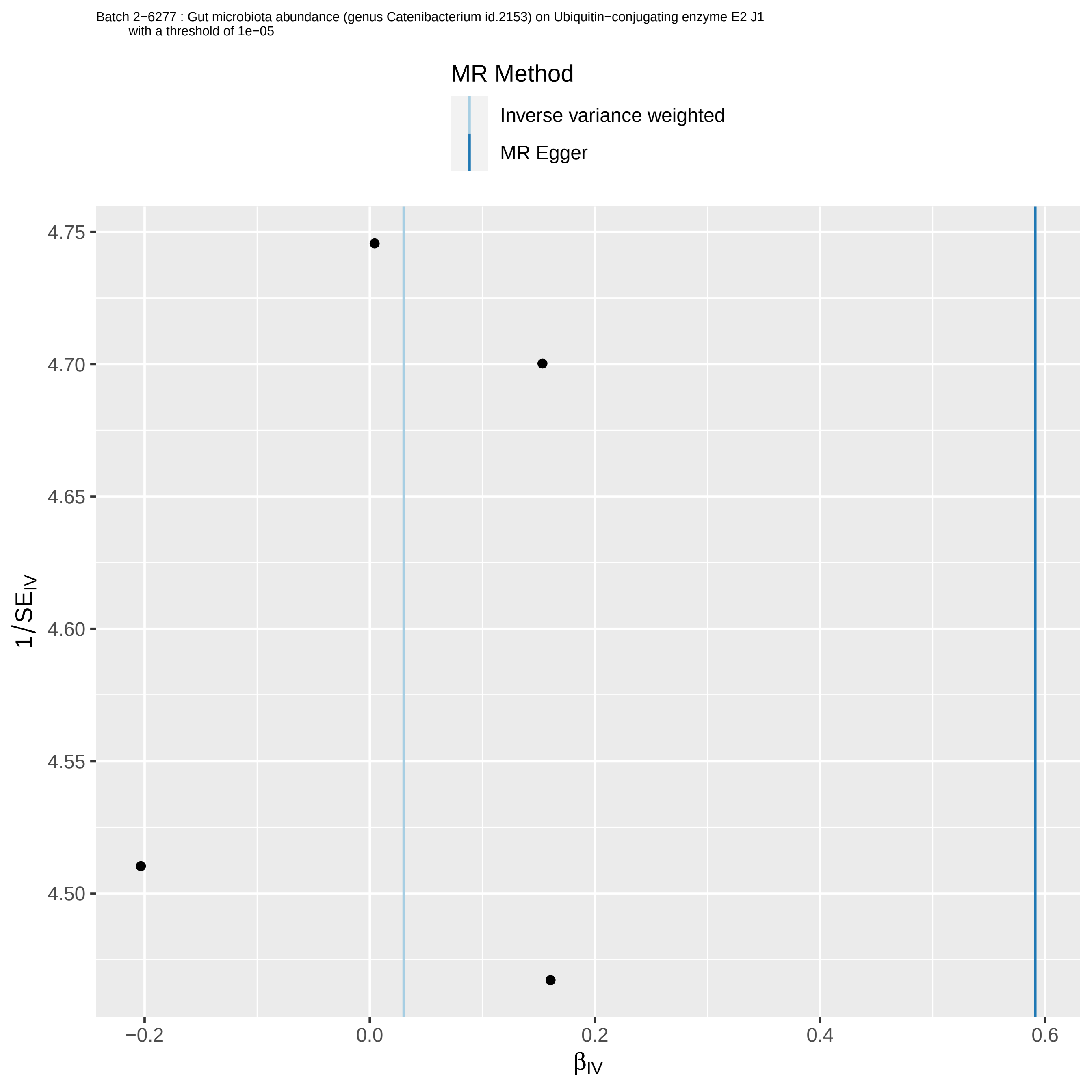
Batch 2−6277 : Gut microbiota abundance (genus Catenibacterium id.2153) on Ubiquitin−conjugating enzyme E2 J1
with a threshold of 1e−05
MR Method
Inverse variance weighted
MR Egger
4.75
4.70
4.65
4.60
4.55
4.50
1/SE_IV
−0.2
0.0
0.2
0.4
0.6
β_IV

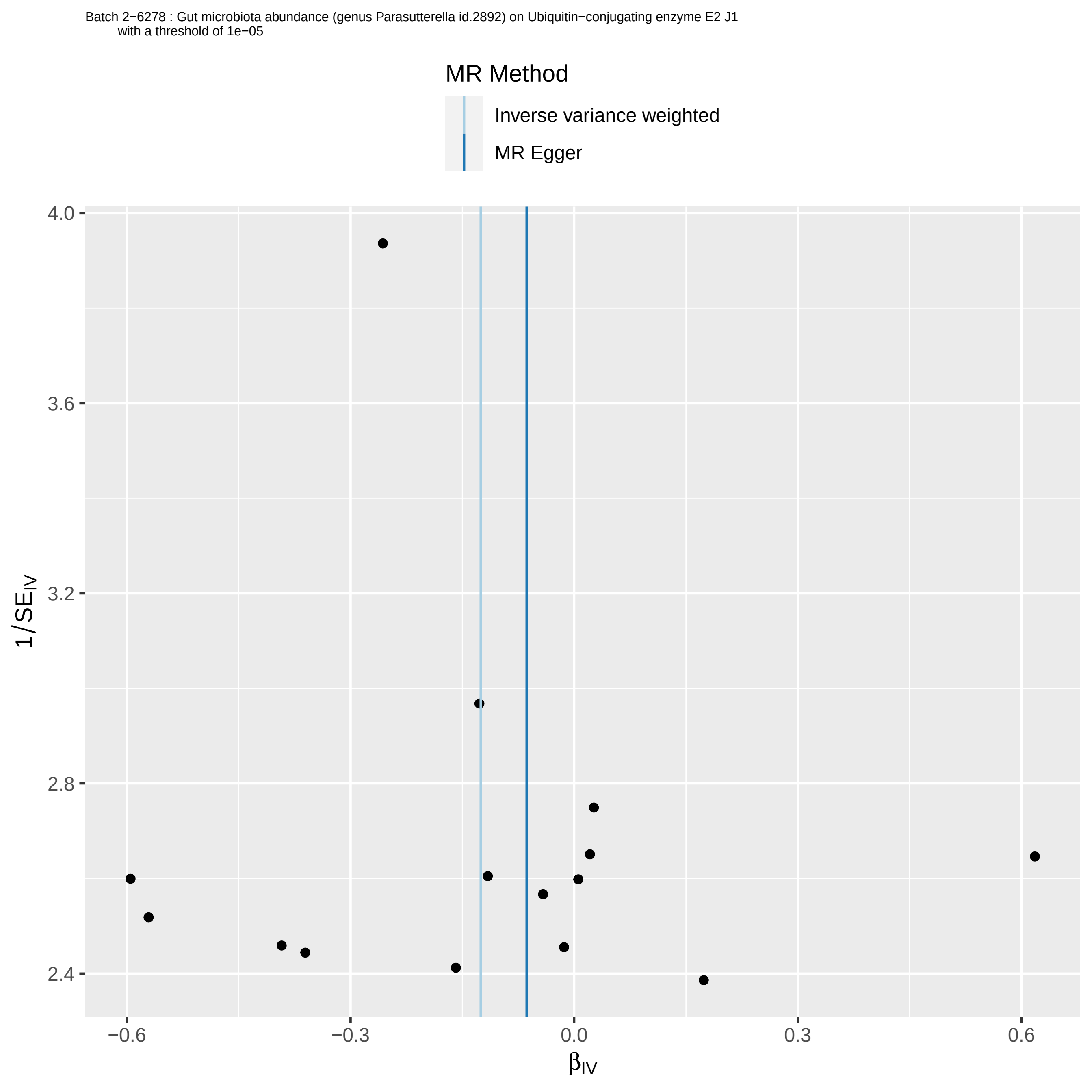
Batch 2−6278 : Gut microbiota abundance (genus Parasutterella id.2892) on Ubiquitin−conjugating enzyme E2 J1
with a threshold of 1e−05
MR Method
Inverse variance weighted
MR Egger
4.0
3.6
3.2
2.8
2.4
1/SE$_{IV}$
−0.6
−0.3
0.0
0.3
0.6
β$_{IV}$

Batch 2−6279 : Gut microbiota abundance (genus Catenibacterium id.2153) on Ubiquitin−conjugating enzyme E2 J2
with a threshold of 1e−05

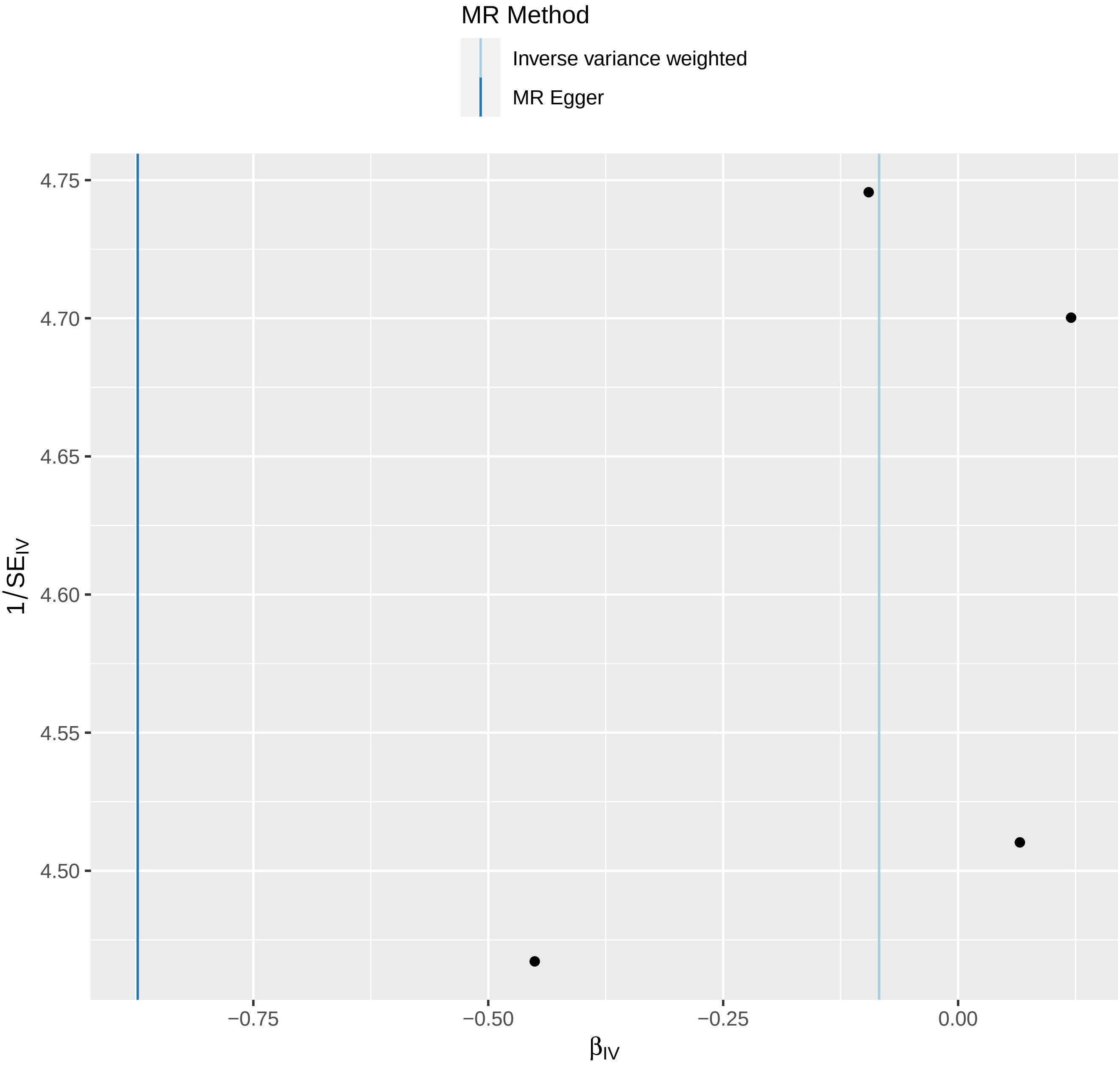

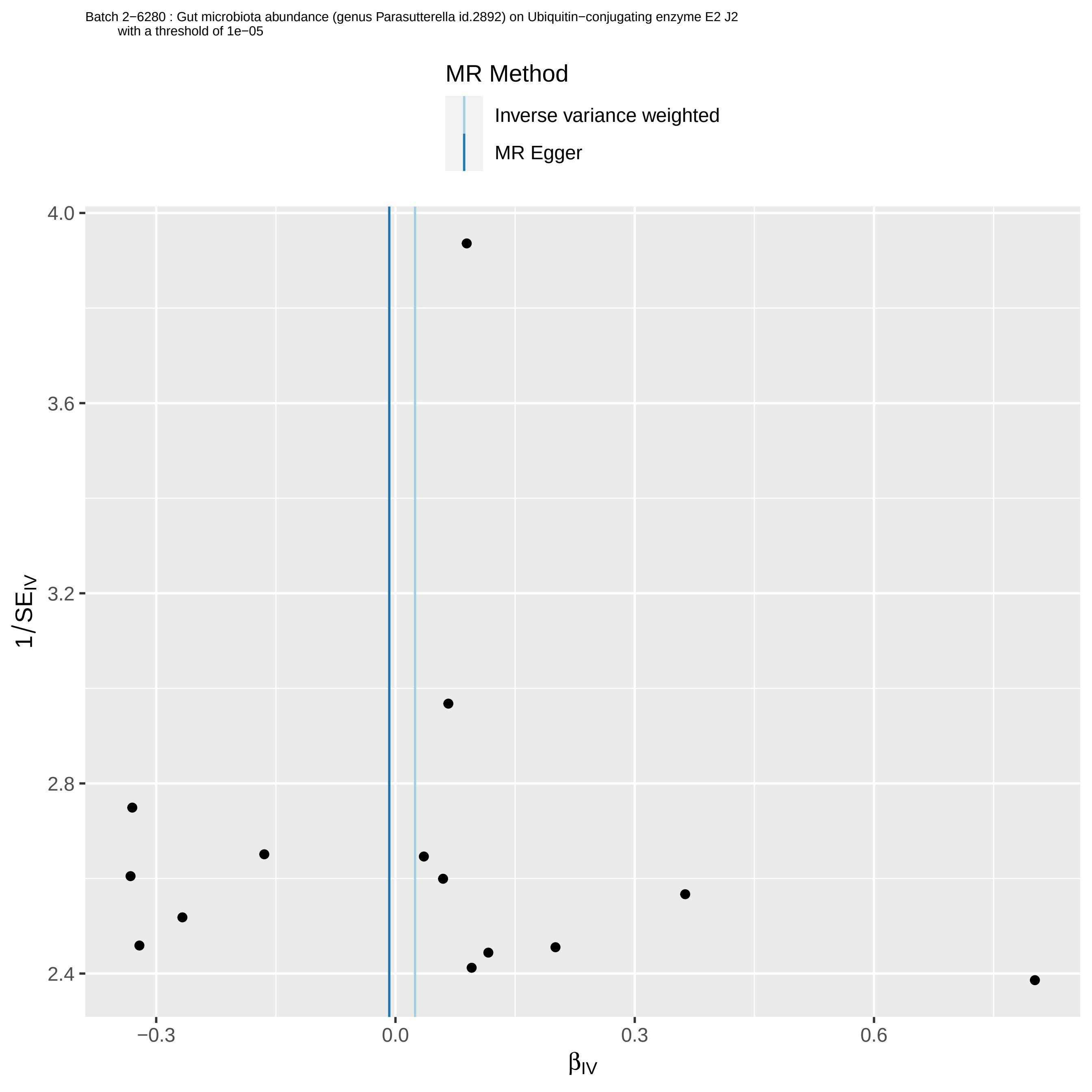
Batch 2−6280 : Gut microbiota abundance (genus Parasutterella id.2892) on Ubiquitin−conjugating enzyme E2 J2
with a threshold of 1e−05
MR Method
Inverse variance weighted
MR Egger
4.0
3.6
3.2
2.8
2.4
1/SE_IV
−0.3
0.0
0.3
0.6
β_IV

Batch 2−6281 : Gut microbiota abundance (genus Catenibacterium id.2153) on Ubiquitin−conjugating enzyme E2 J2
with a threshold of 1e−05

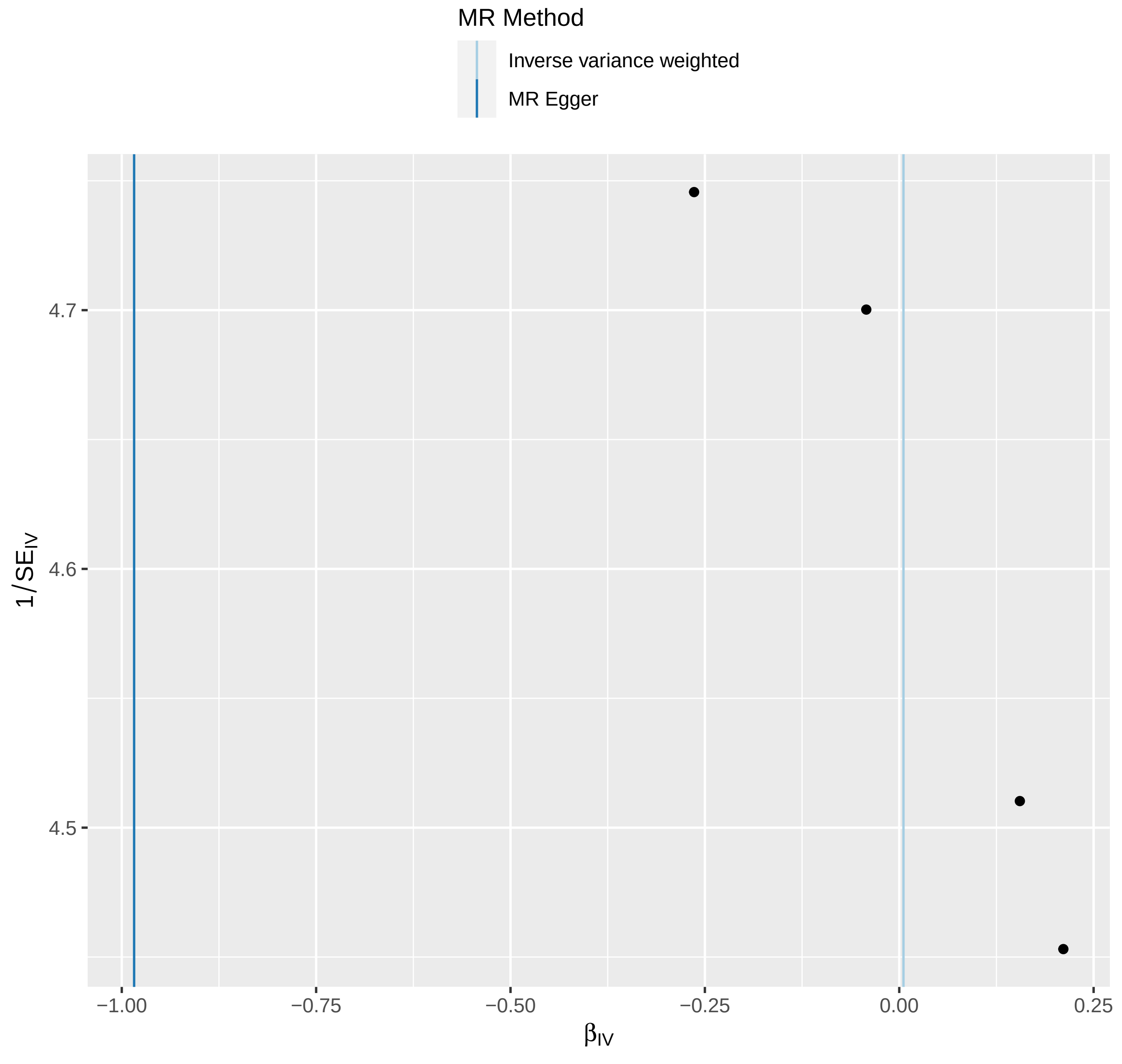

Batch 2−6282 : Gut microbiota abundance (genus Parasutterella id.2892) on Ubiquitin−conjugating enzyme E2 J2
with a threshold of 1e−05

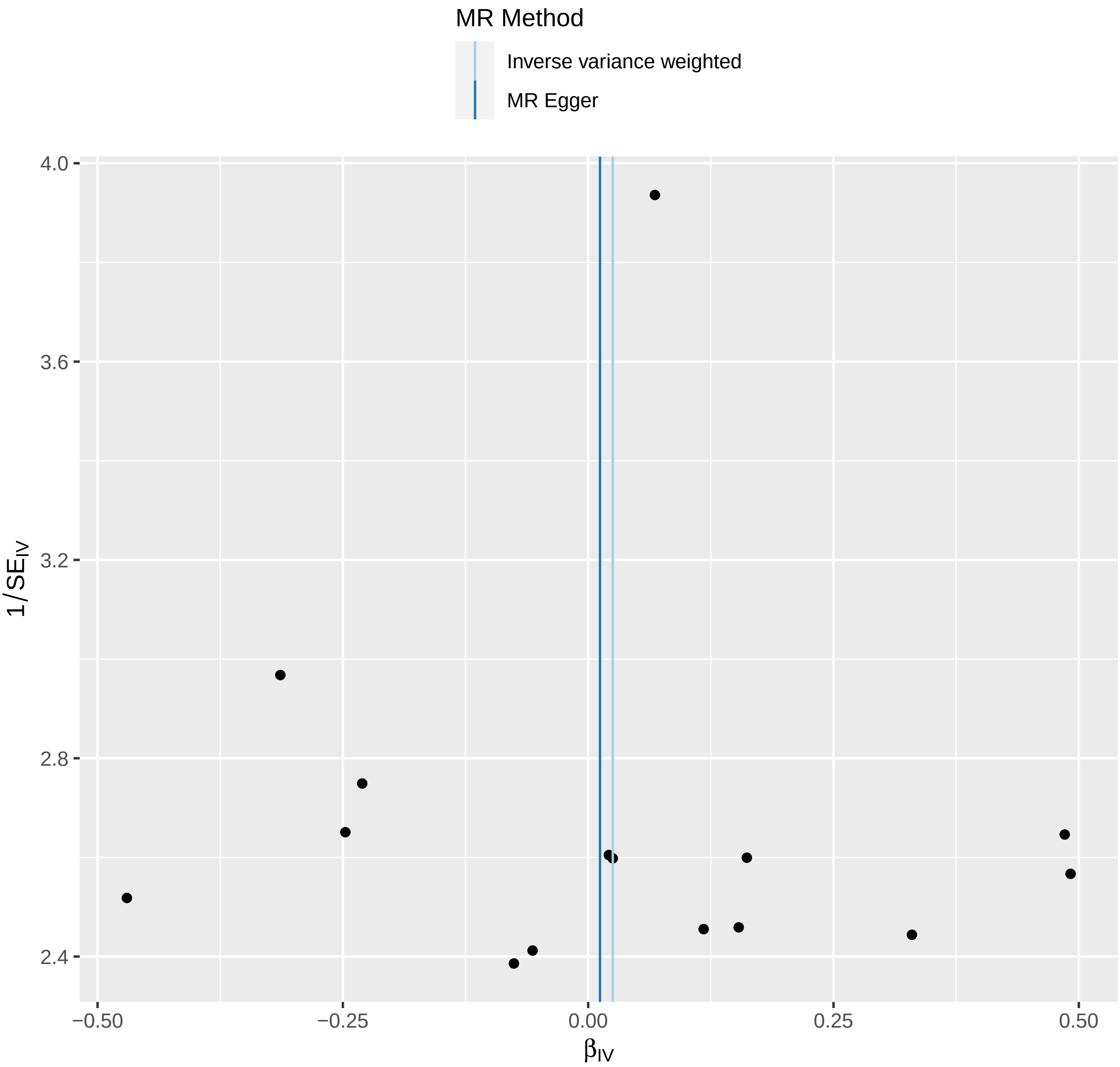

Batch 2−6283 : Gut microbiota abundance (genus Catenibacterium id.2153) on Ubiquitin−conjugating enzyme E2 L3
with a threshold of 1e−05

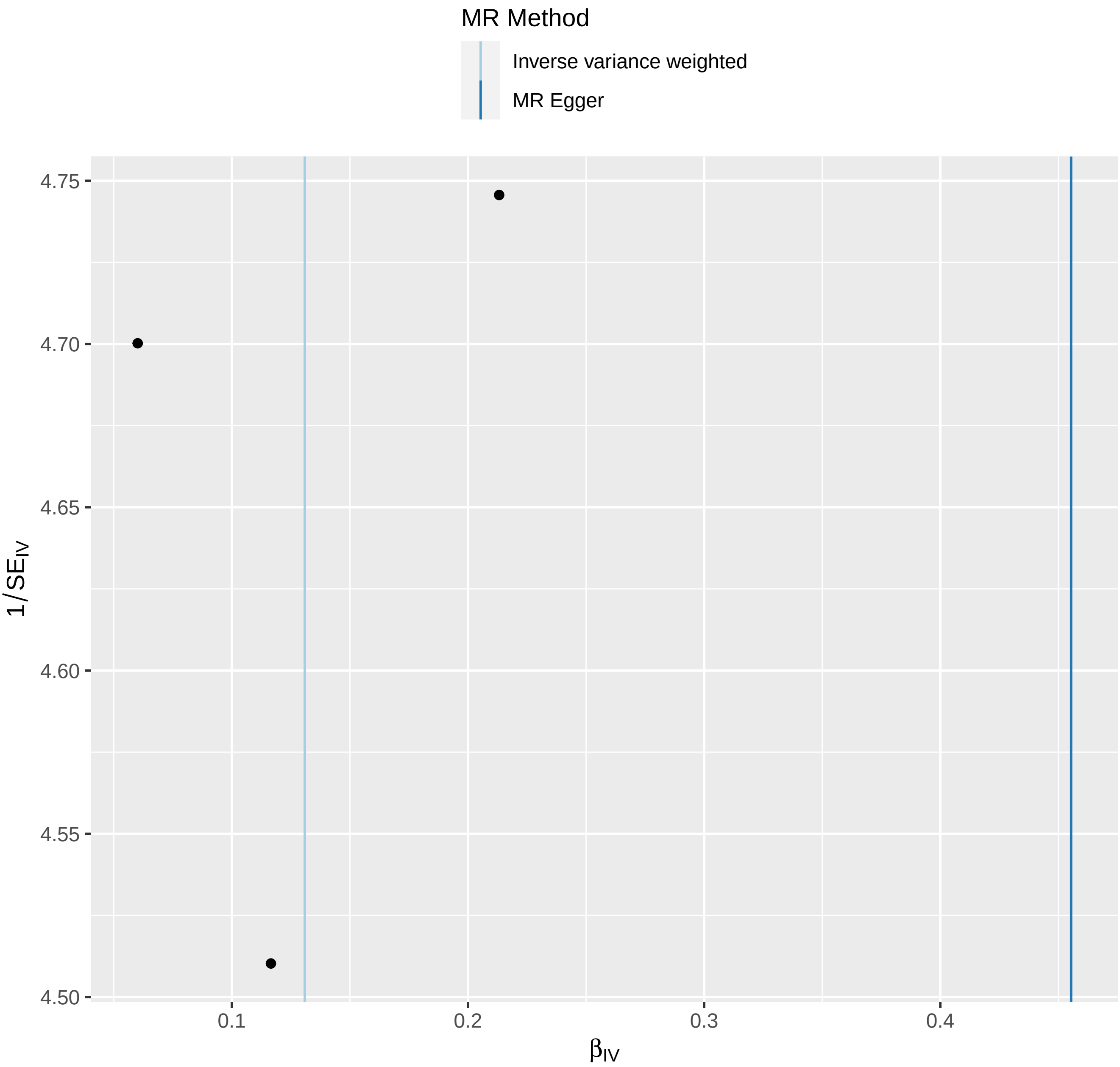

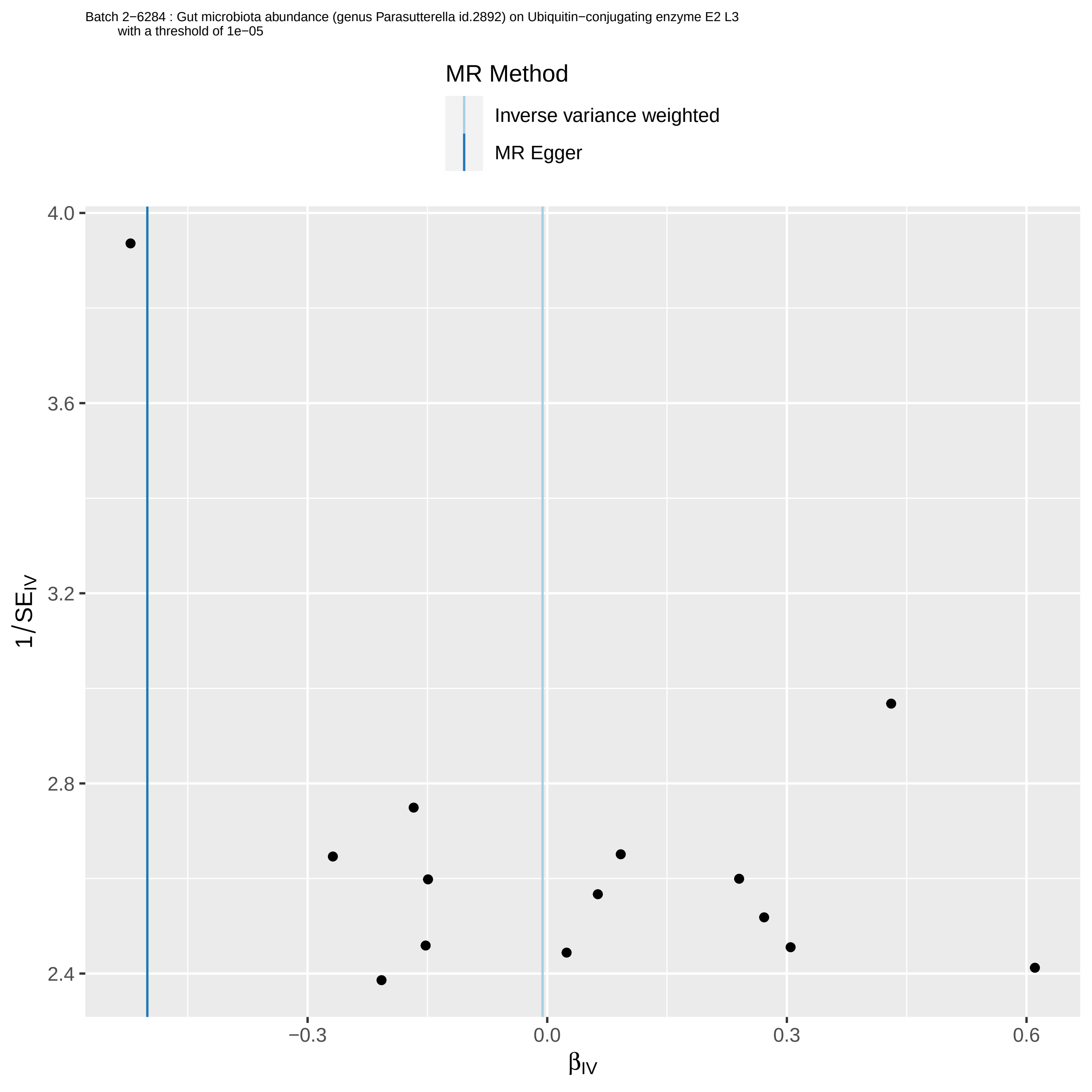
Batch 2−6284 : Gut microbiota abundance (genus Parasutterella id.2892) on Ubiquitin−conjugating enzyme E2 L3
with a threshold of 1e−05
MR Method
Inverse variance weighted
MR Egger
4.0
3.6
3.2
2.8
2.4
1/SE_IV
−0.3
0.0
0.3
0.6
β_IV

Batch 2−6285 : Gut microbiota abundance (genus Catenibacterium id.2153) on Ubiquitin−conjugating enzyme E2 N
with a threshold of 1e−05

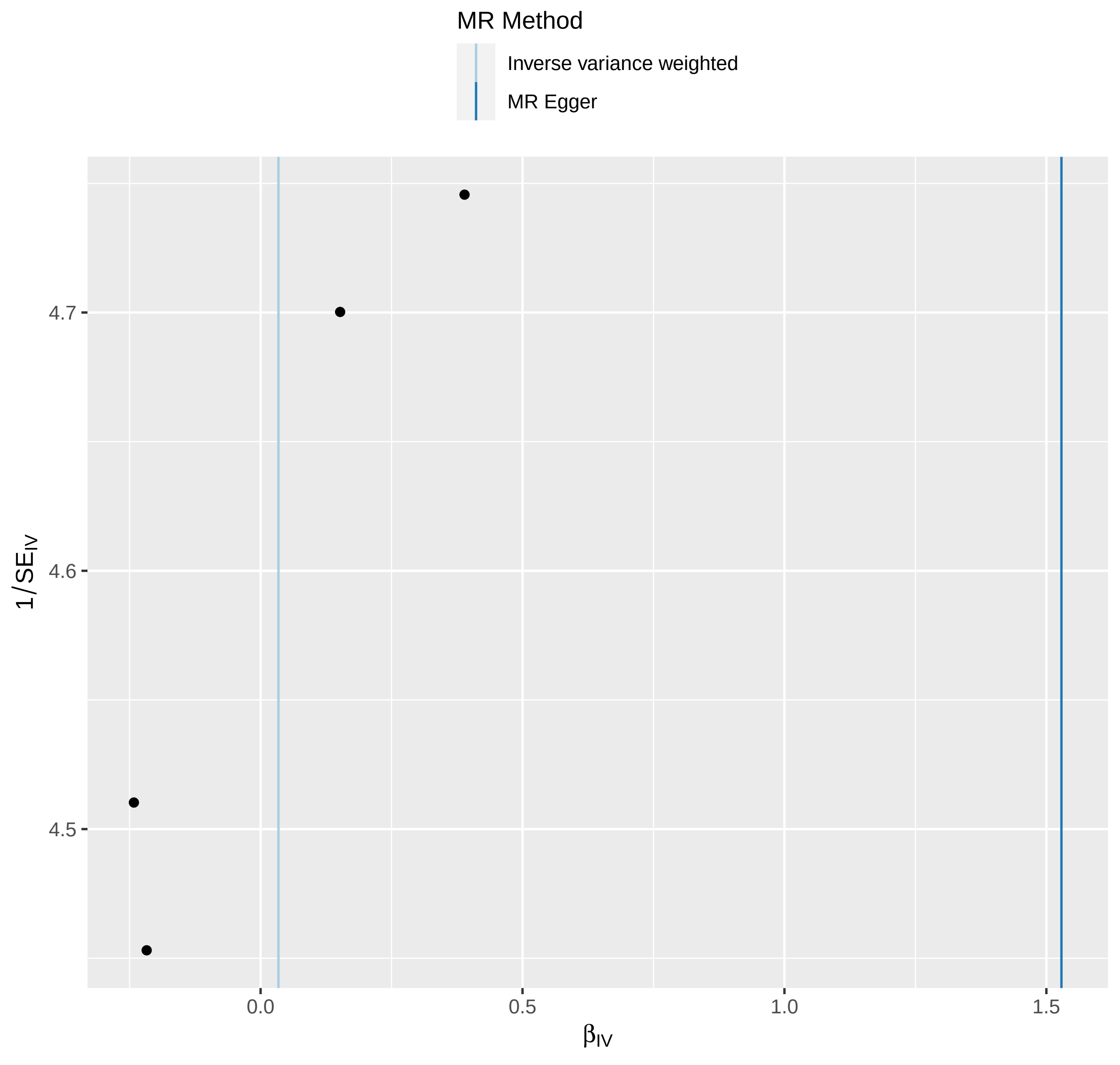

Batch 2−6286 : Gut microbiota abundance (genus Parasutterella id.2892) on Ubiquitin−conjugating enzyme E2 N
with a threshold of 1e−05

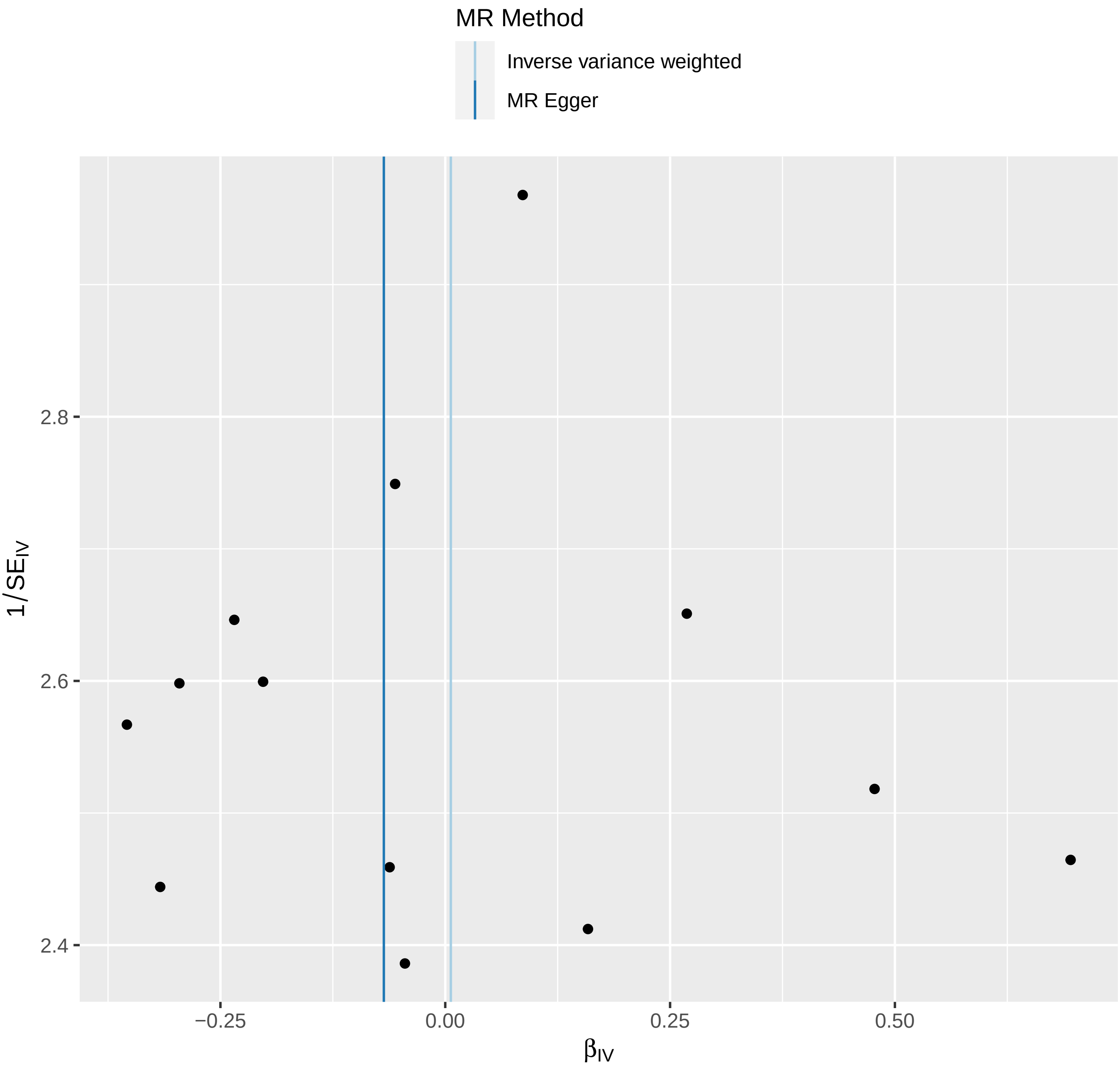

Batch 2−6287 : Gut microbiota abundance (genus Catenibacterium id.2153) on Ubiquitin−conjugating enzyme E2 T
with a threshold of 1e−05

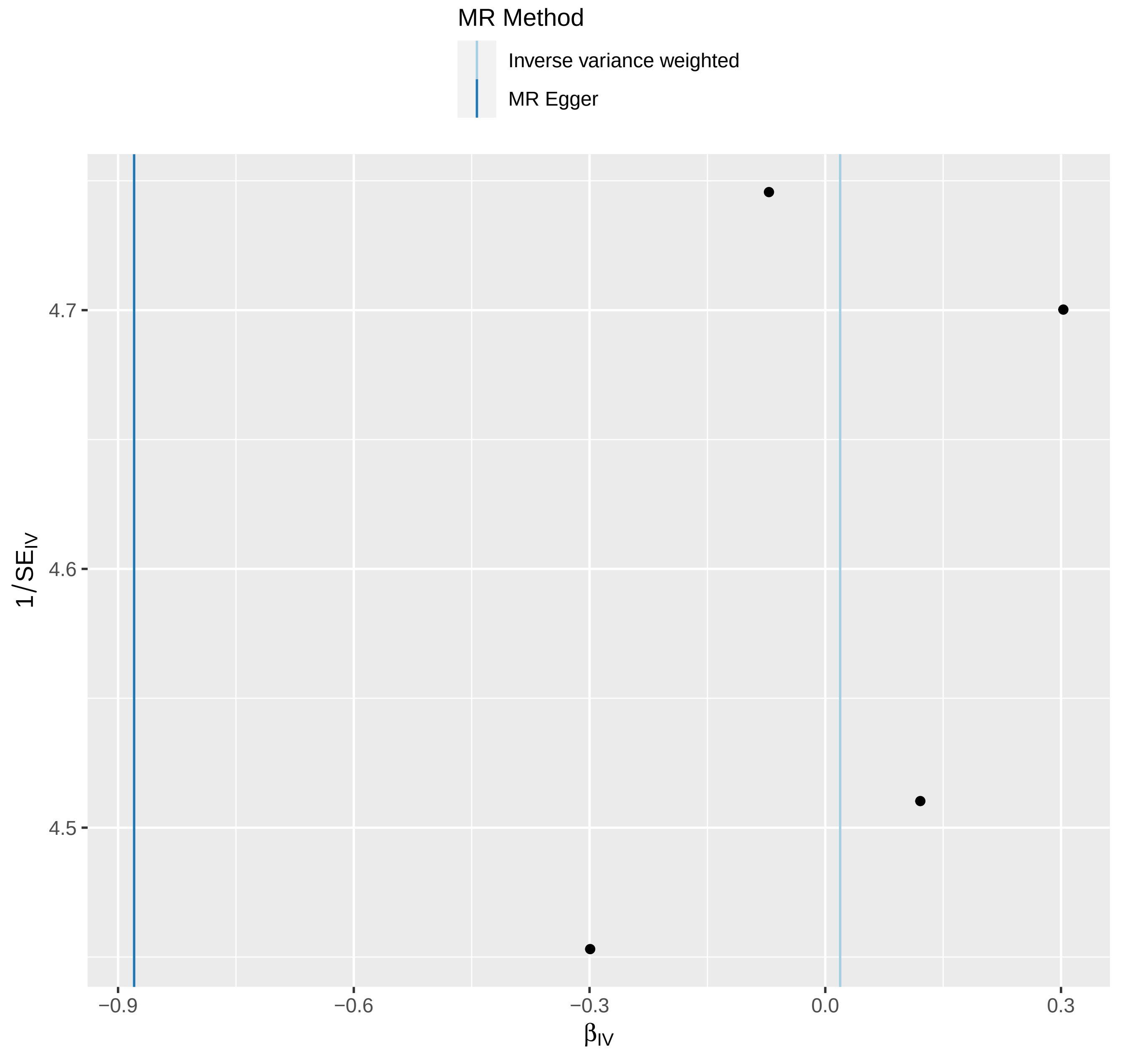

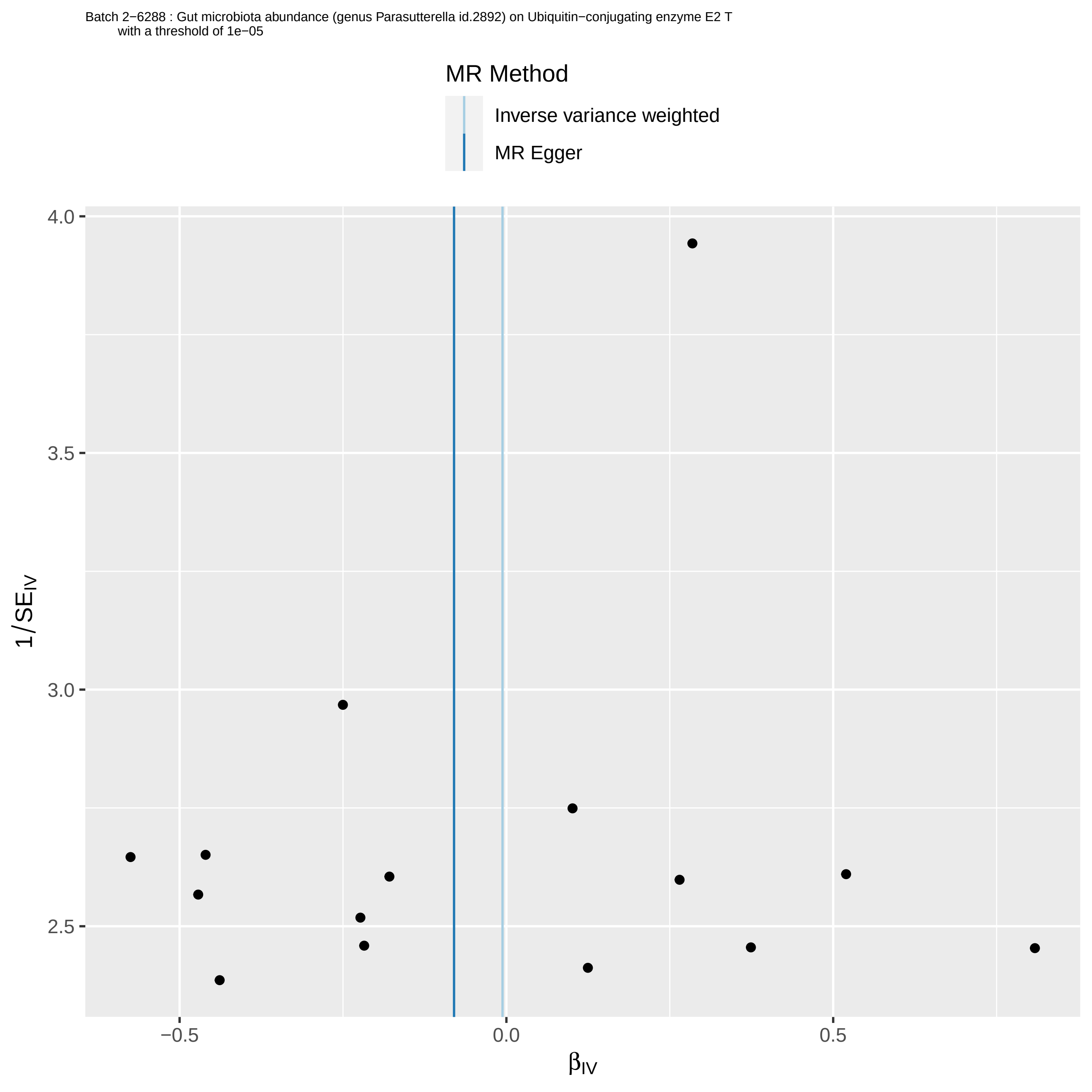
Batch 2−6288 : Gut microbiota abundance (genus Parasutterella id.2892) on Ubiquitin−conjugating enzyme E2 T
with a threshold of 1e−05
MR Method
Inverse variance weighted
MR Egger
4.0
3.5
3.0
2.5
1/SE_IV
−0.5
0.0
0.5
β_IV

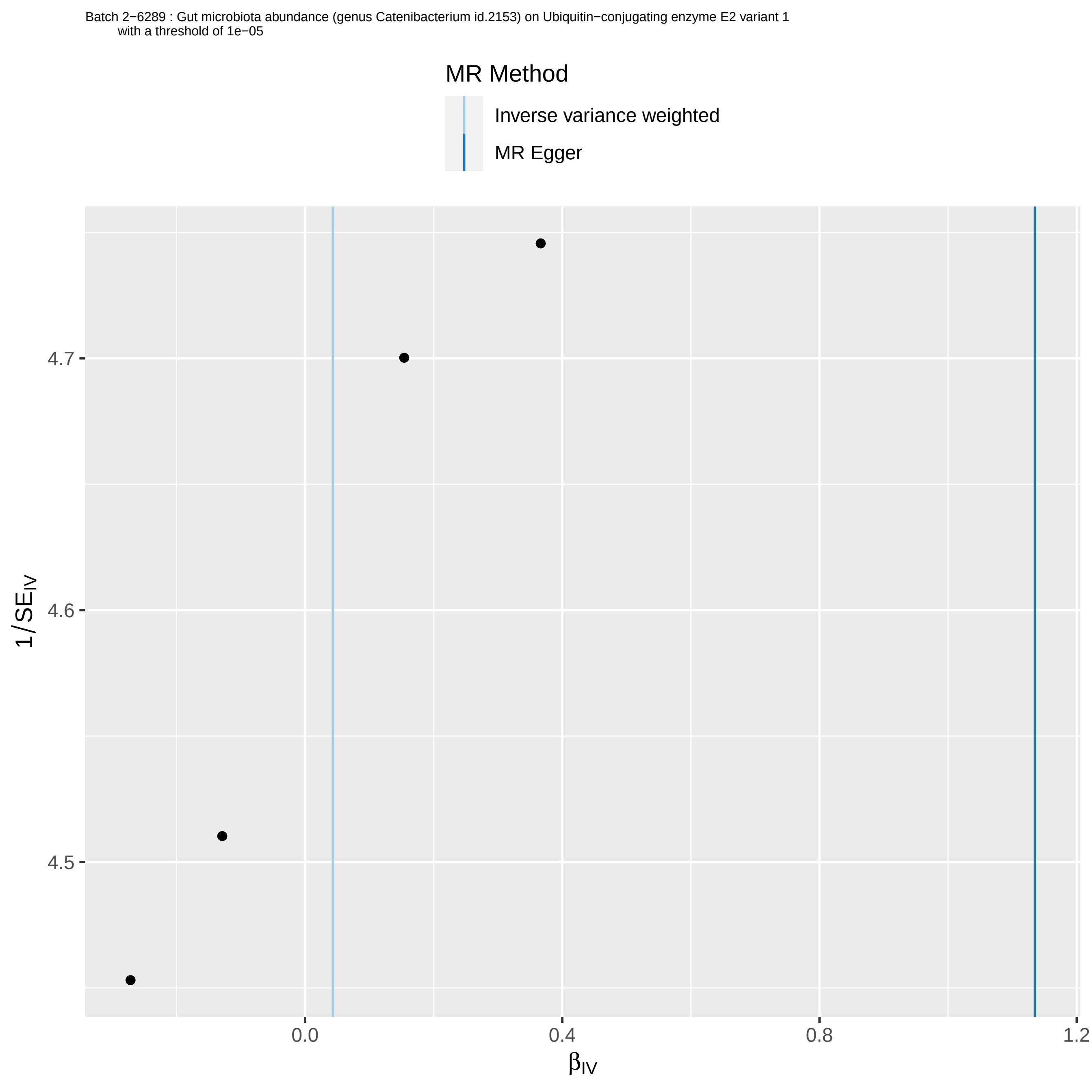
Batch 2−6289 : Gut microbiota abundance (genus Catenibacterium id.2153) on Ubiquitin−conjugating enzyme E2 variant 1
with a threshold of 1e−05
MR Method
Inverse variance weighted
MR Egger
4.7
4.6
4.5
1/SE_IV
0.0
0.4
0.8
1.2
β_IV

Batch 2−6290 : Gut microbiota abundance (genus Parasutterella id.2892) on Ubiquitin−conjugating enzyme E2 variant 1
with a threshold of 1e−05

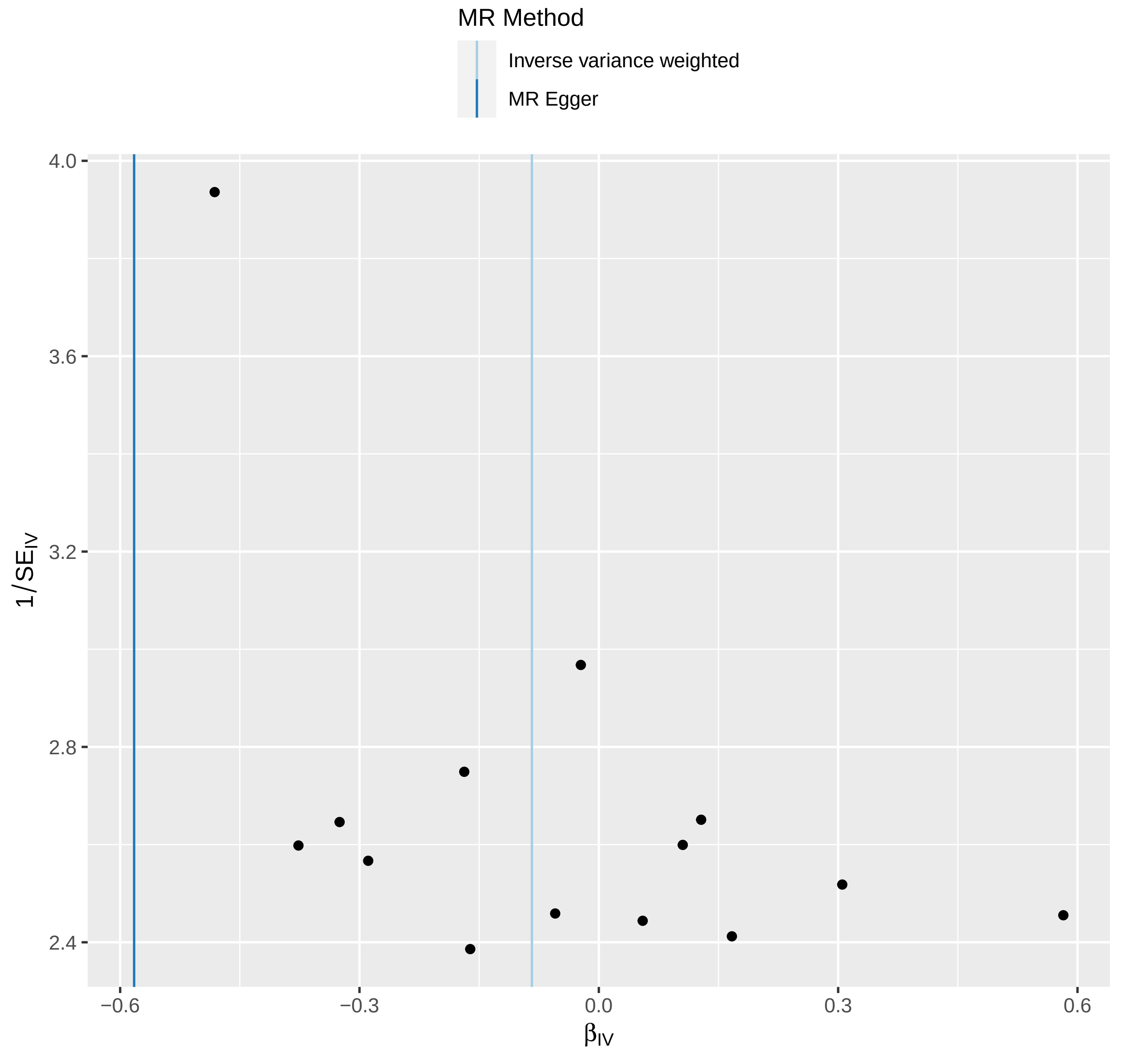

Batch 2−6291 : Gut microbiota abundance (genus Catenibacterium id.2153) on Ubiquitin−like protein 4A
with a threshold of 1e−05

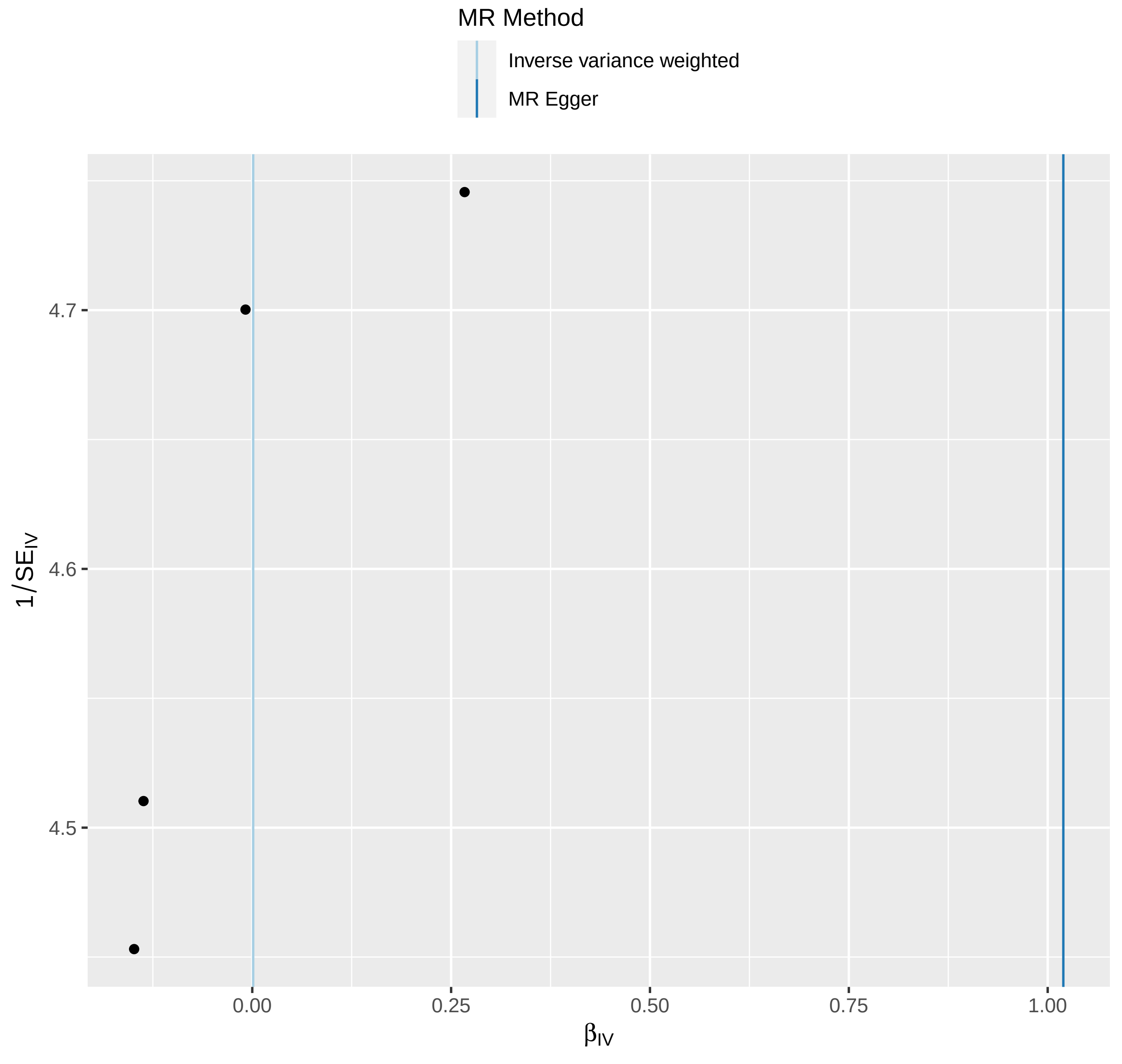

Batch 2−6292 : Gut microbiota abundance (genus Parasutterella id.2892) on Ubiquitin−like protein 4A
with a threshold of 1e−05

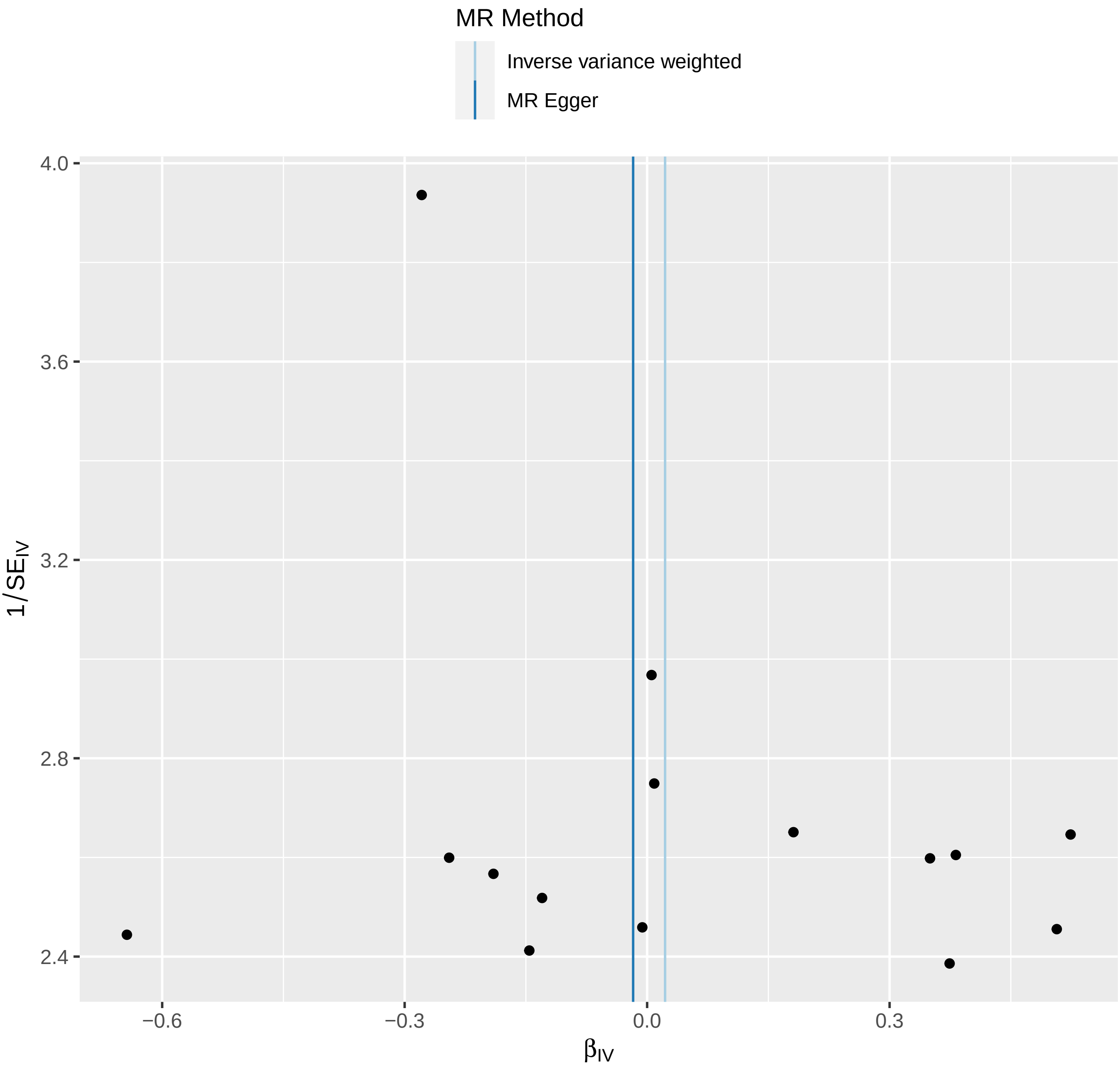

Batch 2−6293 : Gut microbiota abundance (genus Catenibacterium id.2153) on Ubiquilin−4
with a threshold of 1e−05

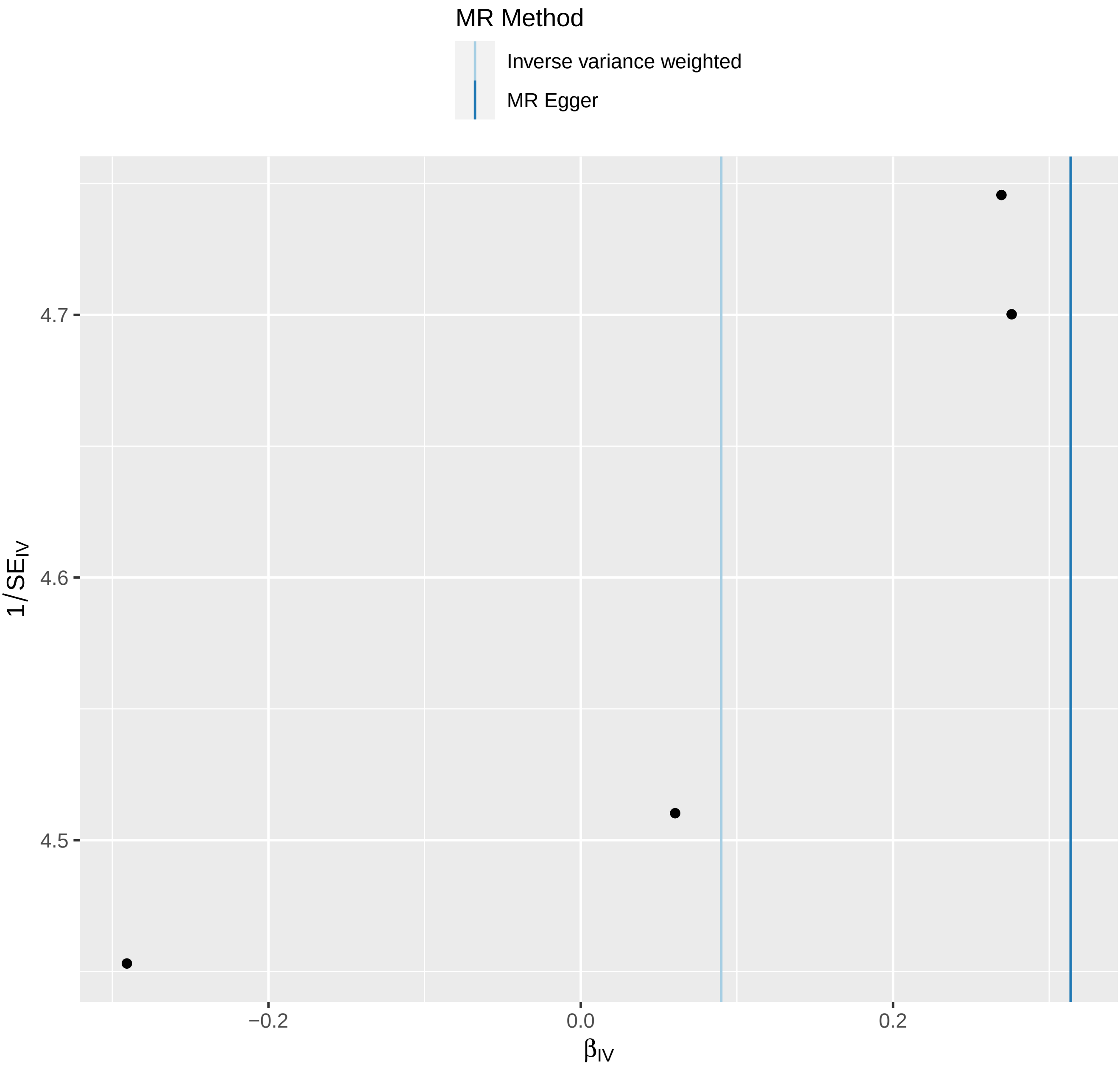

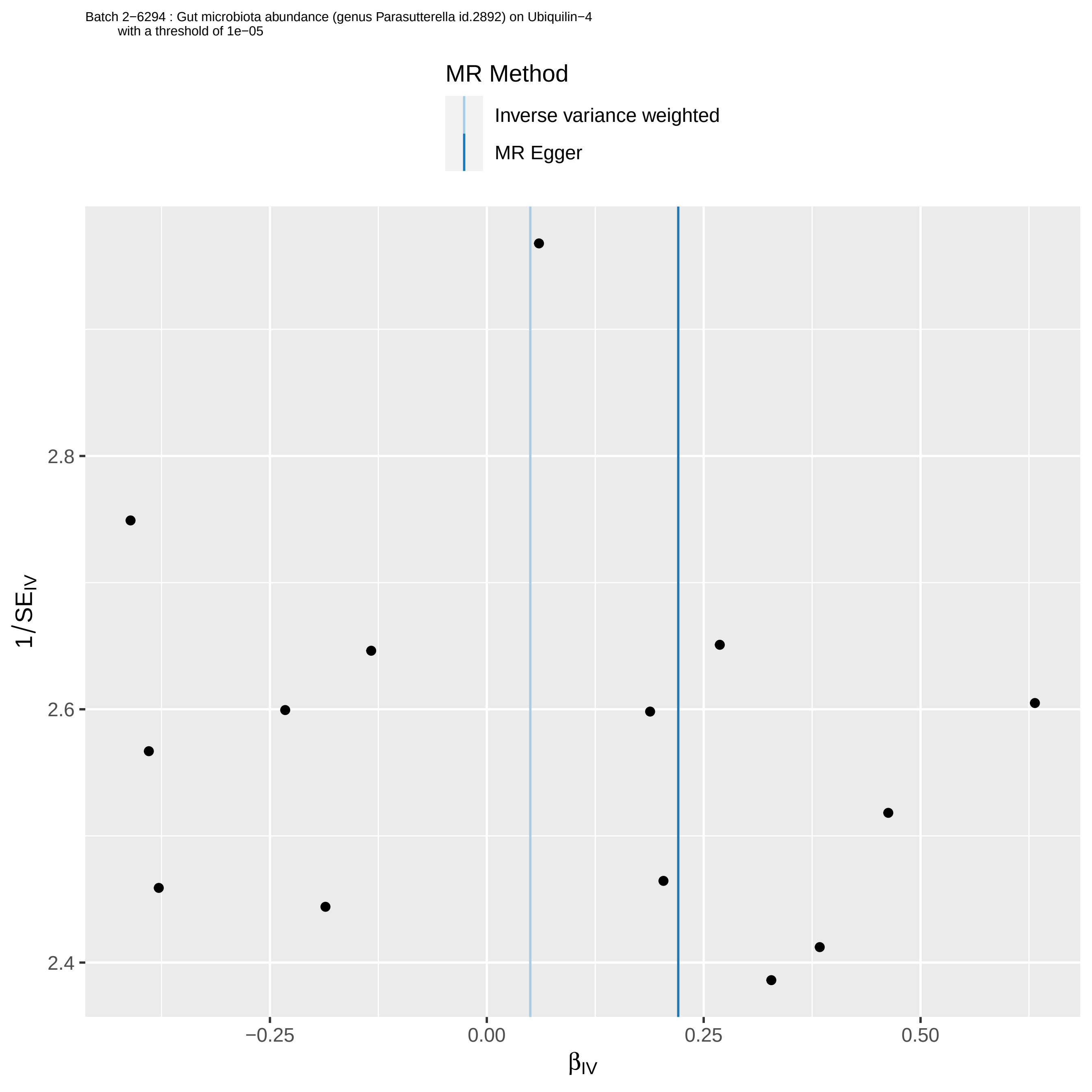

Batch 2−6294 : Gut microbiota abundance (genus Parasutterella id.2892) on Ubiquilin−4
with a threshold of 1e−05
MR Method
Inverse variance weighted
MR Egger
1/SE_IV
2.8
2.6
2.4
−0.25
0.00
0.25
0.50
β_IV

Batch 2−6295 : Gut microbiota abundance (genus Catenibacterium id.2153) on Ubiquitin domain−containing protein 2
with a threshold of 1e−05

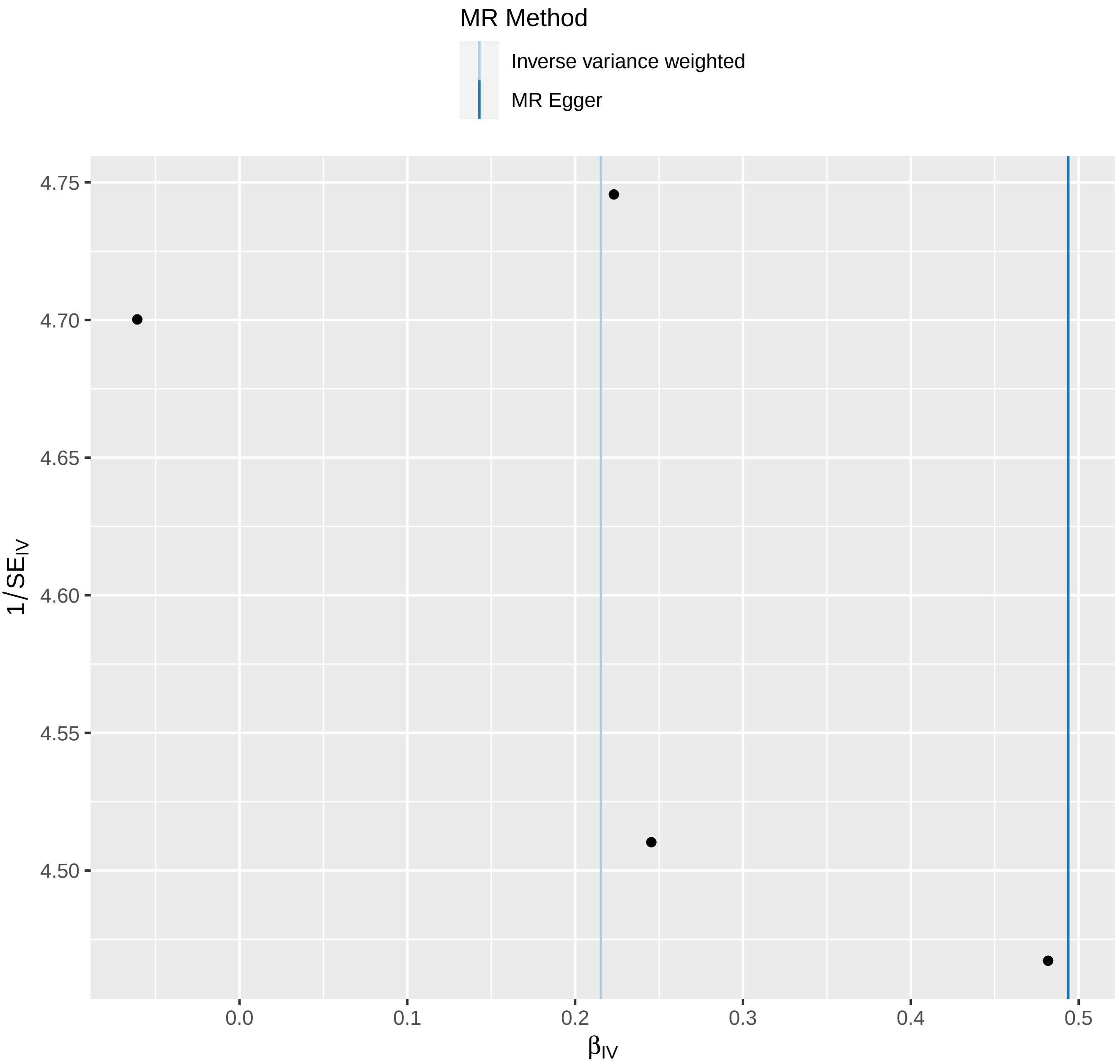

Batch 2−6296 : Gut microbiota abundance (genus Parasutterella id.2892) on Ubiquitin domain−containing protein 2
with a threshold of 1e−05

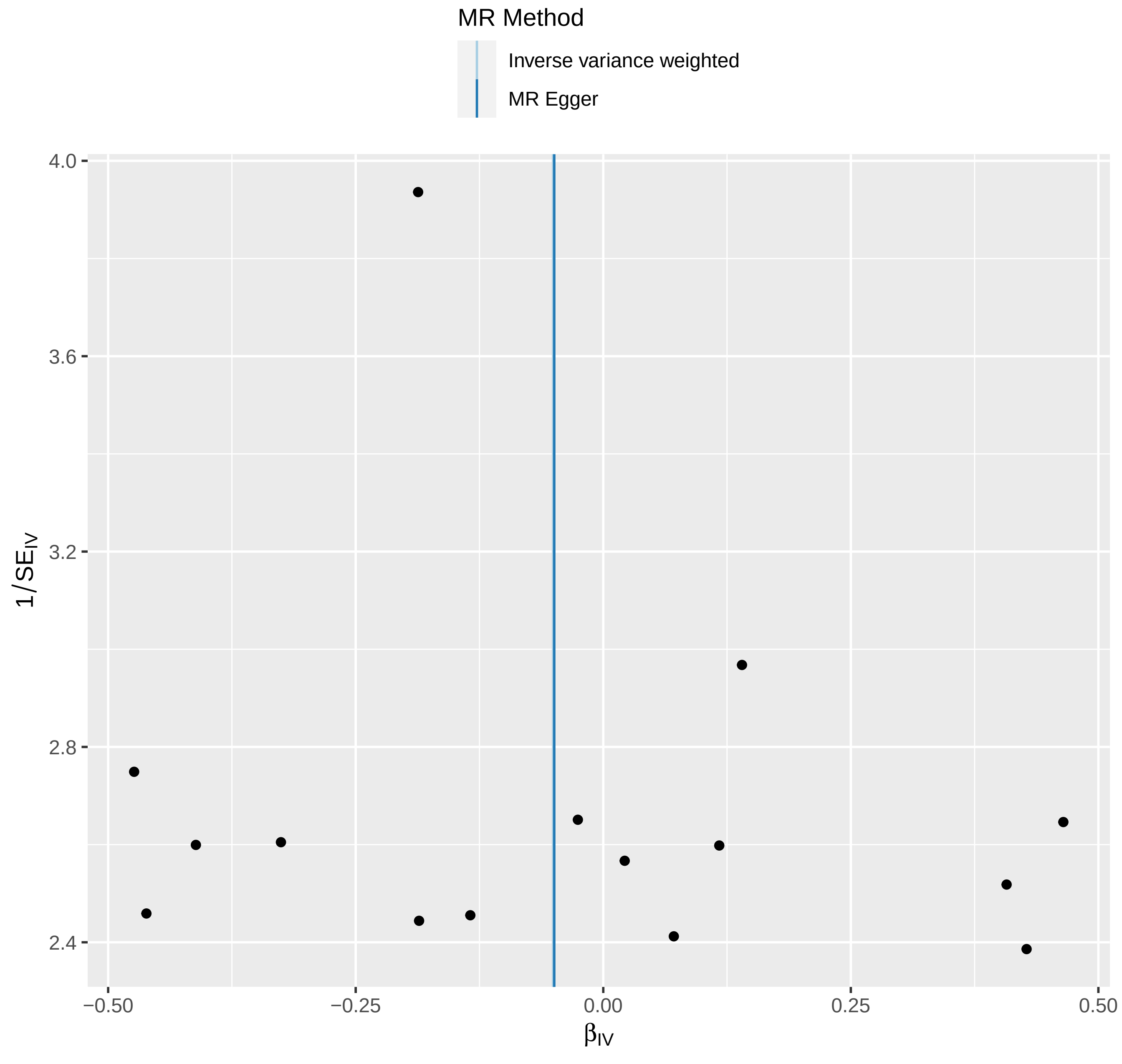

Batch 2−6297 : Gut microbiota abundance (genus Catenibacterium id.2153) on UBX domain−containing protein 4
with a threshold of 1e−05

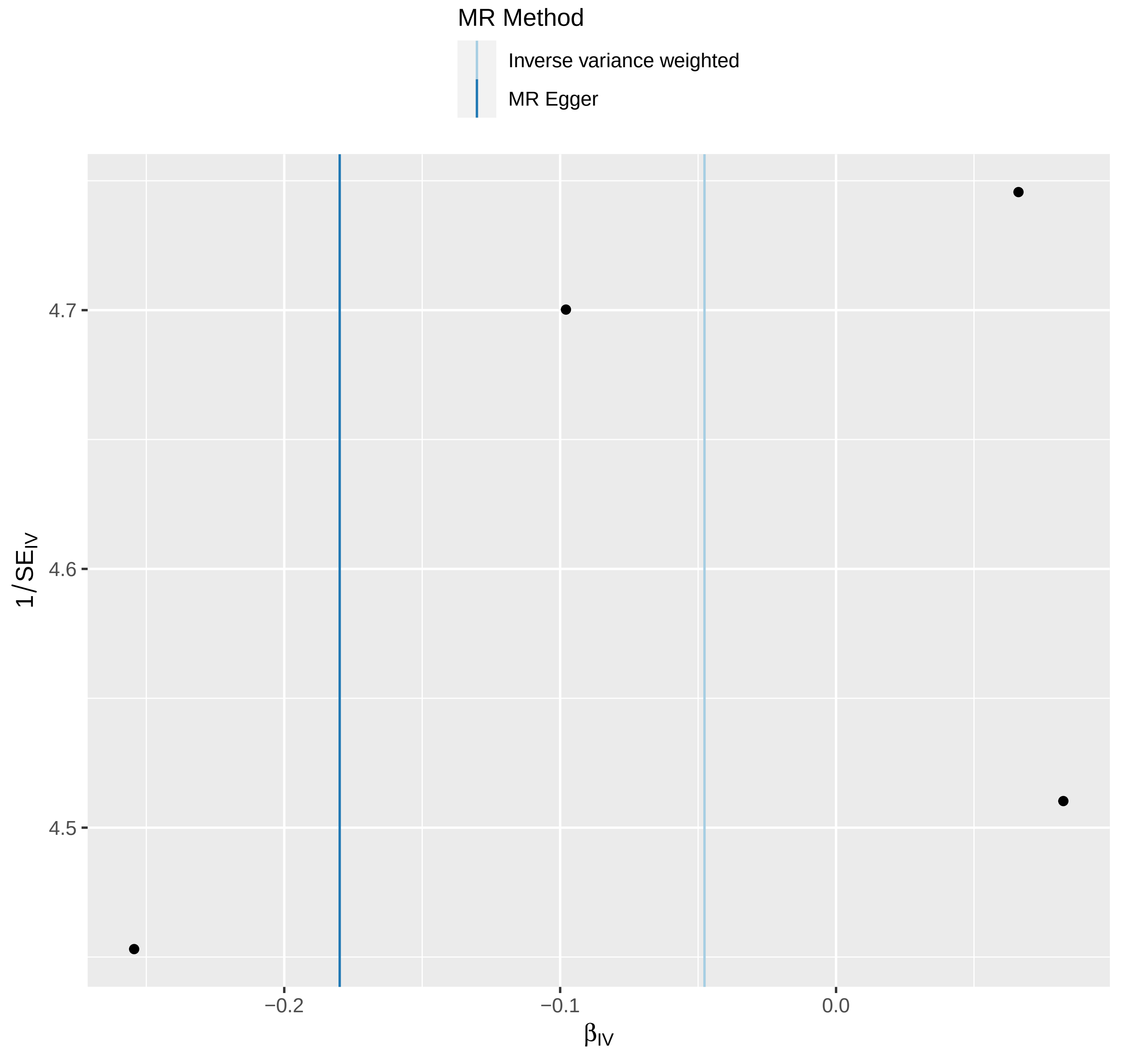

Batch 2−6298 : Gut microbiota abundance (genus Parasutterella id.2892) on UBX domain−containing protein 4
with a threshold of 1e−05

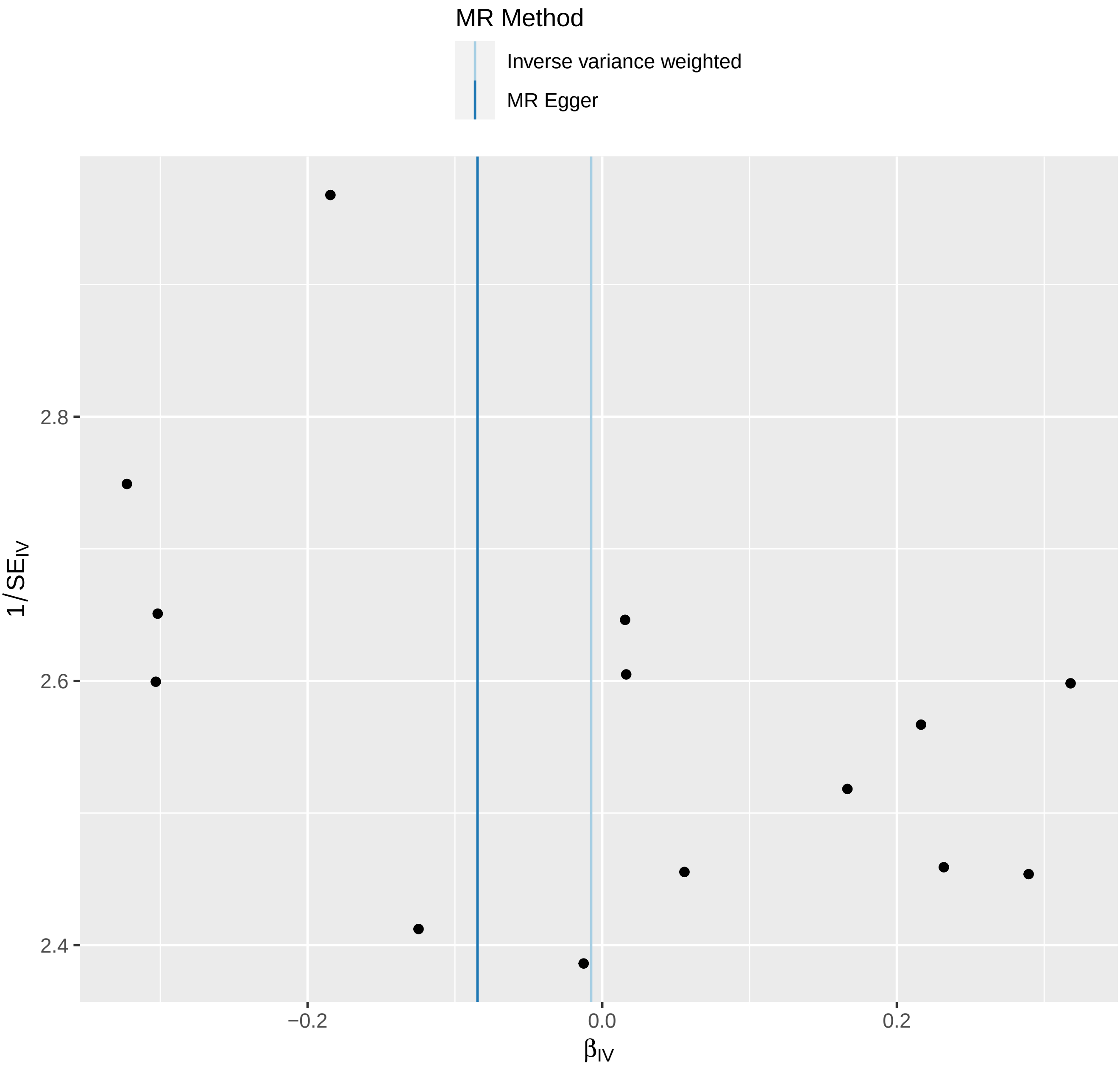

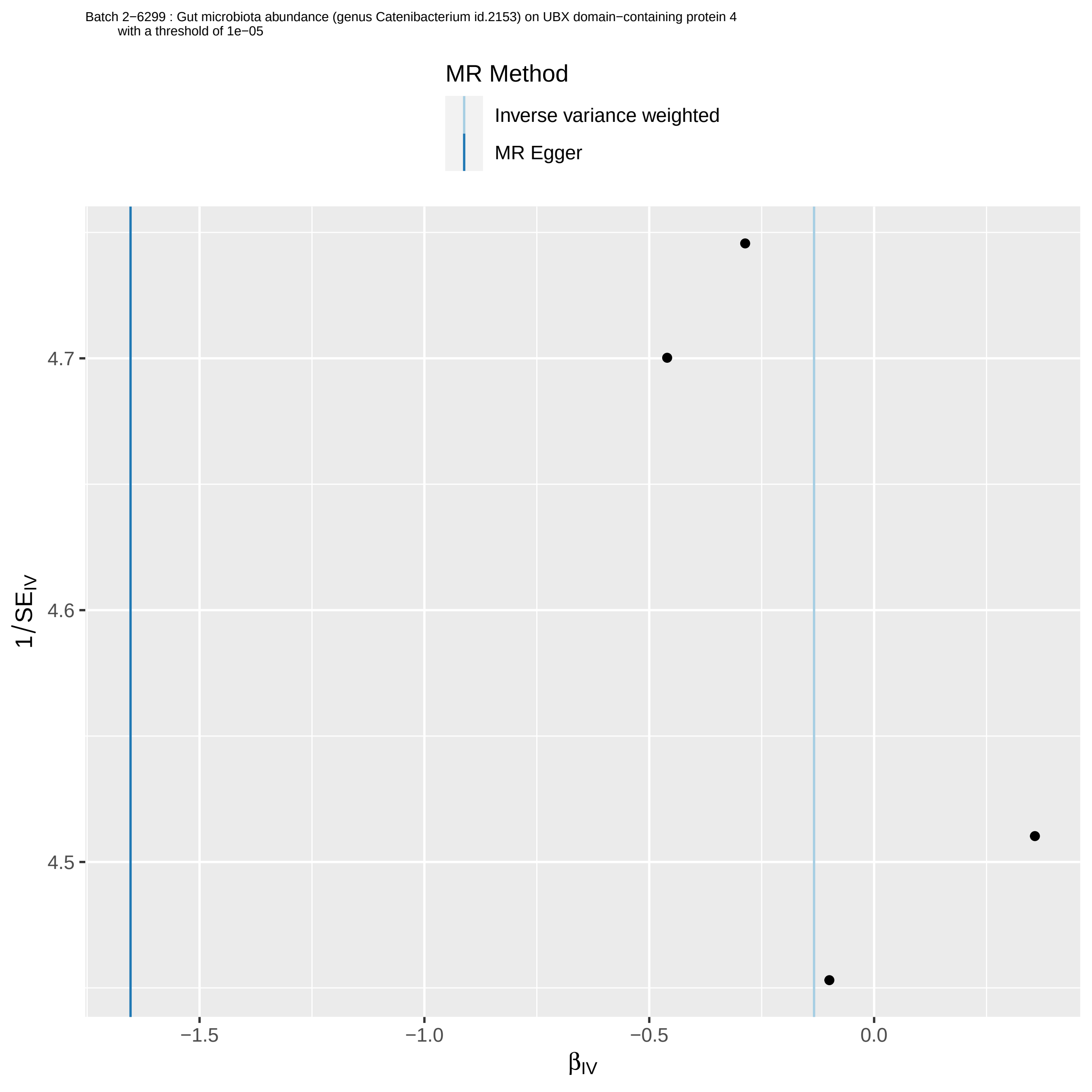

Batch 2−6299 : Gut microbiota abundance (genus Catenibacterium id.2153) on UBX domain−containing protein 4
with a threshold of 1e−05
MR Method
Inverse variance weighted
MR Egger
4.7
4.6
4.5
1/SE_IV
−1.5
−1.0
−0.5
0.0
β_IV

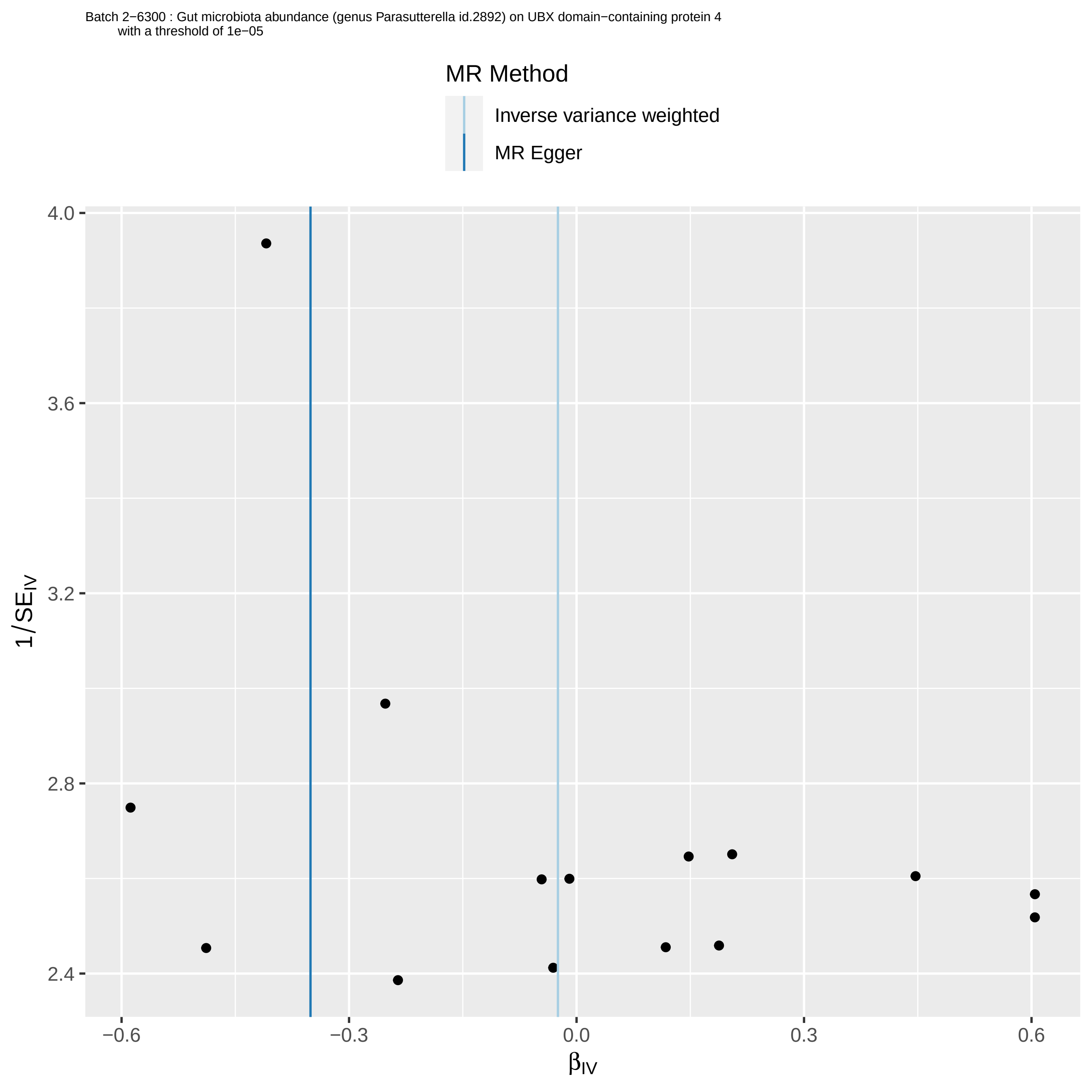
Batch 2−6300 : Gut microbiota abundance (genus Parasutterella id.2892) on UBX domain−containing protein 4
with a threshold of 1e−05
MR Method
Inverse variance weighted
MR Egger
4.0
3.6
3.2
2.8
2.4
1/SE$_{IV}$
−0.6
−0.3
0.0
0.3
0.6
β$_{IV}$

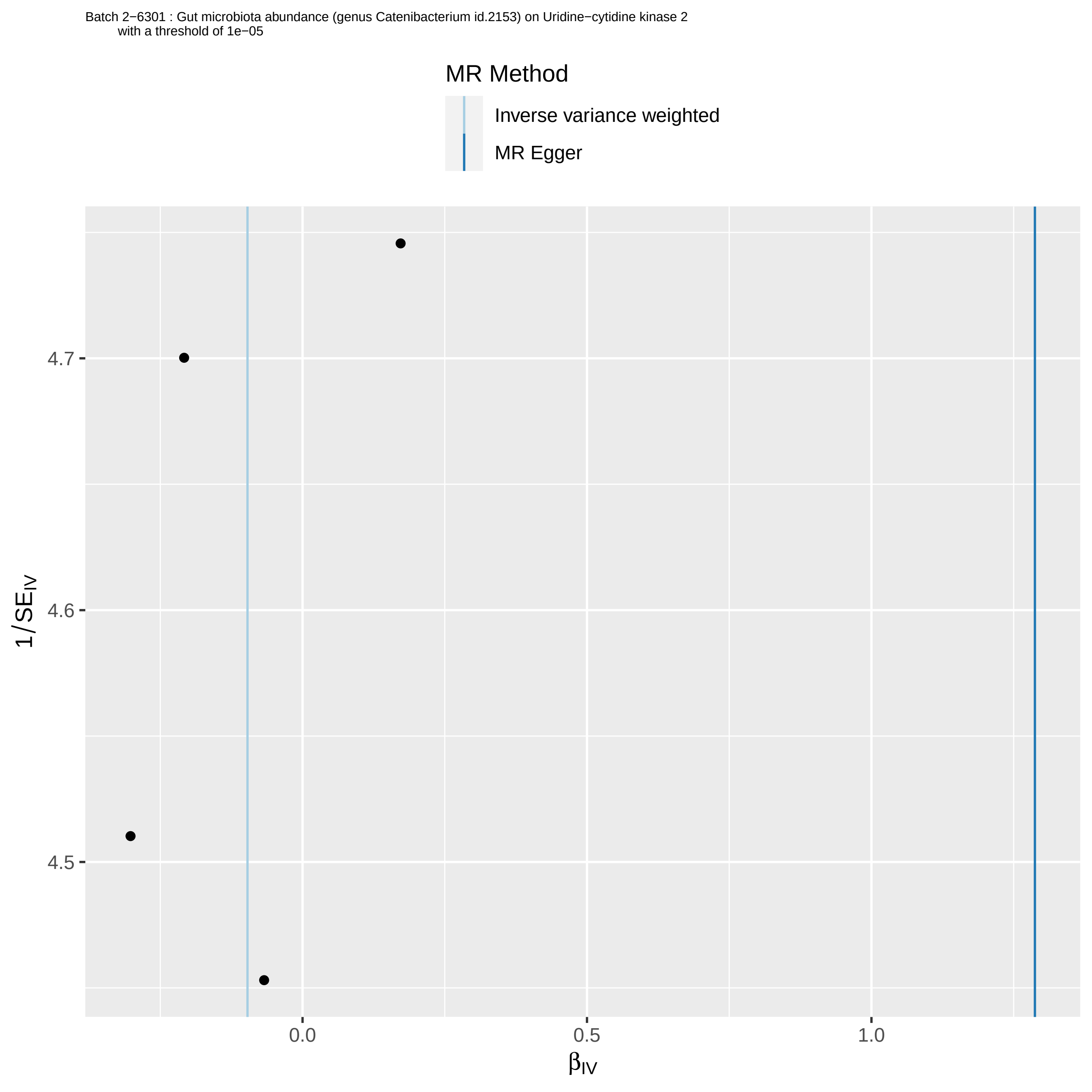

Batch 2−6301 : Gut microbiota abundance (genus Catenibacterium id.2153) on Uridine−cytidine kinase 2
with a threshold of 1e−05
MR Method
Inverse variance weighted
MR Egger
4.7
4.6
4.5
1/SE_IV
0.0
0.5
1.0
β_IV

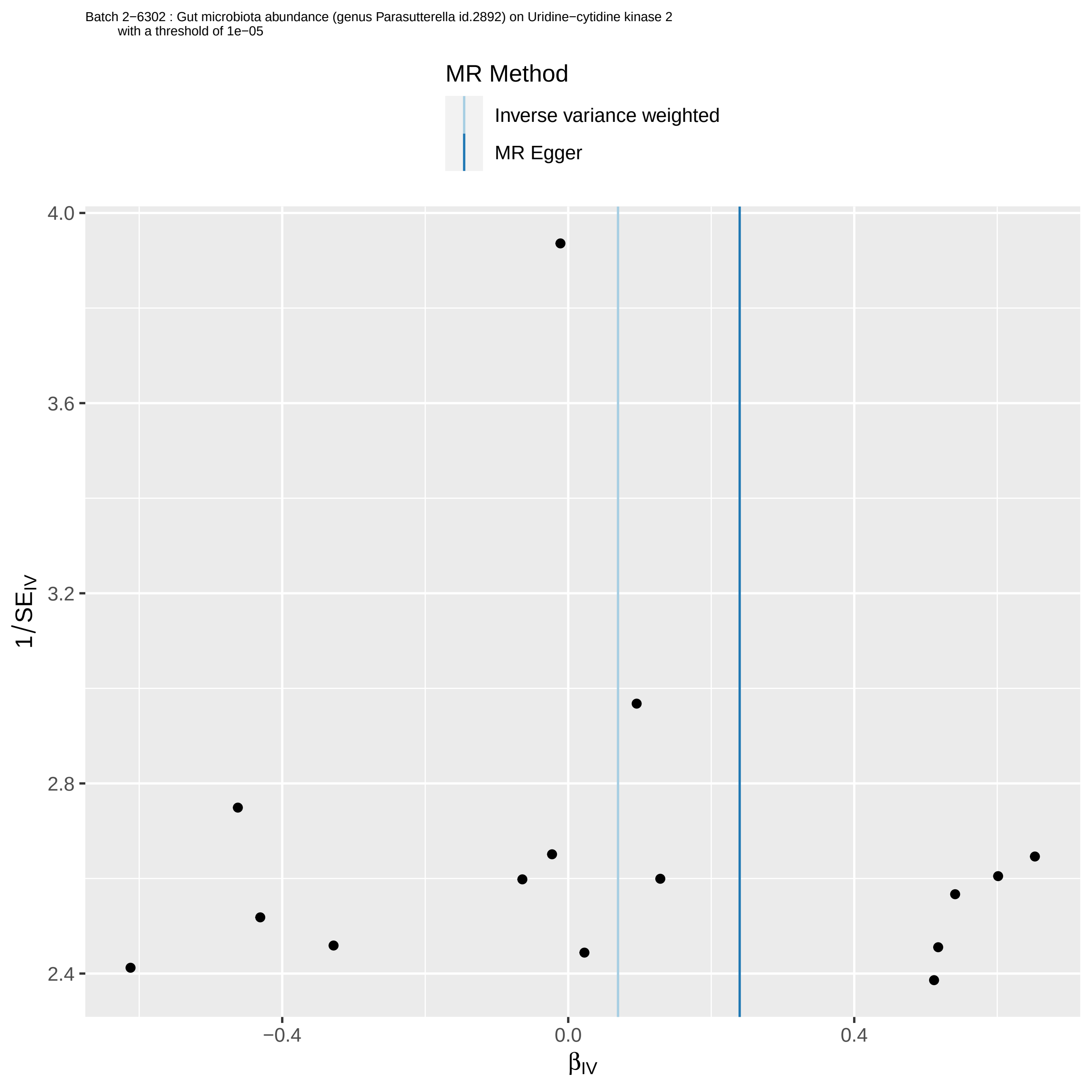
Batch 2−6302 : Gut microbiota abundance (genus Parasutterella id.2892) on Uridine−cytidine kinase 2
with a threshold of 1e−05
MR Method
Inverse variance weighted
MR Egger
4.0
3.6
3.2
2.8
2.4
1/SE_IV
−0.4
0.0
0.4
β_IV

Batch 2−6303 : Gut microbiota abundance (genus Catenibacterium id.2153) on Unique cartilage matrix−associated protein
with a threshold of 1e−05

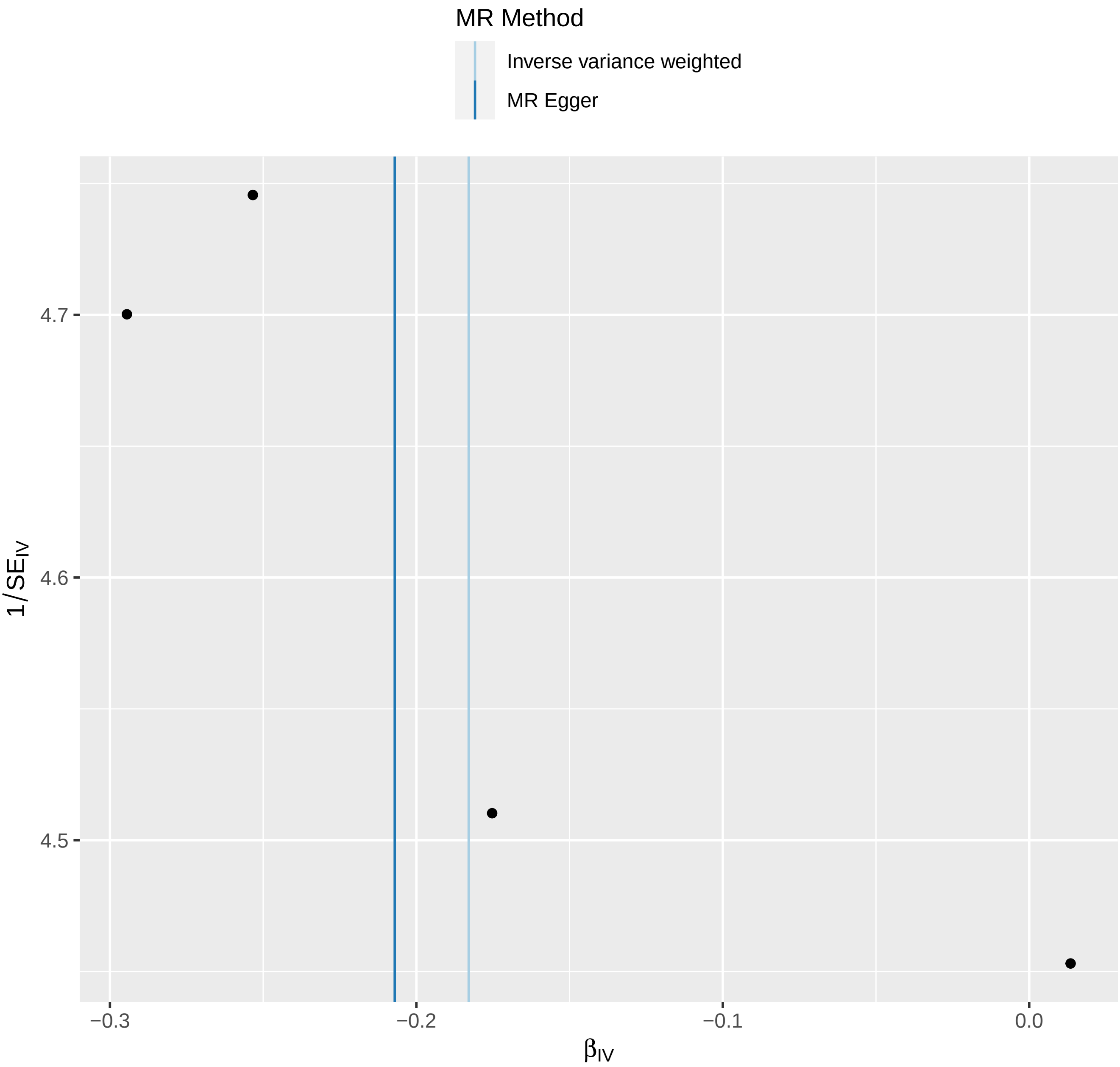

Batch 2−6304 : Gut microbiota abundance (genus Parasutterella id.2892) on Unique cartilage matrix−associated protein
with a threshold of 1e−05

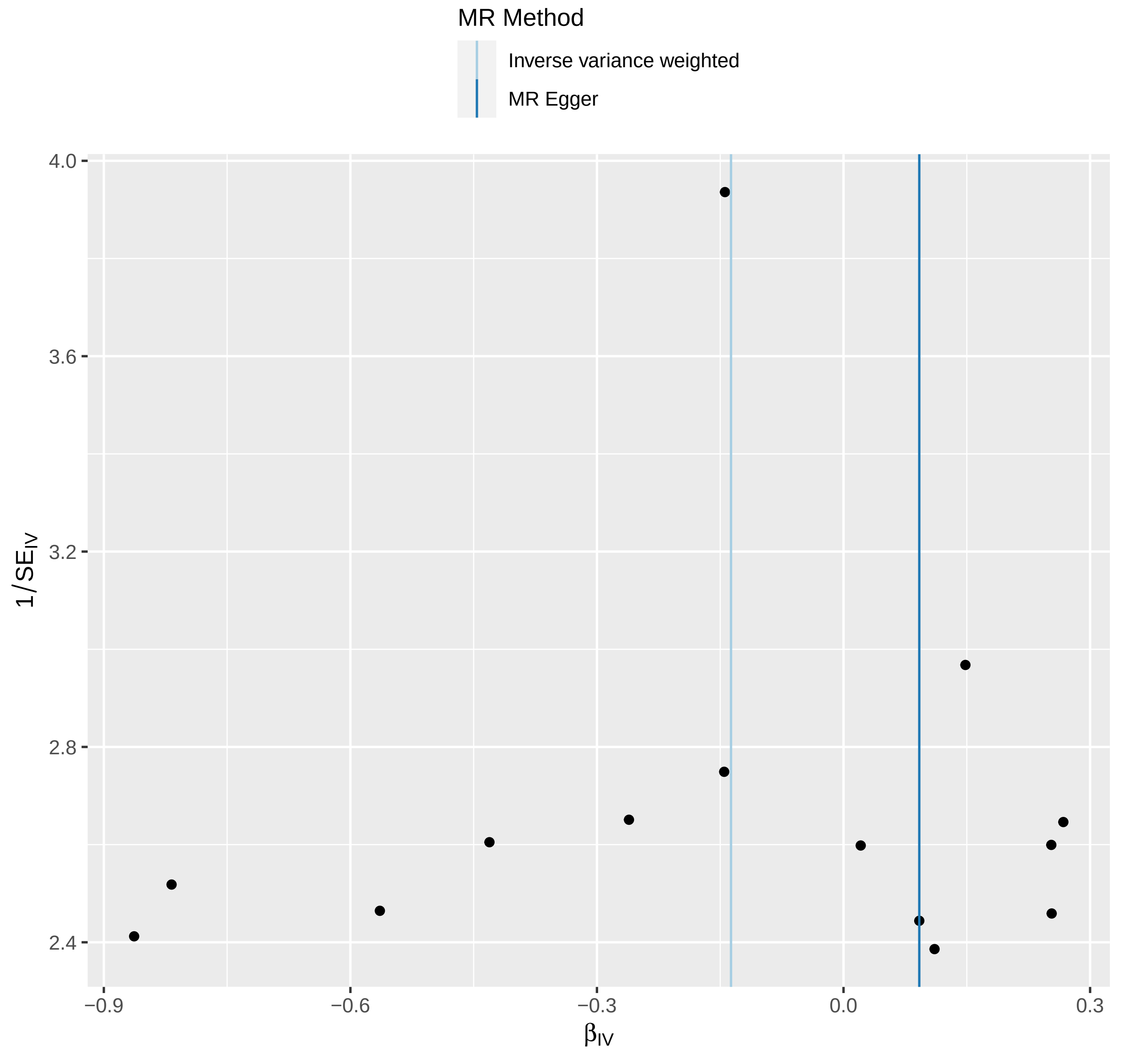

Batch 2−6305 : Gut microbiota abundance (genus Catenibacterium id.2153) on Ubiquitin−fold modifier−conjugating enzyme 1 with a threshold of 1e−05

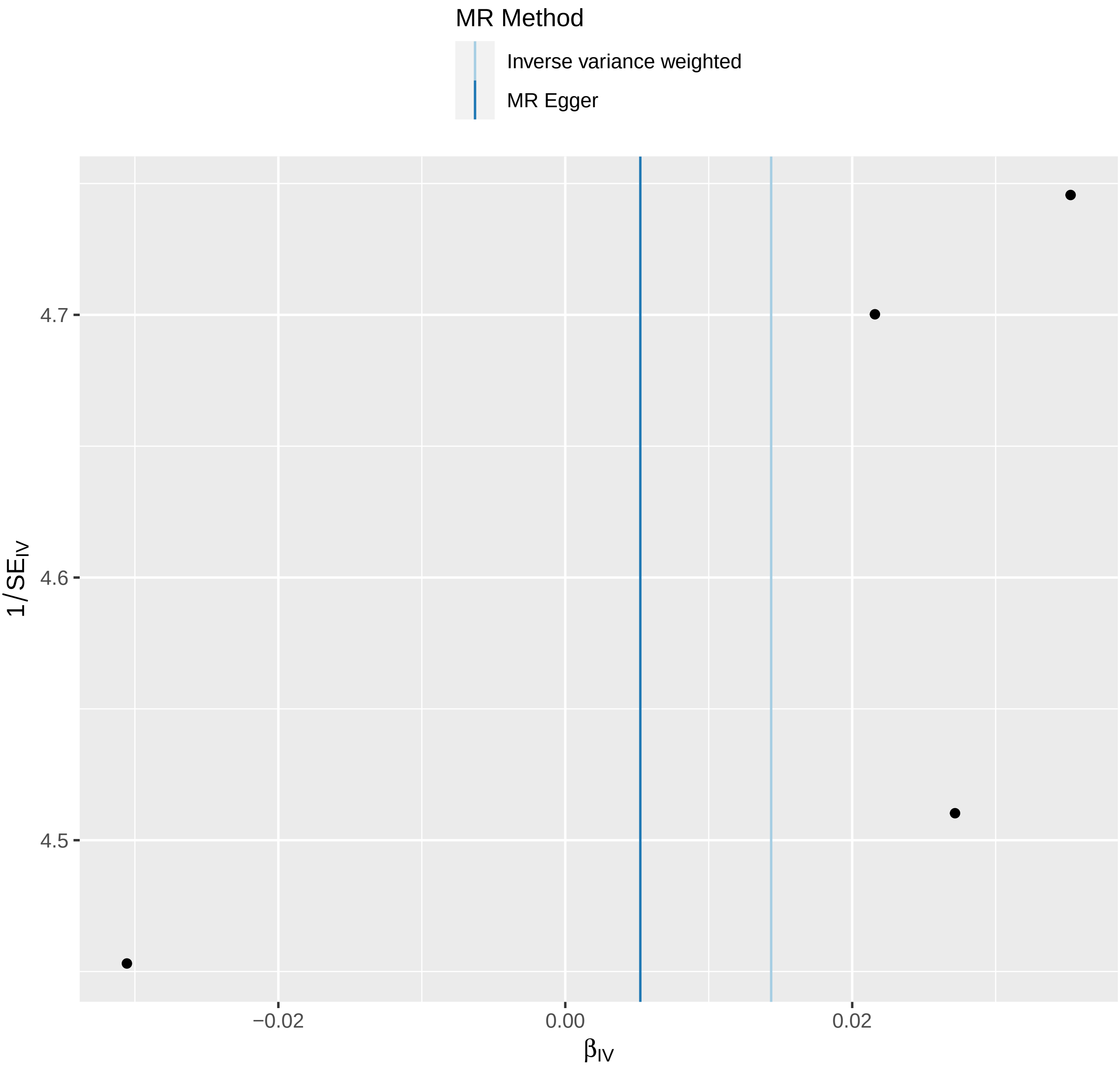

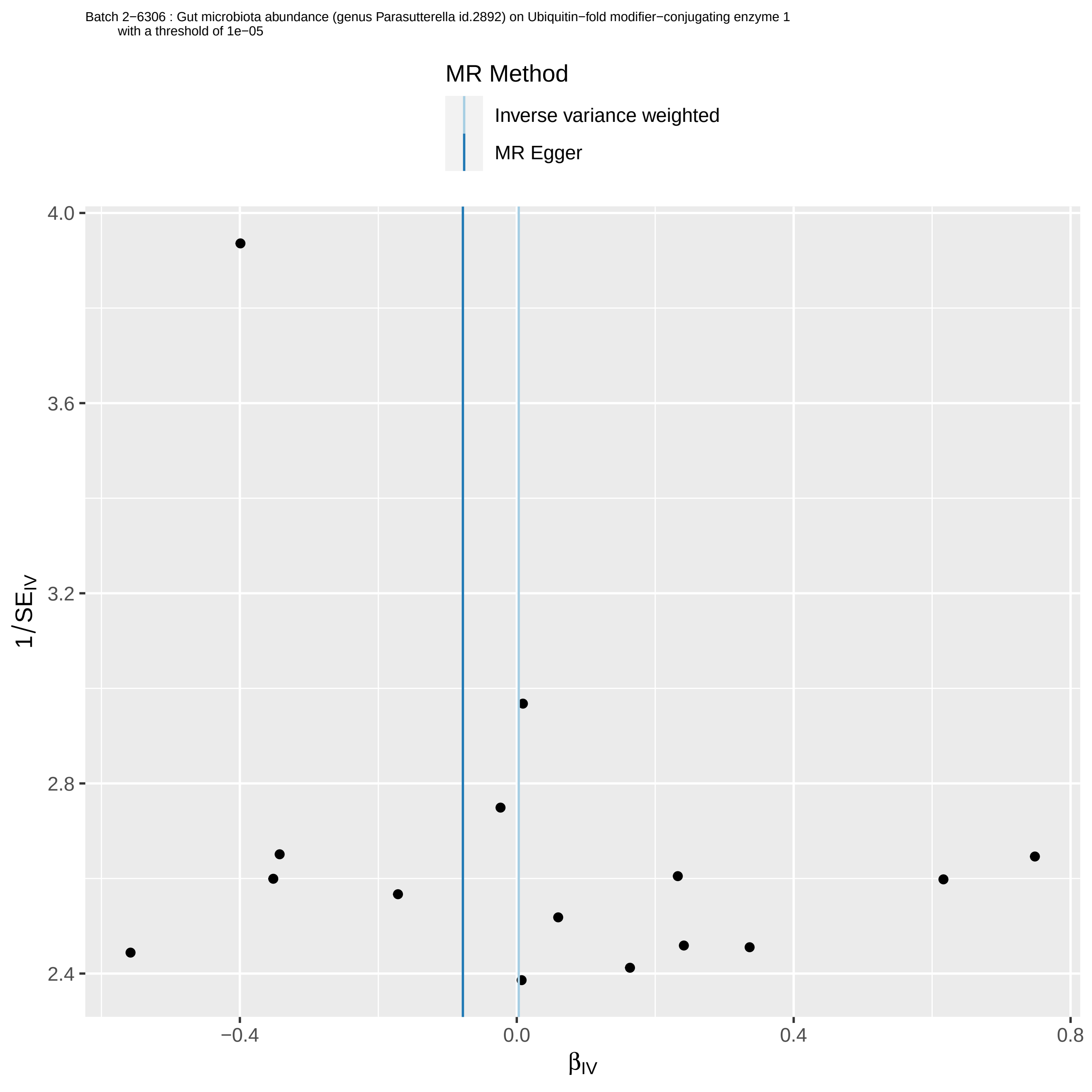
Batch 2−6306 : Gut microbiota abundance (genus Parasutterella id.2892) on Ubiquitin−fold modifier−conjugating enzyme 1
with a threshold of 1e−05
MR Method
Inverse variance weighted
MR Egger
4.0
3.6
3.2
2.8
2.4
1/SE$_{IV}$
−0.4
0.0
0.4
0.8
β$_{IV}$

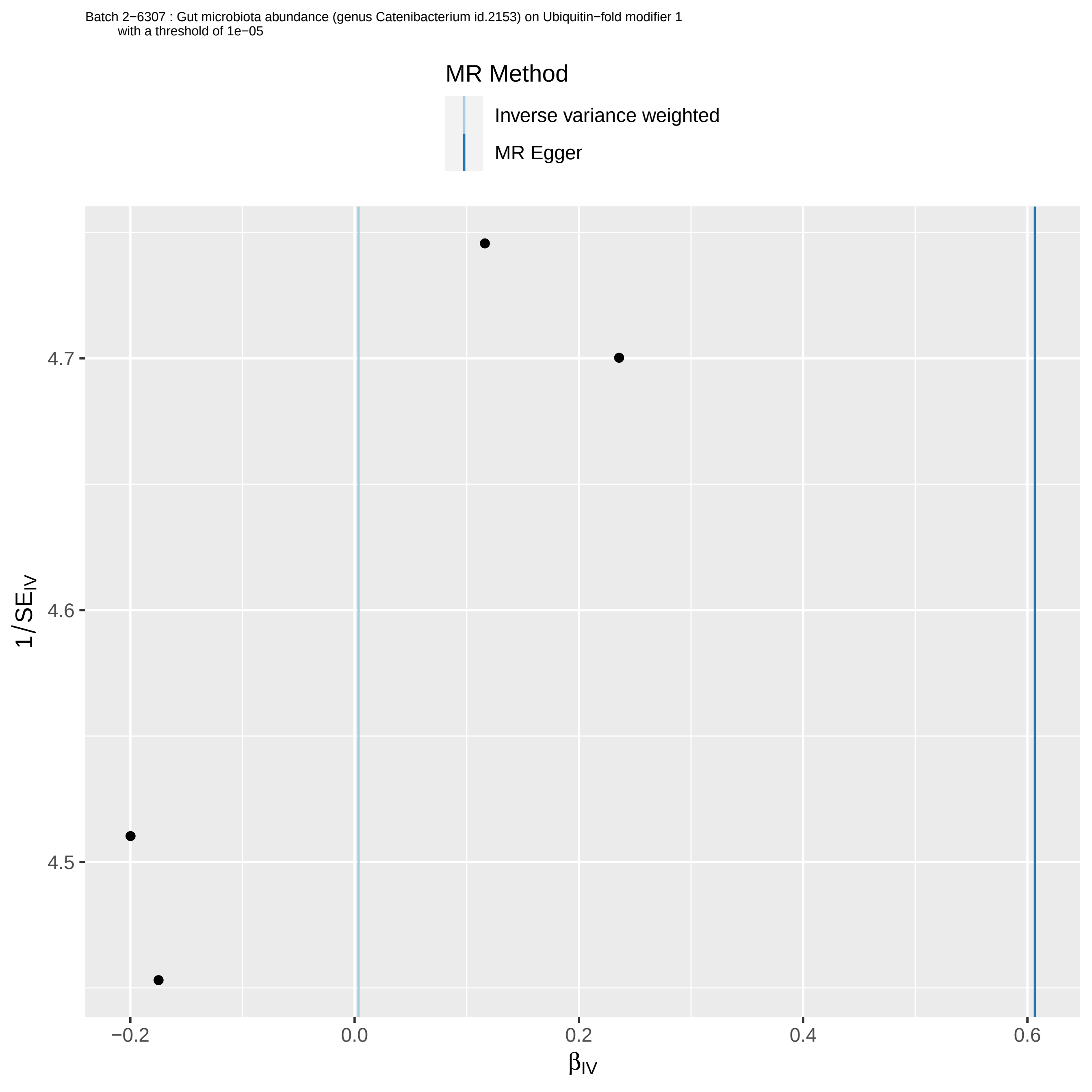

Batch 2−6307 : Gut microbiota abundance (genus Catenibacterium id.2153) on Ubiquitin−fold modifier 1
with a threshold of 1e−05
MR Method
Inverse variance weighted
MR Egger
4.7
4.6
4.5
$1/SE_{IV}$
−0.2
0.0
0.2
0.4
0.6
β_{IV}

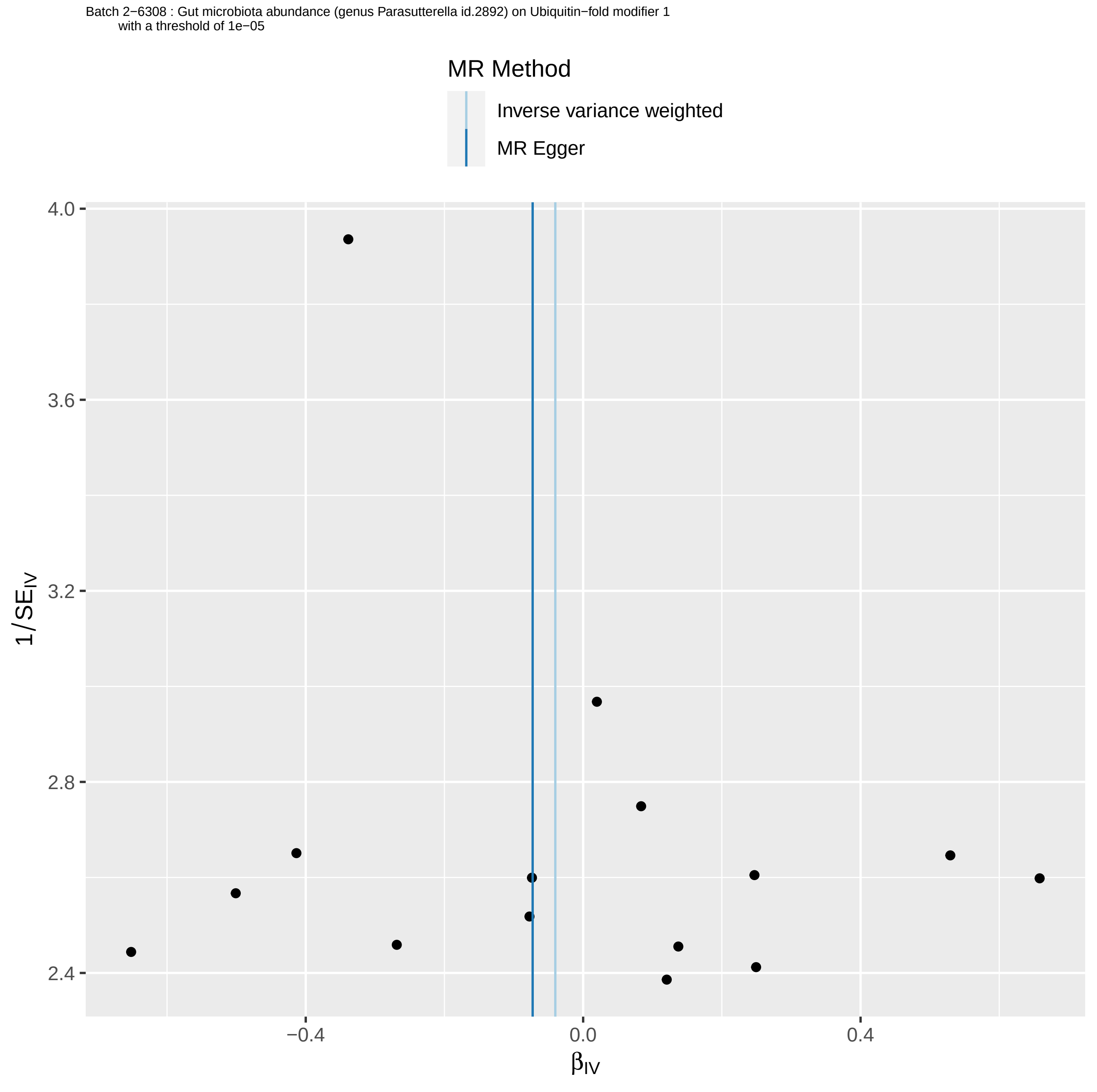

Batch 2−6308 : Gut microbiota abundance (genus Parasutterella id.2892) on Ubiquitin−fold modifier 1
with a threshold of 1e−05
MR Method
Inverse variance weighted
MR Egger
4.0
3.6
3.2
2.8
2.4
1/SE_IV
−0.4
0.0
0.4
β_IV

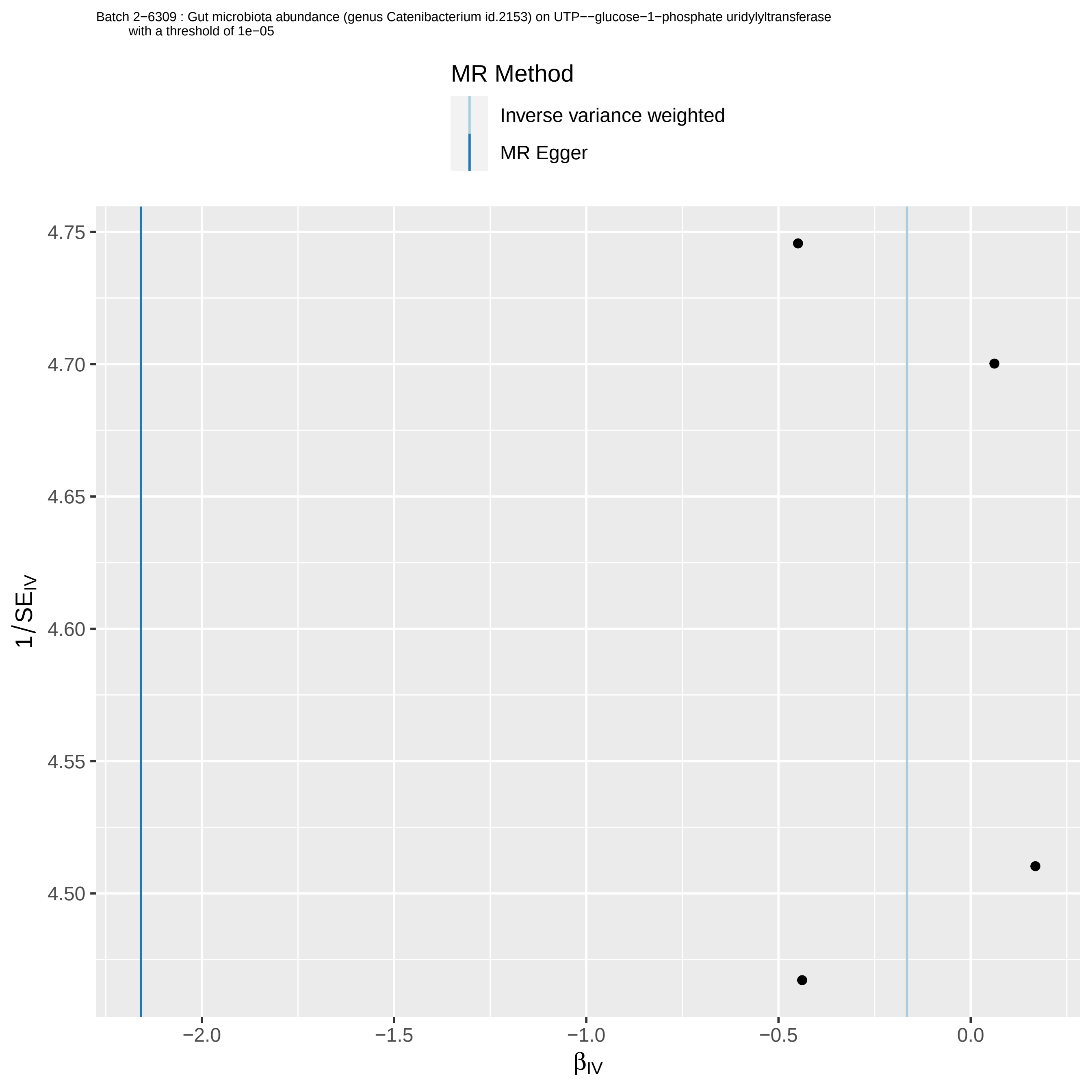

Batch 2−6309 : Gut microbiota abundance (genus Catenibacterium id.2153) on UTP−−glucose−1−phosphate uridylyltransferase
with a threshold of 1e−05
MR Method
Inverse variance weighted
MR Egger
4.75
4.70
4.65
4.60
4.55
4.50
1/SE_IV
−2.0
−1.5
−1.0
−0.5
0.0
β_IV

Batch 2−6310 : Gut microbiota abundance (genus Parasutterella id.2892) on UTP−−glucose−1−phosphate uridylyltransferase with a threshold of 1e−05

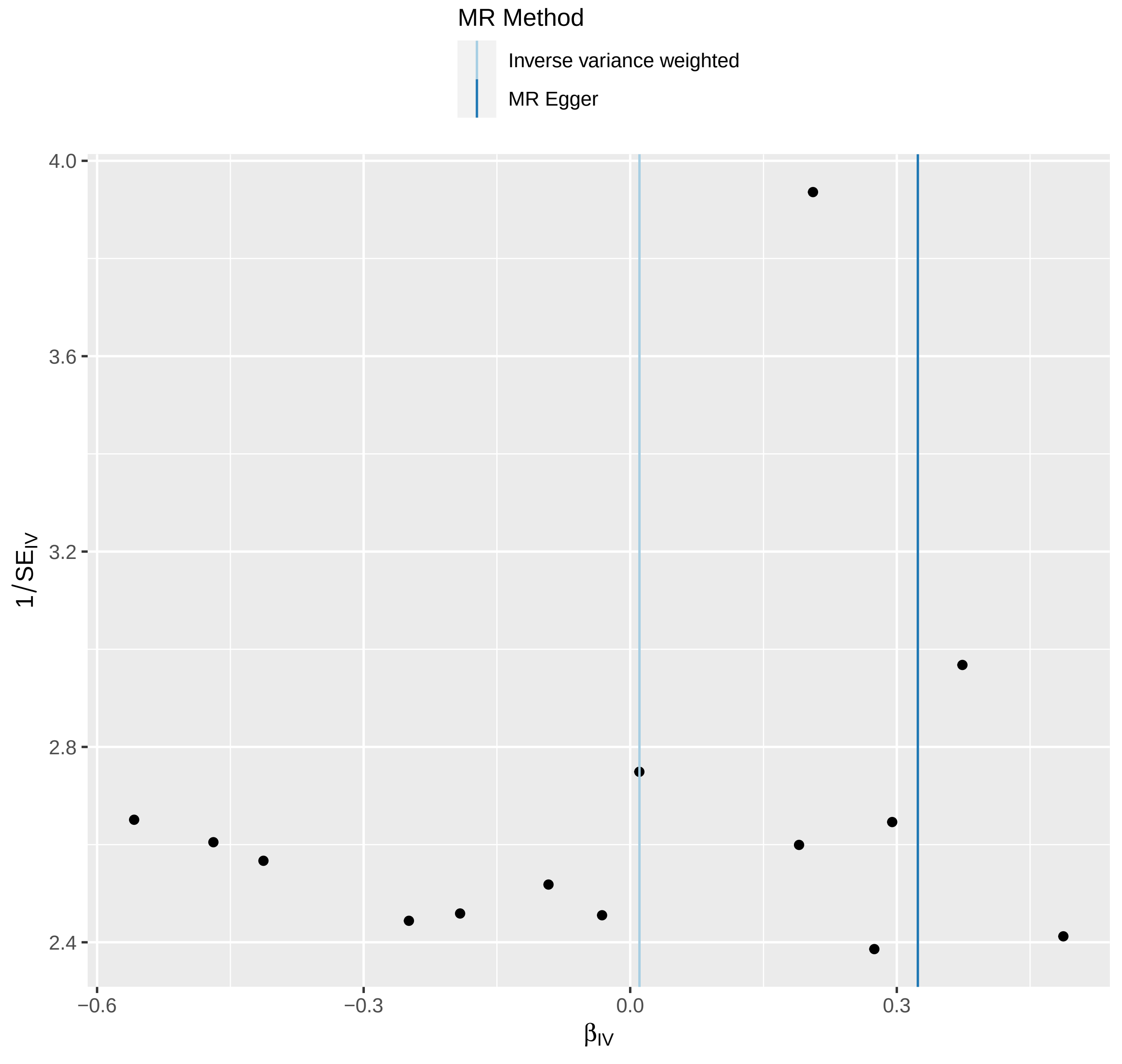

Batch 2−6311 : Gut microbiota abundance (genus Catenibacterium id.2153) on UDP−glucuronosyltransferase 1−6
with a threshold of 1e−05

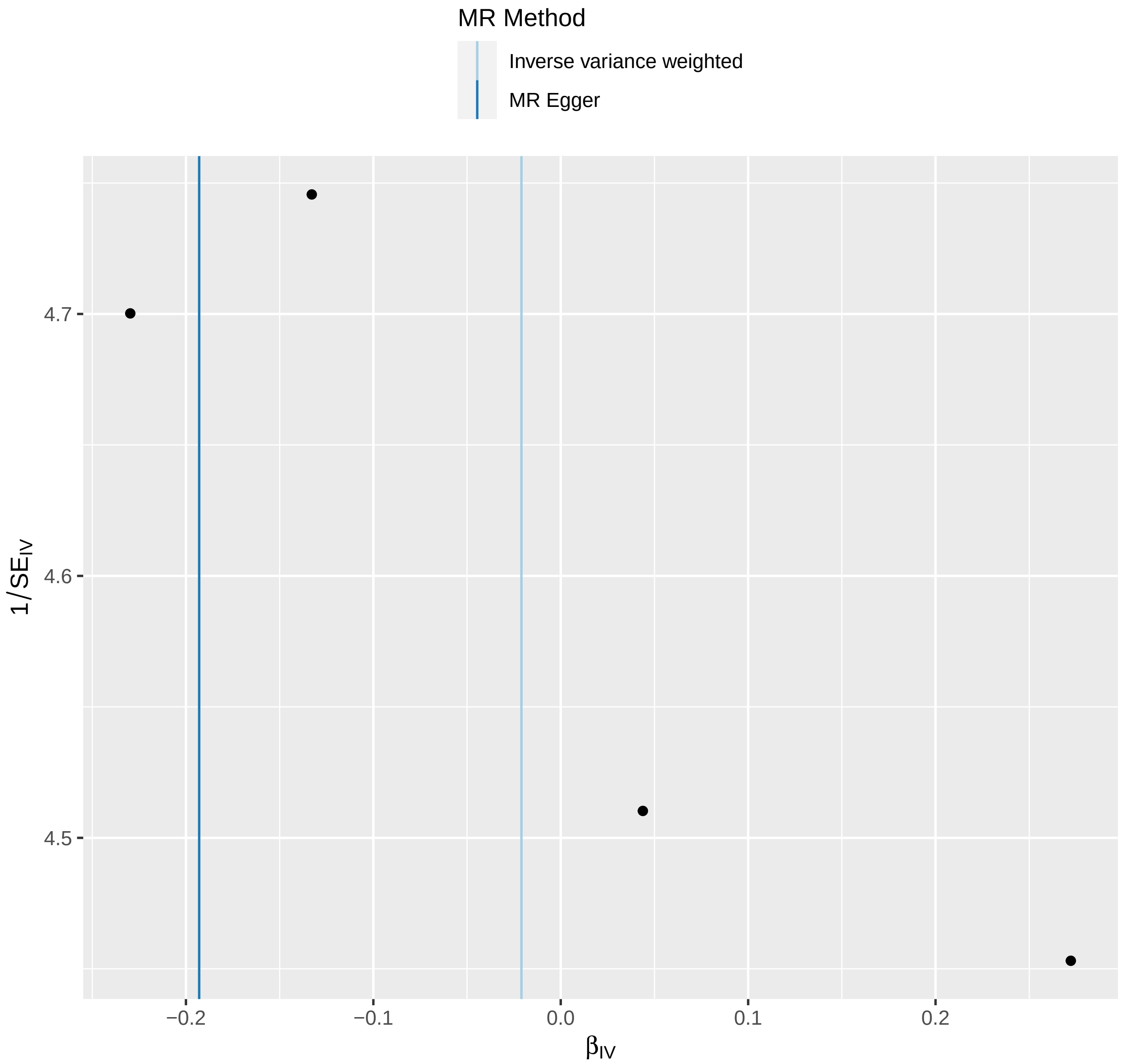

Batch 2−6312 : Gut microbiota abundance (genus Parasutterella id.2892) on UDP−glucuronosyltransferase 1−6
with a threshold of 1e−05

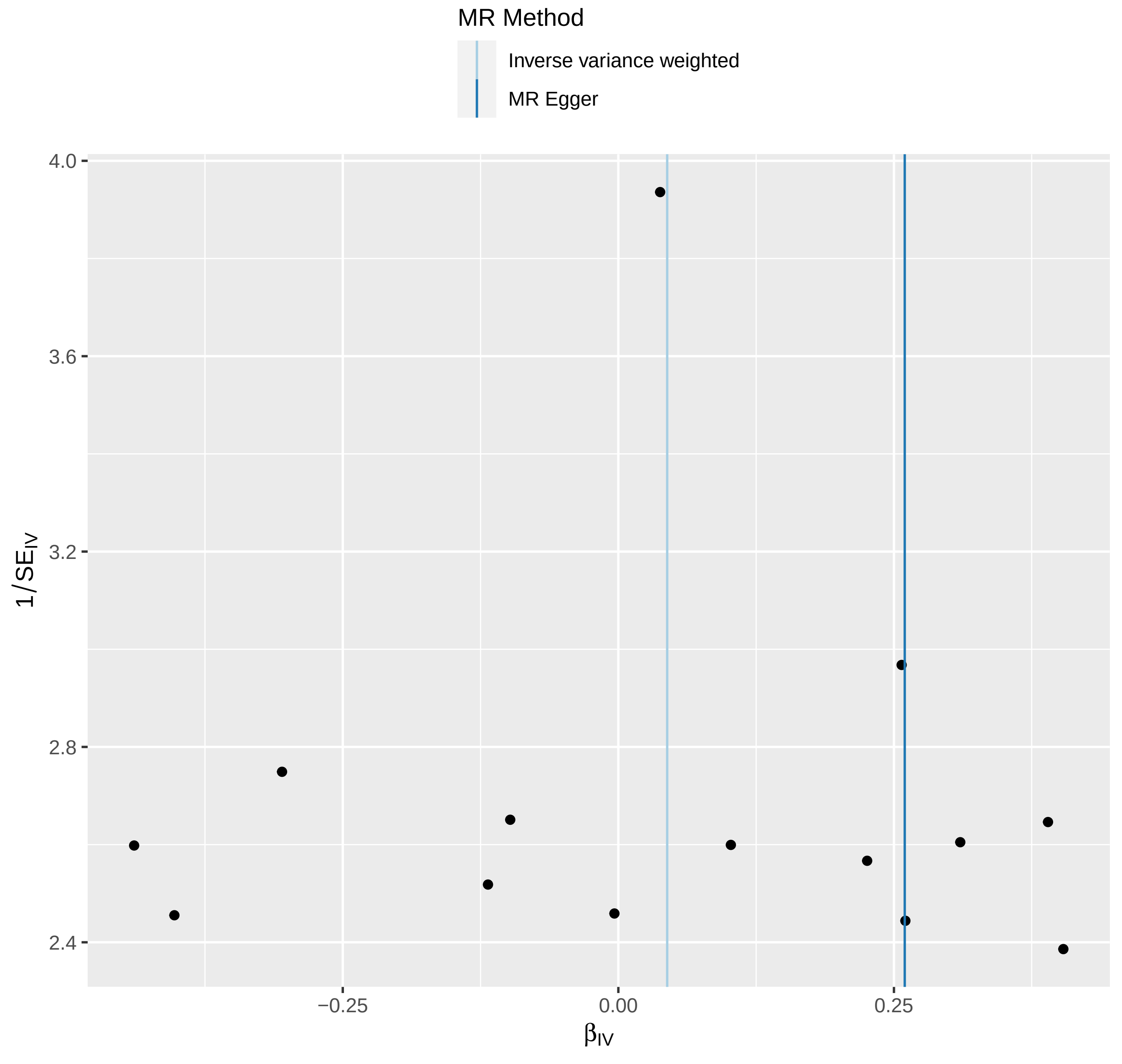

Batch 2−6313 : Gut microbiota abundance (genus Catenibacterium id.2153) on UDP−glucuronosyltransferase 1−8
with a threshold of 1e−05

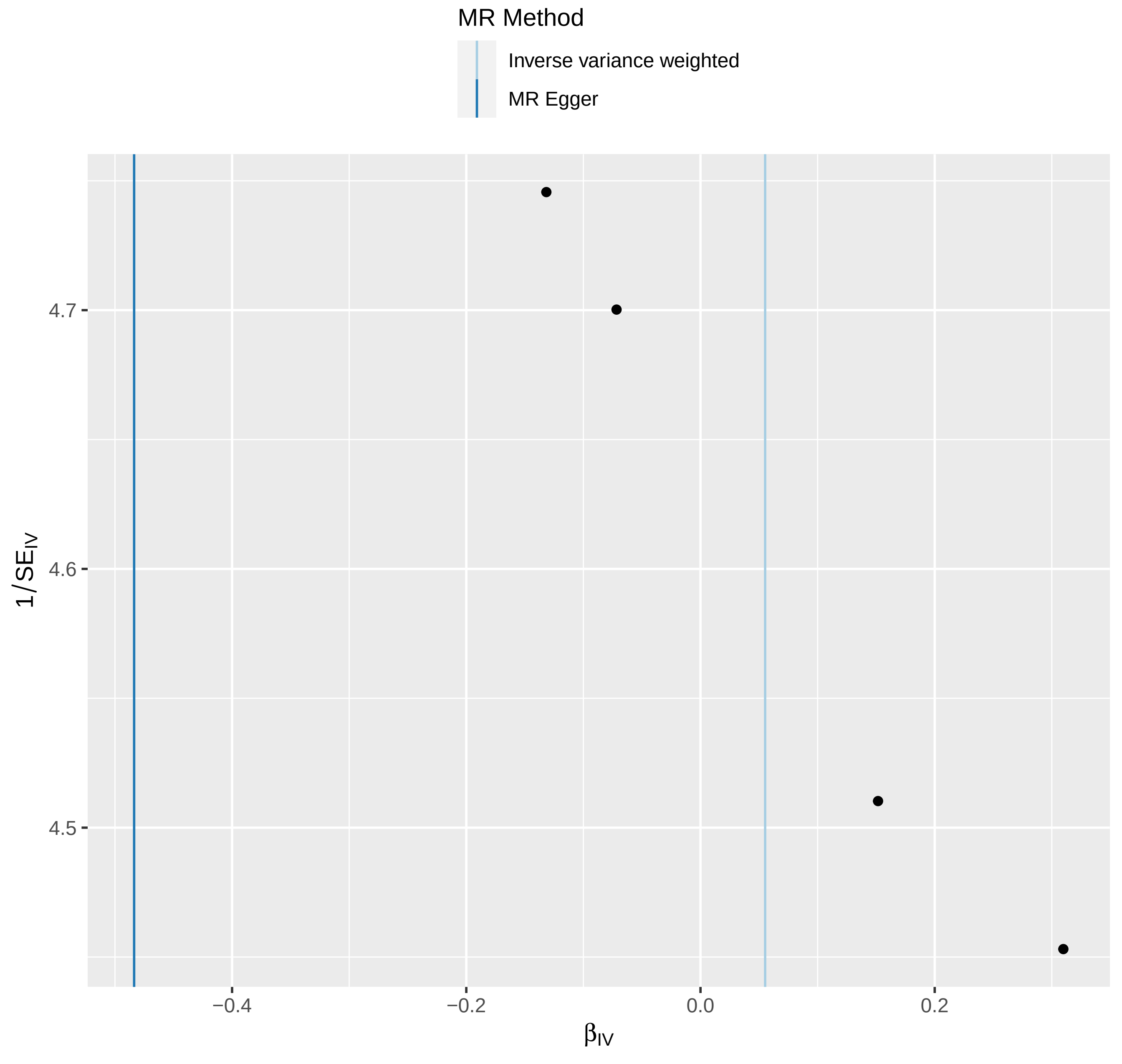

Batch 2−6314 : Gut microbiota abundance (genus Parasutterella id.2892) on UDP−glucuronosyltransferase 1−8
with a threshold of 1e−05

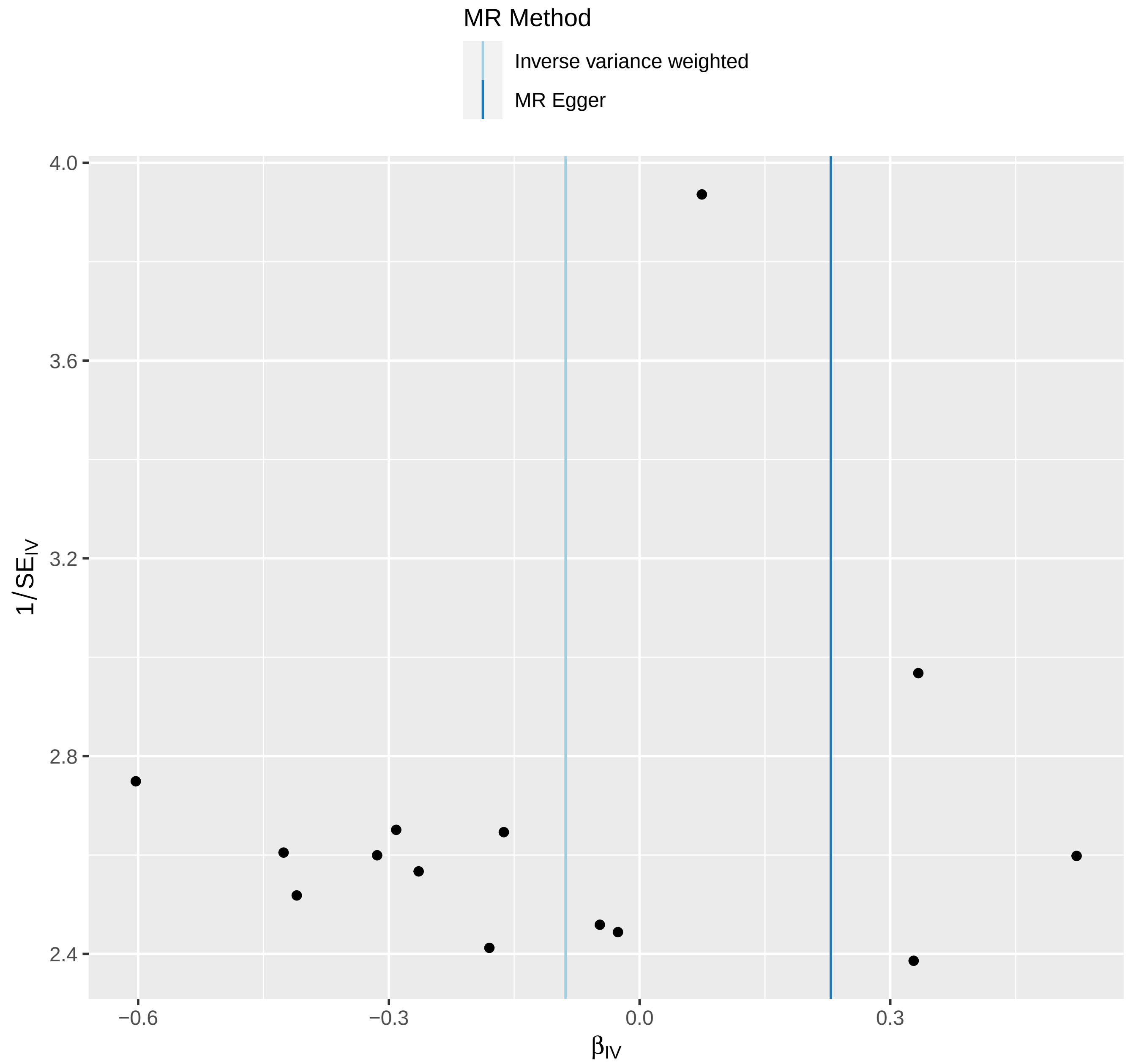

Batch 2−6315 : Gut microbiota abundance (genus Catenibacterium id.2153) on UDP−glucuronosyltransferase 2A1
with a threshold of 1e−05

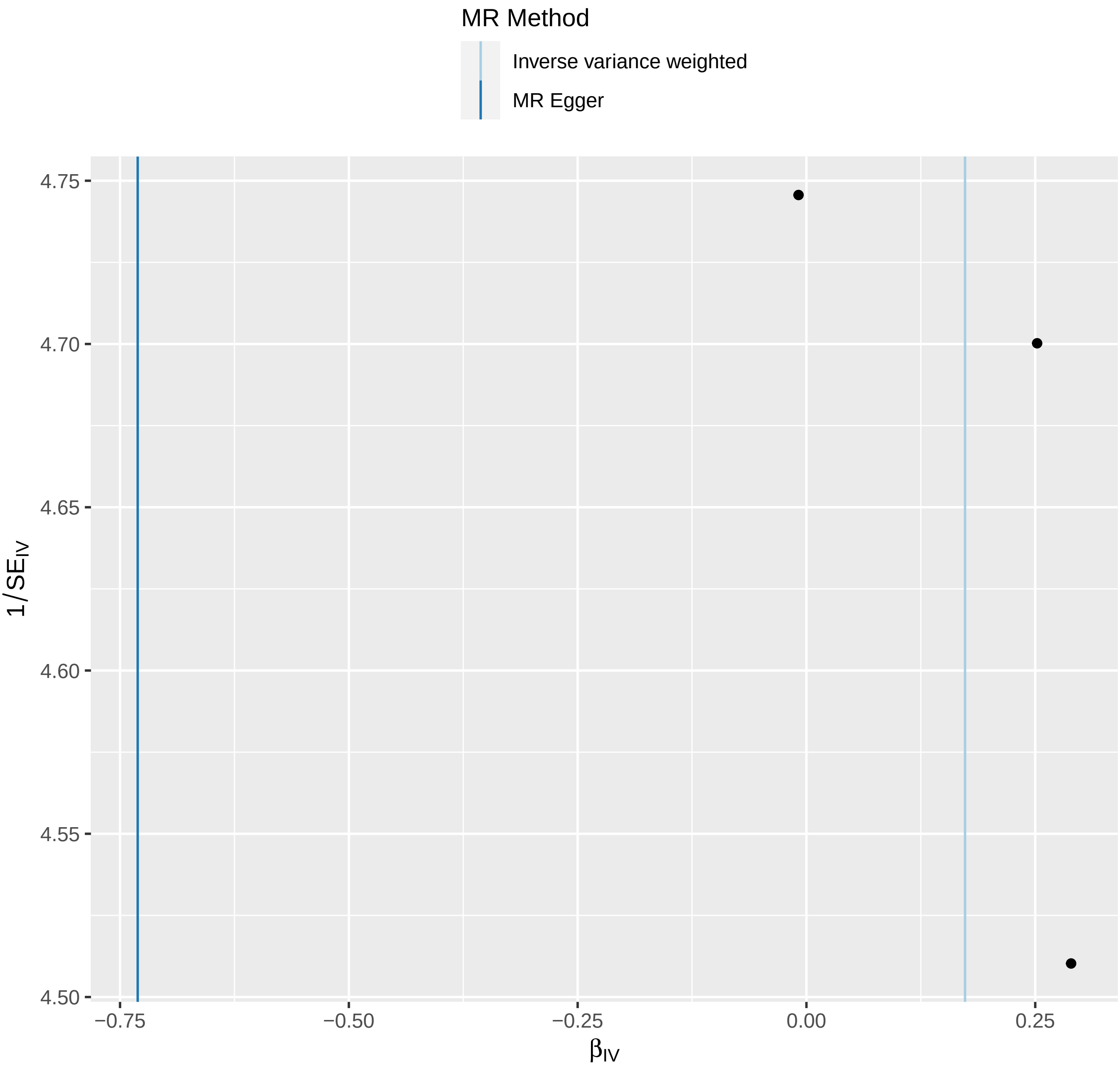

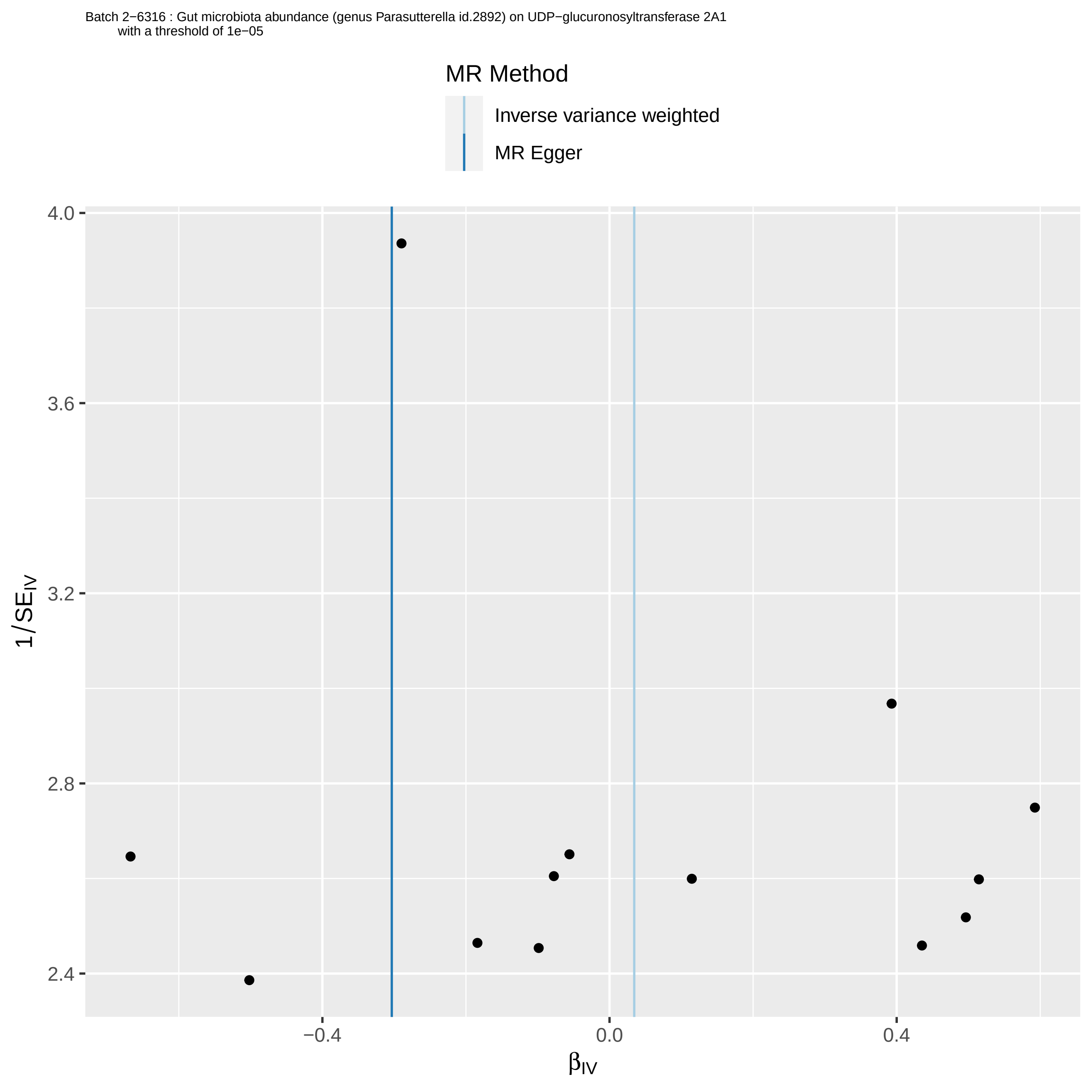

Batch 2−6316 : Gut microbiota abundance (genus Parasutterella id.2892) on UDP−glucuronosyltransferase 2A1
with a threshold of 1e−05
MR Method
Inverse variance weighted
MR Egger
4.0
3.6
3.2
2.8
2.4
1/SE_IV
−0.4
0.0
0.4
β_IV

Batch 2−6317 : Gut microbiota abundance (genus Catenibacterium id.2153) on NKG2D ligand 1
with a threshold of 1e−05

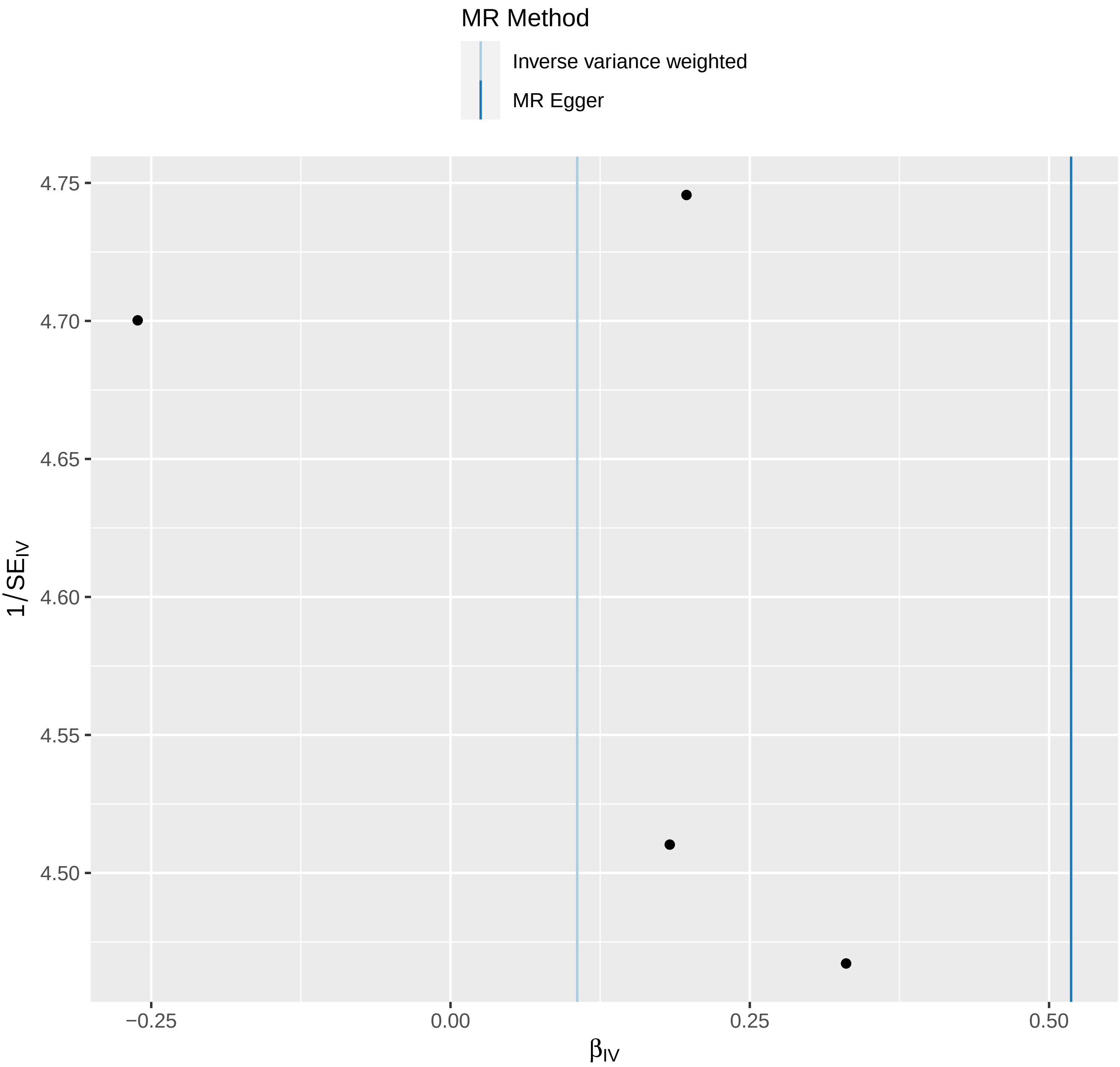

Batch 2−6318 : Gut microbiota abundance (genus Parasutterella id.2892) on NKG2D ligand 1
with a threshold of 1e−05

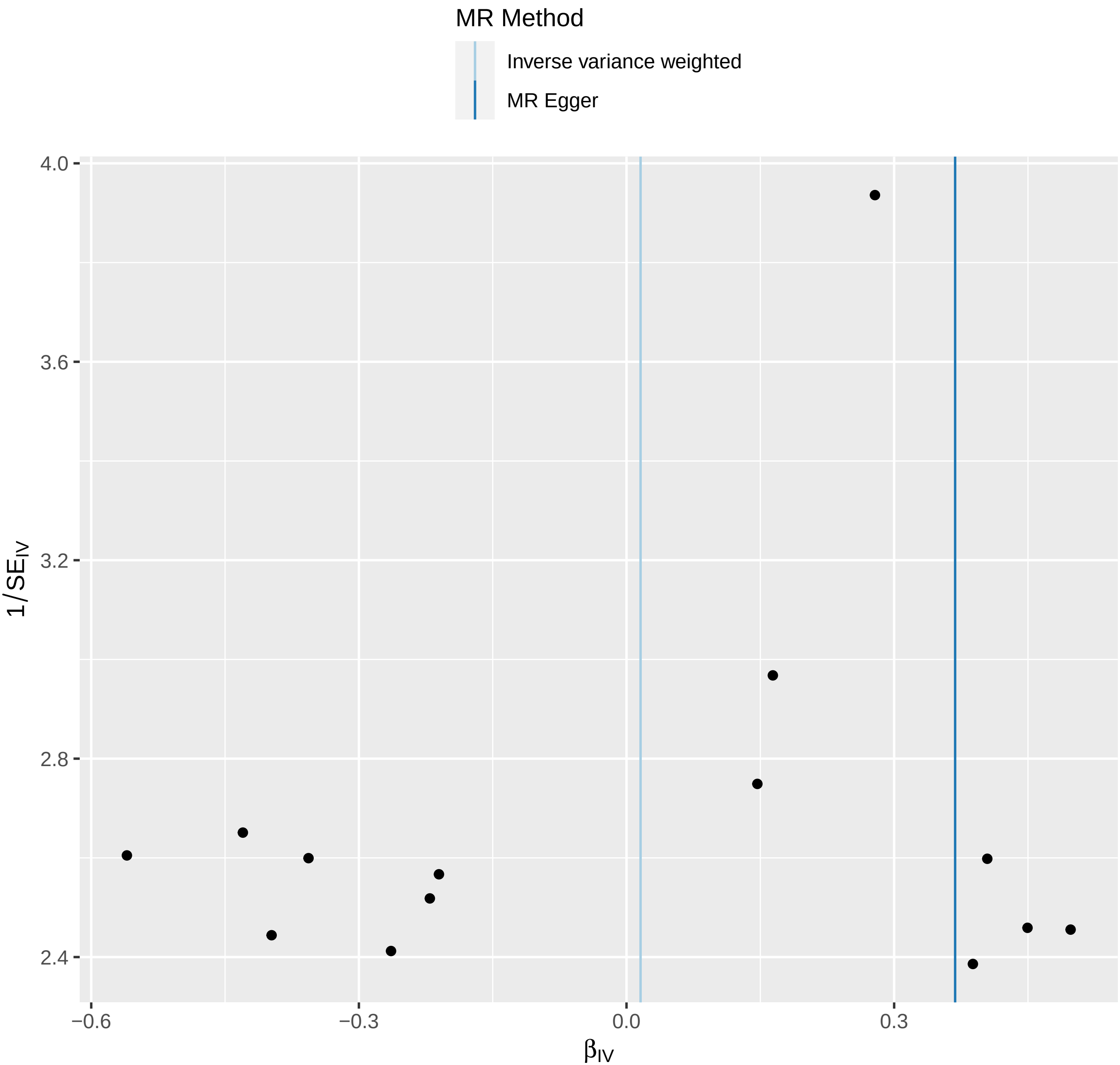

Batch 2−6319 : Gut microbiota abundance (genus Catenibacterium id.2153) on NKG2D ligand 1
with a threshold of 1e−05

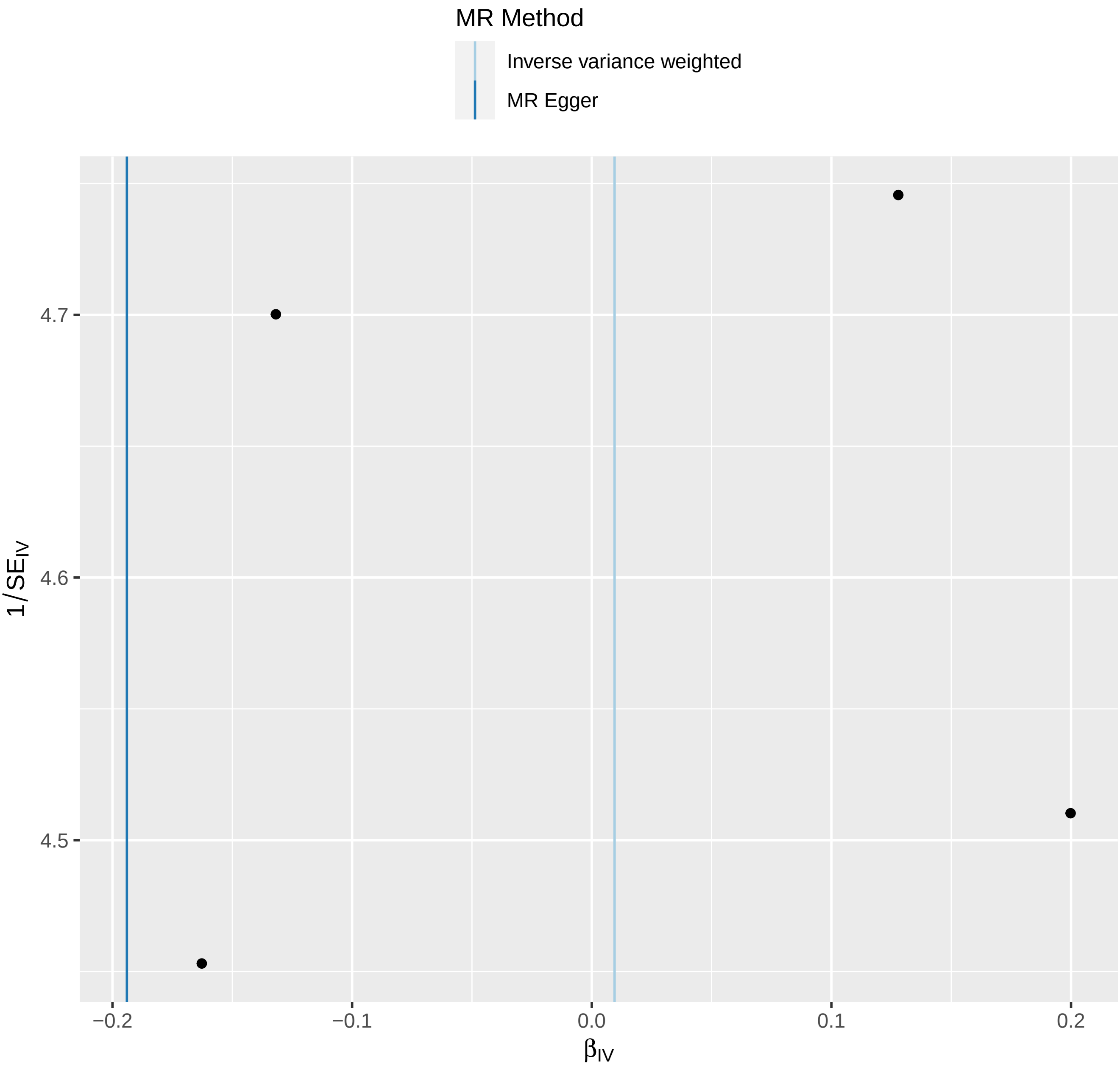

Batch 2−6320 : Gut microbiota abundance (genus Parasutterella id.2892) on NKG2D ligand 1
with a threshold of 1e−05

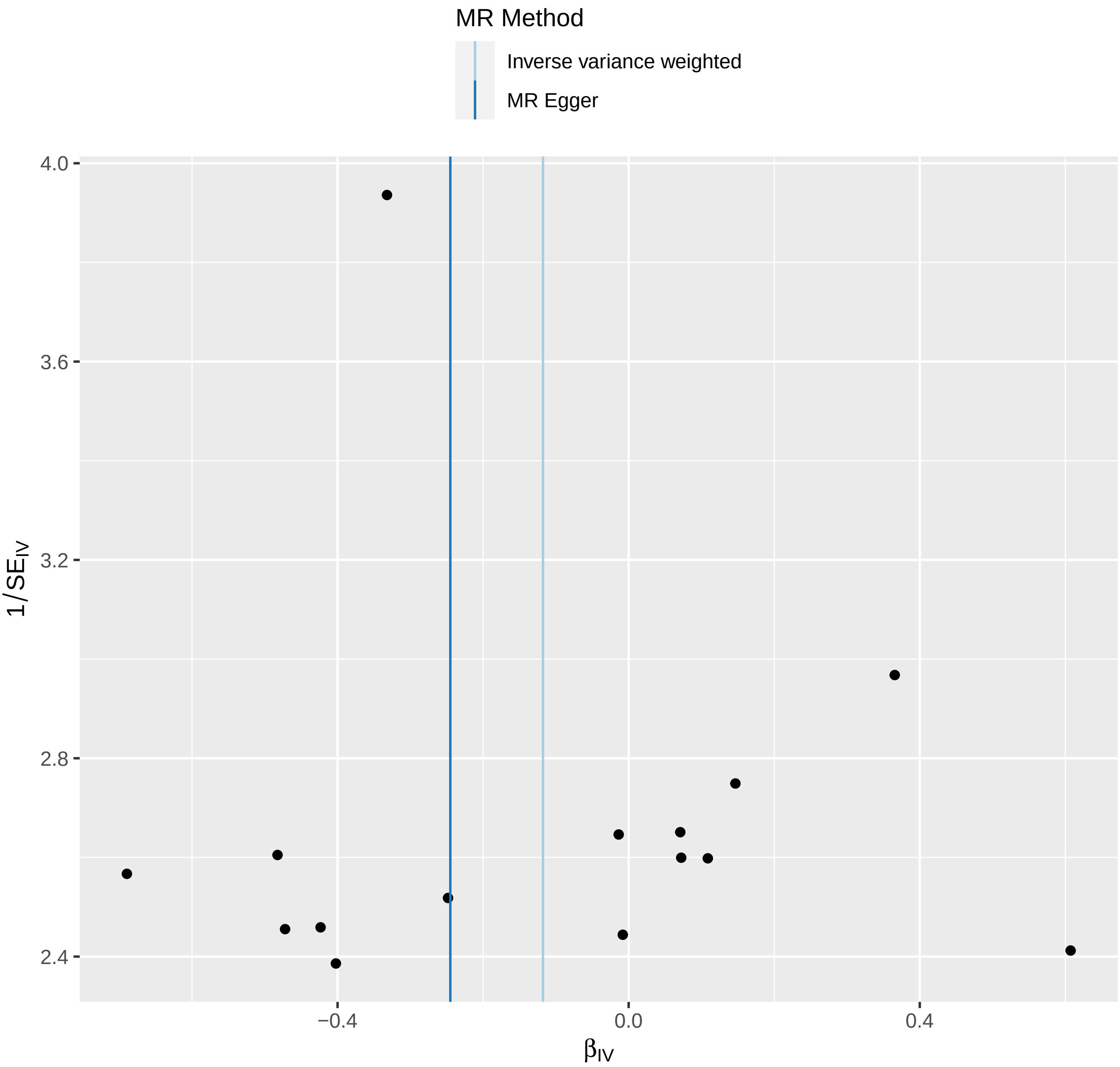

Batch 2−6321 : Gut microbiota abundance (genus Catenibacterium id.2153) on NKG2D ligand 2
with a threshold of 1e−05

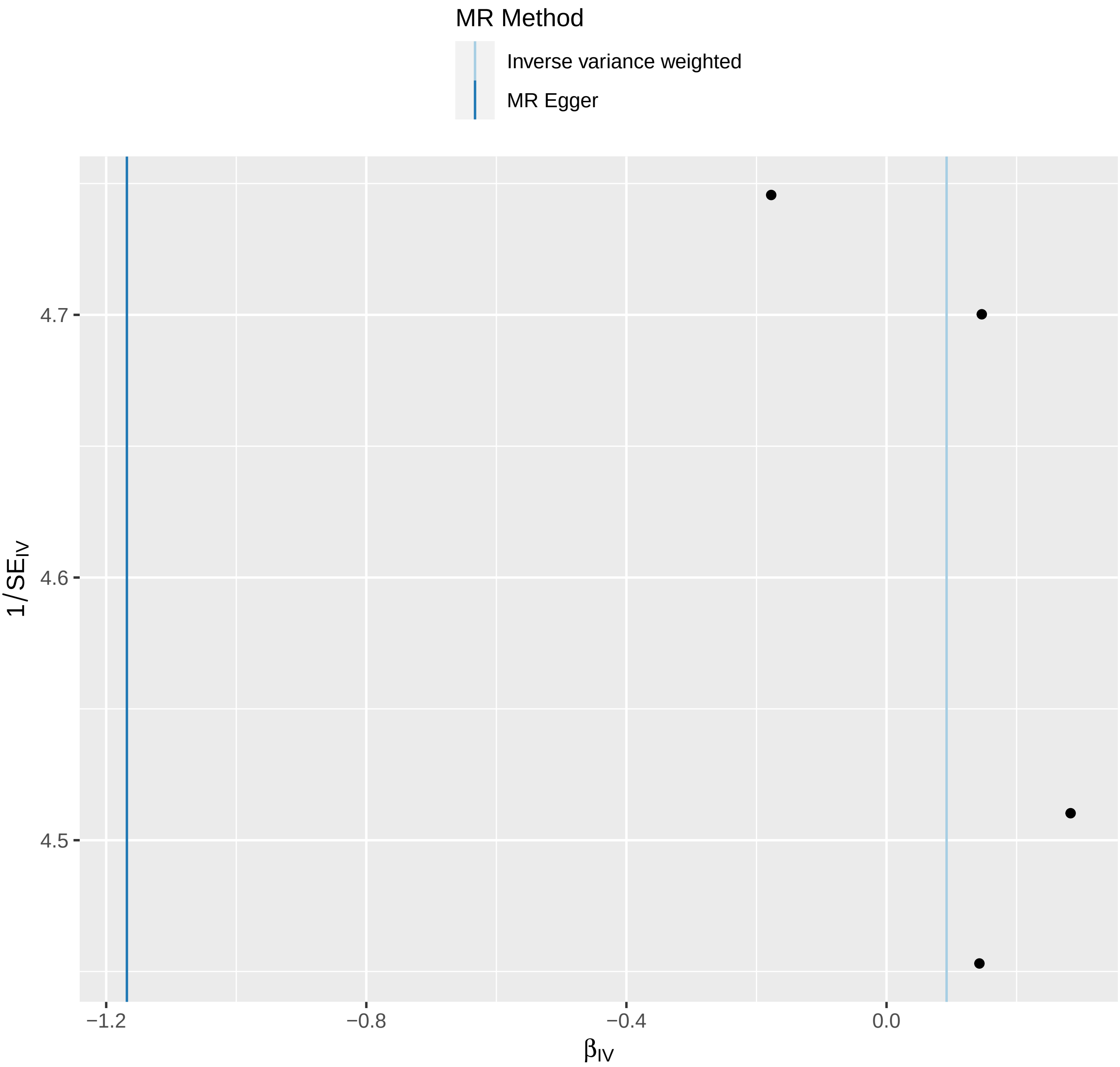

Batch 2−6322 : Gut microbiota abundance (genus Parasutterella id.2892) on NKG2D ligand 2
with a threshold of 1e−05

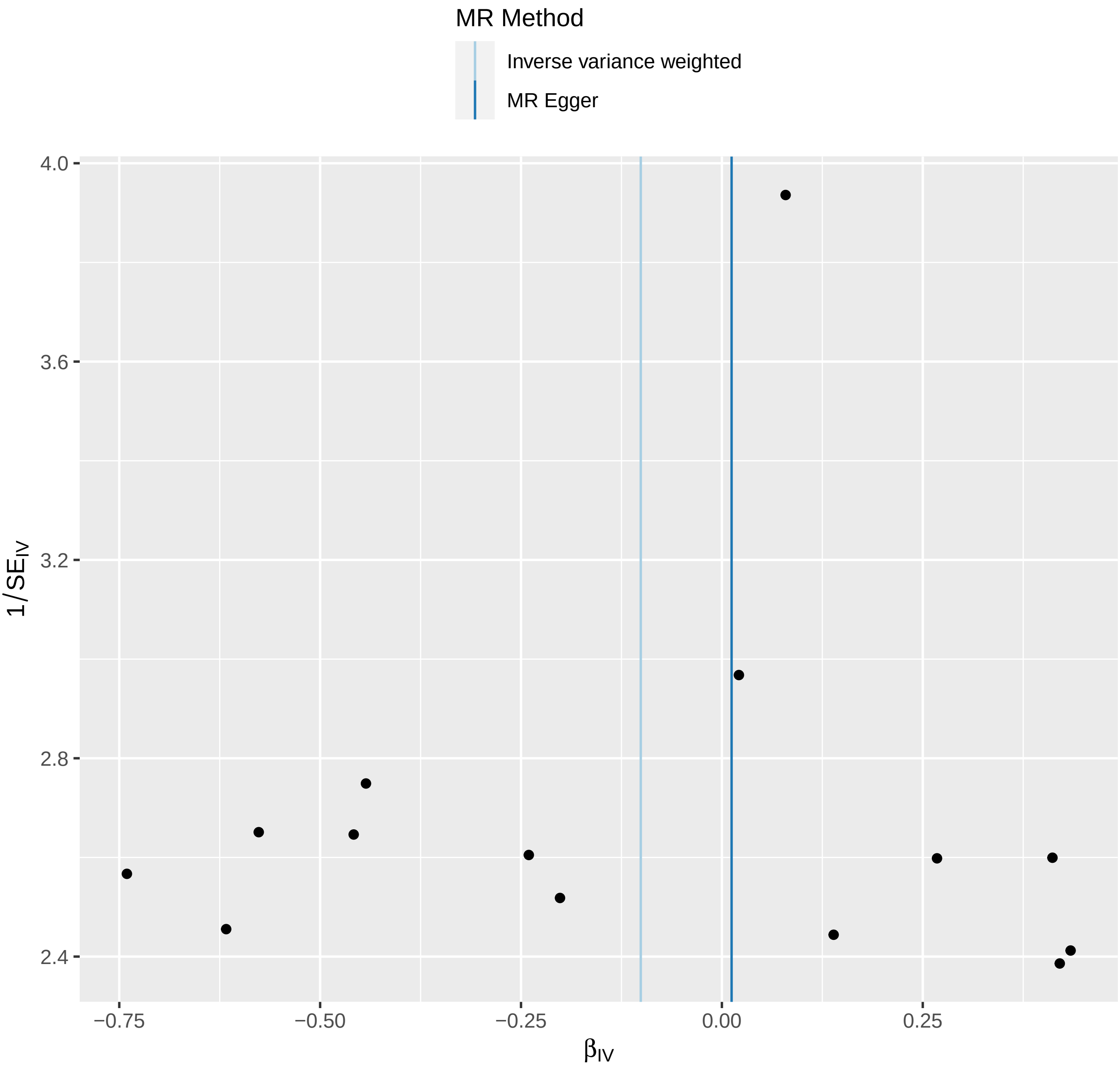

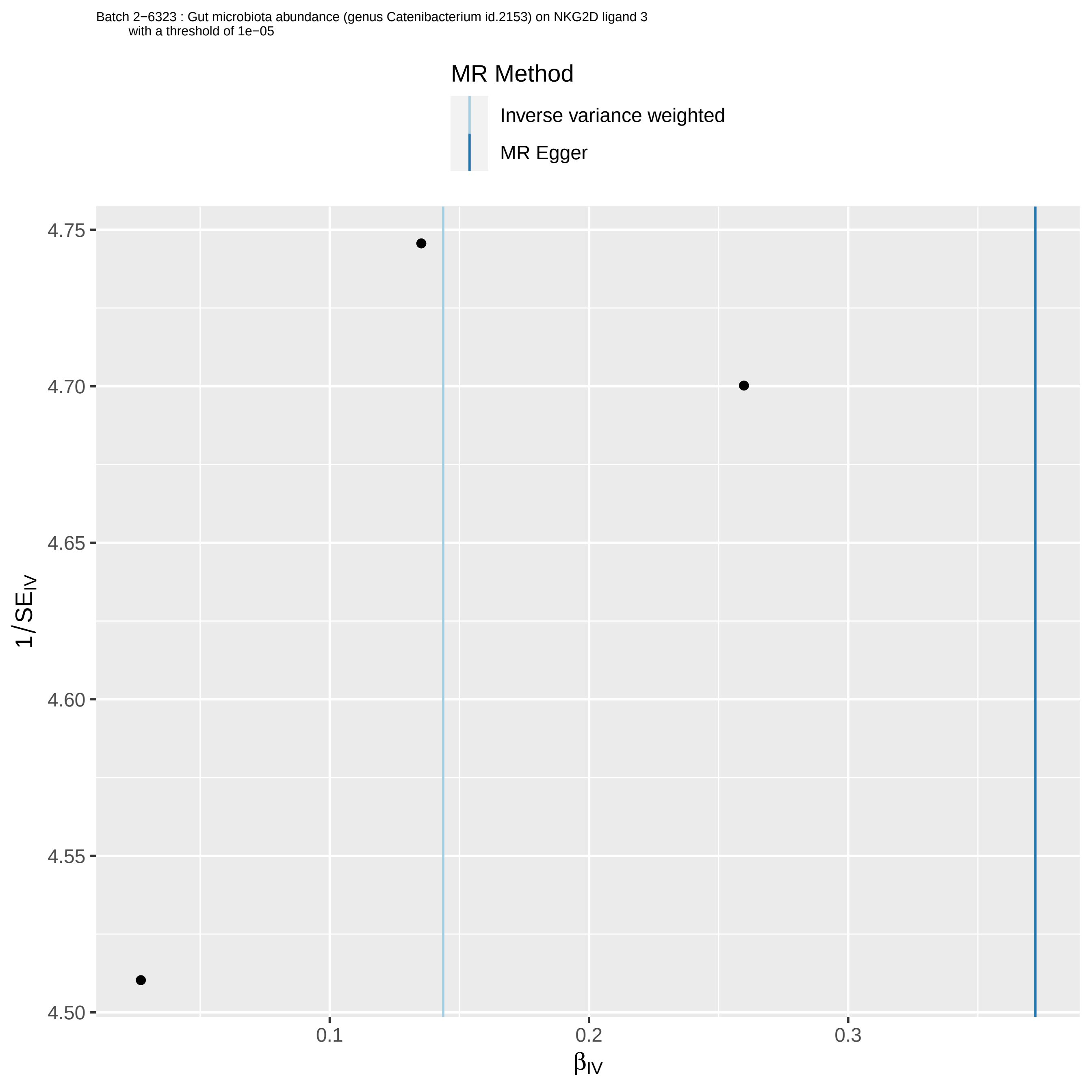

Batch 2−6323 : Gut microbiota abundance (genus Catenibacterium id.2153) on NKG2D ligand 3
with a threshold of 1e−05
MR Method
Inverse variance weighted
MR Egger
4.75
4.70
4.65
4.60
4.55
4.50
1/SE_IV
0.1
0.2
0.3
β_IV

Batch 2−6324 : Gut microbiota abundance (genus Parasutterella id.2892) on NKG2D ligand 3
with a threshold of 1e−05

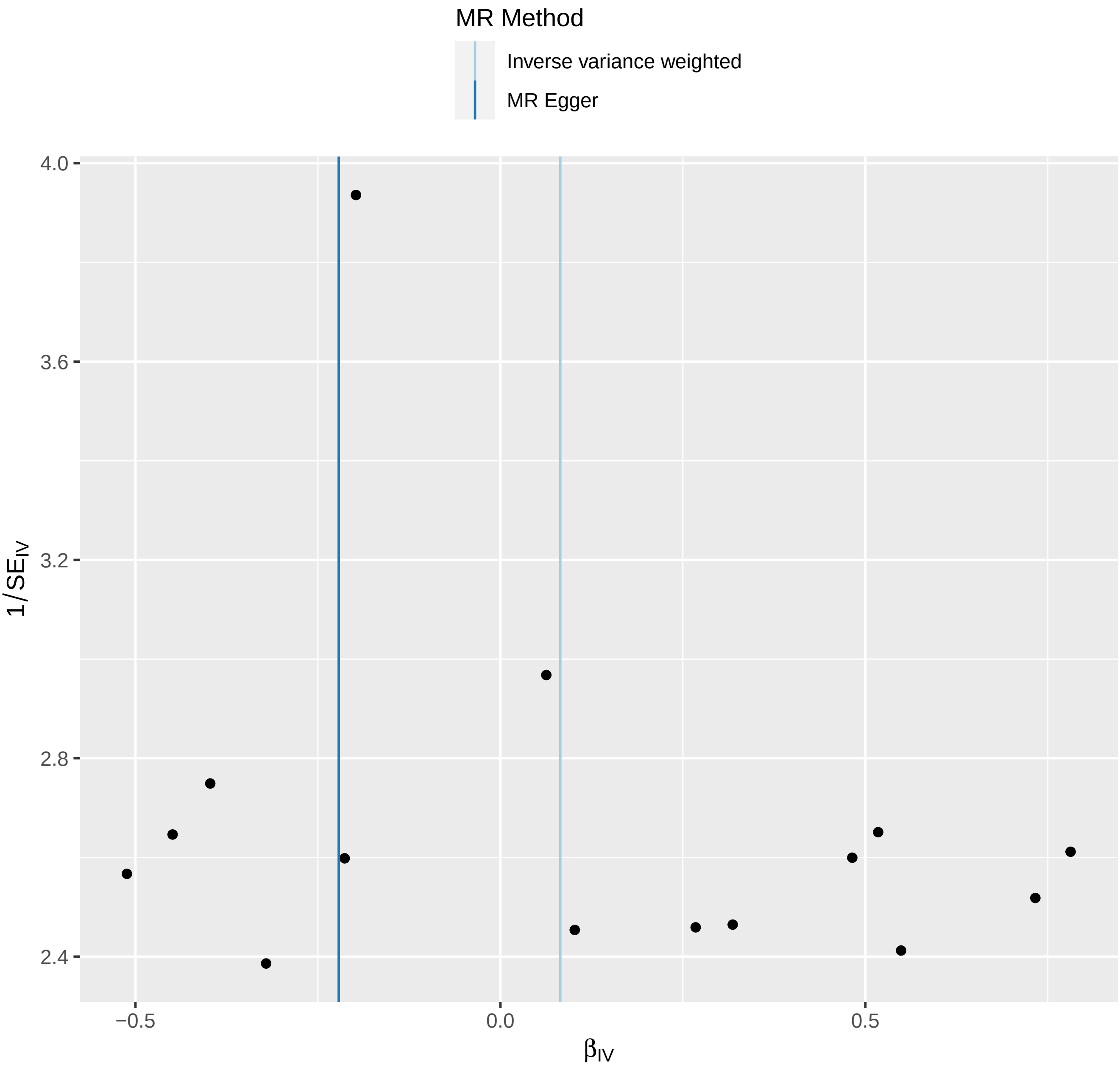

Batch 2−6325 : Gut microbiota abundance (genus Catenibacterium id.2153) on Serine/threonine−protein kinase ULK3 with a threshold of 1e−05

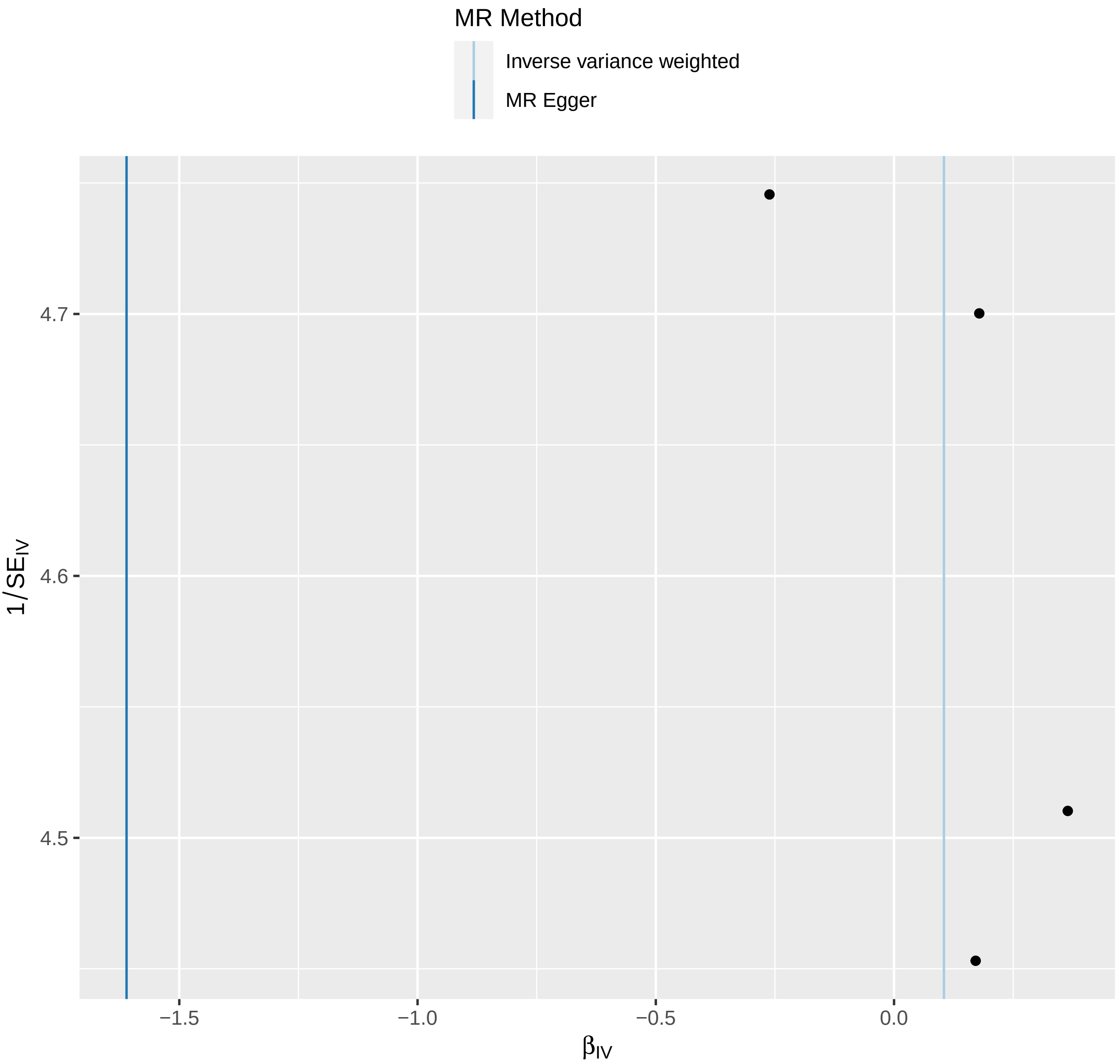

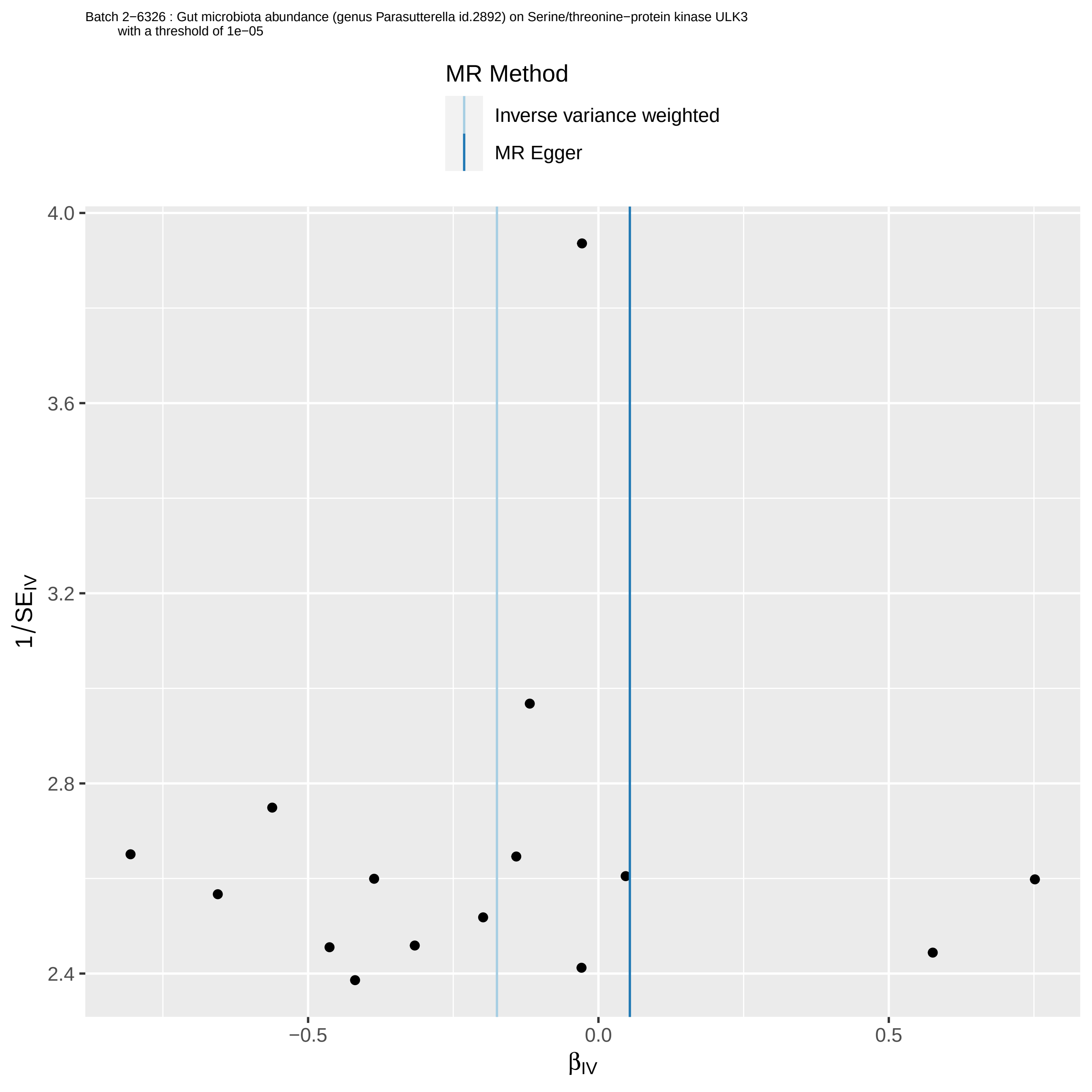

Batch 2−6326 : Gut microbiota abundance (genus Parasutterella id.2892) on Serine/threonine−protein kinase ULK3
with a threshold of 1e−05
MR Method
Inverse variance weighted
MR Egger
4.0
3.6
3.2
2.8
2.4
1/SE_IV
−0.5
0.0
0.5
β_IV

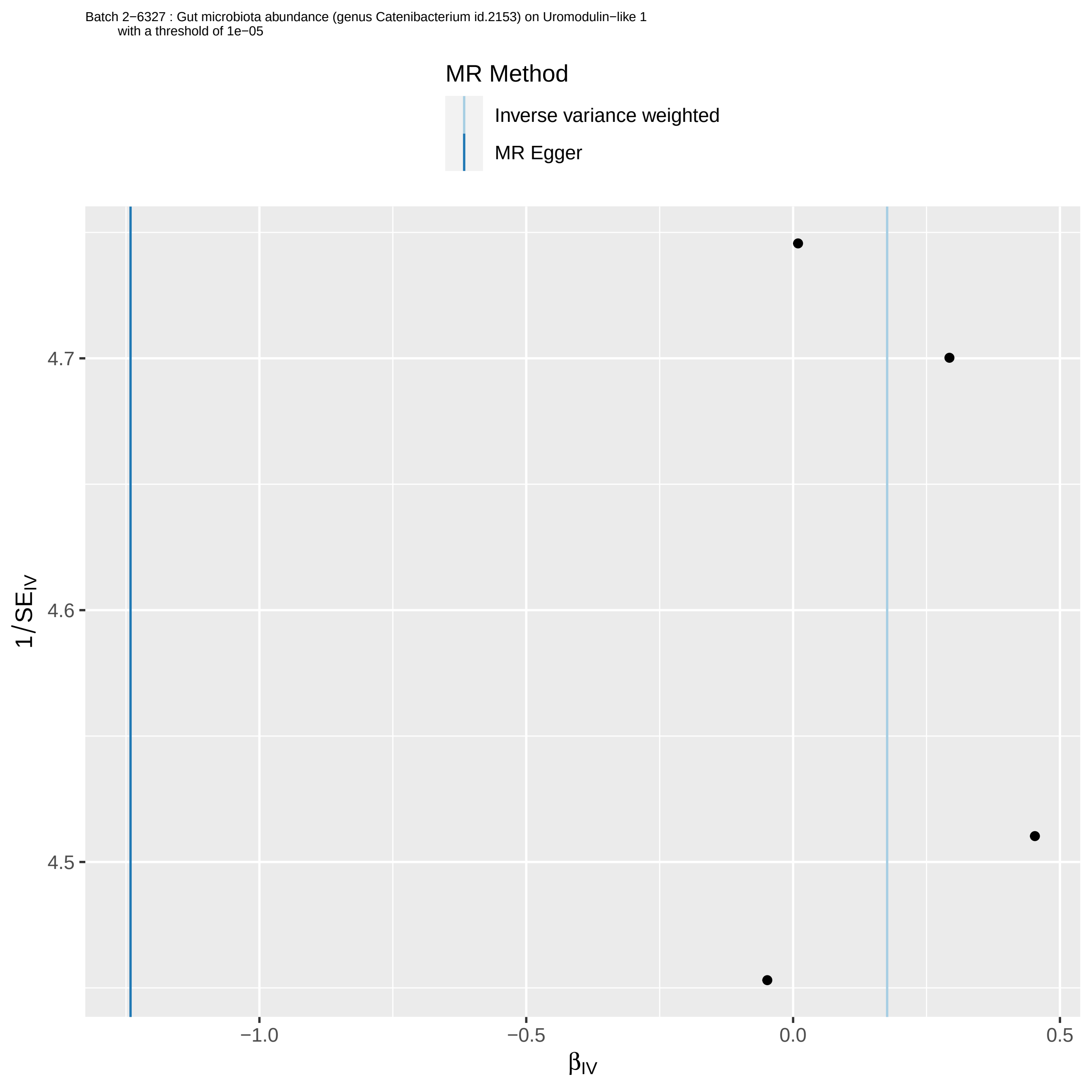

Batch 2−6327 : Gut microbiota abundance (genus Catenibacterium id.2153) on Uromodulin−like 1
with a threshold of 1e−05
MR Method
Inverse variance weighted
MR Egger
4.7
4.6
4.5
$1/SE_{IV}$
−1.0
−0.5
0.0
0.5
β_{IV}

Batch 2−6328 : Gut microbiota abundance (genus Parasutterella id.2892) on Uromodulin−like 1
with a threshold of 1e−05

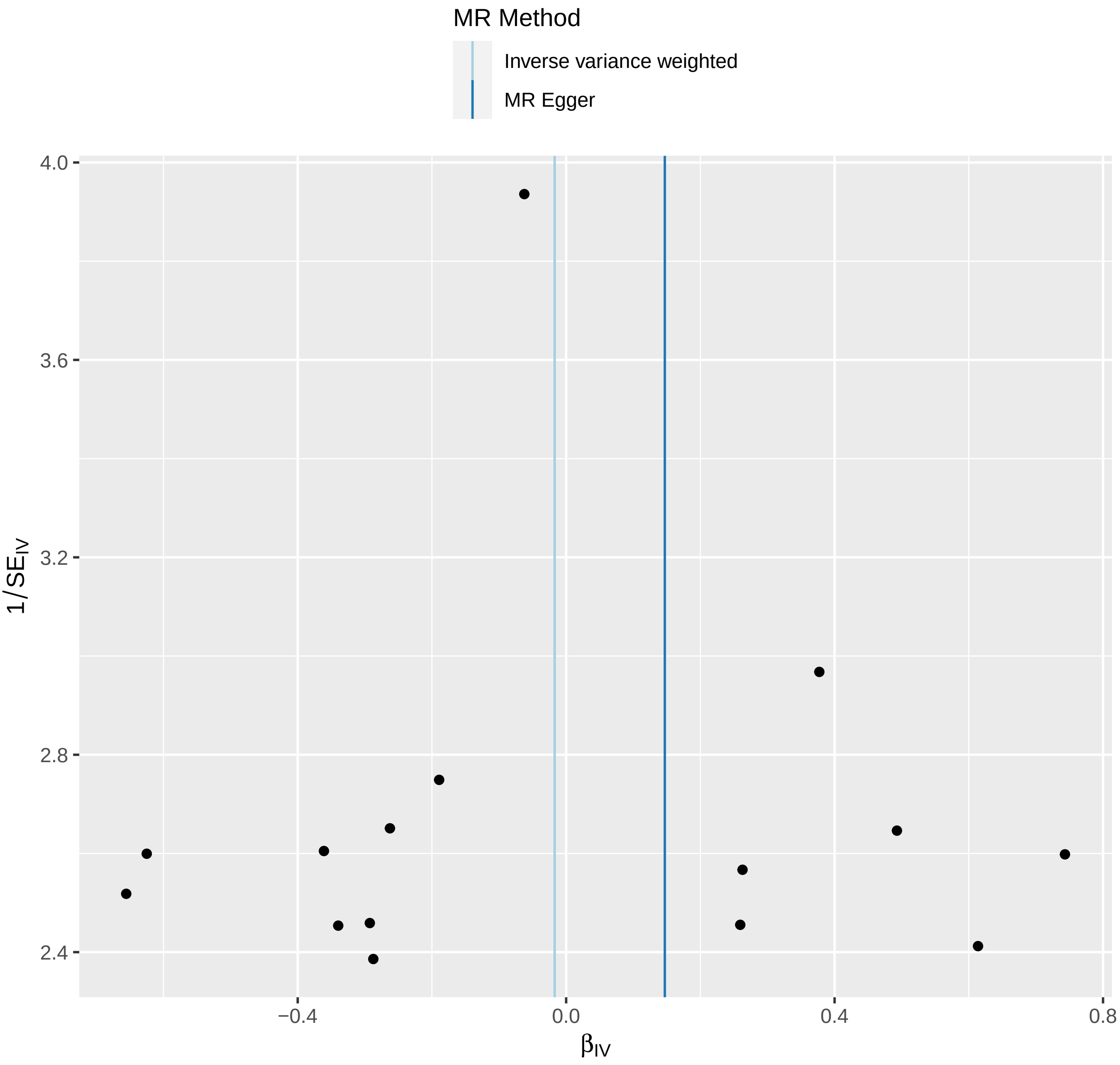

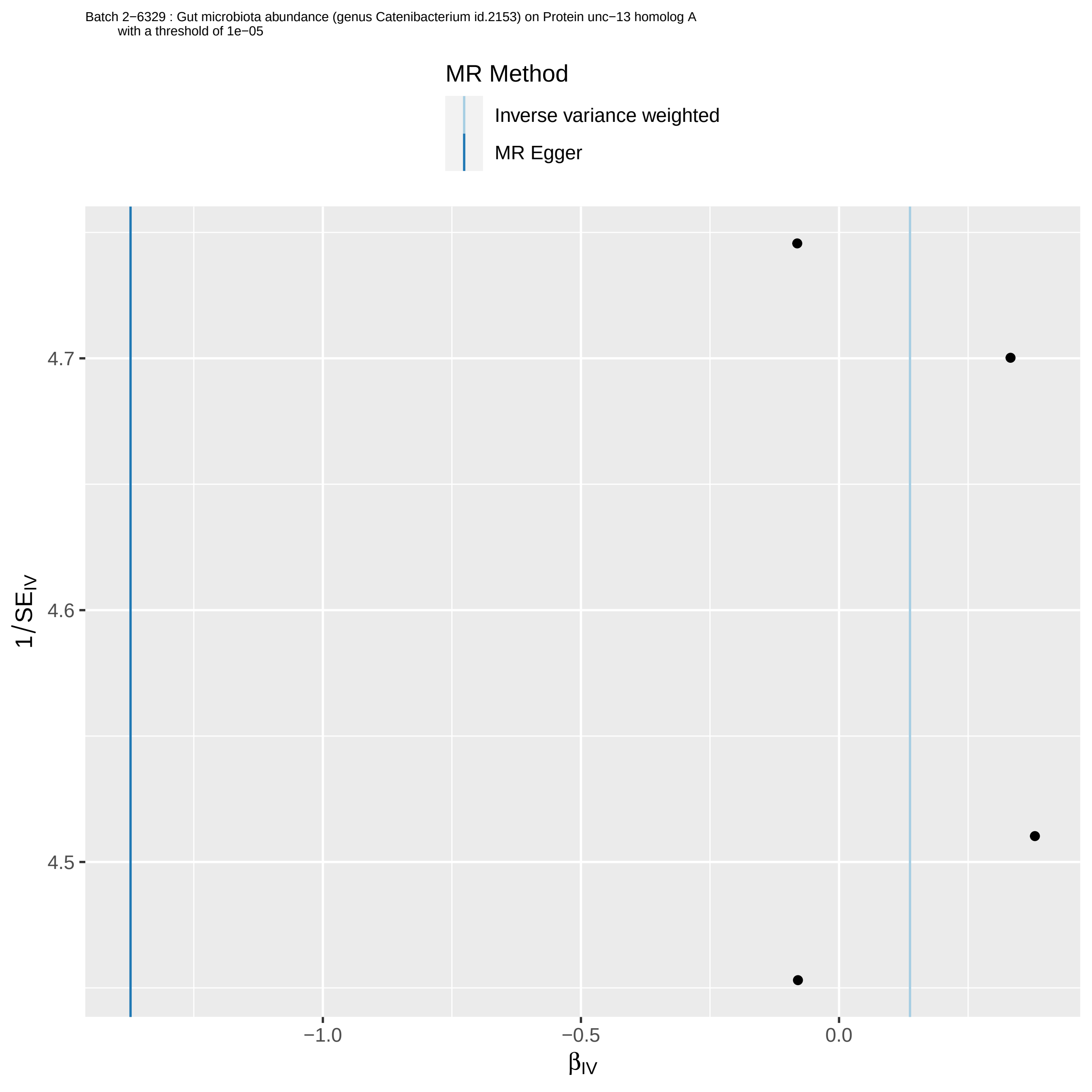
Batch 2−6329 : Gut microbiota abundance (genus Catenibacterium id.2153) on Protein unc−13 homolog A
with a threshold of 1e−05
MR Method
Inverse variance weighted
MR Egger
4.7
4.6
4.5
1/SE_IV
−1.0
−0.5
0.0
β_IV

Batch 2−6330 : Gut microbiota abundance (genus Parasutterella id.2892) on Protein unc−13 homolog A
with a threshold of 1e−05

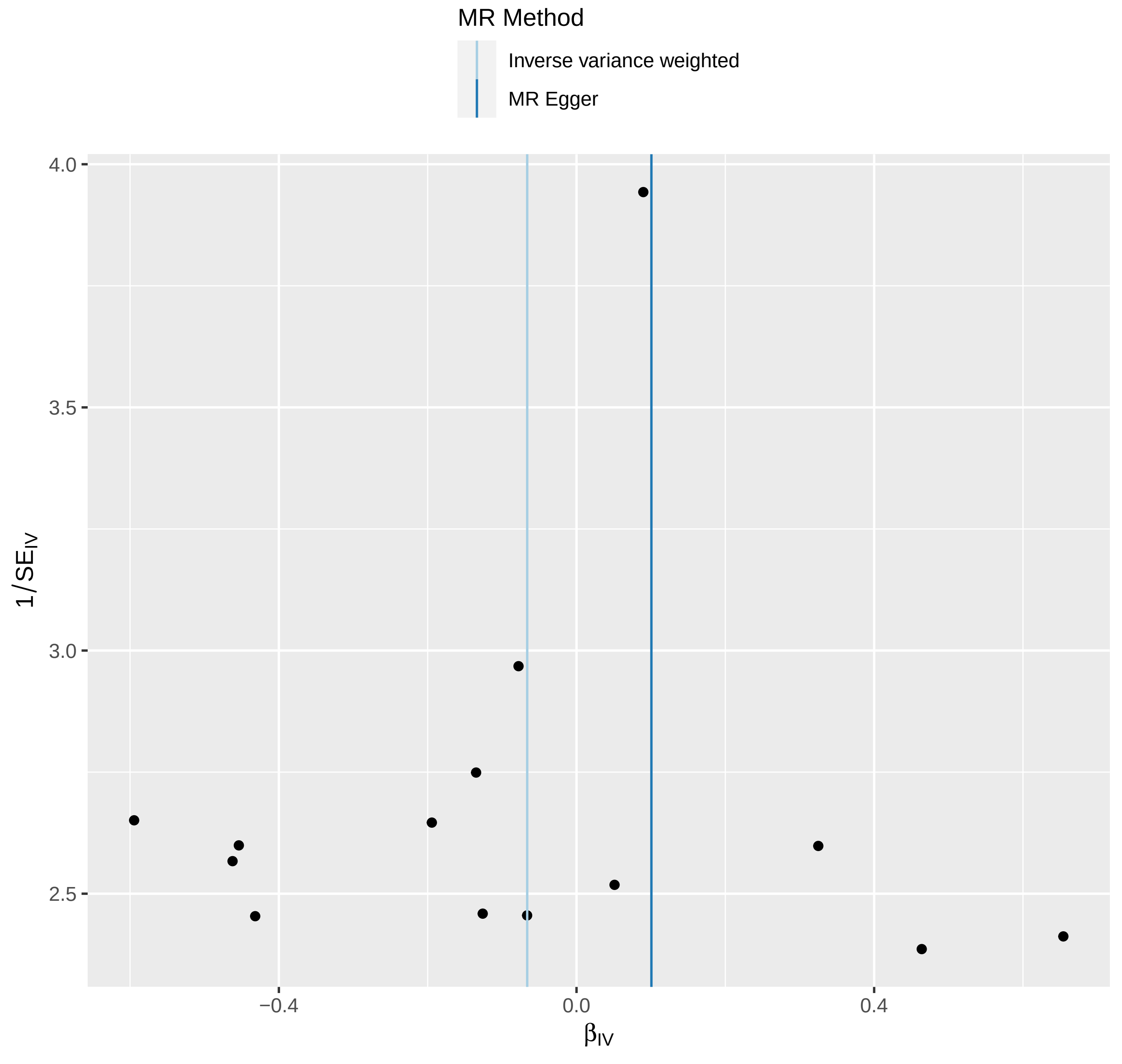

Batch 2−6331 : Gut microbiota abundance (genus Catenibacterium id.2153) on Netrin receptor UNC5A
with a threshold of 1e−05

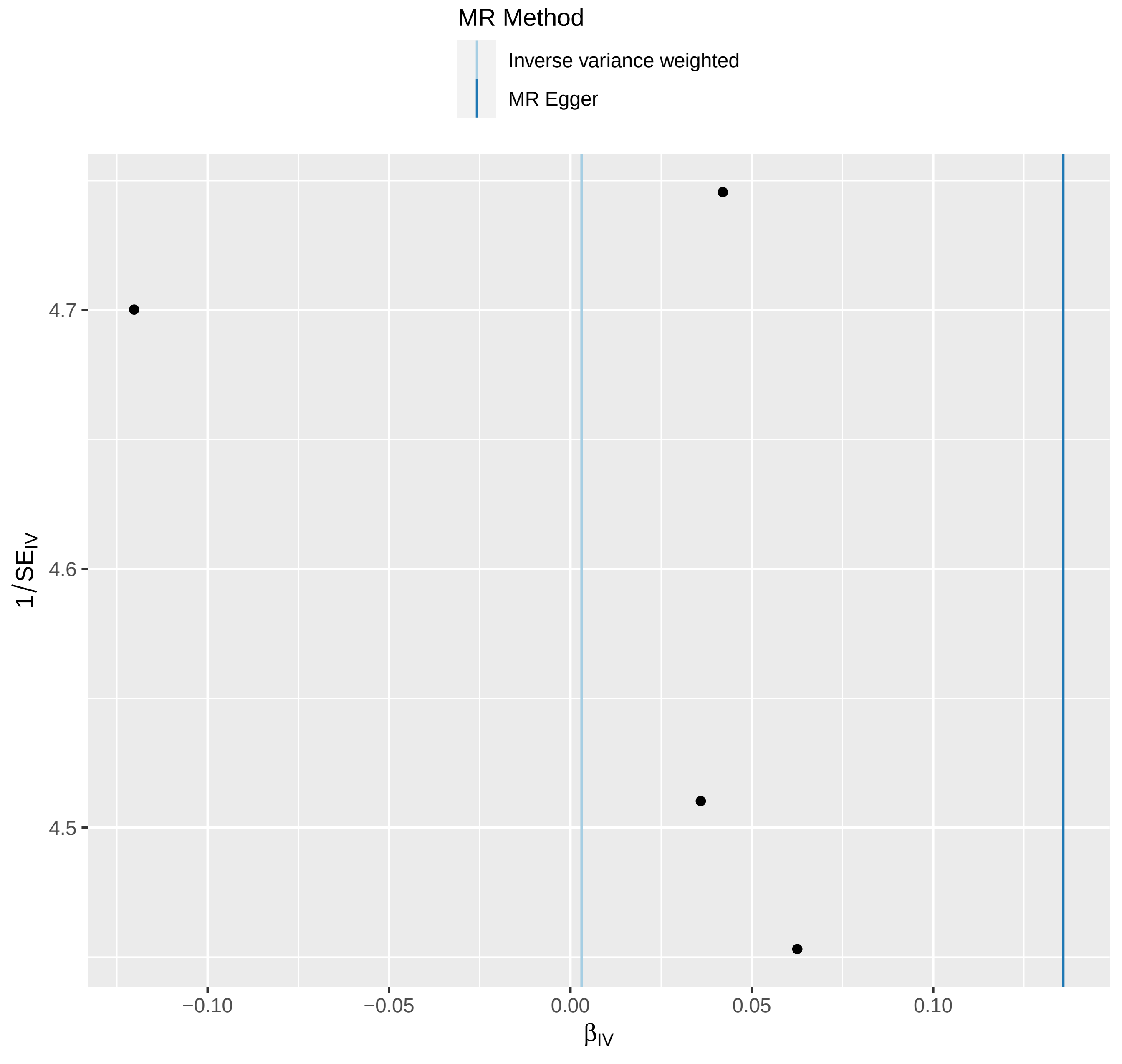

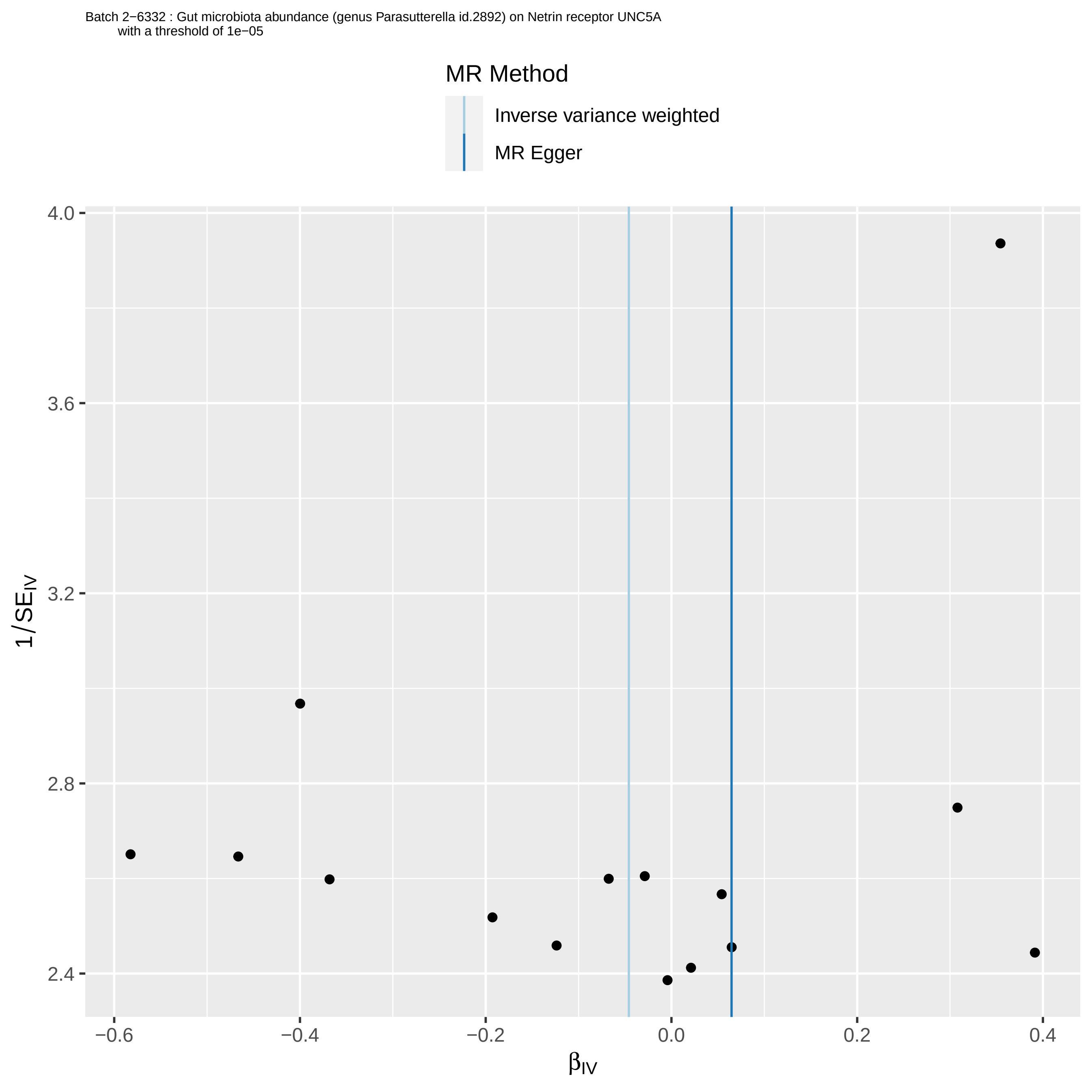
Batch 2−6332 : Gut microbiota abundance (genus Parasutterella id.2892) on Netrin receptor UNC5A
with a threshold of 1e−05
MR Method
Inverse variance weighted
MR Egger
4.0
3.6
3.2
2.8
2.4
1/SE$_{IV}$
−0.6
−0.4
−0.2
0.0
0.2
0.4
β_{IV}

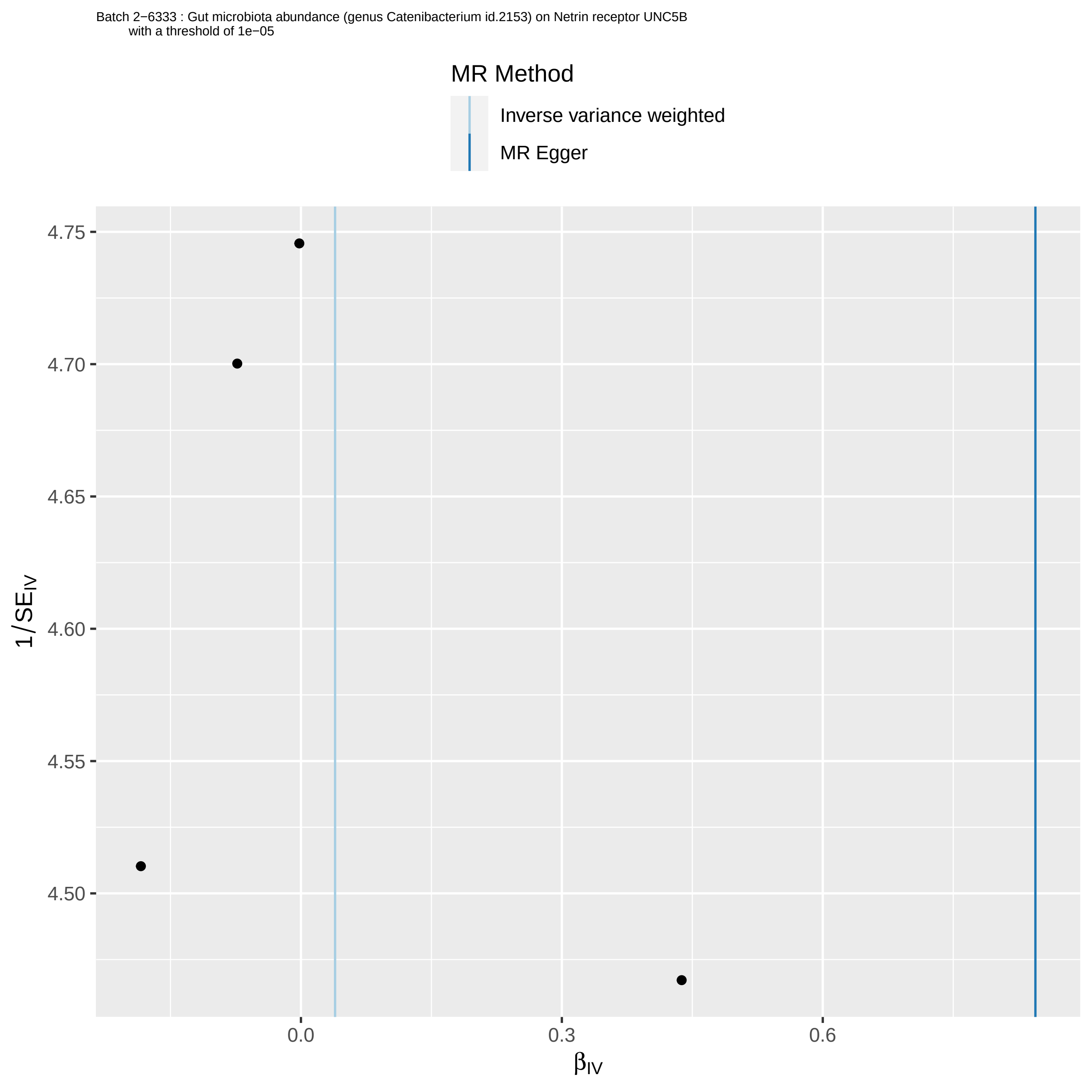
Batch 2−6333 : Gut microbiota abundance (genus Catenibacterium id.2153) on Netrin receptor UNC5B
with a threshold of 1e−05
MR Method
Inverse variance weighted
MR Egger
4.75
4.70
4.65
4.60
4.55
4.50
1/SE_IV
0.0
0.3
0.6
β_IV

Batch 2−6334 : Gut microbiota abundance (genus Parasutterella id.2892) on Netrin receptor UNC5B
with a threshold of 1e−05

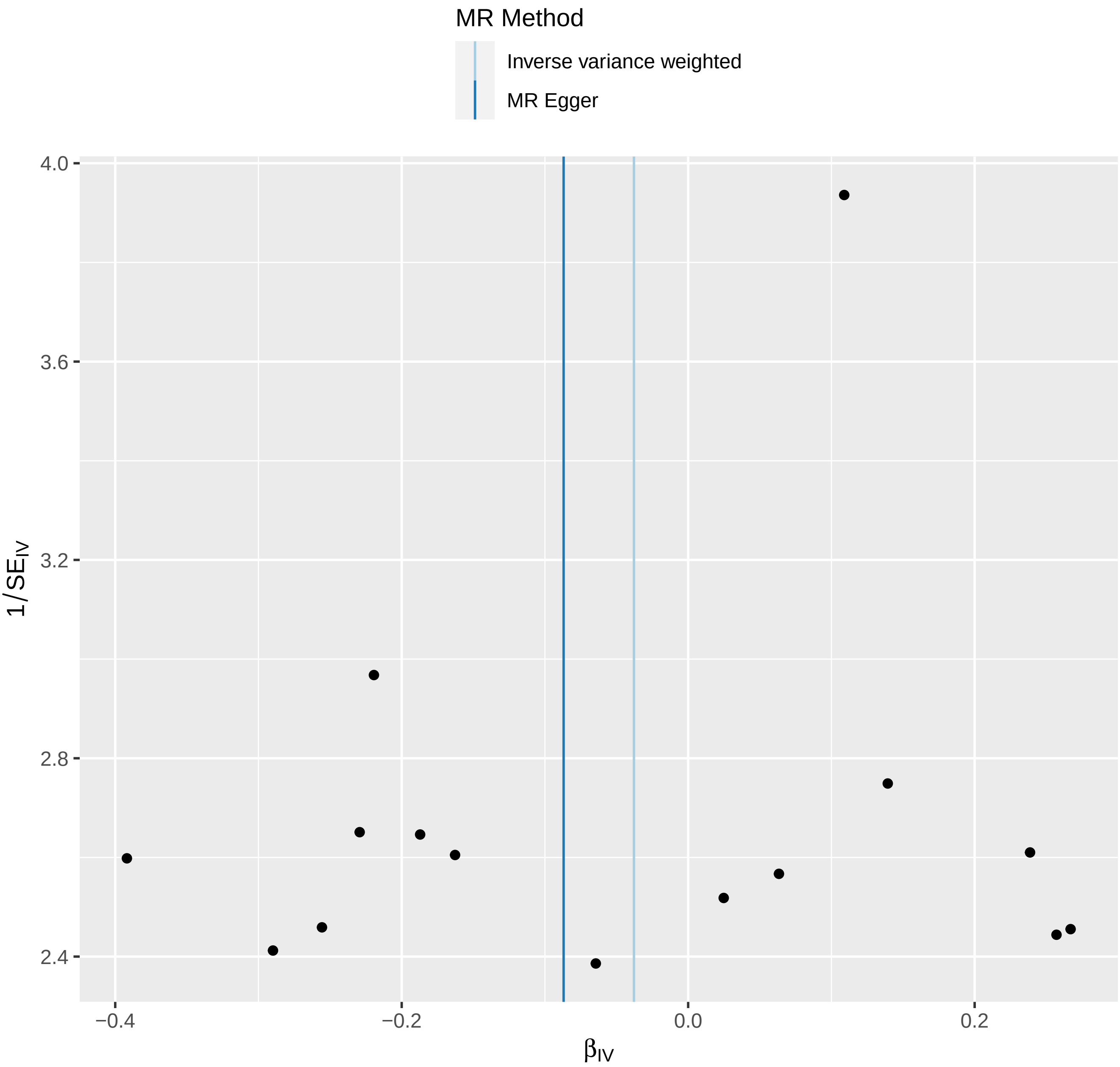

Batch 2−6335 : Gut microbiota abundance (genus Catenibacterium id.2153) on Netrin receptor UNC5C
with a threshold of 1e−05

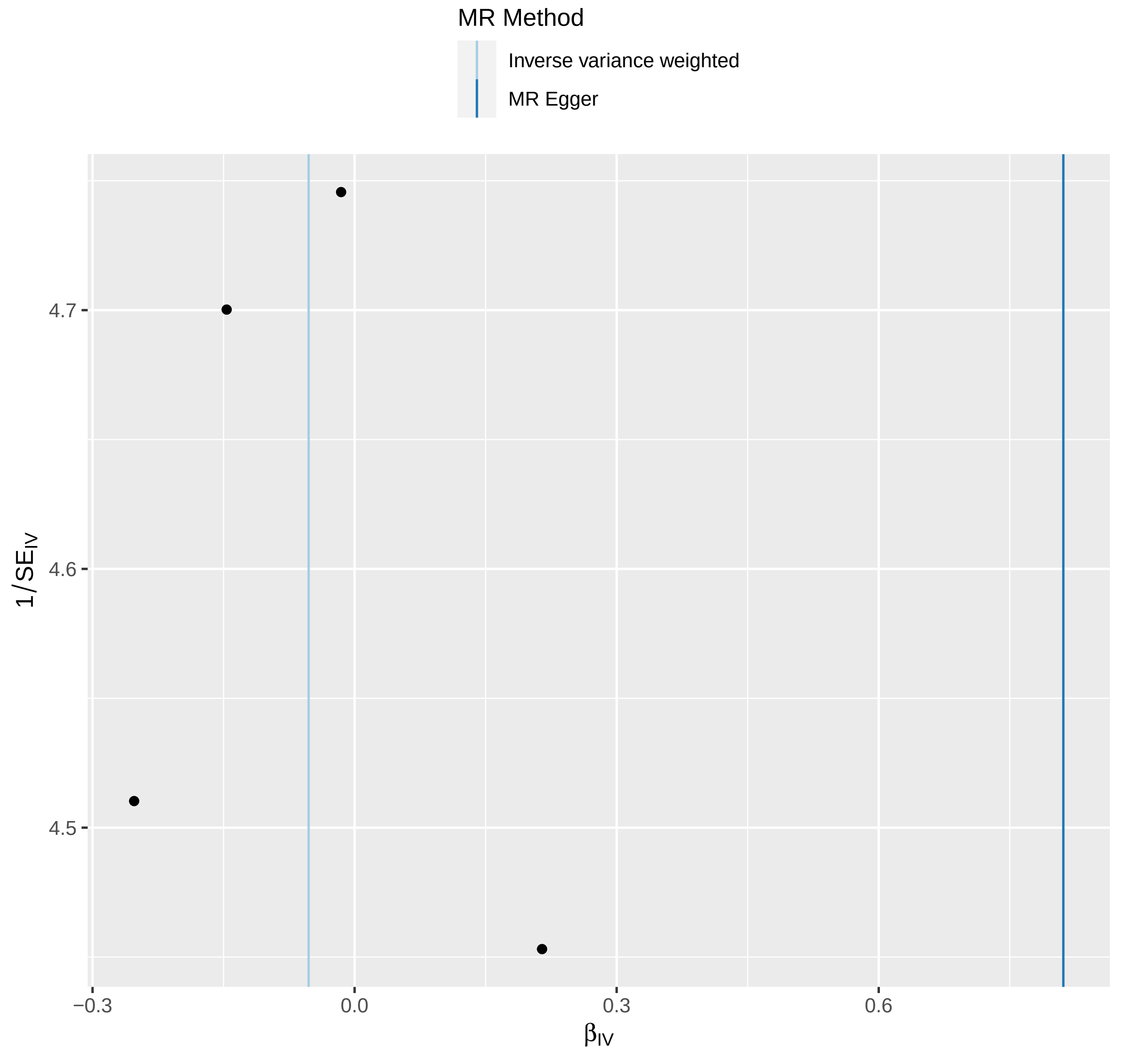

Batch 2−6336 : Gut microbiota abundance (genus Parasutterella id.2892) on Netrin receptor UNC5C
with a threshold of 1e−05

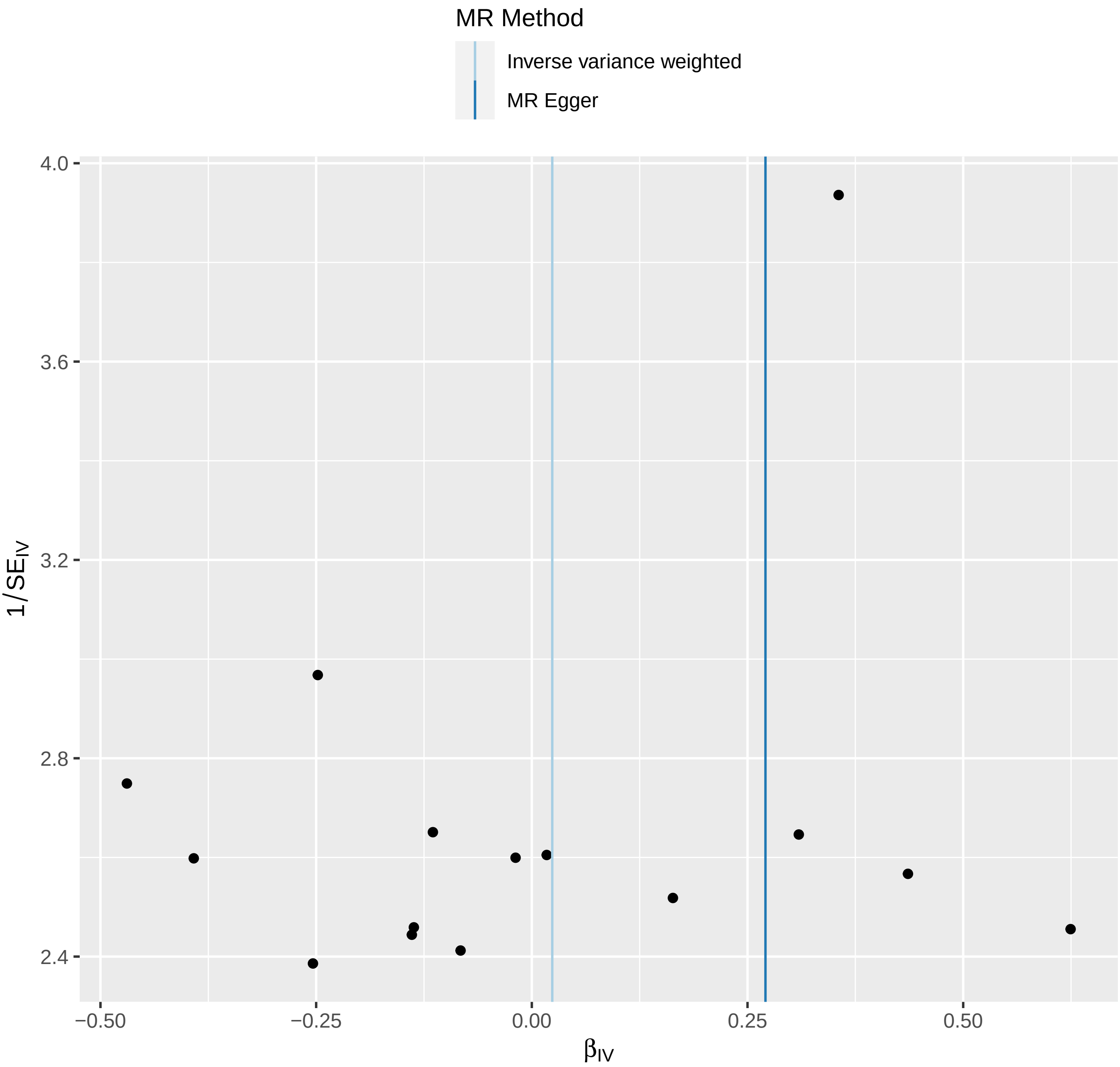

Batch 2−6337 : Gut microbiota abundance (genus Catenibacterium id.2153) on Netrin receptor UNC5D
with a threshold of 1e−05

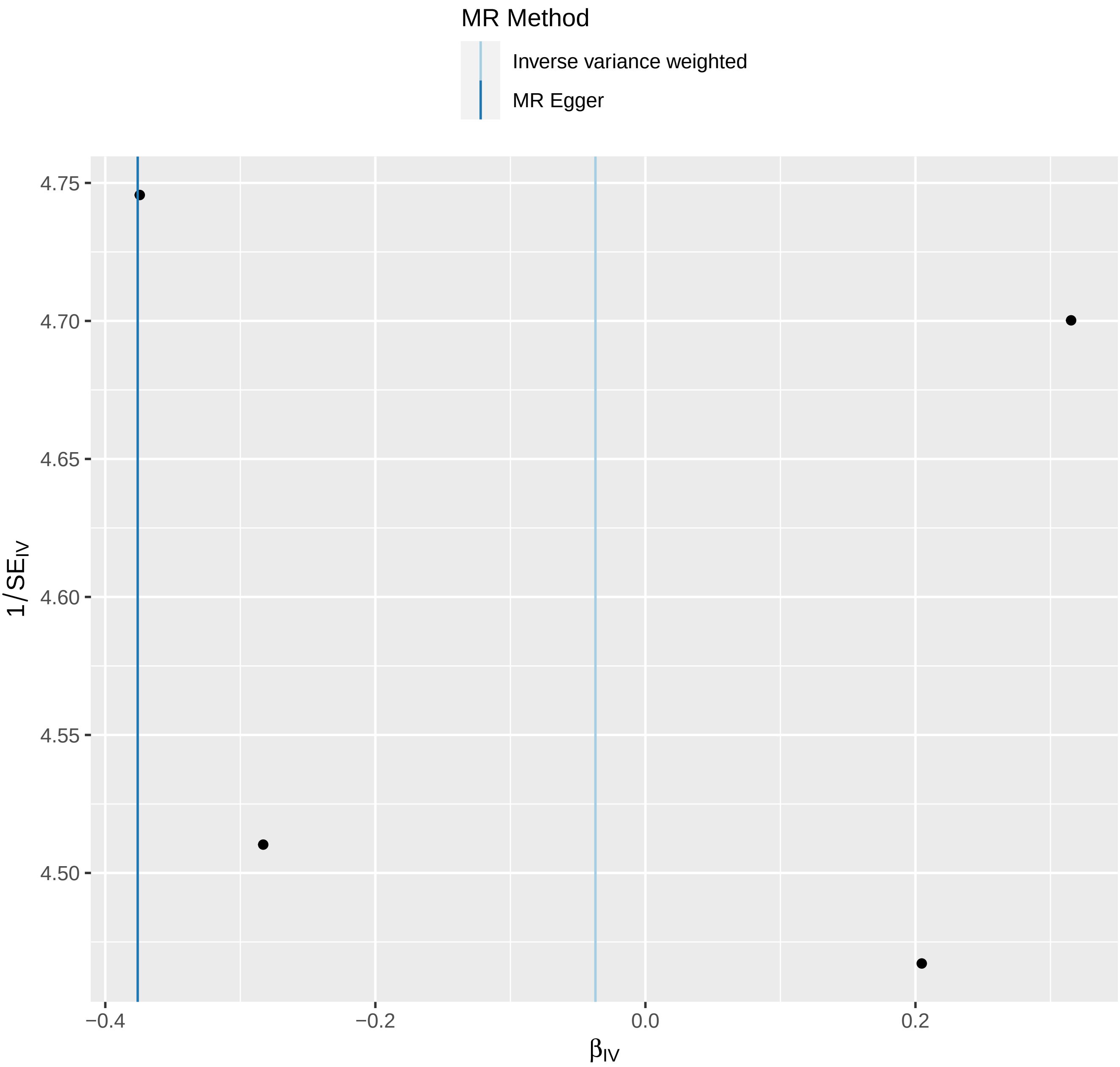

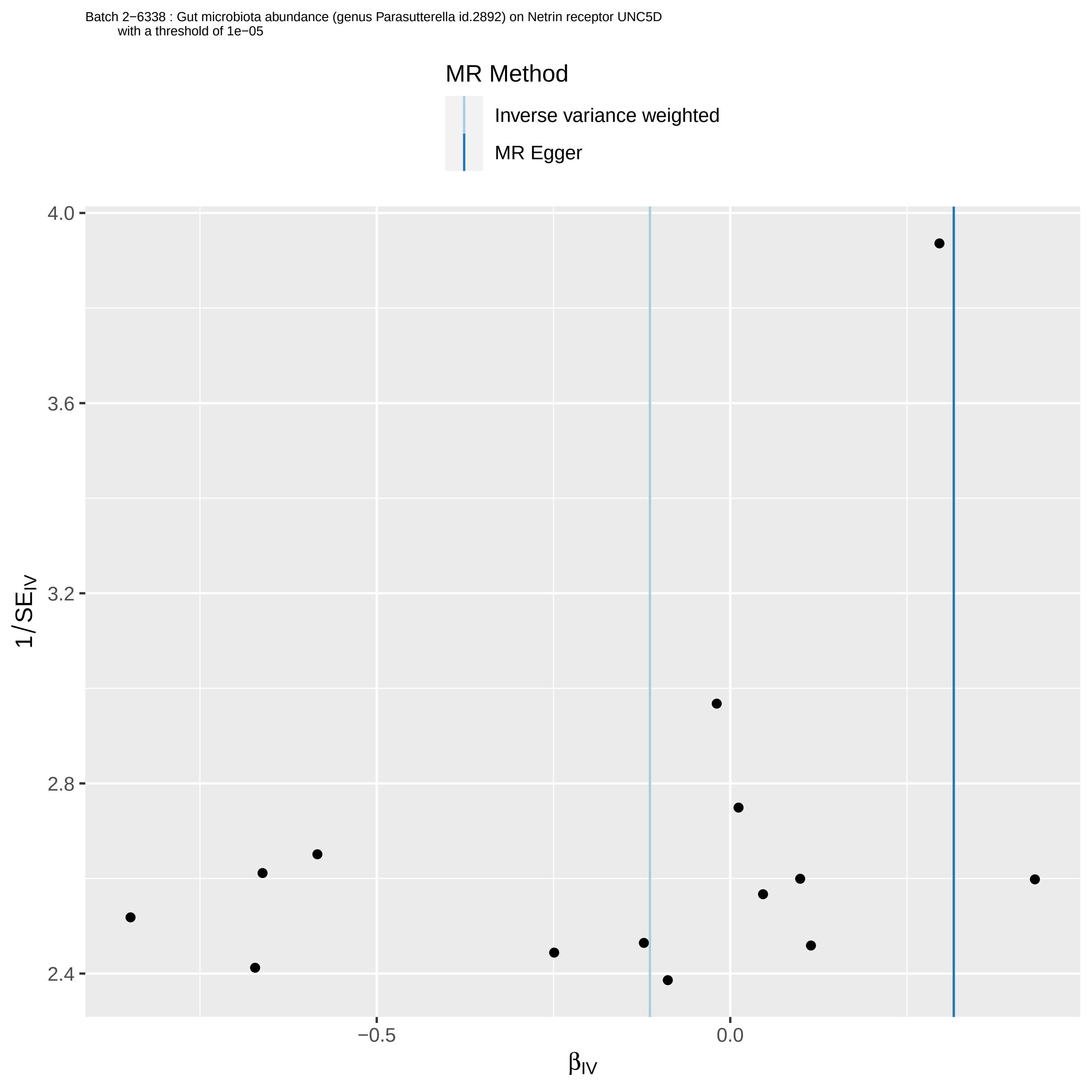
Batch 2−6338 : Gut microbiota abundance (genus Parasutterella id.2892) on Netrin receptor UNC5D
with a threshold of 1e−05
MR Method
Inverse variance weighted
MR Egger
4.0
3.6
3.2
2.8
2.4
1/SE_IV
−0.5
0.0
β_IV

Batch 2−6339 : Gut microbiota abundance (genus Catenibacterium id.2153) on Netrin receptor UNC5D
with a threshold of 1e−05

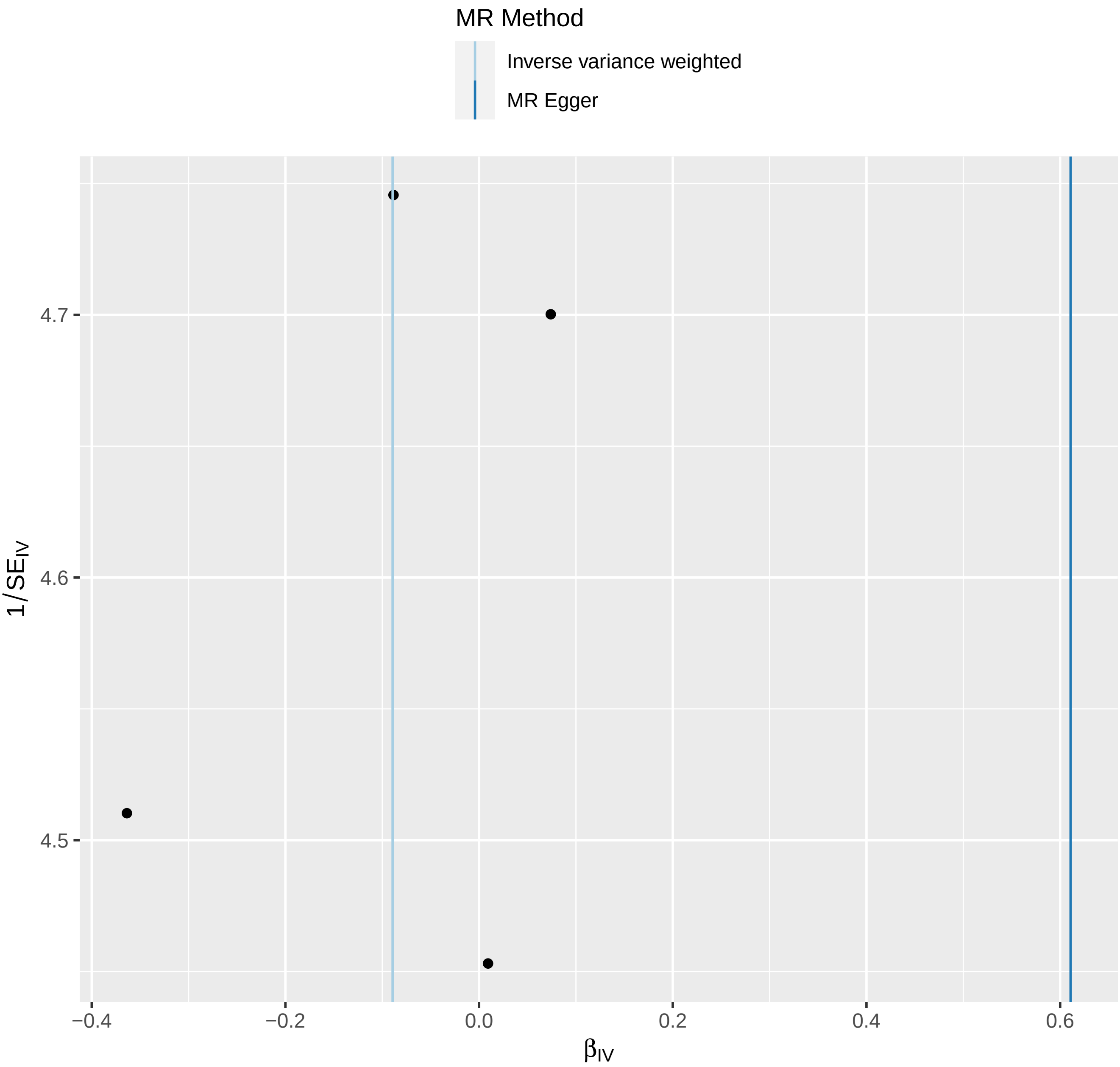

Batch 2−6340 : Gut microbiota abundance (genus Parasutterella id.2892) on Netrin receptor UNC5D
with a threshold of 1e−05

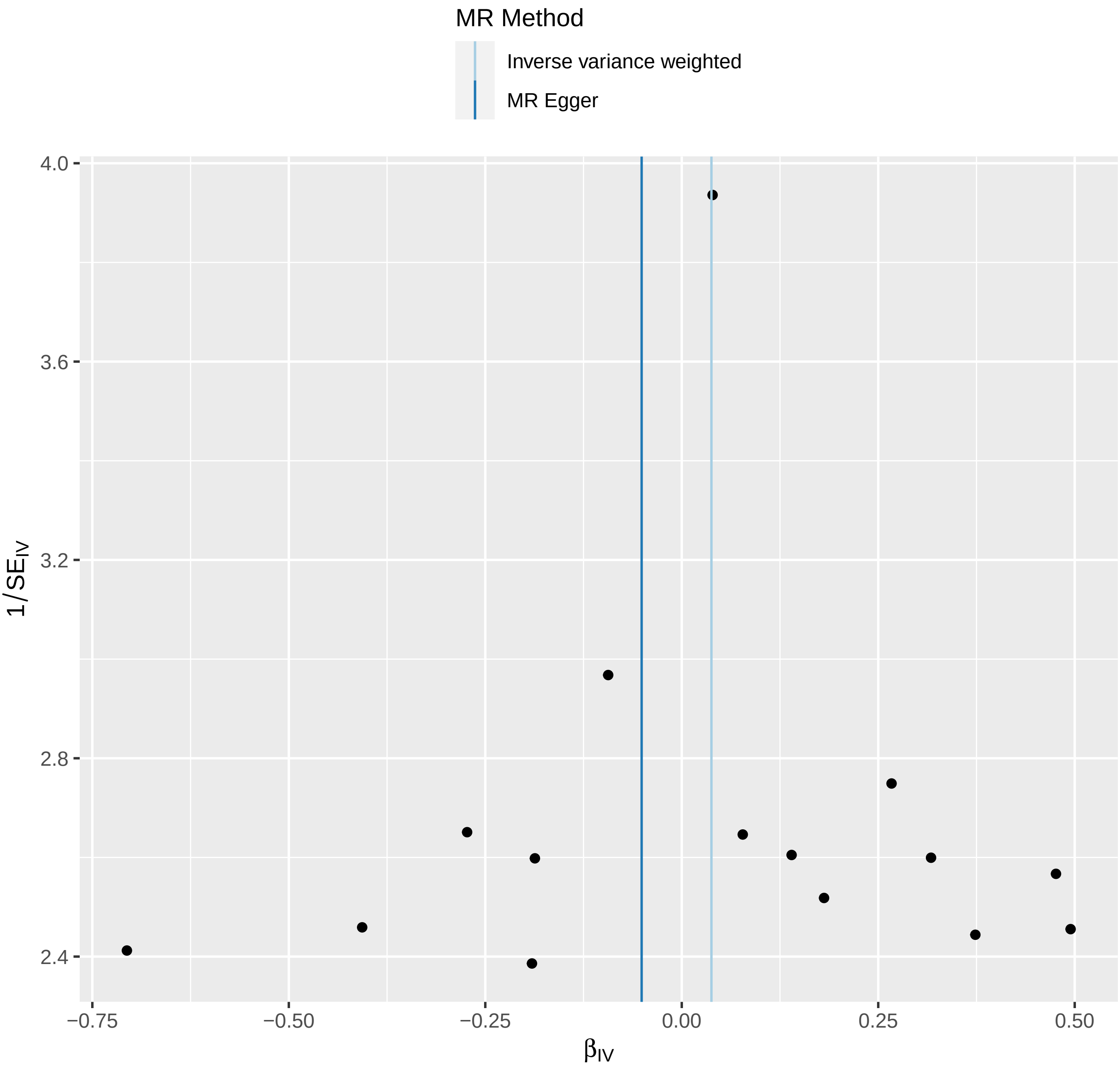

Batch 2−6341 : Gut microbiota abundance (genus Catenibacterium id.2153) on Protein unc−93 homolog B1
with a threshold of 1e−05

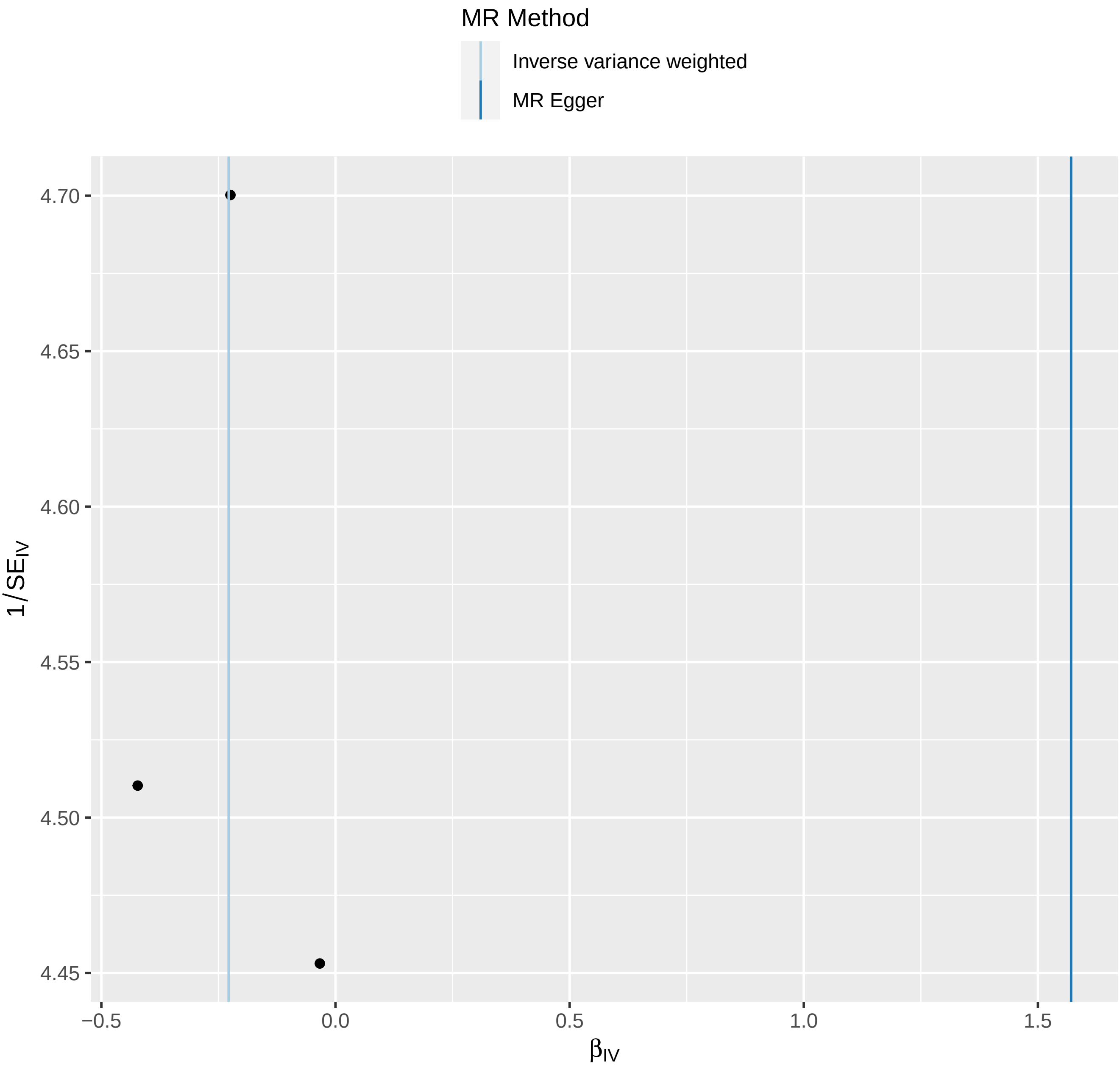

Batch 2−6342 : Gut microbiota abundance (genus Parasutterella id.2892) on Protein unc−93 homolog B1
with a threshold of 1e−05

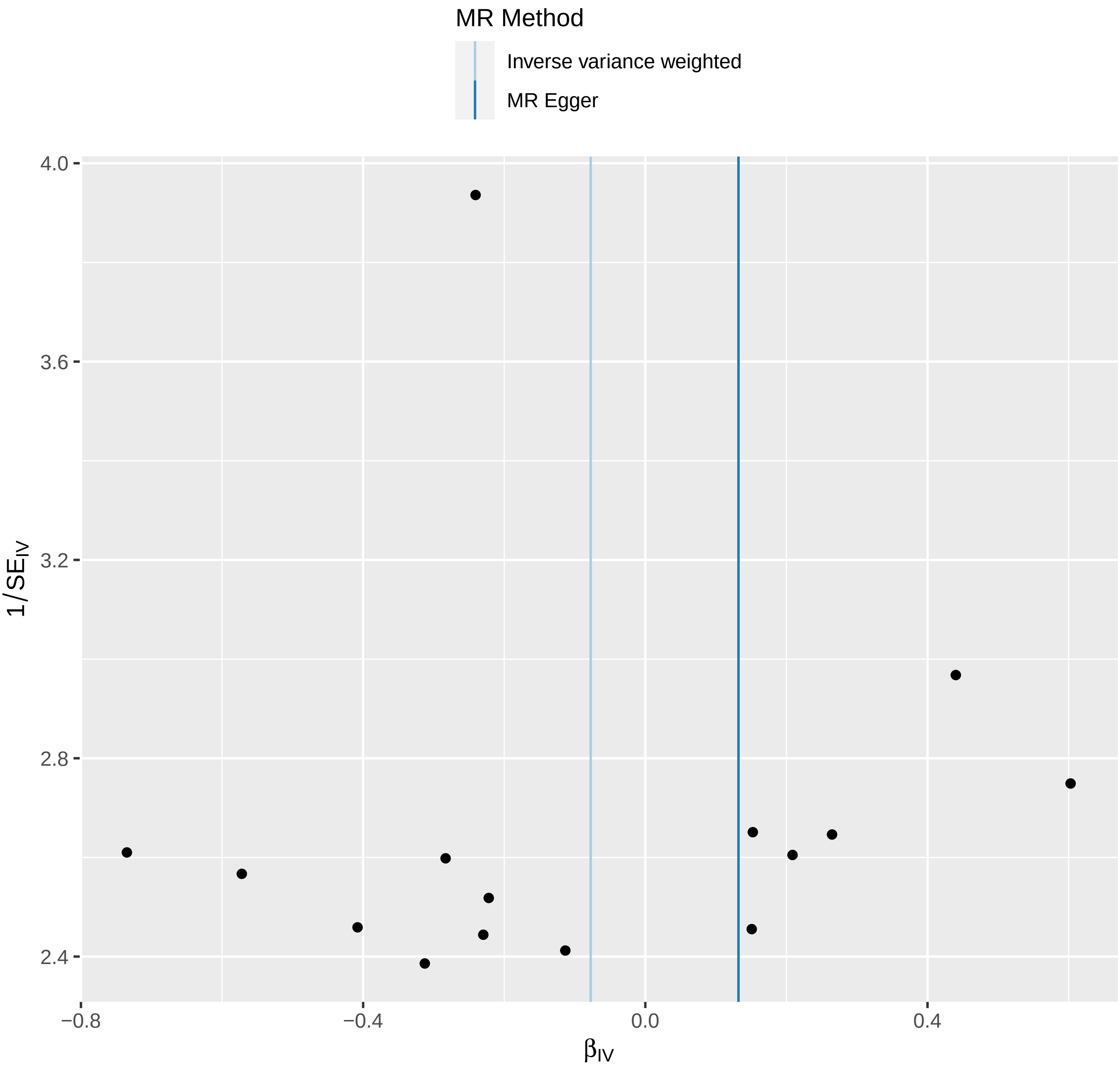

Batch 2−6343 : Gut microbiota abundance (genus Catenibacterium id.2153) on Uroplakin−3b
with a threshold of 1e−05

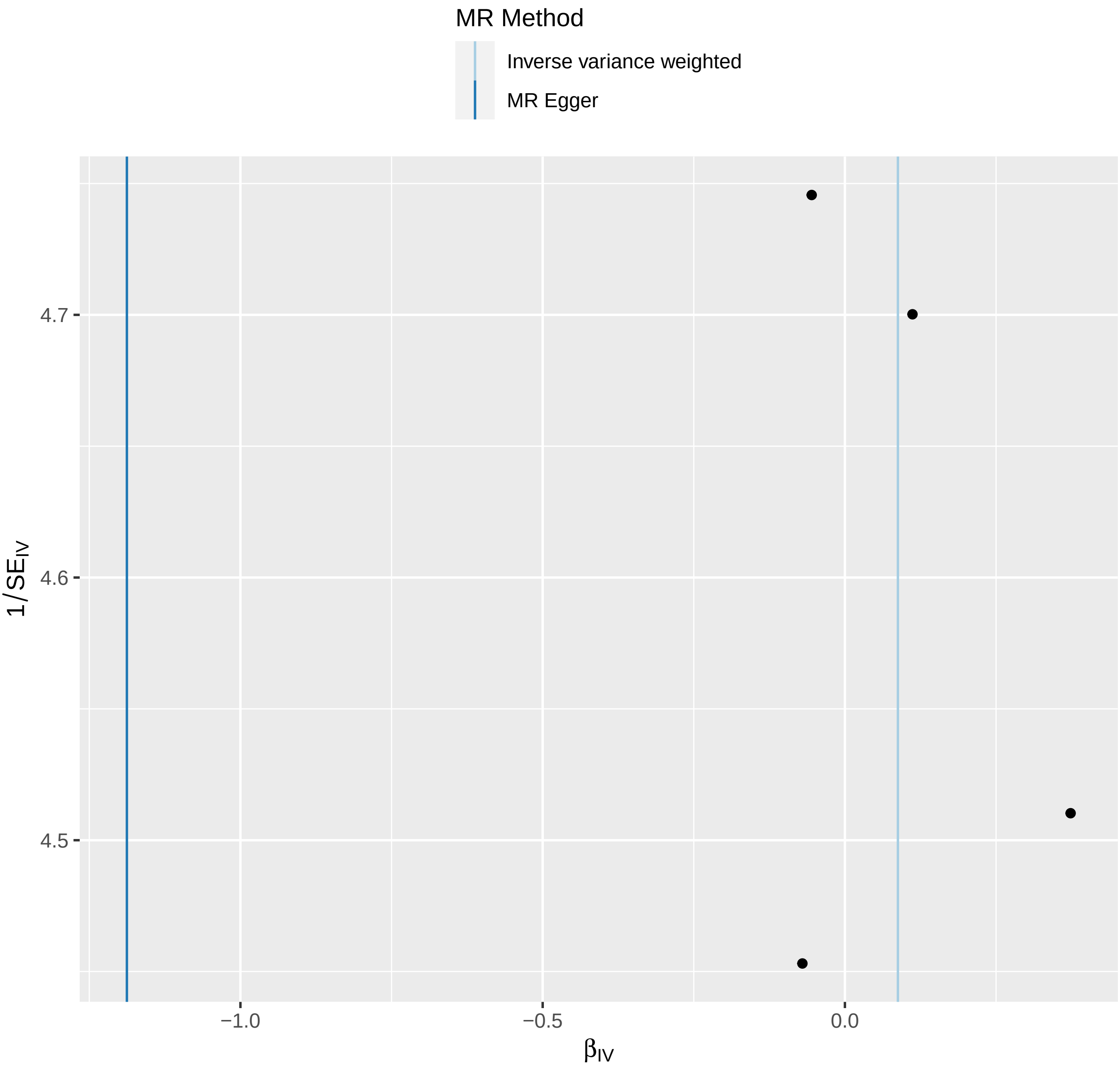

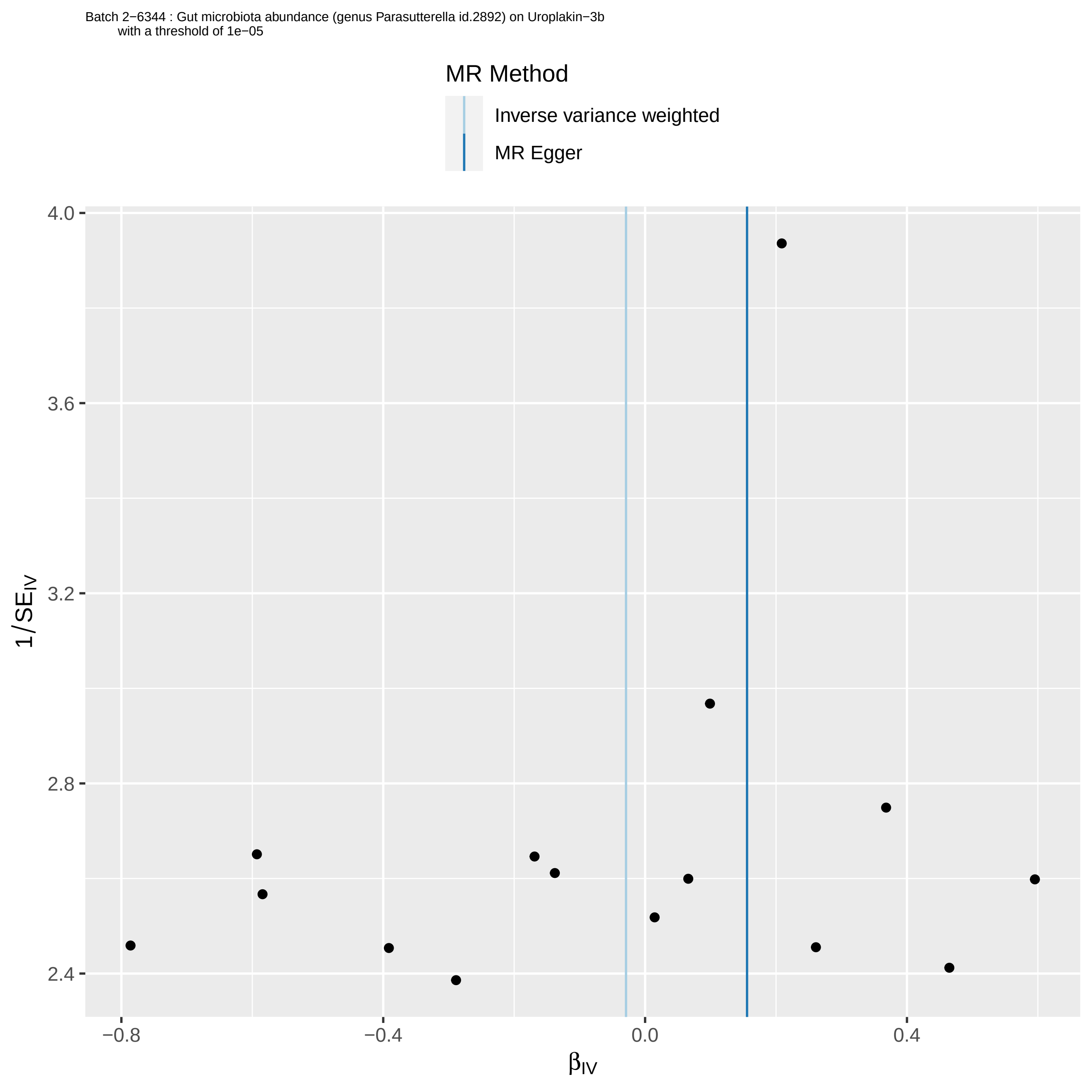

Batch 2−6344 : Gut microbiota abundance (genus Parasutterella id.2892) on Uroplakin−3b
with a threshold of 1e−05
MR Method
Inverse variance weighted
MR Egger
4.0
3.6
3.2
2.8
2.4
1/SE_IV
−0.8
−0.4
0.0
0.4
β_IV

Batch 2−6345 : Gut microbiota abundance (genus Catenibacterium id.2153) on Uroplakin−3b−like protein
with a threshold of 1e−05

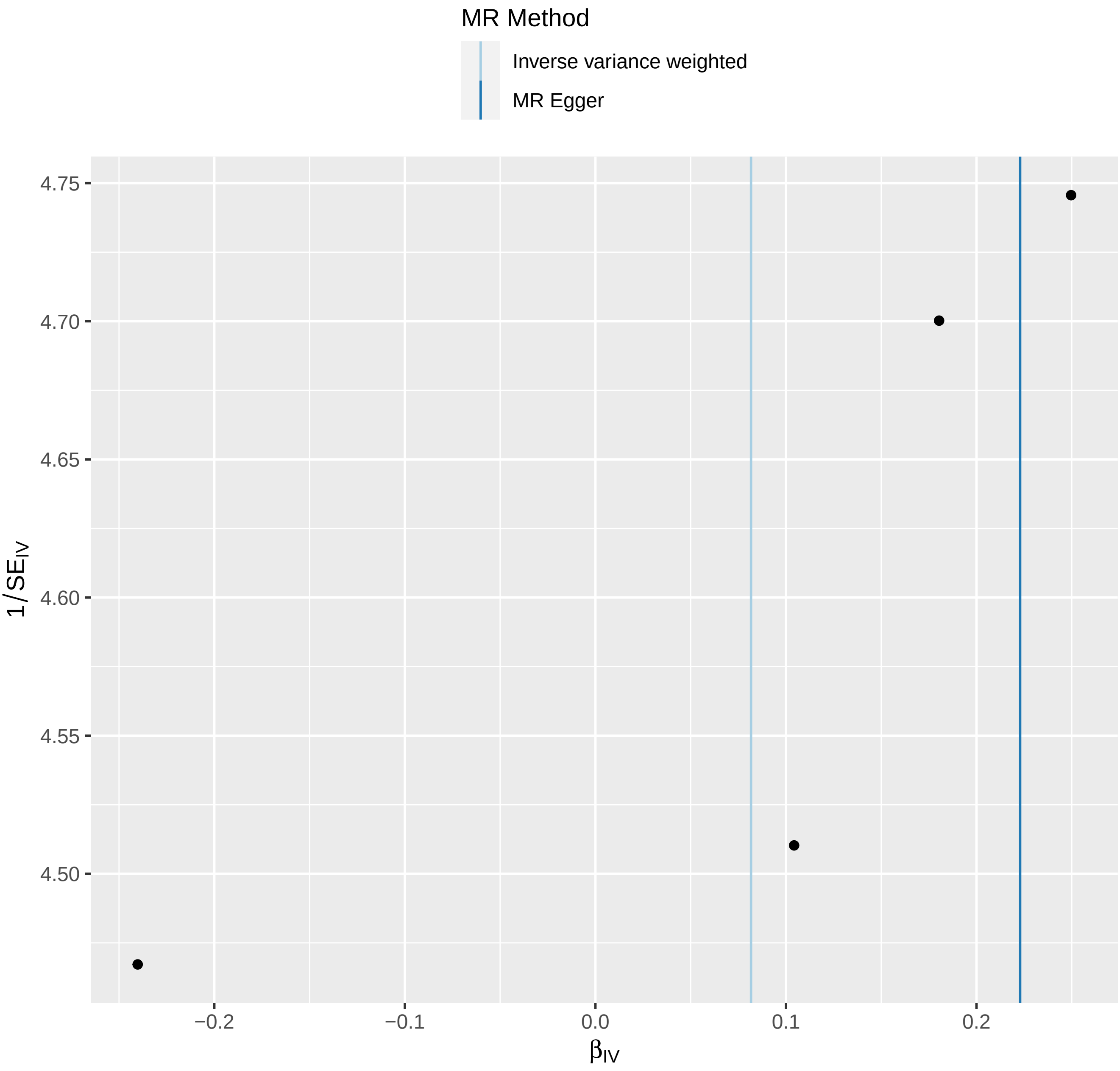

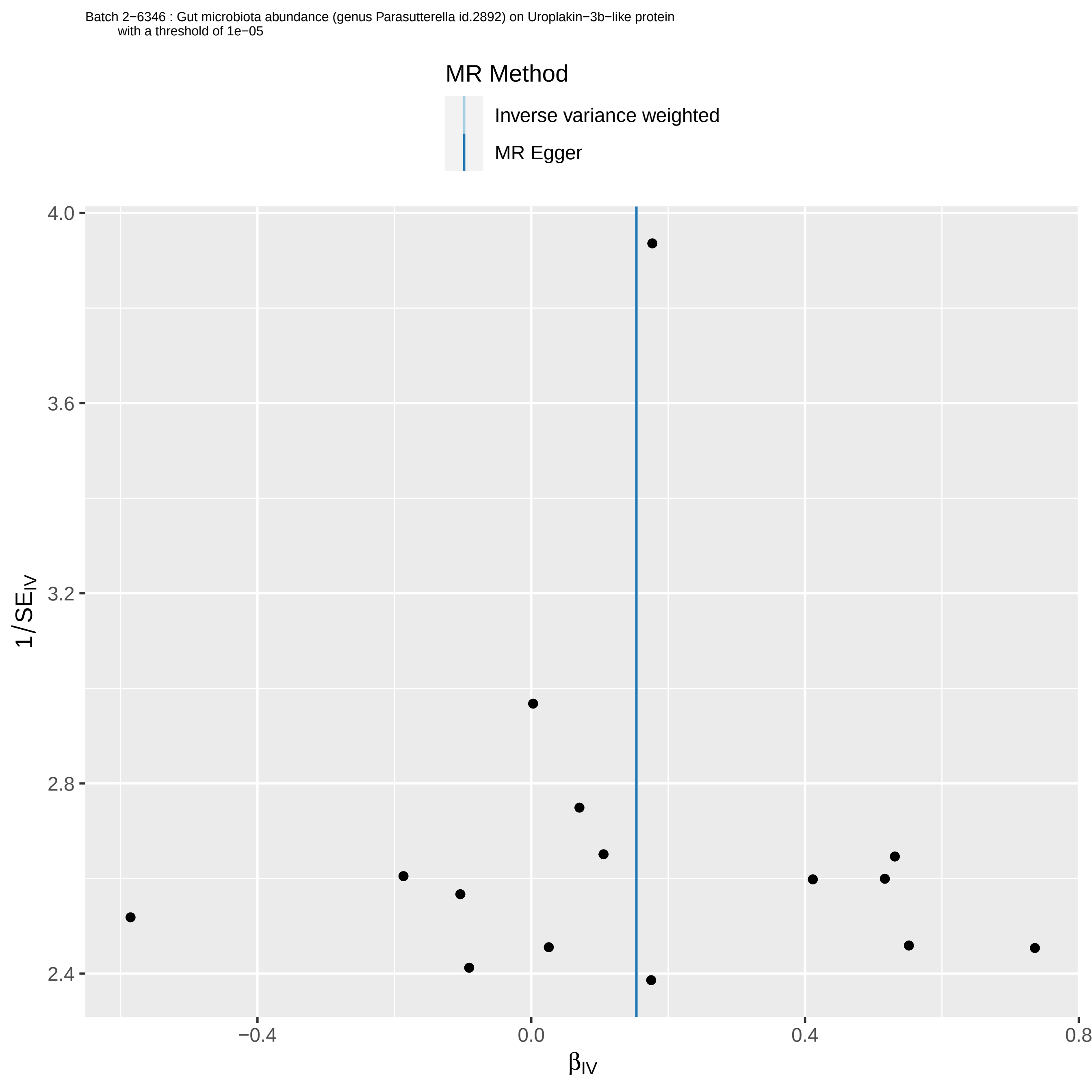
Batch 2−6346 : Gut microbiota abundance (genus Parasutterella id.2892) on Uroplakin−3b−like protein
with a threshold of 1e−05
MR Method
Inverse variance weighted
MR Egger
4.0
3.6
3.2
2.8
2.4
1/SE$_{IV}$
−0.4
0.0
0.4
0.8
β_{IV}

Batch 2−6347 : Gut microbiota abundance (genus Catenibacterium id.2153) on Ubiquinol−cytochrome−c reductase complex assembly factor 3 with a threshold of 1e−05

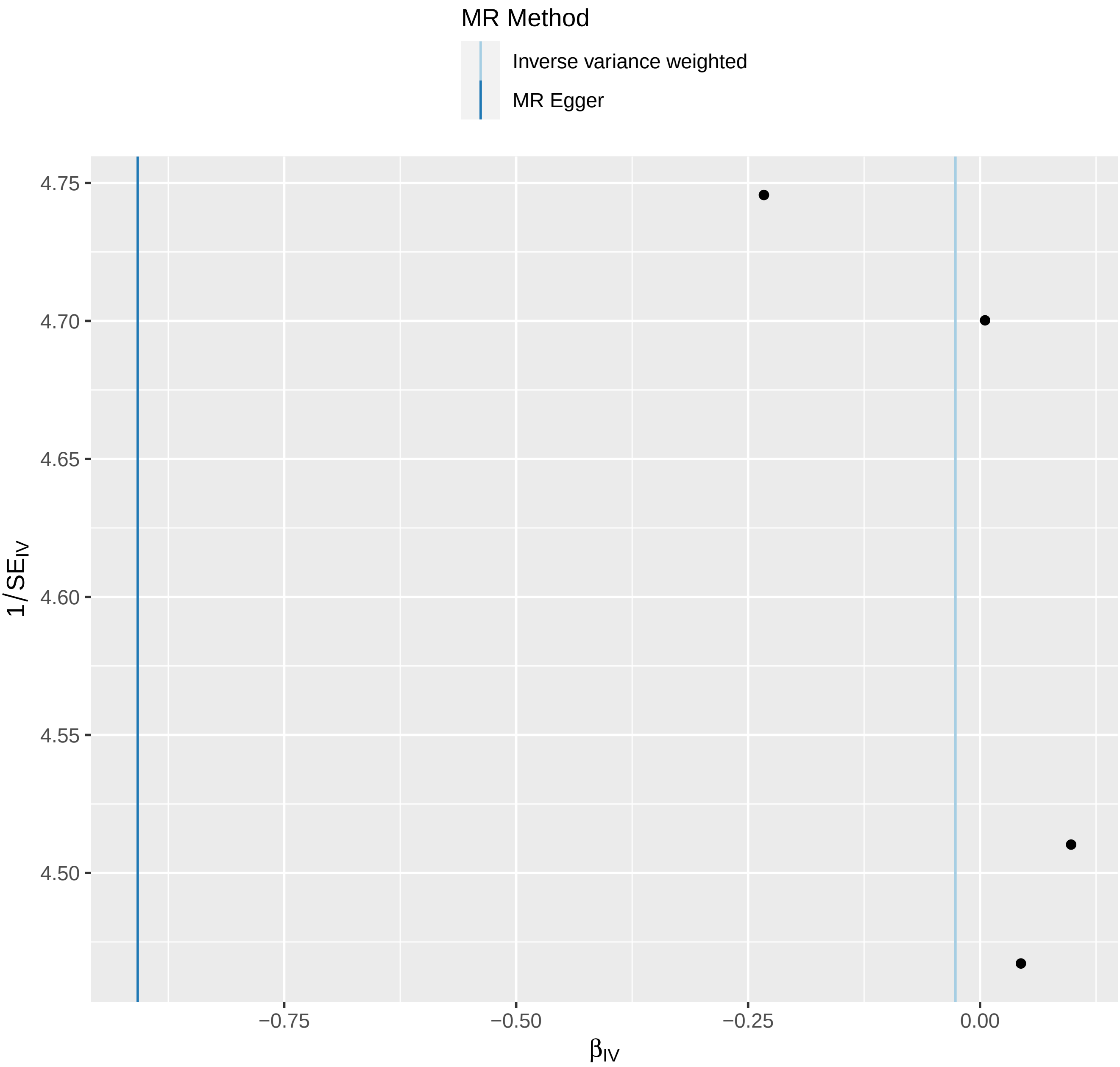

Batch 2−6348 : Gut microbiota abundance (genus Parasutterella id.2892) on Ubiquinol−cytochrome−c reductase complex assembly factor 3 with a threshold of 1e−05

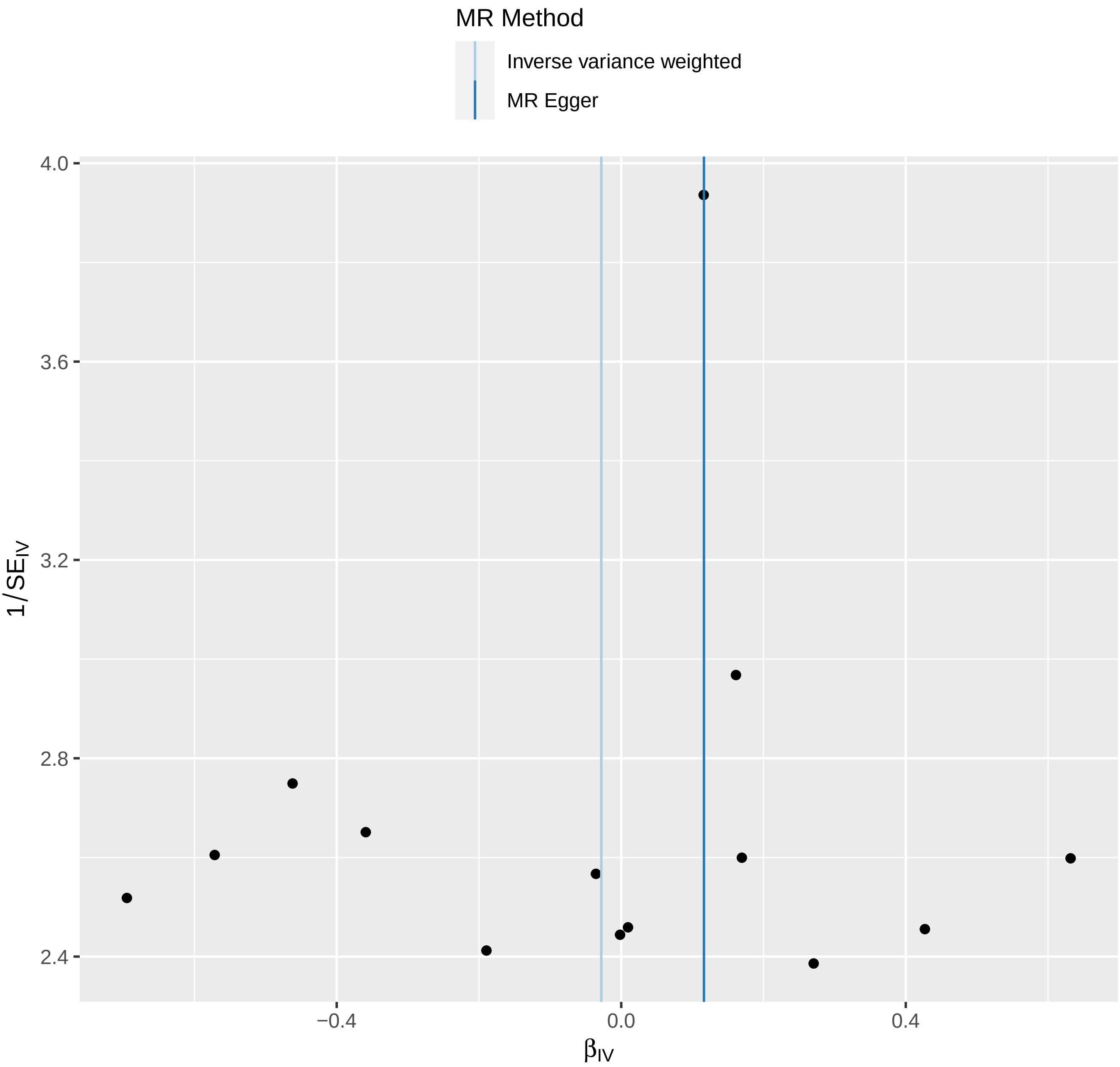

Batch 2−6349 : Gut microbiota abundance (genus Catenibacterium id.2153) on Cytochrome b−c1 complex subunit 7
with a threshold of 1e−05

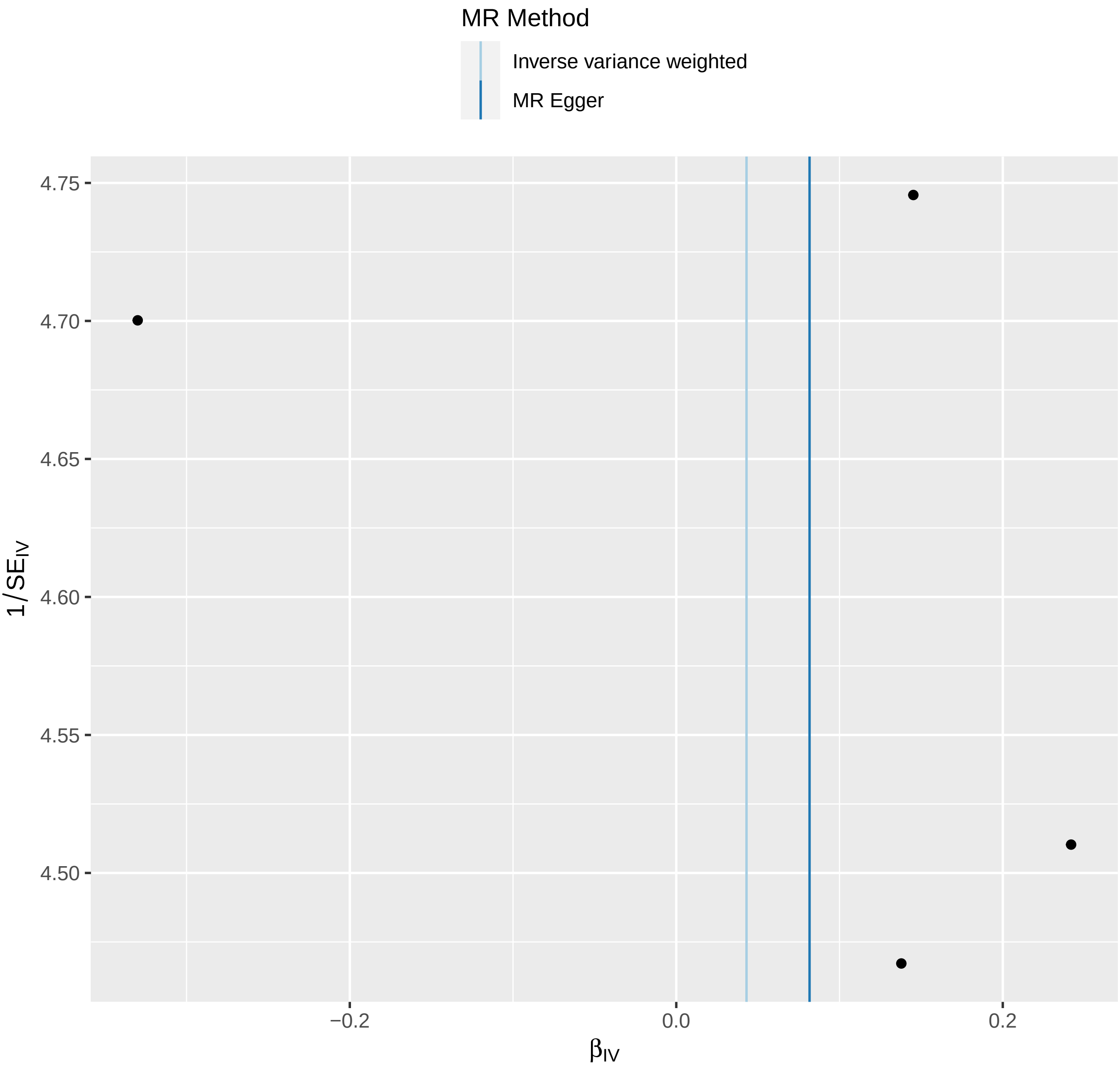

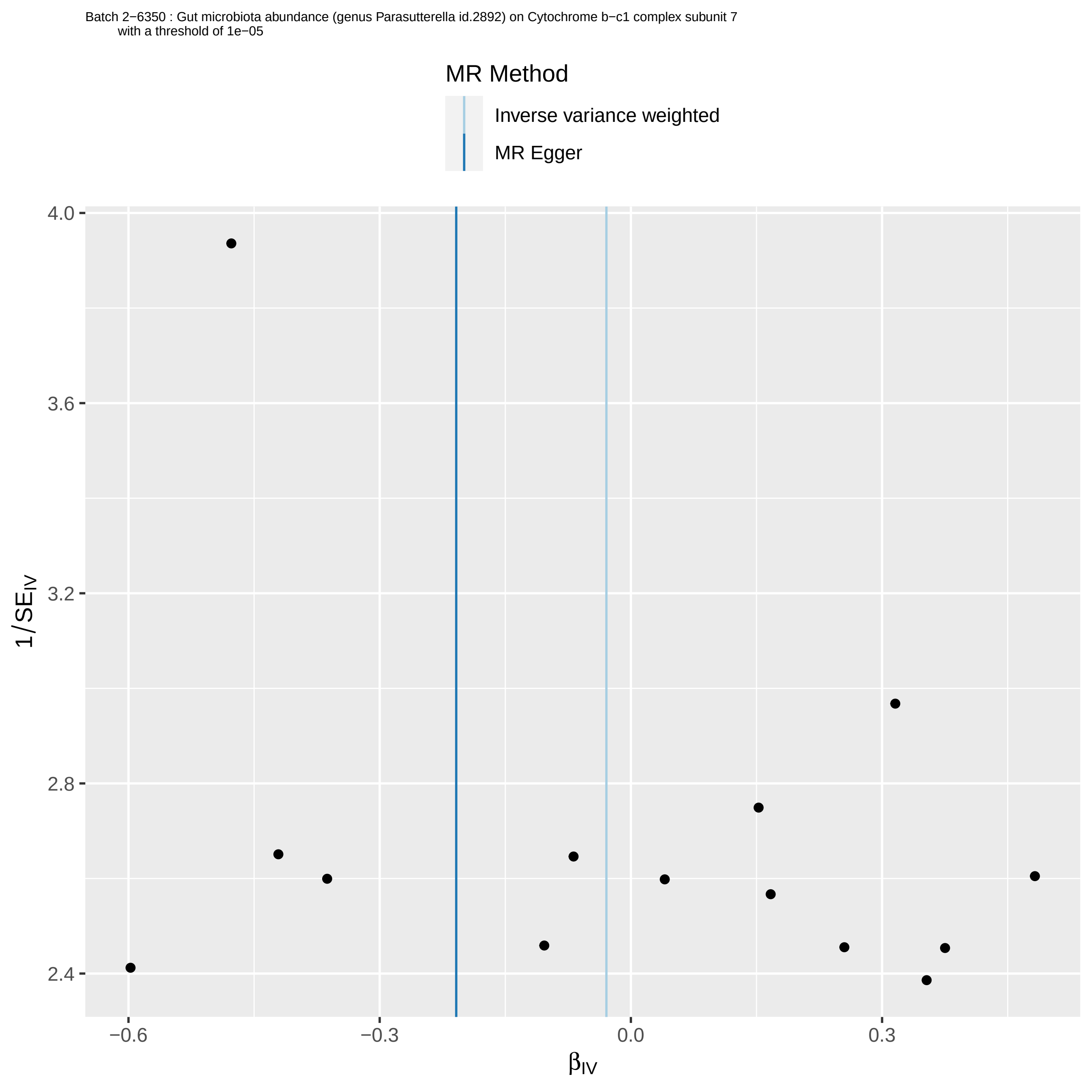

Batch 2−6350 : Gut microbiota abundance (genus Parasutterella id.2892) on Cytochrome b−c1 complex subunit 7
with a threshold of 1e−05
MR Method
Inverse variance weighted
MR Egger
4.0
3.6
3.2
2.8
2.4
1/SE_IV
−0.6
−0.3
0.0
0.3
β_IV

Batch 2−6351 : Gut microbiota abundance (genus Catenibacterium id.2153) on Uroporphyrinogen−III synthase
with a threshold of 1e−05

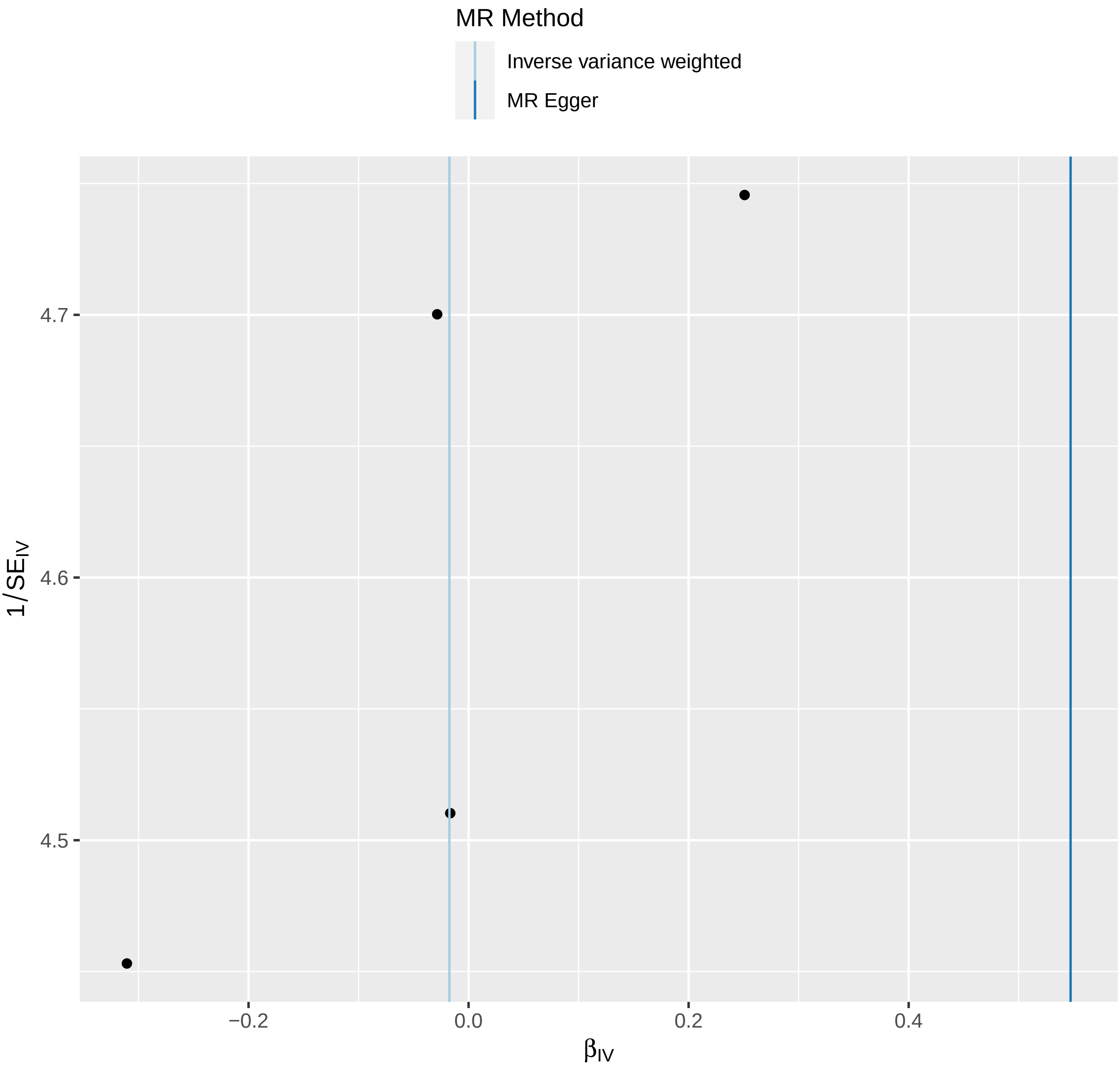

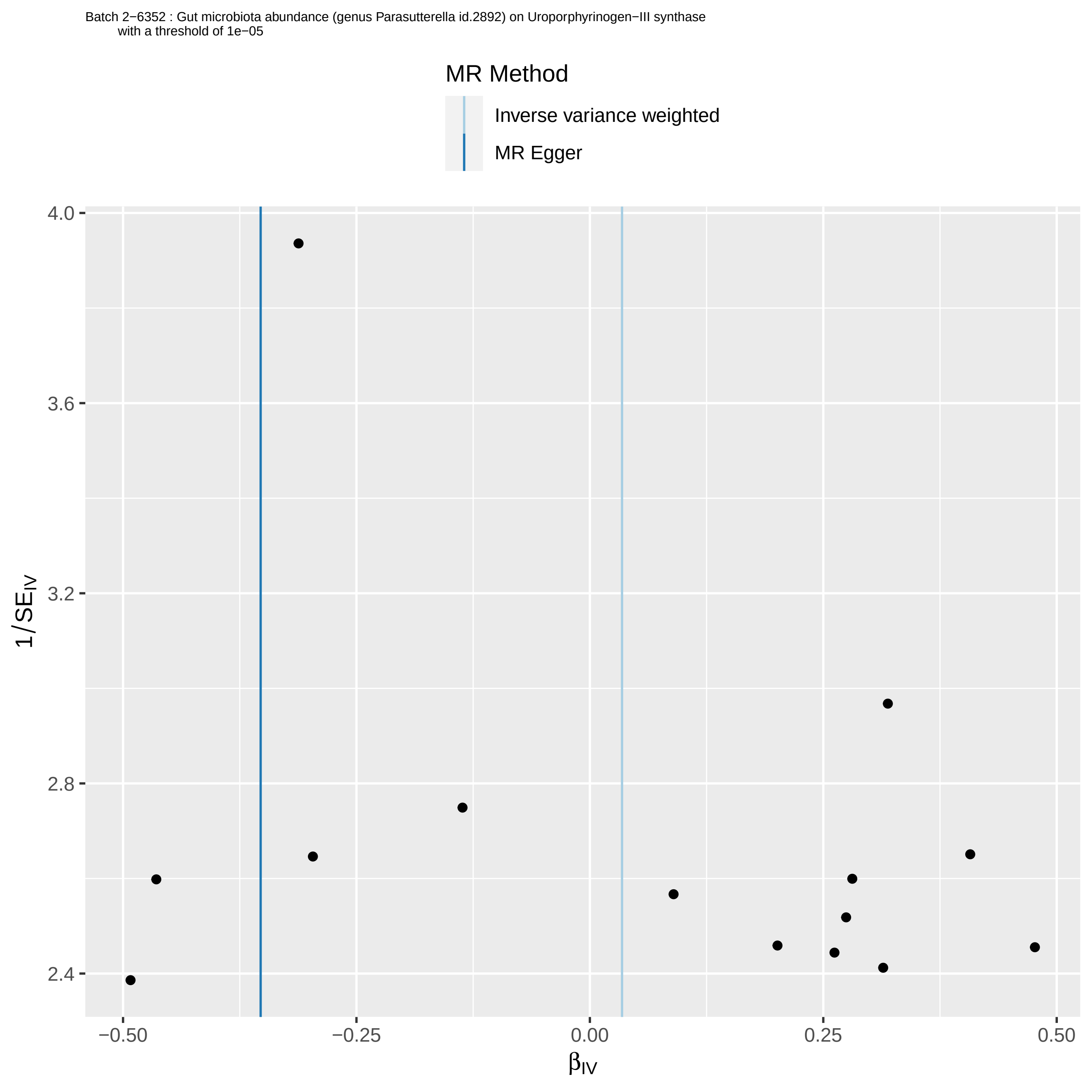

Batch 2−6352 : Gut microbiota abundance (genus Parasutterella id.2892) on Uroporphyrinogen−III synthase
with a threshold of 1e−05
MR Method
Inverse variance weighted
MR Egger
4.0
3.6
3.2
2.8
2.4
1/SE$_{IV}$
−0.50
−0.25
0.00
0.25
0.50
β_{IV}

Batch 2−6353 : Gut microbiota abundance (genus Catenibacterium id.2153) on U6 snRNA phosphodiesterase
with a threshold of 1e−05

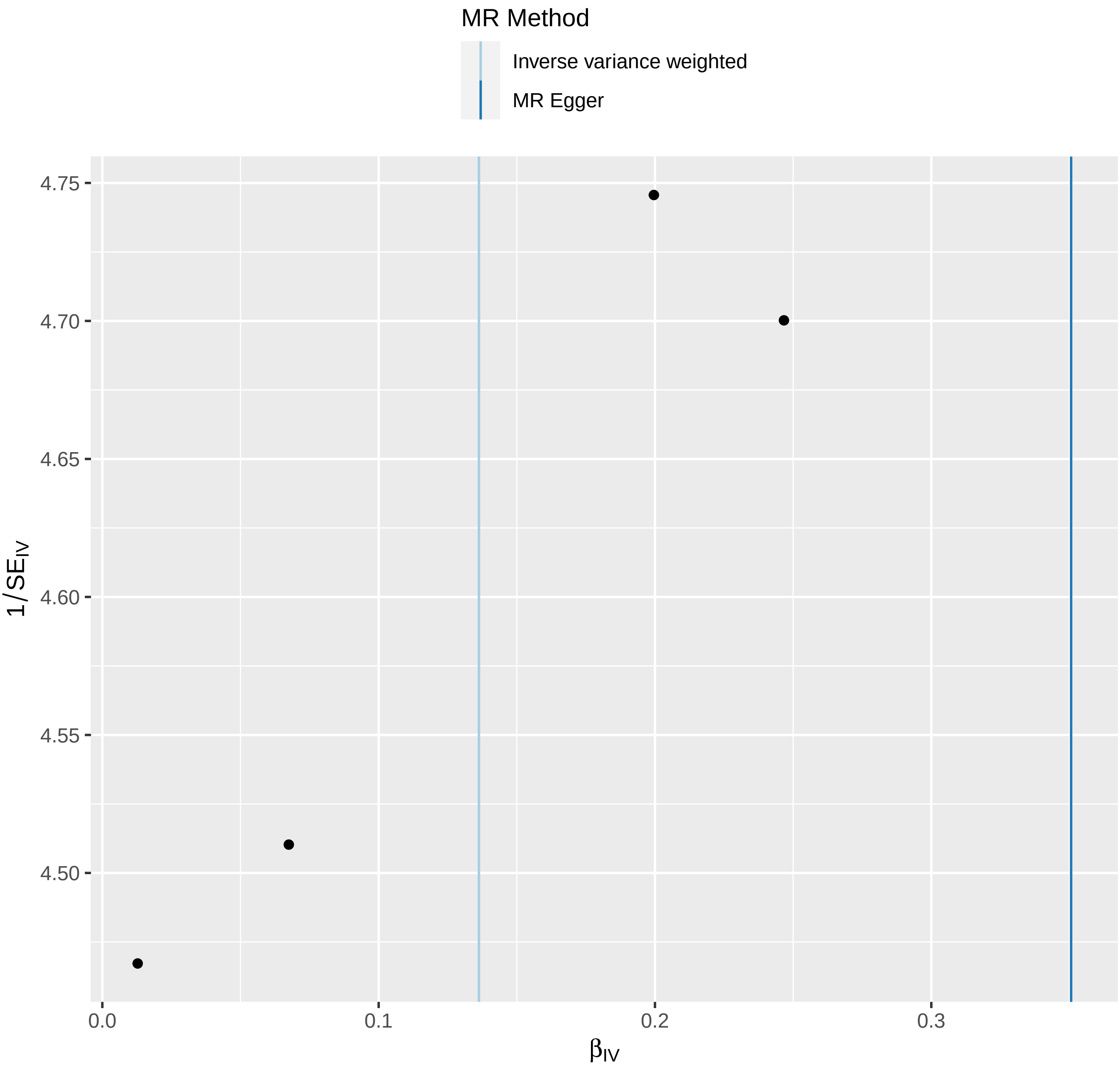

Batch 2−6354 : Gut microbiota abundance (genus Parasutterella id.2892) on U6 snRNA phosphodiesterase
with a threshold of 1e−05

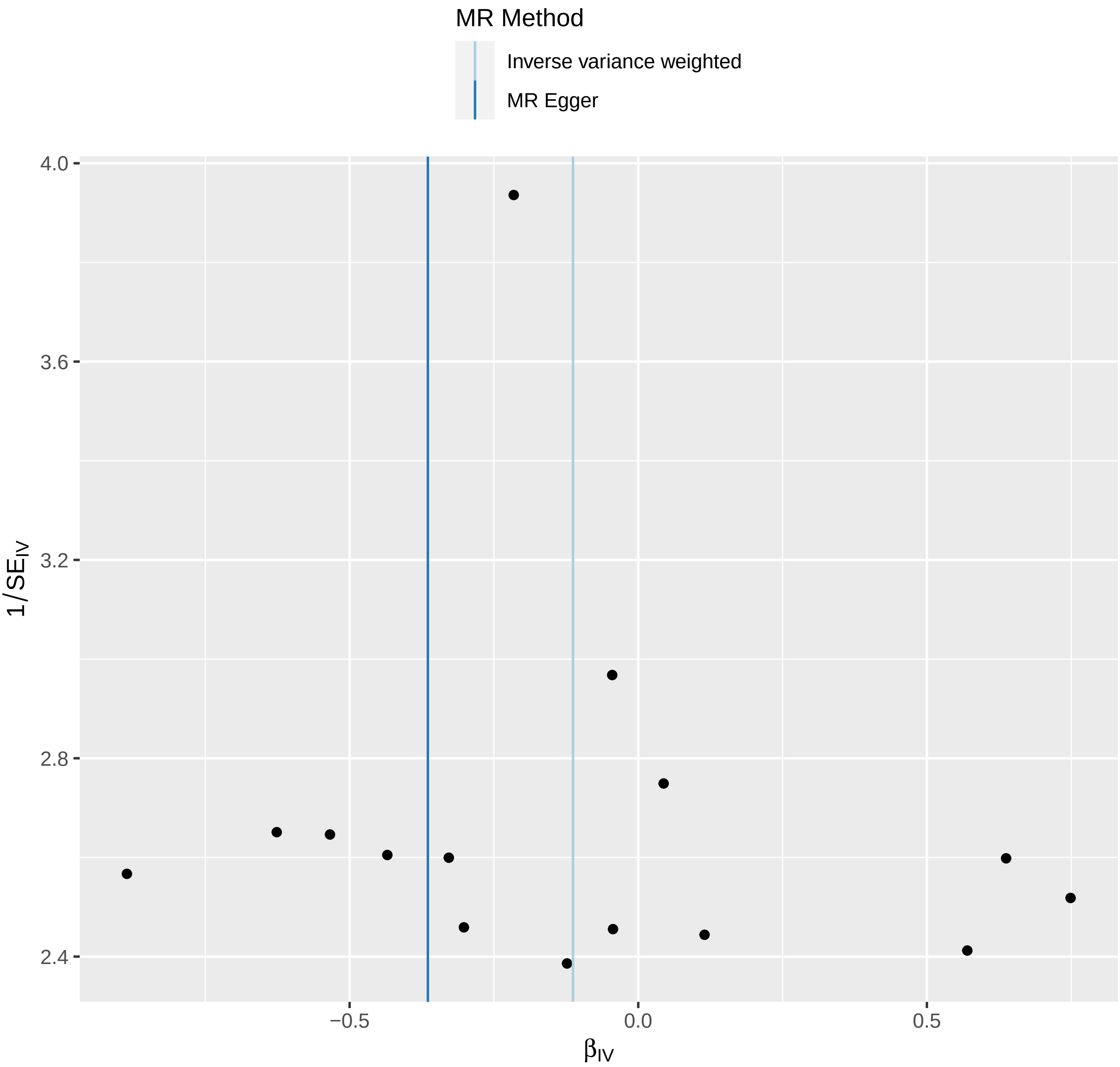

Batch 2−6355 : Gut microbiota abundance (genus Catenibacterium id.2153) on Vesicle transport protein USE1
with a threshold of 1e−05

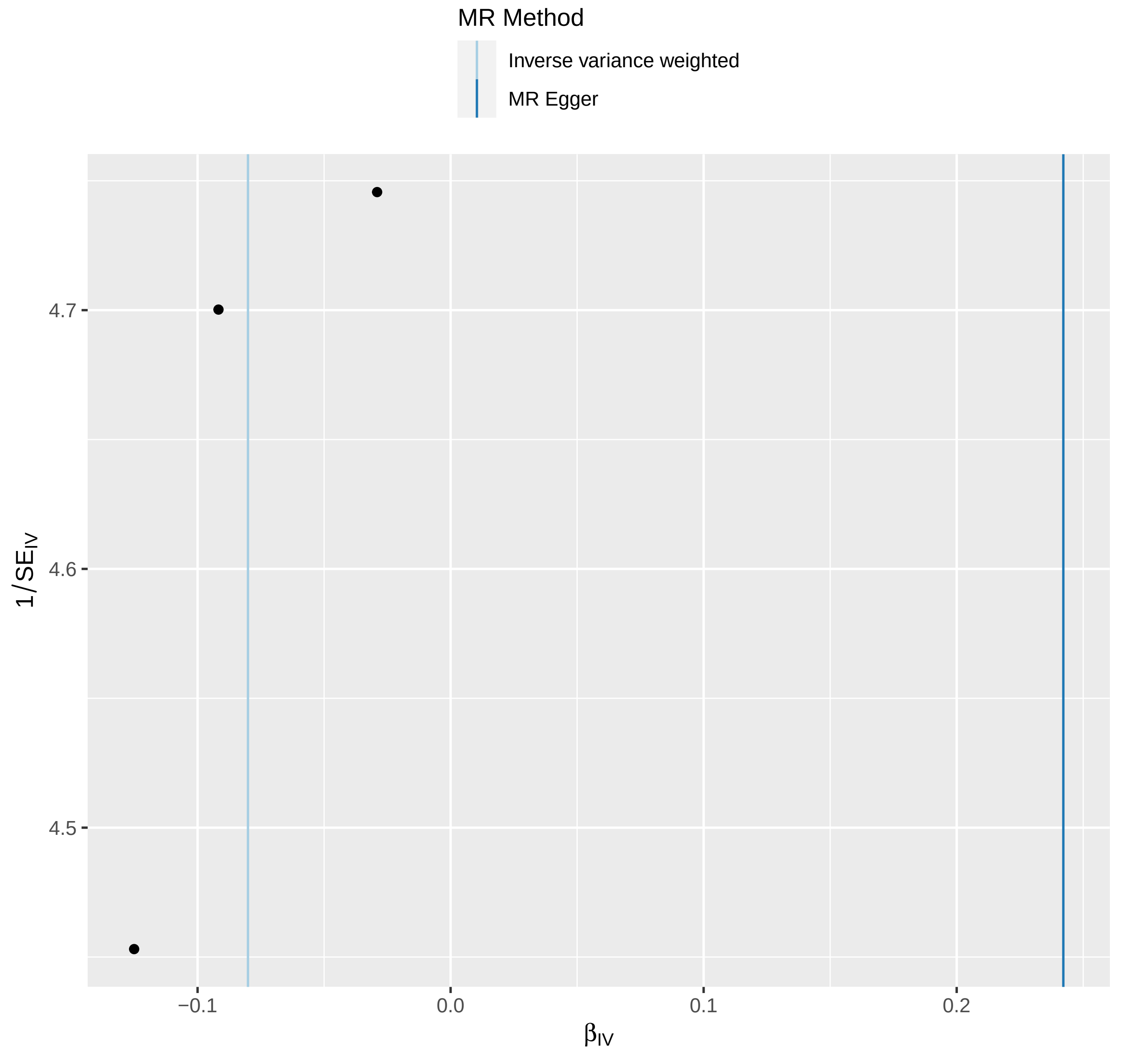

Batch 2−6356 : Gut microbiota abundance (genus Parasutterella id.2892) on Vesicle transport protein USE1
with a threshold of 1e−05

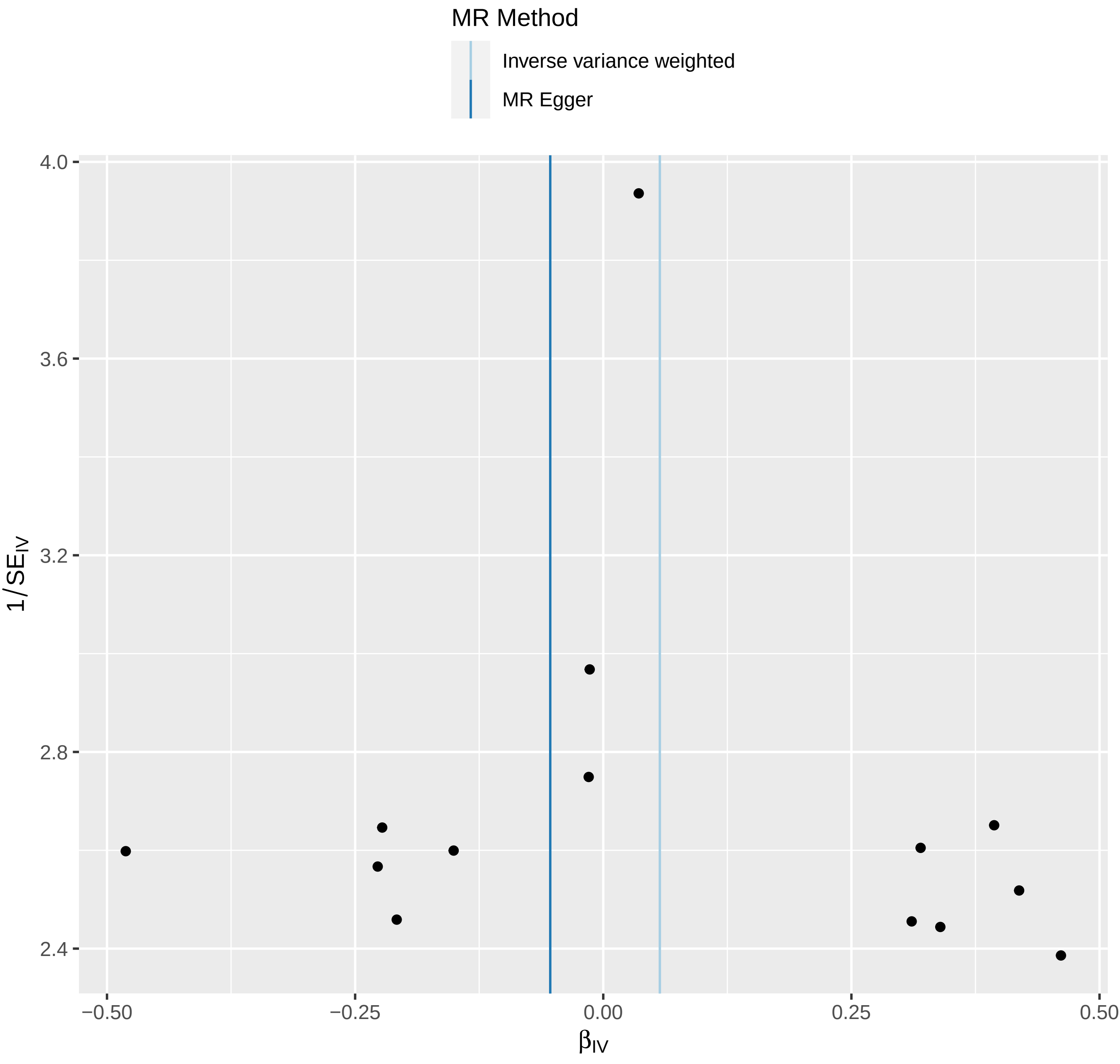

Batch 2−6357 : Gut microbiota abundance (genus Catenibacterium id.2153) on Ubiquitin carboxyl−terminal hydrolase 21
with a threshold of 1e−05

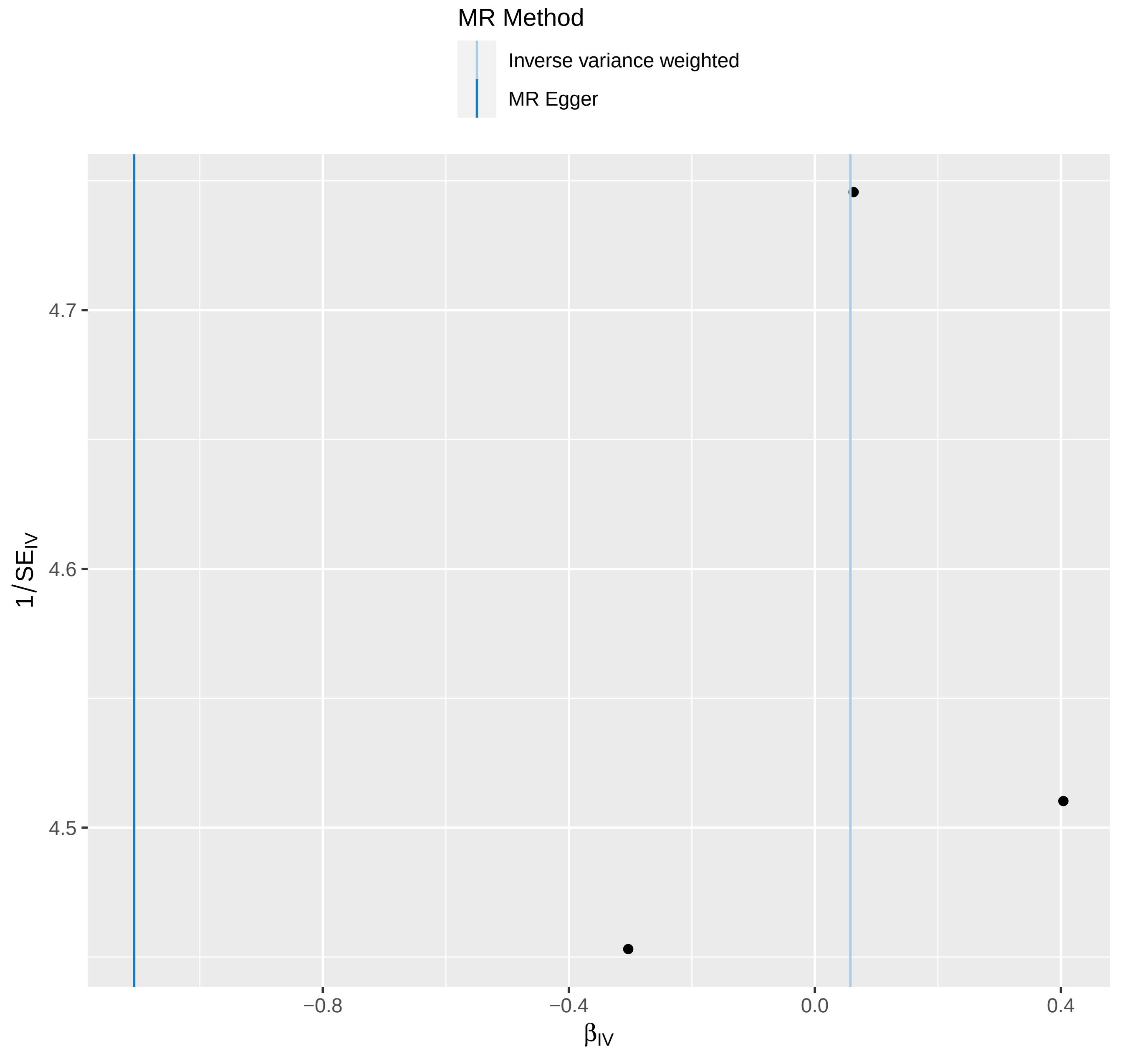

Batch 2−6358 : Gut microbiota abundance (genus Parasutterella id.2892) on Ubiquitin carboxyl−terminal hydrolase 21
with a threshold of 1e−05

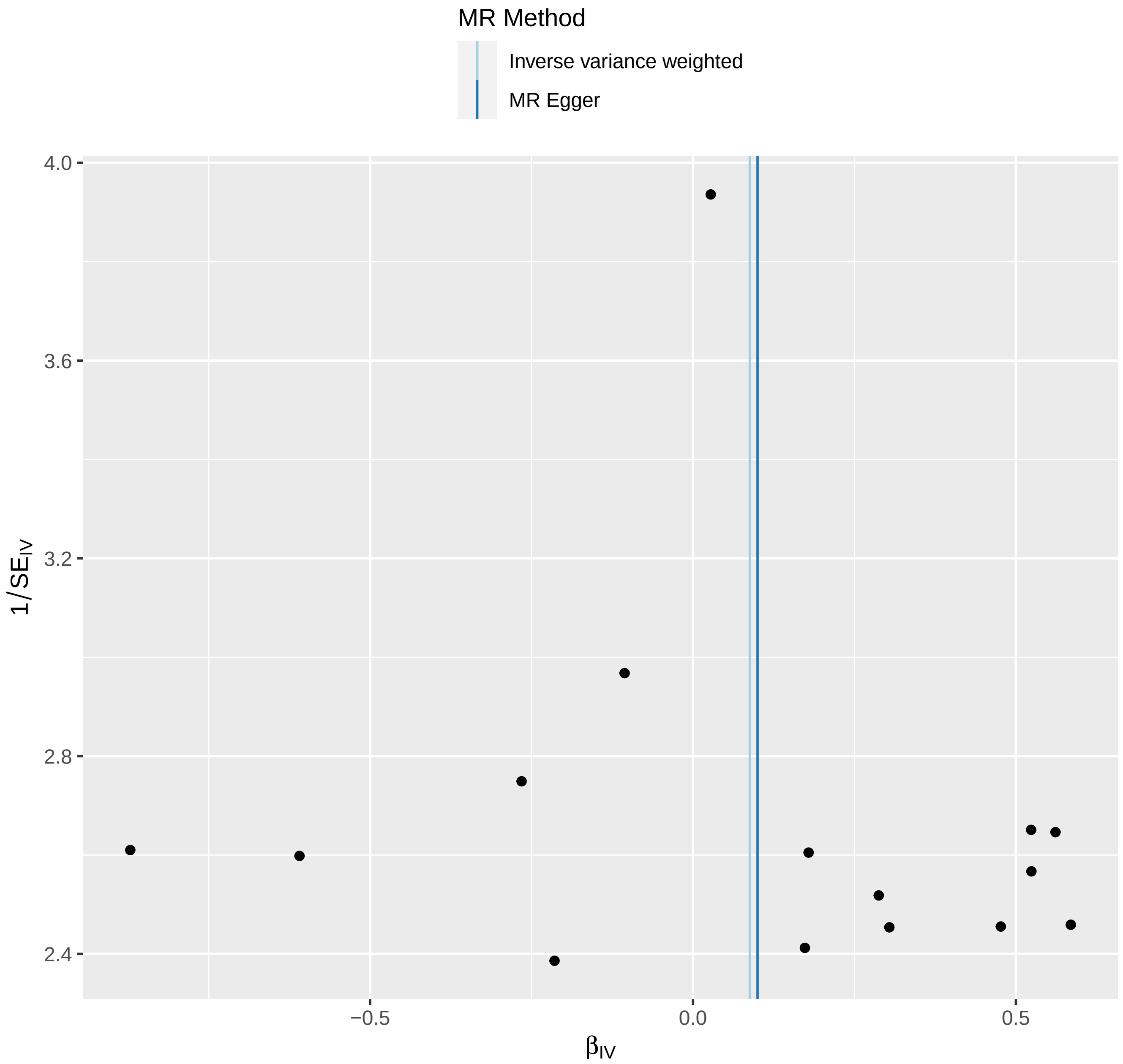

Batch 2−6359 : Gut microbiota abundance (genus Catenibacterium id.2153) on Ubiquitin carboxyl−terminal hydrolase 25
with a threshold of 1e−05

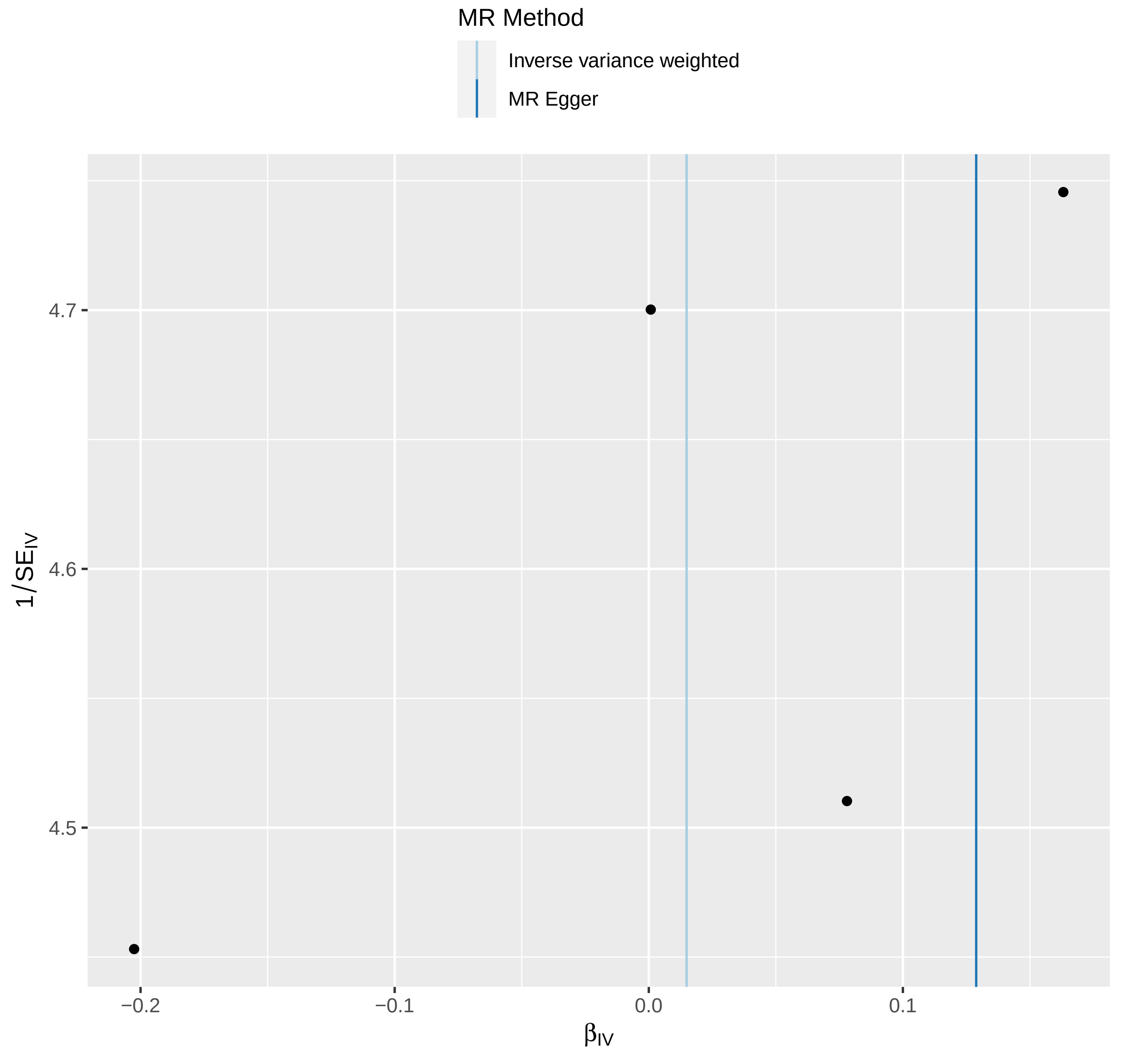

Batch 2−6360 : Gut microbiota abundance (genus Parasutterella id.2892) on Ubiquitin carboxyl−terminal hydrolase 25
with a threshold of 1e−05

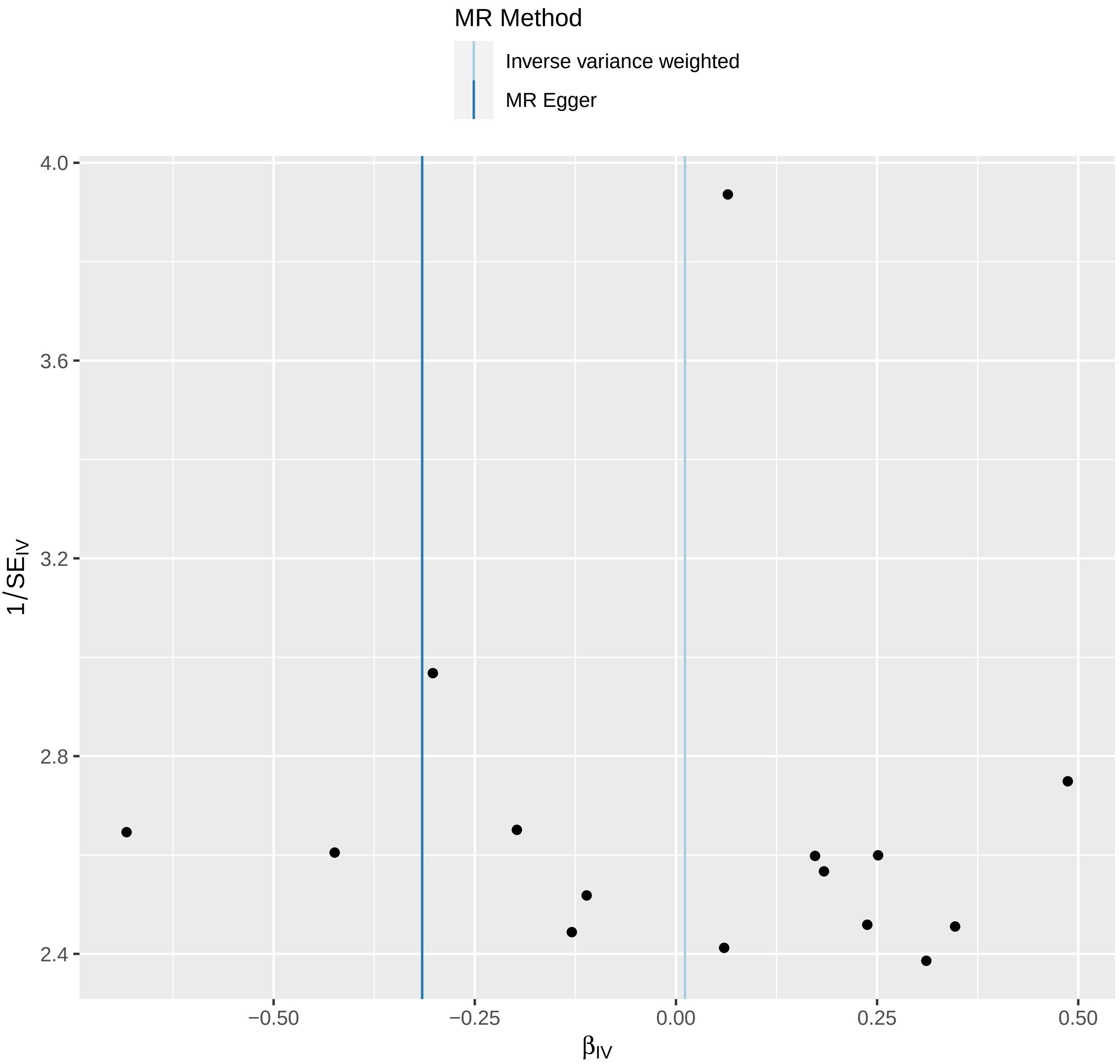

Batch 2−6361 : Gut microbiota abundance (genus Catenibacterium id.2153) on Ubiquitin carboxyl−terminal hydrolase 8
with a threshold of 1e−05

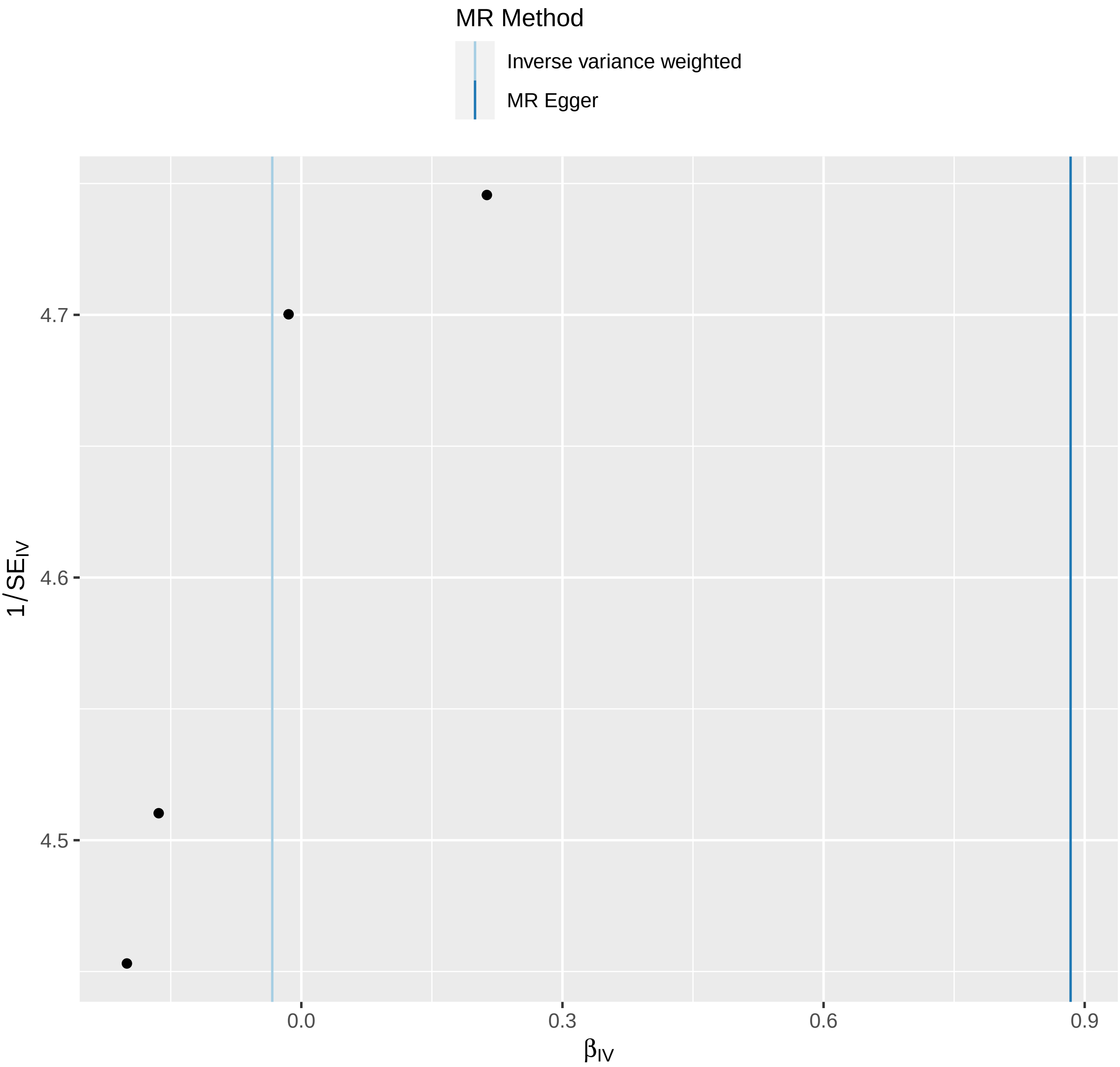

Batch 2−6362 : Gut microbiota abundance (genus Parasutterella id.2892) on Ubiquitin carboxyl−terminal hydrolase 8
with a threshold of 1e−05

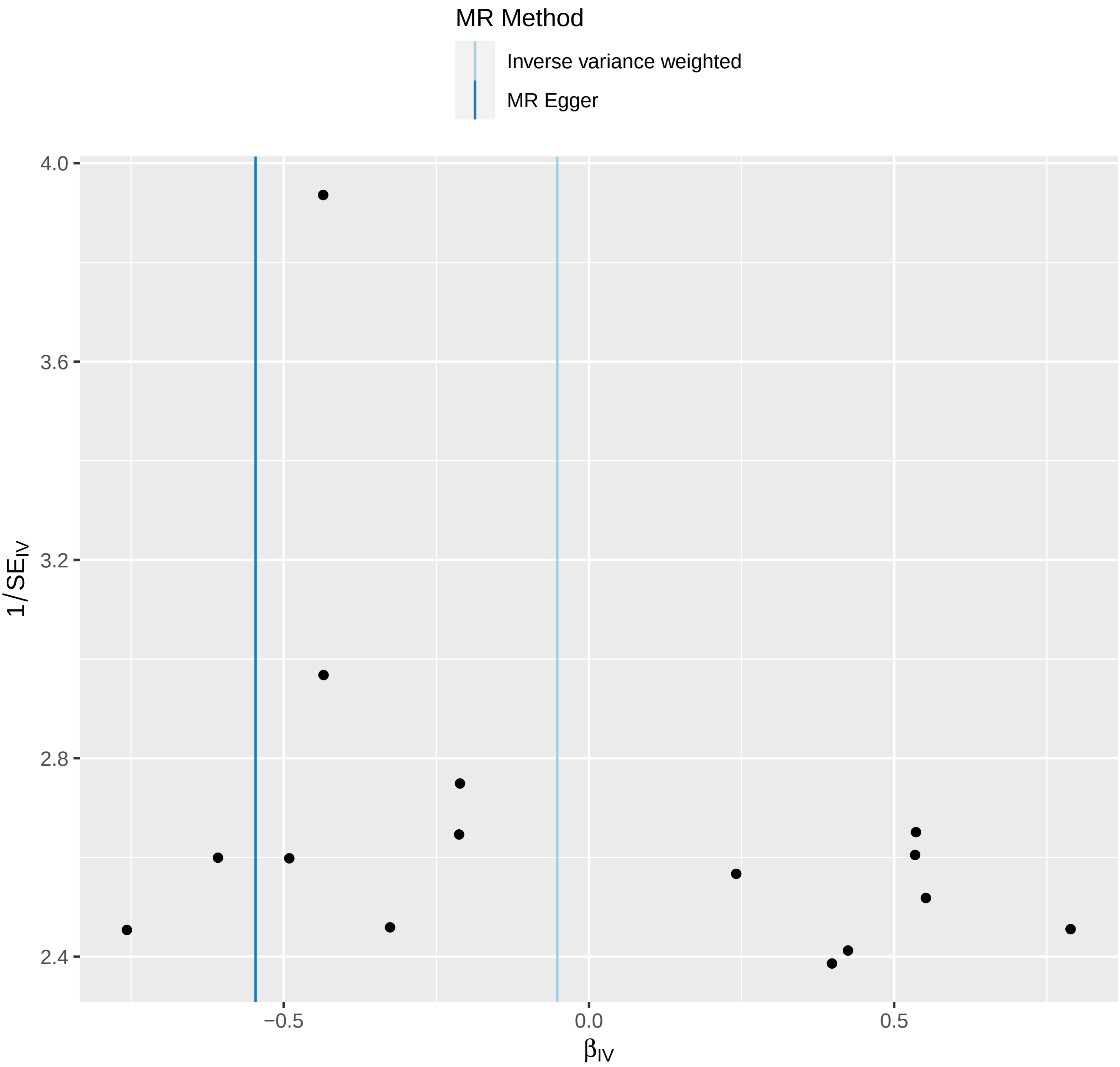

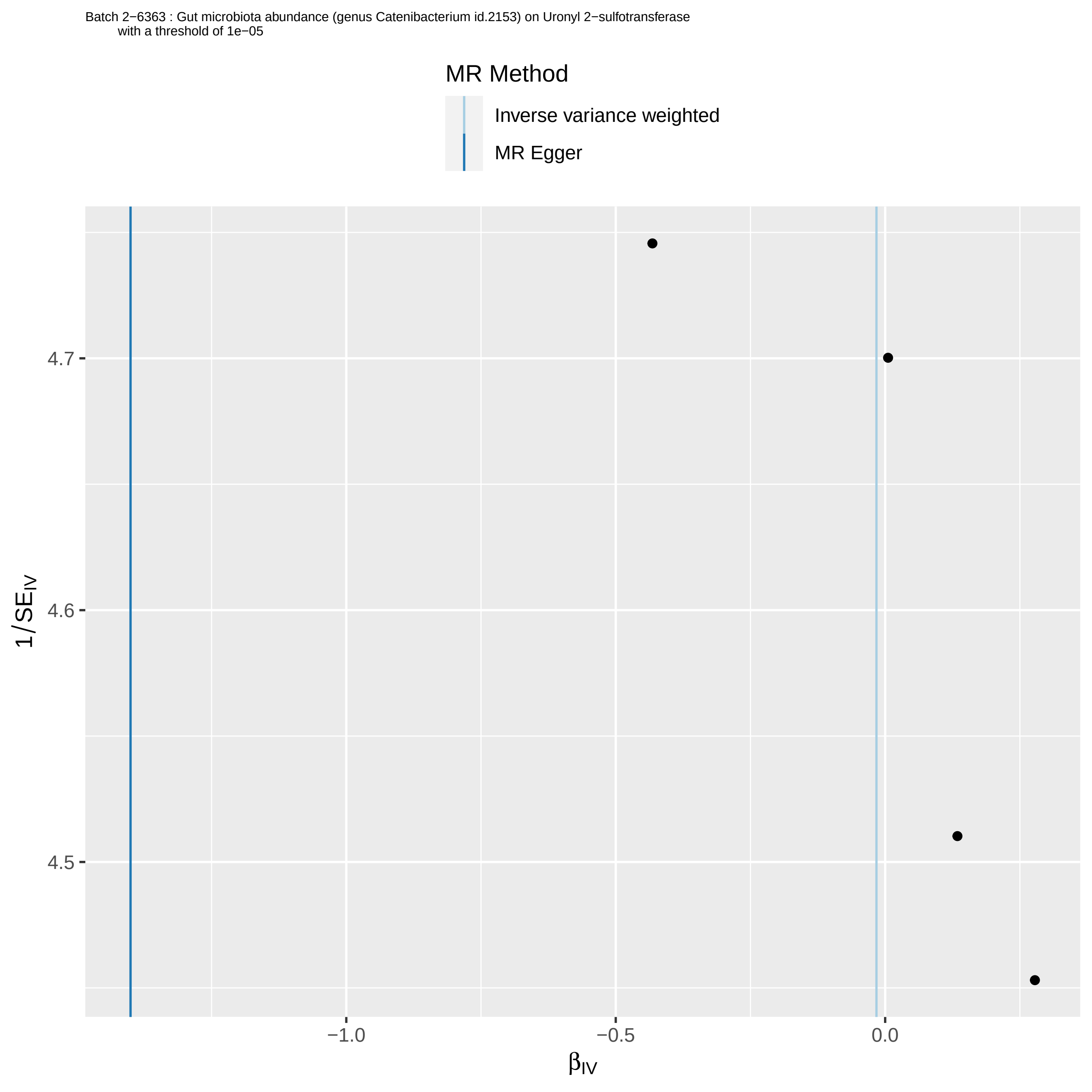

Batch 2−6363 : Gut microbiota abundance (genus Catenibacterium id.2153) on Uronyl 2−sulfotransferase
with a threshold of 1e−05
MR Method
Inverse variance weighted
MR Egger
4.7
4.6
4.5
1/SE_IV
−1.0
−0.5
0.0
β_IV

Batch 2−6364 : Gut microbiota abundance (genus Parasutterella id.2892) on Uronyl 2−sulfotransferase
with a threshold of 1e−05

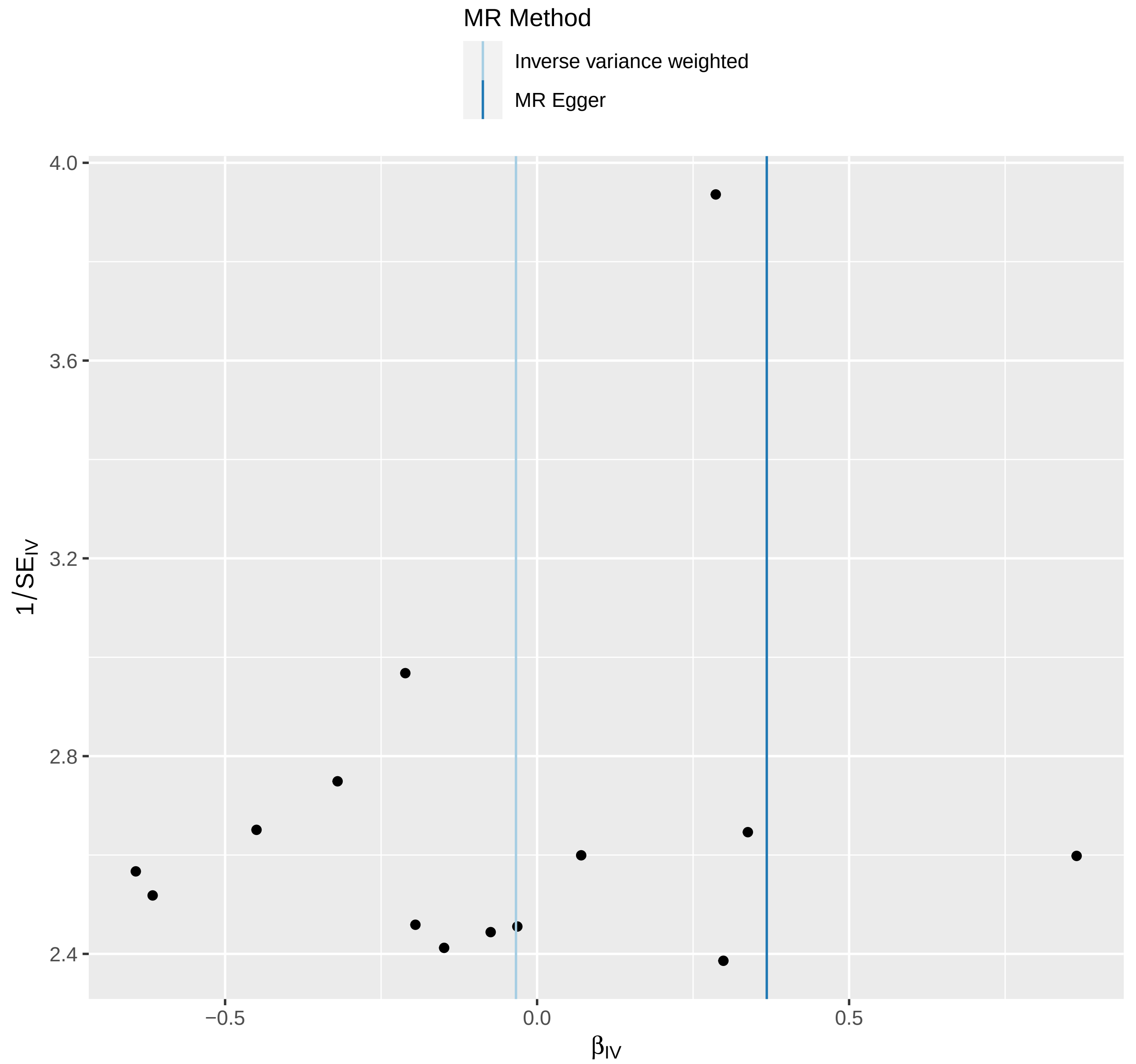

Batch 2−6365 : Gut microbiota abundance (genus Catenibacterium id.2153) on Urotensin−2 receptor
with a threshold of 1e−05

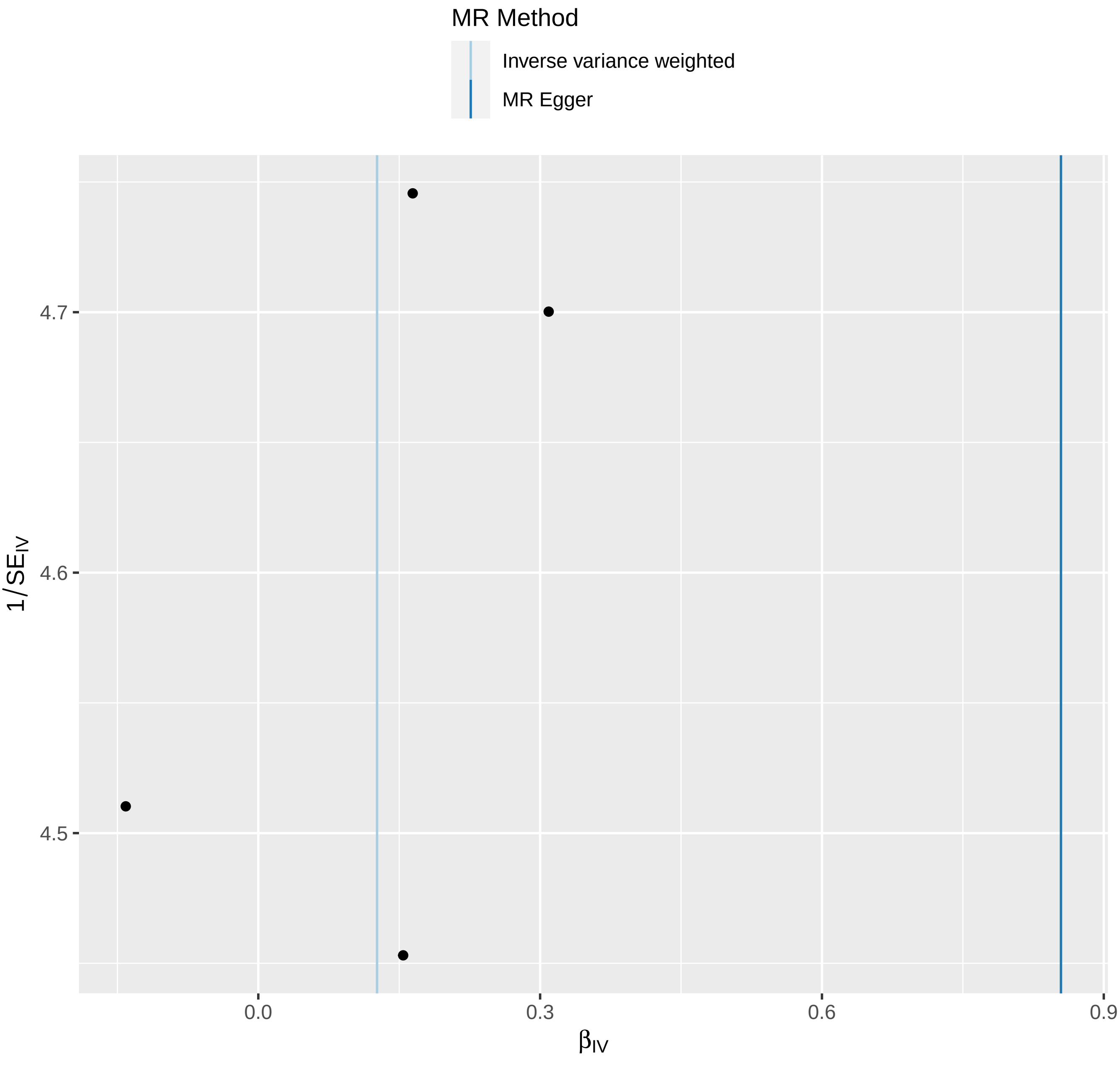

Batch 2−6366 : Gut microbiota abundance (genus Parasutterella id.2892) on Urotensin−2 receptor
with a threshold of 1e−05

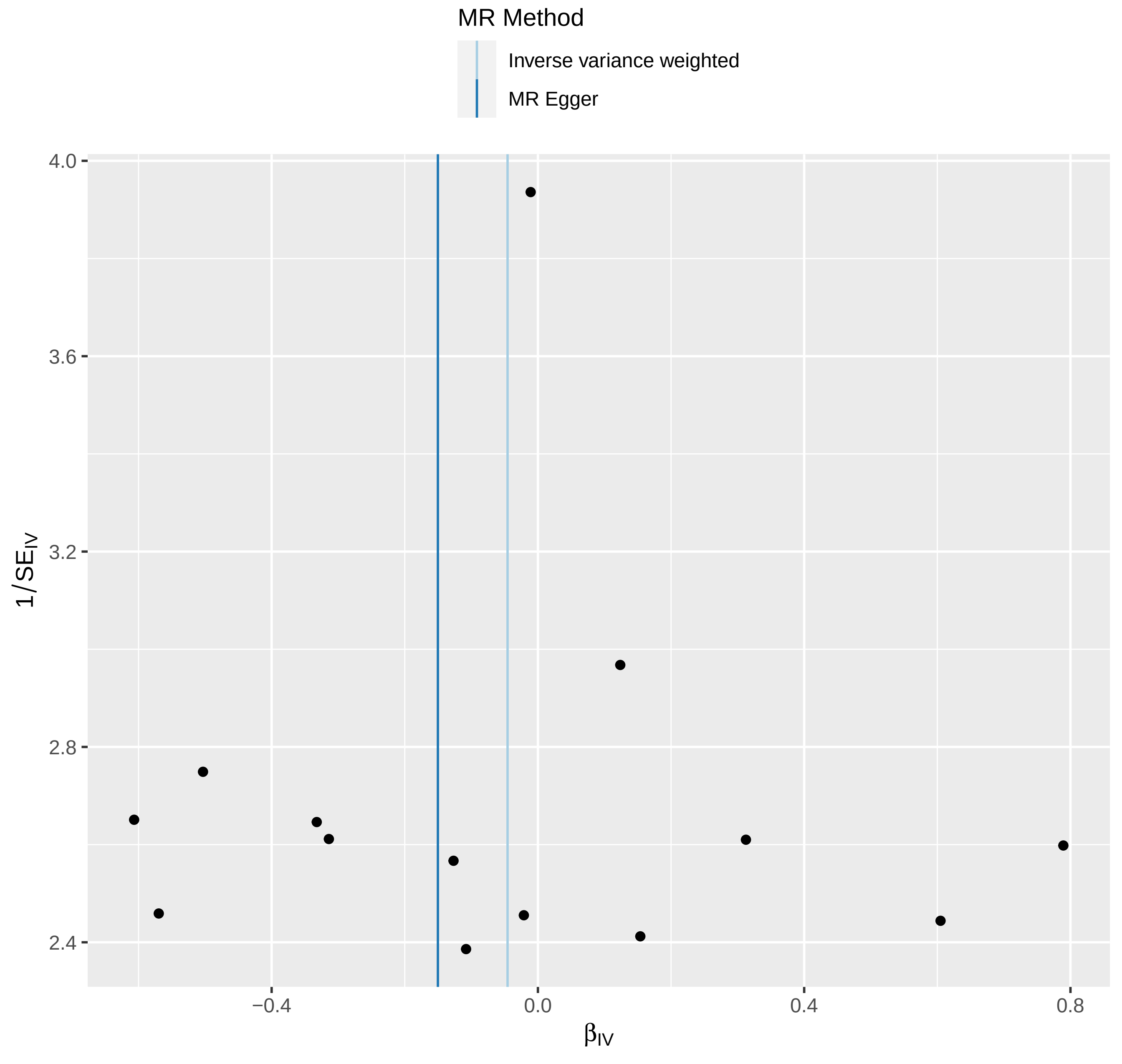

Batch 2−6367 : Gut microbiota abundance (genus Catenibacterium id.2153) on UDP−glucuronic acid decarboxylase 1
with a threshold of 1e−05

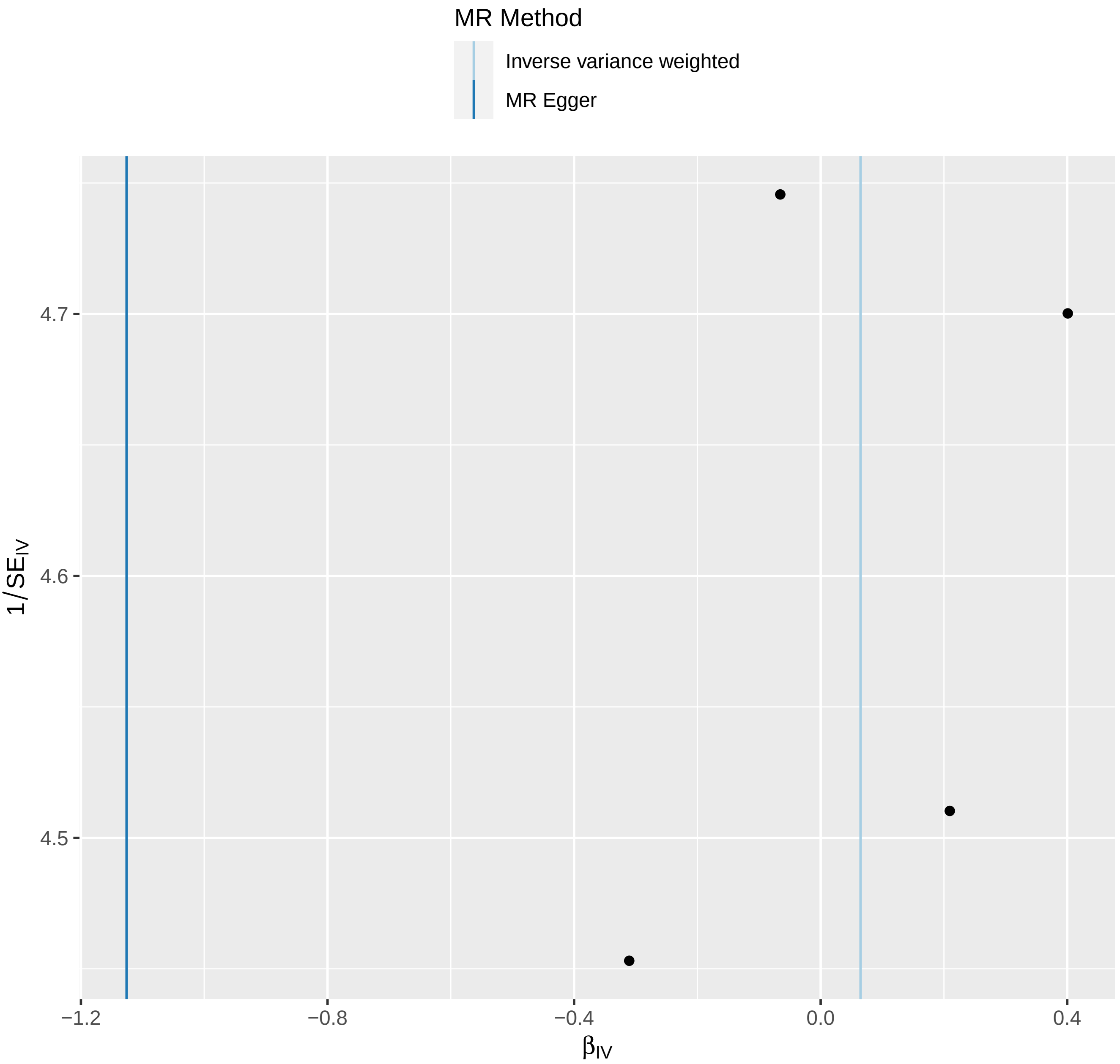

Batch 2−6368 : Gut microbiota abundance (genus Parasutterella id.2892) on UDP−glucuronic acid decarboxylase 1 with a threshold of 1e−05

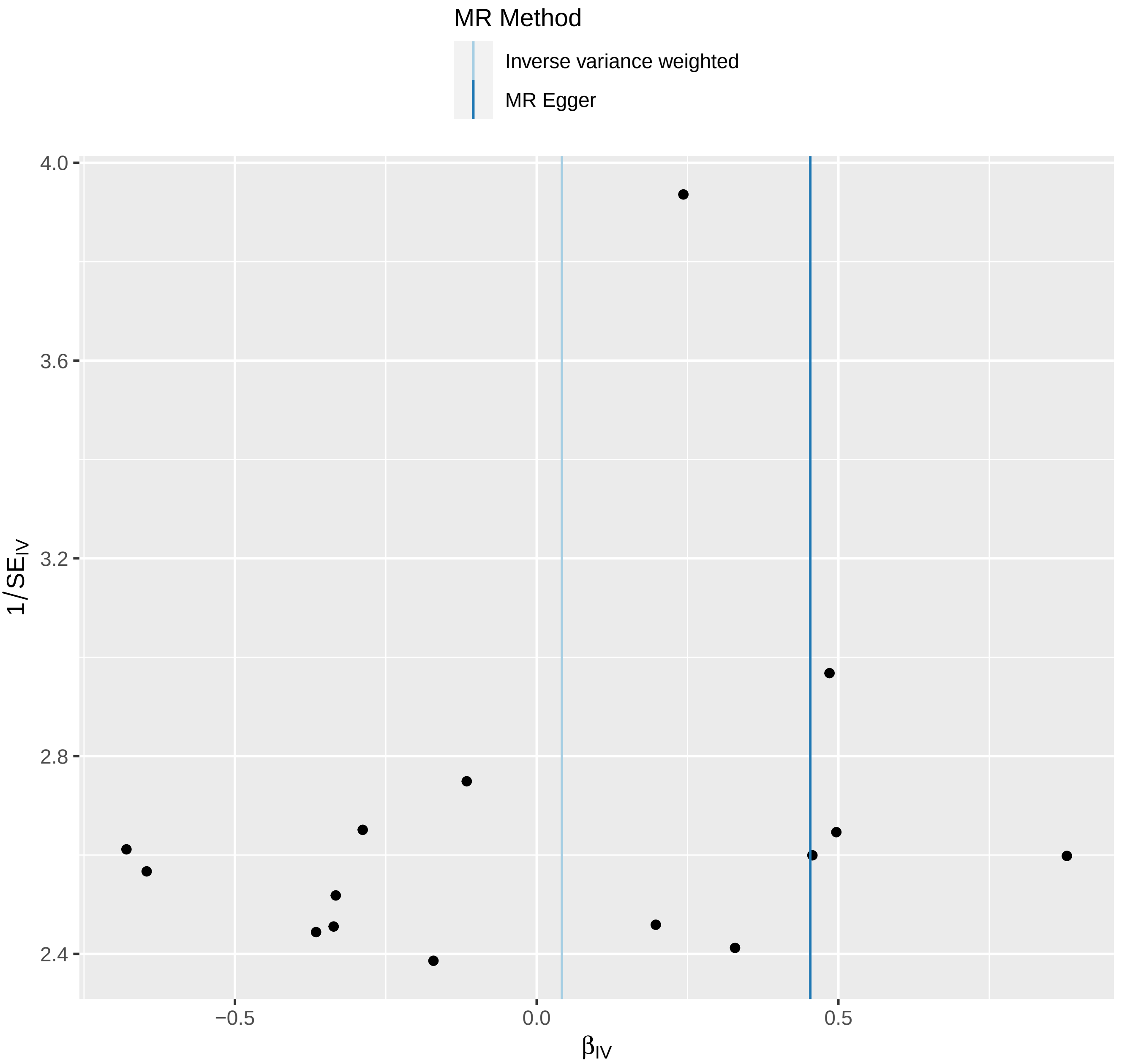

Batch 2−6369 : Gut microbiota abundance (genus Catenibacterium id.2153) on Vesicle−associated membrane protein 3 with a threshold of 1e−05

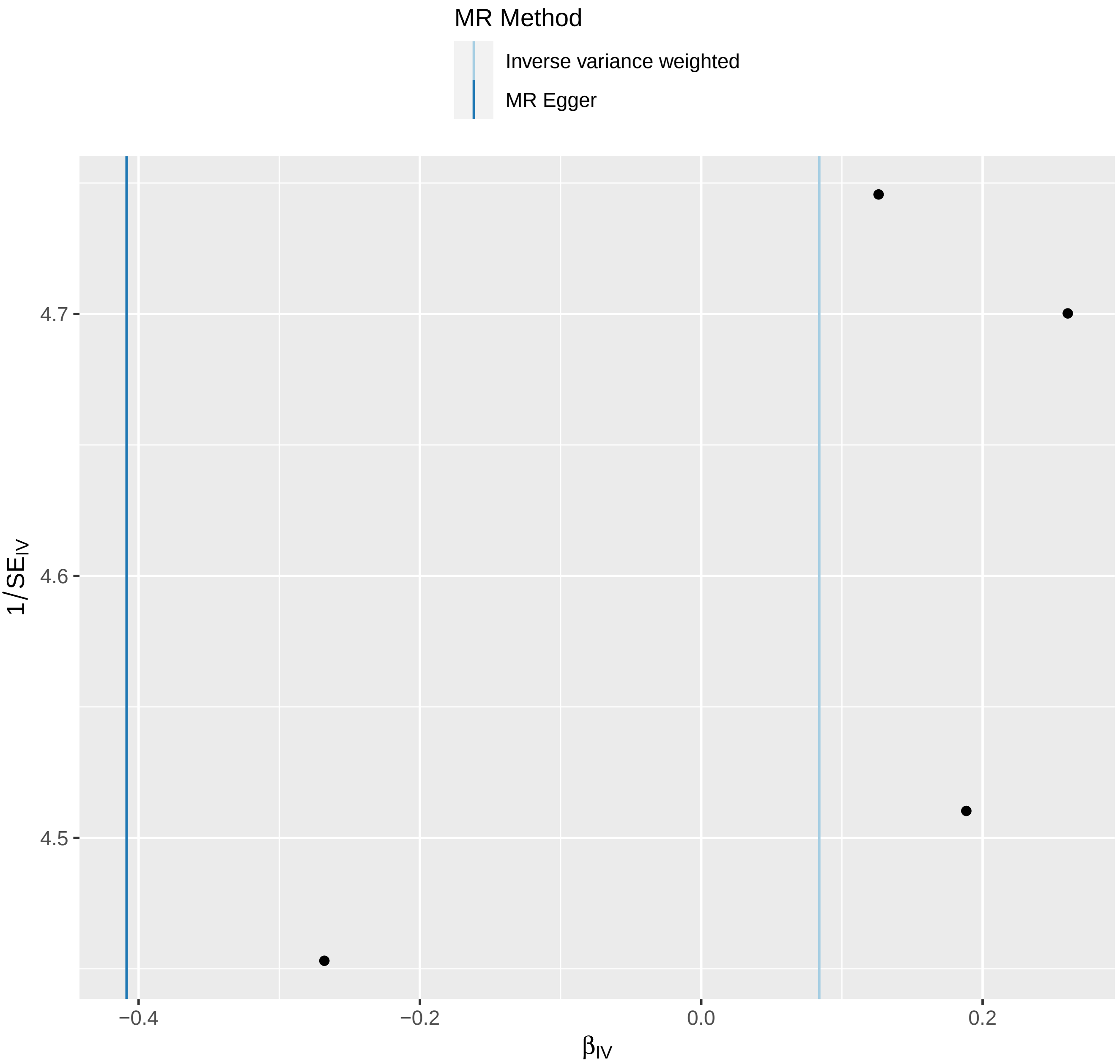

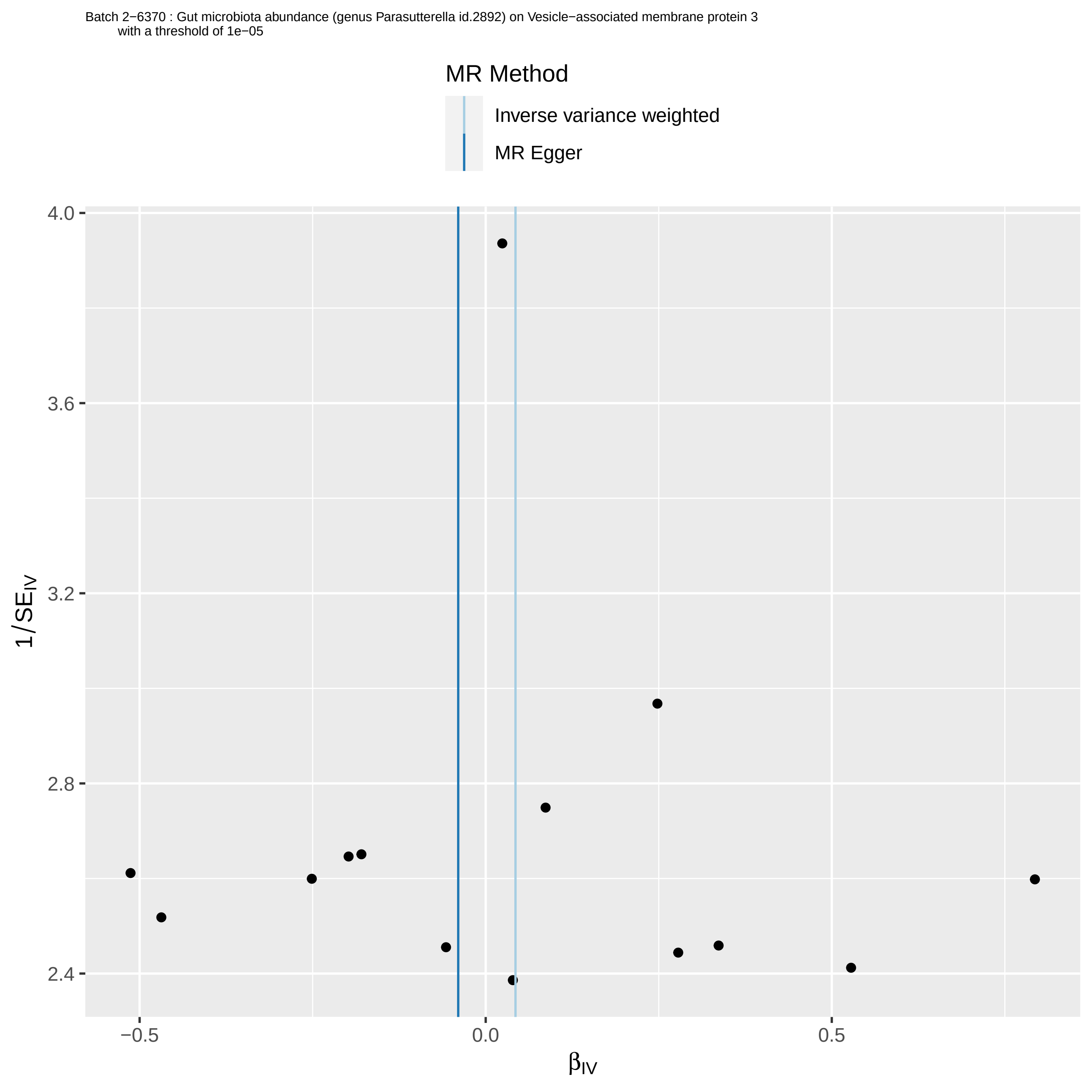
Batch 2−6370 : Gut microbiota abundance (genus Parasutterella id.2892) on Vesicle−associated membrane protein 3
with a threshold of 1e−05
MR Method
Inverse variance weighted
MR Egger
4.0
3.6
3.2
2.8
2.4
1/SE_IV
−0.5
0.0
0.5
β_IV

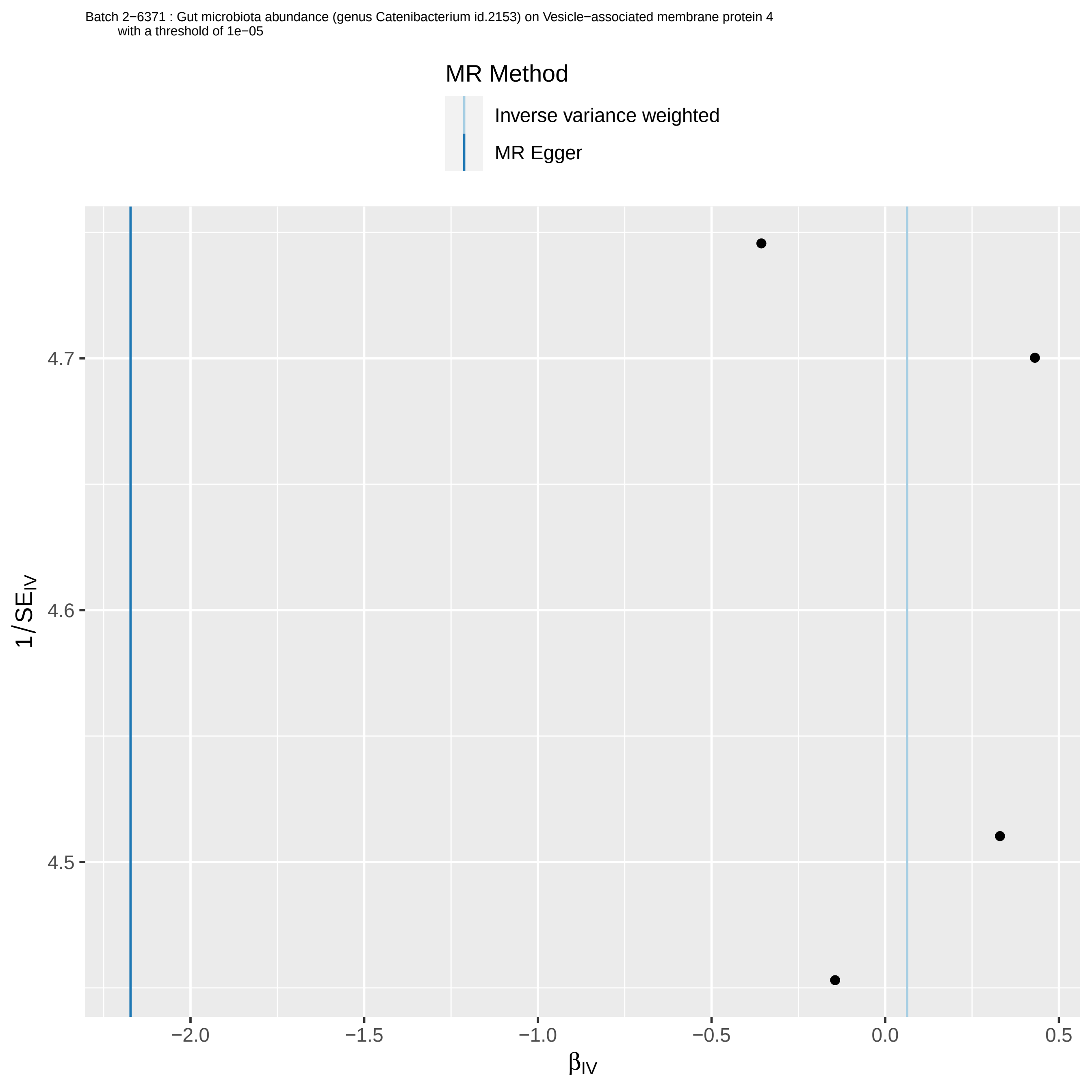

Batch 2−6371 : Gut microbiota abundance (genus Catenibacterium id.2153) on Vesicle−associated membrane protein 4
with a threshold of 1e−05
MR Method
Inverse variance weighted
MR Egger
4.7
4.6
4.5
1/SE_IV
−2.0
−1.5
−1.0
−0.5
0.0
0.5
β_IV

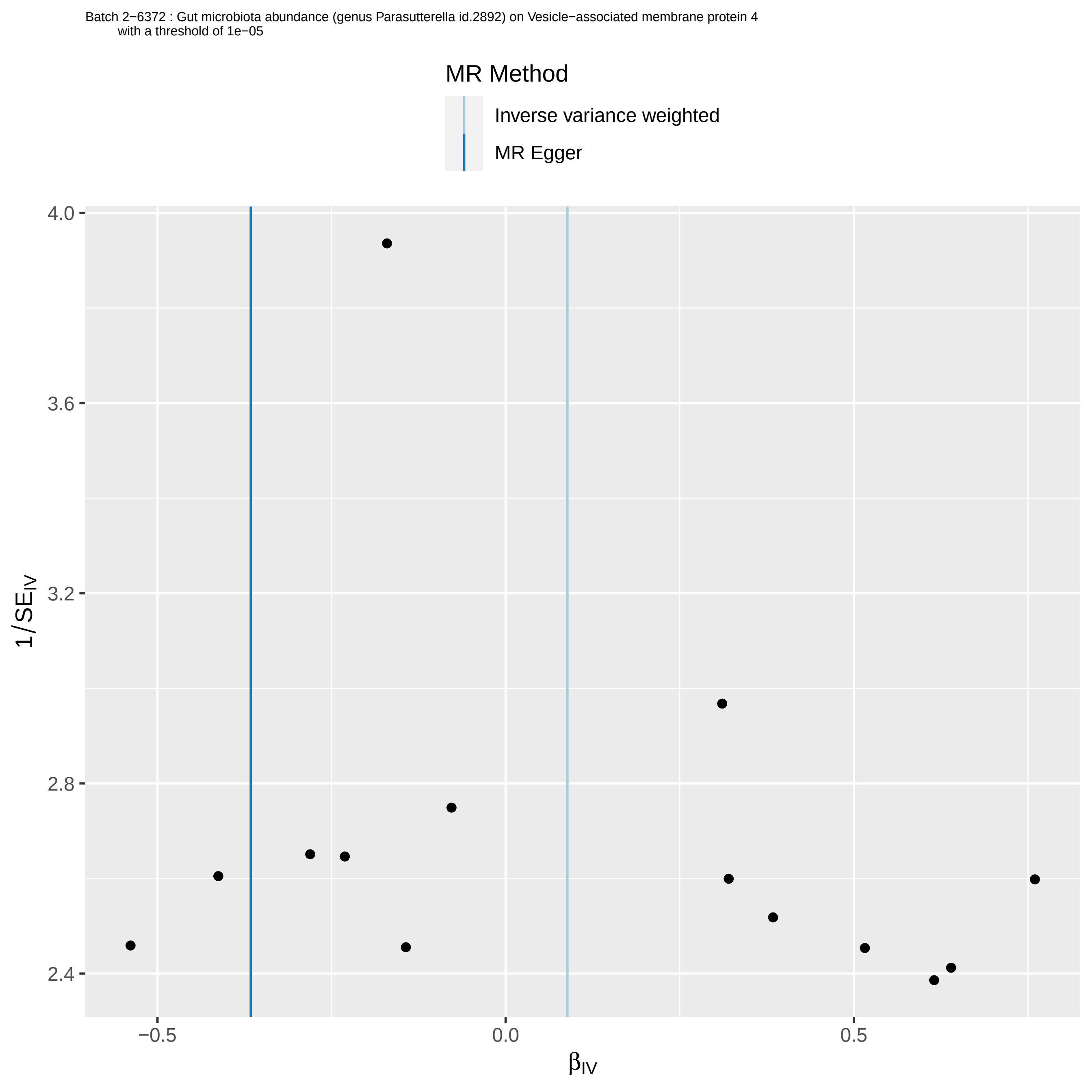
Batch 2−6372 : Gut microbiota abundance (genus Parasutterella id.2892) on Vesicle−associated membrane protein 4
with a threshold of 1e−05
MR Method
Inverse variance weighted
MR Egger
4.0
3.6
3.2
2.8
2.4
1/SE_IV
−0.5
0.0
0.5
β_IV

Batch 2−6373 : Gut microbiota abundance (genus Catenibacterium id.2153) on Vesicle−associated membrane protein 5
with a threshold of 1e−05

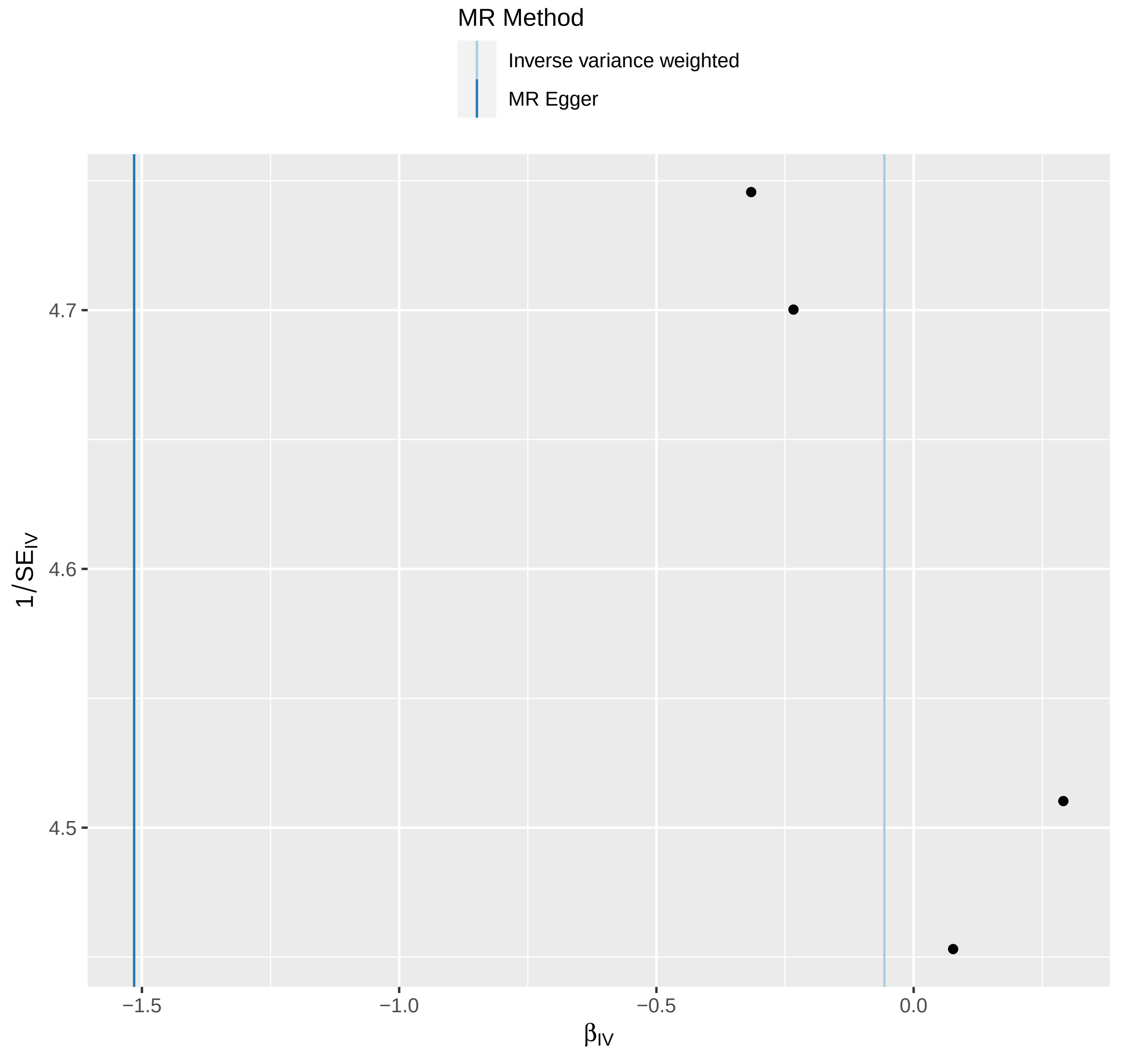

Batch 2−6374 : Gut microbiota abundance (genus Parasutterella id.2892) on Vesicle−associated membrane protein 5 with a threshold of 1e−05

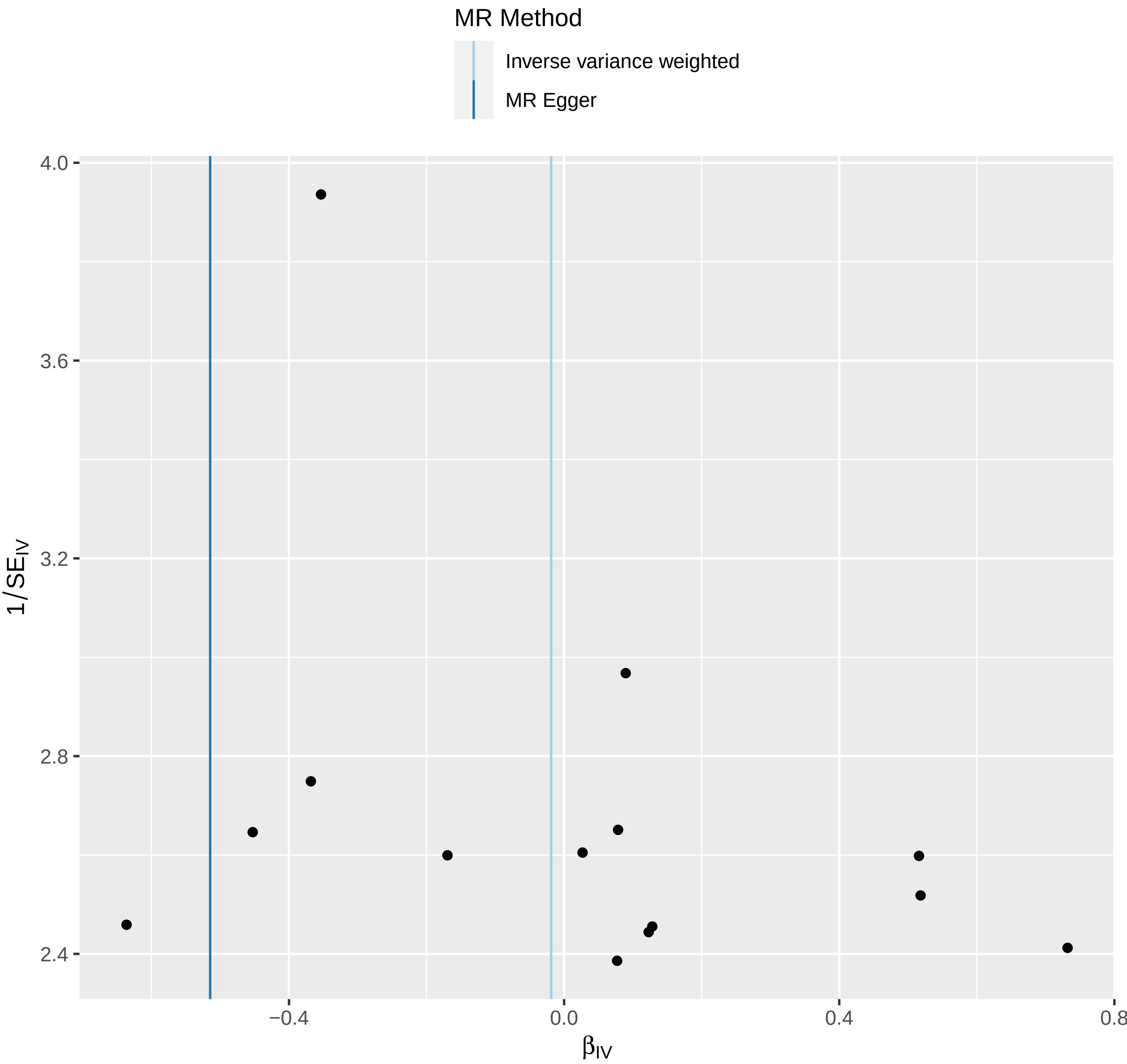

Batch 2−6375 : Gut microbiota abundance (genus Catenibacterium id.2153) on Vesicle−associated membrane protein 8 with a threshold of 1e−05

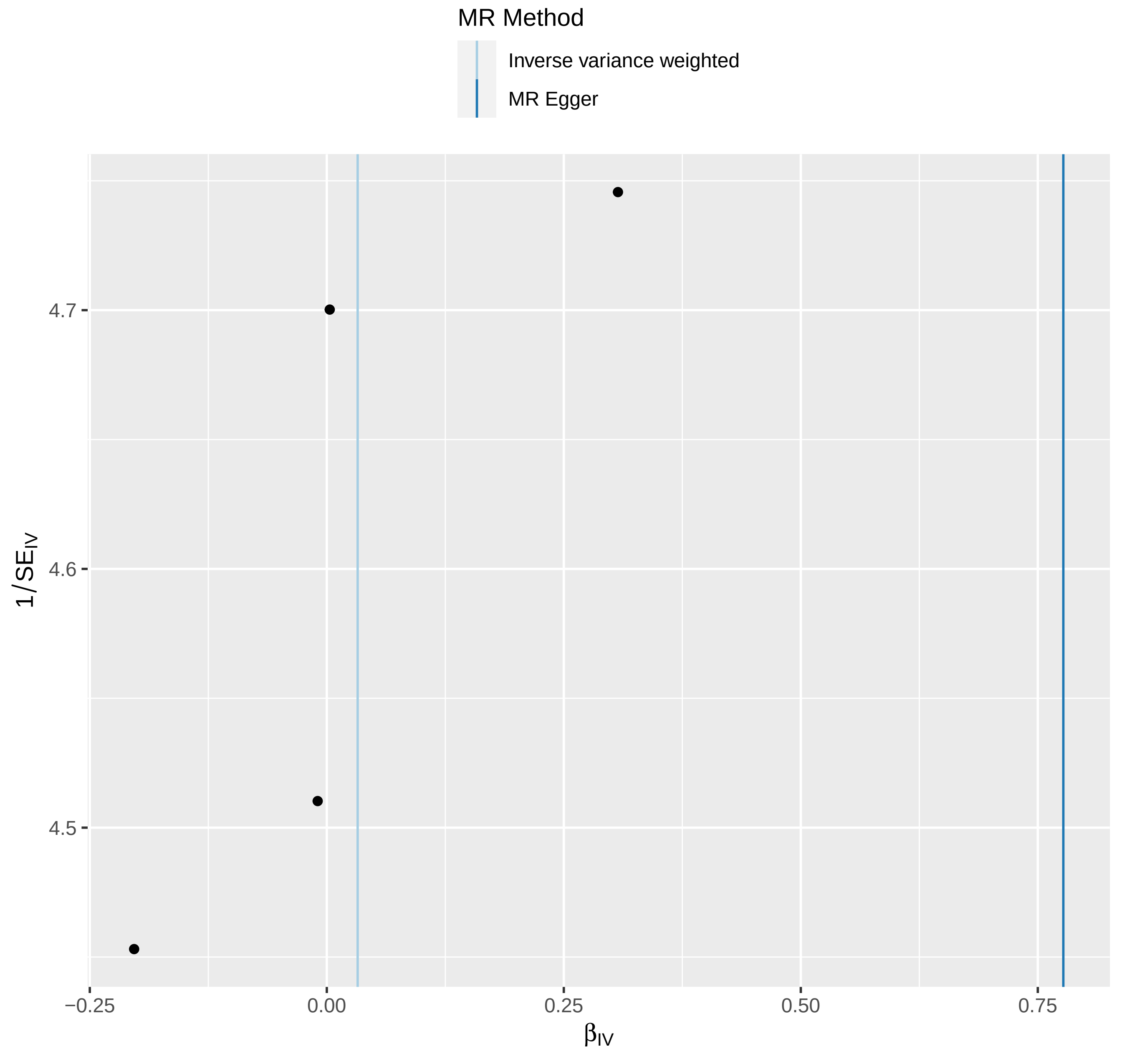

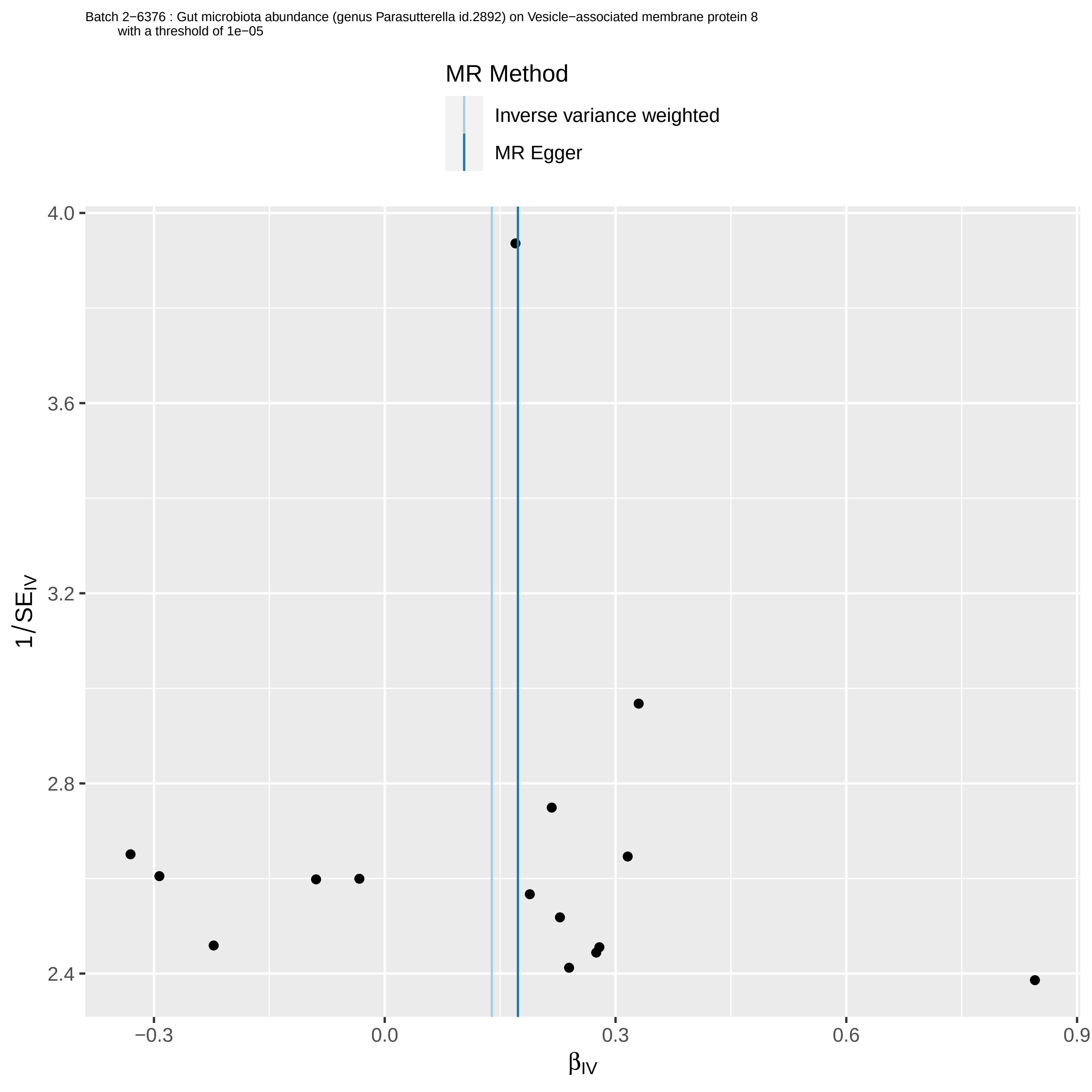
Batch 2−6376 : Gut microbiota abundance (genus Parasutterella id.2892) on Vesicle−associated membrane protein 8
with a threshold of 1e−05
MR Method
Inverse variance weighted
MR Egger
4.0
3.6
3.2
2.8
2.4
1/SE$_{IV}$
−0.3
0.0
0.3
0.6
0.9
β_{IV}

Batch 2−6377 : Gut microbiota abundance (genus Catenibacterium id.2153) on Vesicle−associated membrane protein−associated protein A with a threshold of 1e−05

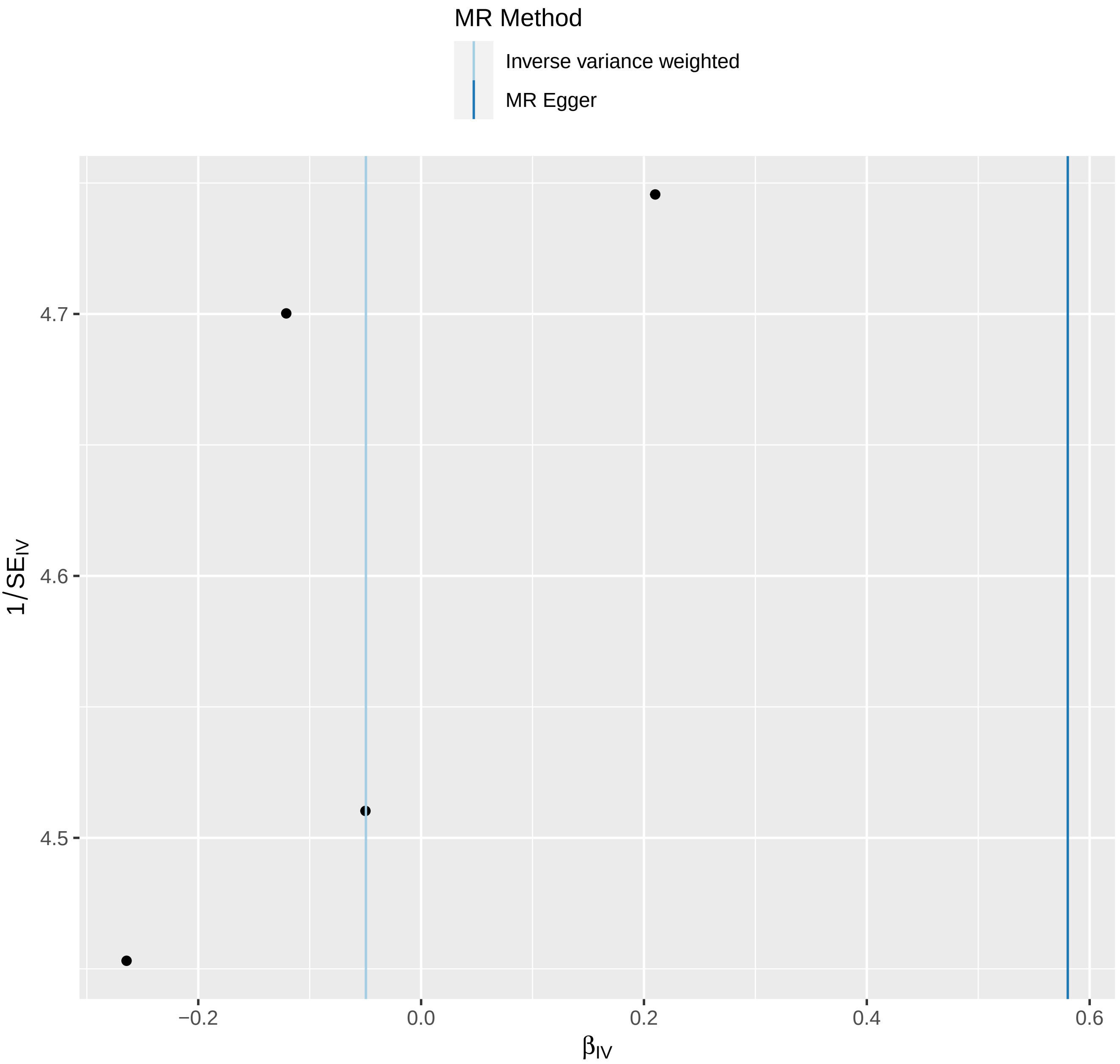

Batch 2−6378 : Gut microbiota abundance (genus Parasutterella id.2892) on Vesicle−associated membrane protein−associated protein A
with a threshold of 1e−05

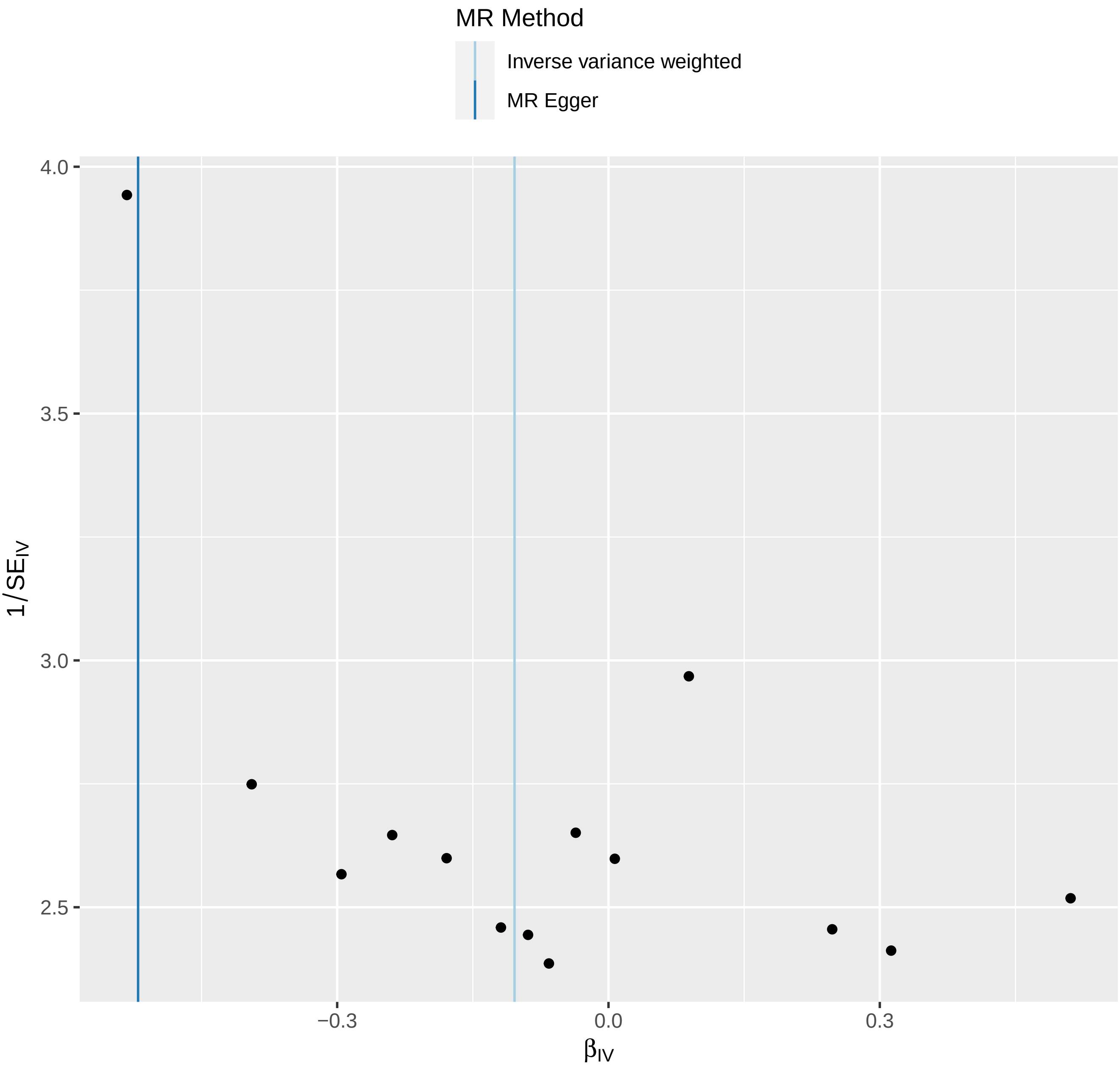

Batch 2−6379 : Gut microbiota abundance (genus Catenibacterium id.2153) on Vesicle−associated membrane protein−associated protein B/C
with a threshold of 1e−05

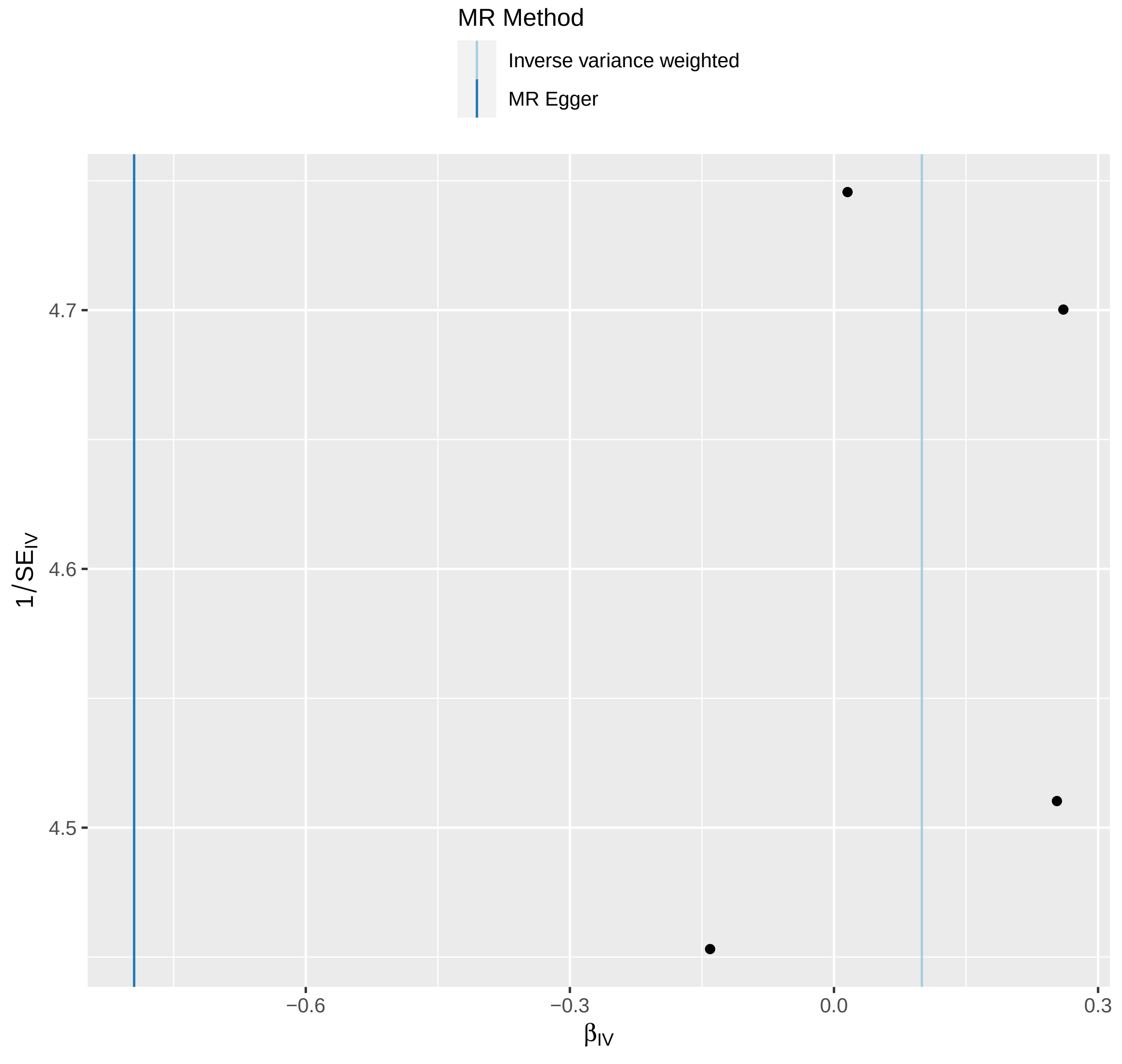

Batch 2−6380 : Gut microbiota abundance (genus Parasutterella id.2892) on Vesicle−associated membrane protein−associated protein B/C
with a threshold of 1e−05

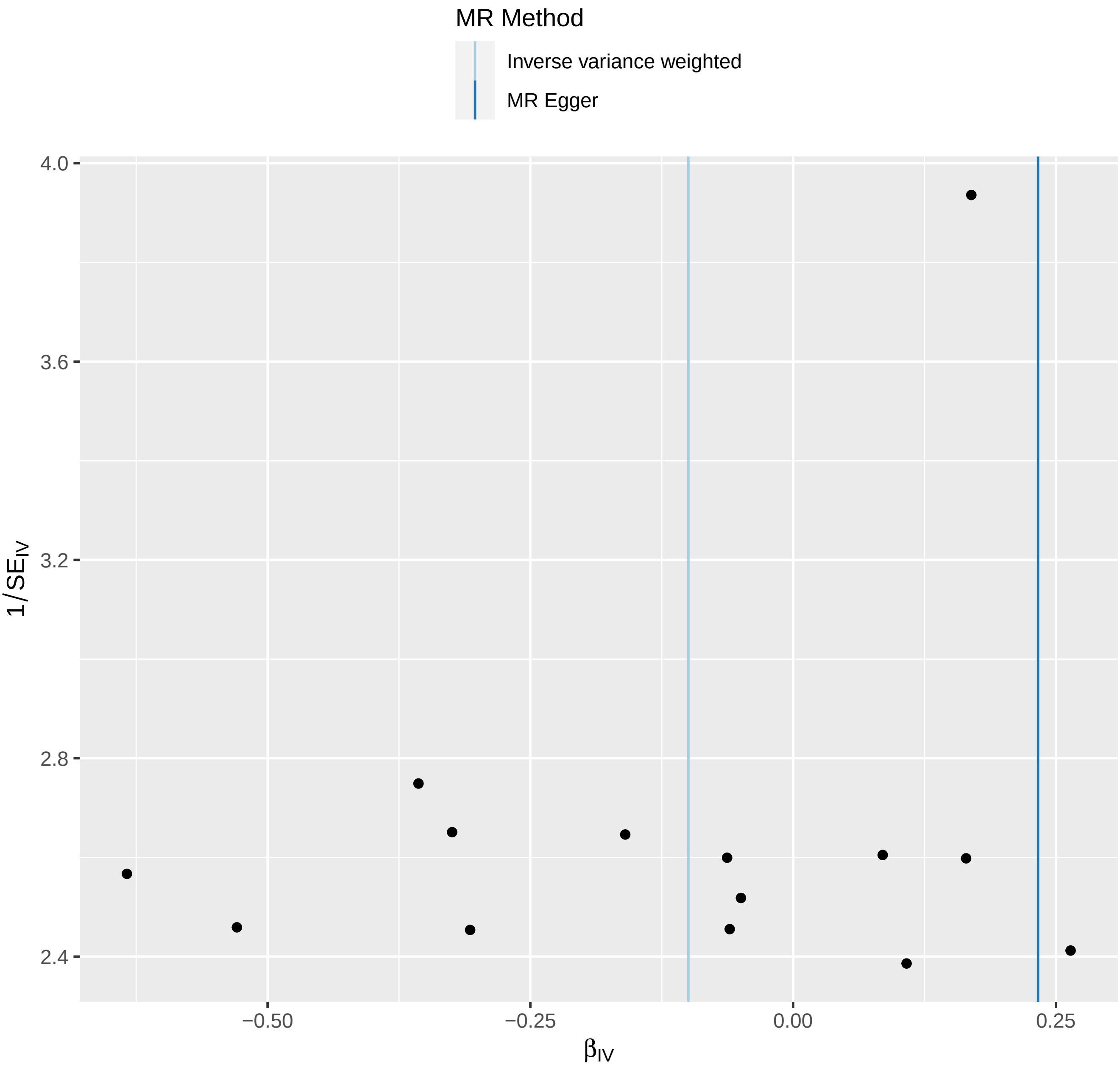

Batch 2−6381 : Gut microbiota abundance (genus Catenibacterium id.2153) on Valine−−tRNA ligase
with a threshold of 1e−05

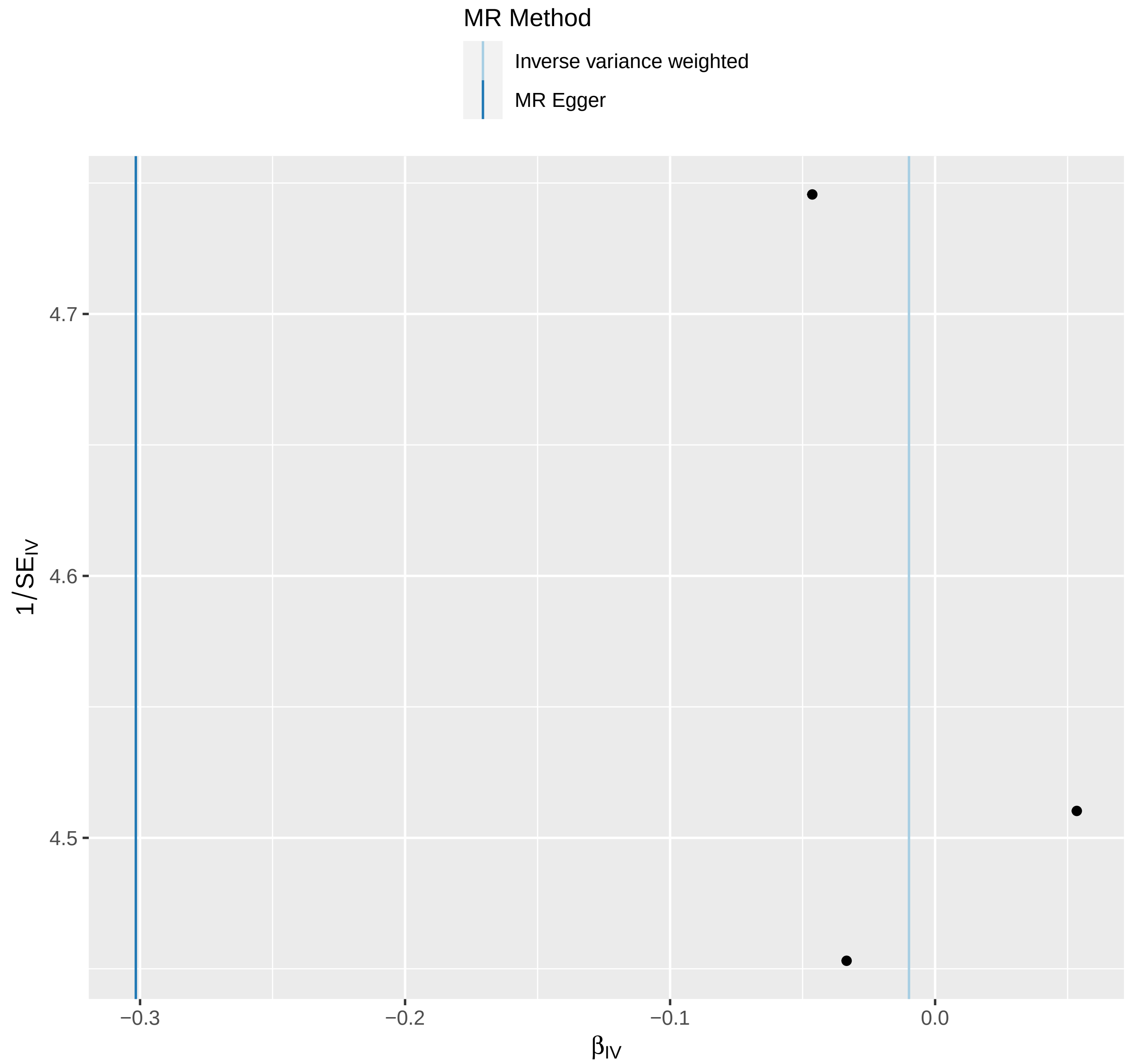

Batch 2−6382 : Gut microbiota abundance (genus Parasutterella id.2892) on Valine−−tRNA ligase
with a threshold of 1e−05

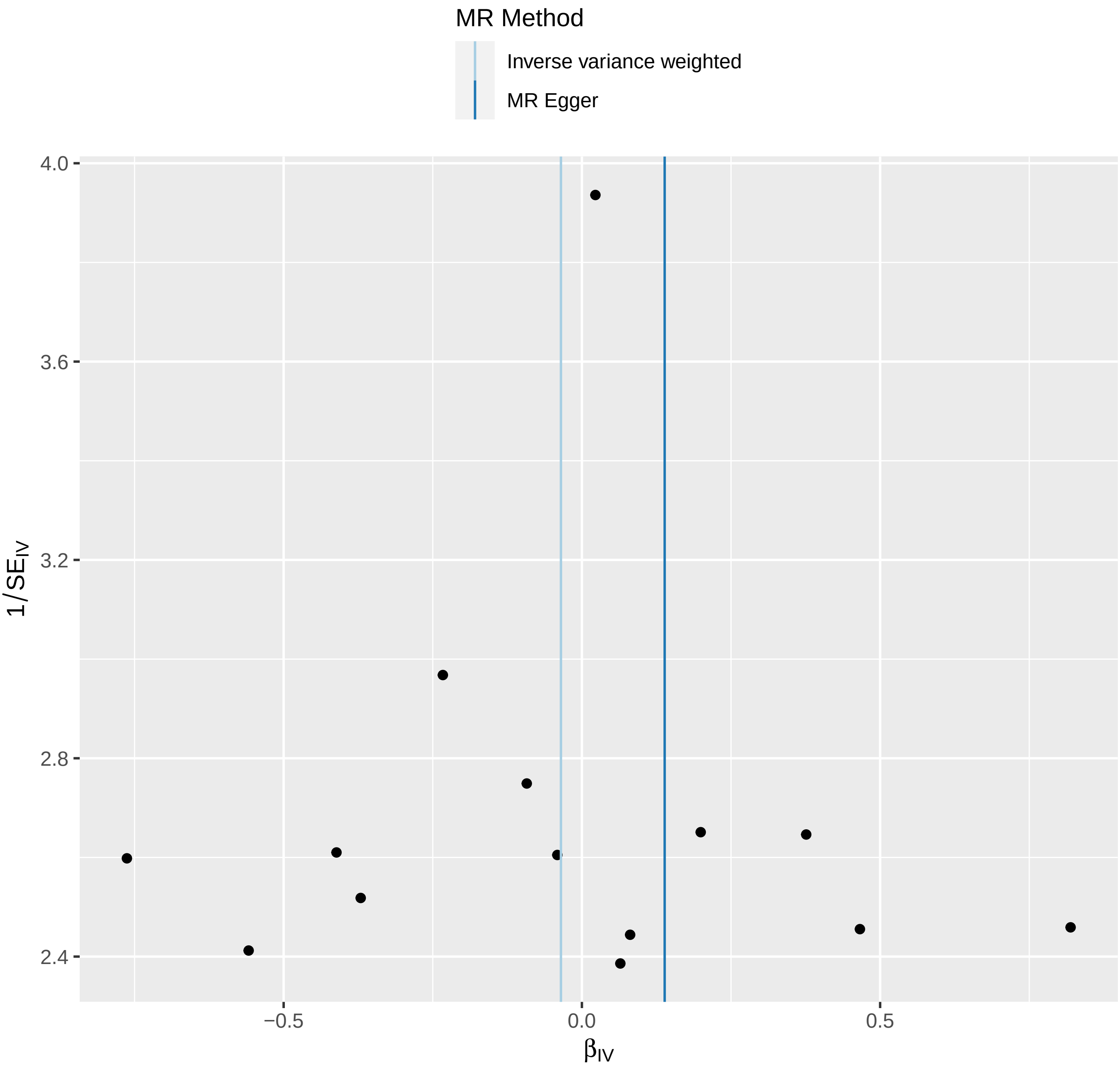

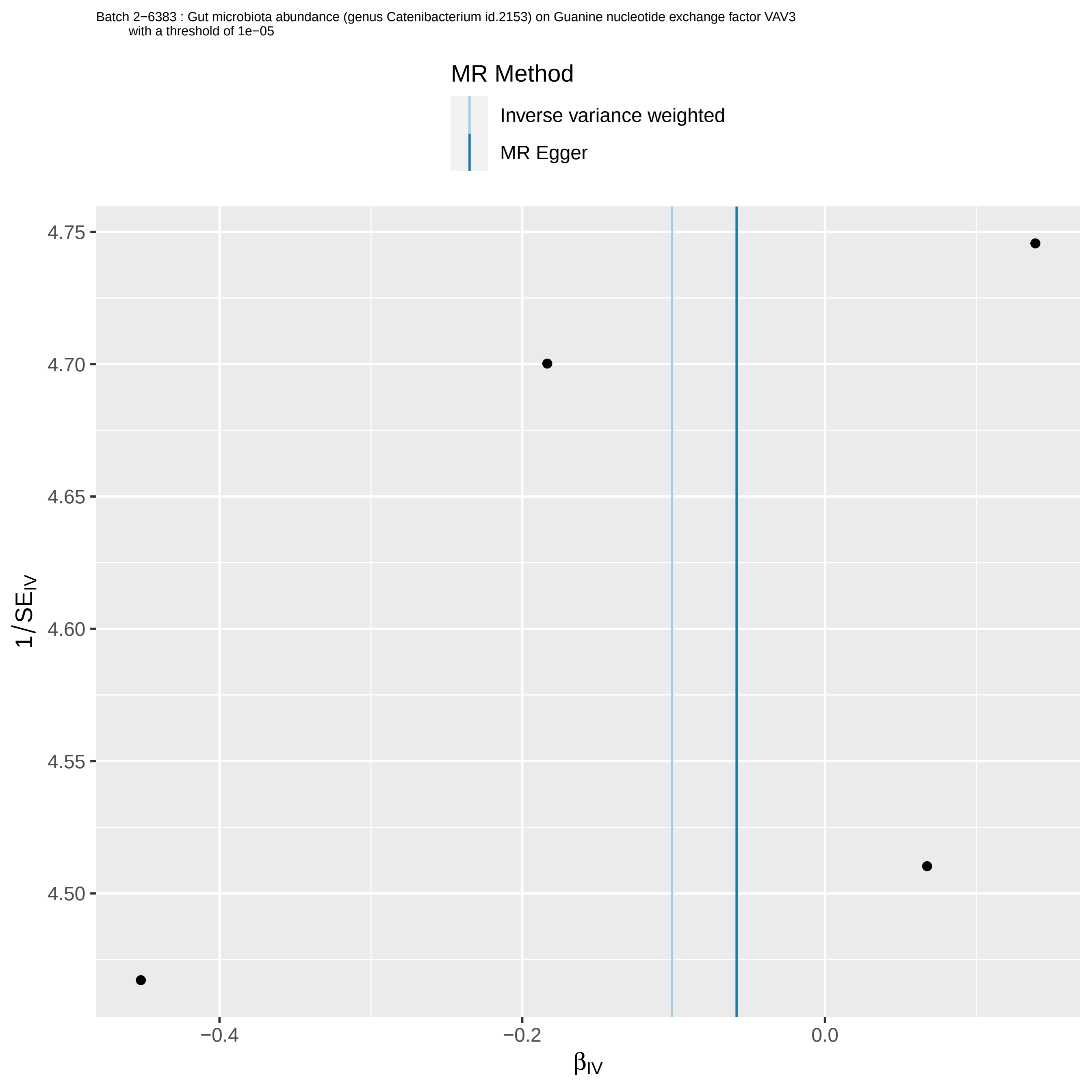
Batch 2−6383 : Gut microbiota abundance (genus Catenibacterium id.2153) on Guanine nucleotide exchange factor VAV3
with a threshold of 1e−05
MR Method
Inverse variance weighted
MR Egger
4.75
4.70
4.65
4.60
4.55
4.50
1/SE_IV
−0.4
−0.2
0.0
β_IV

Batch 2−6384 : Gut microbiota abundance (genus Parasutterella id.2892) on Guanine nucleotide exchange factor VAV3
with a threshold of 1e−05

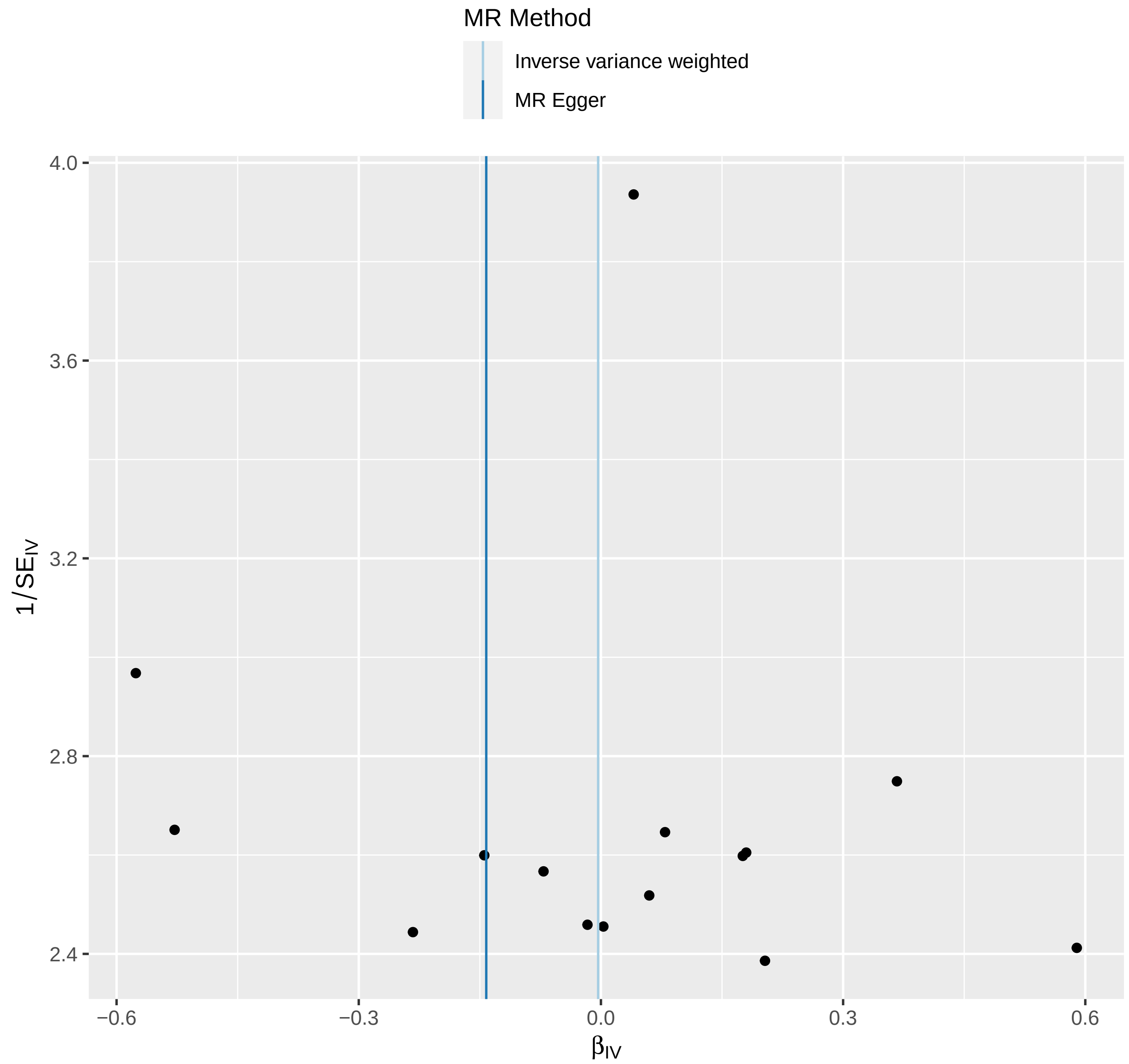

Batch 2−6385 : Gut microbiota abundance (genus Catenibacterium id.2153) on Vascular cell adhesion protein 1 with a threshold of 1e−05

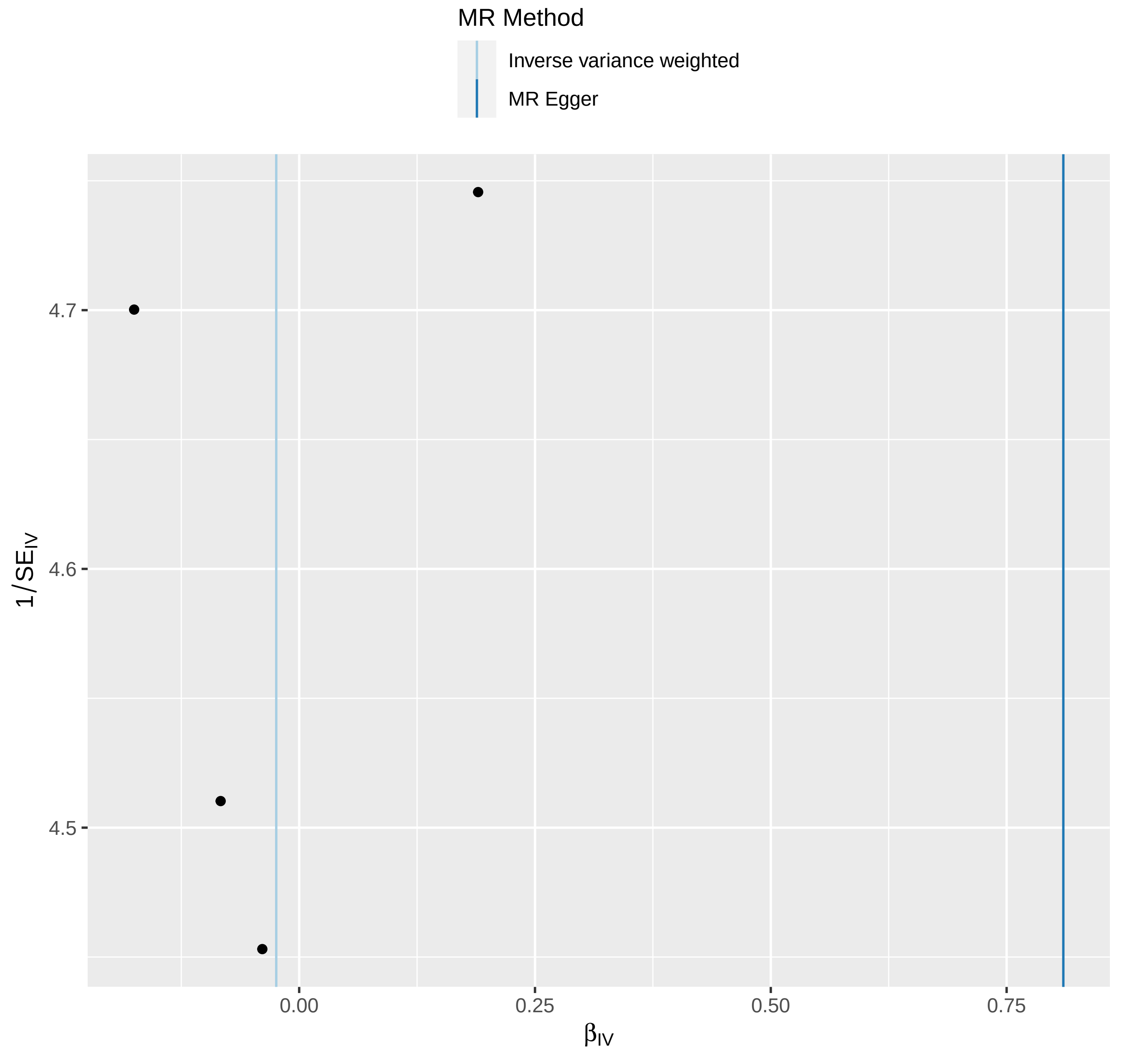

Batch 2−6386 : Gut microbiota abundance (genus Parasutterella id.2892) on Vascular cell adhesion protein 1
with a threshold of 1e−05

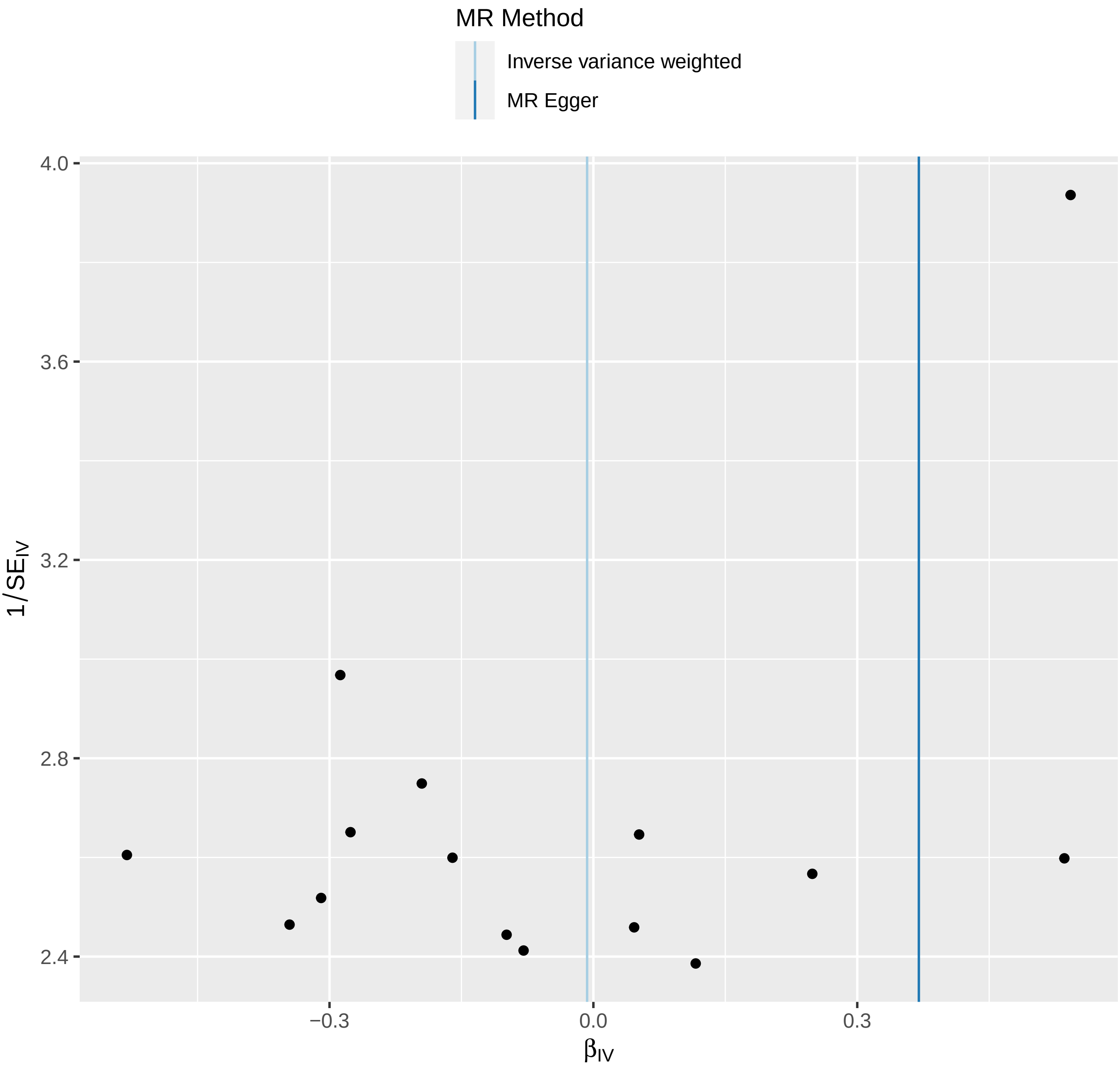

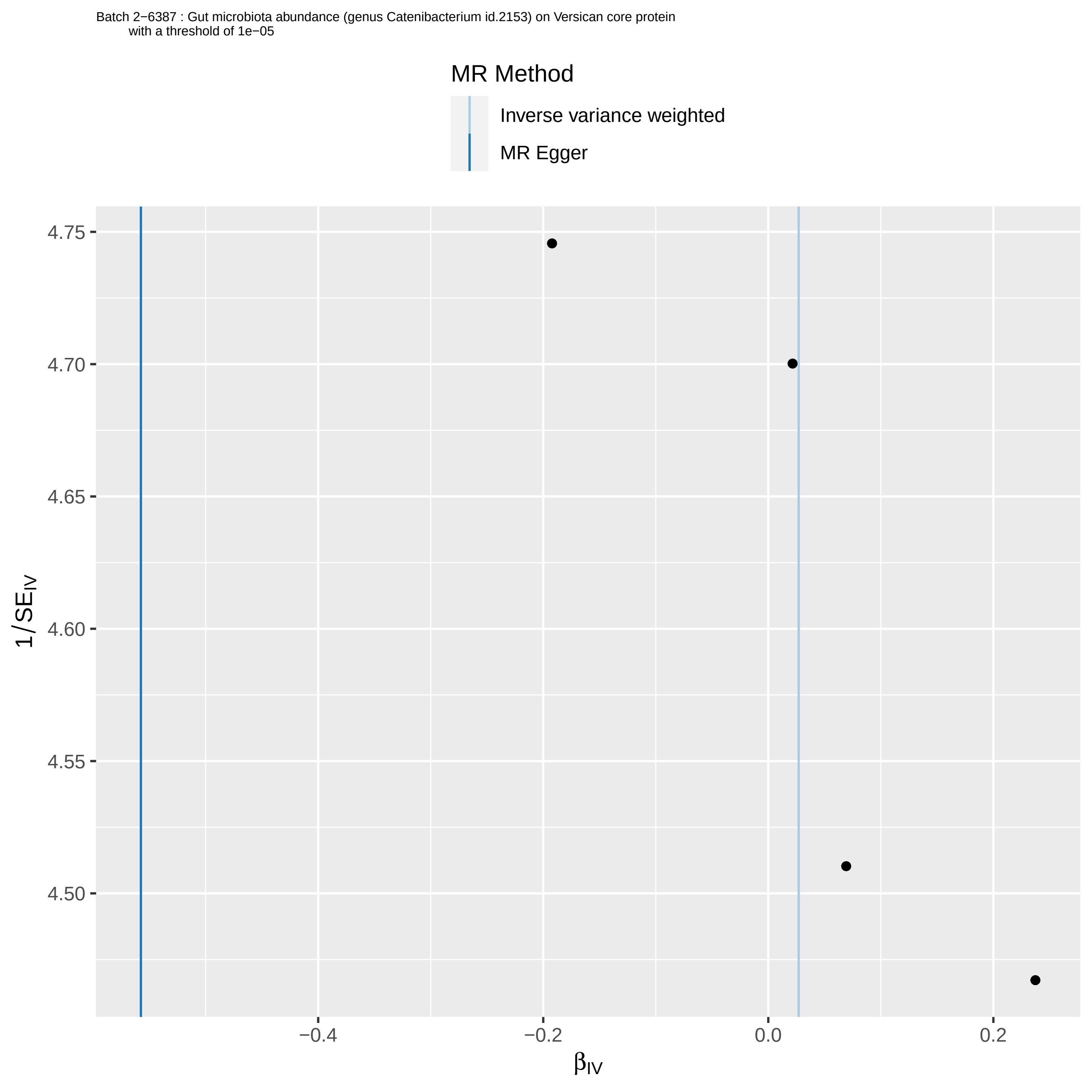

Batch 2−6387 : Gut microbiota abundance (genus Catenibacterium id.2153) on Versican core protein
with a threshold of 1e−05
MR Method
Inverse variance weighted
MR Egger
4.75
4.70
4.65
4.60
4.55
4.50
1/SE_IV
−0.4
−0.2
0.0
0.2
β_IV

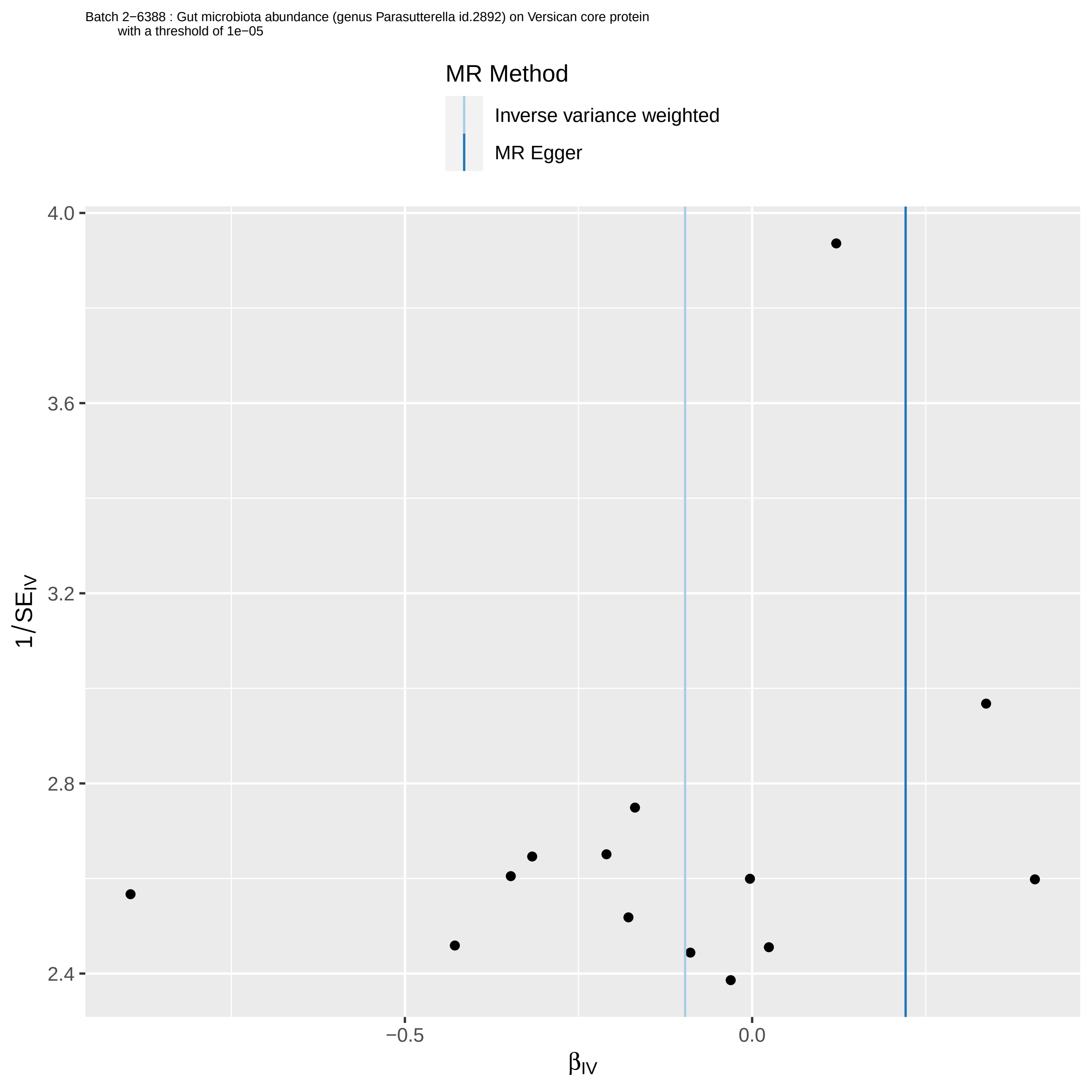
Batch 2−6388 : Gut microbiota abundance (genus Parasutterella id.2892) on Versican core protein
with a threshold of 1e−05
MR Method
Inverse variance weighted
MR Egger
4.0
3.6
3.2
2.8
2.4
1/SE_IV
−0.5
0.0
β_IV

Batch 2−6389 : Gut microbiota abundance (genus Catenibacterium id.2153) on Vinculin
with a threshold of 1e−05

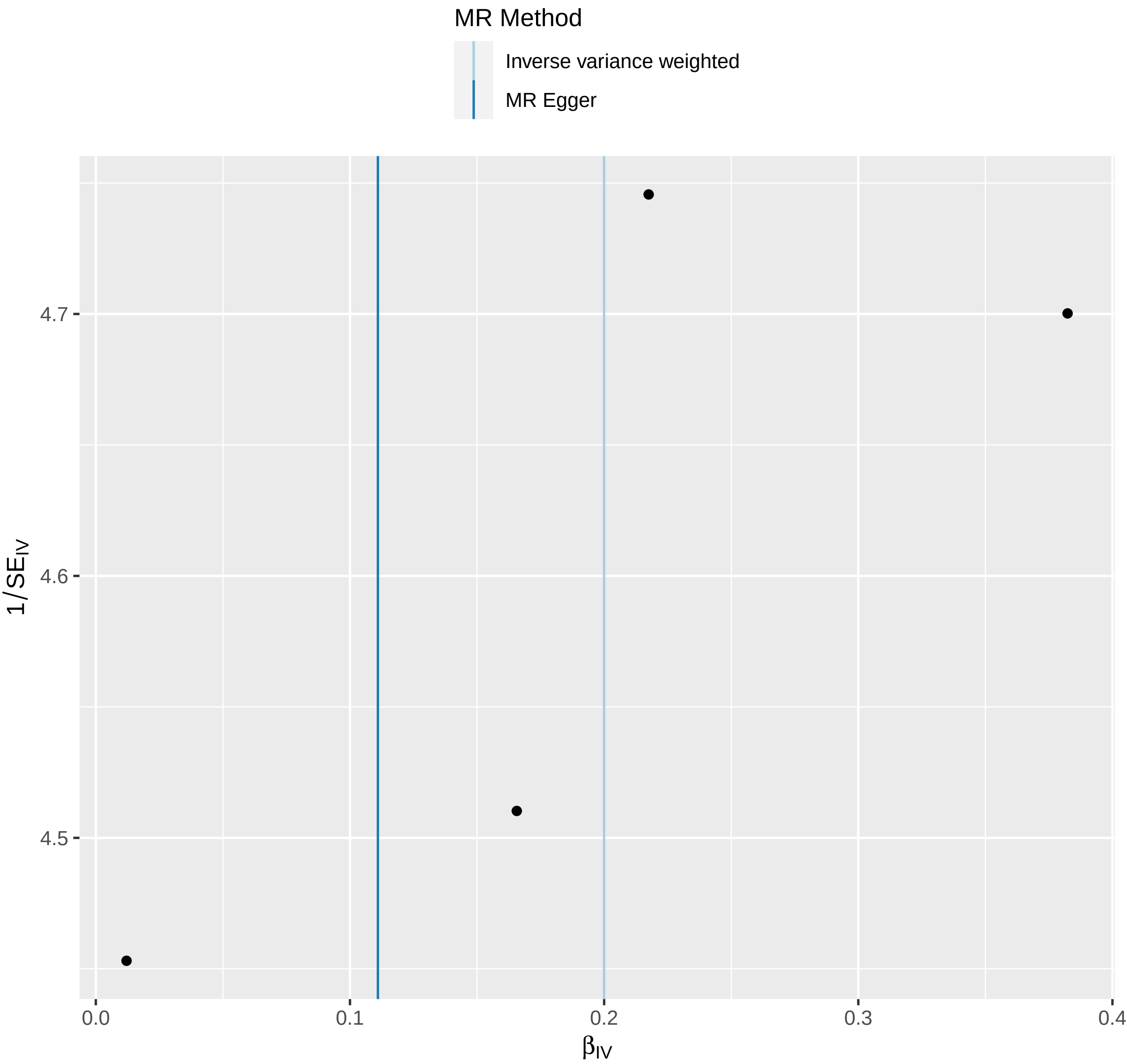

Batch 2−6390 : Gut microbiota abundance (genus Parasutterella id.2892) on Vinculin
with a threshold of 1e−05

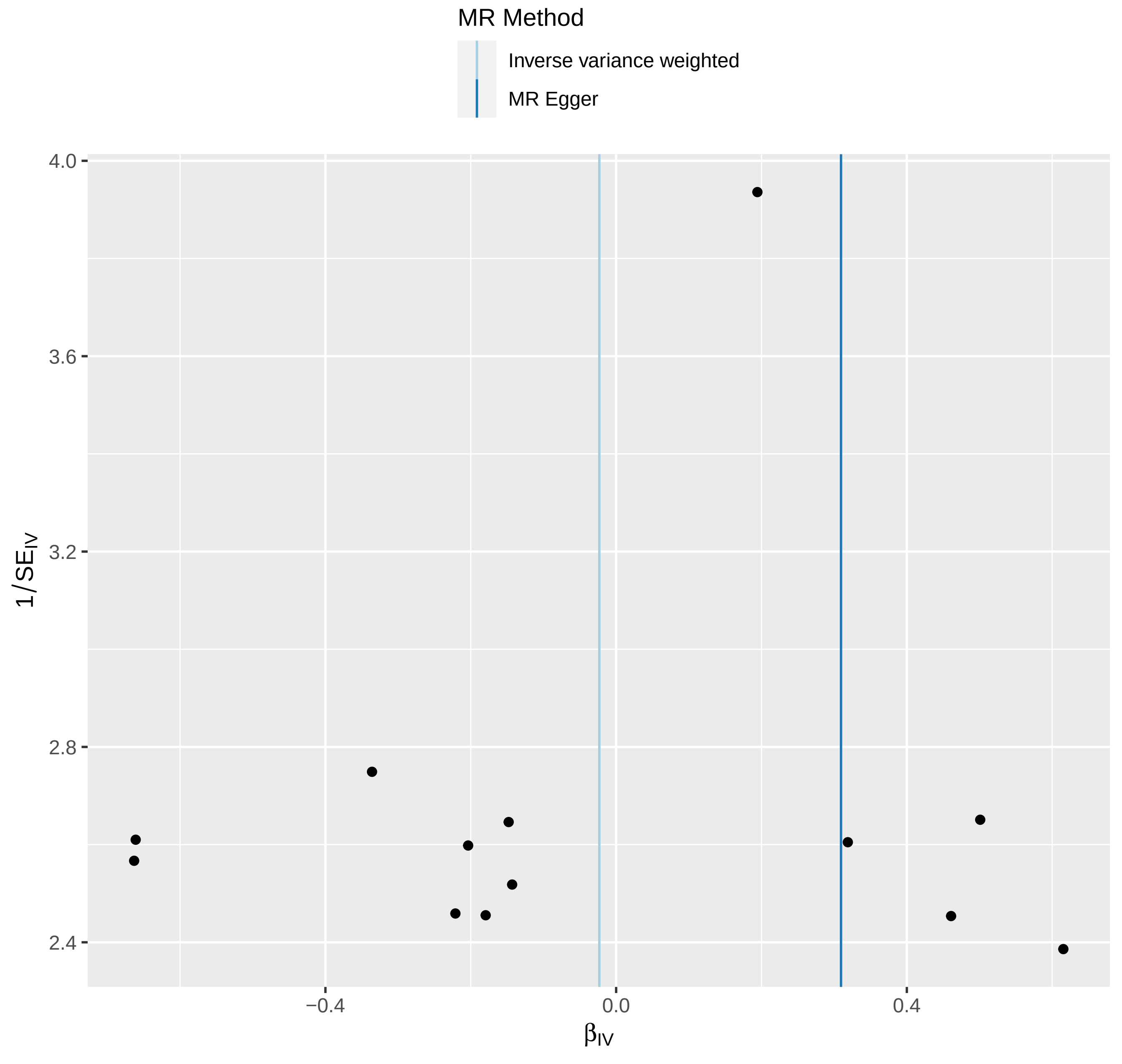

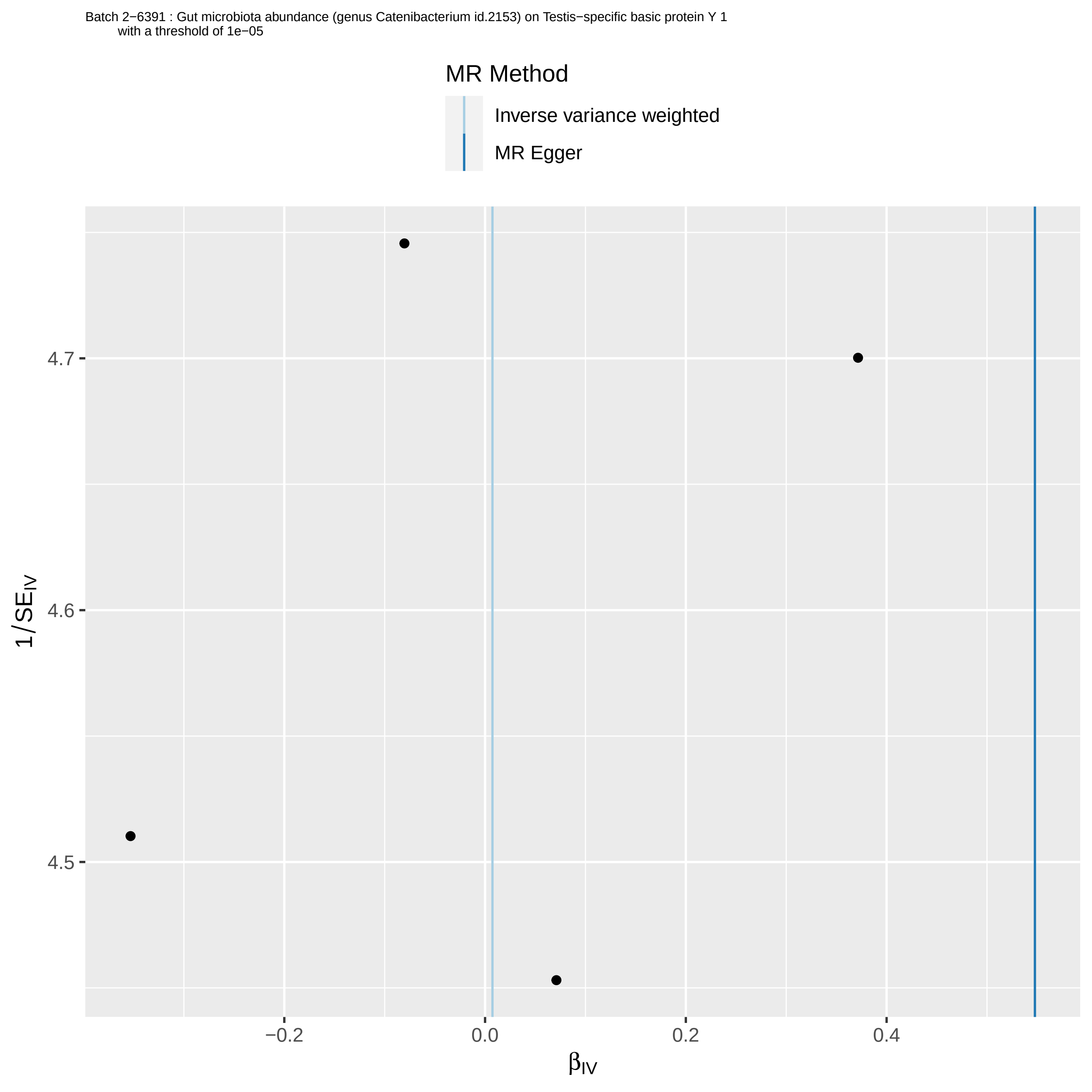

Batch 2−6391 : Gut microbiota abundance (genus Catenibacterium id.2153) on Testis−specific basic protein Y 1
with a threshold of 1e−05
MR Method
Inverse variance weighted
MR Egger
4.7
4.6
4.5
1/SE_IV
−0.2
0.0
0.2
0.4
β_IV

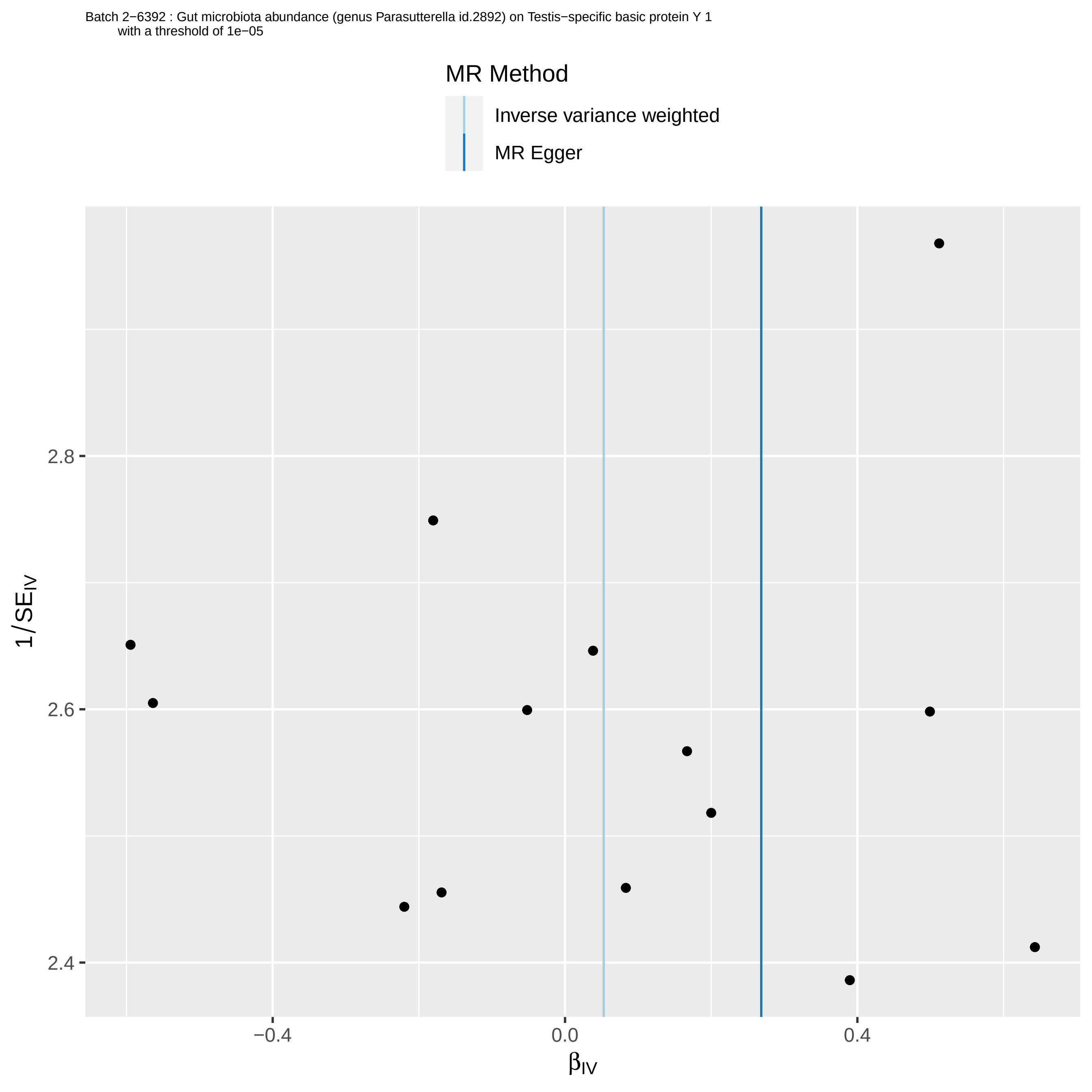

Batch 2−6392 : Gut microbiota abundance (genus Parasutterella id.2892) on Testis−specific basic protein Y 1
with a threshold of 1e−05
MR Method
Inverse variance weighted
MR Egger
2.8
2.6
2.4
1/SE_IV
−0.4
0.0
0.4
β_IV

Batch 2−6393 : Gut microbiota abundance (genus Catenibacterium id.2153) on Vascular endothelial growth factor A, isoform 121
with a threshold of 1e−05

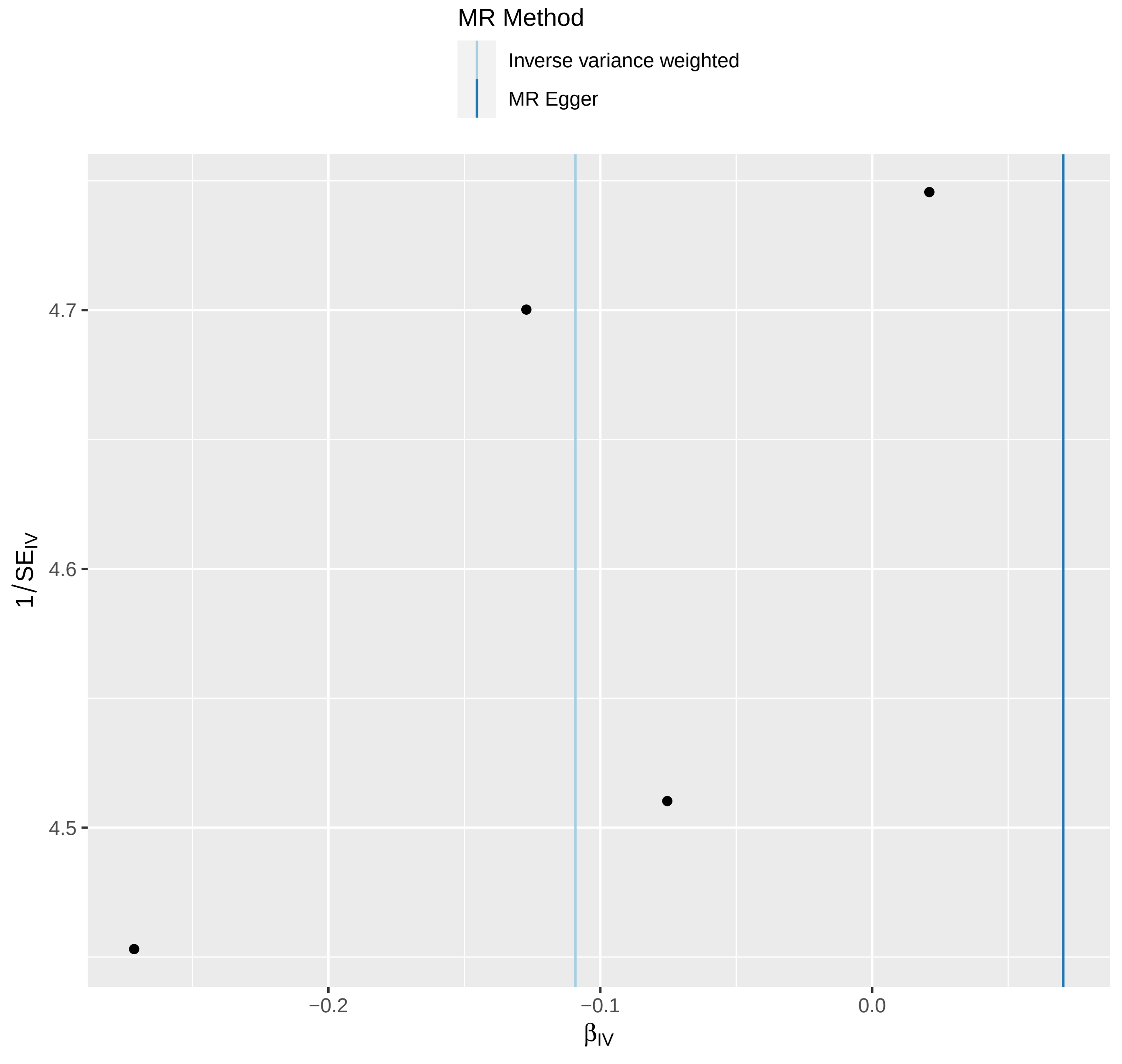

Batch 2−6394 : Gut microbiota abundance (genus Parasutterella id.2892) on Vascular endothelial growth factor A, isoform 121 with a threshold of 1e−05

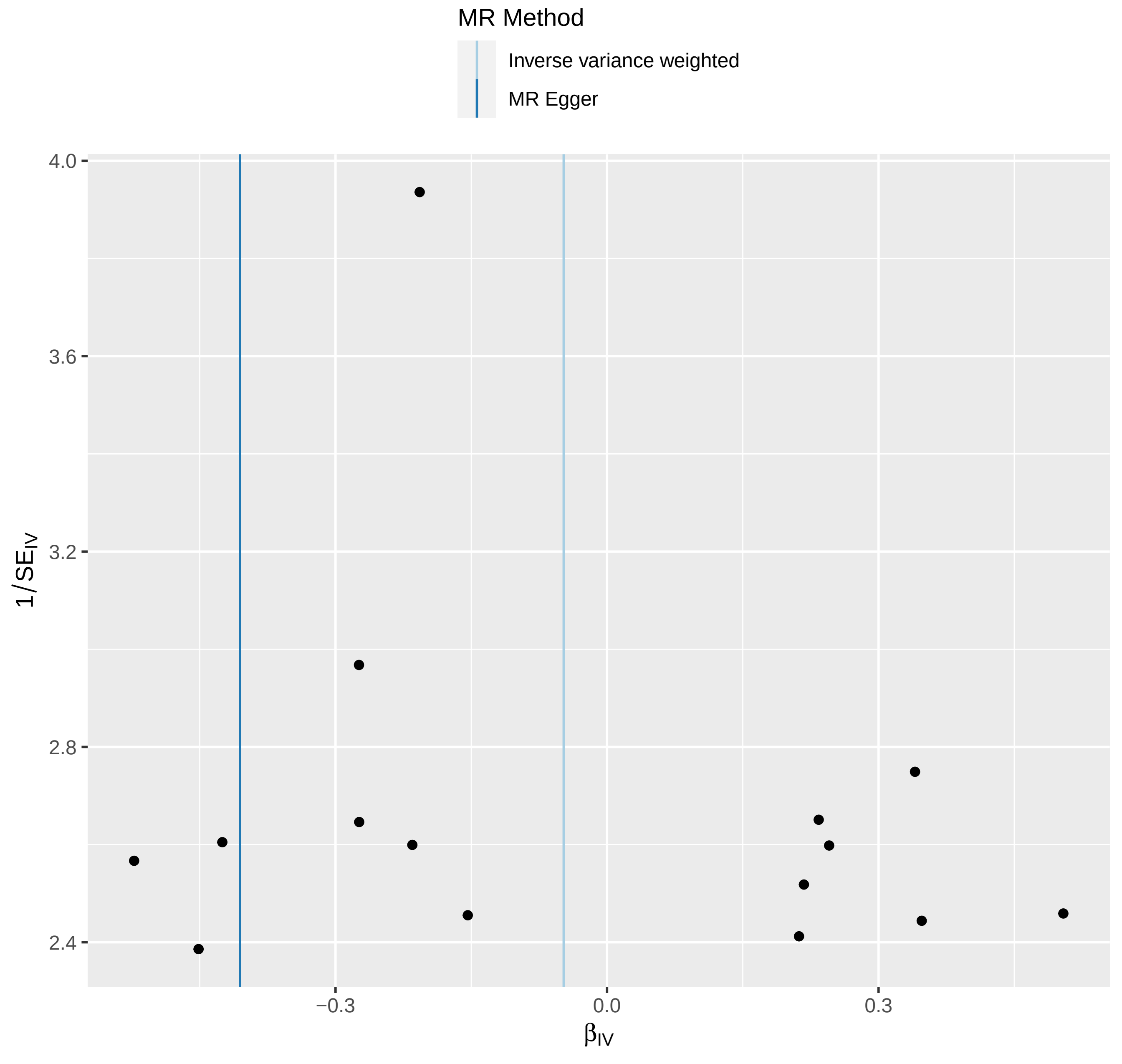

Batch 2−6395 : Gut microbiota abundance (genus Catenibacterium id.2153) on Vascular endothelial growth factor A
with a threshold of 1e−05

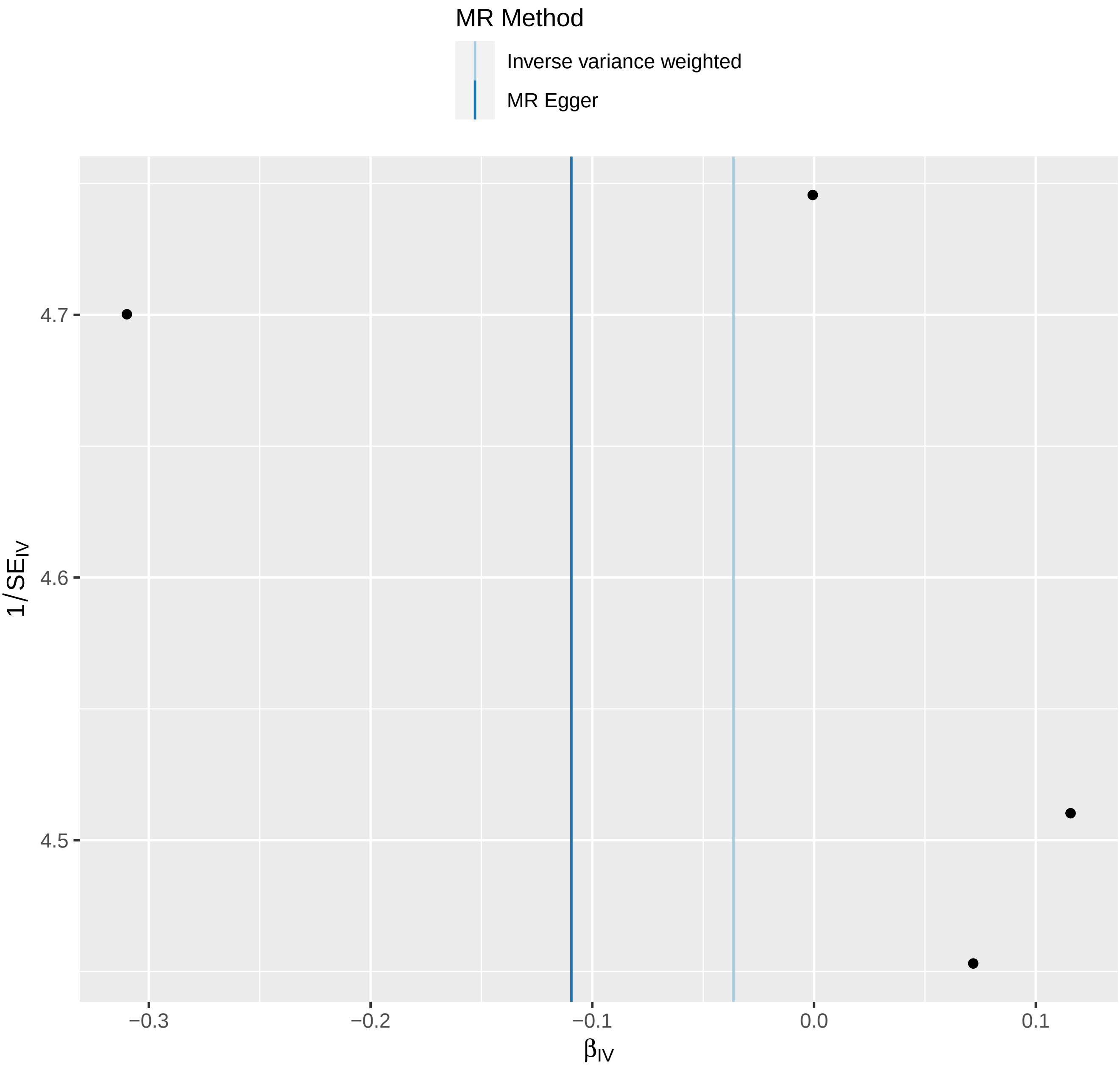

Batch 2−6396 : Gut microbiota abundance (genus Parasutterella id.2892) on Vascular endothelial growth factor A
with a threshold of 1e−05

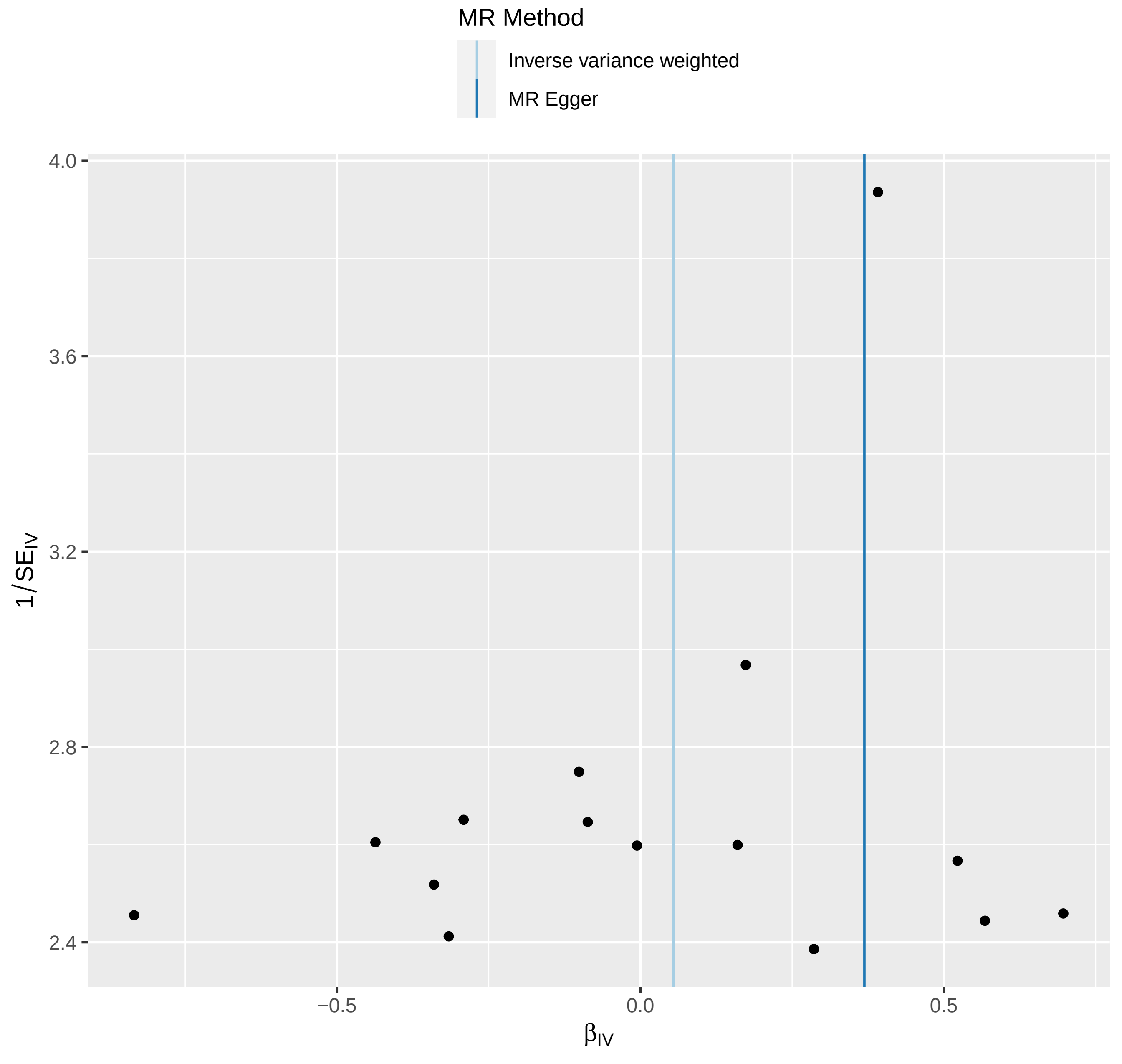

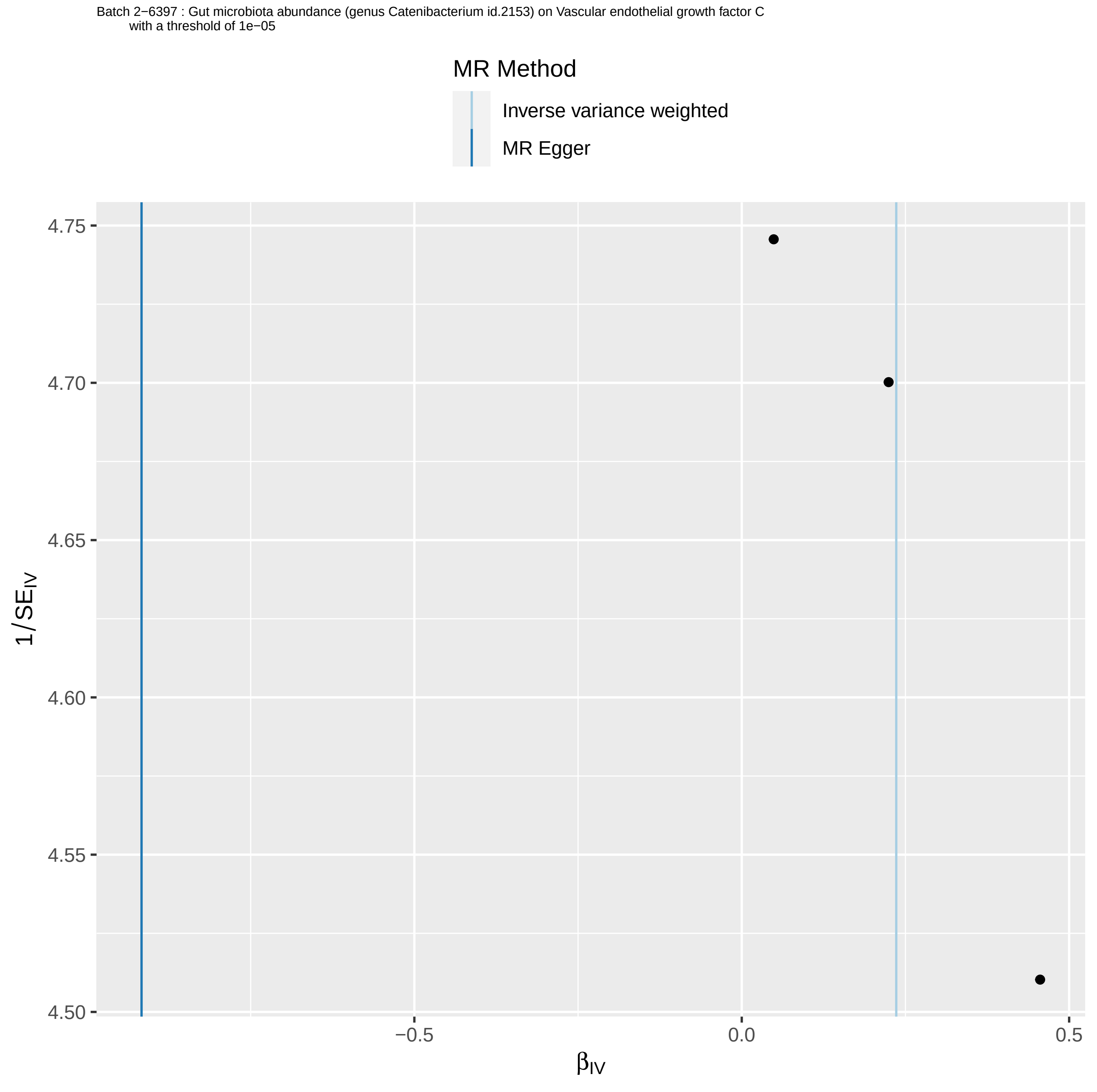

Batch 2−6397 : Gut microbiota abundance (genus Catenibacterium id.2153) on Vascular endothelial growth factor C
with a threshold of 1e−05
MR Method
Inverse variance weighted
MR Egger
4.75
4.70
4.65
4.60
4.55
4.50
1/SE_IV
−0.5
0.0
0.5
β_IV

Batch 2−6398 : Gut microbiota abundance (genus Parasutterella id.2892) on Vascular endothelial growth factor C
with a threshold of 1e−05

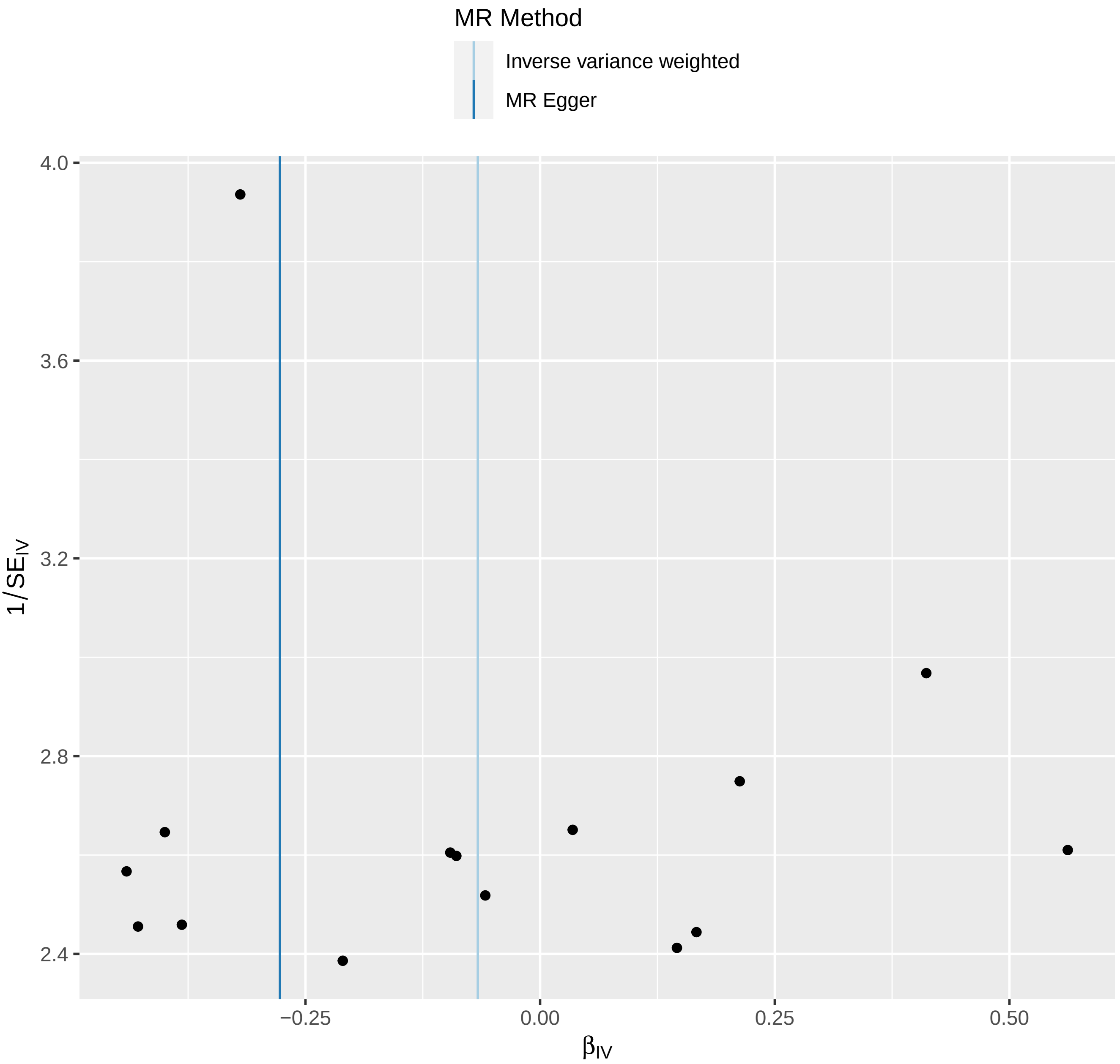

Batch 2−6399 : Gut microbiota abundance (genus Catenibacterium id.2153) on Selenoprotein S
with a threshold of 1e−05

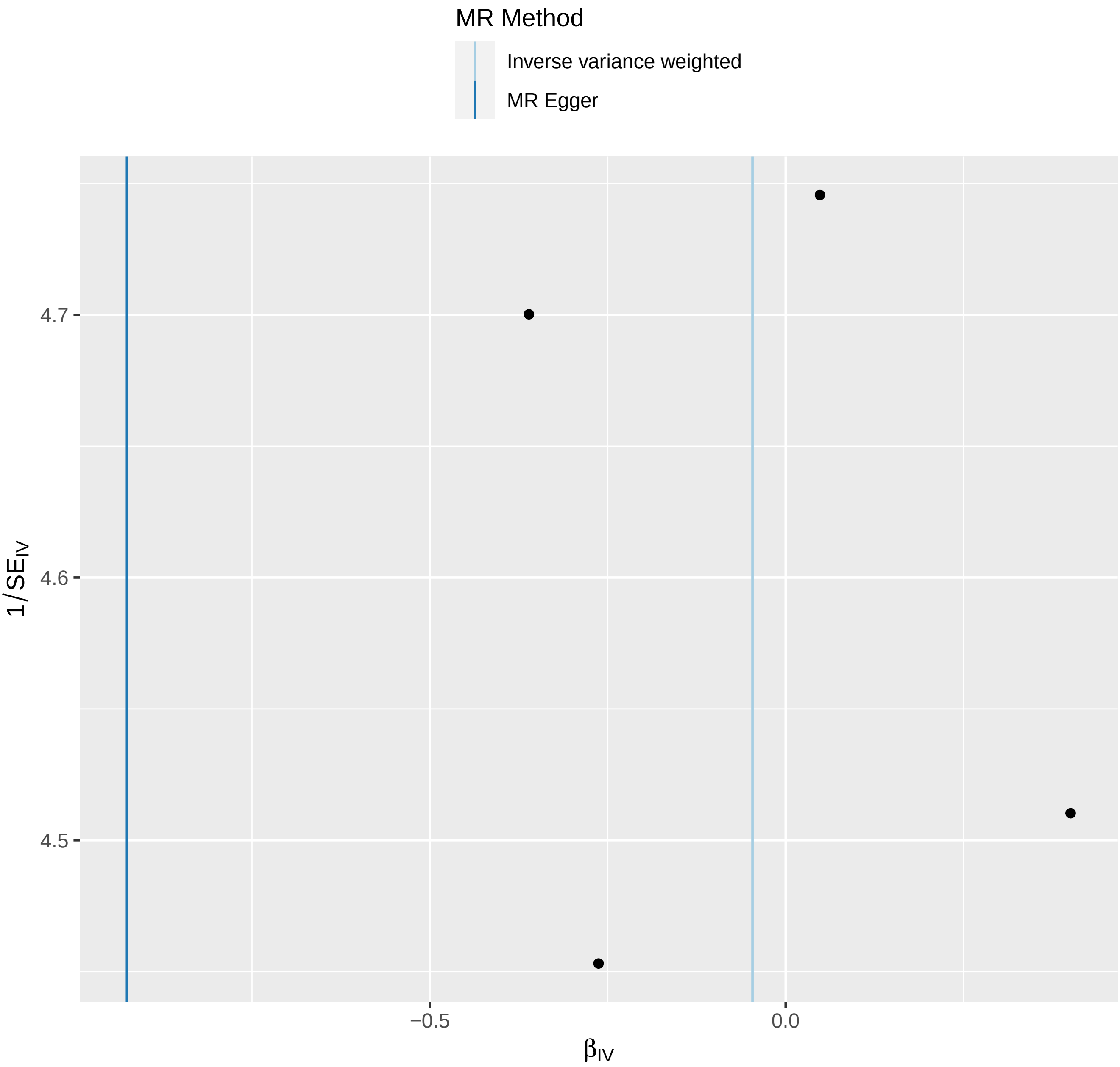

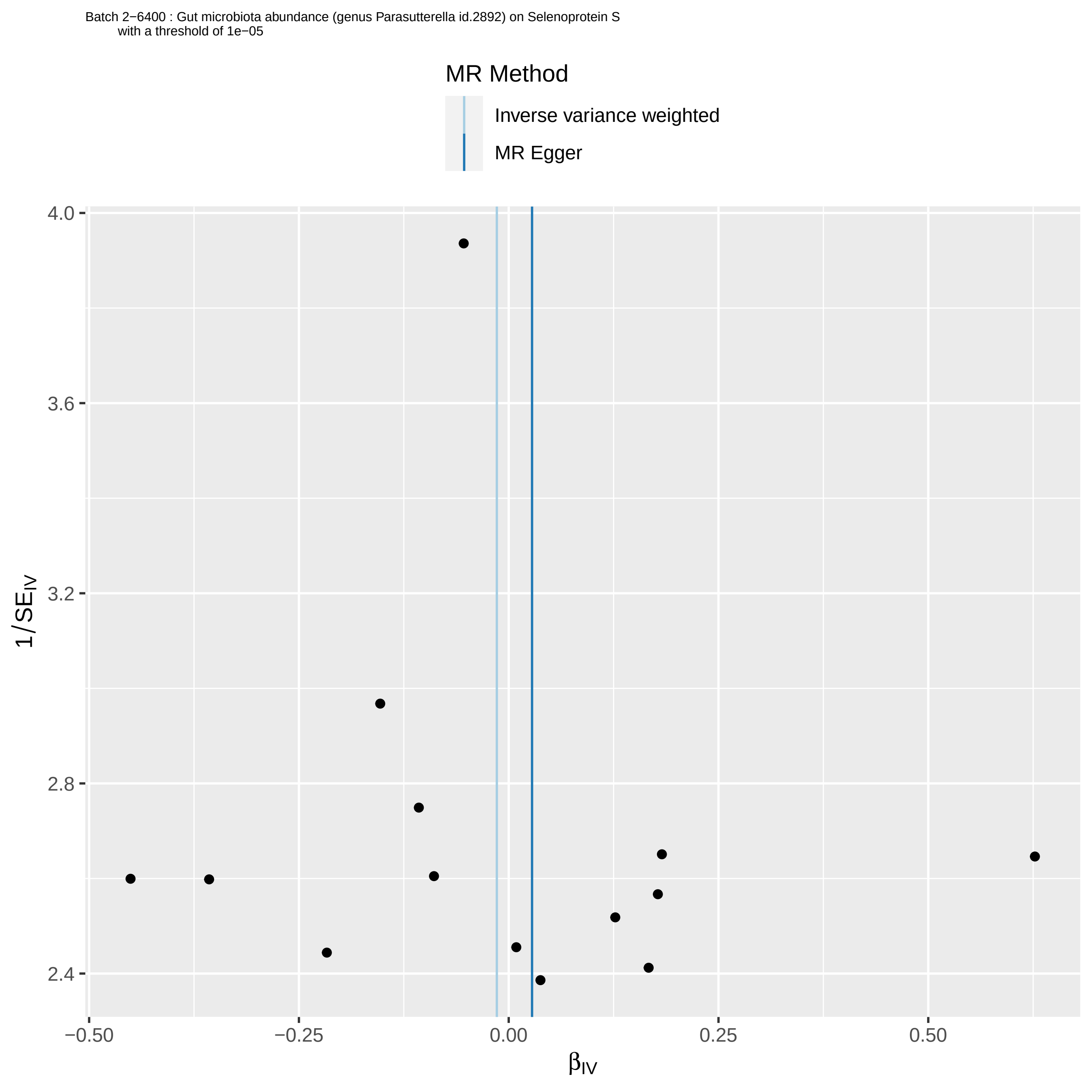
Batch 2−6400 : Gut microbiota abundance (genus Parasutterella id.2892) on Selenoprotein S
with a threshold of 1e−05
MR Method
Inverse variance weighted
MR Egger
4.0
3.6
3.2
2.8
2.4
1/SE_IV
−0.50
−0.25
0.00
0.25
0.50
β_IV

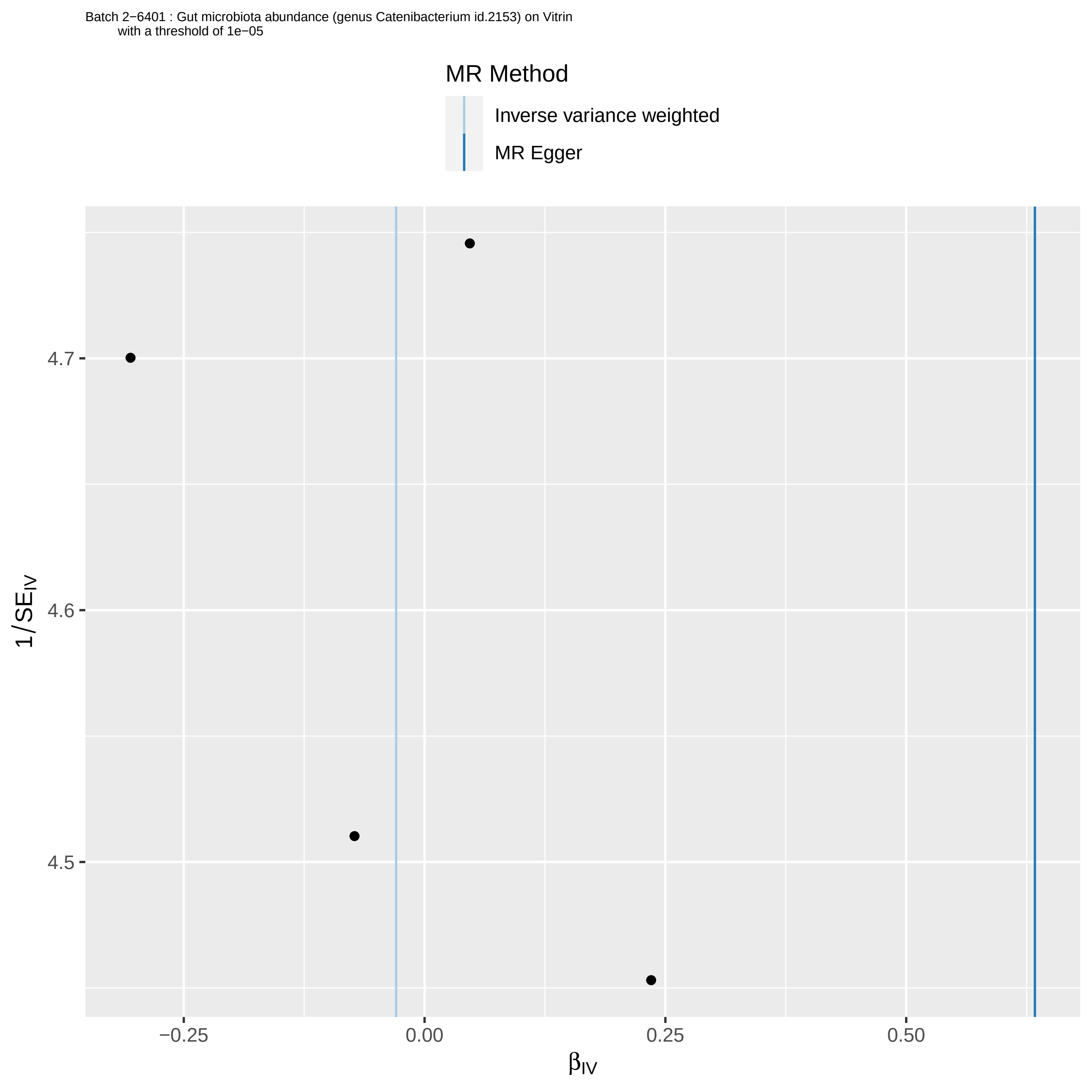
Batch 2−6401 : Gut microbiota abundance (genus Catenibacterium id.2153) on Vitrin
with a threshold of 1e−05
MR Method
Inverse variance weighted
MR Egger
4.7
4.6
4.5
1/SE_IV
−0.25
0.00
0.25
0.50
β_IV

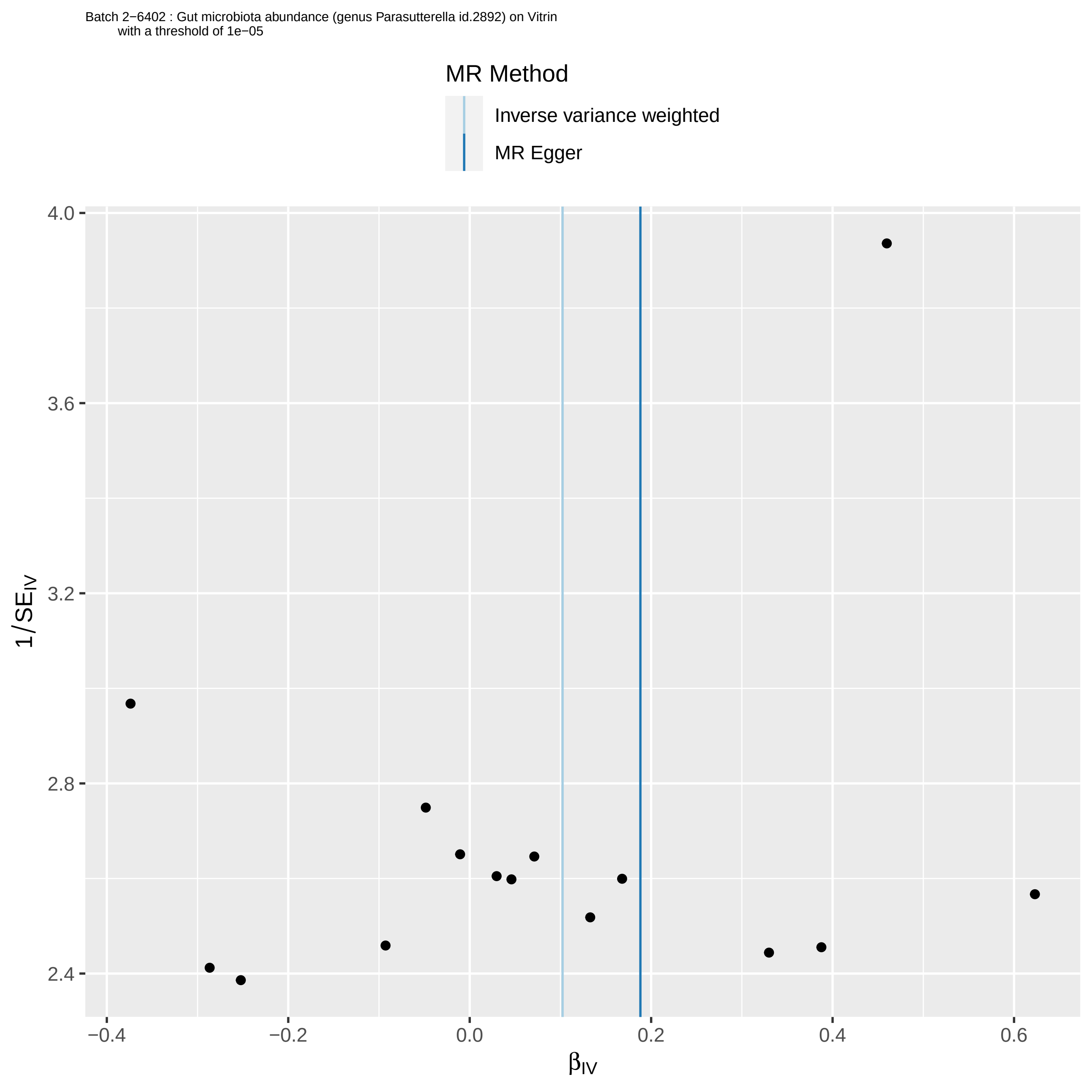

Batch 2−6402 : Gut microbiota abundance (genus Parasutterella id.2892) on Vitrin
with a threshold of 1e−05
MR Method
Inverse variance weighted
MR Egger
4.0
3.6
3.2
2.8
2.4
1/SE_IV
−0.4
−0.2
0.0
0.2
0.4
0.6
β_IV

Batch 2−6403 : Gut microbiota abundance (genus Catenibacterium id.2153) on Charged multivesicular body protein 3
with a threshold of 1e−05

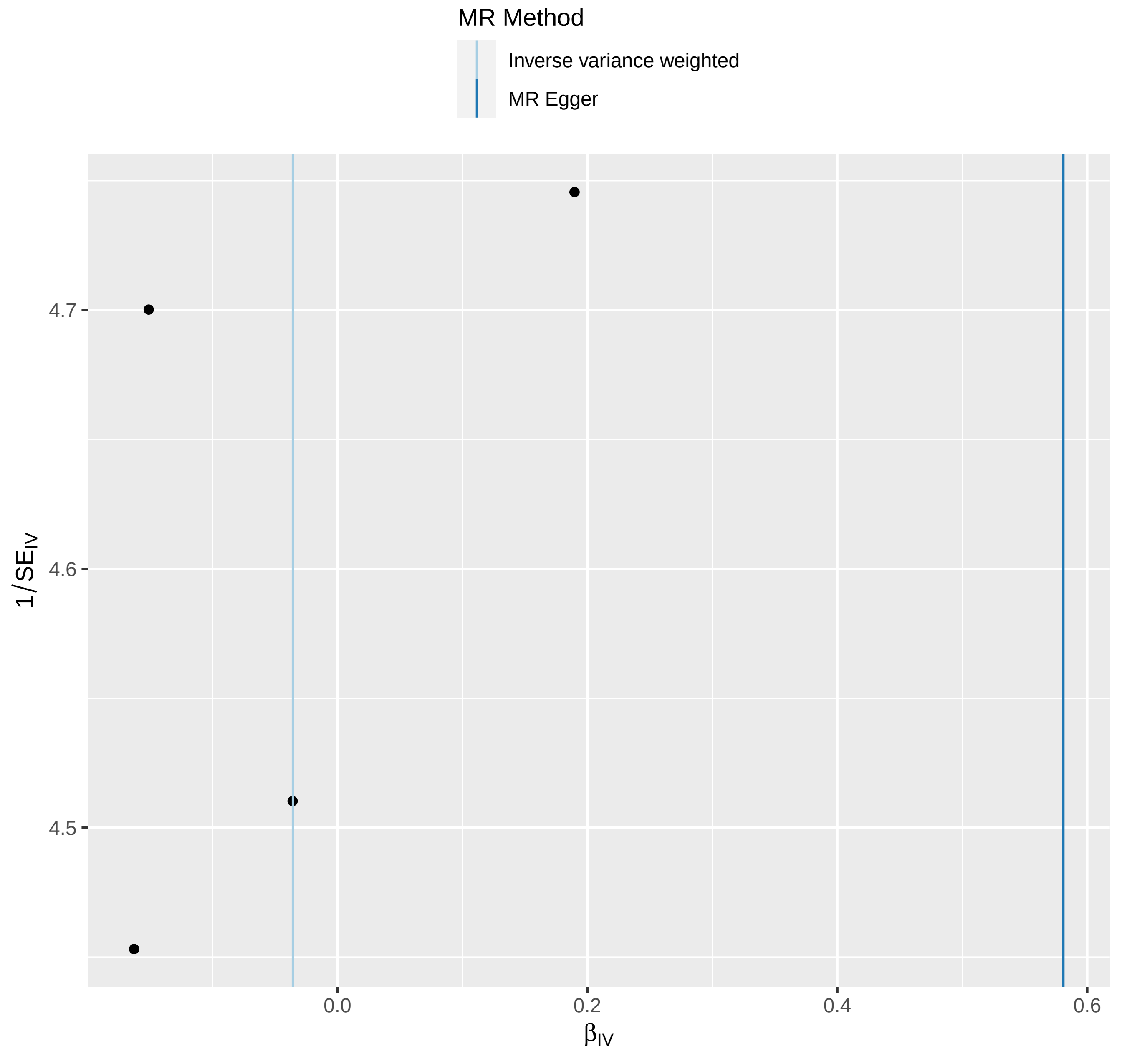

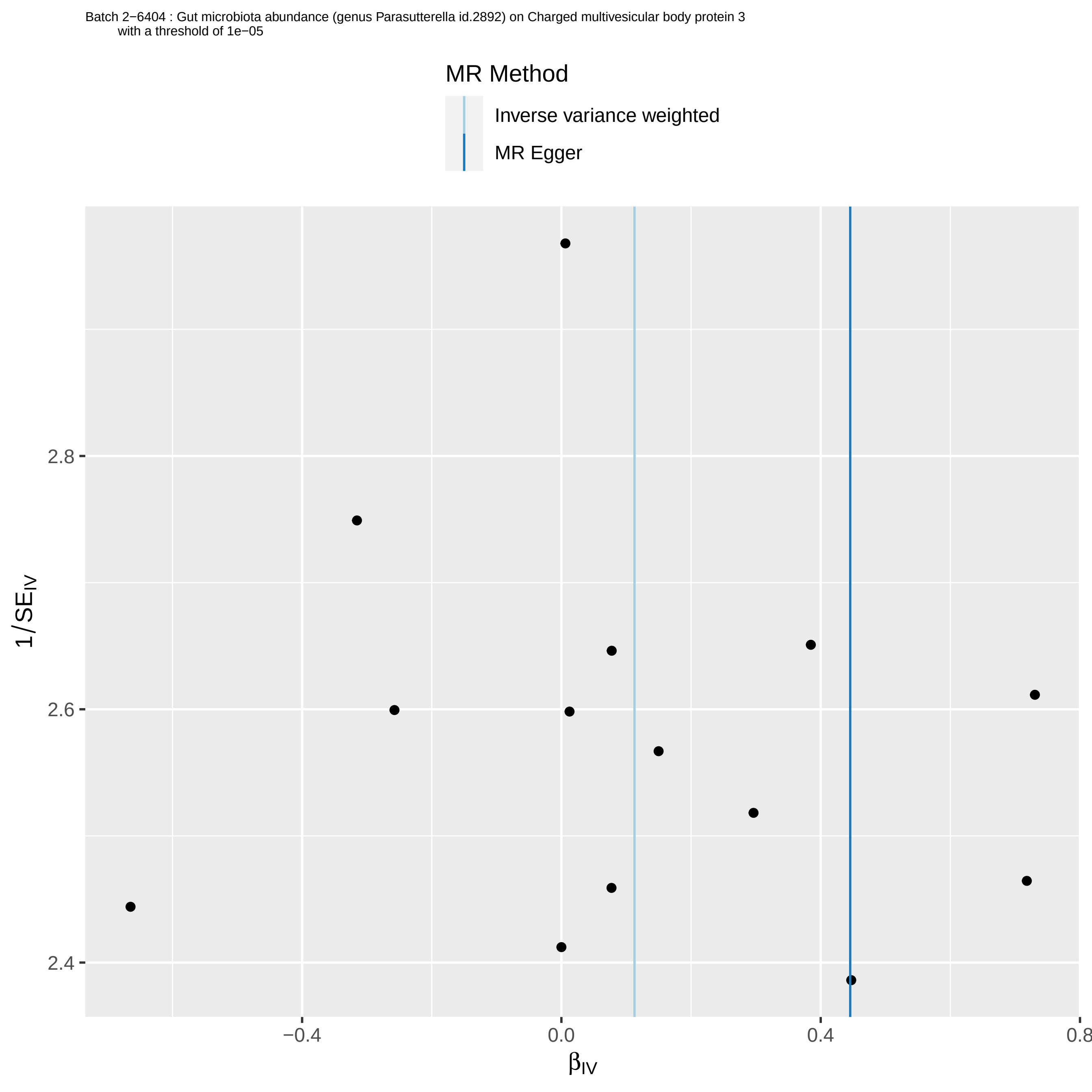

Batch 2−6404 : Gut microbiota abundance (genus Parasutterella id.2892) on Charged multivesicular body protein 3
with a threshold of 1e−05
MR Method
Inverse variance weighted
MR Egger
2.8
2.6
2.4
1/SE_IV
−0.4
0.0
0.4
0.8
β_IV

Batch 2−6405 : Gut microbiota abundance (genus Catenibacterium id.2153) on Vacuolar protein sorting−associated protein 29
with a threshold of 1e−05

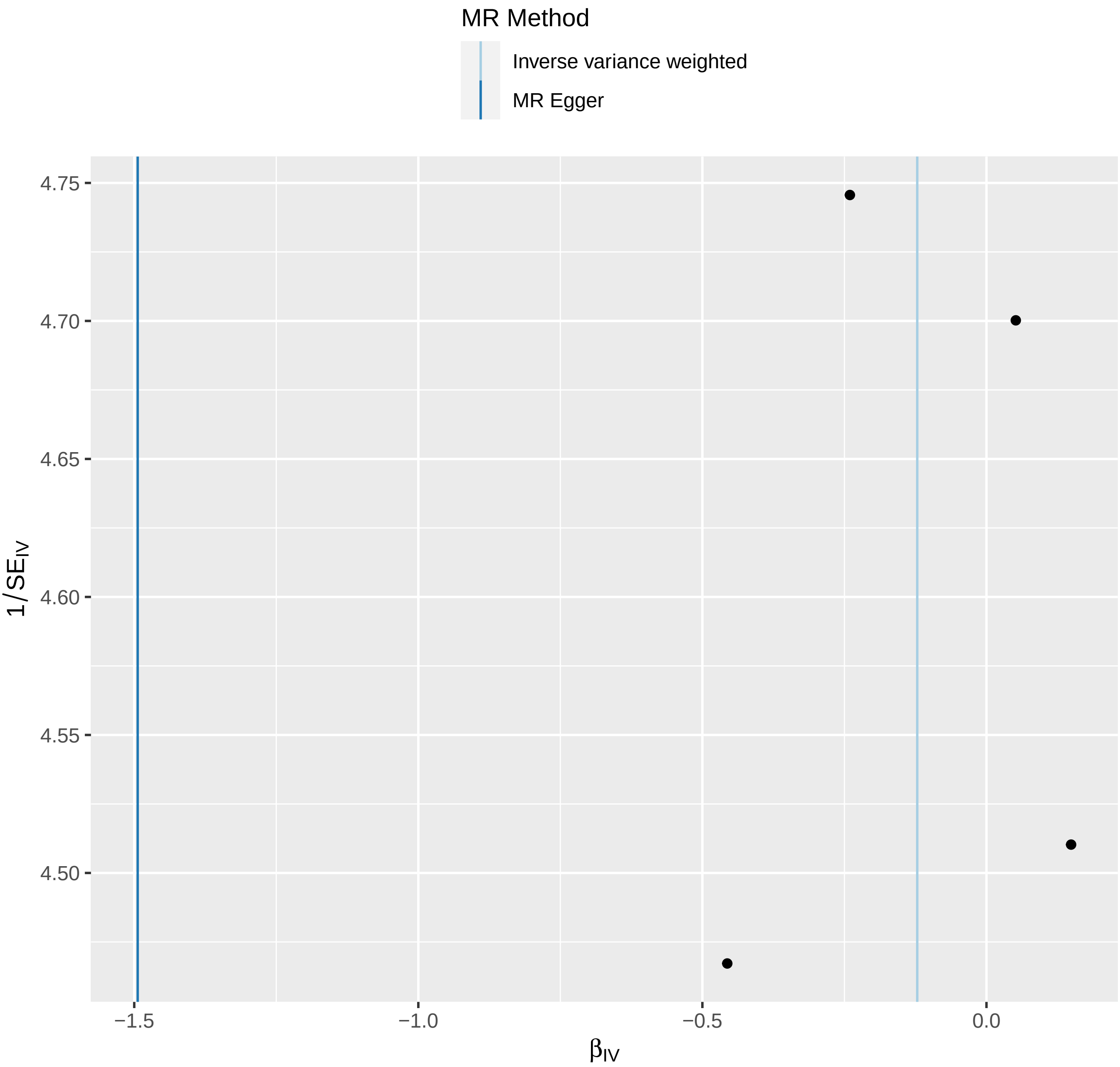

Batch 2−6406 : Gut microbiota abundance (genus Parasutterella id.2892) on Vacuolar protein sorting−associated protein 29
with a threshold of 1e−05

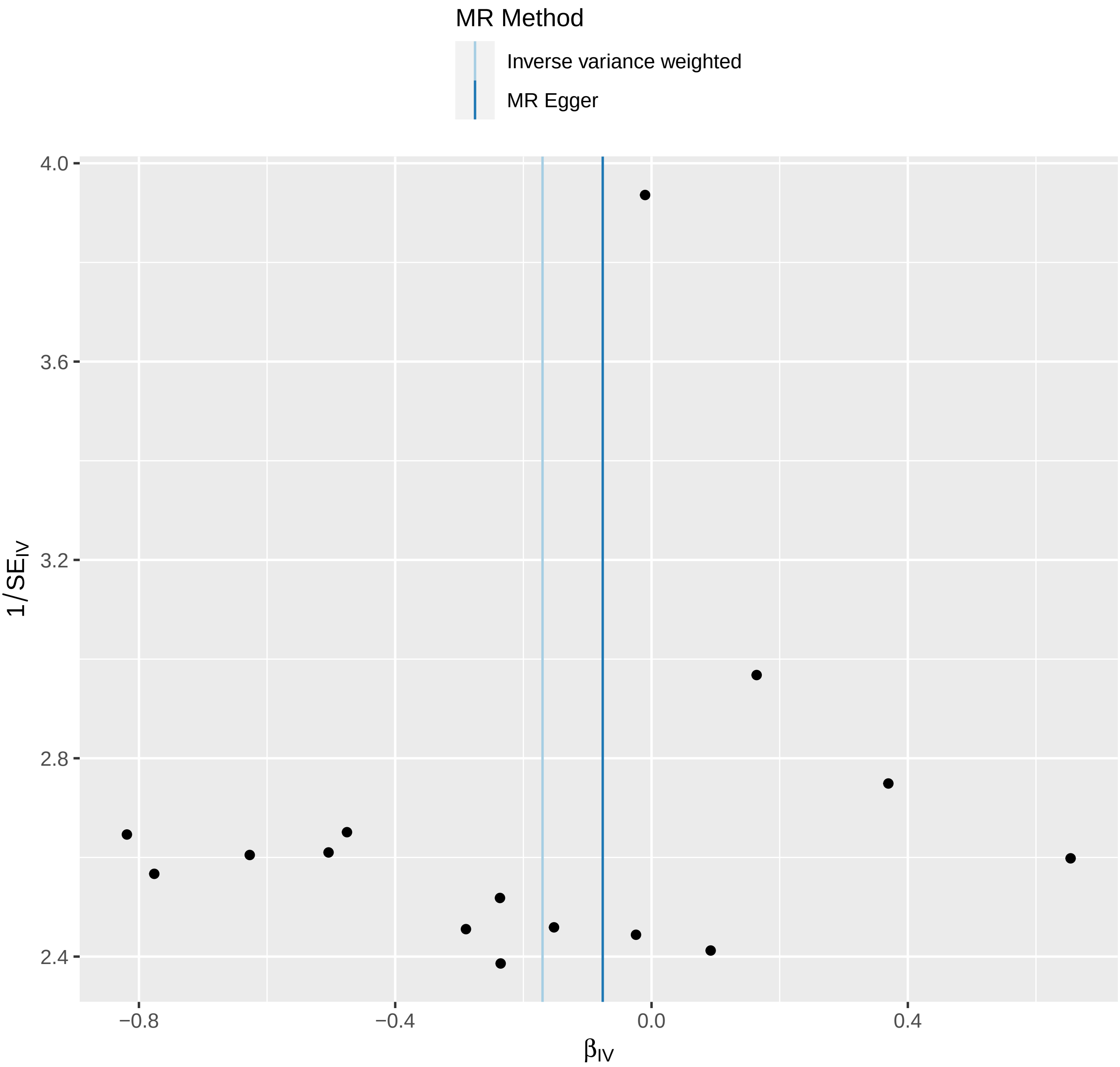

Batch 2−6407 : Gut microbiota abundance (genus Catenibacterium id.2153) on Vacuolar protein sorting−associated protein 4A with a threshold of 1e−05

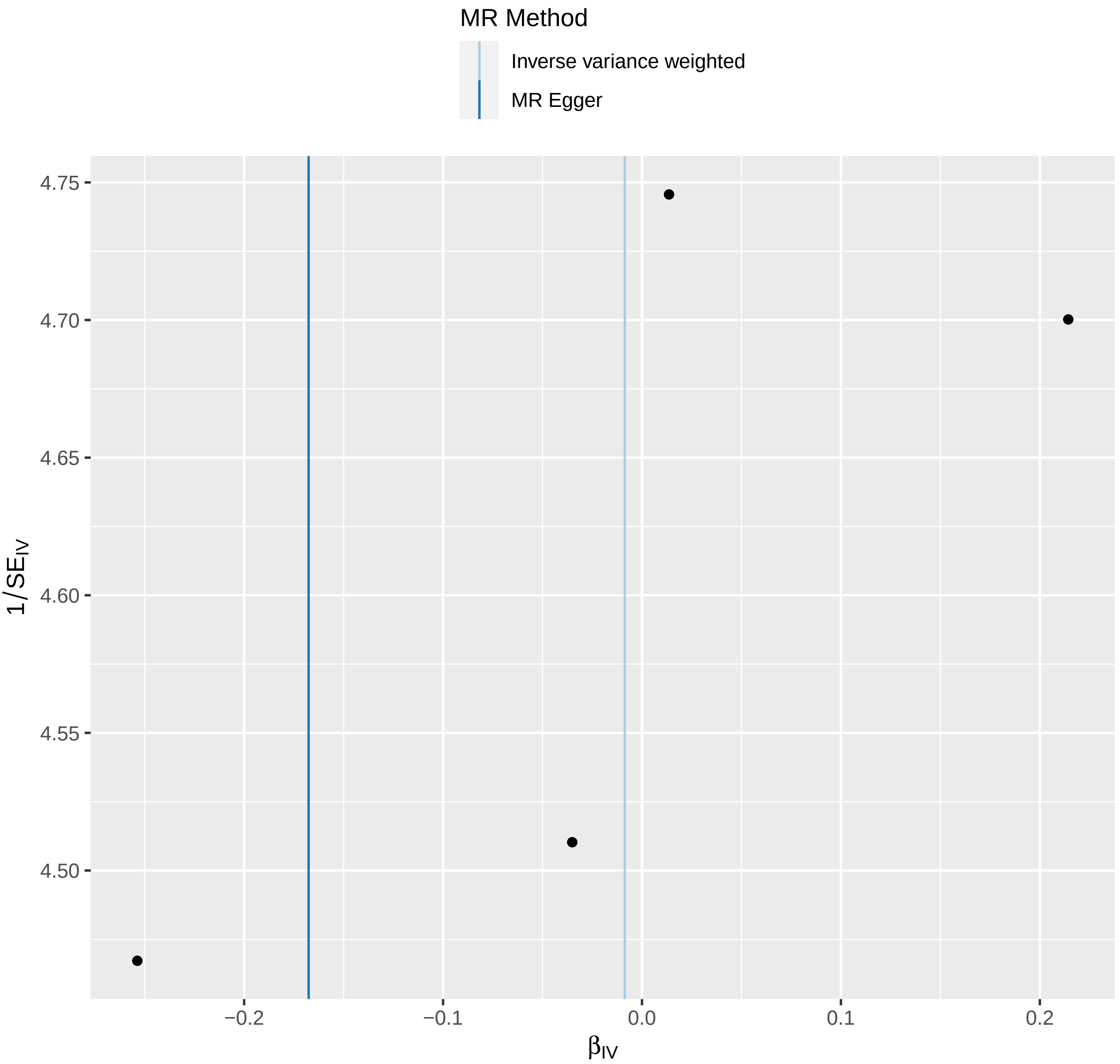

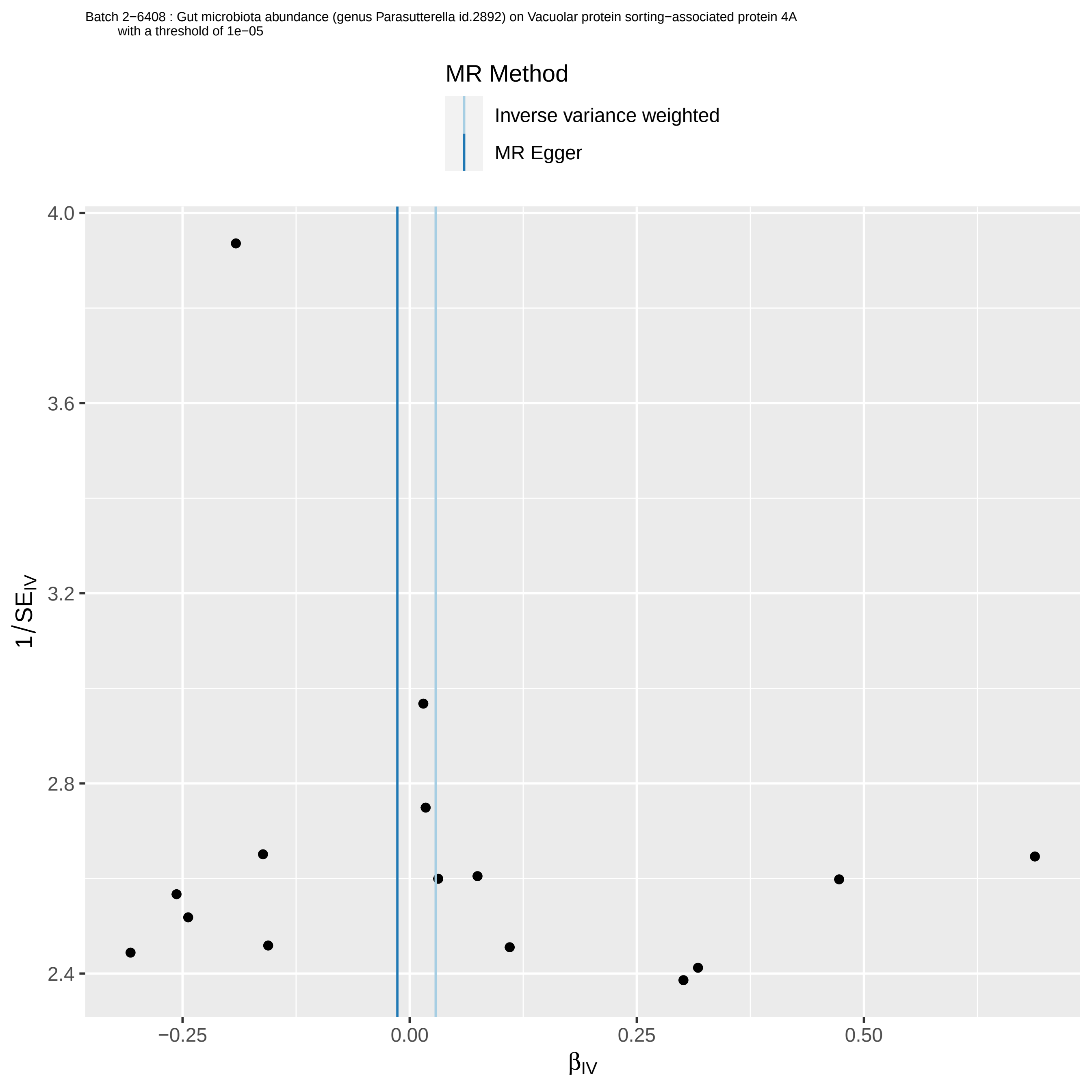
Batch 2−6408 : Gut microbiota abundance (genus Parasutterella id.2892) on Vacuolar protein sorting−associated protein 4A
with a threshold of 1e−05
MR Method
Inverse variance weighted
MR Egger
4.0
3.6
3.2
2.8
2.4
1/SE_IV
−0.25
0.00
0.25
0.50
β_IV

Batch 2−6409 : Gut microbiota abundance (genus Catenibacterium id.2153) on Vacuolar protein sorting−associated protein 4B
with a threshold of 1e−05

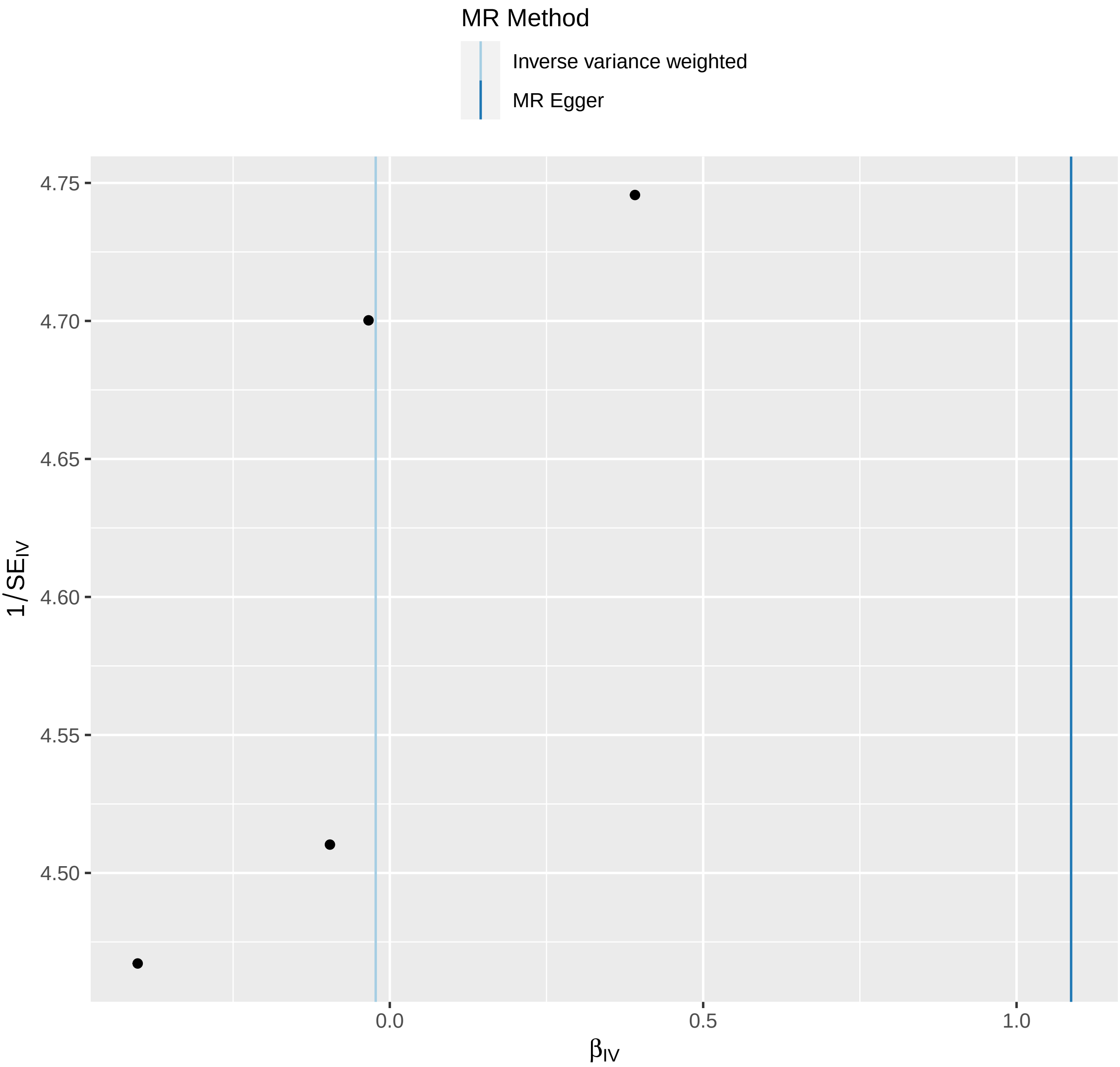

Batch 2−6410 : Gut microbiota abundance (genus Parasutterella id.2892) on Vacuolar protein sorting−associated protein 4B with a threshold of 1e−05

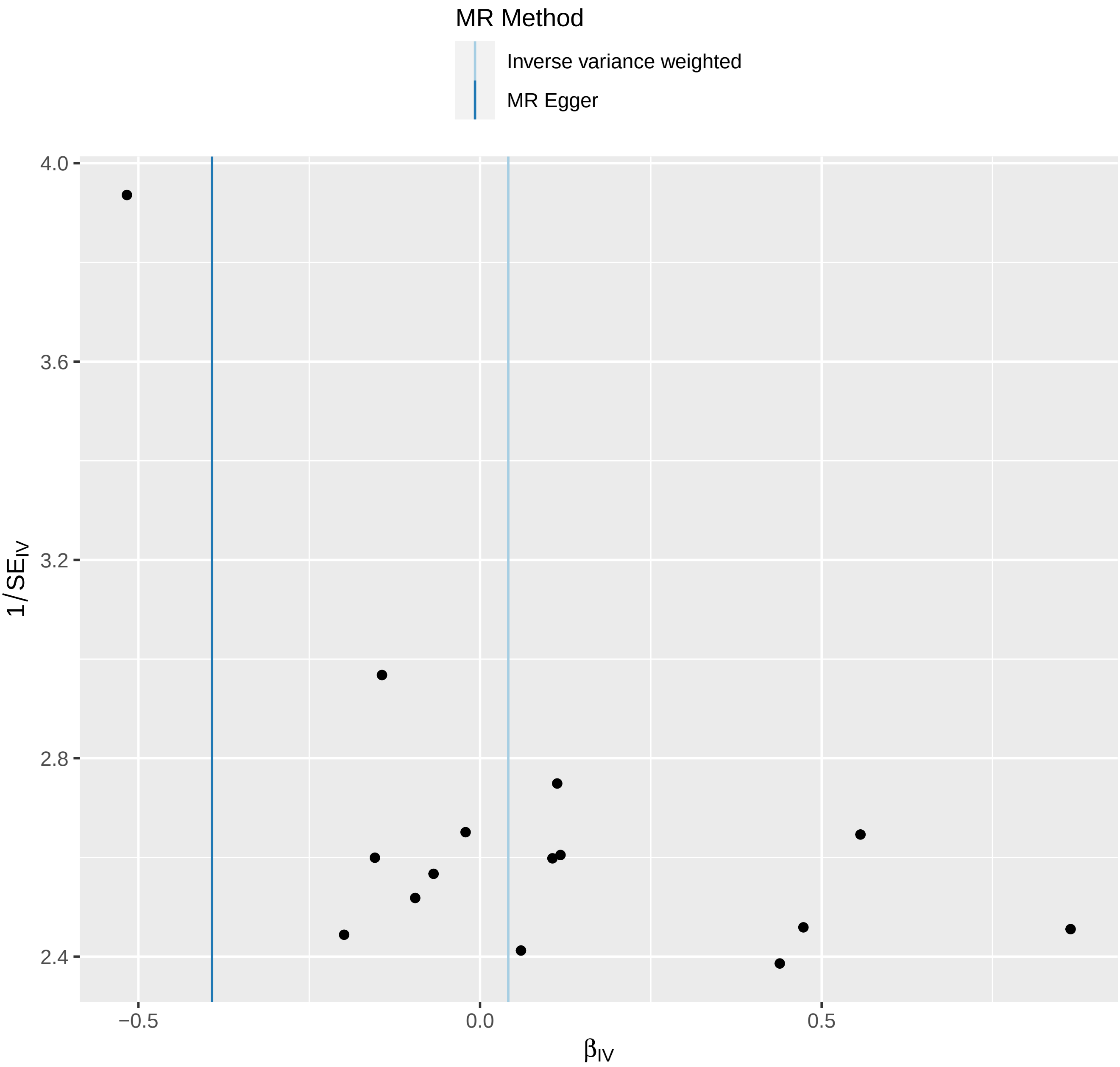

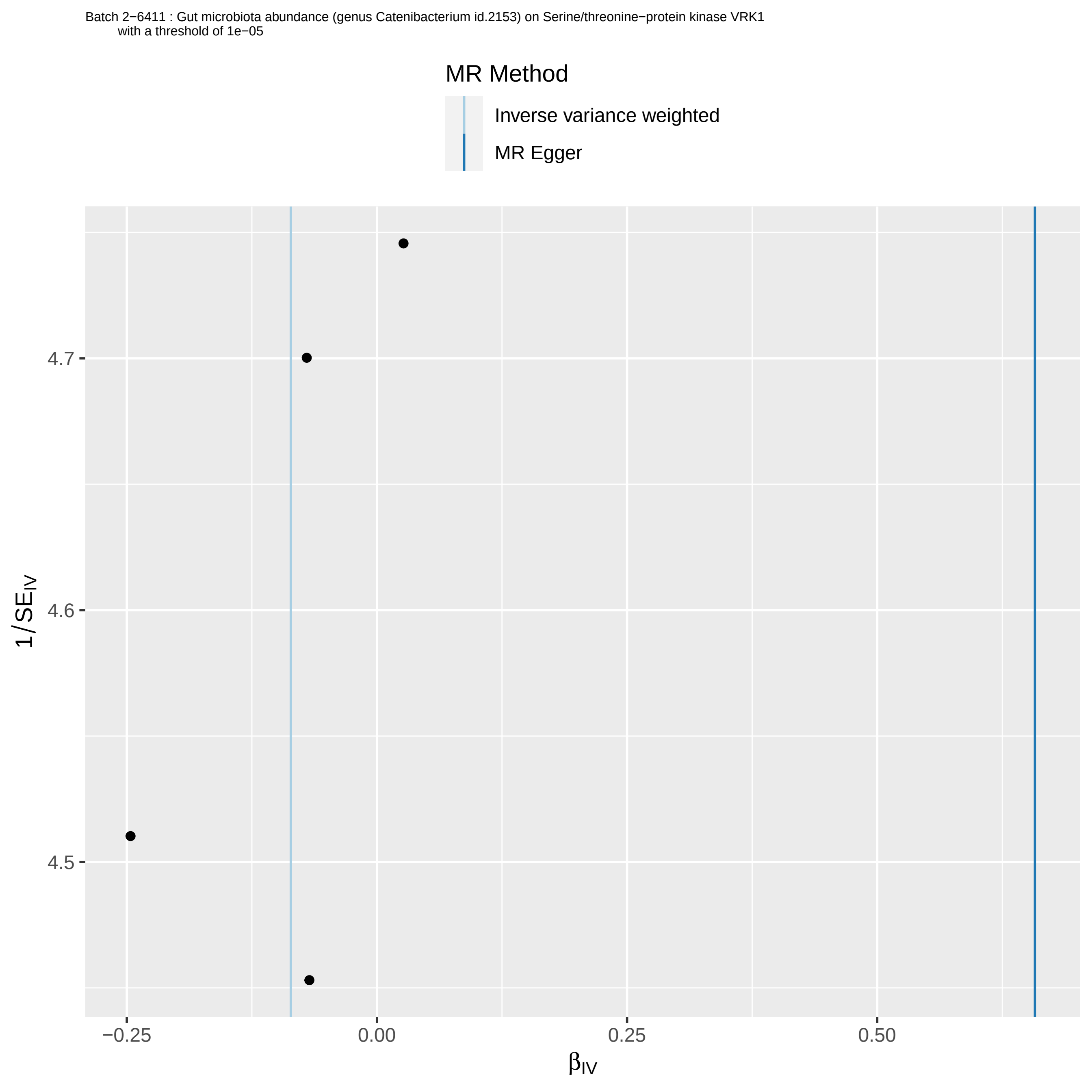

Batch 2−6411 : Gut microbiota abundance (genus Catenibacterium id.2153) on Serine/threonine−protein kinase VRK1
with a threshold of 1e−05
MR Method
Inverse variance weighted
MR Egger
4.7
4.6
4.5
1/SE_IV
−0.25
0.00
0.25
0.50
β_IV

Batch 2−6412 : Gut microbiota abundance (genus Parasutterella id.2892) on Serine/threonine−protein kinase VRK1
with a threshold of 1e−05

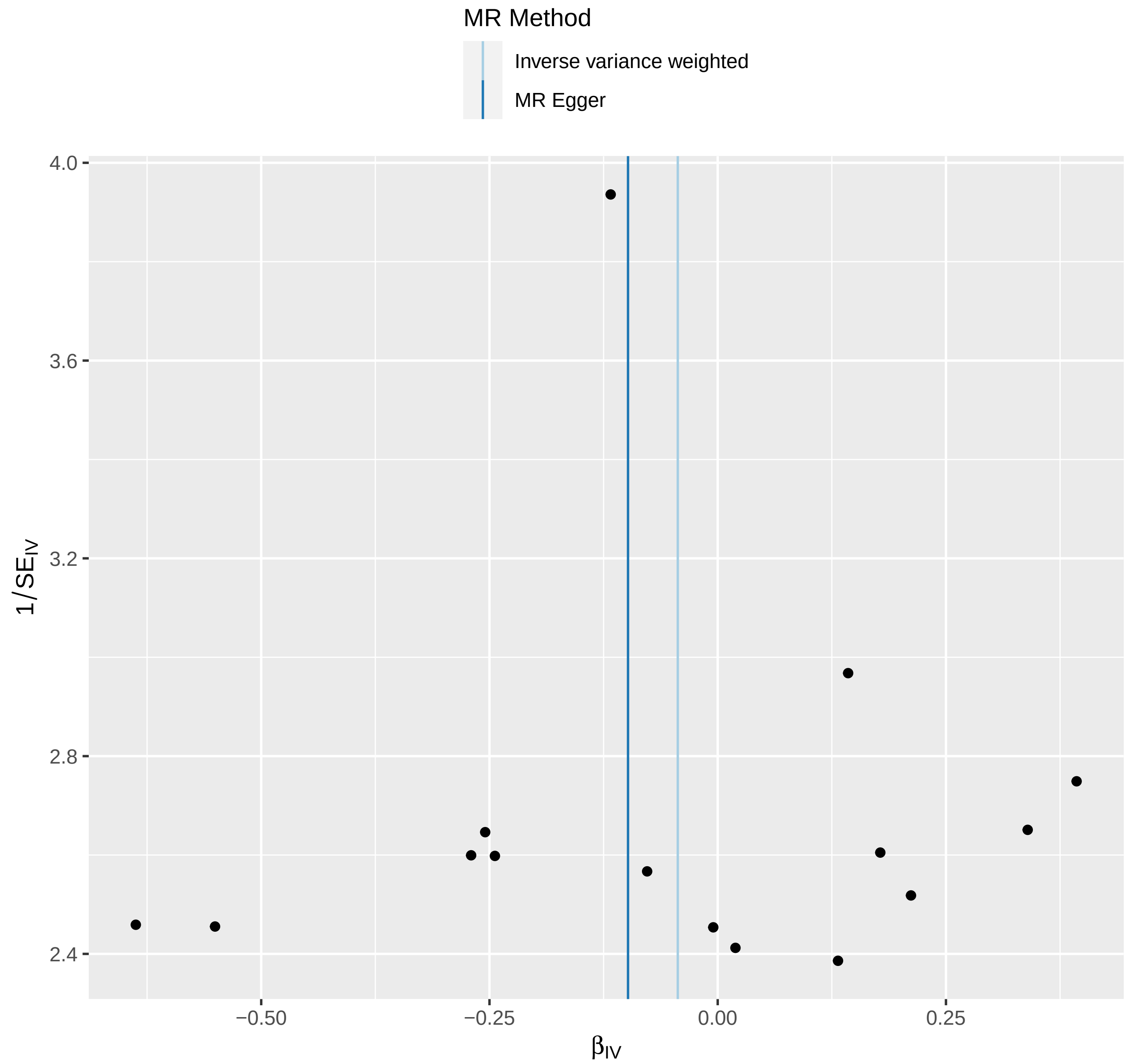

Batch 2−6413 : Gut microbiota abundance (genus Catenibacterium id.2153) on V−set and immunoglobulin domain−containing protein 1 with a threshold of 1e−05

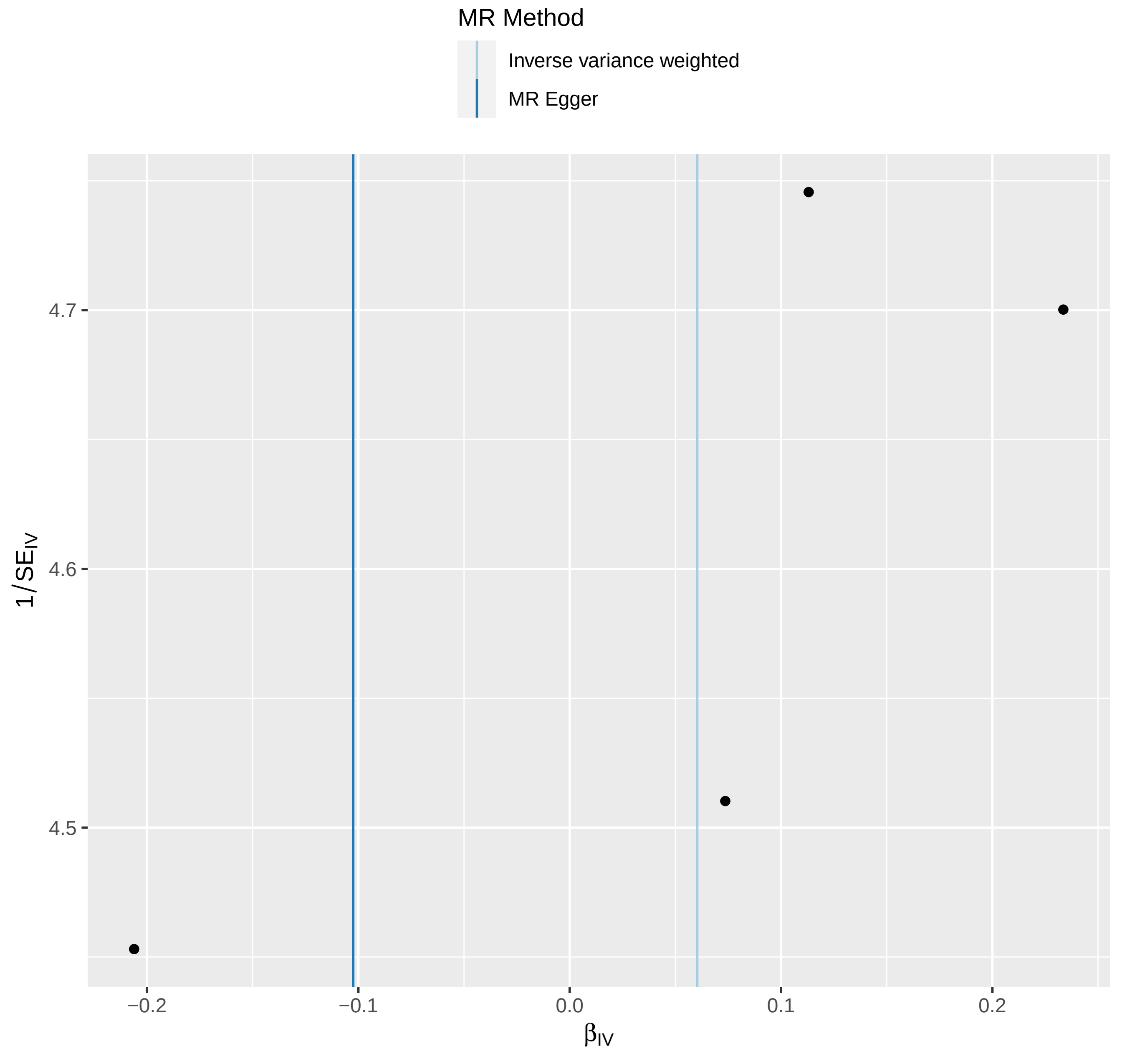

Batch 2−6414 : Gut microbiota abundance (genus Parasutterella id.2892) on V−set and immunoglobulin domain−containing protein 1 with a threshold of 1e−05

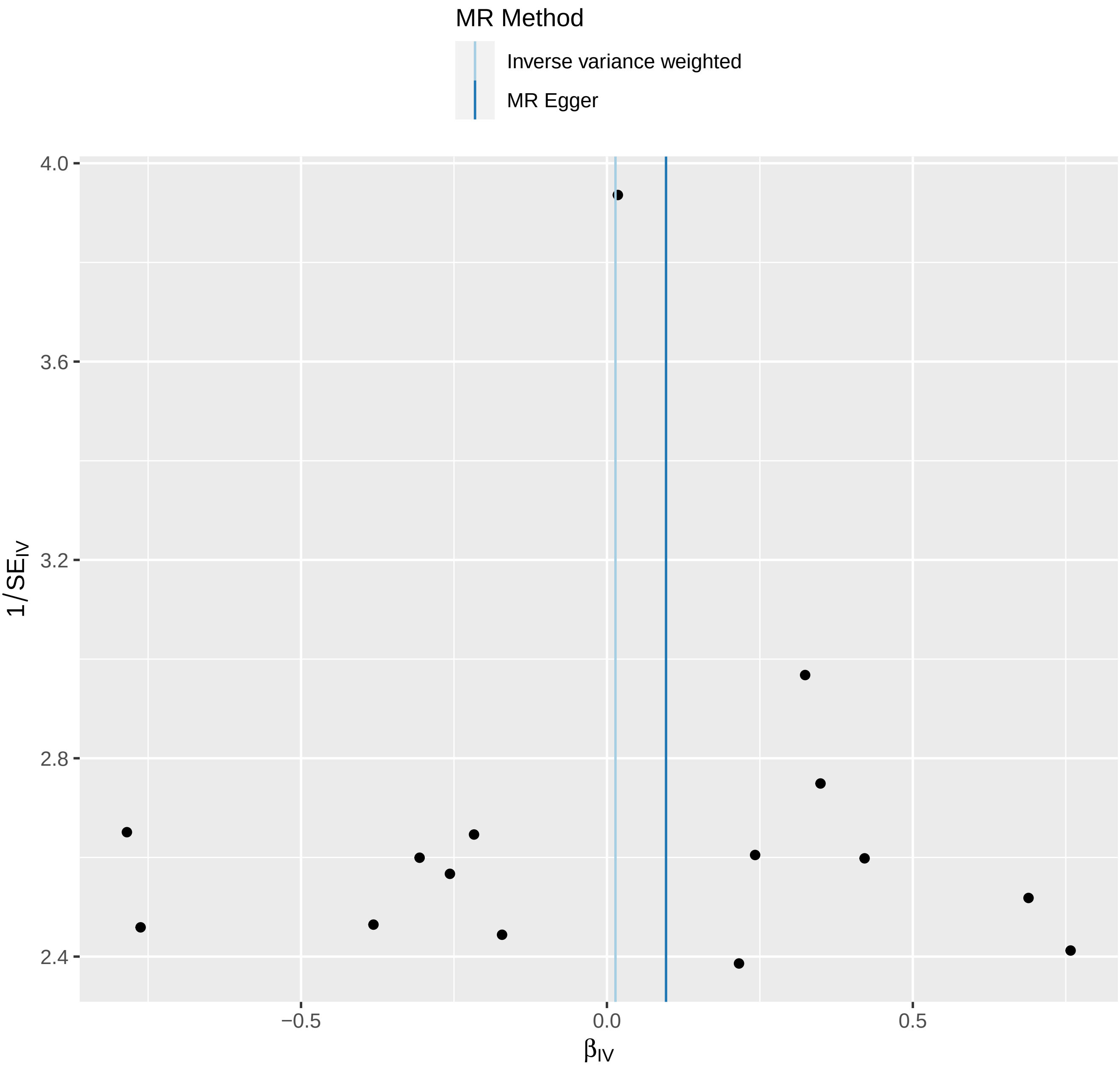

Batch 2−6415 : Gut microbiota abundance (genus Catenibacterium id.2153) on V−set and immunoglobulin domain−containing protein 2 with a threshold of 1e−05

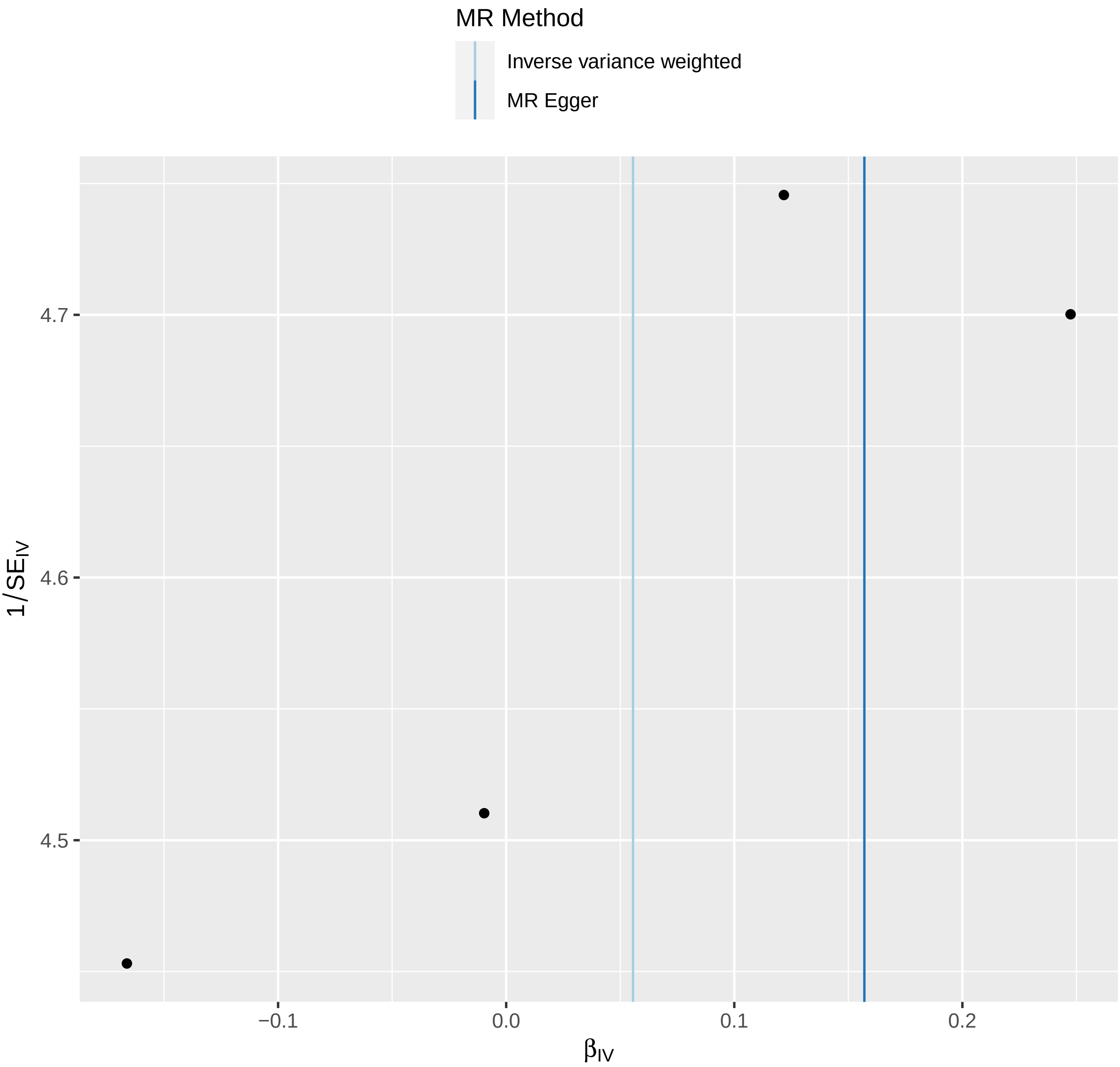

Batch 2−6416 : Gut microbiota abundance (genus Parasutterella id.2892) on V−set and immunoglobulin domain−containing protein 2 with a threshold of 1e−05

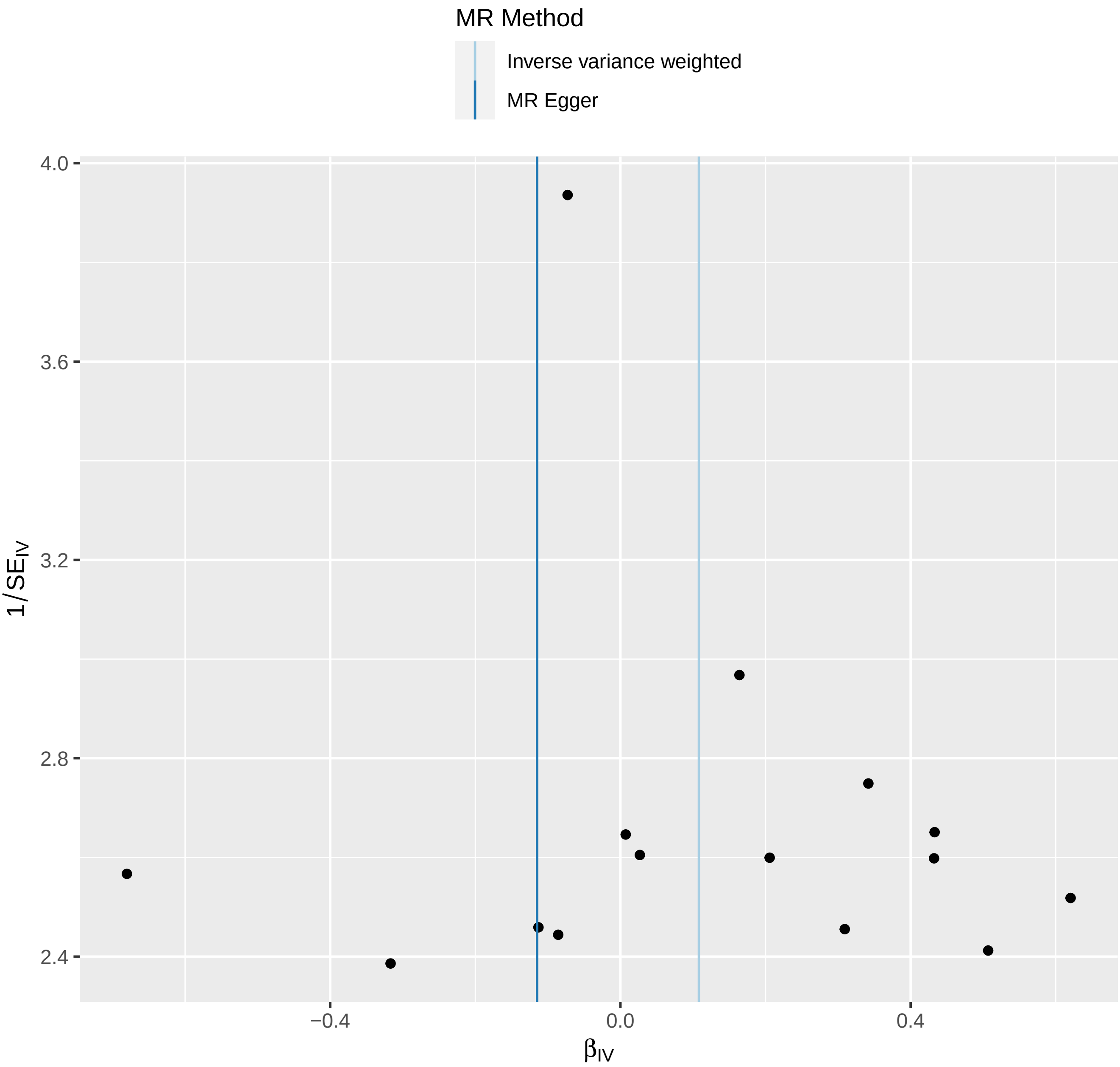

Batch 2−6417 : Gut microbiota abundance (genus Catenibacterium id.2153) on V−set and transmembrane domain−containing protein 2−like protein with a threshold of 1e−05

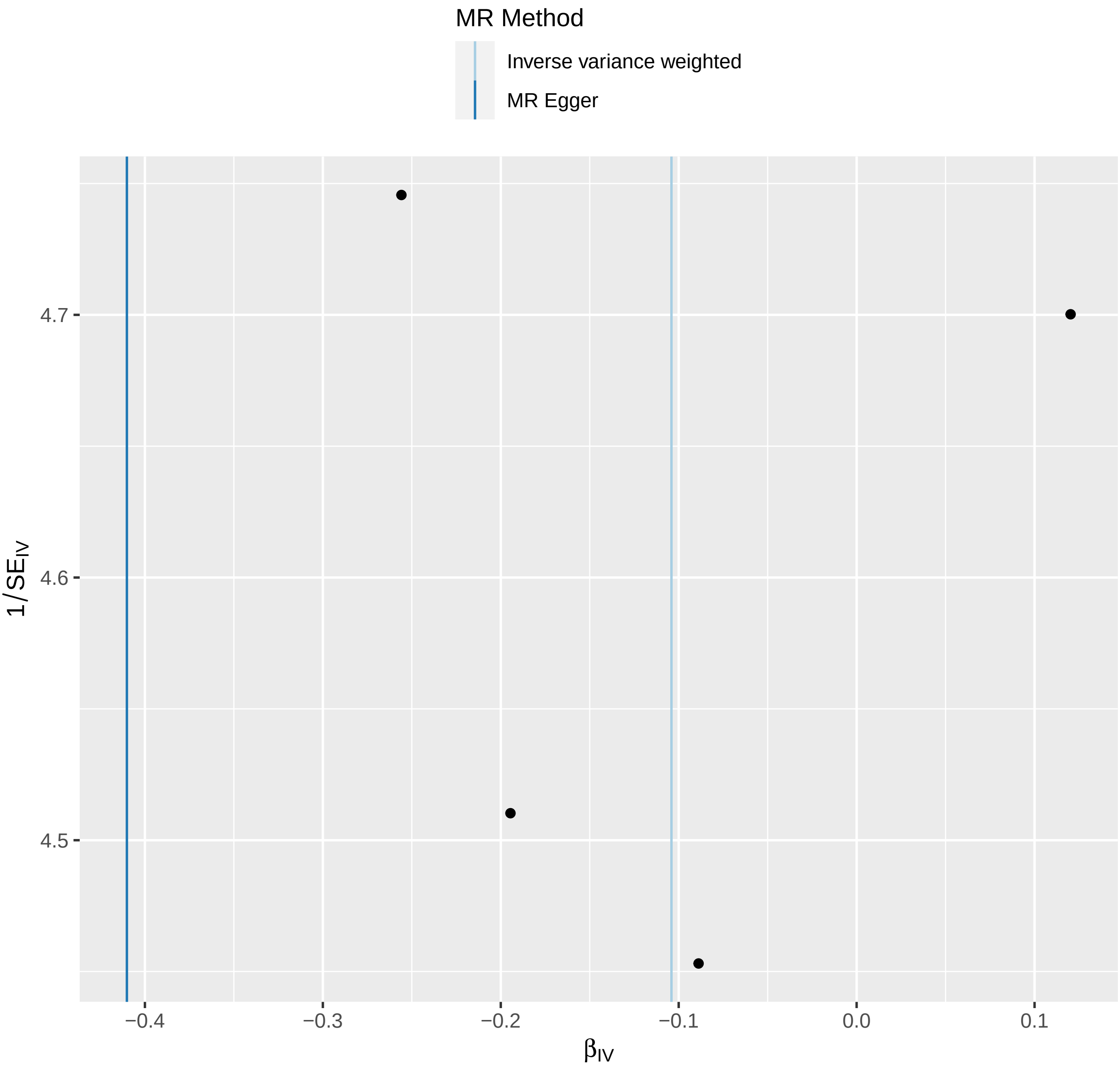

Batch 2−6418 : Gut microbiota abundance (genus Parasutterella id.2892) on V−set and transmembrane domain−containing protein 2−like protein with a threshold of 1e−05

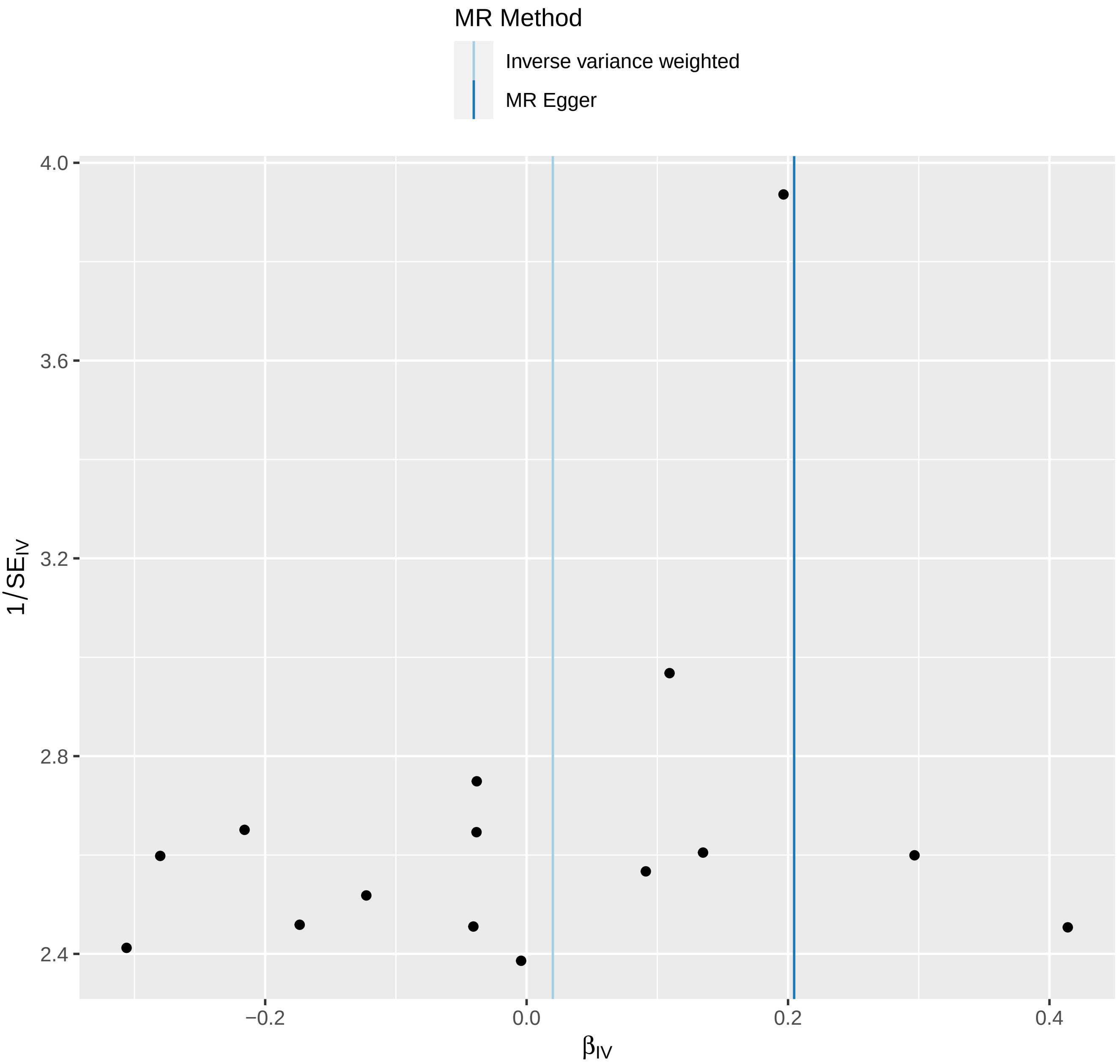

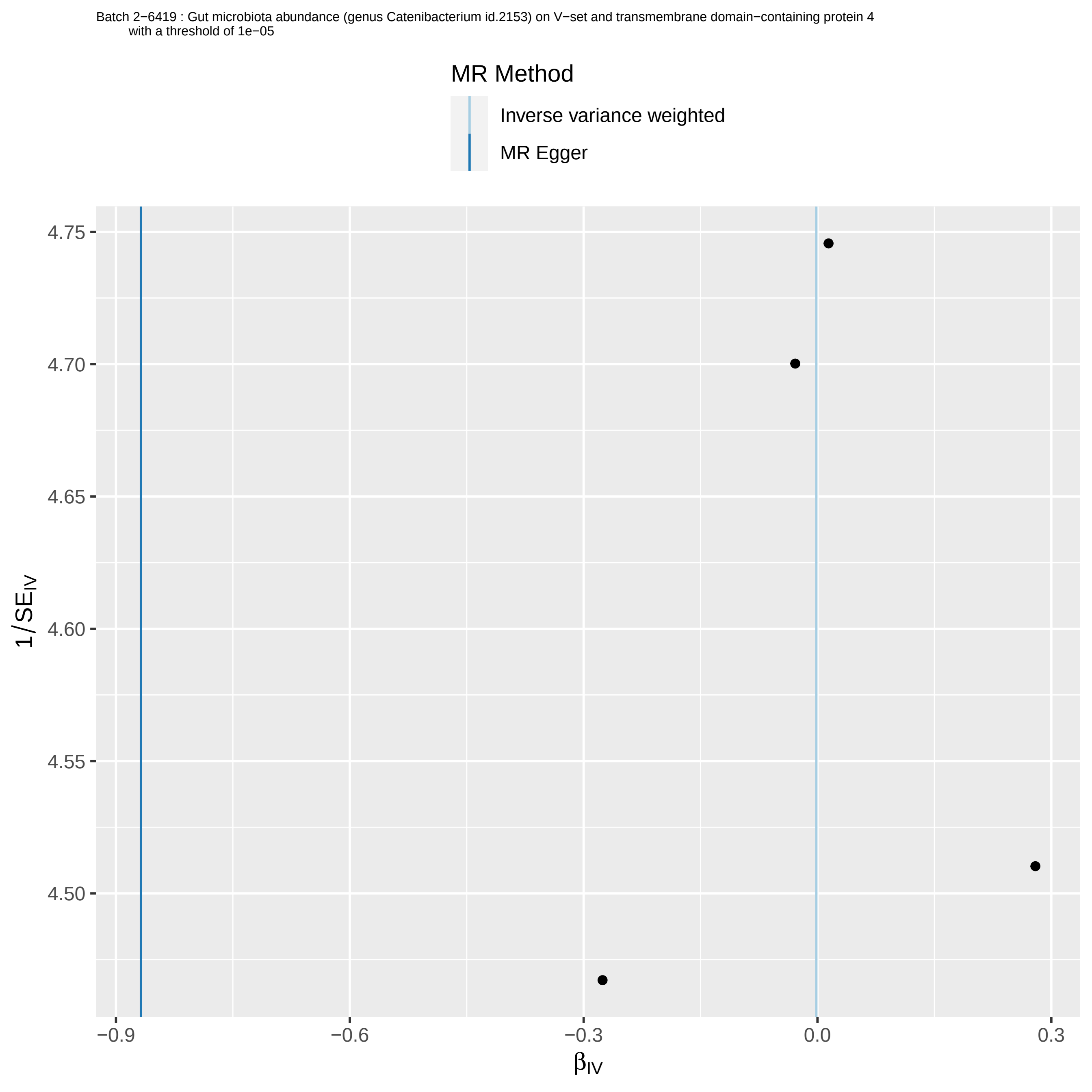
Batch 2−6419 : Gut microbiota abundance (genus Catenibacterium id.2153) on V−set and transmembrane domain−containing protein 4
with a threshold of 1e−05
MR Method
Inverse variance weighted
MR Egger
4.75
4.70
4.65
4.60
4.55
4.50
−0.9
−0.6
−0.3
0.0
0.3
$1/SE_{IV}$
β_{IV}

Batch 2−6420 : Gut microbiota abundance (genus Parasutterella id.2892) on V−set and transmembrane domain−containing protein 4 with a threshold of 1e−05

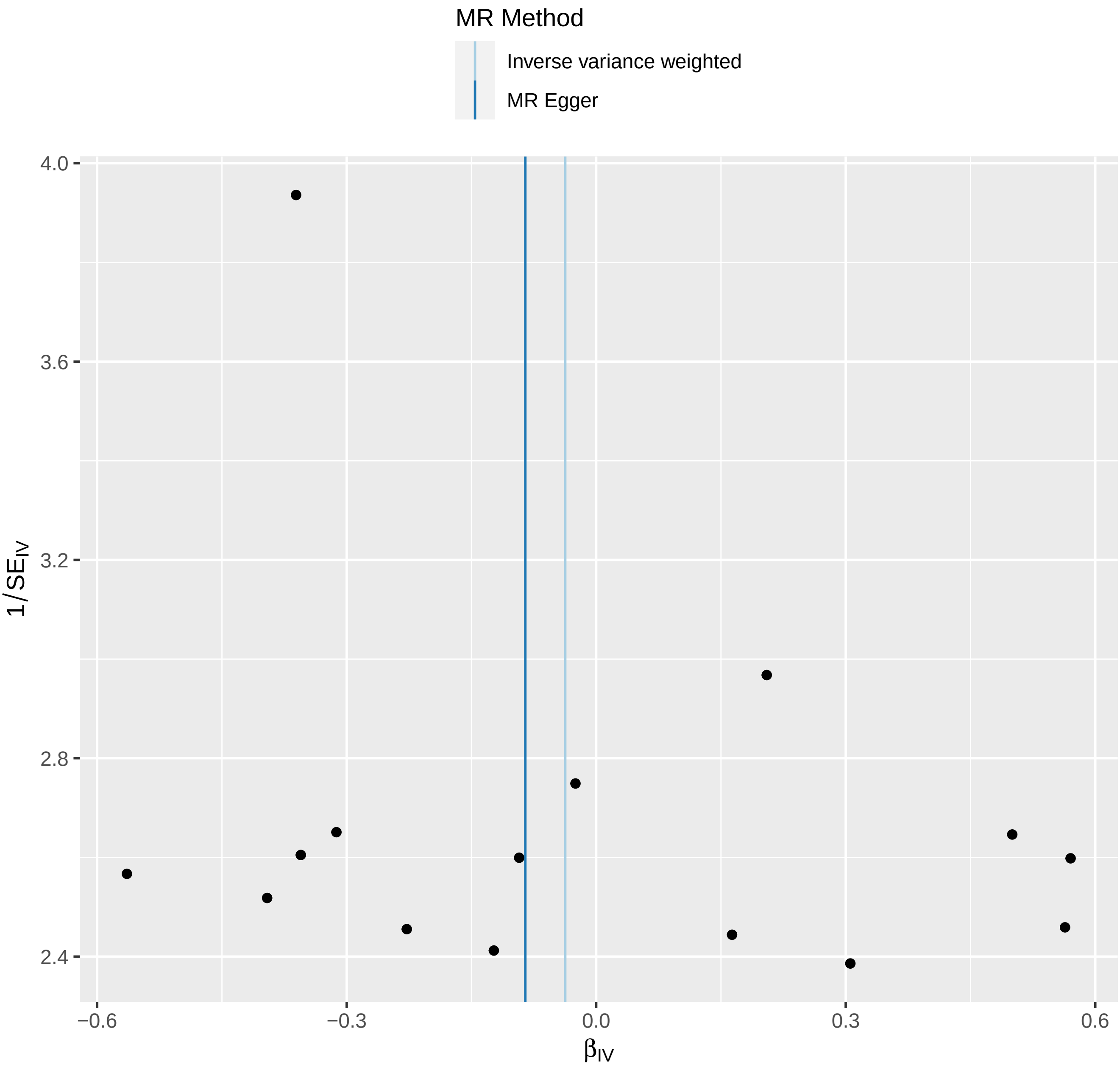

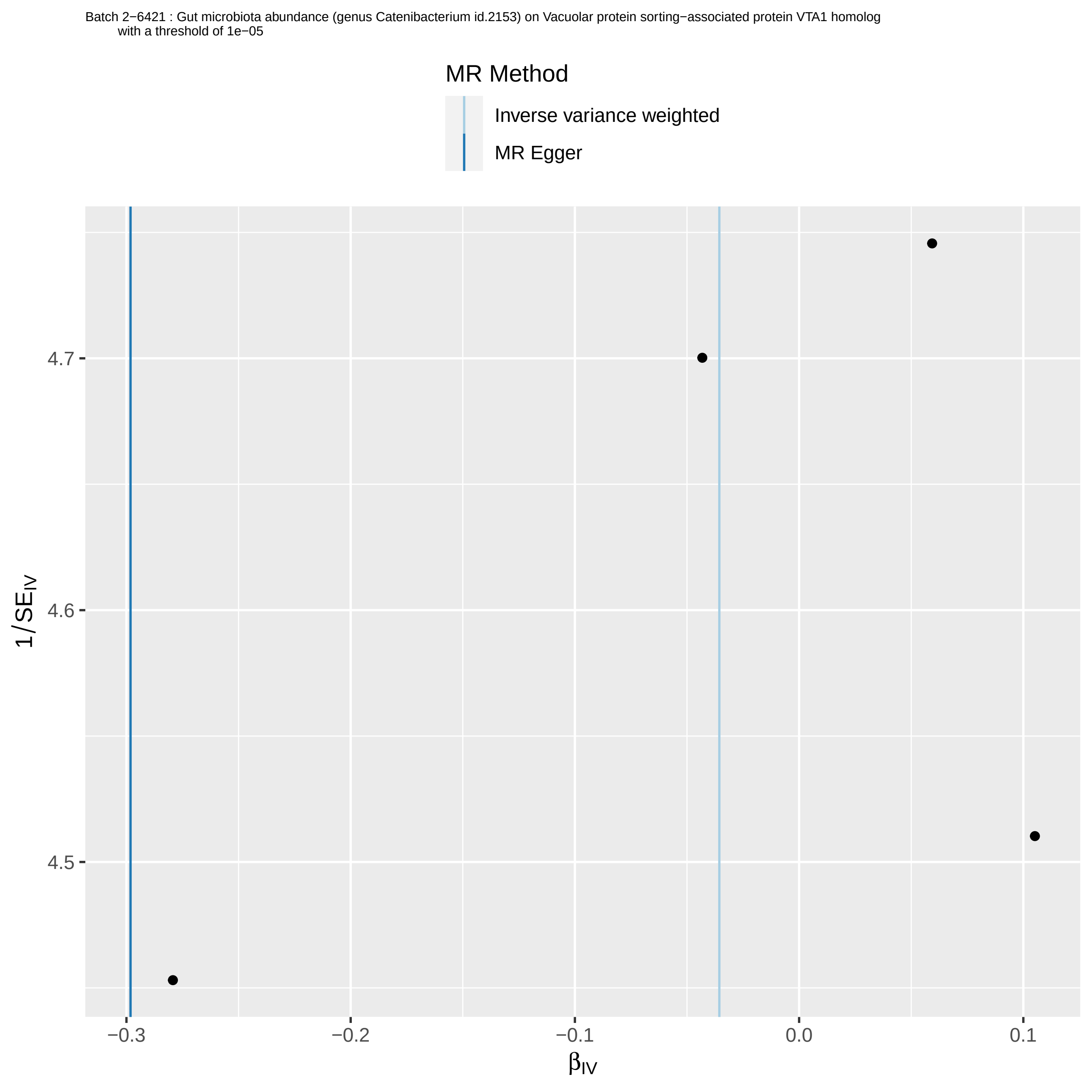
Batch 2−6421 : Gut microbiota abundance (genus Catenibacterium id.2153) on Vacuolar protein sorting−associated protein VTA1 homolog
with a threshold of 1e−05
MR Method
Inverse variance weighted
MR Egger
4.7
4.6
4.5
1/SE_IV
−0.3
−0.2
−0.1
0.0
0.1
β_IV

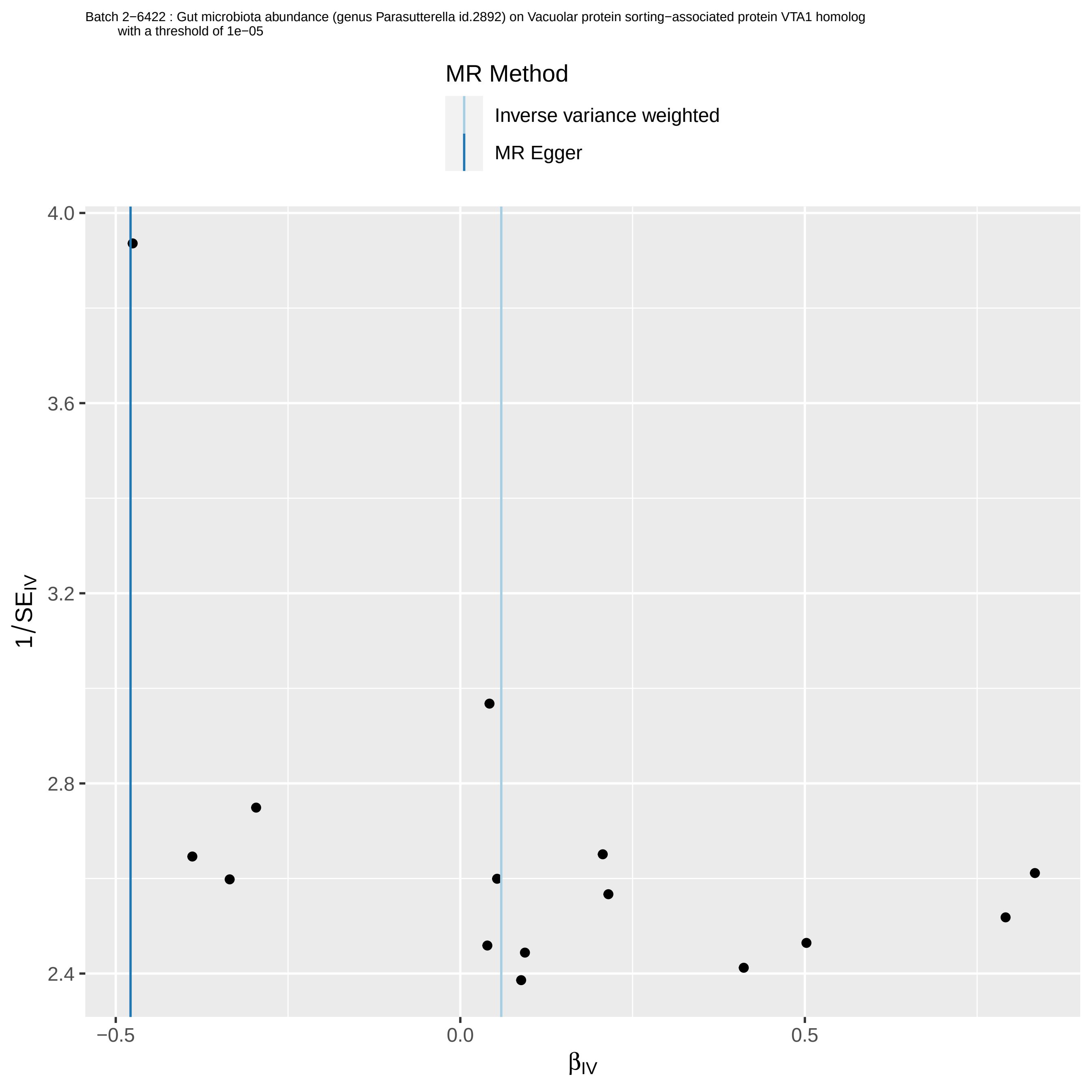
Batch 2−6422 : Gut microbiota abundance (genus Parasutterella id.2892) on Vacuolar protein sorting−associated protein VTA1 homolog
with a threshold of 1e−05
MR Method
Inverse variance weighted
MR Egger
4.0
3.6
3.2
2.8
2.4
1/SE_IV
−0.5
0.0
0.5
β_IV

Batch 2−6423 : Gut microbiota abundance (genus Catenibacterium id.2153) on Vesicle transport through interaction with t−SNAREs homolog 1A with a threshold of 1e−05

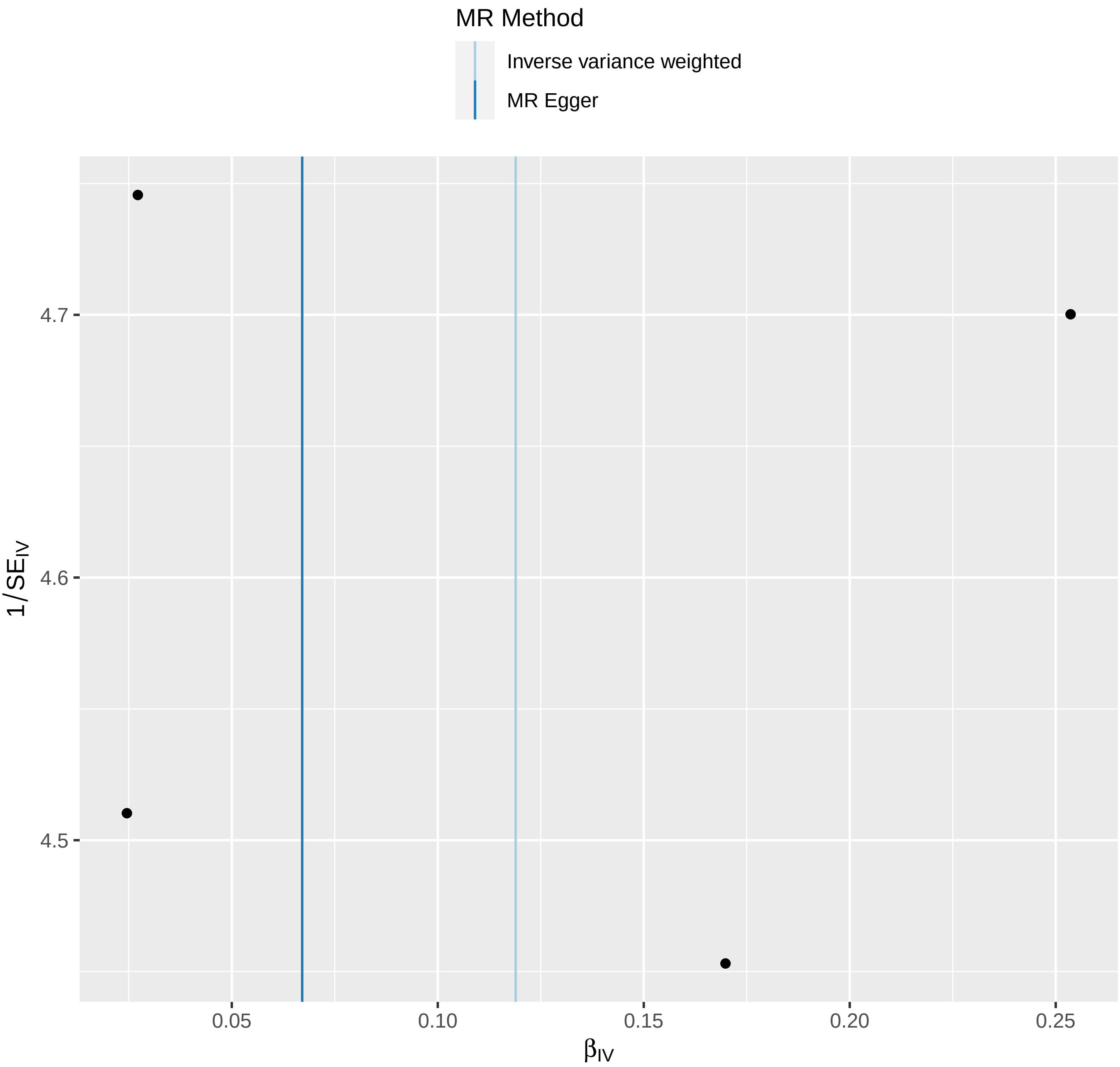

Batch 2−6424 : Gut microbiota abundance (genus Parasutterella id.2892) on Vesicle transport through interaction with t−SNAREs homolog 1A
with a threshold of 1e−05

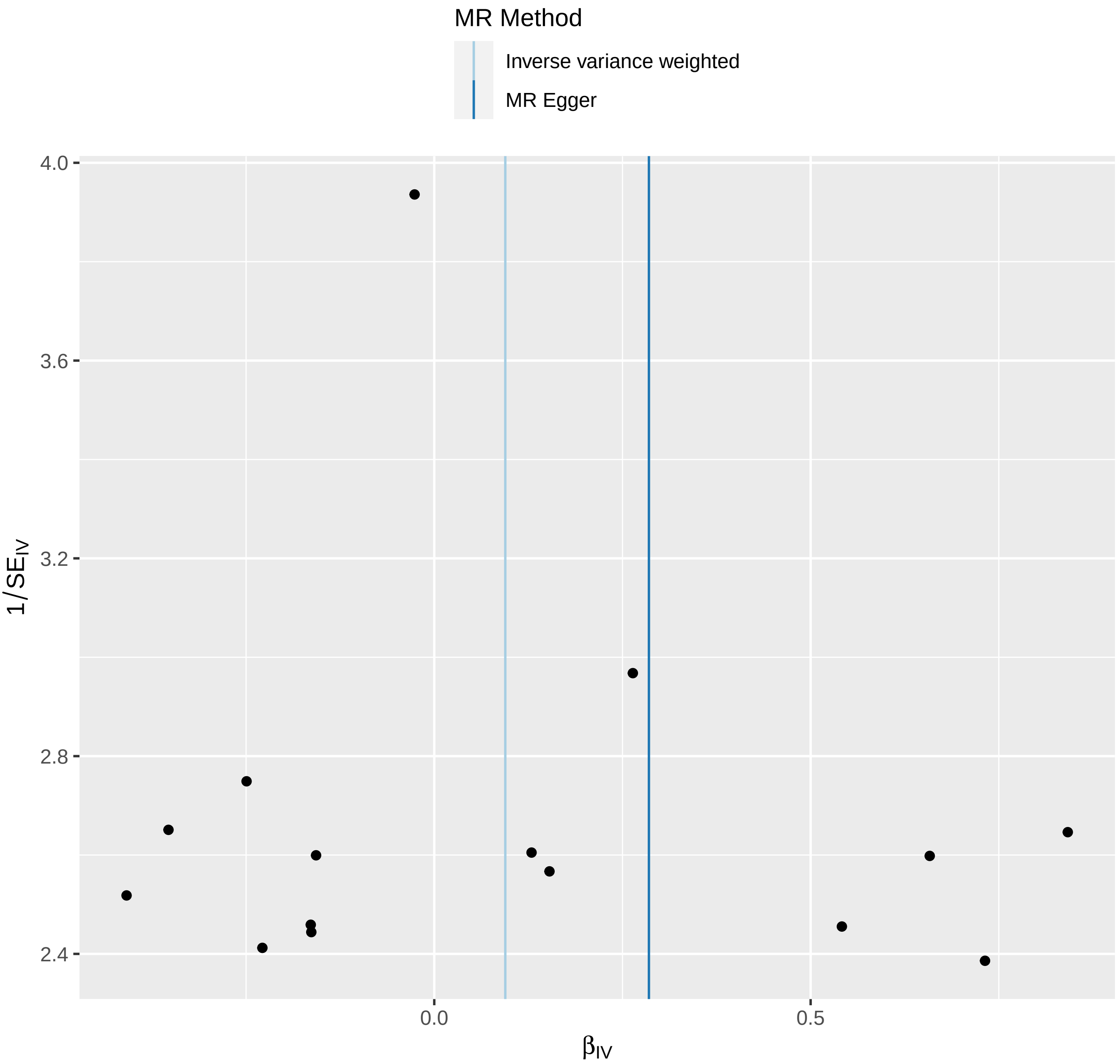

Batch 2−6425 : Gut microbiota abundance (genus Catenibacterium id.2153) on Vesicle transport through interaction with t−SNAREs homolog 1B
with a threshold of 1e−05

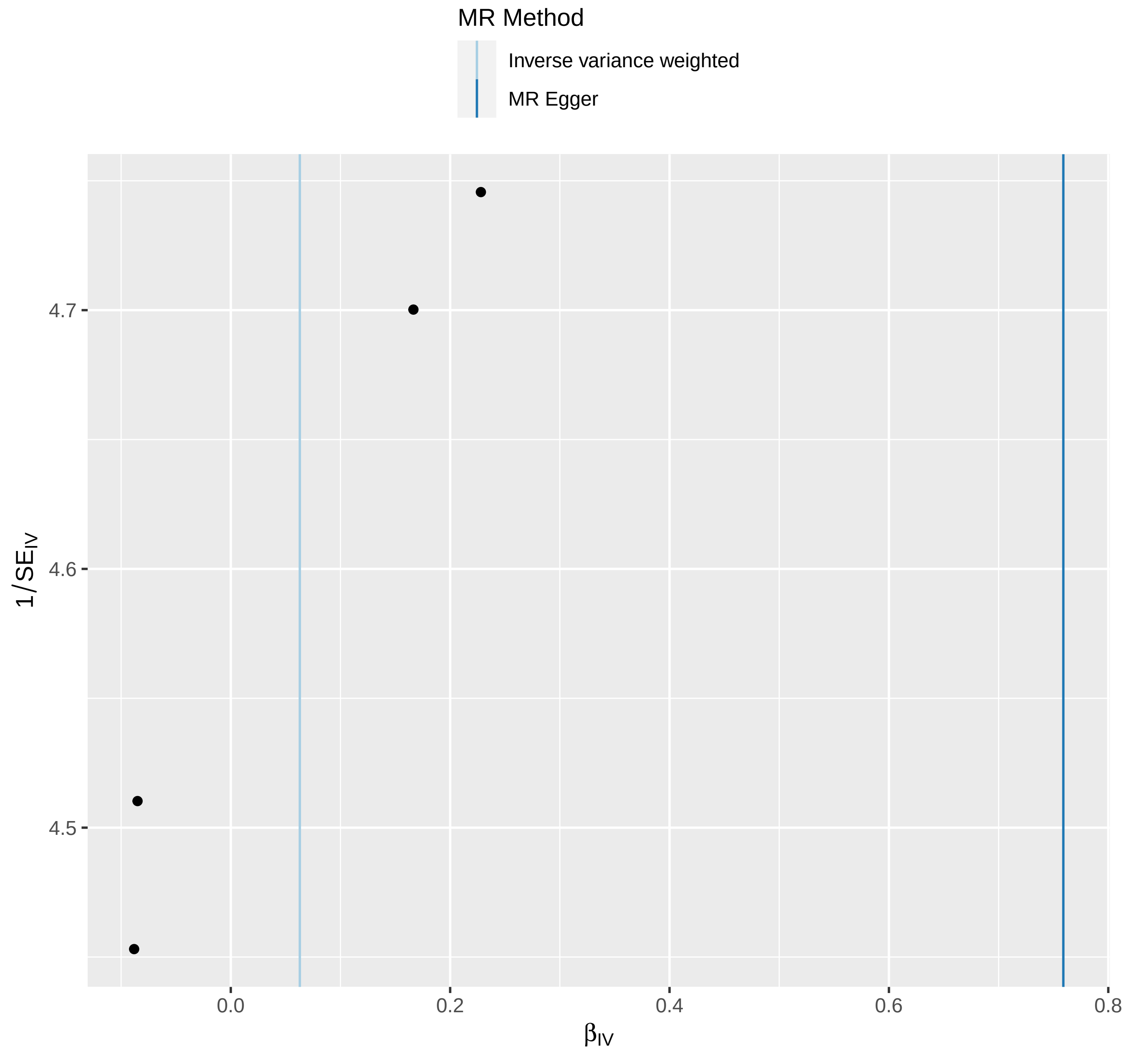

Batch 2−6426 : Gut microbiota abundance (genus Parasutterella id.2892) on Vesicle transport through interaction with t−SNAREs homolog 1B
with a threshold of 1e−05

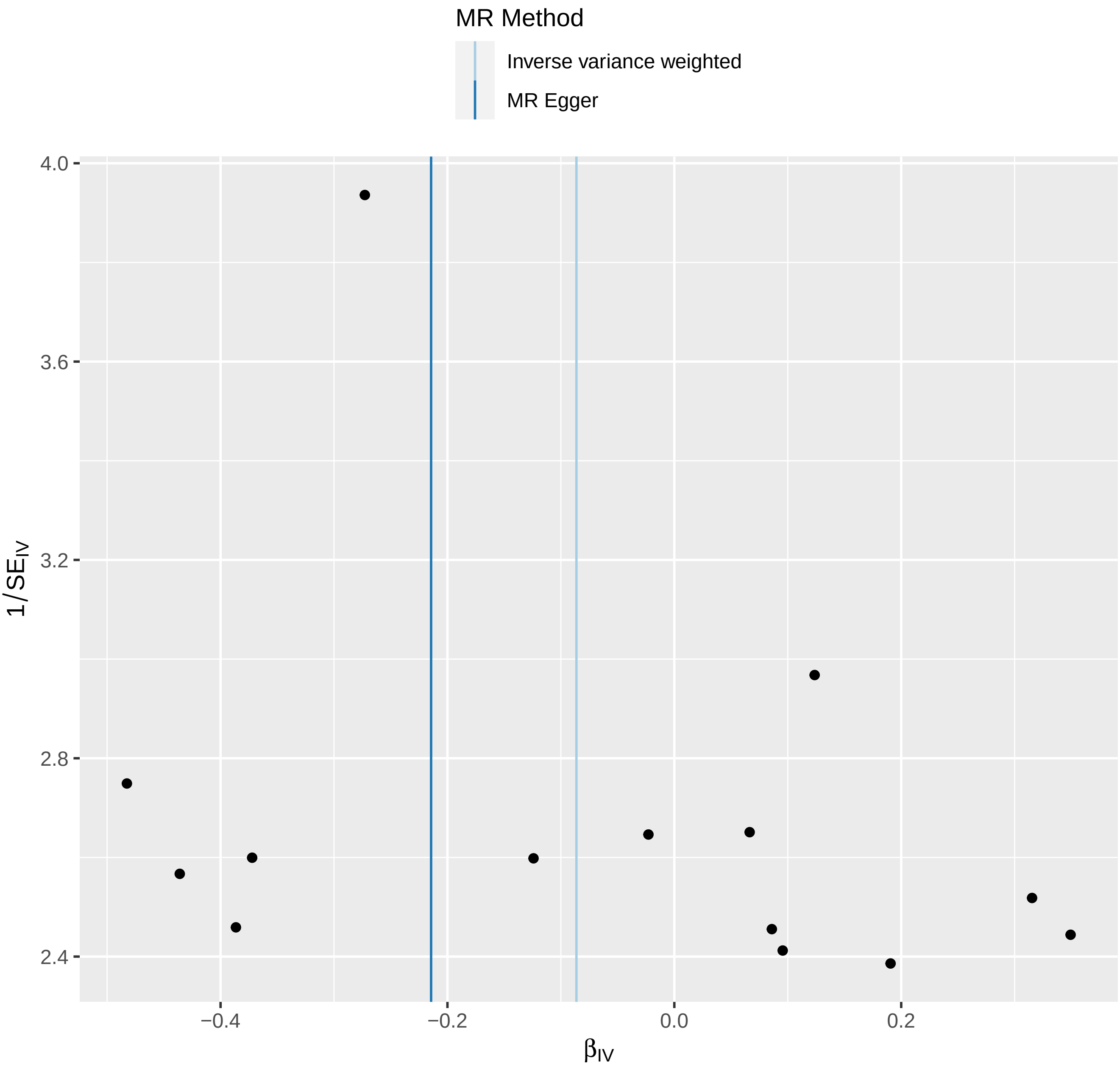

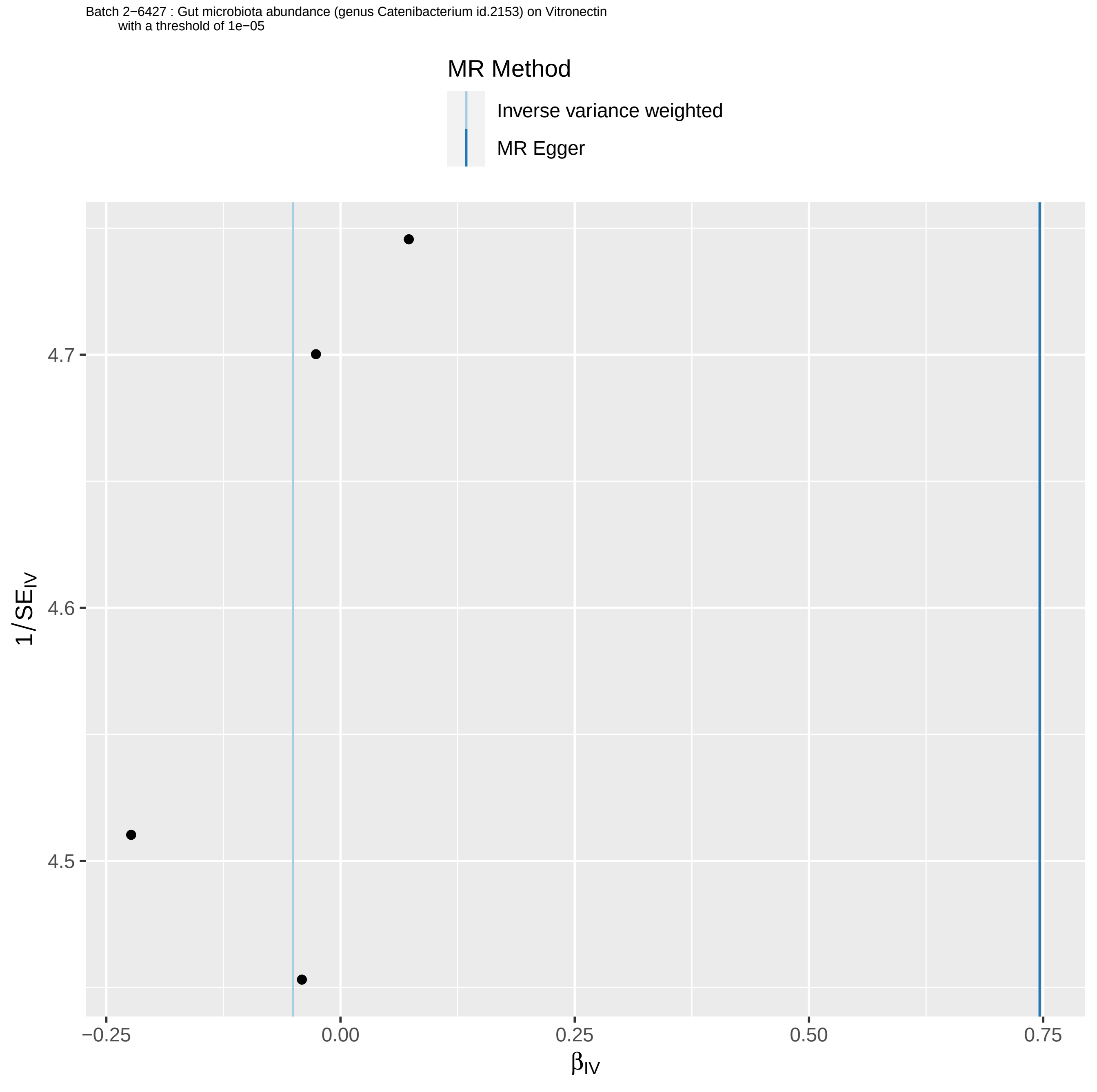

Batch 2−6427 : Gut microbiota abundance (genus Catenibacterium id.2153) on Vitronectin
with a threshold of 1e−05
MR Method
Inverse variance weighted
MR Egger
4.7
4.6
4.5
$1/SE_{IV}$
−0.25
0.00
0.25
0.50
0.75
β_{IV}

Batch 2−6428 : Gut microbiota abundance (genus Parasutterella id.2892) on Vitronectin
with a threshold of 1e−05

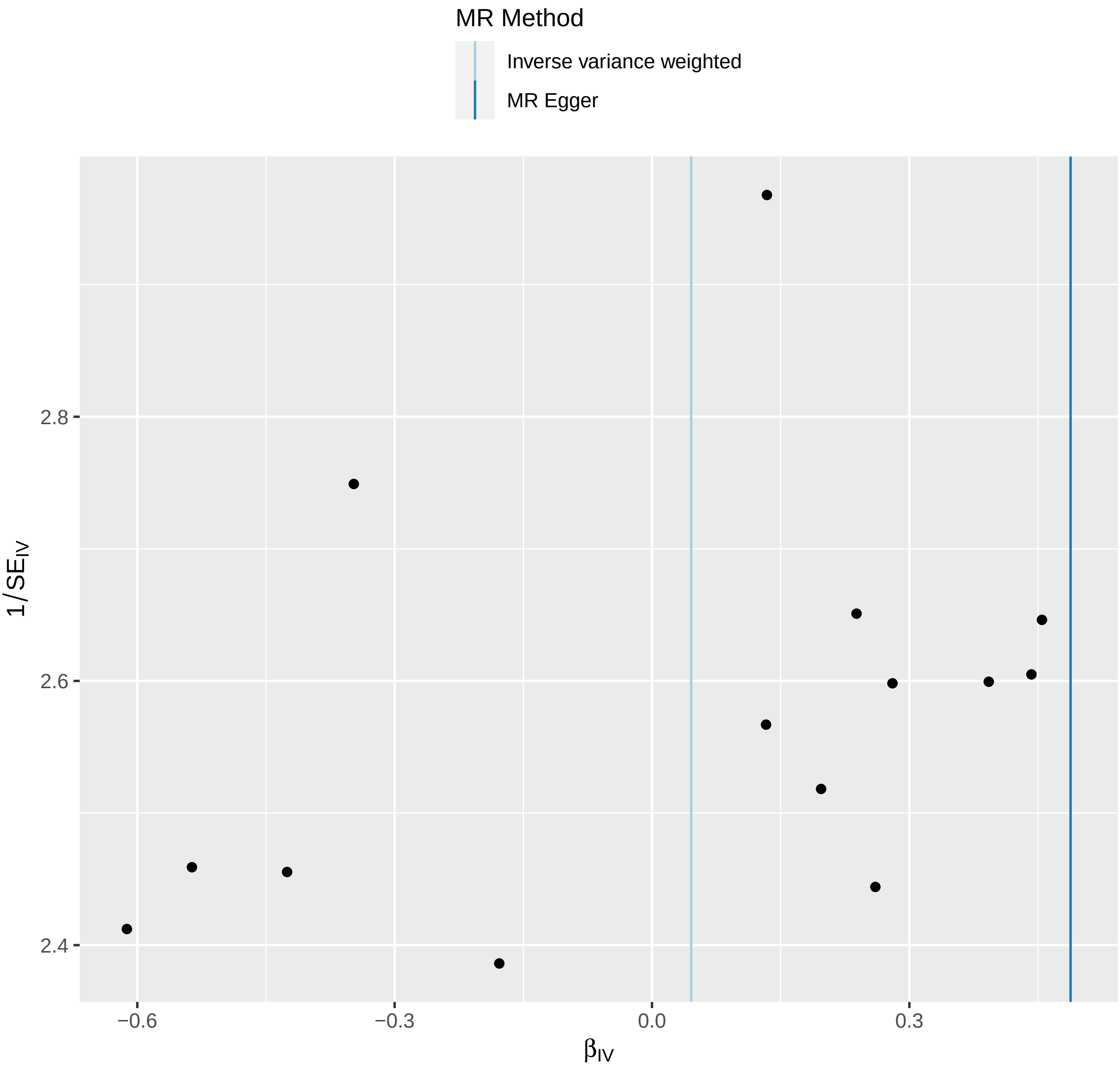

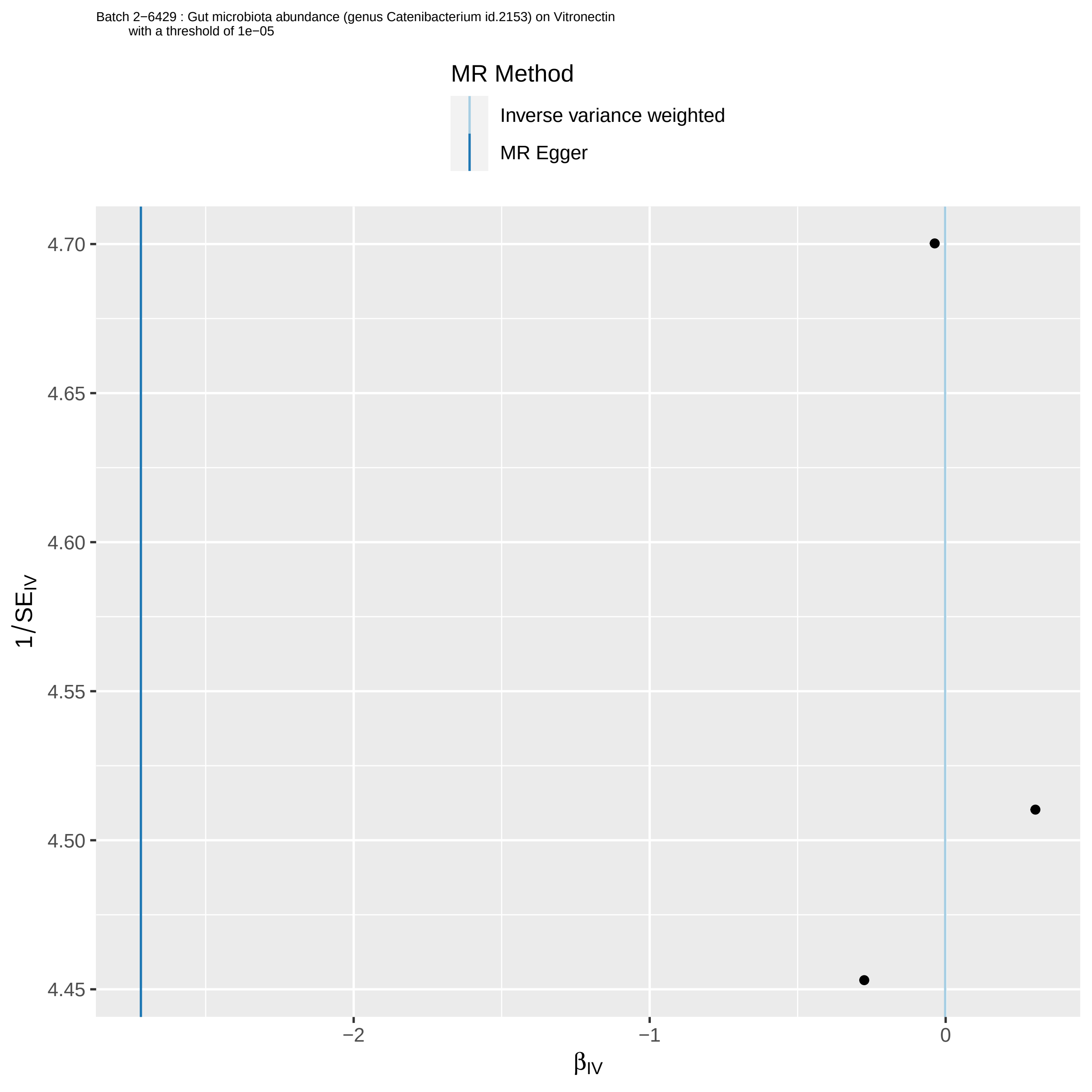
Batch 2−6429 : Gut microbiota abundance (genus Catenibacterium id.2153) on Vitronectin
with a threshold of 1e−05
MR Method
Inverse variance weighted
MR Egger
4.70
4.65
4.60
4.55
4.50
4.45
1/SE_IV
−2
−1
0
β_IV

Batch 2−6430 : Gut microbiota abundance (genus Parasutterella id.2892) on Vitronectin
with a threshold of 1e−05

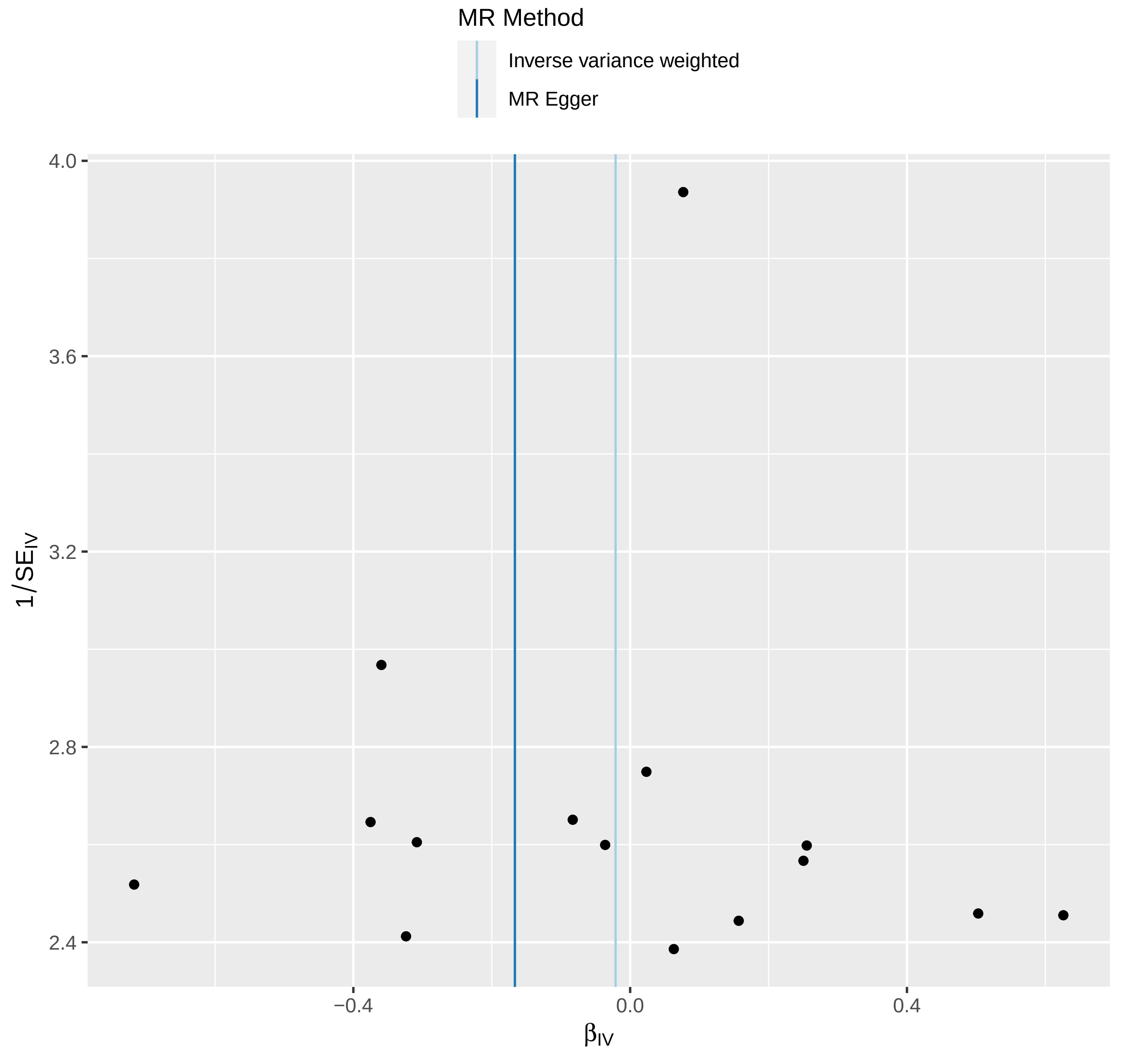

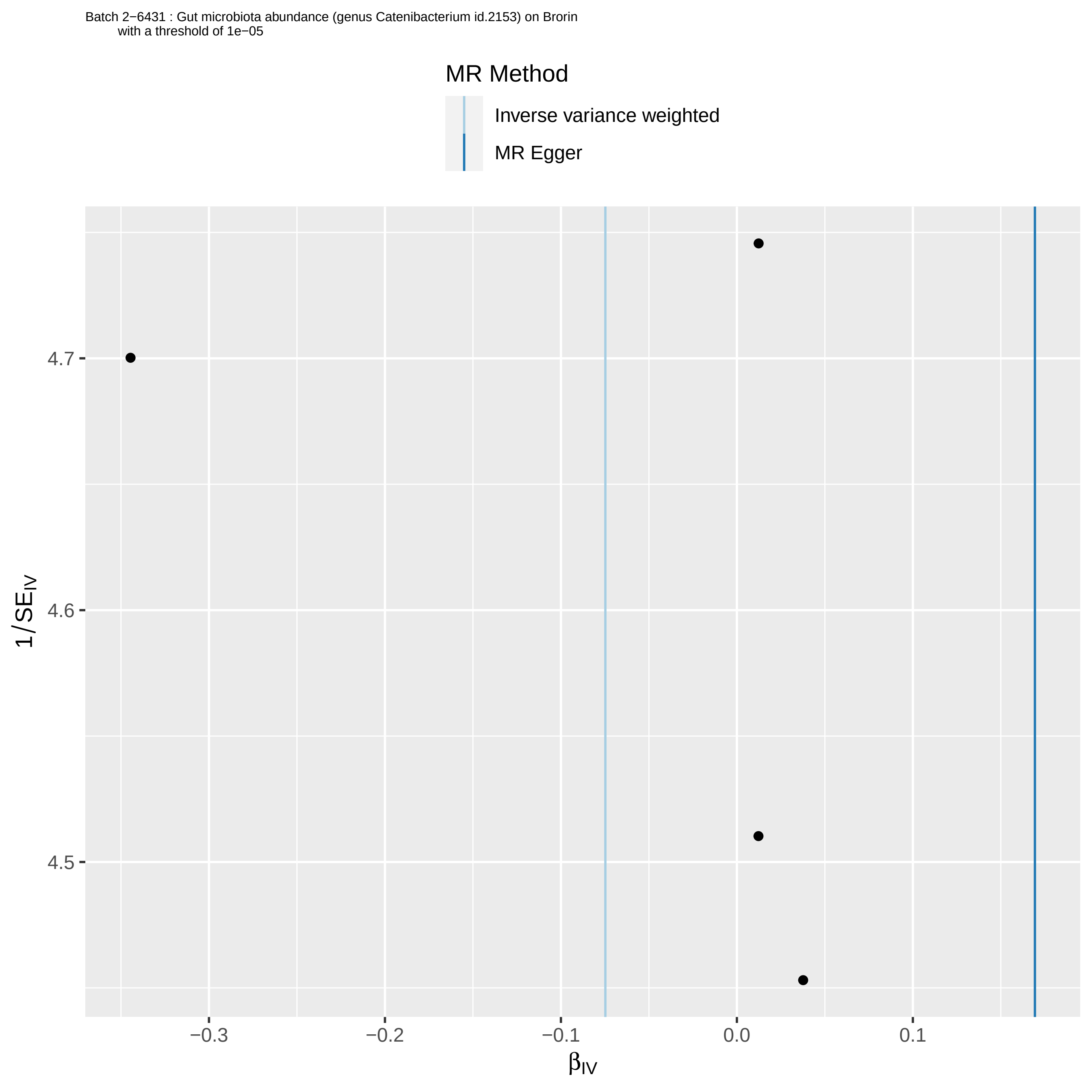

Batch 2−6431 : Gut microbiota abundance (genus Catenibacterium id.2153) on Brorin
with a threshold of 1e−05
MR Method
Inverse variance weighted
MR Egger
4.7
4.6
4.5
1/SE_IV
−0.3
−0.2
−0.1
0.0
0.1
β_IV

Batch 2−6432 : Gut microbiota abundance (genus Parasutterella id.2892) on Brorin
with a threshold of 1e−05

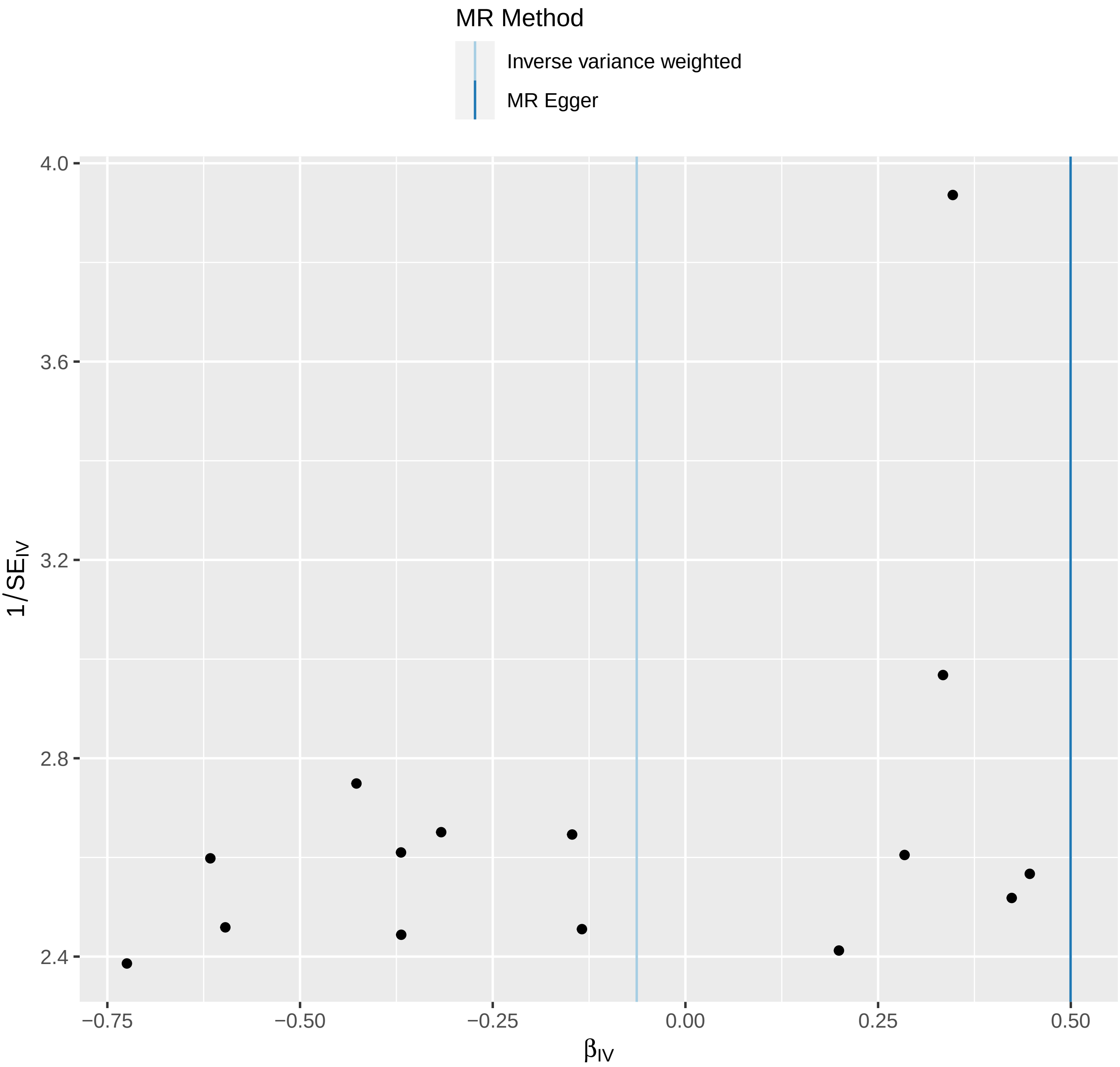

Batch 2−6433 : Gut microbiota abundance (genus Catenibacterium id.2153) on von Willebrand factor C domain−containing protein 2−like with a threshold of 1e−05

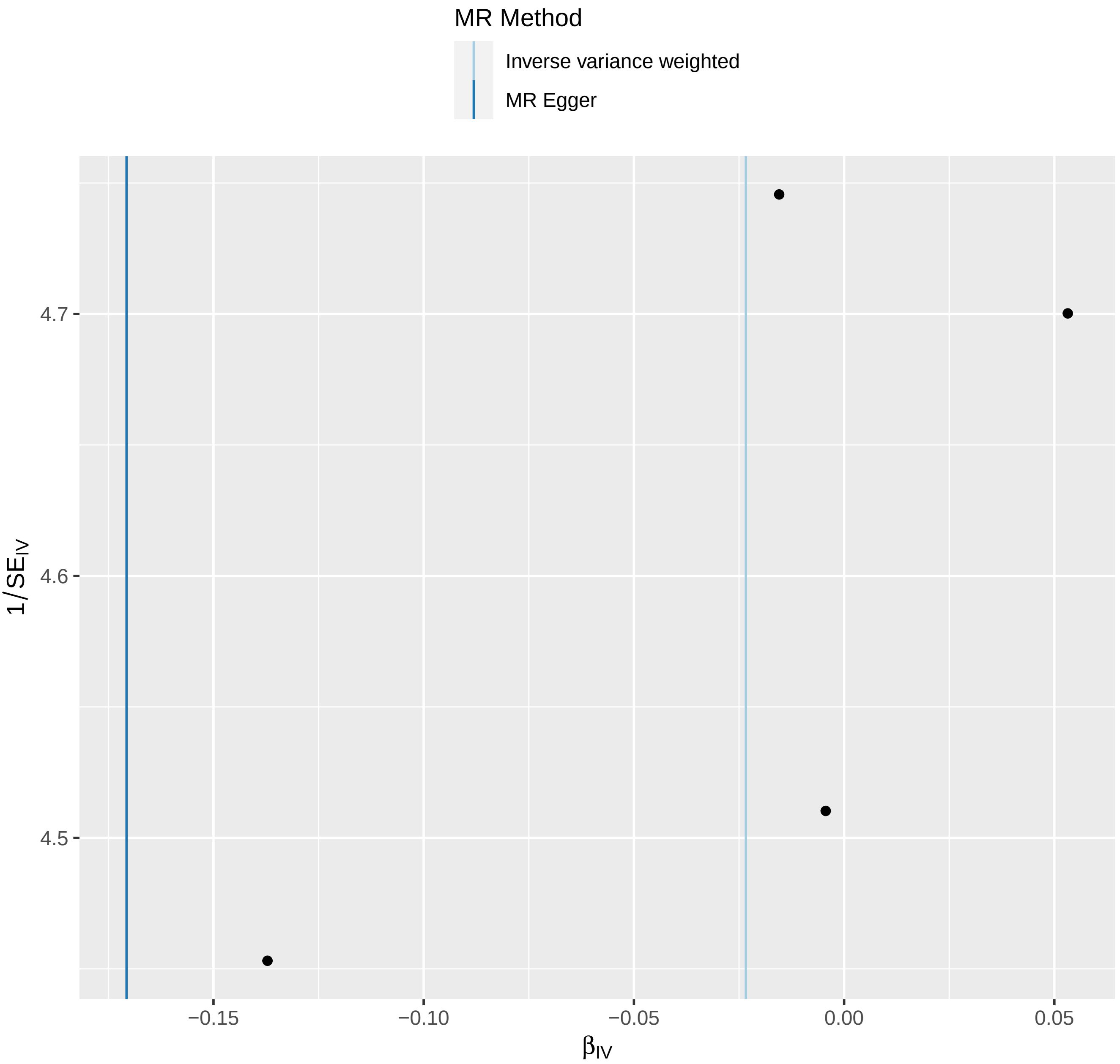

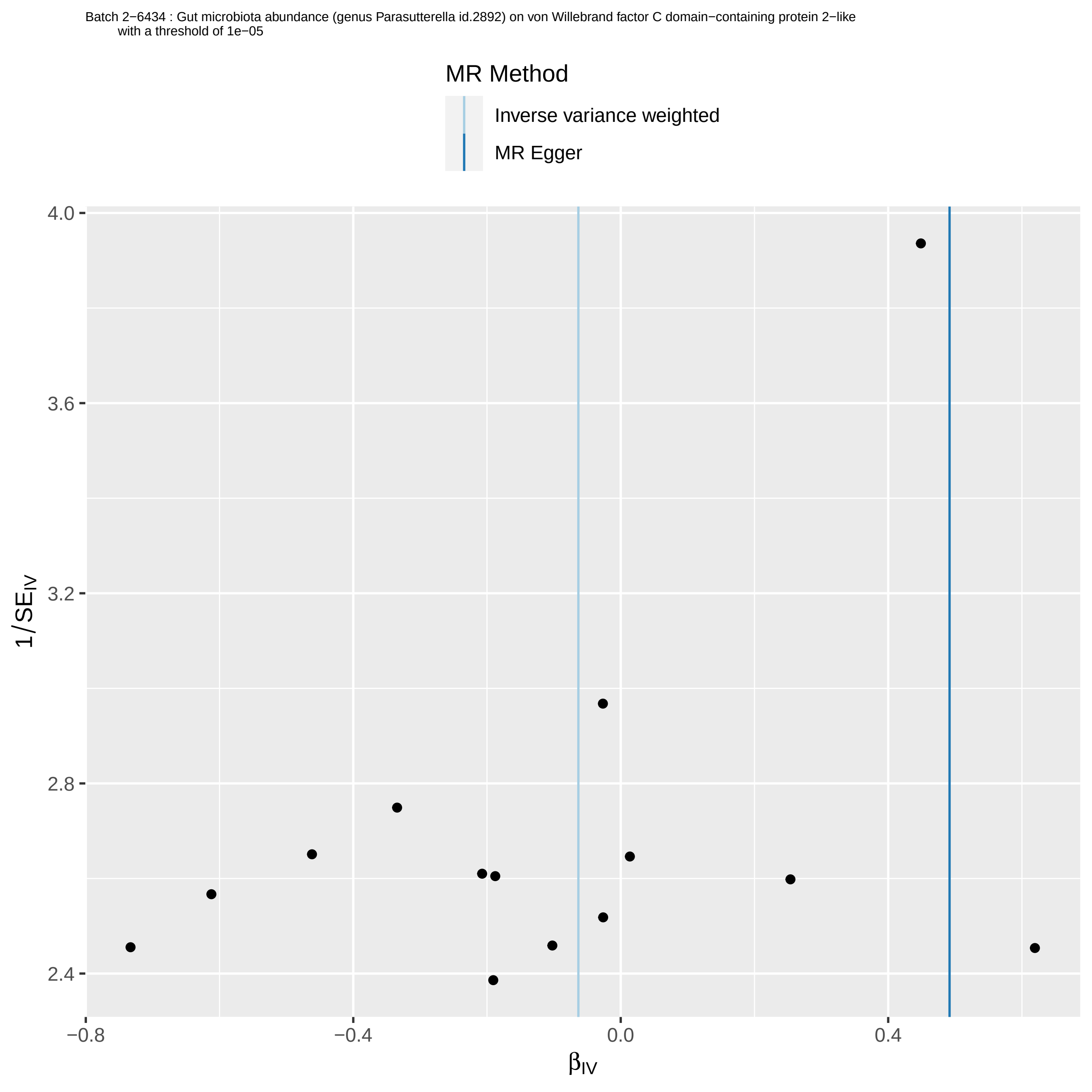

Batch 2−6434 : Gut microbiota abundance (genus Parasutterella id.2892) on von Willebrand factor C domain−containing protein 2−like
with a threshold of 1e−05
MR Method
Inverse variance weighted
MR Egger
4.0
3.6
3.2
2.8
2.4
1/SE_IV
−0.8
−0.4
0.0
0.4
β_IV

Batch 2−6435 : Gut microbiota abundance (genus Catenibacterium id.2153) on WW domain−binding protein 1
with a threshold of 1e−05

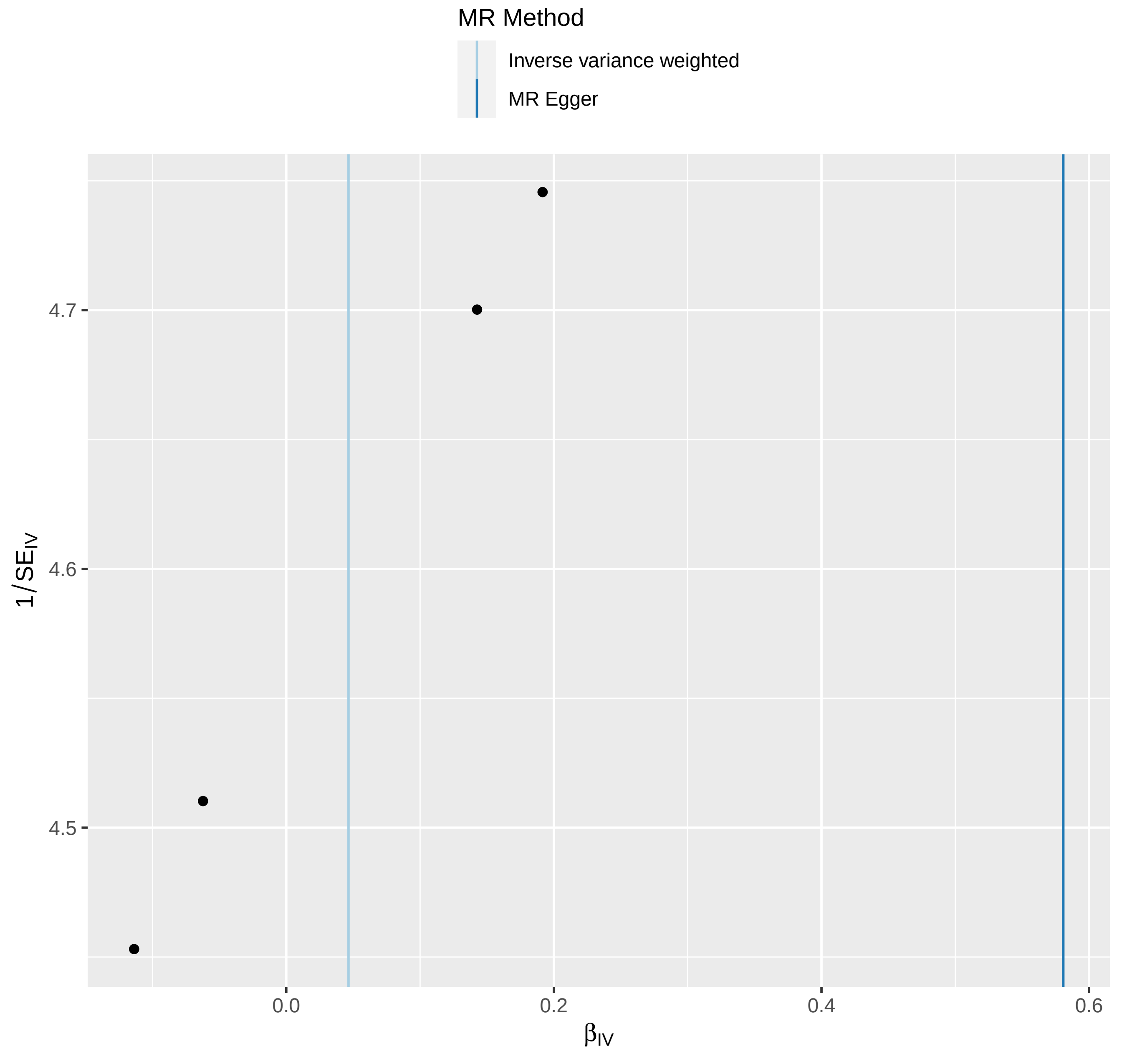

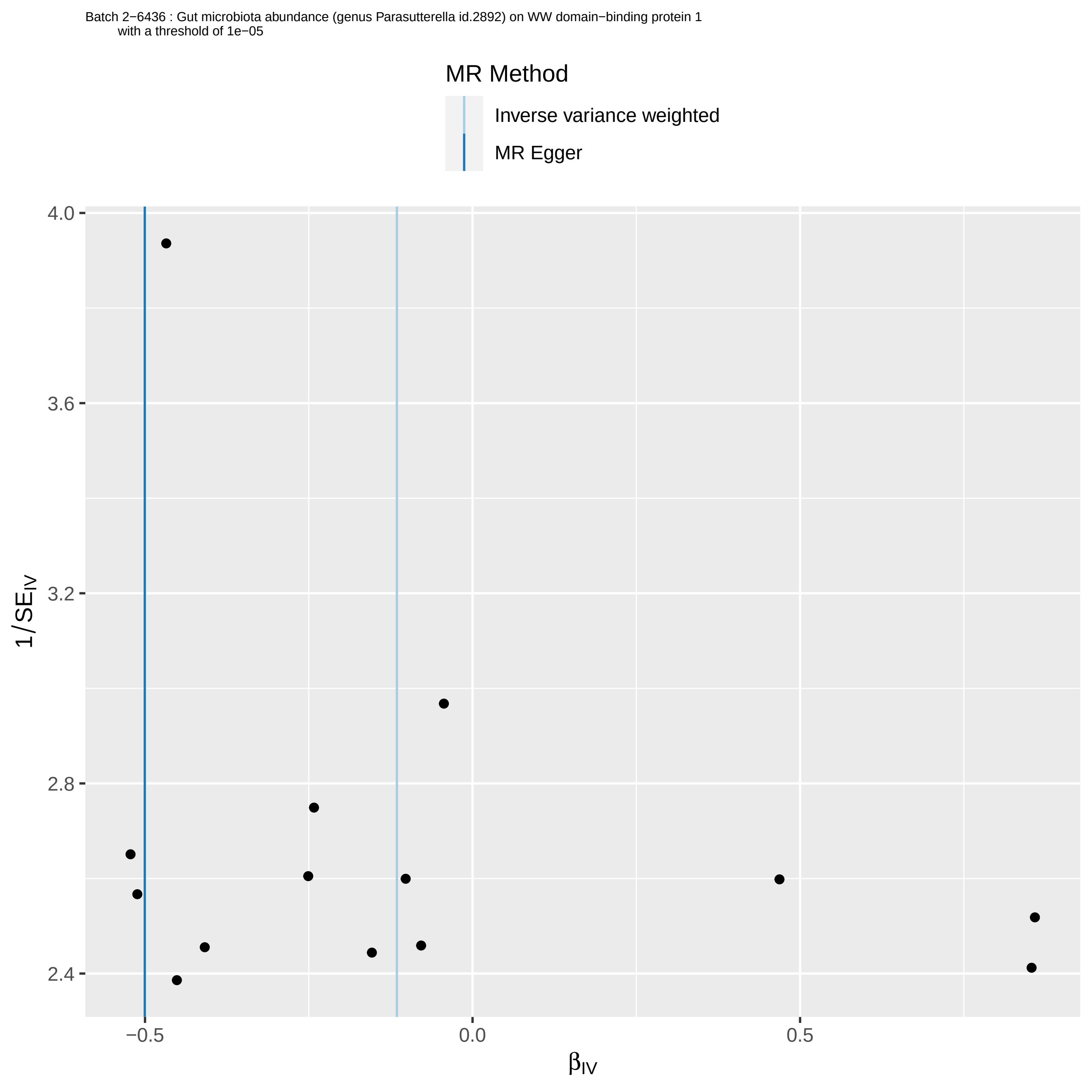
Batch 2−6436 : Gut microbiota abundance (genus Parasutterella id.2892) on WW domain−binding protein 1
with a threshold of 1e−05
MR Method
Inverse variance weighted
MR Egger
4.0
3.6
3.2
2.8
2.4
1/SE$_{IV}$
−0.5
0.0
0.5
β$_{IV}$

Batch 2−6437 : Gut microbiota abundance (genus Catenibacterium id.2153) on WW domain binding protein 1−like
with a threshold of 1e−05

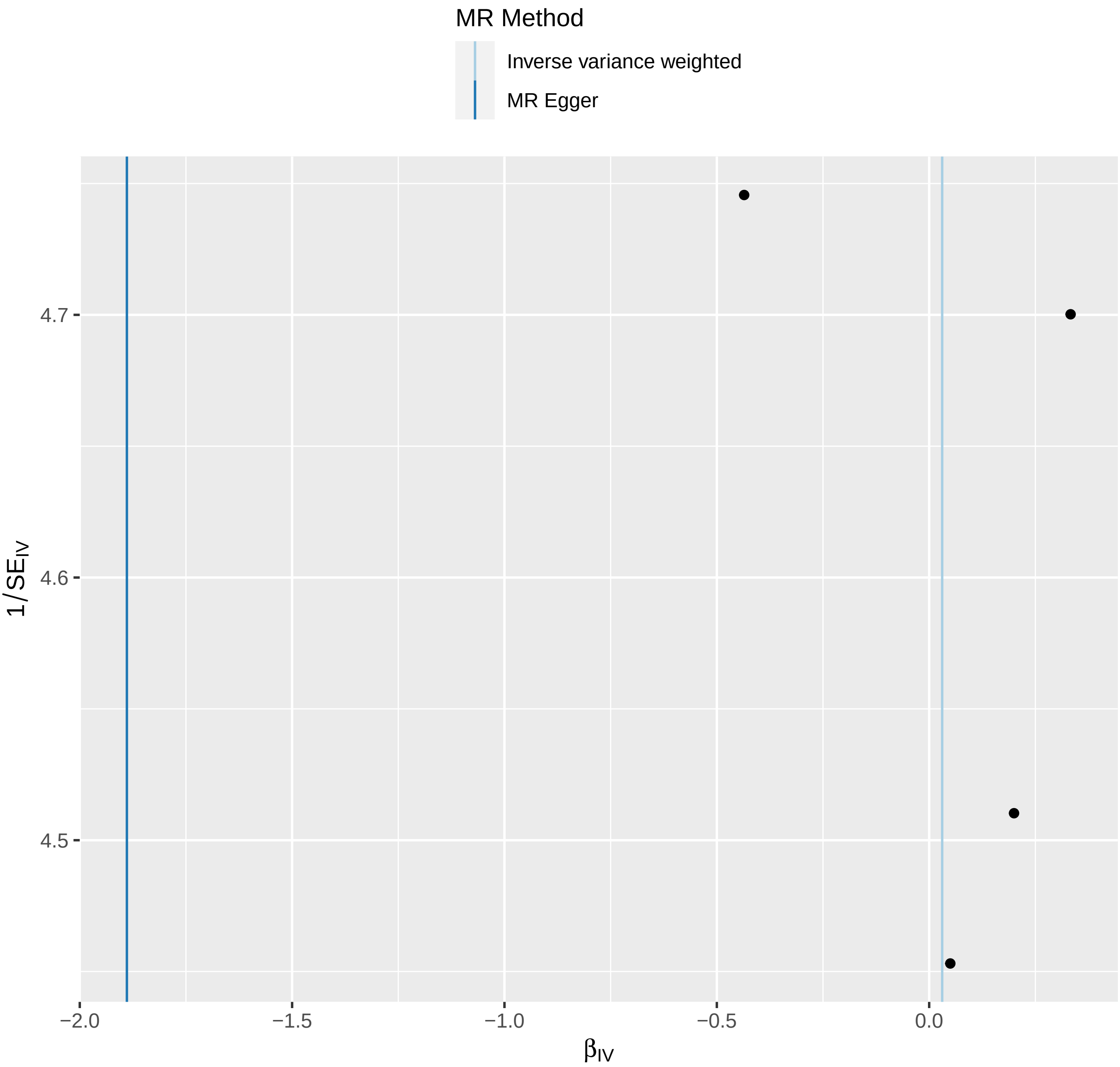

Batch 2−6438 : Gut microbiota abundance (genus Parasutterella id.2892) on WW domain binding protein 1−like with a threshold of 1e−05

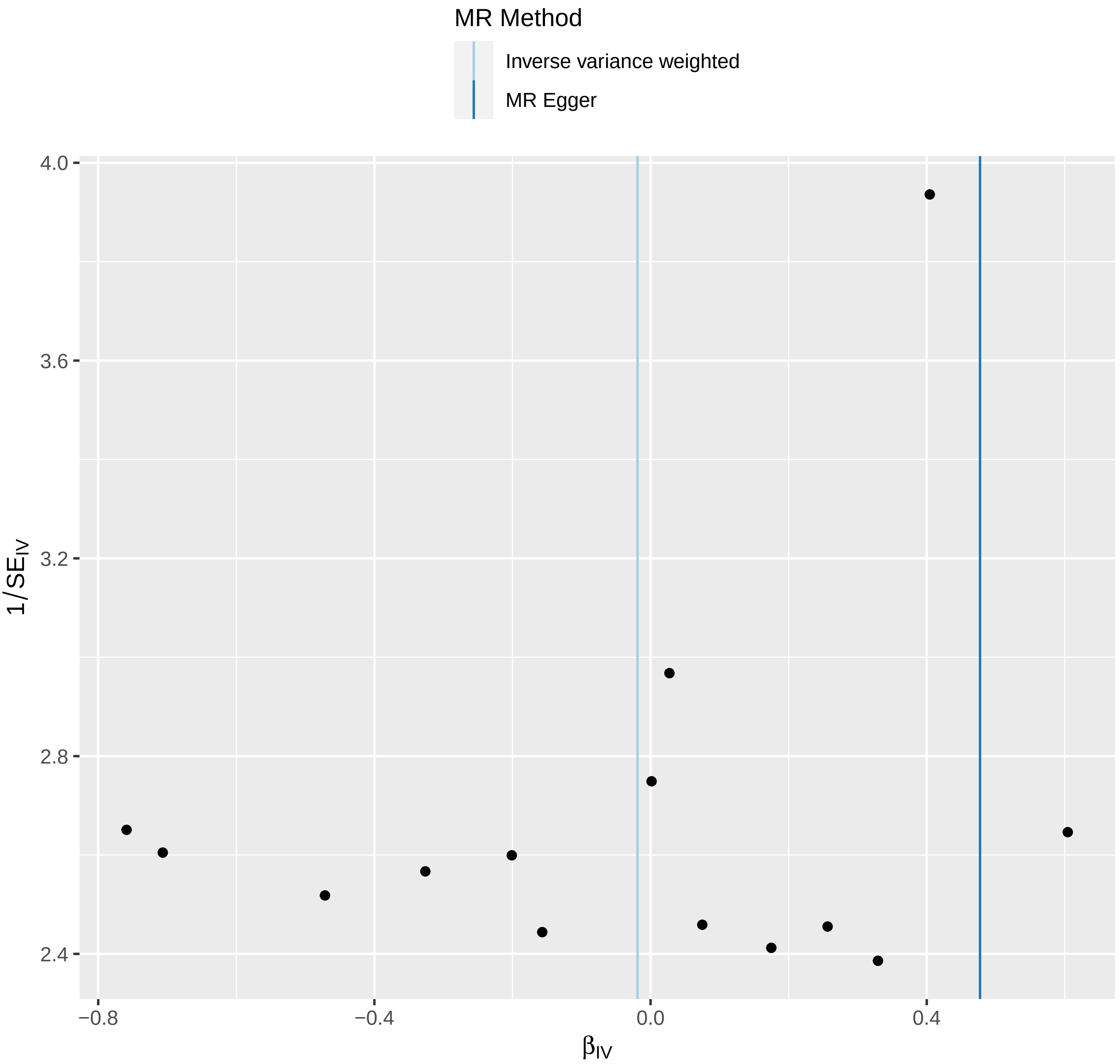

Batch 2−6439 : Gut microbiota abundance (genus Catenibacterium id.2153) on WD repeat−containing protein 1 with a threshold of 1e−05

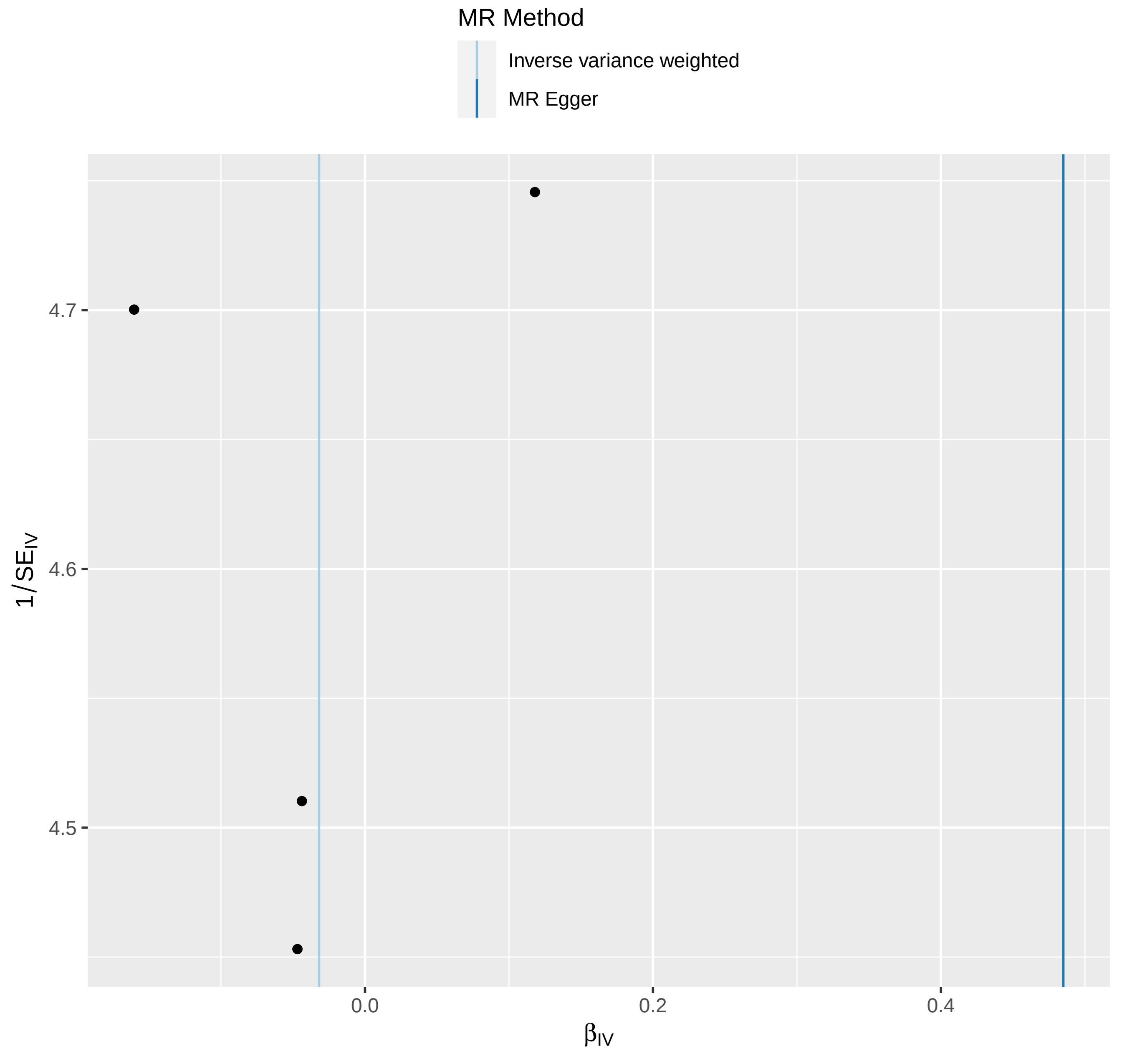

Batch 2−6440 : Gut microbiota abundance (genus Parasutterella id.2892) on WD repeat−containing protein 1
with a threshold of 1e−05

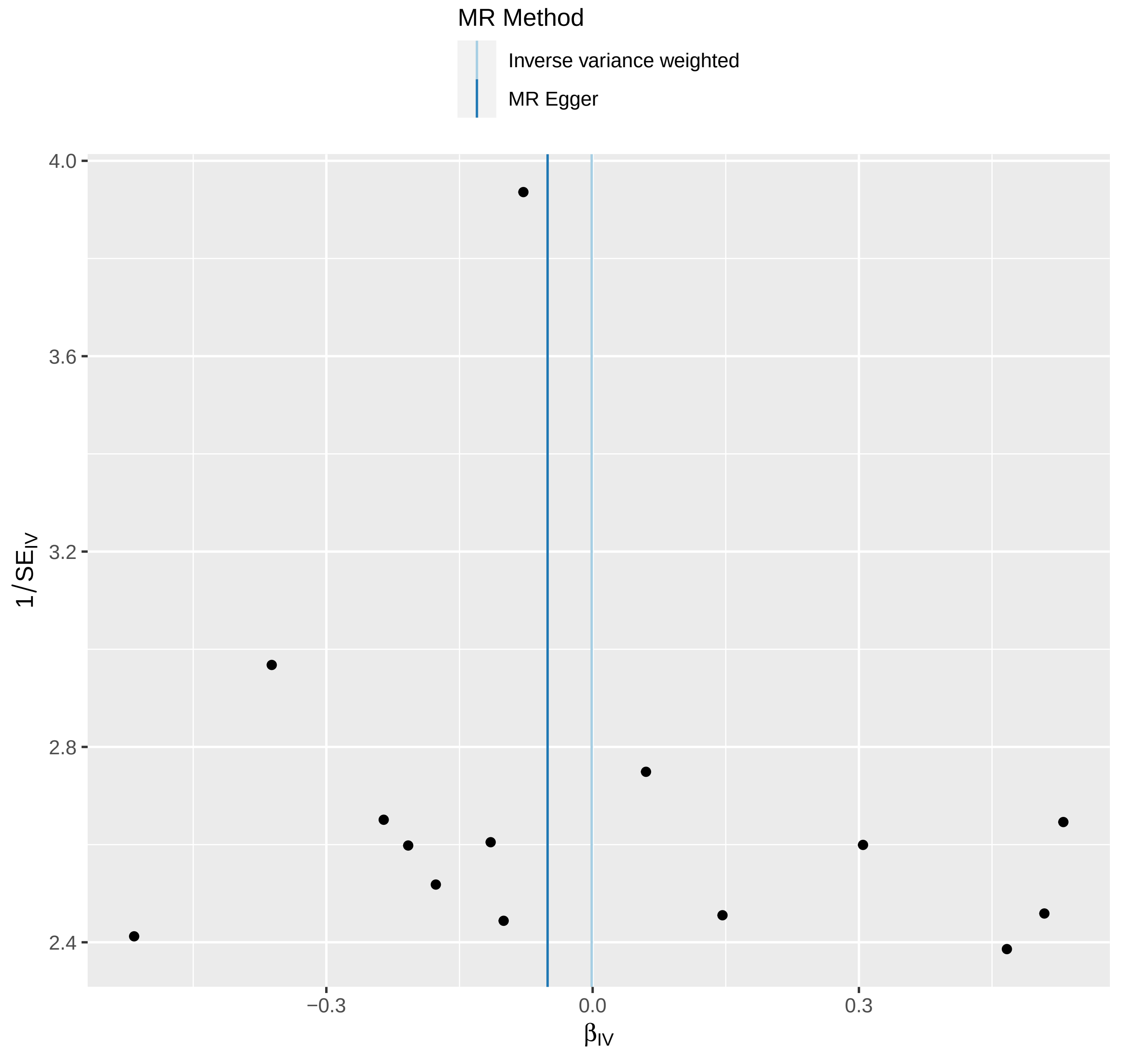

Batch 2−6441 : Gut microbiota abundance (genus Catenibacterium id.2153) on WAP four−disulfide core domain protein 10A
with a threshold of 1e−05

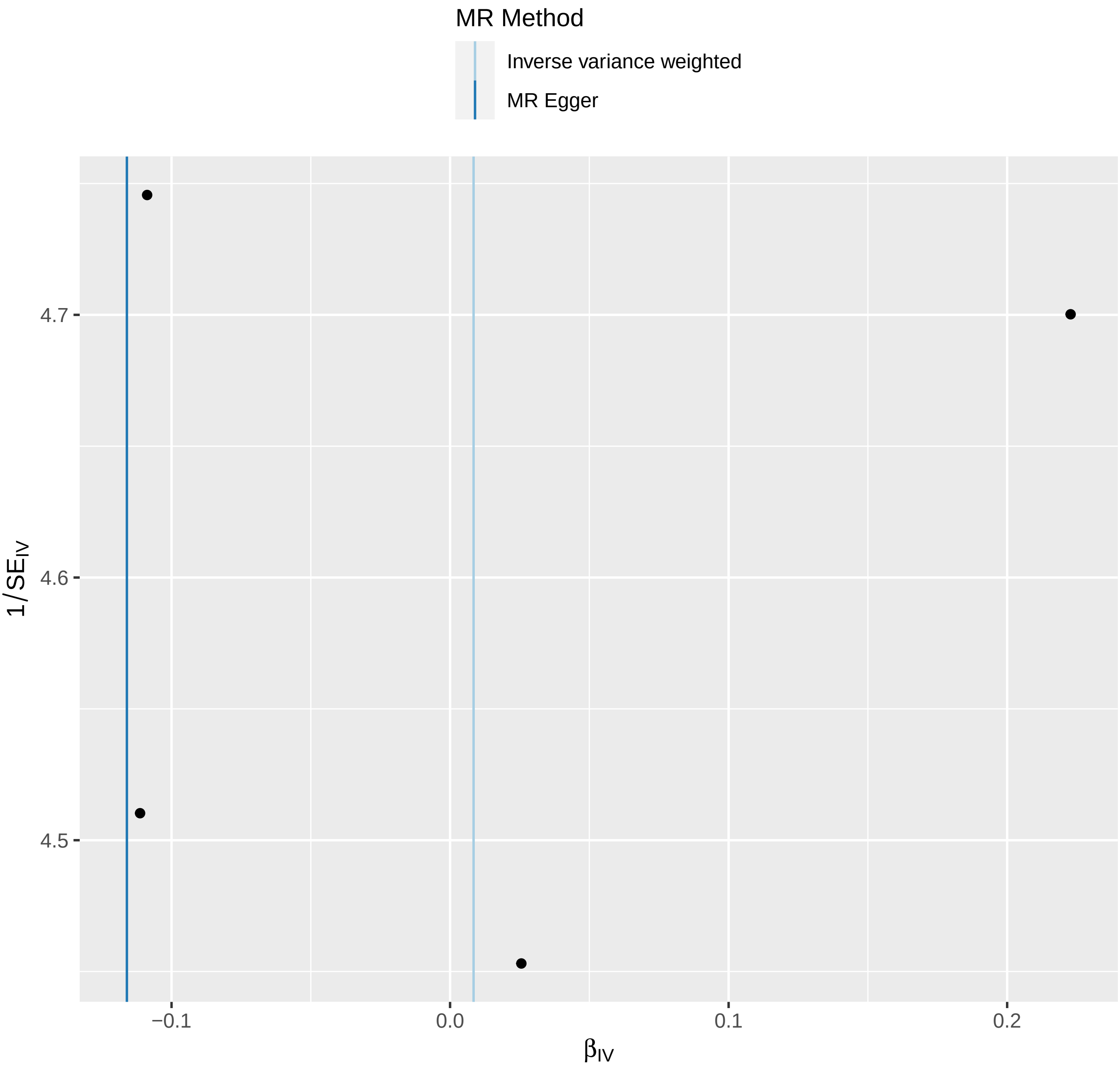

Batch 2−6442 : Gut microbiota abundance (genus Parasutterella id.2892) on WAP four−disulfide core domain protein 10A
with a threshold of 1e−05

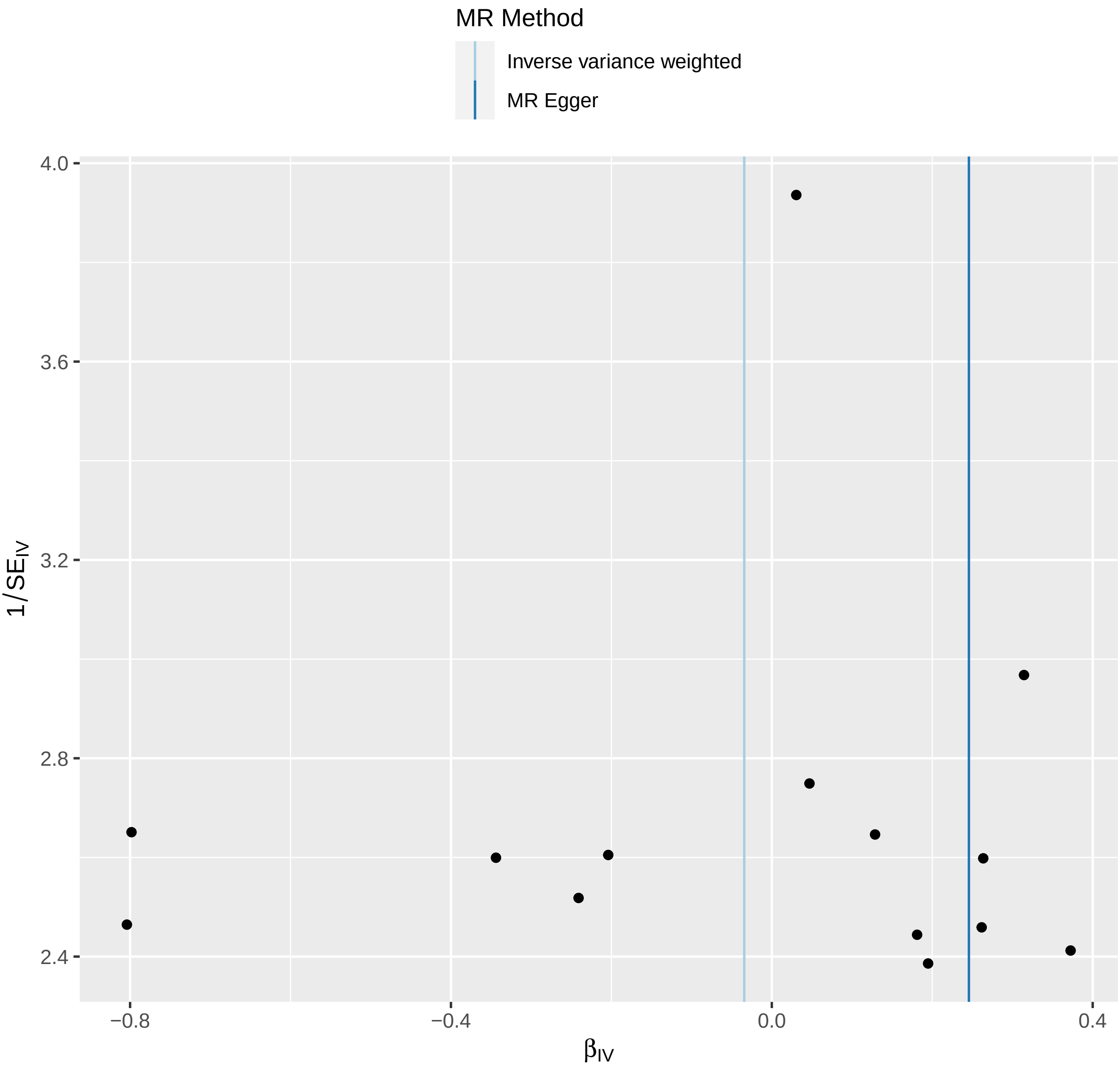

Batch 2−6443 : Gut microbiota abundance (genus Catenibacterium id.2153) on WAP four−disulfide core domain protein 12
with a threshold of 1e−05

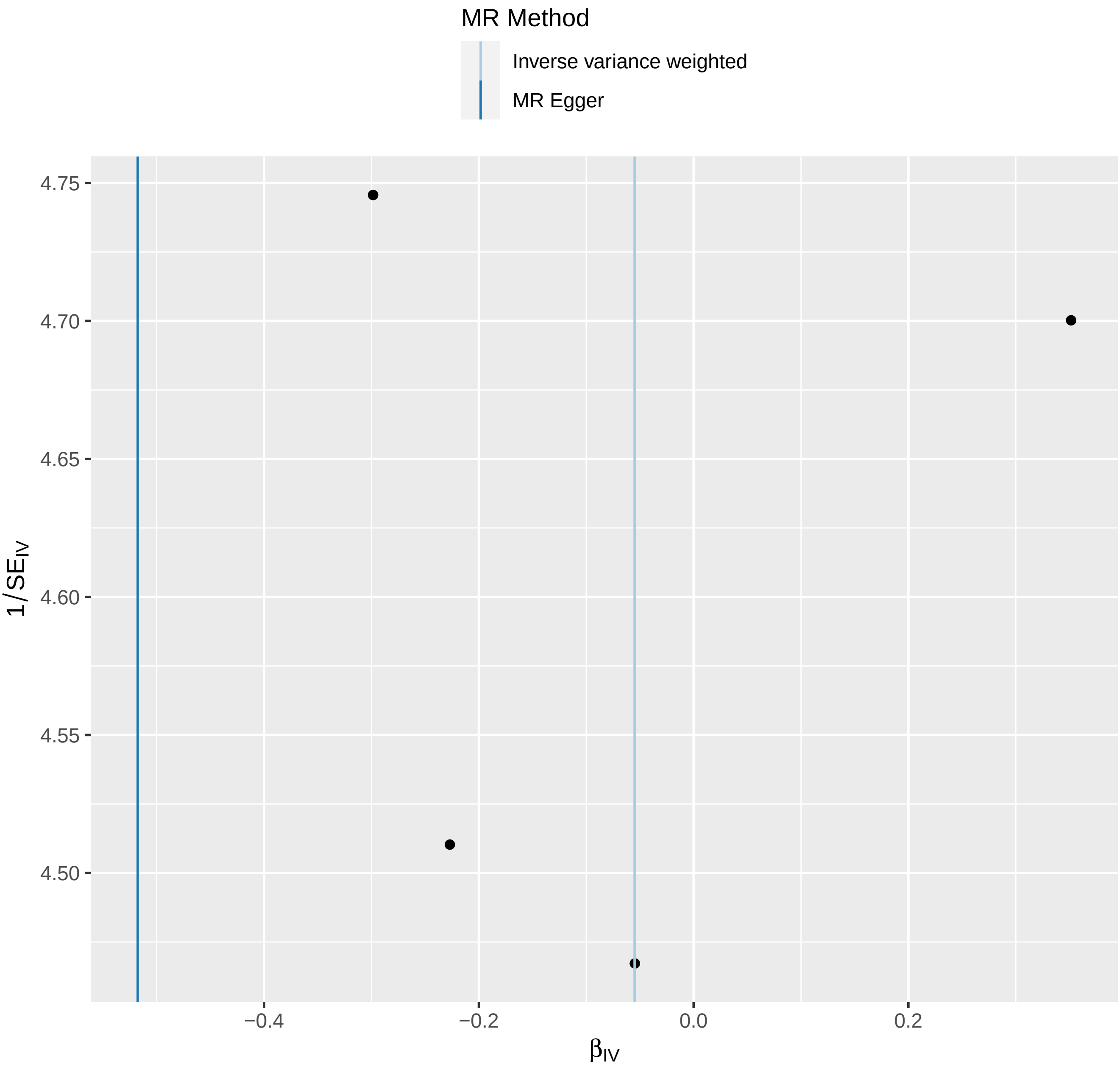

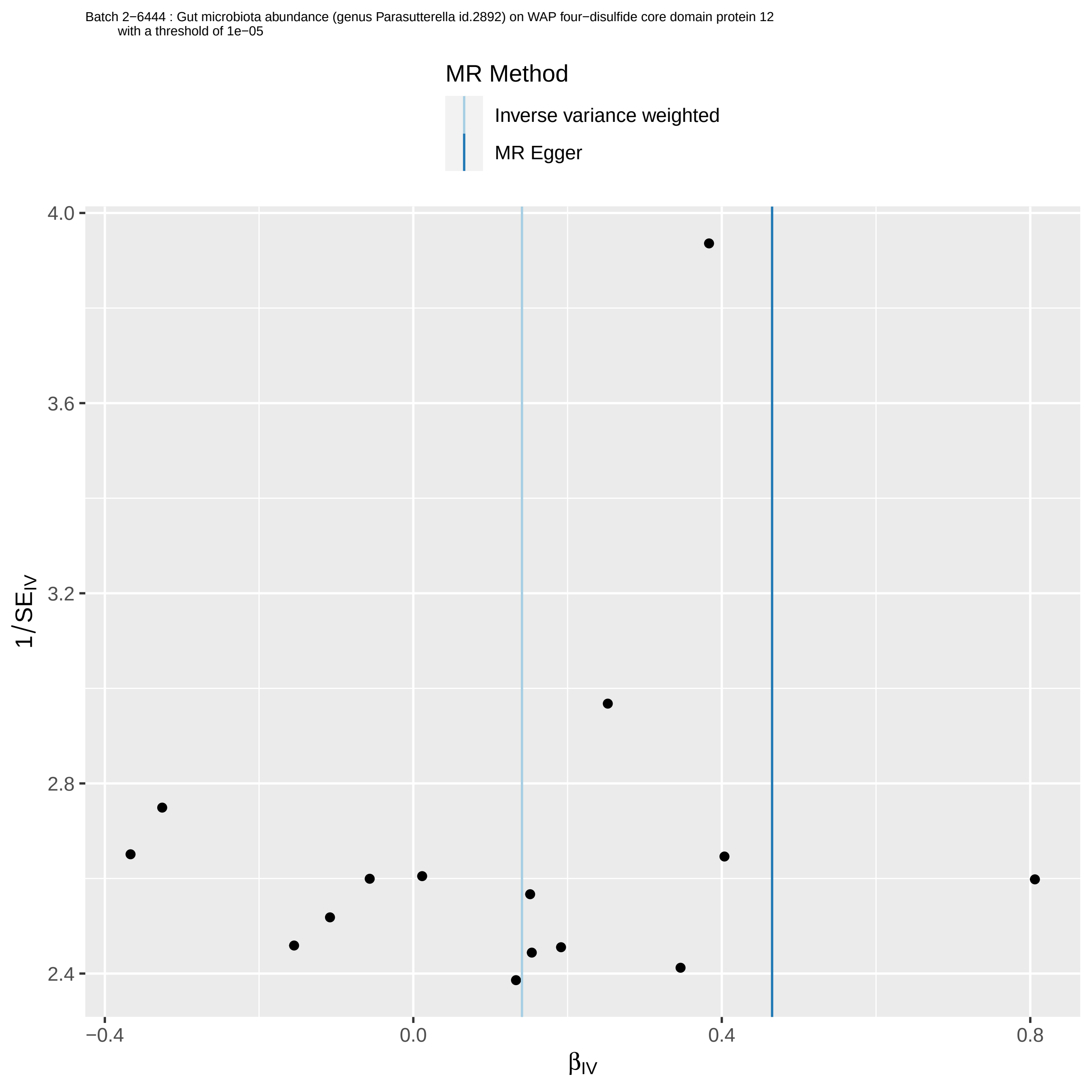

Batch 2−6444 : Gut microbiota abundance (genus Parasutterella id.2892) on WAP four−disulfide core domain protein 12
with a threshold of 1e−05
MR Method
Inverse variance weighted
MR Egger
4.0
3.6
3.2
2.8
2.4
1/SE_IV
−0.4
0.0
0.4
0.8
β_IV

Batch 2−6445 : Gut microbiota abundance (genus Catenibacterium id.2153) on WAP four−disulfide core domain protein 13
with a threshold of 1e−05

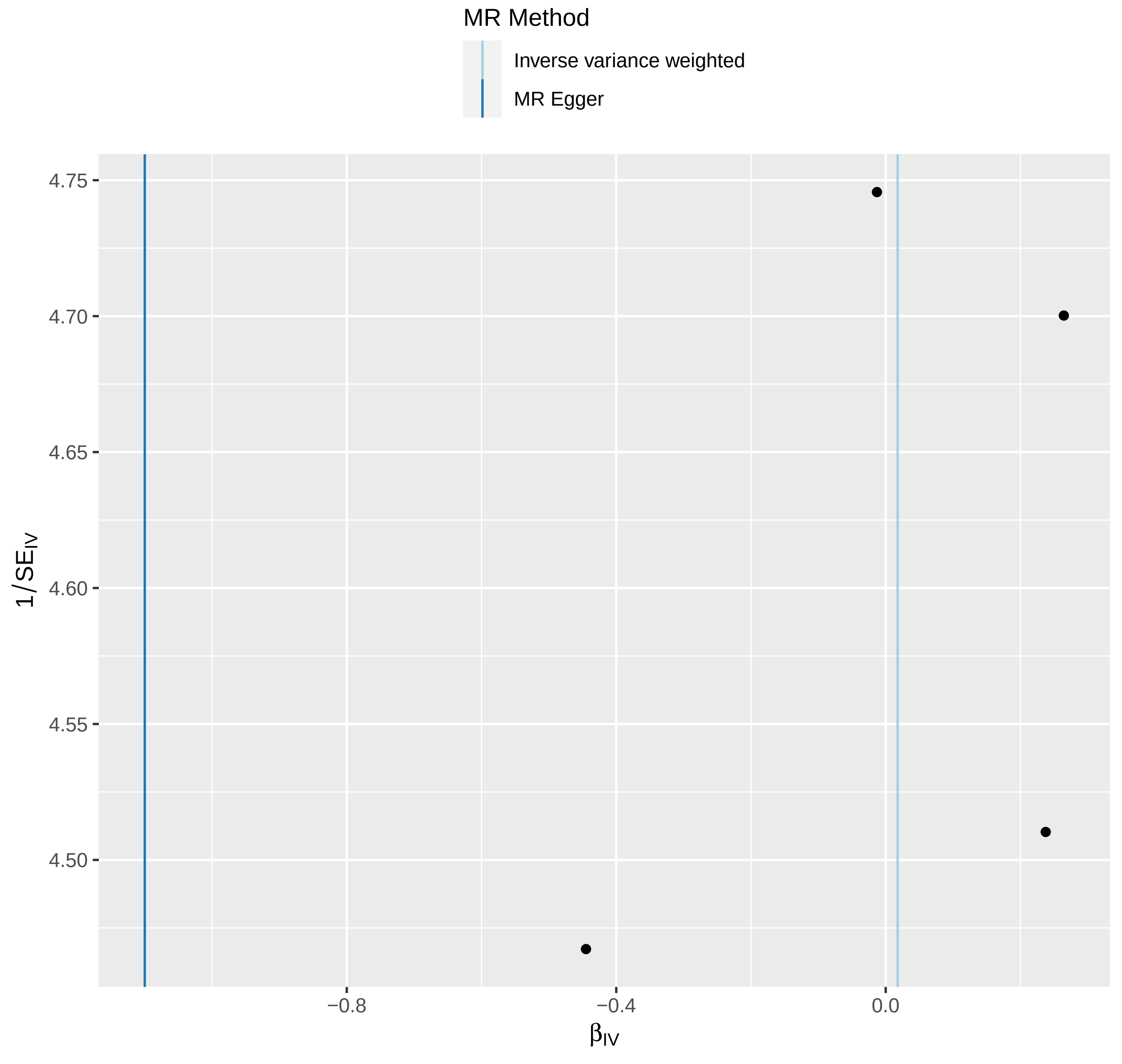

Batch 2−6446 : Gut microbiota abundance (genus Parasutterella id.2892) on WAP four−disulfide core domain protein 13
with a threshold of 1e−05

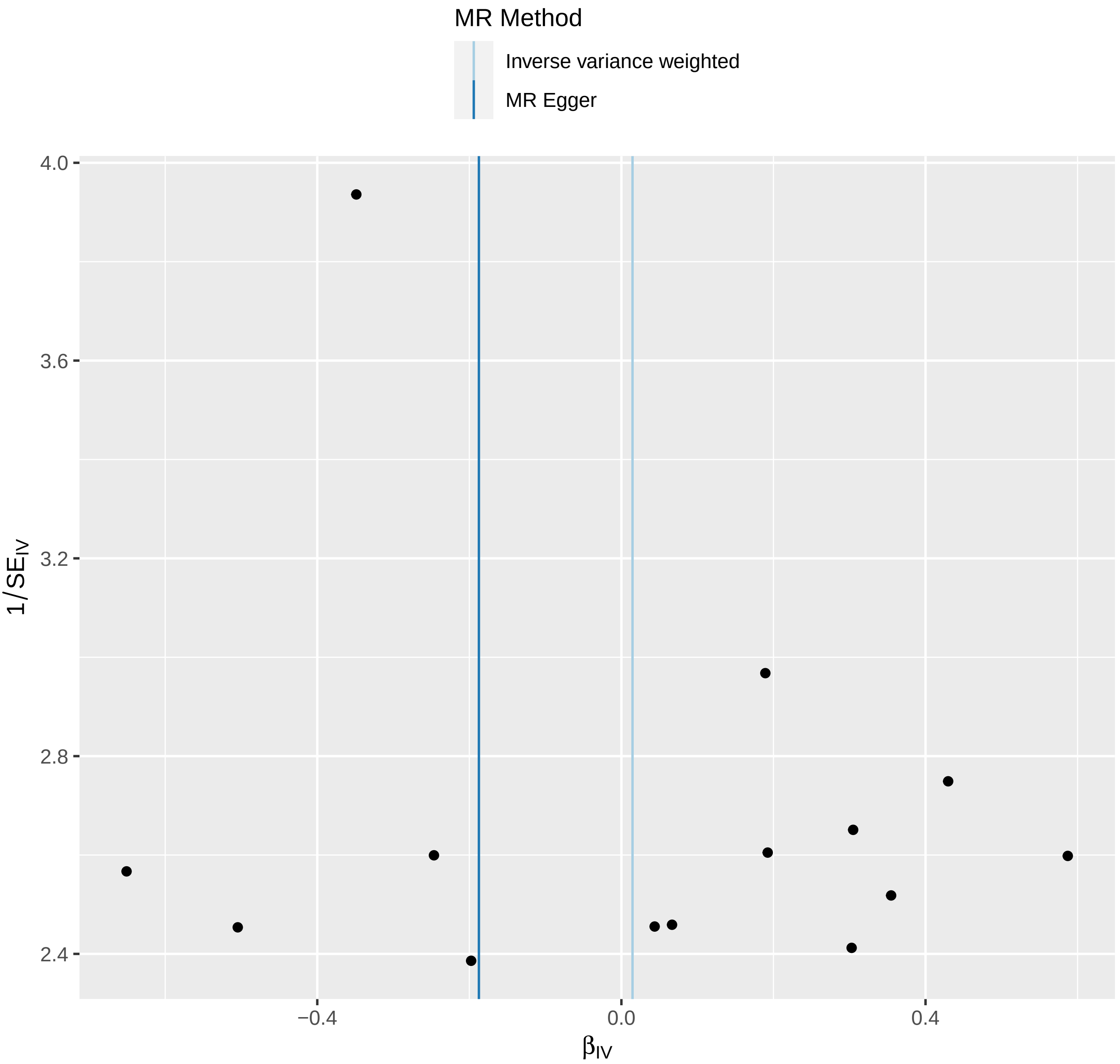

Batch 2−6447 : Gut microbiota abundance (genus Catenibacterium id.2153) on WAP four−disulfide core domain protein 2
with a threshold of 1e−05

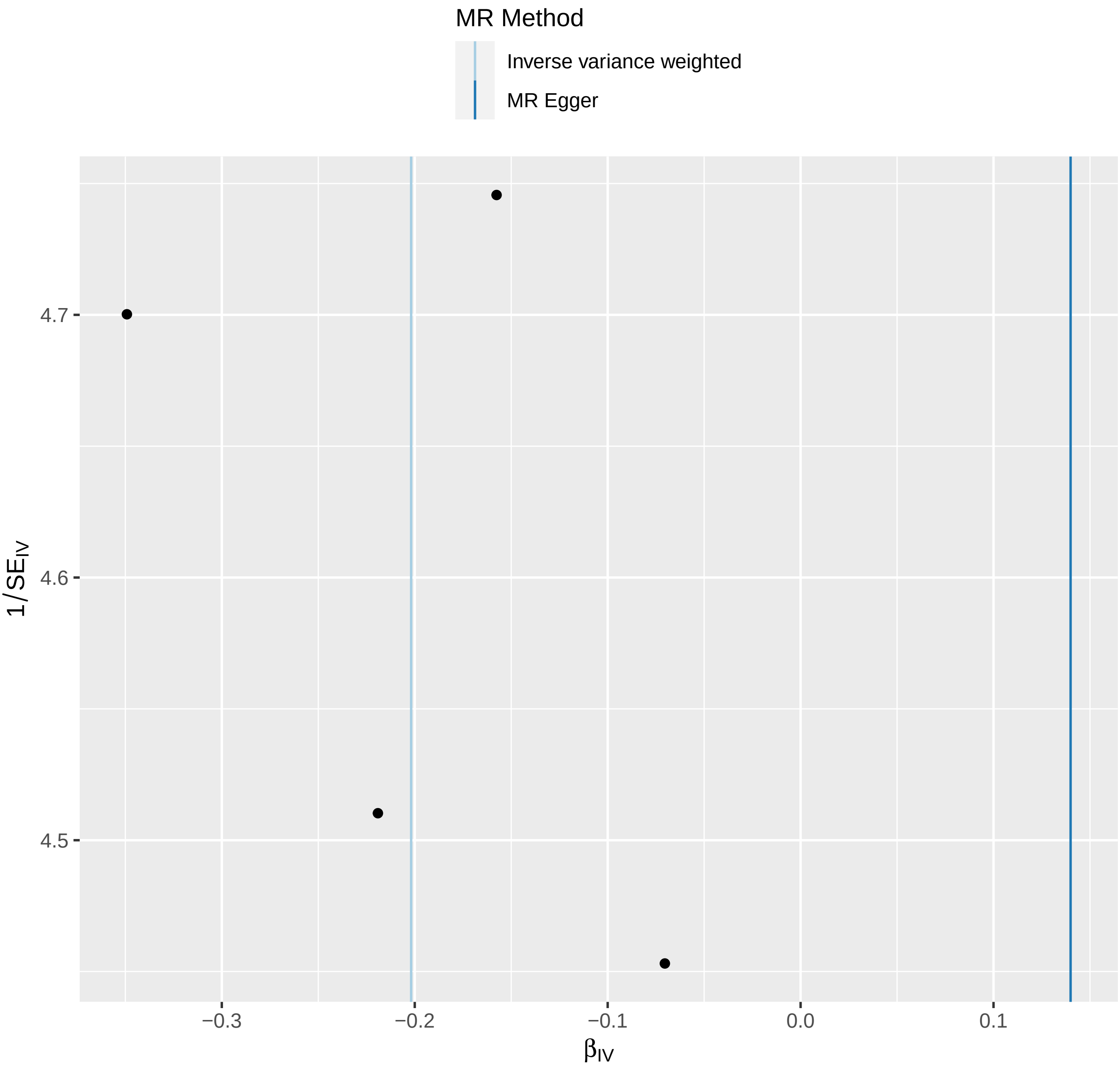

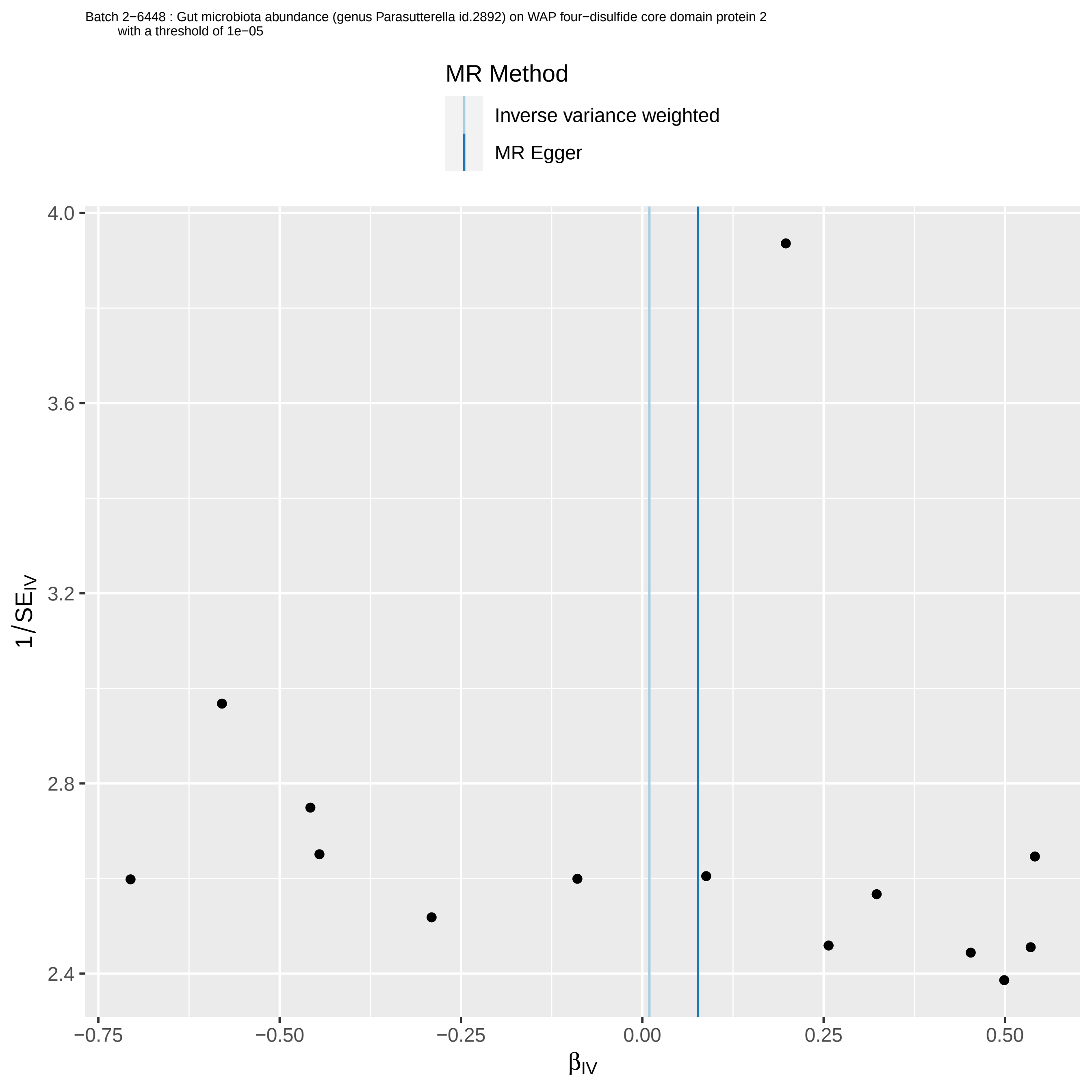
Batch 2−6448 : Gut microbiota abundance (genus Parasutterella id.2892) on WAP four−disulfide core domain protein 2
with a threshold of 1e−05
MR Method
Inverse variance weighted
MR Egger
4.0
3.6
3.2
2.8
2.4
1/SE_IV
−0.75
−0.50
−0.25
0.00
0.25
0.50
β_IV

Batch 2−6449 : Gut microbiota abundance (genus Catenibacterium id.2153) on WAP four−disulfide core domain protein 3
with a threshold of 1e−05

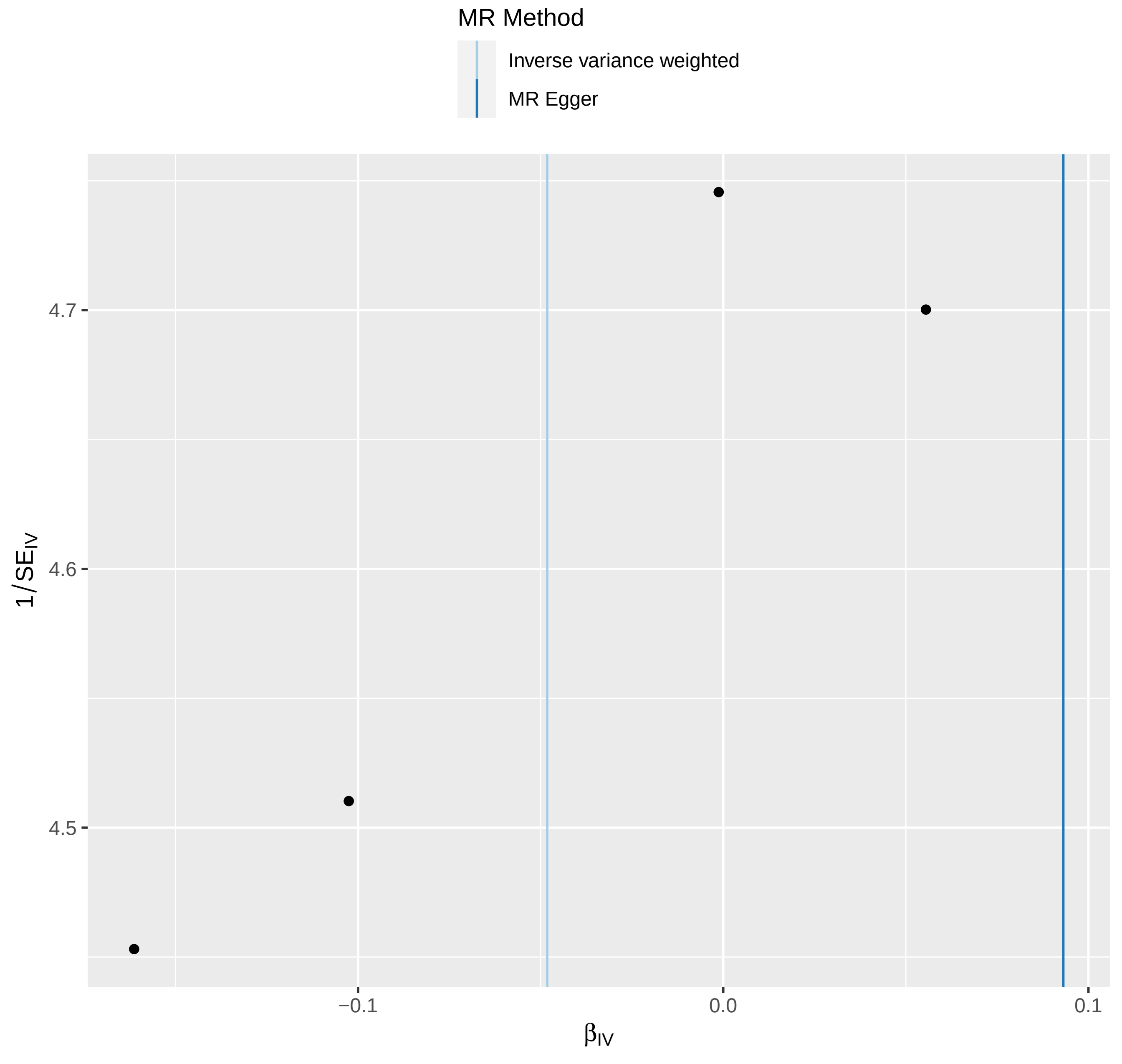

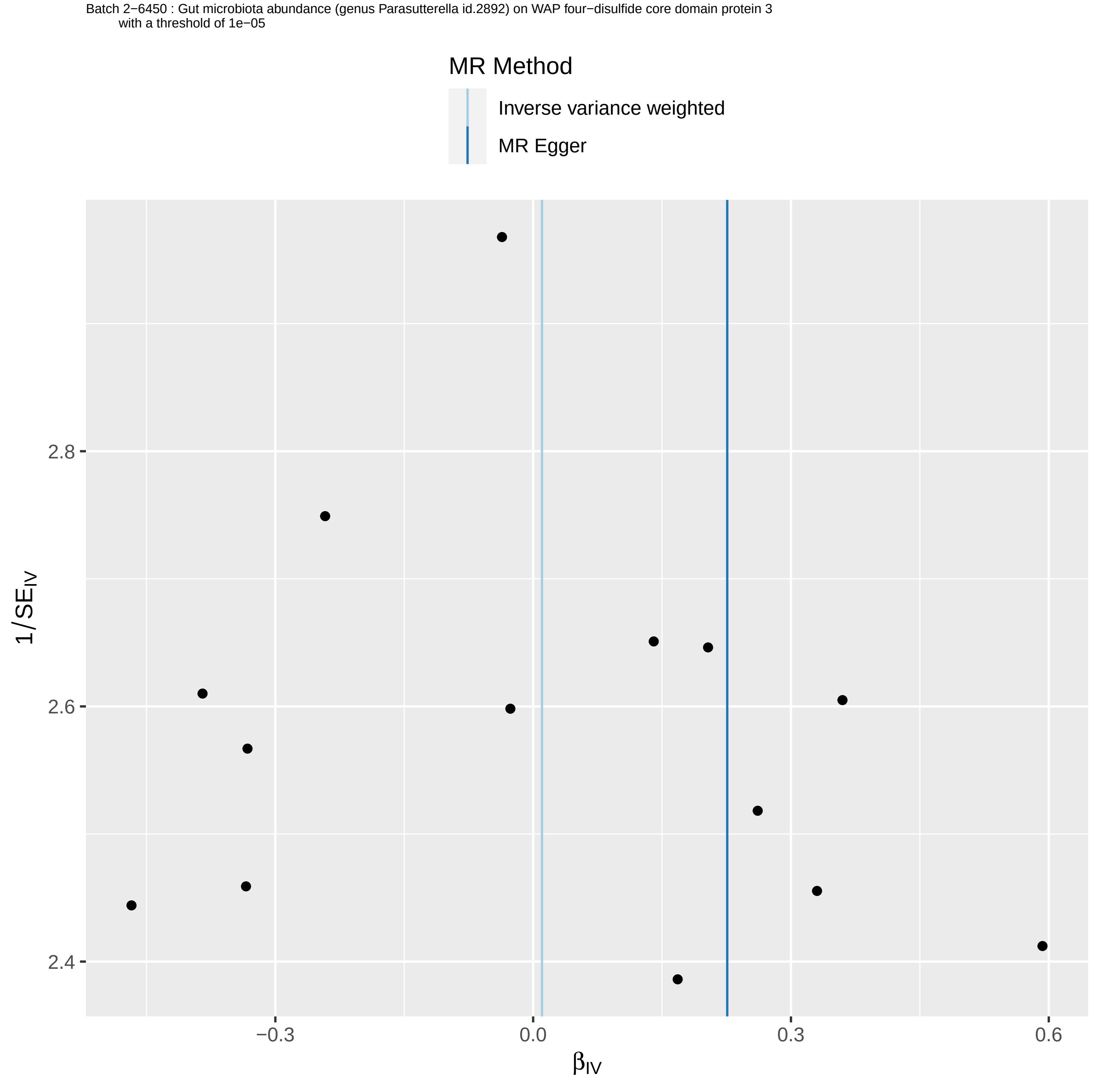

Batch 2−6450 : Gut microbiota abundance (genus Parasutterella id.2892) on WAP four−disulfide core domain protein 3
with a threshold of 1e−05
MR Method
Inverse variance weighted
MR Egger
2.8
2.6
2.4
1/SE$_{IV}$
−0.3
0.0
0.3
0.6
β_{IV}

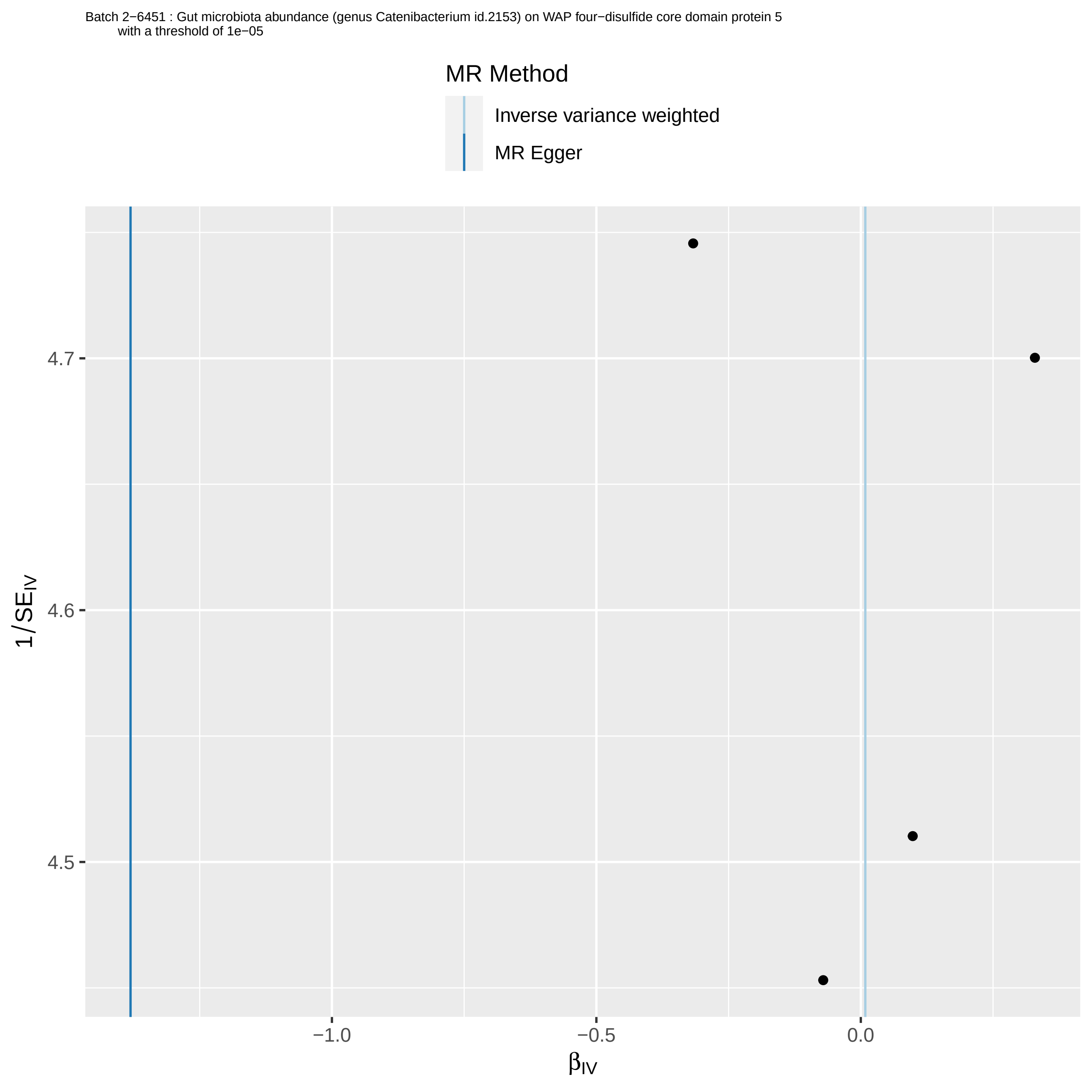

Batch 2−6451 : Gut microbiota abundance (genus Catenibacterium id.2153) on WAP four−disulfide core domain protein 5
with a threshold of 1e−05
MR Method
Inverse variance weighted
MR Egger
4.7
4.6
4.5
1/SE_IV
−1.0
−0.5
0.0
β_IV

Batch 2−6452 : Gut microbiota abundance (genus Parasutterella id.2892) on WAP four−disulfide core domain protein 5
with a threshold of 1e−05

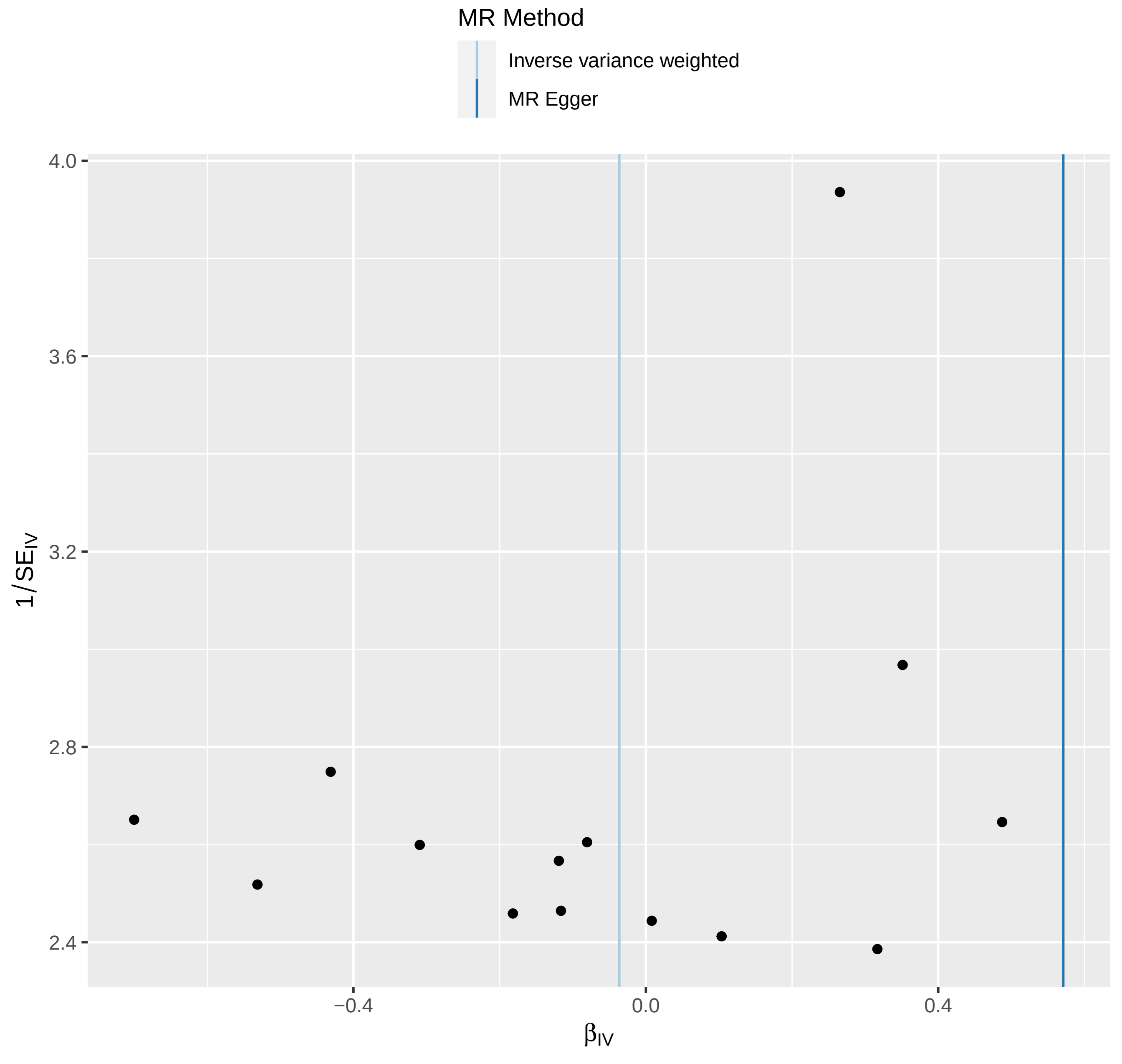

Batch 2−6453 : Gut microbiota abundance (genus Catenibacterium id.2153) on WAP, kazal, immunoglobulin, kunitz and NTR domain−containing protein 1 with a threshold of 1e−05

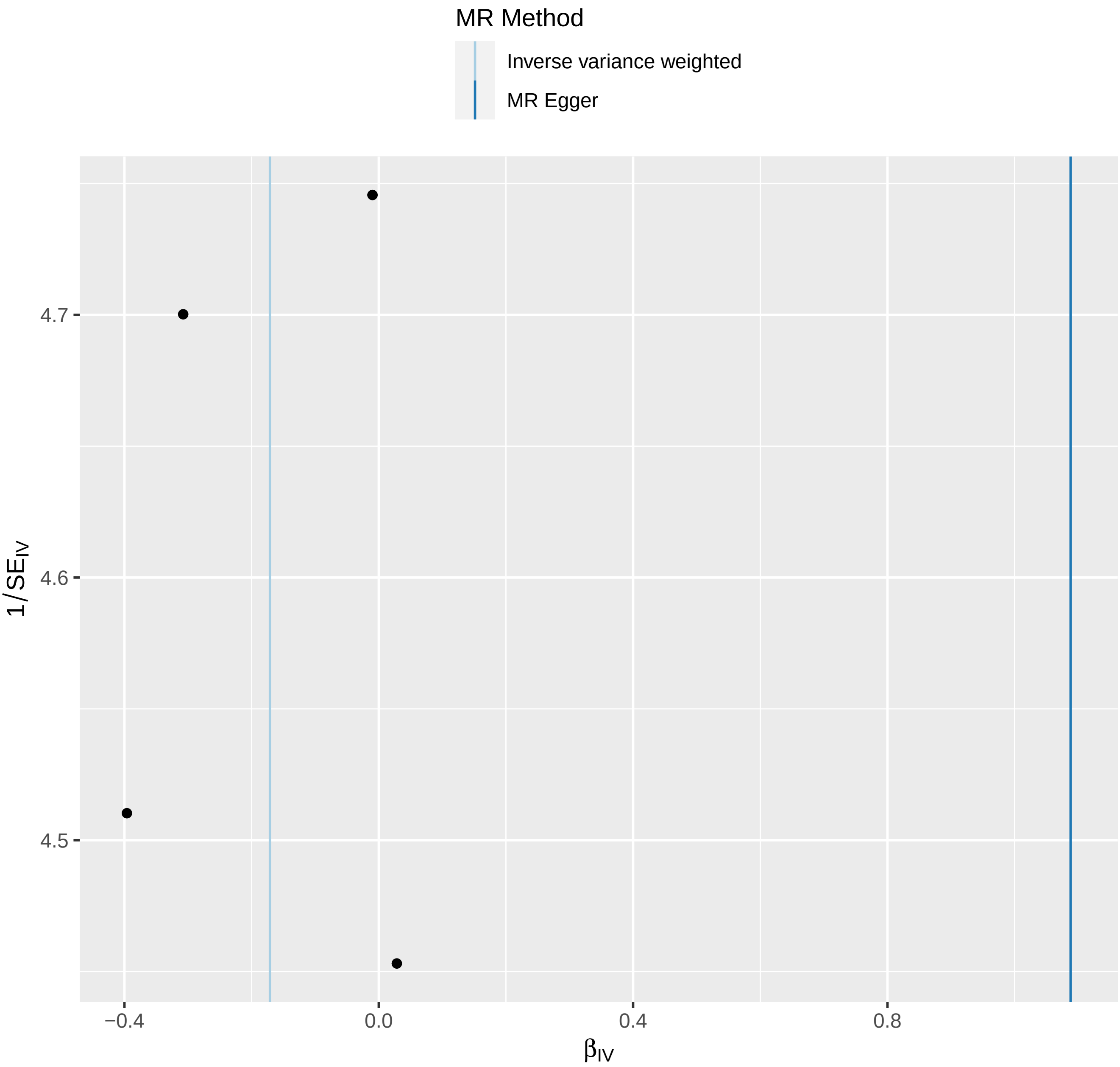

Batch 2−6454 : Gut microbiota abundance (genus Parasutterella id.2892) on WAP, kazal, immunoglobulin, kunitz and NTR domain−containing protein 1 with a threshold of 1e−05

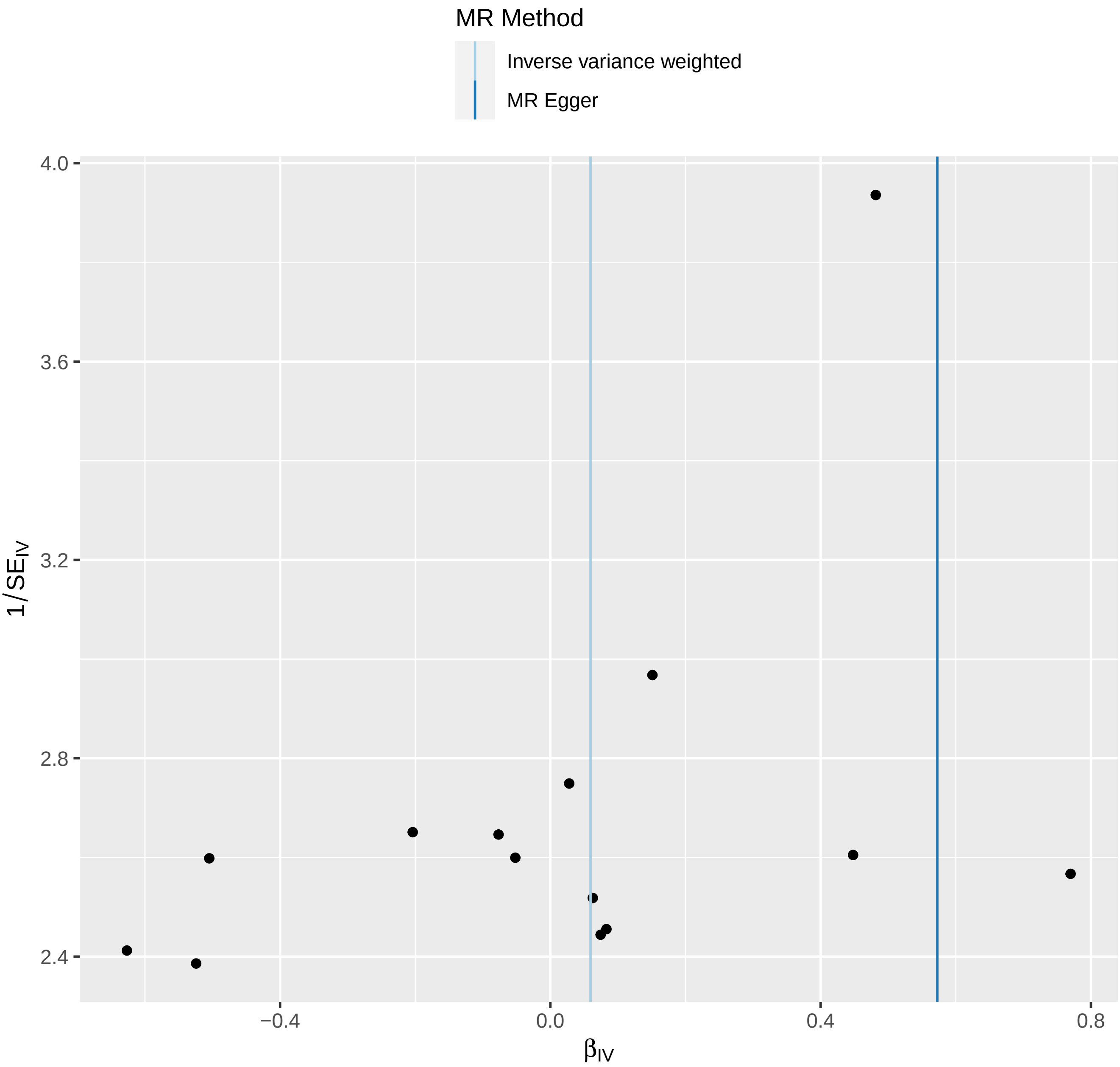

Batch 2−6455 : Gut microbiota abundance (genus Catenibacterium id.2153) on WAP, Kazal, immunoglobulin, Kunitz and NTR domain−containing protein 2 with a threshold of 1e−05

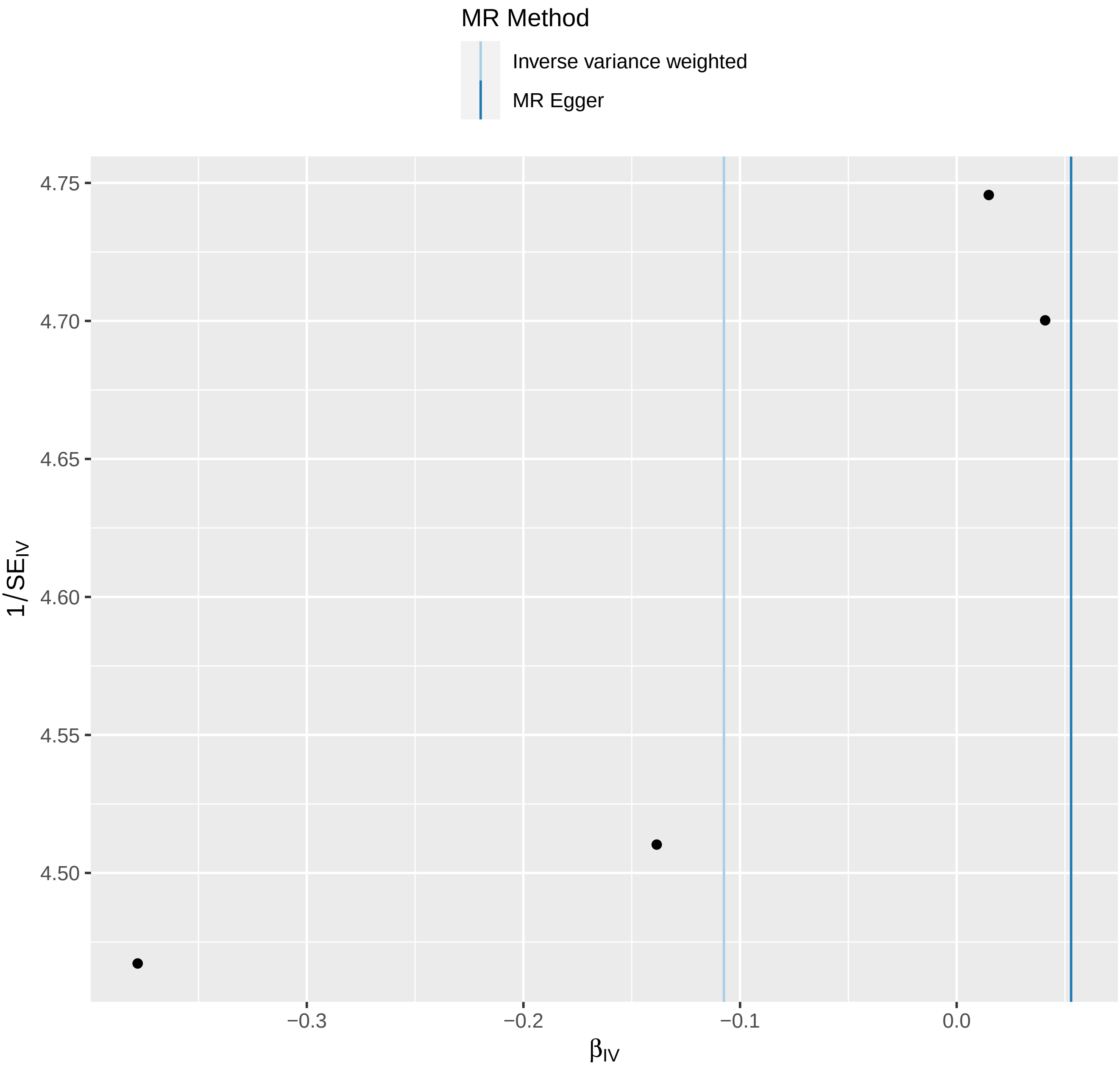

Batch 2−6456 : Gut microbiota abundance (genus Parasutterella id.2892) on WAP, Kazal, immunoglobulin, Kunitz and NTR domain−containing protein 2 with a threshold of 1e−05

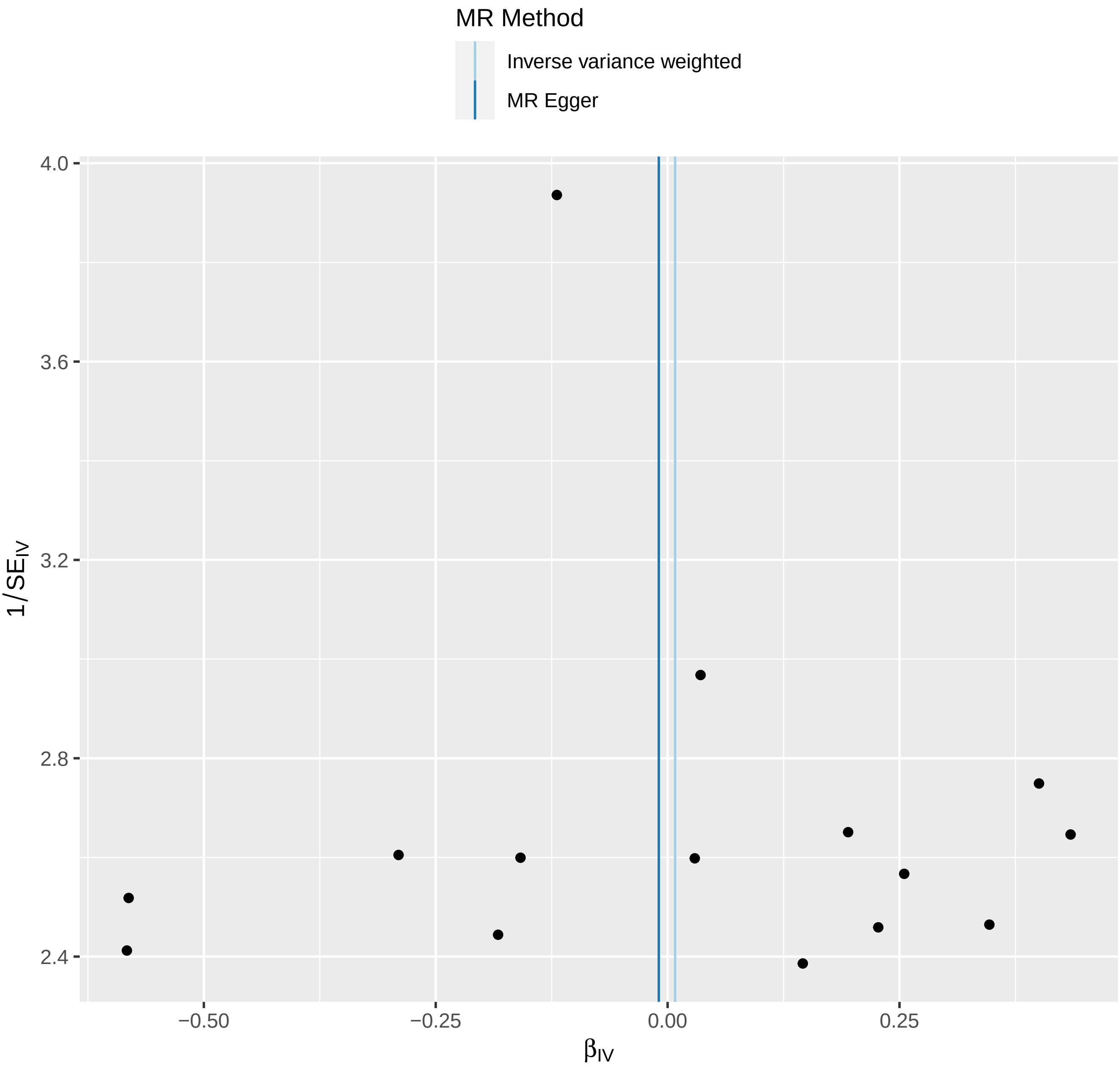

Batch 2−6457 : Gut microbiota abundance (genus Catenibacterium id.2153) on WAP, Kazal, immunoglobulin, Kunitz and NTR domain−containing protein 2 with a threshold of 1e−05

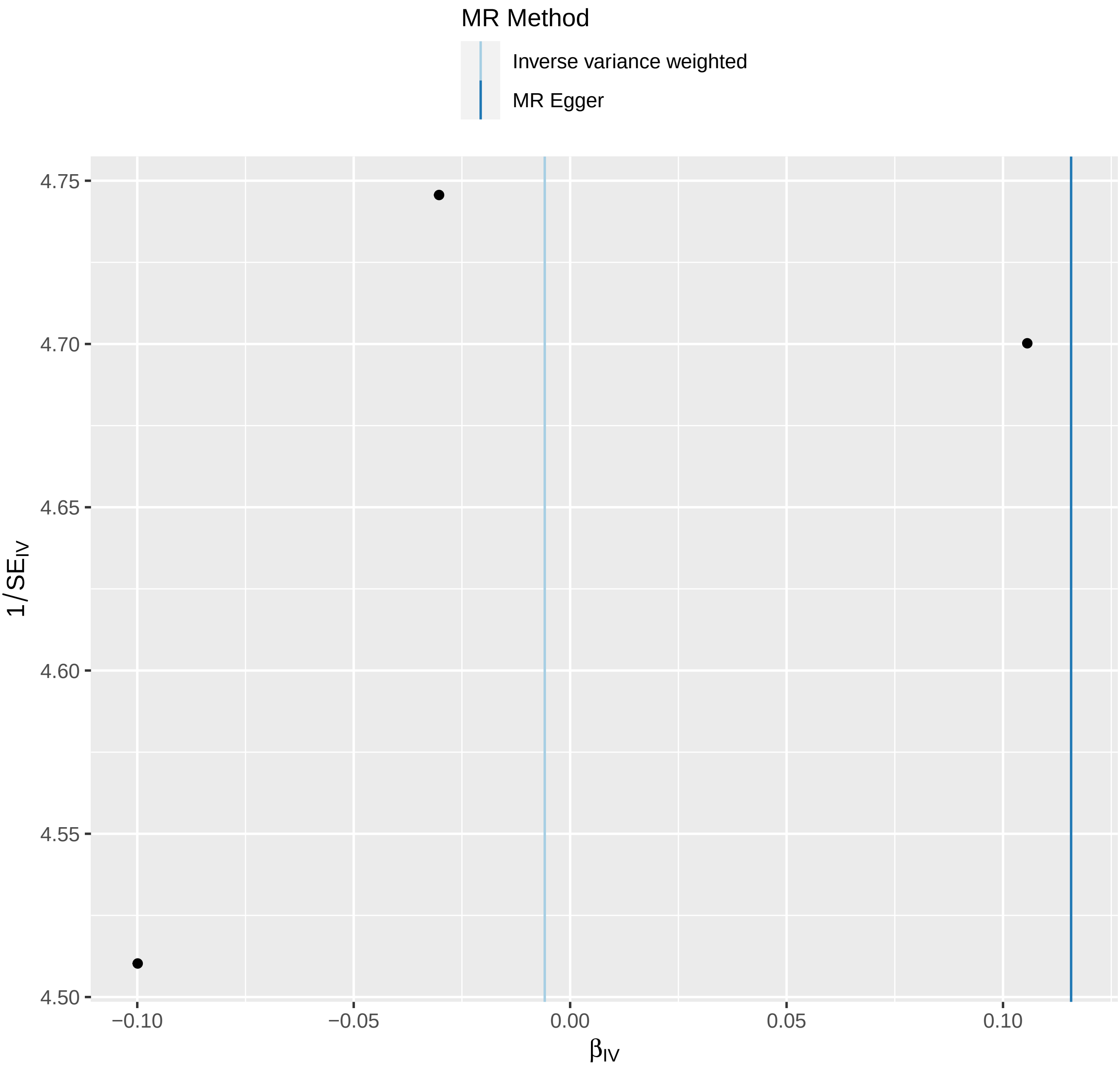

Batch 2−6458 : Gut microbiota abundance (genus Parasutterella id.2892) on WAP, Kazal, immunoglobulin, Kunitz and NTR domain−containing protein 2
with a threshold of 1e−05

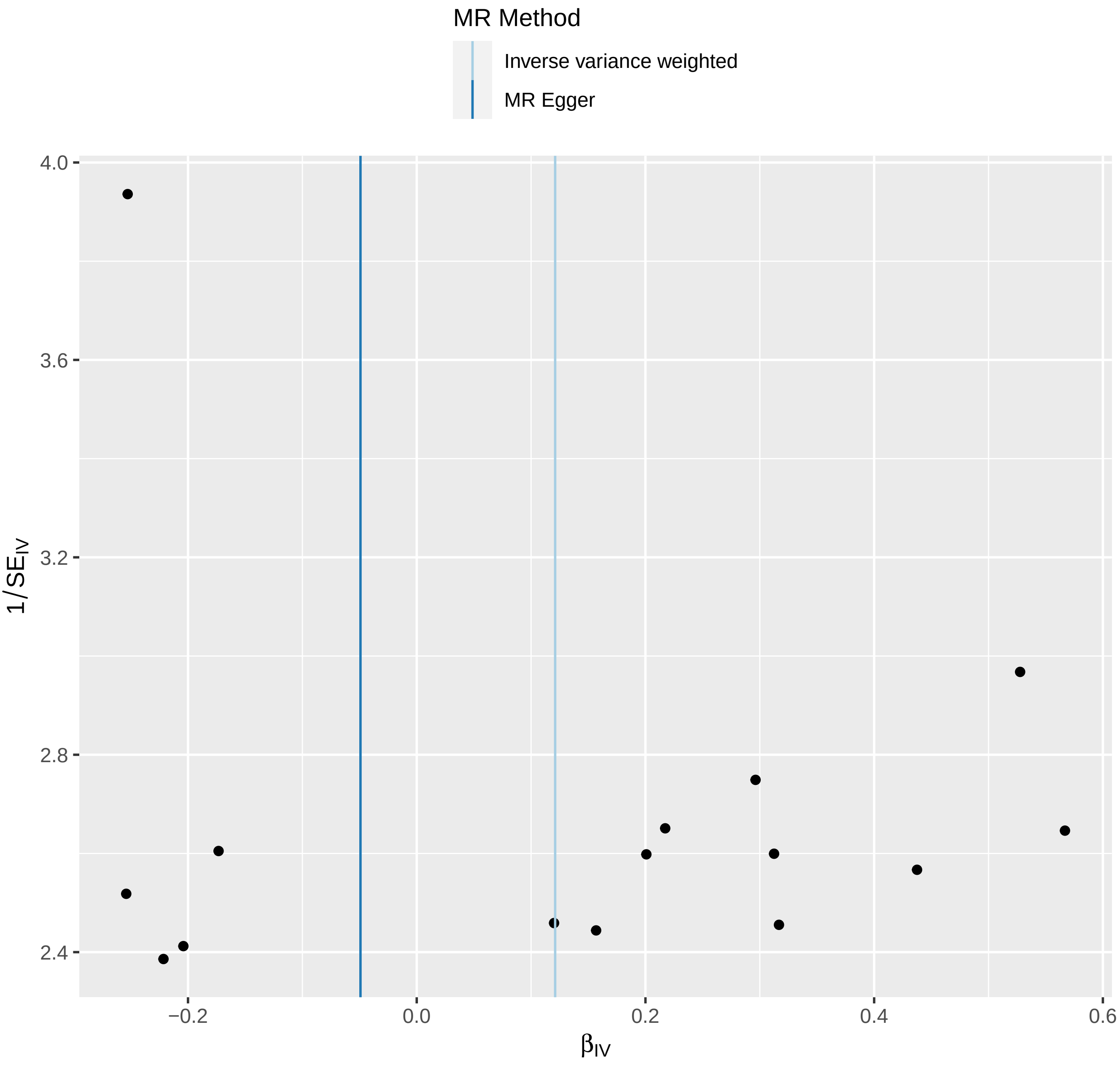

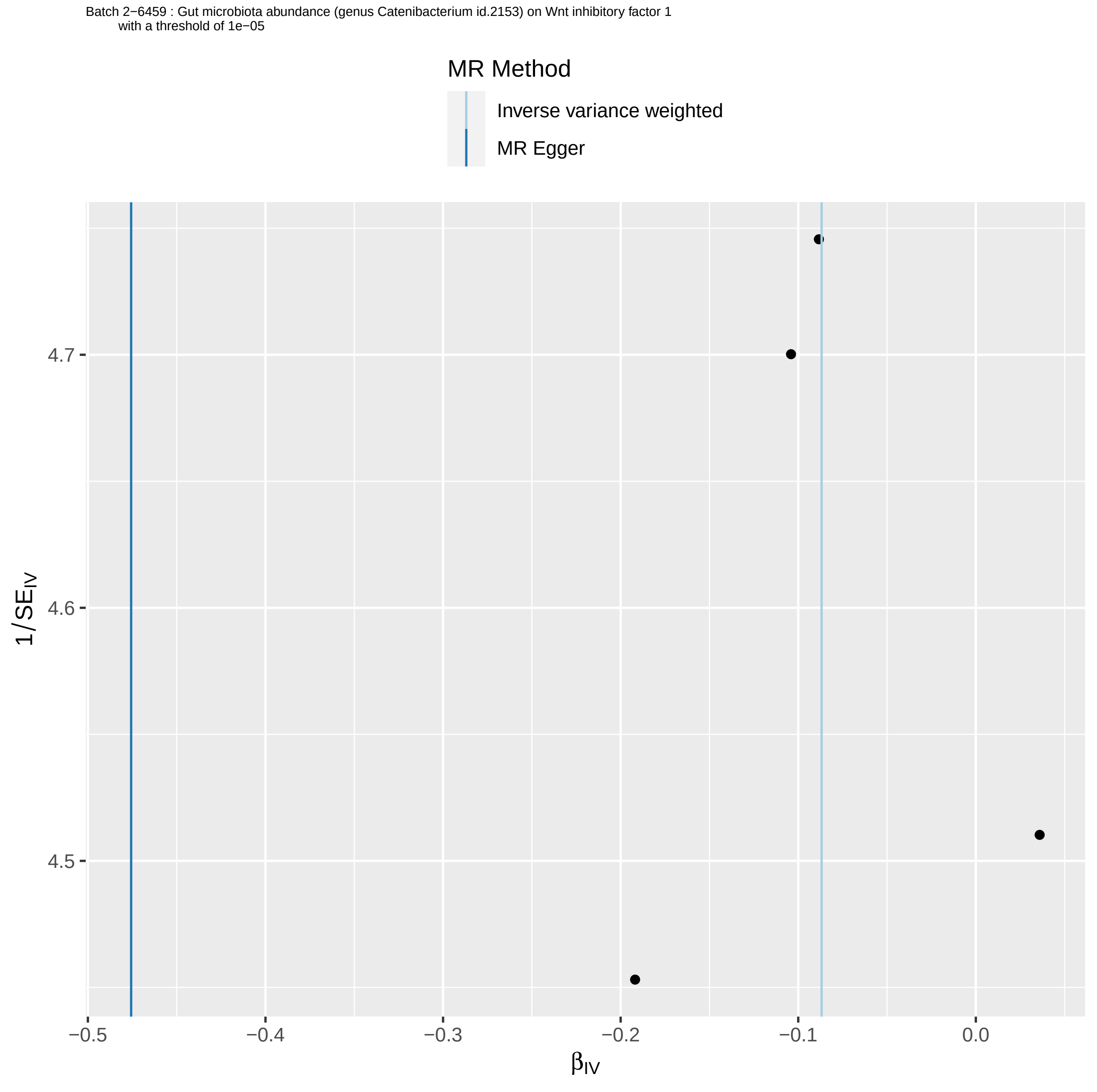

Batch 2−6459 : Gut microbiota abundance (genus Catenibacterium id.2153) on Wnt inhibitory factor 1
with a threshold of 1e−05
MR Method
Inverse variance weighted
MR Egger
4.7
4.6
4.5
1/SE_IV
−0.5
−0.4
−0.3
−0.2
−0.1
0.0
β_IV

Batch 2−6460 : Gut microbiota abundance (genus Parasutterella id.2892) on Wnt inhibitory factor 1
with a threshold of 1e−05

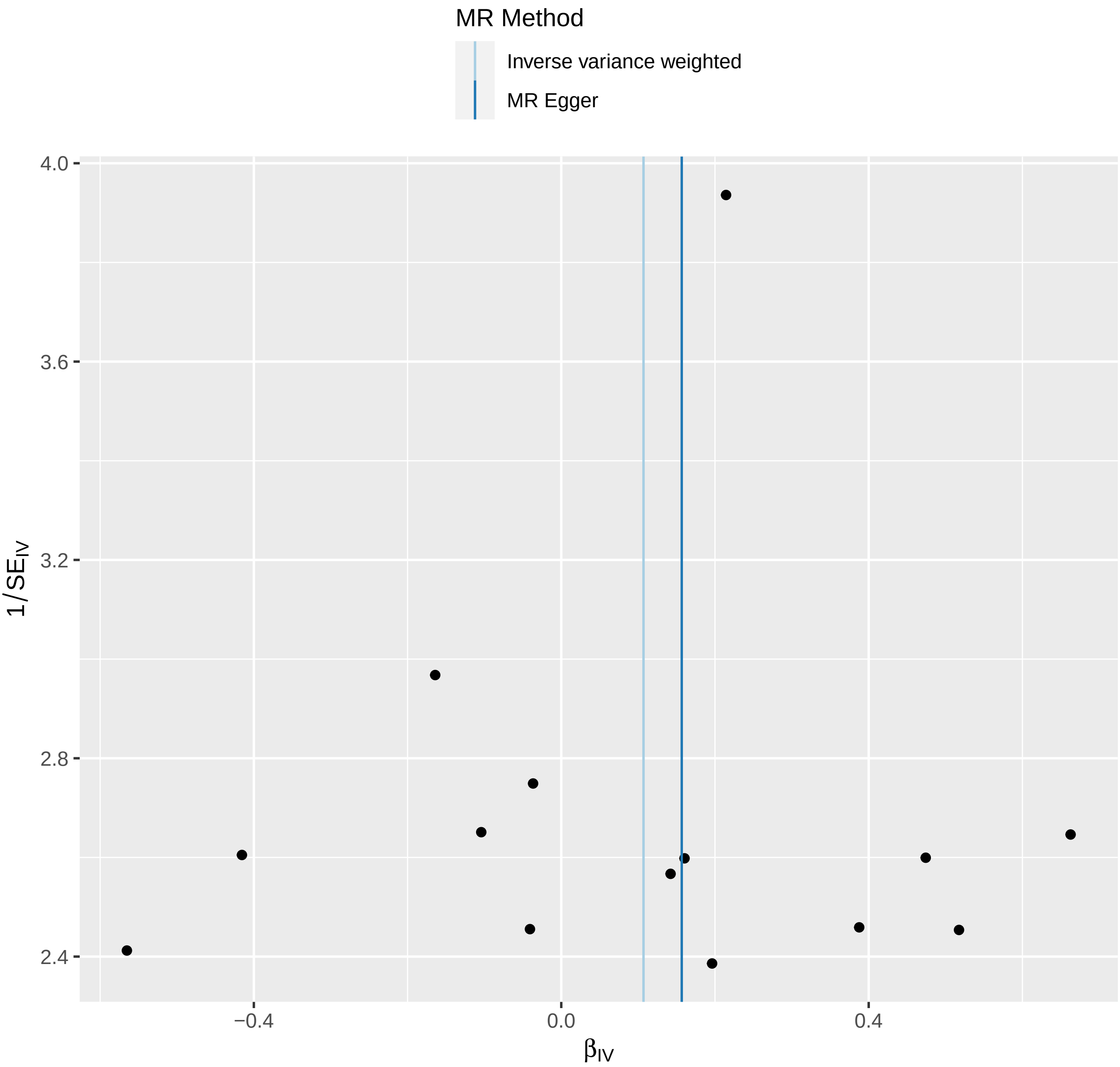

Batch 2−6461 : Gut microbiota abundance (genus Catenibacterium id.2153) on WNT1−inducible−signaling pathway protein 1
with a threshold of 1e−05

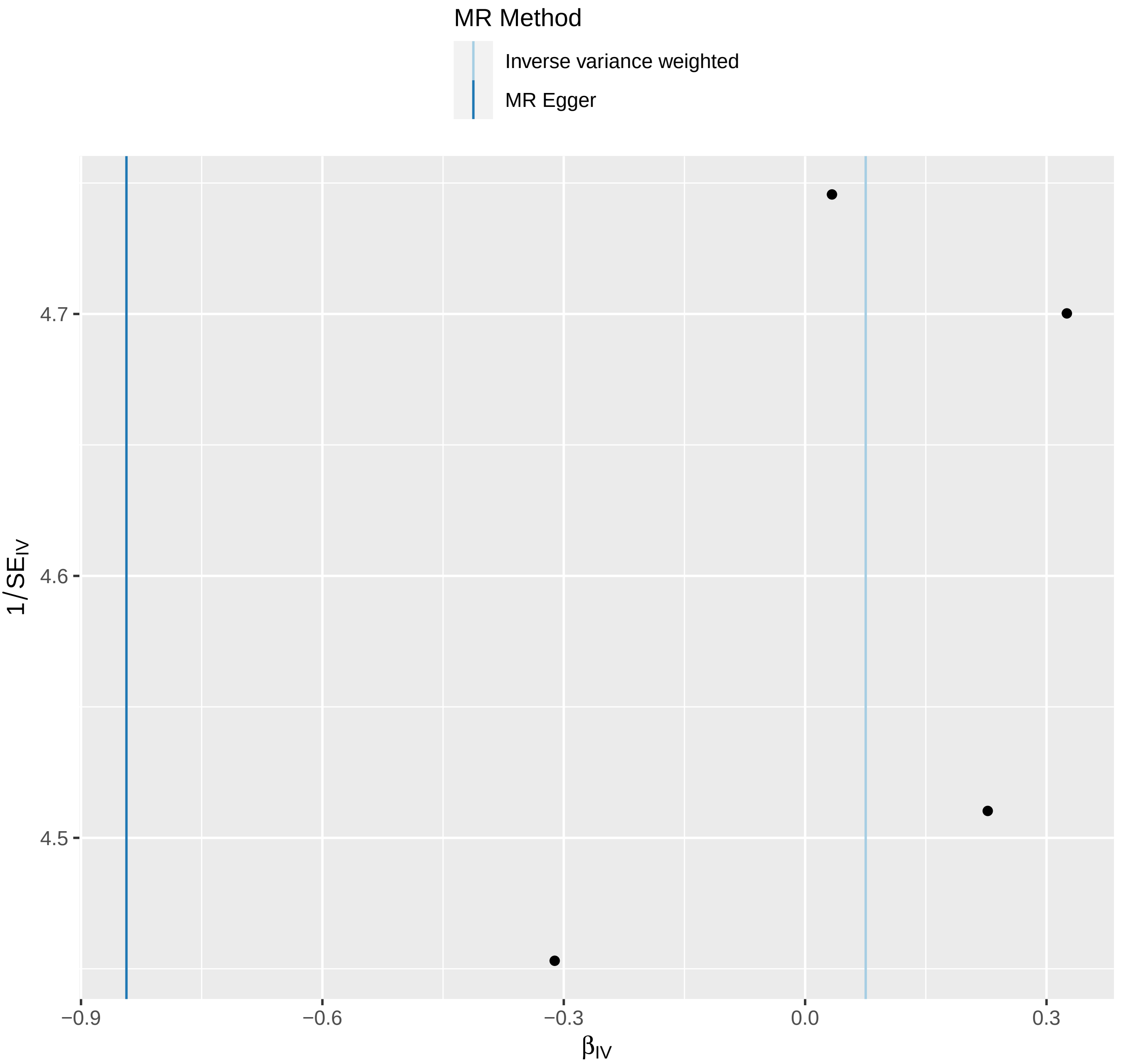

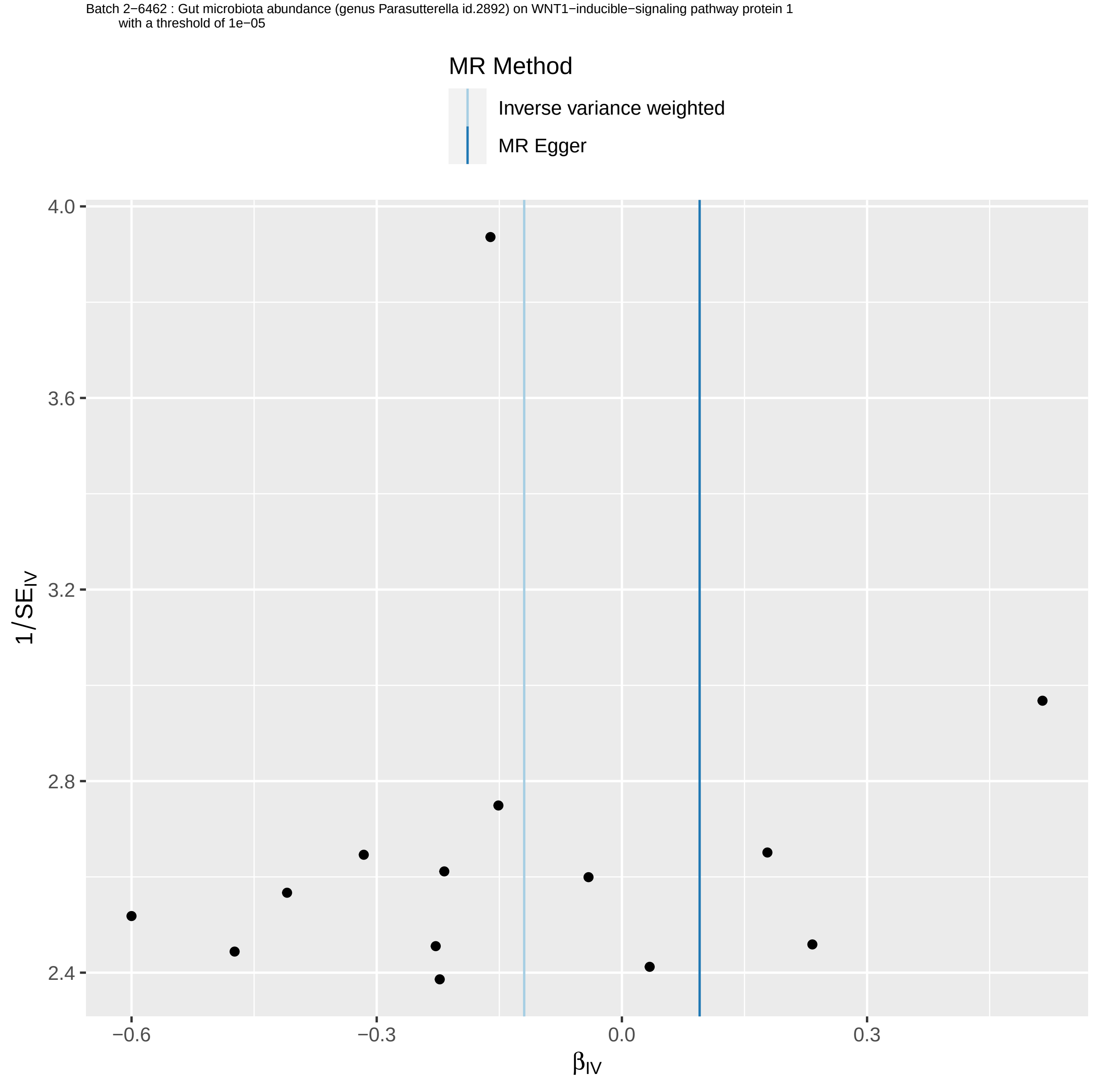

Batch 2−6462 : Gut microbiota abundance (genus Parasutterella id.2892) on WNT1−inducible−signaling pathway protein 1
with a threshold of 1e−05
MR Method
Inverse variance weighted
MR Egger
4.0
3.6
3.2
2.8
2.4
1/SE_IV
−0.6
−0.3
0.0
0.3
β_IV

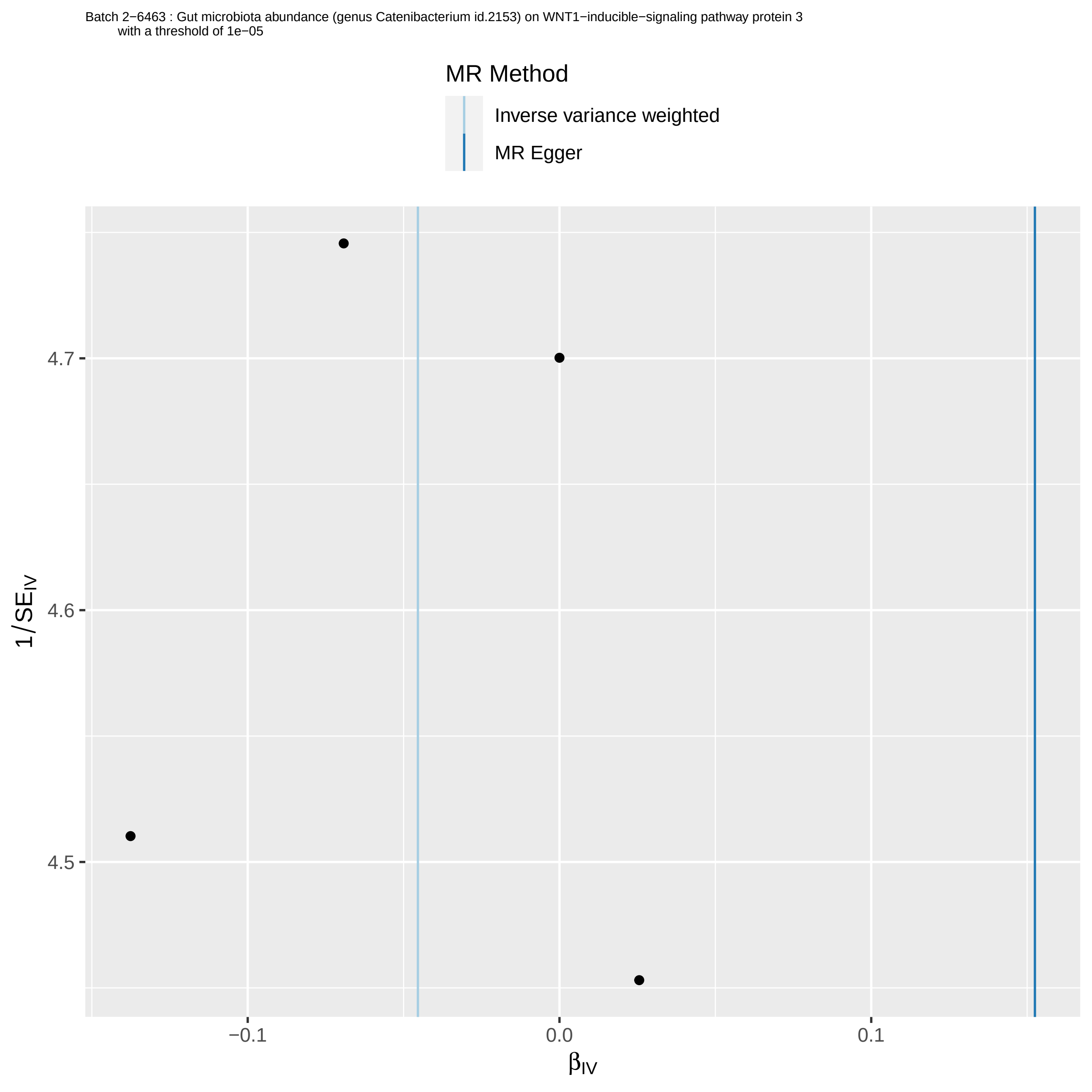
Batch 2−6463 : Gut microbiota abundance (genus Catenibacterium id.2153) on WNT1−inducible−signaling pathway protein 3
with a threshold of 1e−05
MR Method
Inverse variance weighted
MR Egger
4.7
4.6
4.5
1/SE_IV
−0.1
0.0
0.1
β_IV

Batch 2−6464 : Gut microbiota abundance (genus Parasutterella id.2892) on WNT1−inducible−signaling pathway protein 3
with a threshold of 1e−05

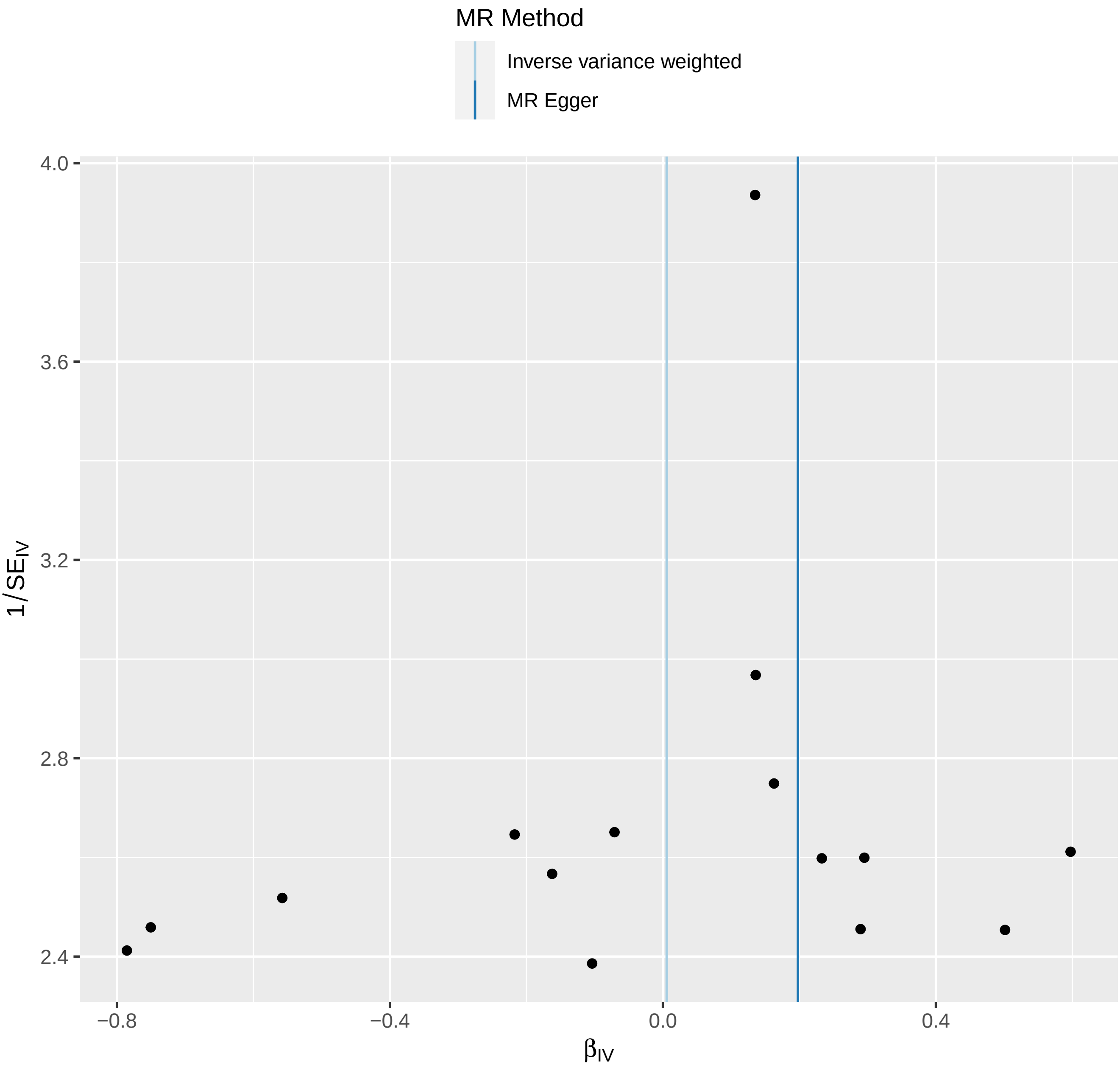

Batch 2−6465 : Gut microbiota abundance (genus Catenibacterium id.2153) on Serine/threonine−protein kinase WNK3
with a threshold of 1e−05

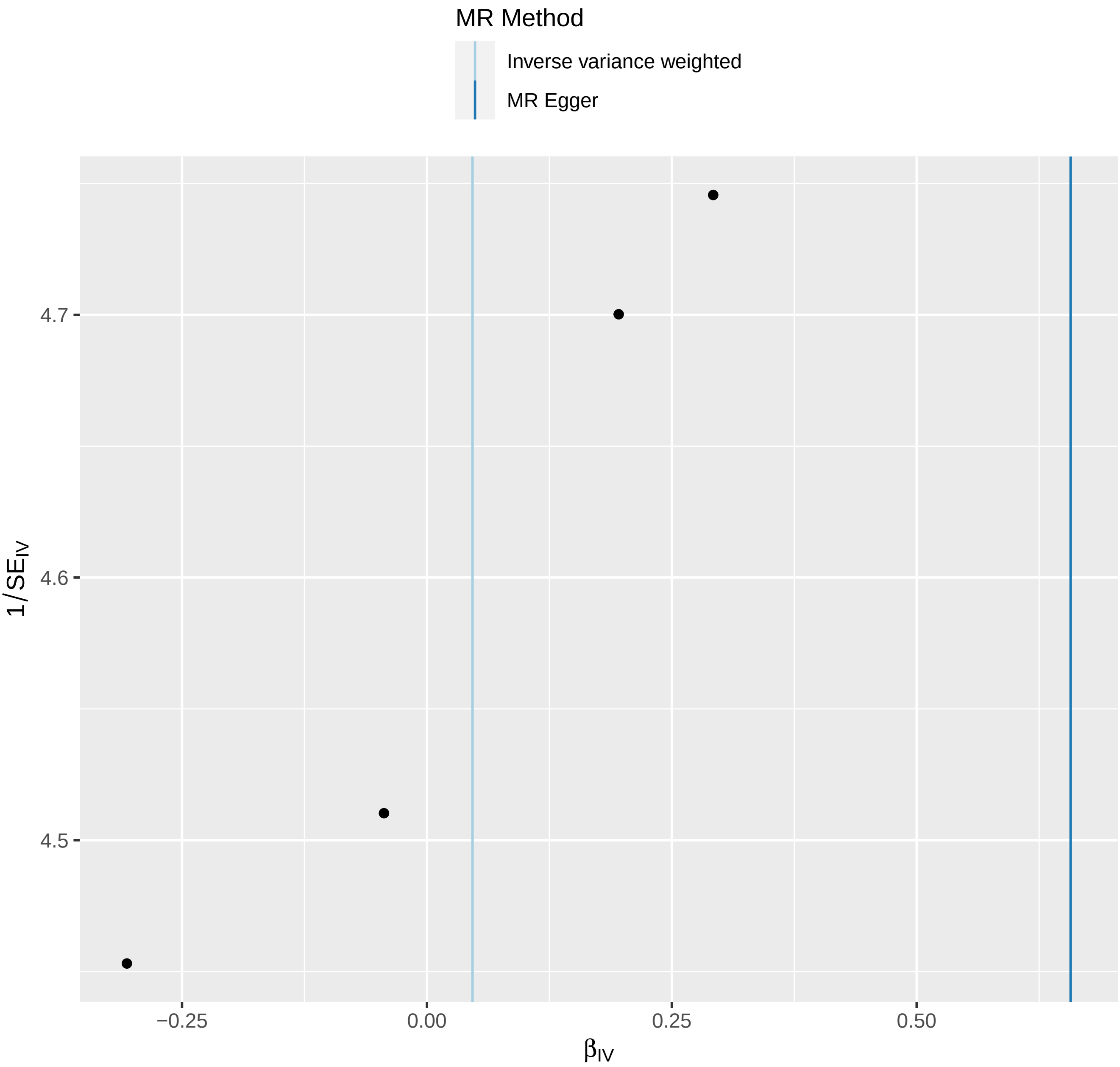

Batch 2−6466 : Gut microbiota abundance (genus Parasutterella id.2892) on Serine/threonine−protein kinase WNK3
with a threshold of 1e−05

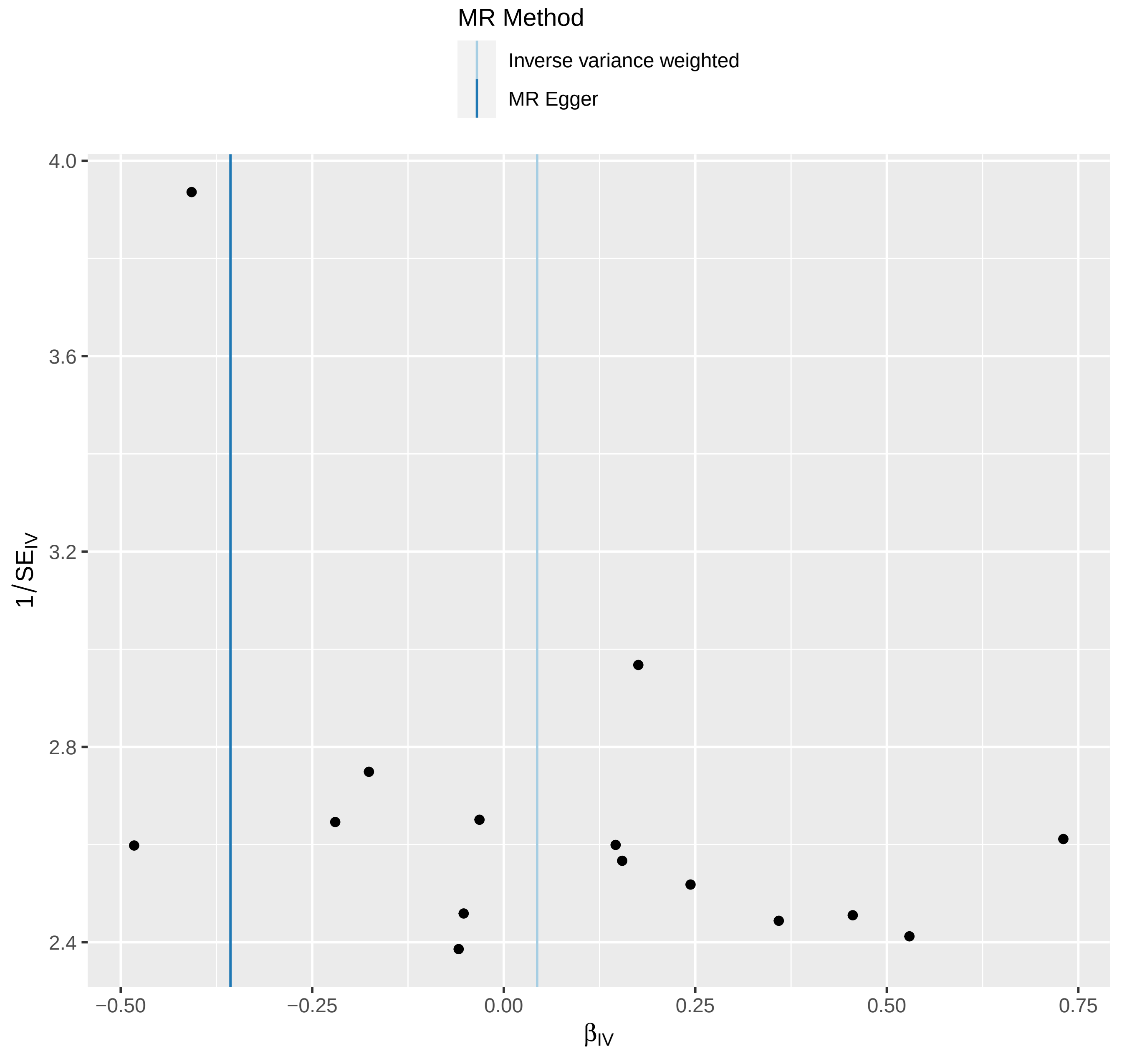

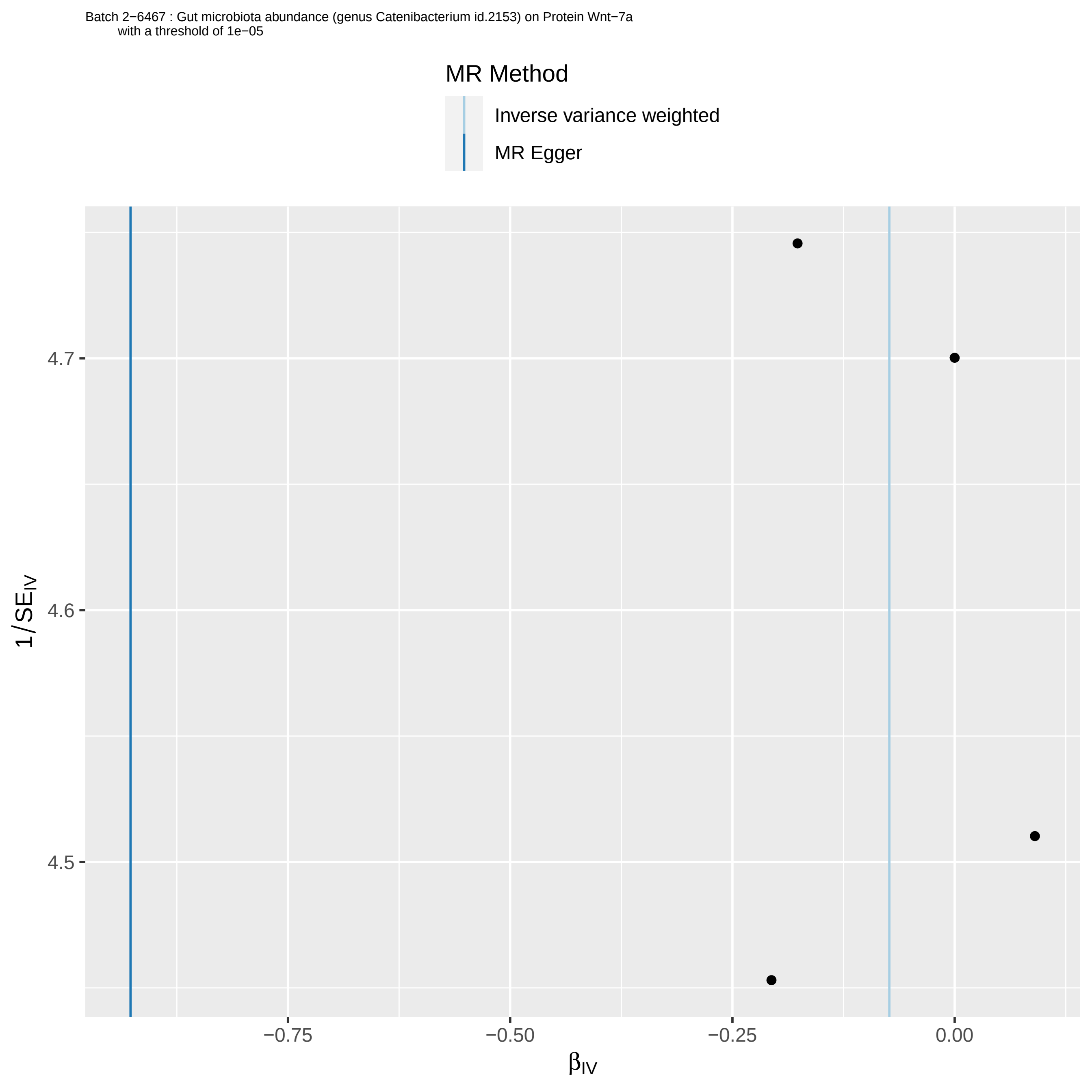
Batch 2−6467 : Gut microbiota abundance (genus Catenibacterium id.2153) on Protein Wnt−7a
with a threshold of 1e−05
MR Method
Inverse variance weighted
MR Egger
4.7
4.6
4.5
1/SE$_{IV}$
−0.75
−0.50
−0.25
0.00
β_{IV}

Batch 2−6468 : Gut microbiota abundance (genus Parasutterella id.2892) on Protein Wnt−7a
with a threshold of 1e−05

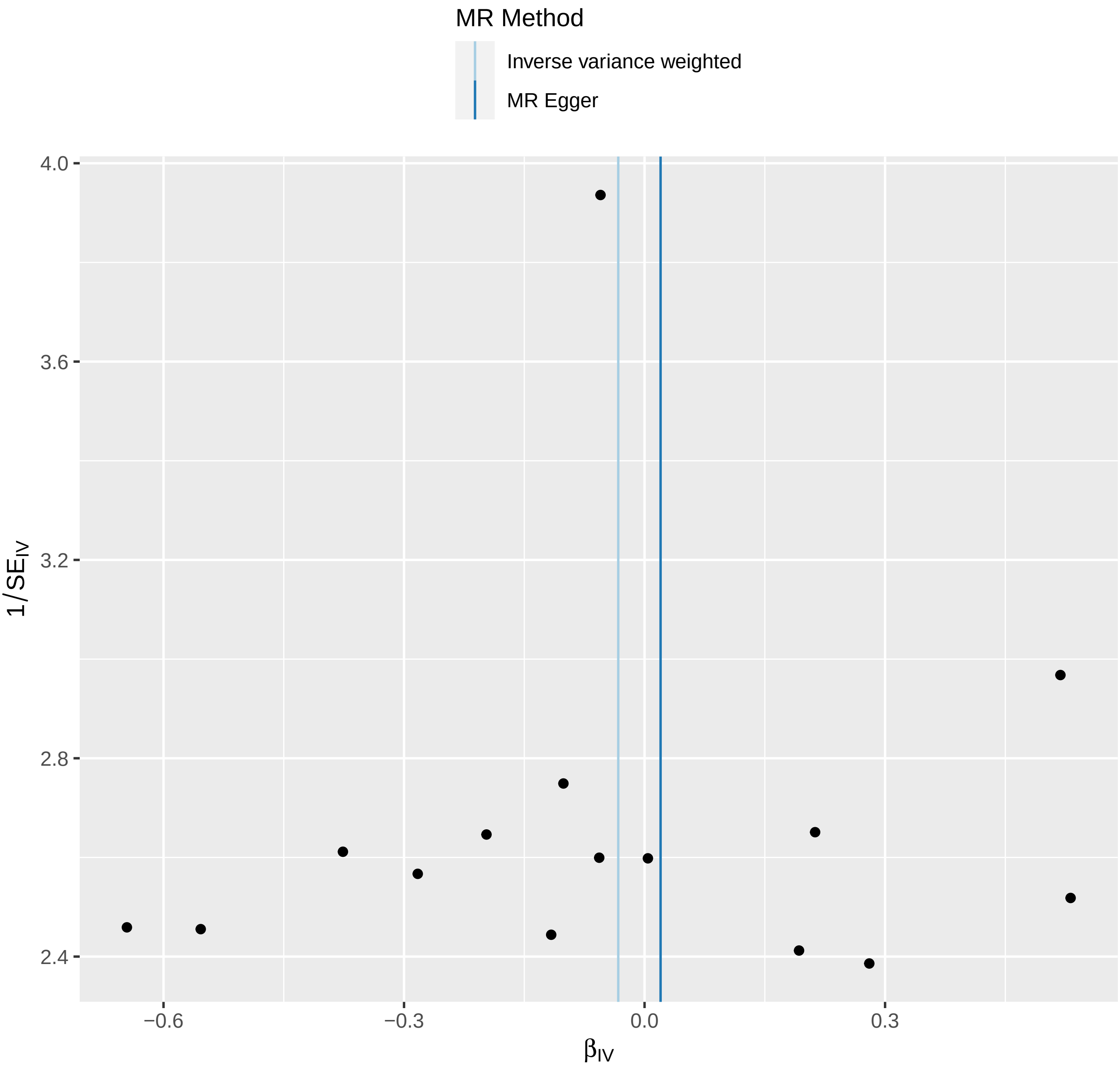

Batch 2−6469 : Gut microbiota abundance (genus Catenibacterium id.2153) on WSC domain−containing protein 2
with a threshold of 1e−05

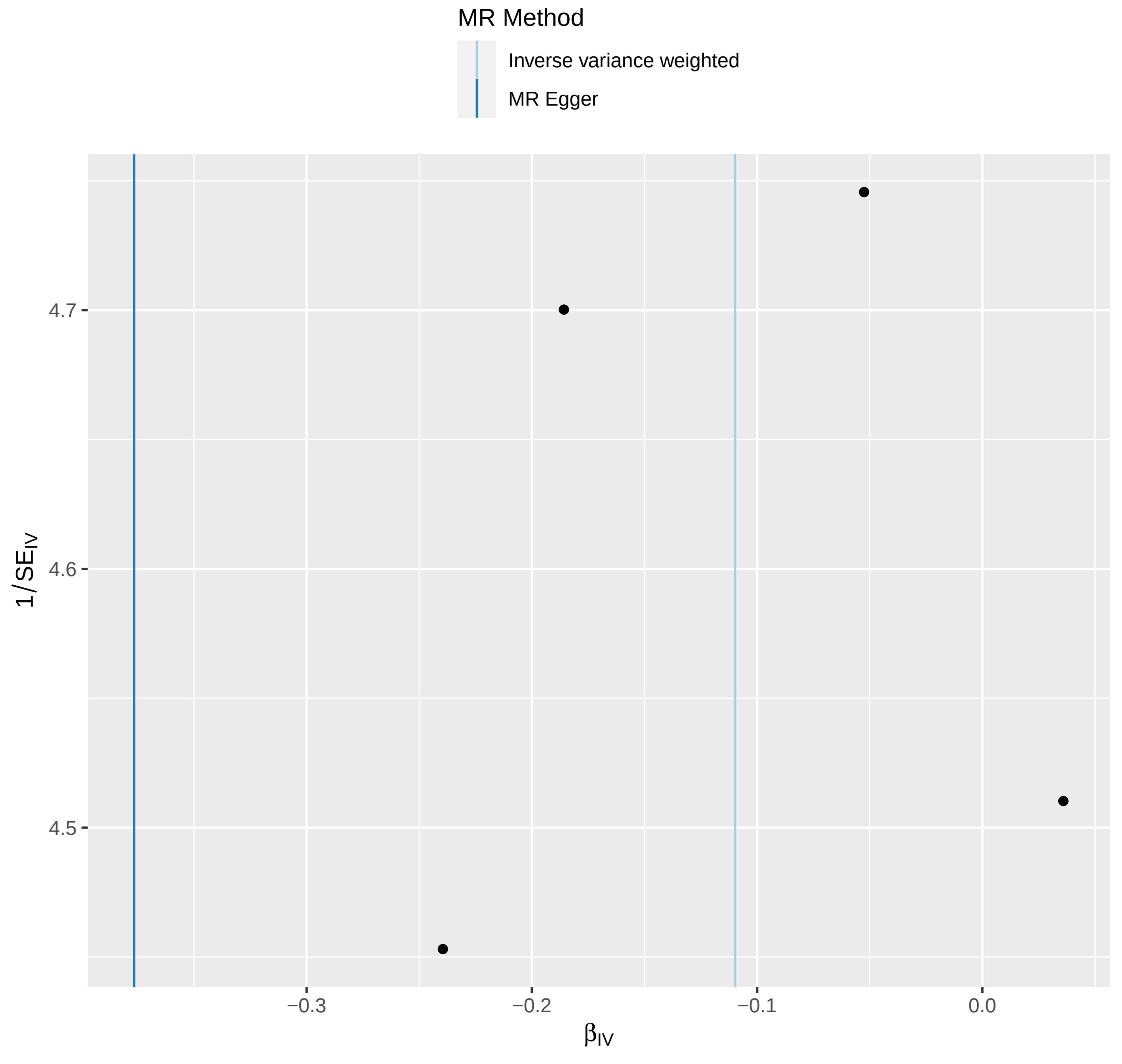

Batch 2−6470 : Gut microbiota abundance (genus Parasutterella id.2892) on WSC domain−containing protein 2
with a threshold of 1e−05

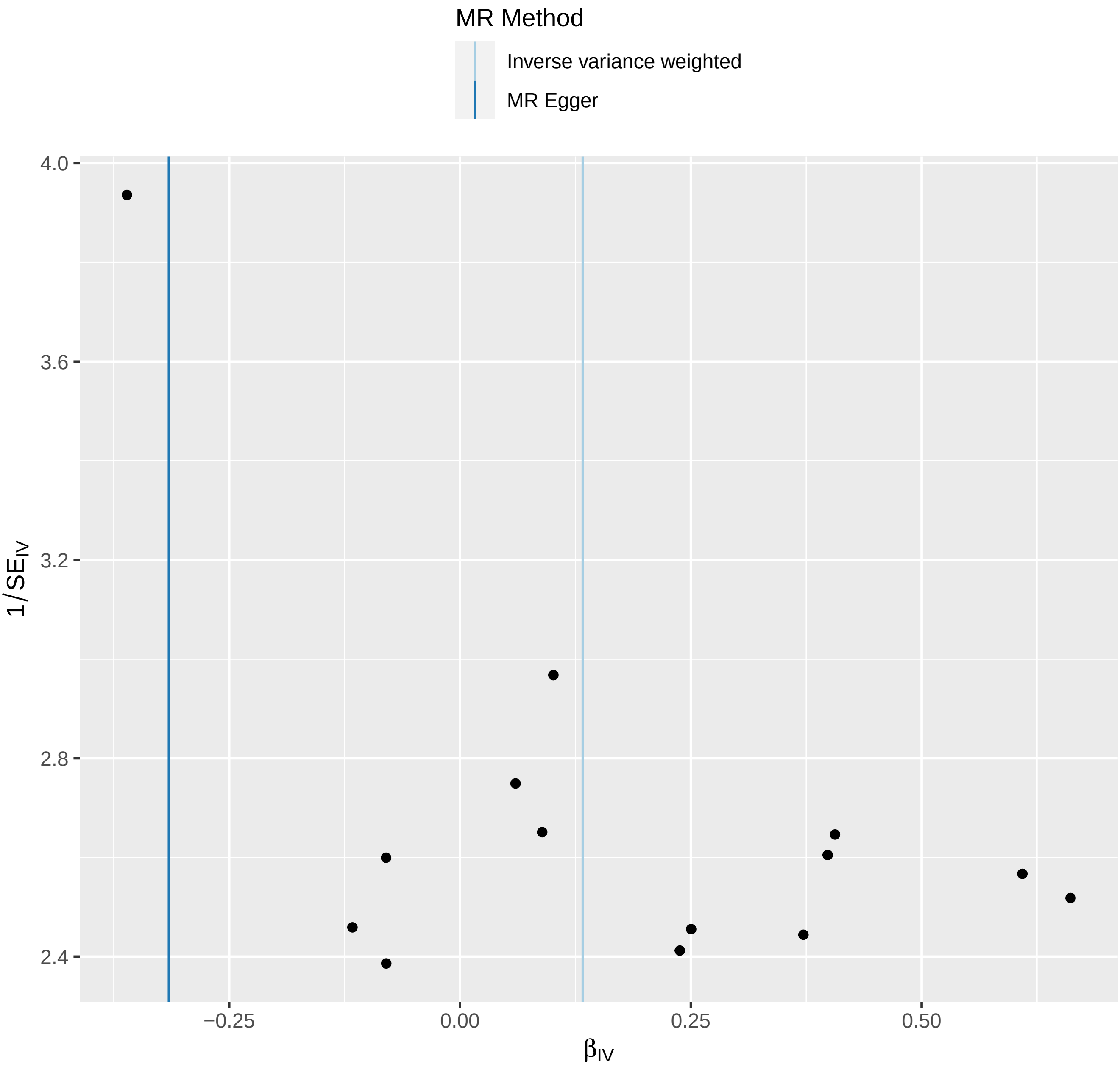

Batch 2−6471 : Gut microbiota abundance (genus Catenibacterium id.2153) on Lymphotactin
with a threshold of 1e−05

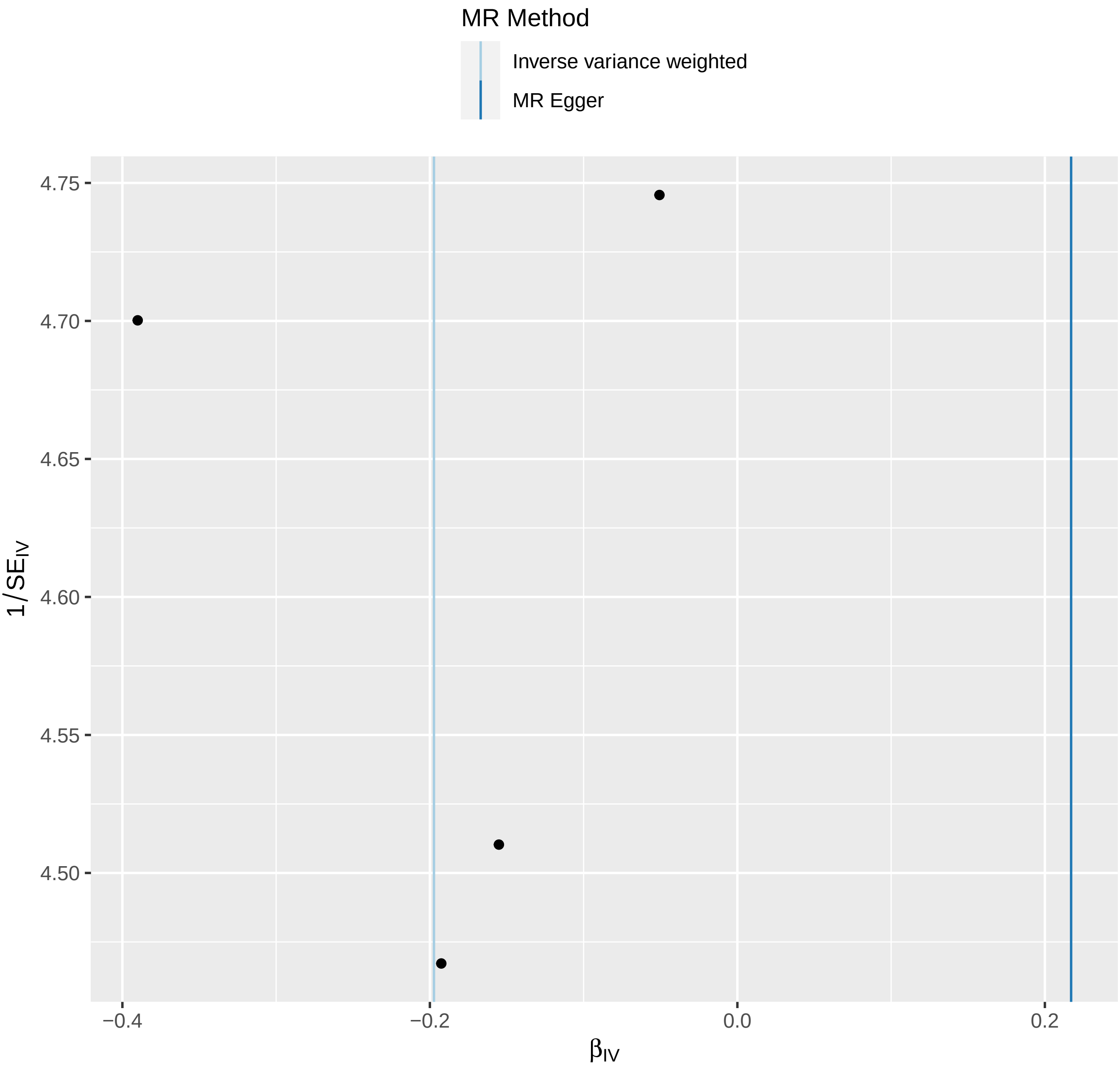

Batch 2−6472 : Gut microbiota abundance (genus Parasutterella id.2892) on Lymphotactin
with a threshold of 1e−05

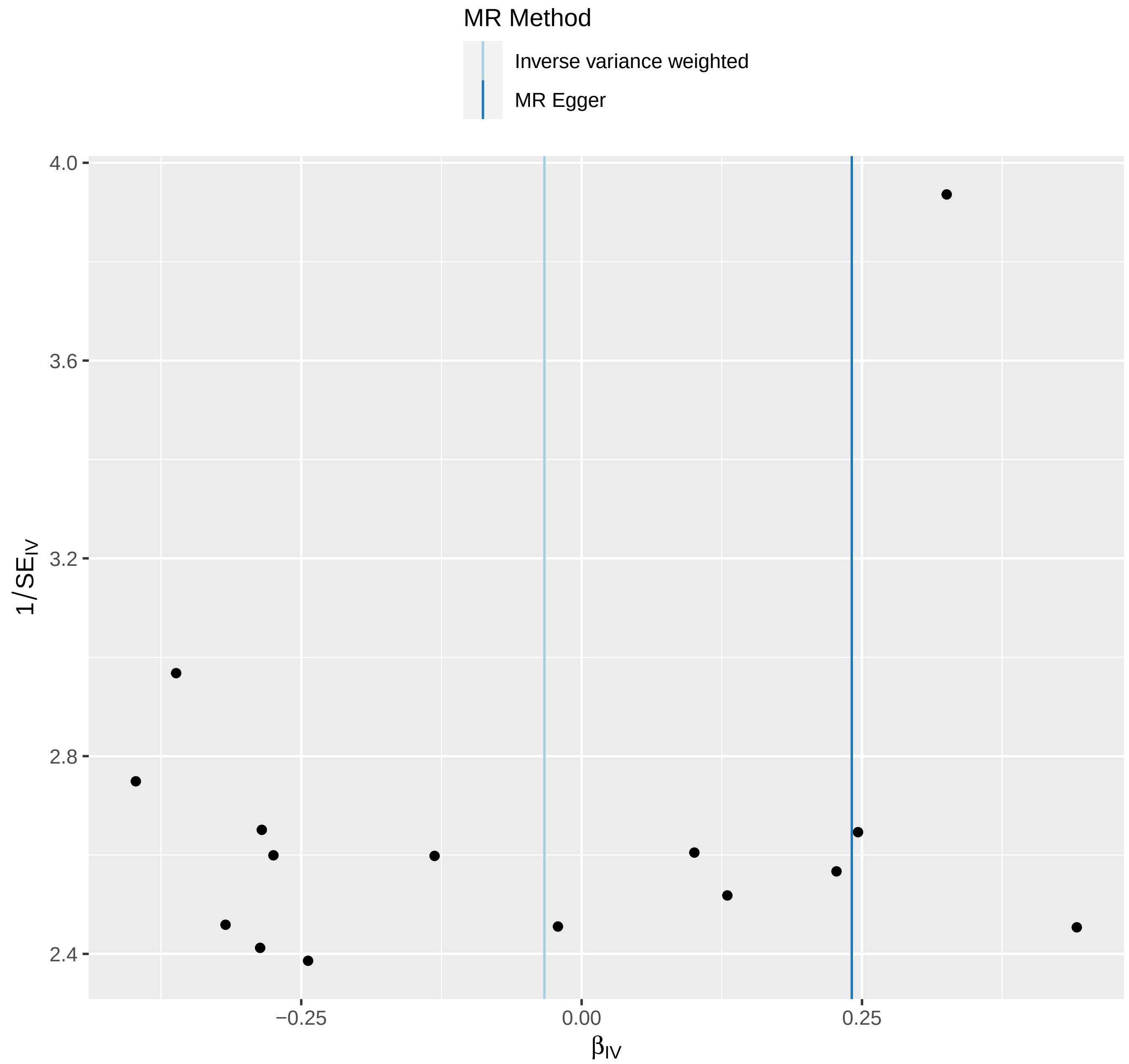

Batch 2−6473 : Gut microbiota abundance (genus Catenibacterium id.2153) on Lymphotactin
with a threshold of 1e−05

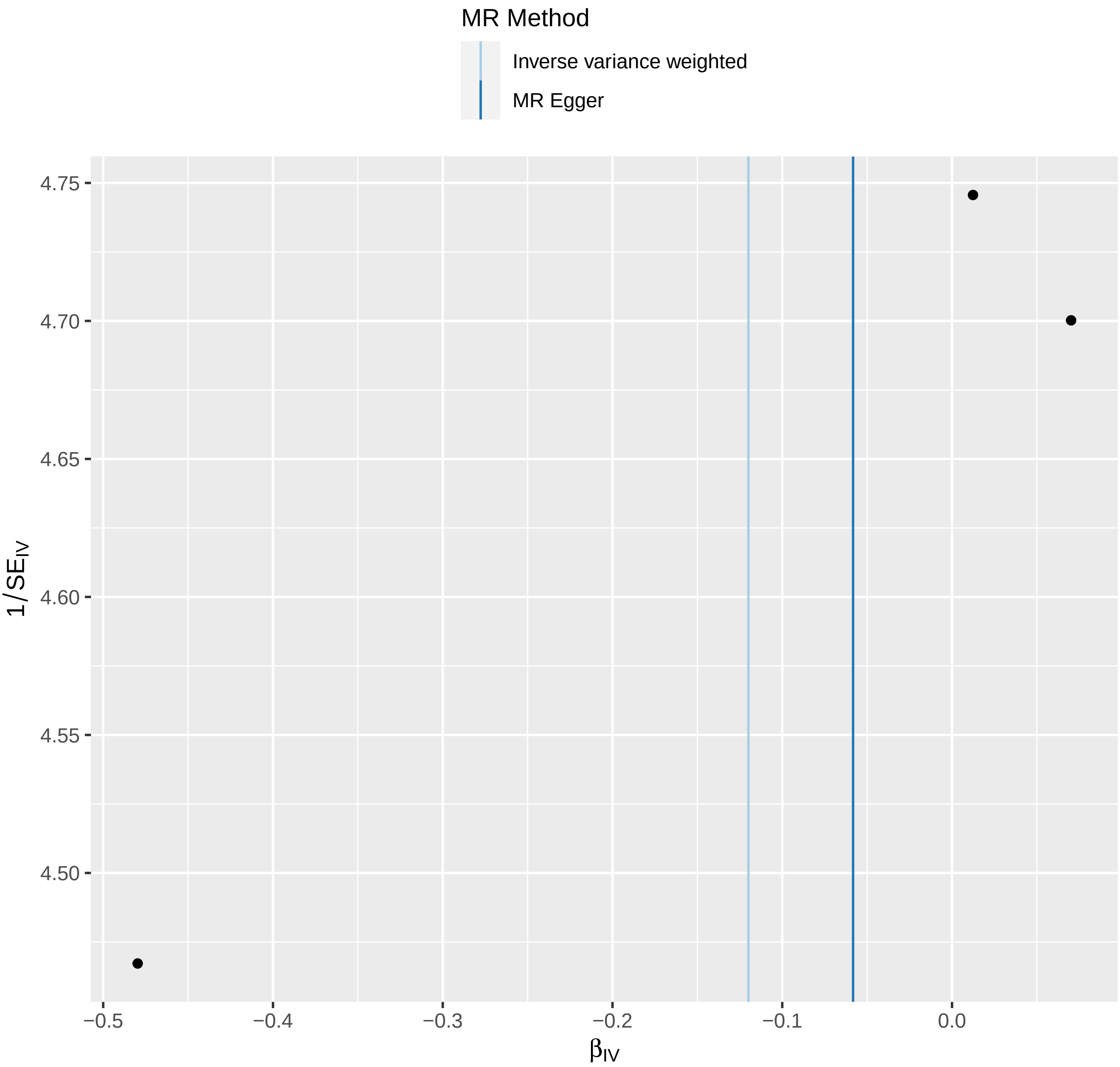

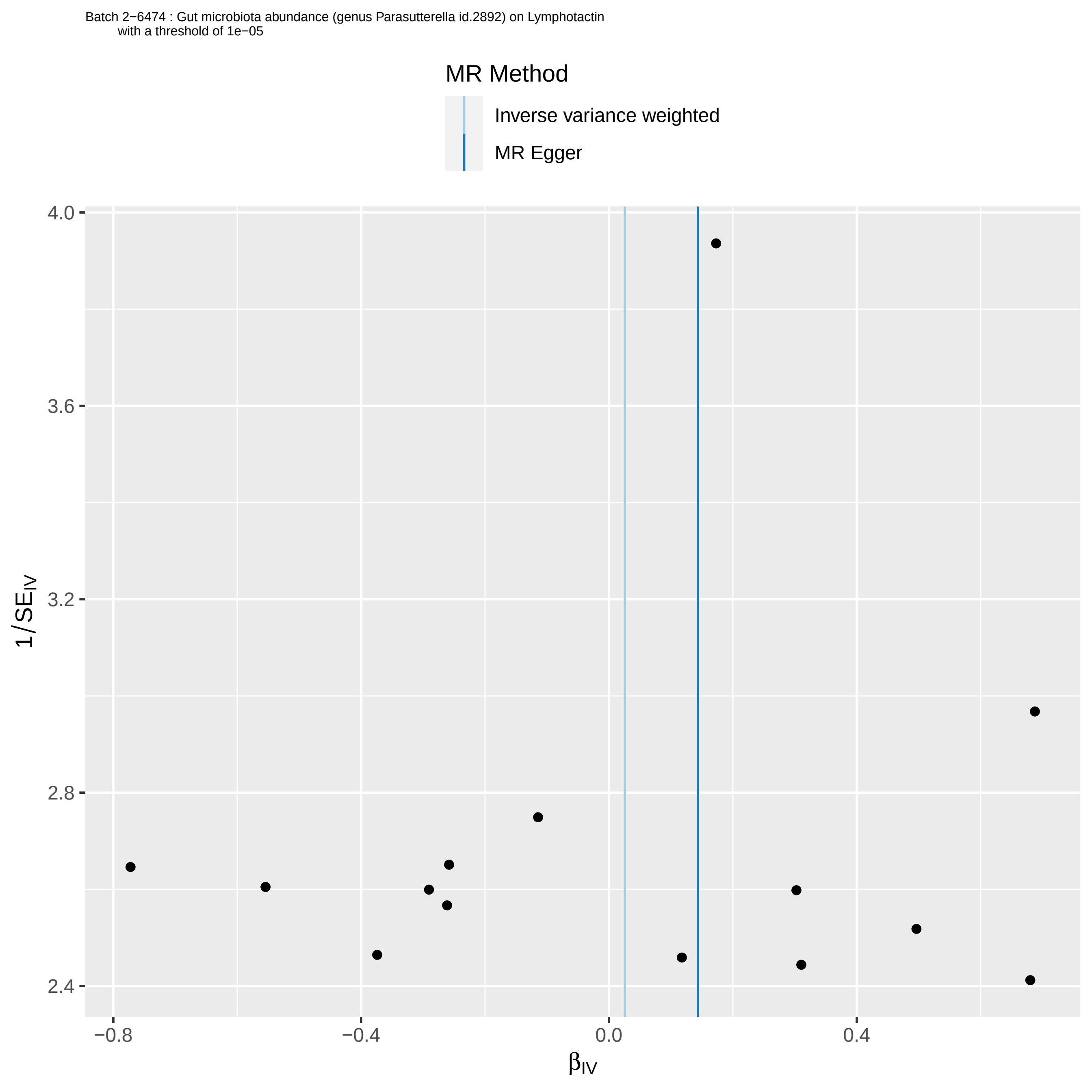

Batch 2−6474 : Gut microbiota abundance (genus Parasutterella id.2892) on Lymphotactin
with a threshold of 1e−05
MR Method
Inverse variance weighted
MR Egger
4.0
3.6
3.2
2.8
2.4
1/SE_IV
−0.8
−0.4
0.0
0.4
β_IV

Batch 2−6475 : Gut microbiota abundance (genus Catenibacterium id.2153) on Xanthine dehydrogenase/oxidase
with a threshold of 1e−05

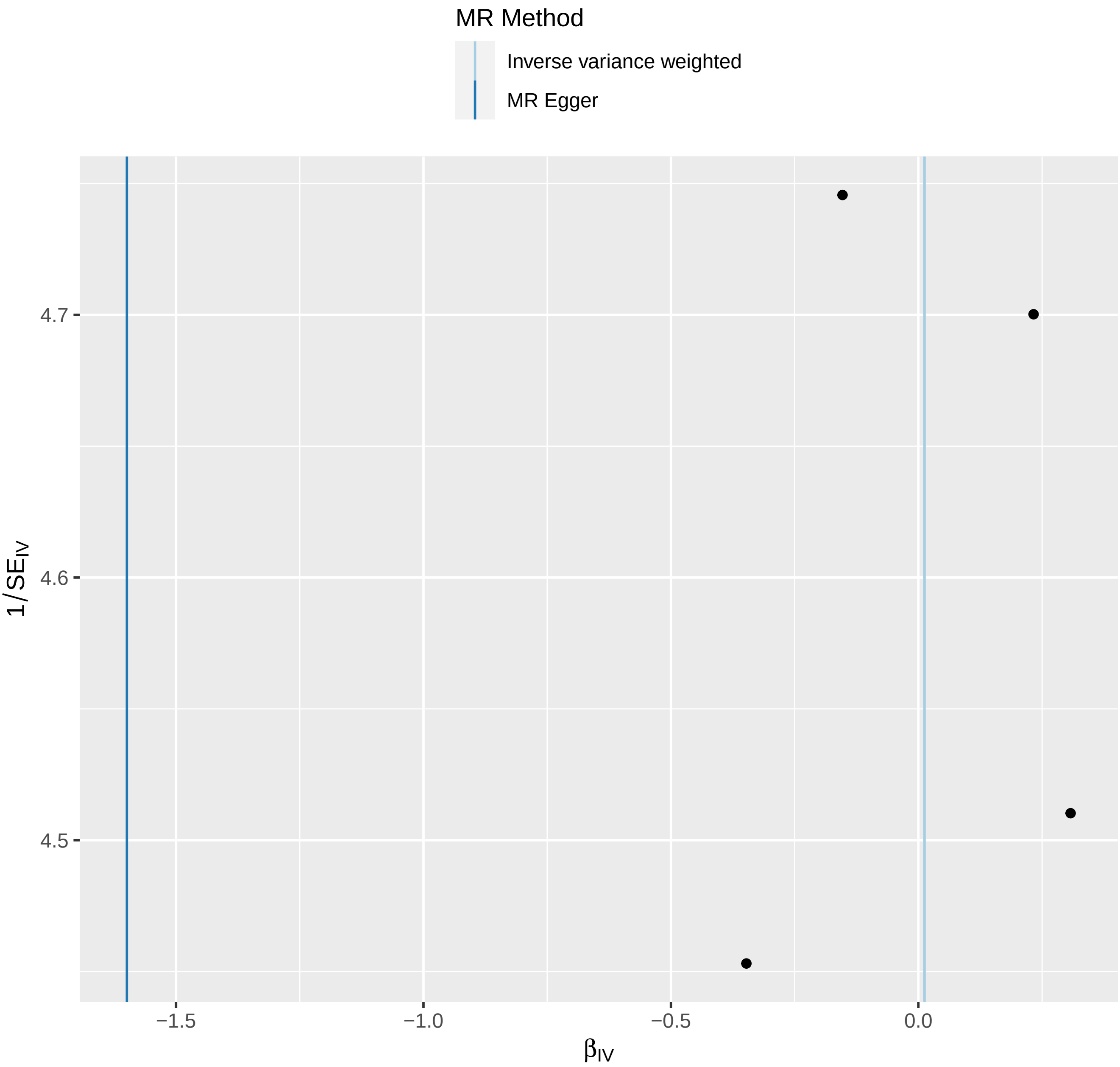

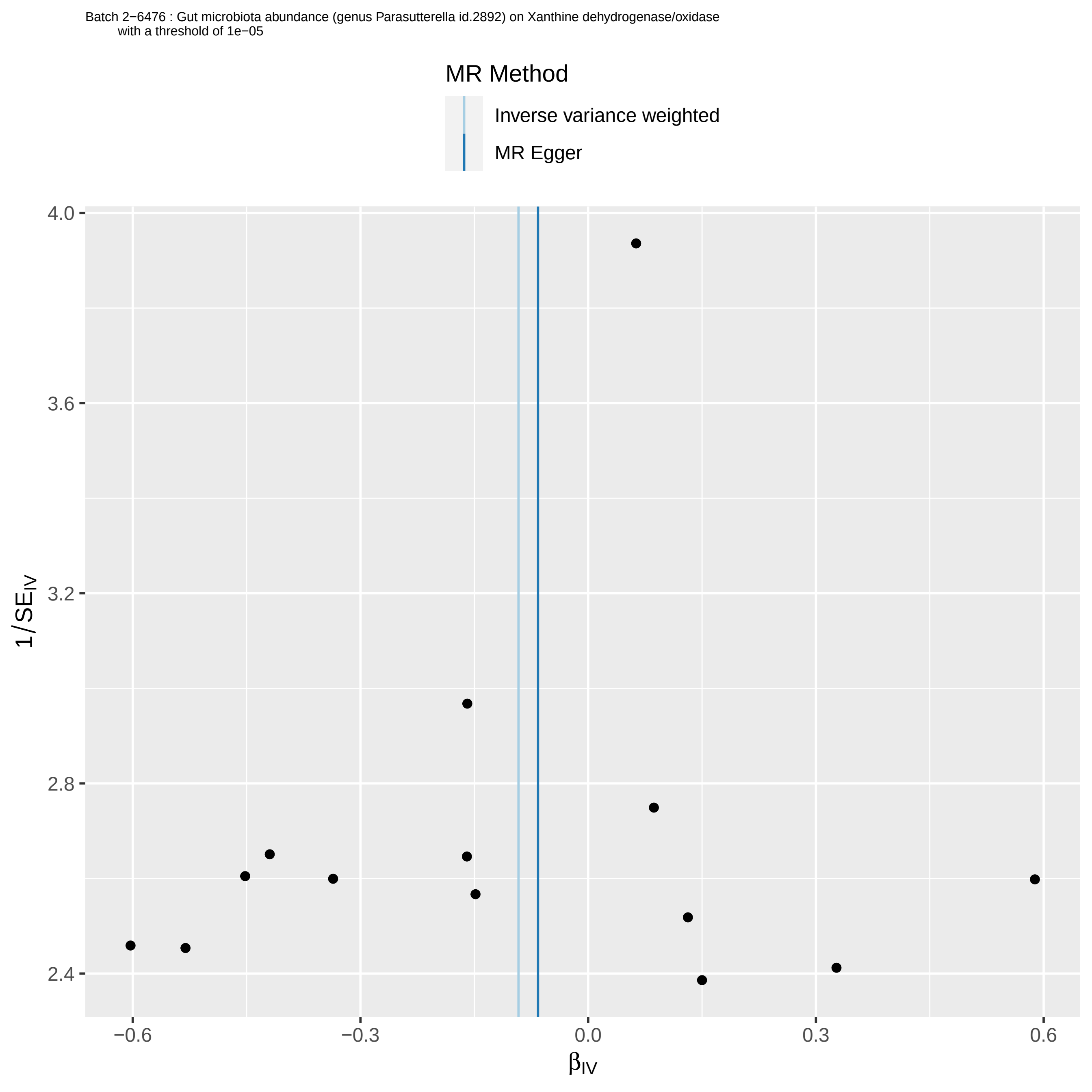

Batch 2−6476 : Gut microbiota abundance (genus Parasutterella id.2892) on Xanthine dehydrogenase/oxidase
with a threshold of 1e−05
MR Method
Inverse variance weighted
MR Egger
4.0
3.6
3.2
2.8
2.4
1/SE_IV
−0.6
−0.3
0.0
0.3
0.6
β_IV

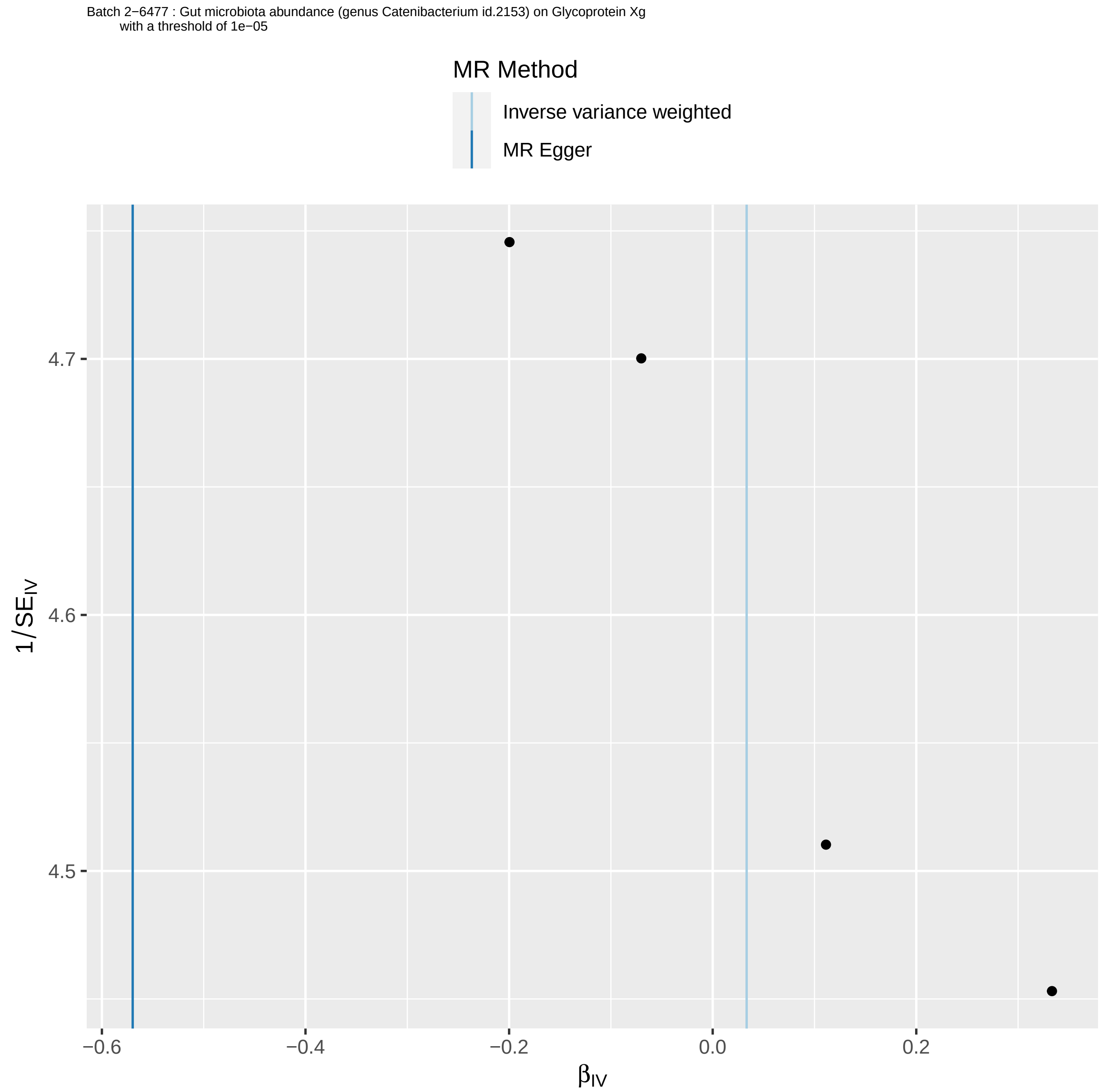

Batch 2−6477 : Gut microbiota abundance (genus Catenibacterium id.2153) on Glycoprotein Xg
with a threshold of 1e−05
MR Method
Inverse variance weighted
MR Egger
4.7
4.6
4.5
1/SE_IV
−0.6
−0.4
−0.2
0.0
0.2
β_IV

Batch 2−6478 : Gut microbiota abundance (genus Parasutterella id.2892) on Glycoprotein Xg
with a threshold of 1e−05

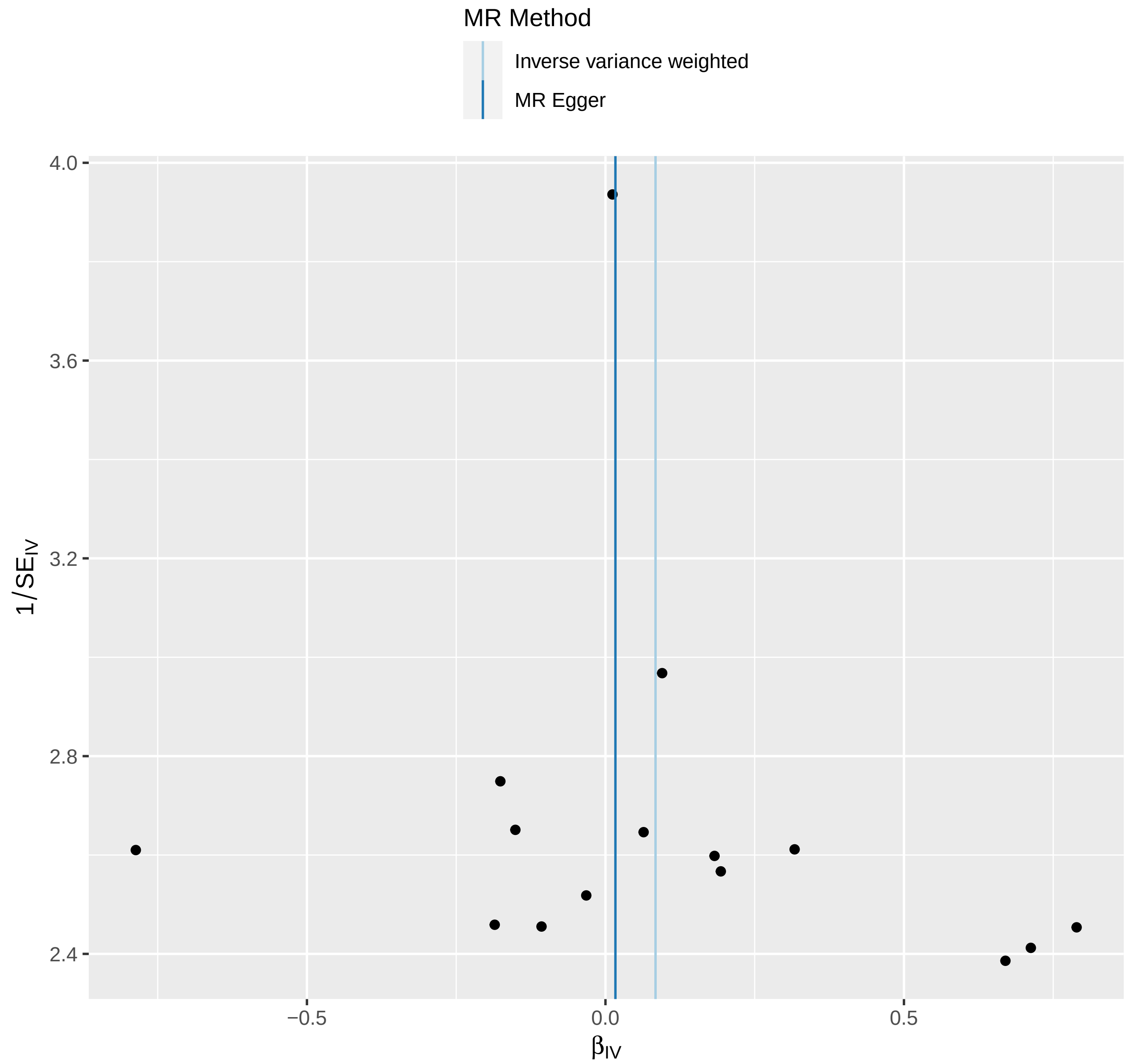

Batch 2−6479 : Gut microbiota abundance (genus Catenibacterium id.2153) on Xaa−Pro aminopeptidase 1
with a threshold of 1e−05

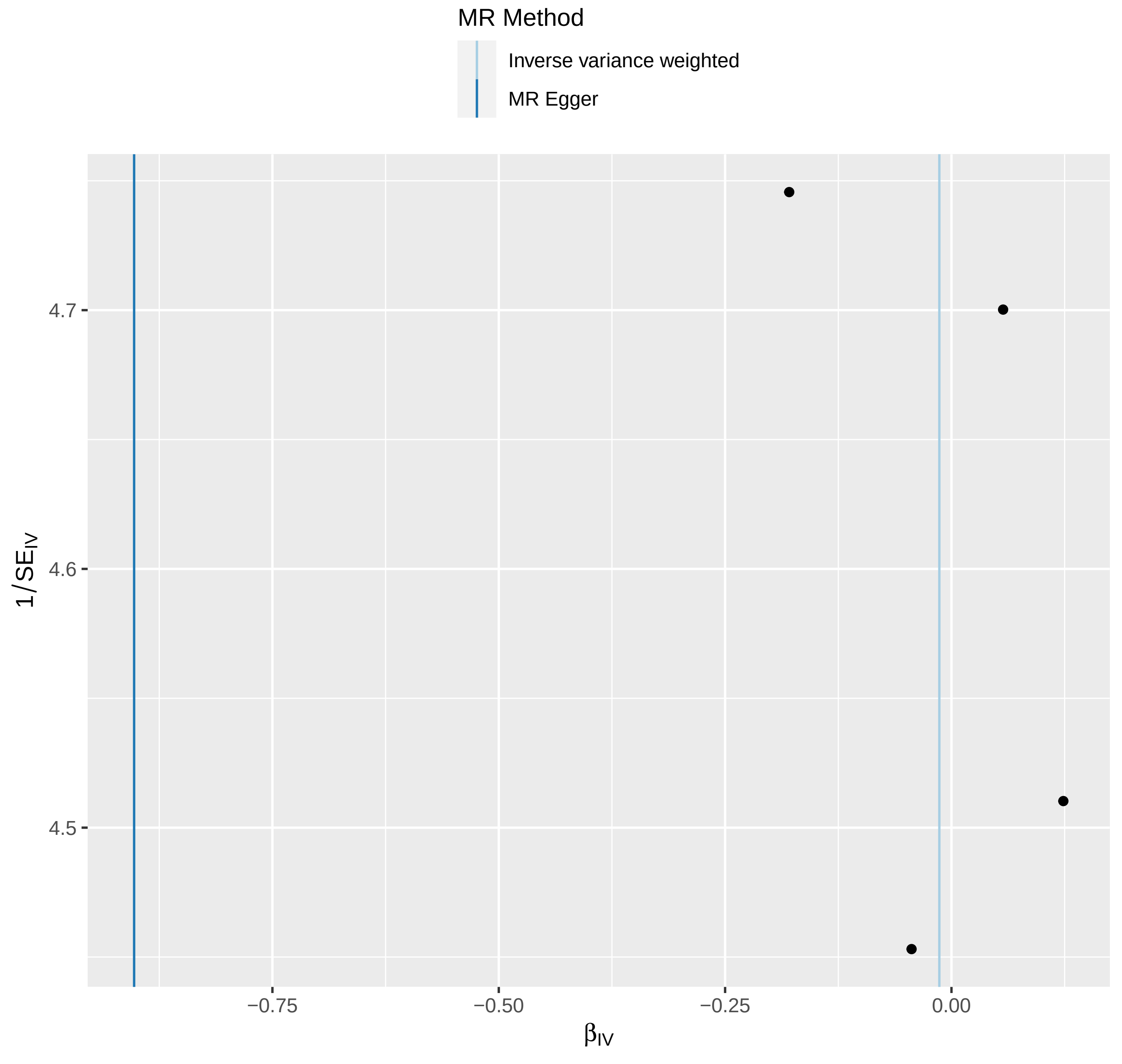

Batch 2−6480 : Gut microbiota abundance (genus Parasutterella id.2892) on Xaa−Pro aminopeptidase 1
with a threshold of 1e−05

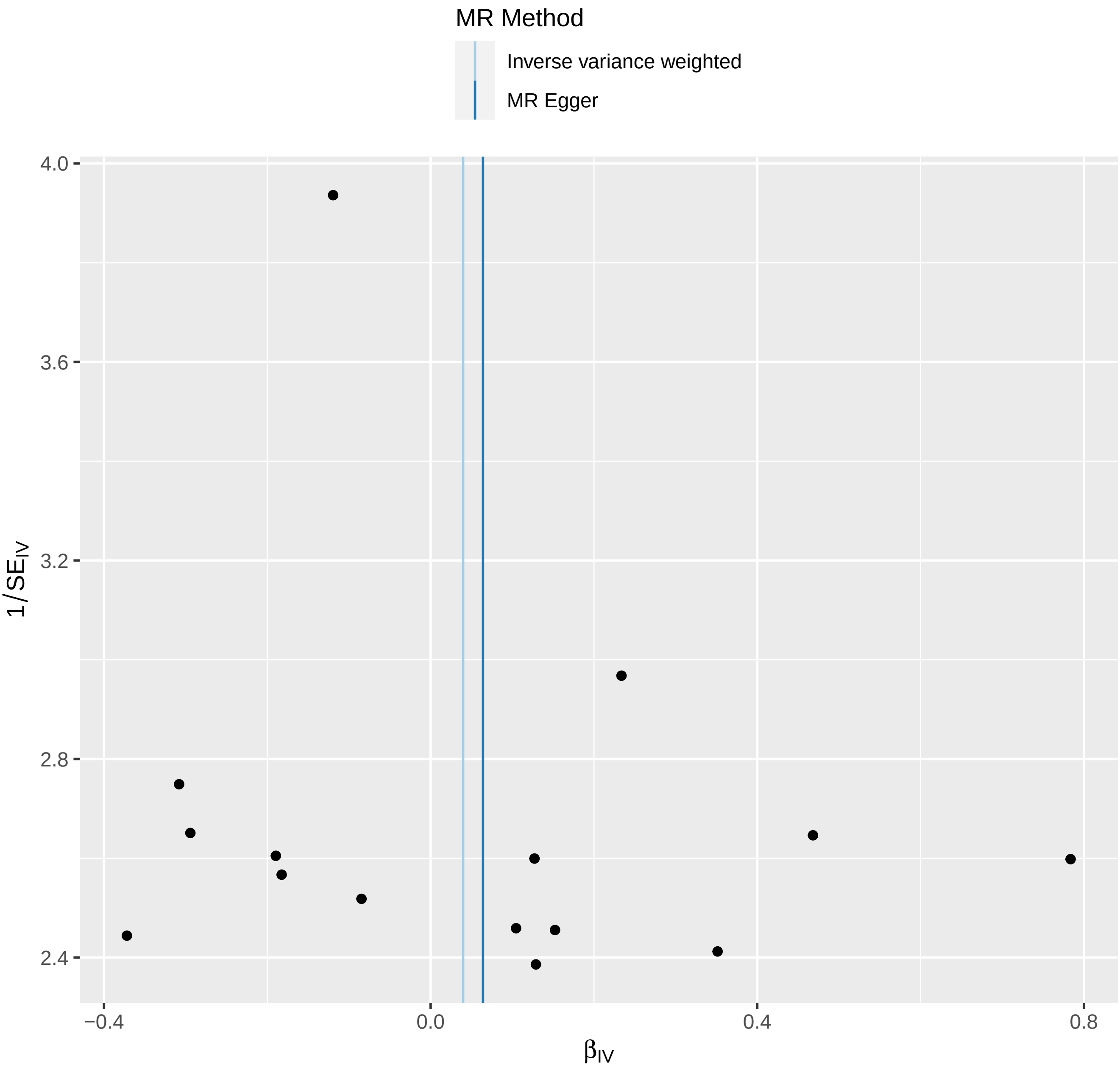

Batch 2−6481 : Gut microbiota abundance (genus Catenibacterium id.2153) on DNA repair protein XRCC1
with a threshold of 1e−05

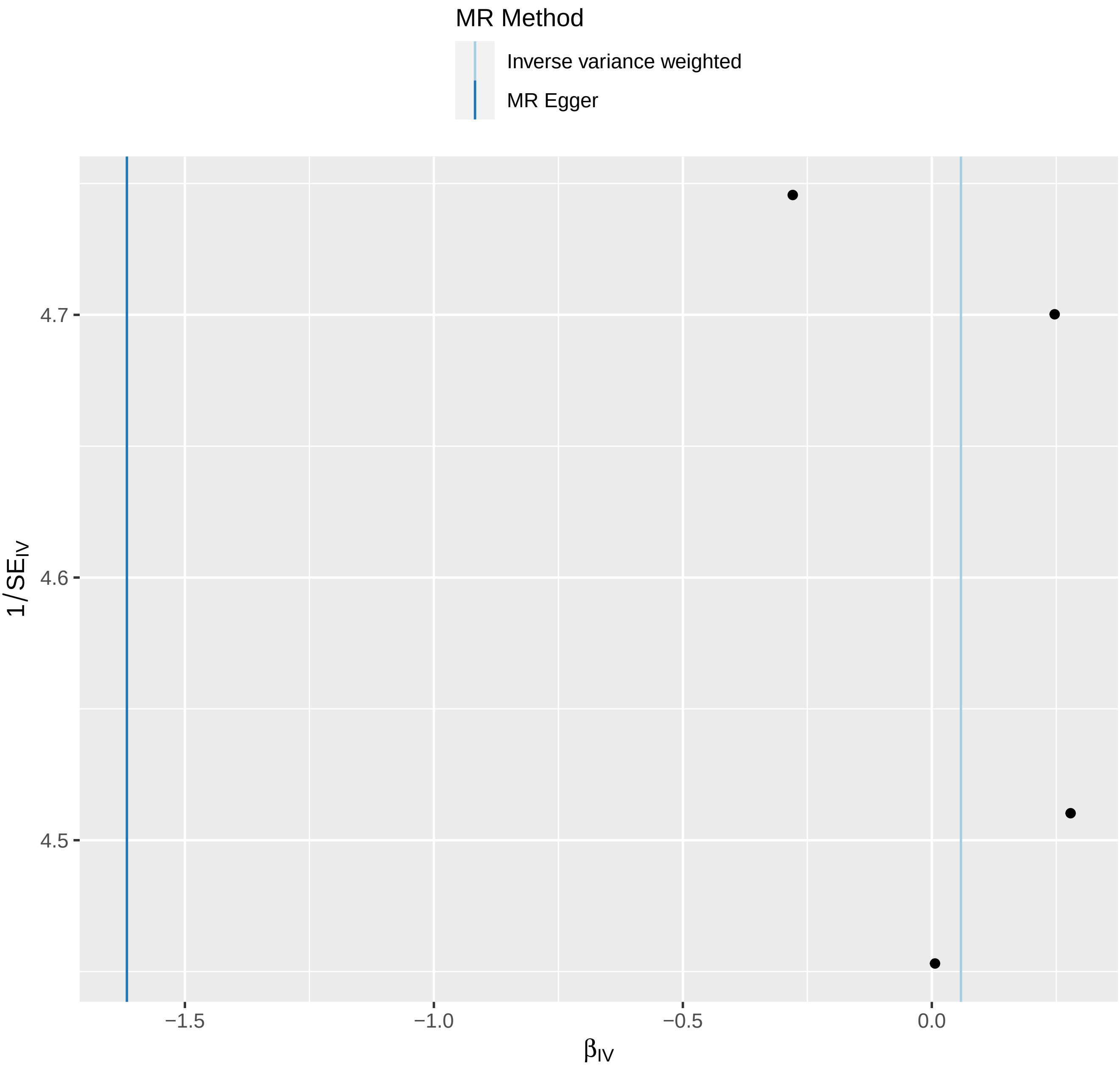

Batch 2−6482 : Gut microbiota abundance (genus Parasutterella id.2892) on DNA repair protein XRCC1
with a threshold of 1e−05

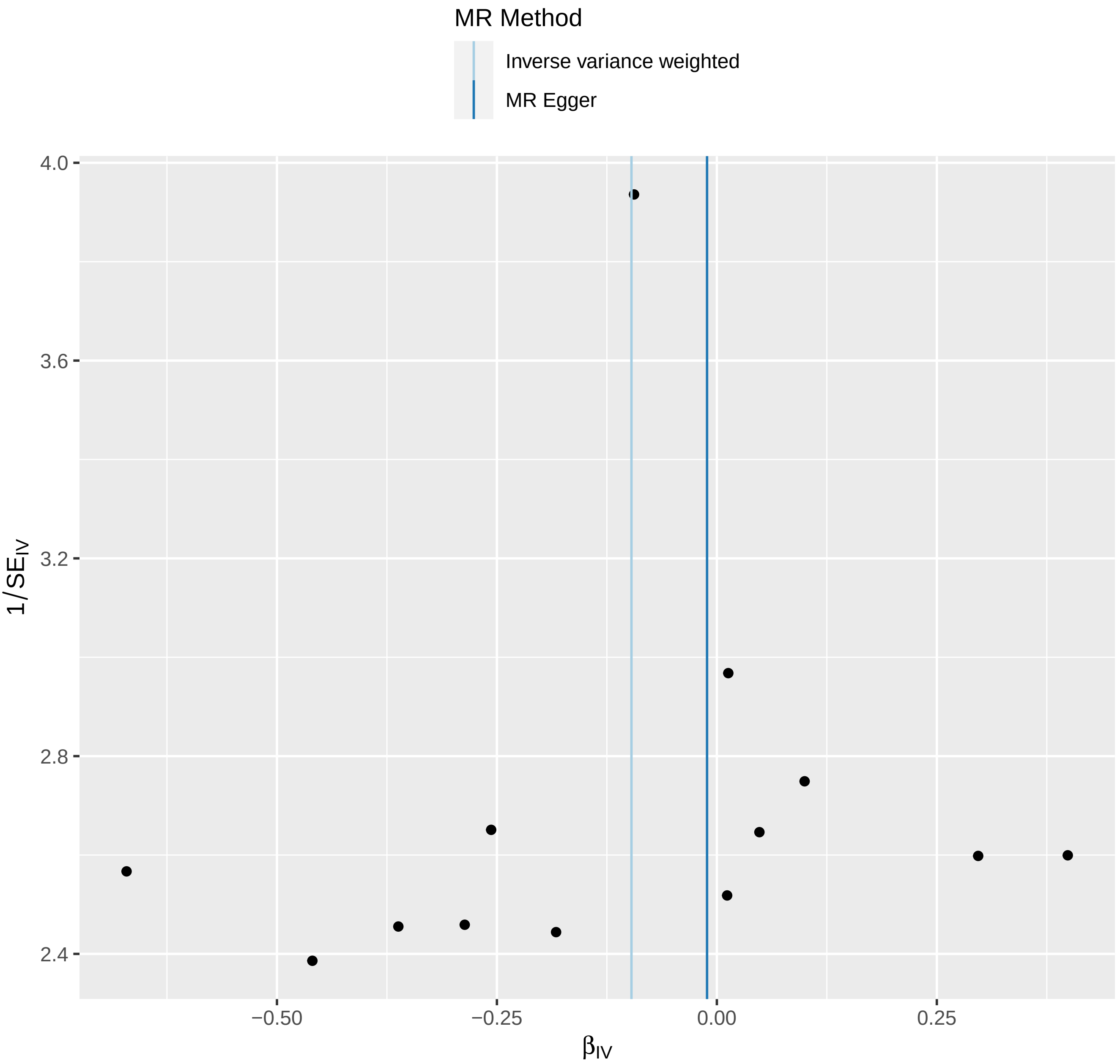

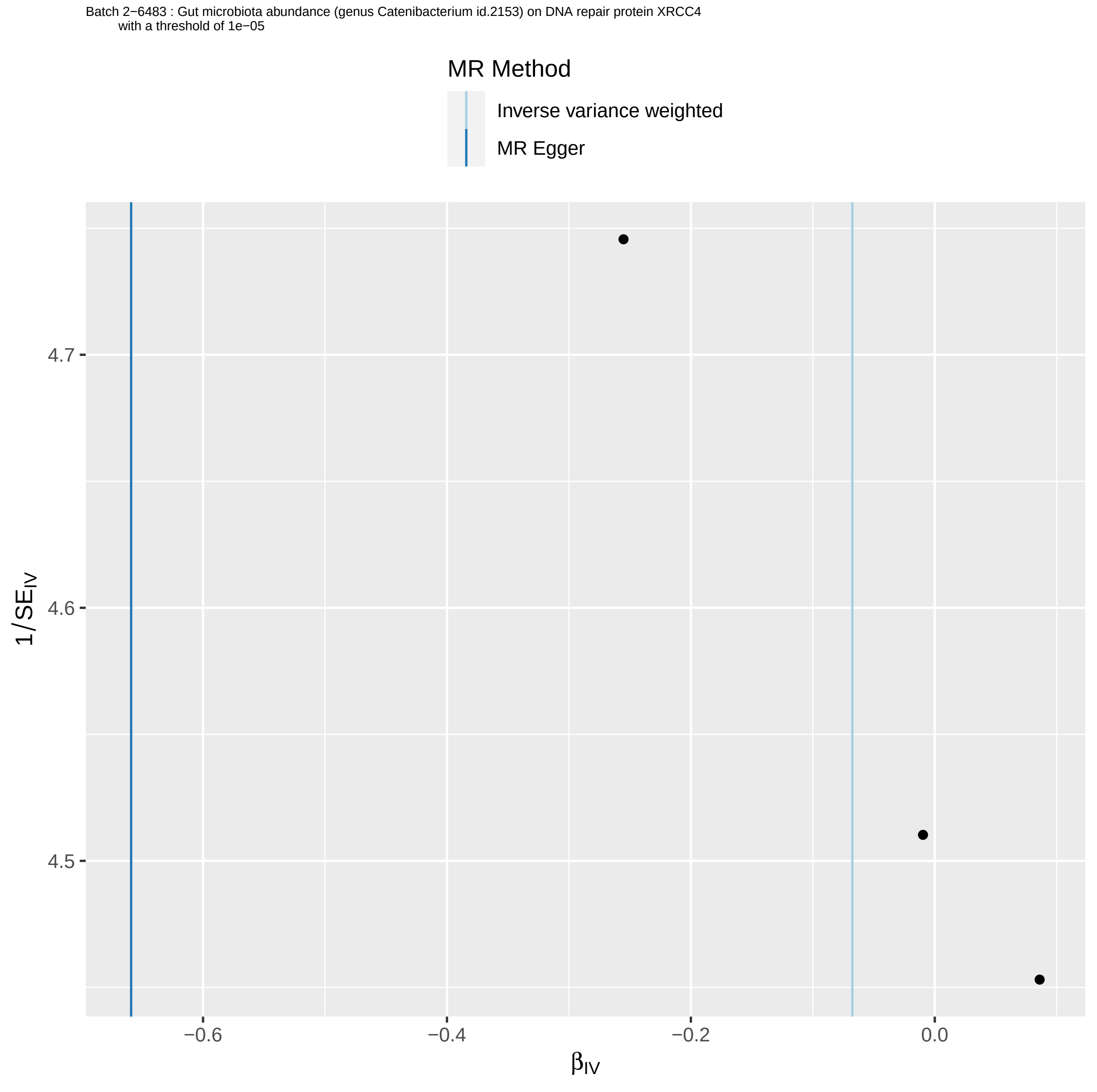
Batch 2−6483 : Gut microbiota abundance (genus Catenibacterium id.2153) on DNA repair protein XRCC4
with a threshold of 1e−05
MR Method
Inverse variance weighted
MR Egger
4.7
4.6
4.5
1/SE_IV
−0.6
−0.4
−0.2
0.0
β_IV

Batch 2−6484 : Gut microbiota abundance (genus Parasutterella id.2892) on DNA repair protein XRCC4
with a threshold of 1e−05

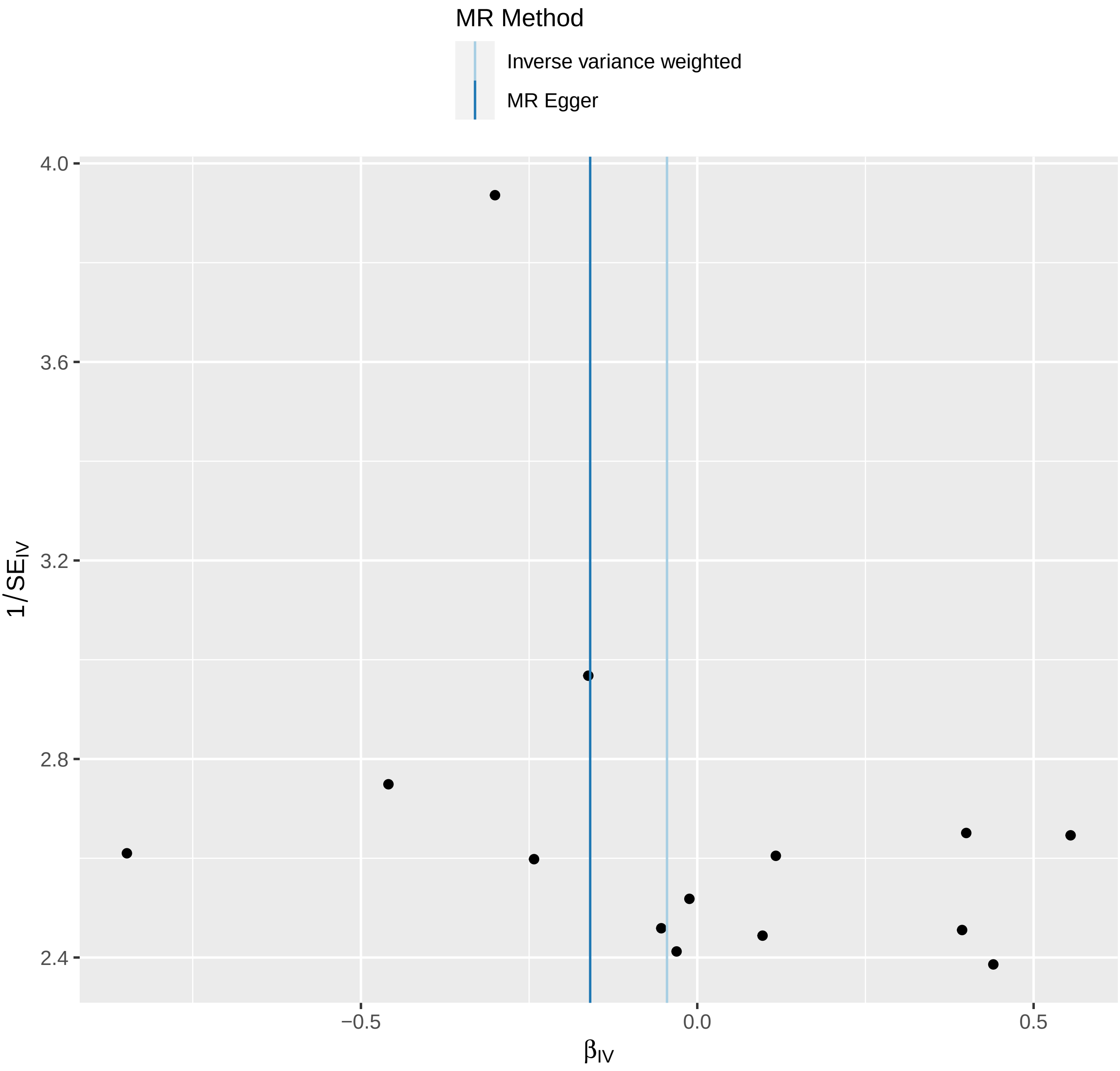

Batch 2−6485 : Gut microbiota abundance (genus Catenibacterium id.2153) on Xyloside xylosyltransferase 1
with a threshold of 1e−05

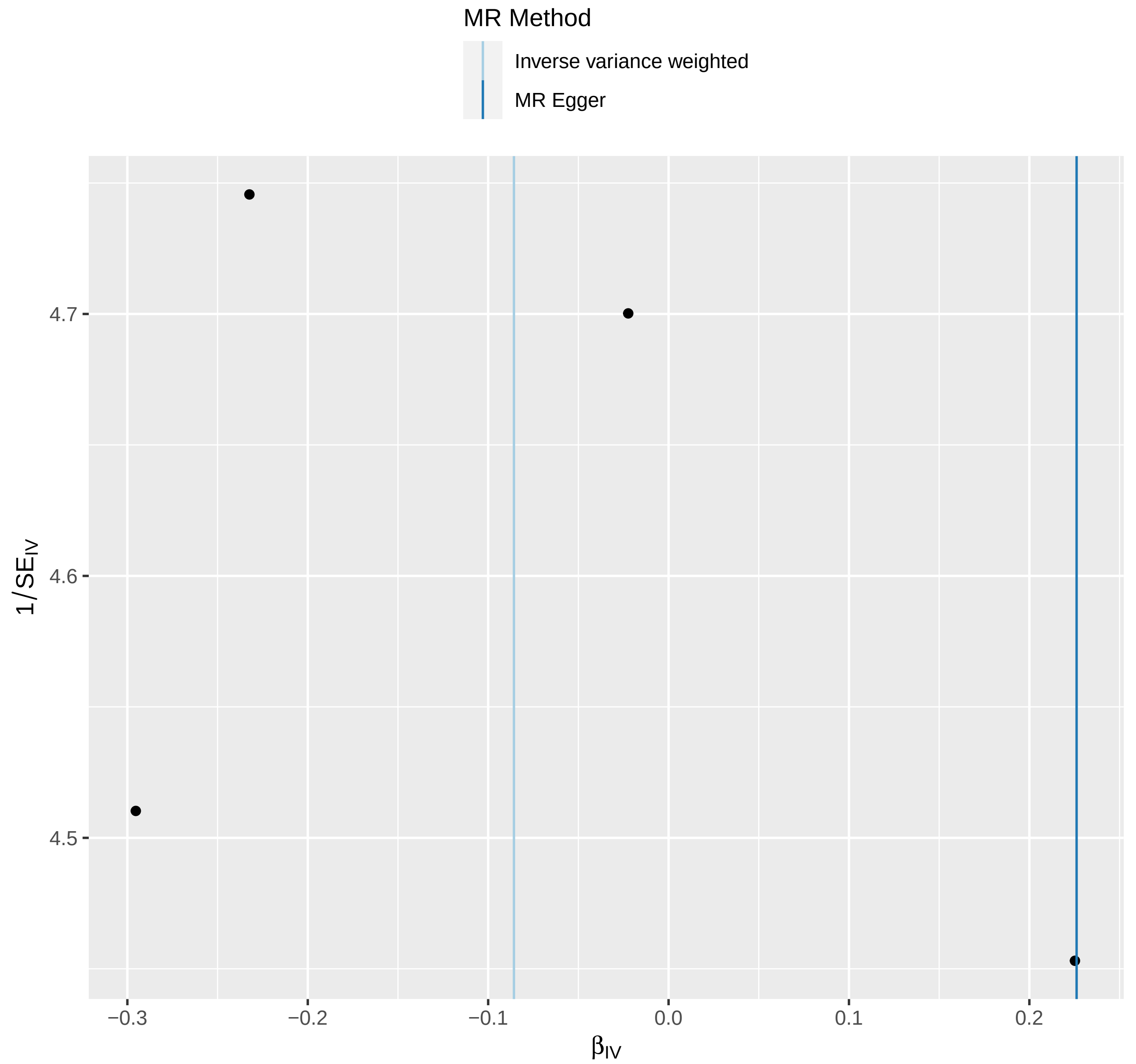

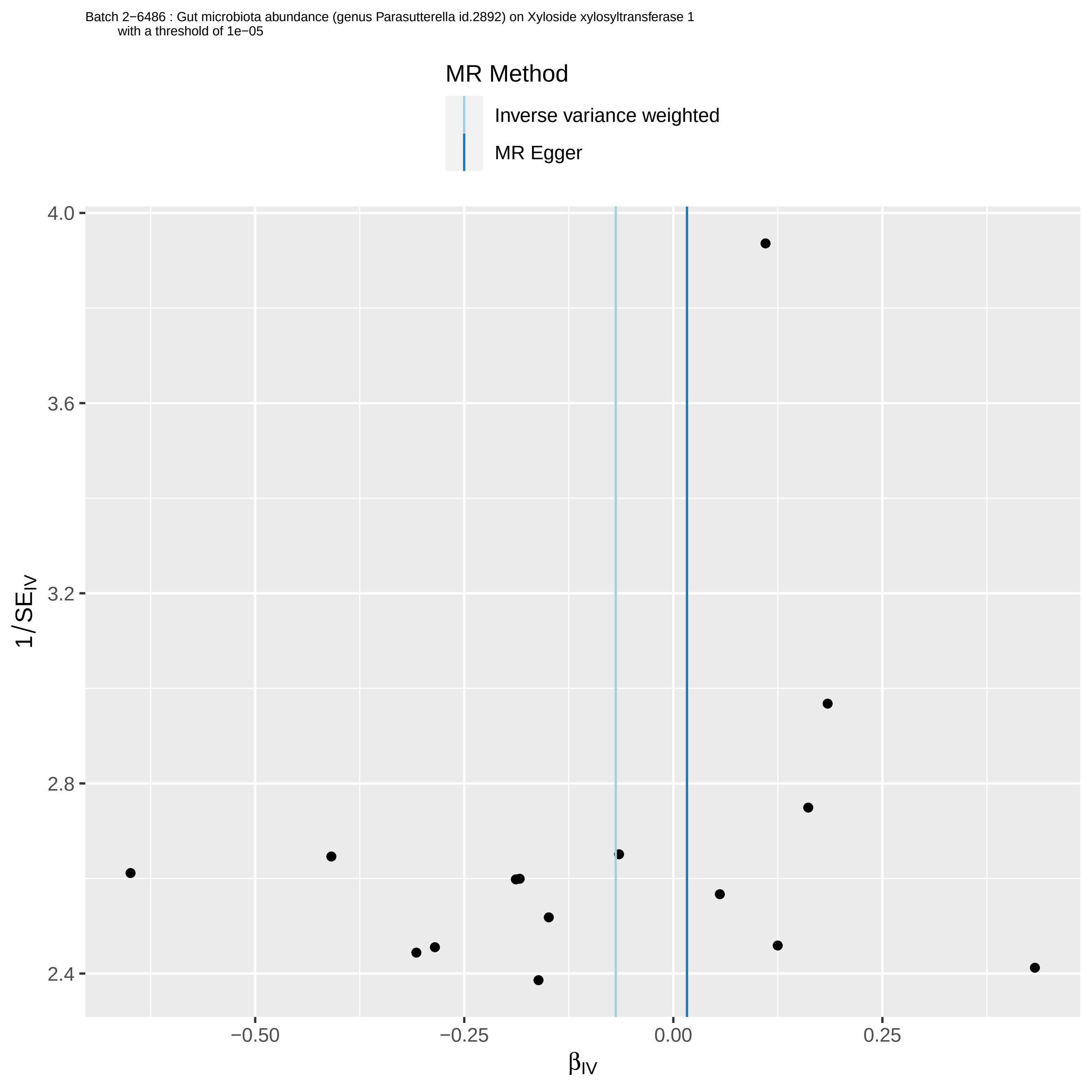

Batch 2−6486 : Gut microbiota abundance (genus Parasutterella id.2892) on Xyloside xylosyltransferase 1
with a threshold of 1e−05
MR Method
Inverse variance weighted
MR Egger
4.0
3.6
3.2
2.8
2.4
1/SE_IV
−0.50
−0.25
0.00
0.25
β_IV

Batch 2−6487 : Gut microbiota abundance (genus Catenibacterium id.2153) on Y−box−binding protein 2
with a threshold of 1e−05

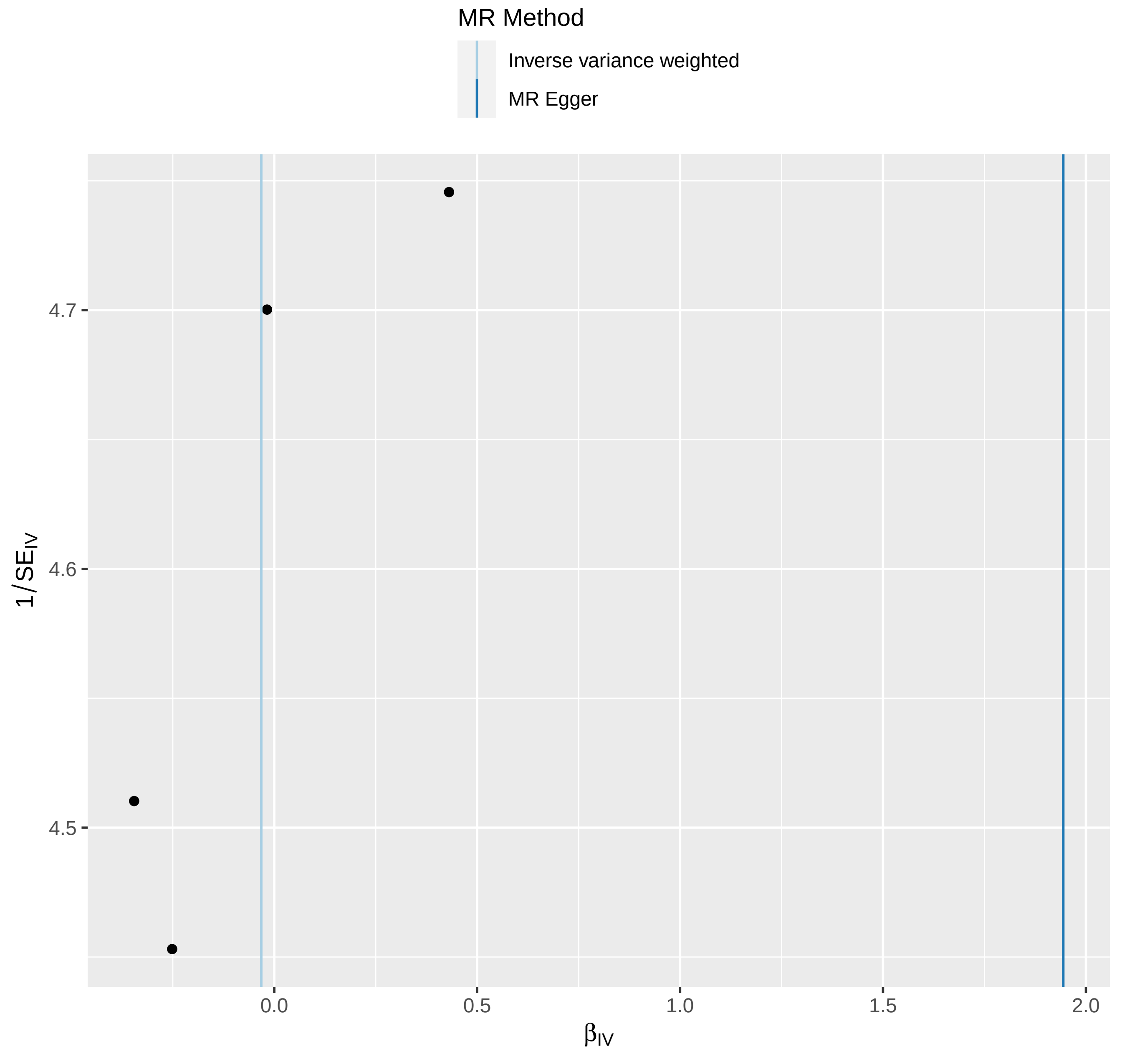

Batch 2−6488 : Gut microbiota abundance (genus Parasutterella id.2892) on Y−box−binding protein 2
with a threshold of 1e−05

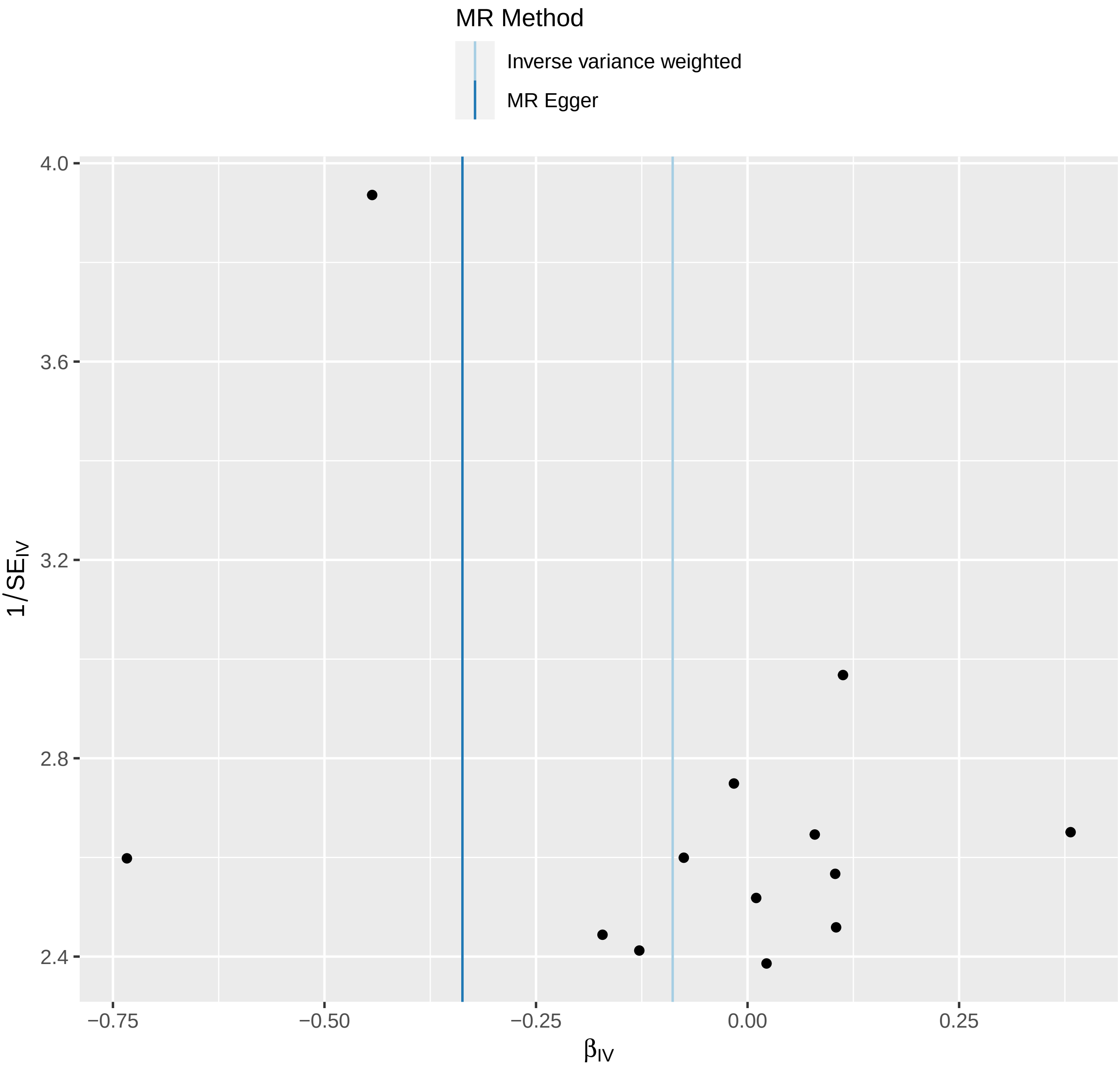

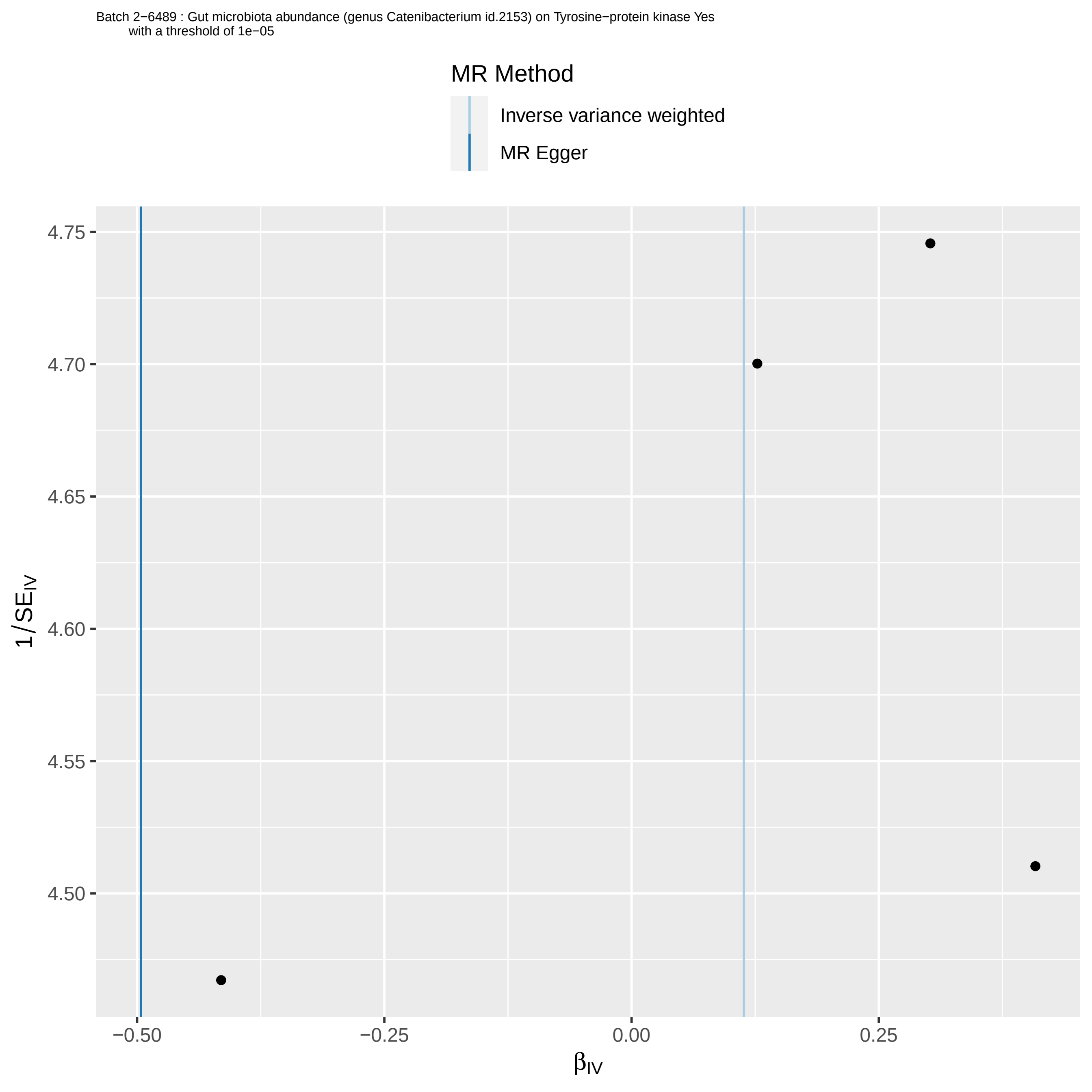
Batch 2−6489 : Gut microbiota abundance (genus Catenibacterium id.2153) on Tyrosine−protein kinase Yes
with a threshold of 1e−05
MR Method
Inverse variance weighted
MR Egger
4.75
4.70
4.65
4.60
4.55
4.50
1/SE_IV
−0.50
−0.25
0.00
0.25
β_IV

Batch 2−6490 : Gut microbiota abundance (genus Parasutterella id.2892) on Tyrosine−protein kinase Yes
with a threshold of 1e−05

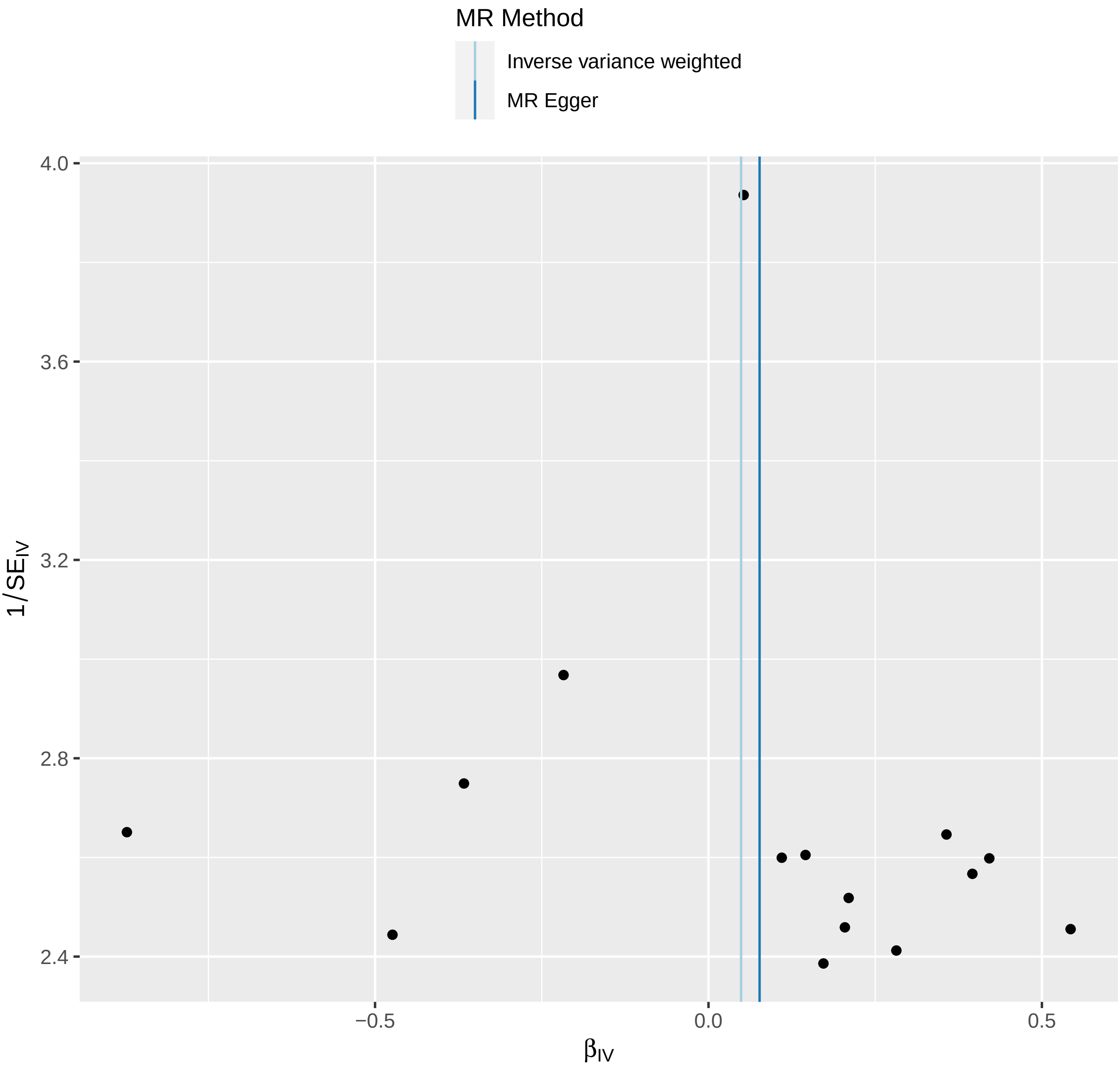

Batch 2−6491 : Gut microbiota abundance (genus Catenibacterium id.2153) on Protein YIPF6
with a threshold of 1e−05

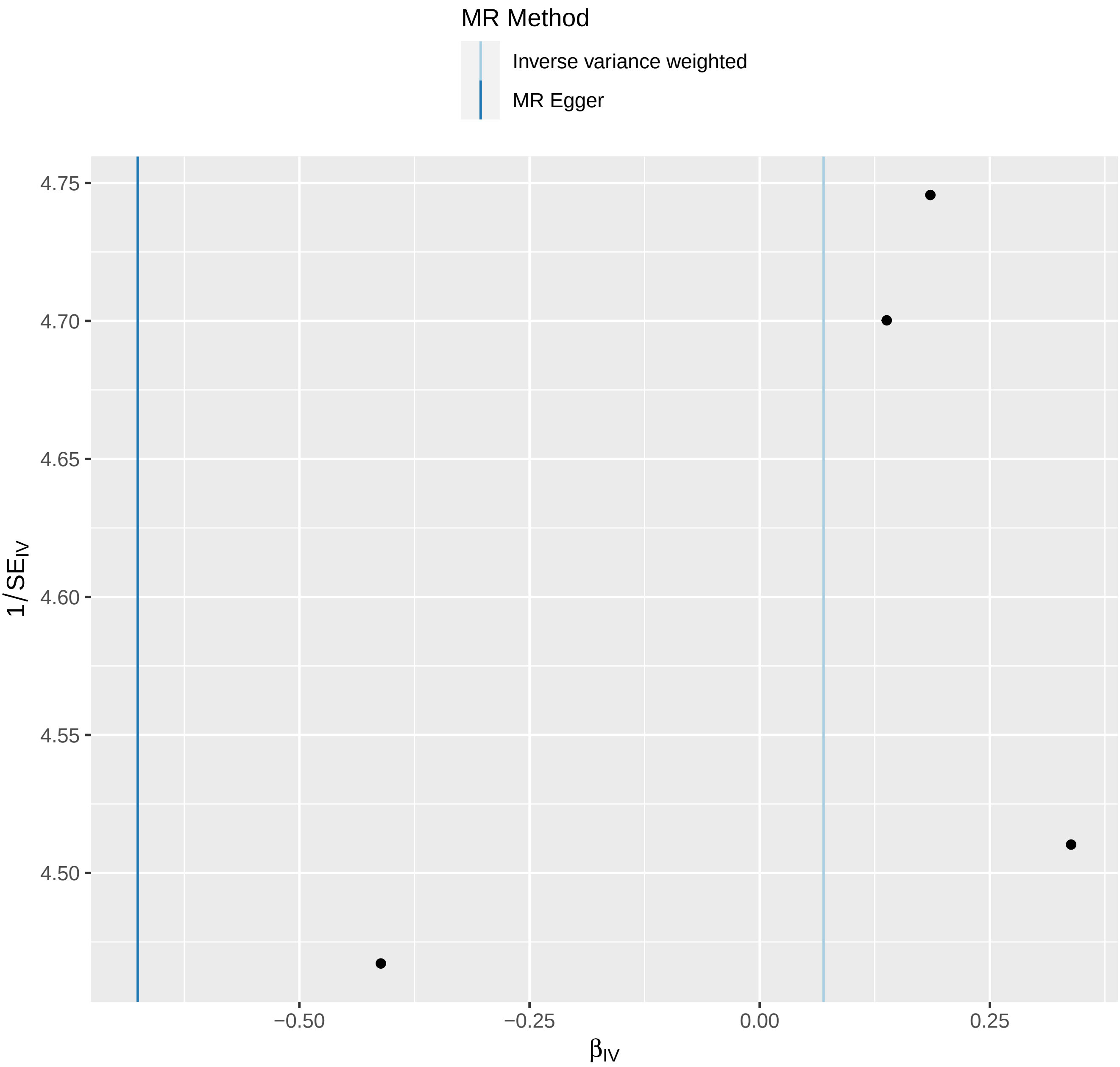

Batch 2−6492 : Gut microbiota abundance (genus Parasutterella id.2892) on Protein YIPF6
with a threshold of 1e−05

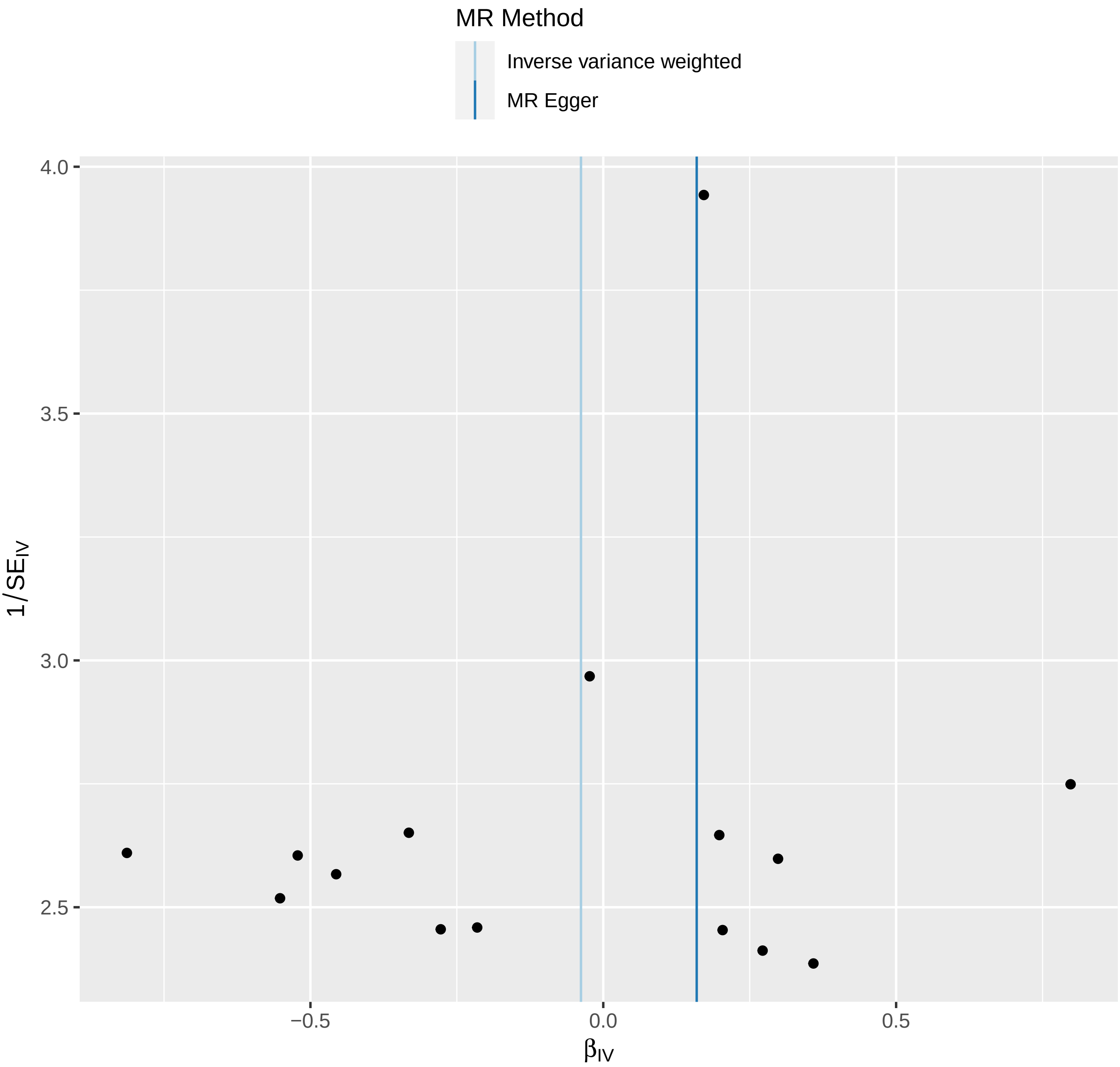

Batch 2−6493 : Gut microbiota abundance (genus Catenibacterium id.2153) on YTH domain−containing protein 1
with a threshold of 1e−05

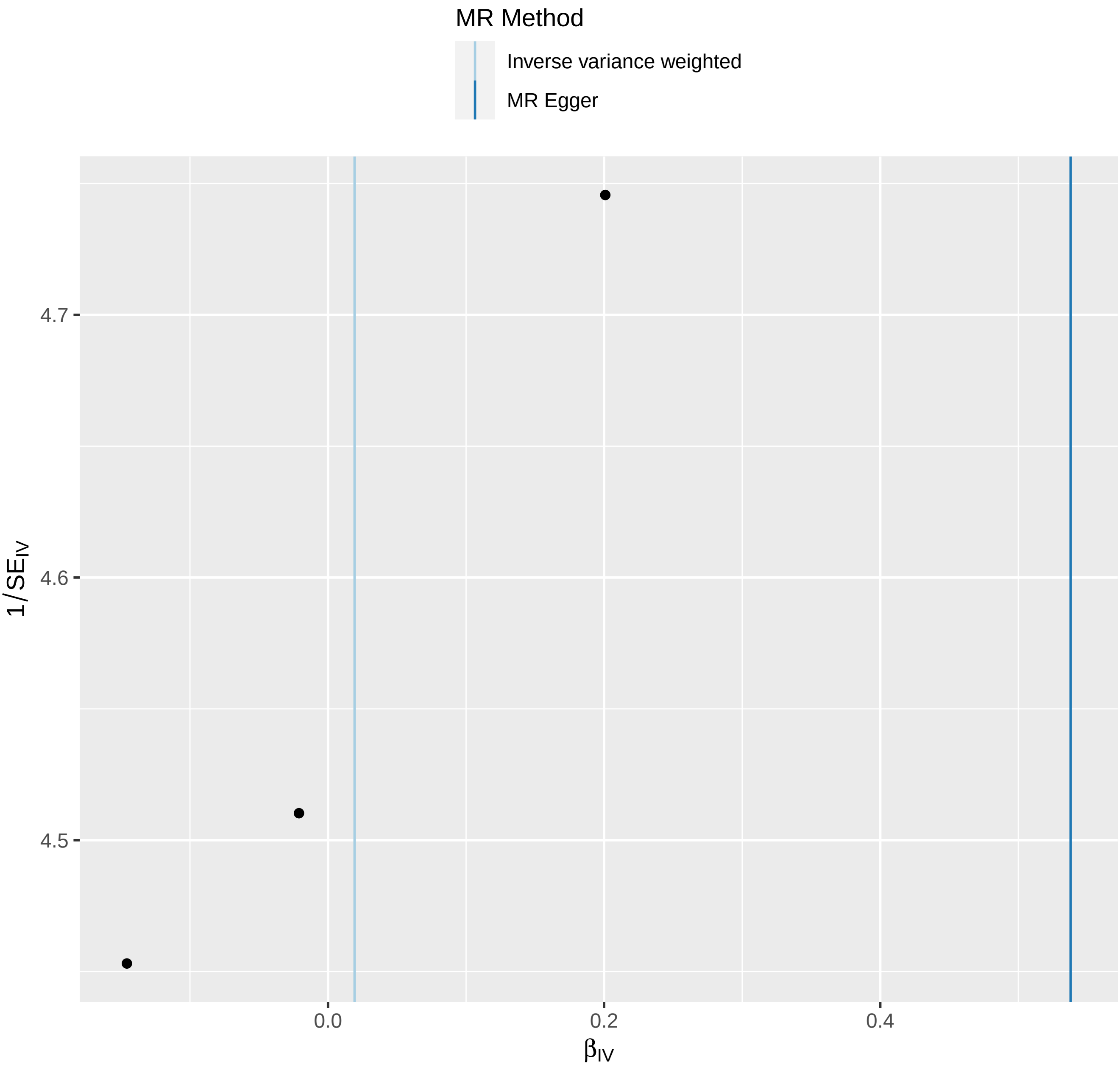

Batch 2−6494 : Gut microbiota abundance (genus Parasutterella id.2892) on YTH domain−containing protein 1
with a threshold of 1e−05

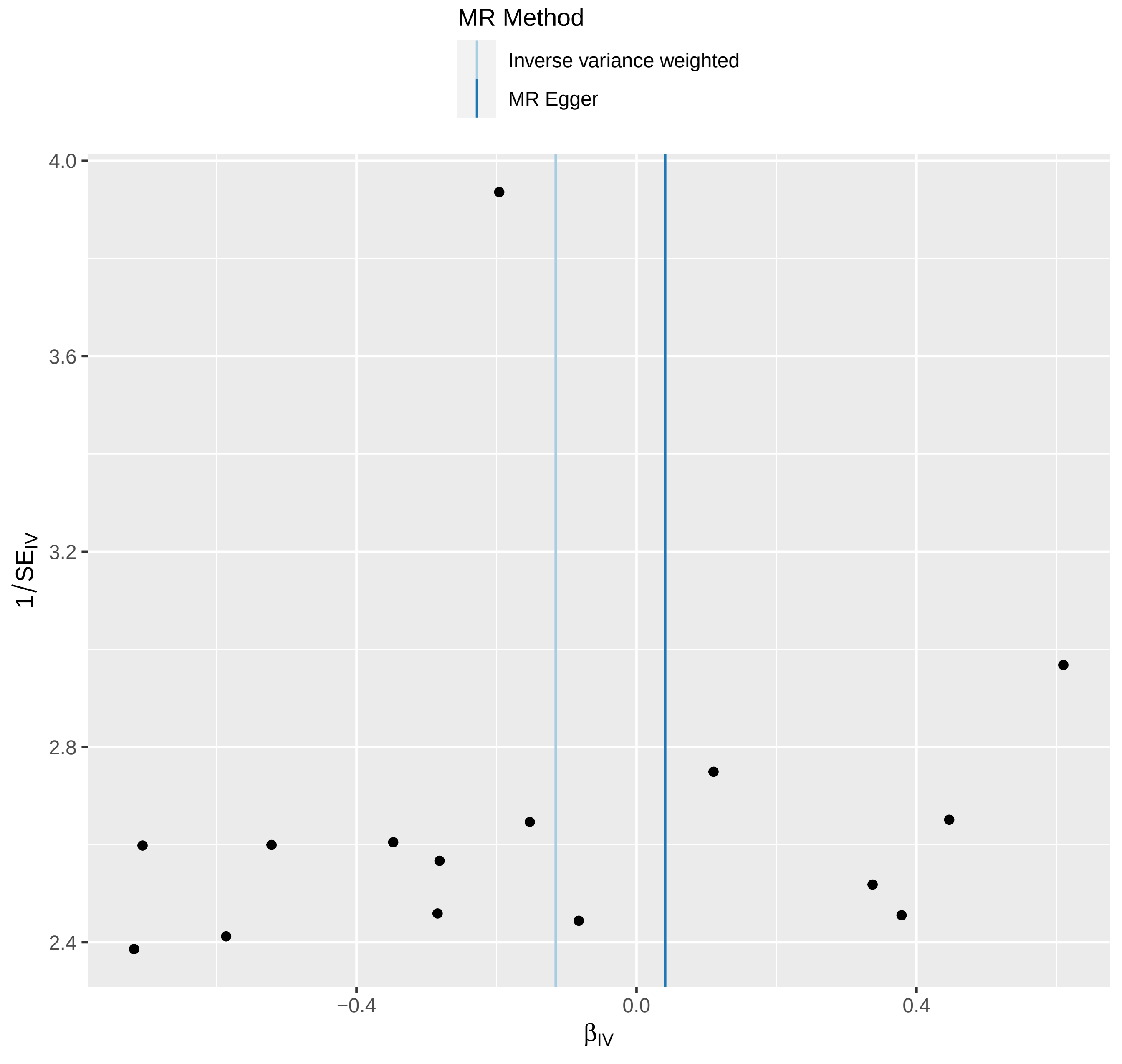

Batch 2−6495 : Gut microbiota abundance (genus Catenibacterium id.2153) on 14−3−3 protein family
with a threshold of 1e−05

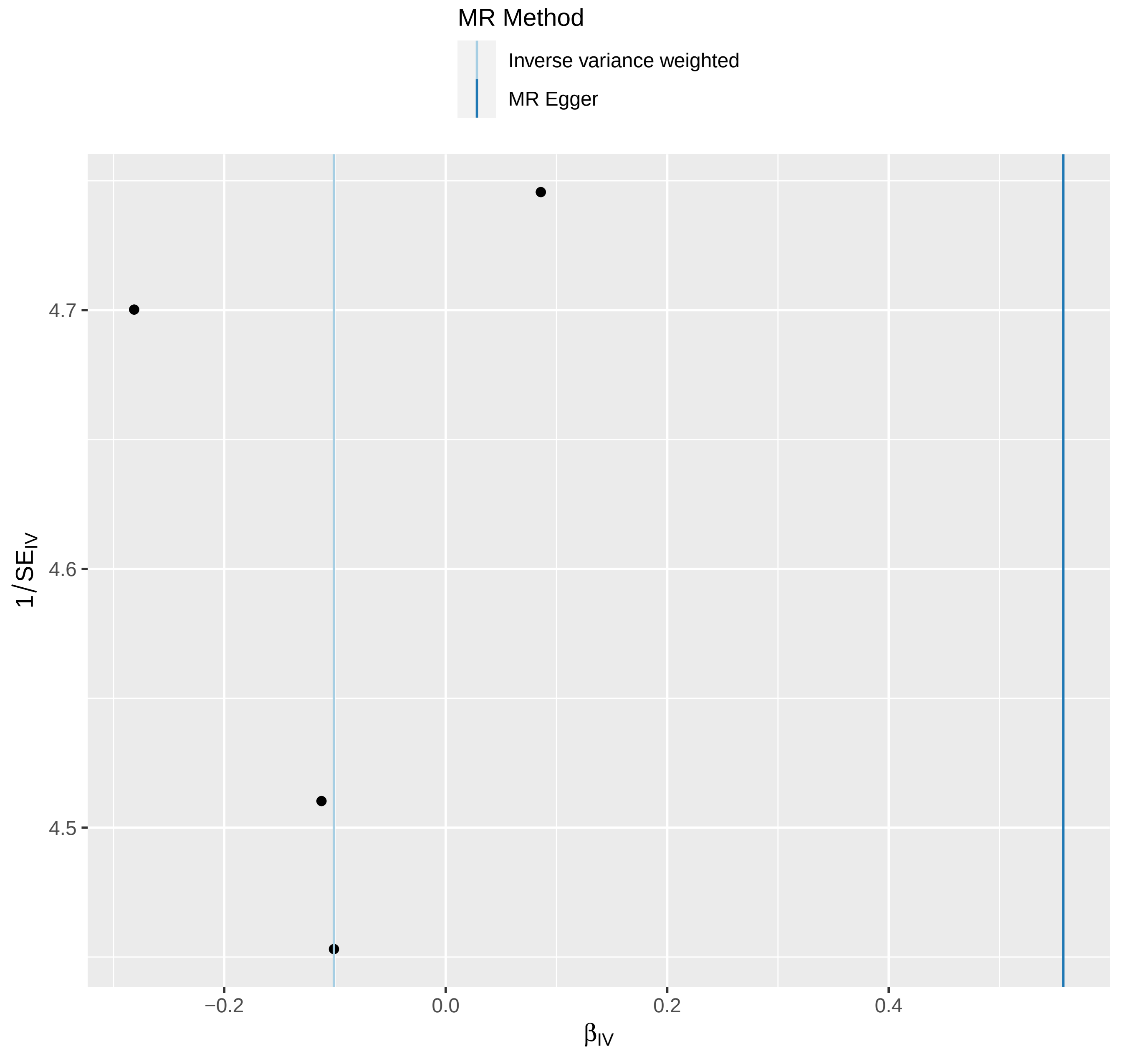

Batch 2−6496 : Gut microbiota abundance (genus Parasutterella id.2892) on 14−3−3 protein family
with a threshold of 1e−05

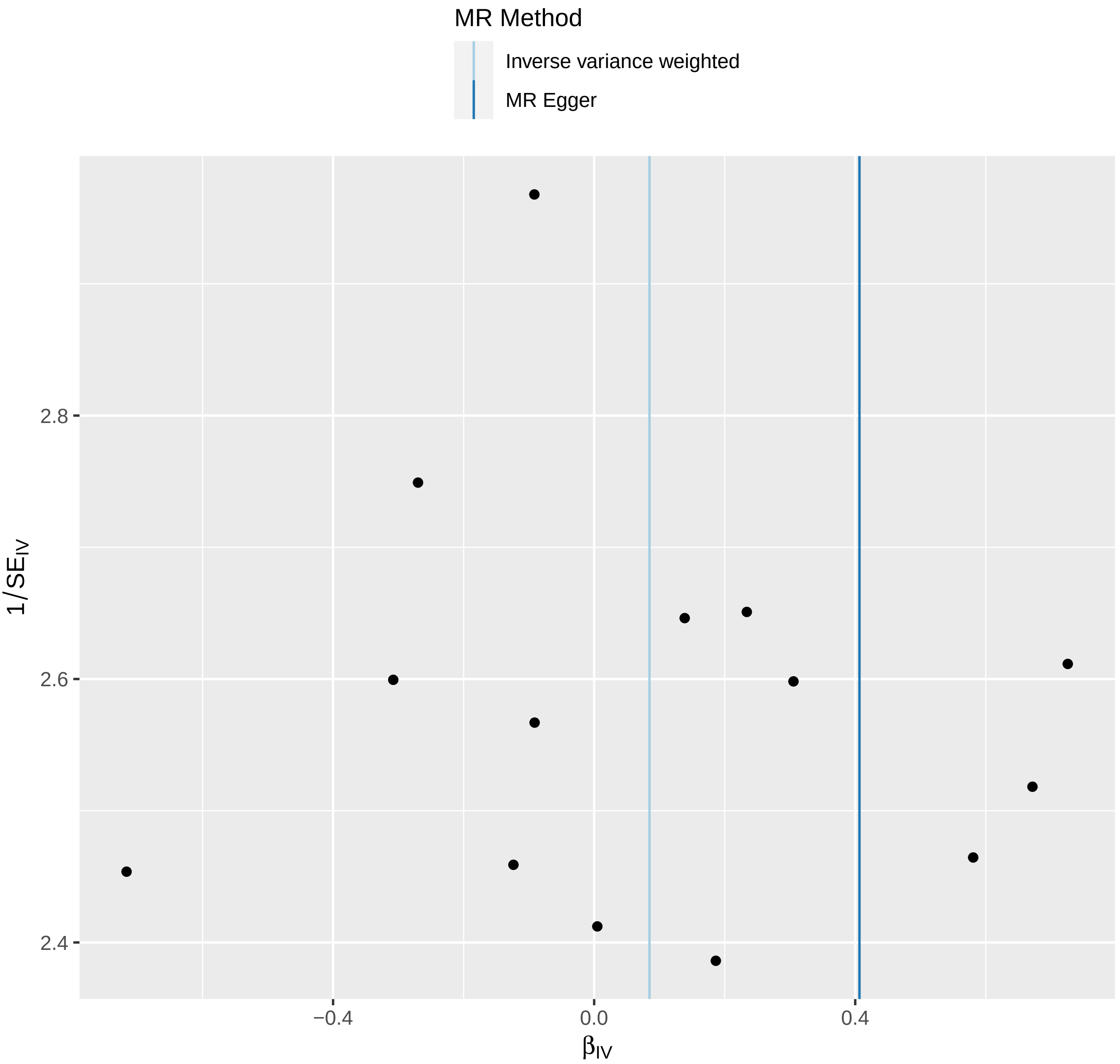

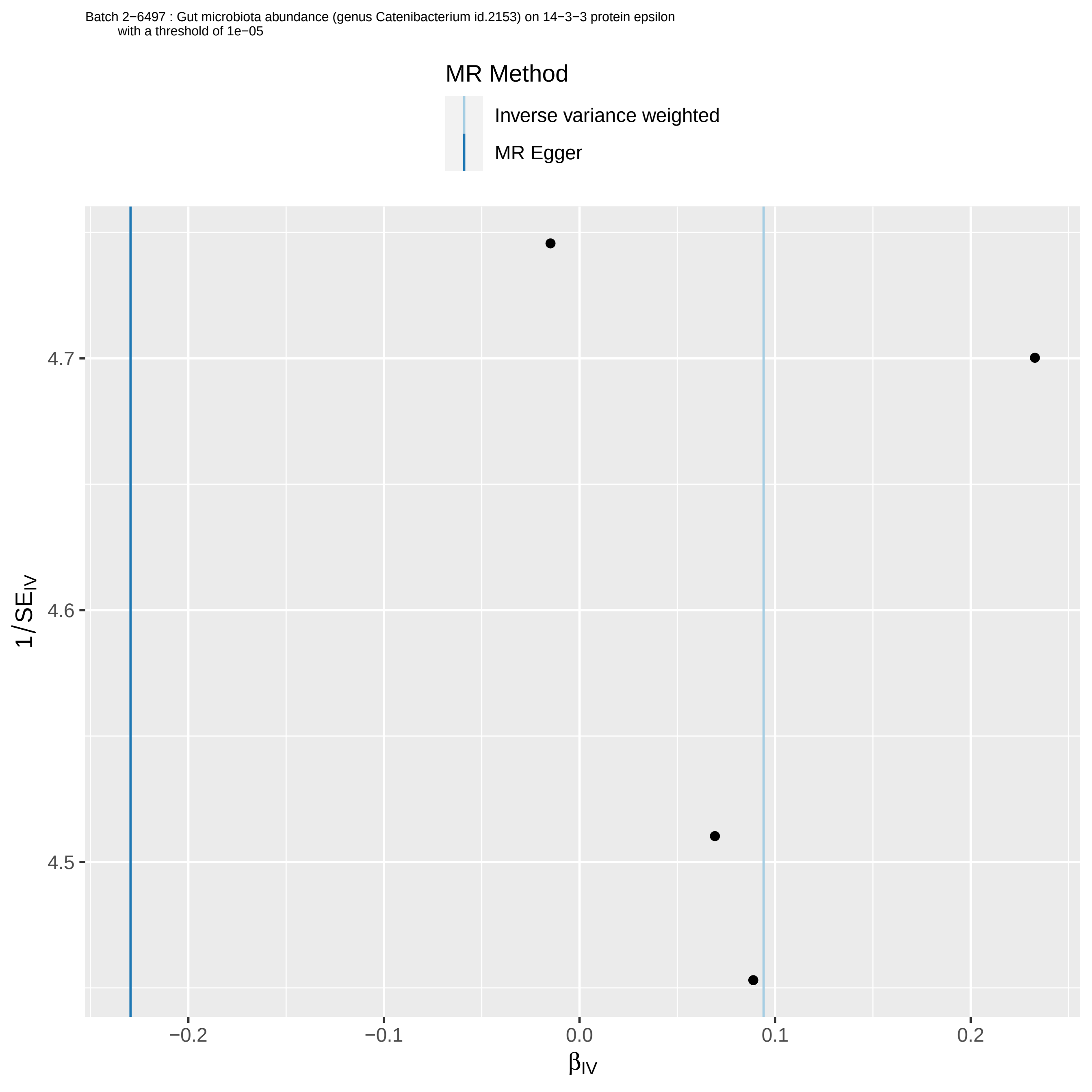

Batch 2−6497 : Gut microbiota abundance (genus Catenibacterium id.2153) on 14−3−3 protein epsilon
with a threshold of 1e−05
MR Method
Inverse variance weighted
MR Egger
4.7
4.6
4.5
1/SE_IV
−0.2
−0.1
0.0
0.1
0.2
β_IV

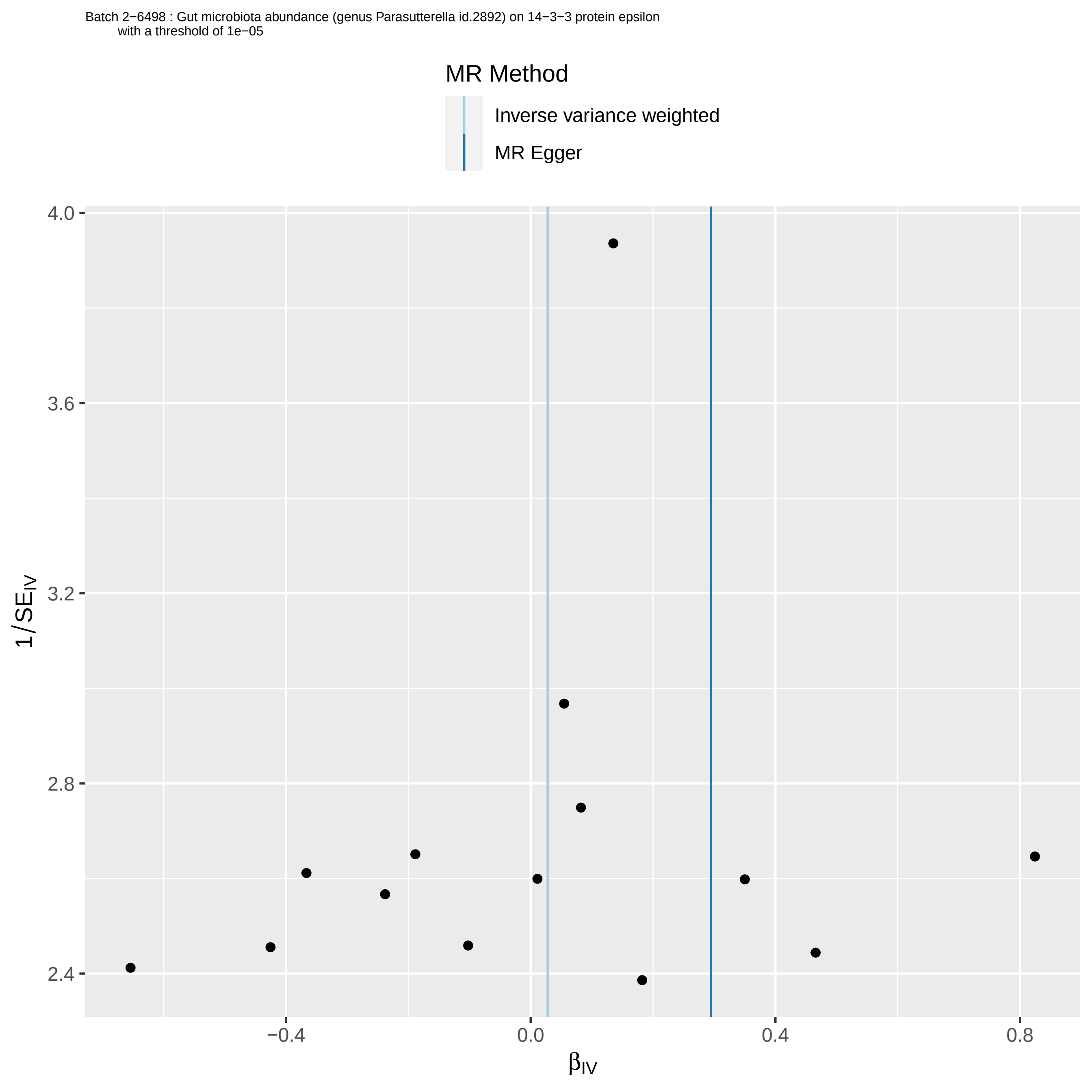
Batch 2−6498 : Gut microbiota abundance (genus Parasutterella id.2892) on 14−3−3 protein epsilon
with a threshold of 1e−05
MR Method
Inverse variance weighted
MR Egger
4.0
3.6
3.2
2.8
2.4
1/SE$_{IV}$
−0.4
0.0
0.4
0.8
β$_{IV}$

Batch 2−6499 : Gut microbiota abundance (genus Catenibacterium id.2153) on 14−3−3 protein theta
with a threshold of 1e−05

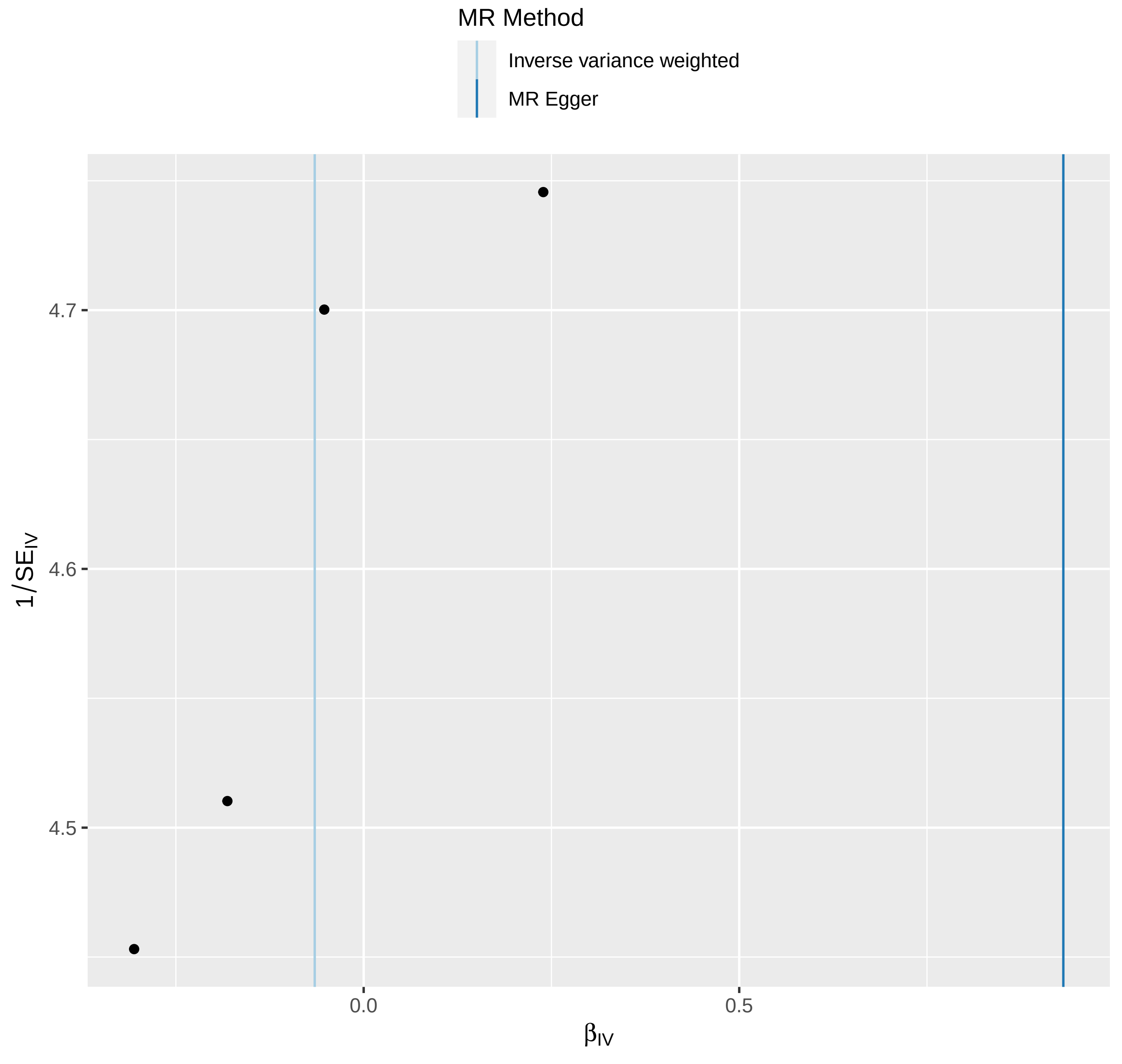

Batch 2−6500 : Gut microbiota abundance (genus Parasutterella id.2892) on 14−3−3 protein theta
with a threshold of 1e−05

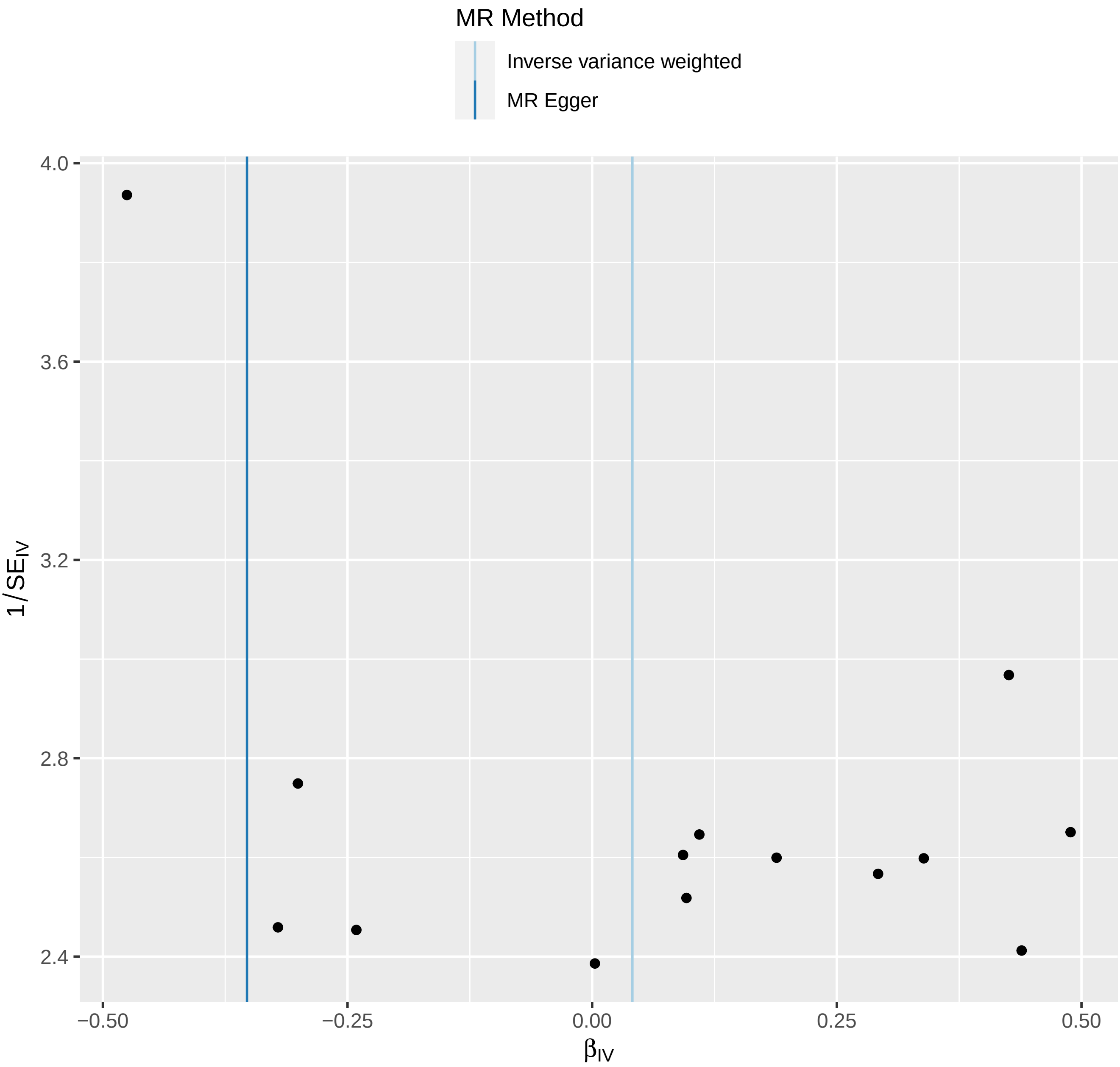

Batch 2−6501 : Gut microbiota abundance (genus Catenibacterium id.2153) on 14−3−3 protein zeta/delta
with a threshold of 1e−05

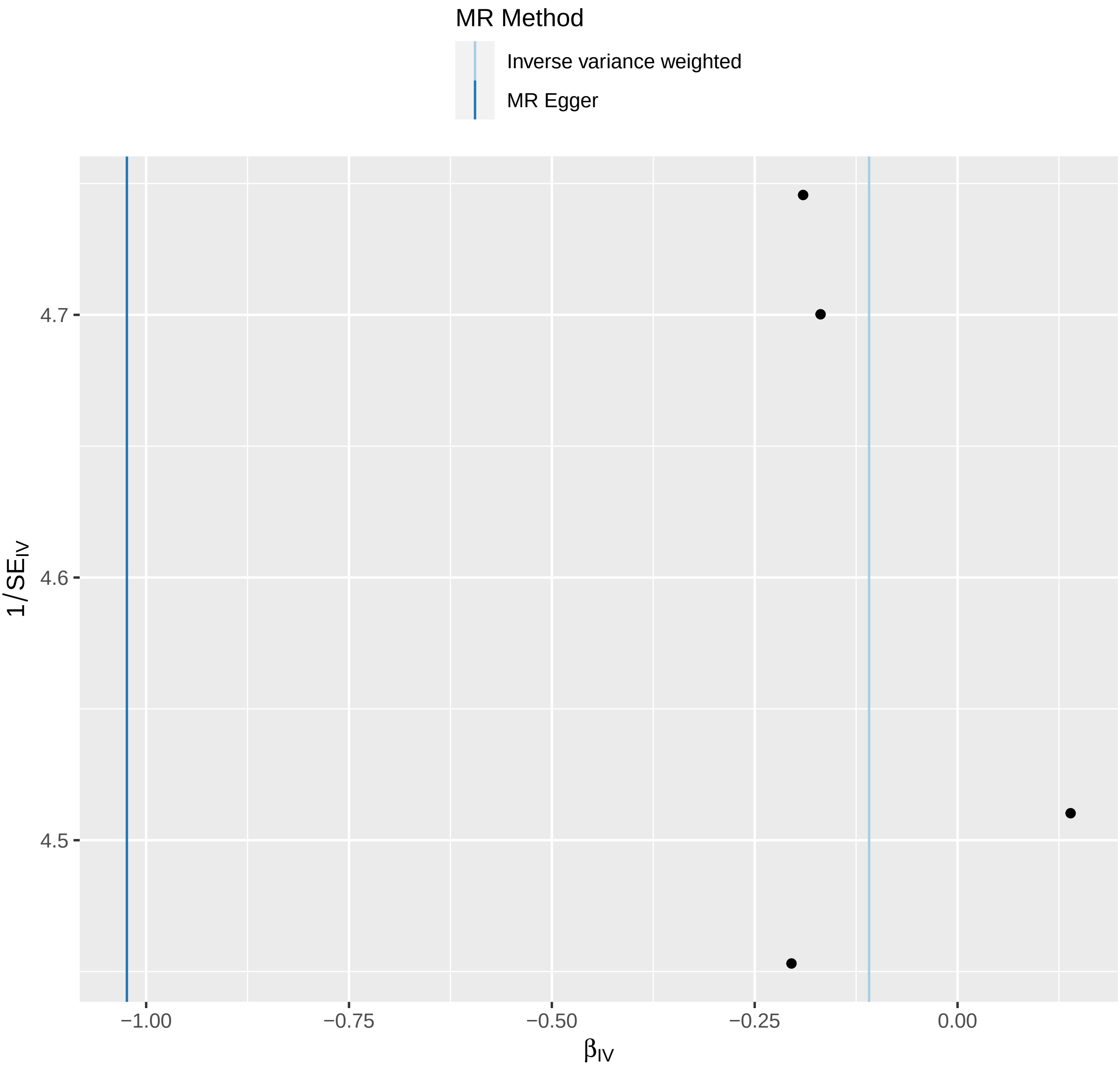

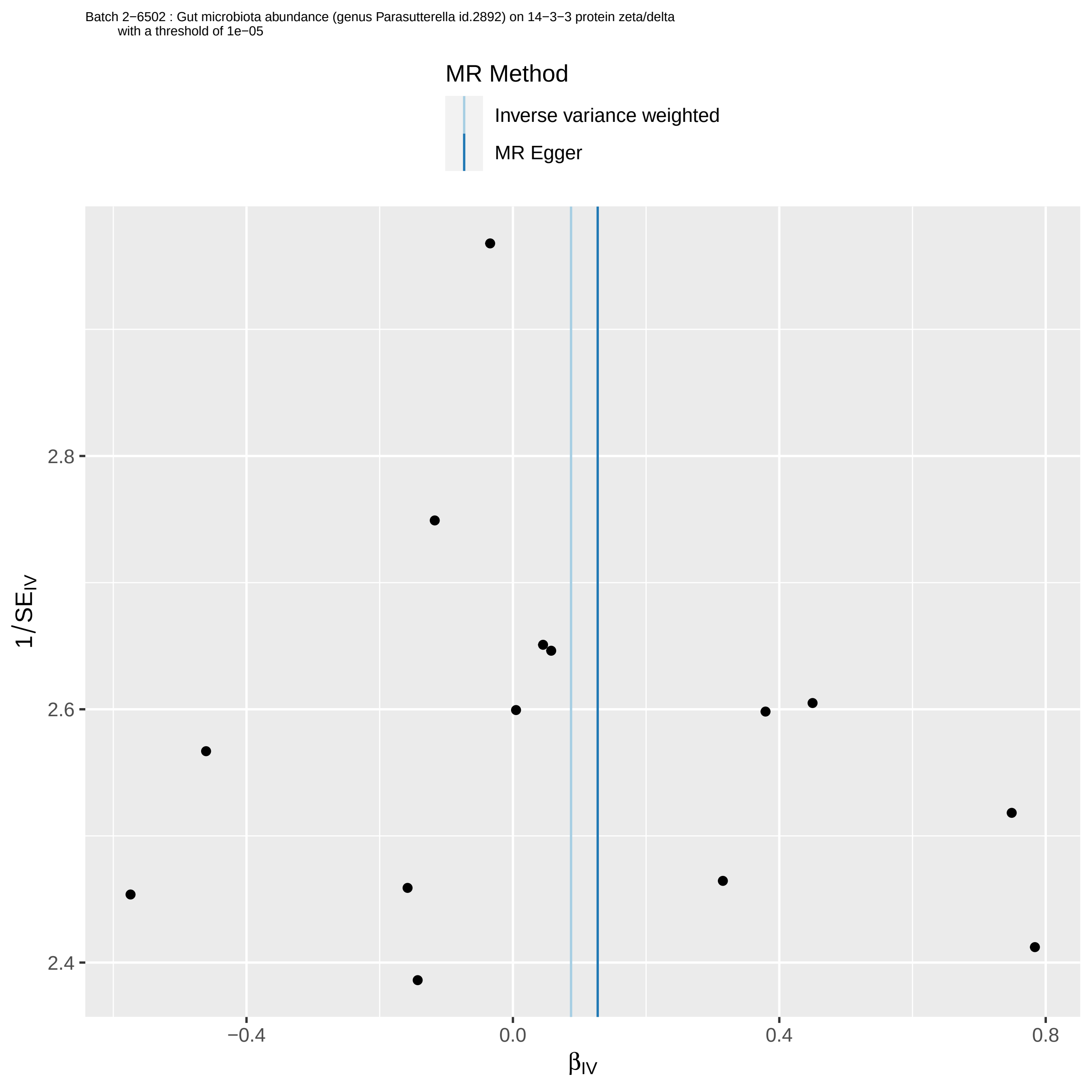
Batch 2−6502 : Gut microbiota abundance (genus Parasutterella id.2892) on 14−3−3 protein zeta/delta
with a threshold of 1e−05
MR Method
Inverse variance weighted
MR Egger
2.8
2.6
2.4
1/SE_IV
−0.4
0.0
0.4
0.8
β_IV

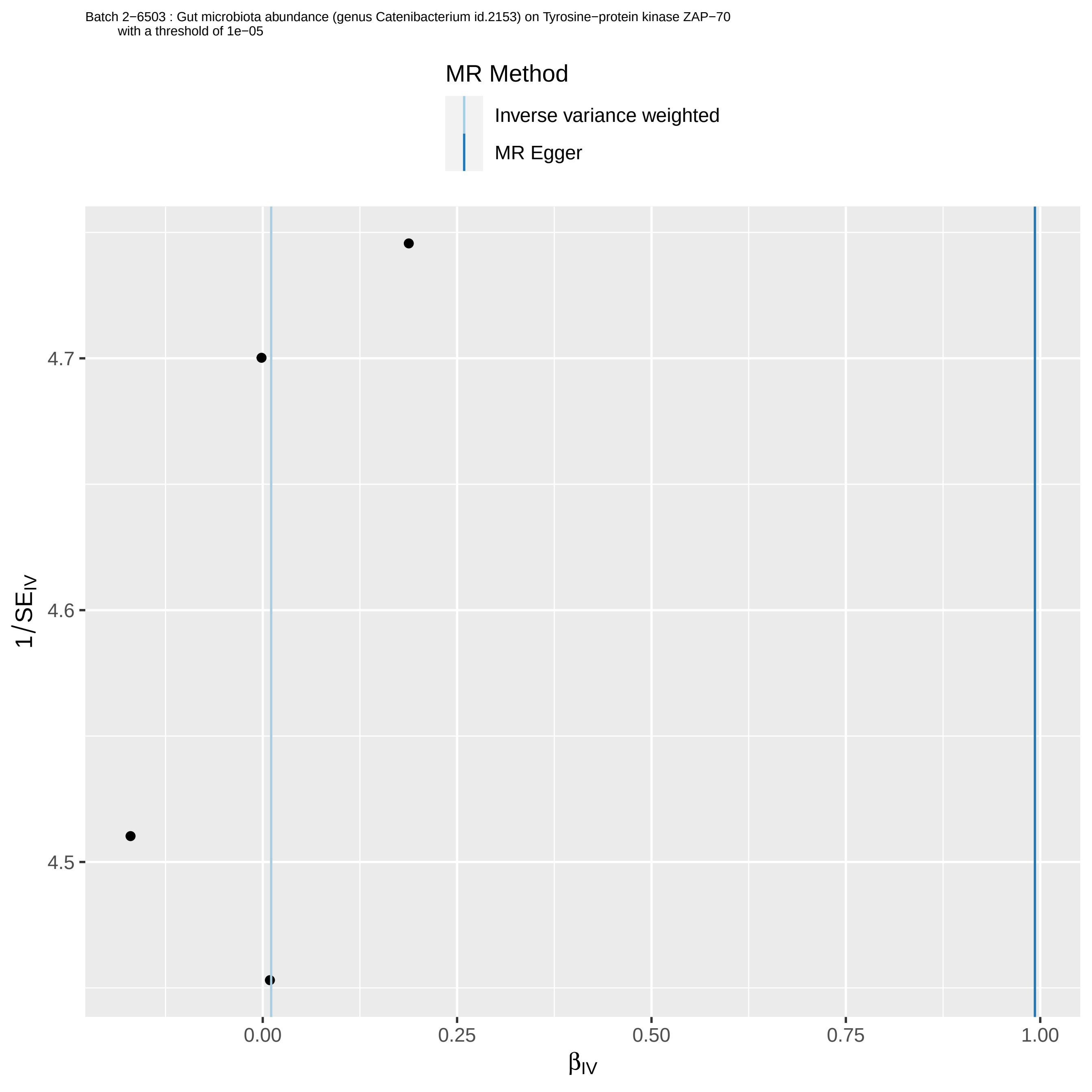
Batch 2−6503 : Gut microbiota abundance (genus Catenibacterium id.2153) on Tyrosine−protein kinase ZAP−70
with a threshold of 1e−05
MR Method
Inverse variance weighted
MR Egger
4.7
4.6
4.5
1/SE_IV
0.00
0.25
0.50
0.75
1.00
β_IV

Batch 2−6504 : Gut microbiota abundance (genus Parasutterella id.2892) on Tyrosine−protein kinase ZAP−70
with a threshold of 1e−05

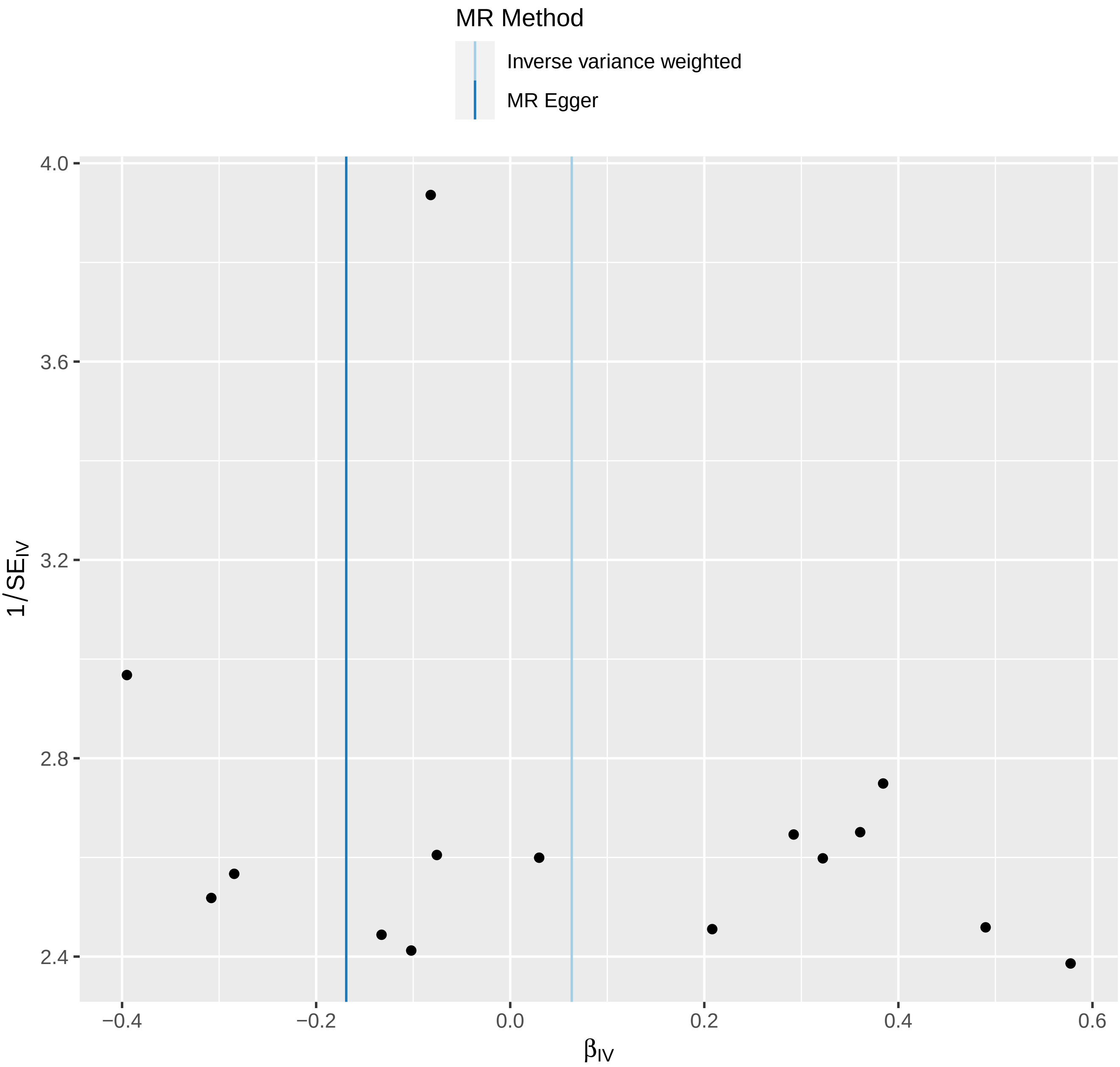

Batch 2−6505 : Gut microbiota abundance (genus Catenibacterium id.2153) on Tyrosine−protein kinase ZAP−70
with a threshold of 1e−05

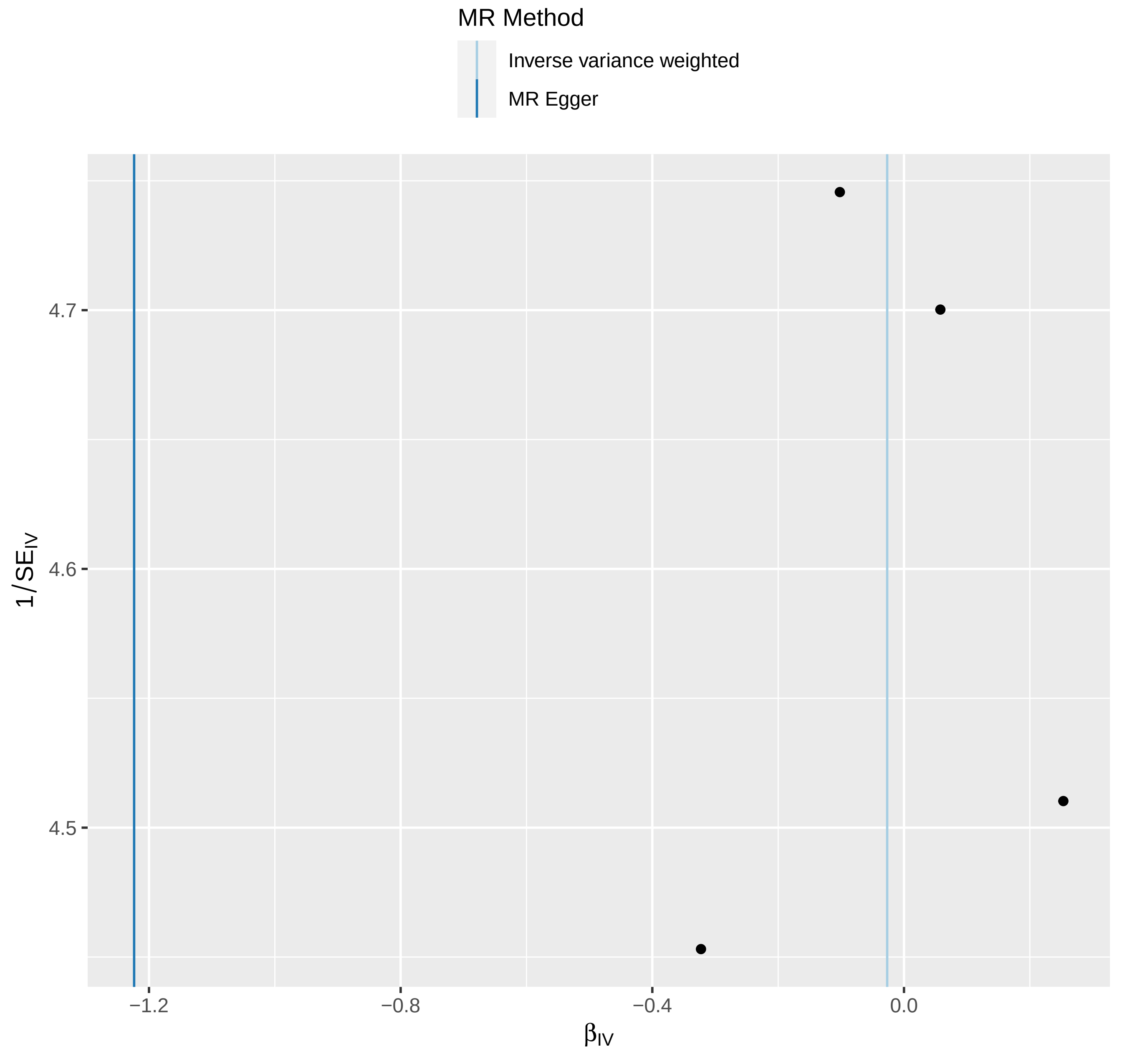

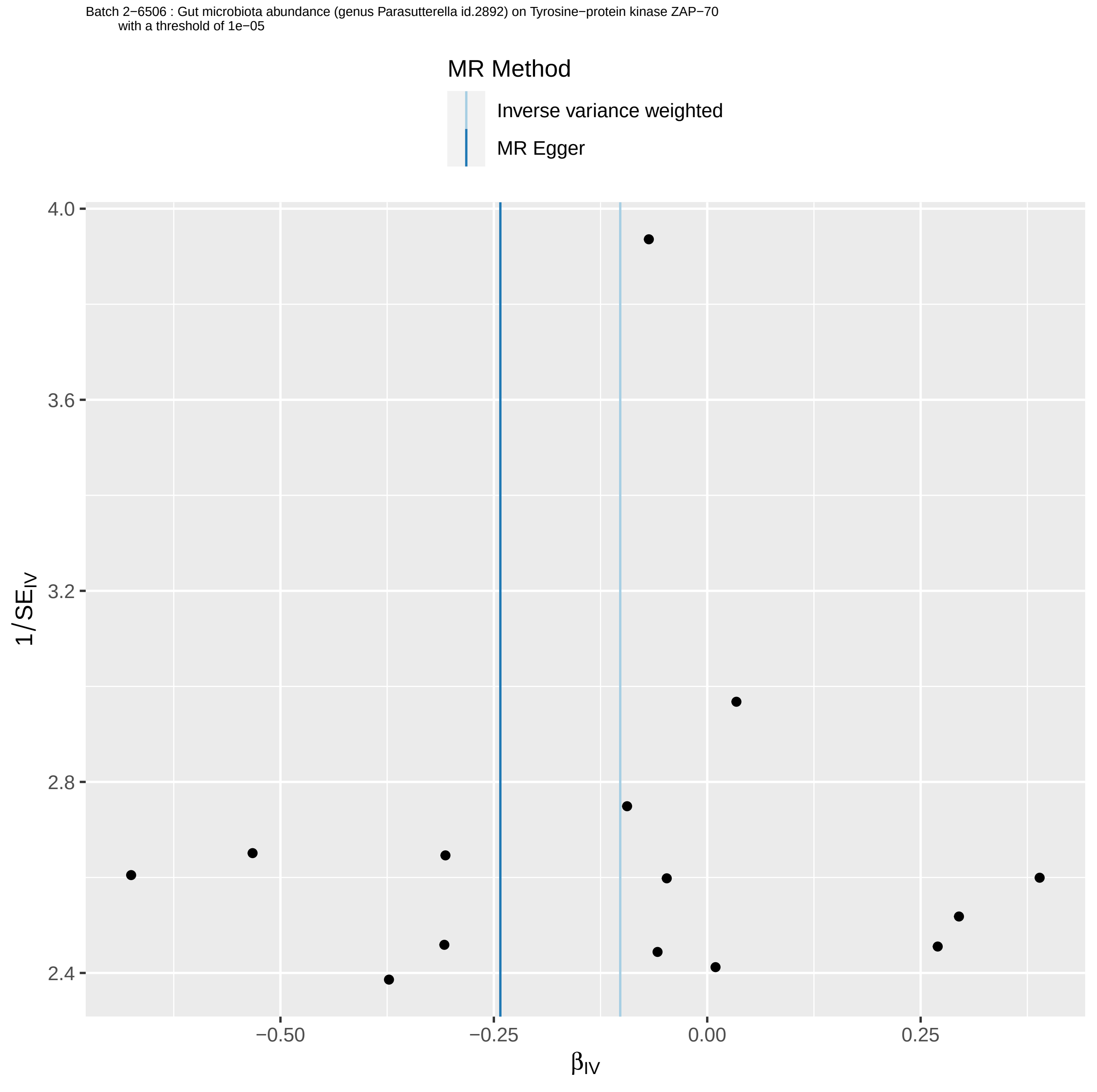

Batch 2−6506 : Gut microbiota abundance (genus Parasutterella id.2892) on Tyrosine−protein kinase ZAP−70
with a threshold of 1e−05
MR Method
Inverse variance weighted
MR Egger
4.0
3.6
3.2
2.8
2.4
1/SE_IV
−0.50
−0.25
0.00
0.25
β_IV

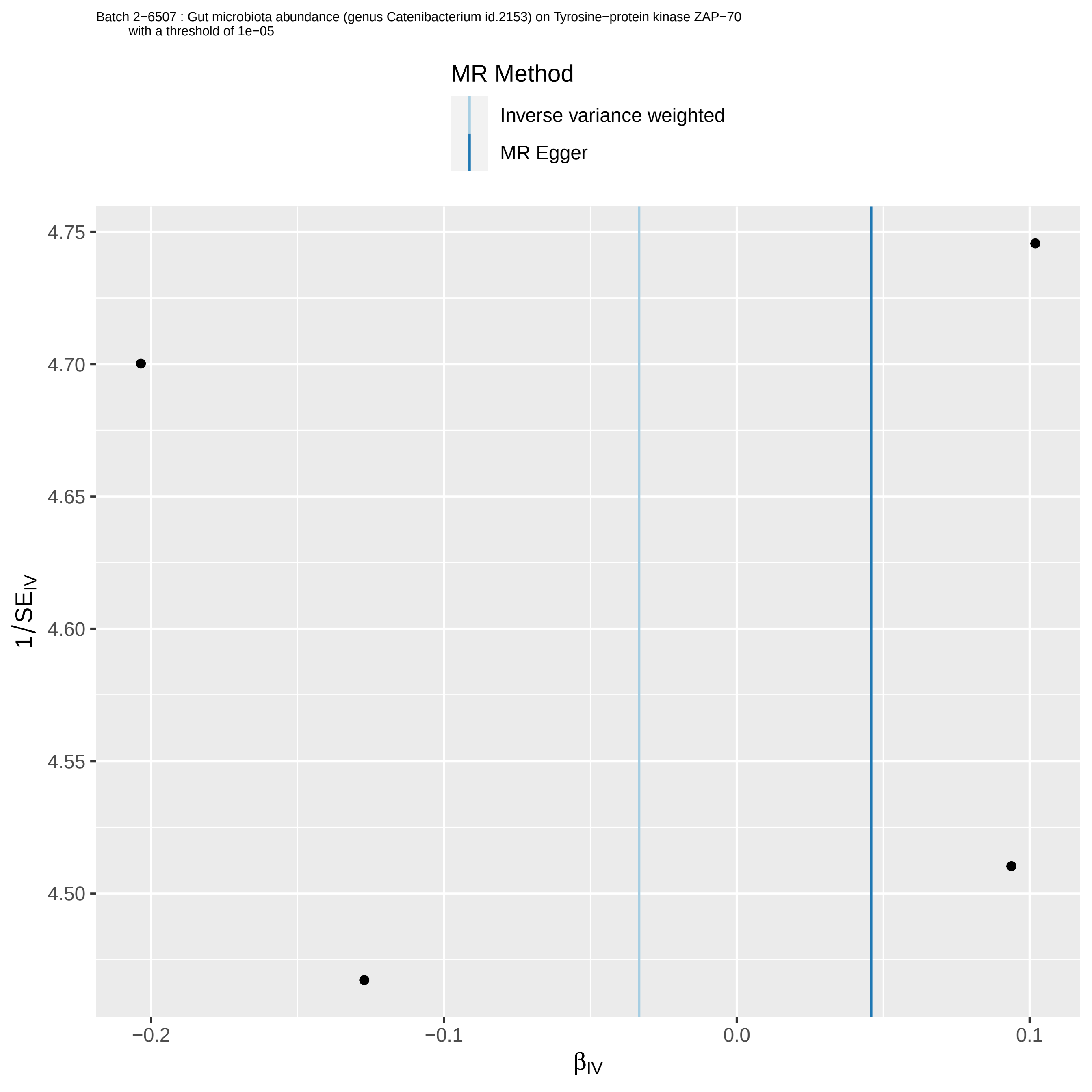
Batch 2−6507 : Gut microbiota abundance (genus Catenibacterium id.2153) on Tyrosine−protein kinase ZAP−70
with a threshold of 1e−05
MR Method
Inverse variance weighted
MR Egger
4.75
4.70
4.65
4.60
4.55
4.50
1/SE_IV
−0.2
−0.1
0.0
0.1
β_IV

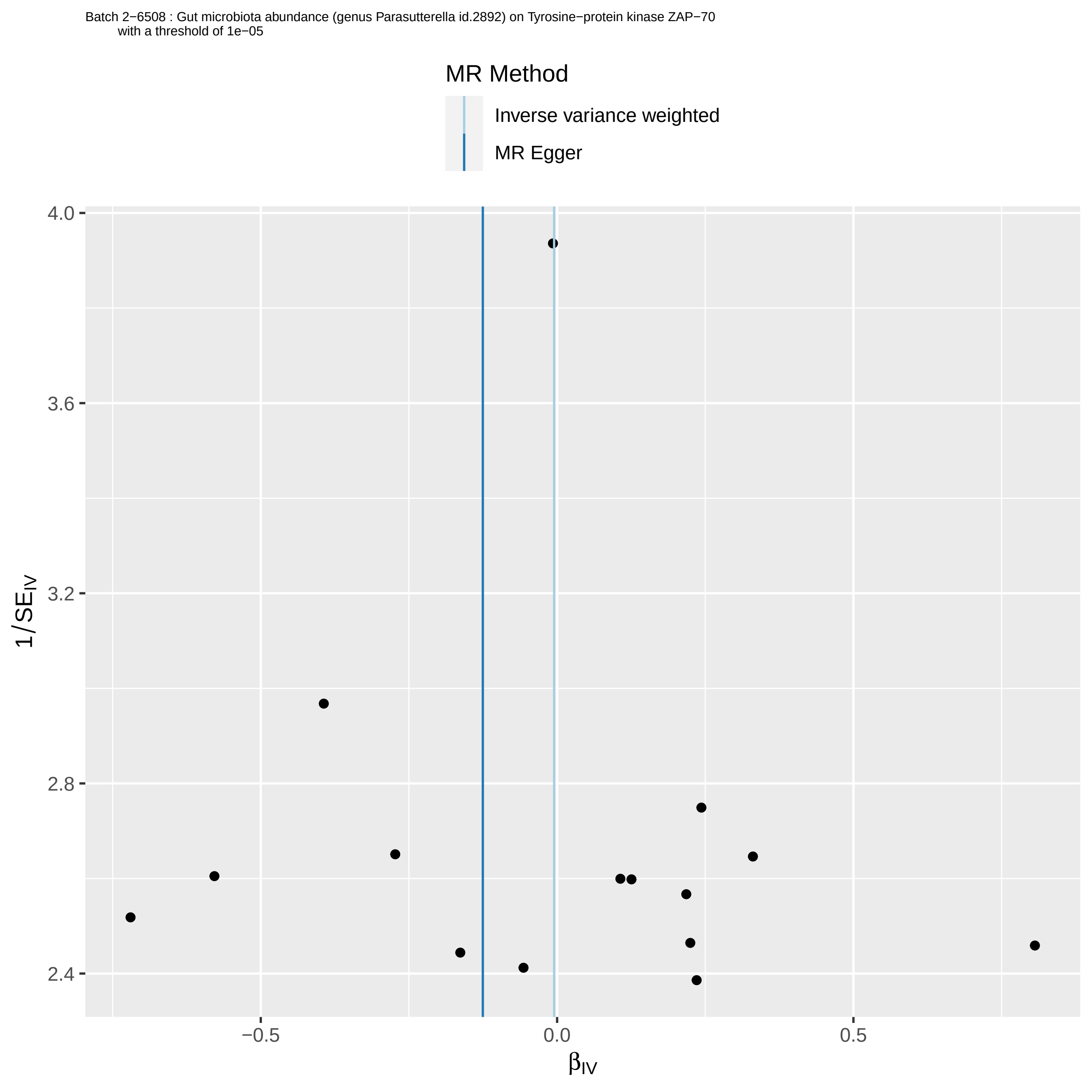

Batch 2−6508 : Gut microbiota abundance (genus Parasutterella id.2892) on Tyrosine−protein kinase ZAP−70
with a threshold of 1e−05
MR Method
Inverse variance weighted
MR Egger
4.0
3.6
3.2
2.8
2.4
1/SE$_{IV}$
−0.5
0.0
0.5
β$_{IV}$

Batch 2−6509 : Gut microbiota abundance (genus Catenibacterium id.2153) on Transcriptional regulator Kaiso
with a threshold of 1e−05

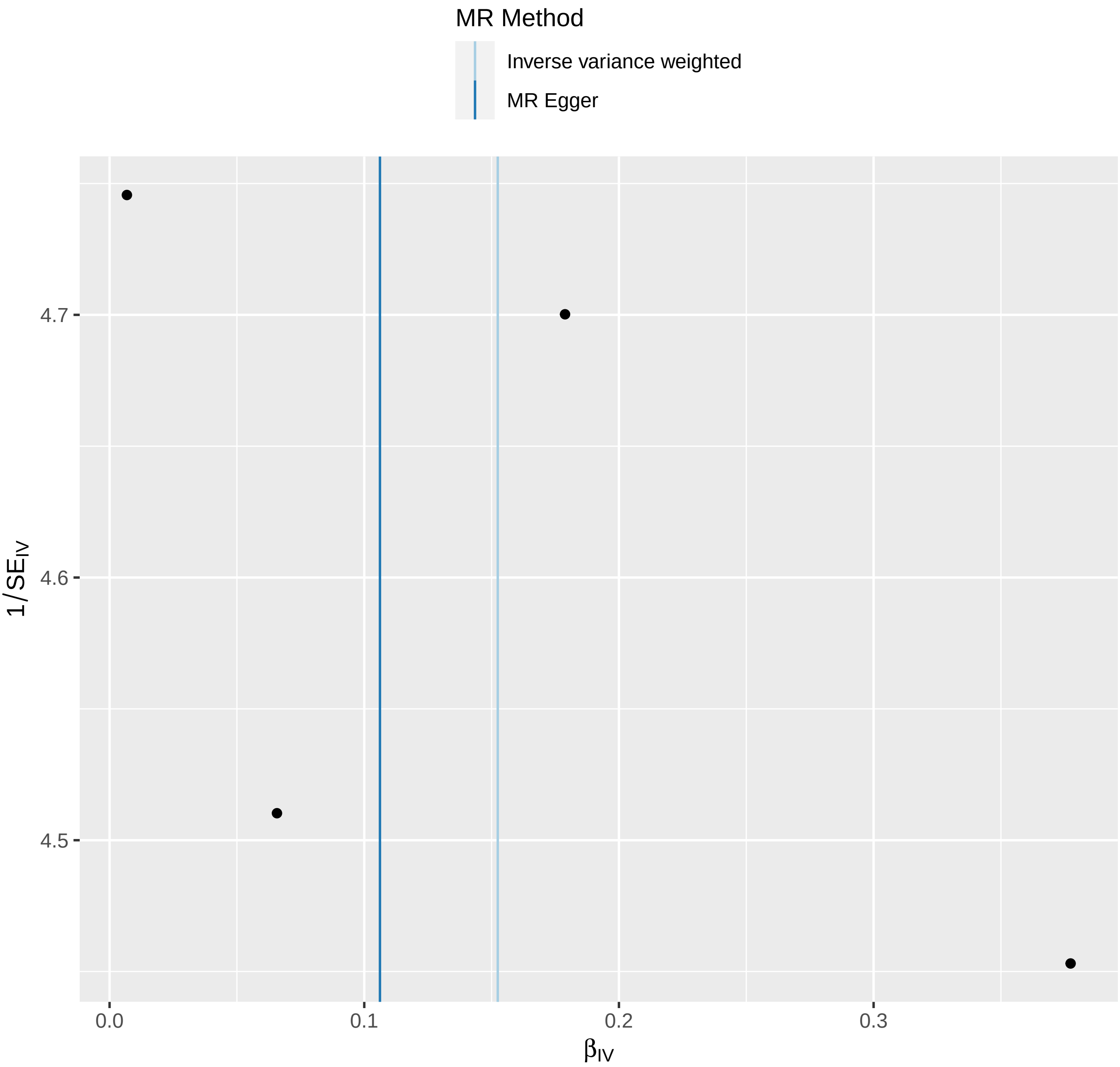

Batch 2−6510 : Gut microbiota abundance (genus Parasutterella id.2892) on Transcriptional regulator Kaiso
with a threshold of 1e−05

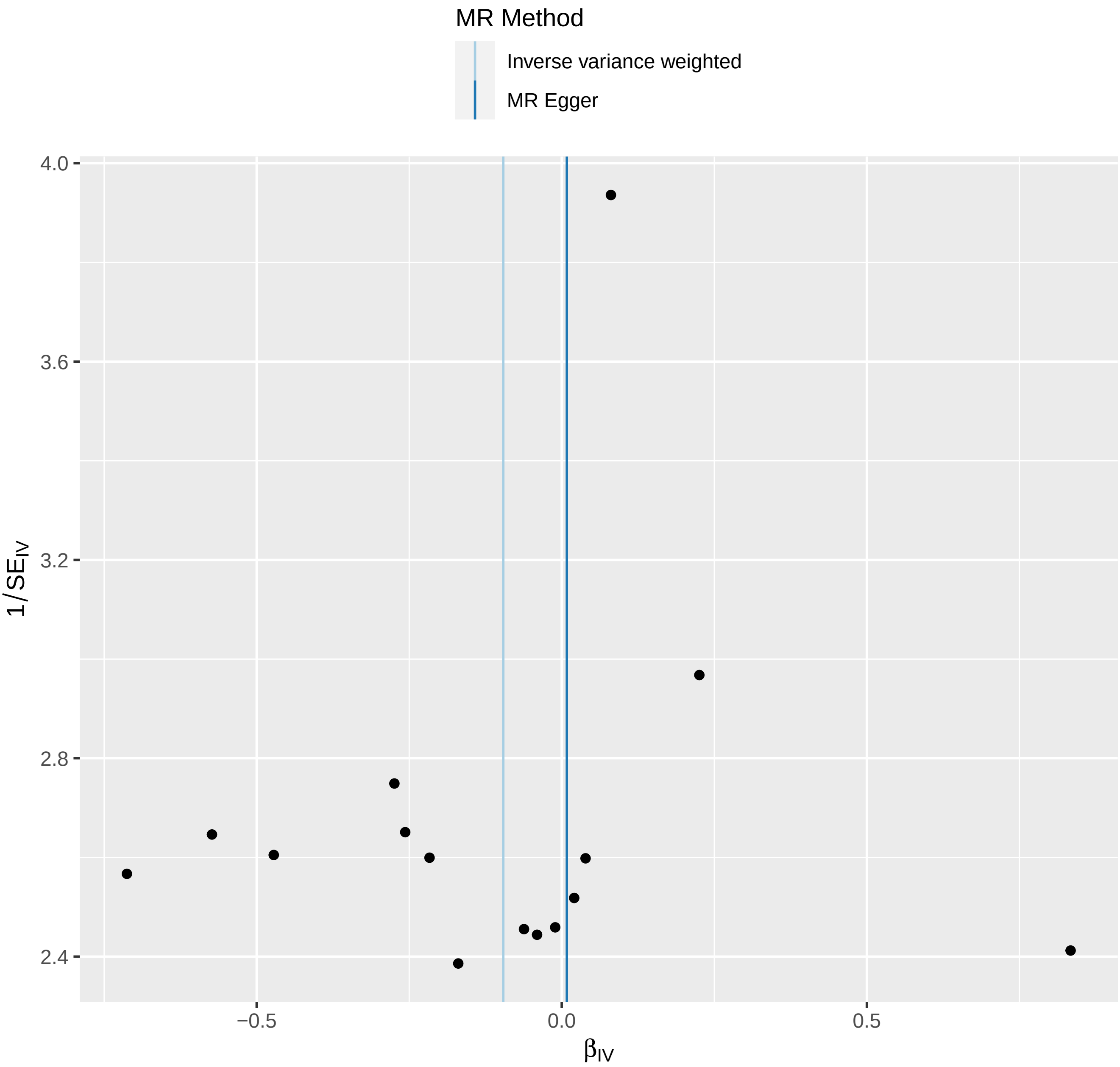

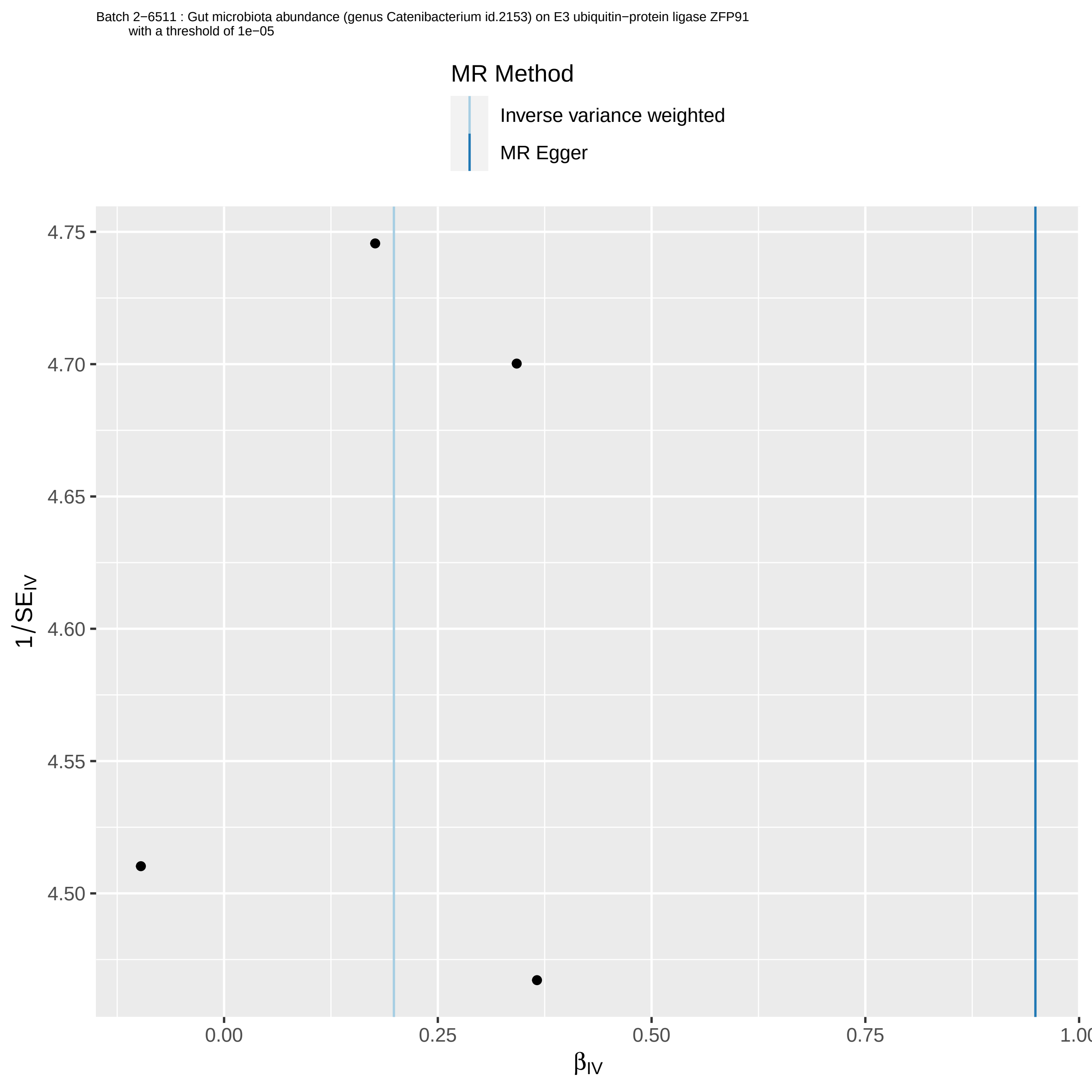

Batch 2−6511 : Gut microbiota abundance (genus Catenibacterium id.2153) on E3 ubiquitin−protein ligase ZFP91
with a threshold of 1e−05
MR Method
Inverse variance weighted
MR Egger
4.75
4.70
4.65
4.60
4.55
4.50
1/SE_IV
0.00
0.25
0.50
0.75
1.00
β_IV

Batch 2−6512 : Gut microbiota abundance (genus Parasutterella id.2892) on E3 ubiquitin−protein ligase ZFP91
with a threshold of 1e−05

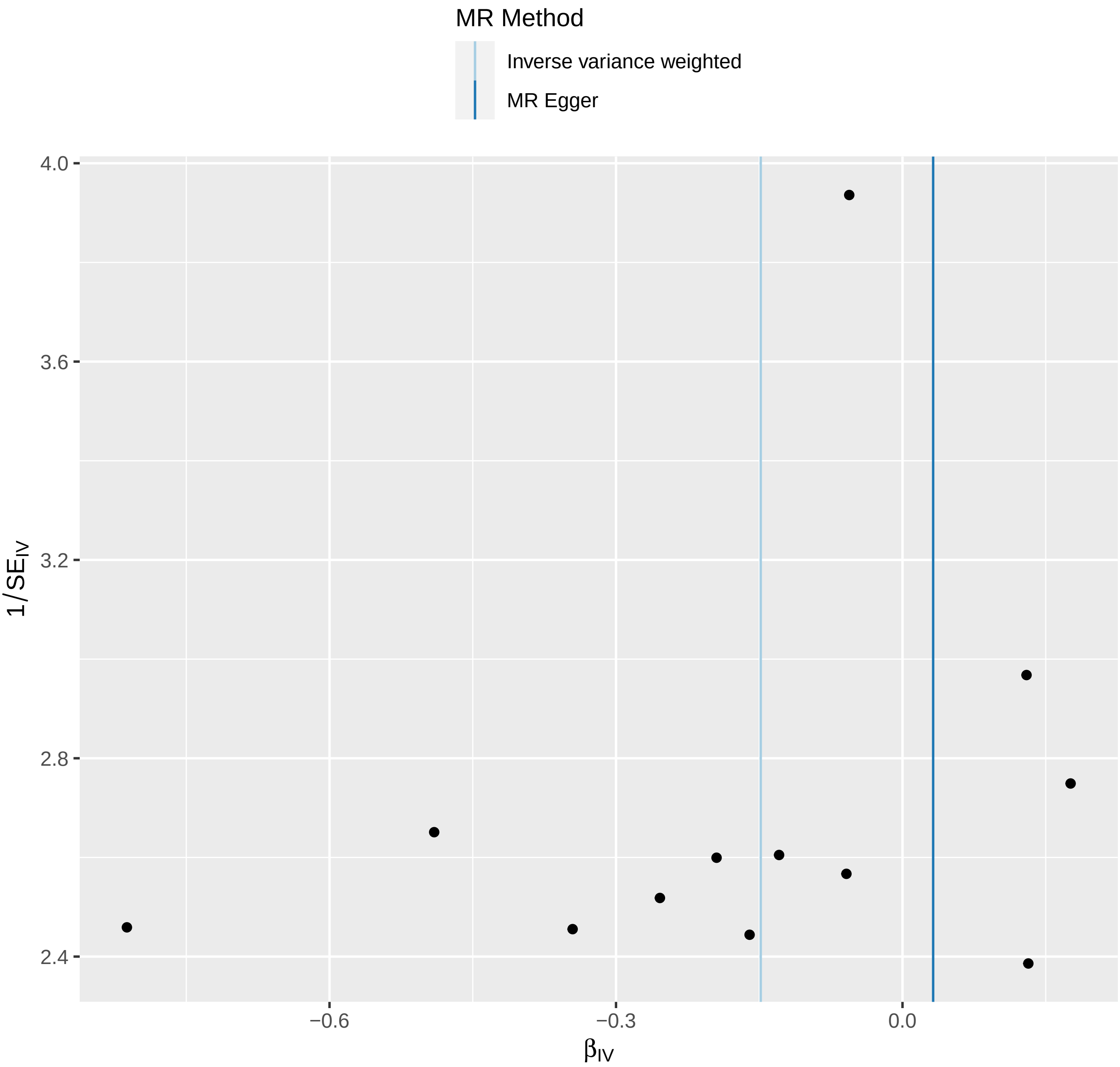

Batch 2−6513 : Gut microbiota abundance (genus Catenibacterium id.2153) on Protrudin
with a threshold of 1e−05

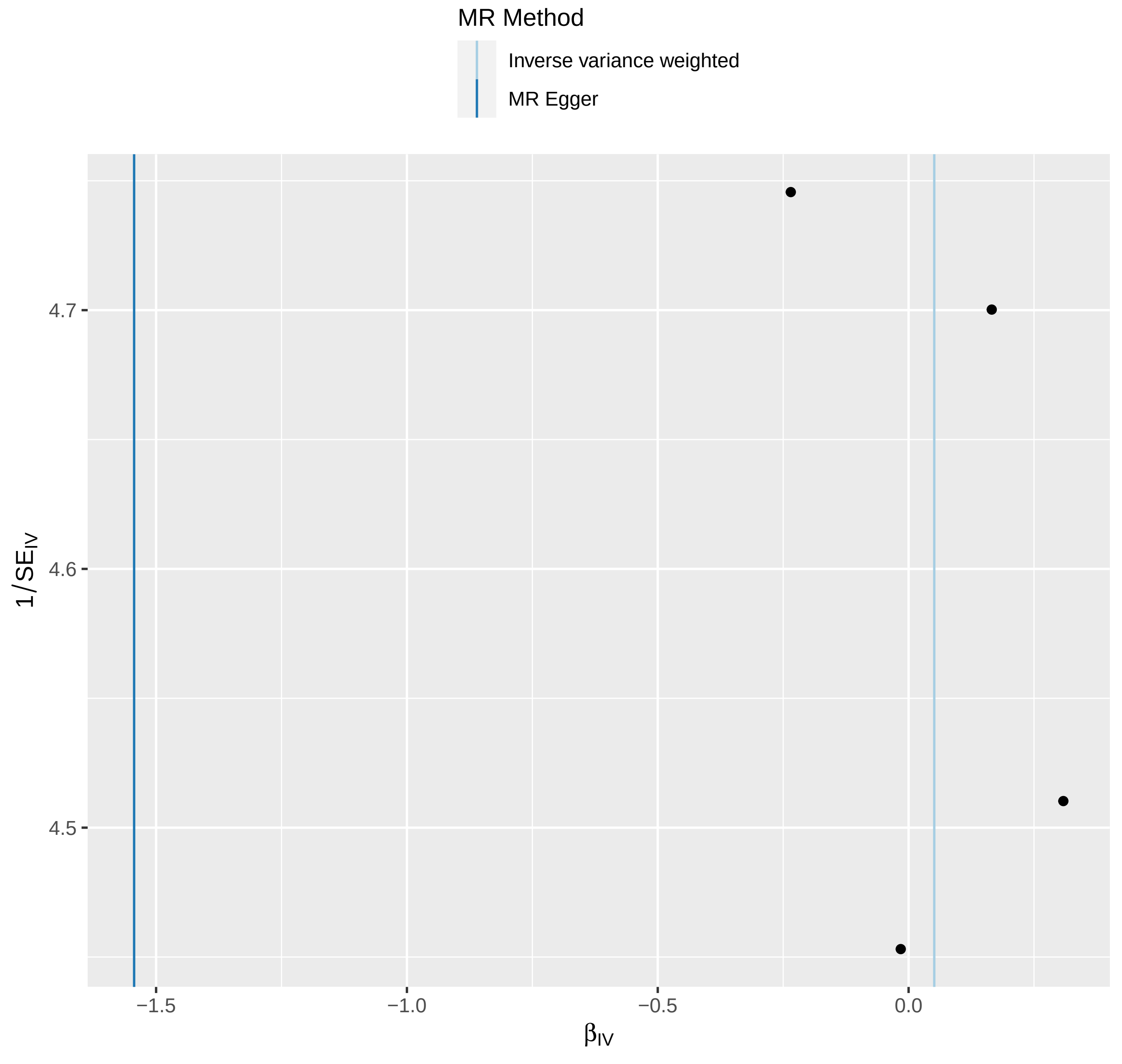

Batch 2−6514 : Gut microbiota abundance (genus Parasutterella id.2892) on Protrudin
with a threshold of 1e−05

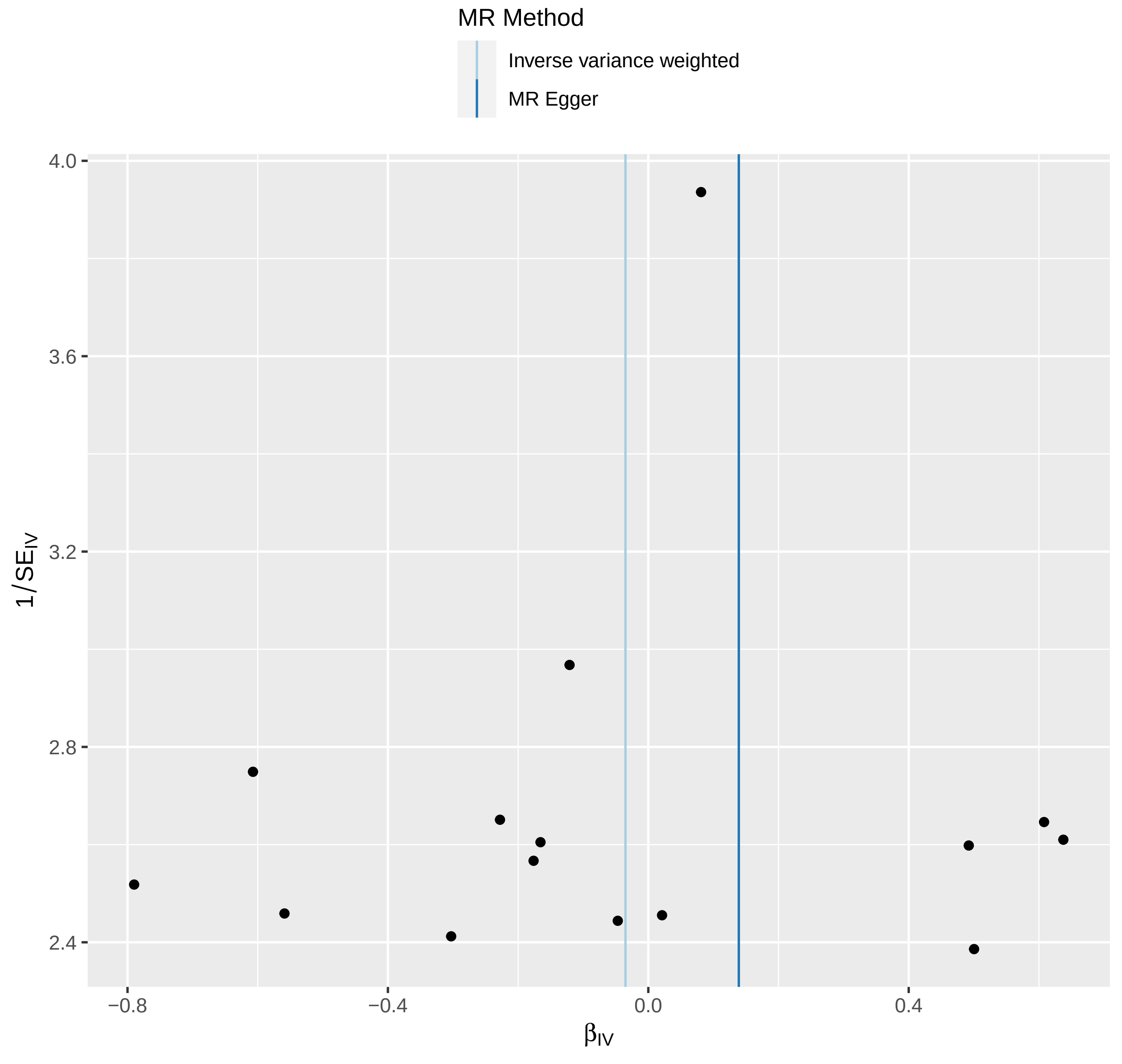

Batch 2−6515 : Gut microbiota abundance (genus Catenibacterium id.2153) on Zinc fingers and homeoboxes protein 3
with a threshold of 1e−05

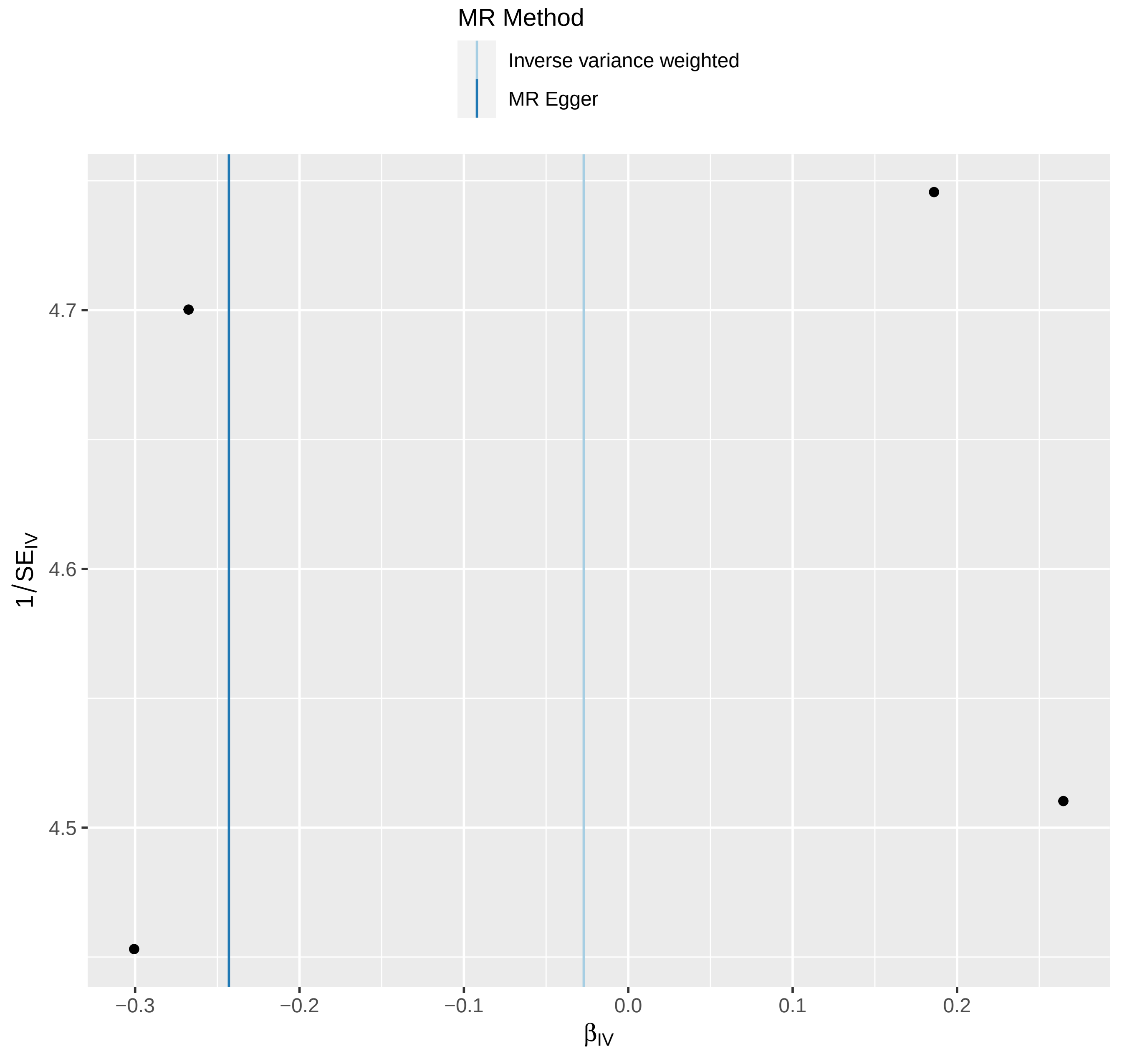

Batch 2−6516 : Gut microbiota abundance (genus Parasutterella id.2892) on Zinc fingers and homeoboxes protein 3
with a threshold of 1e−05

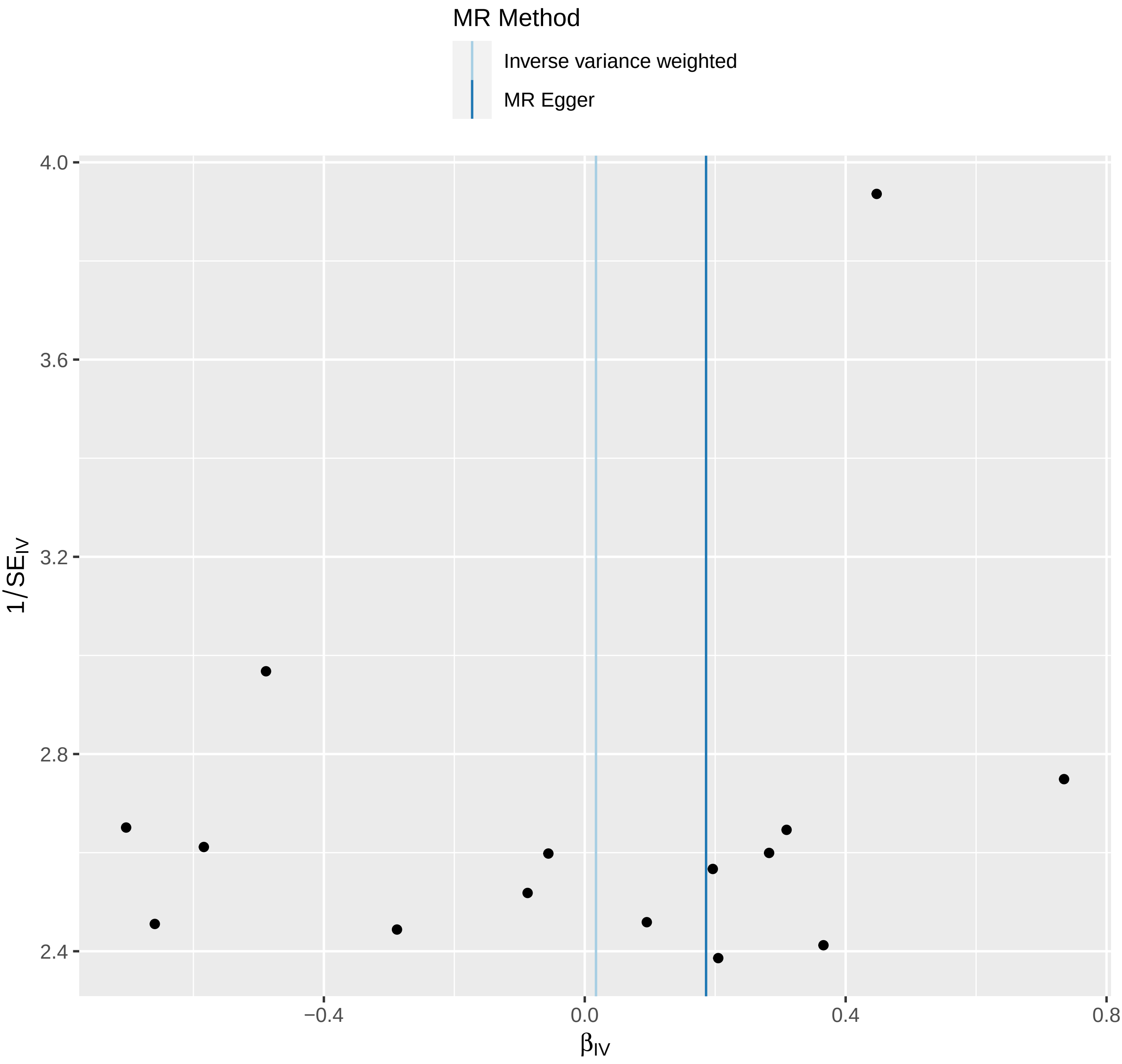

Batch 2−6517 : Gut microbiota abundance (genus Catenibacterium id.2153) on Zinc finger protein 10 with a threshold of 1e−05

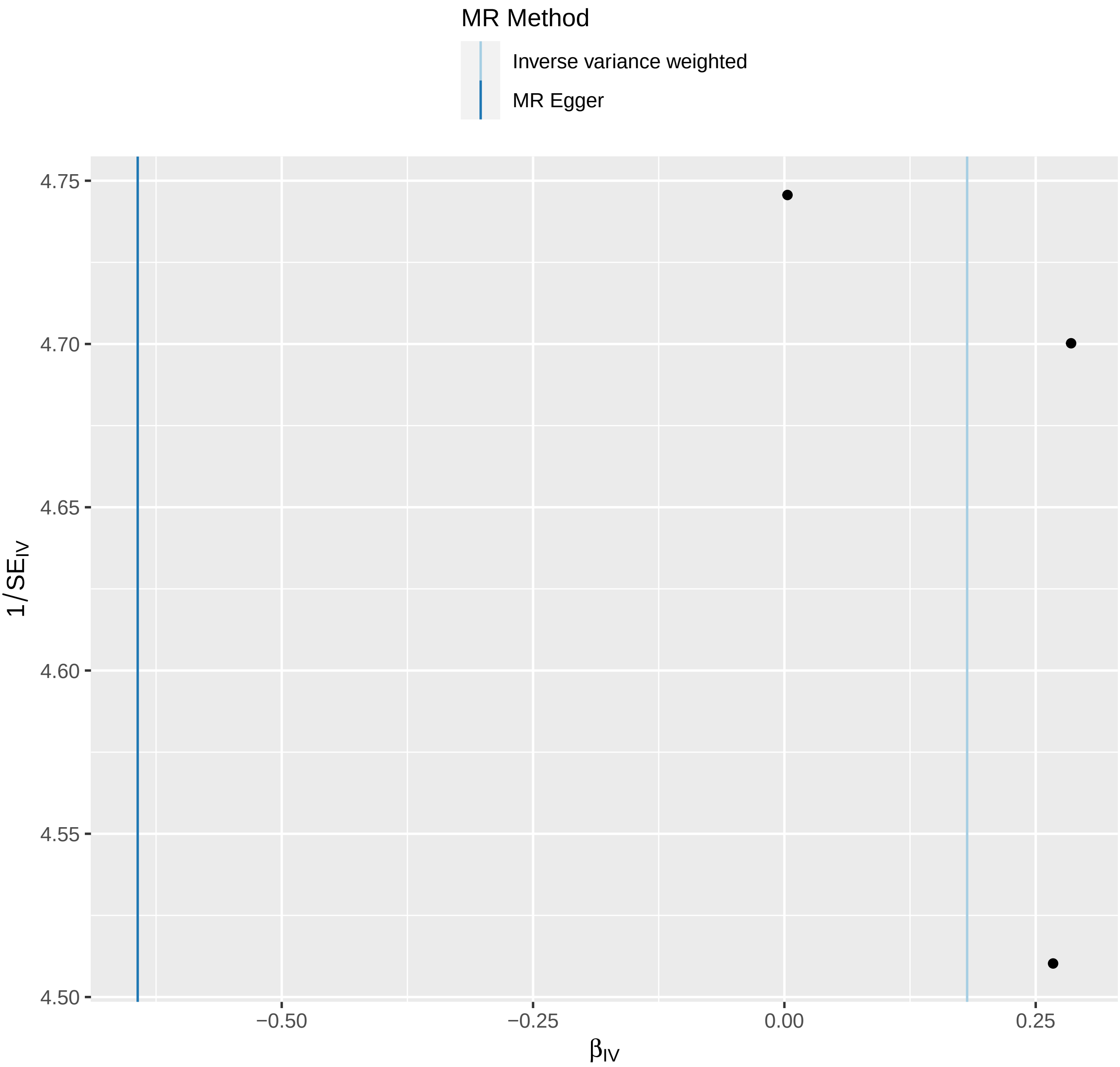

Batch 2−6518 : Gut microbiota abundance (genus Parasutterella id.2892) on Zinc finger protein 10
with a threshold of 1e−05

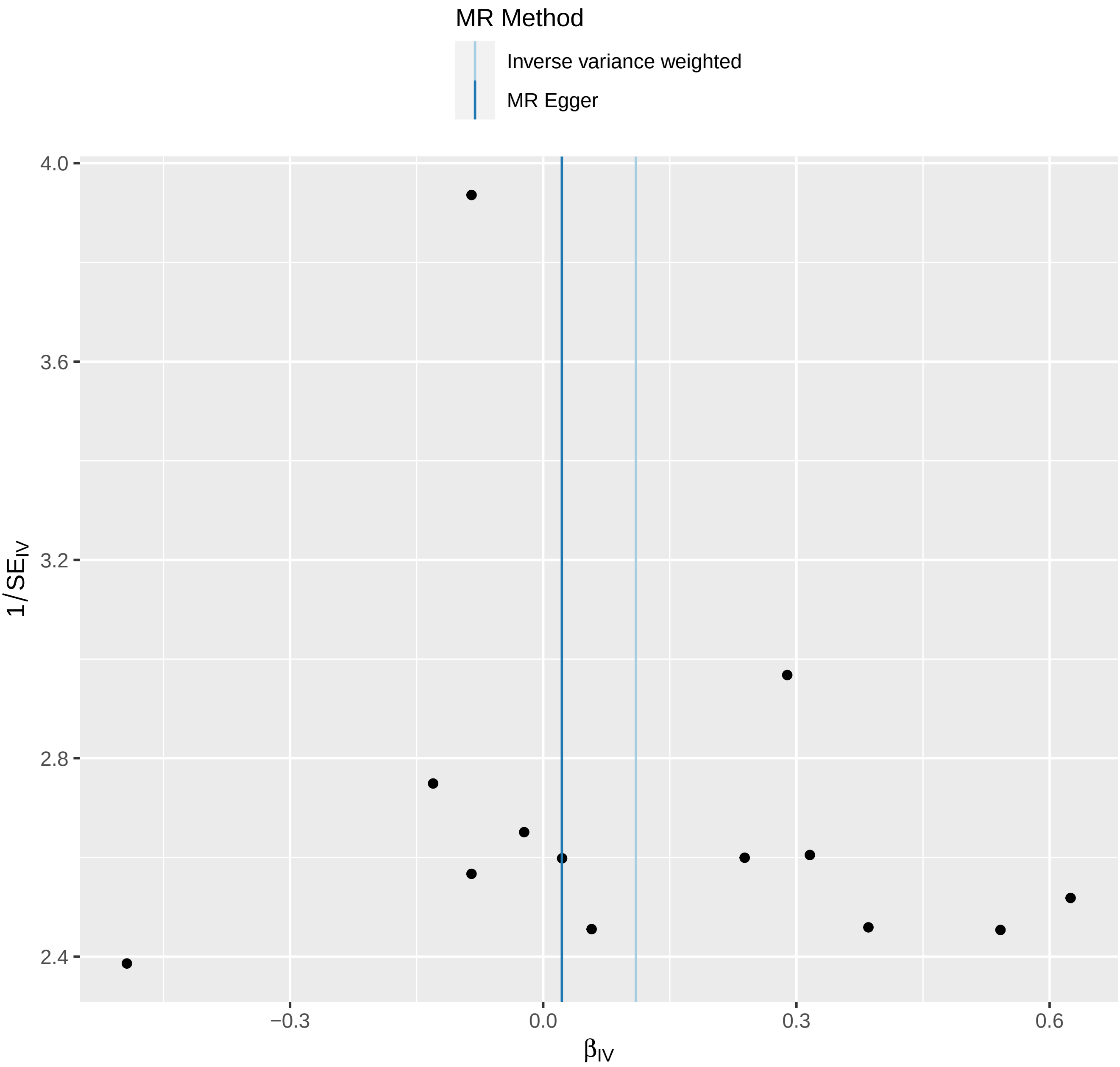

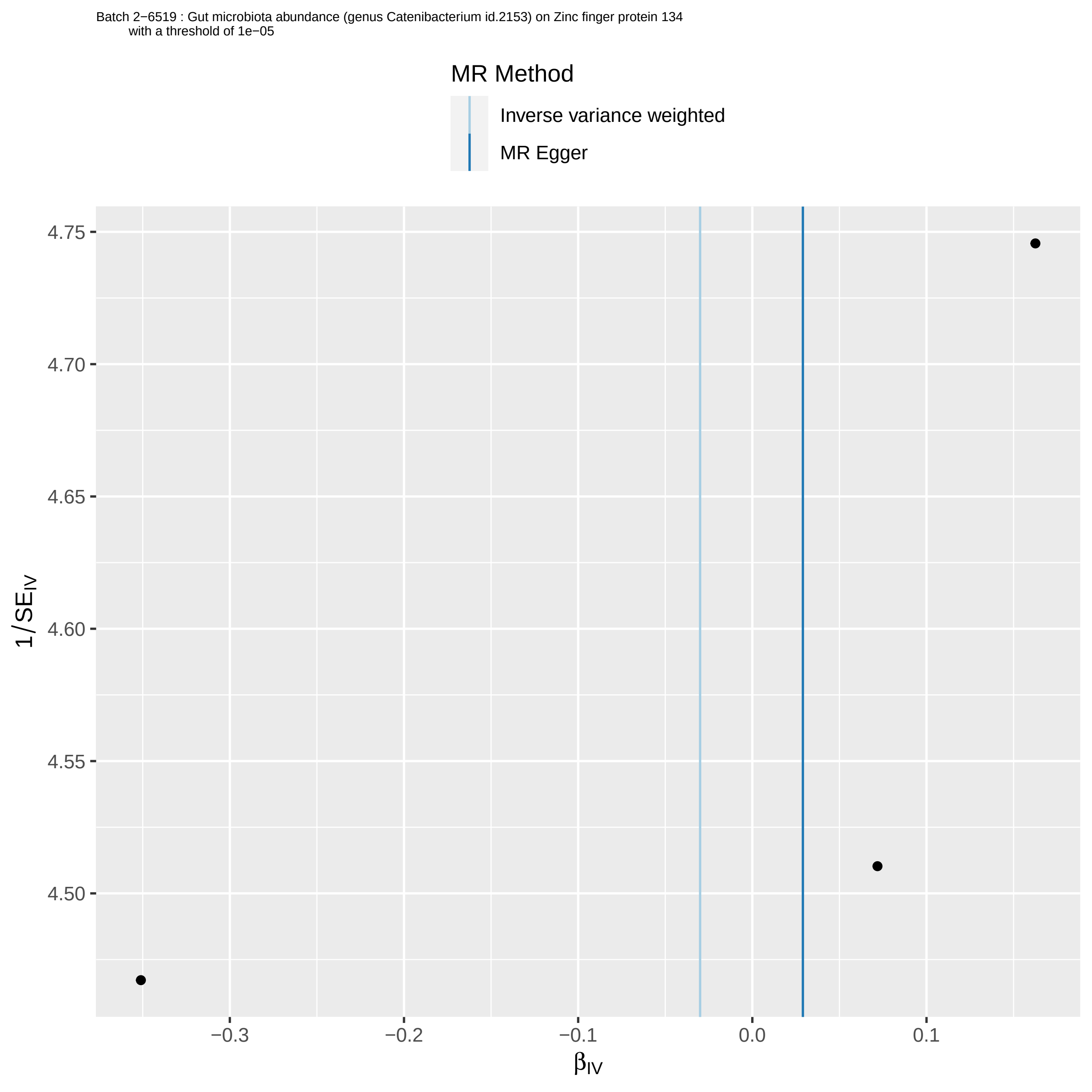
Batch 2−6519 : Gut microbiota abundance (genus Catenibacterium id.2153) on Zinc finger protein 134
with a threshold of 1e−05
MR Method
Inverse variance weighted
MR Egger
4.75
4.70
4.65
4.60
4.55
4.50
1/SE_IV
−0.3
−0.2
−0.1
0.0
0.1
β_IV

Batch 2−6520 : Gut microbiota abundance (genus Parasutterella id.2892) on Zinc finger protein 134
with a threshold of 1e−05

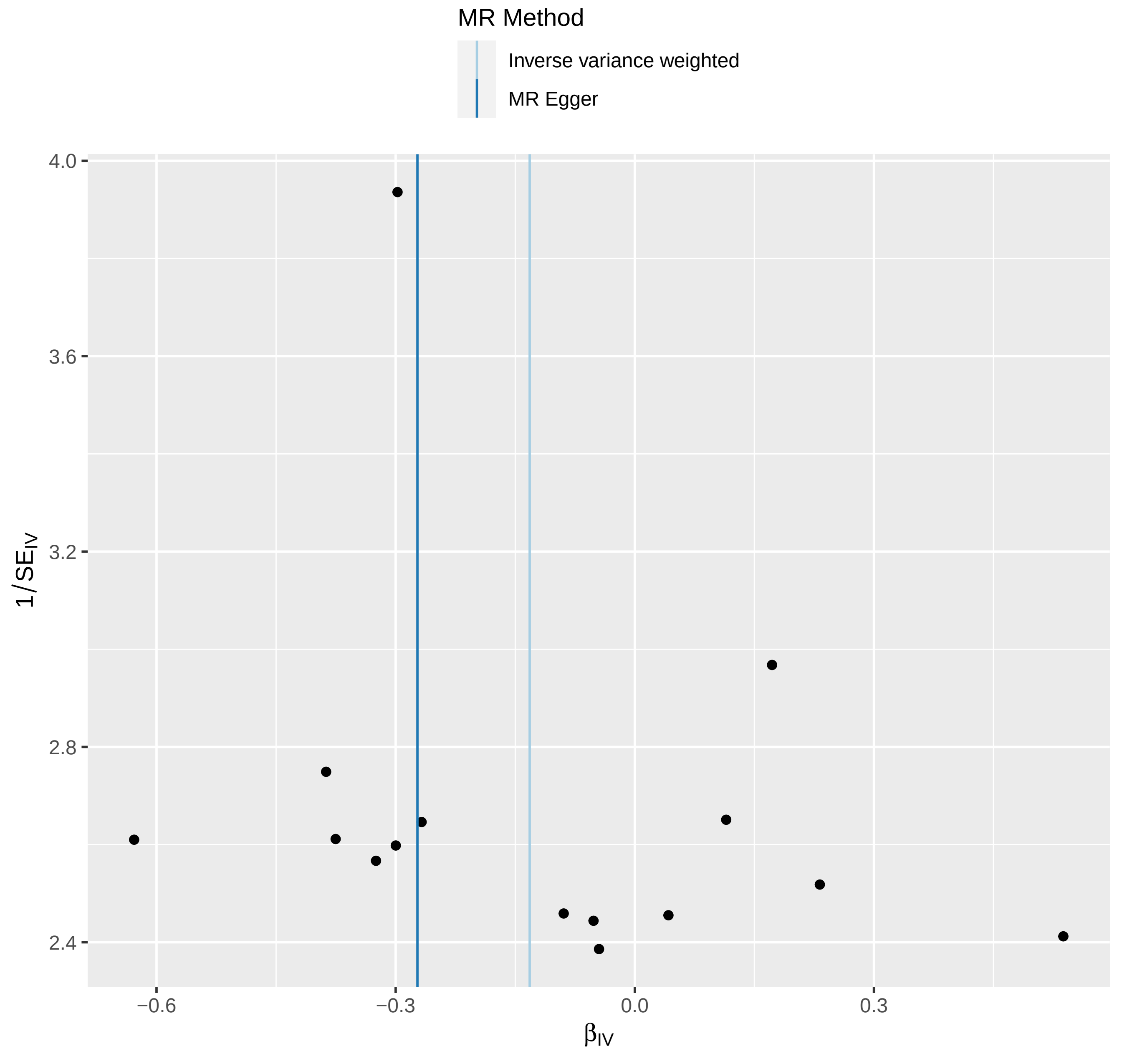

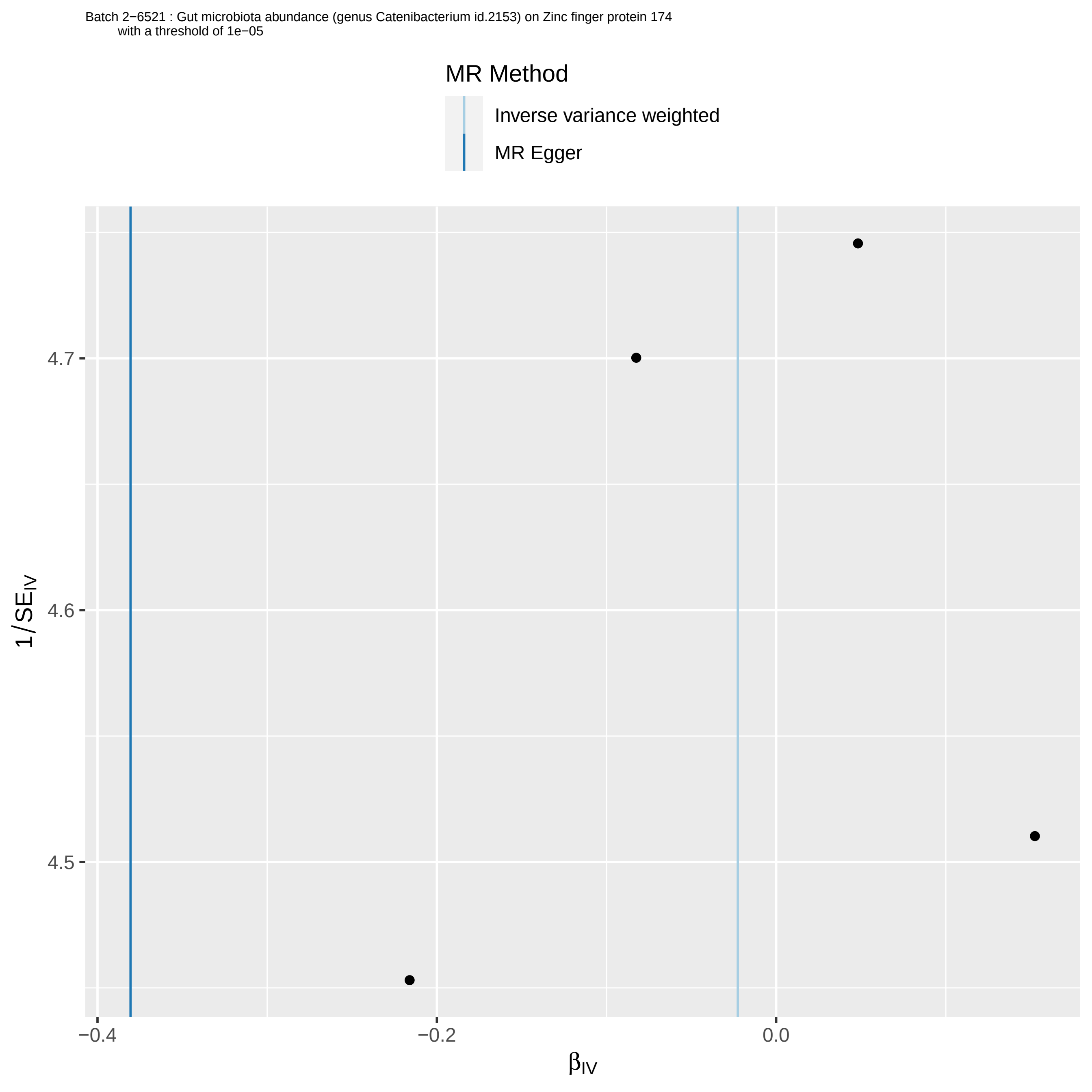

Batch 2−6521 : Gut microbiota abundance (genus Catenibacterium id.2153) on Zinc finger protein 174
with a threshold of 1e−05
MR Method
Inverse variance weighted
MR Egger
4.7
4.6
4.5
1/SE_IV
−0.4
−0.2
0.0
β_IV

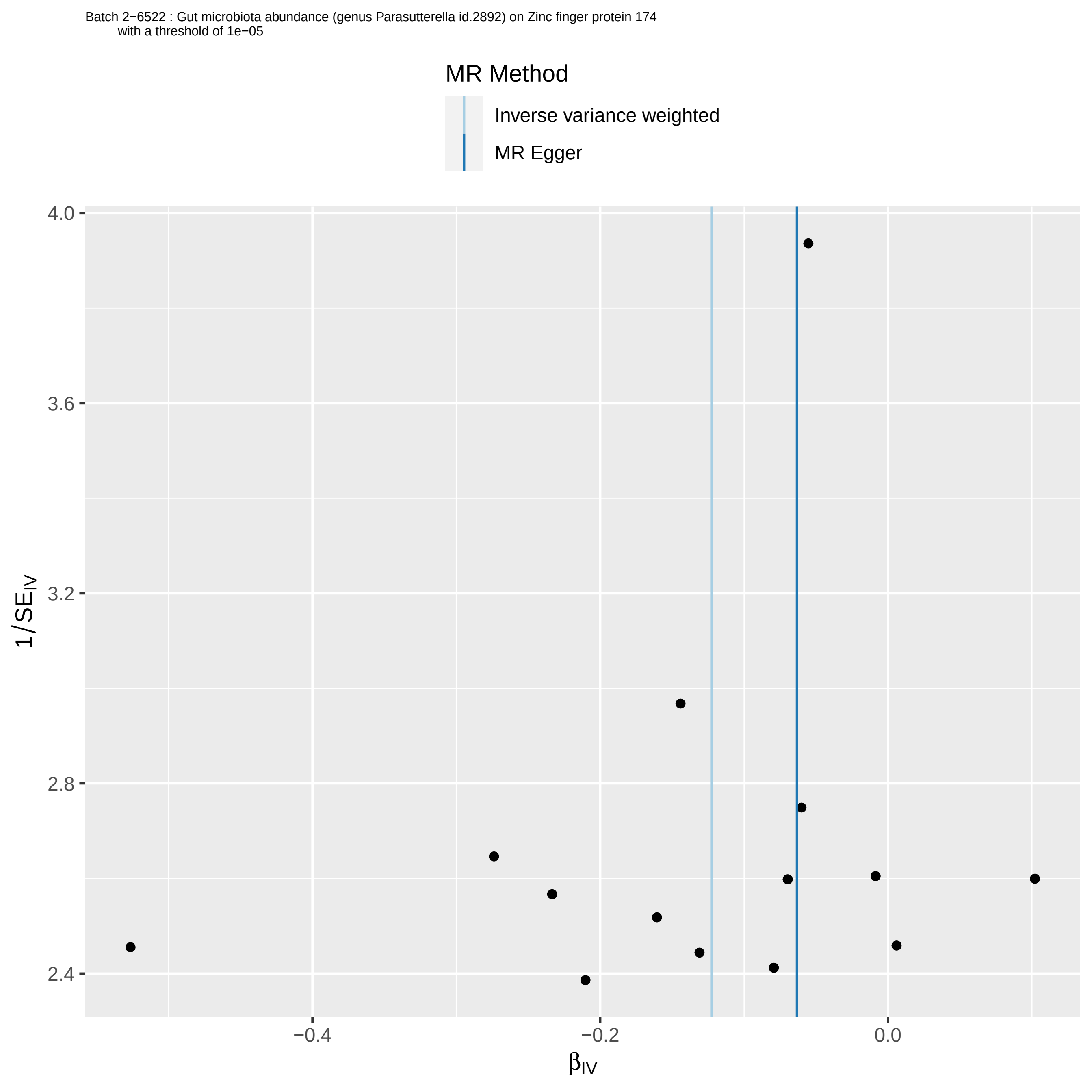

Batch 2−6522 : Gut microbiota abundance (genus Parasutterella id.2892) on Zinc finger protein 174
with a threshold of 1e−05
MR Method
Inverse variance weighted
MR Egger
4.0
3.6
3.2
2.8
2.4
1/SE_IV
−0.4
−0.2
0.0
β_IV

Batch 2−6523 : Gut microbiota abundance (genus Catenibacterium id.2153) on Zinc finger protein 175
with a threshold of 1e−05

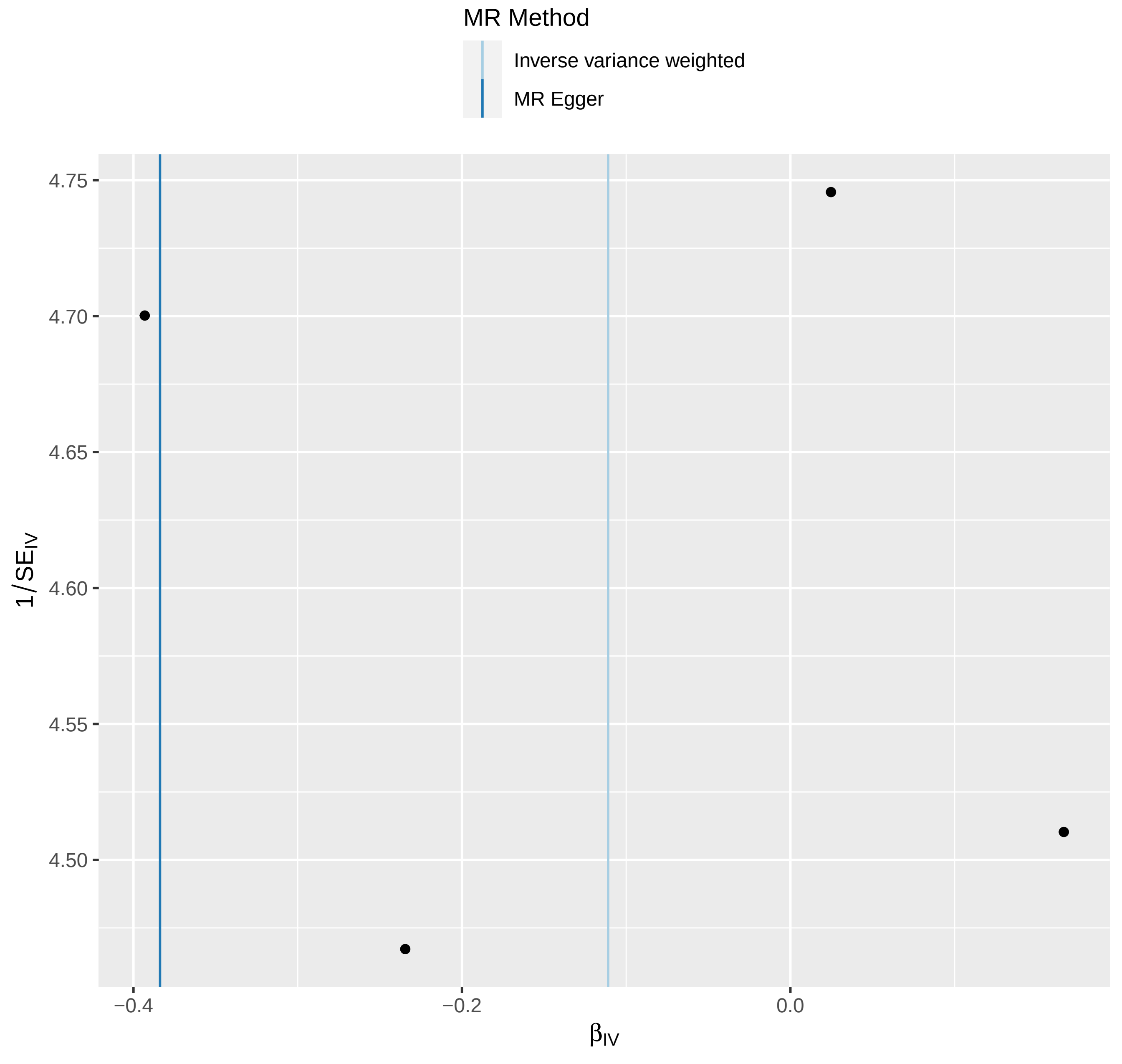

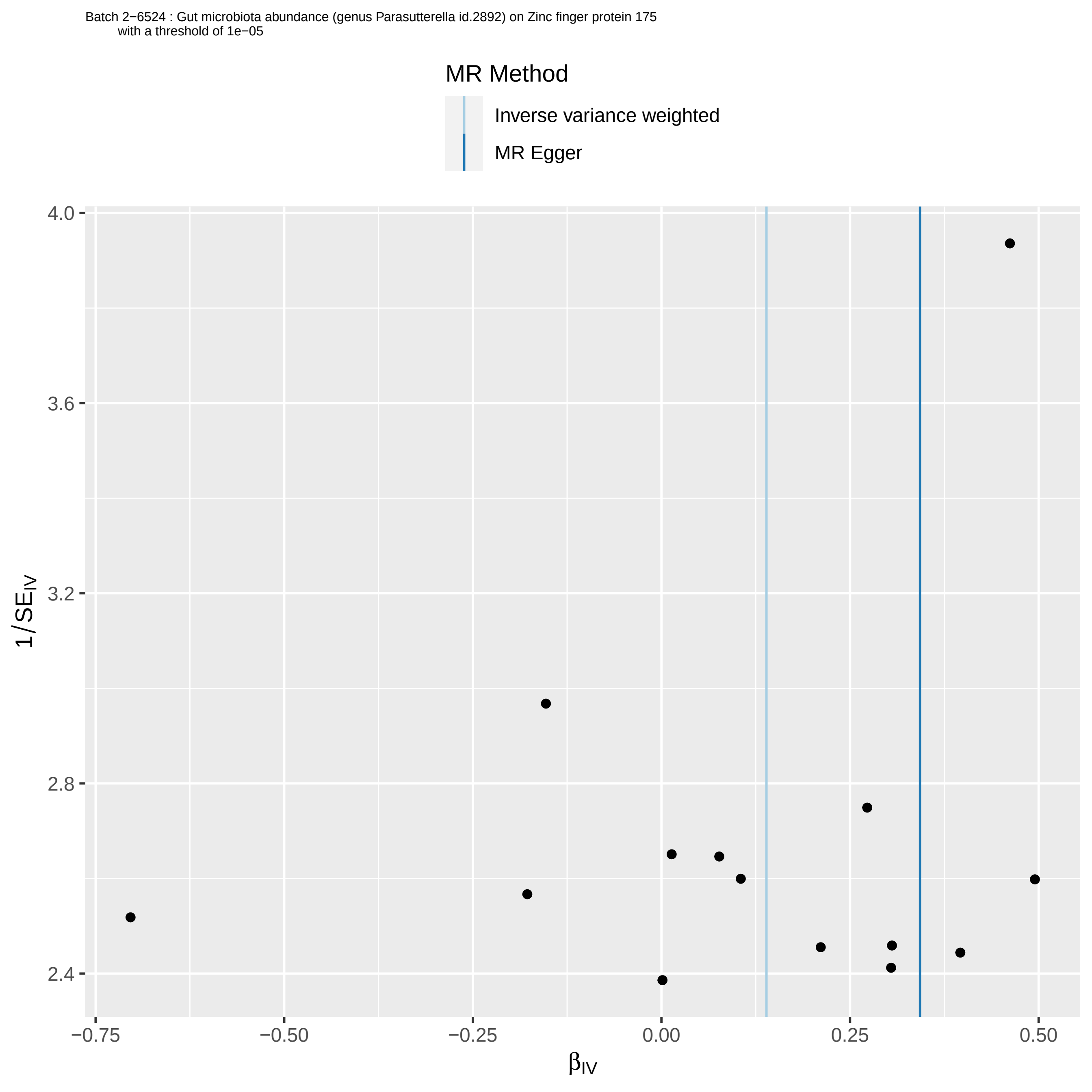

Batch 2−6524 : Gut microbiota abundance (genus Parasutterella id.2892) on Zinc finger protein 175
with a threshold of 1e−05
MR Method
Inverse variance weighted
MR Egger
4.0
3.6
3.2
2.8
2.4
1/SE$_{IV}$
−0.75
−0.50
−0.25
0.00
0.25
0.50
β$_{IV}$

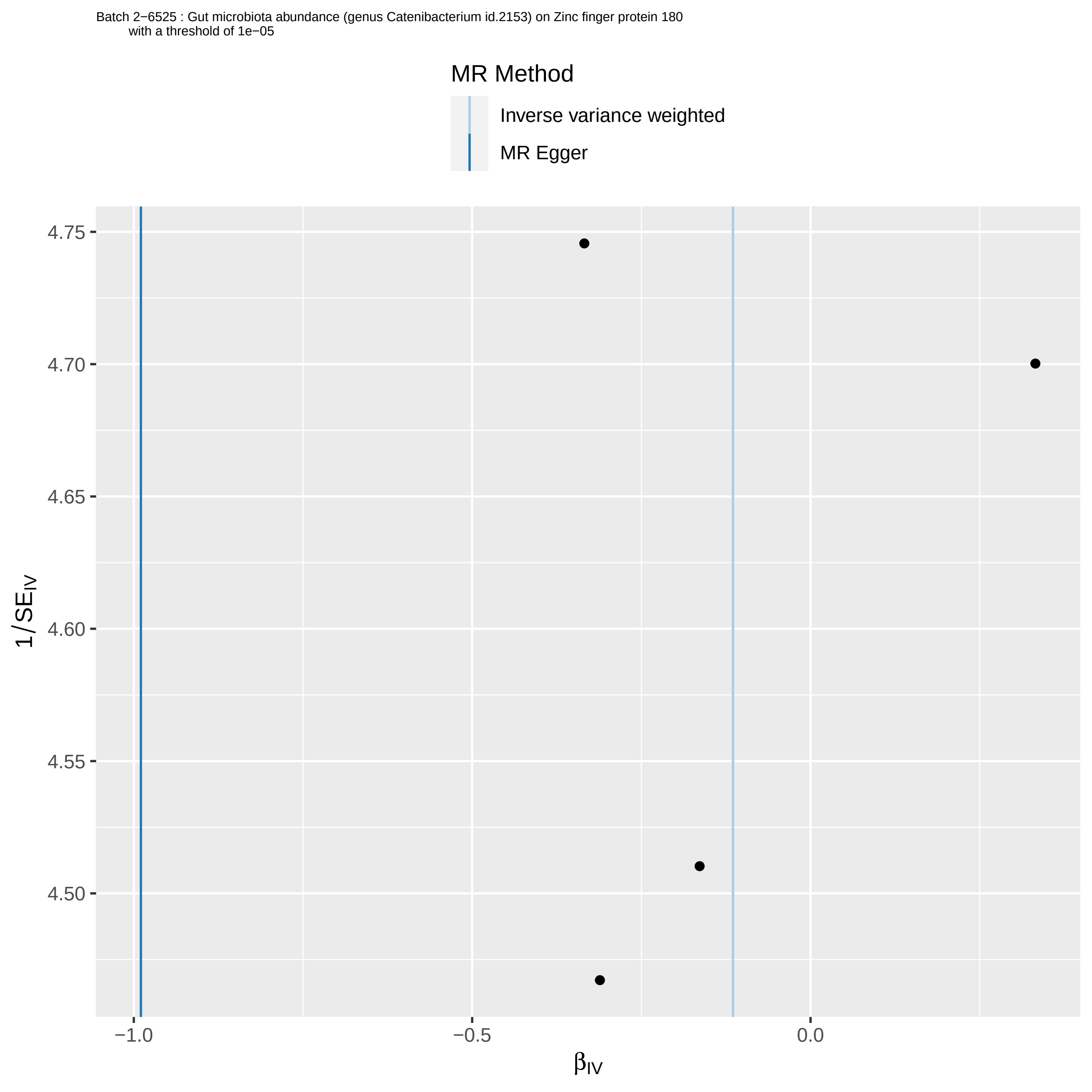

Batch 2−6525 : Gut microbiota abundance (genus Catenibacterium id.2153) on Zinc finger protein 180
with a threshold of 1e−05
MR Method
Inverse variance weighted
MR Egger
4.75
4.70
4.65
4.60
4.55
4.50
1/SE_IV
−1.0
−0.5
0.0
β_IV

Batch 2−6526 : Gut microbiota abundance (genus Parasutterella id.2892) on Zinc finger protein 180
with a threshold of 1e−05

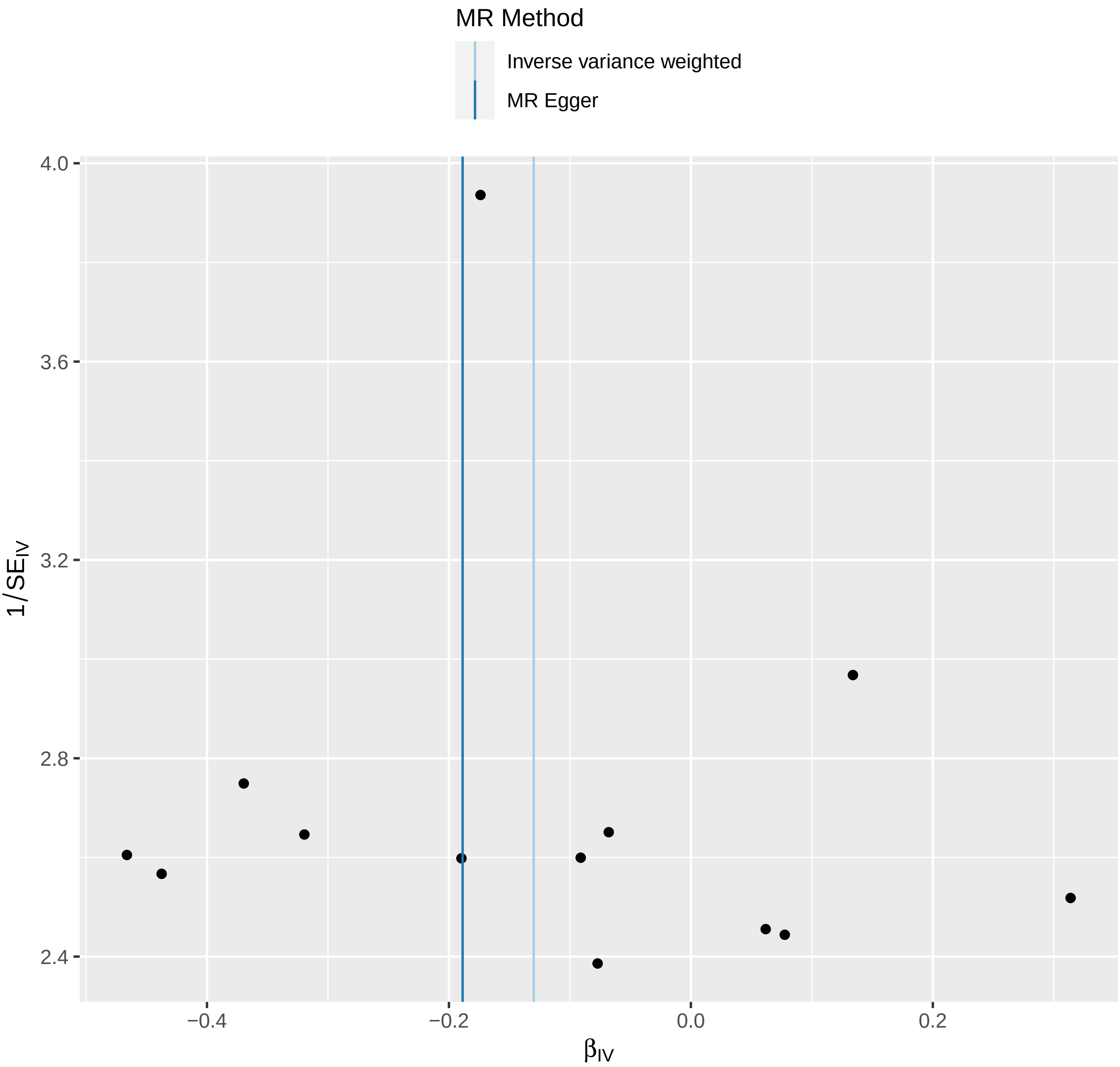

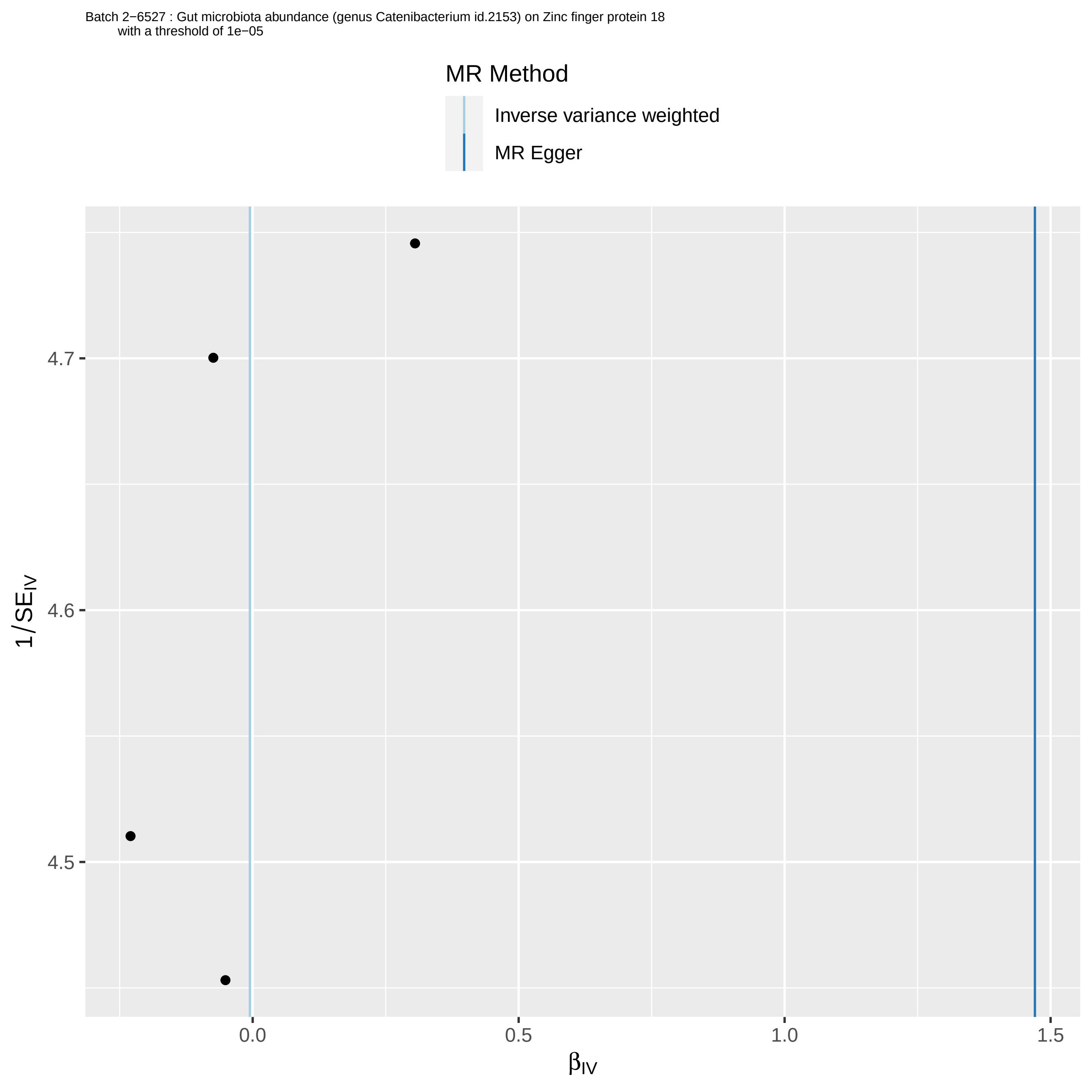
Batch 2−6527 : Gut microbiota abundance (genus Catenibacterium id.2153) on Zinc finger protein 18
with a threshold of 1e−05
MR Method
Inverse variance weighted
MR Egger
4.7
4.6
4.5
1/SE_IV
0.0
0.5
1.0
1.5
β_IV

Batch 2−6528 : Gut microbiota abundance (genus Parasutterella id.2892) on Zinc finger protein 18
with a threshold of 1e−05

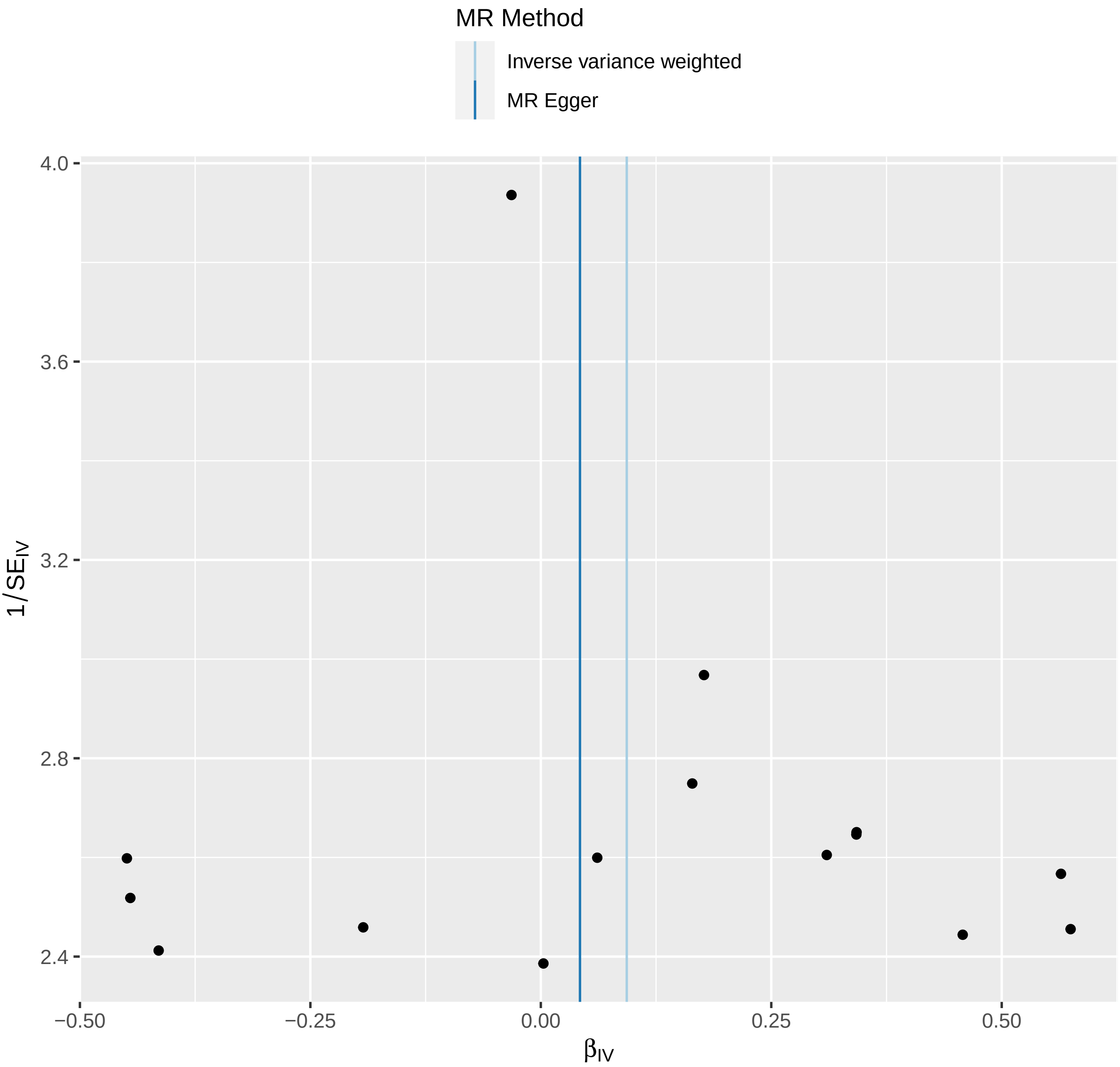

Batch 2−6529 : Gut microbiota abundance (genus Catenibacterium id.2153) on Zinc finger protein 23
with a threshold of 1e−05

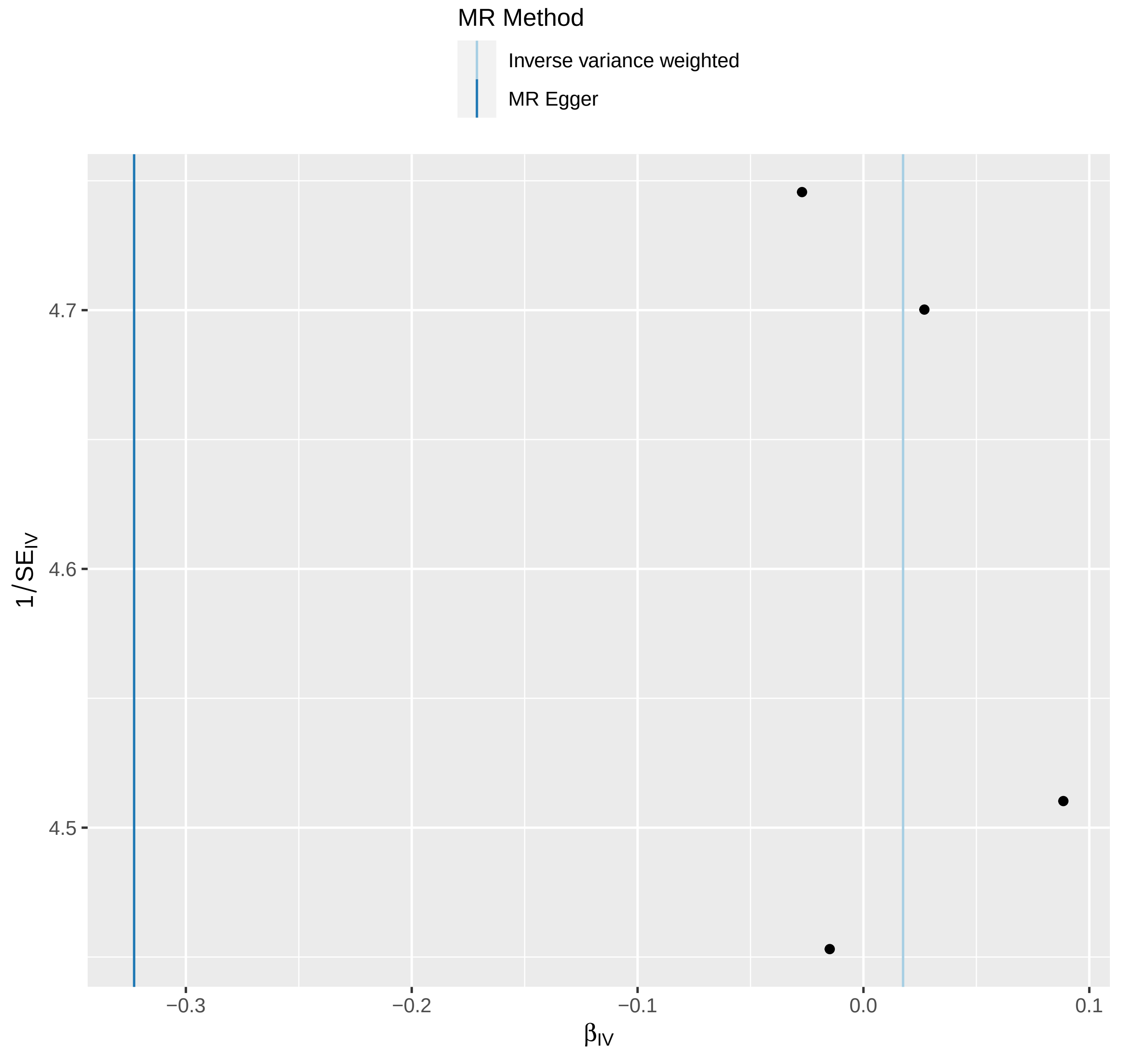

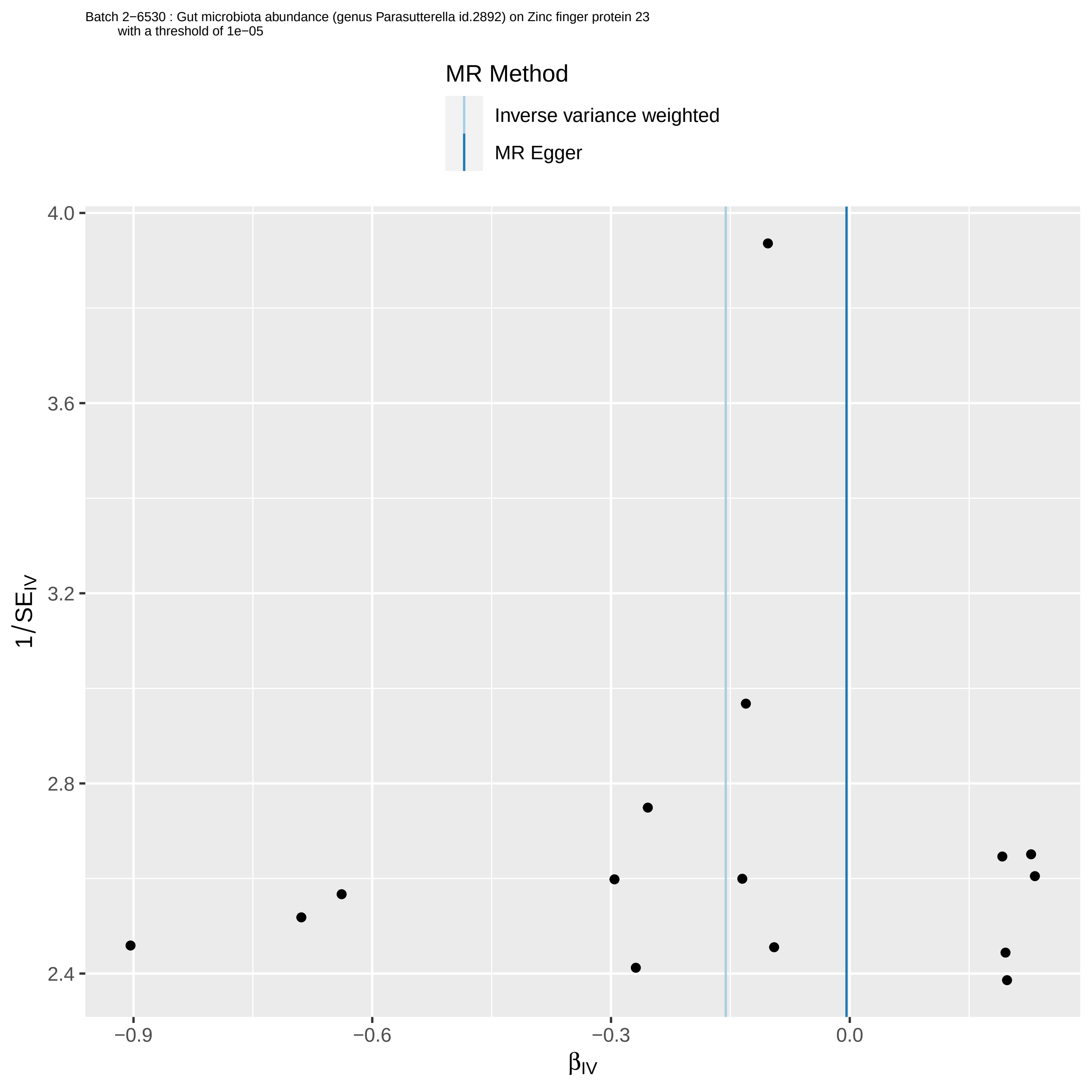

Batch 2−6530 : Gut microbiota abundance (genus Parasutterella id.2892) on Zinc finger protein 23
with a threshold of 1e−05
MR Method
Inverse variance weighted
MR Egger
4.0
3.6
3.2
2.8
2.4
1/SE_IV
−0.9
−0.6
−0.3
0.0
β_IV

Batch 2−6531 : Gut microbiota abundance (genus Catenibacterium id.2153) on Zinc finger protein 264
with a threshold of 1e−05

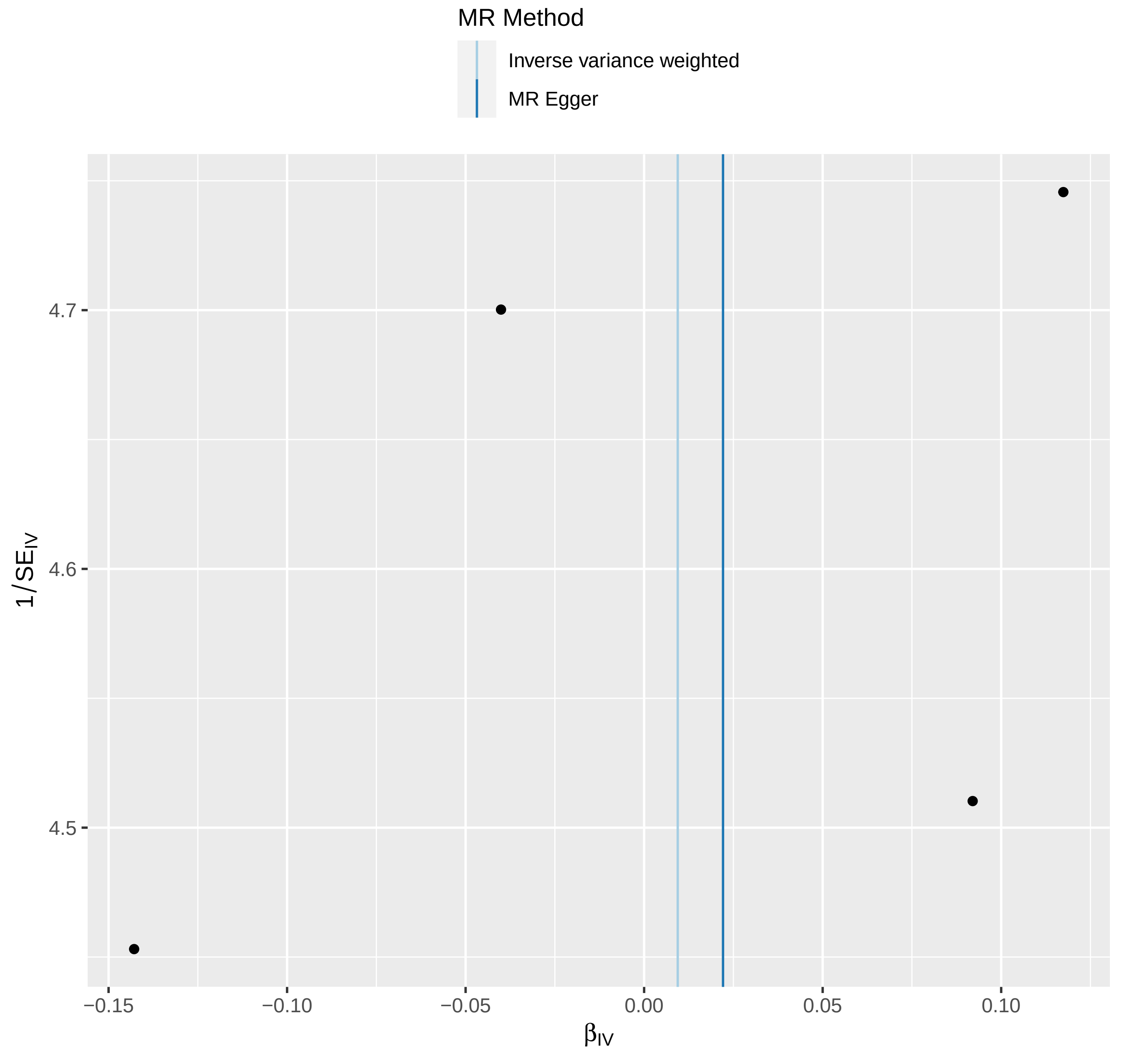

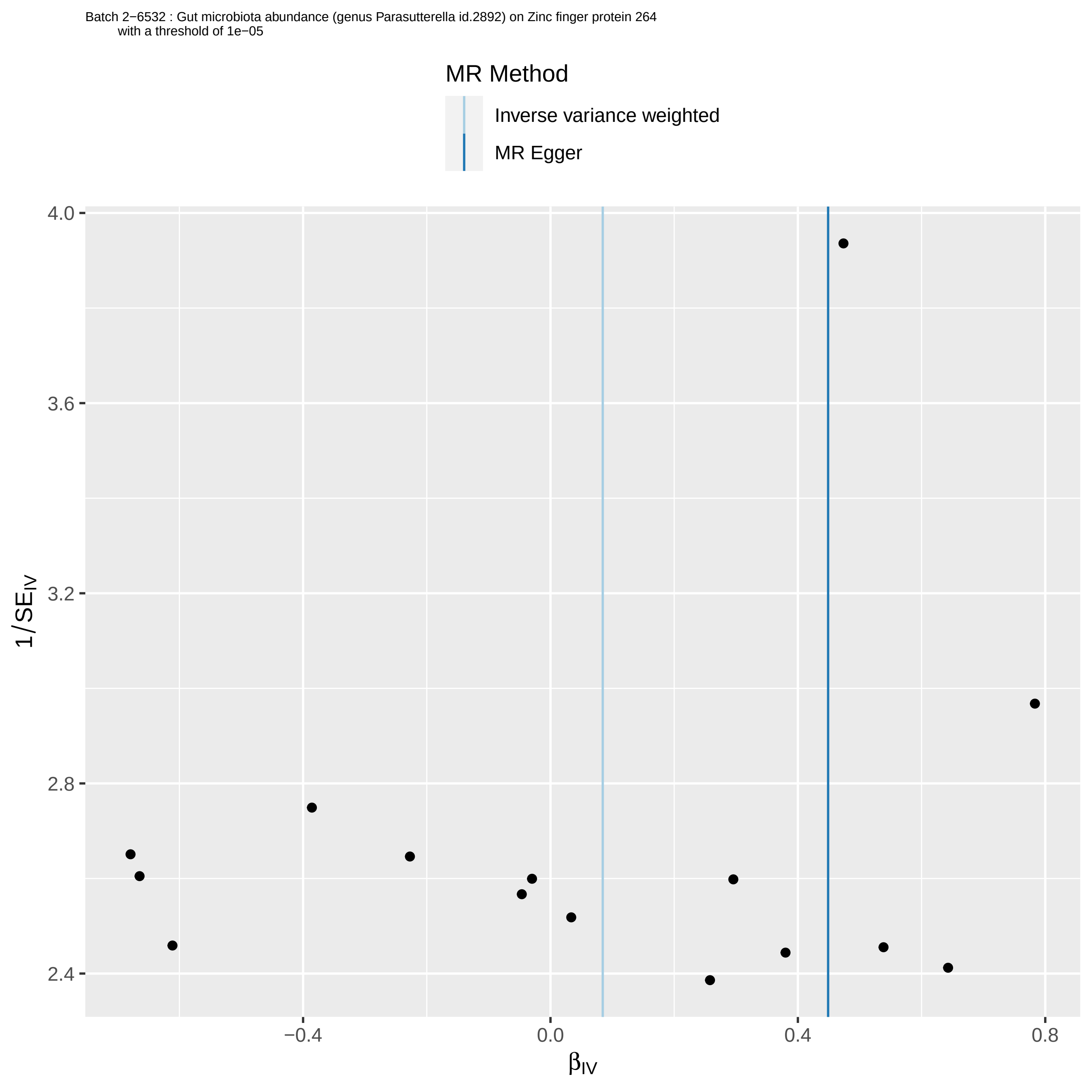

Batch 2−6532 : Gut microbiota abundance (genus Parasutterella id.2892) on Zinc finger protein 264
with a threshold of 1e−05
MR Method
Inverse variance weighted
MR Egger
4.0
3.6
3.2
2.8
2.4
1/SE$_{IV}$
−0.4
0.0
0.4
0.8
β_{IV}

Batch 2−6533 : Gut microbiota abundance (genus Catenibacterium id.2153) on Zinc finger protein 276
with a threshold of 1e−05

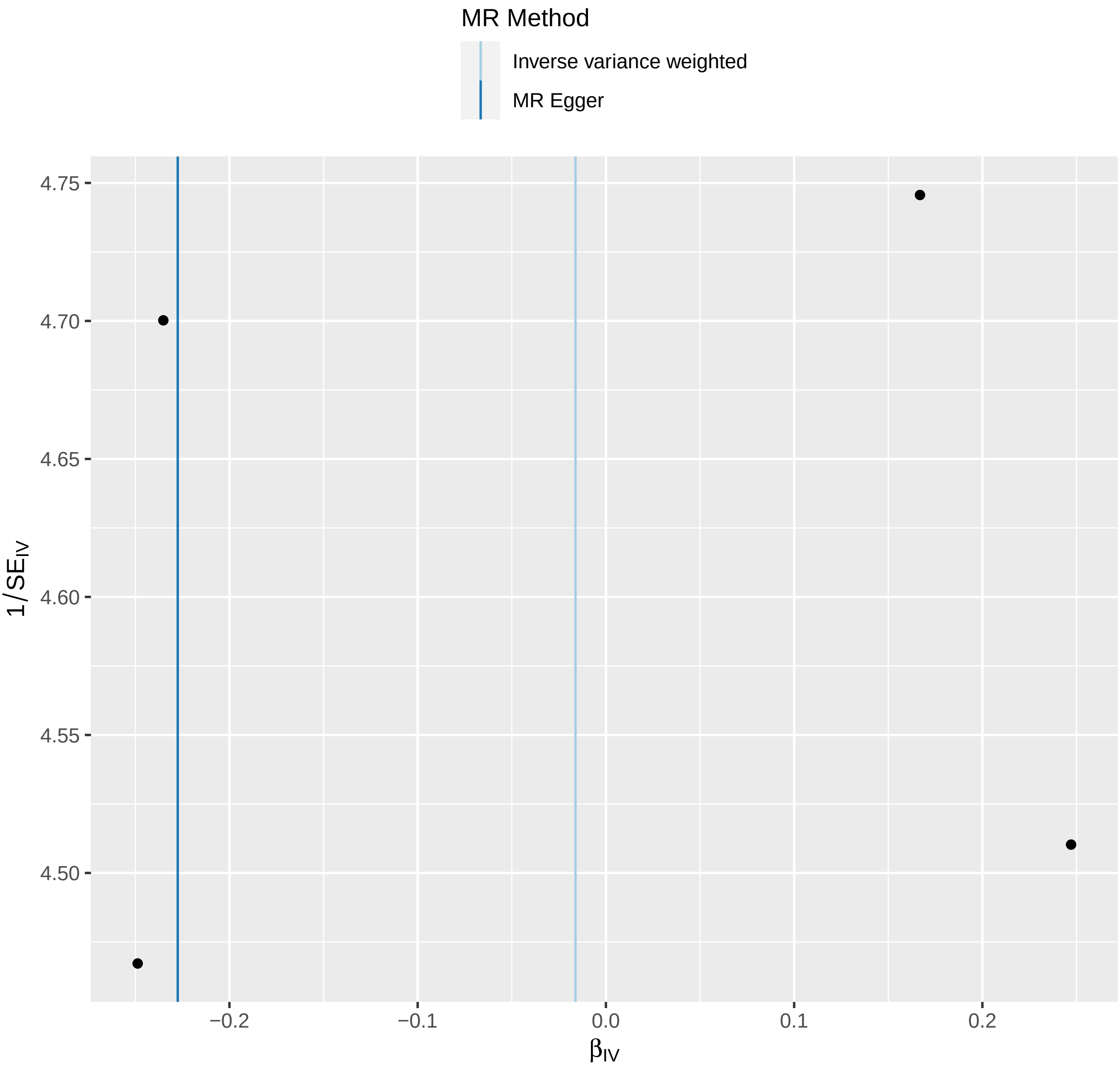

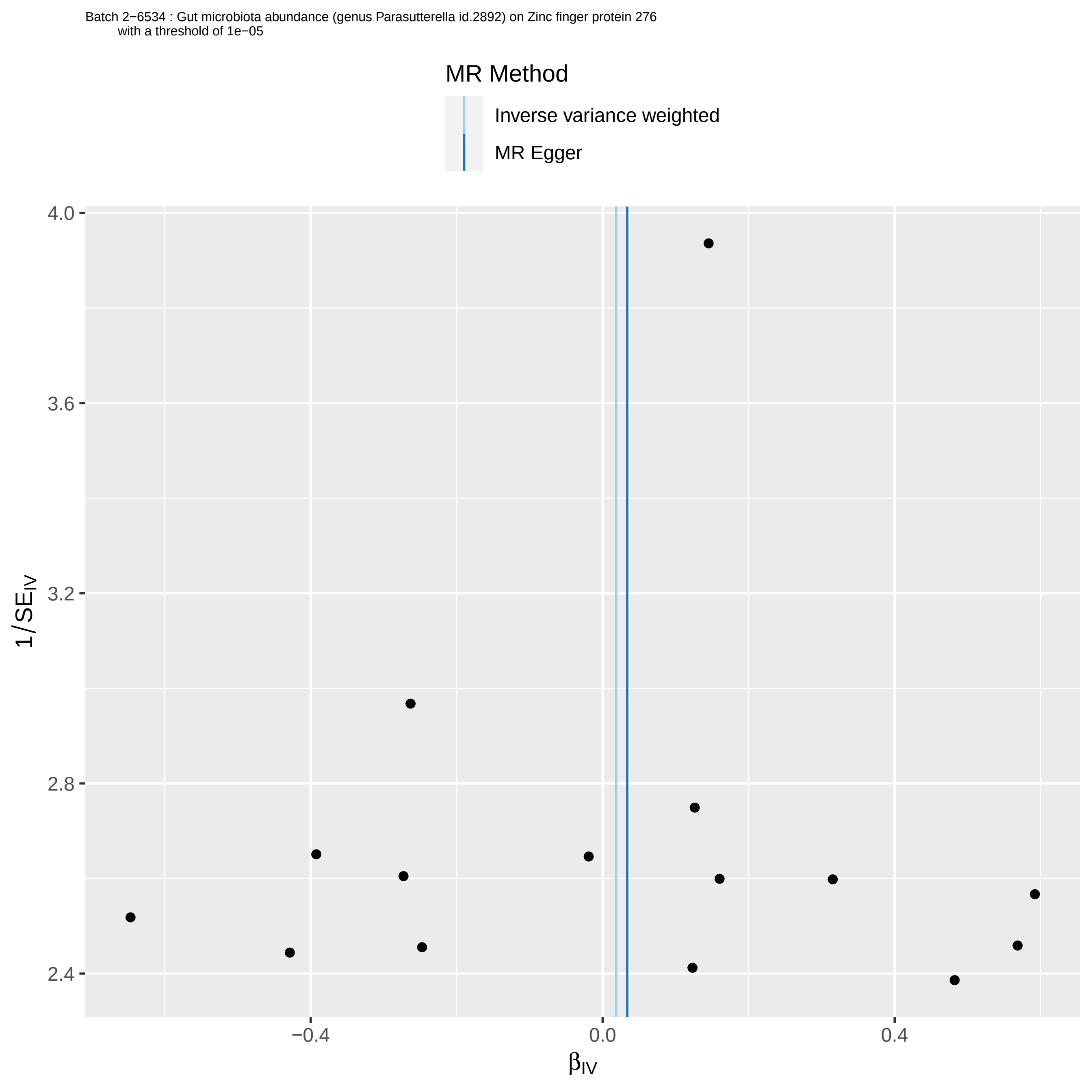

Batch 2−6534 : Gut microbiota abundance (genus Parasutterella id.2892) on Zinc finger protein 276
with a threshold of 1e−05
MR Method
Inverse variance weighted
MR Egger
4.0
3.6
3.2
2.8
2.4
1/SE$_{IV}$
−0.4
0.0
0.4
β_{IV}

Batch 2−6535 : Gut microbiota abundance (genus Catenibacterium id.2153) on Zinc finger protein 329
with a threshold of 1e−05

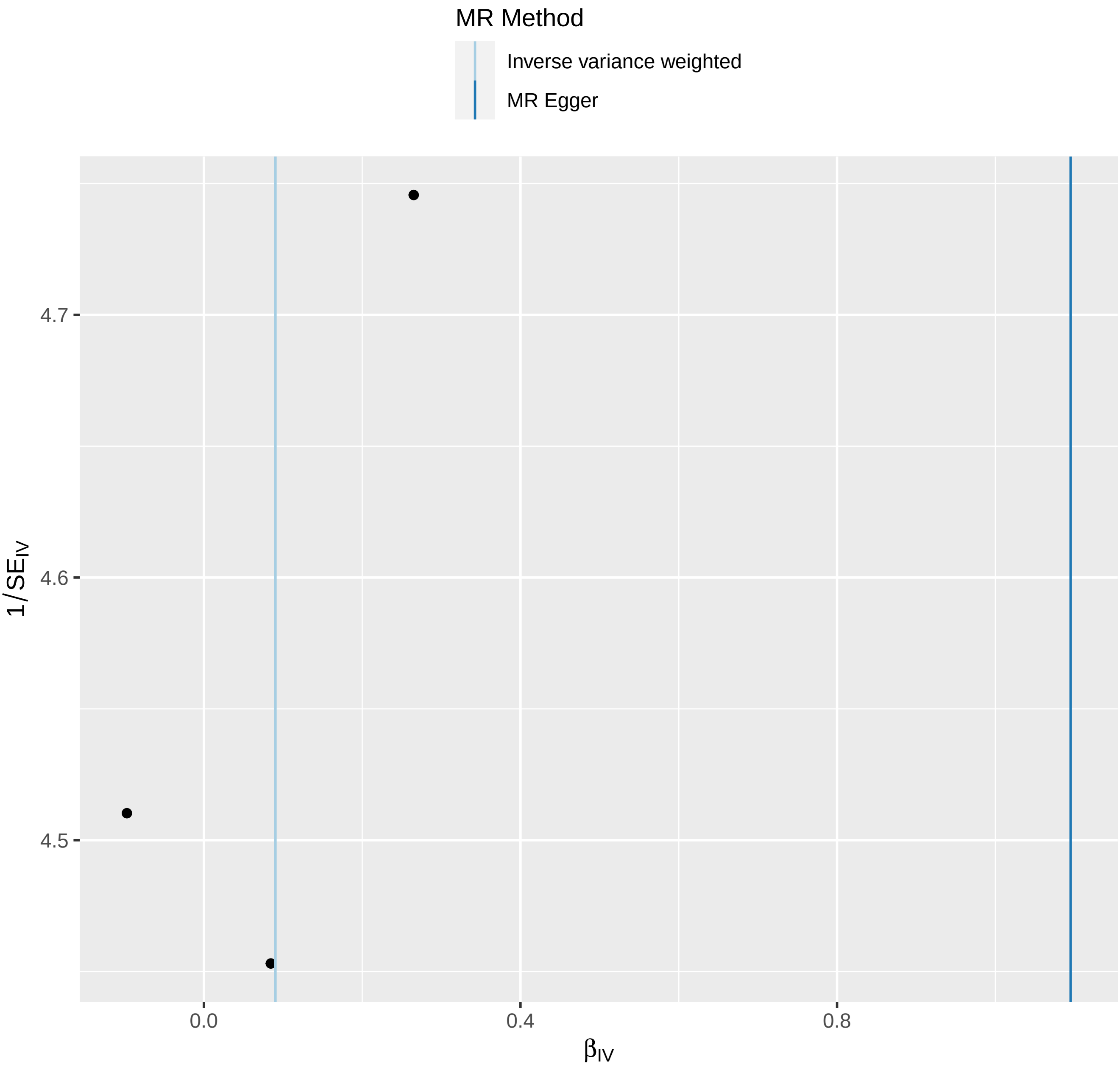

Batch 2−6536 : Gut microbiota abundance (genus Parasutterella id.2892) on Zinc finger protein 329
with a threshold of 1e−05

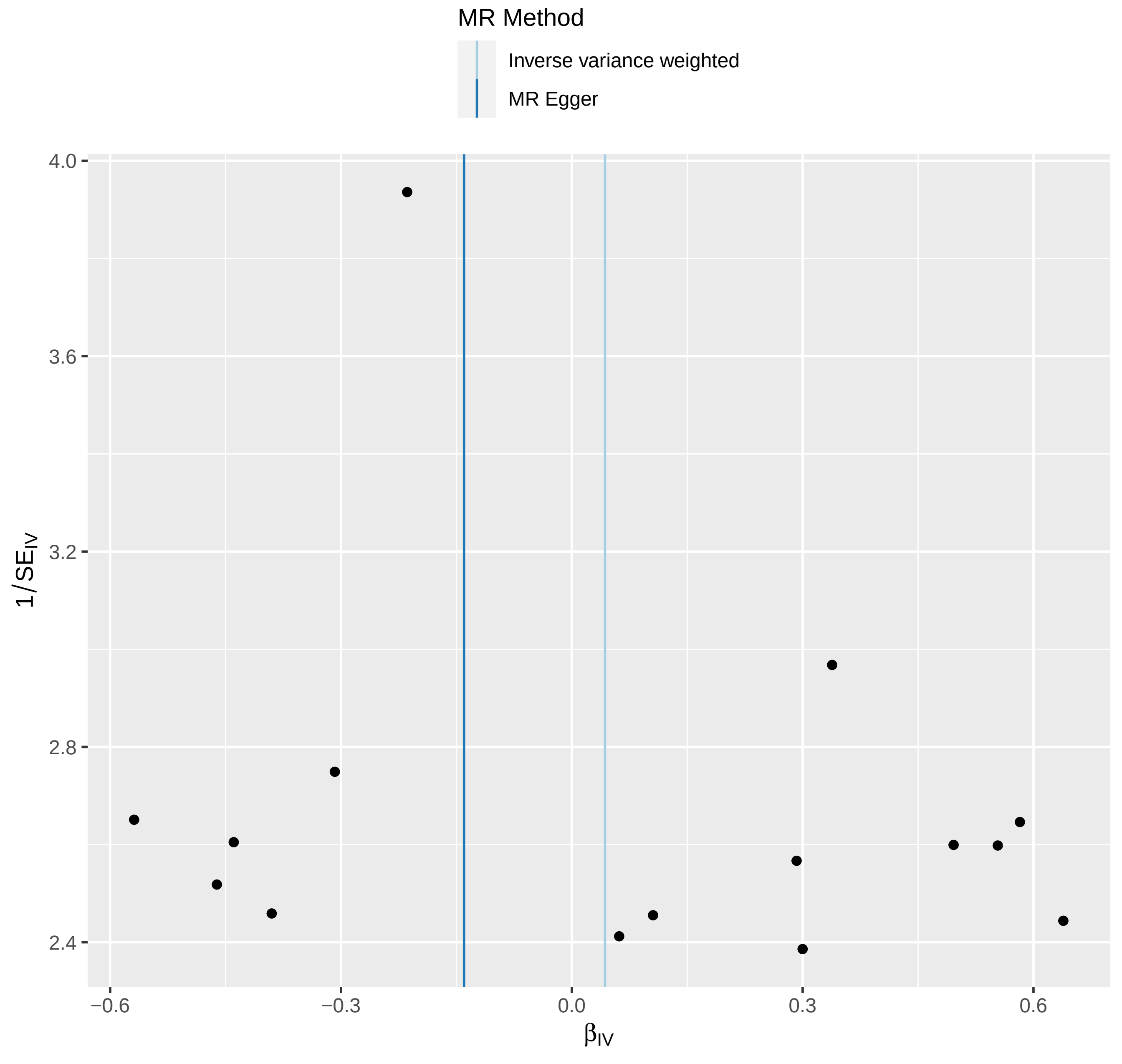

Batch 2−6537 : Gut microbiota abundance (genus Catenibacterium id.2153) on Zinc finger protein 334
with a threshold of 1e−05

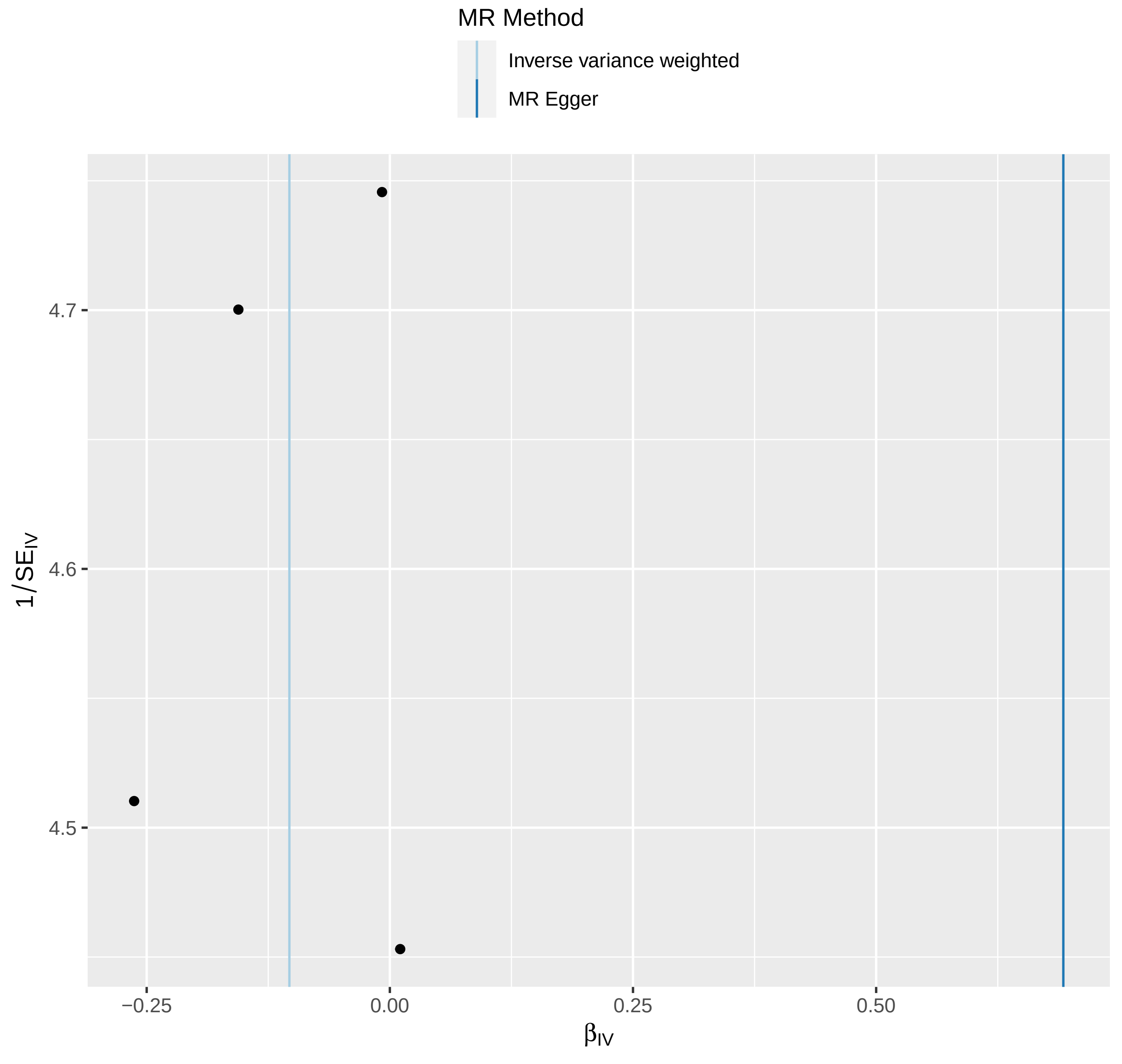

Batch 2−6538 : Gut microbiota abundance (genus Parasutterella id.2892) on Zinc finger protein 334
with a threshold of 1e−05

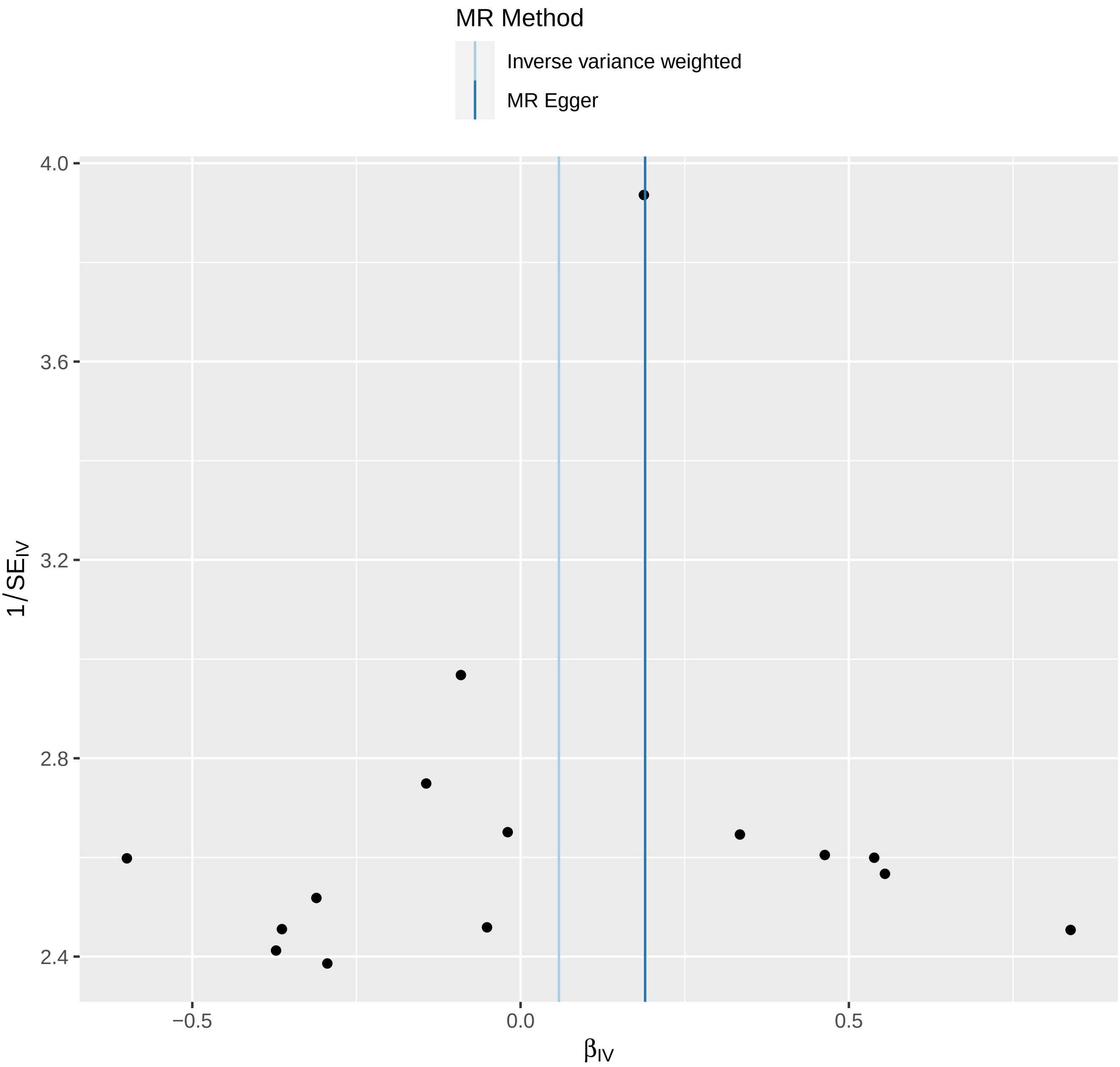

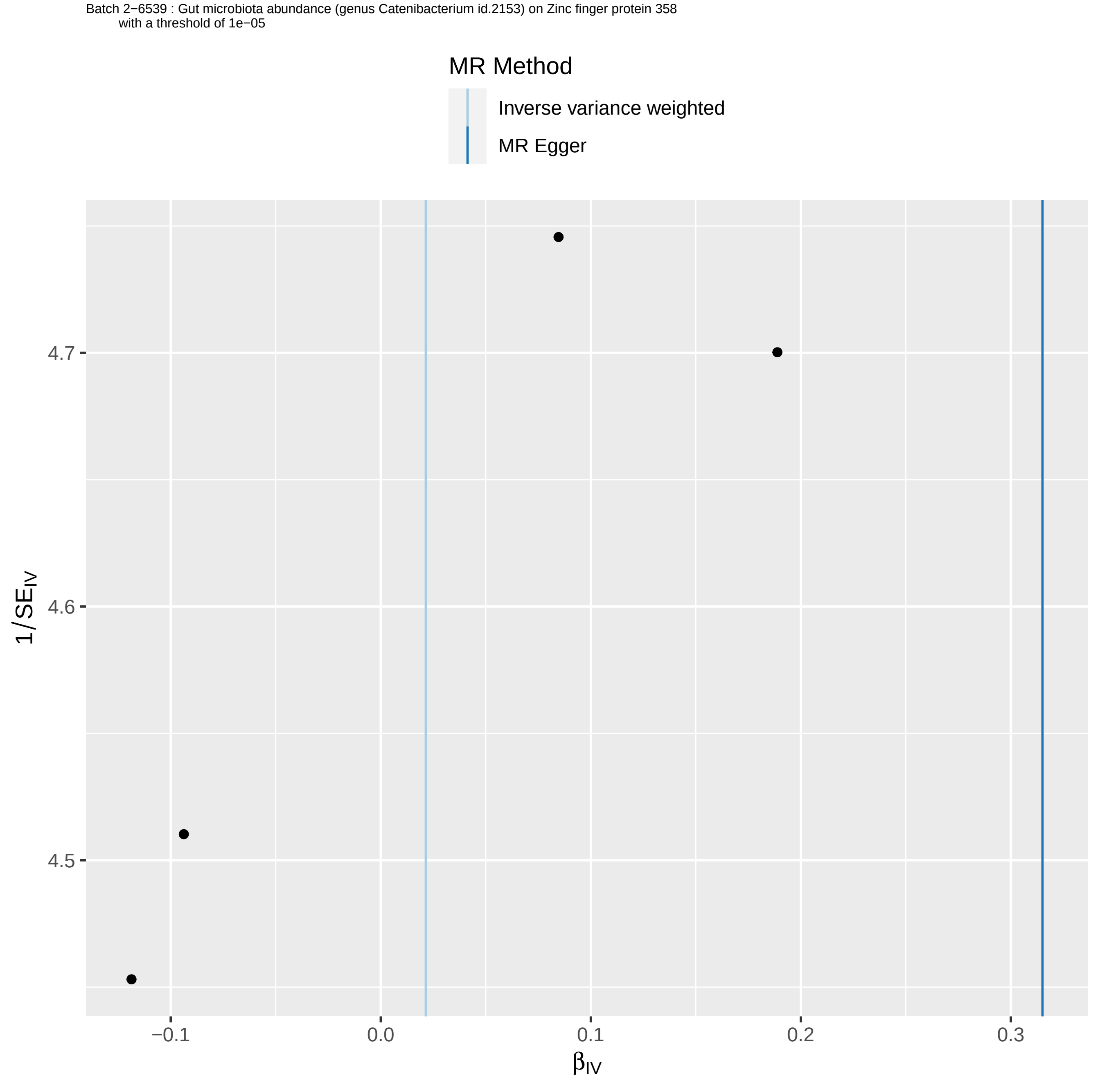
Batch 2−6539 : Gut microbiota abundance (genus Catenibacterium id.2153) on Zinc finger protein 358
with a threshold of 1e−05
MR Method
Inverse variance weighted
MR Egger
4.7
4.6
4.5
1/SE_IV
−0.1
0.0
0.1
0.2
0.3
β_IV

Batch 2−6540 : Gut microbiota abundance (genus Parasutterella id.2892) on Zinc finger protein 358
with a threshold of 1e−05

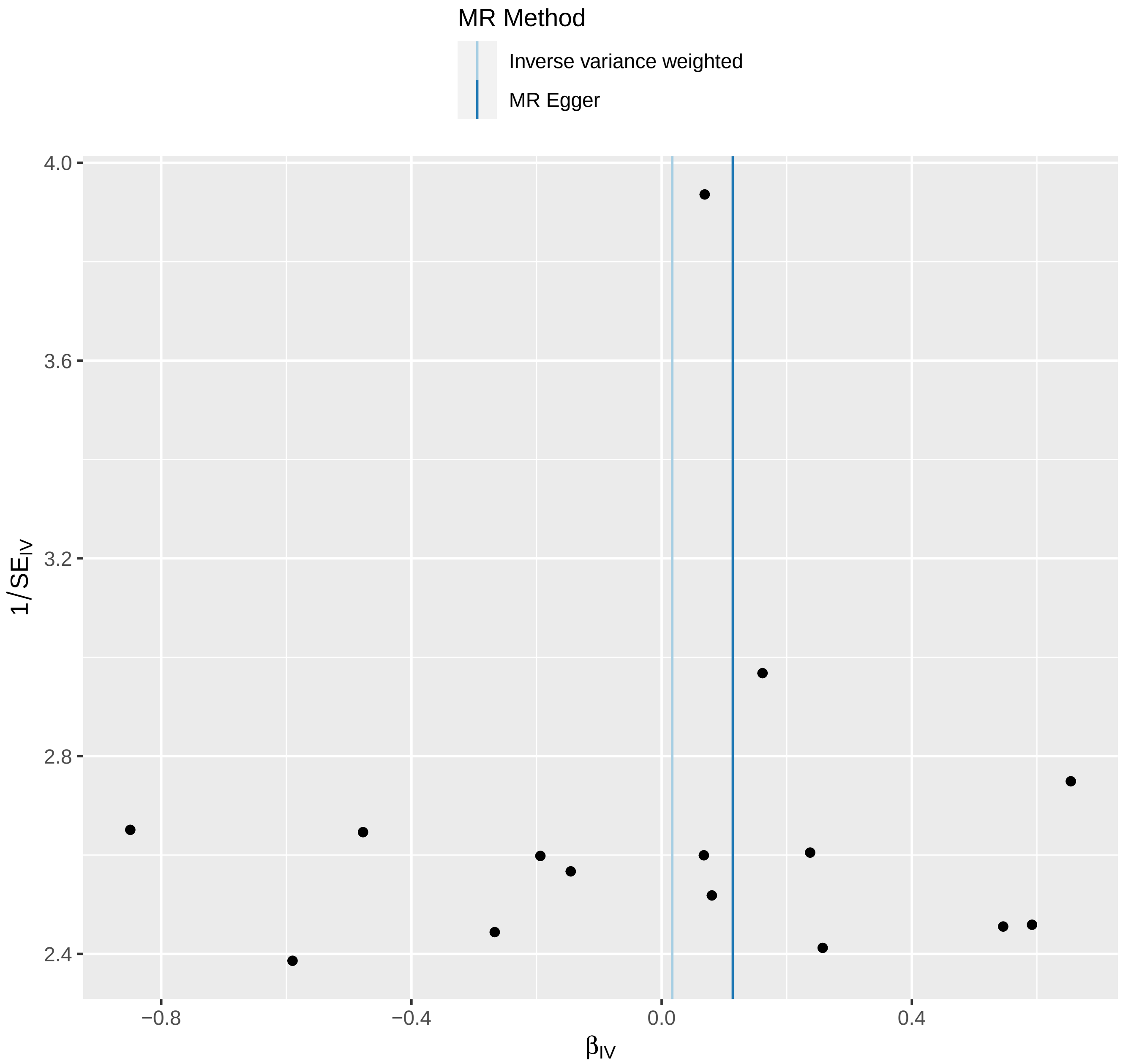

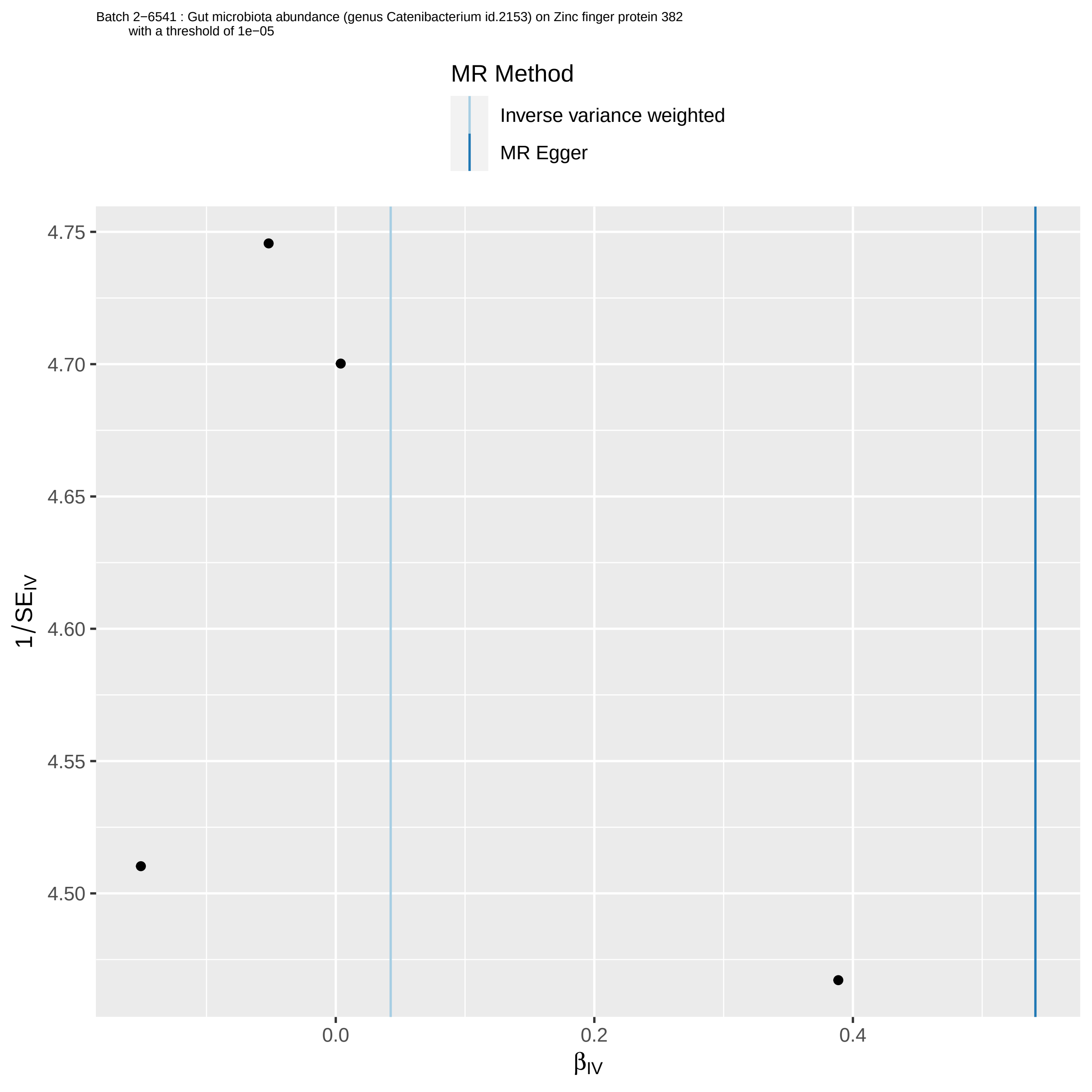
Batch 2−6541 : Gut microbiota abundance (genus Catenibacterium id.2153) on Zinc finger protein 382
with a threshold of 1e−05
MR Method
Inverse variance weighted
MR Egger
4.75
4.70
4.65
4.60
4.55
4.50
1/SE_IV
0.0
0.2
0.4
β_IV

Batch 2−6542 : Gut microbiota abundance (genus Parasutterella id.2892) on Zinc finger protein 382
with a threshold of 1e−05

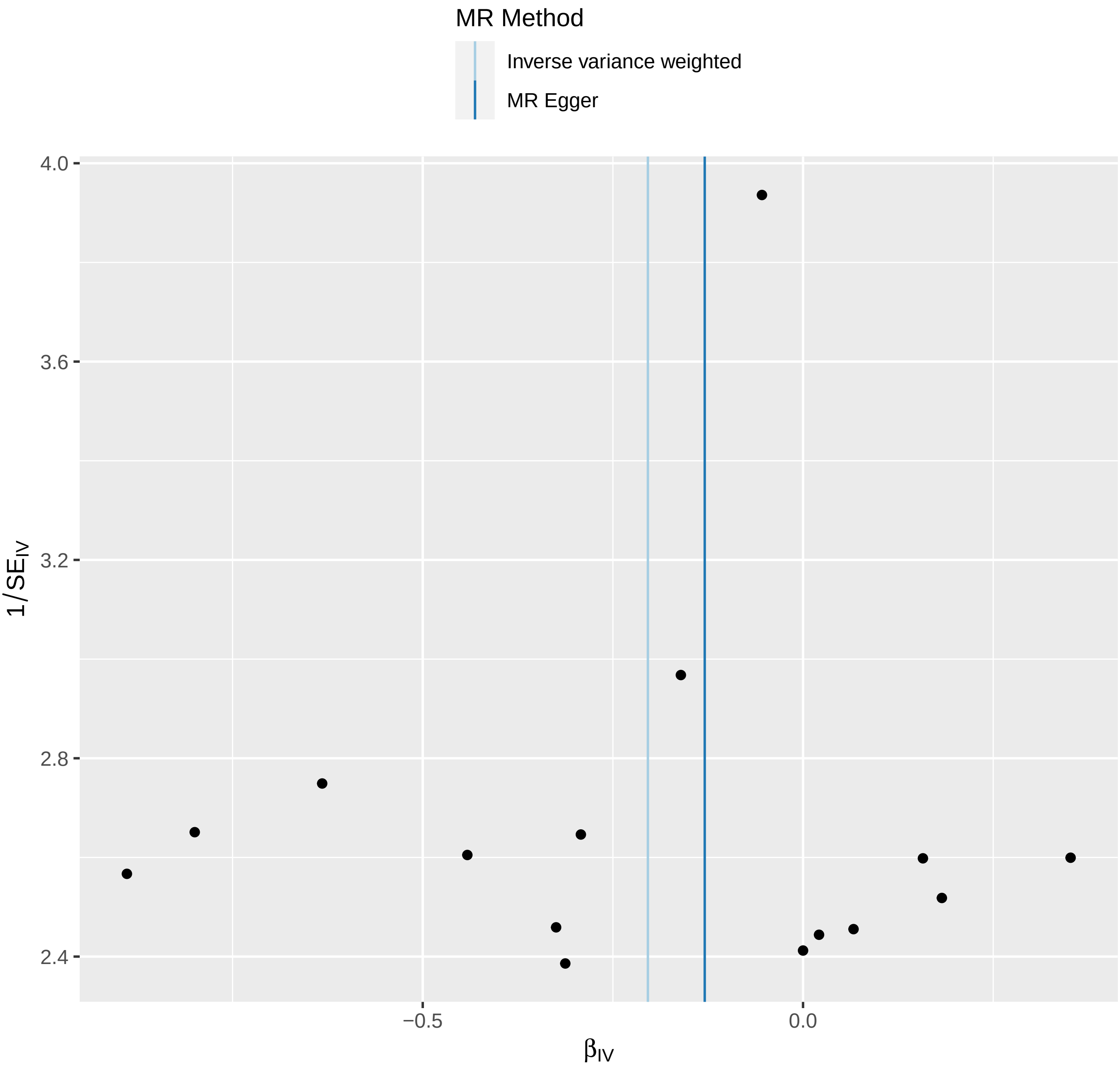

Batch 2−6543 : Gut microbiota abundance (genus Catenibacterium id.2153) on Zinc finger protein 410
with a threshold of 1e−05

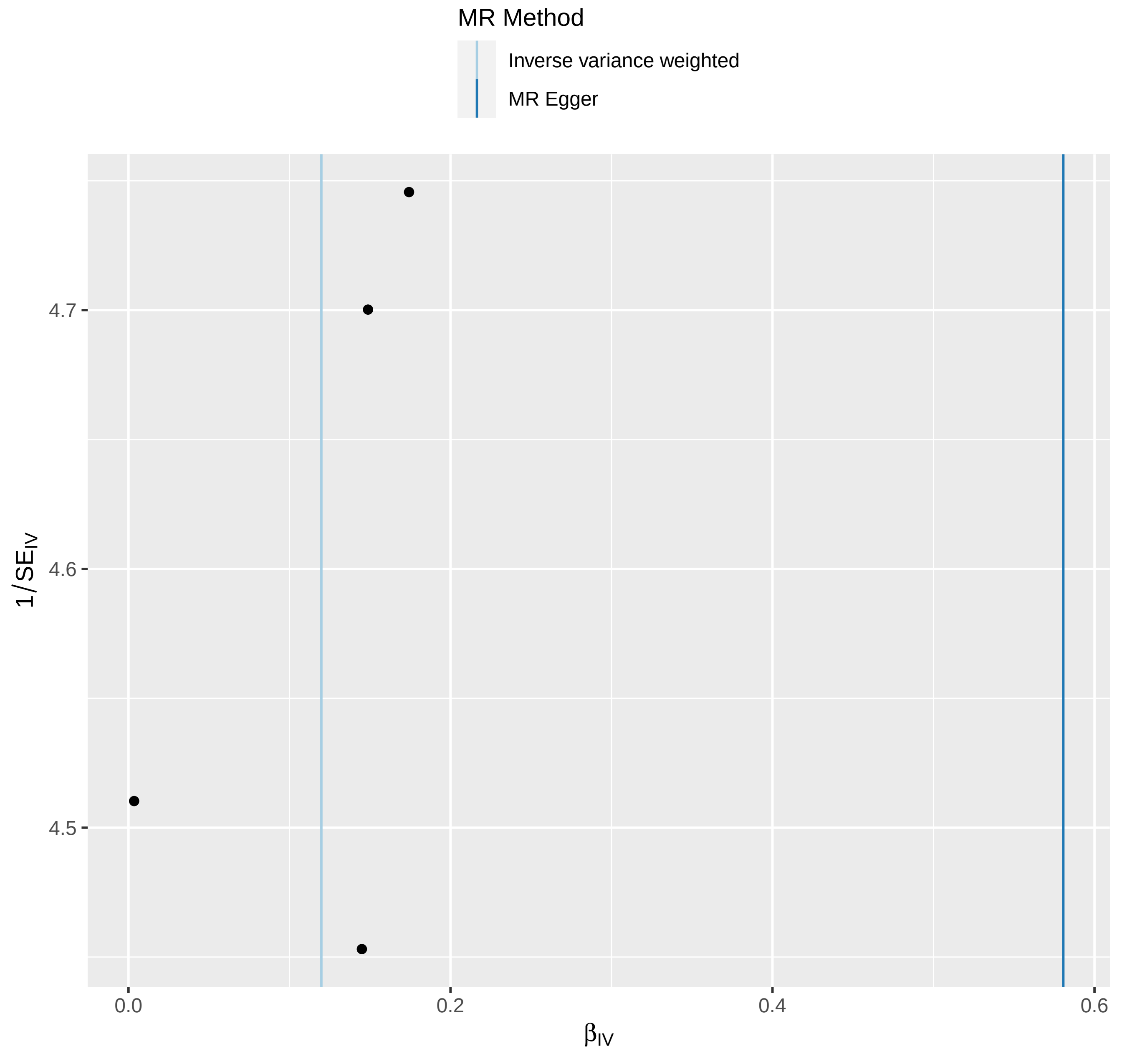

Batch 2−6544 : Gut microbiota abundance (genus Parasutterella id.2892) on Zinc finger protein 410
with a threshold of 1e−05

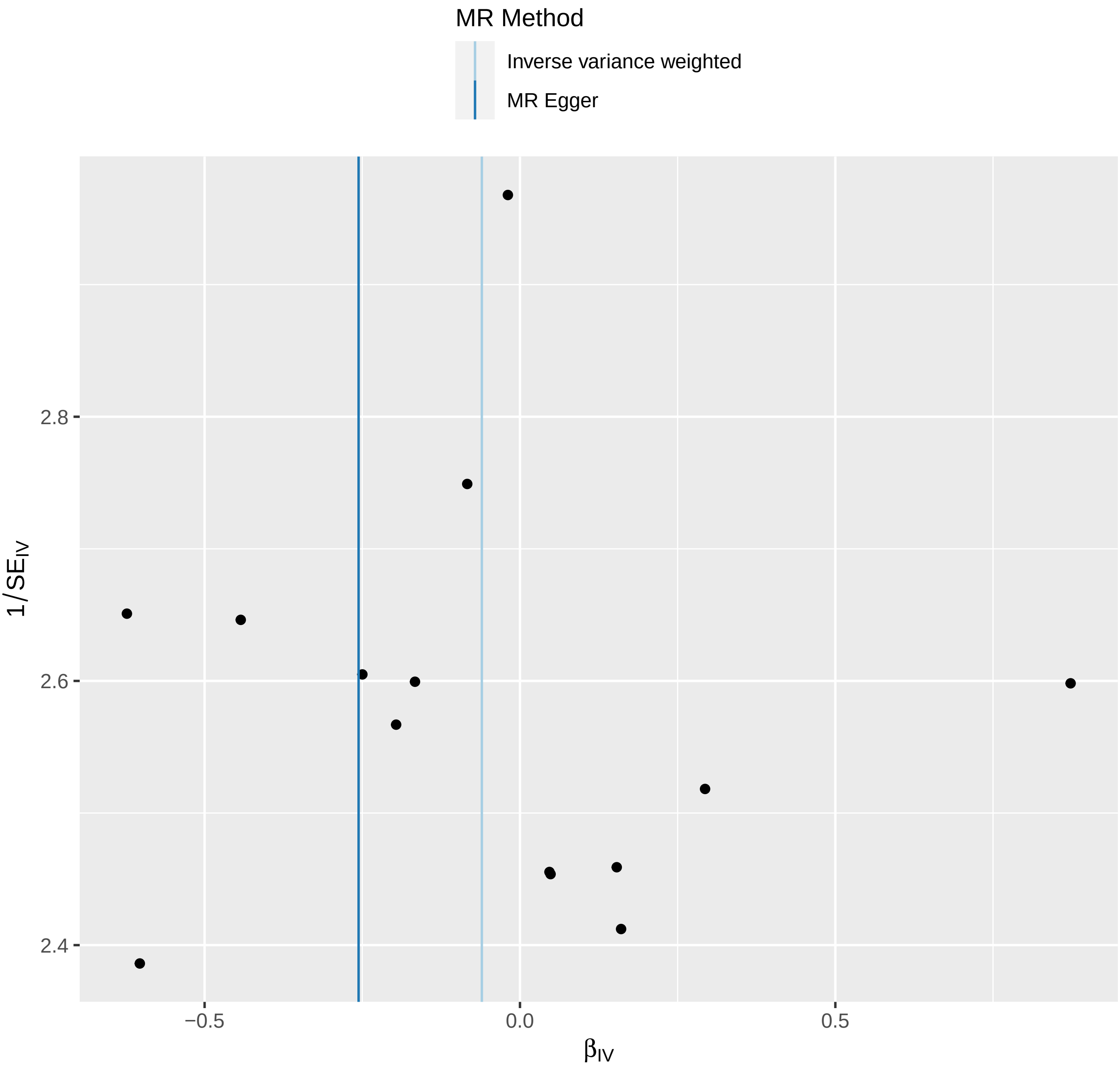

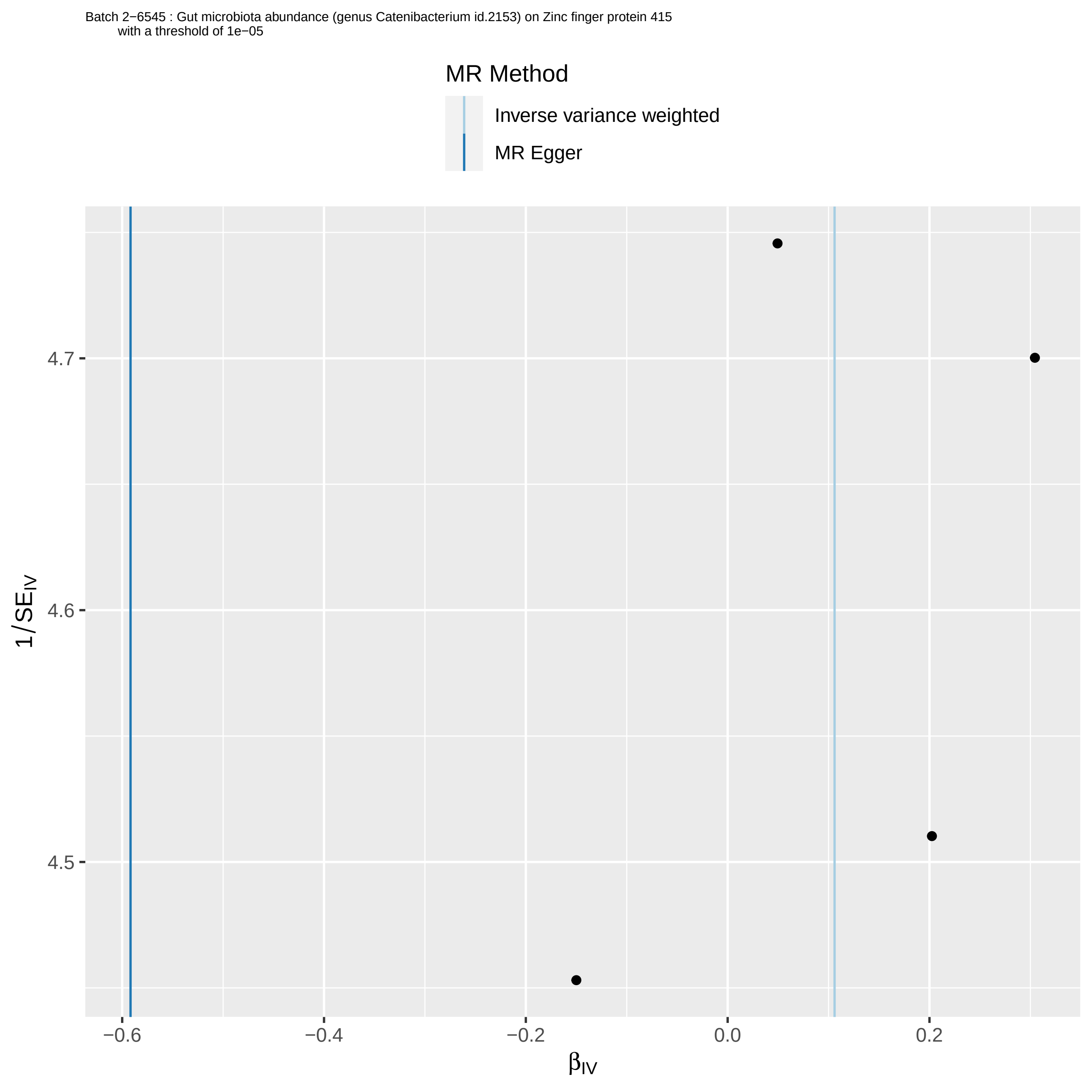
Batch 2−6545 : Gut microbiota abundance (genus Catenibacterium id.2153) on Zinc finger protein 415
with a threshold of 1e−05
MR Method
Inverse variance weighted
MR Egger
4.7
4.6
4.5
1/SE_IV
−0.6
−0.4
−0.2
0.0
0.2
β_IV

Batch 2−6546 : Gut microbiota abundance (genus Parasutterella id.2892) on Zinc finger protein 415
with a threshold of 1e−05

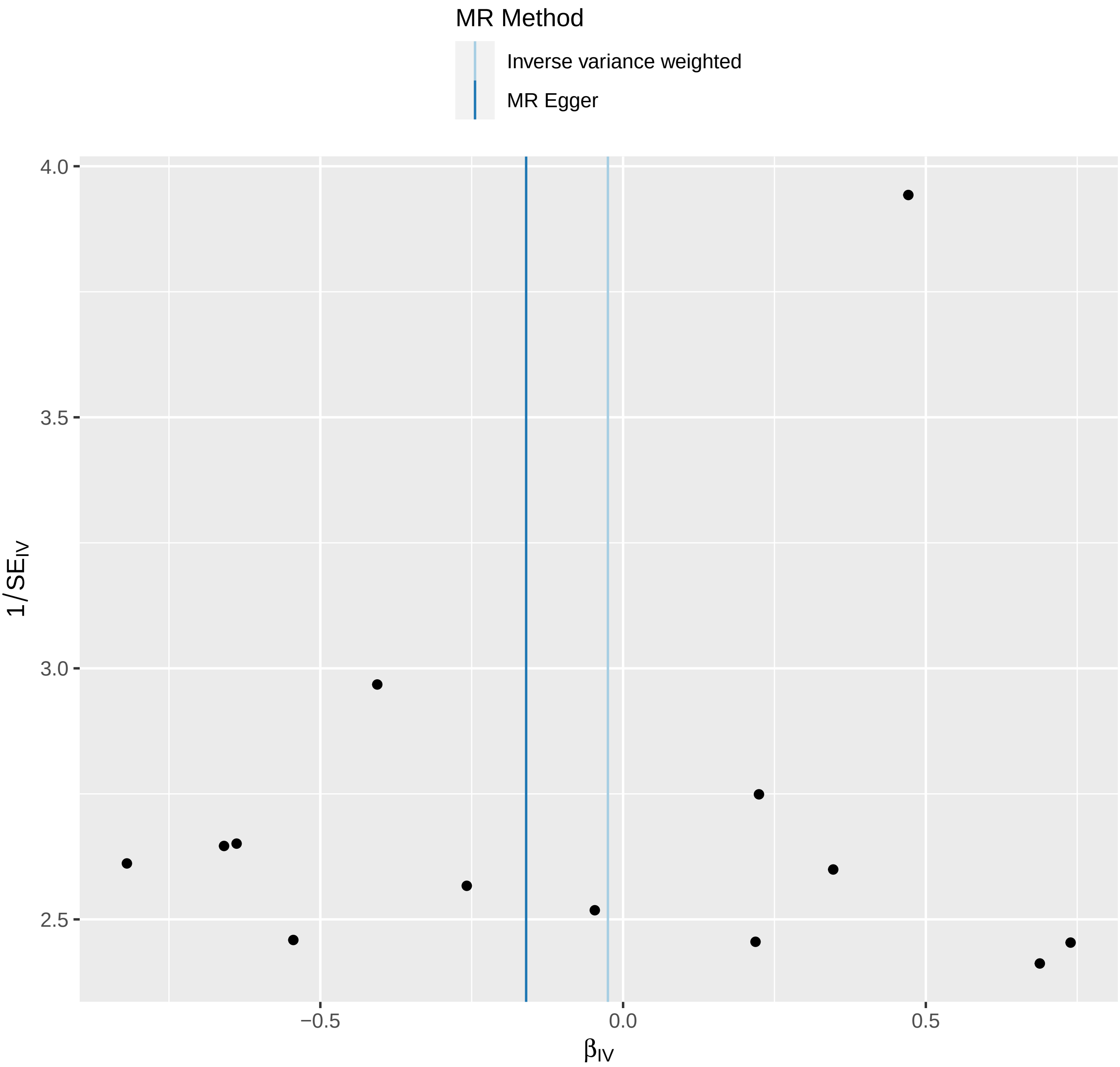

Batch 2−6547 : Gut microbiota abundance (genus Catenibacterium id.2153) on Zinc finger protein 566
with a threshold of 1e−05

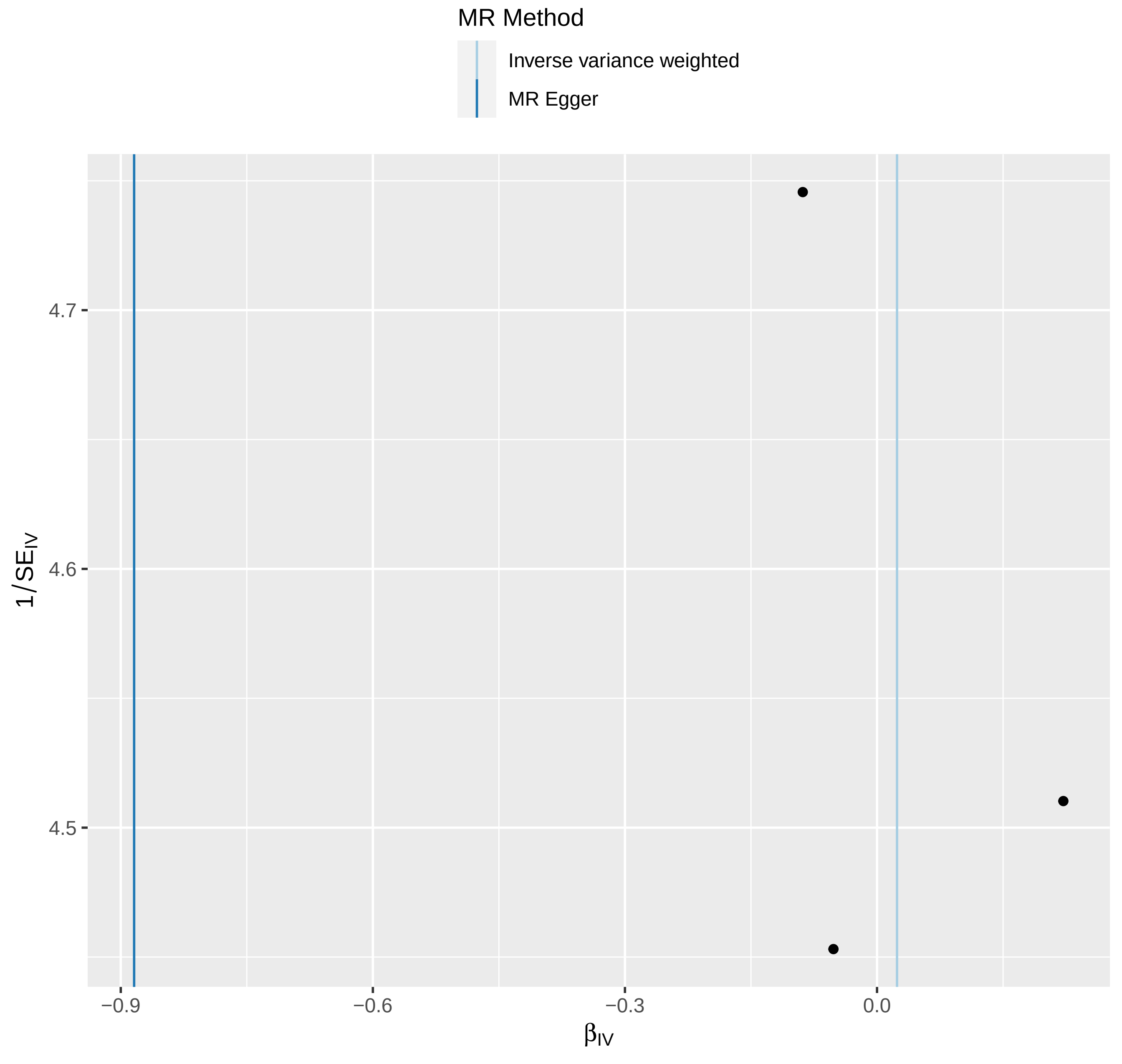

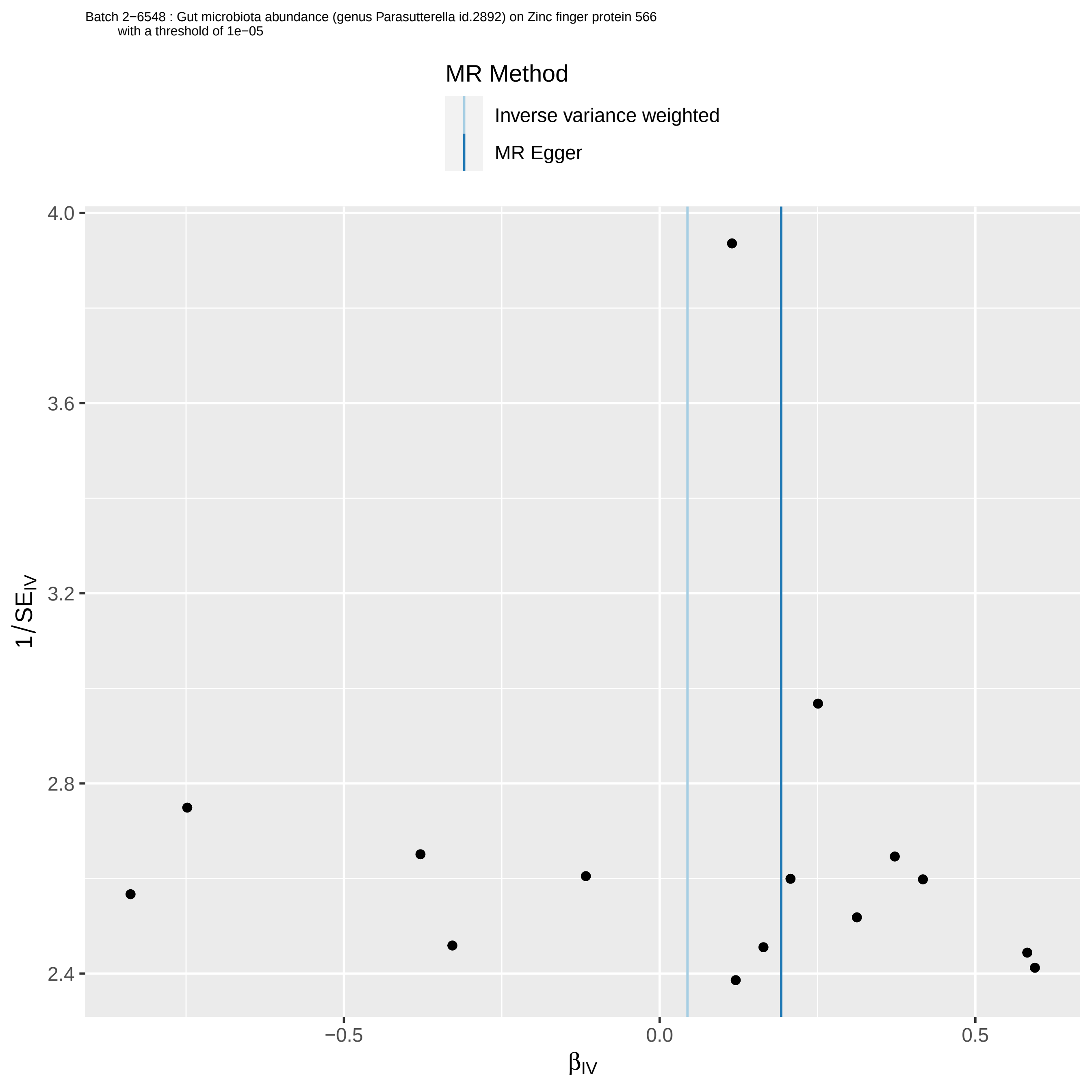

Batch 2−6548 : Gut microbiota abundance (genus Parasutterella id.2892) on Zinc finger protein 566
with a threshold of 1e−05
MR Method
Inverse variance weighted
MR Egger
4.0
3.6
3.2
2.8
2.4
1/SE_IV
−0.5
0.0
0.5
β_IV

Batch 2−6549 : Gut microbiota abundance (genus Catenibacterium id.2153) on Zinc finger protein 75D
with a threshold of 1e−05

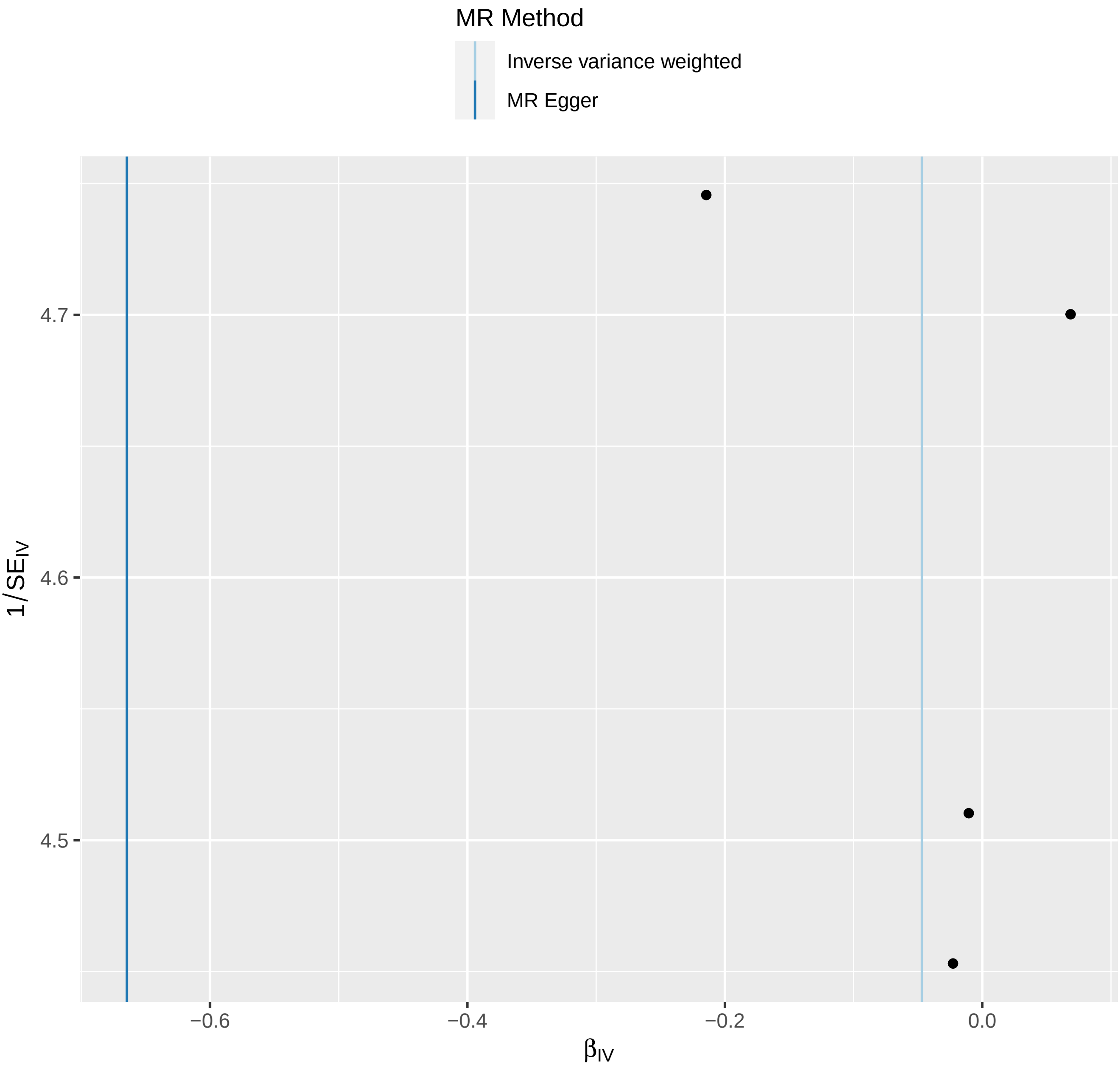

Batch 2−6550 : Gut microbiota abundance (genus Parasutterella id.2892) on Zinc finger protein 75D
with a threshold of 1e−05

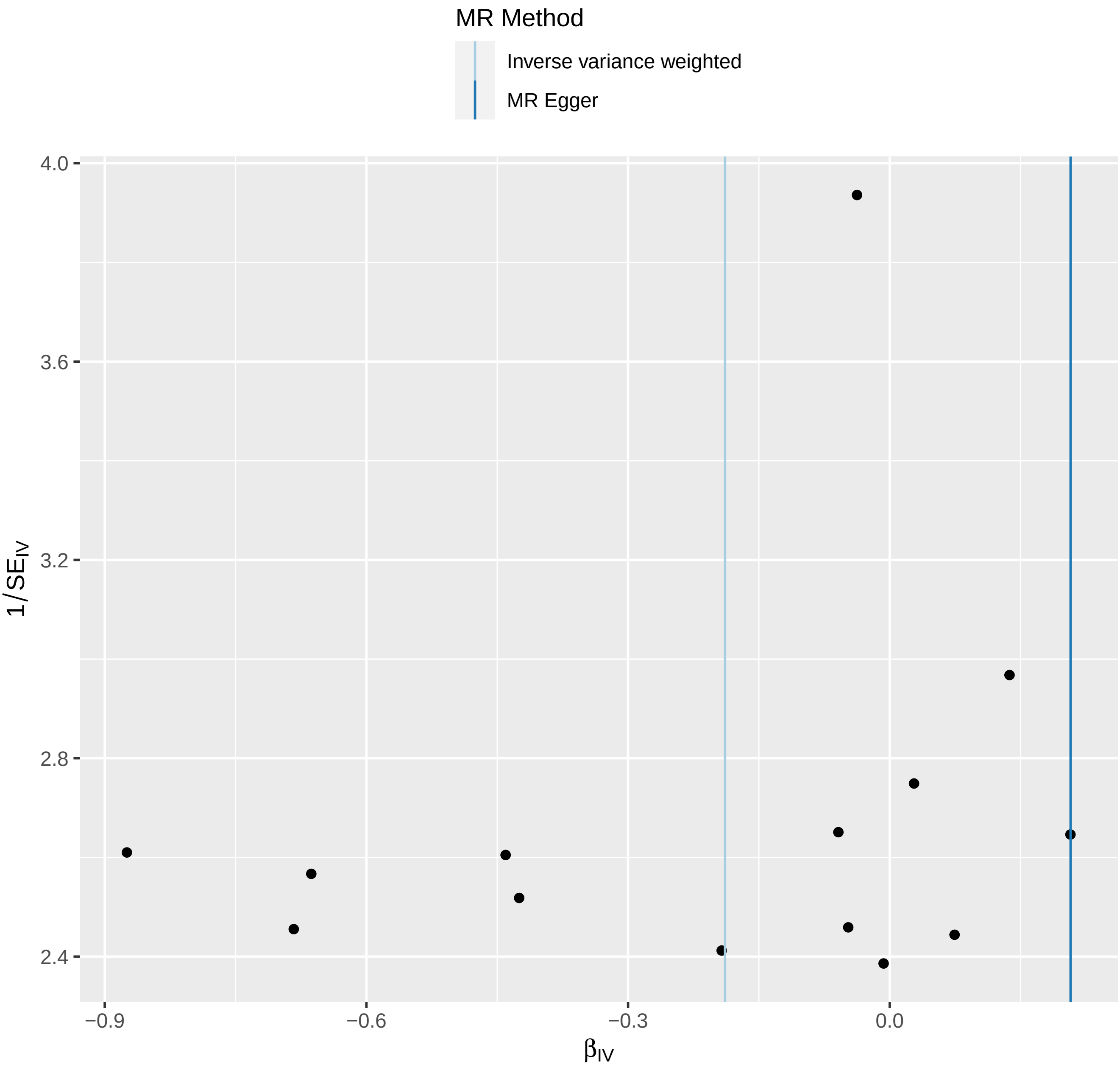

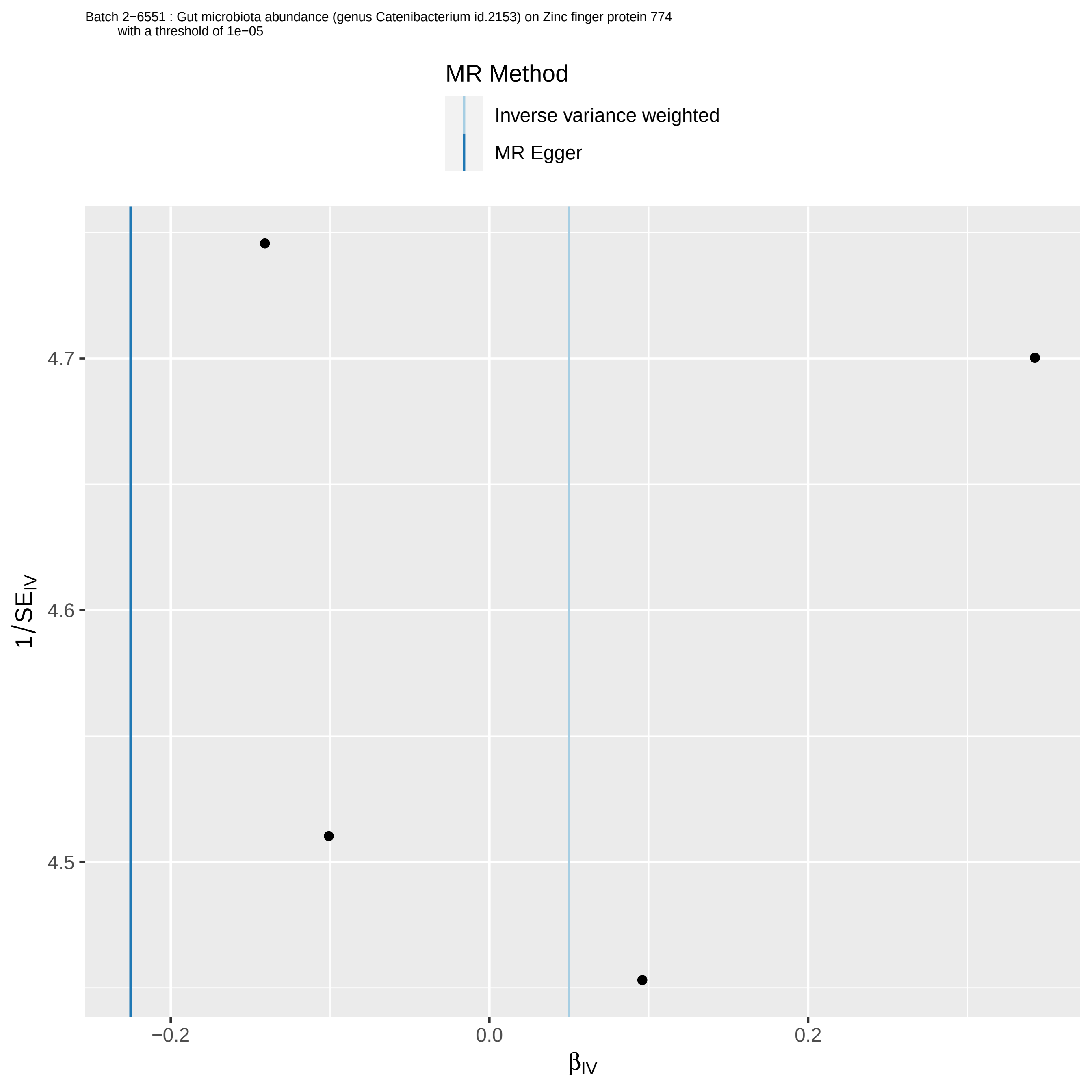
Batch 2−6551 : Gut microbiota abundance (genus Catenibacterium id.2153) on Zinc finger protein 774
with a threshold of 1e−05
MR Method
Inverse variance weighted
MR Egger
4.7
4.6
4.5
1/SE_IV
−0.2
0.0
0.2
β_IV

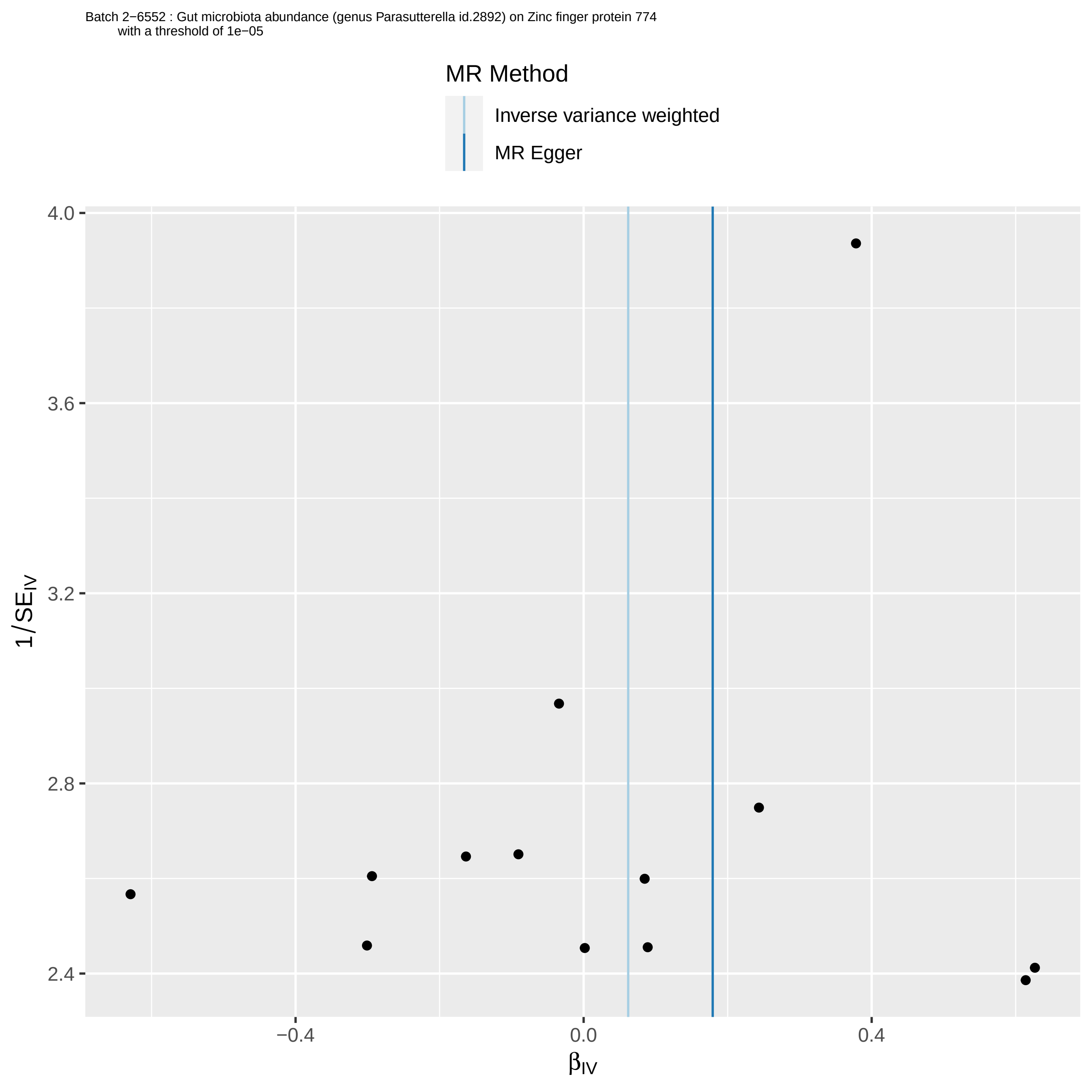

Batch 2−6552 : Gut microbiota abundance (genus Parasutterella id.2892) on Zinc finger protein 774
with a threshold of 1e−05
MR Method
Inverse variance weighted
MR Egger
4.0
3.6
3.2
2.8
2.4
1/SE$_{IV}$
−0.4
0.0
0.4
β$_{IV}$

Batch 2−6553 : Gut microbiota abundance (genus Catenibacterium id.2153) on Zinc finger protein 843
with a threshold of 1e−05

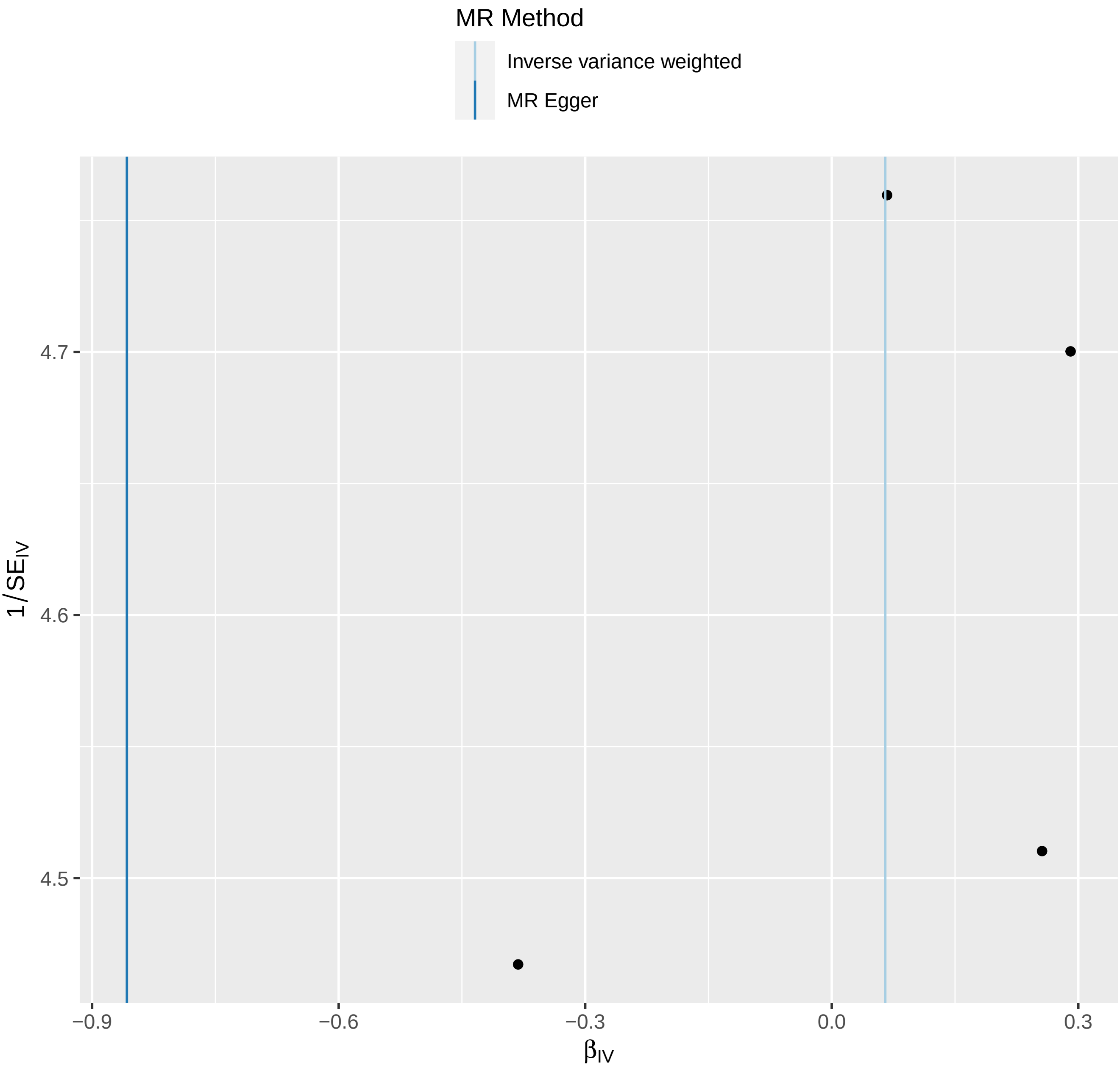

Batch 2−6554 : Gut microbiota abundance (genus Parasutterella id.2892) on Zinc finger protein 843
with a threshold of 1e−05

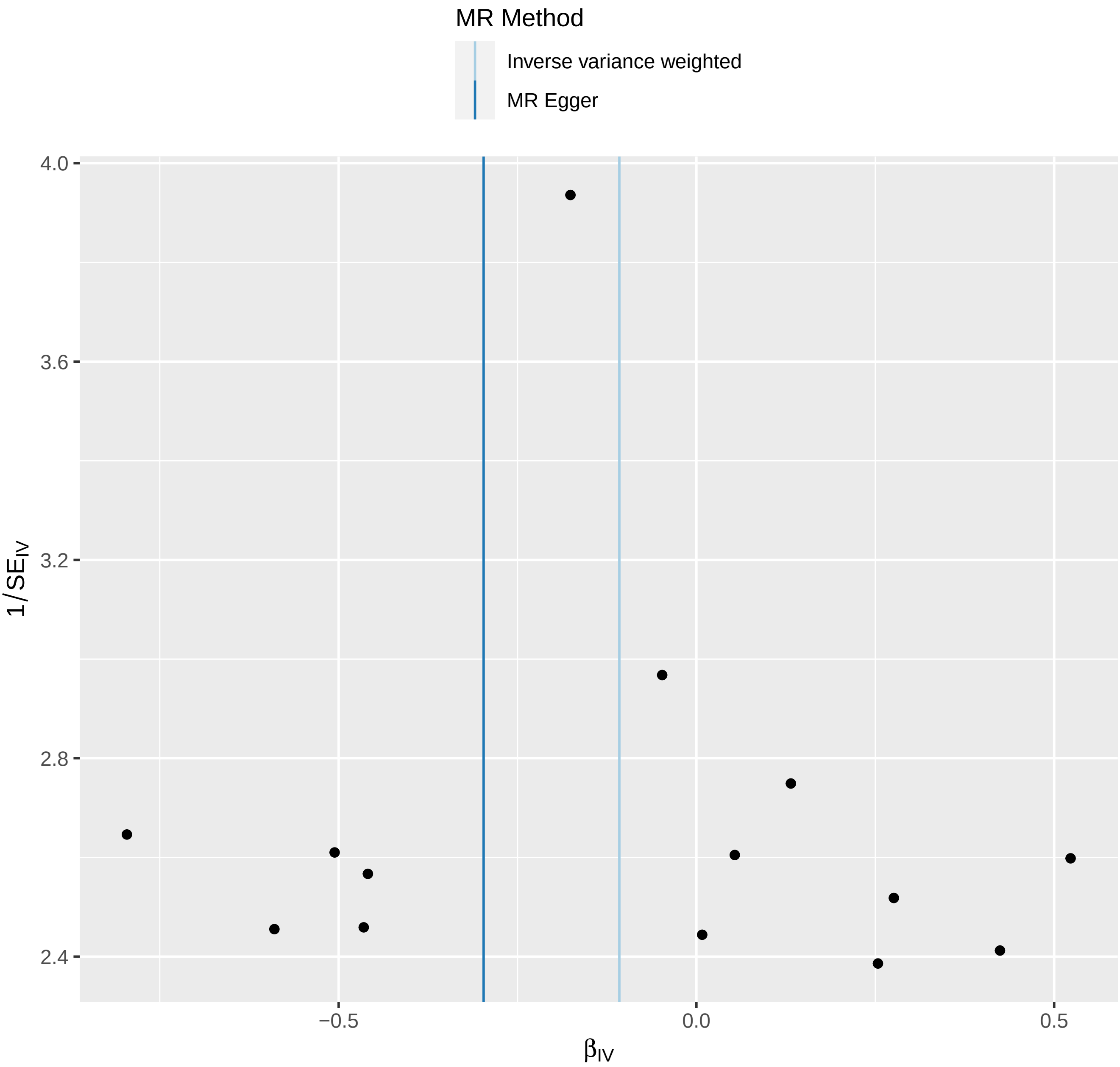

Batch 2−6555 : Gut microbiota abundance (genus Catenibacterium id.2153) on E3 ubiquitin−protein ligase ZNRF3
with a threshold of 1e−05

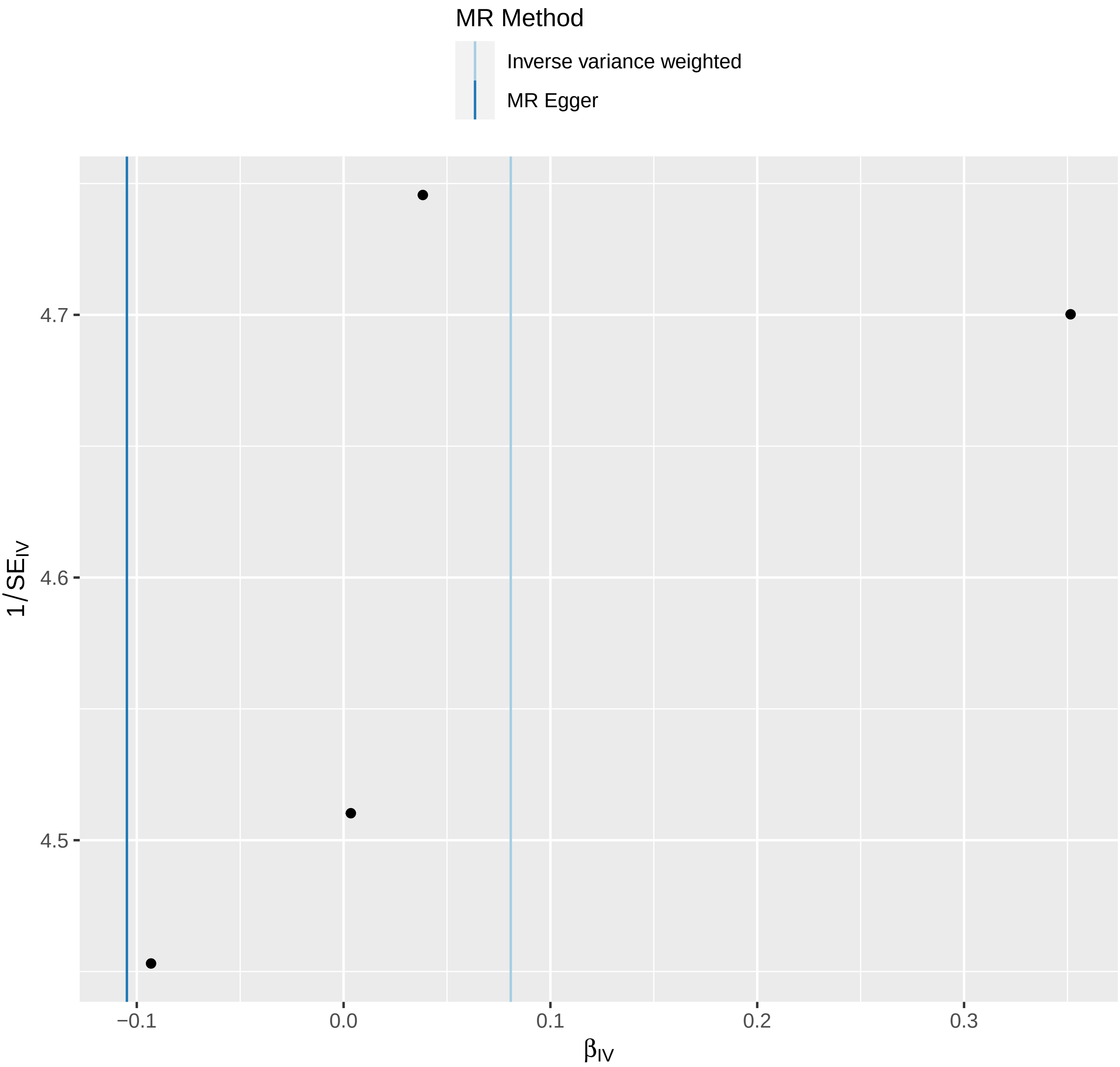

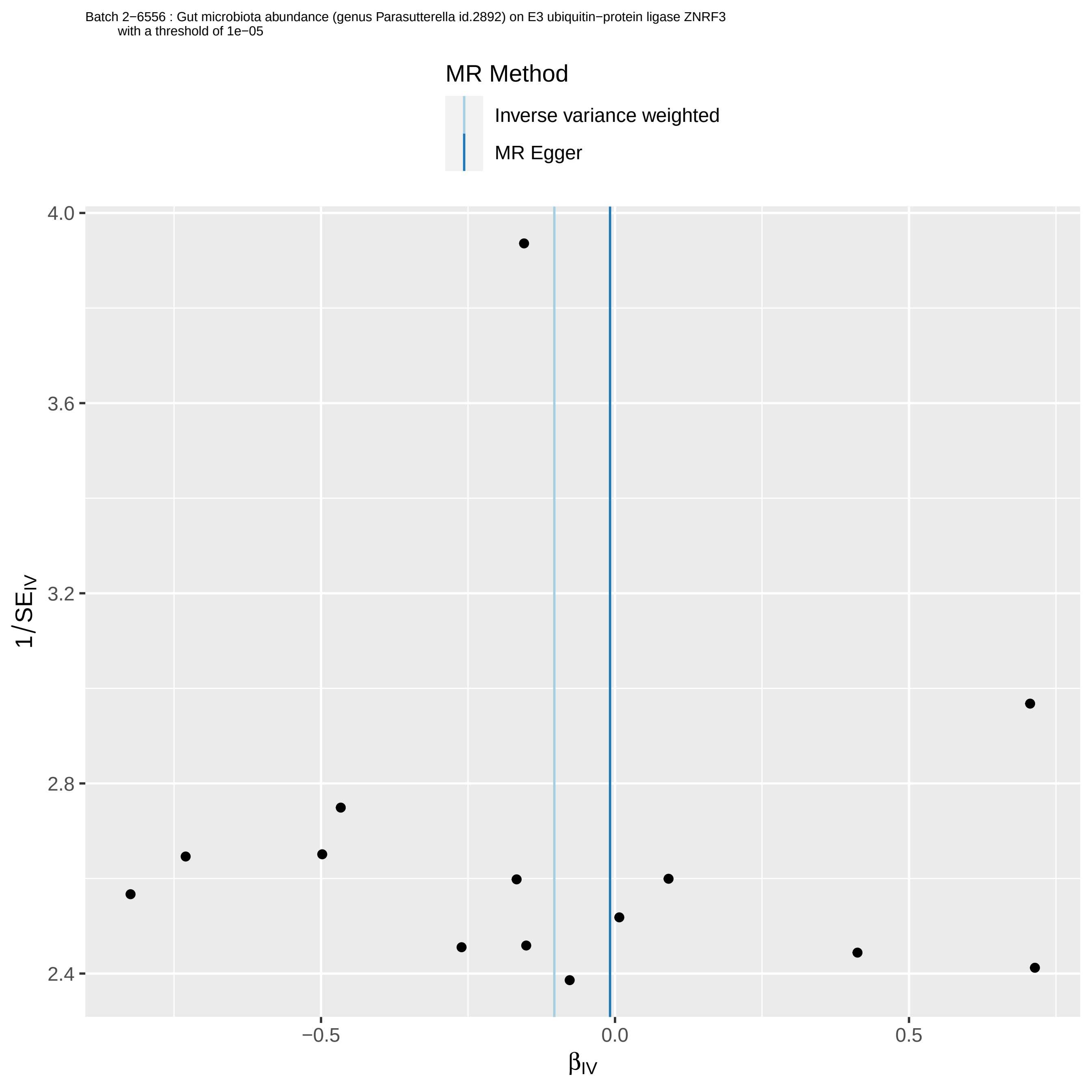

Batch 2−6556 : Gut microbiota abundance (genus Parasutterella id.2892) on E3 ubiquitin−protein ligase ZNRF3
with a threshold of 1e−05
MR Method
Inverse variance weighted
MR Egger
4.0
3.6
3.2
2.8
2.4
1/SE_IV
−0.5
0.0
0.5
β_IV

Batch 2−6557 : Gut microbiota abundance (genus Catenibacterium id.2153) on E3 ubiquitin−protein ligase ZNRF3
with a threshold of 1e−05

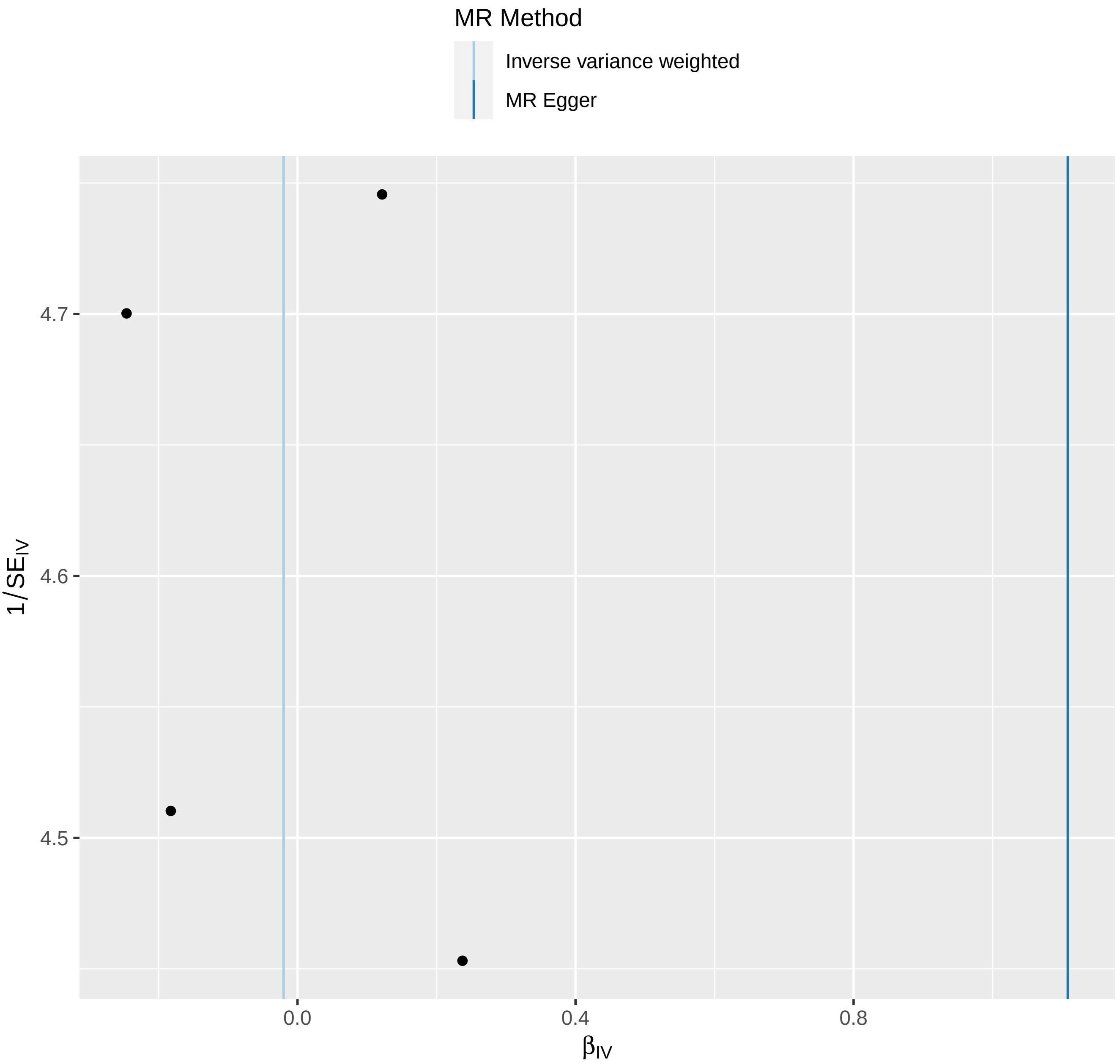

Batch 2−6558 : Gut microbiota abundance (genus Parasutterella id.2892) on E3 ubiquitin−protein ligase ZNRF3
with a threshold of 1e−05

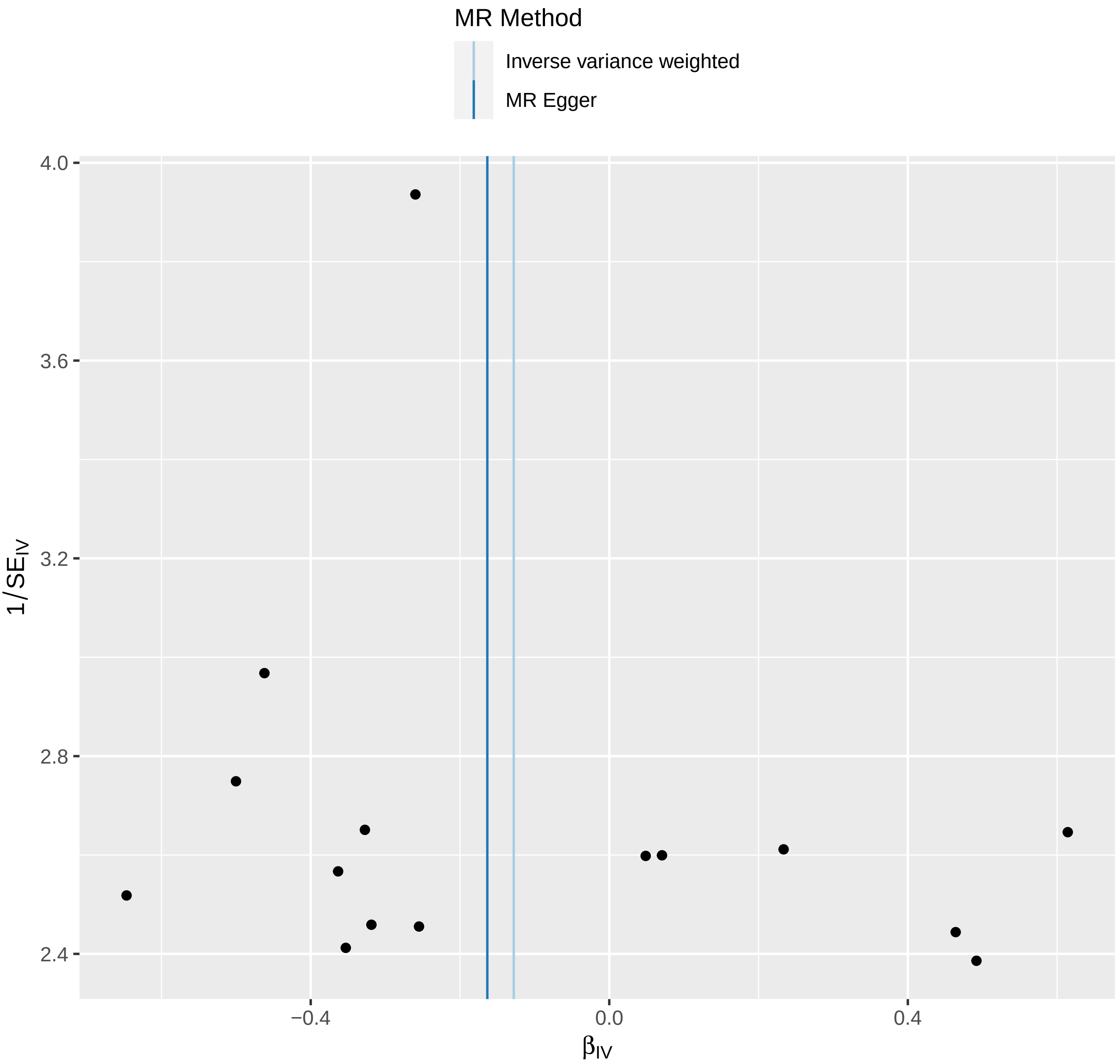

Batch 2−6559 : Gut microbiota abundance (genus Catenibacterium id.2153) on Zinc/RING finger protein 4
with a threshold of 1e−05

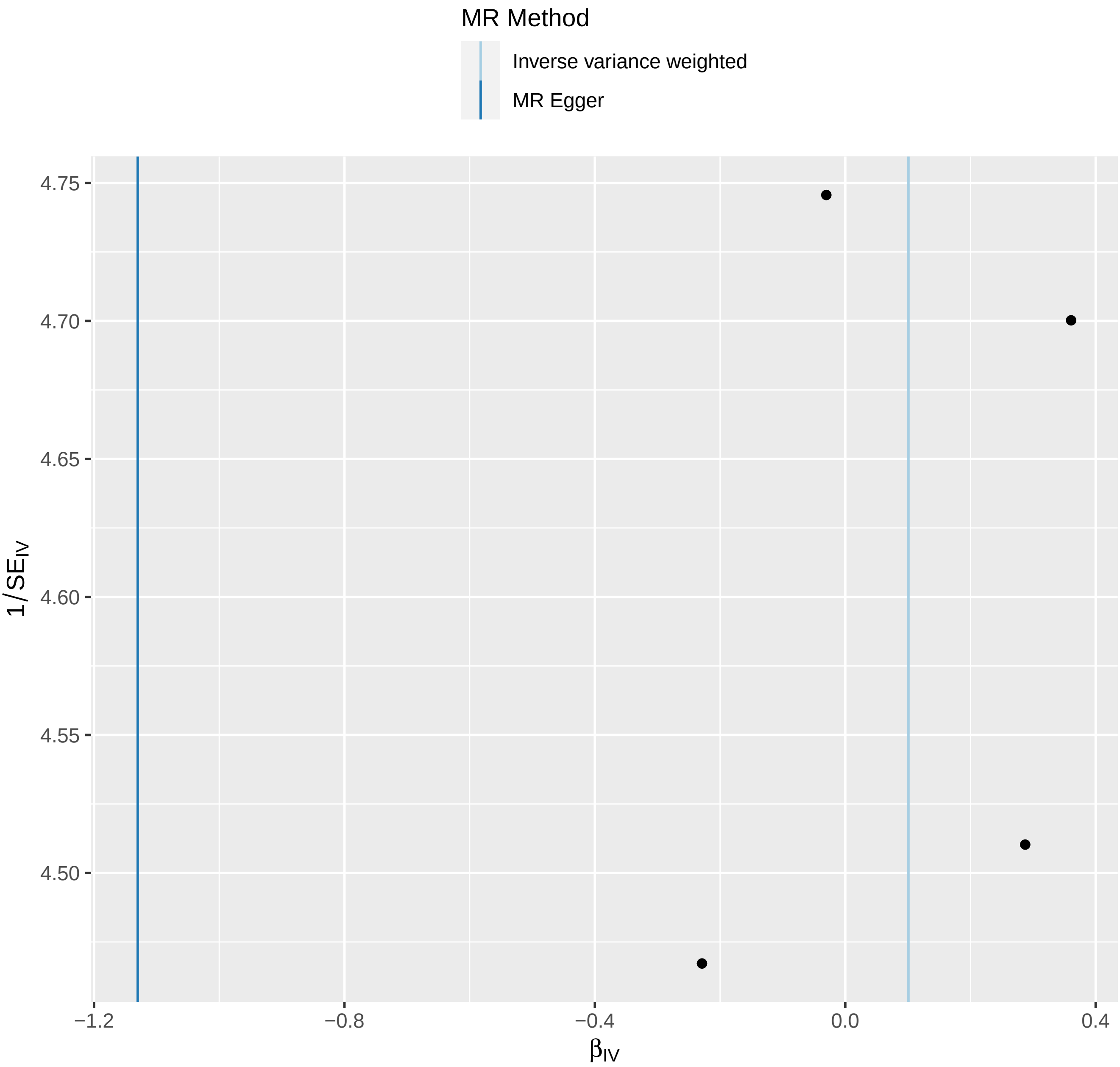

Batch 2−6560 : Gut microbiota abundance (genus Parasutterella id.2892) on Zinc/RING finger protein 4
with a threshold of 1e−05

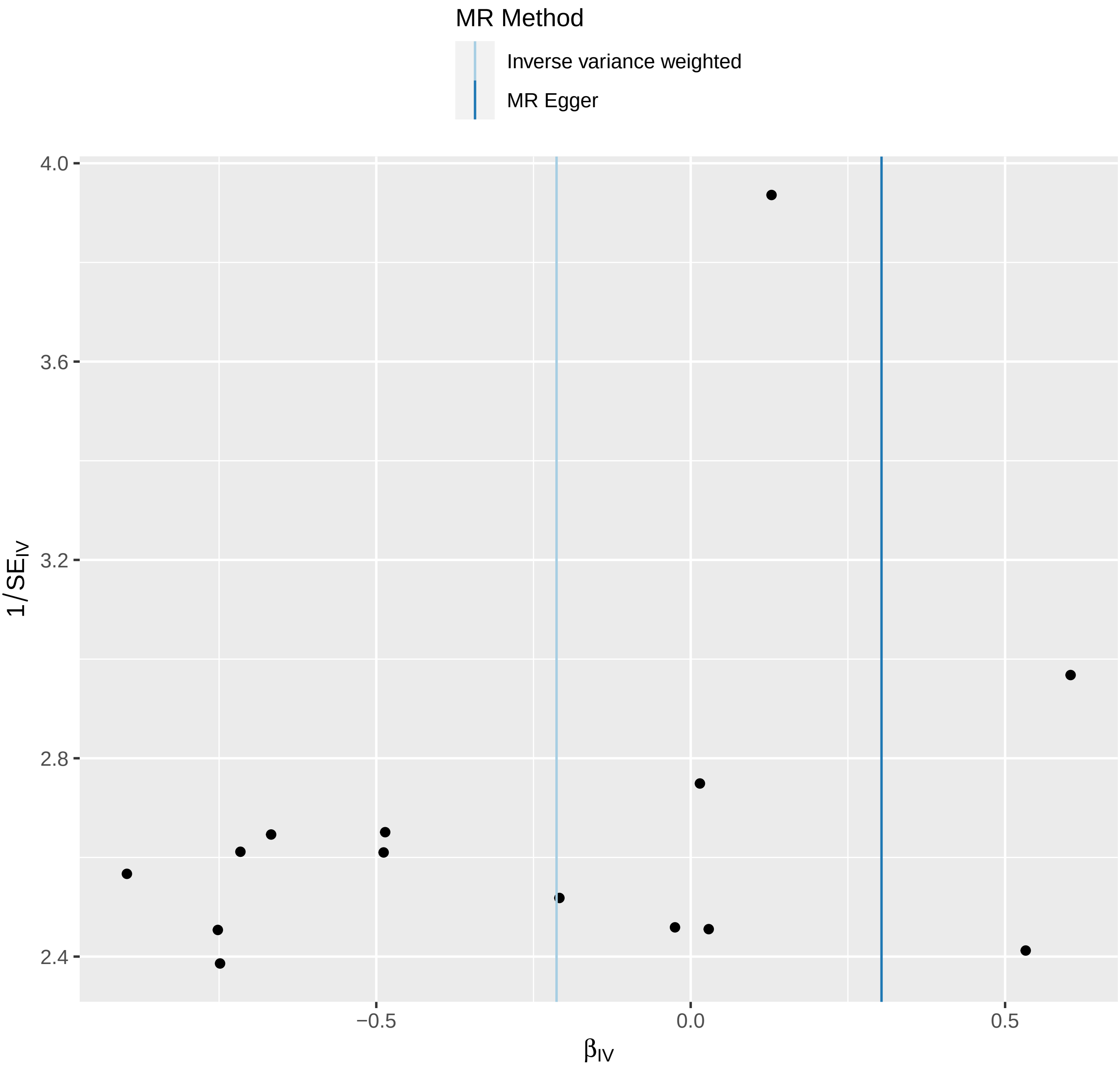

Batch 2−6561 : Gut microbiota abundance (genus Catenibacterium id.2153) on Zona pellucida sperm−binding protein 4 with a threshold of 1e−05

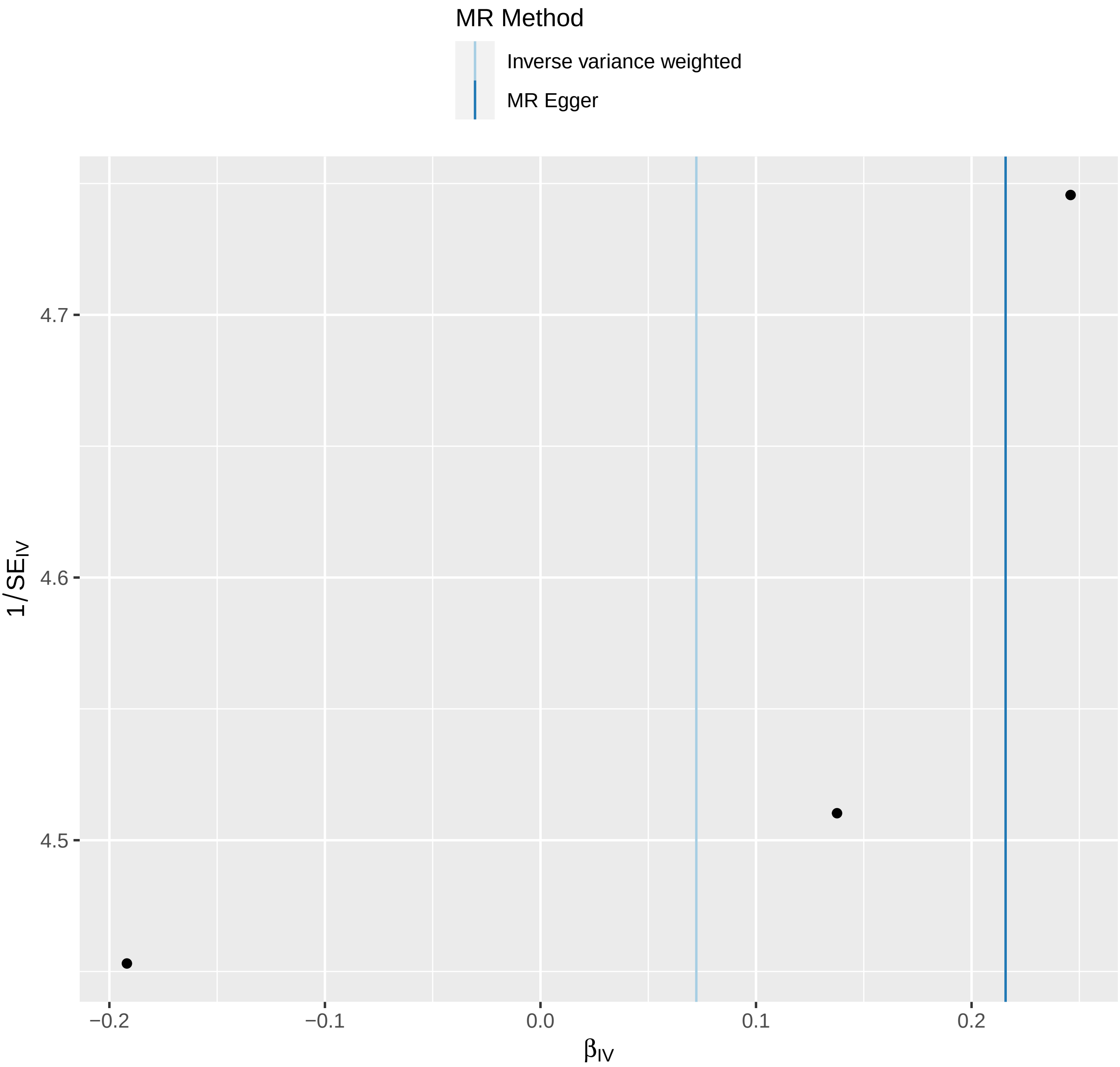

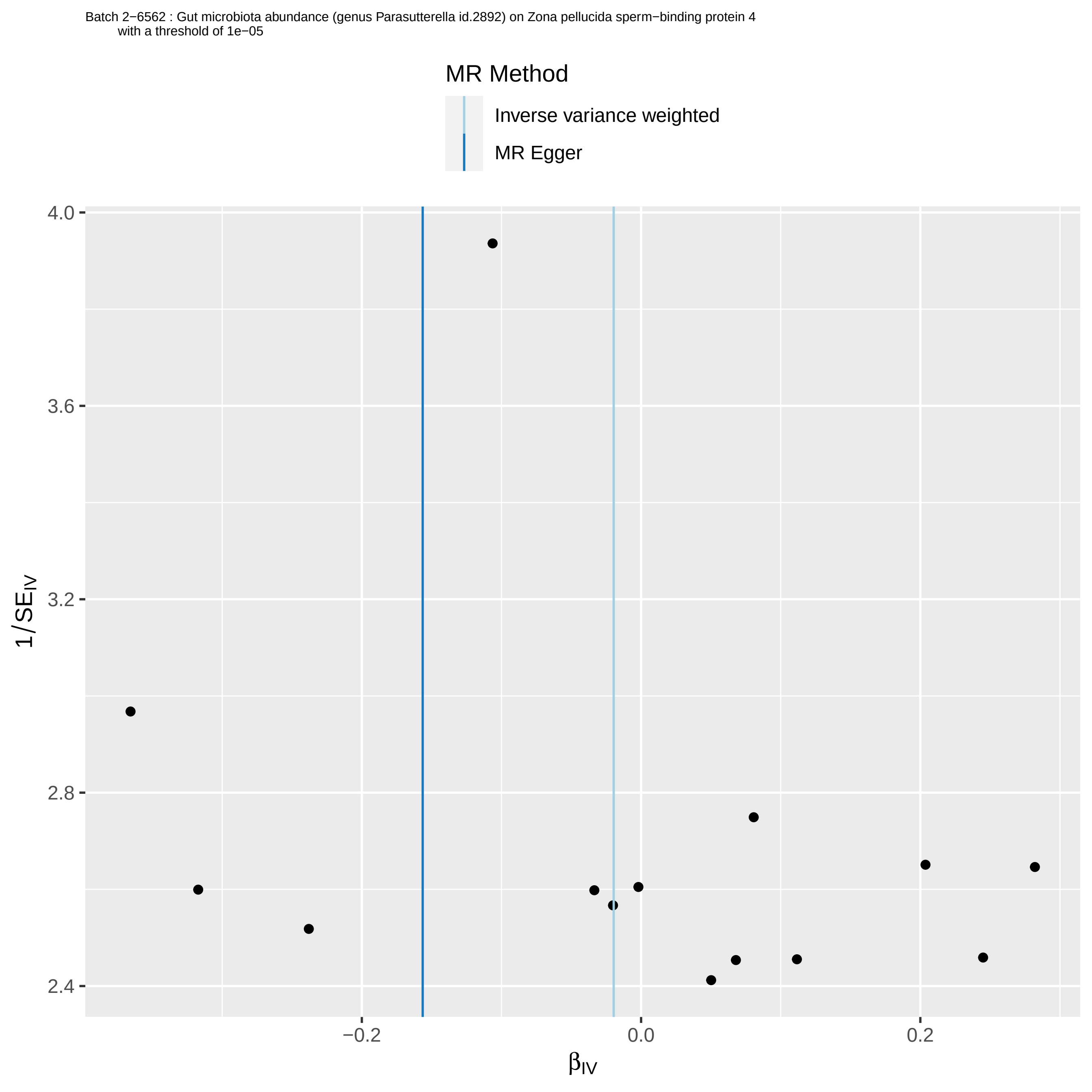
Batch 2−6562 : Gut microbiota abundance (genus Parasutterella id.2892) on Zona pellucida sperm−binding protein 4
with a threshold of 1e−05
MR Method
Inverse variance weighted
MR Egger
4.0
3.6
3.2
2.8
2.4
1/SE_IV
−0.2
0.0
0.2
β_IV

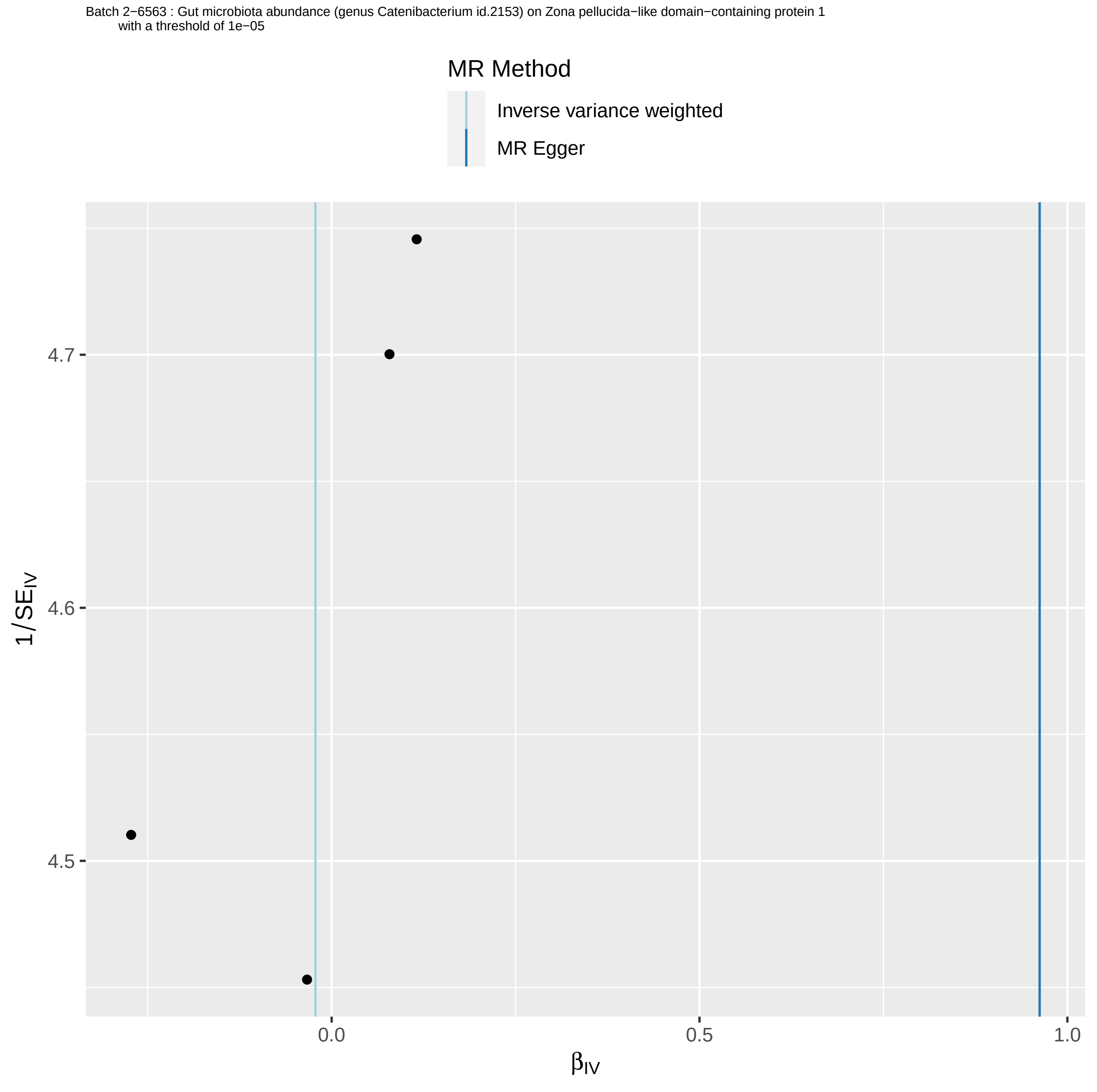
Batch 2−6563 : Gut microbiota abundance (genus Catenibacterium id.2153) on Zona pellucida−like domain−containing protein 1
with a threshold of 1e−05
MR Method
Inverse variance weighted
MR Egger
4.7
4.6
4.5
1/SE_IV
0.0
0.5
1.0
β_IV

Batch 2−6564 : Gut microbiota abundance (genus Parasutterella id.2892) on Zona pellucida−like domain−containing protein 1
with a threshold of 1e−05

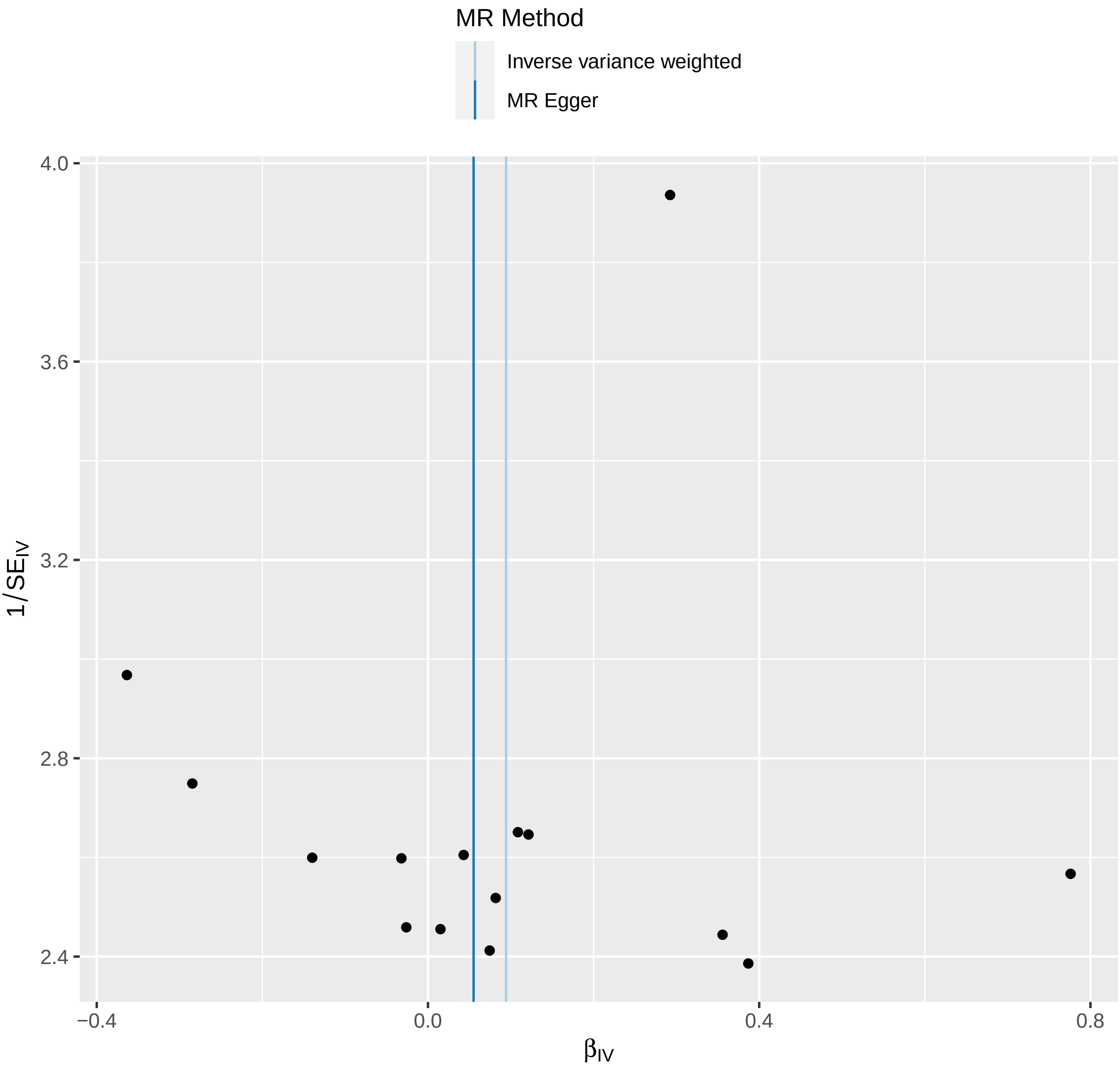

Batch 3−1 : APOBEC1 complementation factor on Diabetic nephropathy (more control exclusions)
with a threshold of 1e−05

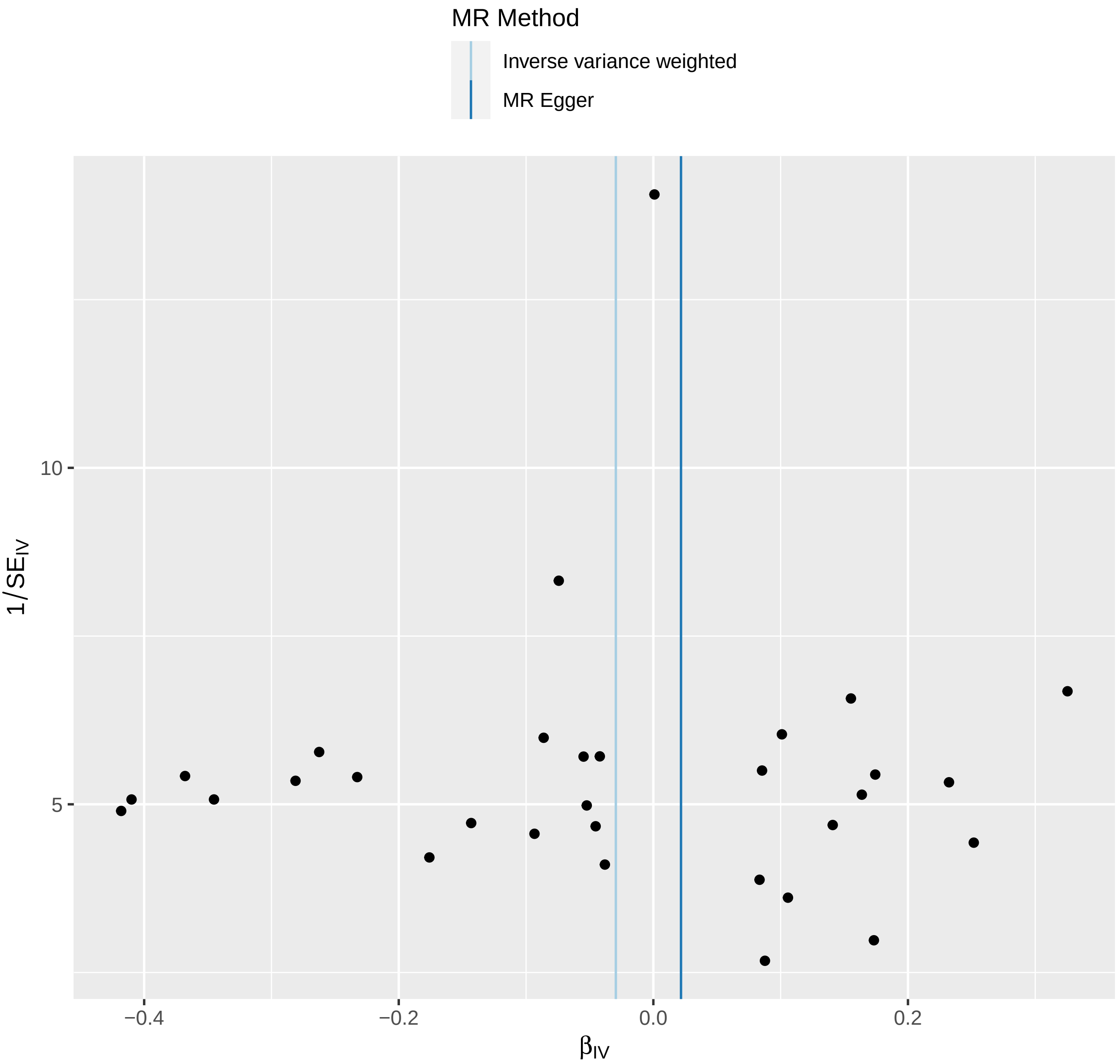

Batch 3−2 : Lactosylceramide 4−alpha−galactosyltransferase on Diabetic nephropathy (more control exclusions)
with a threshold of 1e−05

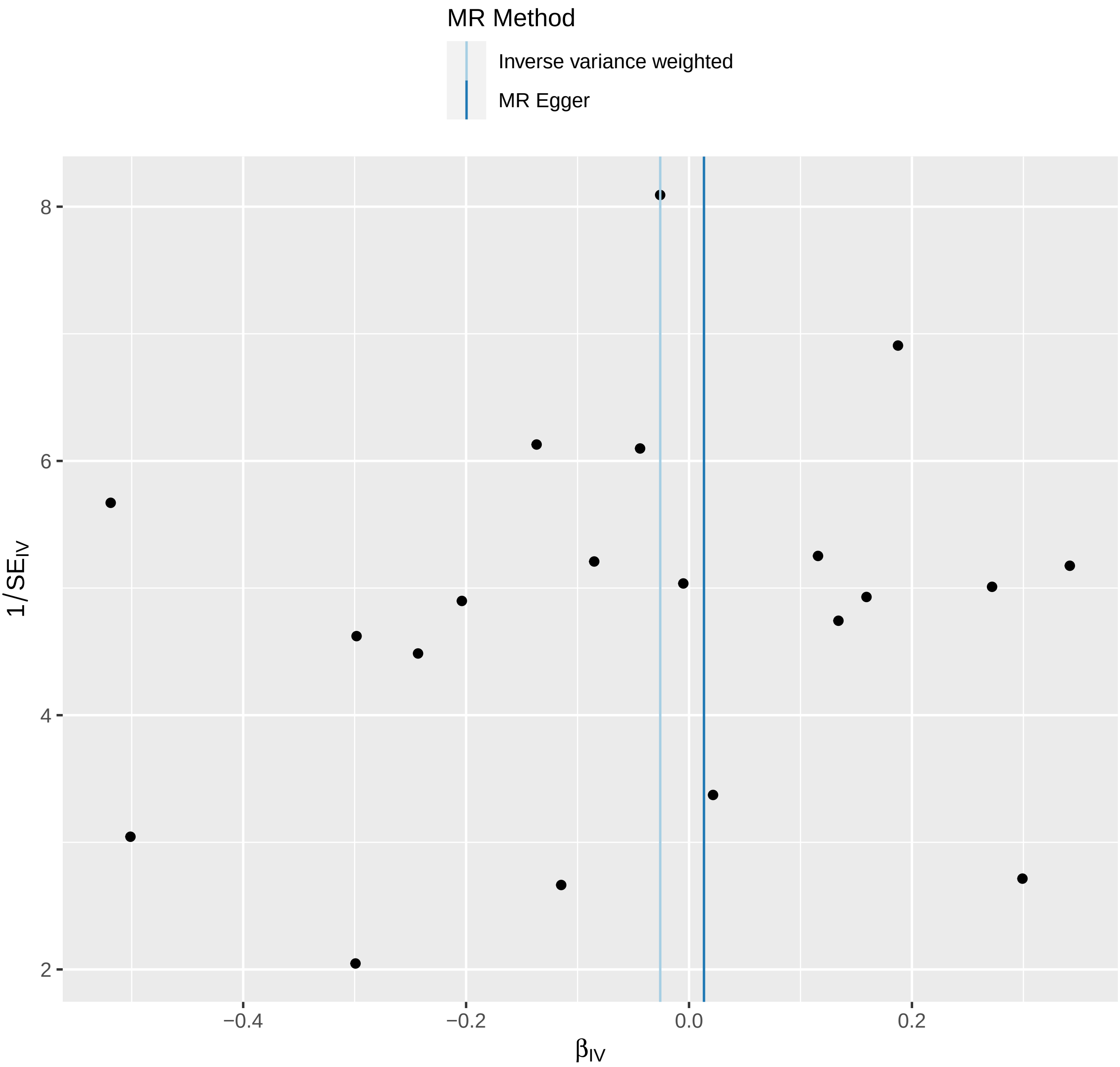

Batch 3−3 : Alanine−−tRNA ligase, cytoplasmic on Diabetic nephropathy (more control exclusions)
with a threshold of 1e−05

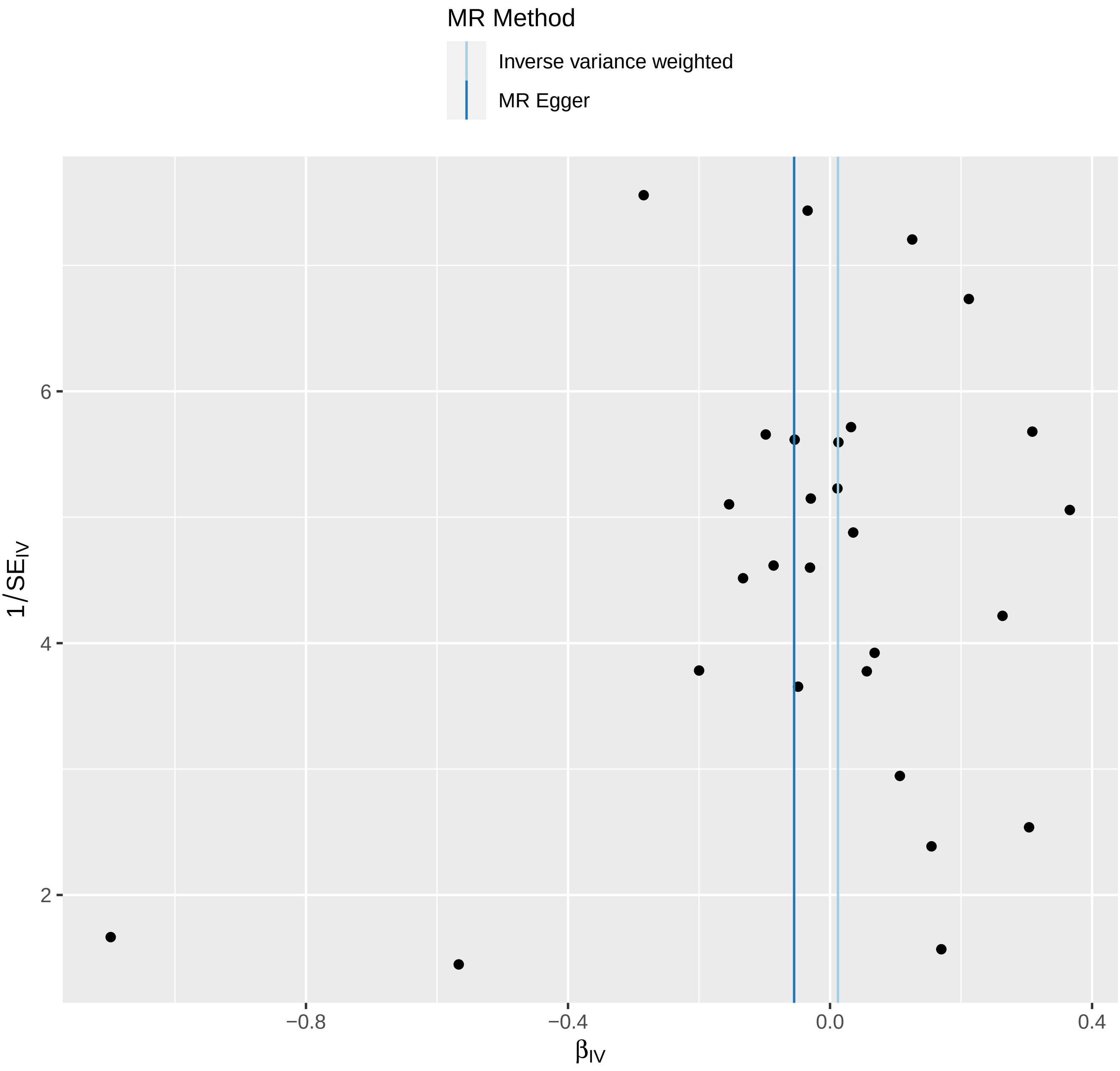

Batch 3−4 : Multidrug resistance−associated protein 6 on Diabetic nephropathy (more control exclusions)
with a threshold of 1e−05

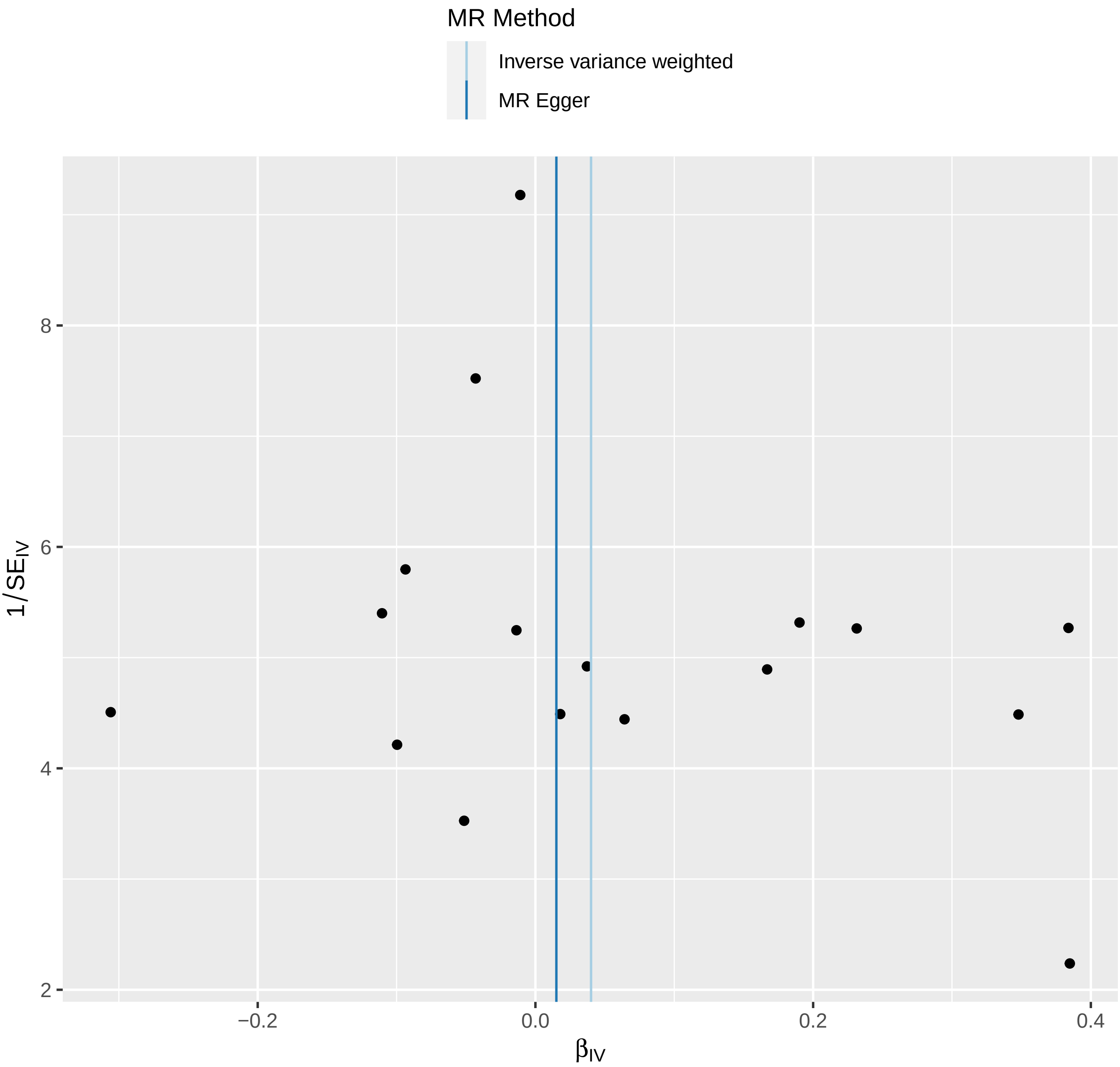

Batch 3−5 : Monoacylglycerol lipase ABHD12 on Diabetic nephropathy (more control exclusions)
with a threshold of 1e−05

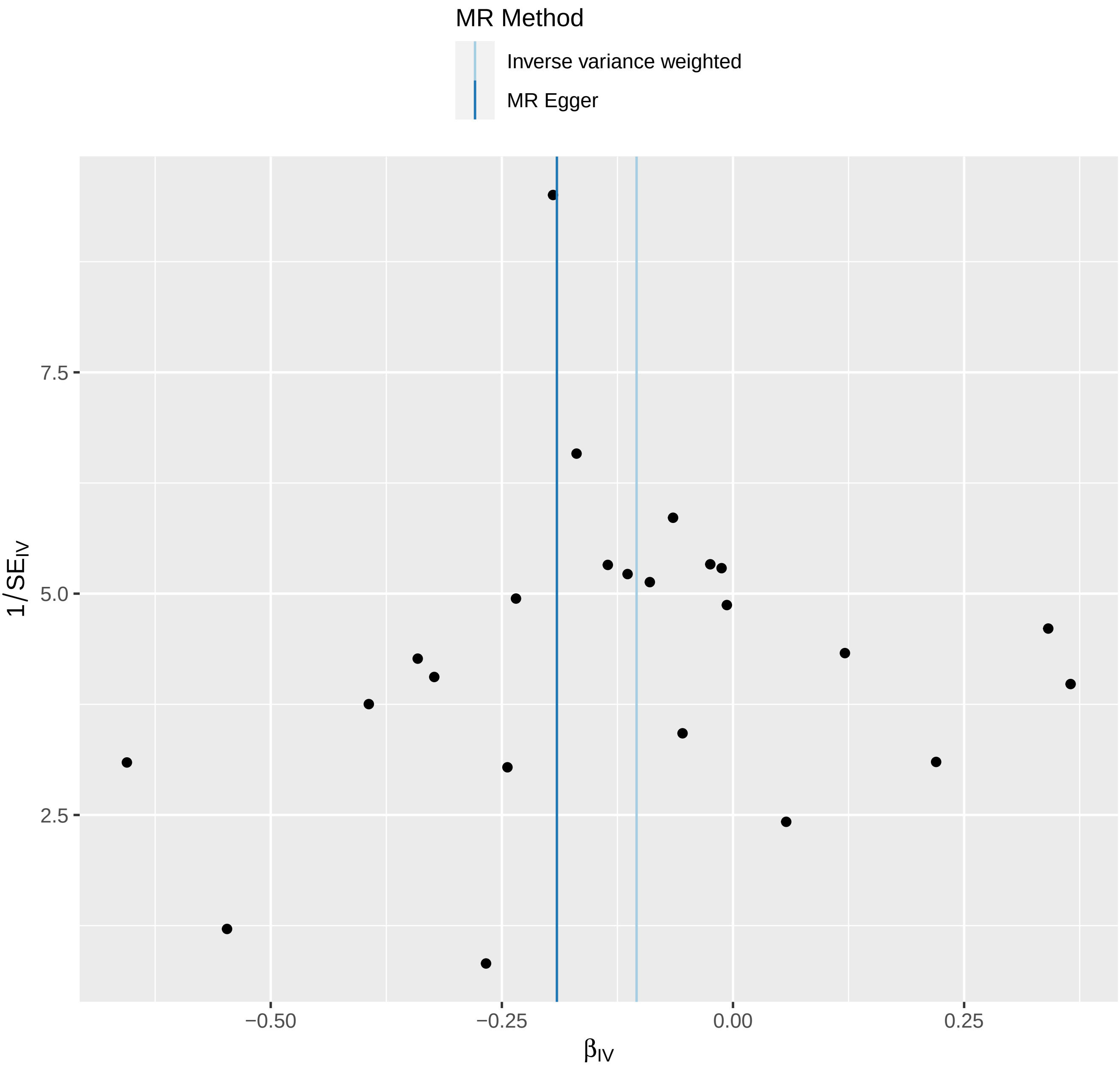

Batch 3−6 : Alpha/beta hydrolase domain−containing protein 14A on Diabetic nephropathy (more control exclusions)
with a threshold of 1e−05

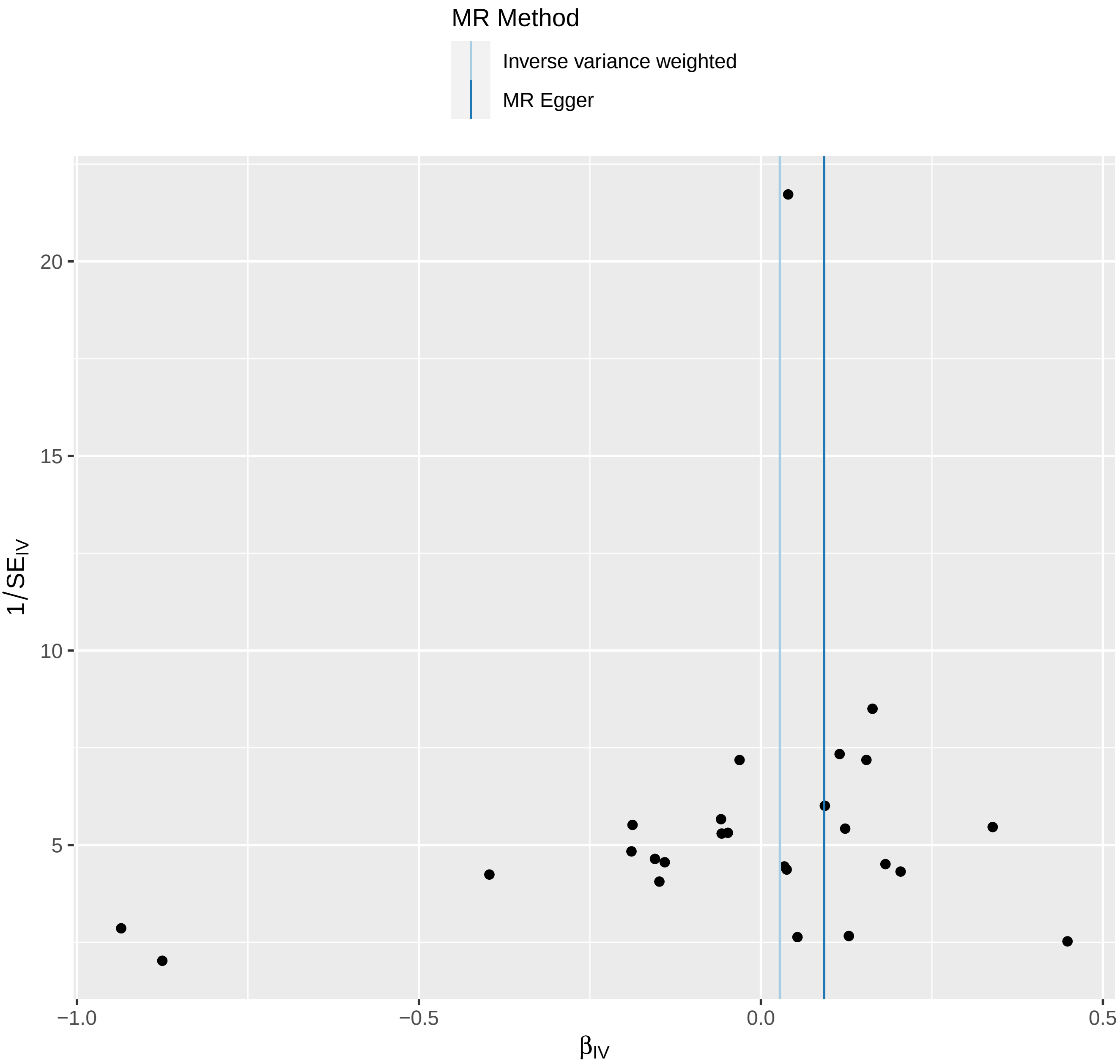

Batch 3−7 : Tyrosine−protein kinase ABL1 on Diabetic nephropathy (more control exclusions)
with a threshold of 1e−05

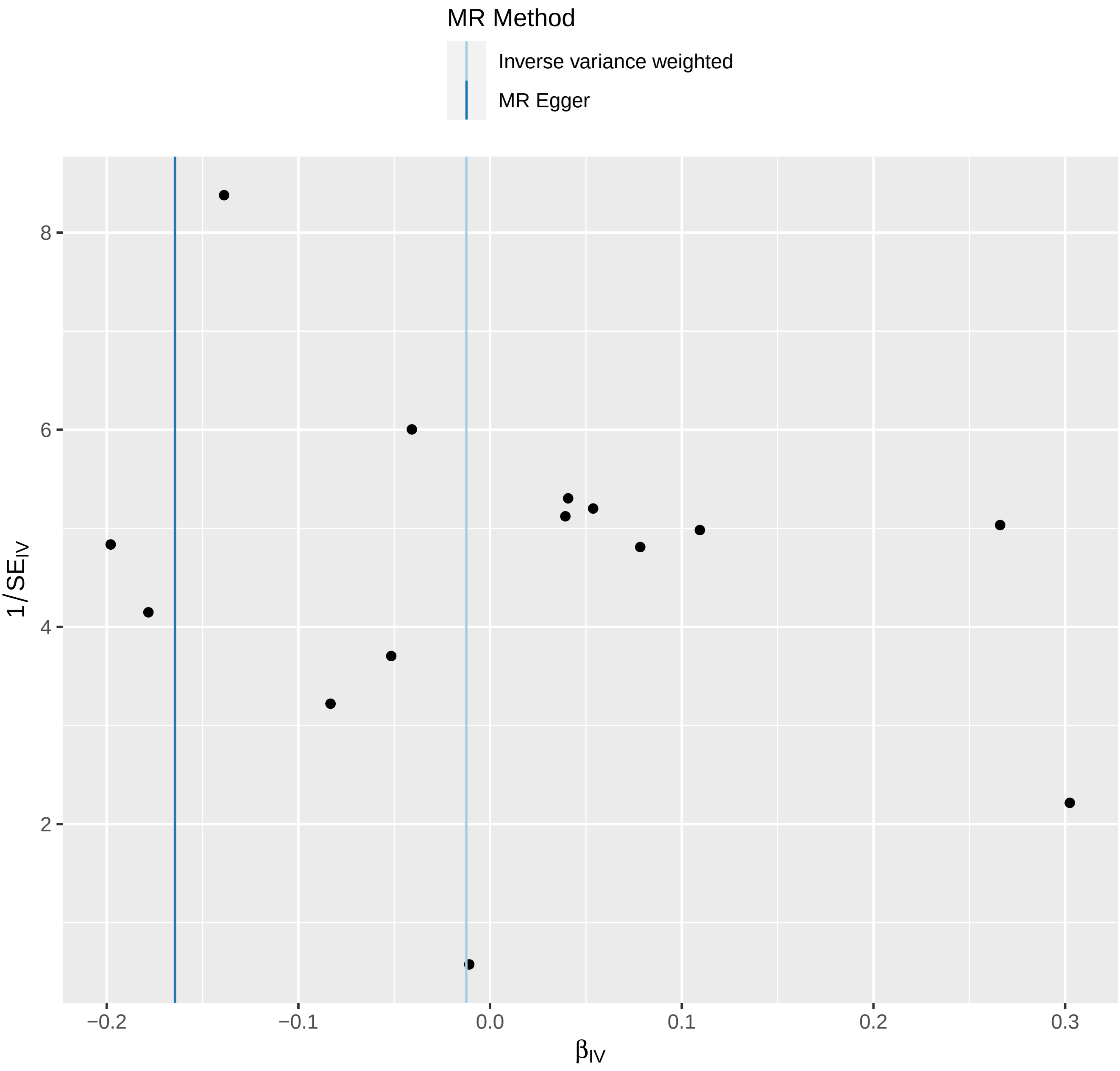

Batch 3−8 : Abelson tyrosine−protein kinase 2 on Diabetic nephropathy (more control exclusions)
with a threshold of 1e−05

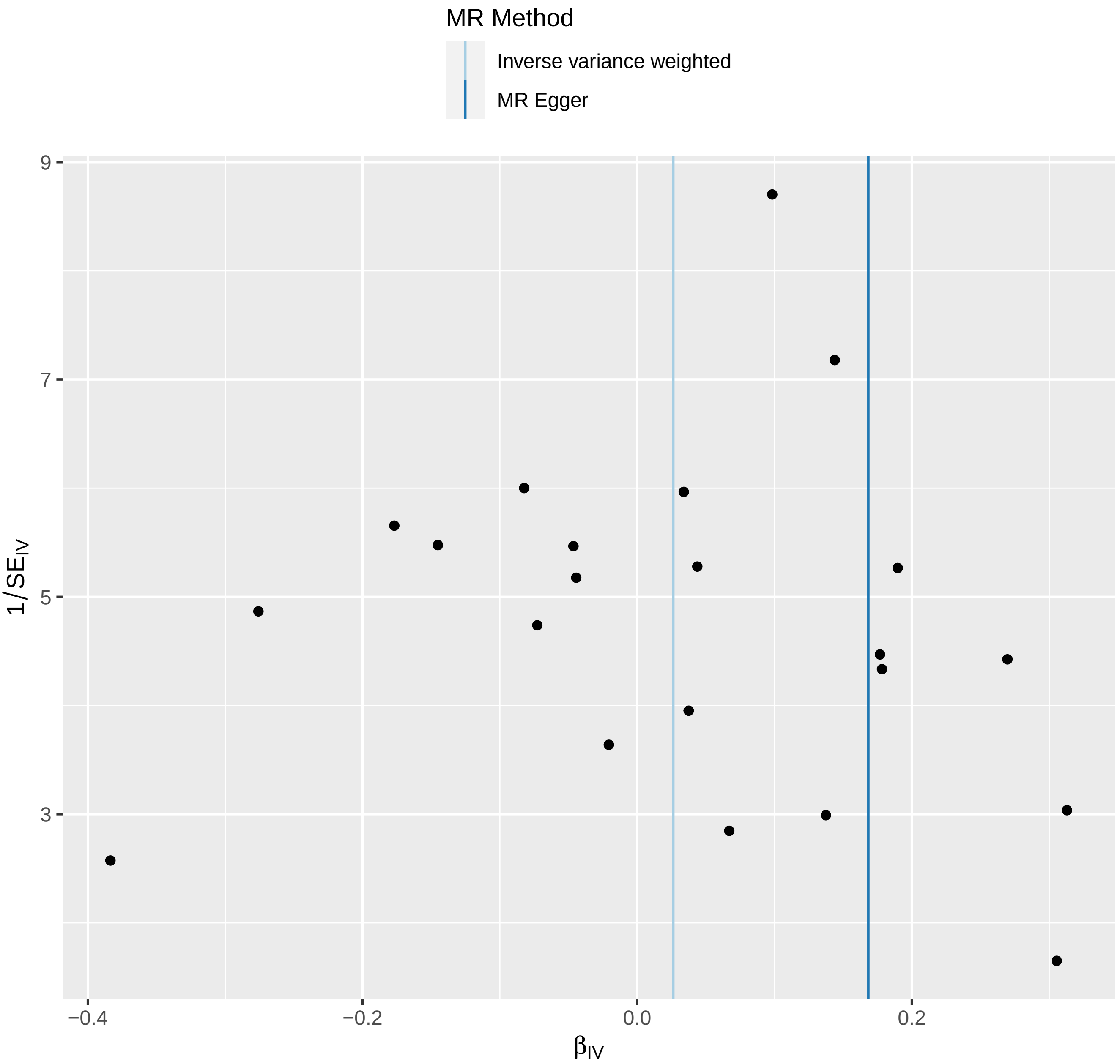

Batch 3−9 : Actin−binding LIM protein 3 on Diabetic nephropathy (more control exclusions)
with a threshold of 1e−05

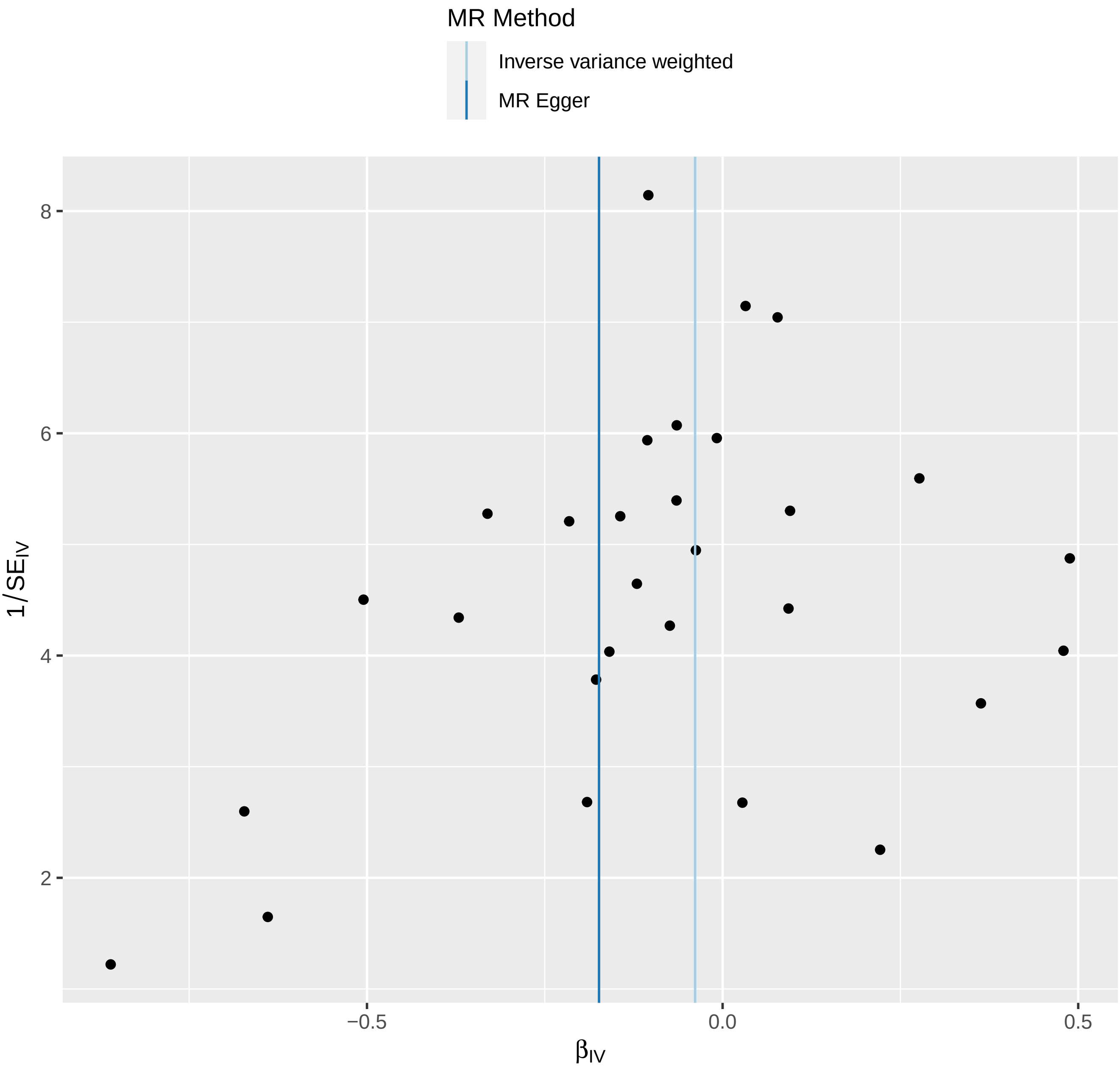

Batch 3−10 : Histo−blood group ABO system transferase on Diabetic nephropathy (more control exclusions)
with a threshold of 1e−05

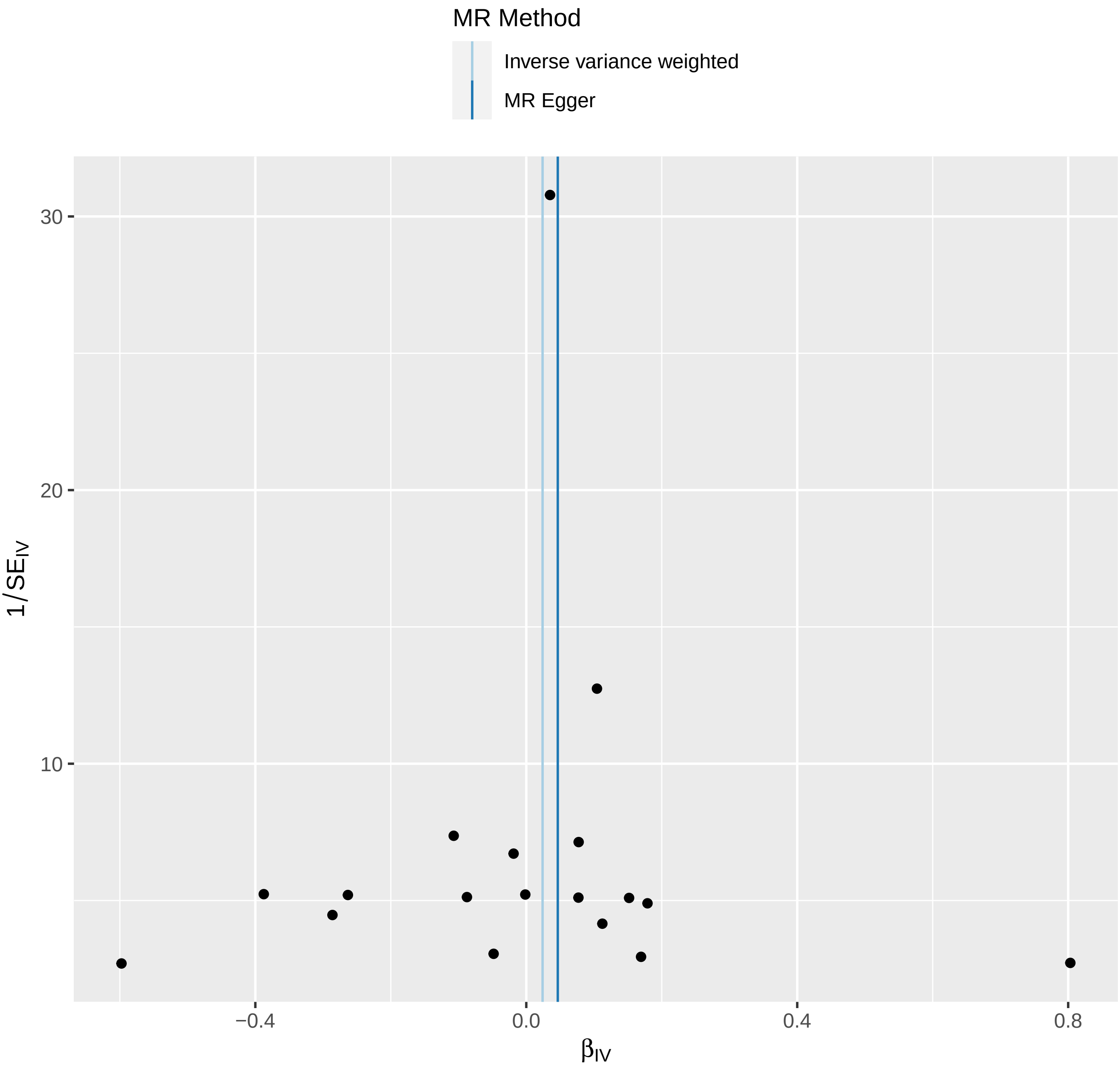

Batch 3−11 : Acetyl−CoA carboxylase 2 on Diabetic nephropathy (more control exclusions)
with a threshold of 1e−05

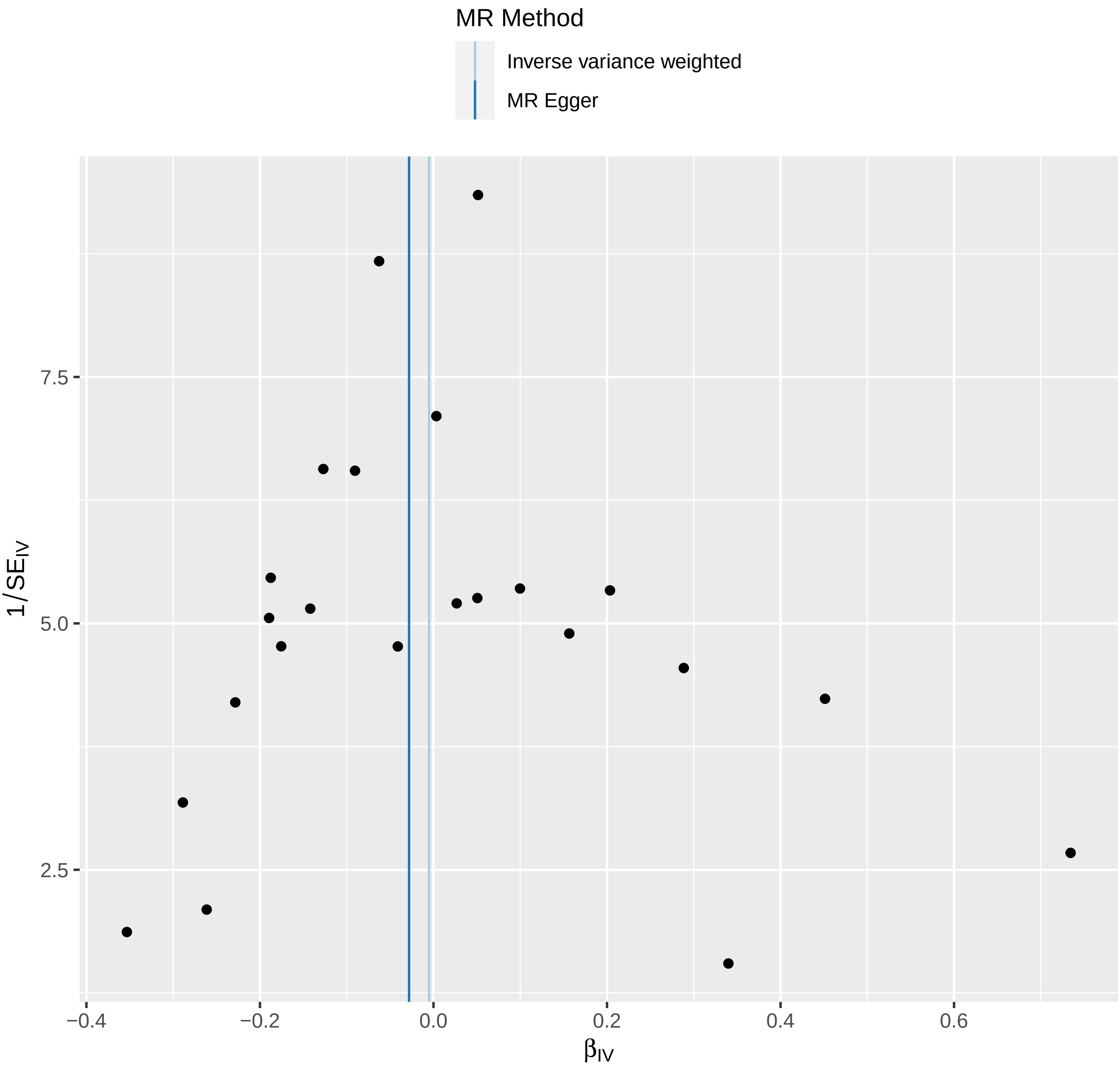

Batch 3−12 : Aggrecan core protein on Diabetic nephropathy (more control exclusions)
with a threshold of 1e−05

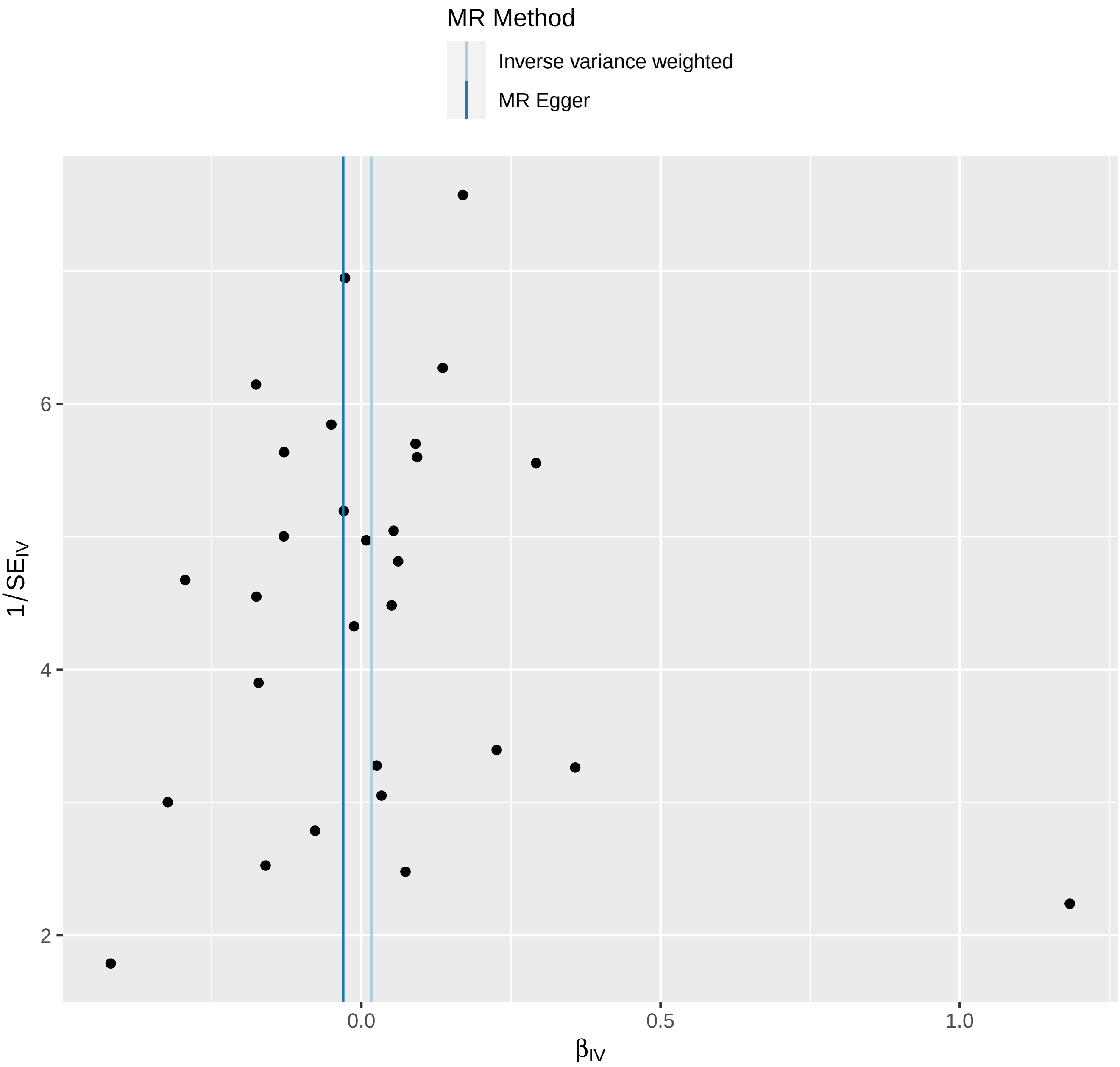

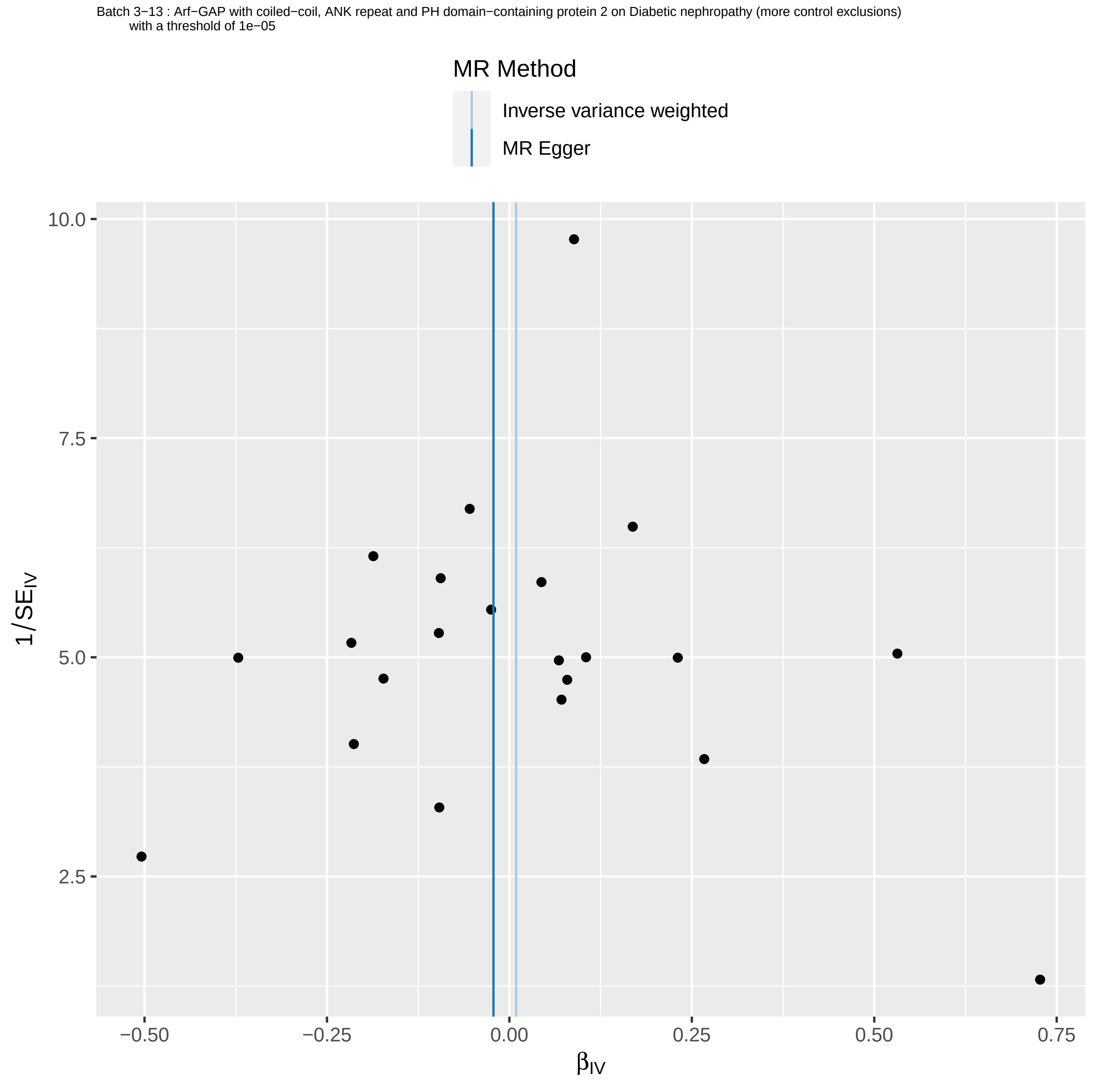

Batch 3−13 : Arf−GAP with coiled−coil, ANK repeat and PH domain−containing protein 2 on Diabetic nephropathy (more control exclusions)
with a threshold of 1e−05
MR Method
Inverse variance weighted
MR Egger
10.0
7.5
5.0
2.5
1/SE_IV
−0.50
−0.25
0.00
0.25
0.50
0.75
β_IV

Batch 3−14 : Acyl−CoA−binding domain−containing protein 6 on Diabetic nephropathy (more control exclusions)
with a threshold of 1e−05

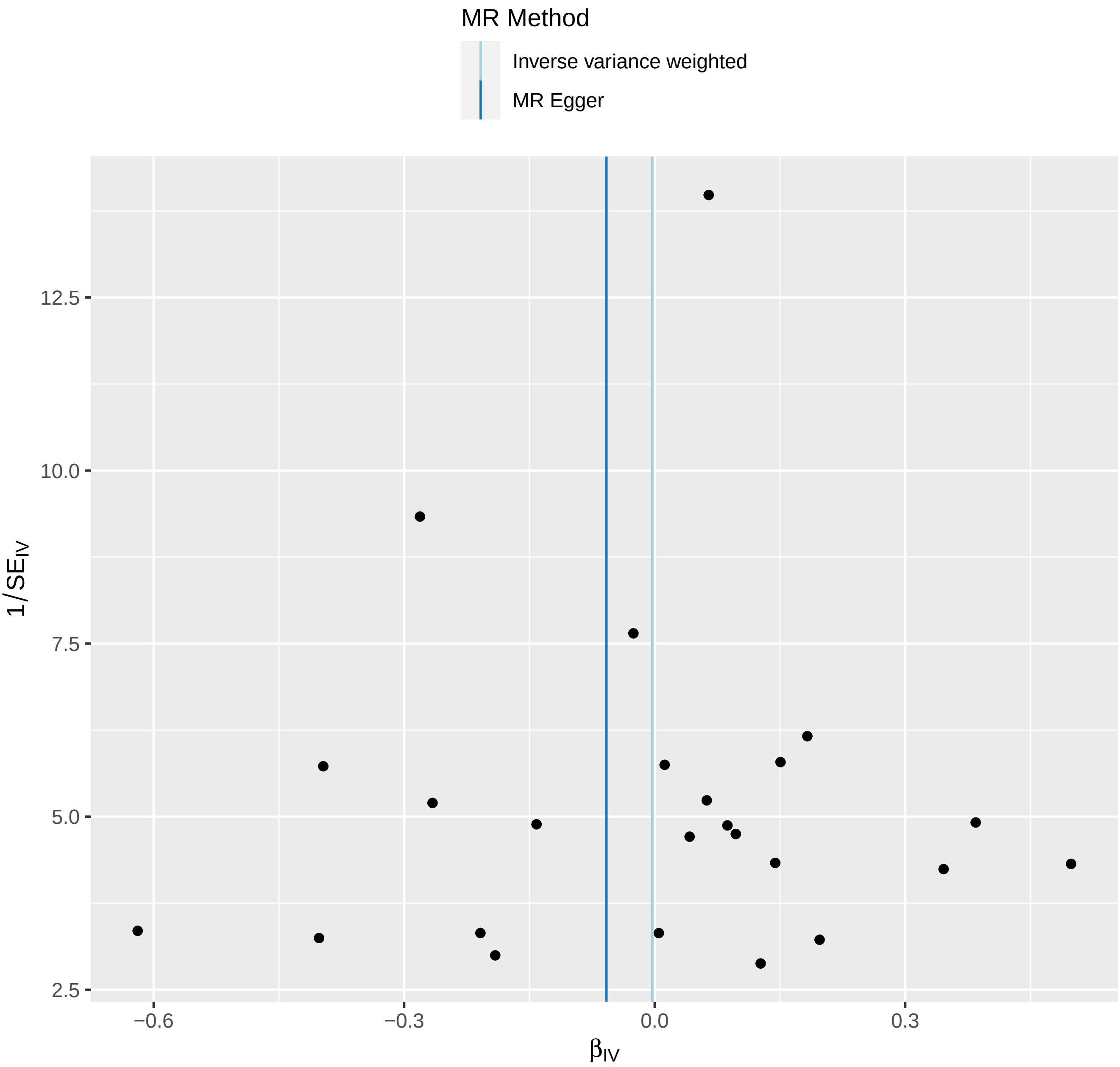

Batch 3−15 : Acyl−CoA−binding domain−containing protein 7 on Diabetic nephropathy (more control exclusions)
with a threshold of 1e−05

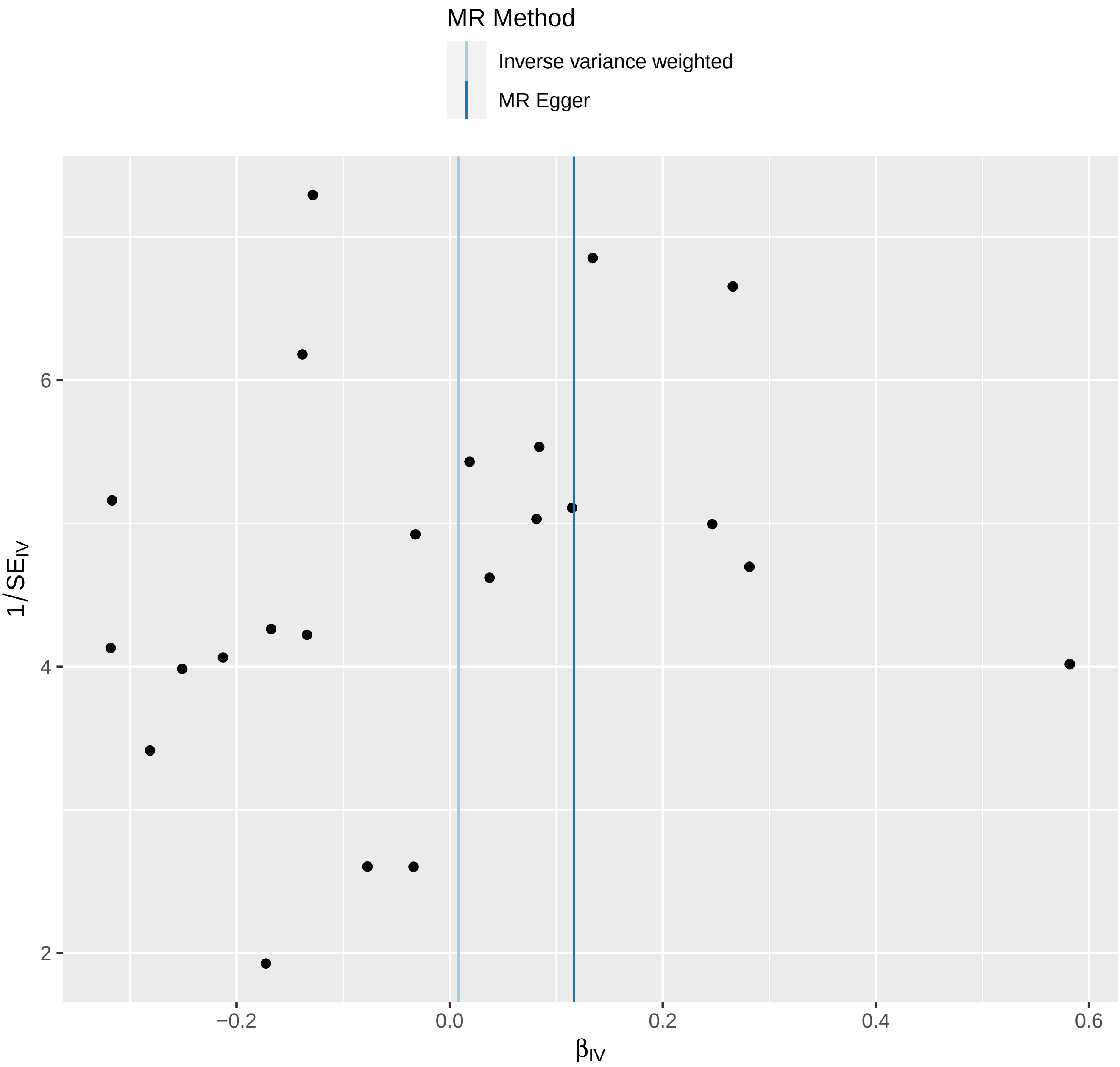

Batch 3−16 : Angiotensin−converting enzyme 2 on Diabetic nephropathy (more control exclusions)
with a threshold of 1e−05

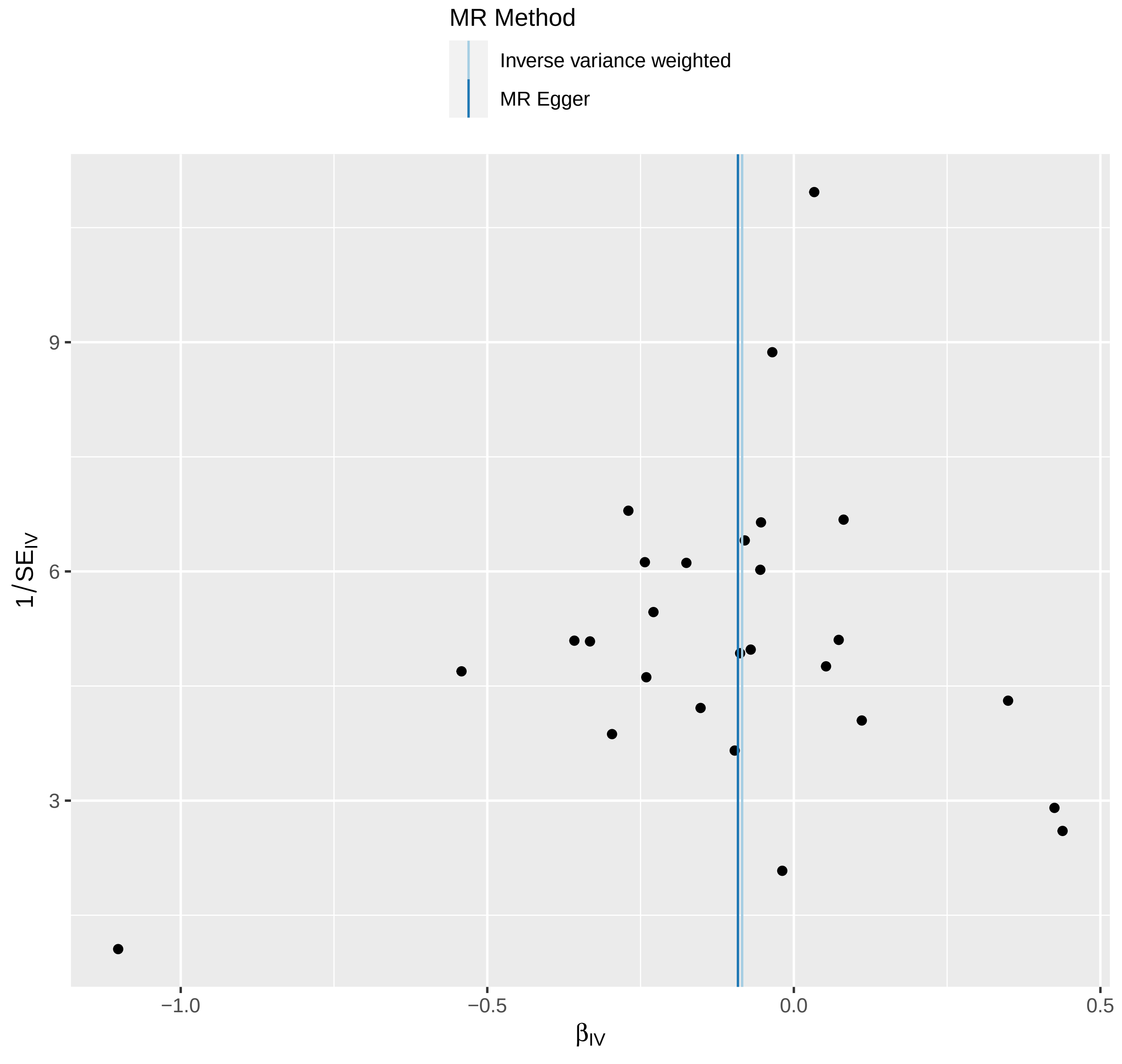

Batch 3−17 : ATP−citrate synthase on Diabetic nephropathy (more control exclusions)
with a threshold of 1e−05

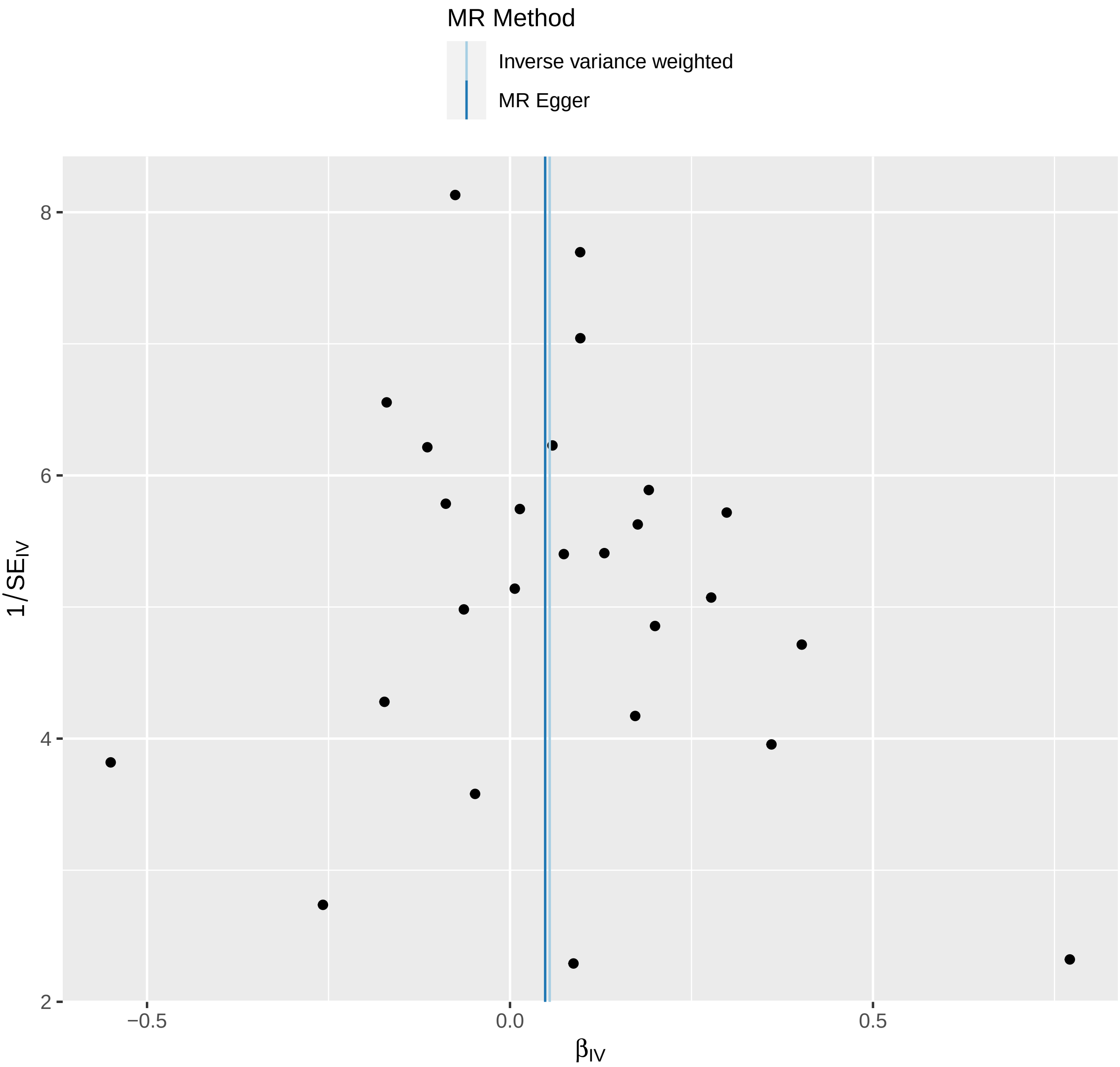

Batch 3−18 : Low molecular weight phosphotyrosine protein phosphatase on Diabetic nephropathy (more control exclusions) with a threshold of 1e−05

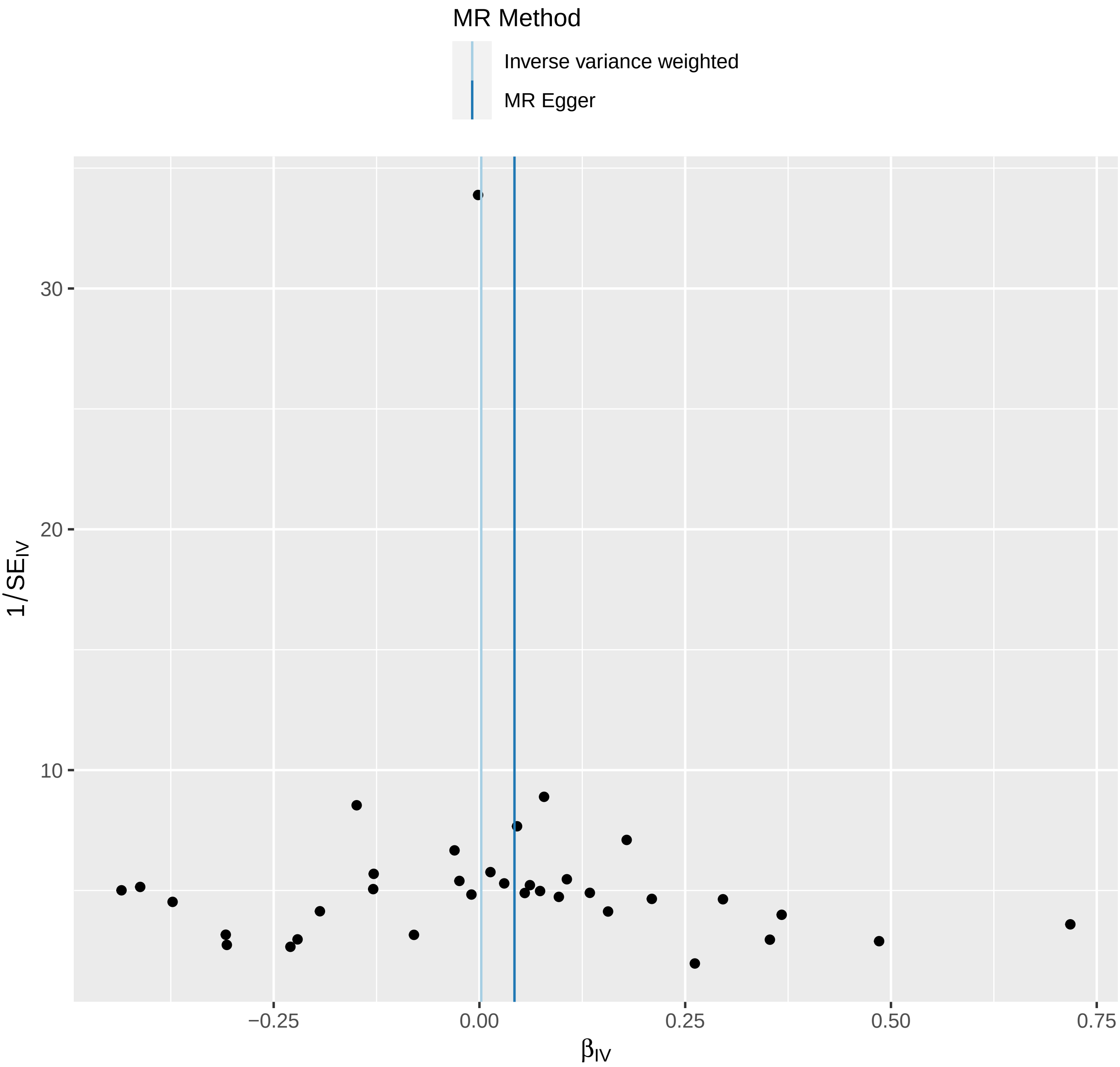

Batch 3−19 : Lysosomal acid phosphatase on Diabetic nephropathy (more control exclusions)
with a threshold of 1e−05

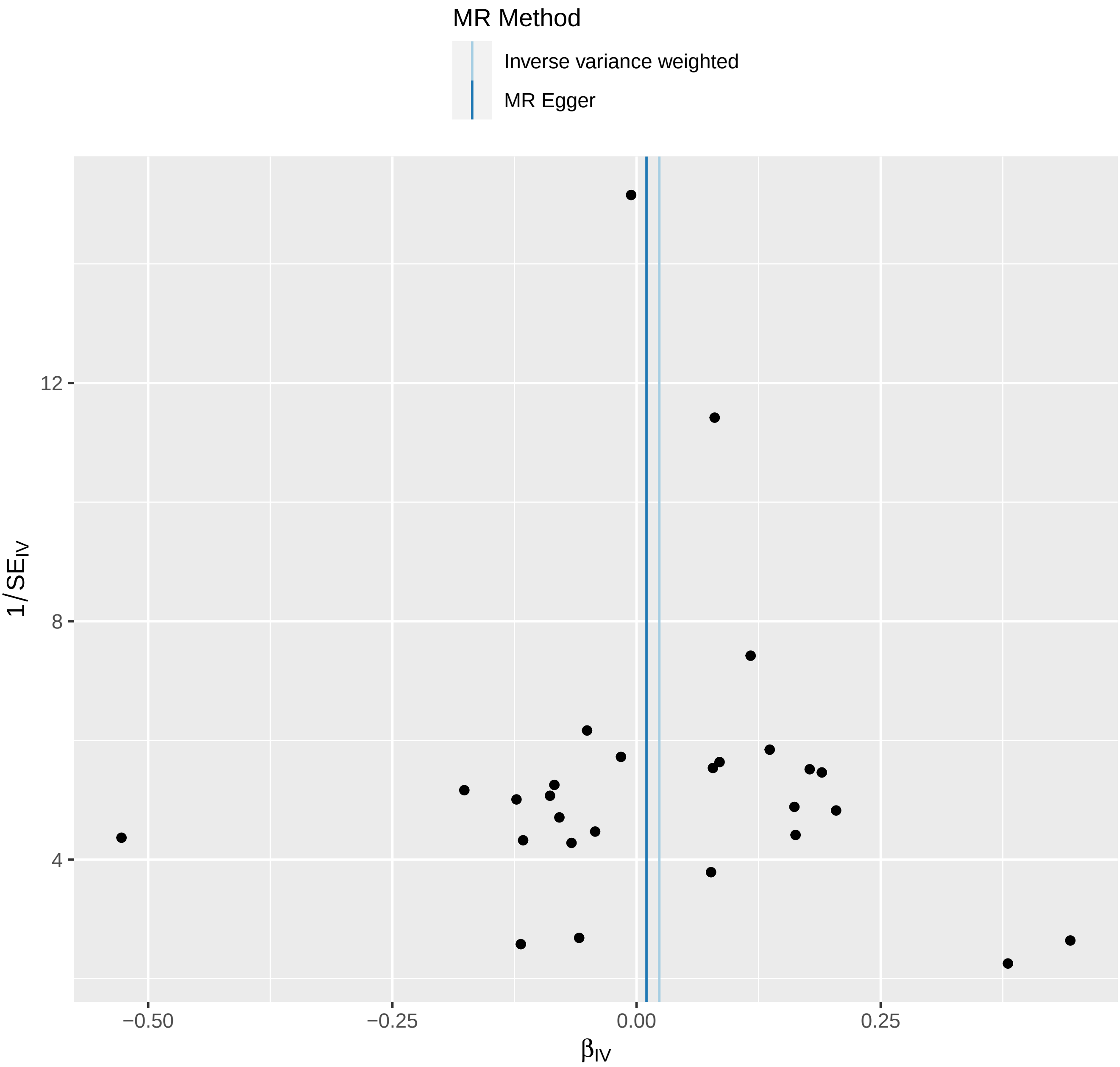

Batch 3−20 : Tartrate−resistant acid phosphatase type 5 on Diabetic nephropathy (more control exclusions)
with a threshold of 1e−05

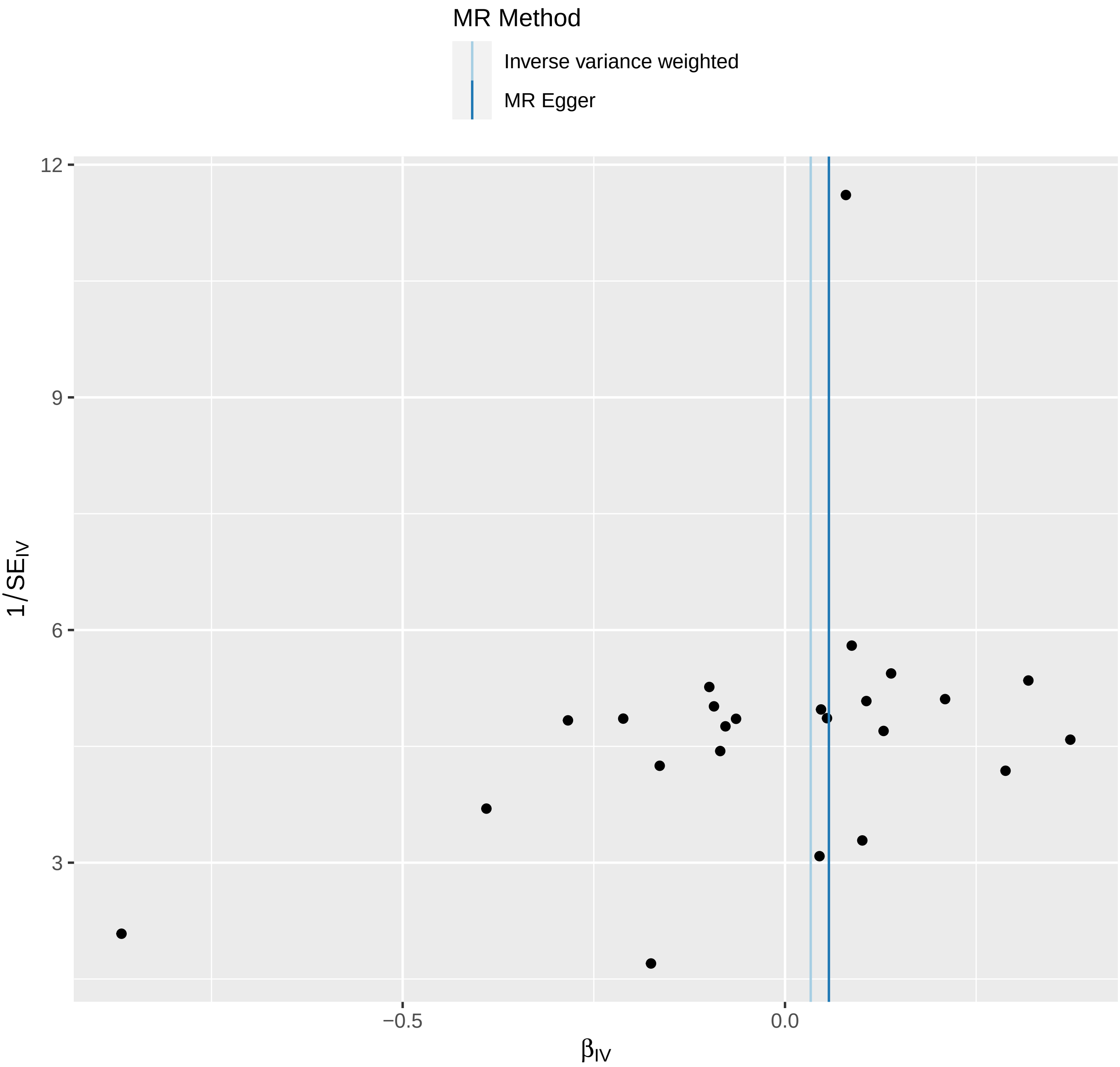

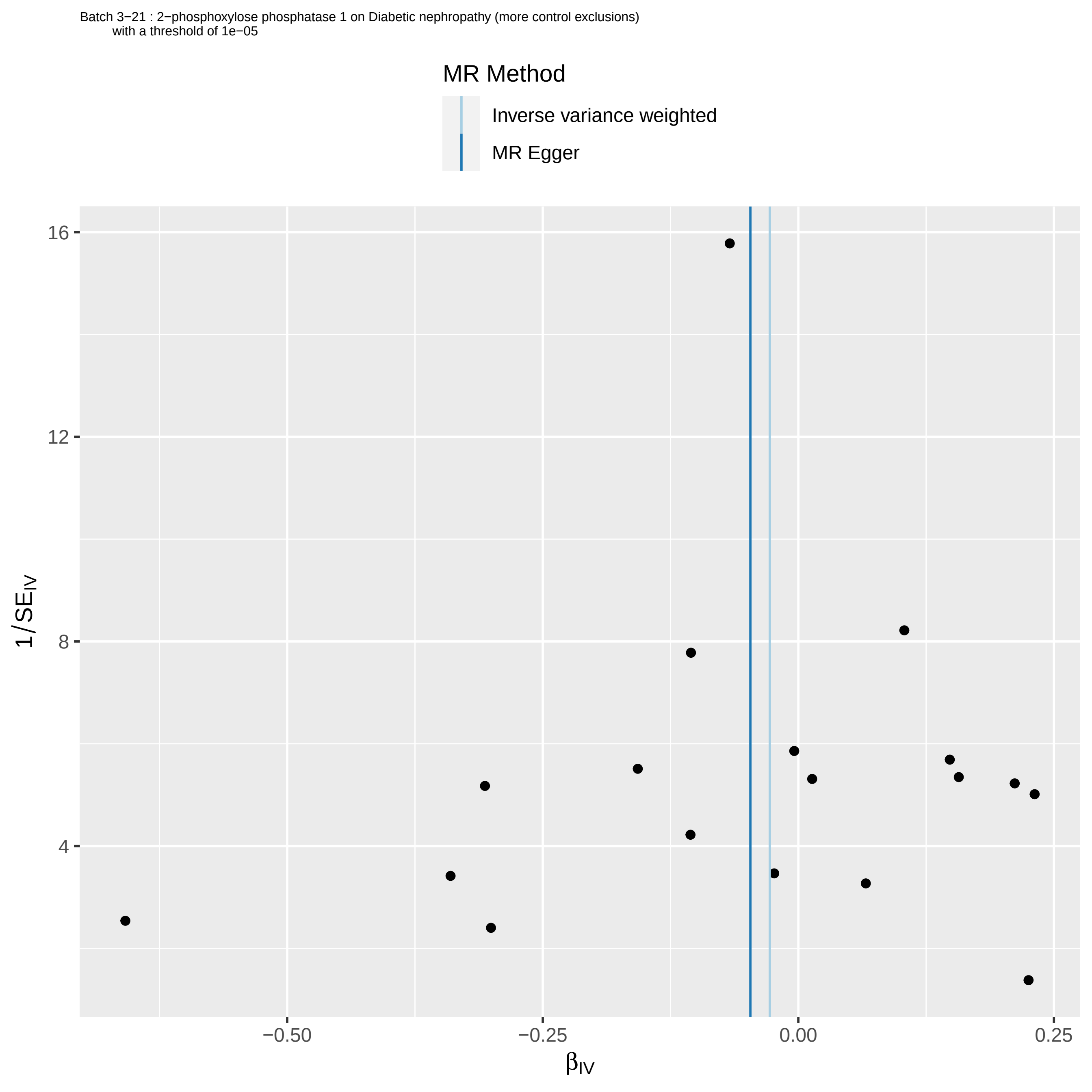
Batch 3−21 : 2−phosphoxylose phosphatase 1 on Diabetic nephropathy (more control exclusions)
with a threshold of 1e−05
MR Method
Inverse variance weighted
MR Egger
16
12
8
4
1/SE_IV
−0.50
−0.25
0.00
0.25
β_IV

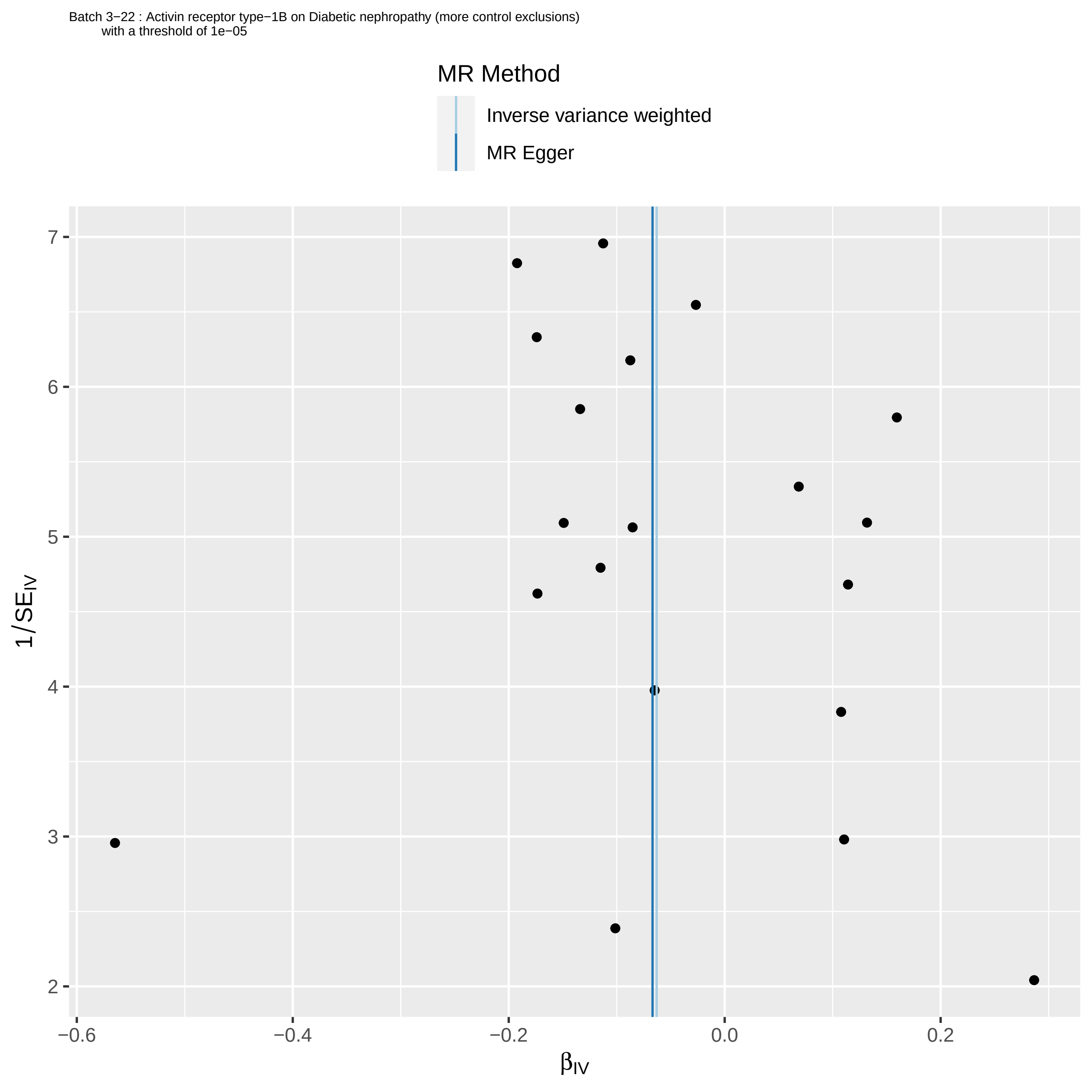

Batch 3−22 : Activin receptor type−1B on Diabetic nephropathy (more control exclusions)
with a threshold of 1e−05
MR Method
Inverse variance weighted
MR Egger
1/SE_IV
β_IV
7
6
5
4
3
2
−0.6
−0.4
−0.2
0.0
0.2

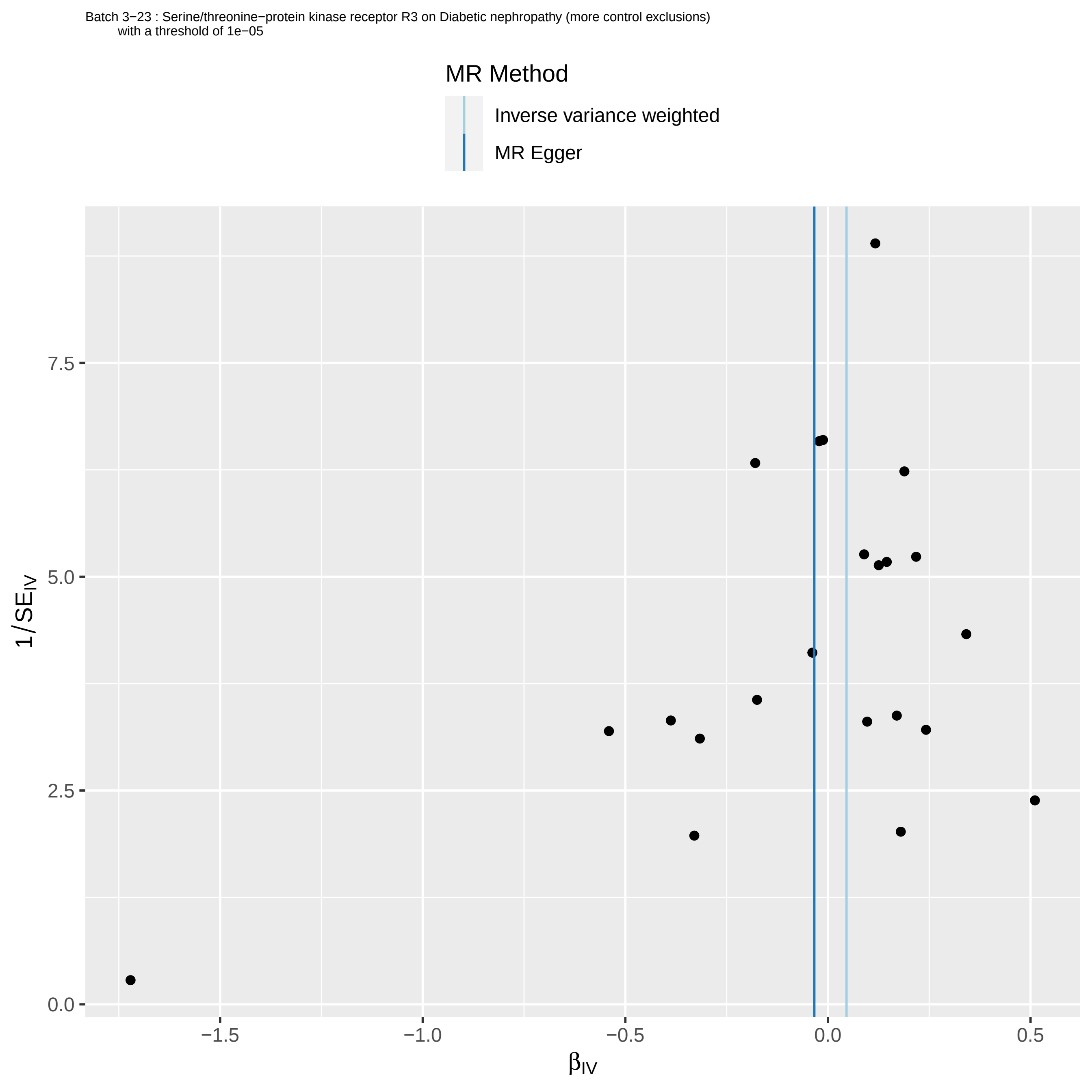
Batch 3−23 : Serine/threonine−protein kinase receptor R3 on Diabetic nephropathy (more control exclusions)
with a threshold of 1e−05
MR Method
Inverse variance weighted
MR Egger
1/SE_IV
7.5
5.0
2.5
0.0
−1.5
−1.0
−0.5
0.0
0.5
β_IV

Batch 3−24 : Acylphosphatase−2 on Diabetic nephropathy (more control exclusions)
with a threshold of 1e−05

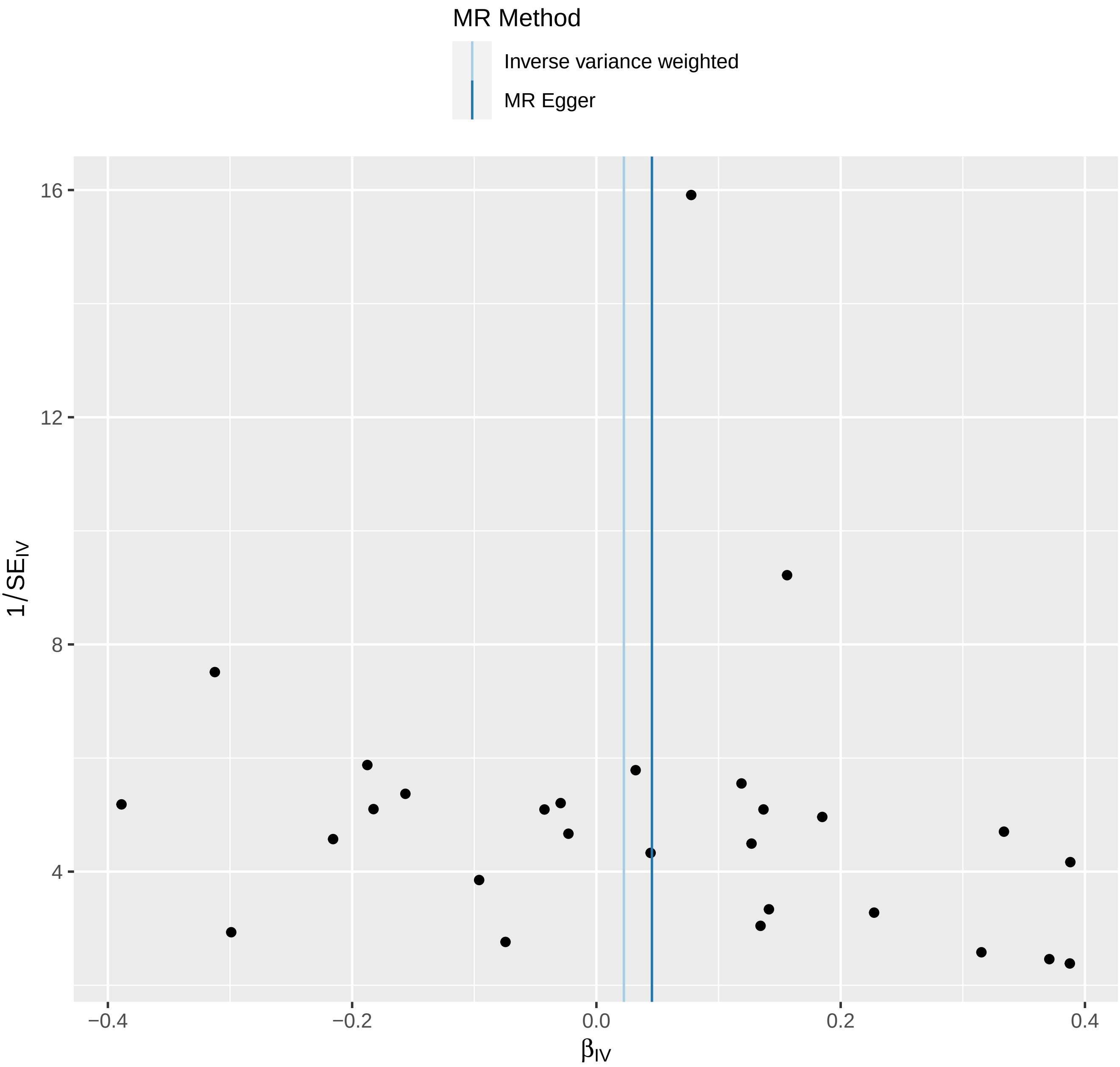

Batch 3−25 : Disintegrin and metalloproteinase domain−containing protein 11 on Diabetic nephropathy (more control exclusions) with a threshold of 1e−05

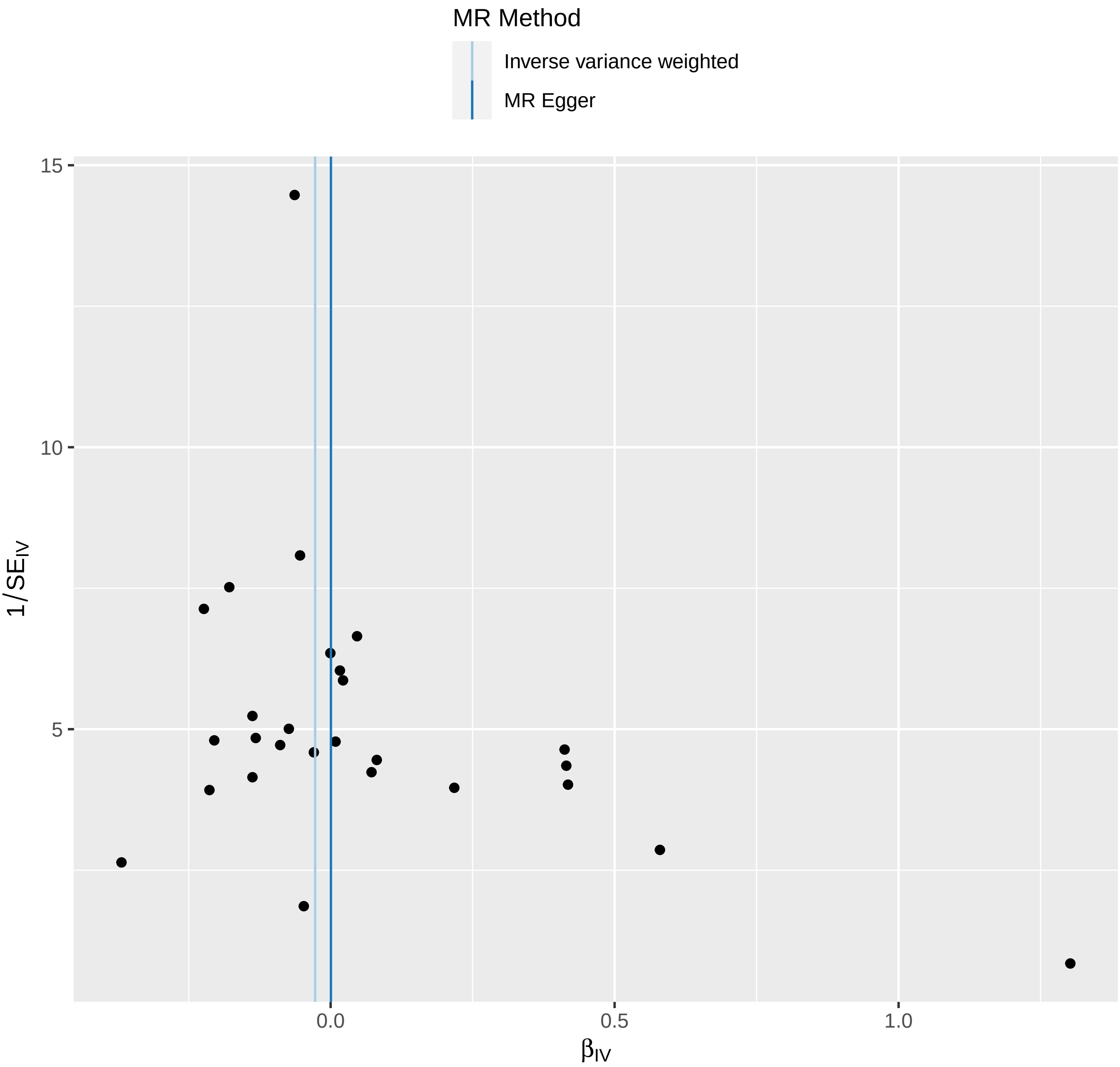

Batch 3−26 : Disintegrin and metalloproteinase domain−containing protein 12 on Diabetic nephropathy (more control exclusions)
with a threshold of 1e−05

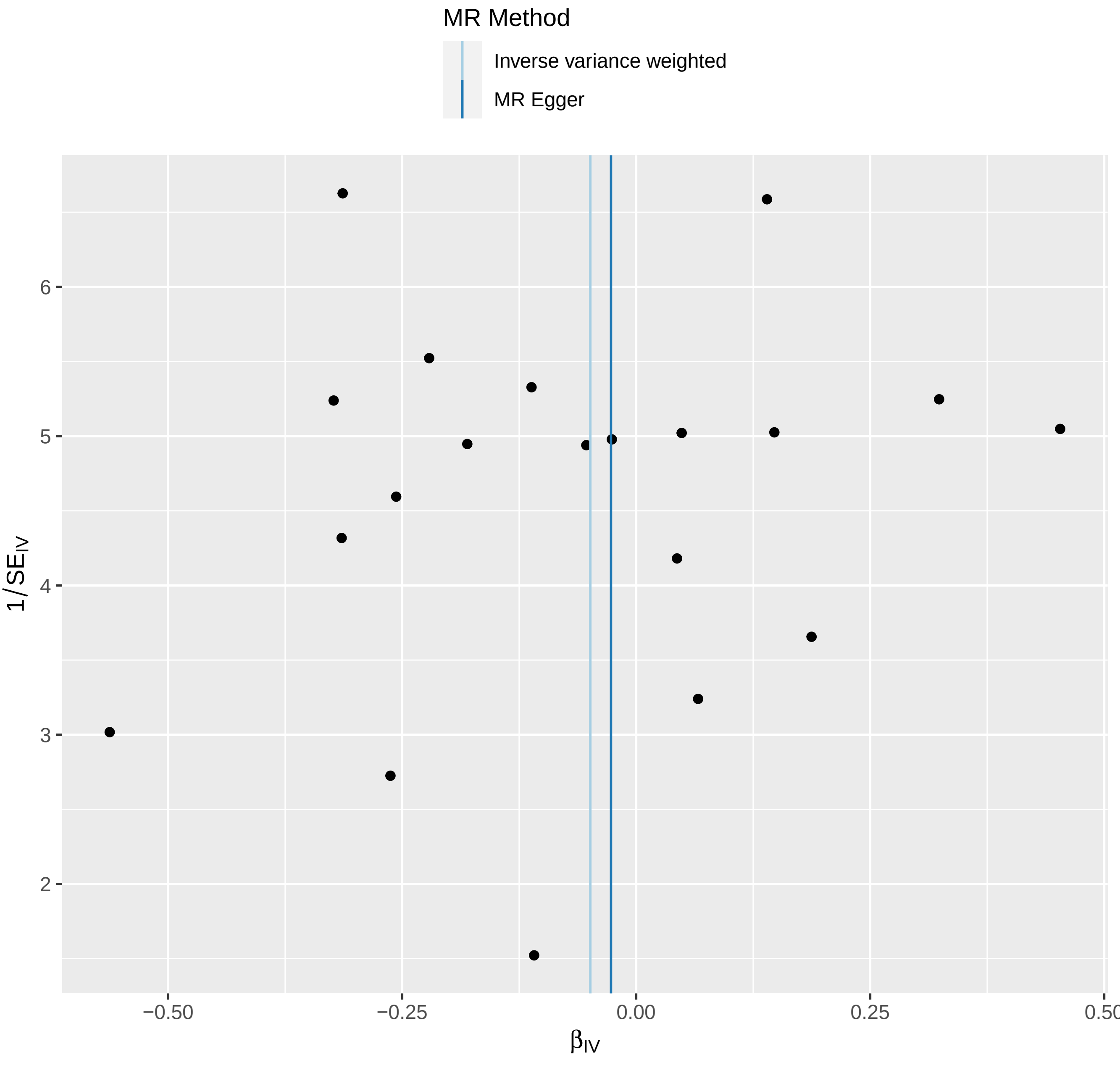

Batch 3−27 : Disintegrin and metalloproteinase domain−containing protein 19 on Diabetic nephropathy (more control exclusions) with a threshold of 1e−05

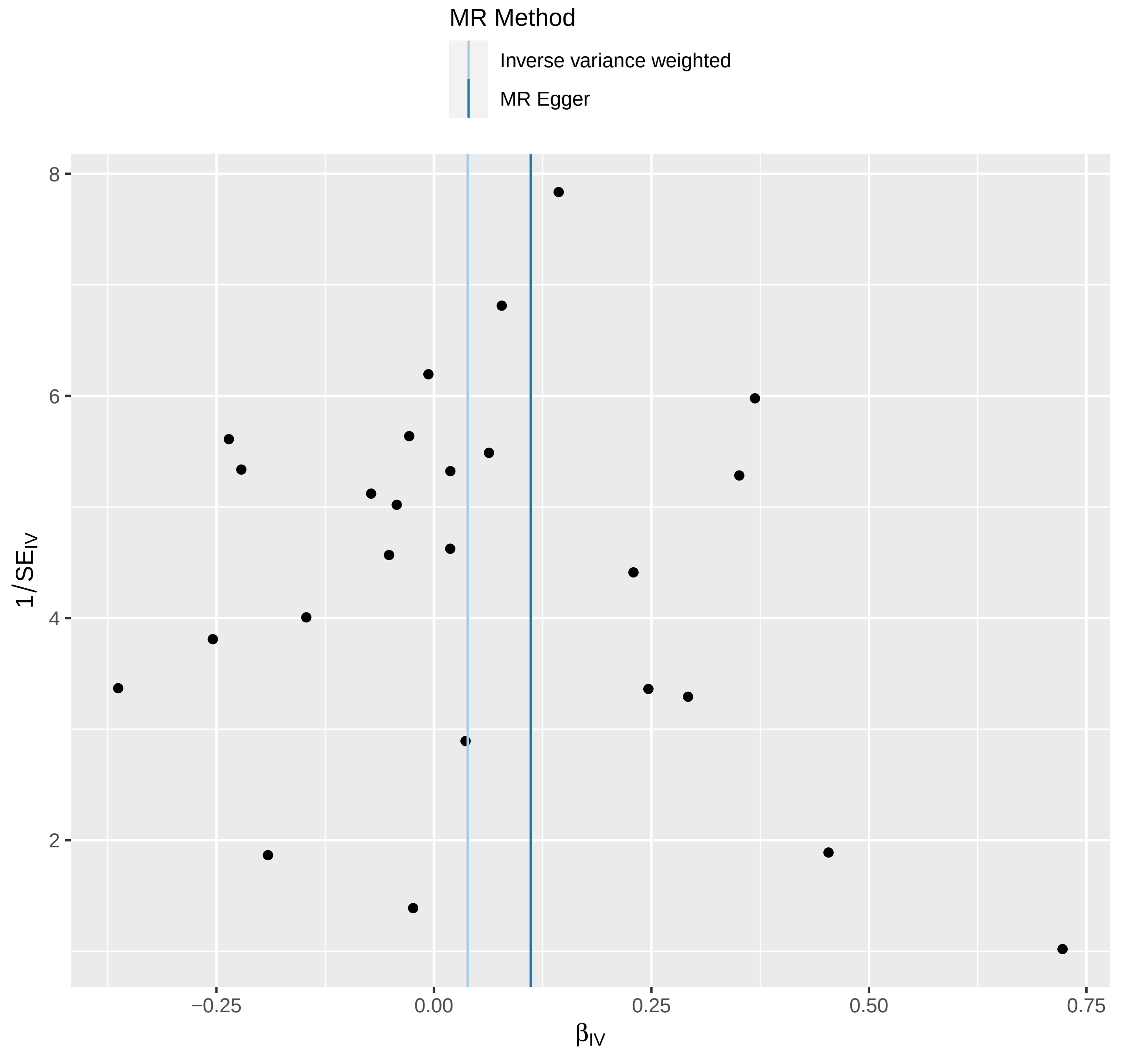

Batch 3−28 : Disintegrin and metalloproteinase domain−containing protein 23 on Diabetic nephropathy (more control exclusions)
with a threshold of 1e−05

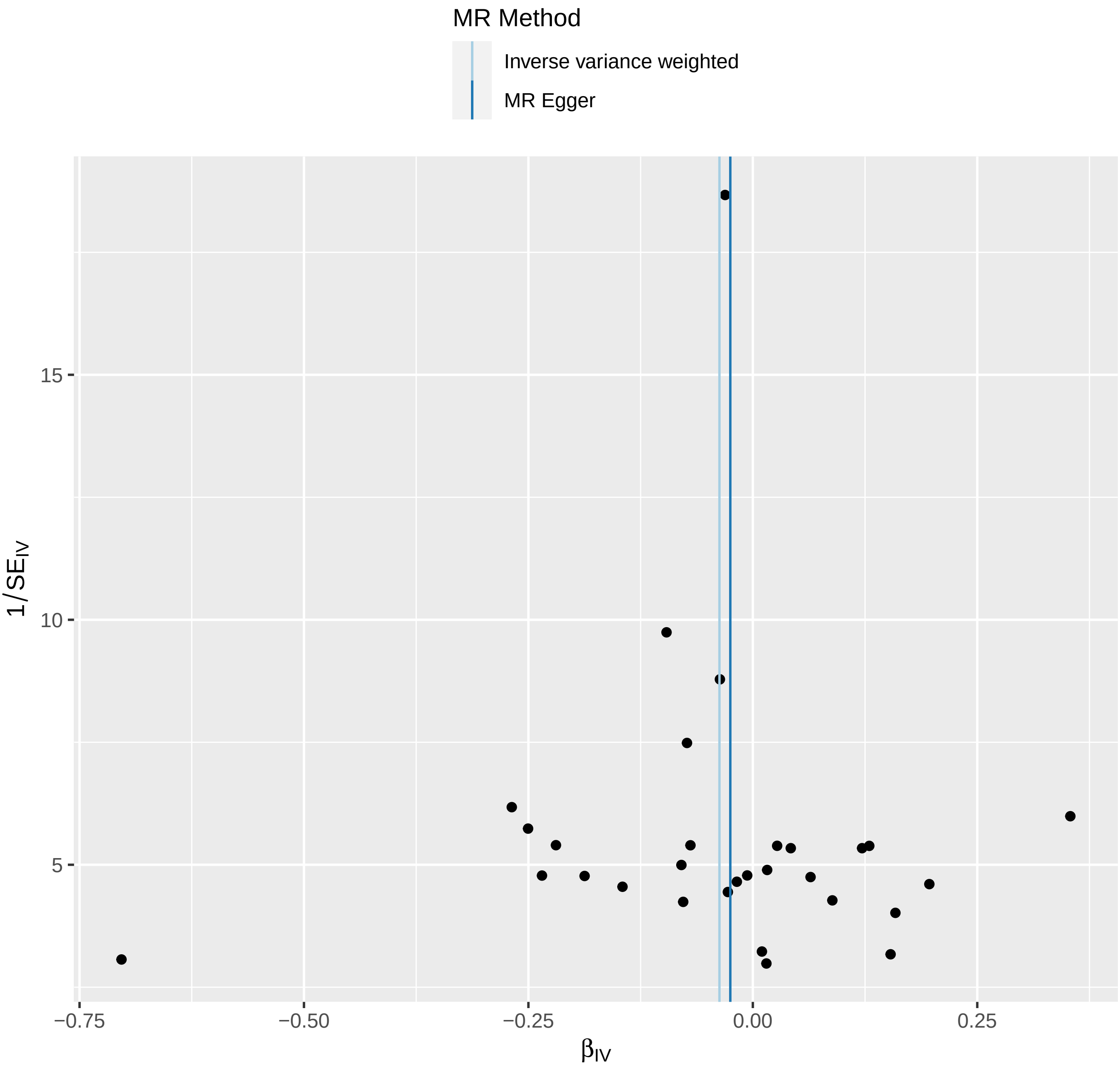

Batch 3−29 : Disintegrin and metalloproteinase domain−containing protein 29 on Diabetic nephropathy (more control exclusions)
with a threshold of 1e−05

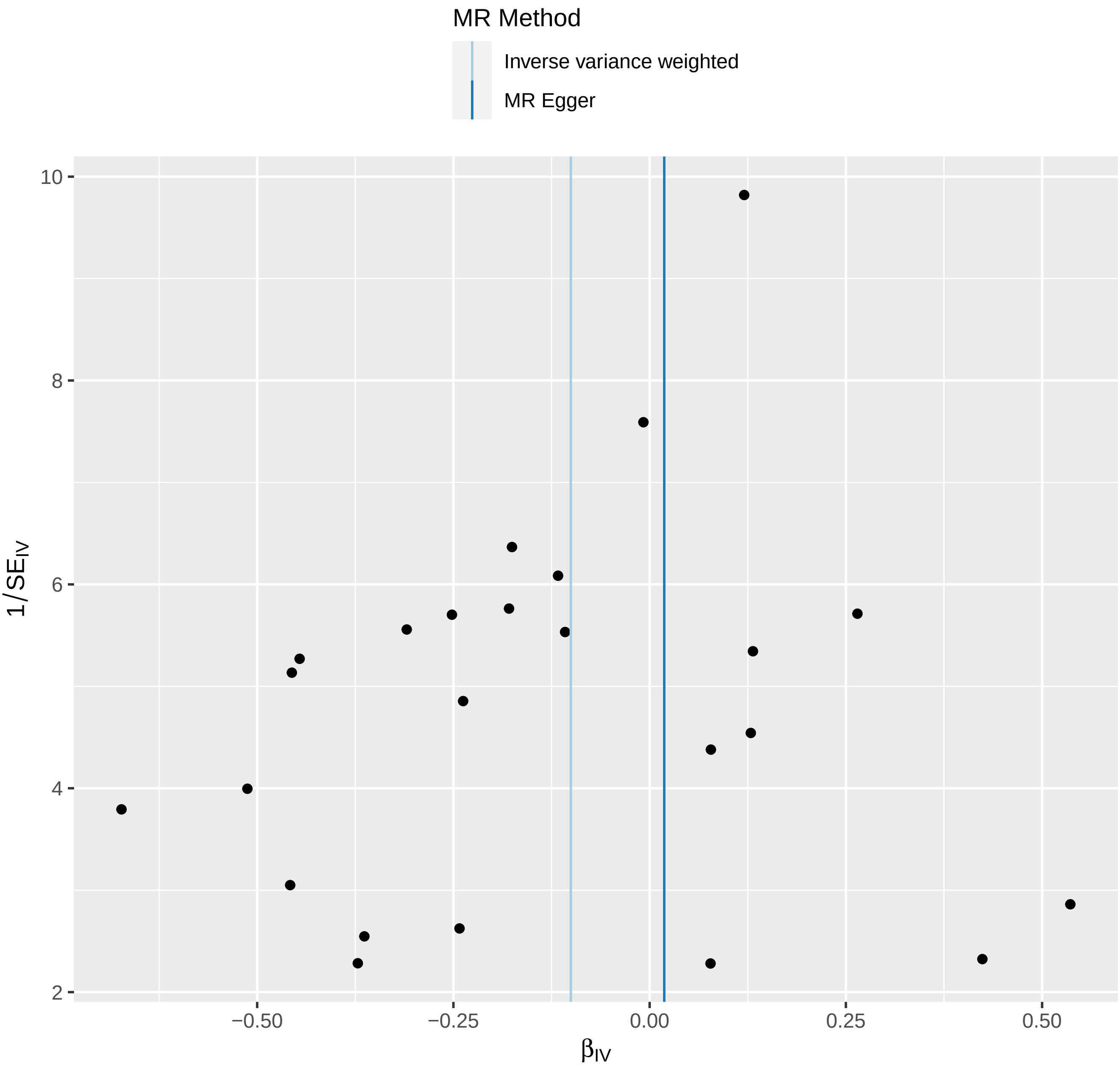

Batch 3−30 : Disintegrin and metalloproteinase domain−containing protein 9 on Diabetic nephropathy (more control exclusions)
with a threshold of 1e−05

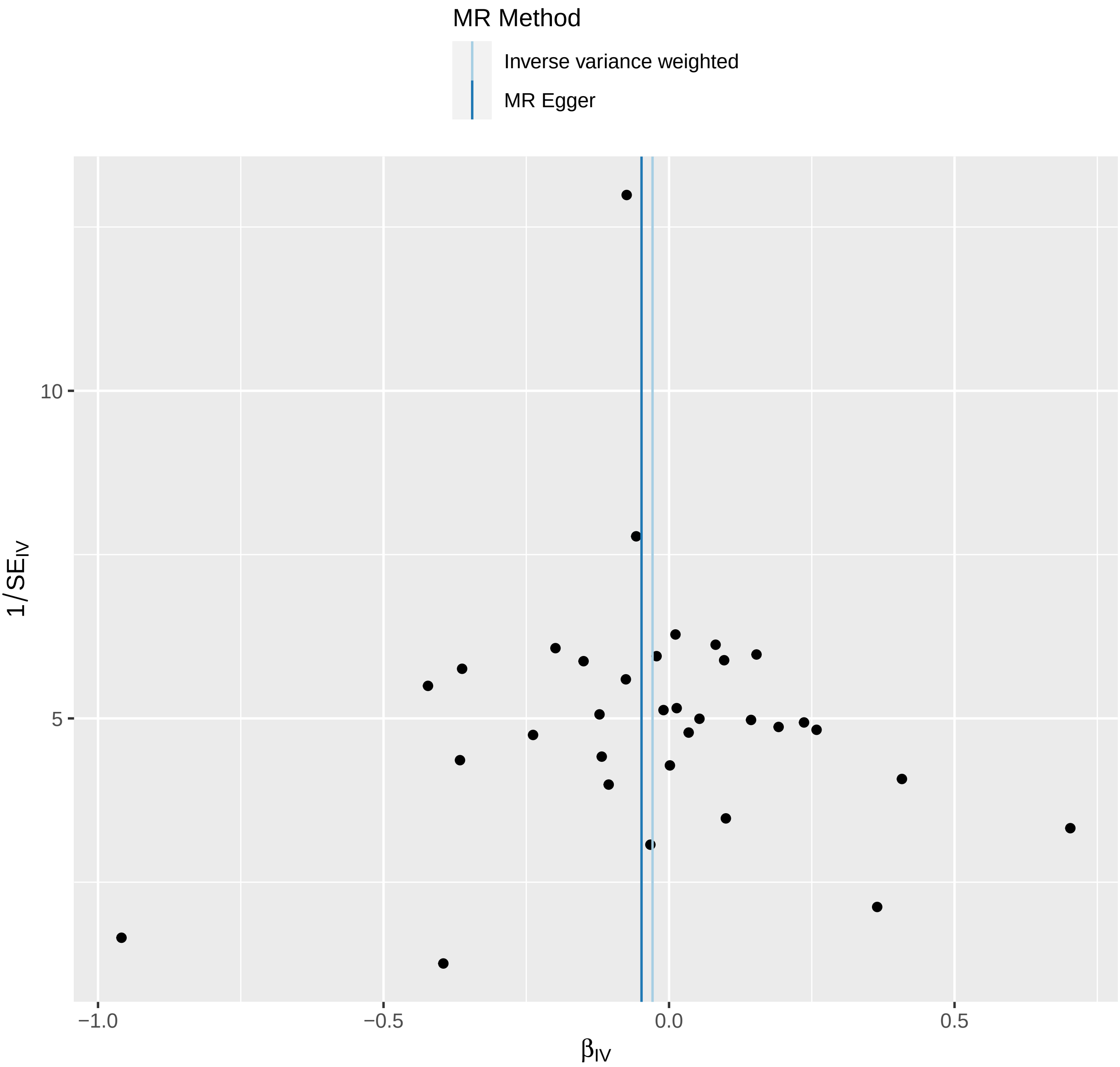

Batch 3−31 : A disintegrin and metalloproteinase with thrombospondin motifs 1 on Diabetic nephropathy (more control exclusions) with a threshold of 1e−05

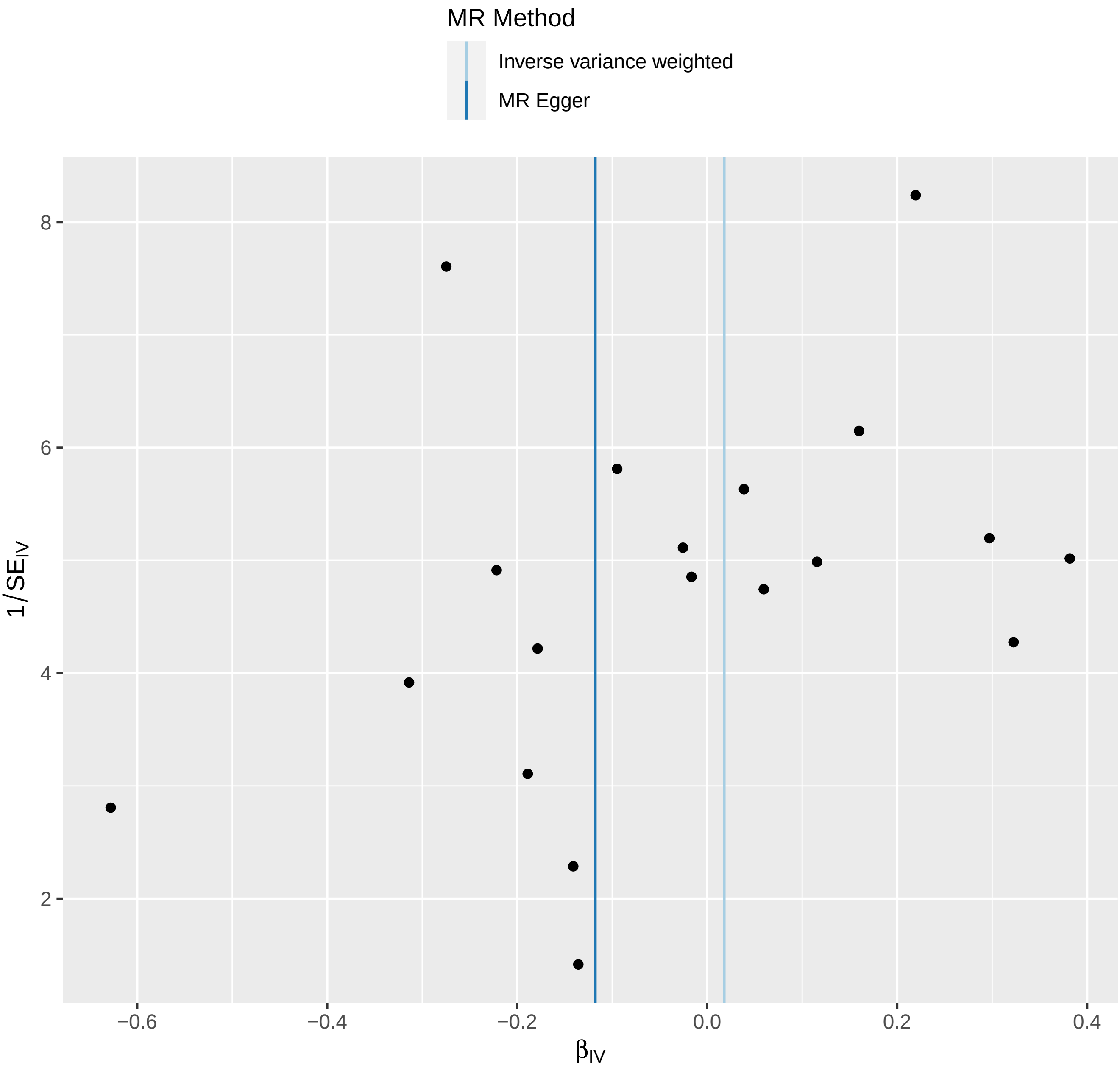

Batch 3−32 : A disintegrin and metalloproteinase with thrombospondin motifs 13 on Diabetic nephropathy (more control exclusions) with a threshold of 1e−05

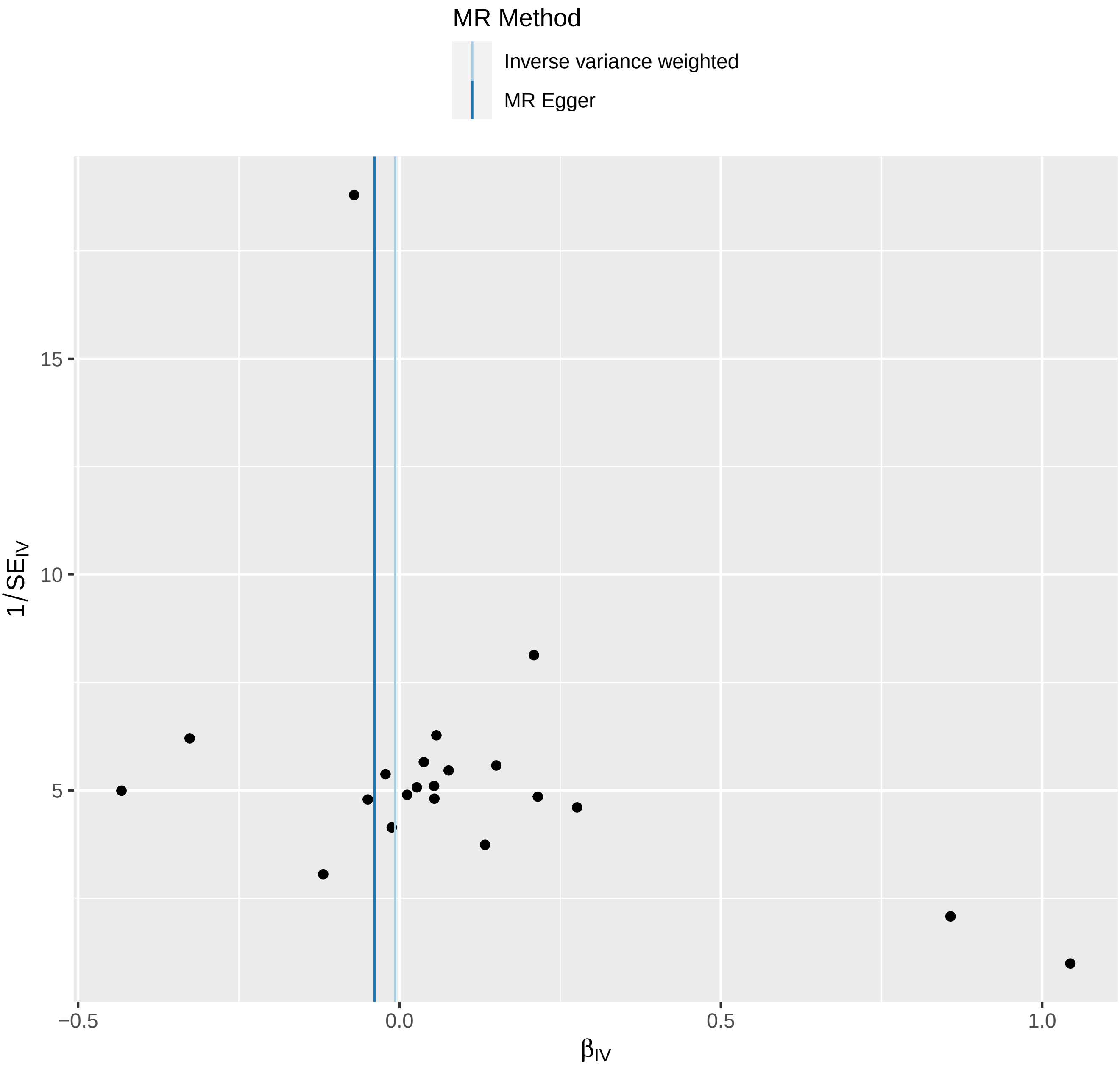

Batch 3−33 : A disintegrin and metalloproteinase with thrombospondin motifs 15 on Diabetic nephropathy (more control exclusions) with a threshold of 1e−05

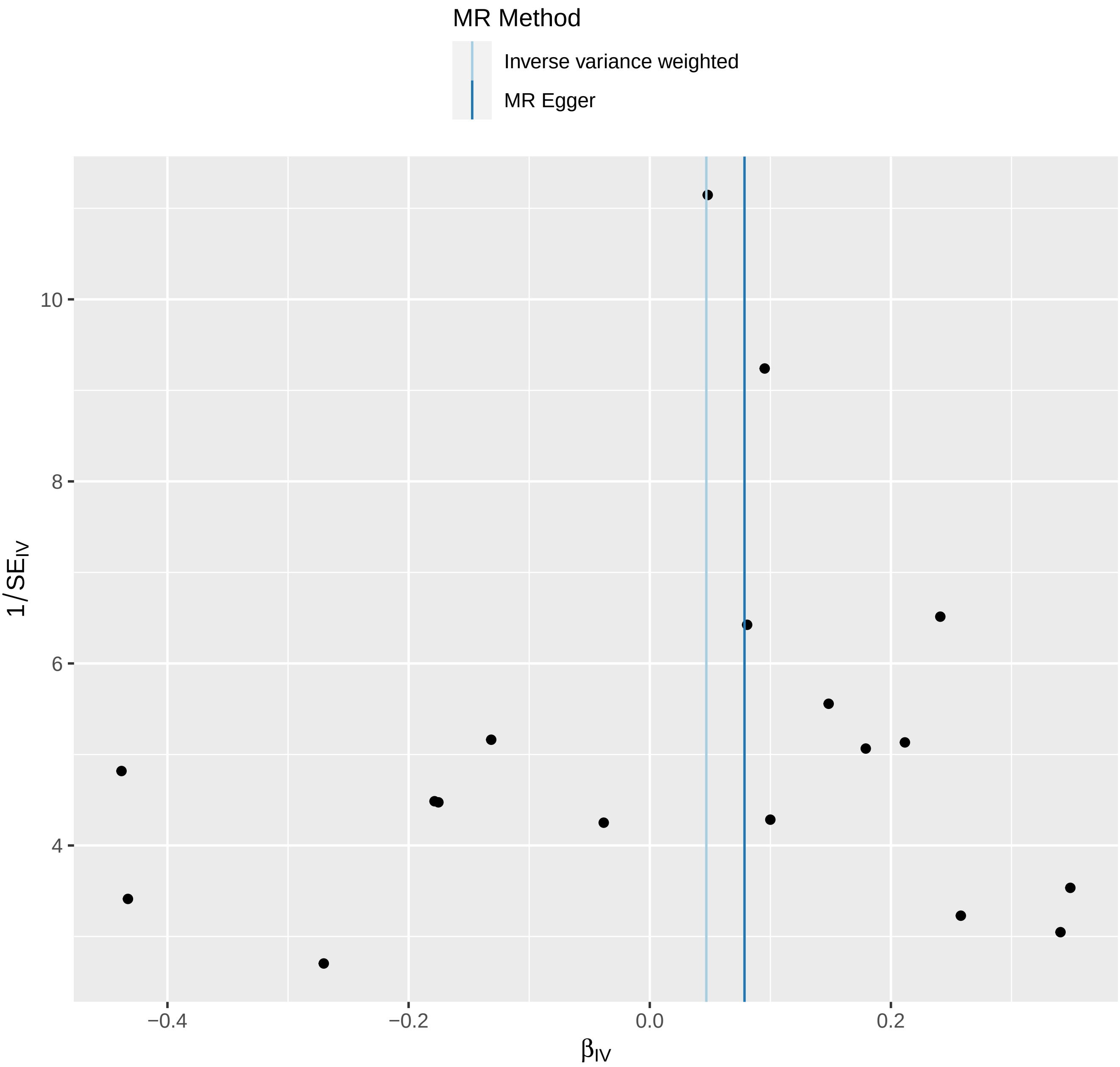

Batch 3−34 : A disintegrin and metalloproteinase with thrombospondin motifs 3 on Diabetic nephropathy (more control exclusions) with a threshold of 1e−05

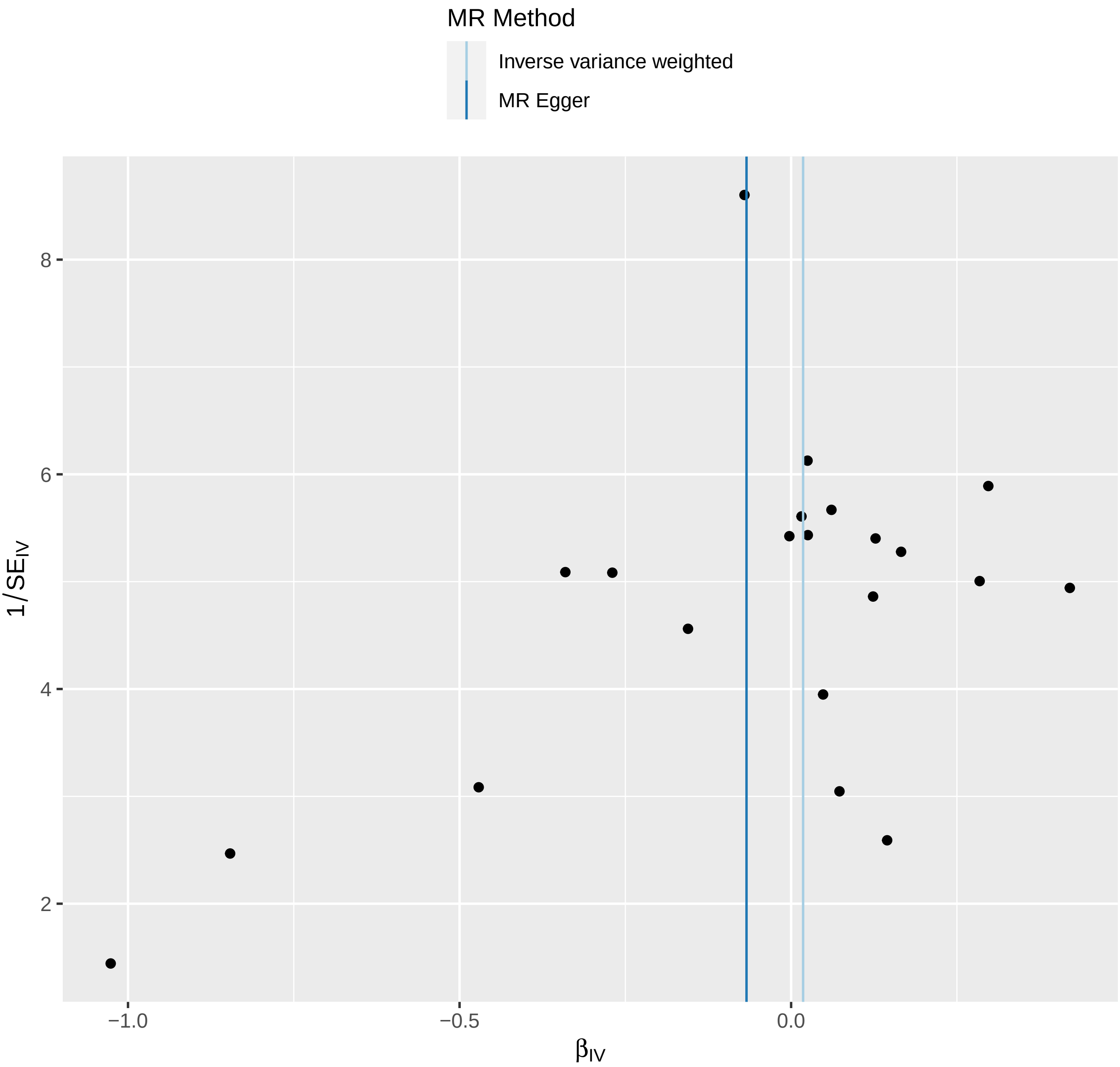

Batch 3−35 : A disintegrin and metalloproteinase with thrombospondin motifs 4 on Diabetic nephropathy (more control exclusions) with a threshold of 1e−05

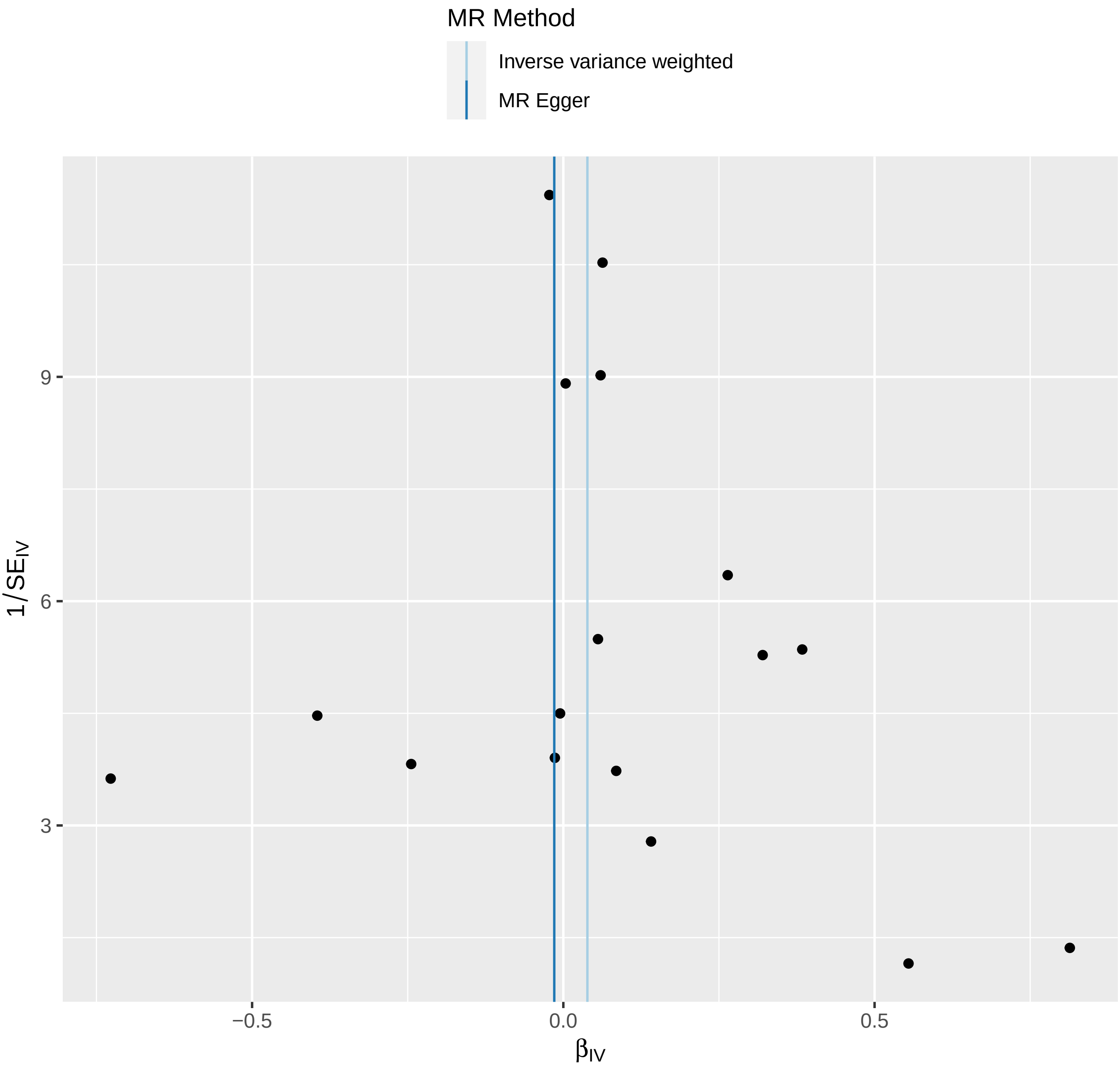

Batch 3−36 : A disintegrin and metalloproteinase with thrombospondin motifs 5 on Diabetic nephropathy (more control exclusions)
with a threshold of 1e−05

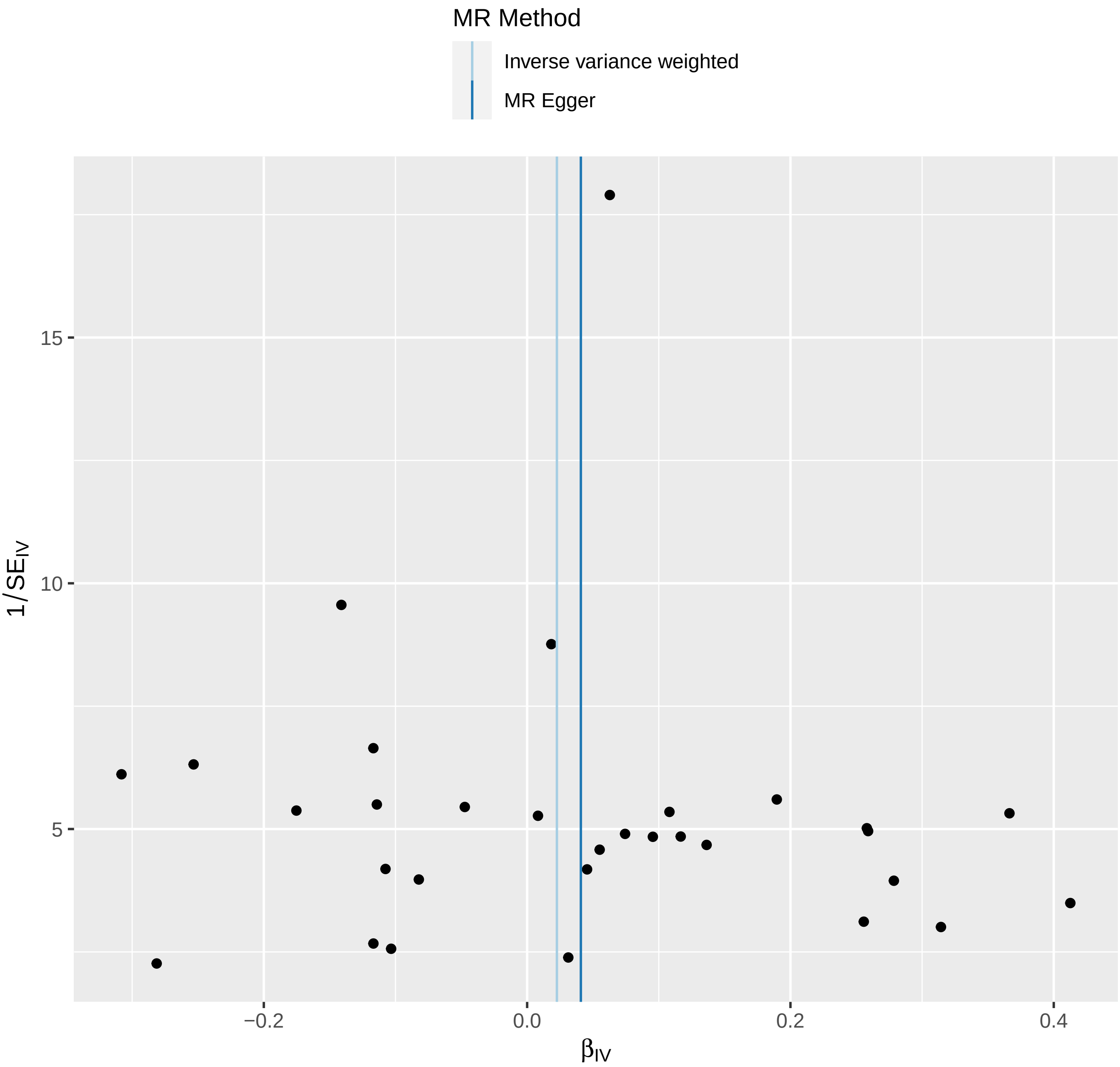

Batch 3−37 : A disintegrin and metalloproteinase with thrombospondin motifs 6 on Diabetic nephropathy (more control exclusions) with a threshold of 1e−05

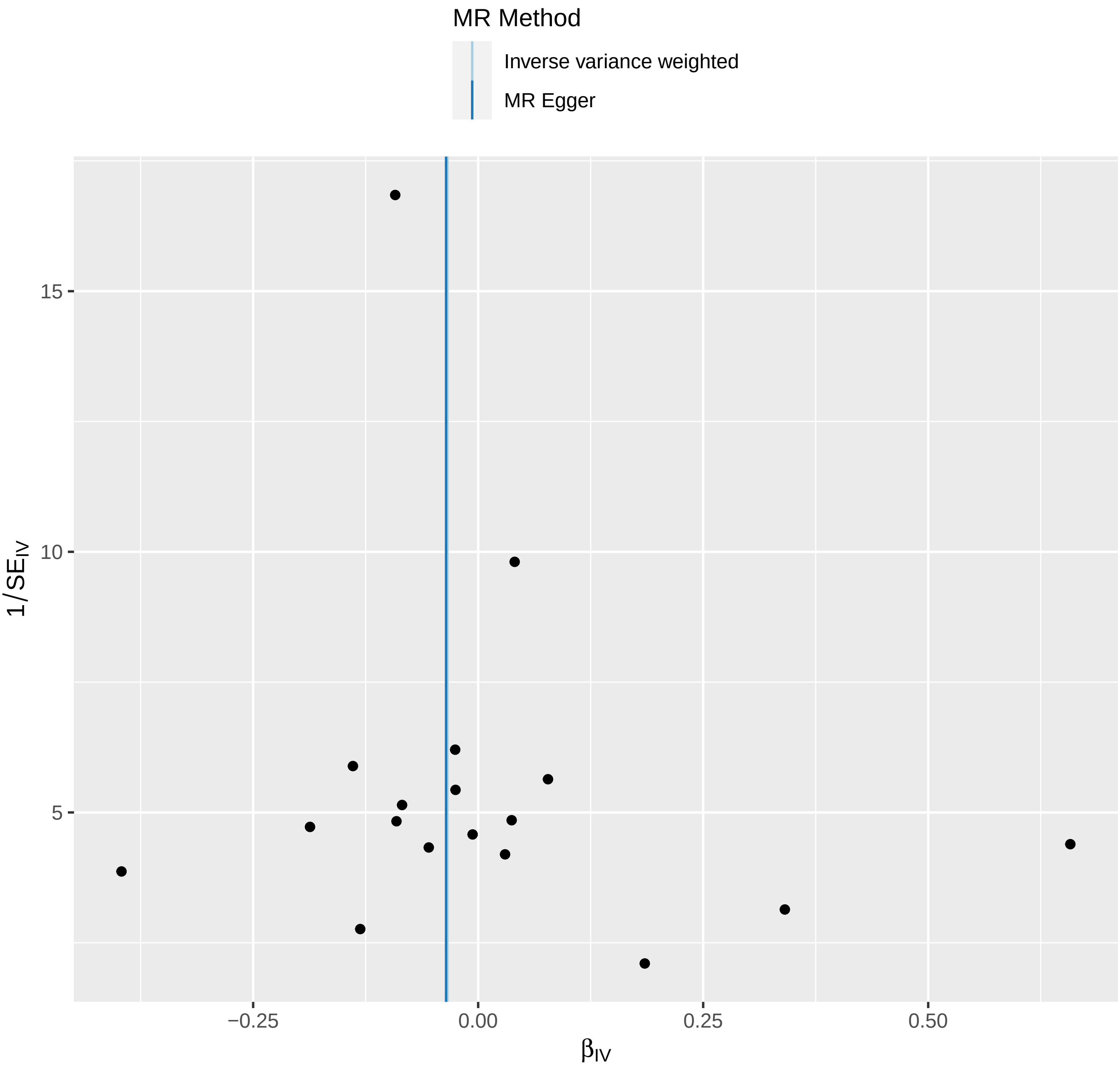

Batch 3−38 : ADAMTS−like protein 1 on Diabetic nephropathy (more control exclusions)
with a threshold of 1e−05

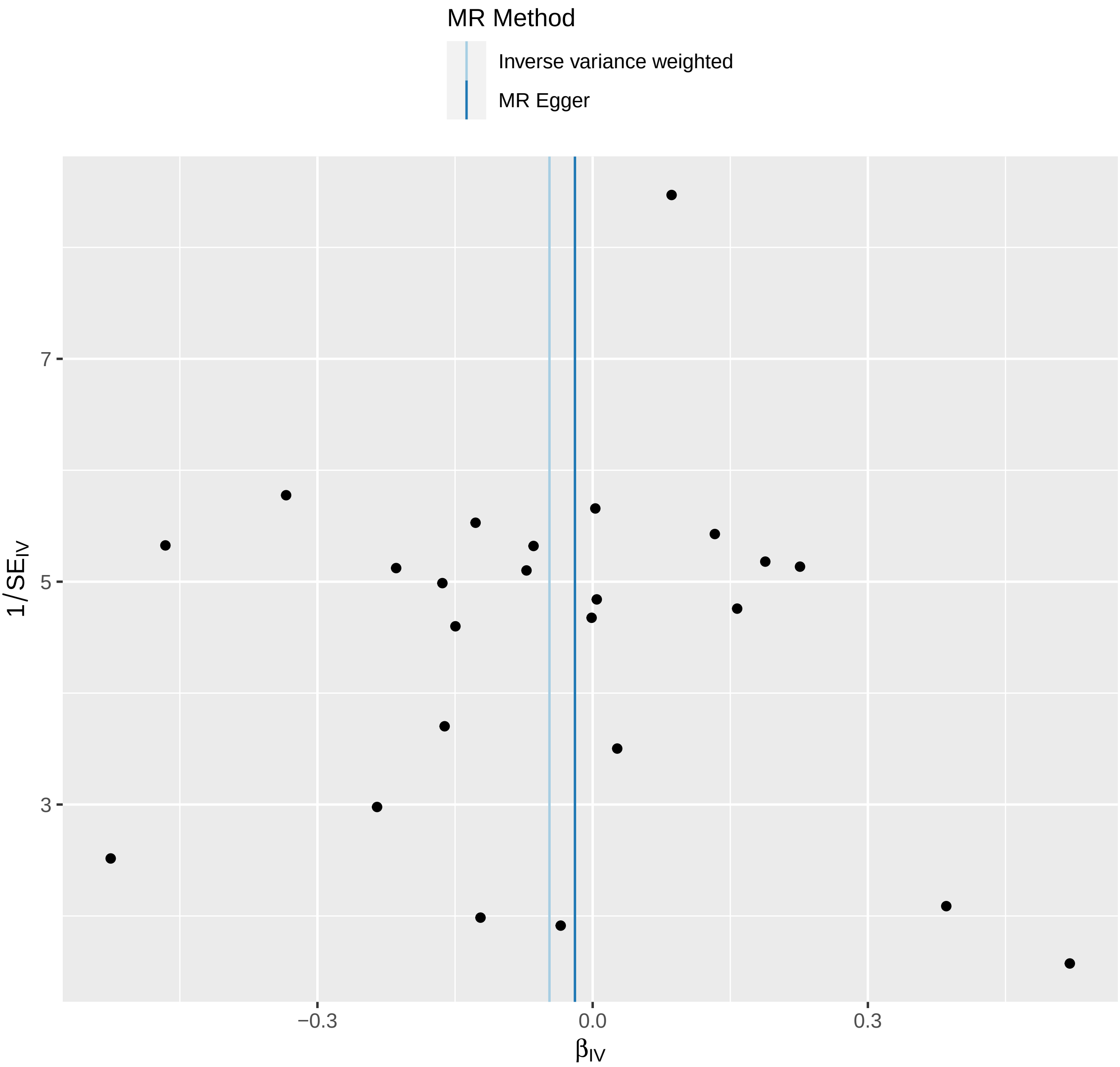

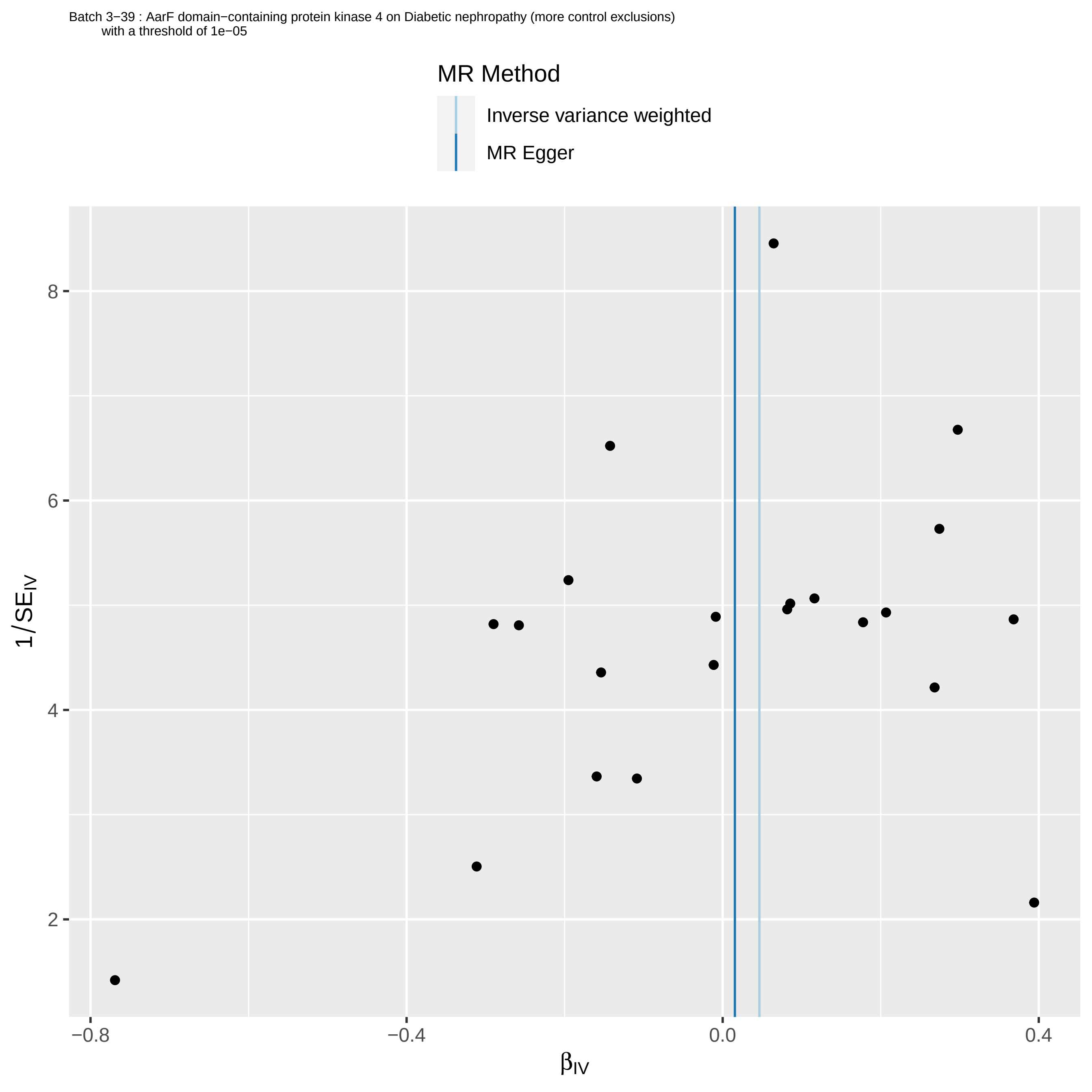
Batch 3−39 : AarF domain−containing protein kinase 4 on Diabetic nephropathy (more control exclusions)
with a threshold of 1e−05
MR Method
Inverse variance weighted
MR Egger
8
6
4
2
1/SE_IV
−0.8
−0.4
0.0
0.4
β_IV

Batch 3−40 : Pituitary adenylate cyclase−activating polypeptide 27 on Diabetic nephropathy (more control exclusions)
with a threshold of 1e−05

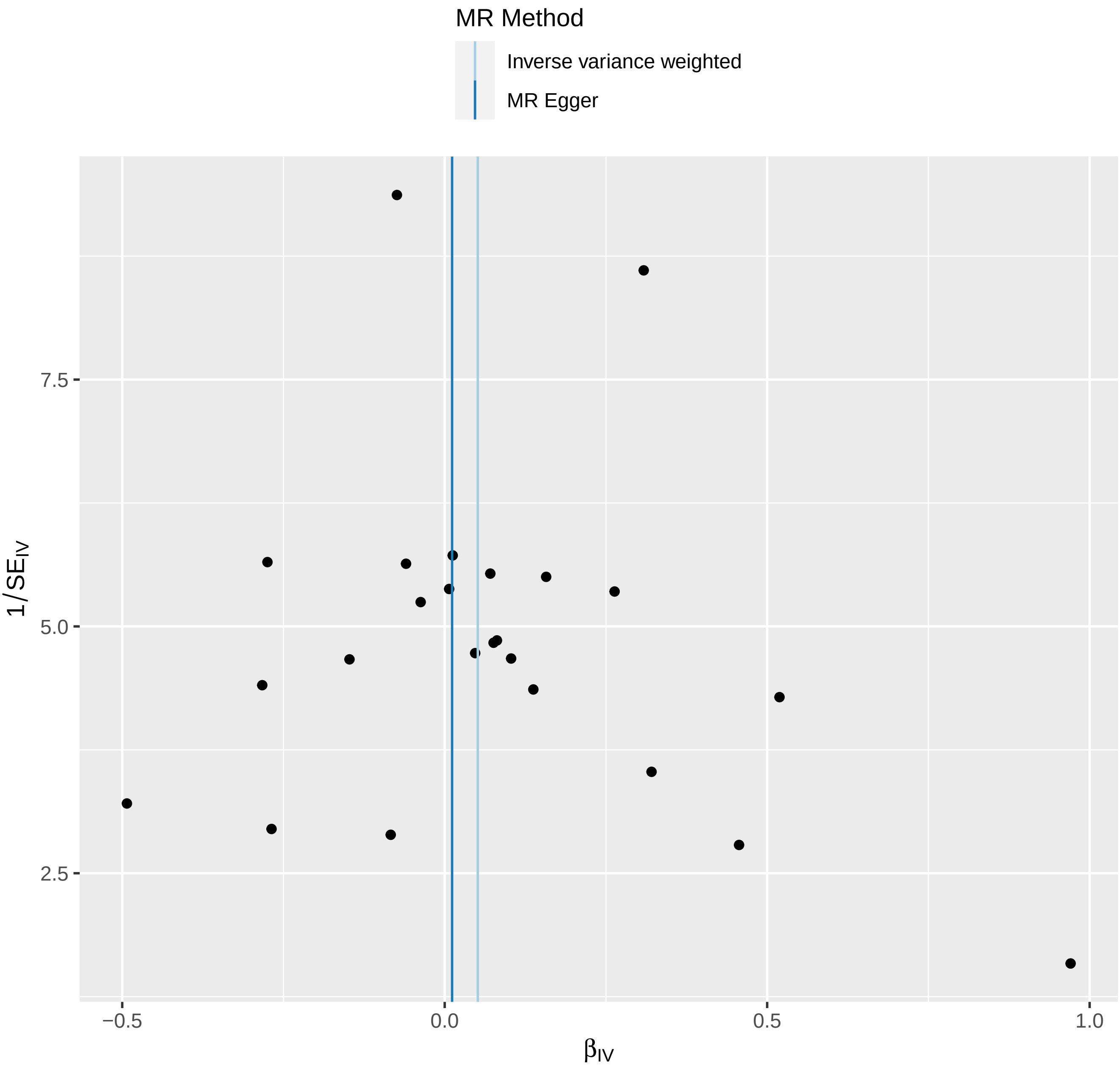

Batch 3−41 : Pituitary adenylate cyclase−activating polypeptide 38 on Diabetic nephropathy (more control exclusions) with a threshold of 1e−05

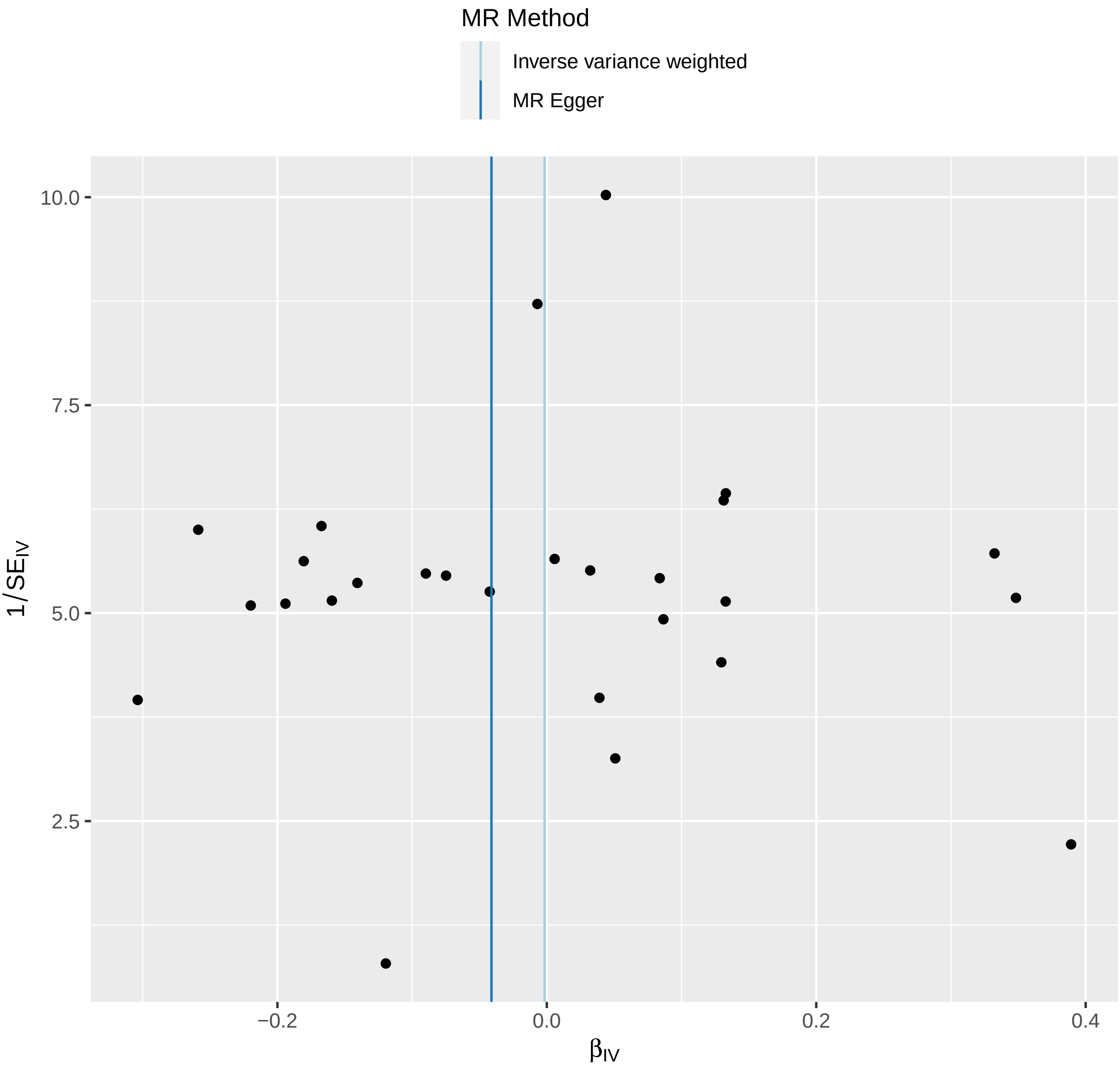

Batch 3−42 : Pituitary adenylate cyclase−activating polypeptide on Diabetic nephropathy (more control exclusions)
with a threshold of 1e−05

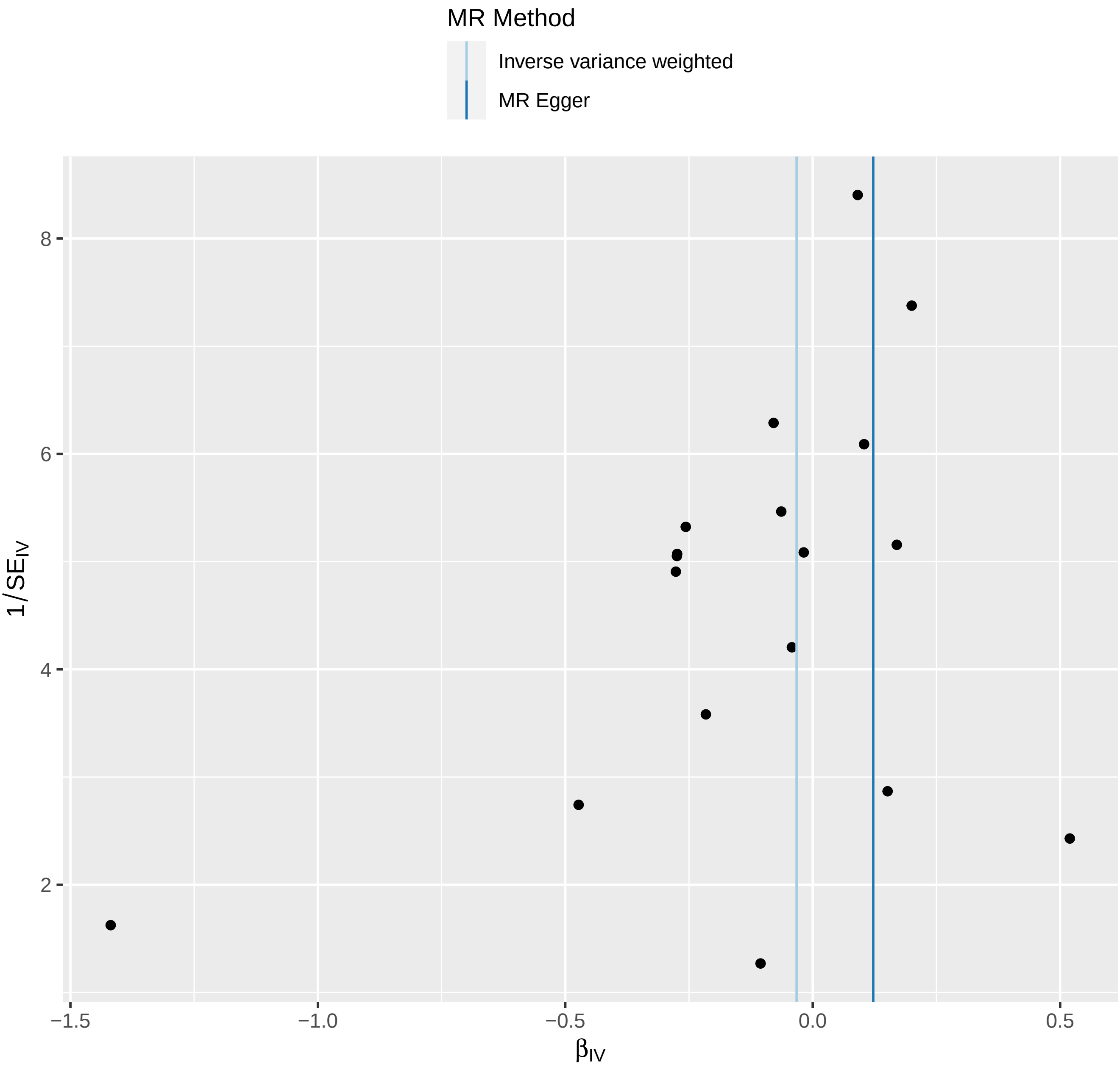

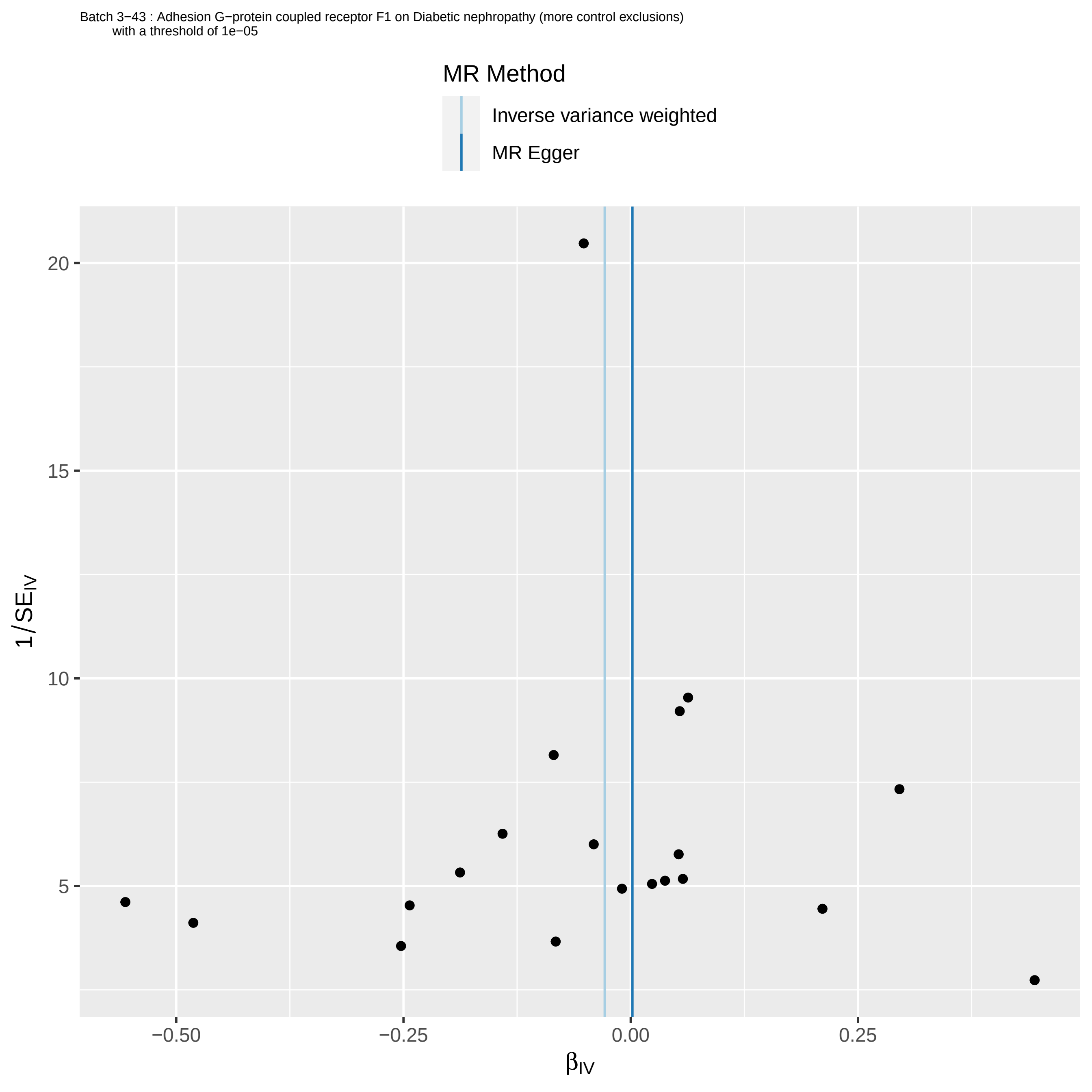
Batch 3−43 : Adhesion G−protein coupled receptor F1 on Diabetic nephropathy (more control exclusions)
with a threshold of 1e−05
MR Method
Inverse variance weighted
MR Egger
20
15
10
5
1/SE$_{IV}$
−0.50
−0.25
0.00
0.25
β_{IV}

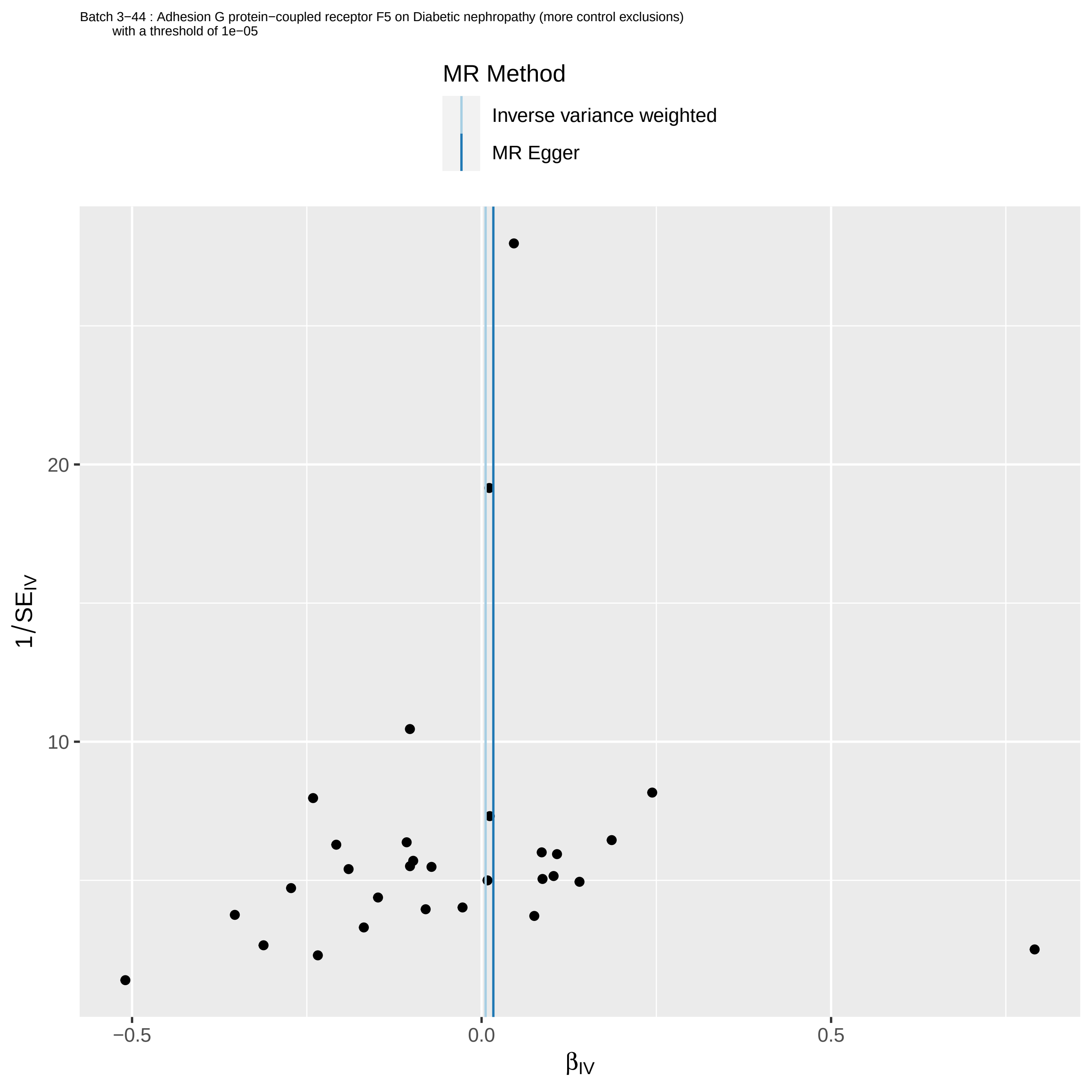
Batch 3−44 : Adhesion G protein−coupled receptor F5 on Diabetic nephropathy (more control exclusions)
with a threshold of 1e−05
MR Method
Inverse variance weighted
MR Egger
20
10
1/SE_IV
−0.5
0.0
0.5
β_IV

Batch 3−45 : Adhesion G−protein coupled receptor G5 on Diabetic nephropathy (more control exclusions)
with a threshold of 1e−05

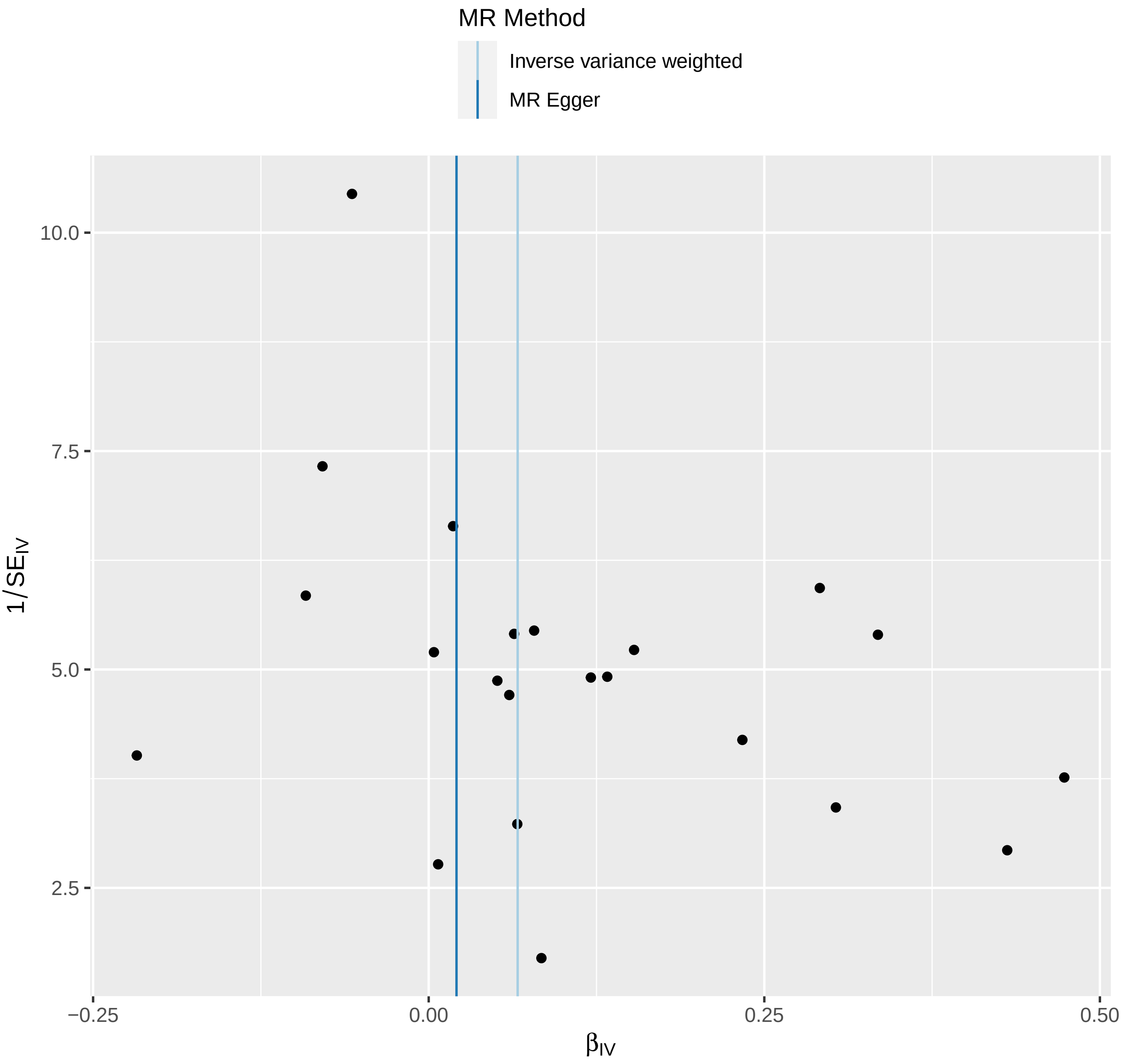

Batch 3−46 : Alcohol dehydrogenase 1B on Diabetic nephropathy (more control exclusions)
with a threshold of 1e−05

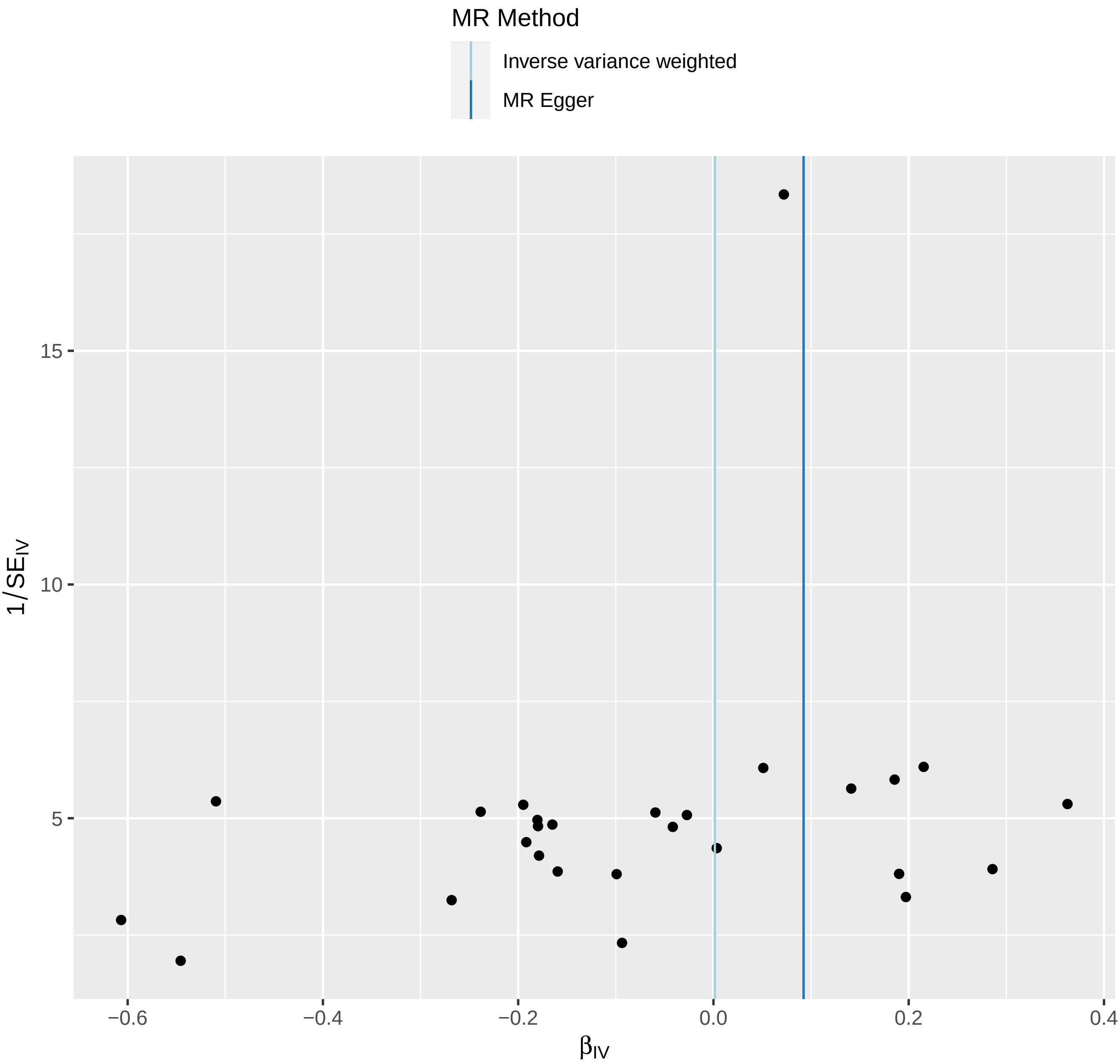

Batch 3−47 : Alcohol dehydrogenase 4 on Diabetic nephropathy (more control exclusions)
with a threshold of 1e−05

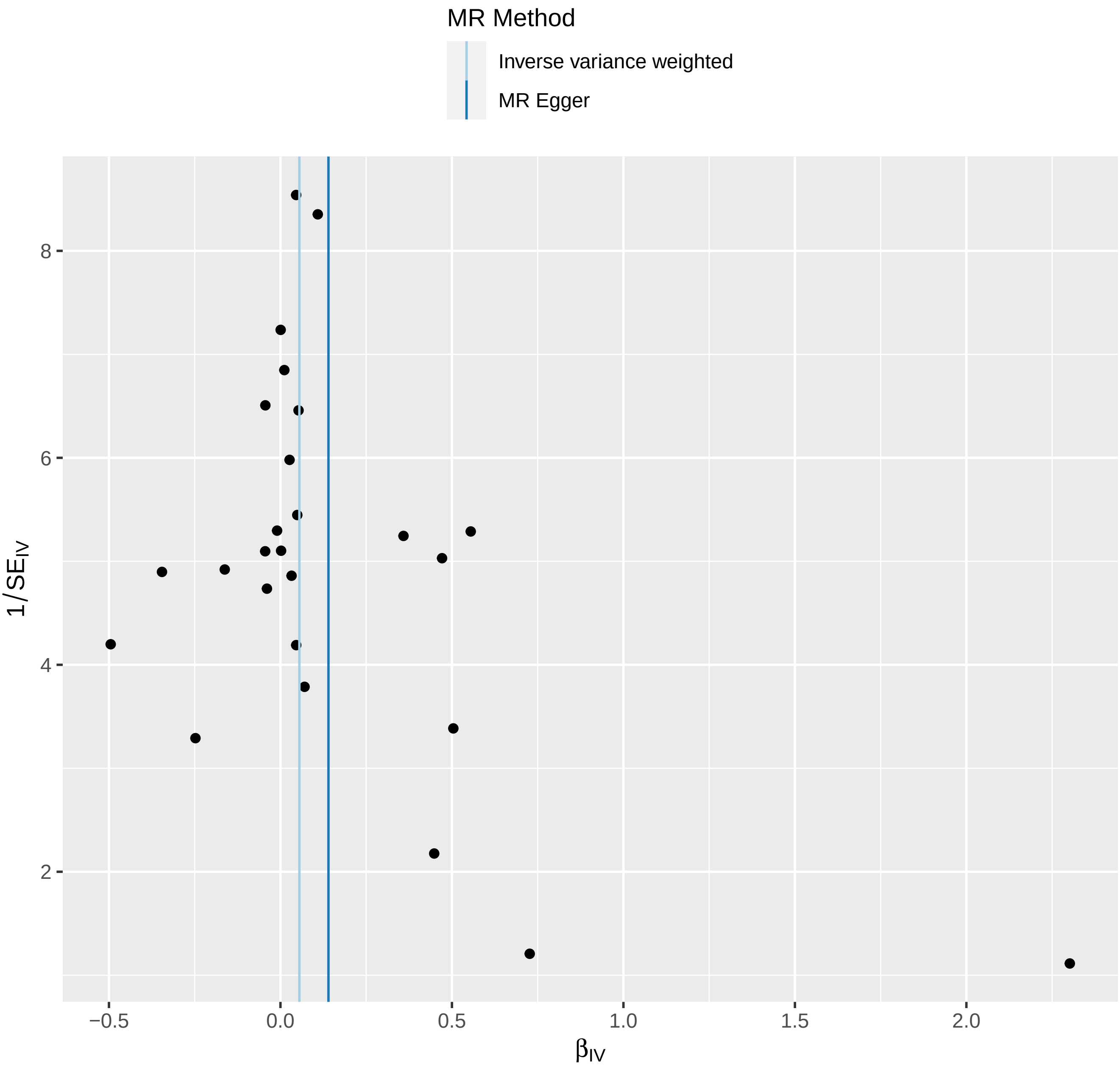

Batch 3−48 : Adrenomedullin on Diabetic nephropathy (more control exclusions)
with a threshold of 1e−05

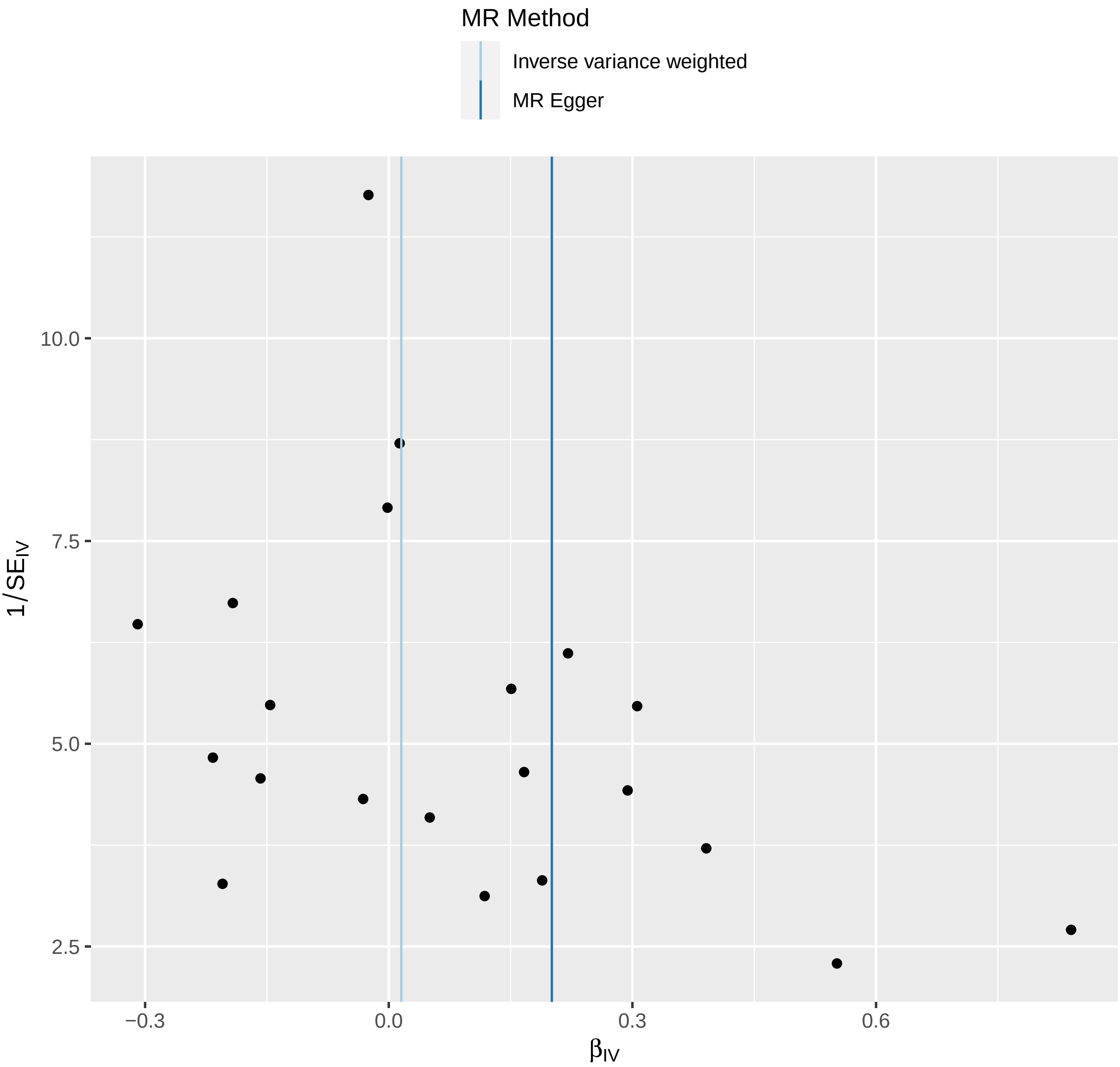

Batch 3−49 : ADP−dependent glucokinase on Diabetic nephropathy (more control exclusions)
with a threshold of 1e−05

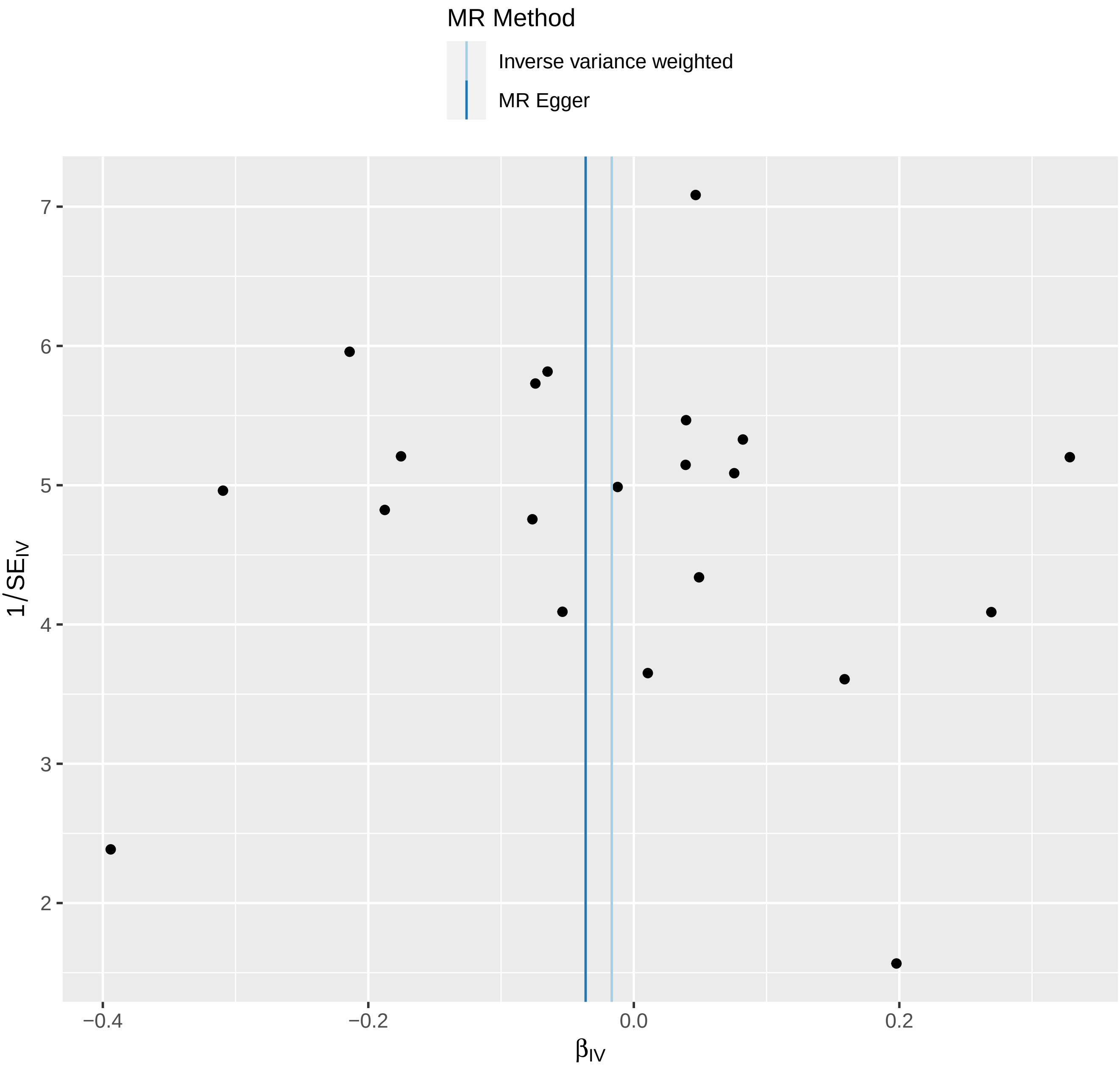

Batch 3−50 : beta−adrenergic receptor kinase 1 on Diabetic nephropathy (more control exclusions)
with a threshold of 1e−05

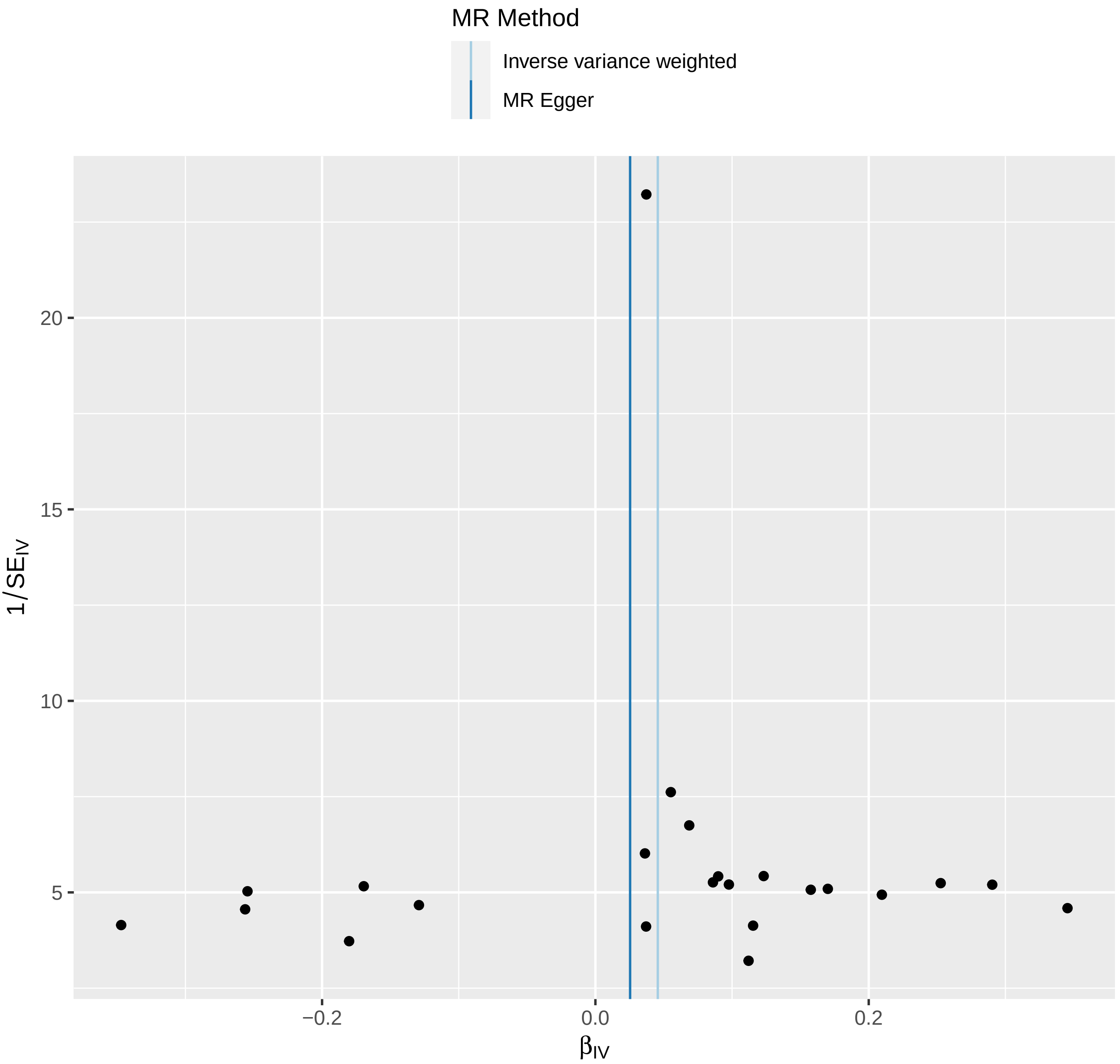

Batch 3−51 : Adenylosuccinate synthetase isozyme 2 on Diabetic nephropathy (more control exclusions)
with a threshold of 1e−05

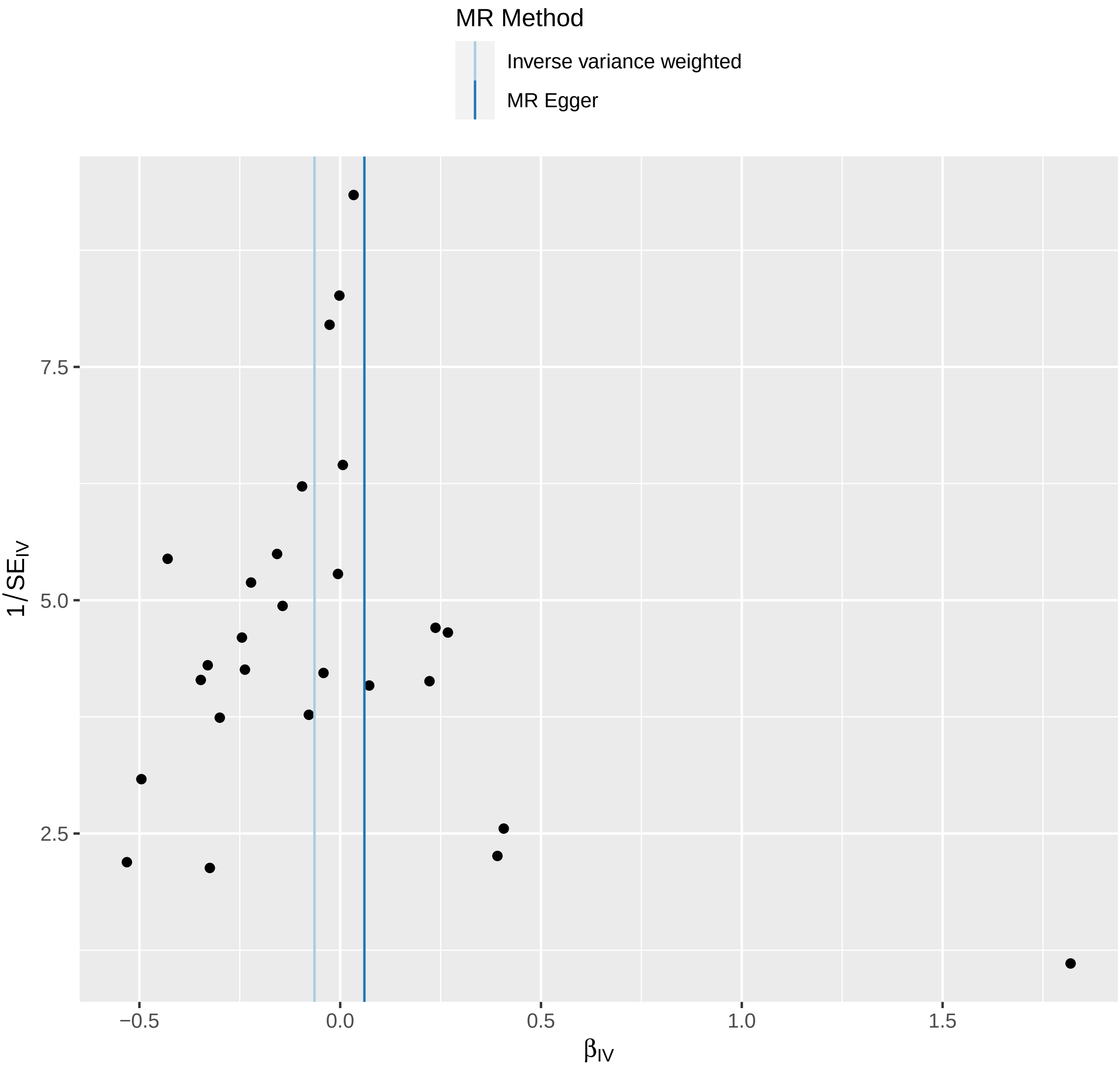

Batch 3−52 : Adenylosuccinate synthetase isozyme 1 on Diabetic nephropathy (more control exclusions)
with a threshold of 1e−05

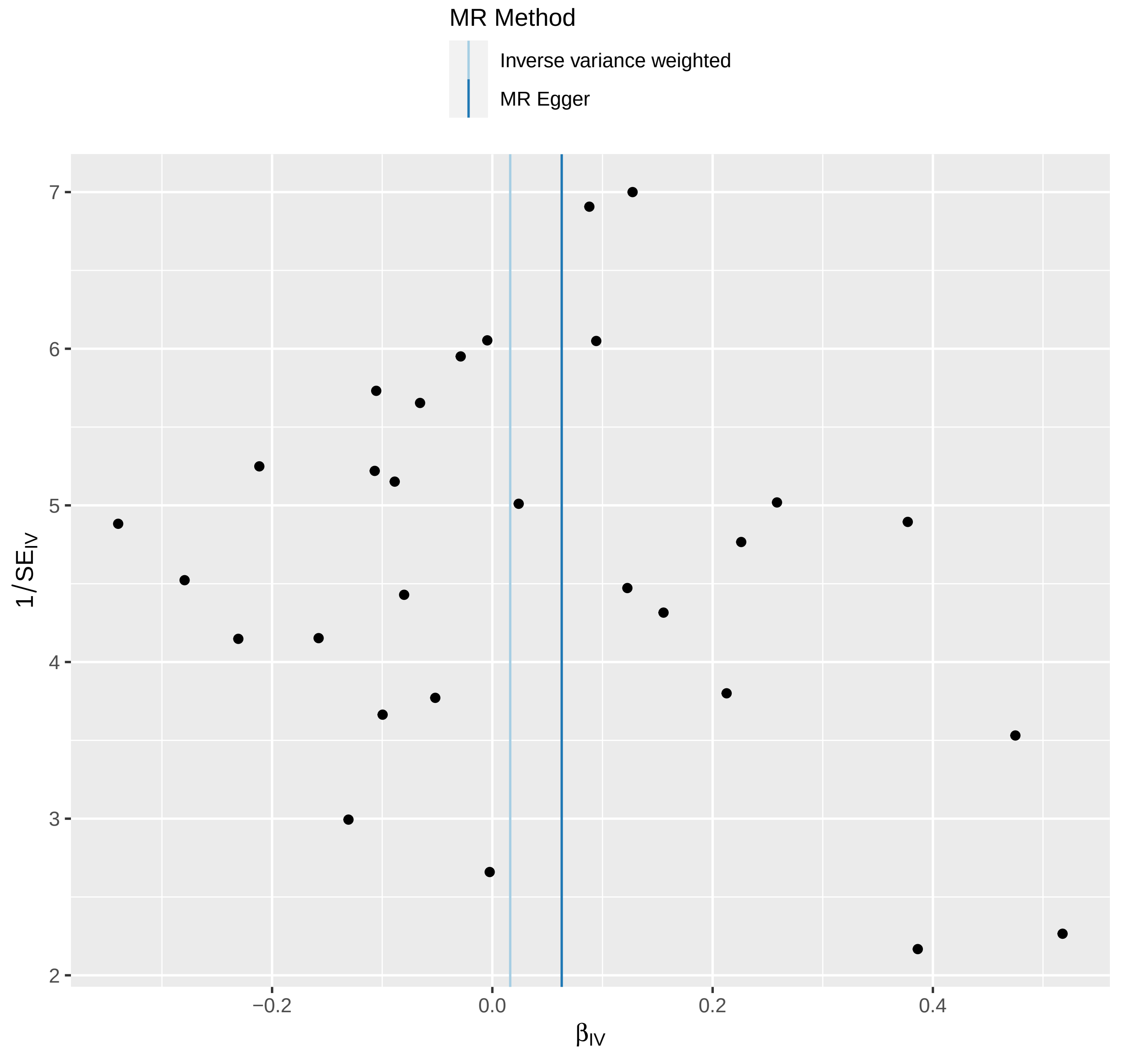

Batch 3−53 : alpha−Fetoprotein on Diabetic nephropathy (more control exclusions)
with a threshold of 1e−05

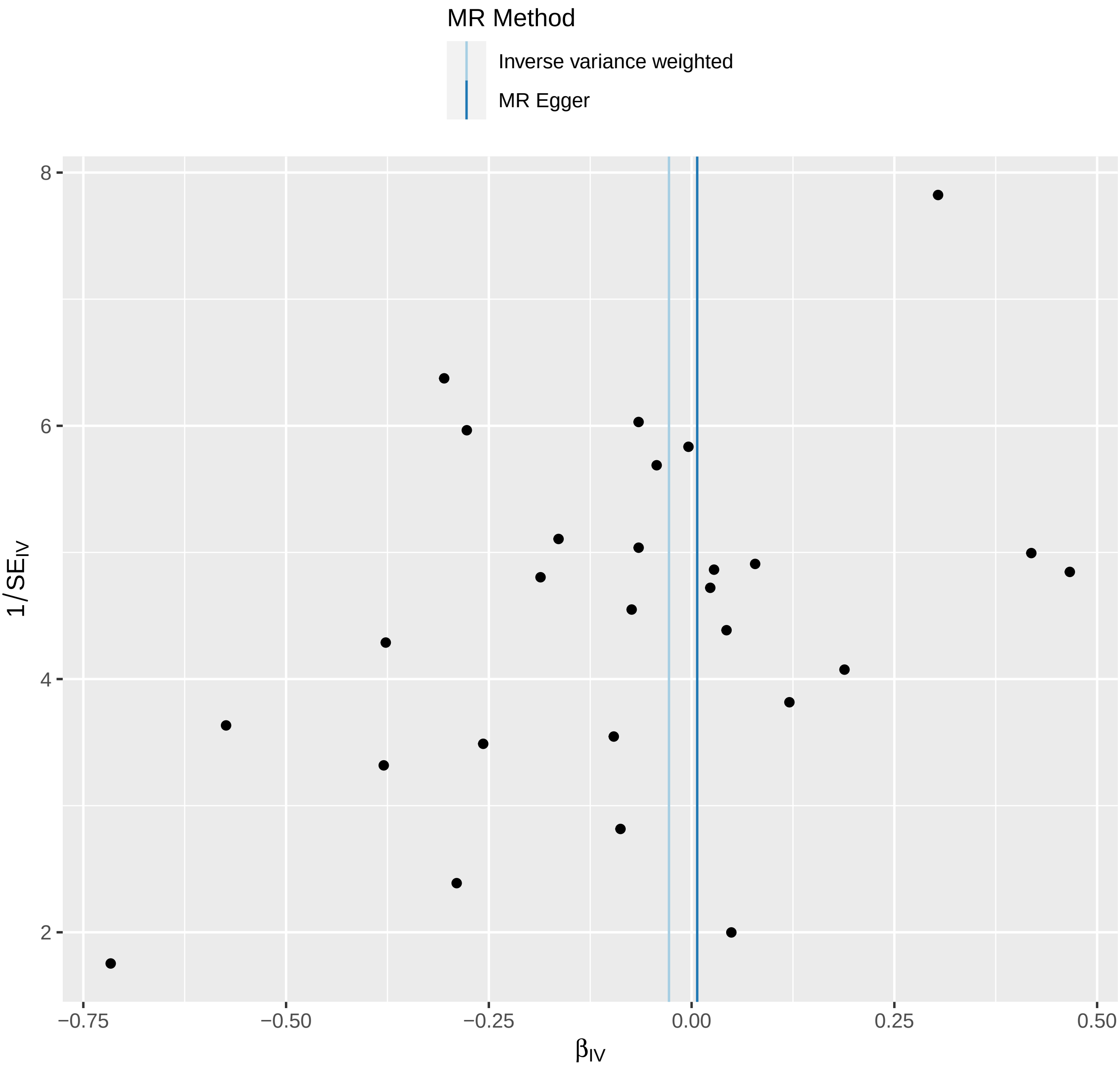

Batch 3−54 : Advanced glycosylation end product−specific receptor, soluble on Diabetic nephropathy (more control exclusions) with a threshold of 1e−05

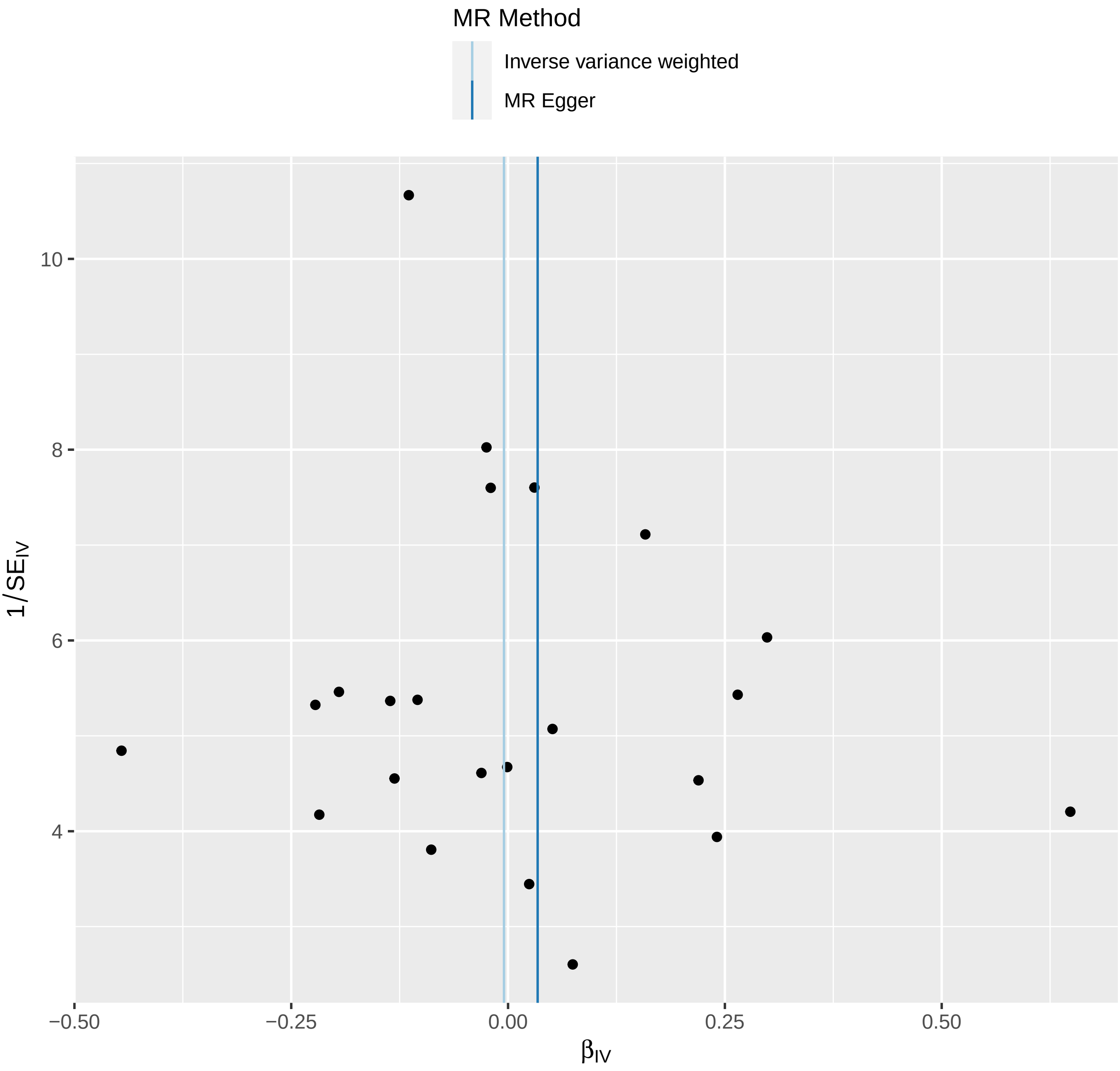

Batch 3−55 : Arf−GAP domain and FG repeat−containing protein 1 on Diabetic nephropathy (more control exclusions) with a threshold of 1e−05

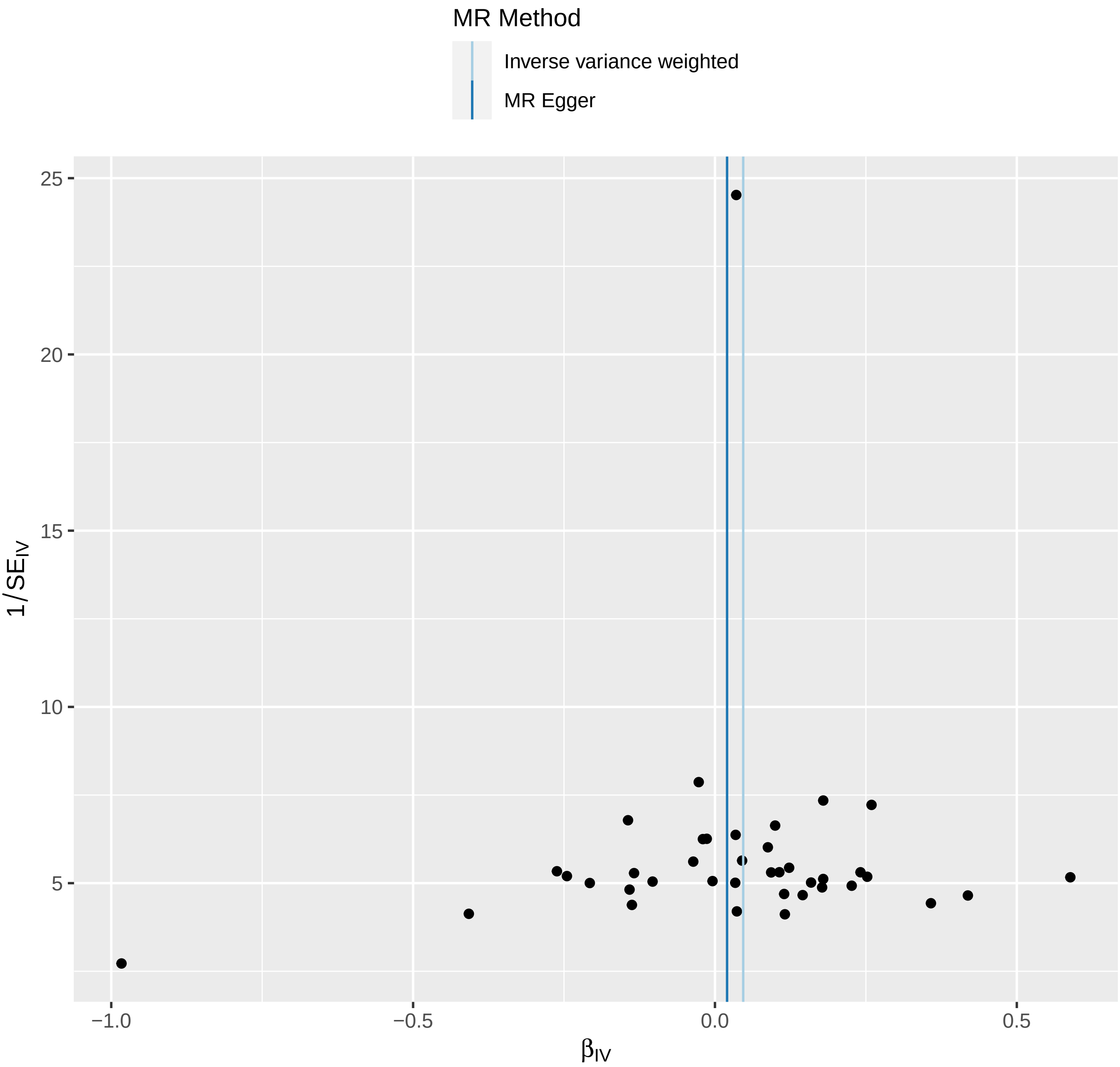

Batch 3−56 : Angiogenic factor with G patch and FHA domains 1 on Diabetic nephropathy (more control exclusions)
with a threshold of 1e−05

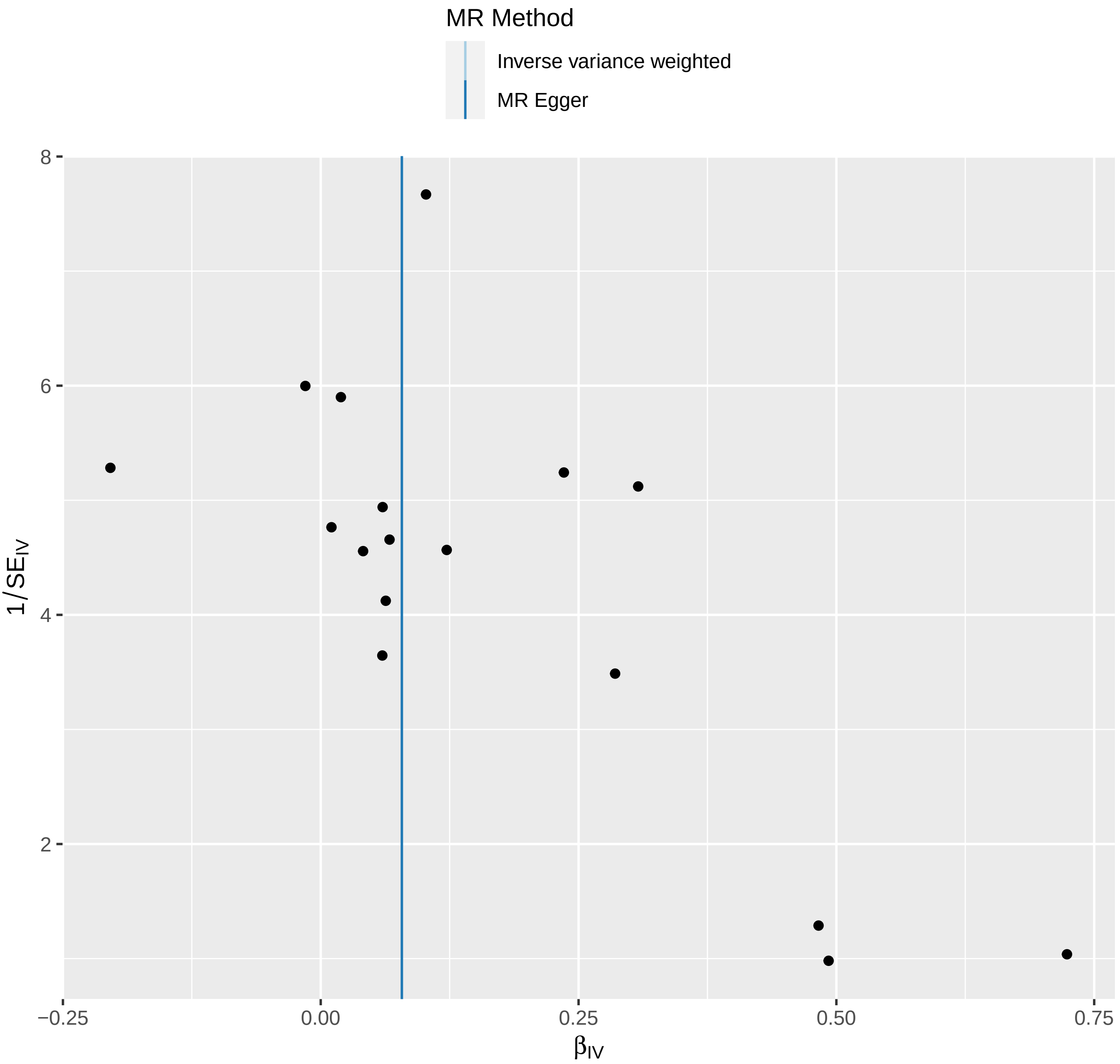

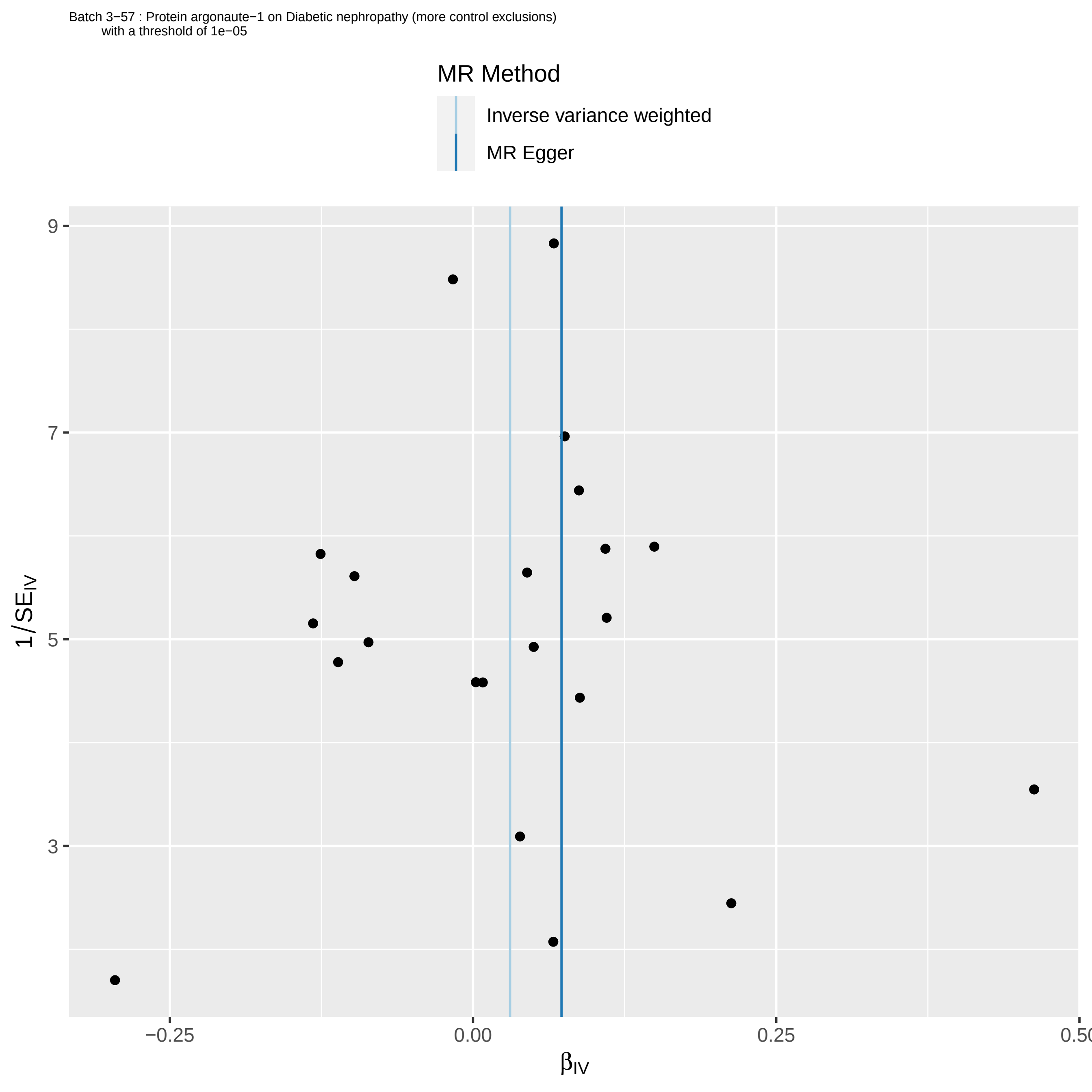
Batch 3−57 : Protein argonaute−1 on Diabetic nephropathy (more control exclusions)
with a threshold of 1e−05
MR Method
Inverse variance weighted
MR Egger
9
7
5
3
1/SE_IV
−0.25
0.00
0.25
0.50
β_IV

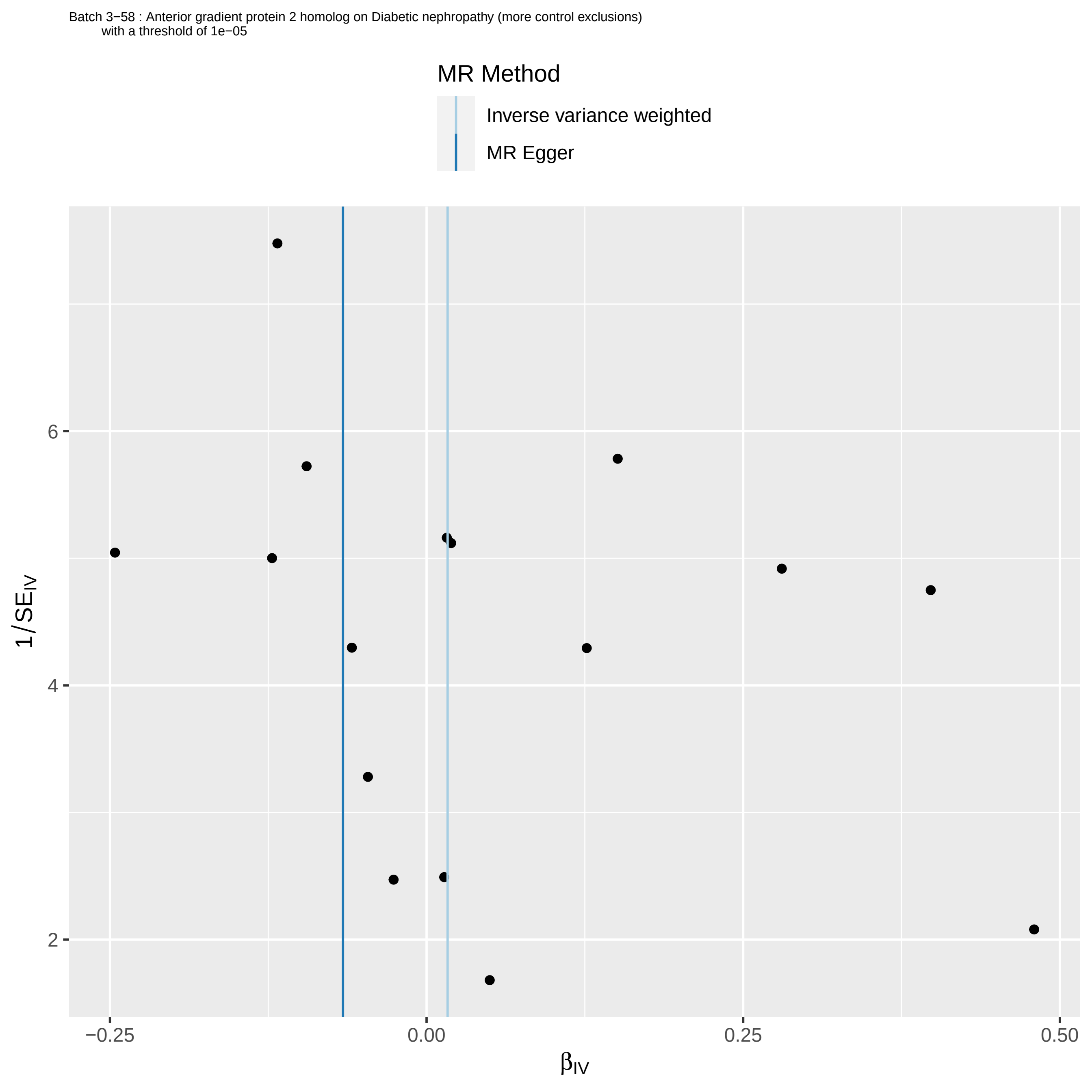

Batch 3−58 : Anterior gradient protein 2 homolog on Diabetic nephropathy (more control exclusions)
with a threshold of 1e−05
MR Method
Inverse variance weighted
MR Egger
6
4
2
1/SE$_{IV}$
−0.25
0.00
0.25
0.50
β_{IV}

Batch 3−59 : Agouti−related protein on Diabetic nephropathy (more control exclusions)
with a threshold of 1e−05

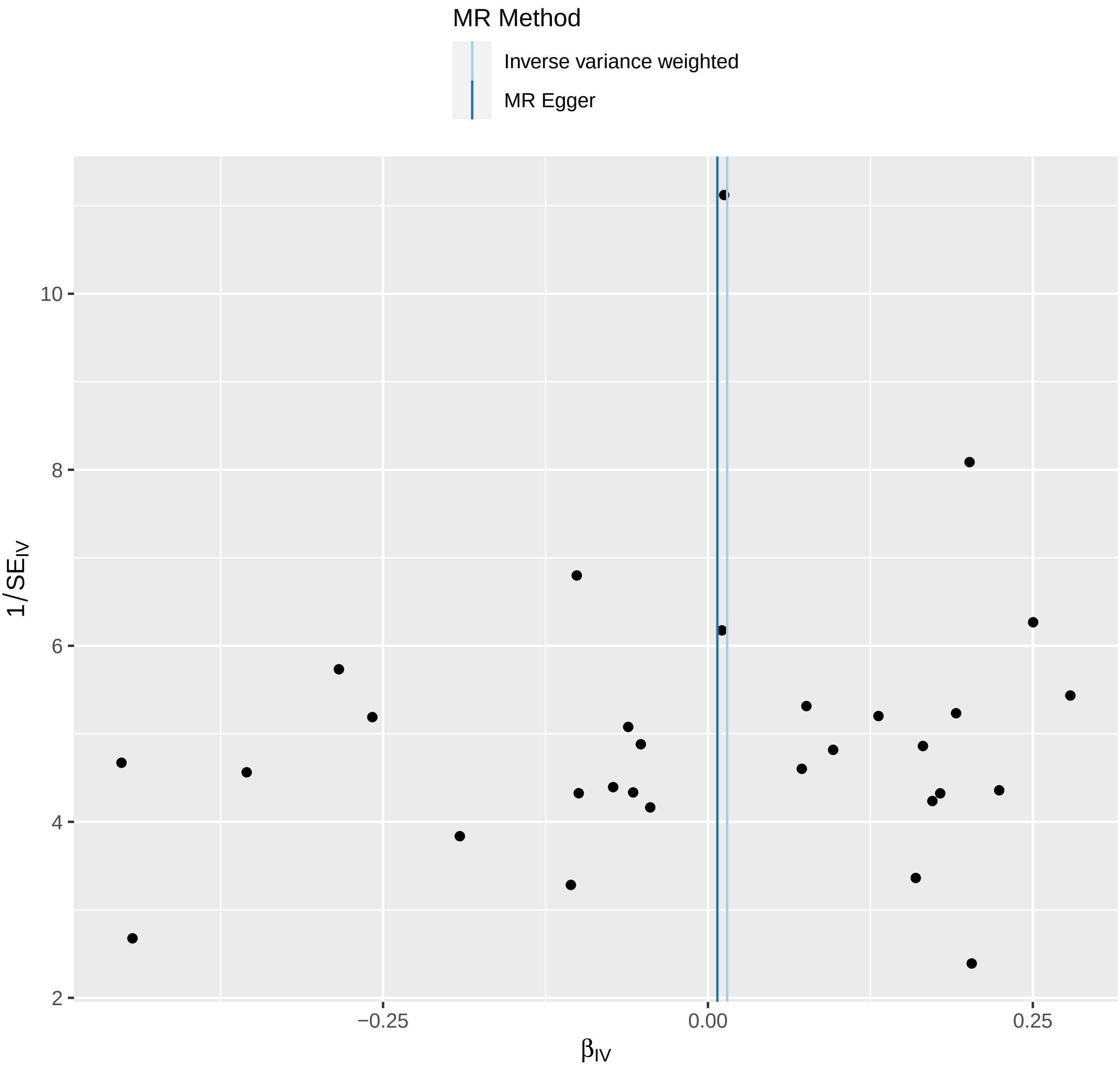

Batch 3−60 : Activator of 90 kDa heat shock protein ATPase homolog 1 on Diabetic nephropathy (more control exclusions)
with a threshold of 1e−05

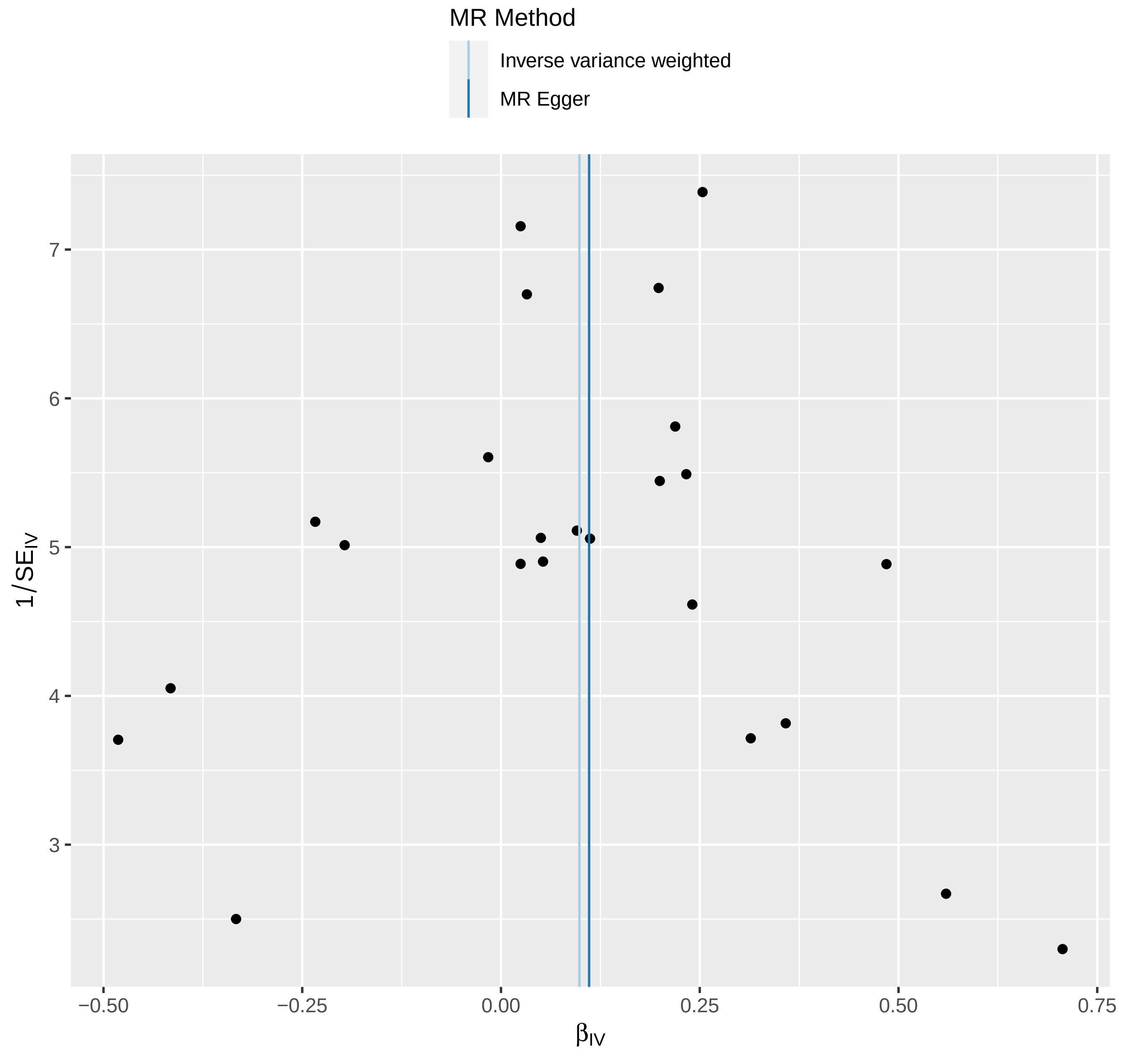

Batch 3−61 : Alpha−2−HS−glycoprotein on Diabetic nephropathy (more control exclusions)
with a threshold of 1e−05

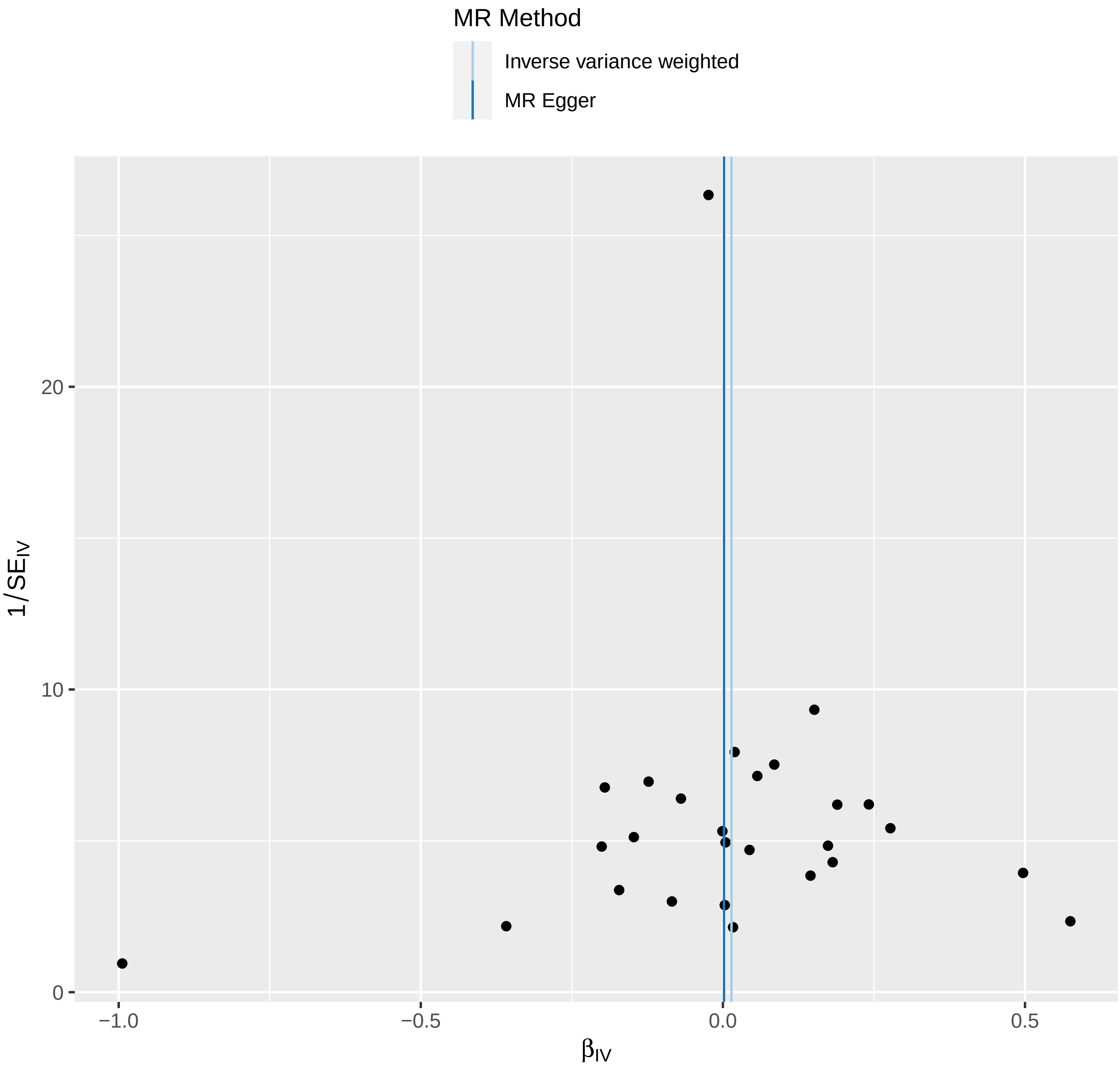

Batch 3−62 : Allograft inflammatory factor 1 on Diabetic nephropathy (more control exclusions)
with a threshold of 1e−05

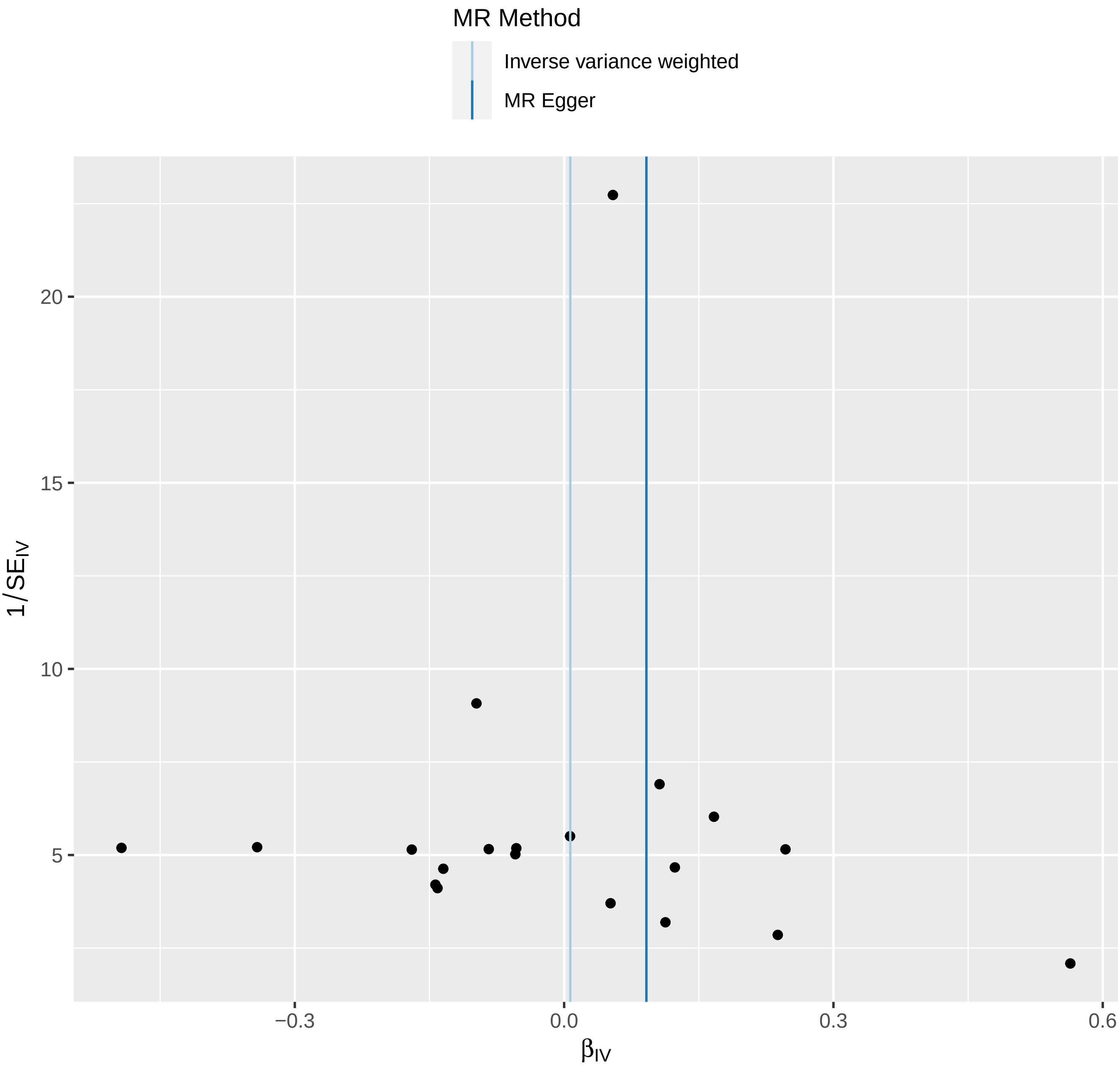

Batch 3−63 : Apoptosis−inducing factor 1, mitochondrial on Diabetic nephropathy (more control exclusions)
with a threshold of 1e−05

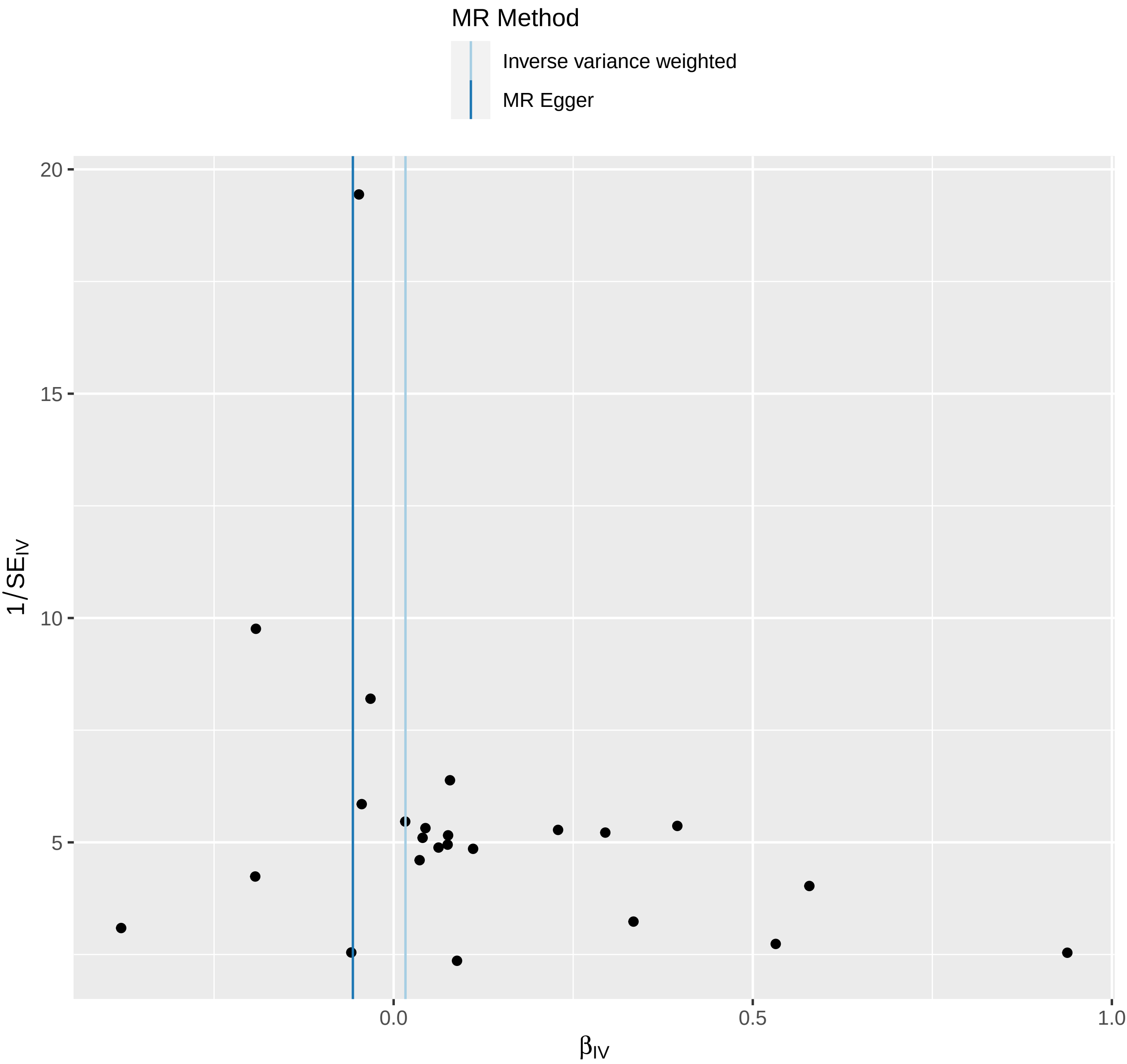

Batch 3−64 : Apoptosis−inducing factor 1, mitochondrial on Diabetic nephropathy (more control exclusions) with a threshold of 1e−05

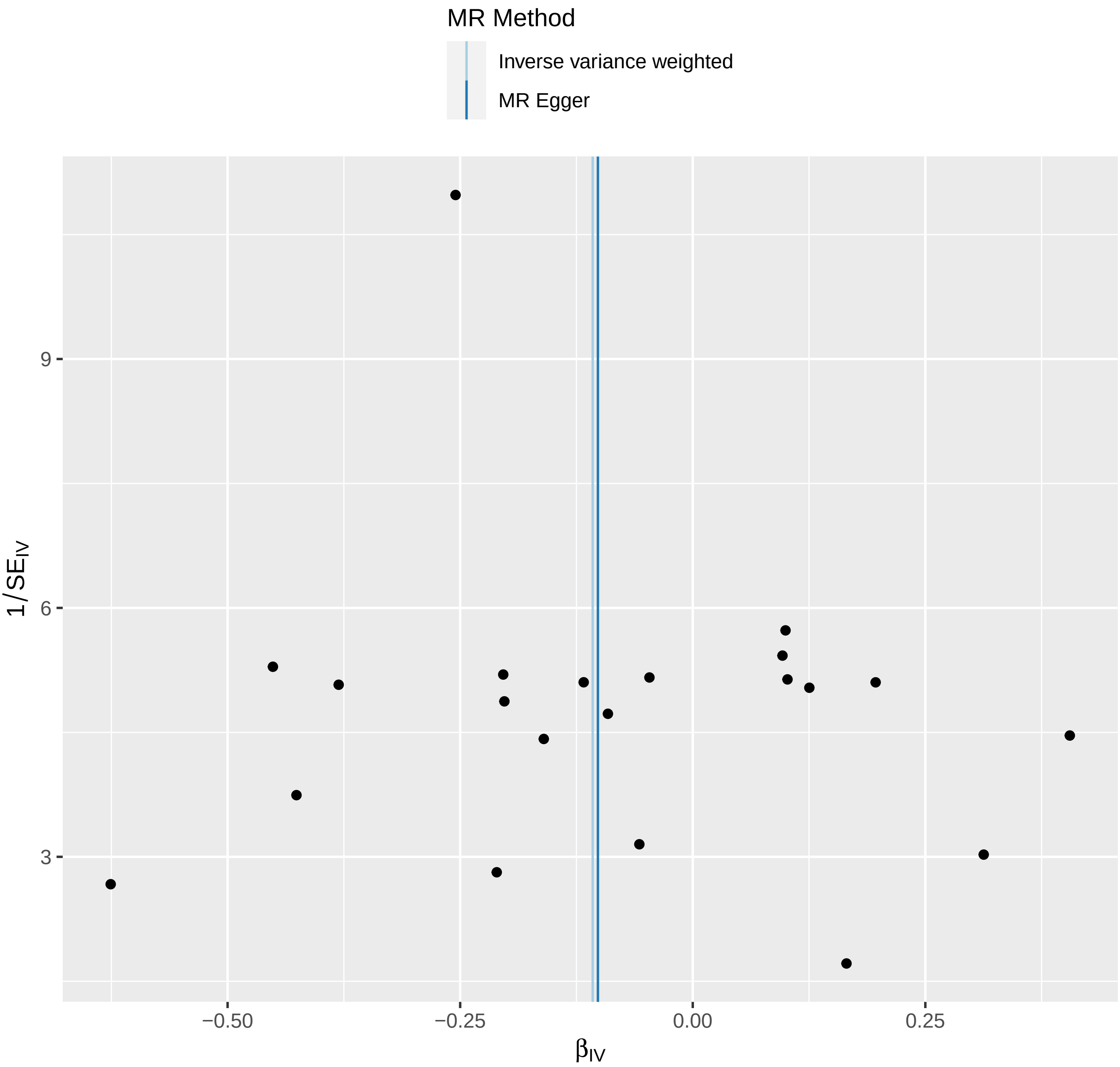

Batch 3−65 : Endothelial monocyte−activating polypeptide 2 on Diabetic nephropathy (more control exclusions)
with a threshold of 1e−05

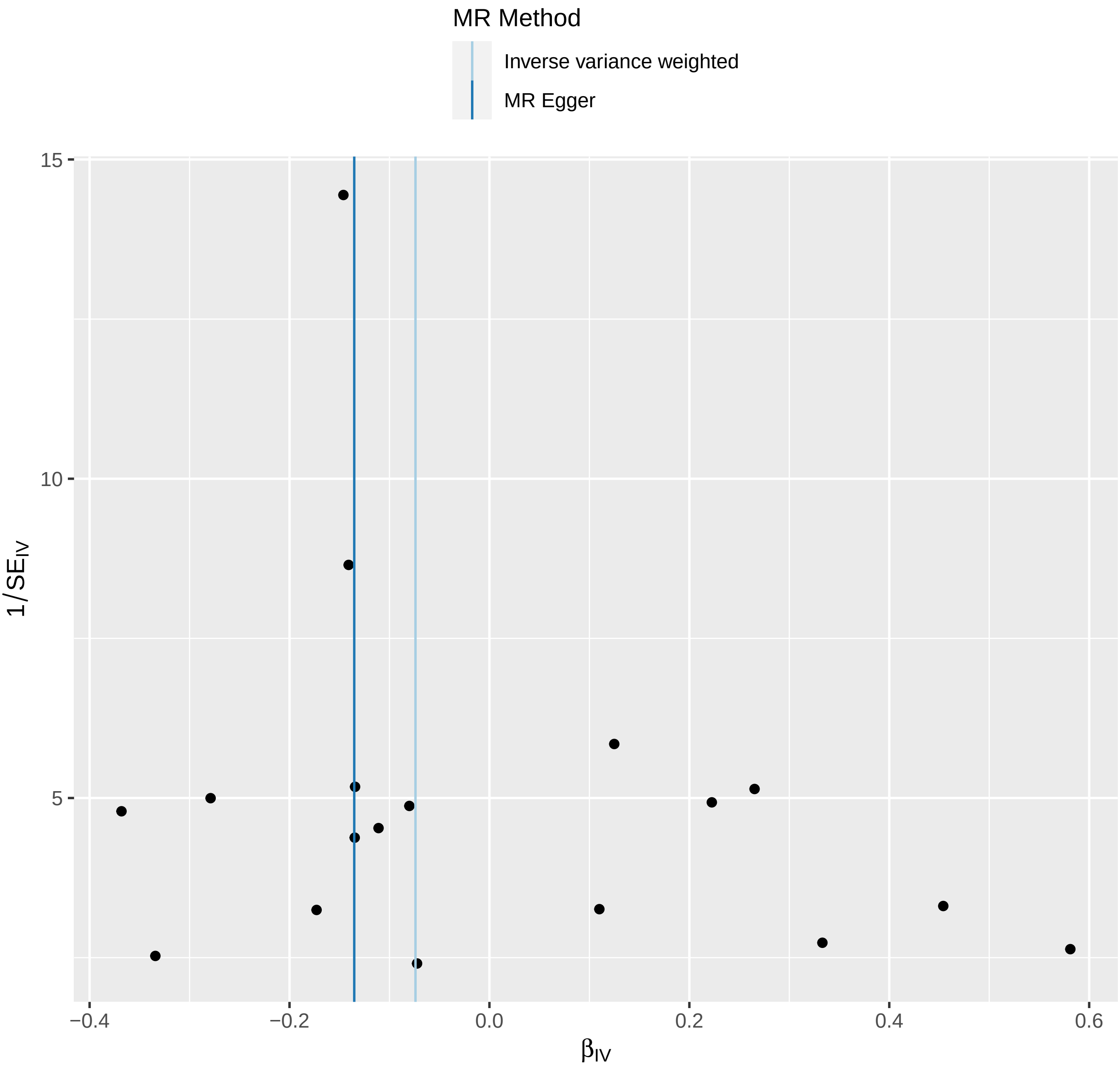

Batch 3−66 : Adenylate kinase isoenzyme 1 on Diabetic nephropathy (more control exclusions)
with a threshold of 1e−05

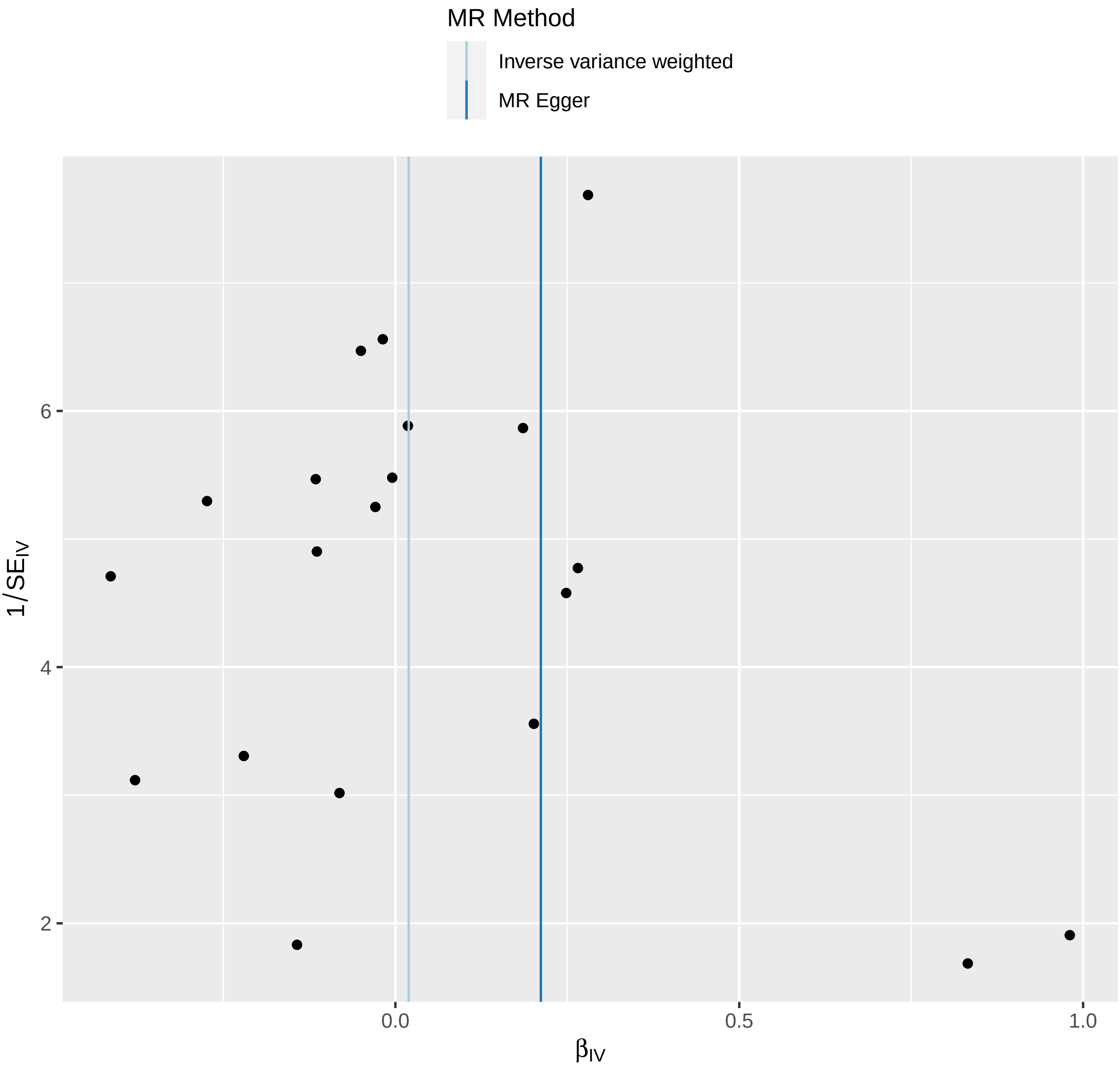

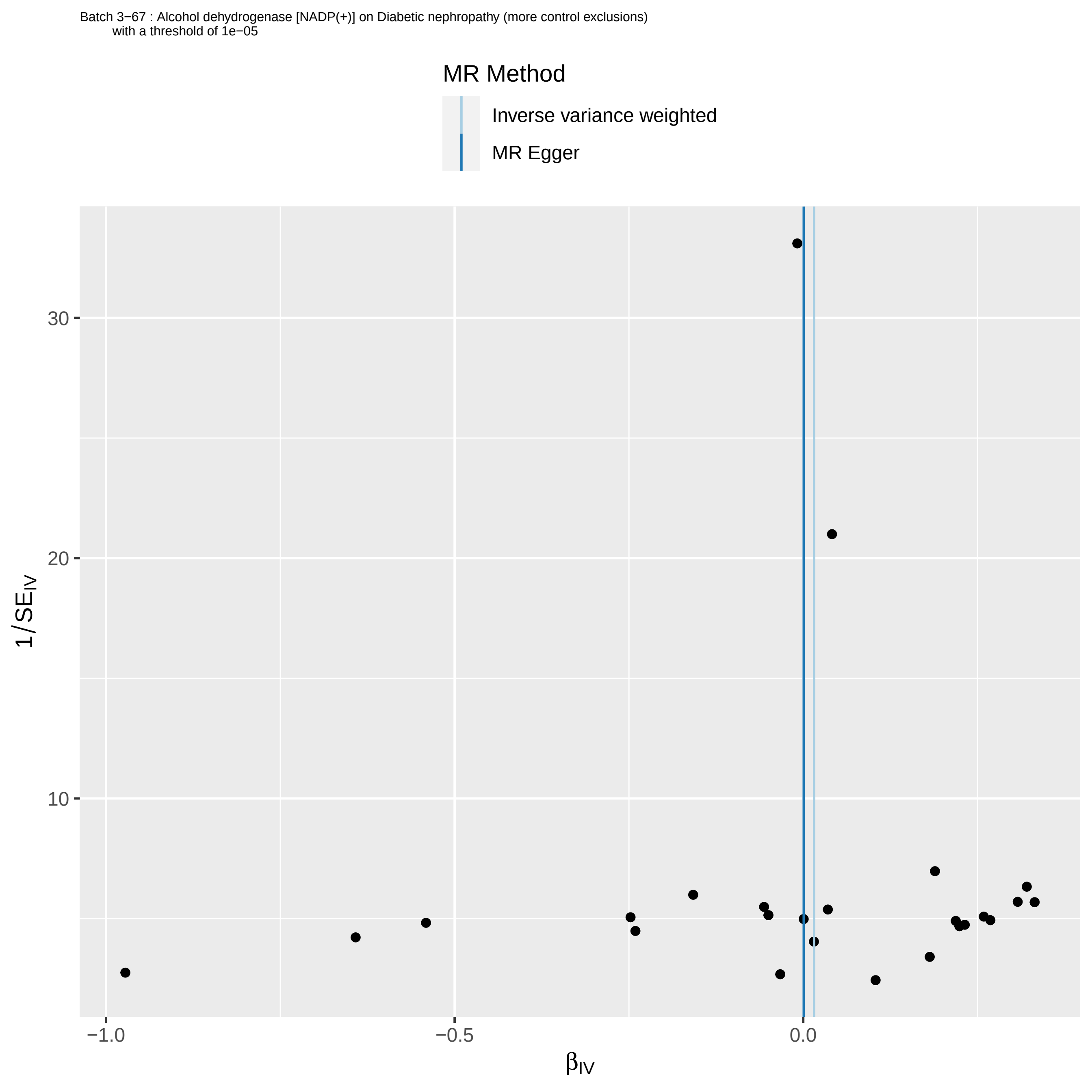
Batch 3−67 : Alcohol dehydrogenase [NADP(+)] on Diabetic nephropathy (more control exclusions)
with a threshold of 1e−05
MR Method
Inverse variance weighted
MR Egger
30
20
10
1/SE$_{IV}$
−1.0
−0.5
0.0
β$_{IV}$

Batch 3−68 : Aldose reductase on Diabetic nephropathy (more control exclusions)
with a threshold of 1e−05

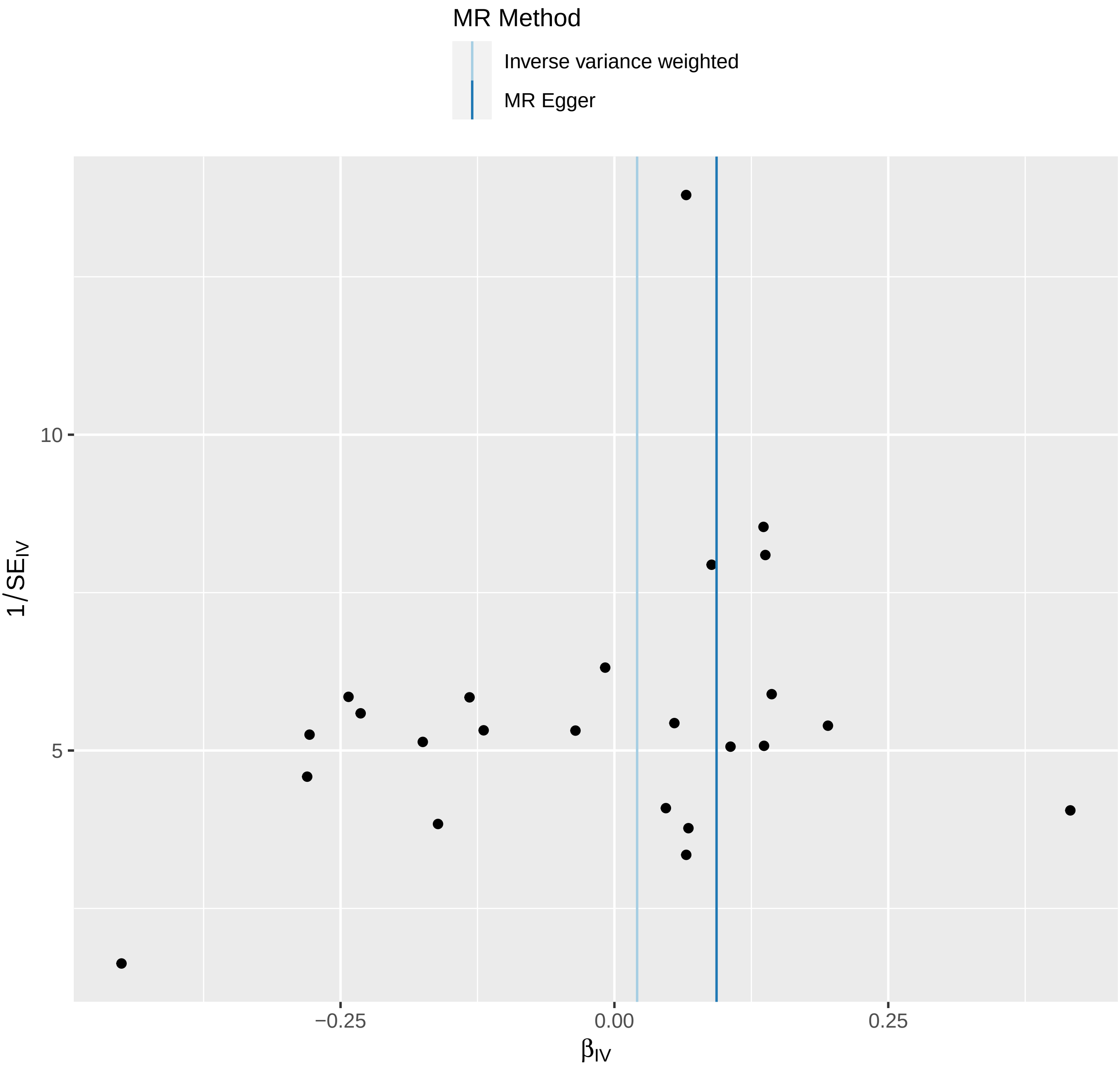

Batch 3−69 : Aldo−keto reductase family 1 member C1 on Diabetic nephropathy (more control exclusions)
with a threshold of 1e−05

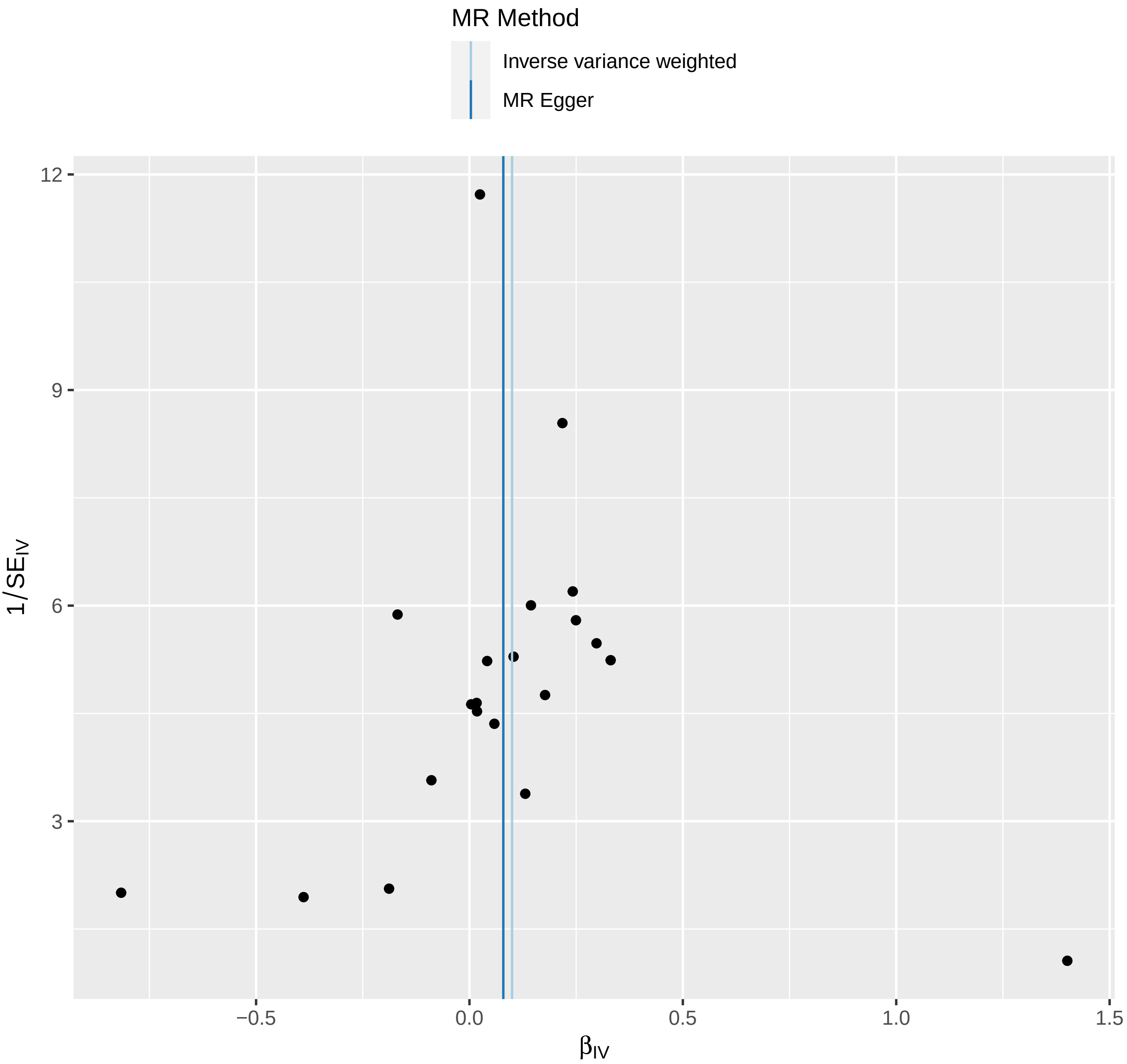

Batch 3−70 : RAC−alpha/beta/gamma serine/threonine−protein kinase on Diabetic nephropathy (more control exclusions)
with a threshold of 1e−05

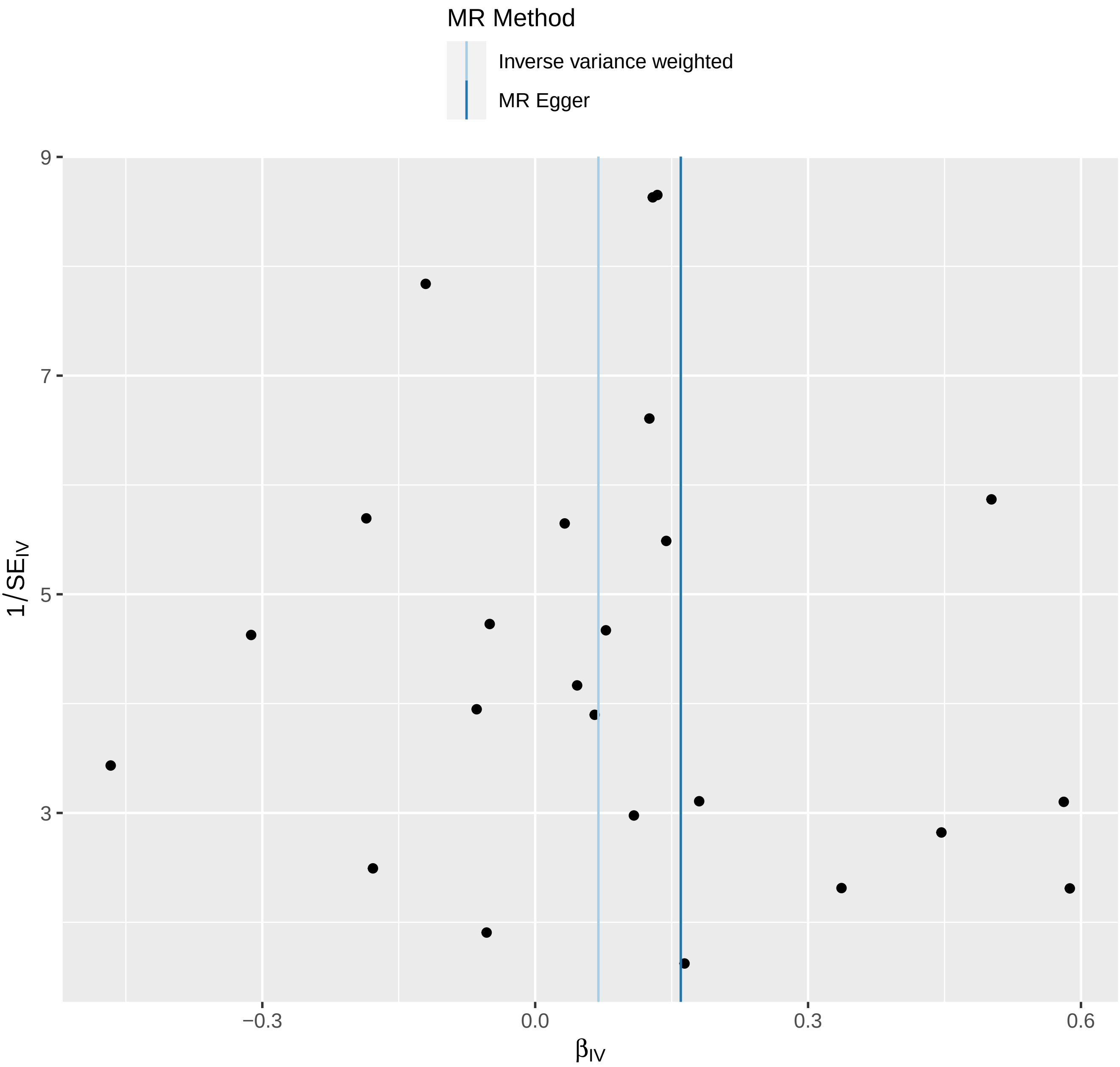

Batch 3−71 : RAC−alpha/beta/gamma serine/threonine−protein kinase on Diabetic nephropathy (more control exclusions) with a threshold of 1e−05

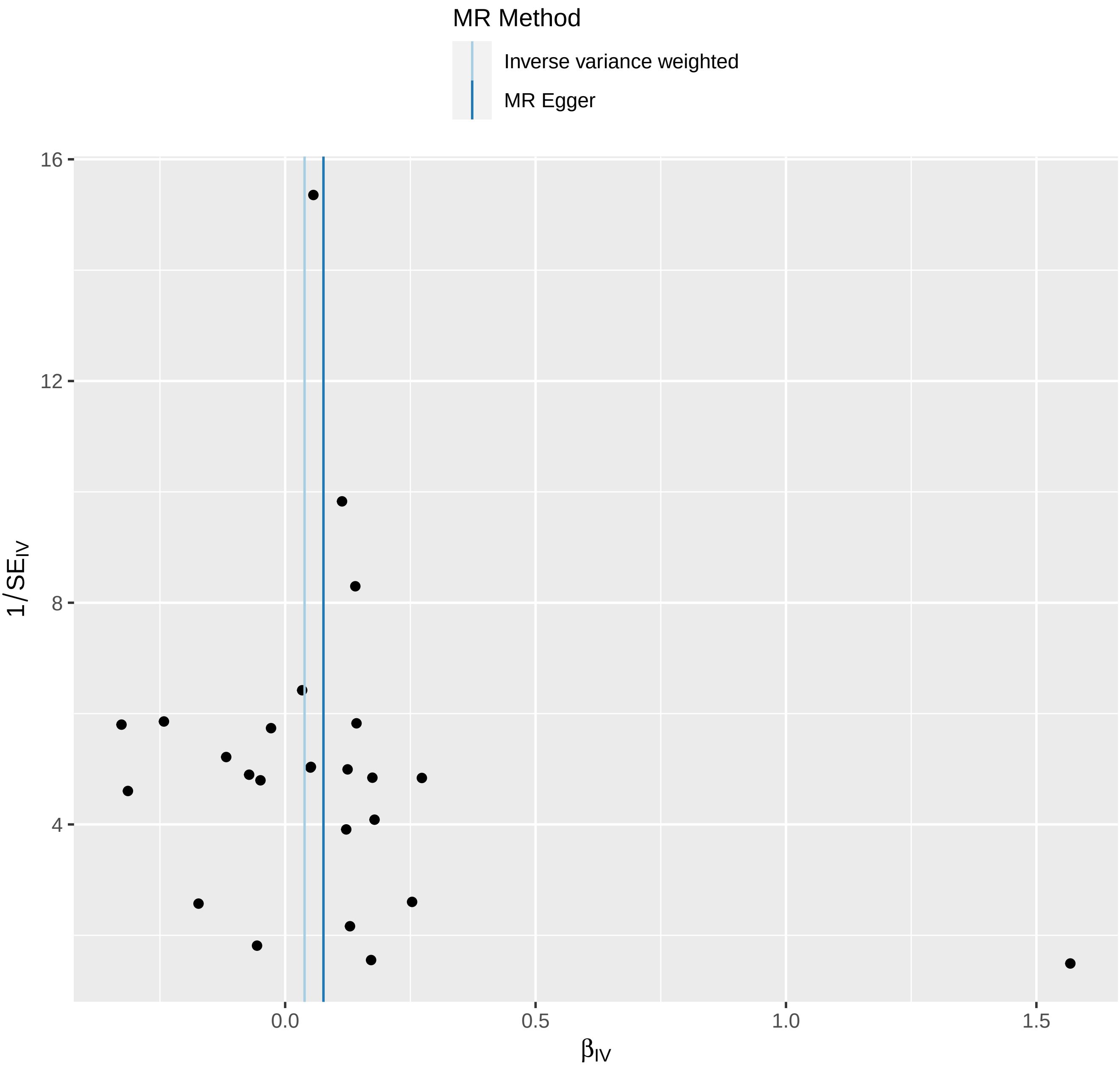

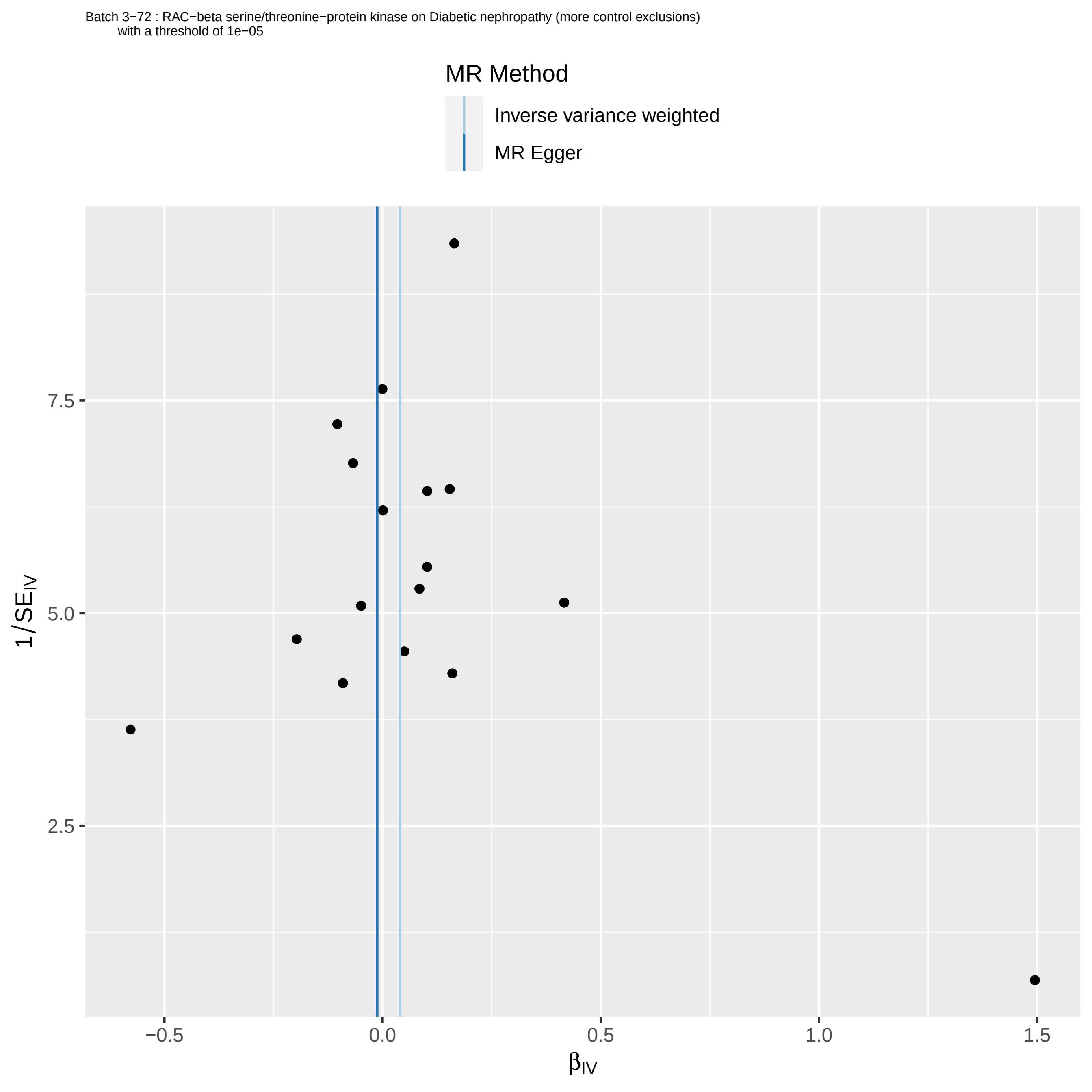
Batch 3−72 : RAC−beta serine/threonine−protein kinase on Diabetic nephropathy (more control exclusions)
with a threshold of 1e−05
MR Method
Inverse variance weighted
MR Egger
7.5
5.0
2.5
1/SE_IV
−0.5
0.0
0.5
1.0
1.5
β_IV

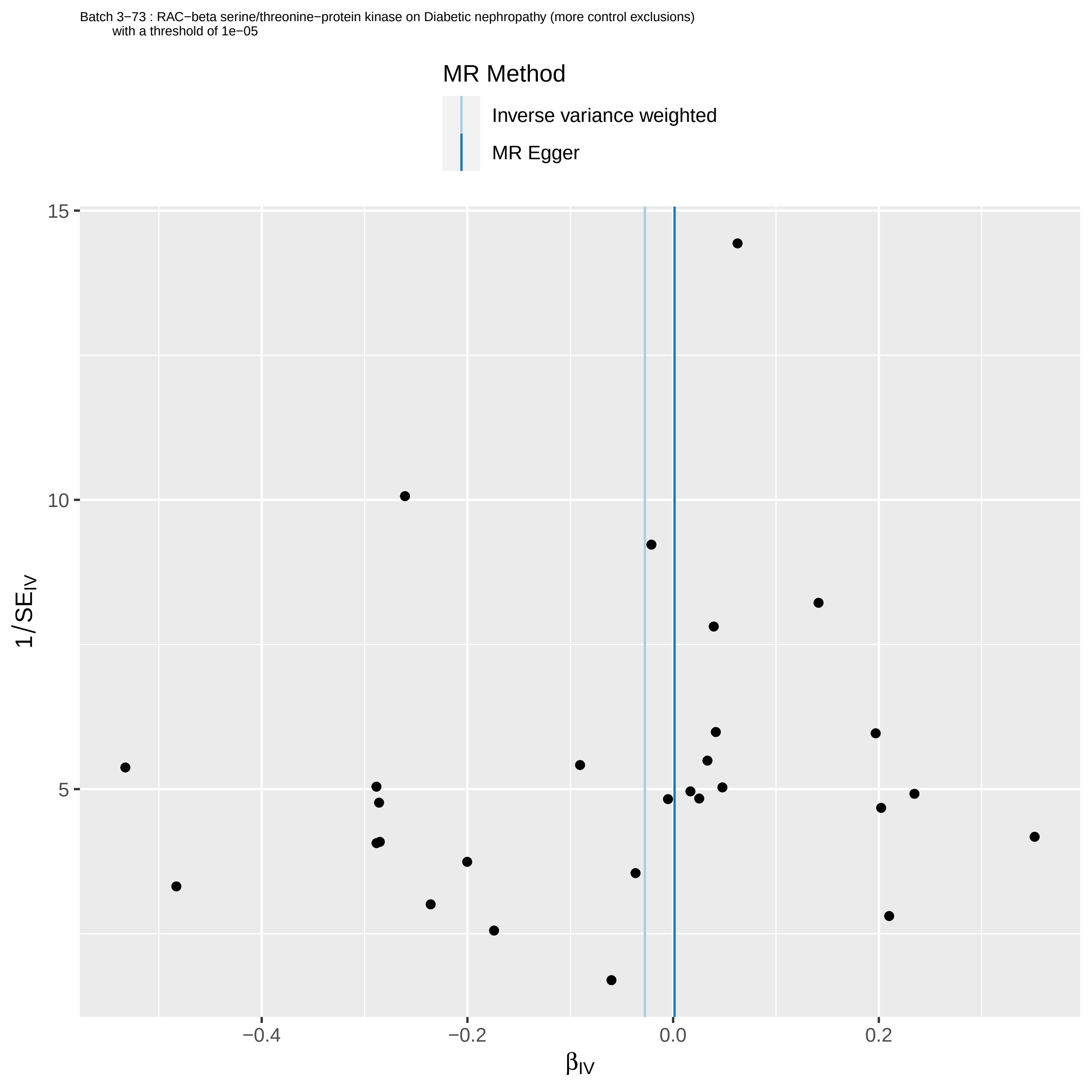

Batch 3−73 : RAC−beta serine/threonine−protein kinase on Diabetic nephropathy (more control exclusions)
with a threshold of 1e−05
MR Method
Inverse variance weighted
MR Egger
15
10
5
1/SE$_{IV}$
−0.4
−0.2
0.0
0.2
β$_{IV}$

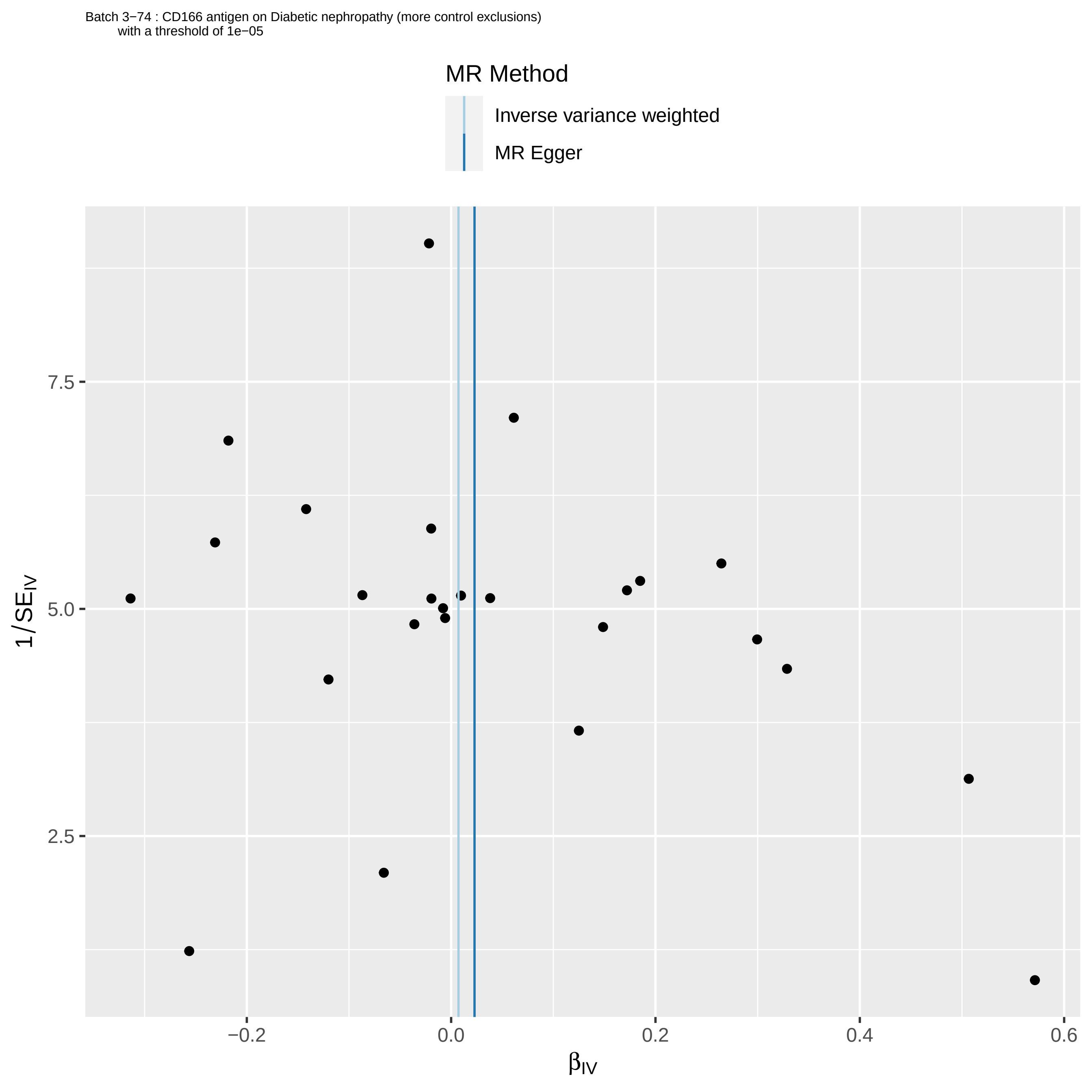

Batch 3−74 : CD166 antigen on Diabetic nephropathy (more control exclusions)
with a threshold of 1e−05
MR Method
Inverse variance weighted
MR Egger
7.5
5.0
2.5
−0.2
0.0
0.2
0.4
0.6
$1/SE_{IV}$
β_{IV}

Batch 3−75 : Aldehyde dehydrogenase, dimeric NADP−preferring on Diabetic nephropathy (more control exclusions) with a threshold of 1e−05

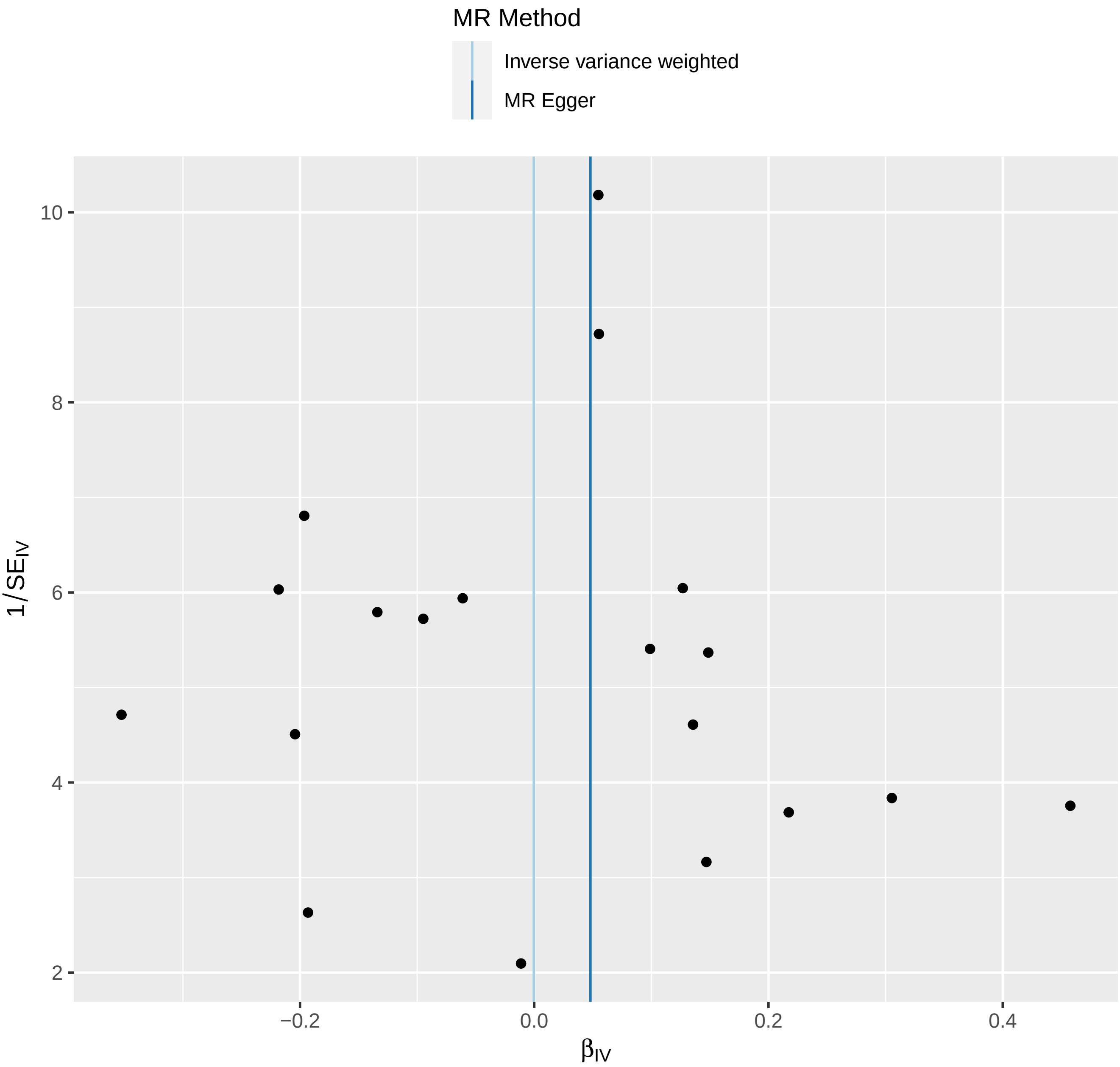

Batch 3−76 : Aldehyde dehydrogenase family 3 member B1 on Diabetic nephropathy (more control exclusions) with a threshold of 1e−05

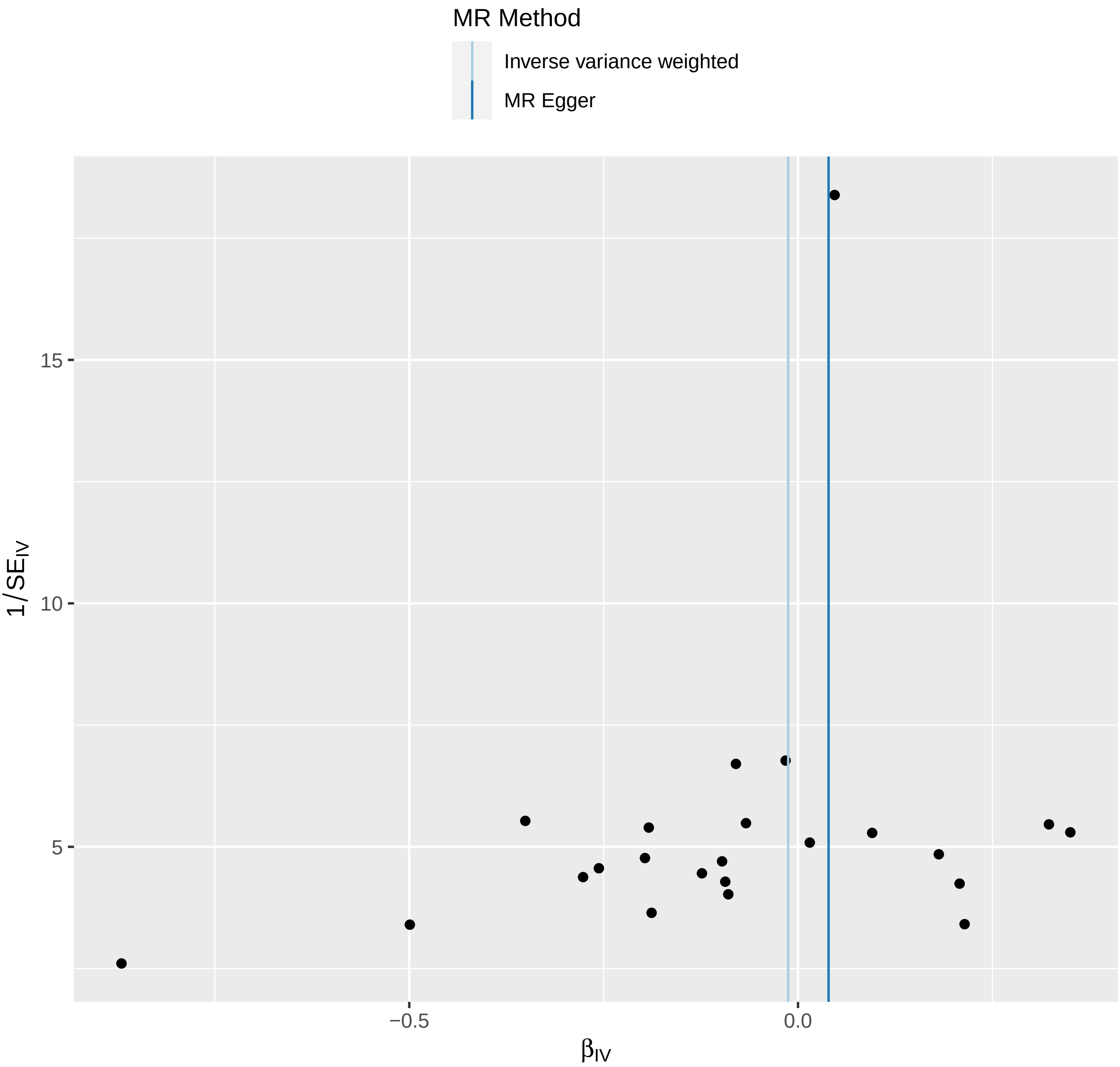

Batch 3−77 : Fructose−bisphosphate aldolase A on Diabetic nephropathy (more control exclusions)
with a threshold of 1e−05

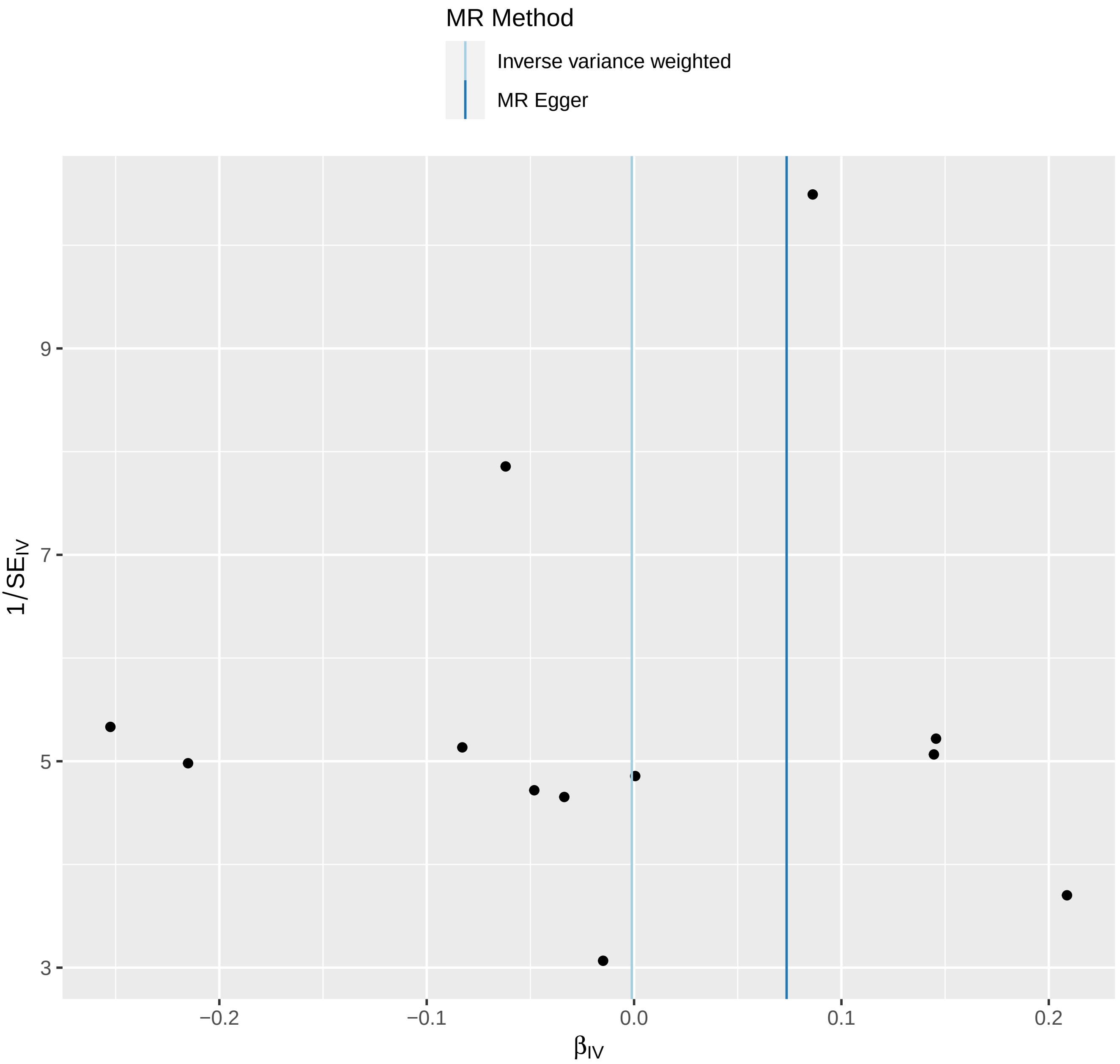

Batch 3−78 : Fructose−bisphosphate aldolase C on Diabetic nephropathy (more control exclusions)
with a threshold of 1e−05

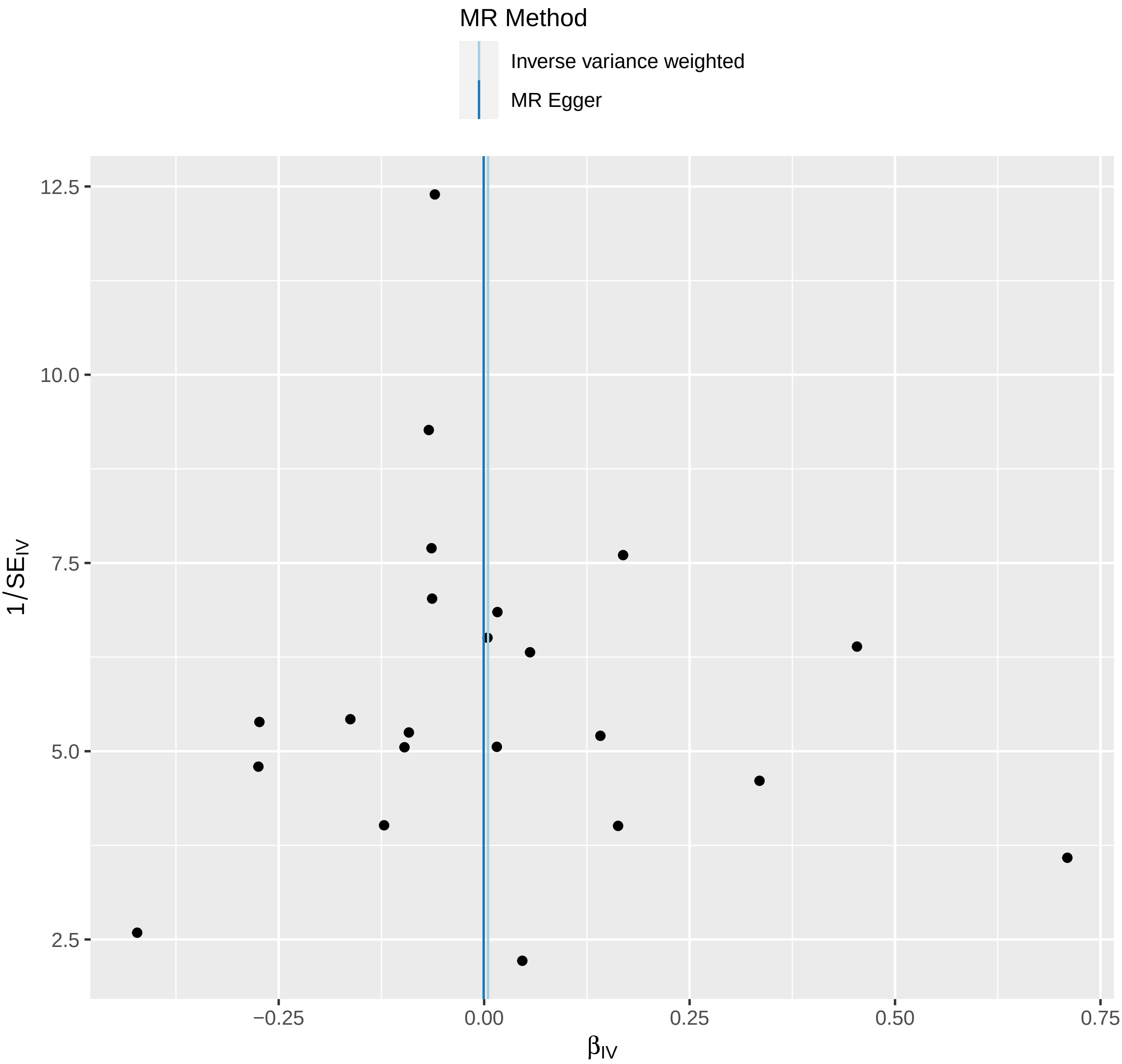

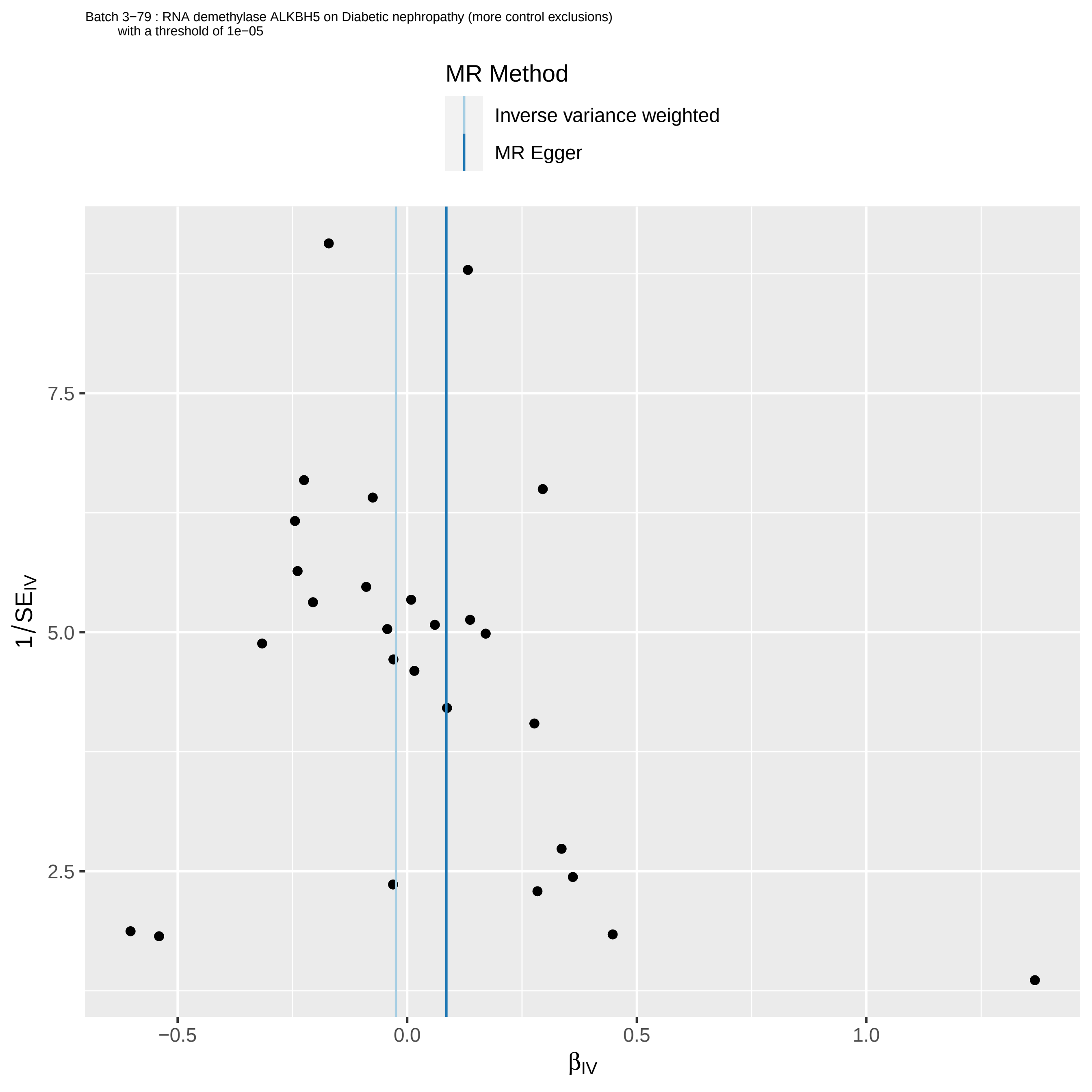
Batch 3−79 : RNA demethylase ALKBH5 on Diabetic nephropathy (more control exclusions)
with a threshold of 1e−05
MR Method
Inverse variance weighted
MR Egger
7.5
5.0
2.5
1/SE_IV
−0.5
0.0
0.5
1.0
β_IV

Batch 3−80 : Arachidonate 15−lipoxygenase B on Diabetic nephropathy (more control exclusions)
with a threshold of 1e−05

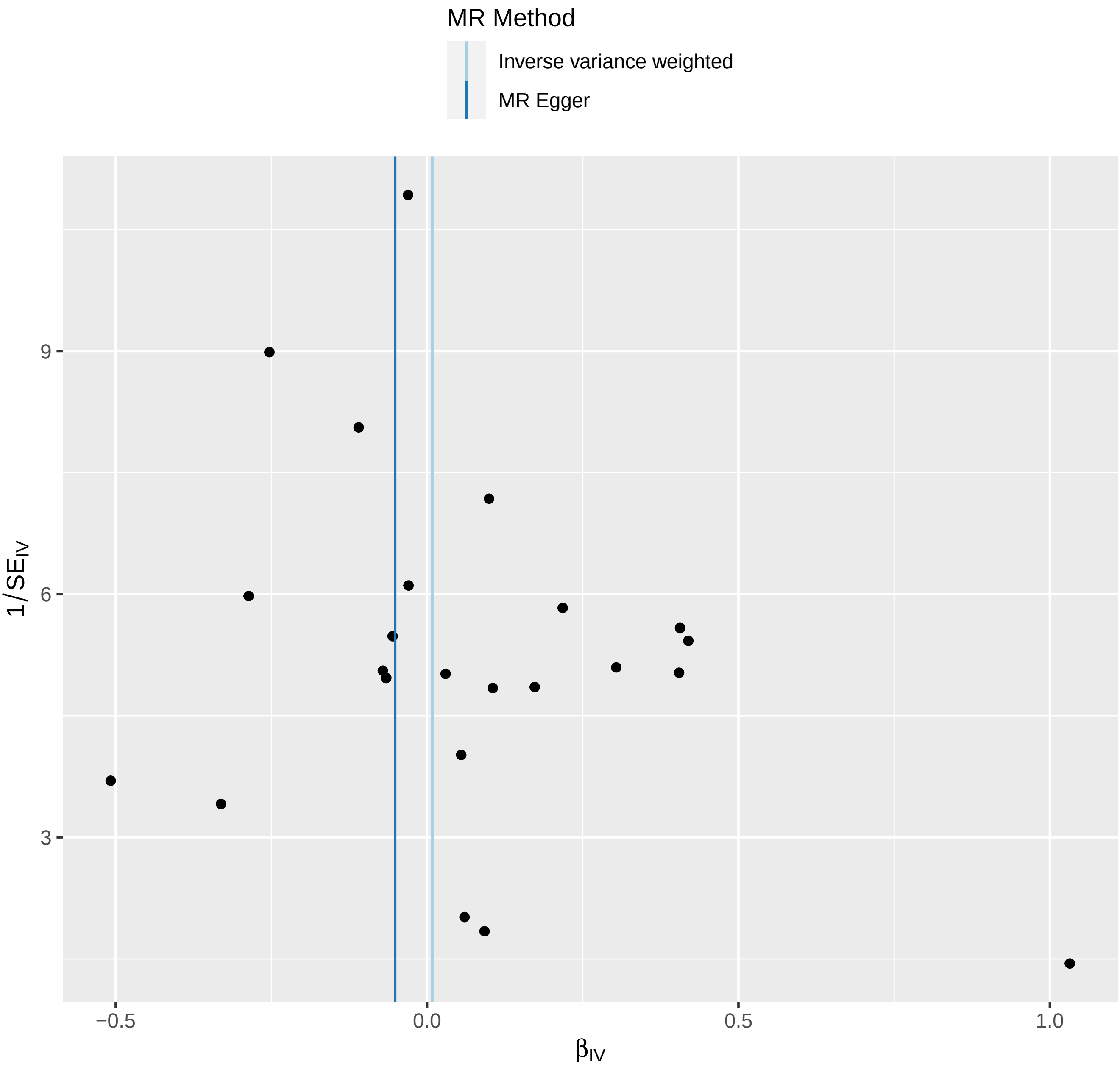

Batch 3−81 : Intestinal−type alkaline phosphatase on Diabetic nephropathy (more control exclusions)
with a threshold of 1e−05

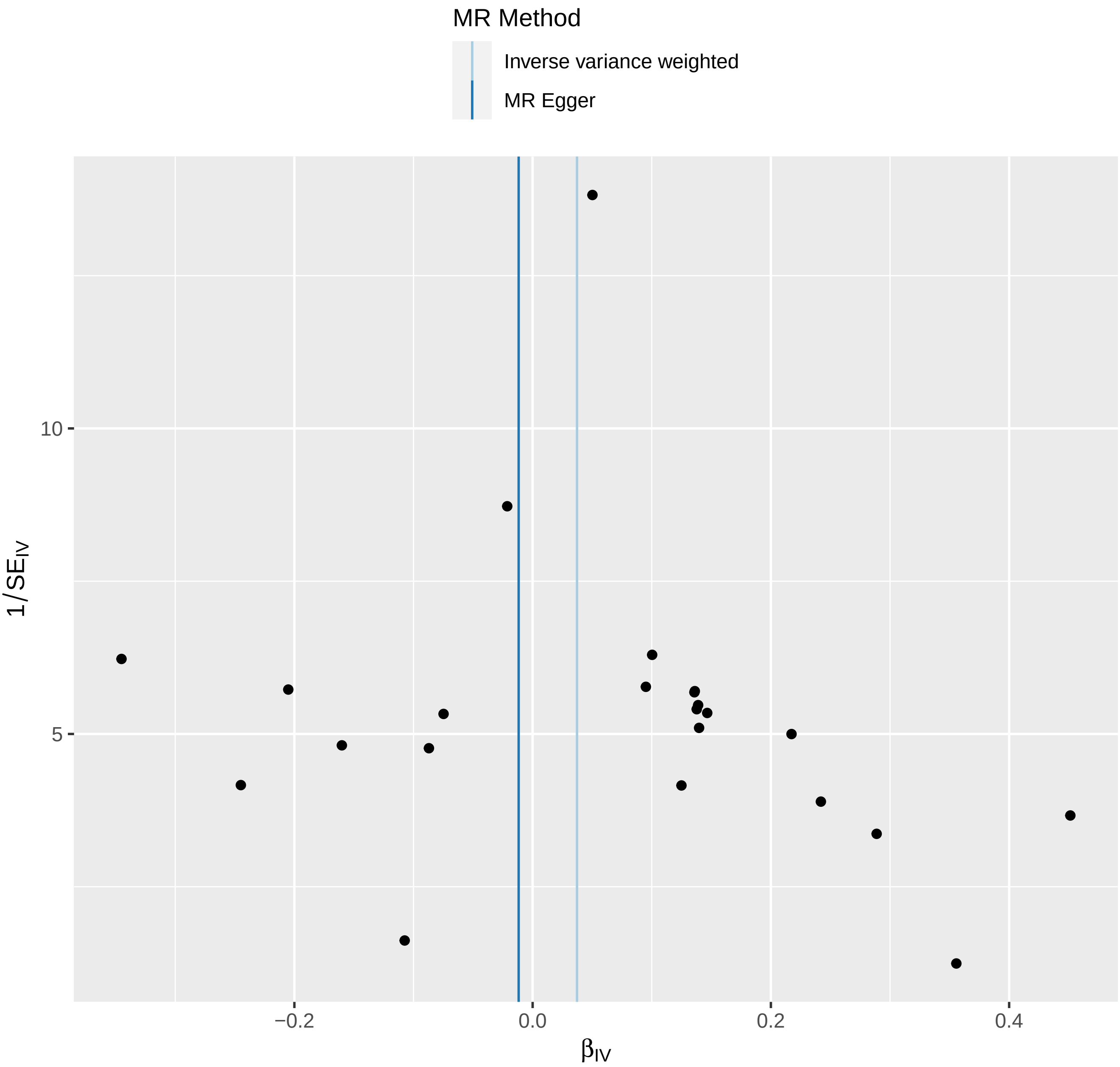

Batch 3−82 : Ameloblastin on Diabetic nephropathy (more control exclusions)
with a threshold of 1e−05

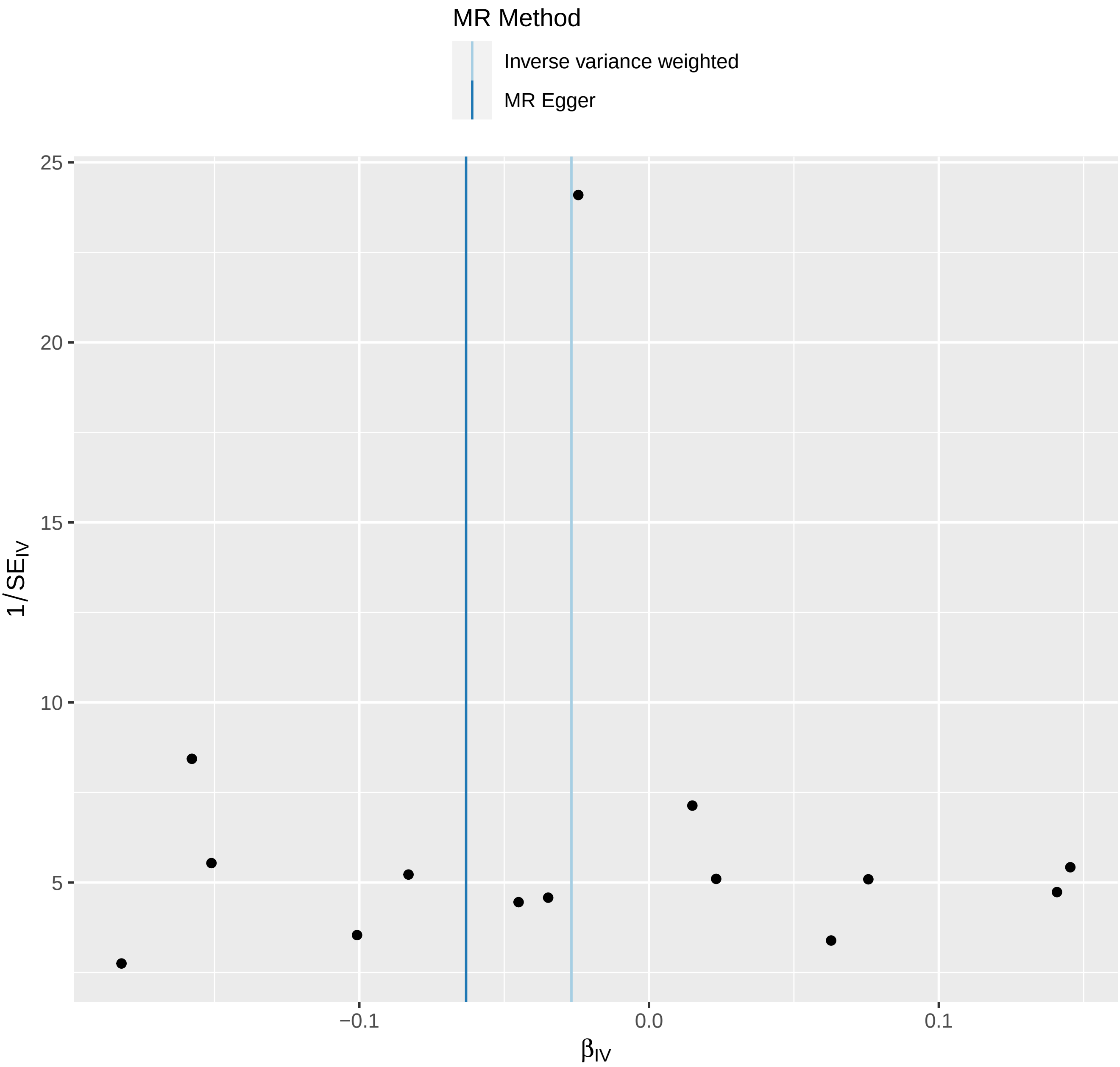

Batch 3−83 : Amelogenin, X isoform on Diabetic nephropathy (more control exclusions)
with a threshold of 1e−05

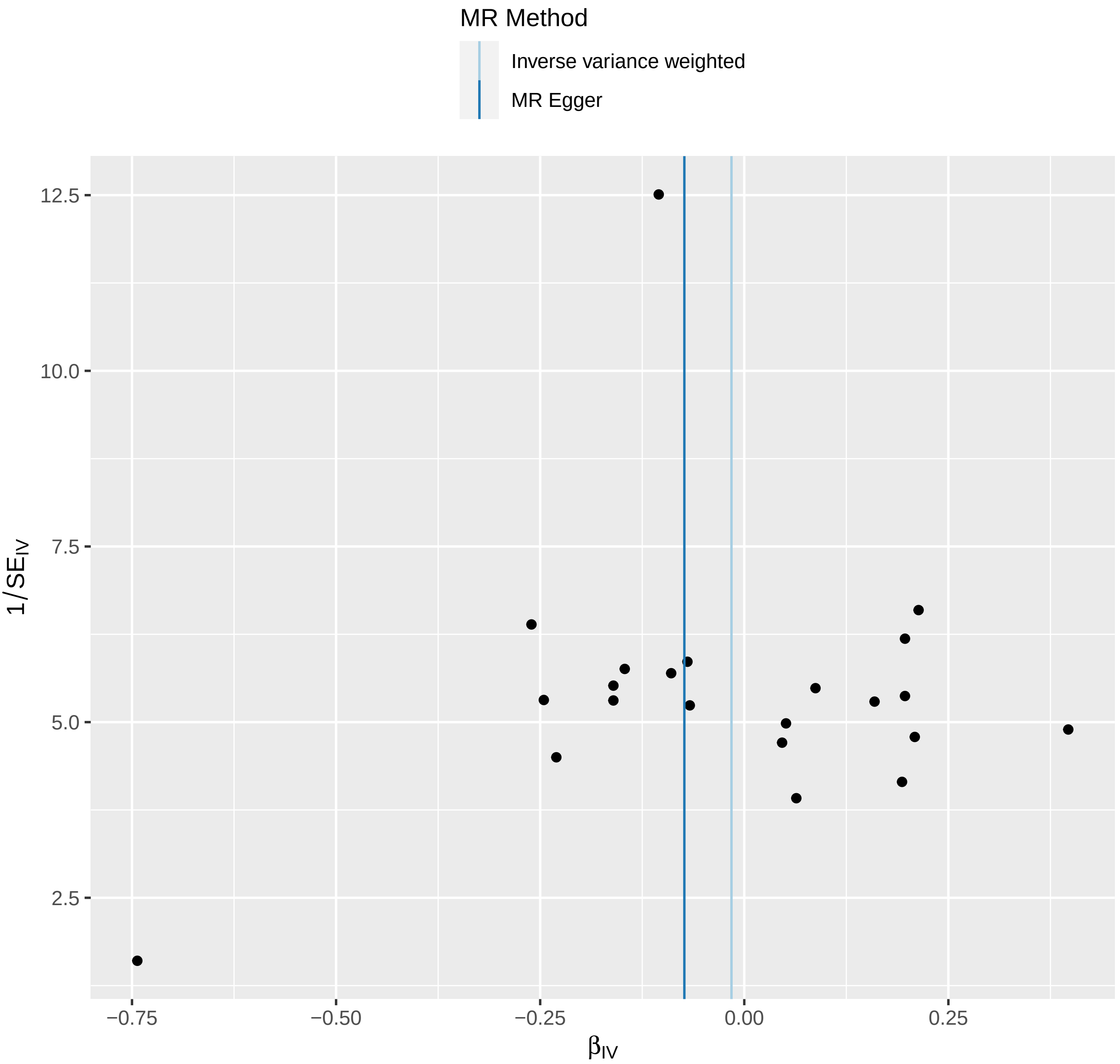

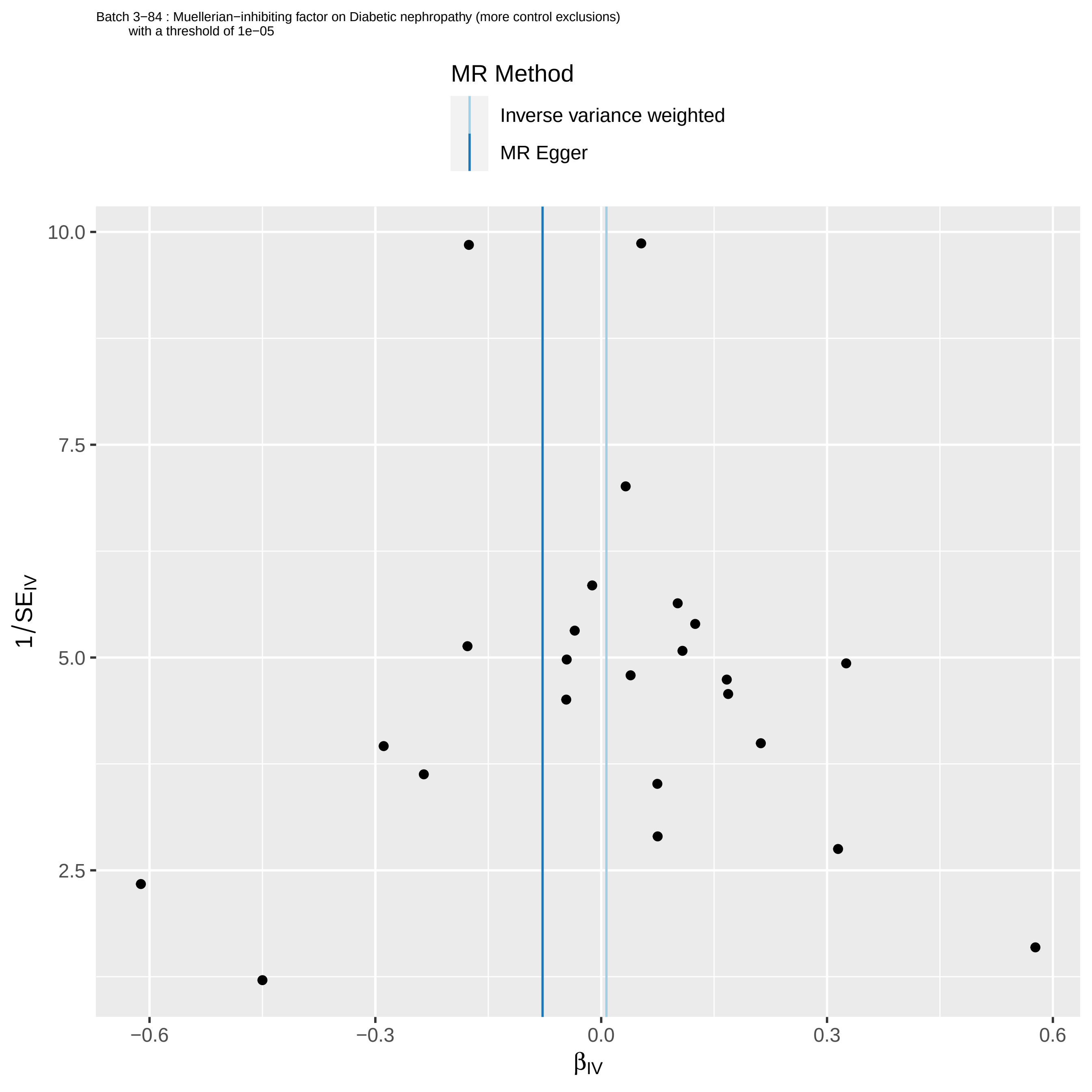
Batch 3−84 : Muellerian−inhibiting factor on Diabetic nephropathy (more control exclusions)
with a threshold of 1e−05
MR Method
Inverse variance weighted
MR Egger
1/SE_IV
10.0
7.5
5.0
2.5
−0.6
−0.3
0.0
0.3
0.6
β_IV

Batch 3−85 : Junctional adhesion molecule−like on Diabetic nephropathy (more control exclusions)
with a threshold of 1e−05

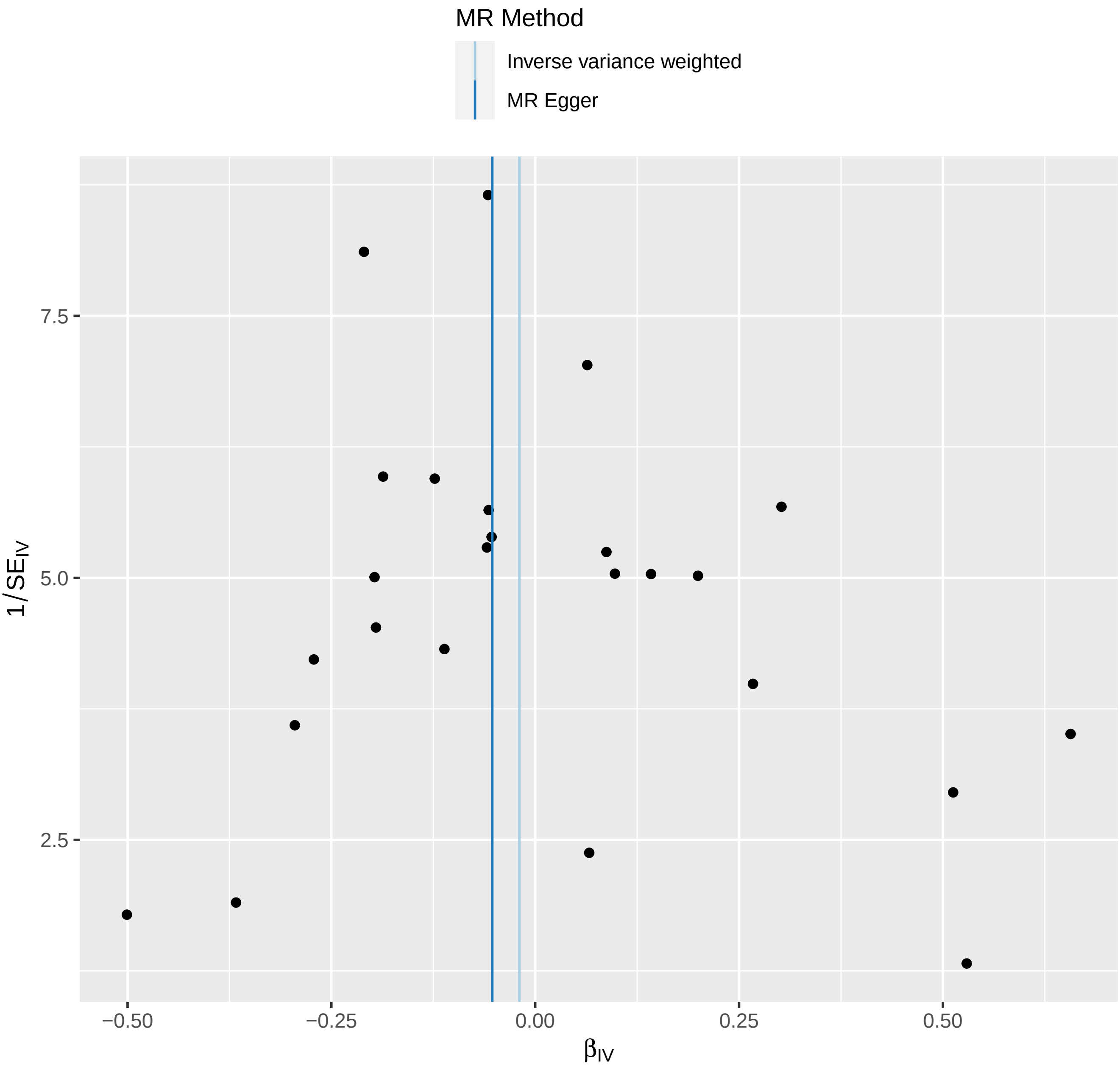

Batch 3−86 : Junctional adhesion molecule−like on Diabetic nephropathy (more control exclusions)
with a threshold of 1e−05

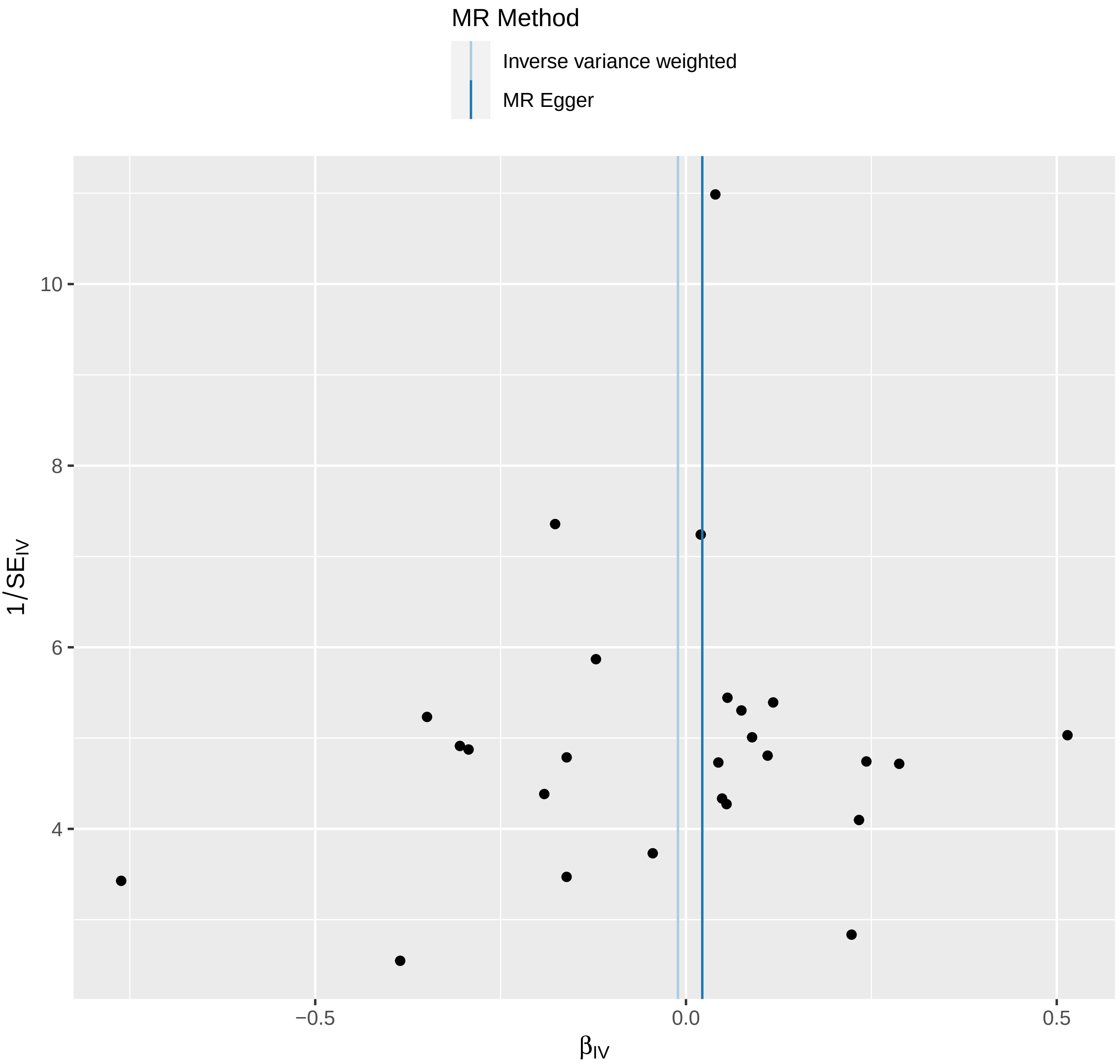

Batch 3−87 : Amphoterin−induced protein 1 on Diabetic nephropathy (more control exclusions)
with a threshold of 1e−05

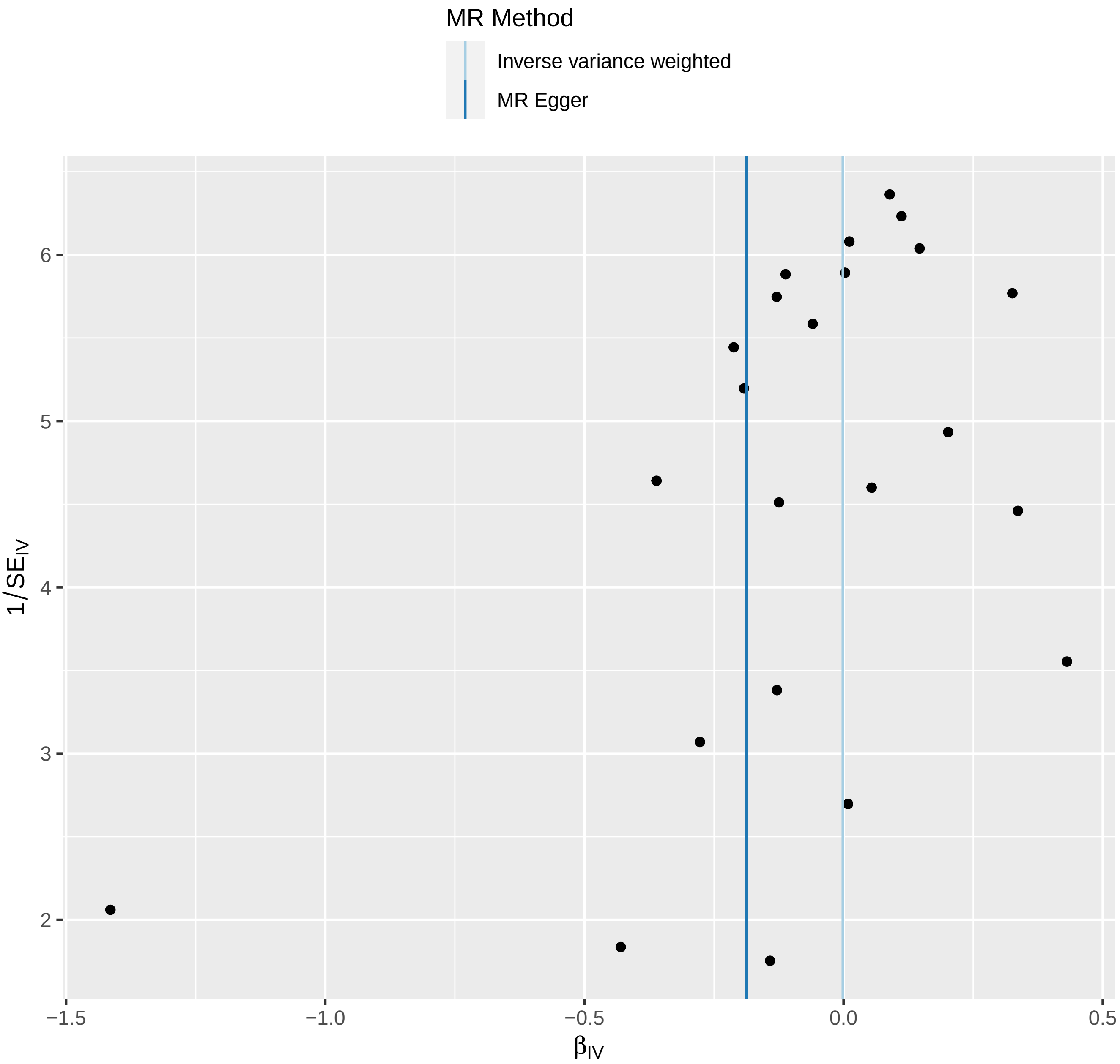

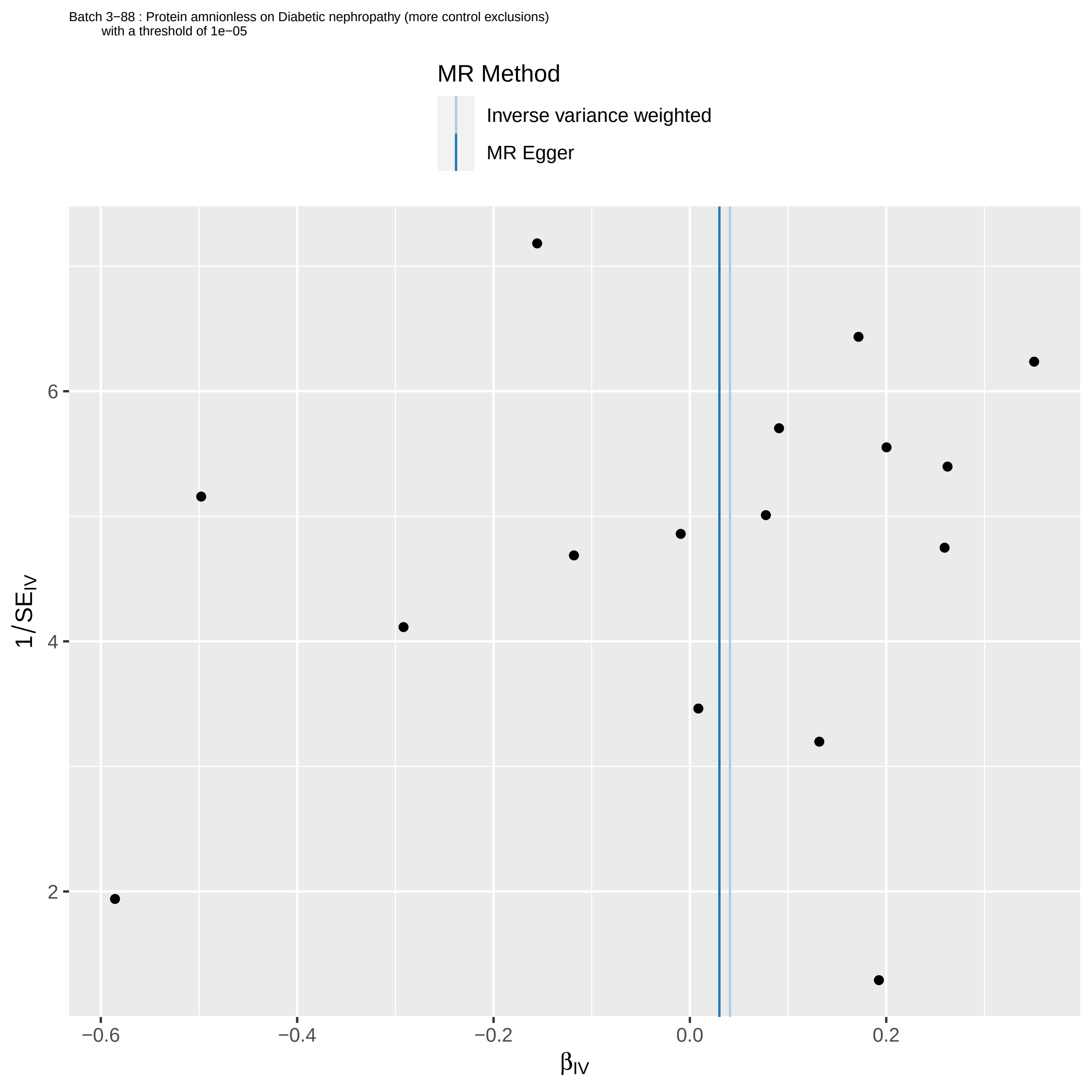

Batch 3−88 : Protein amnionless on Diabetic nephropathy (more control exclusions)
with a threshold of 1e−05
MR Method
Inverse variance weighted
MR Egger
6
4
2
1/SE$_{IV}$
−0.6
−0.4
−0.2
0.0
0.2
β$_{IV}$

Batch 3−89 : Alpha−amylase 1 on Diabetic nephropathy (more control exclusions)
with a threshold of 1e−05

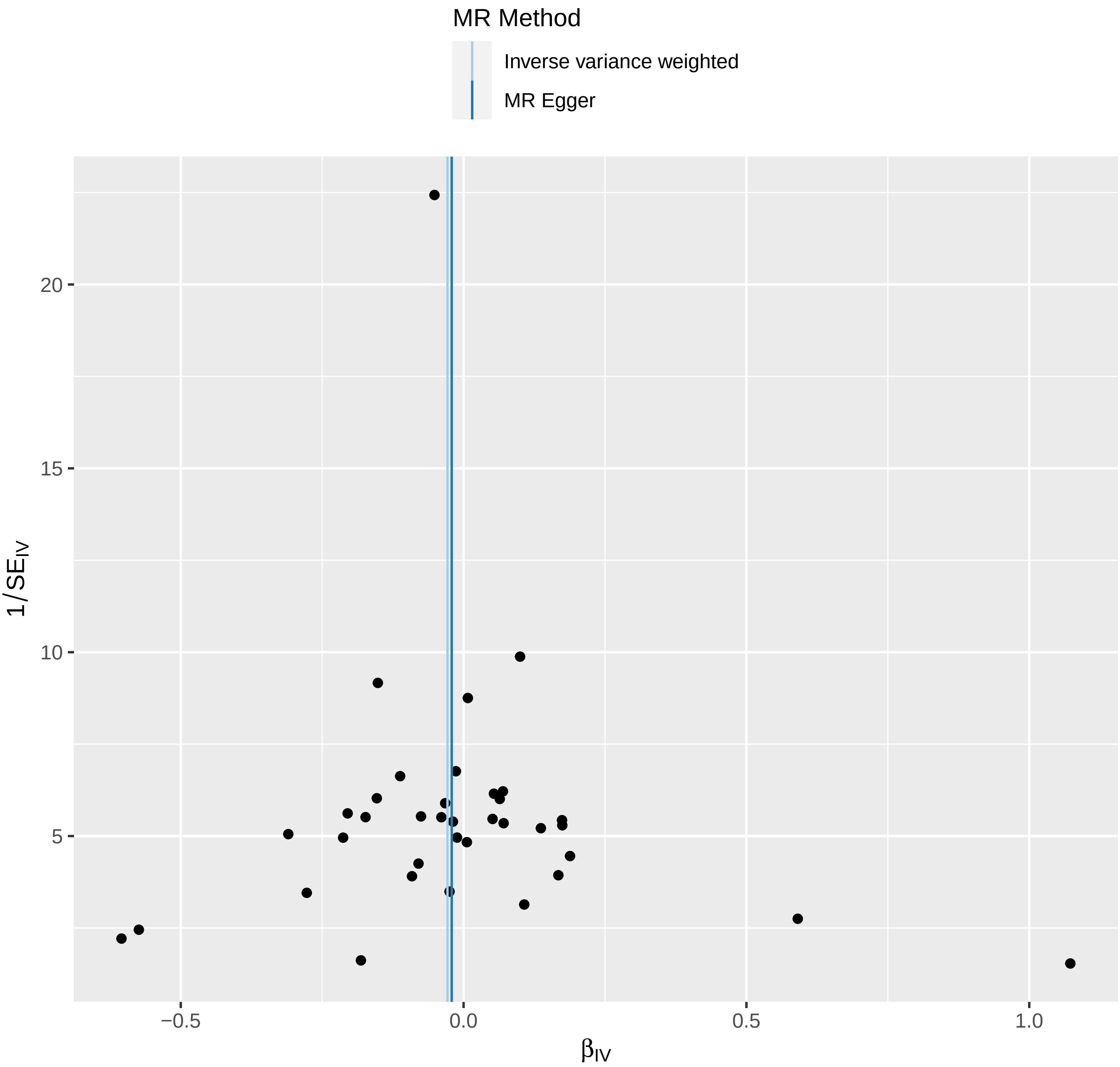

Batch 3−90 : Anaphase−promoting complex subunit 10 on Diabetic nephropathy (more control exclusions)
with a threshold of 1e−05

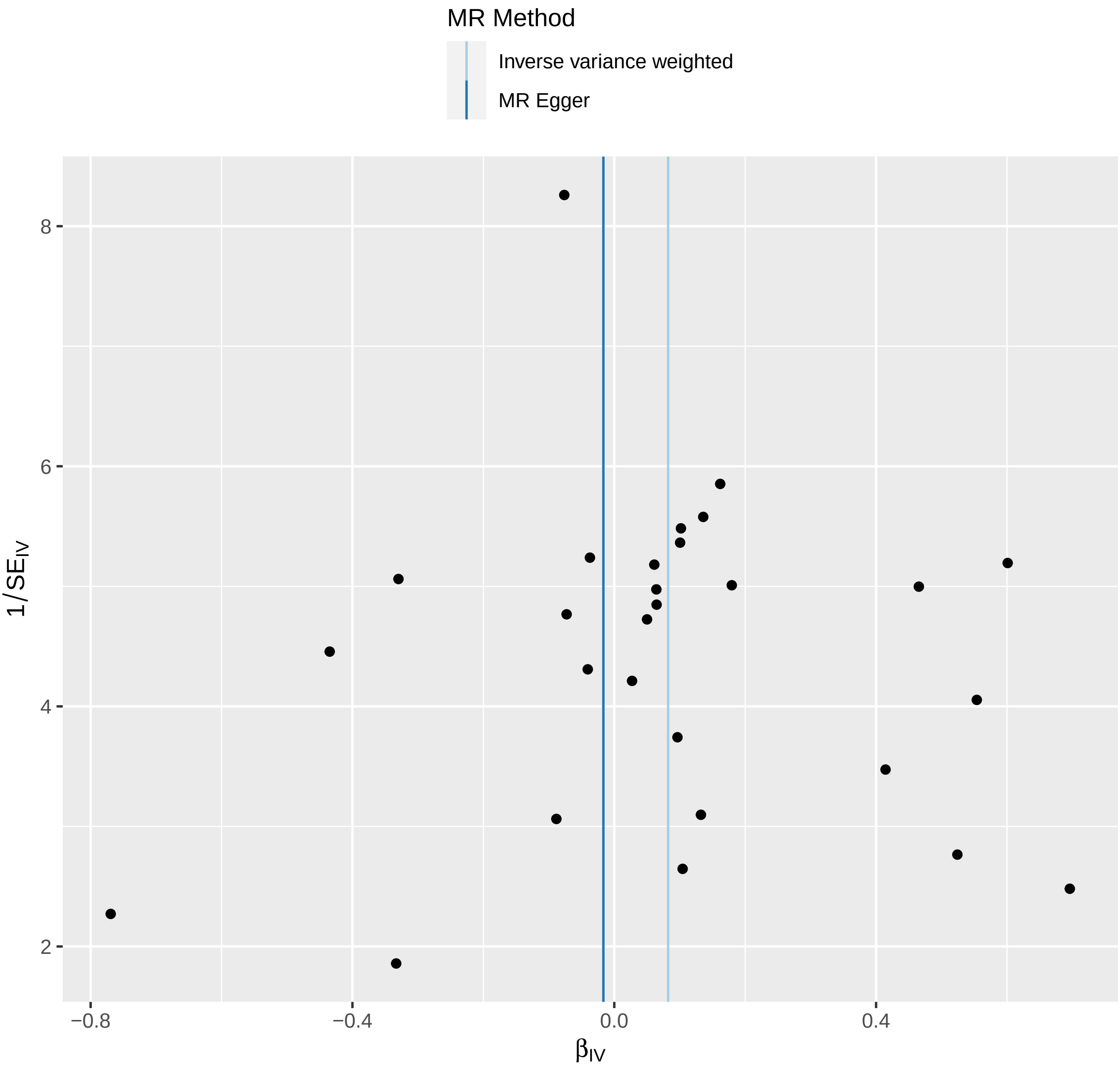

Batch 3−91 : Anaphase−promoting complex subunit 7 on Diabetic nephropathy (more control exclusions)
with a threshold of 1e−05

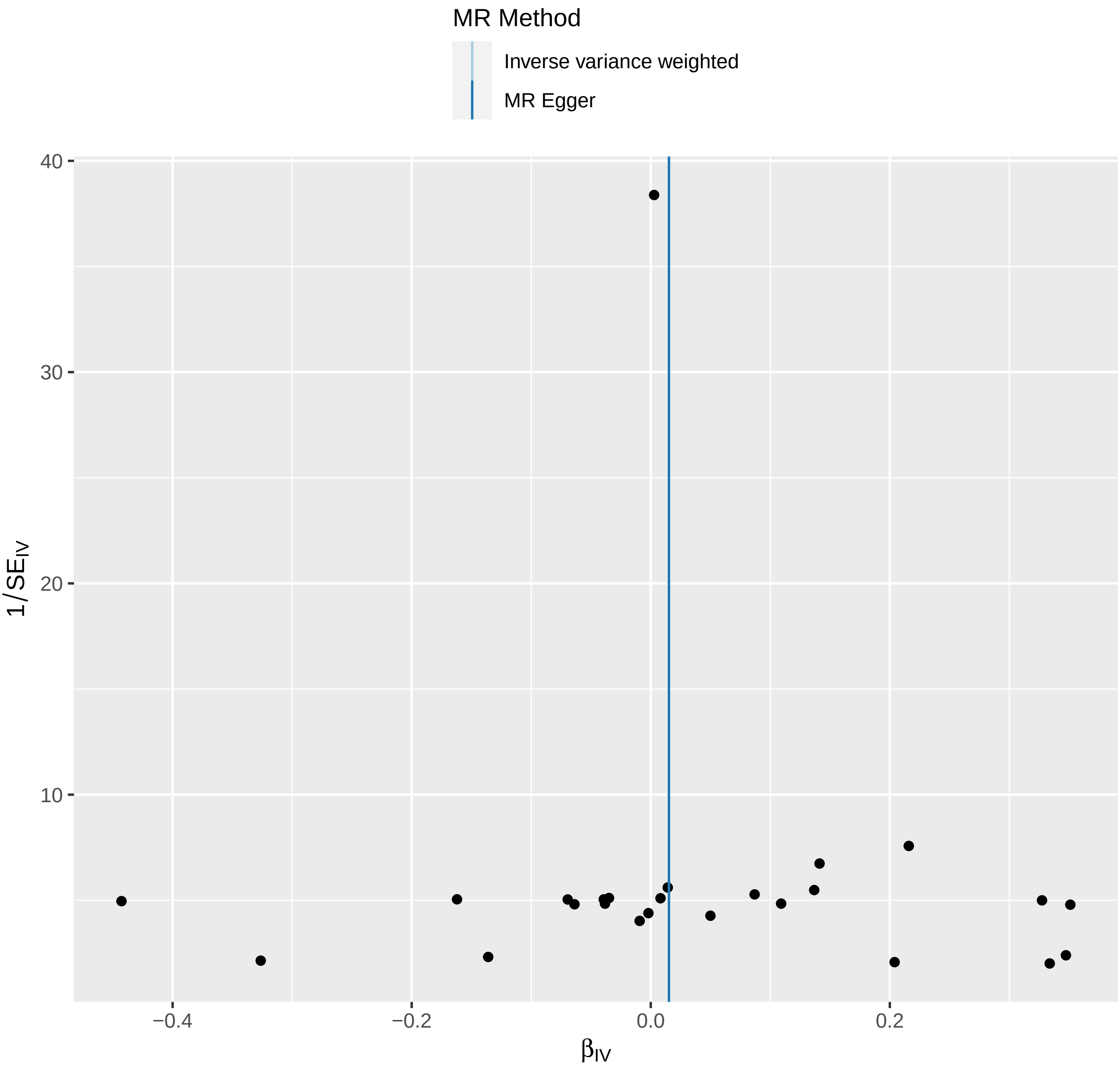

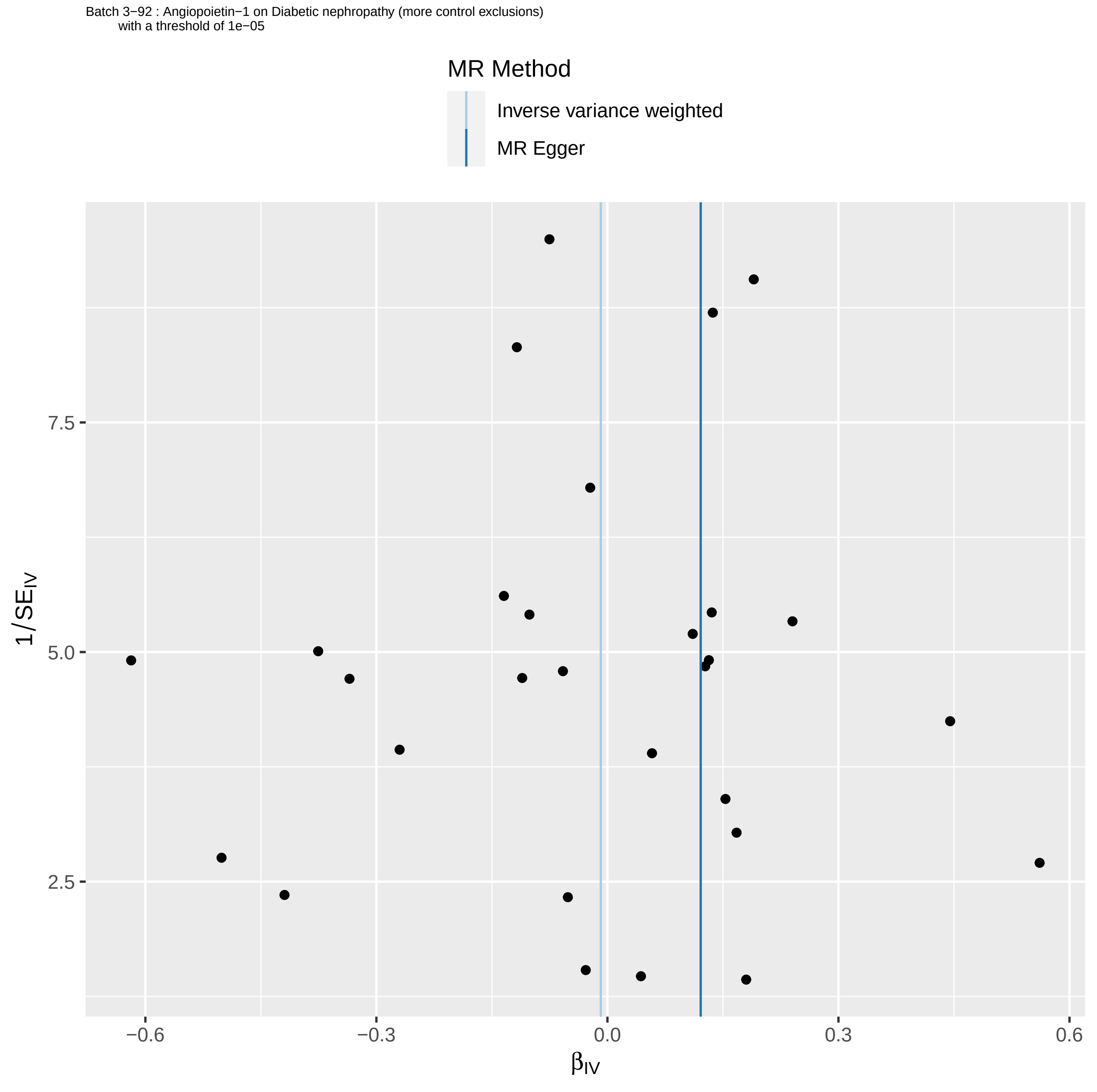
Batch 3−92 : Angiopoietin−1 on Diabetic nephropathy (more control exclusions)
with a threshold of 1e−05
MR Method
Inverse variance weighted
MR Egger
1/SE_IV
7.5
5.0
2.5
−0.6
−0.3
0.0
0.3
0.6
β_IV

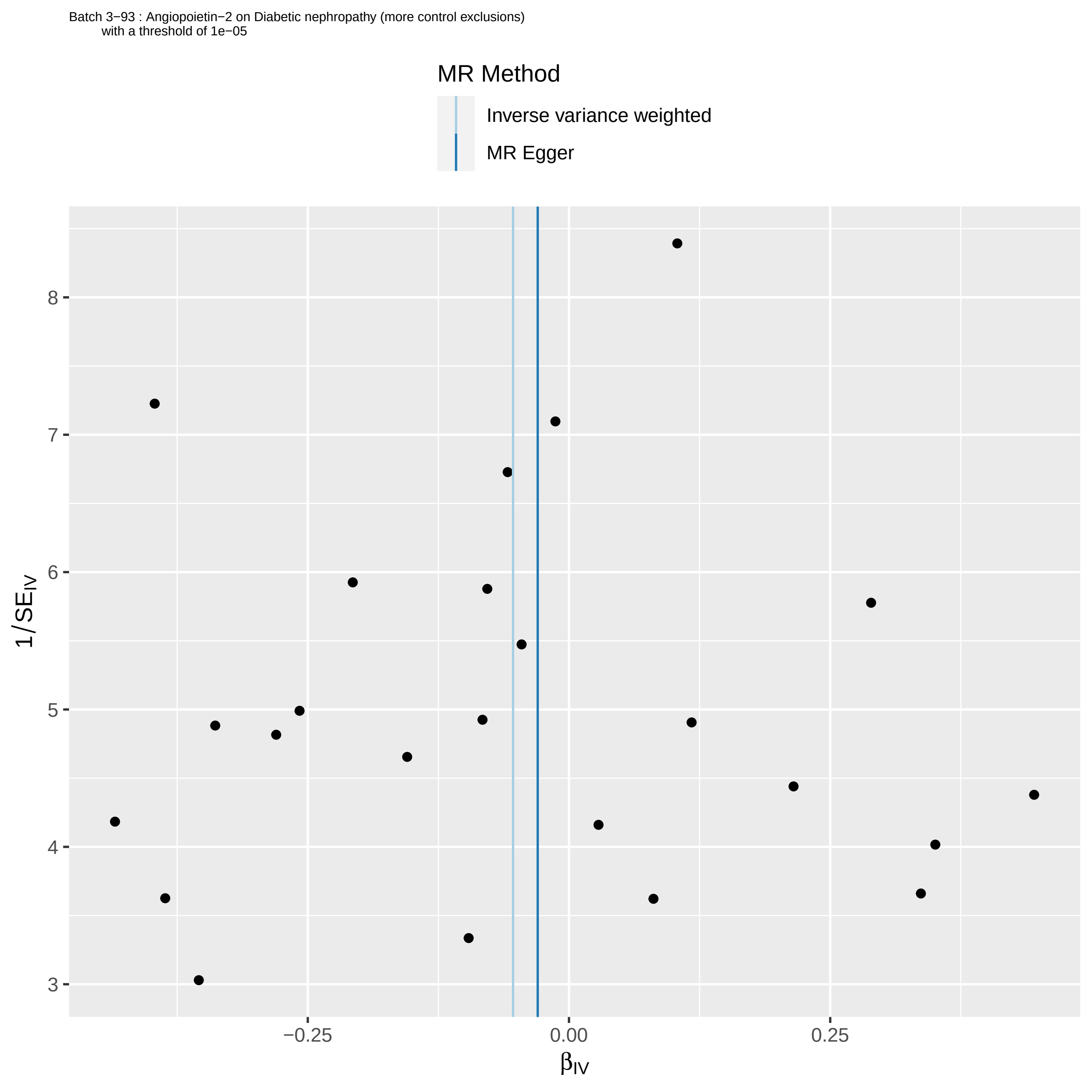

Batch 3−93 : Angiopoietin−2 on Diabetic nephropathy (more control exclusions)
with a threshold of 1e−05
MR Method
Inverse variance weighted
MR Egger
8
7
6
5
4
3
1/SE$_{IV}$
−0.25
0.00
0.25
β_{IV}

Batch 3−94 : Angiopoietin−2 on Diabetic nephropathy (more control exclusions)
with a threshold of 1e−05

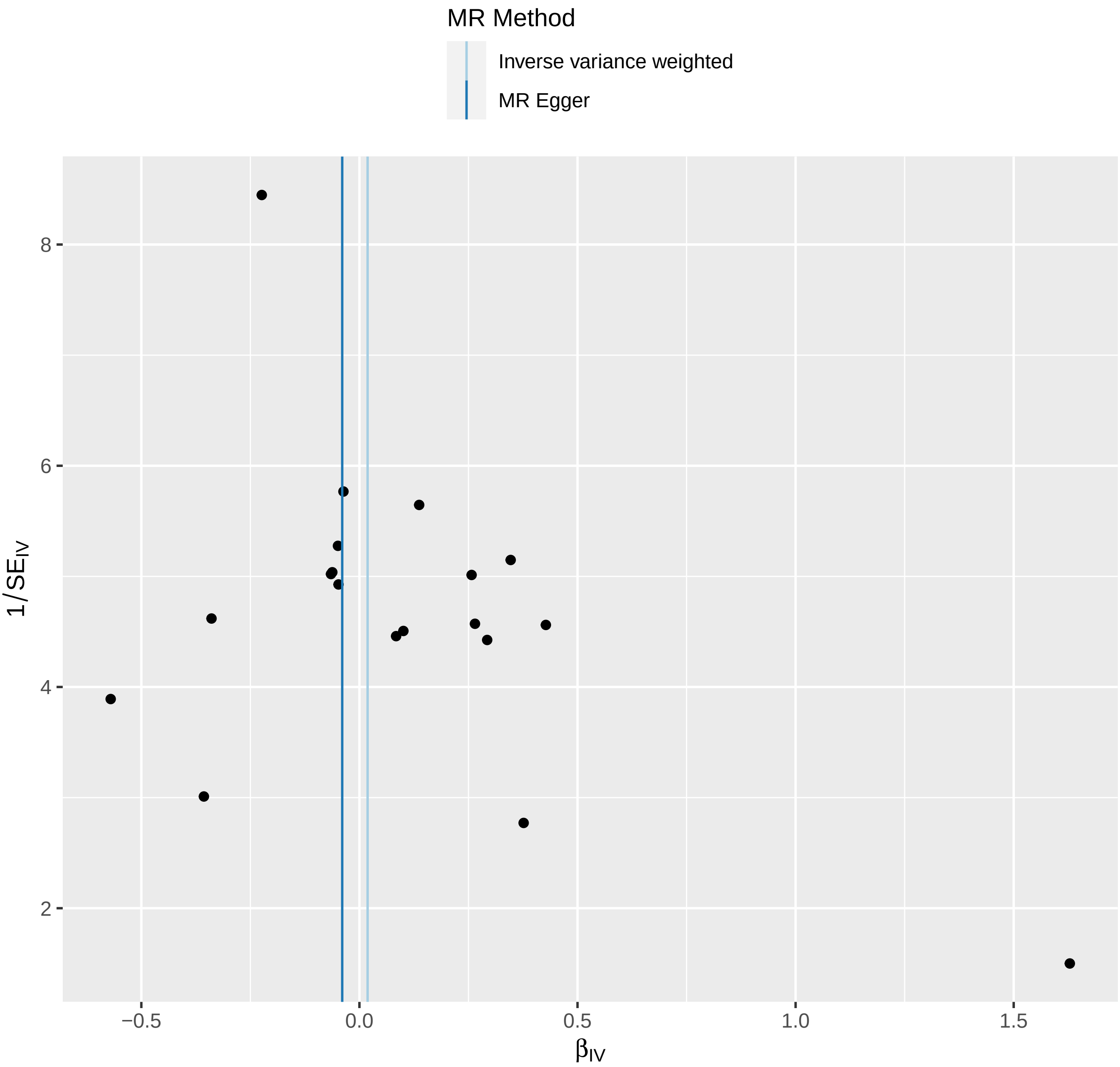

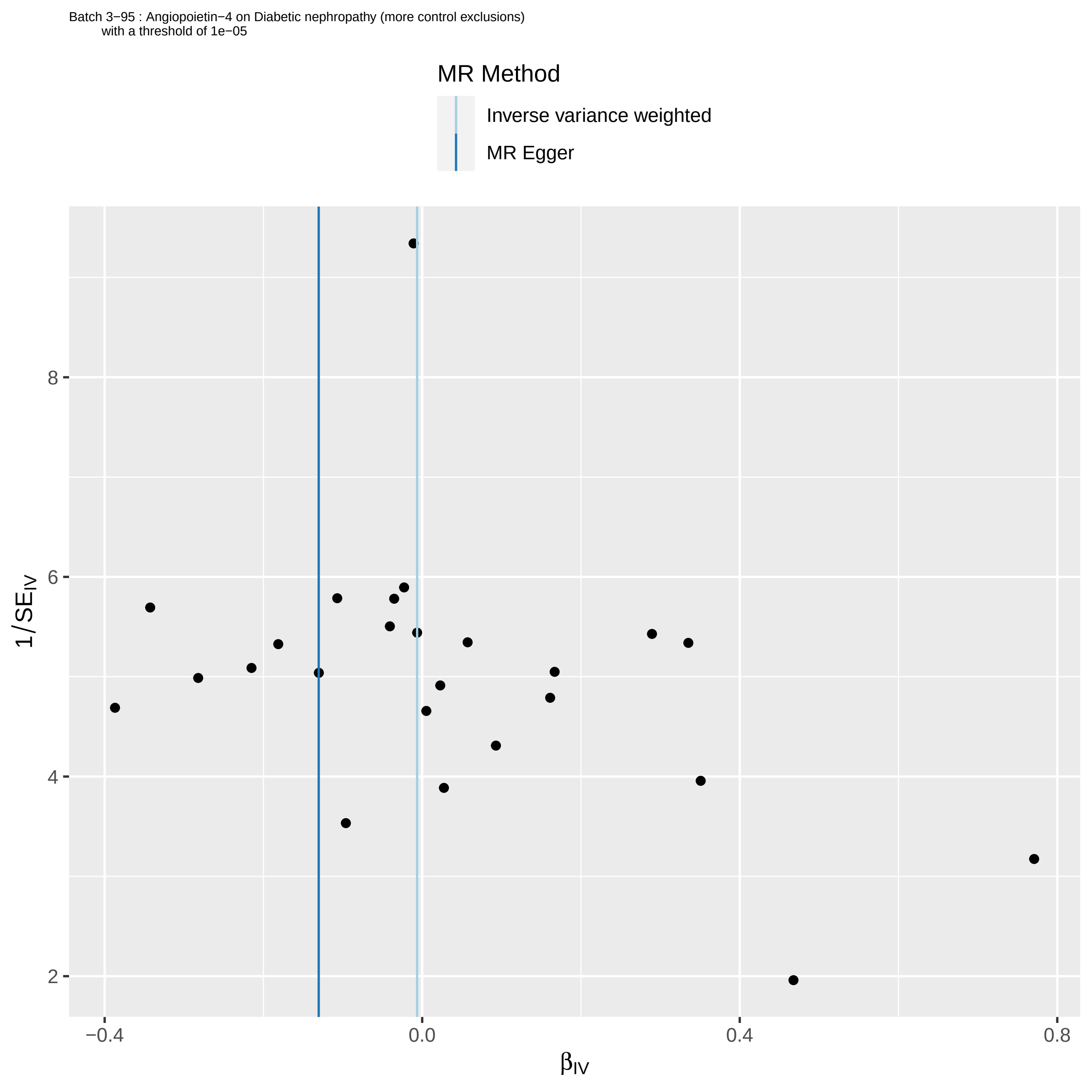

Batch 3−95 : Angiopoietin−4 on Diabetic nephropathy (more control exclusions)
with a threshold of 1e−05
MR Method
Inverse variance weighted
MR Egger
1/SE_IV
8
6
4
2
−0.4
0.0
0.4
0.8
β_IV

Batch 3−96 : Angiopoietin−related protein 1 on Diabetic nephropathy (more control exclusions)
with a threshold of 1e−05

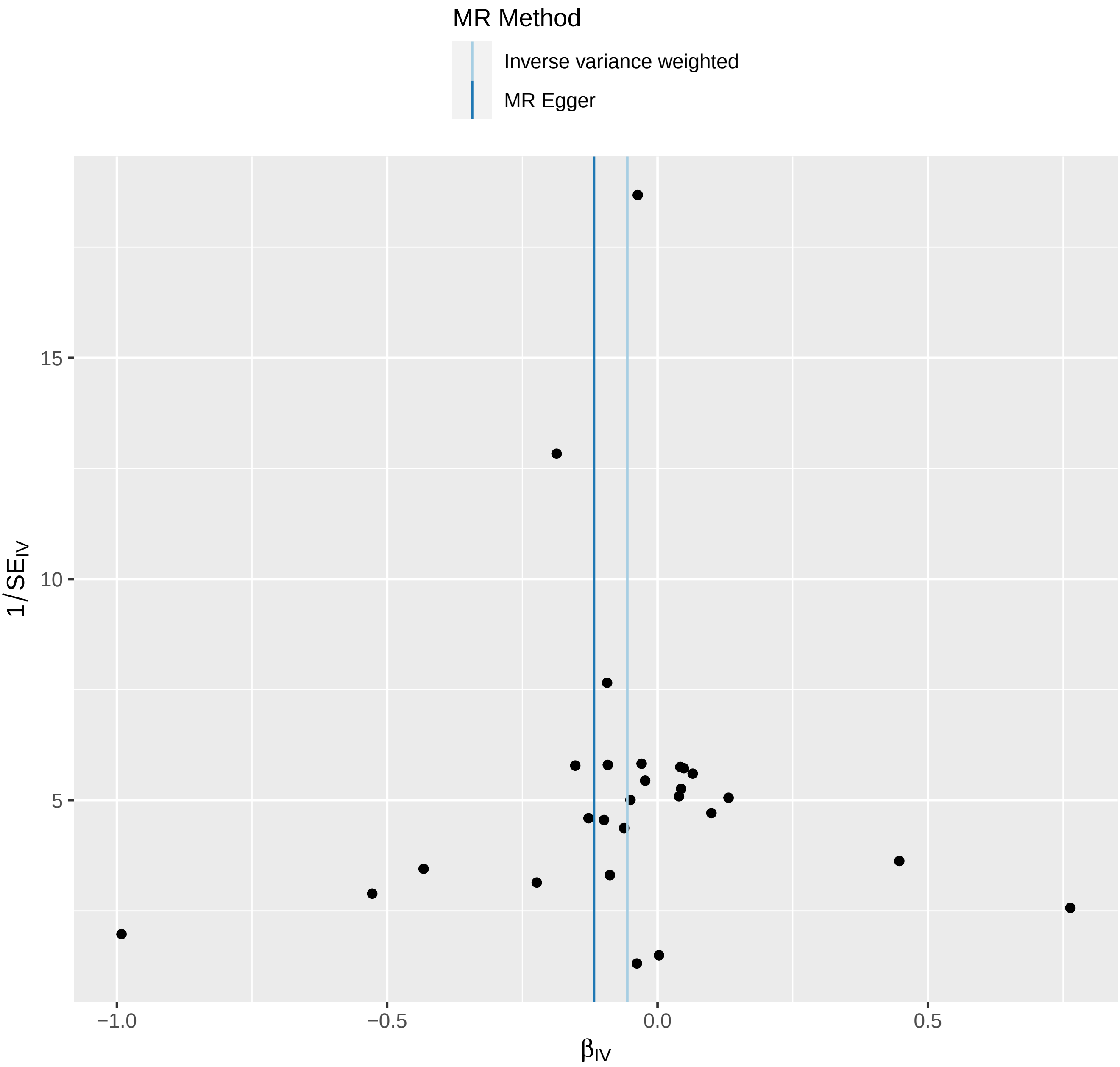

Batch 3−97 : Angiopoietin−related protein 1 on Diabetic nephropathy (more control exclusions)
with a threshold of 1e−05

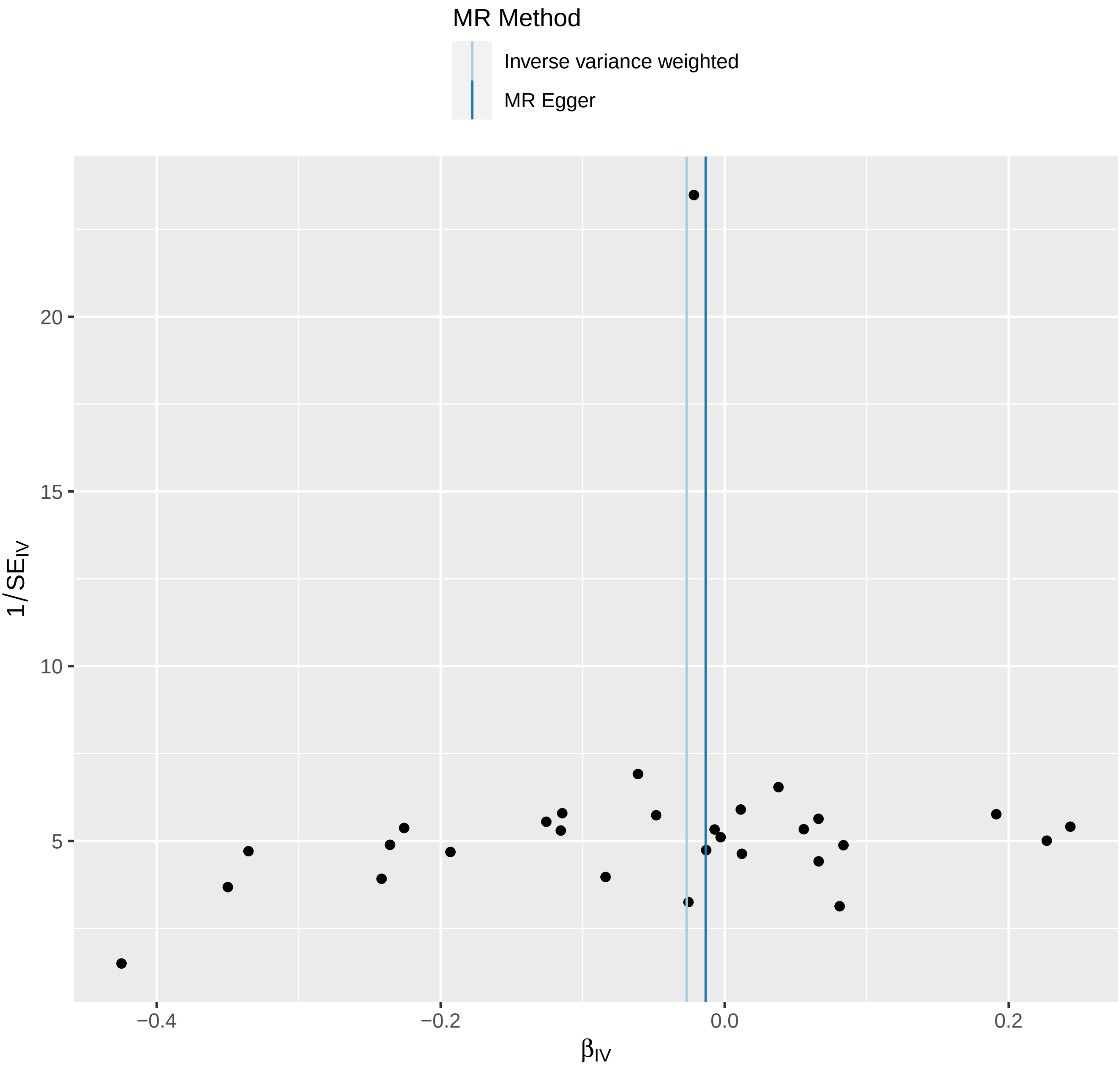

Batch 3−98 : Angiopoietin−related protein 3 on Diabetic nephropathy (more control exclusions)
with a threshold of 1e−05

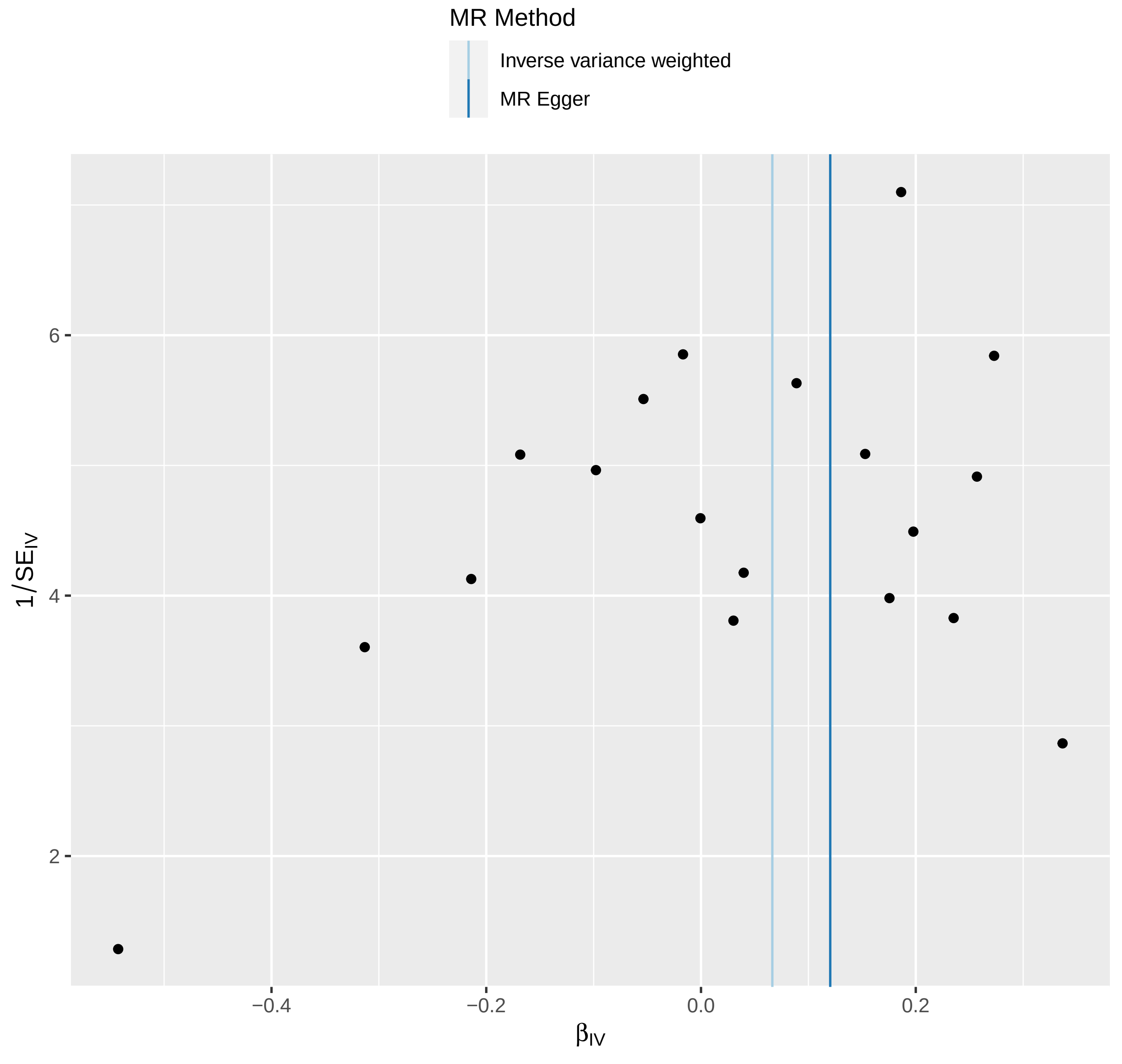

Batch 3−99 : Angiopoietin−related protein 4 on Diabetic nephropathy (more control exclusions)
with a threshold of 1e−05

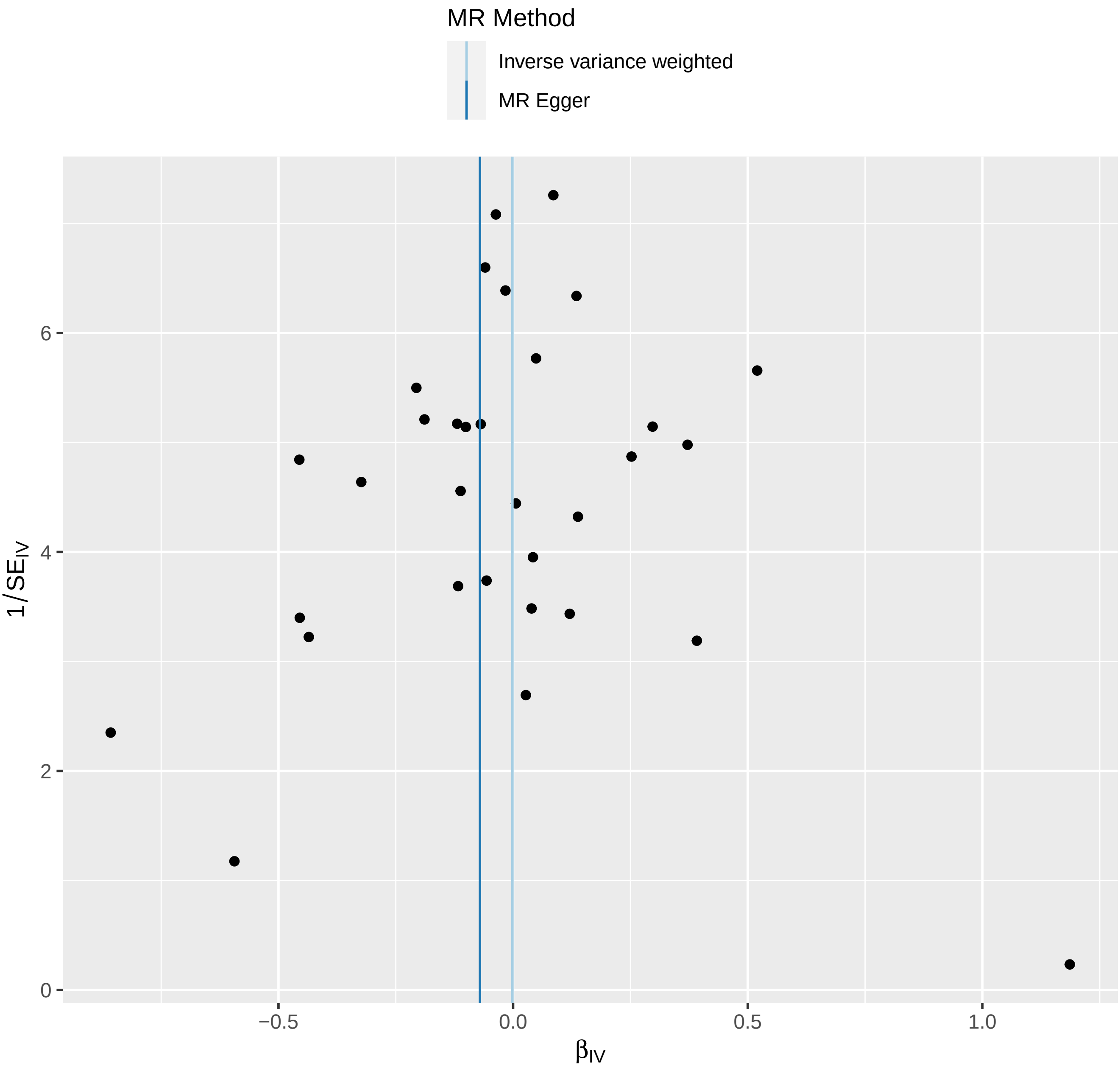

Batch 3−100 : Angiopoietin−related protein 7 on Diabetic nephropathy (more control exclusions)
with a threshold of 1e−05

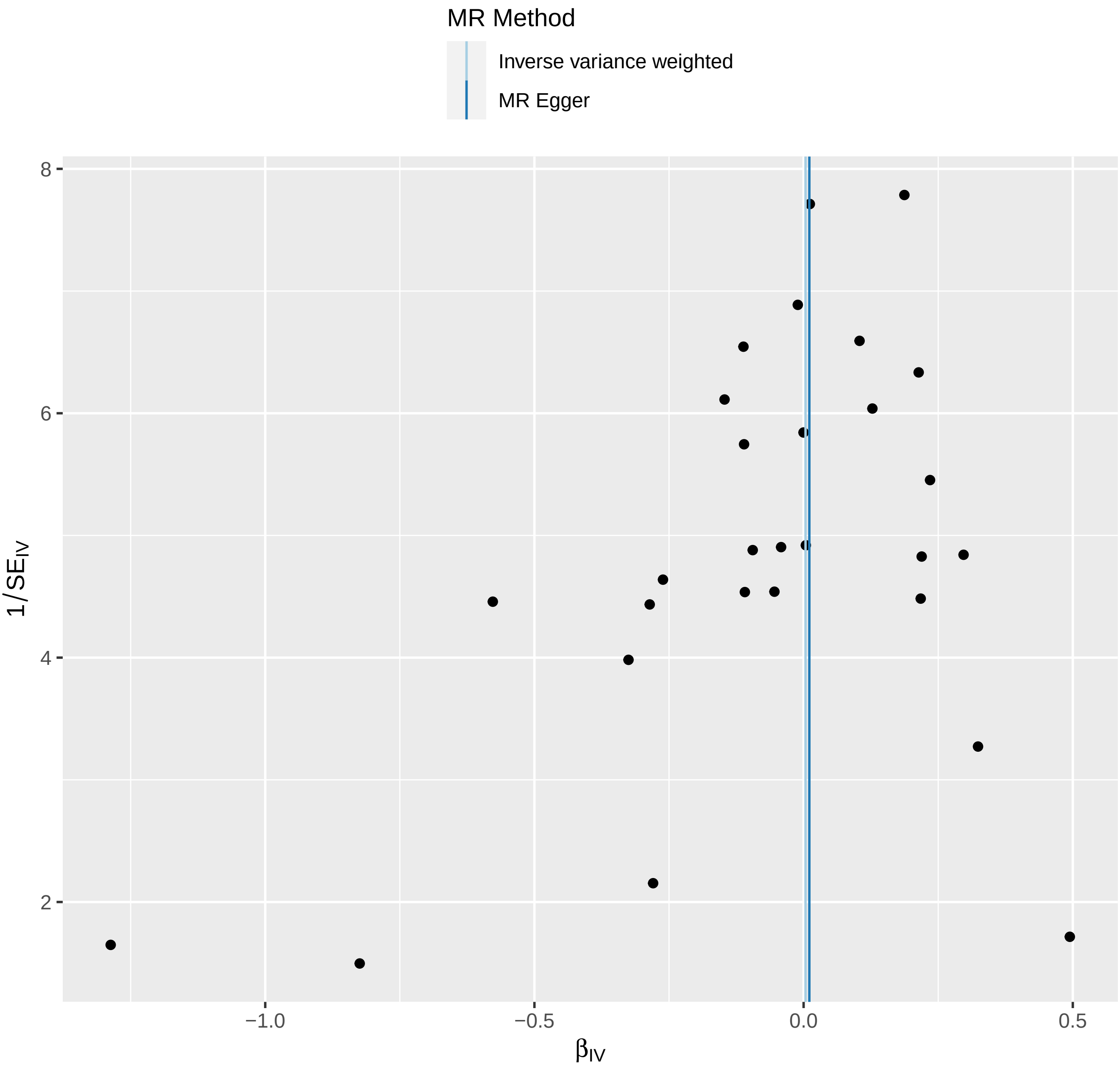

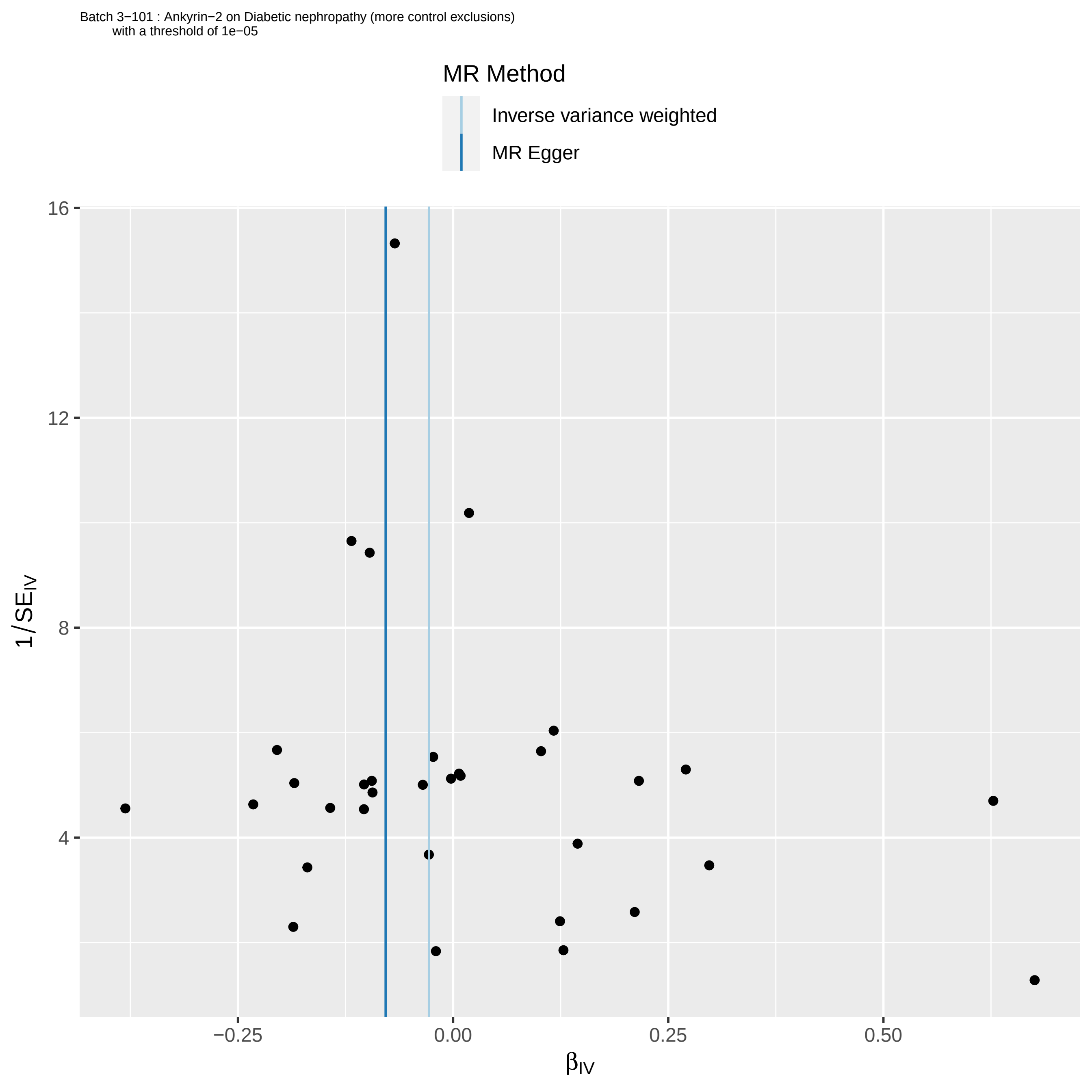

Batch 3−101 : Ankyrin−2 on Diabetic nephropathy (more control exclusions)
with a threshold of 1e−05
MR Method
Inverse variance weighted
MR Egger
16
12
8
4
1/SE_IV
−0.25
0.00
0.25
0.50
β_IV

Batch 3−102 : Ankyrin repeat domain−containing protein 27 on Diabetic nephropathy (more control exclusions) with a threshold of 1e−05

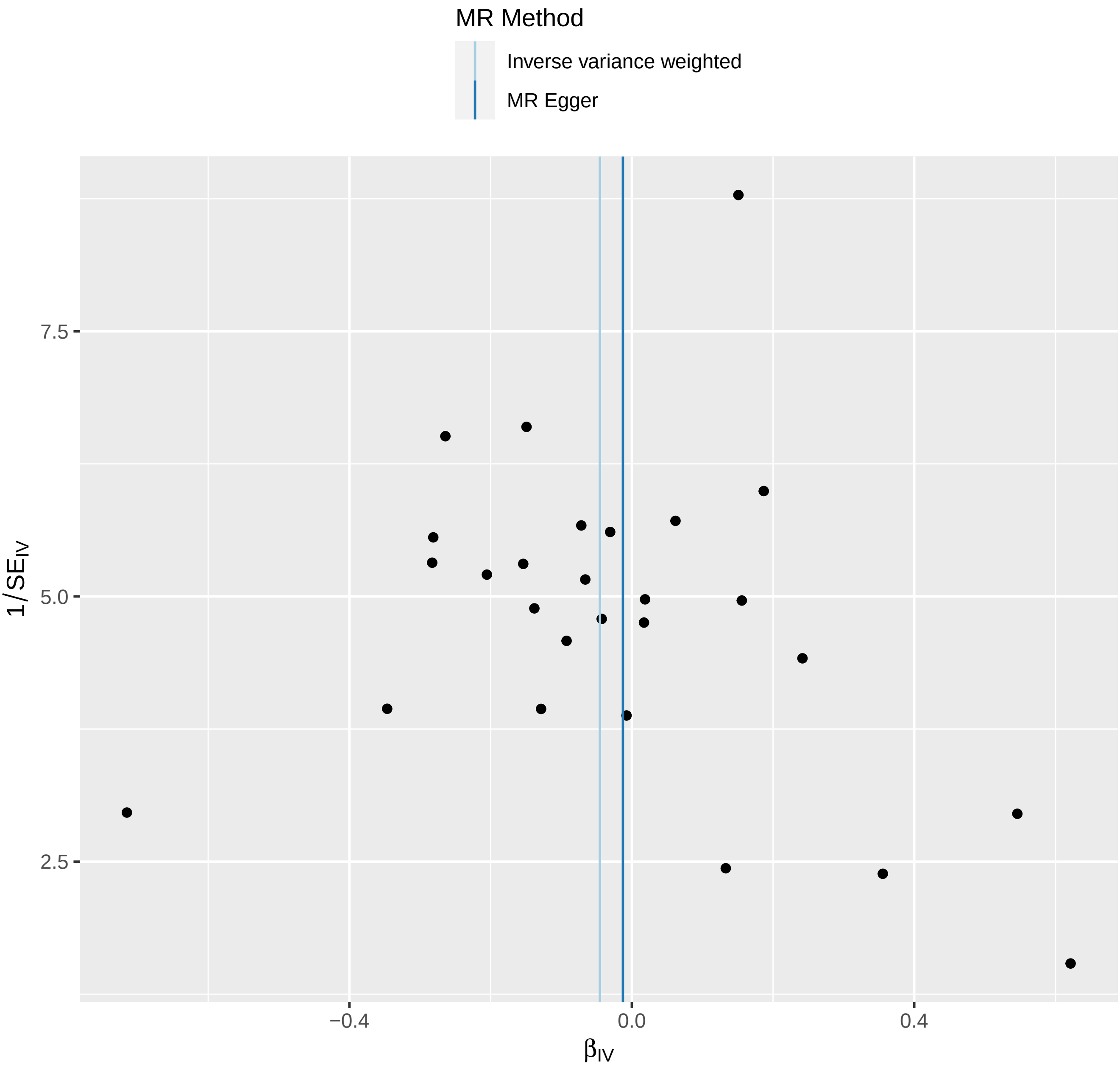

Batch 3−103 : Ankyrin repeat domain−containing protein 46 on Diabetic nephropathy (more control exclusions)
with a threshold of 1e−05

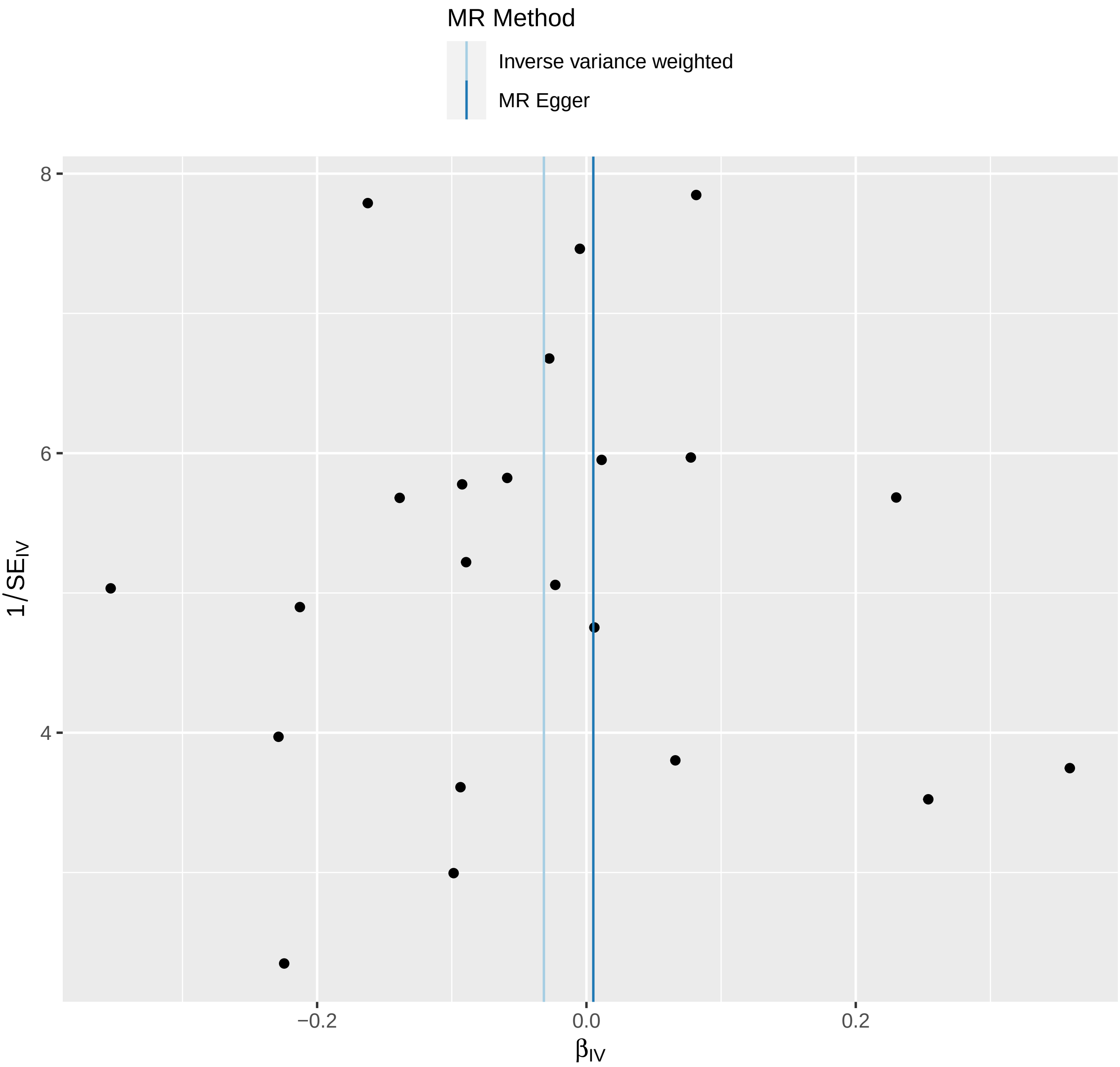

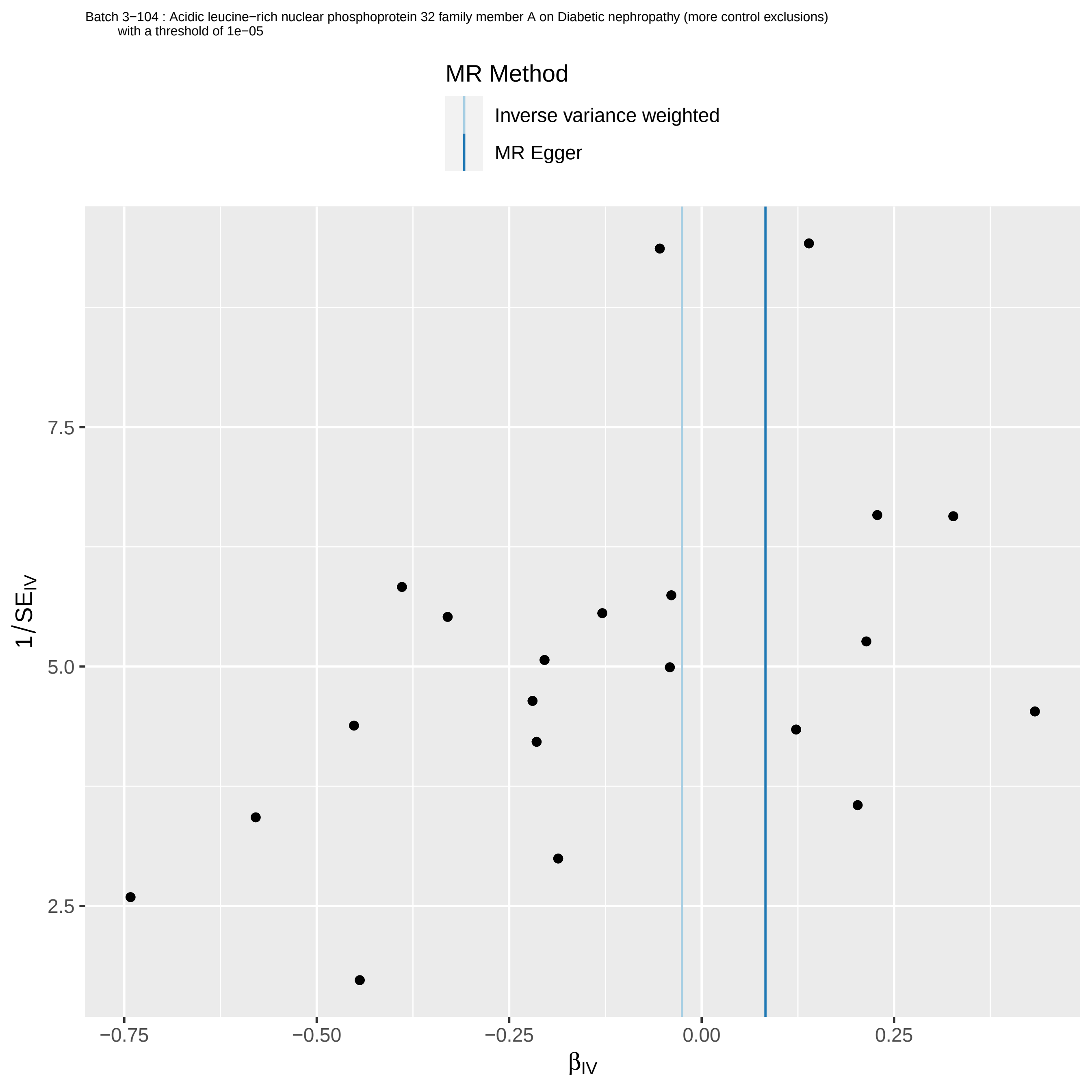
Batch 3−104 : Acidic leucine−rich nuclear phosphoprotein 32 family member A on Diabetic nephropathy (more control exclusions)
with a threshold of 1e−05
MR Method
Inverse variance weighted
MR Egger
7.5
5.0
2.5
1/SE_IV
−0.75
−0.50
−0.25
0.00
0.25
β_IV

Batch 3−105 : Acidic leucine−rich nuclear phosphoprotein 32 family member B on Diabetic nephropathy (more control exclusions) with a threshold of 1e−05

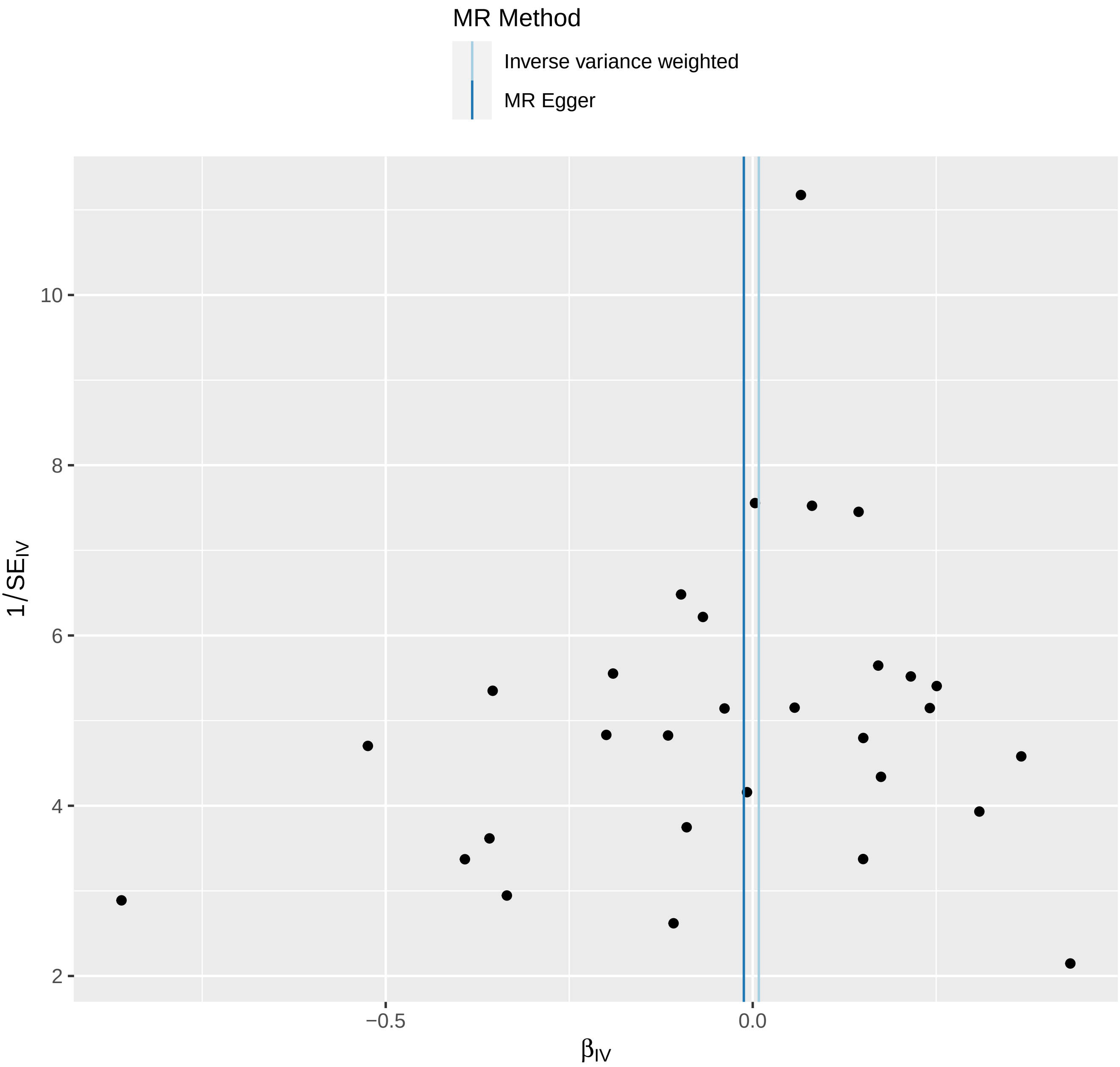

Batch 3−106 : Annexin A10 on Diabetic nephropathy (more control exclusions)
with a threshold of 1e−05

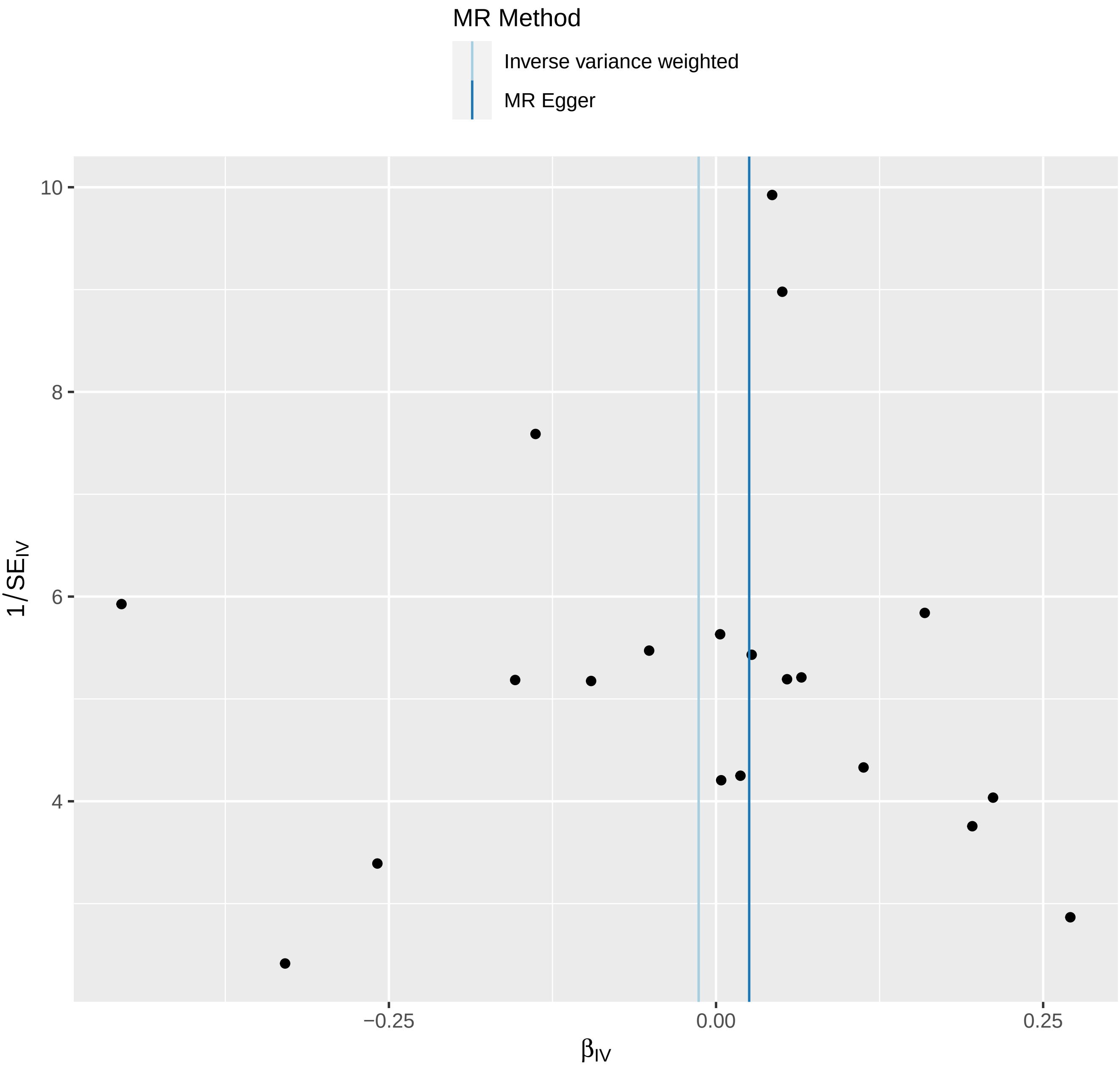

Batch 3−107 : Annexin A1 on Diabetic nephropathy (more control exclusions)
with a threshold of 1e−05

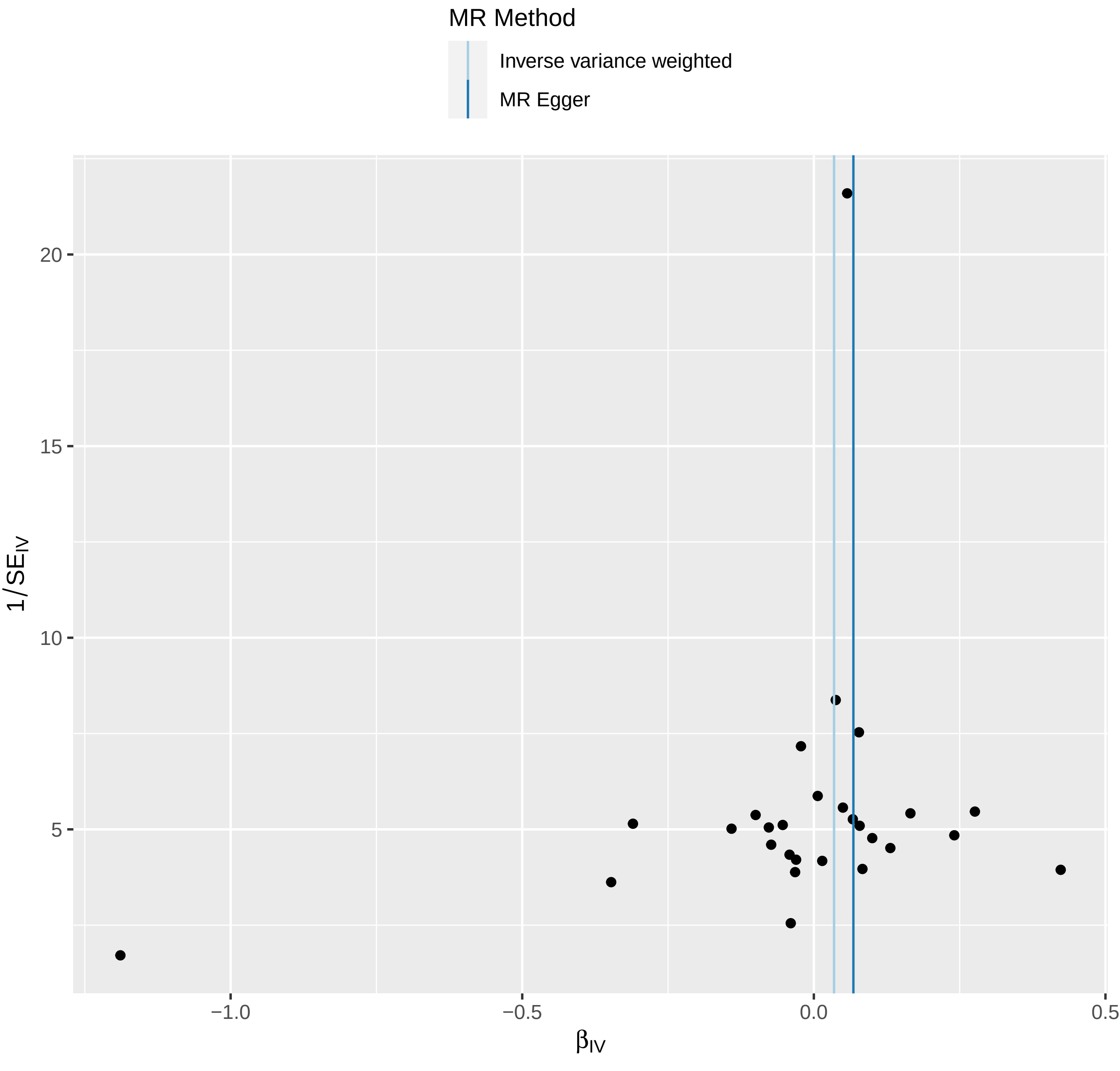

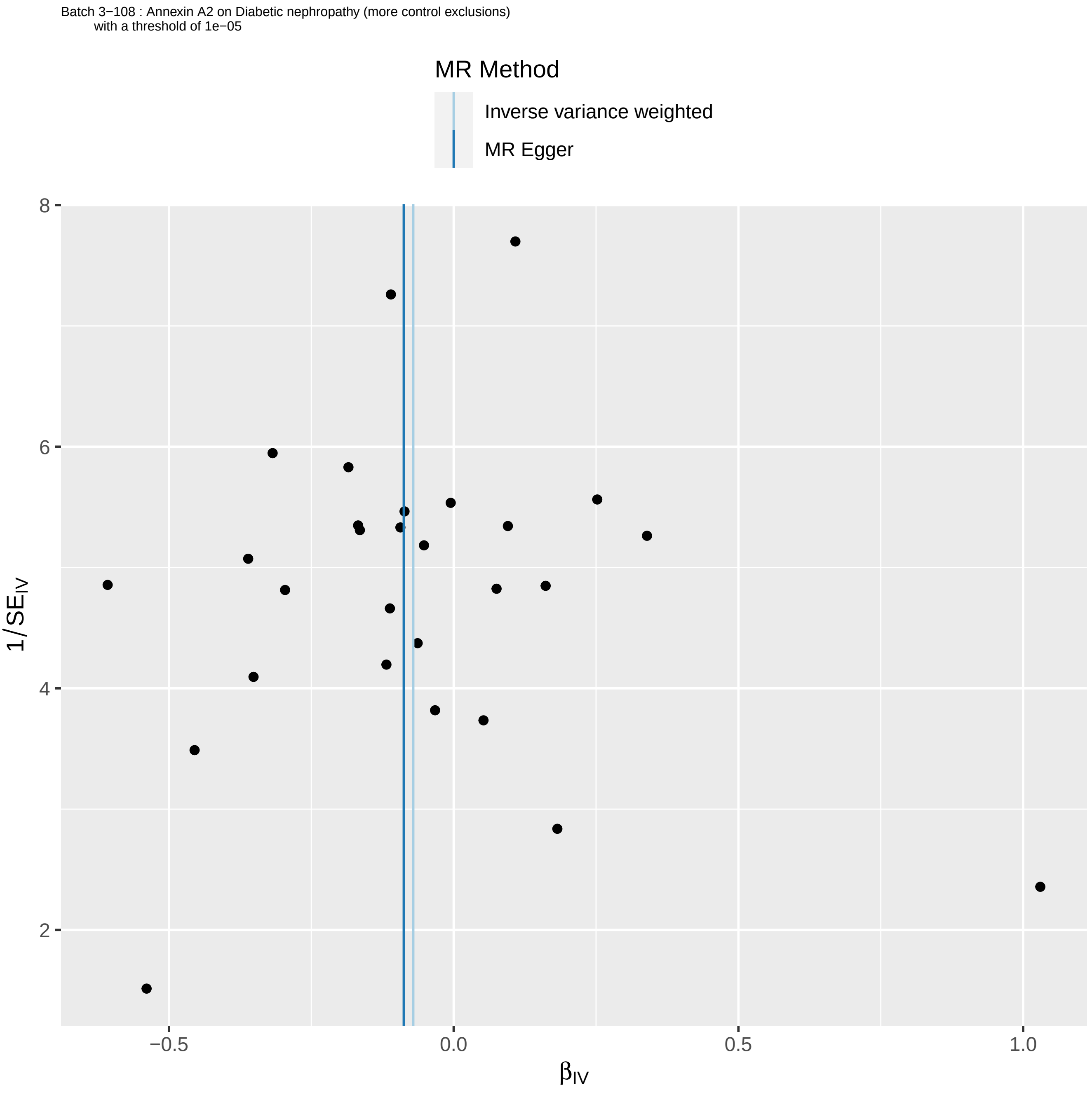

Batch 3−108 : Annexin A2 on Diabetic nephropathy (more control exclusions)
with a threshold of 1e−05
MR Method
Inverse variance weighted
MR Egger
8
6
4
2
1/SE_IV
−0.5
0.0
0.5
1.0
β_IV

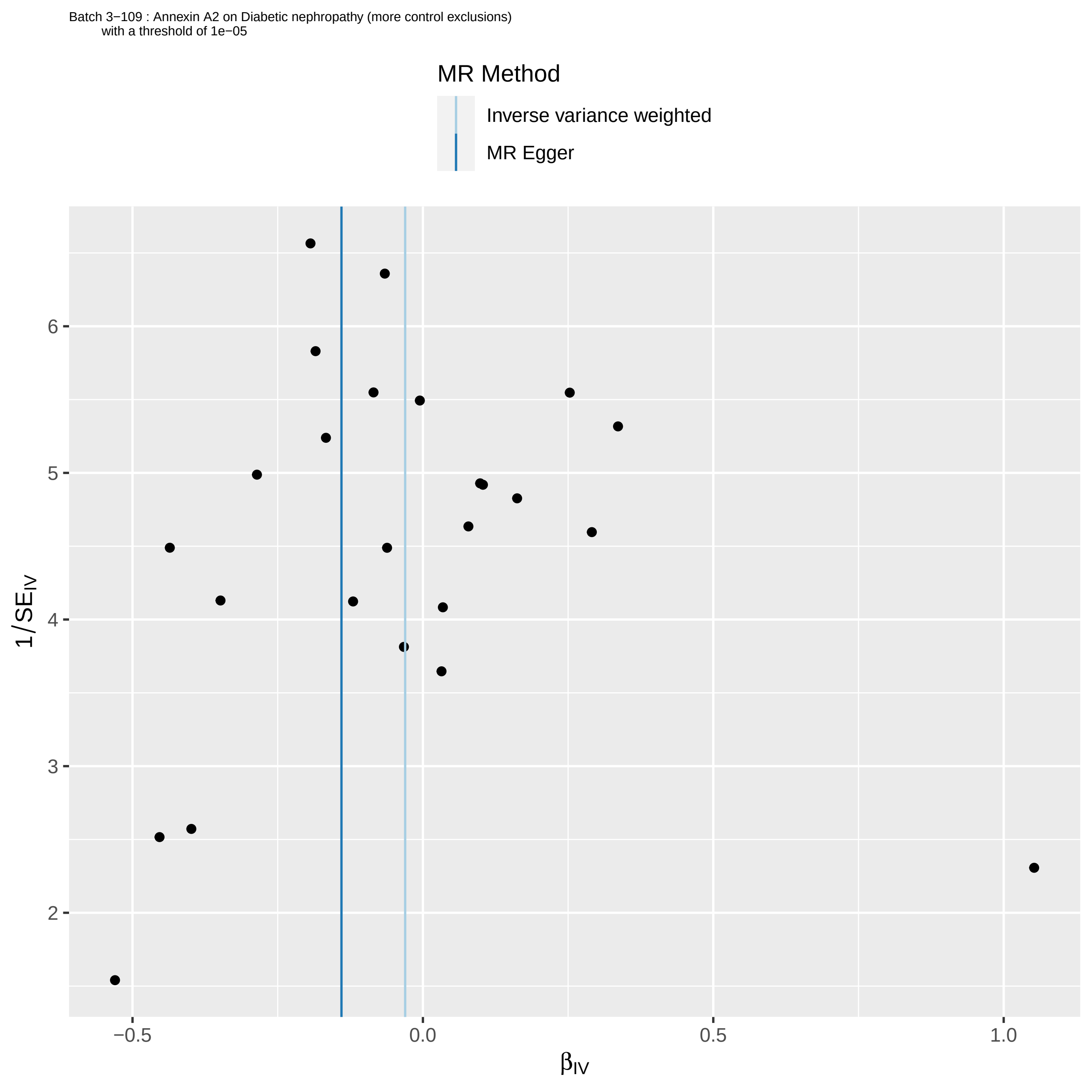
Batch 3−109 : Annexin A2 on Diabetic nephropathy (more control exclusions)
with a threshold of 1e−05
MR Method
Inverse variance weighted
MR Egger
6
5
4
3
2
$1/SE_{IV}$
−0.5
0.0
0.5
1.0
β_{IV}

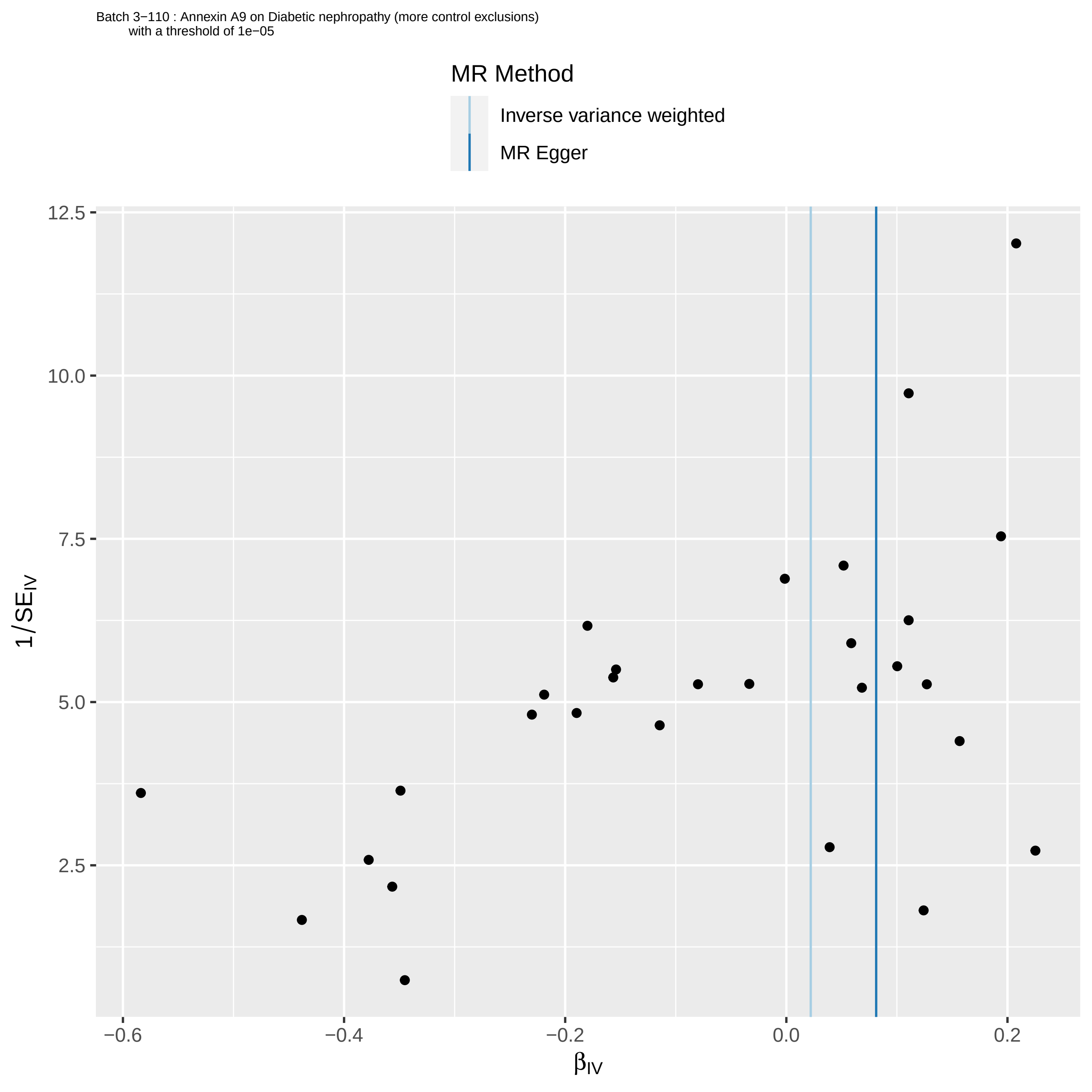
Batch 3−110 : Annexin A9 on Diabetic nephropathy (more control exclusions)
with a threshold of 1e−05
MR Method
Inverse variance weighted
MR Egger
12.5
10.0
7.5
5.0
2.5
1/SE$_{IV}$
−0.6
−0.4
−0.2
0.0
0.2
β$_{IV}$

Batch 3−111 : Retina−specific copper amine oxidase on Diabetic nephropathy (more control exclusions)
with a threshold of 1e−05

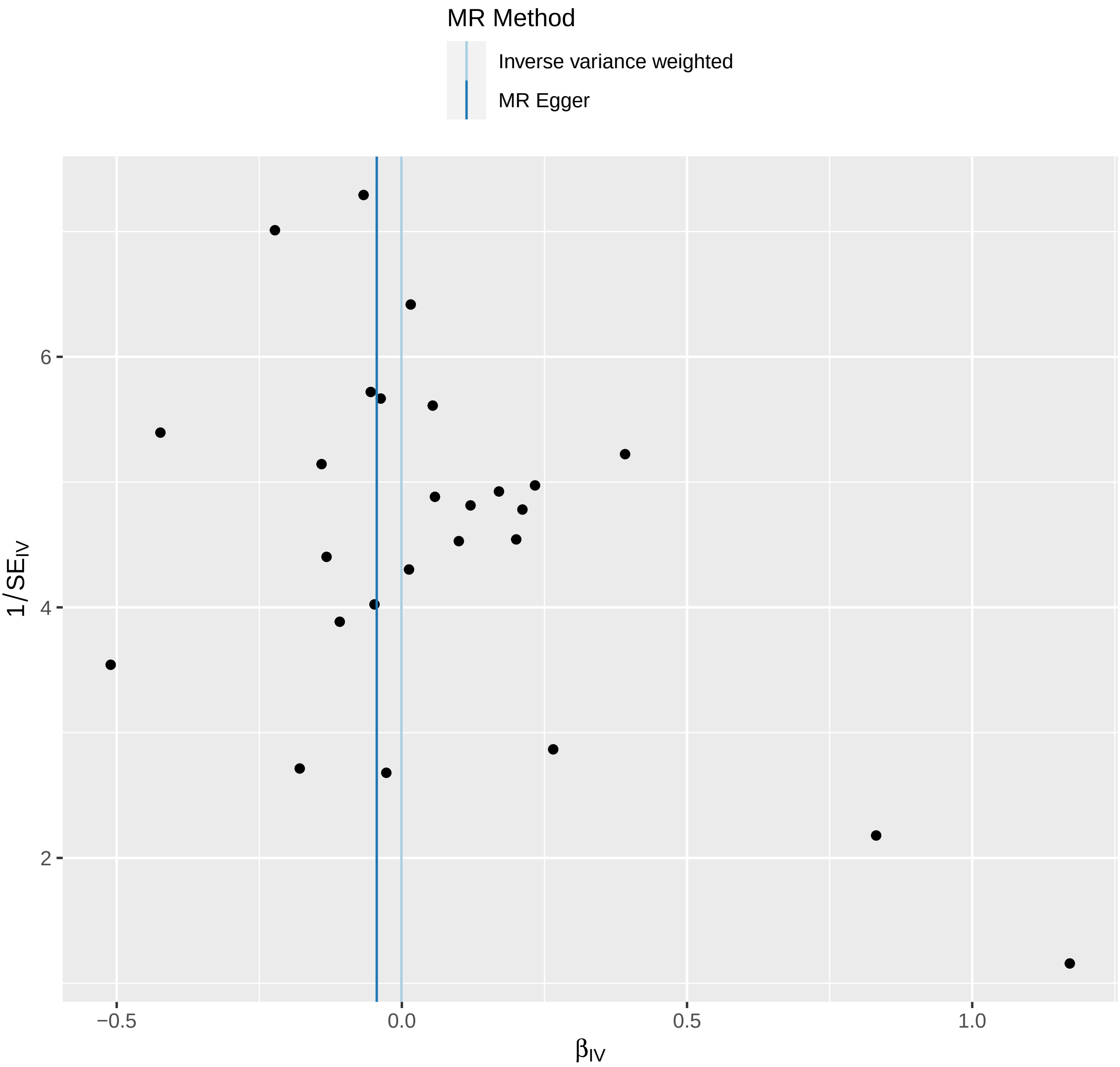

Batch 3−112 : AP−4 complex subunit mu−1 on Diabetic nephropathy (more control exclusions)
with a threshold of 1e−05

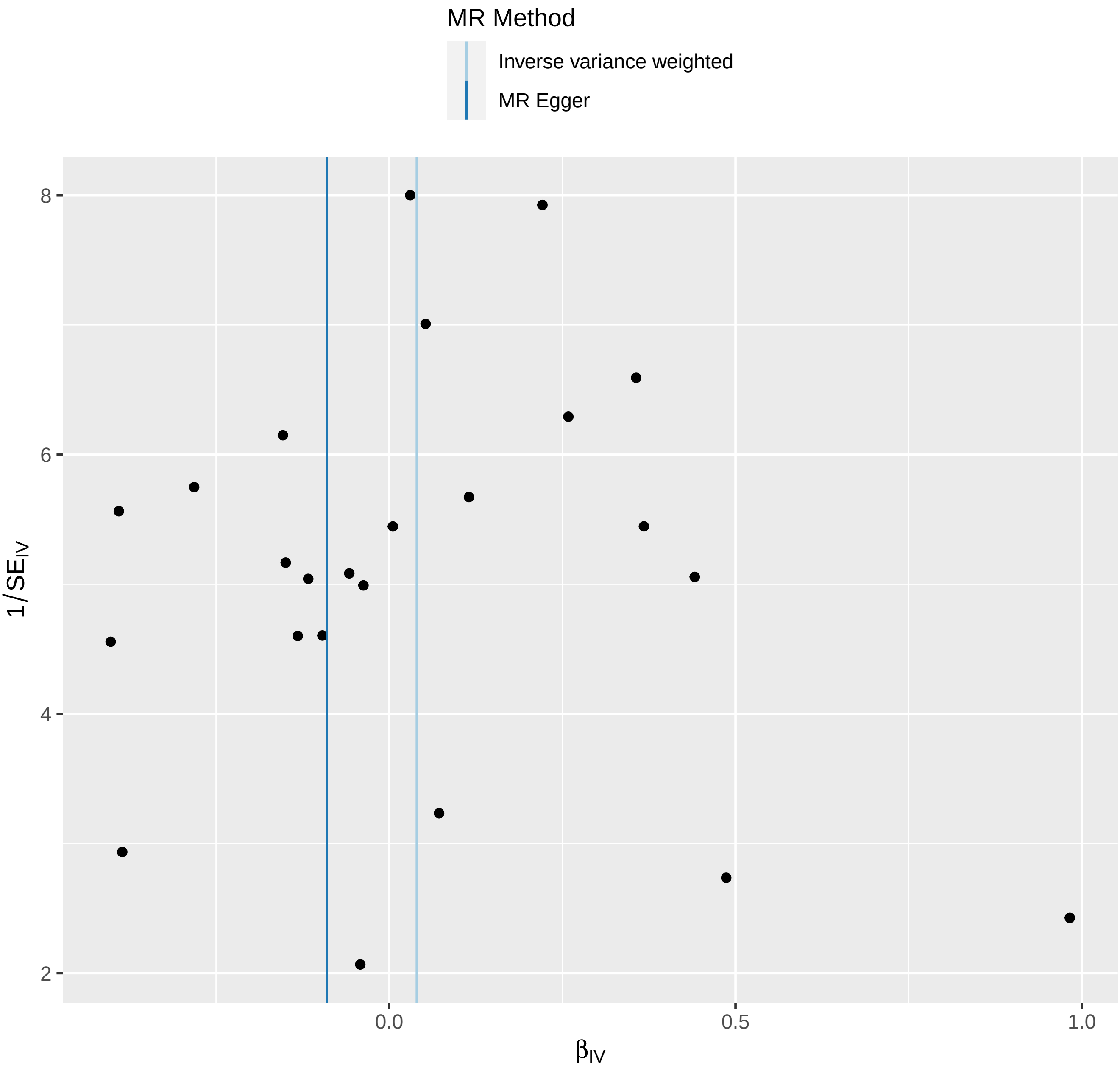

Batch 3−113 : Apoptotic protease−activating factor 1 on Diabetic nephropathy (more control exclusions)
with a threshold of 1e−05

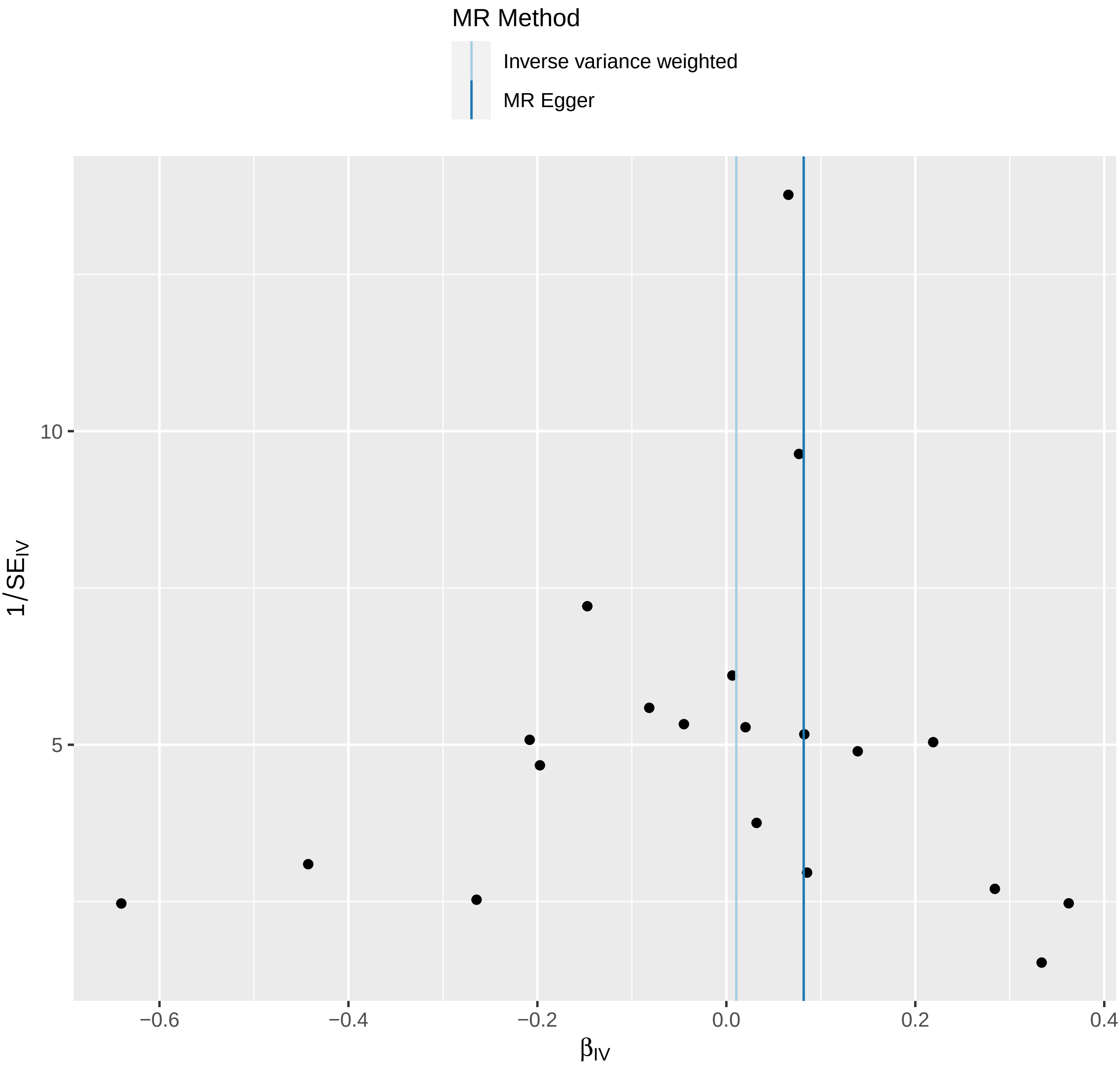

Batch 3−114 : Amyloid beta A4 precursor protein−binding family B member 1 on Diabetic nephropathy (more control exclusions)
with a threshold of 1e−05

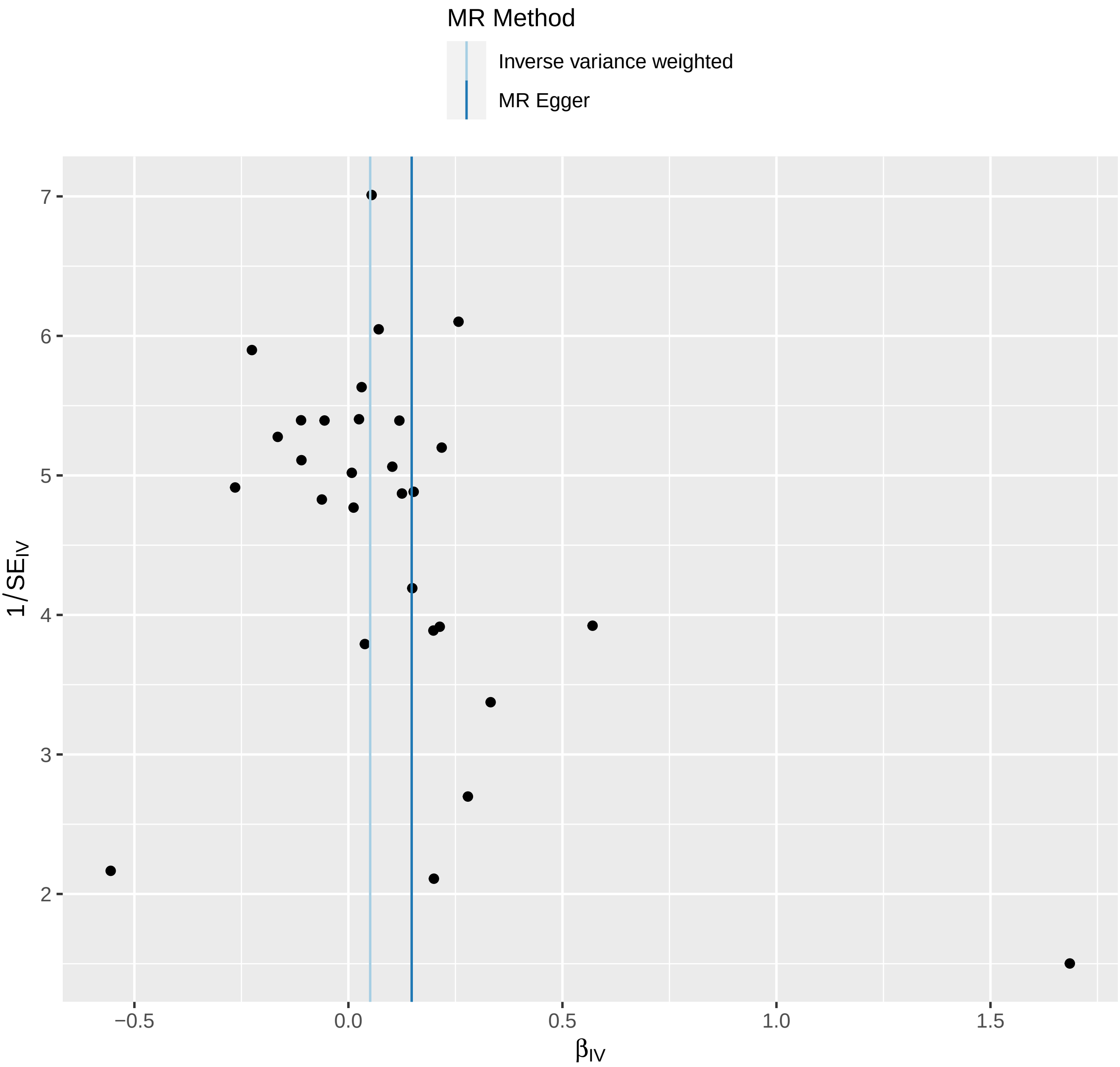

Batch 3−115 : Amyloid beta A4 precursor protein−binding family B member 1 on Diabetic nephropathy (more control exclusions) with a threshold of 1e−05

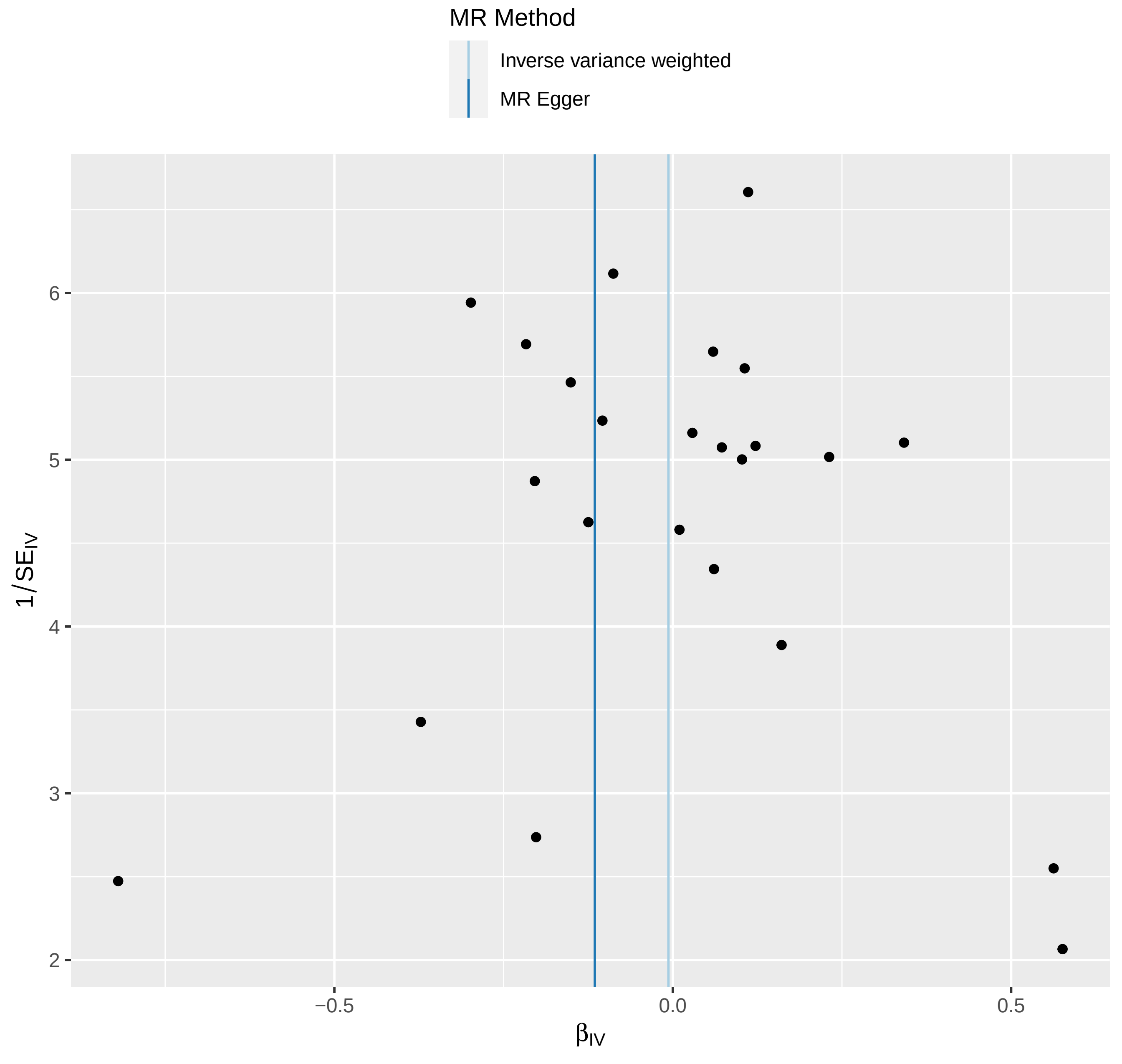

Batch 3−116 : Amyloid beta A4 precursor protein−binding family B member 2 on Diabetic nephropathy (more control exclusions) with a threshold of 1e−05

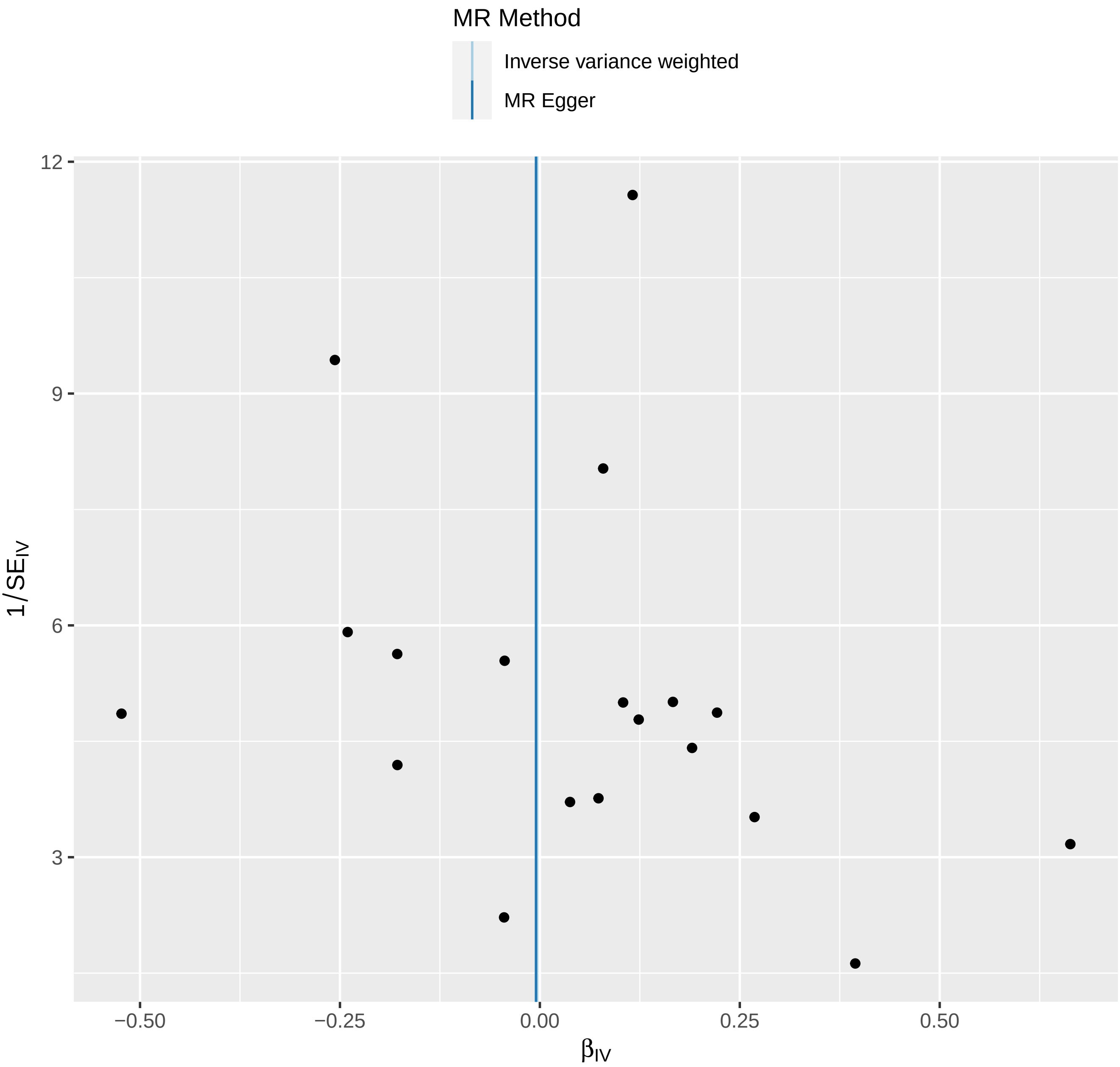

Batch 3−117 : Amyloid beta A4 precursor protein−binding family B member 2 on Diabetic nephropathy (more control exclusions) with a threshold of 1e−05

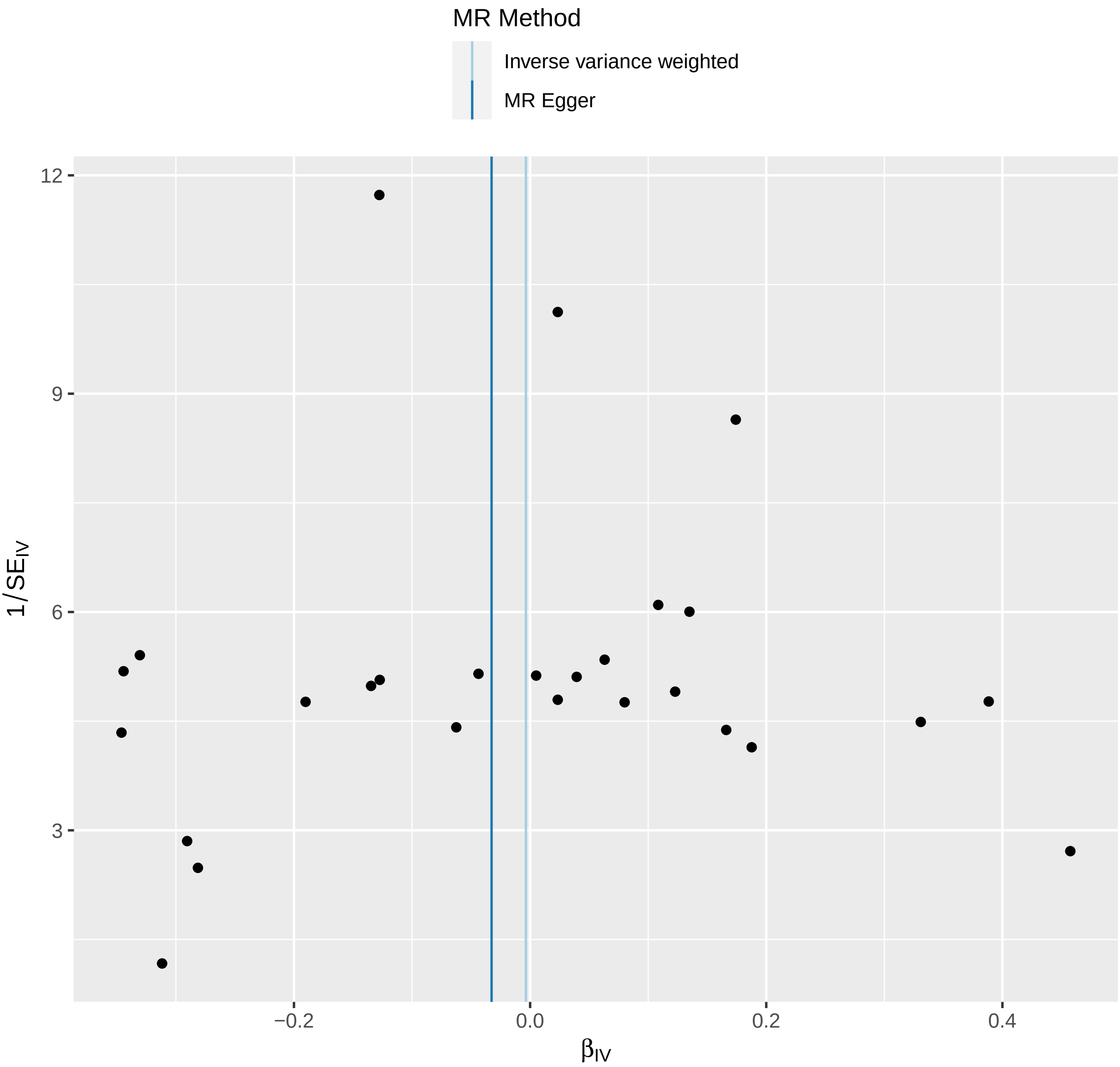

Batch 3−118 : Amyloid beta A4 precursor protein−binding family B member 3 on Diabetic nephropathy (more control exclusions) with a threshold of 1e−05

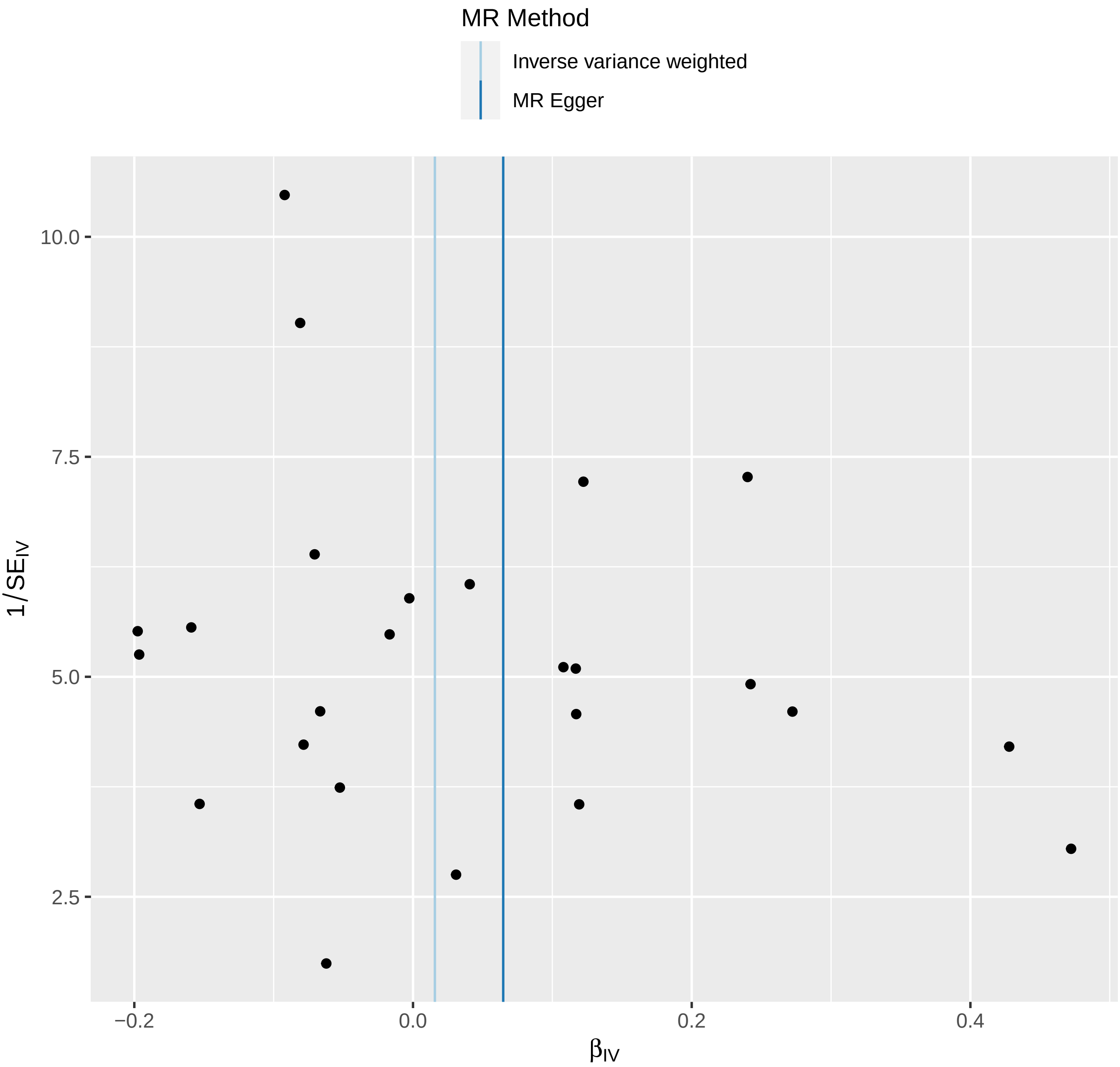

Batch 3−119 : Protein APCDD1 on Diabetic nephropathy (more control exclusions)
with a threshold of 1e−05

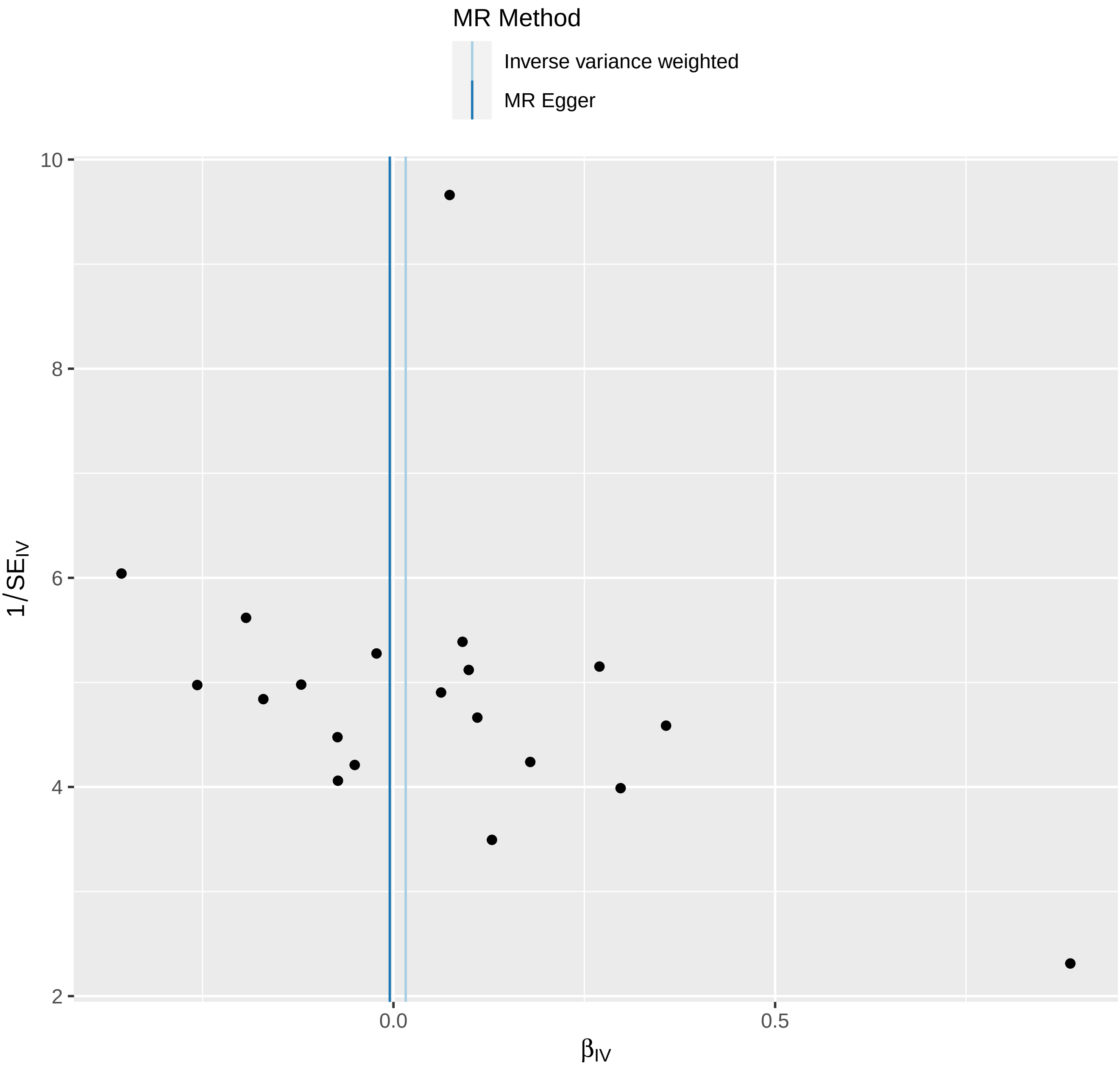

Batch 3−120 : Serum amyloid P−component on Diabetic nephropathy (more control exclusions)
with a threshold of 1e−05

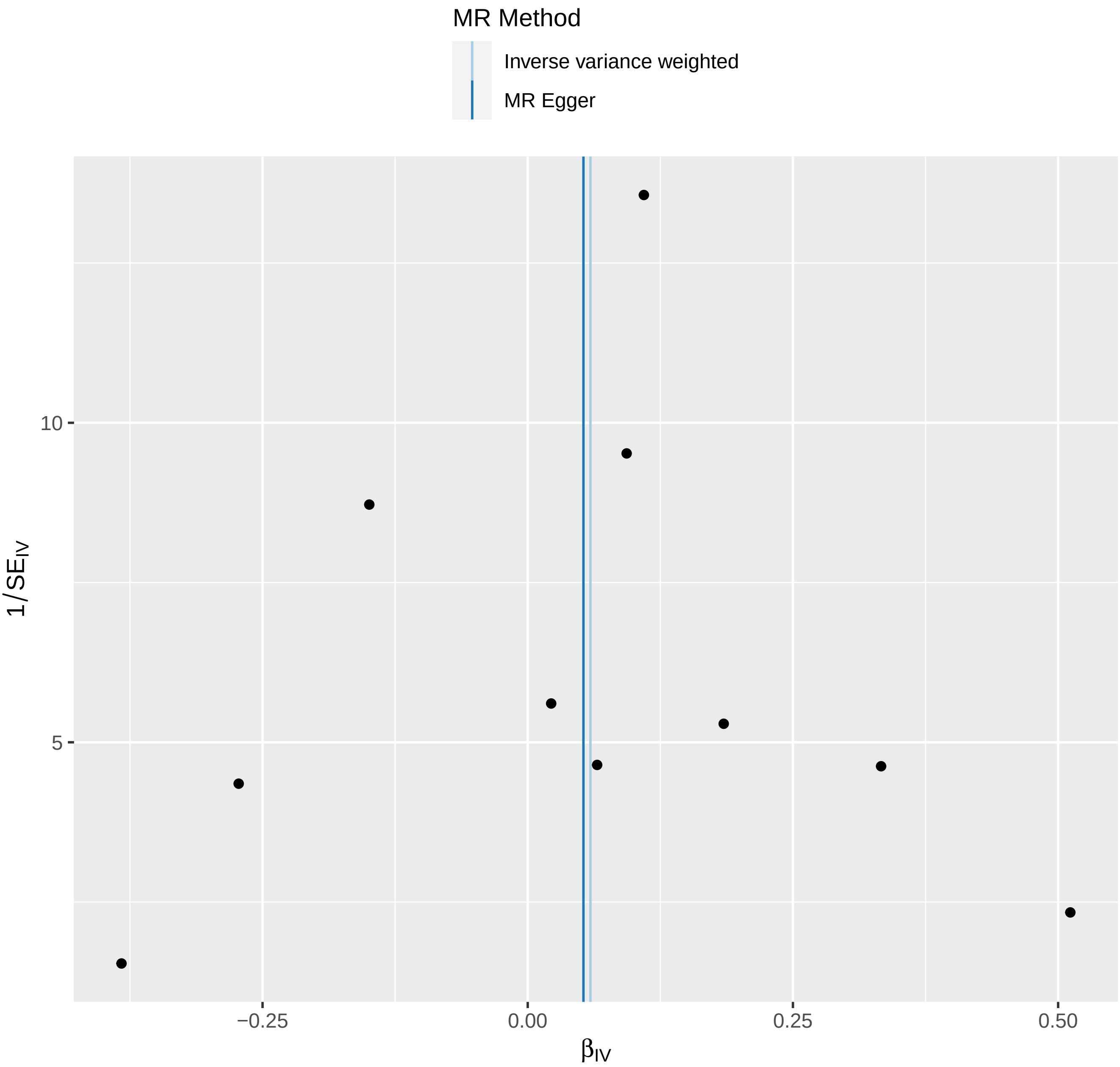

Batch 3−121 : DNA−(apurinic or apyrimidinic site) lyase on Diabetic nephropathy (more control exclusions)
with a threshold of 1e−05

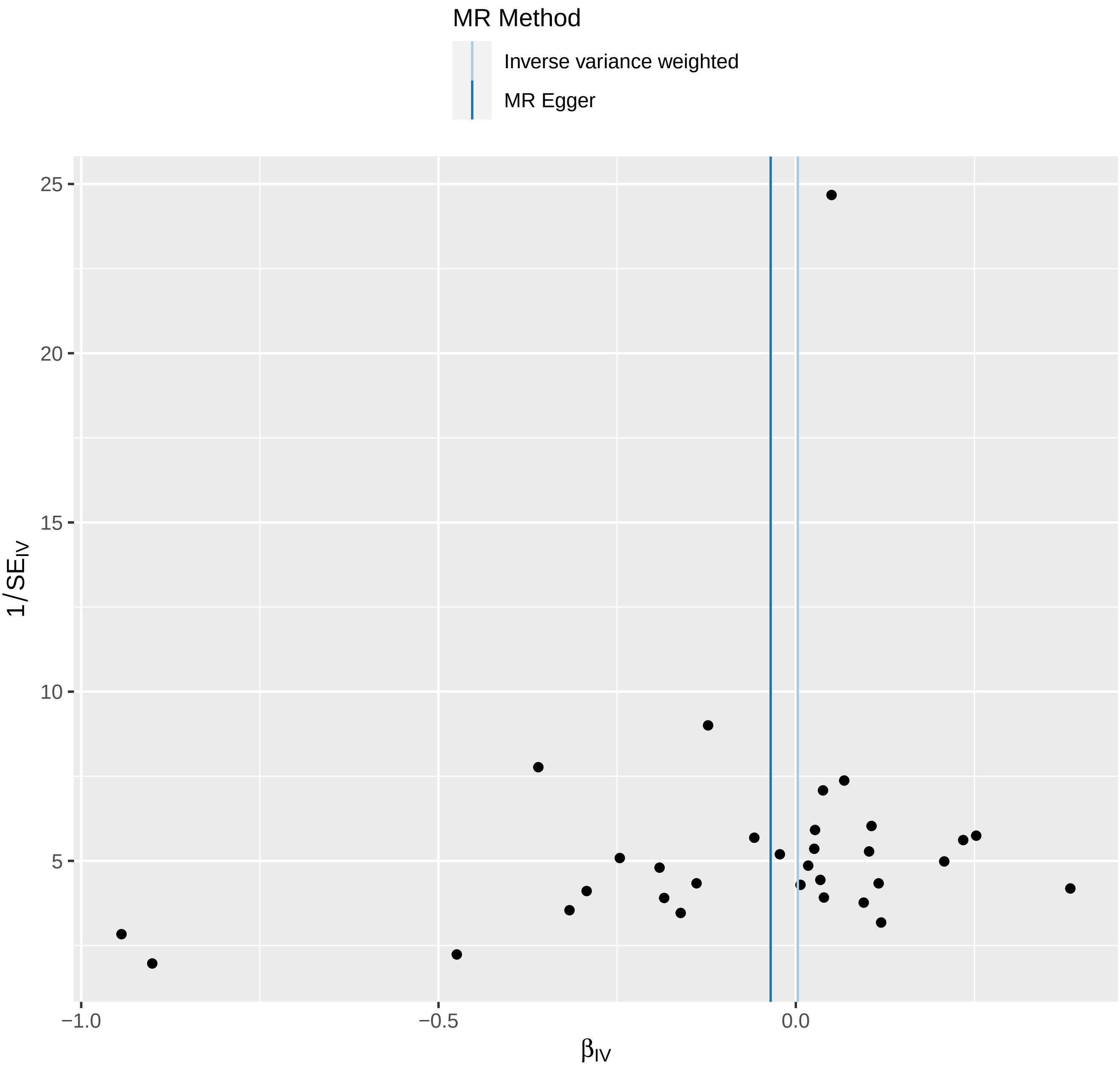

Batch 3−122 : Amyloid−like protein 2 on Diabetic nephropathy (more control exclusions)
with a threshold of 1e−05

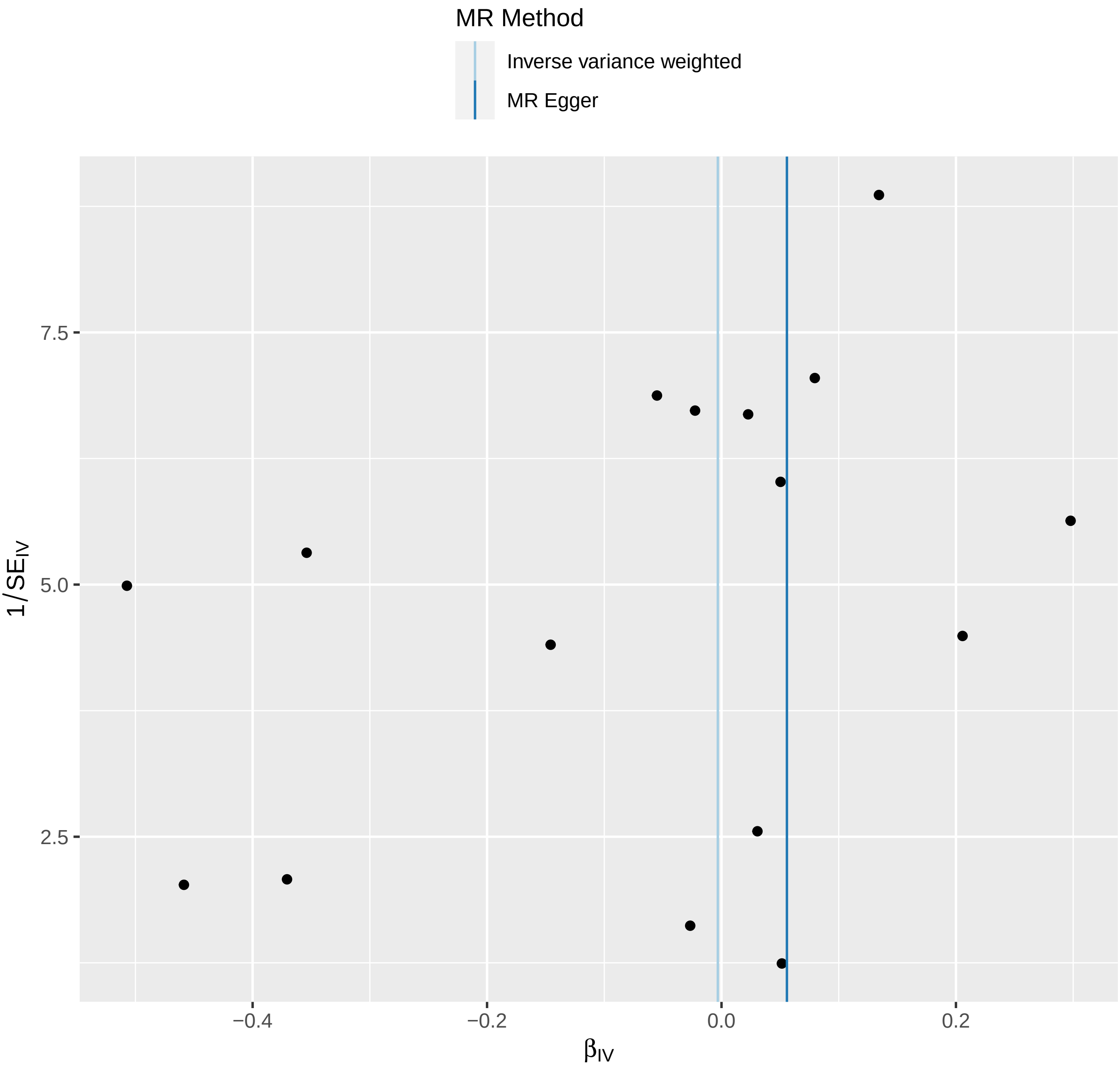

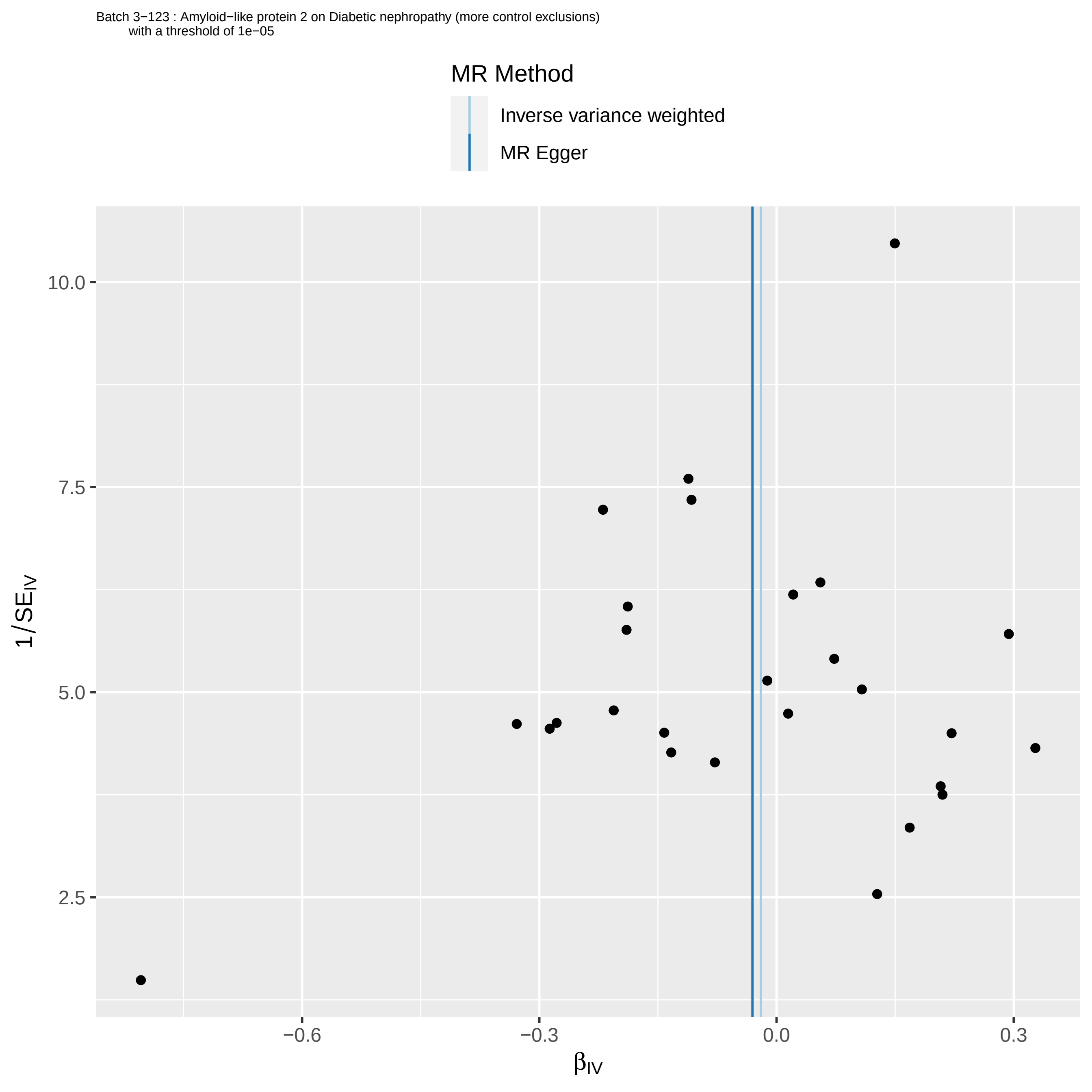
Batch 3−123 : Amyloid−like protein 2 on Diabetic nephropathy (more control exclusions)
with a threshold of 1e−05
MR Method
Inverse variance weighted
MR Egger
10.0
7.5
5.0
2.5
1/SE_IV
−0.6
−0.3
0.0
0.3
β_IV

Batch 3−124 : Adipocyte plasma membrane−associated protein on Diabetic nephropathy (more control exclusions)
with a threshold of 1e−05

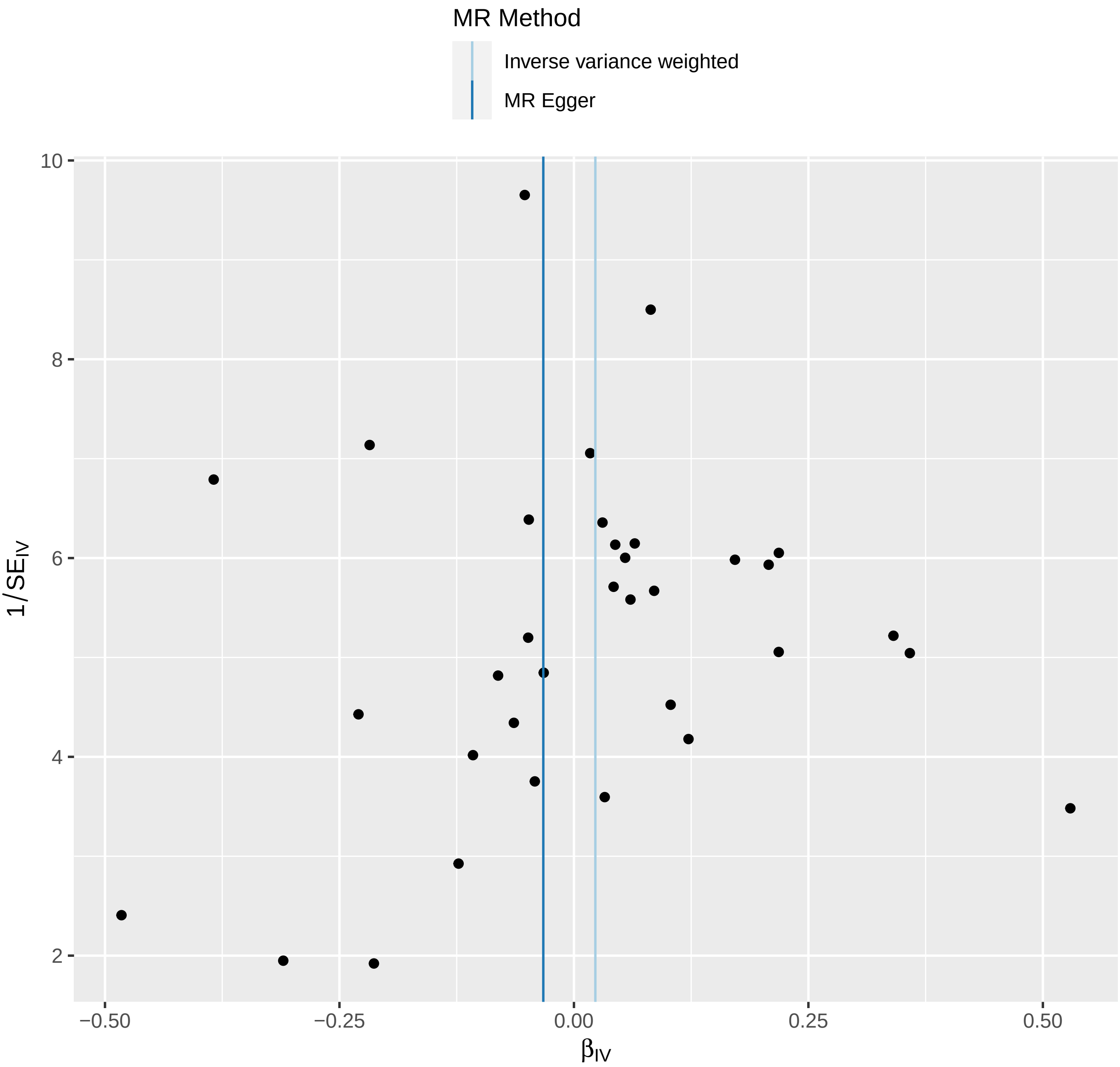

Batch 3−125 : Apolipoprotein A−V on Diabetic nephropathy (more control exclusions)
with a threshold of 1e−05

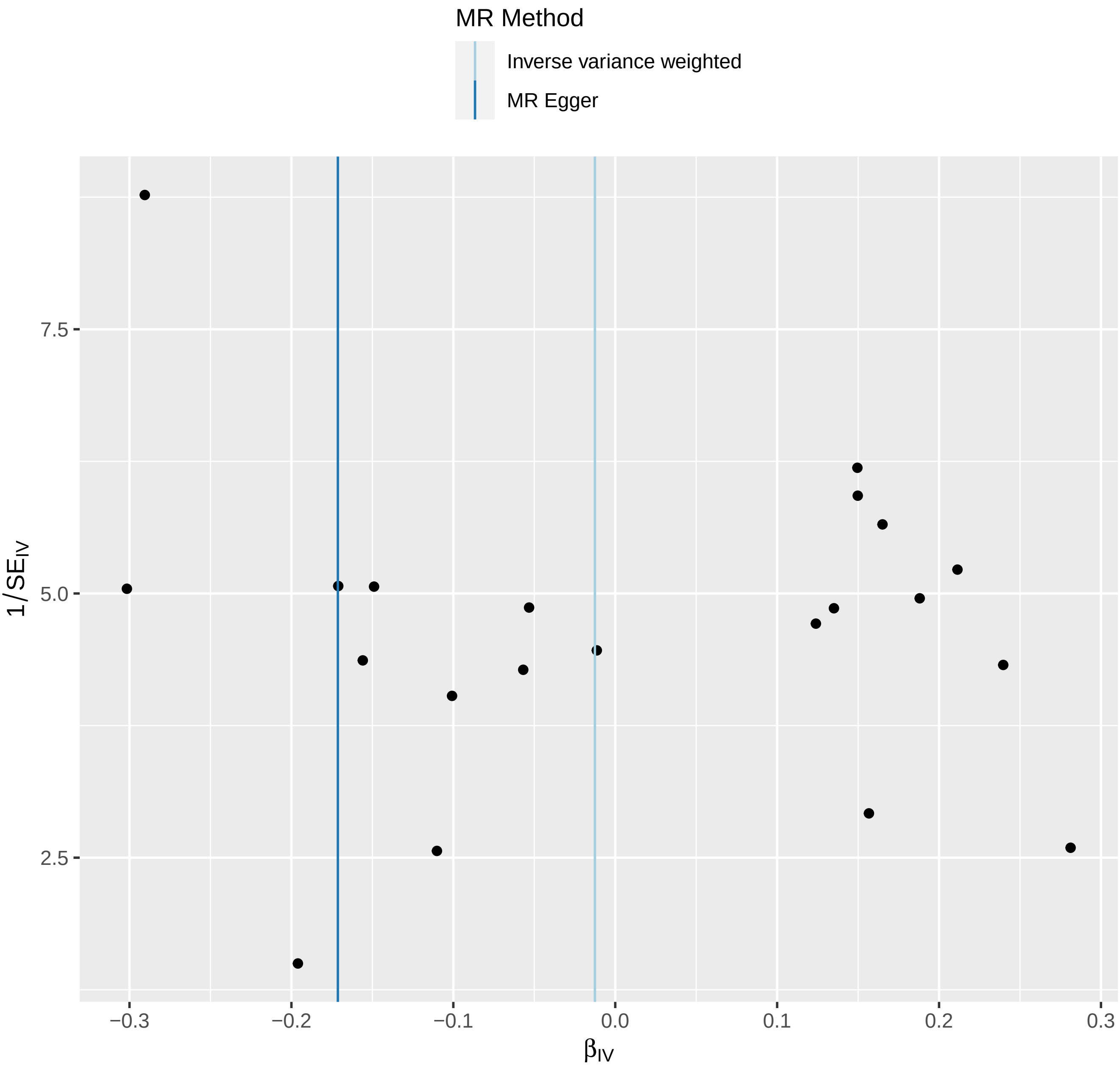

Batch 3−126 : Apolipoprotein A−V on Diabetic nephropathy (more control exclusions)
with a threshold of 1e−05

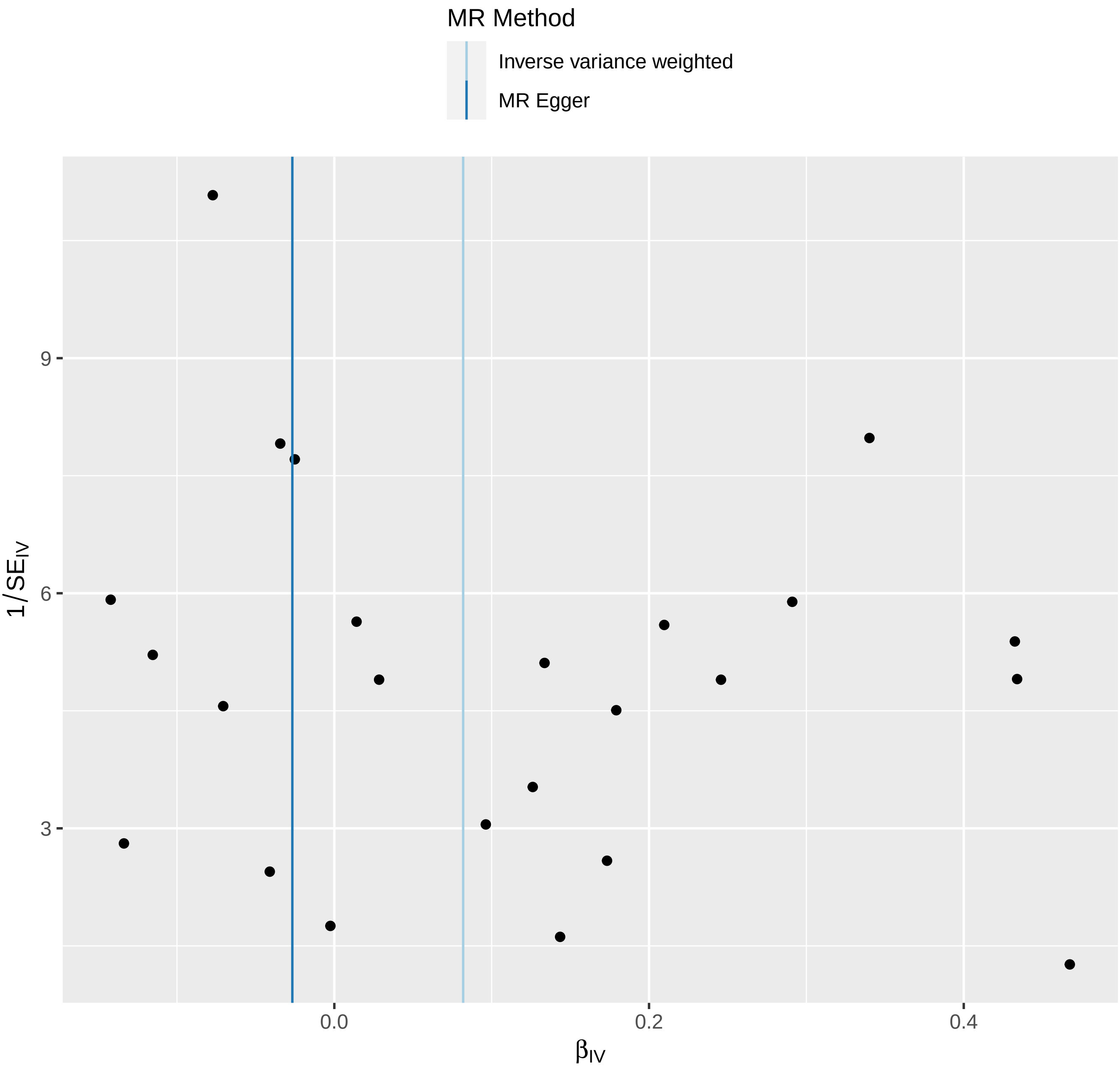

Batch 3−127 : Apolipoprotein B on Diabetic nephropathy (more control exclusions)
with a threshold of 1e−05

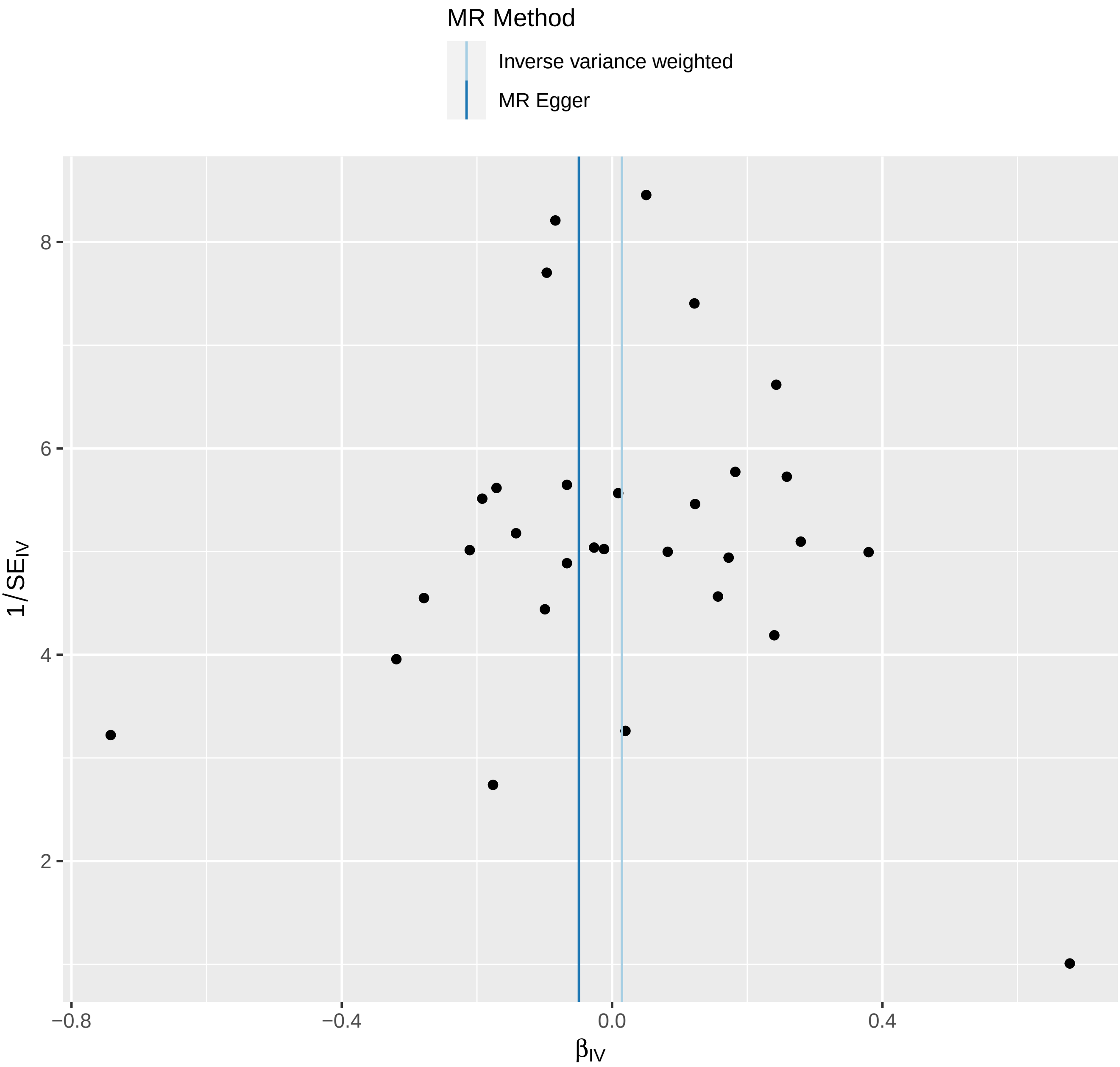

Batch 3−128 : DNA dC−>dU−editing enzyme APOBEC−3G on Diabetic nephropathy (more control exclusions)
with a threshold of 1e−05

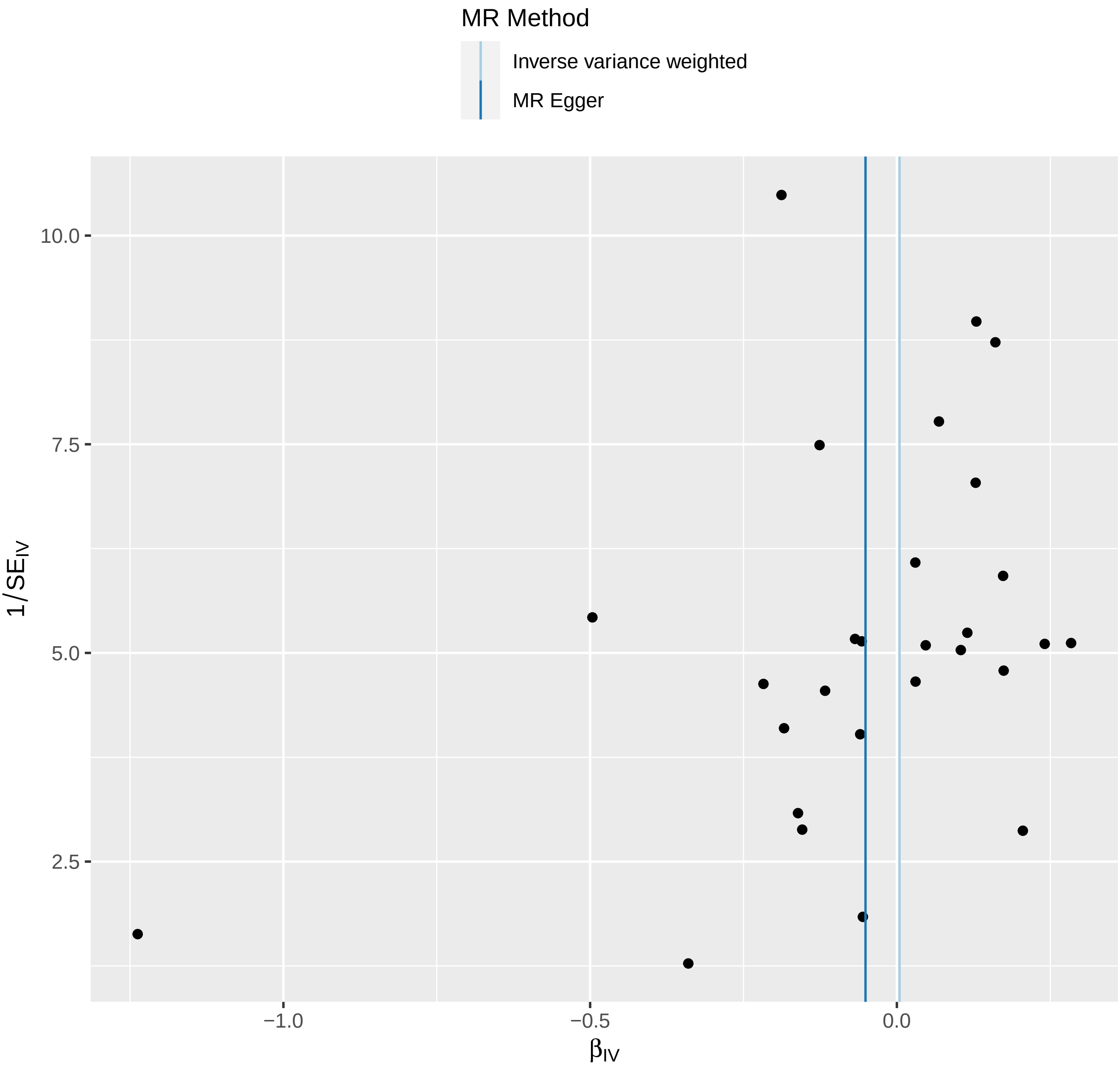

Batch 3−129 : Apolipoprotein D on Diabetic nephropathy (more control exclusions)
with a threshold of 1e−05

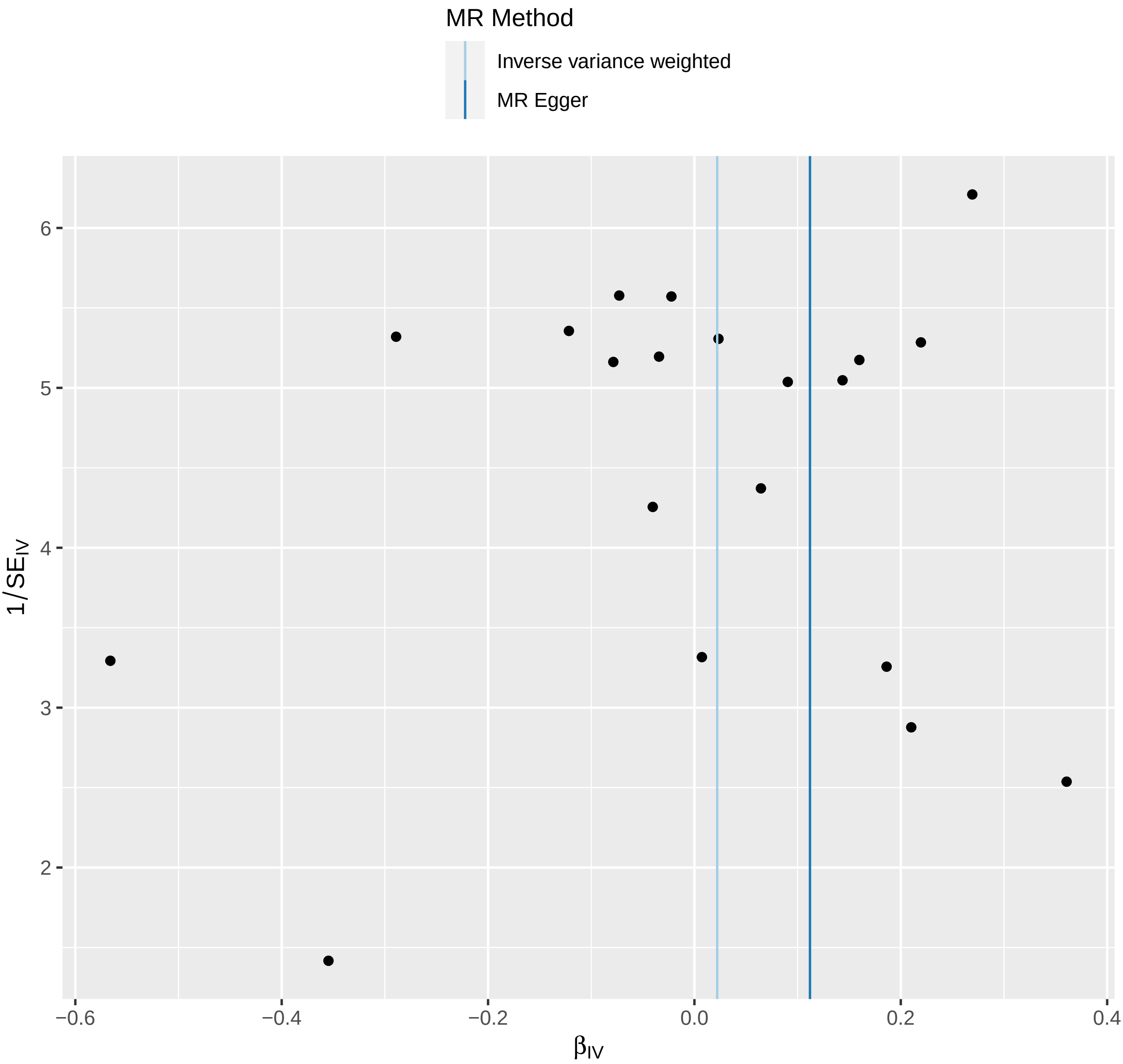

Batch 3−130 : Apolipoprotein D on Diabetic nephropathy (more control exclusions)
with a threshold of 1e−05

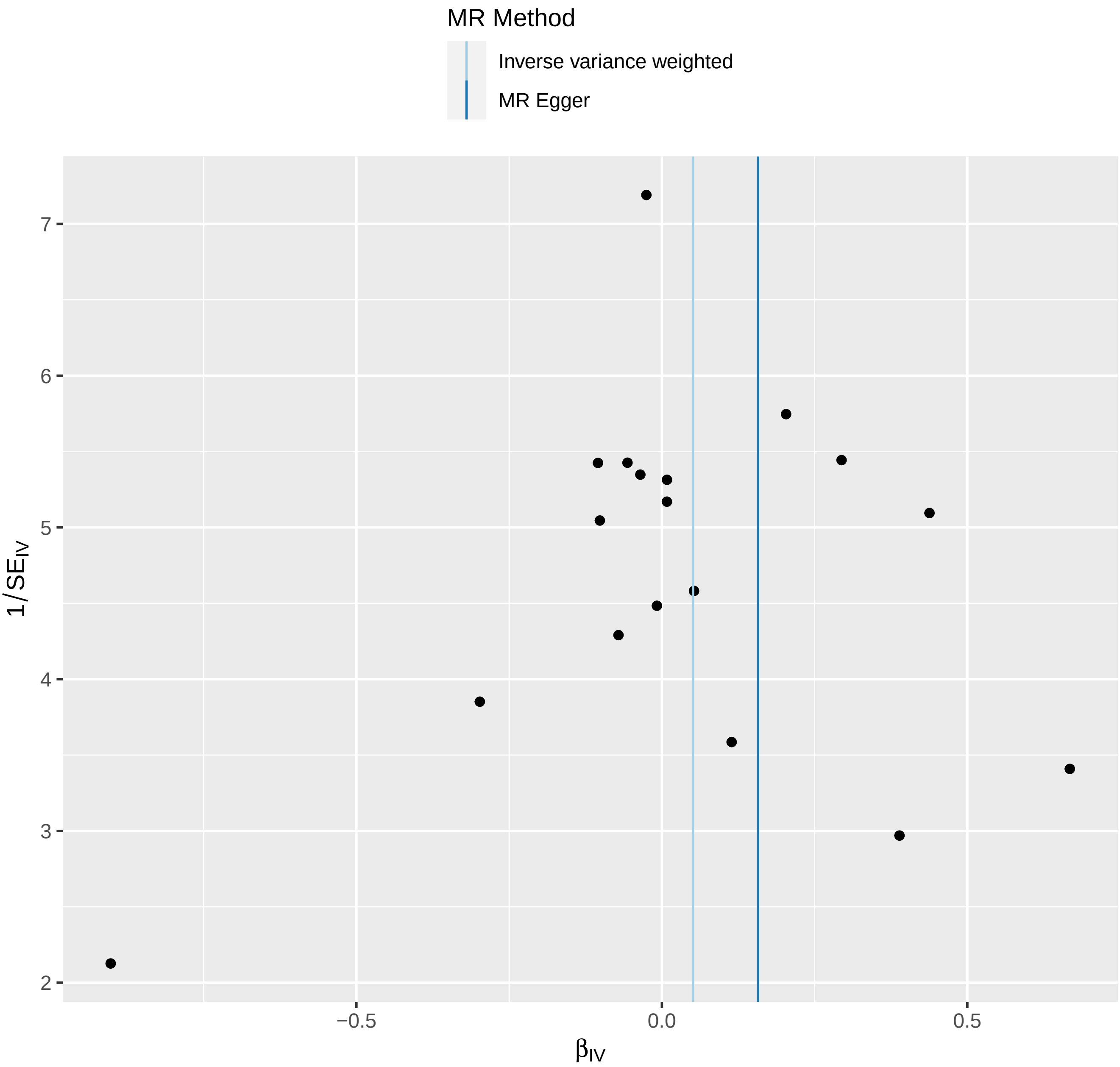

Batch 3−131 : Apolipoprotein E (isoform E3) on Diabetic nephropathy (more control exclusions) with a threshold of 1e−05

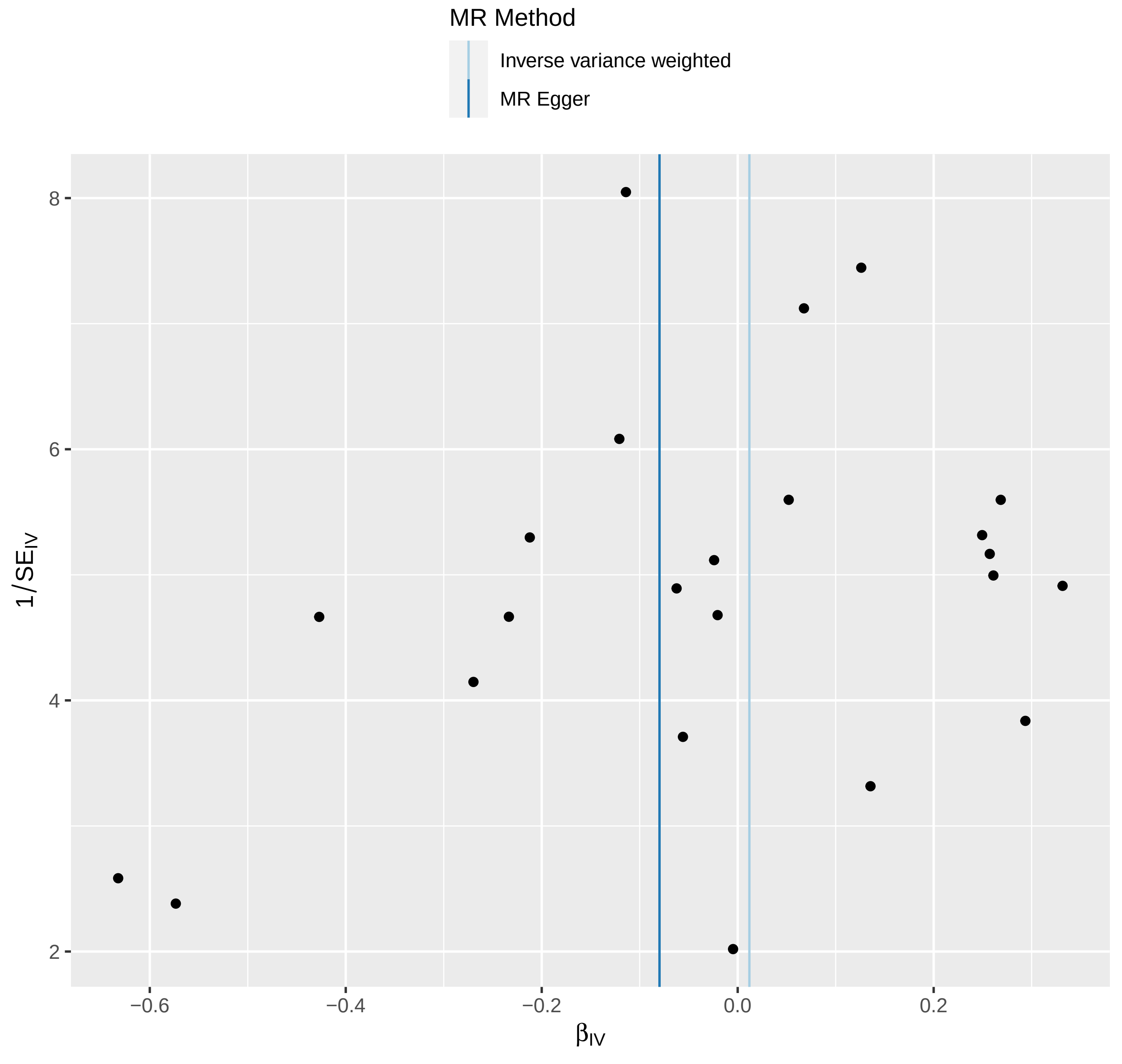

Batch 3−132 : Apolipoprotein E (isoform E2) on Diabetic nephropathy (more control exclusions)
with a threshold of 1e−05

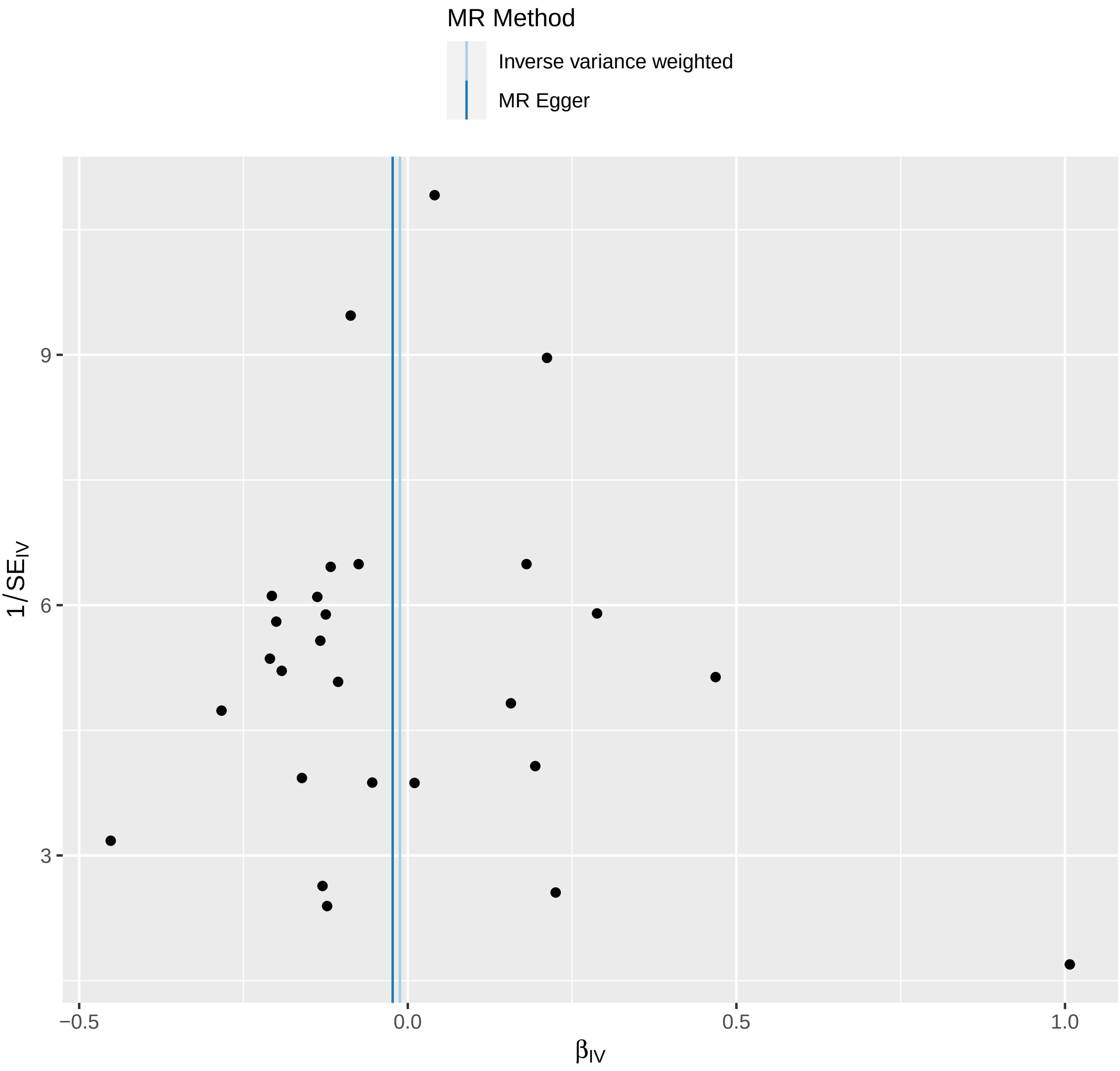

Batch 3−133 : Apolipoprotein F on Diabetic nephropathy (more control exclusions)
with a threshold of 1e−05

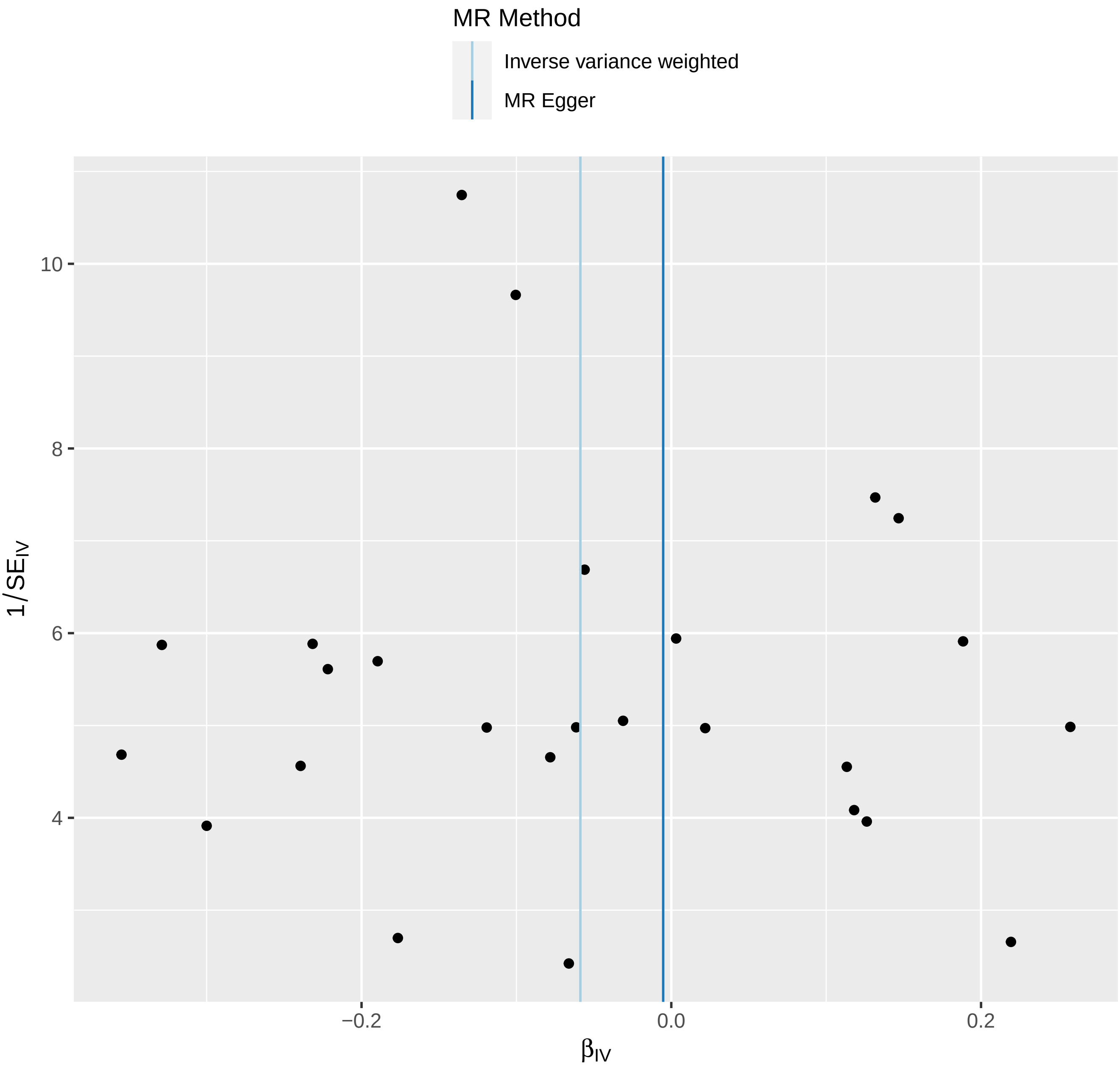

Batch 3−134 : Apolipoprotein L1 on Diabetic nephropathy (more control exclusions)
with a threshold of 1e−05

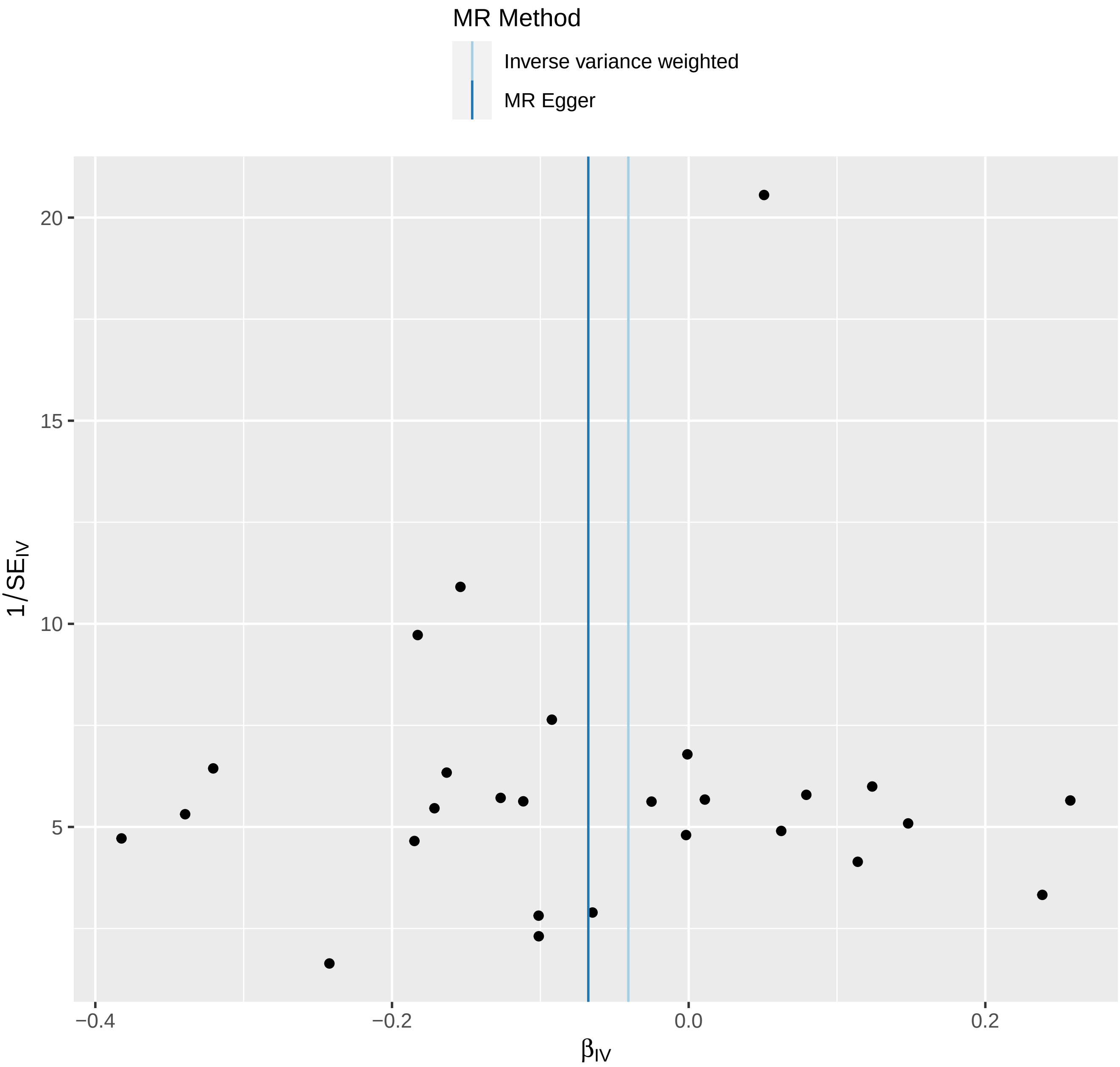

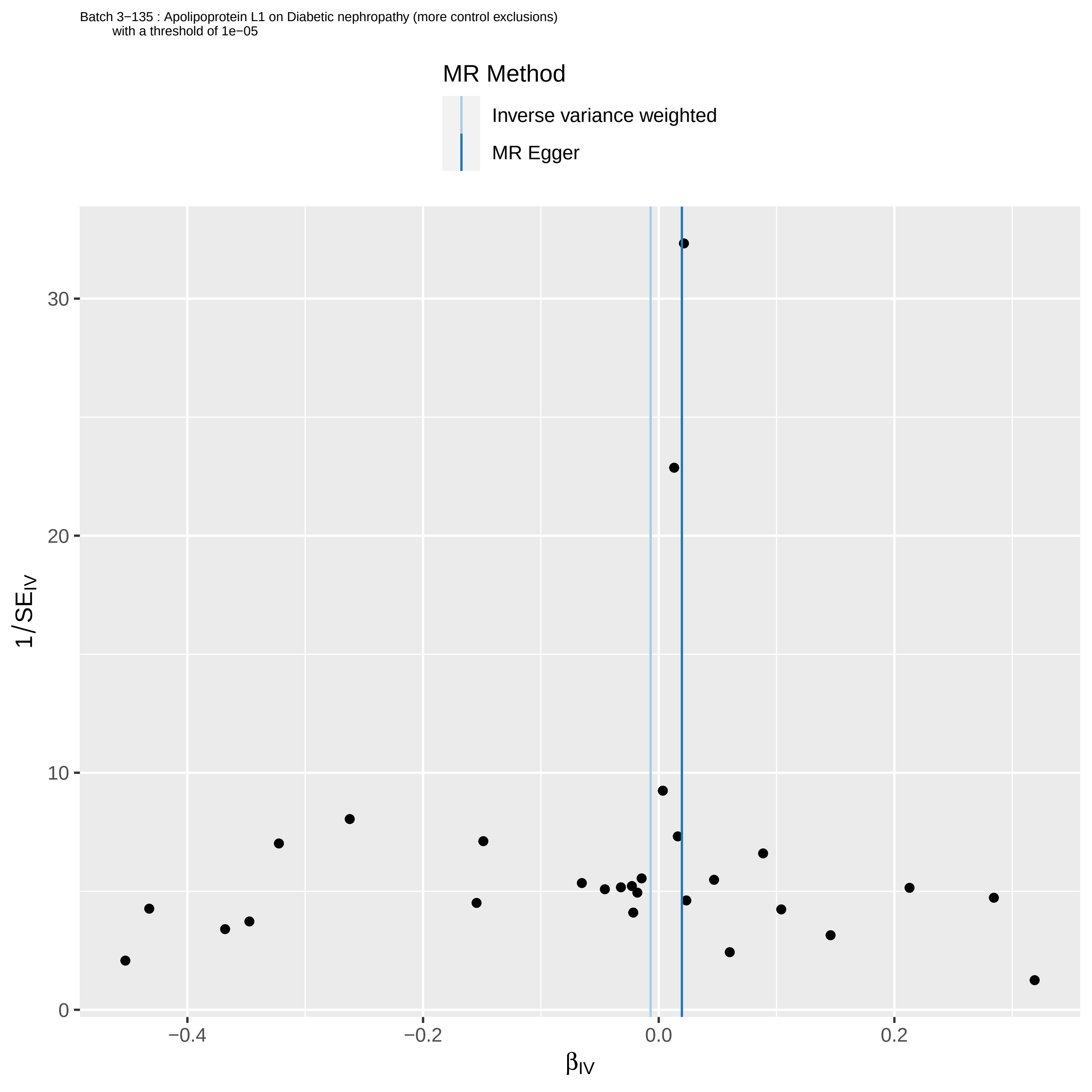

Batch 3−135 : Apolipoprotein L1 on Diabetic nephropathy (more control exclusions)
with a threshold of 1e−05
MR Method
Inverse variance weighted
MR Egger
30
20
10
0
−0.4
−0.2
0.0
0.2
1/SE_IV
β_IV

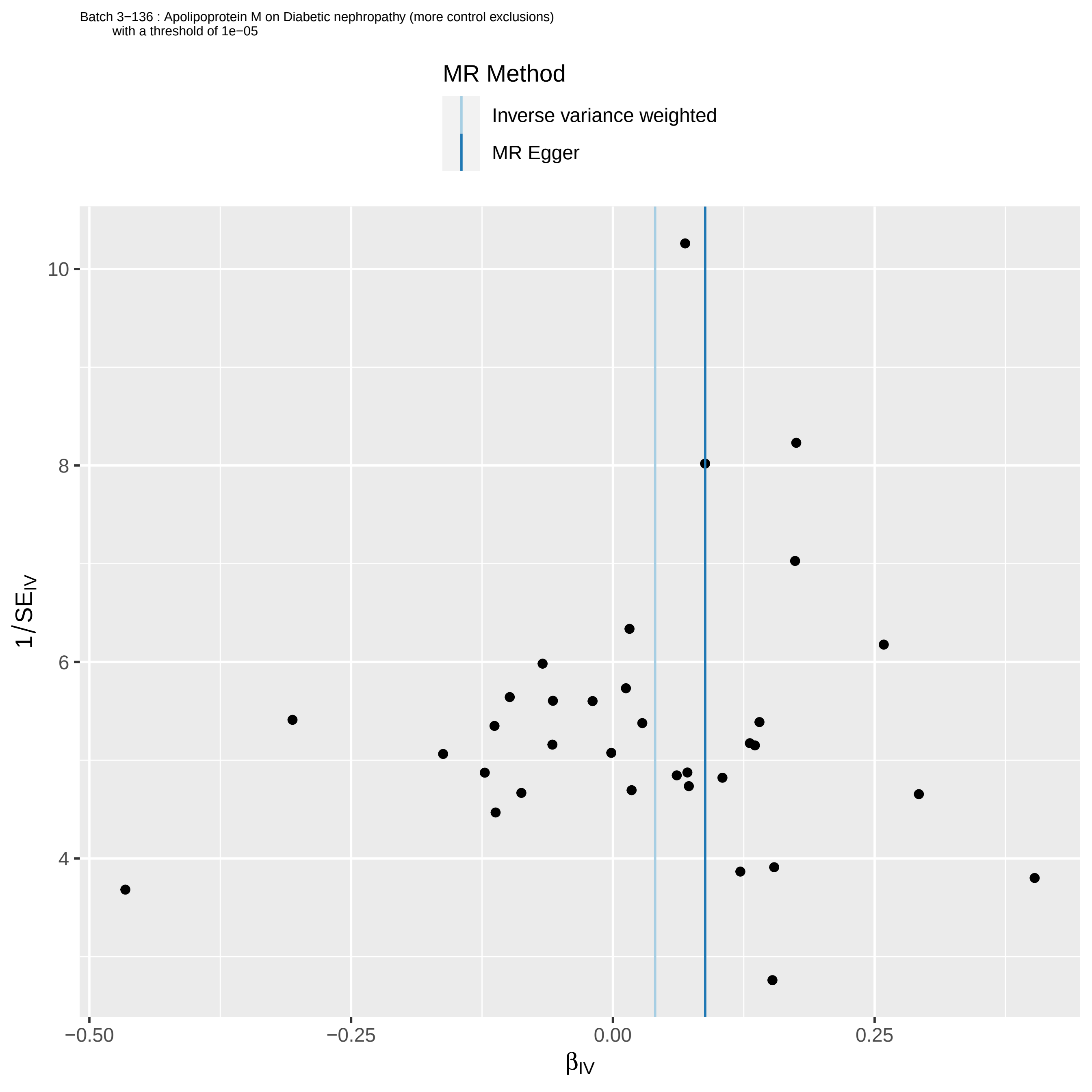

Batch 3−136 : Apolipoprotein M on Diabetic nephropathy (more control exclusions)
with a threshold of 1e−05
MR Method
Inverse variance weighted
MR Egger
10
8
6
4
$1/SE_{IV}$
−0.50
−0.25
0.00
0.25
β_{IV}

Batch 3−137 : Apolipoprotein M on Diabetic nephropathy (more control exclusions)
with a threshold of 1e−05

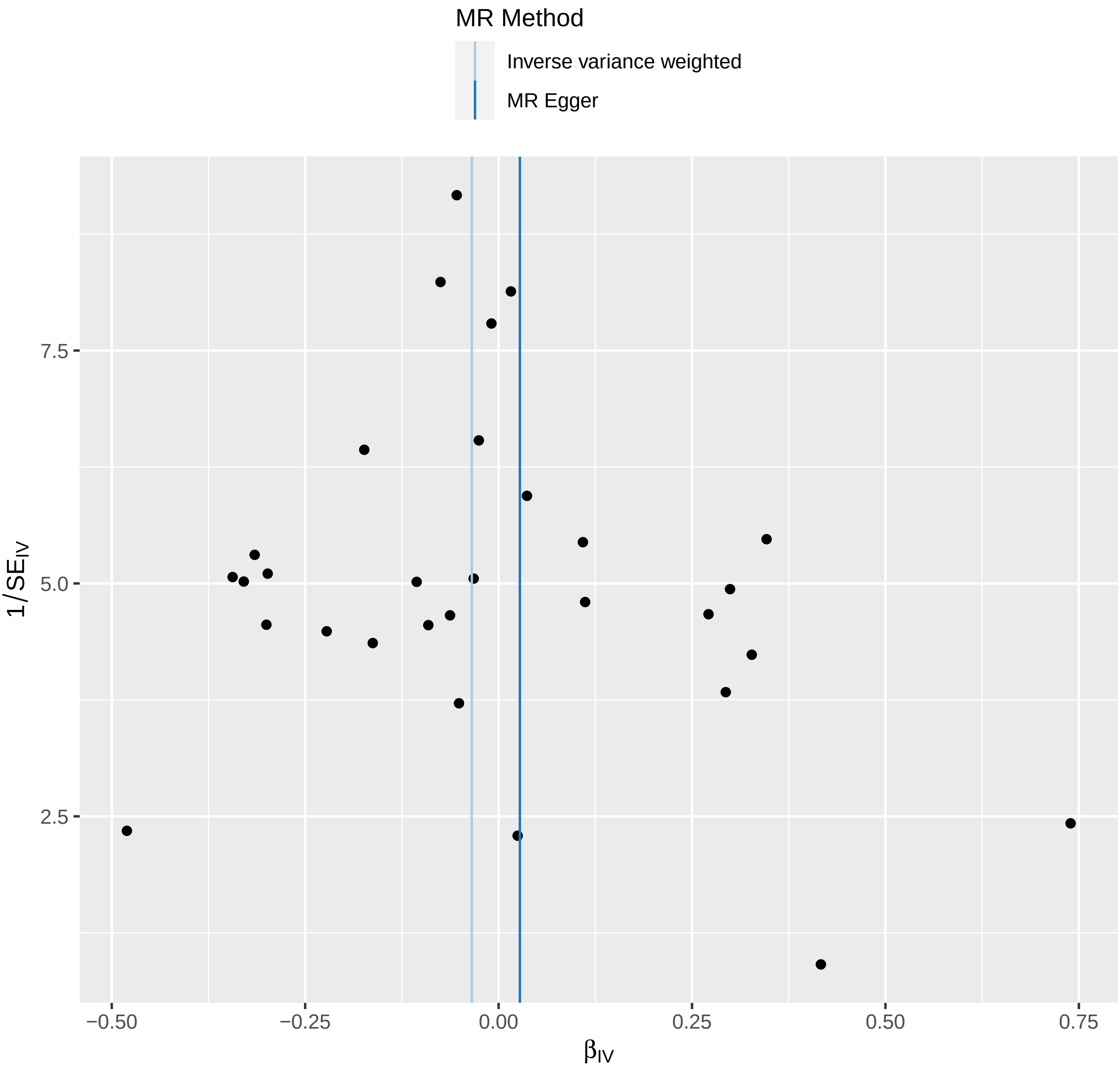

Batch 3−138 : MICOS complex subunit MIC26 on Diabetic nephropathy (more control exclusions)
with a threshold of 1e−05

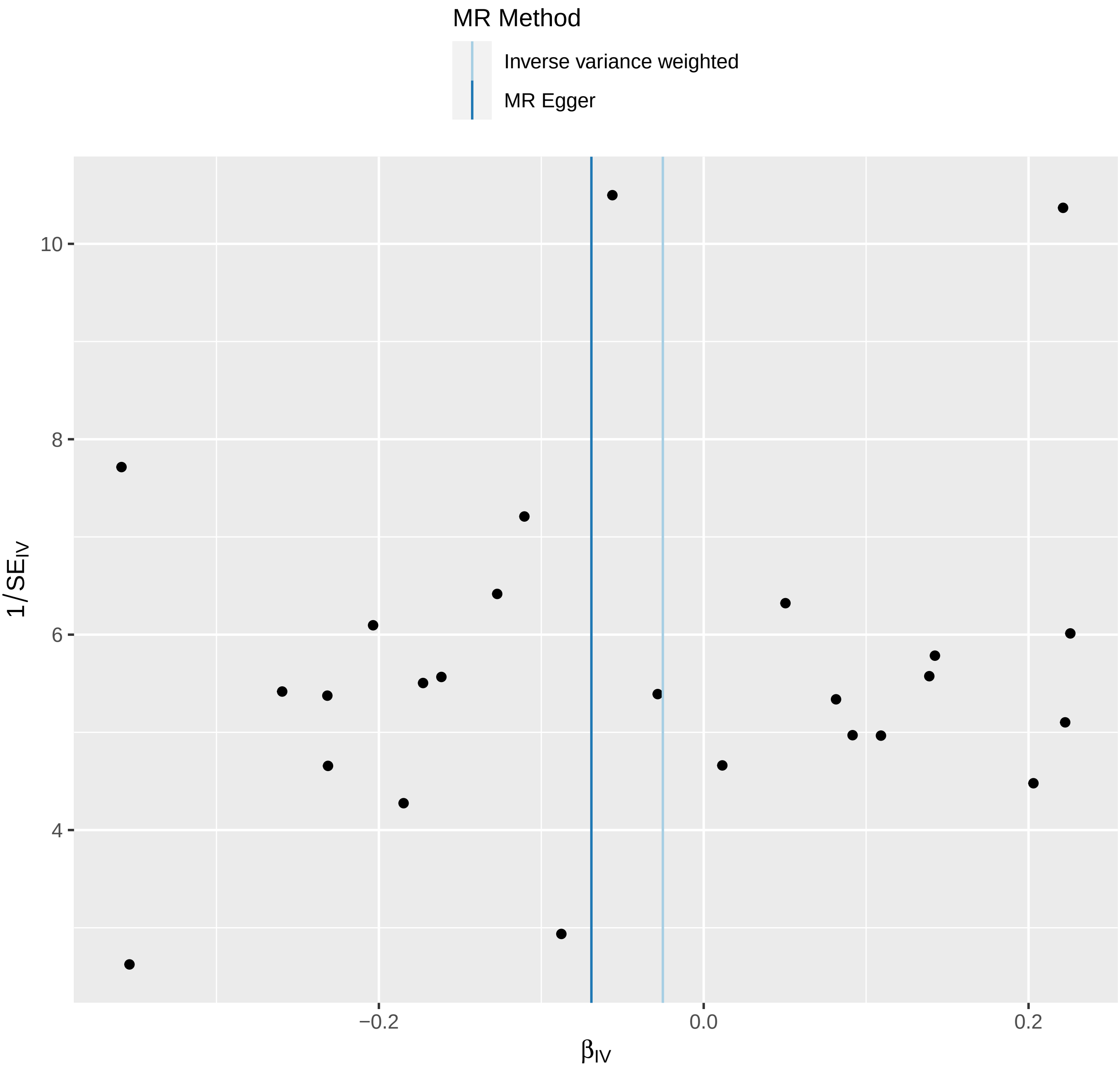

Batch 3−139 : Amyloid beta A4 protein on Diabetic nephropathy (more control exclusions)
with a threshold of 1e−05

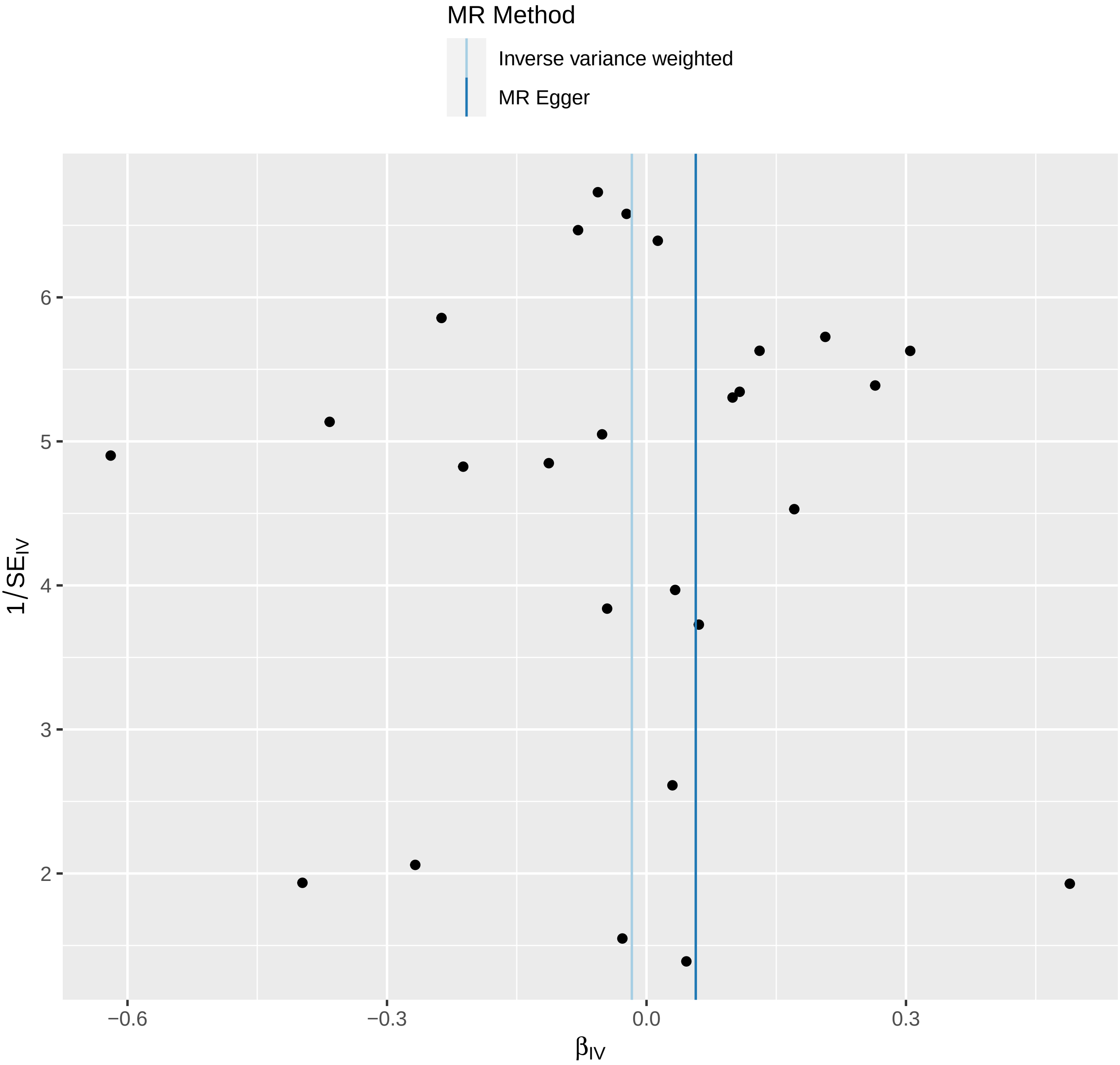

Batch 3−140 : DCC−interacting protein 13−alpha on Diabetic nephropathy (more control exclusions)
with a threshold of 1e−05

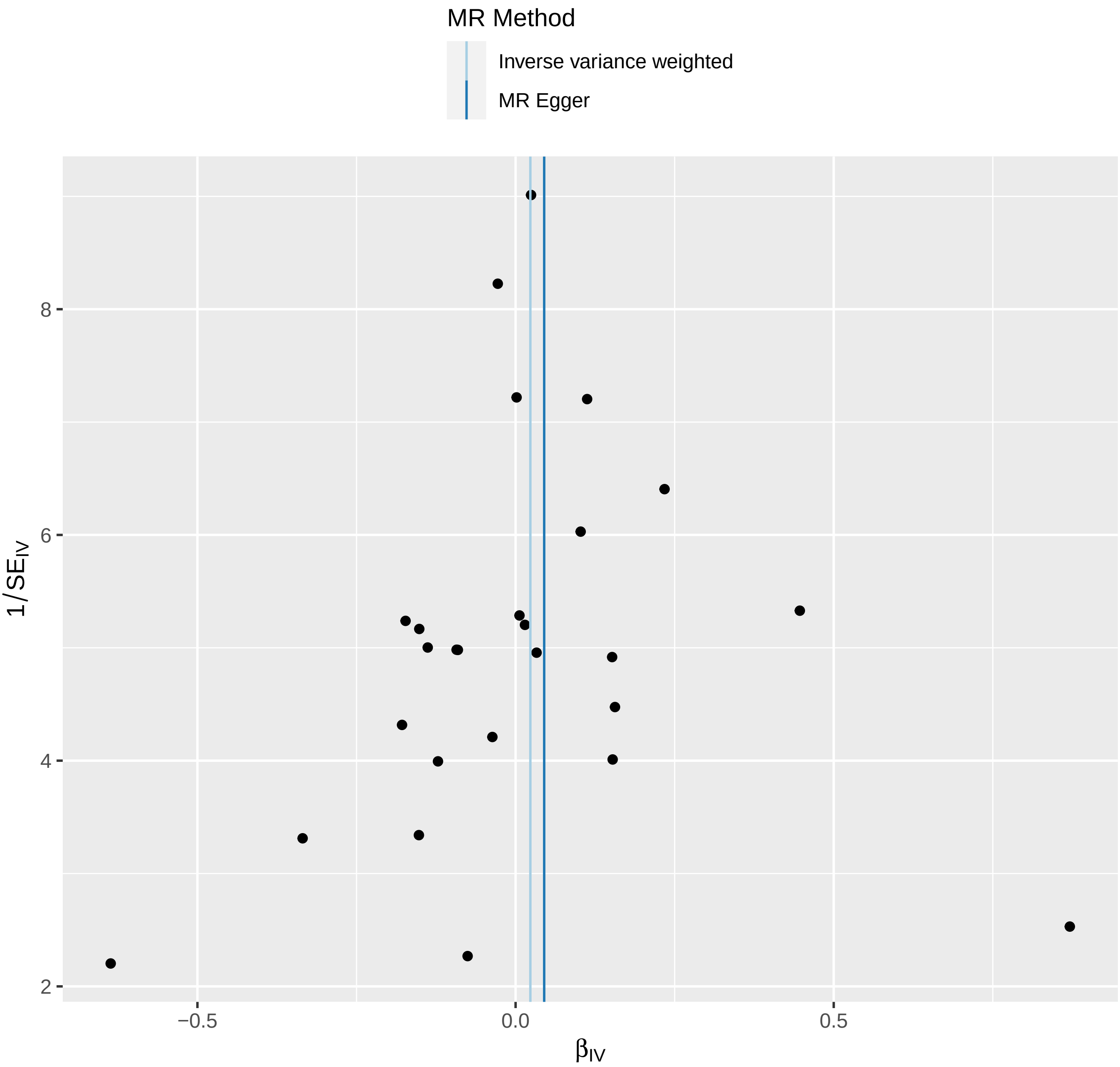

Batch 3−141 : Amphiregulin on Diabetic nephropathy (more control exclusions)
with a threshold of 1e−05

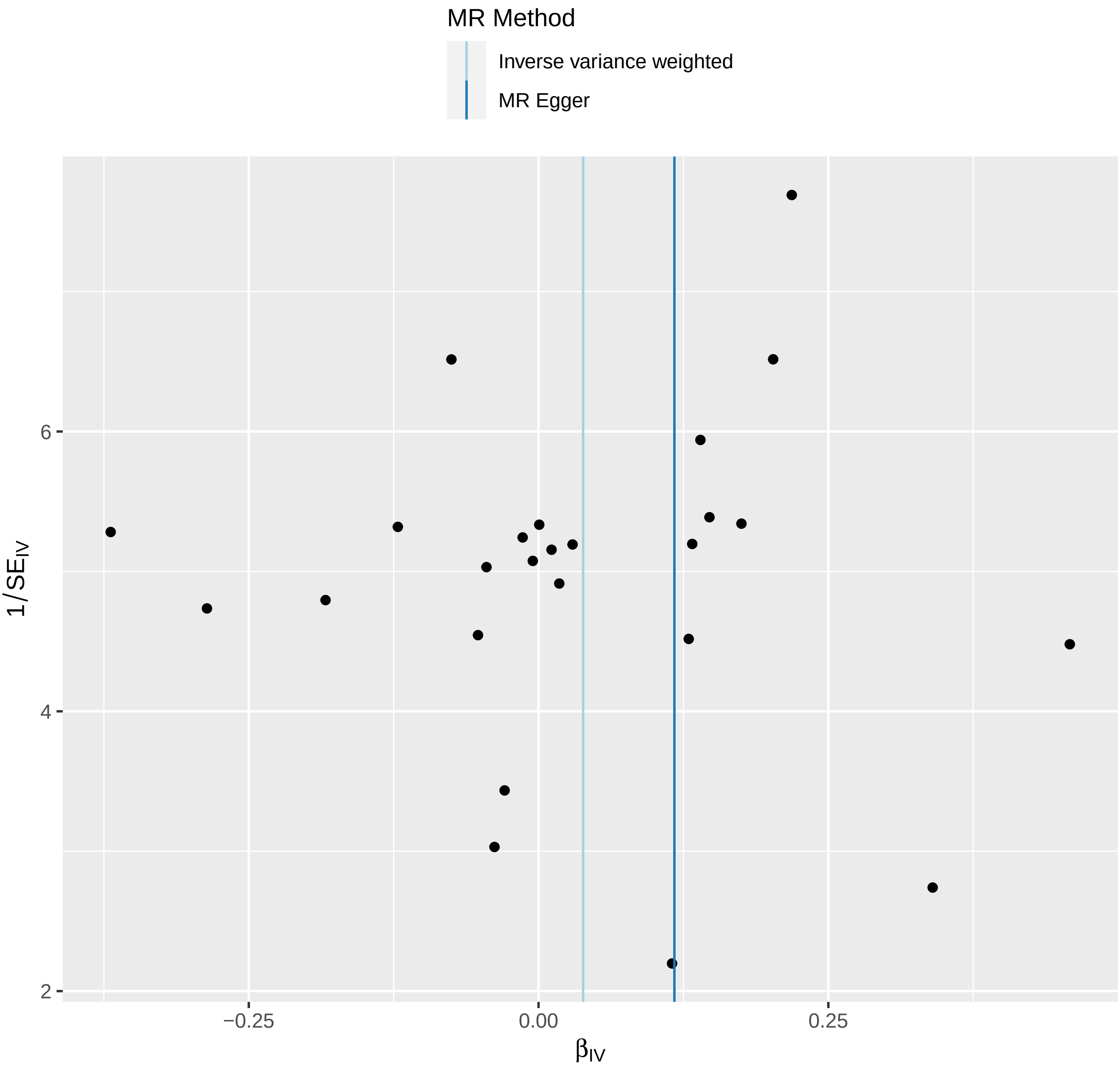

Batch 3−142 : ADP−ribosylation factor 3 on Diabetic nephropathy (more control exclusions)
with a threshold of 1e−05

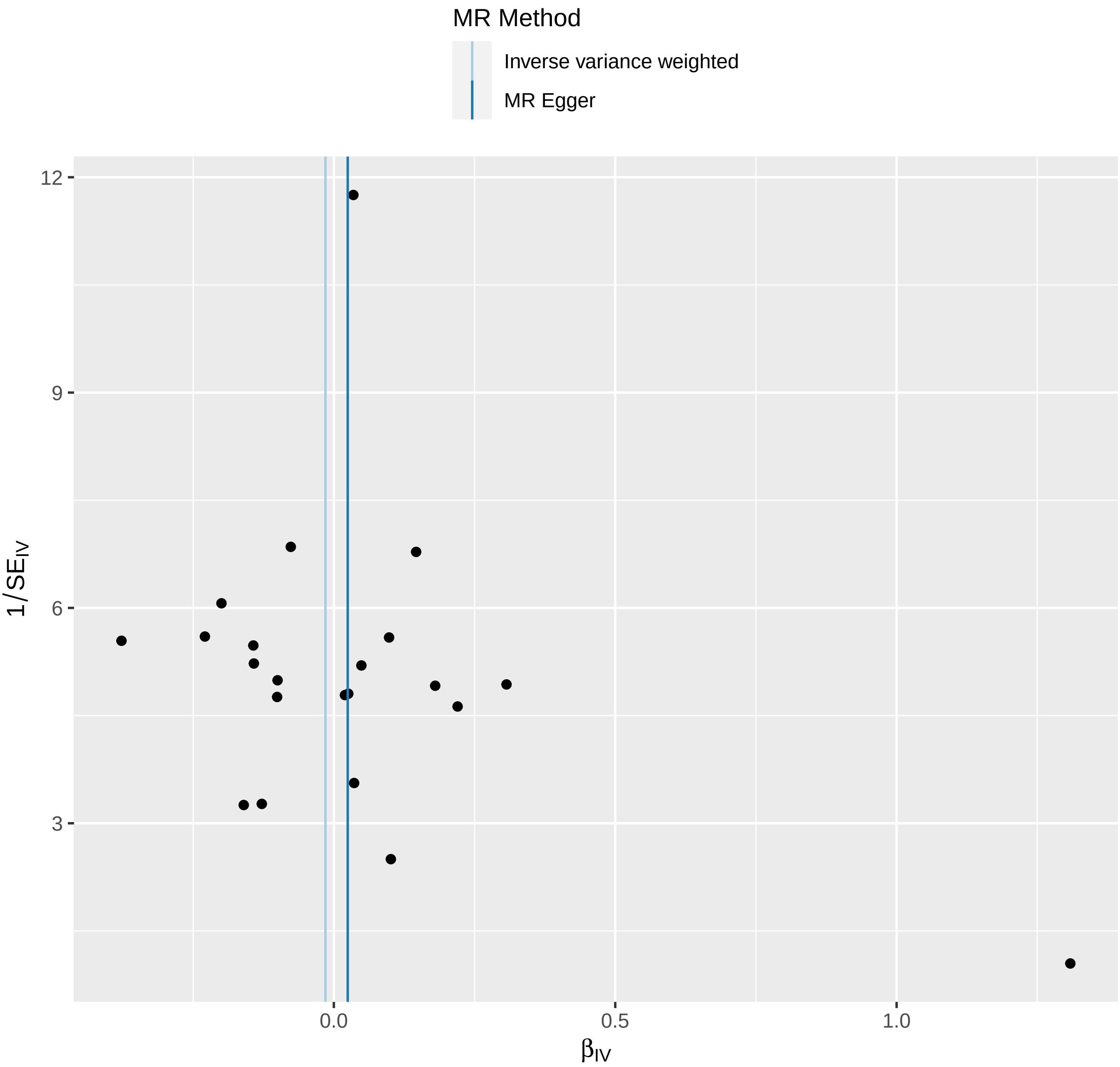

Batch 3−143 : ADP−ribosylation factor 6 on Diabetic nephropathy (more control exclusions)
with a threshold of 1e−05

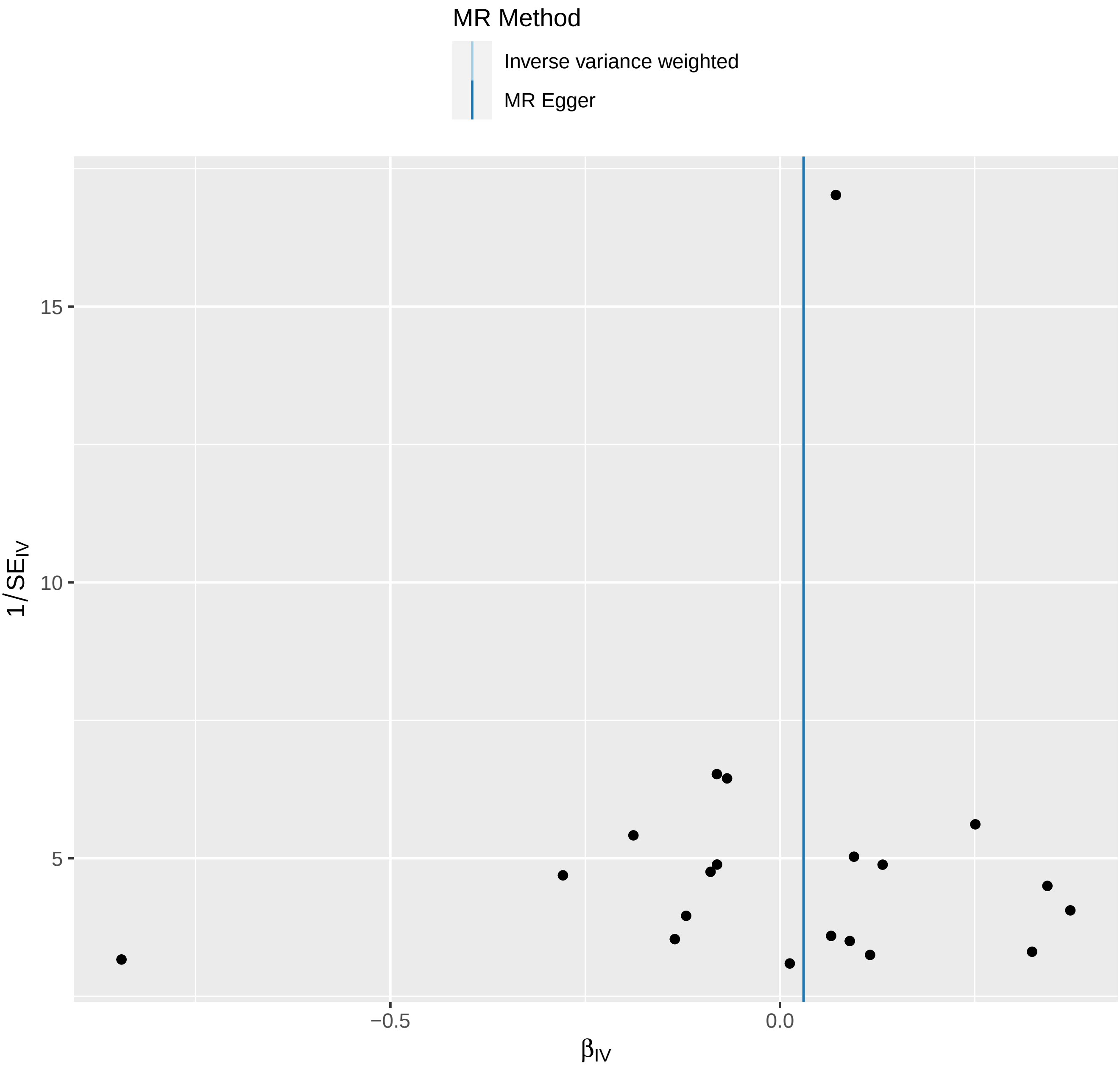

Batch 3−144 : ADP−ribosylation factor GTPase−activating protein 2 on Diabetic nephropathy (more control exclusions)
with a threshold of 1e−05

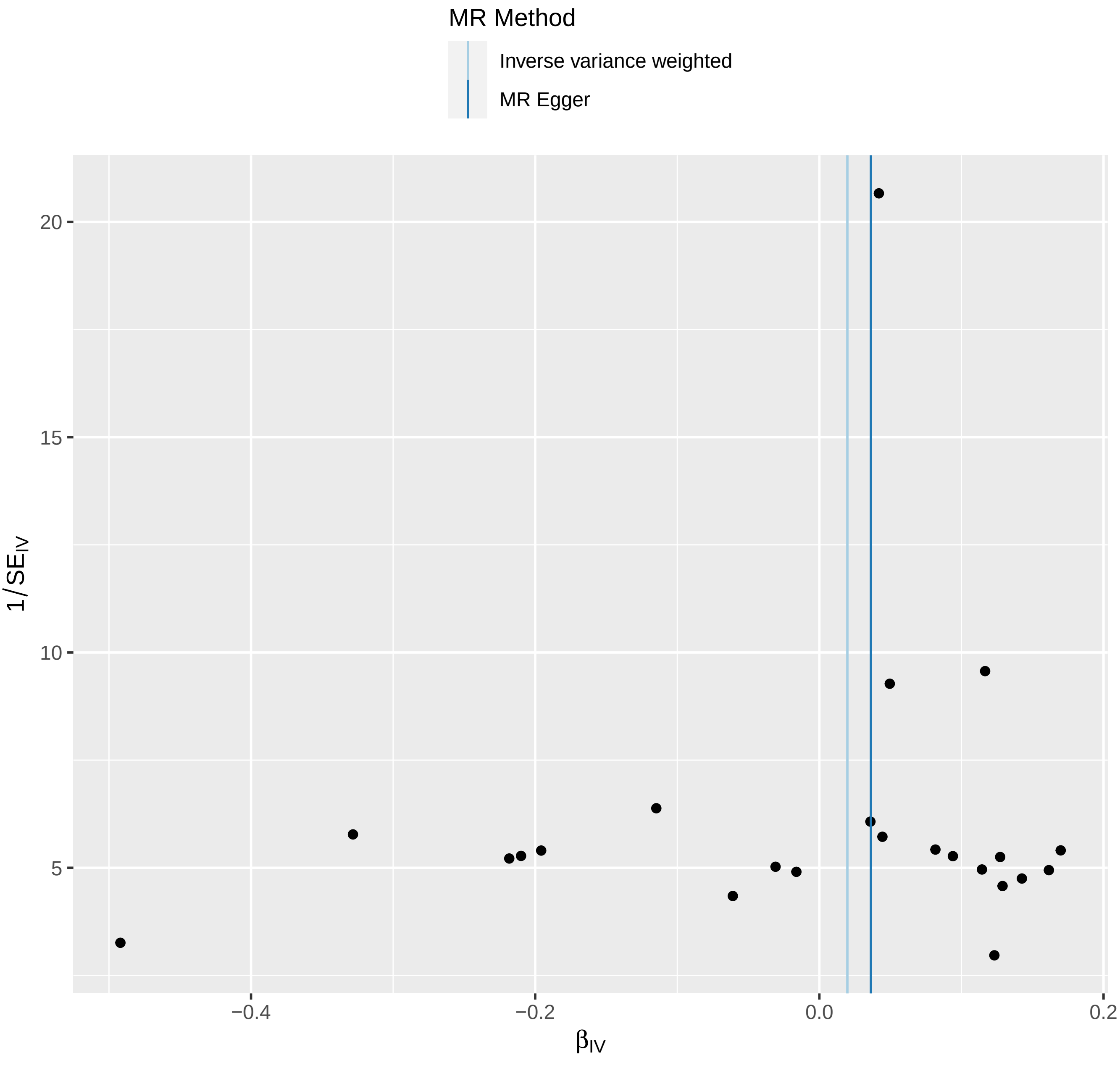

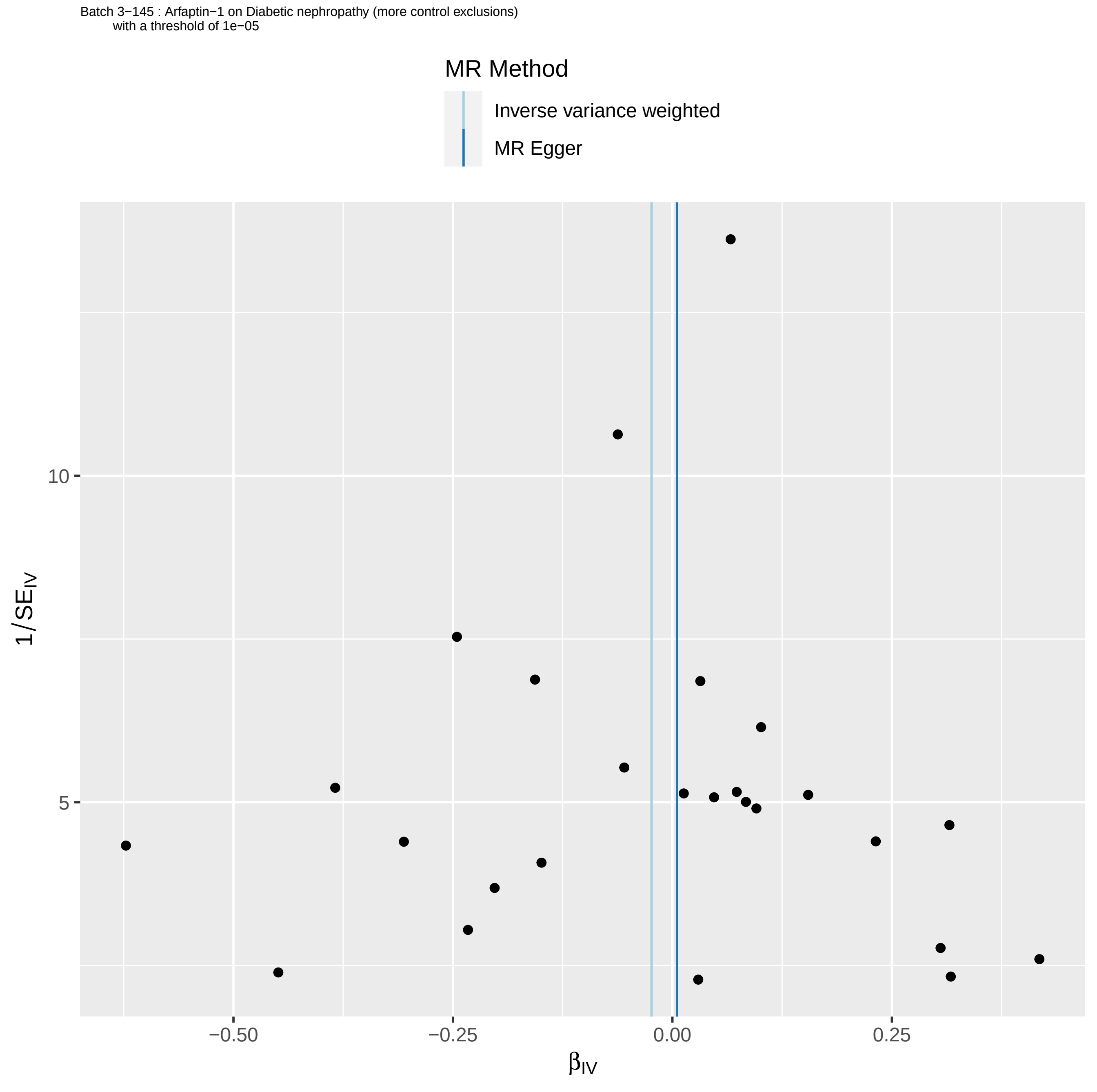

Batch 3−145 : Arfaptin−1 on Diabetic nephropathy (more control exclusions)
with a threshold of 1e−05
MR Method
Inverse variance weighted
MR Egger
10
5
1/SE$_{IV}$
−0.50
−0.25
0.00
0.25
β_{IV}

Batch 3−146 : Arfaptin−2 on Diabetic nephropathy (more control exclusions)
with a threshold of 1e−05

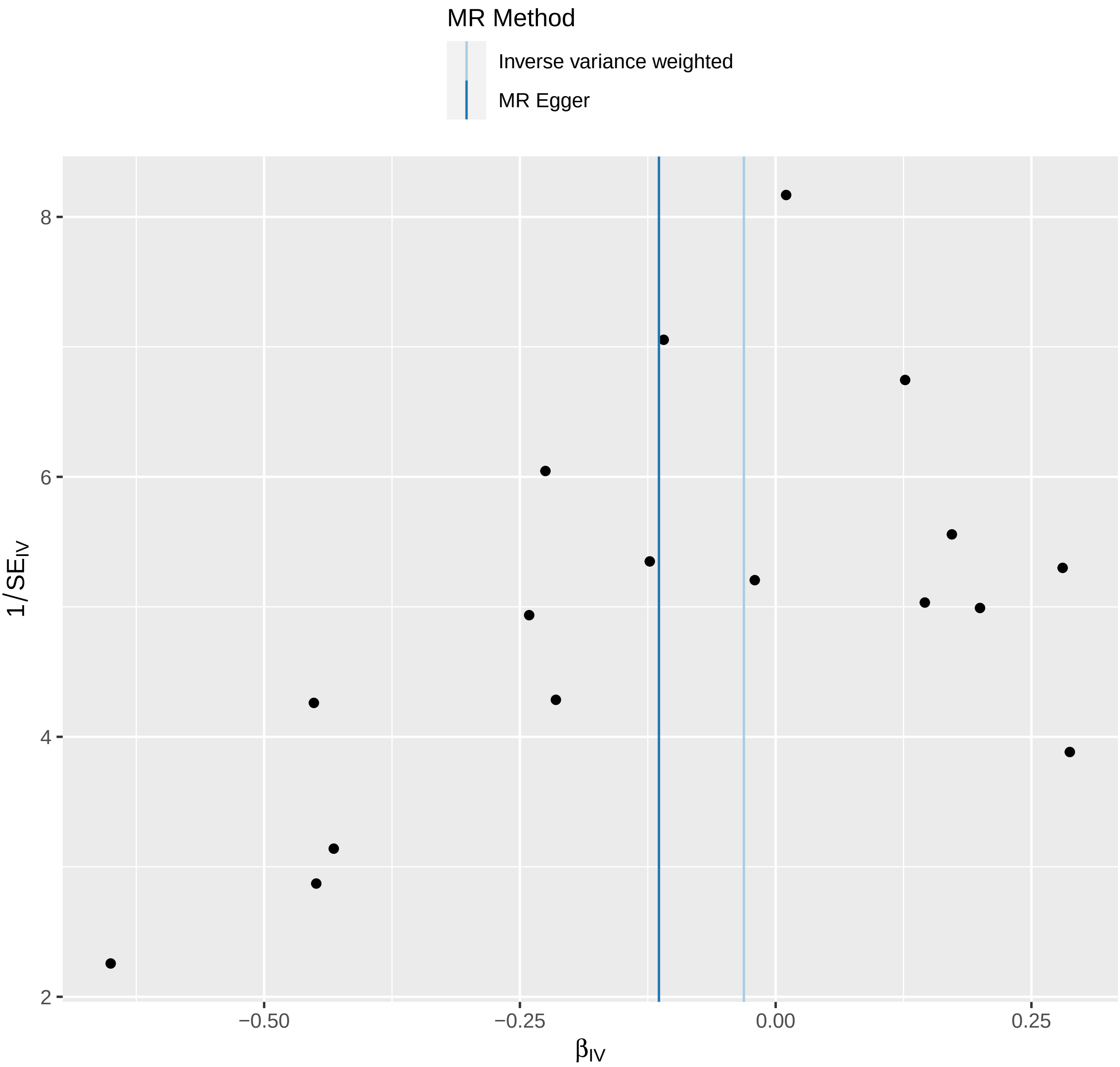

Batch 3−147 : Rho GTPase−activating protein 1 on Diabetic nephropathy (more control exclusions)
with a threshold of 1e−05

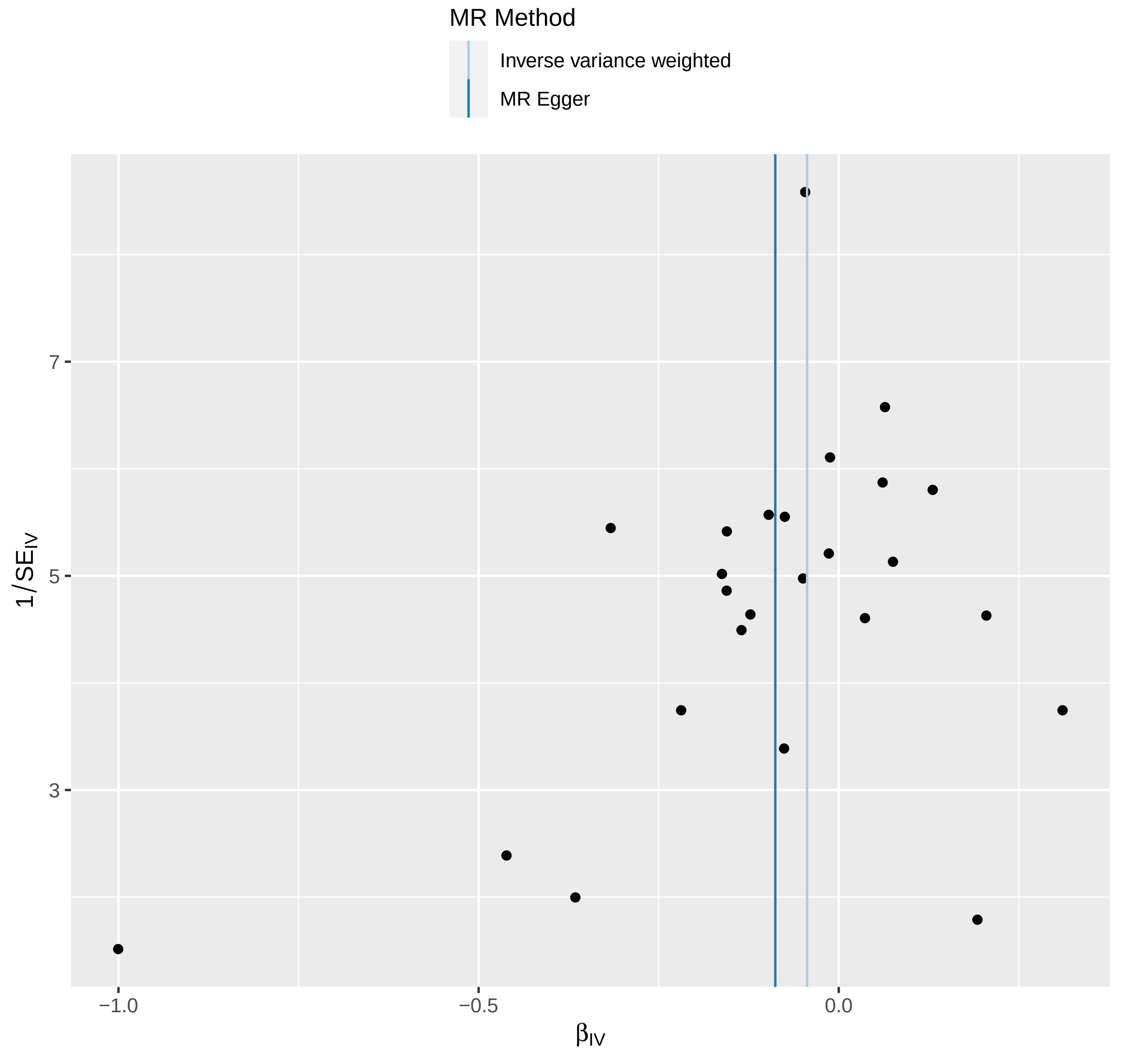

Batch 3−148 : Rho GTPase−activating protein 25 on Diabetic nephropathy (more control exclusions)
with a threshold of 1e−05

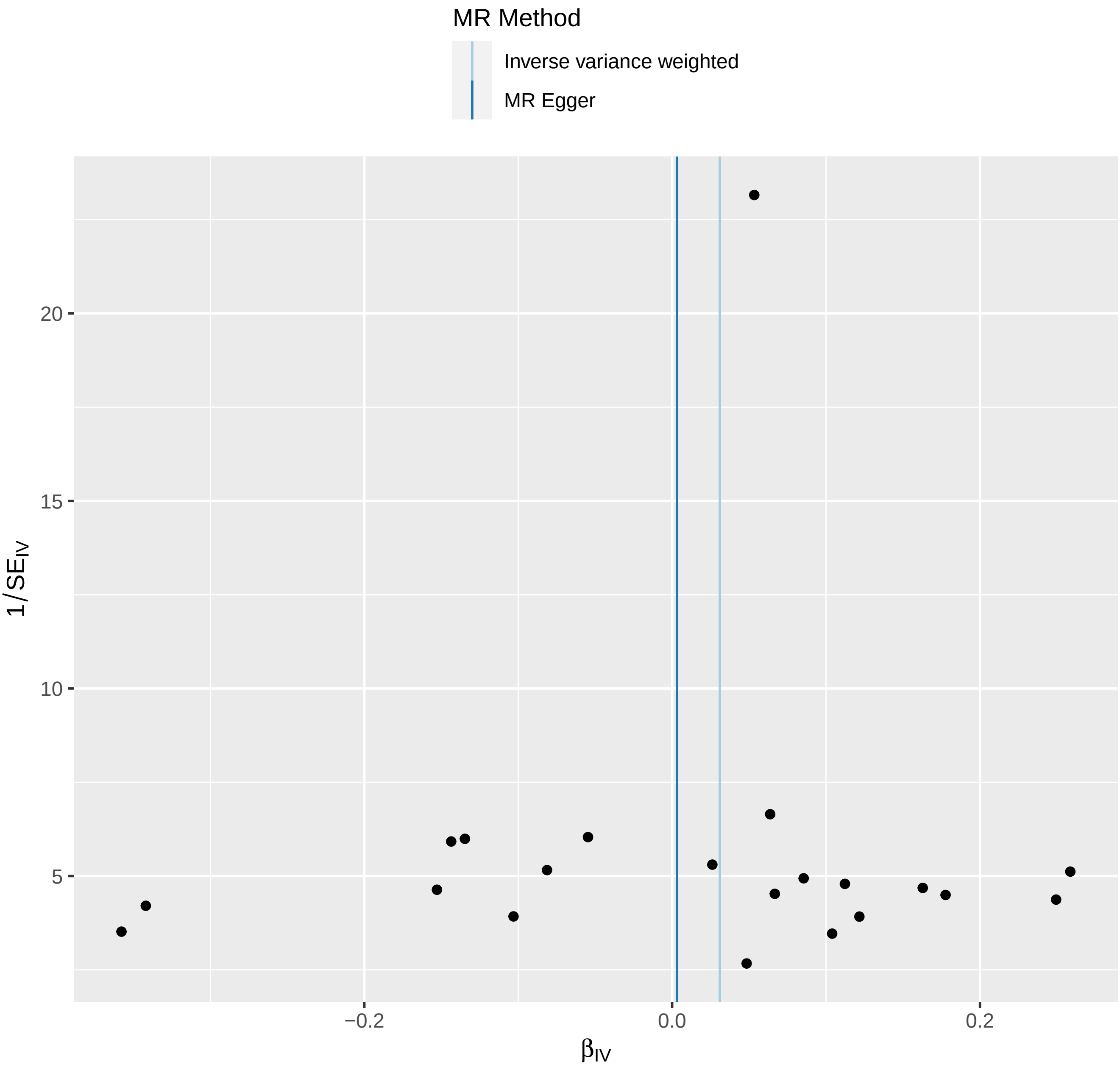

Batch 3−149 : Rho GTPase−activating protein 30 on Diabetic nephropathy (more control exclusions)
with a threshold of 1e−05

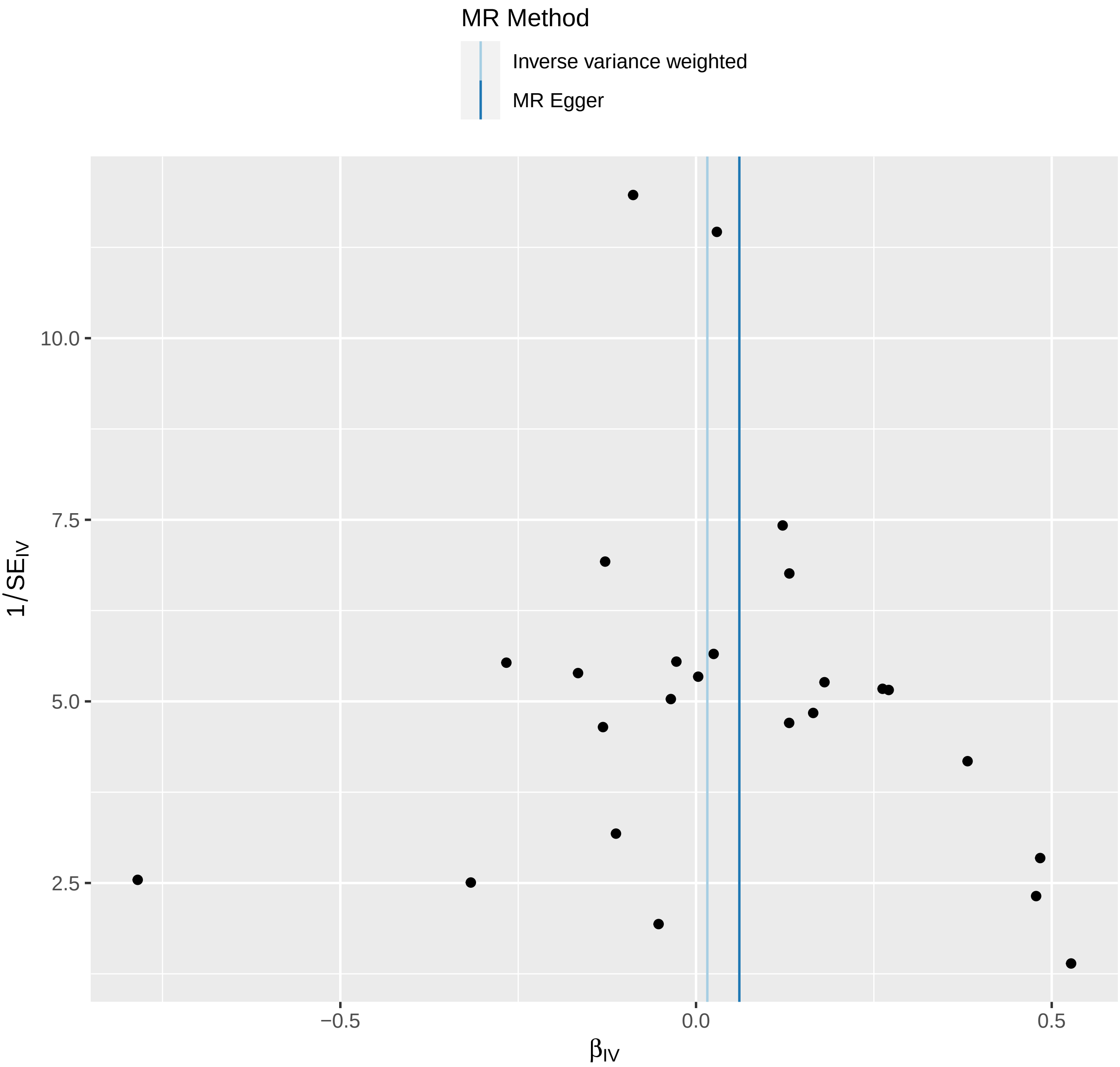

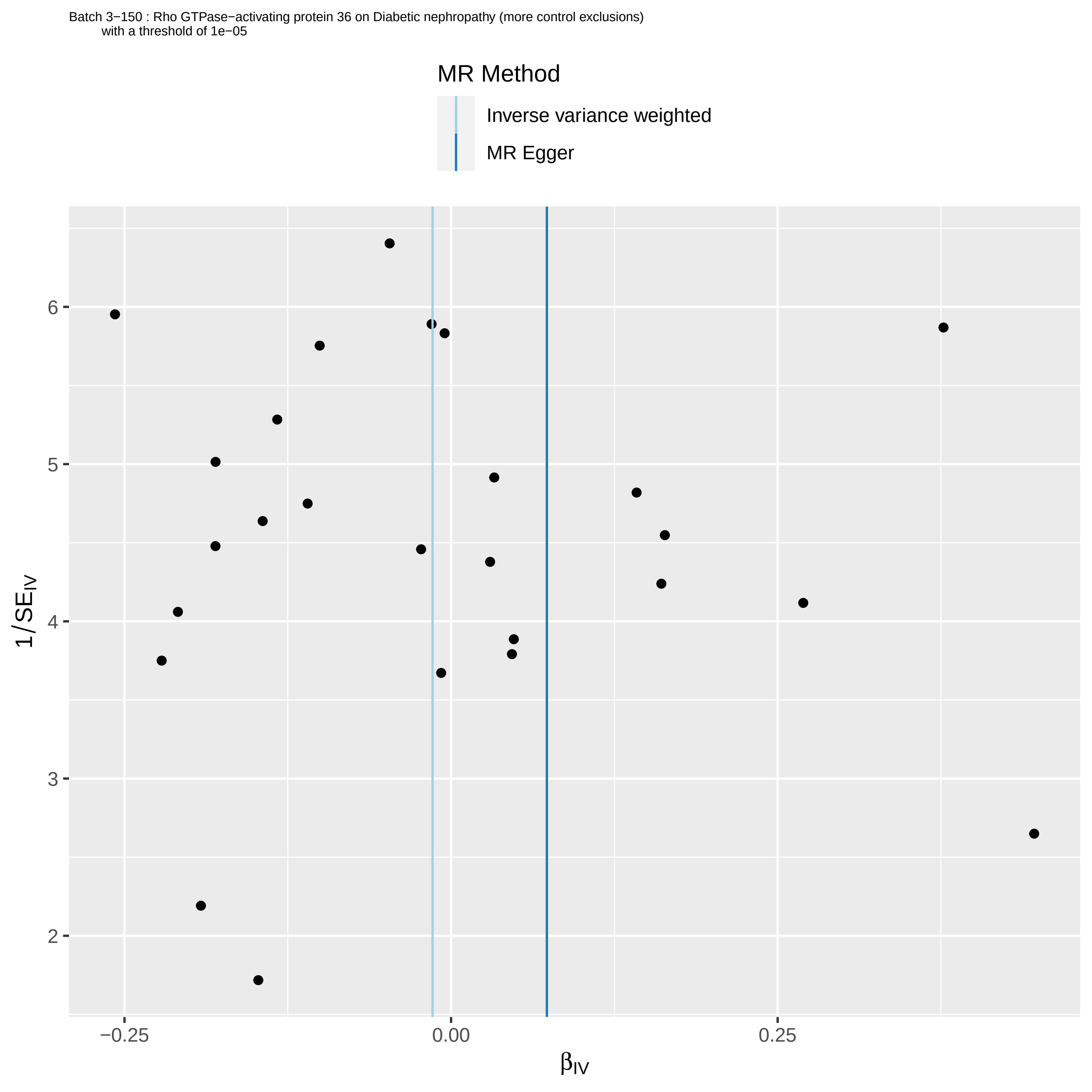

Batch 3−150 : Rho GTPase−activating protein 36 on Diabetic nephropathy (more control exclusions)
with a threshold of 1e−05
MR Method
Inverse variance weighted
MR Egger
1/SE_IV
β_IV
6
5
4
3
2
−0.25
0.00
0.25

Batch 3−151 : Rho GTPase−activating protein 5 on Diabetic nephropathy (more control exclusions)
with a threshold of 1e−05

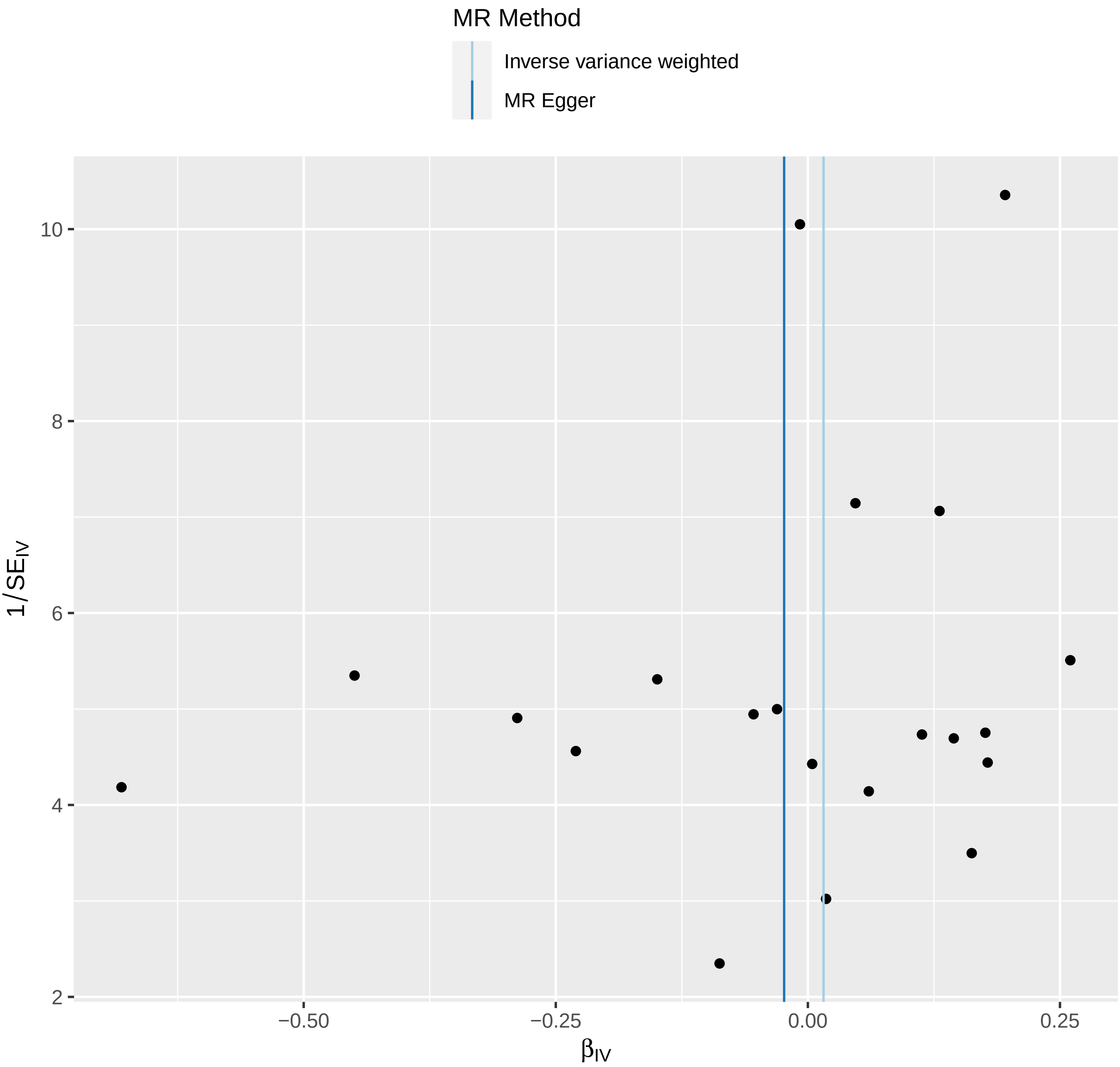

Batch 3−152 : Rho GDP−dissociation inhibitor 1 on Diabetic nephropathy (more control exclusions)
with a threshold of 1e−05

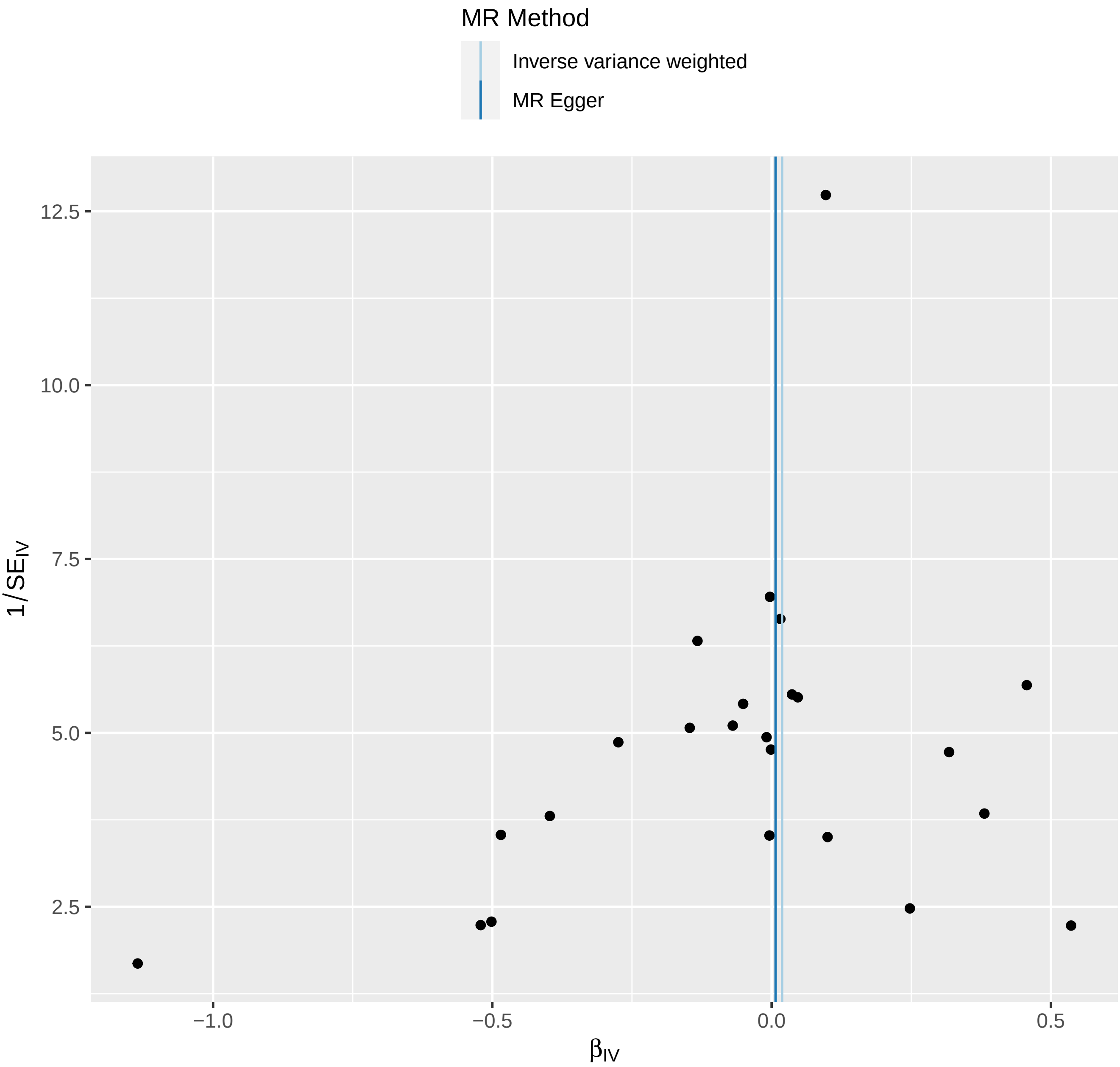

Batch 3−153 : Rho GDP−dissociation inhibitor 2 on Diabetic nephropathy (more control exclusions)
with a threshold of 1e−05

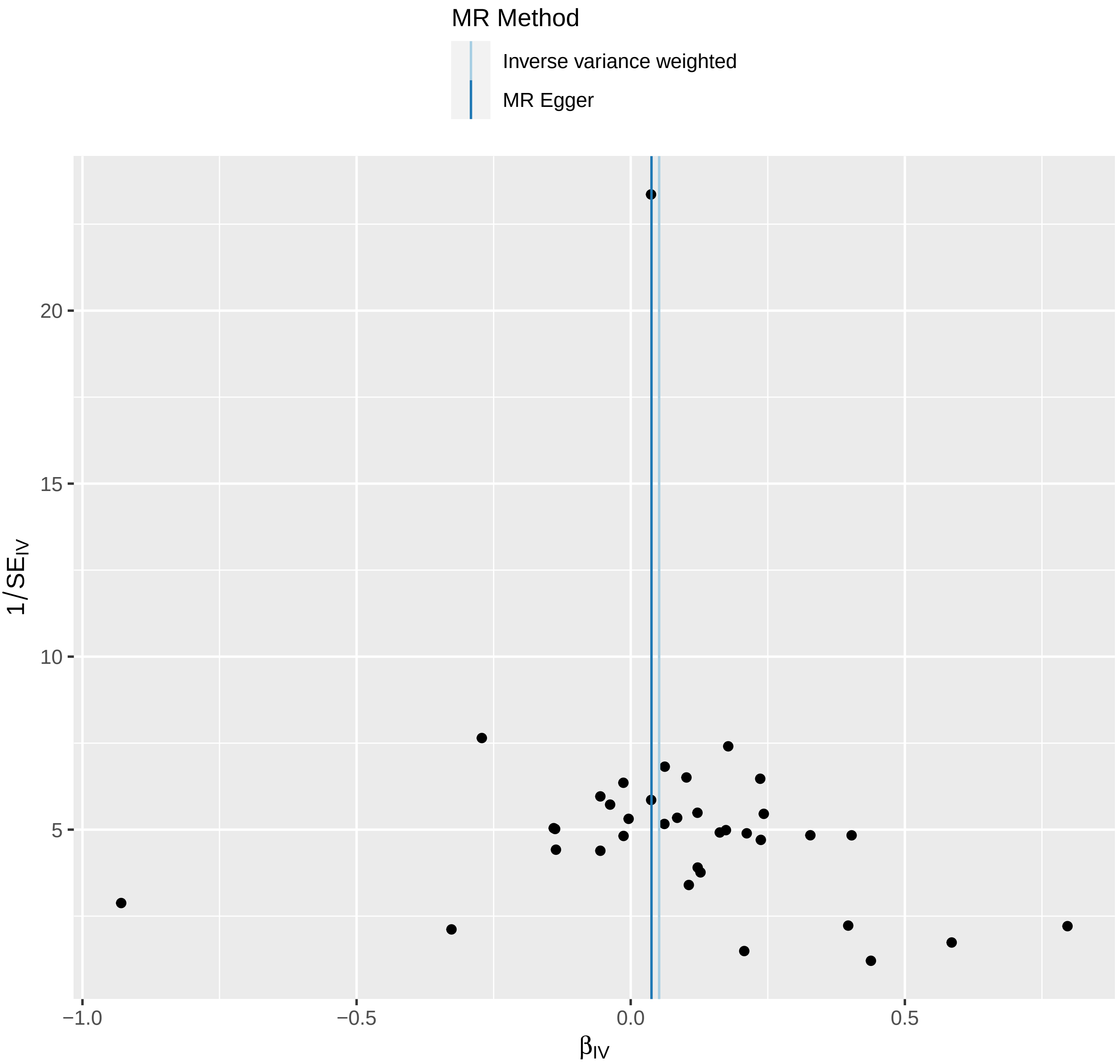

Batch 3−154 : Rho guanine nucleotide exchange factor 10 on Diabetic nephropathy (more control exclusions) with a threshold of 1e−05

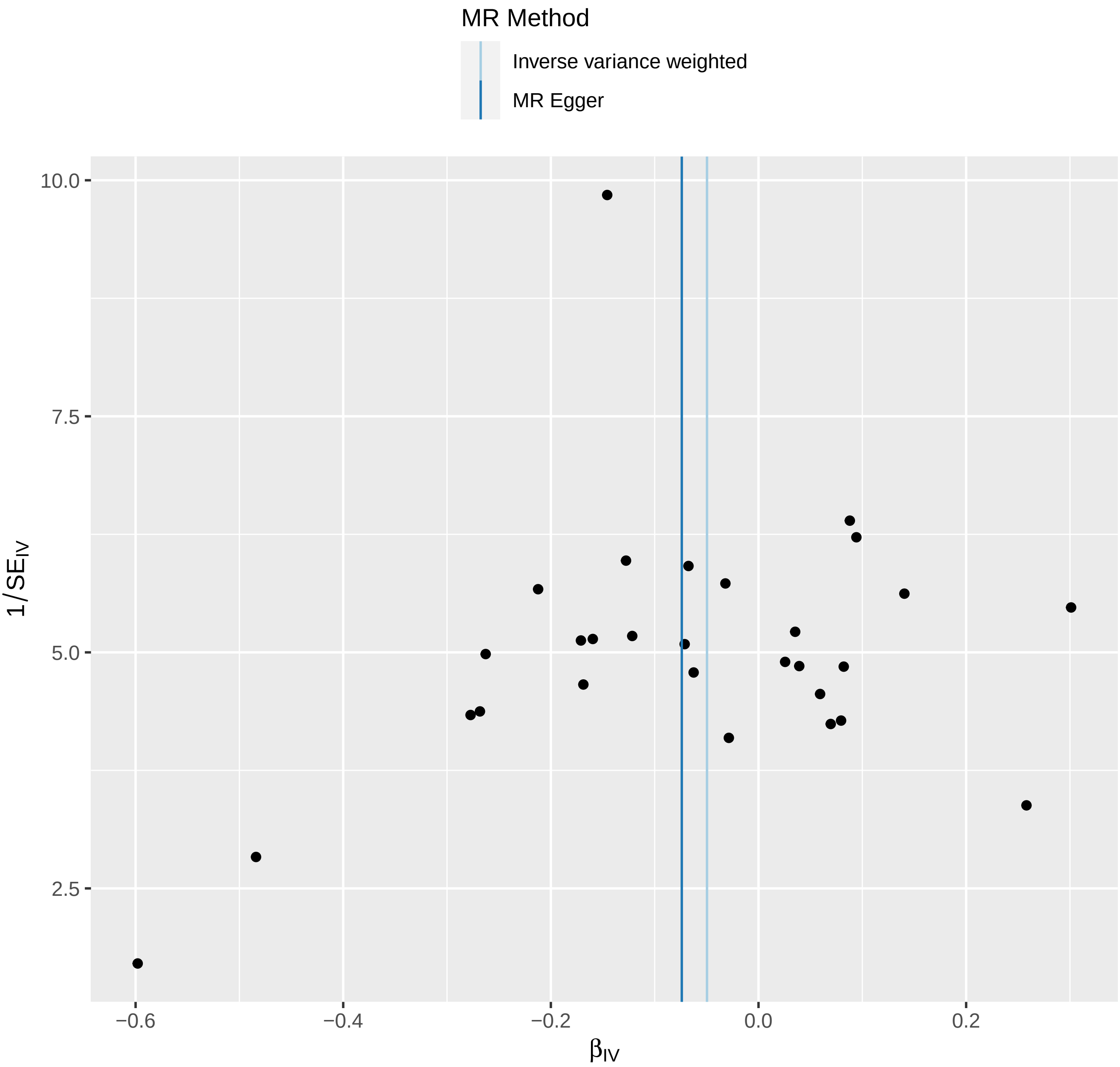

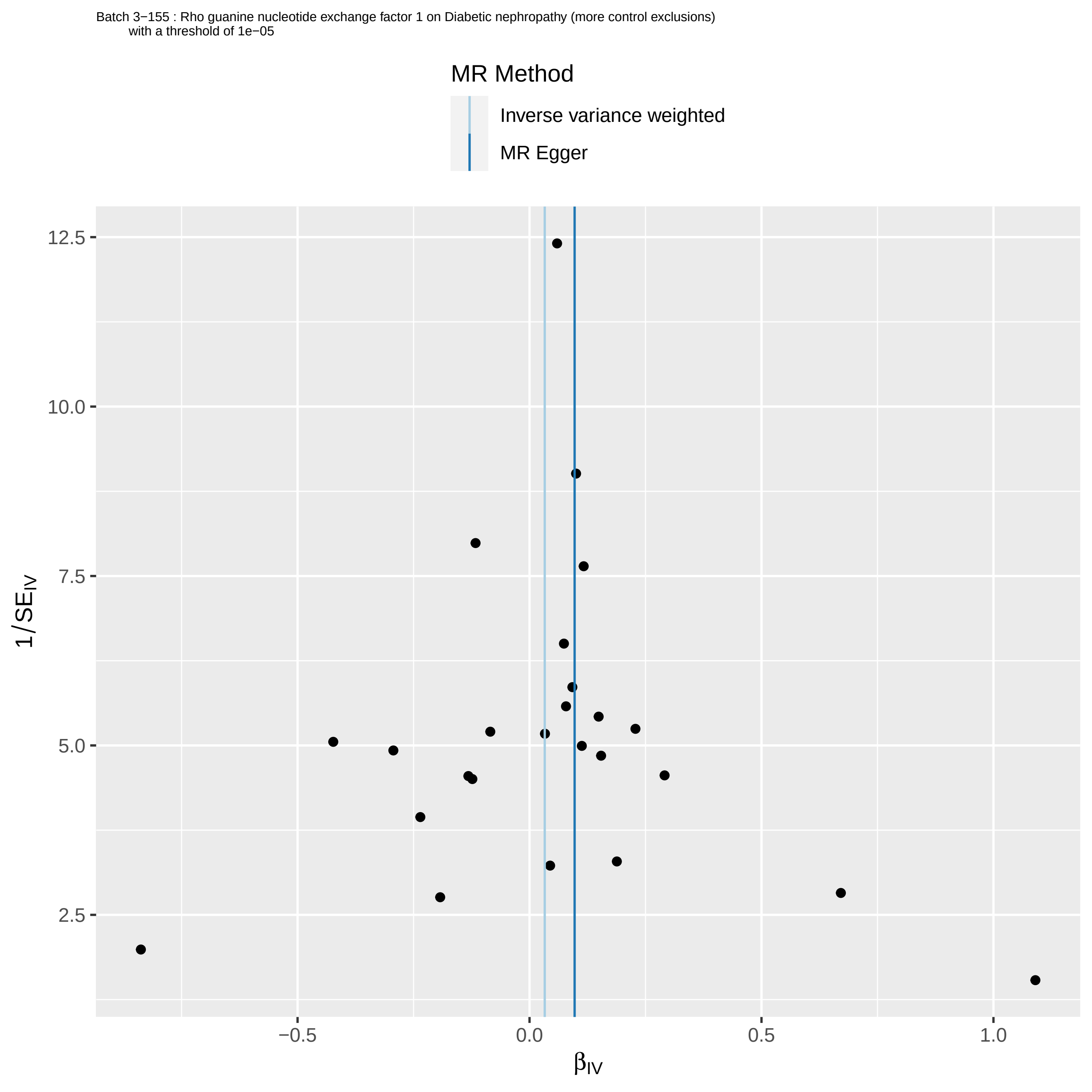

Batch 3−155 : Rho guanine nucleotide exchange factor 1 on Diabetic nephropathy (more control exclusions)
with a threshold of 1e−05
MR Method
Inverse variance weighted
MR Egger
12.5
10.0
7.5
5.0
2.5
1/SE_IV
−0.5
0.0
0.5
1.0
β_IV

Batch 3−156 : Rho guanine nucleotide exchange factor 2 on Diabetic nephropathy (more control exclusions) with a threshold of 1e−05

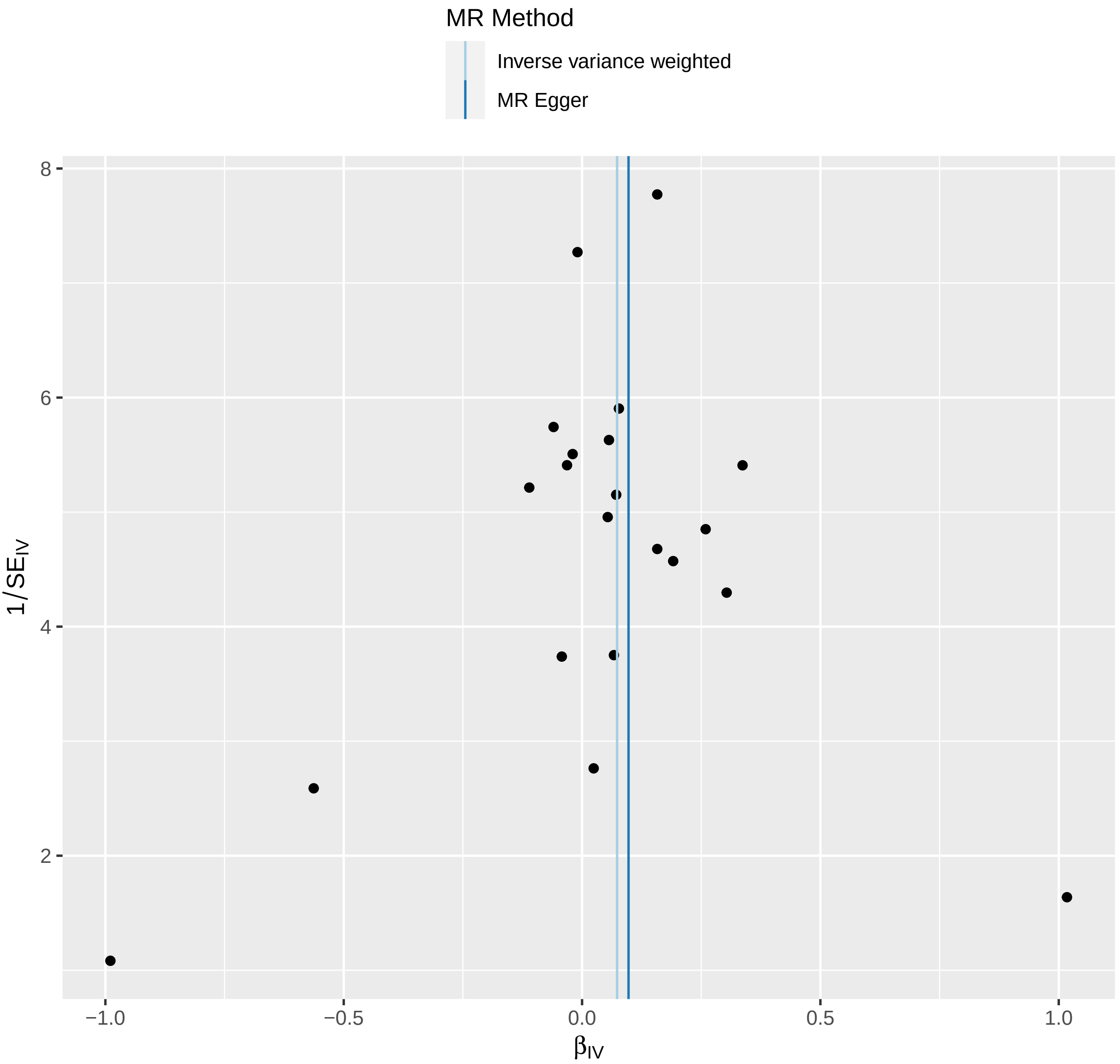

Batch 3−157 : Rho guanine nucleotide exchange factor 25 on Diabetic nephropathy (more control exclusions) with a threshold of 1e−05

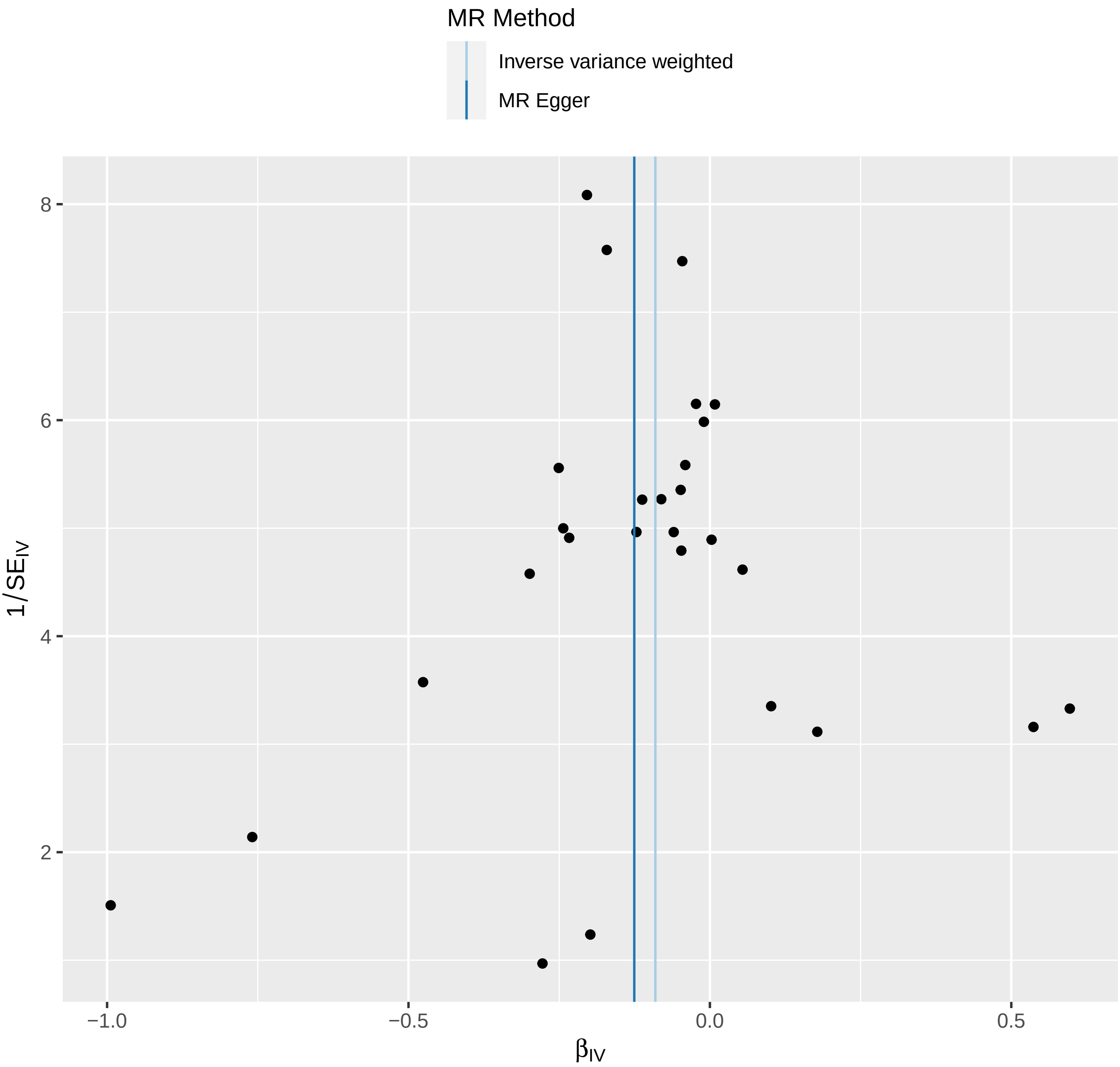

Batch 3−158 : Rho guanine nucleotide exchange factor 7 on Diabetic nephropathy (more control exclusions)
with a threshold of 1e−05

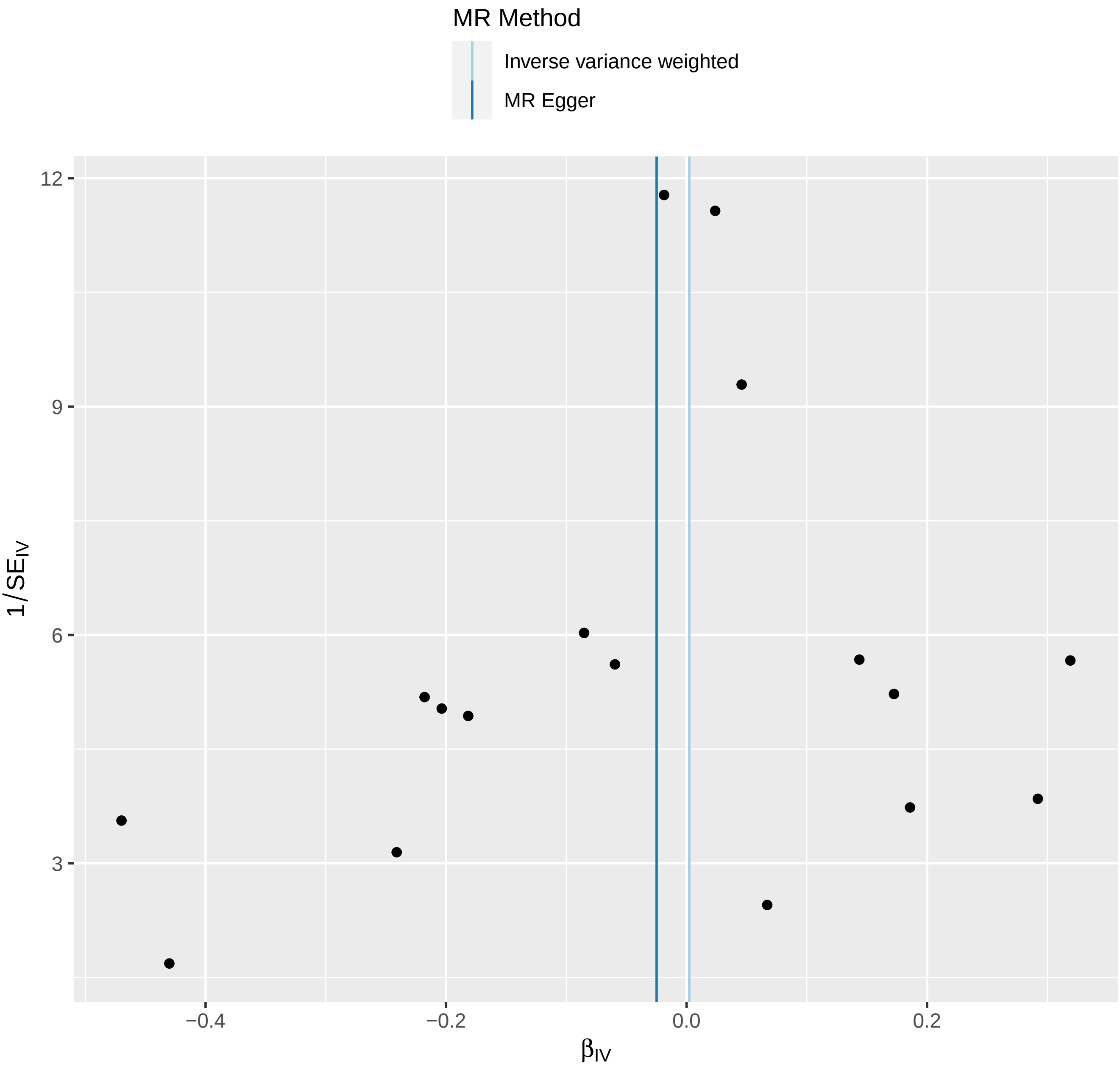

Batch 3−159 : AT−rich interactive domain−containing protein 3A on Diabetic nephropathy (more control exclusions) with a threshold of 1e−05

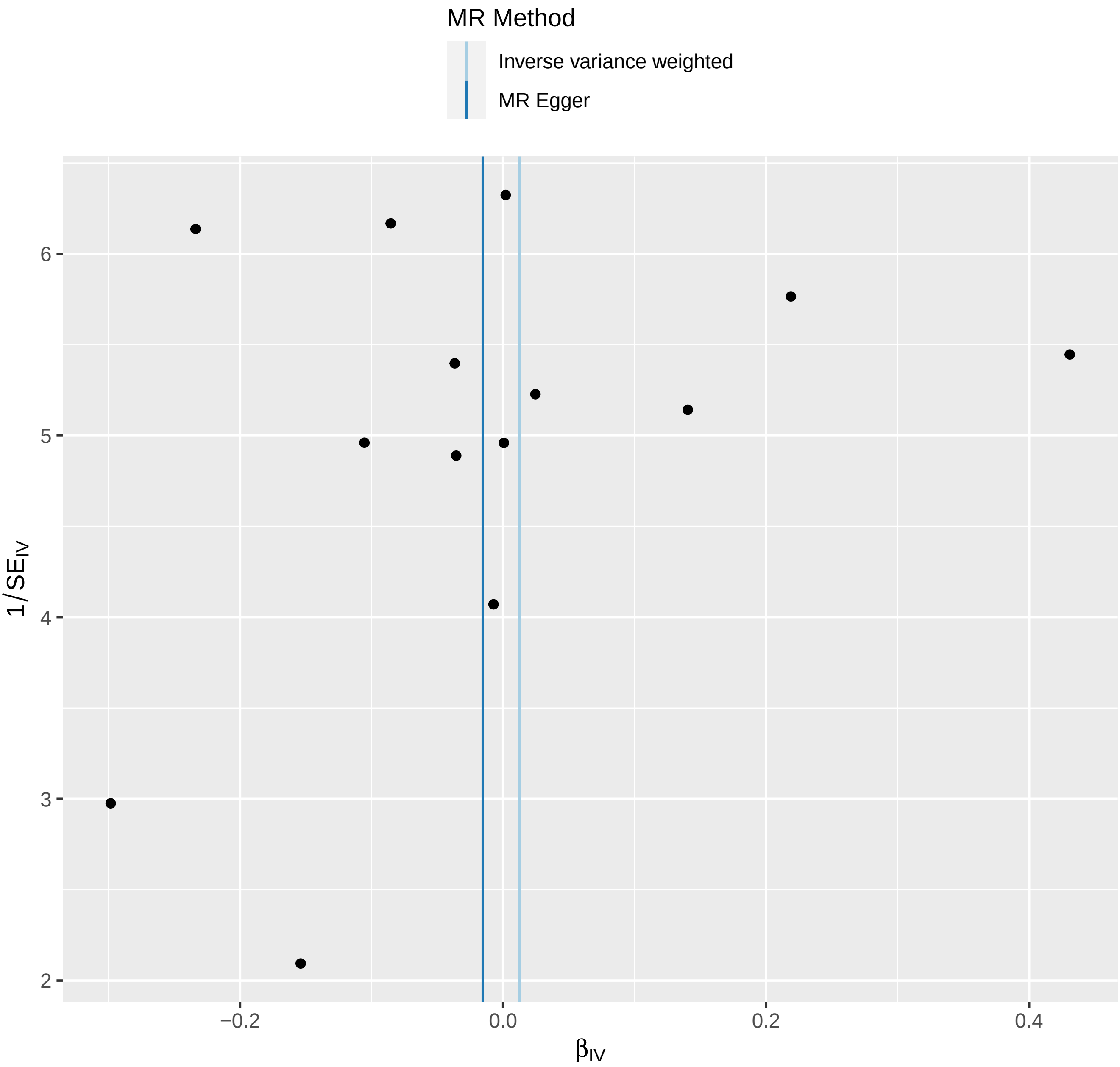

Batch 3−160 : ADP−ribosylation factor−like protein 11 on Diabetic nephropathy (more control exclusions)
with a threshold of 1e−05

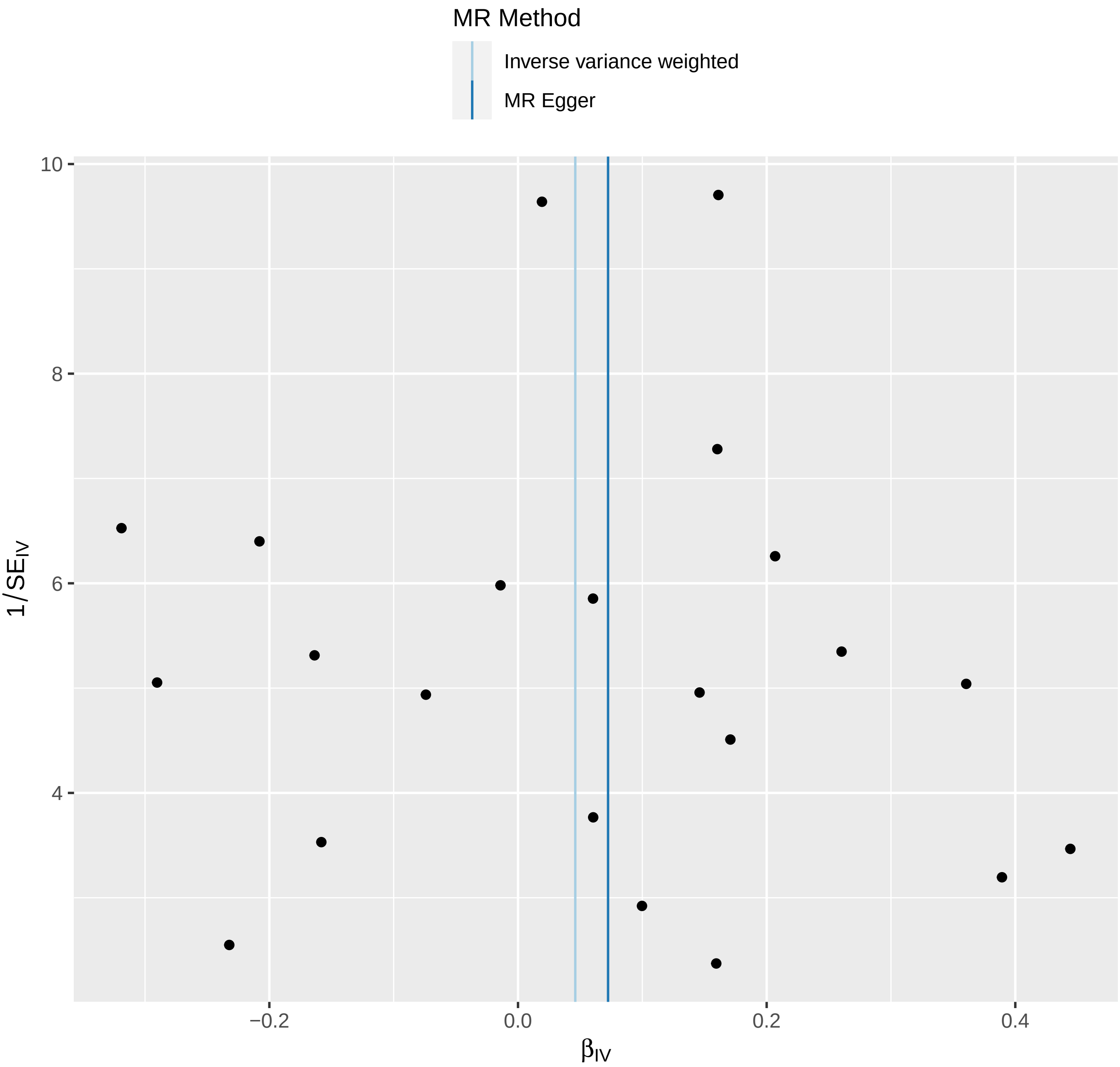

Batch 3−161 : ADP−ribosylation factor−like protein 1 on Diabetic nephropathy (more control exclusions)
with a threshold of 1e−05

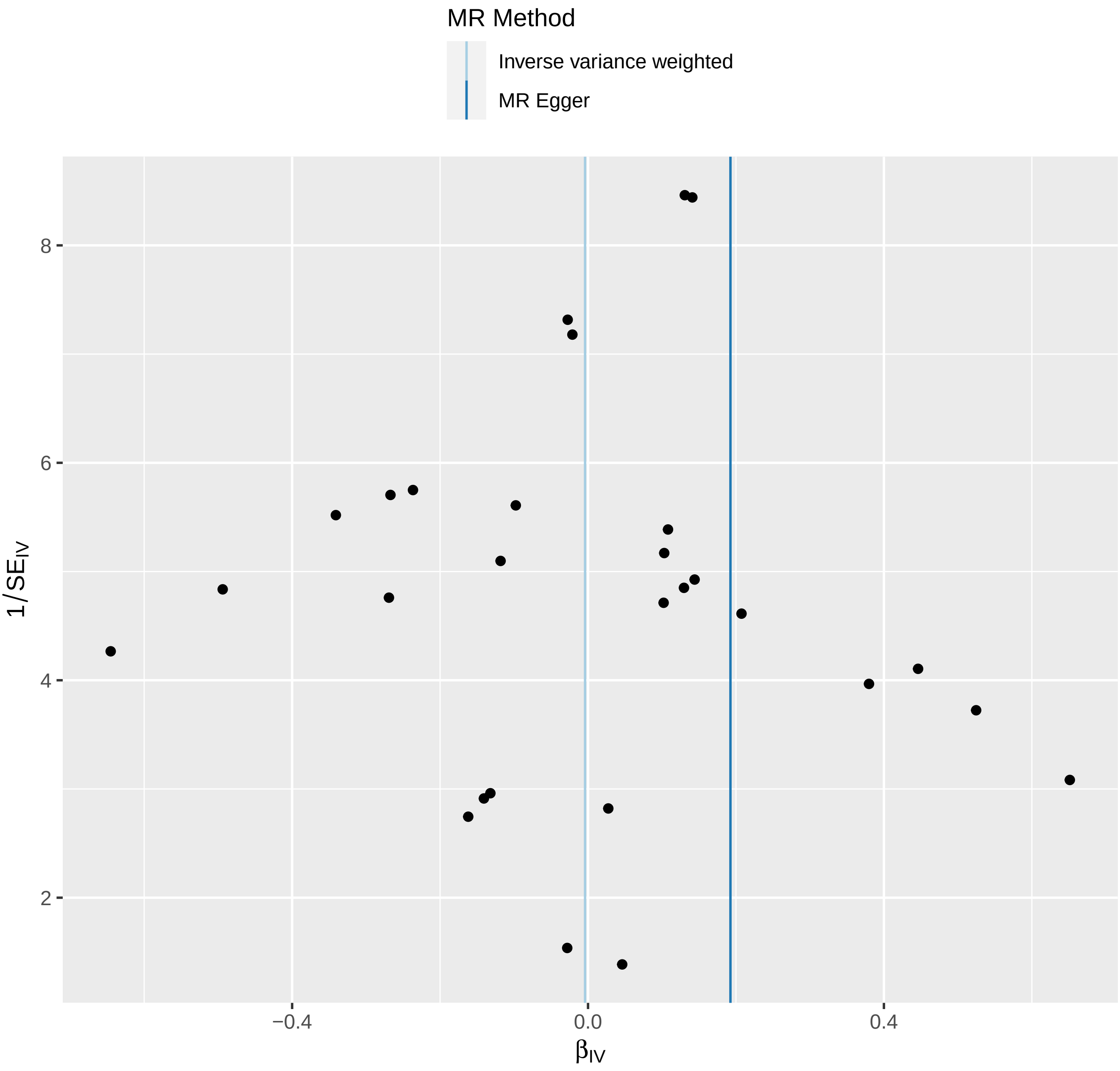

Batch 3−162 : ADP−ribosylation factor−like protein 3 on Diabetic nephropathy (more control exclusions) with a threshold of 1e−05

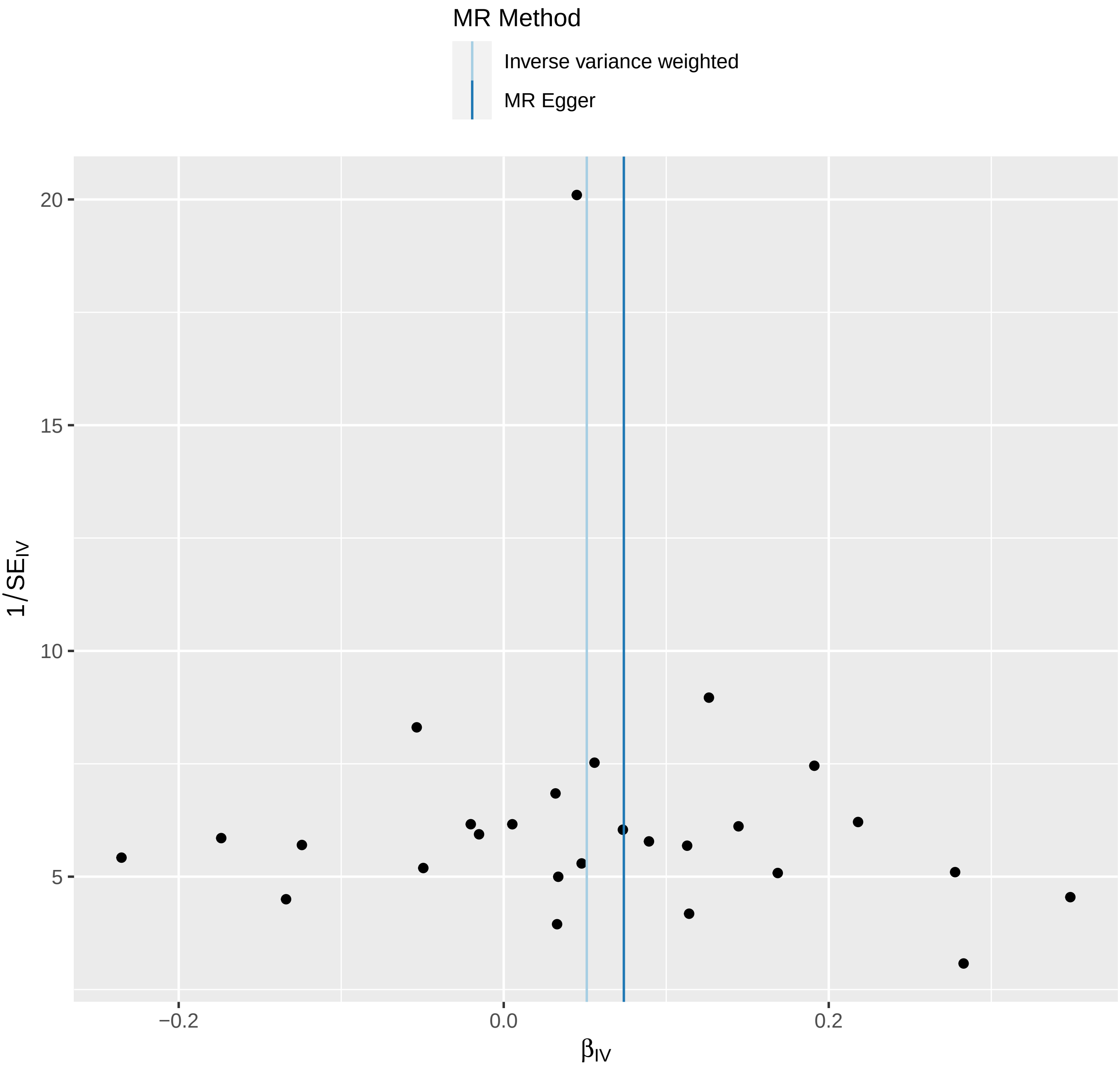

Batch 3−163 : Armadillo repeat−containing protein 5 on Diabetic nephropathy (more control exclusions)
with a threshold of 1e−05

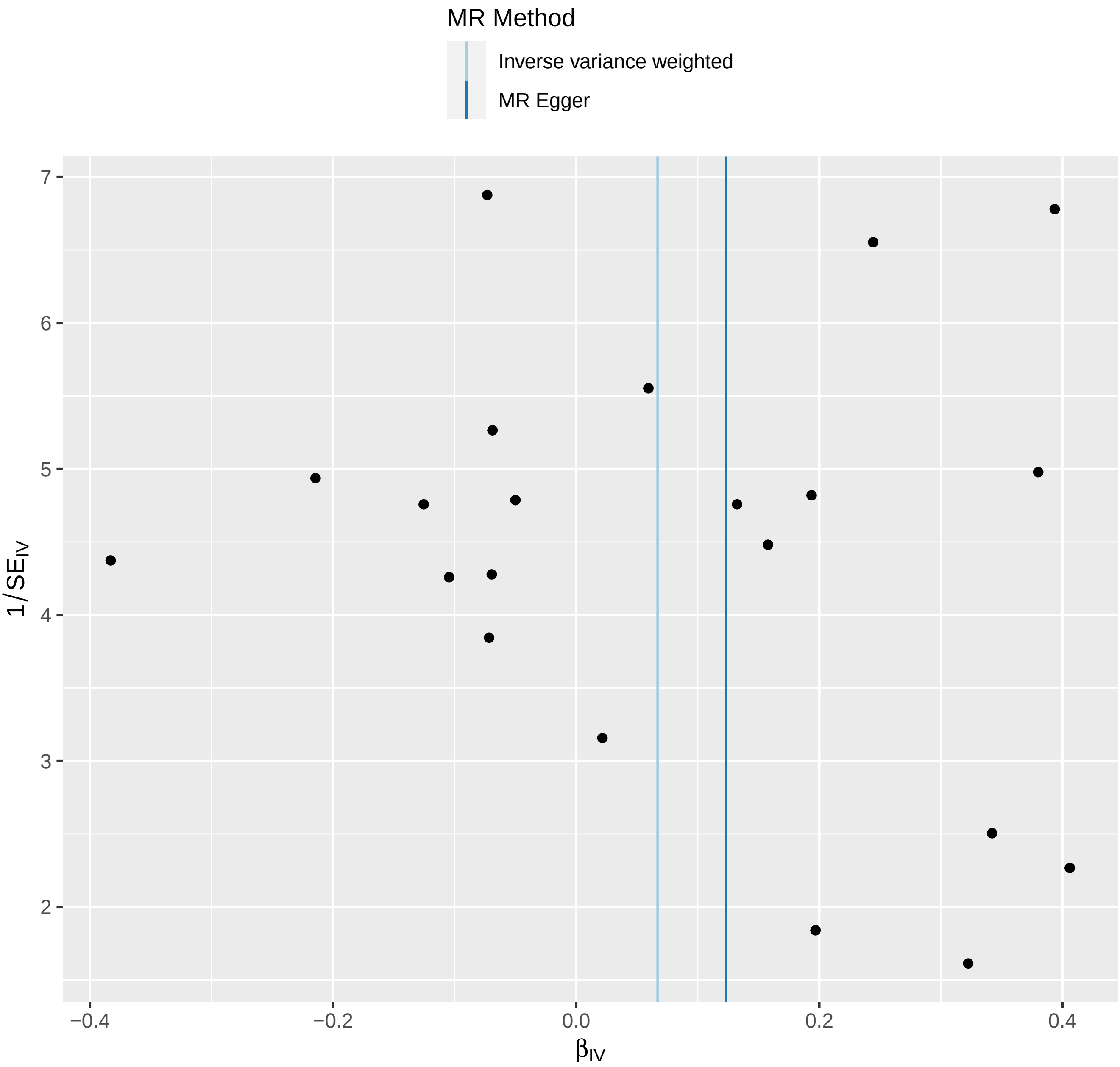

Batch 3−164 : Armadillo repeat−containing protein 5 on Diabetic nephropathy (more control exclusions)
with a threshold of 1e−05

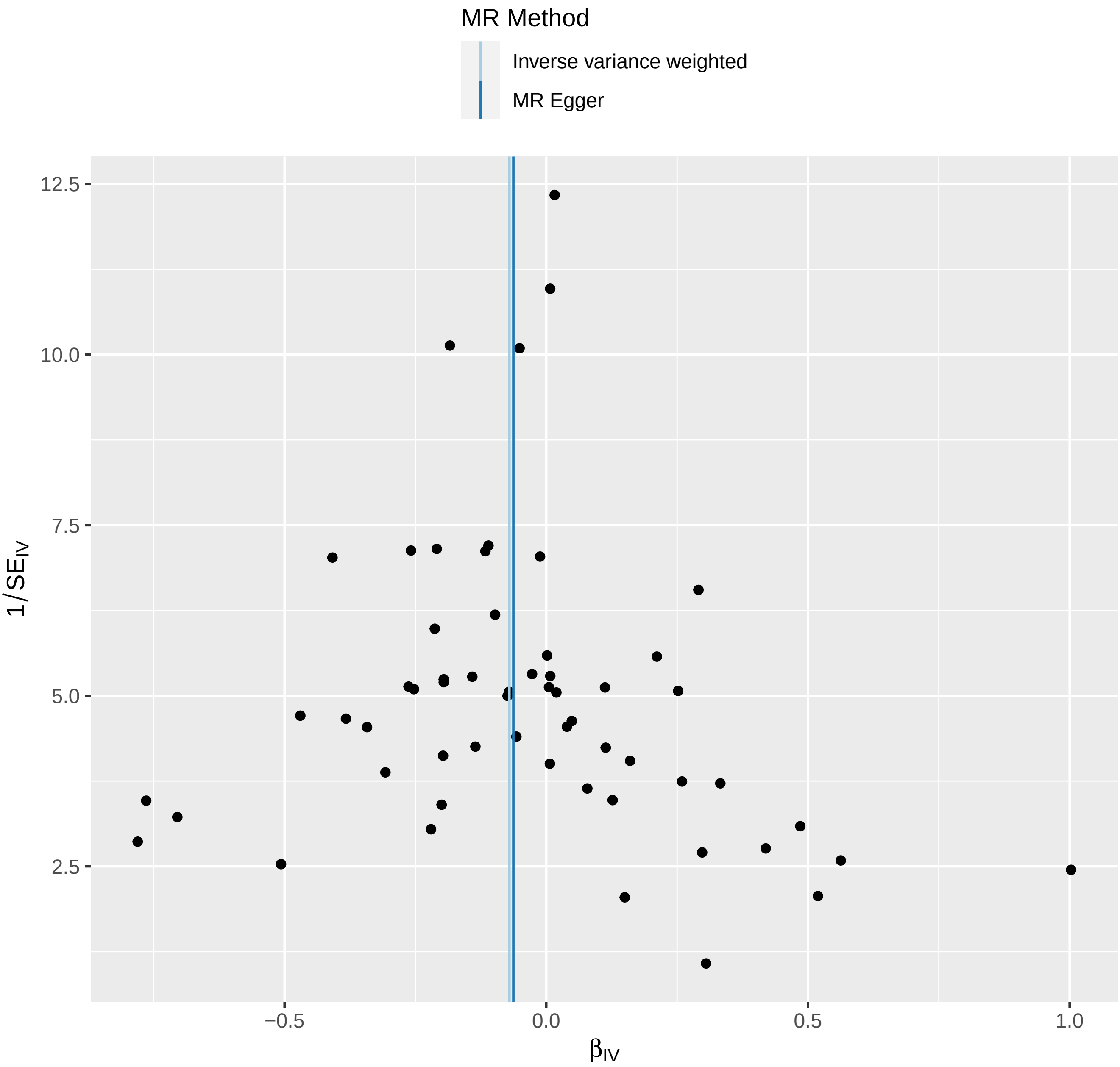

Batch 3−165 : Armadillo repeat−containing protein 5 on Diabetic nephropathy (more control exclusions)
with a threshold of 1e−05

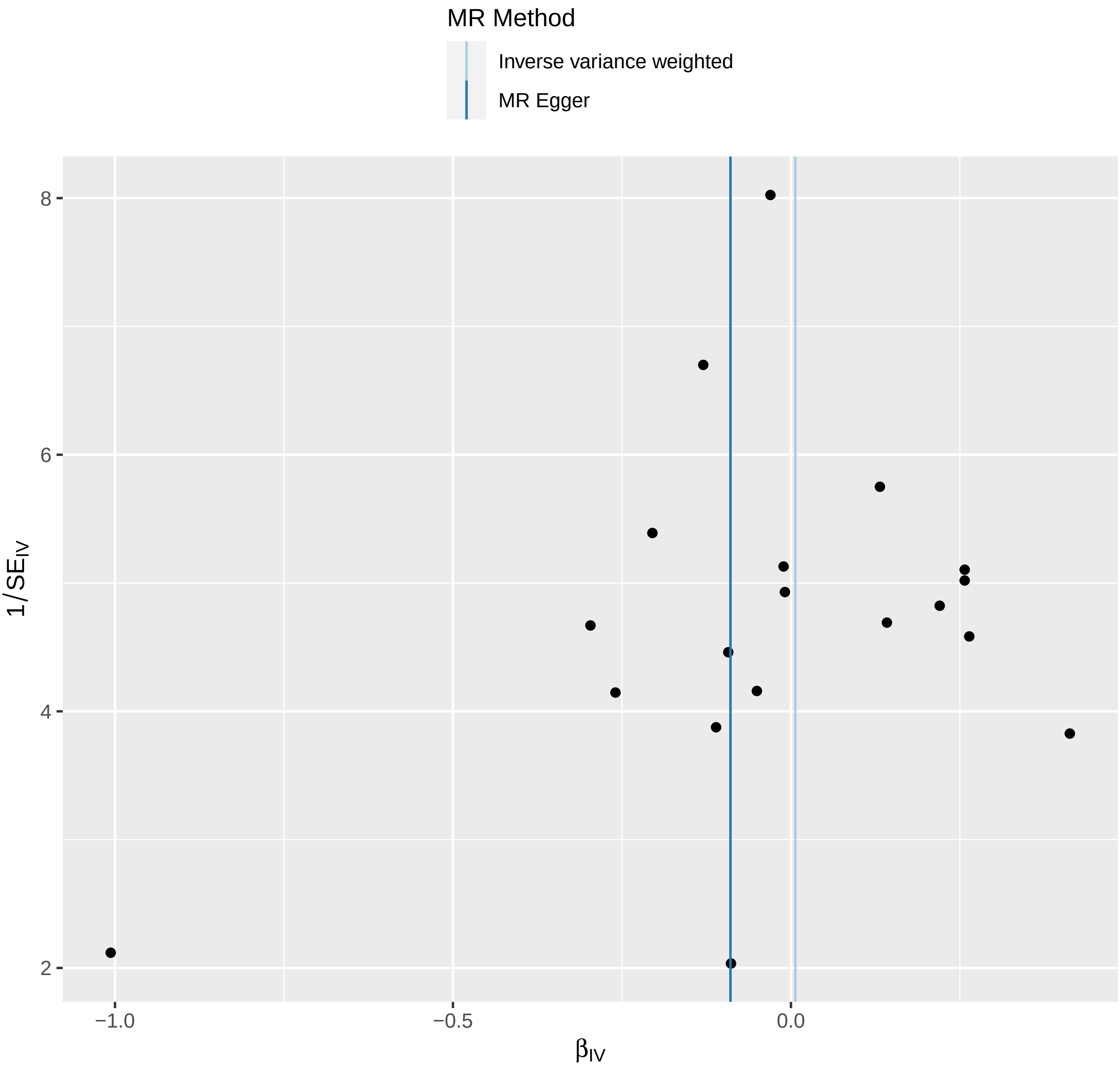

Batch 3−166 : Actin−related protein 2/3 complex subunit 3 on Diabetic nephropathy (more control exclusions)
with a threshold of 1e−05

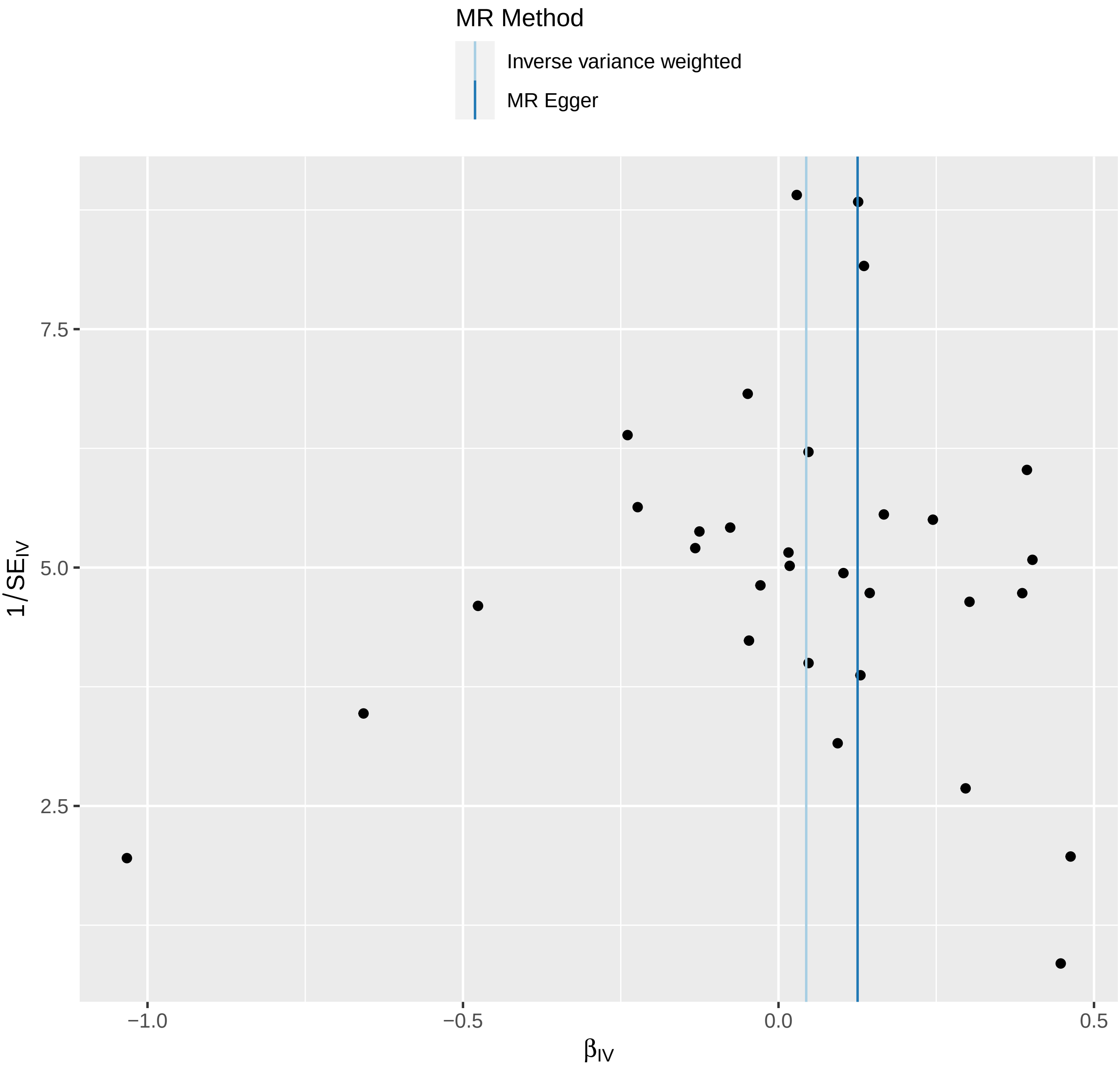

Batch 3−167 : cAMP−regulated phosphoprotein 19 on Diabetic nephropathy (more control exclusions)
with a threshold of 1e−05

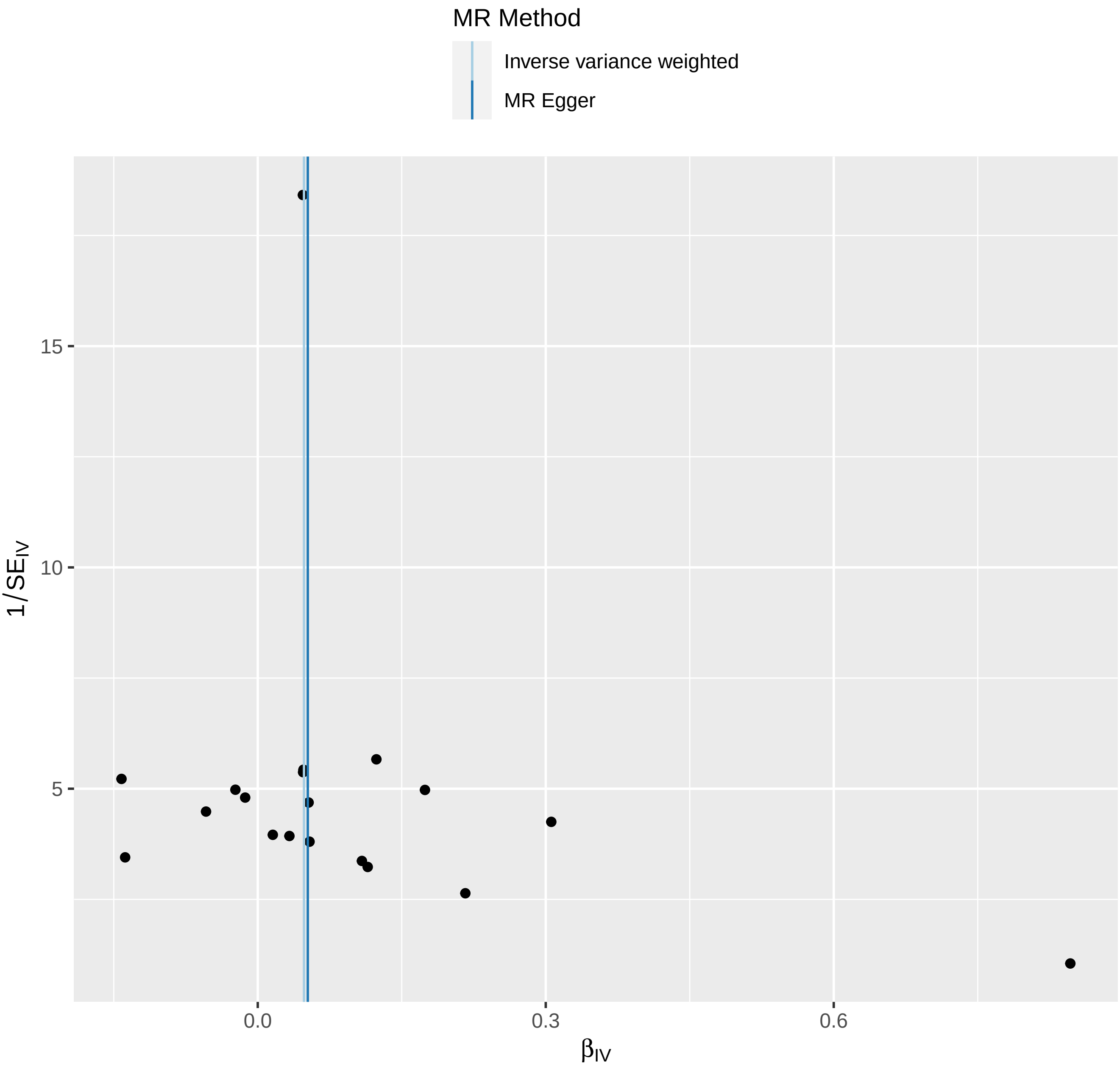

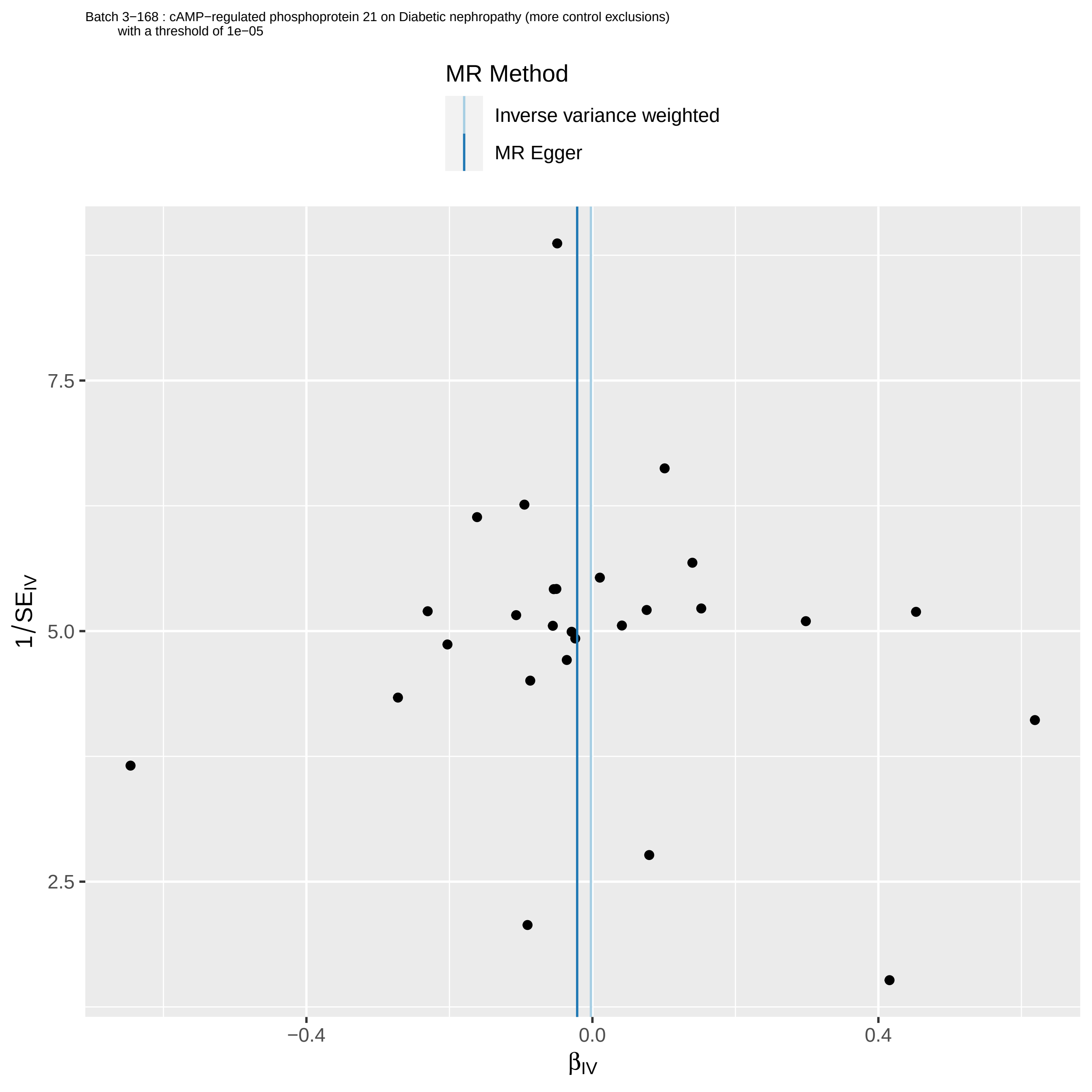
Batch 3−168 : cAMP−regulated phosphoprotein 21 on Diabetic nephropathy (more control exclusions)
with a threshold of 1e−05
MR Method
Inverse variance weighted
MR Egger
7.5
5.0
2.5
1/SE_IV
−0.4
0.0
0.4
β_IV

Batch 3−169 : Beta−arrestin−1 on Diabetic nephropathy (more control exclusions)
with a threshold of 1e−05

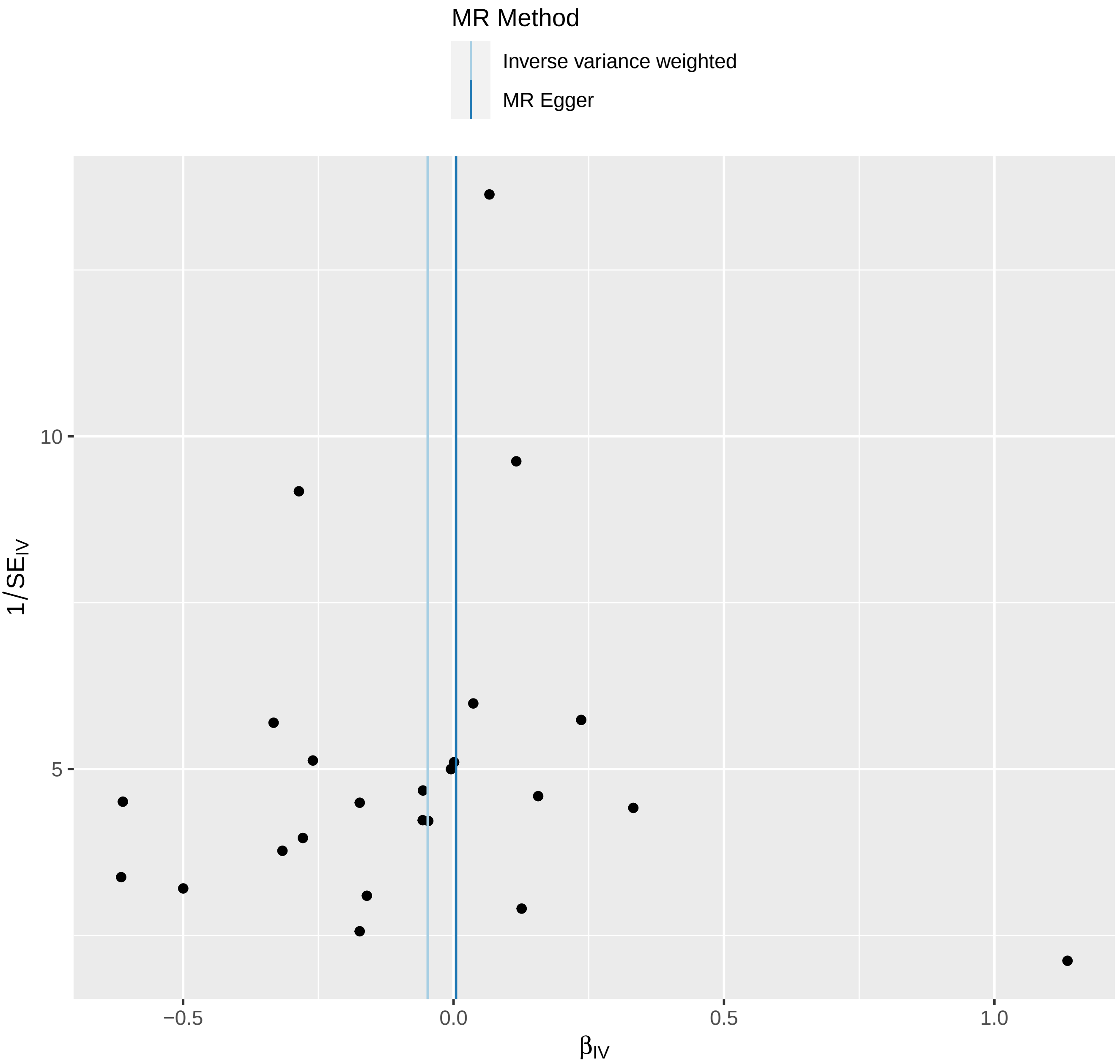

Batch 3−170 : Arrestin domain−containing protein 3 on Diabetic nephropathy (more control exclusions)
with a threshold of 1e−05

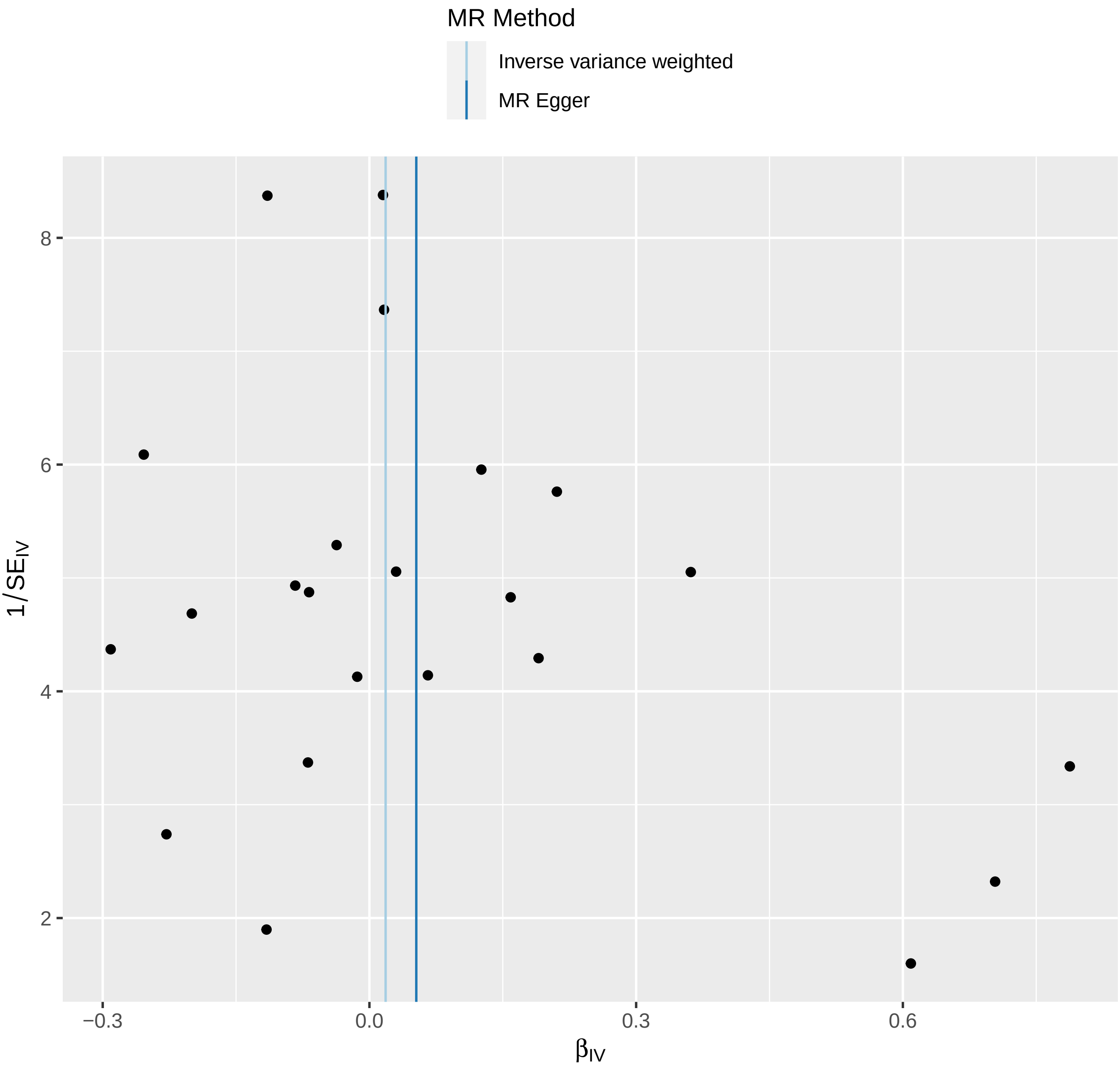

Batch 3−171 : Arylsulfatase A on Diabetic nephropathy (more control exclusions)
with a threshold of 1e−05

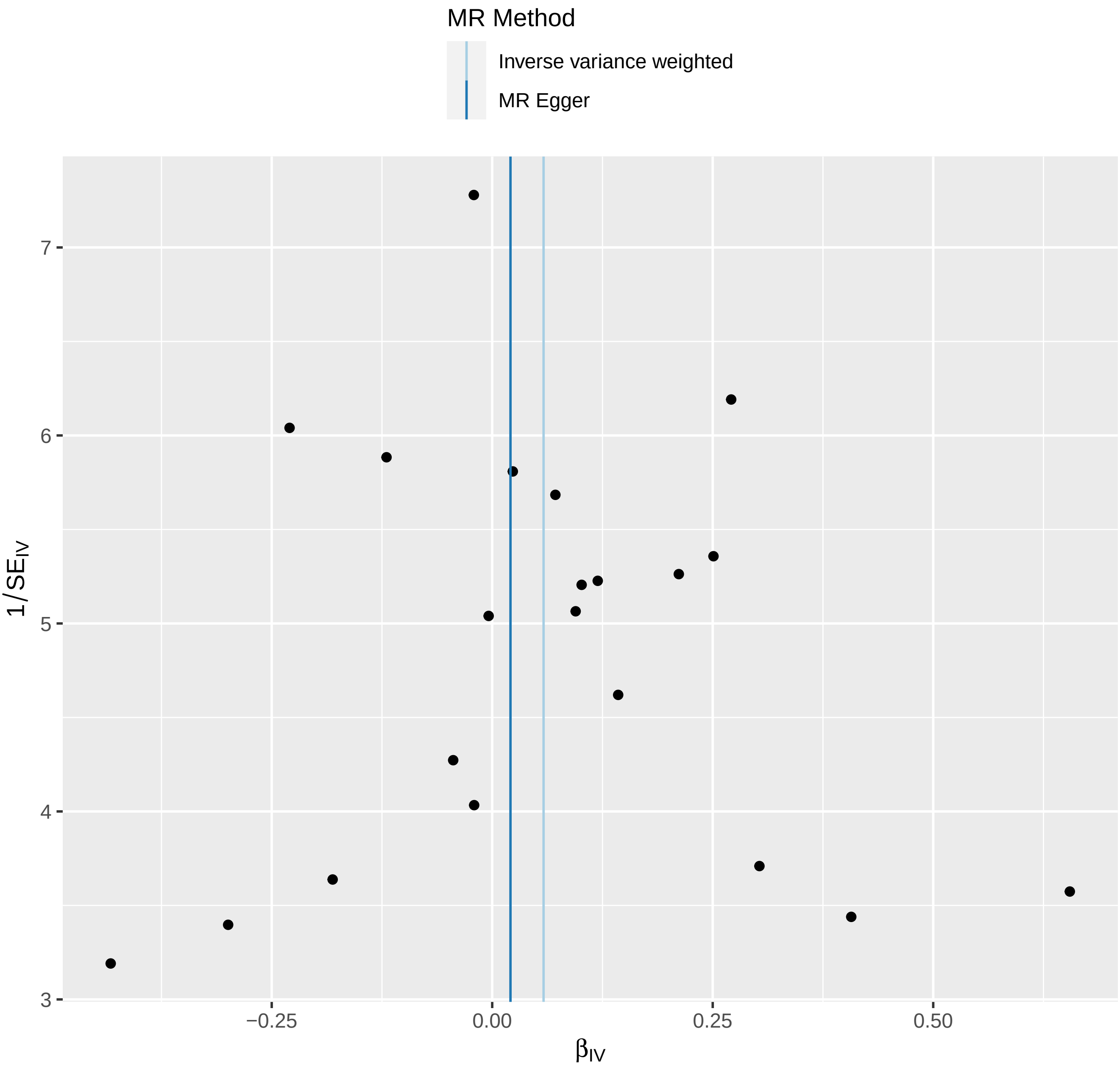

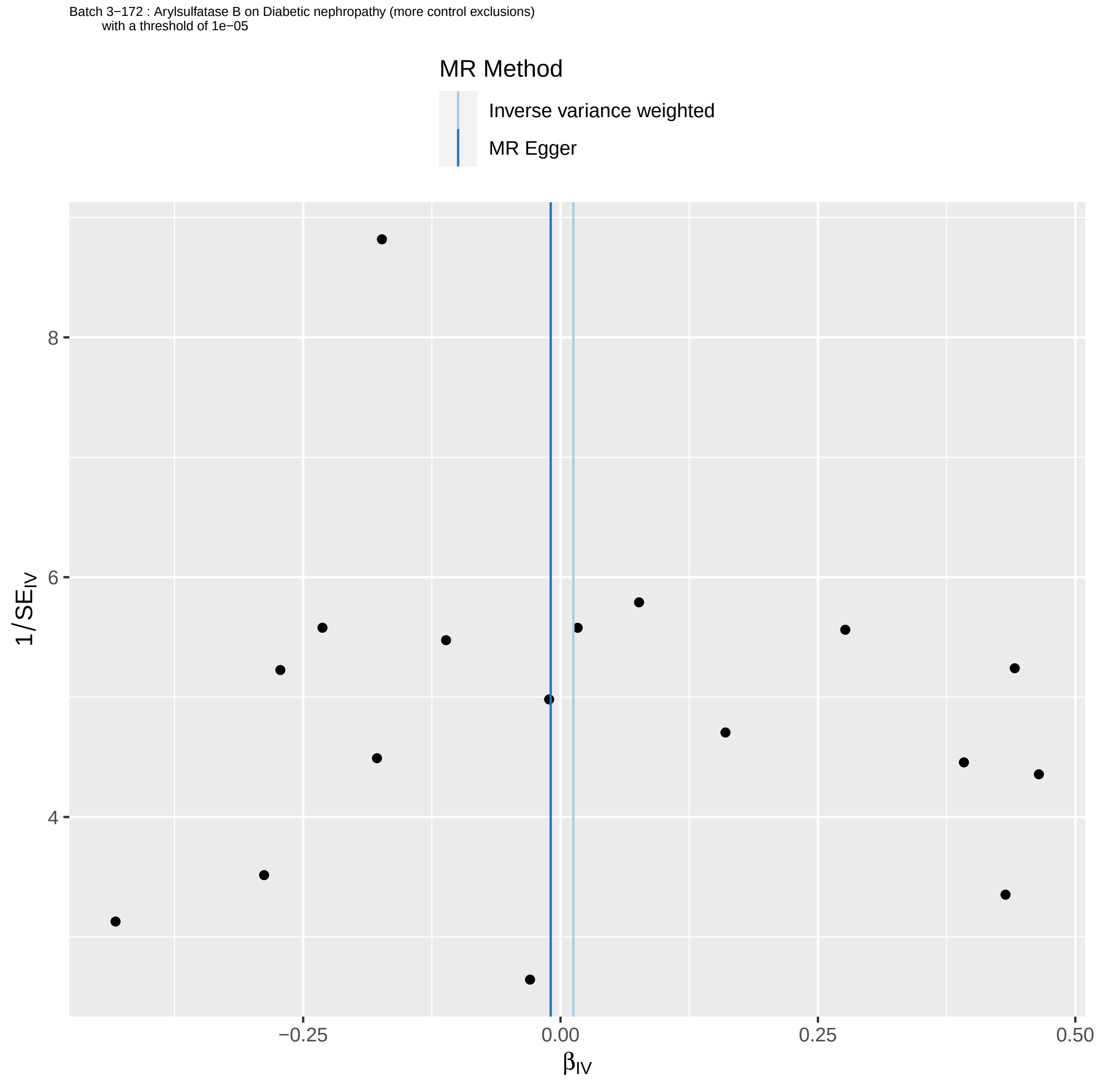
Batch 3−172 : Arylsulfatase B on Diabetic nephropathy (more control exclusions)
with a threshold of 1e−05
MR Method
Inverse variance weighted
MR Egger
8
6
4
1/SE$_{IV}$
−0.25
0.00
0.25
0.50
β_{IV}

Batch 3−173 : Arylsulfatase K on Diabetic nephropathy (more control exclusions)
with a threshold of 1e−05

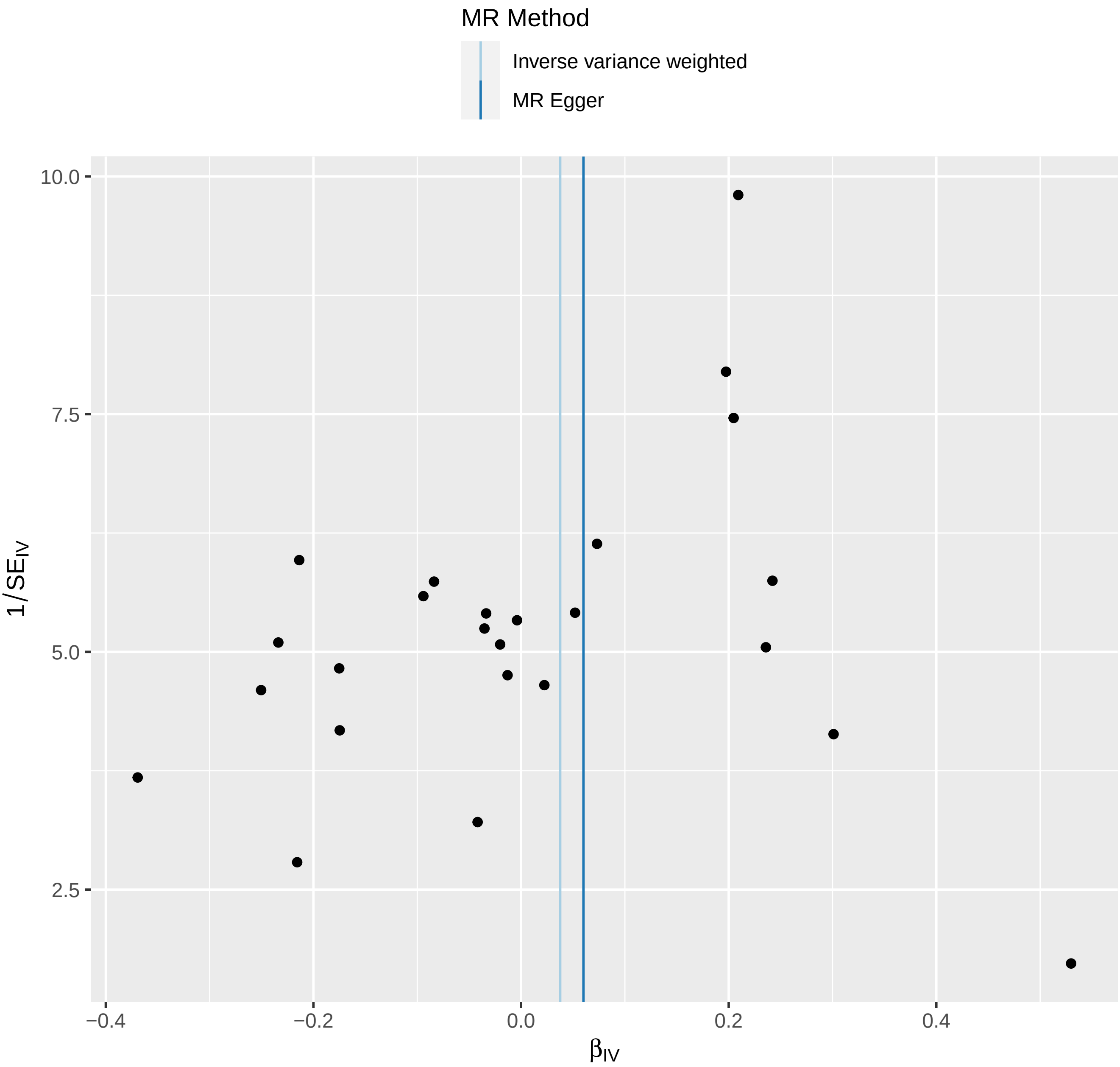

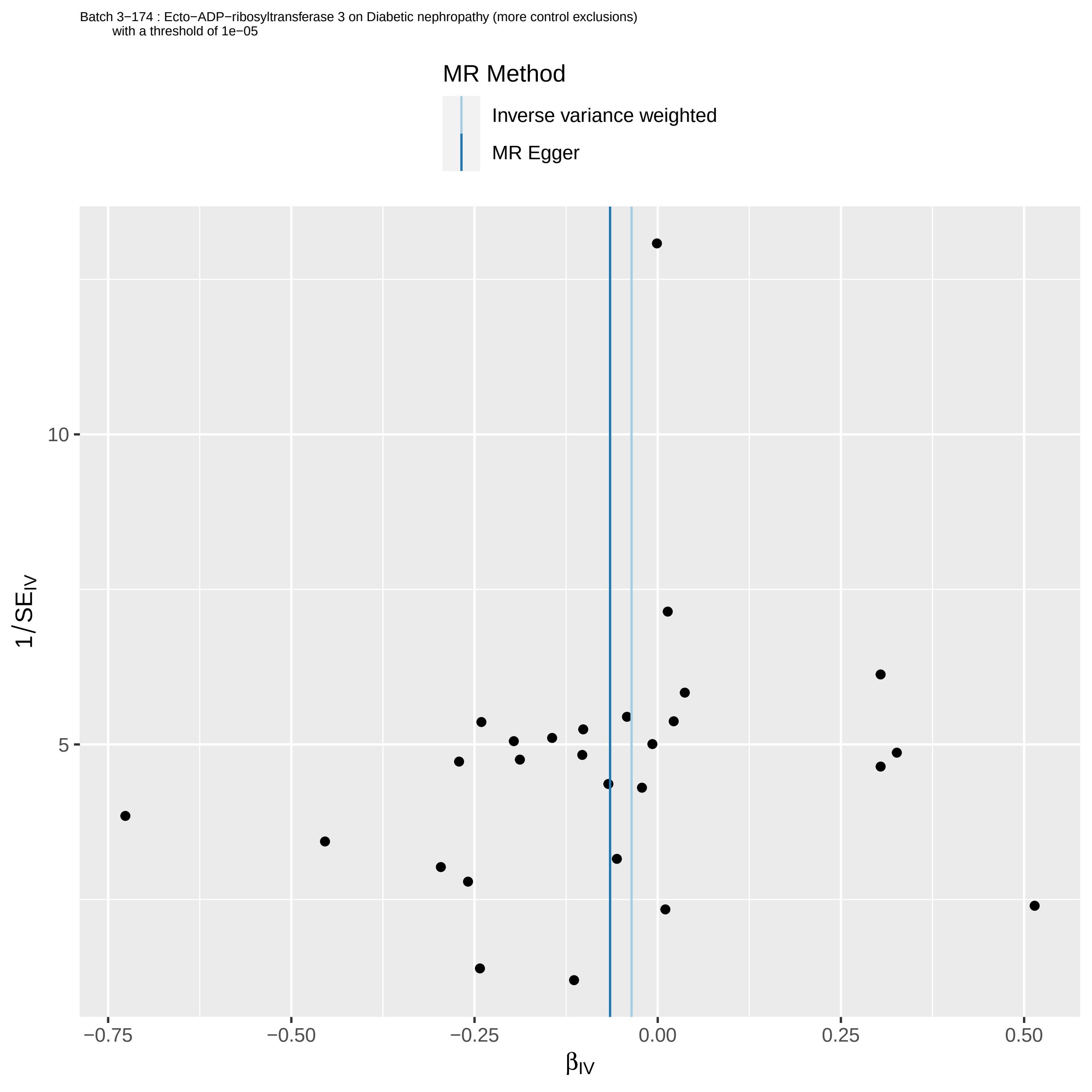
Batch 3−174 : Ecto−ADP−ribosyltransferase 3 on Diabetic nephropathy (more control exclusions)
with a threshold of 1e−05
MR Method
Inverse variance weighted
MR Egger
10
5
1/SE_IV
−0.75
−0.50
−0.25
0.00
0.25
0.50
β_IV

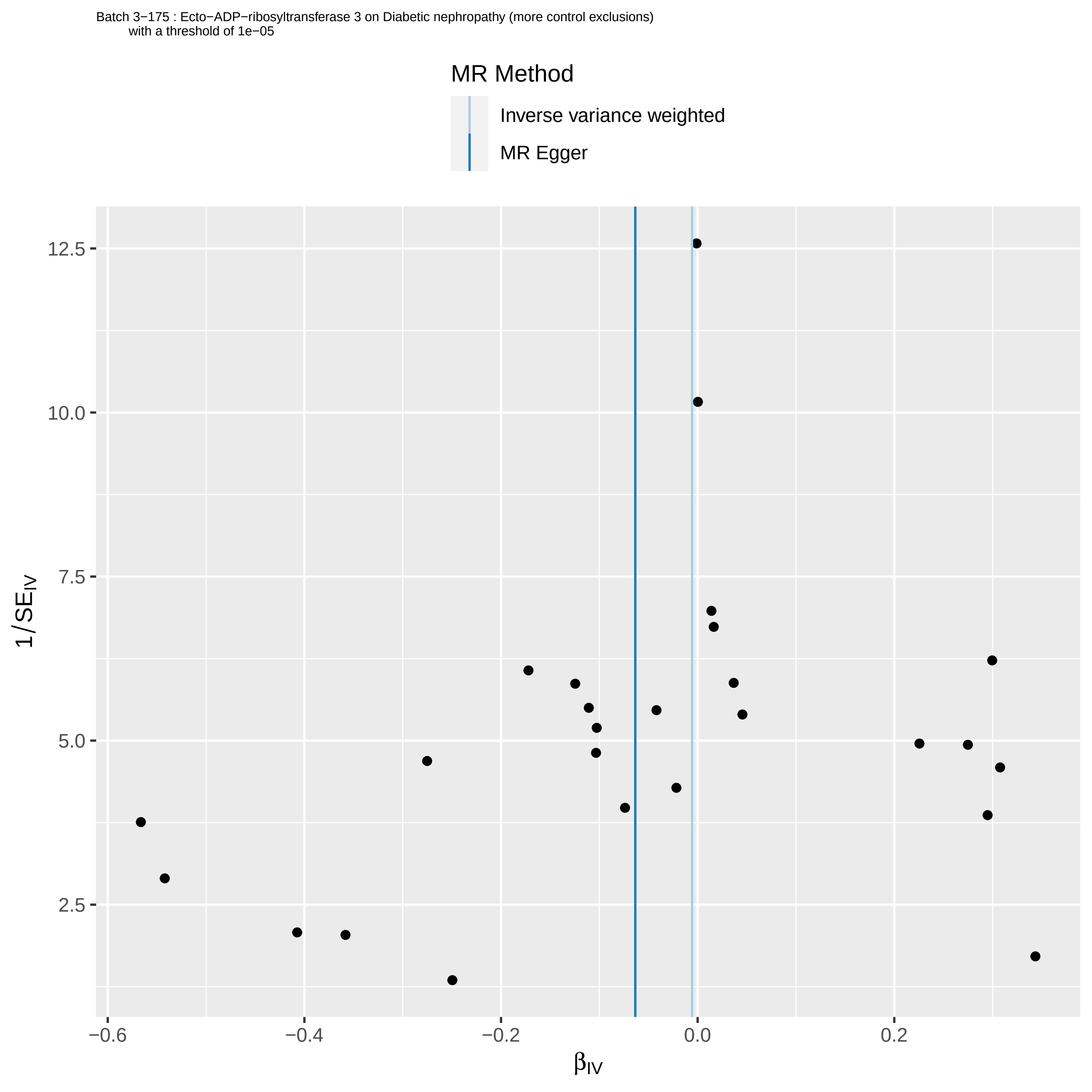

Batch 3−175 : Ecto−ADP−ribosyltransferase 3 on Diabetic nephropathy (more control exclusions)
with a threshold of 1e−05
MR Method
Inverse variance weighted
MR Egger
12.5
10.0
7.5
5.0
2.5
1/SE_IV
−0.6
−0.4
−0.2
0.0
0.2
β_IV

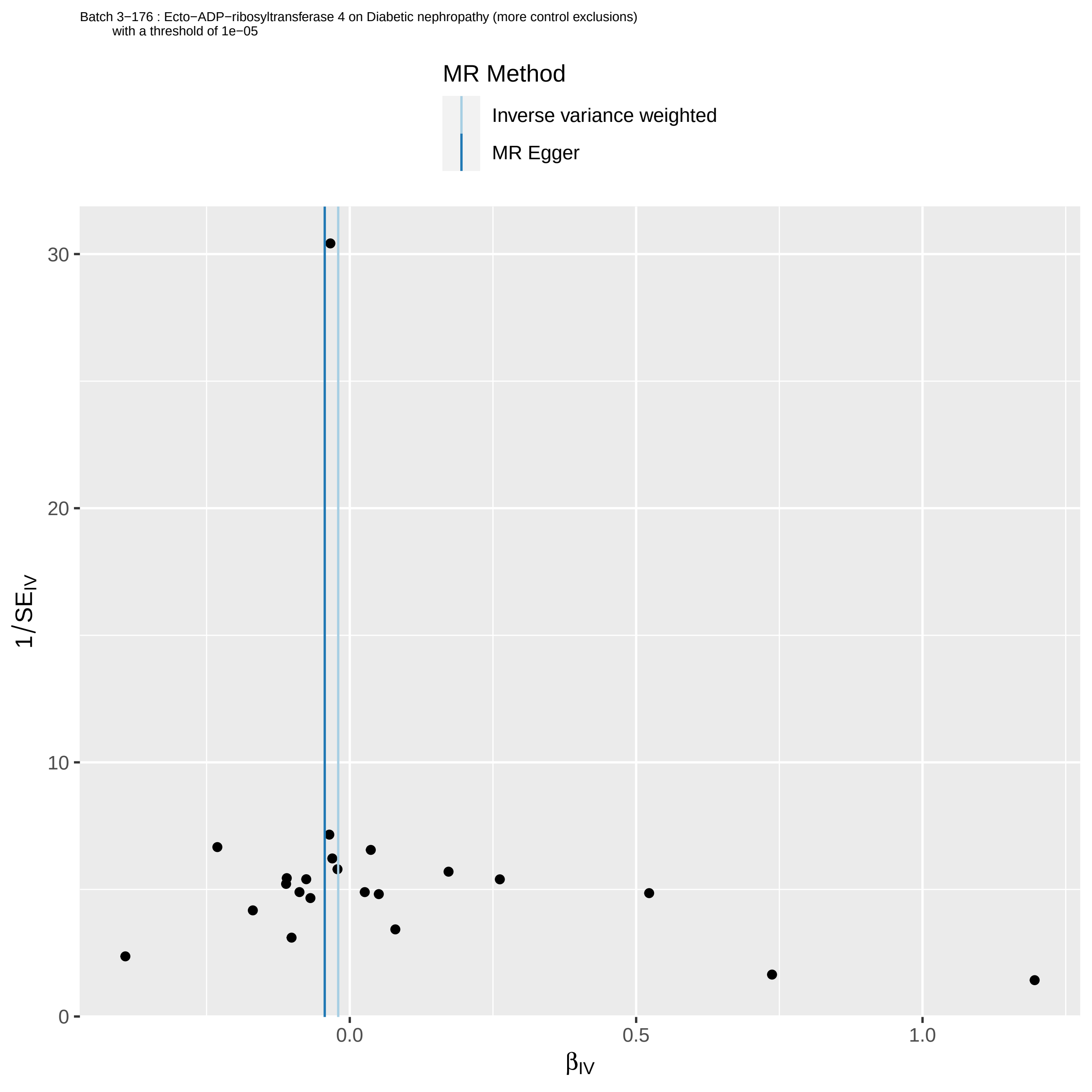
Batch 3−176 : Ecto−ADP−ribosyltransferase 4 on Diabetic nephropathy (more control exclusions)
with a threshold of 1e−05
MR Method
Inverse variance weighted
MR Egger
30
20
10
0
0.0
0.5
1.0
1/SE_IV
β_IV

Batch 3−177 : Artemin on Diabetic nephropathy (more control exclusions)
with a threshold of 1e−05

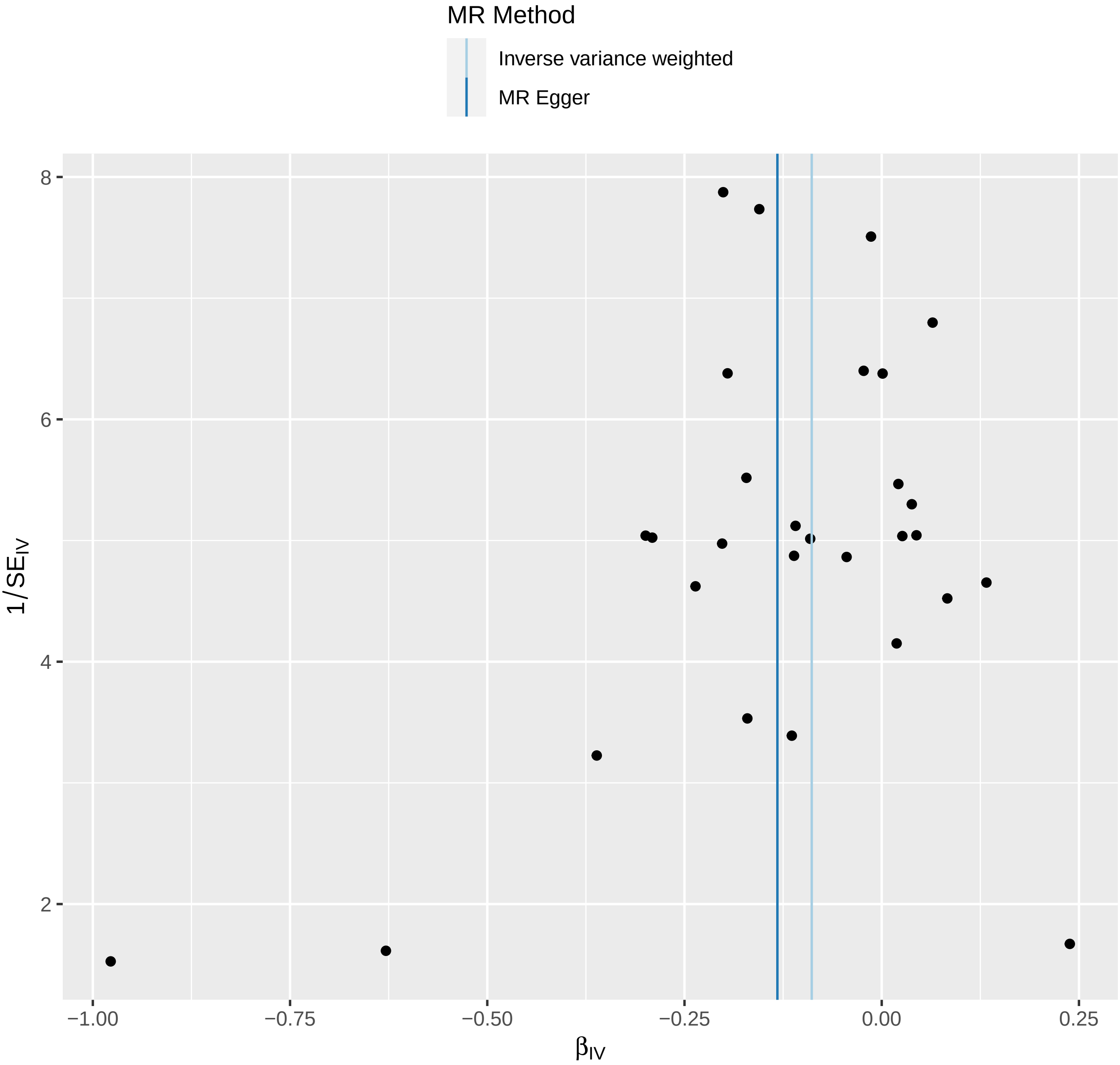

Batch 3−178 : Acid ceramidase on Diabetic nephropathy (more control exclusions)
with a threshold of 1e−05

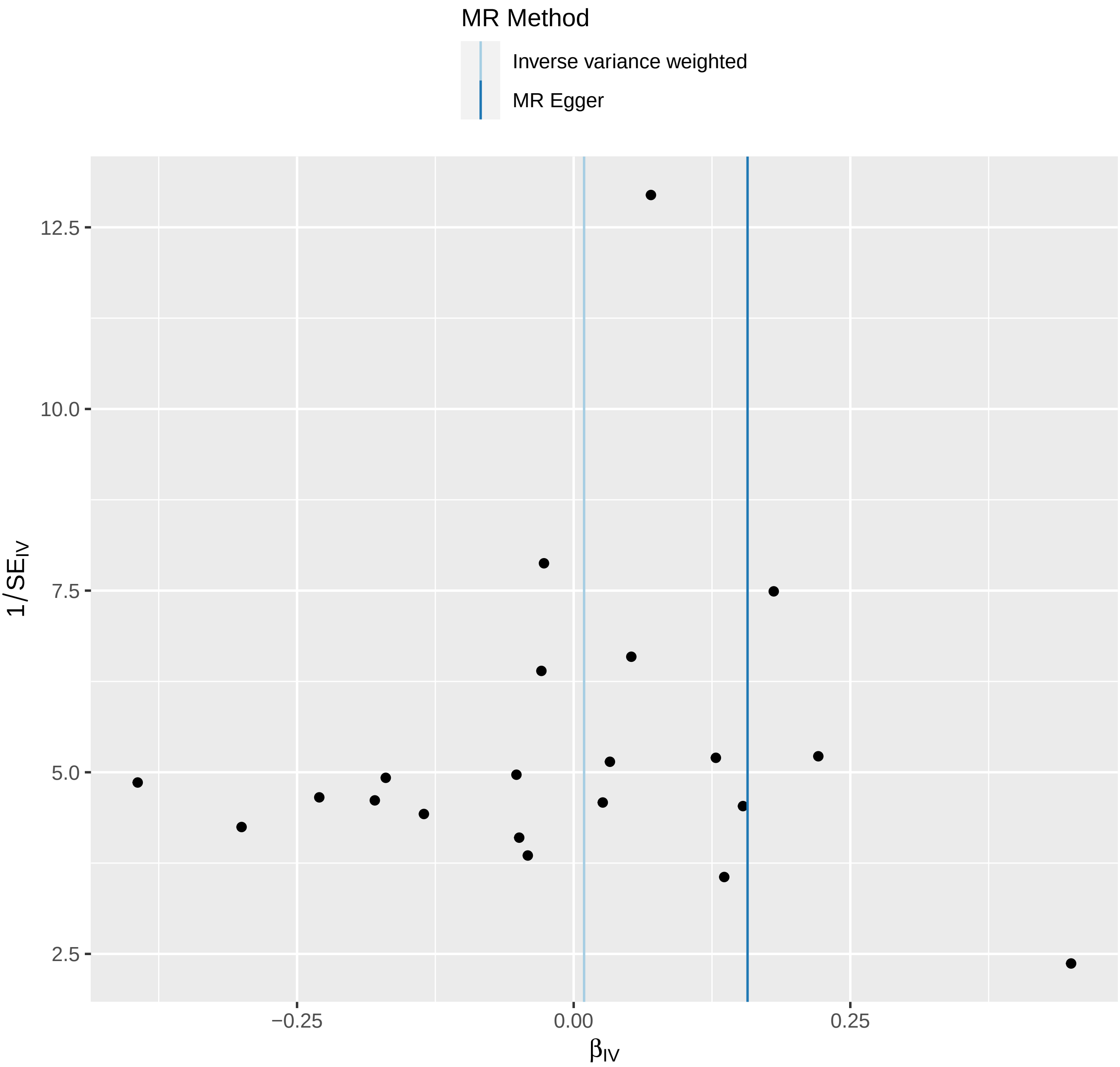

Batch 3−179 : Neutral ceramidase on Diabetic nephropathy (more control exclusions)
with a threshold of 1e−05

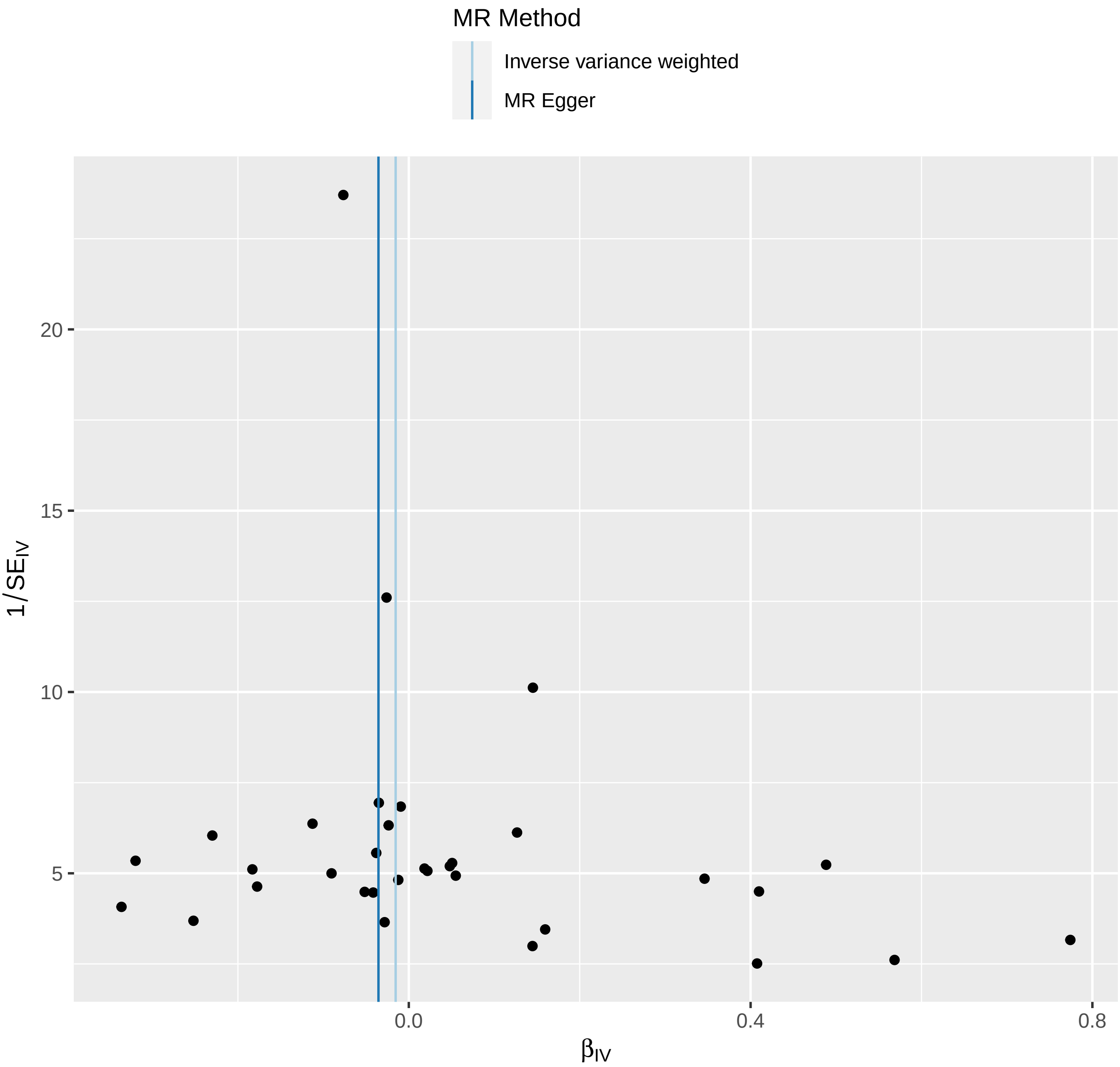

Batch 3−180 : Arf−GAP with SH3 domain, ANK repeat and PH domain−containing protein 2 on Diabetic nephropathy (more control exclusions) with a threshold of 1e−05

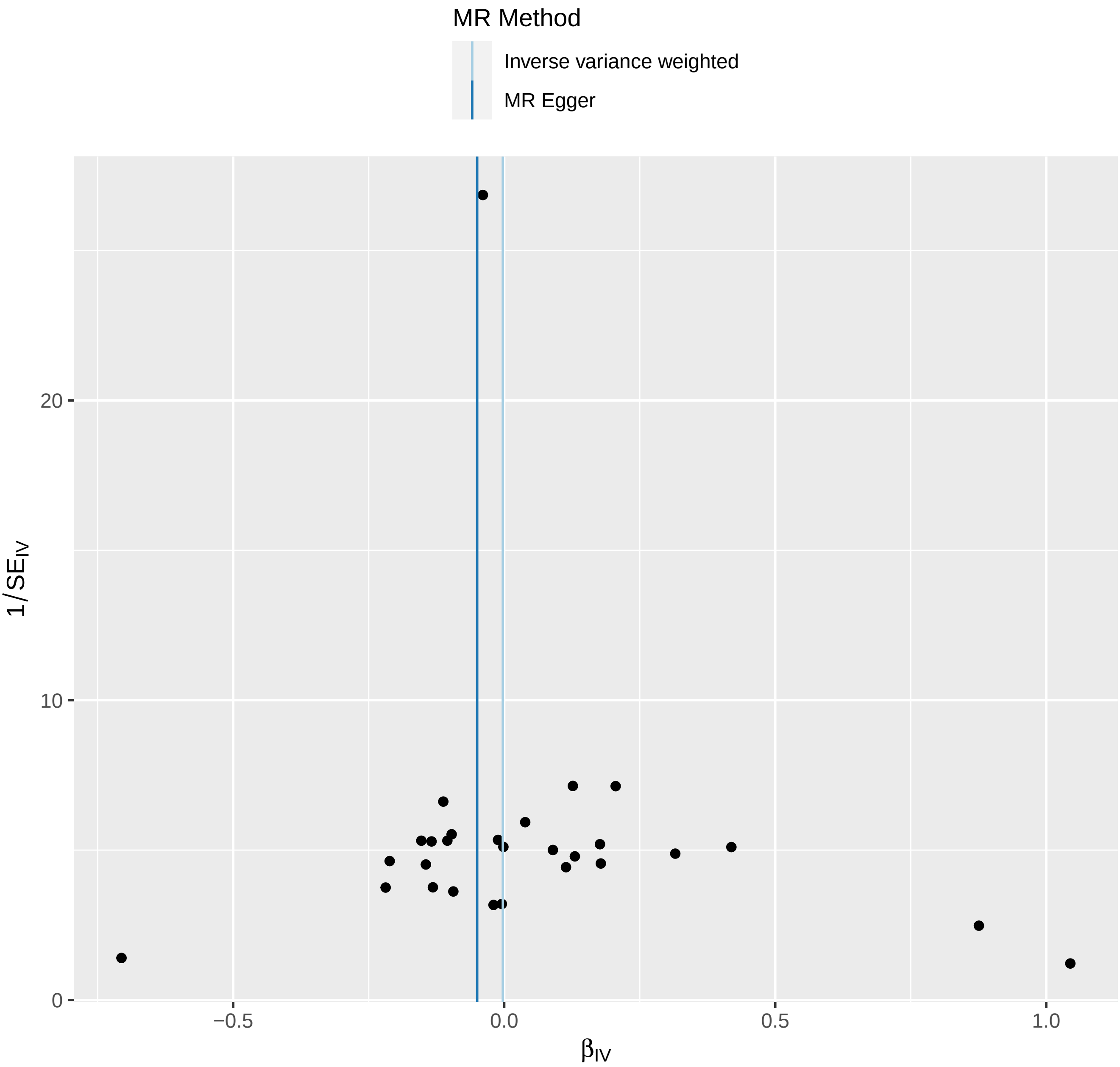

Batch 3−181 : Activating signal cointegrator 1 complex subunit 1 on Diabetic nephropathy (more control exclusions) with a threshold of 1e−05

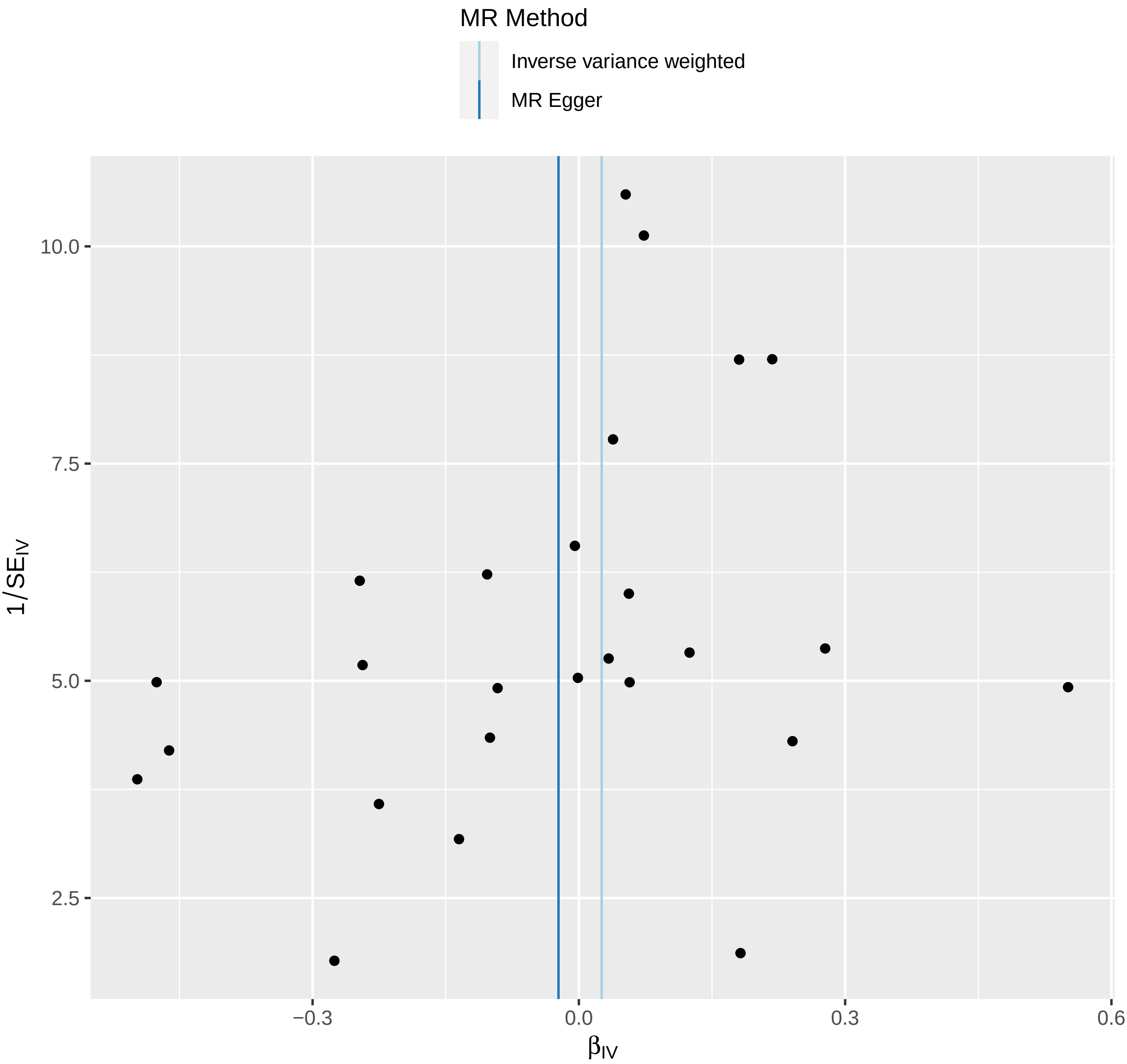

Batch 3−182 : Asialoglycoprotein receptor 1 on Diabetic nephropathy (more control exclusions)
with a threshold of 1e−05

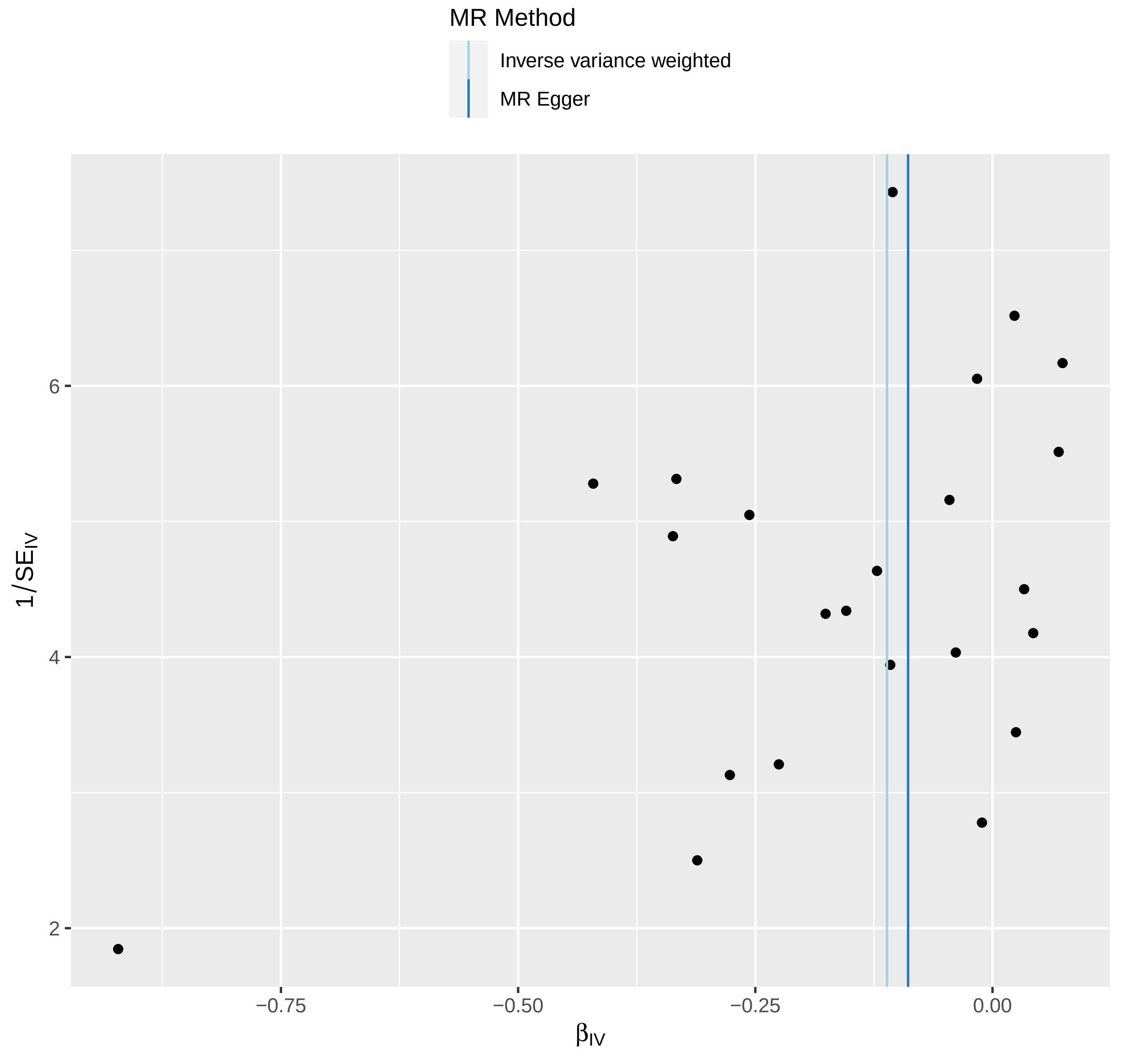

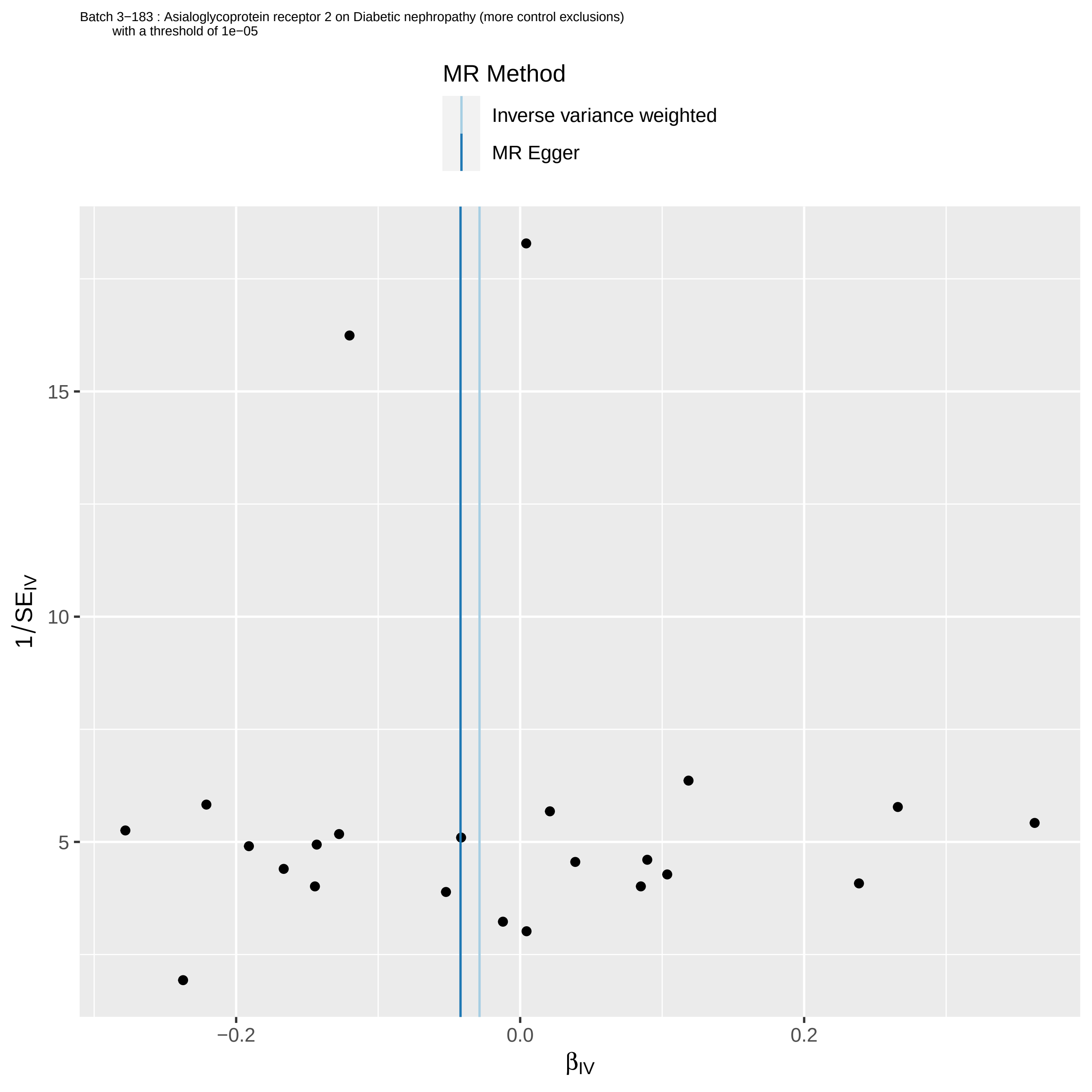
Batch 3−183 : Asialoglycoprotein receptor 2 on Diabetic nephropathy (more control exclusions)
with a threshold of 1e−05
MR Method
Inverse variance weighted
MR Egger
15
10
5
1/SE$_{IV}$
−0.2
0.0
0.2
β$_{IV}$

Batch 3−184 : Histone−lysine N−methyltransferase ASH1L on Diabetic nephropathy (more control exclusions)
with a threshold of 1e−05

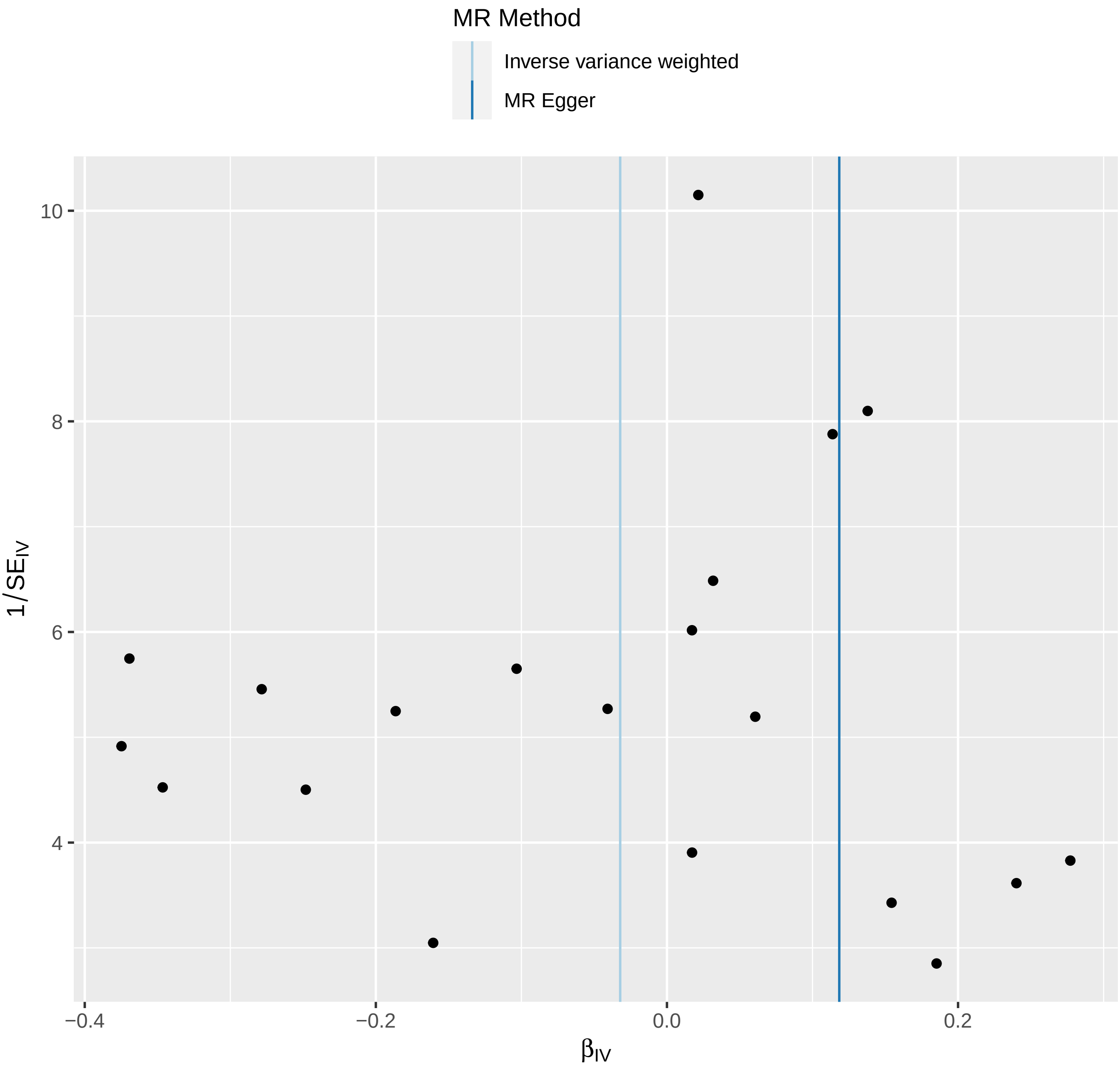

Batch 3−185 : Set1/Ash2 histone methyltransferase complex subunit ASH2 on Diabetic nephropathy (more control exclusions) with a threshold of 1e−05

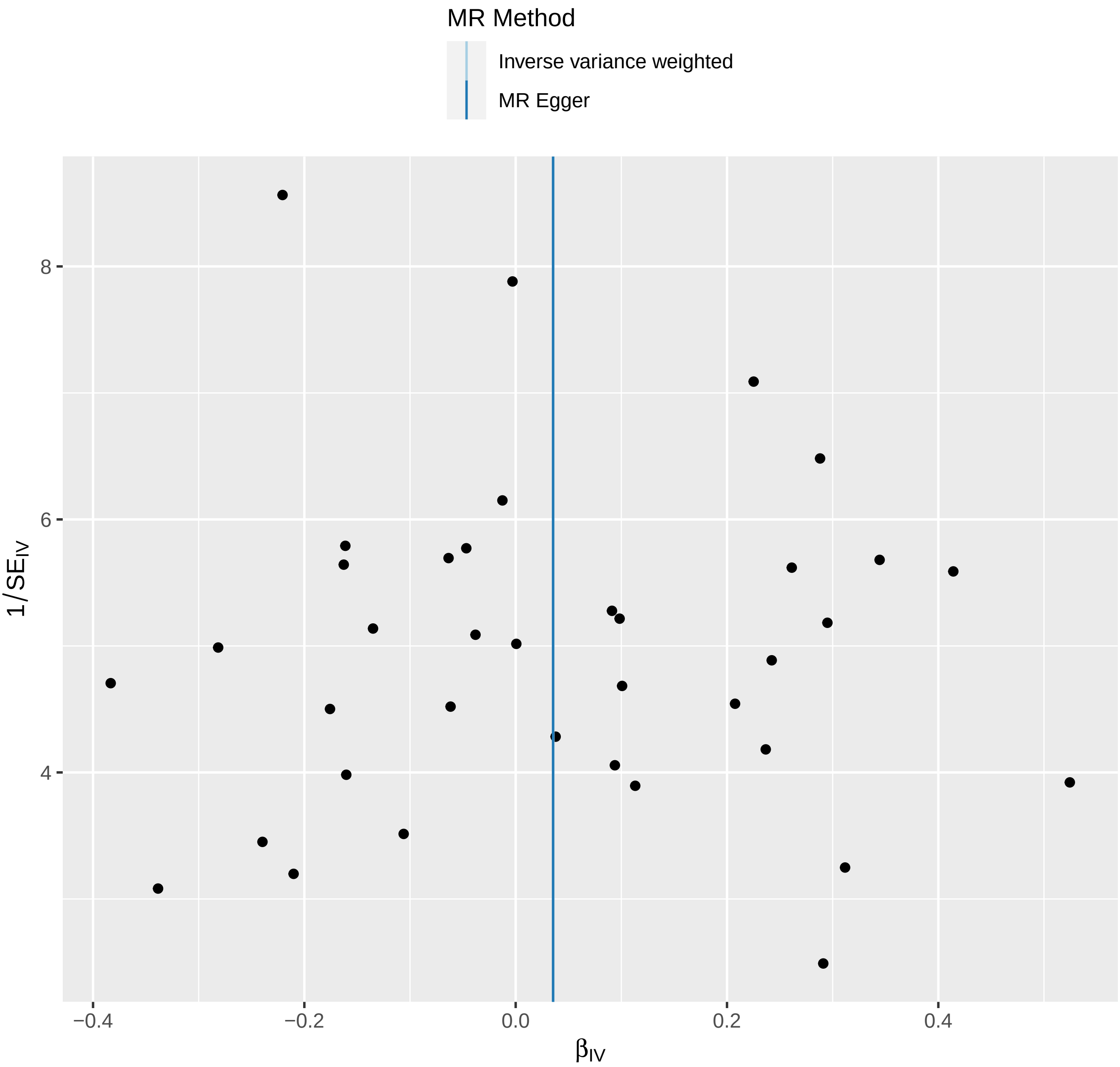

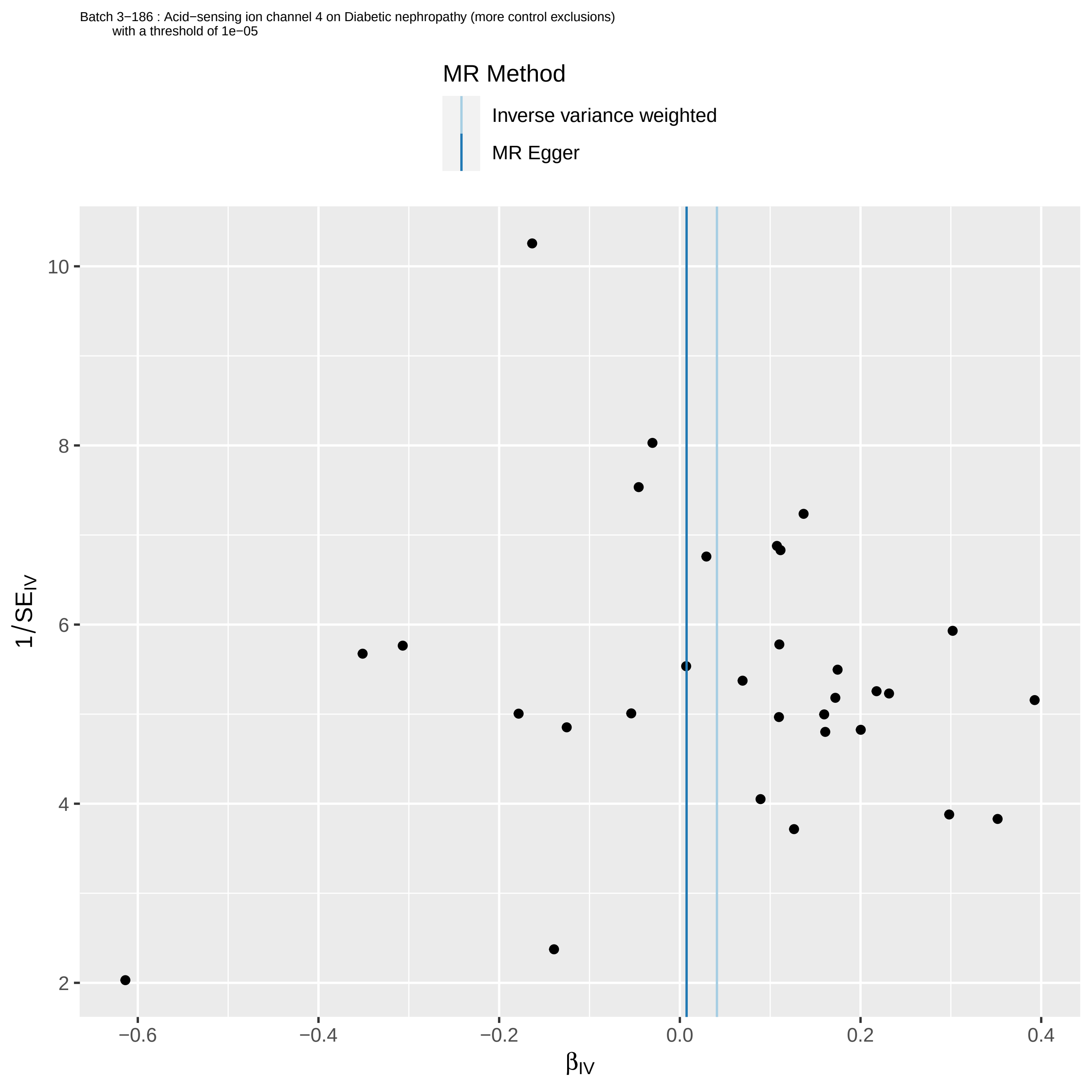
Batch 3−186 : Acid−sensing ion channel 4 on Diabetic nephropathy (more control exclusions)
with a threshold of 1e−05
MR Method
Inverse variance weighted
MR Egger
10
8
6
4
2
1/SE_IV
−0.6
−0.4
−0.2
0.0
0.2
0.4
β_IV

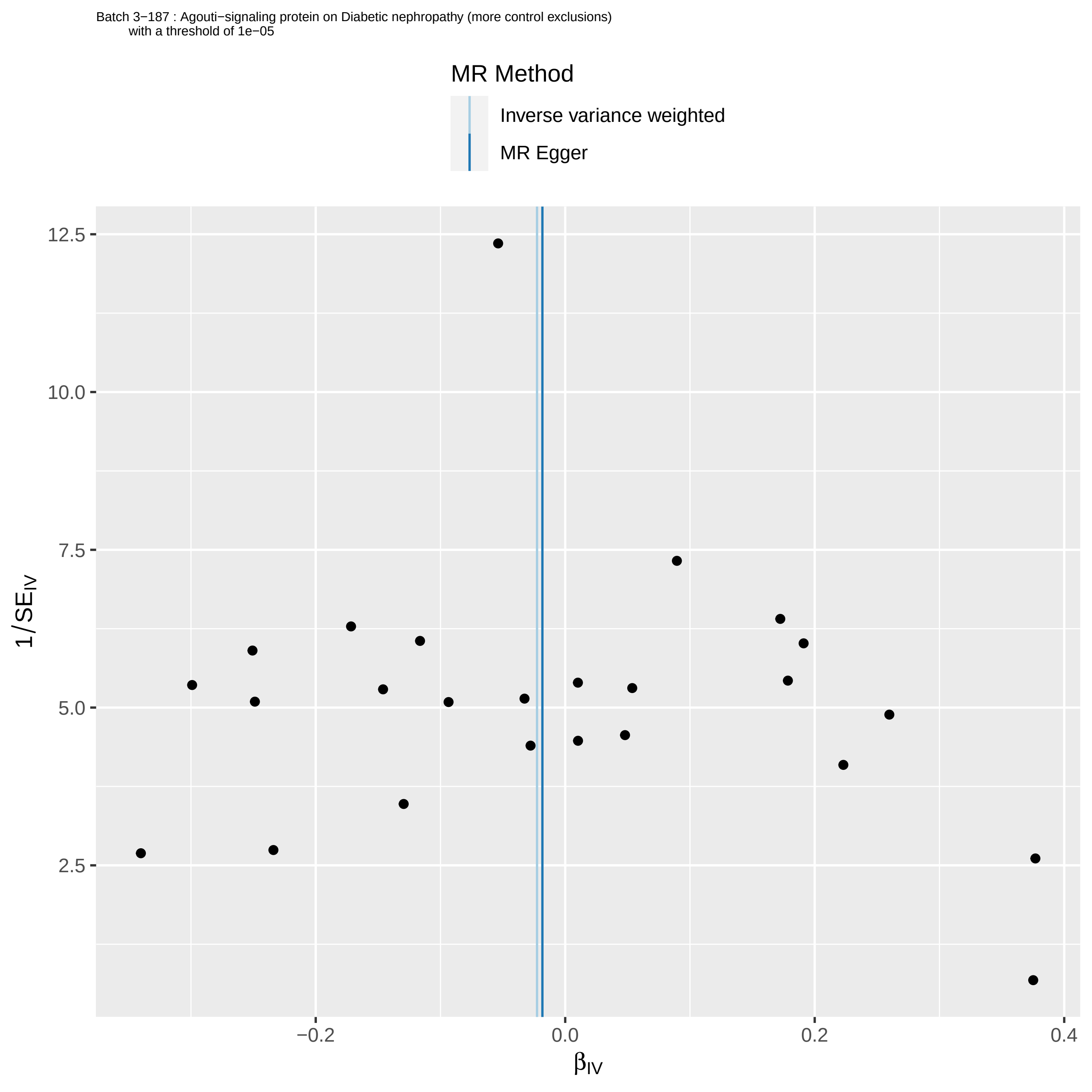

Batch 3−187 : Agouti−signaling protein on Diabetic nephropathy (more control exclusions)
with a threshold of 1e−05
MR Method
Inverse variance weighted
MR Egger
12.5
10.0
7.5
5.0
2.5
1/SE$_{IV}$
−0.2
0.0
0.2
0.4
β_{IV}

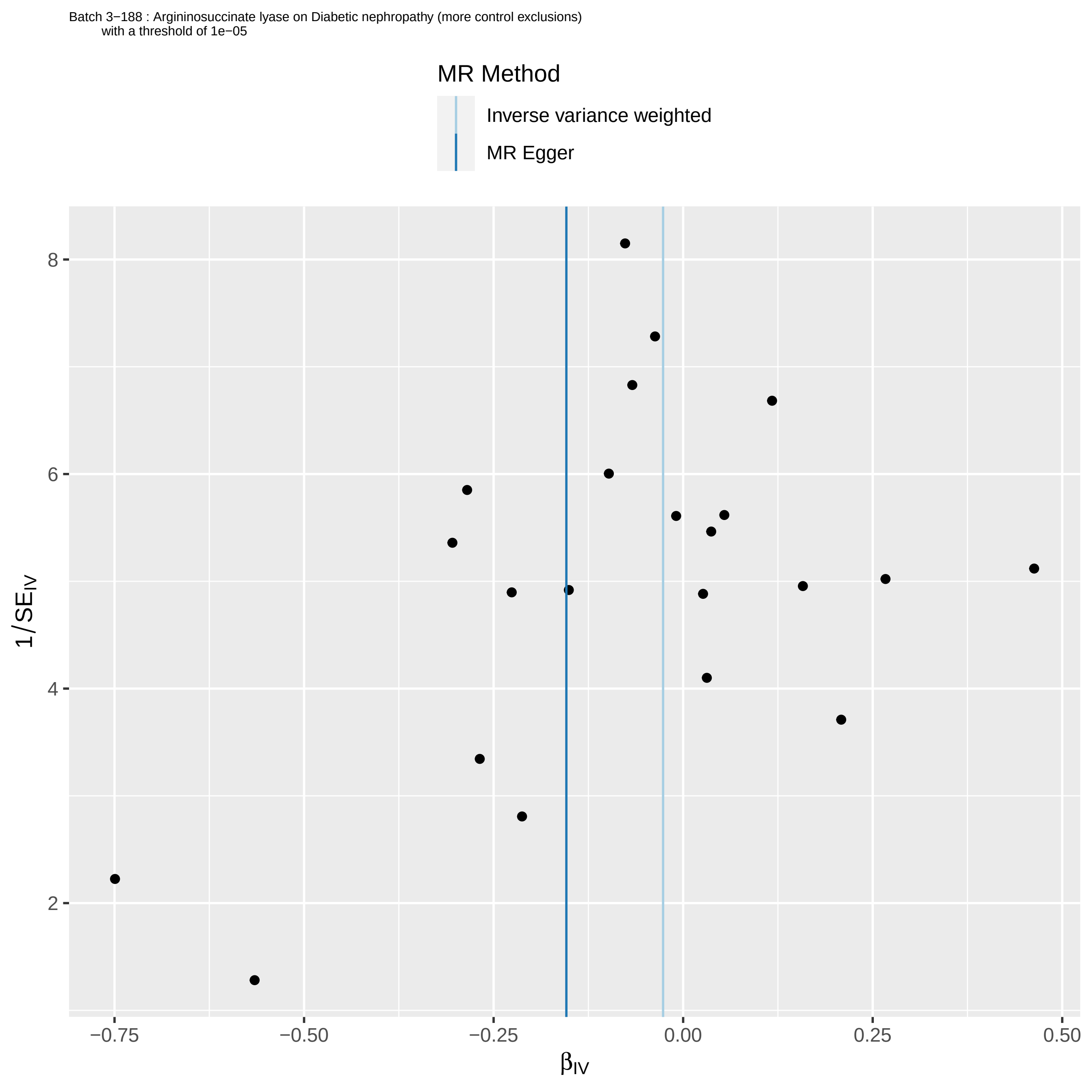

Batch 3−188 : Argininosuccinate lyase on Diabetic nephropathy (more control exclusions) with a threshold of 1e−05
MR Method
Inverse variance weighted
MR Egger
8
6
4
2
1/SE_IV
−0.75
−0.50
−0.25
0.00
0.25
0.50
β_IV

Batch 3−189 : N−acetylserotonin O−methyltransferase−like protein on Diabetic nephropathy (more control exclusions) with a threshold of 1e−05

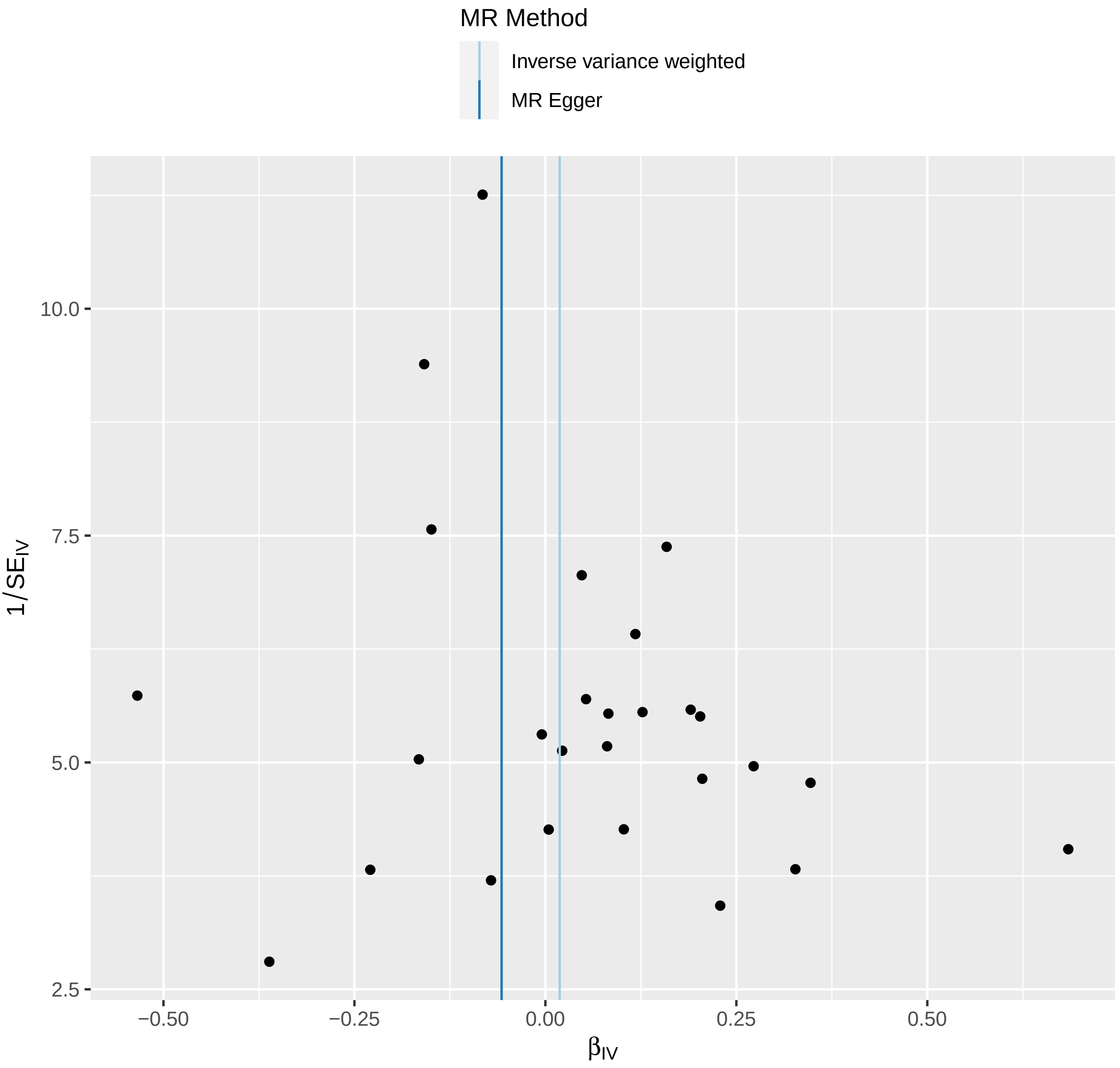

Batch 3−190 : ATPase ASNA1 on Diabetic nephropathy (more control exclusions)
with a threshold of 1e−05

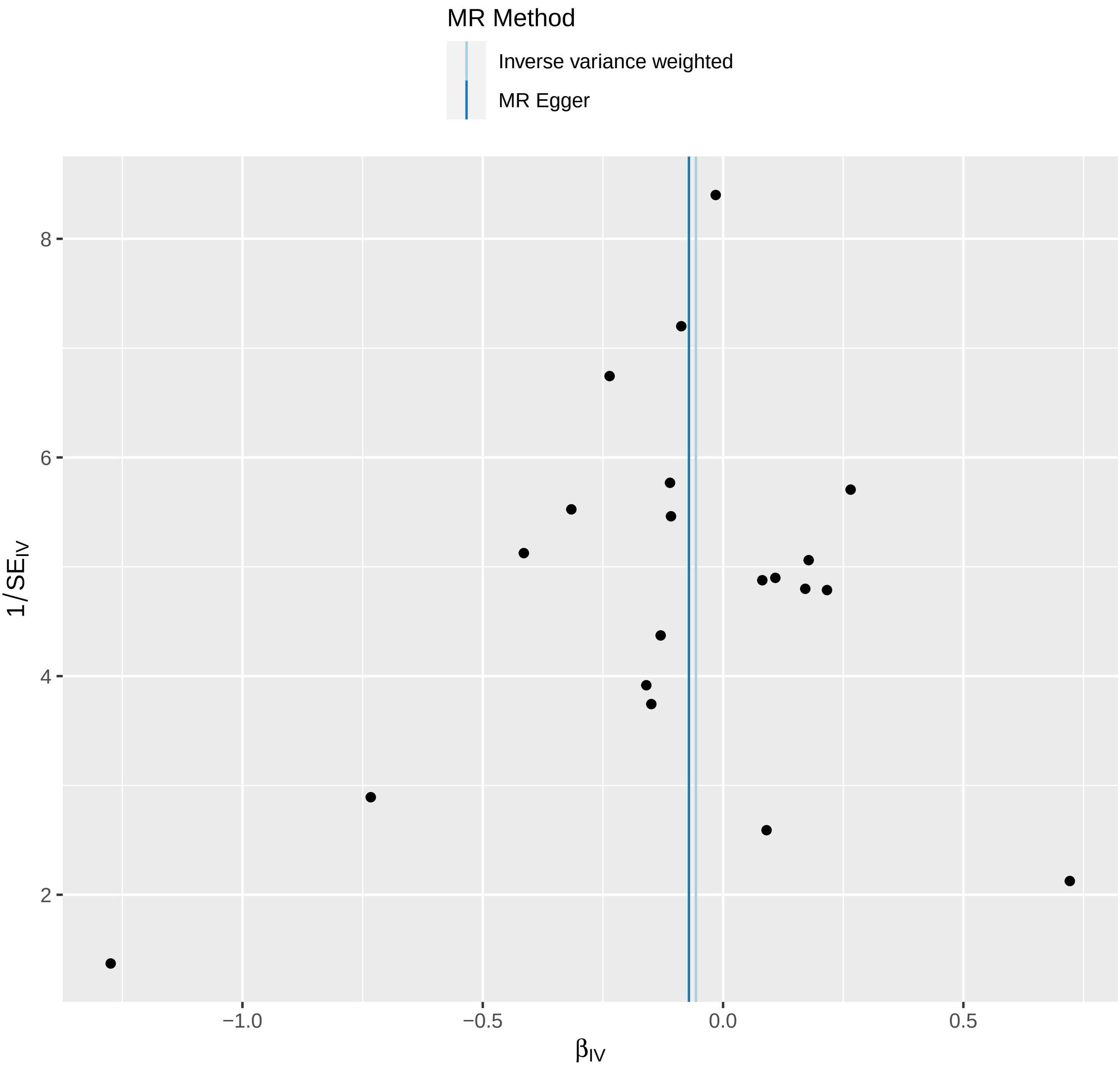

Batch 3−191 : Aspartyl/asparaginyl beta−hydroxylase on Diabetic nephropathy (more control exclusions)
with a threshold of 1e−05

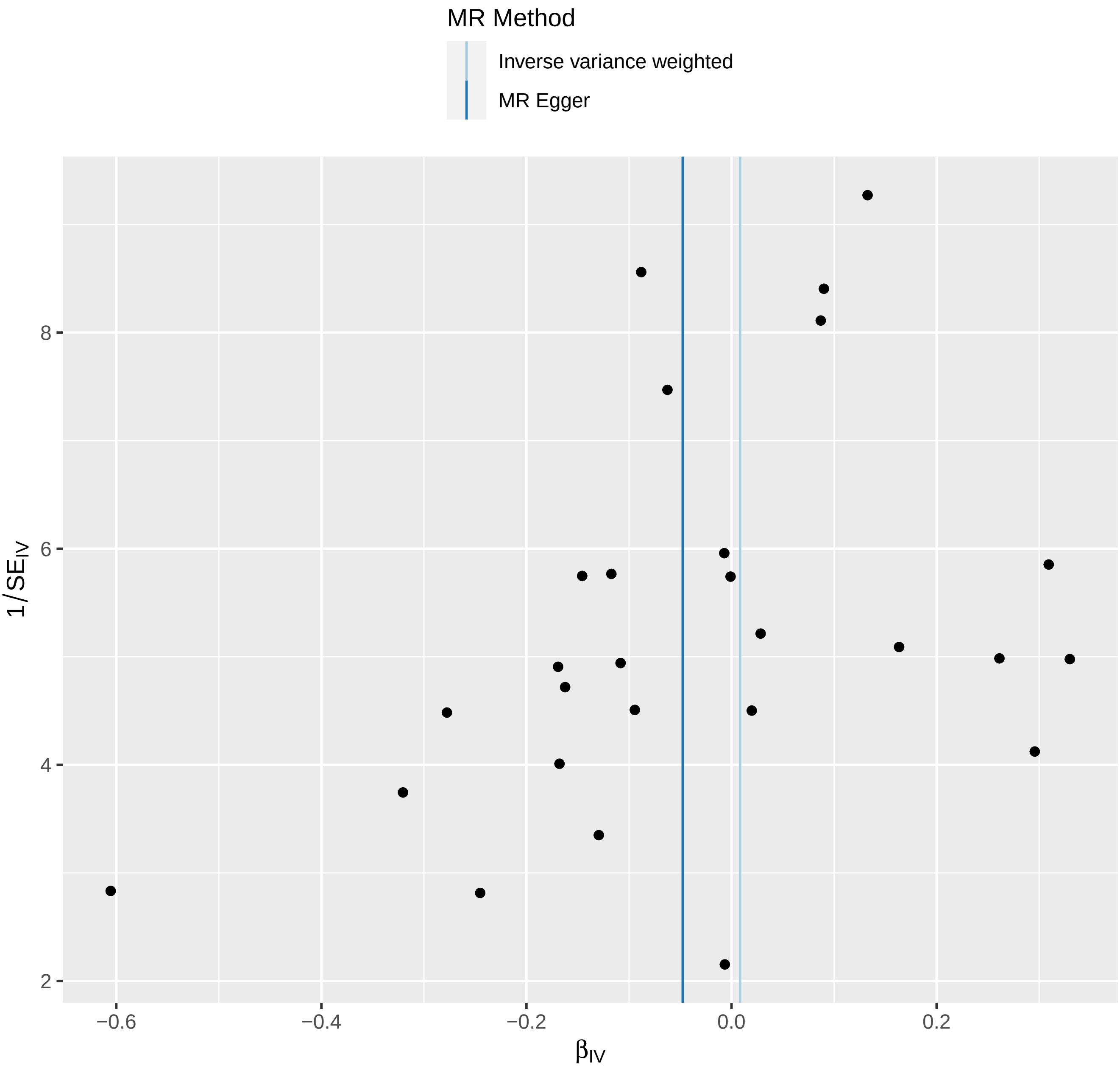

Batch 3−192 : Asporin on Diabetic nephropathy (more control exclusions)
with a threshold of 1e−05

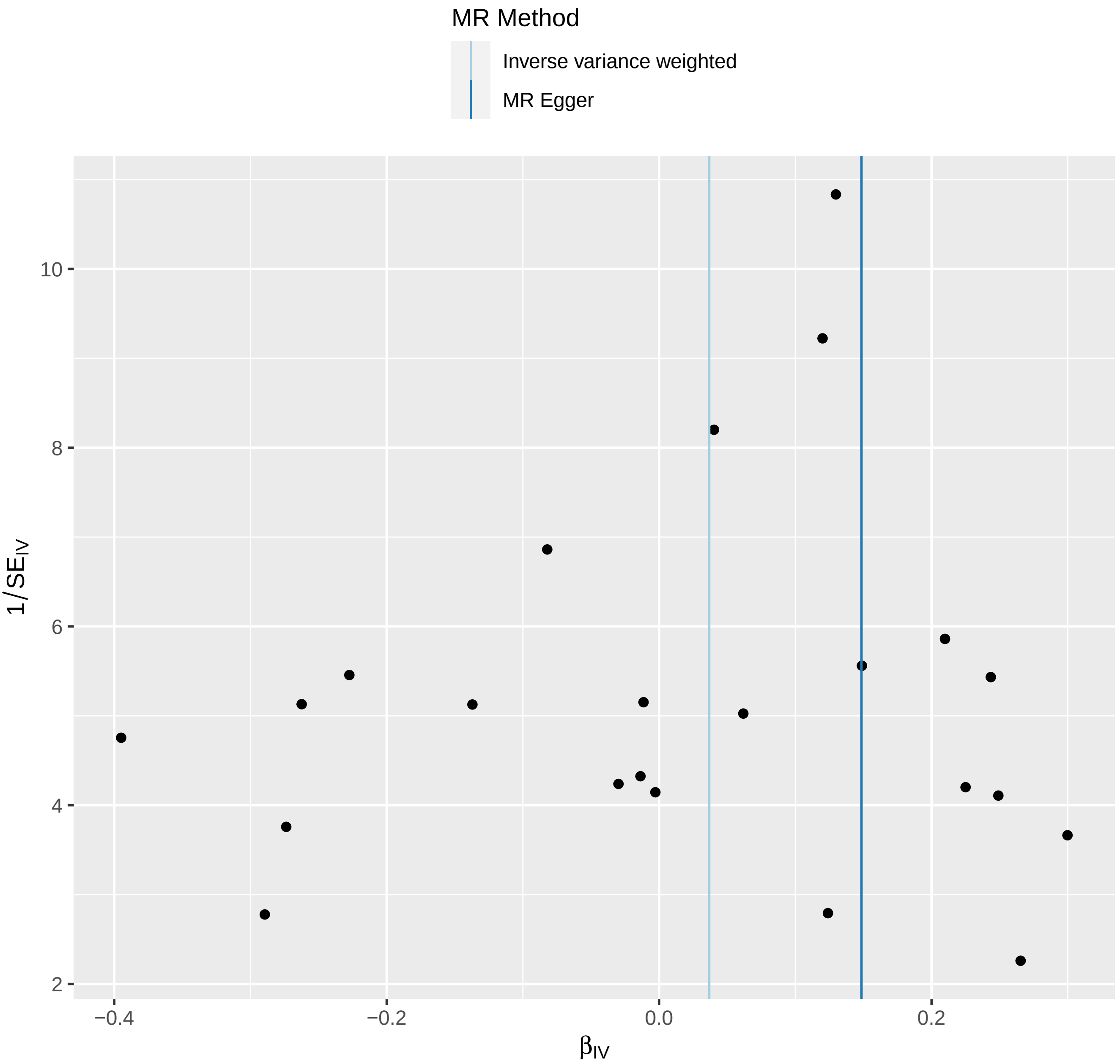

Batch 3−193 : Retroviral−like aspartic protease 1 on Diabetic nephropathy (more control exclusions)
with a threshold of 1e−05

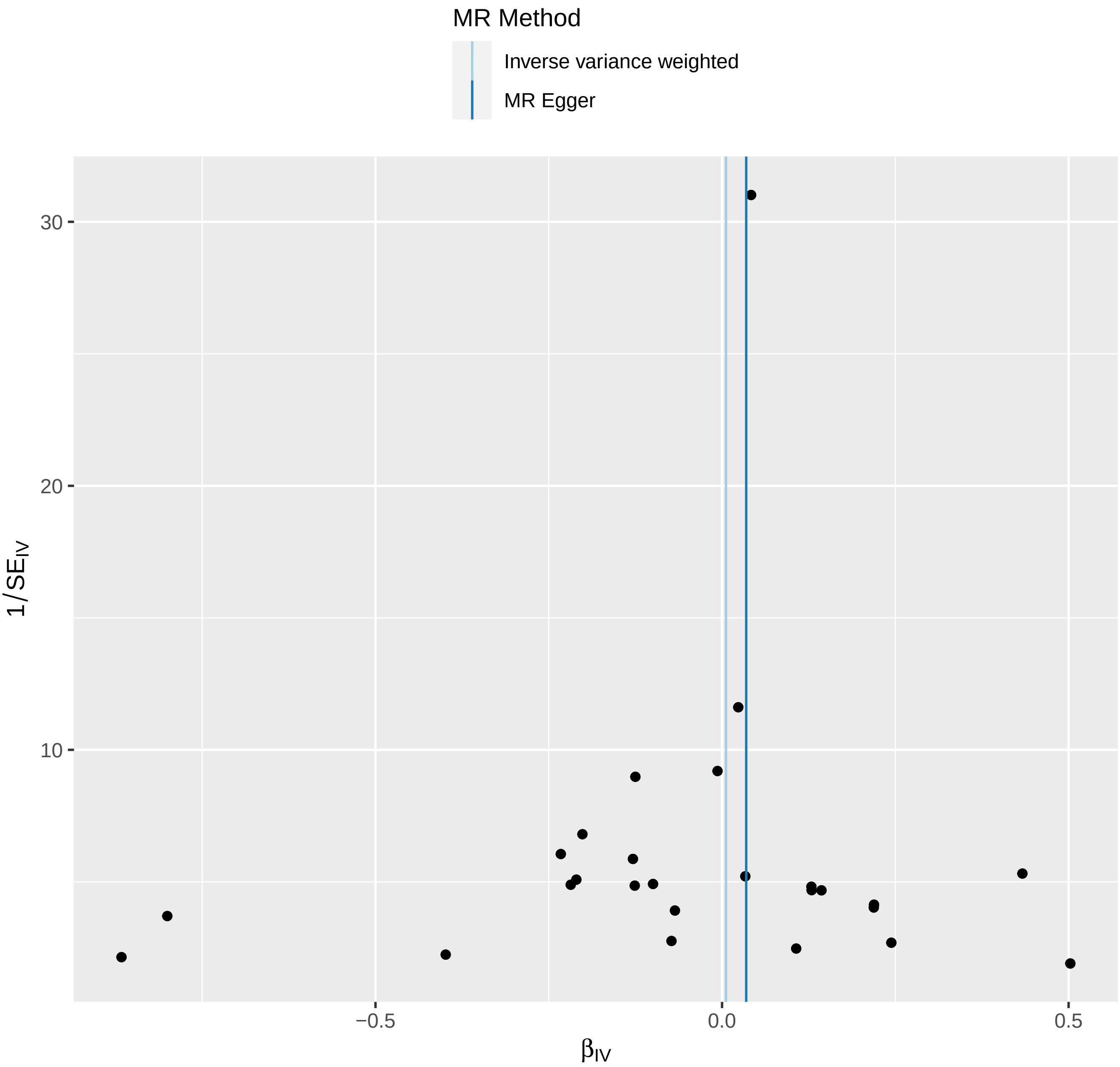

Batch 3−194 : Astacin−like metalloendopeptidase on Diabetic nephropathy (more control exclusions) with a threshold of 1e−05

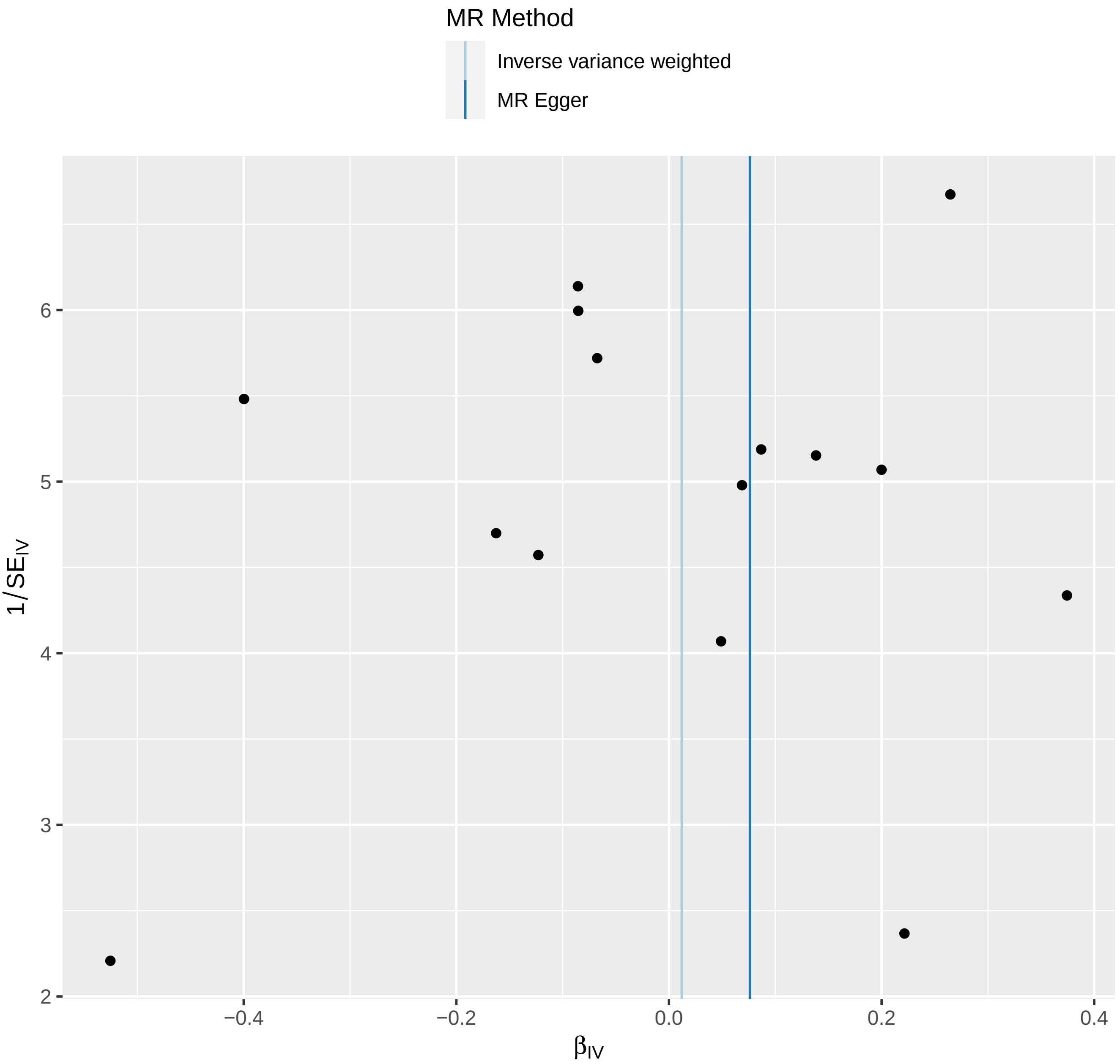

Batch 3−195 : ATPase family AAA domain−containing protein 1 on Diabetic nephropathy (more control exclusions)
with a threshold of 1e−05

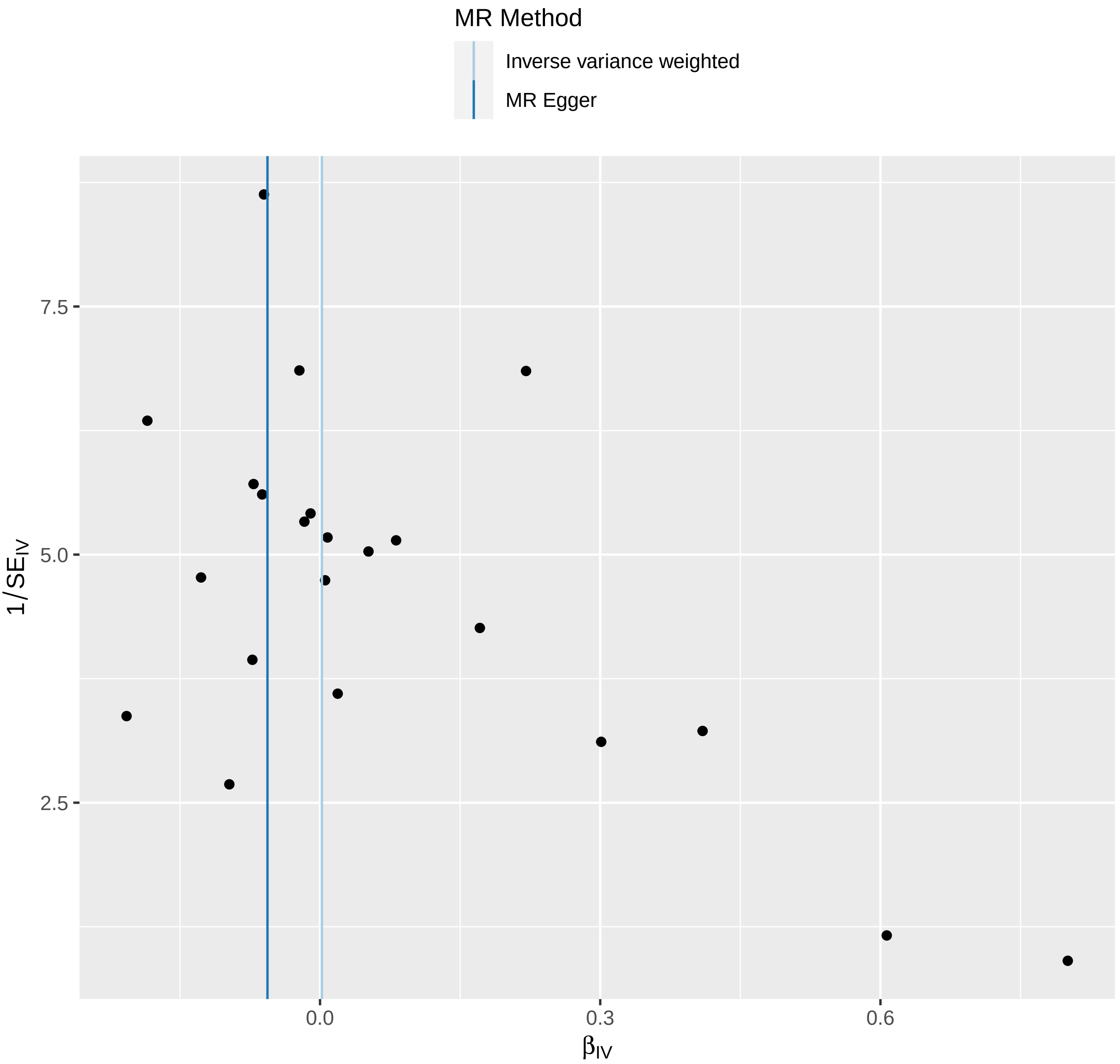

Batch 3−196 : ATPase family AAA domain−containing protein 2 on Diabetic nephropathy (more control exclusions)
with a threshold of 1e−05

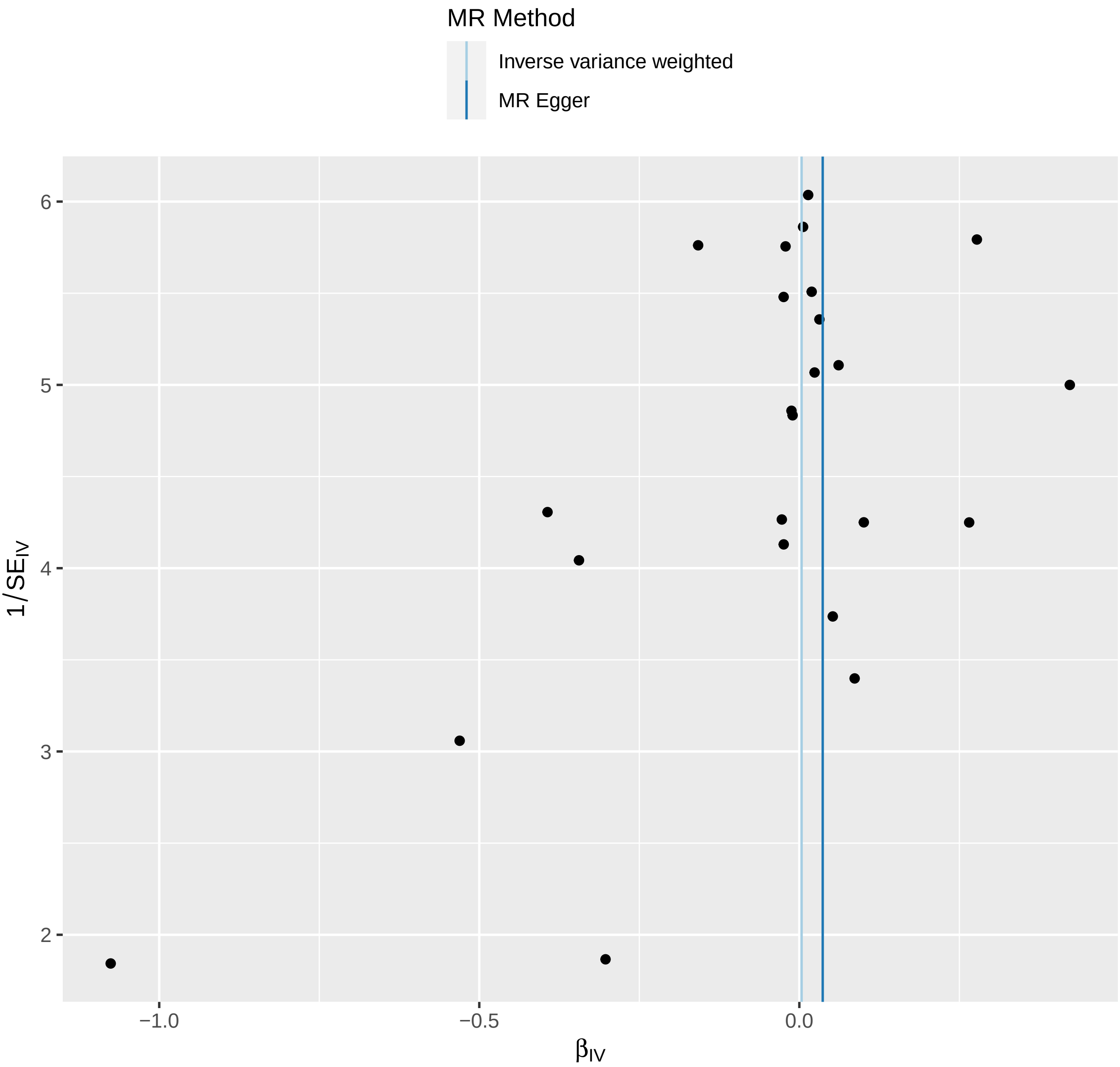

Batch 3−197 : Cyclic AMP−dependent transcription factor ATF−6 alpha on Diabetic nephropathy (more control exclusions) with a threshold of 1e−05

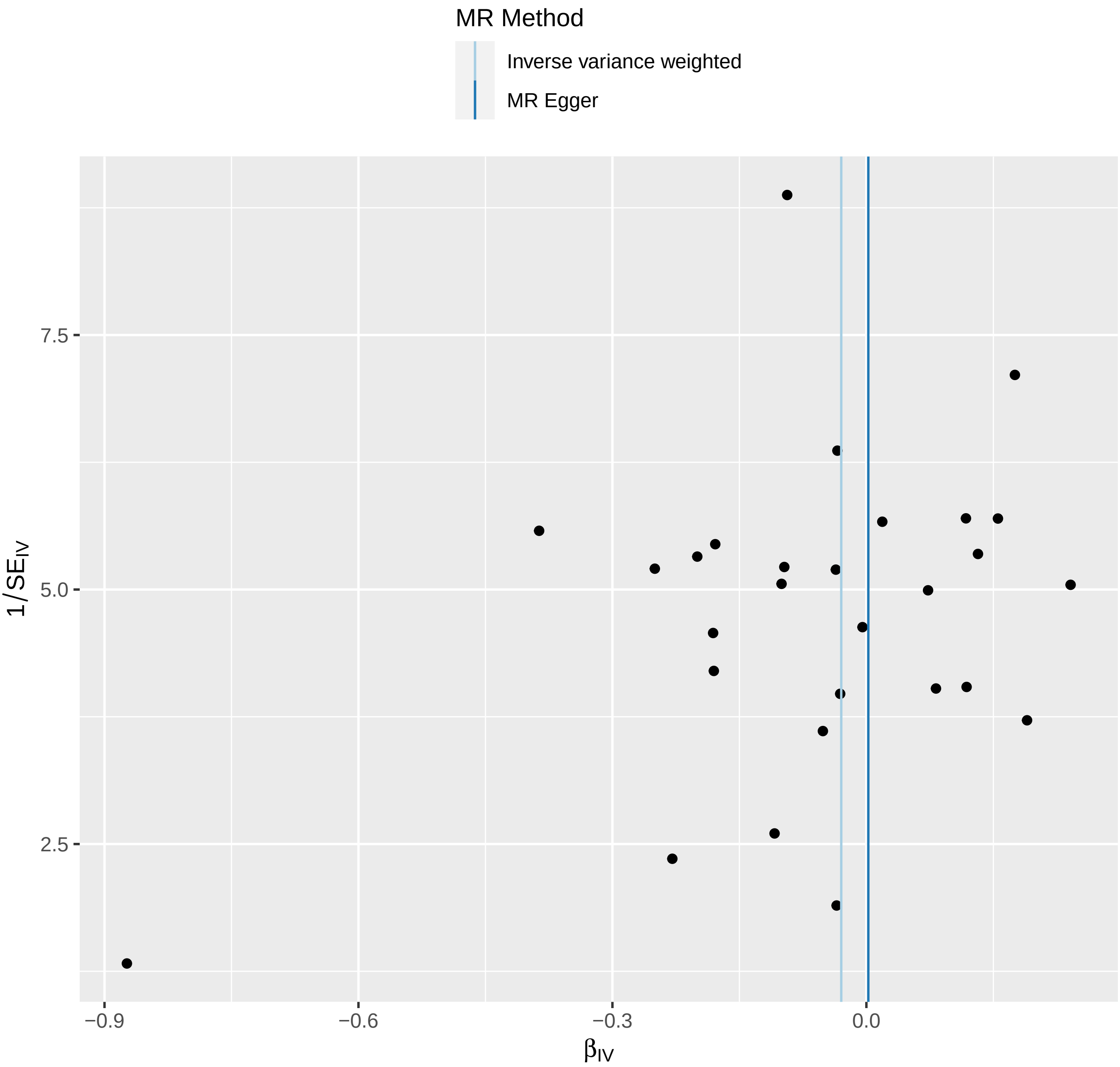

Batch 3−198 : Ubiquitin−like modifier−activating enzyme ATG7 on Diabetic nephropathy (more control exclusions) with a threshold of 1e−05

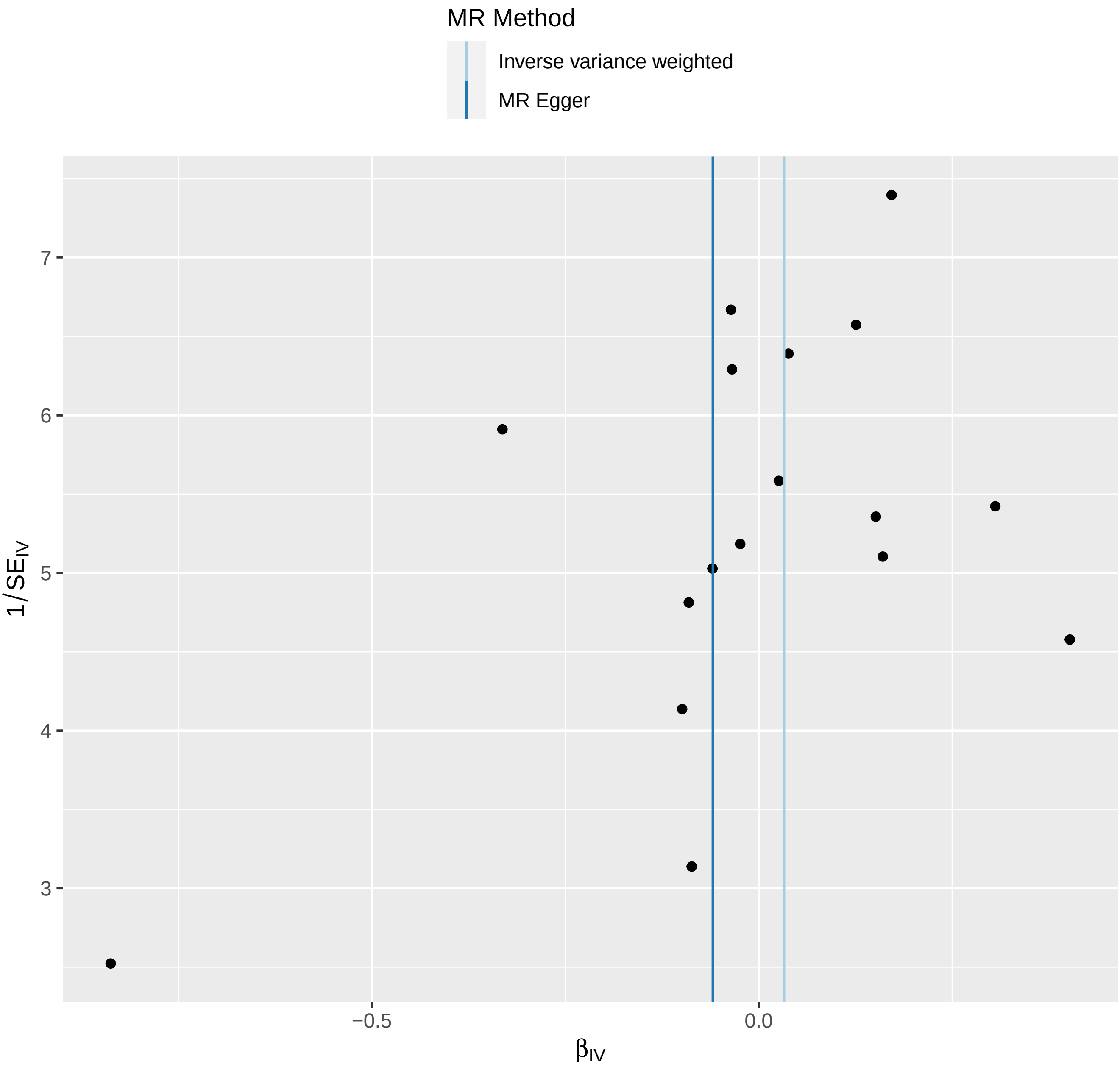

Batch 3−199 : Sodium/potassium−transporting ATPase subunit alpha−1 on Diabetic nephropathy (more control exclusions)
with a threshold of 1e−05

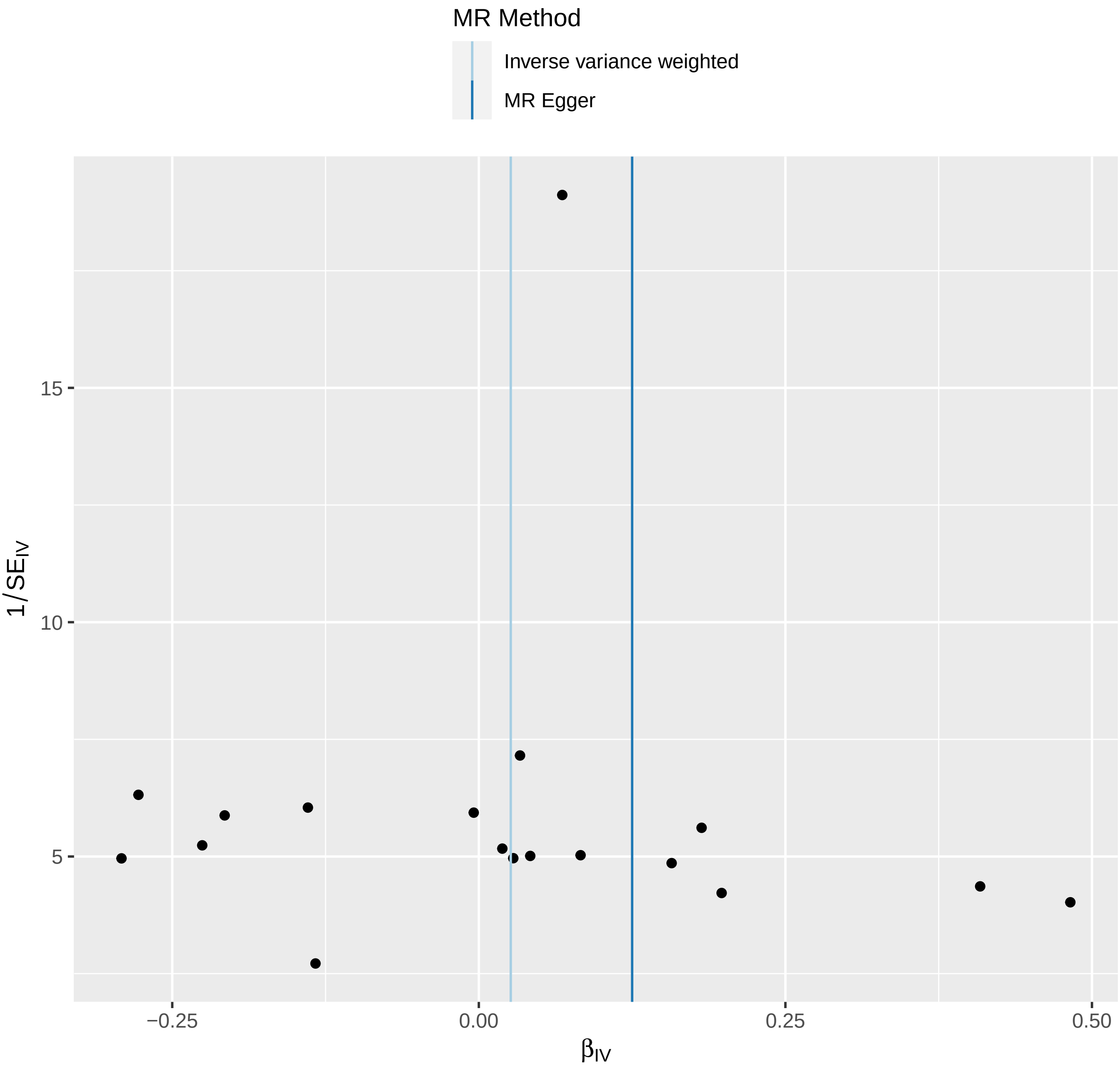

Batch 3−200 : Sodium/potassium−transporting ATPase subunit beta−2 on Diabetic nephropathy (more control exclusions)
with a threshold of 1e−05

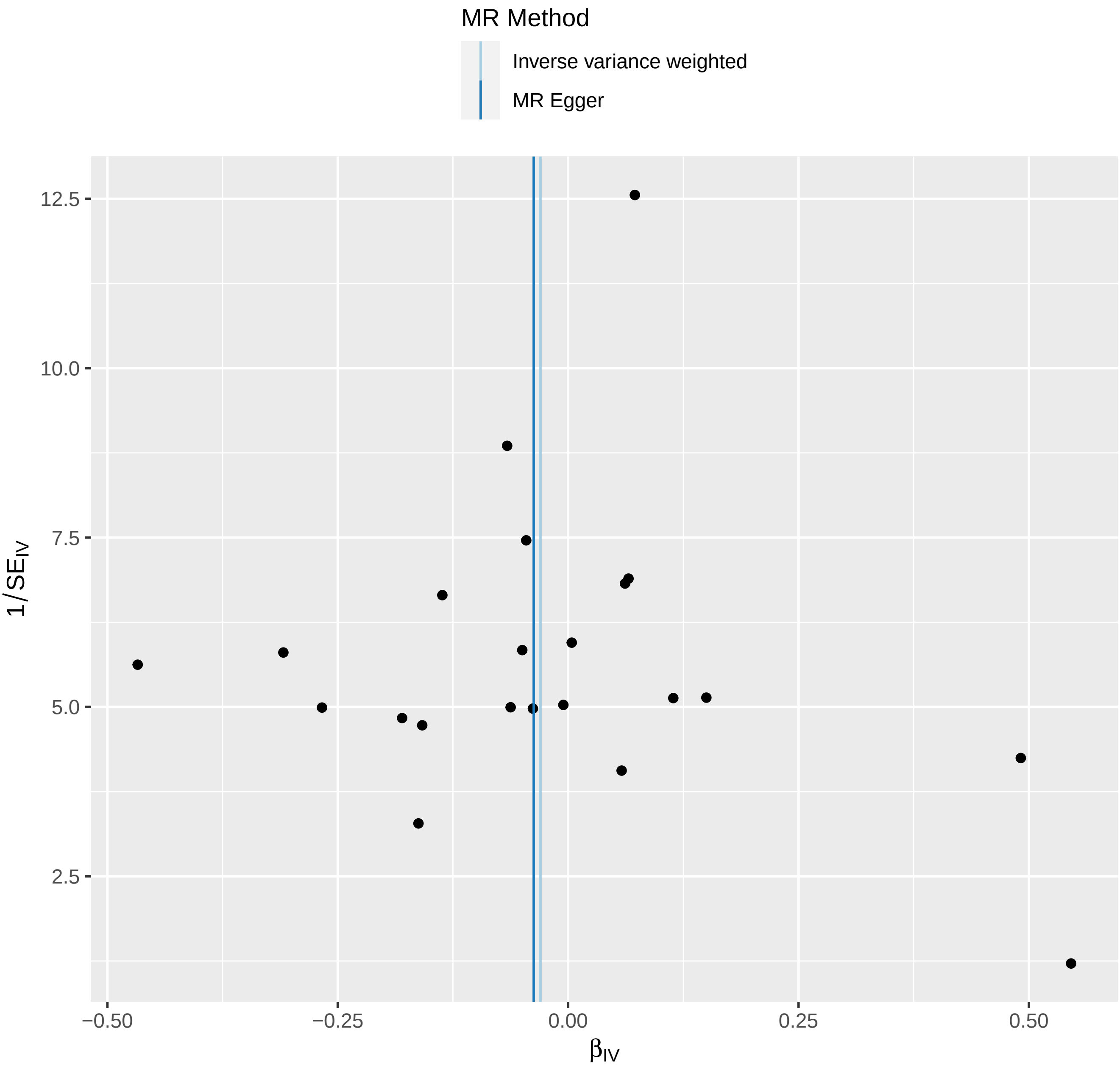

Batch 3−201 : Sarcoplasmic/endoplasmic reticulum calcium ATPase 3 on Diabetic nephropathy (more control exclusions)
with a threshold of 1e−05

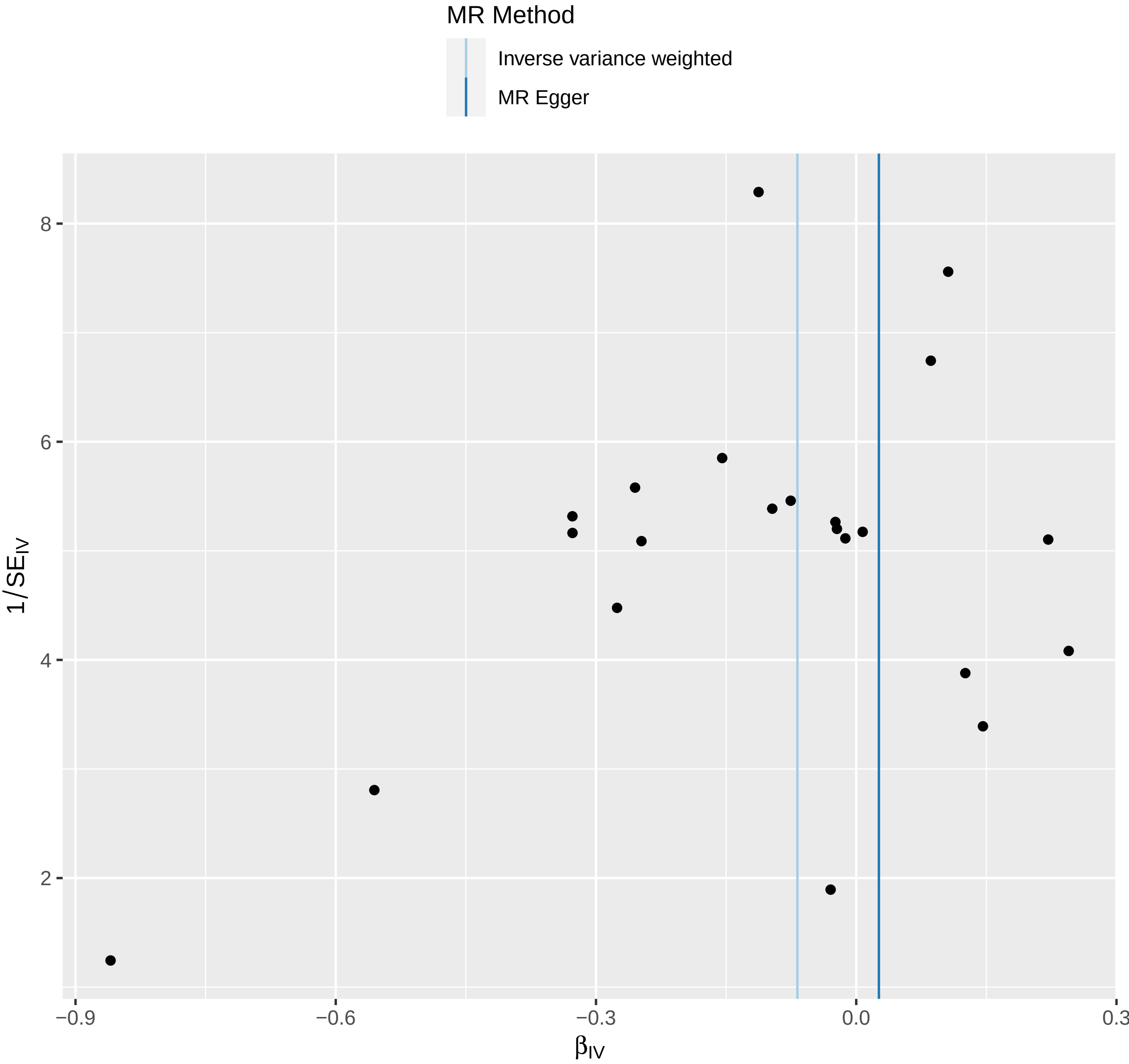

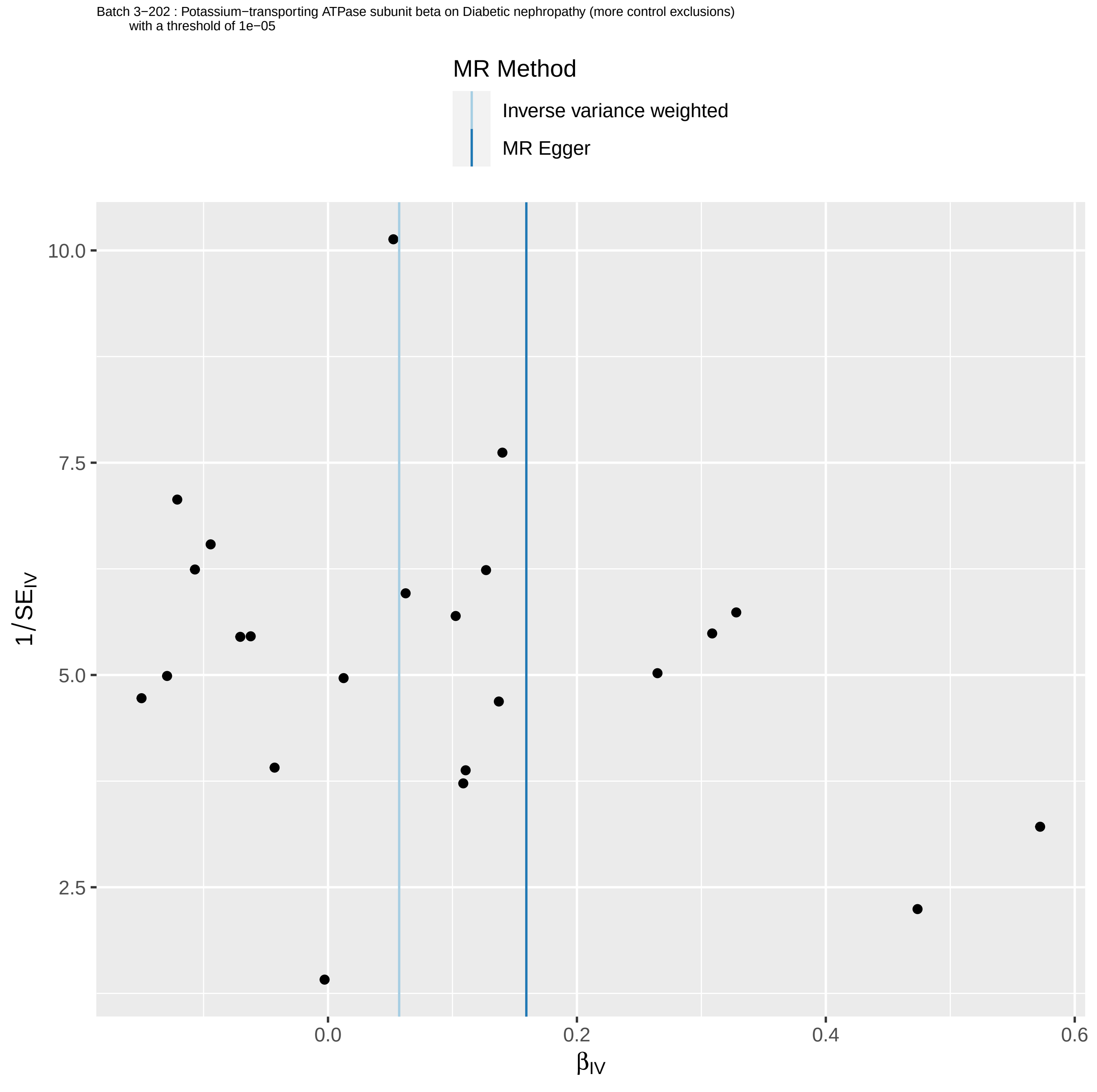
Batch 3−202 : Potassium−transporting ATPase subunit beta on Diabetic nephropathy (more control exclusions)
with a threshold of 1e−05
MR Method
Inverse variance weighted
MR Egger
10.0
7.5
5.0
2.5
1/SE_IV
0.0
0.2
0.4
0.6
β_IV

Batch 3−203 : ATP synthase subunit beta, mitochondrial on Diabetic nephropathy (more control exclusions)
with a threshold of 1e−05

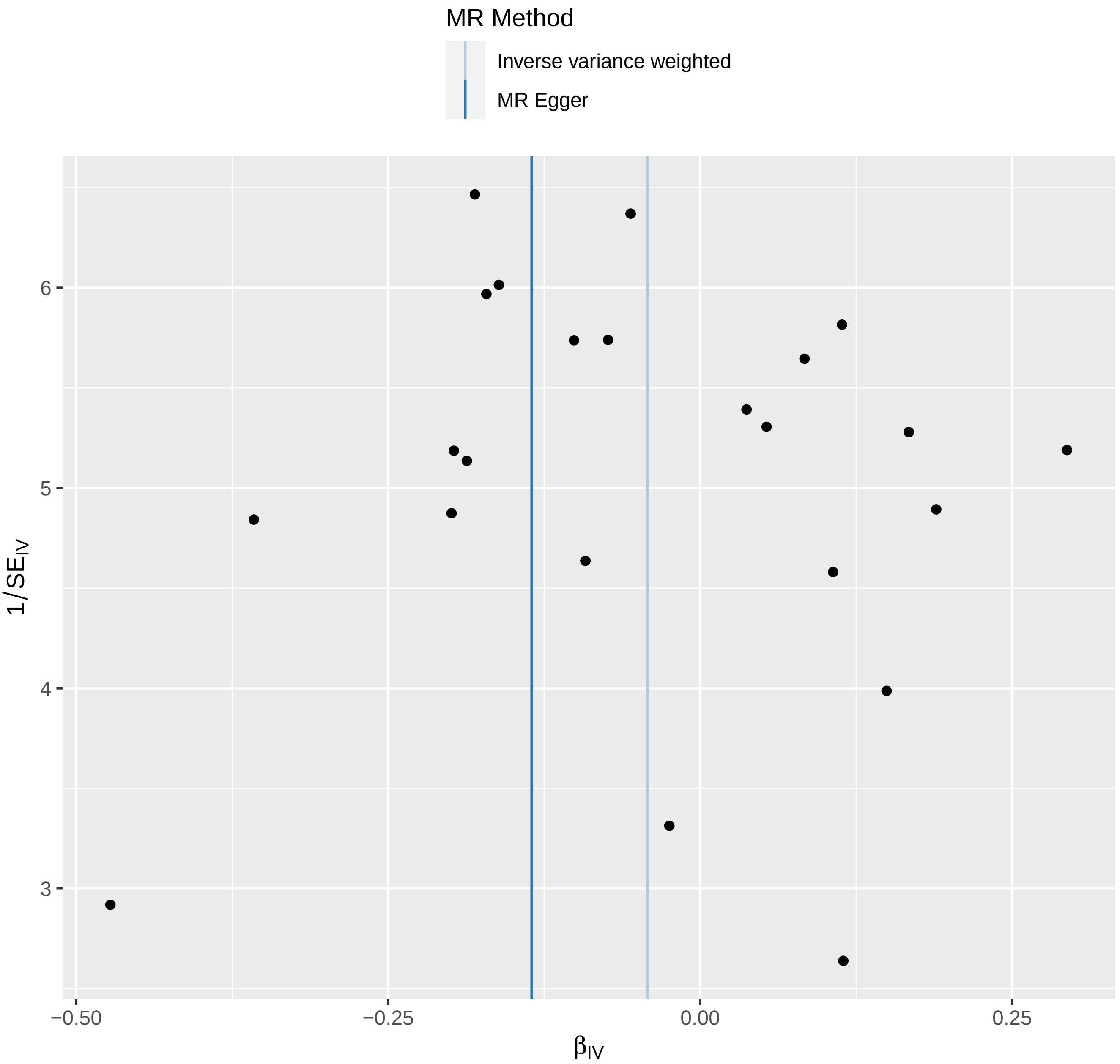

Batch 3−204 : Aurora kinase A on Diabetic nephropathy (more control exclusions)
with a threshold of 1e−05

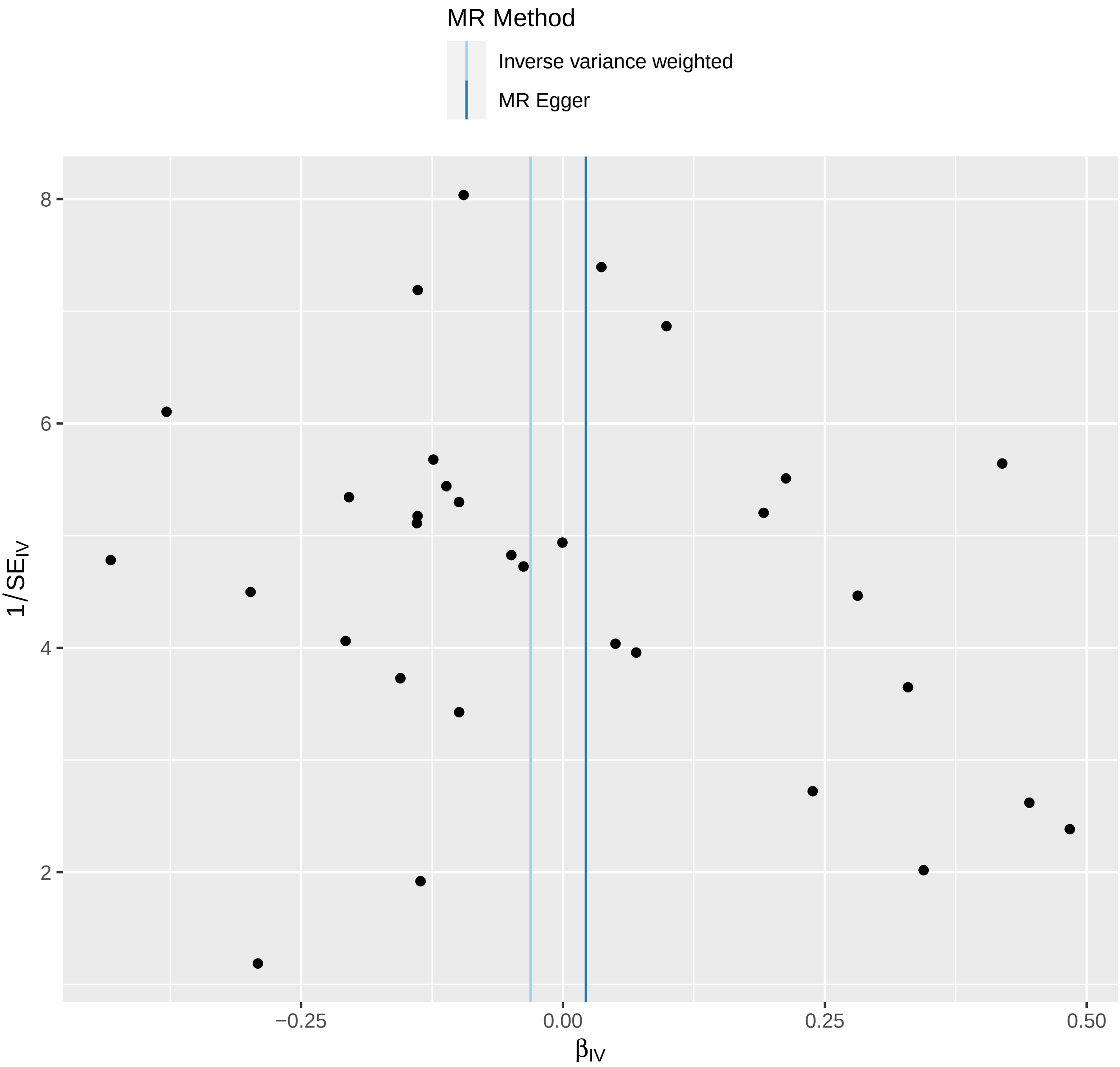

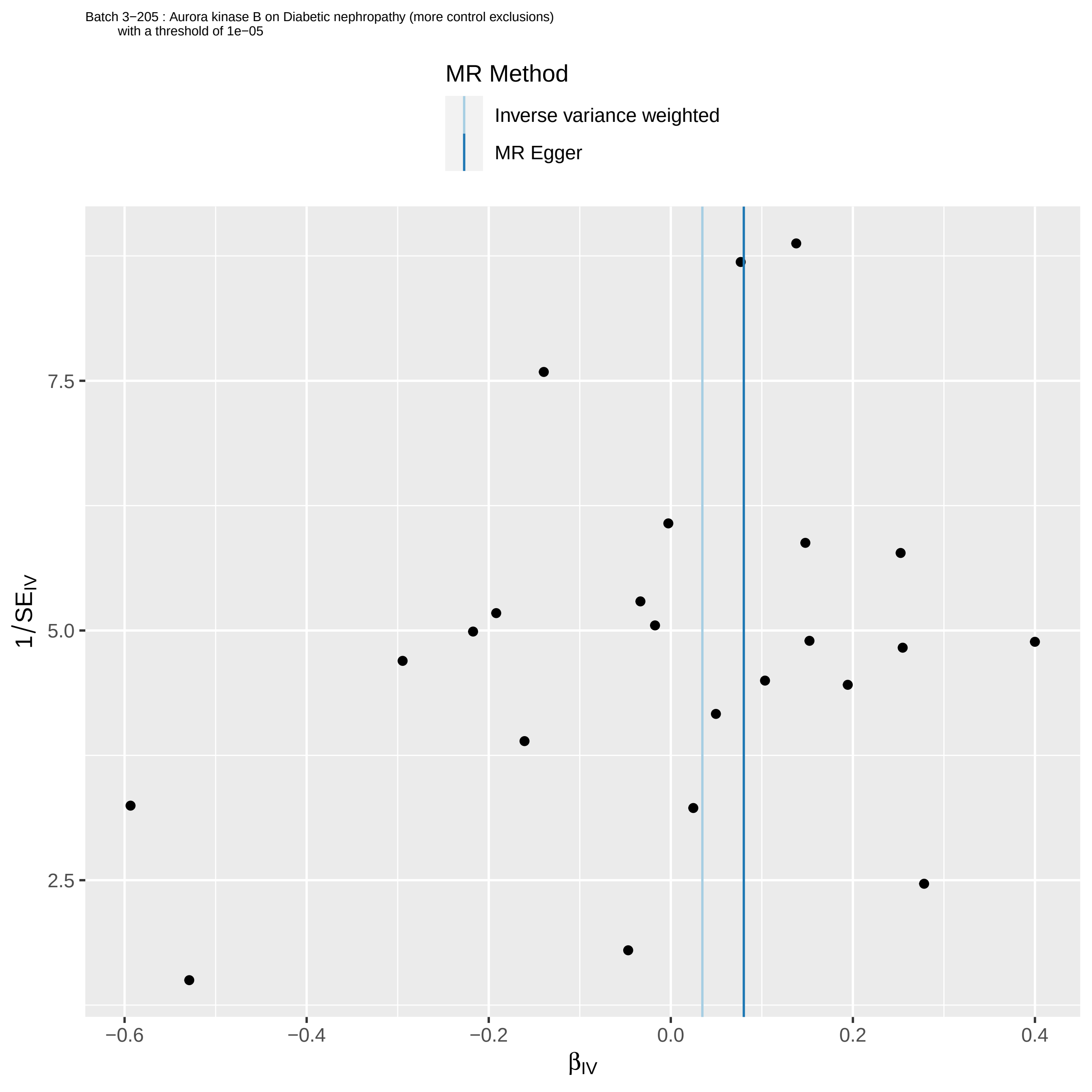

Batch 3−205 : Aurora kinase B on Diabetic nephropathy (more control exclusions)
with a threshold of 1e−05
MR Method
Inverse variance weighted
MR Egger
7.5
5.0
2.5
1/SE_IV
−0.6
−0.4
−0.2
0.0
0.2
0.4
β_IV

Batch 3−206 : Axin−2 on Diabetic nephropathy (more control exclusions)
with a threshold of 1e−05

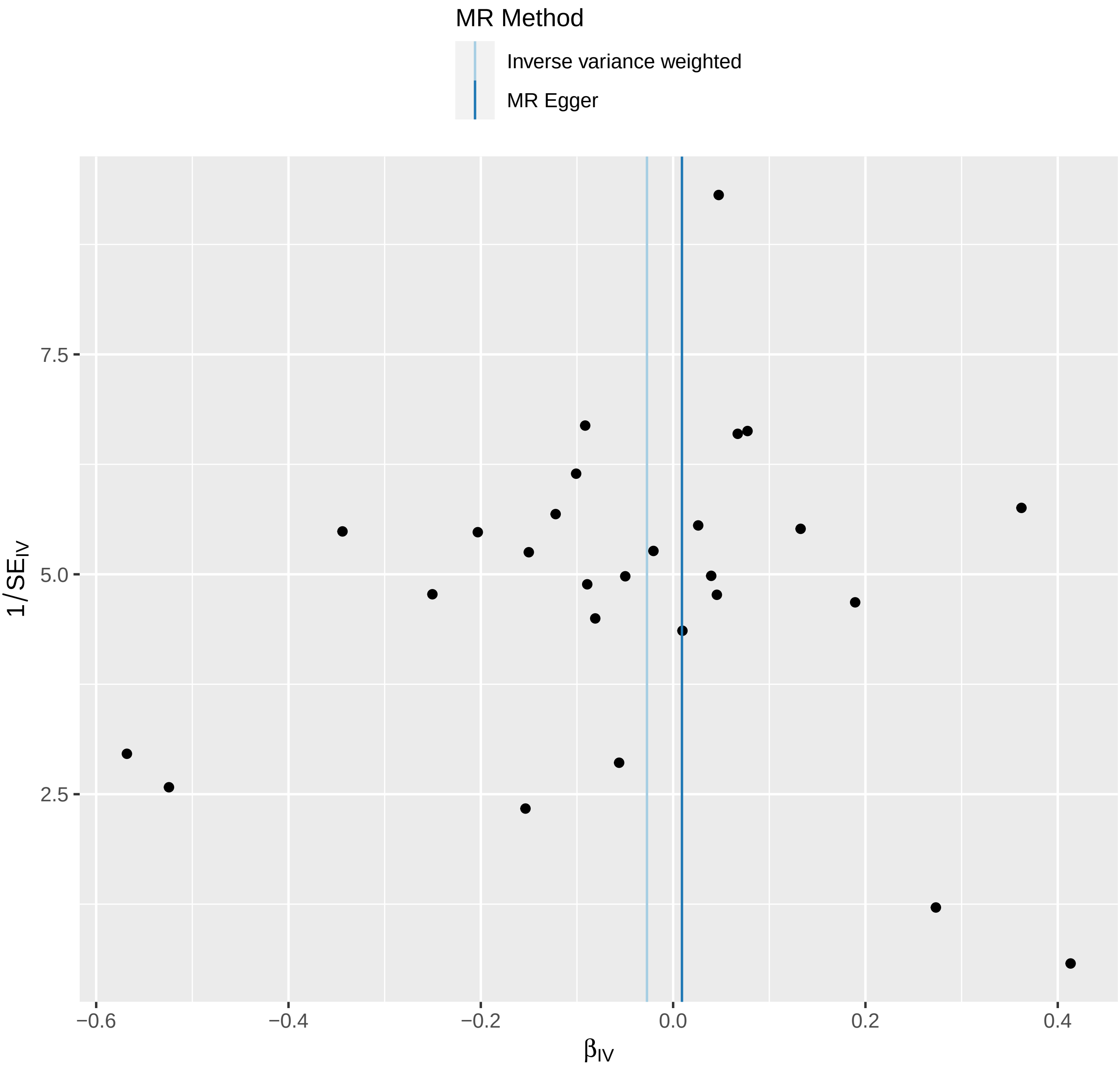

Batch 3−207 : Axin−2 on Diabetic nephropathy (more control exclusions)
with a threshold of 1e−05

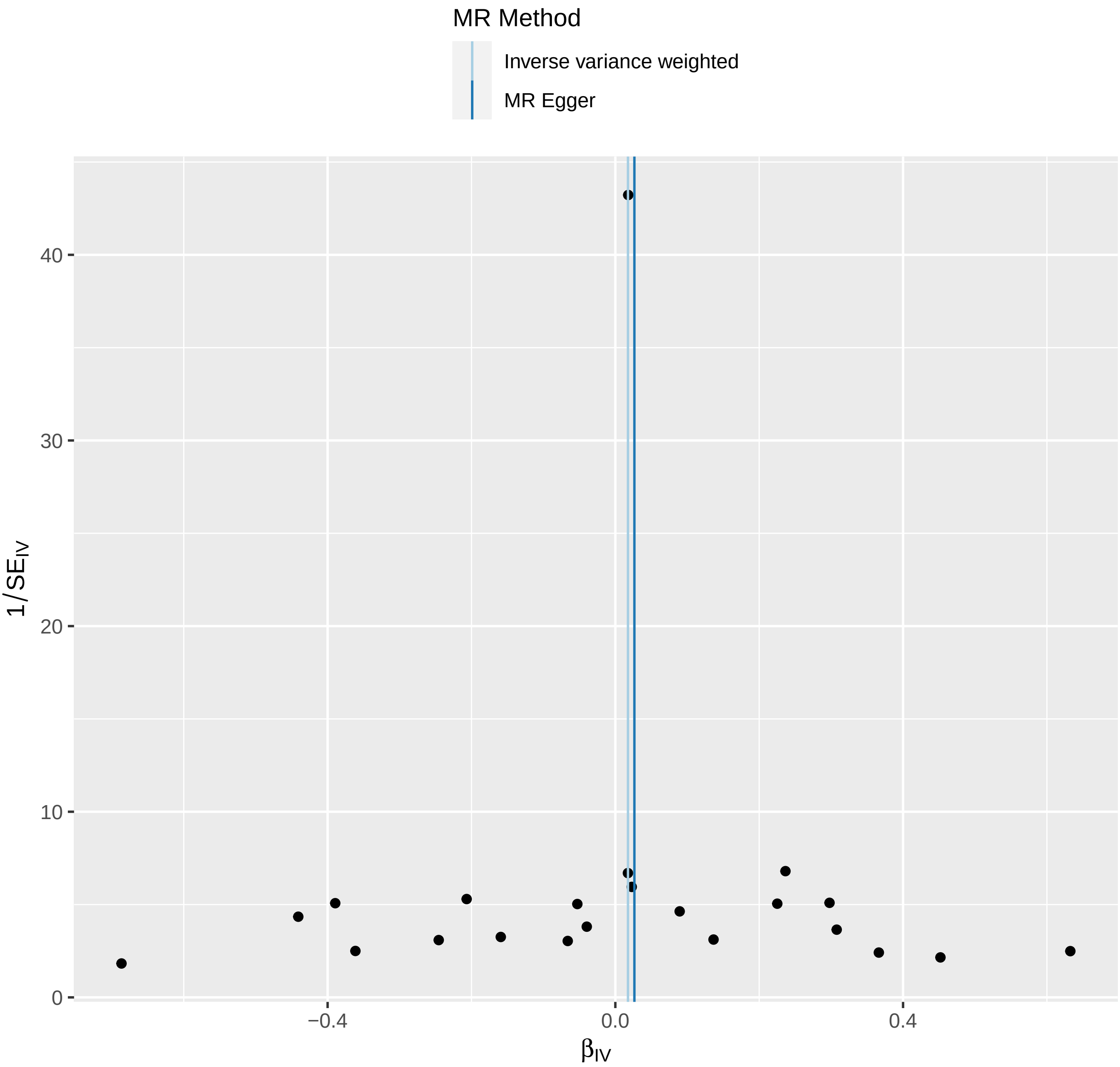

Batch 3−208 : Zinc−alpha−2−glycoprotein on Diabetic nephropathy (more control exclusions)
with a threshold of 1e−05

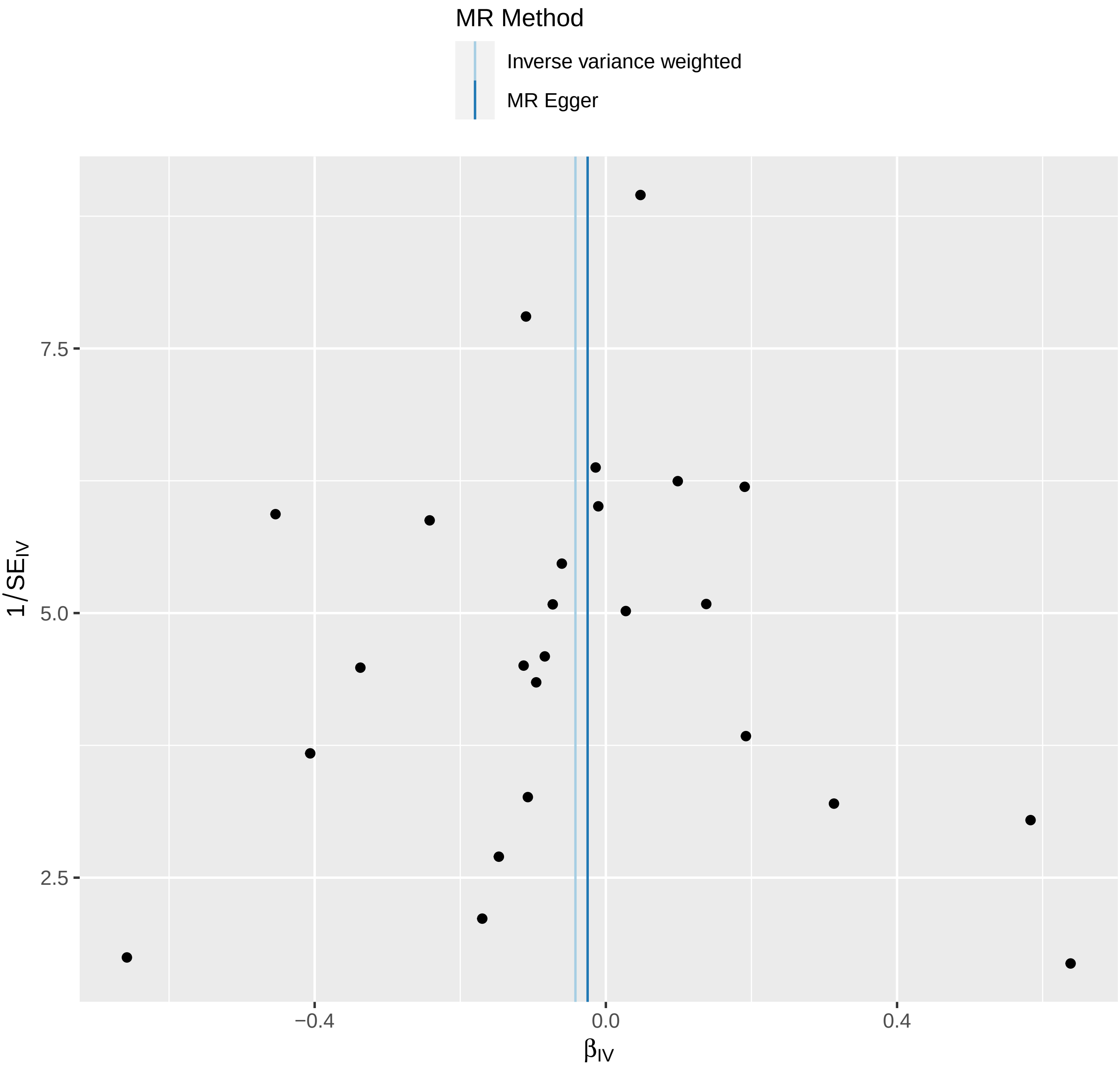

Batch 3−209 : Beta−1,3−galactosyltransferase 6 on Diabetic nephropathy (more control exclusions)
with a threshold of 1e−05

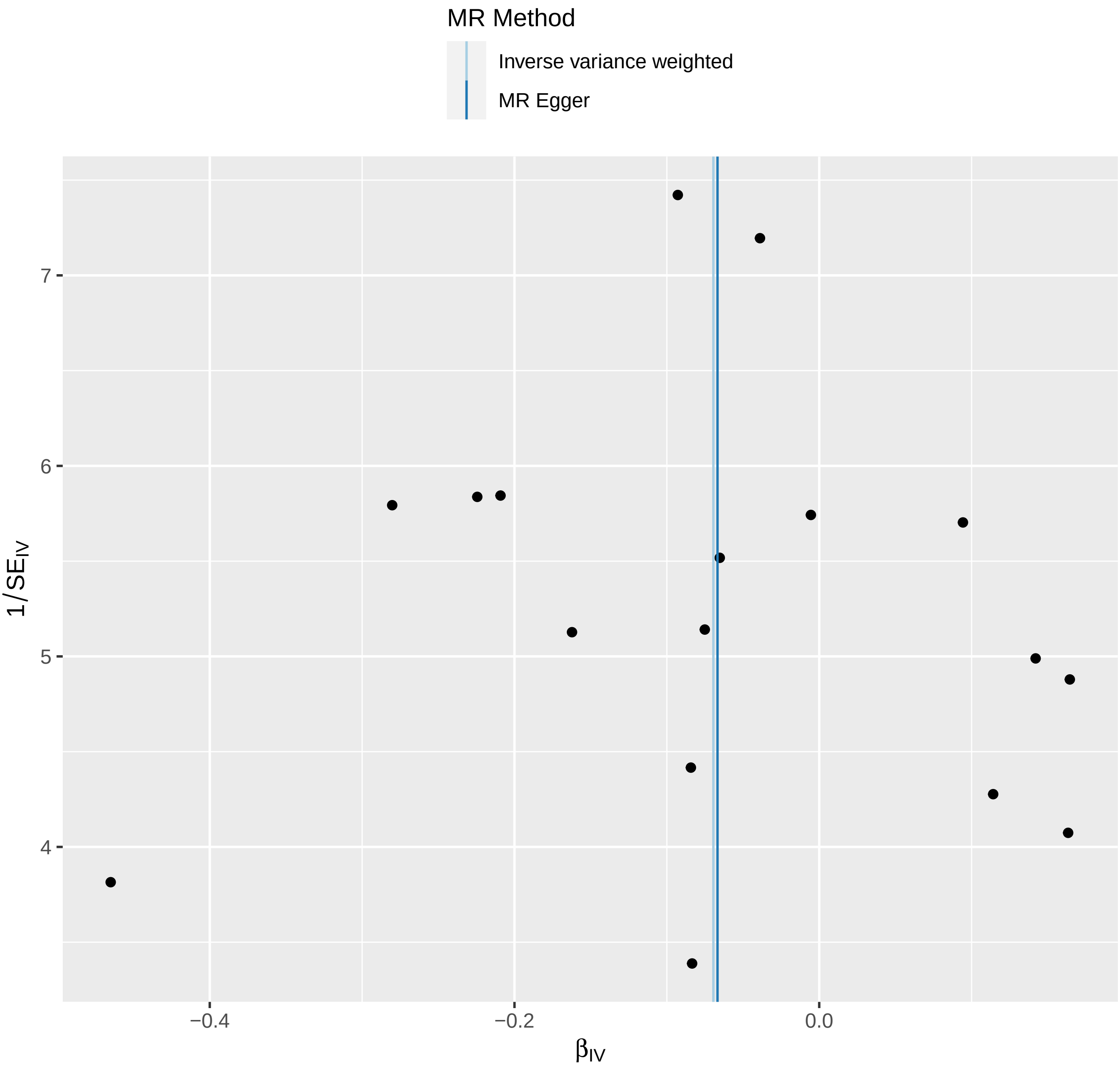

Batch 3−210 : Beta−1,3−glucosyltransferase on Diabetic nephropathy (more control exclusions)
with a threshold of 1e−05

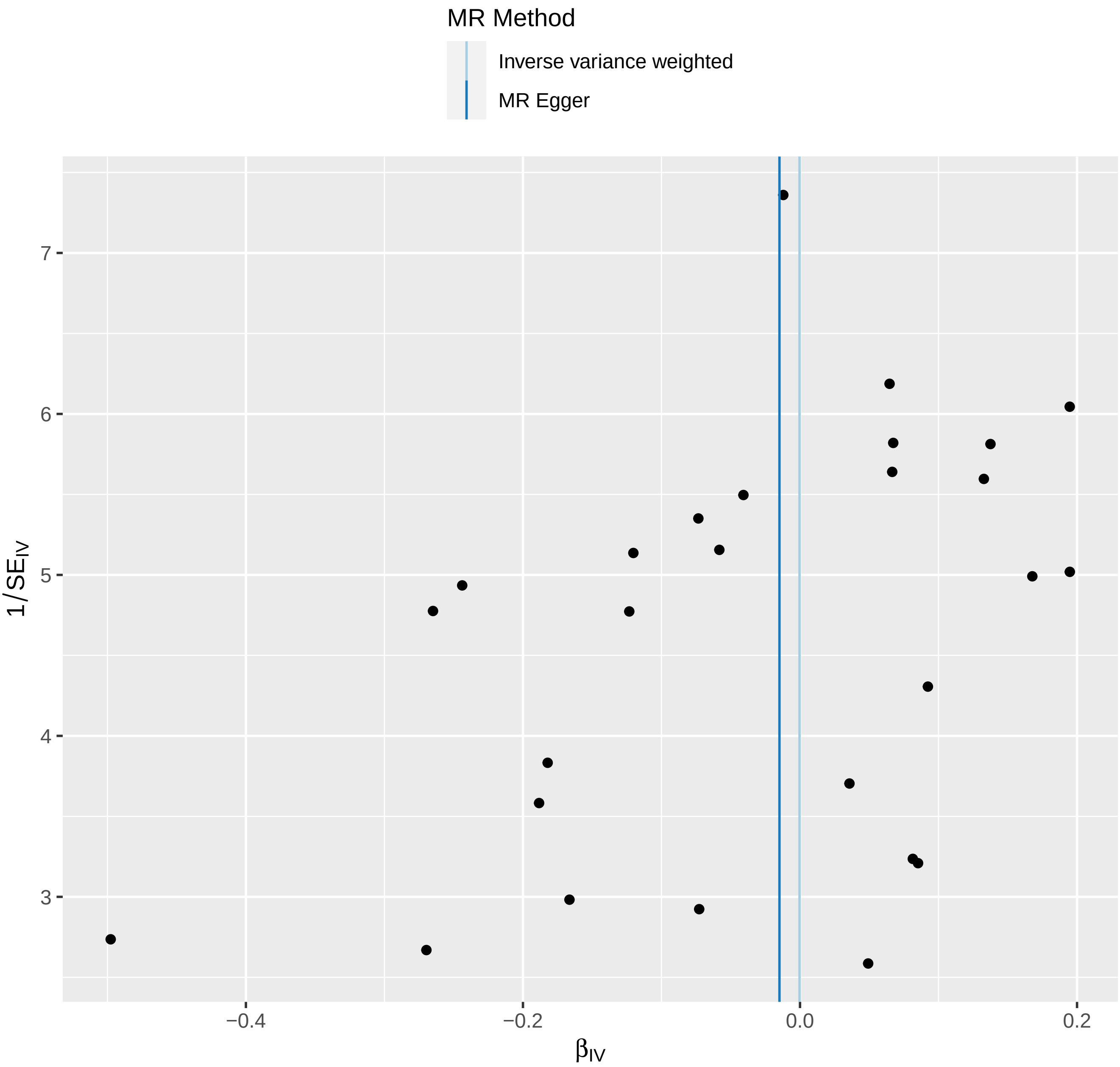

Batch 3−211 : Galactosylgalactosylxylosylprotein 3−beta−glucuronosyltransferase 3 on Diabetic nephropathy (more control exclusions)
with a threshold of 1e−05

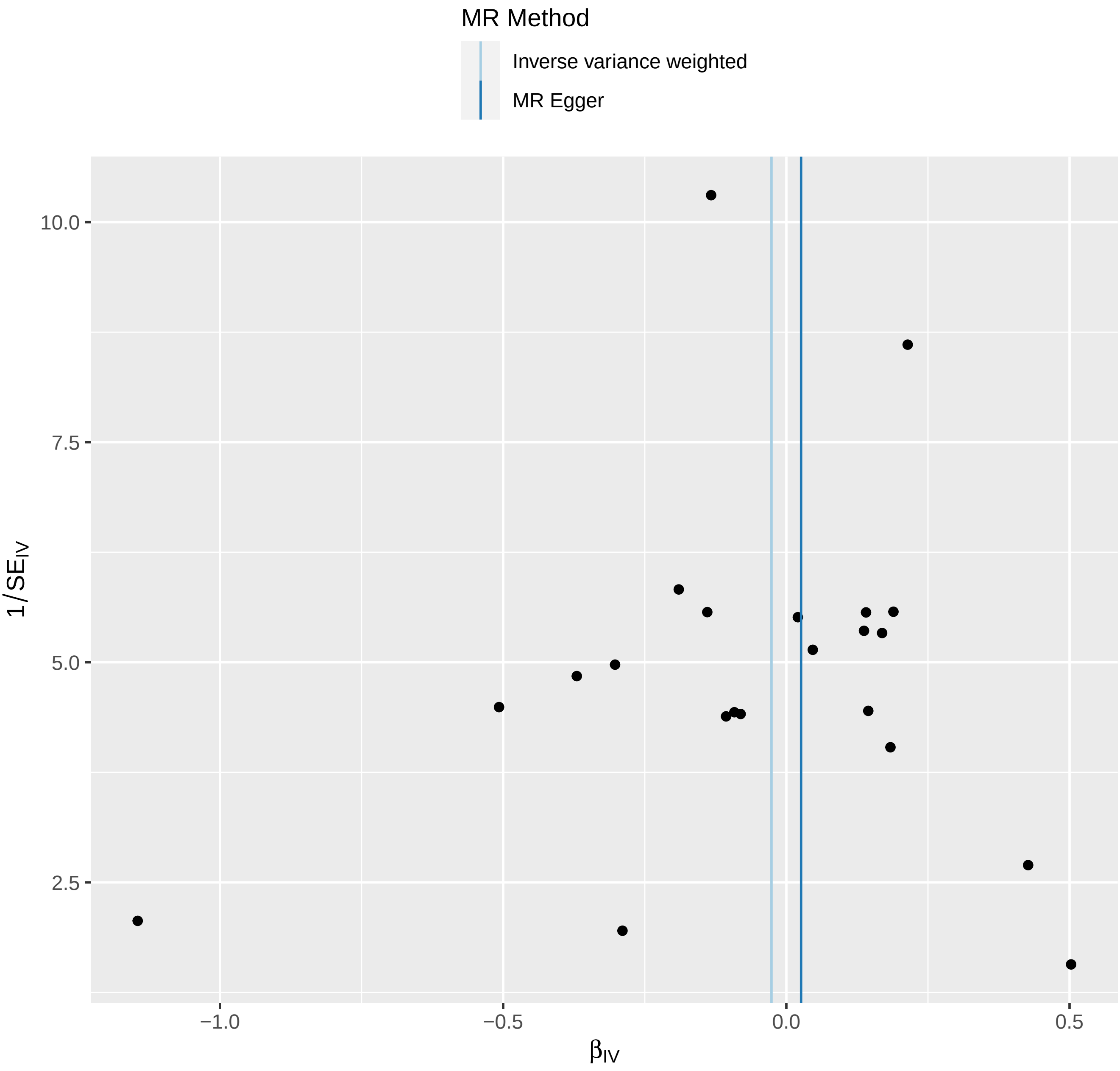

Batch 3−212 : Beta−1,4−glucuronyltransferase 1 on Diabetic nephropathy (more control exclusions)
with a threshold of 1e−05

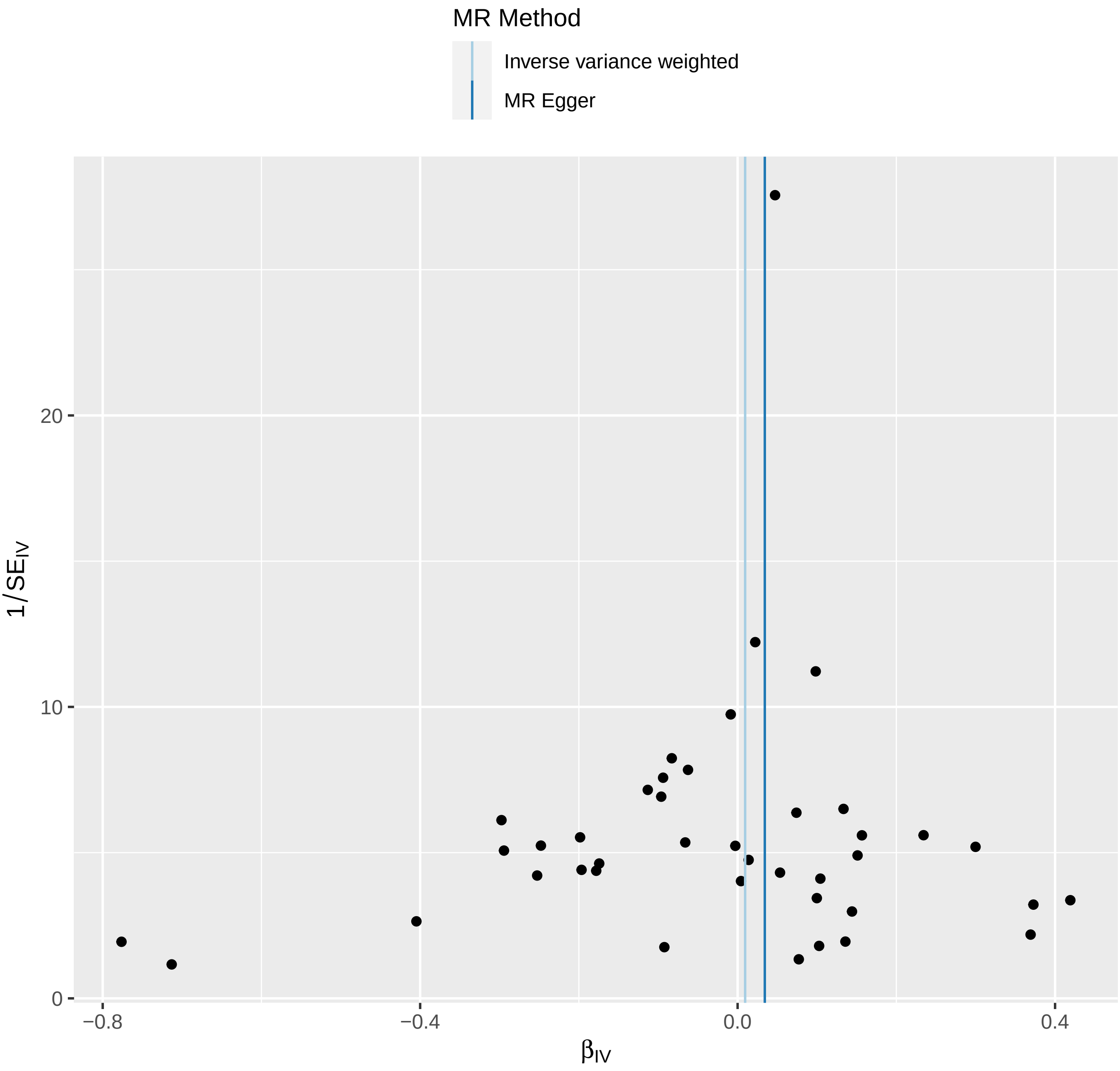

Batch 3−213 : N−acetyllactosaminide beta−1,3−N−acetylglucosaminyltransferase 2 on Diabetic nephropathy (more control exclusions) with a threshold of 1e−05

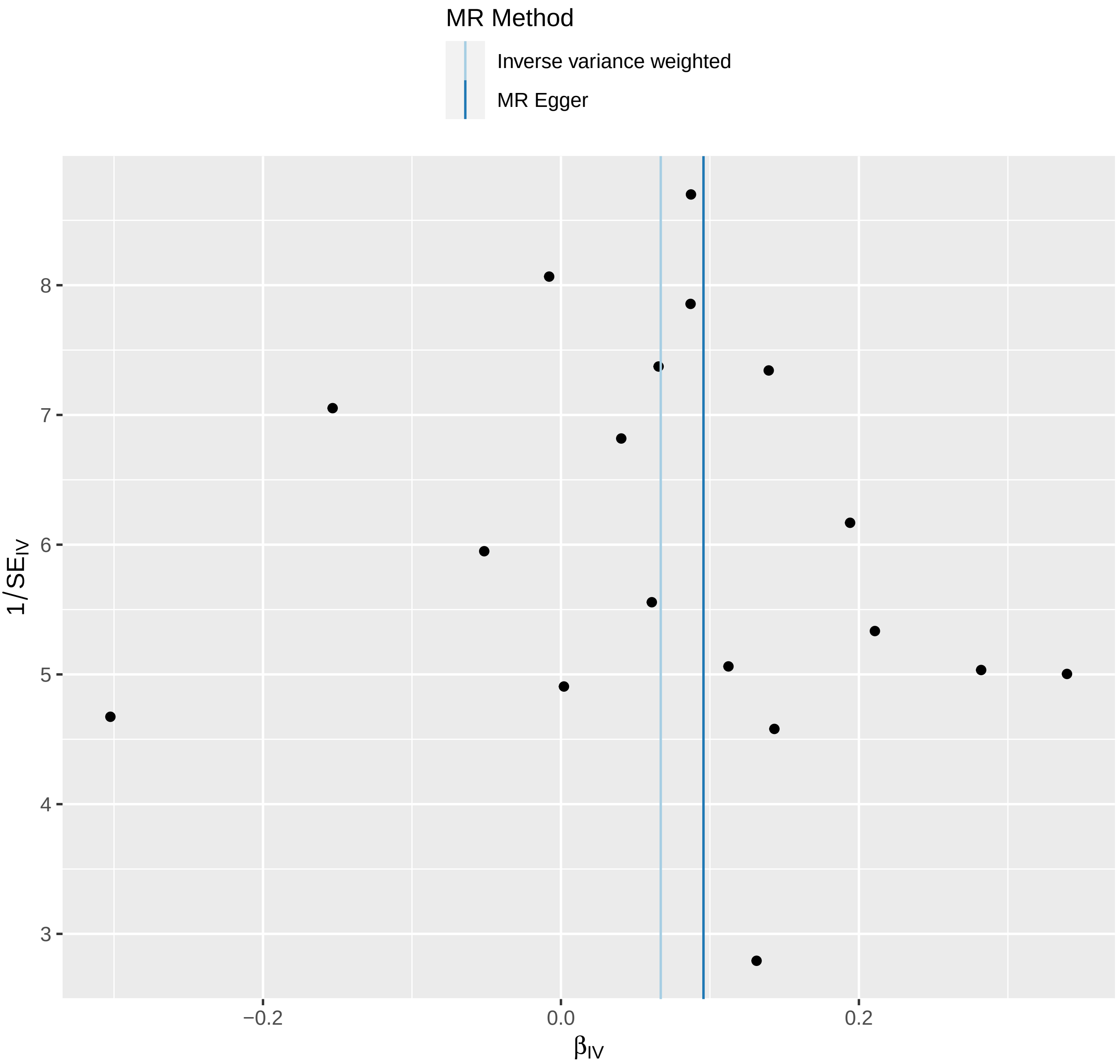

Batch 3−214 : UDP−GlcNAc:betaGal beta−1,3−N−acetylglucosaminyltransferase 6 on Diabetic nephropathy (more control exclusions)
with a threshold of 1e−05

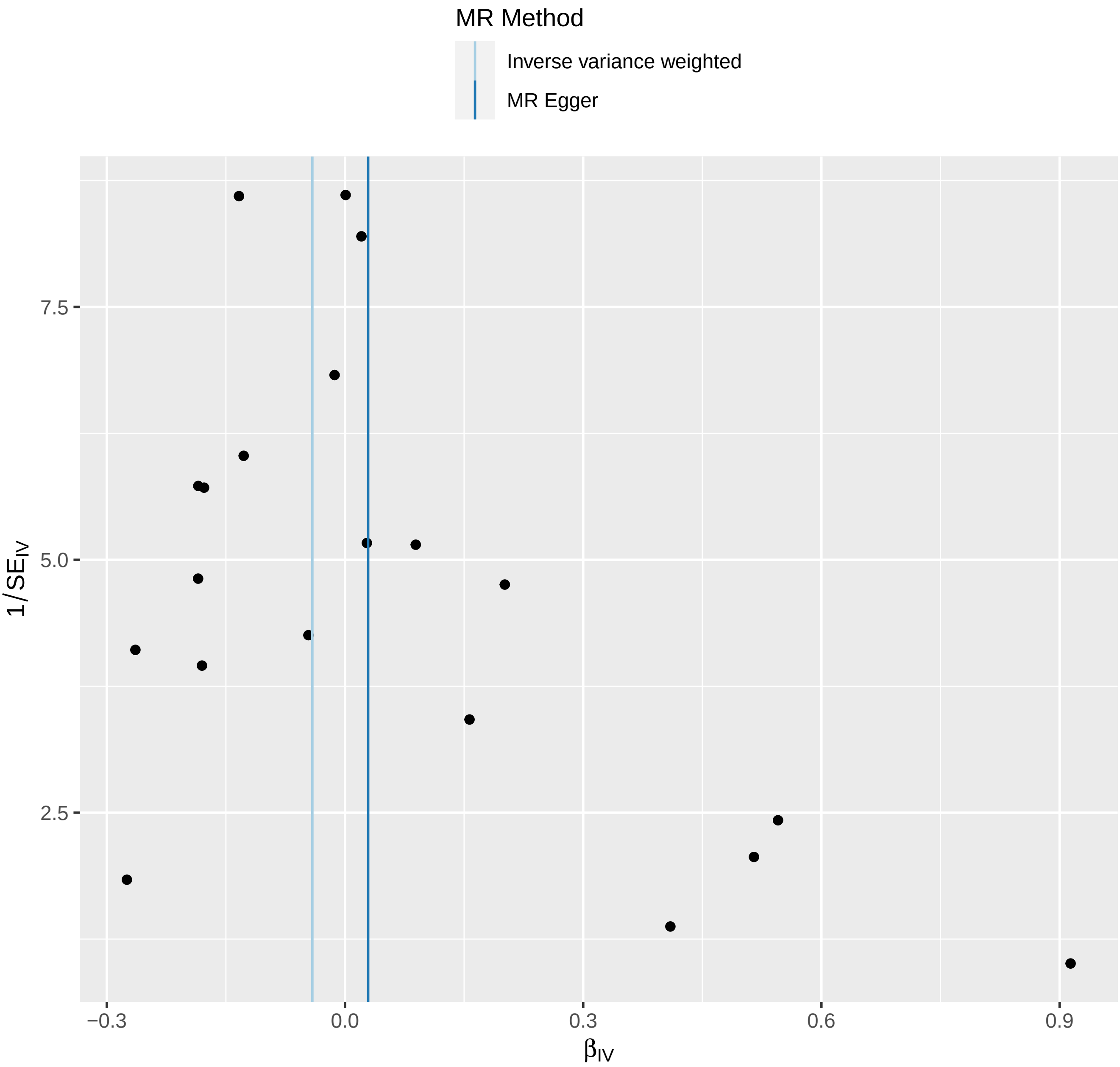

Batch 3−215 : UDP−GlcNAc:betaGal beta−1,3−N−acetylglucosaminyltransferase 8 on Diabetic nephropathy (more control exclusions) with a threshold of 1e−05

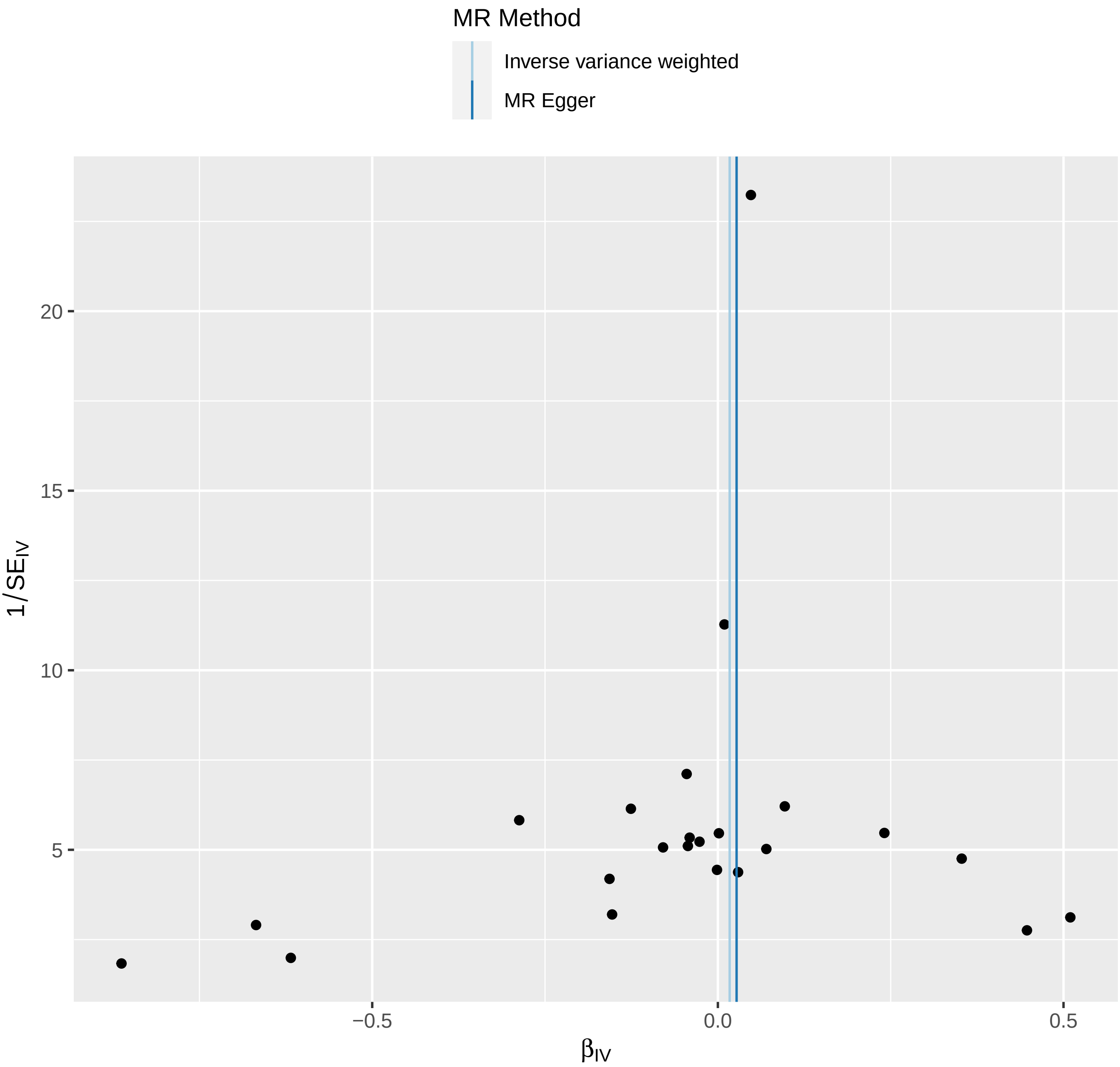

Batch 3−216 : Beta−1,4−galactosyltransferase 1 on Diabetic nephropathy (more control exclusions)
with a threshold of 1e−05

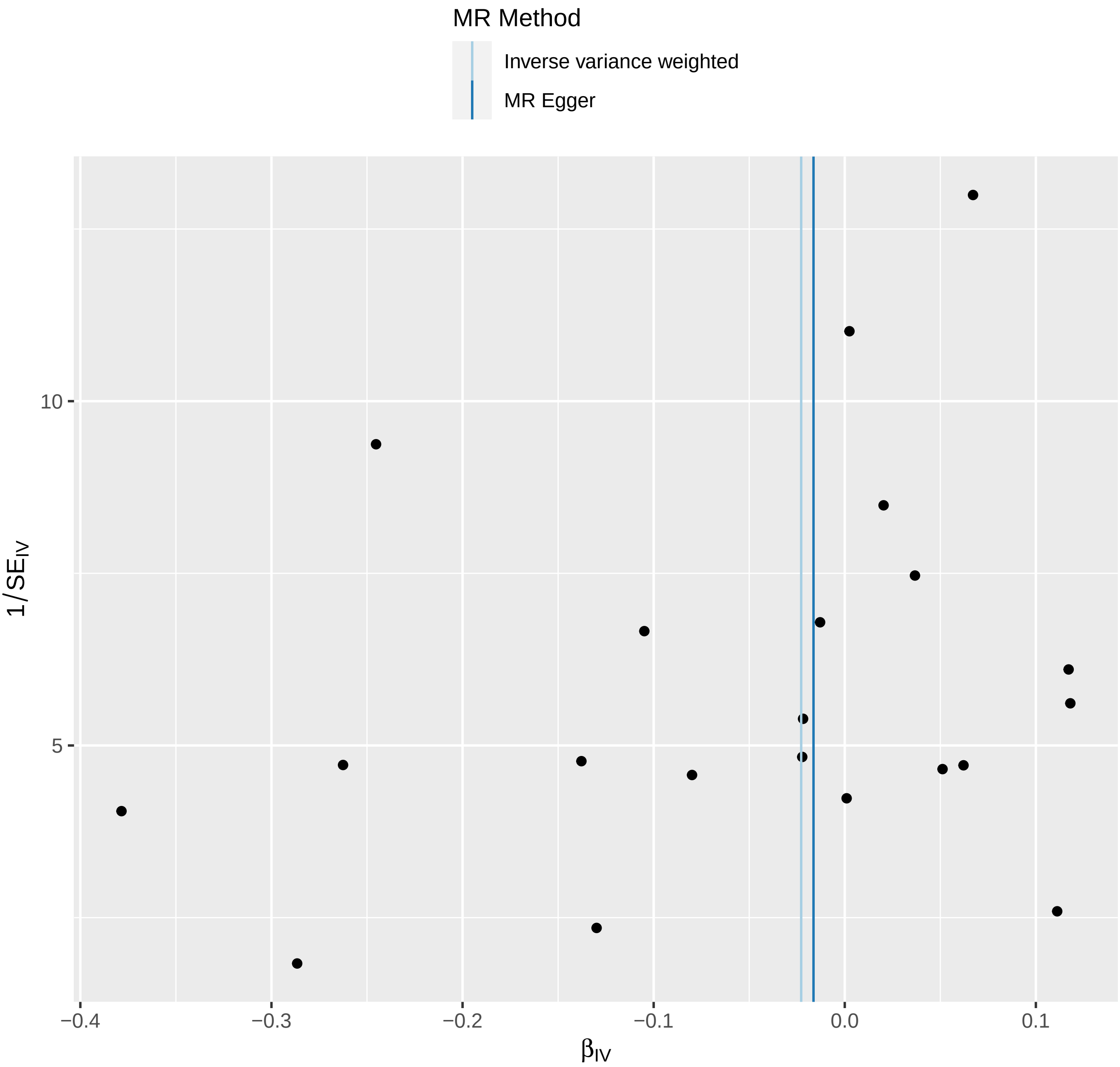

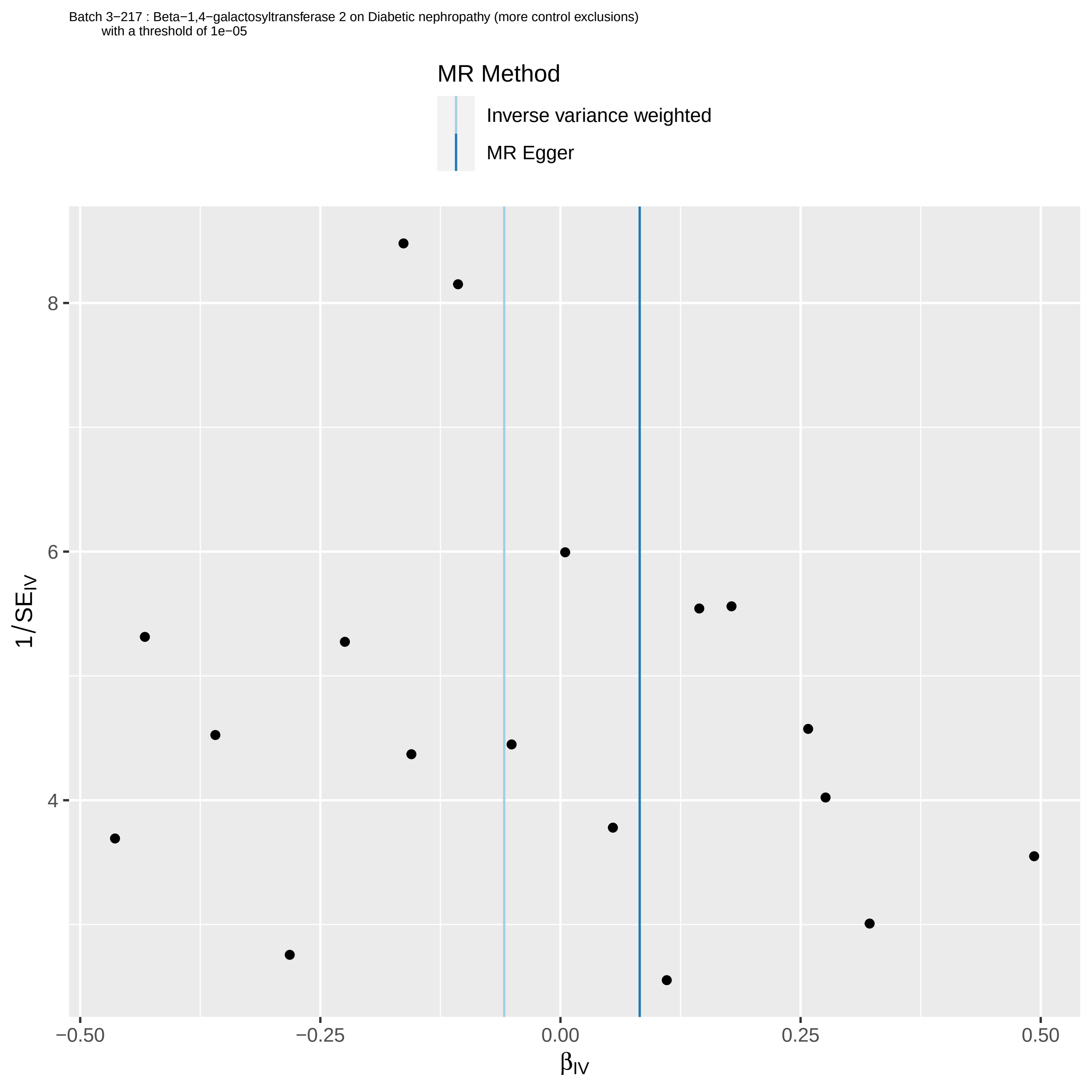

Batch 3−217 : Beta−1,4−galactosyltransferase 2 on Diabetic nephropathy (more control exclusions)
with a threshold of 1e−05
MR Method
Inverse variance weighted
MR Egger
8
6
4
1/SE$_{IV}$
−0.50
−0.25
0.00
0.25
0.50
β$_{IV}$

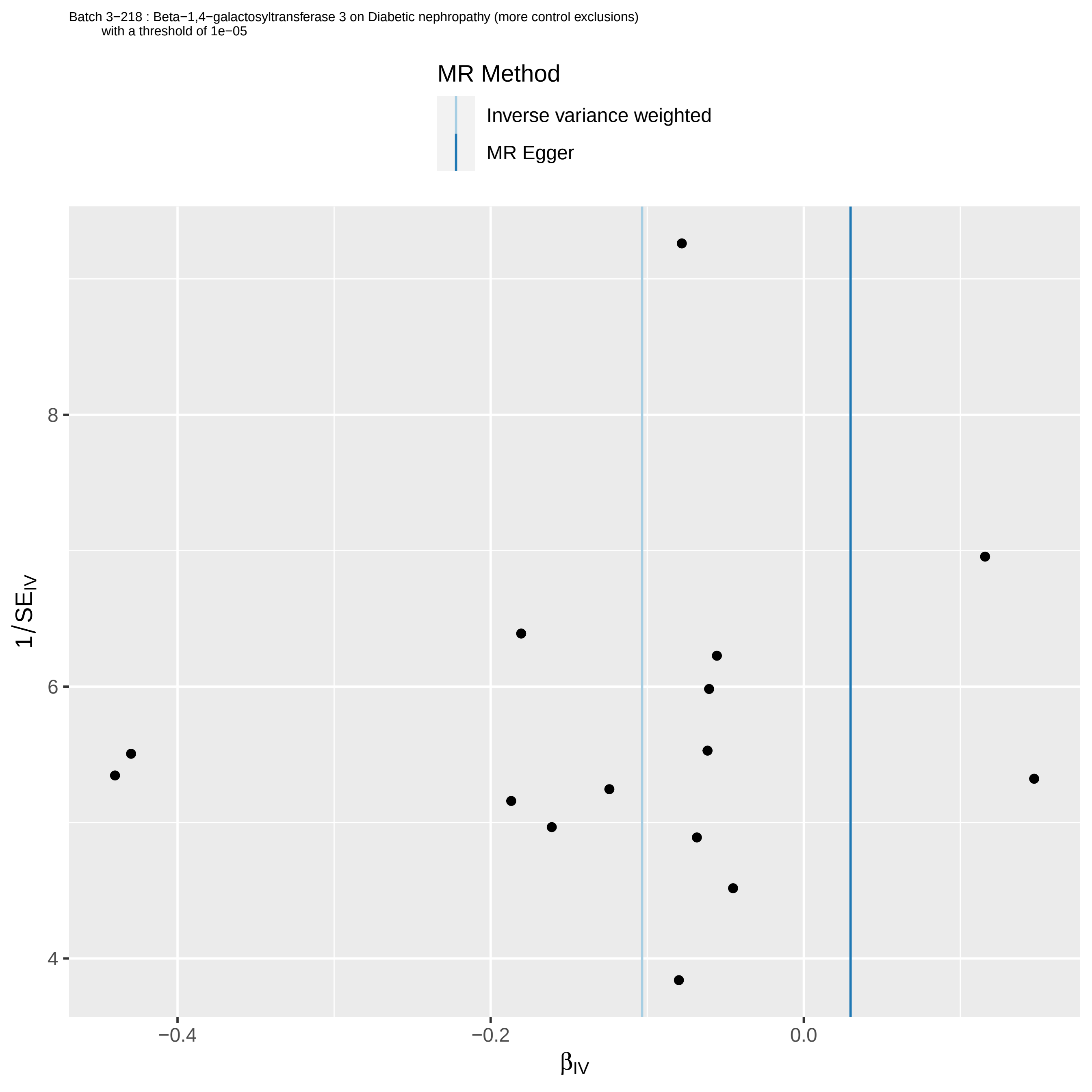

Batch 3−218 : Beta−1,4−galactosyltransferase 3 on Diabetic nephropathy (more control exclusions)
with a threshold of 1e−05
MR Method
Inverse variance weighted
MR Egger
1/SE$_{IV}$
8
6
4
−0.4
−0.2
0.0
β_{IV}

Batch 3−219 : Beta−1,4−galactosyltransferase 5 on Diabetic nephropathy (more control exclusions)
with a threshold of 1e−05

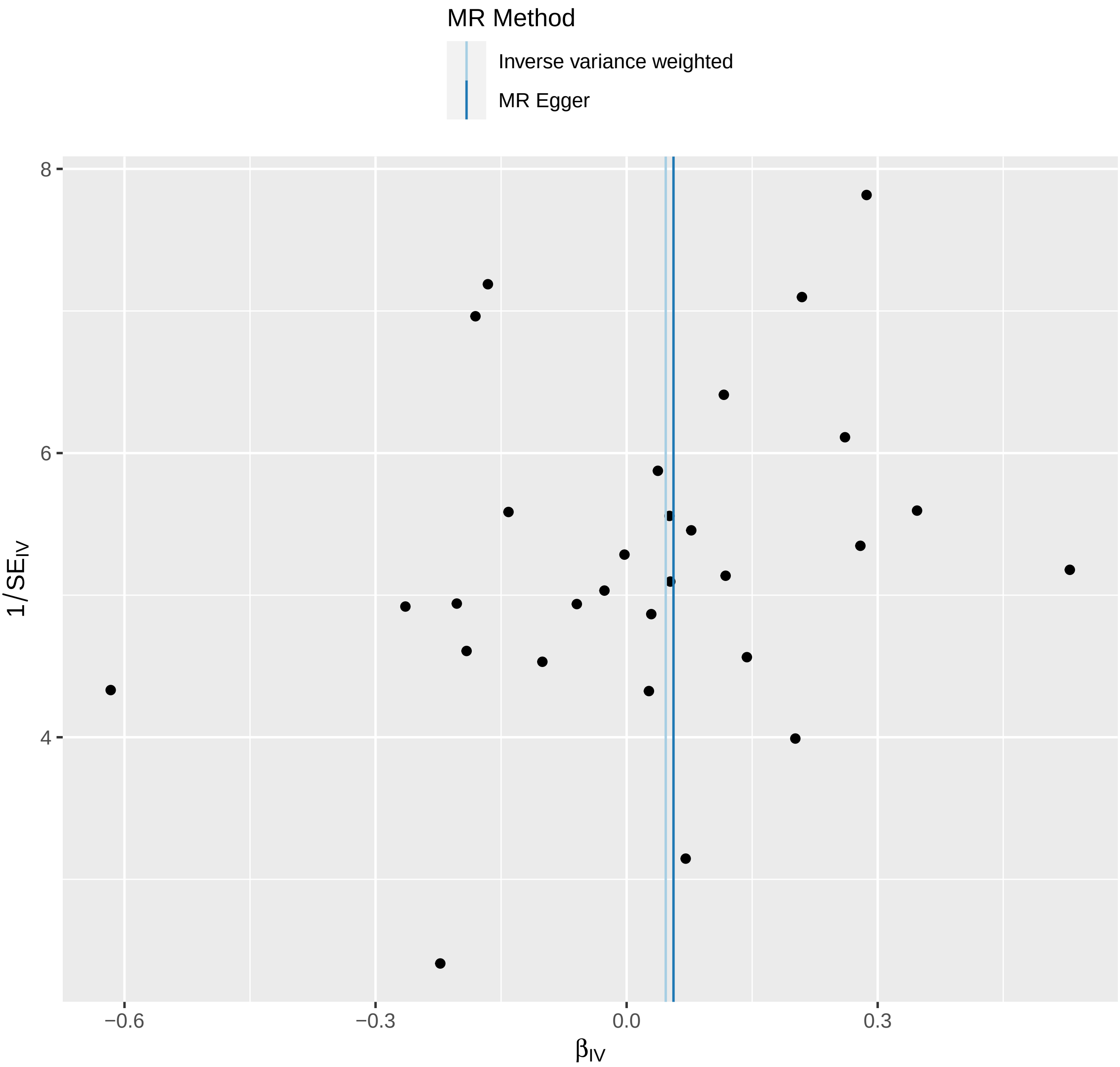

Batch 3−220 : Beta−1,4−galactosyltransferase 6 on Diabetic nephropathy (more control exclusions) with a threshold of 1e−05

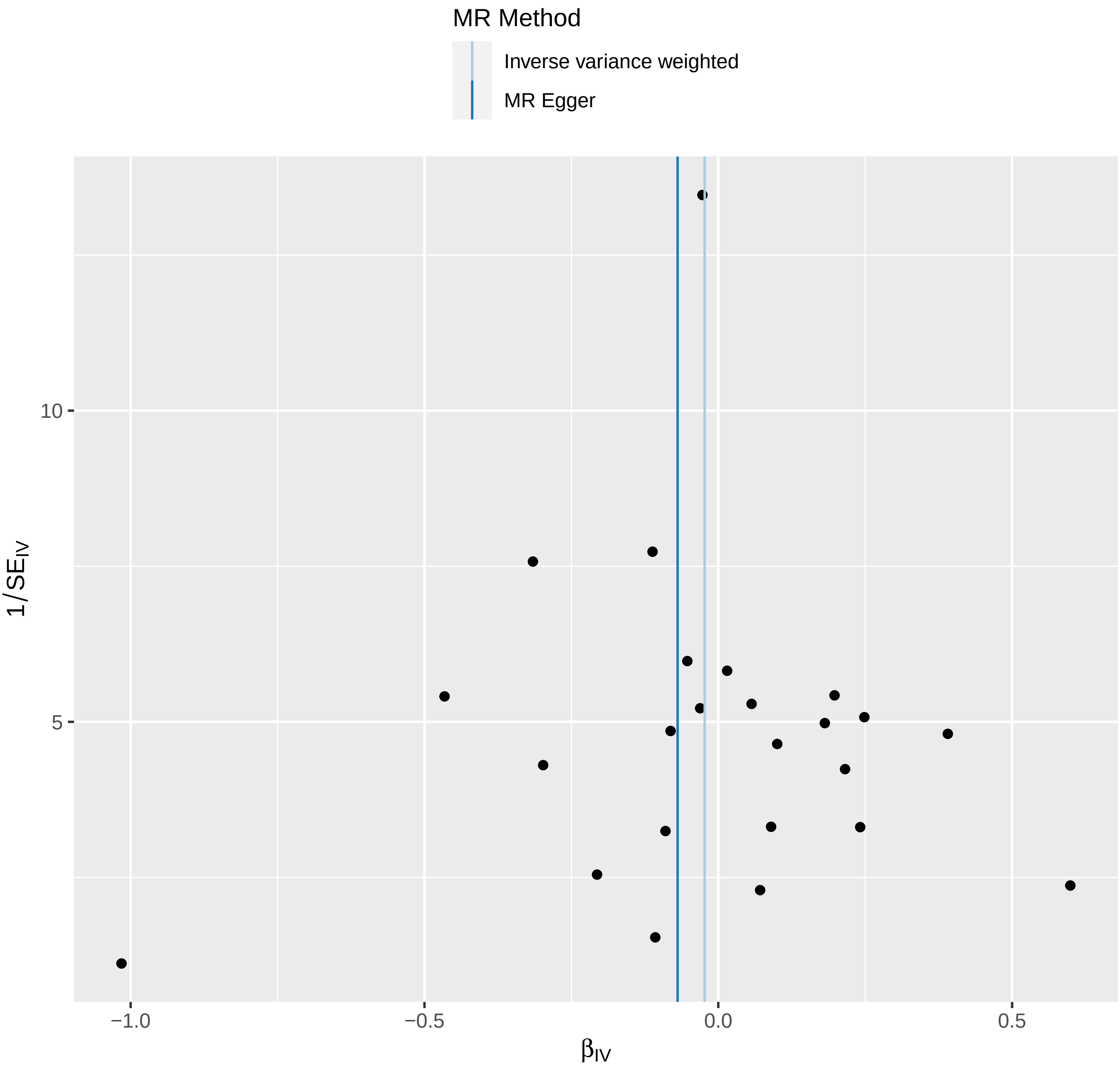

Batch 3−221 : Beta−1,4−galactosyltransferase 7 on Diabetic nephropathy (more control exclusions)
with a threshold of 1e−05

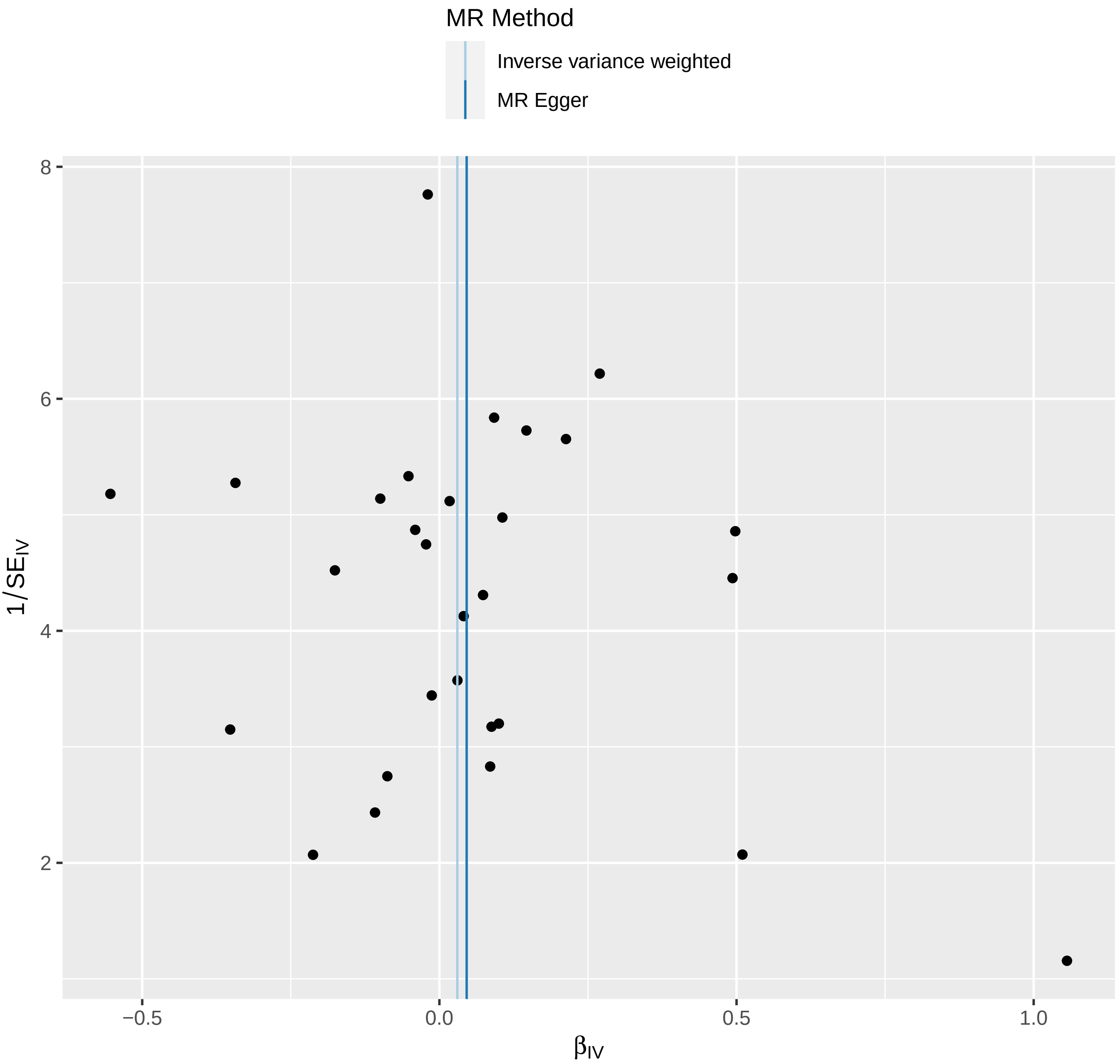

Batch 3−222 : Transcription regulator protein BACH1 on Diabetic nephropathy (more control exclusions)
with a threshold of 1e−05

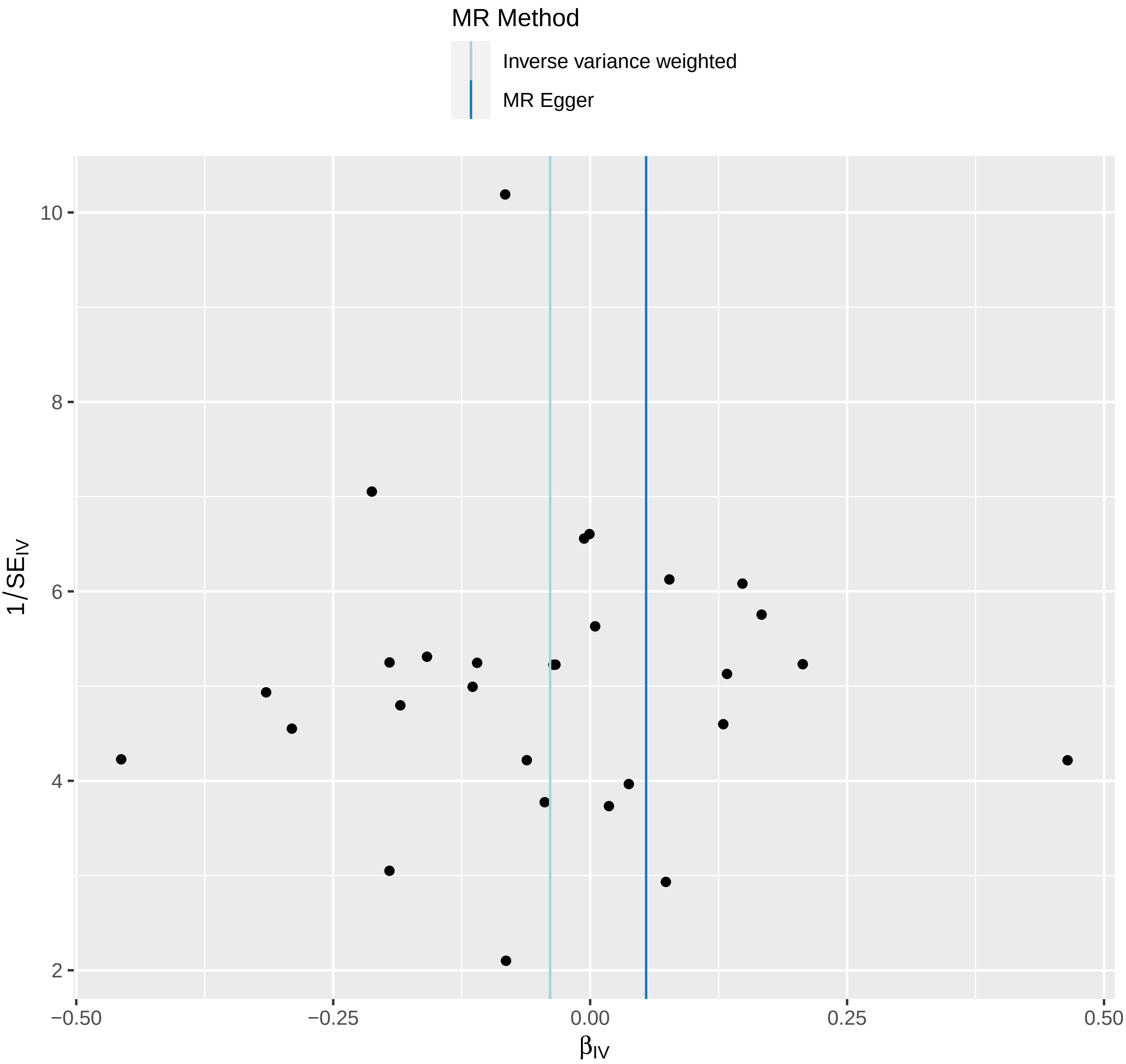

Batch 3−223 : Transcription regulator protein BACH2 on Diabetic nephropathy (more control exclusions)
with a threshold of 1e−05

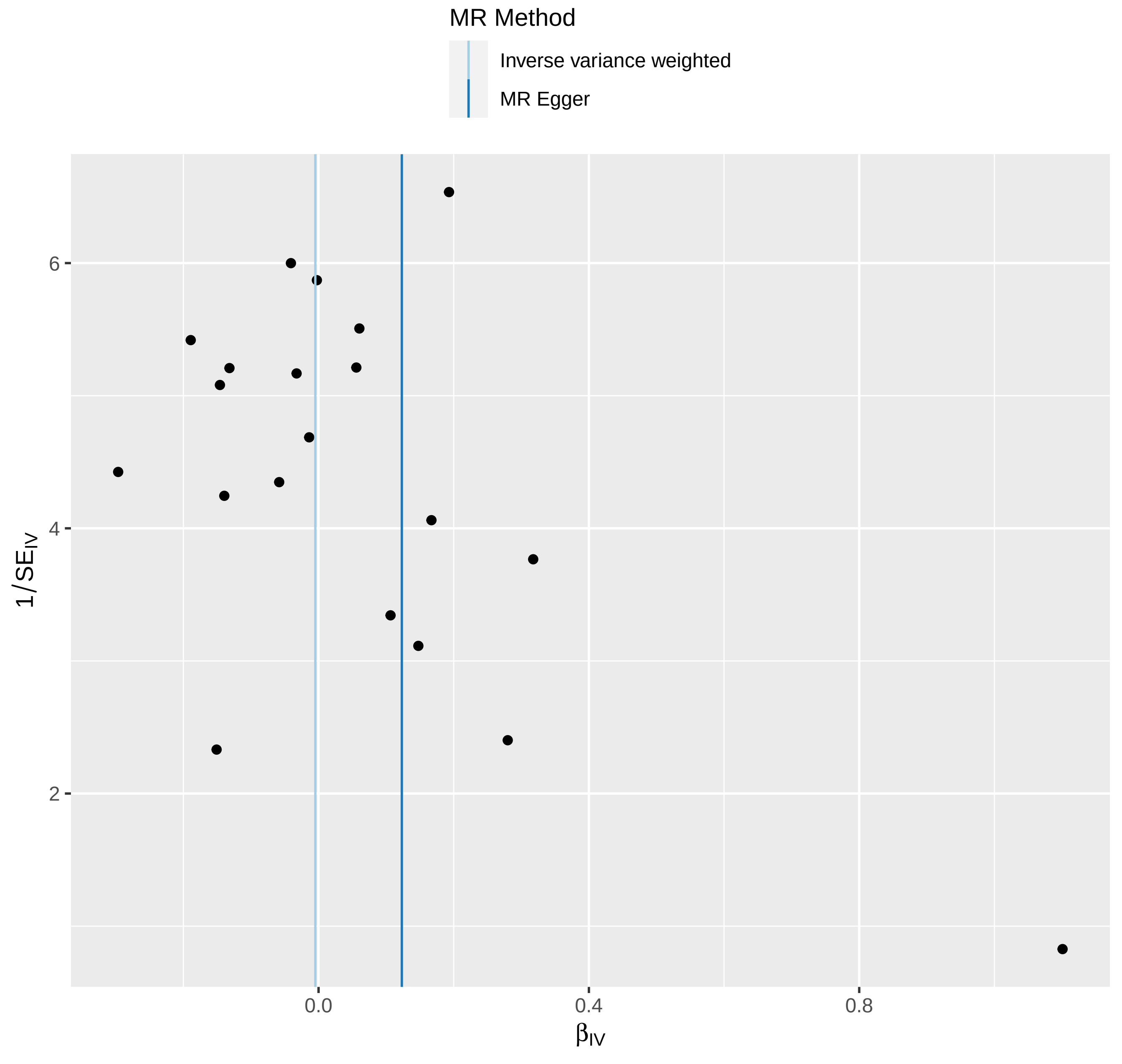

Batch 3−224 : Bcl2−associated agonist of cell death on Diabetic nephropathy (more control exclusions)
with a threshold of 1e−05

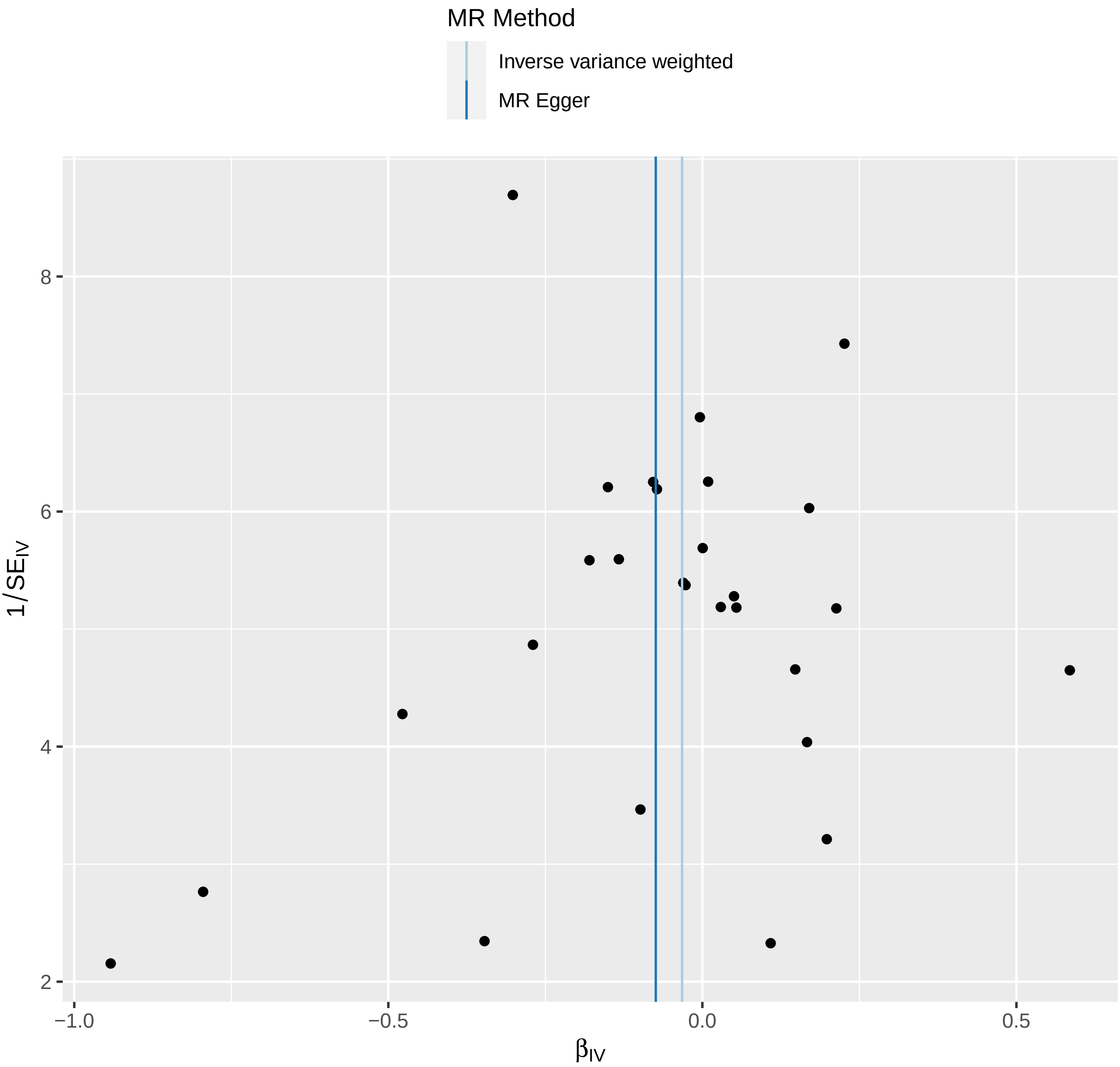

Batch 3−225 : BAG family molecular chaperone regulator 3 on Diabetic nephropathy (more control exclusions) with a threshold of 1e−05

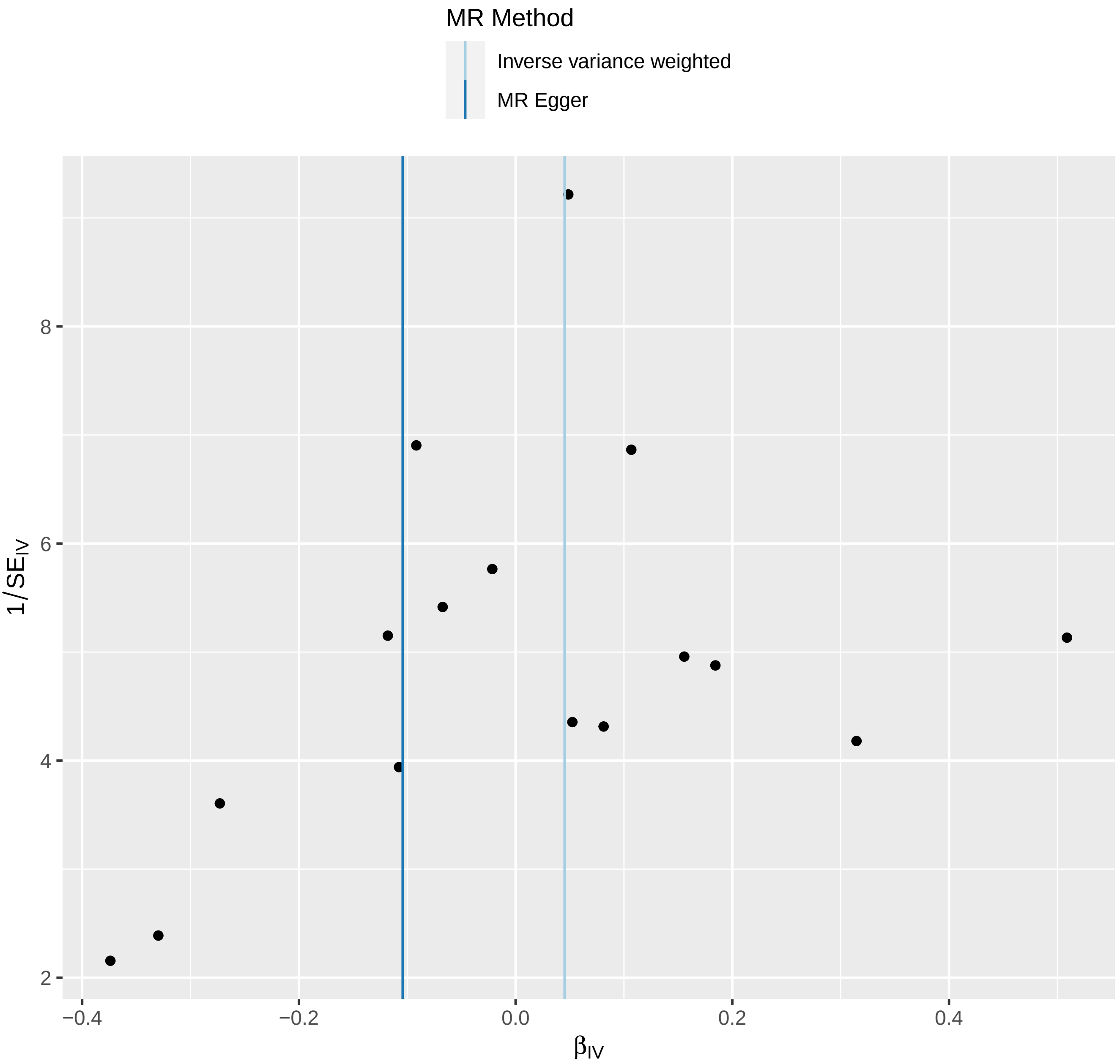

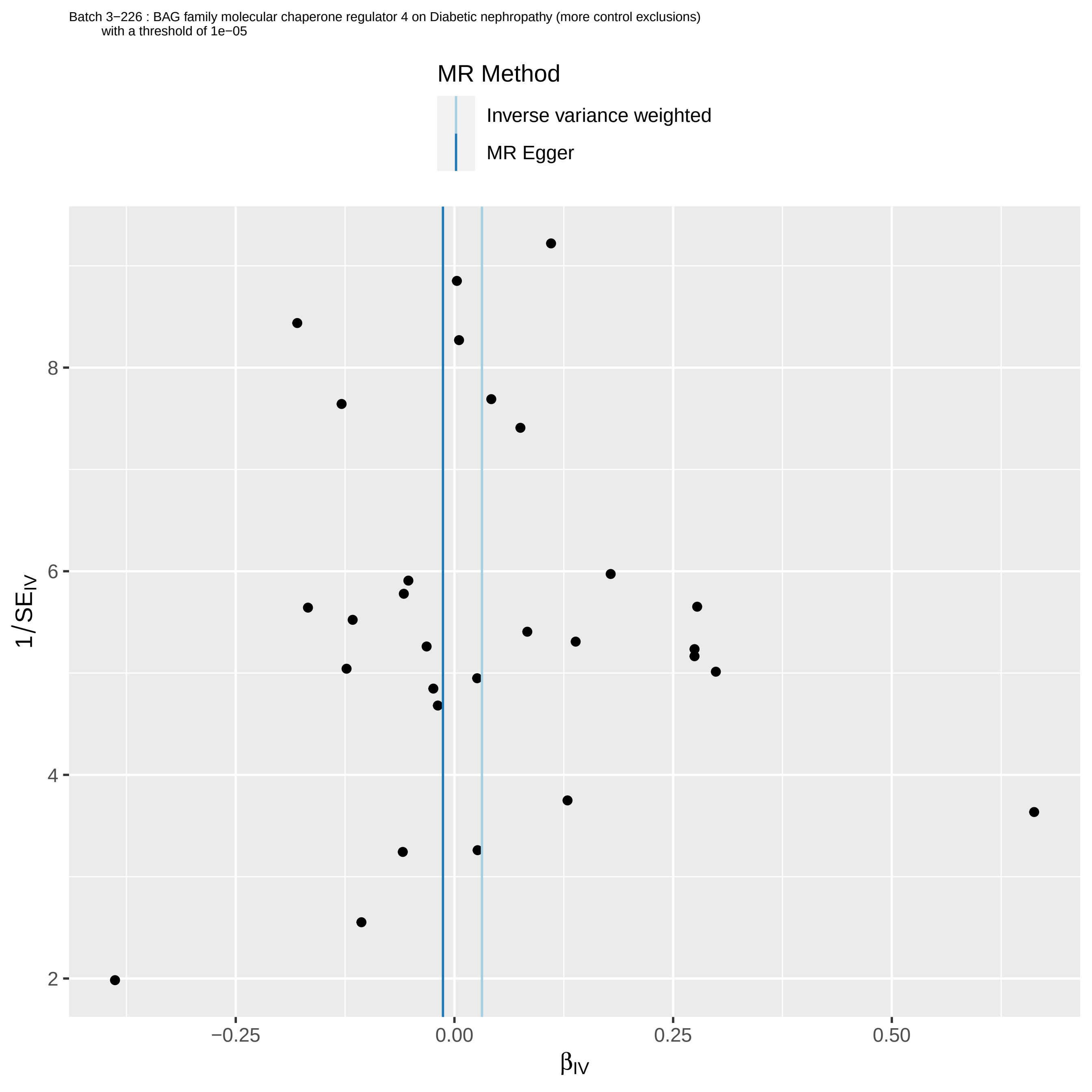

Batch 3−226 : BAG family molecular chaperone regulator 4 on Diabetic nephropathy (more control exclusions)
with a threshold of 1e−05
MR Method
Inverse variance weighted
MR Egger
1/SE_IV
β_IV
8
6
4
2
−0.25
0.00
0.25
0.50

Batch 3−227 : B melanoma antigen 2 on Diabetic nephropathy (more control exclusions)
with a threshold of 1e−05

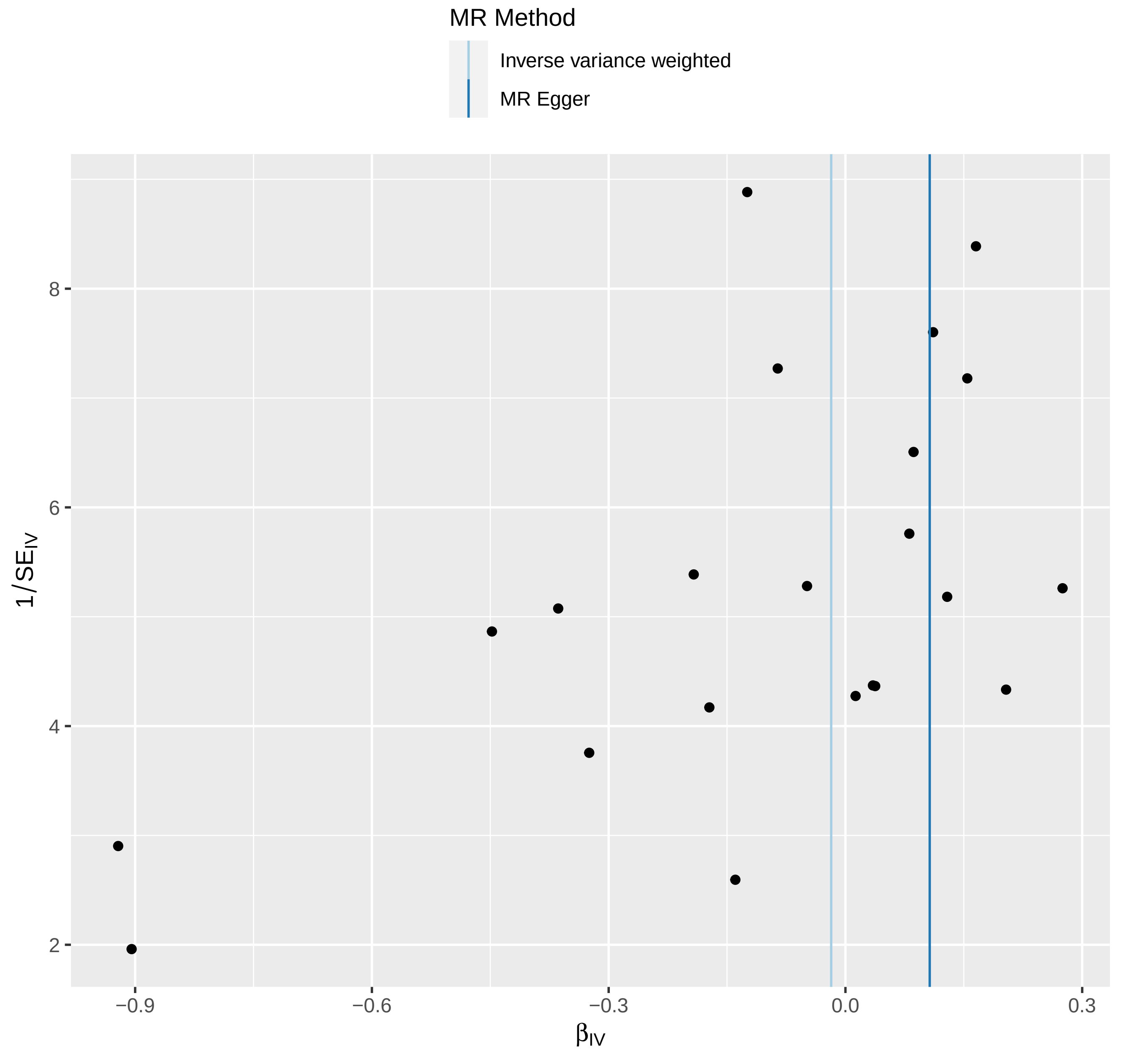

Batch 3−228 : B melanoma antigen 3 on Diabetic nephropathy (more control exclusions)
with a threshold of 1e−05

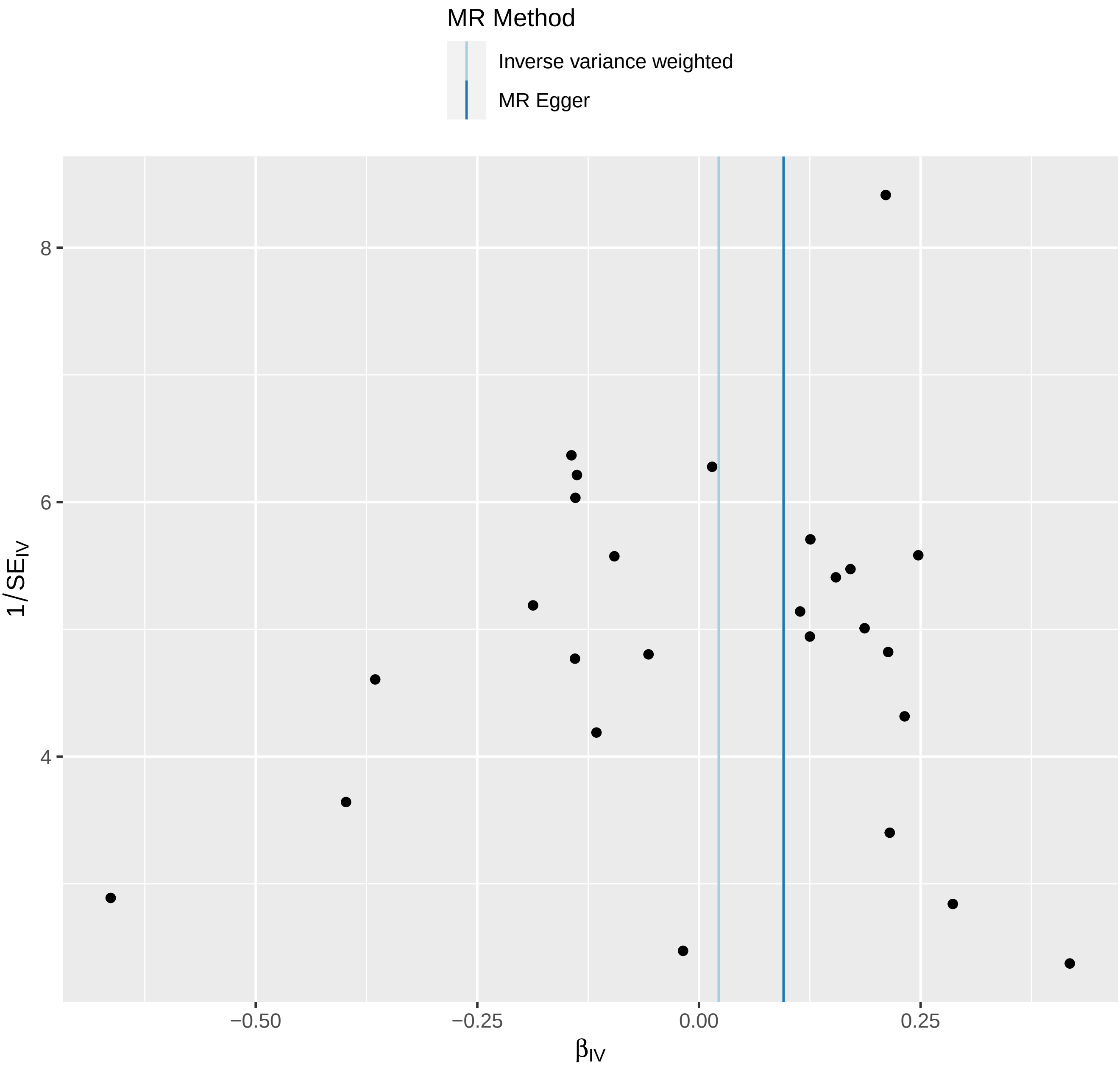

Batch 3−229 : BRCA1−associated RING domain protein 1 on Diabetic nephropathy (more control exclusions) with a threshold of 1e−05

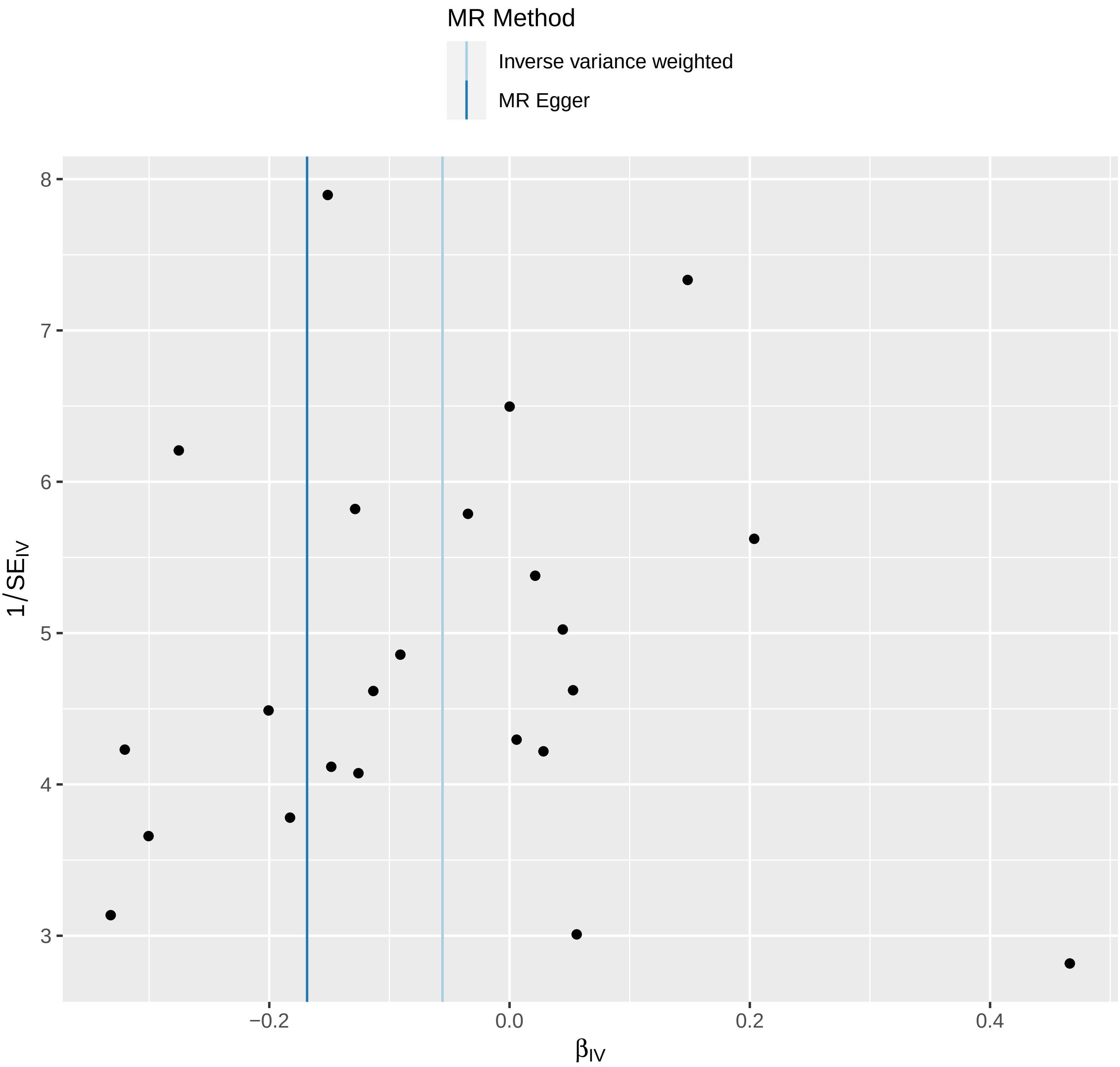

Batch 3−230 : Basic leucine zipper transcriptional factor ATF−like 3 on Diabetic nephropathy (more control exclusions)
with a threshold of 1e−05

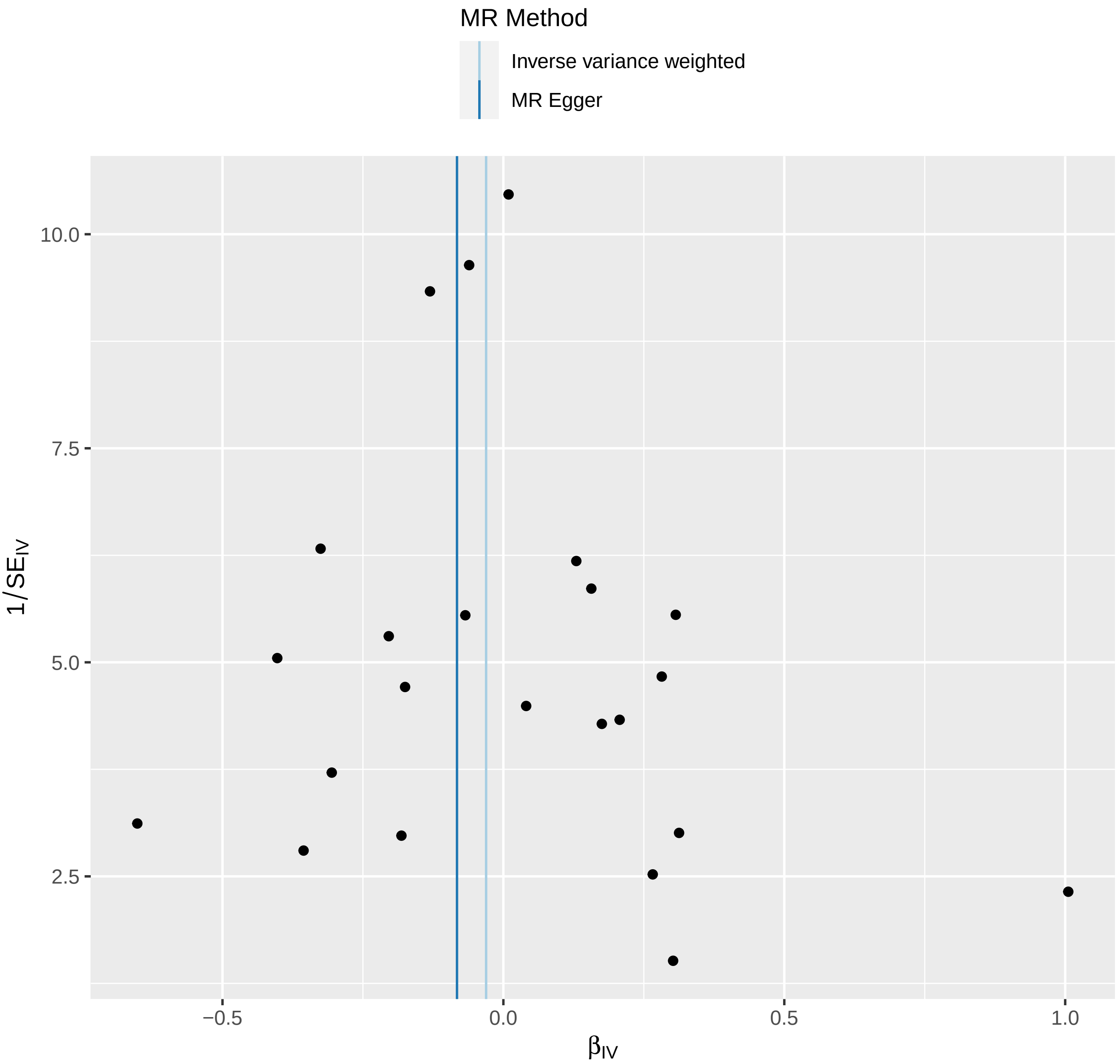

Batch 3−231 : Basal Cell Adhesion Molecule on Diabetic nephropathy (more control exclusions)
with a threshold of 1e−05

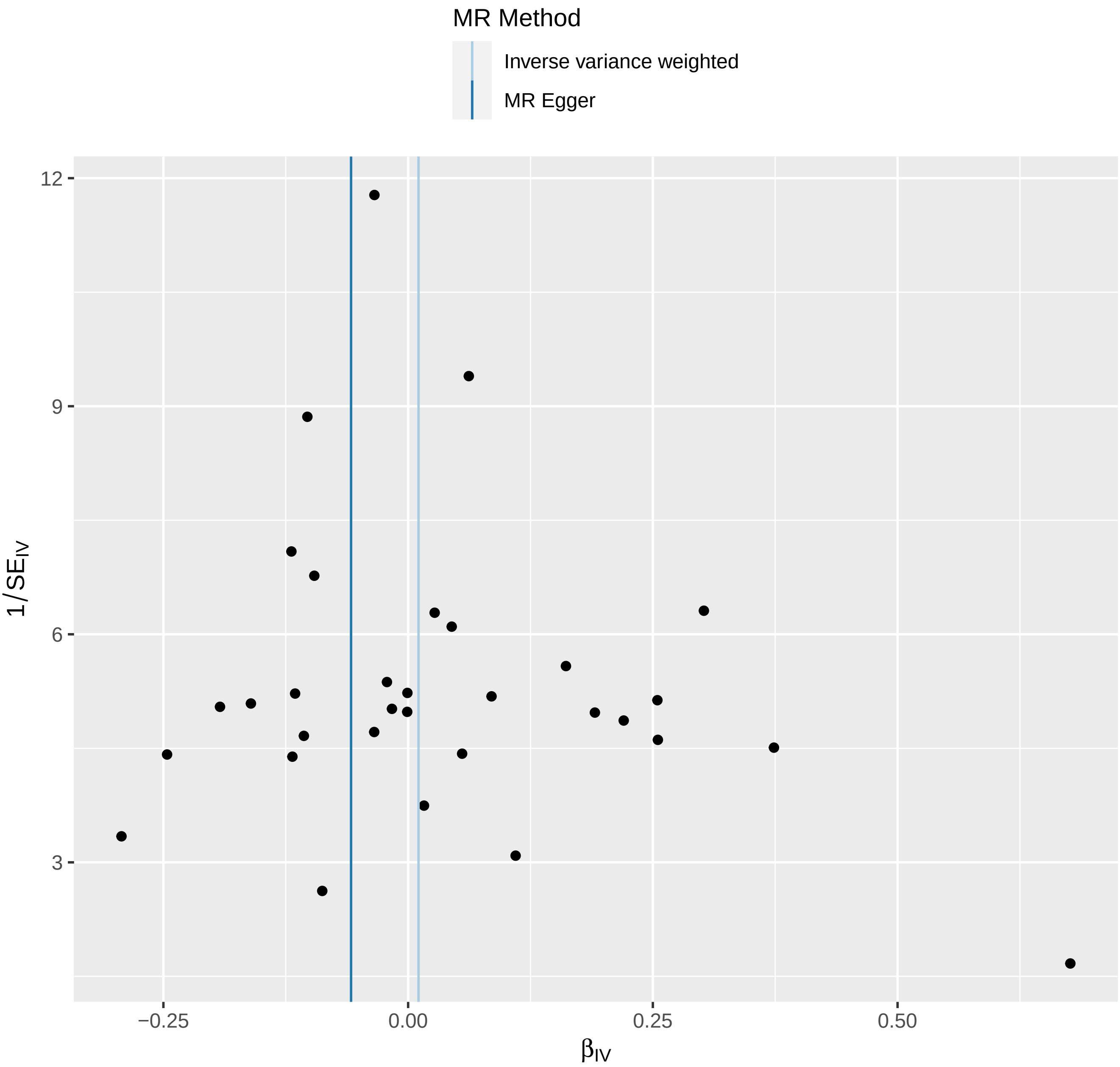

Batch 3−232 : Brevican core protein on Diabetic nephropathy (more control exclusions)
with a threshold of 1e−05

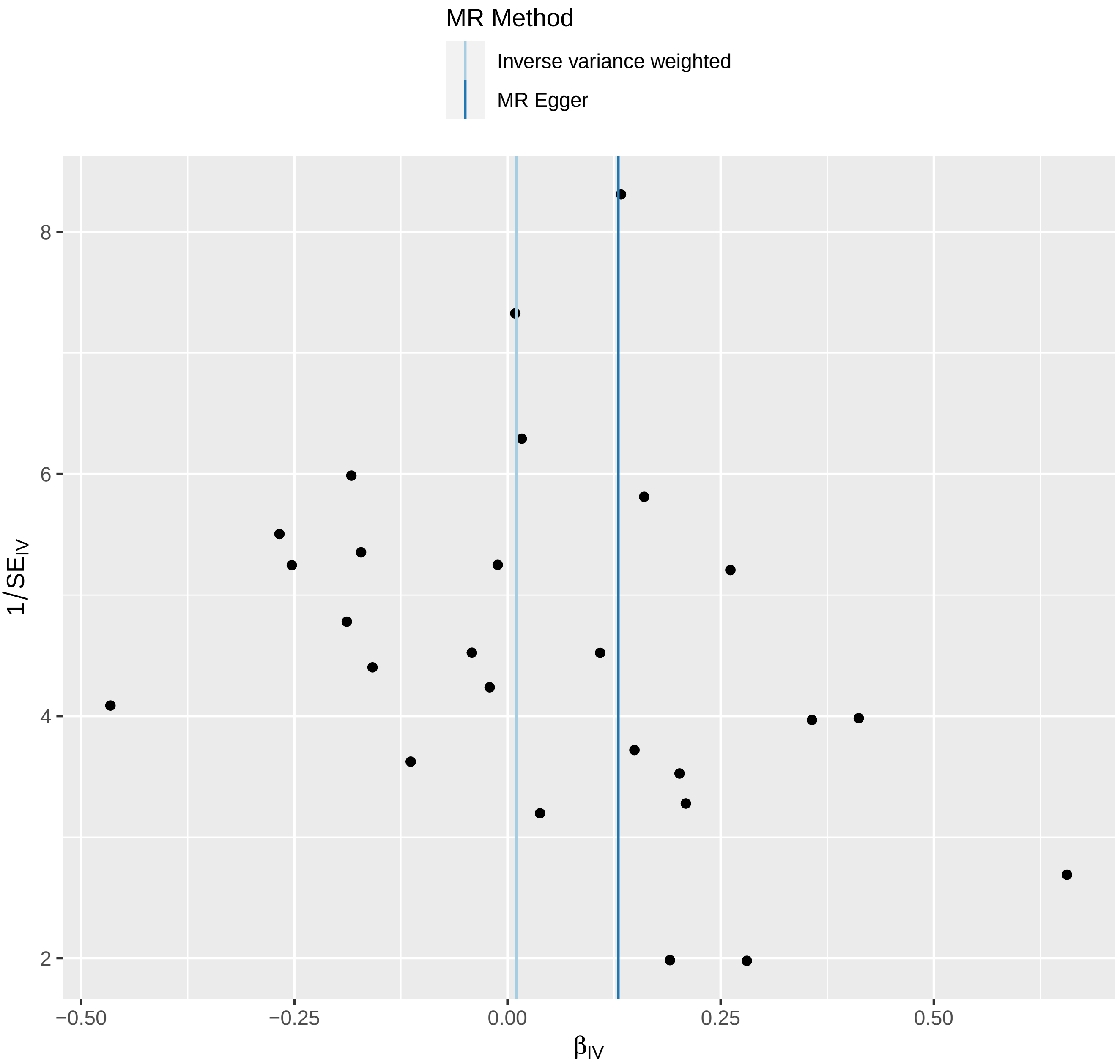

Batch 3−233 : B−cell receptor−associated protein 29 on Diabetic nephropathy (more control exclusions)
with a threshold of 1e−05

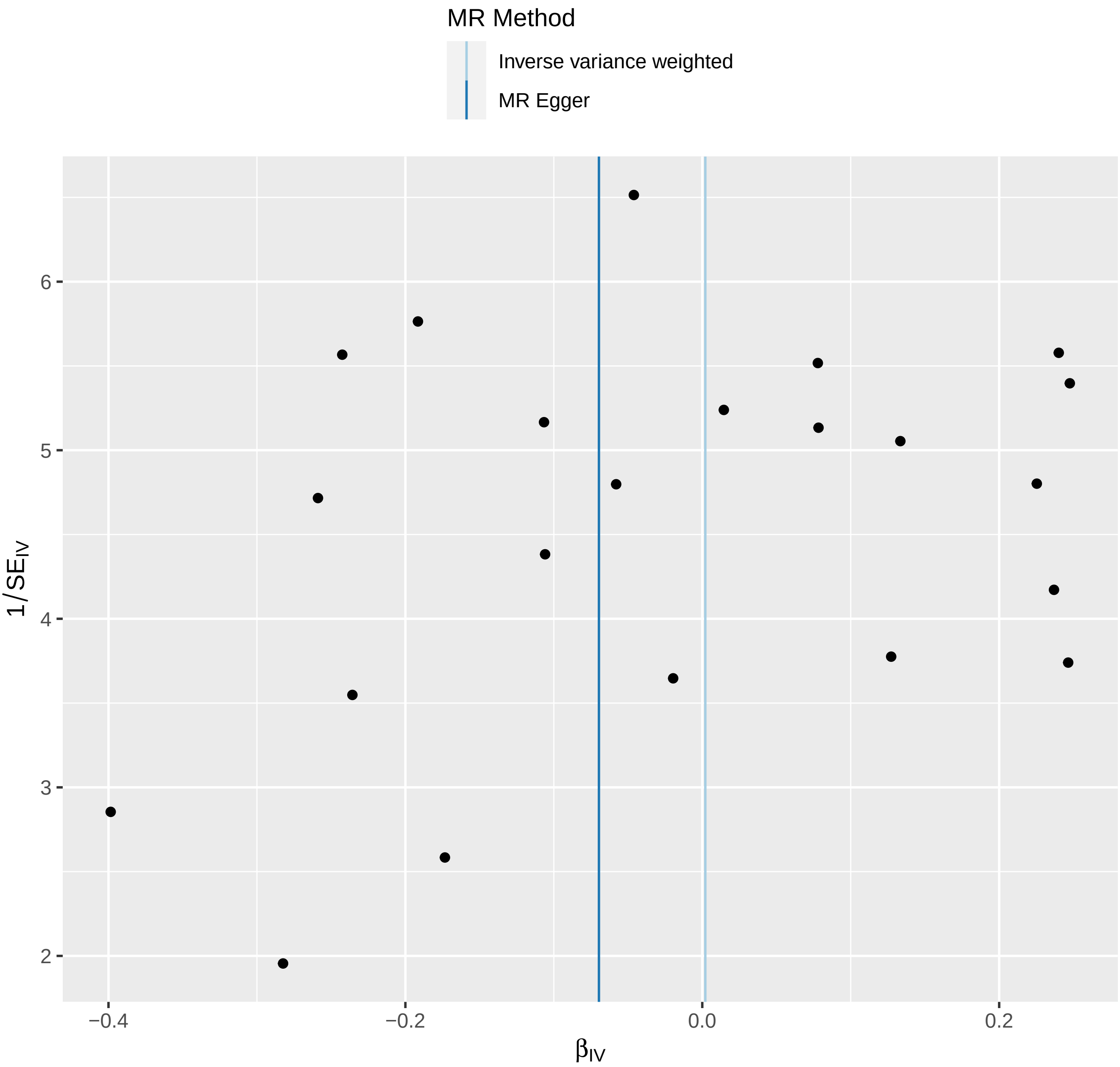

Batch 3−234 : Breast cancer anti−estrogen resistance protein 3 on Diabetic nephropathy (more control exclusions)
with a threshold of 1e−05

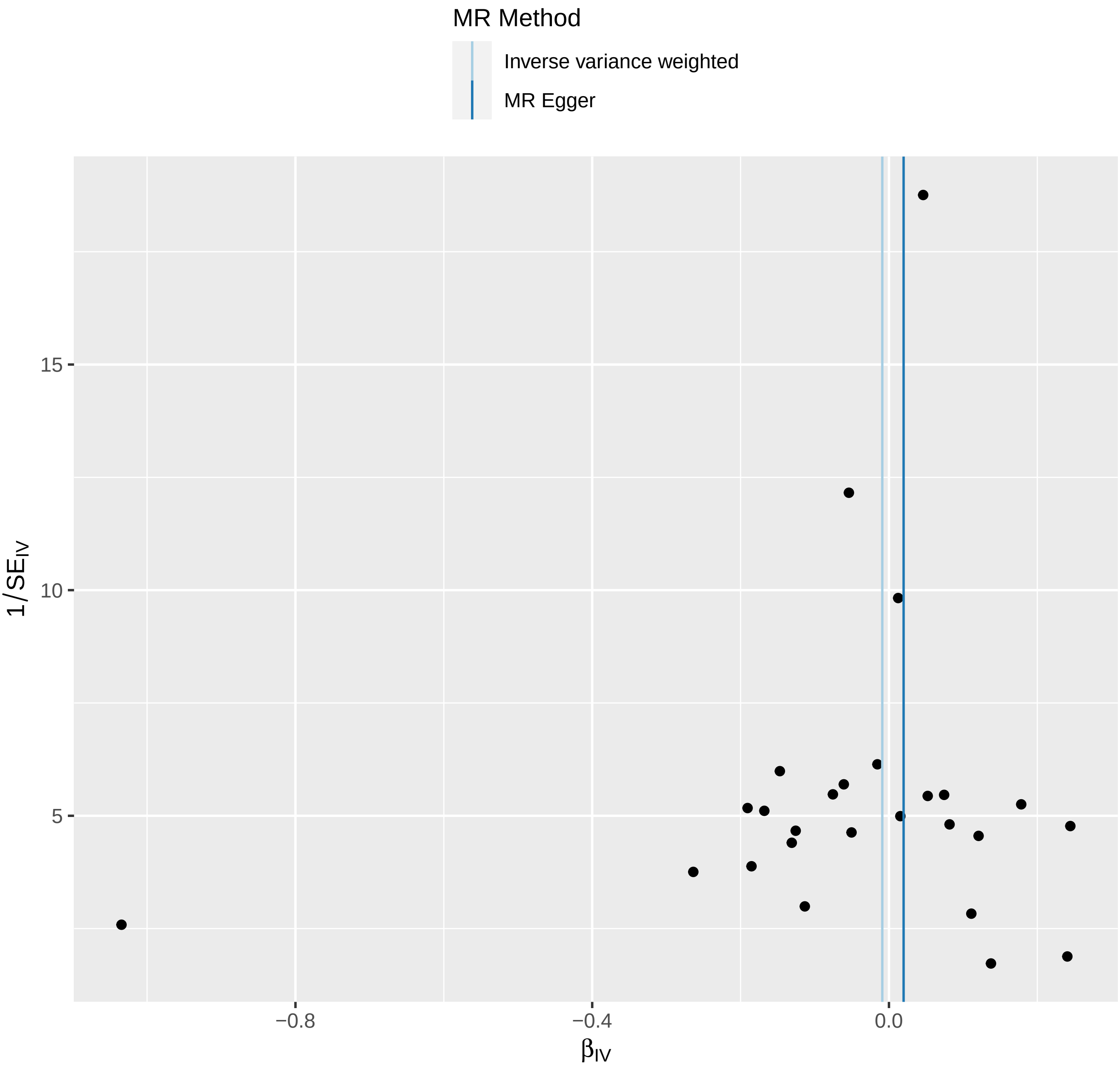

Batch 3−235 : B−cell lymphoma/leukemia 10 on Diabetic nephropathy (more control exclusions)
with a threshold of 1e−05

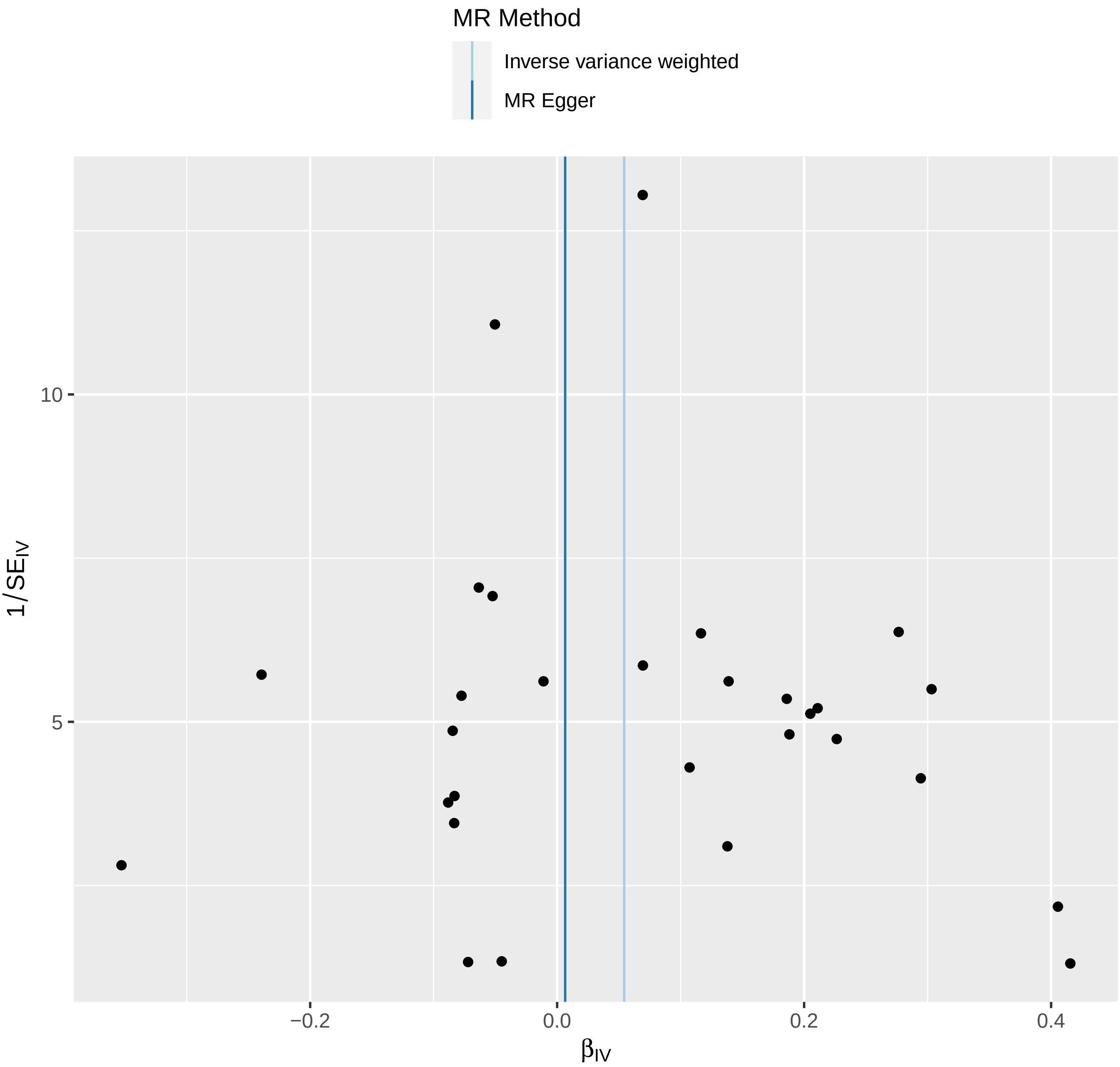

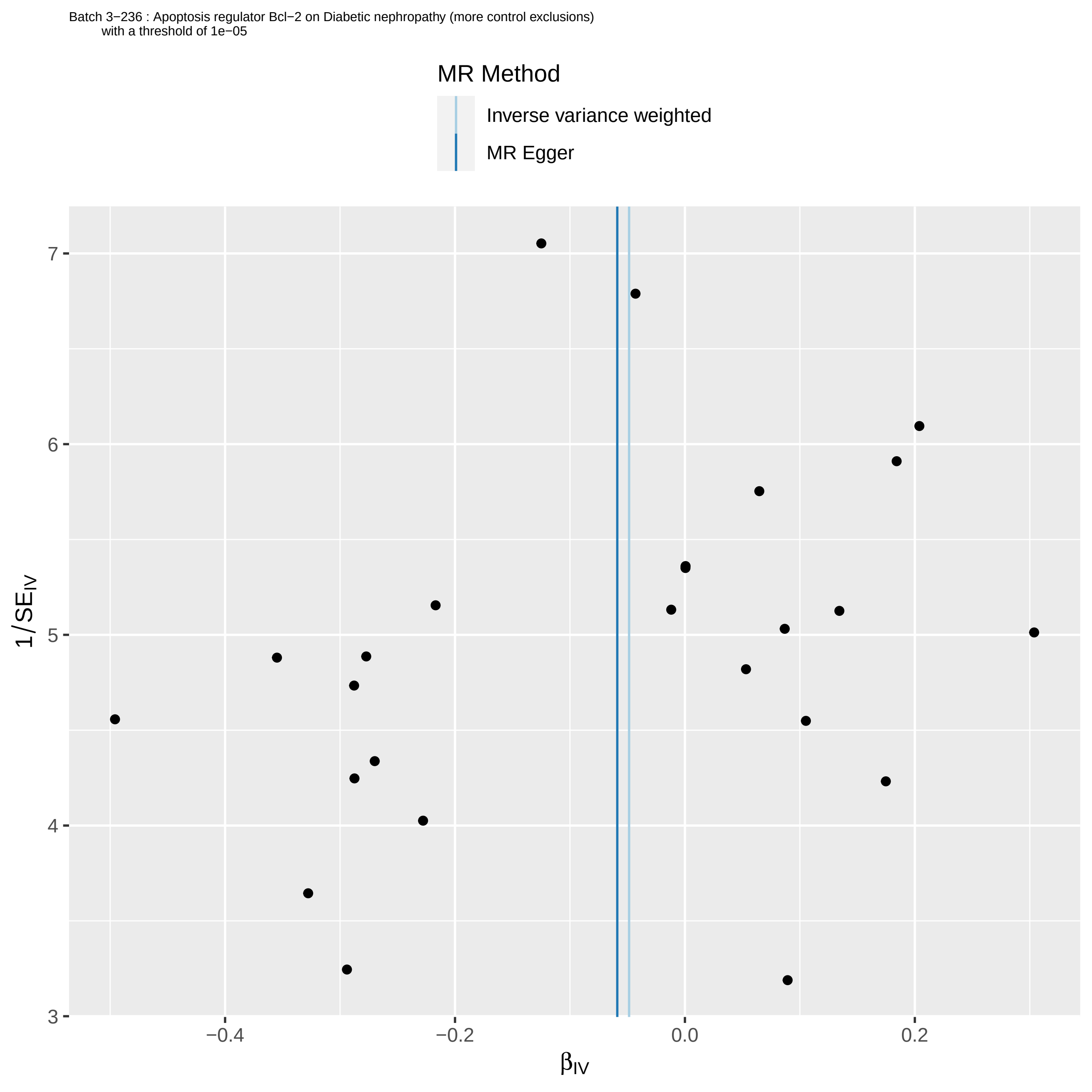
Batch 3−236 : Apoptosis regulator Bcl−2 on Diabetic nephropathy (more control exclusions)
with a threshold of 1e−05
MR Method
Inverse variance weighted
MR Egger
7
6
5
4
3
1/SE_IV
−0.4
−0.2
0.0
0.2
β_IV

Batch 3−237 : Bcl−2−related protein A1 on Diabetic nephropathy (more control exclusions)
with a threshold of 1e−05

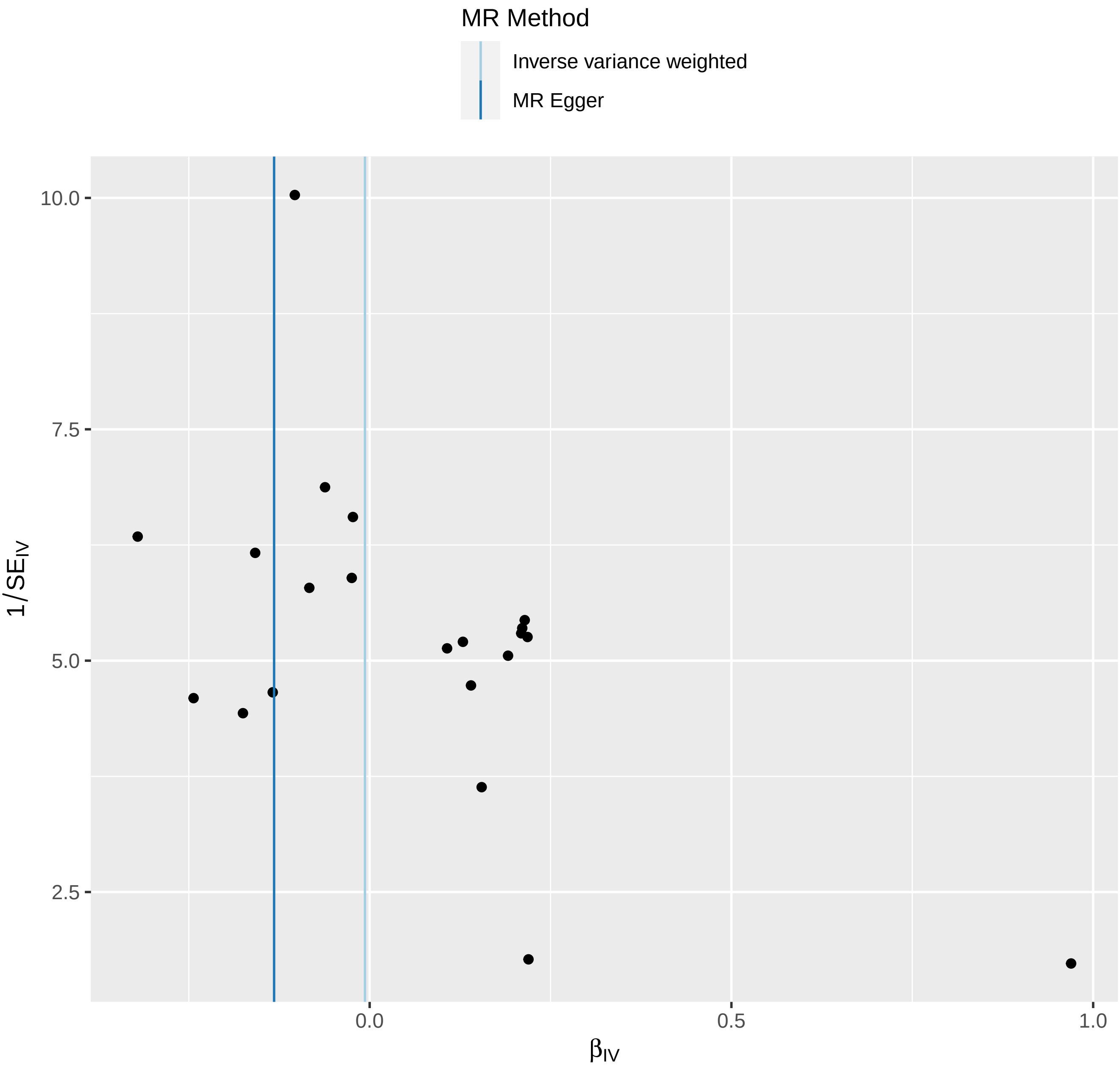

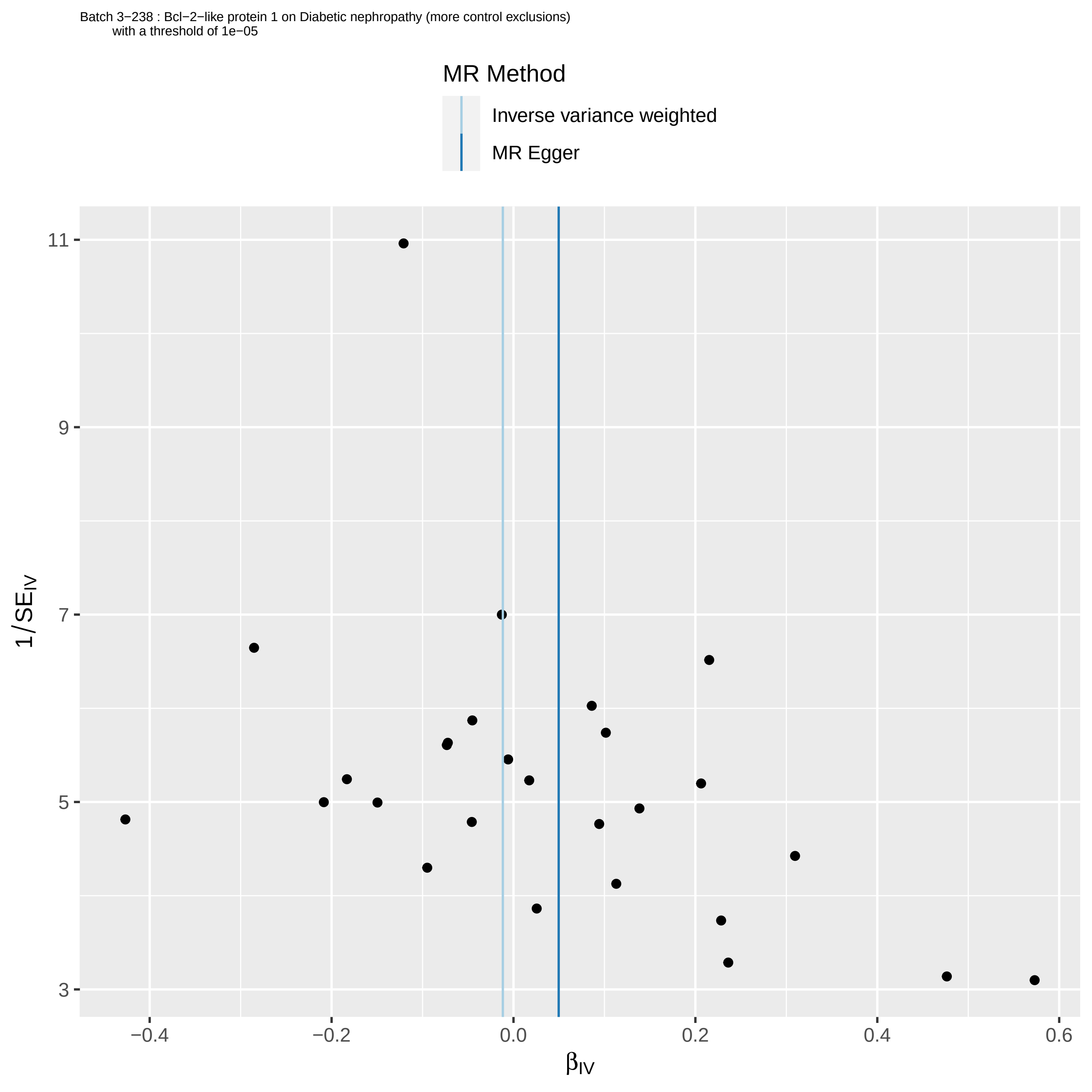

Batch 3−238 : Bcl−2−like protein 1 on Diabetic nephropathy (more control exclusions)
with a threshold of 1e−05
MR Method
Inverse variance weighted
MR Egger
1/SE_IV
β_IV
11
9
7
5
3
−0.4
−0.2
0.0
0.2
0.4
0.6

Batch 3−239 : Bcl−2−like protein 2 on Diabetic nephropathy (more control exclusions)
with a threshold of 1e−05

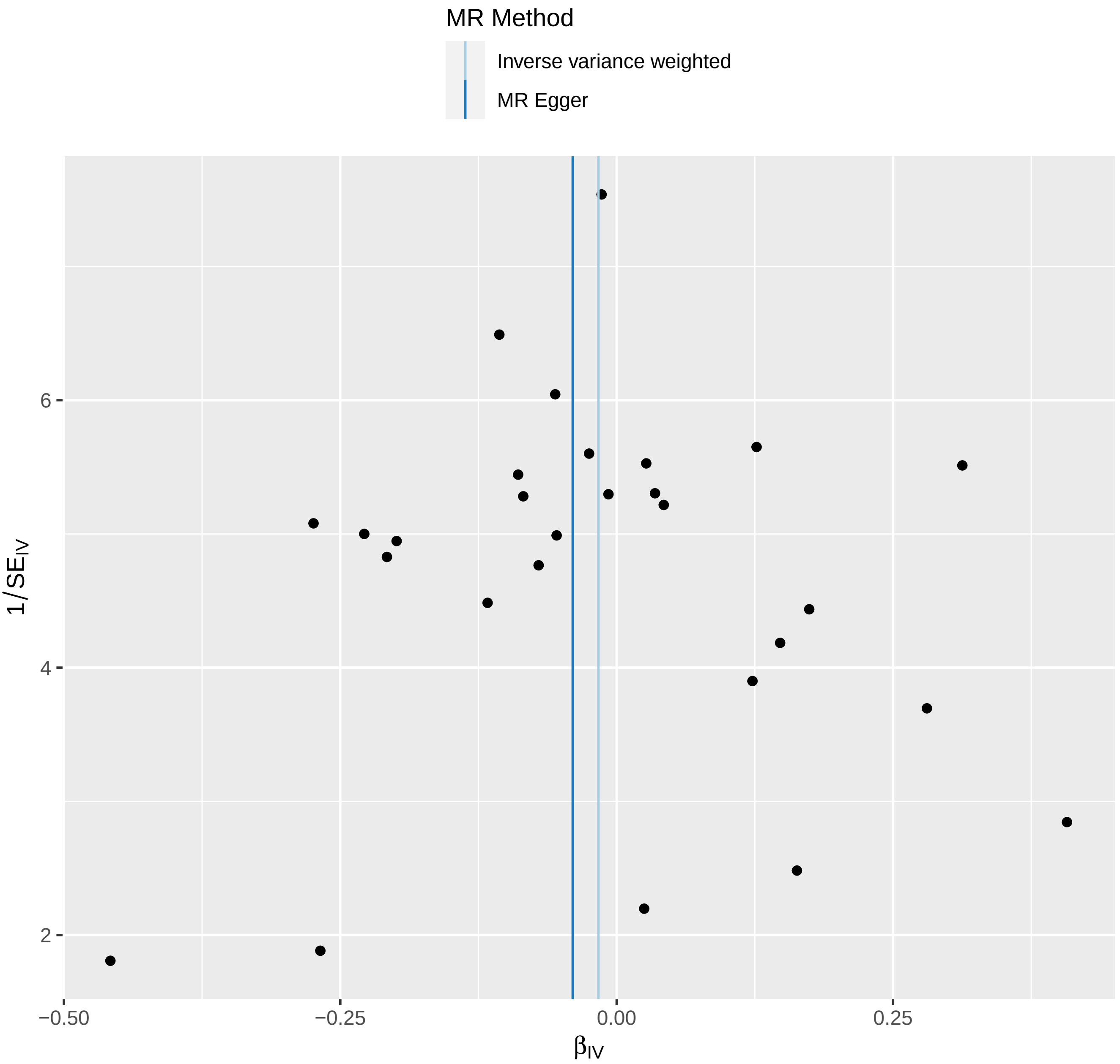

Batch 3−240 : Bcl−2−like protein 2 on Diabetic nephropathy (more control exclusions)
with a threshold of 1e−05

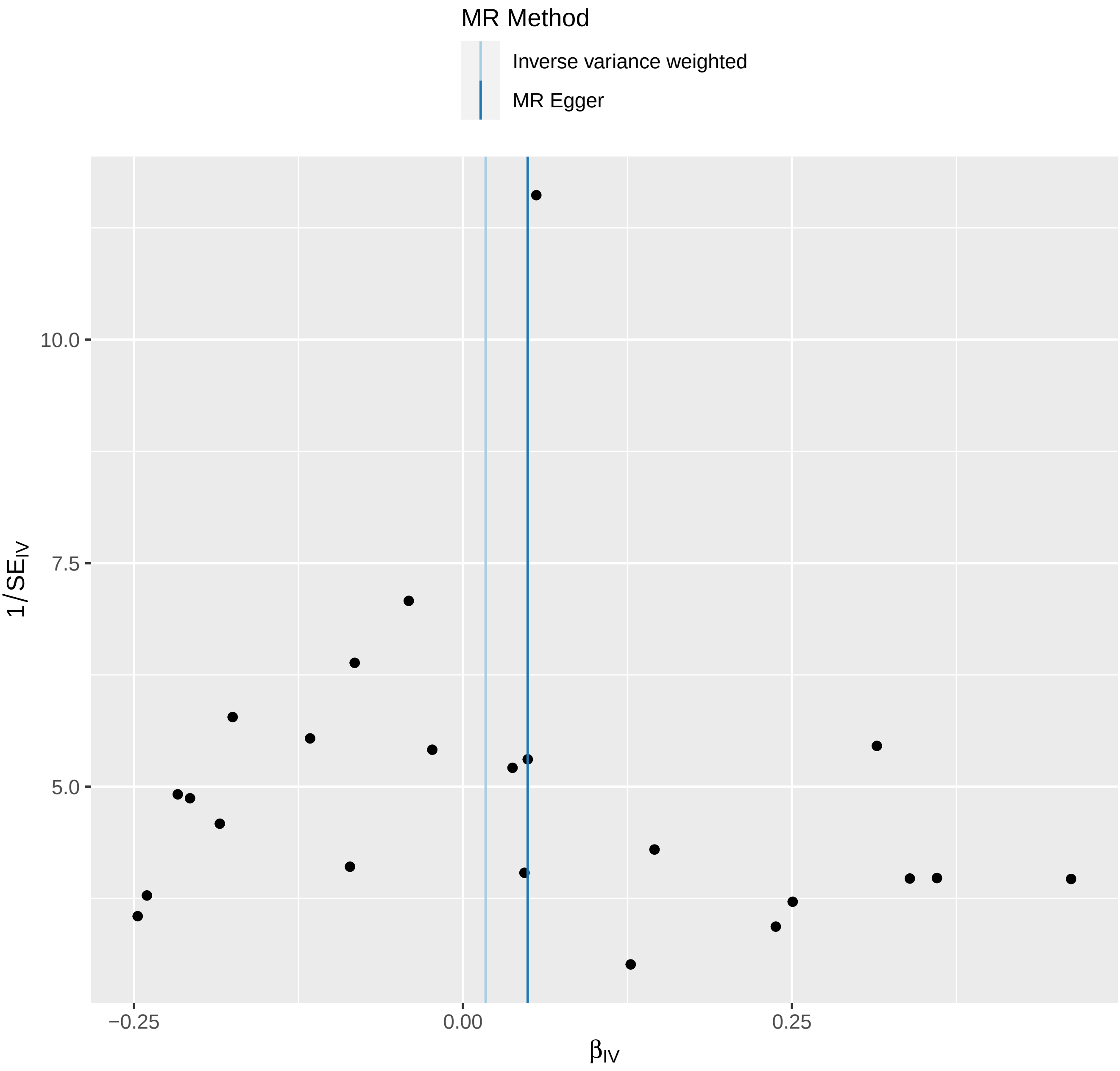

Batch 3−241 : B−cell lymphoma 6 protein on Diabetic nephropathy (more control exclusions)
with a threshold of 1e−05

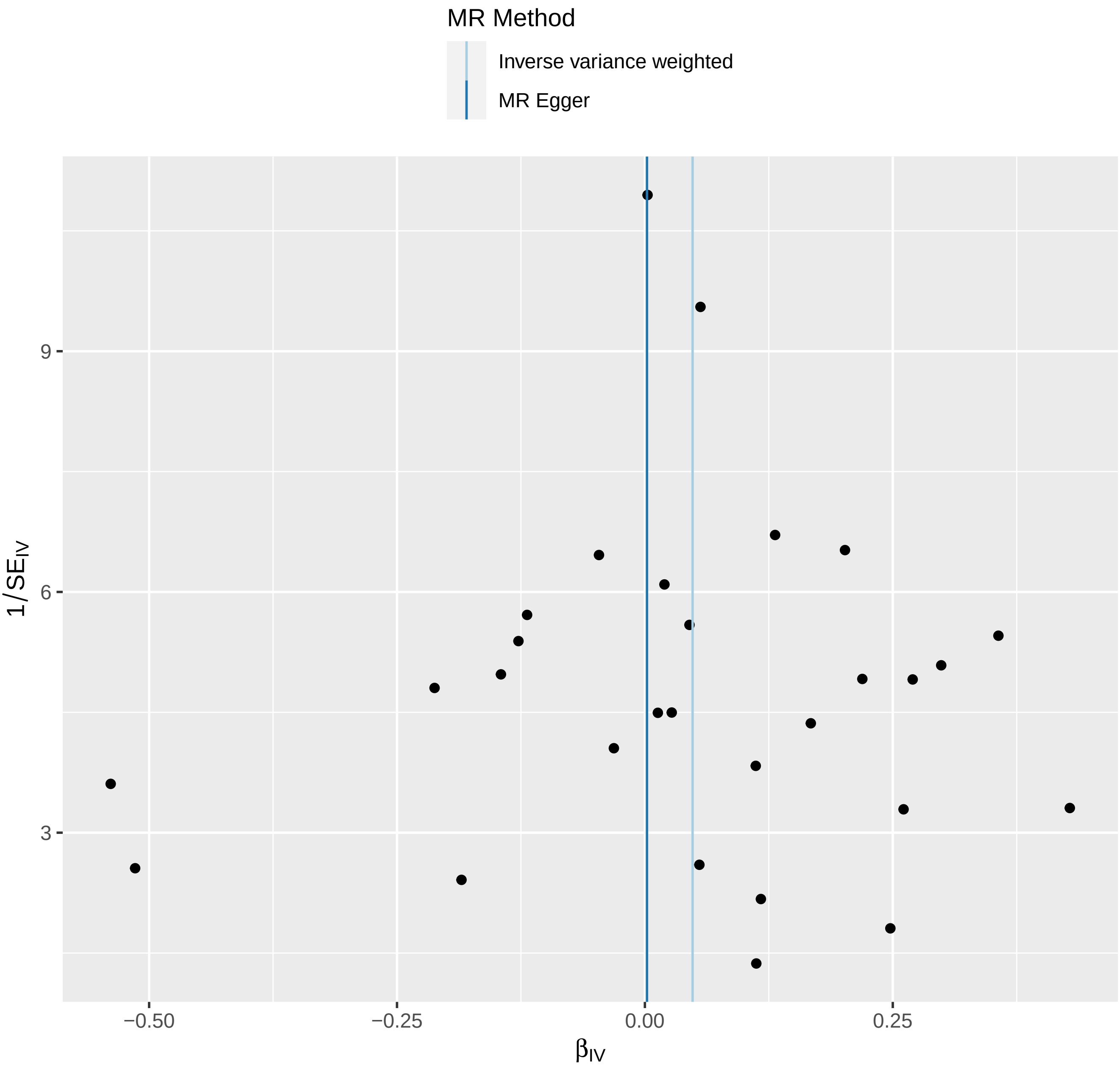

Batch 3−242 : Brain−derived neurotrophic factor on Diabetic nephropathy (more control exclusions)
with a threshold of 1e−05

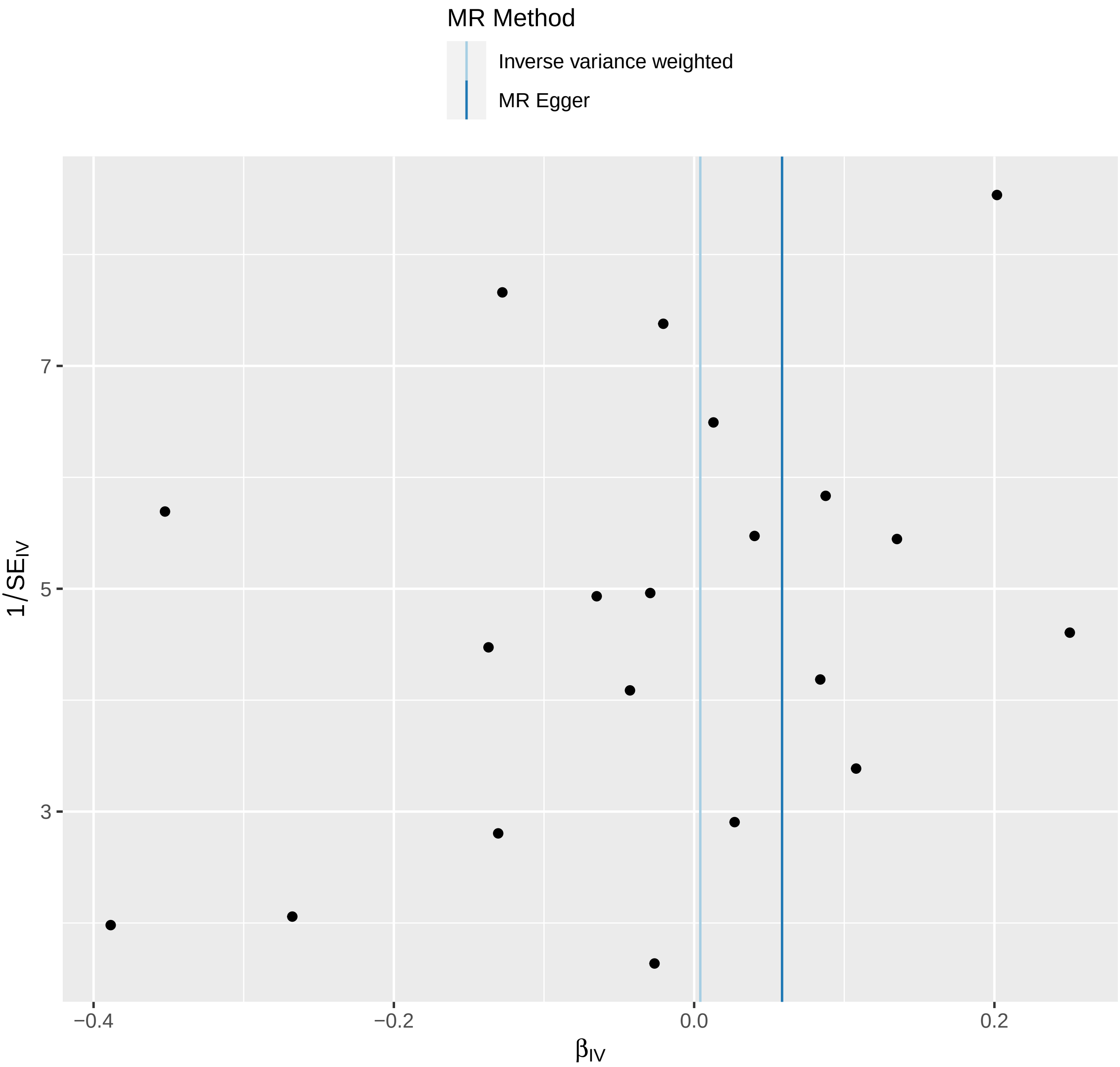

Batch 3−243 : Transcription factor TFIIIB component B" homolog on Diabetic nephropathy (more control exclusions) with a threshold of 1e−05

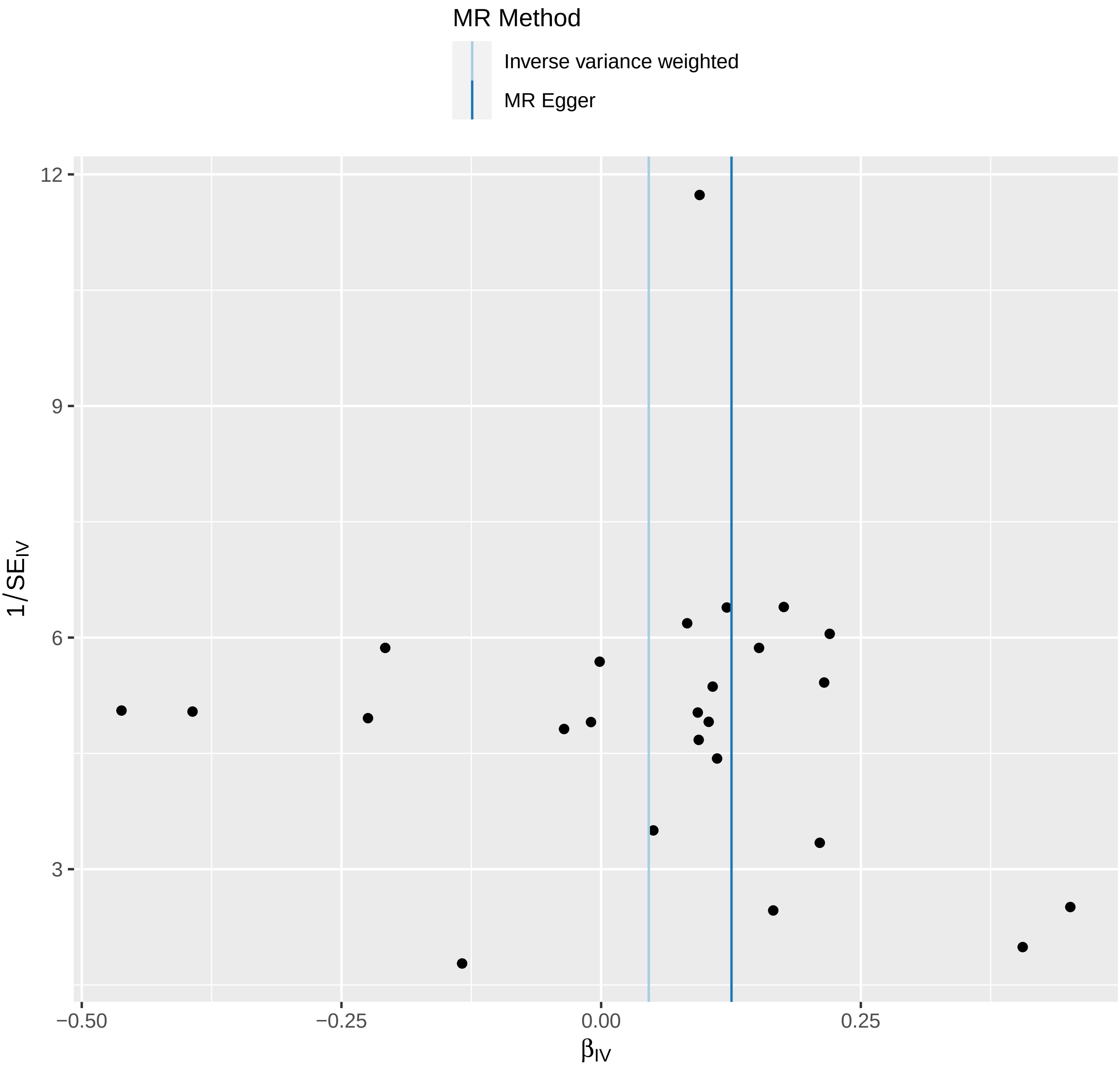

Batch 3−244 : Beclin−1 on Diabetic nephropathy (more control exclusions)
with a threshold of 1e−05

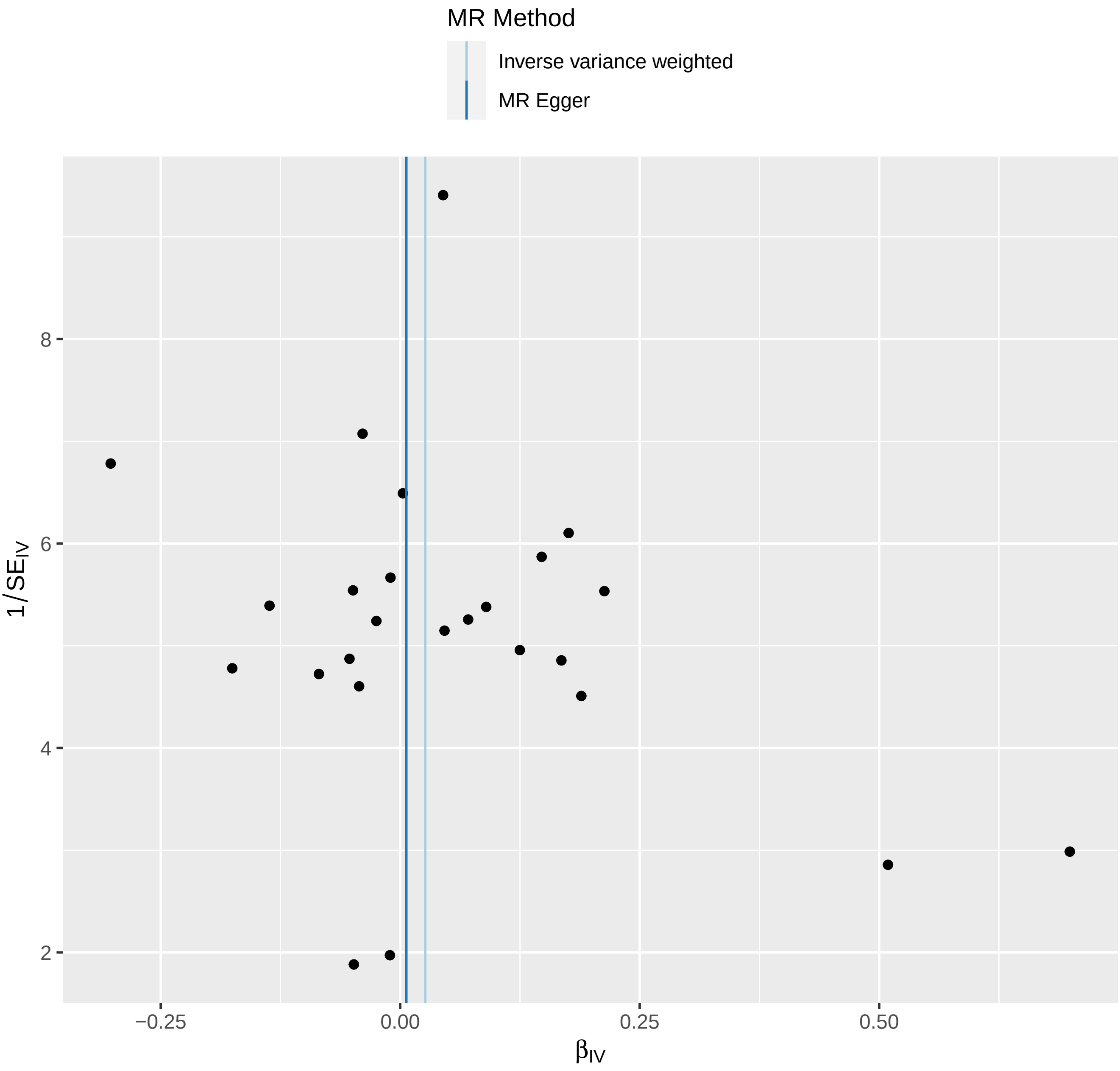

Batch 3−245 : BET1−like protein on Diabetic nephropathy (more control exclusions)
with a threshold of 1e−05

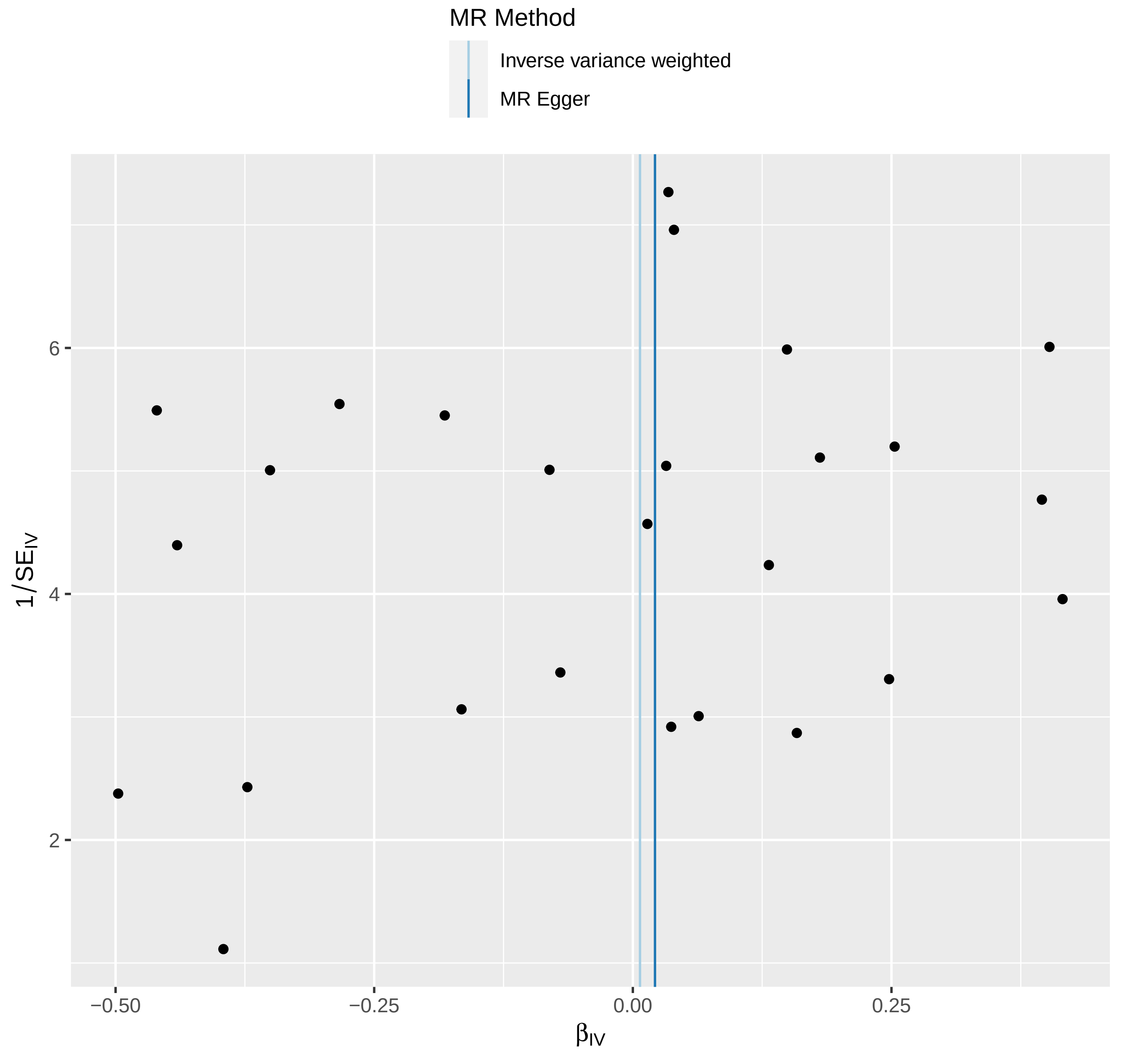

Batch 3−246 : Osteocalcin on Diabetic nephropathy (more control exclusions)
with a threshold of 1e−05

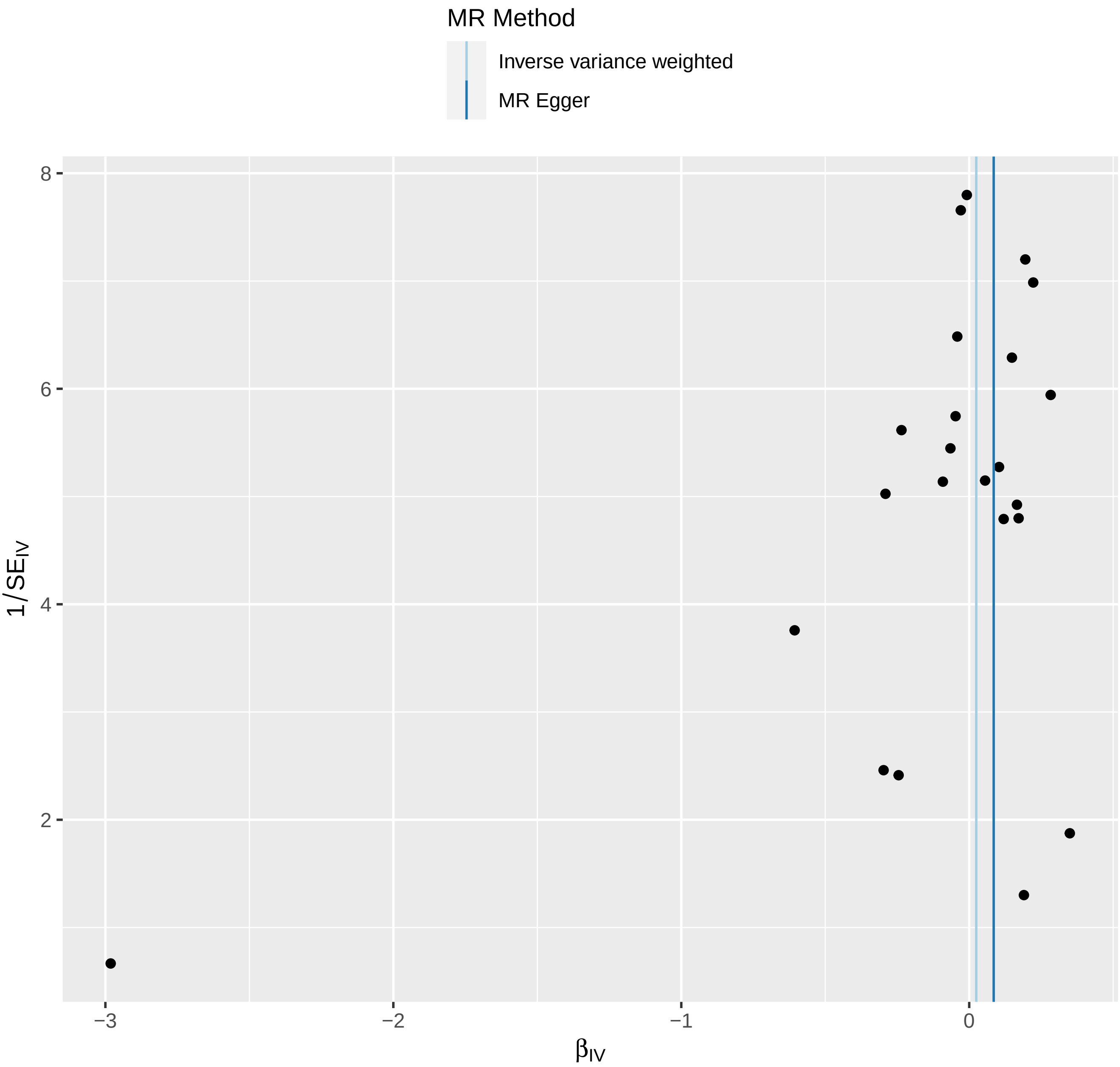

Batch 3−247 : Biglycan on Diabetic nephropathy (more control exclusions)
with a threshold of 1e−05

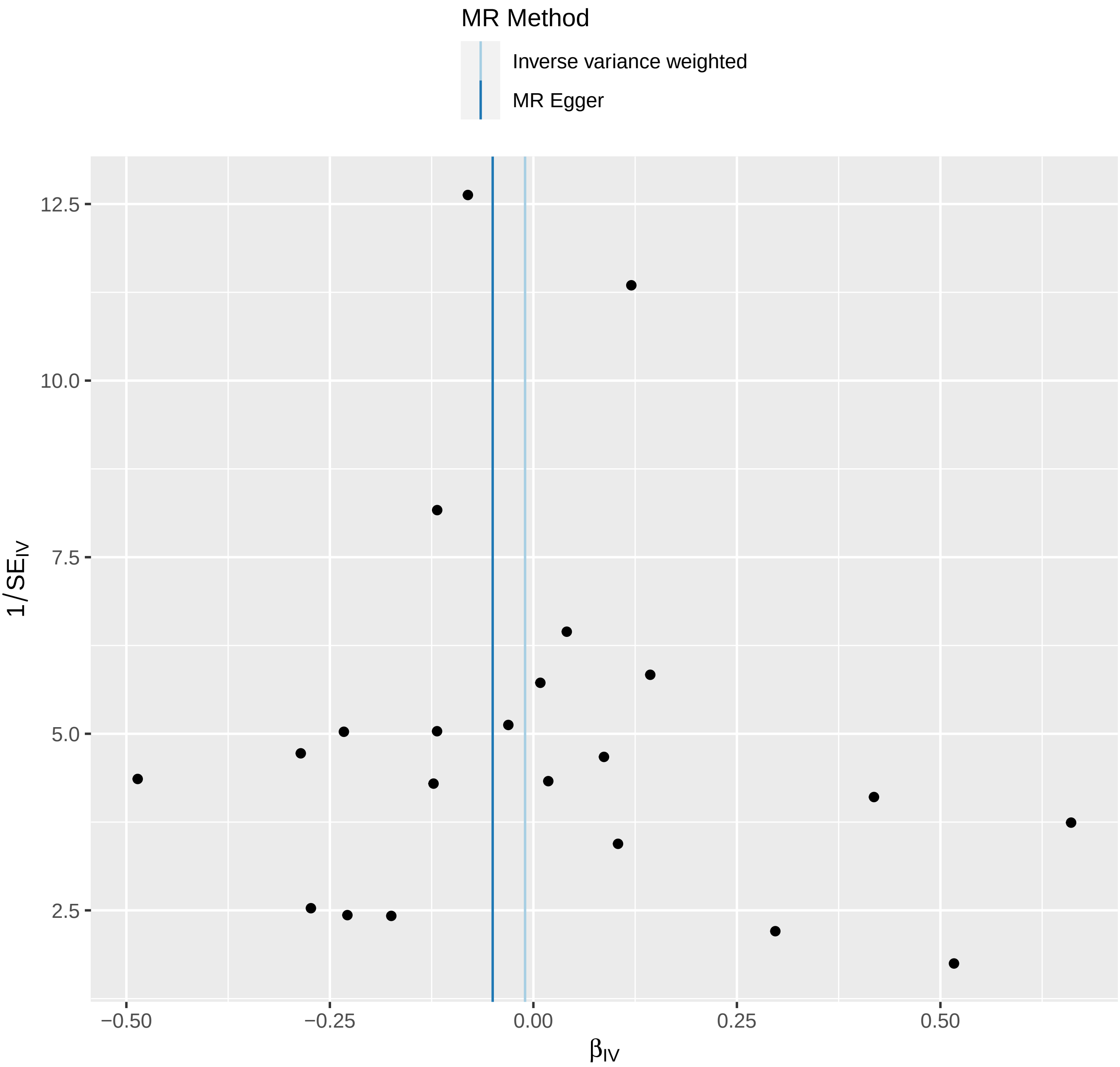

Batch 3−248 : Biglycan on Diabetic nephropathy (more control exclusions)
with a threshold of 1e−05

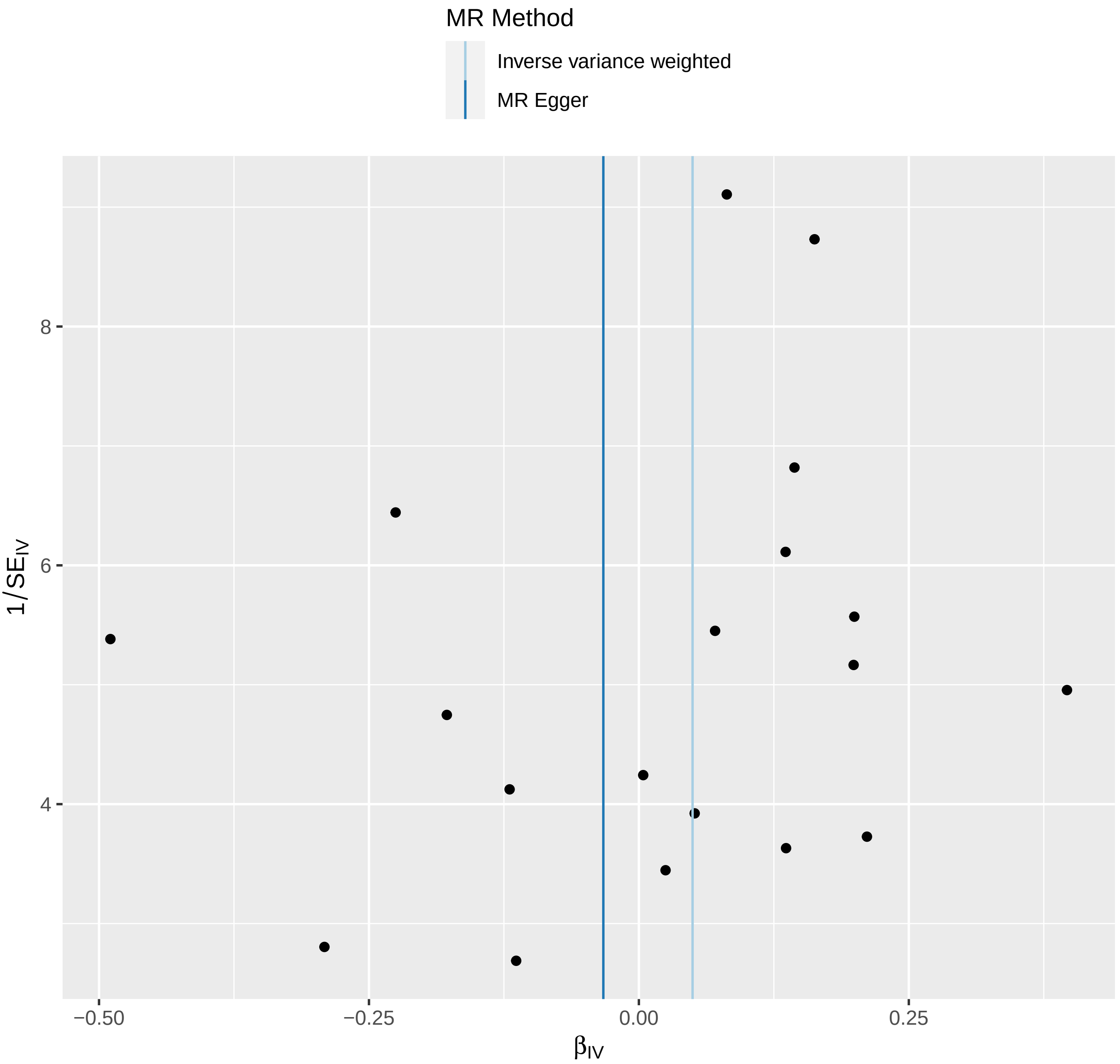

Batch 3−249 : BH3−interacting domain death agonist on Diabetic nephropathy (more control exclusions)
with a threshold of 1e−05

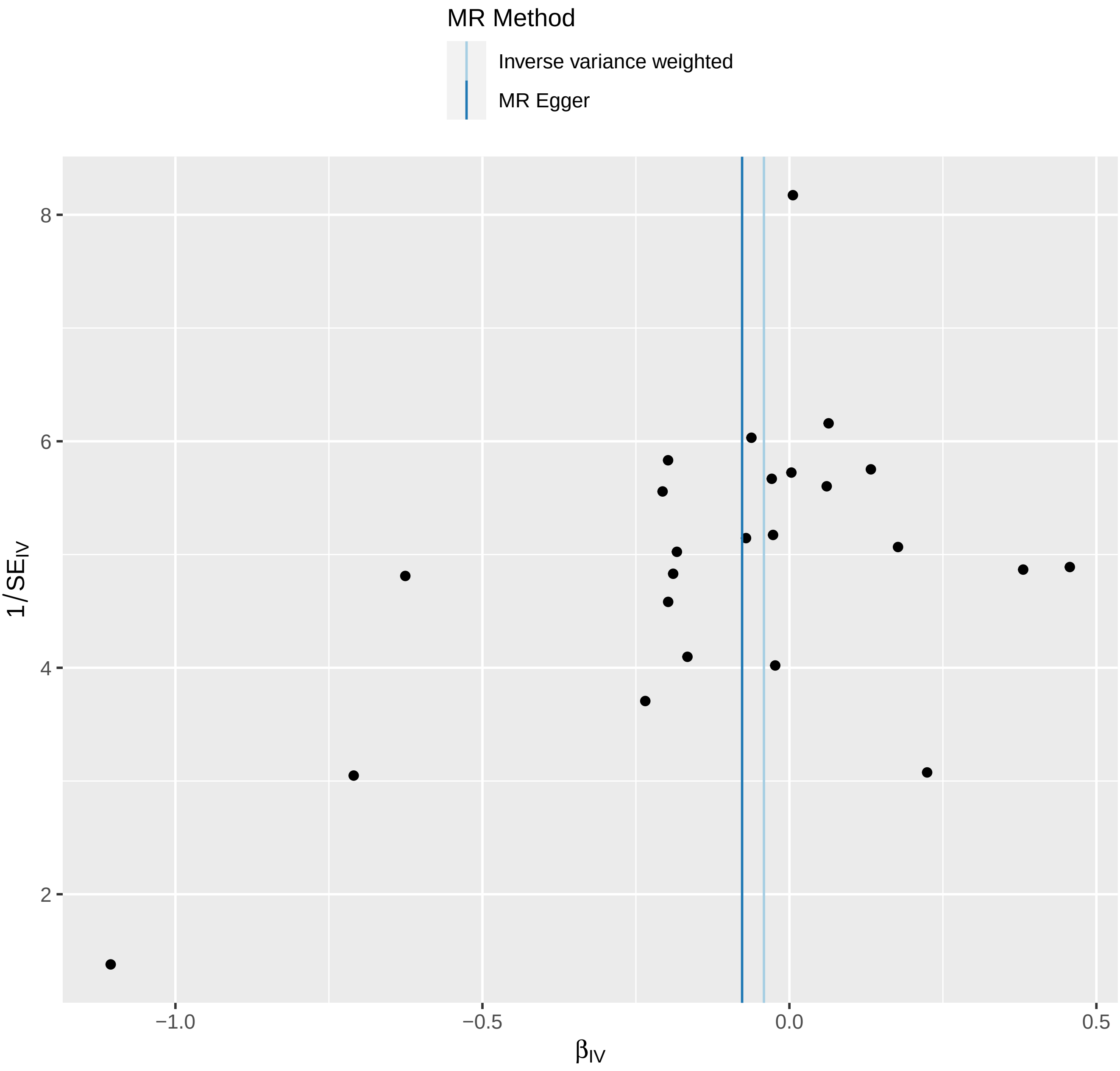

Batch 3−250 : Myc box−dependent−interacting protein 1 on Diabetic nephropathy (more control exclusions) with a threshold of 1e−05

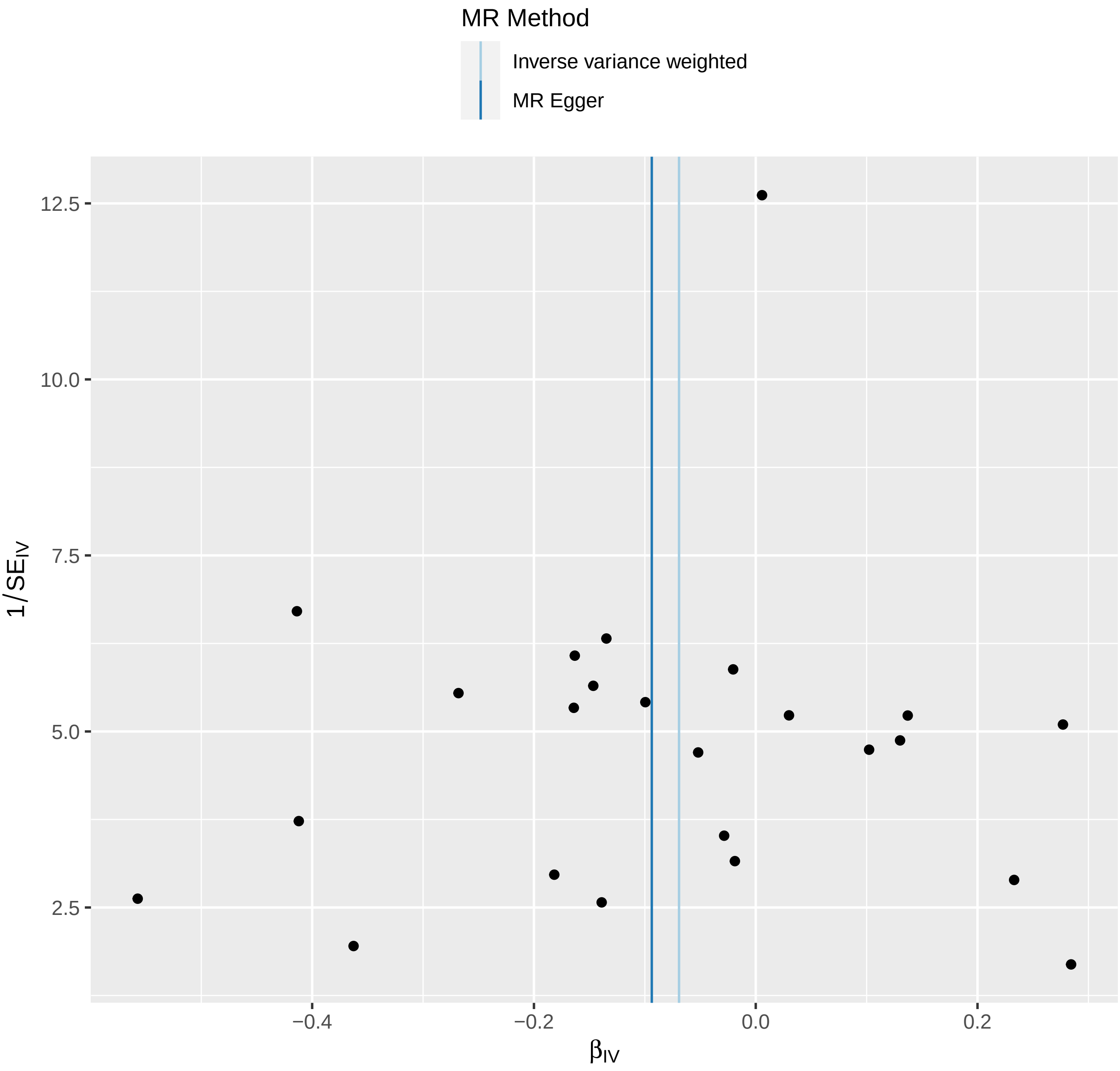

Batch 3−251 : Baculoviral IAP repeat−containing protein 3 on Diabetic nephropathy (more control exclusions)
with a threshold of 1e−05

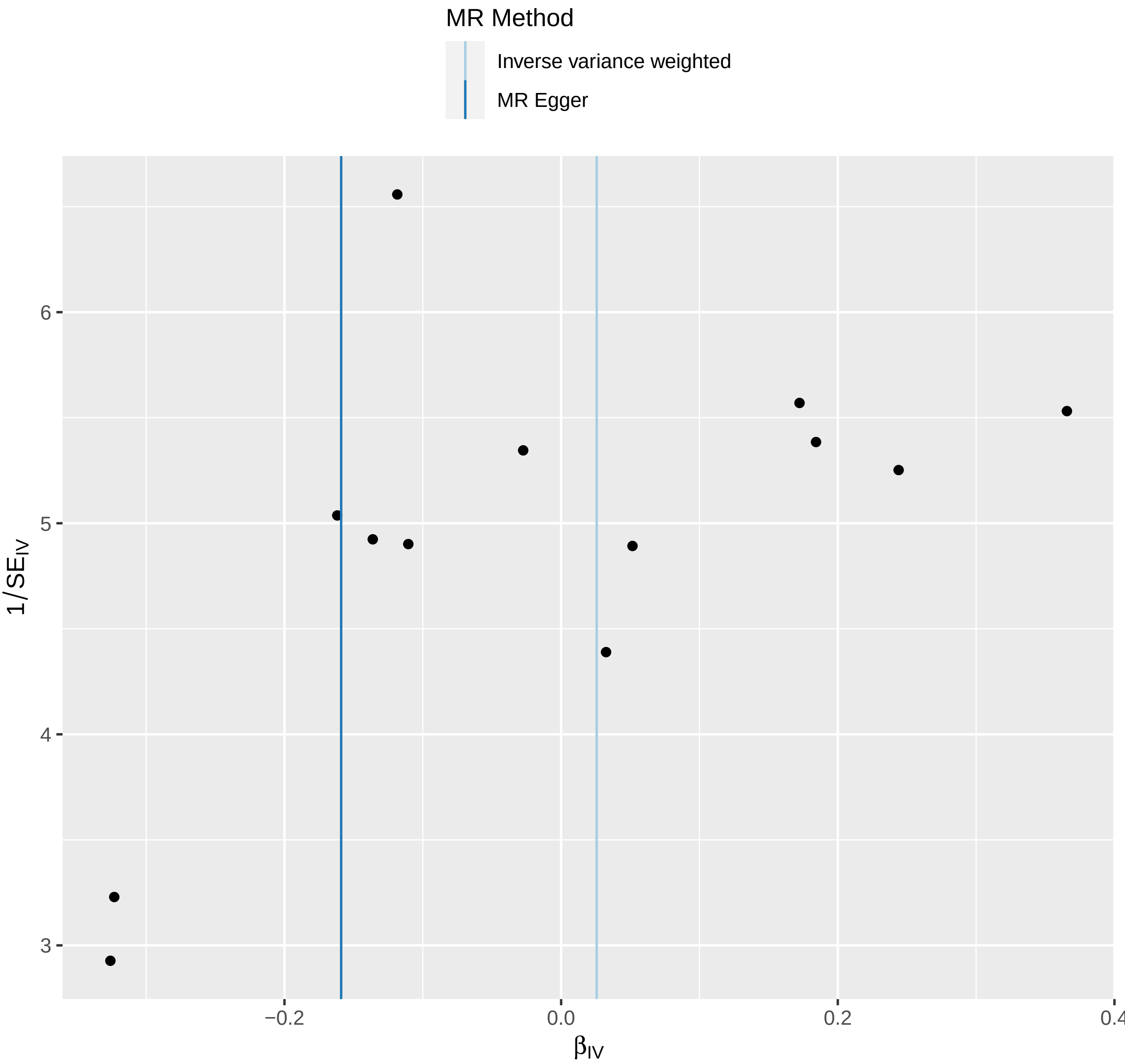

Batch 3−252 : Baculoviral IAP repeat−containing protein 5 on Diabetic nephropathy (more control exclusions)
with a threshold of 1e−05

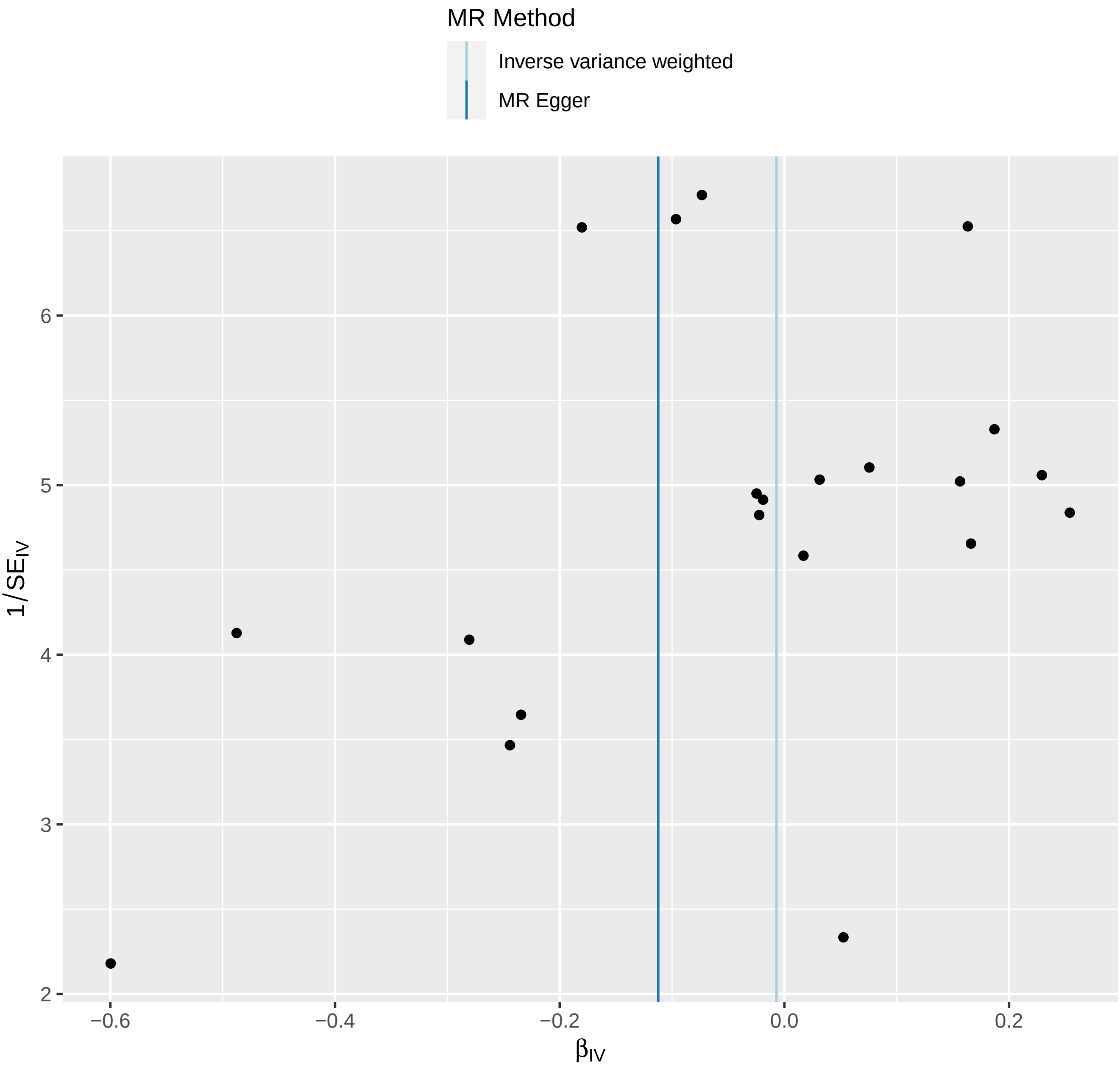

Batch 3−253 : Biliverdin reductase A on Diabetic nephropathy (more control exclusions)
with a threshold of 1e−05

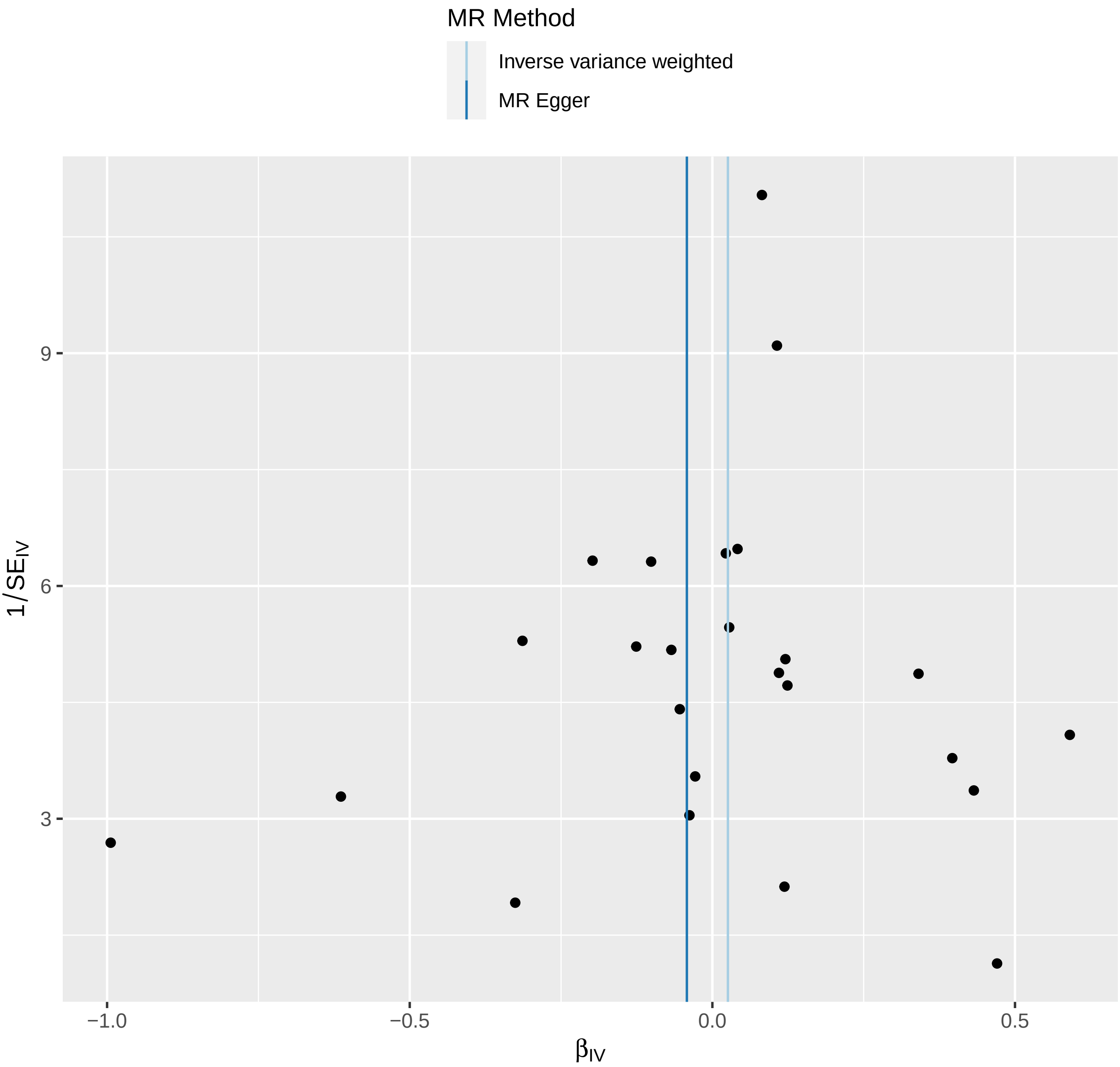

Batch 3−254 : Bone morphogenetic protein 10 on Diabetic nephropathy (more control exclusions)
with a threshold of 1e−05

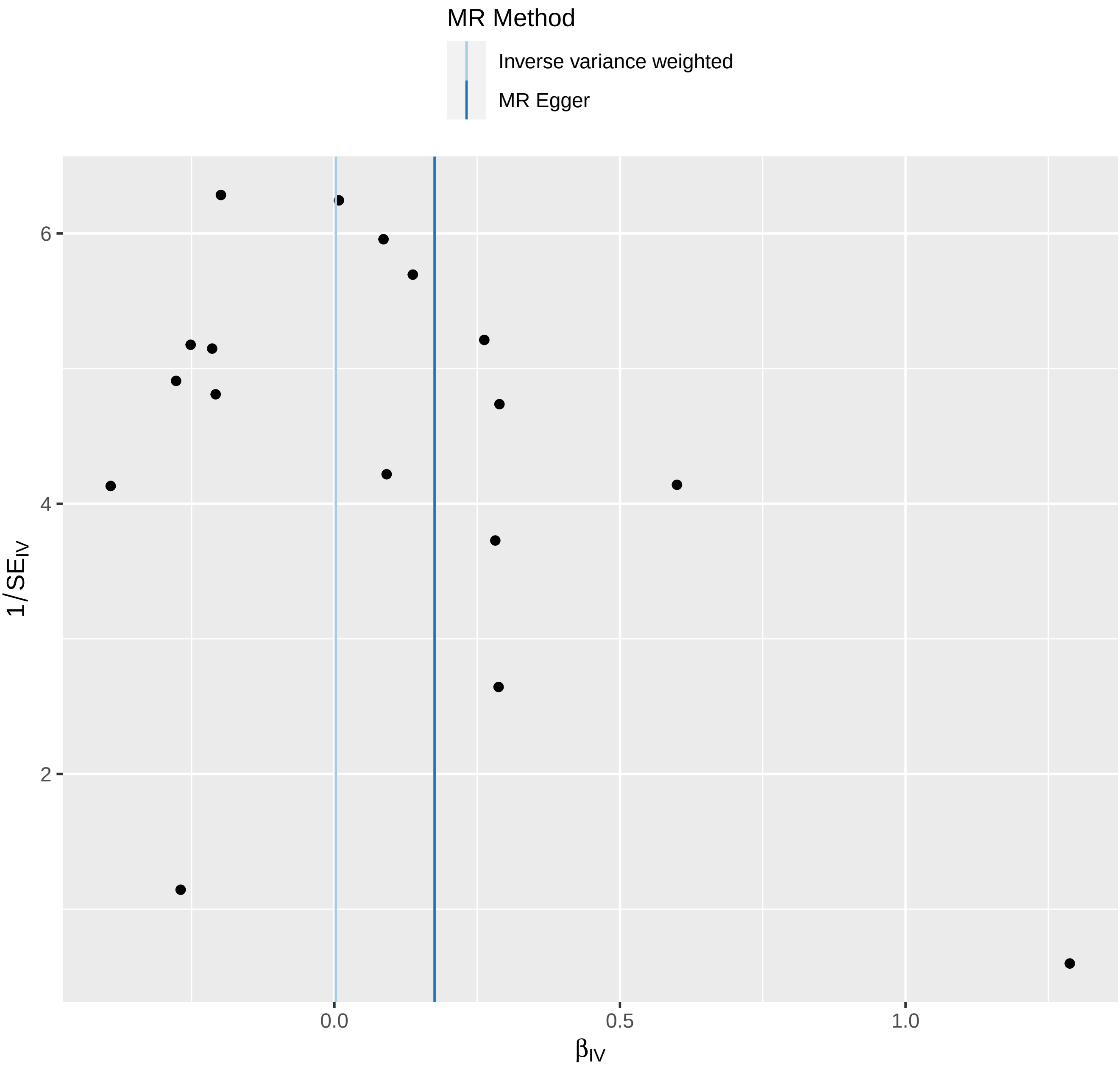

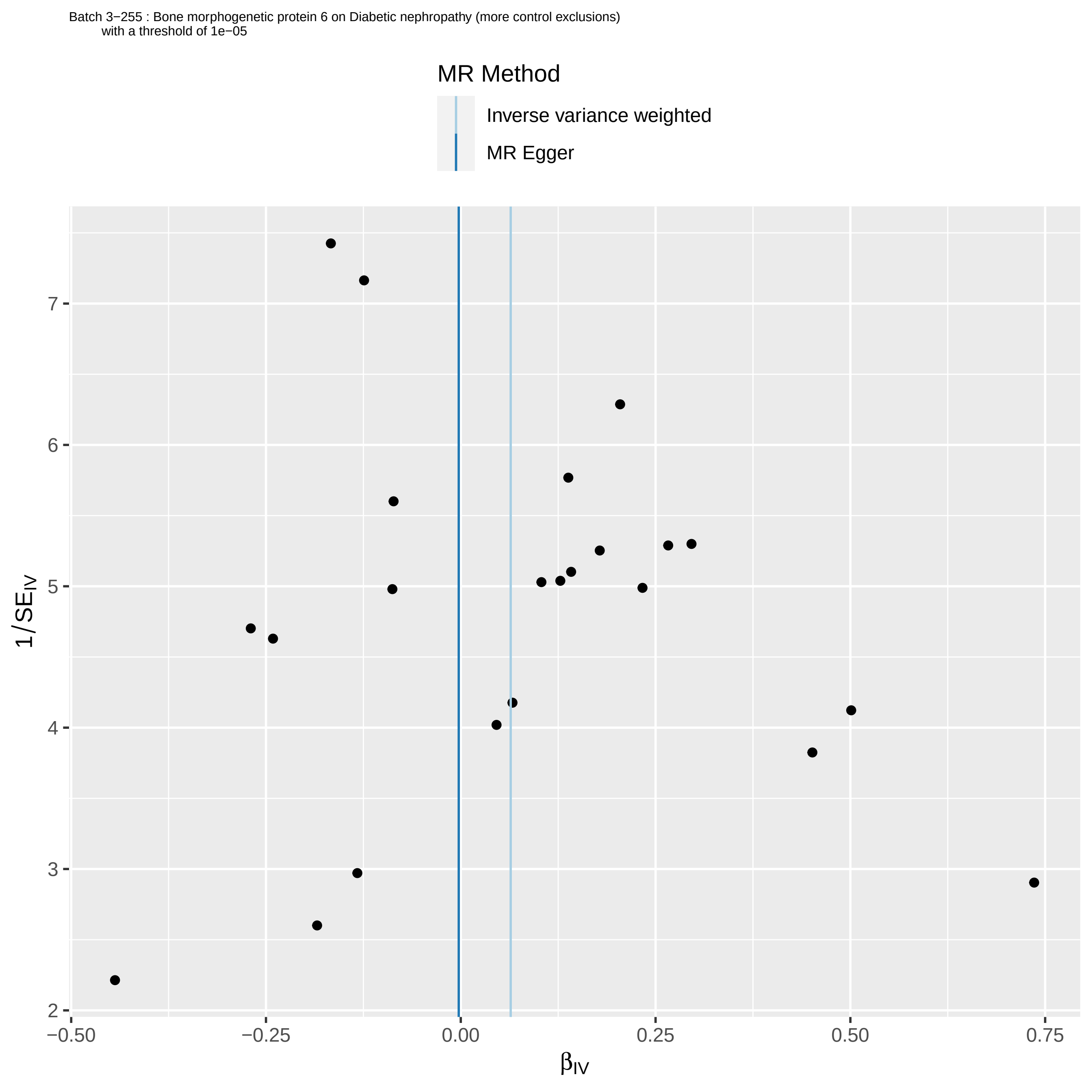
Batch 3−255 : Bone morphogenetic protein 6 on Diabetic nephropathy (more control exclusions)
with a threshold of 1e−05
MR Method
Inverse variance weighted
MR Egger
1/SE_IV
β_IV
7
6
5
4
3
2
−0.50
−0.25
0.00
0.25
0.50
0.75

Batch 3−256 : Bone morphogenetic protein 6 on Diabetic nephropathy (more control exclusions)
with a threshold of 1e−05

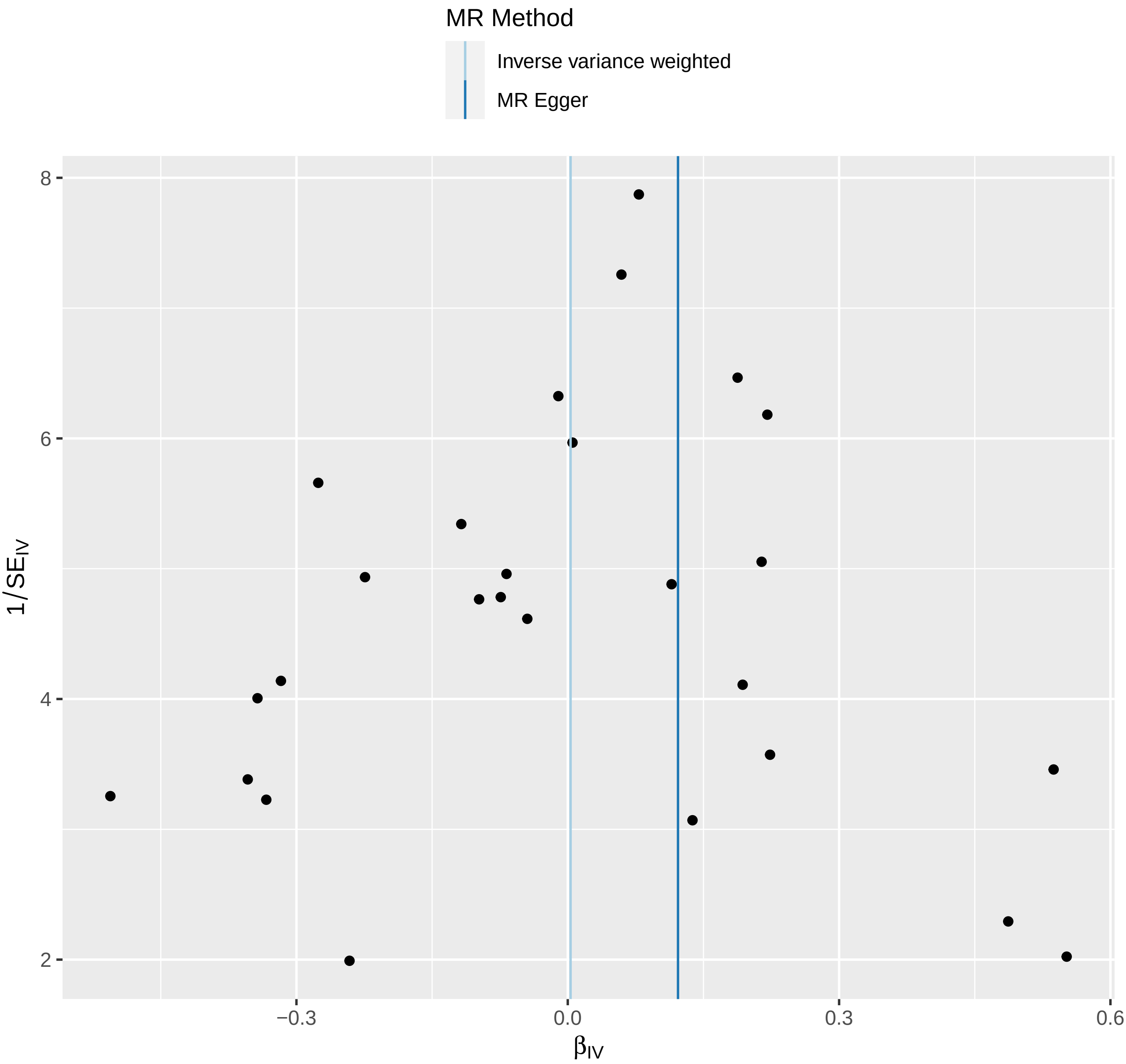

Batch 3−257 : Bone morphogenetic protein 7 on Diabetic nephropathy (more control exclusions)
with a threshold of 1e−05

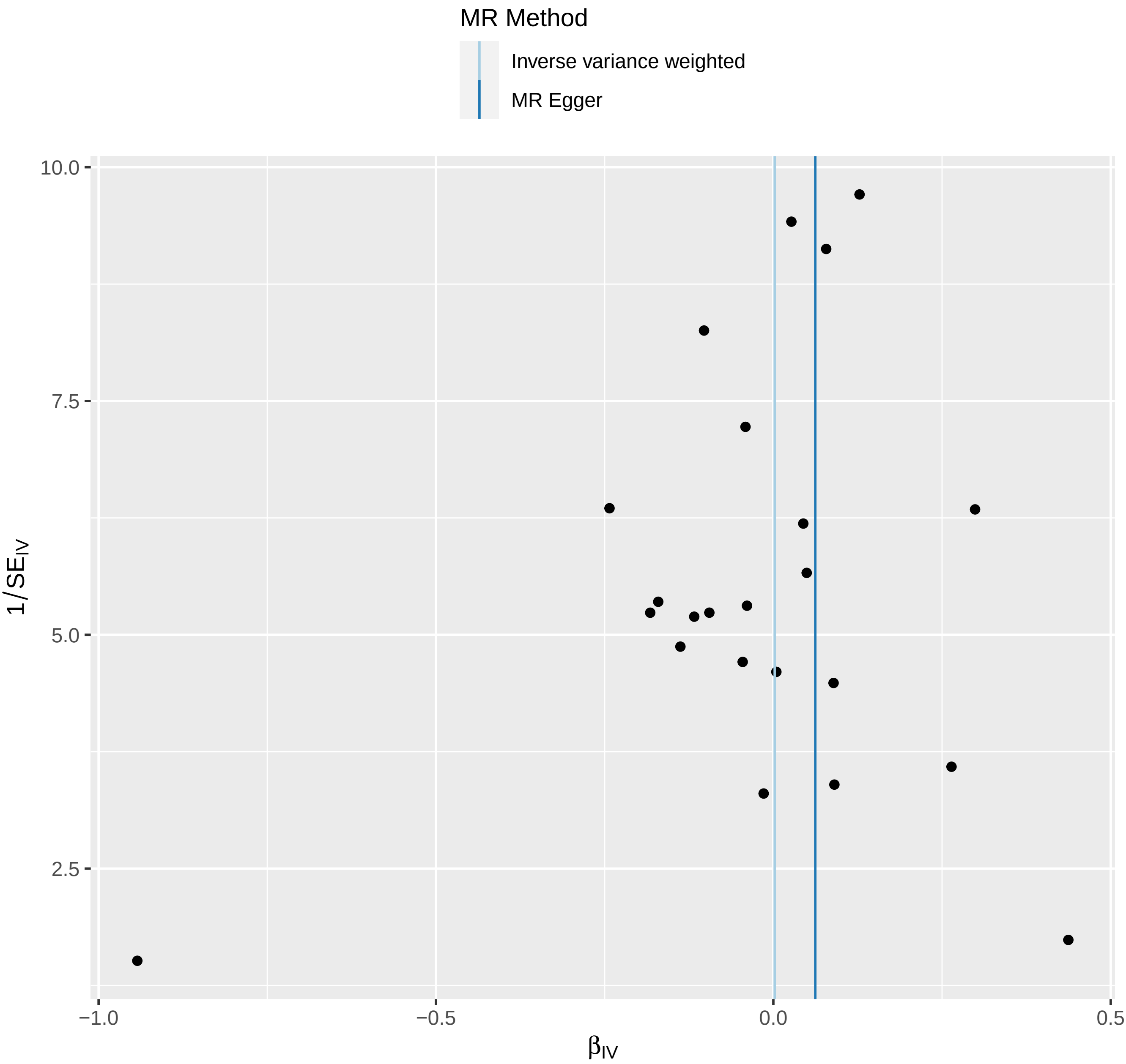

Batch 3−258 : BMP−binding endothelial regulator protein on Diabetic nephropathy (more control exclusions)
with a threshold of 1e−05

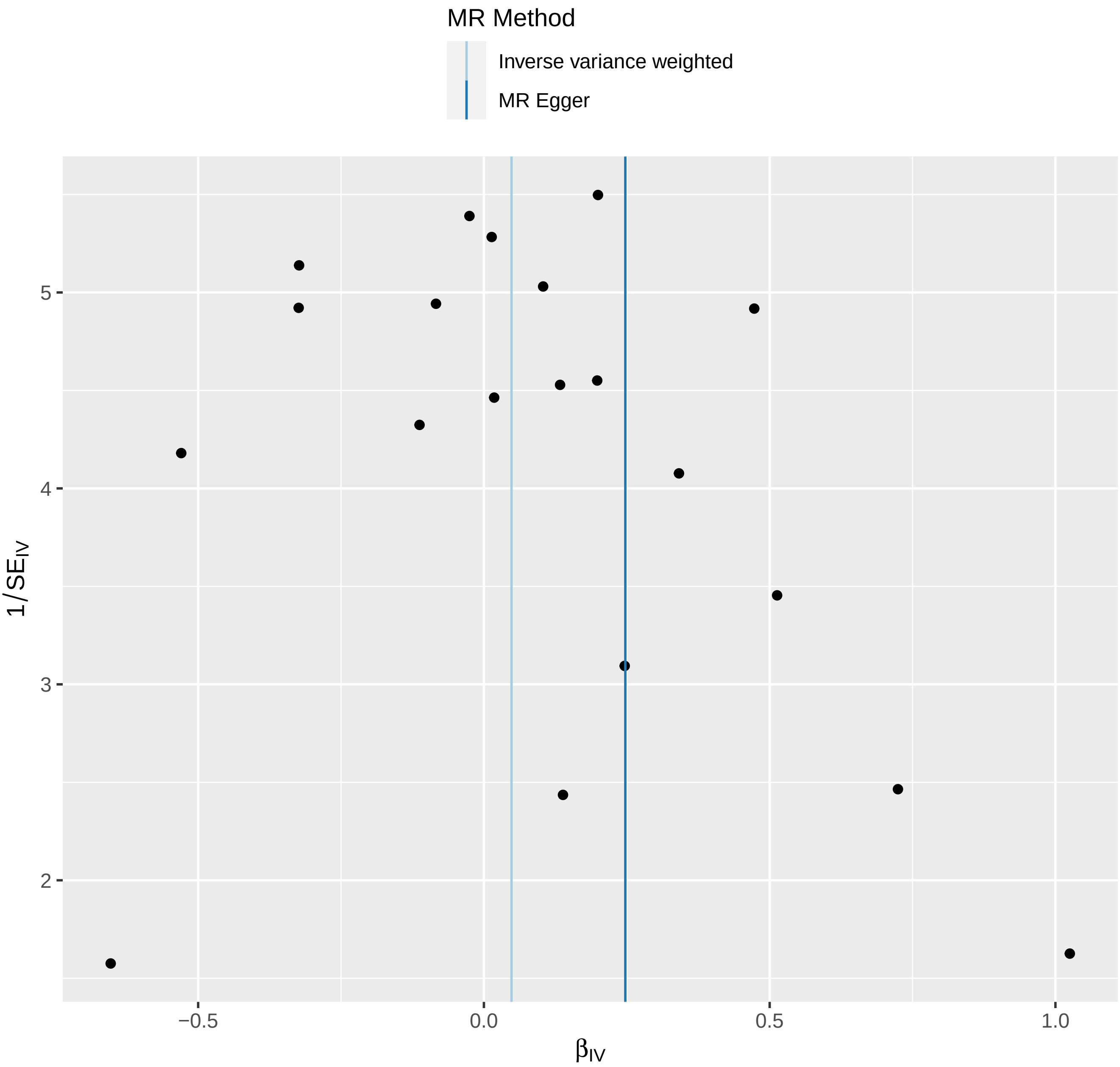

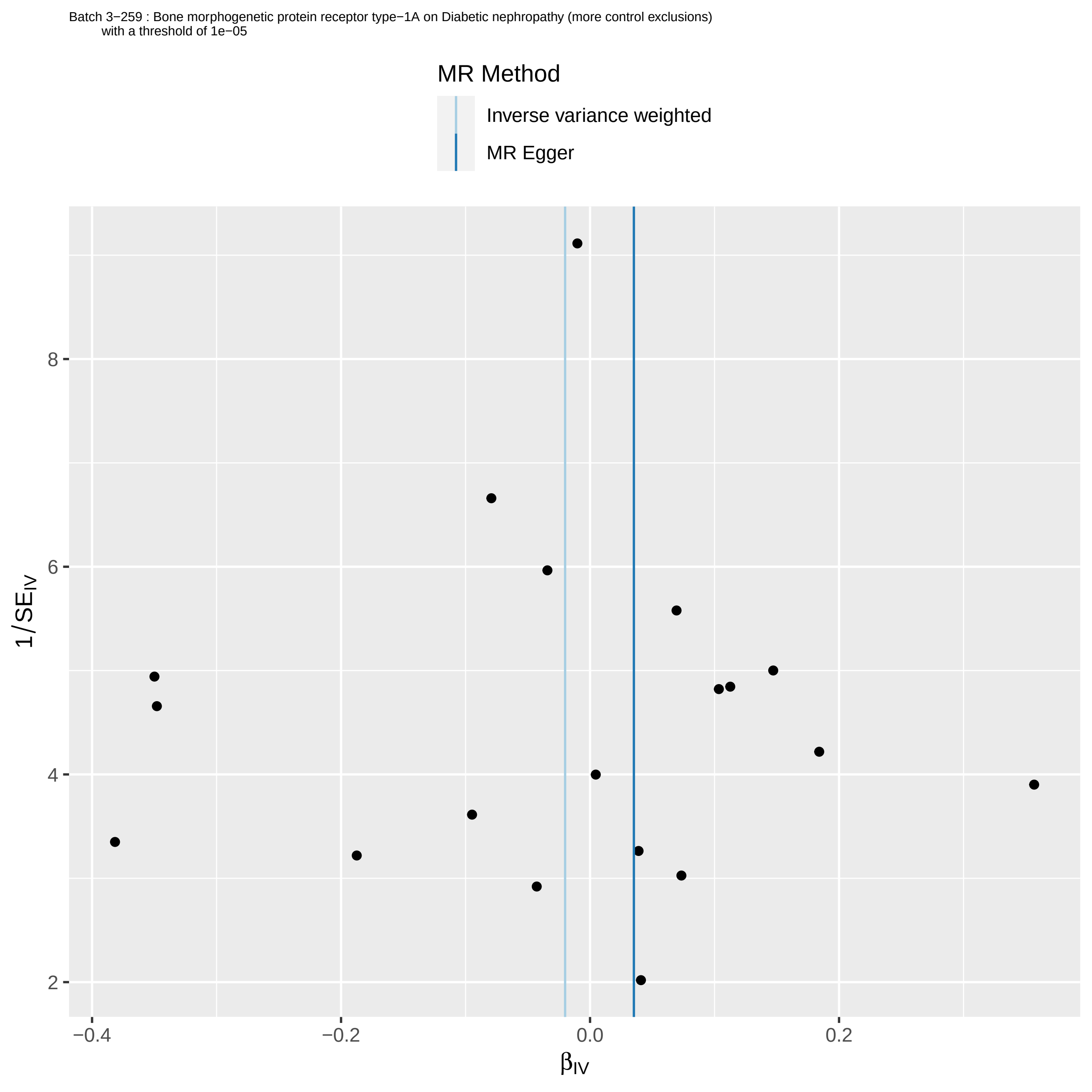
Batch 3−259 : Bone morphogenetic protein receptor type−1A on Diabetic nephropathy (more control exclusions)
with a threshold of 1e−05
MR Method
Inverse variance weighted
MR Egger
1/SE$_{IV}$
8
6
4
2
−0.4
−0.2
0.0
0.2
β$_{IV}$

Batch 3−260 : Cytoplasmic tyrosine−protein kinase BMX on Diabetic nephropathy (more control exclusions) with a threshold of 1e−05

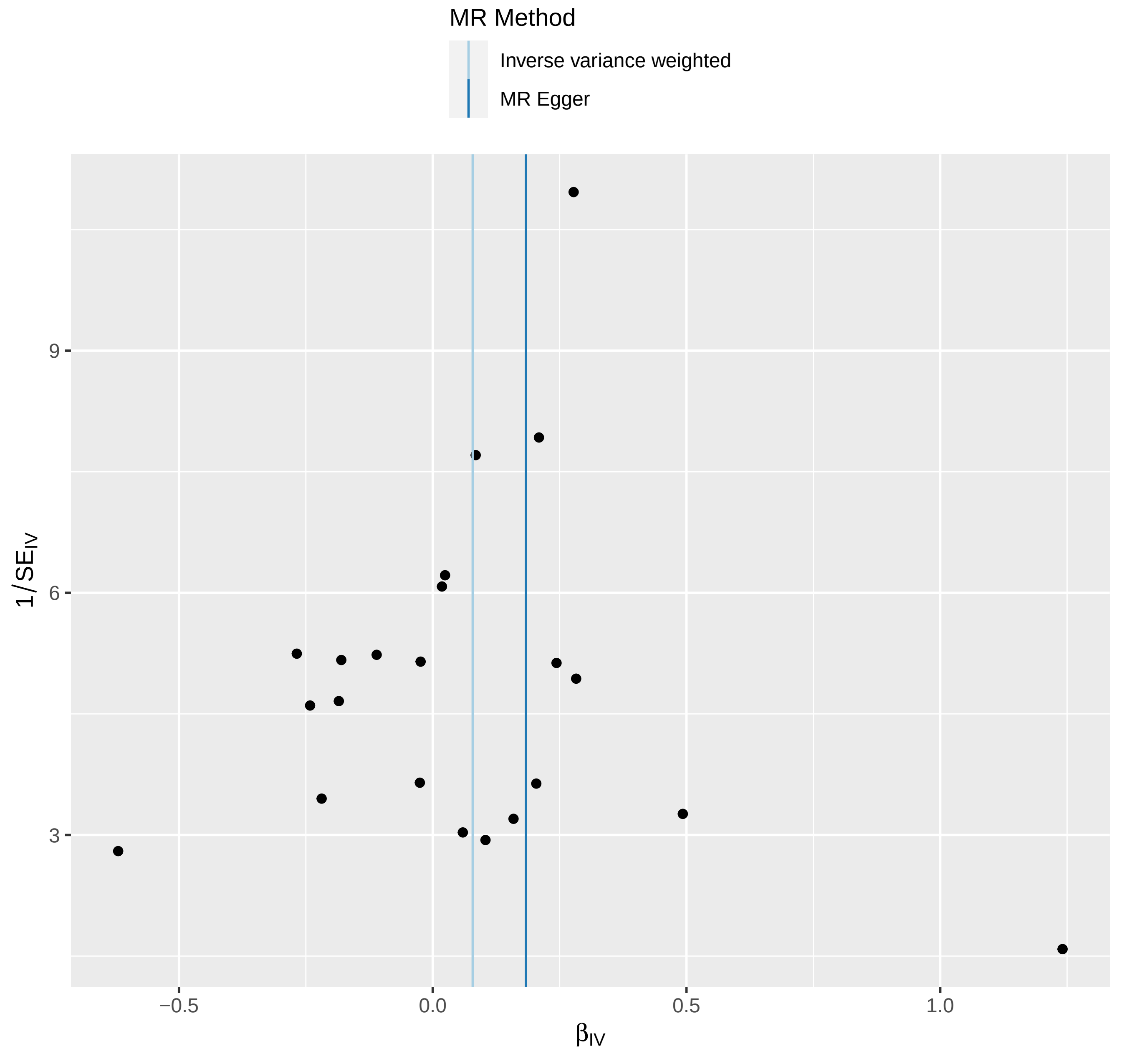

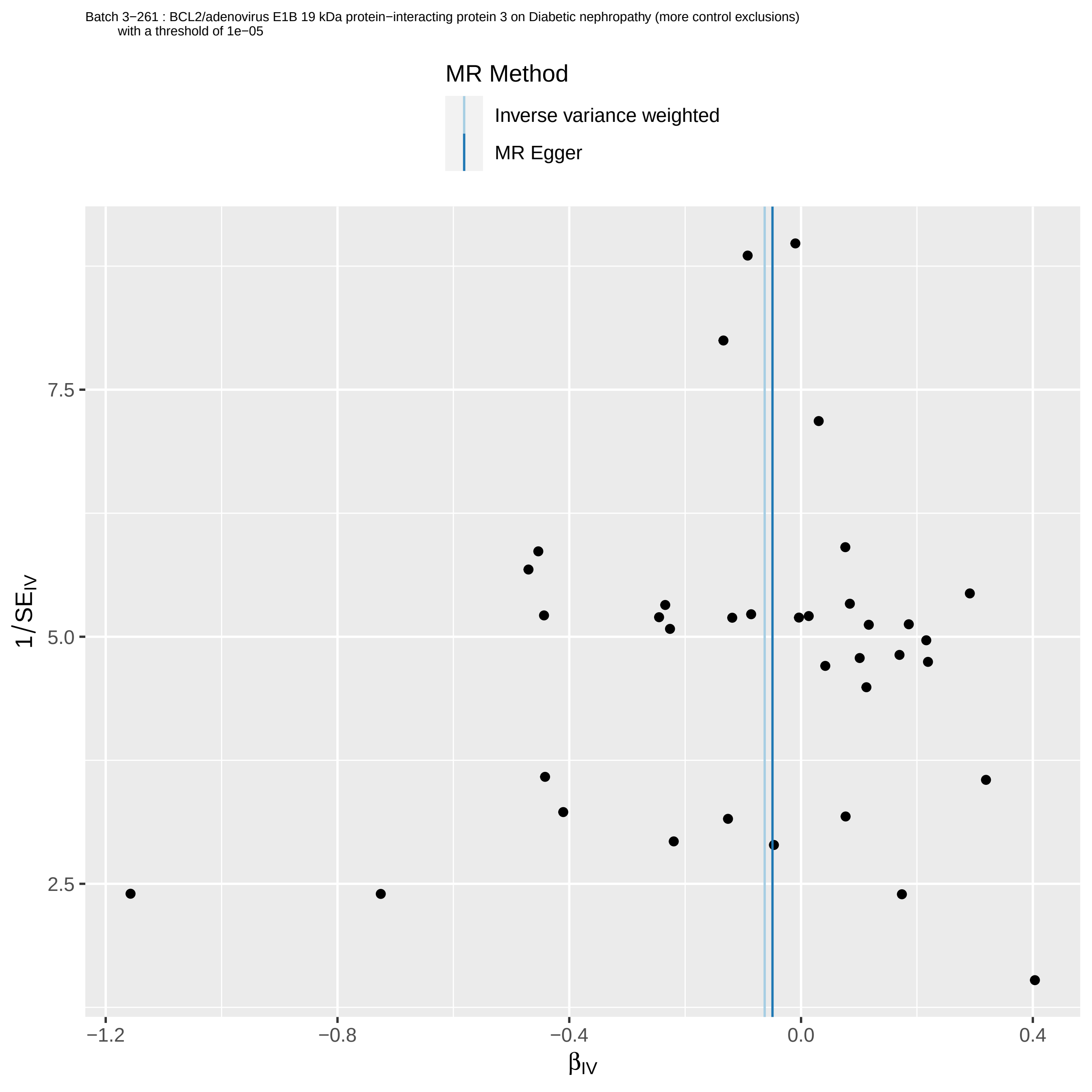

Batch 3−261 : BCL2/adenovirus E1B 19 kDa protein−interacting protein 3 on Diabetic nephropathy (more control exclusions)
with a threshold of 1e−05
MR Method
Inverse variance weighted
MR Egger
7.5
5.0
2.5
−1.2
−0.8
−0.4
0.0
0.4
$1/SE_{IV}$
β_{IV}

Batch 3−262 : BCL2/adenovirus E1B 19 kDa protein−interacting protein 3−like on Diabetic nephropathy (more control exclusions) with a threshold of 1e−05

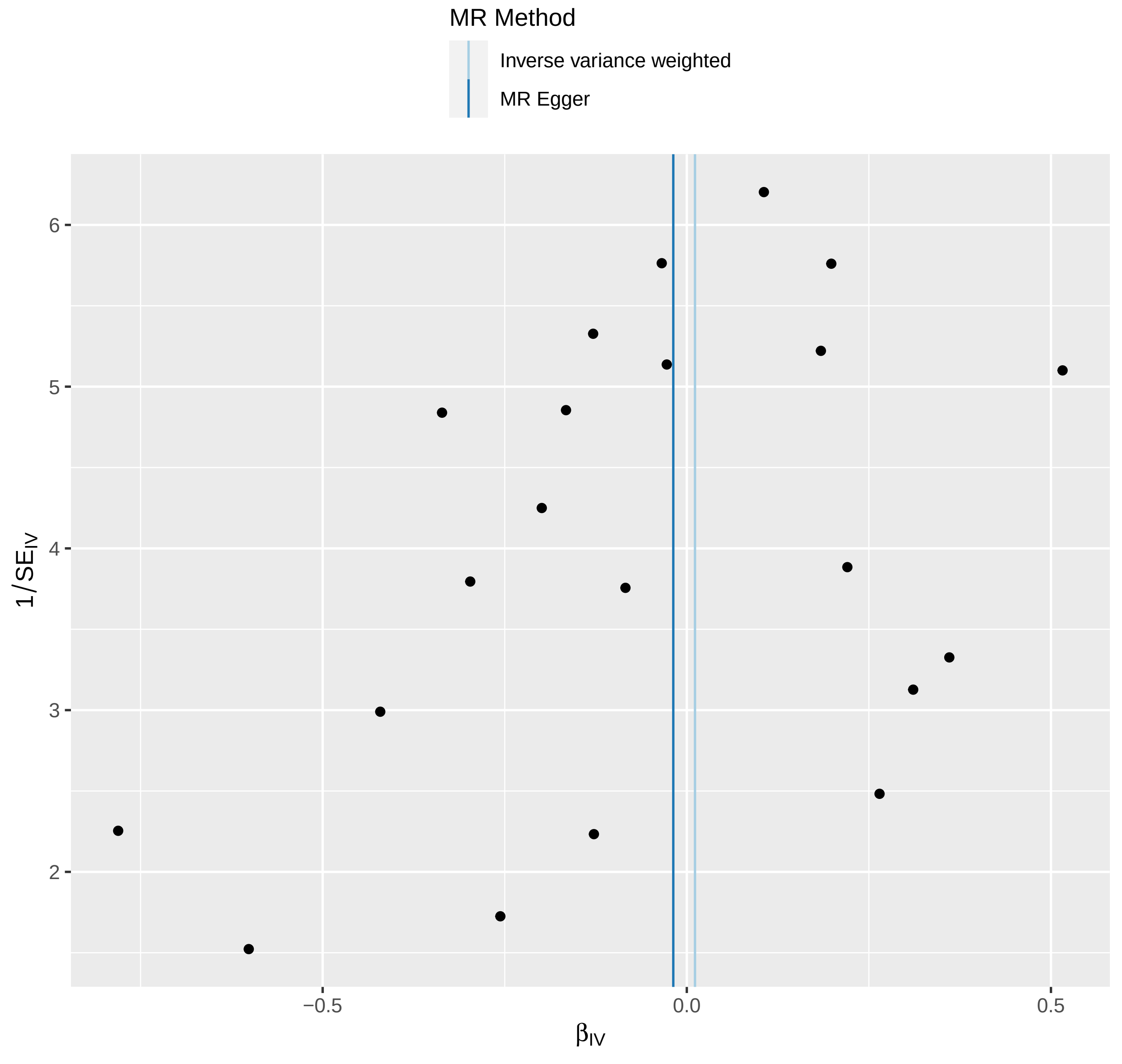

Batch 3−263 : Brother of CDO on Diabetic nephropathy (more control exclusions)
with a threshold of 1e−05

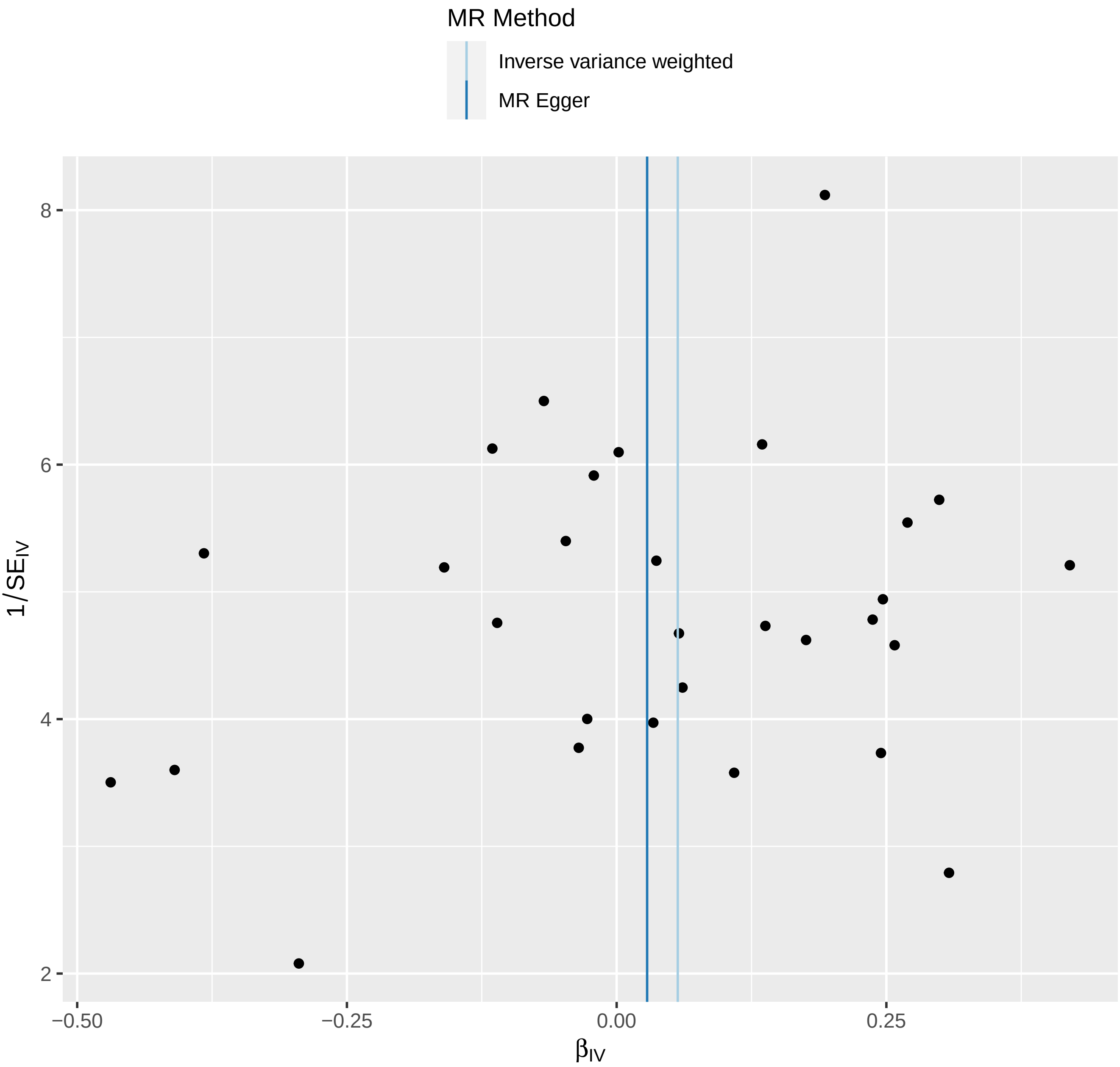

Batch 3−264 : BolA−like protein 2 on Diabetic nephropathy (more control exclusions)
with a threshold of 1e−05

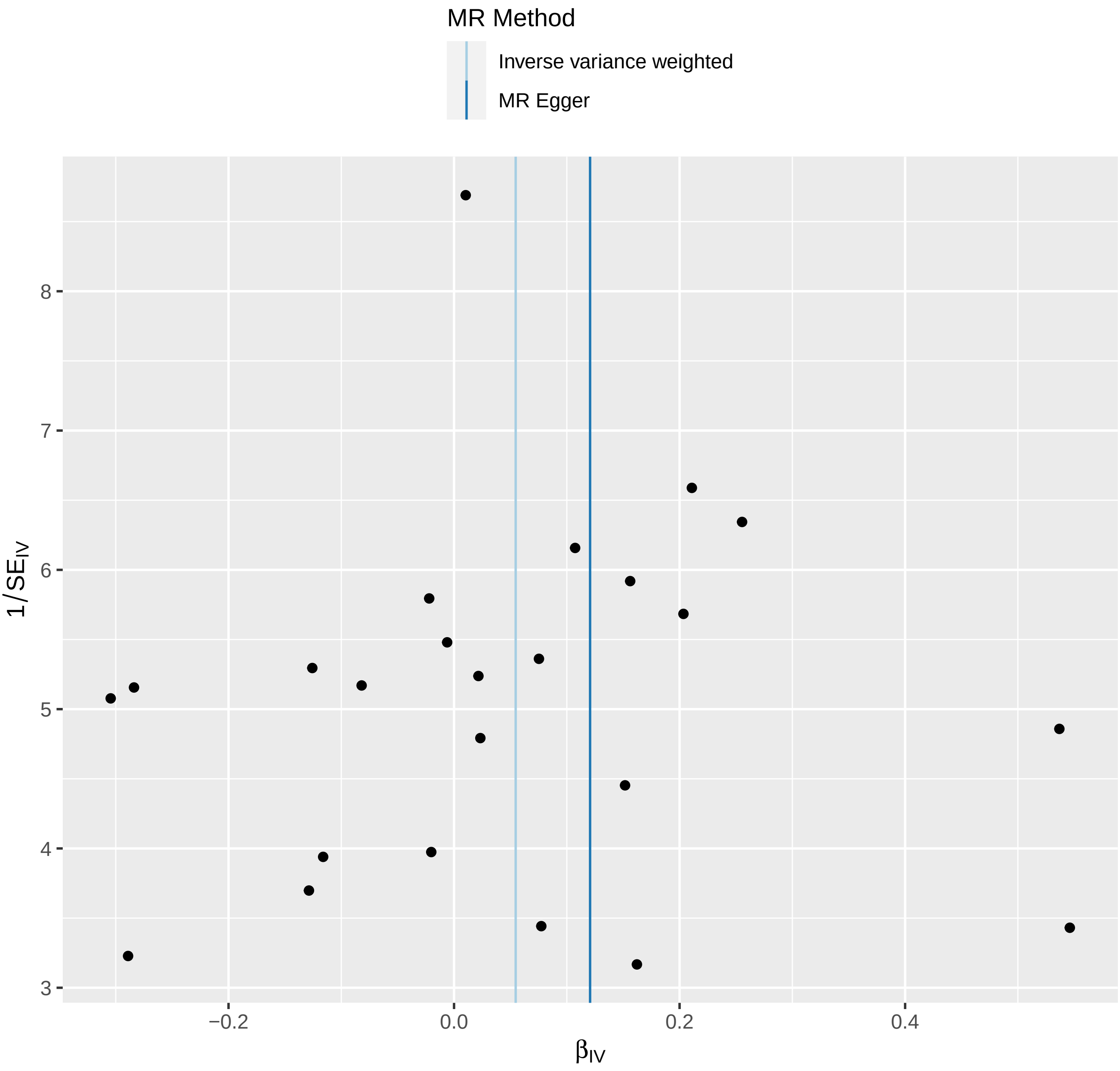

Batch 3−265 : Bisphosphoglycerate mutase on Diabetic nephropathy (more control exclusions) with a threshold of 1e−05

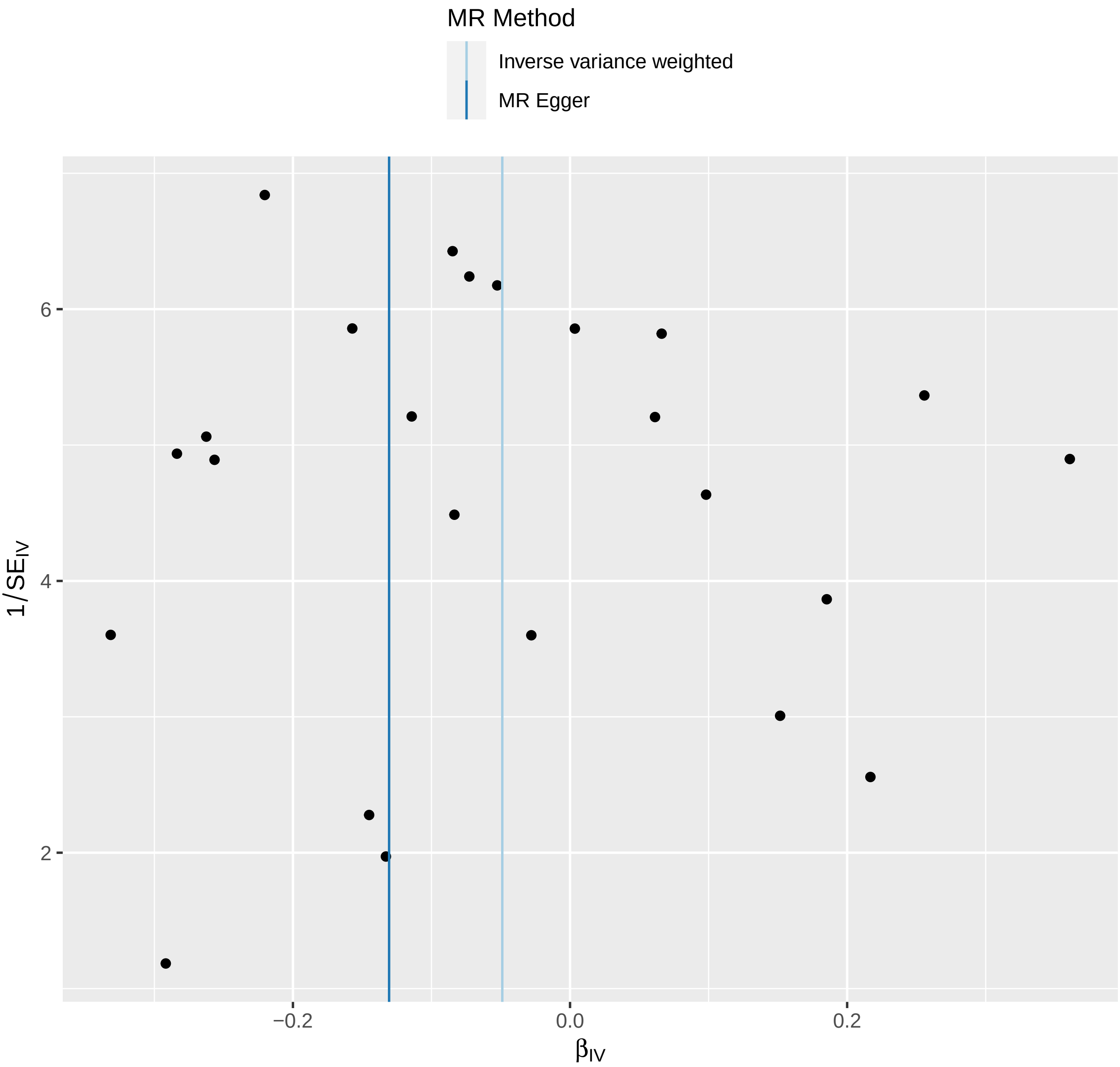

Batch 3−266 : Bactericidal permeability−increasing protein on Diabetic nephropathy (more control exclusions)
with a threshold of 1e−05

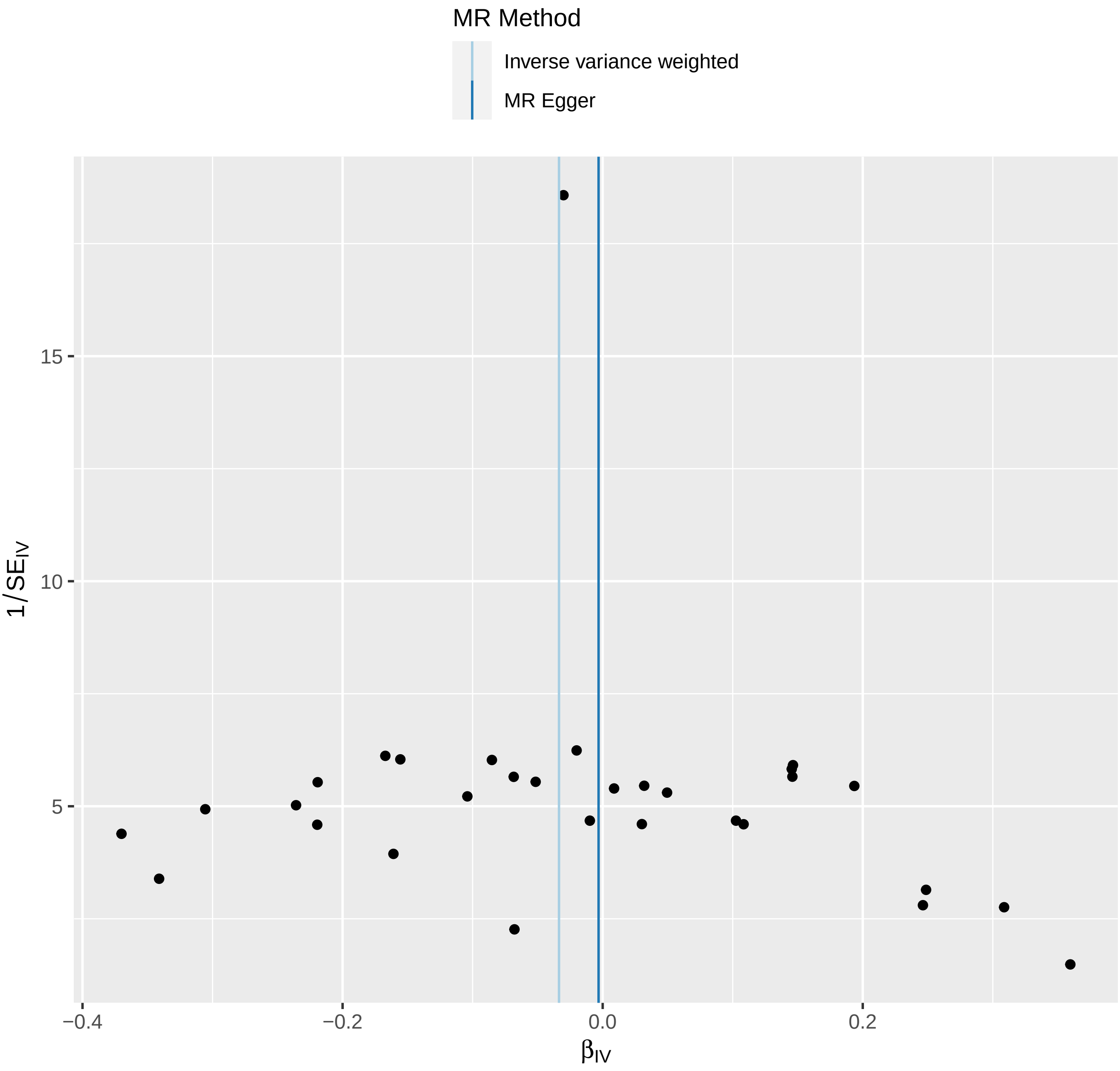

Batch 3−267 : BPI fold−containing family A member 1 on Diabetic nephropathy (more control exclusions)
with a threshold of 1e−05

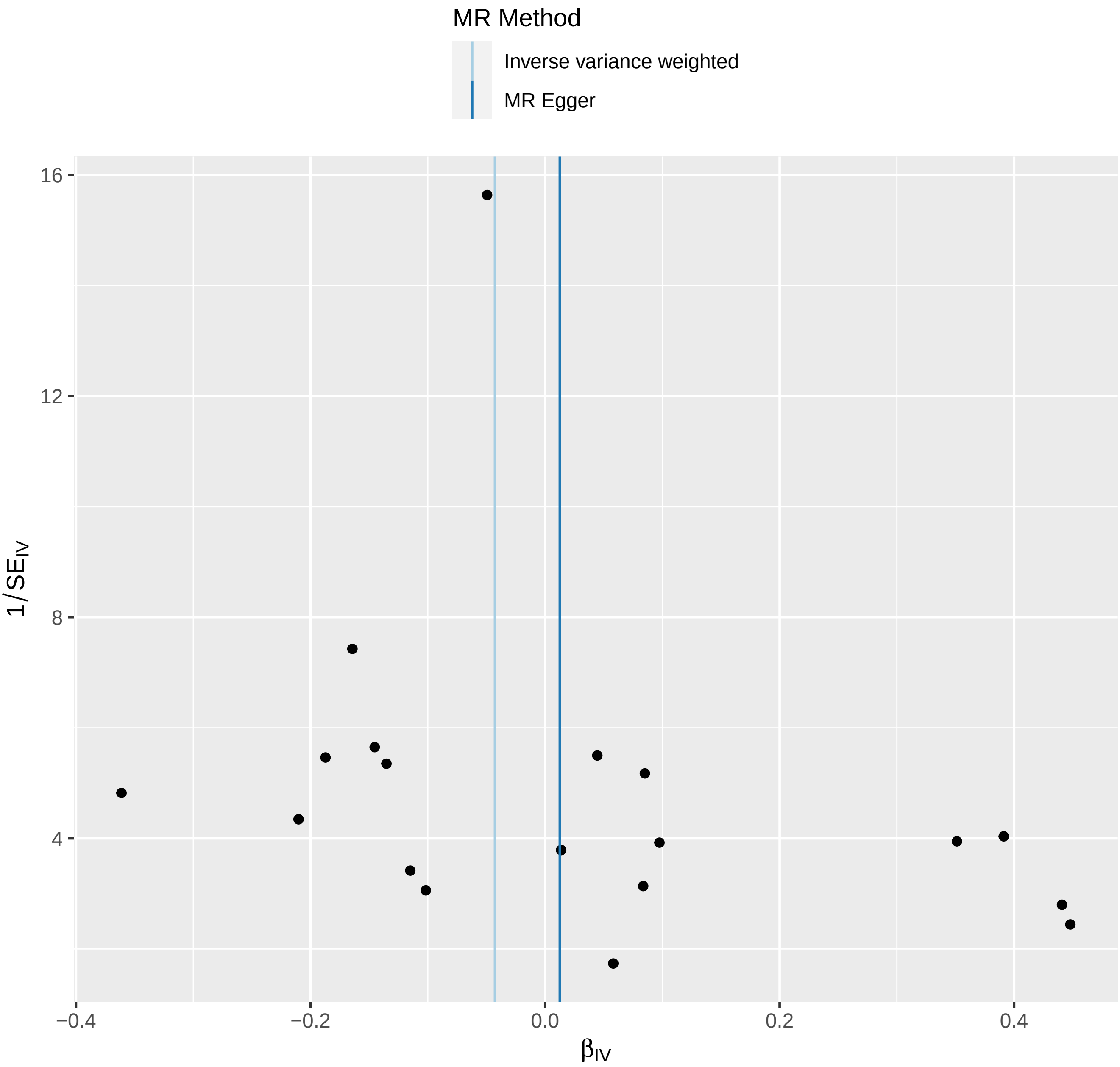

Batch 3−268 : BPI fold−containing family B member 1 on Diabetic nephropathy (more control exclusions)
with a threshold of 1e−05

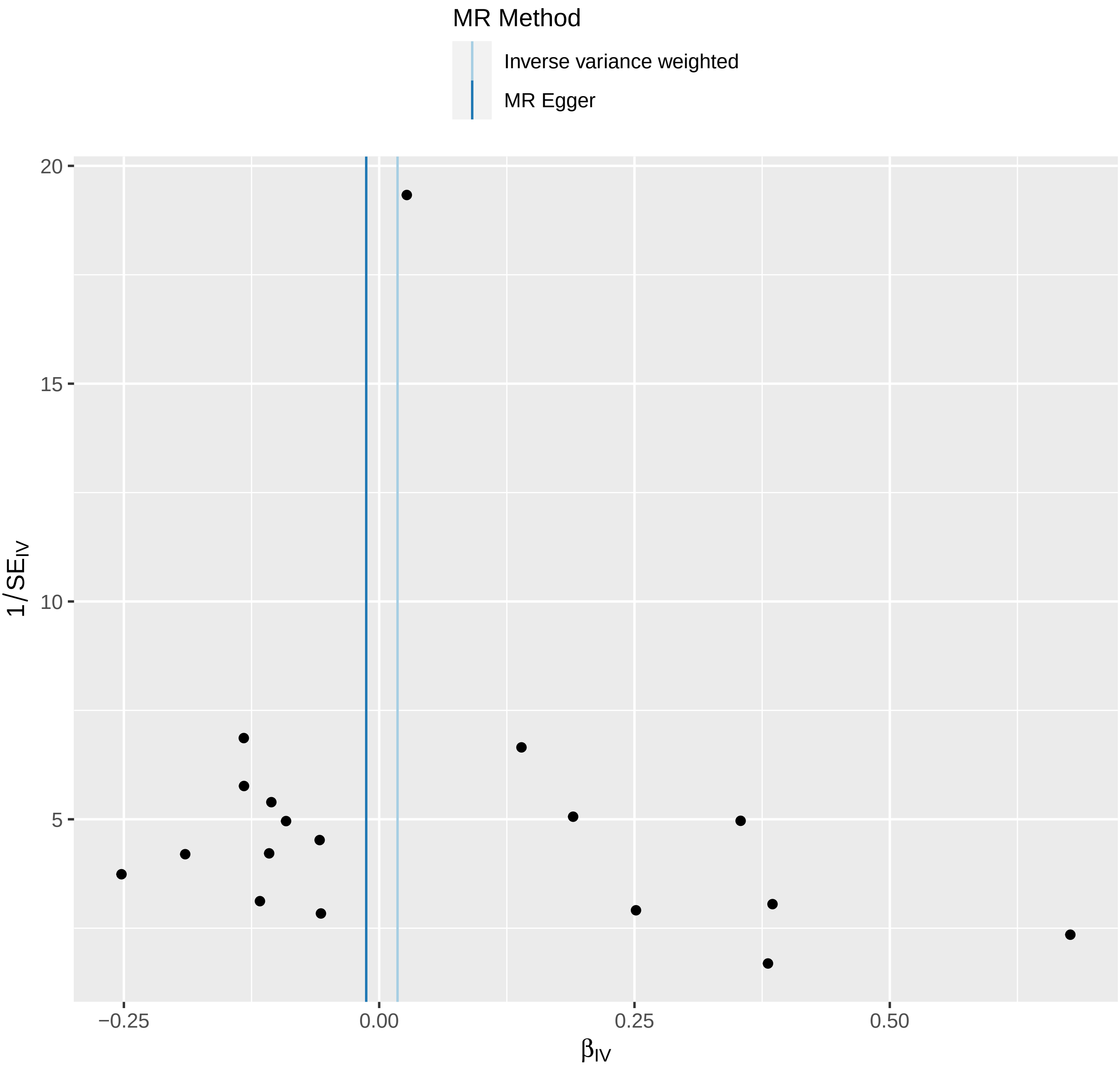

Batch 3−269 : Bromodomain−containing protein 1 on Diabetic nephropathy (more control exclusions) with a threshold of 1e−05

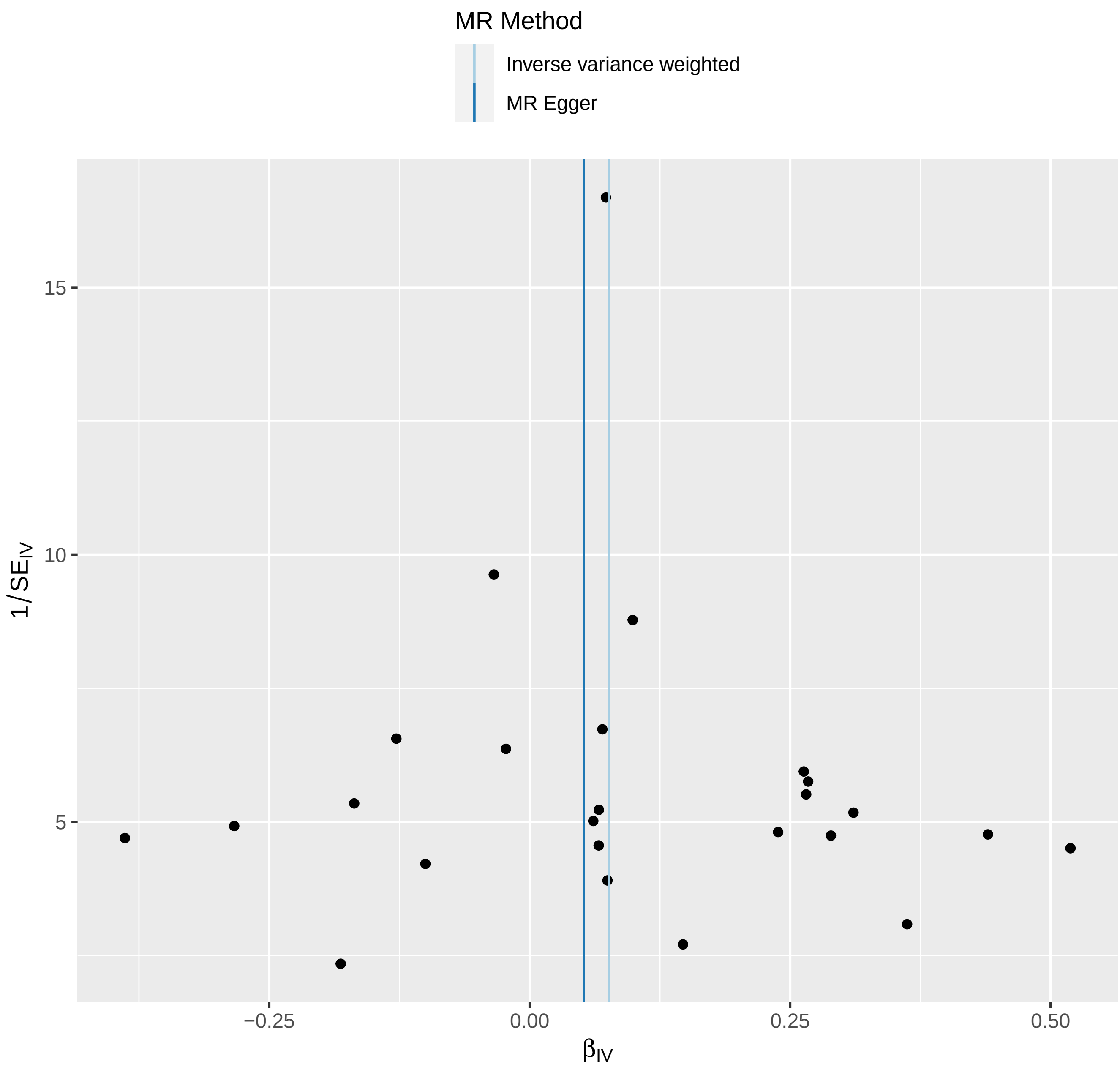

Batch 3−270 : Bromodomain testis−specific protein on Diabetic nephropathy (more control exclusions)
with a threshold of 1e−05

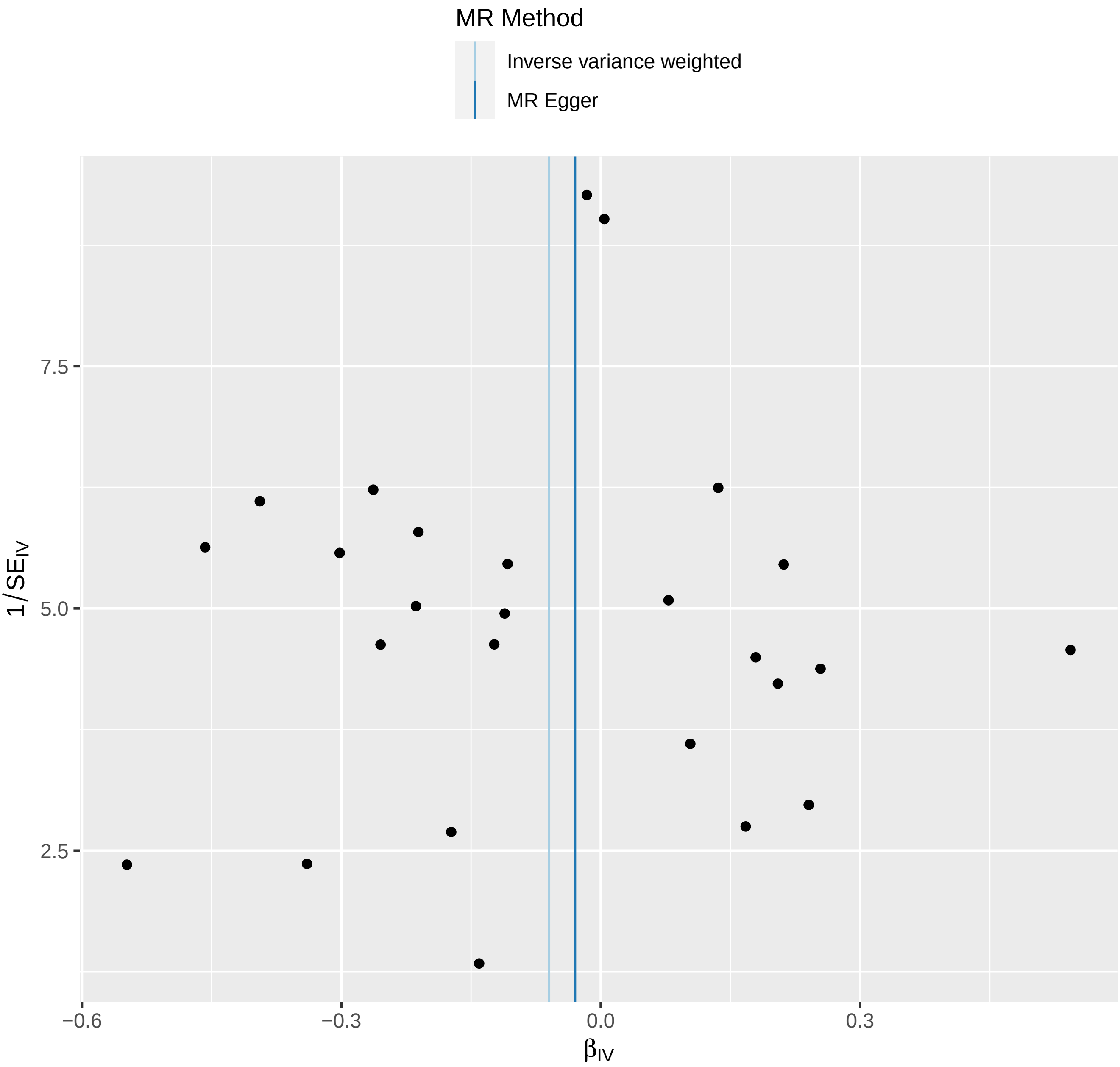

Batch 3−271 : Transcription factor IIIB 90 kDa subunit on Diabetic nephropathy (more control exclusions)
with a threshold of 1e−05

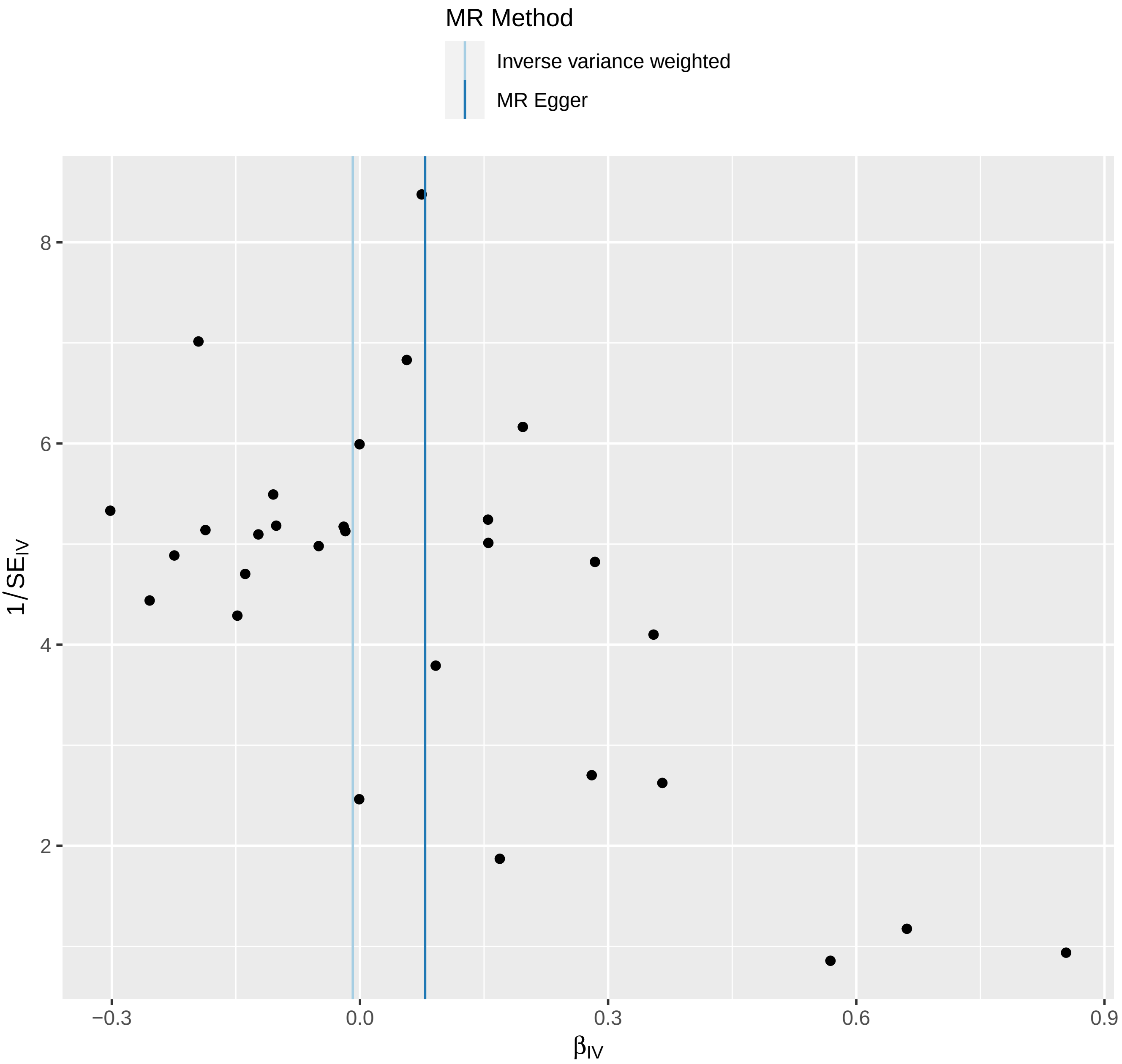

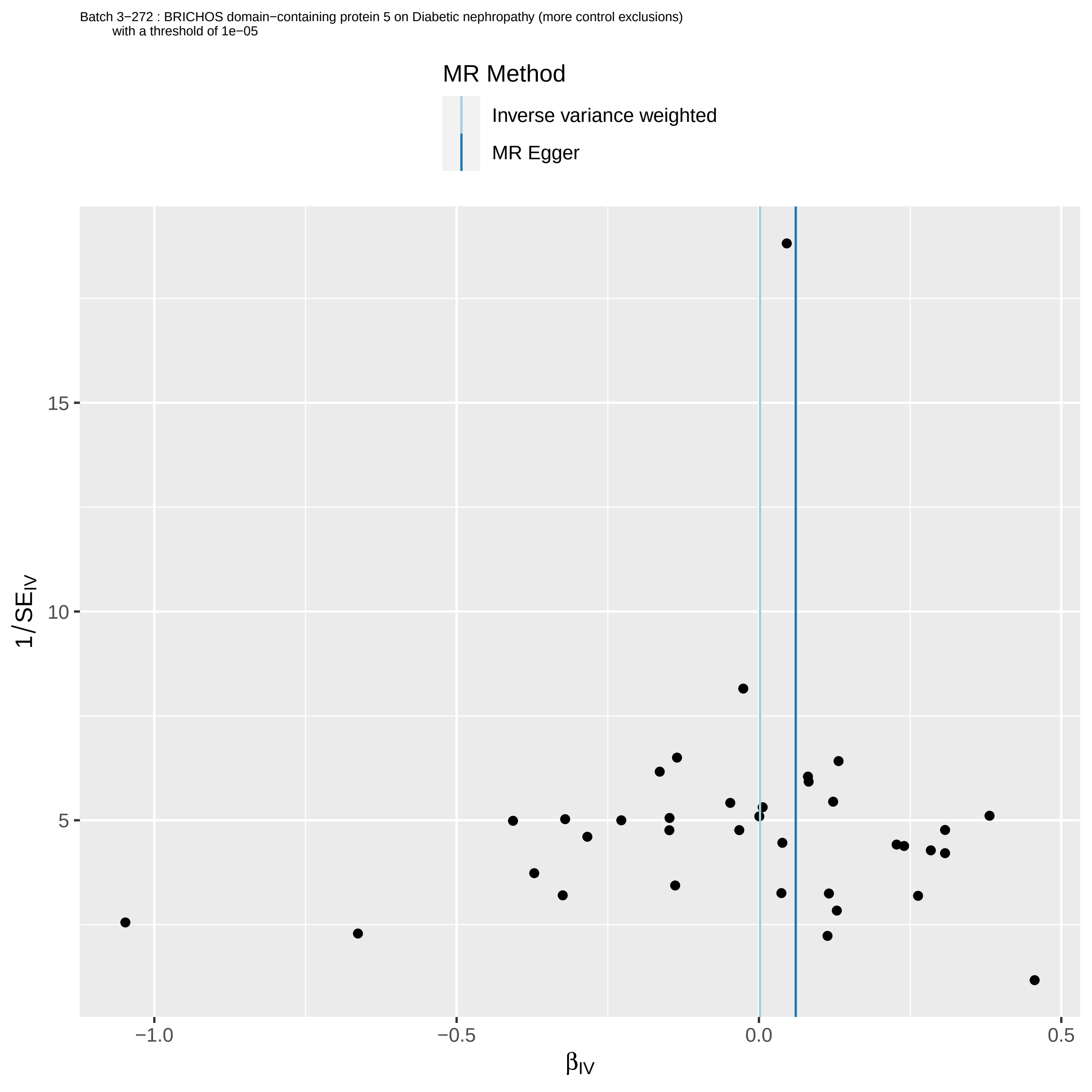
Batch 3−272 : BRICHOS domain−containing protein 5 on Diabetic nephropathy (more control exclusions)
with a threshold of 1e−05
MR Method
Inverse variance weighted
MR Egger
15
10
5
$1/SE_{IV}$
−1.0
−0.5
0.0
0.5
β_{IV}

Batch 3−273 : Peregrin on Diabetic nephropathy (more control exclusions)
with a threshold of 1e−05

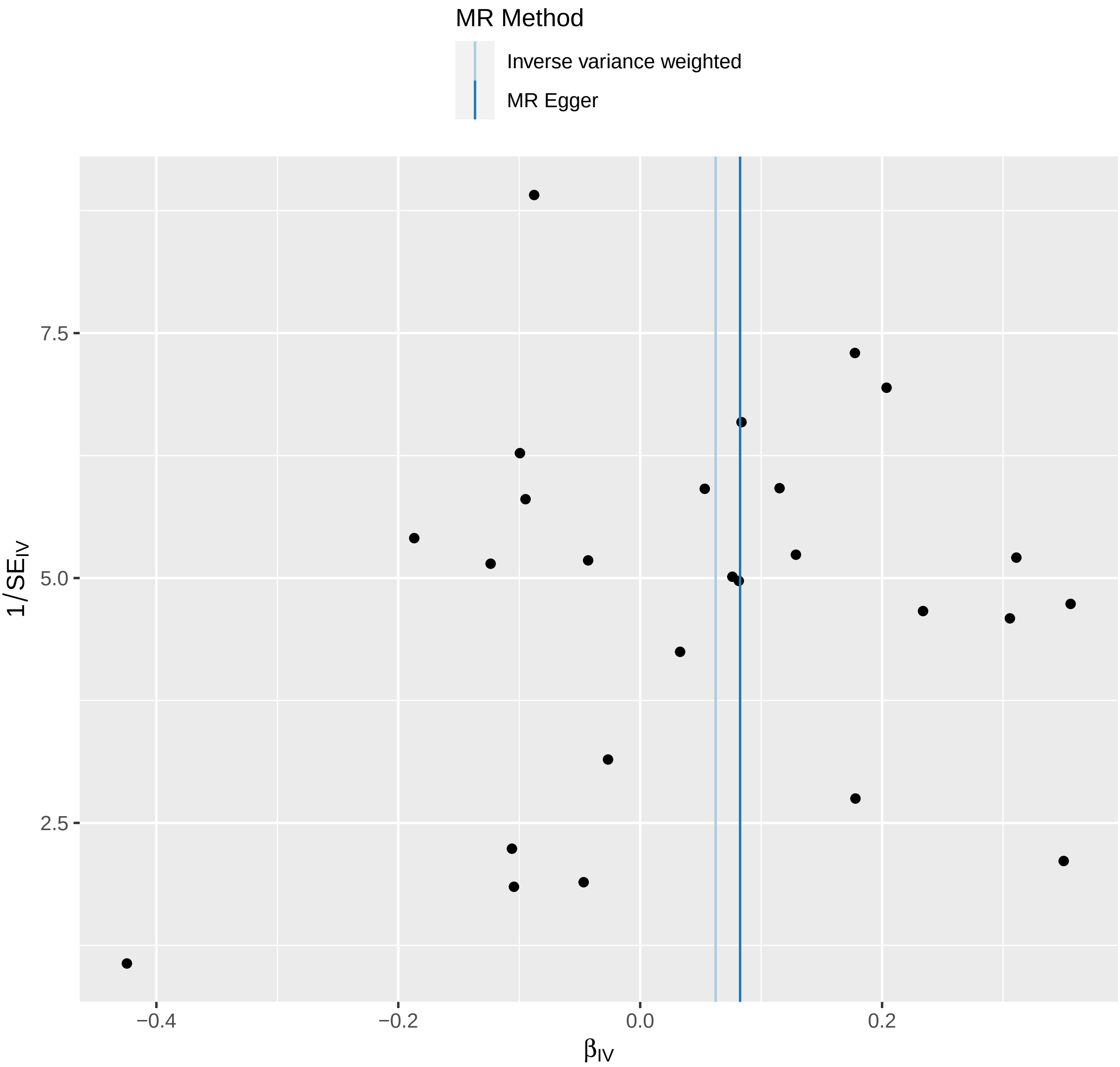

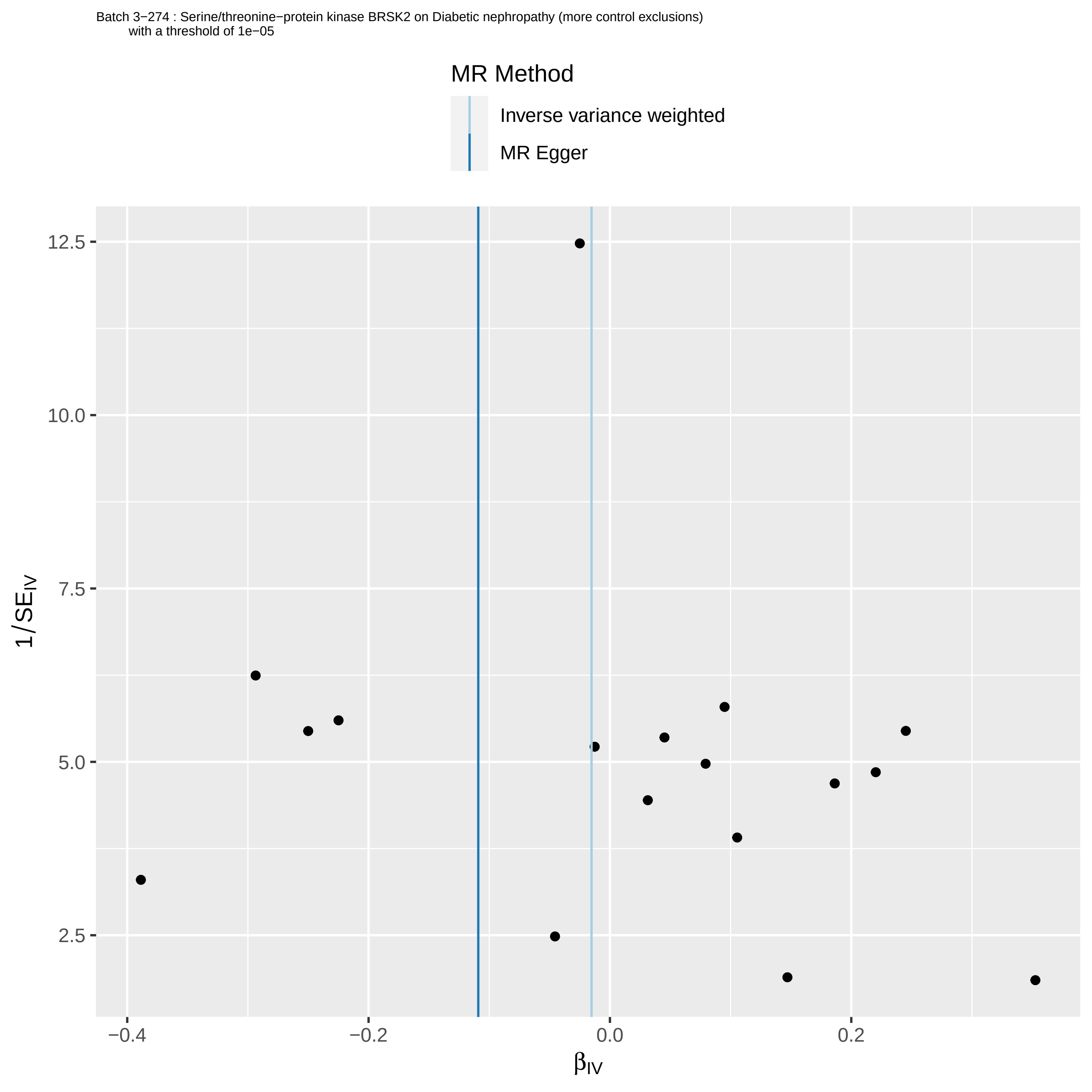
Batch 3−274 : Serine/threonine−protein kinase BRSK2 on Diabetic nephropathy (more control exclusions)
with a threshold of 1e−05
MR Method
Inverse variance weighted
MR Egger
12.5
10.0
7.5
5.0
2.5
1/SE_IV
−0.4
−0.2
0.0
0.2
β_IV

Batch 3−275 : Basigin on Diabetic nephropathy (more control exclusions)
with a threshold of 1e−05

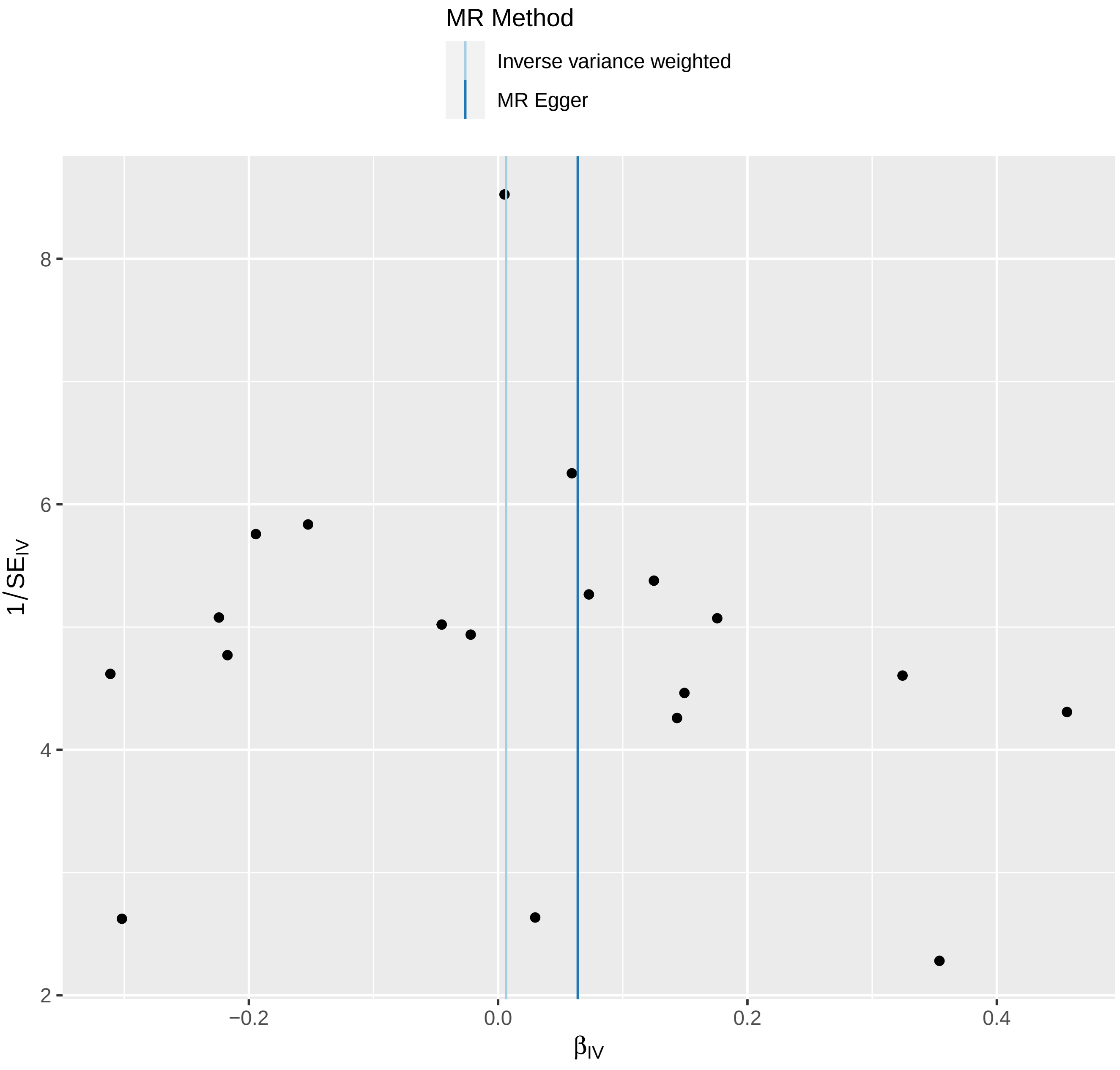

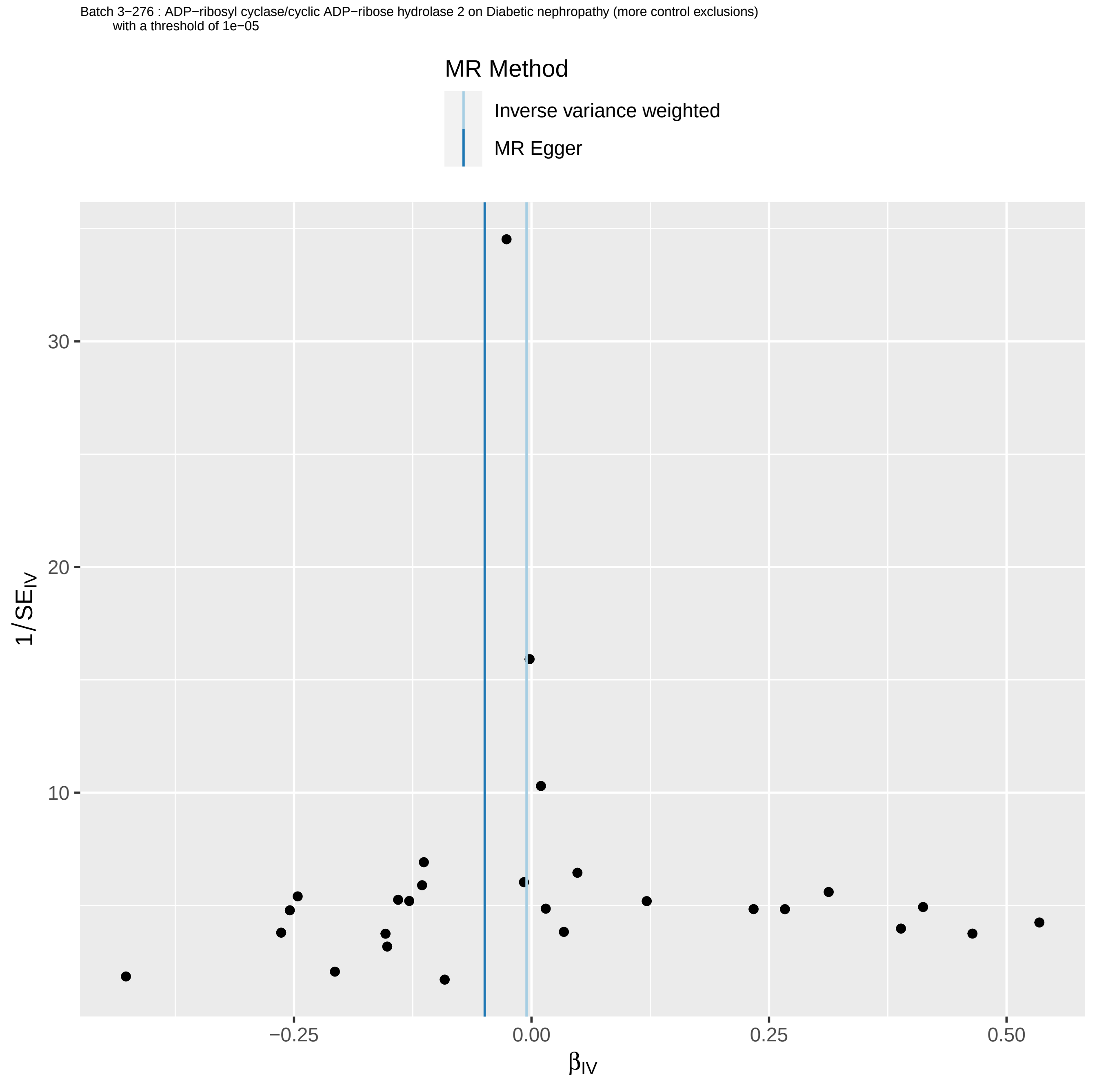

Batch 3−276 : ADP−ribosyl cyclase/cyclic ADP−ribose hydrolase 2 on Diabetic nephropathy (more control exclusions)
with a threshold of 1e−05
MR Method
Inverse variance weighted
MR Egger
30
20
10
1/SE_IV
−0.25
0.00
0.25
0.50
β_IV

Batch 3−277 : Tyrosine−protein kinase BTK on Diabetic nephropathy (more control exclusions)
with a threshold of 1e−05

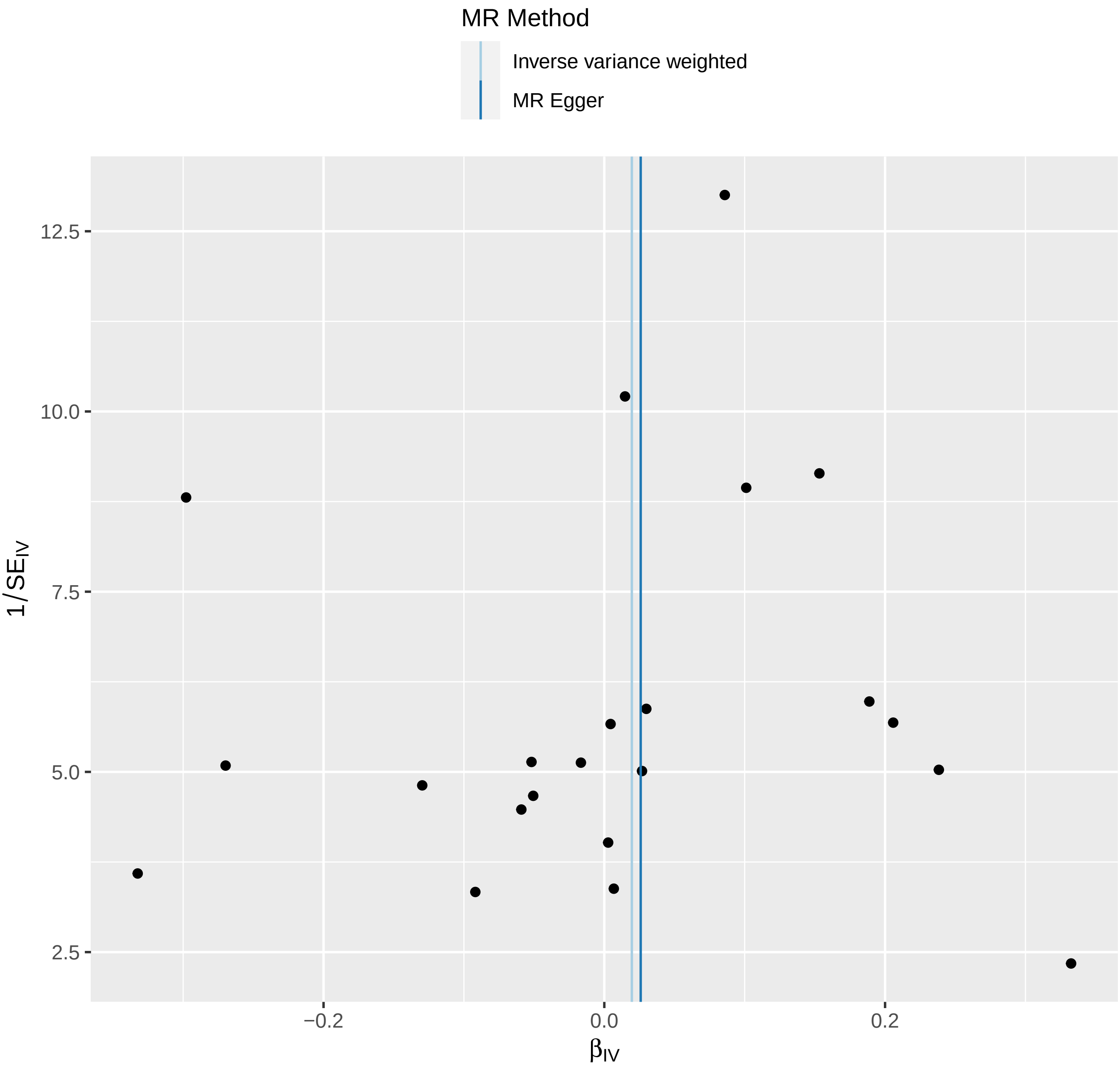

Batch 3−278 : Butyrophilin−like protein 9 on Diabetic nephropathy (more control exclusions)
with a threshold of 1e−05

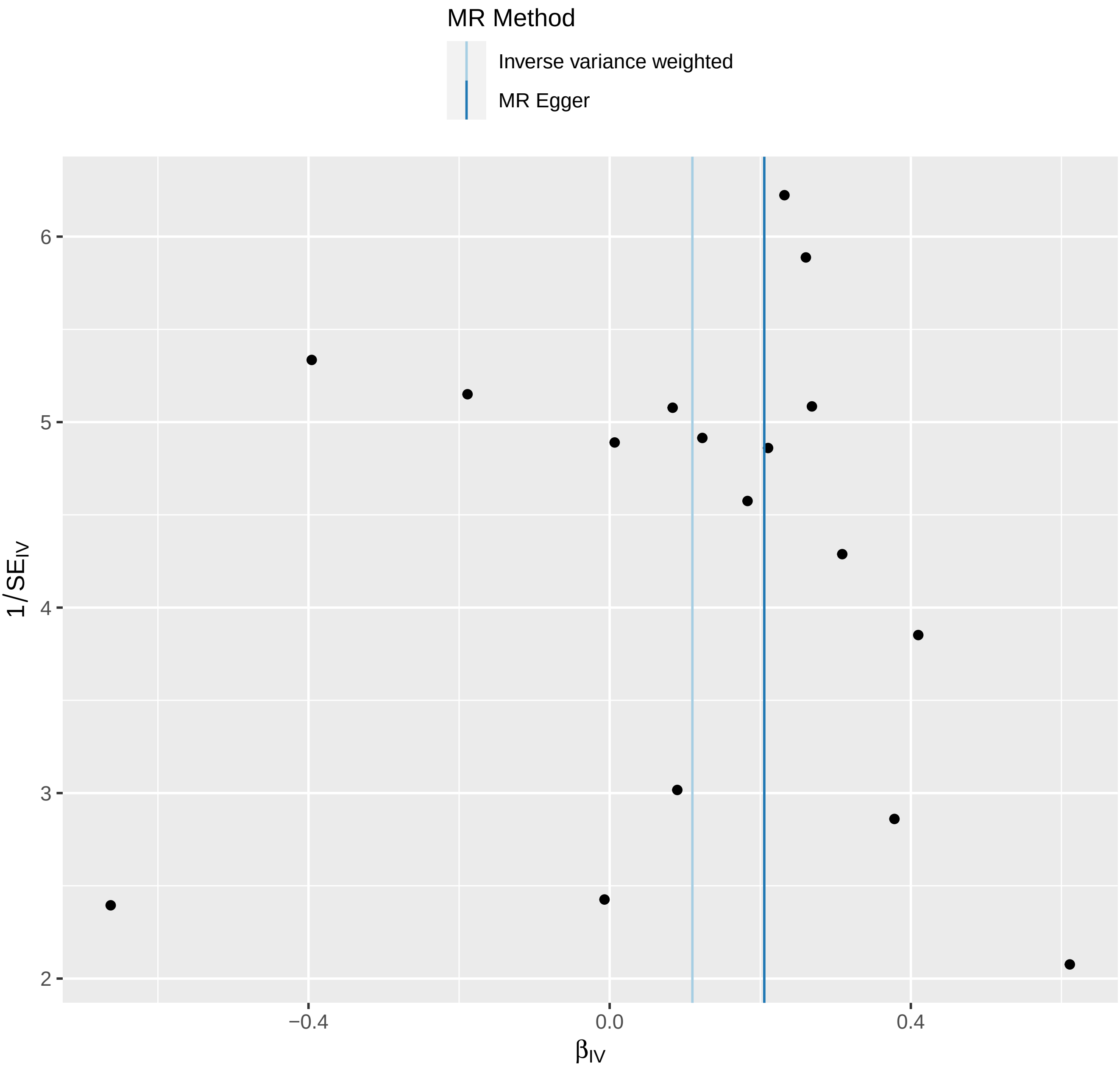

Batch 3−279 : Mitotic checkpoint serine/threonine−protein kinase BUB1 on Diabetic nephropathy (more control exclusions) with a threshold of 1e−05

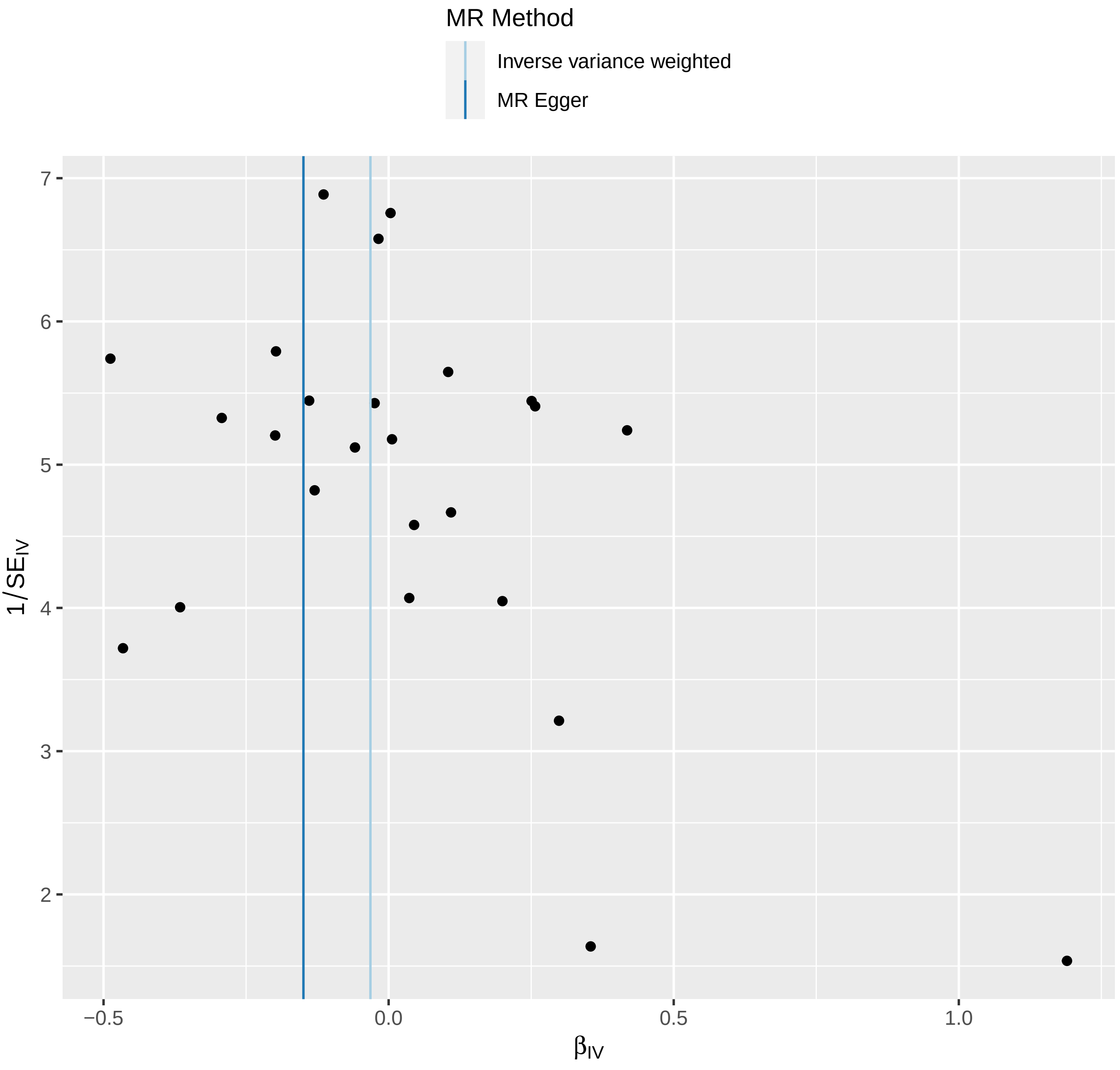

Batch 3−280 : Uncharacterized protein C10orf35 on Diabetic nephropathy (more control exclusions)
with a threshold of 1e−05

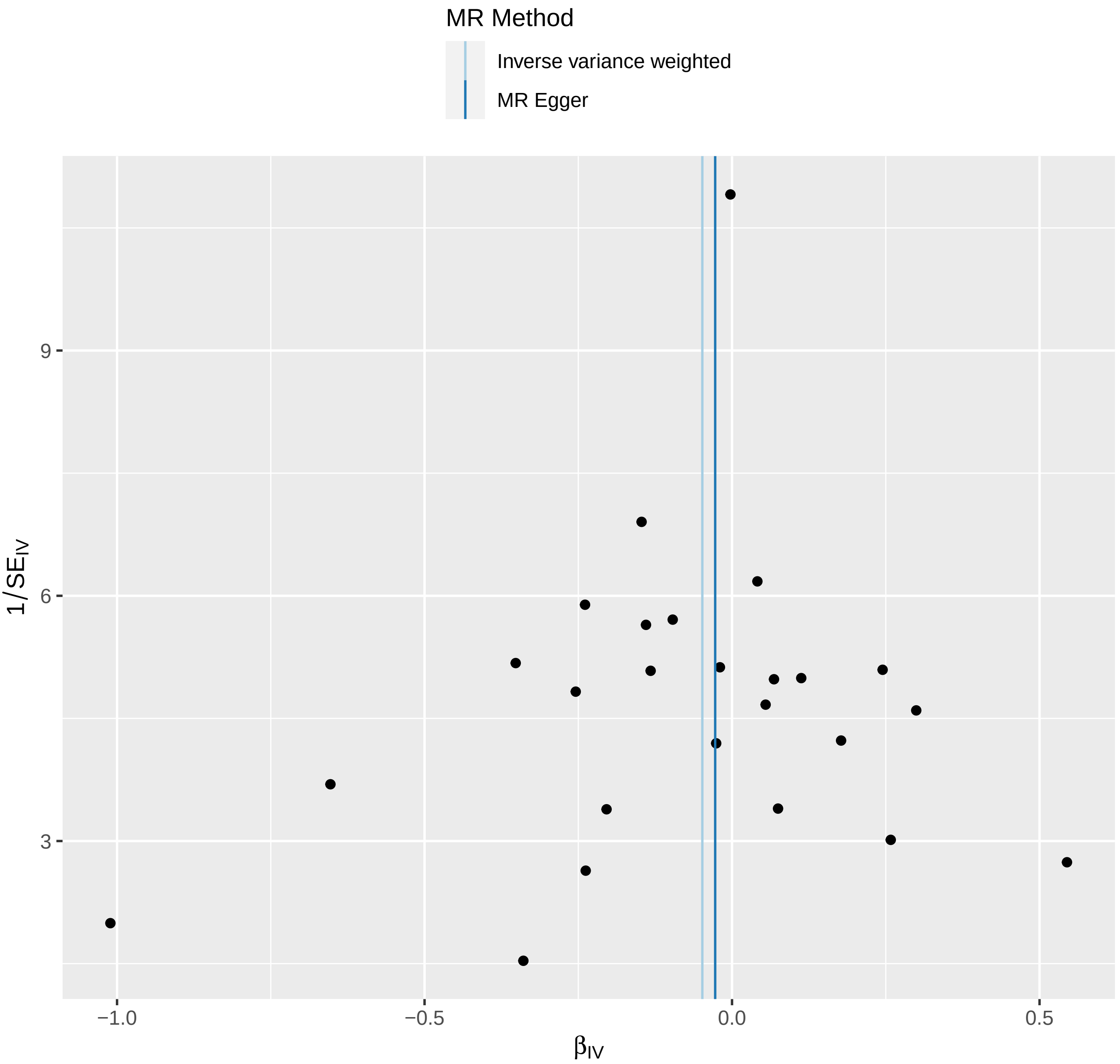

Batch 3−281 : Platelet receptor Gi24 on Diabetic nephropathy (more control exclusions)
with a threshold of 1e−05

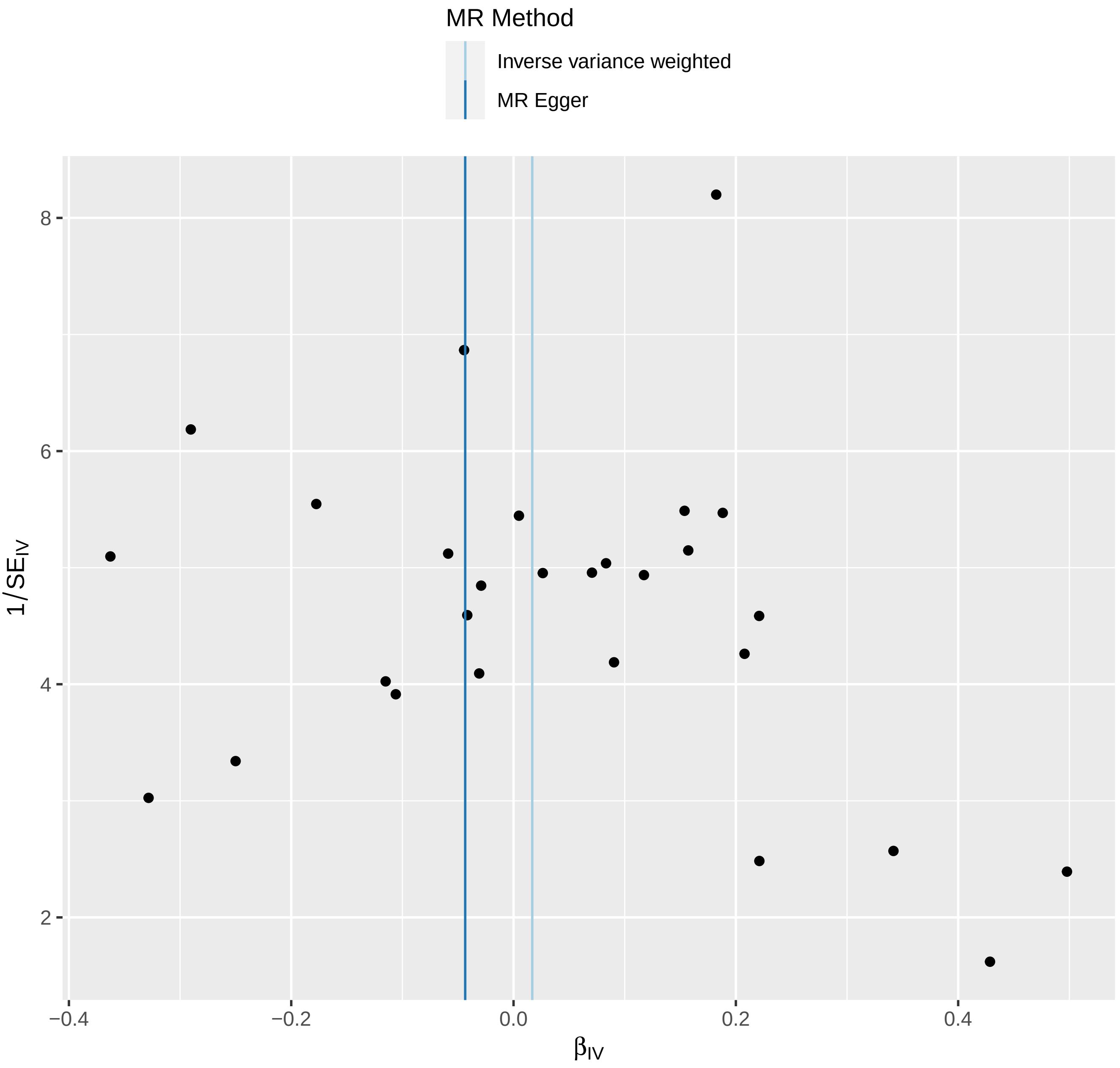

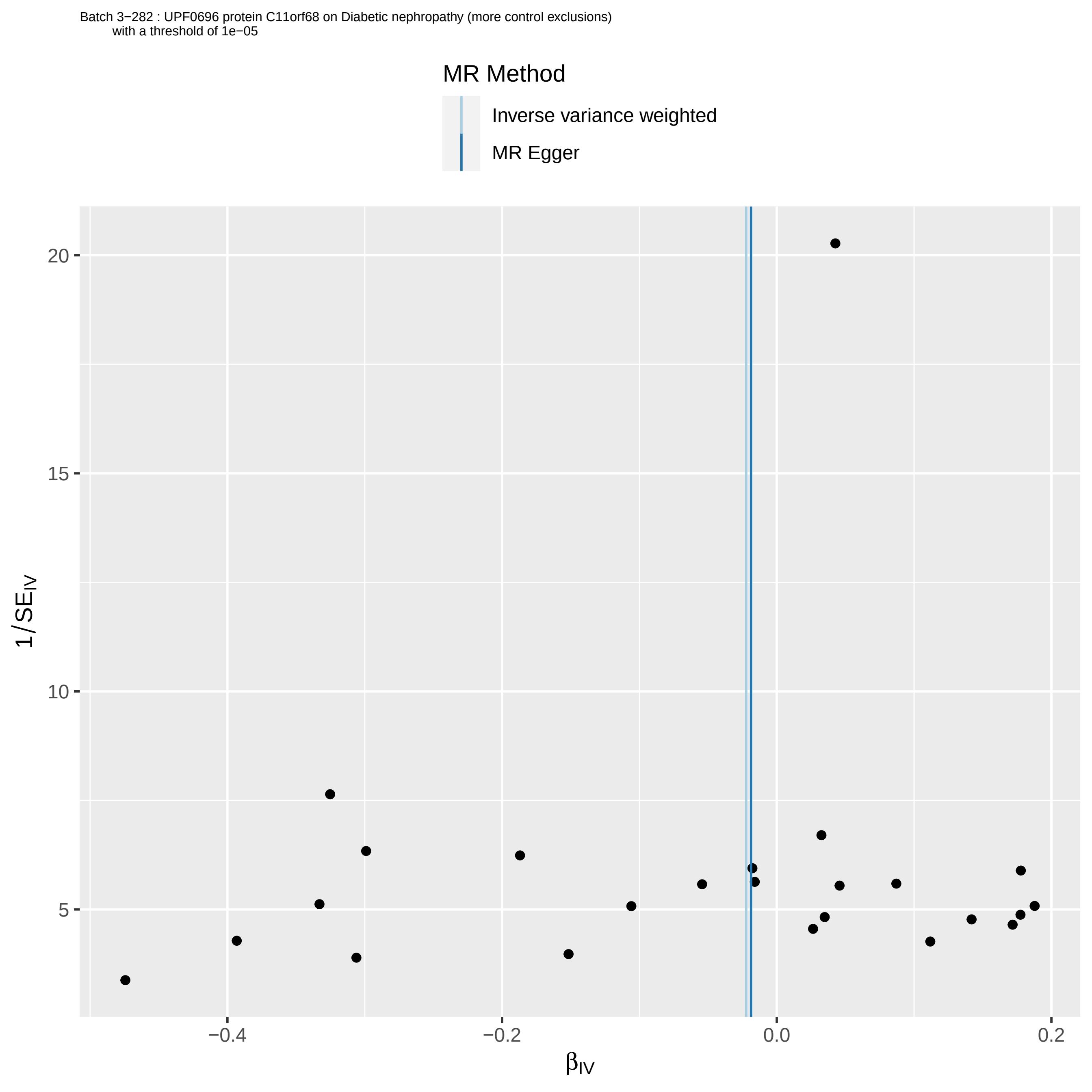
Batch 3−282 : UPF0696 protein C11orf68 on Diabetic nephropathy (more control exclusions)
with a threshold of 1e−05
MR Method
Inverse variance weighted
MR Egger
20
15
10
5
1/SE_IV
−0.4
−0.2
0.0
0.2
β_IV

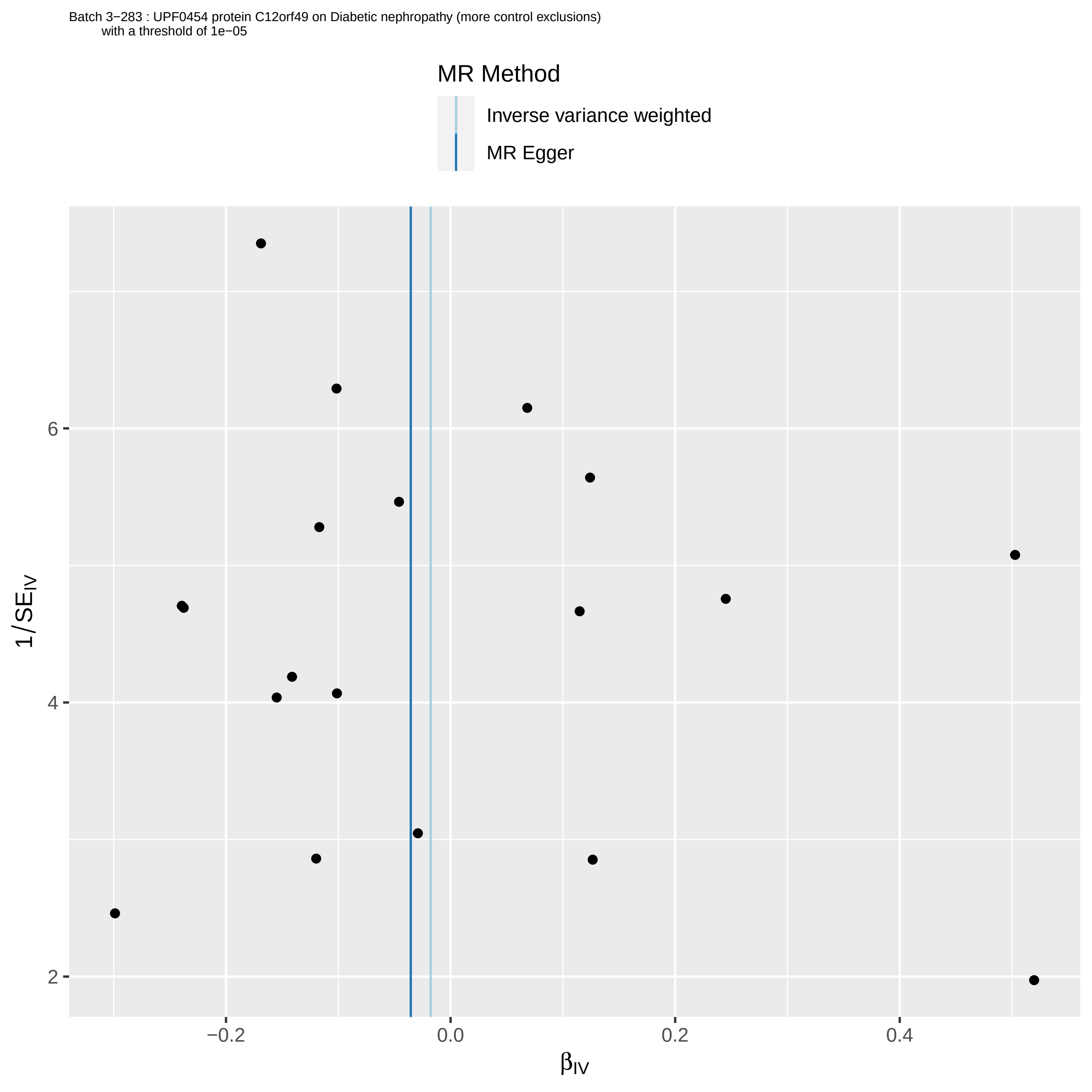

Batch 3−283 : UPF0454 protein C12orf49 on Diabetic nephropathy (more control exclusions)
with a threshold of 1e−05
MR Method
Inverse variance weighted
MR Egger
6
4
2
1/SE$_{IV}$
−0.2
0.0
0.2
0.4
β_{IV}

Batch 3−284 : Uncharacterized protein C14orf93 on Diabetic nephropathy (more control exclusions)
with a threshold of 1e−05

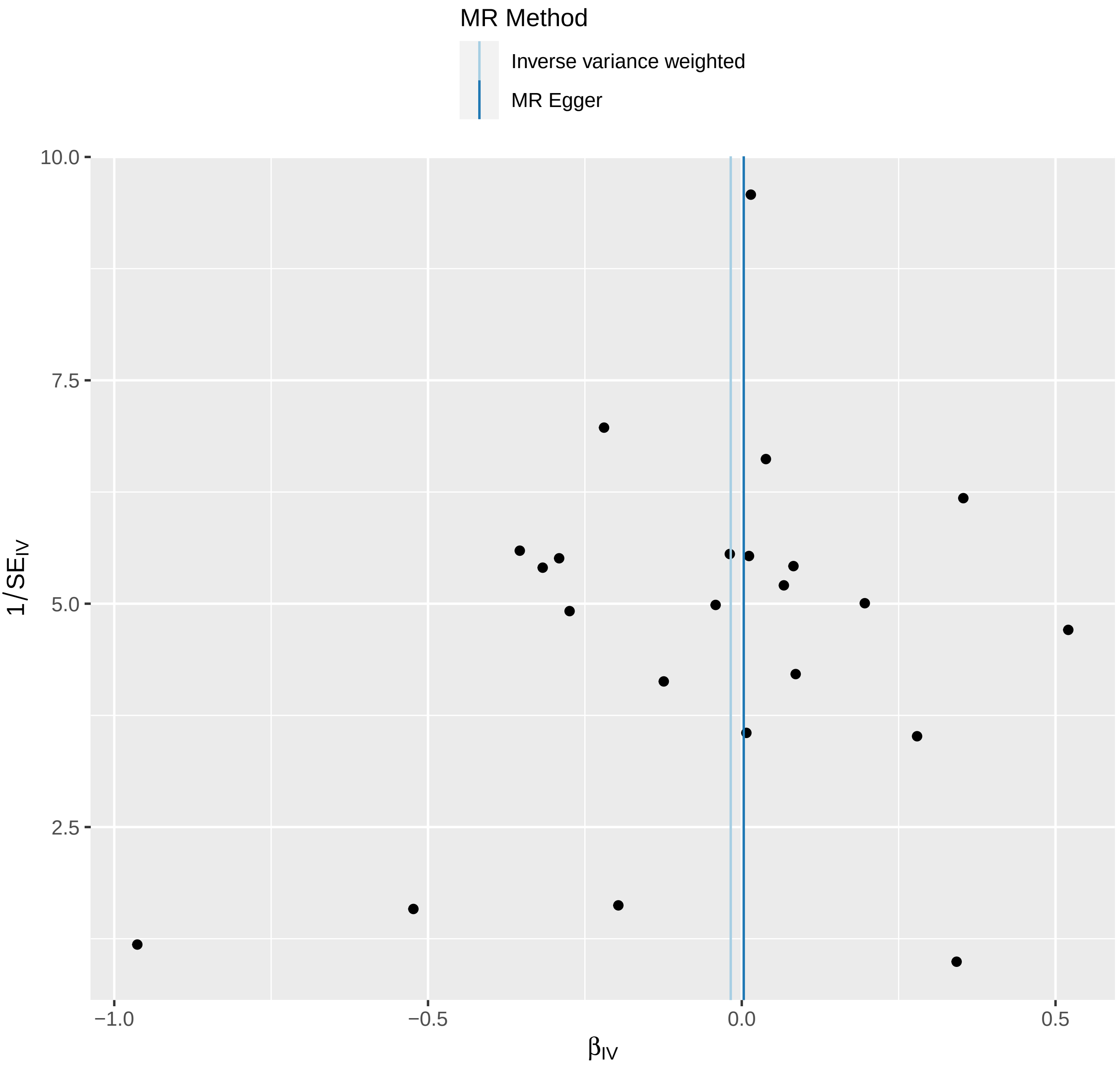

Batch 3−285 : Transmembrane protein C16orf54 on Diabetic nephropathy (more control exclusions) with a threshold of 1e−05

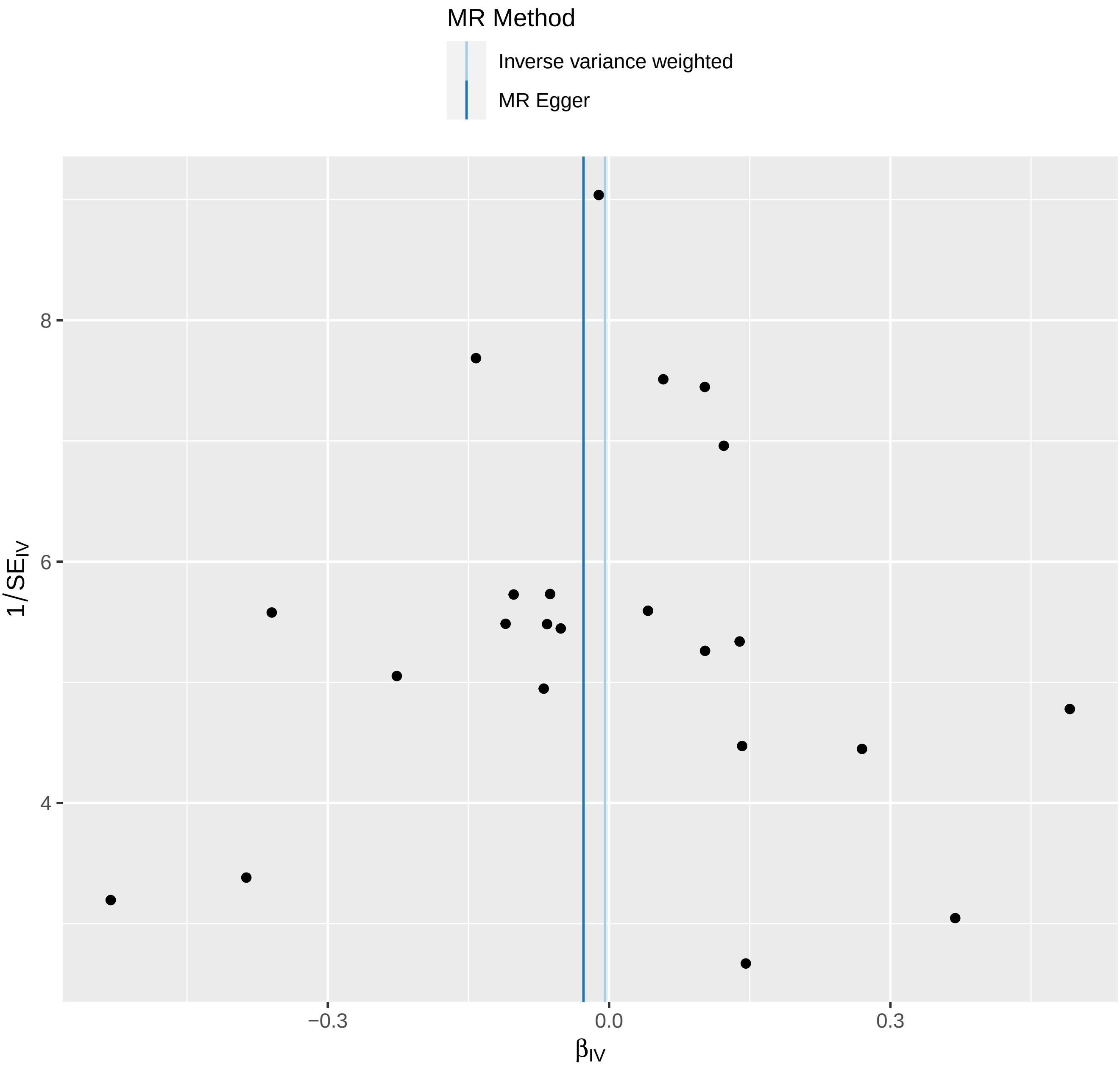

Batch 3−286 : Uncharacterized protein C17orf67 on Diabetic nephropathy (more control exclusions)
with a threshold of 1e−05

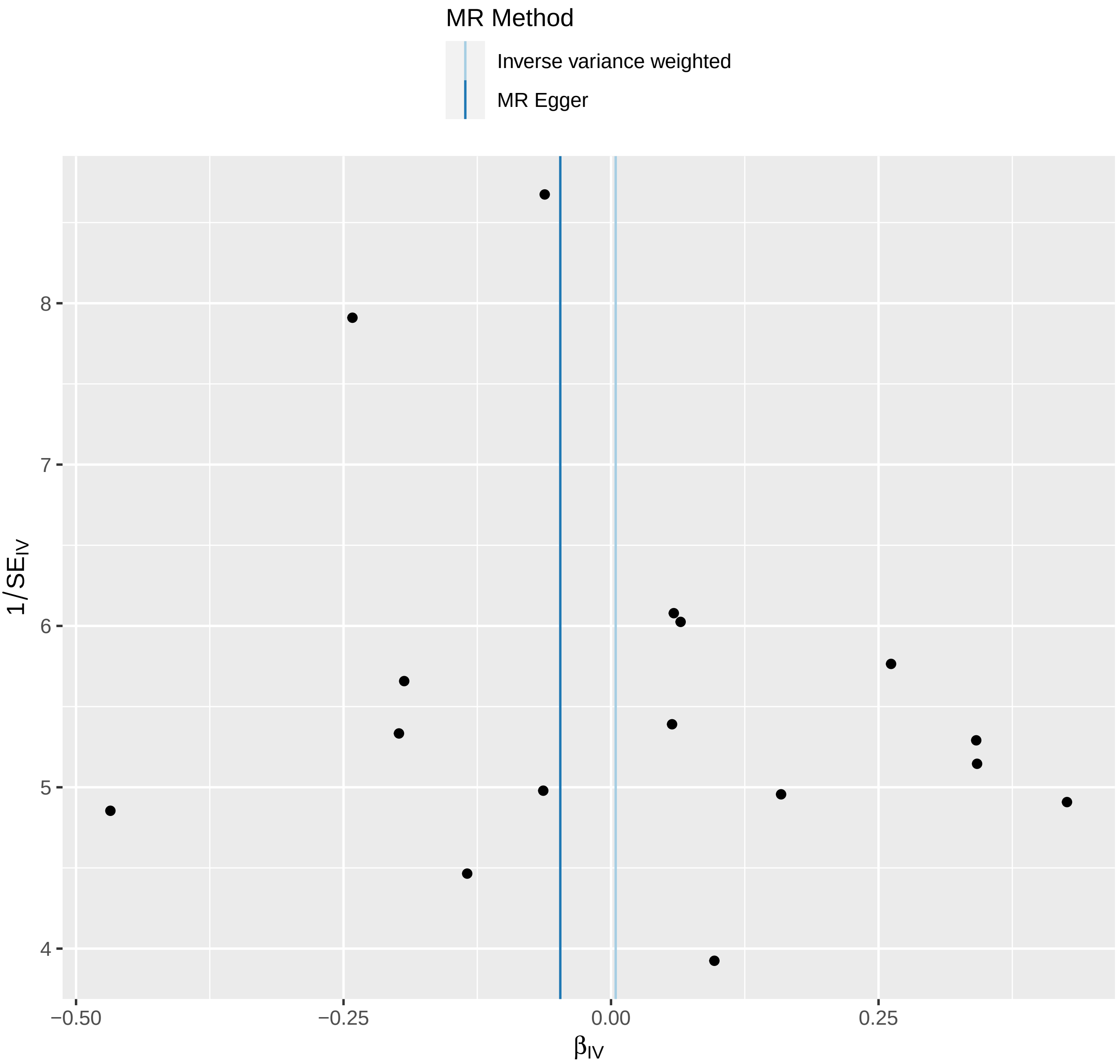

Batch 3−287 : Uncharacterized protein C17orf78 on Diabetic nephropathy (more control exclusions)
with a threshold of 1e−05

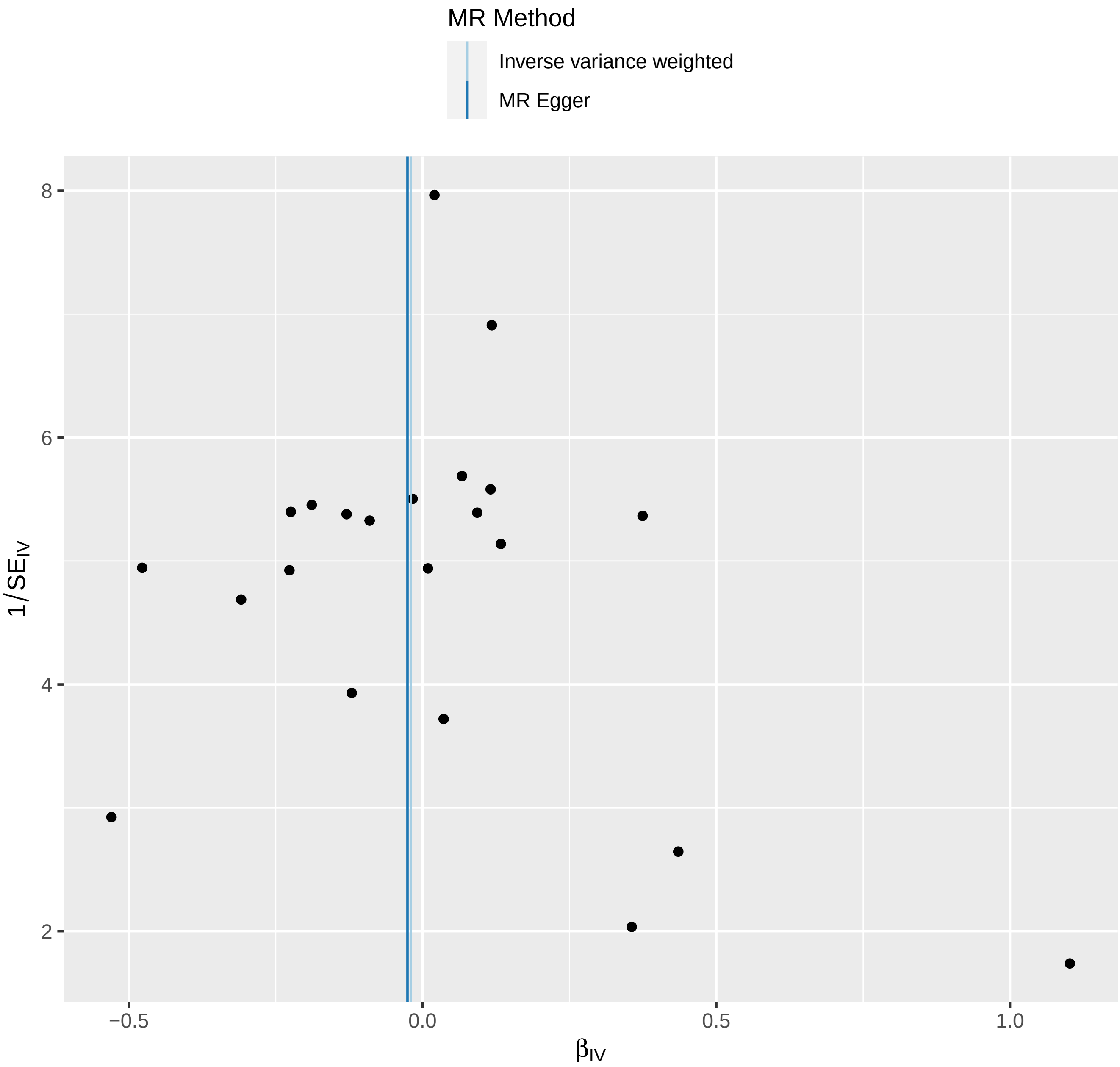

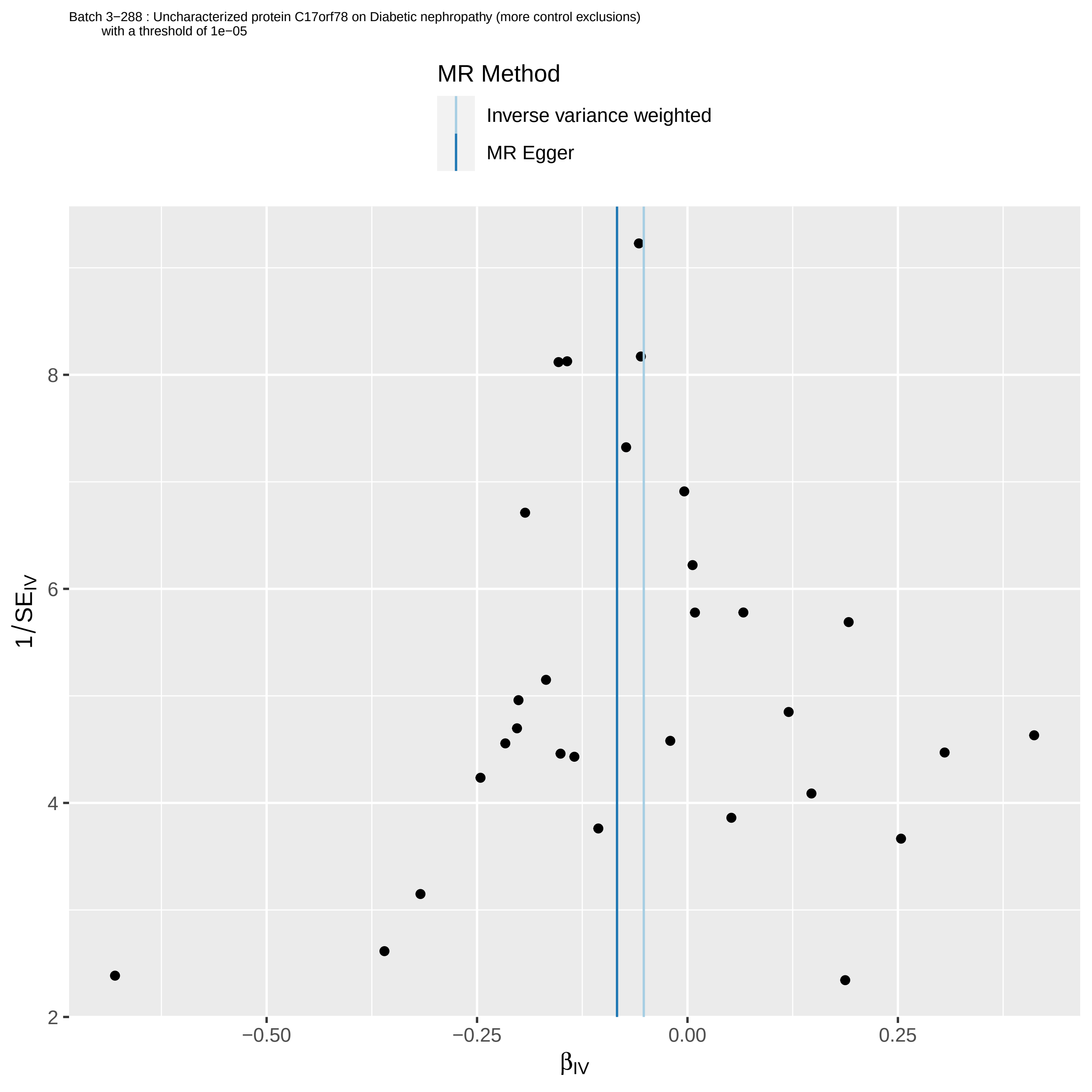

Batch 3−288 : Uncharacterized protein C17orf78 on Diabetic nephropathy (more control exclusions)
with a threshold of 1e−05
MR Method
Inverse variance weighted
MR Egger
8
6
4
2
1/SE_IV
−0.50
−0.25
0.00
0.25
β_IV

Batch 3−289 : Uncharacterized protein C17orf89 on Diabetic nephropathy (more control exclusions)
with a threshold of 1e−05

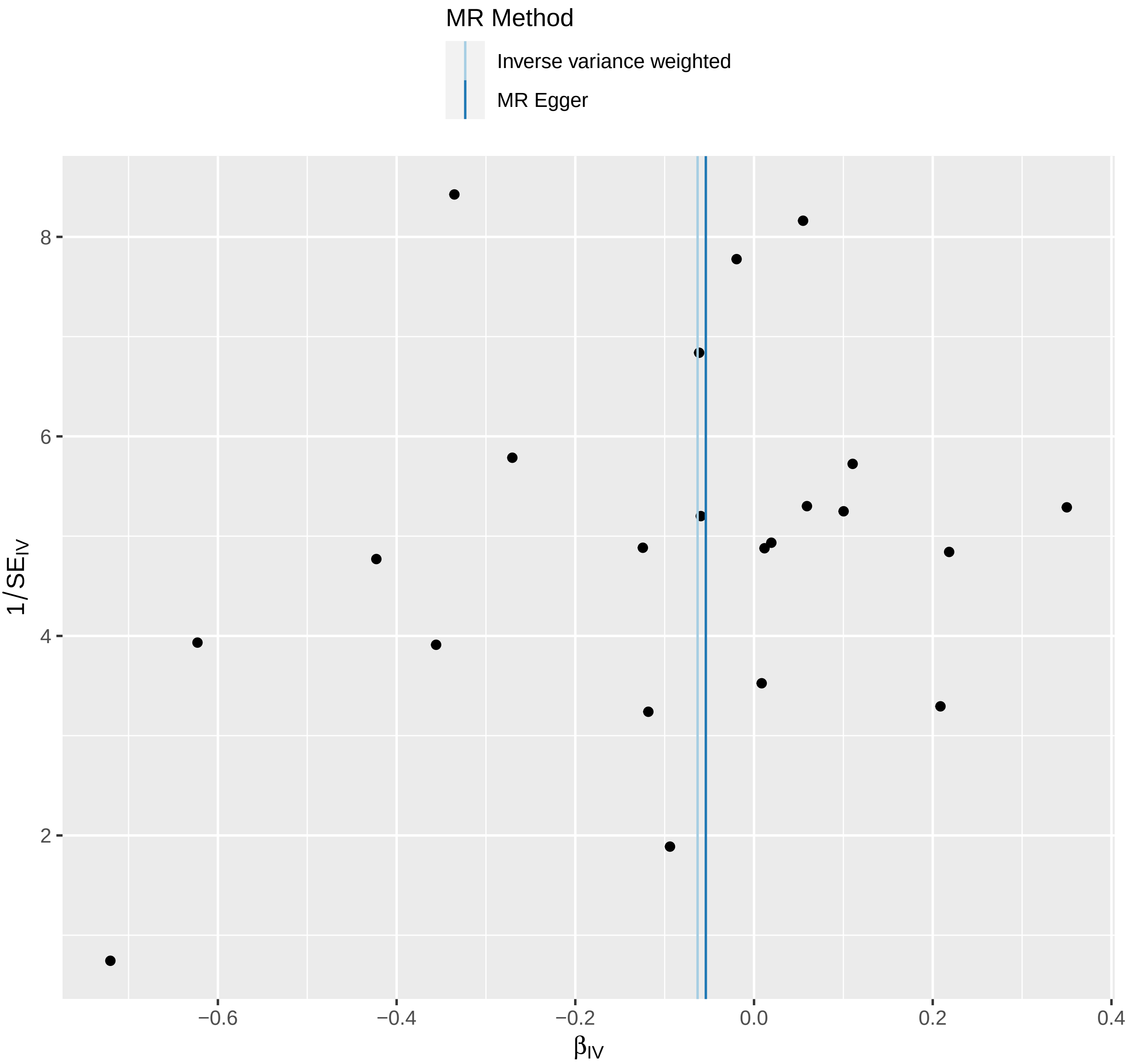

Batch 3−290 : UPF0729 protein C18orf32 on Diabetic nephropathy (more control exclusions)
with a threshold of 1e−05

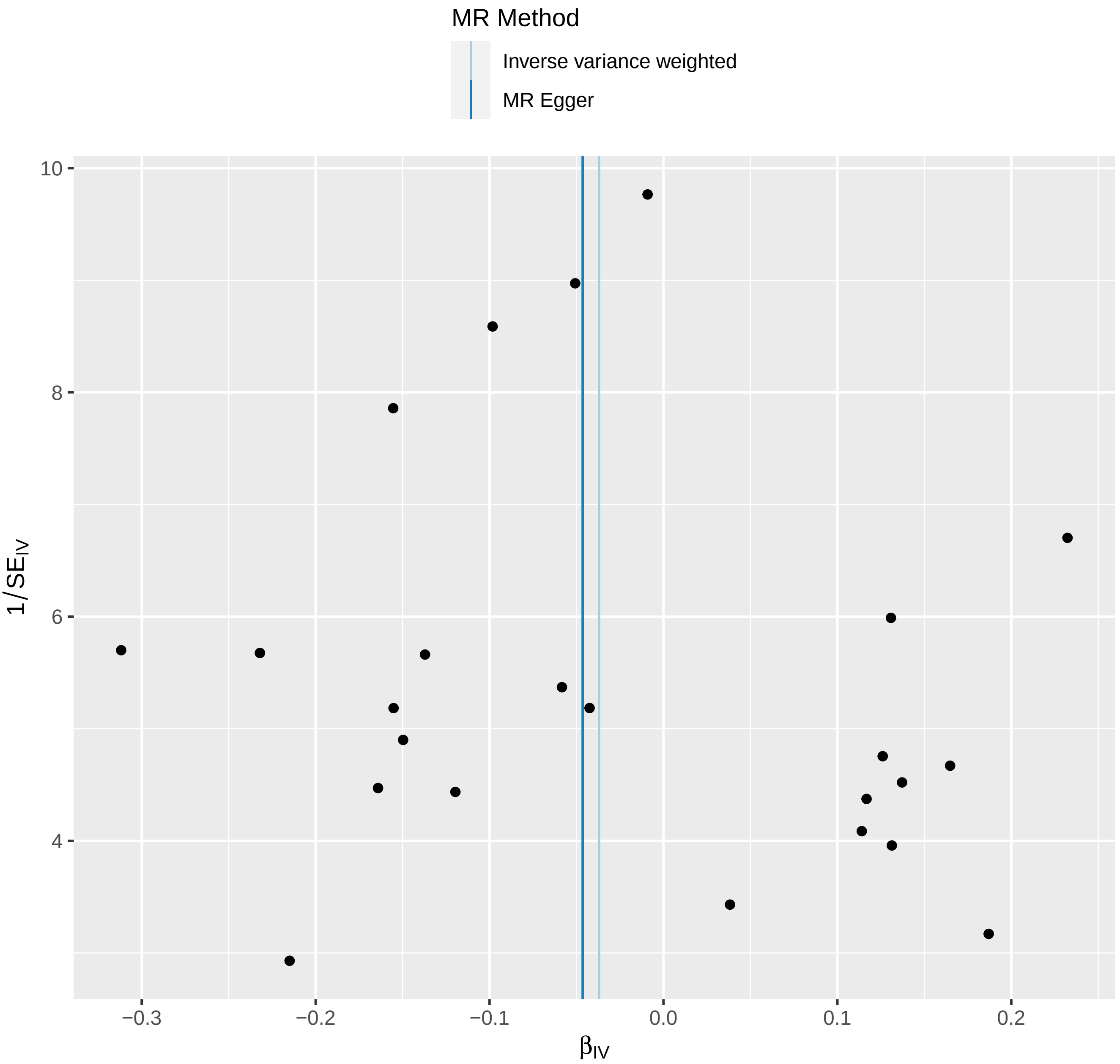

Batch 3−291 : Uncharacterized protein C19orf18 on Diabetic nephropathy (more control exclusions) with a threshold of 1e−05

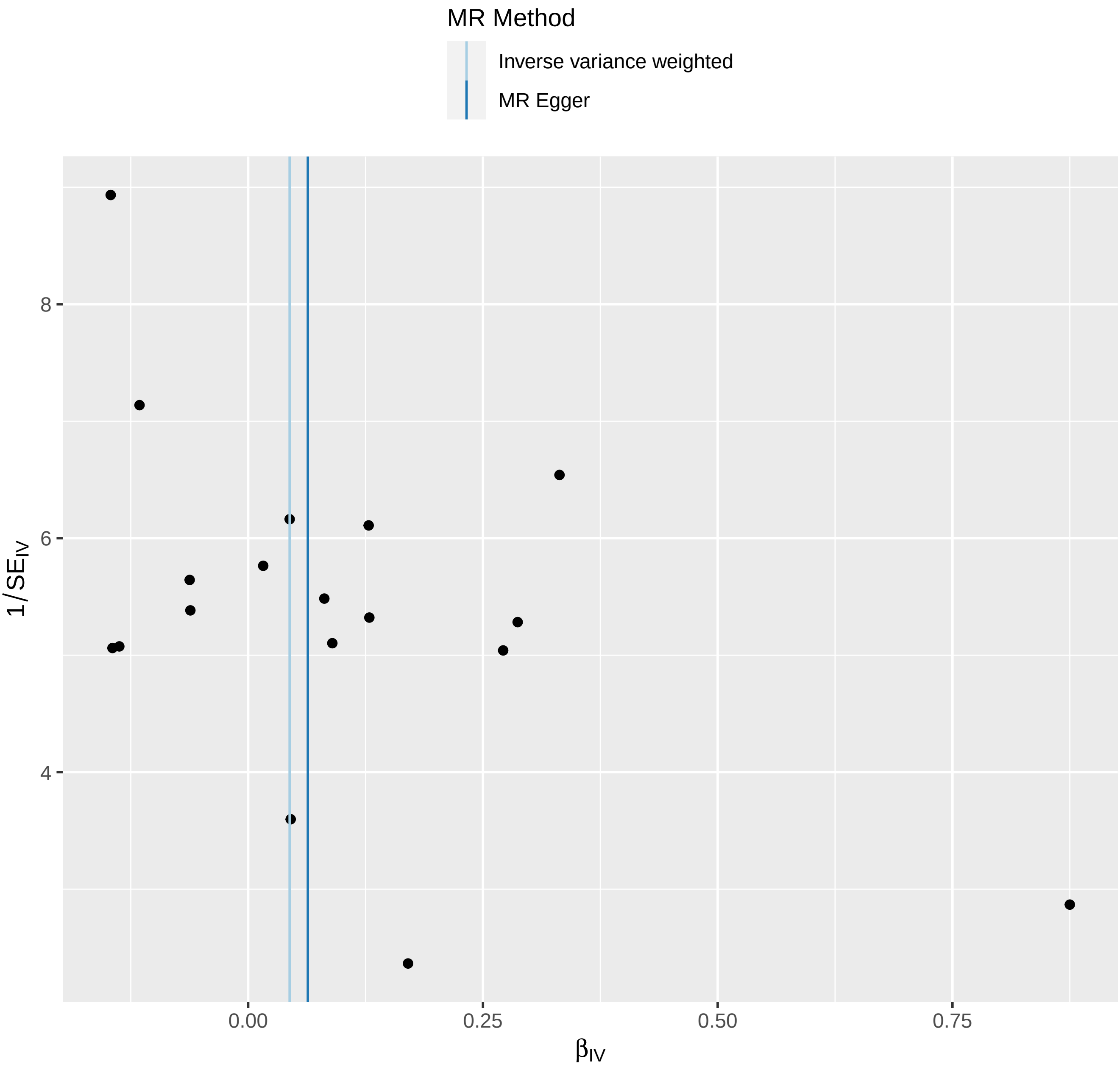

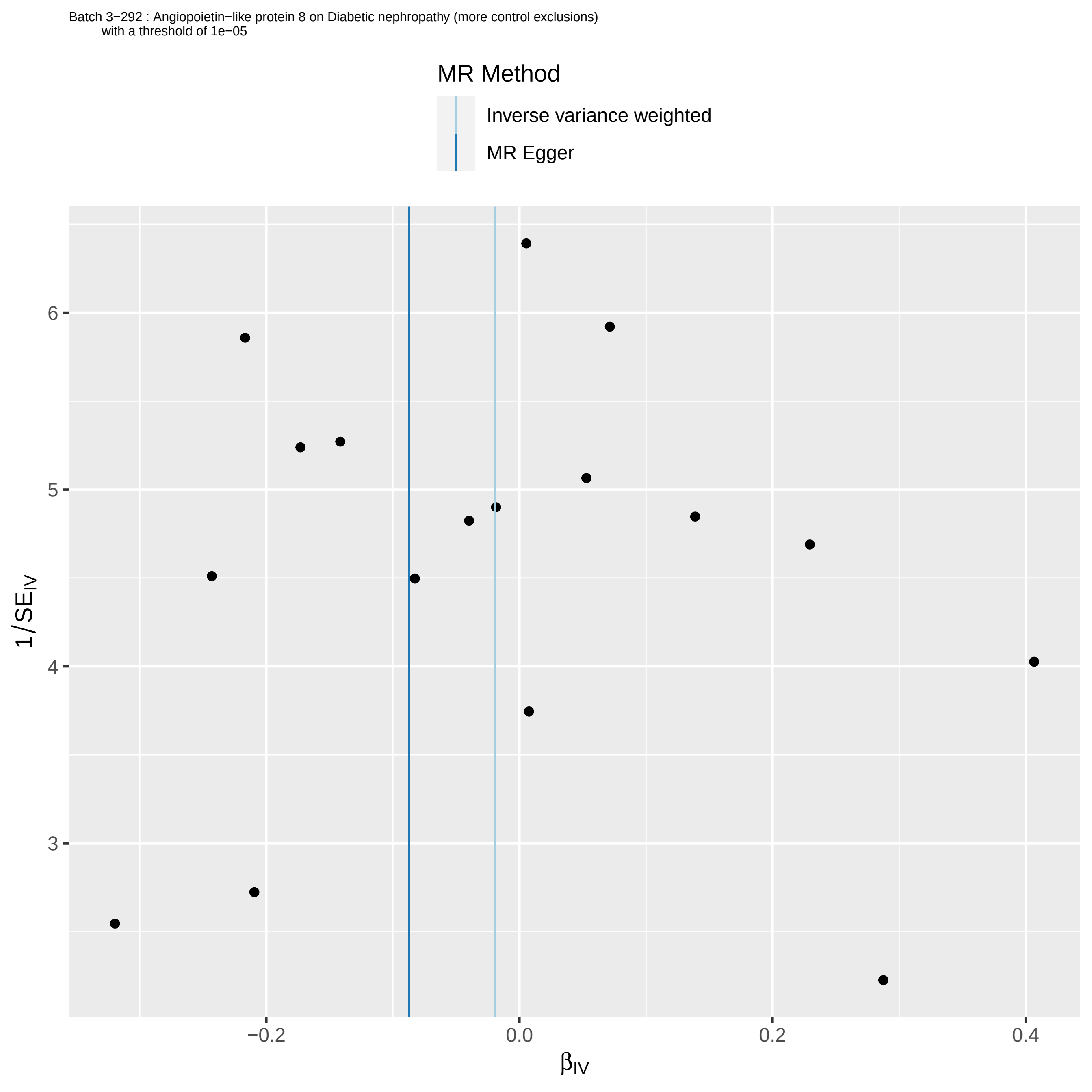
Batch 3−292 : Angiopoietin−like protein 8 on Diabetic nephropathy (more control exclusions)
with a threshold of 1e−05
MR Method
Inverse variance weighted
MR Egger
6
5
4
3
1/SE_IV
−0.2
0.0
0.2
0.4
β_IV

Batch 3−293 : Nuclear nucleic acid−binding protein C1D on Diabetic nephropathy (more control exclusions)
with a threshold of 1e−05

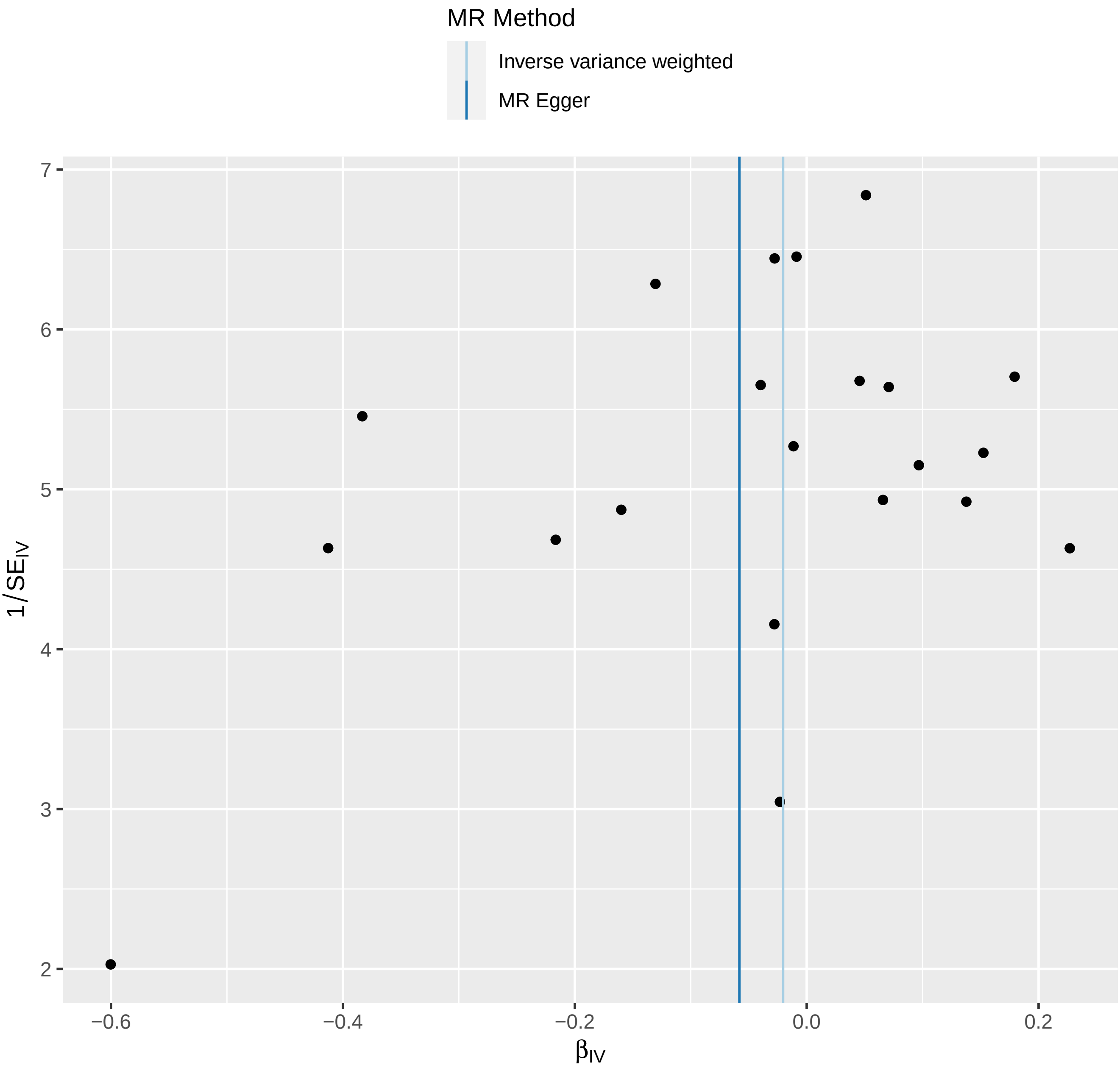

Batch 3−294 : C1GALT1−specific chaperone 1 on Diabetic nephropathy (more control exclusions)
with a threshold of 1e−05

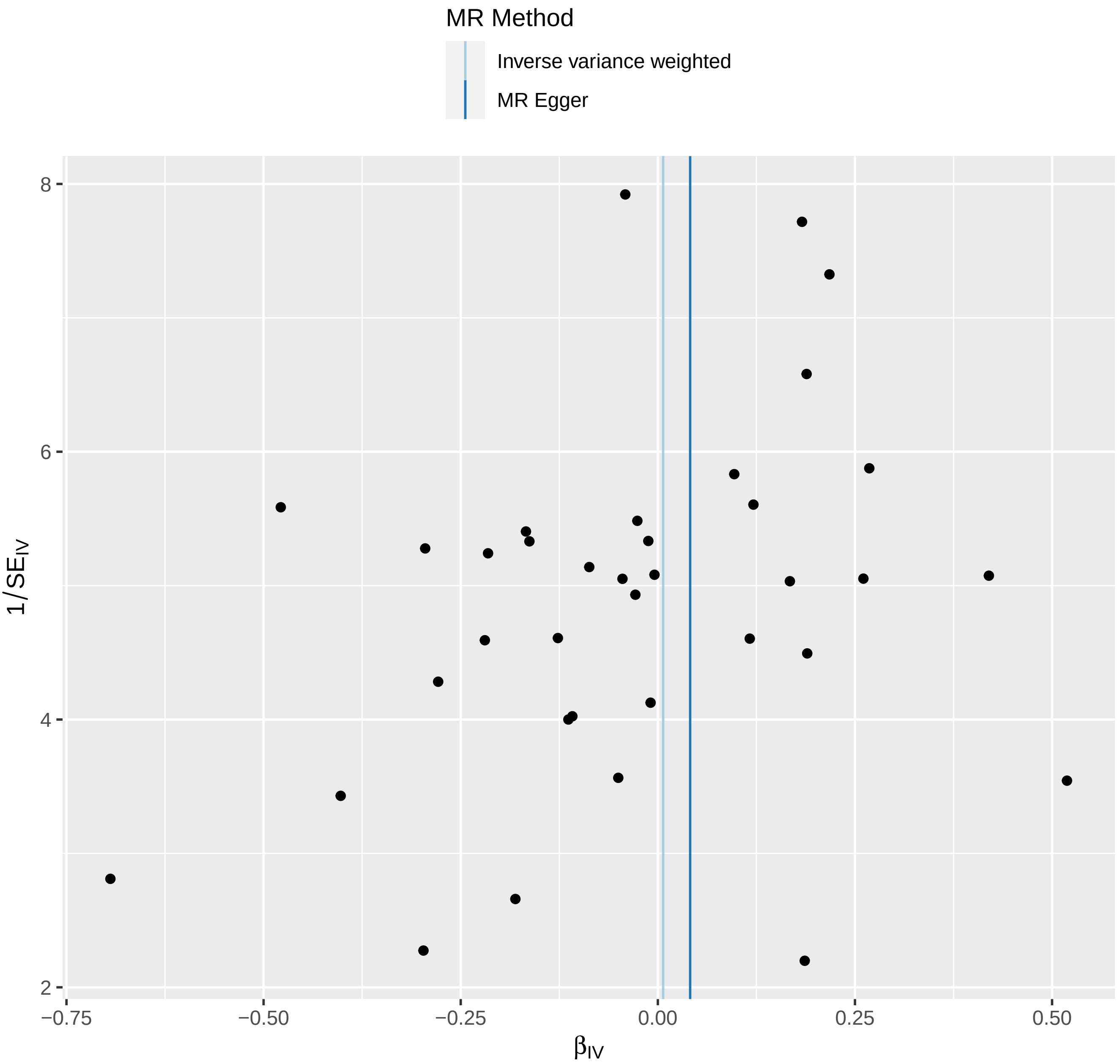

Batch 3−295 : Uncharacterized protein C1orf115 on Diabetic nephropathy (more control exclusions)
with a threshold of 1e−05

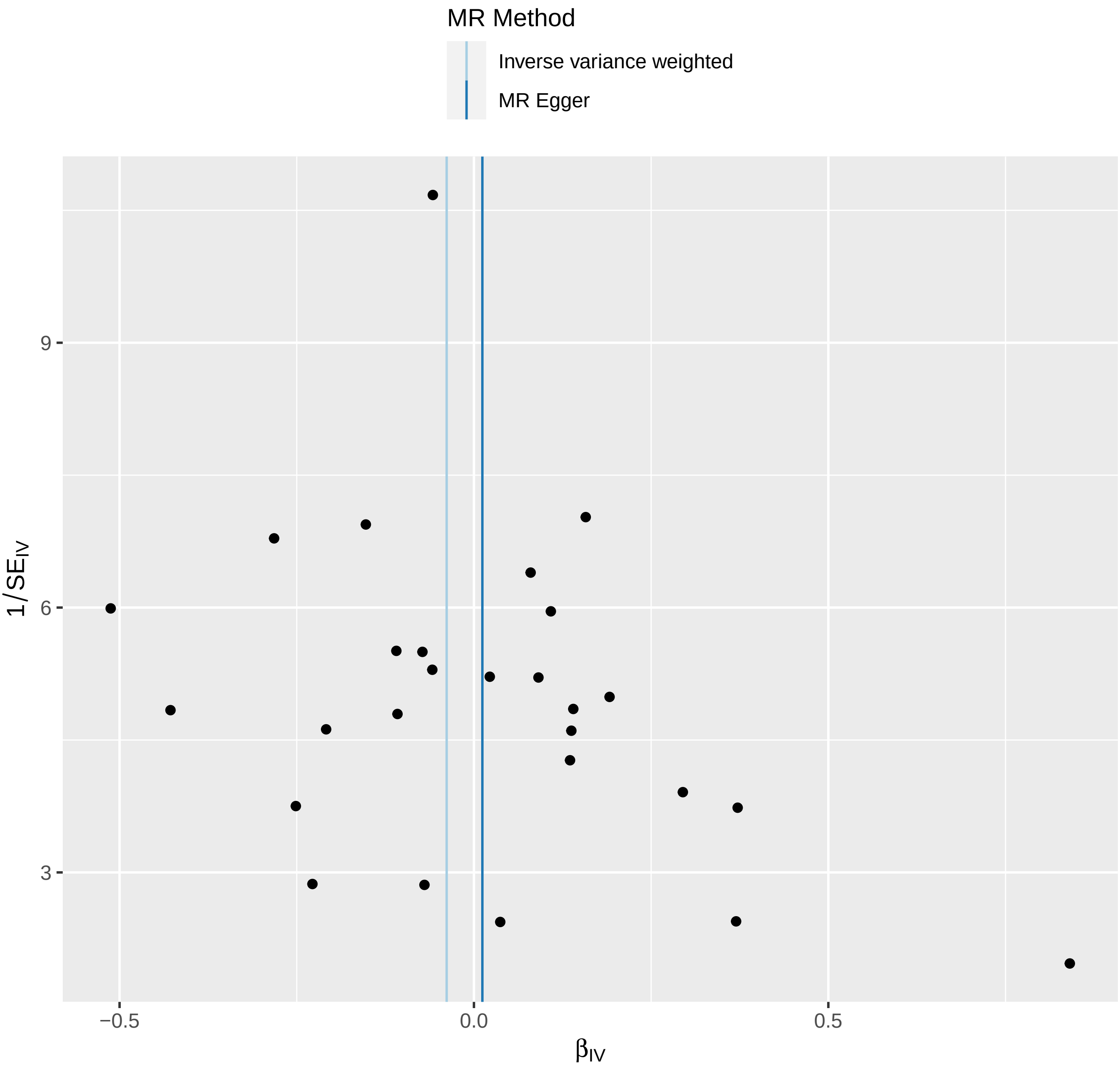

Batch 3−296 : Uncharacterized protein C1orf185 on Diabetic nephropathy (more control exclusions)
with a threshold of 1e−05

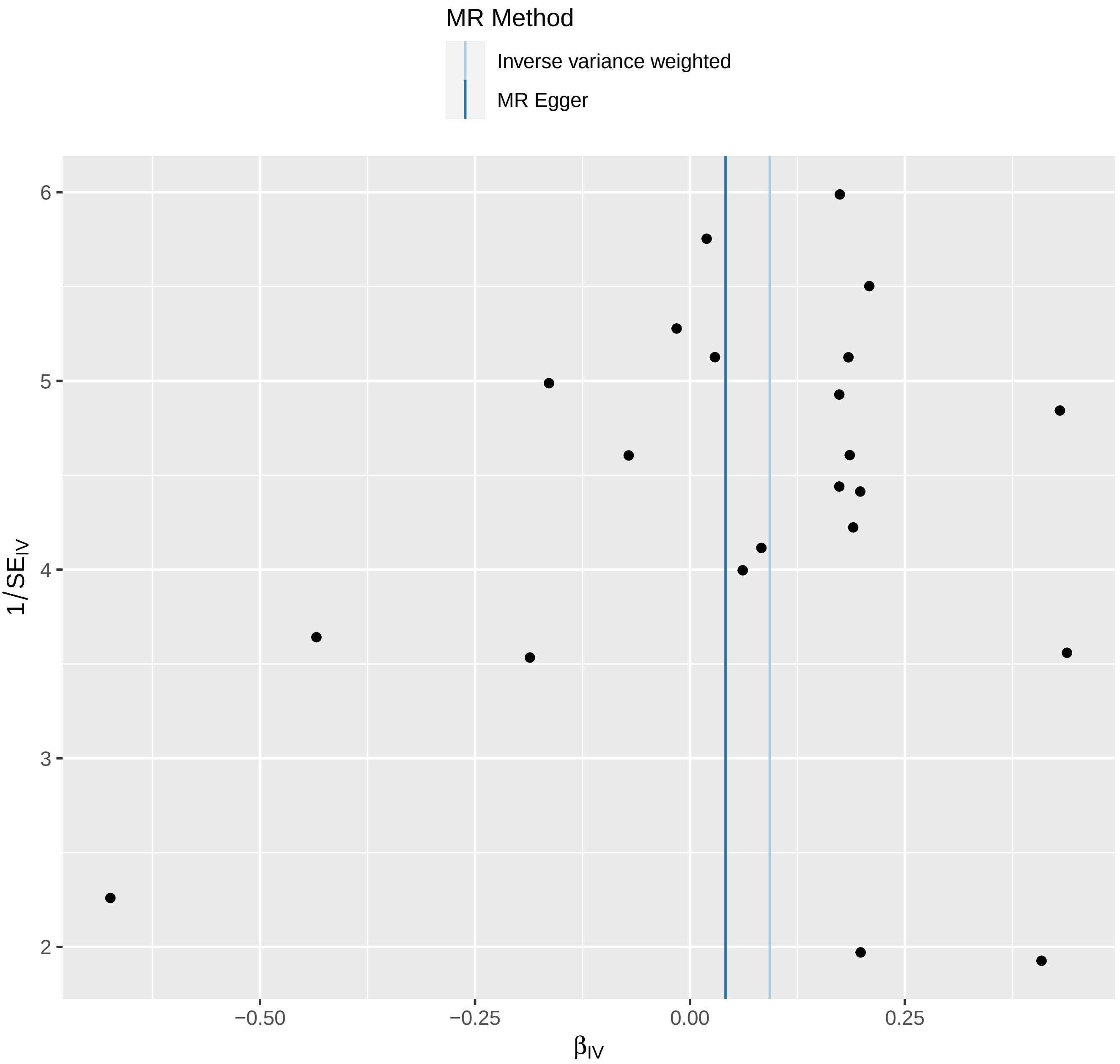

Batch 3−297 : Uncharacterized protein C1orf198 on Diabetic nephropathy (more control exclusions)
with a threshold of 1e−05

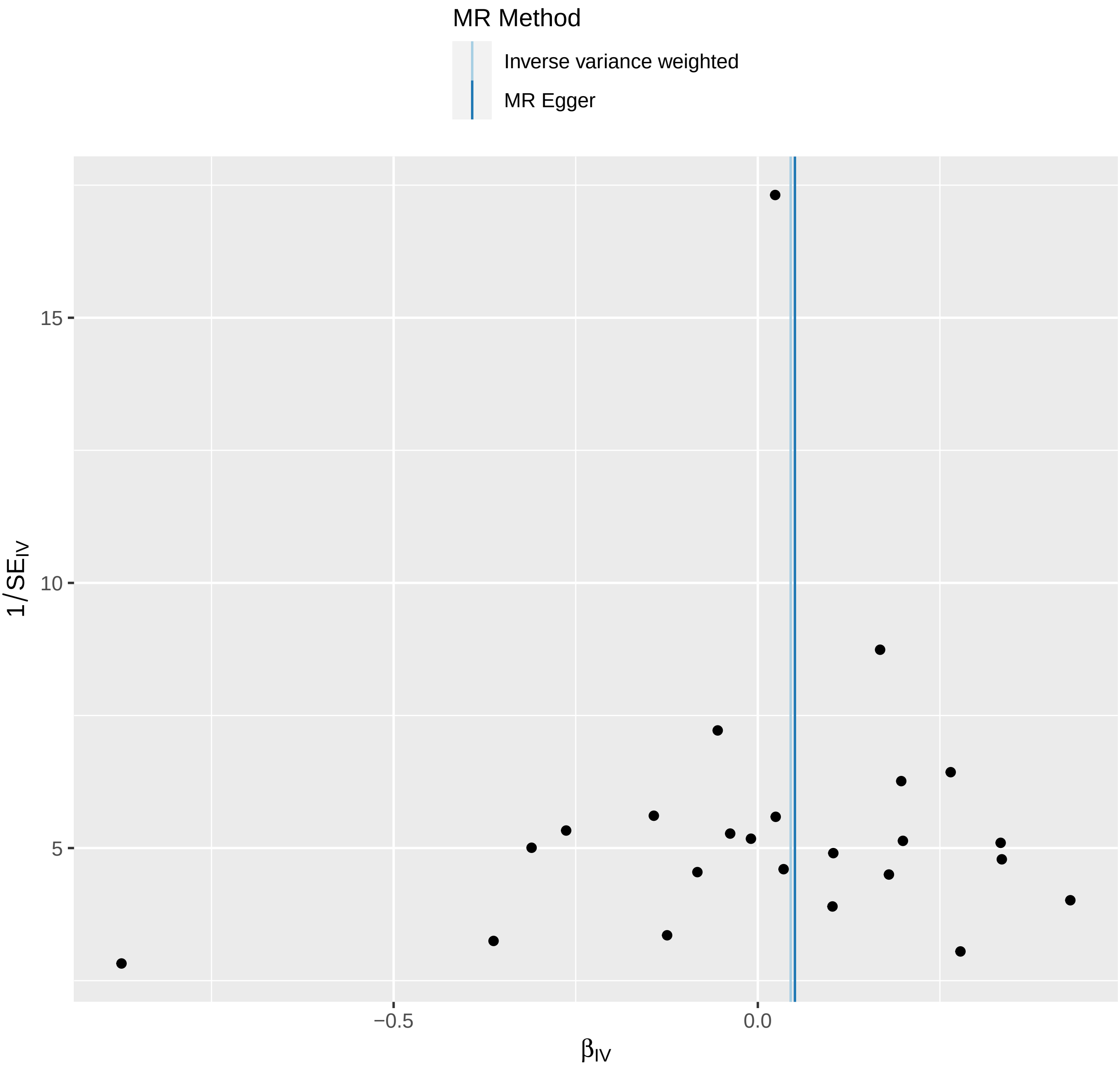

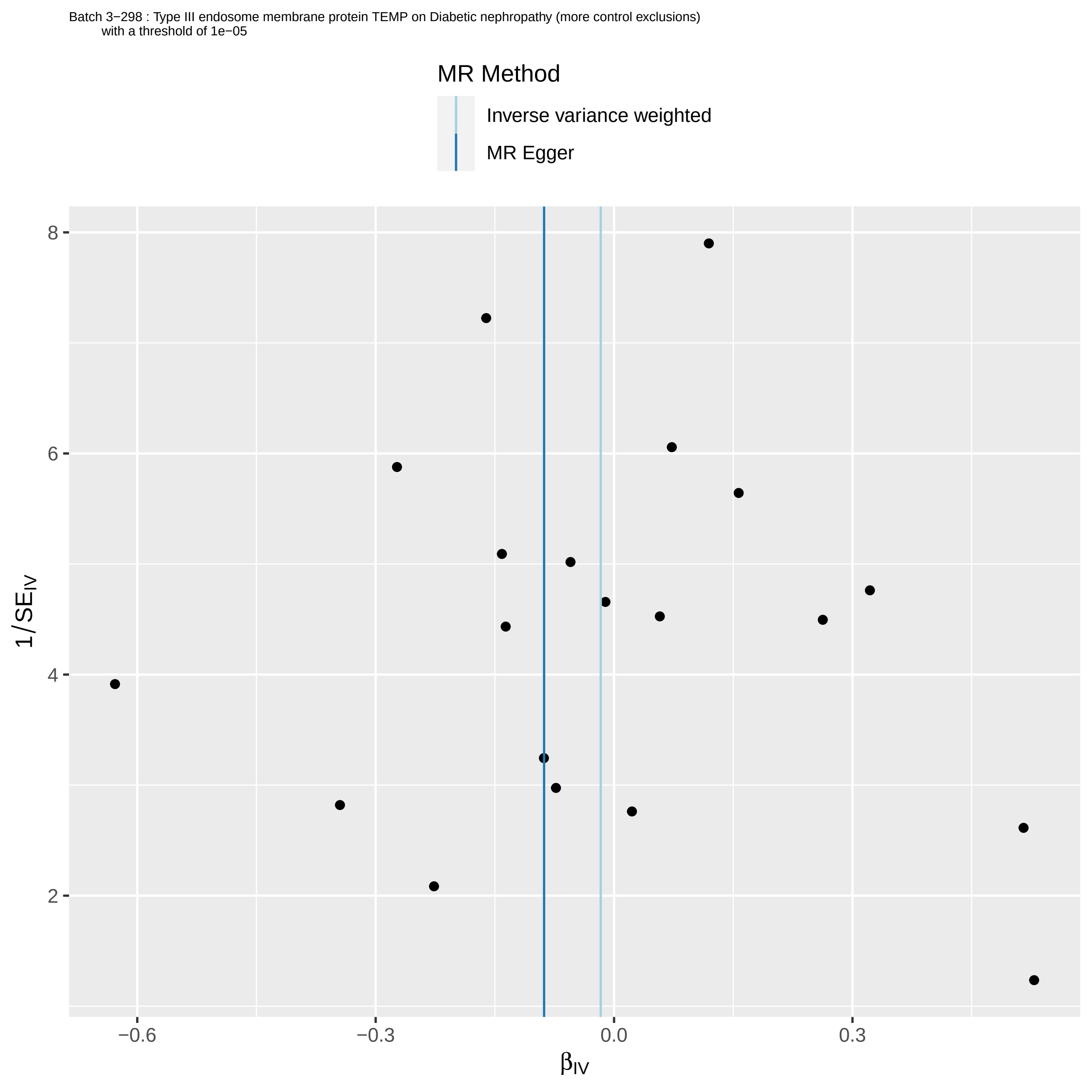
Batch 3−298 : Type III endosome membrane protein TEMP on Diabetic nephropathy (more control exclusions)
with a threshold of 1e−05
MR Method
Inverse variance weighted
MR Egger
8
6
4
2
$1/SE_{IV}$
−0.6
−0.3
0.0
0.3
β_{IV}

Batch 3−299 : Uncharacterized protein C1orf226 on Diabetic nephropathy (more control exclusions)
with a threshold of 1e−05

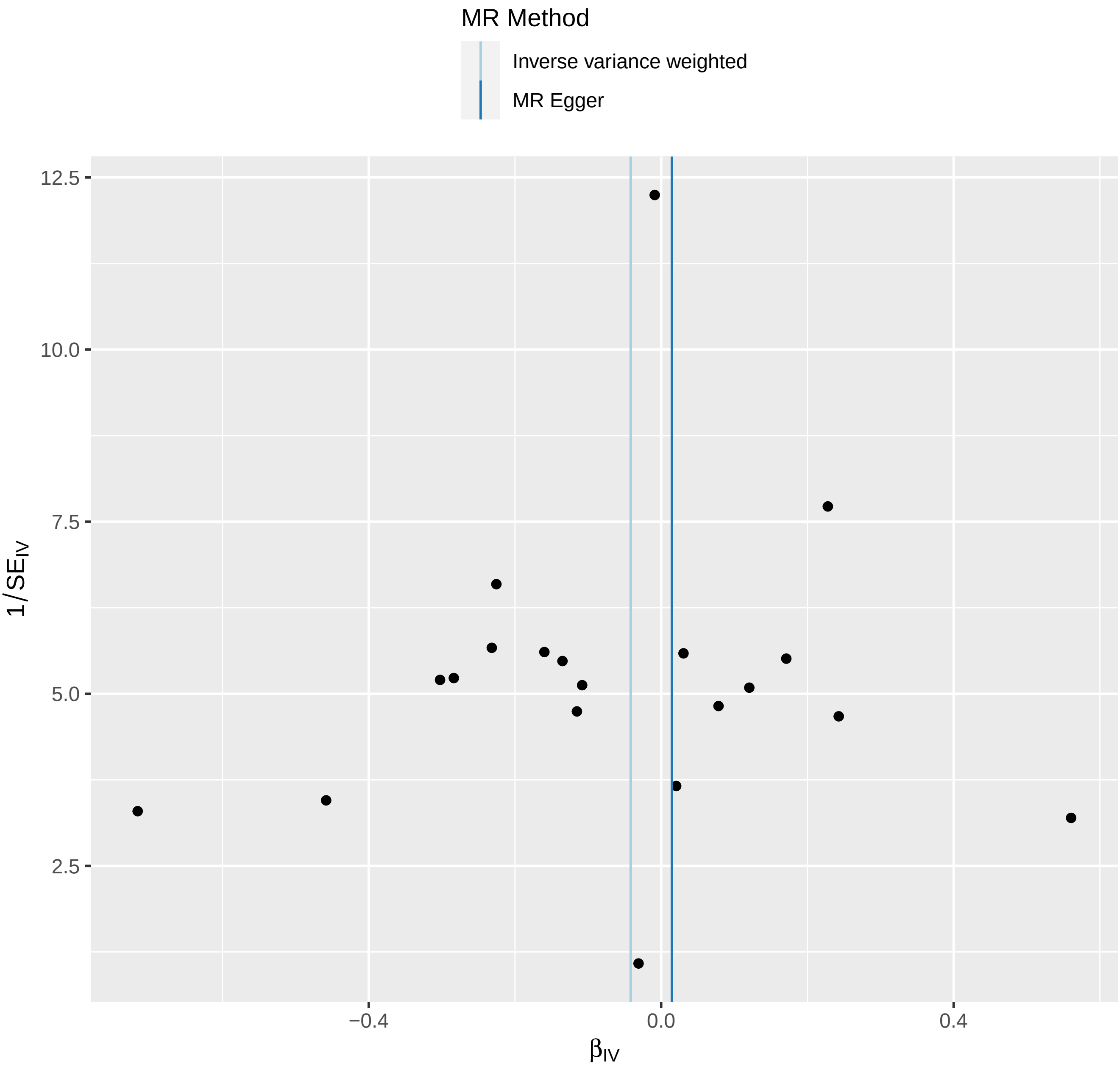

Batch 3−300 : Complement component 1 Q subcomponent−binding protein, mitochondrial on Diabetic nephropathy (more control exclusions)
with a threshold of 1e−05

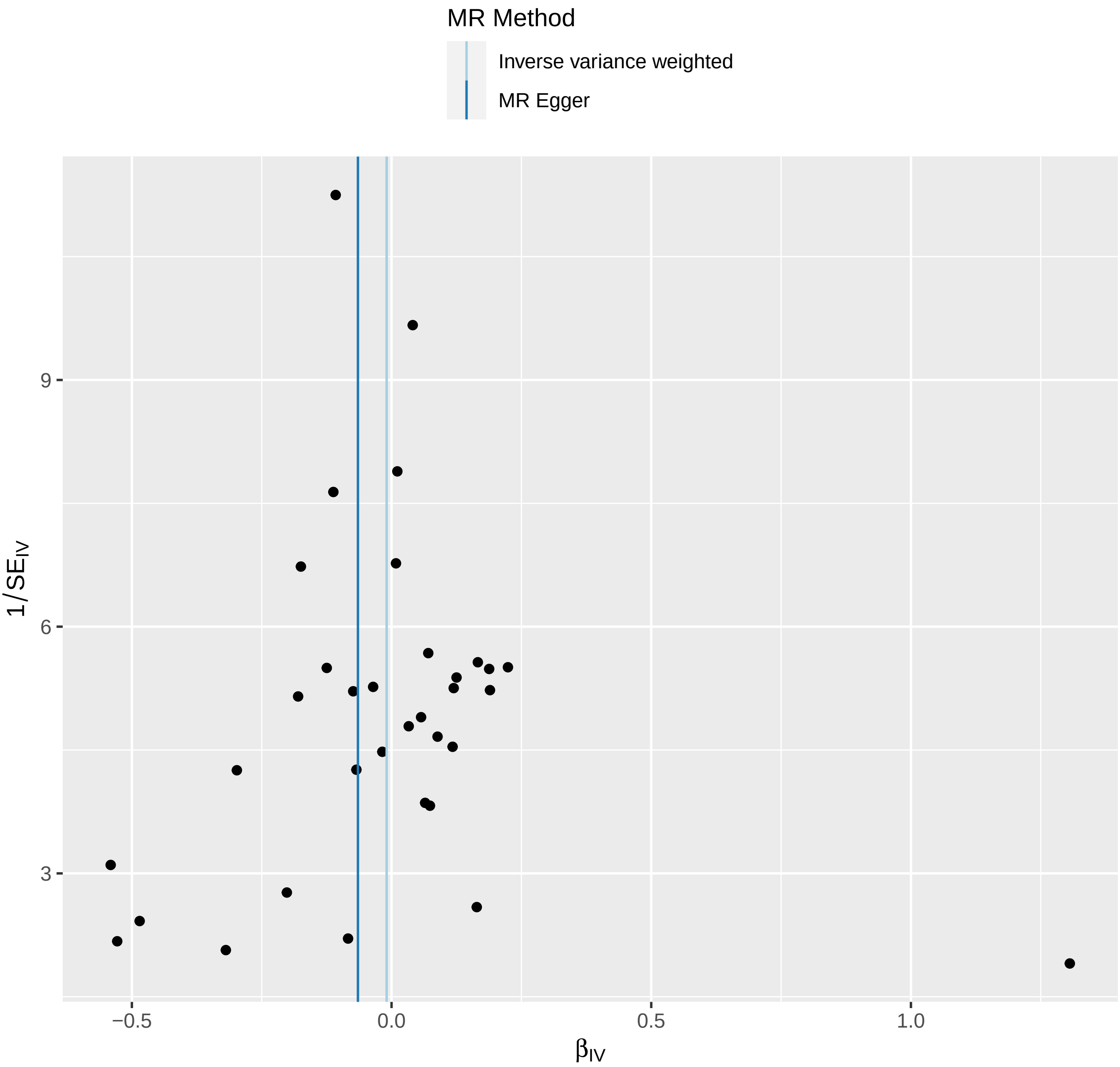

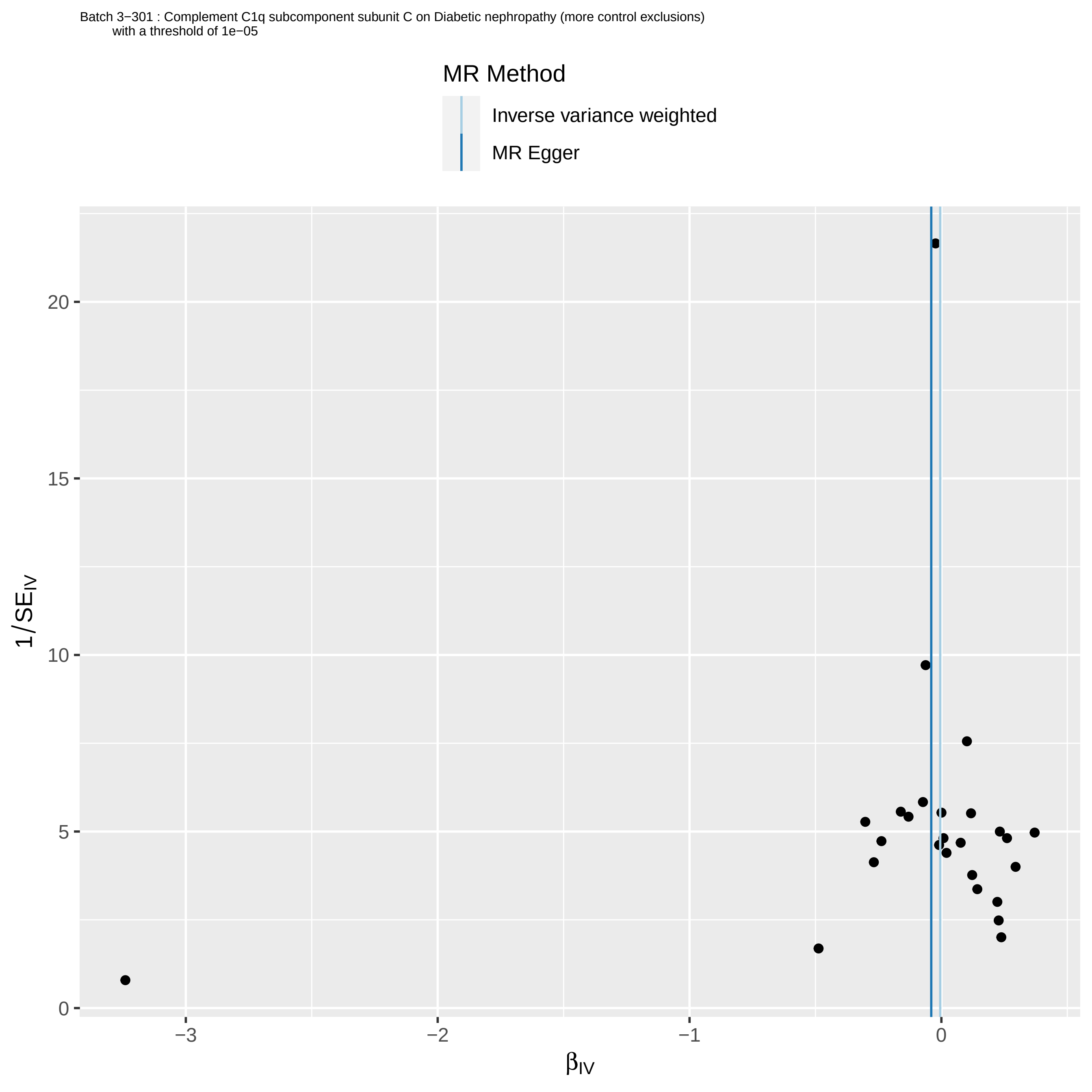

Batch 3−301 : Complement C1q subcomponent subunit C on Diabetic nephropathy (more control exclusions)
with a threshold of 1e−05
MR Method
Inverse variance weighted
MR Egger
1/SE_IV
β_IV
20
15
10
5
0
−3
−2
−1
0

Batch 3−302 : Complement C1q−like protein 4 on Diabetic nephropathy (more control exclusions)
with a threshold of 1e−05

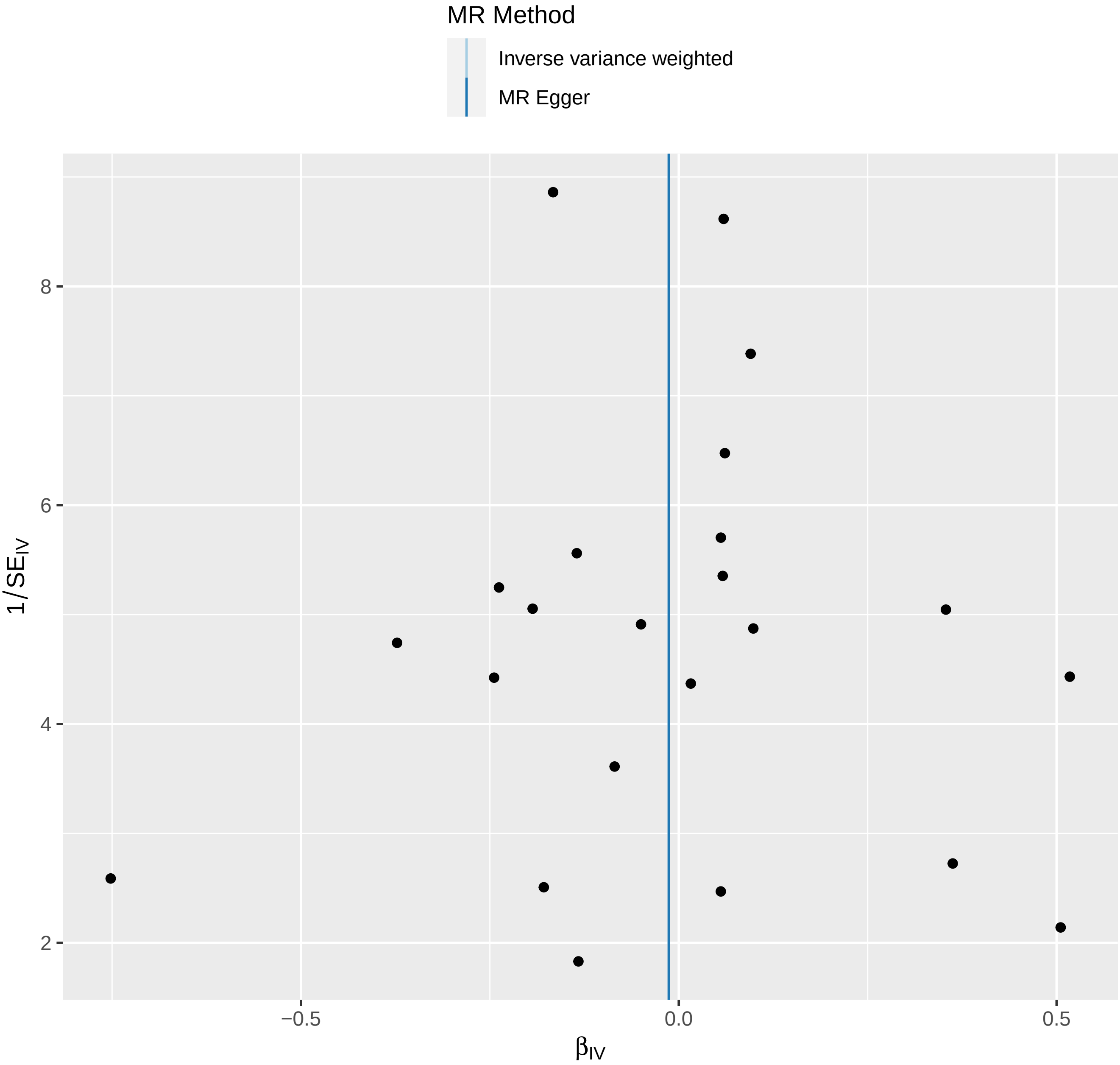

Batch 3−303 : Complement C1q tumor necrosis factor−related protein 1 on Diabetic nephropathy (more control exclusions) with a threshold of 1e−05

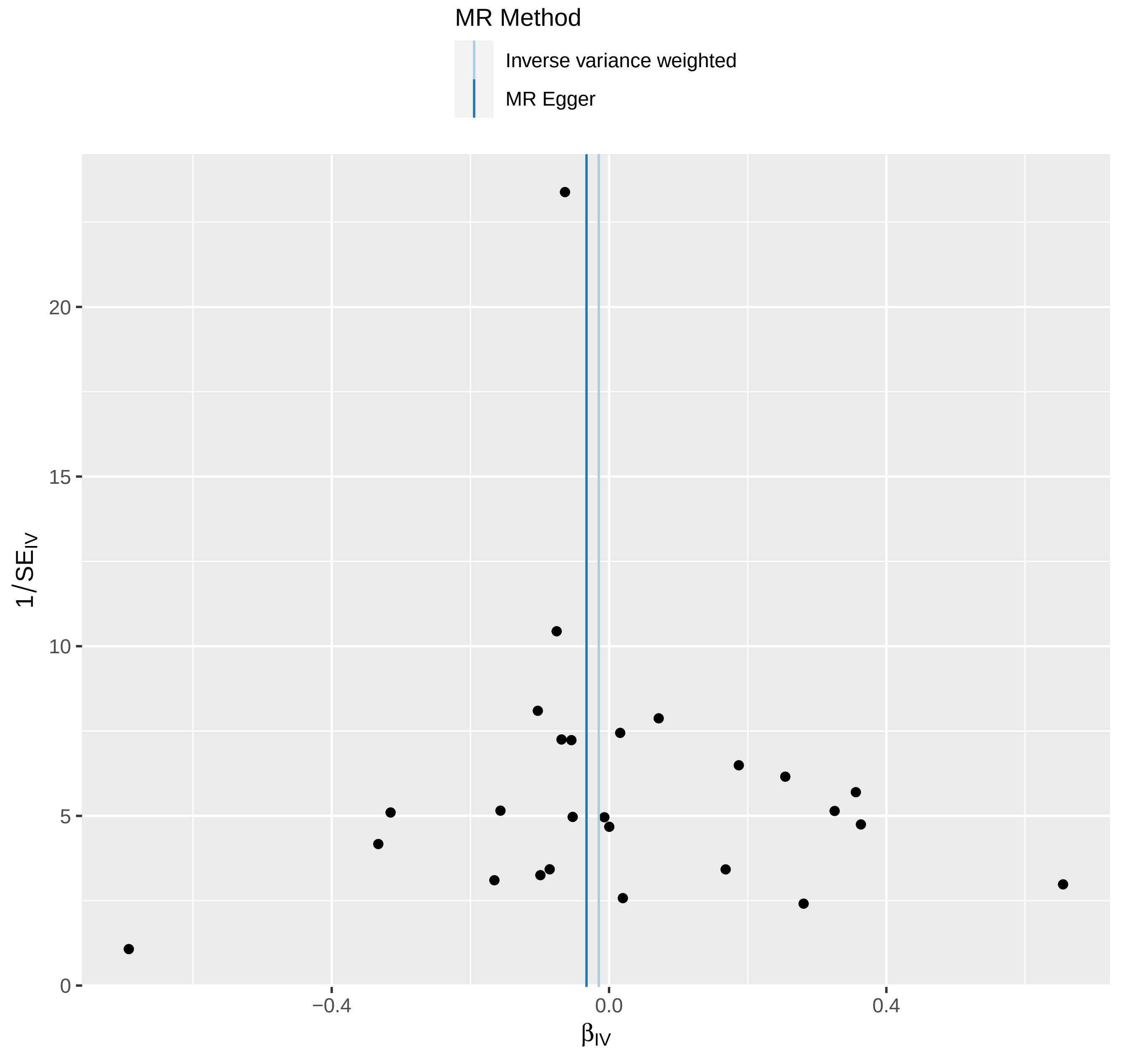

Batch 3−304 : Complement C1q tumor necrosis factor−related protein 3 on Diabetic nephropathy (more control exclusions)
with a threshold of 1e−05

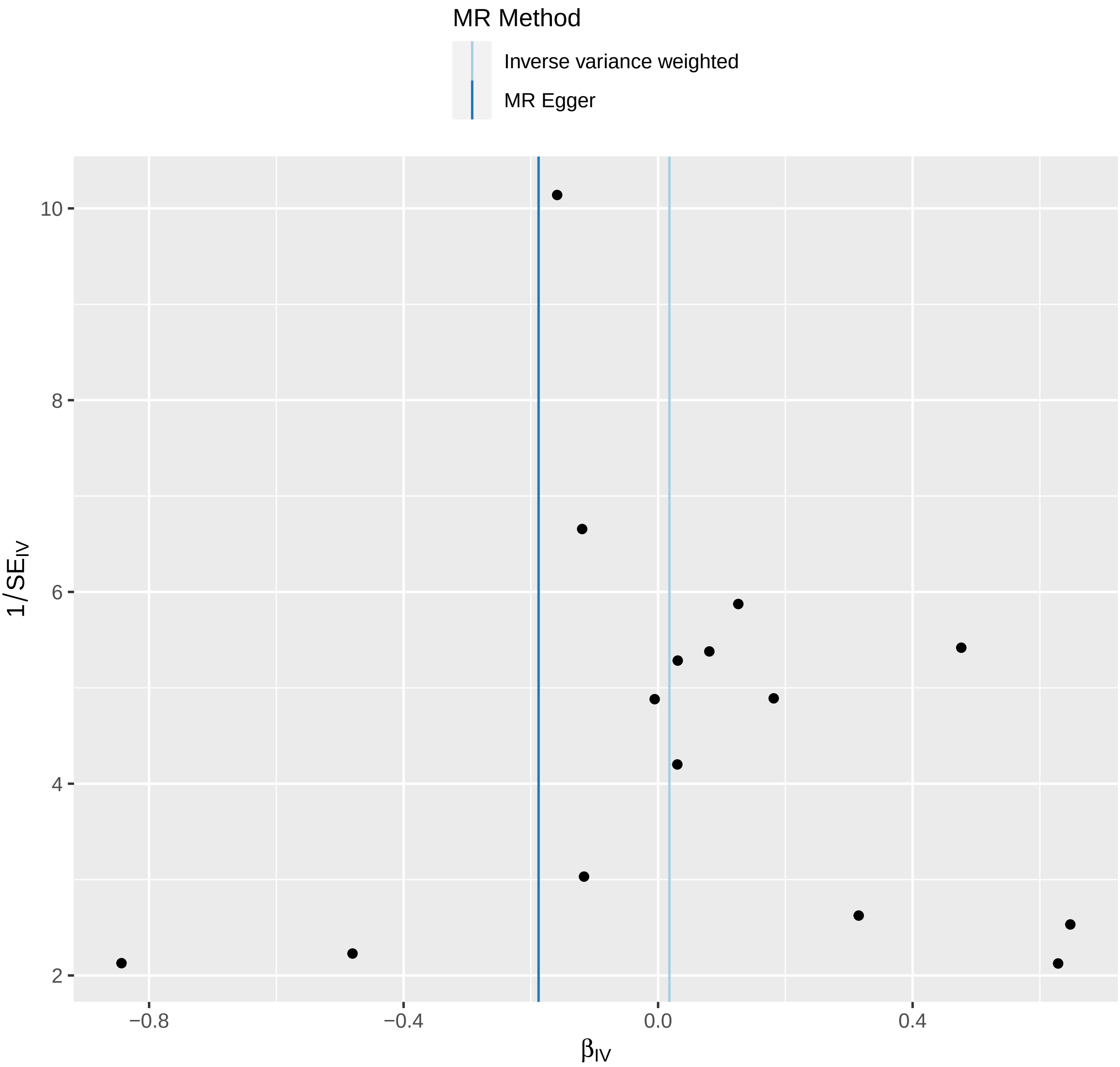

Batch 3−305 : Complement C1q tumor necrosis factor−related protein 5 on Diabetic nephropathy (more control exclusions)
with a threshold of 1e−05

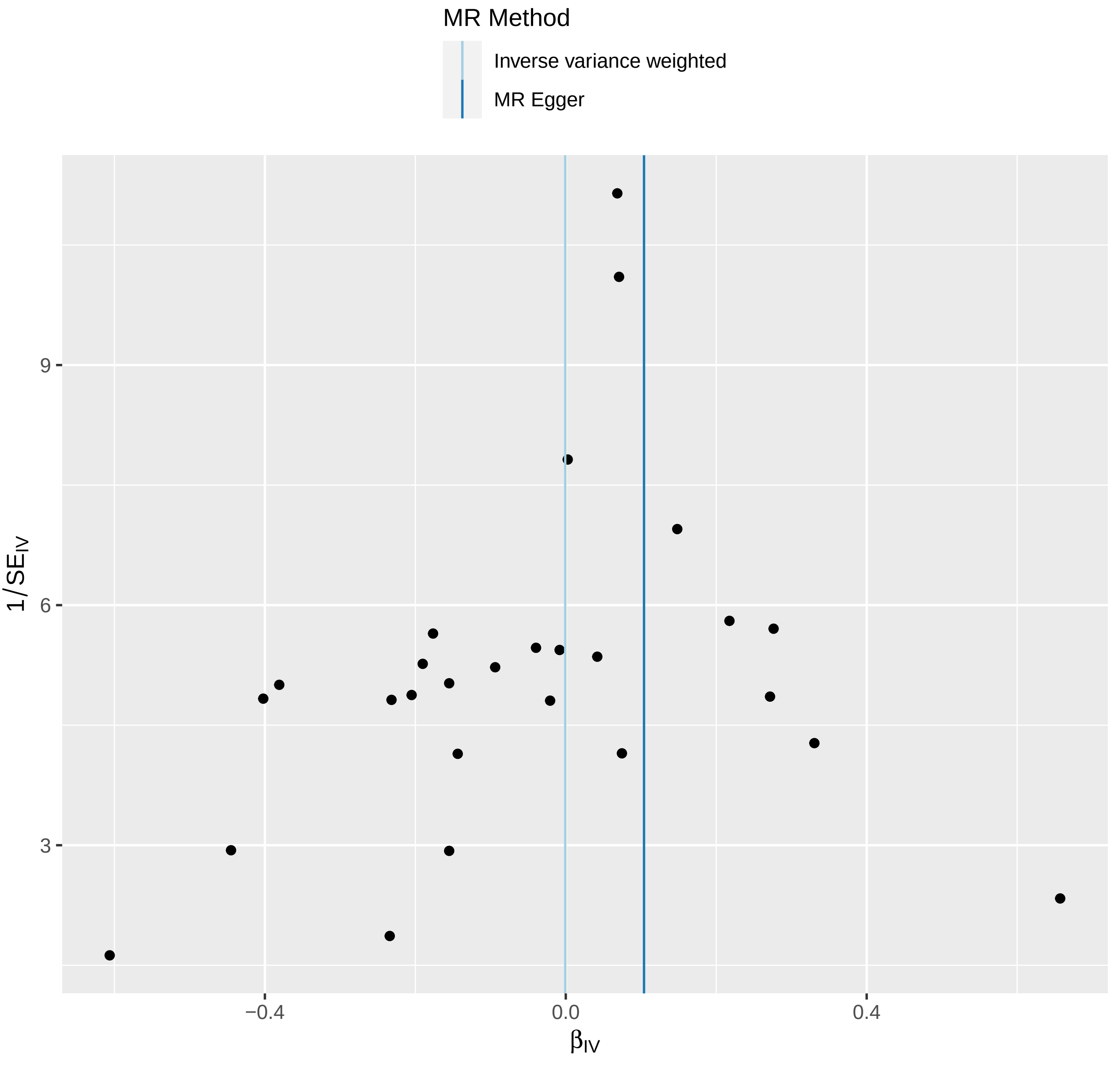

Batch 3−306 : Complement C1q and tumor necrosis factor−related protein 9A on Diabetic nephropathy (more control exclusions) with a threshold of 1e−05

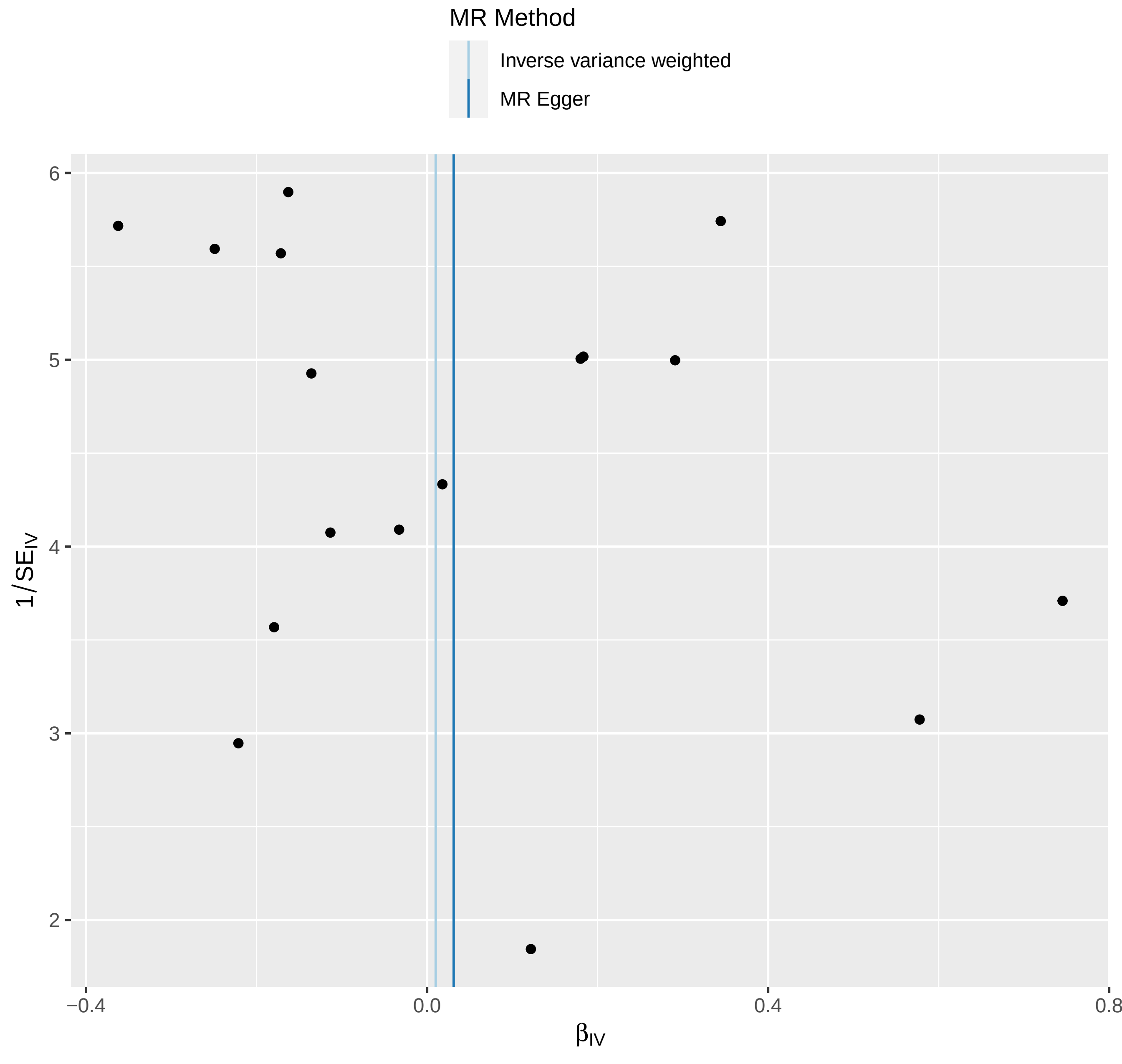

Batch 3−307 : Complement C1r subcomponent−like protein on Diabetic nephropathy (more control exclusions)
with a threshold of 1e−05

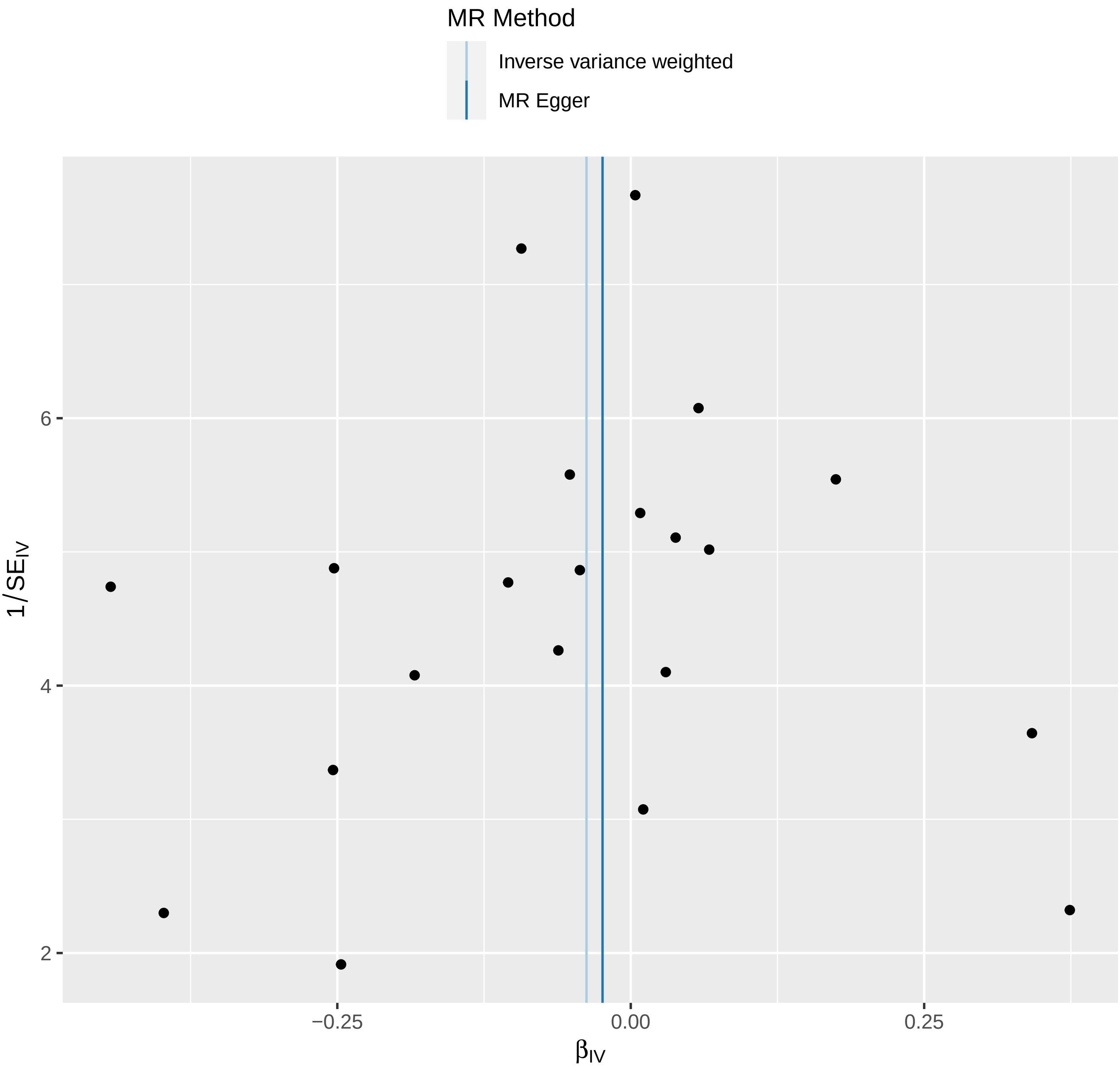

Batch 3−308 : ES1 protein homolog, mitochondrial on Diabetic nephropathy (more control exclusions)
with a threshold of 1e−05

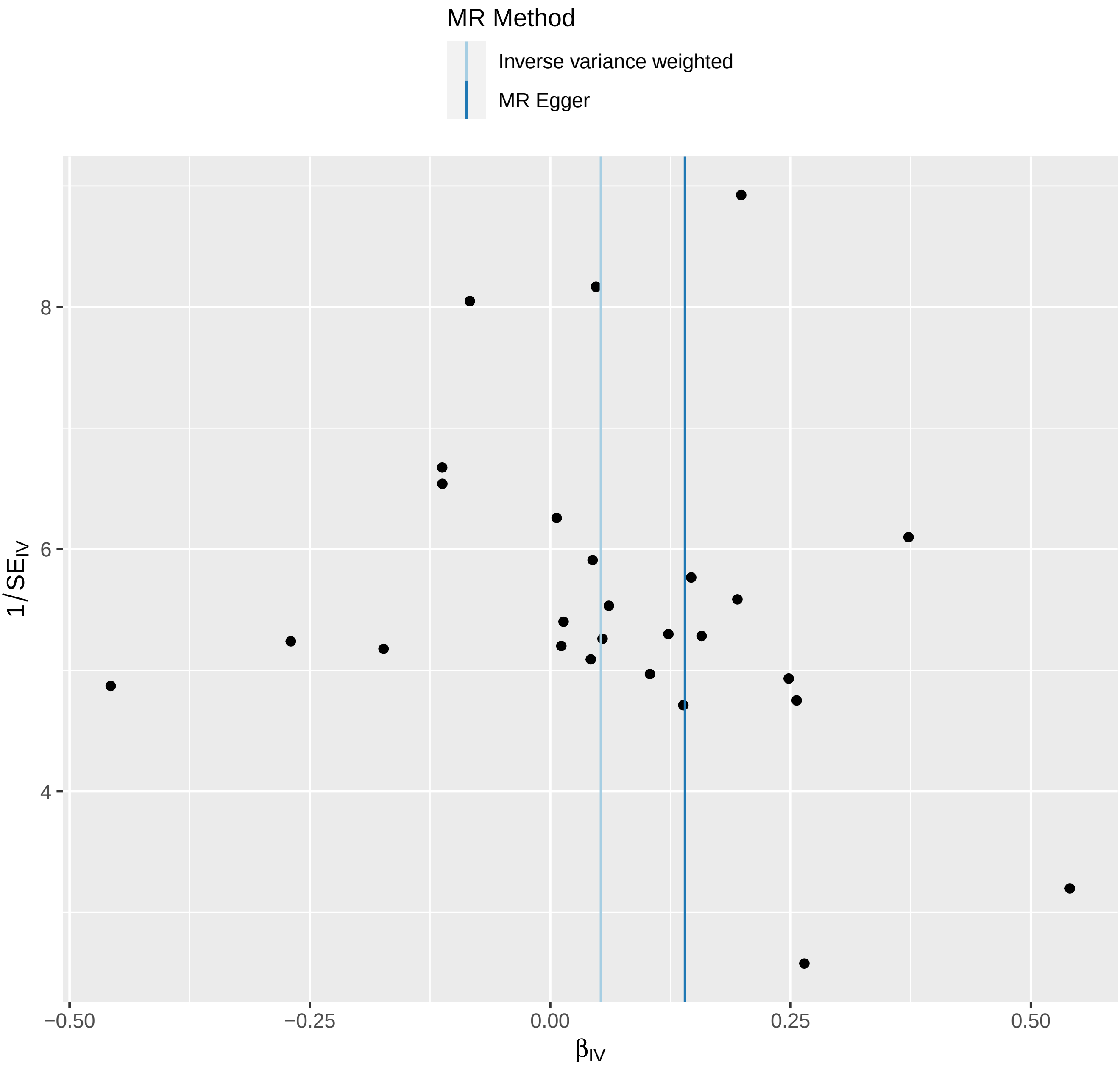

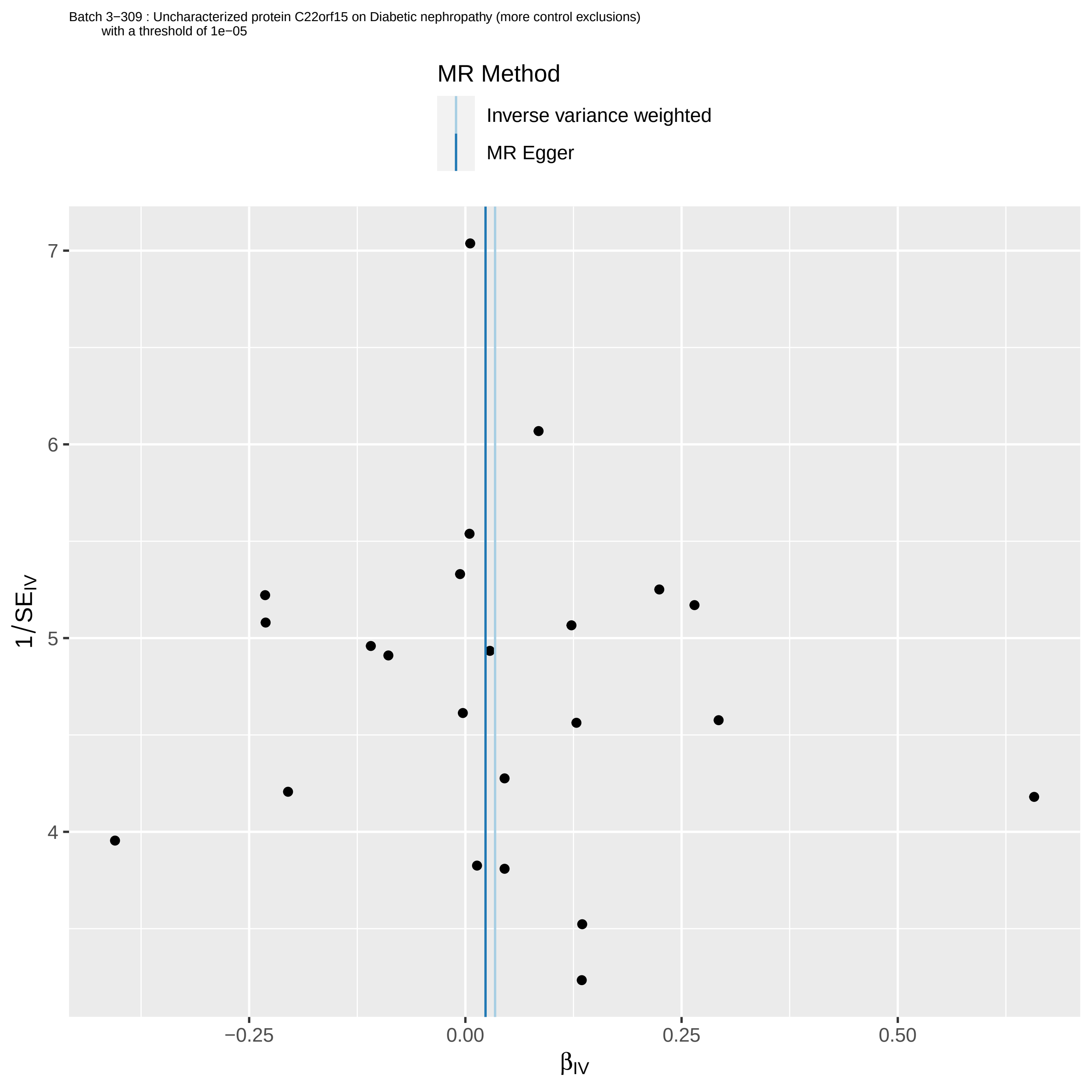

Batch 3−309 : Uncharacterized protein C22orf15 on Diabetic nephropathy (more control exclusions)
with a threshold of 1e−05
MR Method
Inverse variance weighted
MR Egger
7
6
5
4
1/SE_IV
−0.25
0.00
0.25
0.50
β_IV

Batch 3−310 : Augurin on Diabetic nephropathy (more control exclusions)
with a threshold of 1e−05

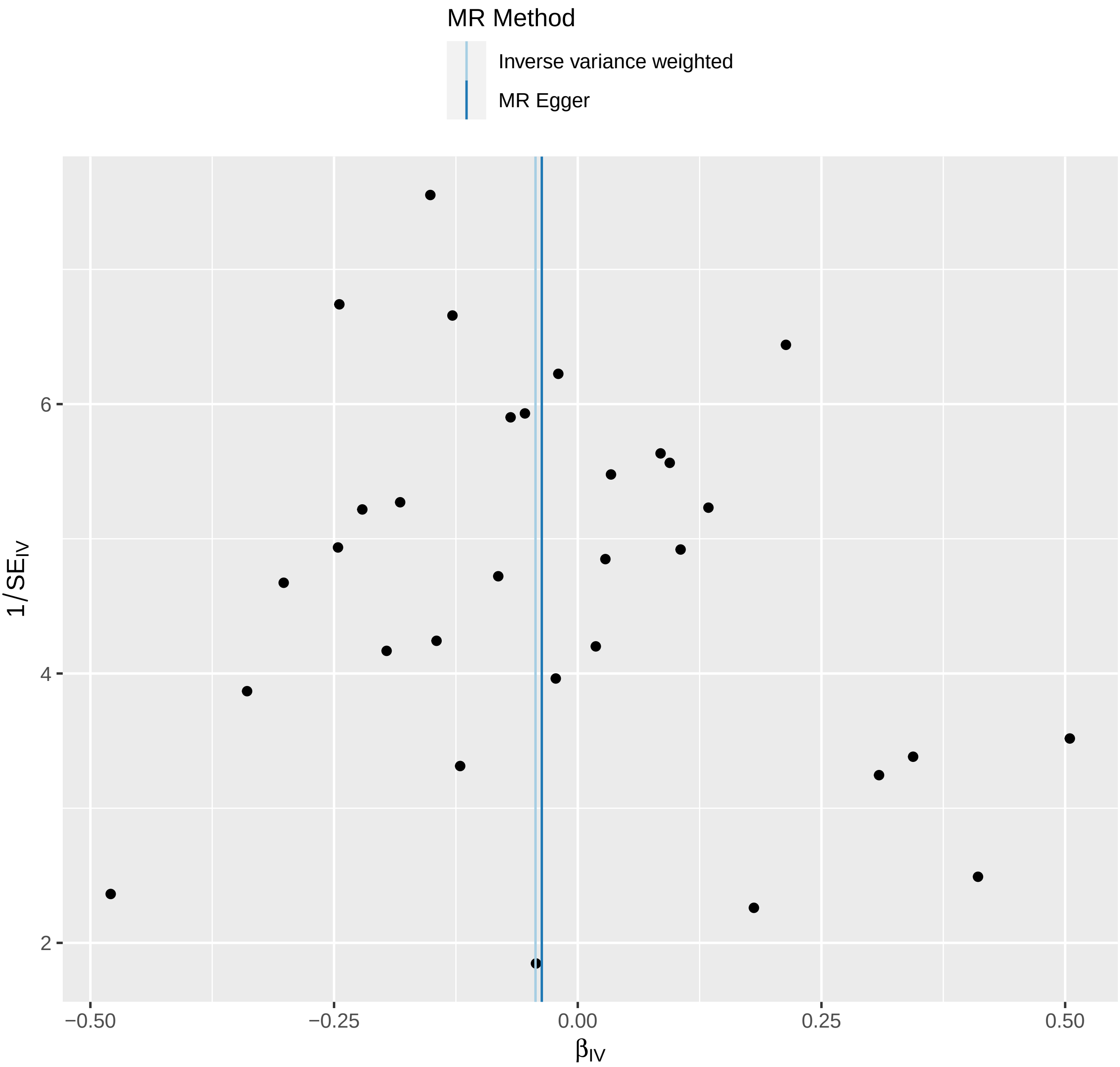

Batch 3−311 : Uncharacterized protein C2orf66 on Diabetic nephropathy (more control exclusions)
with a threshold of 1e−05

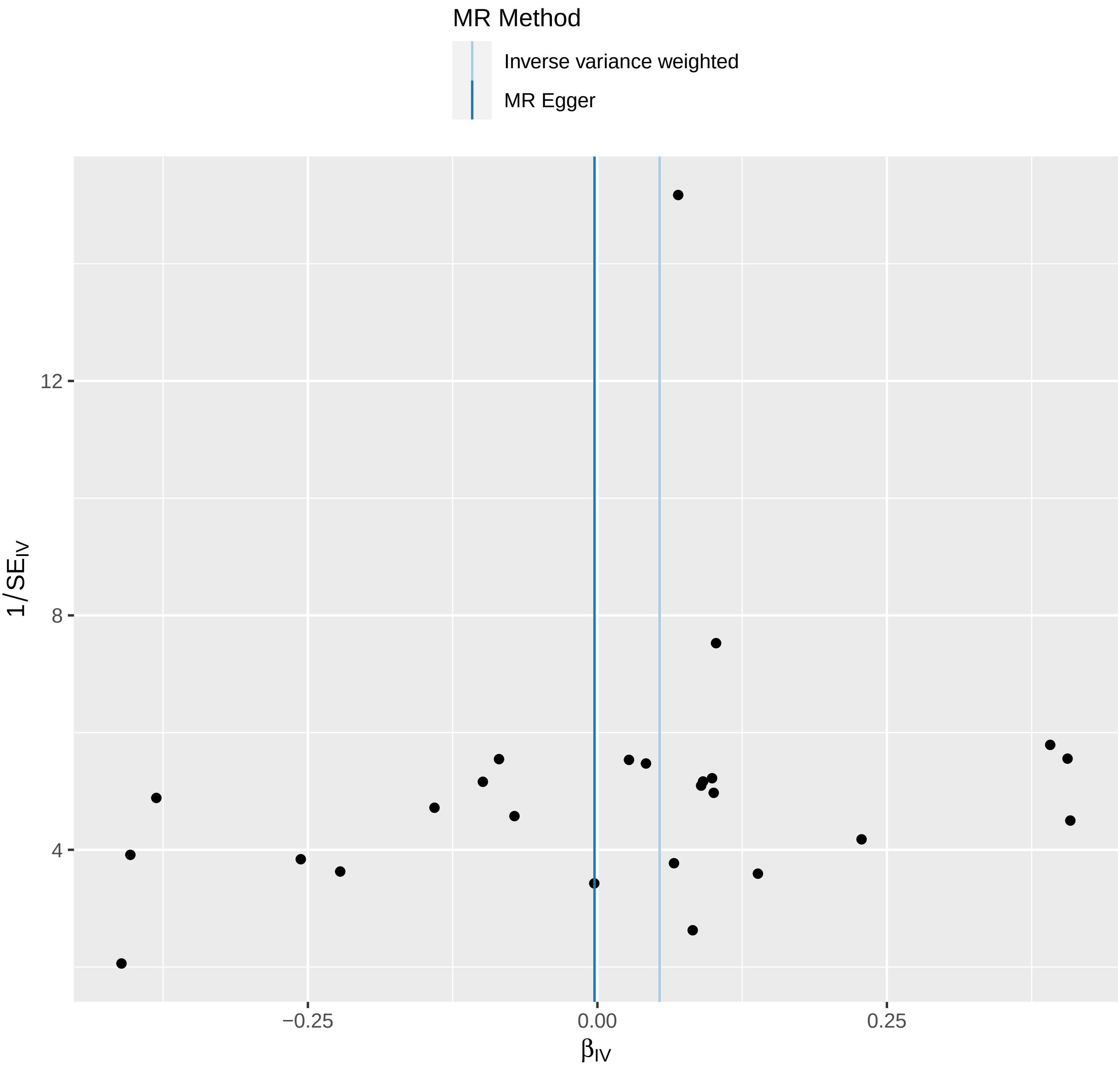

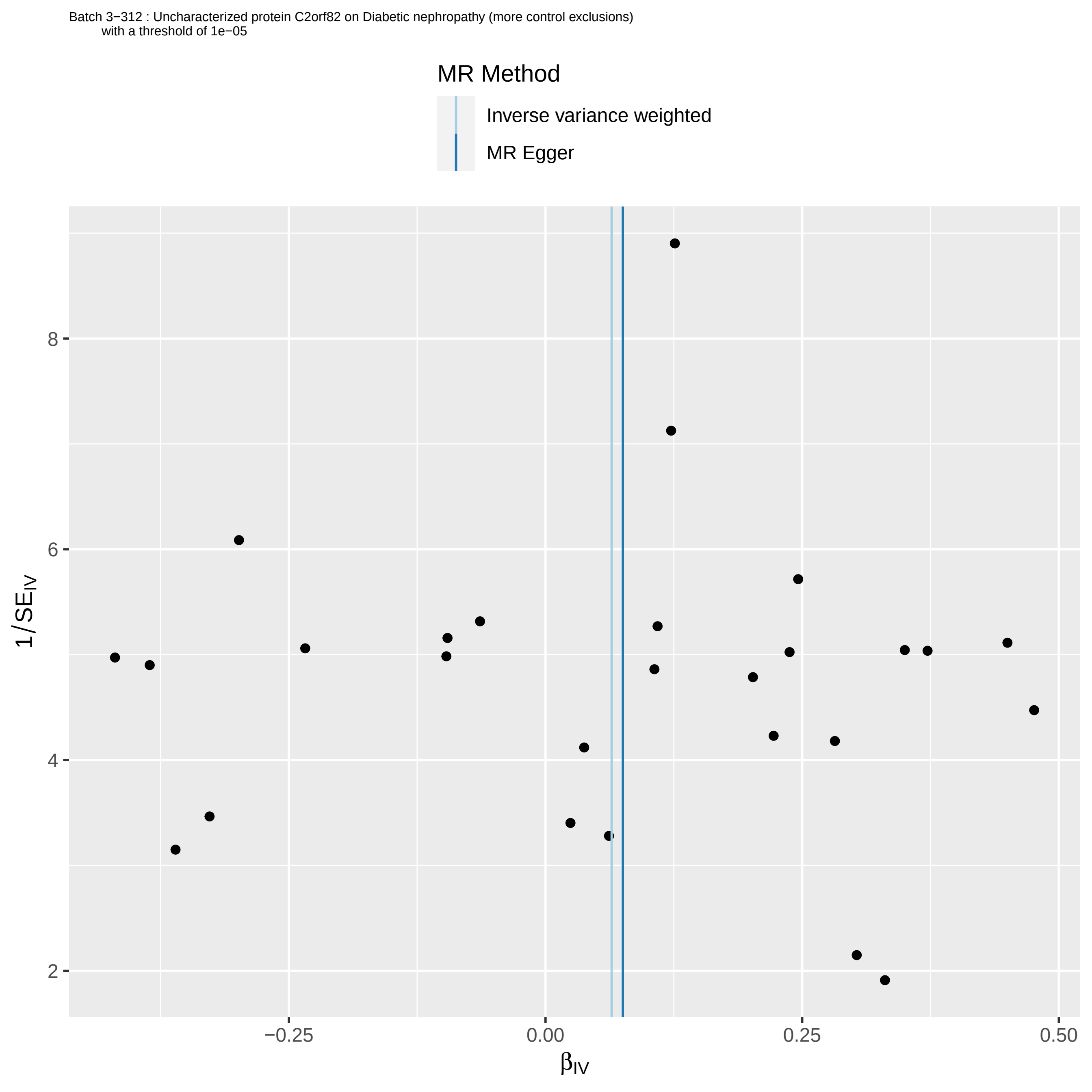

Batch 3−312 : Uncharacterized protein C2orf82 on Diabetic nephropathy (more control exclusions)
with a threshold of 1e−05
MR Method
Inverse variance weighted
MR Egger
8
6
4
2
1/SE_IV
−0.25
0.00
0.25
0.50
β_IV

Batch 3−313 : C3a anaphylatoxin des Arginine on Diabetic nephropathy (more control exclusions)
with a threshold of 1e−05

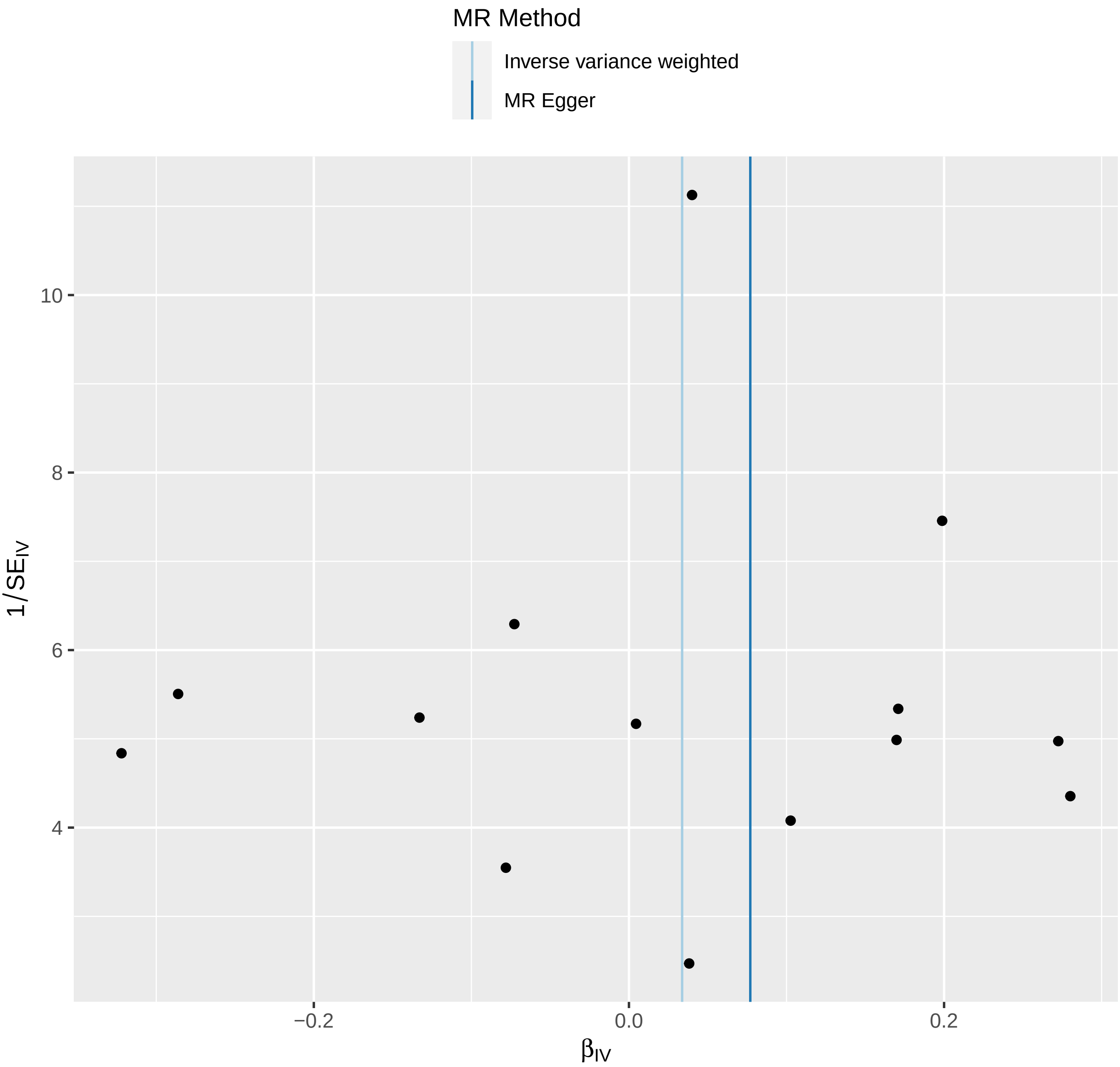

Batch 3−314 : Uncharacterized protein C3orf18 on Diabetic nephropathy (more control exclusions)
with a threshold of 1e−05

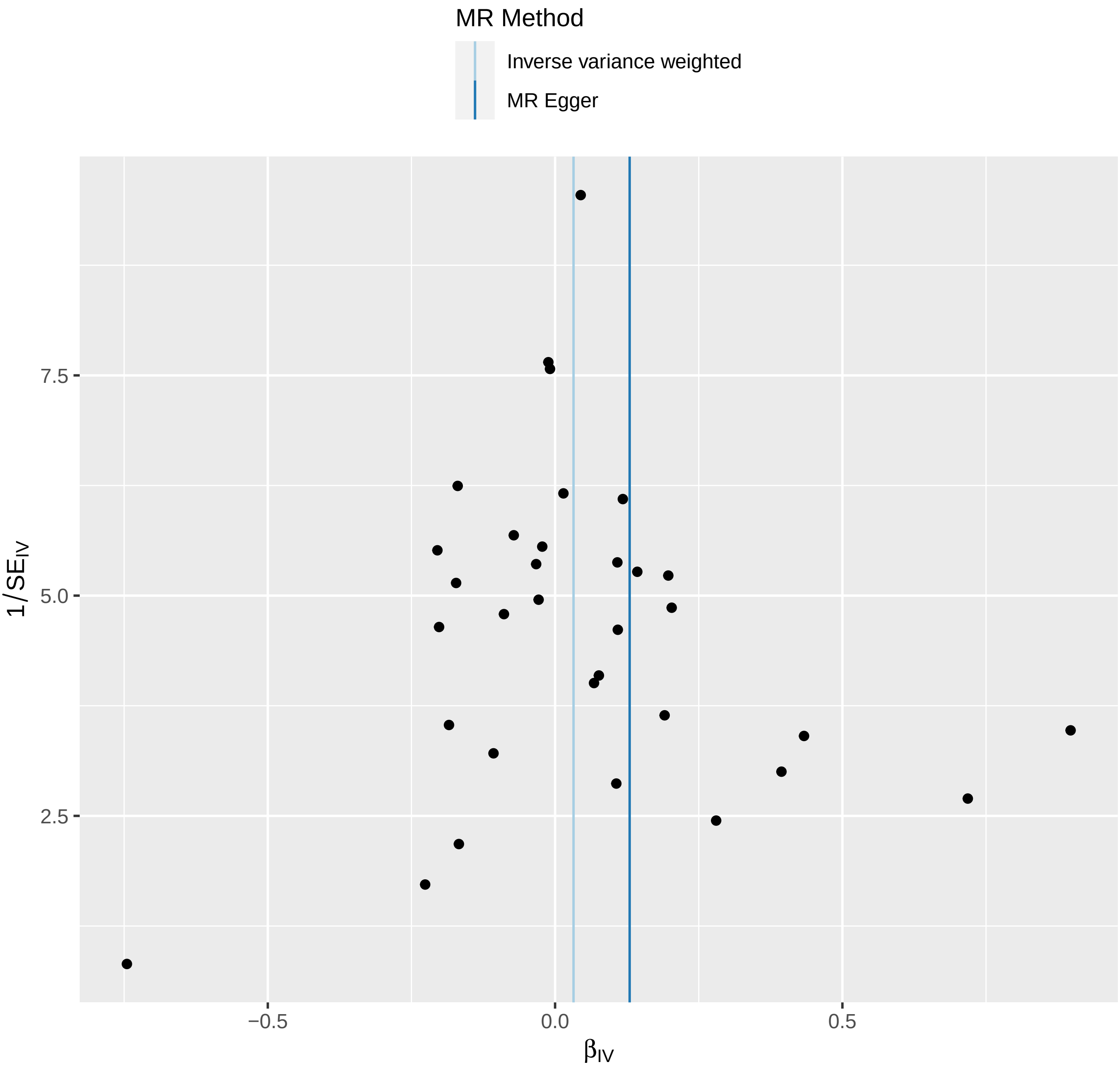

Batch 3−315 : Complement C4 on Diabetic nephropathy (more control exclusions)
with a threshold of 1e−05

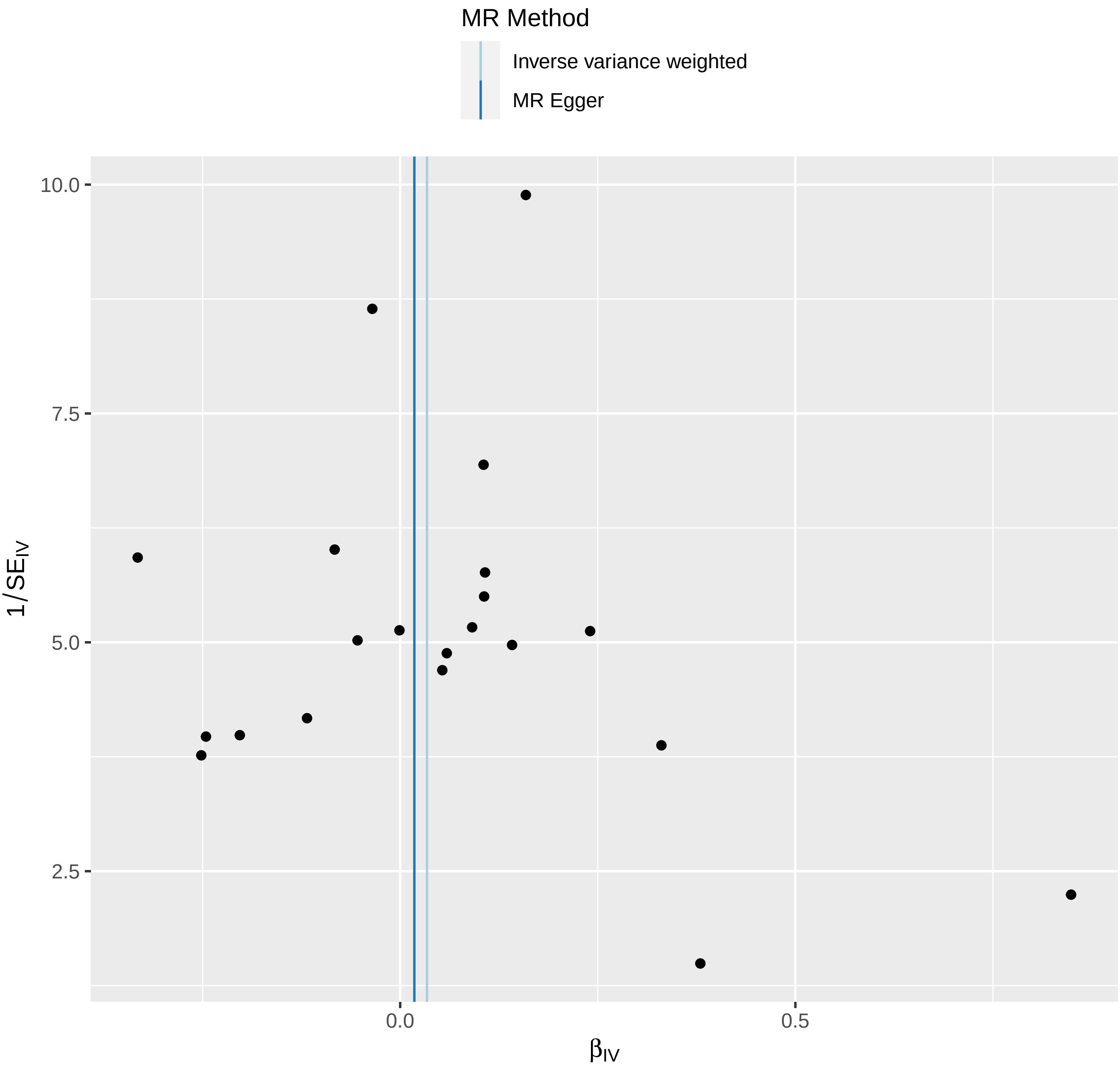

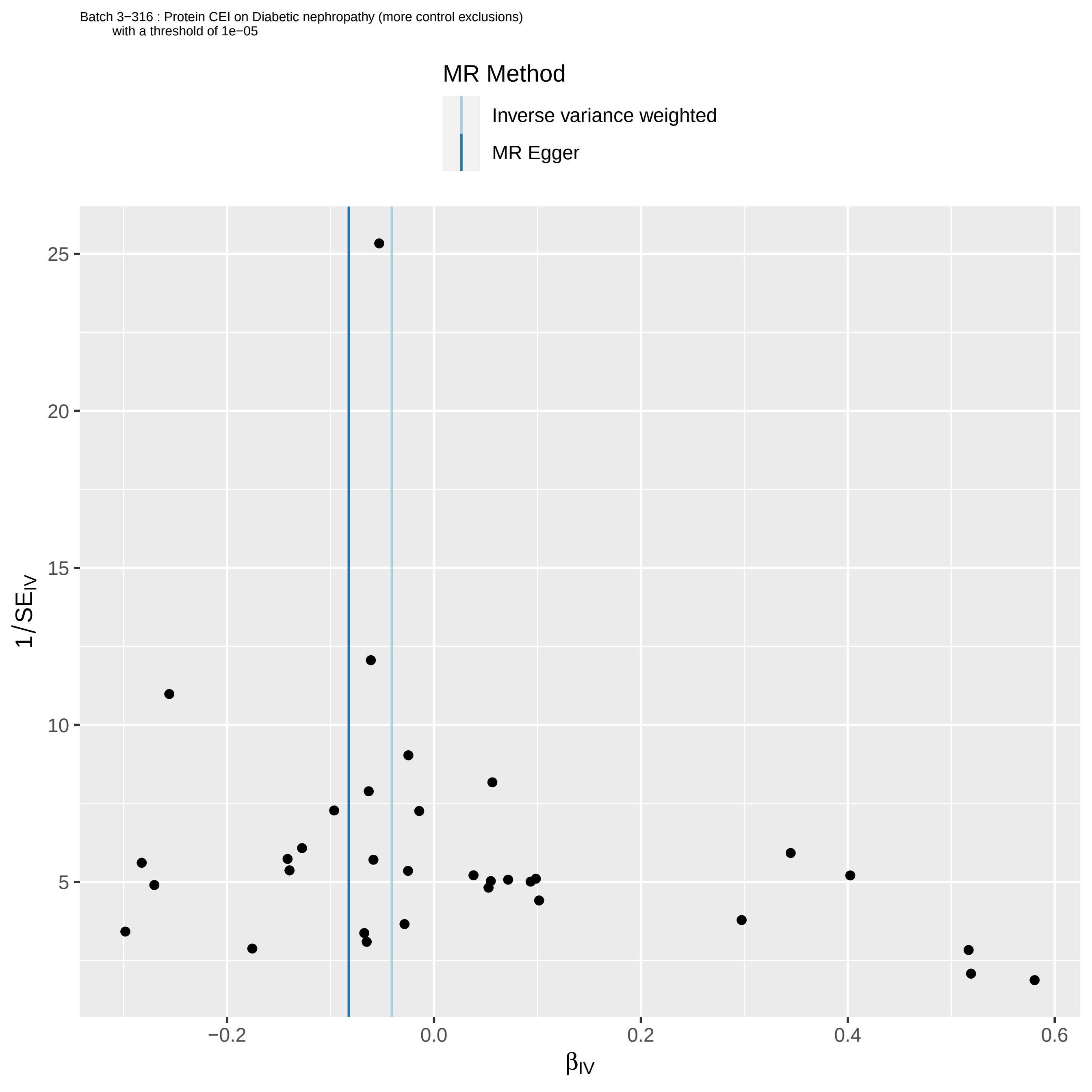
Batch 3−316 : Protein CEI on Diabetic nephropathy (more control exclusions)
with a threshold of 1e−05
MR Method
Inverse variance weighted
MR Egger
25
20
15
10
5
$1/SE_{IV}$
−0.2
0.0
0.2
0.4
0.6
β_{IV}

Batch 3−317 : Glutaredoxin−like protein C5orf63 on Diabetic nephropathy (more control exclusions)
with a threshold of 1e−05

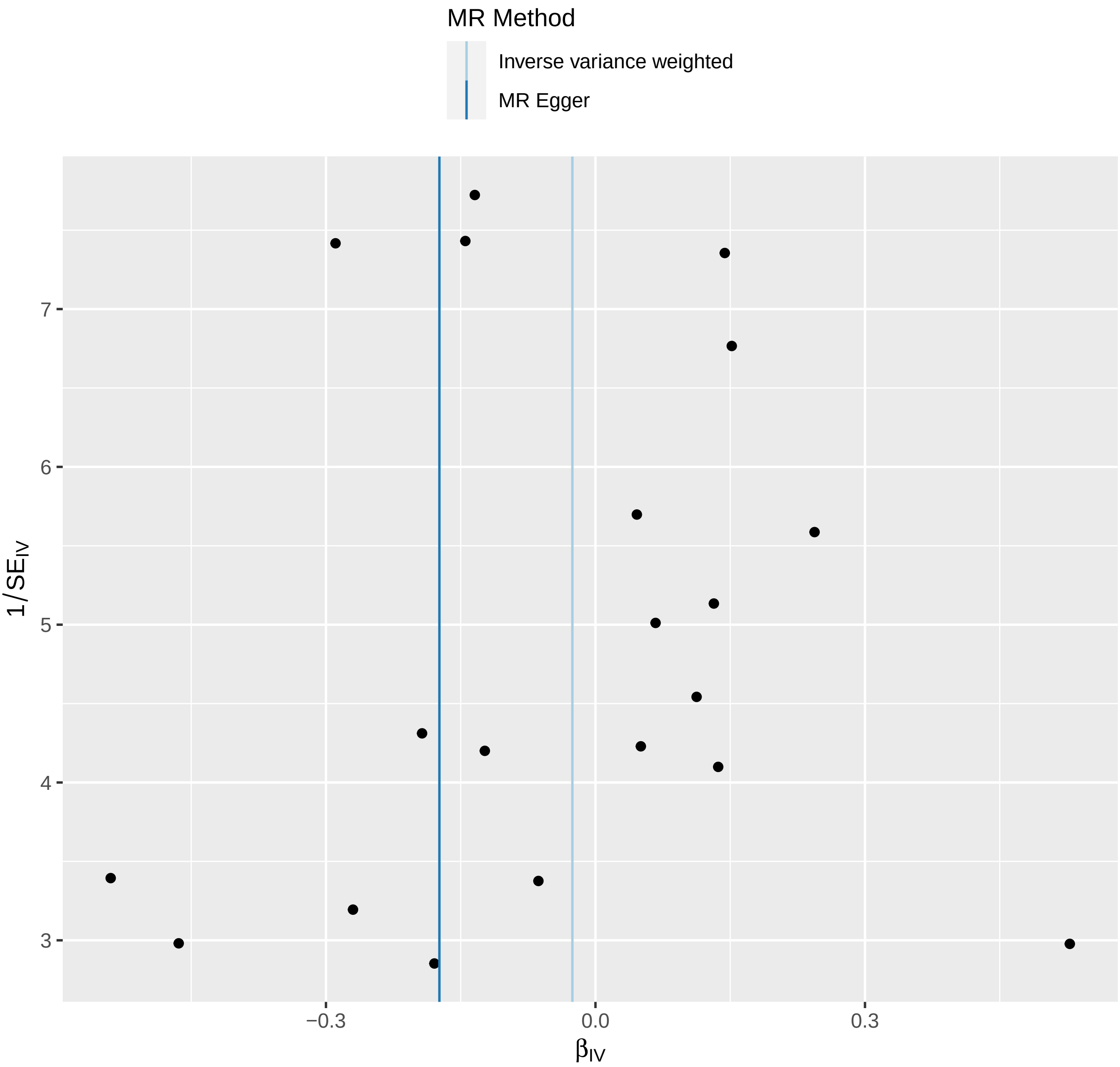

Batch 3−318 : Complement component C6 on Diabetic nephropathy (more control exclusions)
with a threshold of 1e−05

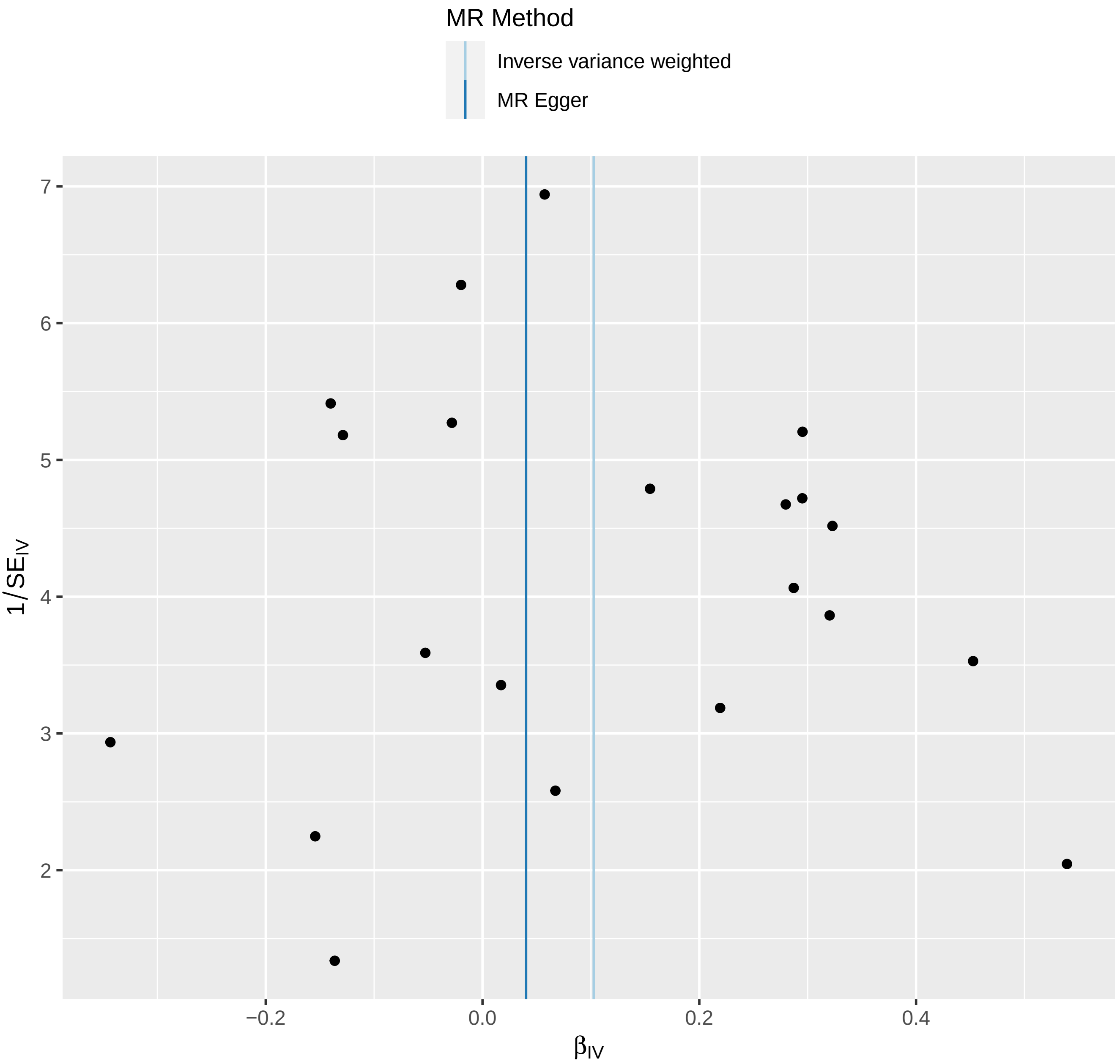

Batch 3−319 : Uncharacterized protein C6orf226 on Diabetic nephropathy (more control exclusions)
with a threshold of 1e−05

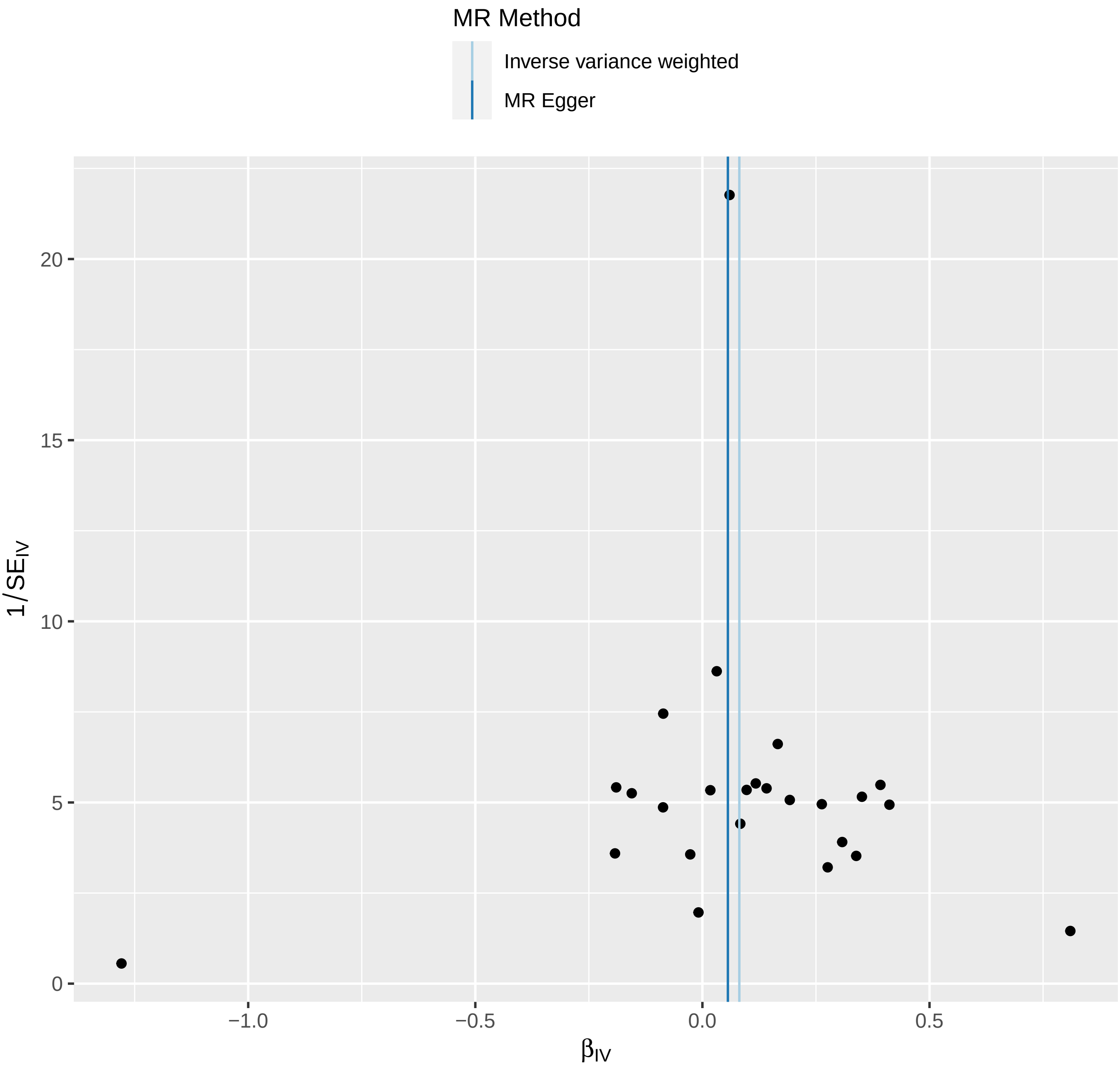

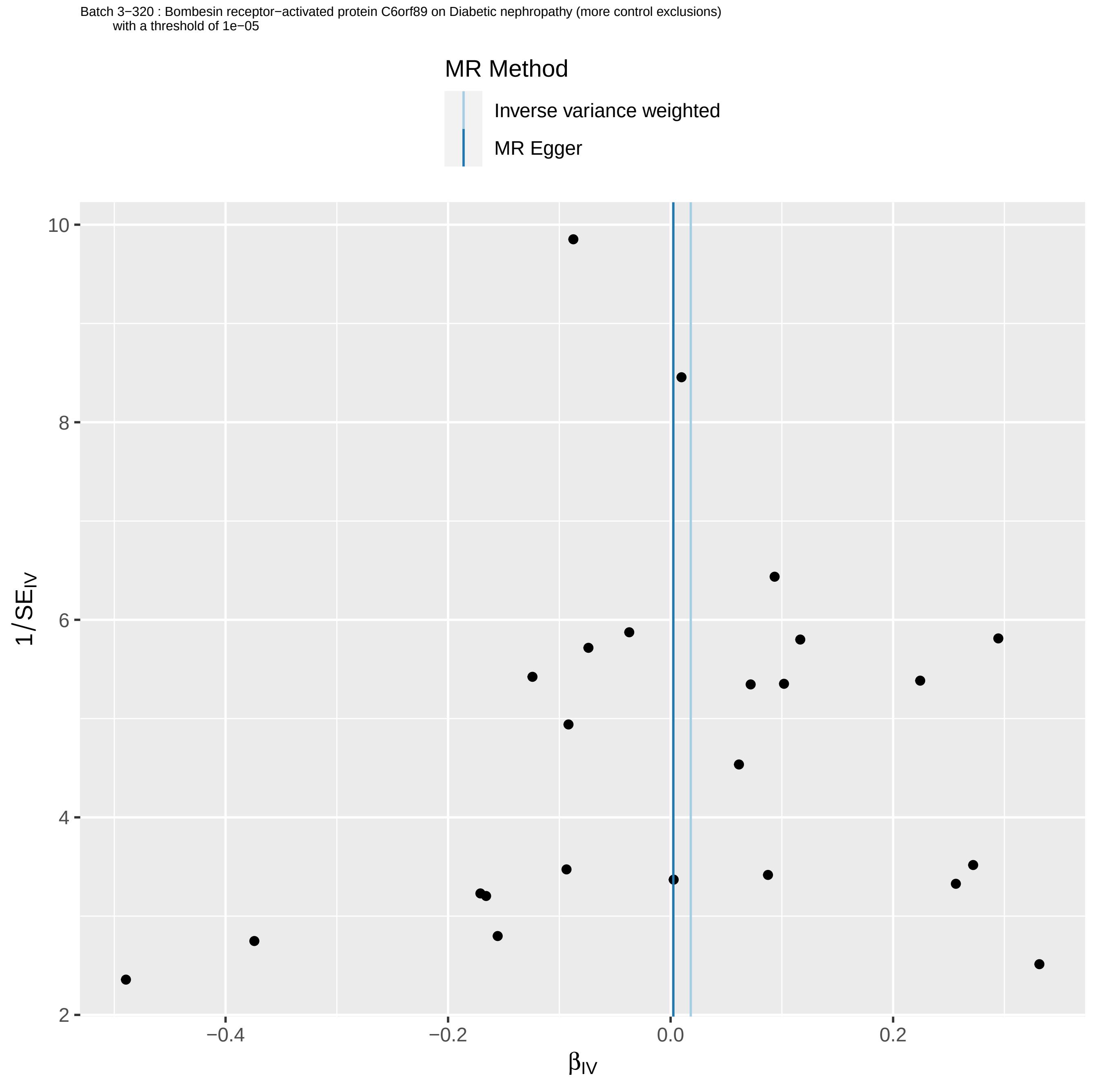

Batch 3−320 : Bombesin receptor−activated protein C6orf89 on Diabetic nephropathy (more control exclusions)
with a threshold of 1e−05
MR Method
Inverse variance weighted
MR Egger
10
8
6
4
2
1/SE_IV
−0.4
−0.2
0.0
0.2
β_IV

Batch 3−321 : Uncharacterized protein C7orf69 on Diabetic nephropathy (more control exclusions)
with a threshold of 1e−05

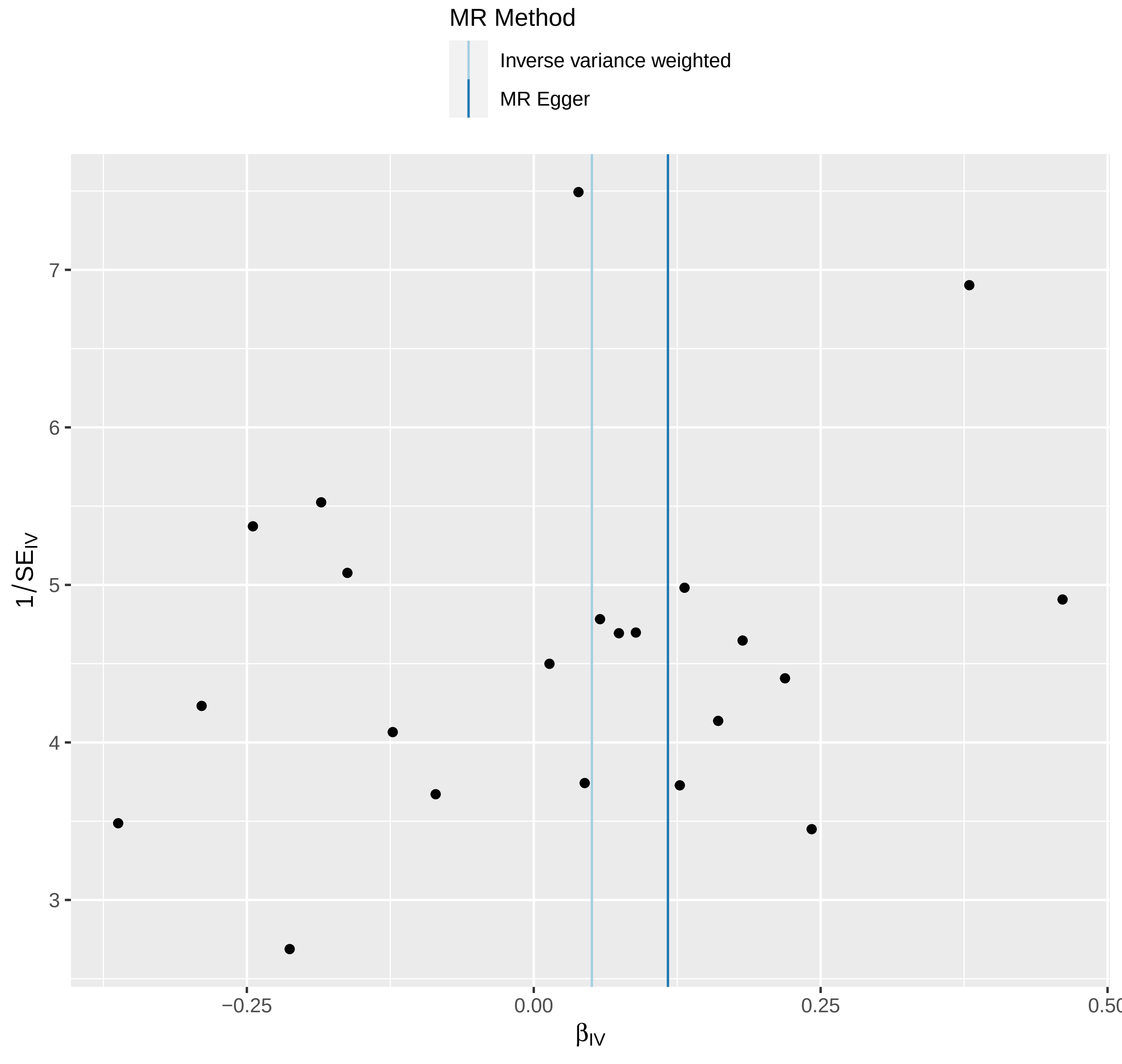

Batch 3−322 : Complement component C8 on Diabetic nephropathy (more control exclusions)
with a threshold of 1e−05

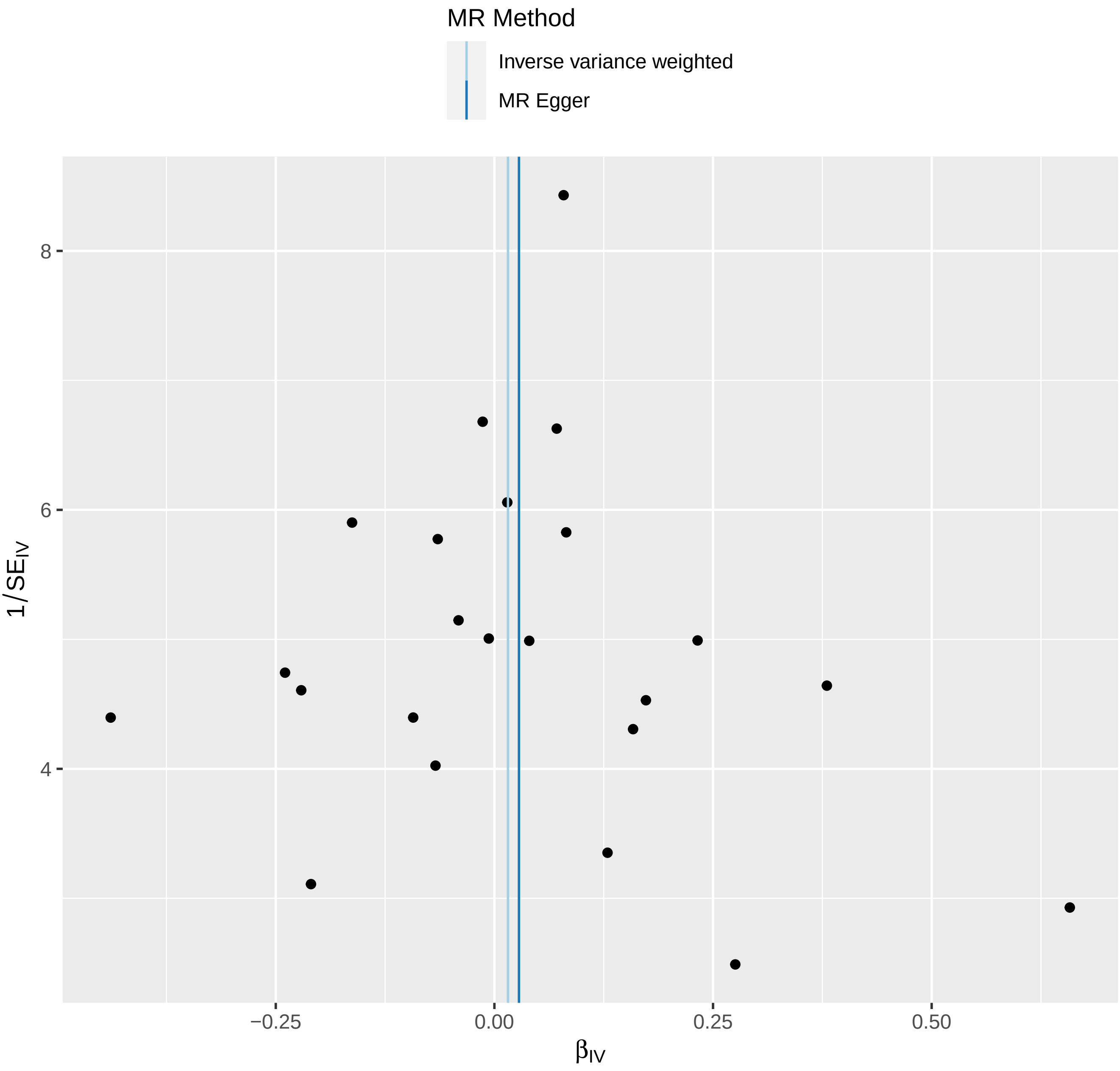

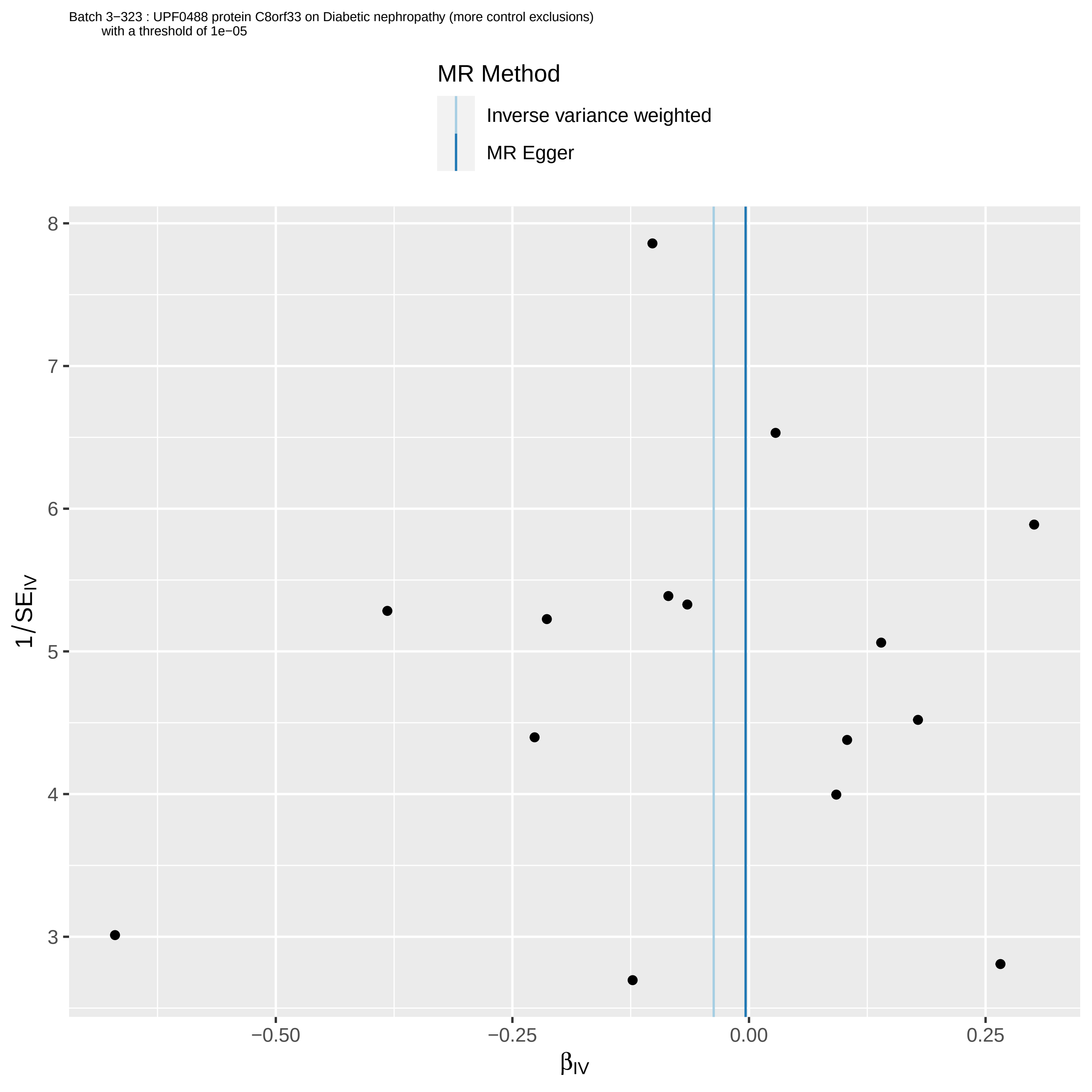

Batch 3−323 : UPF0488 protein C8orf33 on Diabetic nephropathy (more control exclusions)
with a threshold of 1e−05
MR Method
Inverse variance weighted
MR Egger
8
7
6
5
4
3
1/SE_IV
−0.50
−0.25
0.00
0.25
β_IV

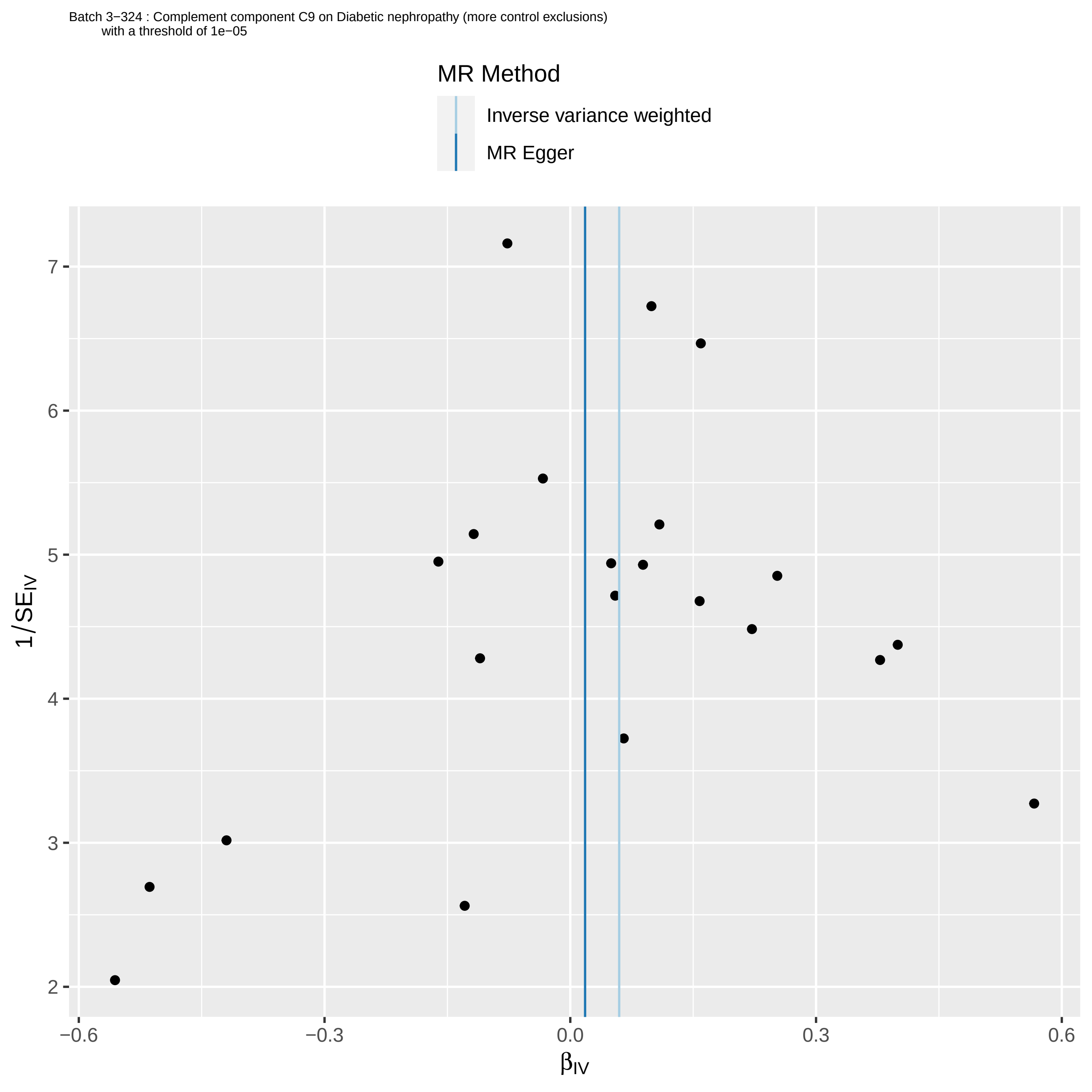

Batch 3−324 : Complement component C9 on Diabetic nephropathy (more control exclusions)
with a threshold of 1e−05
MR Method
Inverse variance weighted
MR Egger
1/SE_IV
β_IV
7
6
5
4
3
2
−0.6
−0.3
0.0
0.3
0.6

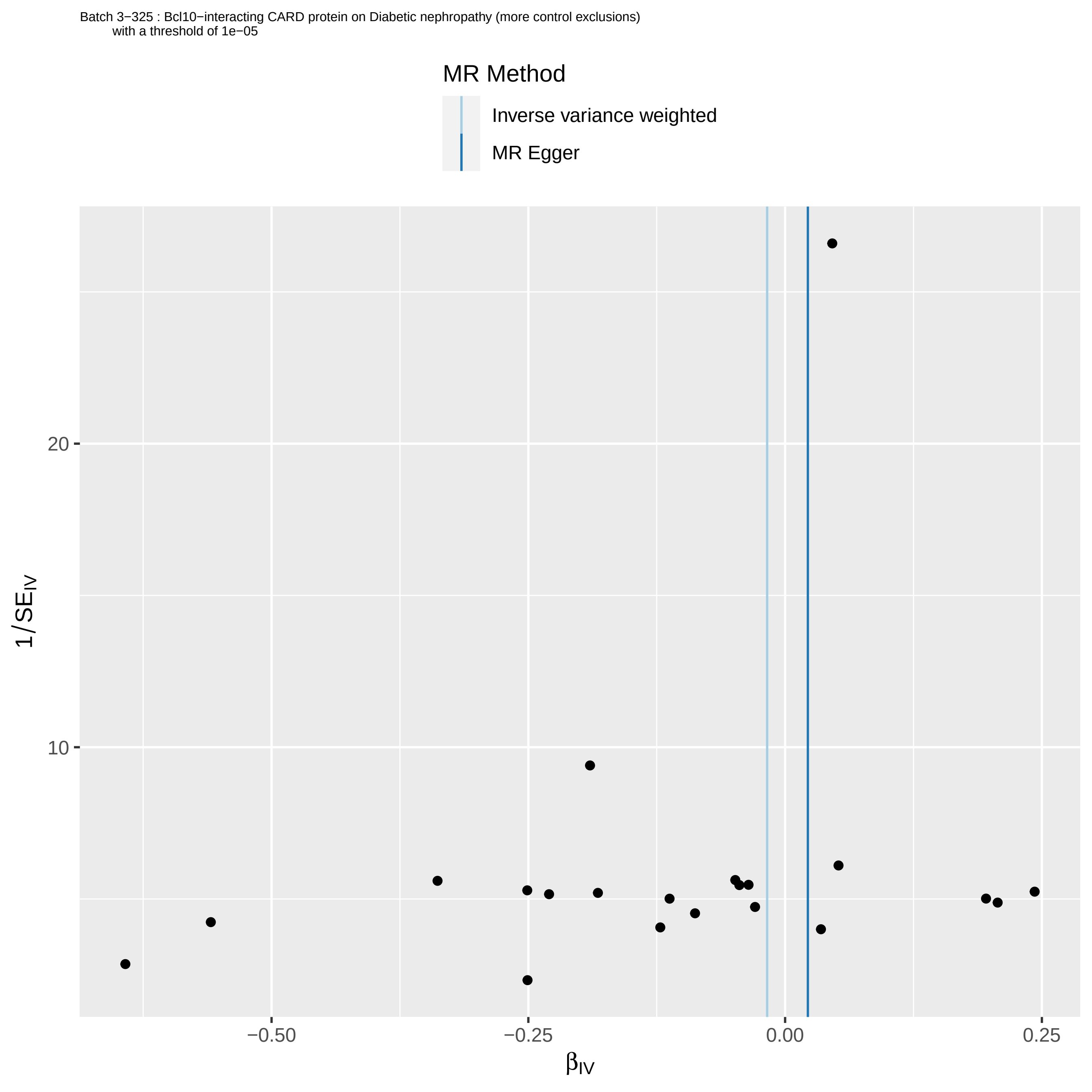
Batch 3−325 : Bcl10−interacting CARD protein on Diabetic nephropathy (more control exclusions)
with a threshold of 1e−05
MR Method
Inverse variance weighted
MR Egger
20
10
1/SE_IV
−0.50
−0.25
0.00
0.25
β_IV

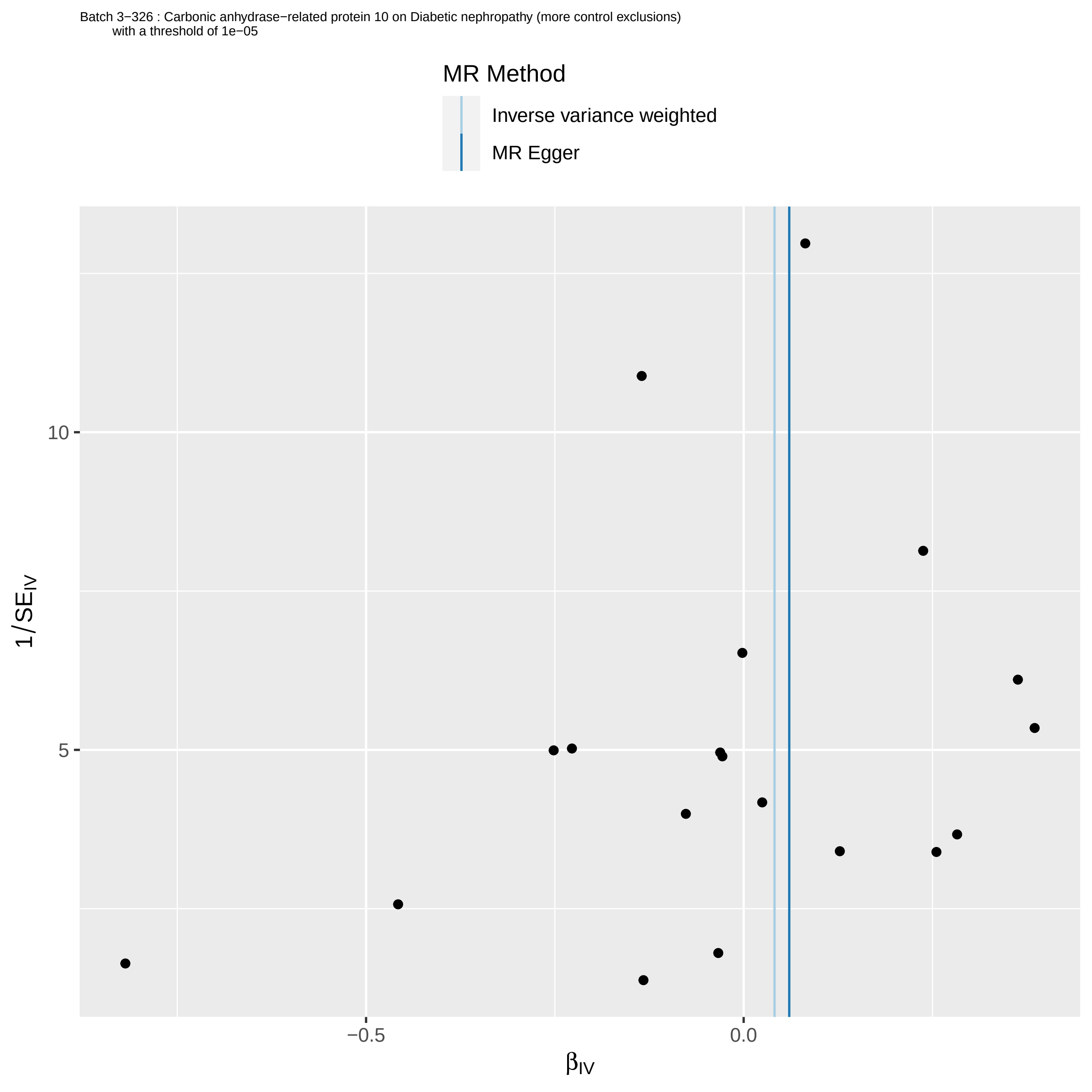

Batch 3−326 : Carbonic anhydrase−related protein 10 on Diabetic nephropathy (more control exclusions)
with a threshold of 1e−05
MR Method
Inverse variance weighted
MR Egger
10
5
1/SE_IV
−0.5
0.0
β_IV

Batch 3−327 : Carbonic anhydrase−related protein 10 on Diabetic nephropathy (more control exclusions)
with a threshold of 1e−05

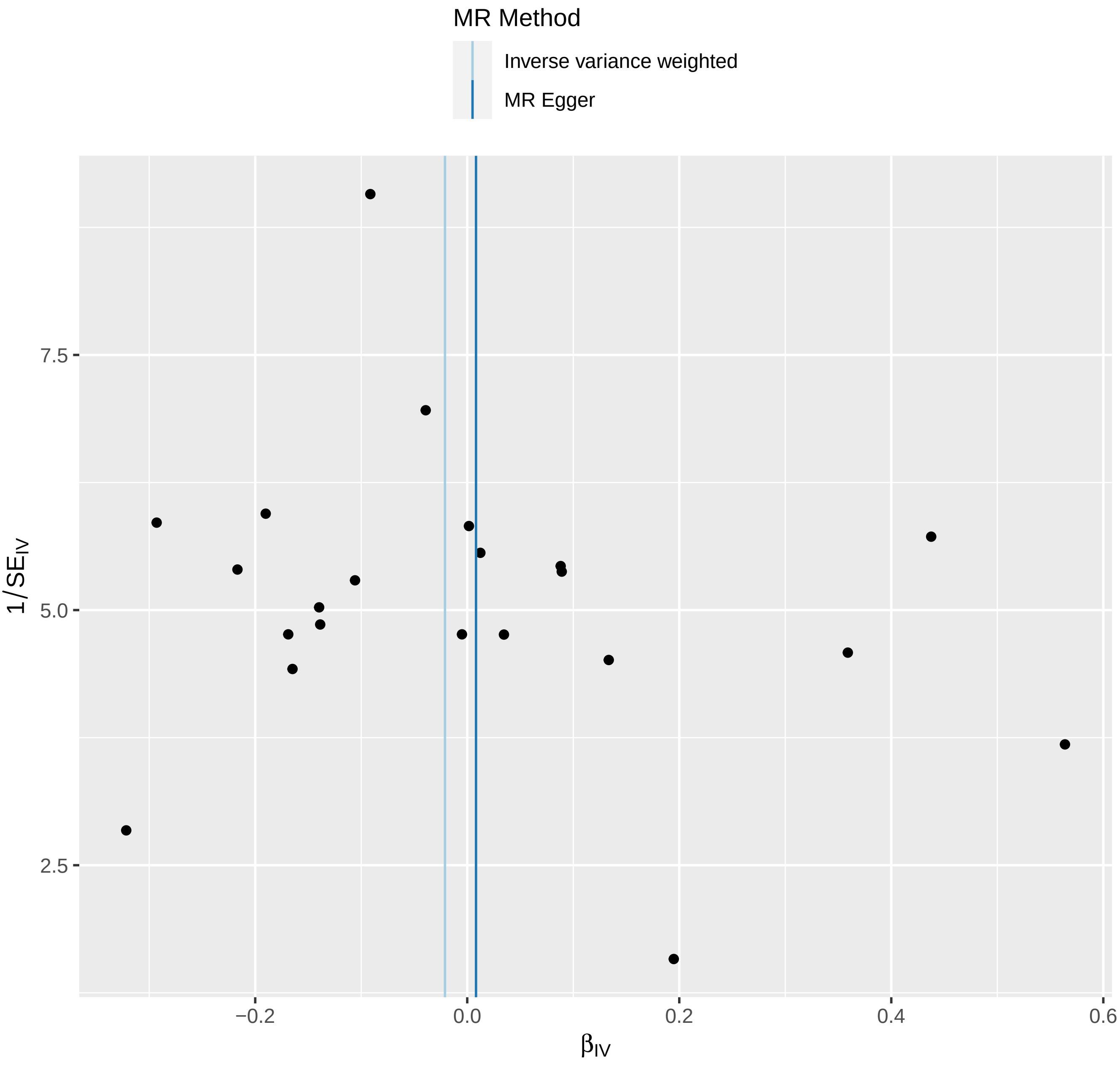

Batch 3−328 : Carbonic anhydrase 1 on Diabetic nephropathy (more control exclusions)
with a threshold of 1e−05

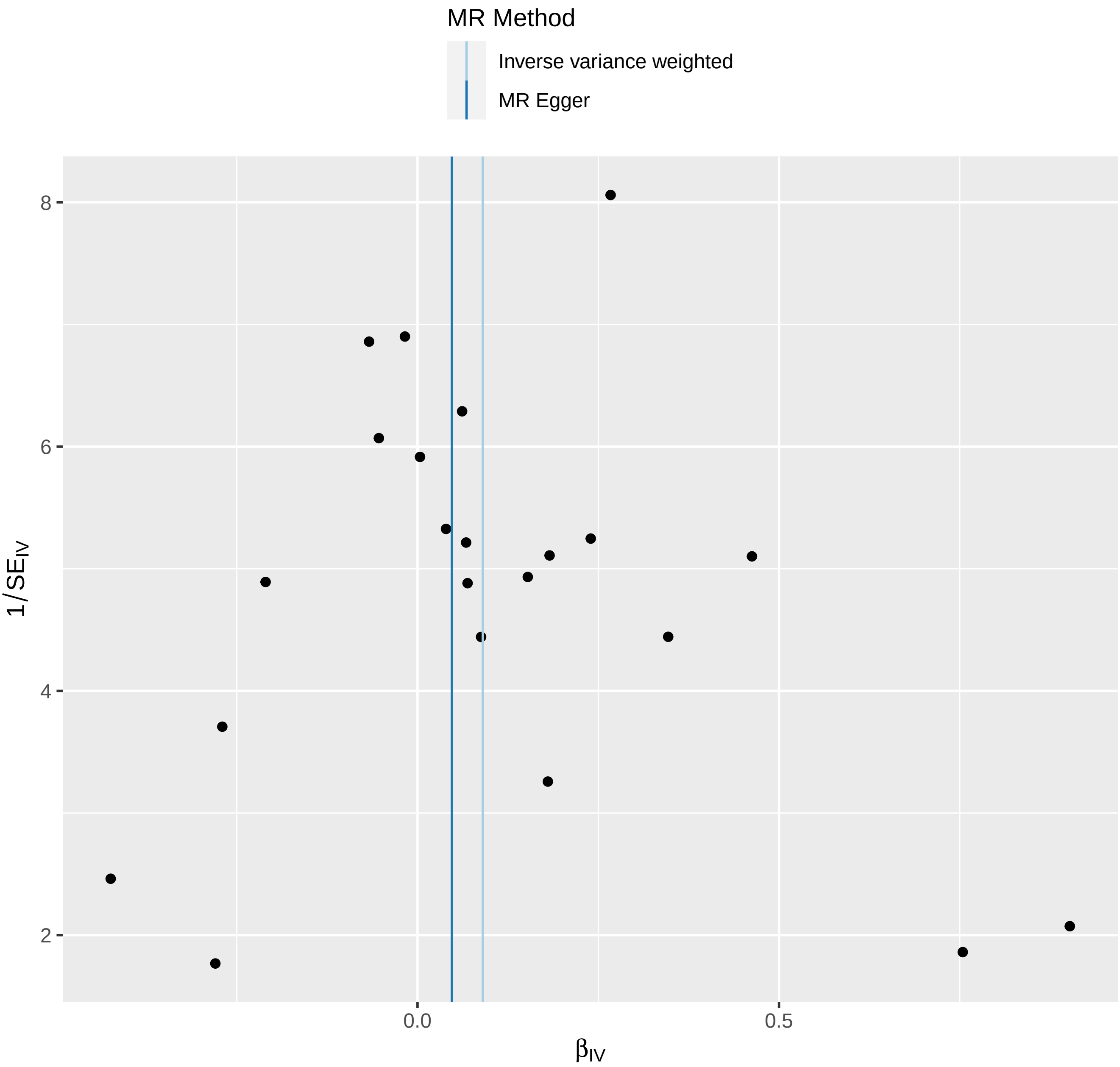

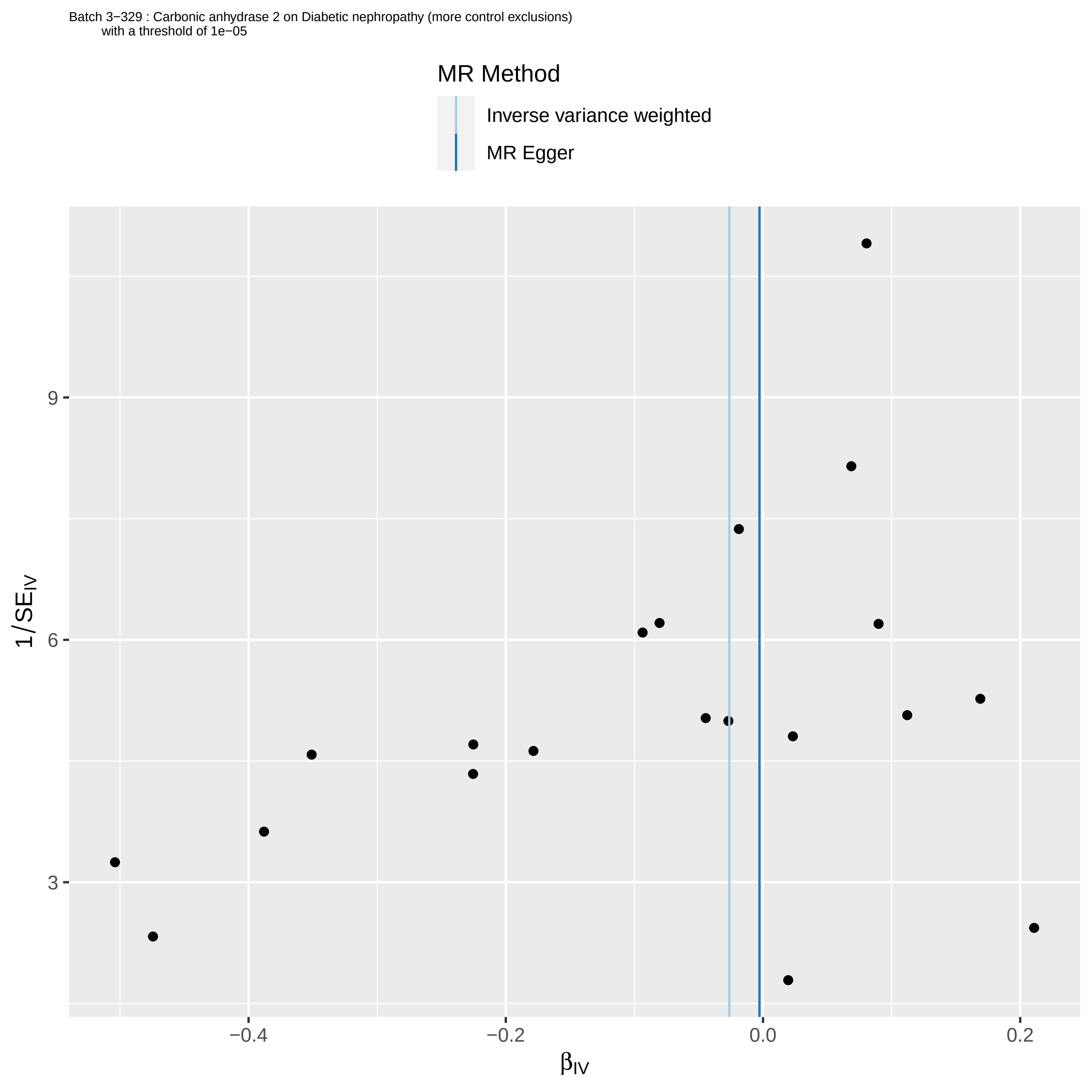

Batch 3−329 : Carbonic anhydrase 2 on Diabetic nephropathy (more control exclusions)
with a threshold of 1e−05
MR Method
Inverse variance weighted
MR Egger
9
6
3
1/SE_IV
−0.4
−0.2
0.0
0.2
β_IV

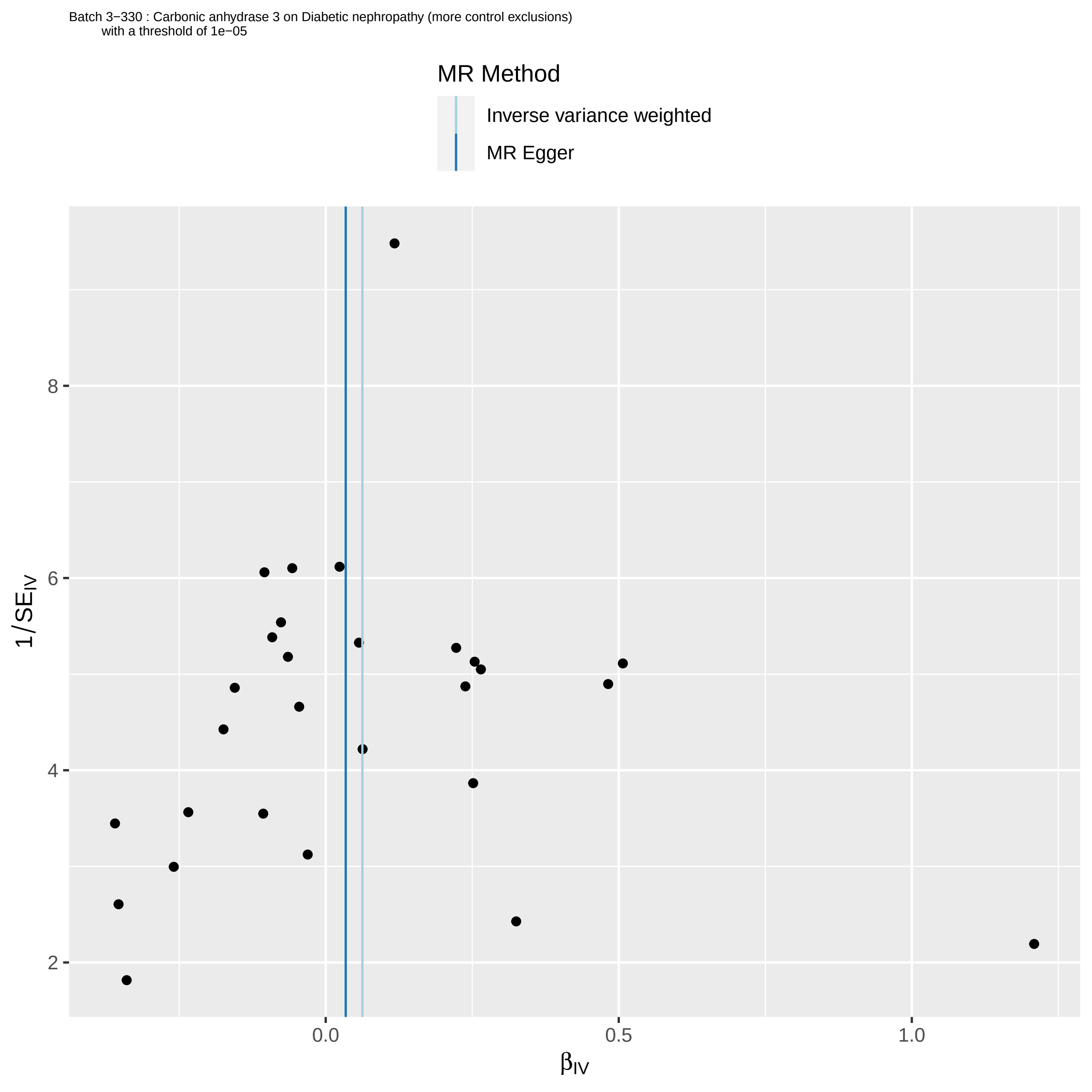
Batch 3−330 : Carbonic anhydrase 3 on Diabetic nephropathy (more control exclusions)
with a threshold of 1e−05
MR Method
Inverse variance weighted
MR Egger
8
6
4
2
1/SE$_{IV}$
0.0
0.5
1.0
β$_{IV}$

Batch 3−331 : Carbonic anhydrase 4 on Diabetic nephropathy (more control exclusions)
with a threshold of 1e−05

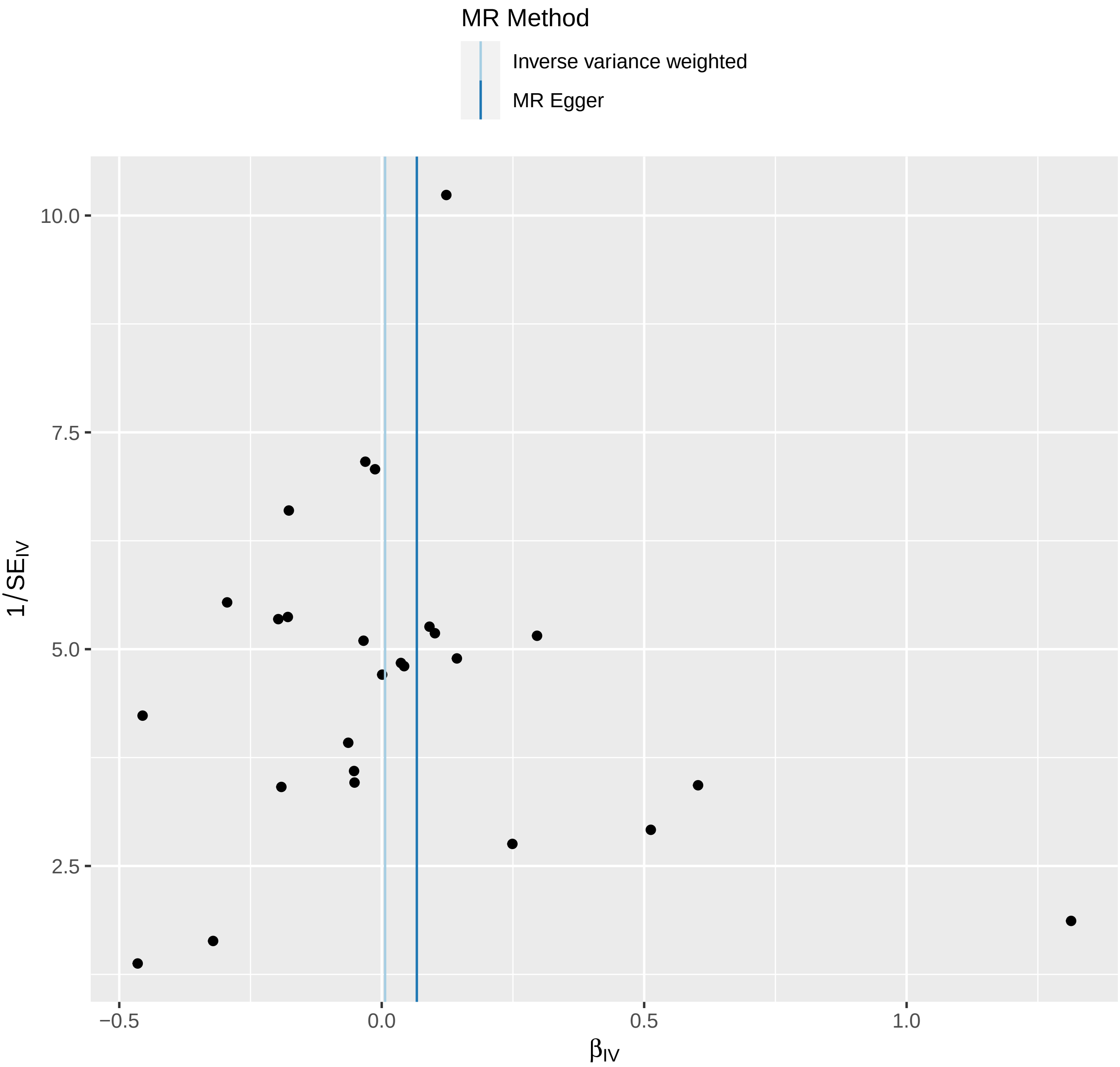

Batch 3−332 : Carbonic anhydrase 5A, mitochondrial on Diabetic nephropathy (more control exclusions)
with a threshold of 1e−05

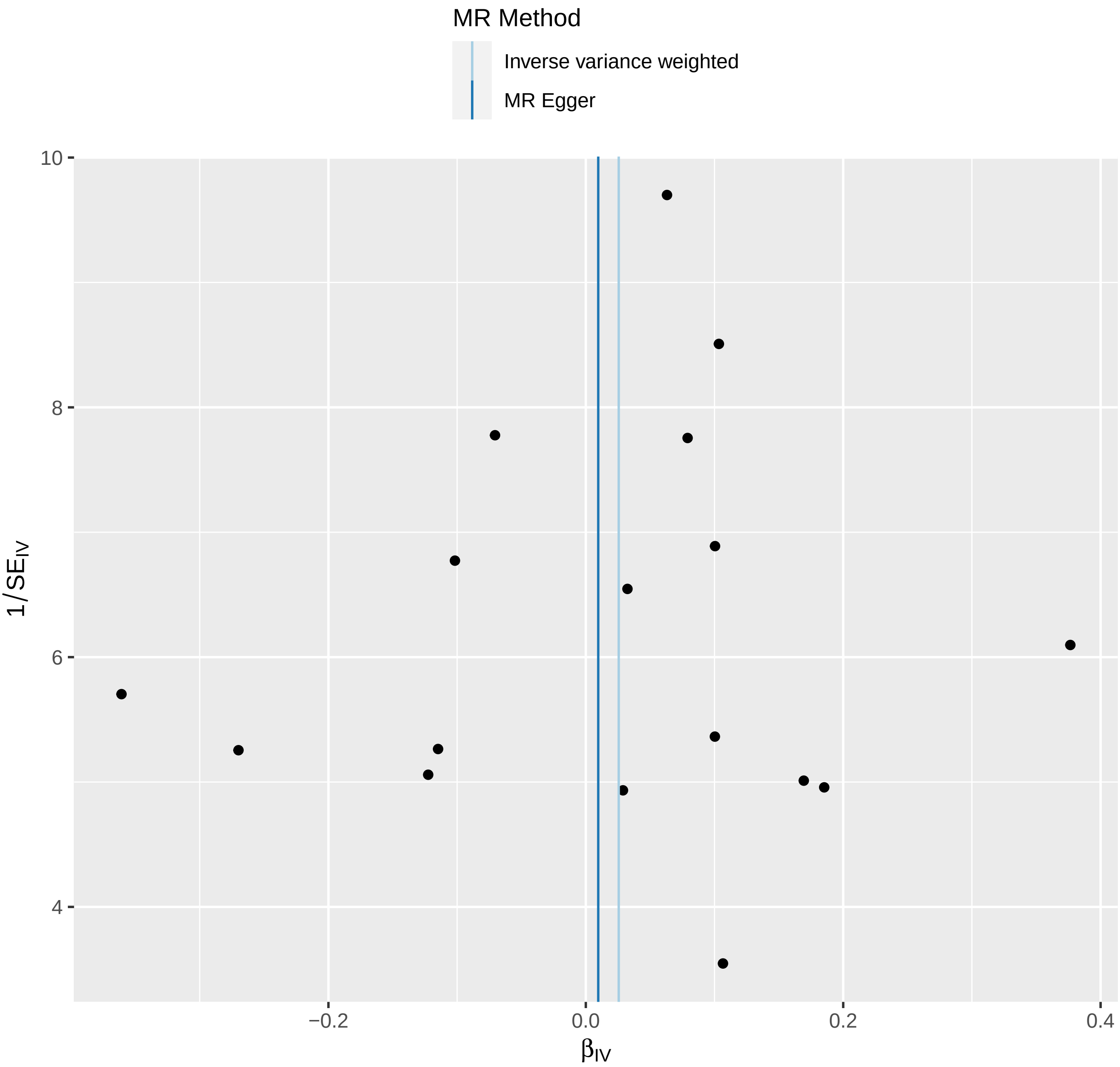

Batch 3−333 : Carbonic anhydrase 6 on Diabetic nephropathy (more control exclusions)
with a threshold of 1e−05

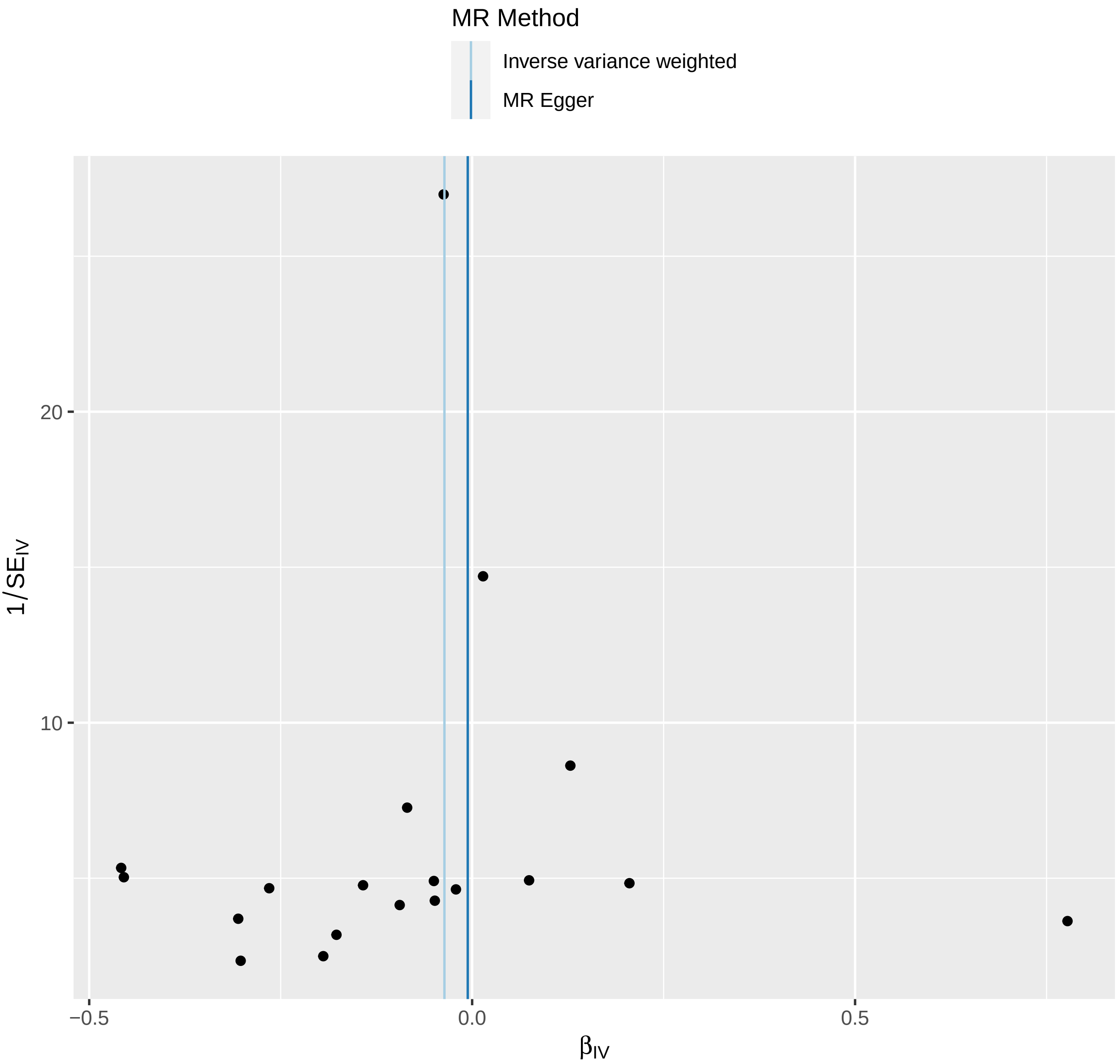

Batch 3−334 : Carbonic anhydrase 9 on Diabetic nephropathy (more control exclusions)
with a threshold of 1e−05

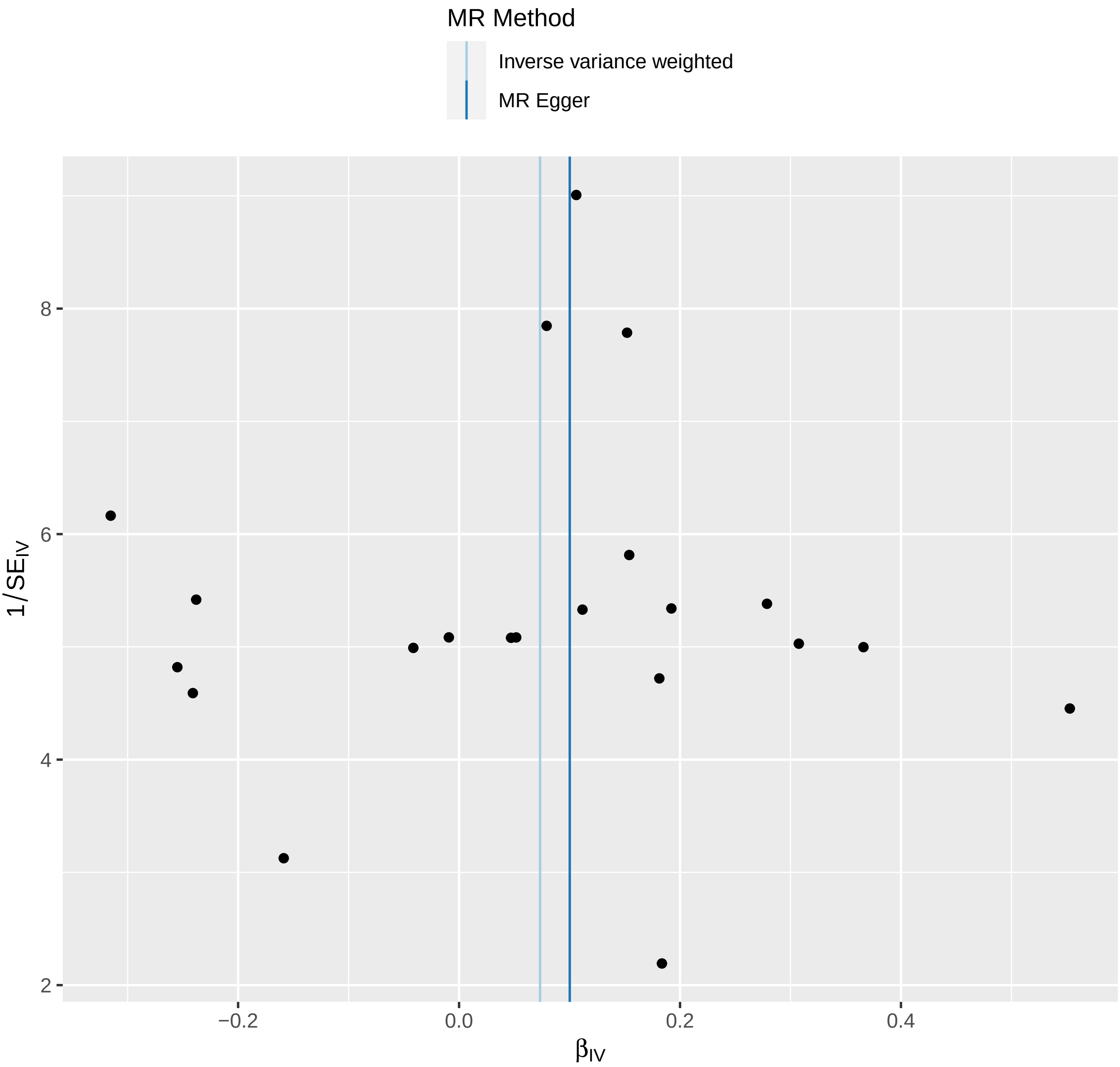

Batch 3−335 : Voltage−dependent calcium channel subunit alpha−2/delta−3 on Diabetic nephropathy (more control exclusions) with a threshold of 1e−05

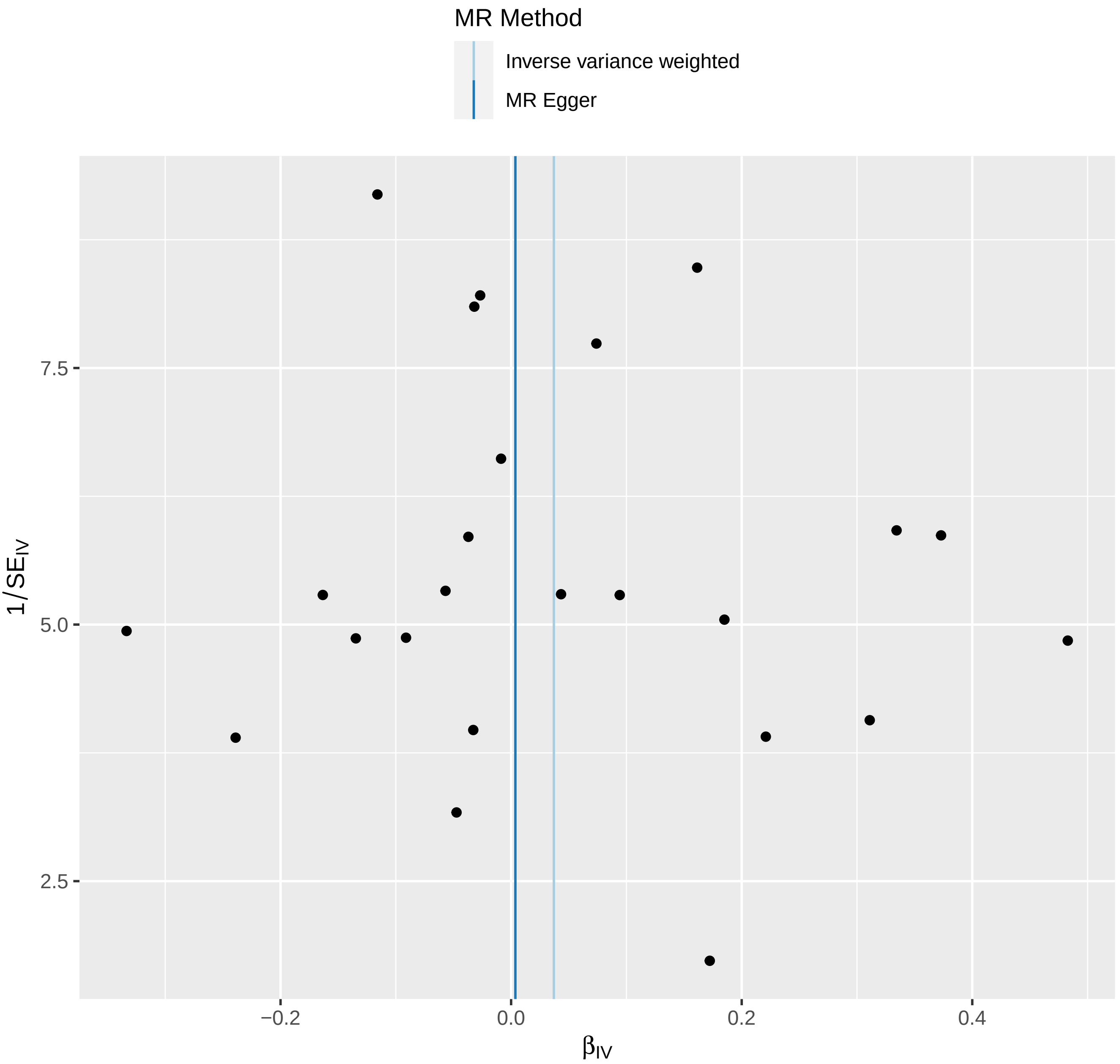

Batch 3−336 : Calcyclin−binding protein on Diabetic nephropathy (more control exclusions)
with a threshold of 1e−05

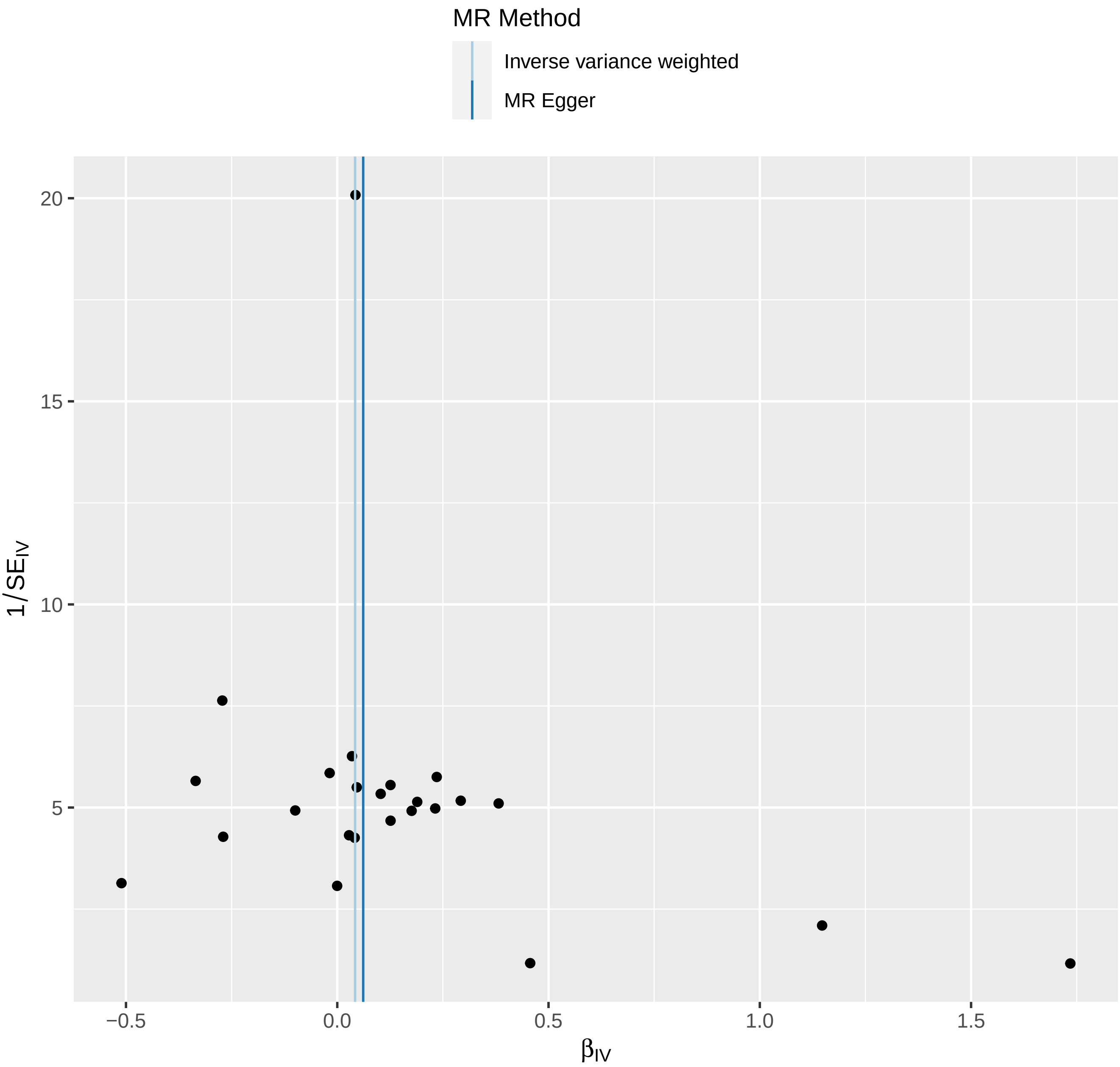

Batch 3−337 : Cell adhesion molecule 1 on Diabetic nephropathy (more control exclusions)
with a threshold of 1e−05

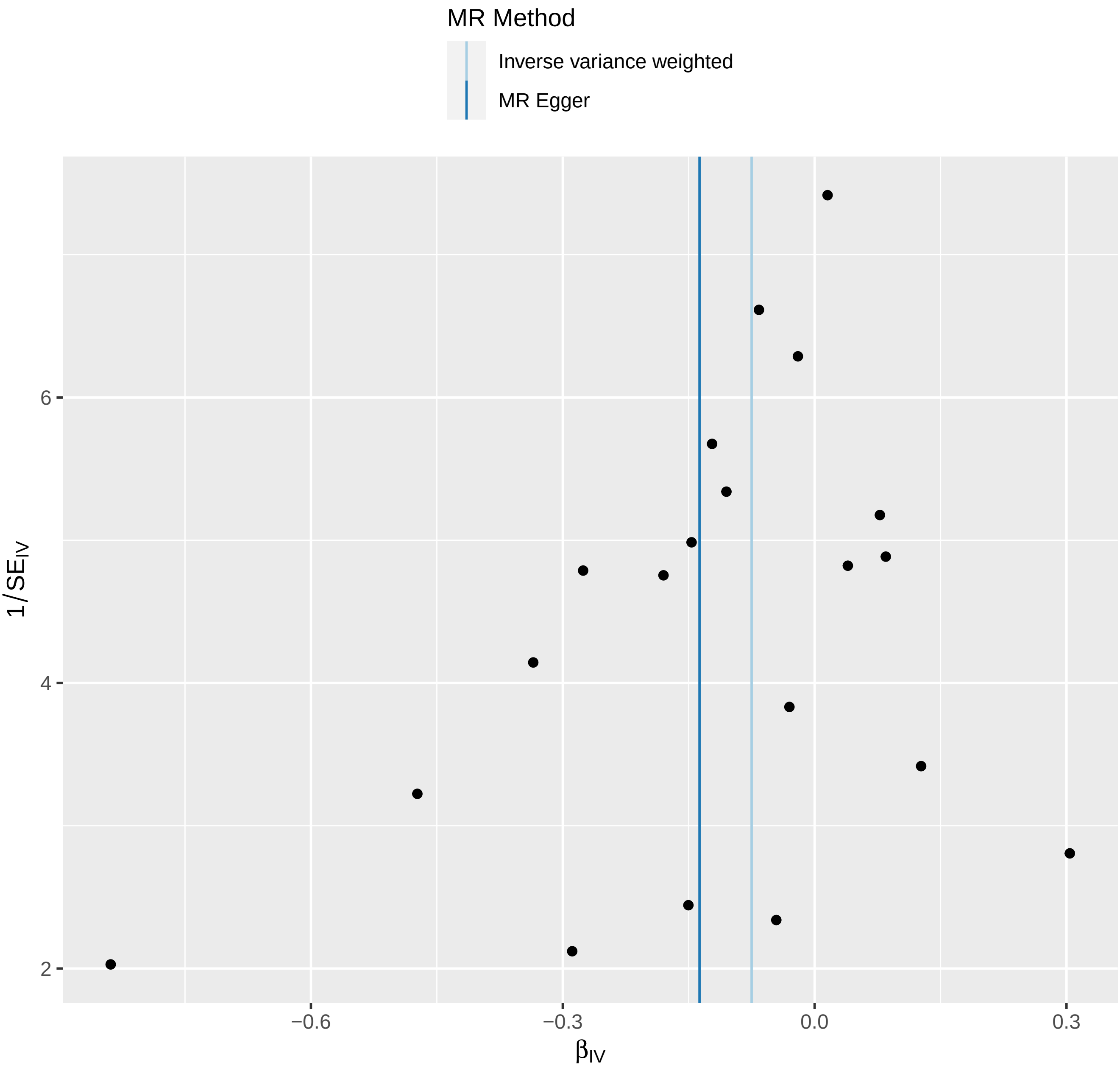

Batch 3−338 : Cell adhesion molecule 3 on Diabetic nephropathy (more control exclusions) with a threshold of 1e−05

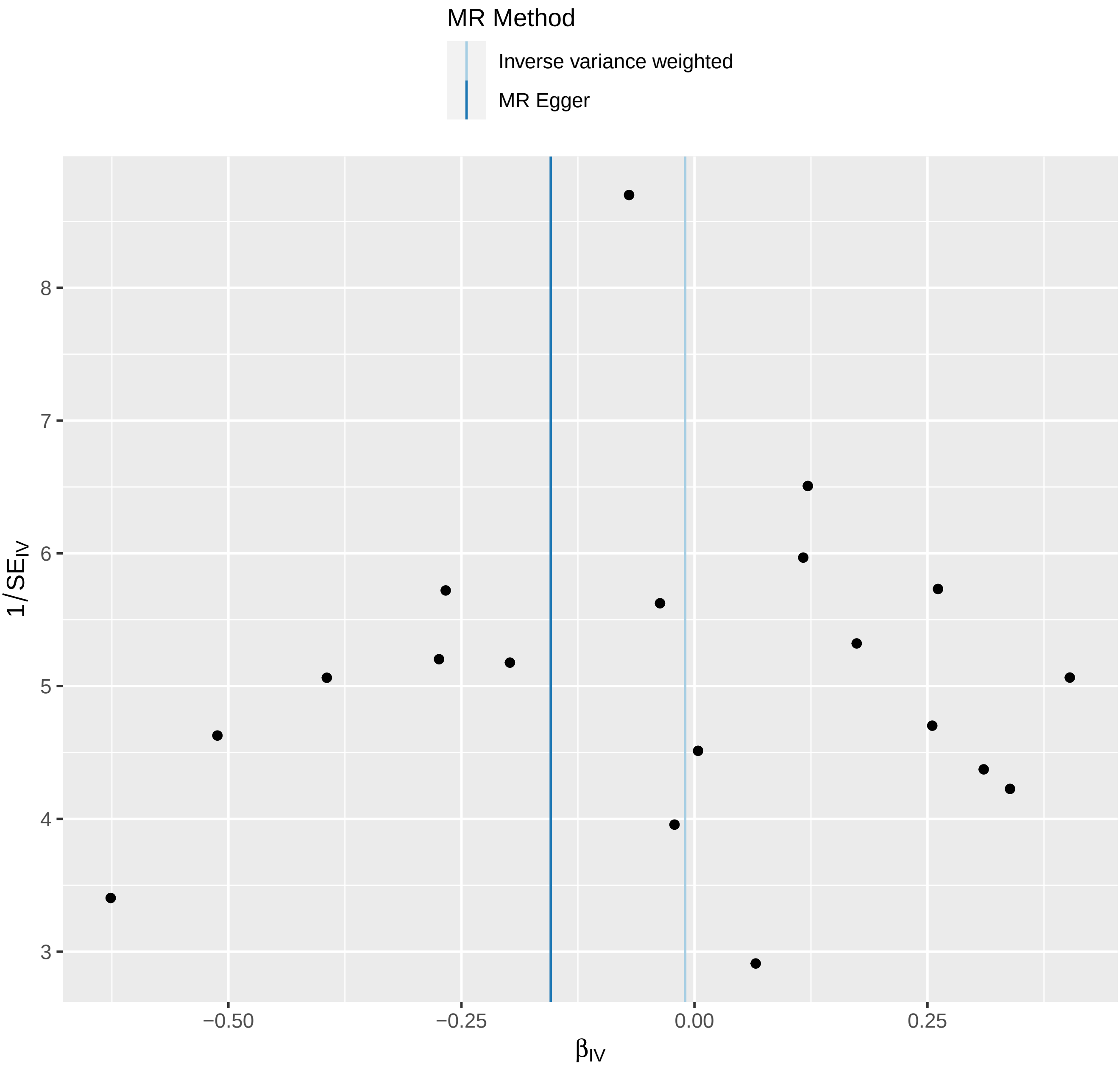

Batch 3−339 : Cell adhesion molecule 4 on Diabetic nephropathy (more control exclusions)
with a threshold of 1e−05

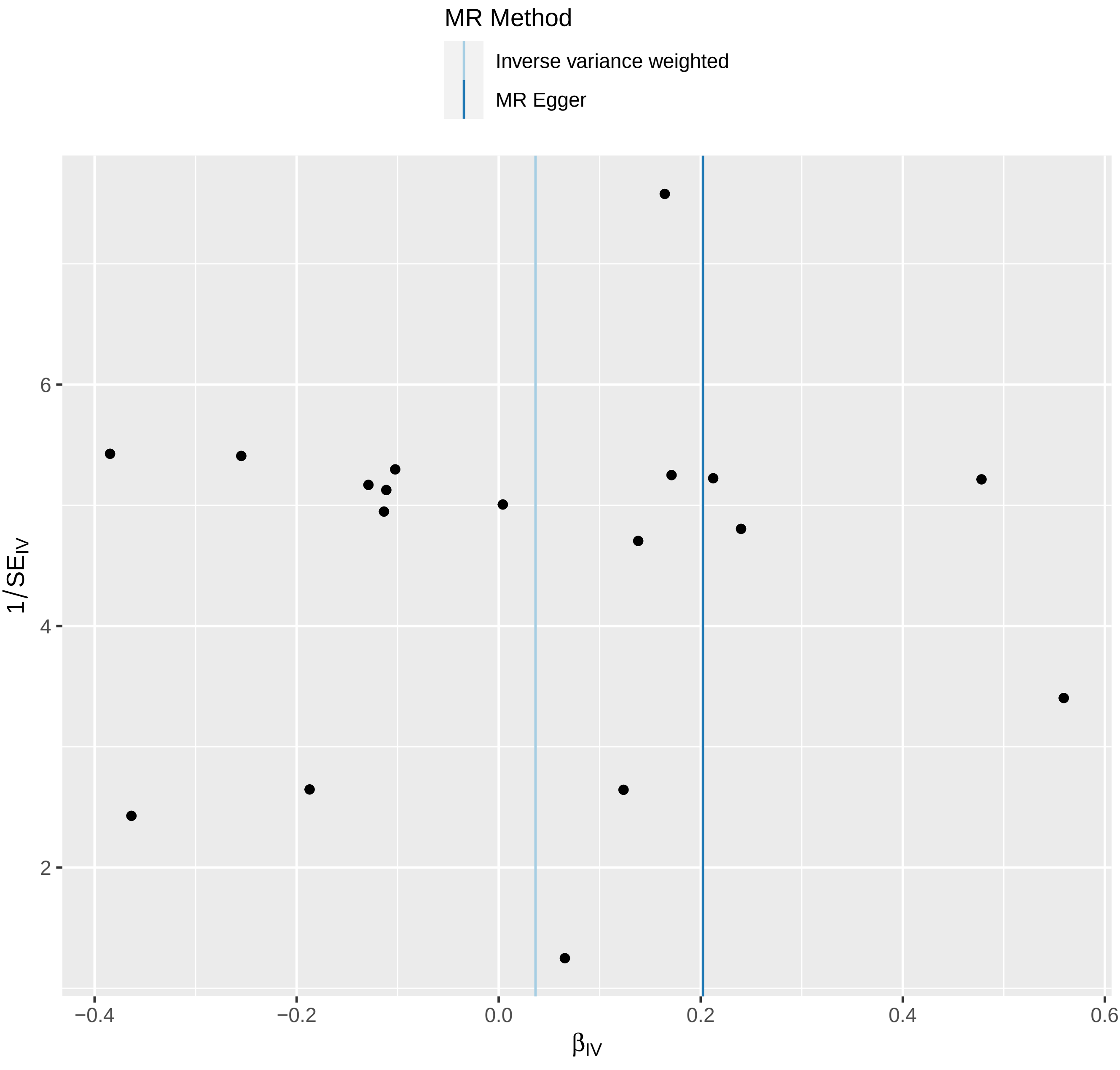

Batch 3−340 : Calbindin on Diabetic nephropathy (more control exclusions)
with a threshold of 1e−05

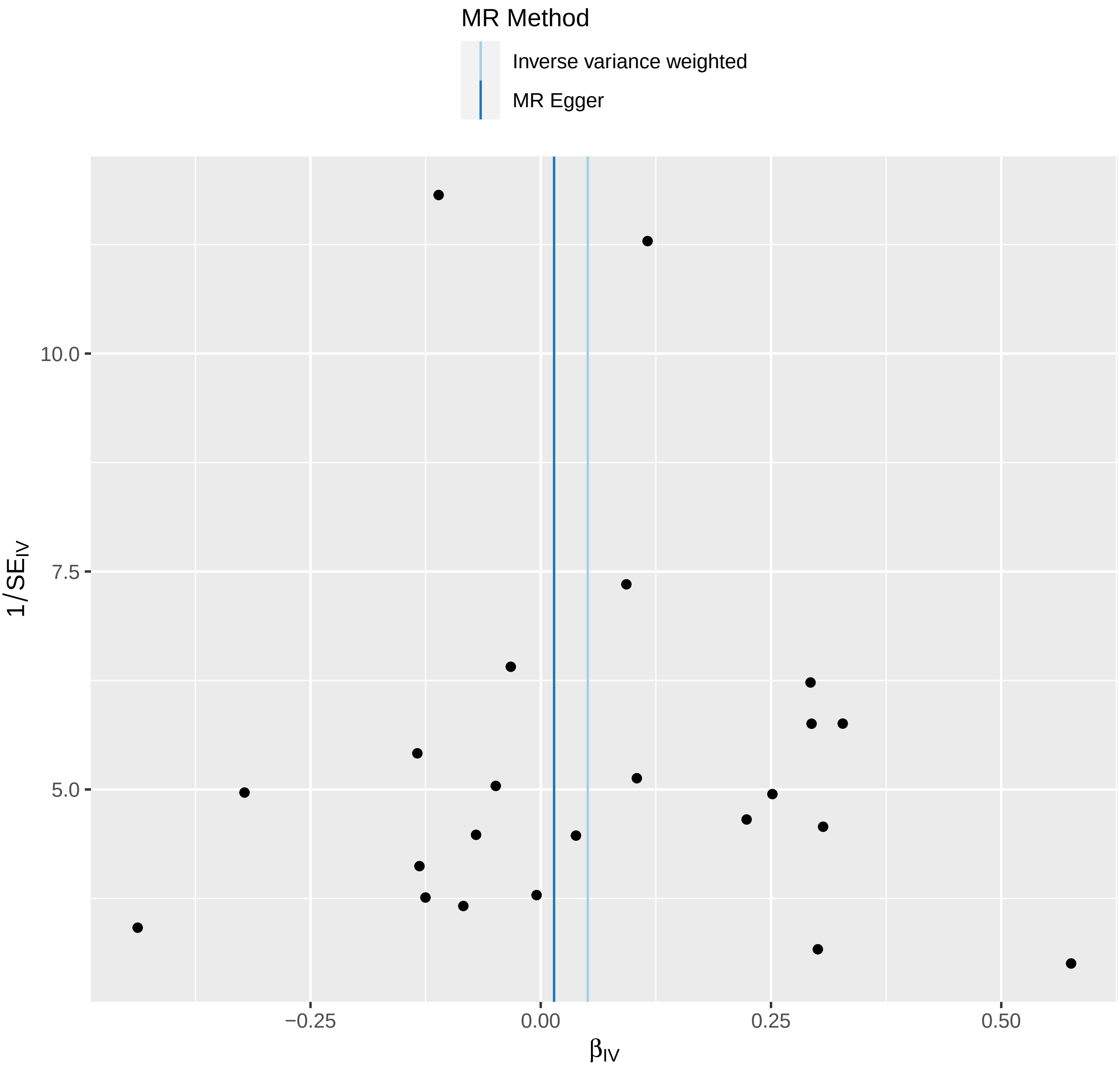

Batch 3−341 : Procalcitonin on Diabetic nephropathy (more control exclusions)
with a threshold of 1e−05

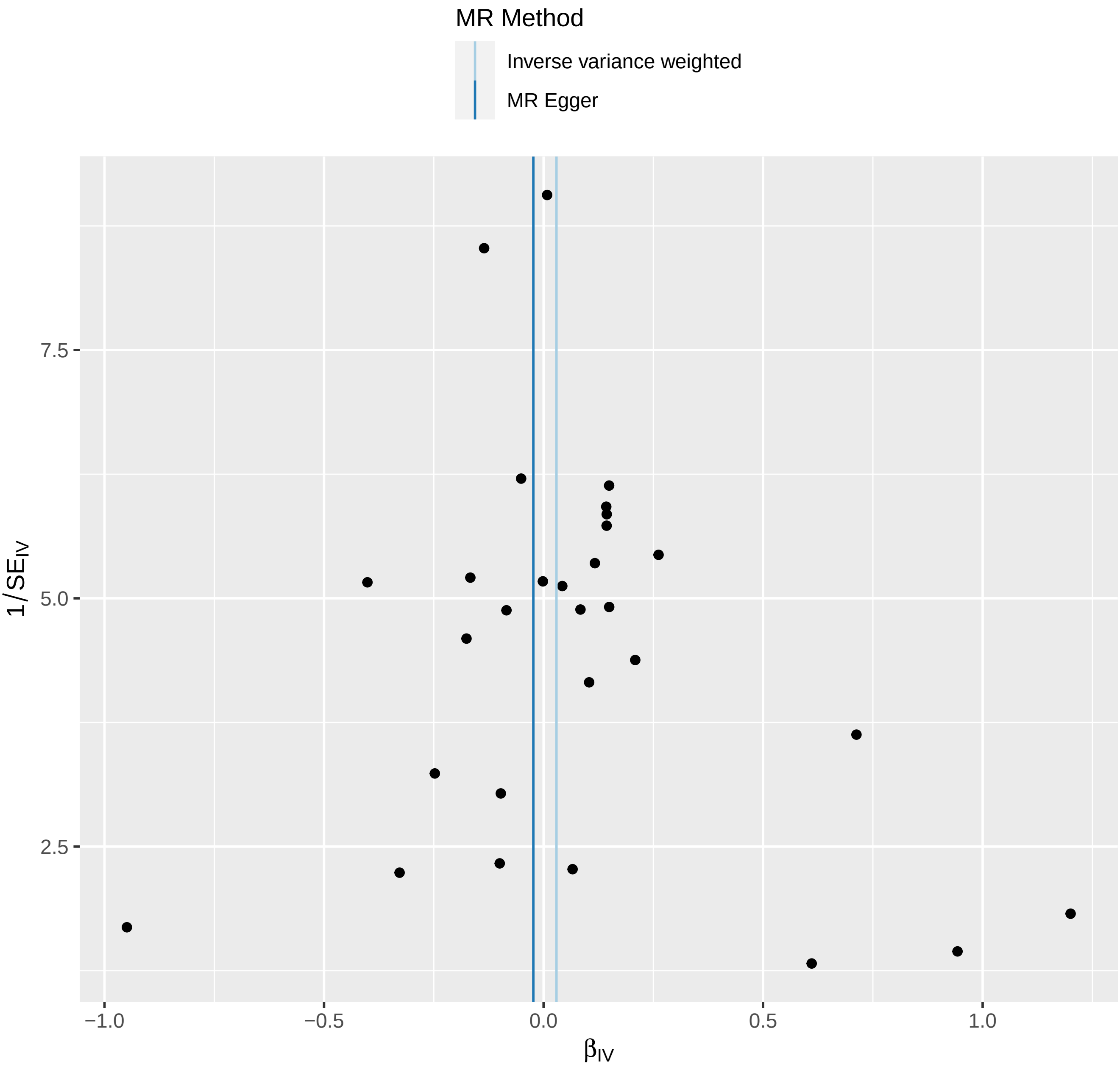

Batch 3−342 : Calcium−binding and coiled−coil domain−containing protein 2 on Diabetic nephropathy (more control exclusions) with a threshold of 1e−05

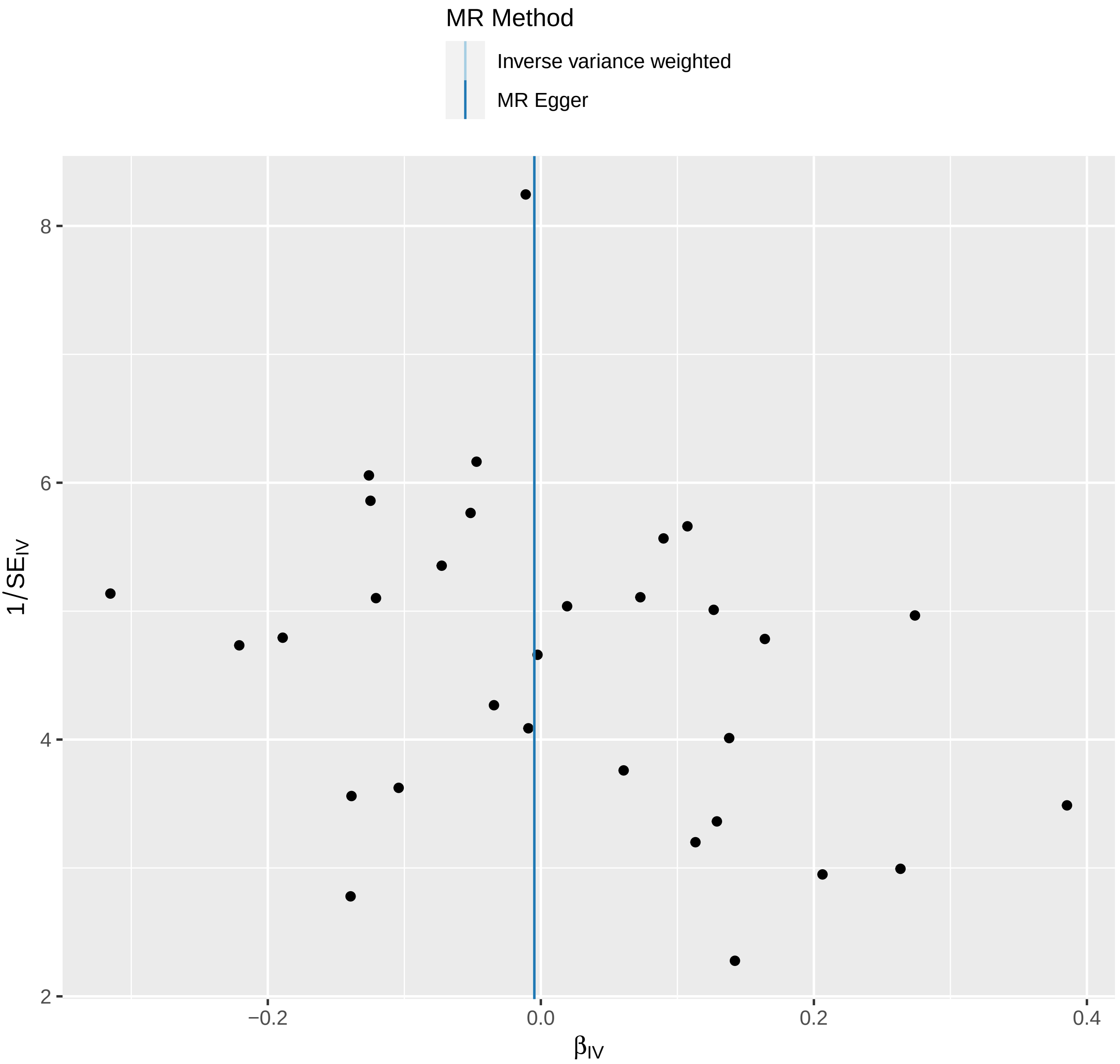

Batch 3−343 : Calcium−binding protein 8 on Diabetic nephropathy (more control exclusions)
with a threshold of 1e−05

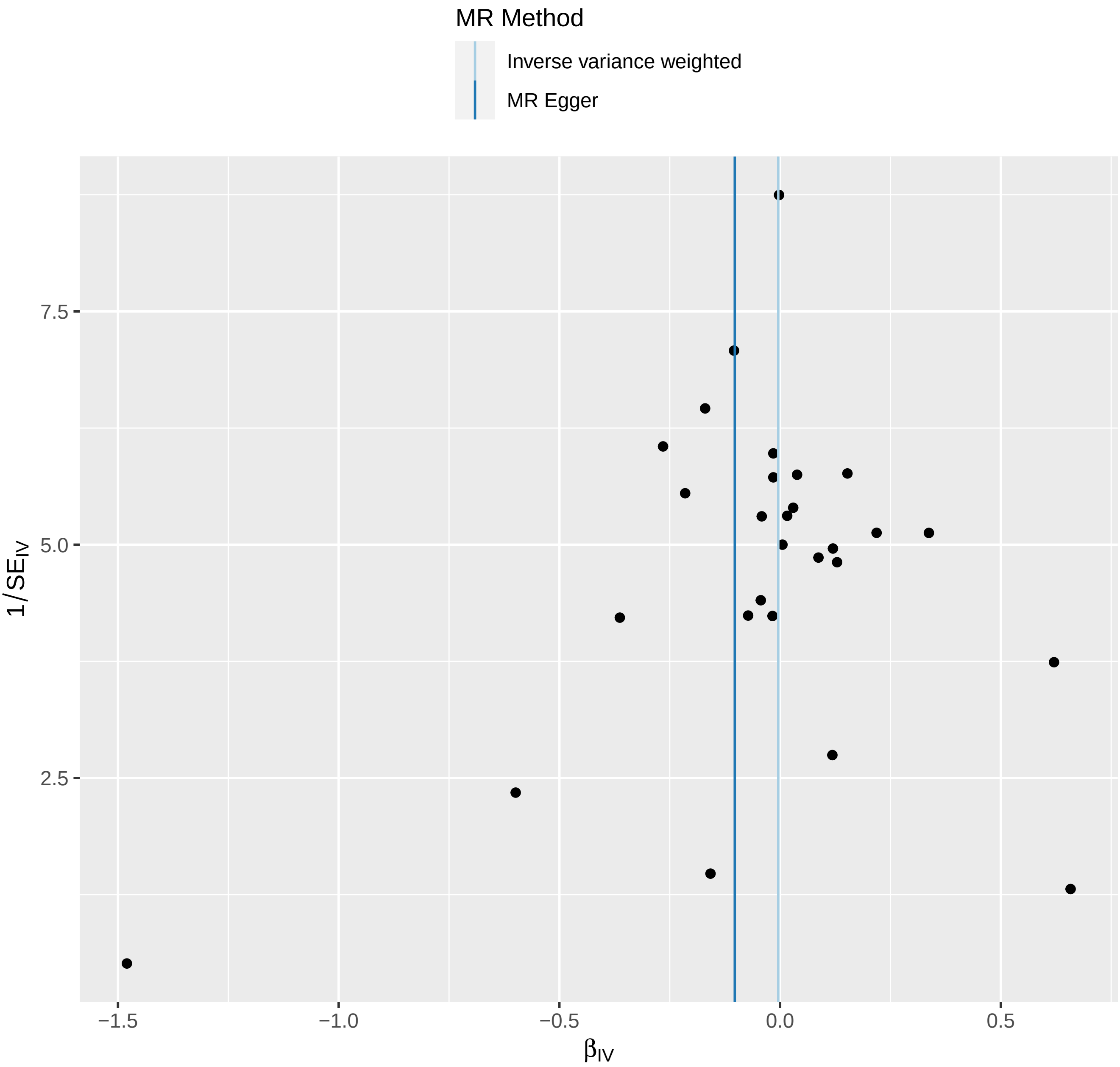

Batch 3−344 : Calreticulin on Diabetic nephropathy (more control exclusions)
with a threshold of 1e−05

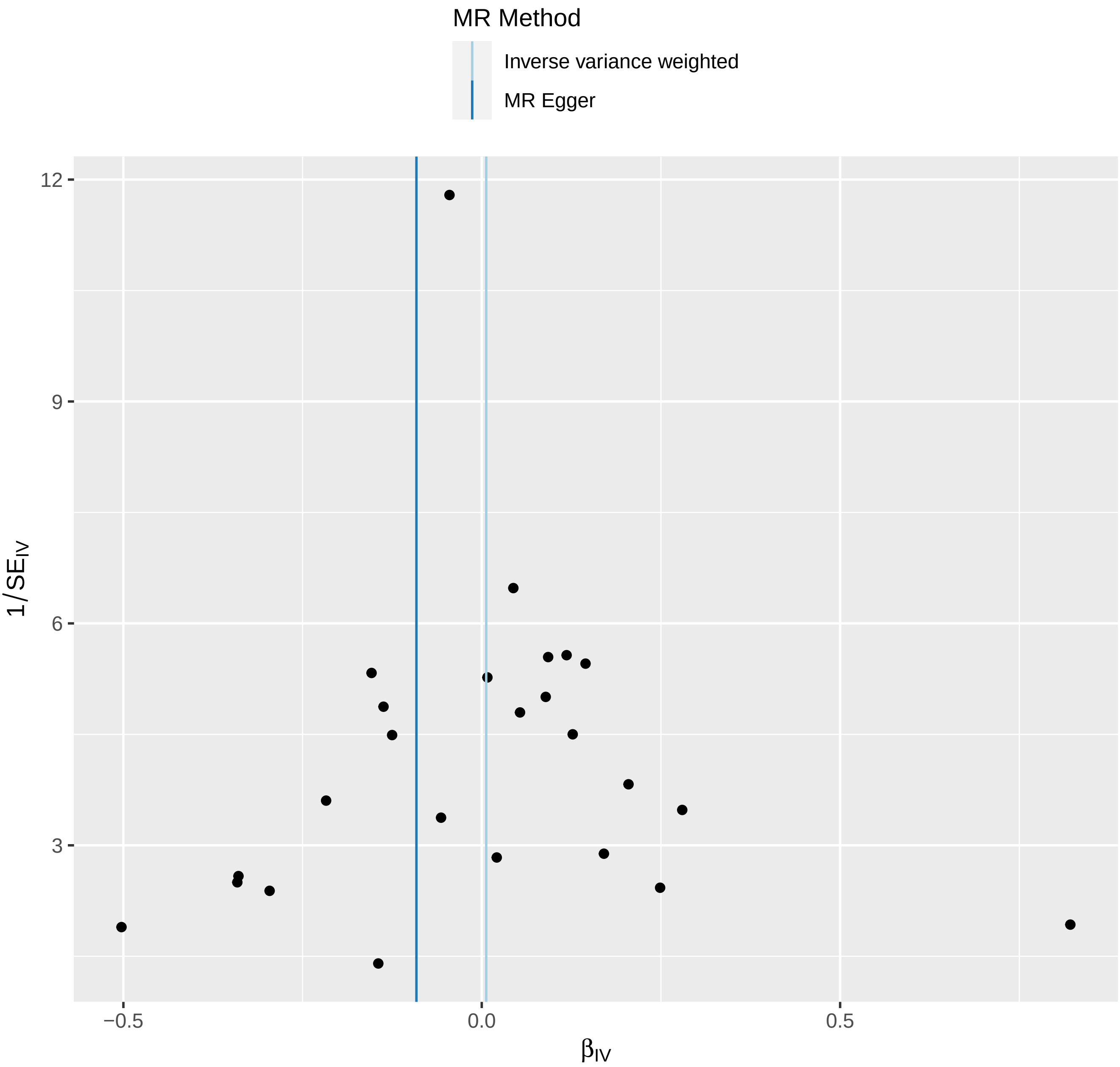

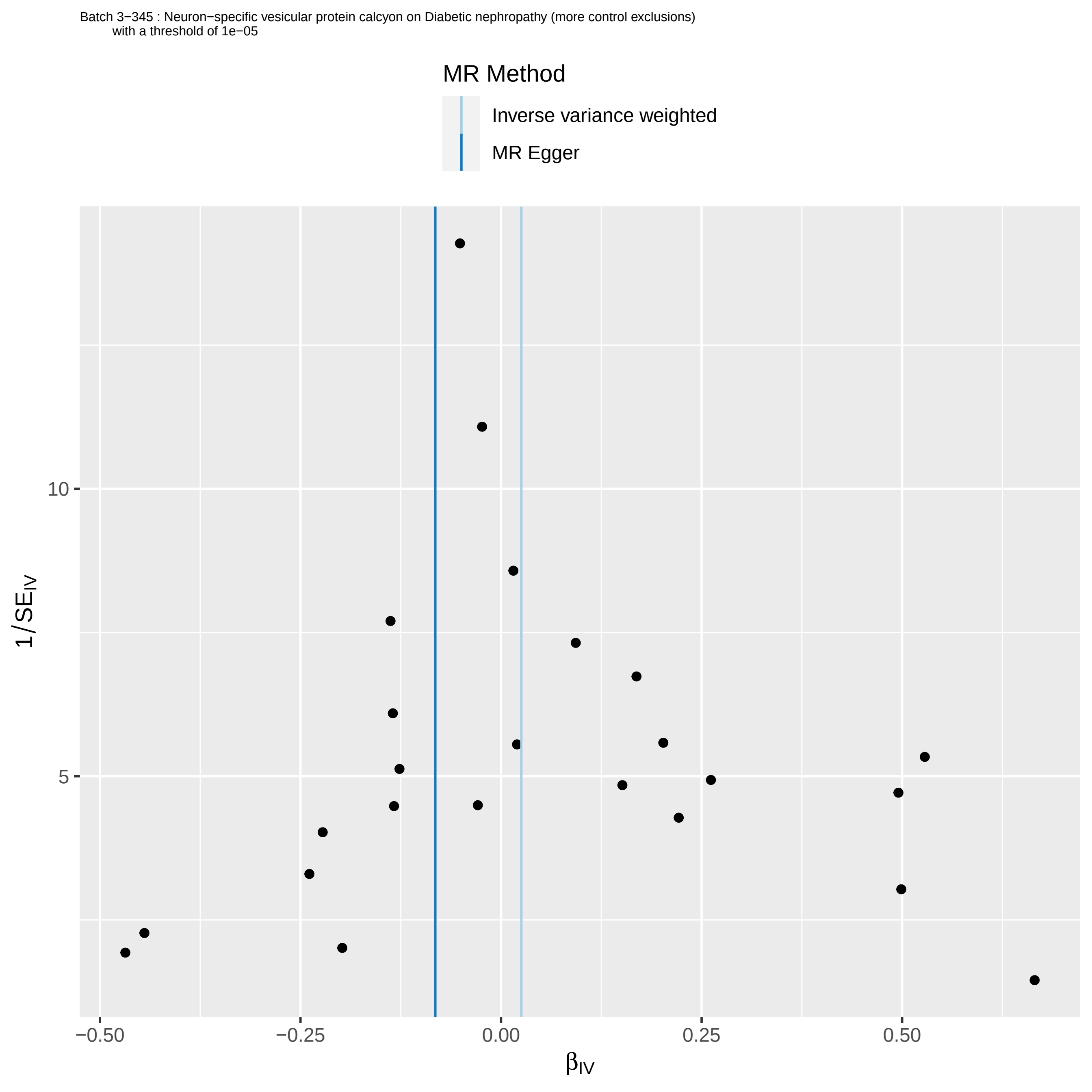
Batch 3−345 : Neuron−specific vesicular protein calcyon on Diabetic nephropathy (more control exclusions)
with a threshold of 1e−05
MR Method
Inverse variance weighted
MR Egger
1/SE$_{IV}$
10
5
−0.50
−0.25
0.00
0.25
0.50
β$_{IV}$

Batch 3−346 : Calcium/calmodulin−dependent protein kinase type 1 on Diabetic nephropathy (more control exclusions)
with a threshold of 1e−05

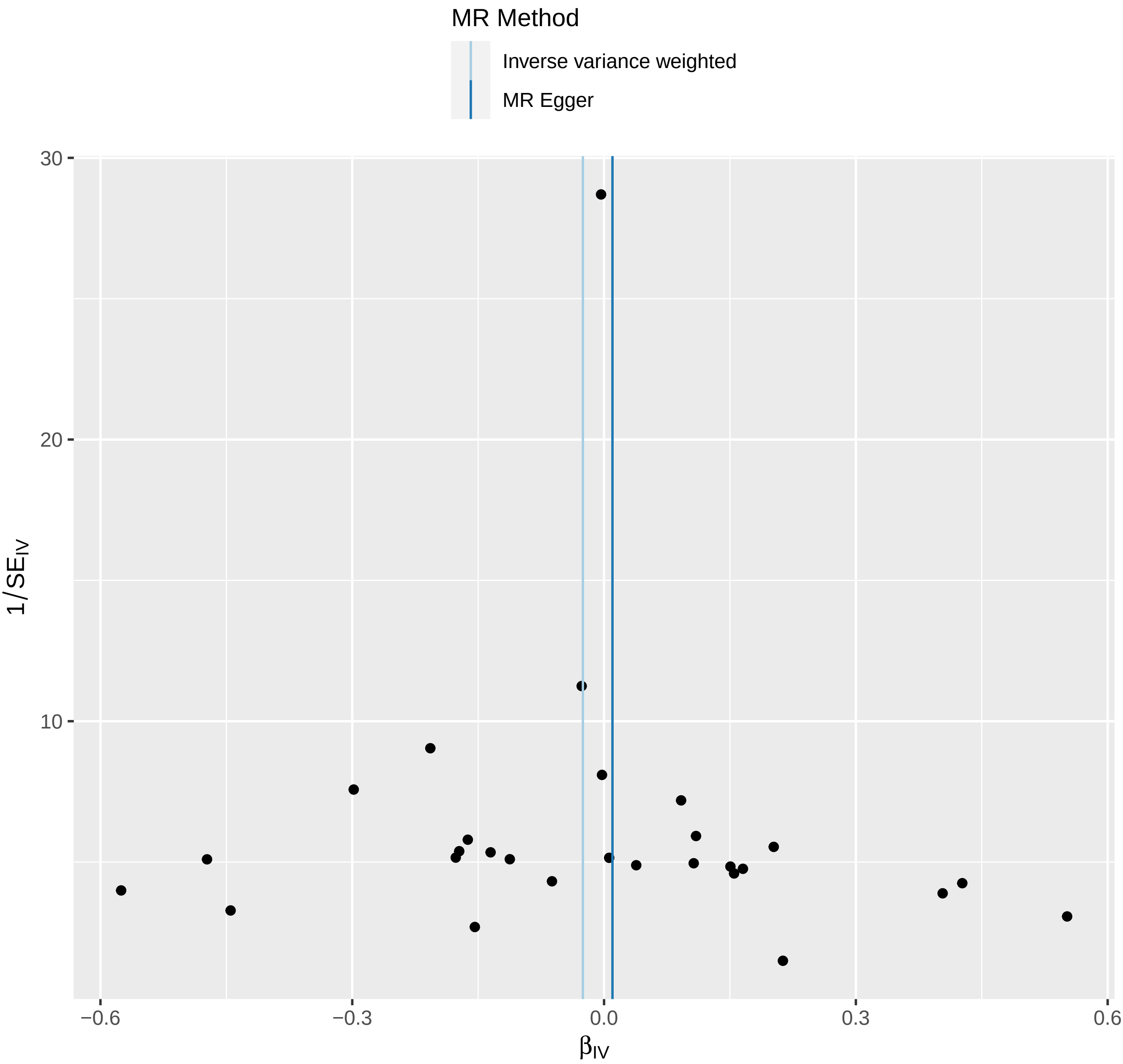

Batch 3−347 : Calcium/calmodulin−dependent protein kinase type 1D on Diabetic nephropathy (more control exclusions)
with a threshold of 1e−05

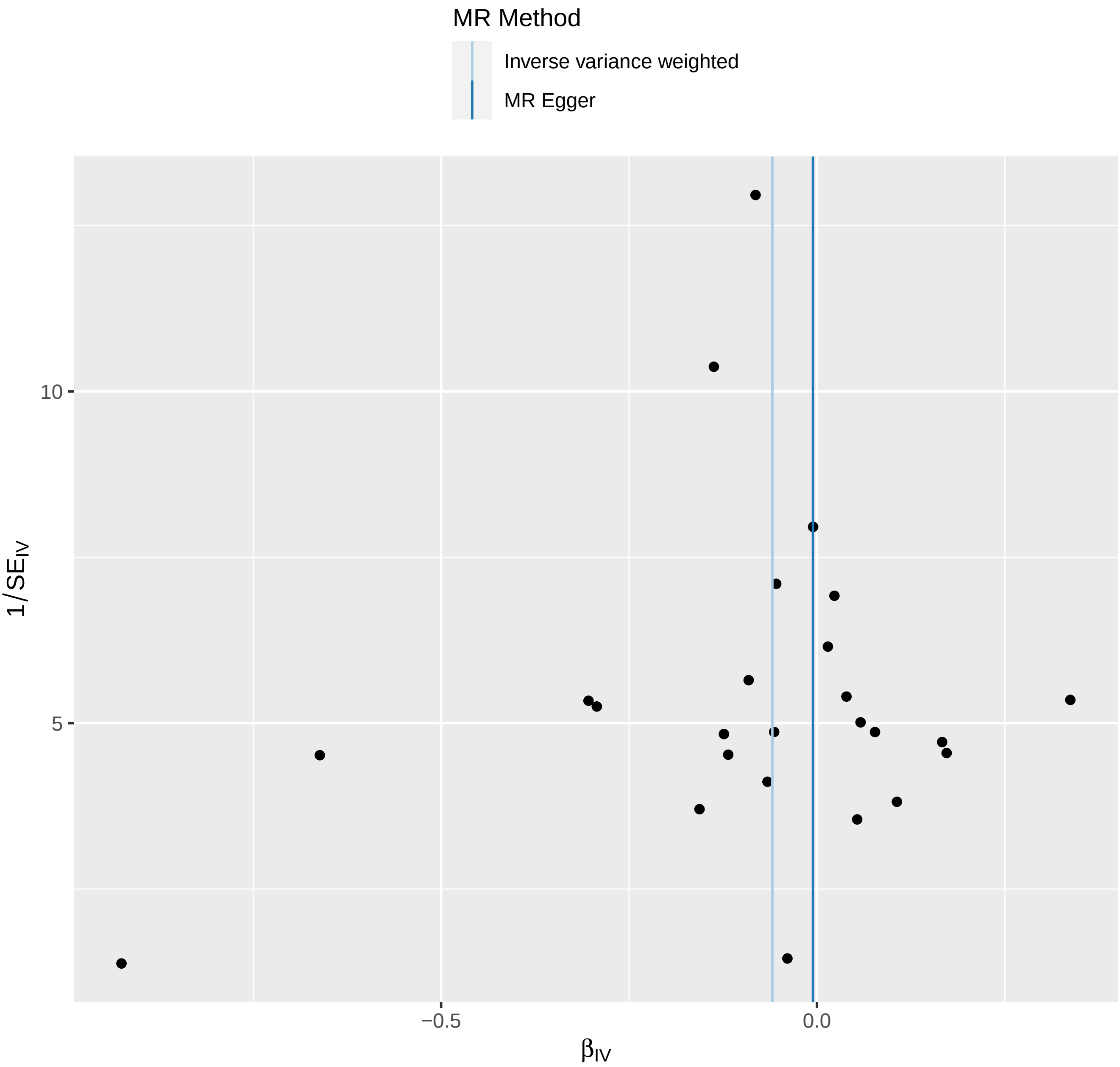

Batch 3−348 : Calcium/calmodulin−dependent protein kinase type II subunit alpha on Diabetic nephropathy (more control exclusions)
with a threshold of 1e−05

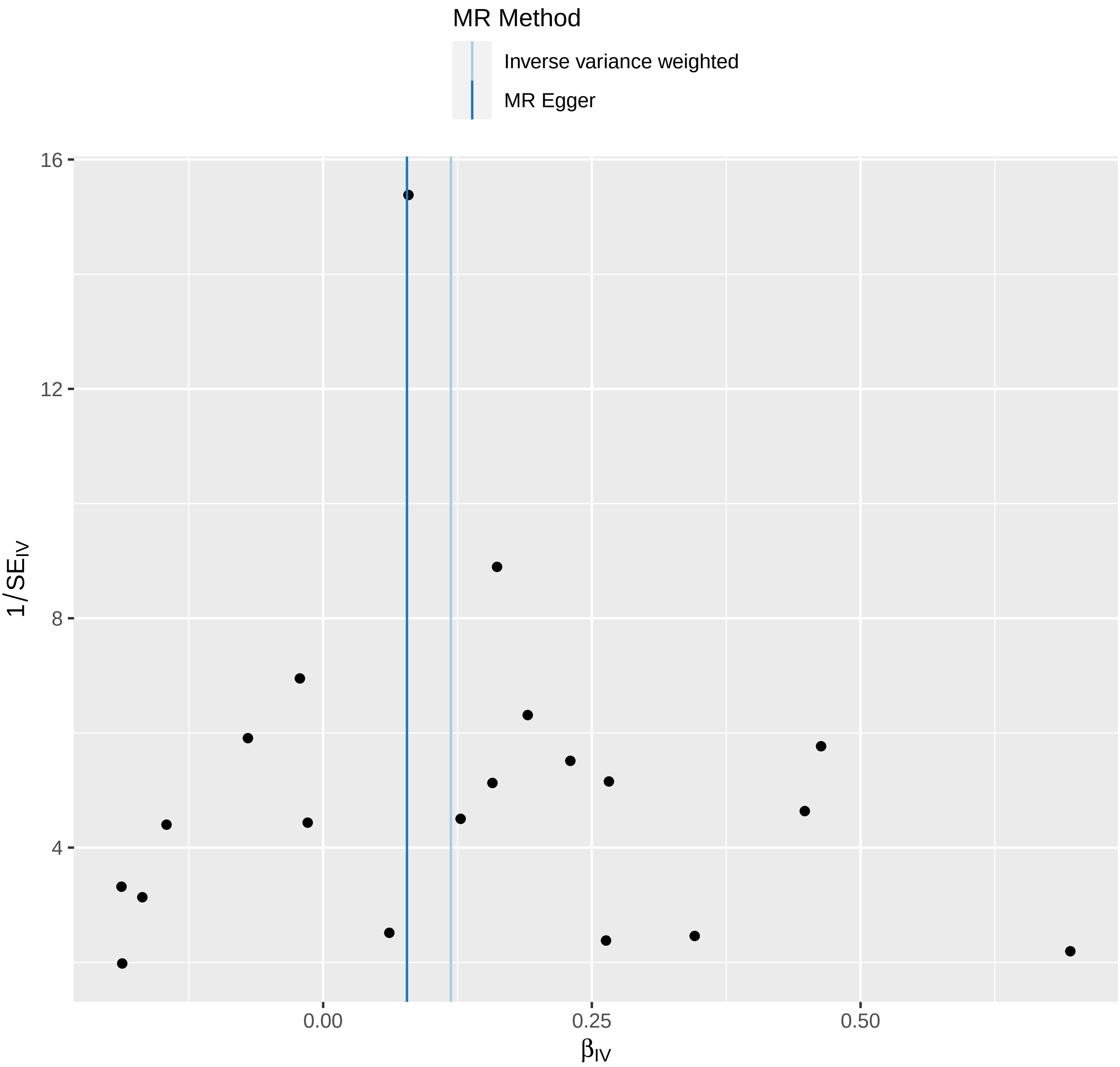

Batch 3−349 : Calcium/calmodulin−dependent protein kinase type II subunit beta on Diabetic nephropathy (more control exclusions)
with a threshold of 1e−05

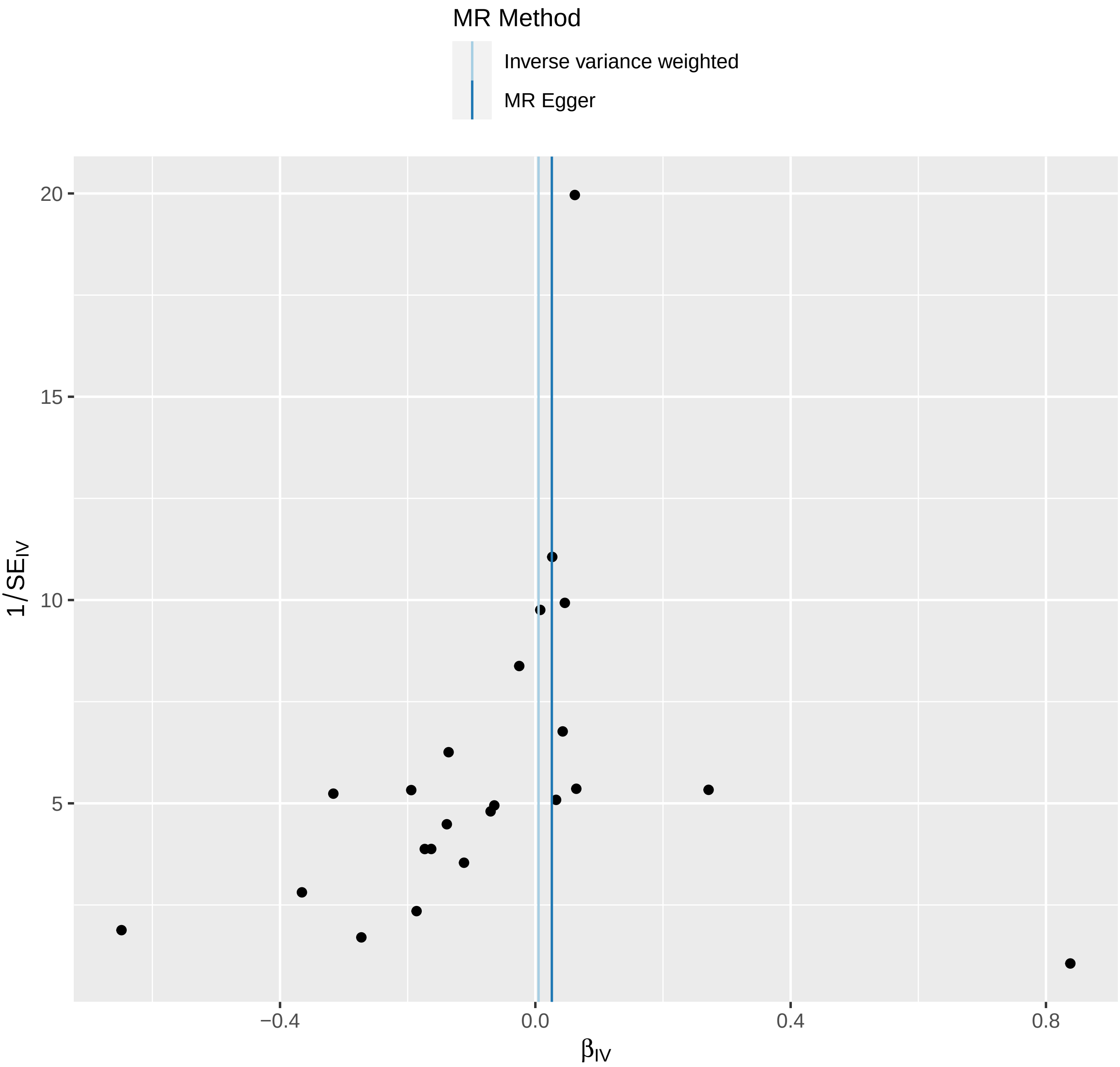

Batch 3−350 : Calcium/calmodulin−dependent protein kinase kinase 1 on Diabetic nephropathy (more control exclusions)
with a threshold of 1e−05

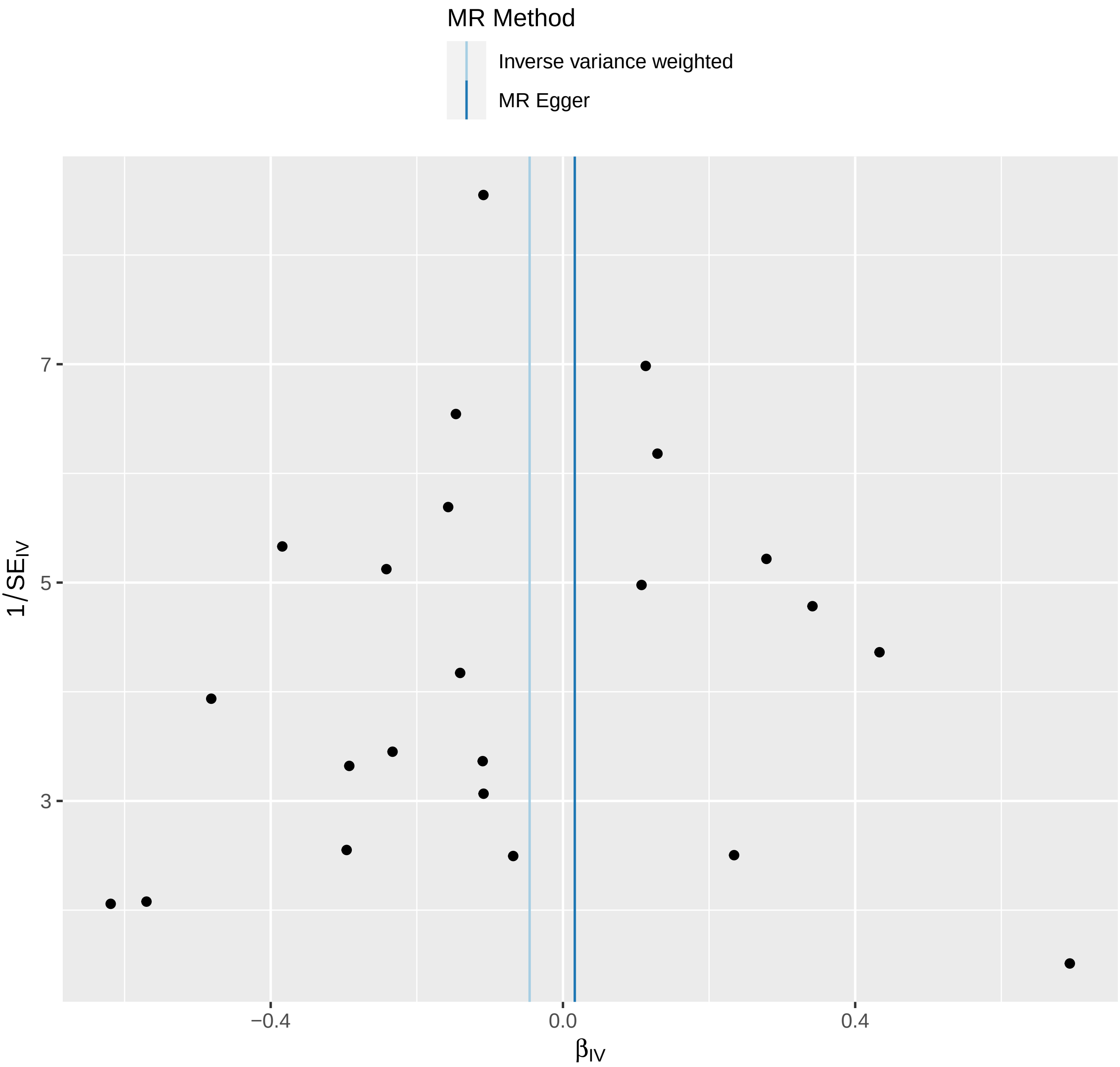

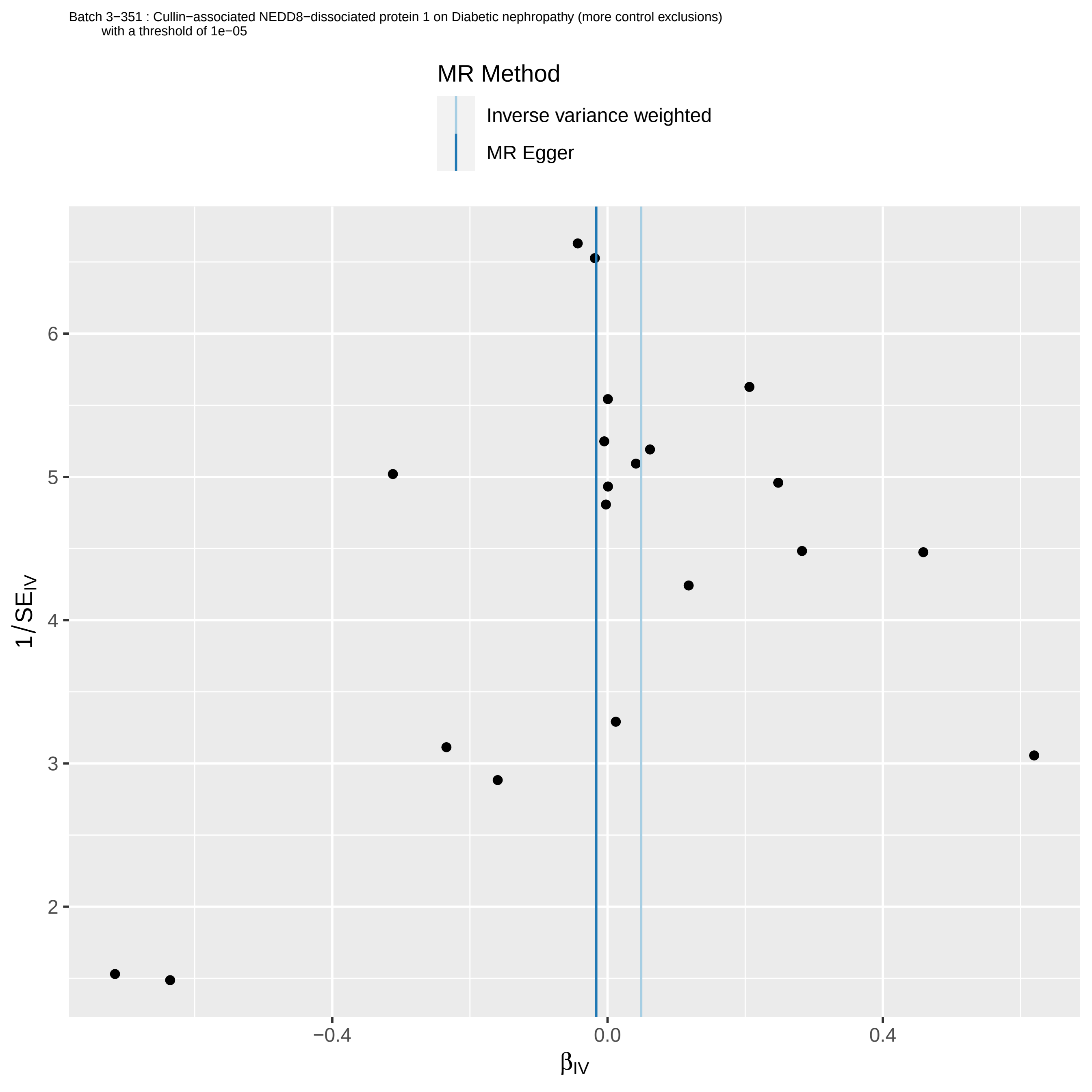

Batch 3−351 : Cullin−associated NEDD8−dissociated protein 1 on Diabetic nephropathy (more control exclusions)
with a threshold of 1e−05
MR Method
Inverse variance weighted
MR Egger
6
5
4
3
2
1/SE_IV
−0.4
0.0
0.4
β_IV

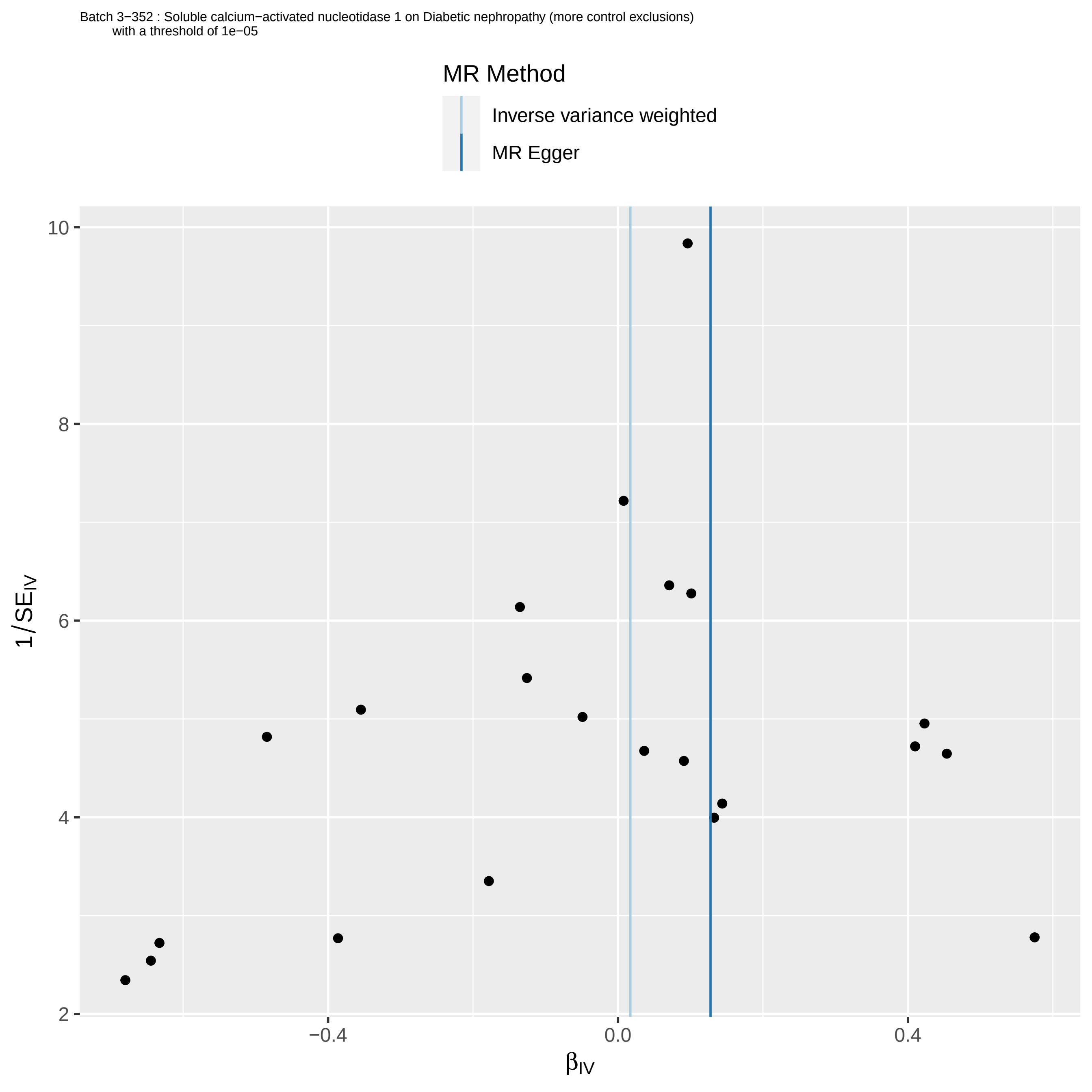
Batch 3−352 : Soluble calcium−activated nucleotidase 1 on Diabetic nephropathy (more control exclusions)
with a threshold of 1e−05
MR Method
Inverse variance weighted
MR Egger
10
8
6
4
2
−0.4
0.0
0.4
1/SE_IV
β_IV

Batch 3−353 : Calnexin on Diabetic nephropathy (more control exclusions)
with a threshold of 1e−05

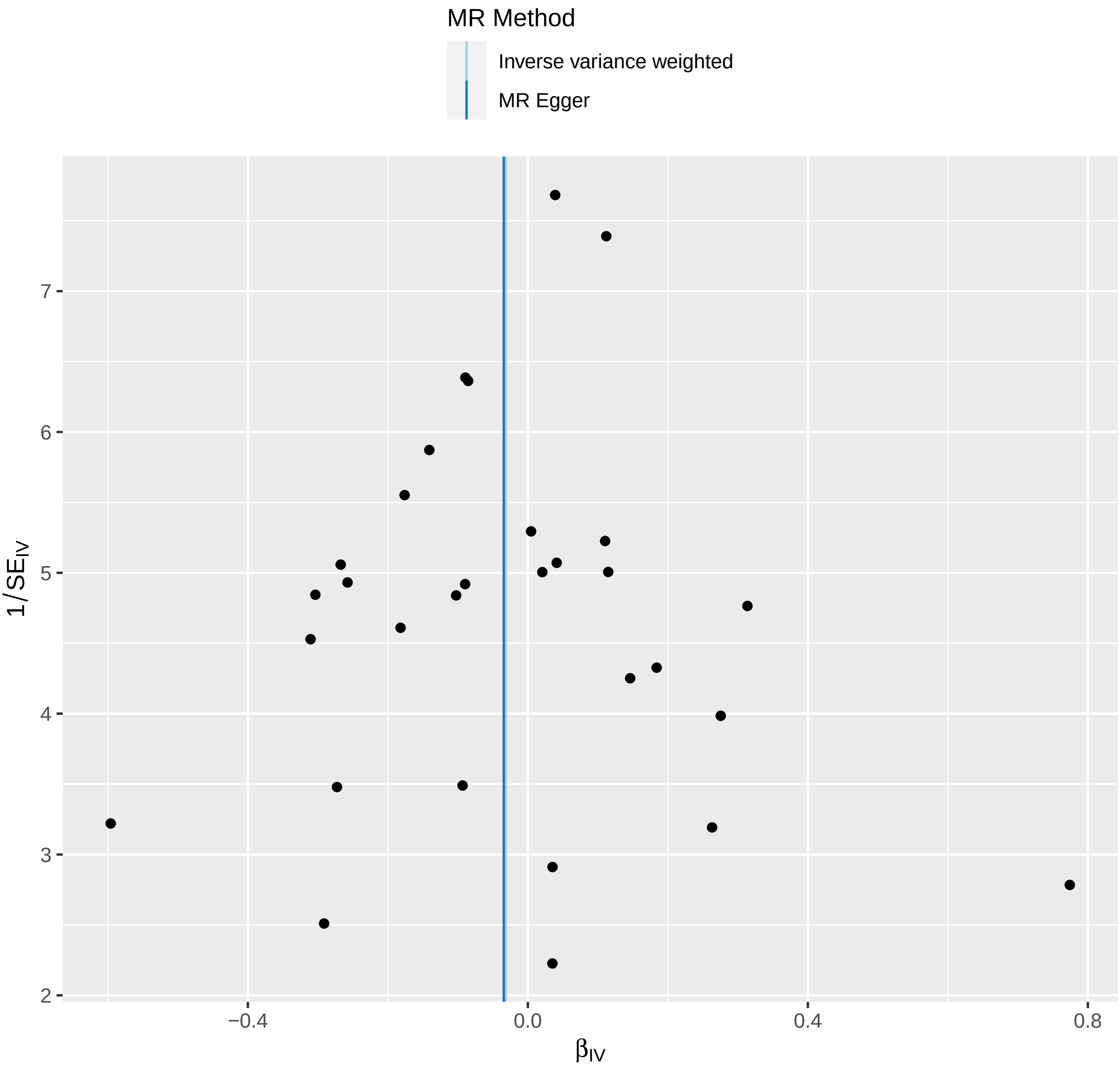

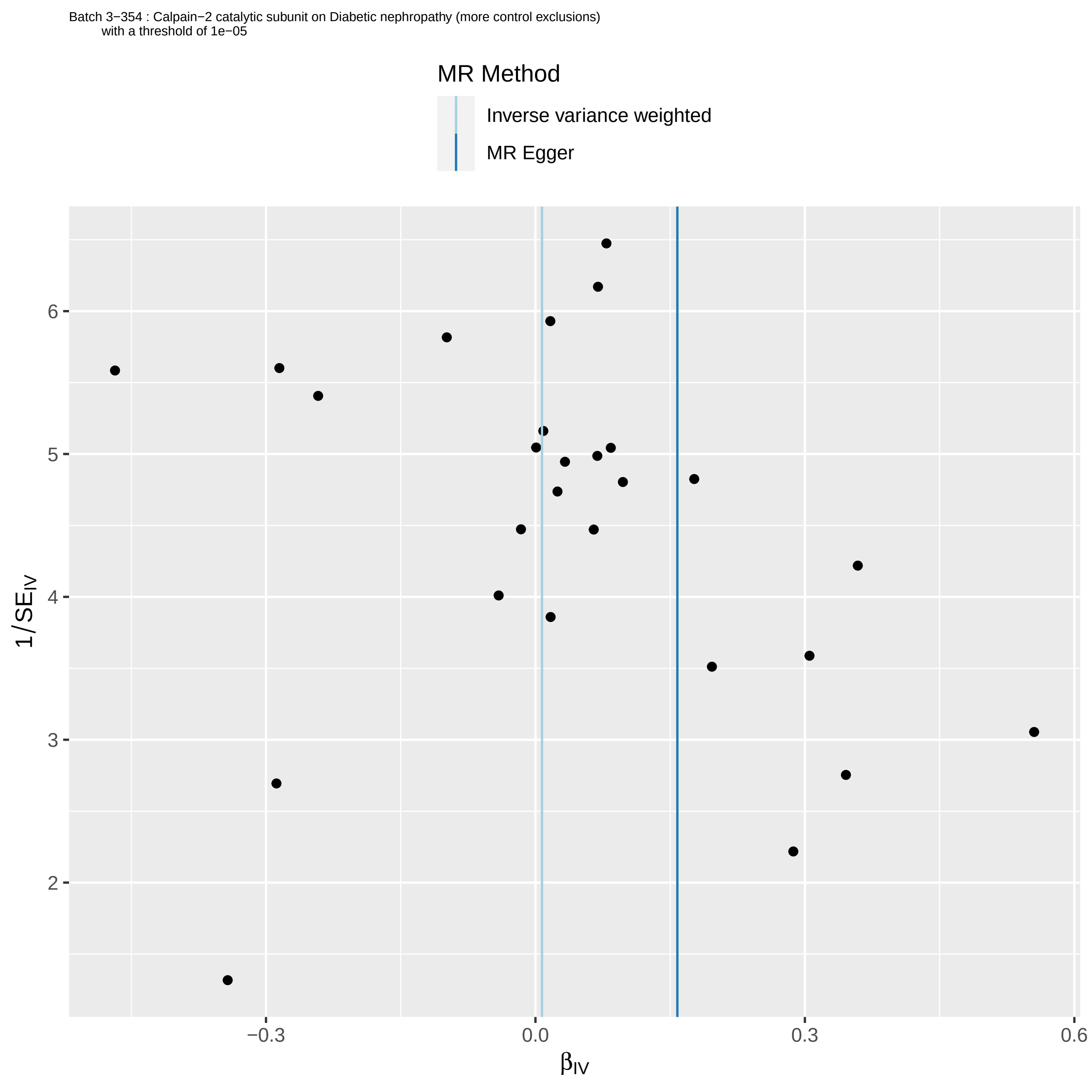
Batch 3−354 : Calpain−2 catalytic subunit on Diabetic nephropathy (more control exclusions)
with a threshold of 1e−05
MR Method
Inverse variance weighted
MR Egger
1/SE$_{IV}$
β_{IV}
−0.3
0.0
0.3
0.6
2
3
4
5
6

Batch 3−355 : Calpain−3 on Diabetic nephropathy (more control exclusions)
with a threshold of 1e−05

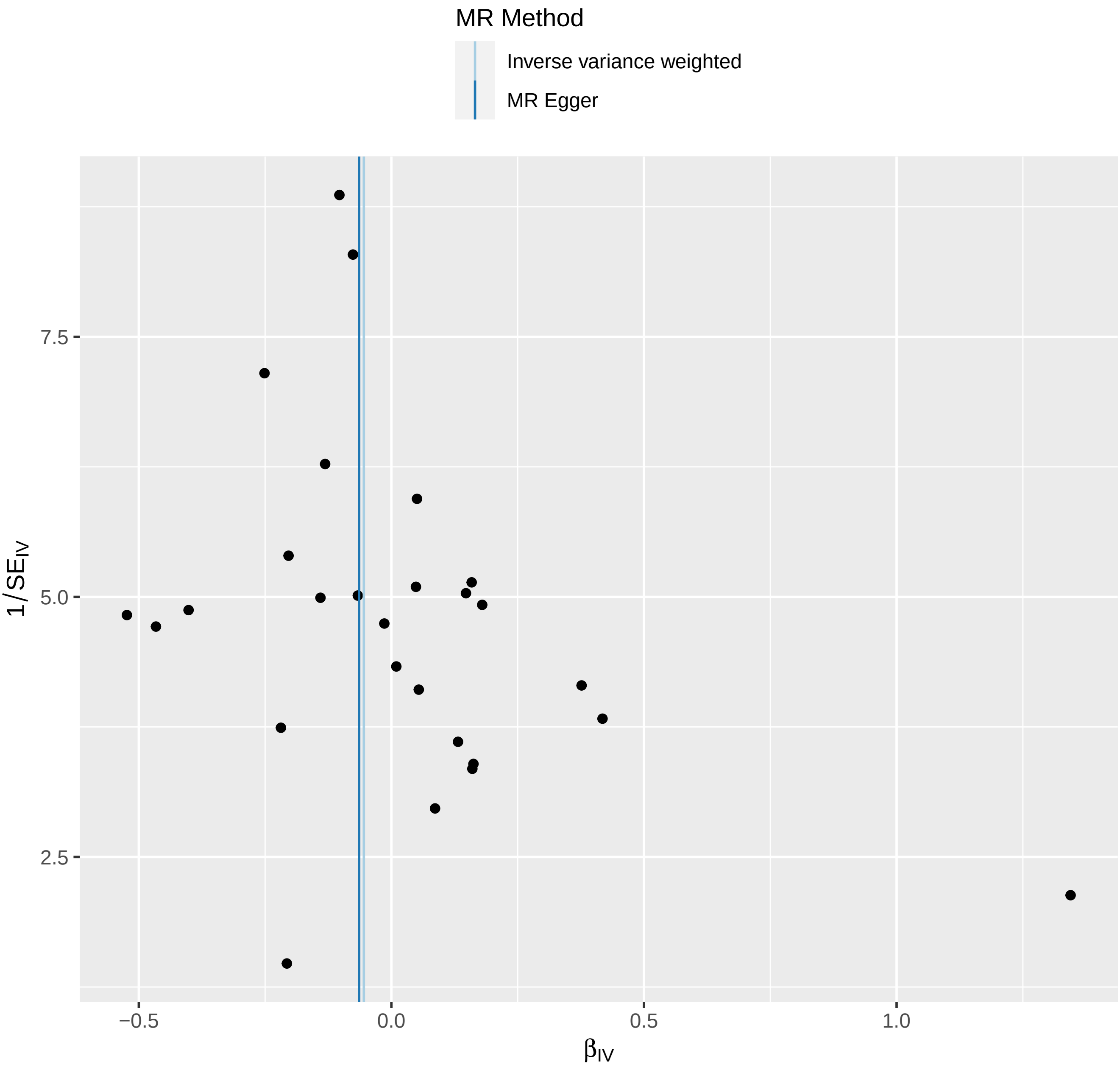

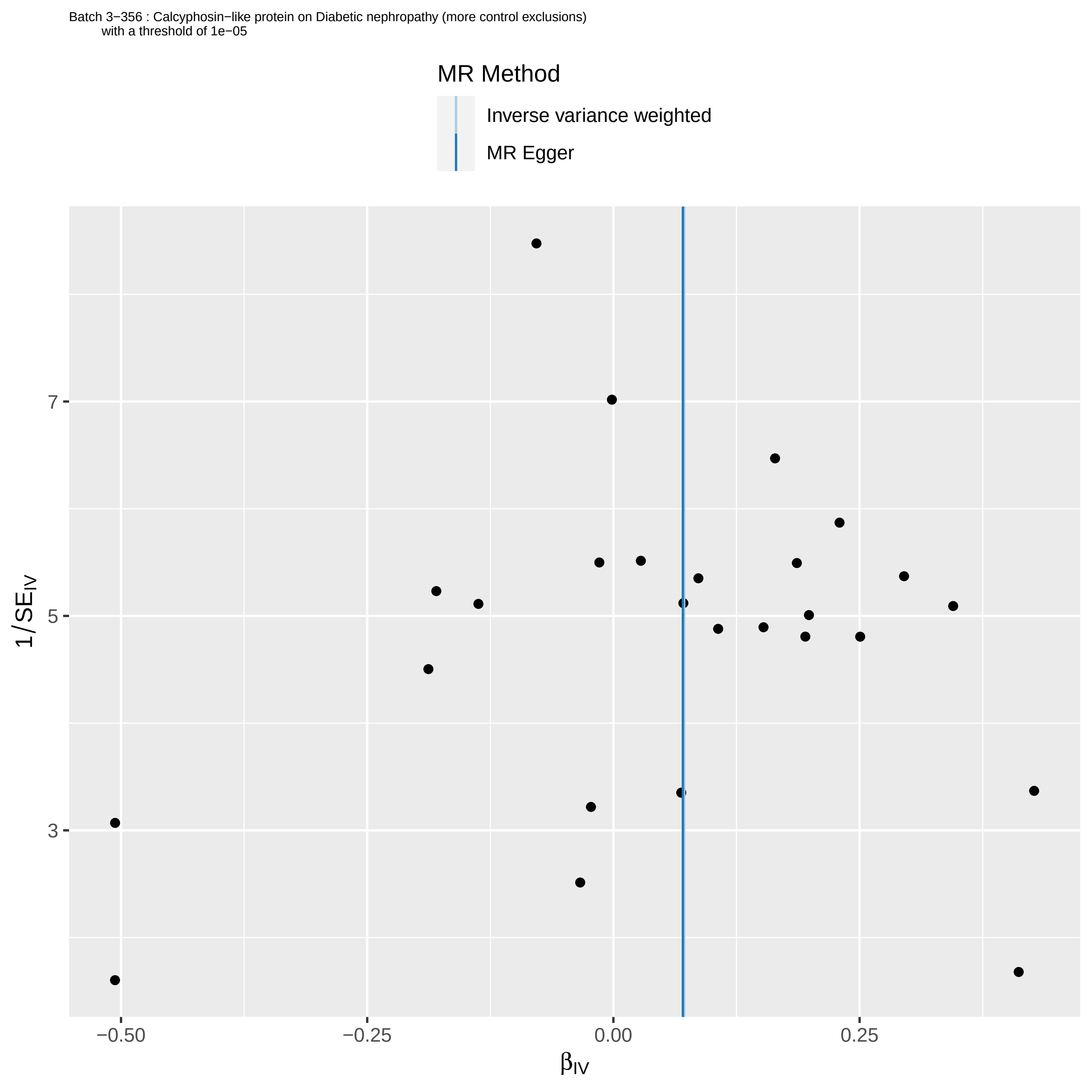
Batch 3−356 : Calcyphosin−like protein on Diabetic nephropathy (more control exclusions)
with a threshold of 1e−05
MR Method
Inverse variance weighted
MR Egger
7
5
3
1/SE_IV
−0.50
−0.25
0.00
0.25
β_IV

Batch 3−357 : Calcium−regulated heat stable protein 1 on Diabetic nephropathy (more control exclusions)
with a threshold of 1e−05

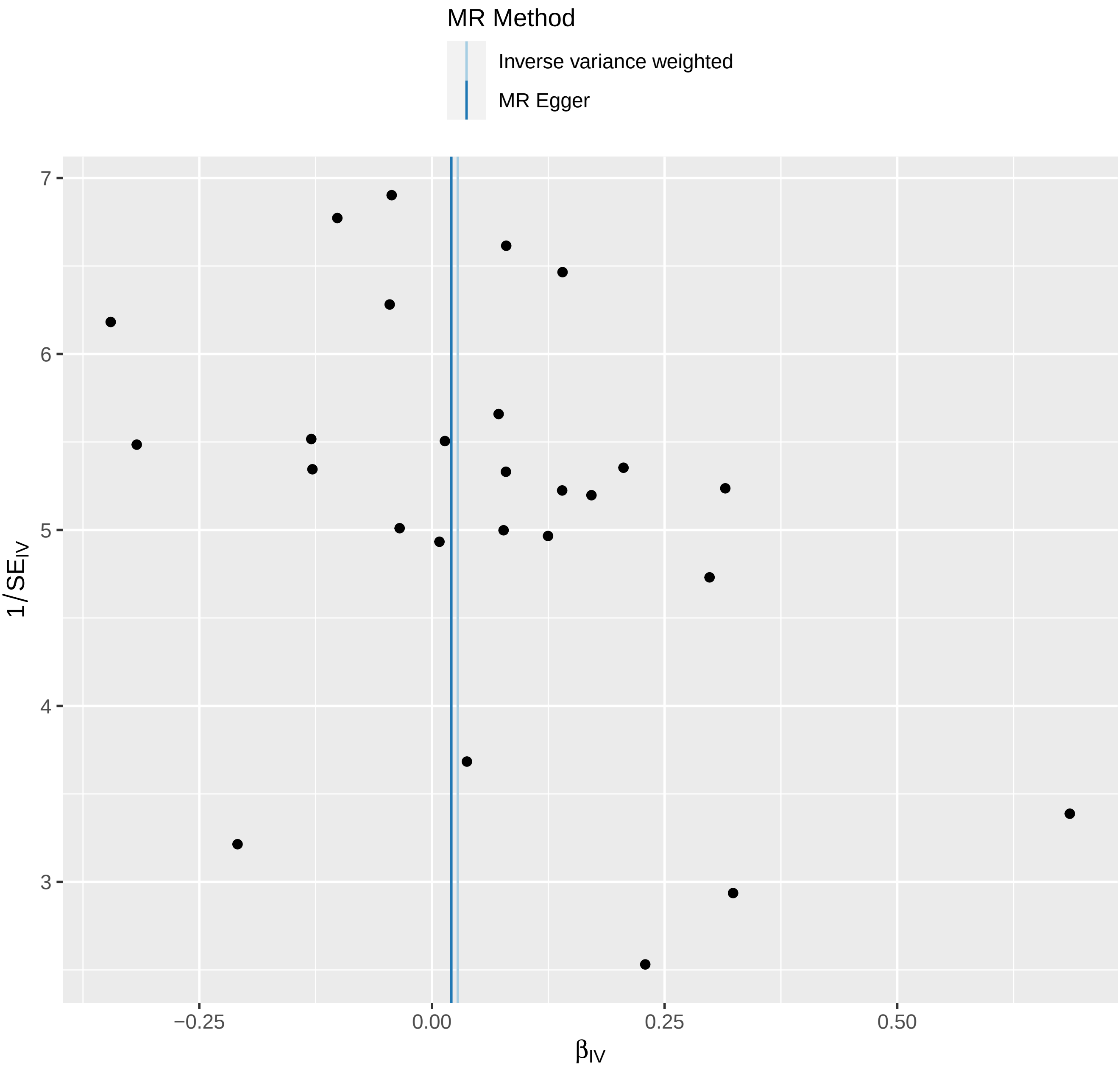

Batch 3−358 : Cysteine−−tRNA ligase, cytoplasmic on Diabetic nephropathy (more control exclusions)
with a threshold of 1e−05

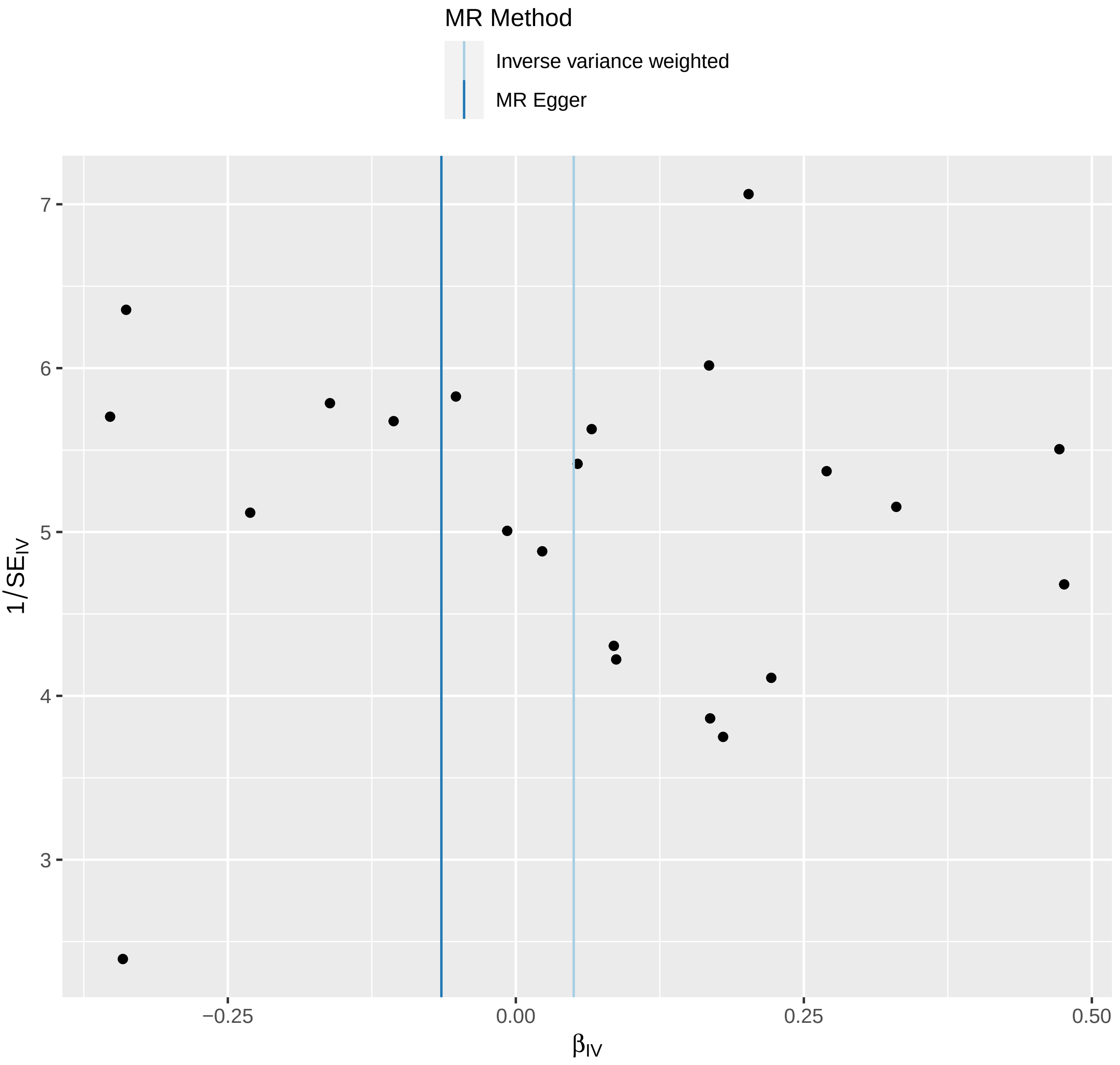

Batch 3−359 : Cocaine− and amphetamine−regulated transcript protein on Diabetic nephropathy (more control exclusions) with a threshold of 1e−05

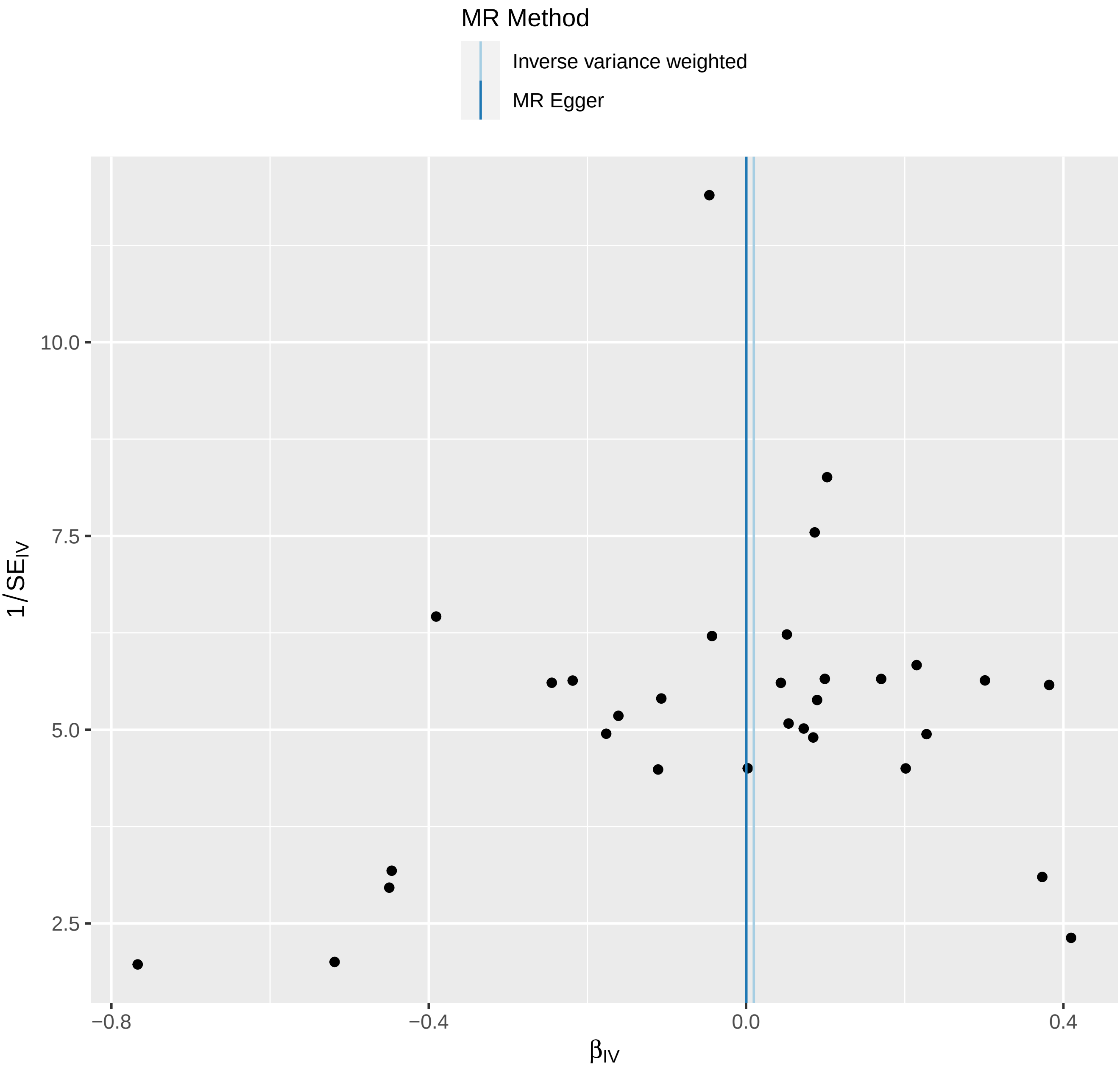

Batch 3−360 : Protein CASC4 on Diabetic nephropathy (more control exclusions)
with a threshold of 1e−05

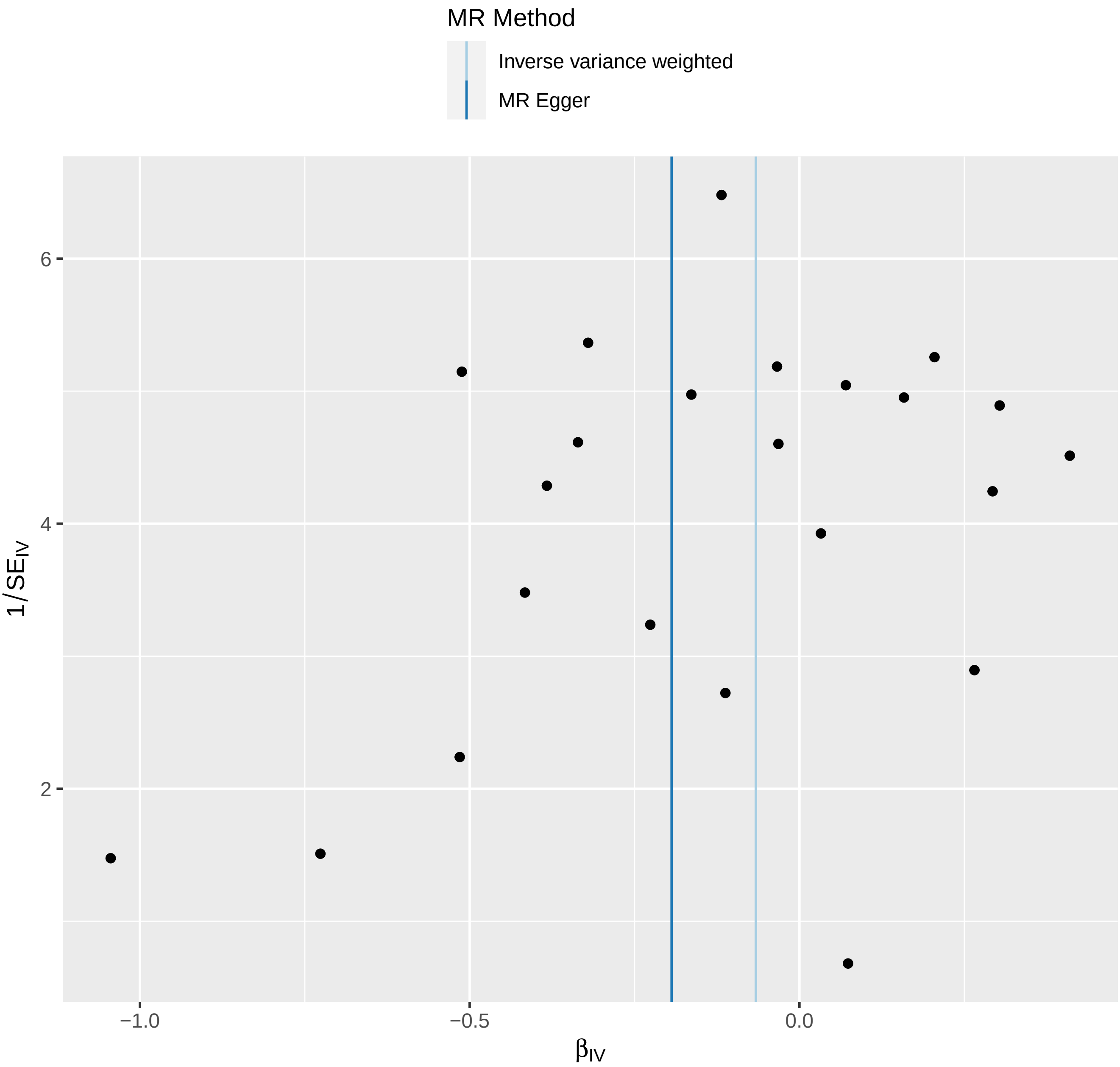

Batch 3−361 : Protein CASC4 on Diabetic nephropathy (more control exclusions)
with a threshold of 1e−05

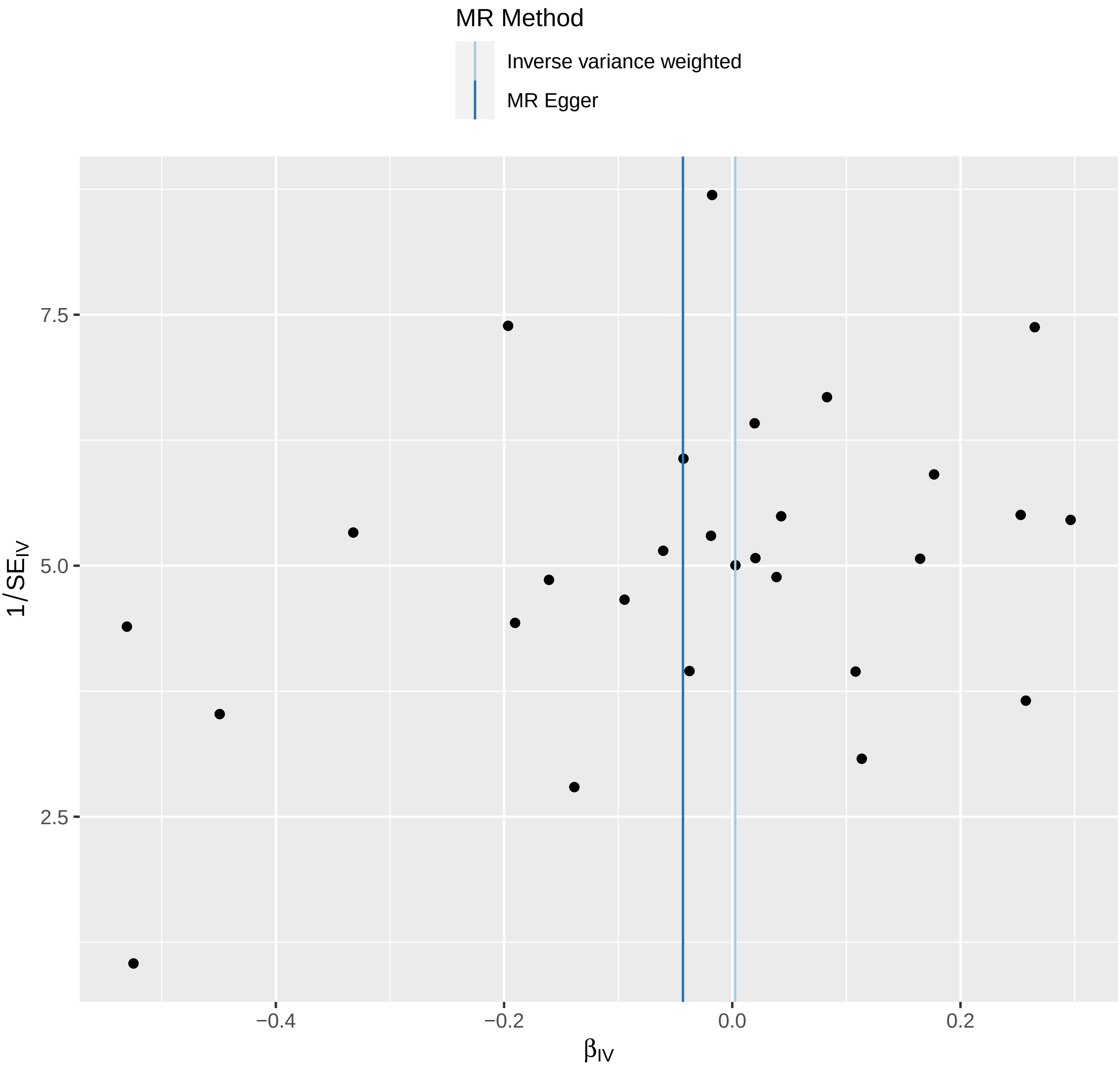

Batch 3−362 : Caspase−10 on Diabetic nephropathy (more control exclusions) with a threshold of 1e−05

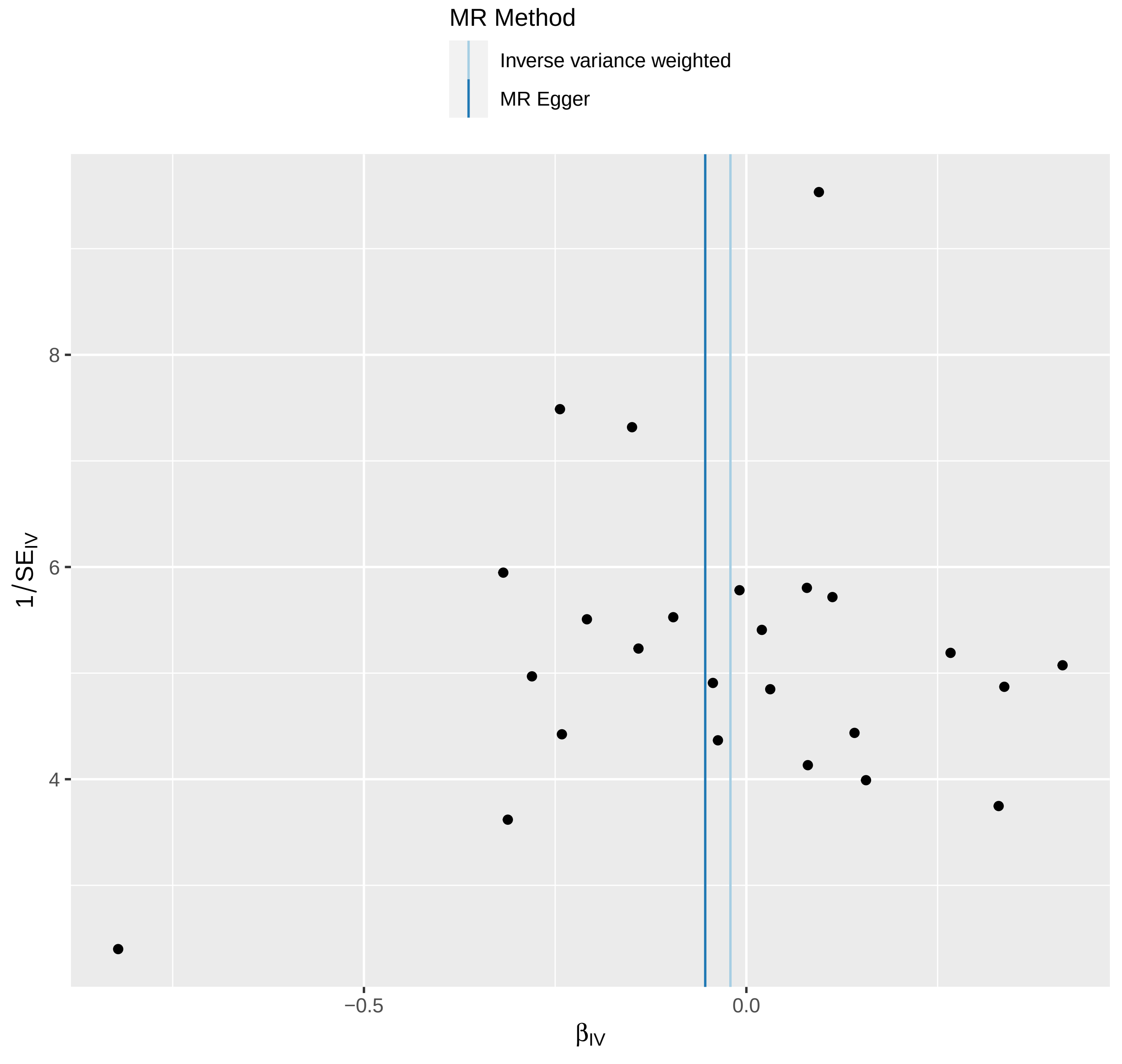

Batch 3−363 : Caspase−2 on Diabetic nephropathy (more control exclusions)
with a threshold of 1e−05

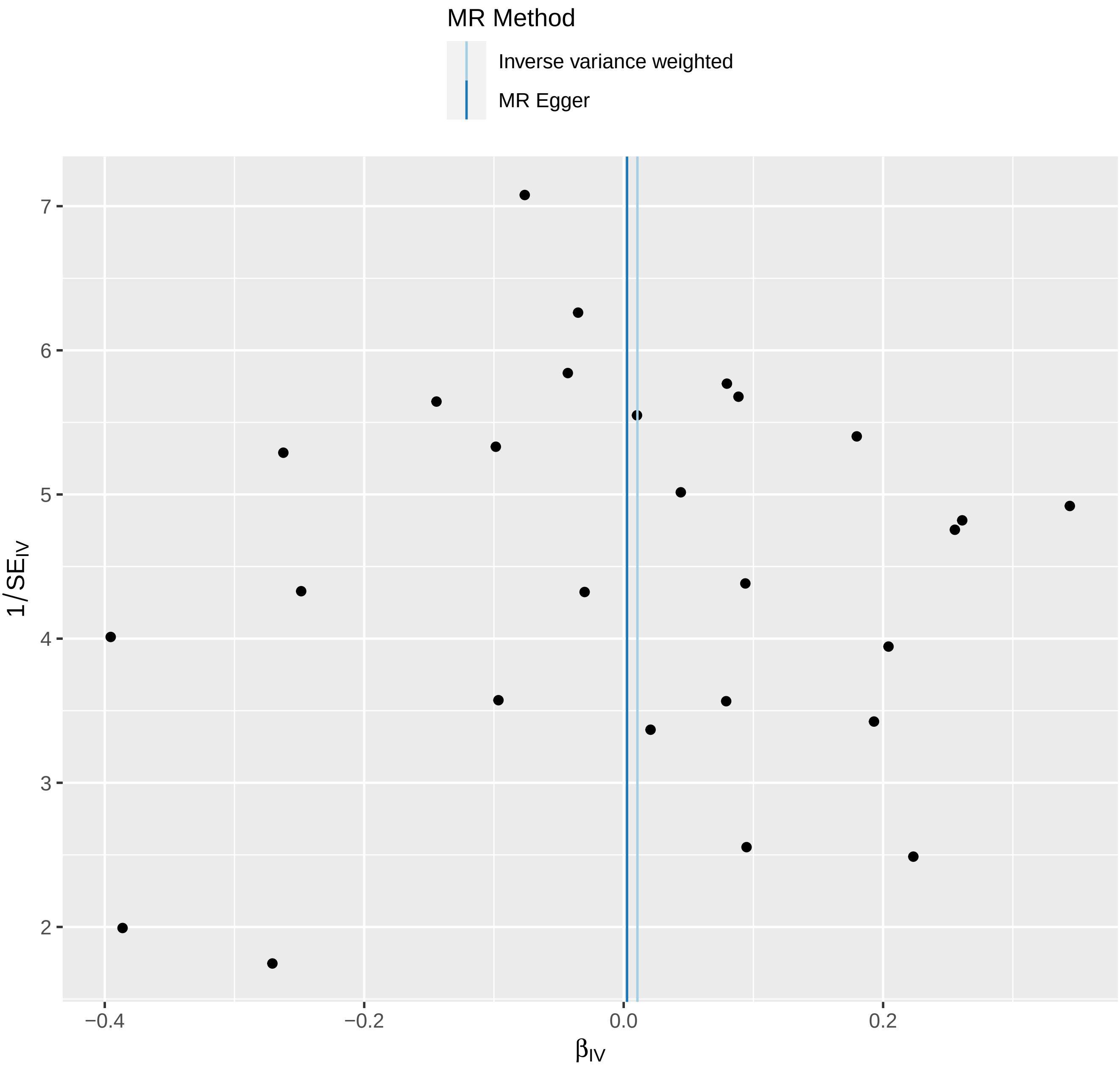

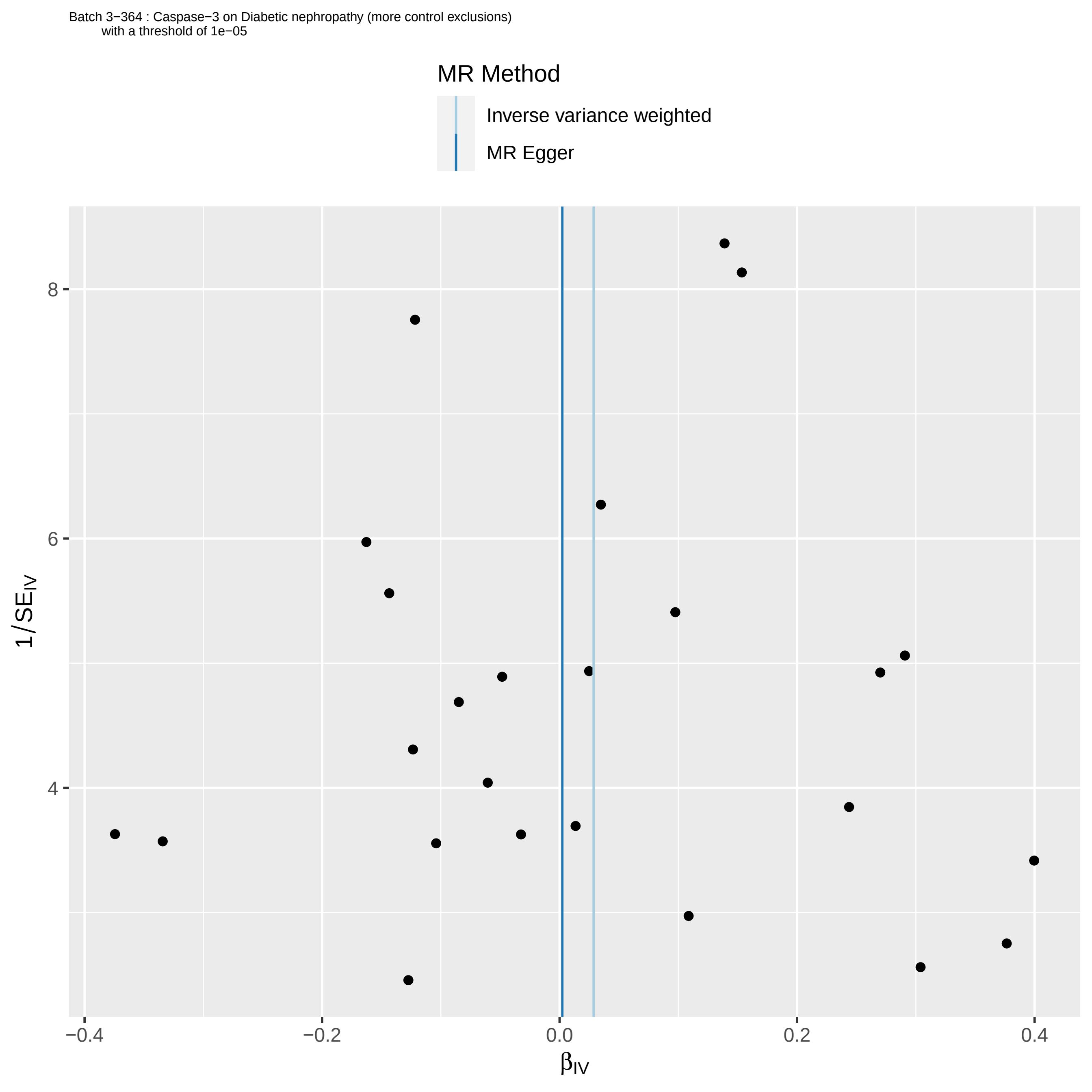
Batch 3−364 : Caspase−3 on Diabetic nephropathy (more control exclusions)
with a threshold of 1e−05
MR Method
Inverse variance weighted
MR Egger
8
6
4
1/SE$_{IV}$
−0.4
−0.2
0.0
0.2
0.4
β_{IV}

Batch 3−365 : Cas scaffolding protein family member 4 on Diabetic nephropathy (more control exclusions)
with a threshold of 1e−05

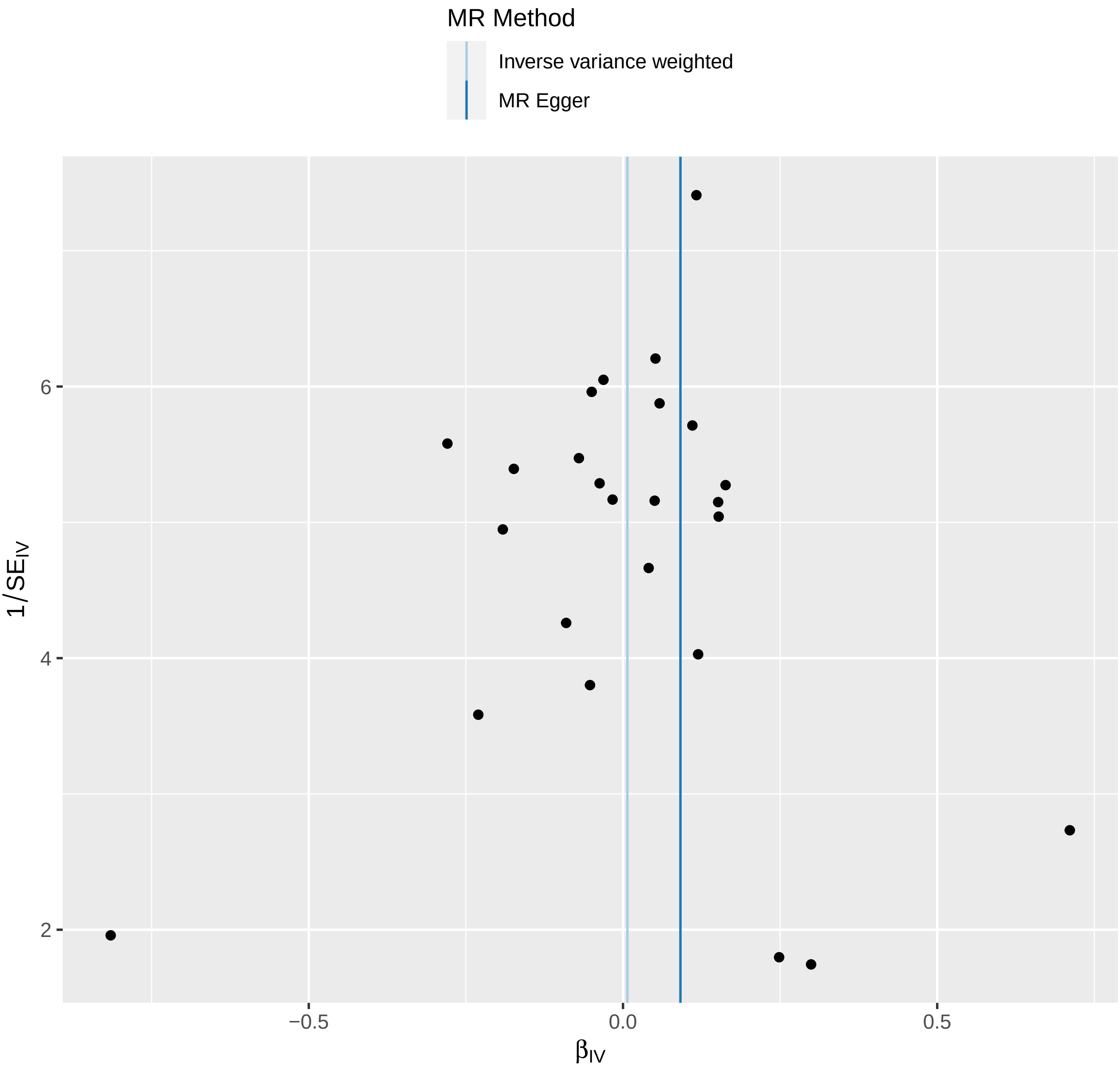

Batch 3−366 : Calpastatin on Diabetic nephropathy (more control exclusions)
with a threshold of 1e−05

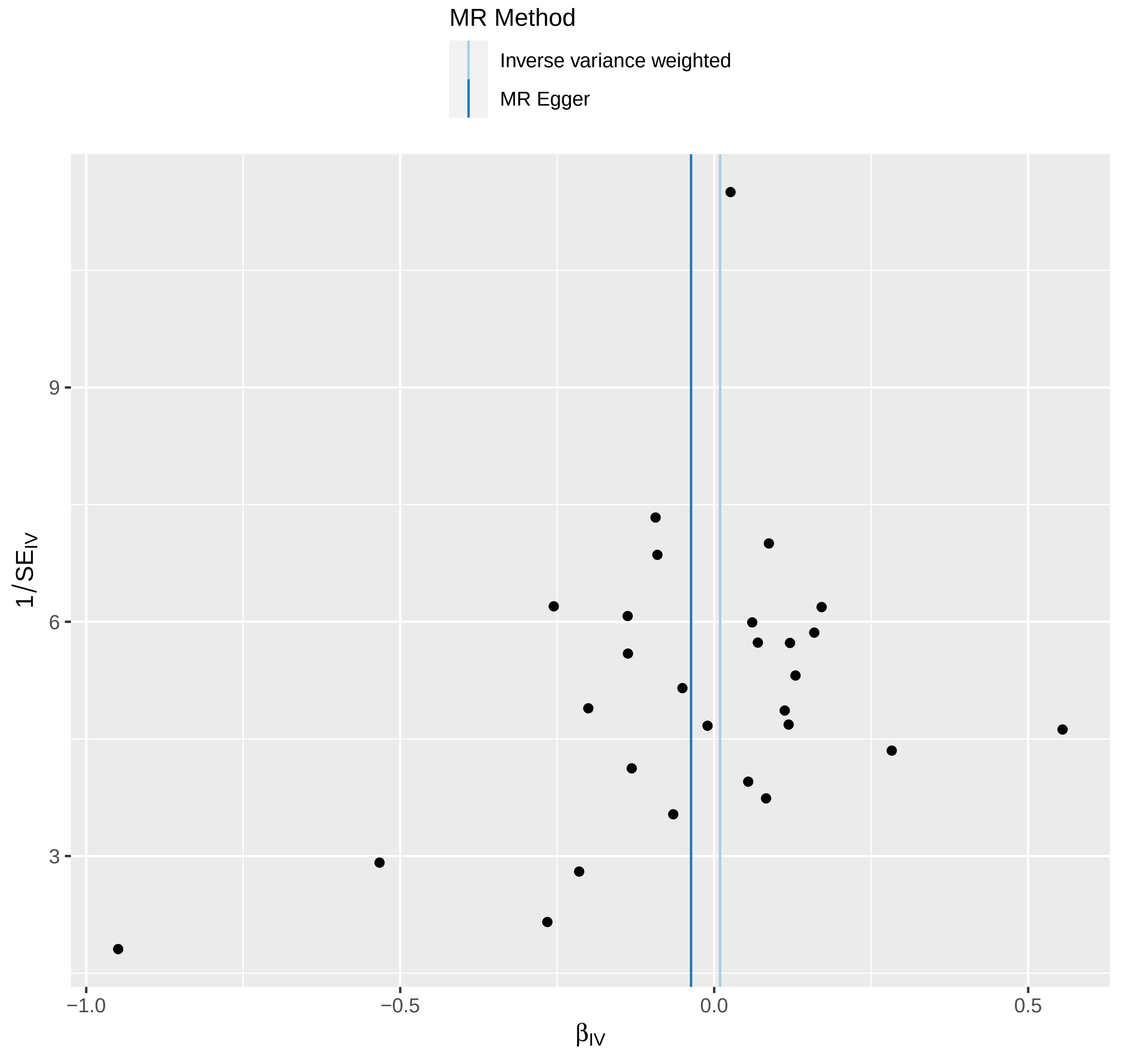

Batch 3−367 : Catalase on Diabetic nephropathy (more control exclusions)
with a threshold of 1e−05

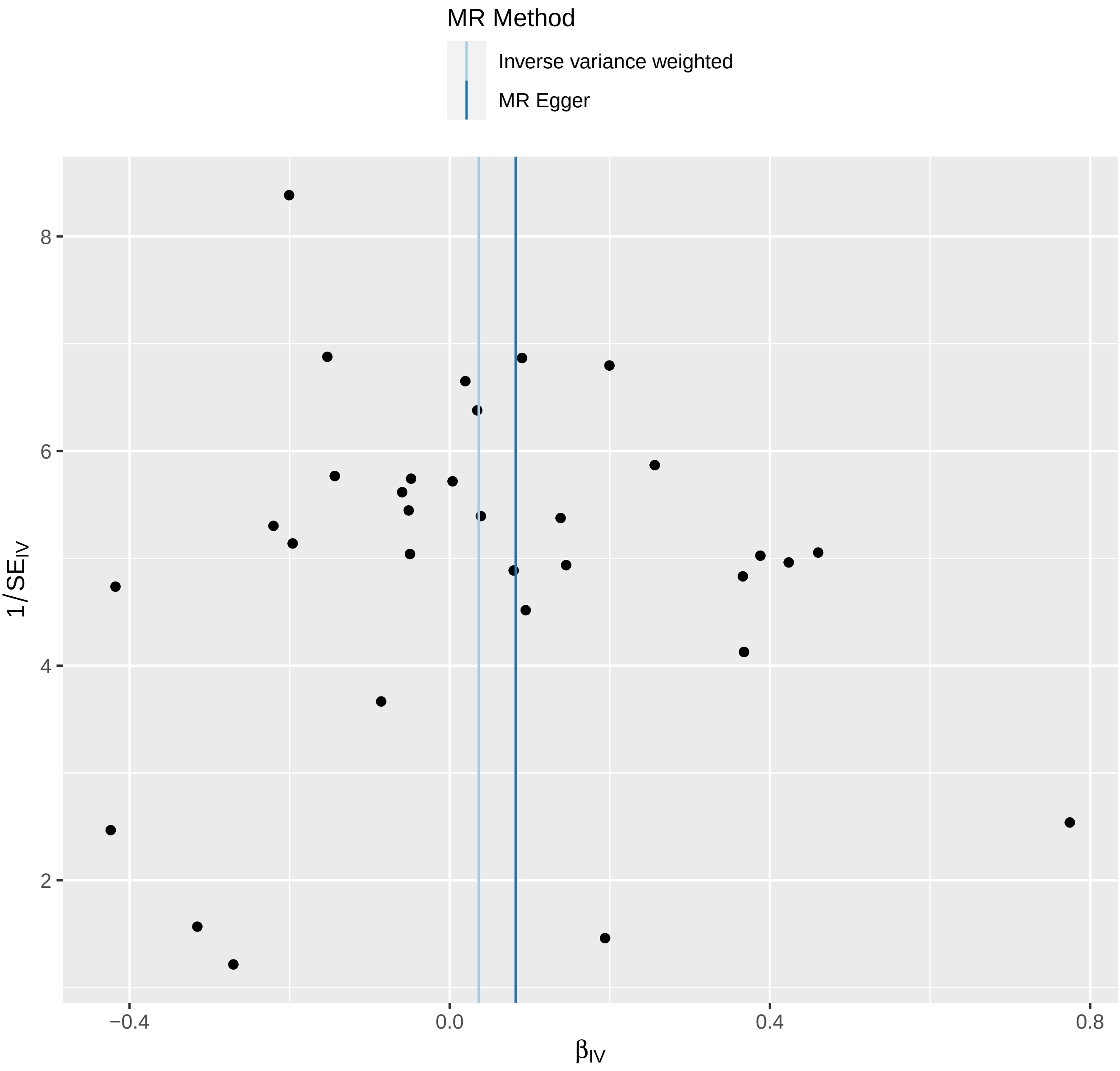

Batch 3−368 : Caveolin−2 on Diabetic nephropathy (more control exclusions)
with a threshold of 1e−05

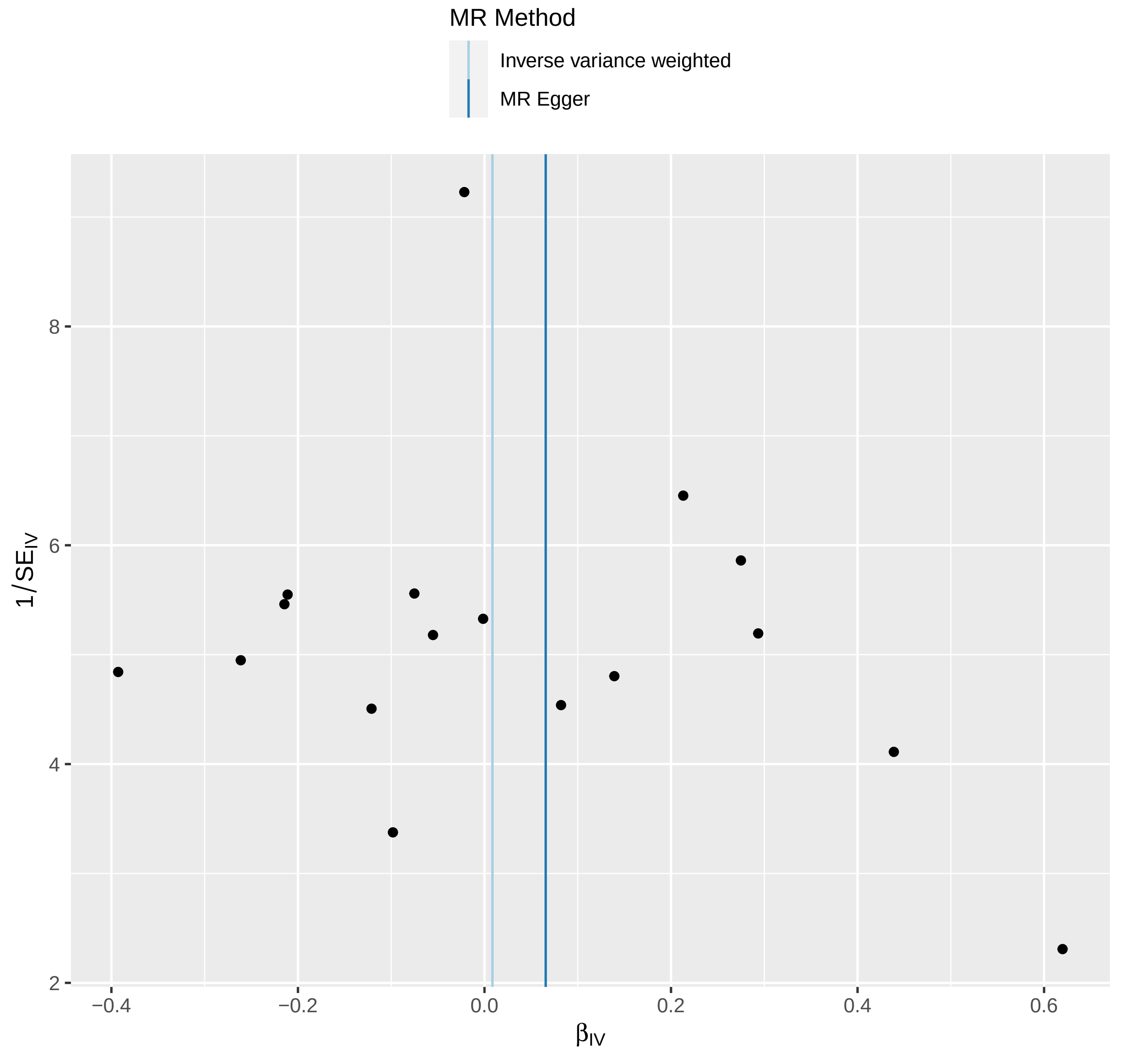

Batch 3−369 : Core−binding factor subunit beta on Diabetic nephropathy (more control exclusions)
with a threshold of 1e−05

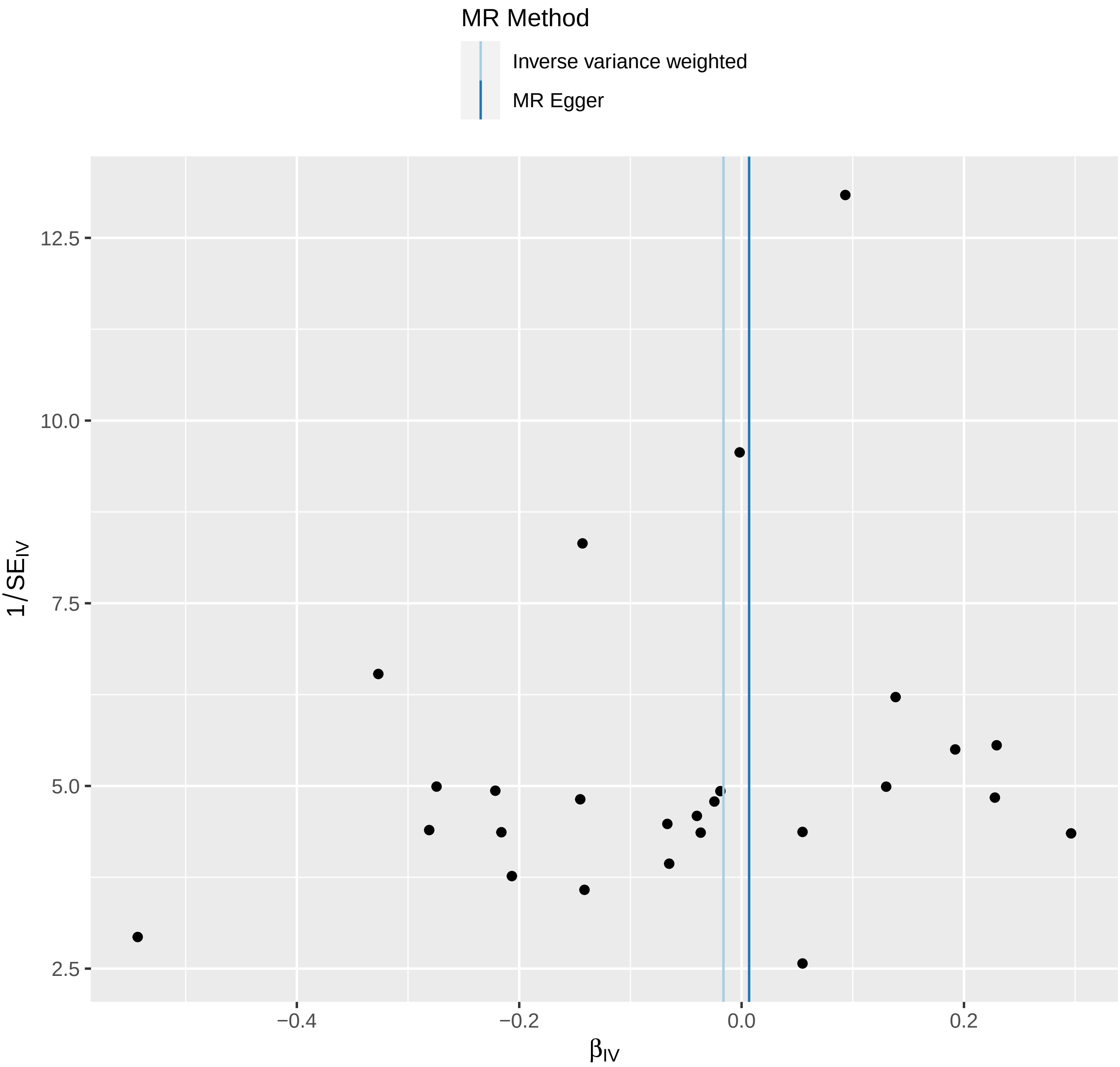

Batch 3−370 : E3 ubiquitin−protein ligase CBL on Diabetic nephropathy (more control exclusions)
with a threshold of 1e−05

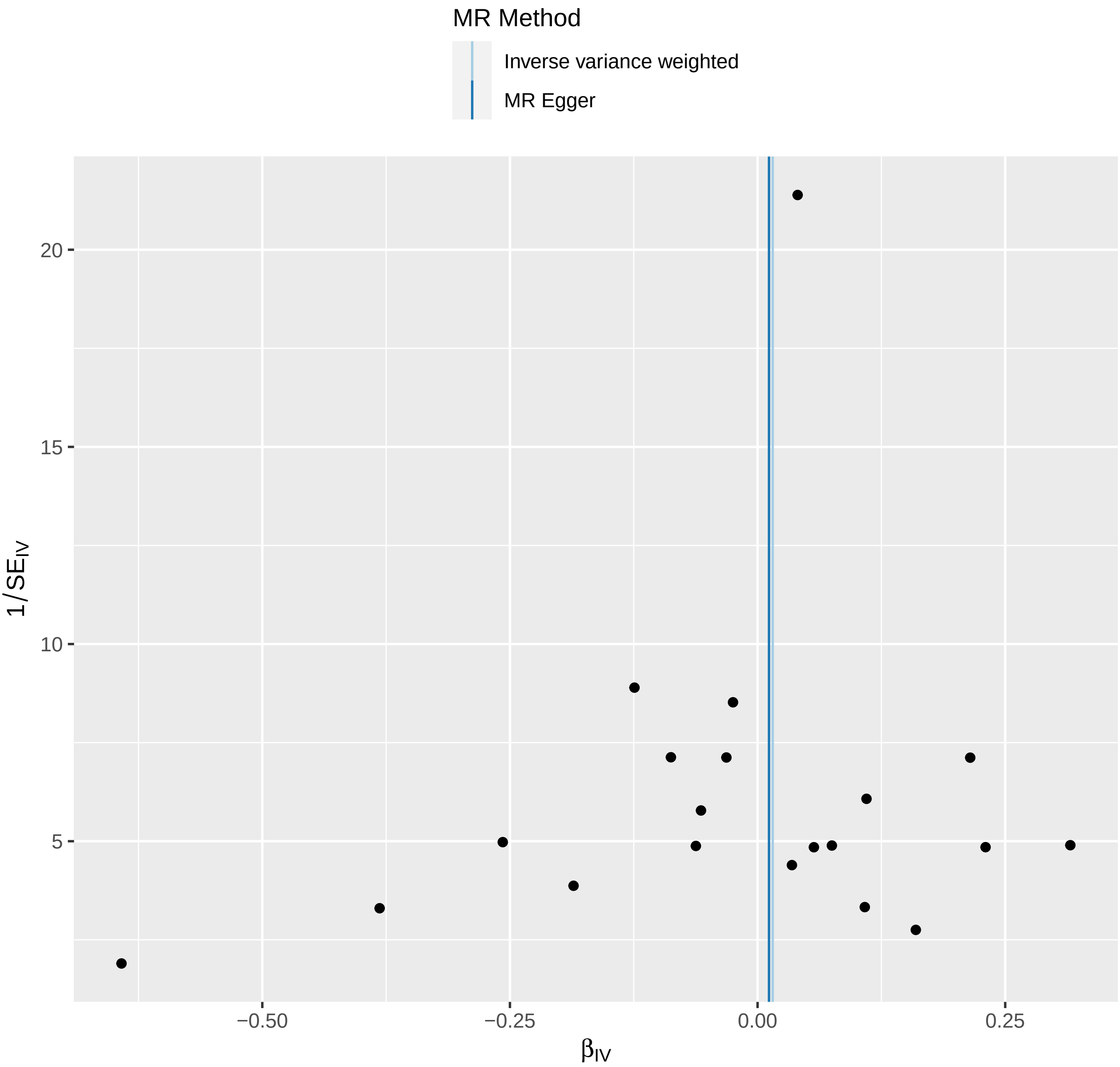

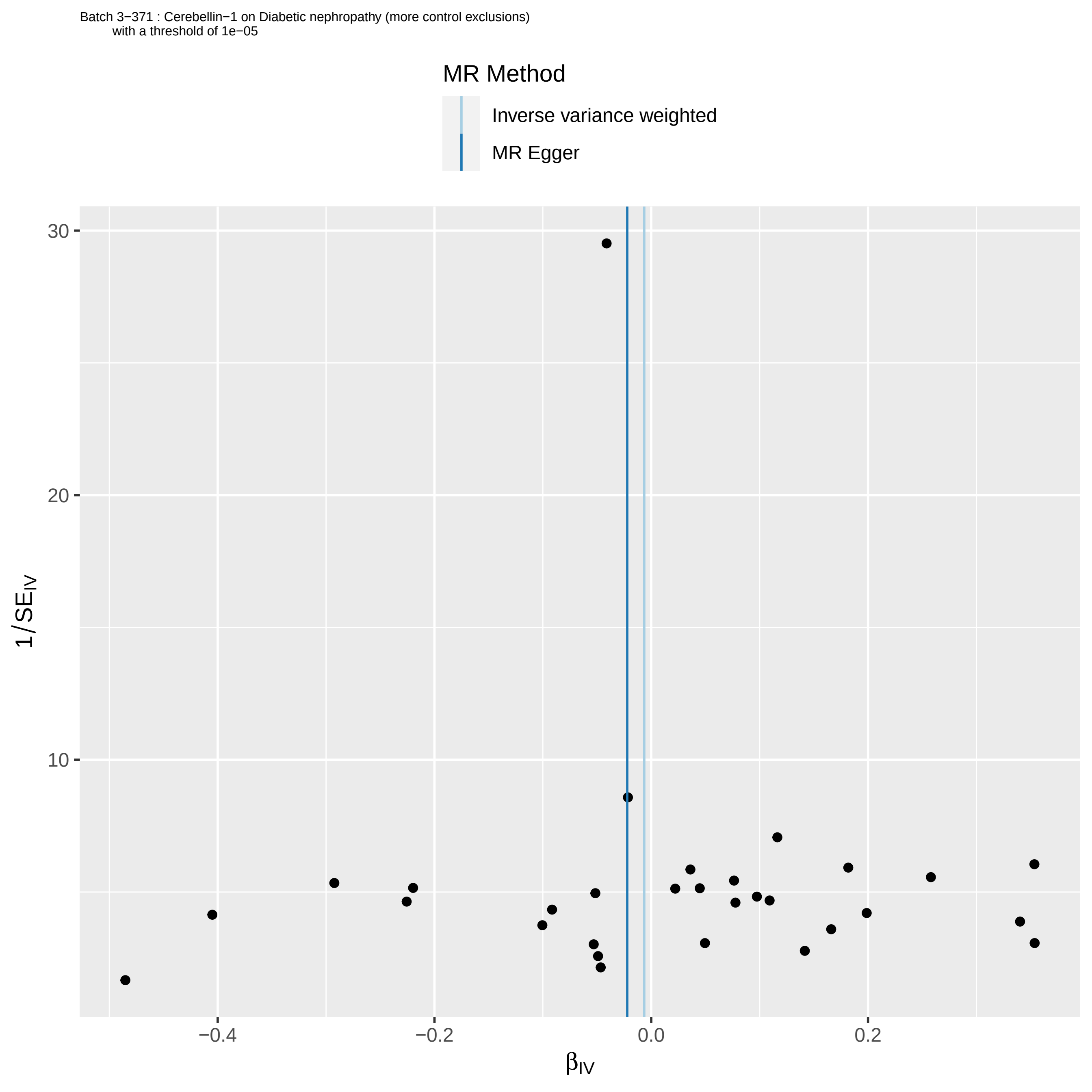
Batch 3−371 : Cerebellin−1 on Diabetic nephropathy (more control exclusions)
with a threshold of 1e−05
MR Method
Inverse variance weighted
MR Egger
30
20
10
1/SE_IV
−0.4
−0.2
0.0
0.2
β_IV

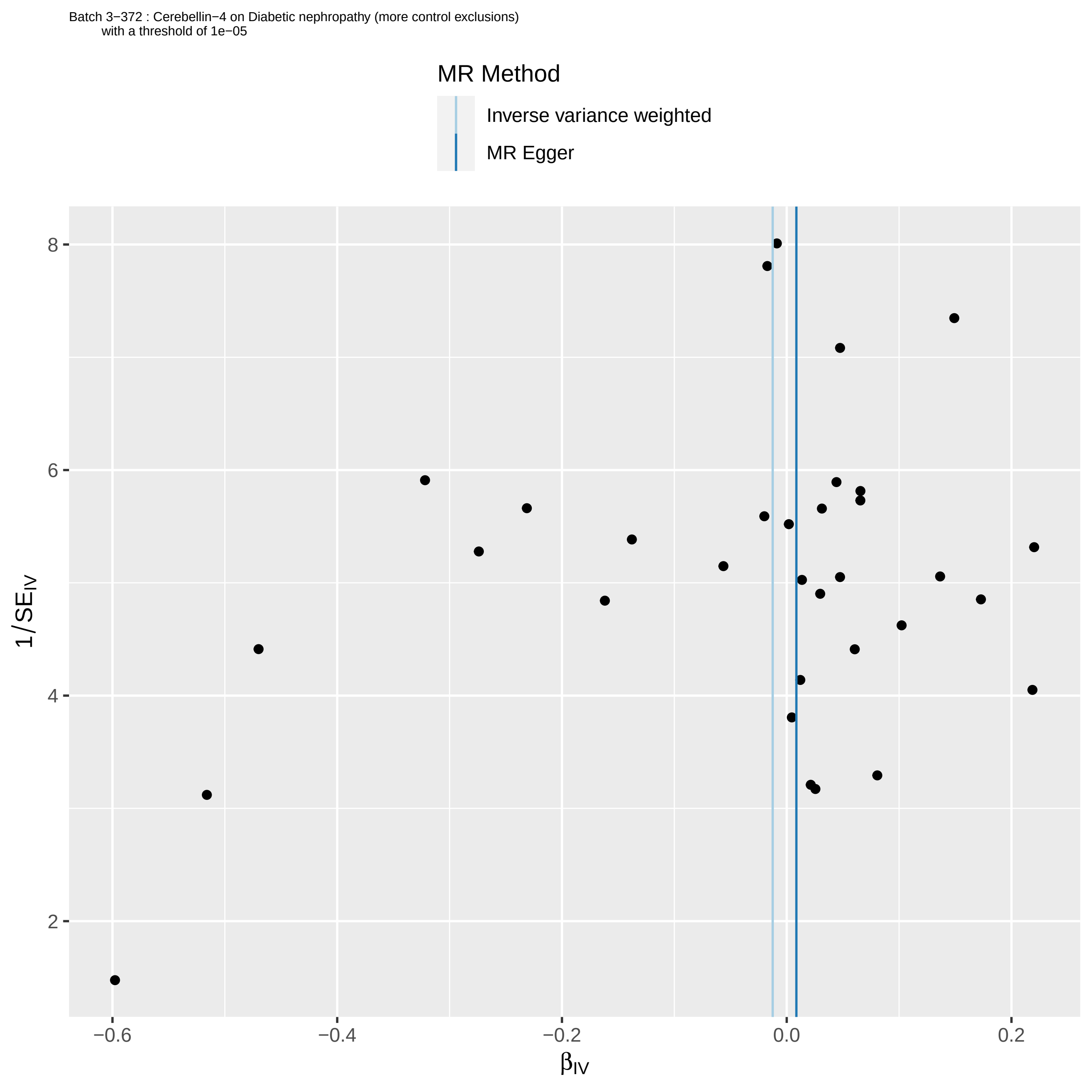
Batch 3−372 : Cerebellin−4 on Diabetic nephropathy (more control exclusions)
with a threshold of 1e−05
MR Method
Inverse variance weighted
MR Egger
8
6
4
2
1/SE$_{IV}$
−0.6
−0.4
−0.2
0.0
0.2
β$_{IV}$

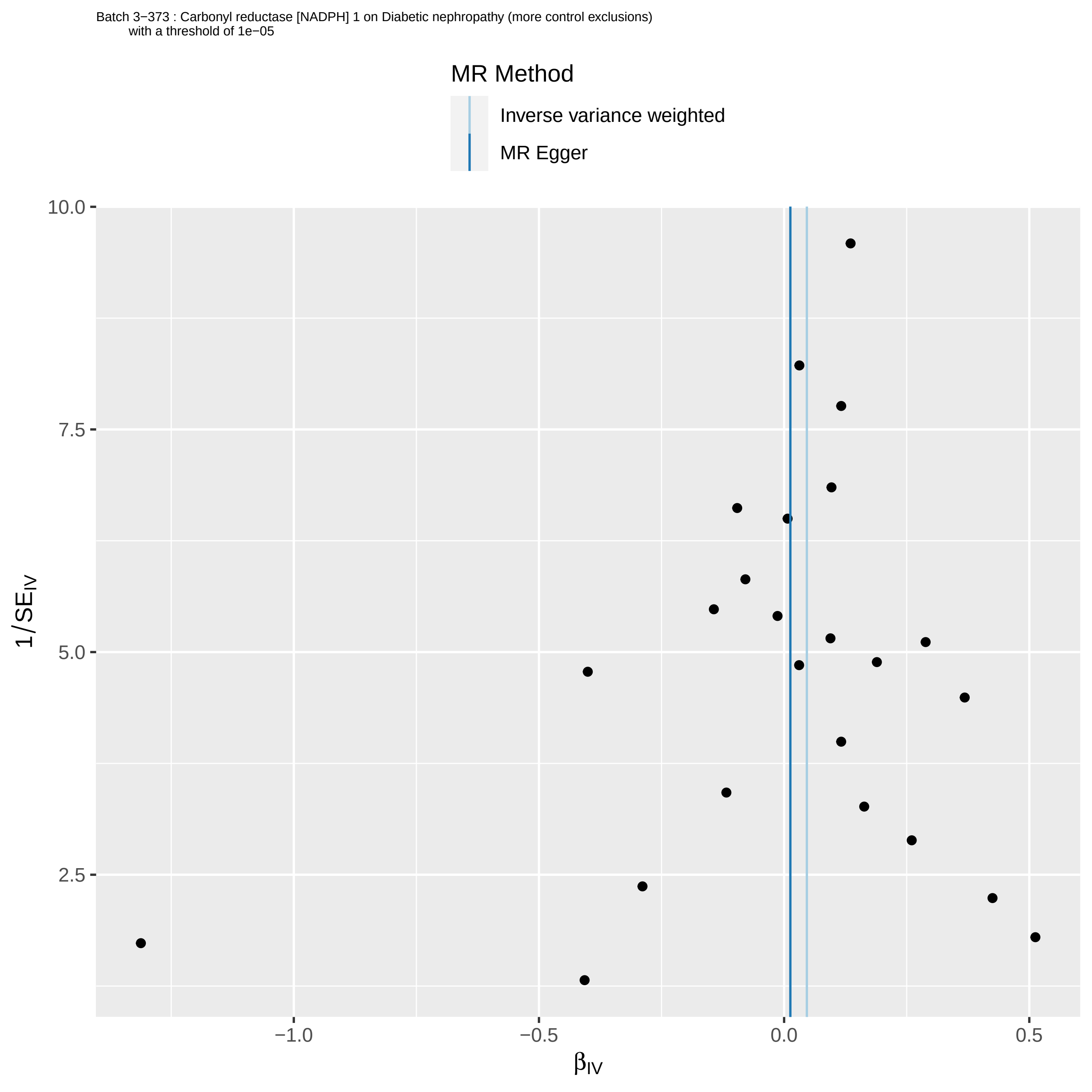
Batch 3−373 : Carbonyl reductase [NADPH] 1 on Diabetic nephropathy (more control exclusions)
with a threshold of 1e−05
MR Method
Inverse variance weighted
MR Egger
10.0
7.5
5.0
2.5
1/SE_IV
−1.0
−0.5
0.0
0.5
β_IV

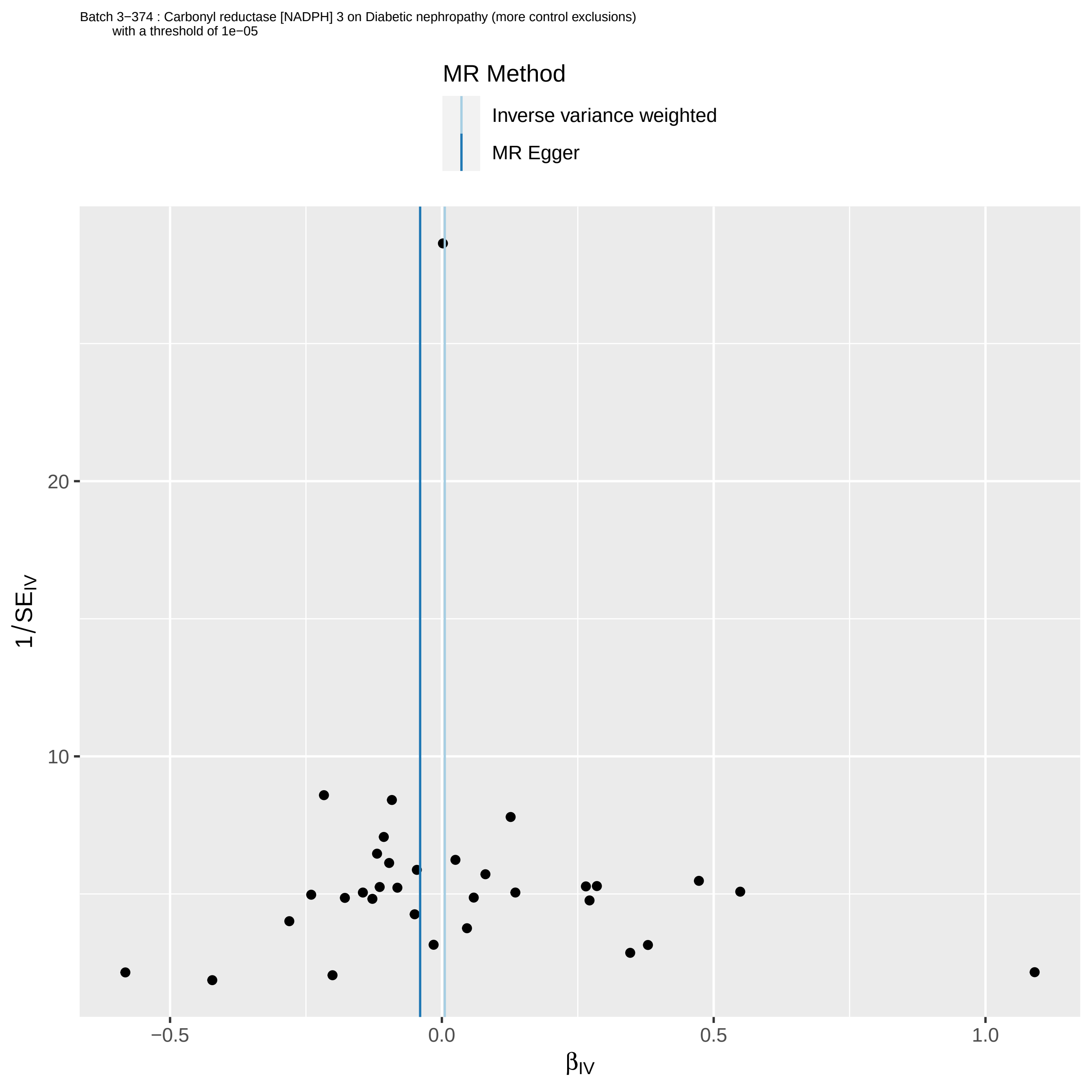

Batch 3−374 : Carbonyl reductase [NADPH] 3 on Diabetic nephropathy (more control exclusions)
with a threshold of 1e−05
MR Method
Inverse variance weighted
MR Egger
20
10
1/SE_IV
−0.5
0.0
0.5
1.0
β_IV

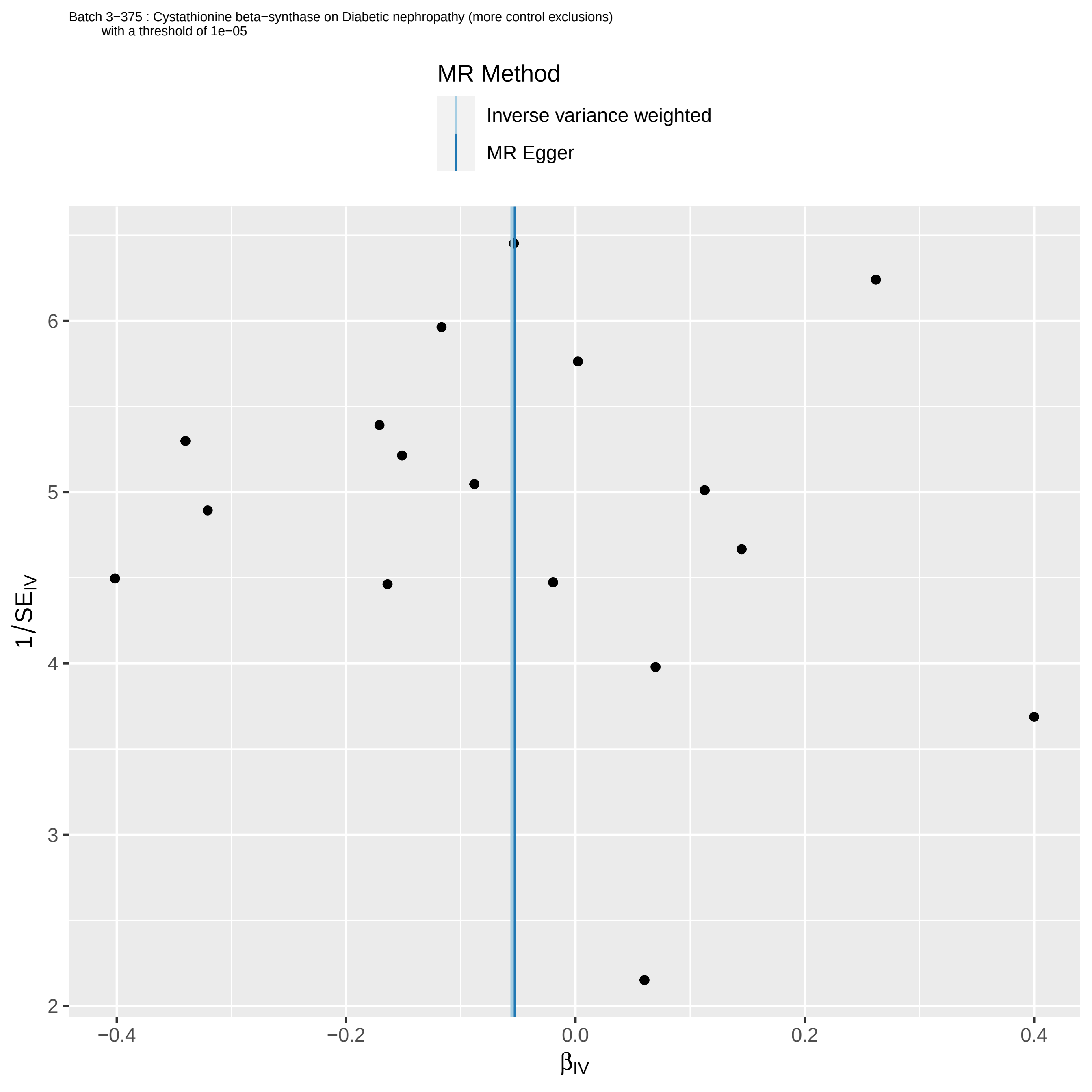
Batch 3−375 : Cystathionine beta−synthase on Diabetic nephropathy (more control exclusions)
with a threshold of 1e−05
MR Method
Inverse variance weighted
MR Egger
1/SE_IV
β_IV
6
5
4
3
2
−0.4
−0.2
0.0
0.2
0.4

Batch 3−376 : Chromobox protein homolog 5 on Diabetic nephropathy (more control exclusions)
with a threshold of 1e−05

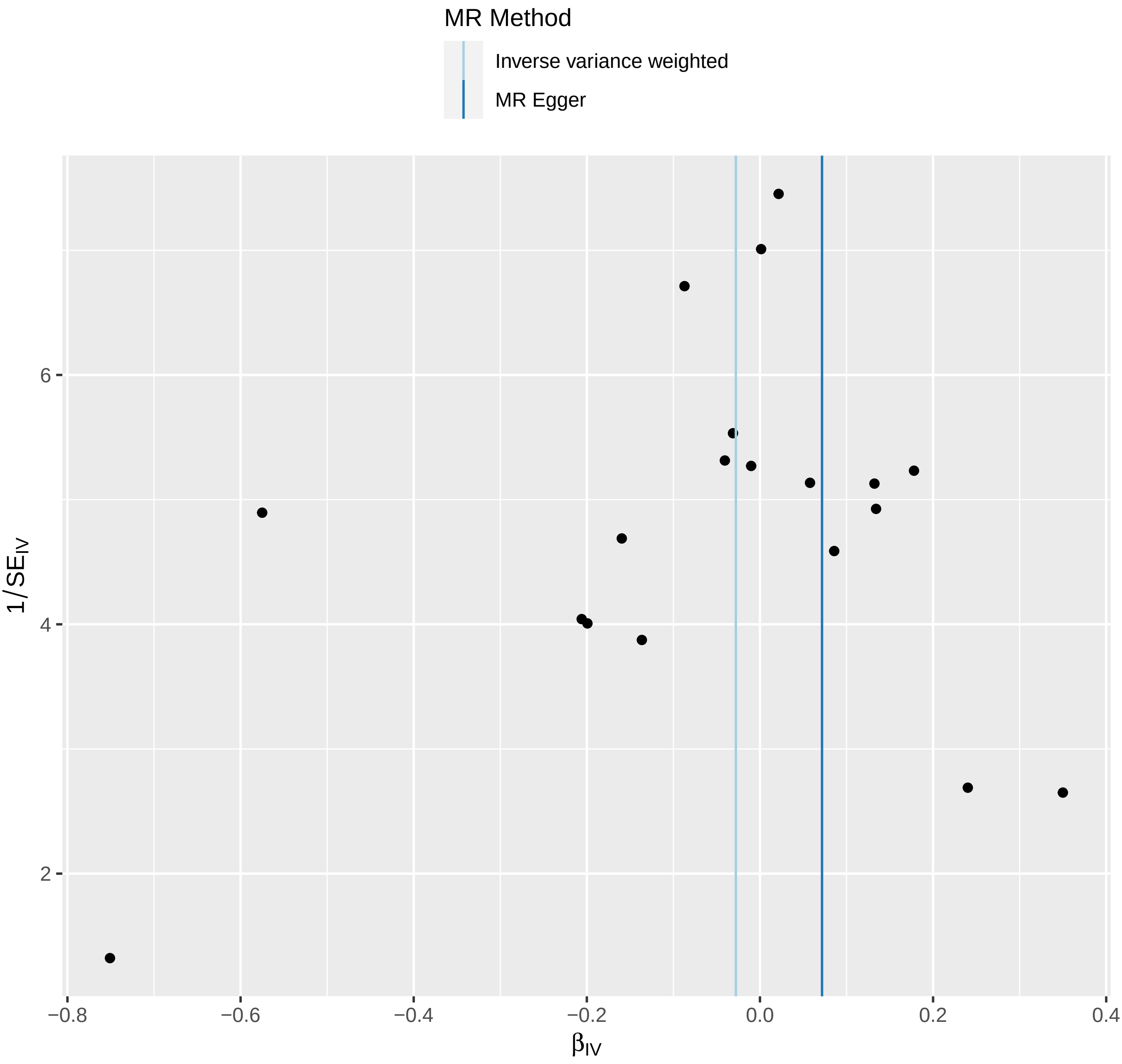

Batch 3−377 : Chromobox protein homolog 7 on Diabetic nephropathy (more control exclusions)
with a threshold of 1e−05

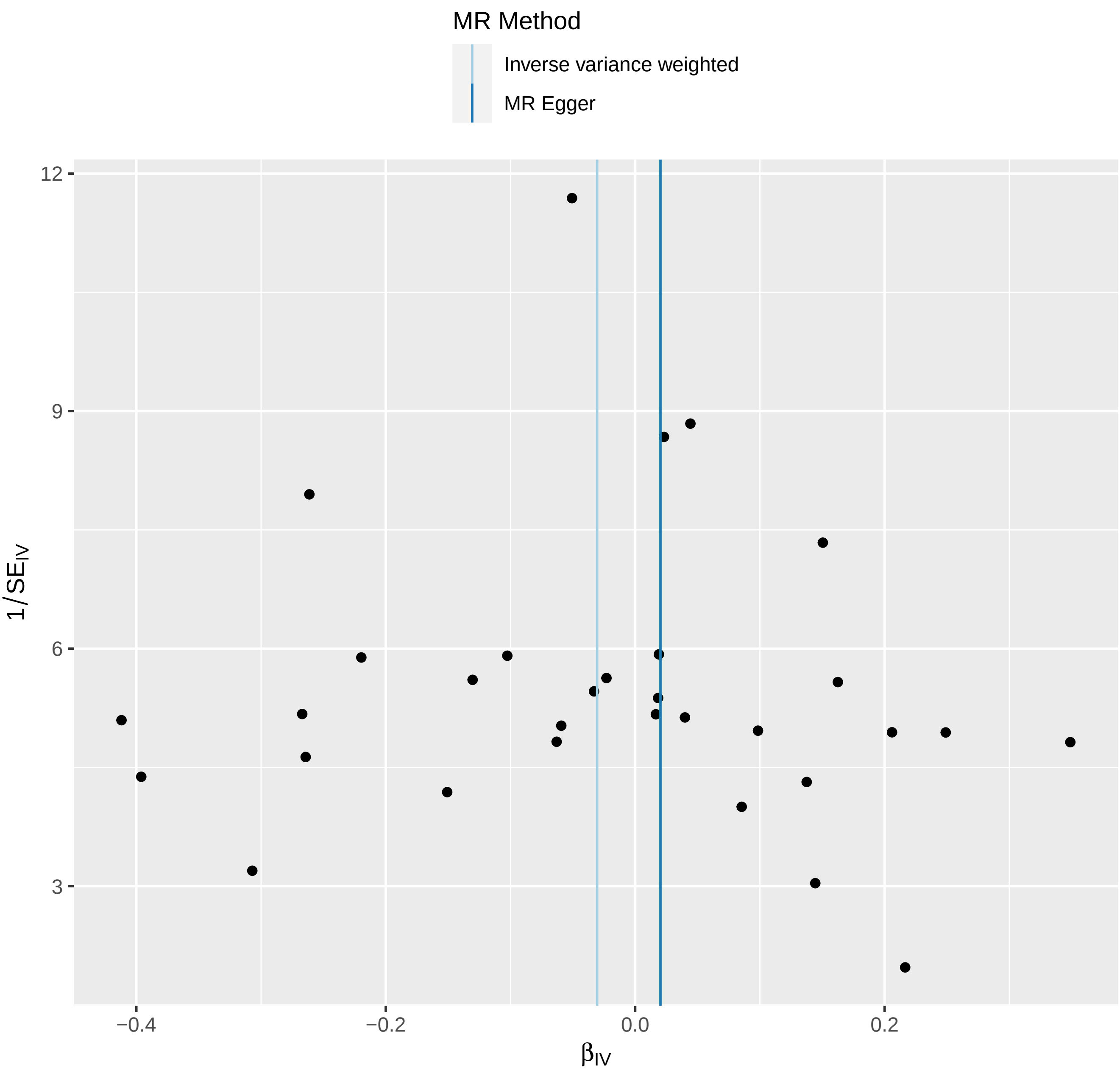

Batch 3−378 : Kynurenine−−oxoglutarate transaminase 3 on Diabetic nephropathy (more control exclusions)
with a threshold of 1e−05

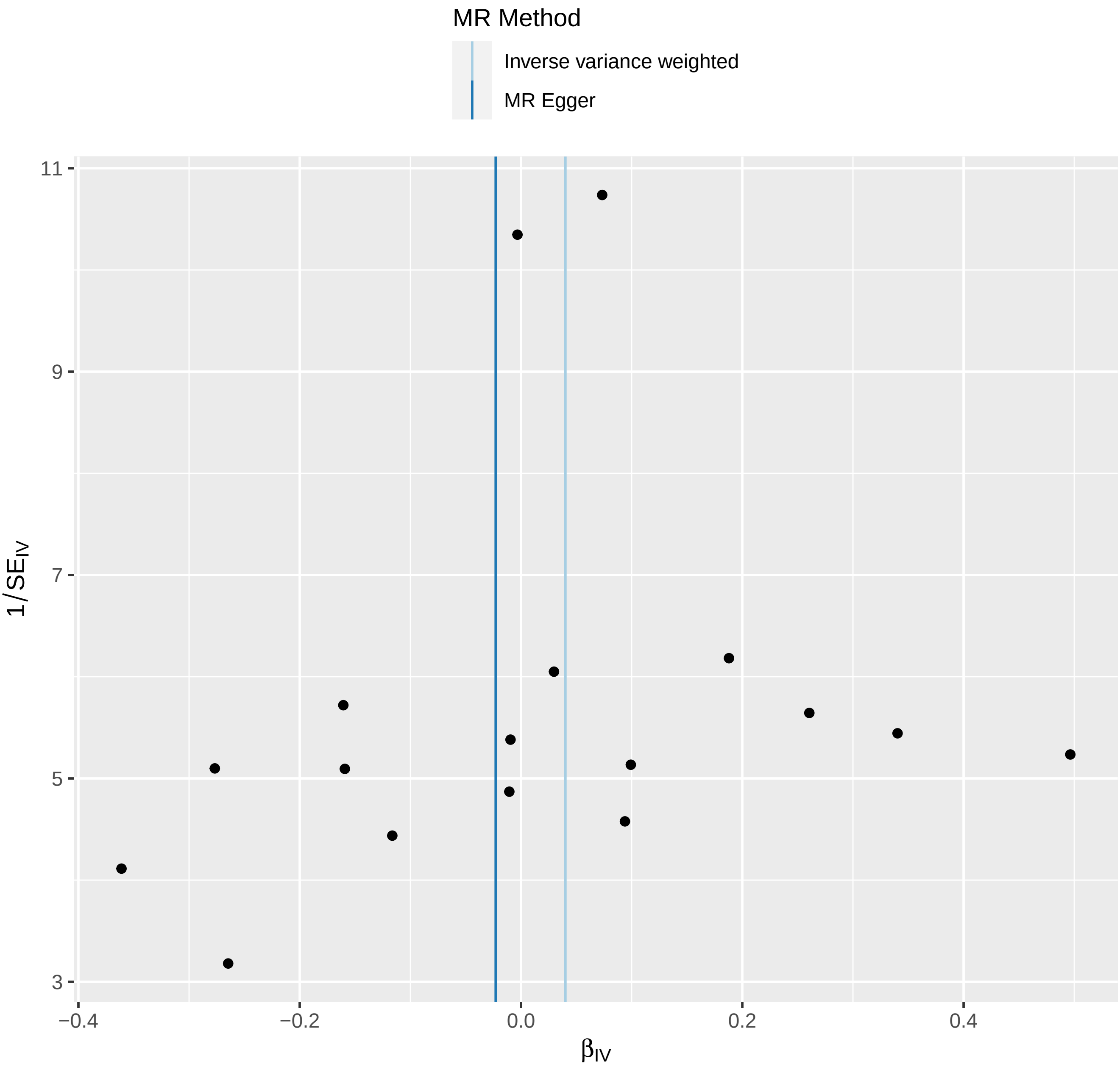

Batch 3−379 : SAGA−associated factor 29 homolog on Diabetic nephropathy (more control exclusions) with a threshold of 1e−05

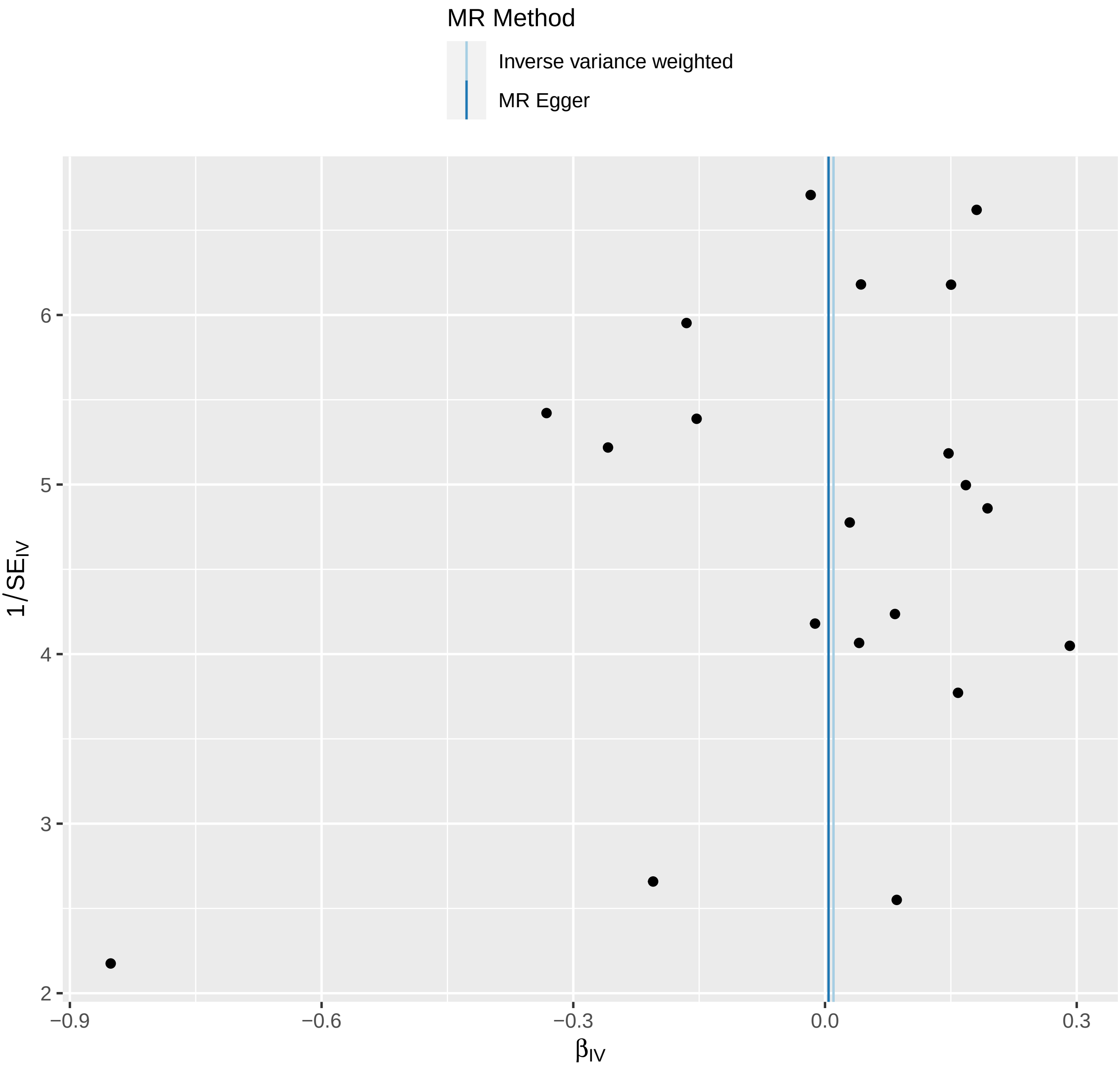

Batch 3−380 : Coiled−coil domain−containing protein 126 on Diabetic nephropathy (more control exclusions) with a threshold of 1e−05

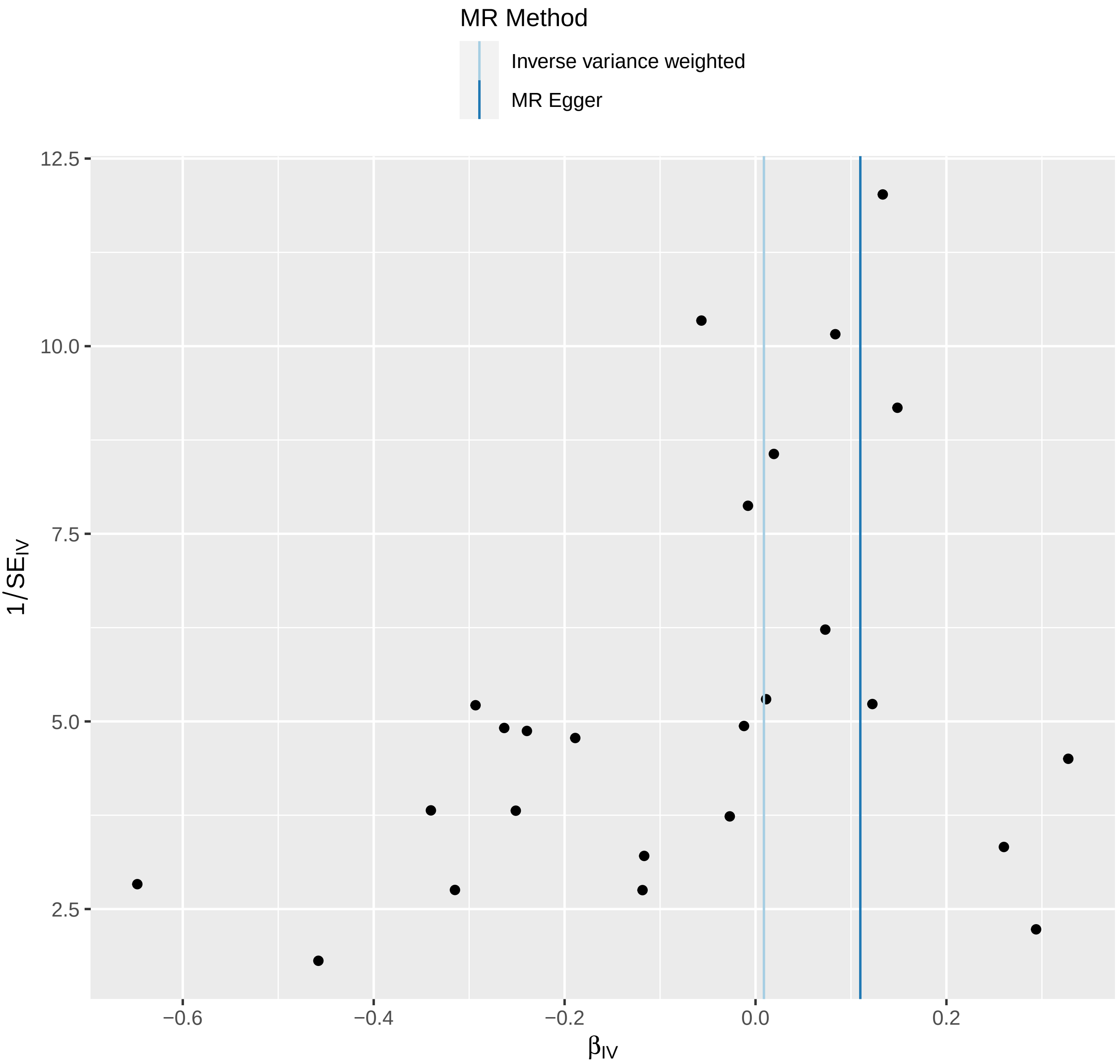

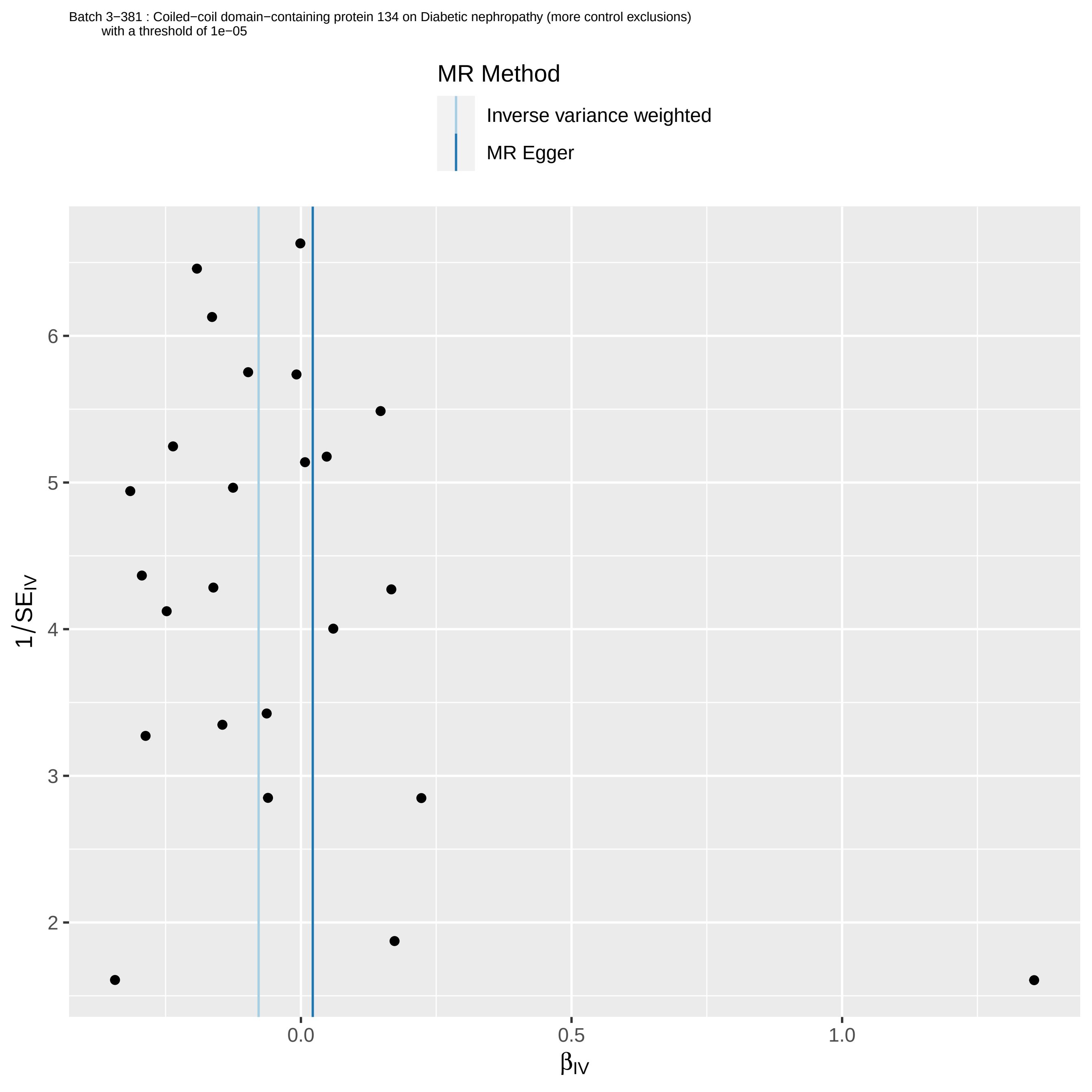
Batch 3−381 : Coiled−coil domain−containing protein 134 on Diabetic nephropathy (more control exclusions)
with a threshold of 1e−05
MR Method
Inverse variance weighted
MR Egger
6
5
4
3
2
1/SE_IV
0.0
0.5
1.0
β_IV

Batch 3−382 : Coiled−coil domain−containing protein 167 on Diabetic nephropathy (more control exclusions)
with a threshold of 1e−05

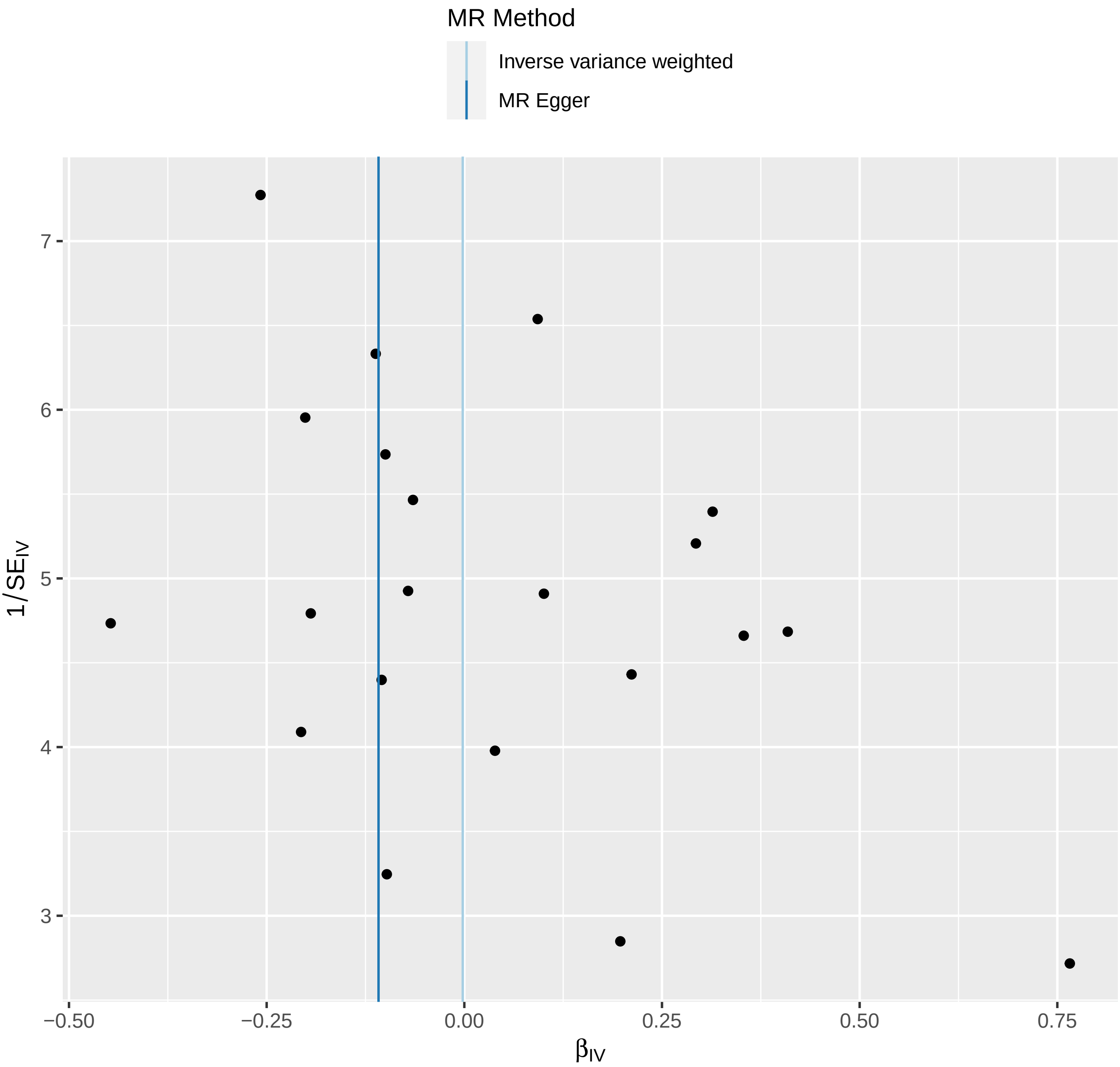

Batch 3−383 : Coiled−coil domain−containing protein 51 on Diabetic nephropathy (more control exclusions)
with a threshold of 1e−05

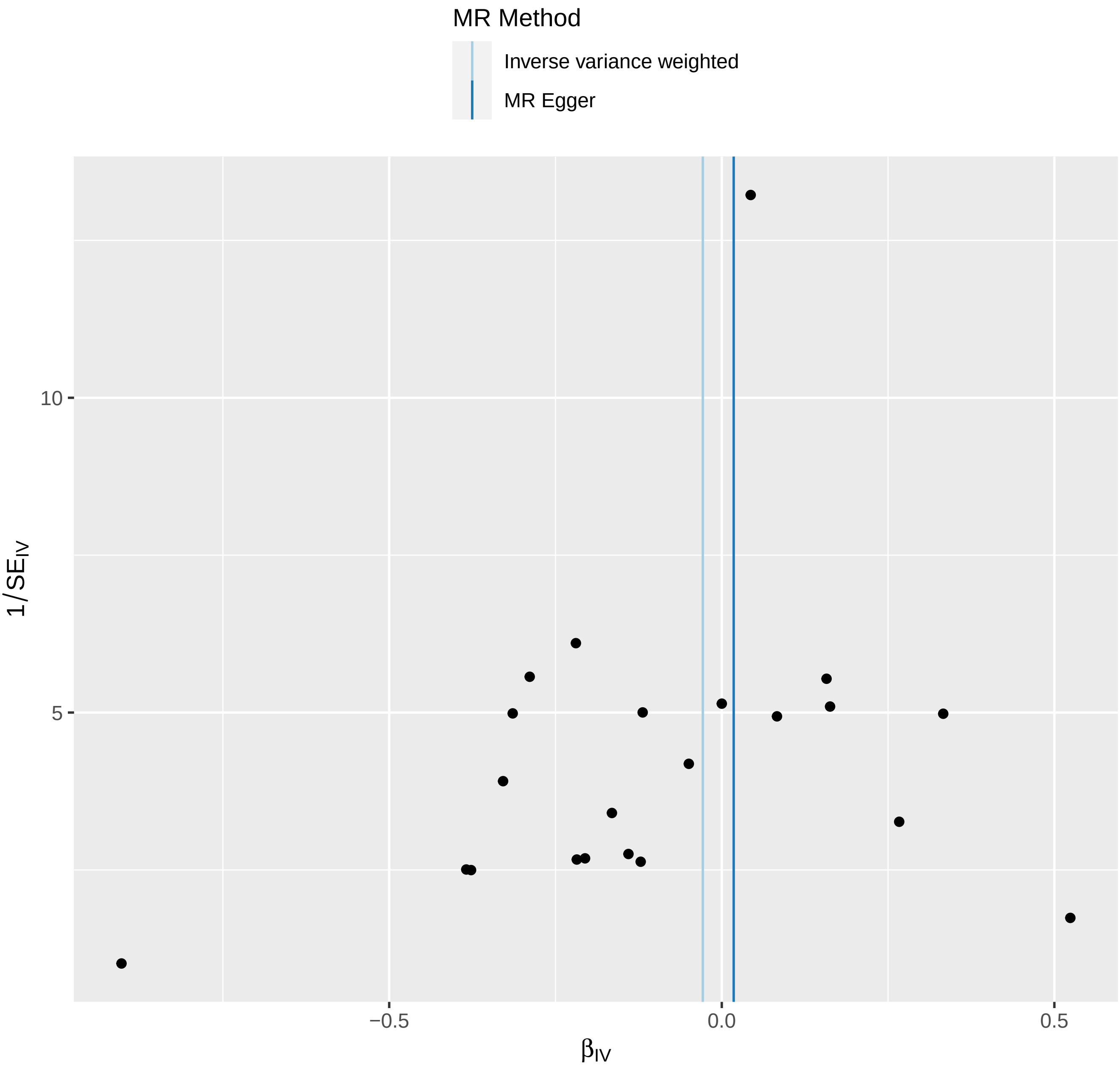

Batch 3−384 : Coiled−coil domain−containing protein 80 on Diabetic nephropathy (more control exclusions) with a threshold of 1e−05

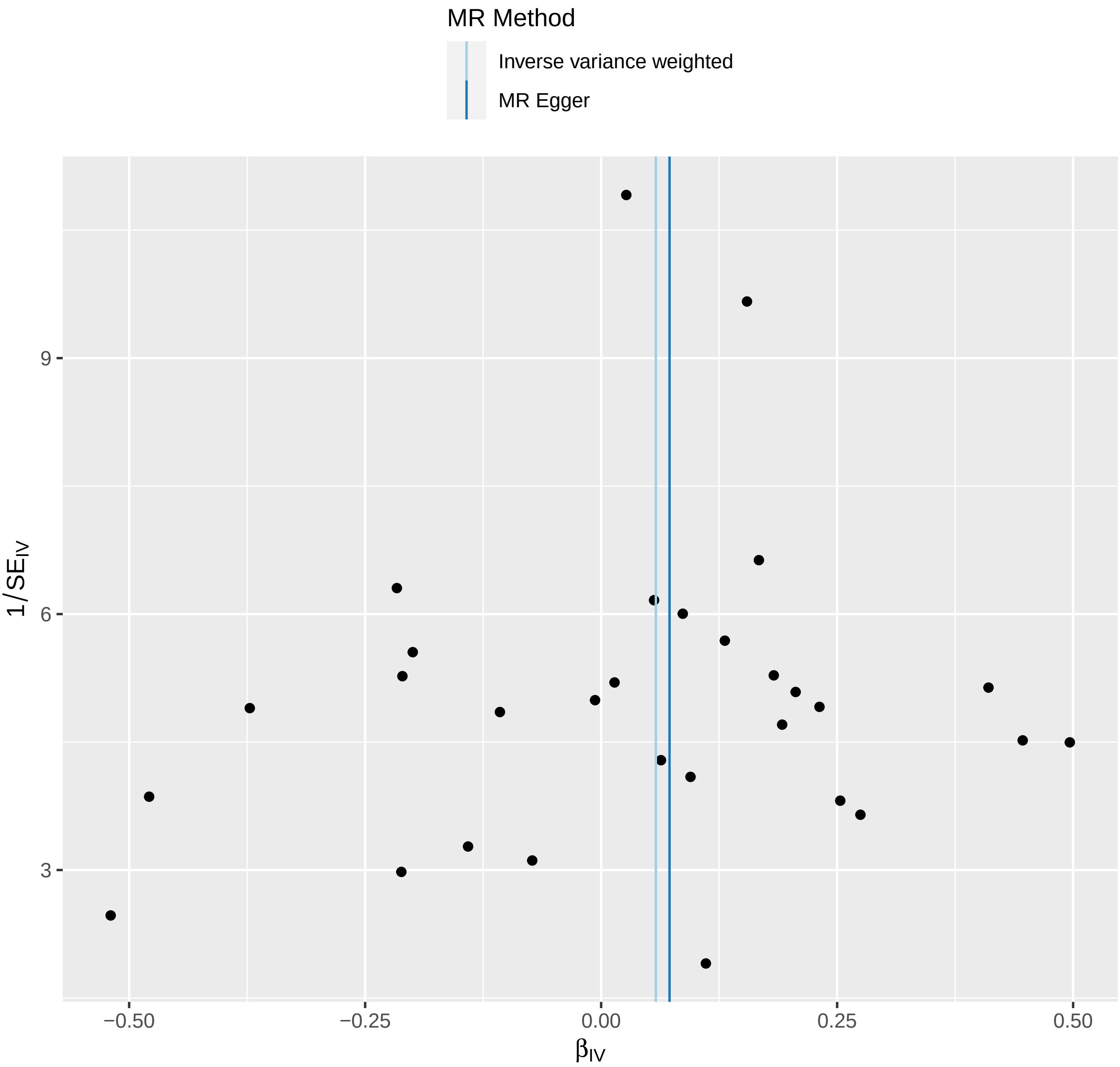

Batch 3−385 : Coiled−coil domain−containing protein 90B, mitochondrial on Diabetic nephropathy (more control exclusions) with a threshold of 1e−05

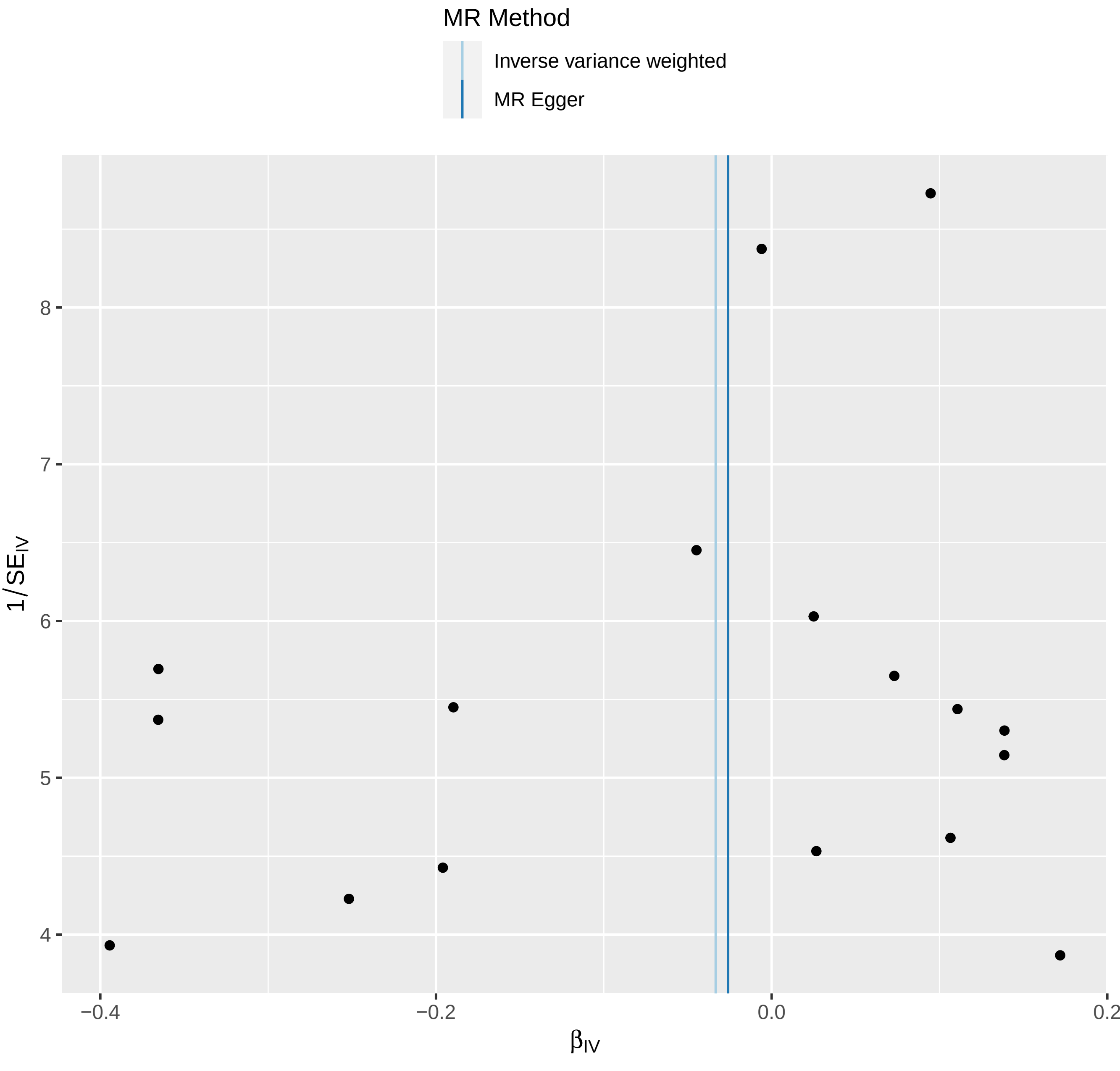

Batch 3−386 : C−C motif chemokine 1 on Diabetic nephropathy (more control exclusions) with a threshold of 1e−05

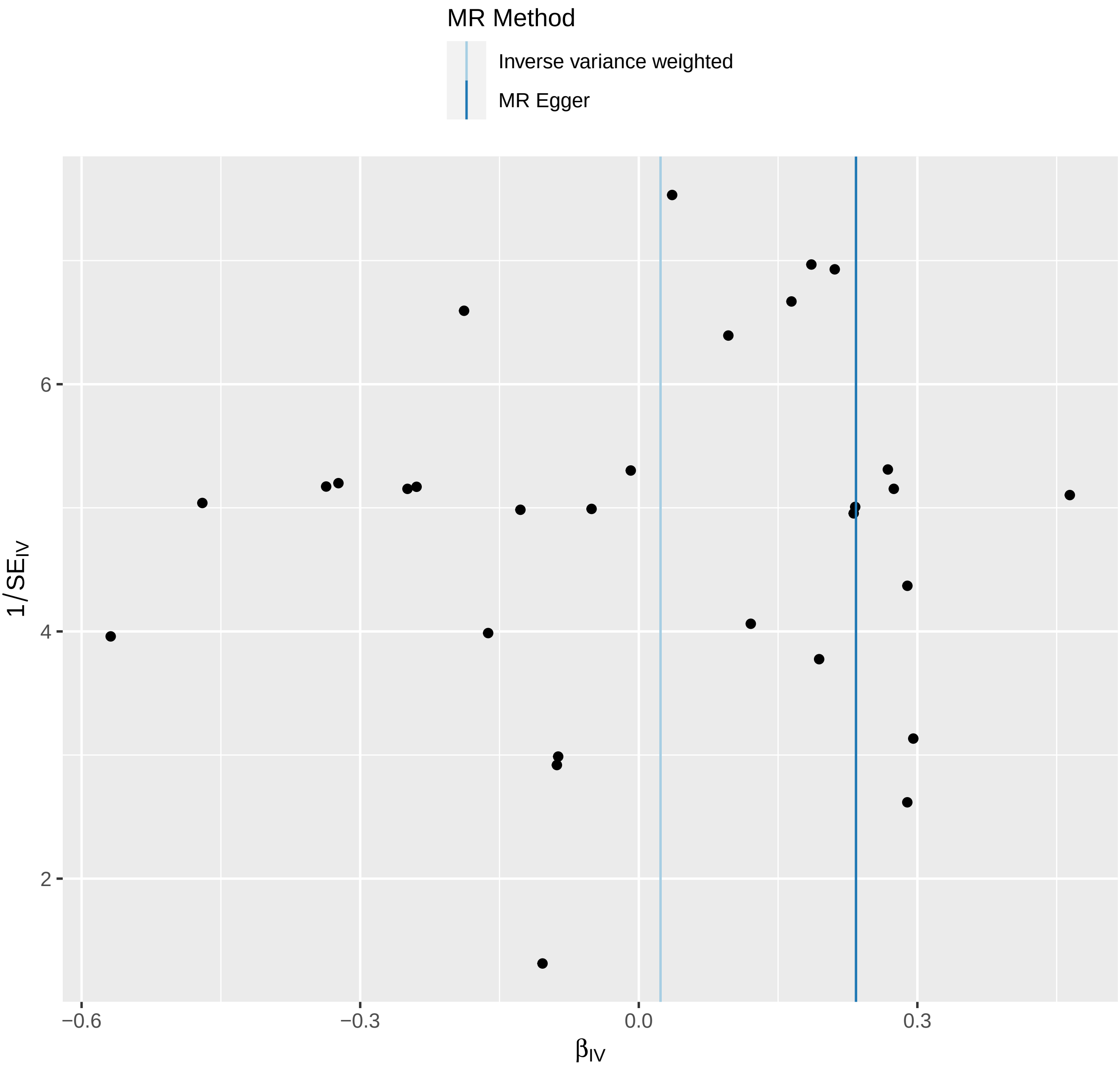

Batch 3−387 : Eotaxin on Diabetic nephropathy (more control exclusions)
with a threshold of 1e−05

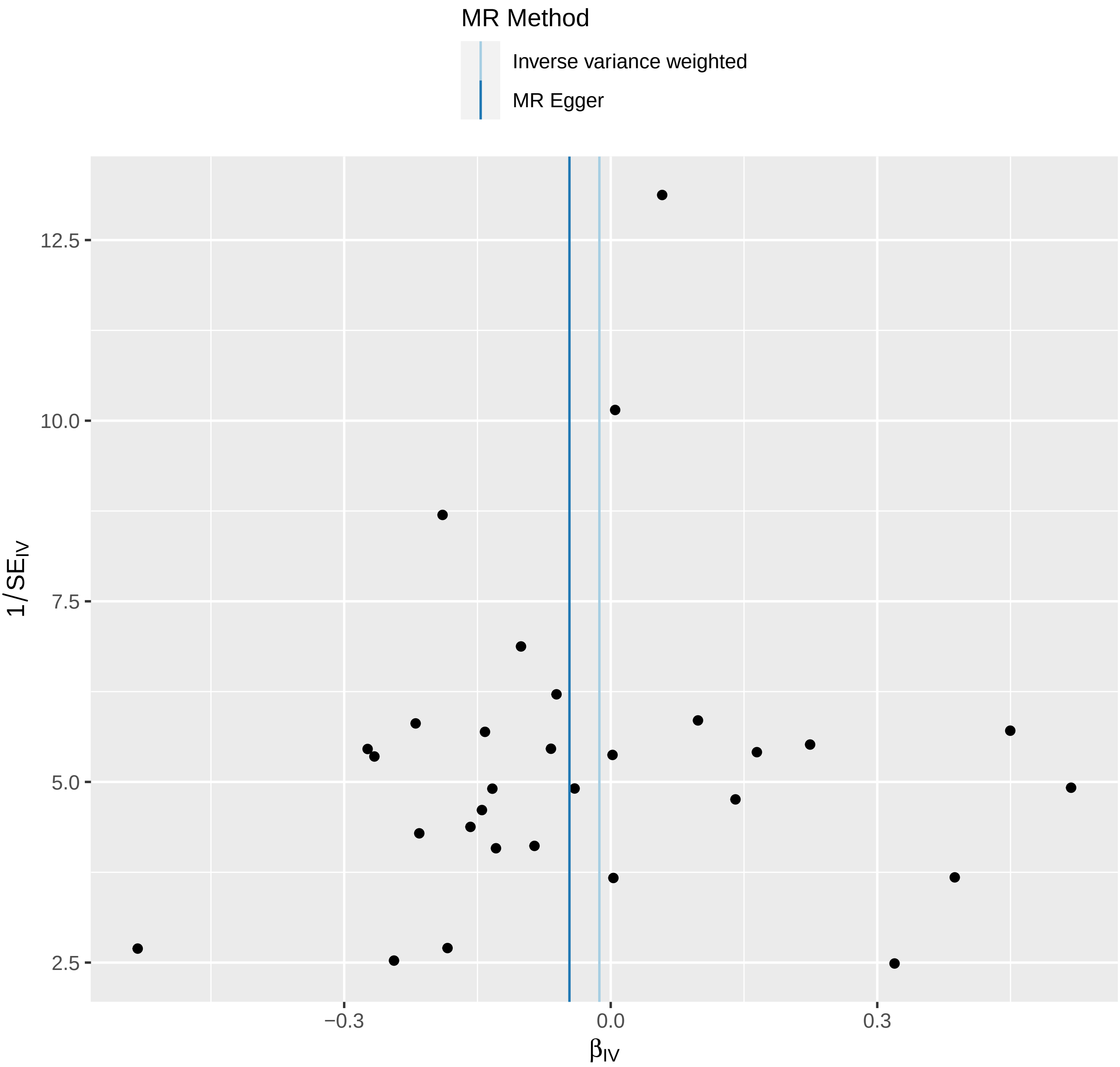

Batch 3−388 : C−C motif chemokine 1 on Diabetic nephropathy (more control exclusions)
with a threshold of 1e−05

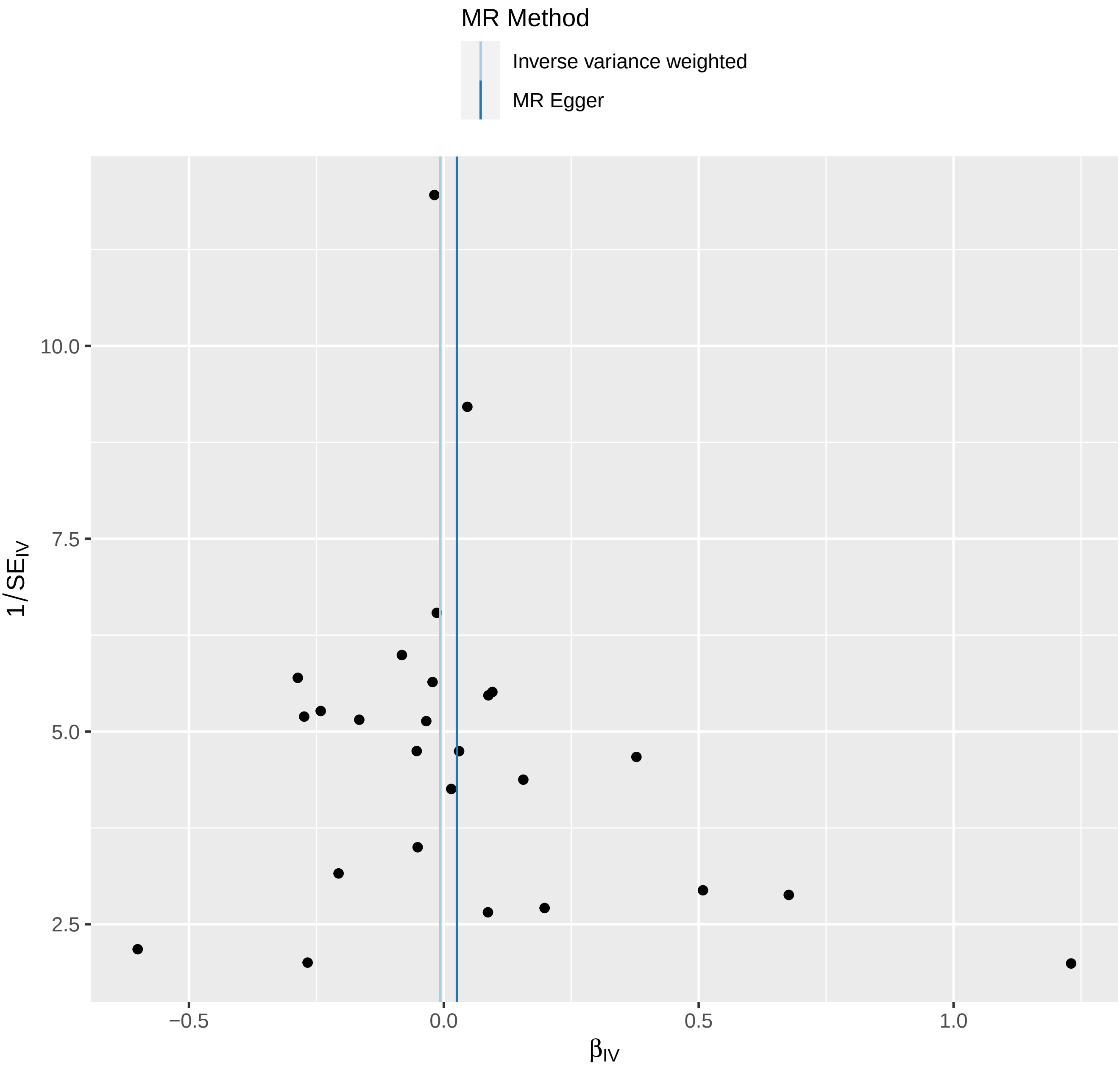

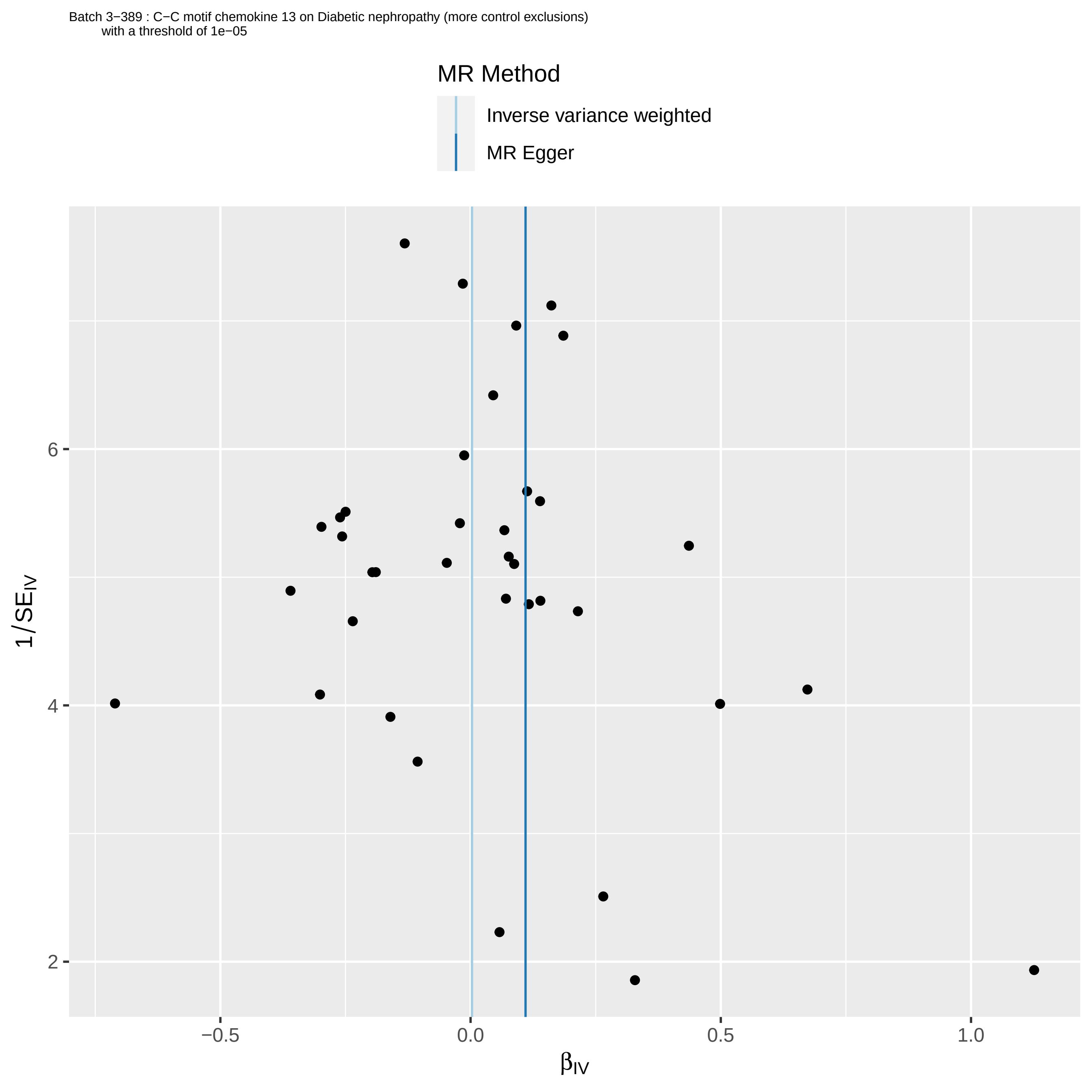
Batch 3−389 : C−C motif chemokine 13 on Diabetic nephropathy (more control exclusions)
with a threshold of 1e−05
MR Method
Inverse variance weighted
MR Egger
1/SE$_{IV}$
6
4
2
−0.5
0.0
0.5
1.0
β$_{IV}$

Batch 3−390 : C−C motif chemokine 14 on Diabetic nephropathy (more control exclusions)
with a threshold of 1e−05

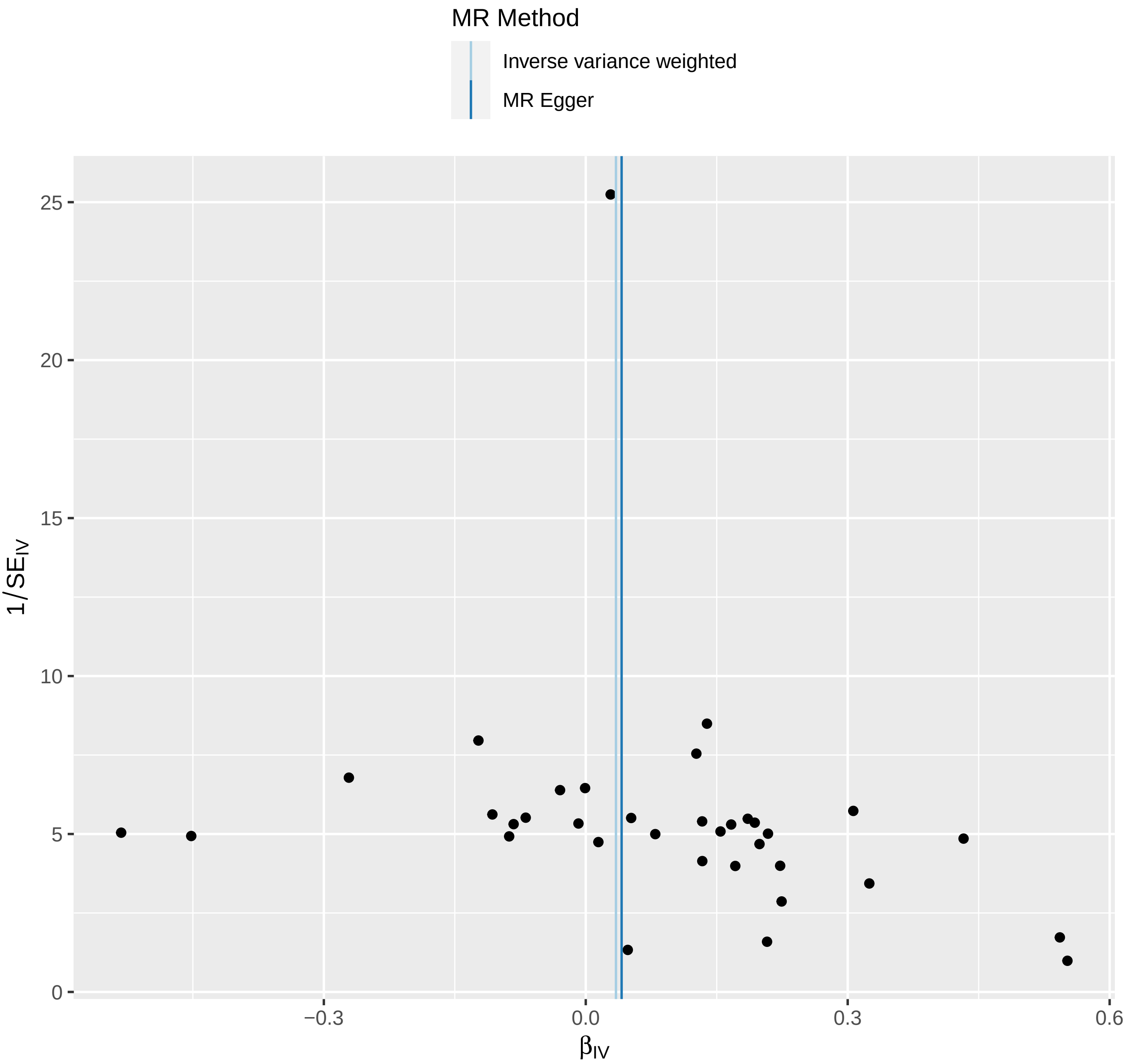

Batch 3−391 : C−C motif chemokine 15 on Diabetic nephropathy (more control exclusions)
with a threshold of 1e−05

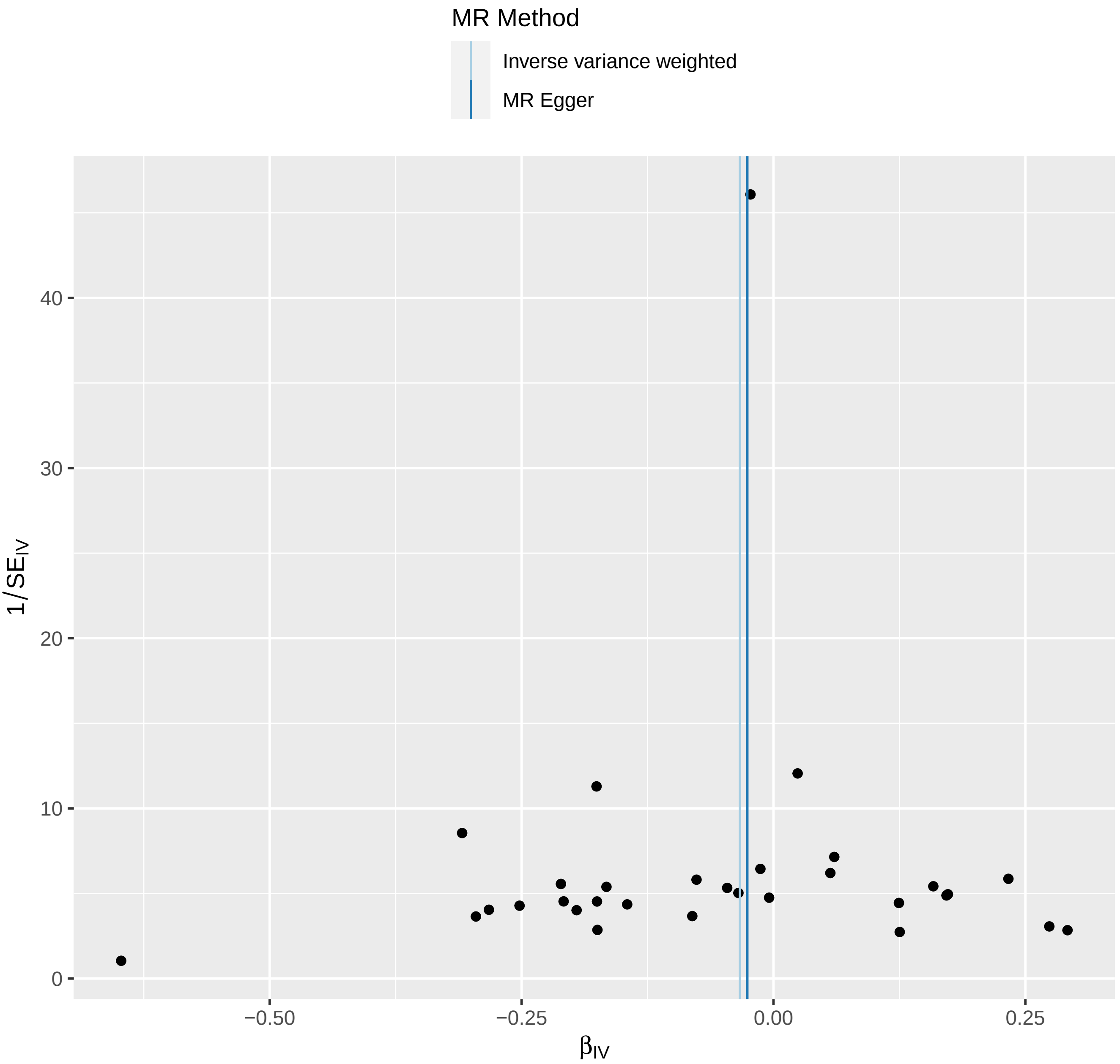

Batch 3−392 : C−C motif chemokine 15 on Diabetic nephropathy (more control exclusions)
with a threshold of 1e−05

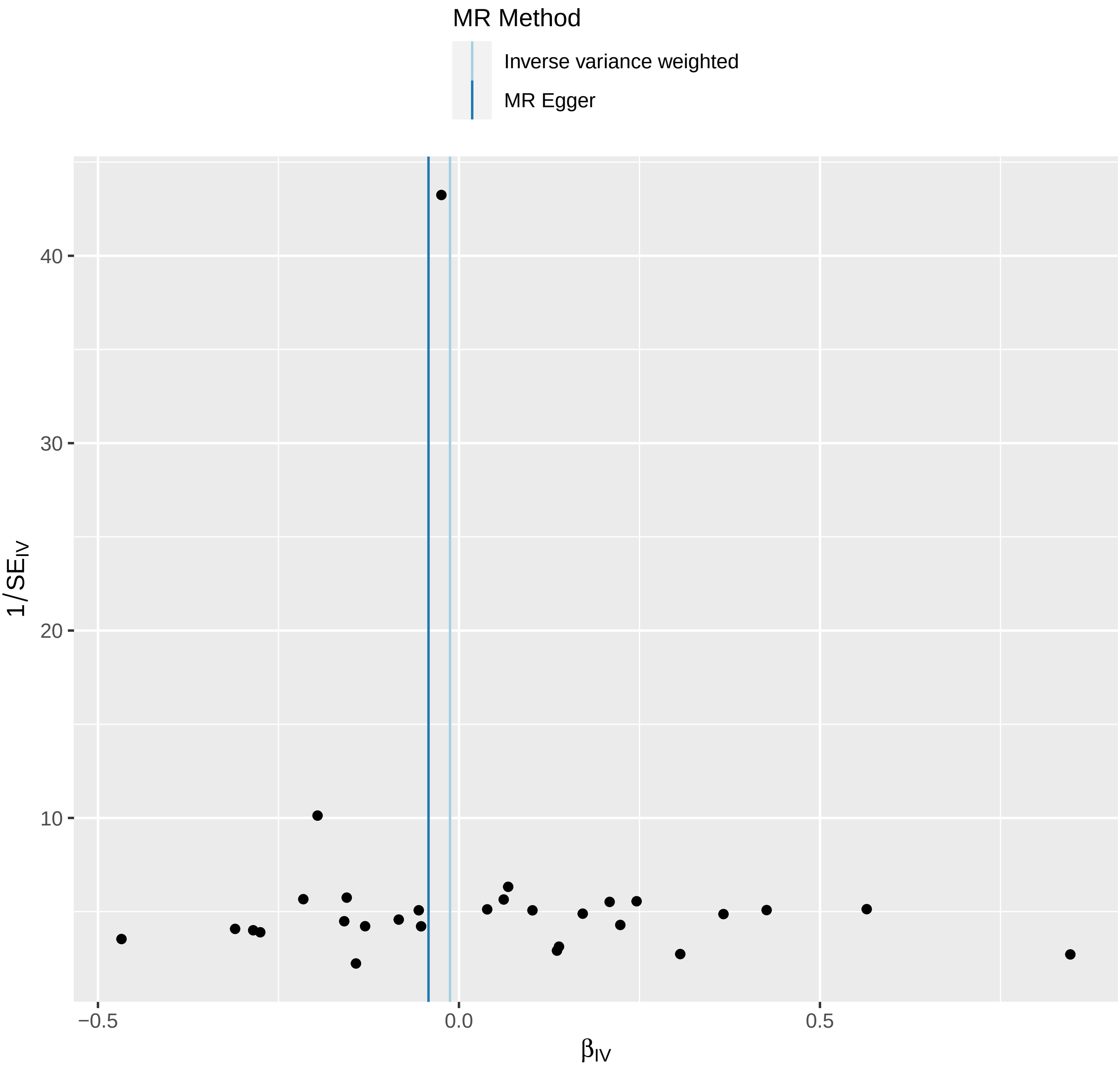

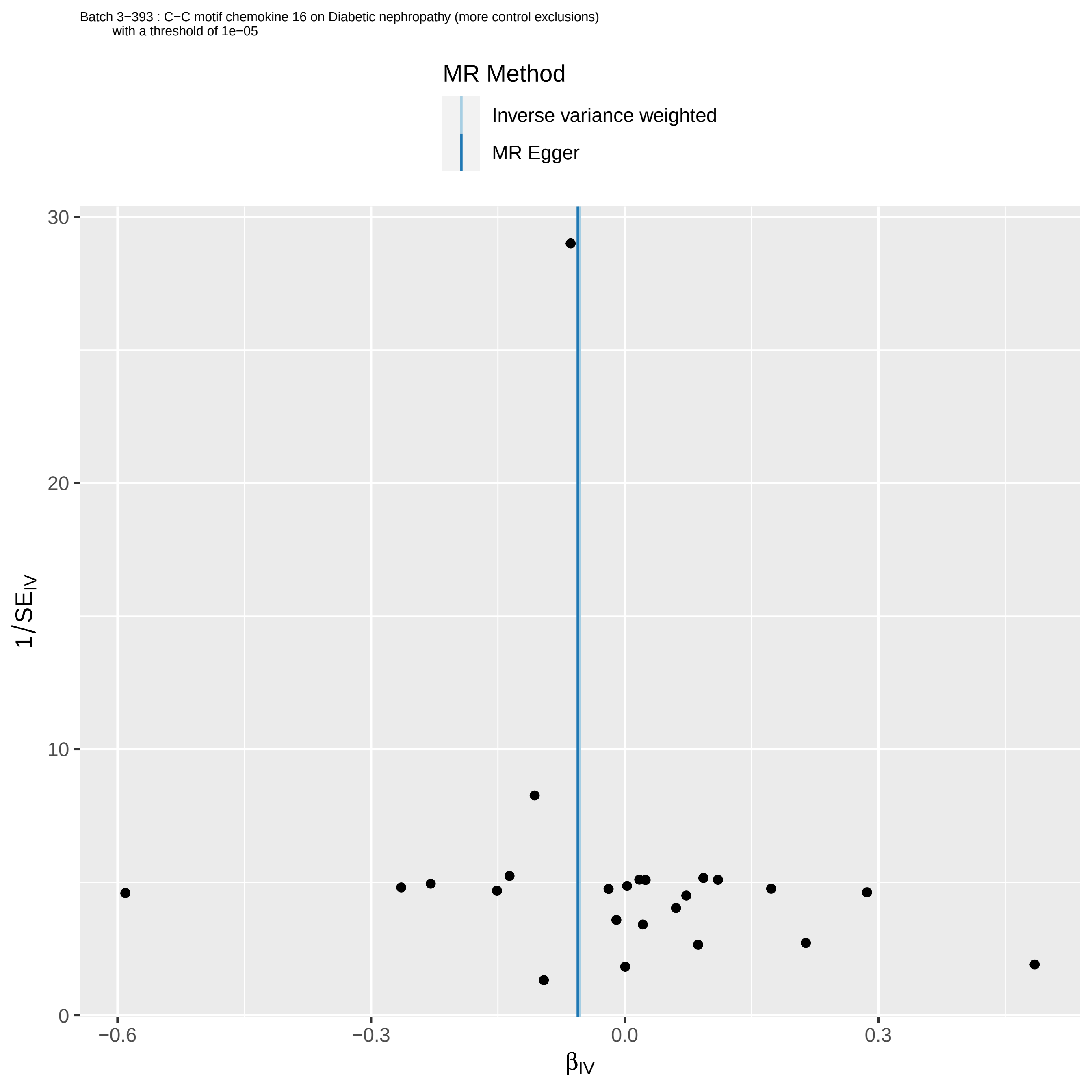
Batch 3−393 : C−C motif chemokine 16 on Diabetic nephropathy (more control exclusions)
with a threshold of 1e−05
MR Method
Inverse variance weighted
MR Egger
30
20
10
0
1/SE$_{IV}$
−0.6
−0.3
0.0
0.3
β_{IV}

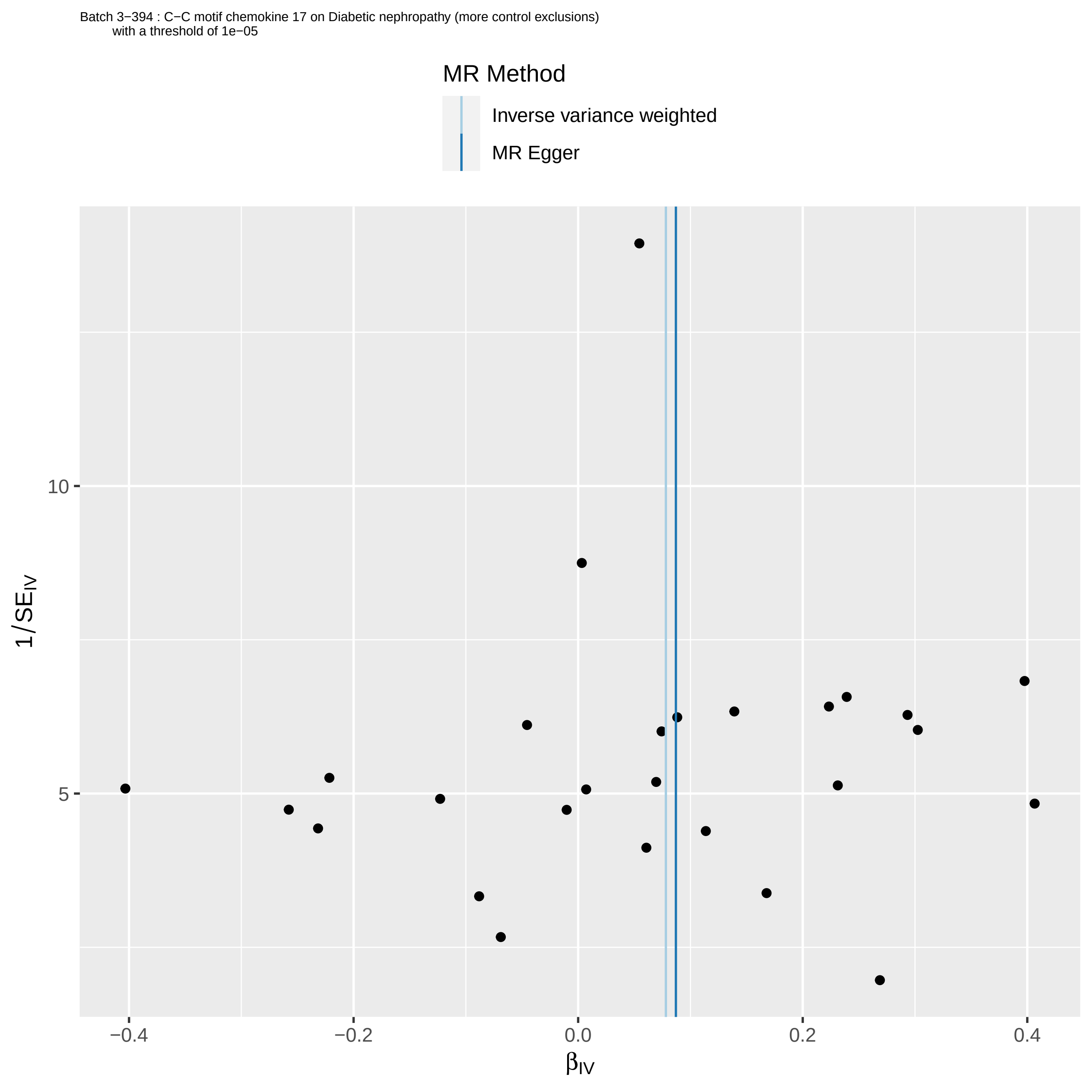
Batch 3−394 : C−C motif chemokine 17 on Diabetic nephropathy (more control exclusions)
with a threshold of 1e−05
MR Method
Inverse variance weighted
MR Egger
10
5
1/SE_IV
−0.4
−0.2
0.0
0.2
0.4
β_IV

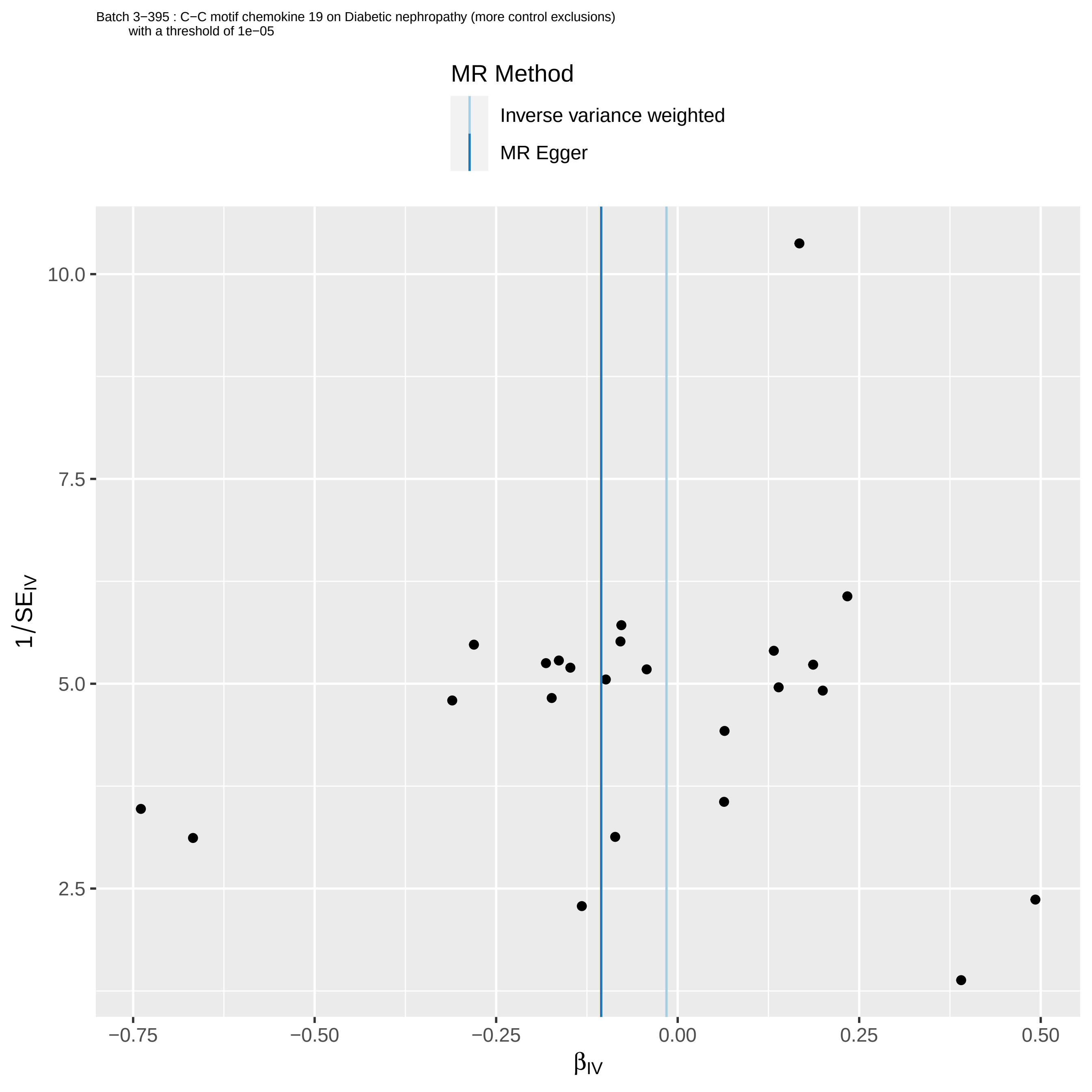
Batch 3−395 : C−C motif chemokine 19 on Diabetic nephropathy (more control exclusions)
with a threshold of 1e−05
MR Method
Inverse variance weighted
MR Egger
10.0
7.5
5.0
2.5
1/SE_IV
−0.75
−0.50
−0.25
0.00
0.25
0.50
β_IV

Batch 3−396 : C−C motif chemokine 20 on Diabetic nephropathy (more control exclusions)
with a threshold of 1e−05

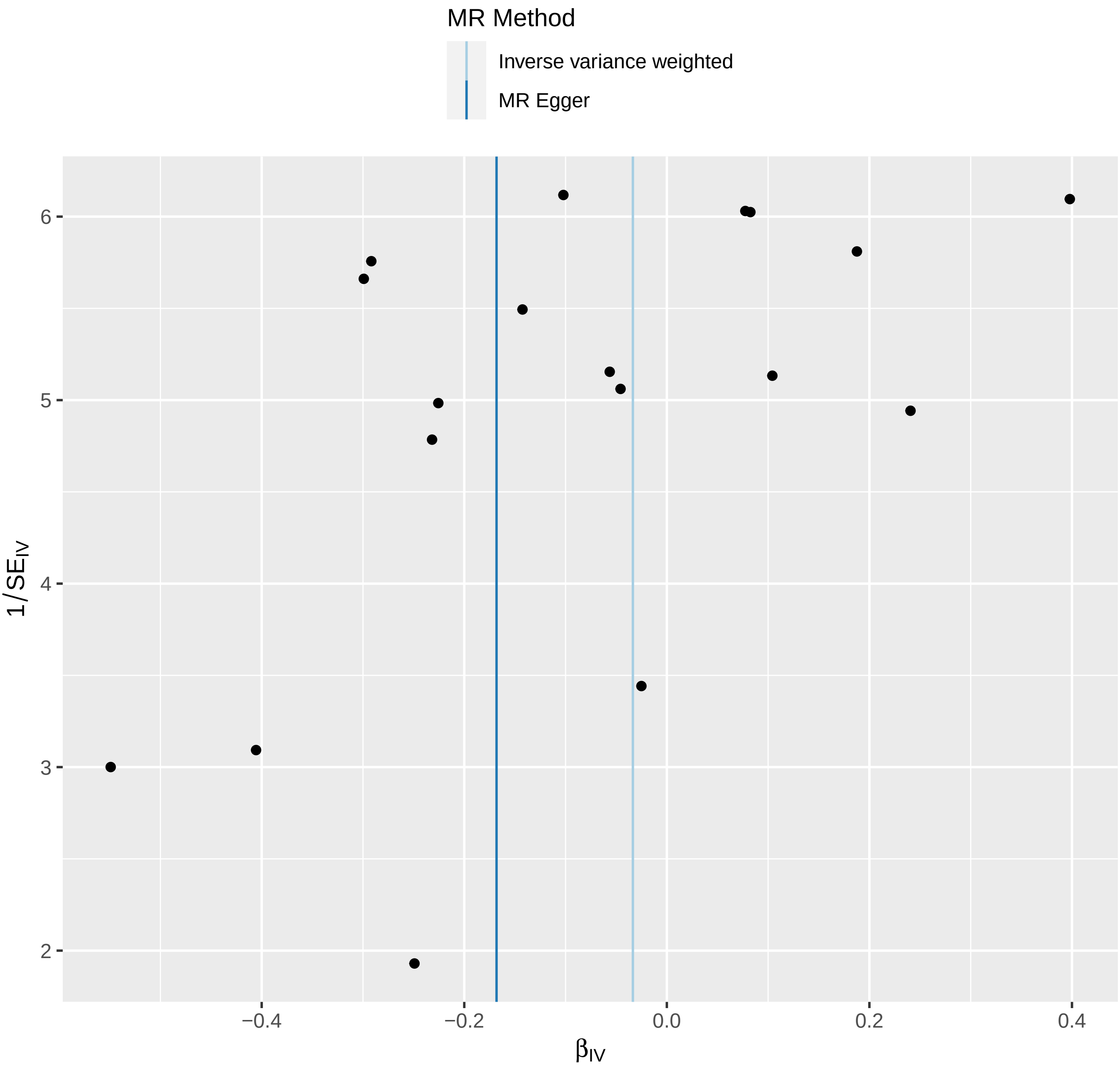

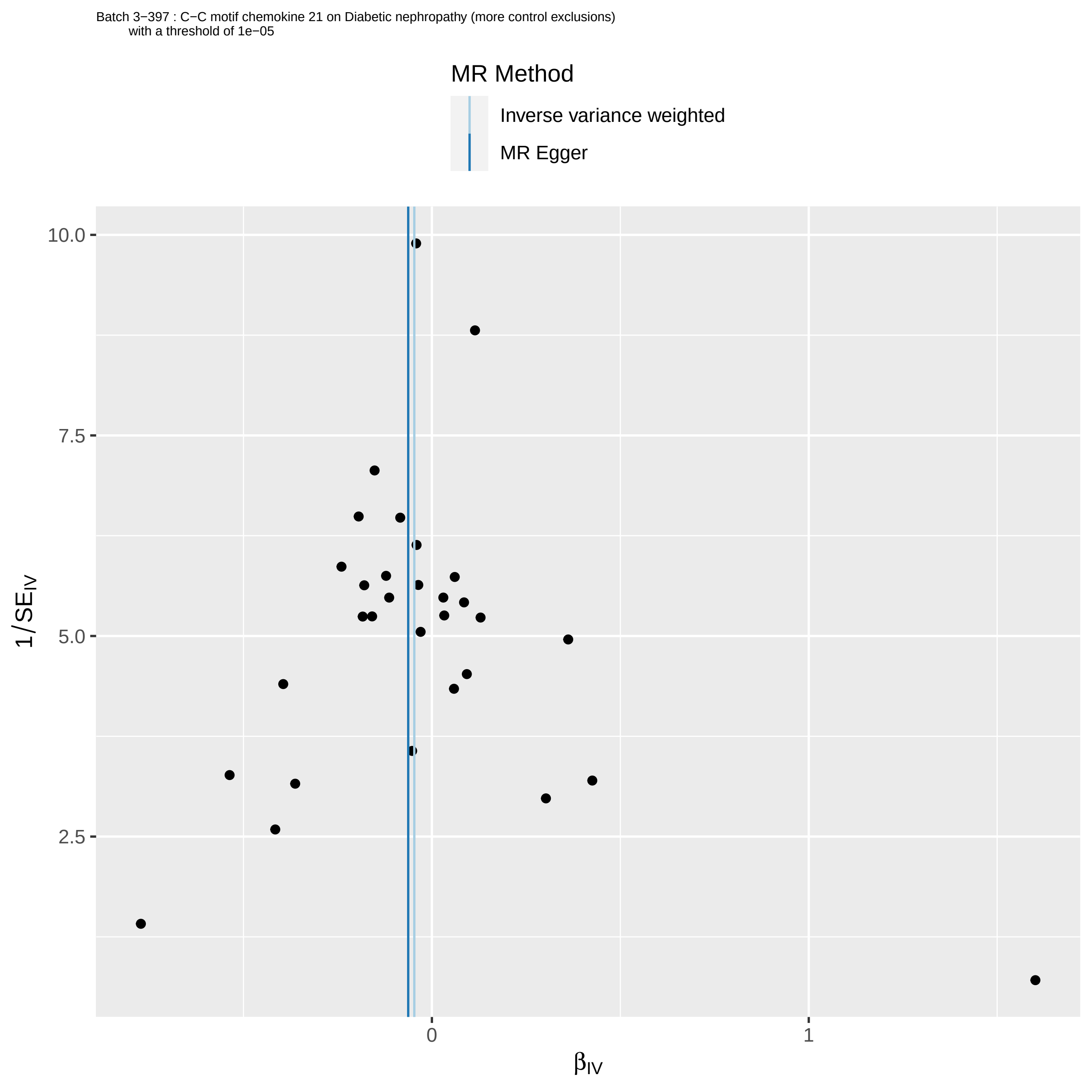

Batch 3−397 : C−C motif chemokine 21 on Diabetic nephropathy (more control exclusions)
with a threshold of 1e−05
MR Method
Inverse variance weighted
MR Egger
10.0
7.5
5.0
2.5
1/SE$_{IV}$
0
1
β$_{IV}$

Batch 3−398 : C−C motif chemokine 22 on Diabetic nephropathy (more control exclusions)
with a threshold of 1e−05

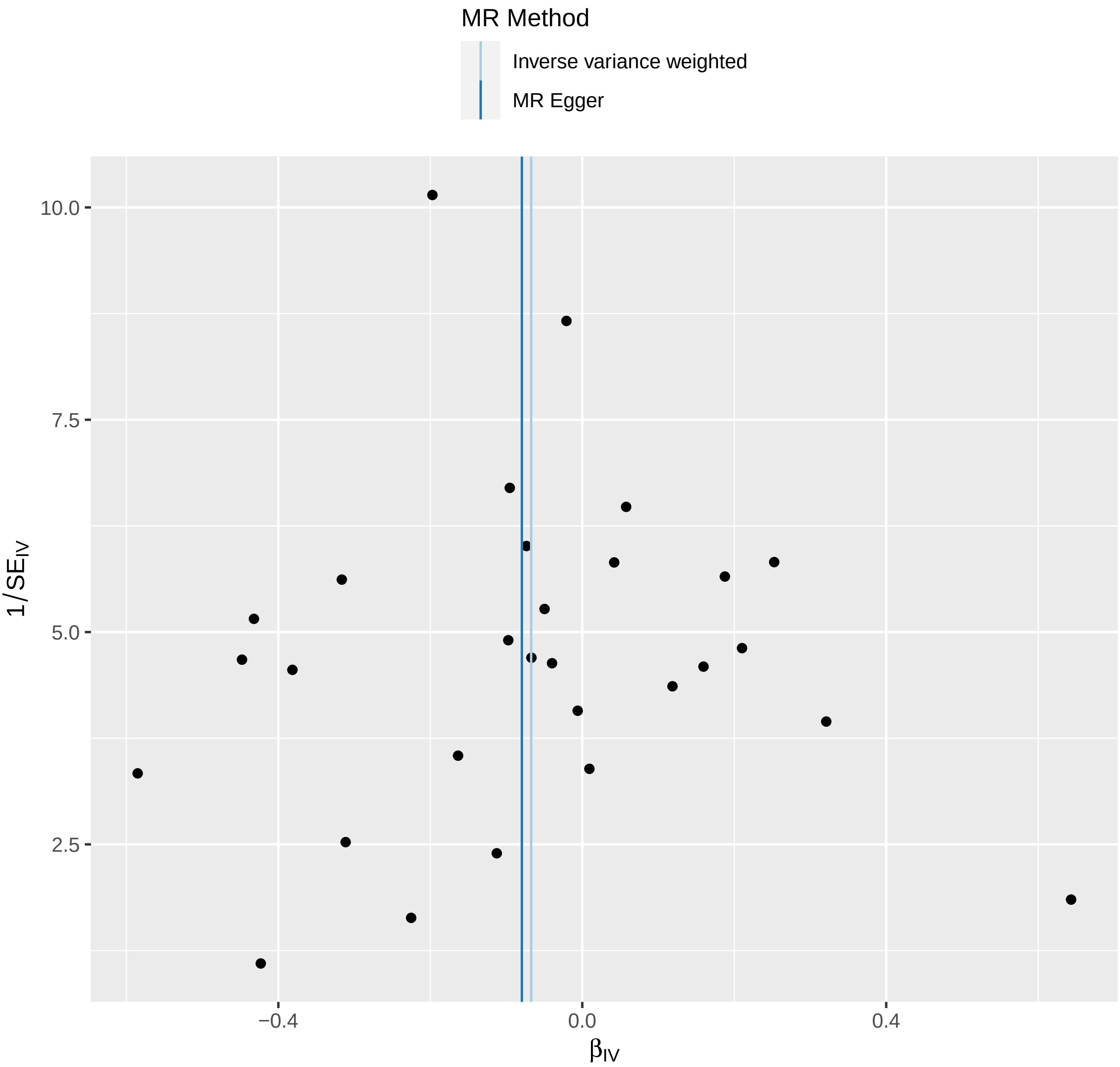

Batch 3−399 : C−C motif chemokine 23 on Diabetic nephropathy (more control exclusions)
with a threshold of 1e−05

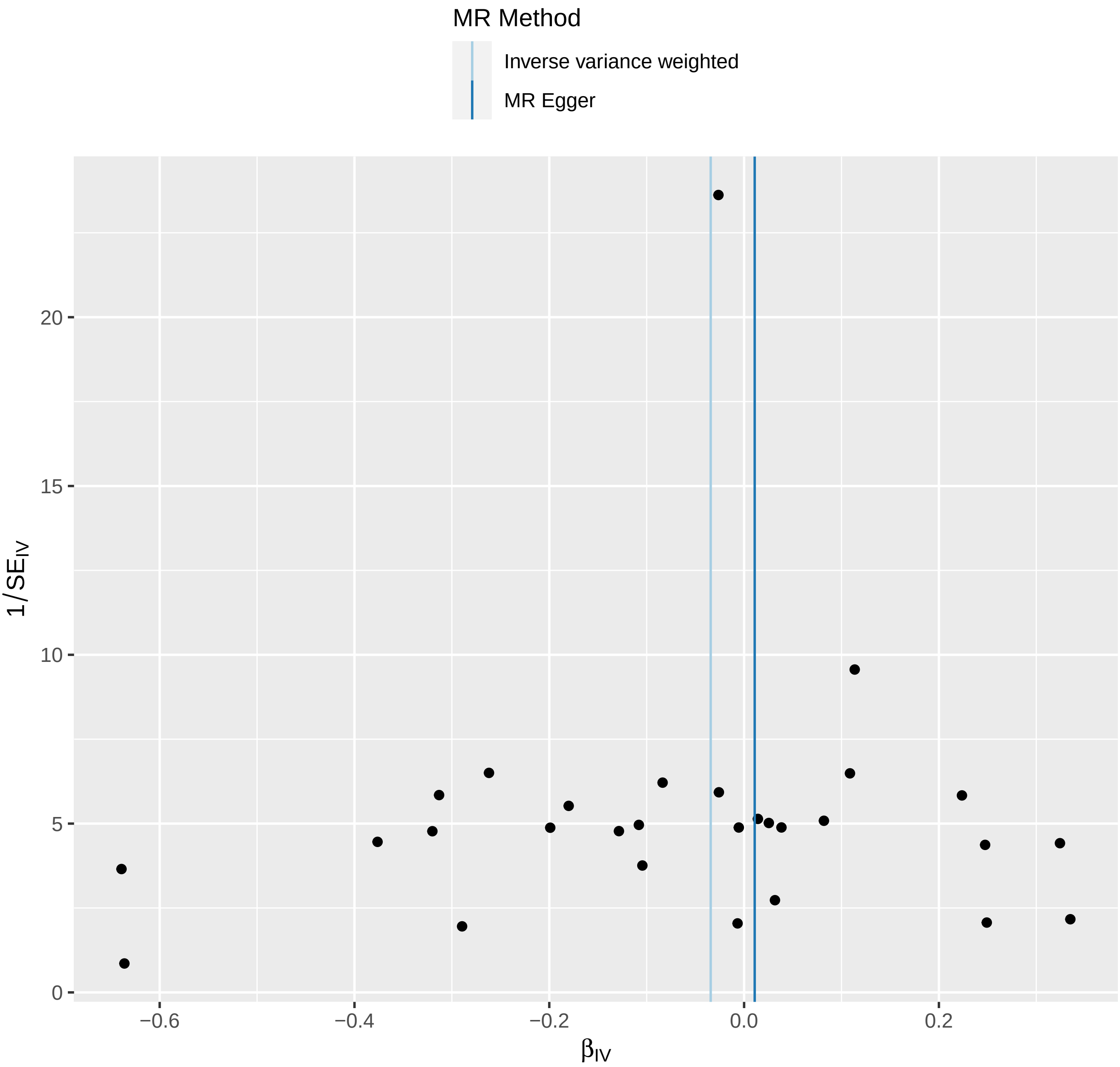

Batch 3−400 : Ck−beta−8−1 on Diabetic nephropathy (more control exclusions)
with a threshold of 1e−05

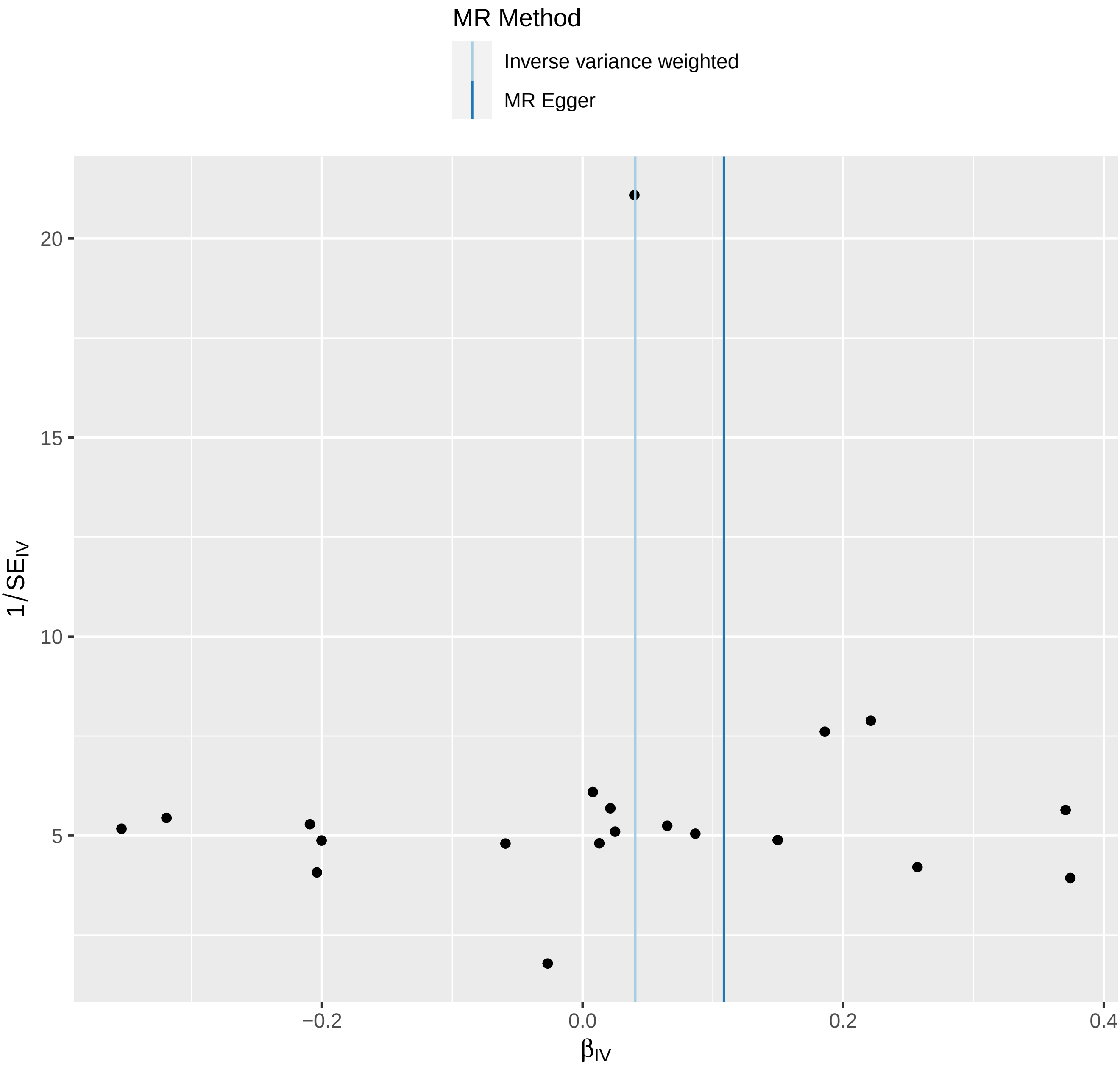

Batch 3−401 : C−C motif chemokine 24 on Diabetic nephropathy (more control exclusions)
with a threshold of 1e−05

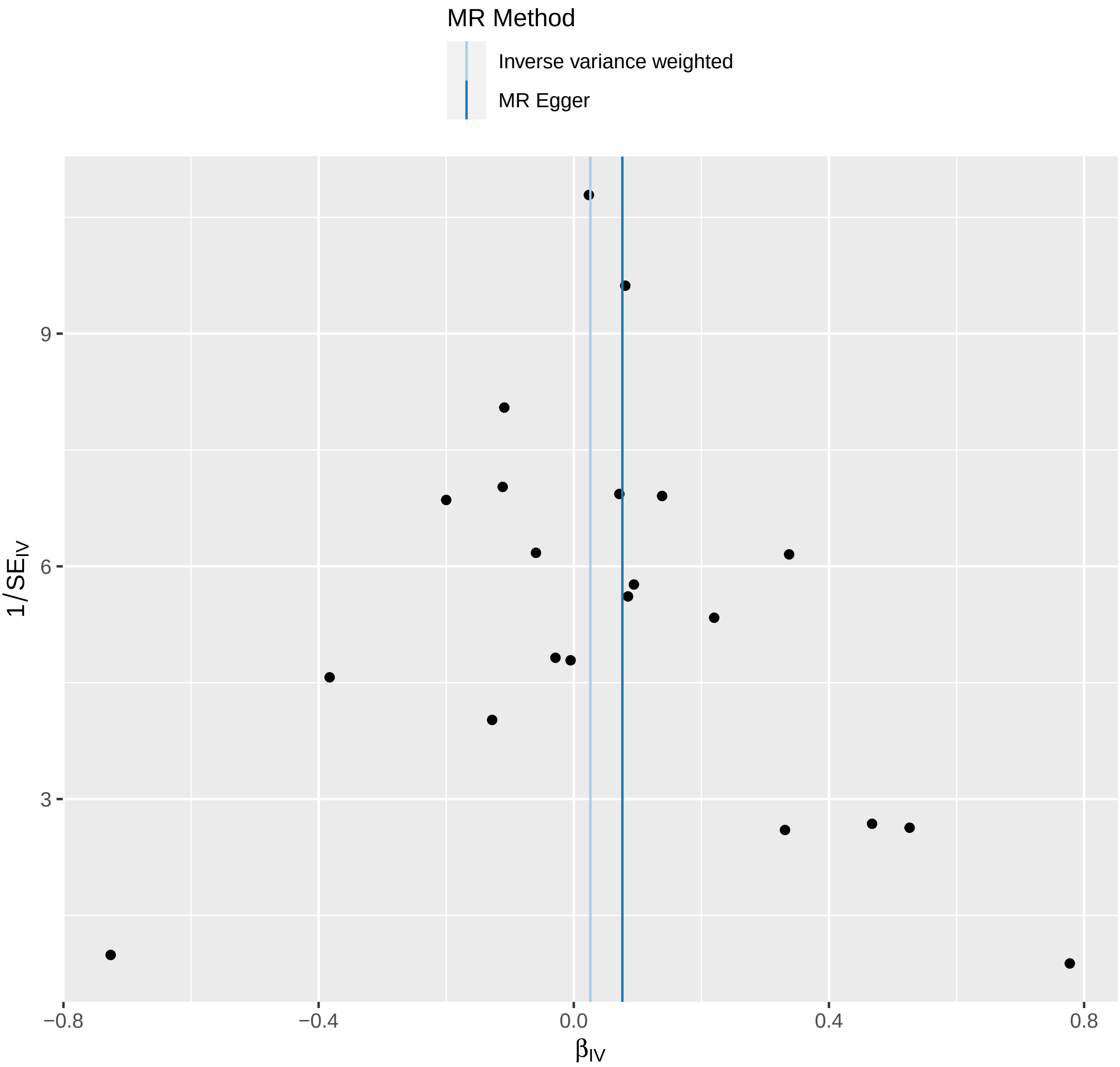

Batch 3−402 : C−C motif chemokine 25 on Diabetic nephropathy (more control exclusions)
with a threshold of 1e−05

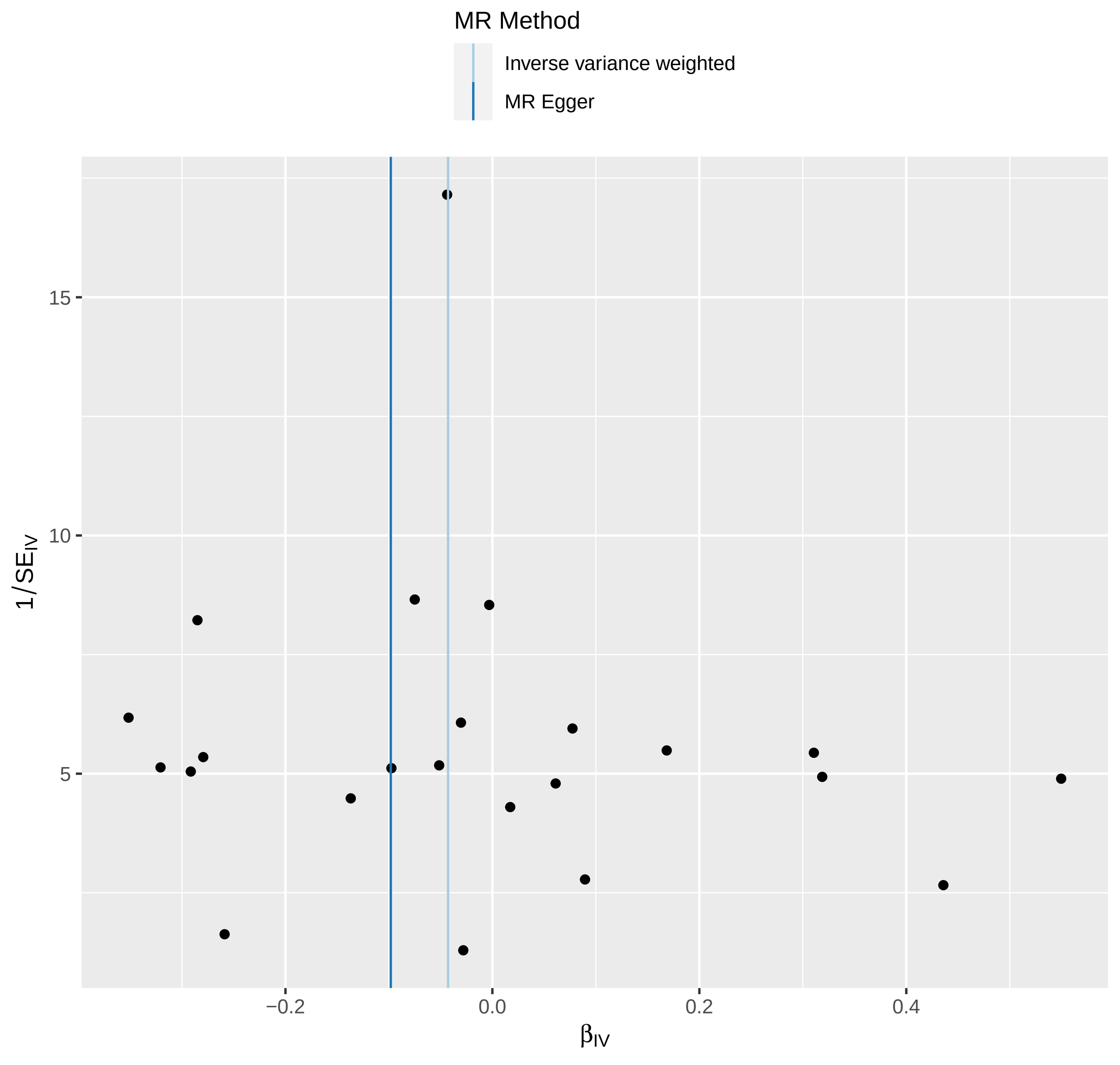

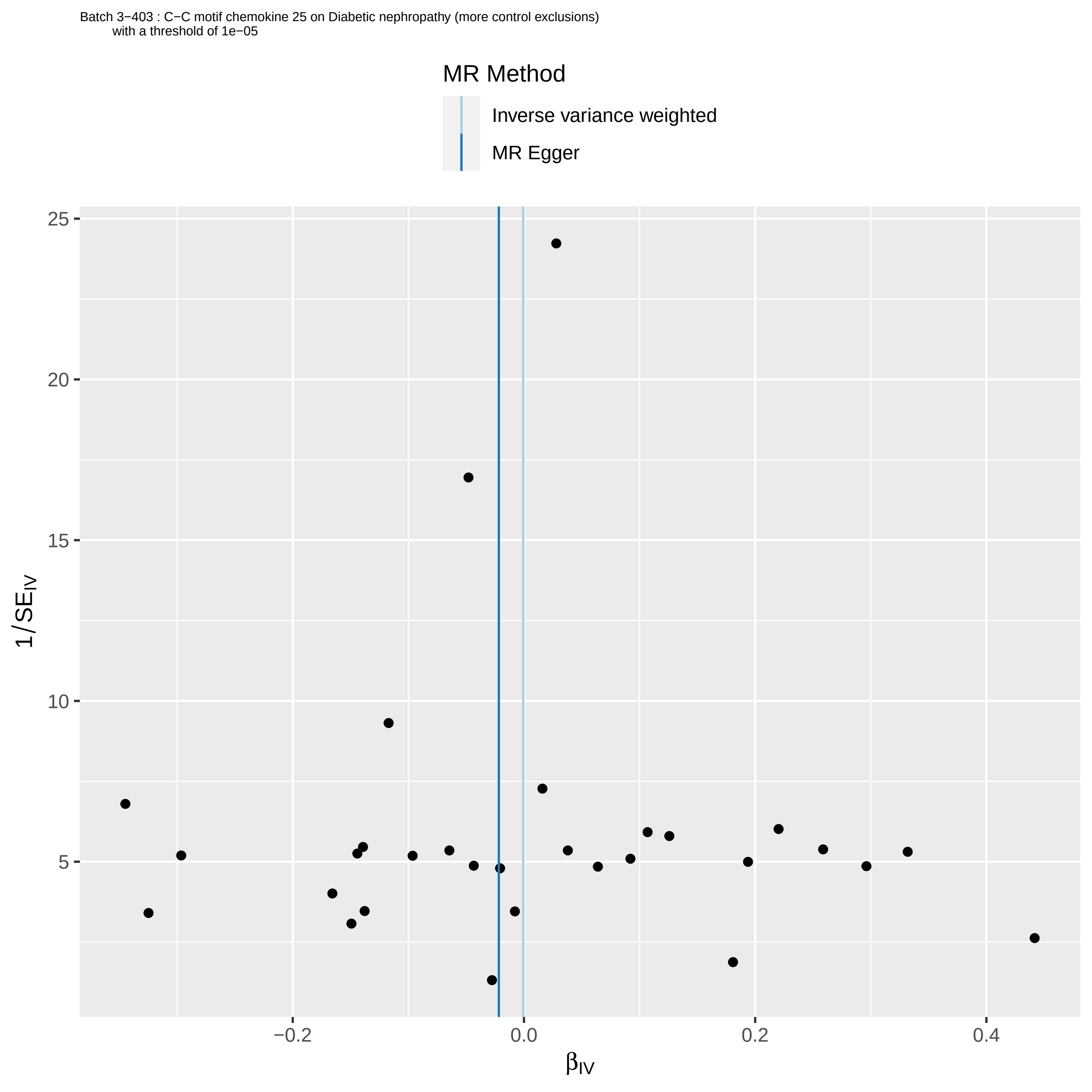

Batch 3−403 : C−C motif chemokine 25 on Diabetic nephropathy (more control exclusions)
with a threshold of 1e−05
MR Method
Inverse variance weighted
MR Egger
25
20
15
10
5
1/SE_IV
−0.2
0.0
0.2
0.4
β_IV

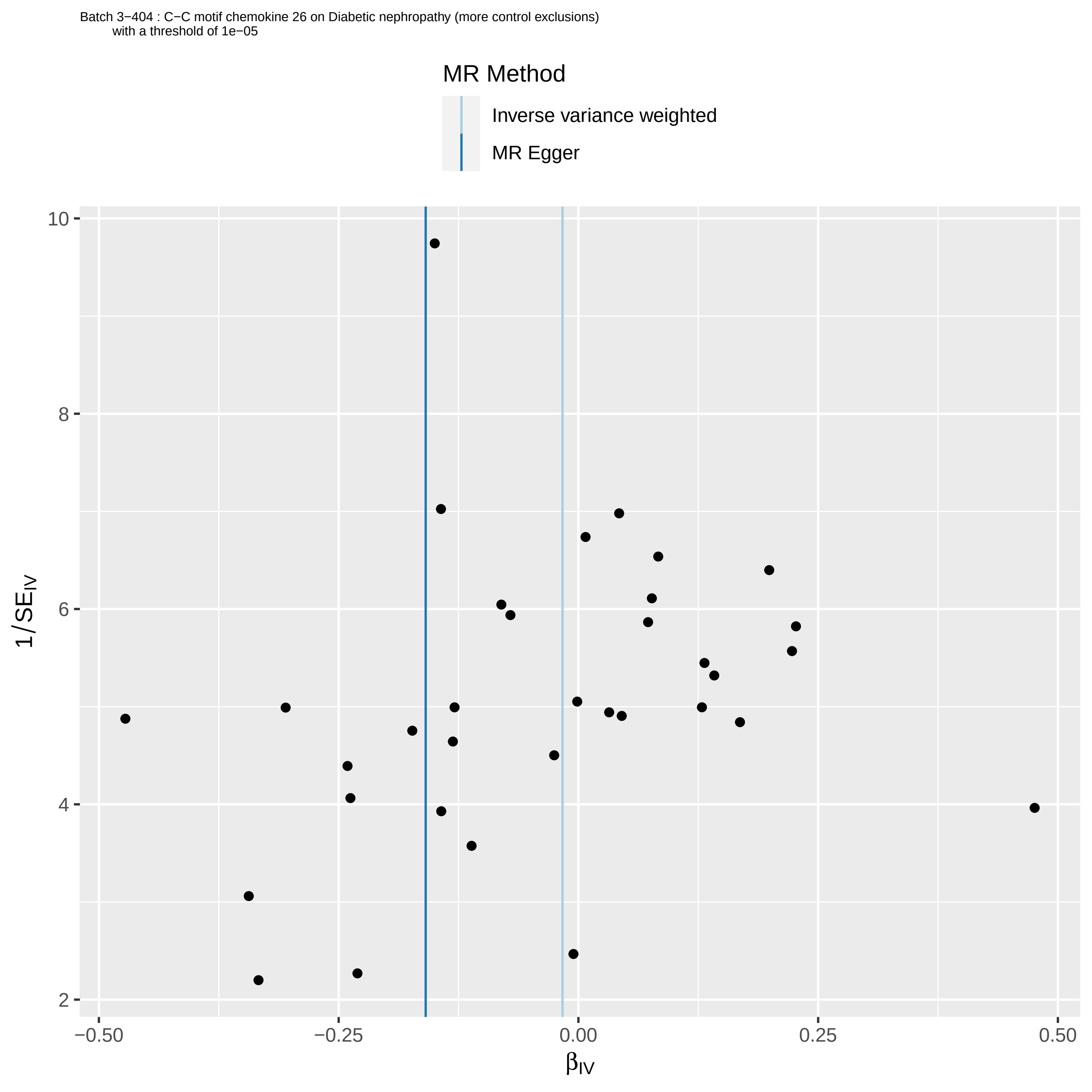

Batch 3−404 : C−C motif chemokine 26 on Diabetic nephropathy (more control exclusions)
with a threshold of 1e−05
MR Method
Inverse variance weighted
MR Egger
10
8
6
4
2
1/SE_IV
−0.50
−0.25
0.00
0.25
0.50
β_IV

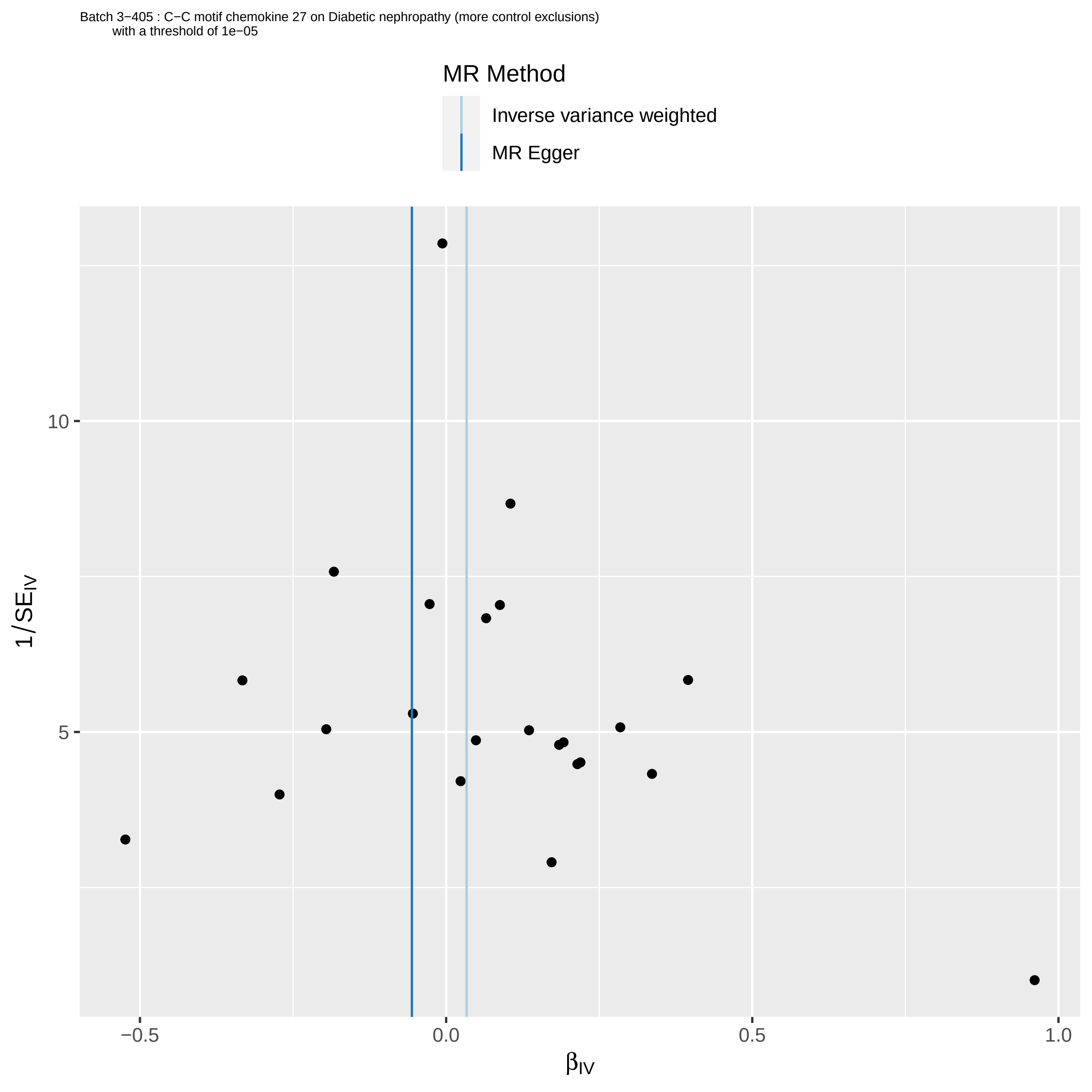

Batch 3−405 : C−C motif chemokine 27 on Diabetic nephropathy (more control exclusions)
with a threshold of 1e−05
MR Method
Inverse variance weighted
MR Egger
10
5
1/SE_IV
−0.5
0.0
0.5
1.0
β_IV

Batch 3−406 : C−C motif chemokine 28 on Diabetic nephropathy (more control exclusions)
with a threshold of 1e−05

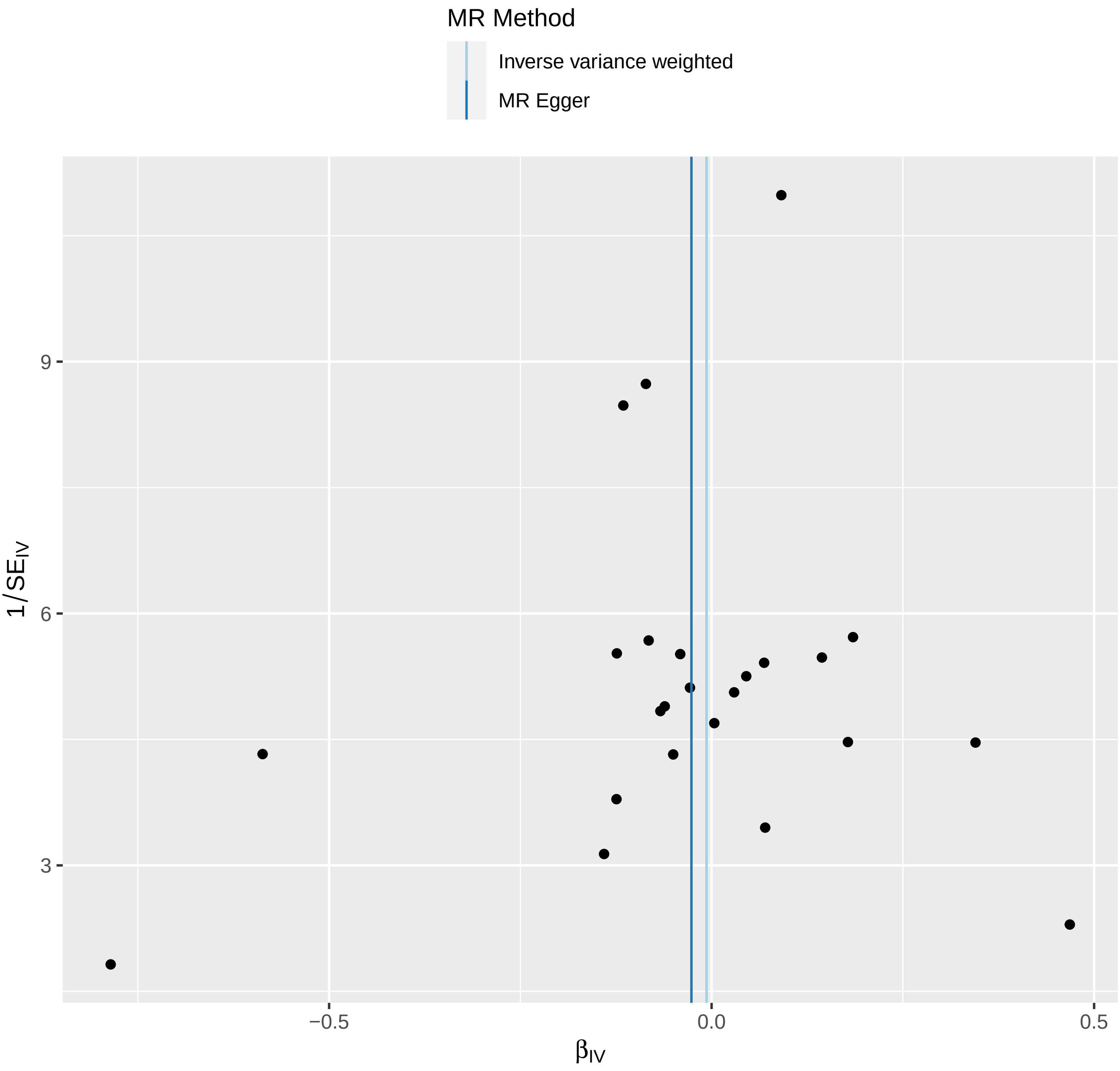

Batch 3−407 : C−C motif chemokine 3 on Diabetic nephropathy (more control exclusions)
with a threshold of 1e−05

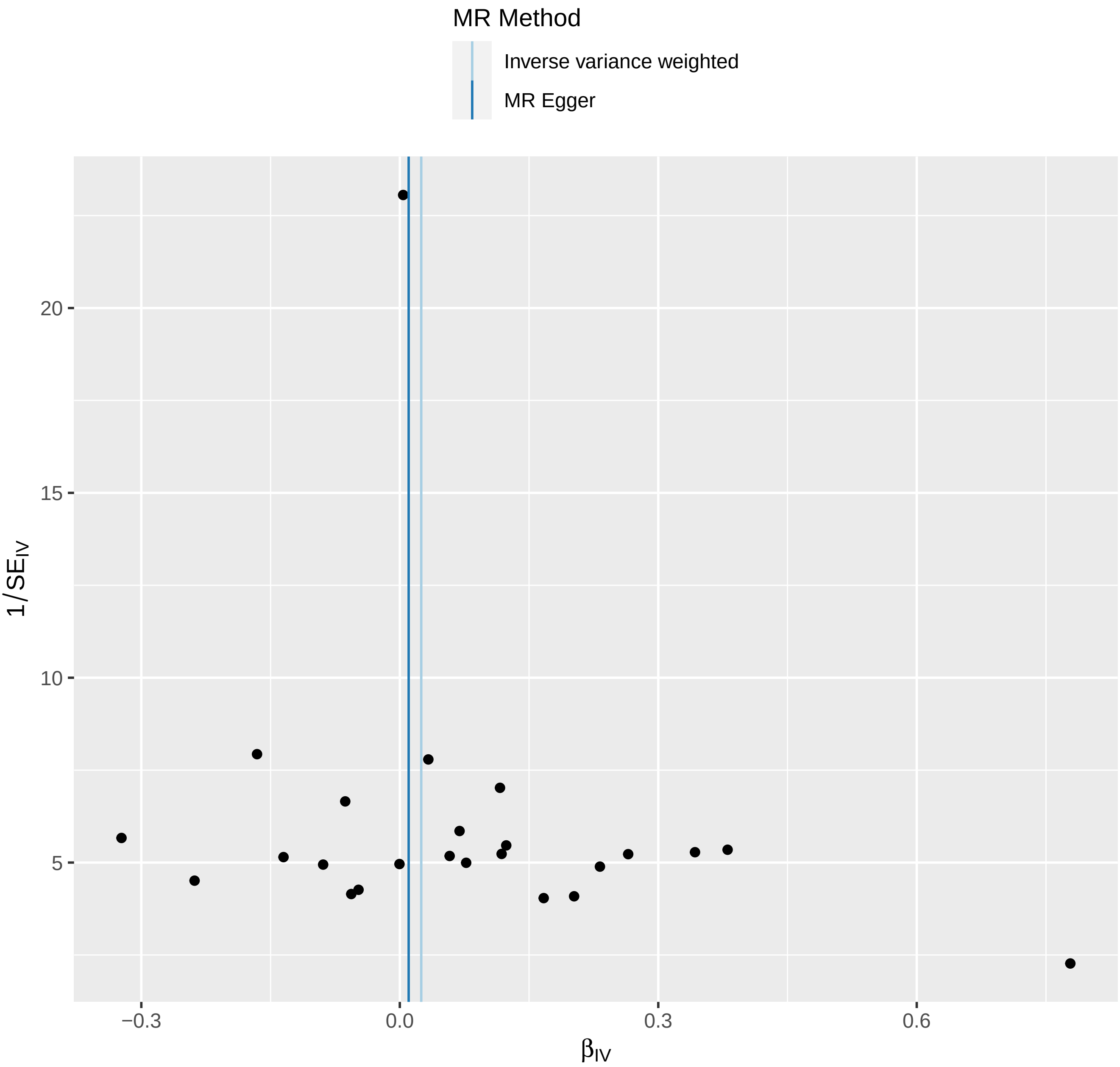

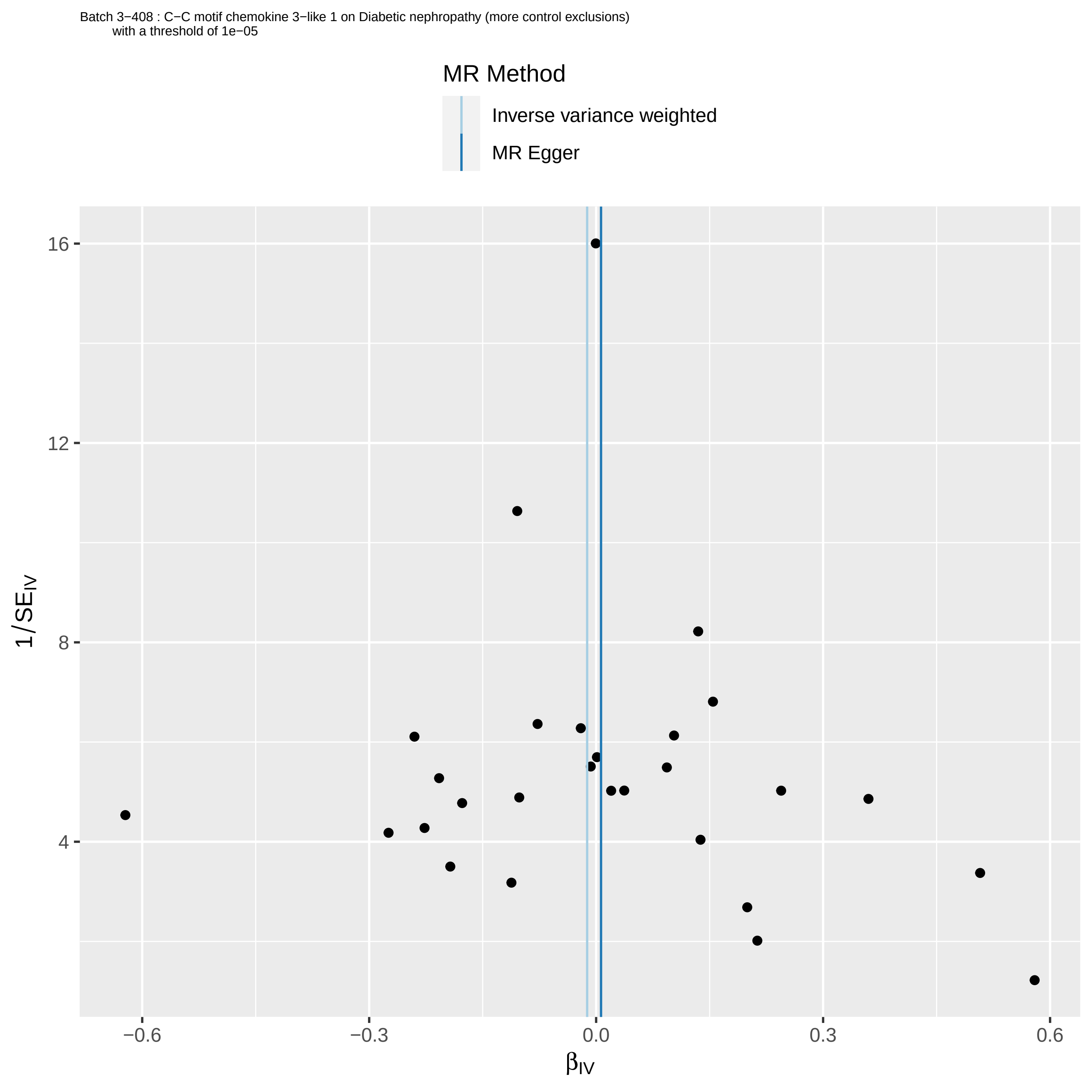

Batch 3−408 : C−C motif chemokine 3−like 1 on Diabetic nephropathy (more control exclusions)
with a threshold of 1e−05
MR Method
Inverse variance weighted
MR Egger
16
12
8
4
−0.6
−0.3
0.0
0.3
0.6
$1/SE_{IV}$
β_{IV}

Batch 3−409 : C−C motif chemokine 5 on Diabetic nephropathy (more control exclusions)
with a threshold of 1e−05

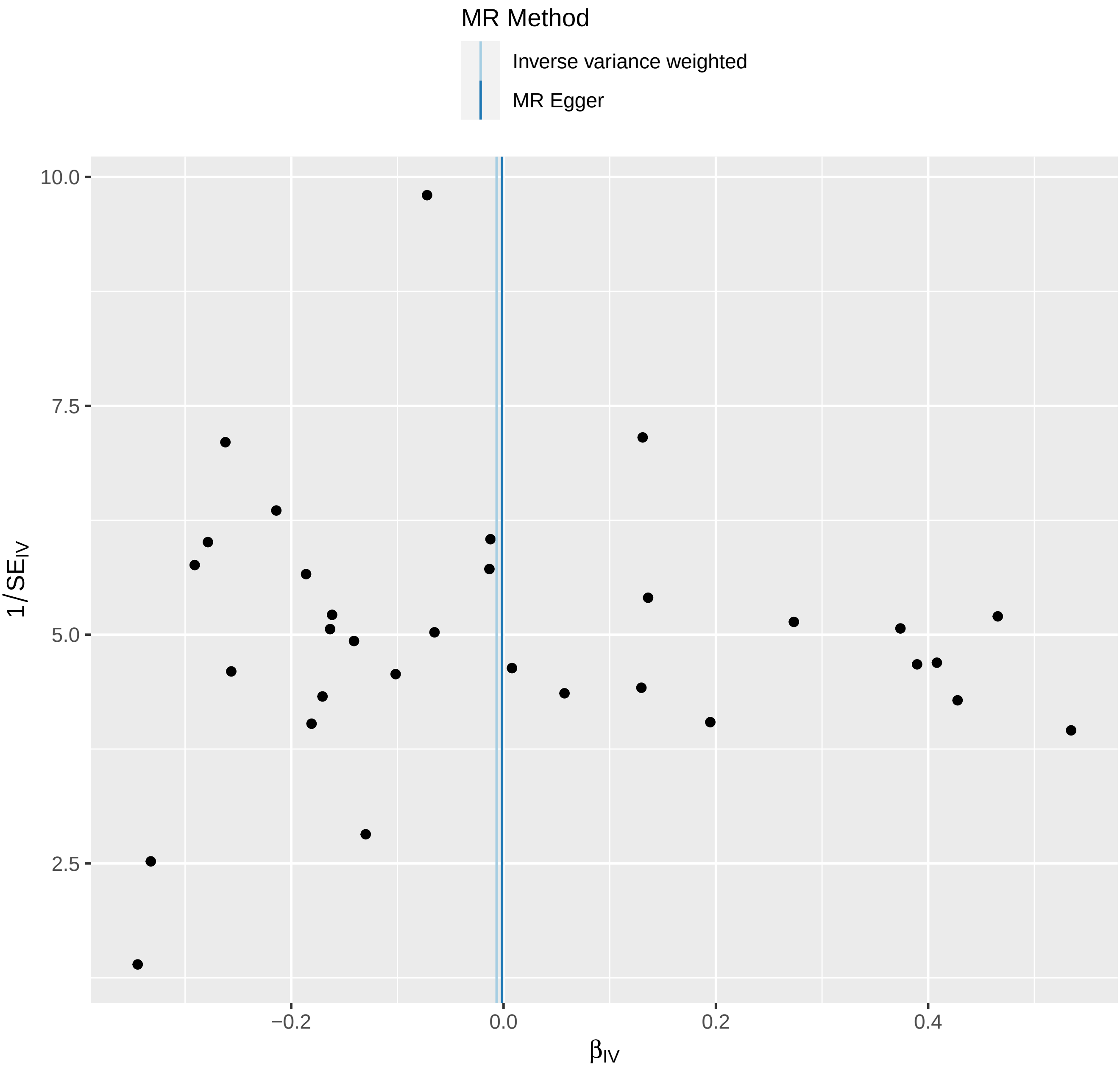

Batch 3−410 : C−C motif chemokine 7 on Diabetic nephropathy (more control exclusions)
with a threshold of 1e−05

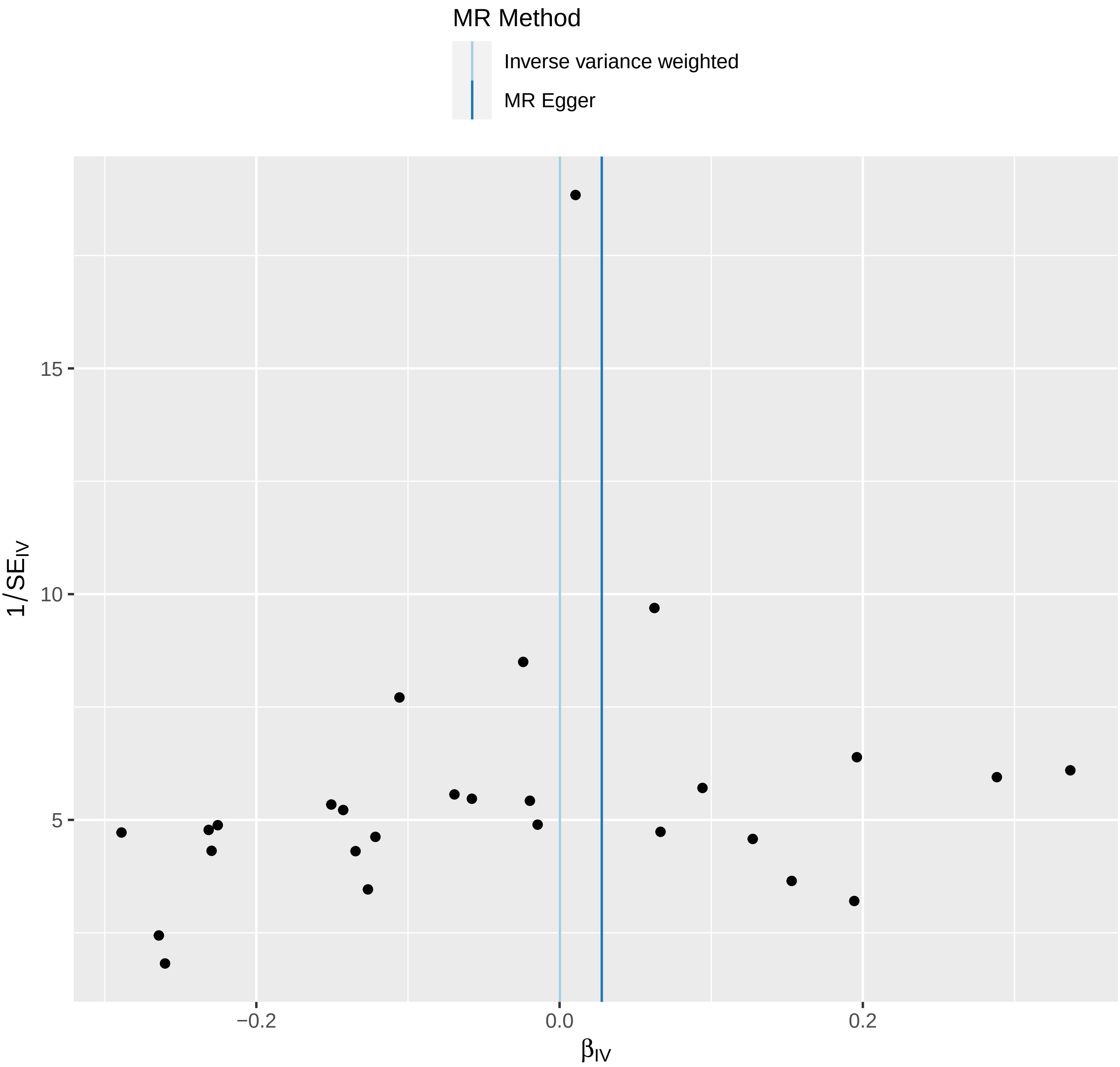

Batch 3−411 : C−C motif chemokine 8 on Diabetic nephropathy (more control exclusions)
with a threshold of 1e−05

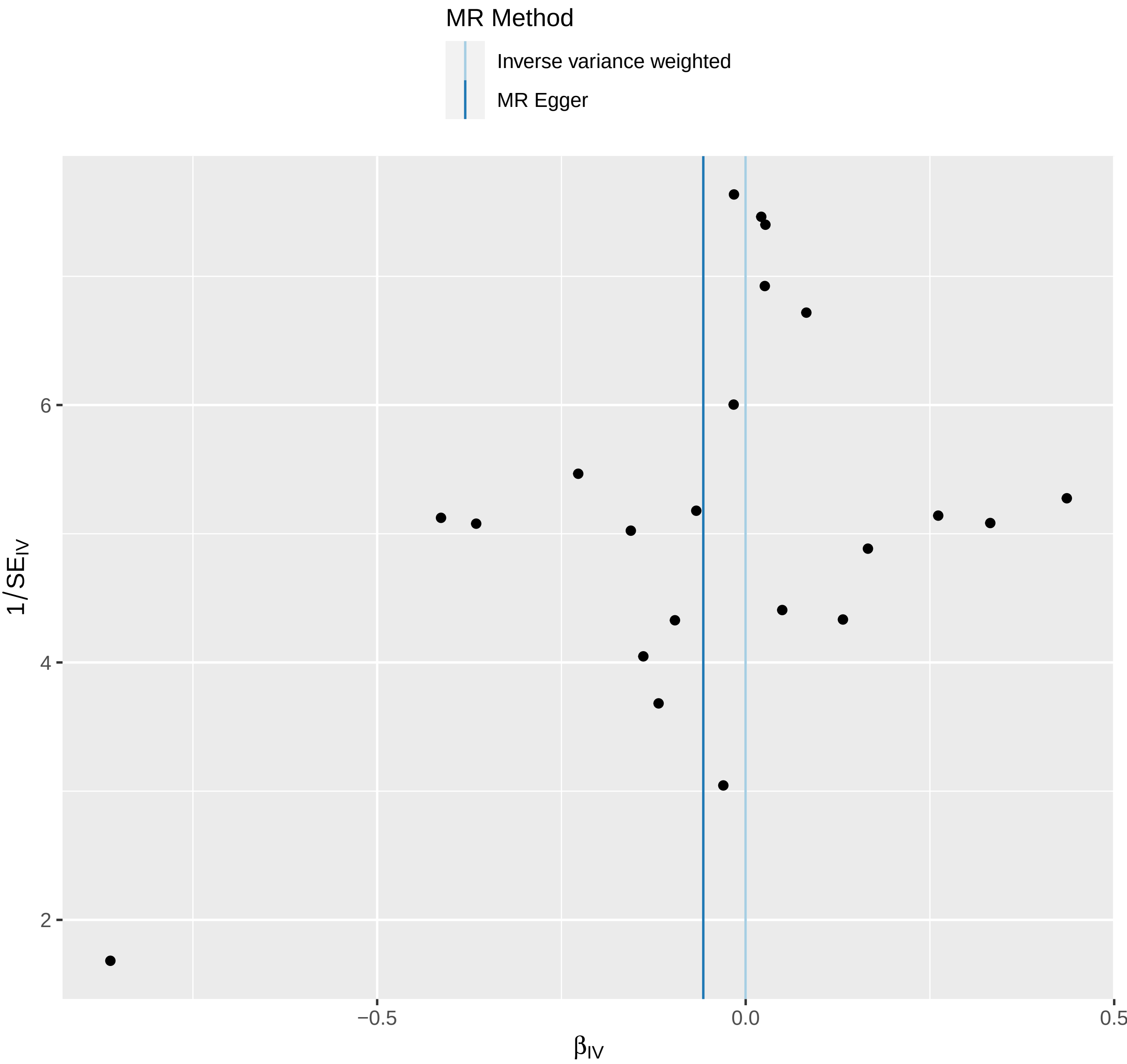

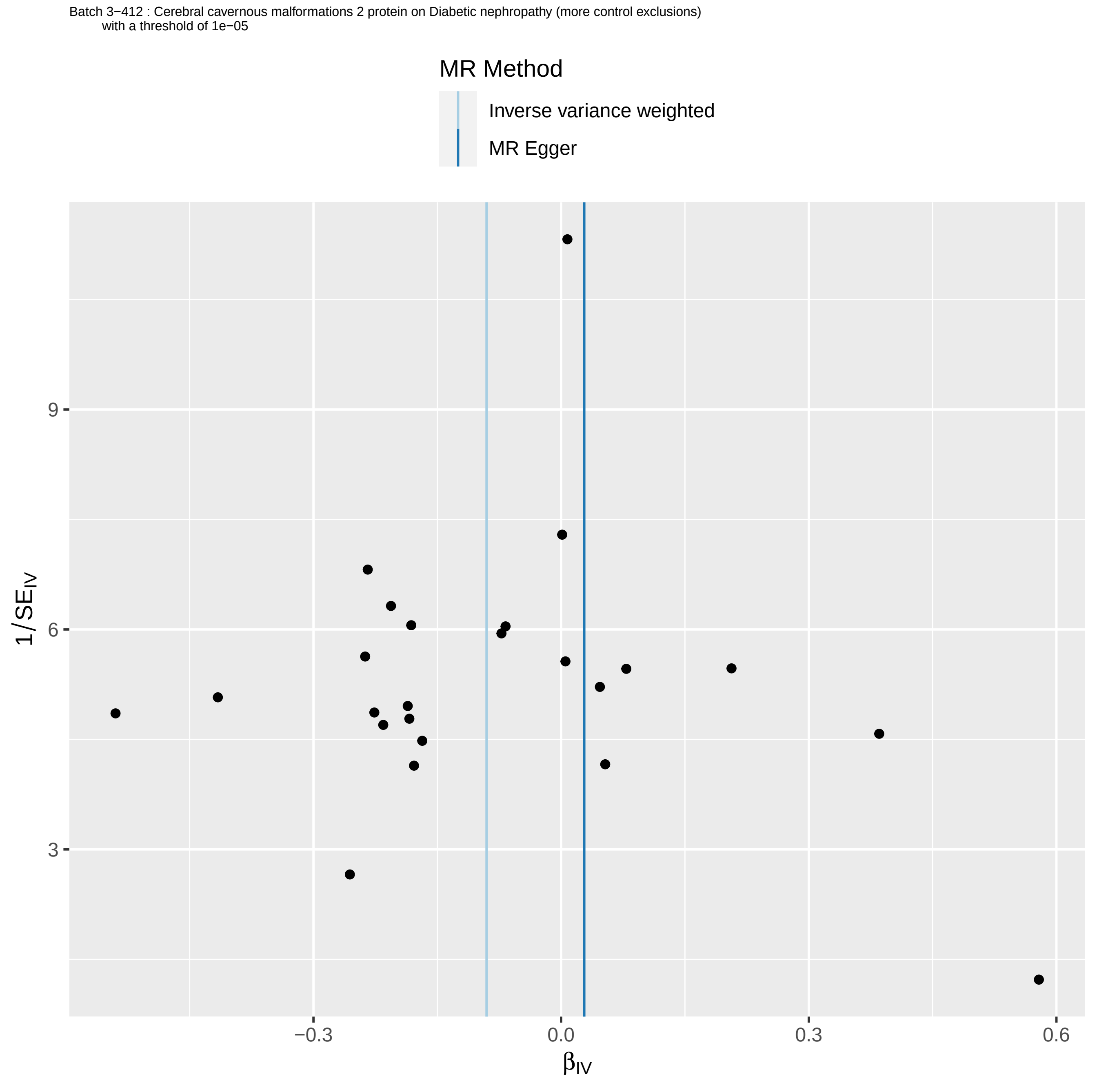
Batch 3−412 : Cerebral cavernous malformations 2 protein on Diabetic nephropathy (more control exclusions)
with a threshold of 1e−05
MR Method
Inverse variance weighted
MR Egger
9
6
3
1/SE$_{IV}$
−0.3
0.0
0.3
0.6
β$_{IV}$

Batch 3−413 : G2/mitotic−specific cyclin−B1 on Diabetic nephropathy (more control exclusions)
with a threshold of 1e−05

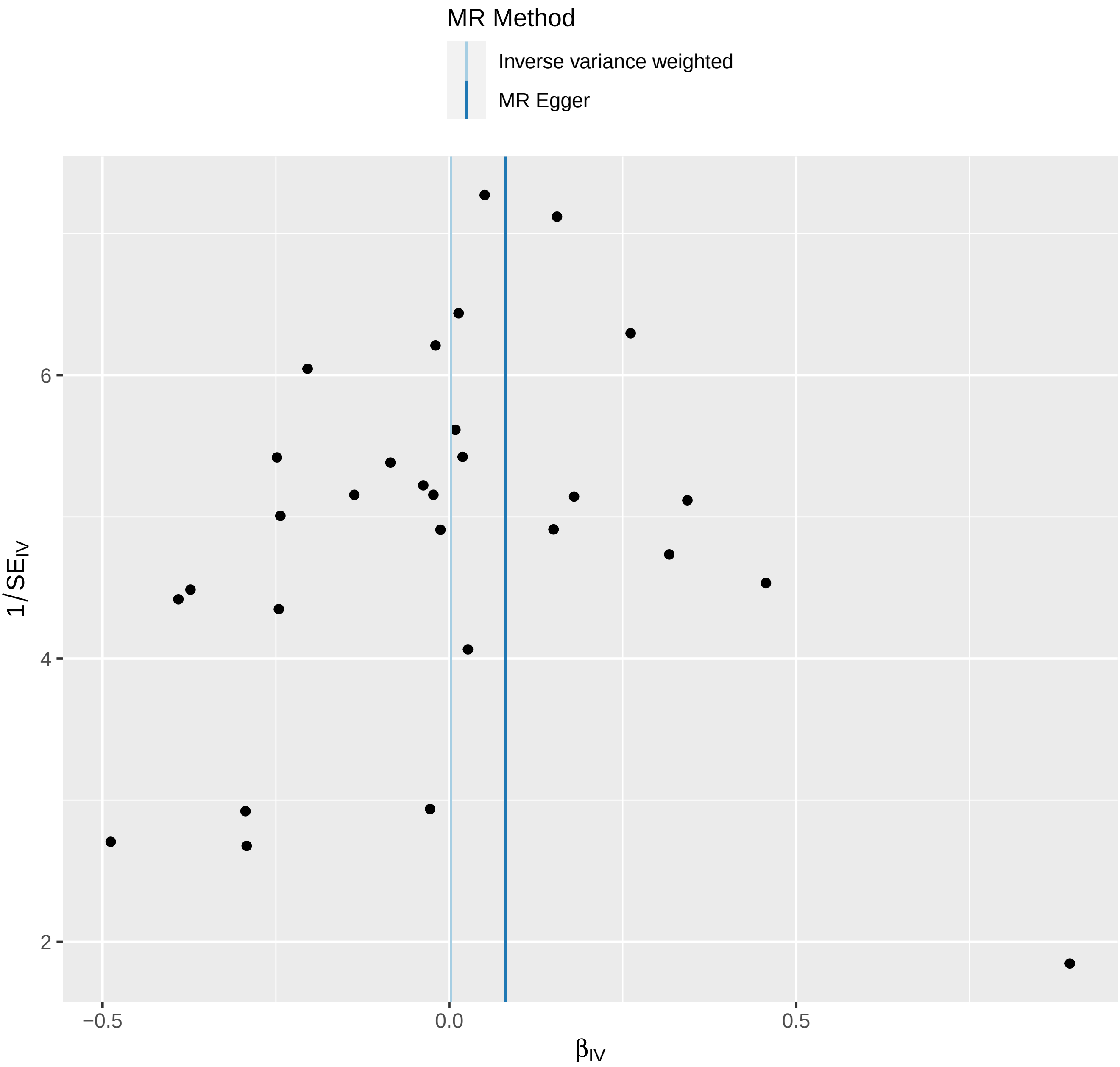

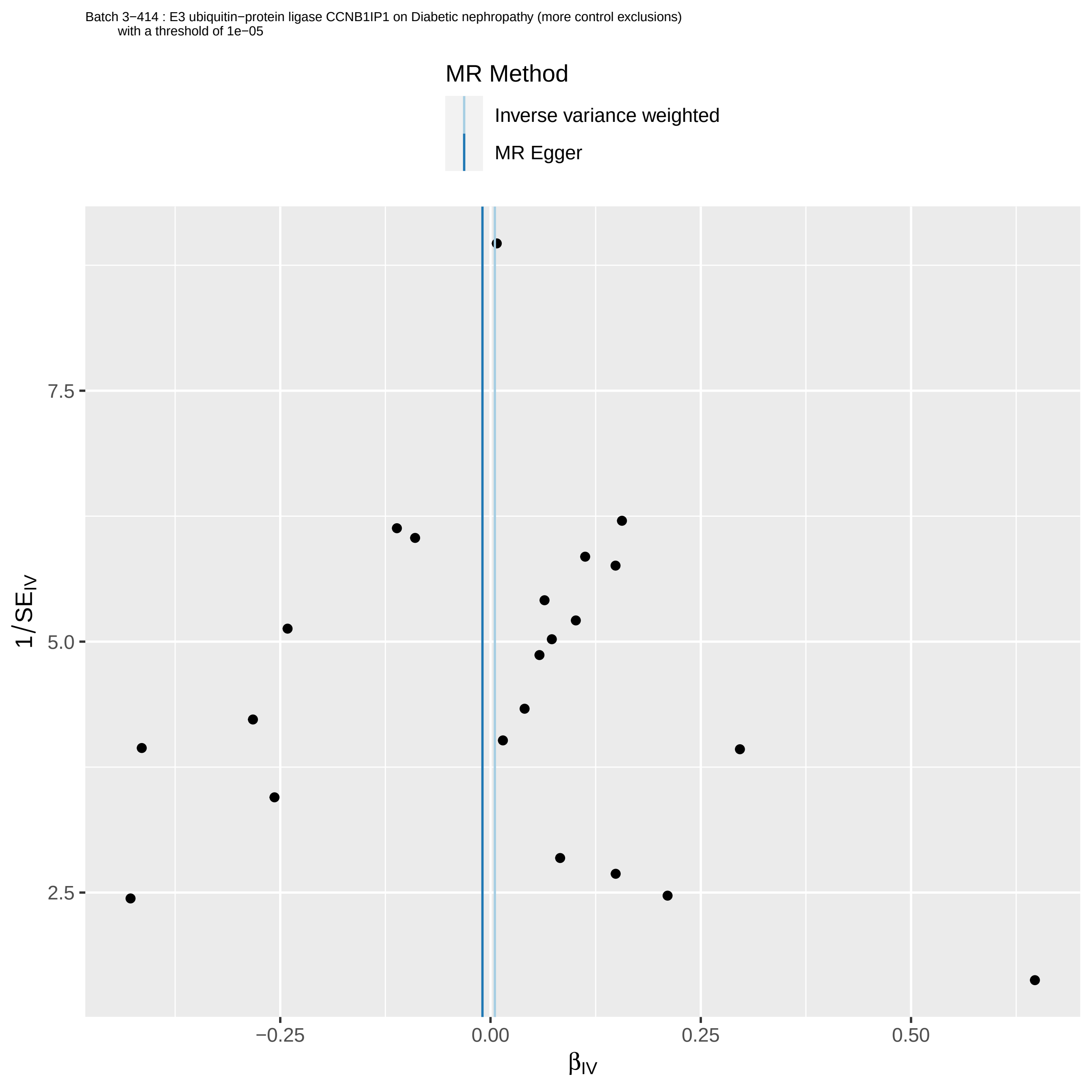
Batch 3−414 : E3 ubiquitin−protein ligase CCNB1IP1 on Diabetic nephropathy (more control exclusions)
with a threshold of 1e−05
MR Method
Inverse variance weighted
MR Egger
7.5
5.0
2.5
$1/SE_{IV}$
−0.25
0.00
0.25
0.50
β_{IV}

Batch 3−415 : Cyclin−C on Diabetic nephropathy (more control exclusions)
with a threshold of 1e−05

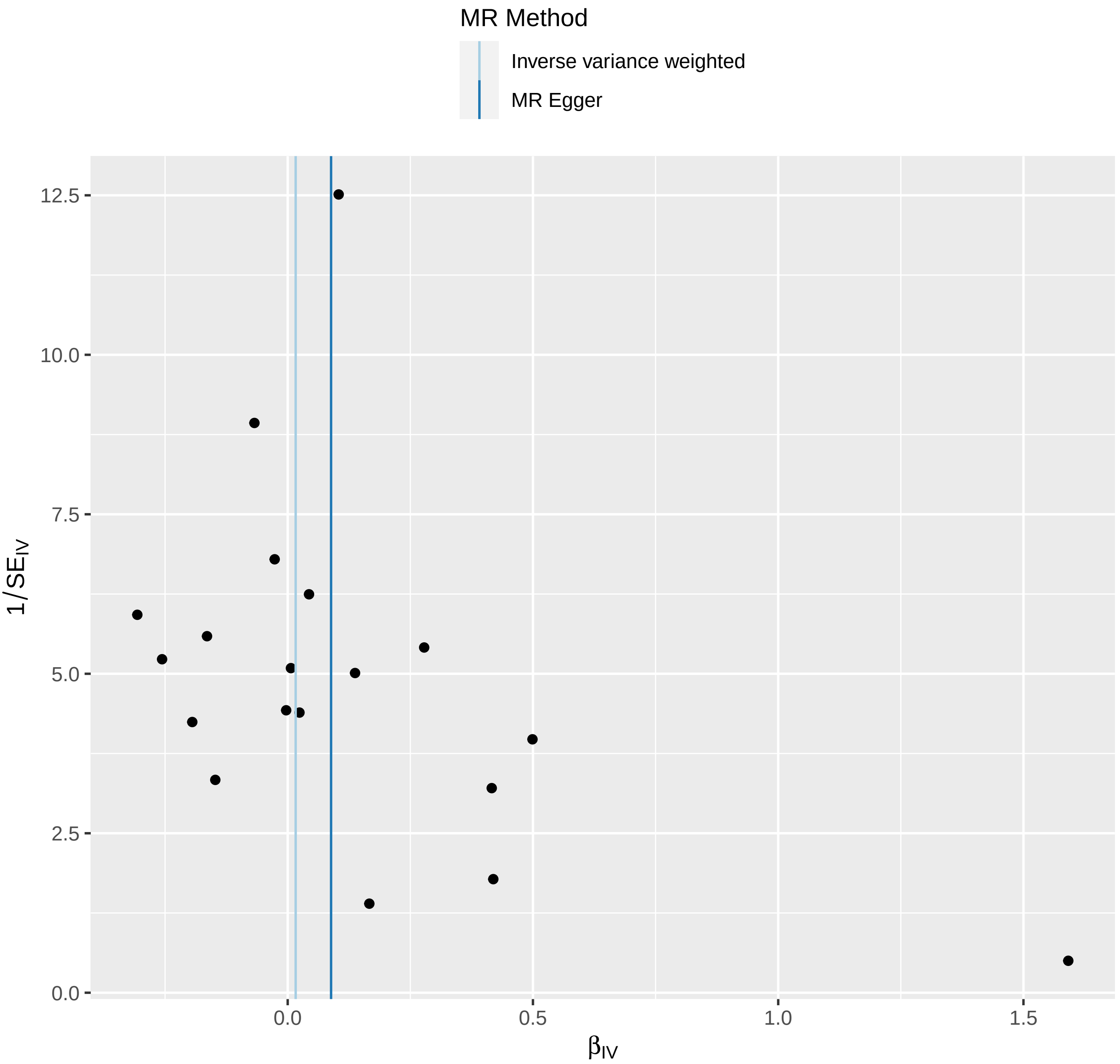

Batch 3−416 : Cyclin−H on Diabetic nephropathy (more control exclusions)
with a threshold of 1e−05

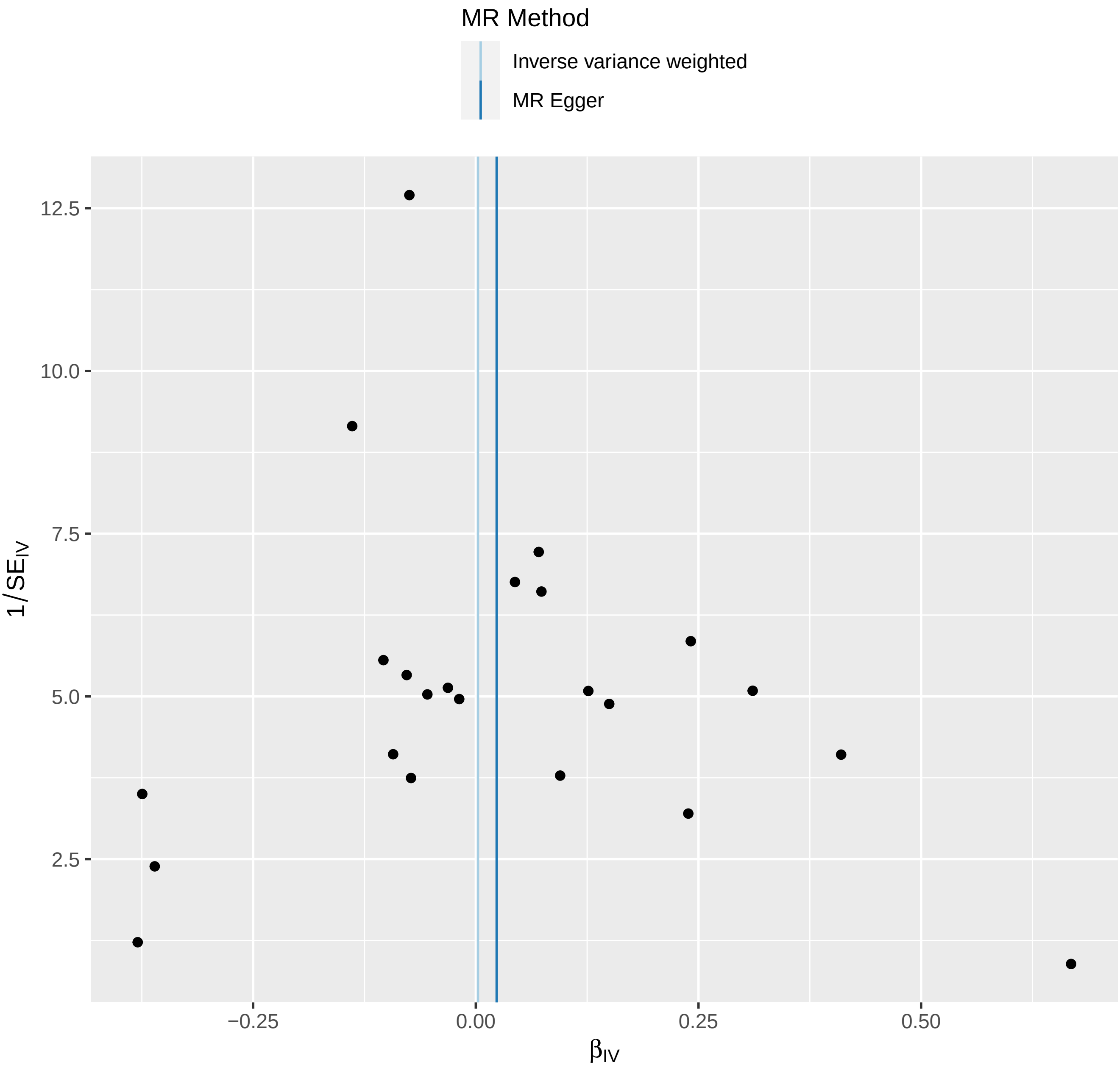

Batch 3−417 : Cell cycle progression protein 1 on Diabetic nephropathy (more control exclusions)
with a threshold of 1e−05

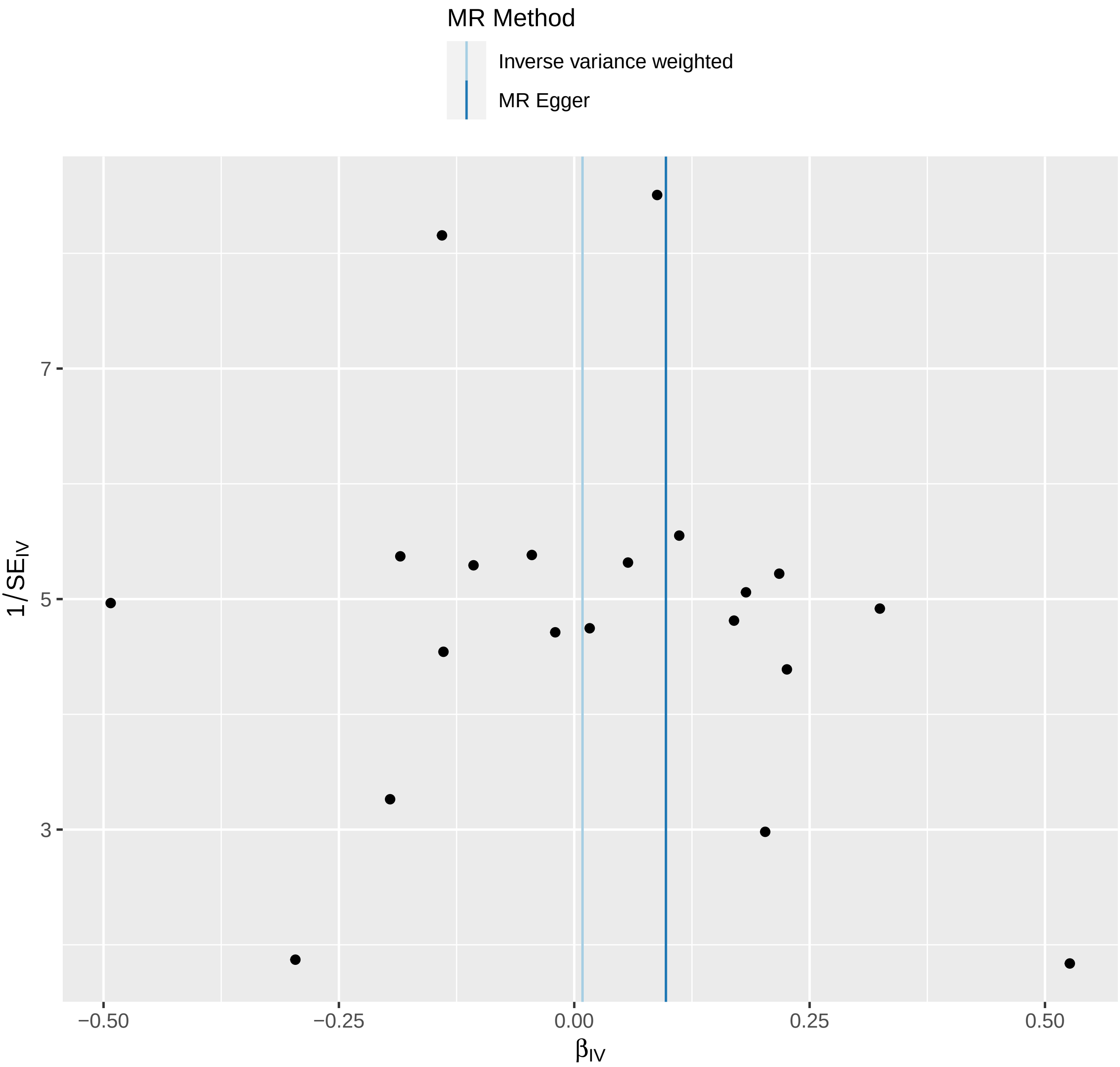

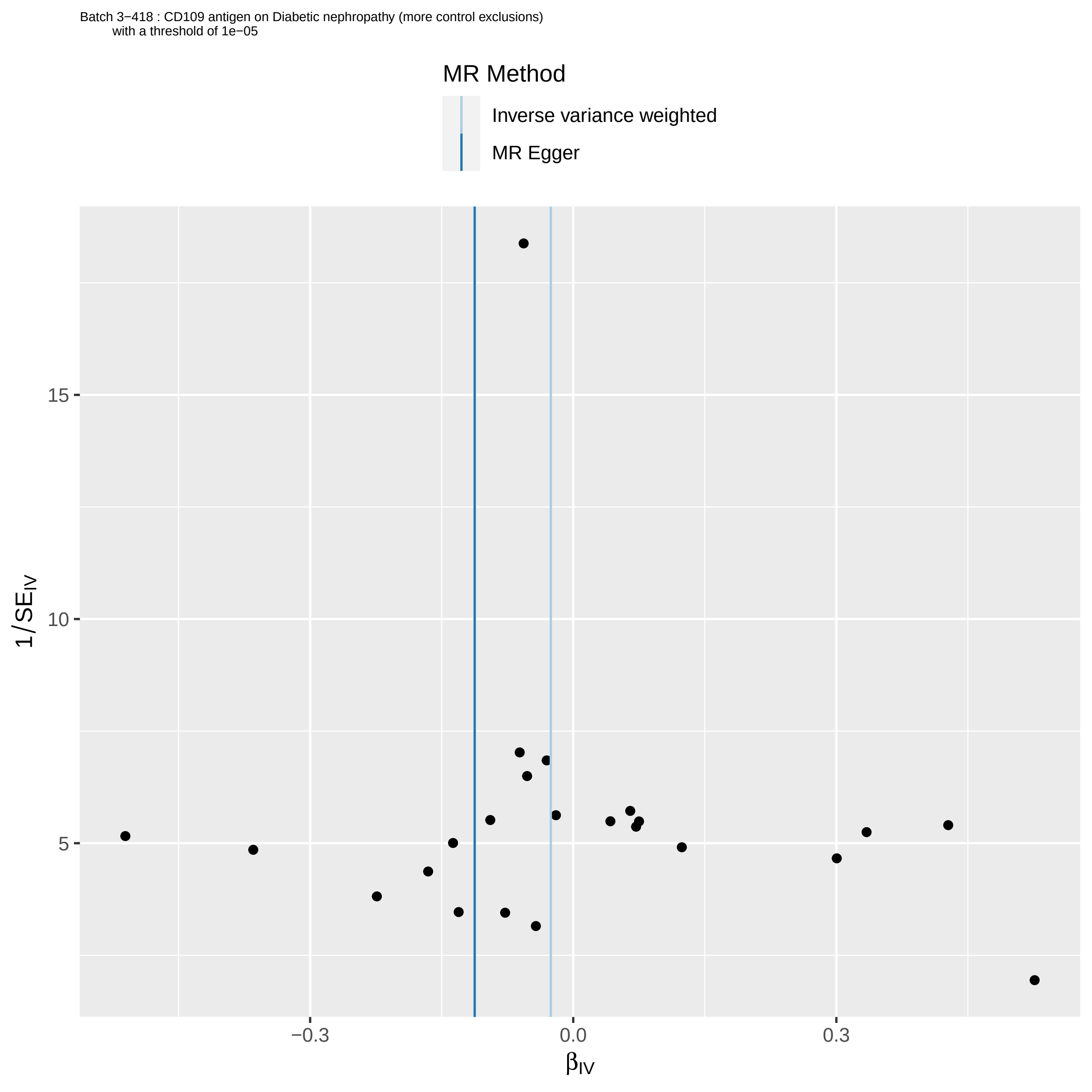

Batch 3−418 : CD109 antigen on Diabetic nephropathy (more control exclusions)
with a threshold of 1e−05
MR Method
Inverse variance weighted
MR Egger
15
10
5
$1/SE_{IV}$
−0.3
0.0
0.3
β_{IV}

Batch 3−419 : Scavenger receptor cysteine−rich type 1 protein M130 on Diabetic nephropathy (more control exclusions) with a threshold of 1e−05

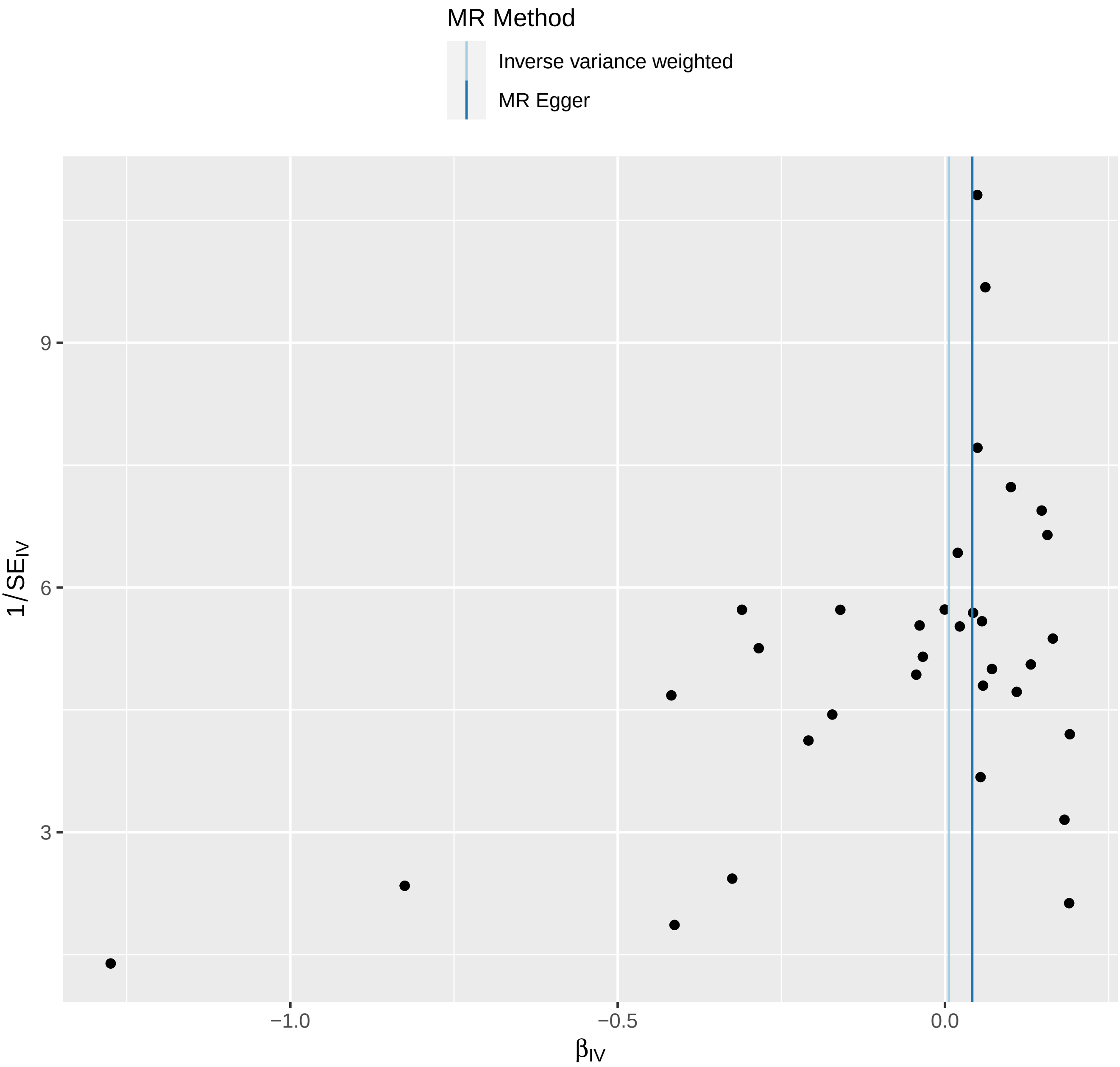

Batch 3−420 : CD177 antigen on Diabetic nephropathy (more control exclusions)
with a threshold of 1e−05

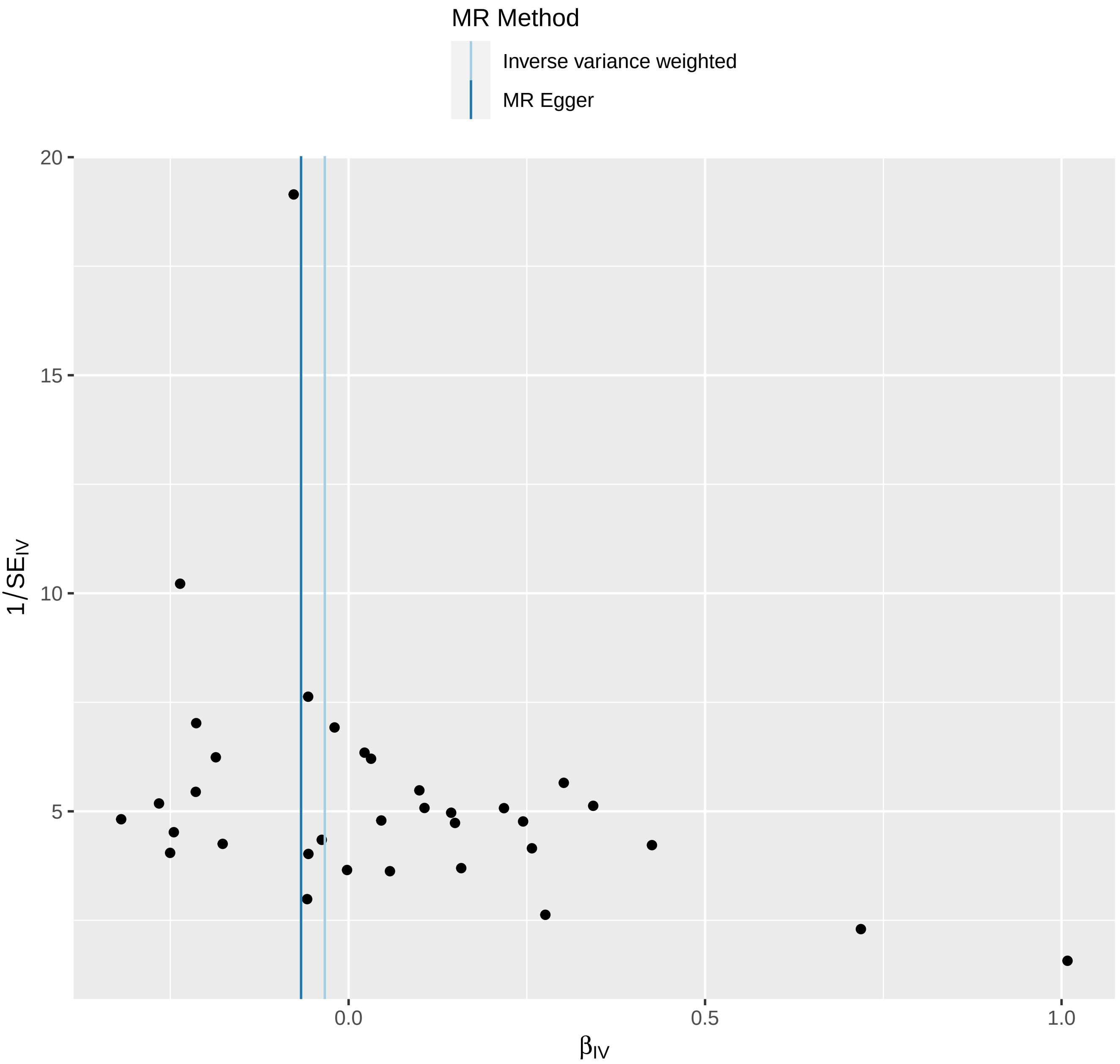

Batch 3−421 : Antigen−presenting glycoprotein CD1d on Diabetic nephropathy (more control exclusions)
with a threshold of 1e−05

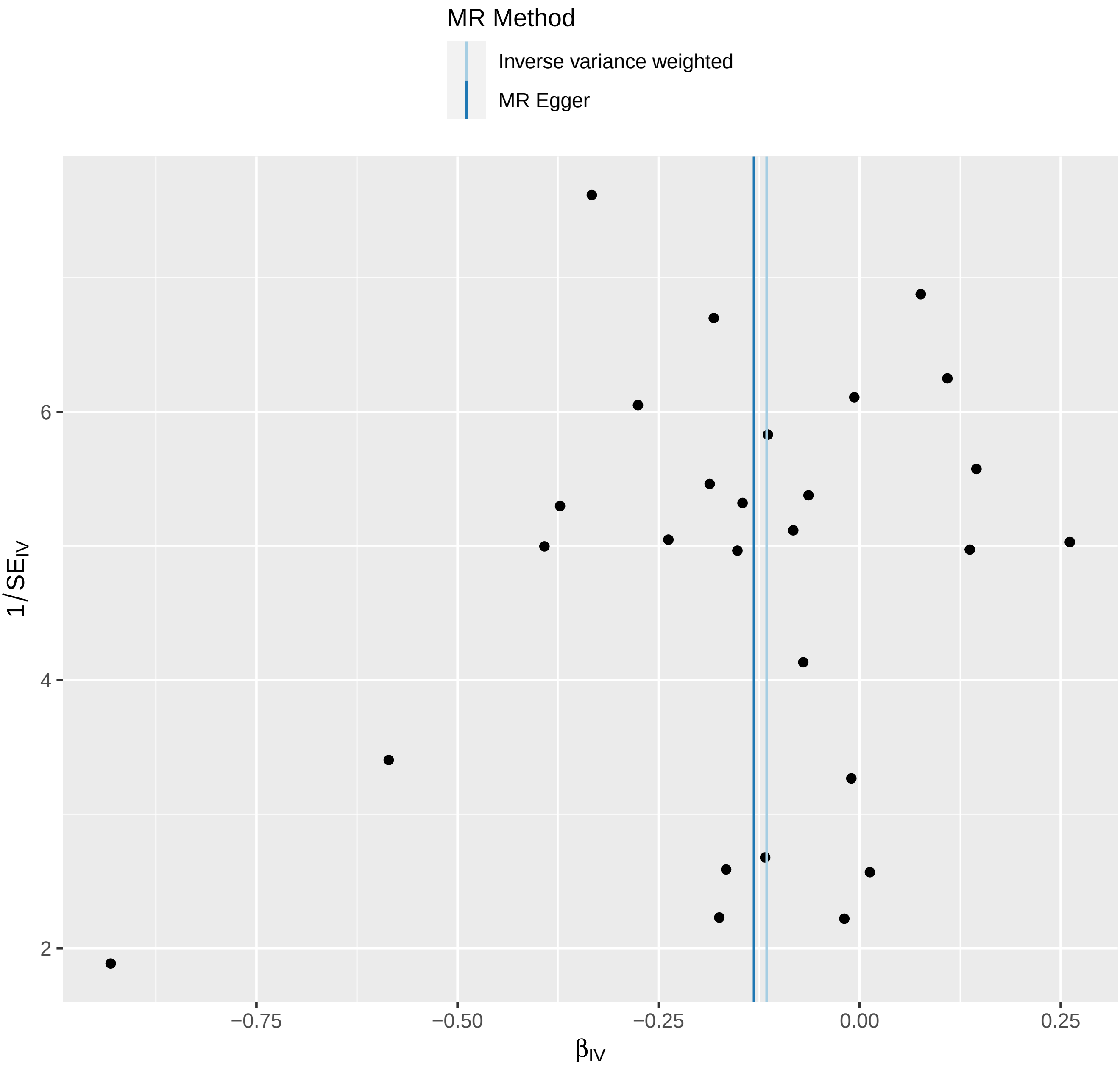

Batch 3−422 : OX−2 membrane glycoprotein on Diabetic nephropathy (more control exclusions)
with a threshold of 1e−05

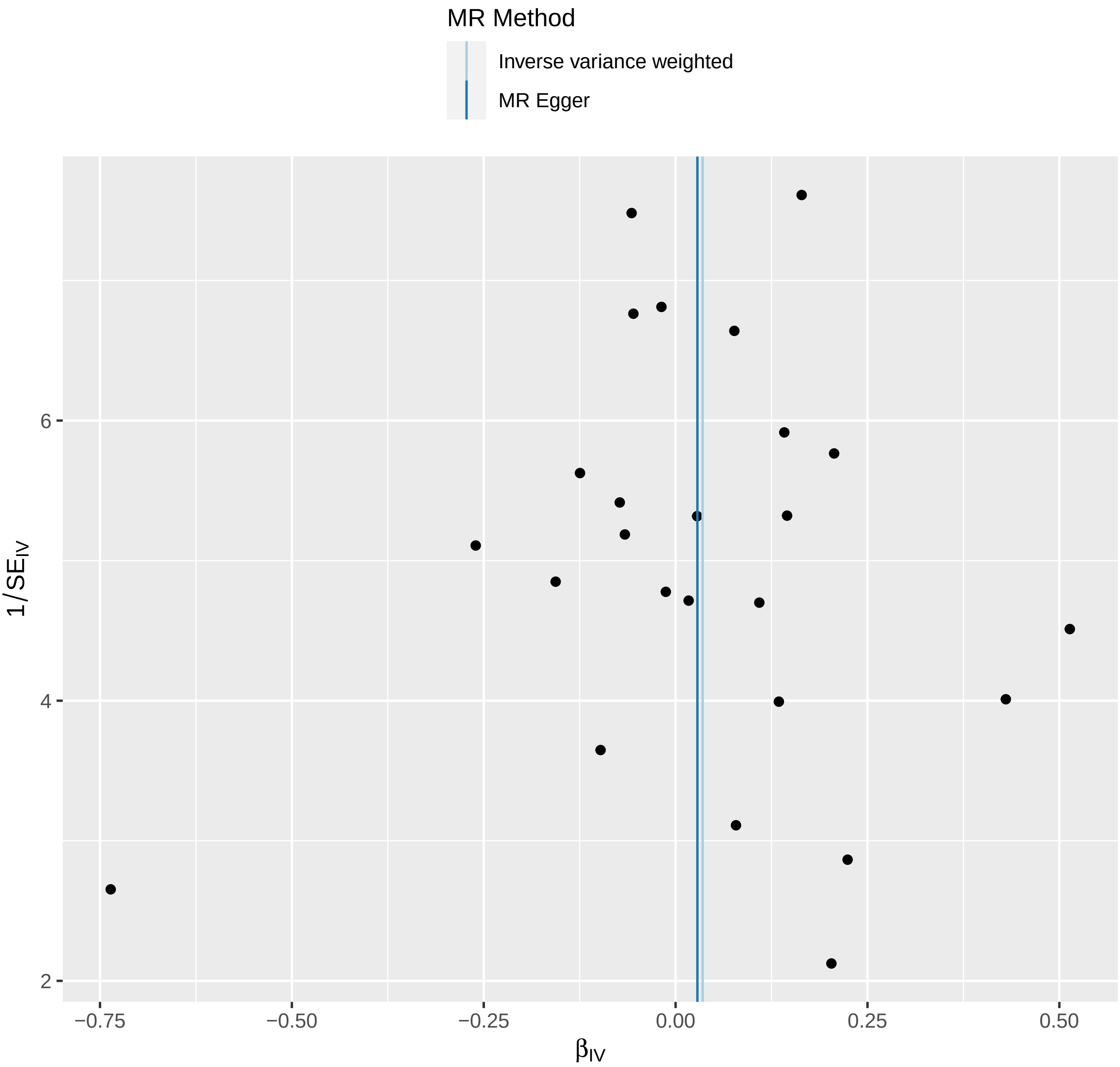

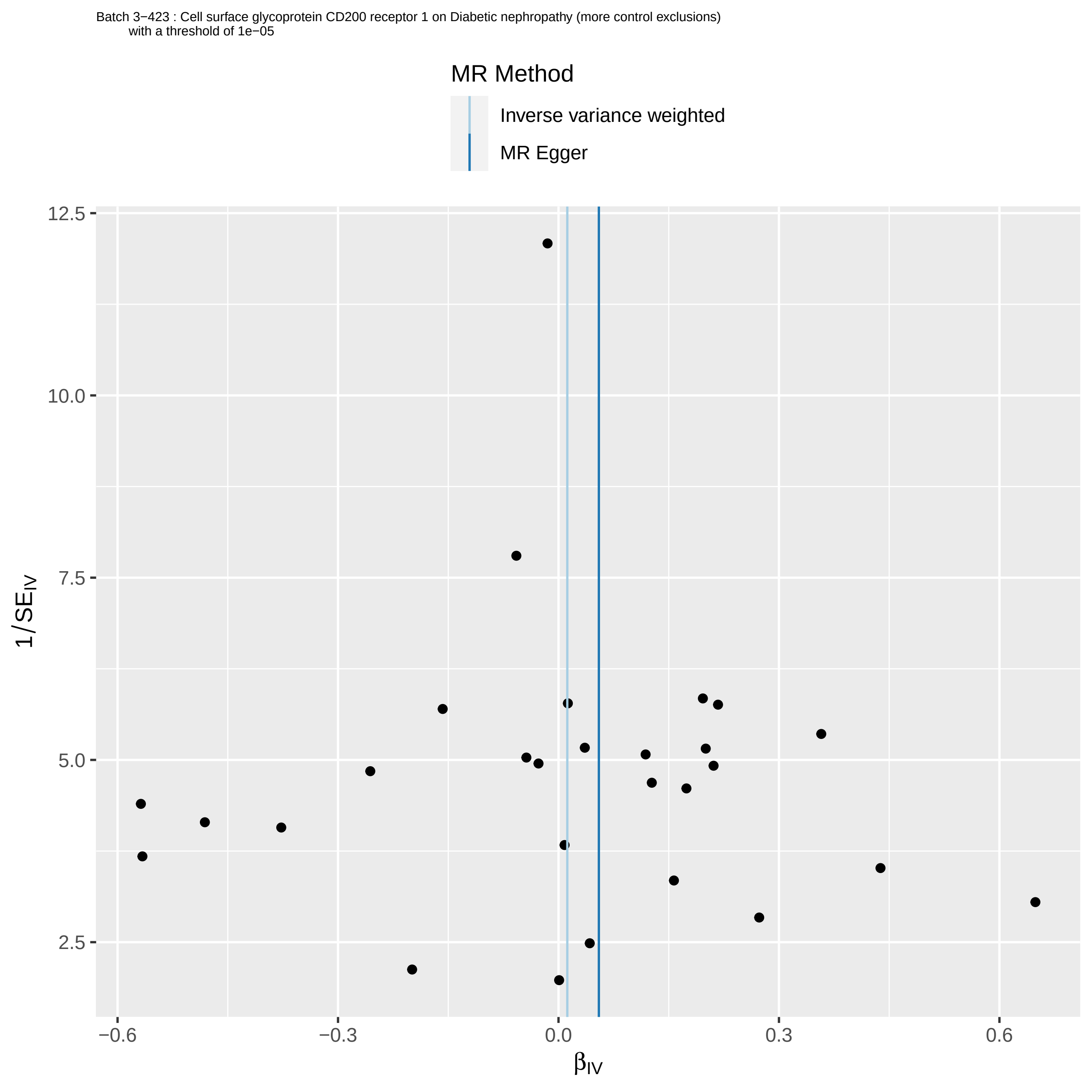

Batch 3−423 : Cell surface glycoprotein CD200 receptor 1 on Diabetic nephropathy (more control exclusions)
with a threshold of 1e−05
MR Method
Inverse variance weighted
MR Egger
12.5
10.0
7.5
5.0
2.5
−0.6
−0.3
0.0
0.3
0.6
1/SE_IV
β_IV

Batch 3−424 : Cell surface glycoprotein CD200 receptor 2 on Diabetic nephropathy (more control exclusions)
with a threshold of 1e−05

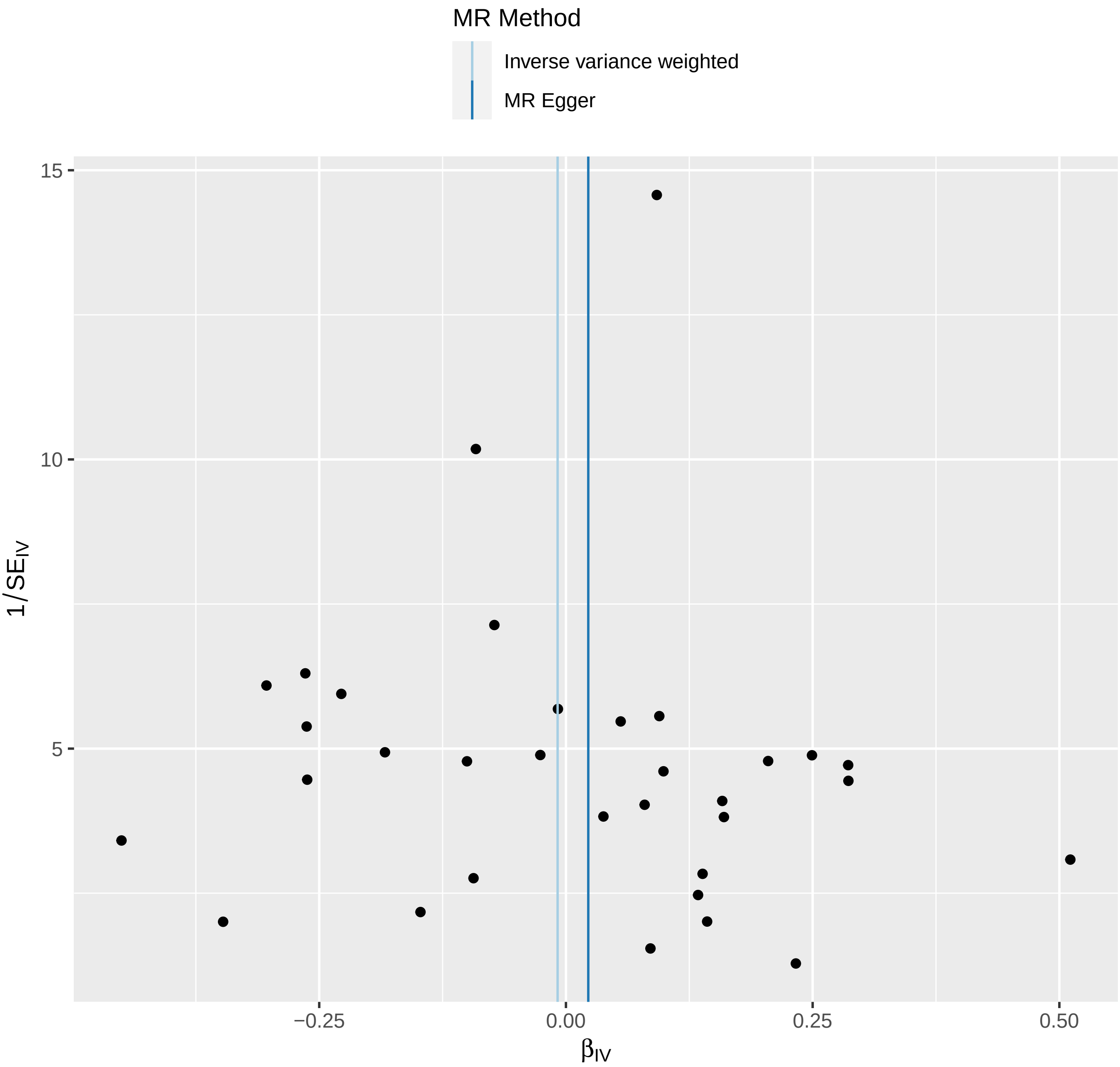

Batch 3−425 : C−type lectin domain family 4 member K on Diabetic nephropathy (more control exclusions)
with a threshold of 1e−05

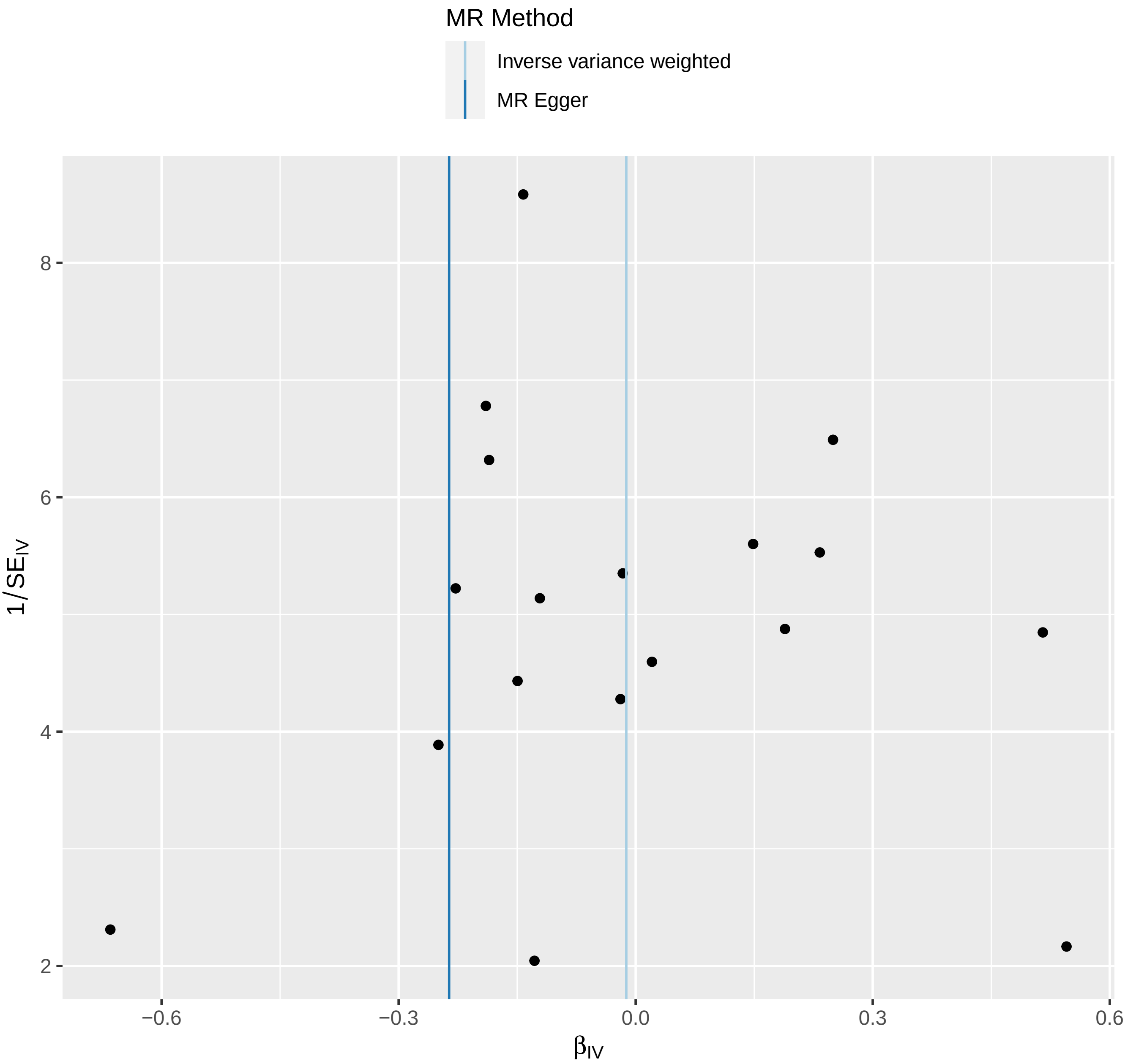

Batch 3−426 : CD209 antigen on Diabetic nephropathy (more control exclusions)
with a threshold of 1e−05

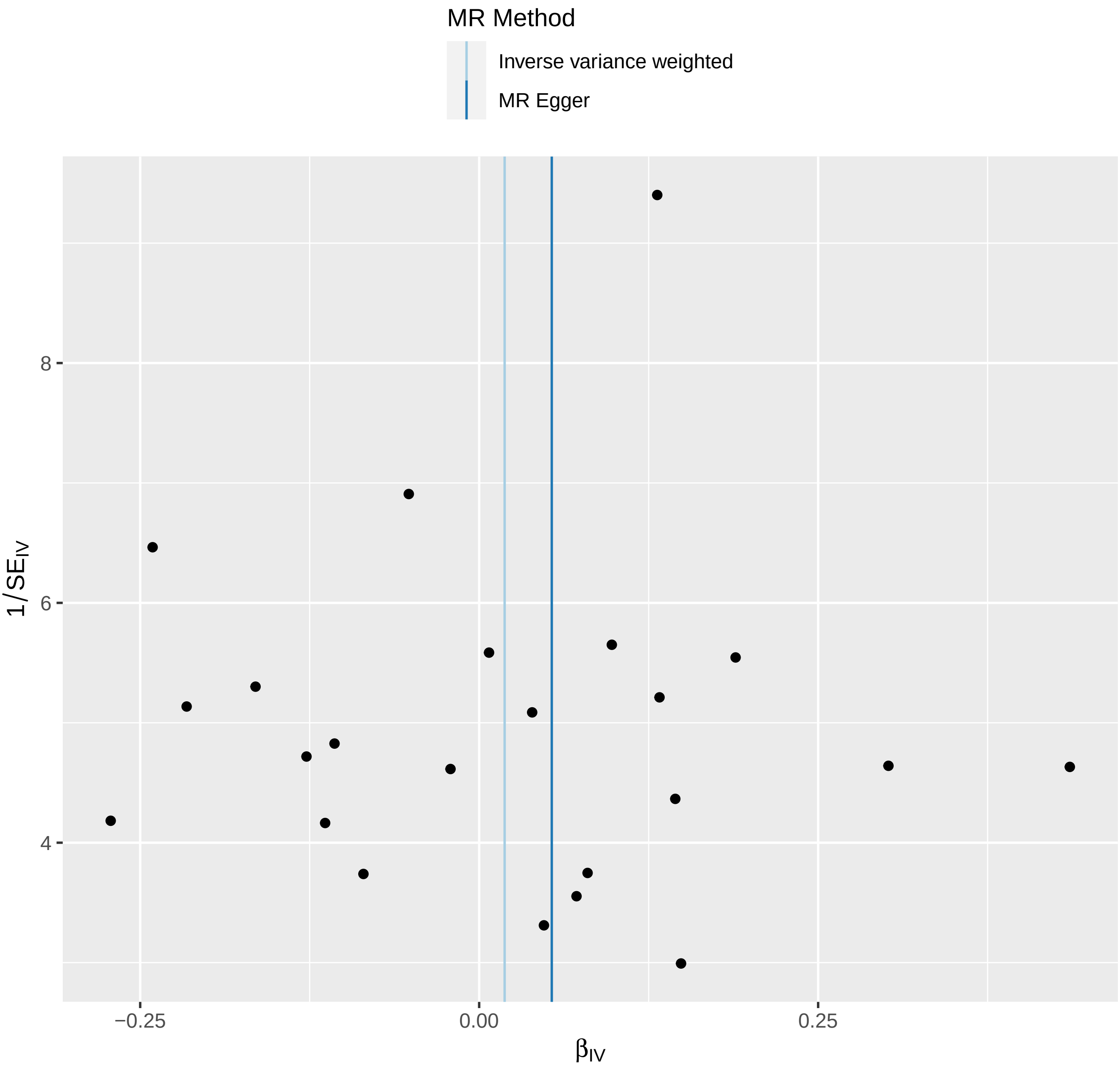

Batch 3−427 : B−cell receptor CD22 on Diabetic nephropathy (more control exclusions)
with a threshold of 1e−05

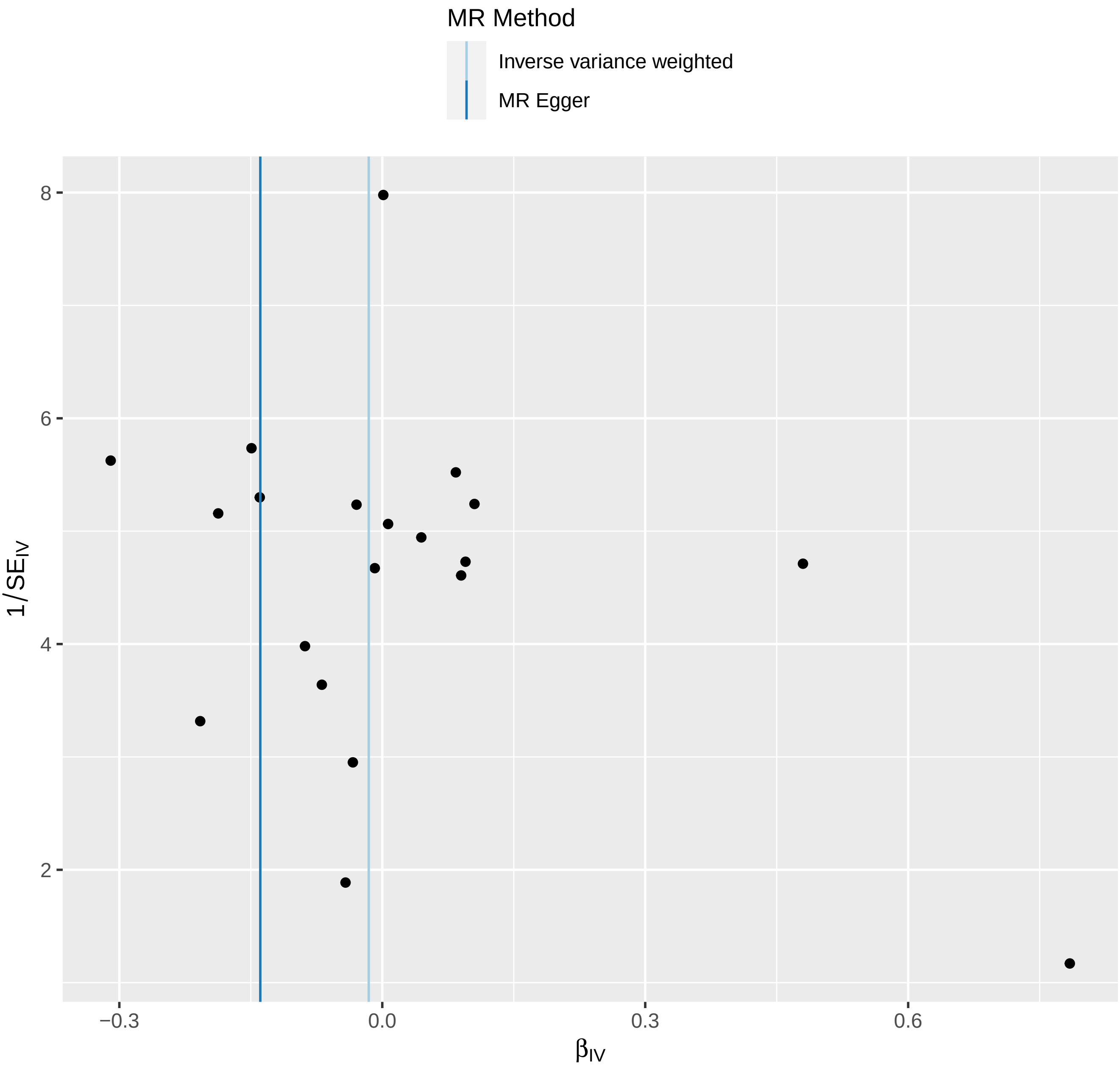

Batch 3−428 : CD226 antigen on Diabetic nephropathy (more control exclusions)
with a threshold of 1e−05

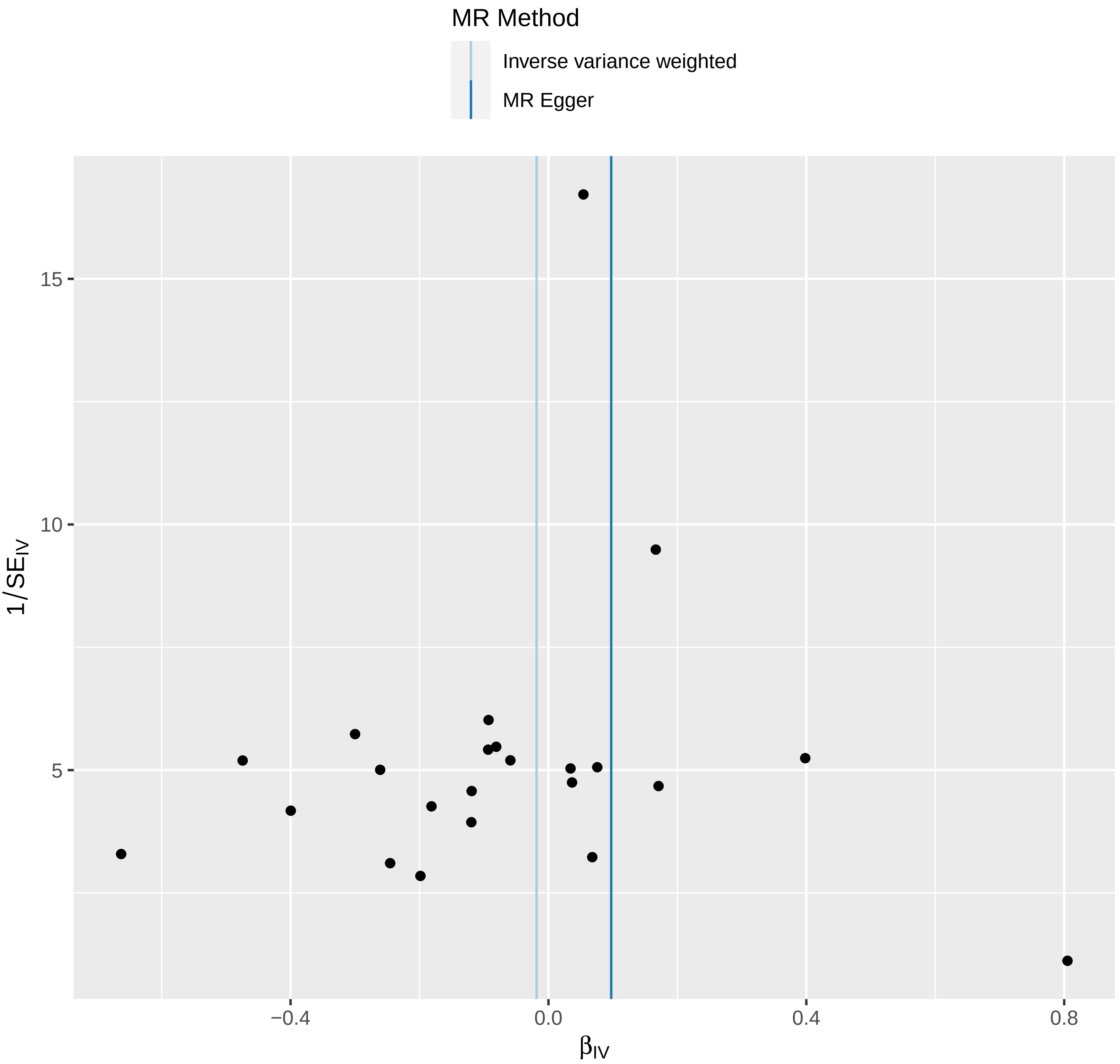

Batch 3−429 : CD226 antigen on Diabetic nephropathy (more control exclusions)
with a threshold of 1e−05

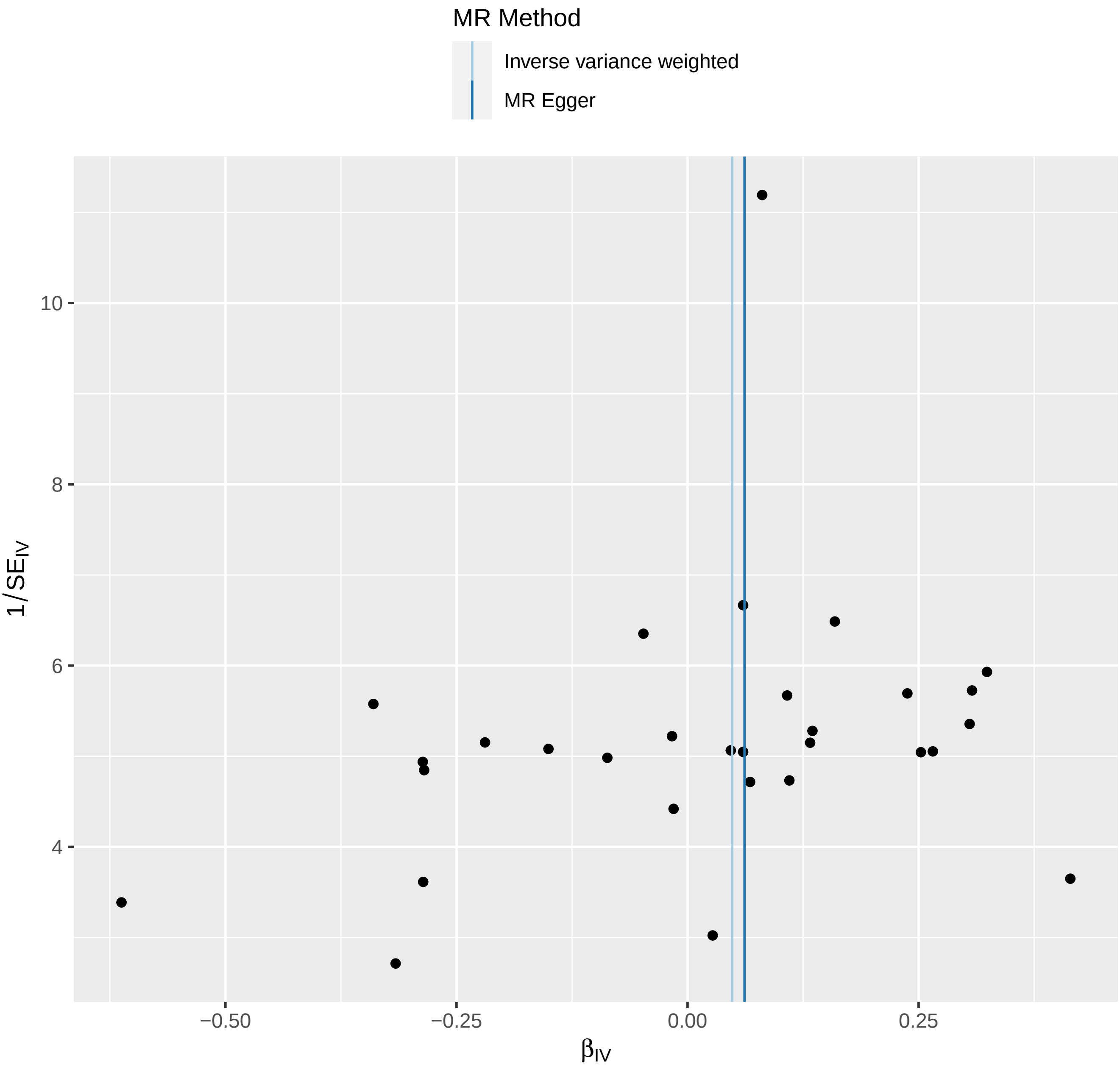

Batch 3−430 : T−cell surface antigen CD2 on Diabetic nephropathy (more control exclusions)
with a threshold of 1e−05

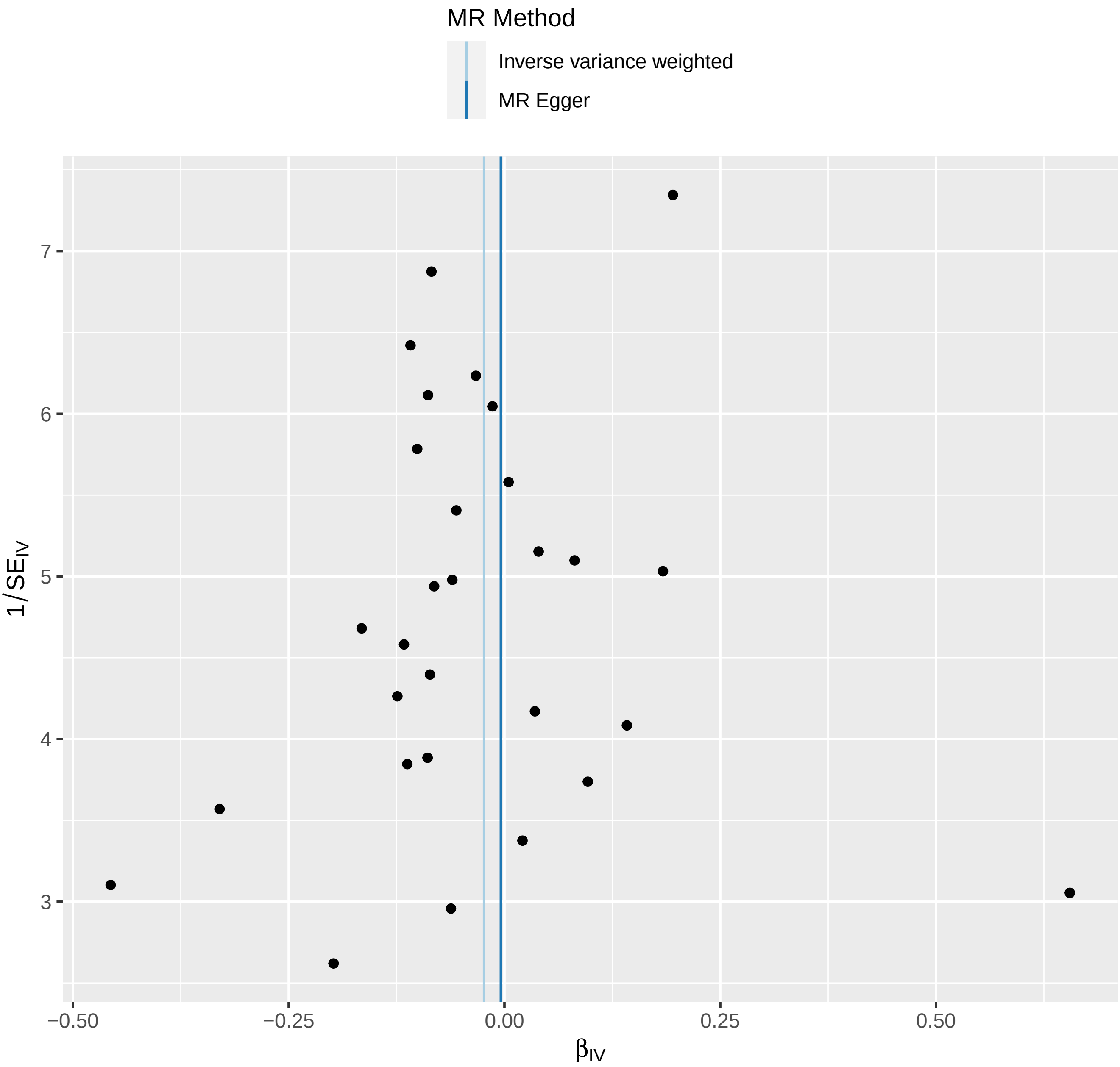

Batch 3−431 : Programmed cell death 1 ligand 1 on Diabetic nephropathy (more control exclusions)
with a threshold of 1e−05

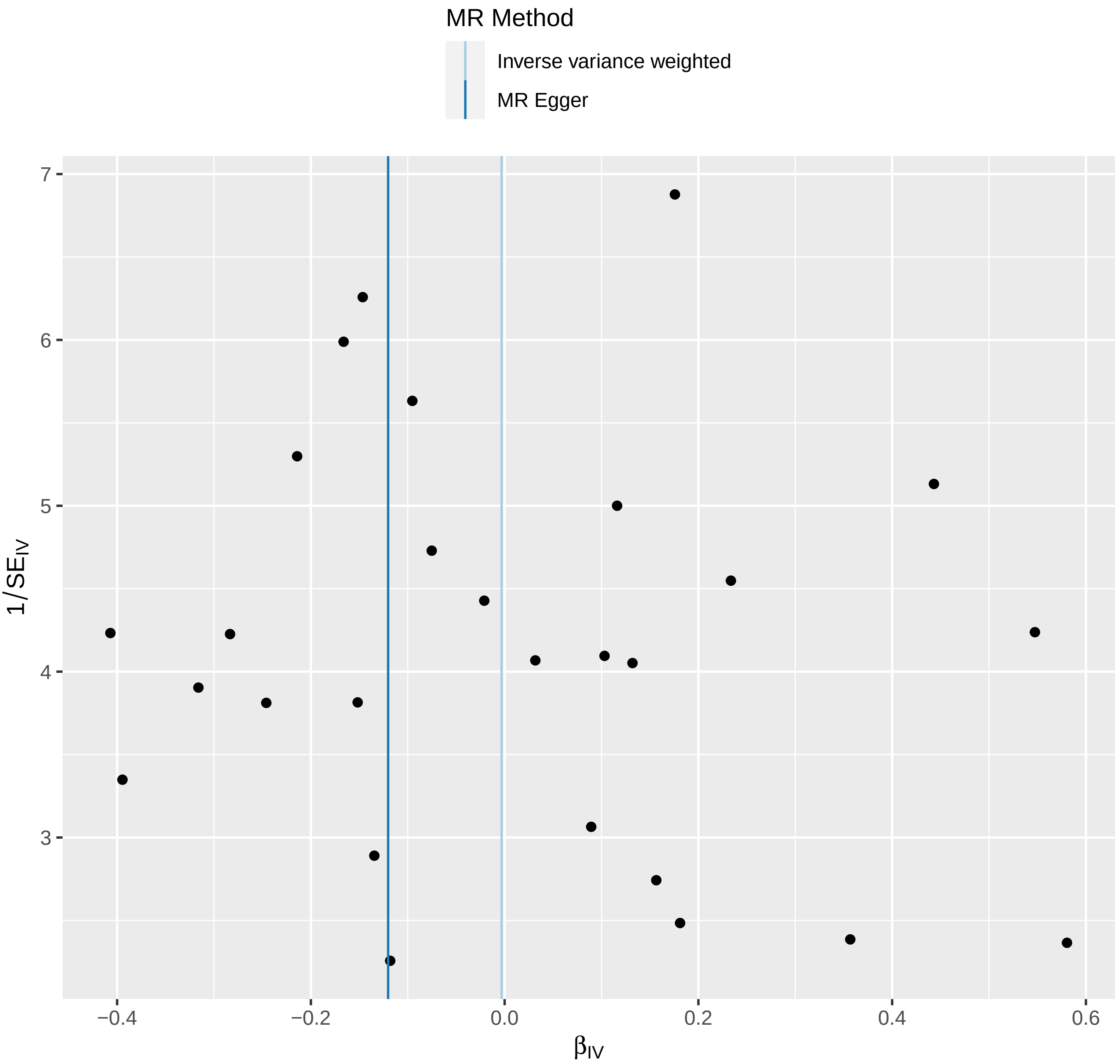

Batch 3−432 : CMRF35−like molecule 8 on Diabetic nephropathy (more control exclusions)
with a threshold of 1e−05

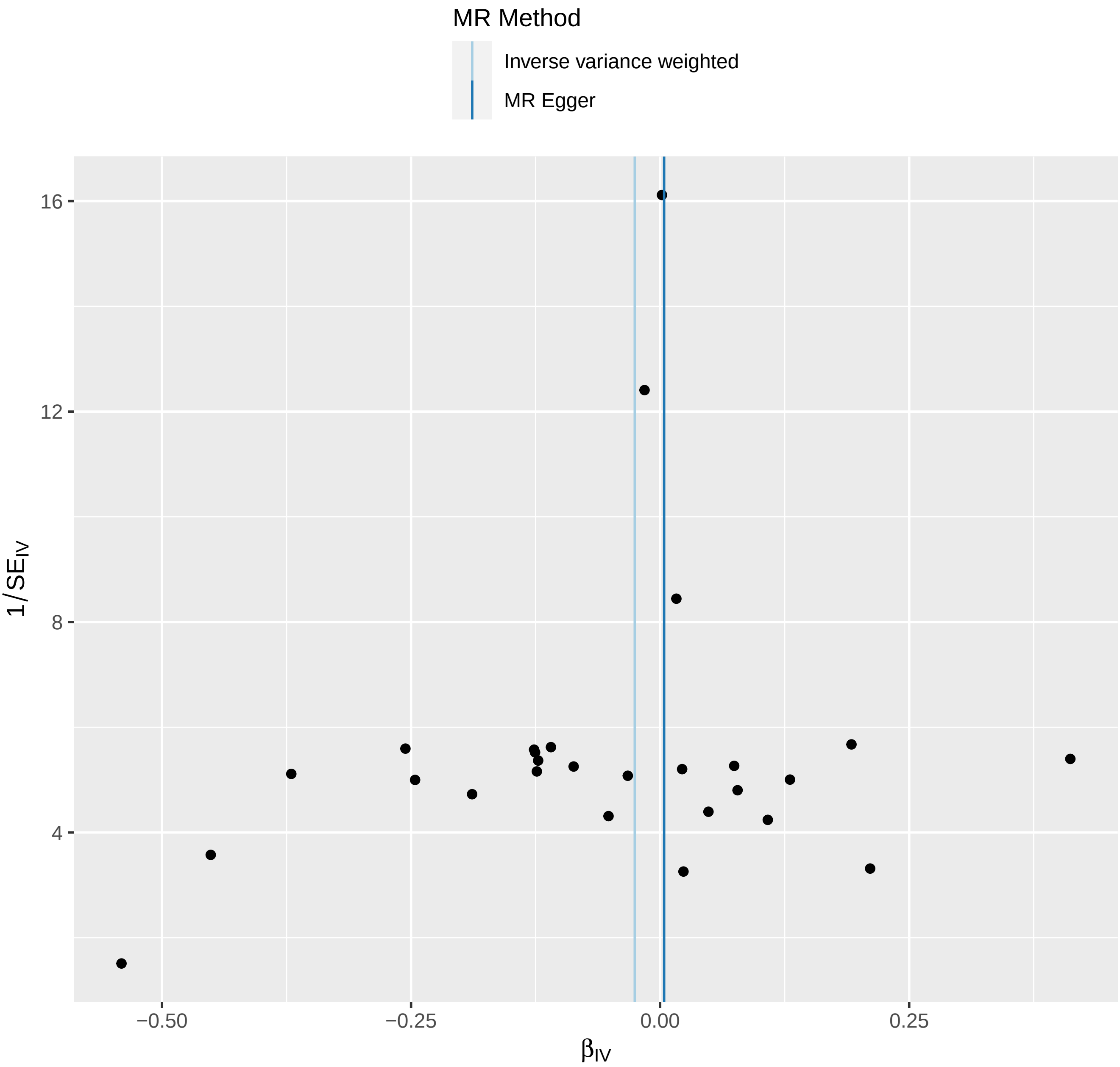

Batch 3−433 : CMRF35−like molecule 6 on Diabetic nephropathy (more control exclusions)
with a threshold of 1e−05

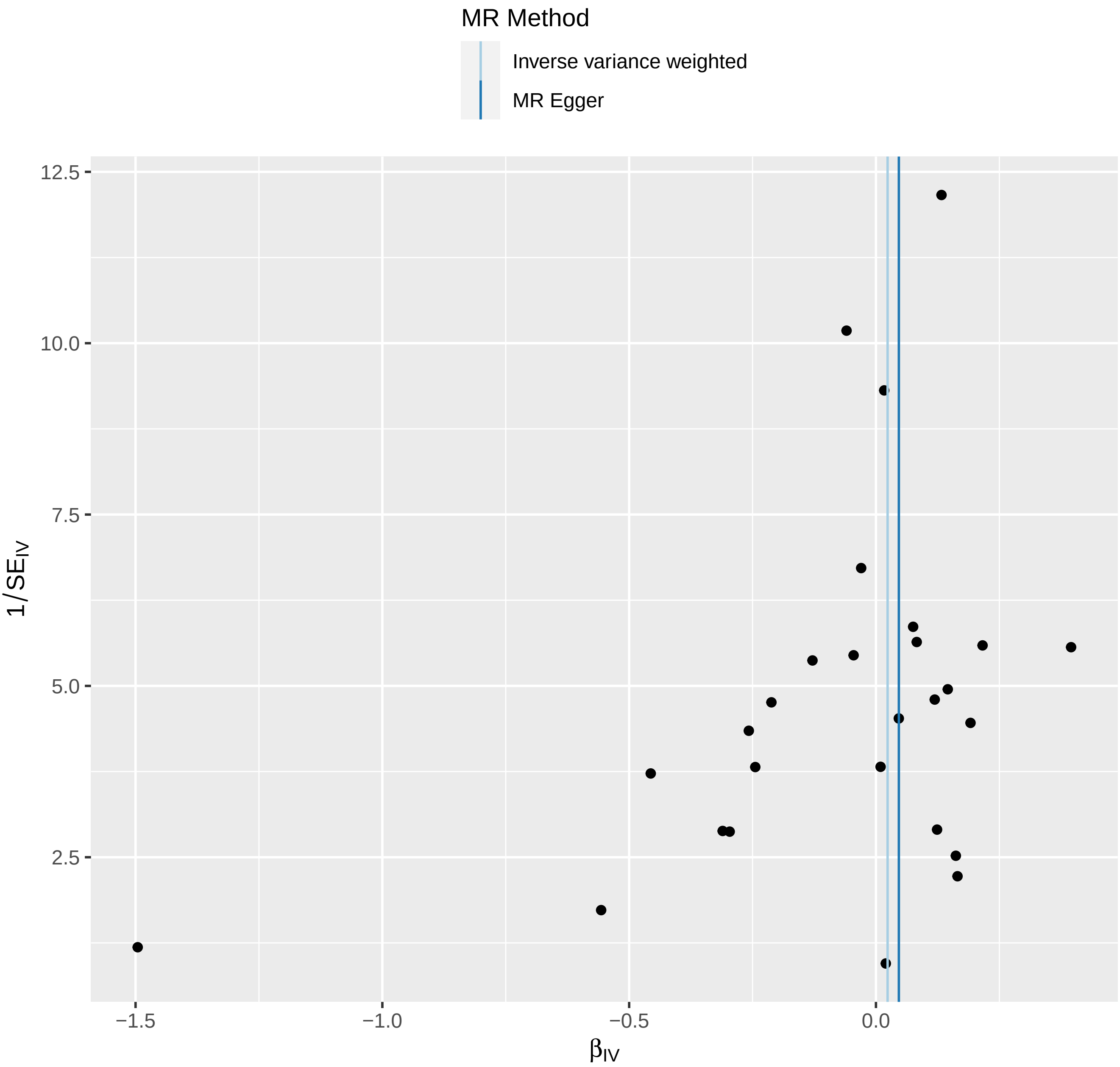

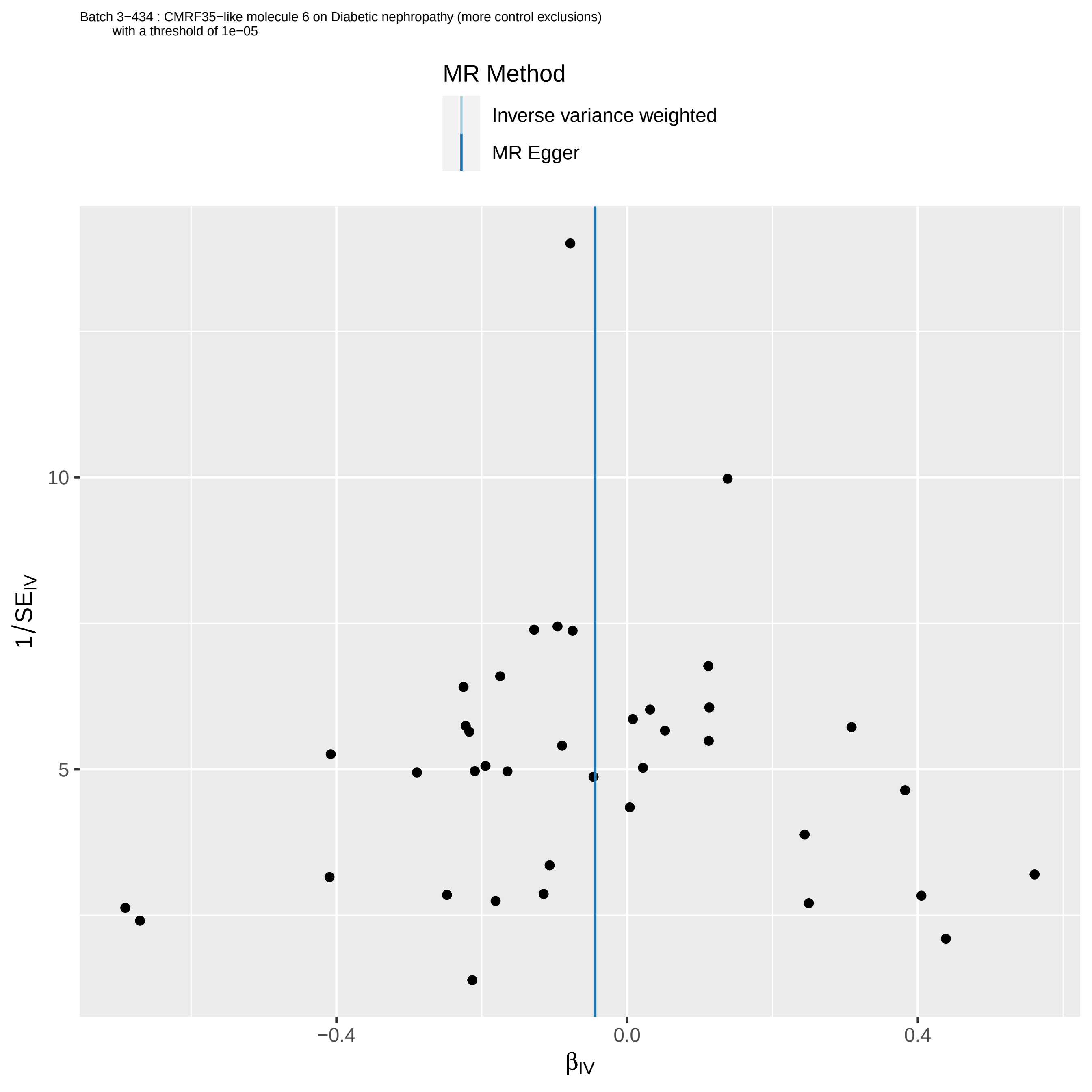

Batch 3−434 : CMRF35−like molecule 6 on Diabetic nephropathy (more control exclusions)
with a threshold of 1e−05
MR Method
Inverse variance weighted
MR Egger
10
5
1/SE_IV
−0.4
0.0
0.4
β_IV

Batch 3−435 : CMRF35−like molecule 2 on Diabetic nephropathy (more control exclusions)
with a threshold of 1e−05

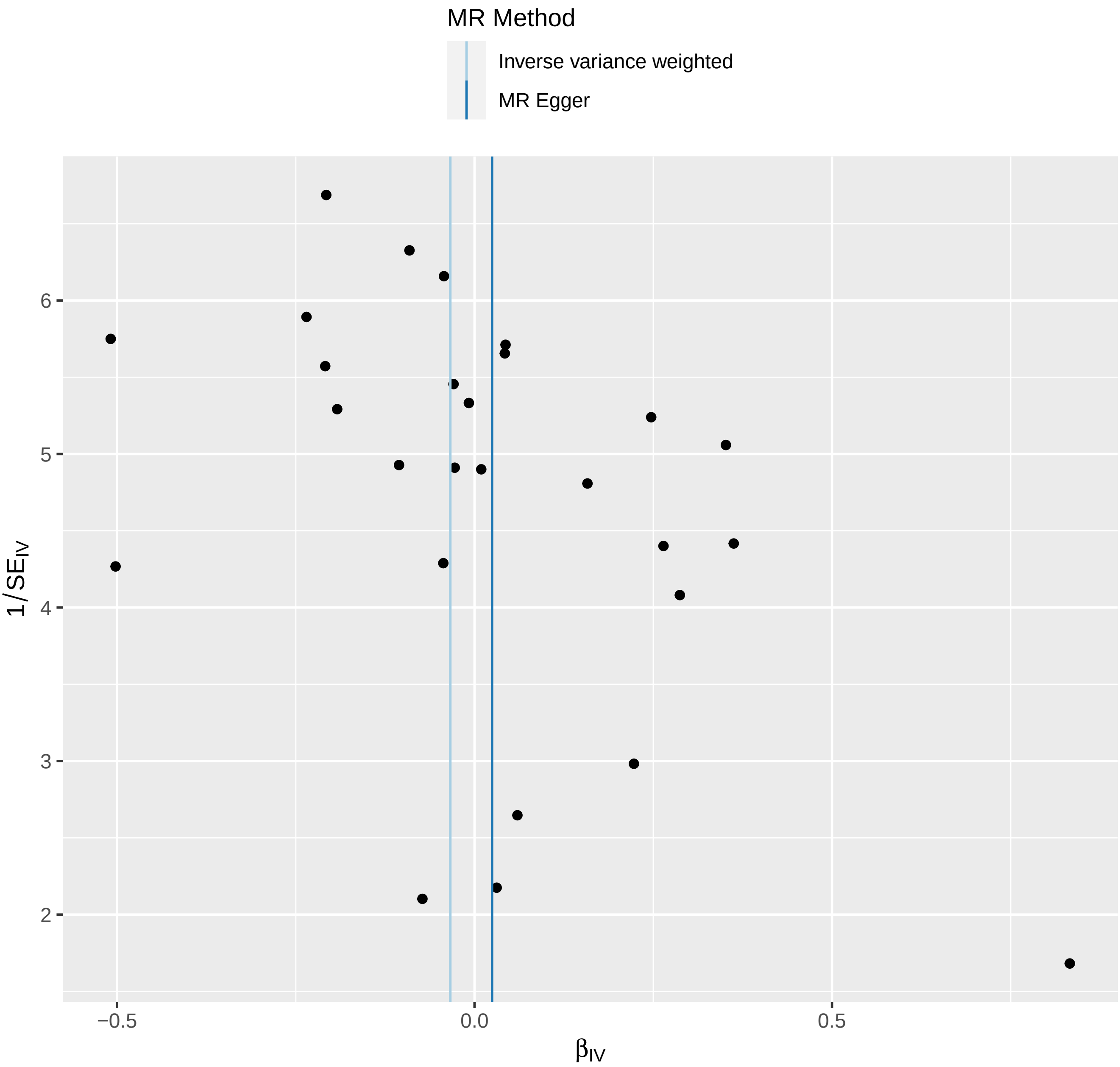

Batch 3−436 : CMRF35−like molecule 7 on Diabetic nephropathy (more control exclusions)
with a threshold of 1e−05

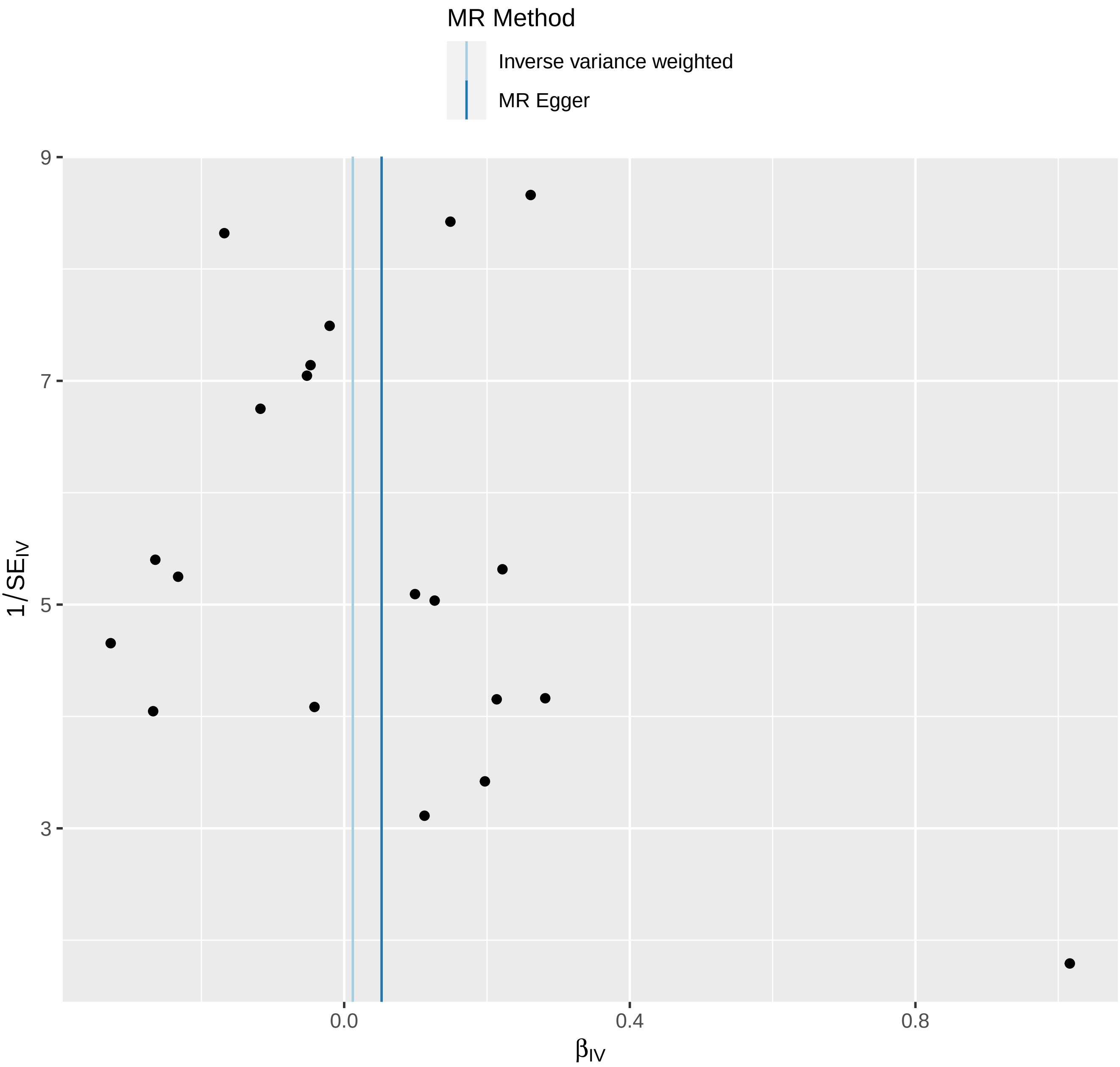

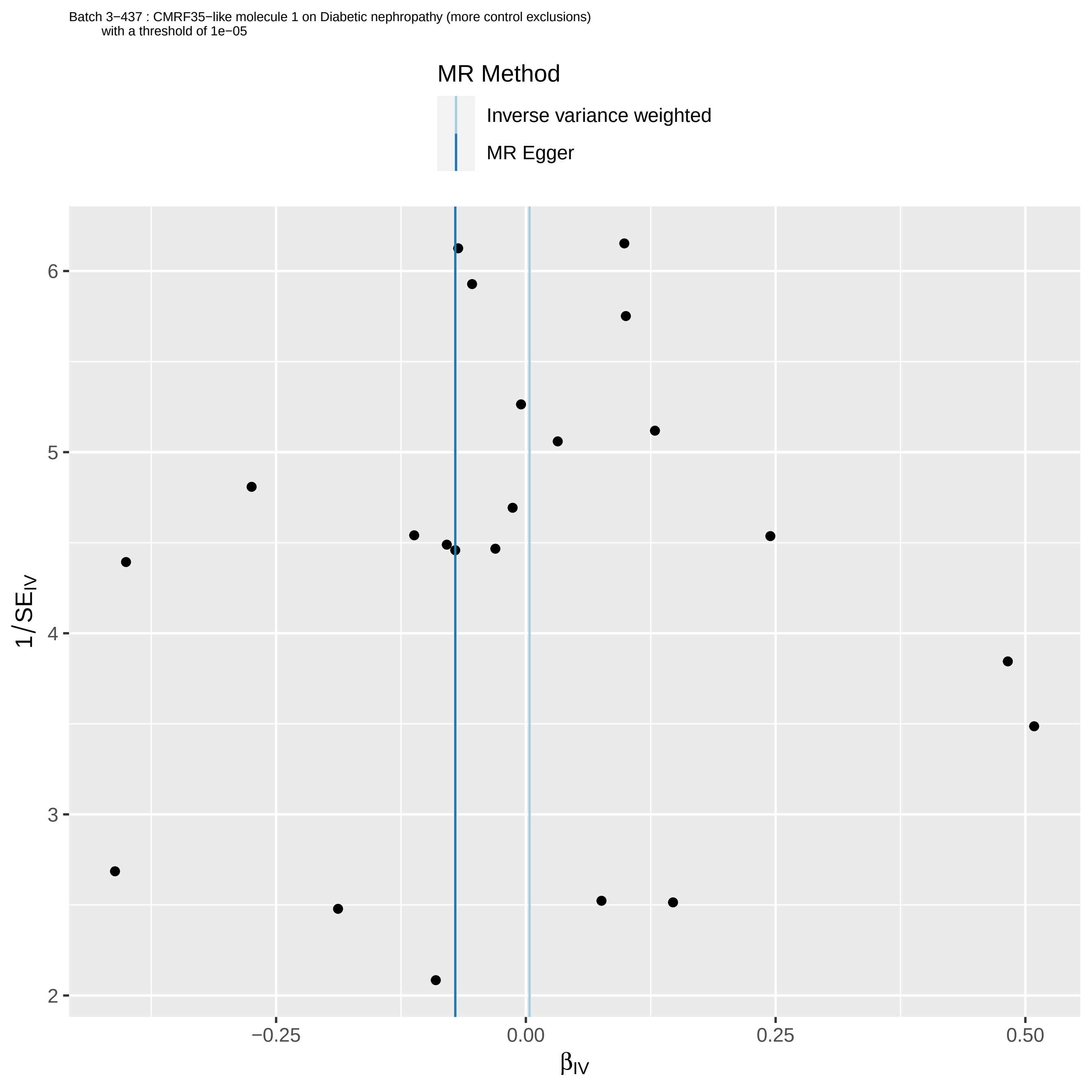
Batch 3−437 : CMRF35−like molecule 1 on Diabetic nephropathy (more control exclusions)
with a threshold of 1e−05
MR Method
Inverse variance weighted
MR Egger
1/SE$_{IV}$
6
5
4
3
2
−0.25
0.00
0.25
0.50
β$_{IV}$

Batch 3−438 : CMRF35−like molecule 9 on Diabetic nephropathy (more control exclusions)
with a threshold of 1e−05

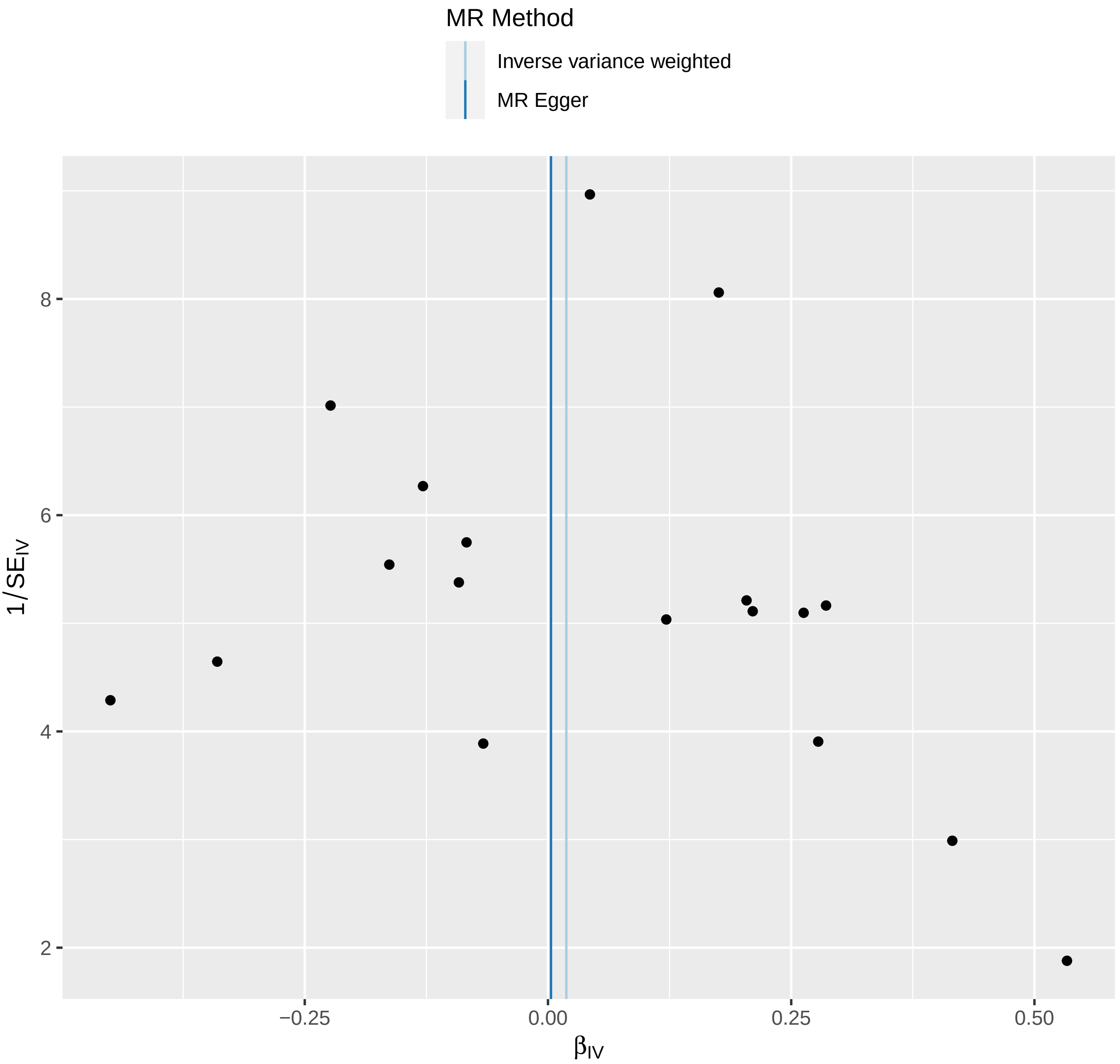

Batch 3−439 : Myeloid cell surface antigen CD33 on Diabetic nephropathy (more control exclusions)
with a threshold of 1e−05

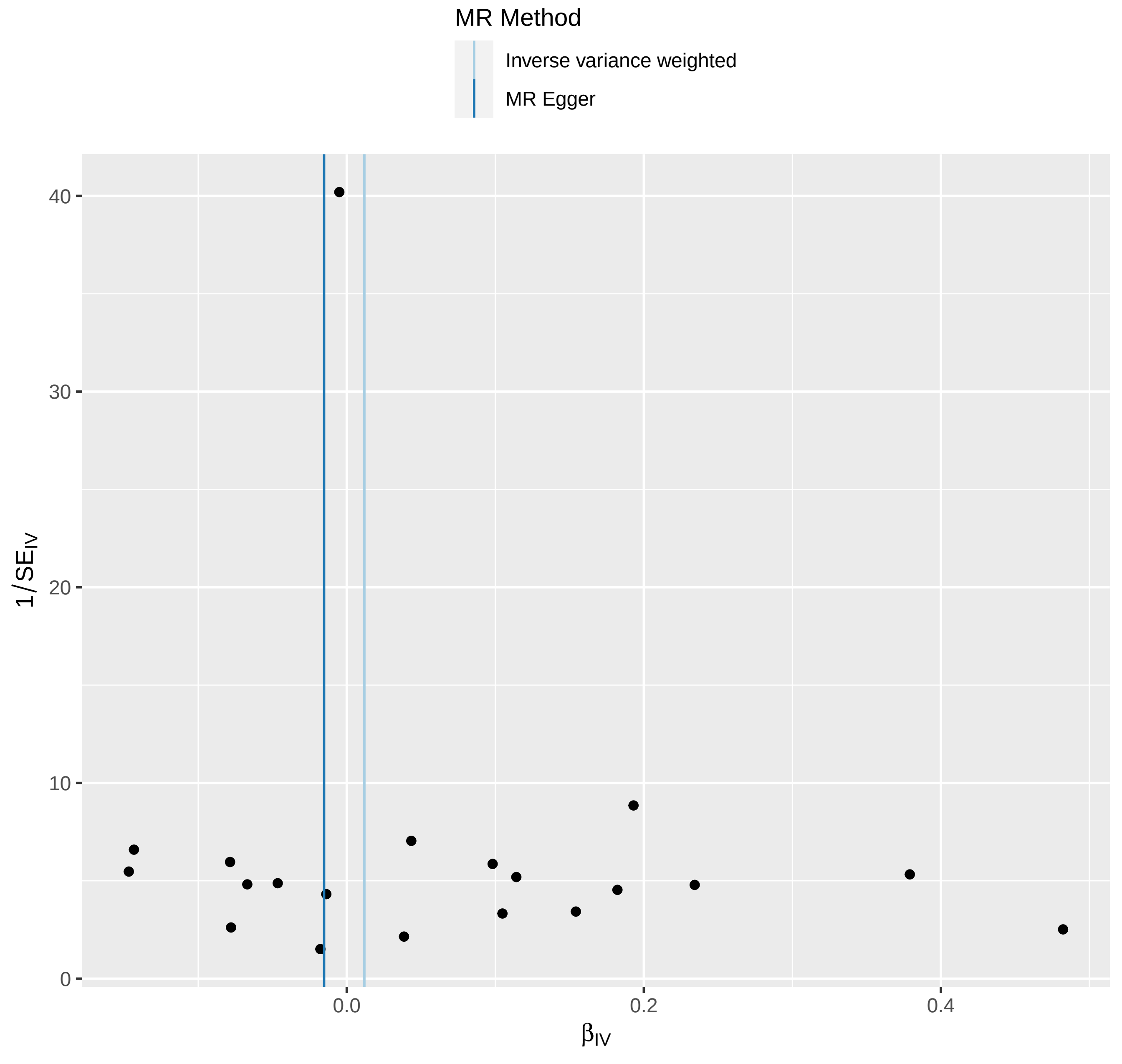

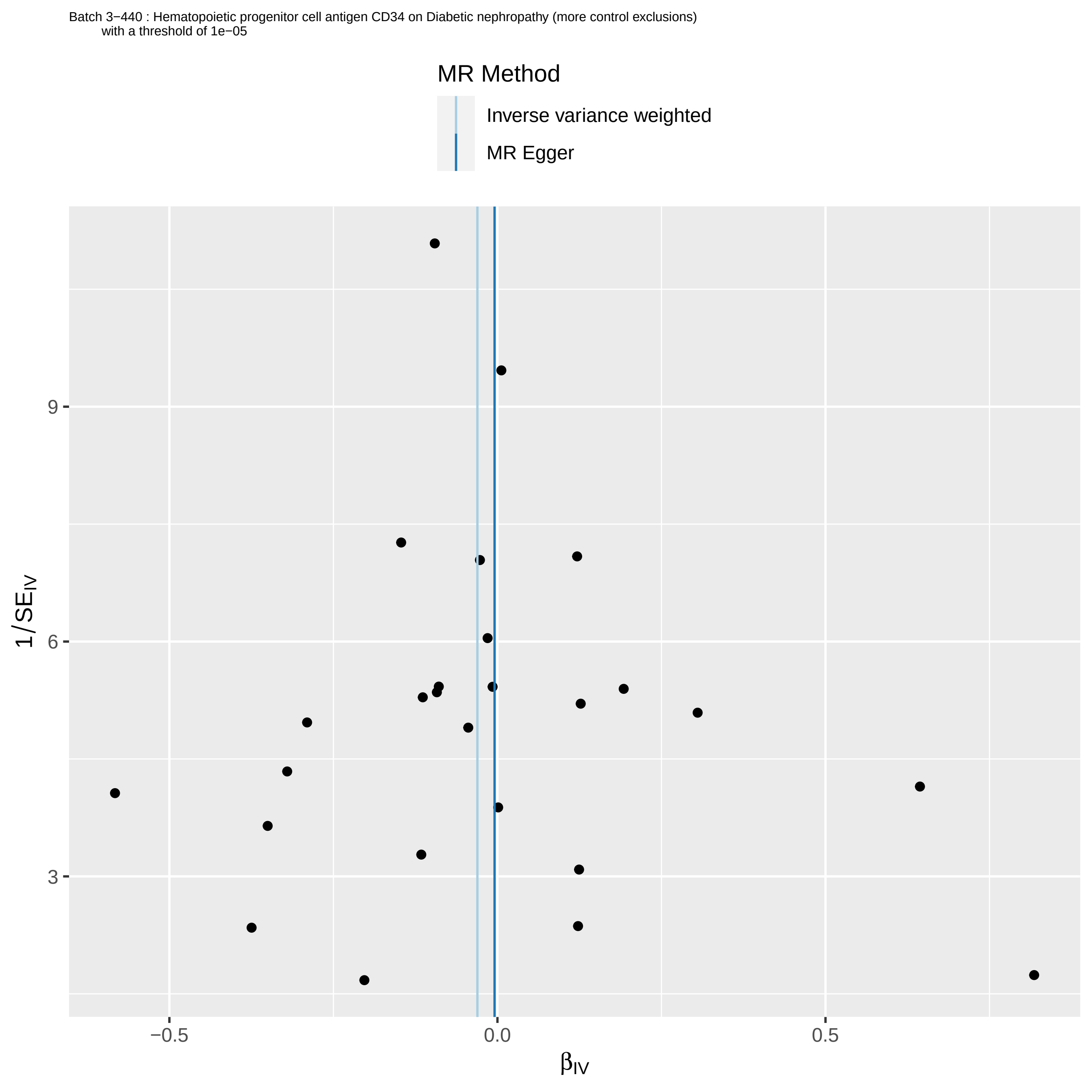
Batch 3−440 : Hematopoietic progenitor cell antigen CD34 on Diabetic nephropathy (more control exclusions)
with a threshold of 1e−05
MR Method
Inverse variance weighted
MR Egger
9
6
3
$1/SE_{IV}$
−0.5
0.0
0.5
β_{IV}

Batch 3−441 : Hematopoietic progenitor cell antigen CD34 on Diabetic nephropathy (more control exclusions)
with a threshold of 1e−05

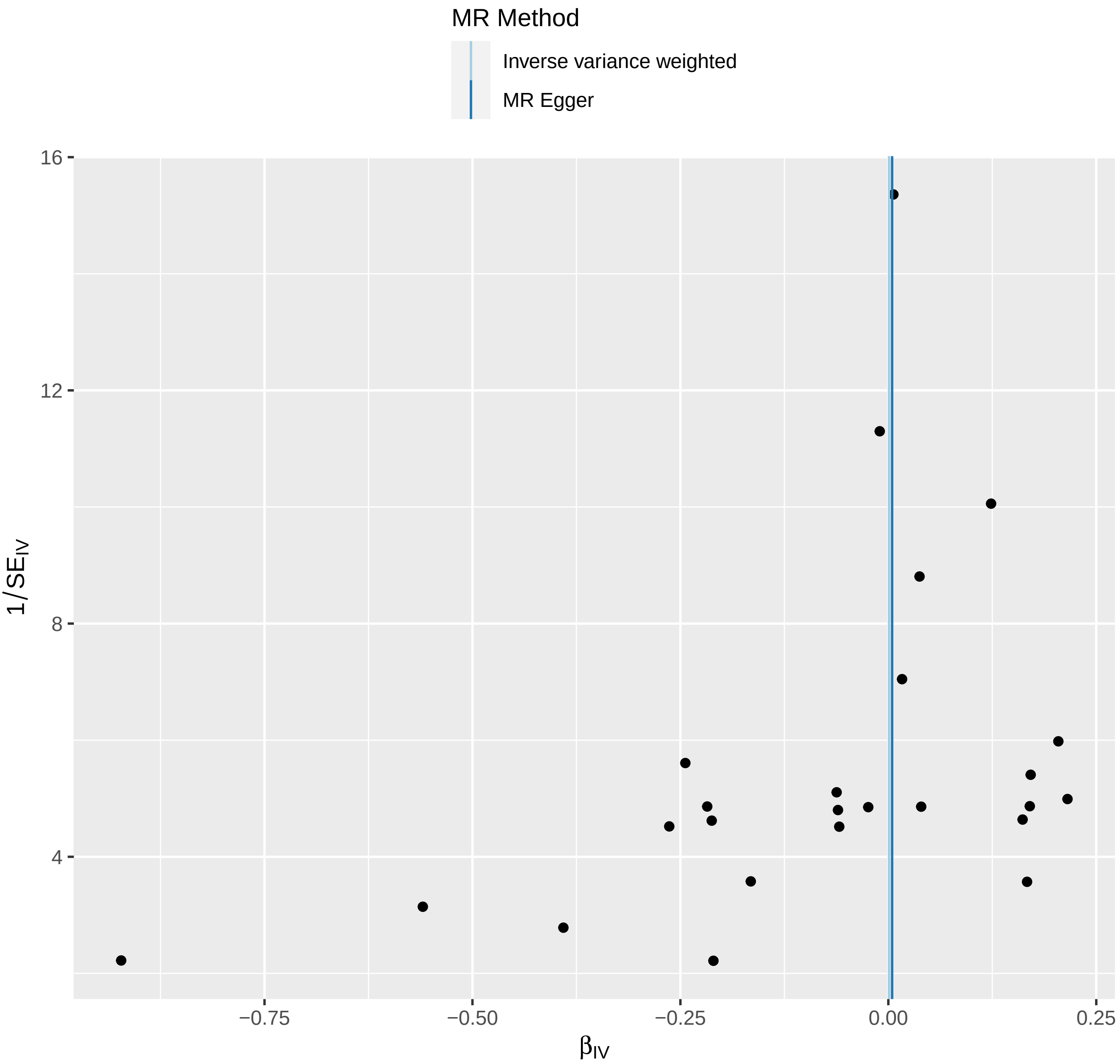

Batch 3−442 : Platelet glycoprotein 4 on Diabetic nephropathy (more control exclusions)
with a threshold of 1e−05

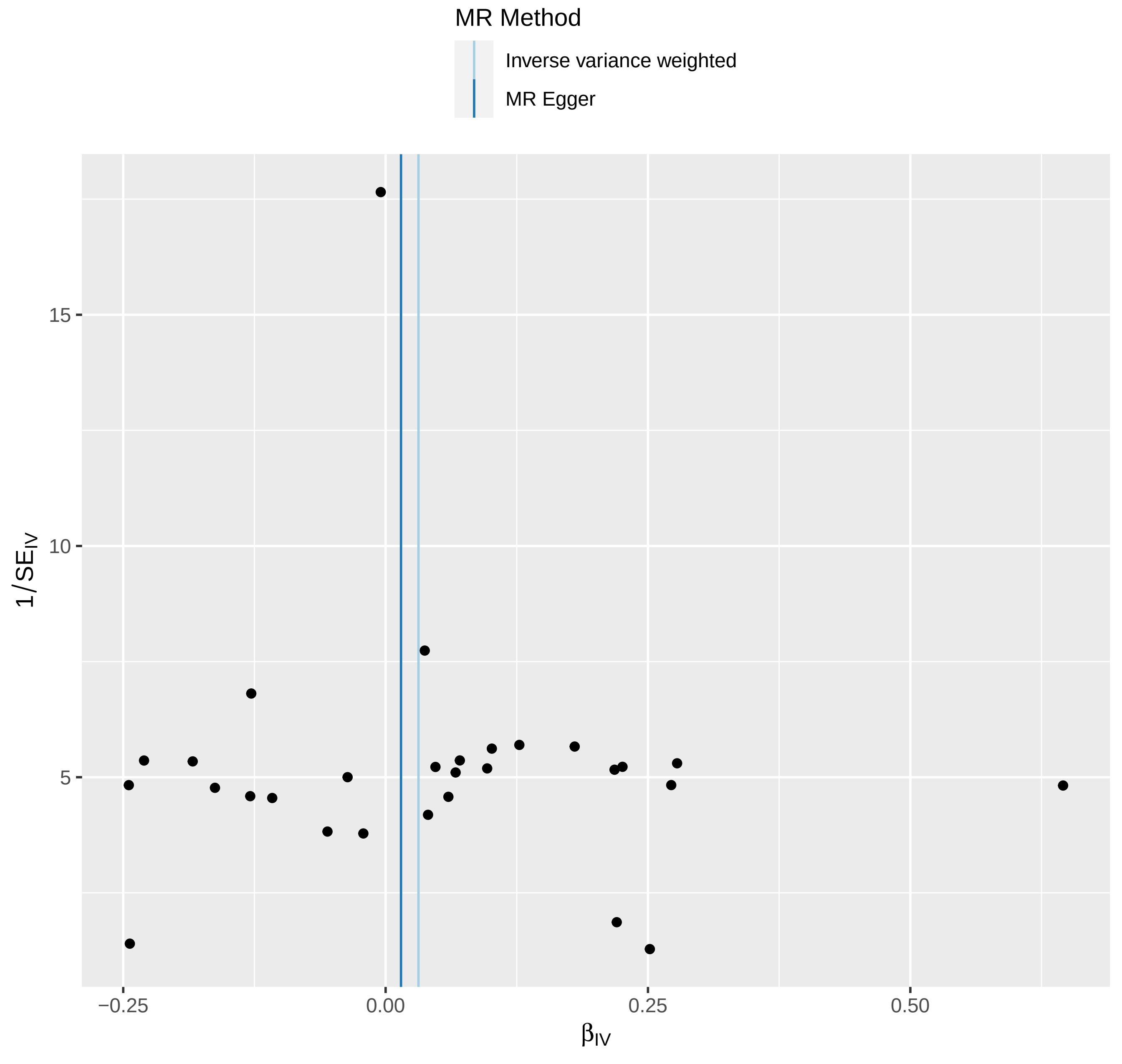

Batch 3−443 : ADP−ribosyl cyclase/cyclic ADP−ribose hydrolase 1 on Diabetic nephropathy (more control exclusions) with a threshold of 1e−05

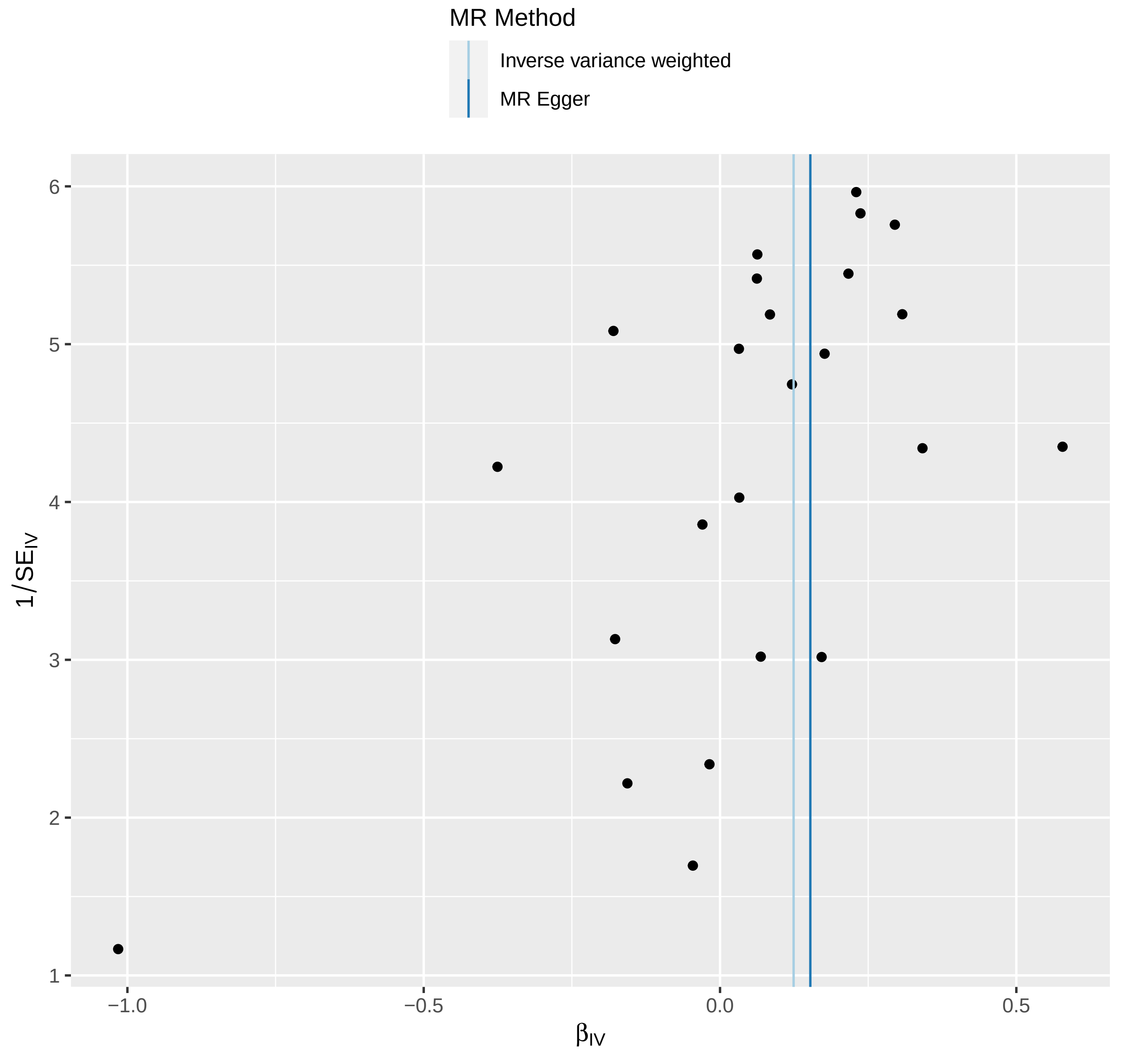

Batch 3−444 : ADP−ribosyl cyclase/cyclic ADP−ribose hydrolase 1 on Diabetic nephropathy (more control exclusions)
with a threshold of 1e−05

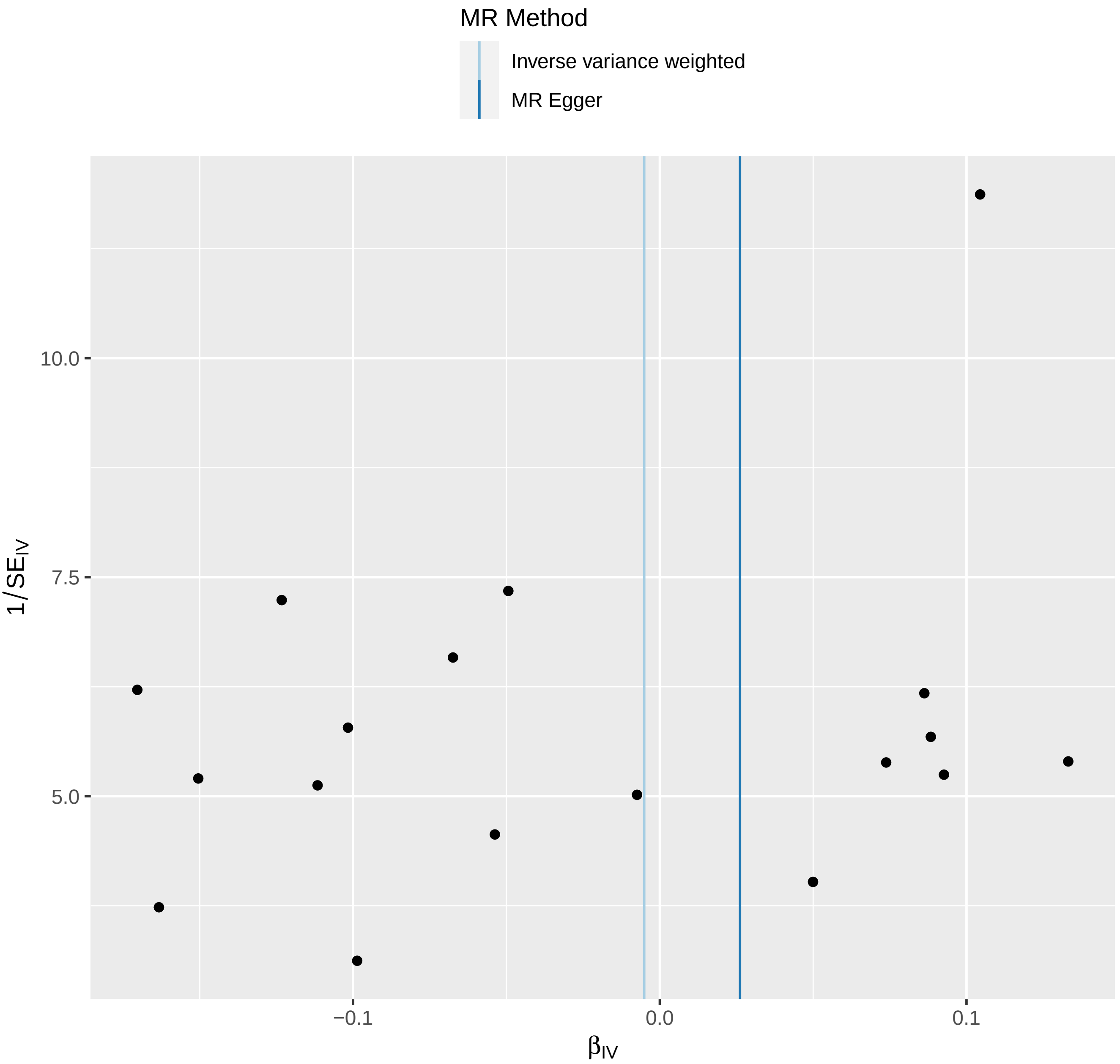

Batch 3−445 : T−cell surface glycoprotein CD3 epsilon chain on Diabetic nephropathy (more control exclusions) with a threshold of 1e−05

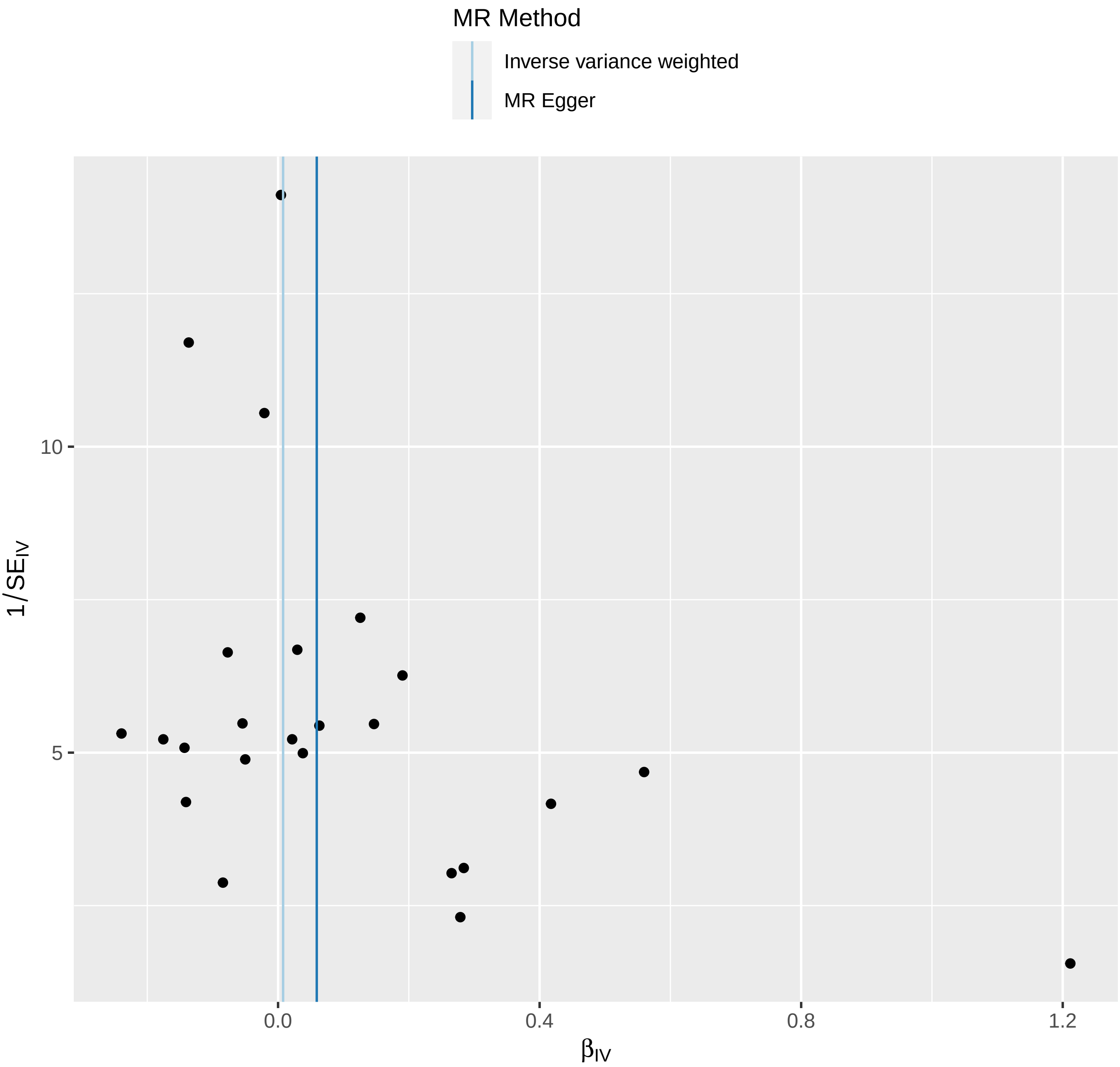

Batch 3−446 : T−cell surface glycoprotein CD3 gamma chain on Diabetic nephropathy (more control exclusions)
with a threshold of 1e−05

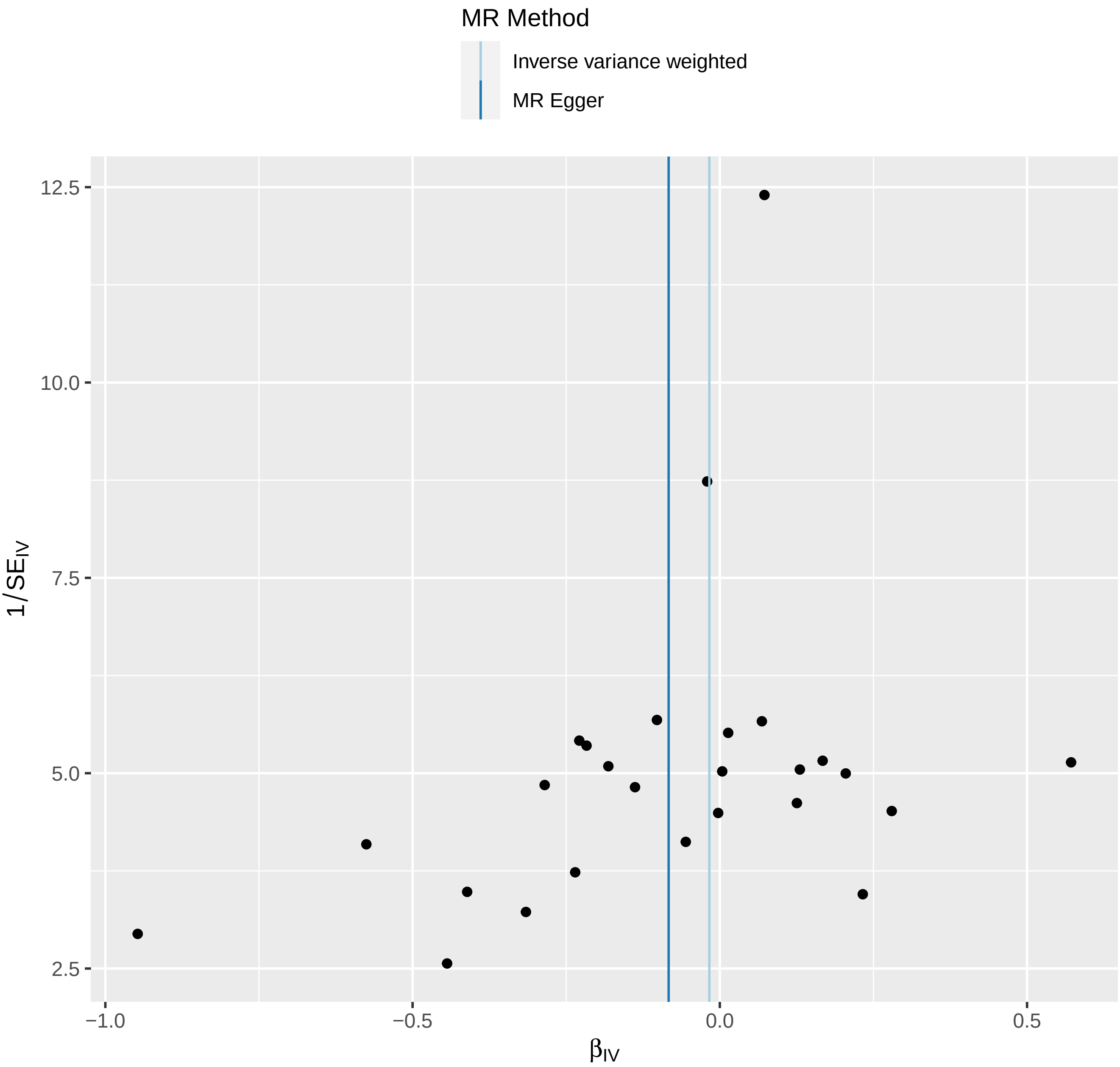

Batch 3−447 : CD40 ligand on Diabetic nephropathy (more control exclusions)
with a threshold of 1e−05

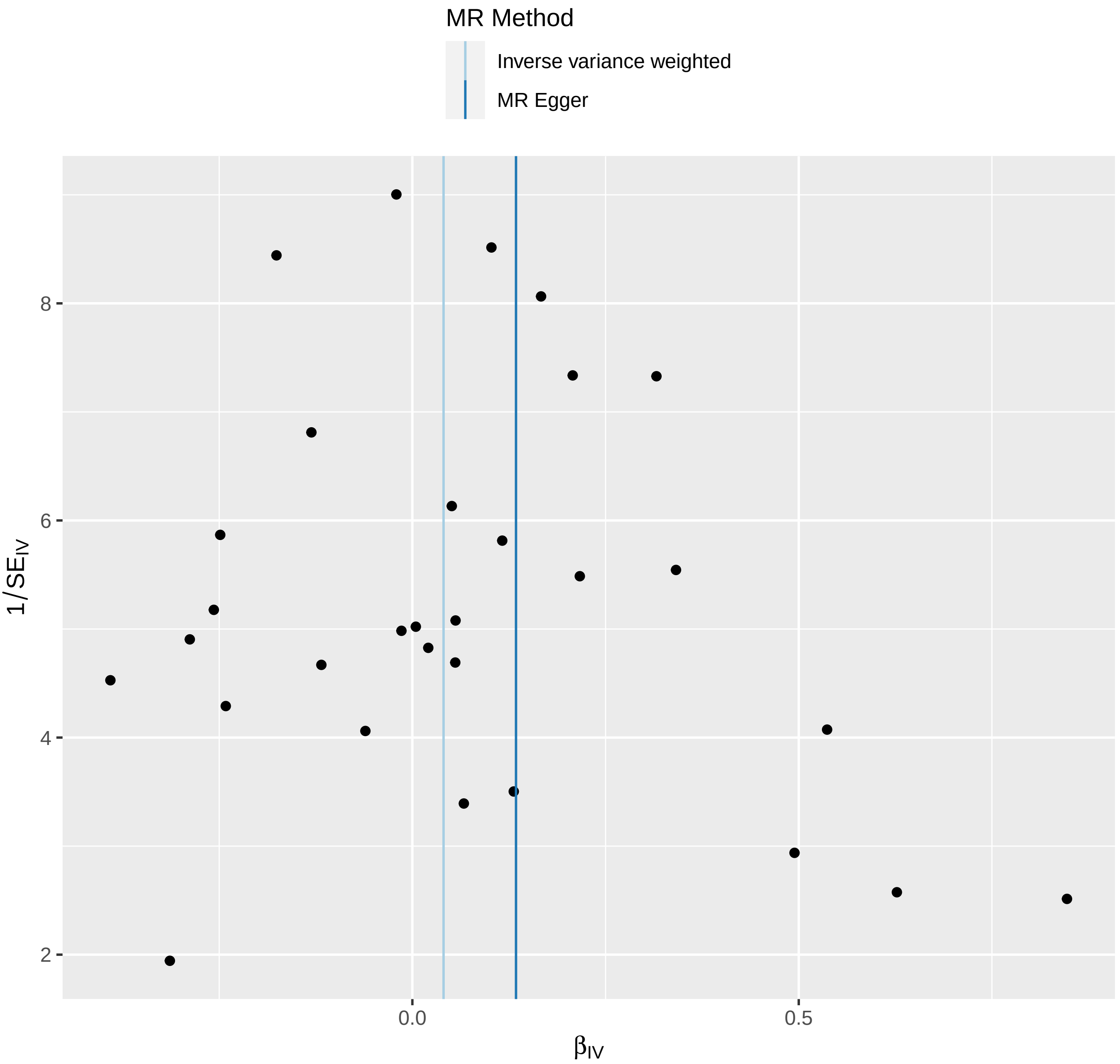

Batch 3−448 : T−cell surface glycoprotein CD4 on Diabetic nephropathy (more control exclusions) with a threshold of 1e−05

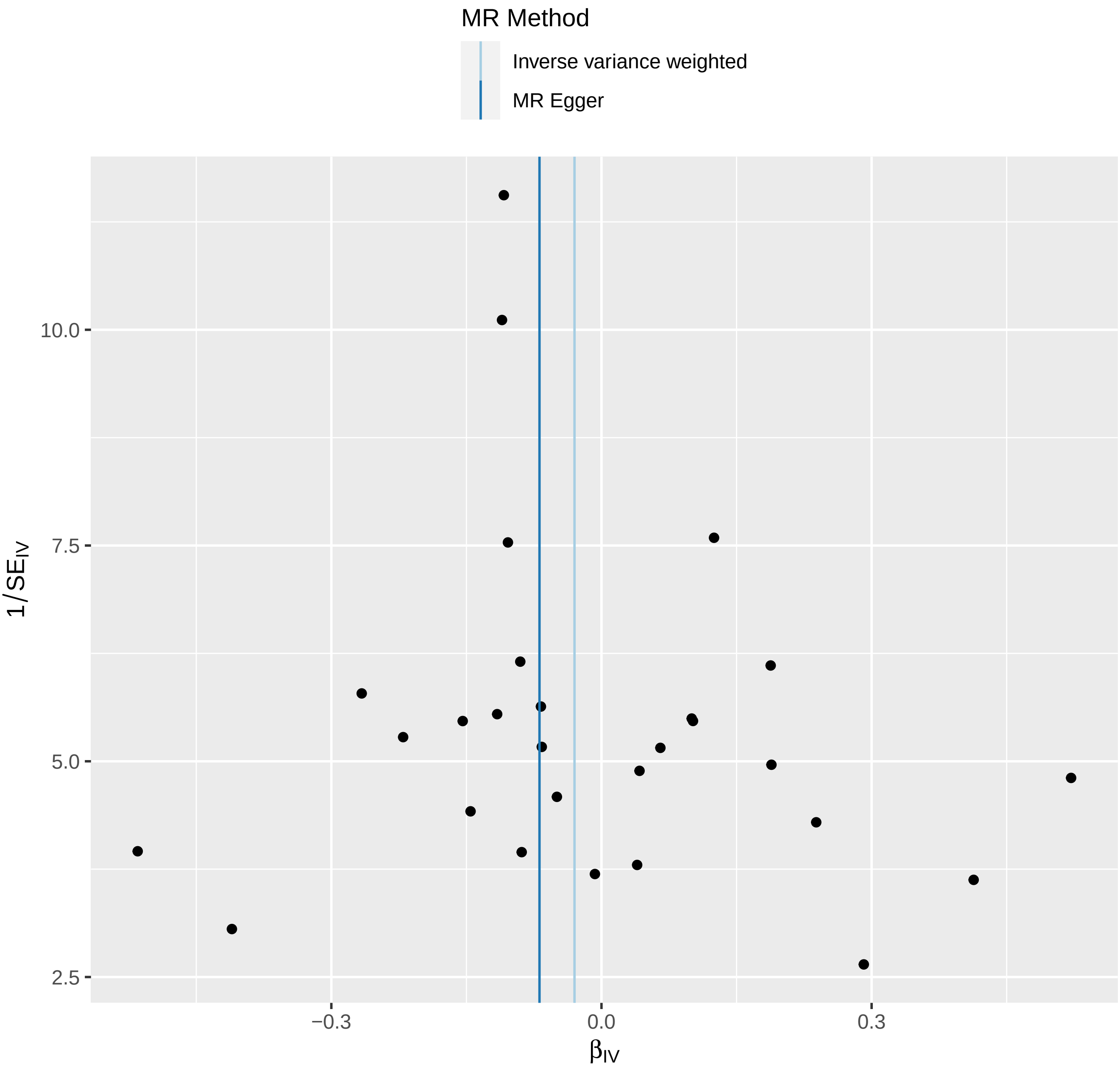

Batch 3−449 : CD44 antigen on Diabetic nephropathy (more control exclusions)
with a threshold of 1e−05

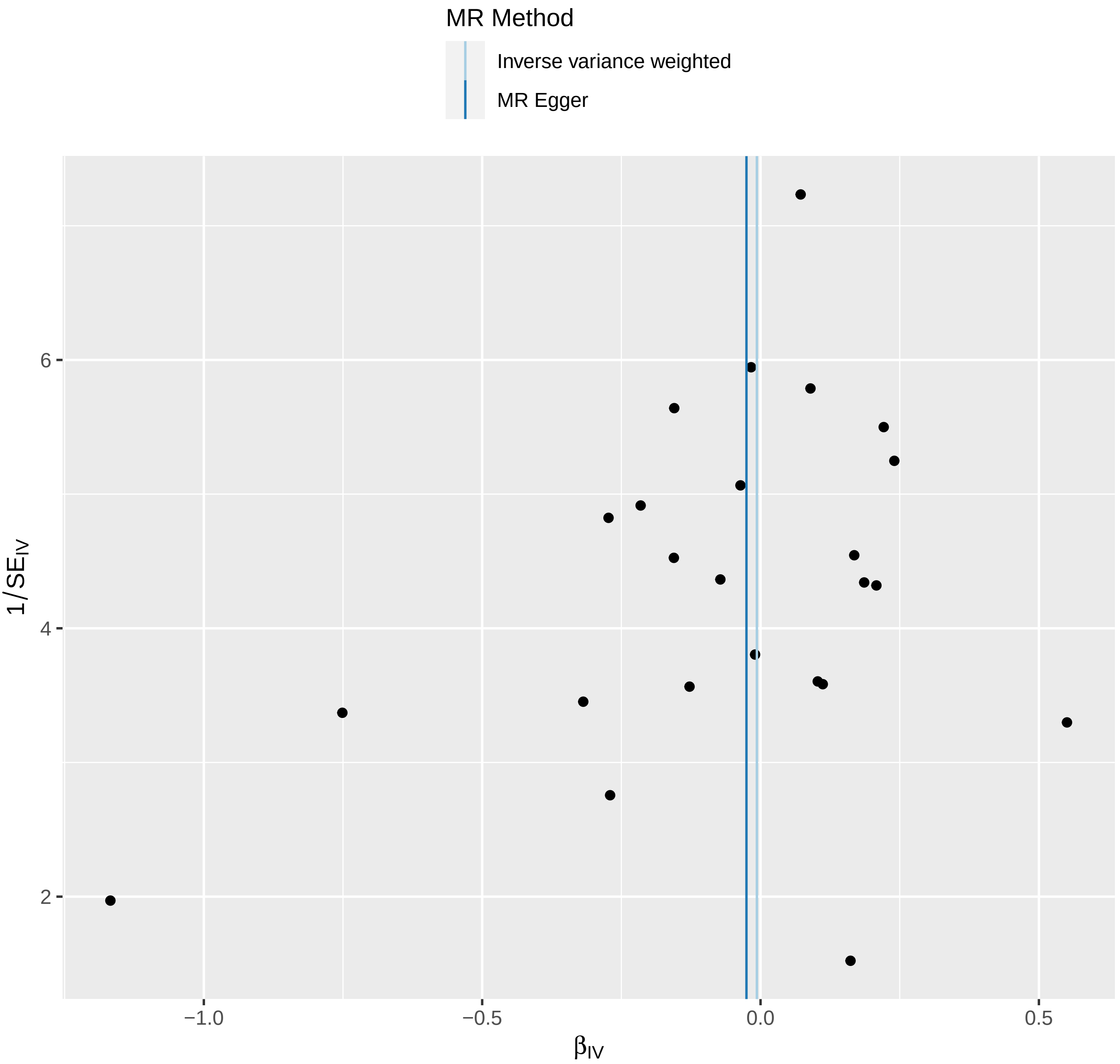

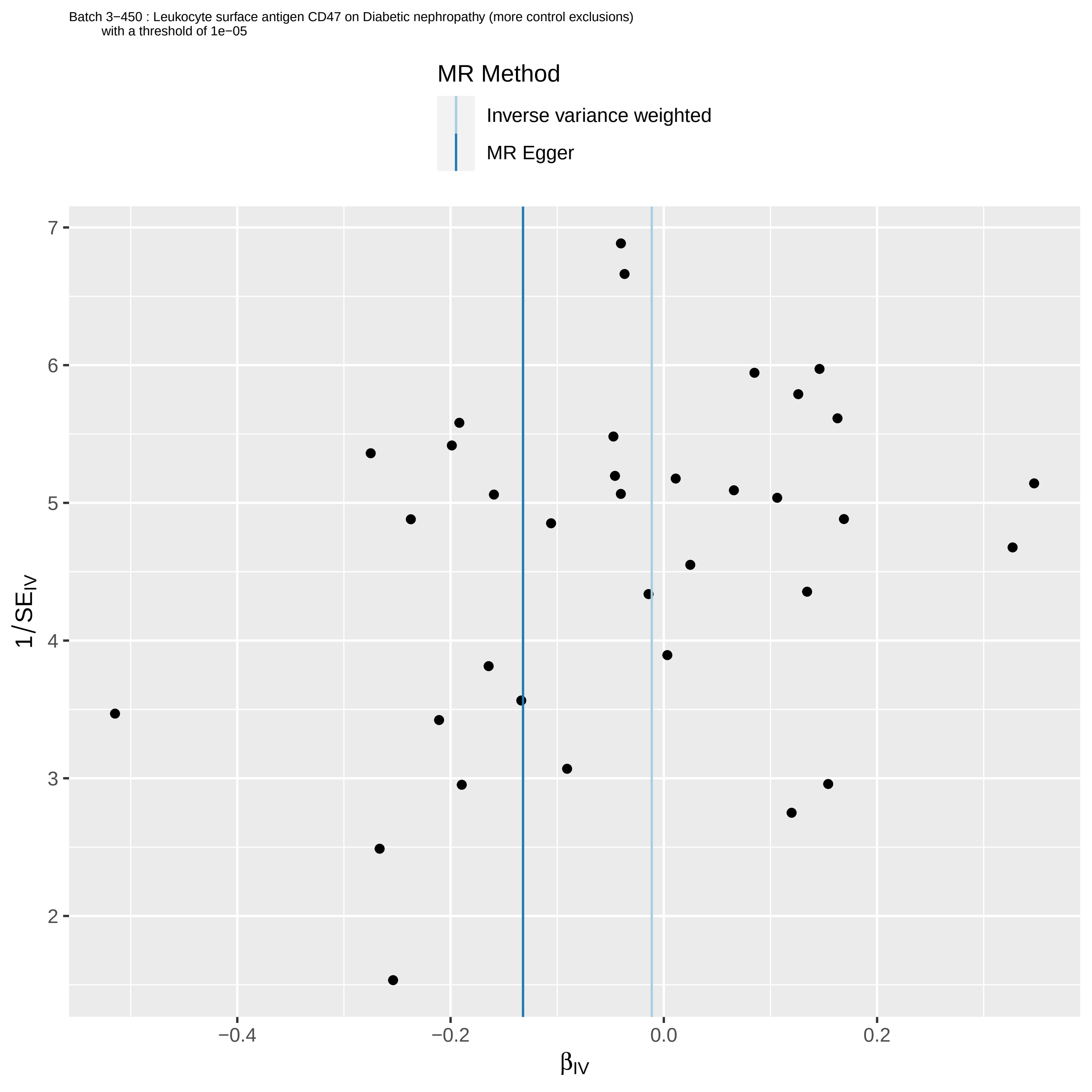
Batch 3−450 : Leukocyte surface antigen CD47 on Diabetic nephropathy (more control exclusions)
with a threshold of 1e−05
MR Method
Inverse variance weighted
MR Egger
1/SE$_{IV}$
7
6
5
4
3
2
−0.4
−0.2
0.0
0.2
β$_{IV}$

Batch 3−451 : CD48 antigen on Diabetic nephropathy (more control exclusions)
with a threshold of 1e−05

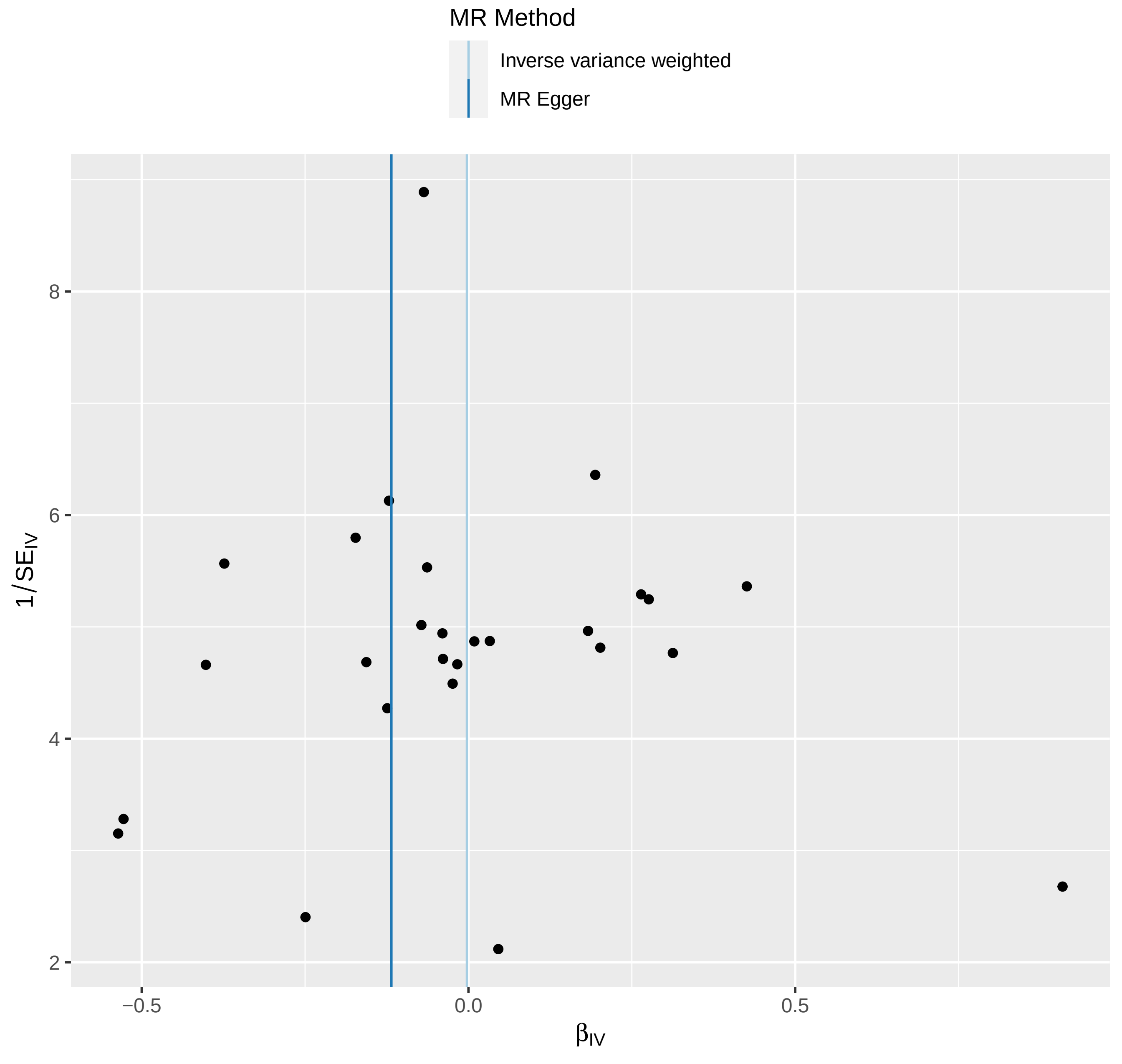

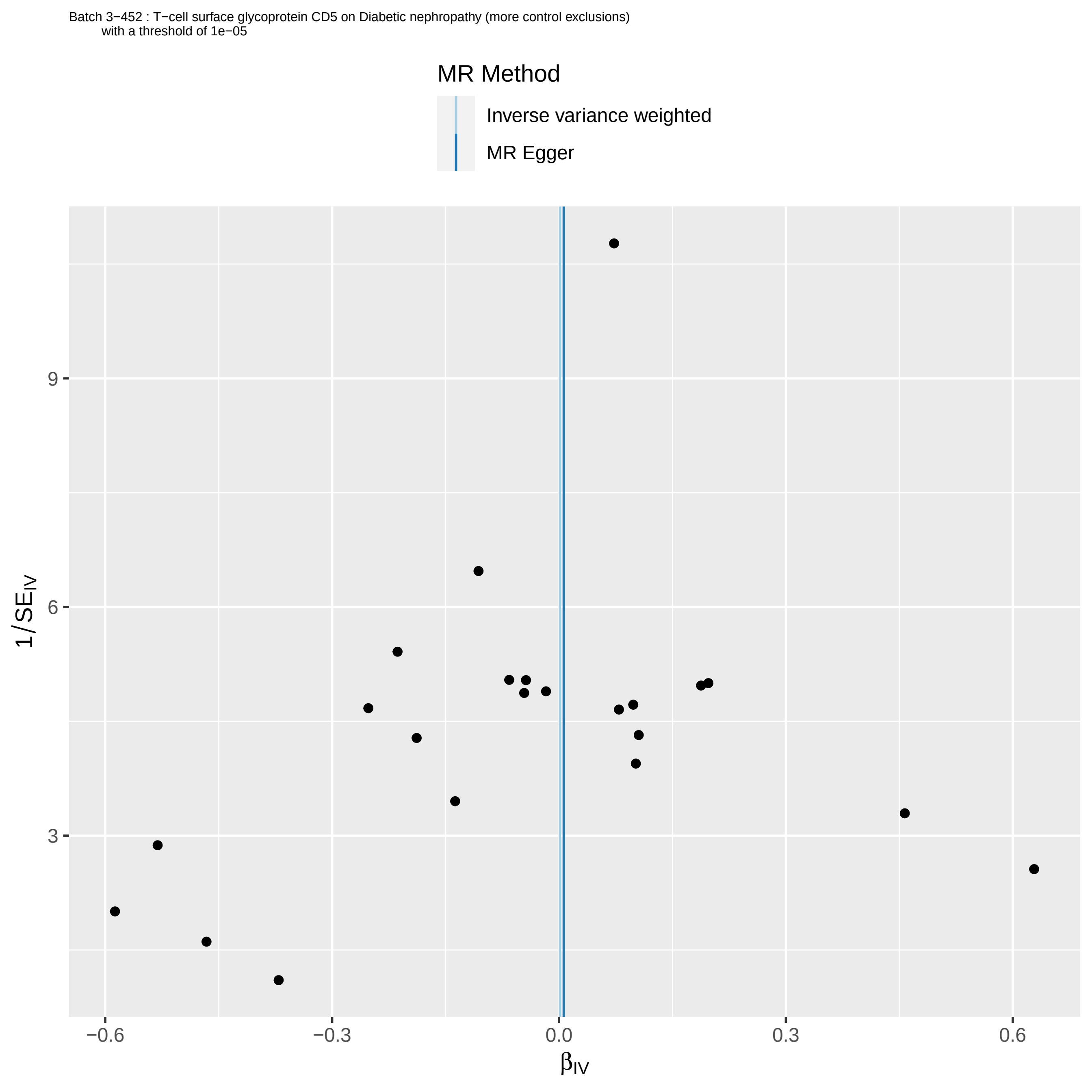
Batch 3−452 : T−cell surface glycoprotein CD5 on Diabetic nephropathy (more control exclusions)
with a threshold of 1e−05
MR Method
Inverse variance weighted
MR Egger
9
6
3
1/SE_IV
−0.6
−0.3
0.0
0.3
0.6
β_IV

Batch 3−453 : Complement decay−accelerating factor on Diabetic nephropathy (more control exclusions)
with a threshold of 1e−05

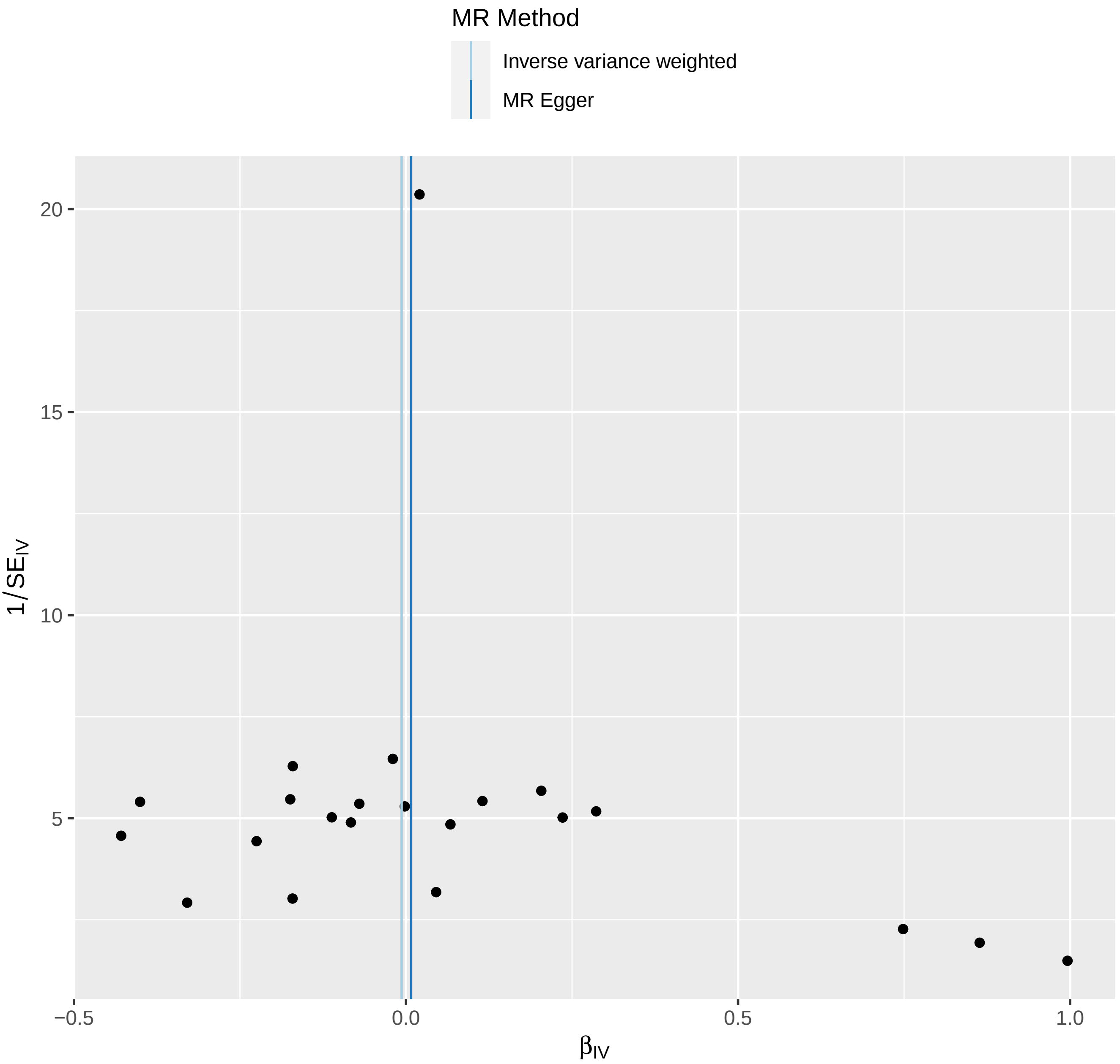

Batch 3−454 : Lymphocyte function−associated antigen 3 on Diabetic nephropathy (more control exclusions)
with a threshold of 1e−05

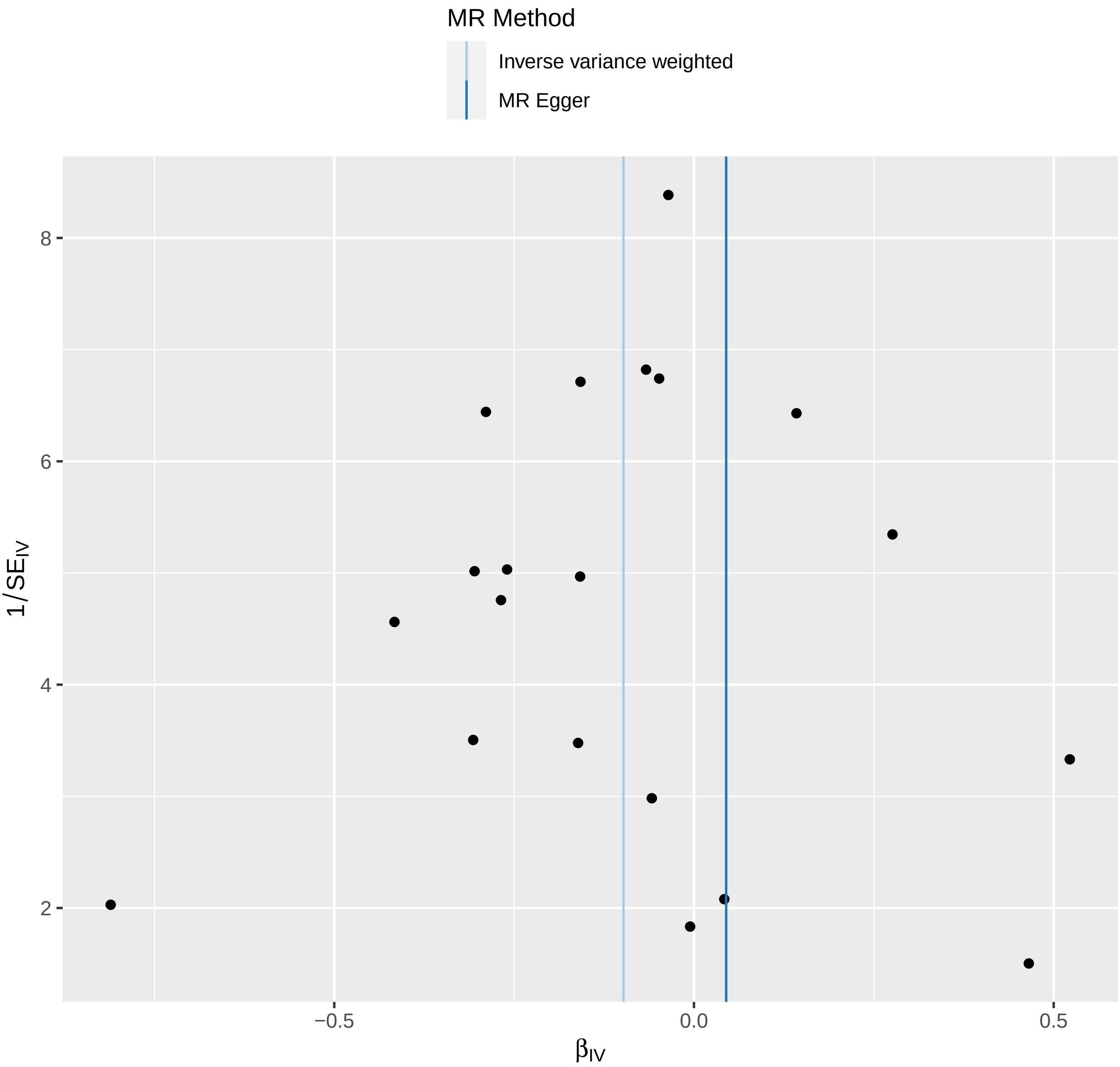

Batch 3−455 : CD59 glycoprotein on Diabetic nephropathy (more control exclusions)
with a threshold of 1e−05

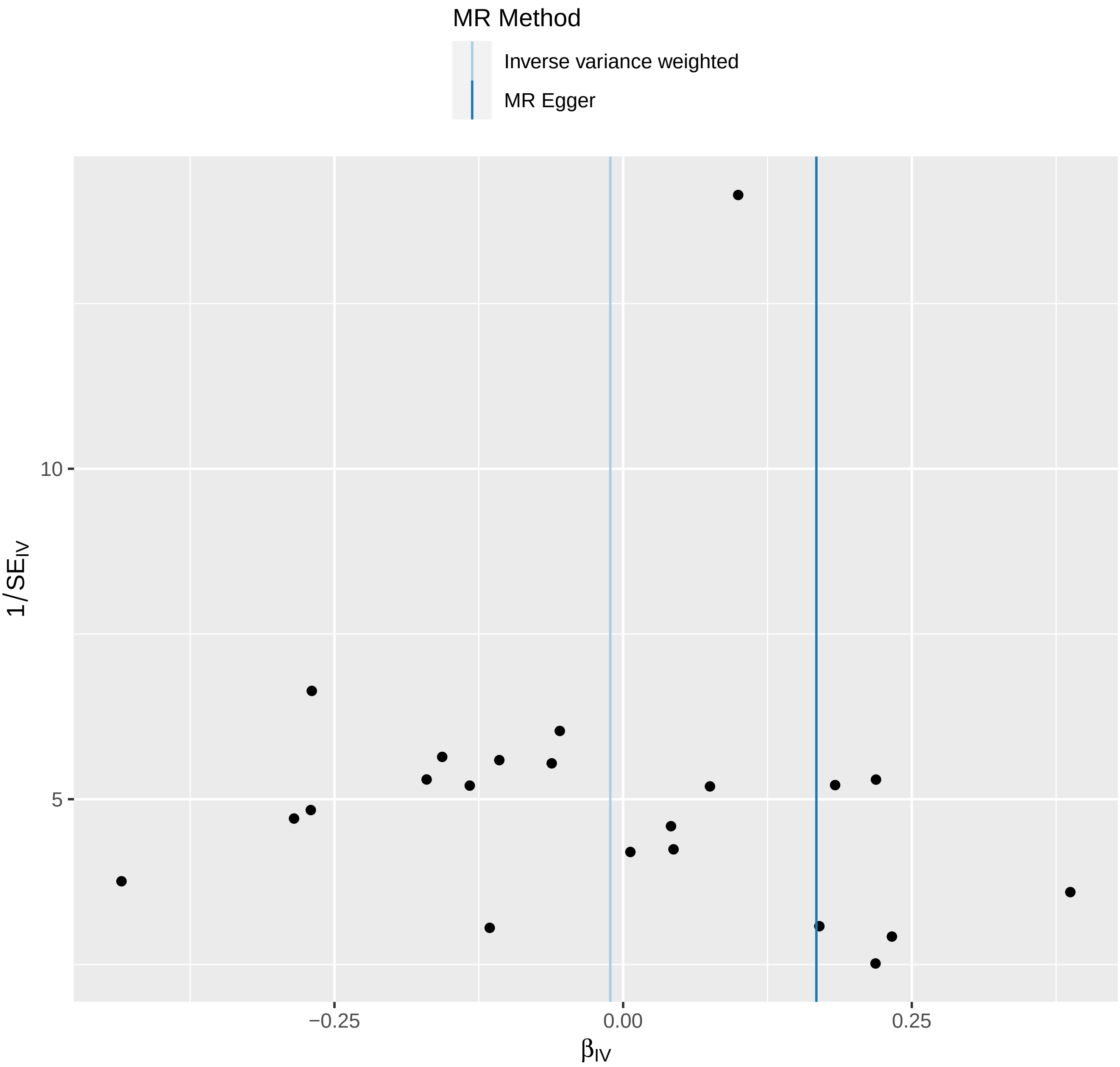

Batch 3−456 : CD63 antigen on Diabetic nephropathy (more control exclusions)
with a threshold of 1e−05

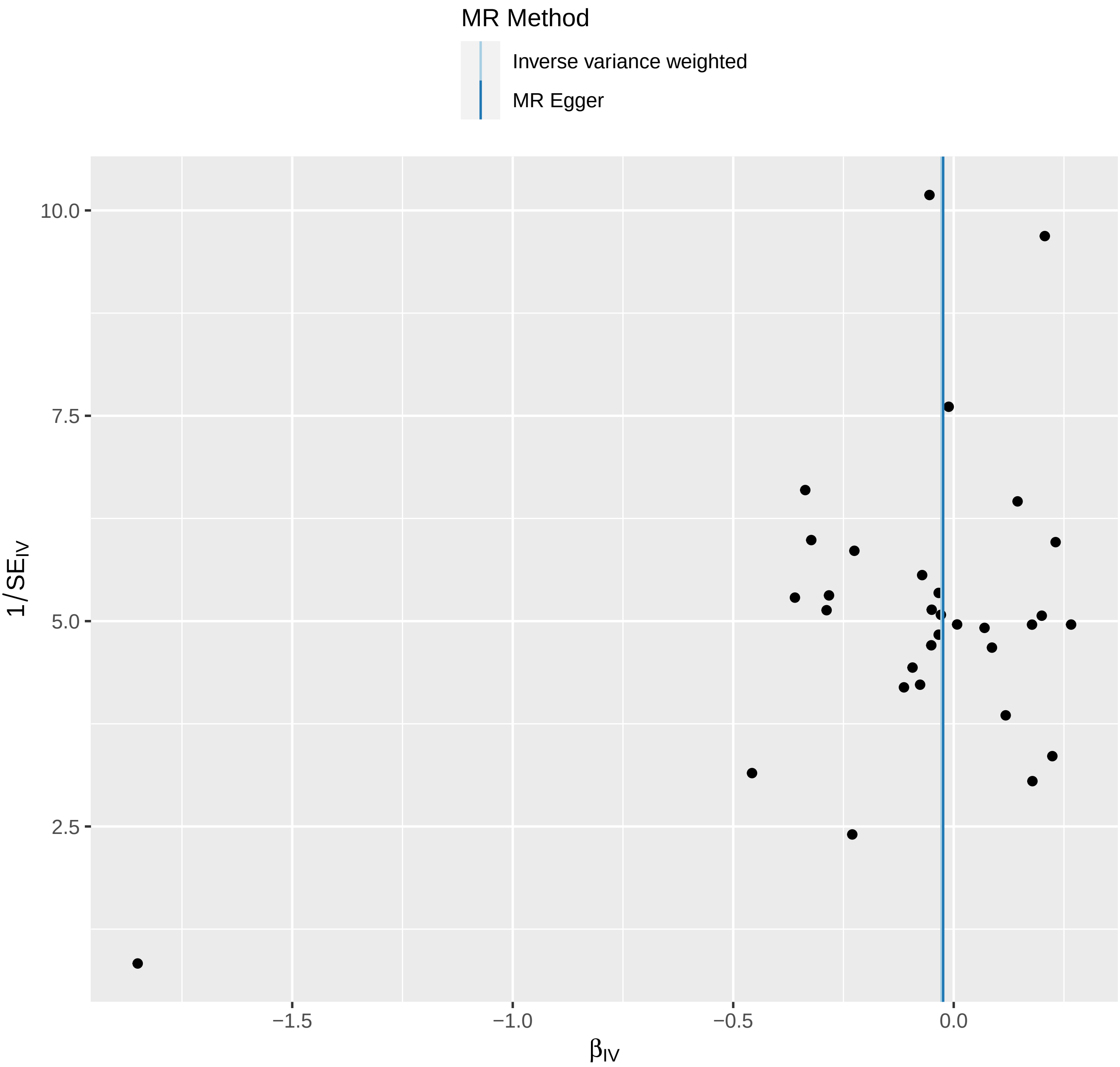

Batch 3−457 : CD70 antigen on Diabetic nephropathy (more control exclusions)
with a threshold of 1e−05

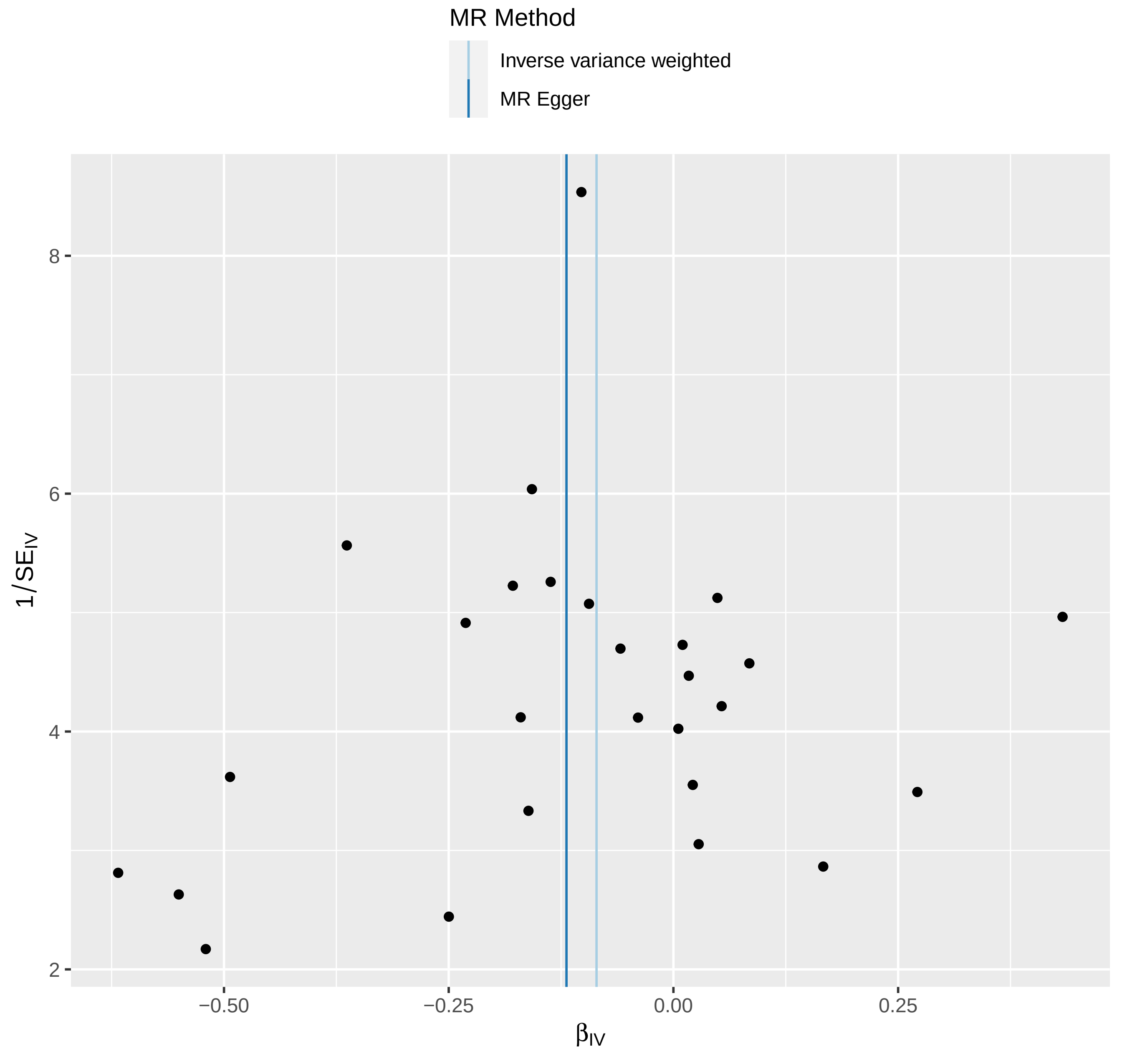

Batch 3−458 : CD70 antigen on Diabetic nephropathy (more control exclusions)
with a threshold of 1e−05

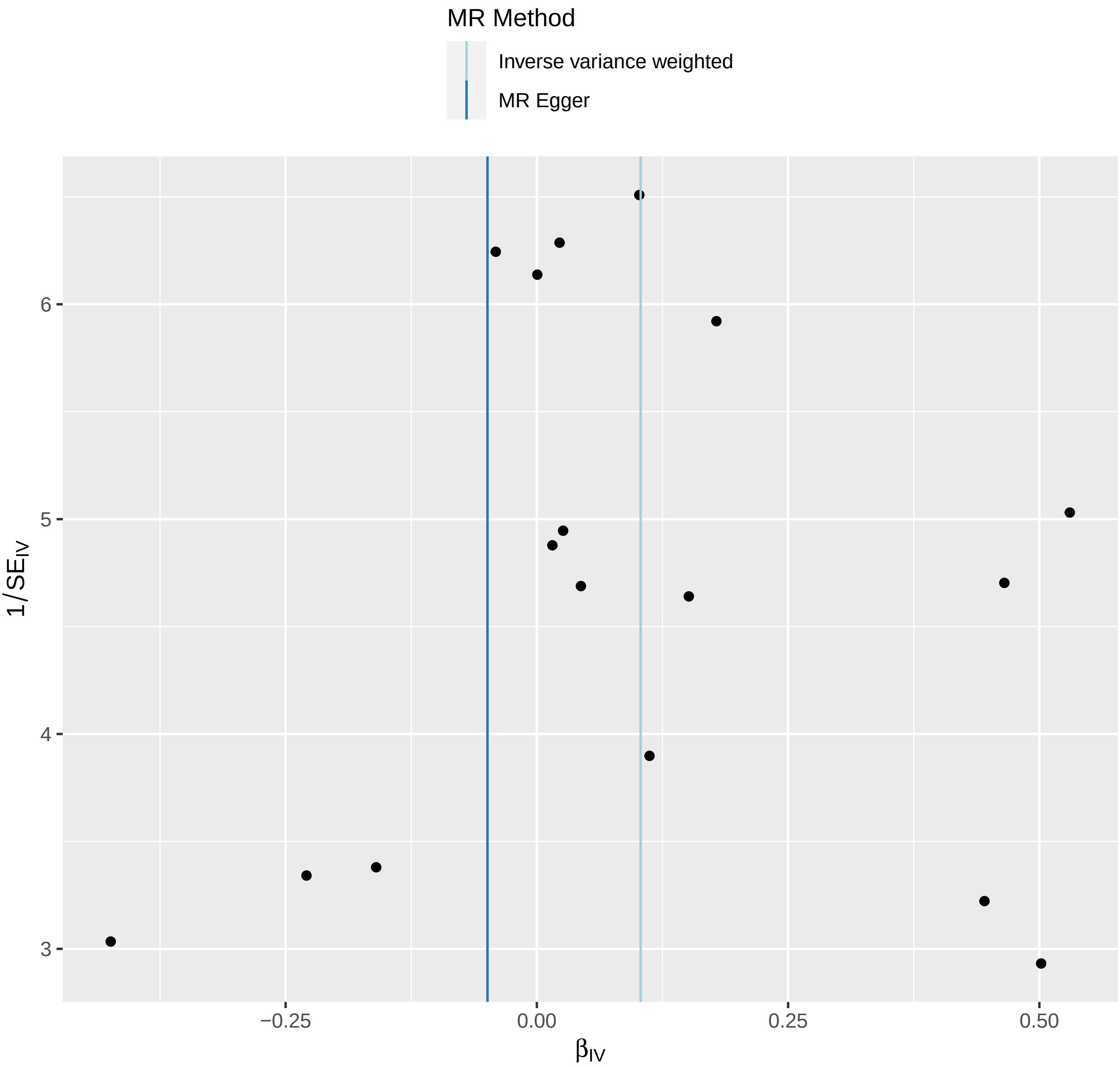

Batch 3−459 : HLA class II histocompatibility antigen gamma chain on Diabetic nephropathy (more control exclusions)
with a threshold of 1e−05

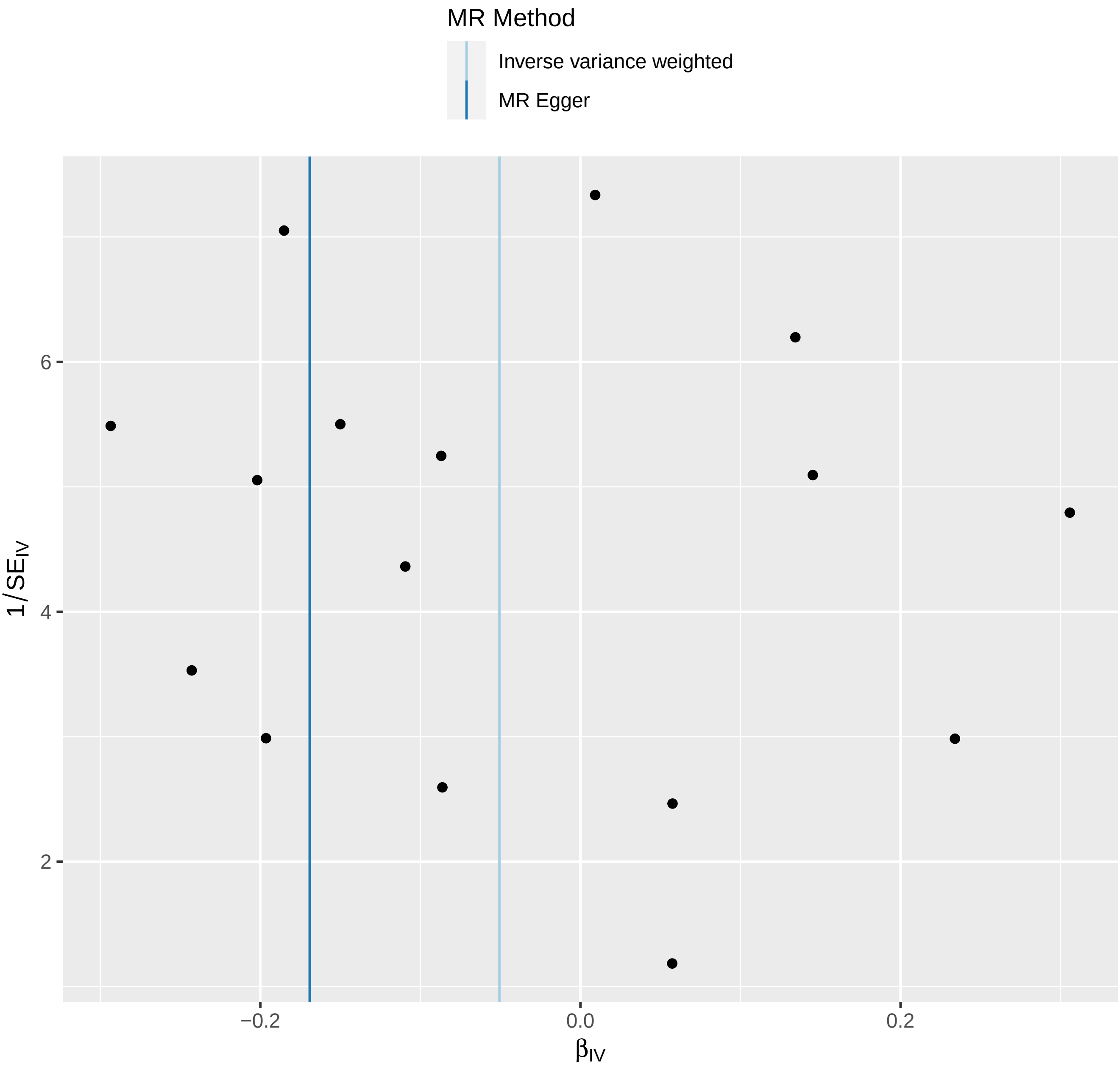

Batch 3−460 : HLA class II histocompatibility antigen gamma chain on Diabetic nephropathy (more control exclusions) with a threshold of 1e−05

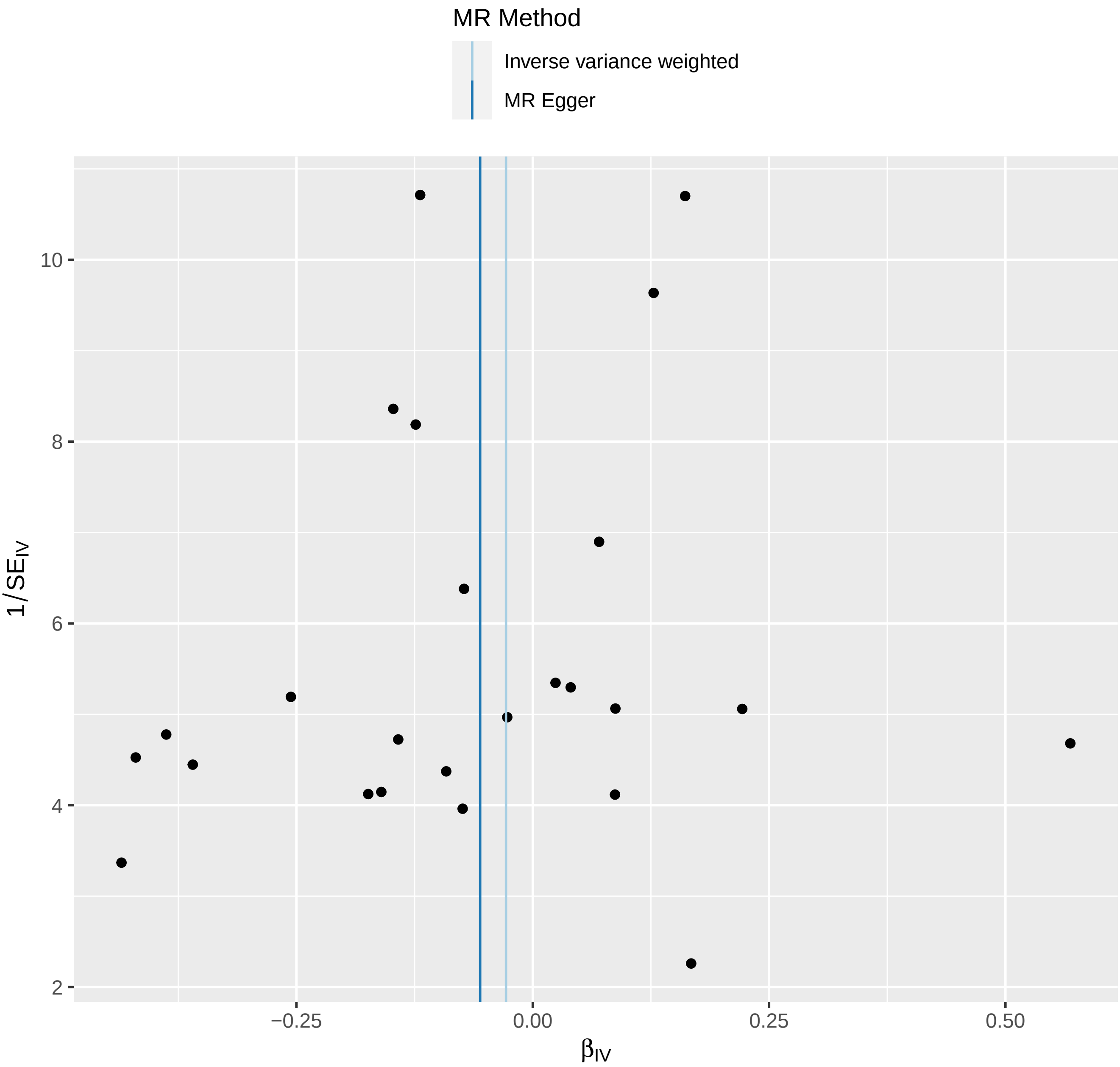

Batch 3−461 : B−cell antigen receptor complex−associated protein alpha chain on Diabetic nephropathy (more control exclusions)
with a threshold of 1e−05

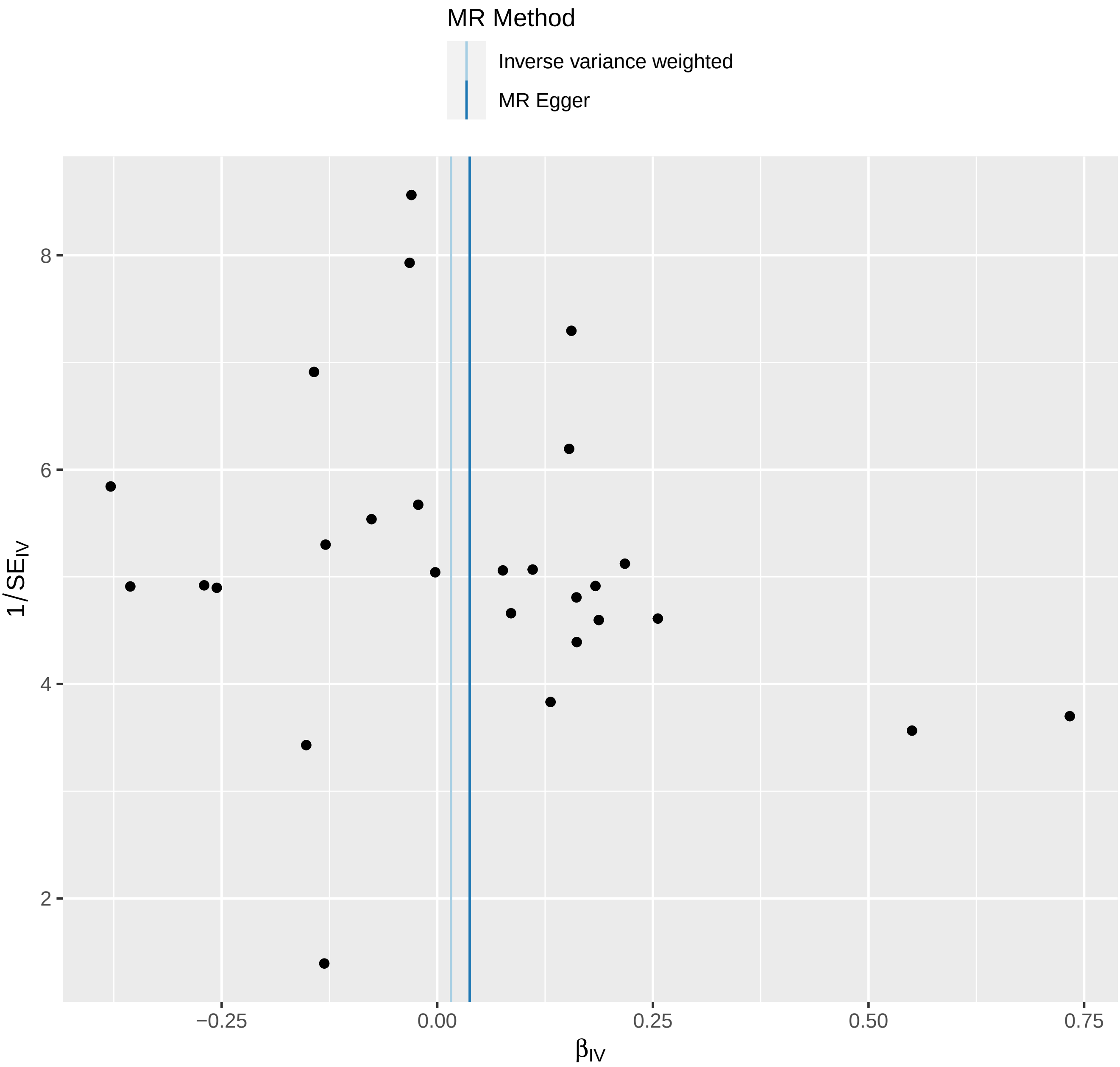

Batch 3−462 : B−cell antigen receptor complex−associated protein beta chain on Diabetic nephropathy (more control exclusions) with a threshold of 1e−05

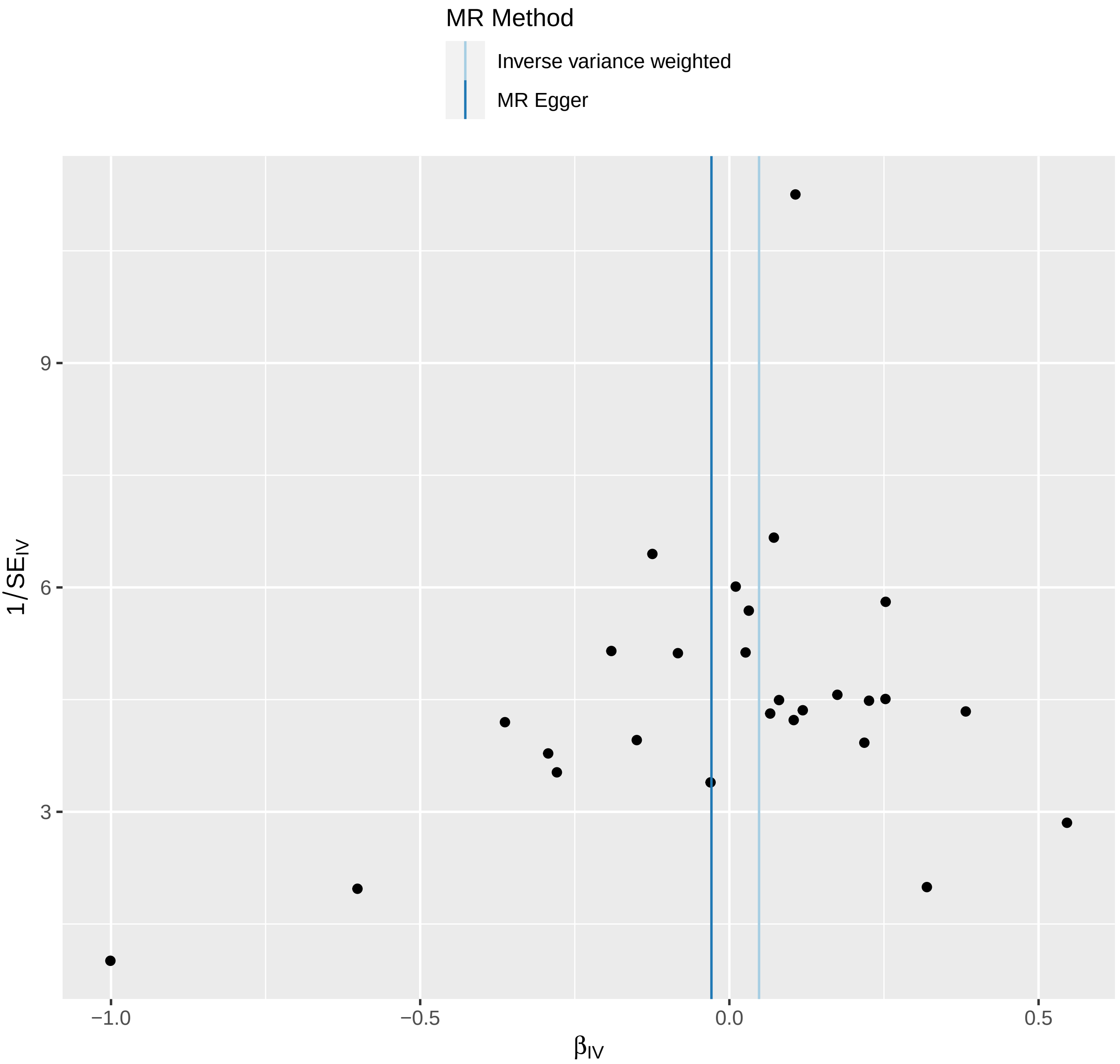

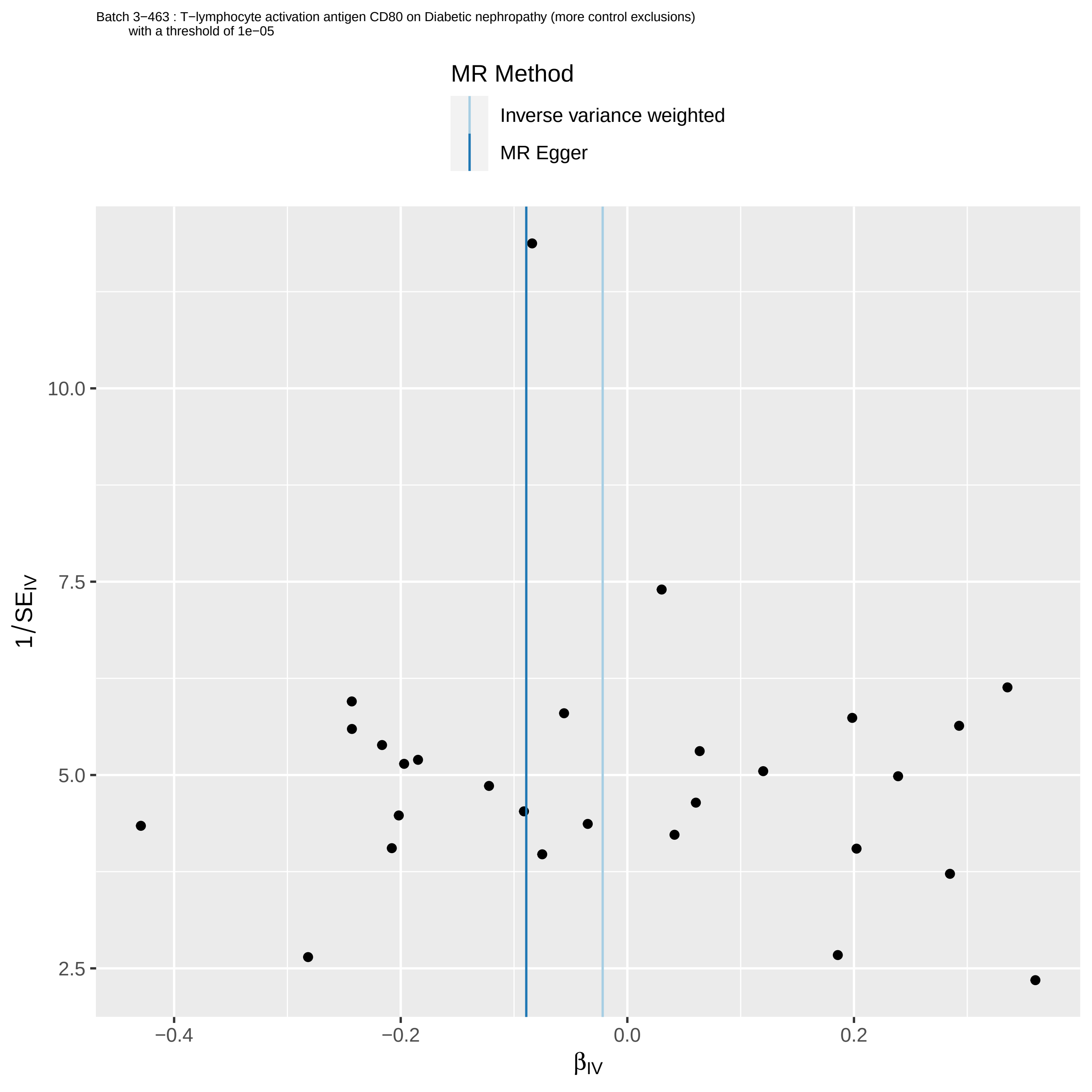
Batch 3−463 : T−lymphocyte activation antigen CD80 on Diabetic nephropathy (more control exclusions)
with a threshold of 1e−05
MR Method
Inverse variance weighted
MR Egger
10.0
7.5
5.0
2.5
1/SE_IV
−0.4
−0.2
0.0
0.2
β_IV

Batch 3−464 : SLAM family member 5 on Diabetic nephropathy (more control exclusions)
with a threshold of 1e−05

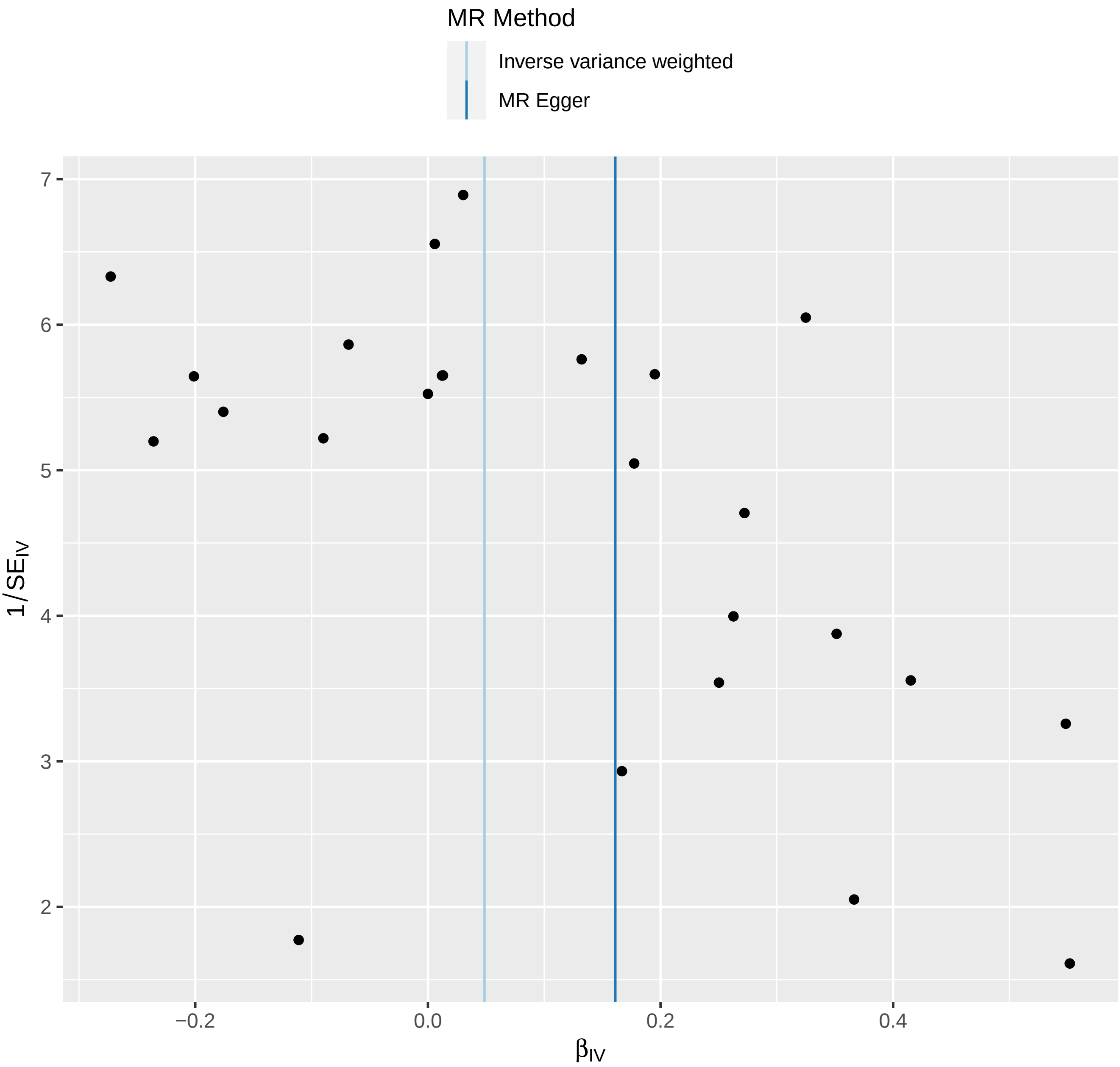

Batch 3−465 : SLAM family member 5 on Diabetic nephropathy (more control exclusions)
with a threshold of 1e−05

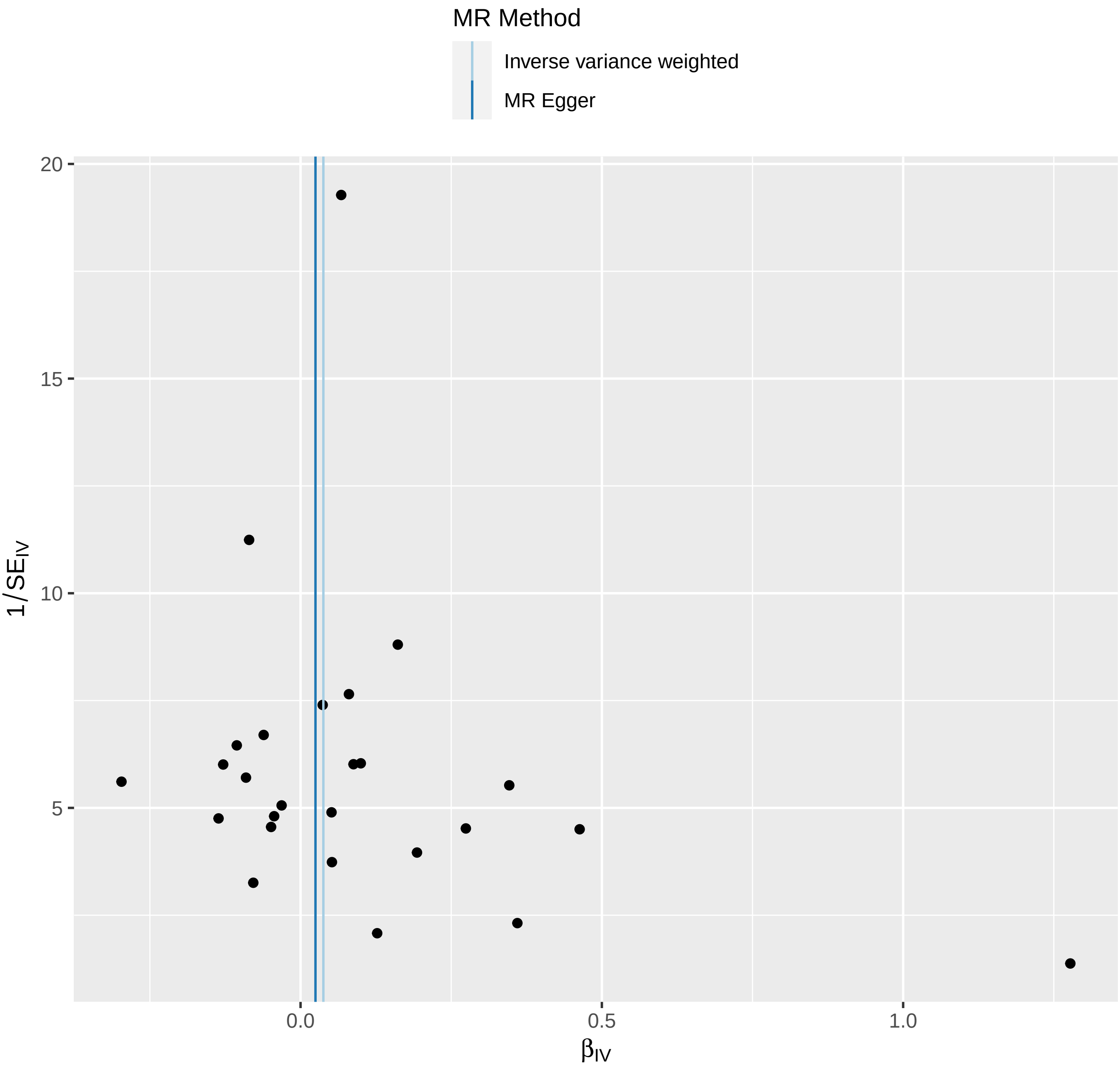

Batch 3−466 : T−lymphocyte activation antigen CD86 on Diabetic nephropathy (more control exclusions)
with a threshold of 1e−05

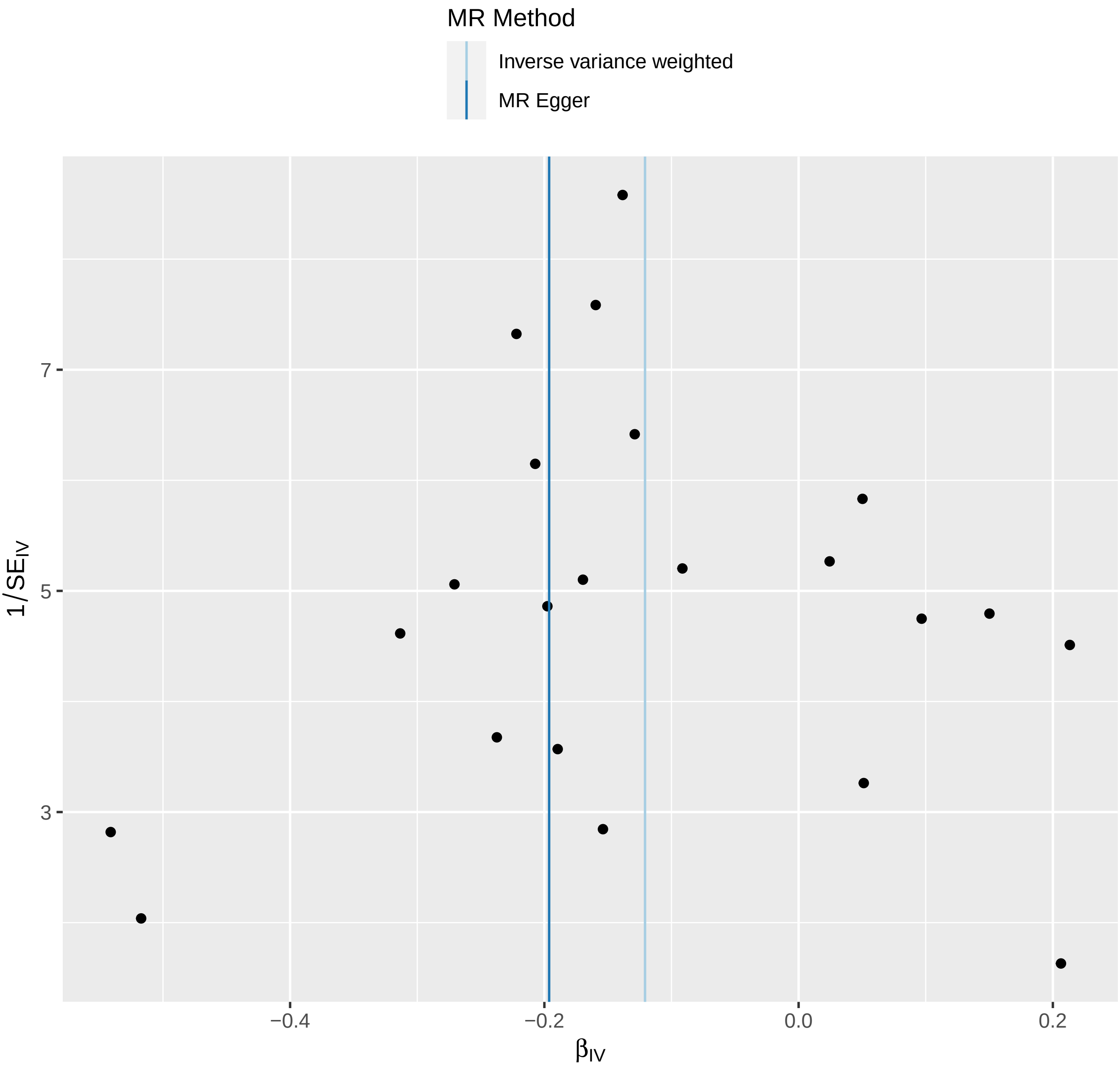

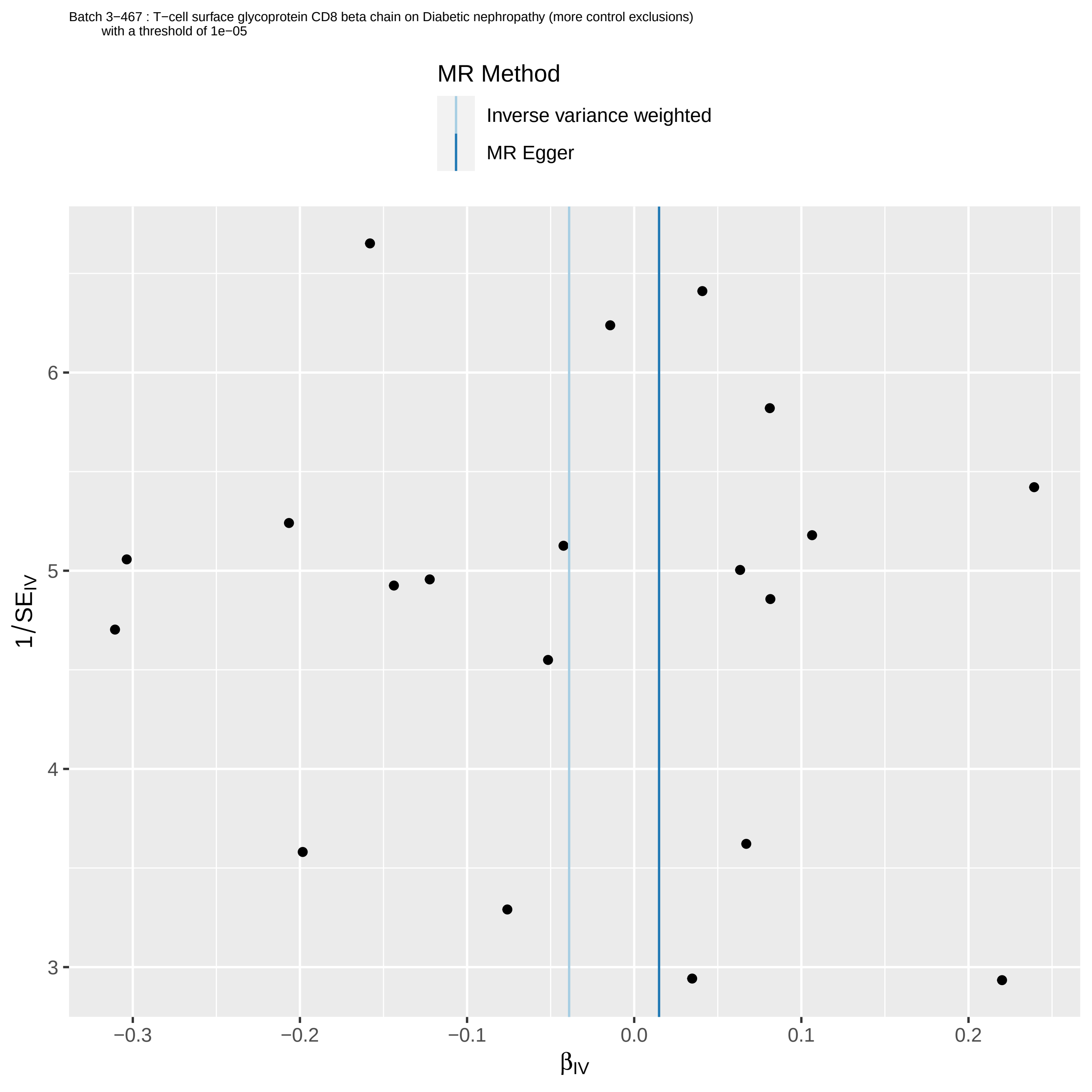

Batch 3−467 : T−cell surface glycoprotein CD8 beta chain on Diabetic nephropathy (more control exclusions)
with a threshold of 1e−05
MR Method
Inverse variance weighted
MR Egger
6
5
4
3
1/SE_IV
−0.3
−0.2
−0.1
0.0
0.1
0.2
β_IV

Batch 3−468 : Complement component C1q receptor on Diabetic nephropathy (more control exclusions)
with a threshold of 1e−05

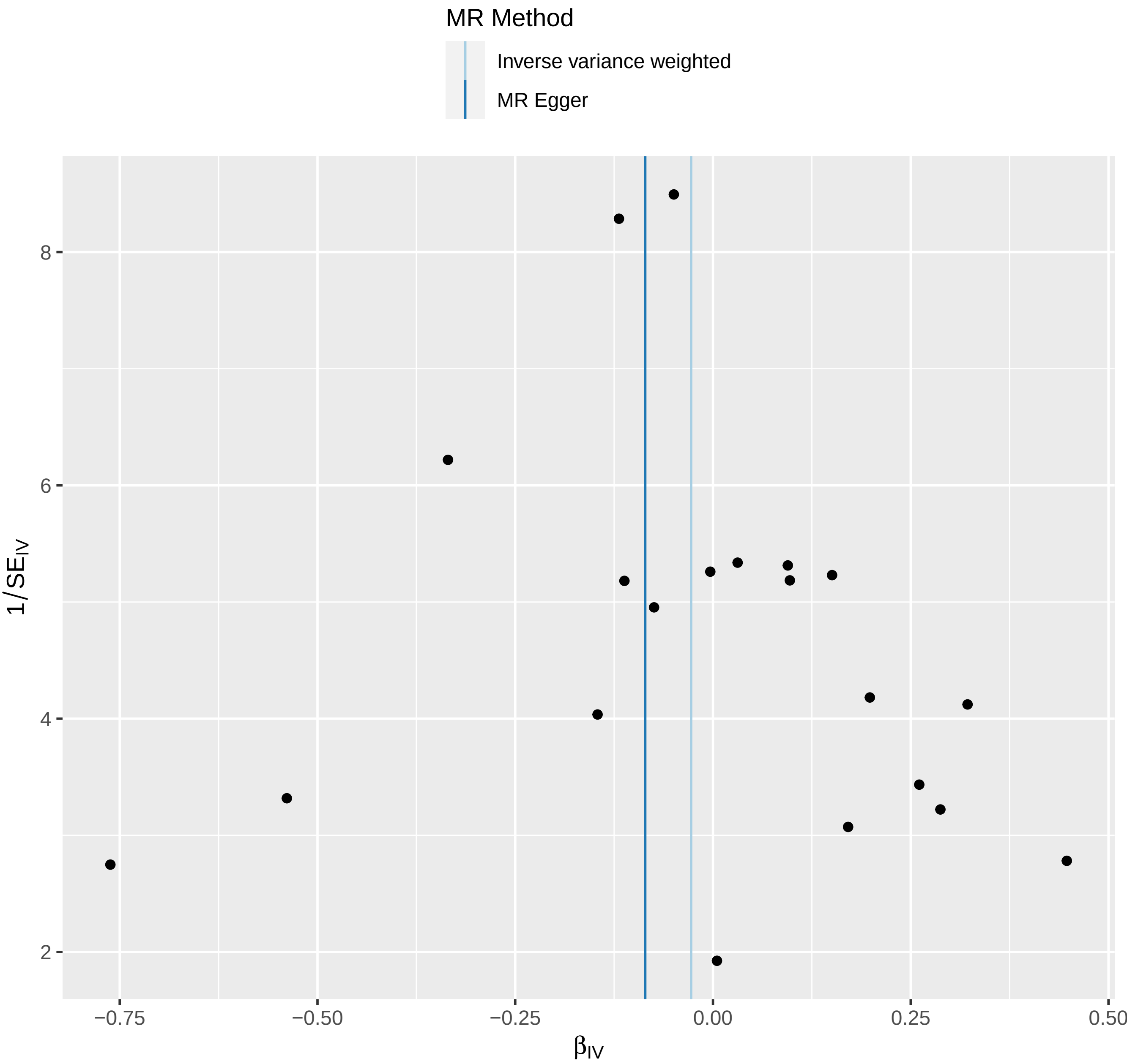

Batch 3−469 : Complement component C1q receptor on Diabetic nephropathy (more control exclusions)
with a threshold of 1e−05

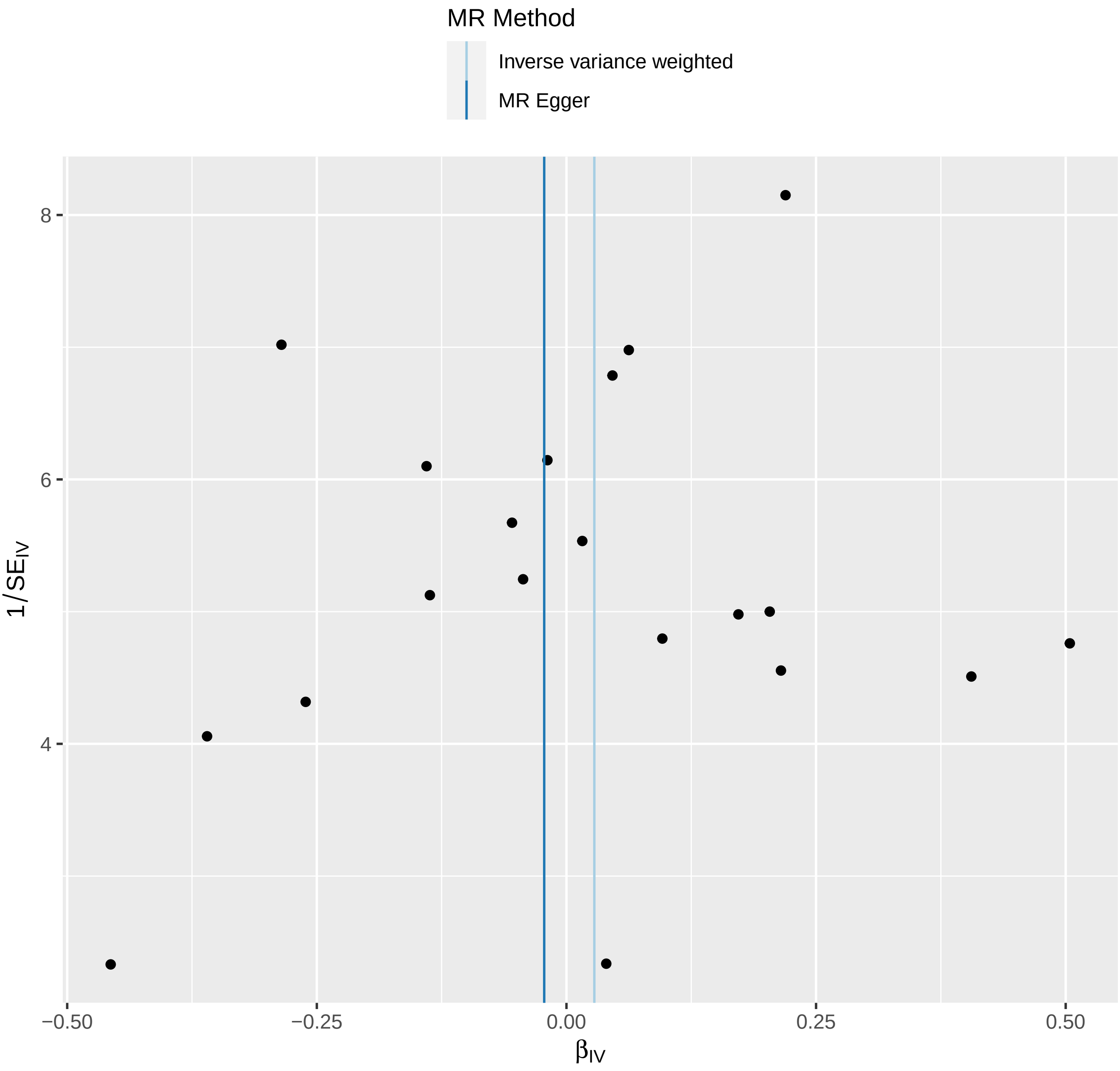

Batch 3−470 : T−cell surface protein tactile on Diabetic nephropathy (more control exclusions)
with a threshold of 1e−05

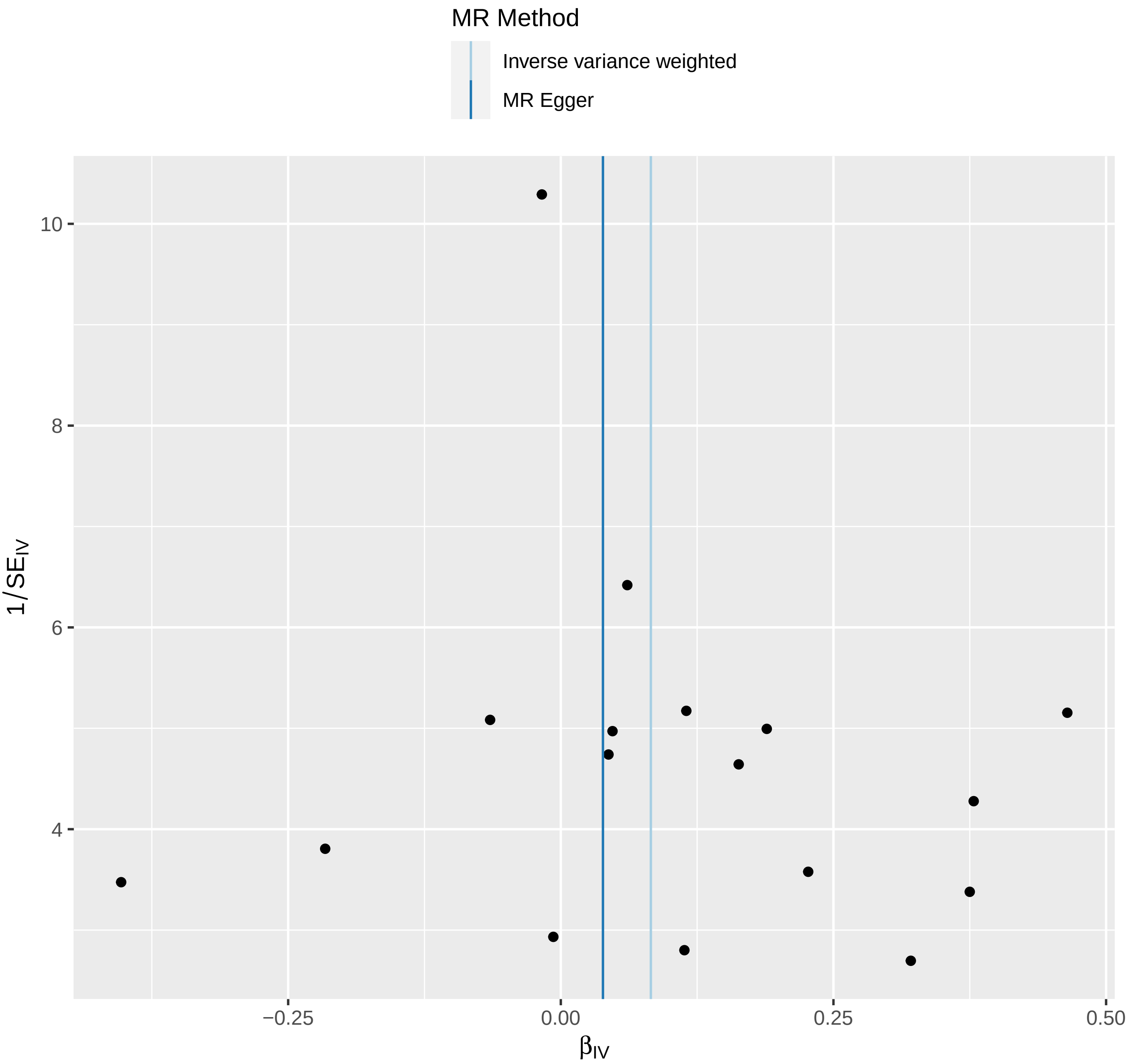

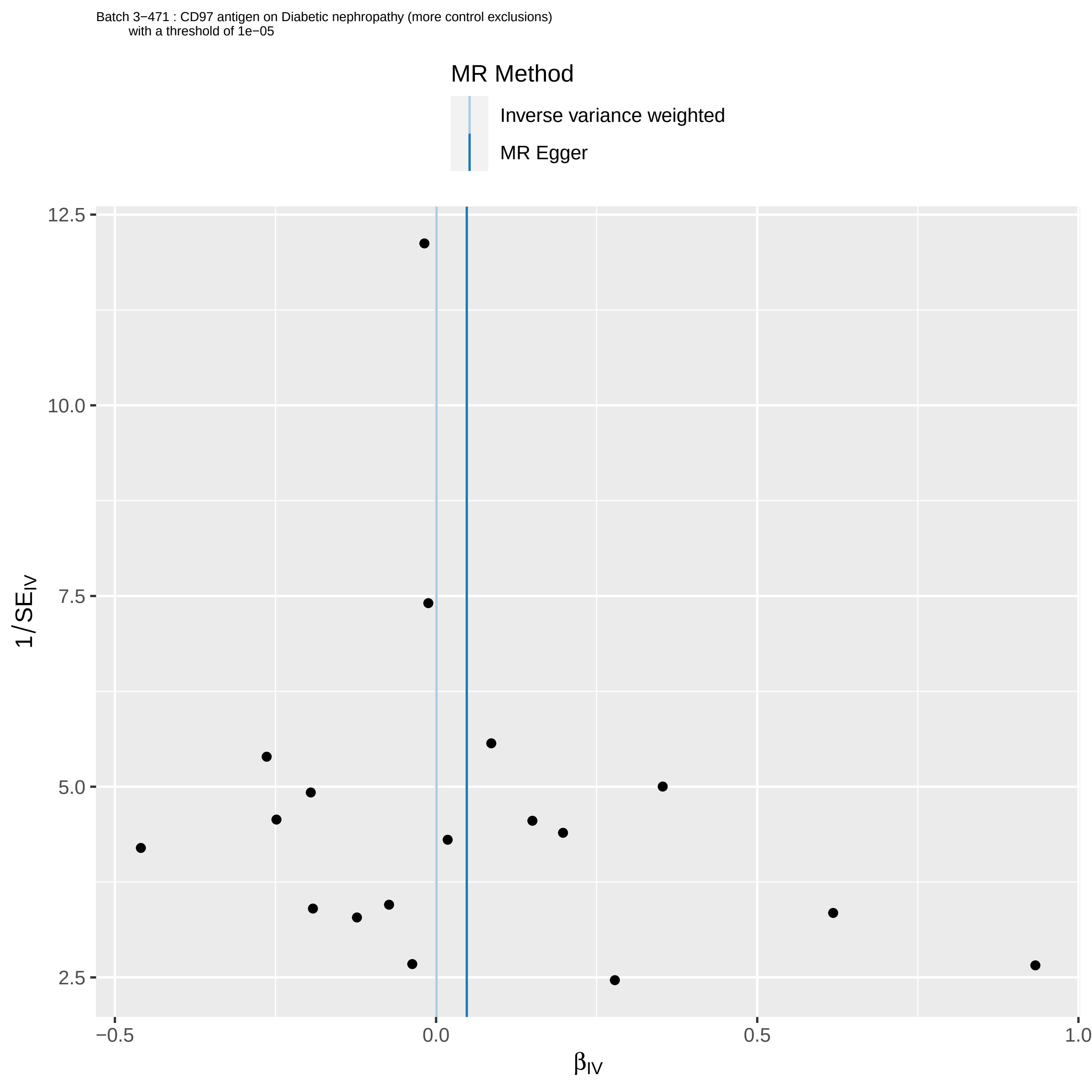

Batch 3−471 : CD97 antigen on Diabetic nephropathy (more control exclusions)
with a threshold of 1e−05
MR Method
Inverse variance weighted
MR Egger
12.5
10.0
7.5
5.0
2.5
1/SE_IV
−0.5
0.0
0.5
1.0
β_IV

Batch 3−472 : M−phase inducer phosphatase 2 on Diabetic nephropathy (more control exclusions)
with a threshold of 1e−05

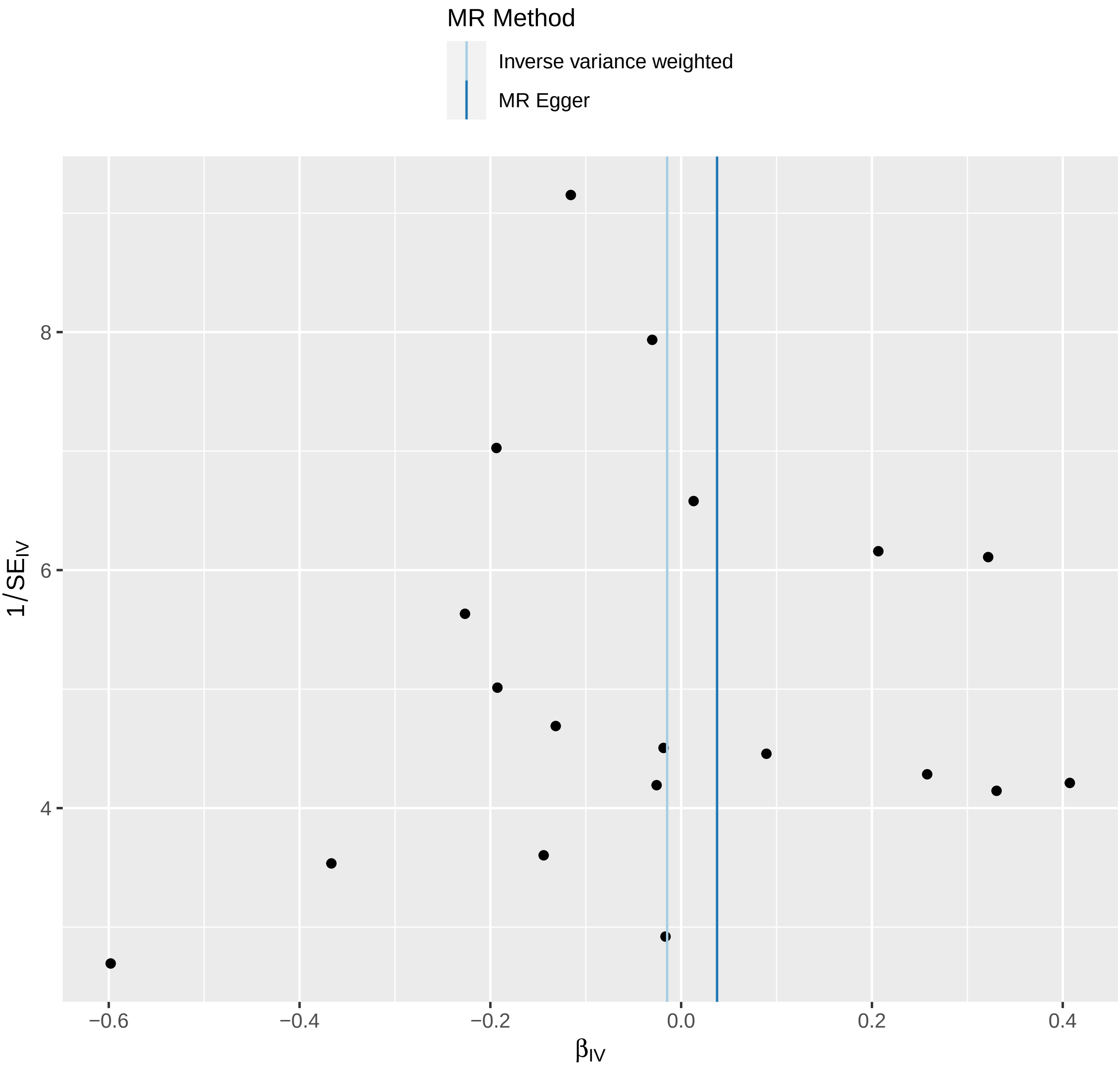

Batch 3−473 : Cyclin−dependent kinase 1:G2/mitotic−specific cyclin−B1 complex on Diabetic nephropathy (more control exclusions)
with a threshold of 1e−05

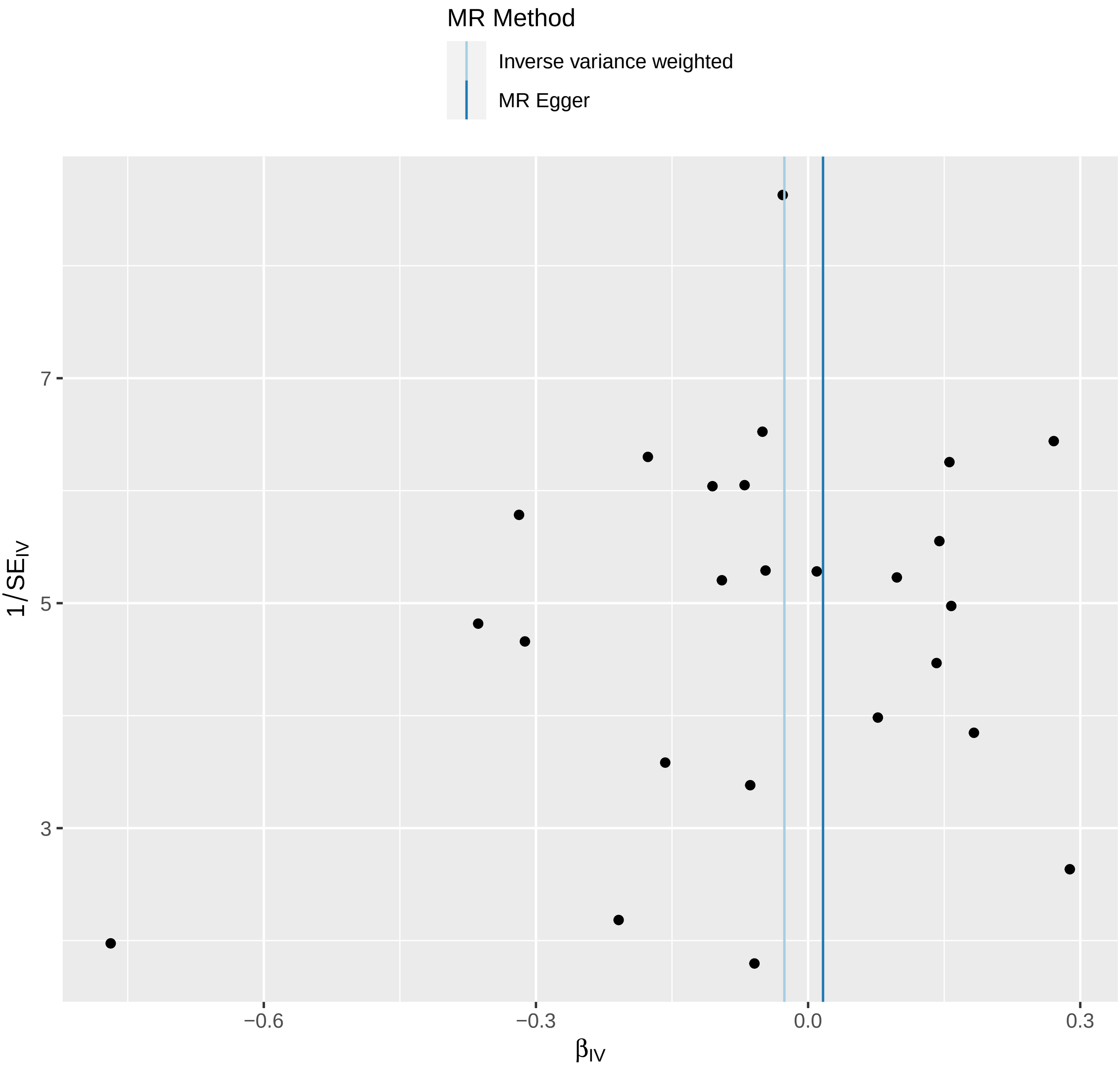

Batch 3−474 : Cell division control protein 42 homolog on Diabetic nephropathy (more control exclusions)
with a threshold of 1e−05

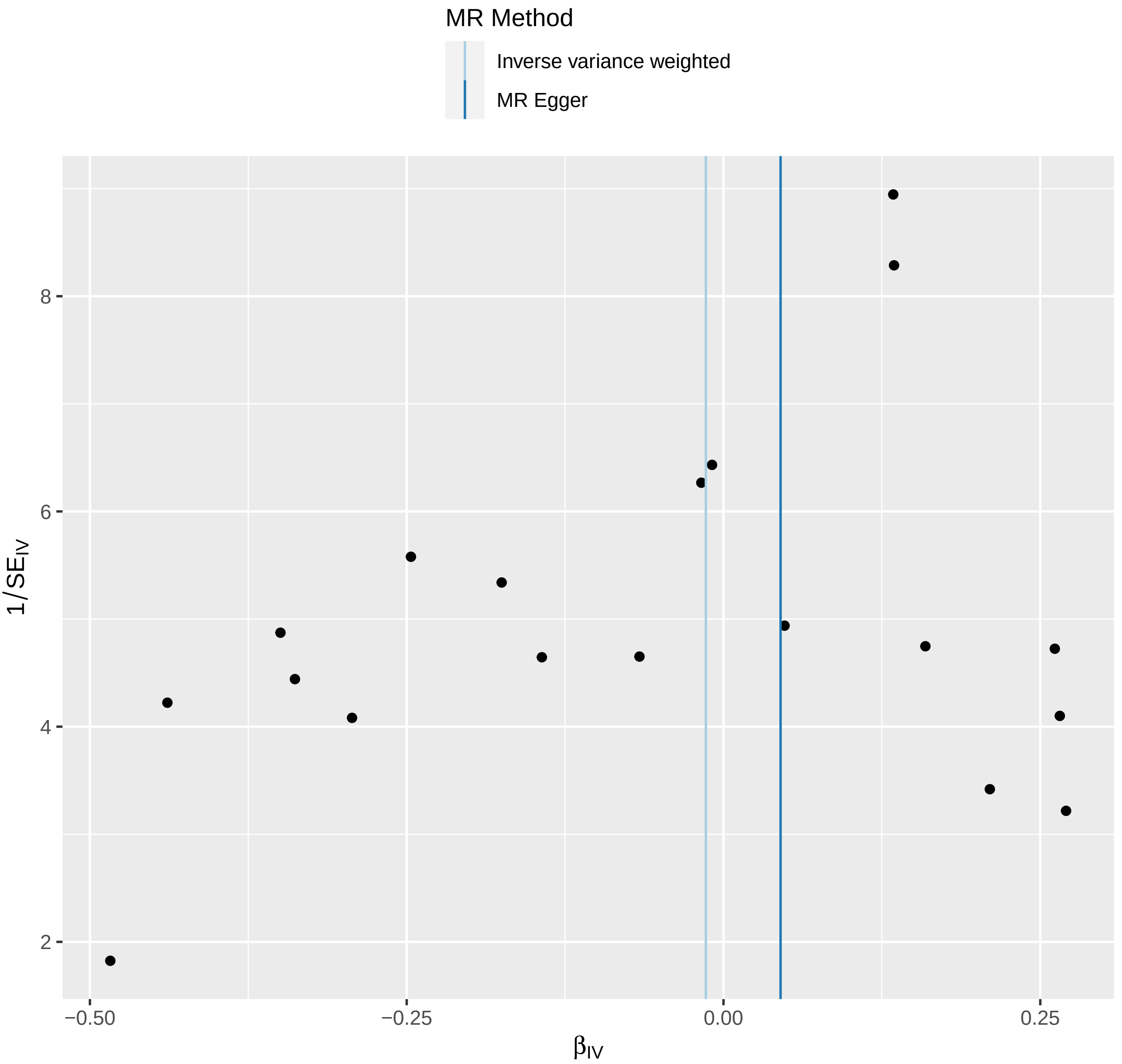

Batch 3−475 : Serine/threonine−protein kinase MRCK alpha on Diabetic nephropathy (more control exclusions)
with a threshold of 1e−05

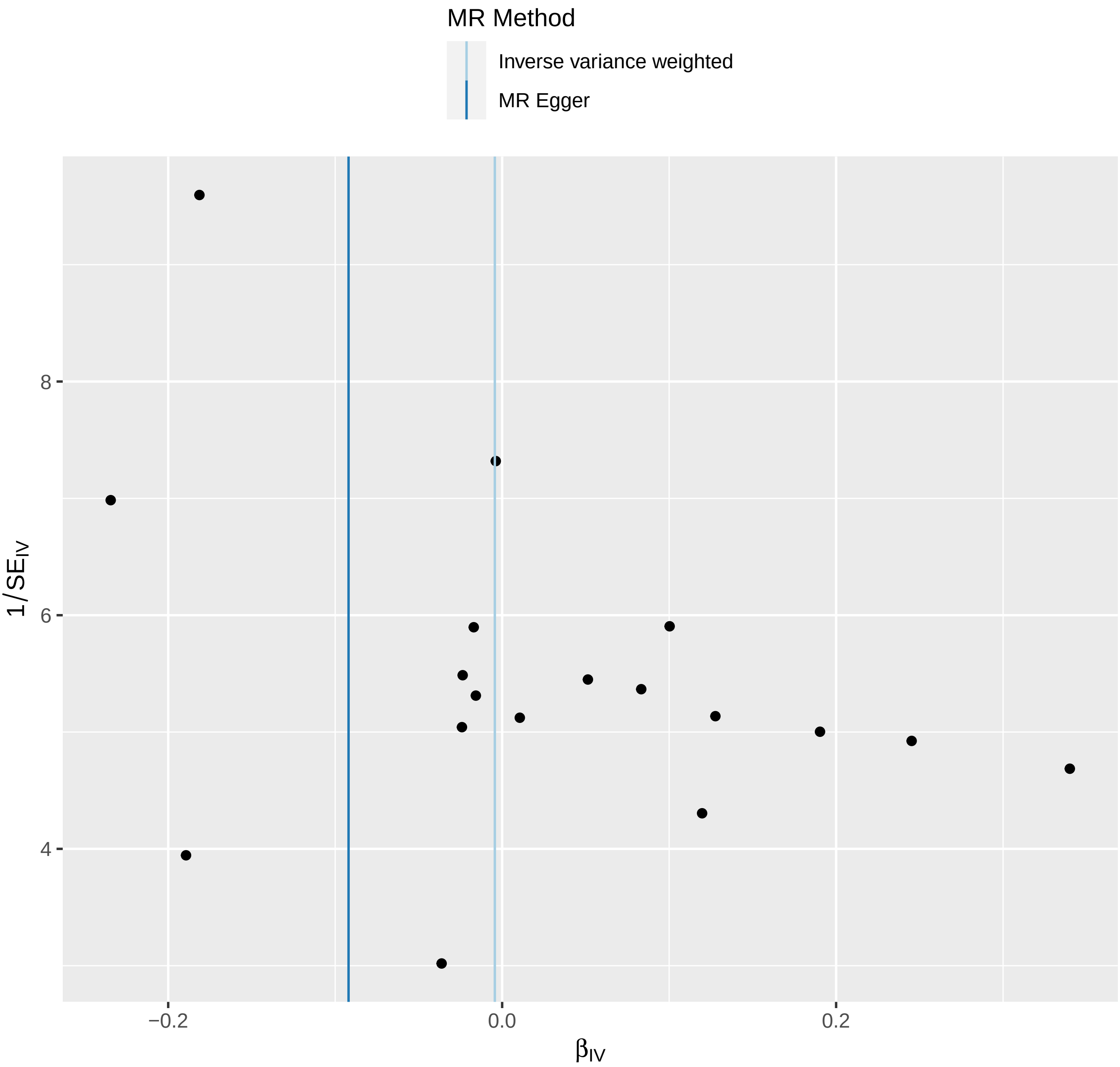

Batch 3−476 : Serine/threonine−protein kinase MRCK beta on Diabetic nephropathy (more control exclusions)
with a threshold of 1e−05

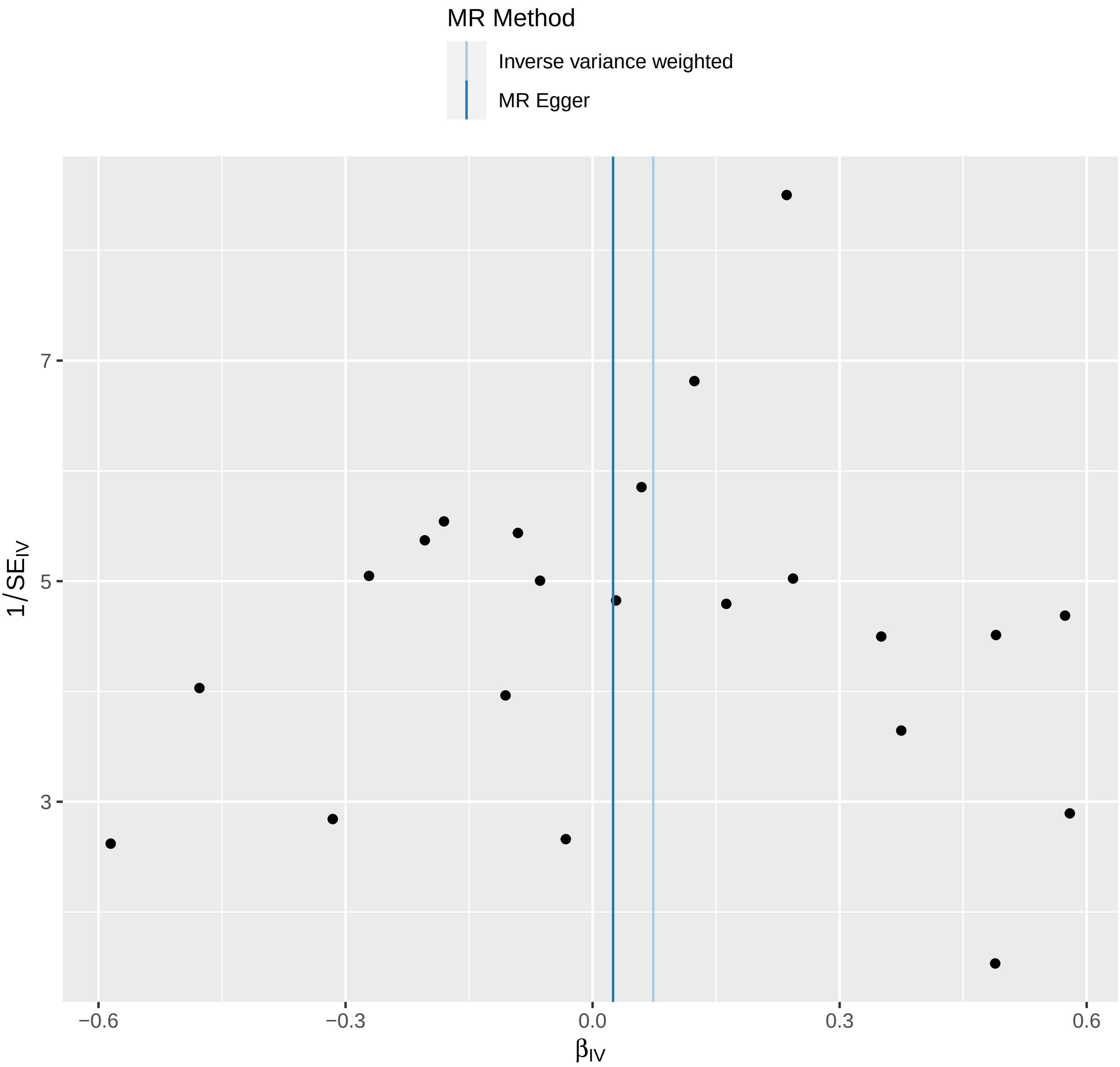

Batch 3−477 : CUB domain−containing protein 1 on Diabetic nephropathy (more control exclusions) with a threshold of 1e−05

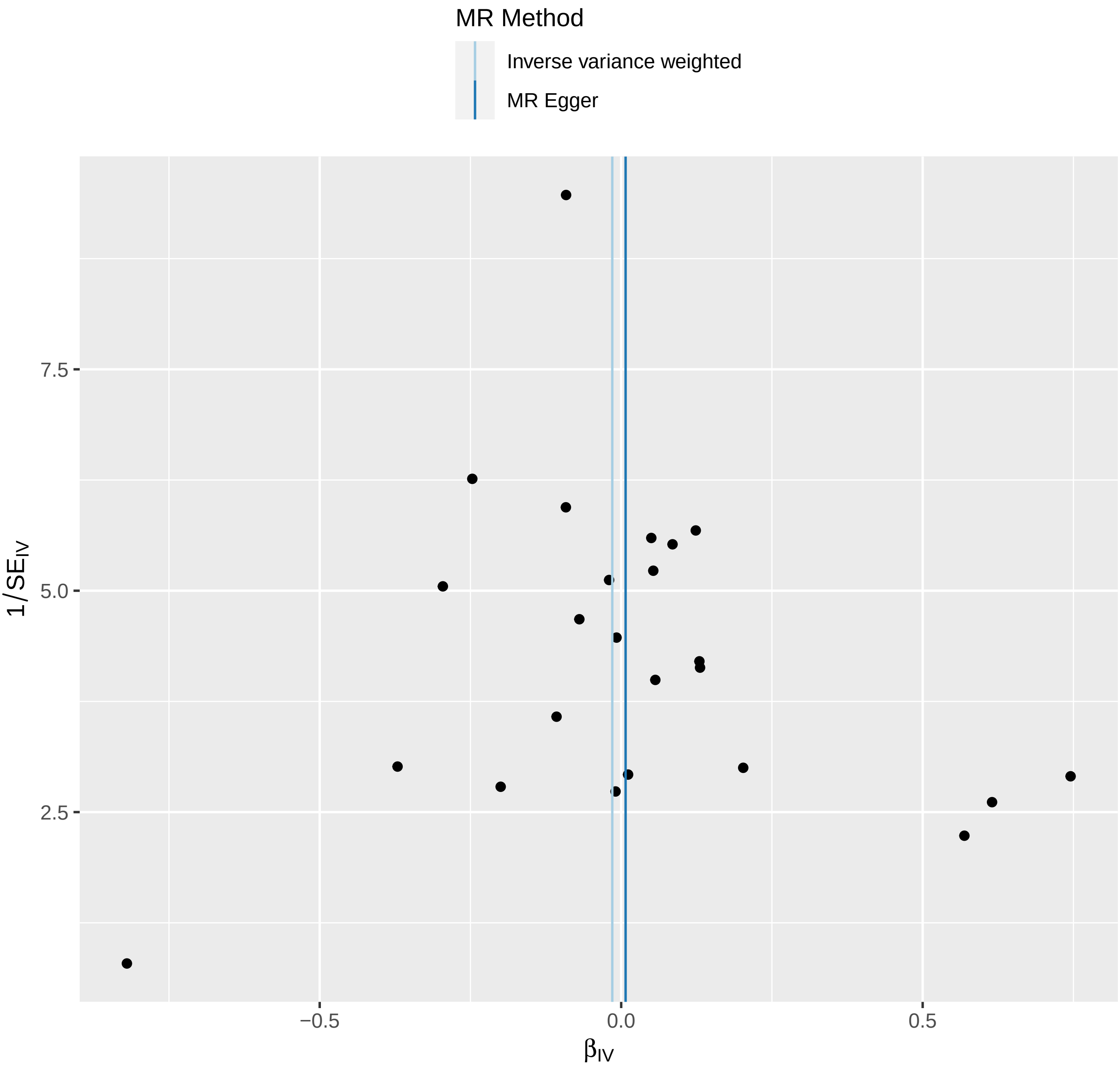

Batch 3−478 : Cadherin−12 on Diabetic nephropathy (more control exclusions)
with a threshold of 1e−05

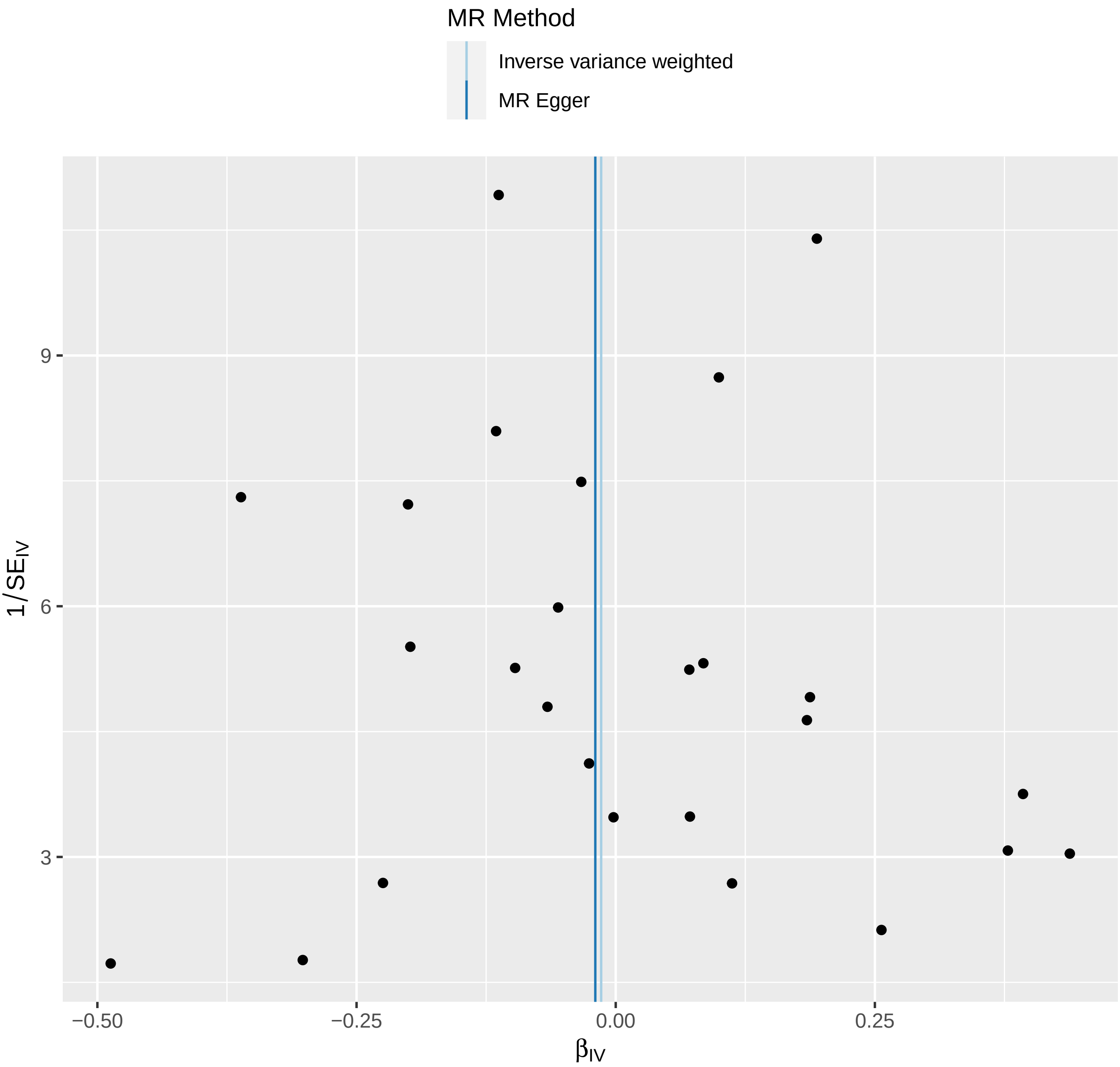

Batch 3−479 : Cadherin−12 on Diabetic nephropathy (more control exclusions)
with a threshold of 1e−05

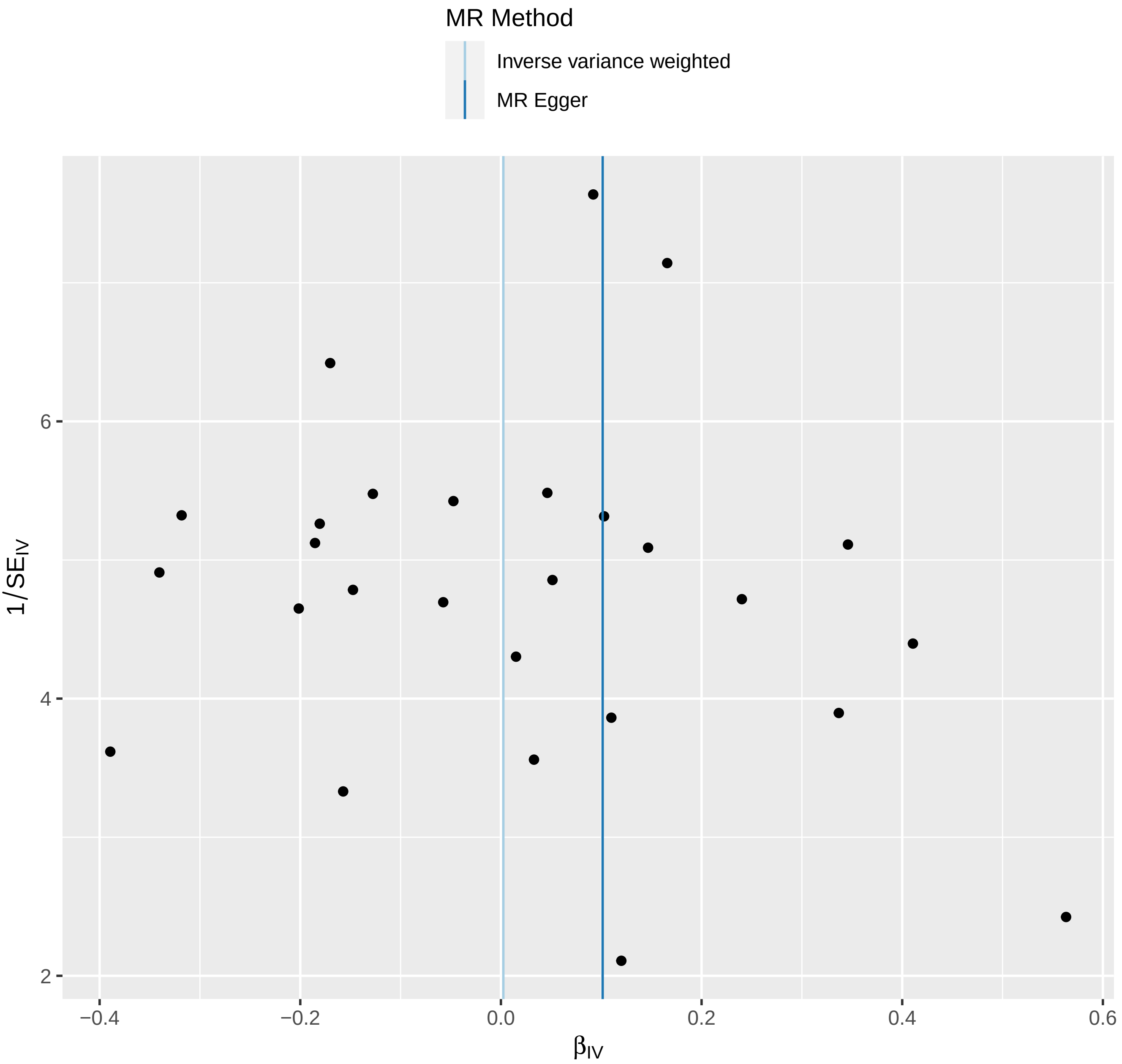

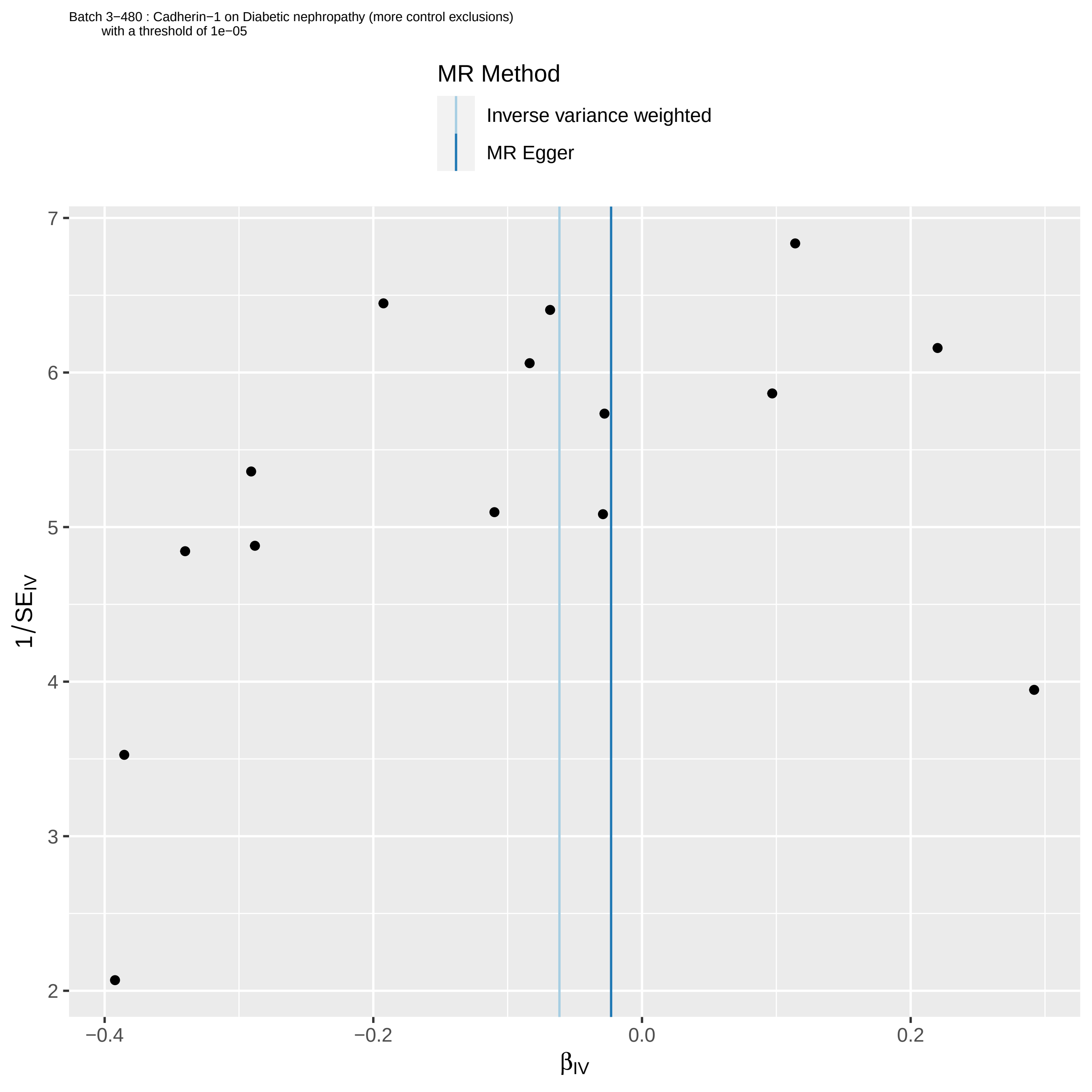
Batch 3−480 : Cadherin−1 on Diabetic nephropathy (more control exclusions)
with a threshold of 1e−05
MR Method
Inverse variance weighted
MR Egger
1/SE_IV
β_IV
7
6
5
4
3
2
−0.4
−0.2
0.0
0.2

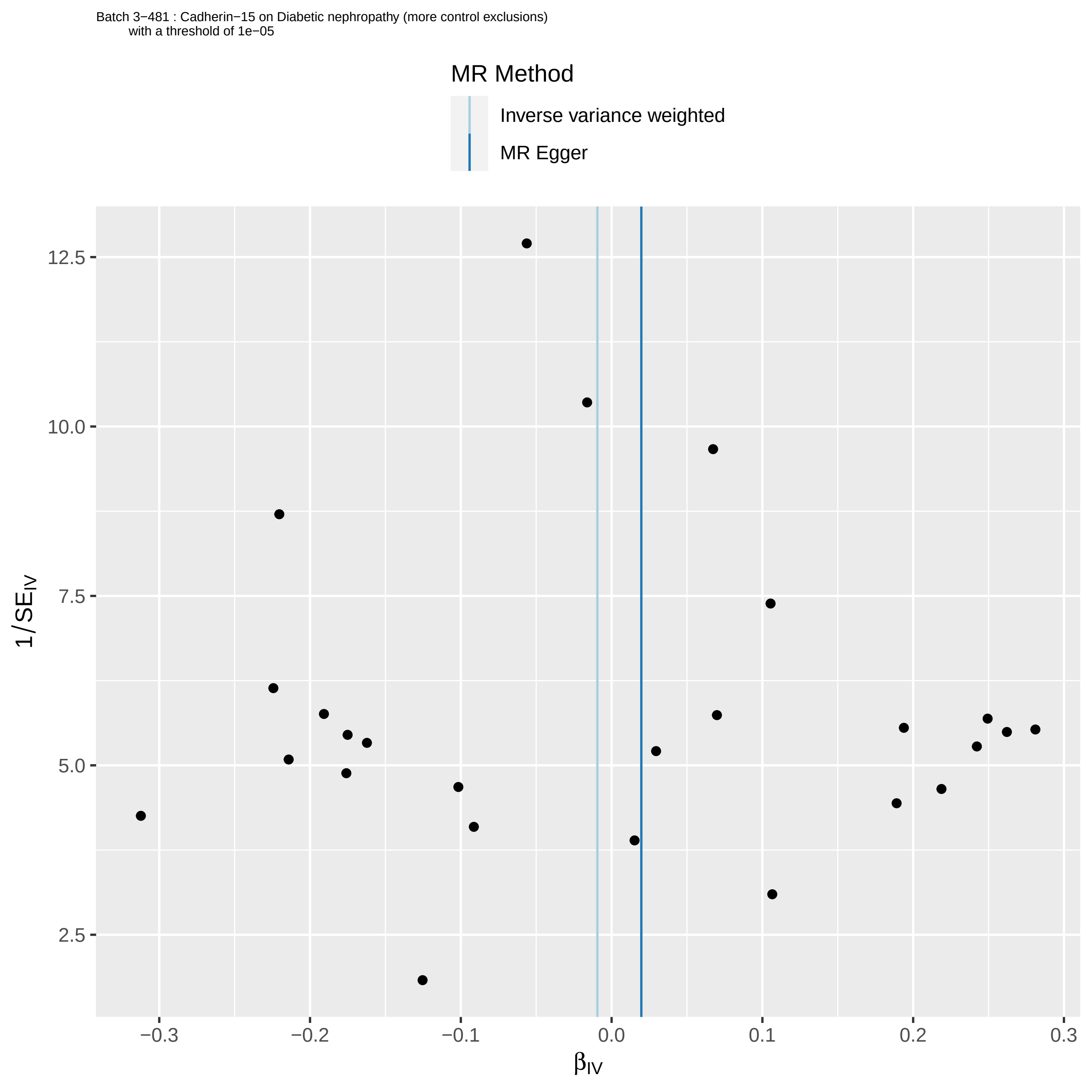

Batch 3−481 : Cadherin−15 on Diabetic nephropathy (more control exclusions)
with a threshold of 1e−05
MR Method
Inverse variance weighted
MR Egger
12.5
10.0
7.5
5.0
2.5
1/SE_IV
−0.3
−0.2
−0.1
0.0
0.1
0.2
0.3
β_IV

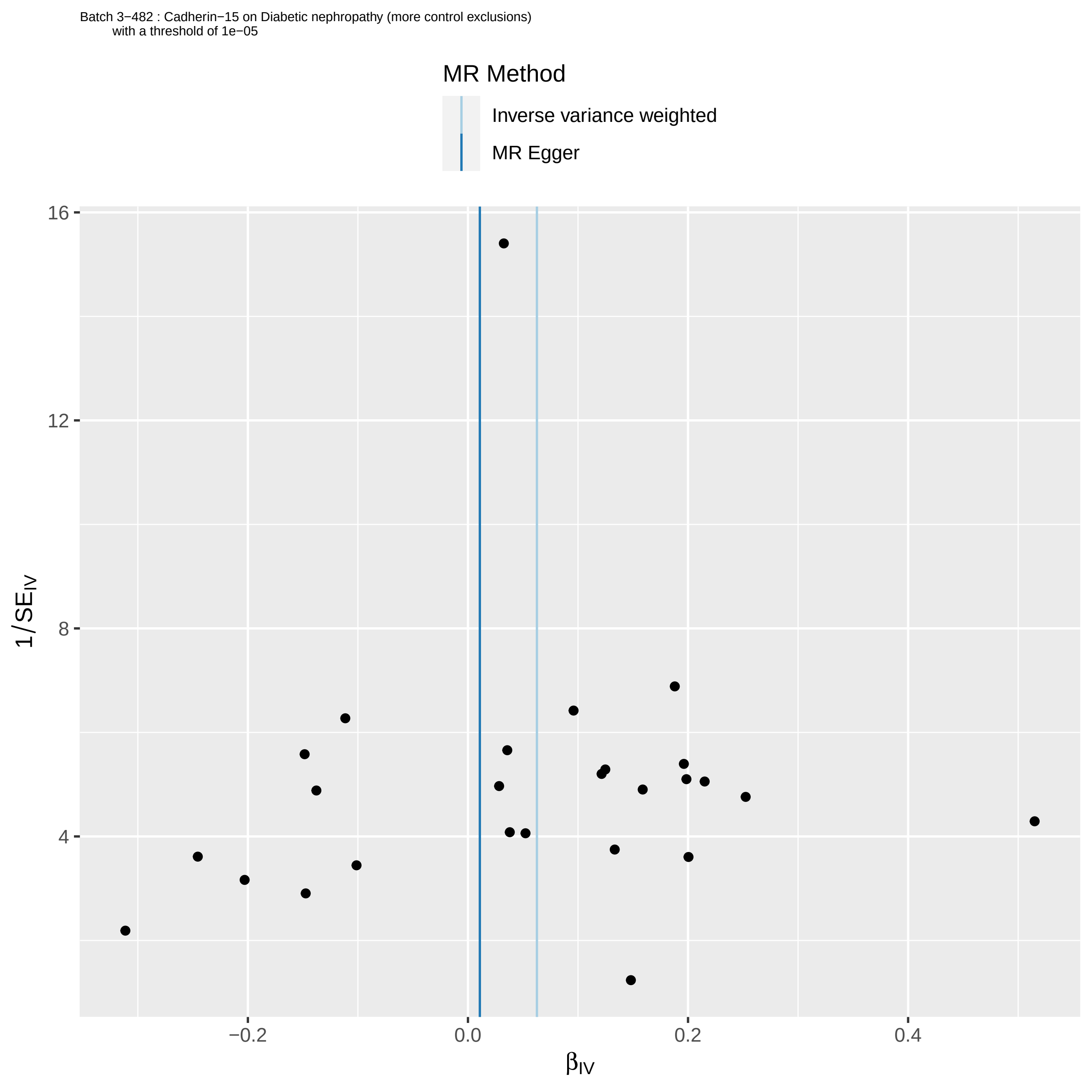
Batch 3−482 : Cadherin−15 on Diabetic nephropathy (more control exclusions)
with a threshold of 1e−05
MR Method
Inverse variance weighted
MR Egger
16
12
8
4
$1/SE_{IV}$
−0.2
0.0
0.2
0.4
β_{IV}

Batch 3−483 : Cadherin−2 on Diabetic nephropathy (more control exclusions)
with a threshold of 1e−05

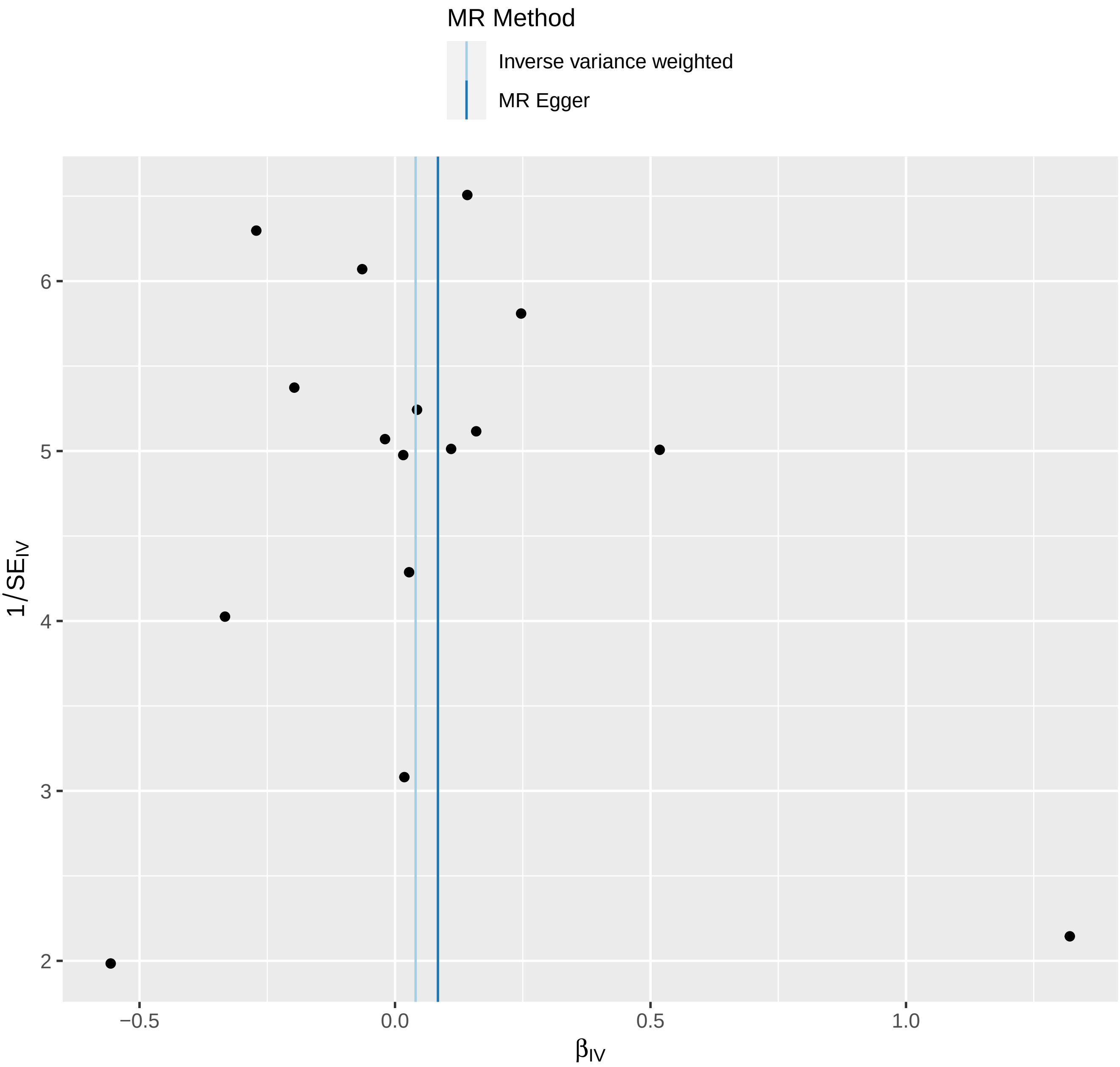

Batch 3−484 : Cadherin−3 on Diabetic nephropathy (more control exclusions)
with a threshold of 1e−05

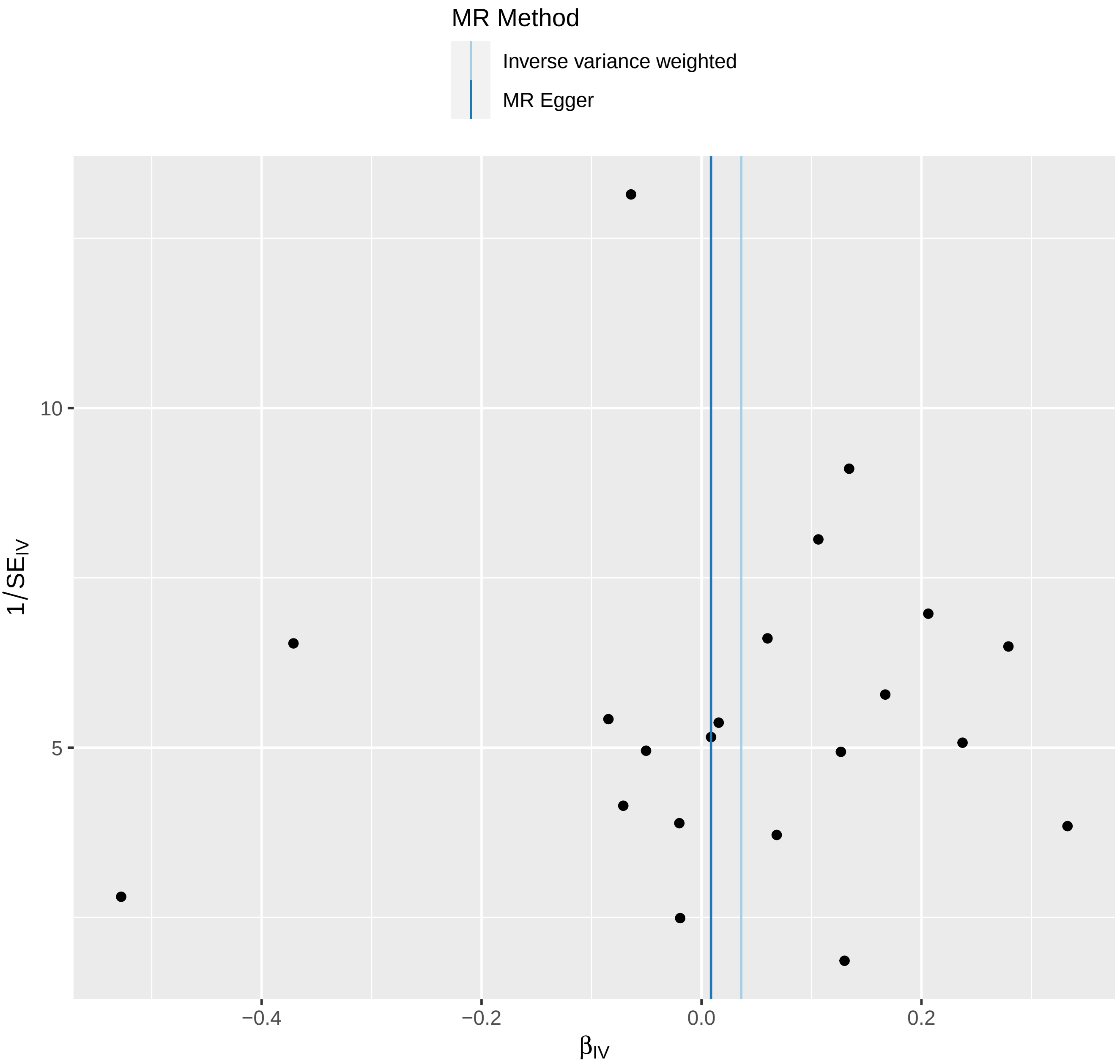

Batch 3−485 : Cadherin−5 on Diabetic nephropathy (more control exclusions)
with a threshold of 1e−05

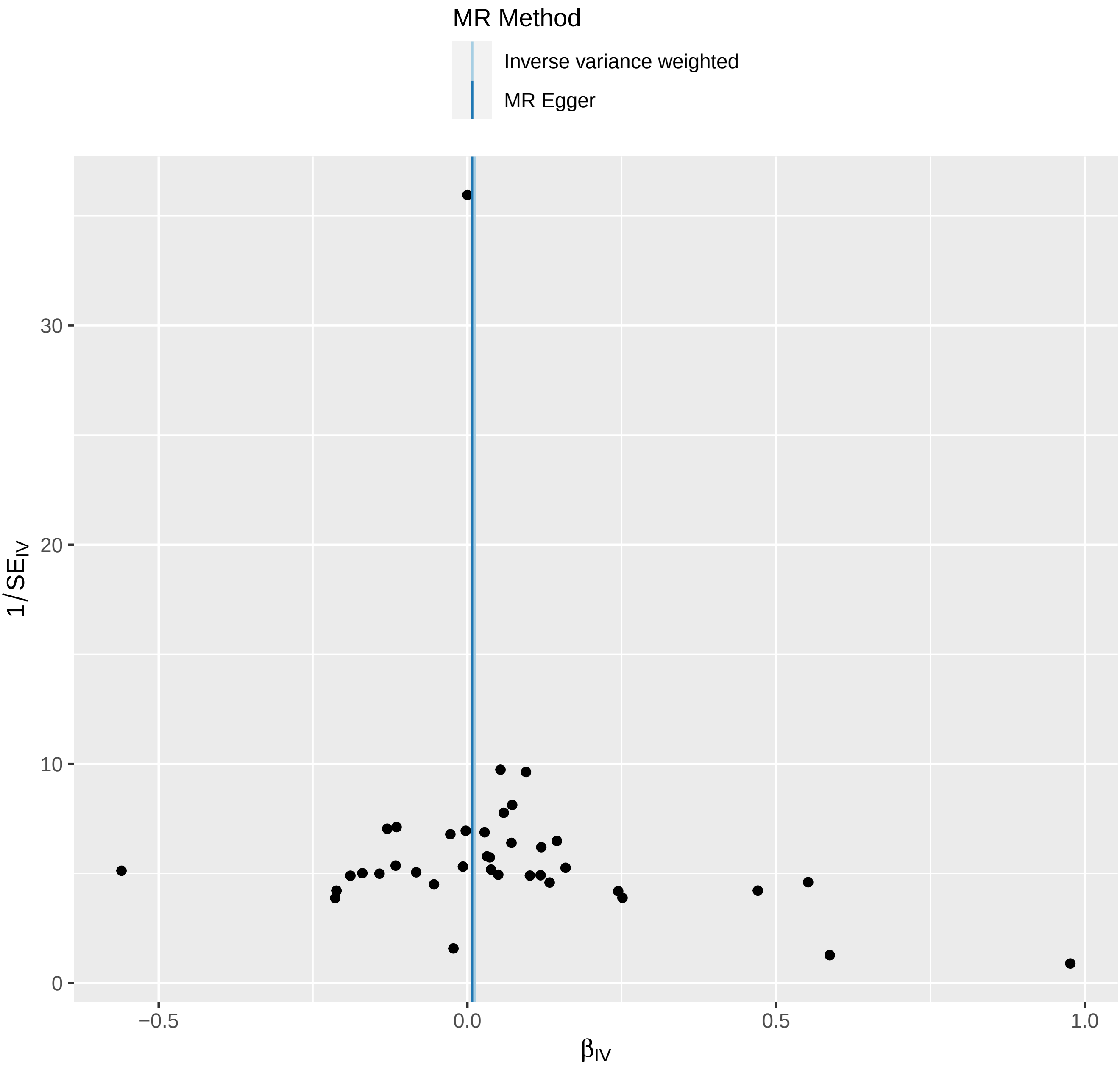

Batch 3−486 : Cadherin−7 on Diabetic nephropathy (more control exclusions)
with a threshold of 1e−05

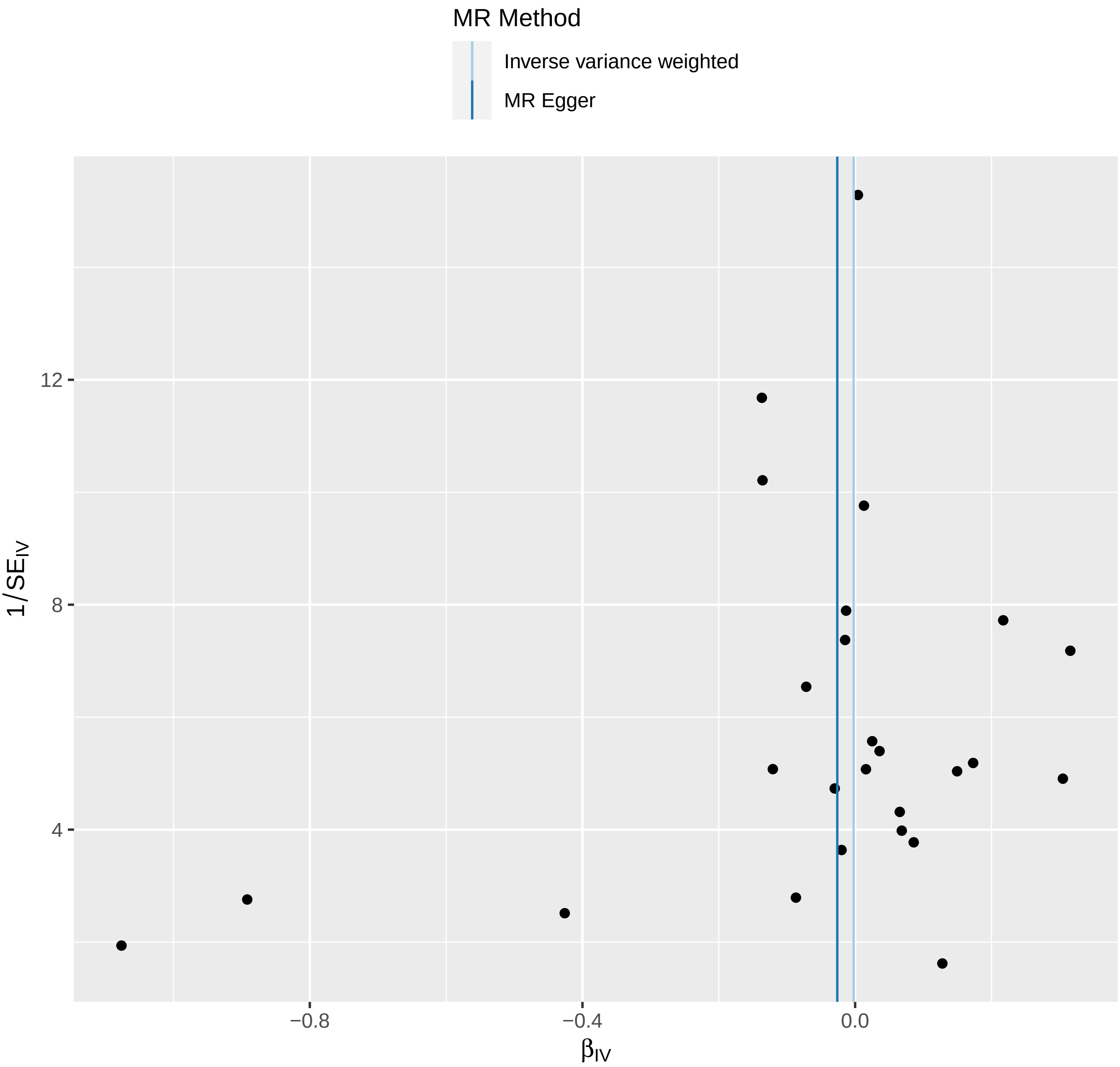

Batch 3−487 : Cadherin−related family member 1 on Diabetic nephropathy (more control exclusions)
with a threshold of 1e−05

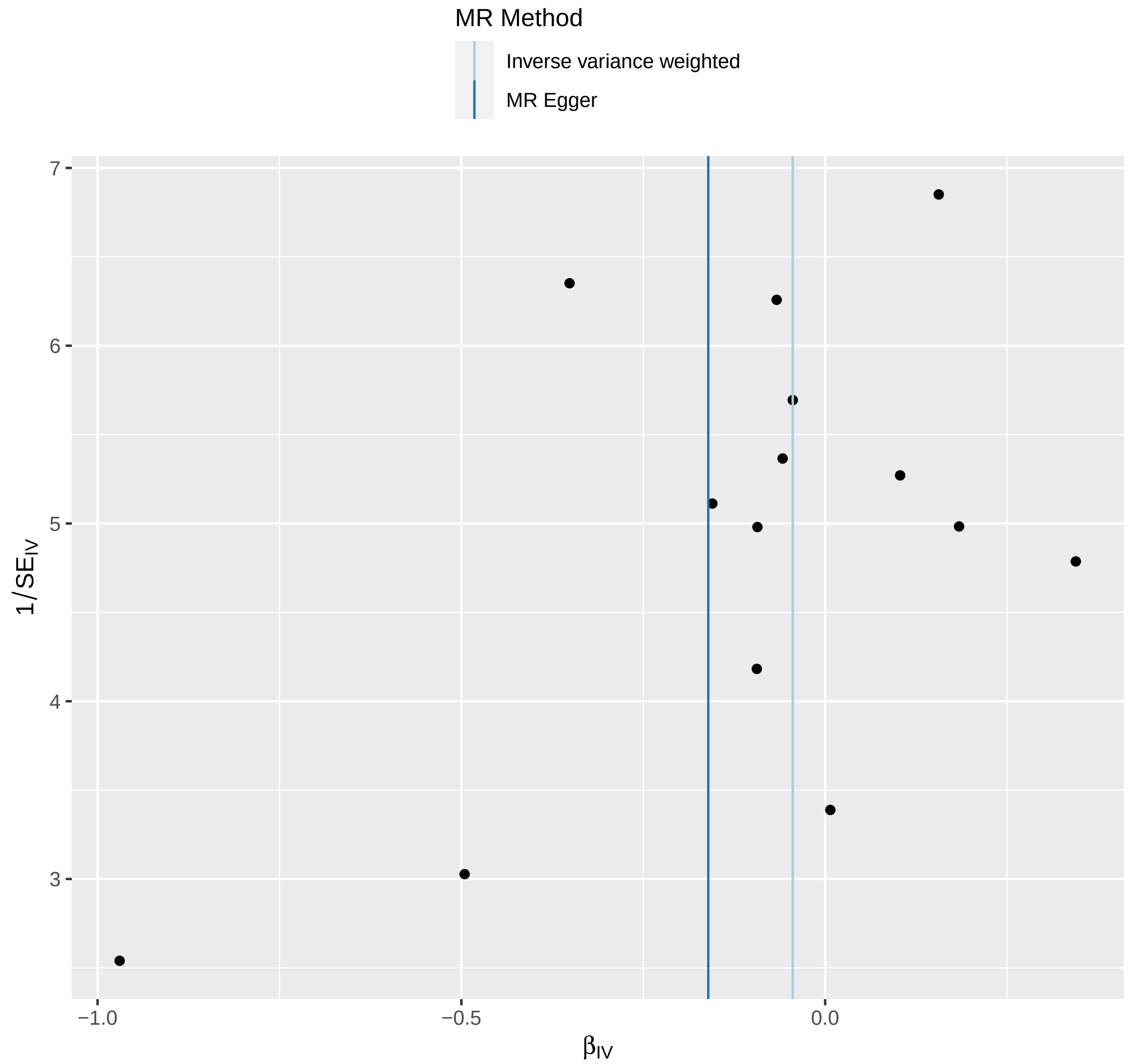

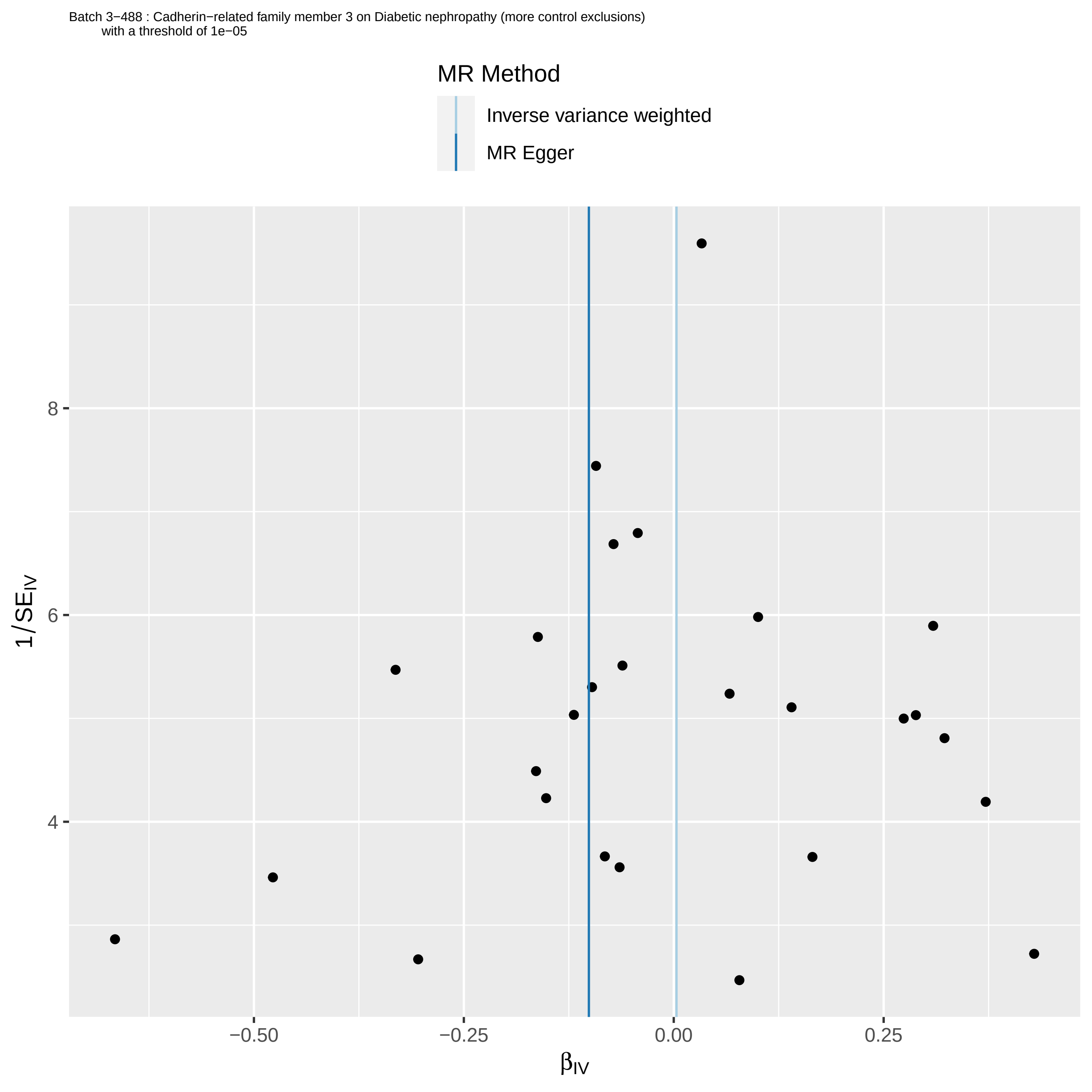
Batch 3−488 : Cadherin−related family member 3 on Diabetic nephropathy (more control exclusions)
with a threshold of 1e−05
MR Method
Inverse variance weighted
MR Egger
8
6
4
−0.50
−0.25
0.00
0.25
1/SE_IV
β_IV

Batch 3−489 : Cadherin−related family member 3 on Diabetic nephropathy (more control exclusions) with a threshold of 1e−05

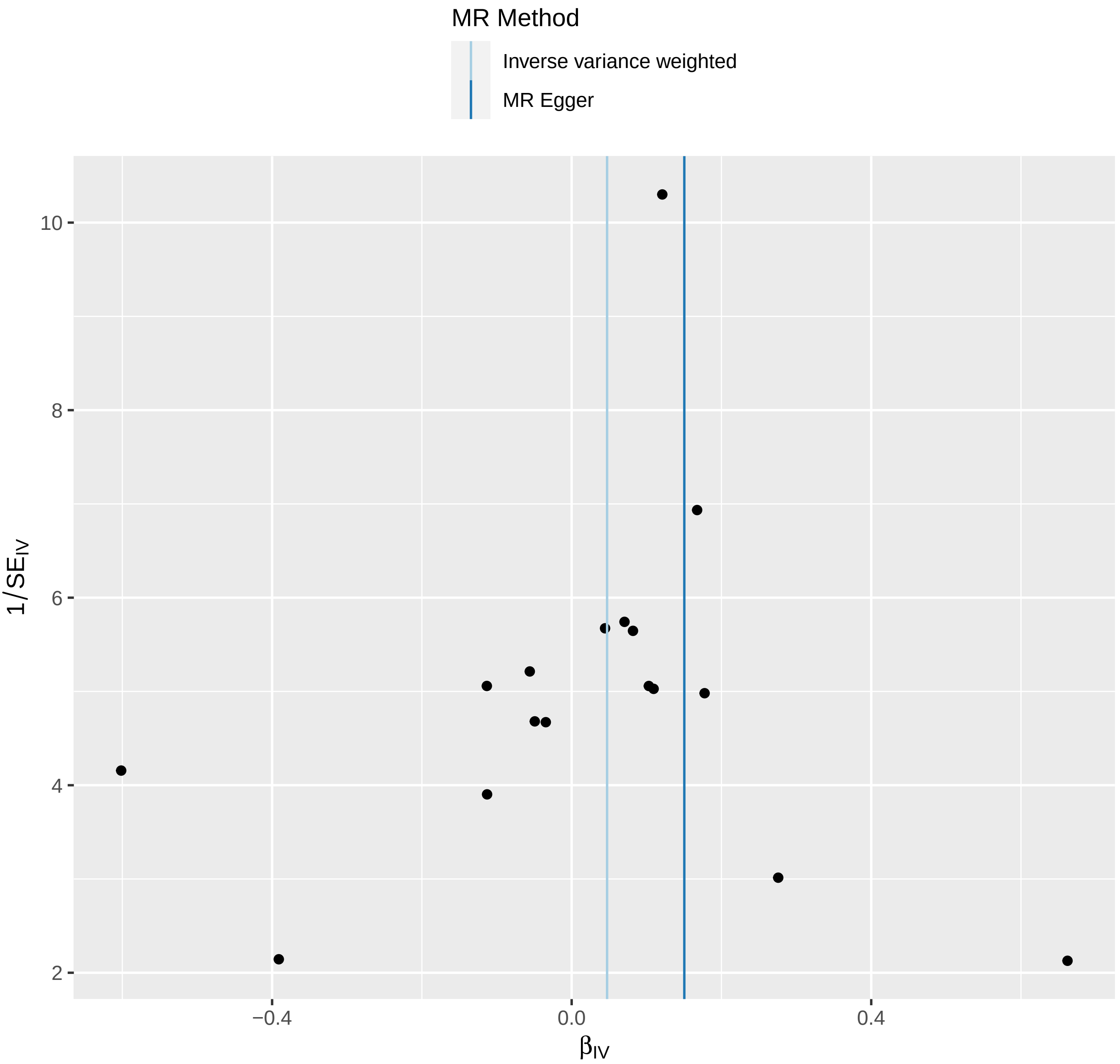

Batch 3−490 : Cadherin−related family member 5 on Diabetic nephropathy (more control exclusions)
with a threshold of 1e−05

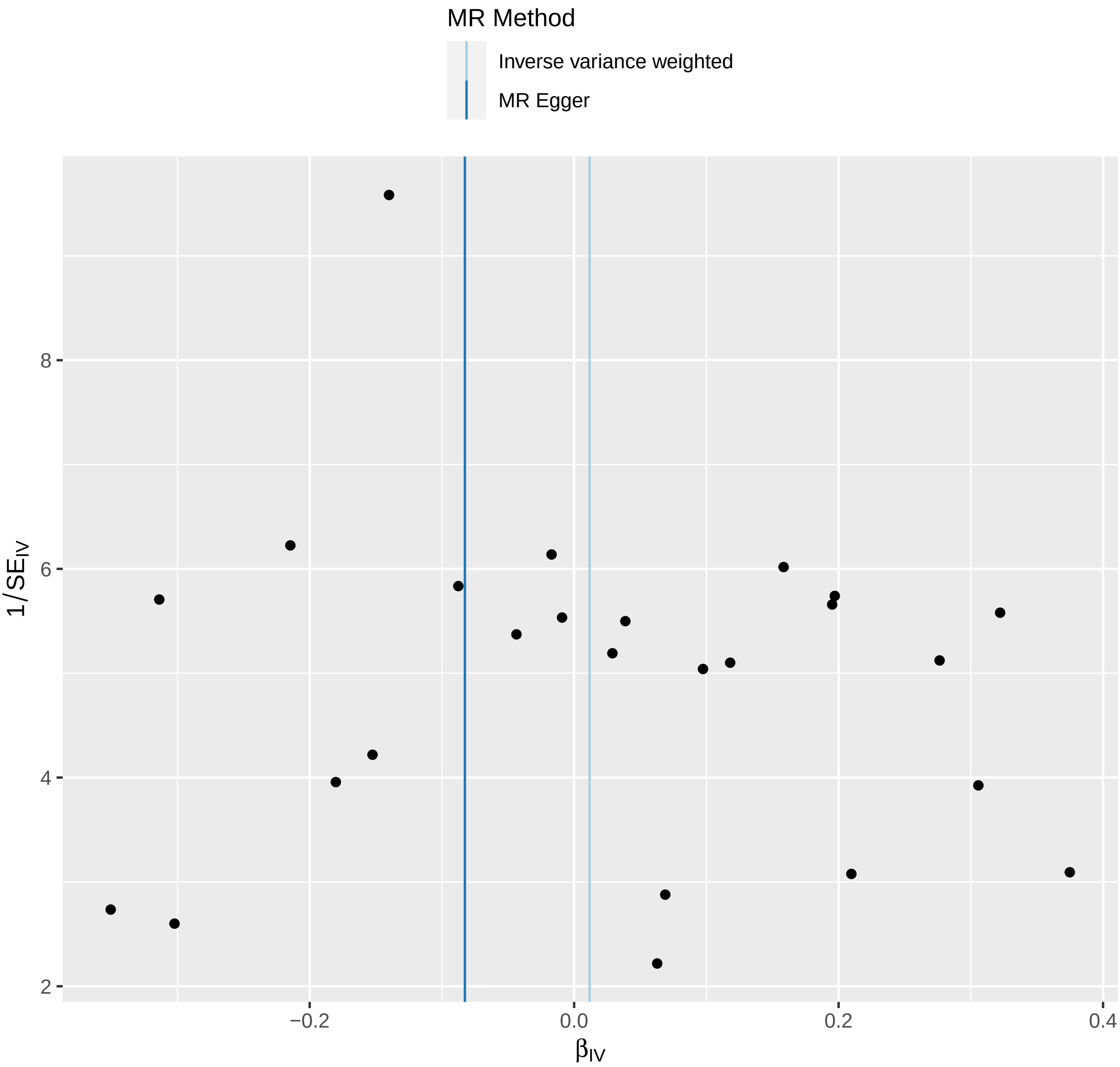

Batch 3−491 : Cyclin−dependent kinase 2−associated protein 1 on Diabetic nephropathy (more control exclusions) with a threshold of 1e−05

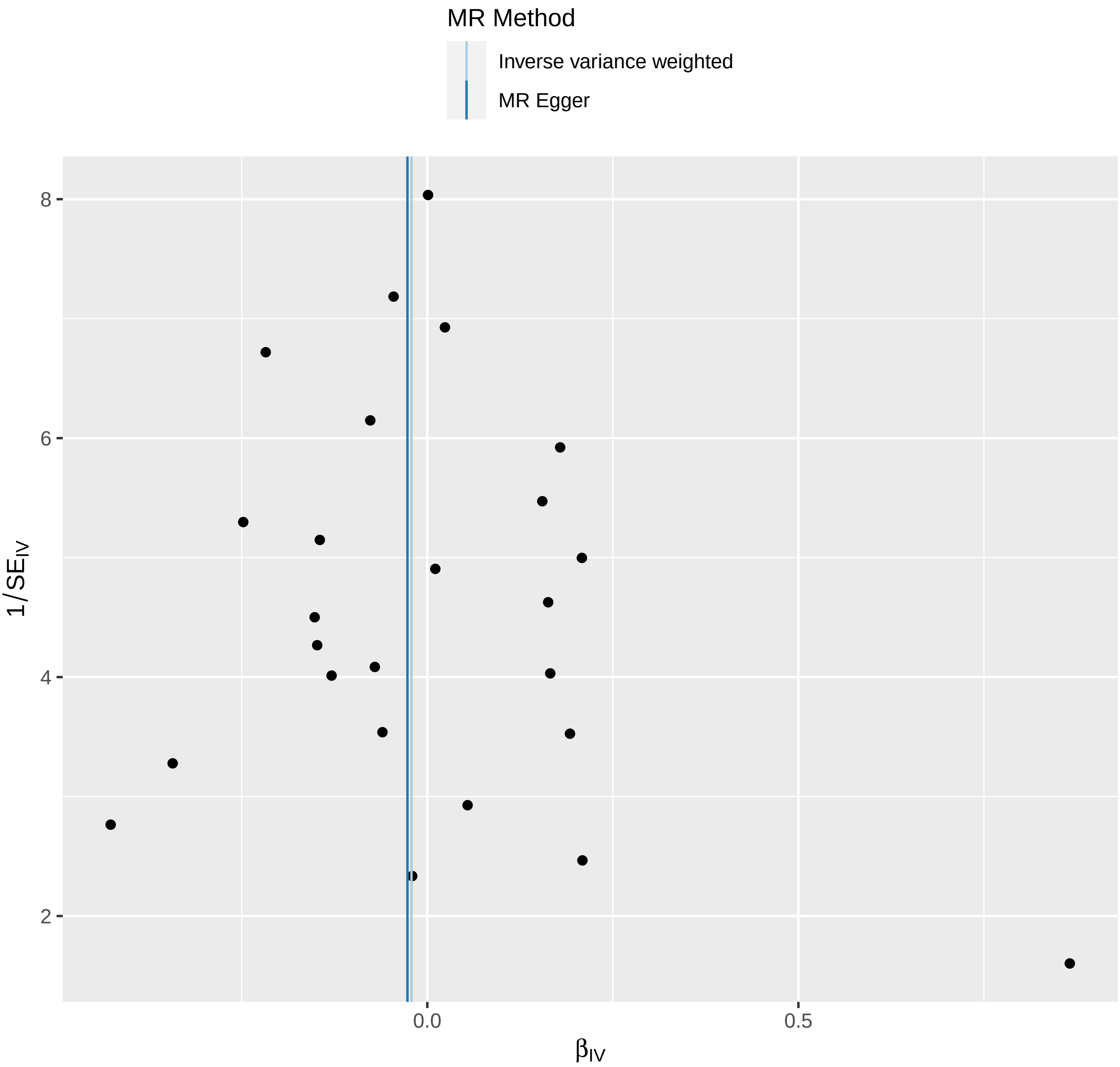

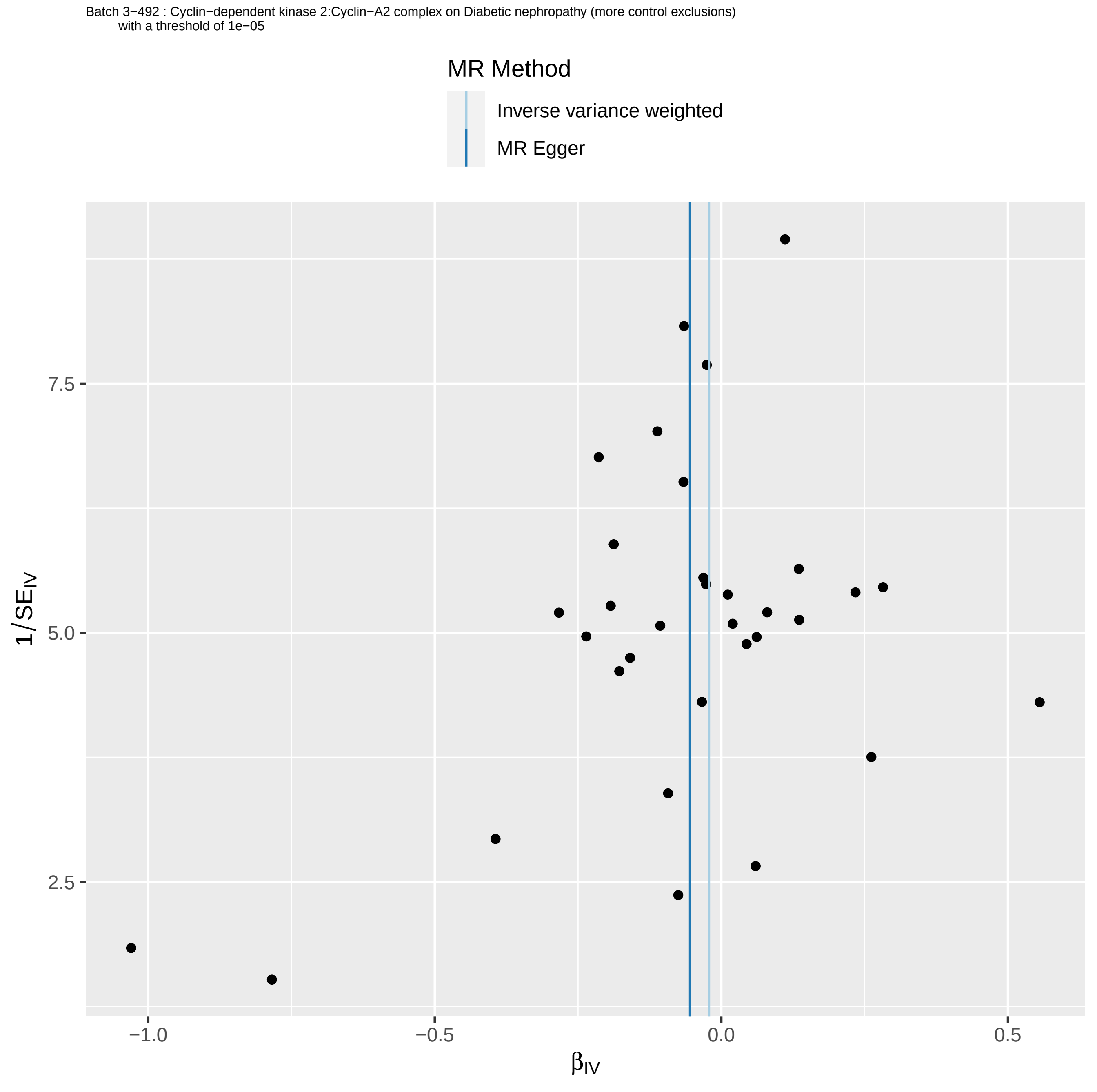

Batch 3−492 : Cyclin−dependent kinase 2:Cyclin−A2 complex on Diabetic nephropathy (more control exclusions)
with a threshold of 1e−05
MR Method
Inverse variance weighted
MR Egger
7.5
5.0
2.5
1/SE_IV
−1.0
−0.5
0.0
0.5
β_IV

Batch 3−493 : Cyclin−dependent kinase 5:Cyclin−dependent kinase 5 activator 1 complex on Diabetic nephropathy (more control exclusions) with a threshold of 1e−05

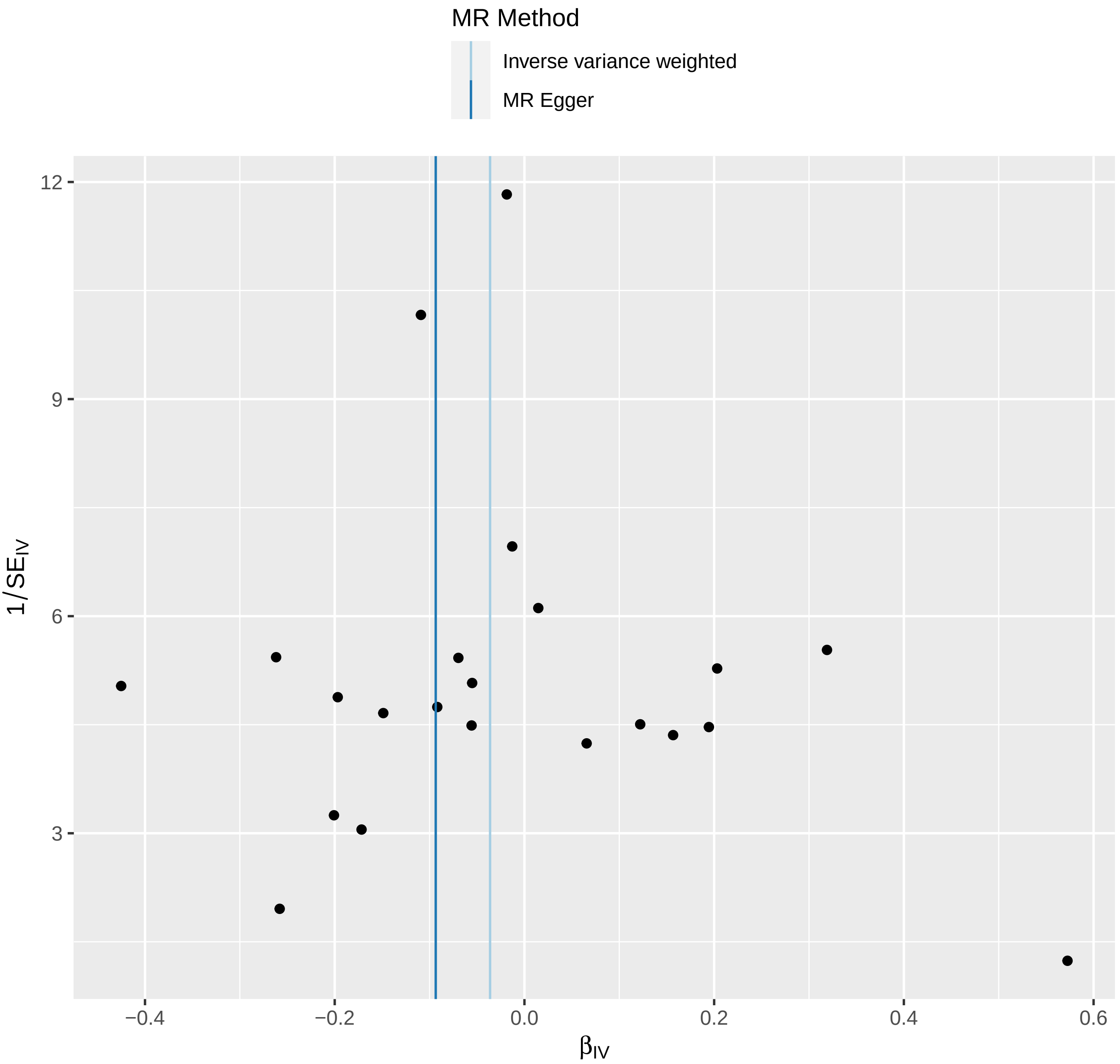

Batch 3−494 : Cyclin−dependent kinase 8:Cyclin−C complex on Diabetic nephropathy (more control exclusions) with a threshold of 1e−05

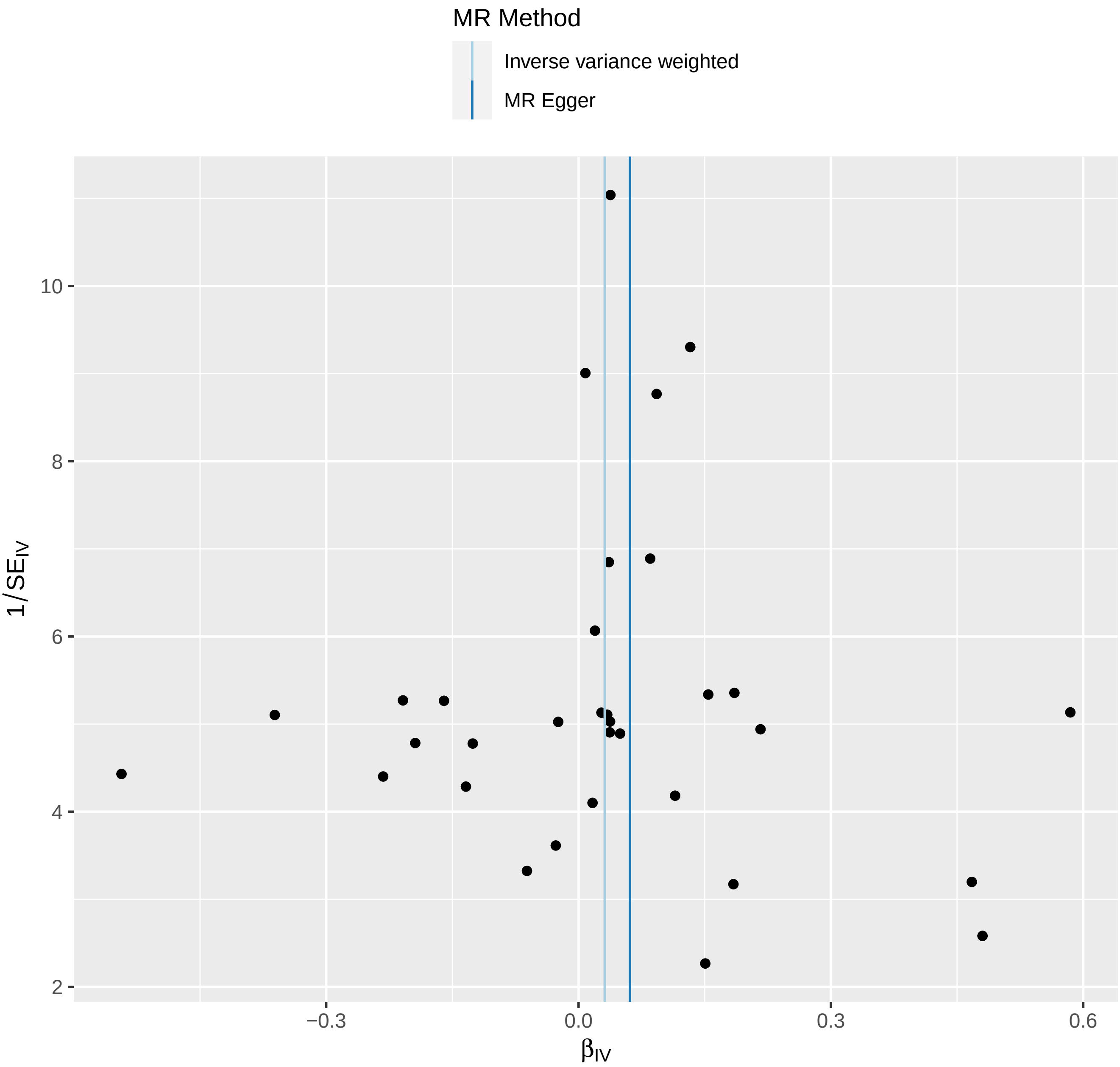

Batch 3−495 : Cyclin−dependent kinase inhibitor 1B on Diabetic nephropathy (more control exclusions)
with a threshold of 1e−05

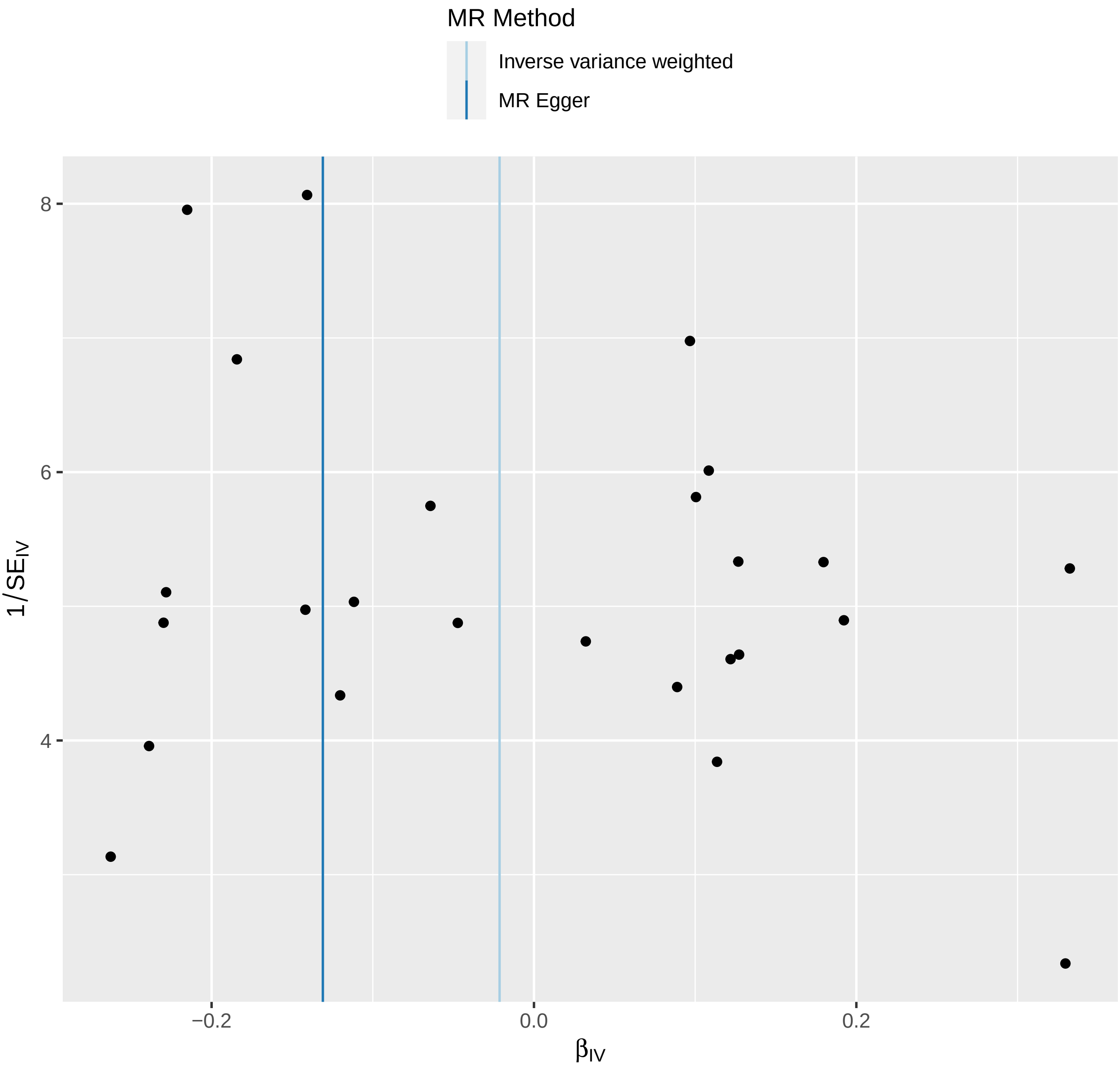

Batch 3−496 : Cyclin−dependent kinase 4 inhibitor B on Diabetic nephropathy (more control exclusions) with a threshold of 1e−05

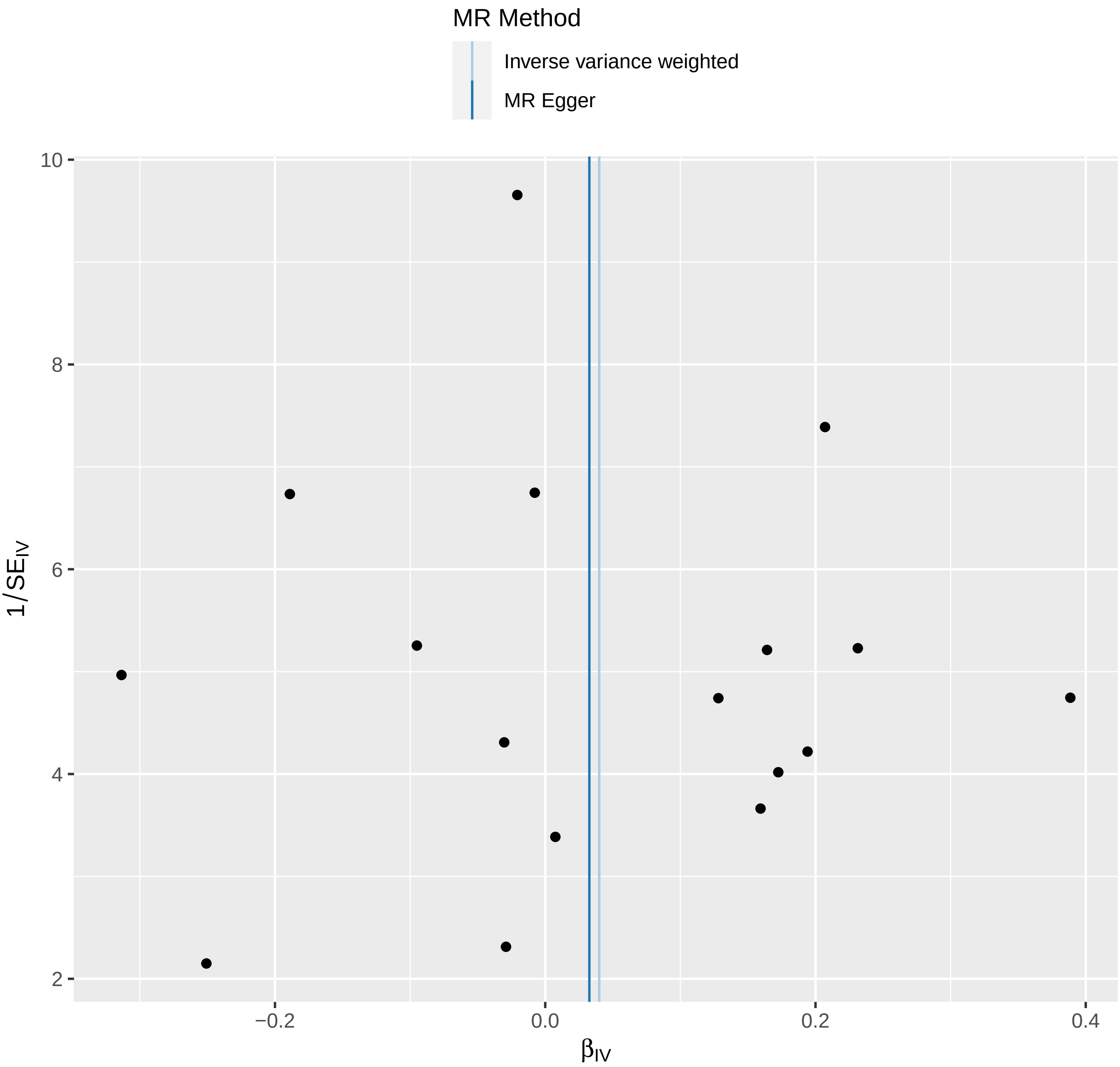

Batch 3−497 : Cyclin−dependent kinase inhibitor 3 on Diabetic nephropathy (more control exclusions)
with a threshold of 1e−05

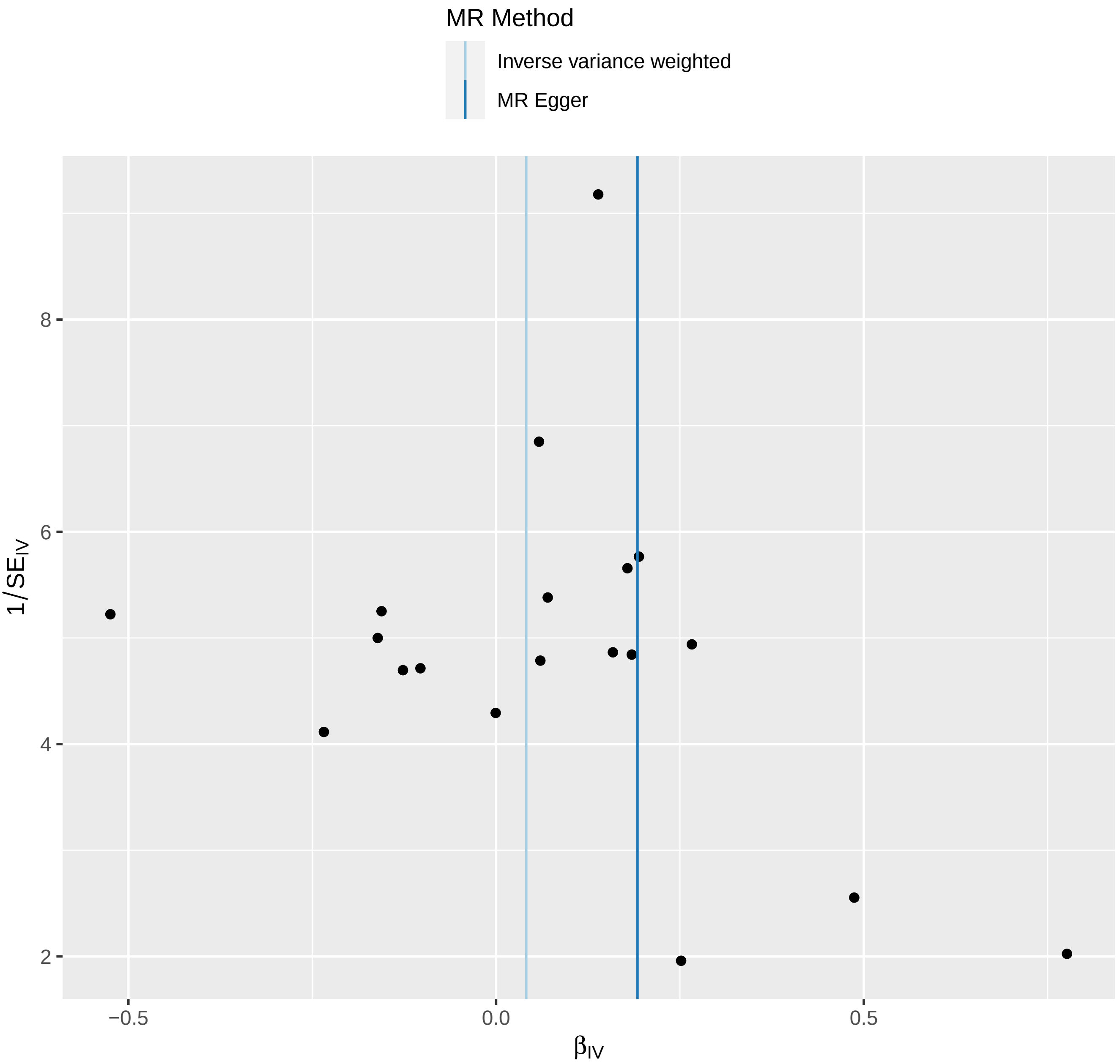

Batch 3−498 : Cerebral dopamine neurotrophic factor on Diabetic nephropathy (more control exclusions) with a threshold of 1e−05

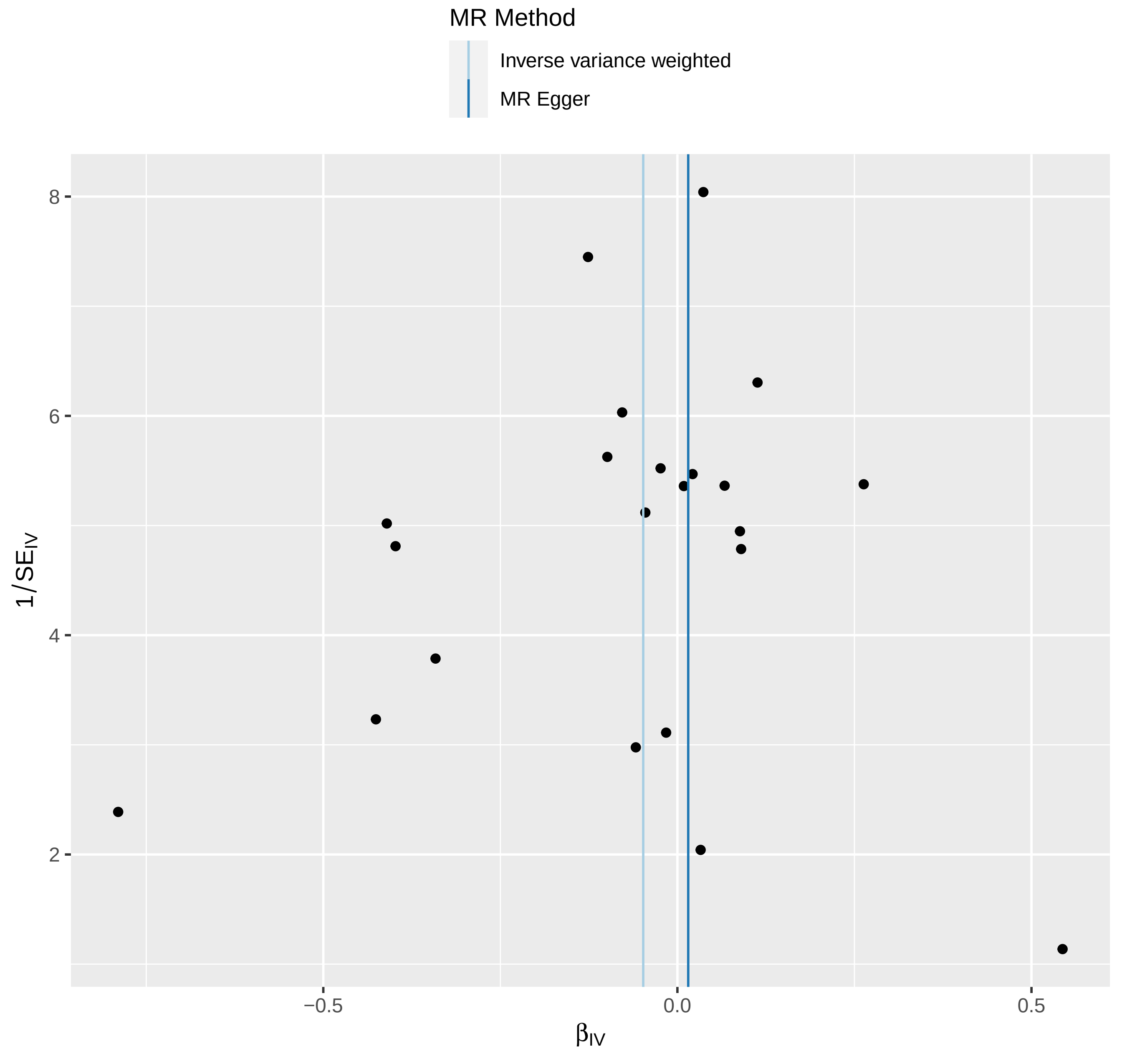

Batch 3−499 : Cell adhesion molecule−related/down−regulated by oncogenes on Diabetic nephropathy (more control exclusions)
with a threshold of 1e−05

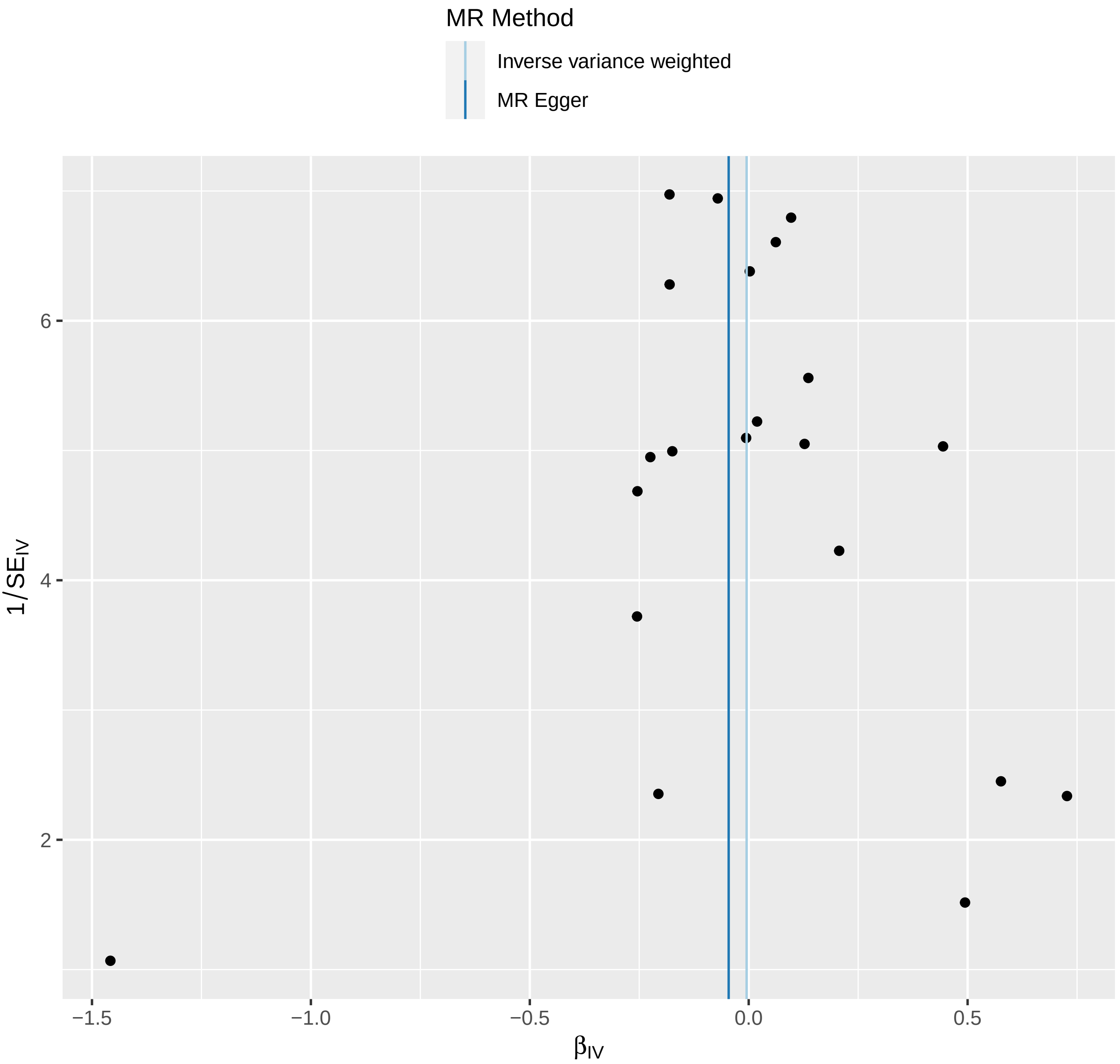

Batch 3−500 : Corneodesmosin on Diabetic nephropathy (more control exclusions)
with a threshold of 1e−05

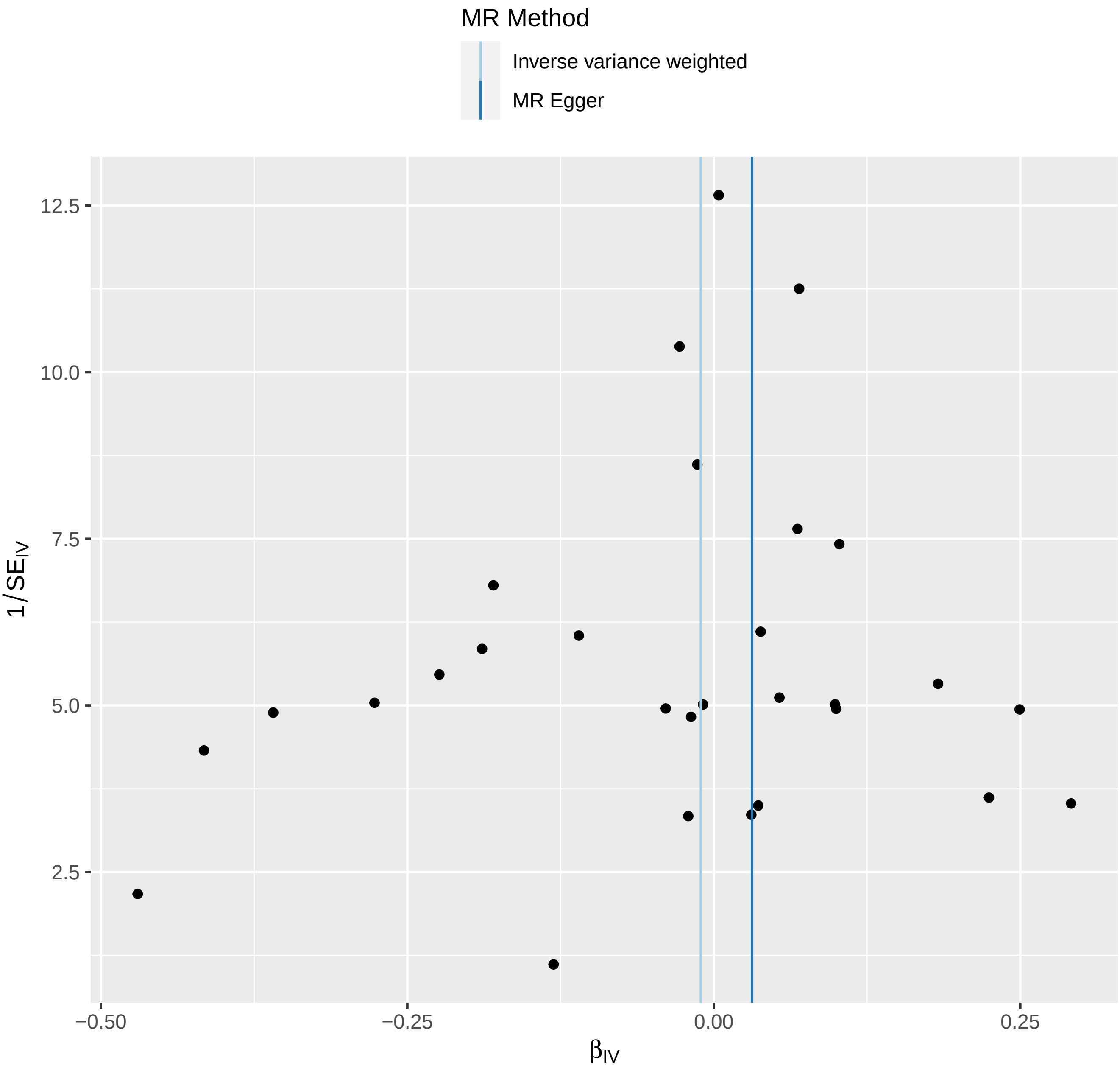

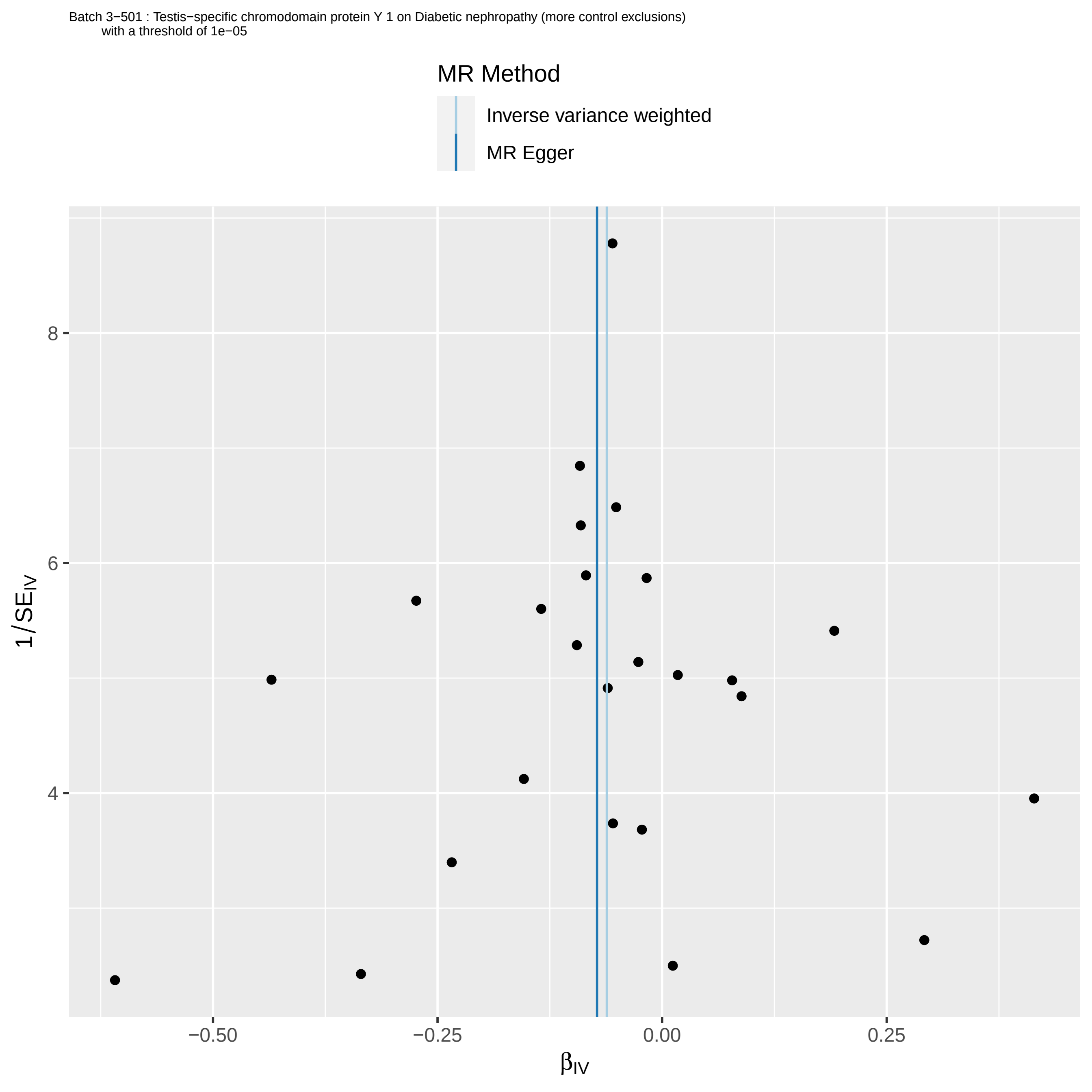

Batch 3−501 : Testis−specific chromodomain protein Y 1 on Diabetic nephropathy (more control exclusions)
with a threshold of 1e−05
MR Method
Inverse variance weighted
MR Egger
8
6
4
1/SE_IV
−0.50
−0.25
0.00
0.25
β_IV

Batch 3−502 : Carcinoembryonic antigen−related cell adhesion molecule 1 on Diabetic nephropathy (more control exclusions)
with a threshold of 1e−05

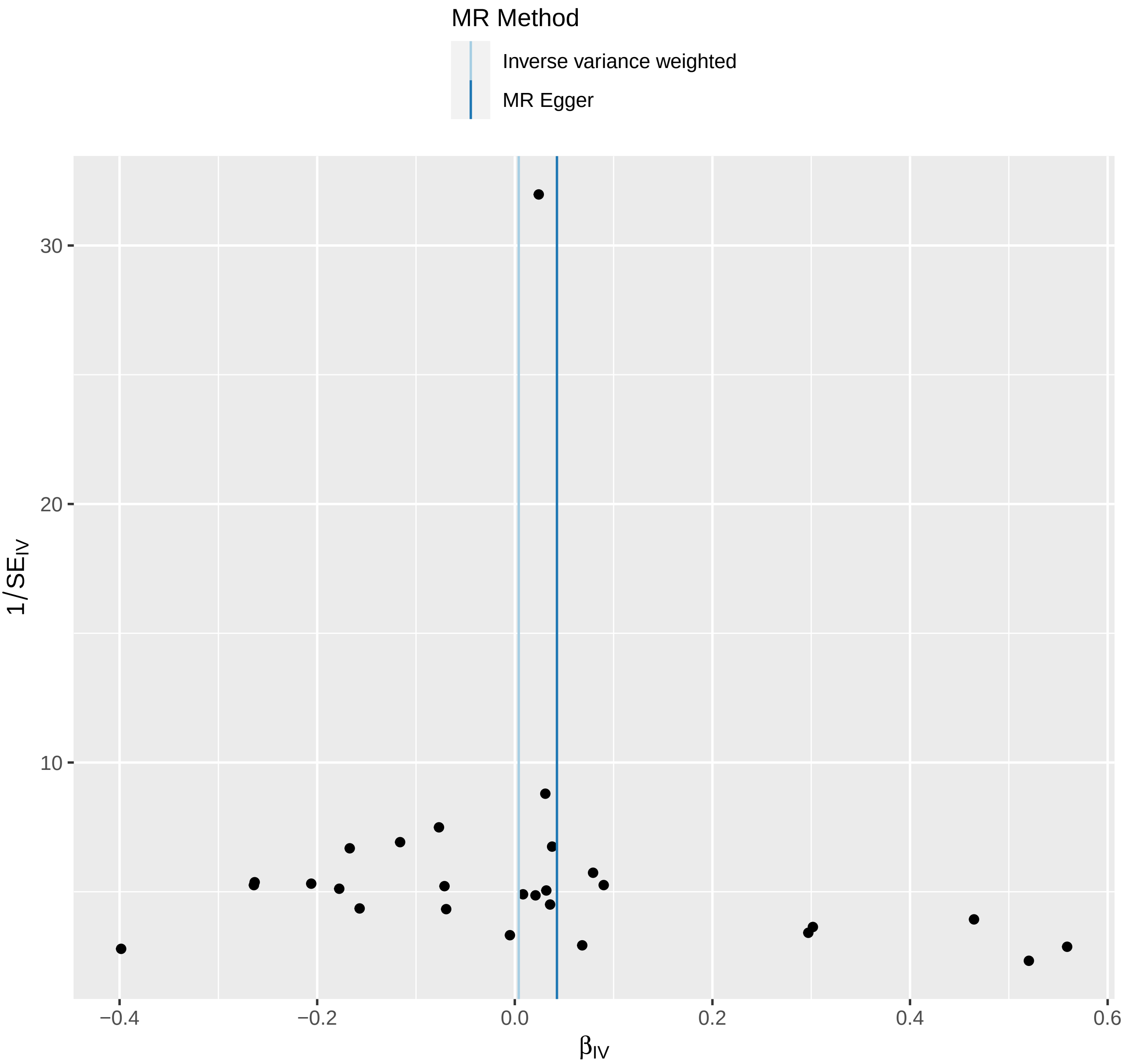

Batch 3−503 : Carcinoembryonic antigen−related cell adhesion molecule 21 on Diabetic nephropathy (more control exclusions) with a threshold of 1e−05

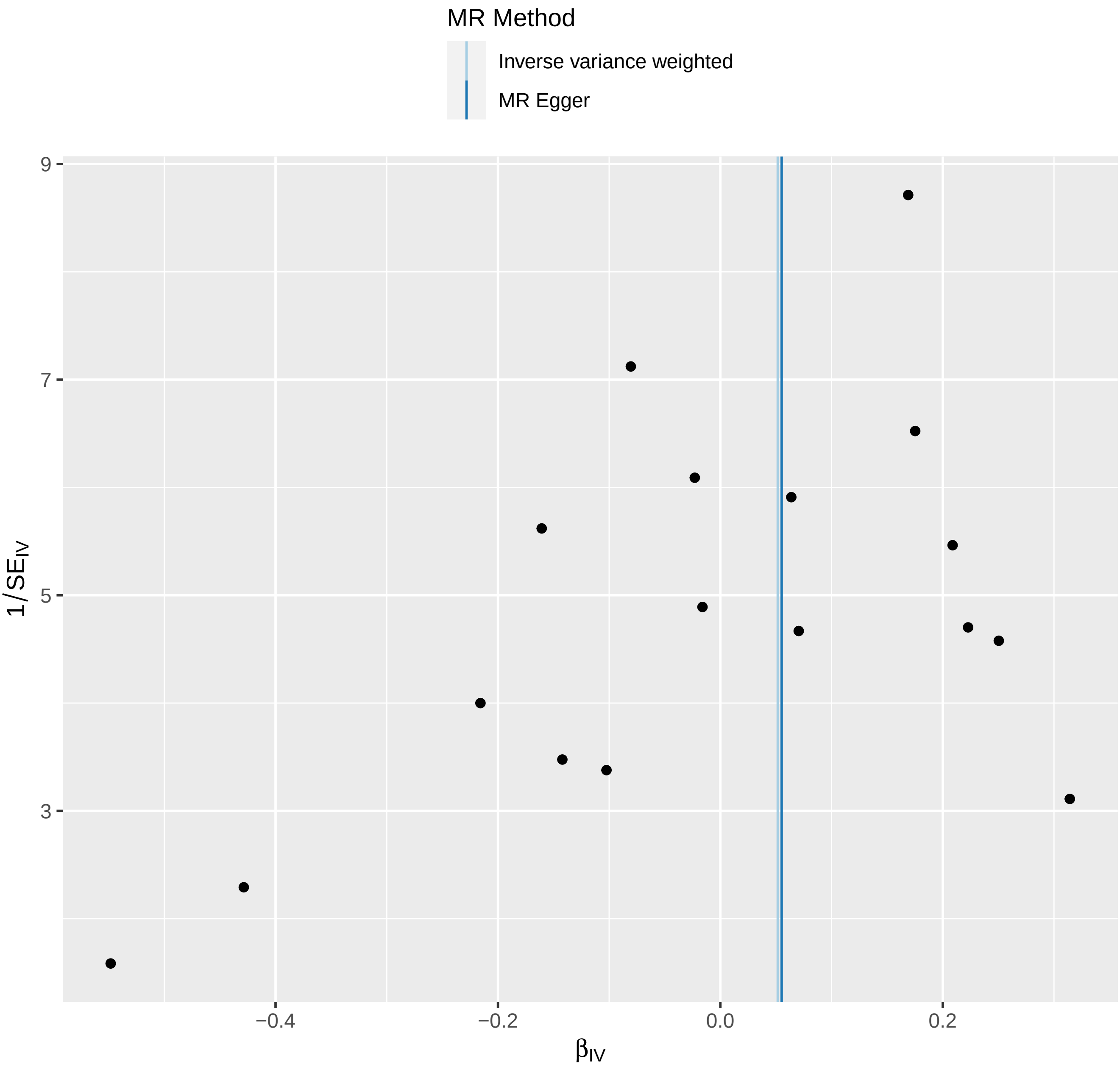

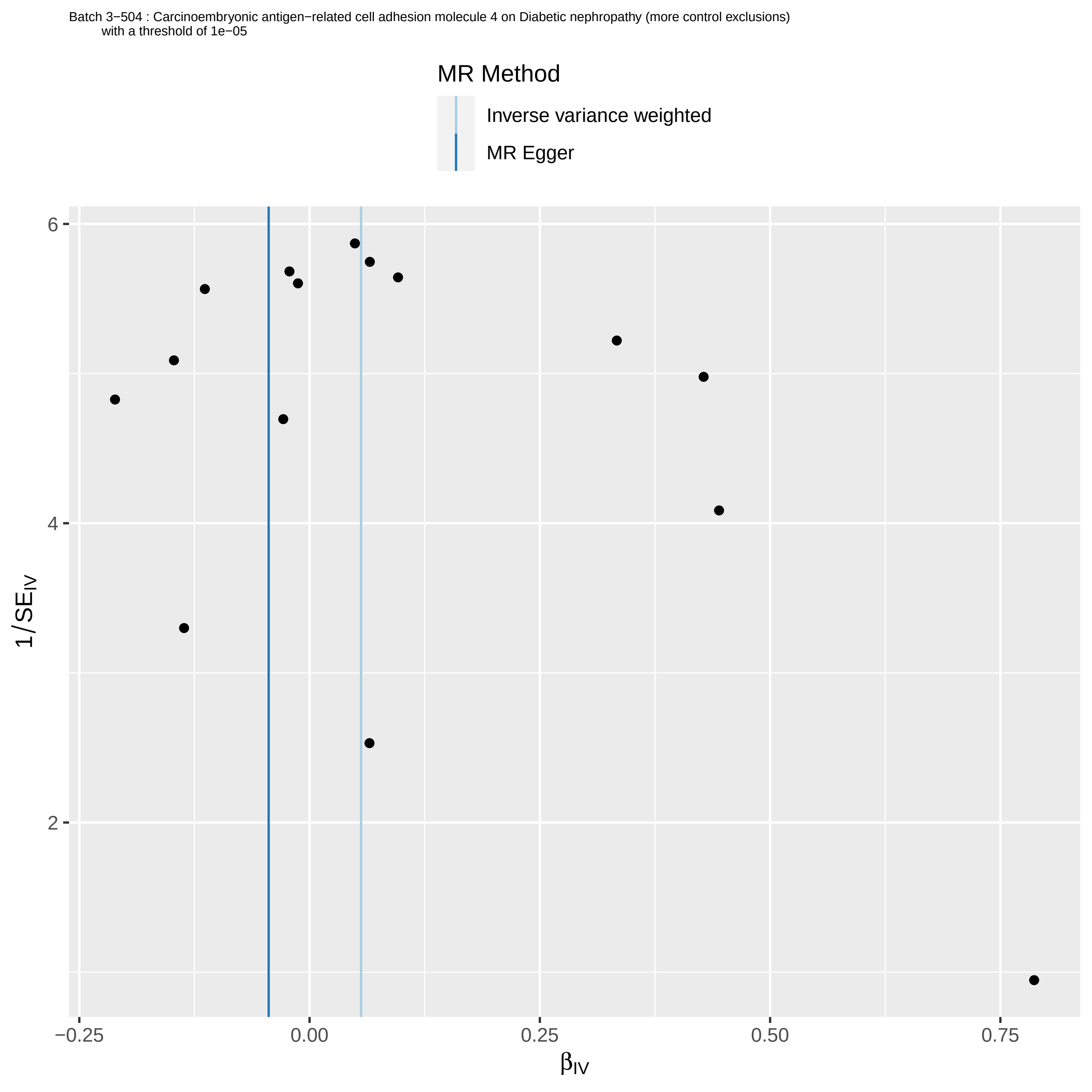
Batch 3−504 : Carcinoembryonic antigen−related cell adhesion molecule 4 on Diabetic nephropathy (more control exclusions)
with a threshold of 1e−05
MR Method
Inverse variance weighted
MR Egger
6
4
2
1/SE_IV
−0.25
0.00
0.25
0.50
0.75
β_IV

Batch 3−505 : CCAAT/enhancer−binding protein beta on Diabetic nephropathy (more control exclusions) with a threshold of 1e−05

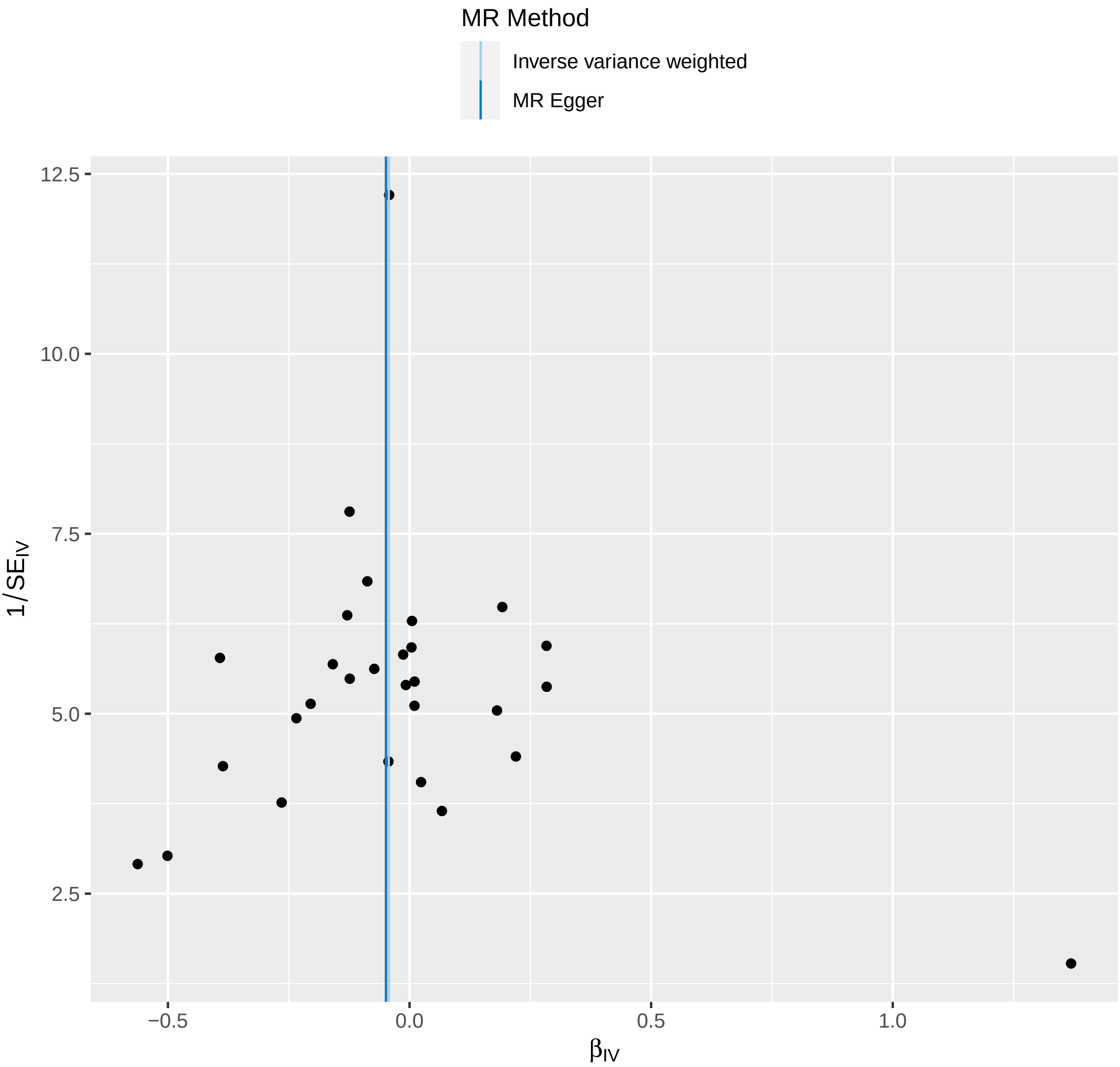

Batch 3−506 : Adenosine deaminase CECR1 on Diabetic nephropathy (more control exclusions)
with a threshold of 1e−05

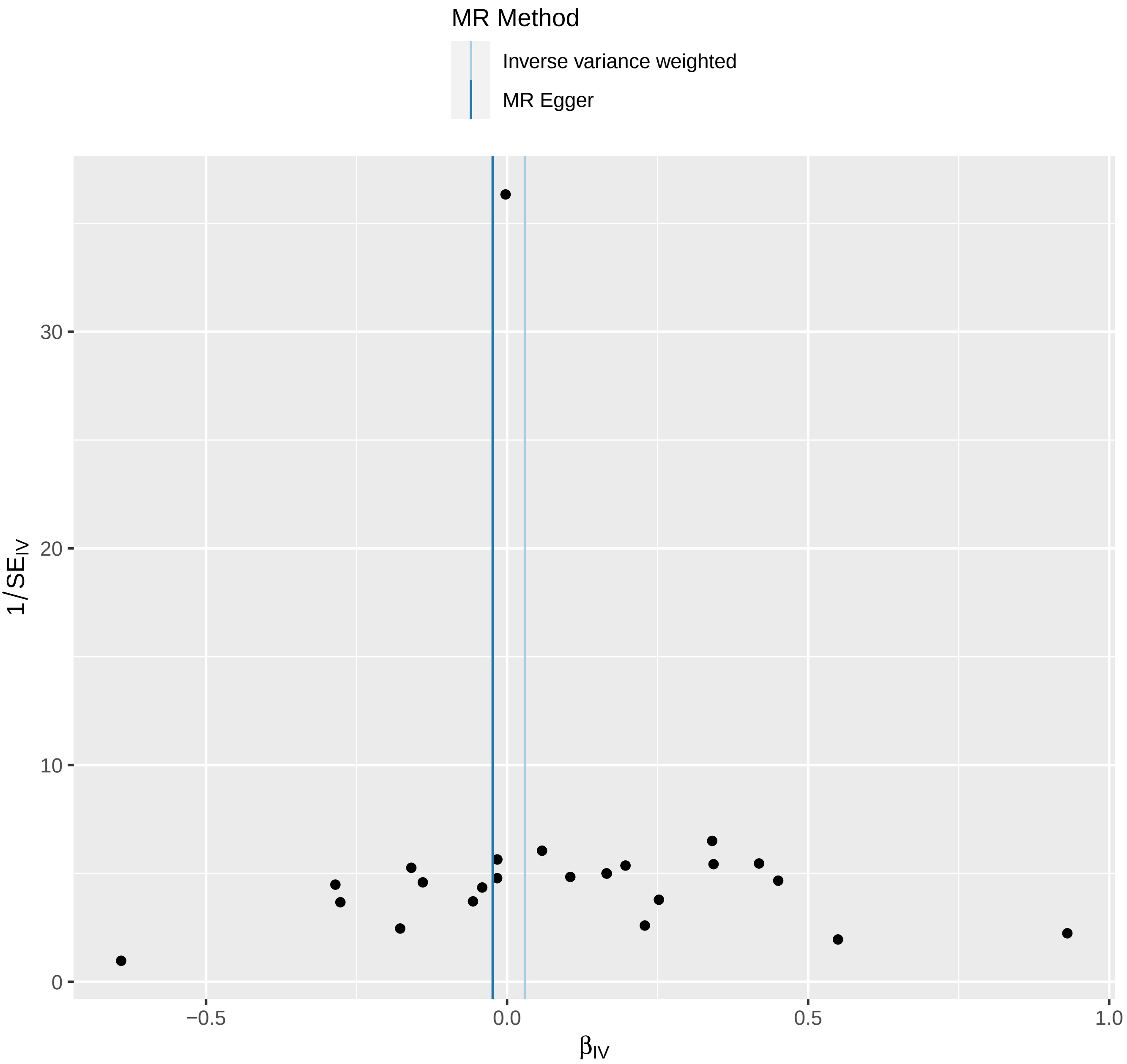

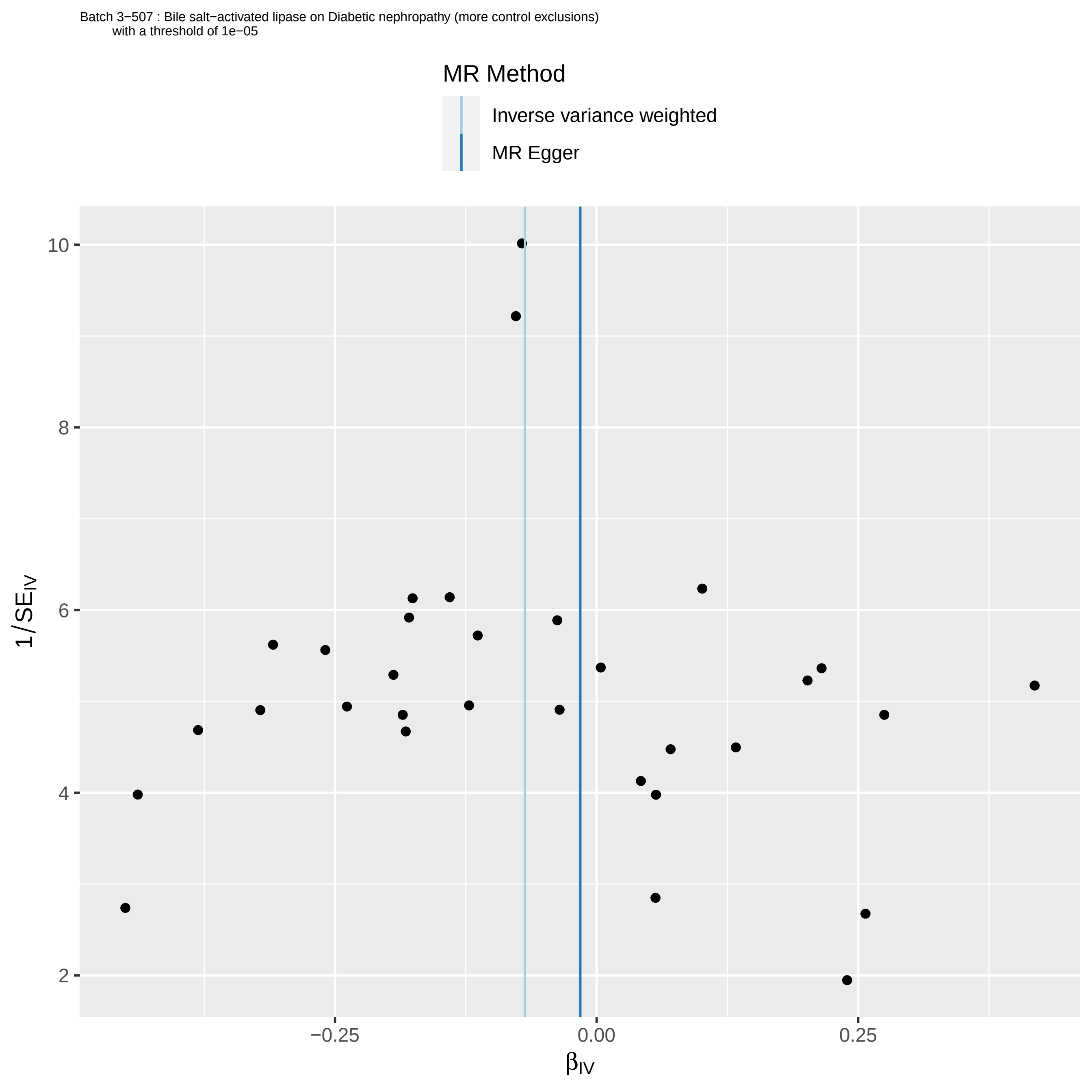

Batch 3−507 : Bile salt−activated lipase on Diabetic nephropathy (more control exclusions)
with a threshold of 1e−05
MR Method
Inverse variance weighted
MR Egger
10
8
6
4
2
1/SE$_{IV}$
−0.25
0.00
0.25
β$_{IV}$

Batch 3−508 : Chymotrypsin−like elastase family member 2A on Diabetic nephropathy (more control exclusions)
with a threshold of 1e−05

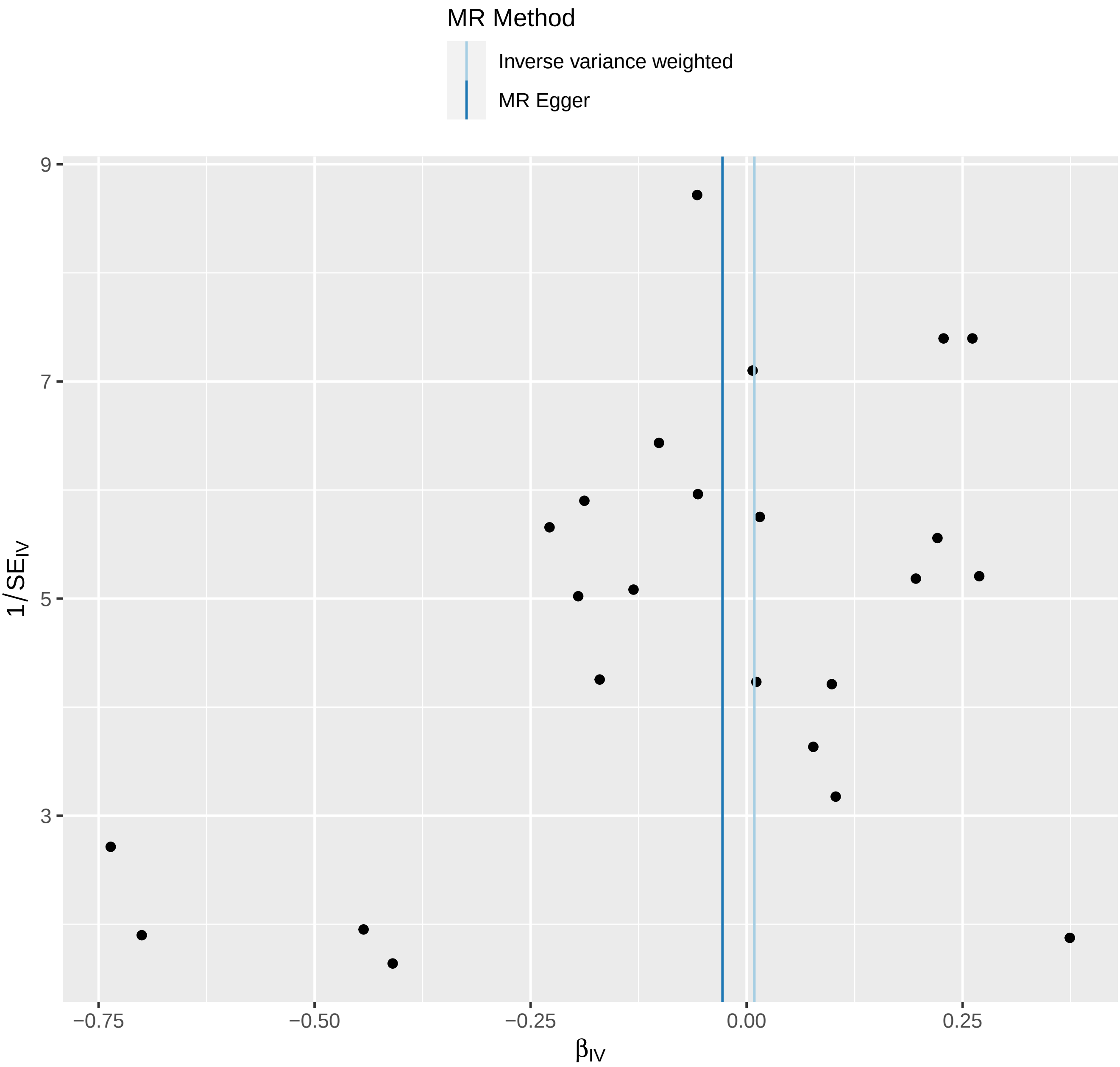

Batch 3−509 : Chymotrypsin−like elastase family member 3B on Diabetic nephropathy (more control exclusions)
with a threshold of 1e−05

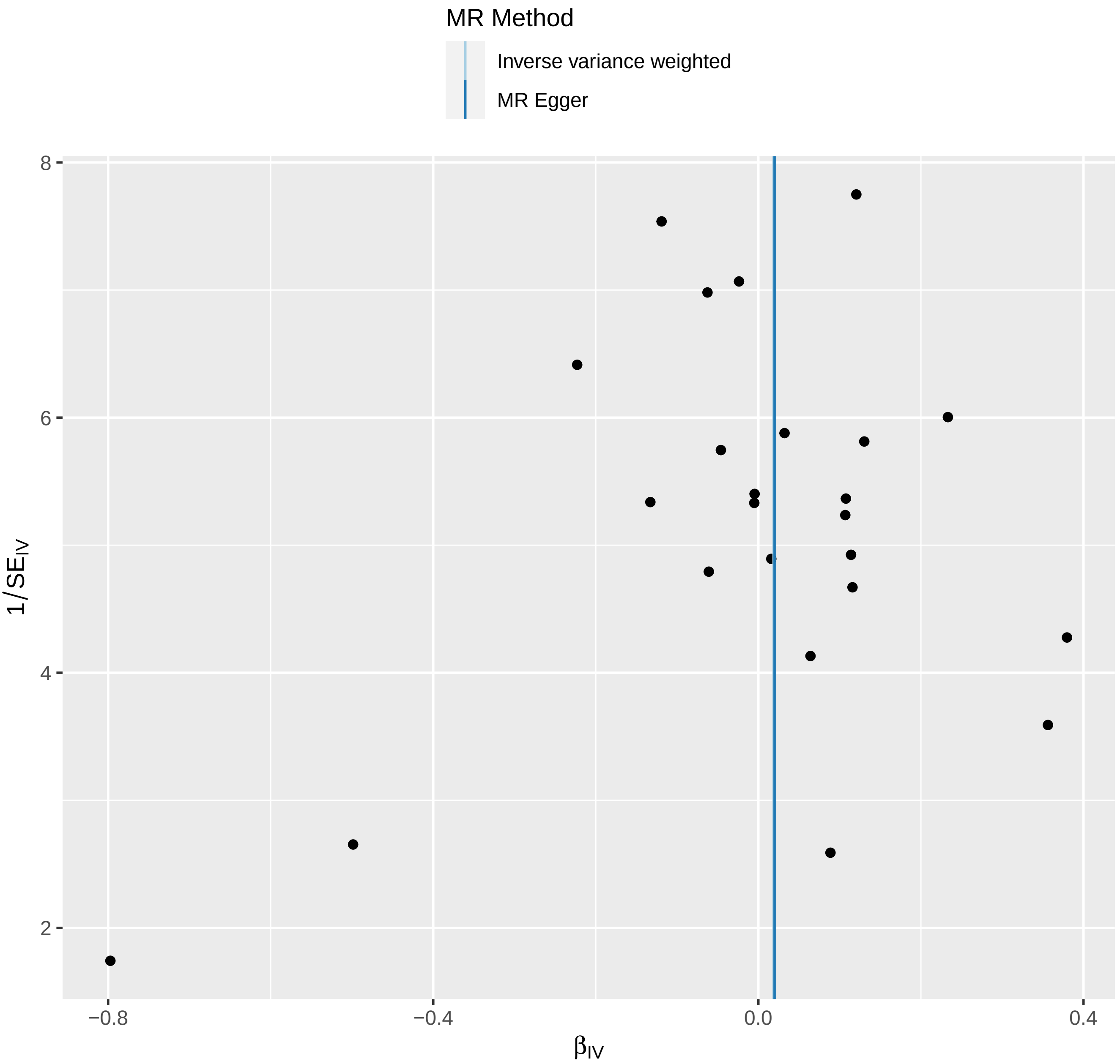

Batch 3−510 : CUGBP Elav−like family member 2 on Diabetic nephropathy (more control exclusions)
with a threshold of 1e−05

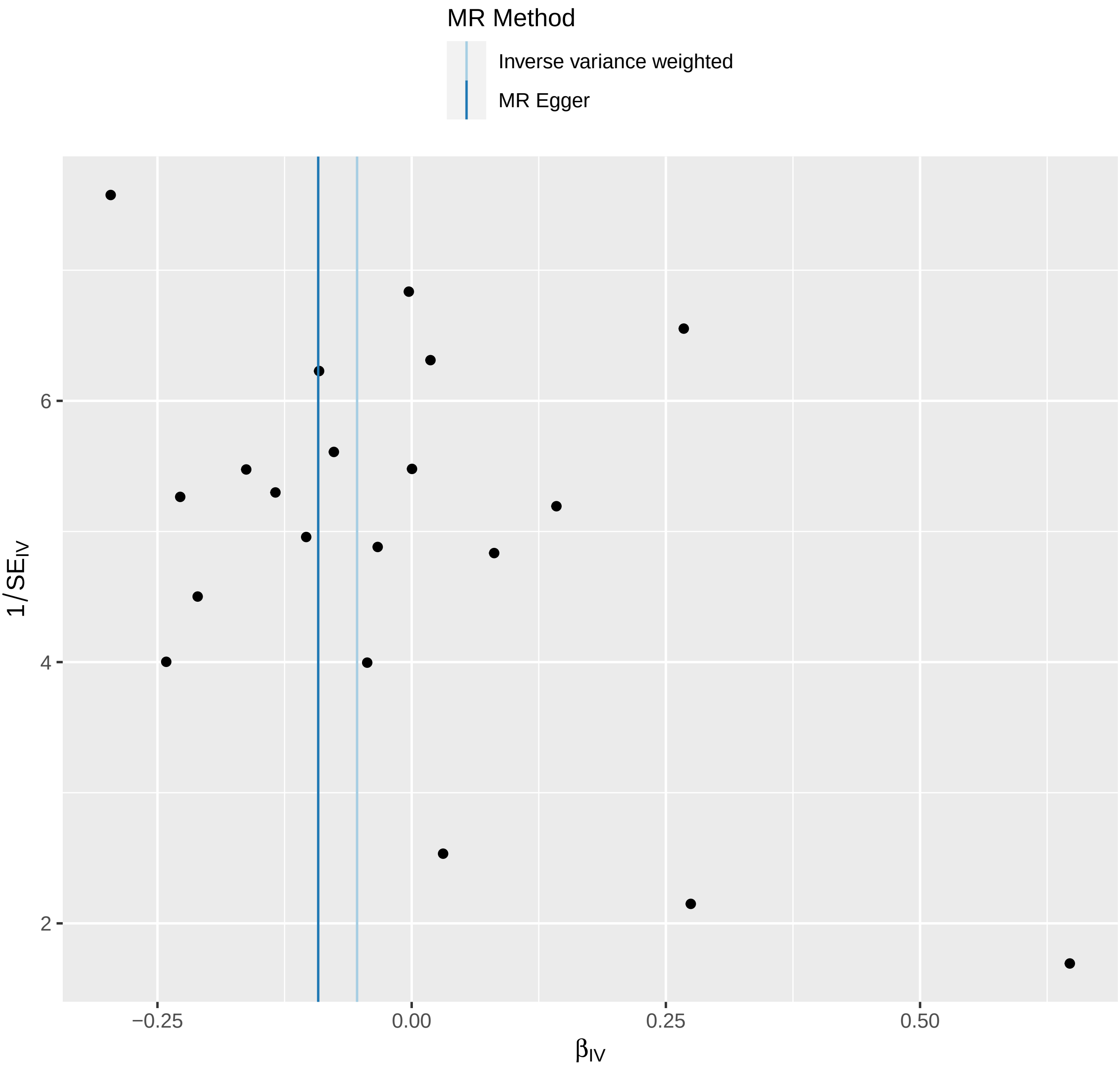

Batch 3−511 : Centromere protein W on Diabetic nephropathy (more control exclusions)
with a threshold of 1e−05

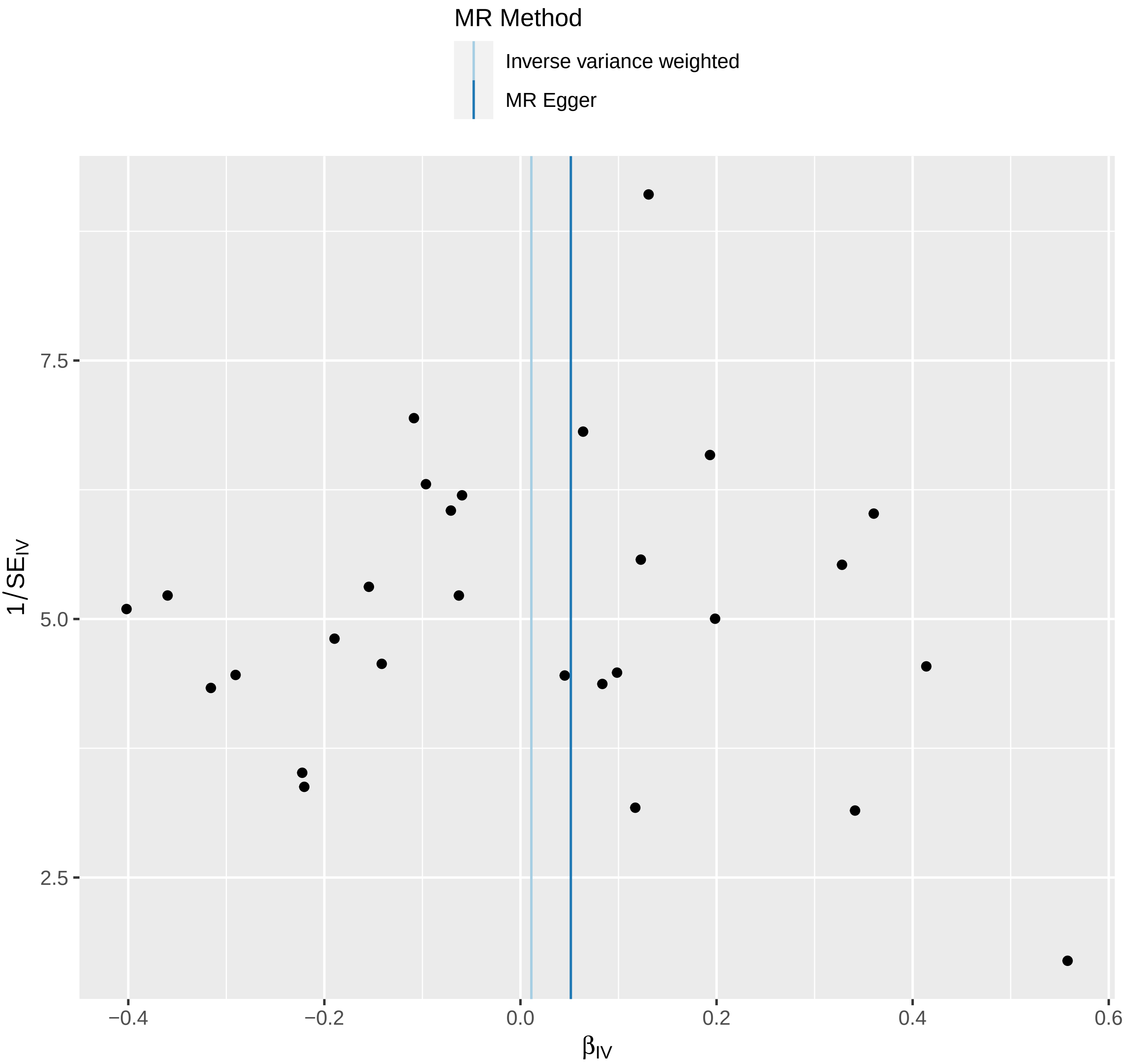

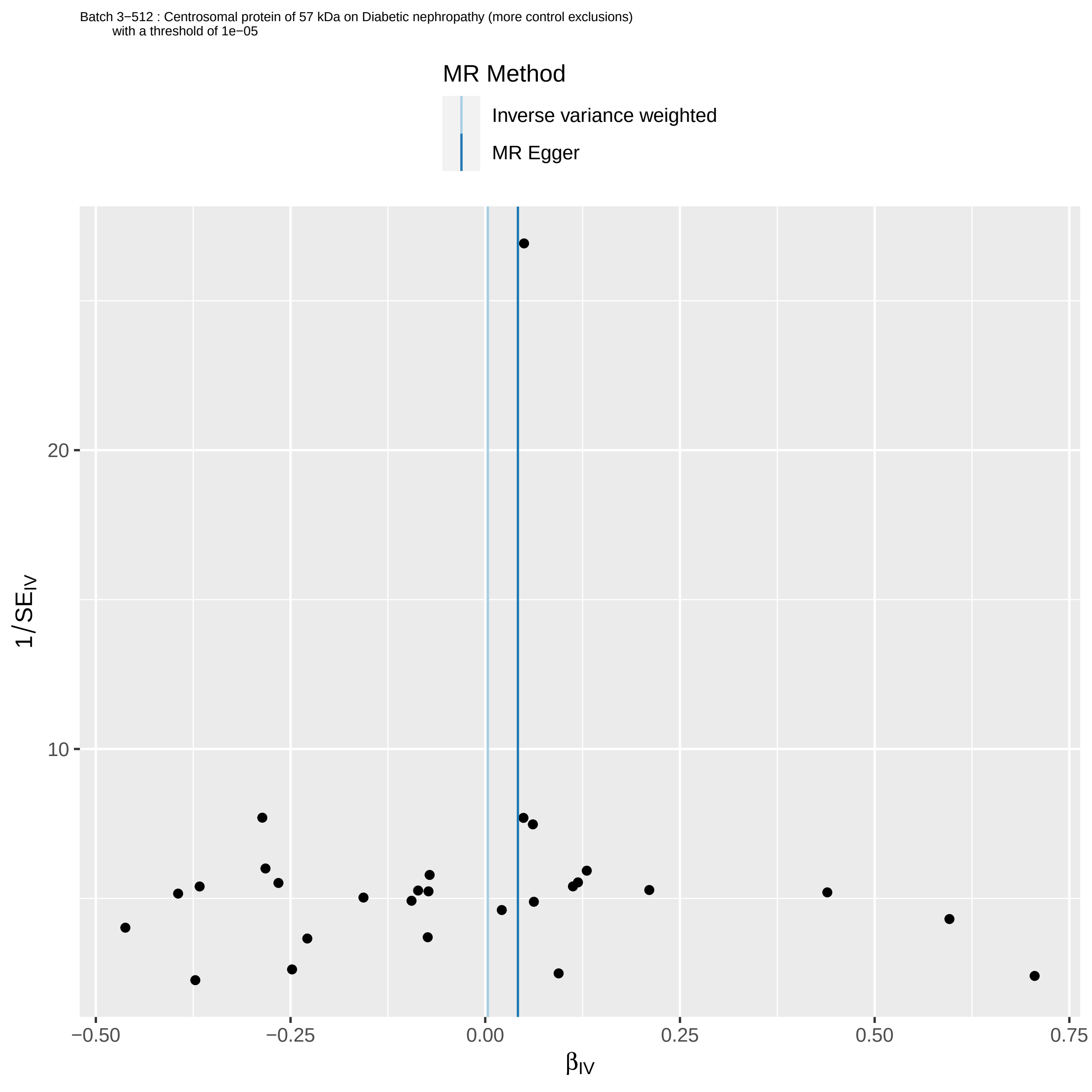

Batch 3−512 : Centrosomal protein of 57 kDa on Diabetic nephropathy (more control exclusions)
with a threshold of 1e−05
MR Method
Inverse variance weighted
MR Egger
20
10
1/SE$_{IV}$
−0.50
−0.25
0.00
0.25
0.50
0.75
β_{IV}

Batch 3−513 : Centrosomal protein of 57 kDa on Diabetic nephropathy (more control exclusions)
with a threshold of 1e−05

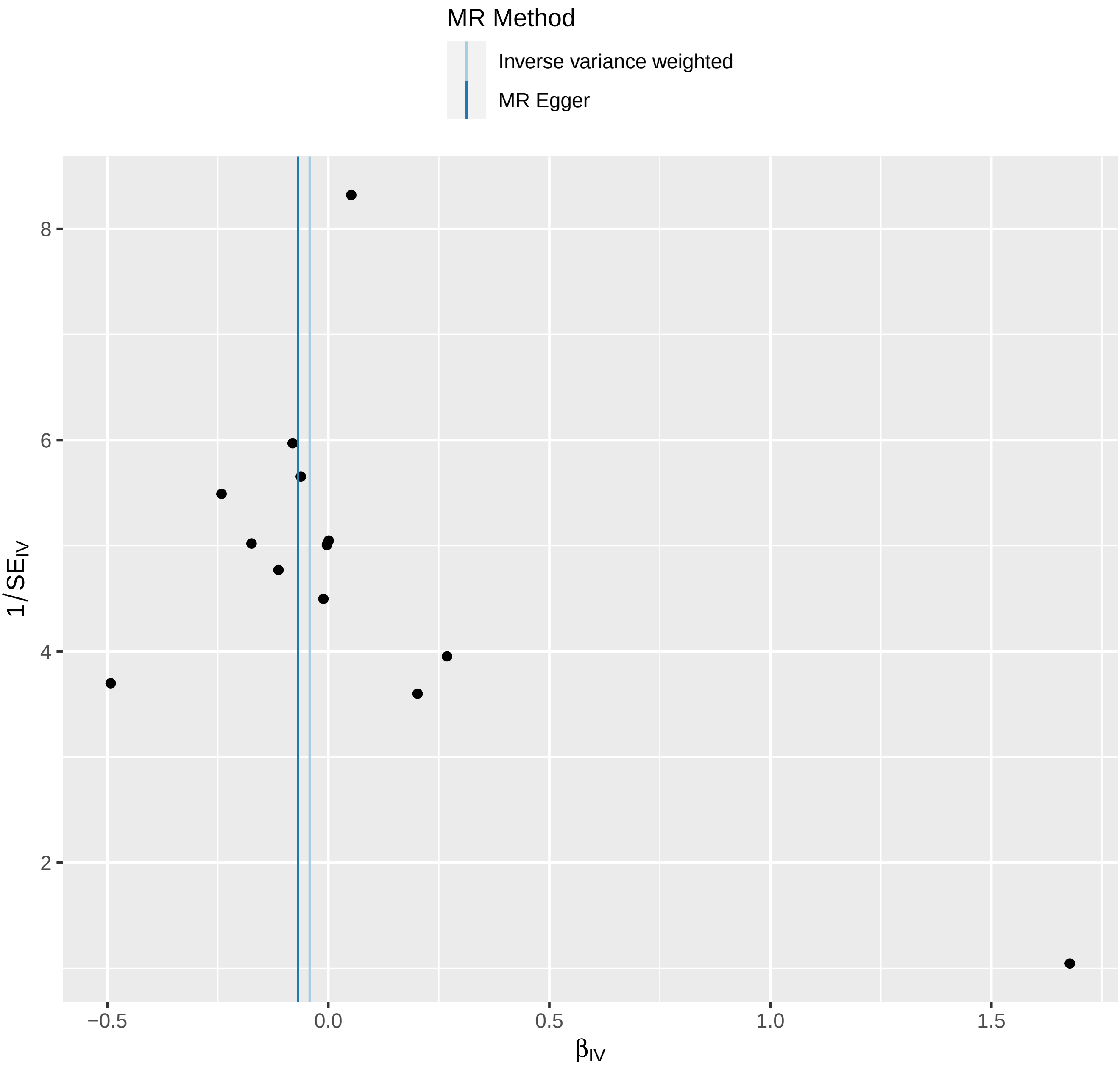

Batch 3−514 : Centrosomal protein of 57 kDa on Diabetic nephropathy (more control exclusions)
with a threshold of 1e−05

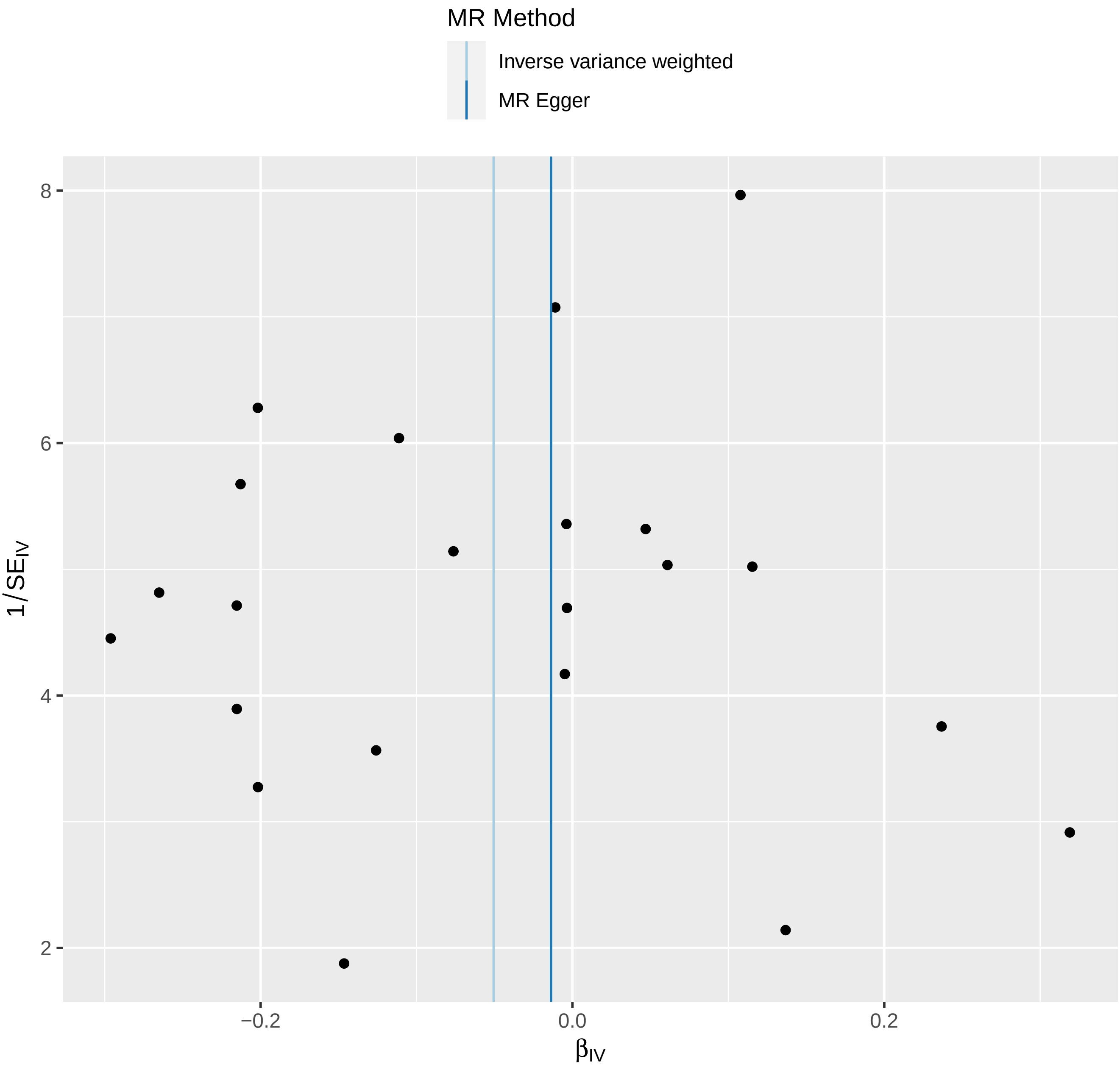

Batch 3−515 : Ceramide synthase 5 on Diabetic nephropathy (more control exclusions)
with a threshold of 1e−05

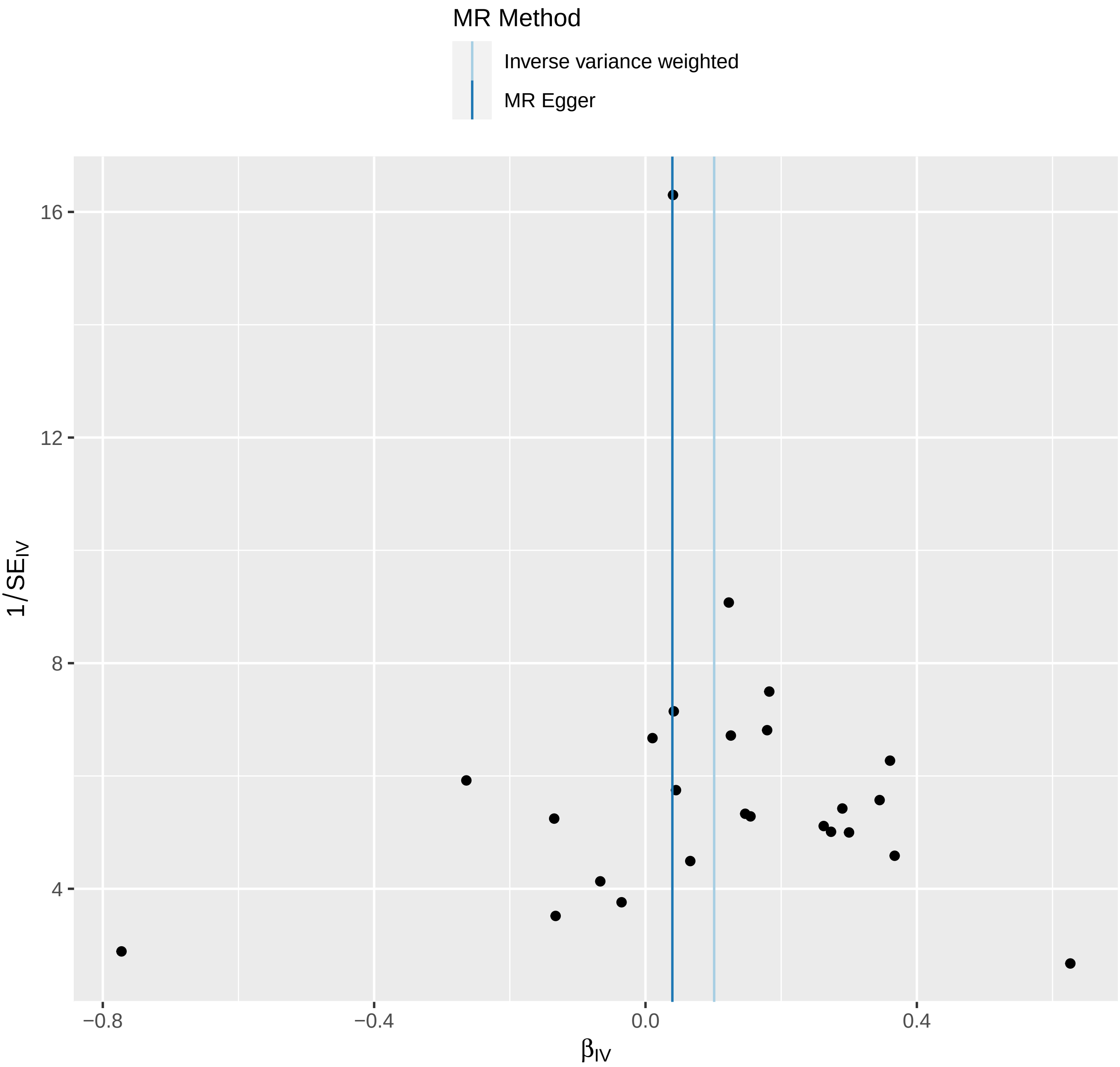

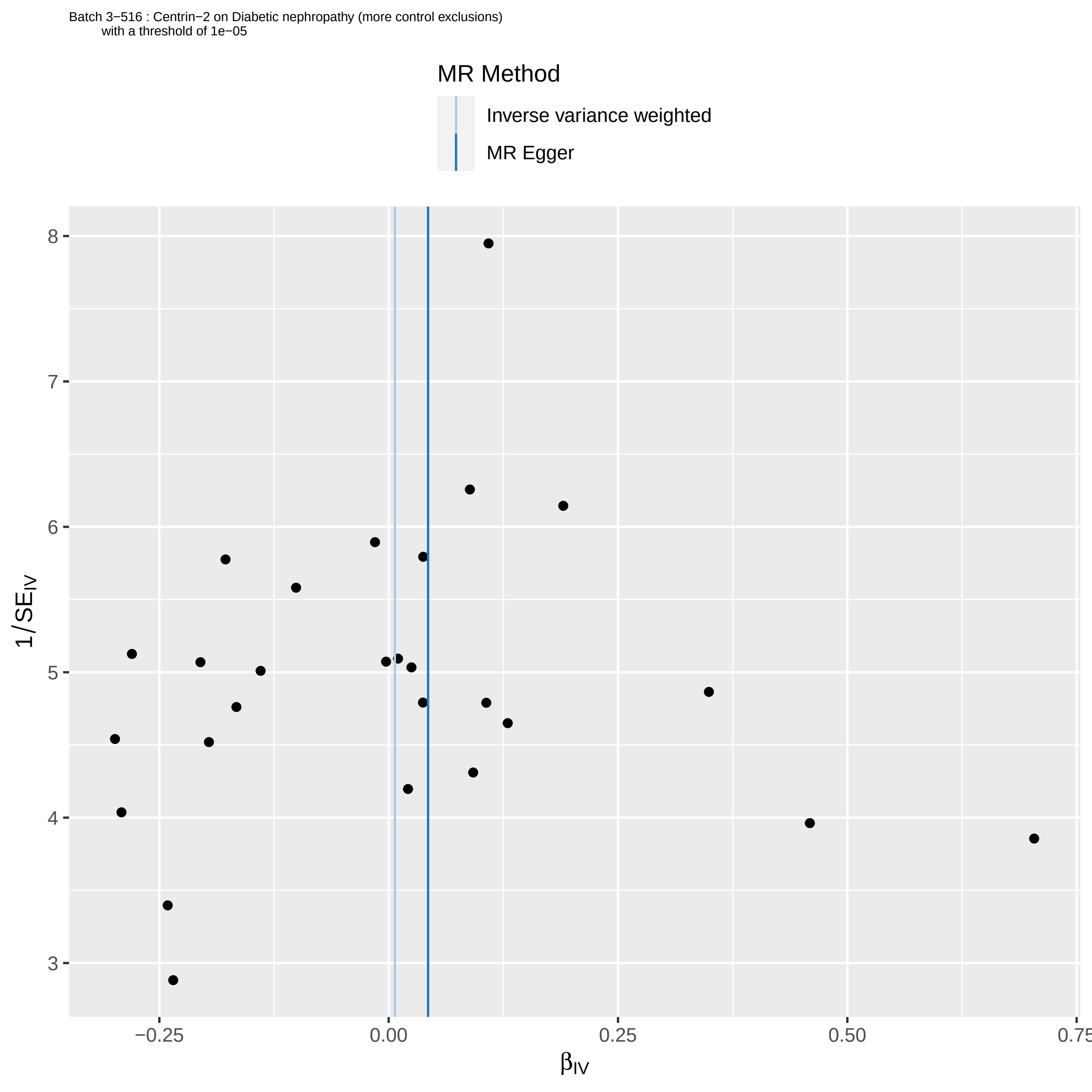

Batch 3−516 : Centrin−2 on Diabetic nephropathy (more control exclusions)
with a threshold of 1e−05
MR Method
Inverse variance weighted
MR Egger
8
7
6
5
4
3
1/SE$_{IV}$
−0.25
0.00
0.25
0.50
0.75
β$_{IV}$

Batch 3−517 : Cilia− and flagella−associated protein 45 on Diabetic nephropathy (more control exclusions)
with a threshold of 1e−05

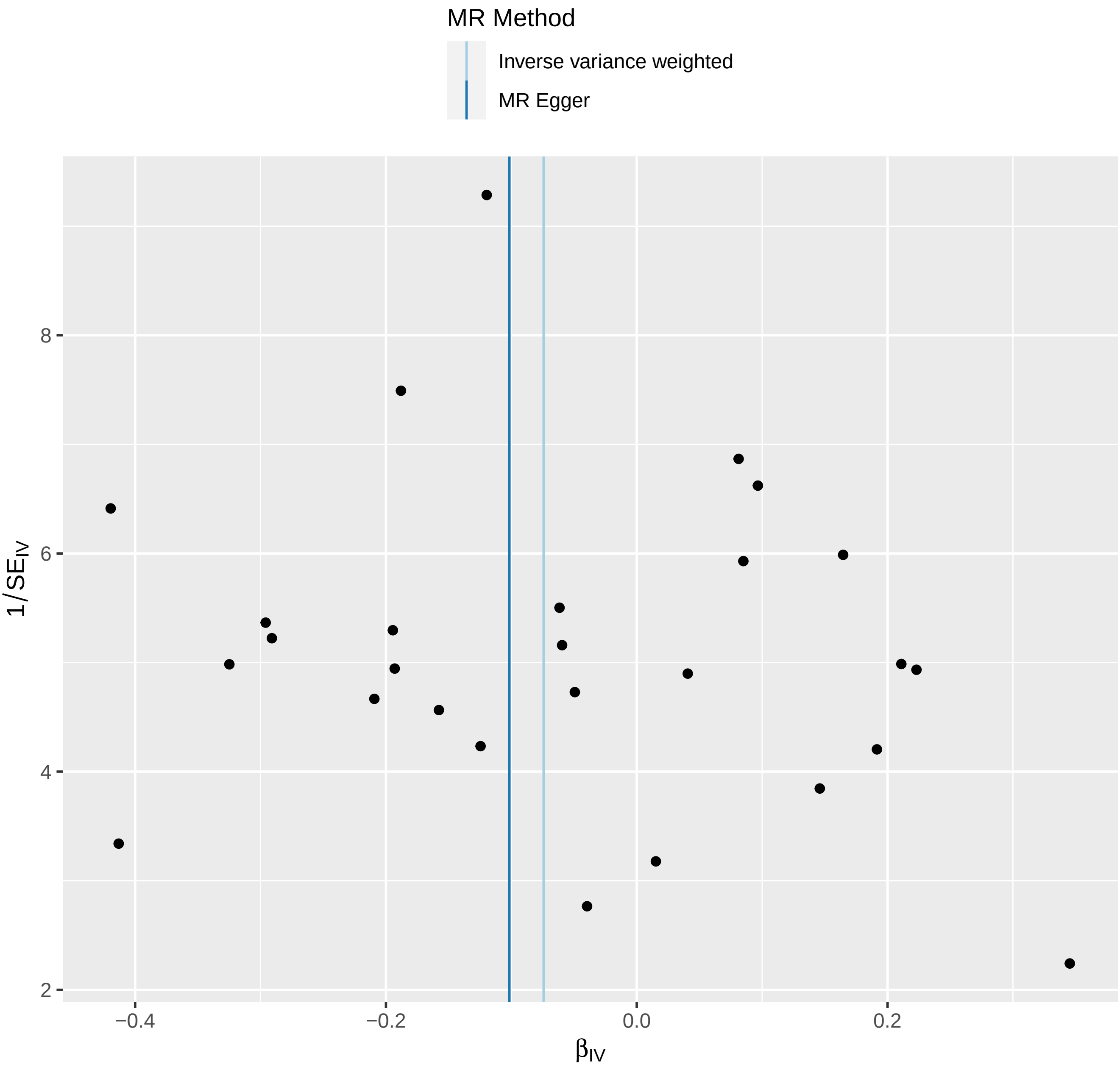

Batch 3−518 : Complement factor B on Diabetic nephropathy (more control exclusions)
with a threshold of 1e−05

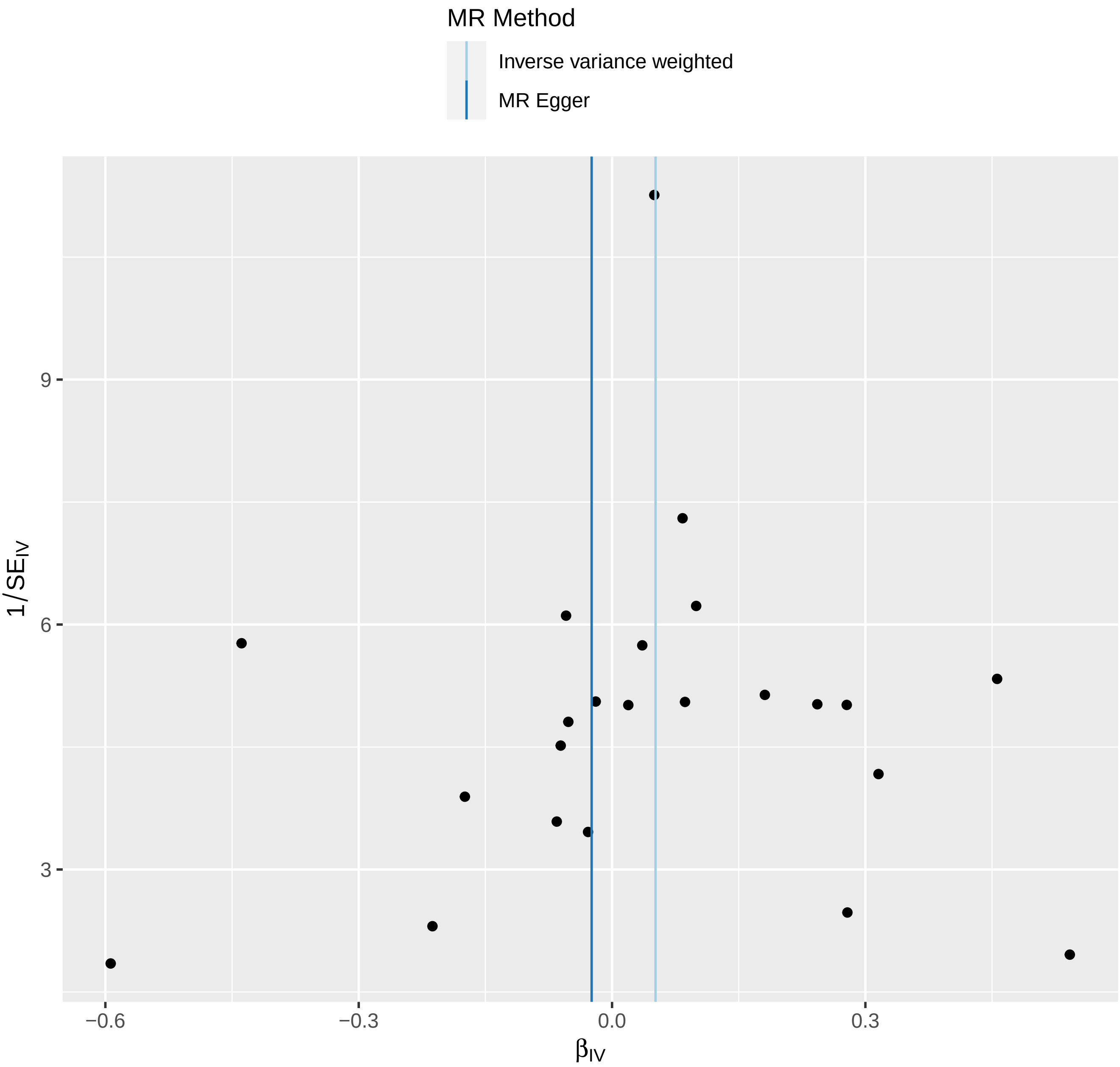

Batch 3−519 : Cryptic protein on Diabetic nephropathy (more control exclusions)
with a threshold of 1e−05

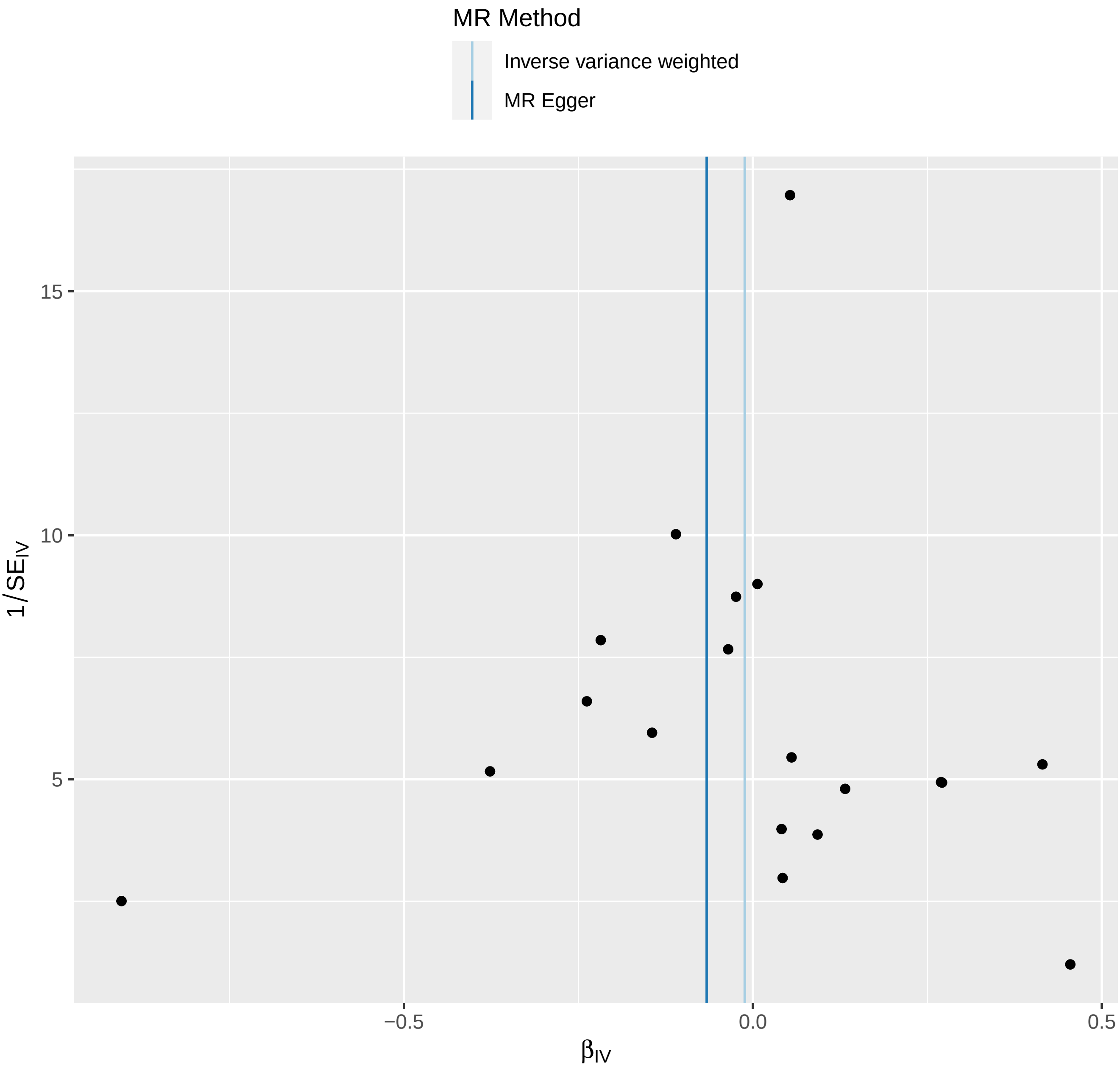

Batch 3−520 : Complement factor H on Diabetic nephropathy (more control exclusions)
with a threshold of 1e−05

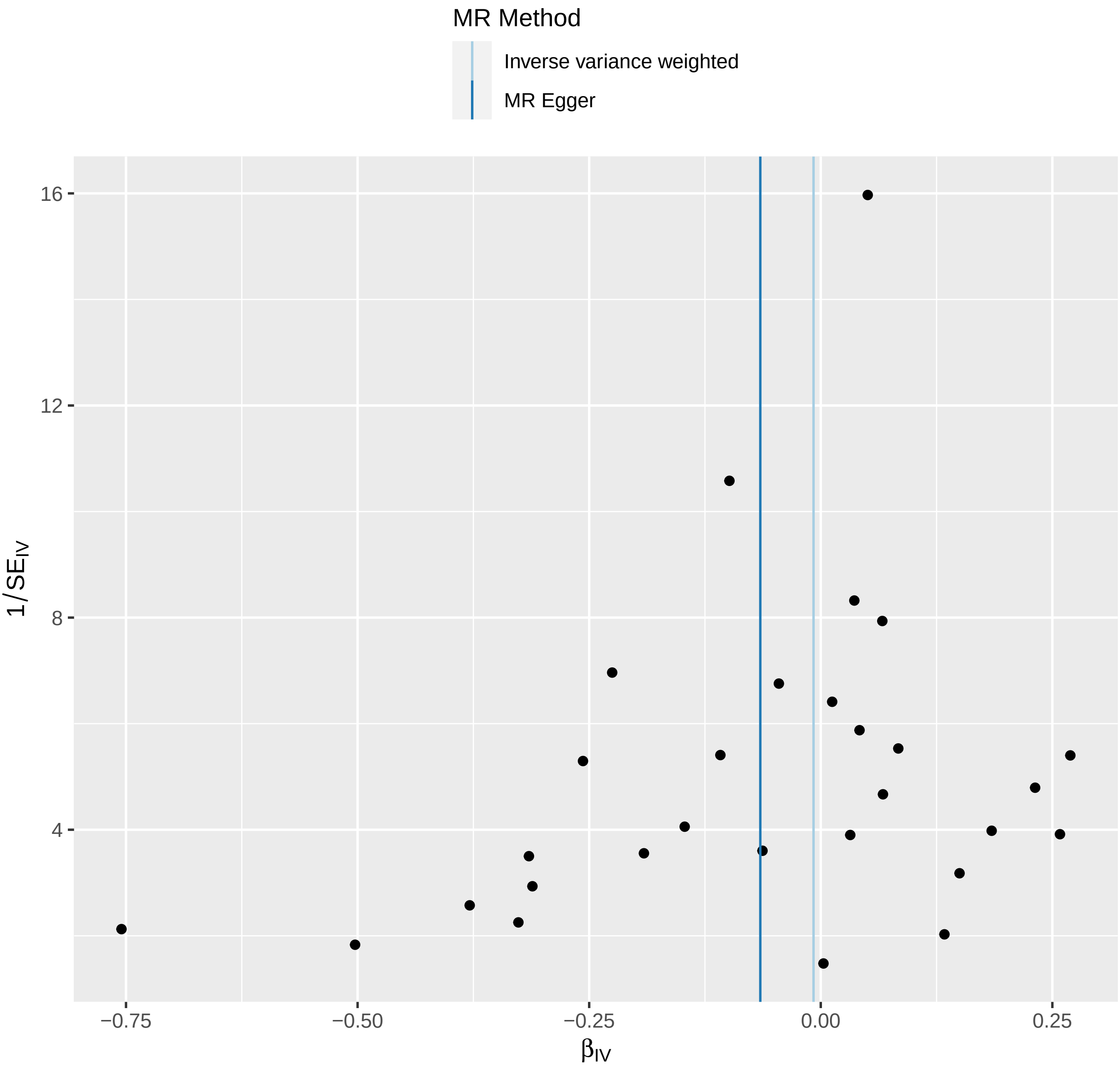

Batch 3−521 : Complement factor H−related protein 5 on Diabetic nephropathy (more control exclusions)
with a threshold of 1e−05

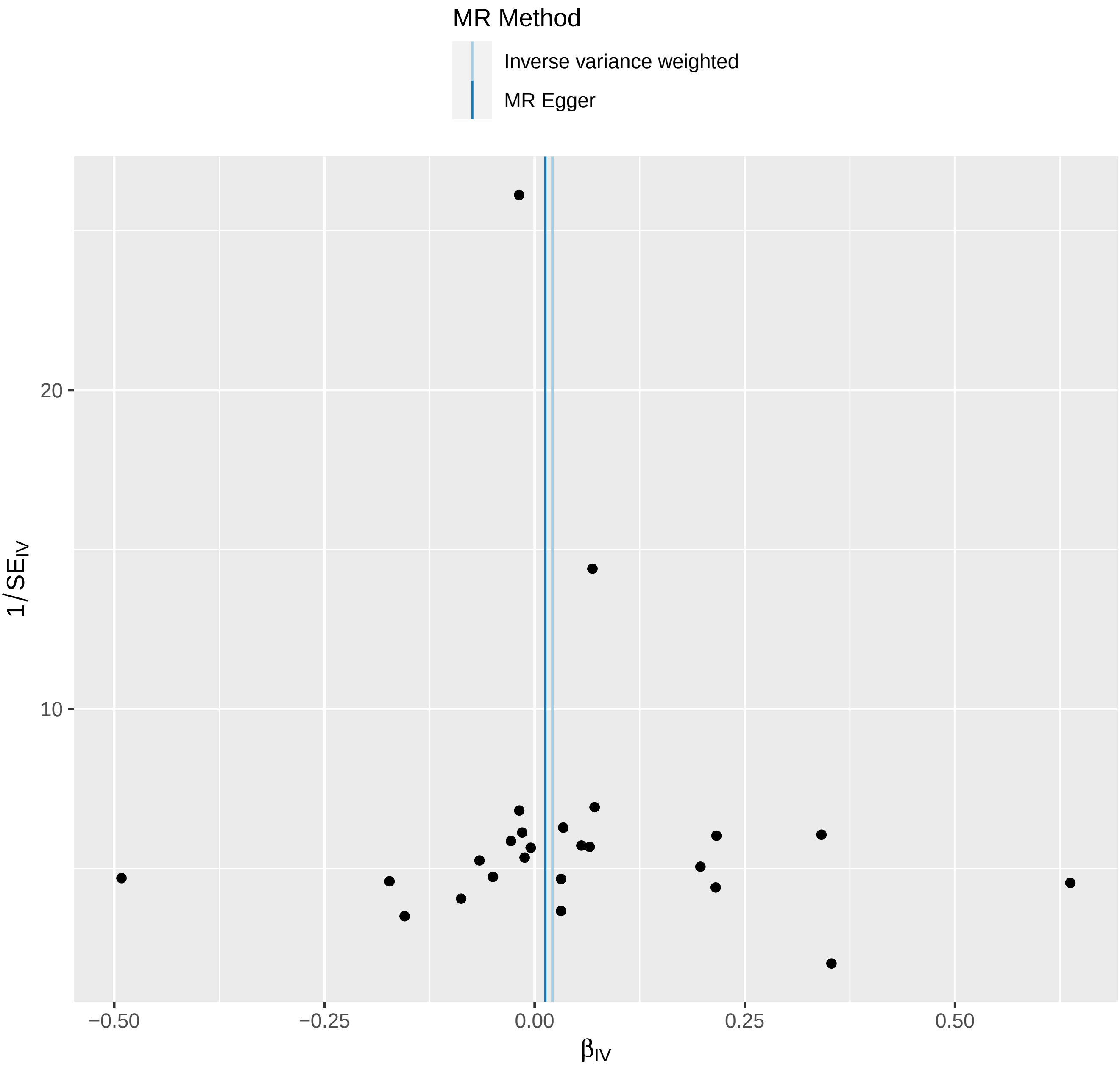

Batch 3−522 : Complement factor H−related protein 5 on Diabetic nephropathy (more control exclusions)
with a threshold of 1e−05

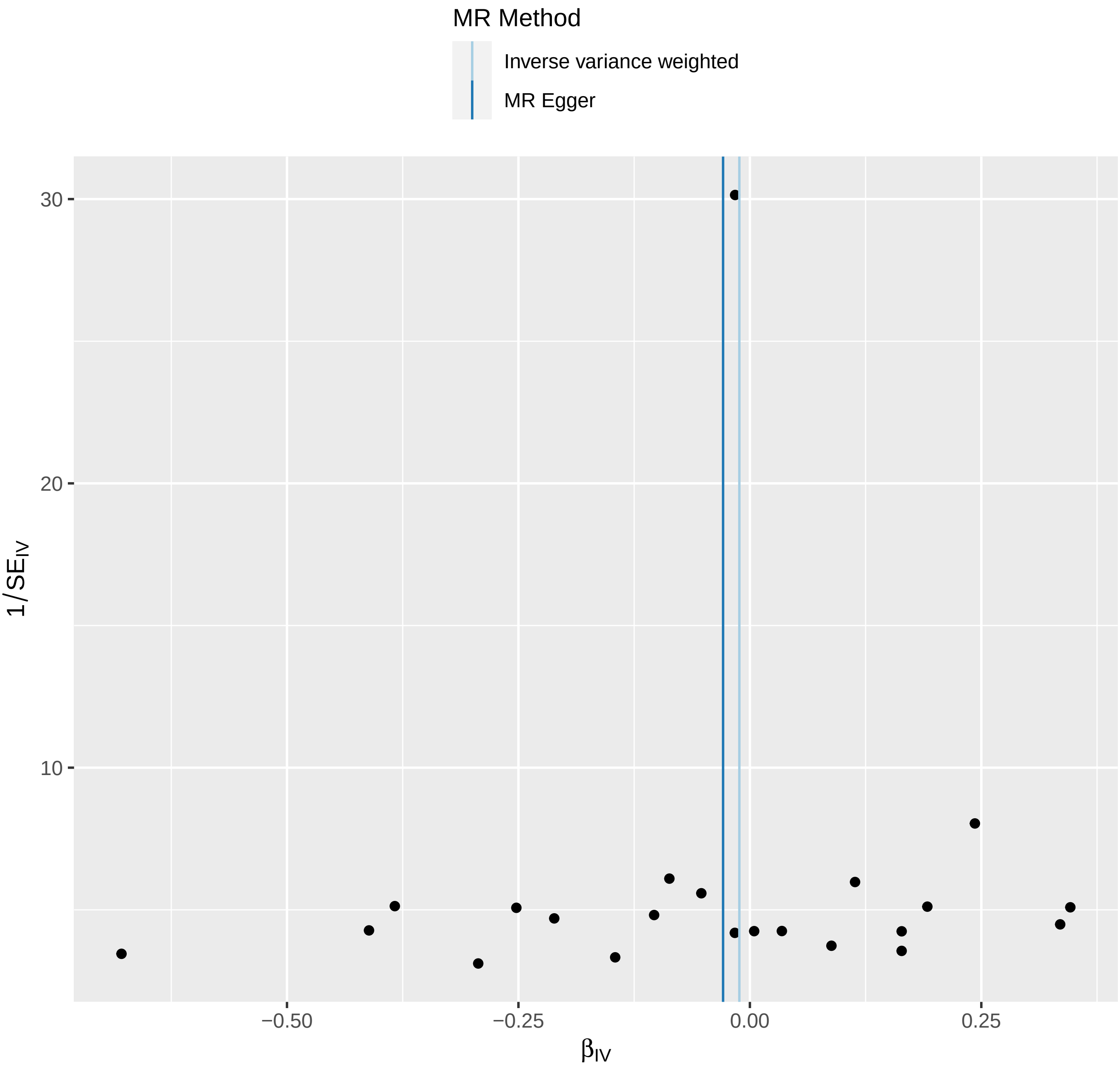

Batch 3−523 : Complement factor I on Diabetic nephropathy (more control exclusions)
with a threshold of 1e−05

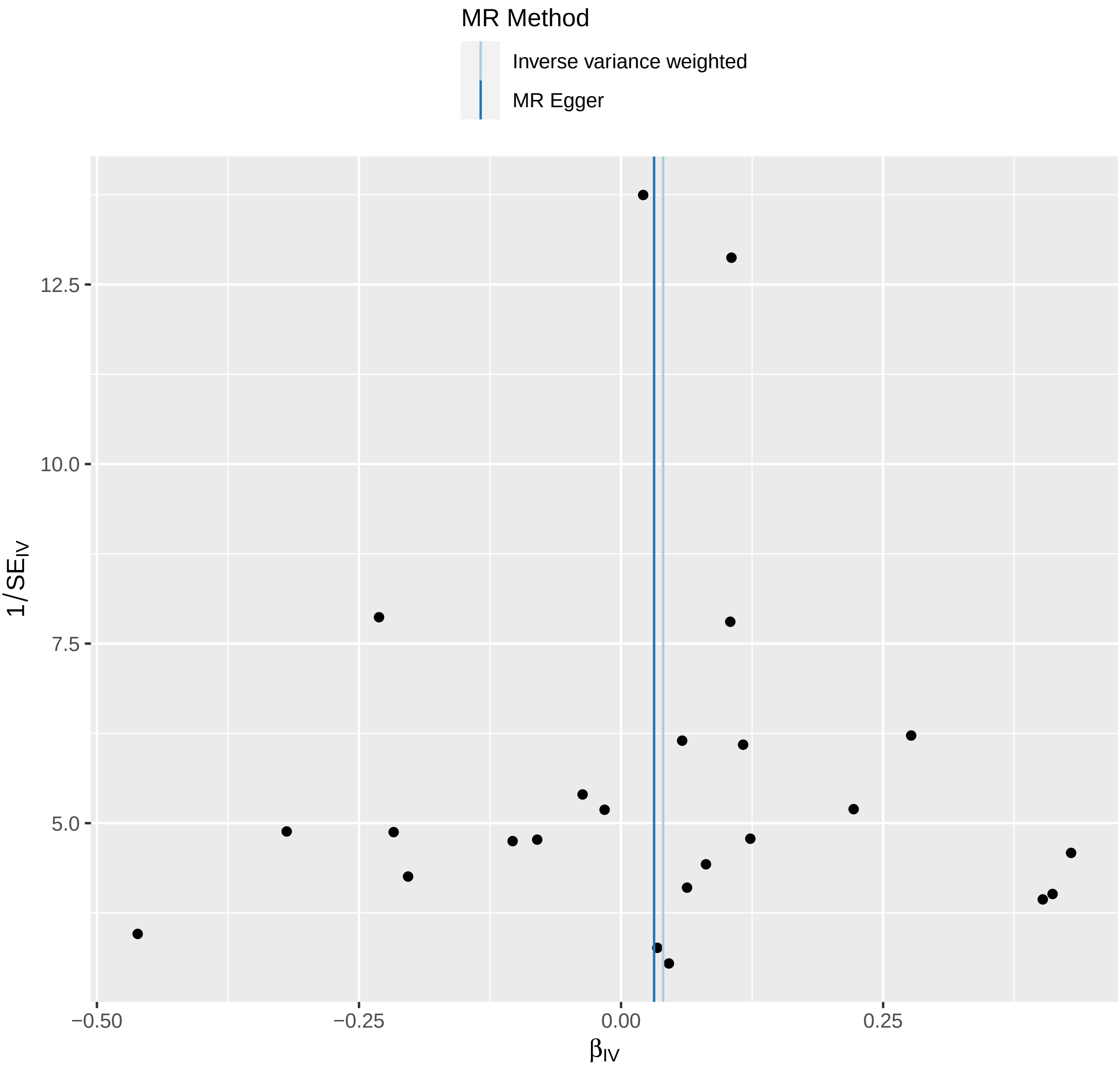

Batch 3−524 : Cofilin−1 on Diabetic nephropathy (more control exclusions)
with a threshold of 1e−05

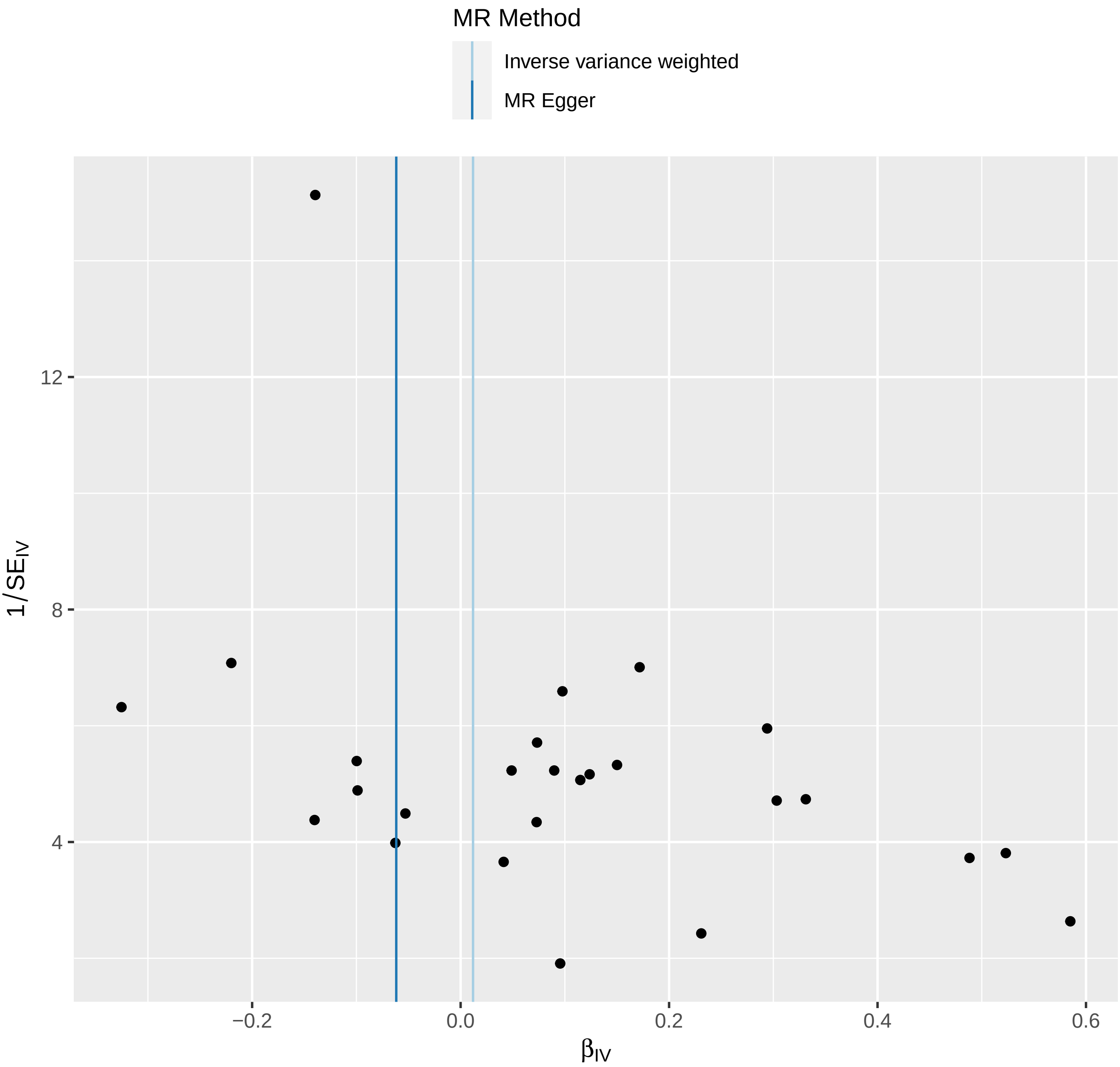

Batch 3−525 : Properdin on Diabetic nephropathy (more control exclusions)
with a threshold of 1e−05

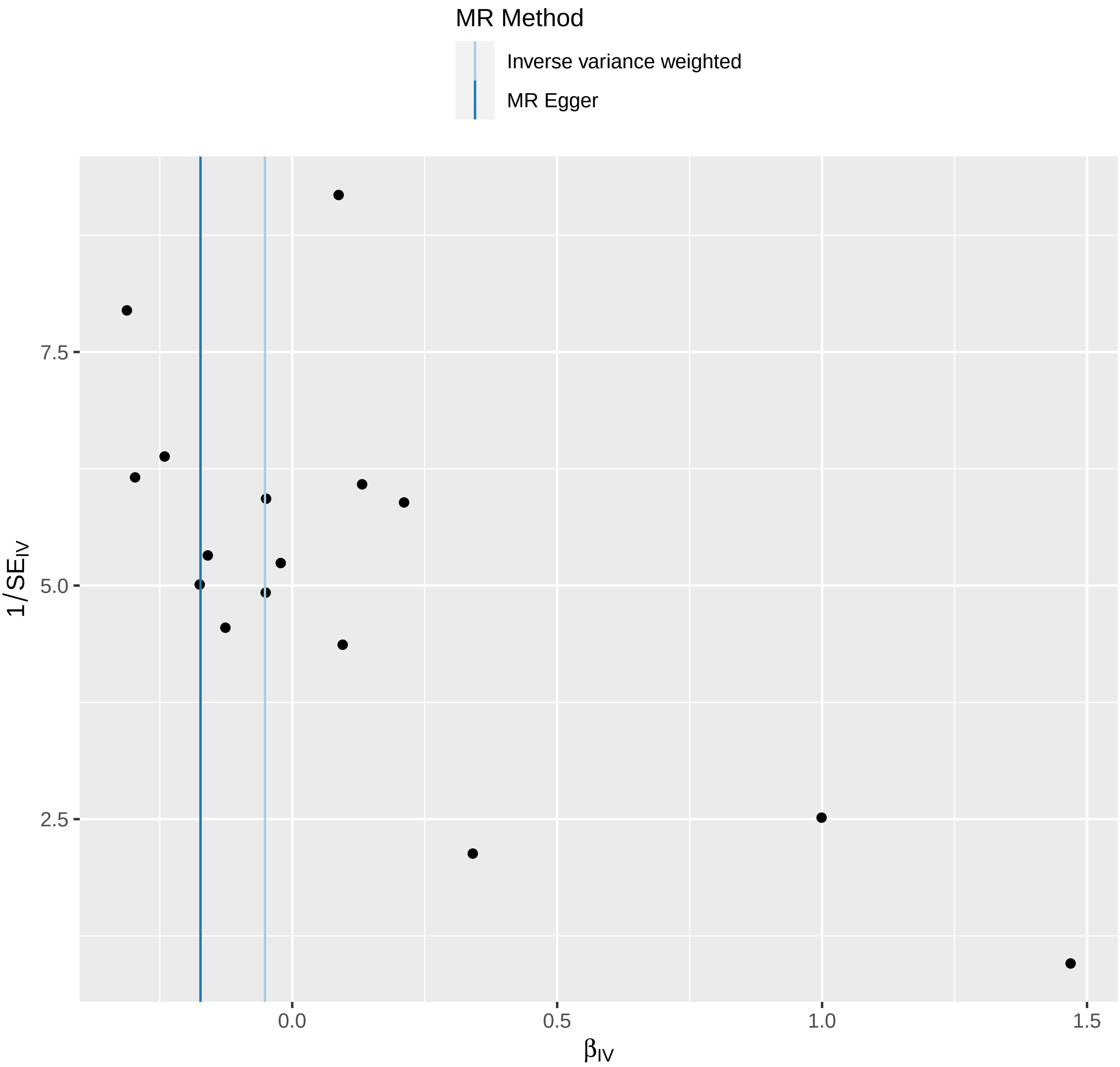

Batch 3−526 : Glycoprotein hormones alpha chain on Diabetic nephropathy (more control exclusions)
with a threshold of 1e−05

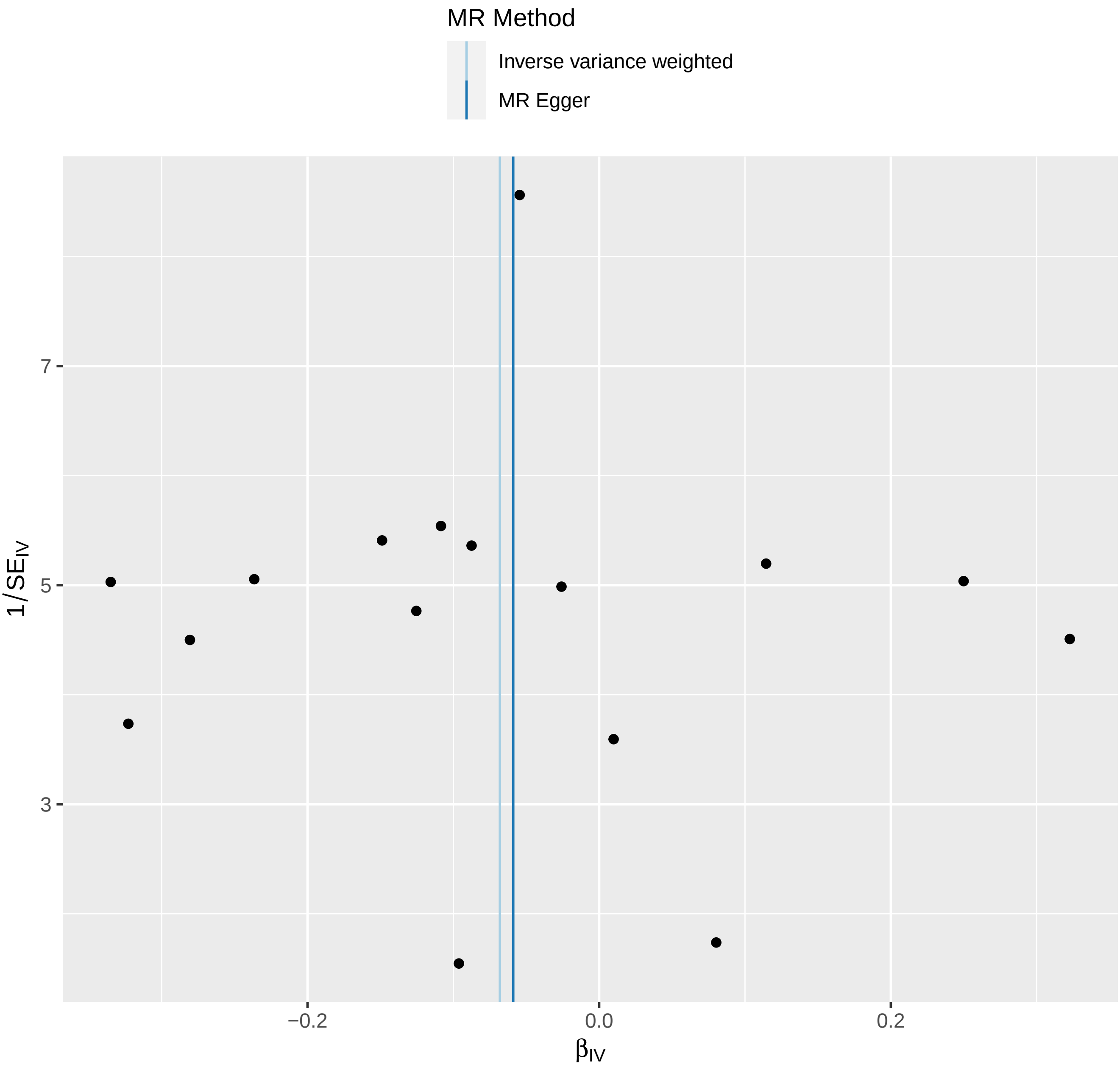

Batch 3−527 : Human Chorionic Gonadotropin on Diabetic nephropathy (more control exclusions)
with a threshold of 1e−05

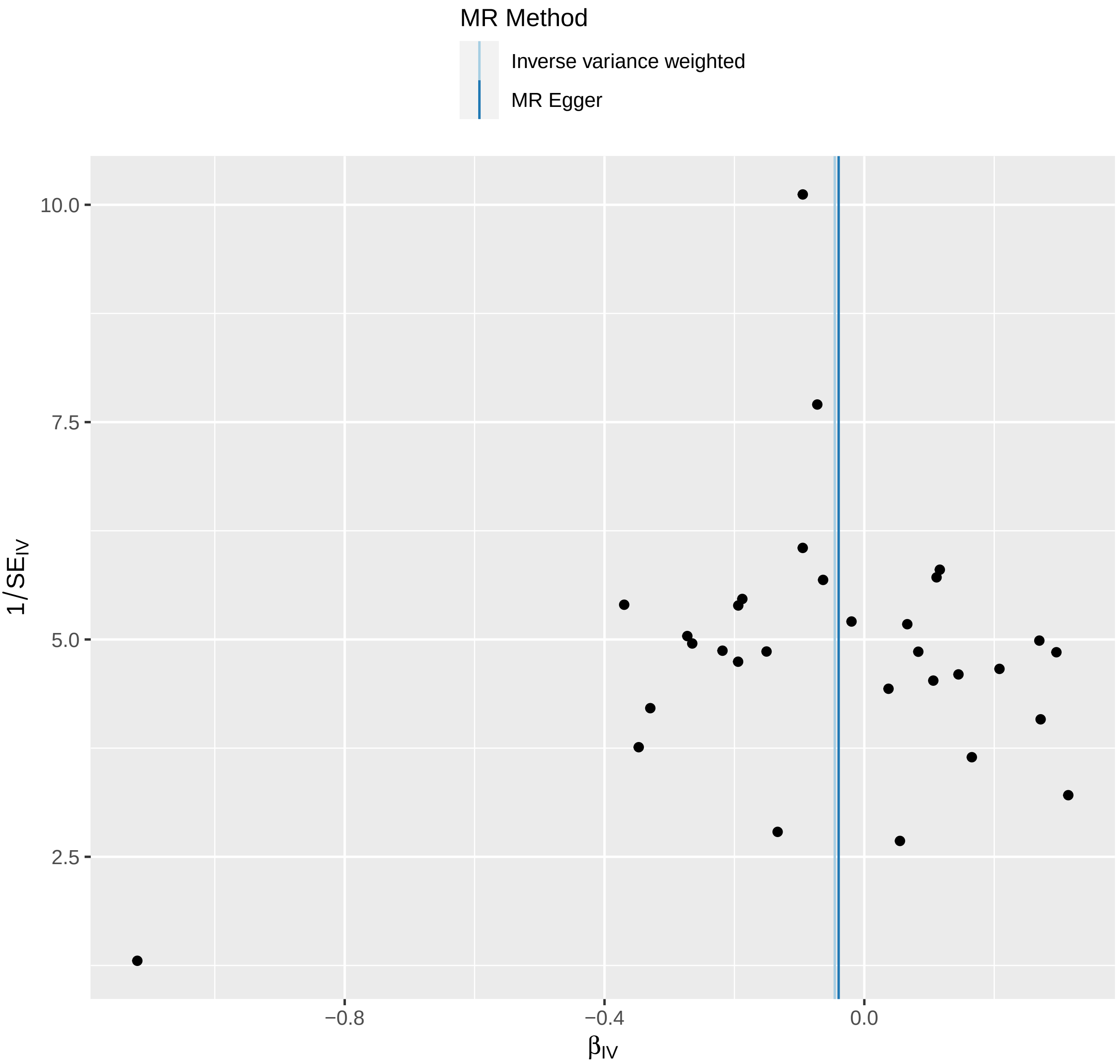

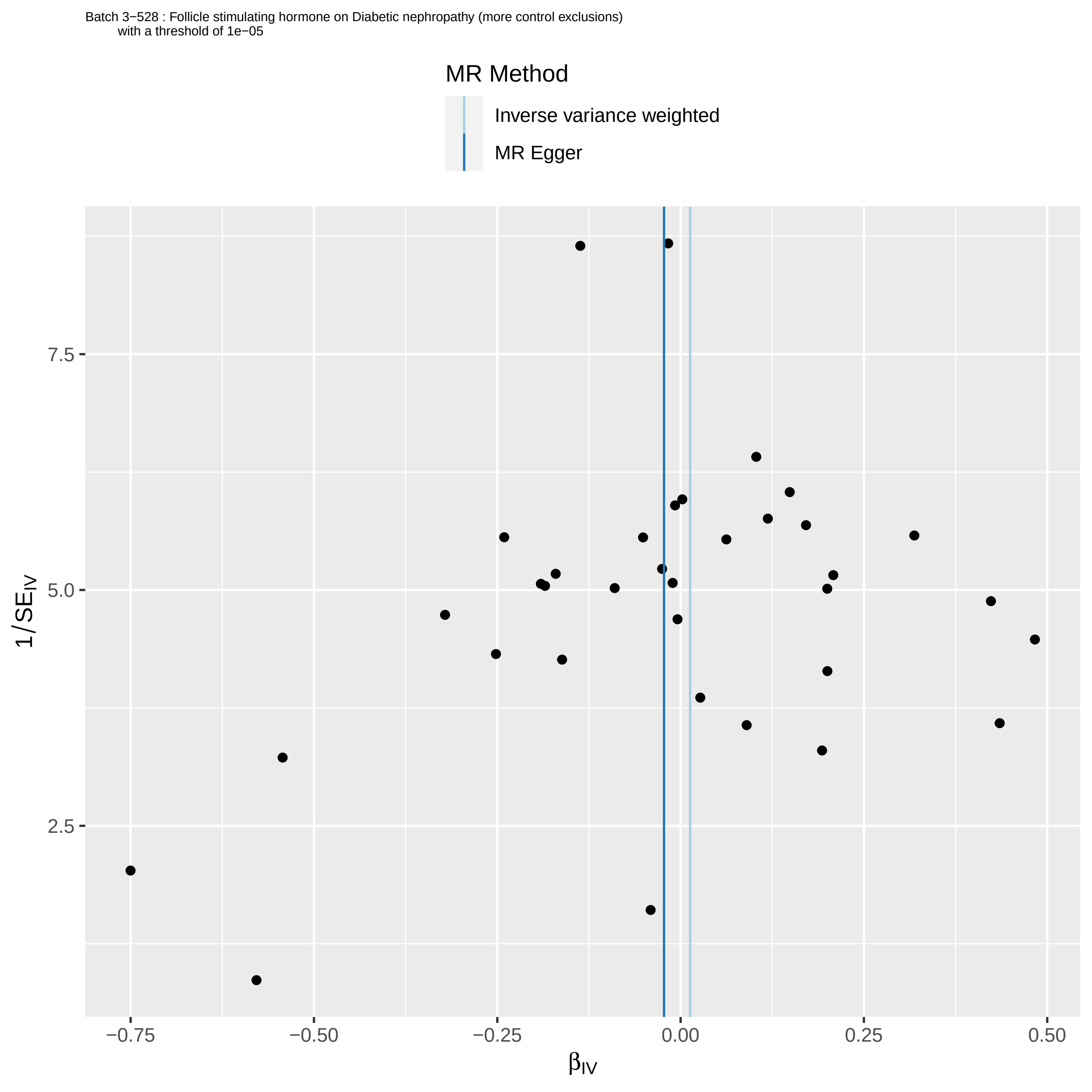

Batch 3−528 : Follicle stimulating hormone on Diabetic nephropathy (more control exclusions)
with a threshold of 1e−05
MR Method
Inverse variance weighted
MR Egger
7.5
5.0
2.5
1/SE_IV
−0.75
−0.50
−0.25
0.00
0.25
0.50
β_IV

Batch 3−529 : Luteinizing hormone on Diabetic nephropathy (more control exclusions)
with a threshold of 1e−05

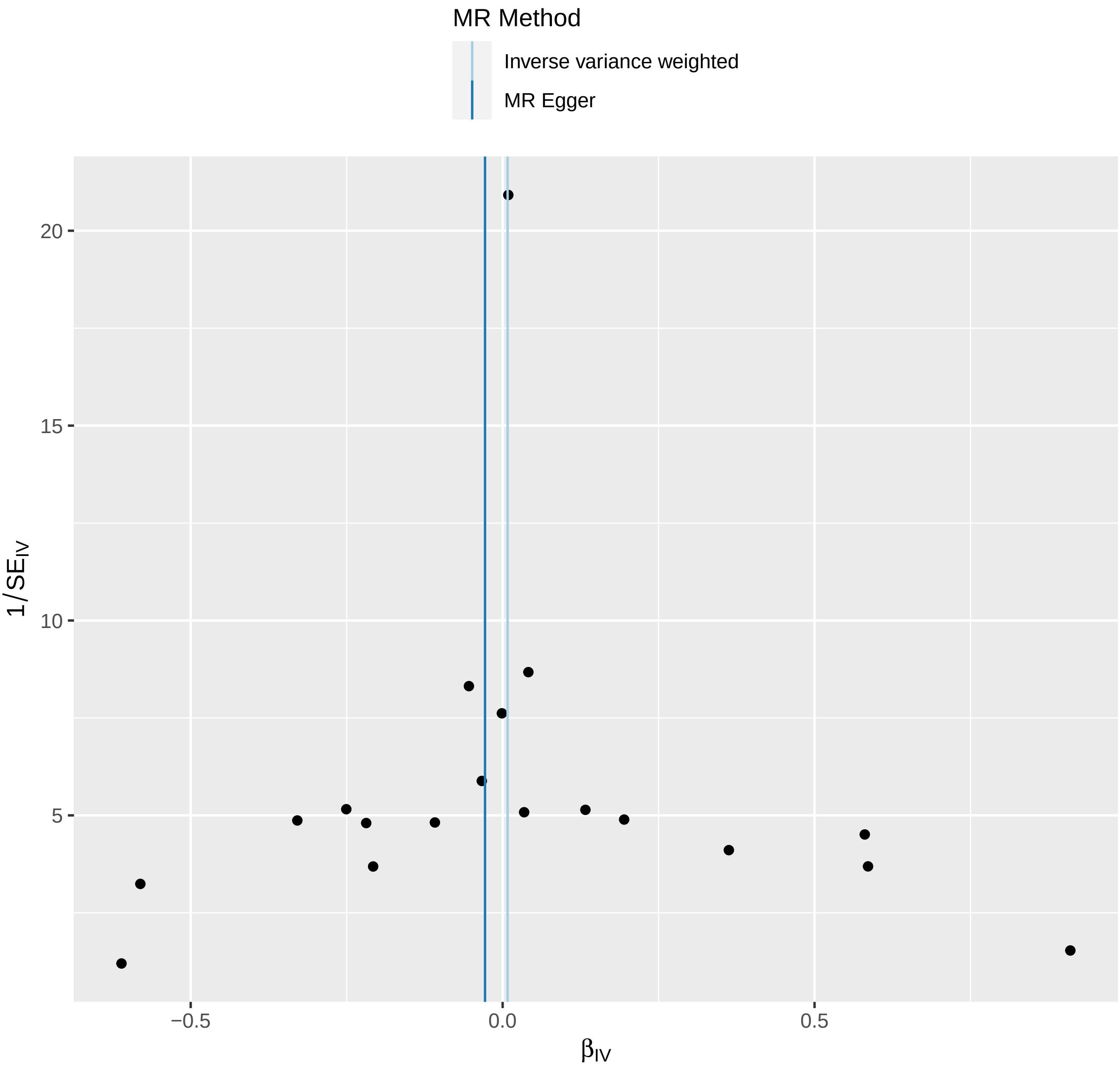

Batch 3−530 : Thyroid Stimulating Hormone on Diabetic nephropathy (more control exclusions)
with a threshold of 1e−05

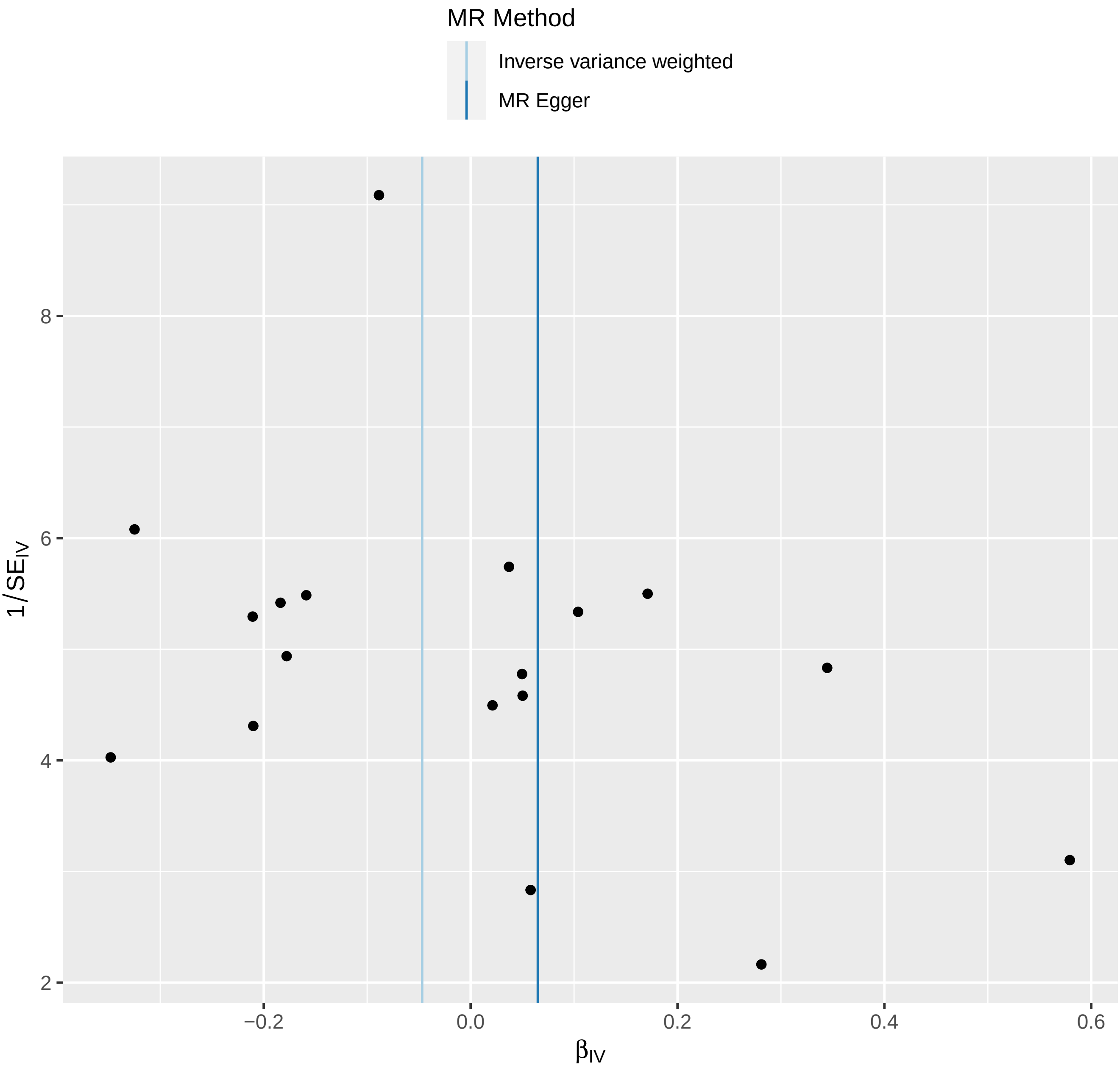

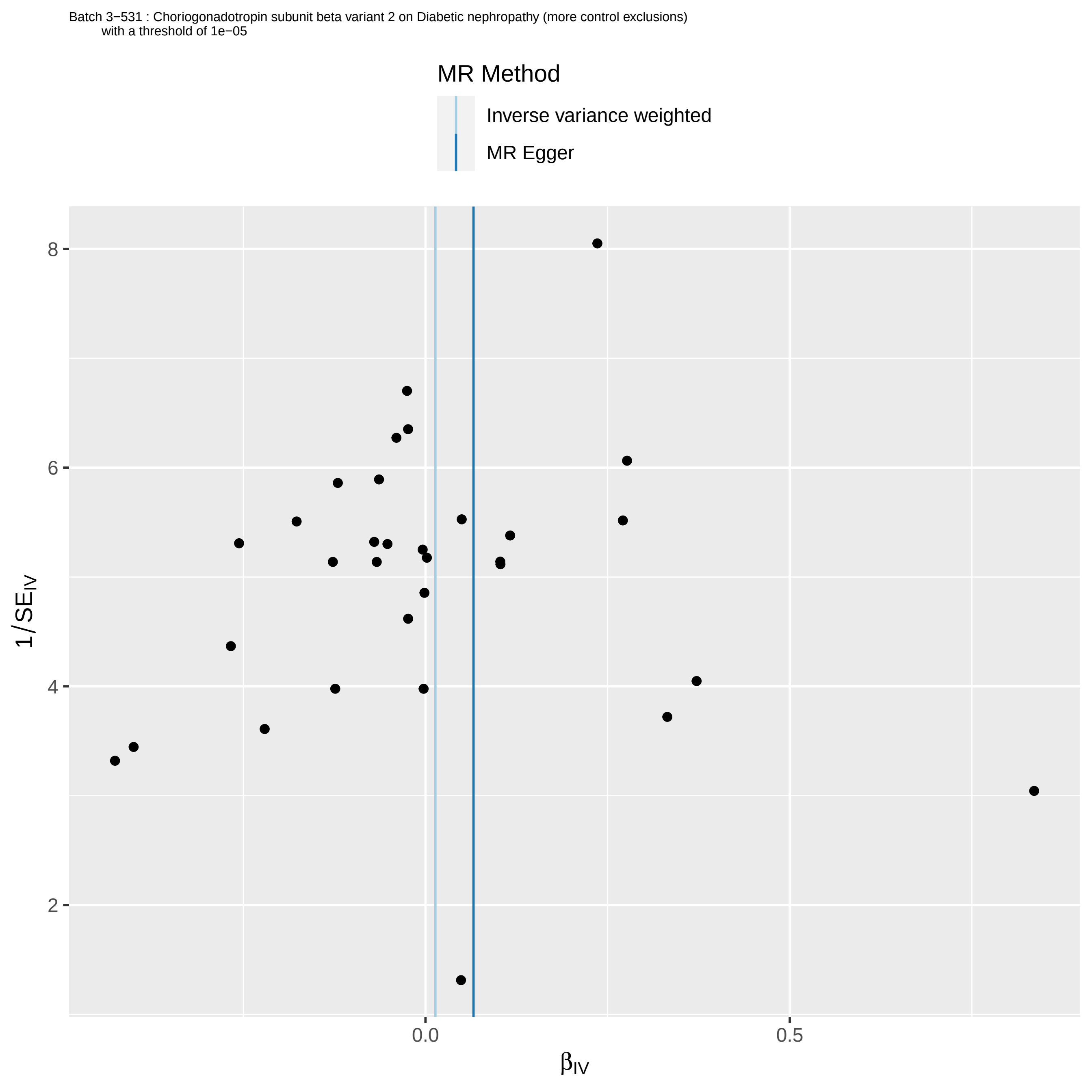
Batch 3−531 : Choriogonadotropin subunit beta variant 2 on Diabetic nephropathy (more control exclusions)
with a threshold of 1e−05
MR Method
Inverse variance weighted
MR Egger
1/SE$_{IV}$
8
6
4
2
0.0
0.5
β$_{IV}$

Batch 3−532 : Cell growth regulator with EF hand domain protein 1 on Diabetic nephropathy (more control exclusions) with a threshold of 1e−05

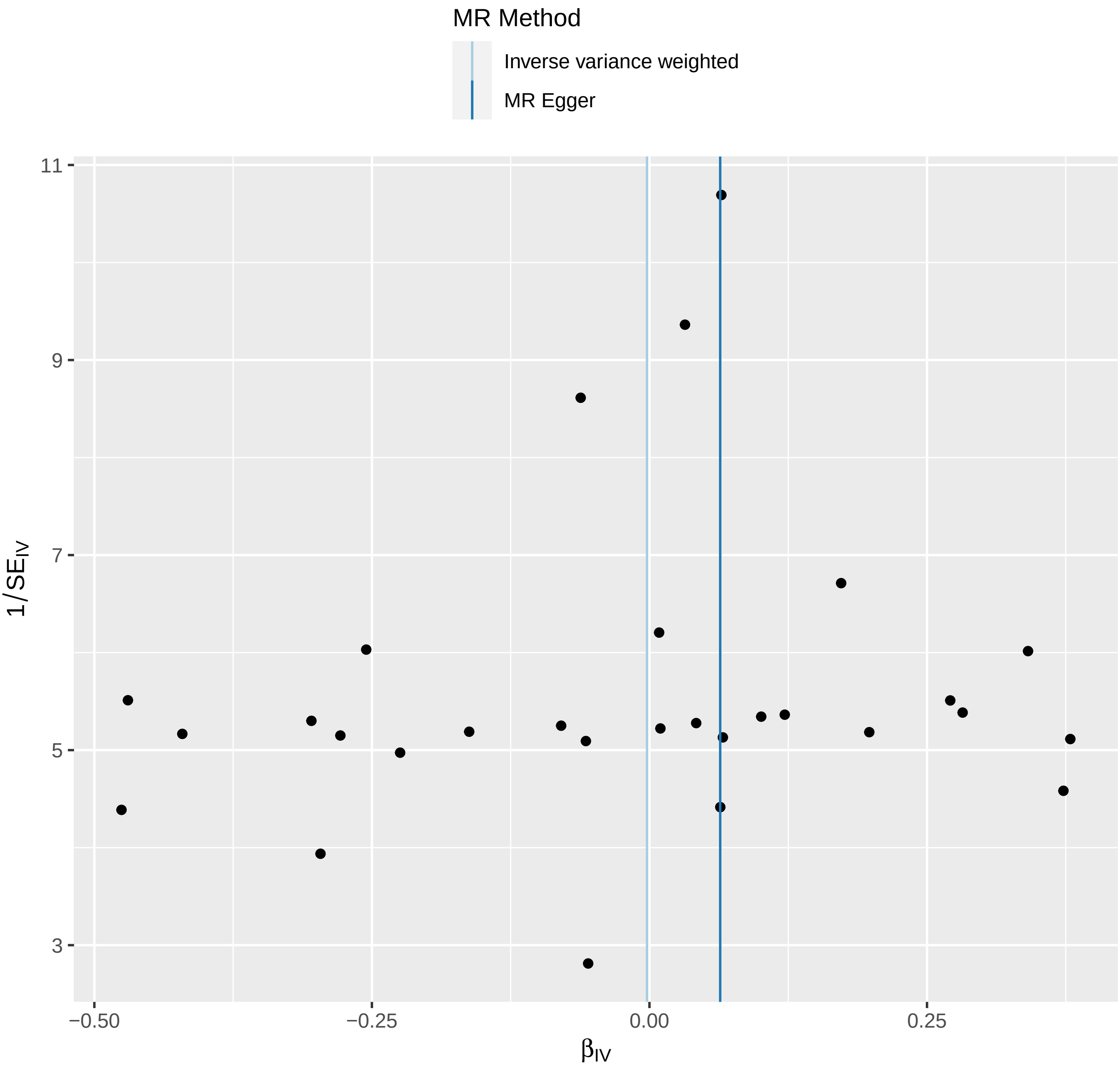

Batch 3−533 : Chondroadherin on Diabetic nephropathy (more control exclusions)
with a threshold of 1e−05

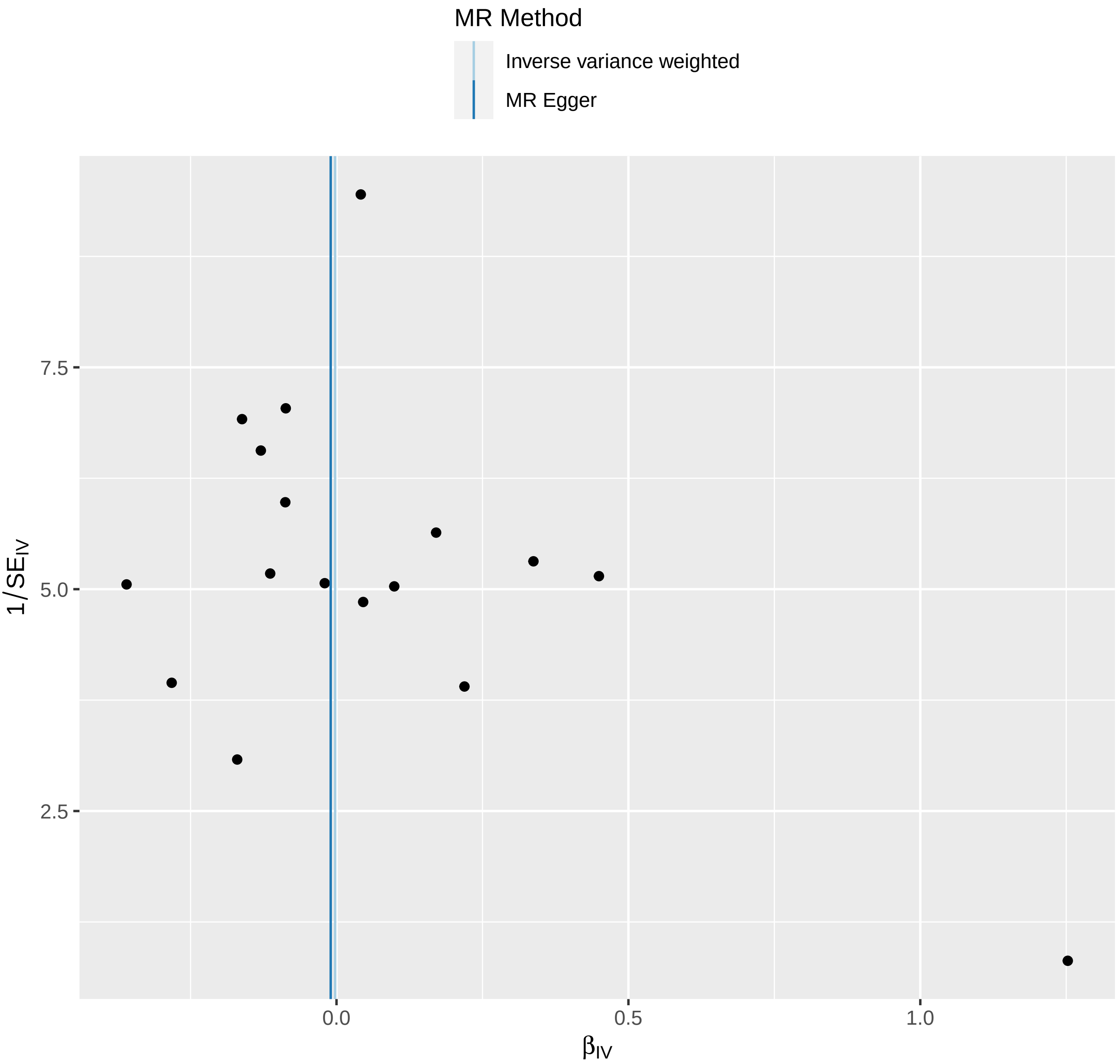

Batch 3−534 : Coiled−coil−helix−coiled−coil−helix domain−containing protein 10, mitochondrial on Diabetic nephropathy (more control exclusions) with a threshold of 1e−05

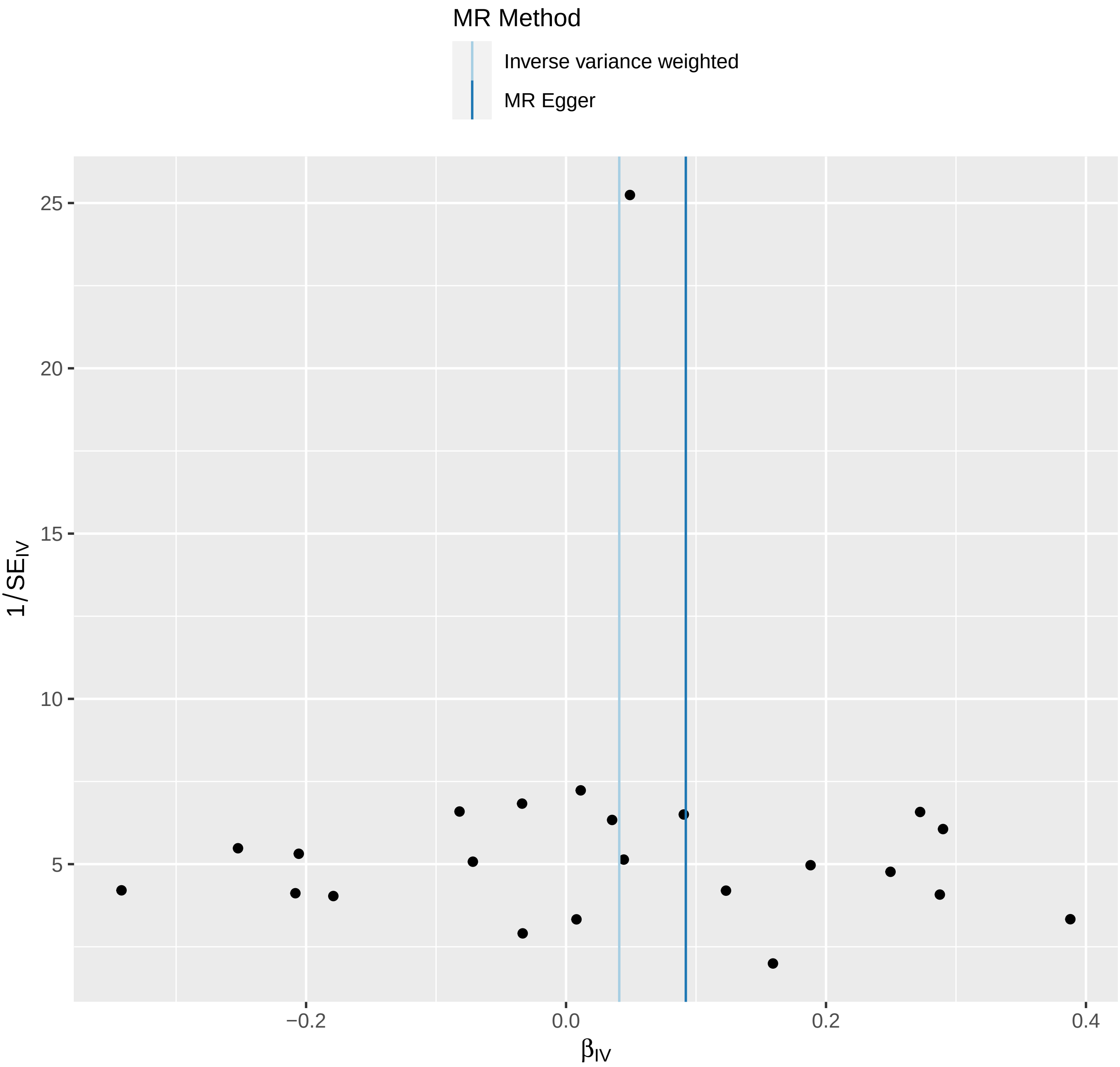

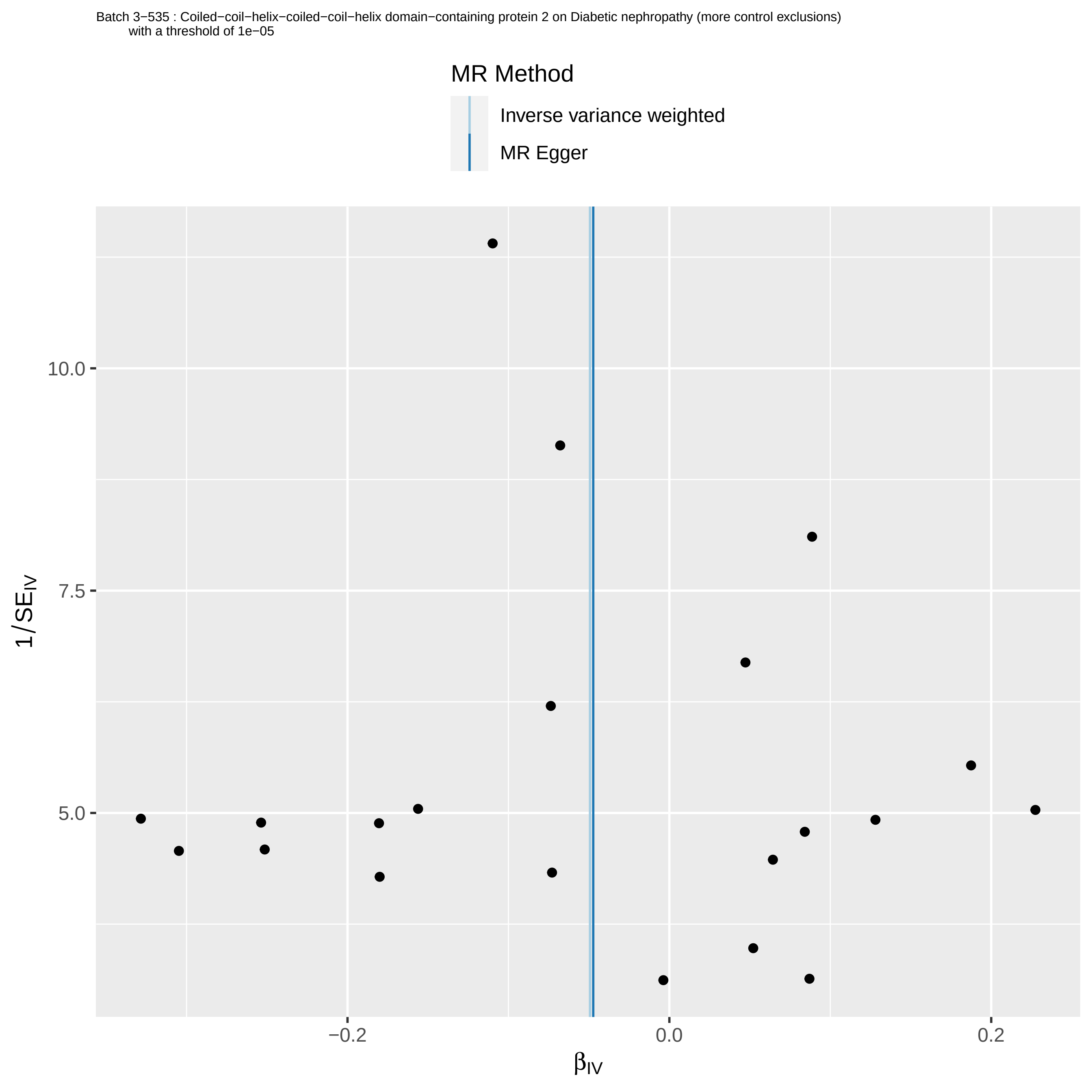
Batch 3−535 : Coiled−coil−helix−coiled−coil−helix domain−containing protein 2 on Diabetic nephropathy (more control exclusions)
with a threshold of 1e−05
MR Method
Inverse variance weighted
MR Egger
10.0
7.5
5.0
1/SE$_{IV}$
−0.2
0.0
0.2
β$_{IV}$

Batch 3−536 : Chromodomain−helicase−DNA−binding protein 7 on Diabetic nephropathy (more control exclusions)
with a threshold of 1e−05

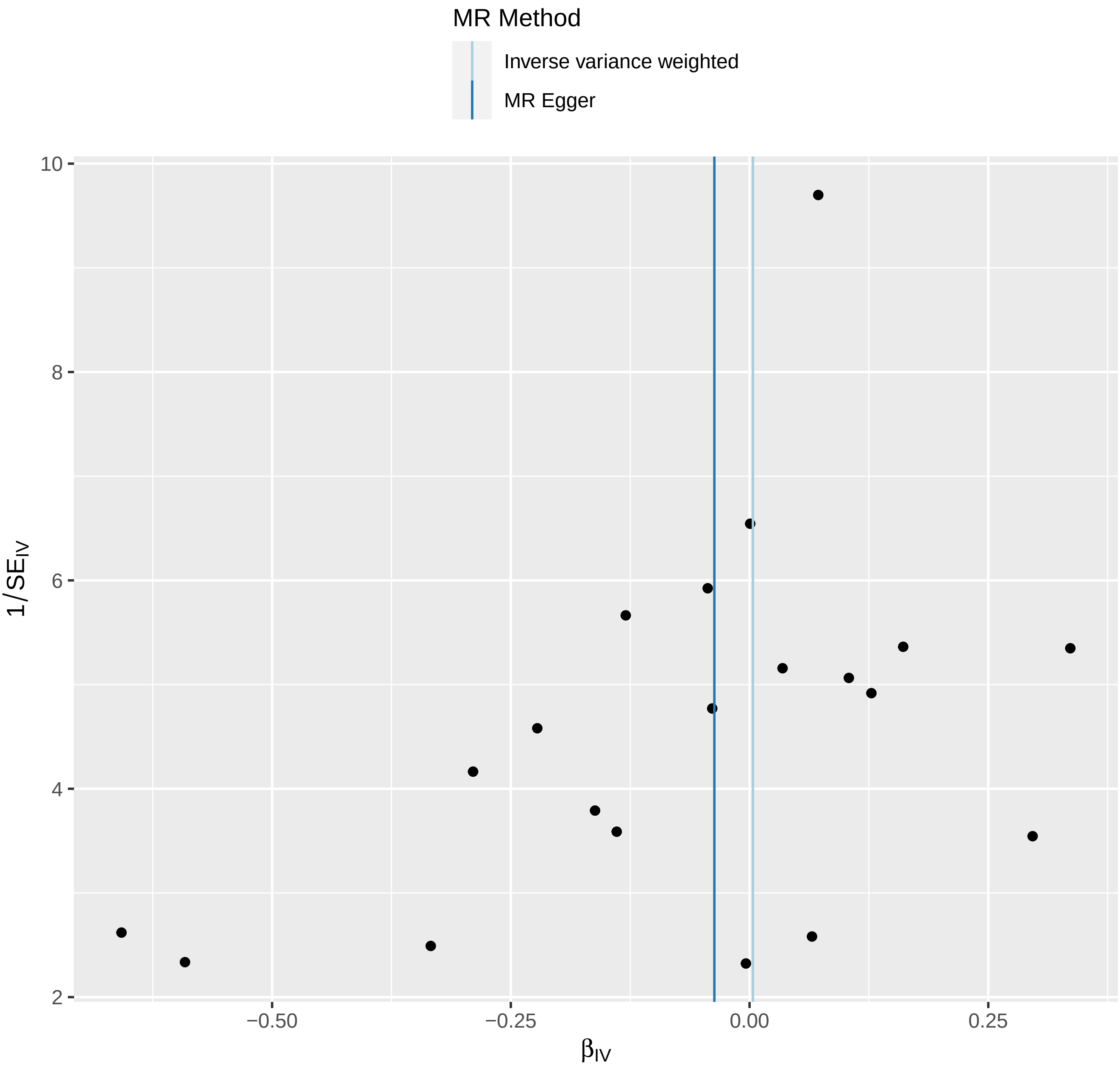

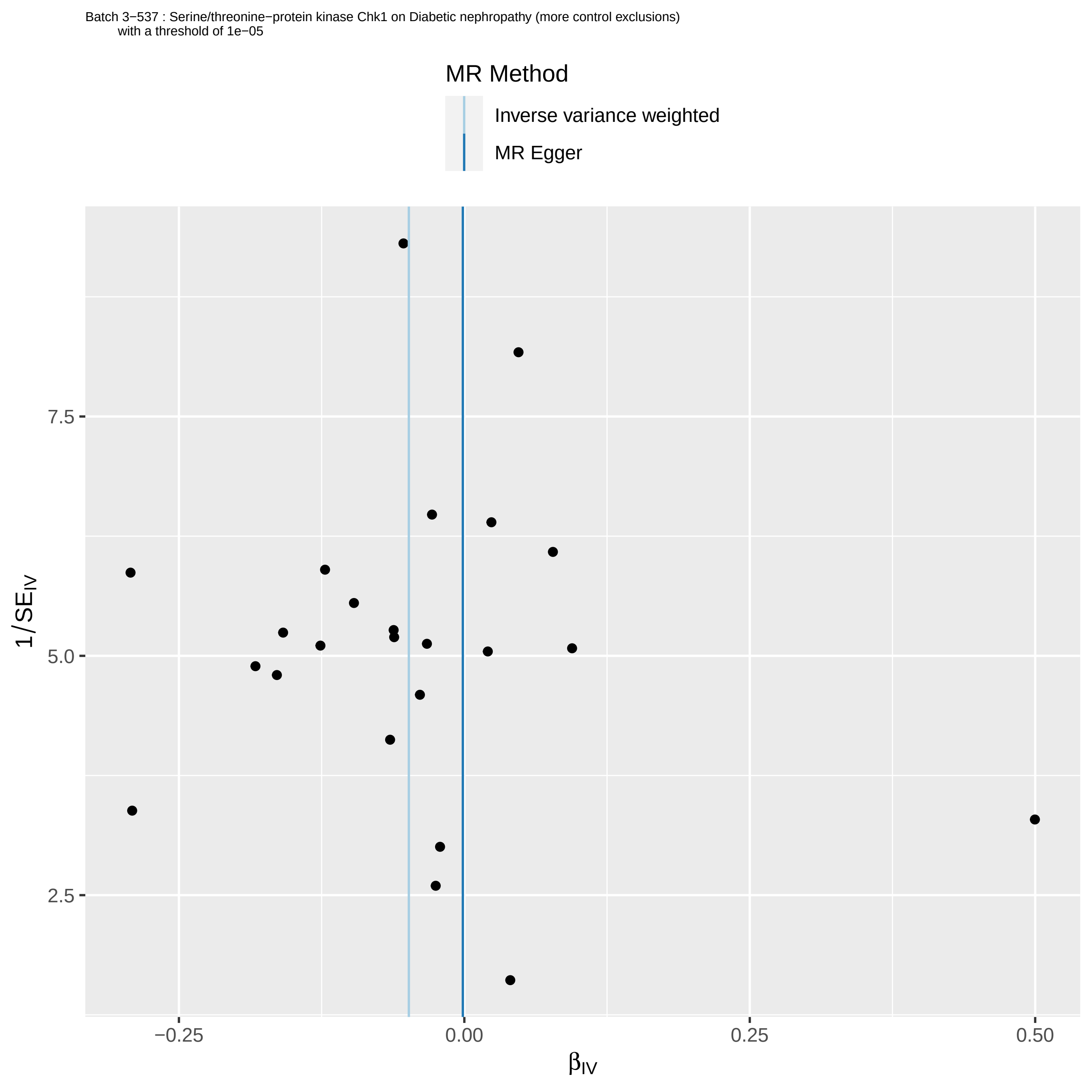

Batch 3−537 : Serine/threonine−protein kinase Chk1 on Diabetic nephropathy (more control exclusions)
with a threshold of 1e−05
MR Method
Inverse variance weighted
MR Egger
7.5
5.0
2.5
1/SE$_{IV}$
−0.25
0.00
0.25
0.50
β$_{IV}$

Batch 3−538 : Chromogranin−A on Diabetic nephropathy (more control exclusions)
with a threshold of 1e−05

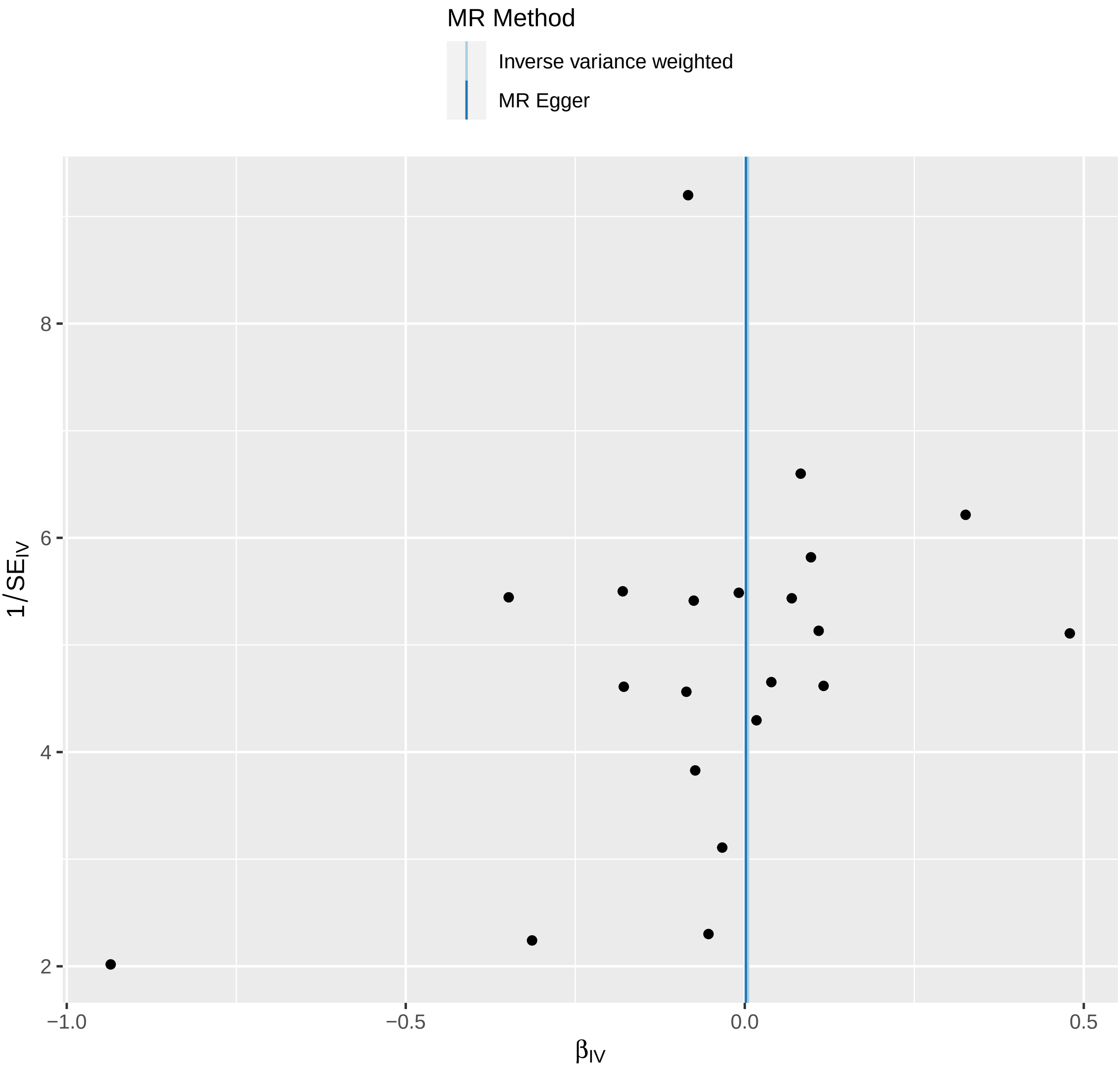

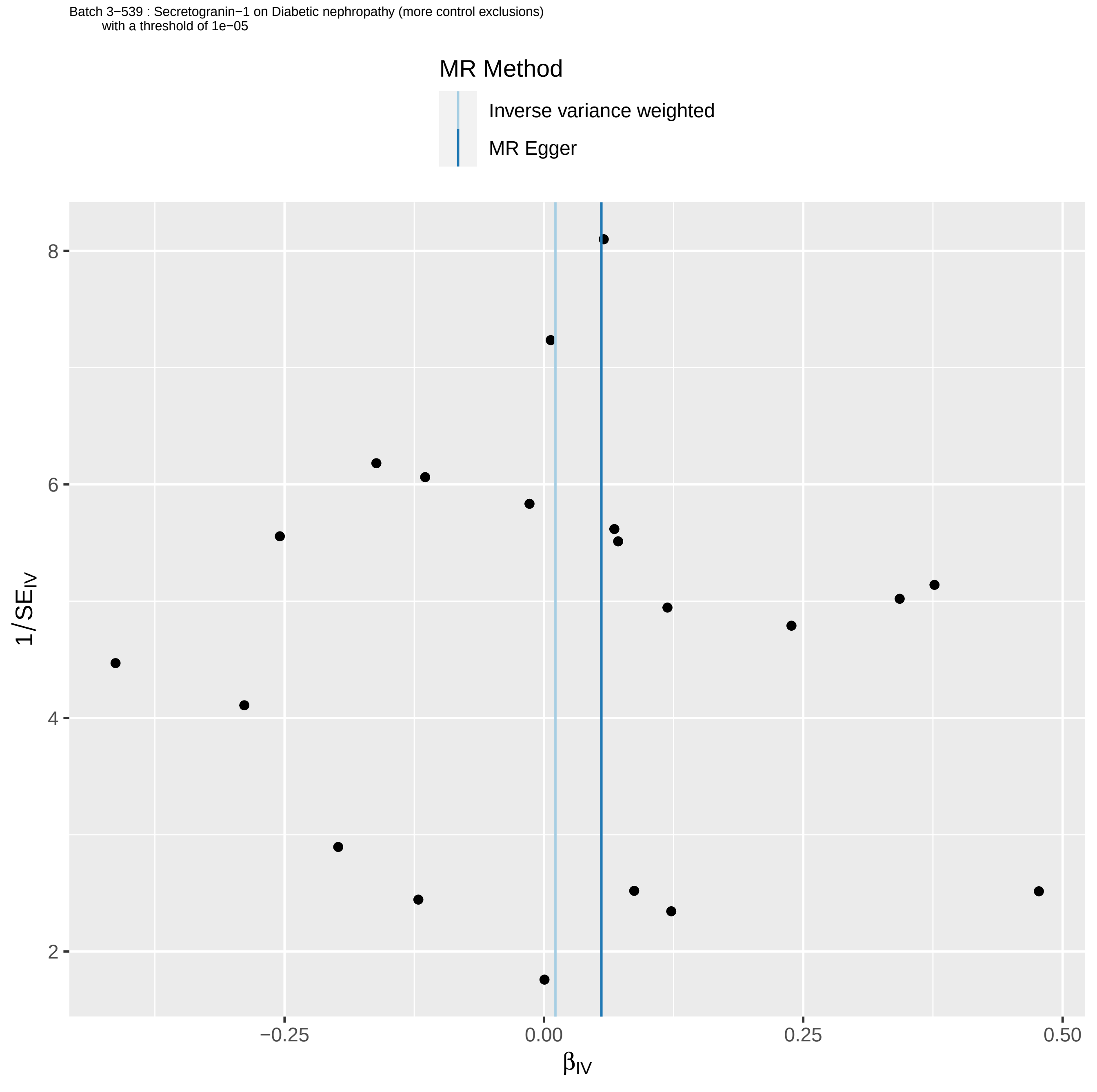
Batch 3−539 : Secretogranin−1 on Diabetic nephropathy (more control exclusions)
with a threshold of 1e−05
MR Method
Inverse variance weighted
MR Egger
8
6
4
2
$1/SE_{IV}$
−0.25
0.00
0.25
0.50
β_{IV}

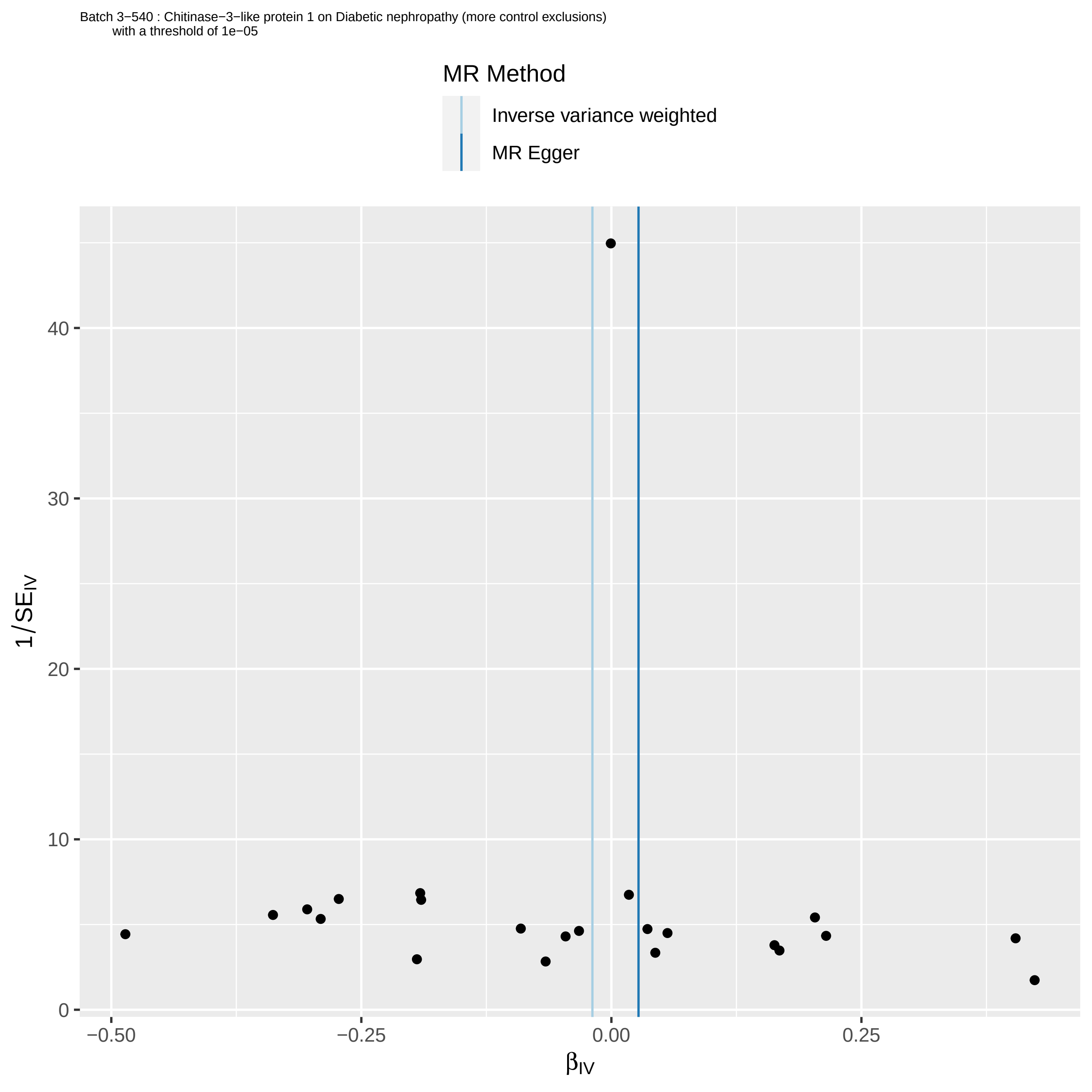

Batch 3−540 : Chitinase−3−like protein 1 on Diabetic nephropathy (more control exclusions)
with a threshold of 1e−05
MR Method
Inverse variance weighted
MR Egger
40
30
20
10
0
1/SE$_{IV}$
−0.50
−0.25
0.00
0.25
β$_{IV}$

Batch 3−541 : Chitinase−3−like protein 2 on Diabetic nephropathy (more control exclusions)
with a threshold of 1e−05

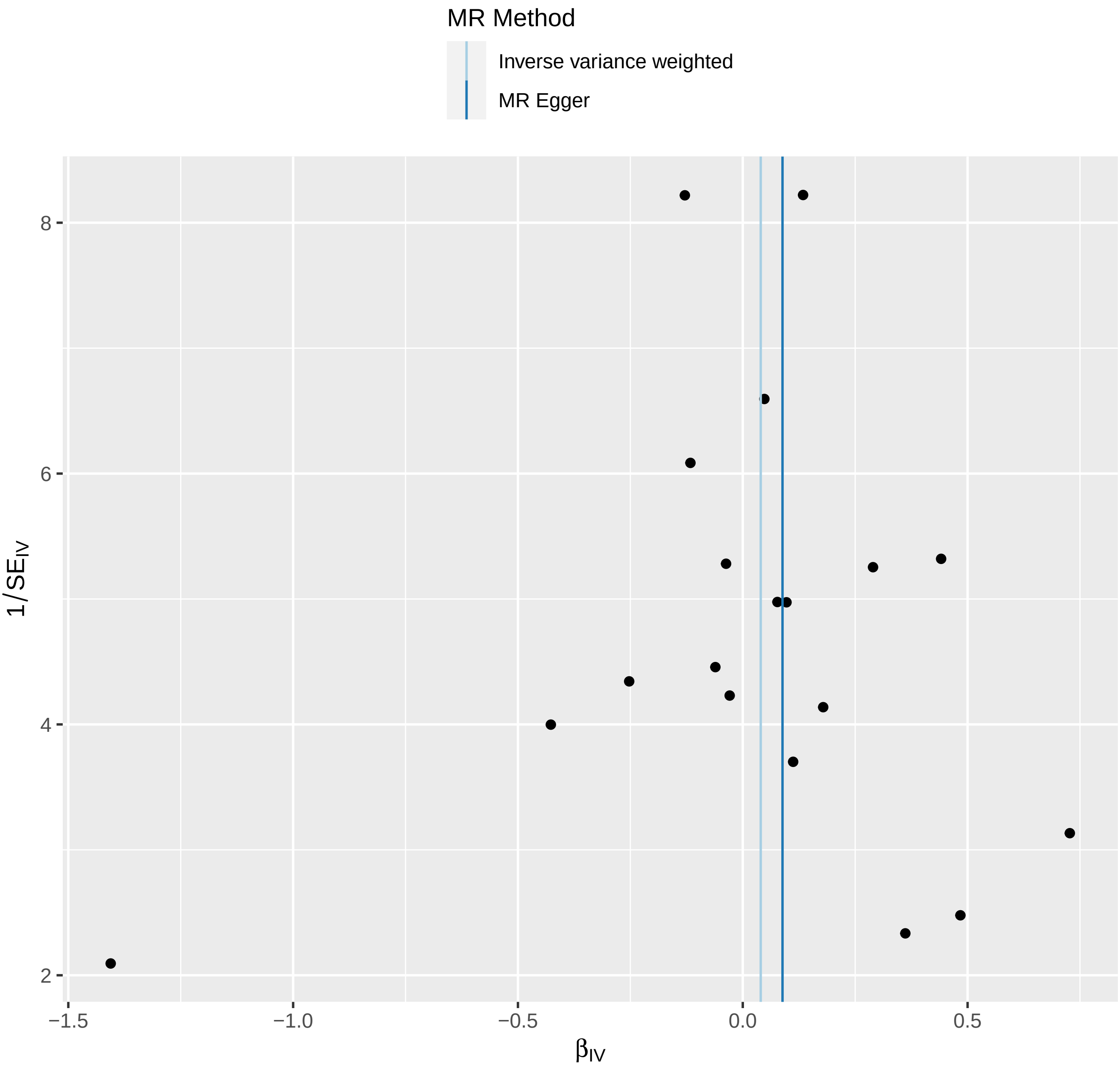

Batch 3−542 : Cysteine−rich hydrophobic domain−containing protein 2 on Diabetic nephropathy (more control exclusions) with a threshold of 1e−05

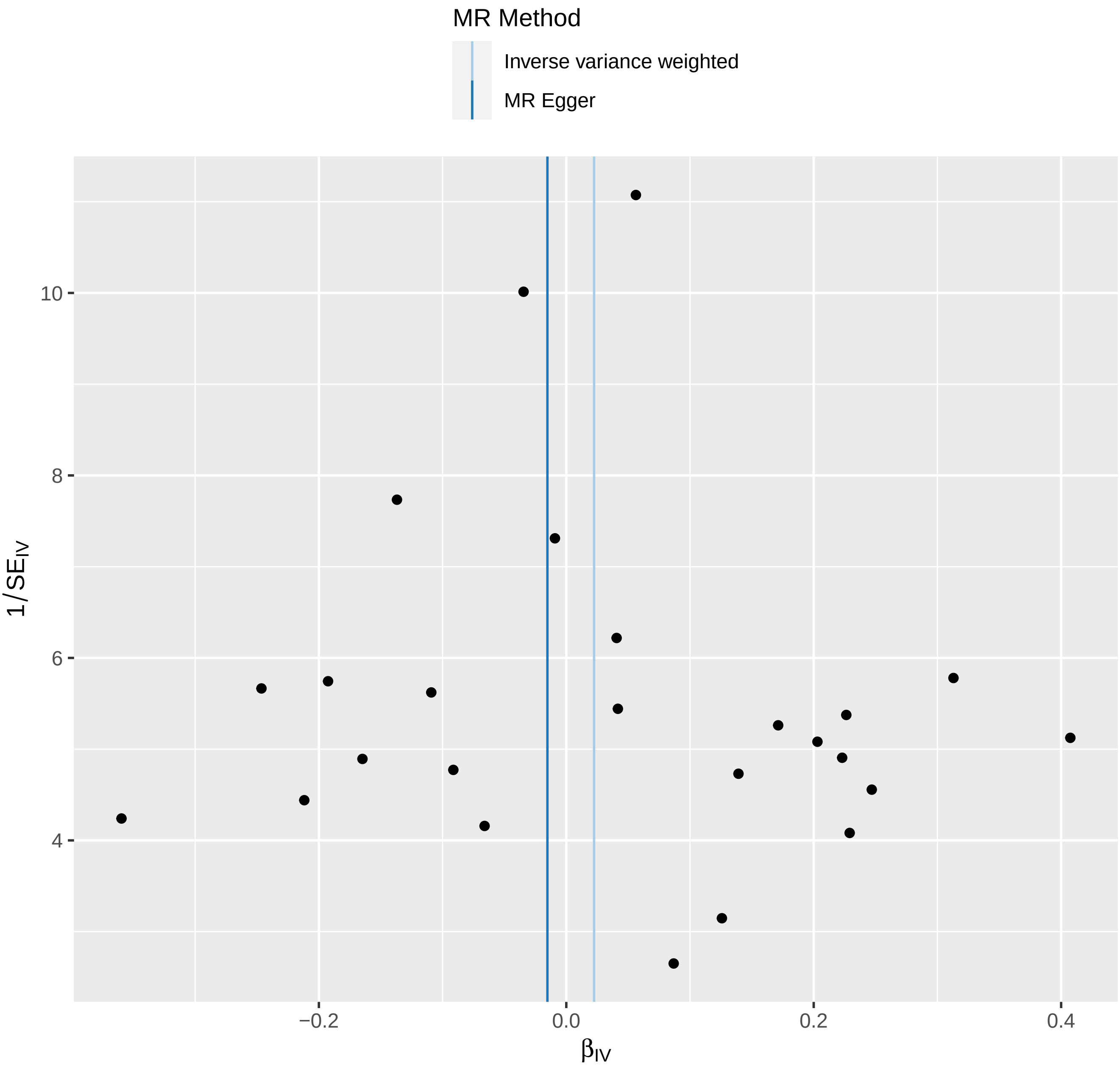

Batch 3−543 : Chitotriosidase−1 on Diabetic nephropathy (more control exclusions)
with a threshold of 1e−05

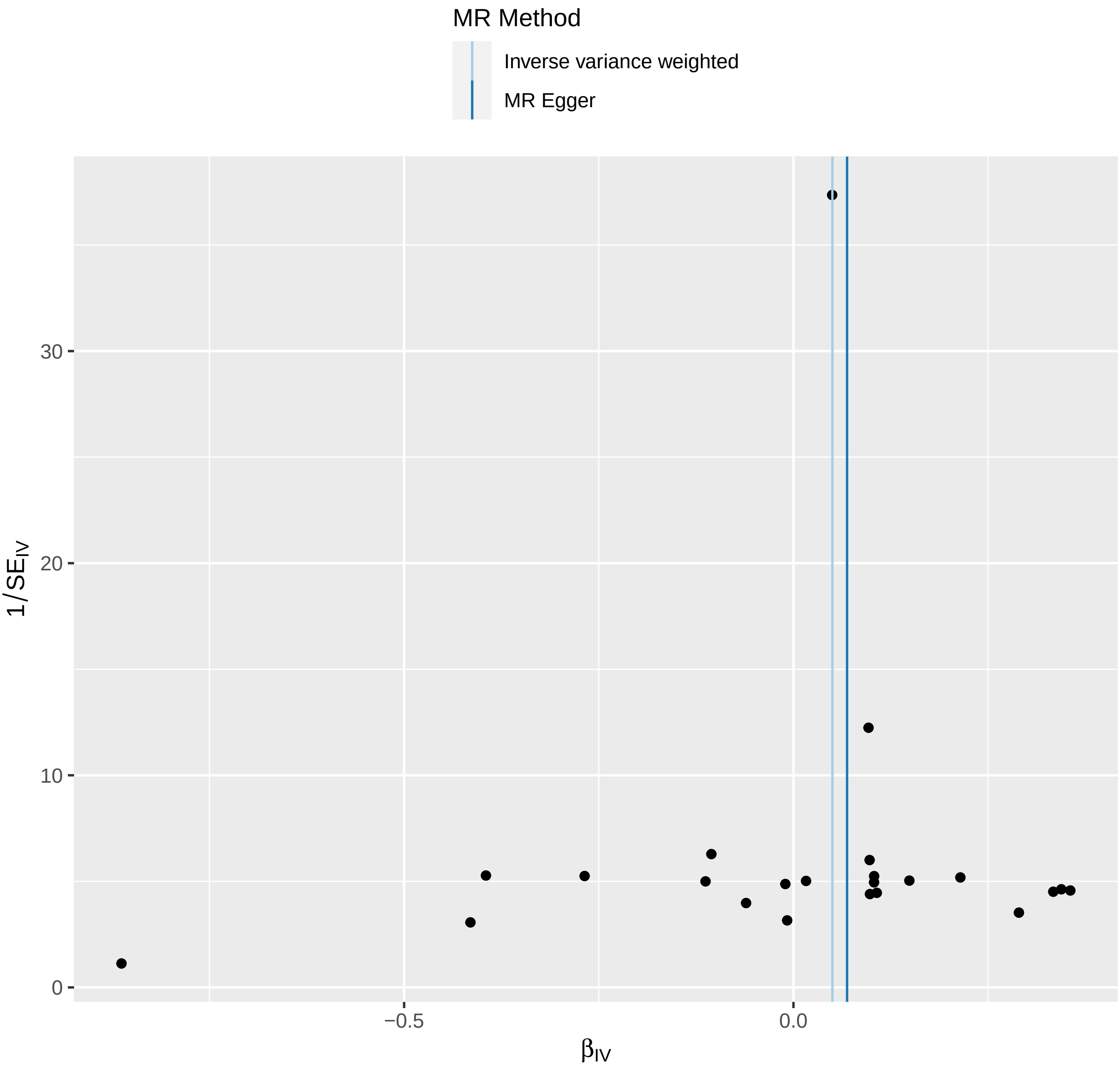

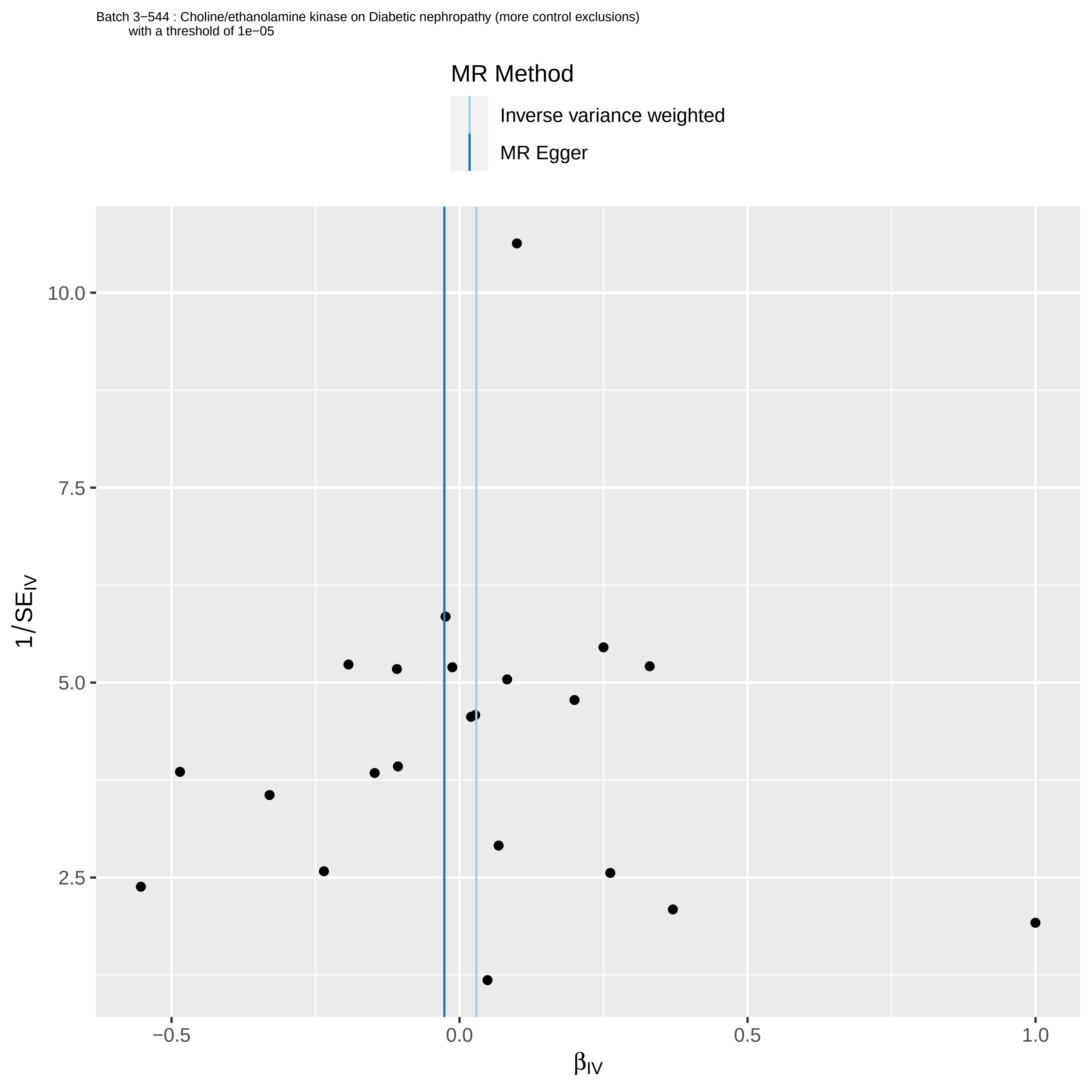
Batch 3−544 : Choline/ethanolamine kinase on Diabetic nephropathy (more control exclusions)
with a threshold of 1e−05
MR Method
Inverse variance weighted
MR Egger
10.0
7.5
5.0
2.5
1/SE_IV
−0.5
0.0
0.5
1.0
β_IV

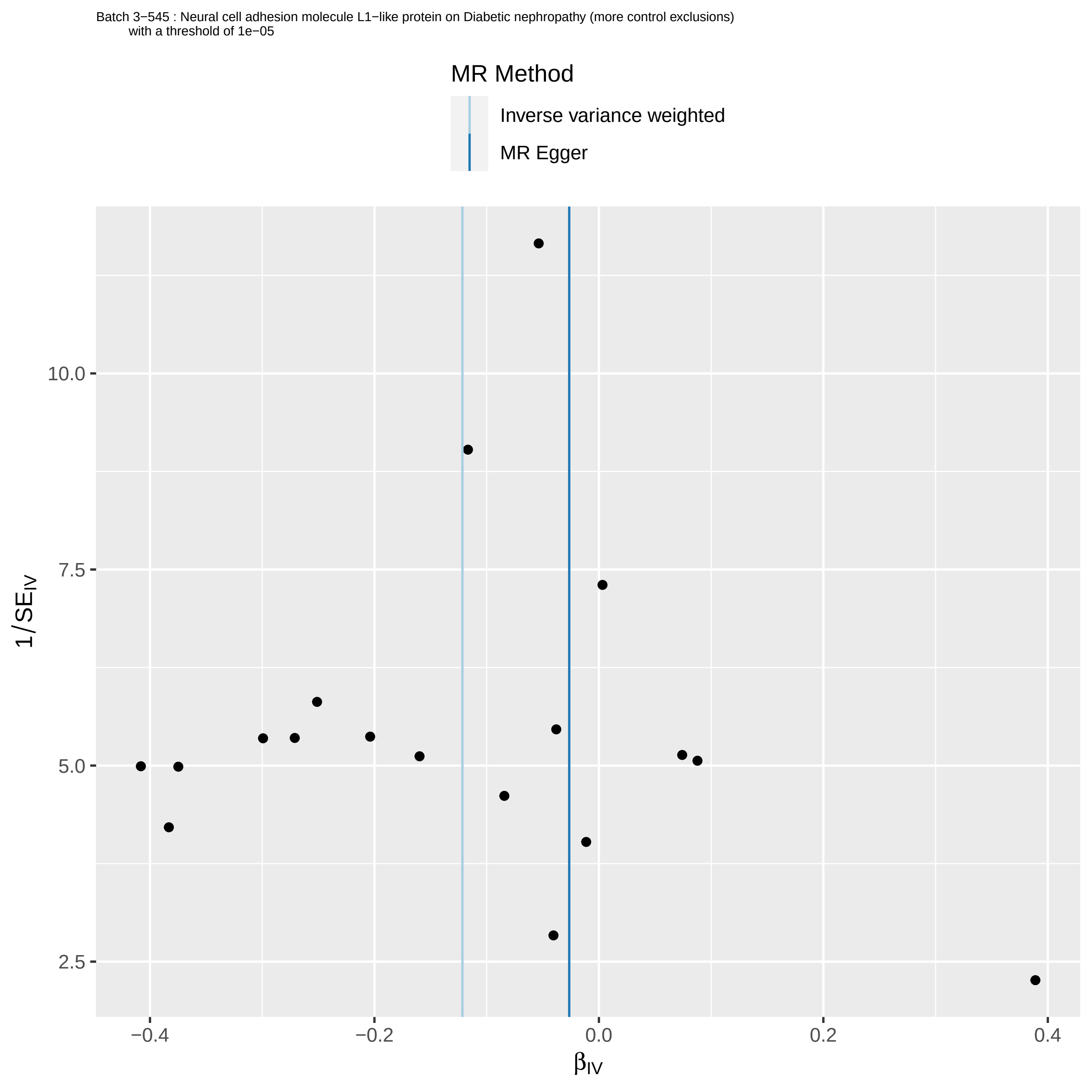
Batch 3−545 : Neural cell adhesion molecule L1−like protein on Diabetic nephropathy (more control exclusions)
with a threshold of 1e−05
MR Method
Inverse variance weighted
MR Egger
10.0
7.5
5.0
2.5
1/SE_IV
−0.4
−0.2
0.0
0.2
0.4
β_IV

Batch 3−546 : Calcineurin B homologous protein 1 on Diabetic nephropathy (more control exclusions)
with a threshold of 1e−05

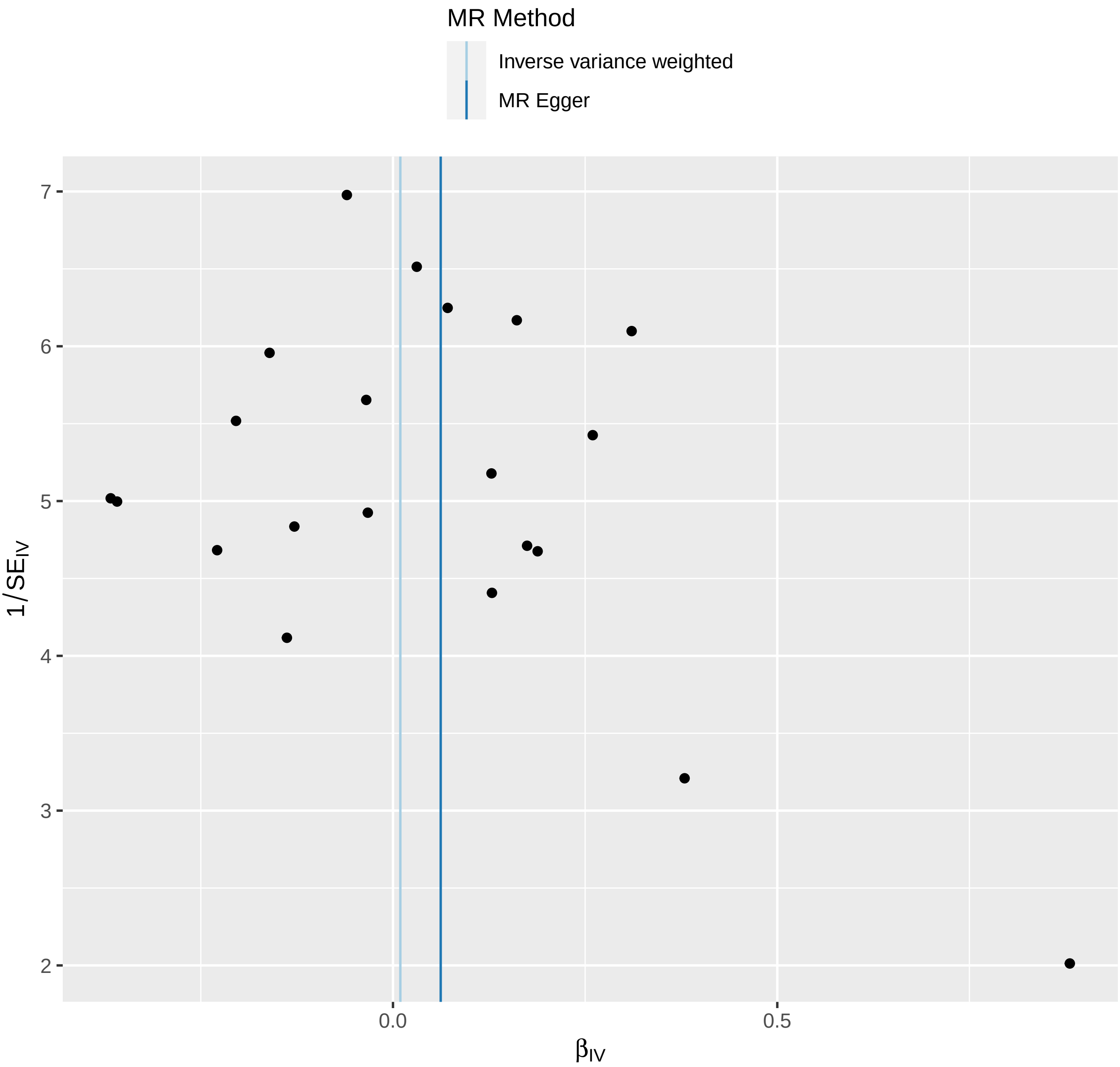

Batch 3−547 : Chordin on Diabetic nephropathy (more control exclusions)
with a threshold of 1e−05

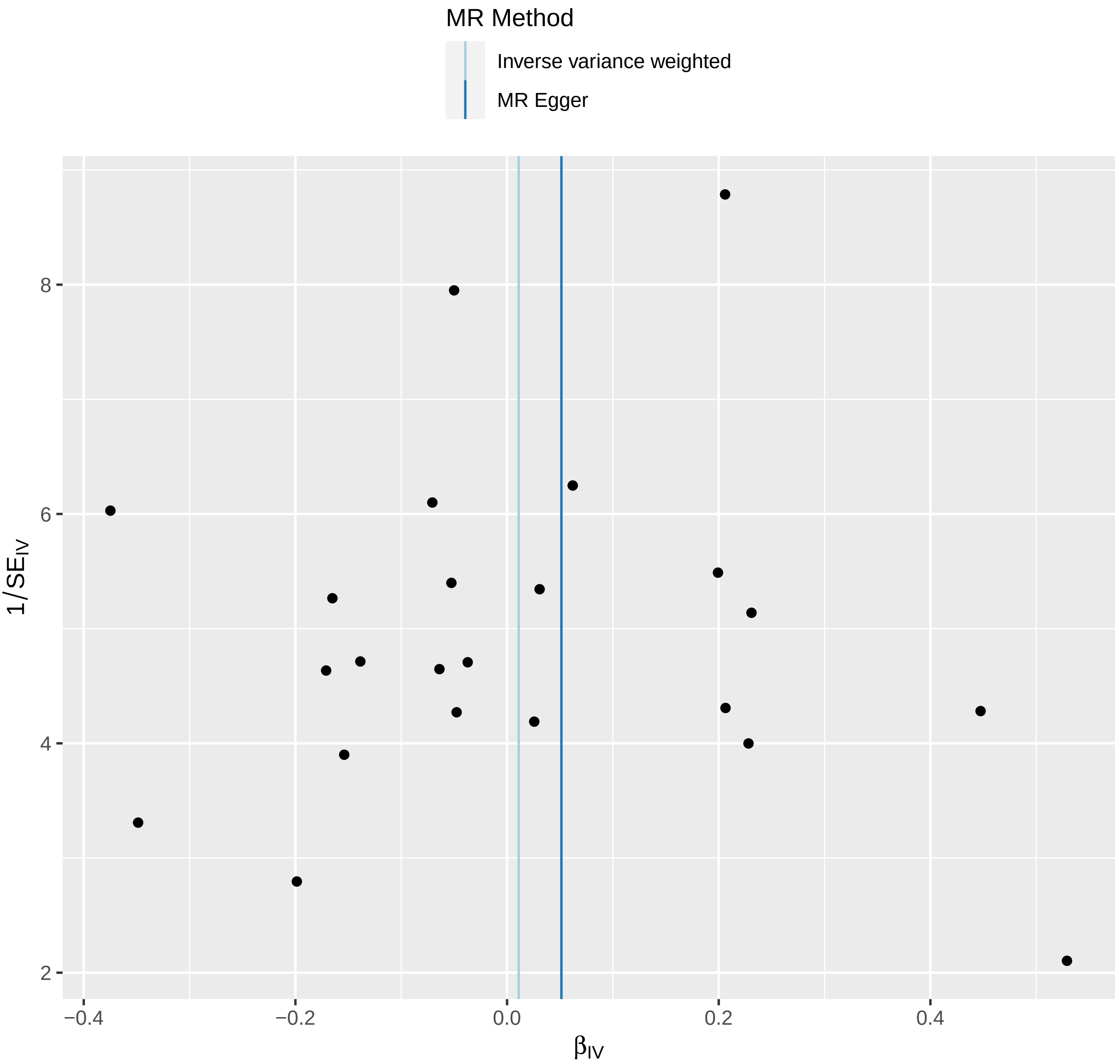

Batch 3−548 : Chordin−like protein 1 on Diabetic nephropathy (more control exclusions)
with a threshold of 1e−05

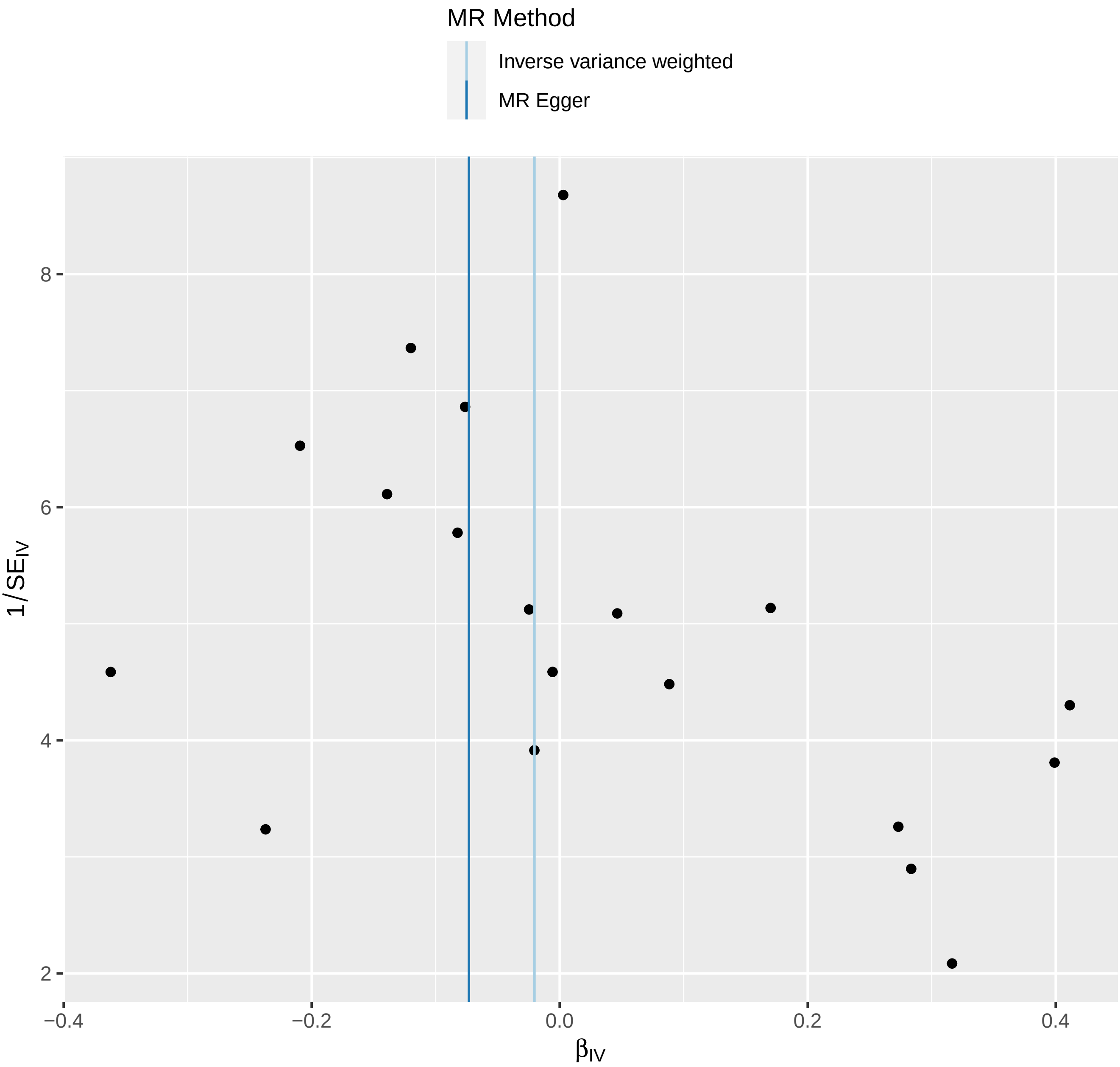

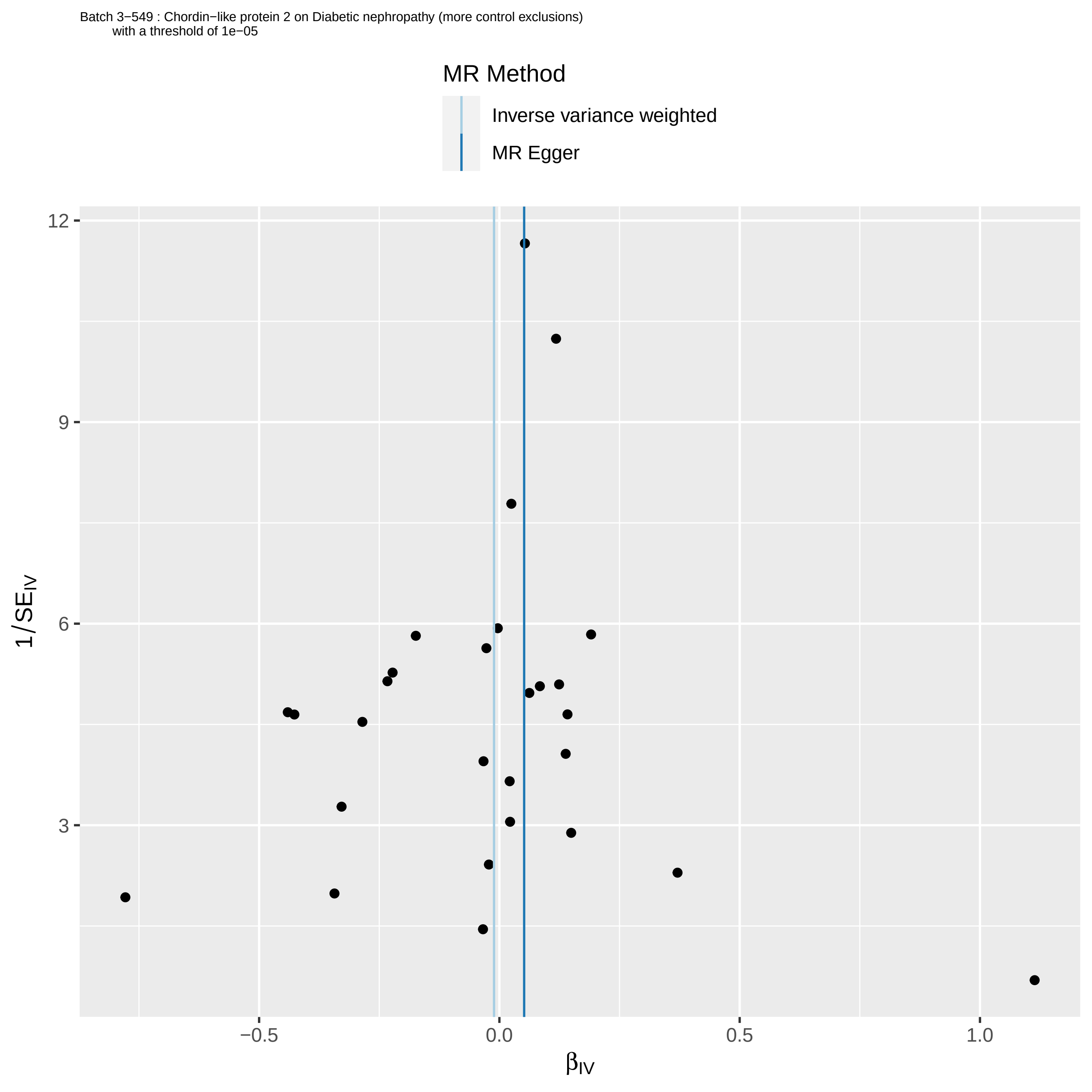

Batch 3−549 : Chordin−like protein 2 on Diabetic nephropathy (more control exclusions)
with a threshold of 1e−05
MR Method
Inverse variance weighted
MR Egger
12
9
6
3
1/SE$_{IV}$
−0.5
0.0
0.5
1.0
β$_{IV}$

Batch 3−550 : Carbohydrate sulfotransferase 11 on Diabetic nephropathy (more control exclusions)
with a threshold of 1e−05

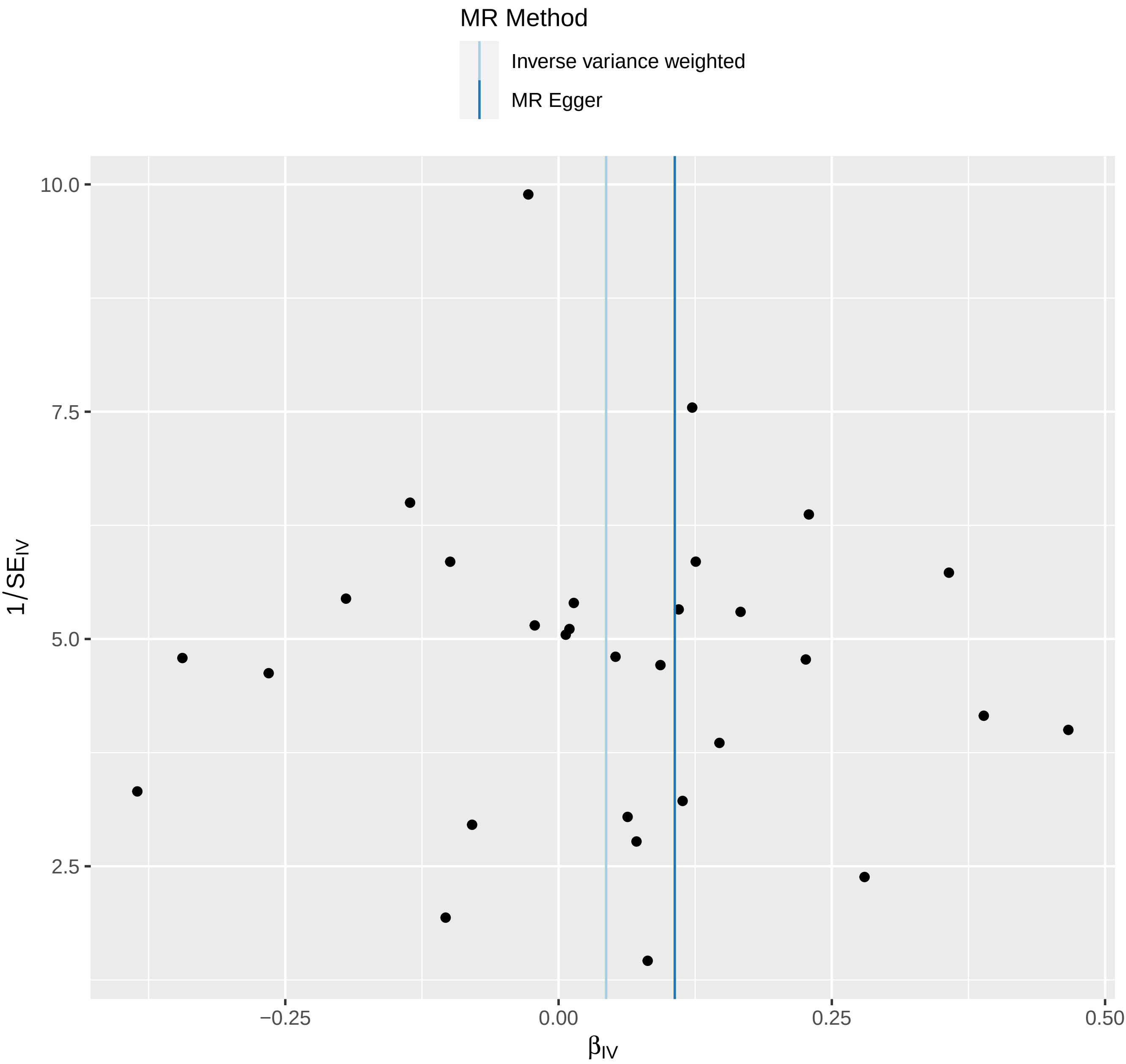

Batch 3−551 : Carbohydrate sulfotransferase 14 on Diabetic nephropathy (more control exclusions)
with a threshold of 1e−05

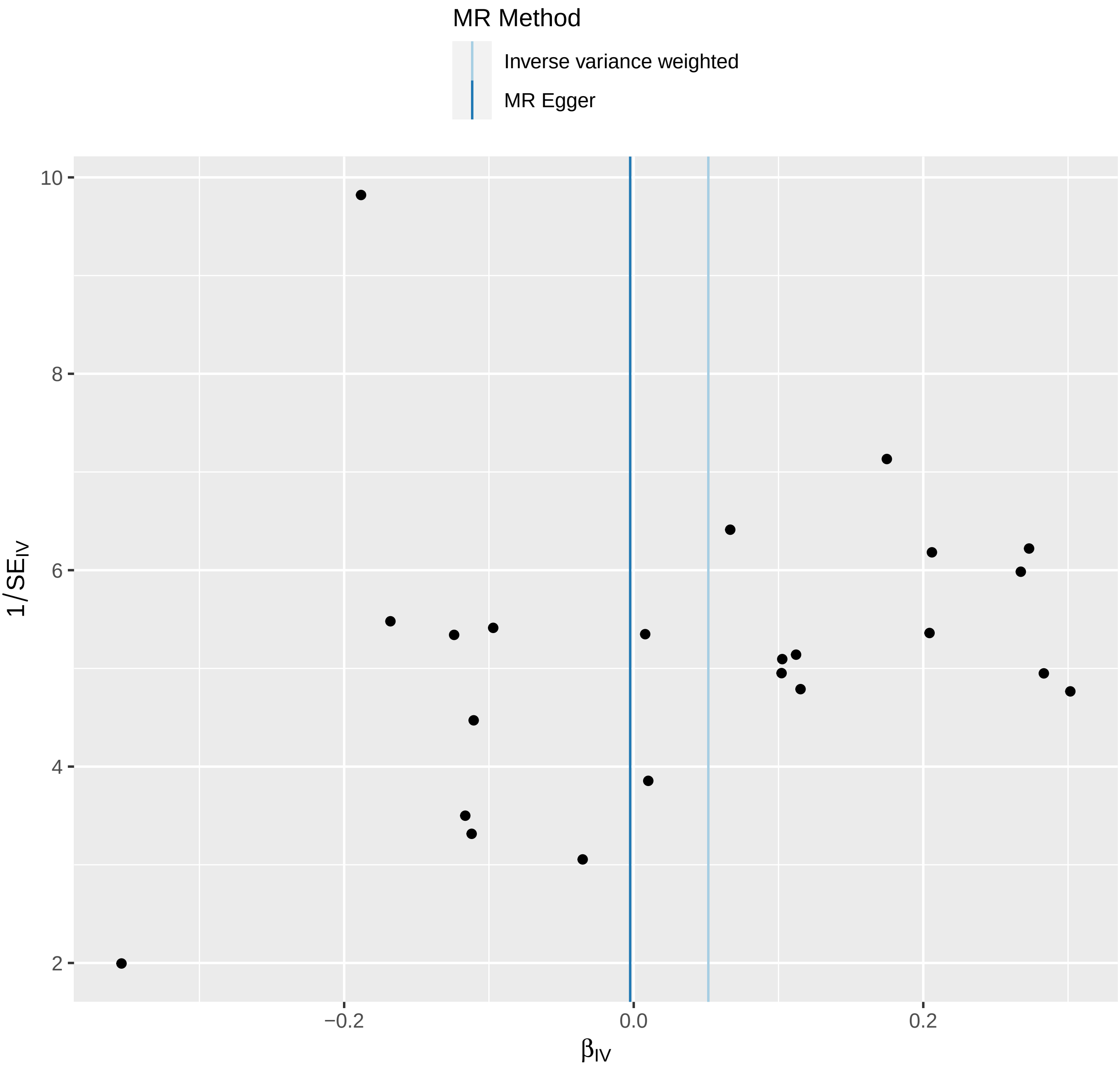

Batch 3−552 : Carbohydrate sulfotransferase 15 on Diabetic nephropathy (more control exclusions)
with a threshold of 1e−05

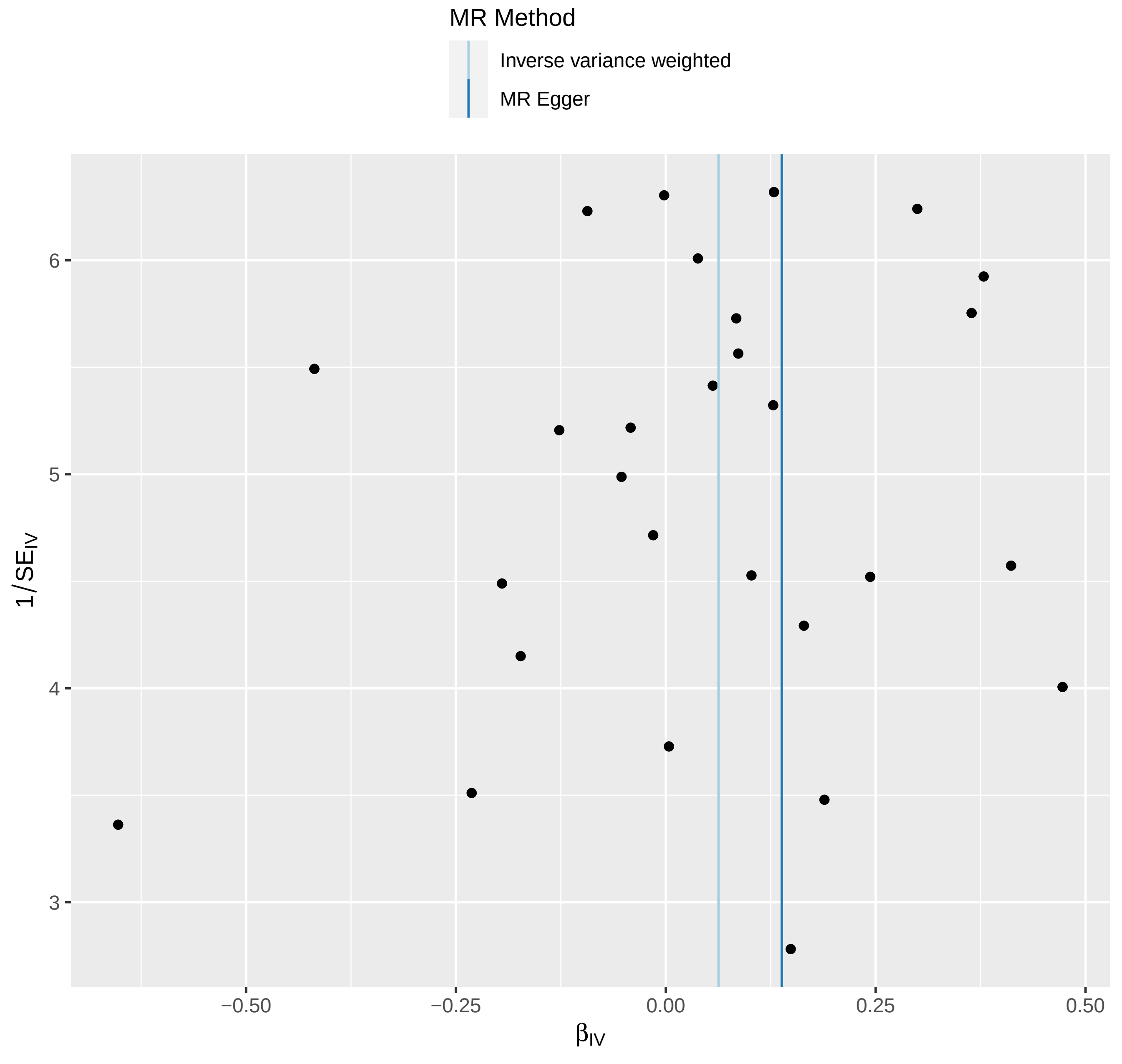

Batch 3−553 : Carbohydrate sulfotransferase 15 on Diabetic nephropathy (more control exclusions)
with a threshold of 1e−05

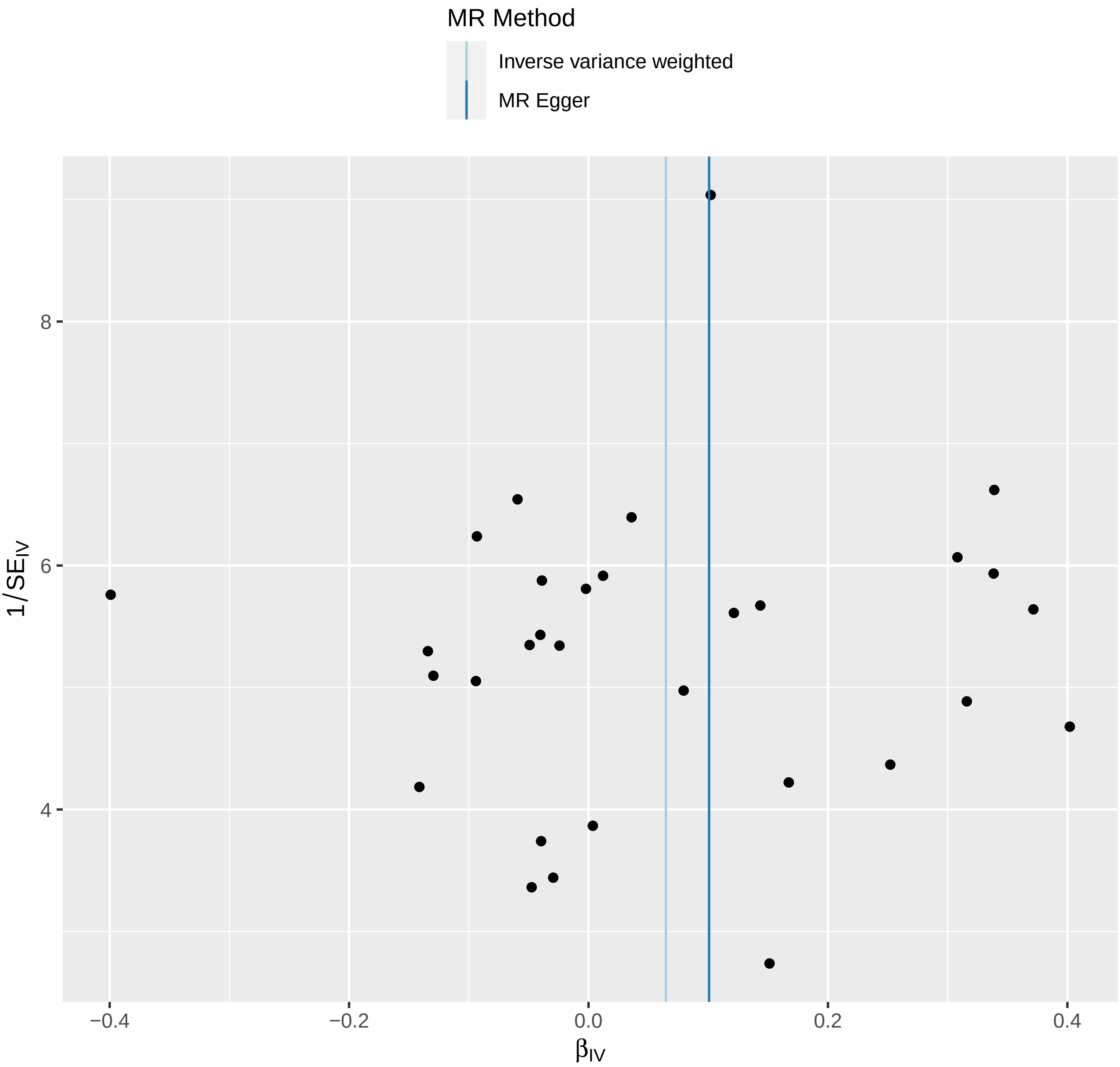

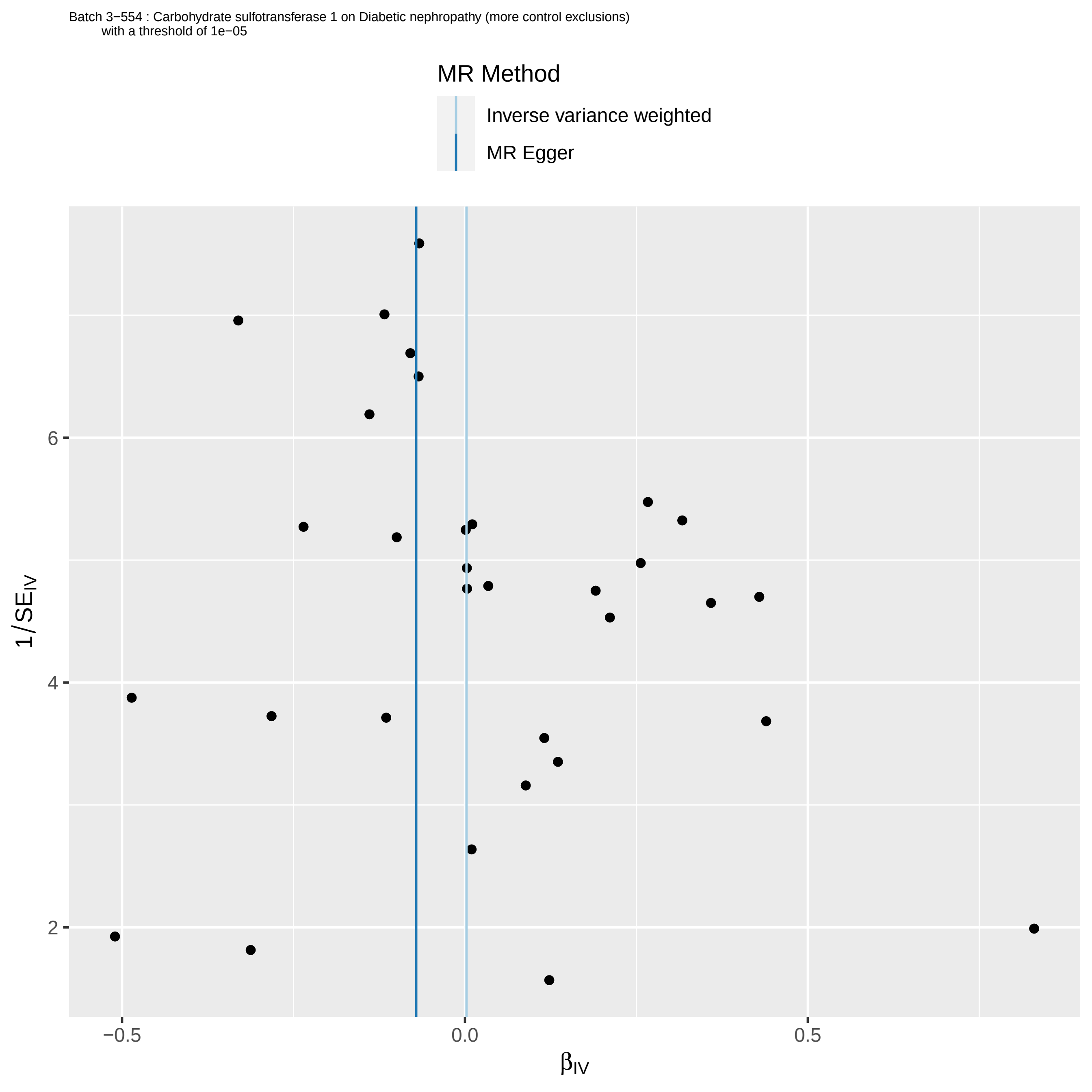

Batch 3−554 : Carbohydrate sulfotransferase 1 on Diabetic nephropathy (more control exclusions)
with a threshold of 1e−05
MR Method
Inverse variance weighted
MR Egger
1/SE_IV
6
4
2
−0.5
0.0
0.5
β_IV

Batch 3−555 : Carbohydrate sulfotransferase 2 on Diabetic nephropathy (more control exclusions)
with a threshold of 1e−05

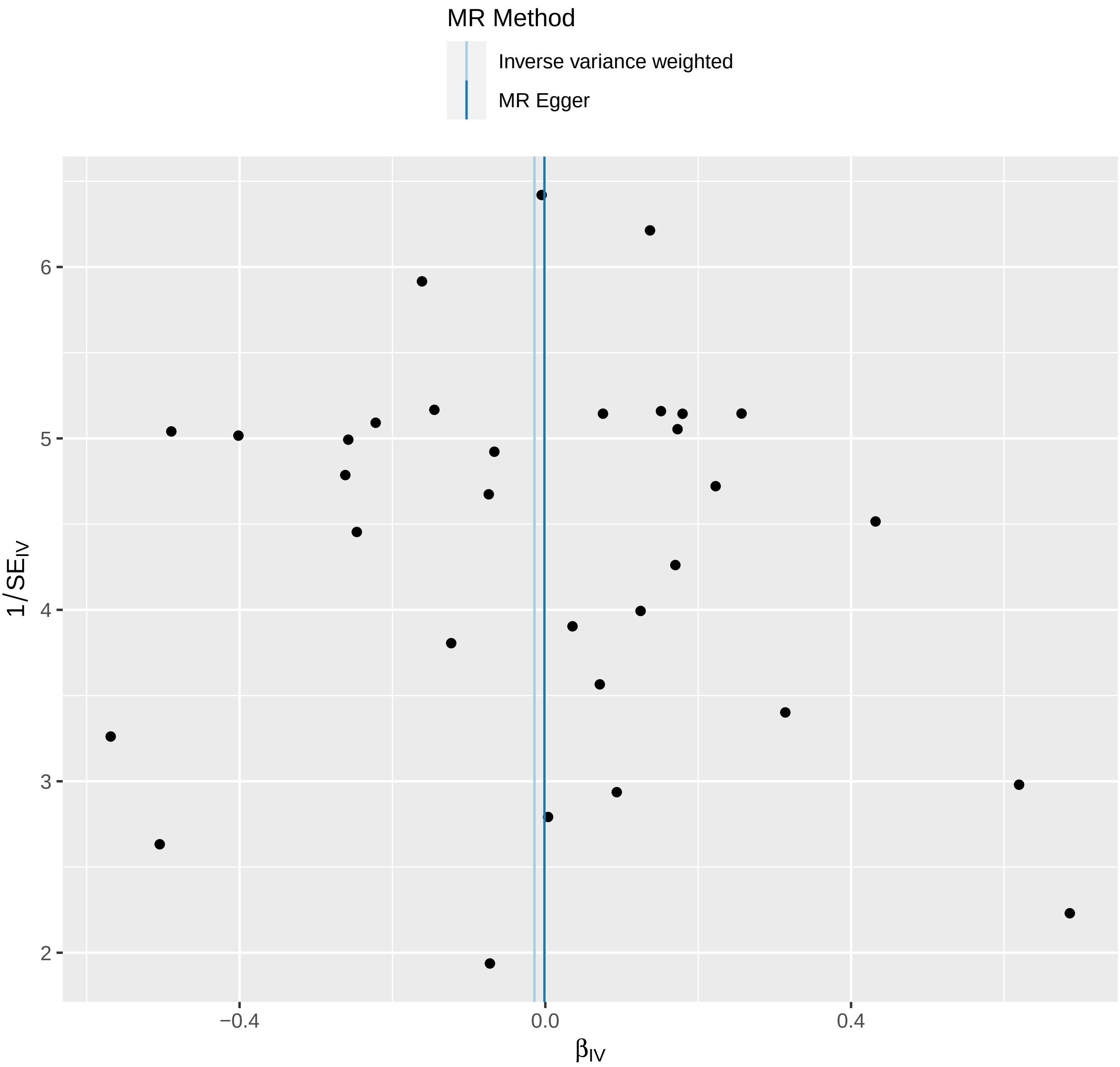

Batch 3−556 : Carbohydrate sulfotransferase 3 on Diabetic nephropathy (more control exclusions)
with a threshold of 1e−05

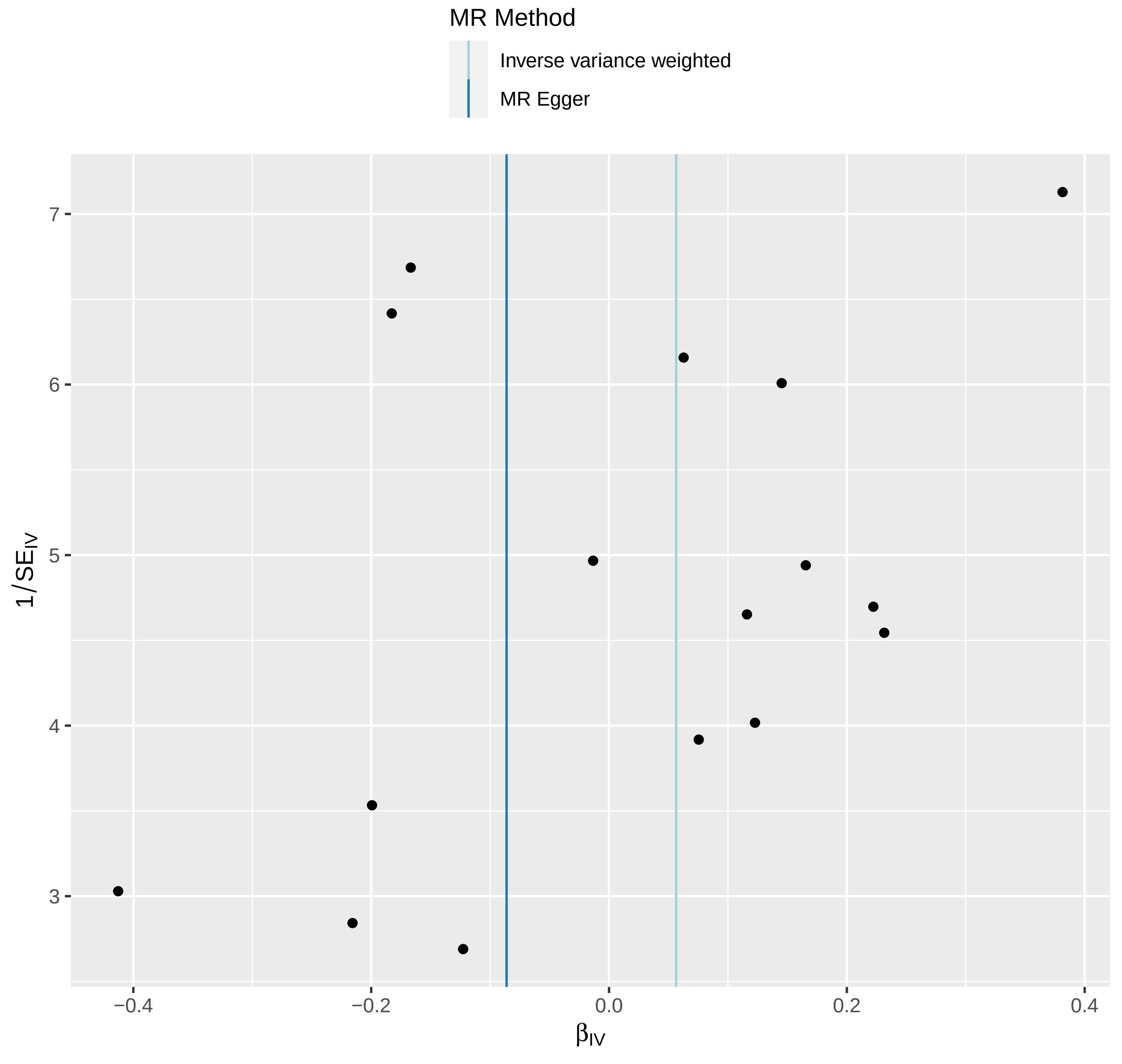

Batch 3−557 : Carbohydrate sulfotransferase 5 on Diabetic nephropathy (more control exclusions)
with a threshold of 1e−05

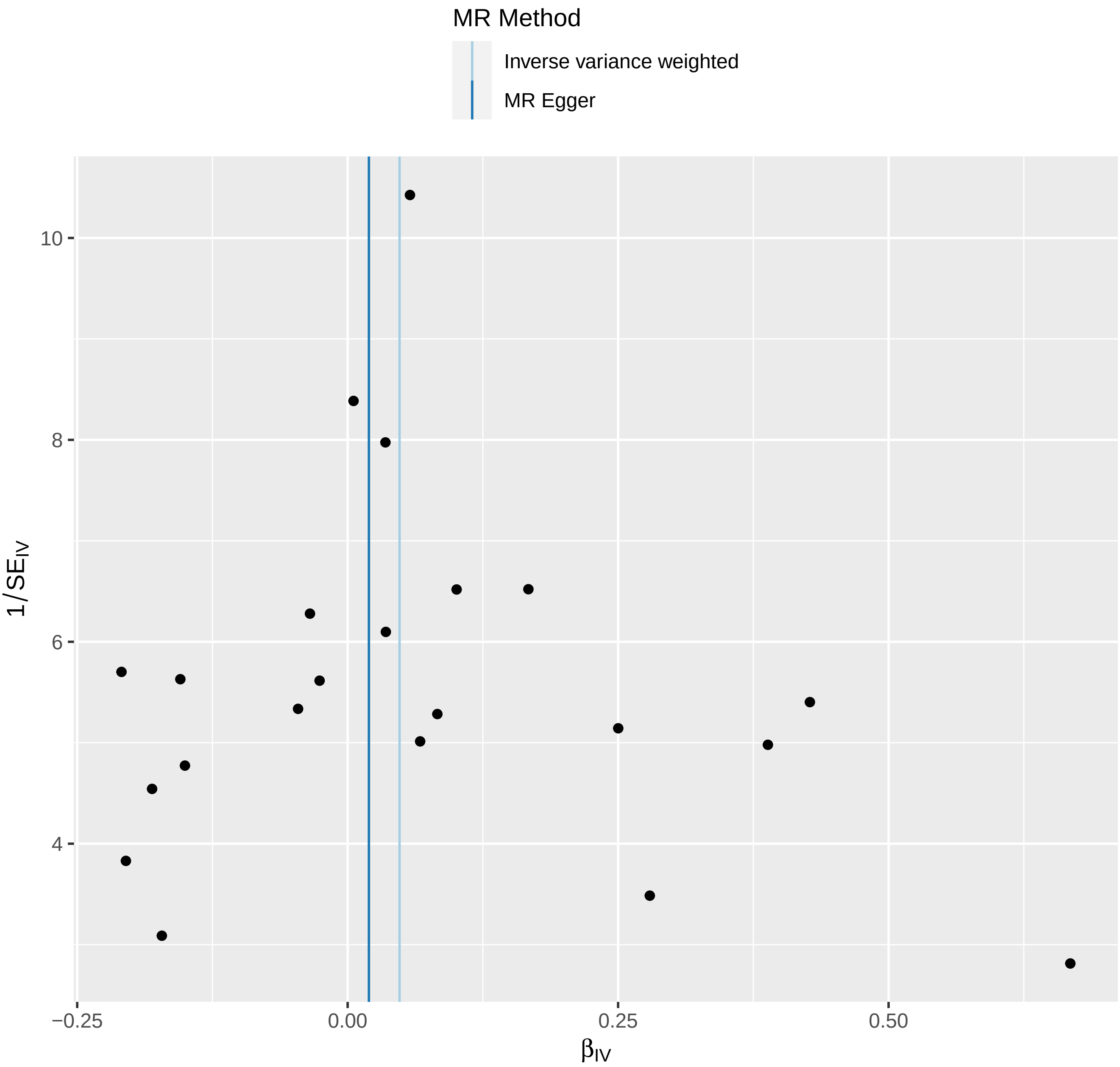

Batch 3−558 : Carbohydrate sulfotransferase 6 on Diabetic nephropathy (more control exclusions)
with a threshold of 1e−05

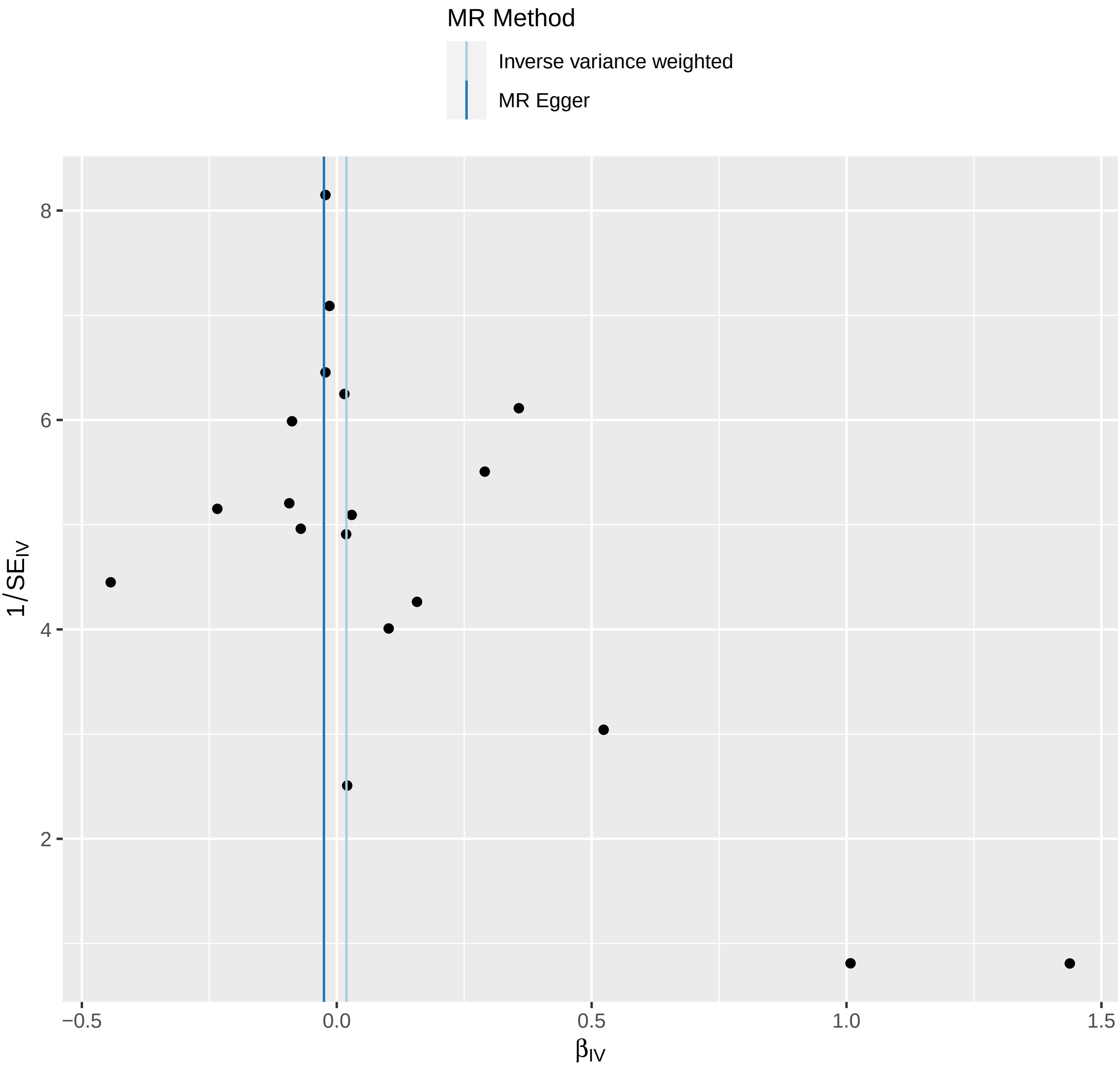

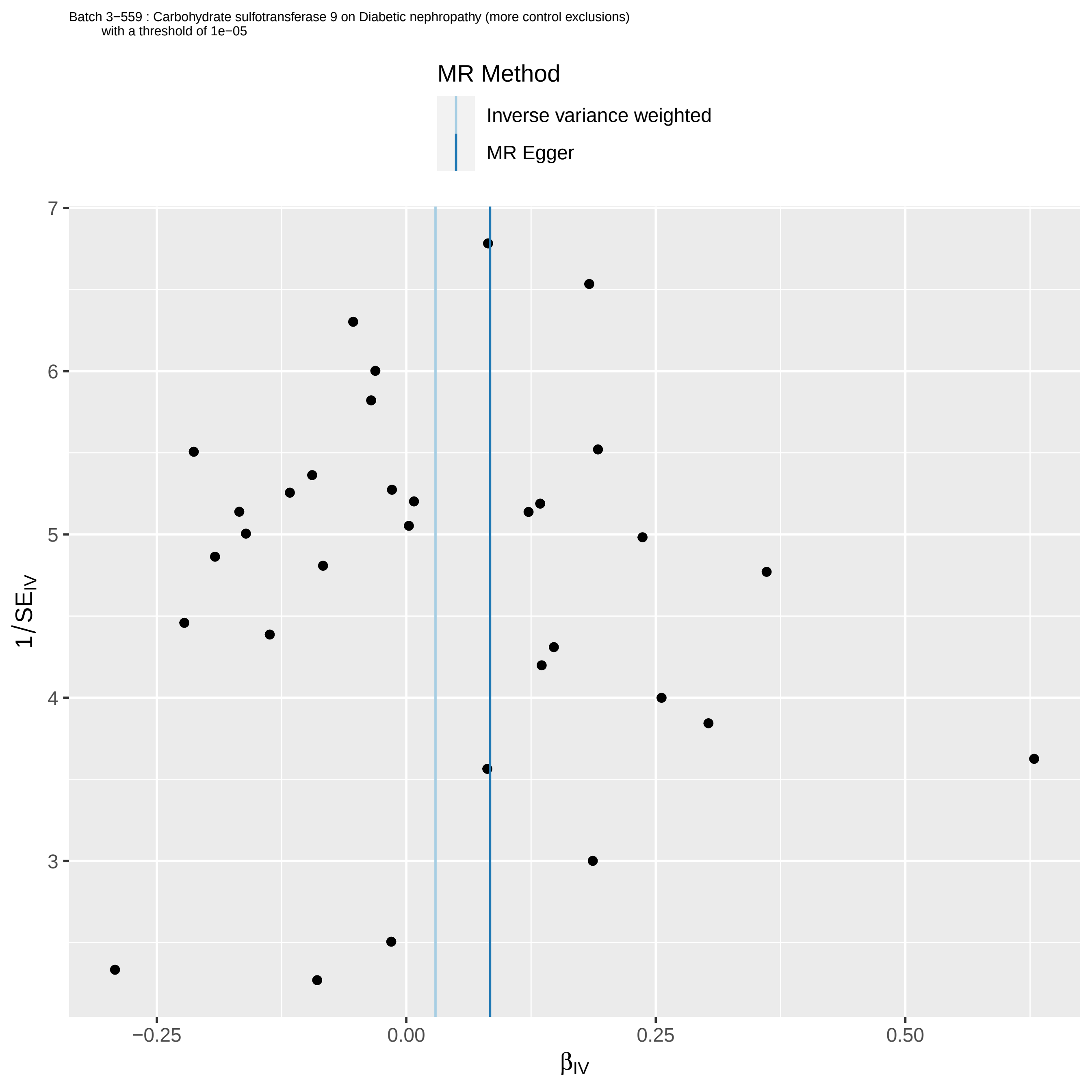
Batch 3−559 : Carbohydrate sulfotransferase 9 on Diabetic nephropathy (more control exclusions)
with a threshold of 1e−05
MR Method
Inverse variance weighted
MR Egger
7
6
5
4
3
1/SE$_{IV}$
−0.25
0.00
0.25
0.50
β$_{IV}$

Batch 3−560 : Cartilage intermediate layer protein 1 on Diabetic nephropathy (more control exclusions) with a threshold of 1e−05

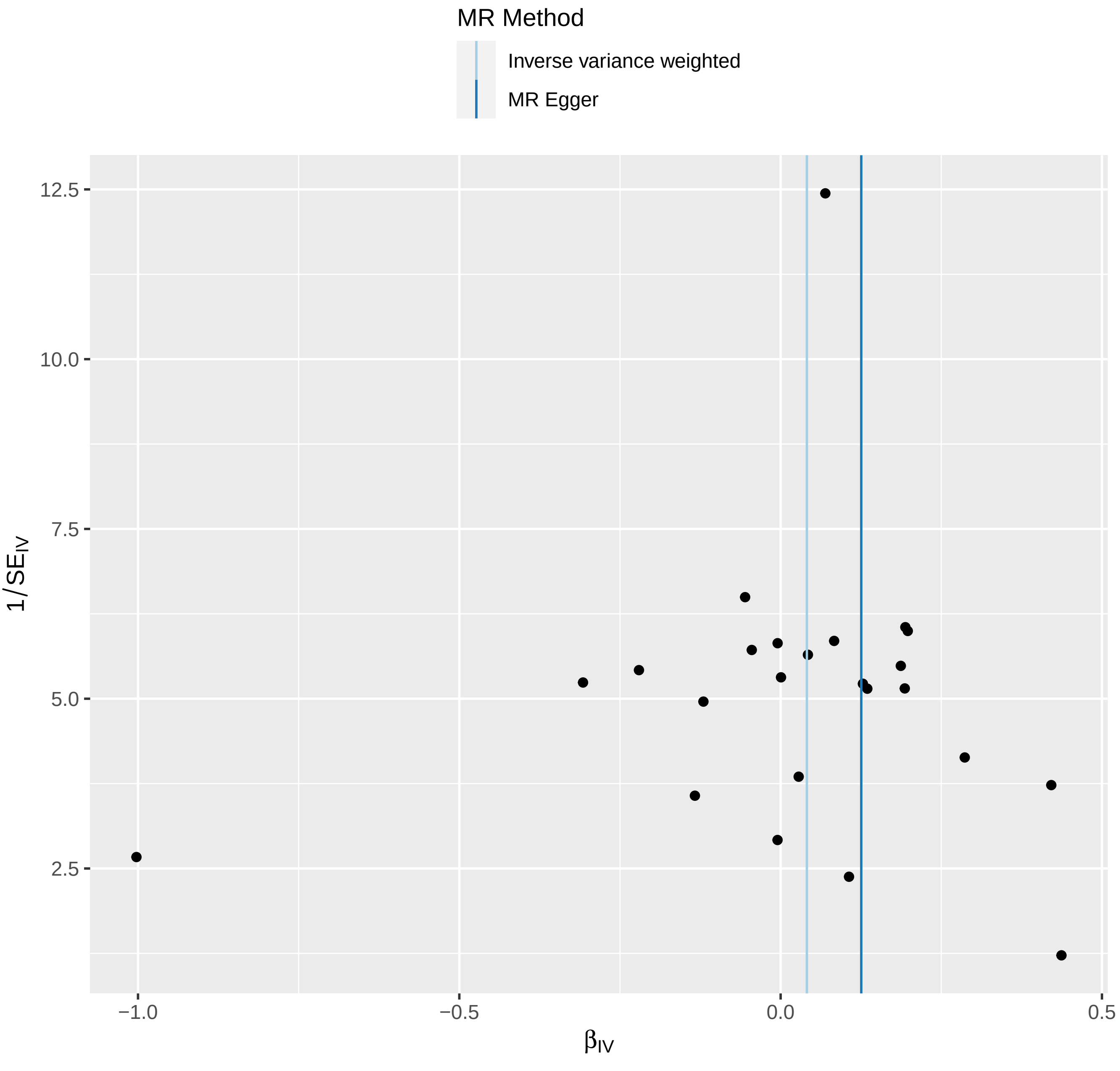

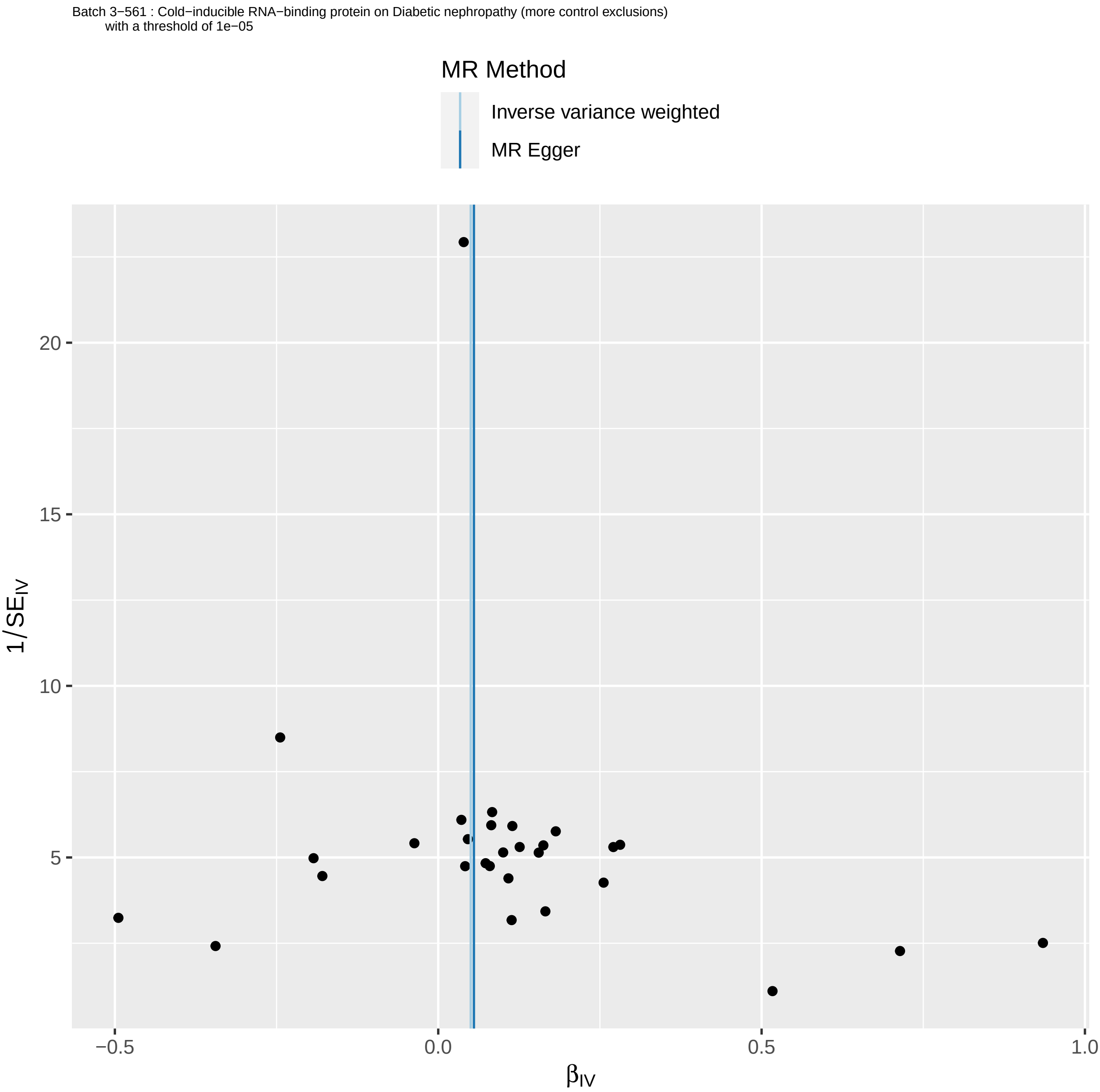
Batch 3−561 : Cold−inducible RNA−binding protein on Diabetic nephropathy (more control exclusions)
with a threshold of 1e−05
MR Method
Inverse variance weighted
MR Egger
20
15
10
5
1/SE_IV
−0.5
0.0
0.5
1.0
β_IV

Batch 3−562 : CDGSH iron−sulfur domain−containing protein 1 on Diabetic nephropathy (more control exclusions)
with a threshold of 1e−05

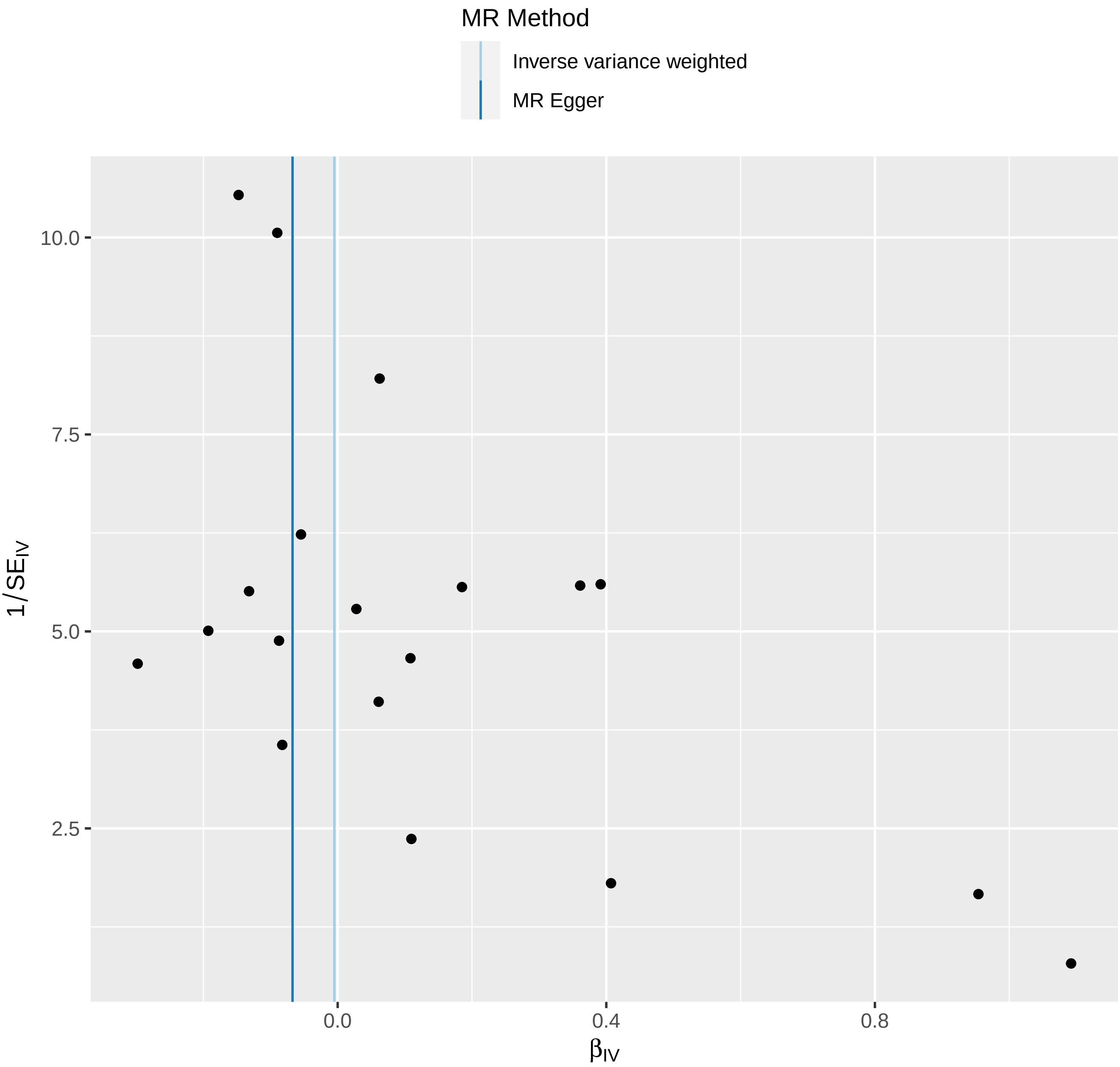

Batch 3−563 : CDGSH iron−sulfur domain−containing protein 2 on Diabetic nephropathy (more control exclusions)
with a threshold of 1e−05

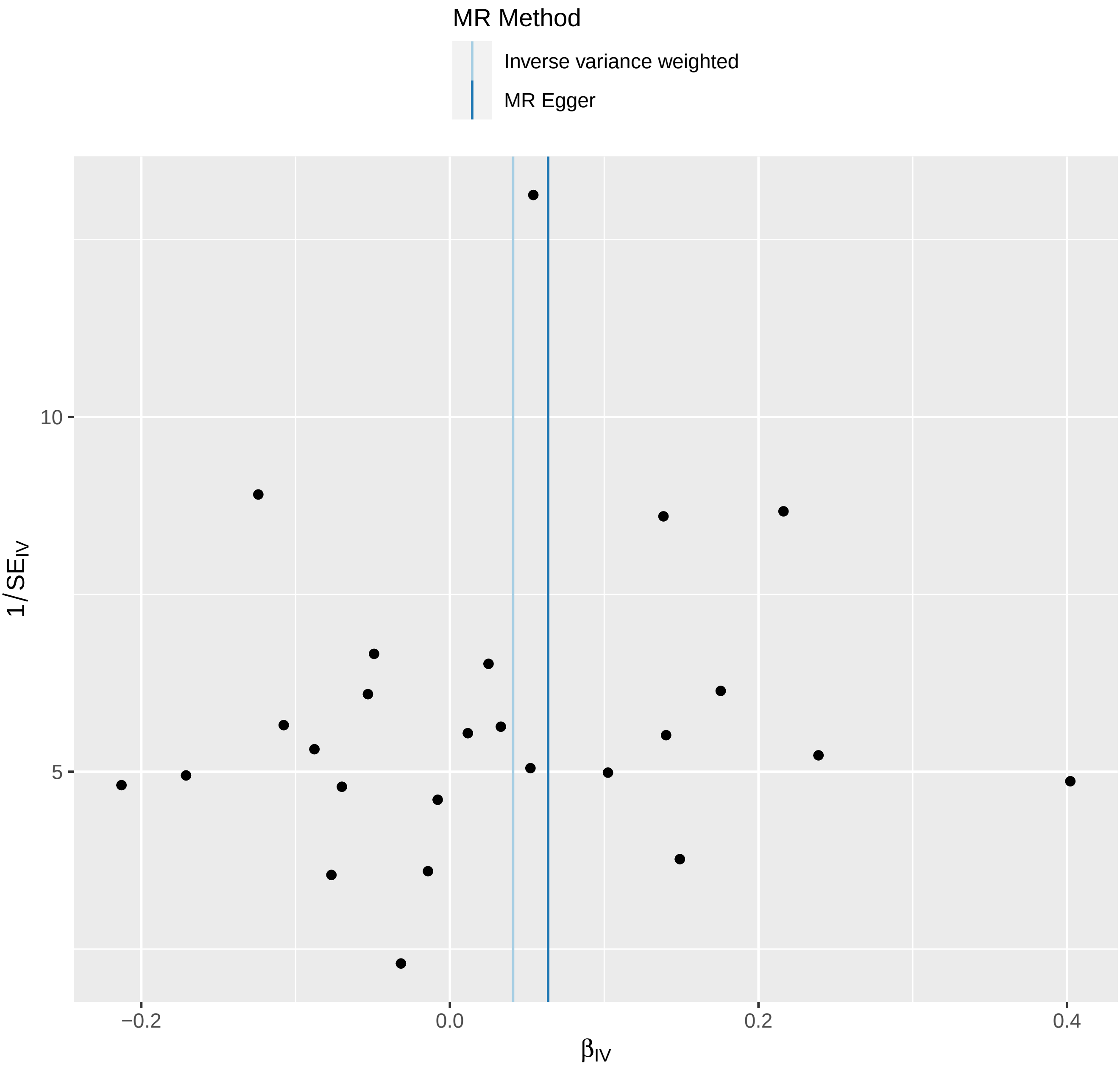

Batch 3−564 : Cytoskeleton−associated protein 2 on Diabetic nephropathy (more control exclusions)
with a threshold of 1e−05

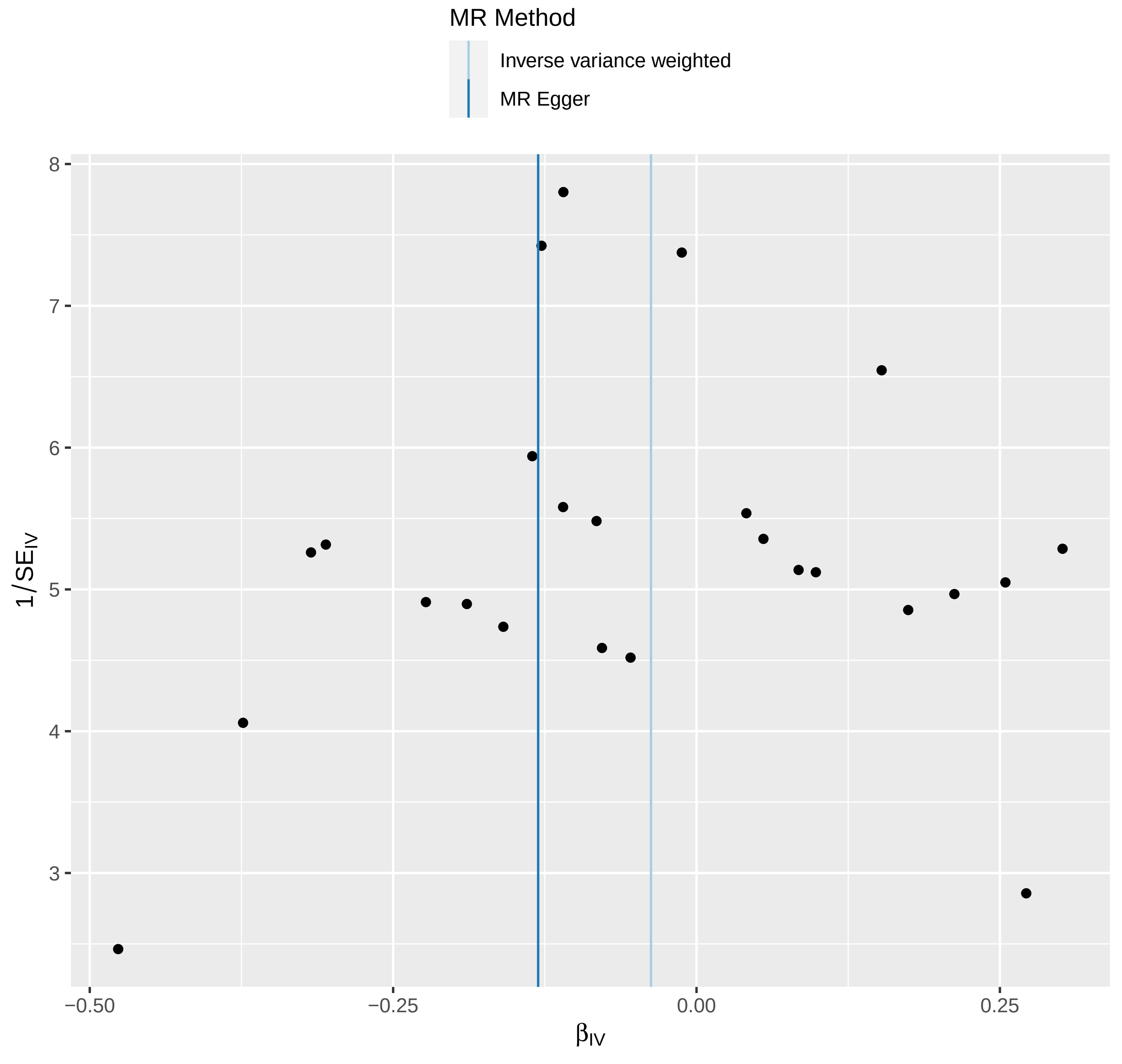

Batch 3−565 : Creatine kinase B−type on Diabetic nephropathy (more control exclusions)
with a threshold of 1e−05

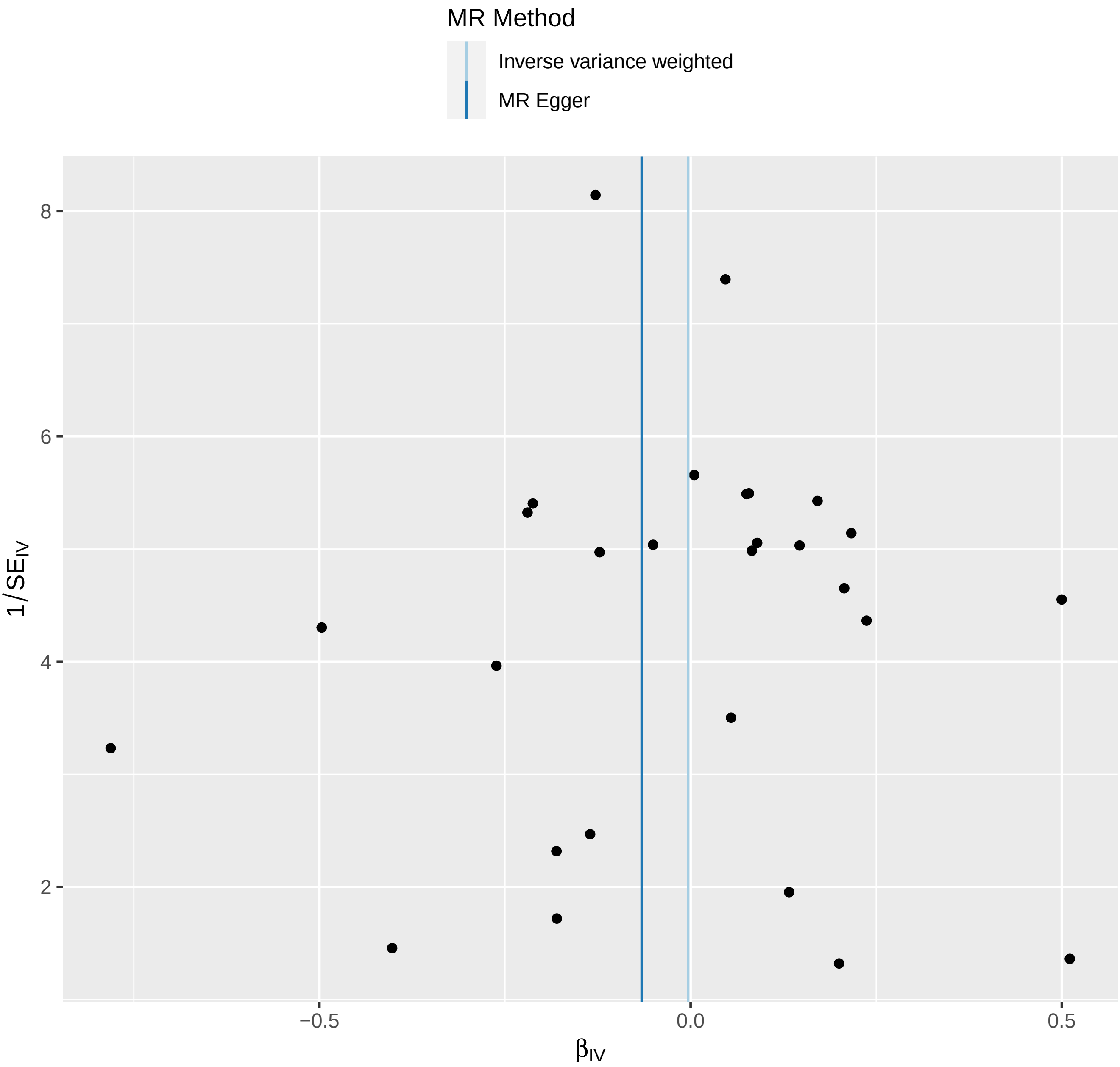

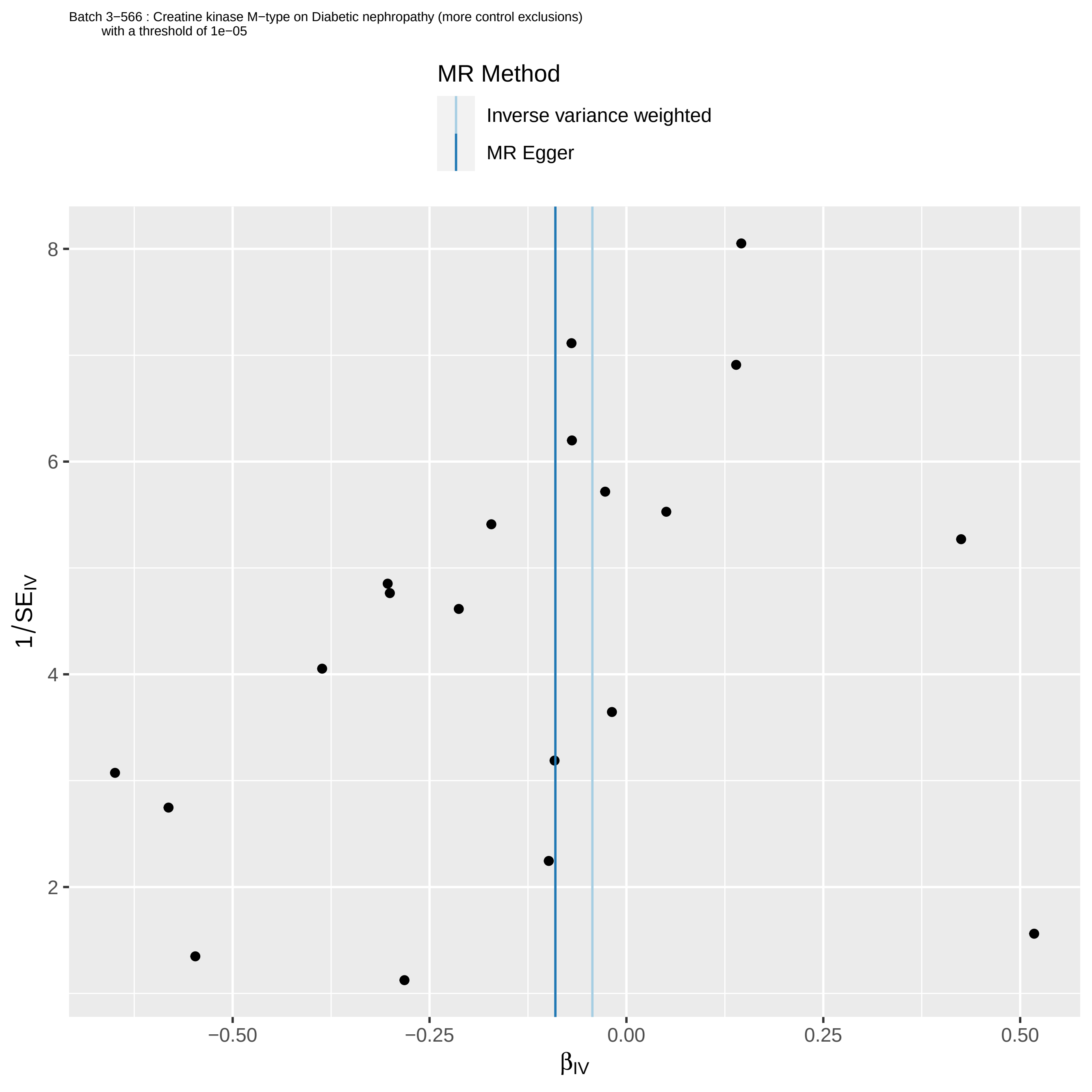
Batch 3−566 : Creatine kinase M−type on Diabetic nephropathy (more control exclusions)
with a threshold of 1e−05
MR Method
Inverse variance weighted
MR Egger
8
6
4
2
1/SE_IV
−0.50
−0.25
0.00
0.25
0.50
β_IV

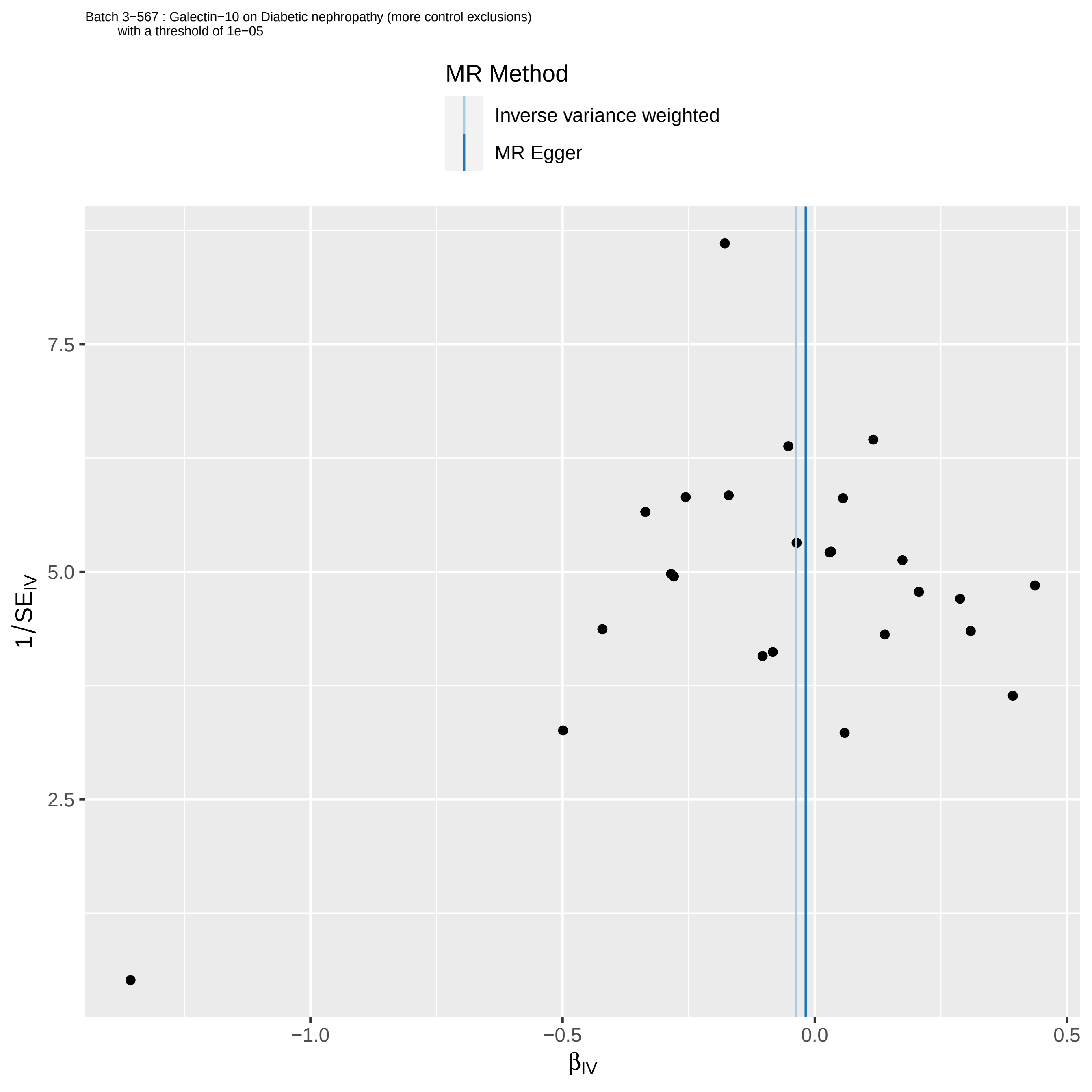

Batch 3−567 : Galectin−10 on Diabetic nephropathy (more control exclusions)
with a threshold of 1e−05
MR Method
Inverse variance weighted
MR Egger
7.5
5.0
2.5
1/SE$_{IV}$
−1.0
−0.5
0.0
0.5
β$_{IV}$

Batch 3−568 : C−type lectin domain family 10 member A on Diabetic nephropathy (more control exclusions)
with a threshold of 1e−05

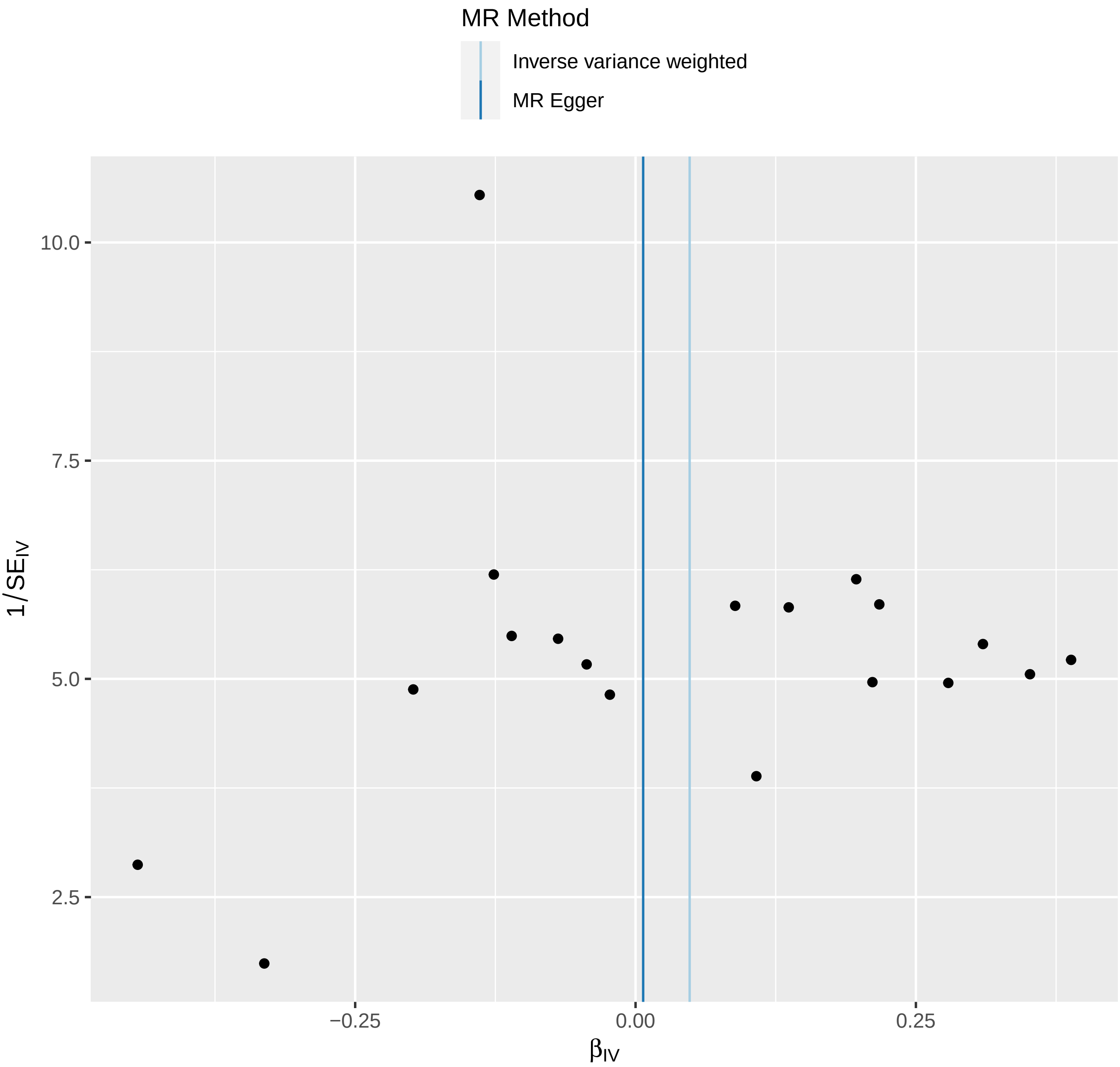

Batch 3−569 : Stem Cell Growth Factor−beta on Diabetic nephropathy (more control exclusions)
with a threshold of 1e−05

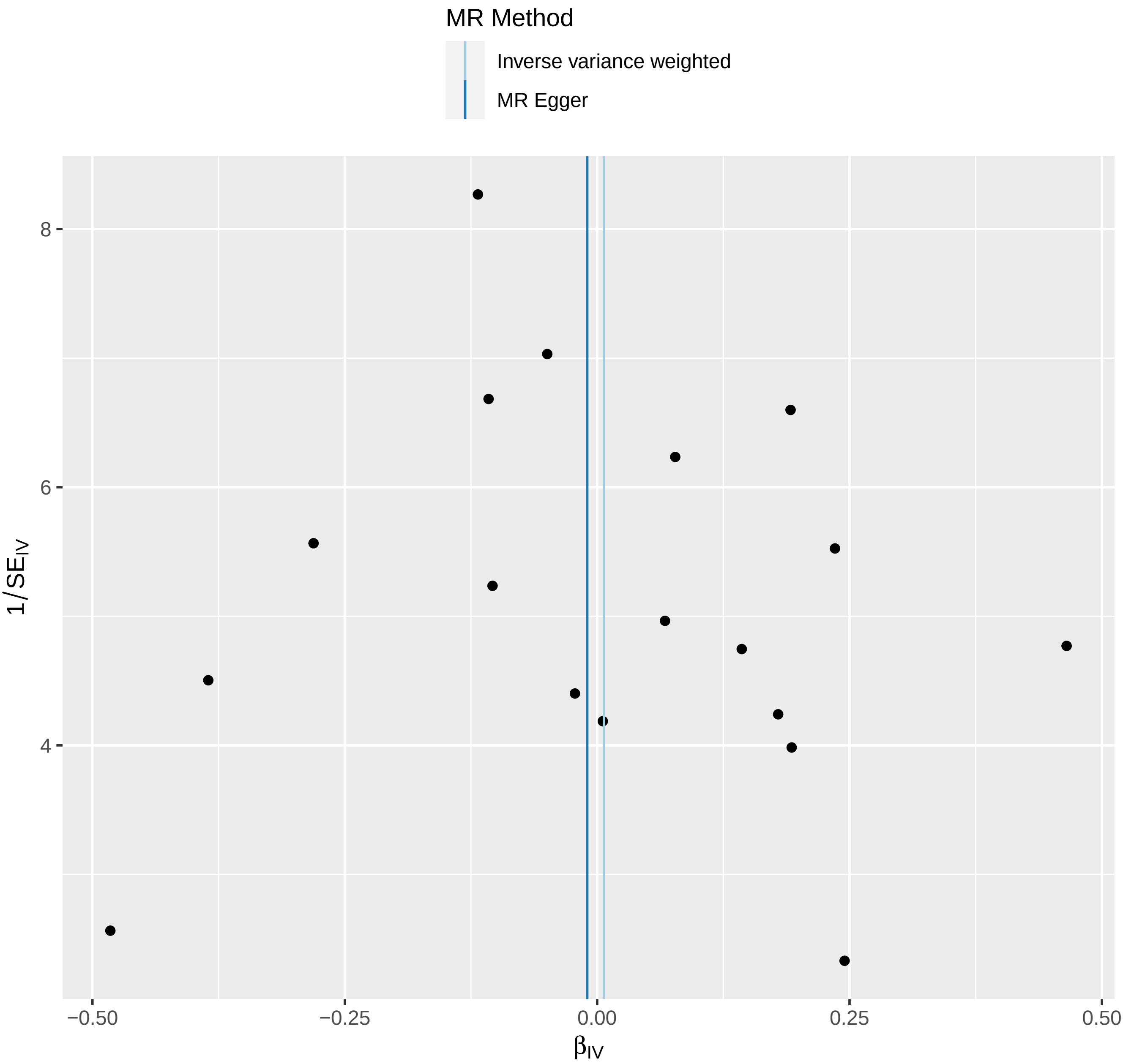

Batch 3−570 : C−type lectin domain family 12 member A on Diabetic nephropathy (more control exclusions)
with a threshold of 1e−05

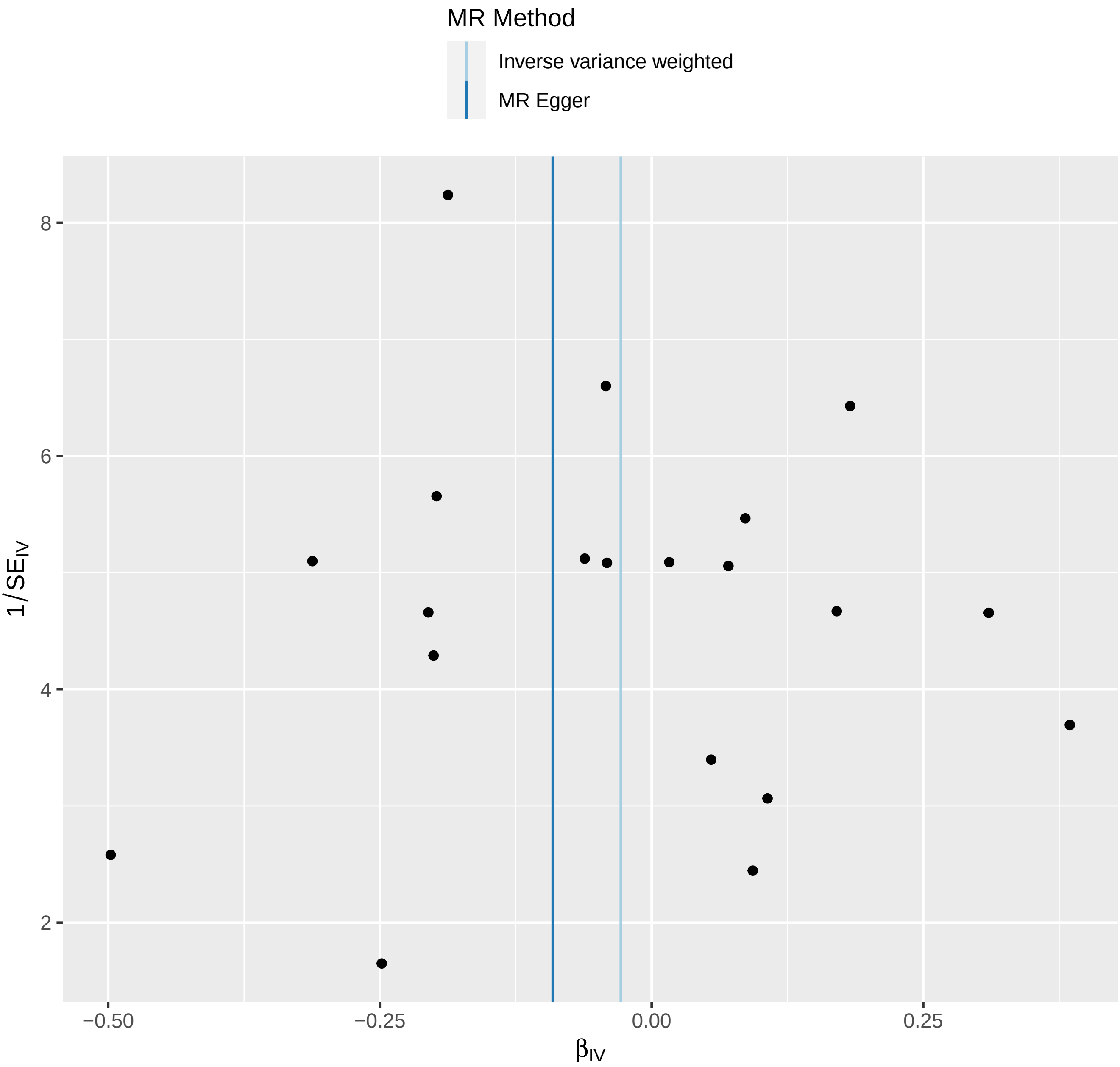

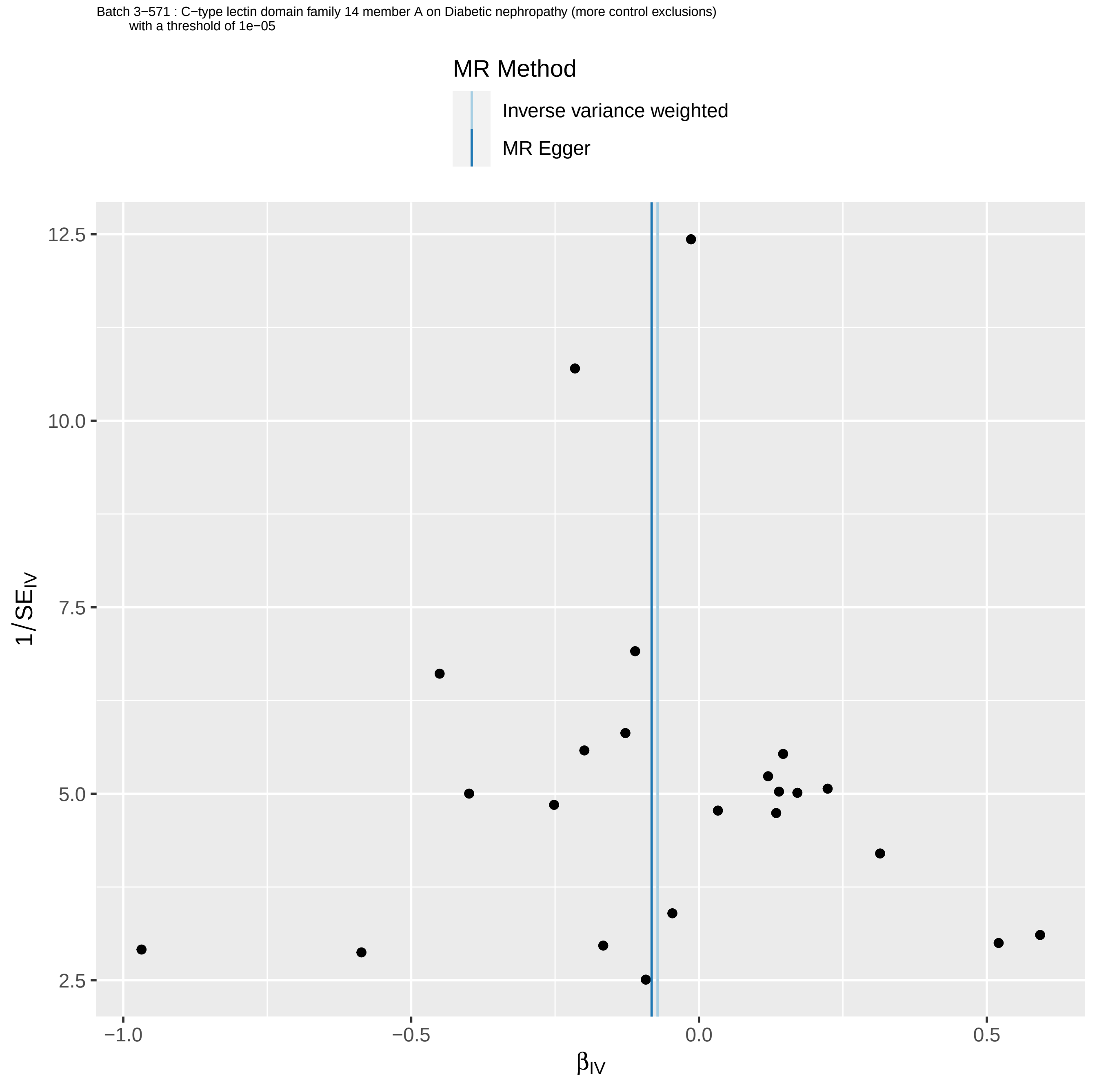

Batch 3−571 : C−type lectin domain family 14 member A on Diabetic nephropathy (more control exclusions)
with a threshold of 1e−05
MR Method
Inverse variance weighted
MR Egger
12.5
10.0
7.5
5.0
2.5
−1.0
−0.5
0.0
0.5
$1/SE_{IV}$
β_{IV}

Batch 3−572 : C−type lectin domain family 2 member A on Diabetic nephropathy (more control exclusions)
with a threshold of 1e−05

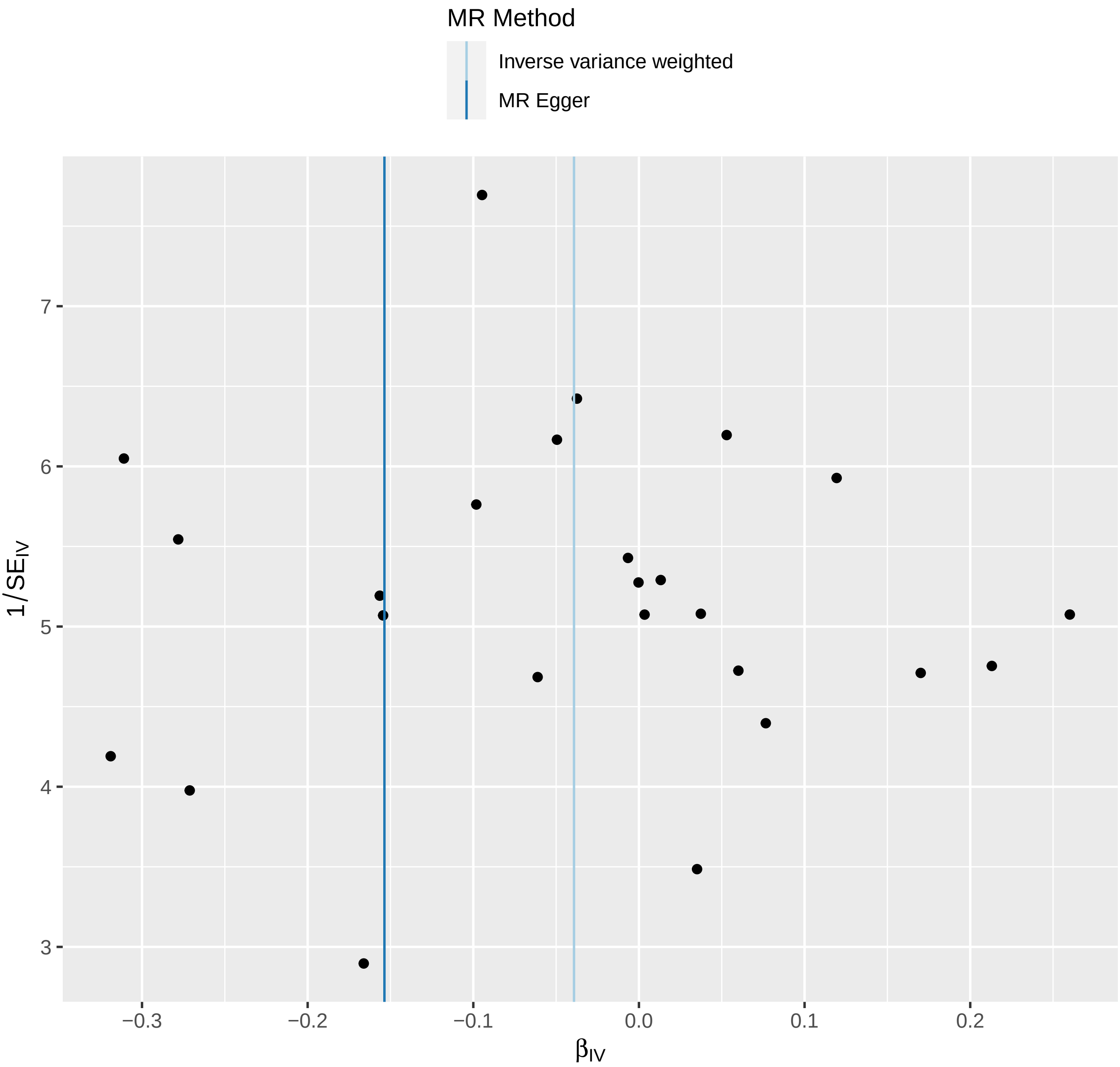

Batch 3−573 : C−type lectin domain family 2 member B on Diabetic nephropathy (more control exclusions)
with a threshold of 1e−05

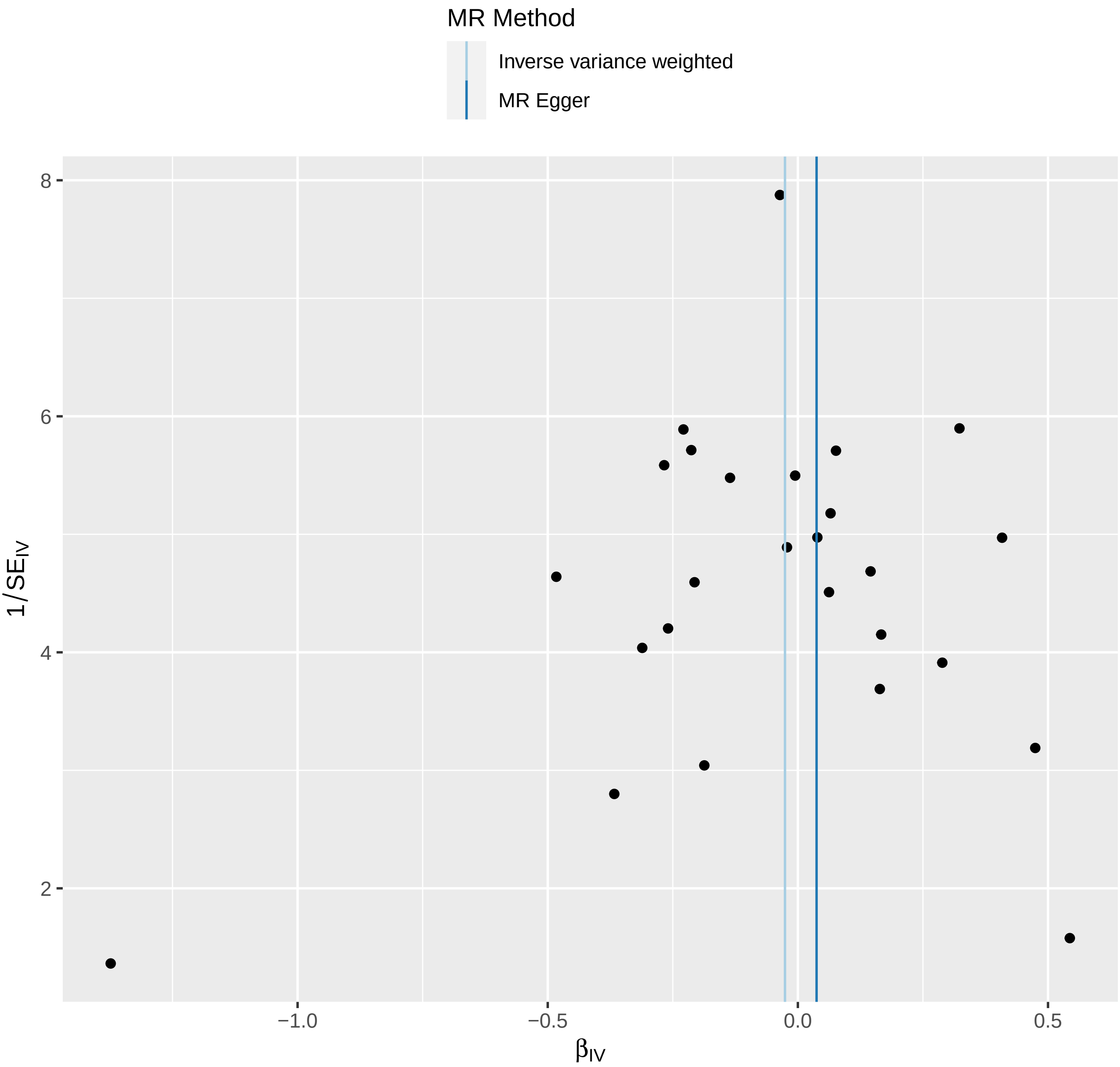

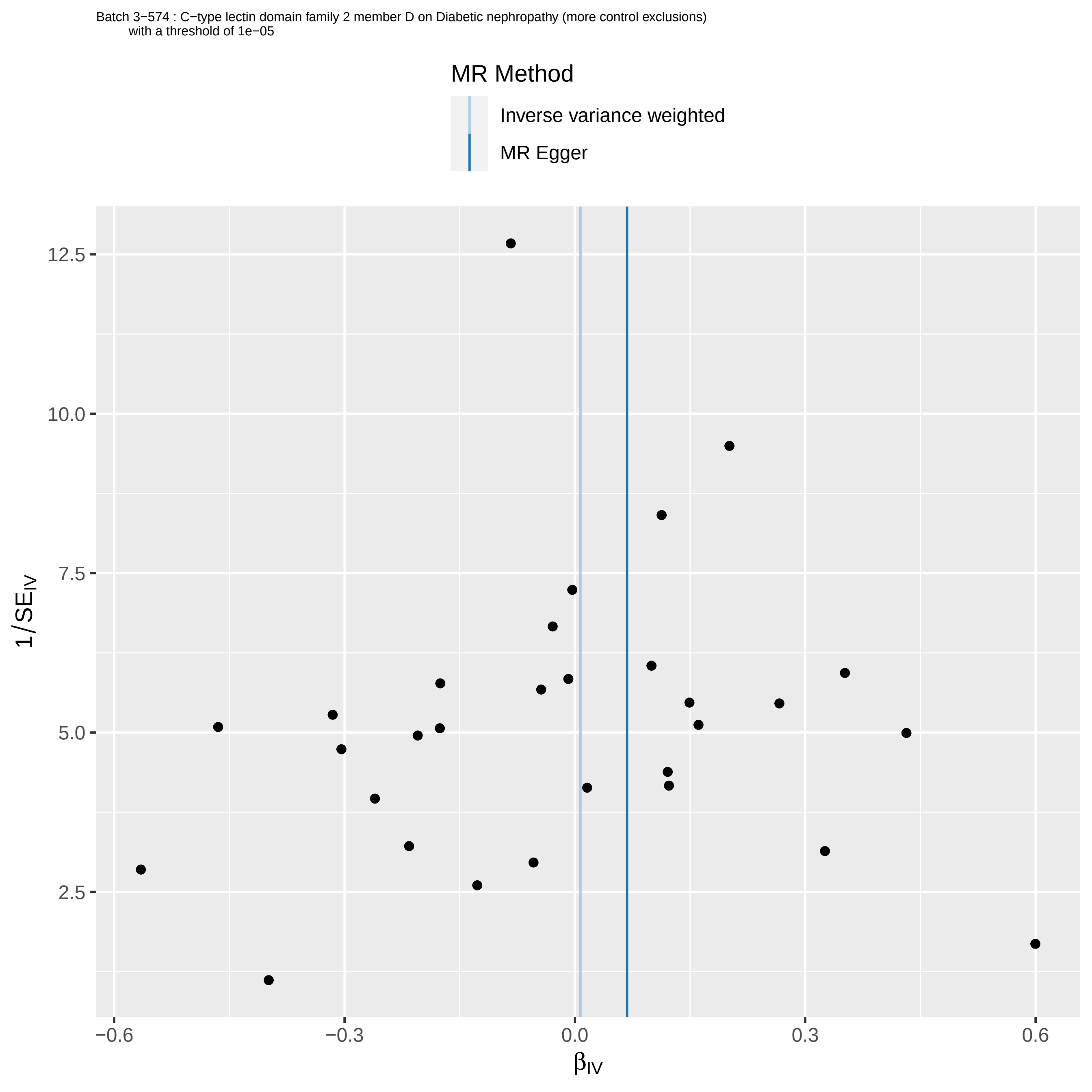
Batch 3−574 : C−type lectin domain family 2 member D on Diabetic nephropathy (more control exclusions)
with a threshold of 1e−05
MR Method
Inverse variance weighted
MR Egger
12.5
10.0
7.5
5.0
2.5
1/SE_IV
−0.6
−0.3
0.0
0.3
0.6
β_IV

Batch 3−575 : C−type lectin domain family 2 member L on Diabetic nephropathy (more control exclusions)
with a threshold of 1e−05

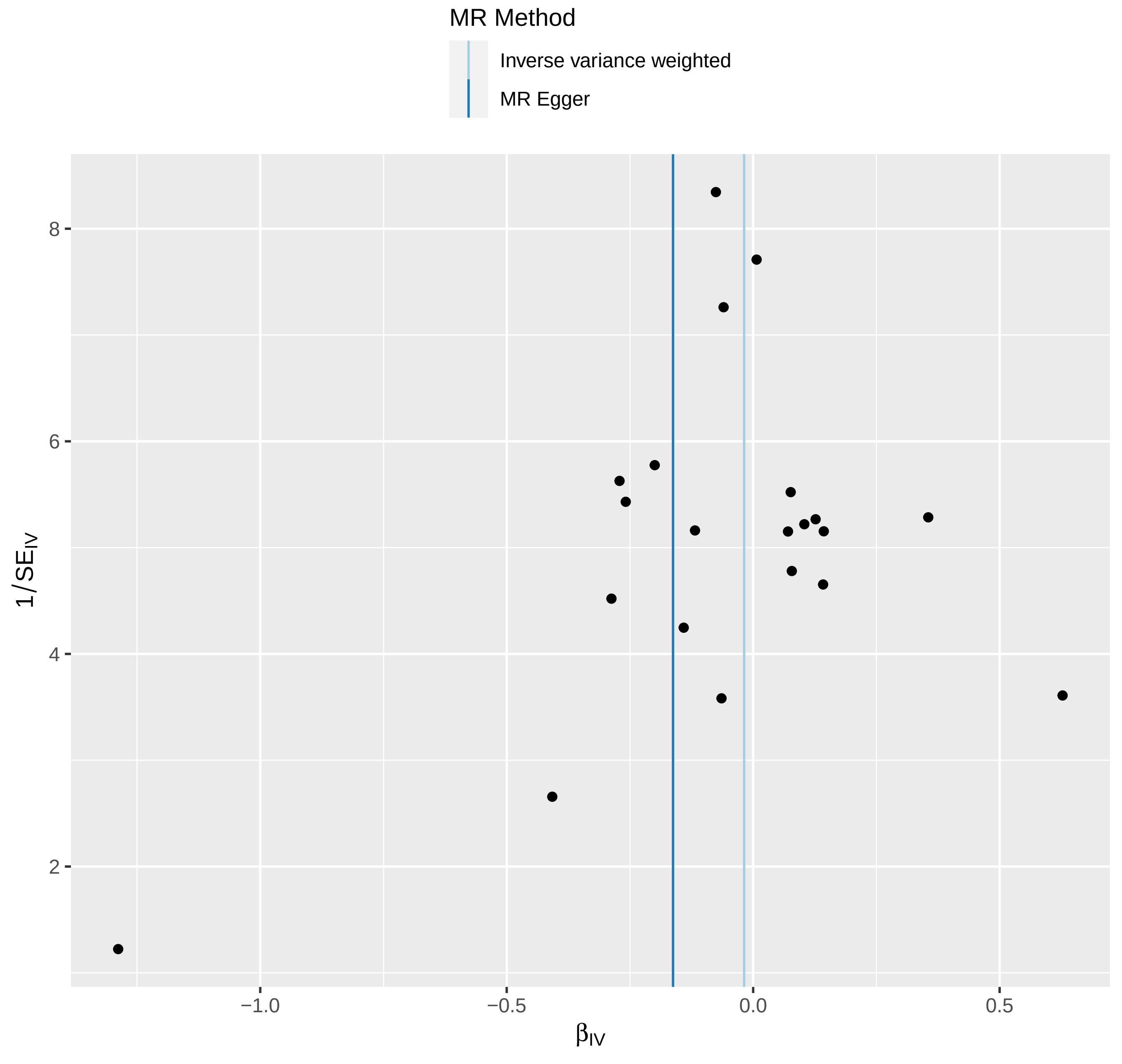

Batch 3−576 : C−type lectin domain family 4 member D on Diabetic nephropathy (more control exclusions)
with a threshold of 1e−05

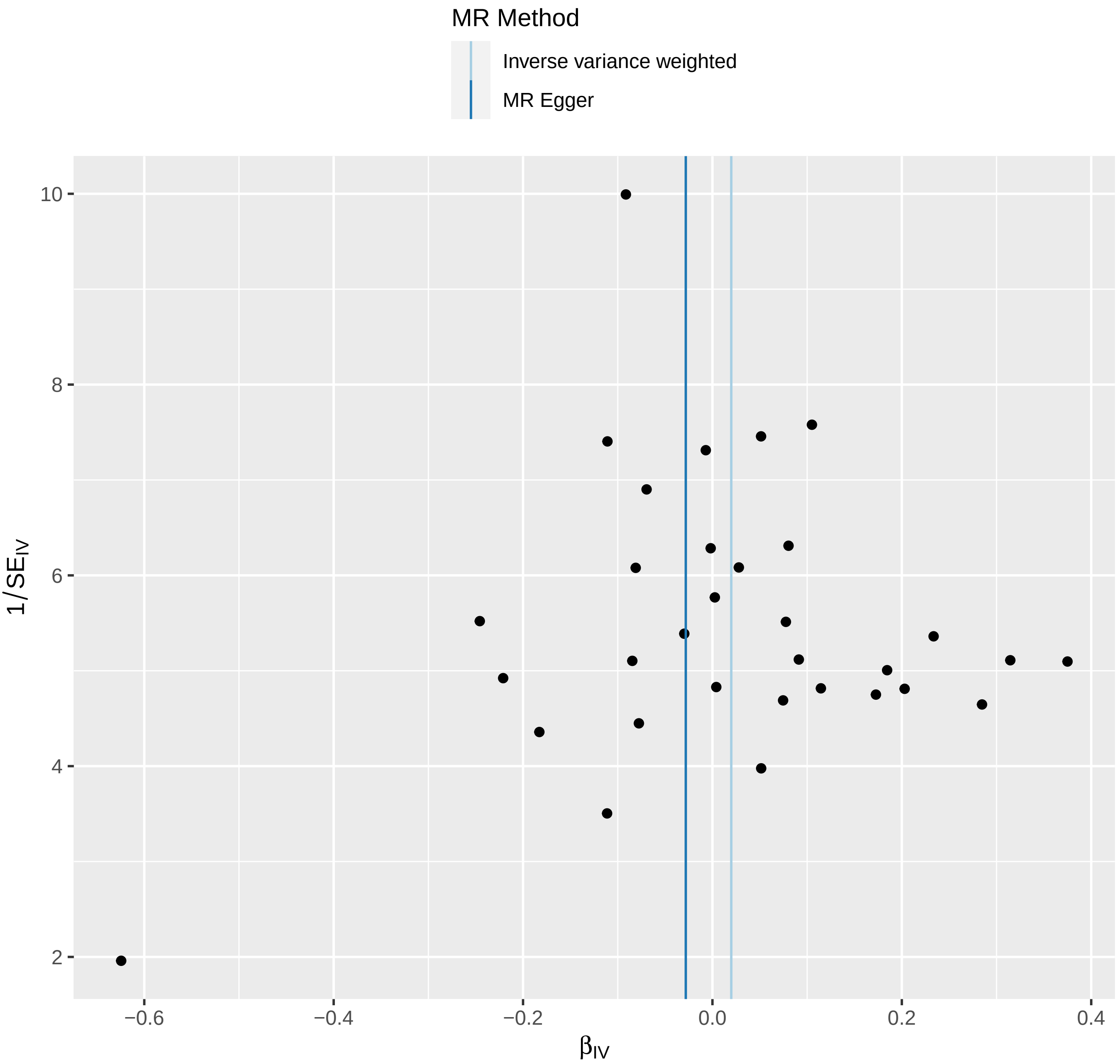

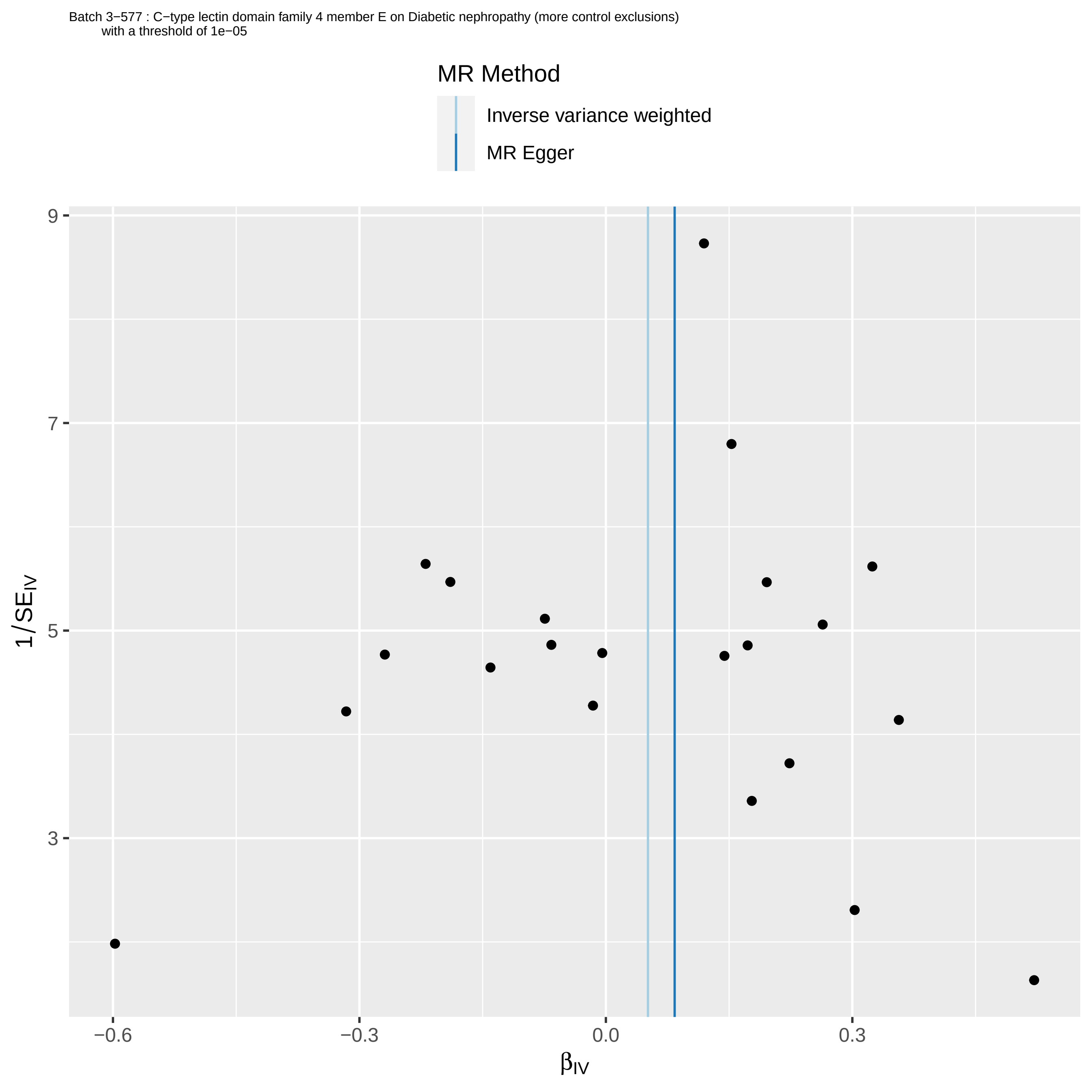

Batch 3−577 : C−type lectin domain family 4 member E on Diabetic nephropathy (more control exclusions)
with a threshold of 1e−05
MR Method
Inverse variance weighted
MR Egger
9
7
5
3
1/SE_IV
−0.6
−0.3
0.0
0.3
β_IV

Batch 3−578 : C−type lectin domain family 4 member M on Diabetic nephropathy (more control exclusions)
with a threshold of 1e−05

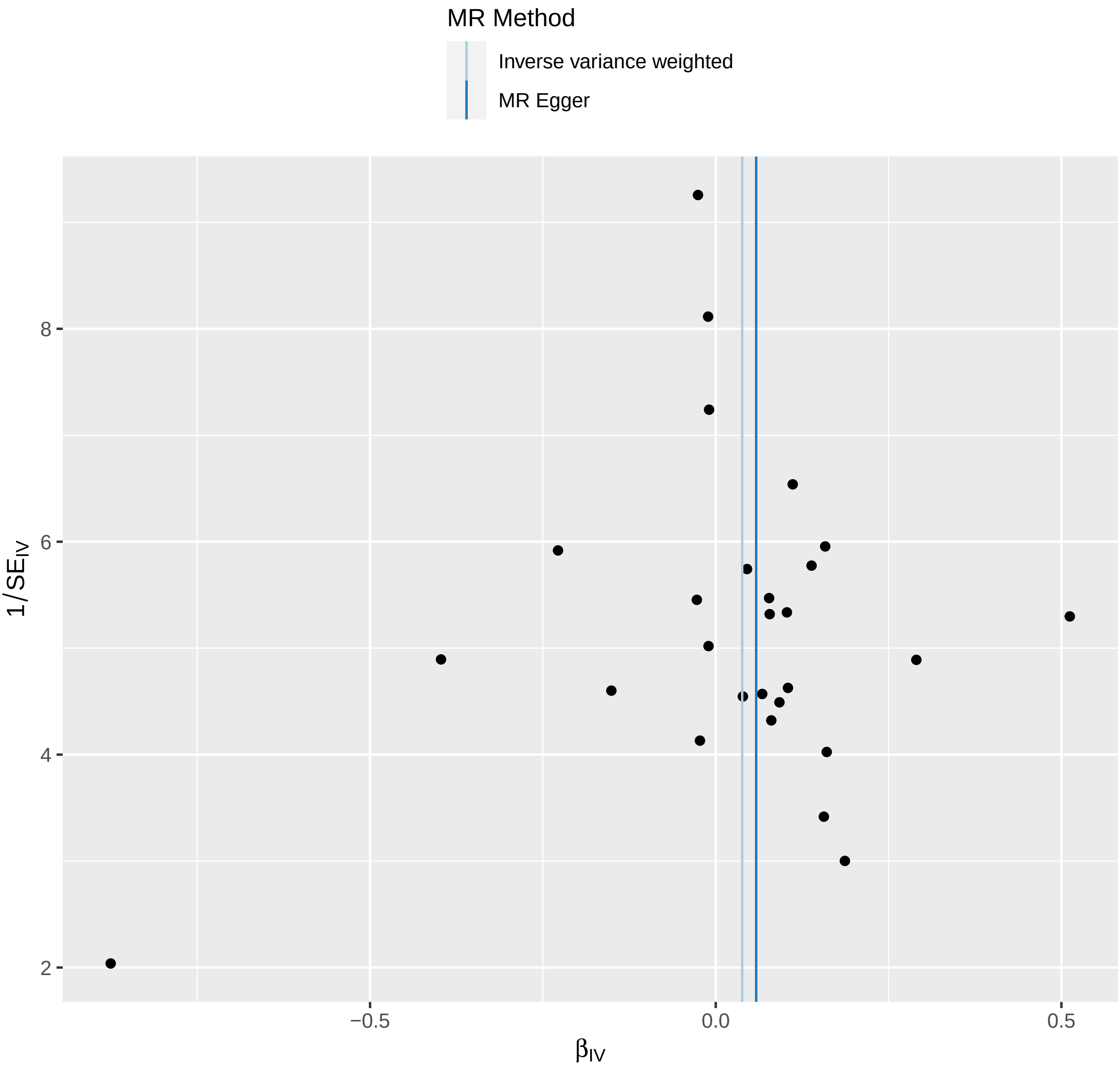

Batch 3−579 : C−type lectin domain family 5 member A on Diabetic nephropathy (more control exclusions)
with a threshold of 1e−05

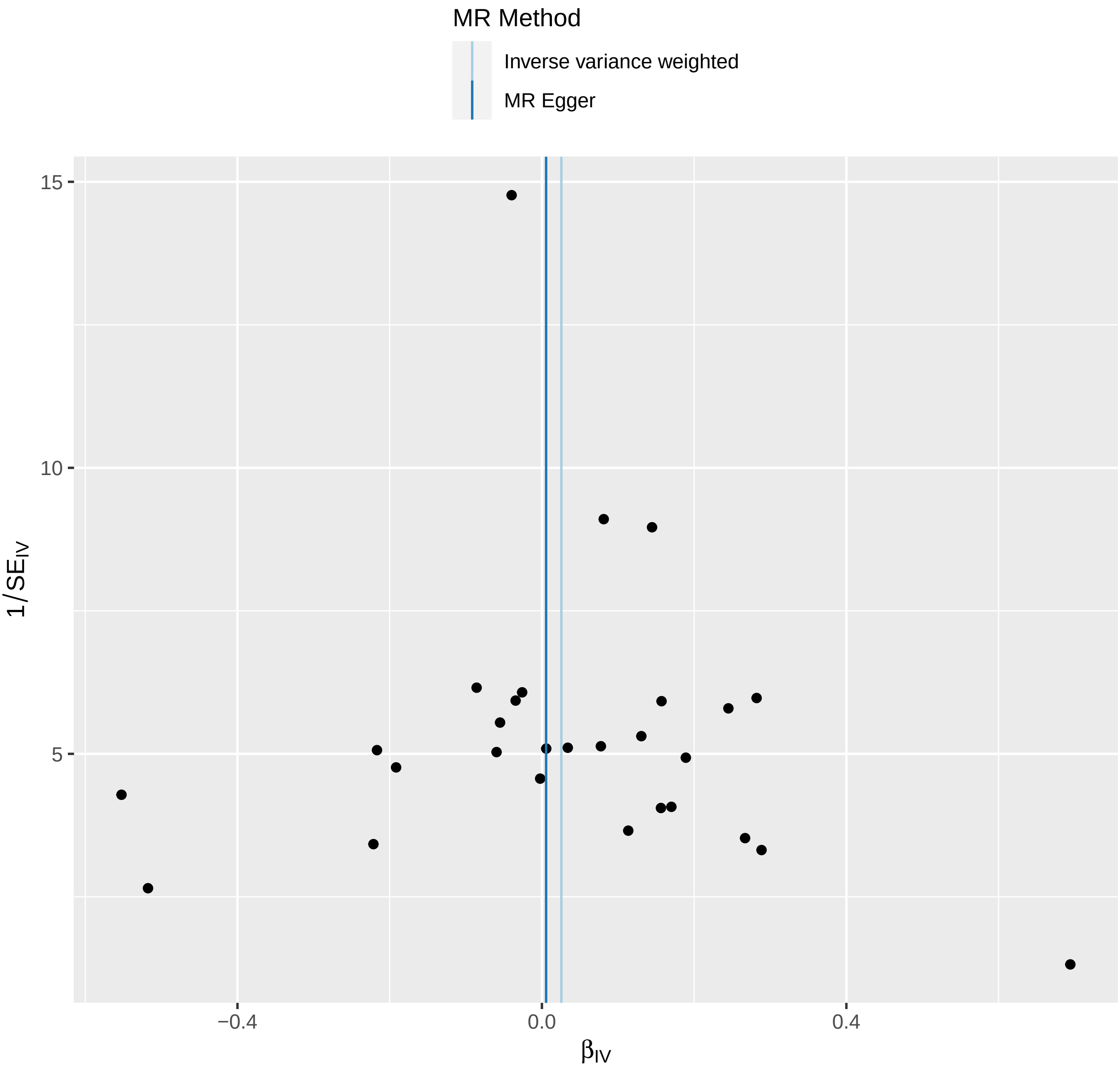

Batch 3−580 : C−type lectin domain family 6 member A on Diabetic nephropathy (more control exclusions)
with a threshold of 1e−05

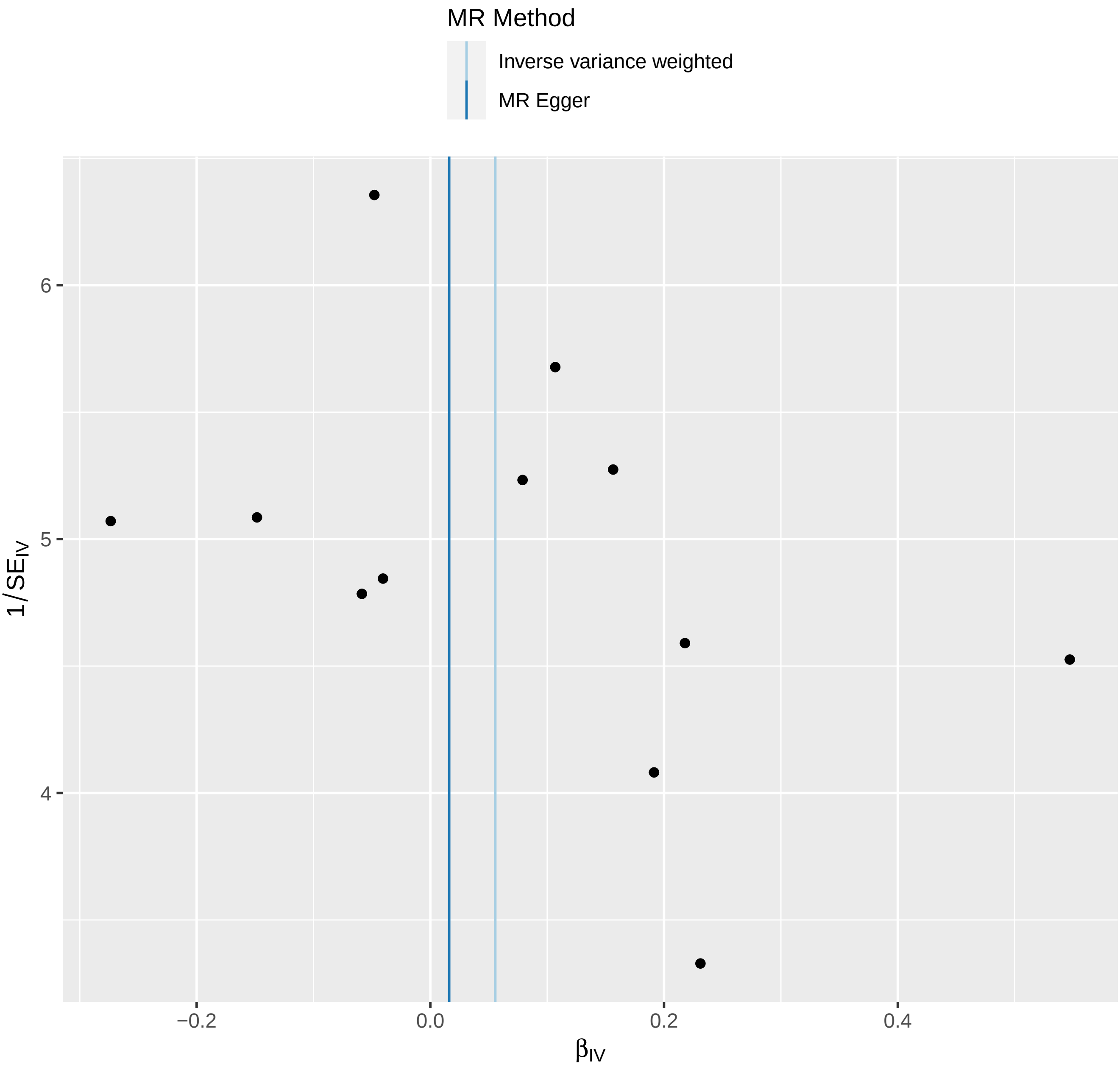

Batch 3−581 : C−type lectin domain family 7 member A on Diabetic nephropathy (more control exclusions)
with a threshold of 1e−05

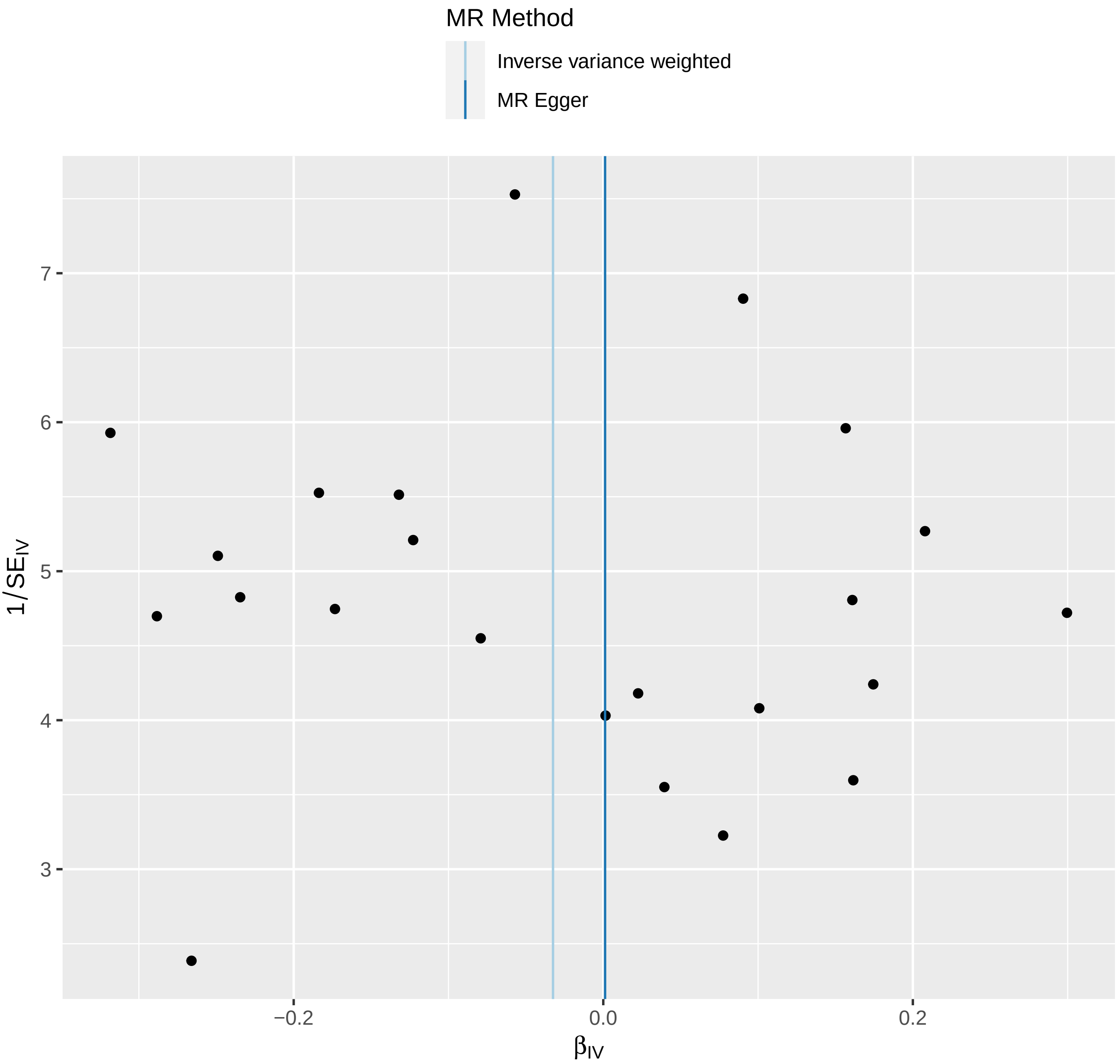

Batch 3−582 : C−type lectin−like domain family 1 on Diabetic nephropathy (more control exclusions)
with a threshold of 1e−05

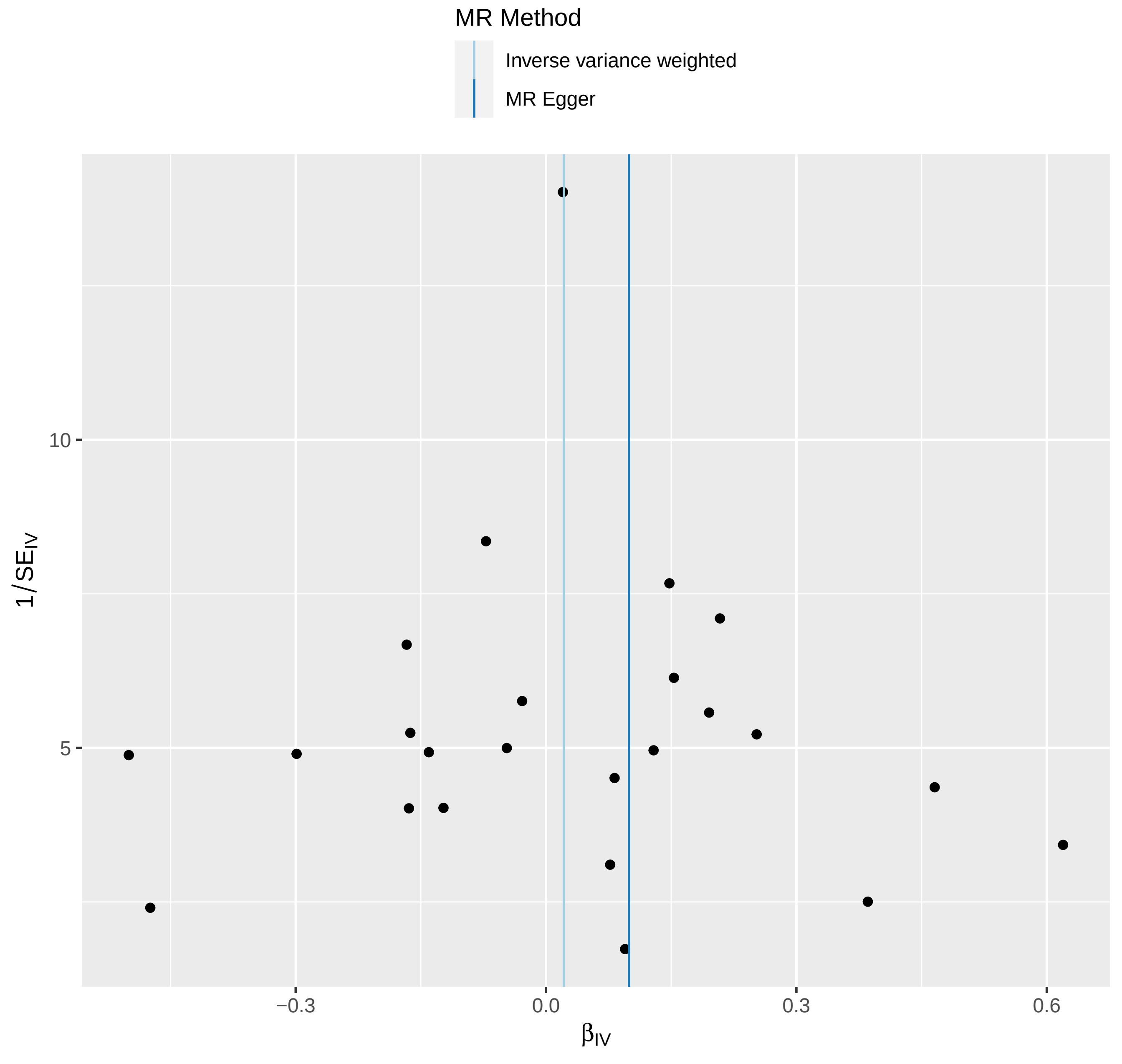

Batch 3−583 : Chloride intracellular channel protein 4 on Diabetic nephropathy (more control exclusions)
with a threshold of 1e−05

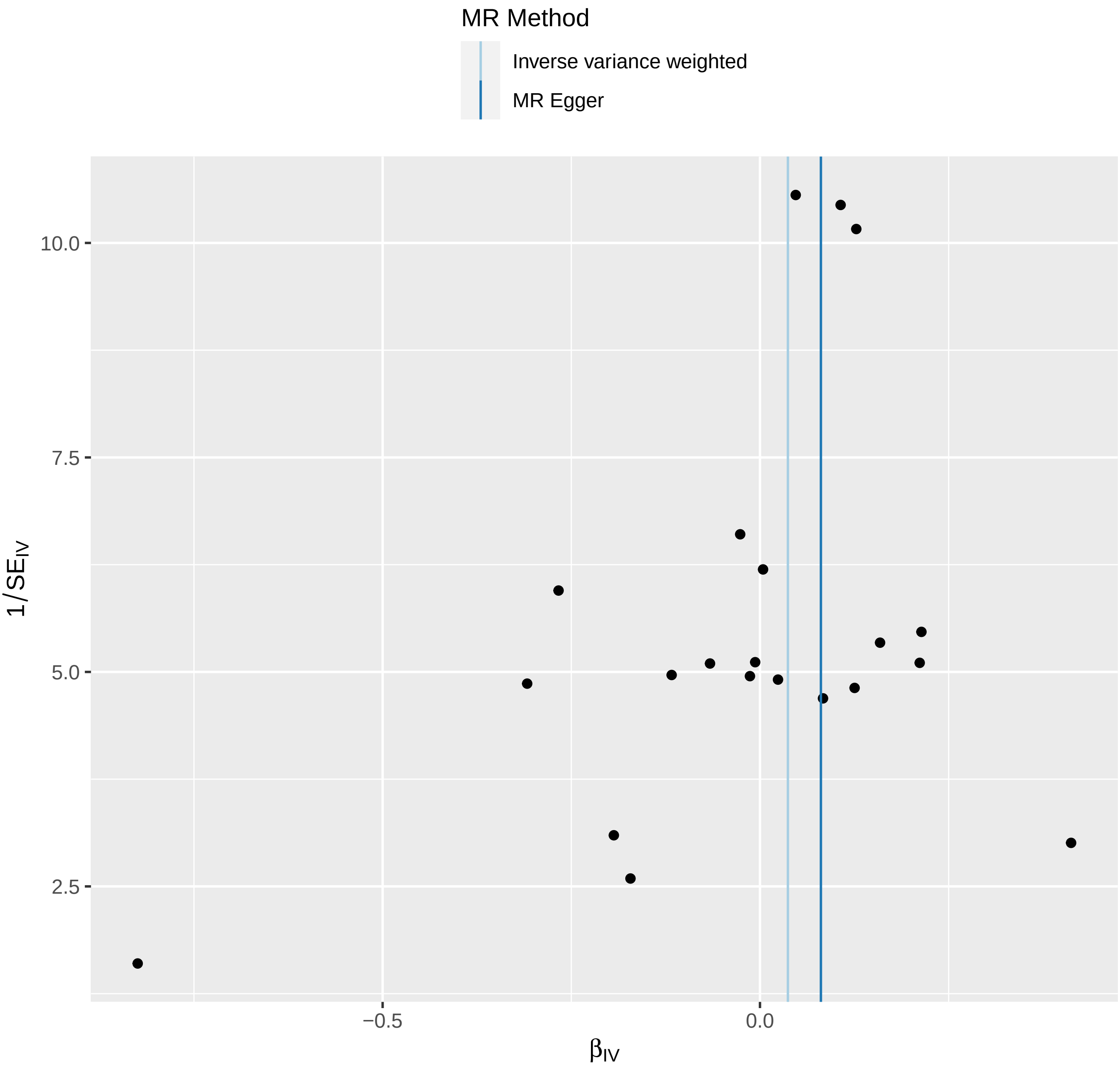

Batch 3−584 : Chloride intracellular channel protein 5 on Diabetic nephropathy (more control exclusions)
with a threshold of 1e−05

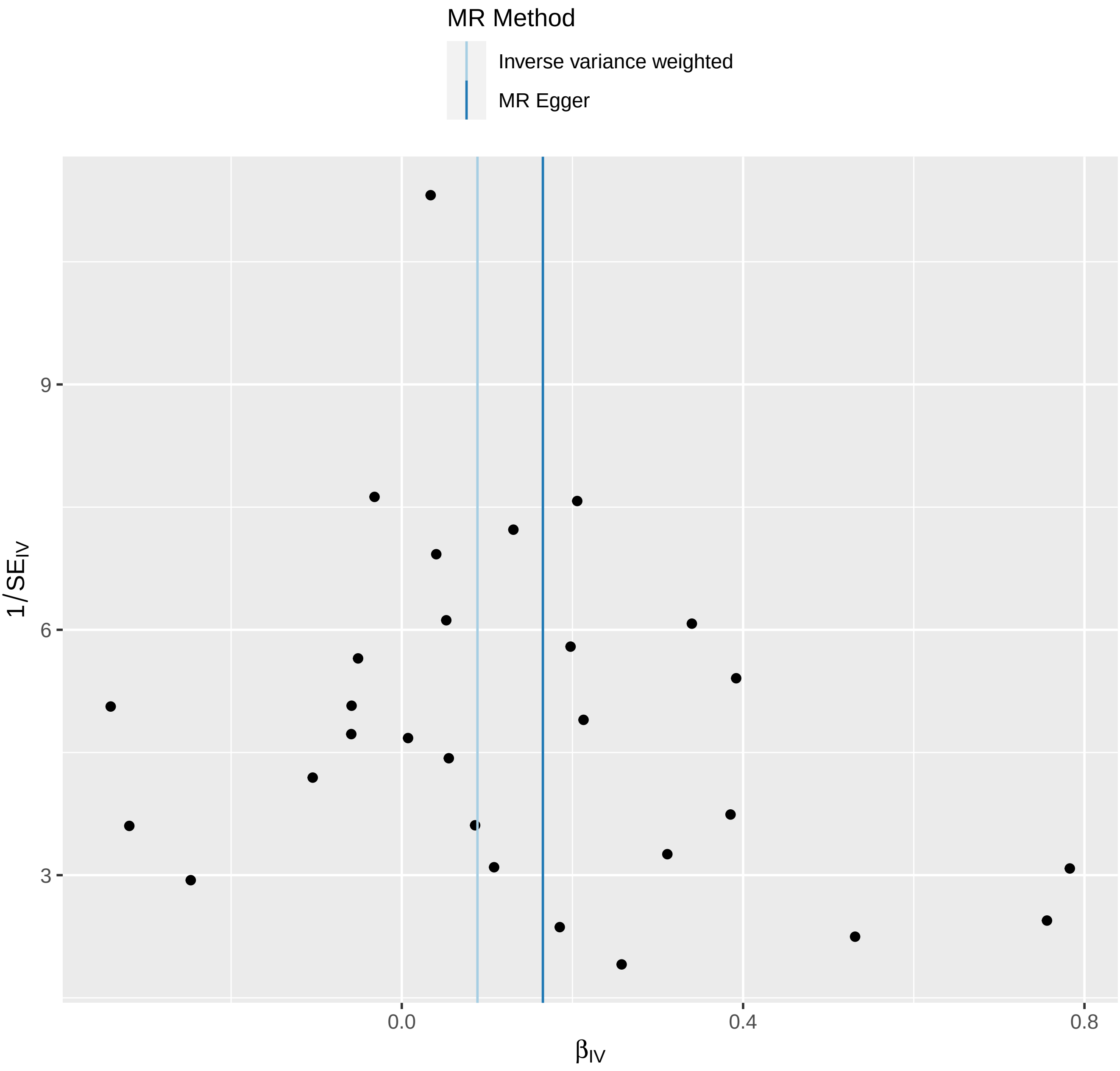

Batch 3−585 : Clathrin interactor 1 on Diabetic nephropathy (more control exclusions)
with a threshold of 1e−05

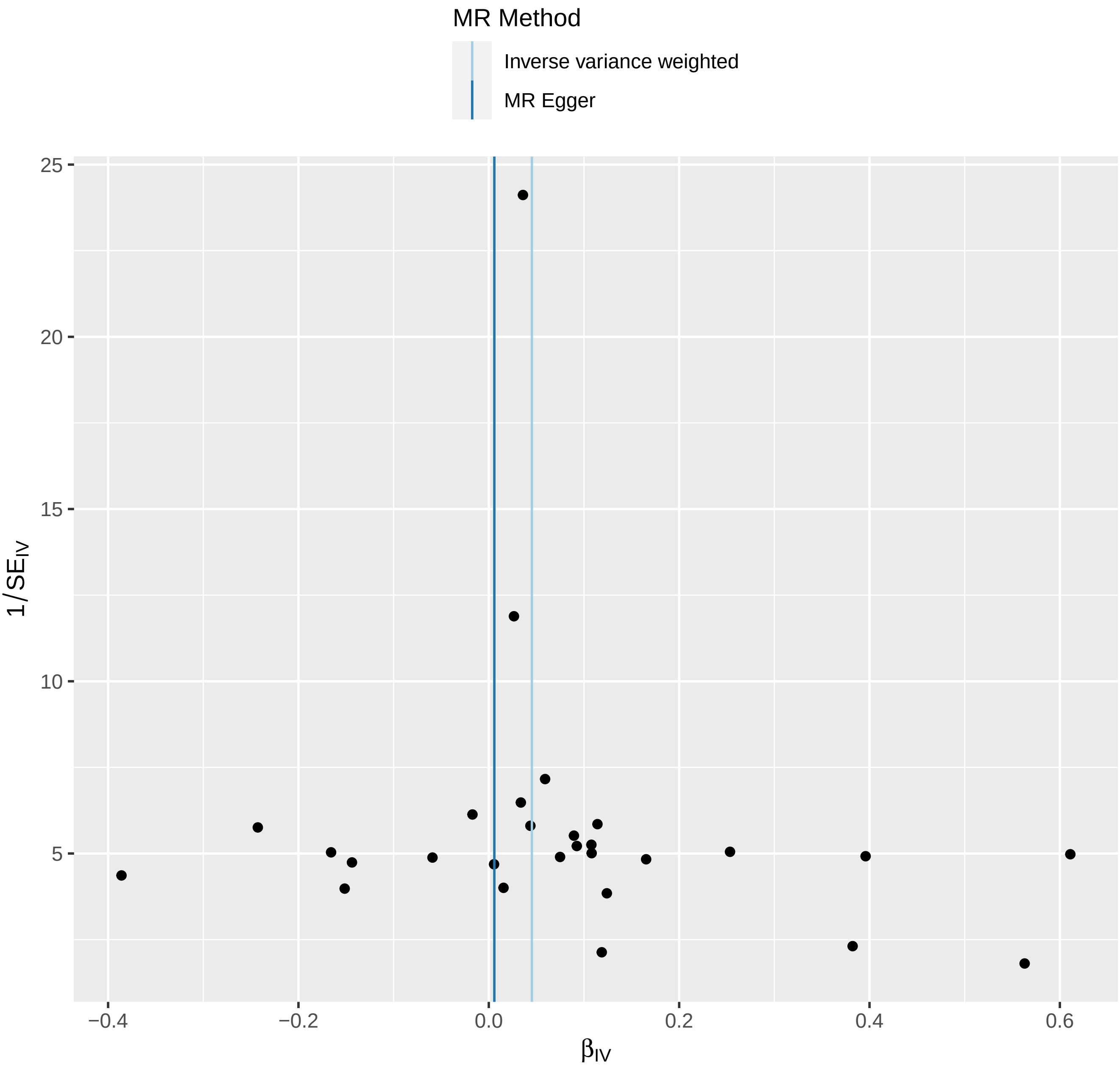

Batch 3−586 : Dual specificity protein kinase CLK2 on Diabetic nephropathy (more control exclusions)
with a threshold of 1e−05

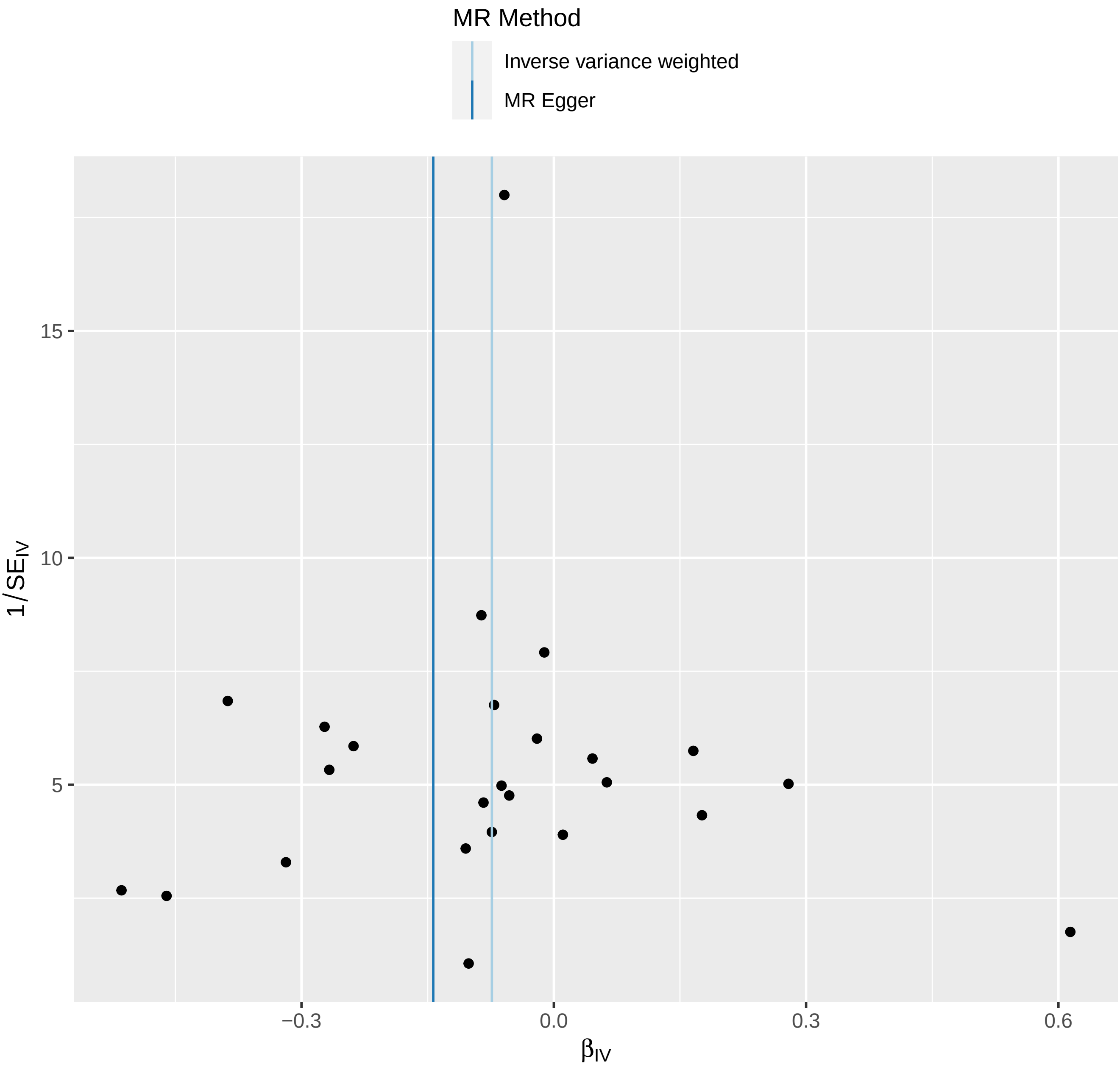

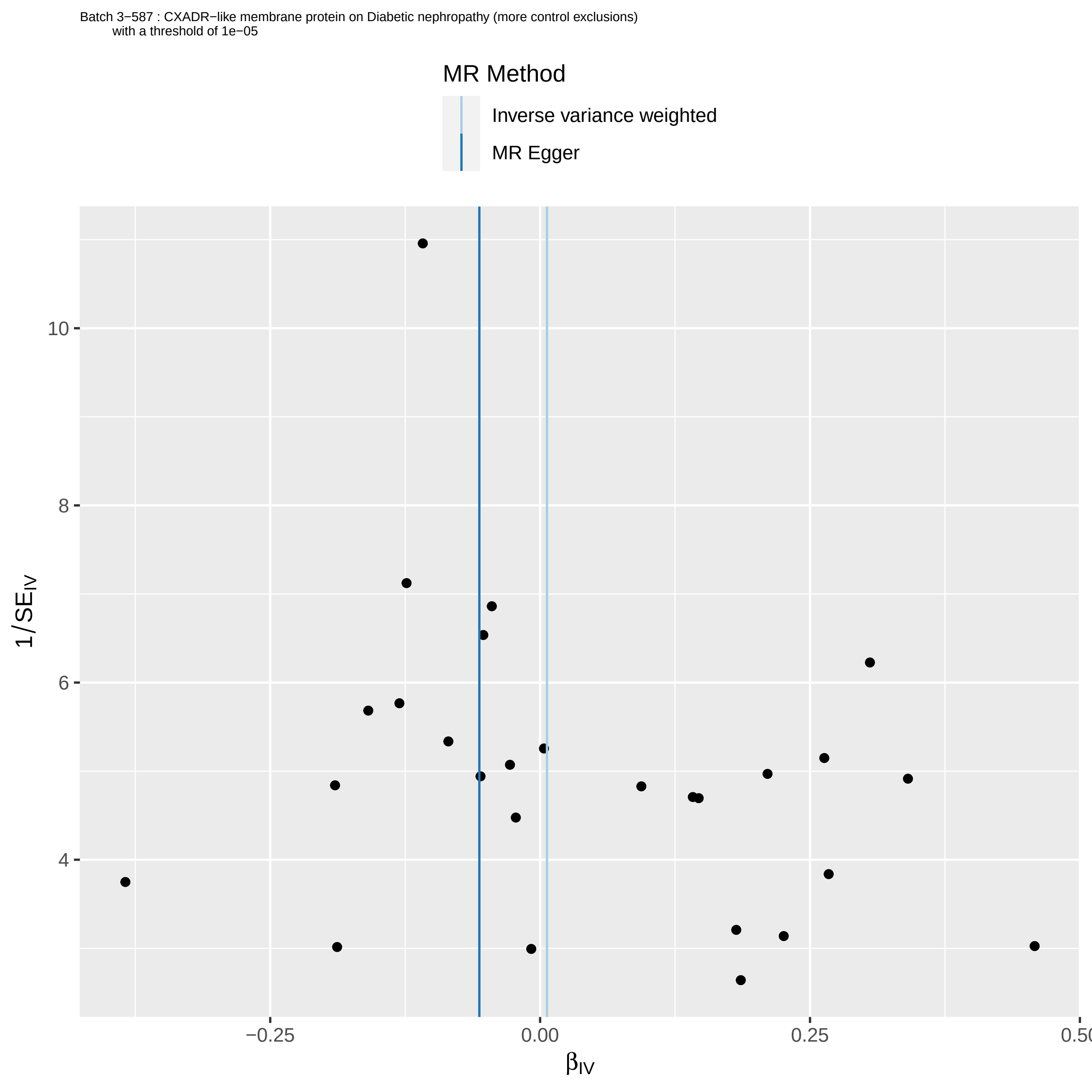
Batch 3−587 : CXADR−like membrane protein on Diabetic nephropathy (more control exclusions)
with a threshold of 1e−05
MR Method
Inverse variance weighted
MR Egger
10
8
6
4
1/SE_IV
−0.25
0.00
0.25
0.50
β_IV

Batch 3−588 : CXADR−like membrane protein on Diabetic nephropathy (more control exclusions)
with a threshold of 1e−05

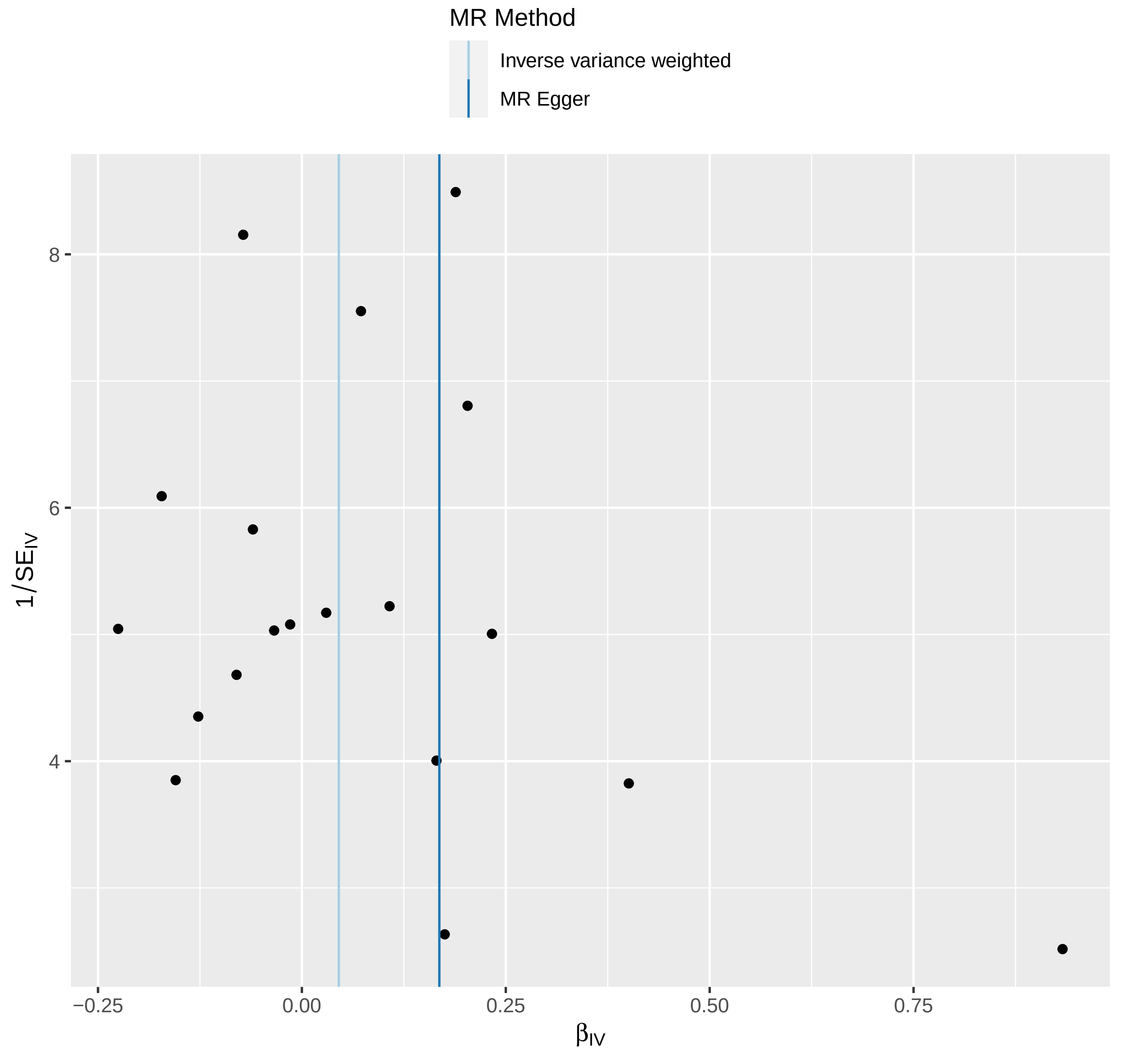

Batch 3−589 : Ceroid−lipofuscinosis neuronal protein 5 on Diabetic nephropathy (more control exclusions)
with a threshold of 1e−05

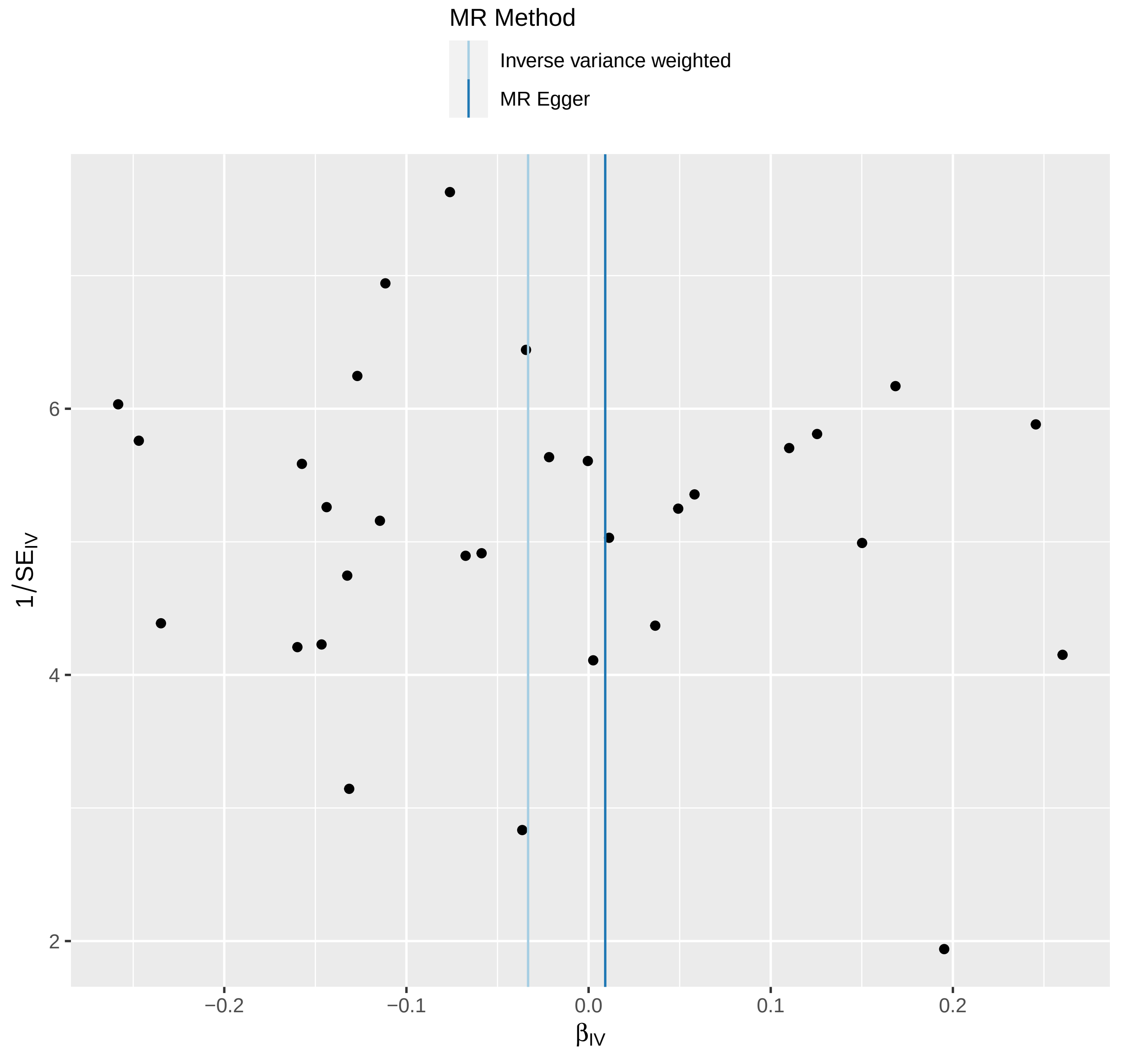

Batch 3−590 : Ceroid−lipofuscinosis neuronal protein 5 on Diabetic nephropathy (more control exclusions)
with a threshold of 1e−05

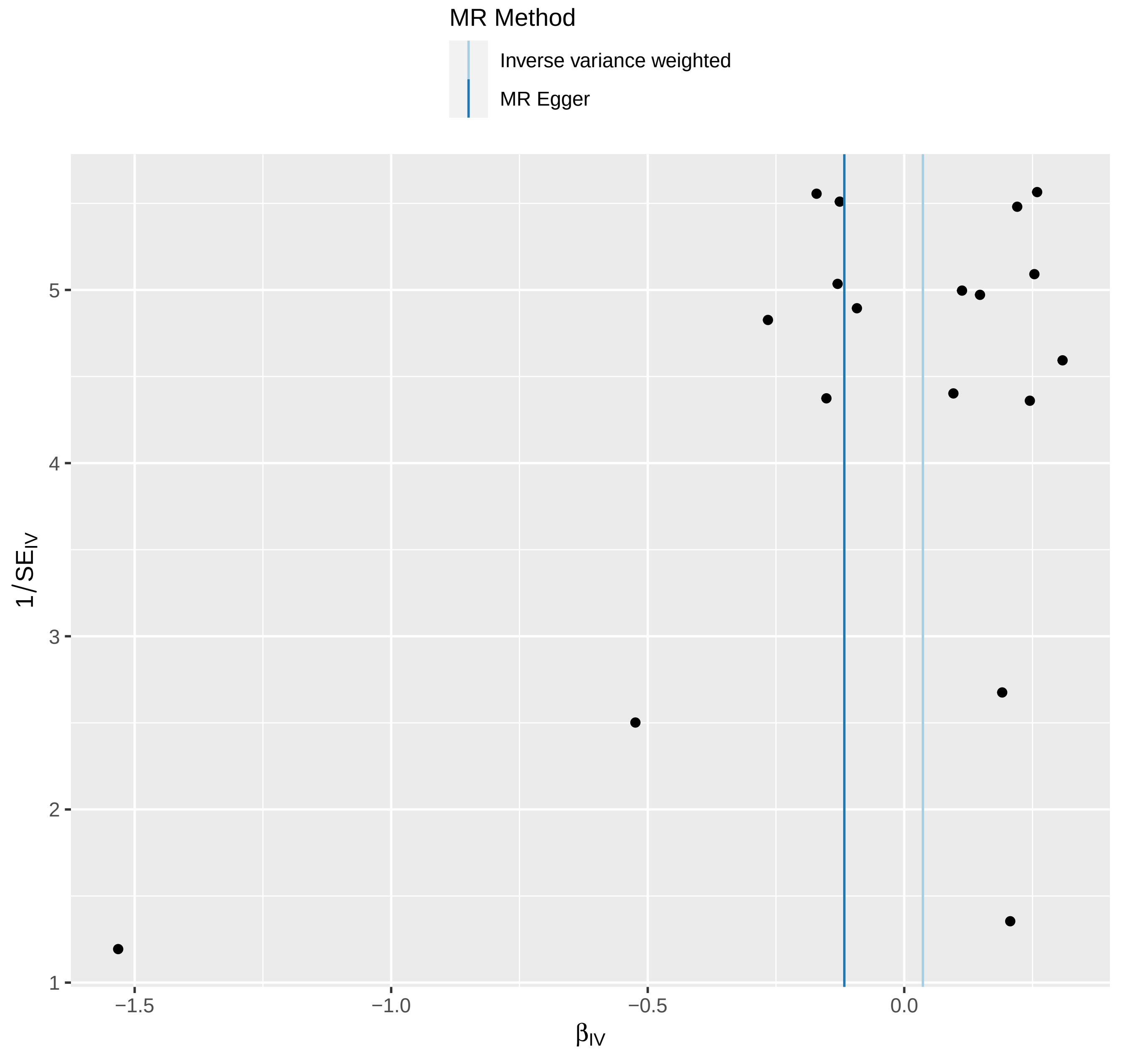

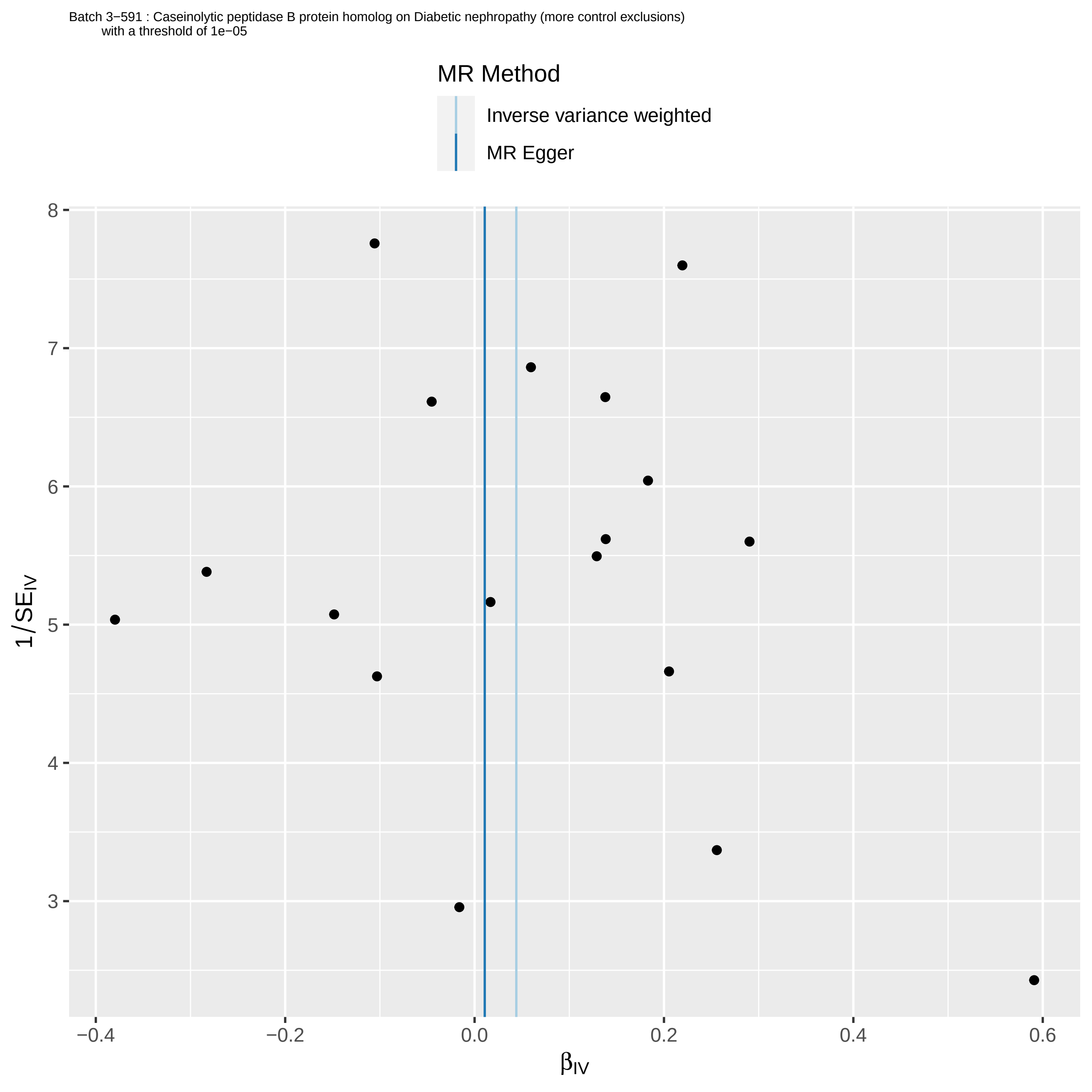
Batch 3−591 : Caseinolytic peptidase B protein homolog on Diabetic nephropathy (more control exclusions)
with a threshold of 1e−05
MR Method
Inverse variance weighted
MR Egger
8
7
6
5
4
3
1/SE_IV
−0.4
−0.2
0.0
0.2
0.4
0.6
β_IV

Batch 3−592 : Colipase on Diabetic nephropathy (more control exclusions)
with a threshold of 1e−05

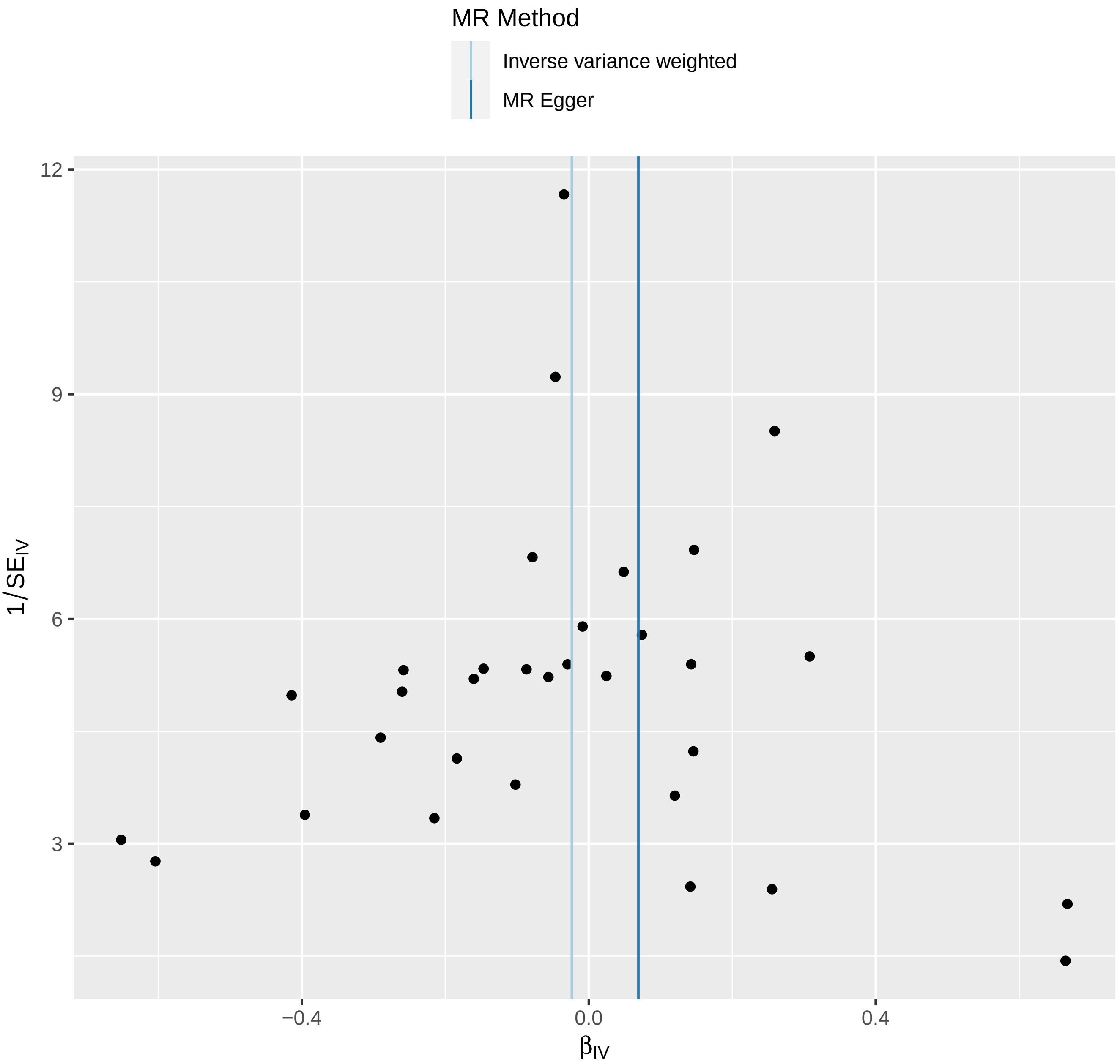

Batch 3−593 : Colipase−like protein 2 on Diabetic nephropathy (more control exclusions)
with a threshold of 1e−05

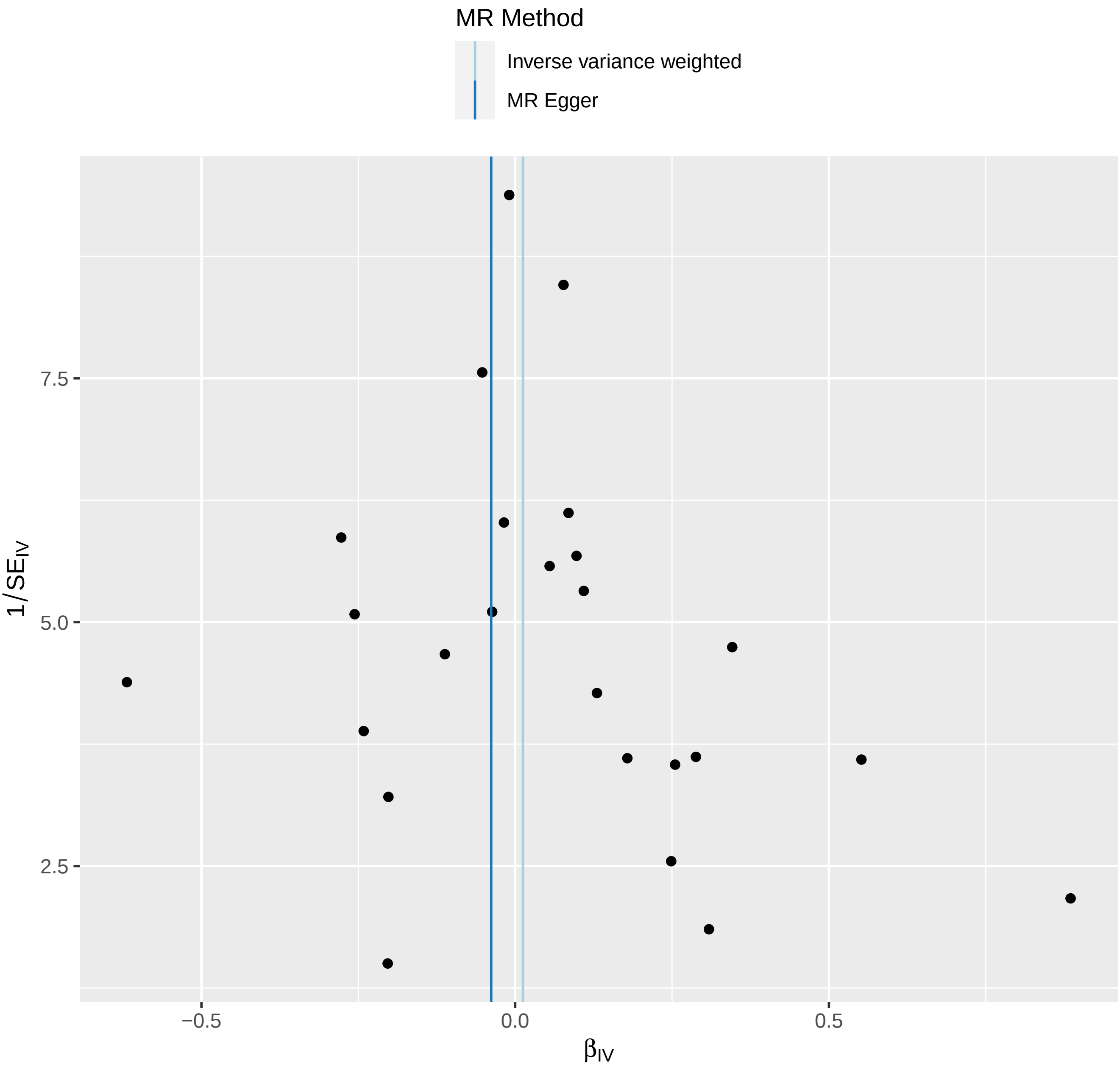

Batch 3−594 : Calsyntenin−3 on Diabetic nephropathy (more control exclusions)
with a threshold of 1e−05

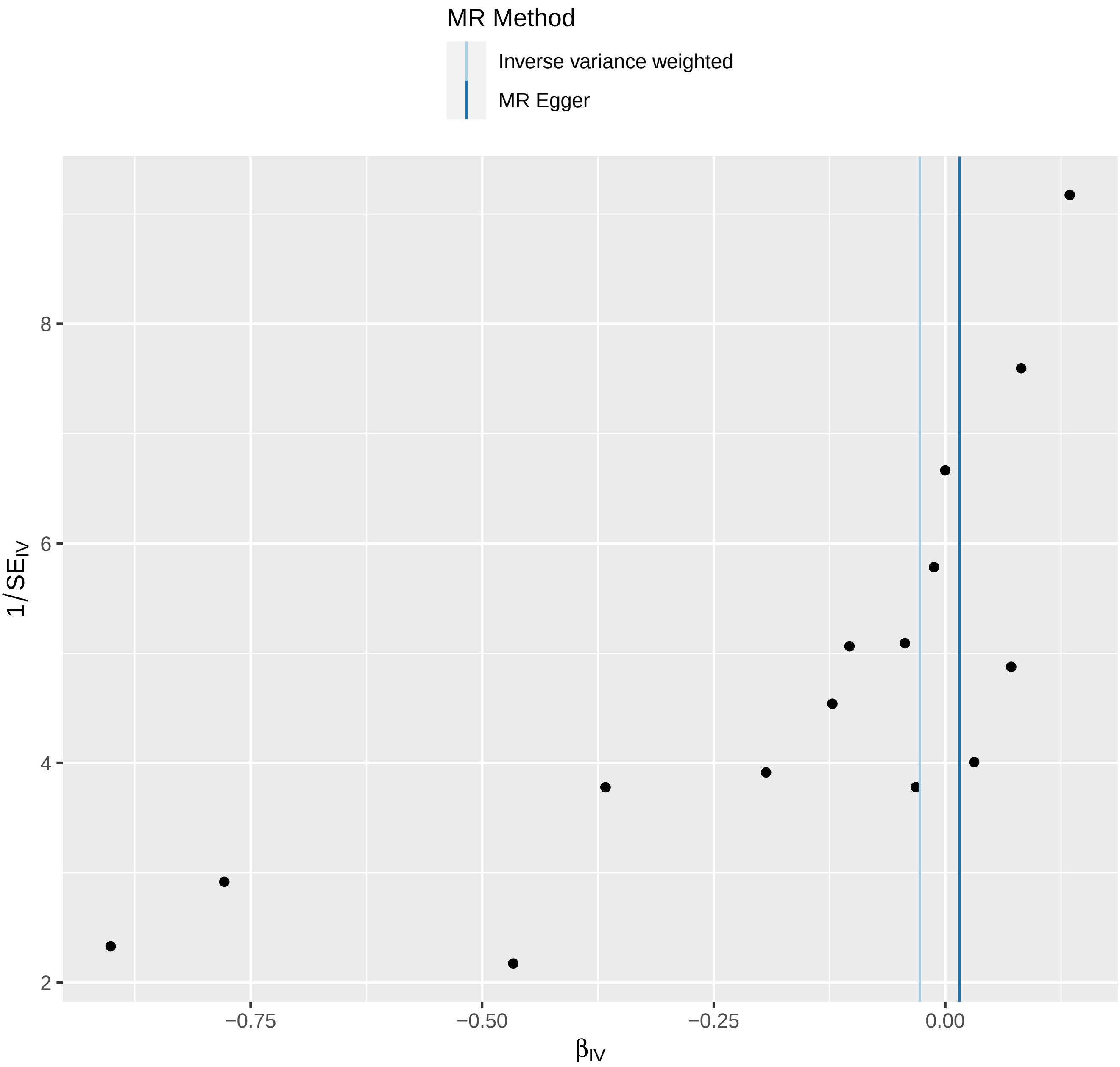

Batch 3−595 : Clathrin heavy chain 1 on Diabetic nephropathy (more control exclusions)
with a threshold of 1e−05

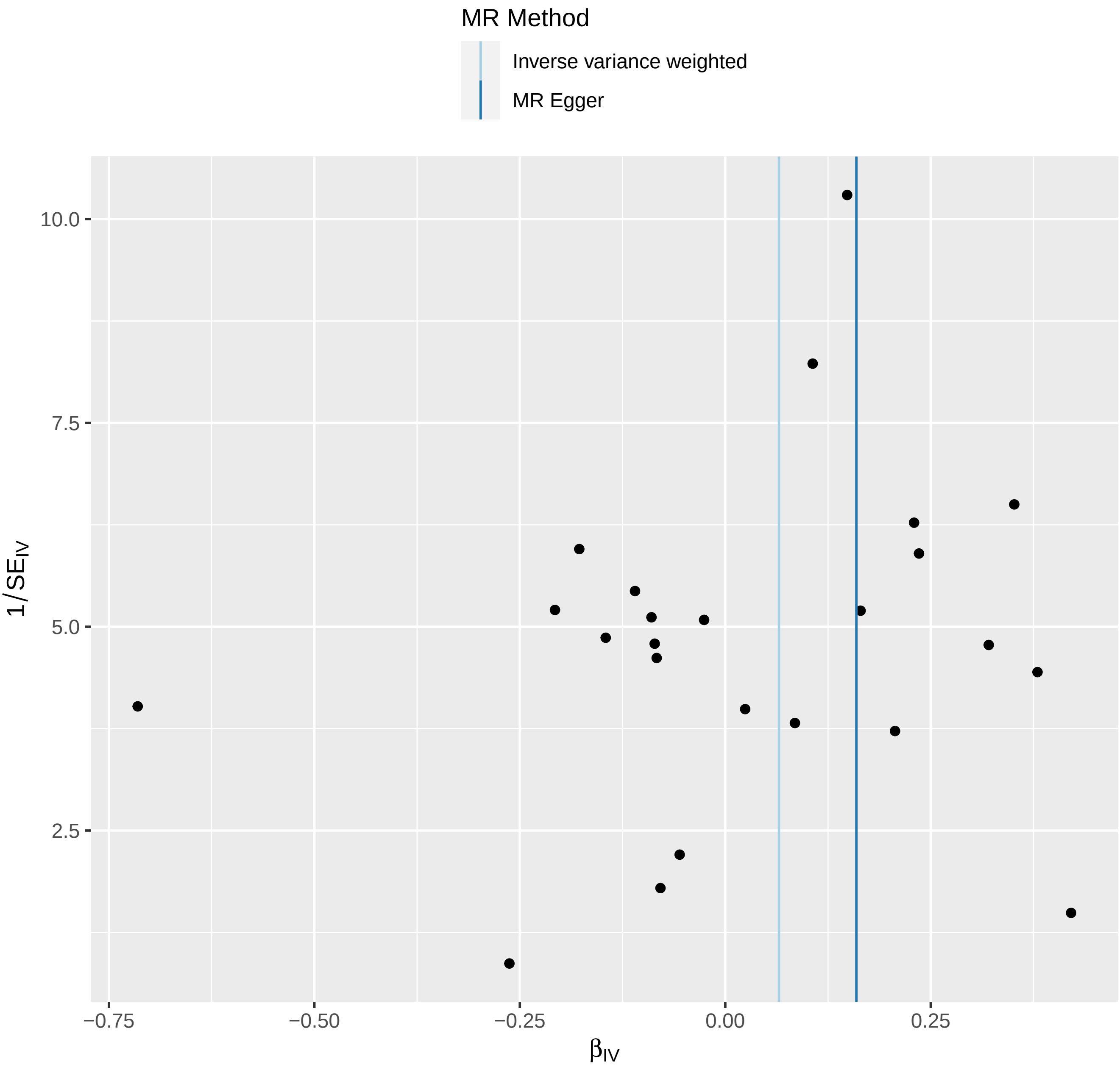

Batch 3−596 : Clusterin on Diabetic nephropathy (more control exclusions)
with a threshold of 1e−05

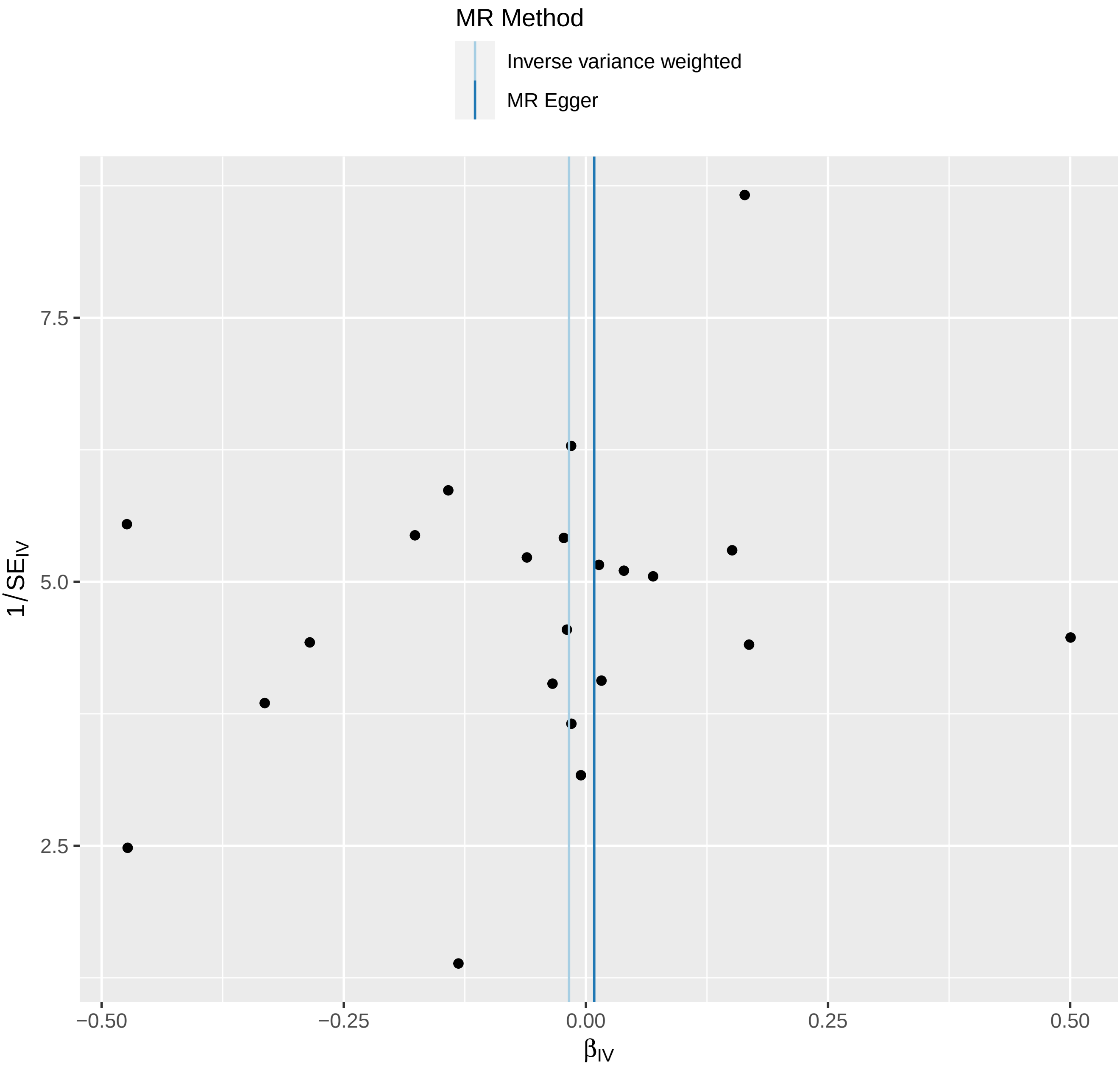

Batch 3−597 : Chymase on Diabetic nephropathy (more control exclusions)
with a threshold of 1e−05

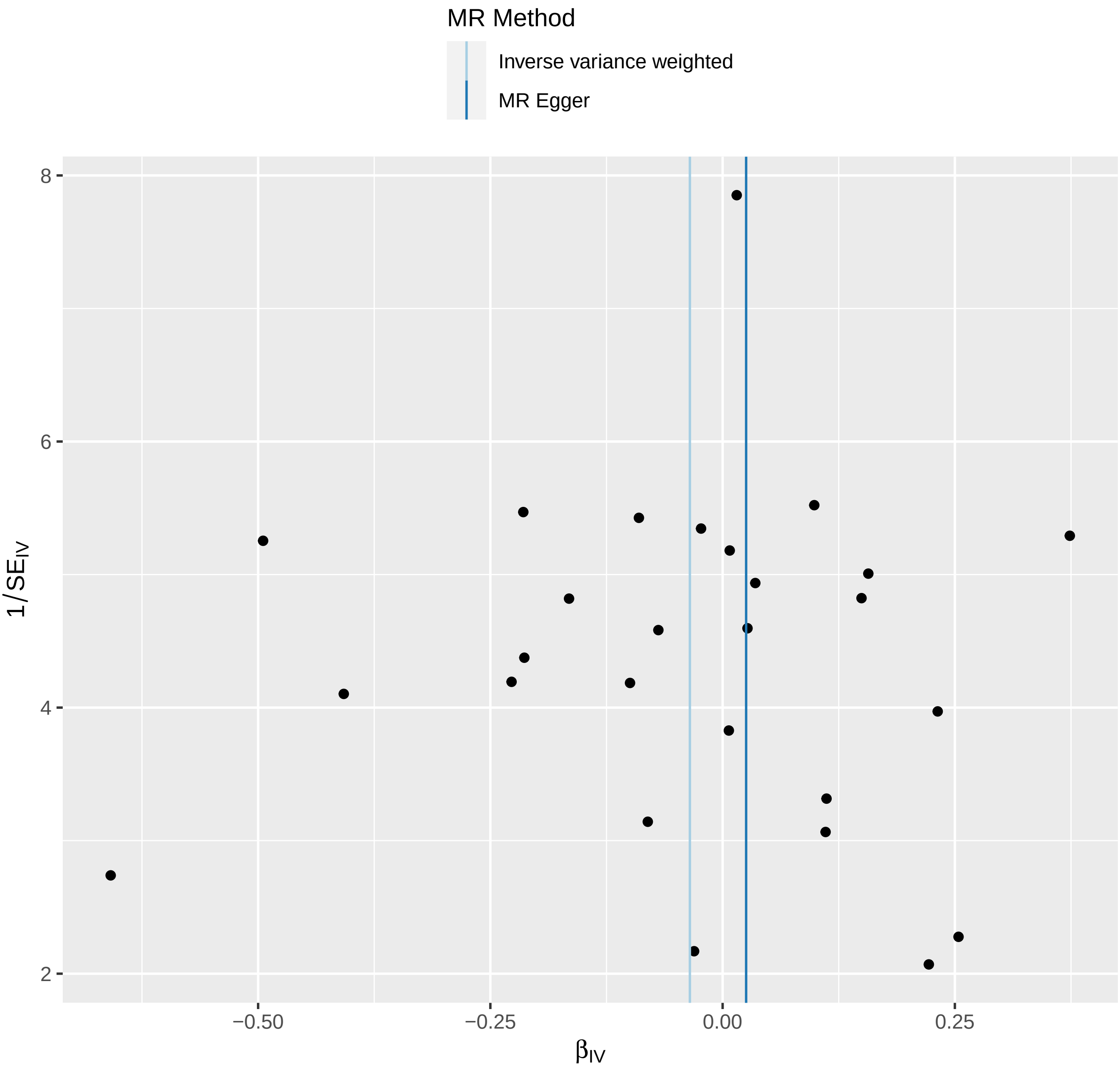

Batch 3−598 : UMP−CMP kinase on Diabetic nephropathy (more control exclusions)
with a threshold of 1e−05

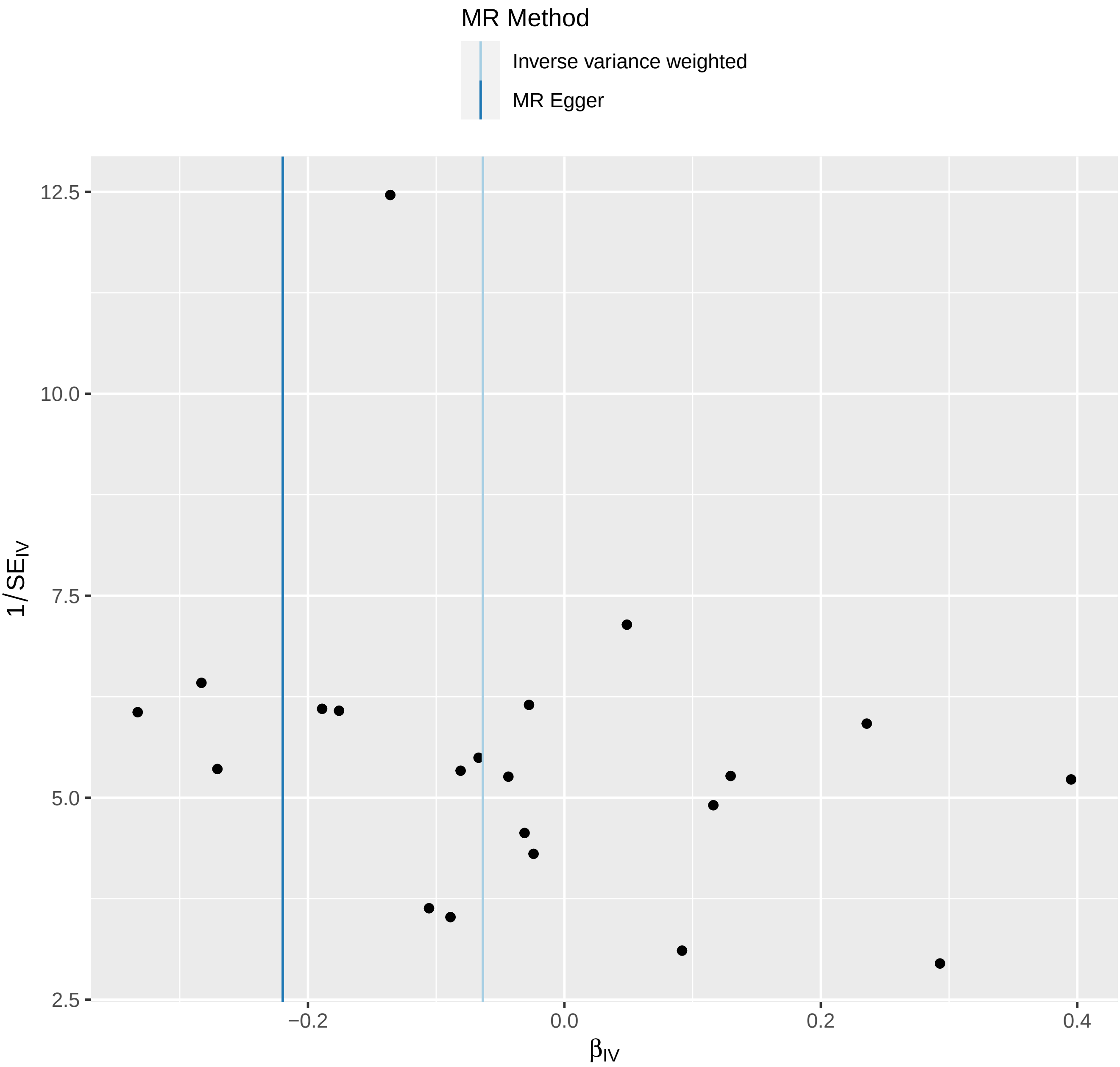

Batch 3−599 : UMP−CMP kinase on Diabetic nephropathy (more control exclusions)
with a threshold of 1e−05

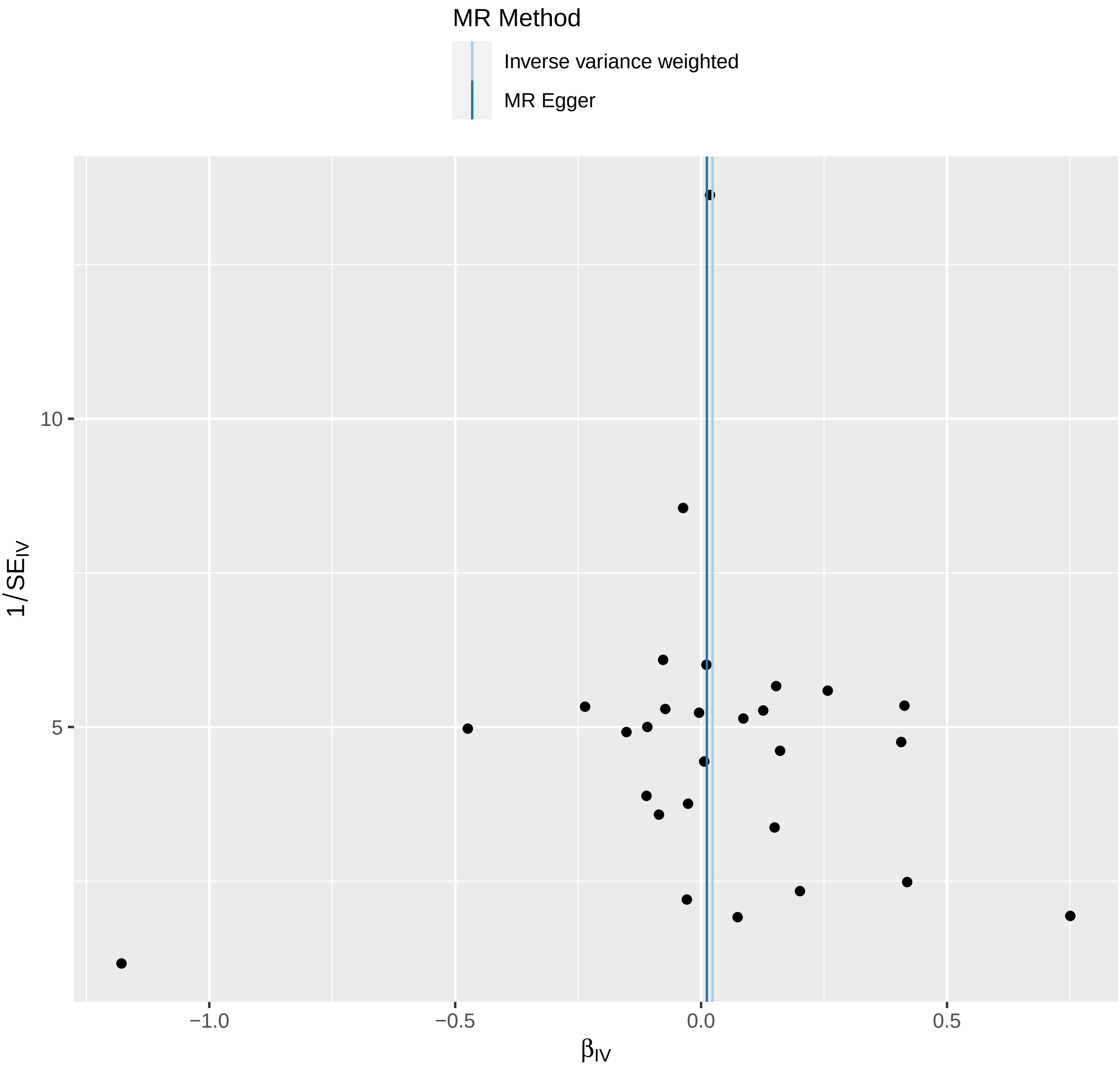

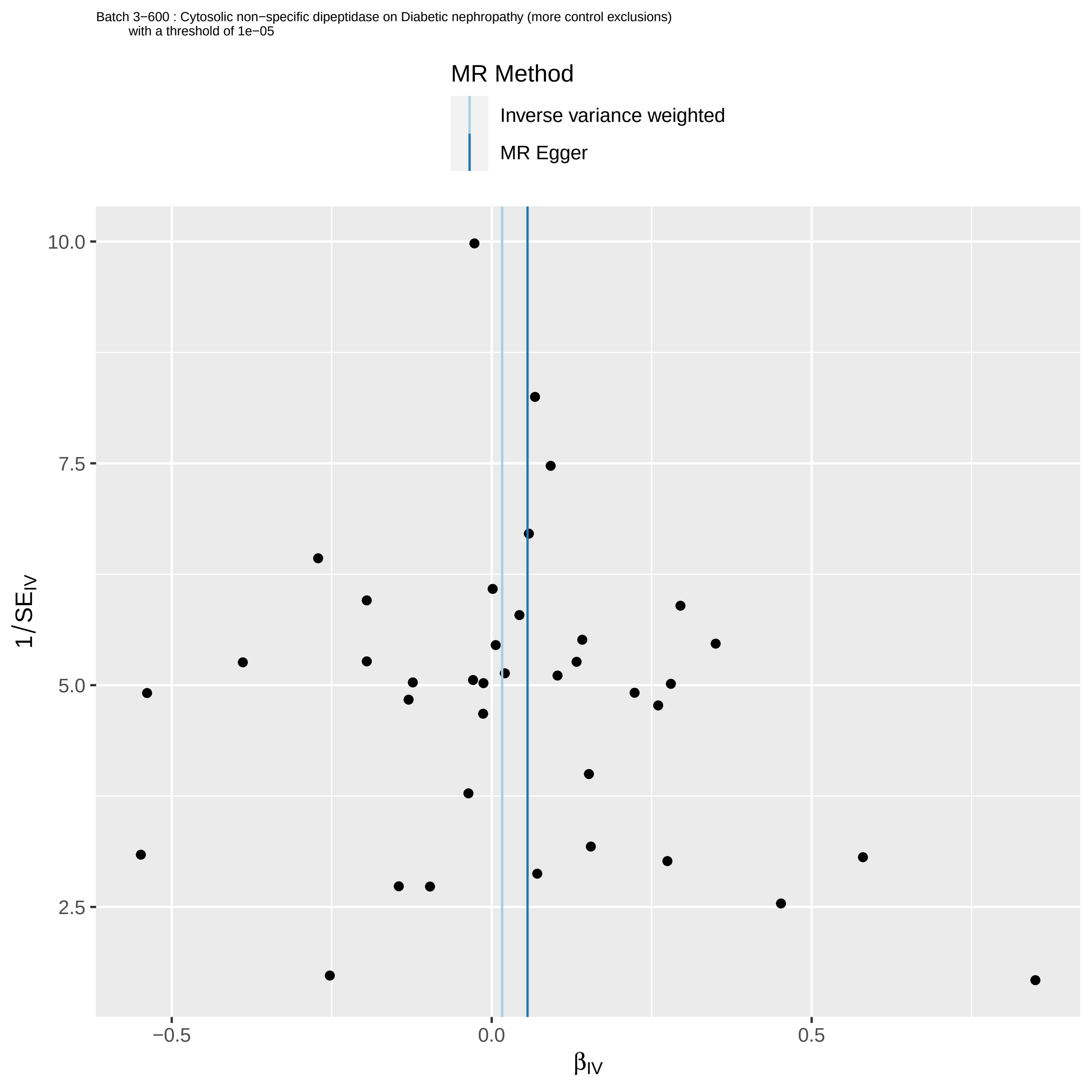
Batch 3−600 : Cytosolic non−specific dipeptidase on Diabetic nephropathy (more control exclusions)
with a threshold of 1e−05
MR Method
Inverse variance weighted
MR Egger
10.0
7.5
5.0
2.5
1/SE_IV
−0.5
0.0
0.5
β_IV

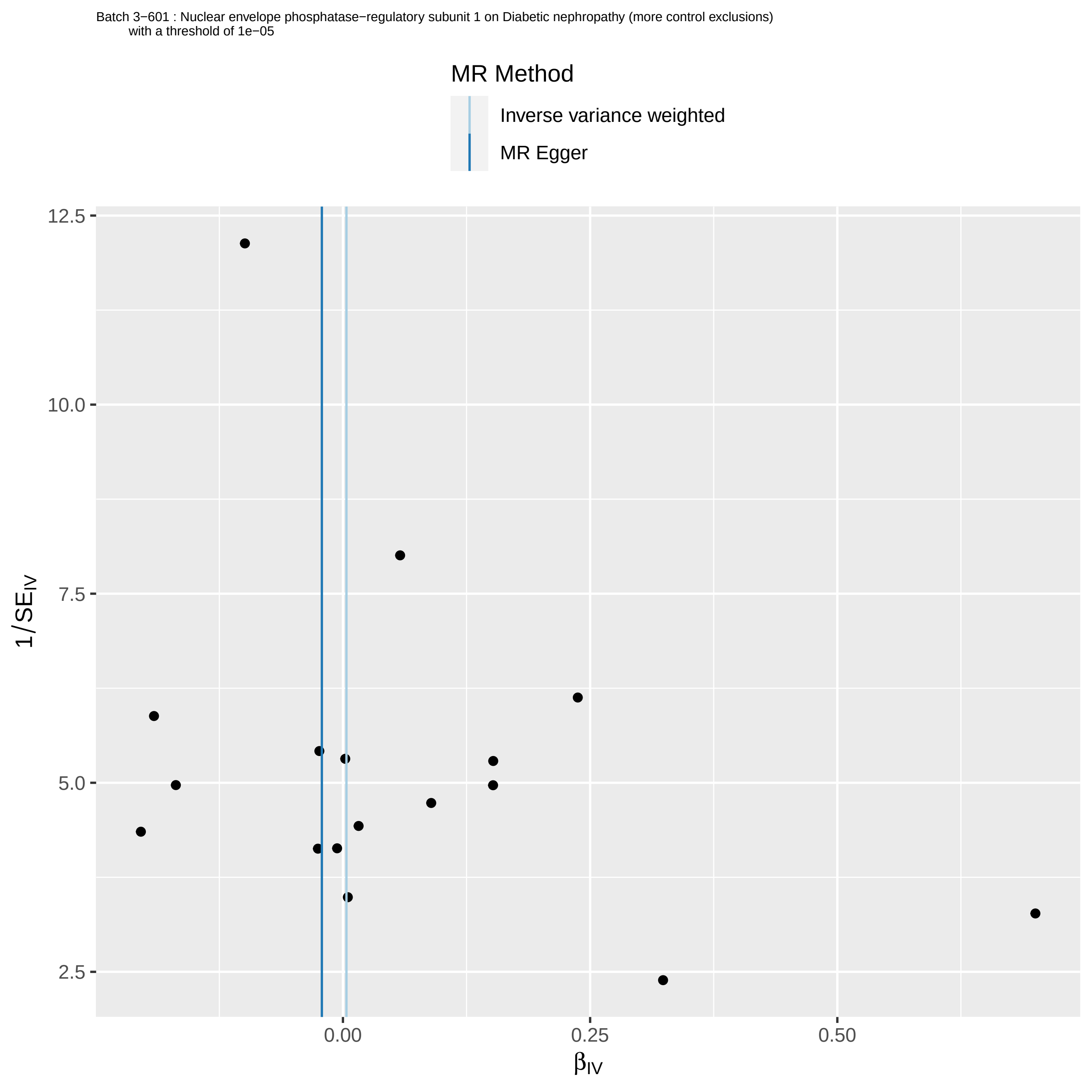

Batch 3−601 : Nuclear envelope phosphatase−regulatory subunit 1 on Diabetic nephropathy (more control exclusions)
with a threshold of 1e−05
MR Method
Inverse variance weighted
MR Egger
12.5
10.0
7.5
5.0
2.5
1/SE$_{IV}$
0.00
0.25
0.50
β$_{IV}$

Batch 3−602 : CCR4−NOT transcription complex subunit 1 on Diabetic nephropathy (more control exclusions)
with a threshold of 1e−05

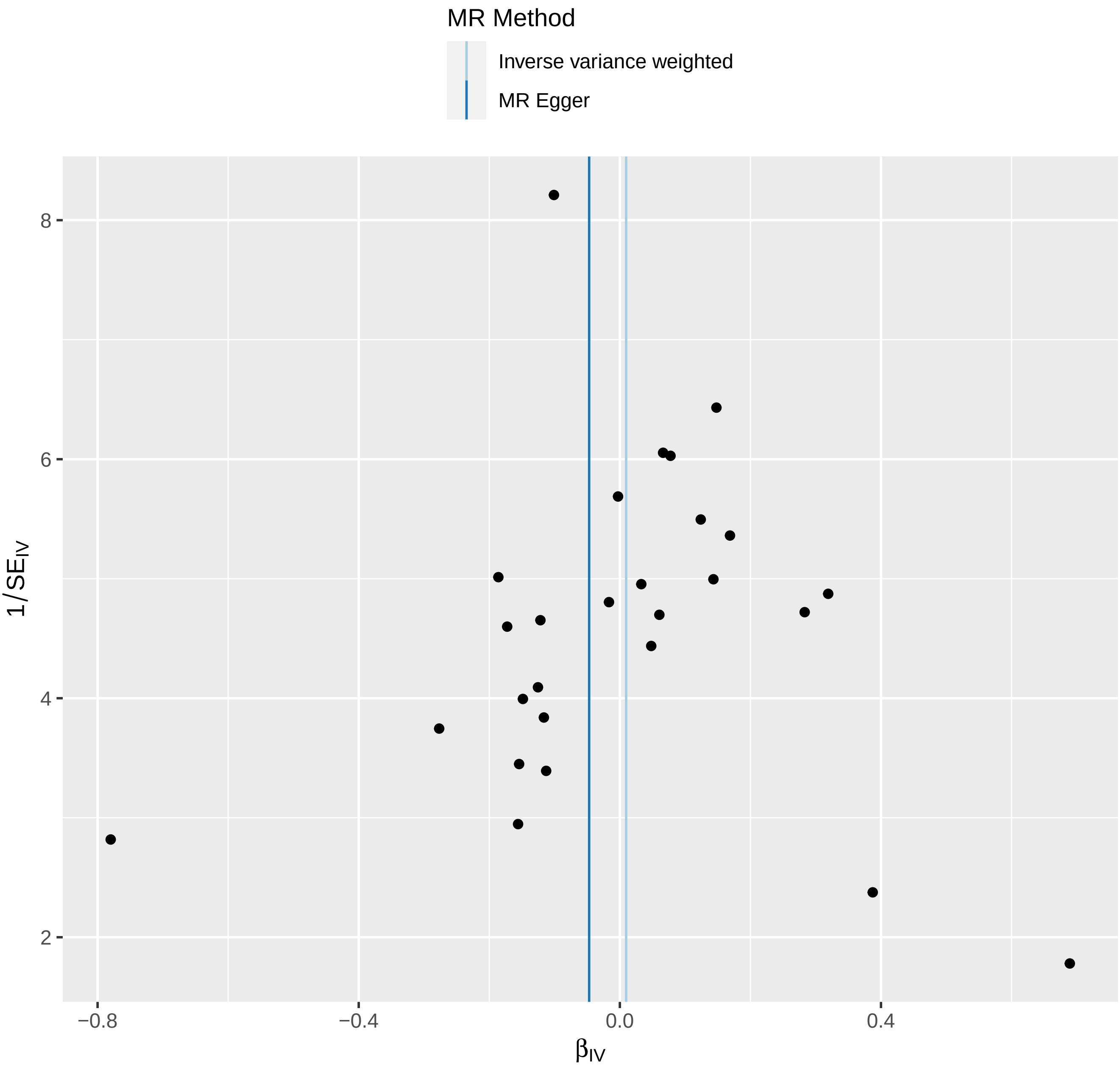

Batch 3−603 : 2',3'−cyclic−nucleotide 3'−phosphodiesterase on Diabetic nephropathy (more control exclusions) with a threshold of 1e−05

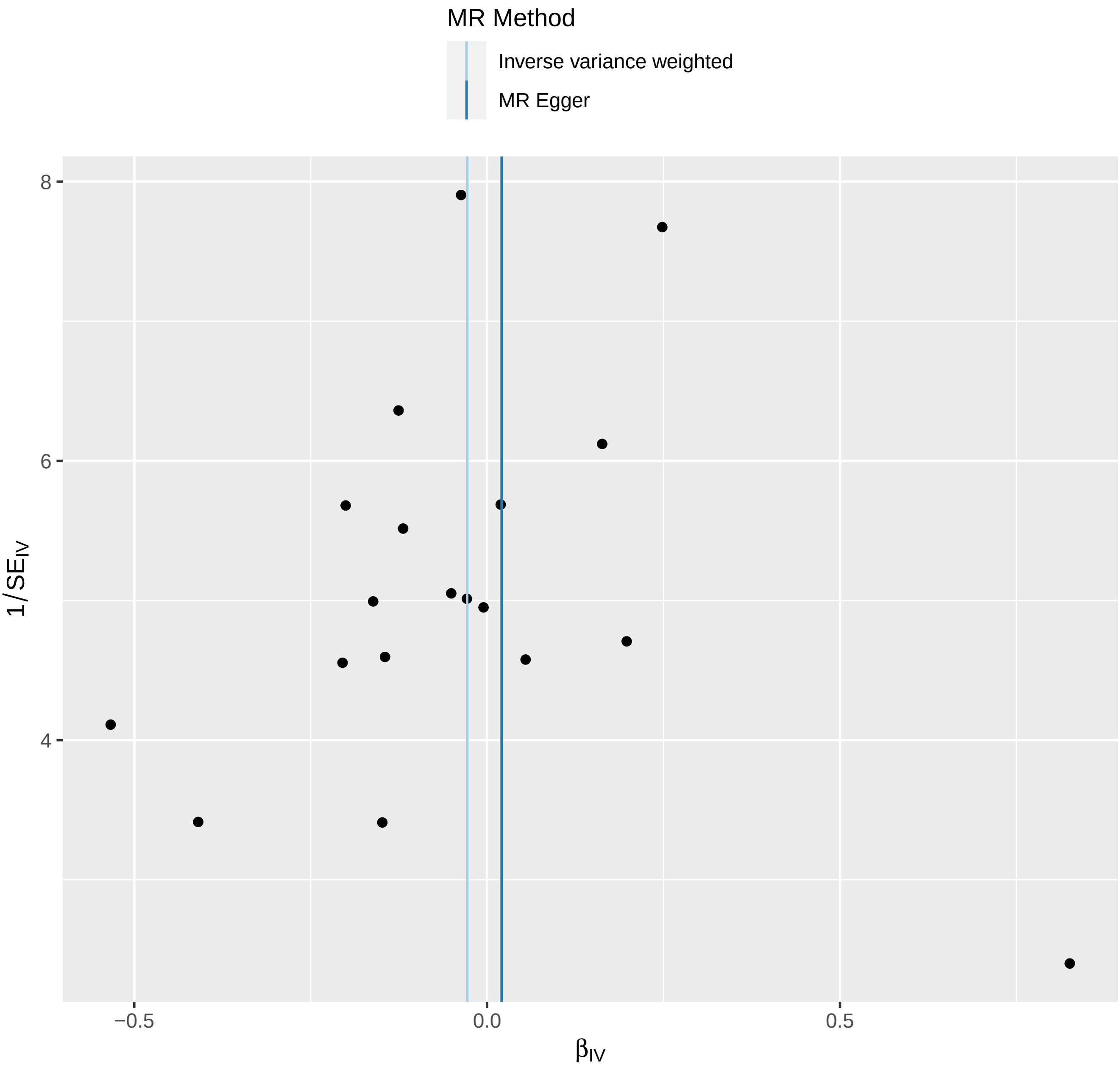

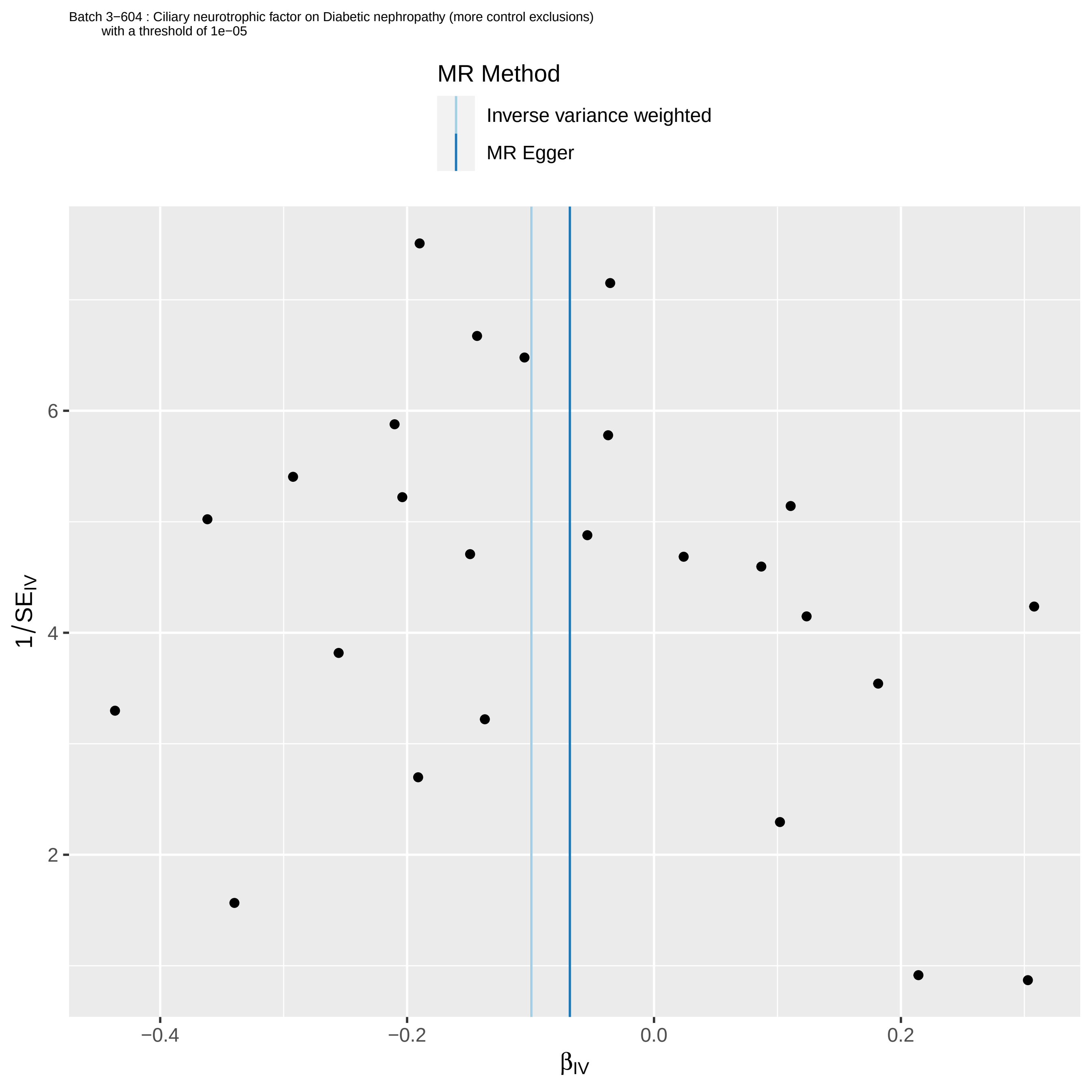

Batch 3−604 : Ciliary neurotrophic factor on Diabetic nephropathy (more control exclusions)
with a threshold of 1e−05
MR Method
Inverse variance weighted
MR Egger
6
4
2
1/SE_IV
−0.4
−0.2
0.0
0.2
β_IV

Batch 3−605 : Ciliary neurotrophic factor receptor subunit alpha on Diabetic nephropathy (more control exclusions)
with a threshold of 1e−05

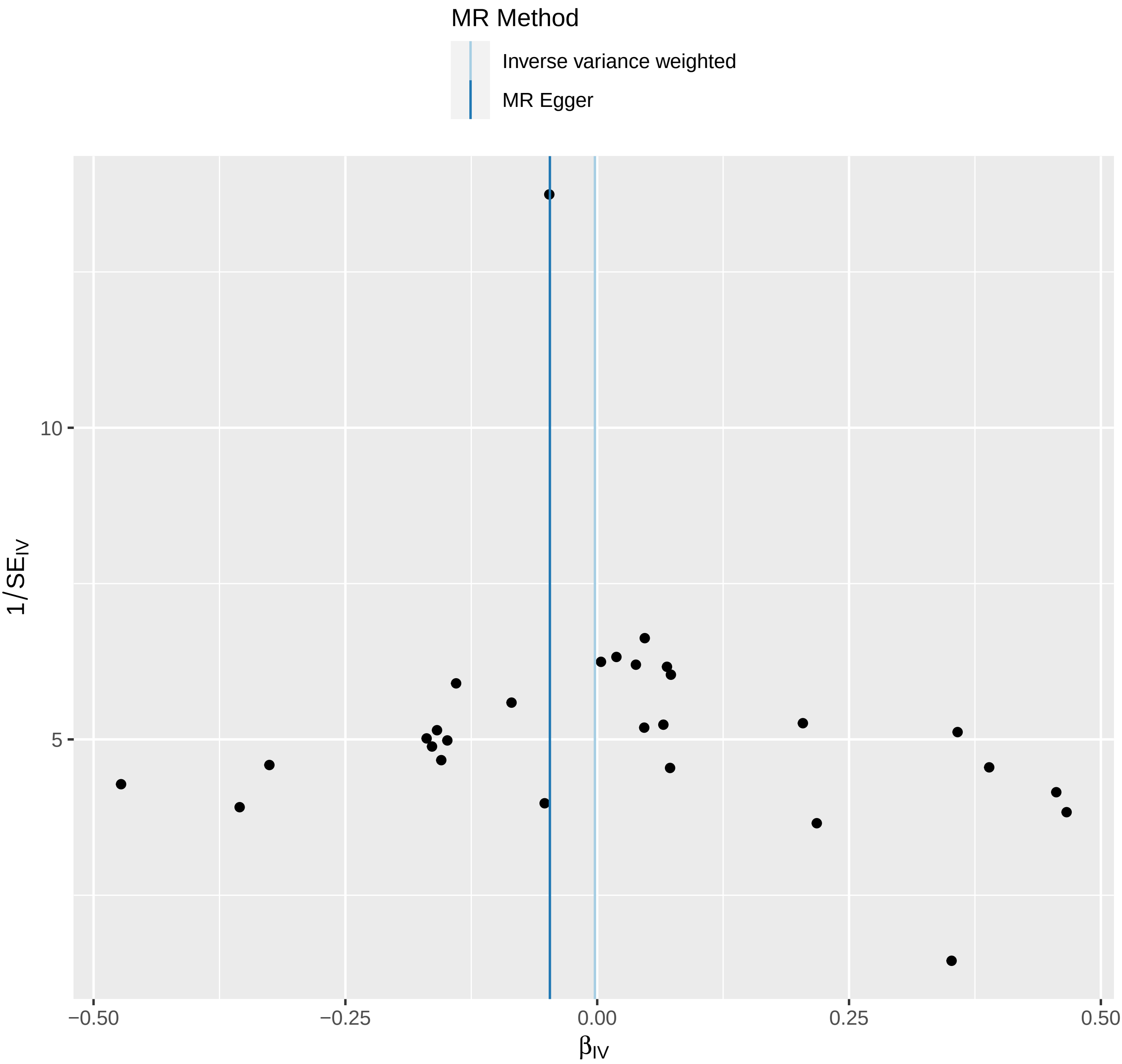

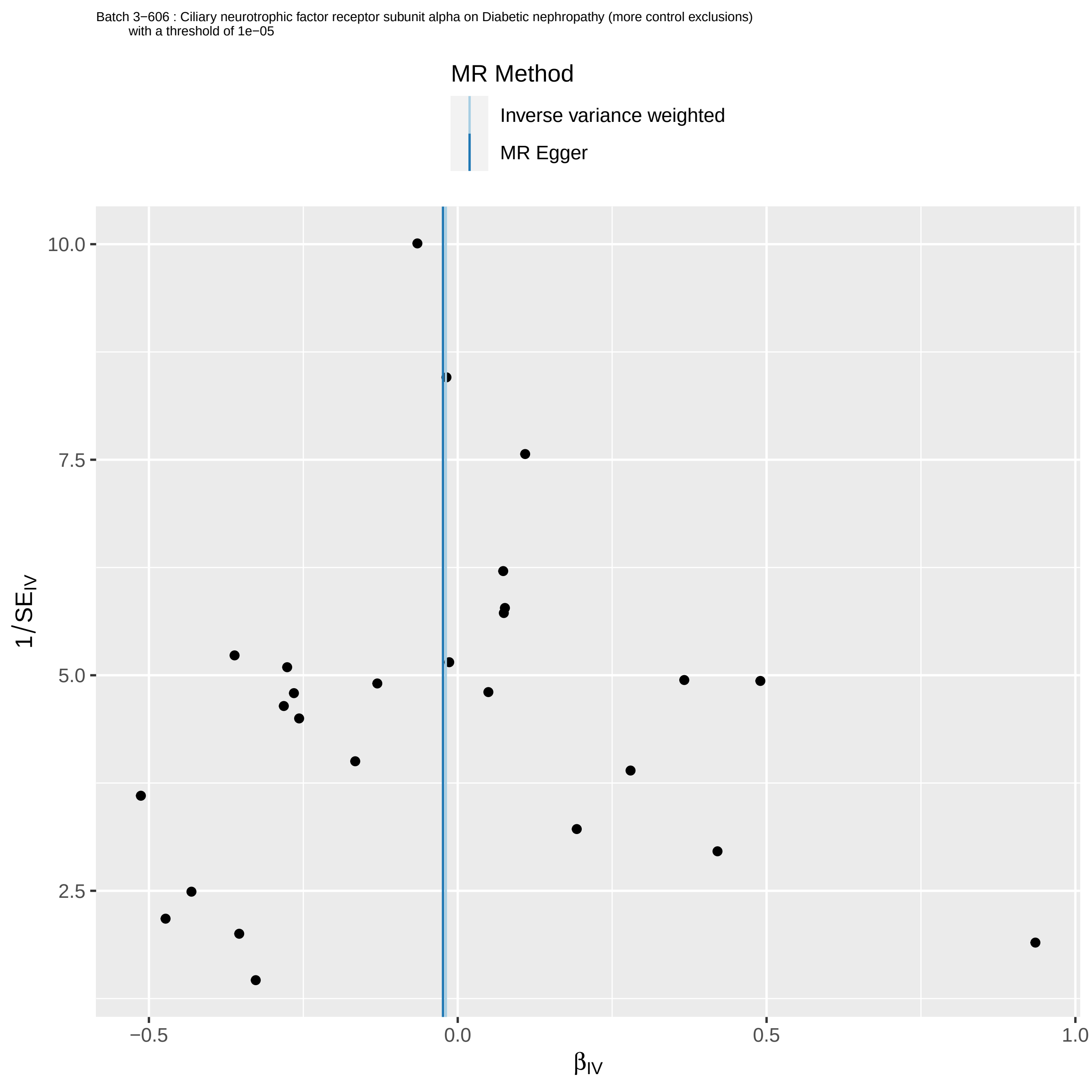

Batch 3−606 : Ciliary neurotrophic factor receptor subunit alpha on Diabetic nephropathy (more control exclusions) with a threshold of 1e−05
MR Method
Inverse variance weighted
MR Egger
10.0
7.5
5.0
2.5
$1/SE_{IV}$
−0.5
0.0
0.5
1.0
β_{IV}

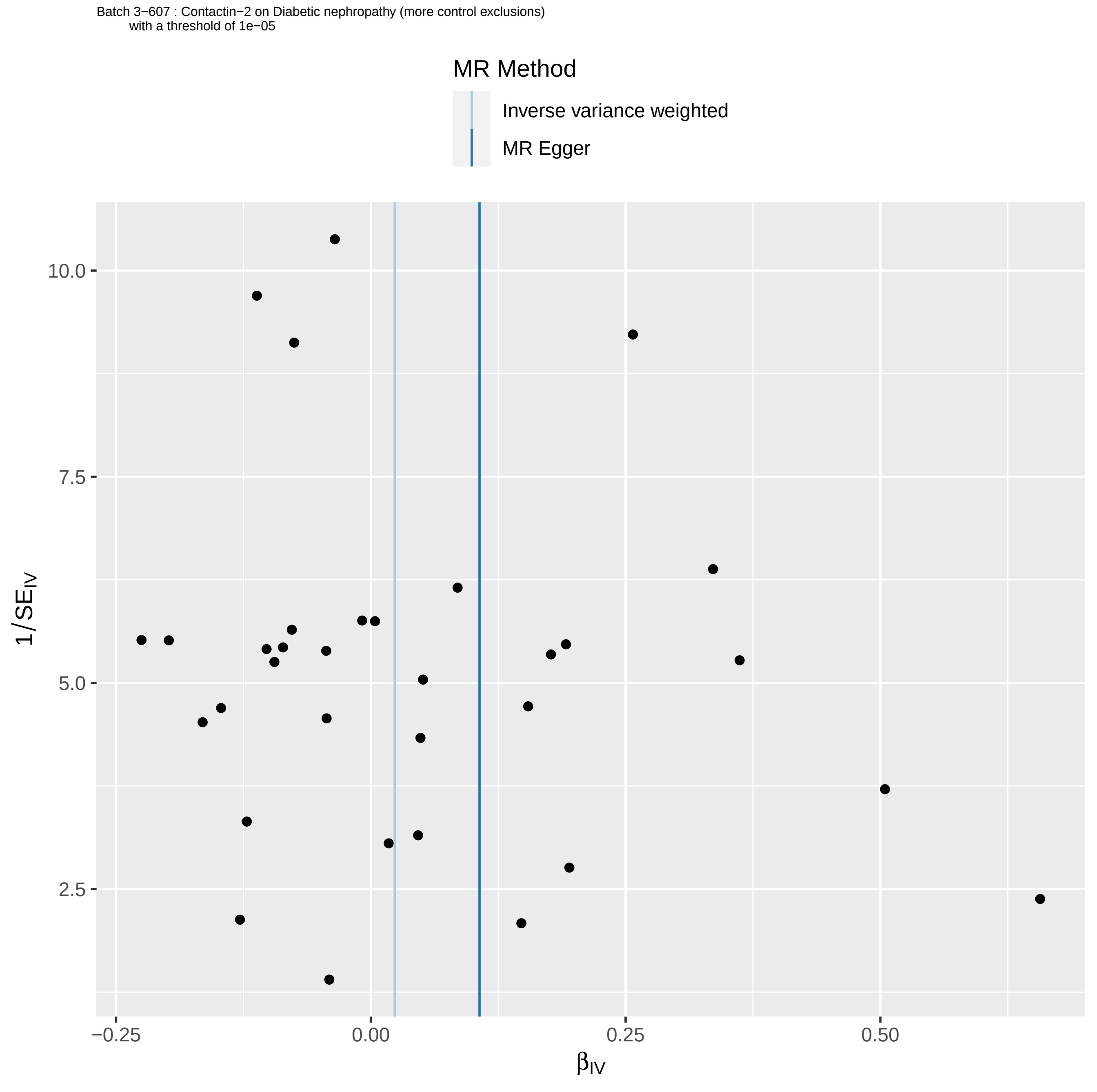

Batch 3−607 : Contactin−2 on Diabetic nephropathy (more control exclusions)
with a threshold of 1e−05
MR Method
Inverse variance weighted
MR Egger
10.0
7.5
5.0
2.5
−0.25
0.00
0.25
0.50
$1/SE_{IV}$
β_{IV}

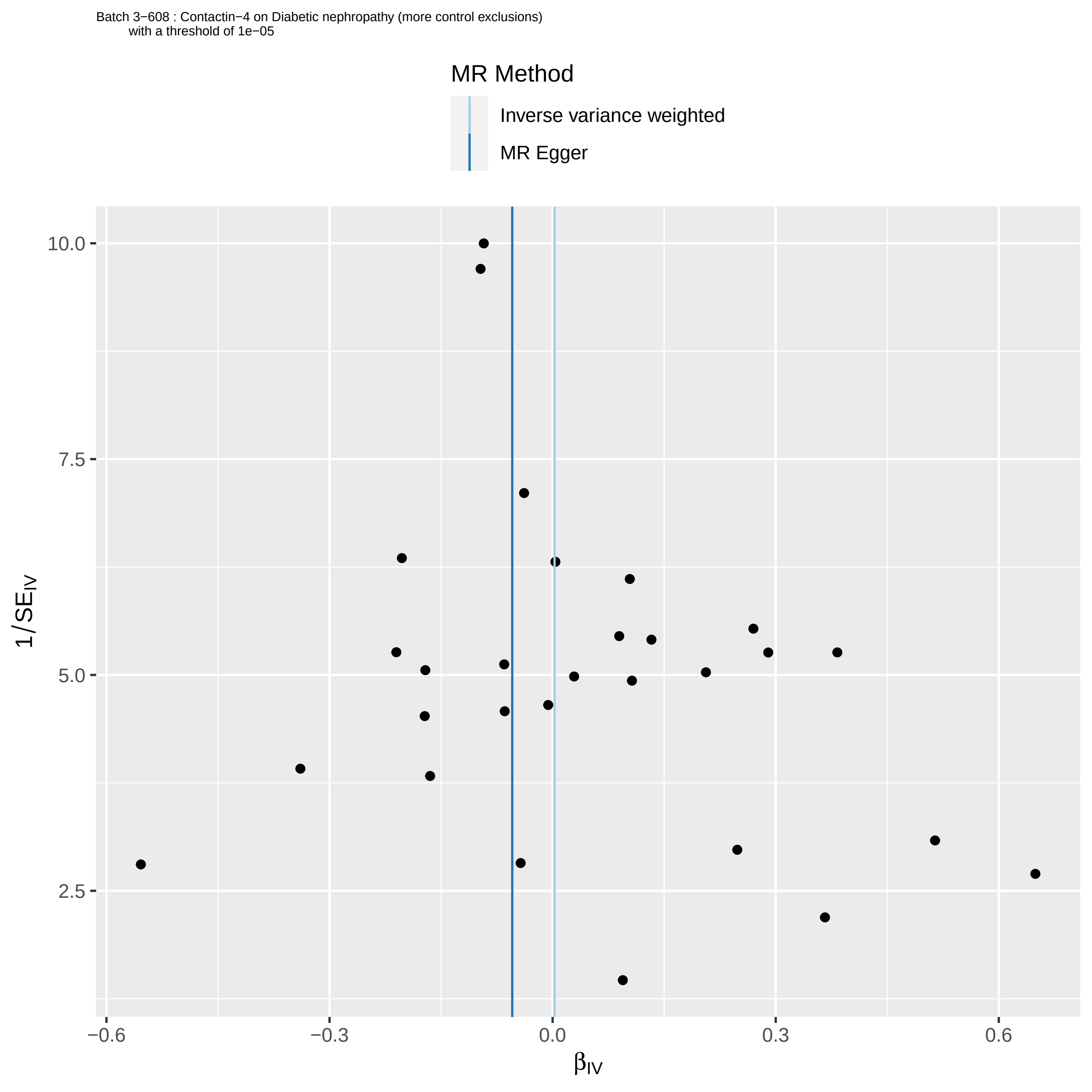

Batch 3−608 : Contactin−4 on Diabetic nephropathy (more control exclusions)
with a threshold of 1e−05
MR Method
Inverse variance weighted
MR Egger
10.0
7.5
5.0
2.5
1/SE_IV
−0.6
−0.3
0.0
0.3
0.6
β_IV

Batch 3−609 : Contactin−5 on Diabetic nephropathy (more control exclusions)
with a threshold of 1e−05

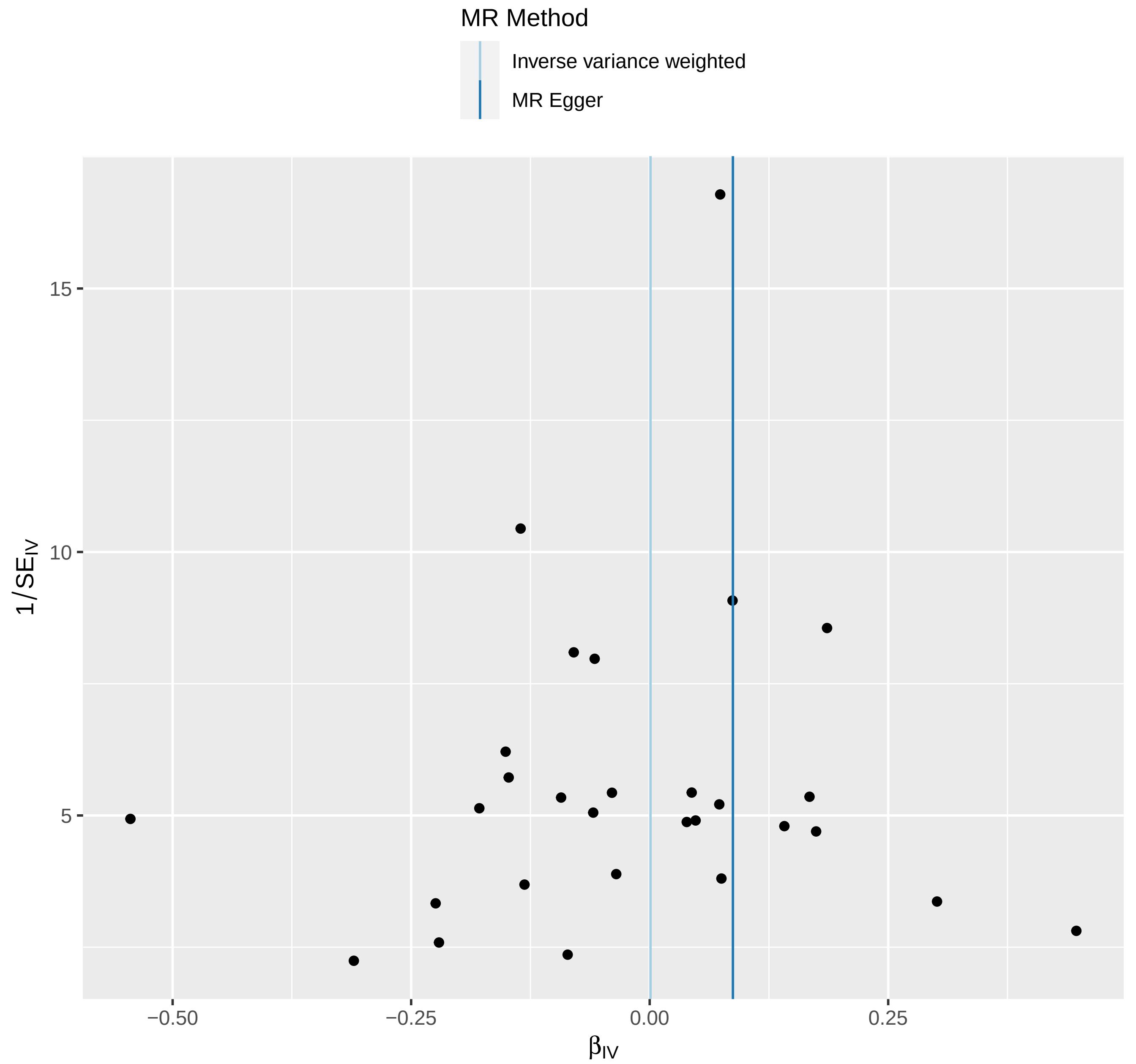

Batch 3−610 : Contactin−associated protein 1 on Diabetic nephropathy (more control exclusions)
with a threshold of 1e−05

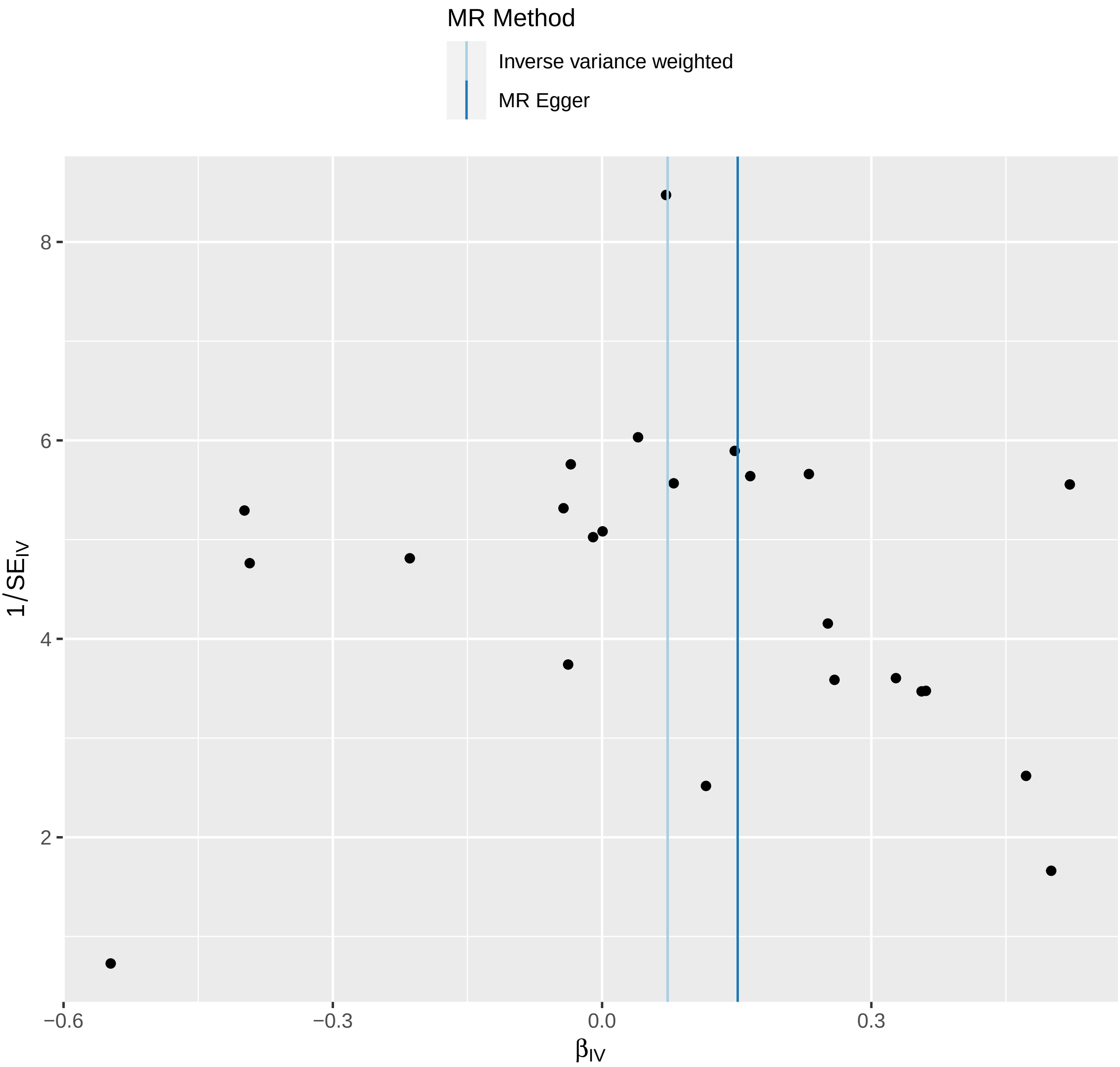

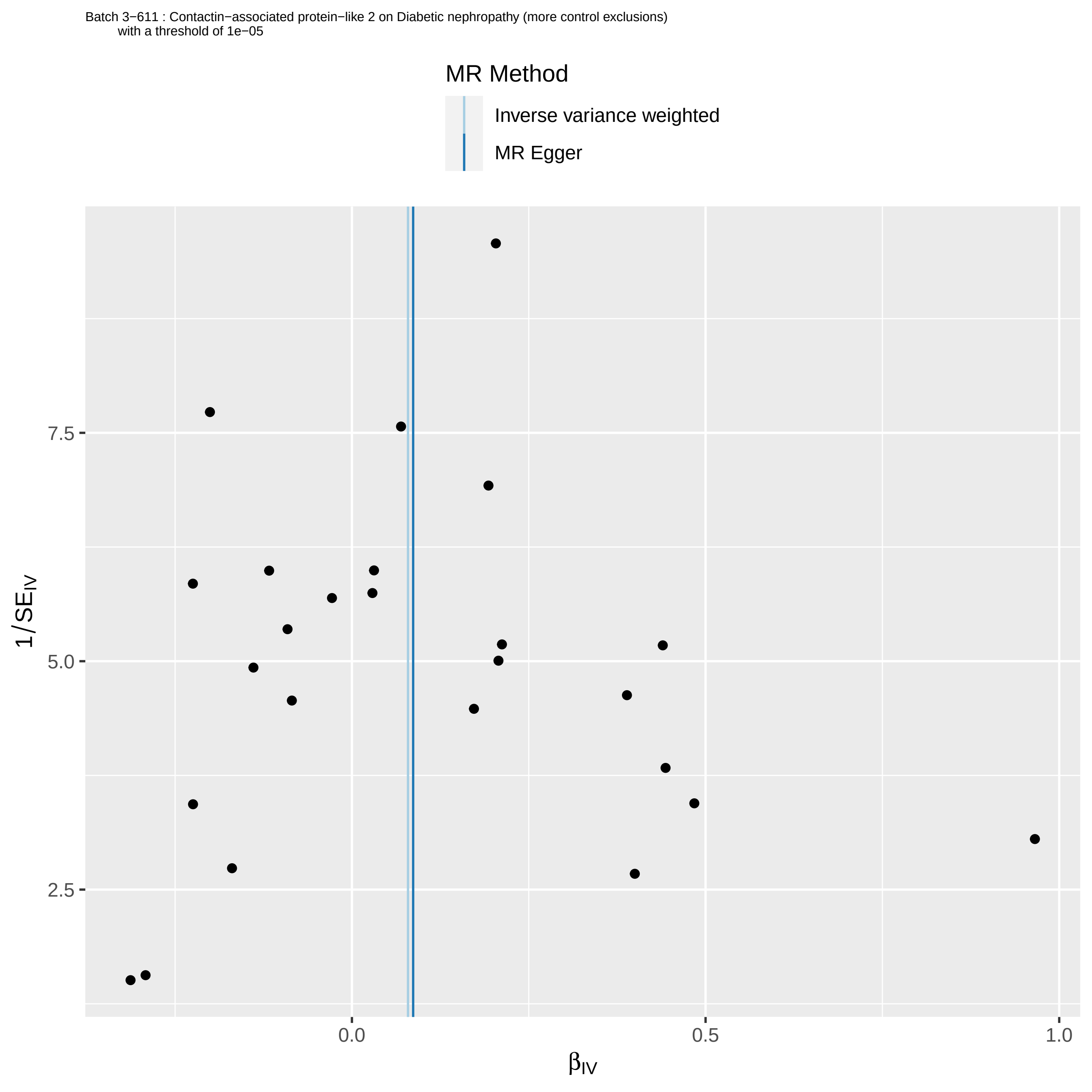

Batch 3−611 : Contactin−associated protein−like 2 on Diabetic nephropathy (more control exclusions)
with a threshold of 1e−05
MR Method
Inverse variance weighted
MR Egger
7.5
5.0
2.5
1/SE$_{IV}$
0.0
0.5
1.0
β_{IV}

Batch 3−612 : Cytochrome c oxidase assembly factor 3 homolog, mitochondrial on Diabetic nephropathy (more control exclusions)
with a threshold of 1e−05

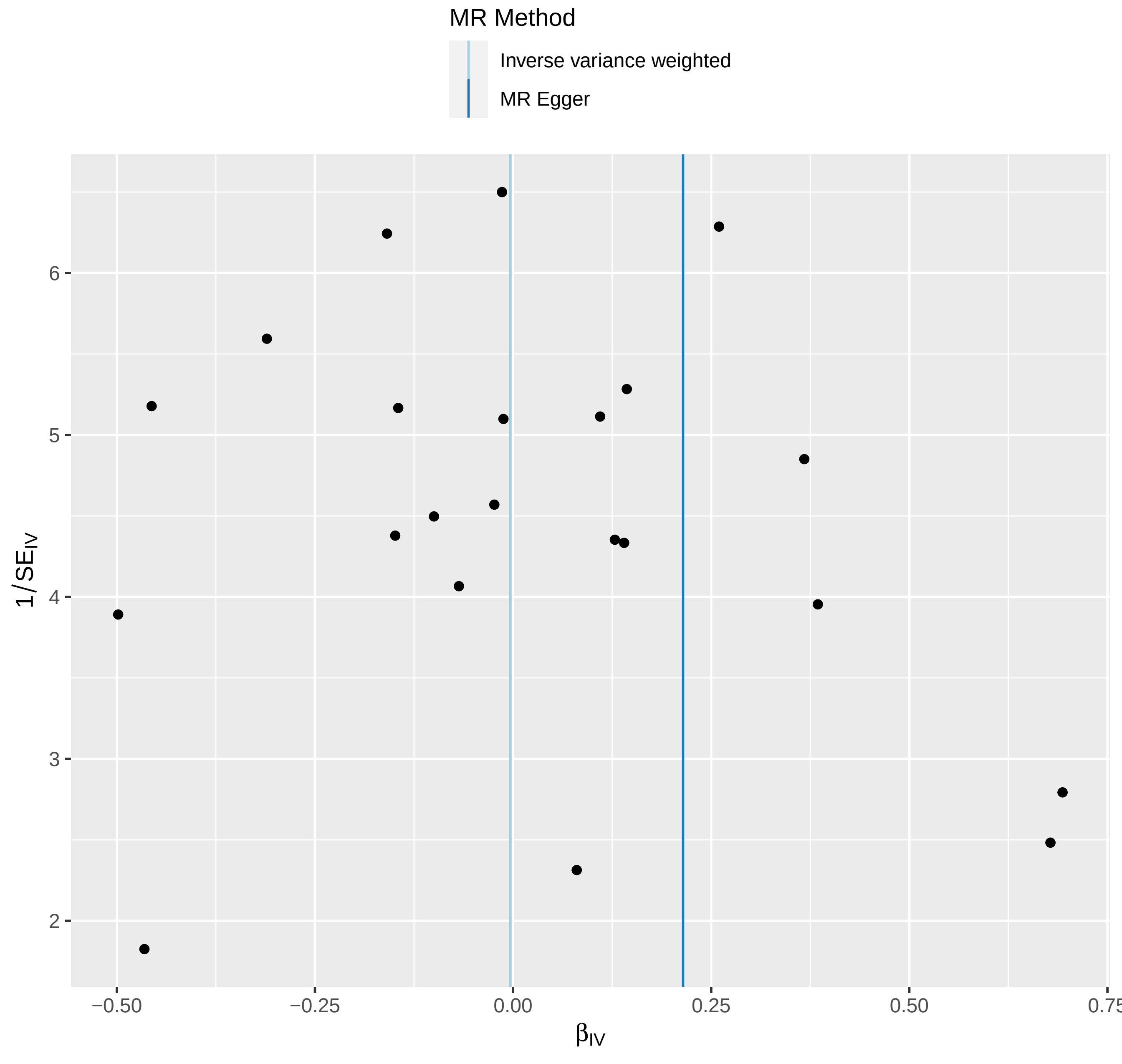

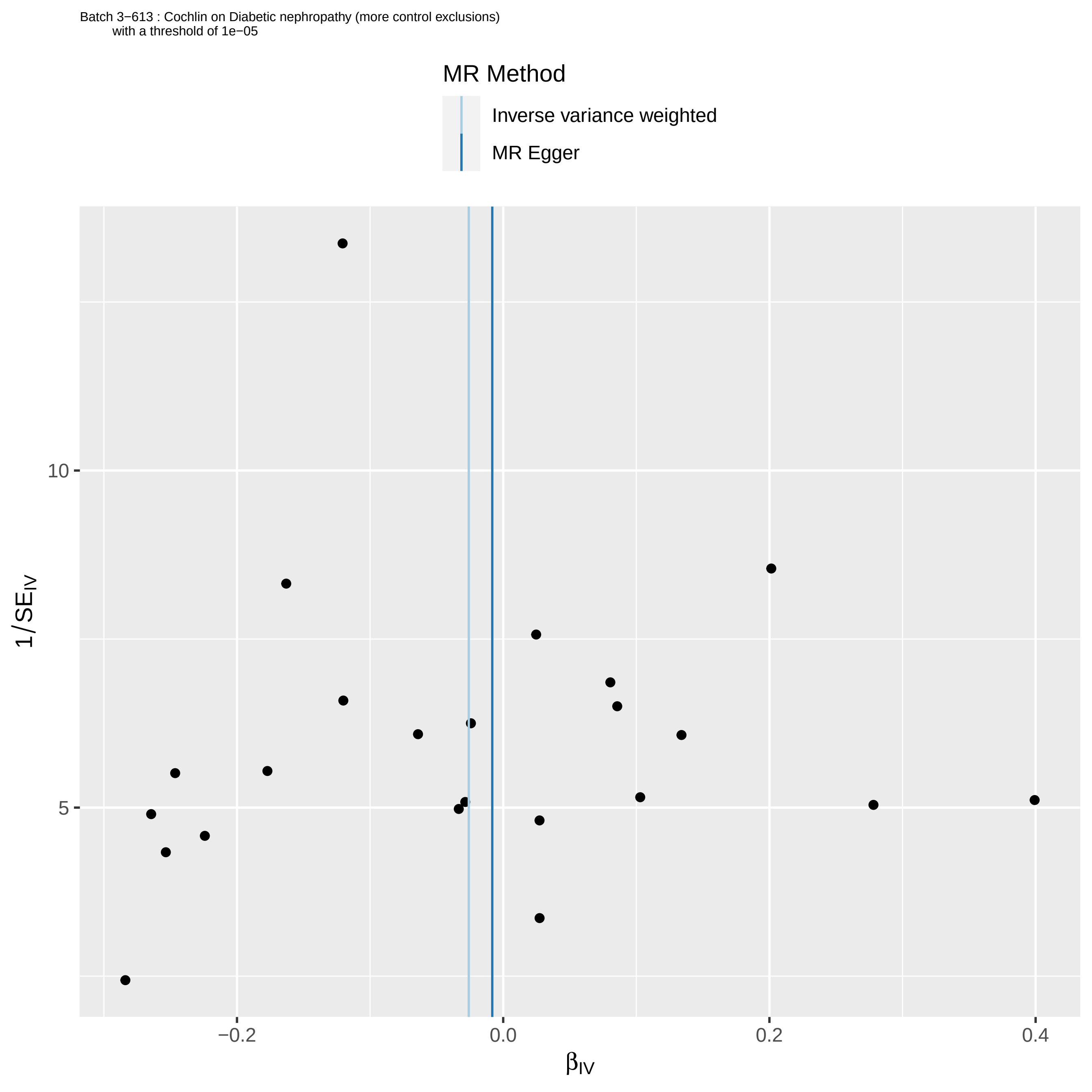

Batch 3−613 : Cochlin on Diabetic nephropathy (more control exclusions)
with a threshold of 1e−05
MR Method
Inverse variance weighted
MR Egger
10
5
1/SE_IV
−0.2
0.0
0.2
0.4
β_IV

Batch 3−614 : Conserved oligomeric Golgi complex subunit 8 on Diabetic nephropathy (more control exclusions)
with a threshold of 1e−05

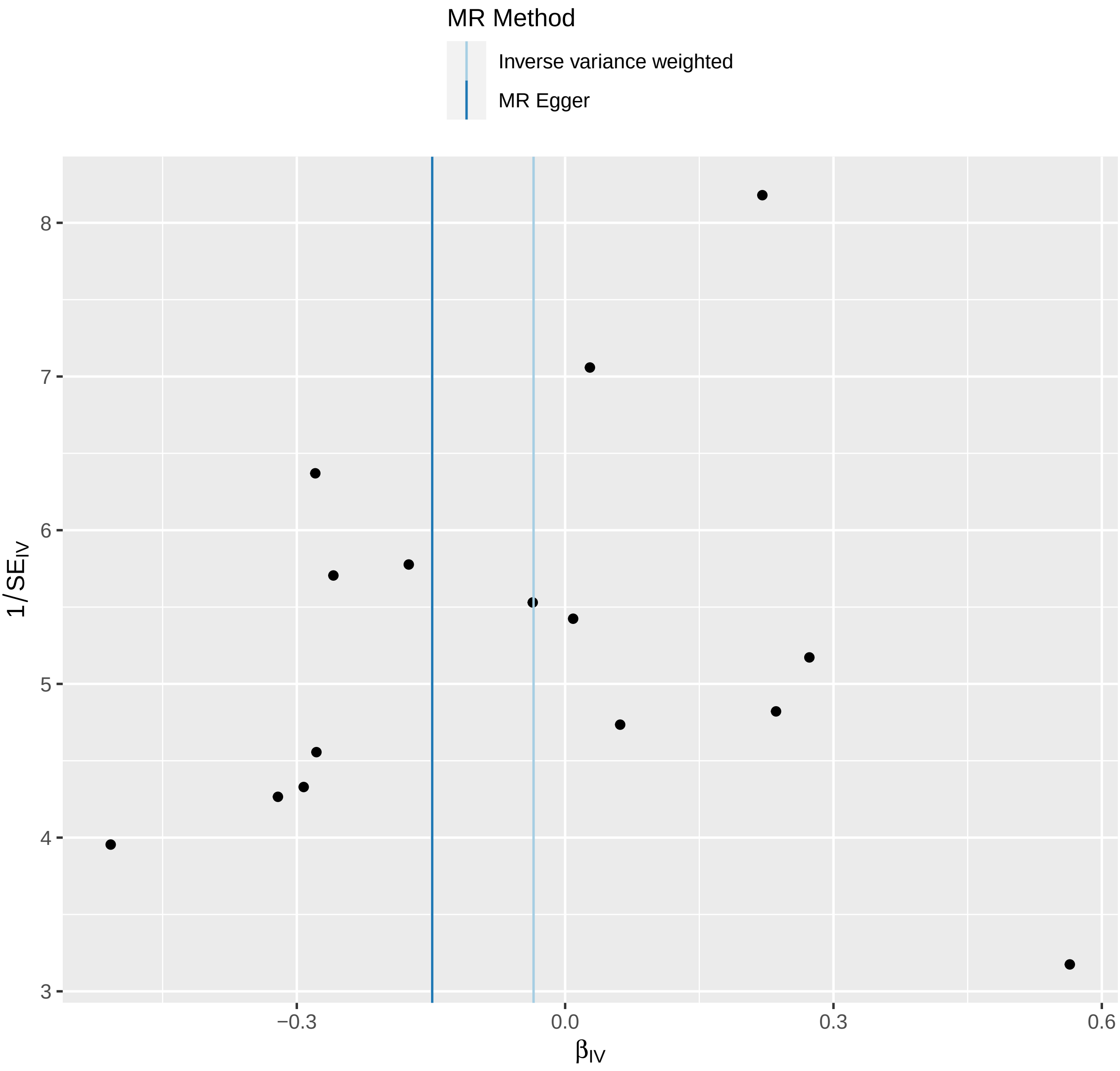

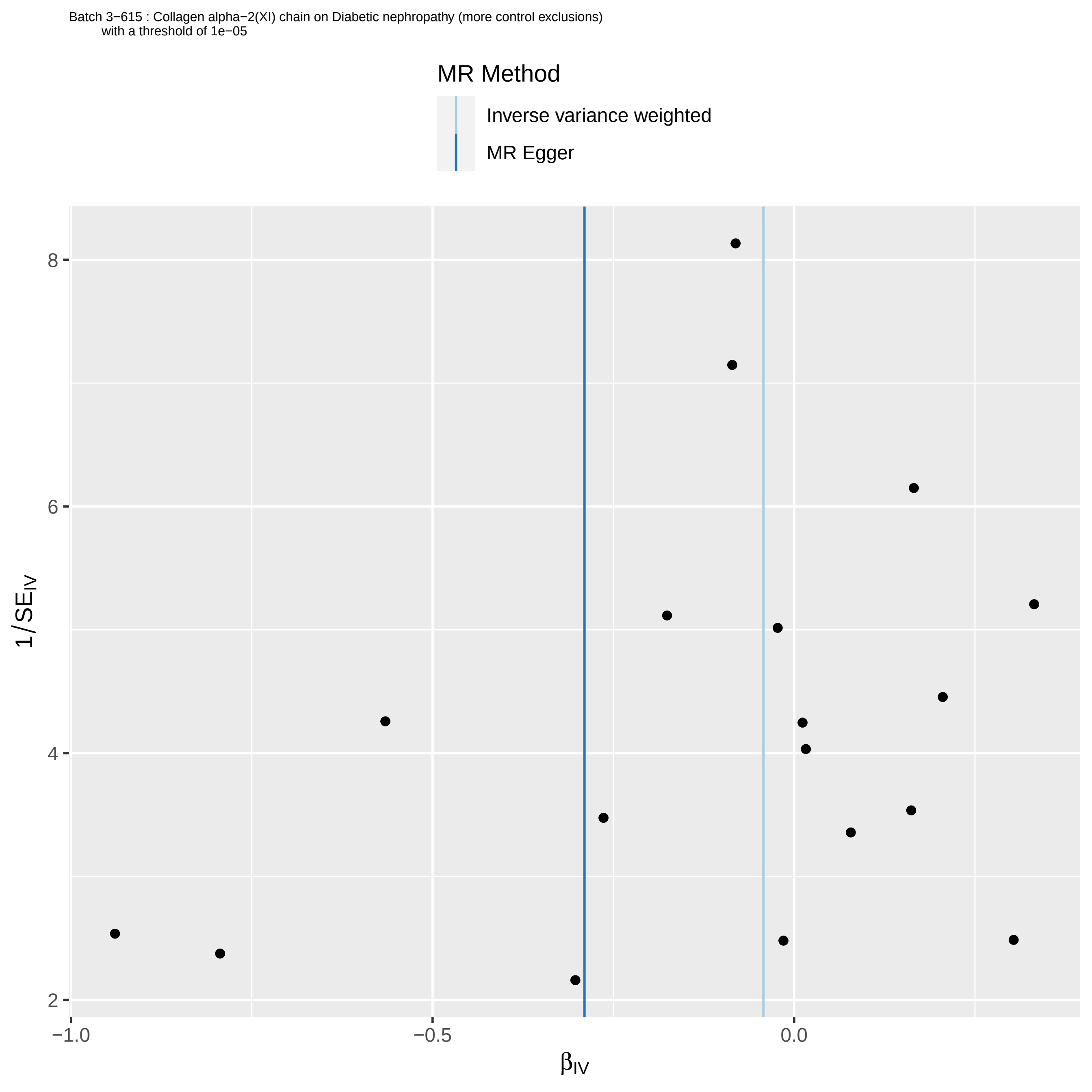
Batch 3−615 : Collagen alpha−2(XI) chain on Diabetic nephropathy (more control exclusions)
with a threshold of 1e−05
MR Method
Inverse variance weighted
MR Egger
8
6
4
2
1/SE_IV
−1.0
−0.5
0.0
β_IV

Batch 3−616 : Collagen alpha−1(XV) chain on Diabetic nephropathy (more control exclusions)
with a threshold of 1e−05

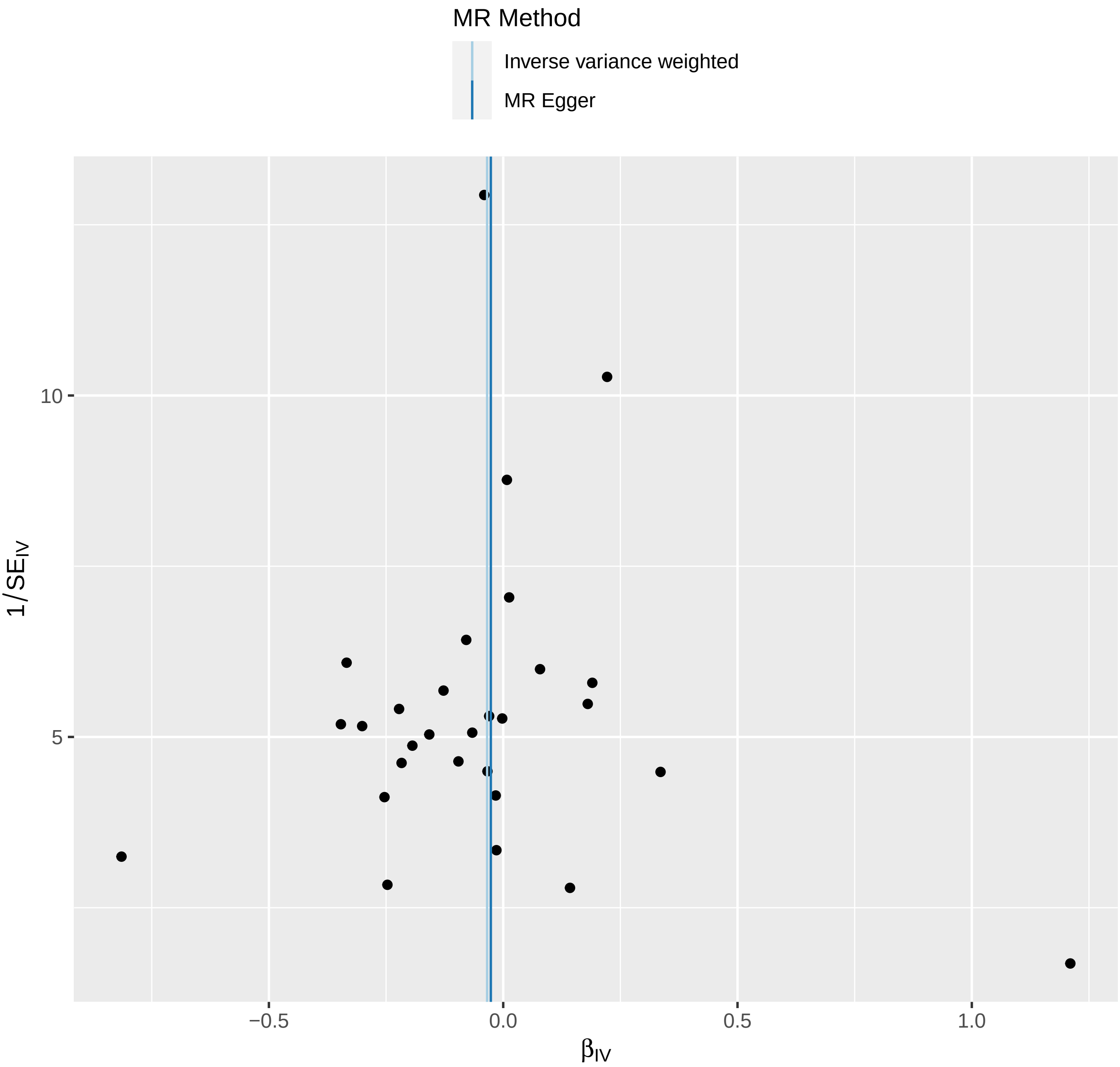

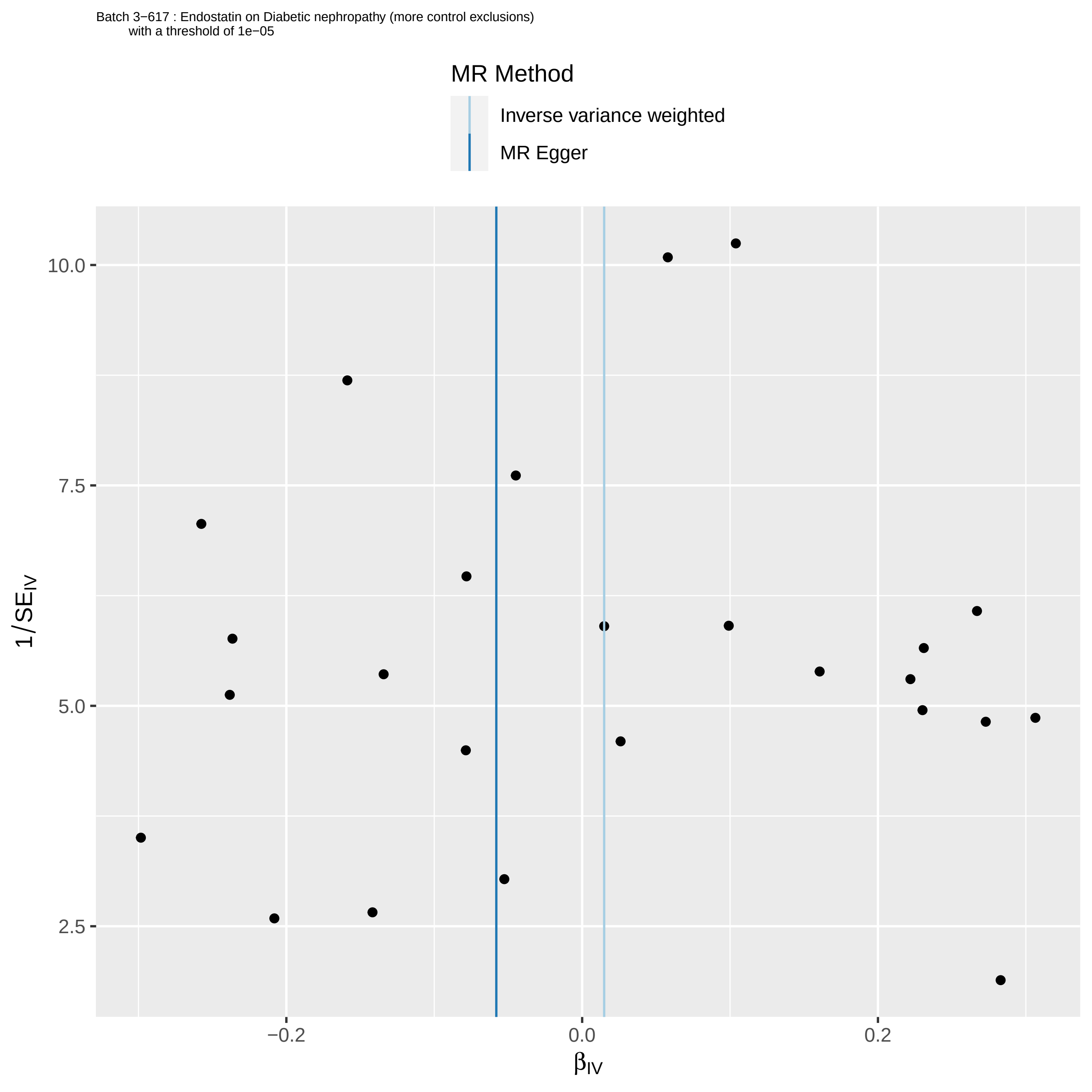

Batch 3−617 : Endostatin on Diabetic nephropathy (more control exclusions)
with a threshold of 1e−05
MR Method
Inverse variance weighted
MR Egger
10.0
7.5
5.0
2.5
1/SE_IV
−0.2
0.0
0.2
β_IV

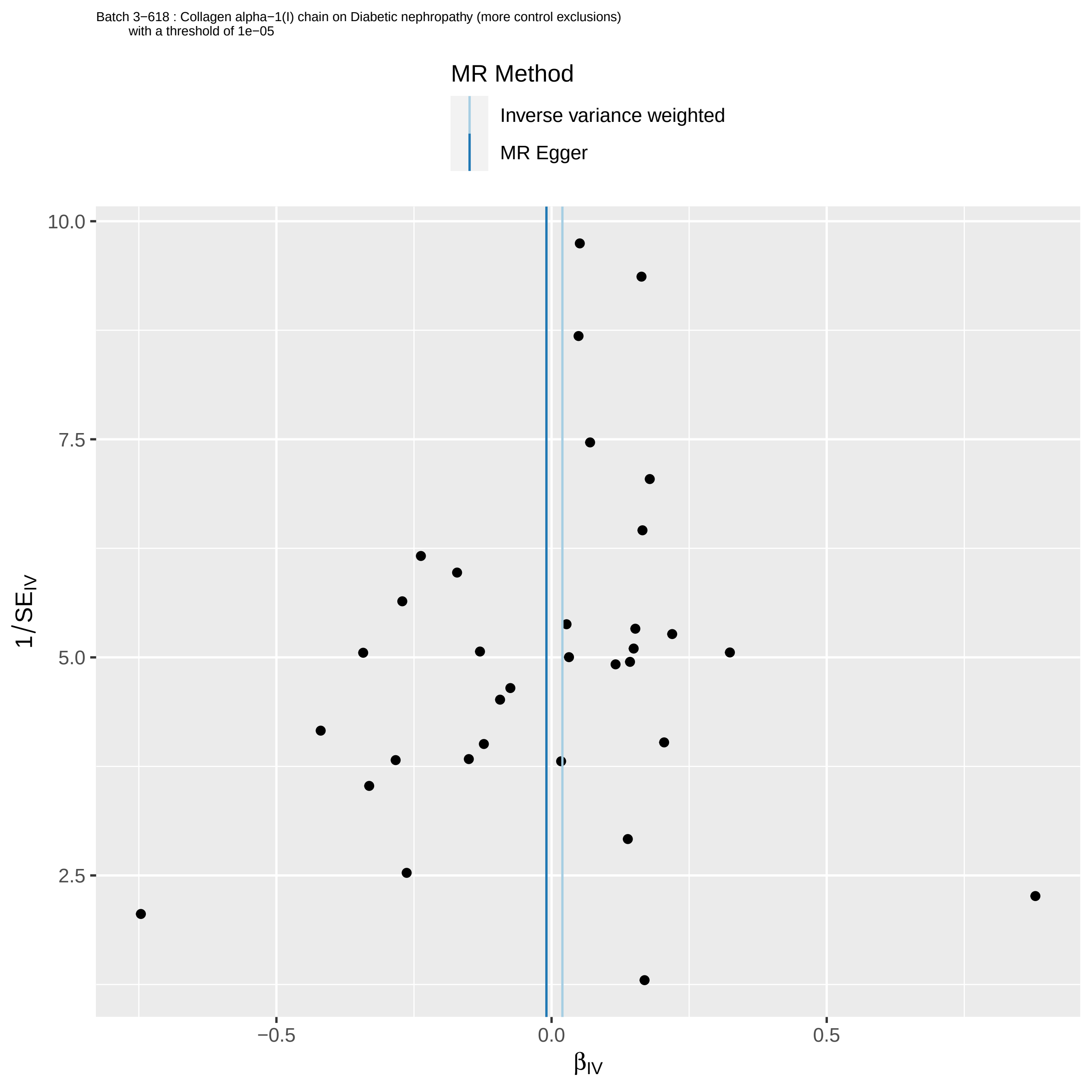

Batch 3−618 : Collagen alpha−1(I) chain on Diabetic nephropathy (more control exclusions)
with a threshold of 1e−05
MR Method
Inverse variance weighted
MR Egger
10.0
7.5
5.0
2.5
1/SE_IV
−0.5
0.0
0.5
β_IV

Batch 3−619 : Collagen alpha−1(XX) chain on Diabetic nephropathy (more control exclusions)
with a threshold of 1e−05

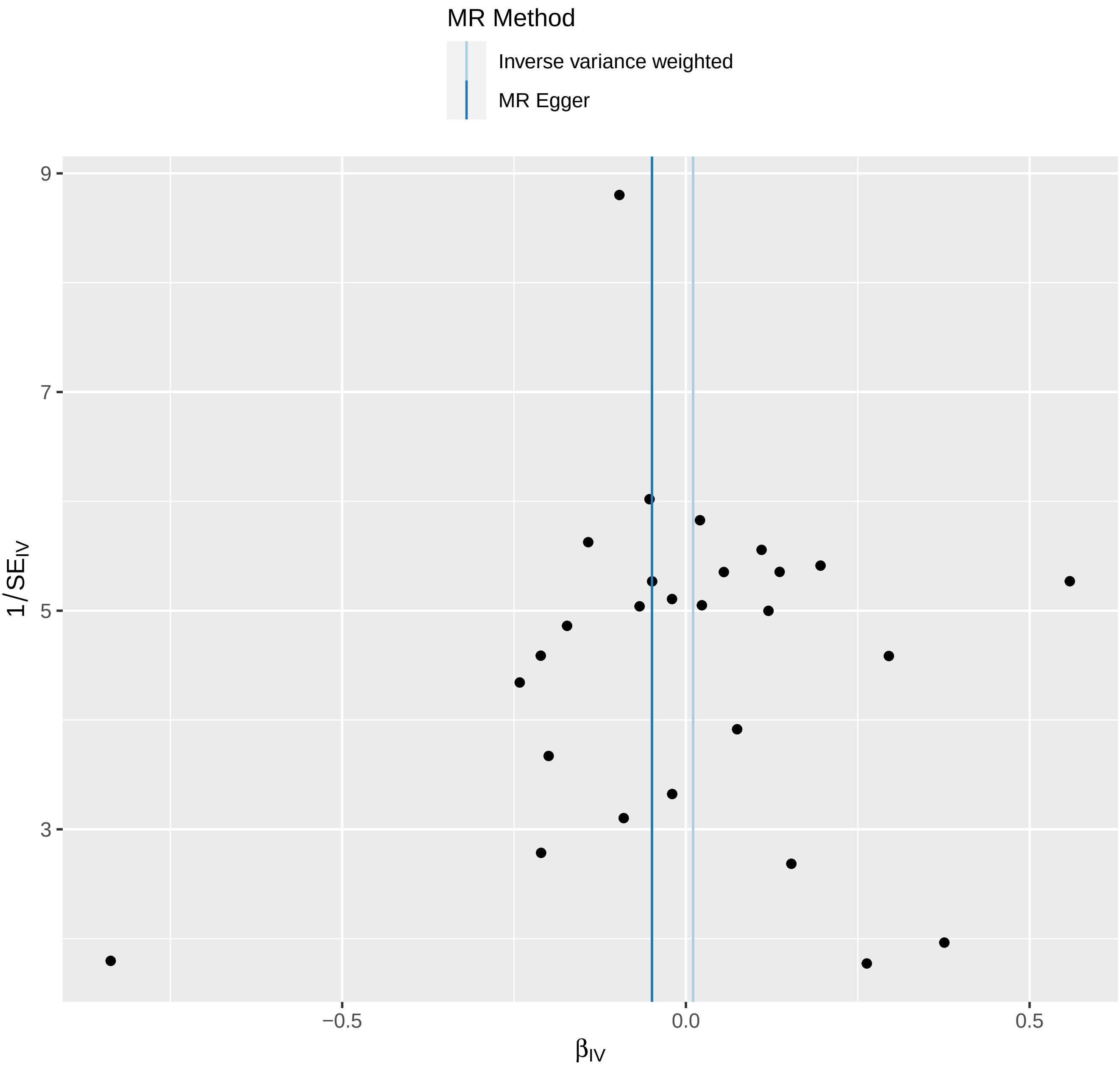

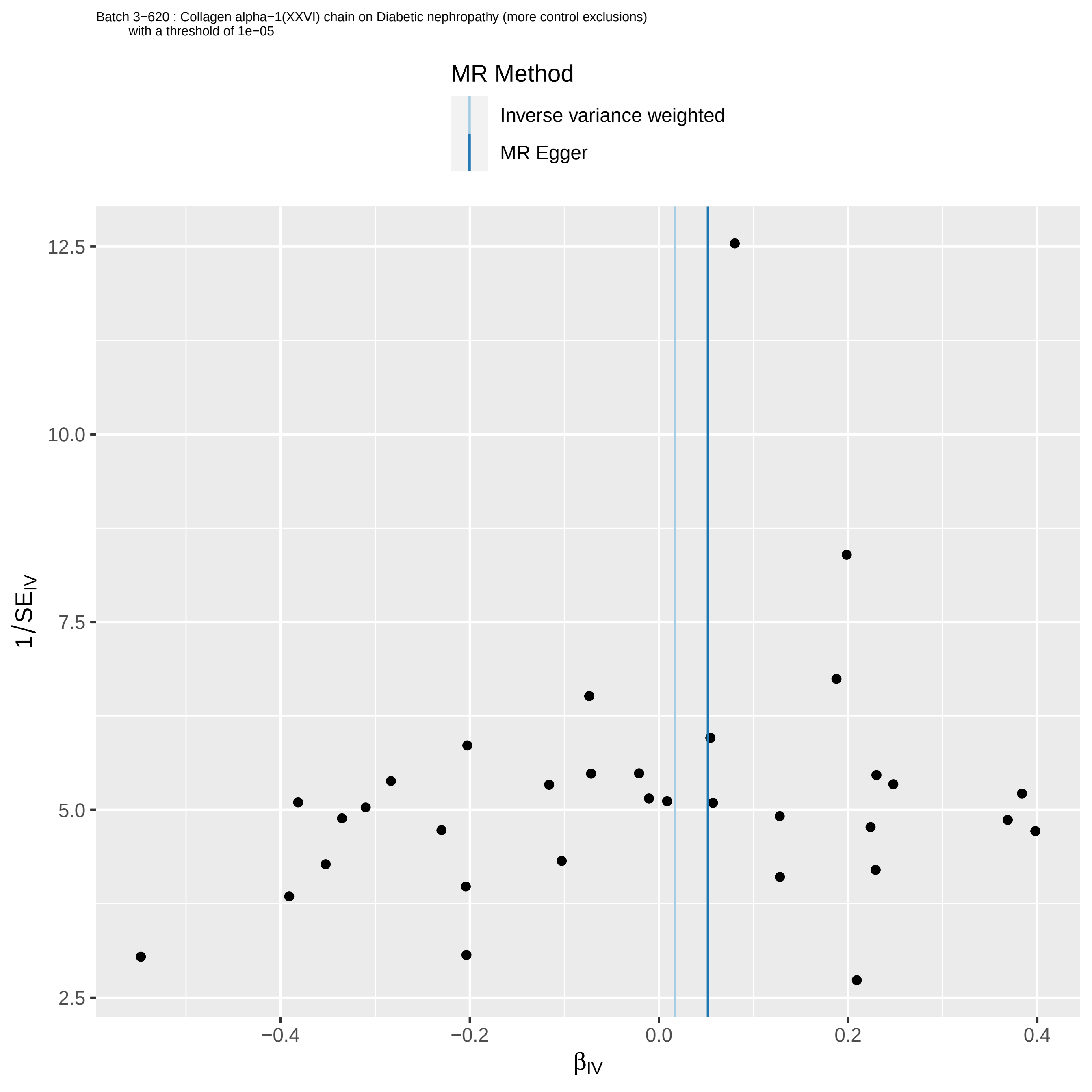

Batch 3−620 : Collagen alpha−1(XXVI) chain on Diabetic nephropathy (more control exclusions)
with a threshold of 1e−05
MR Method
Inverse variance weighted
MR Egger
12.5
10.0
7.5
5.0
2.5
1/SE_IV
−0.4
−0.2
0.0
0.2
0.4
β_IV

Batch 3−621 : Collagen alpha−1(XXVIII) chain on Diabetic nephropathy (more control exclusions)
with a threshold of 1e−05

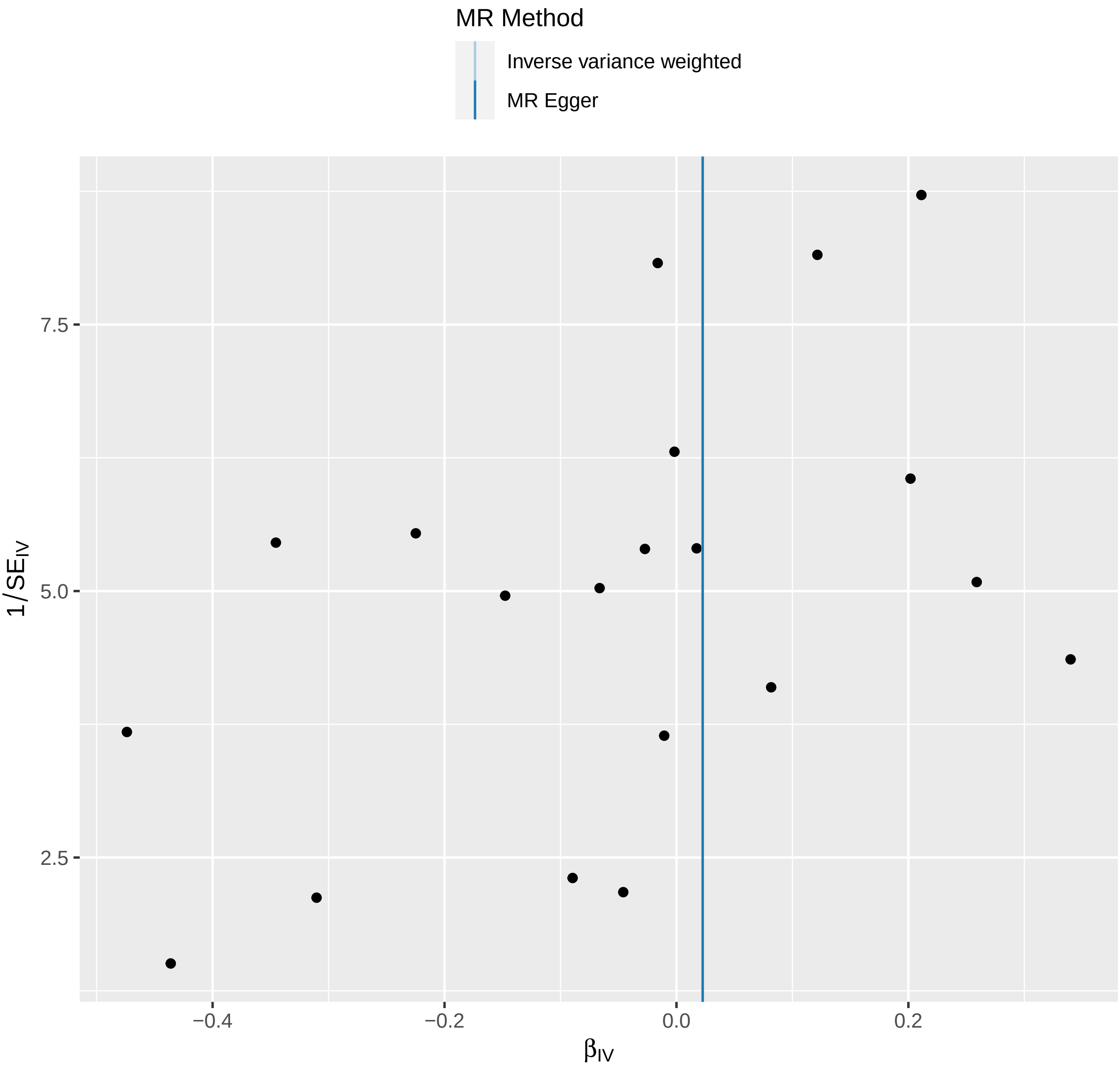

Batch 3−622 : Collagen alpha−1(VI) chain on Diabetic nephropathy (more control exclusions)
with a threshold of 1e−05

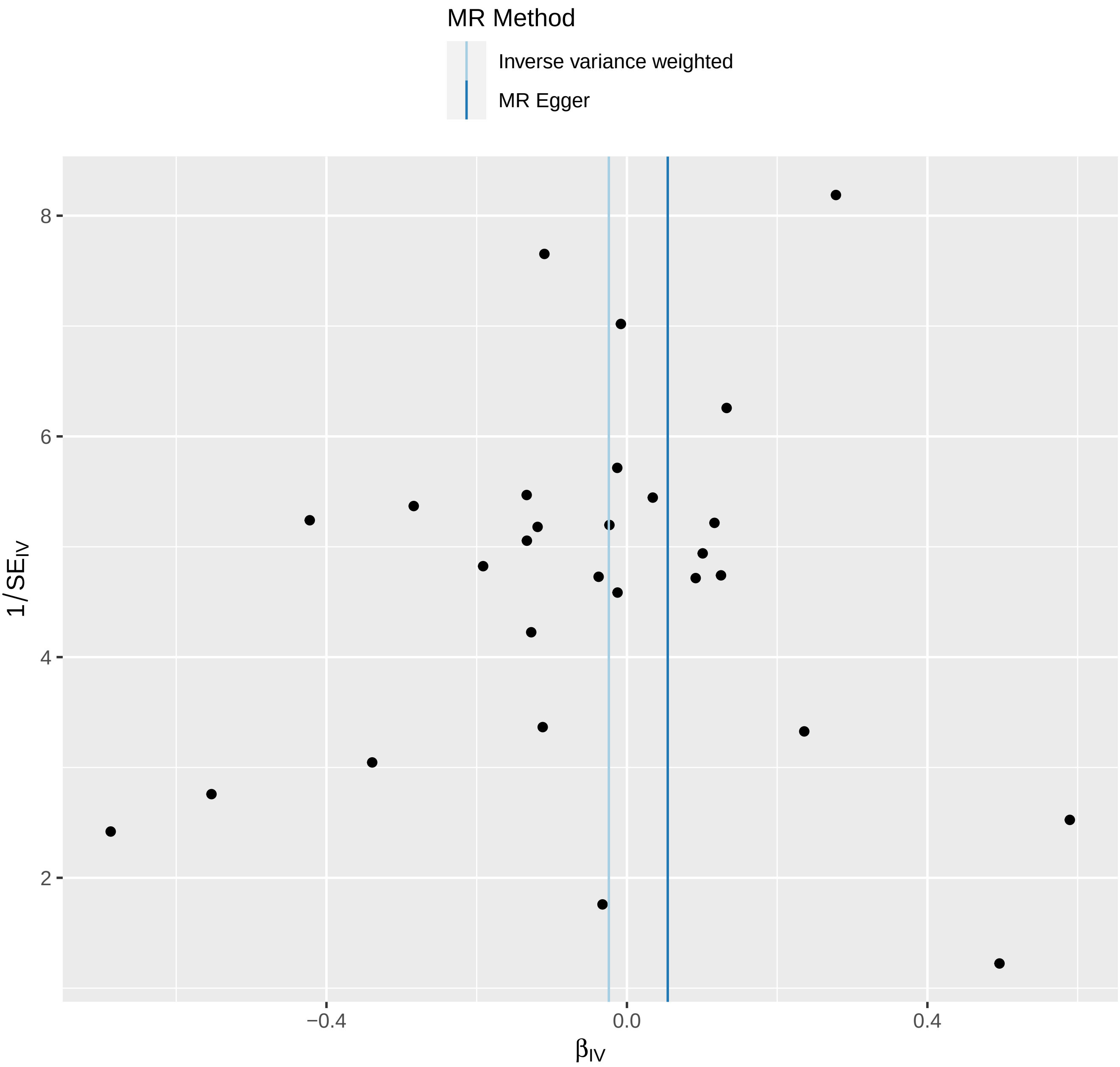

Batch 3−623 : Collagen alpha−3(VI) chain on Diabetic nephropathy (more control exclusions)
with a threshold of 1e−05

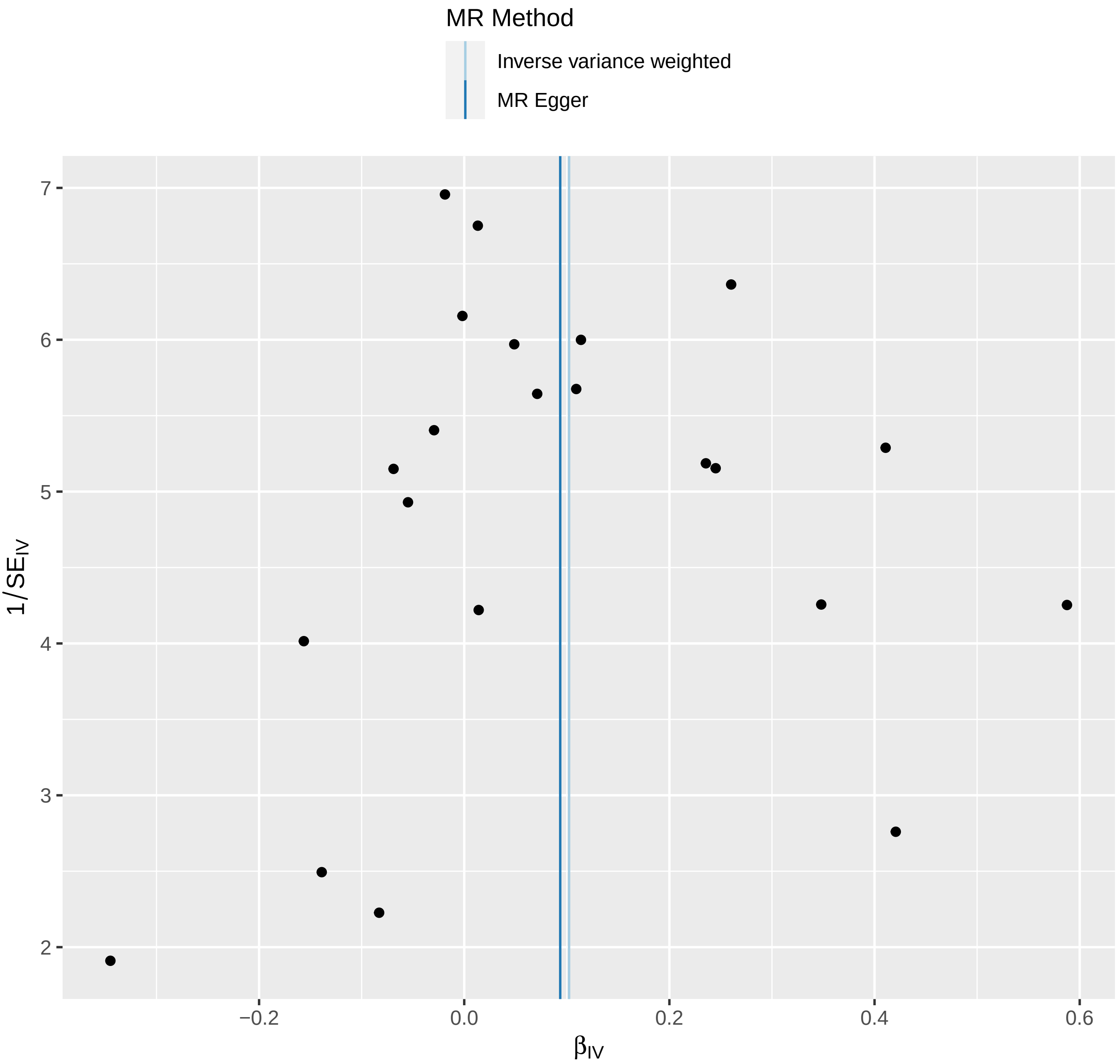

Batch 3−624 : Collagen alpha−5(VI) chain on Diabetic nephropathy (more control exclusions)
with a threshold of 1e−05

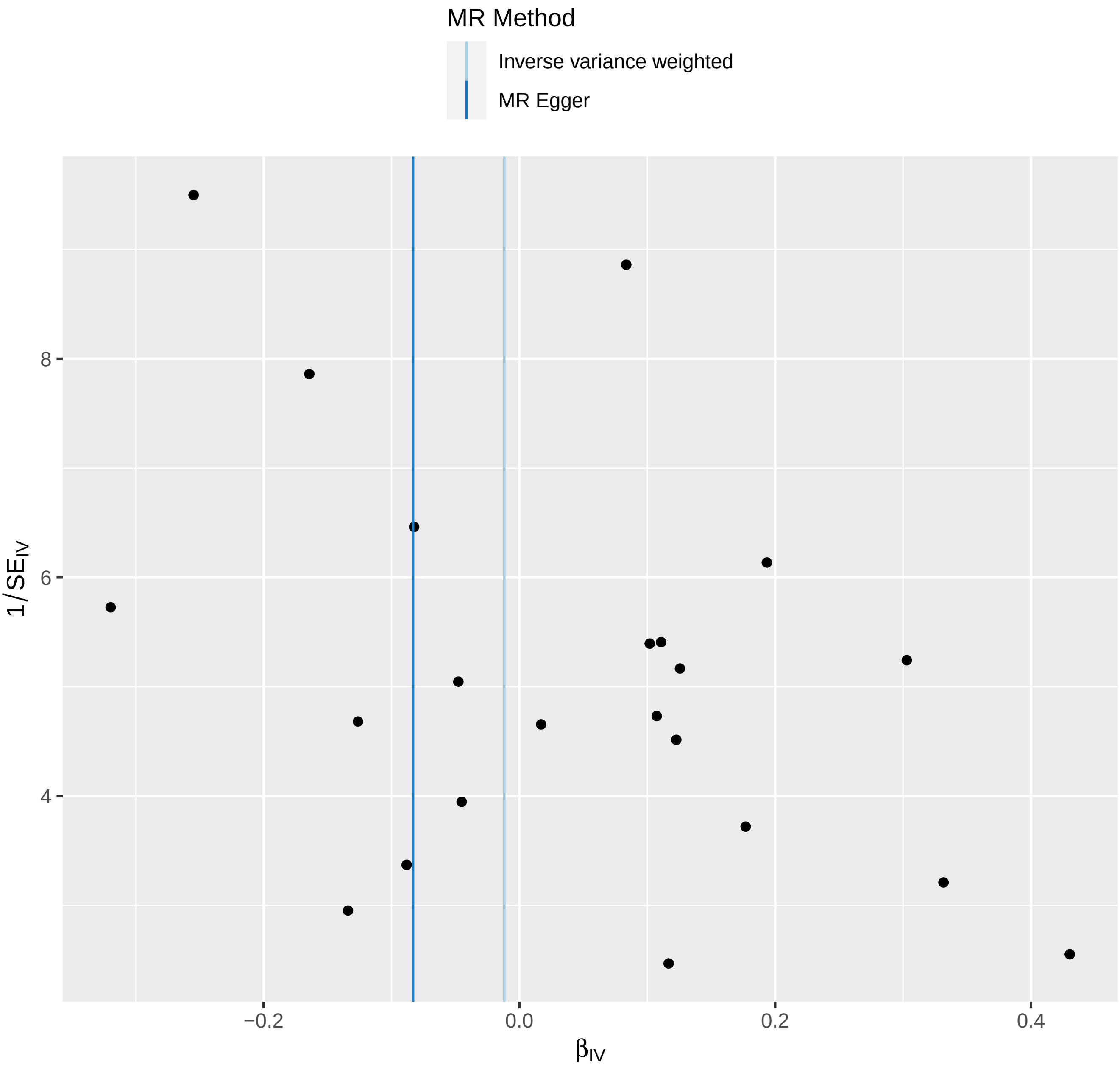

Batch 3−625 : Collagen alpha−1(VIII) chain on Diabetic nephropathy (more control exclusions)
with a threshold of 1e−05

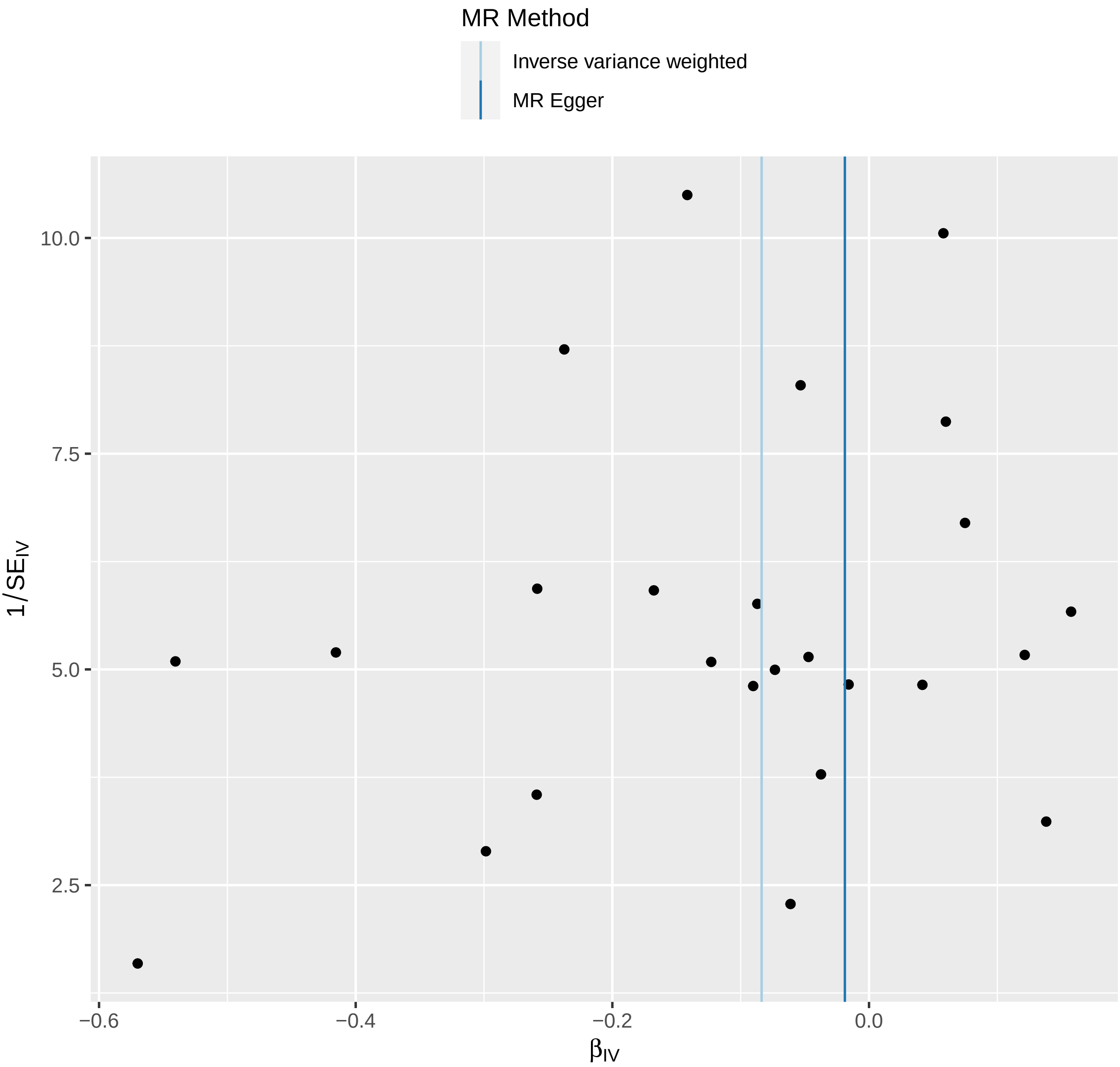

Batch 3−626 : Collagen alpha−2(IX) chain on Diabetic nephropathy (more control exclusions) with a threshold of 1e−05

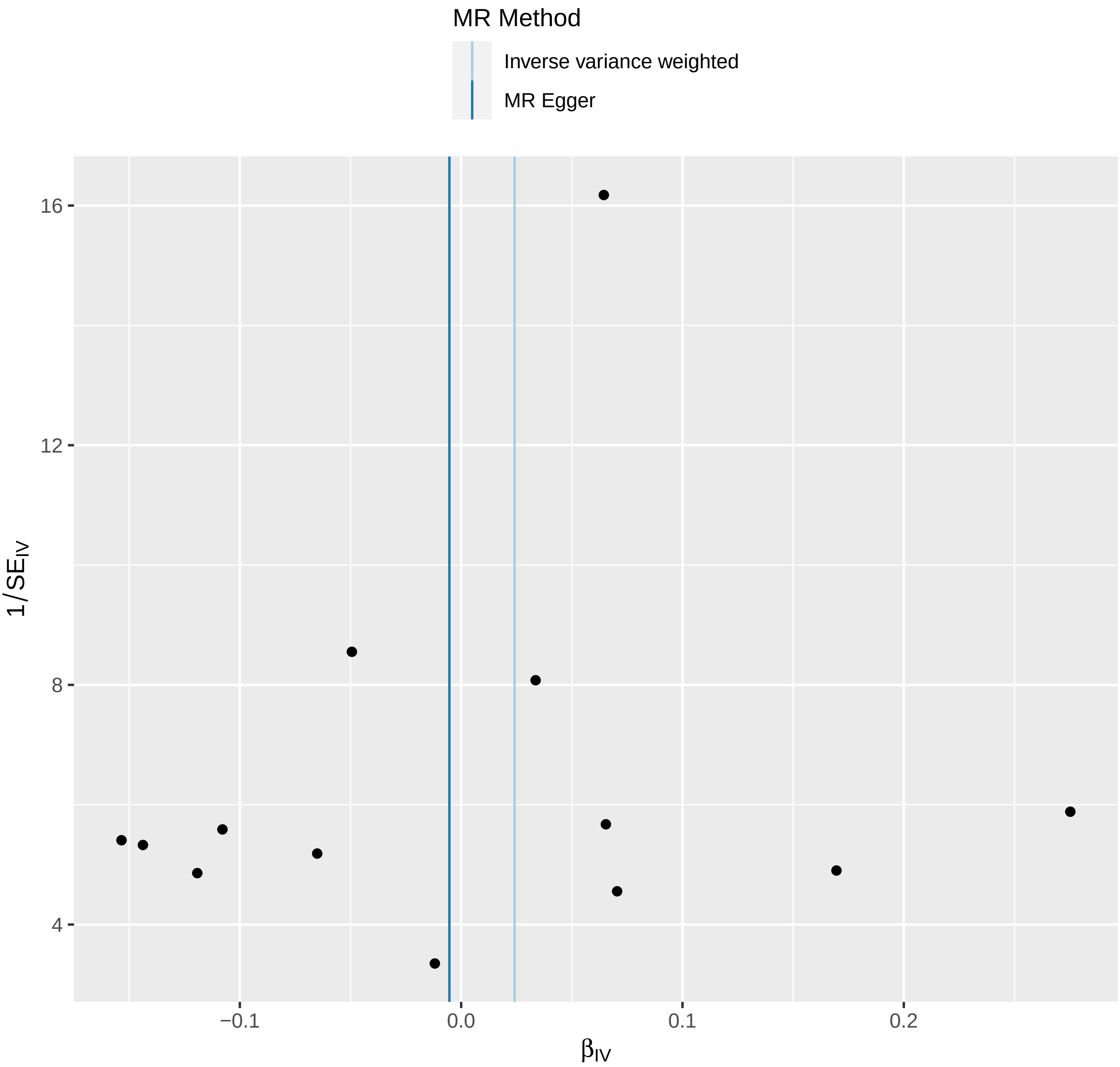

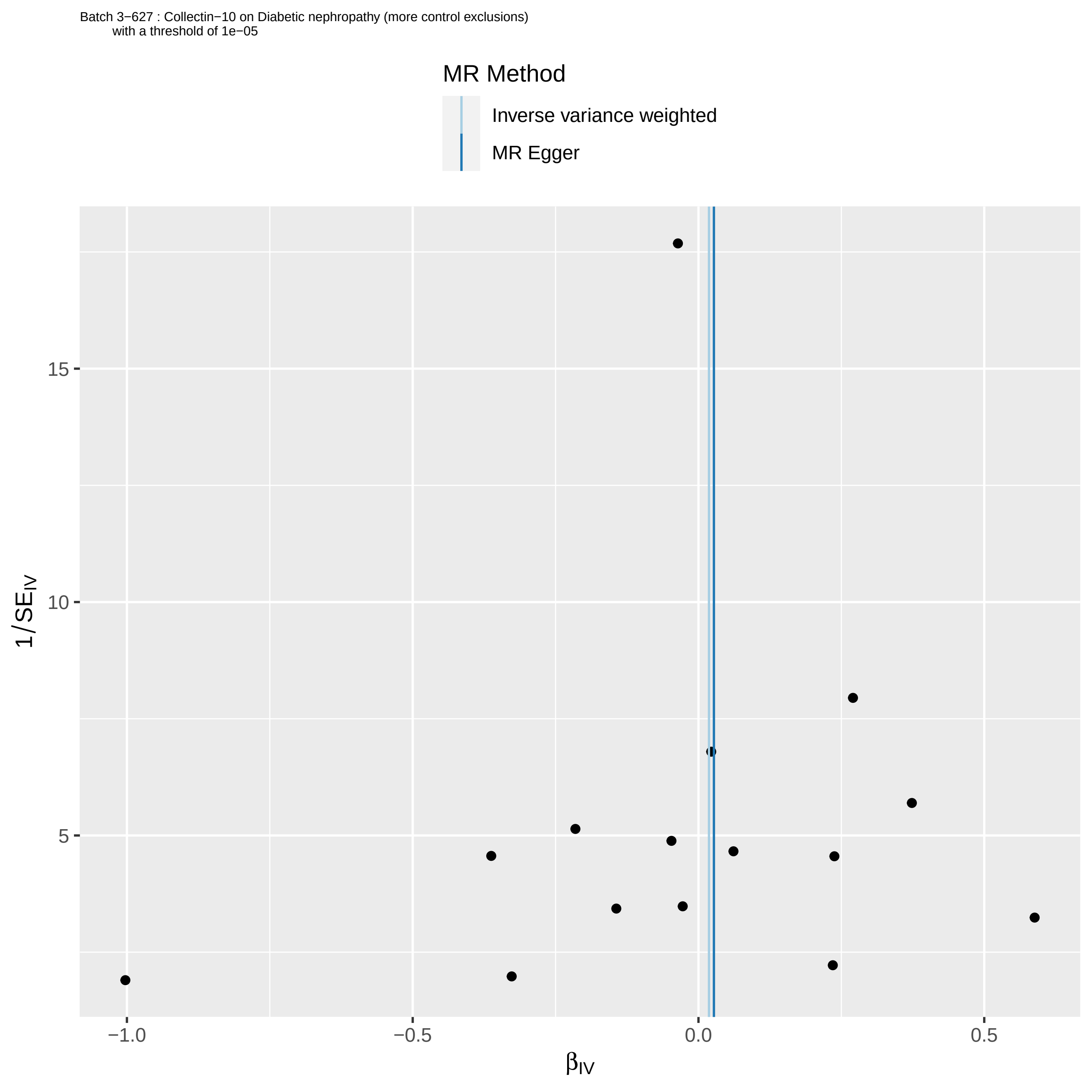

Batch 3−627 : Collectin−10 on Diabetic nephropathy (more control exclusions)
with a threshold of 1e−05
MR Method
Inverse variance weighted
MR Egger
15
10
5
1/SE_IV
−1.0
−0.5
0.0
0.5
β_IV

Batch 3−628 : Collectin−11 on Diabetic nephropathy (more control exclusions)
with a threshold of 1e−05

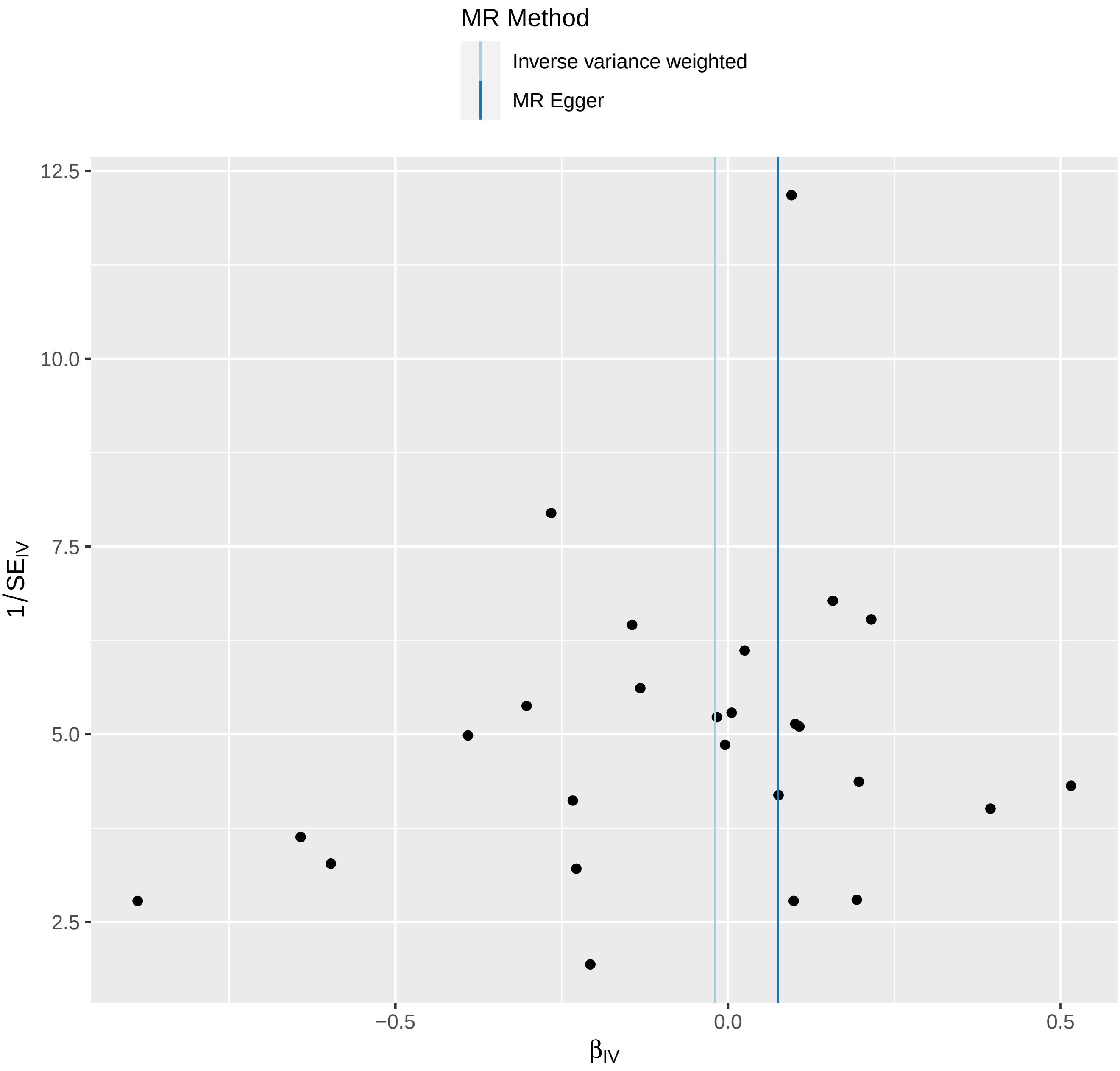

Batch 3−629 : Collectin−12 on Diabetic nephropathy (more control exclusions)
with a threshold of 1e−05

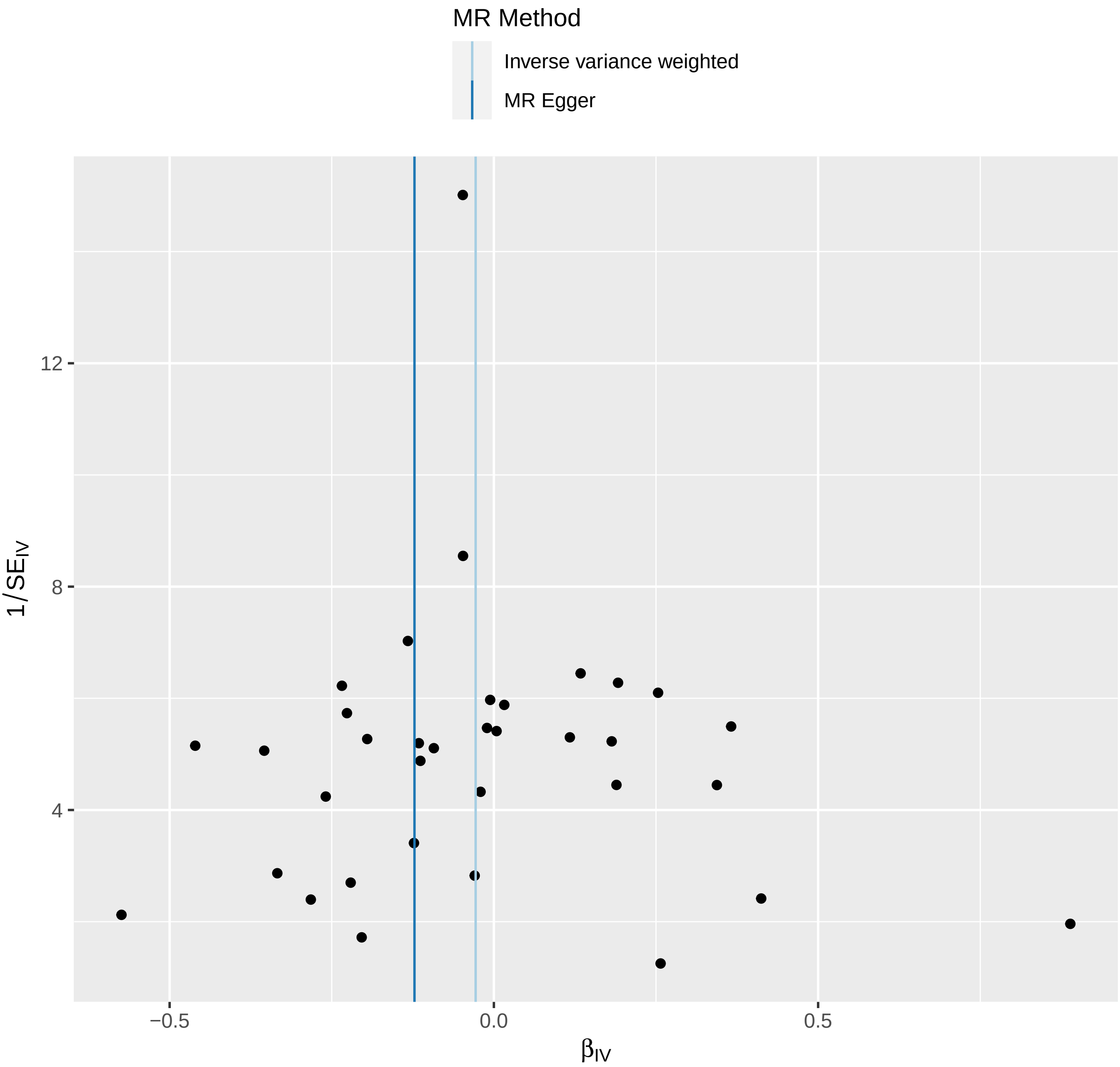

Batch 3−630 : Collectin−12 on Diabetic nephropathy (more control exclusions)
with a threshold of 1e−05

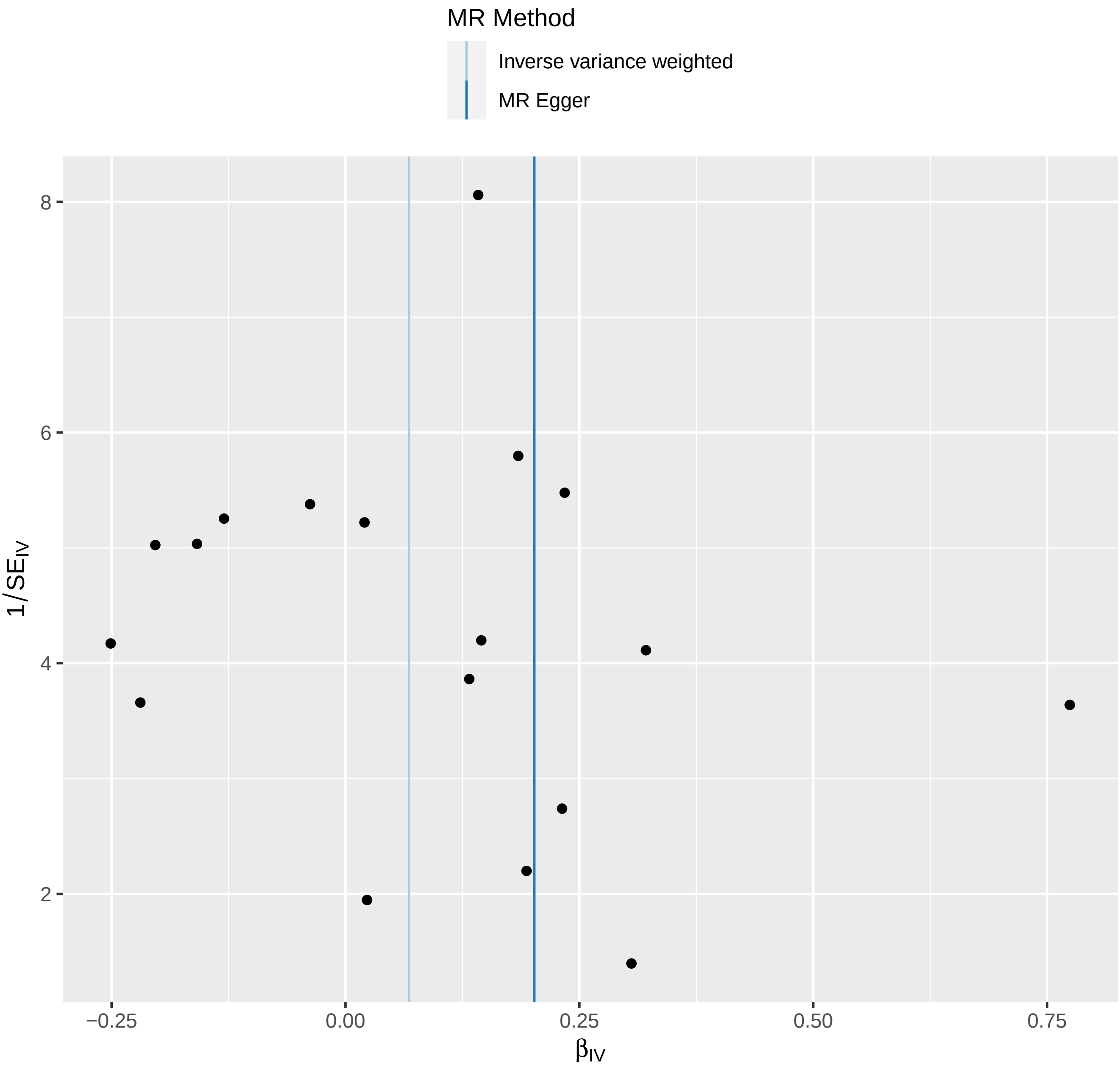

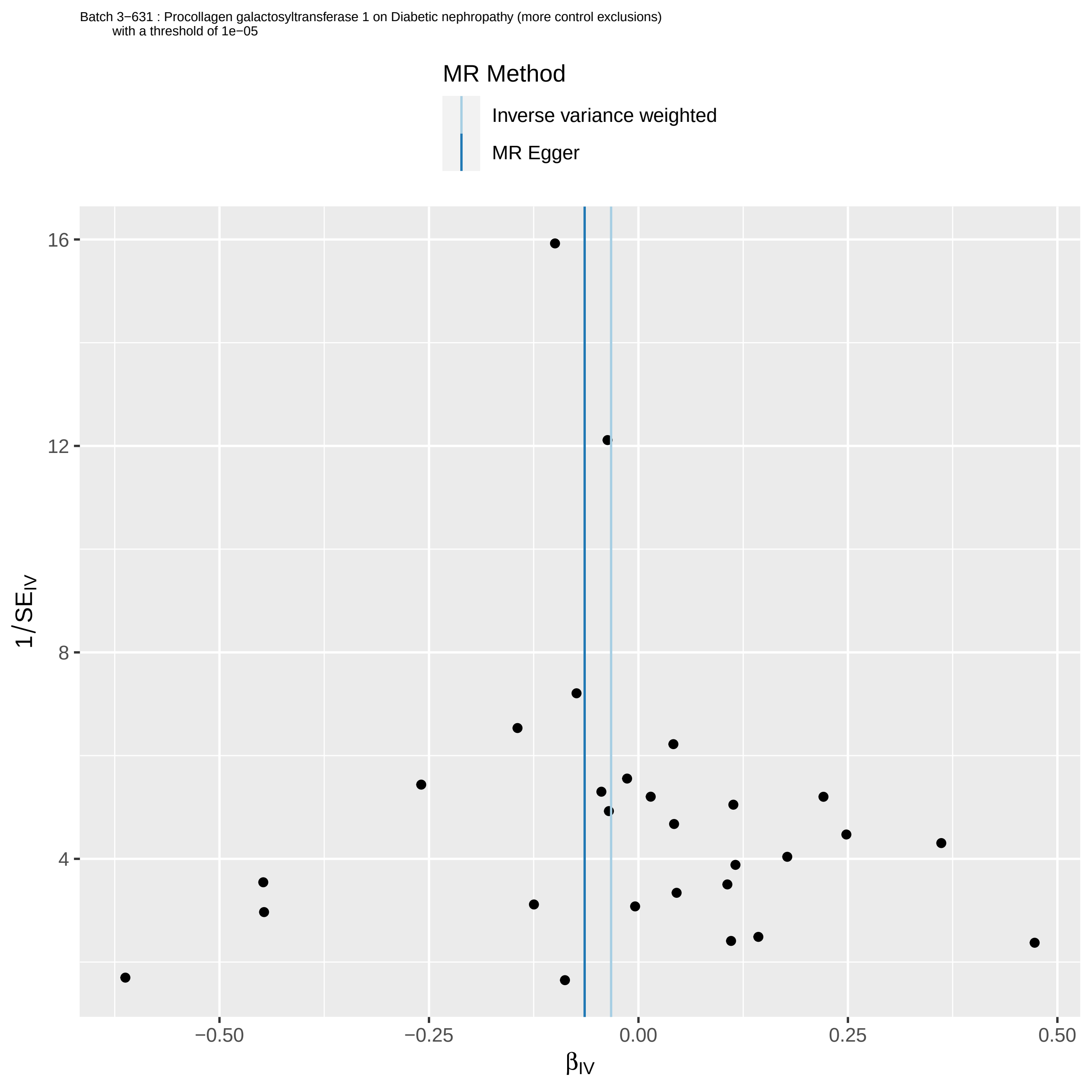
Batch 3−631 : Procollagen galactosyltransferase 1 on Diabetic nephropathy (more control exclusions)
with a threshold of 1e−05
MR Method
Inverse variance weighted
MR Egger
1/SE_IV
16
12
8
4
−0.50
−0.25
0.00
0.25
0.50
β_IV

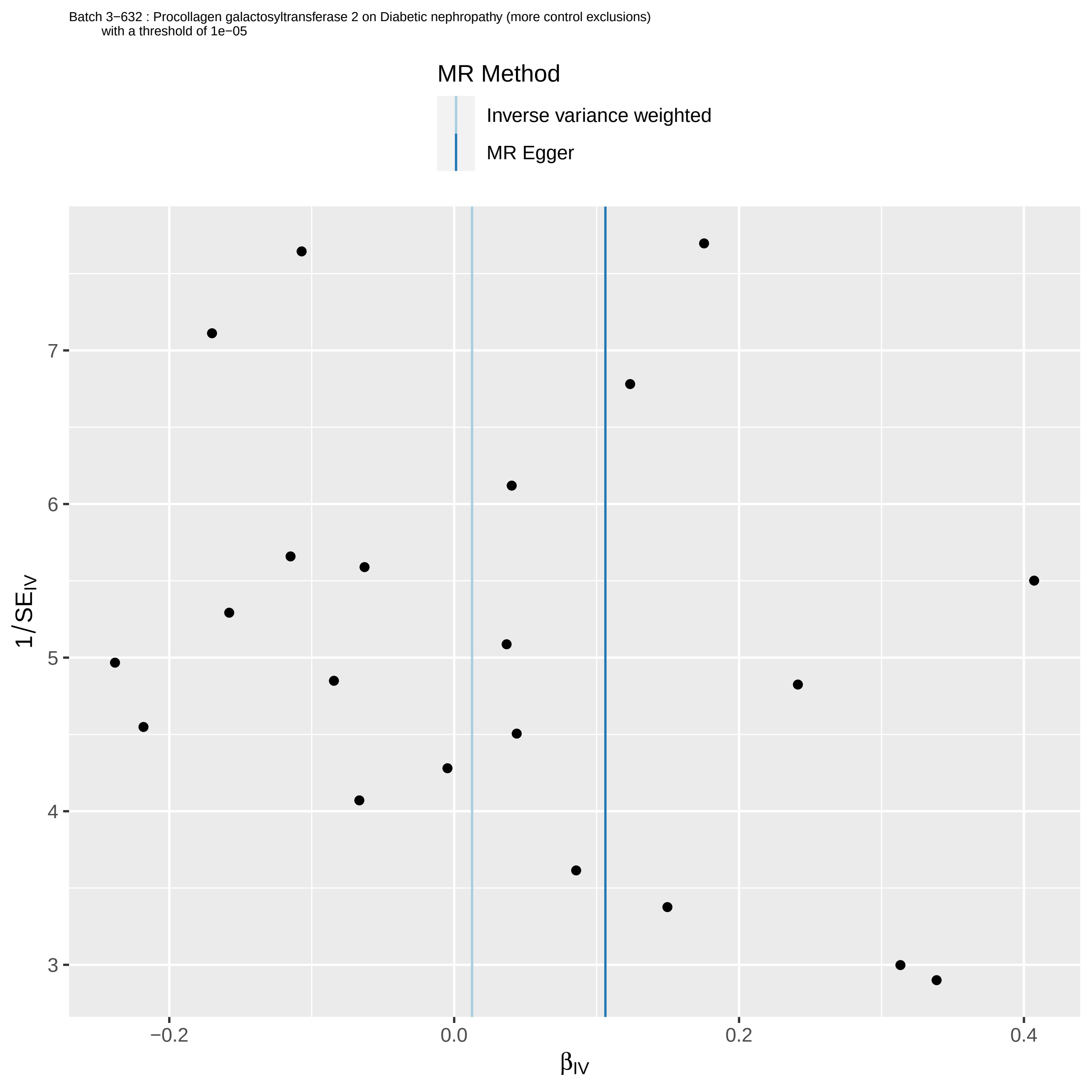

Batch 3−632 : Procollagen galactosyltransferase 2 on Diabetic nephropathy (more control exclusions)
with a threshold of 1e−05
MR Method
Inverse variance weighted
MR Egger
7
6
5
4
3
1/SE$_{IV}$
−0.2
0.0
0.2
0.4
β$_{IV}$

Batch 3−633 : COMM domain−containing protein 1 on Diabetic nephropathy (more control exclusions)
with a threshold of 1e−05

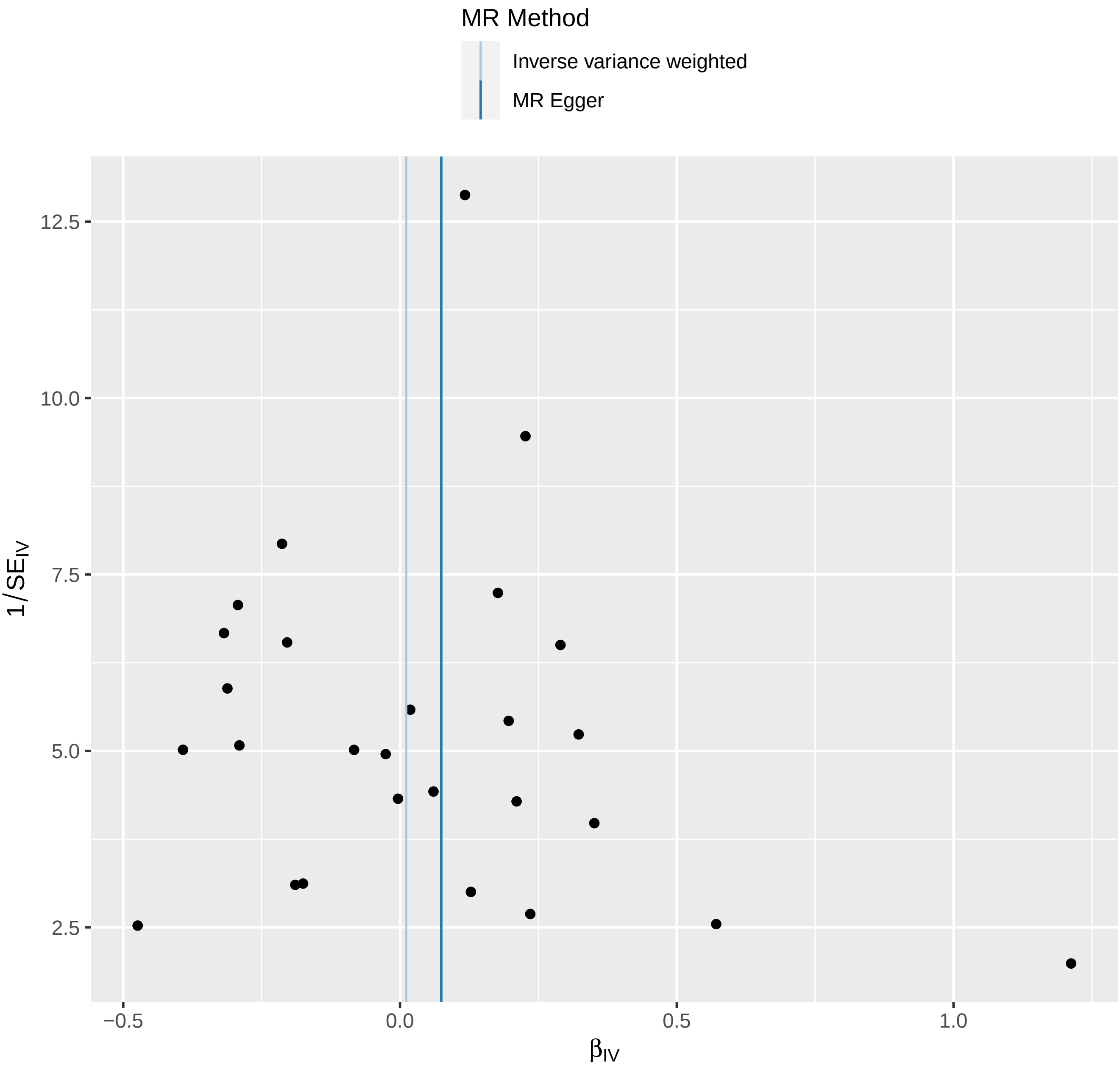

Batch 3−634 : COMM domain−containing protein 7 on Diabetic nephropathy (more control exclusions)
with a threshold of 1e−05

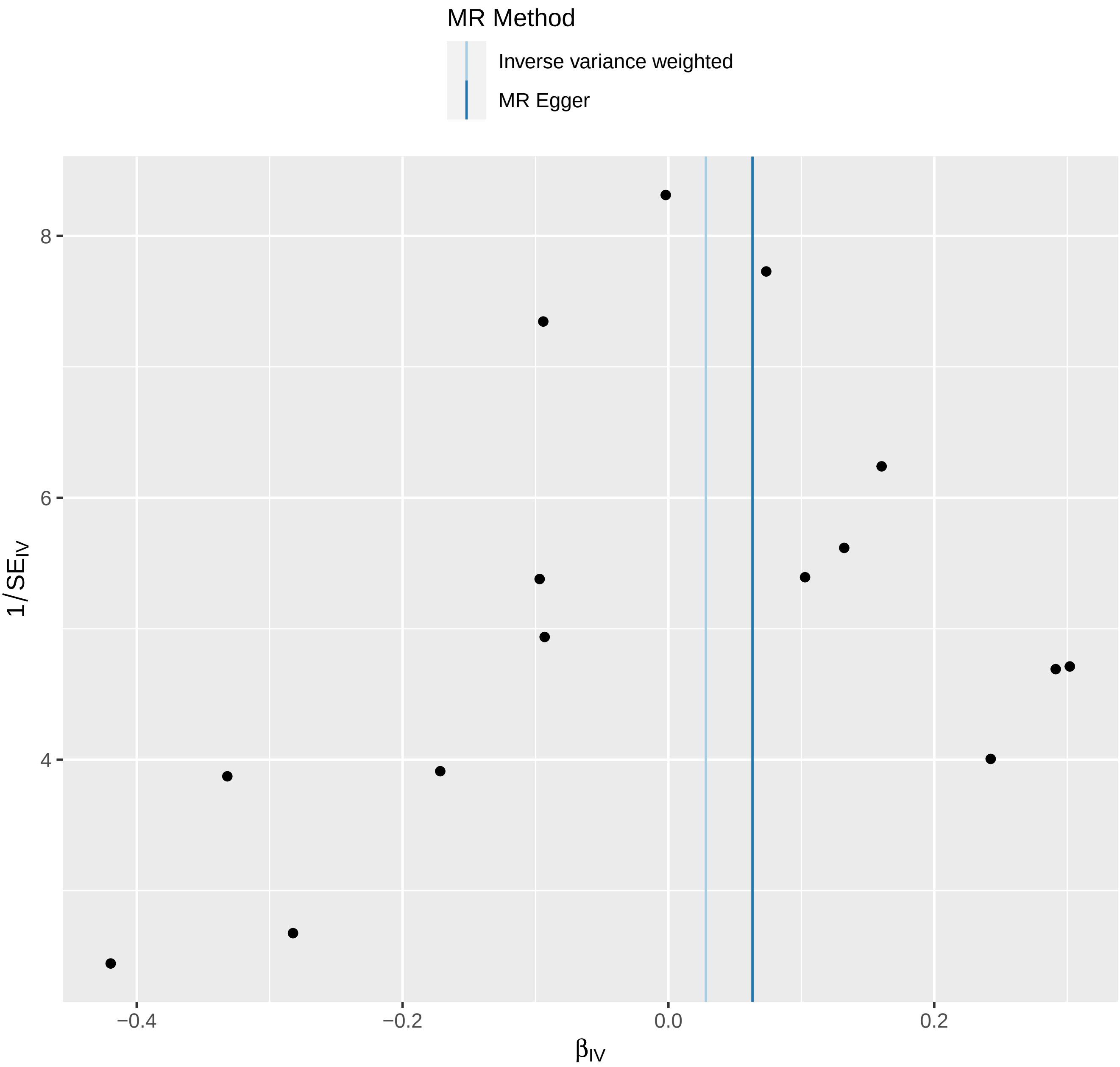

Batch 3−635 : COP9 signalosome complex subunit 2 on Diabetic nephropathy (more control exclusions)
with a threshold of 1e−05

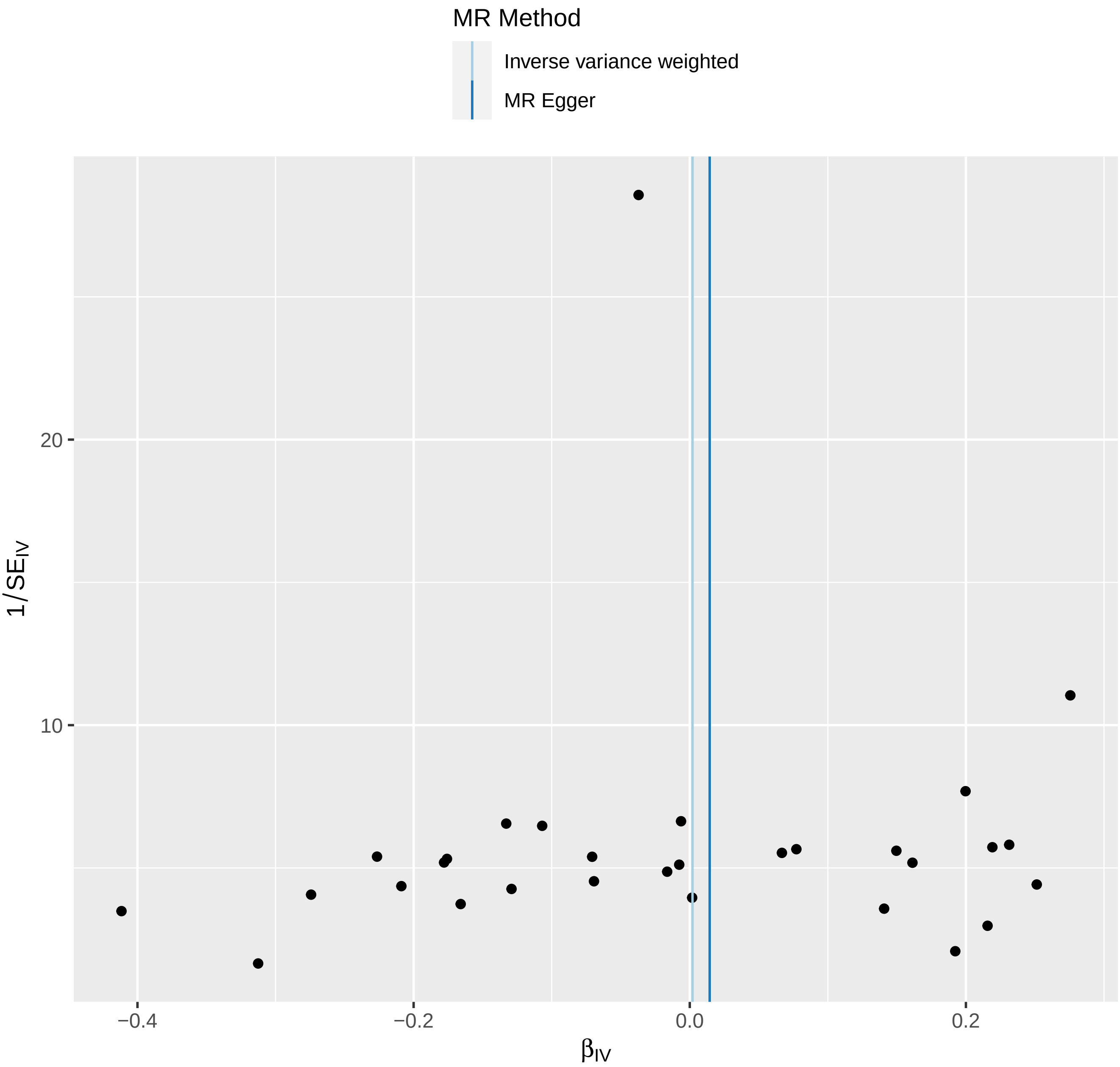

Batch 3−636 : Coactosin−like protein on Diabetic nephropathy (more control exclusions)
with a threshold of 1e−05

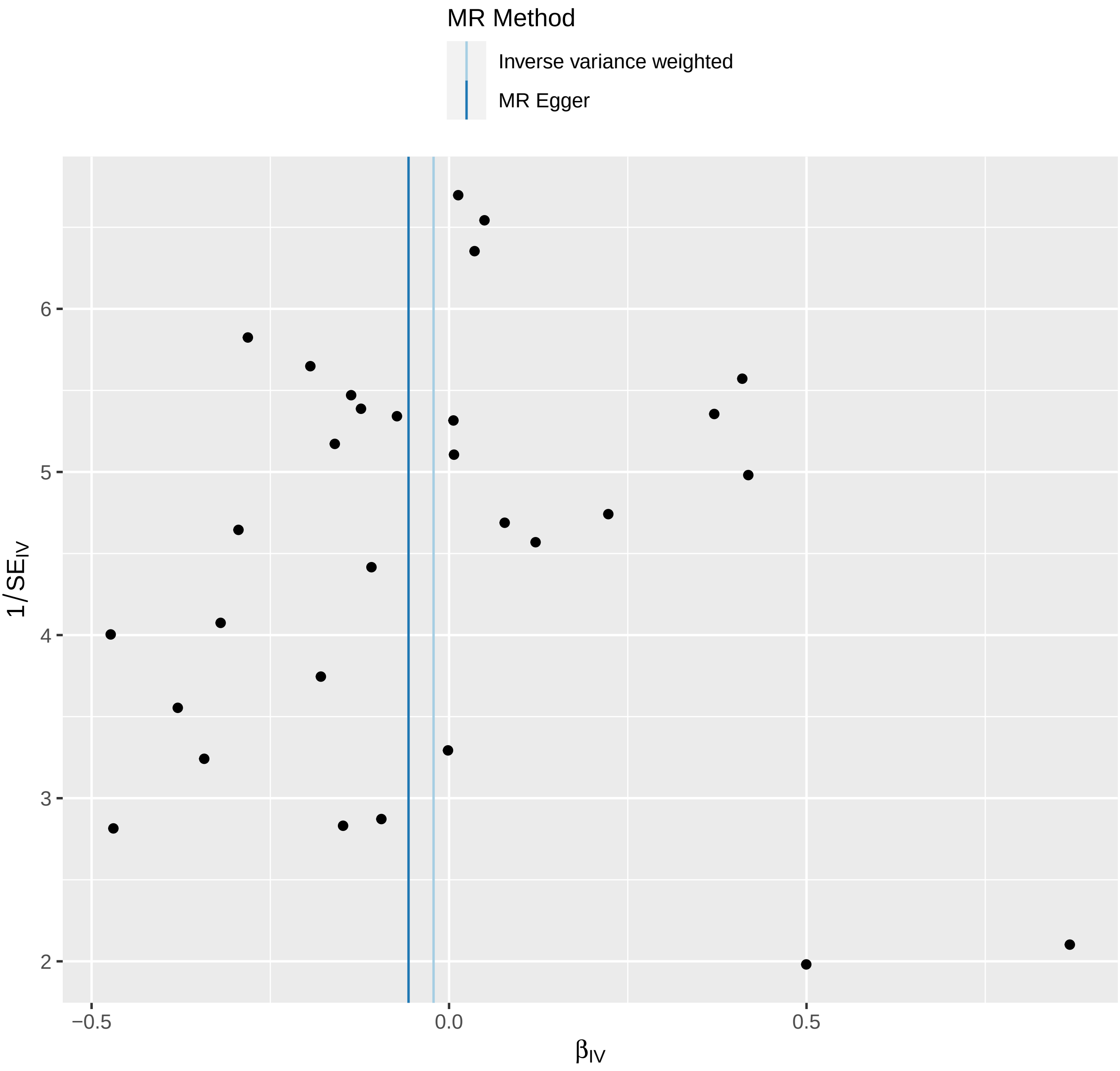

Batch 3−637 : Cytochrome c oxidase subunit 4 isoform 2, mitochondrial on Diabetic nephropathy (more control exclusions) with a threshold of 1e−05

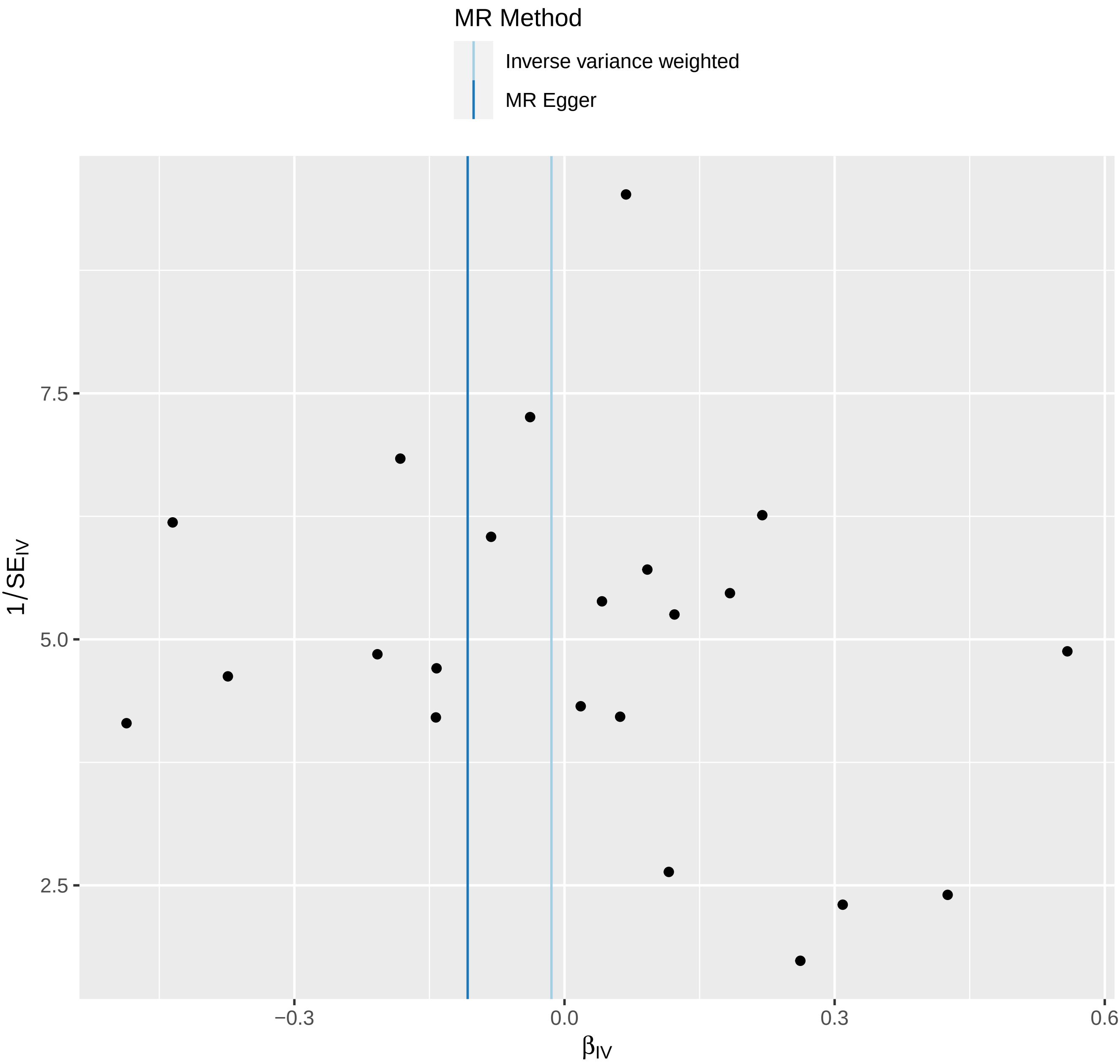

Batch 3−638 : Cytochrome c oxidase subunit 5B, mitochondrial on Diabetic nephropathy (more control exclusions)
with a threshold of 1e−05

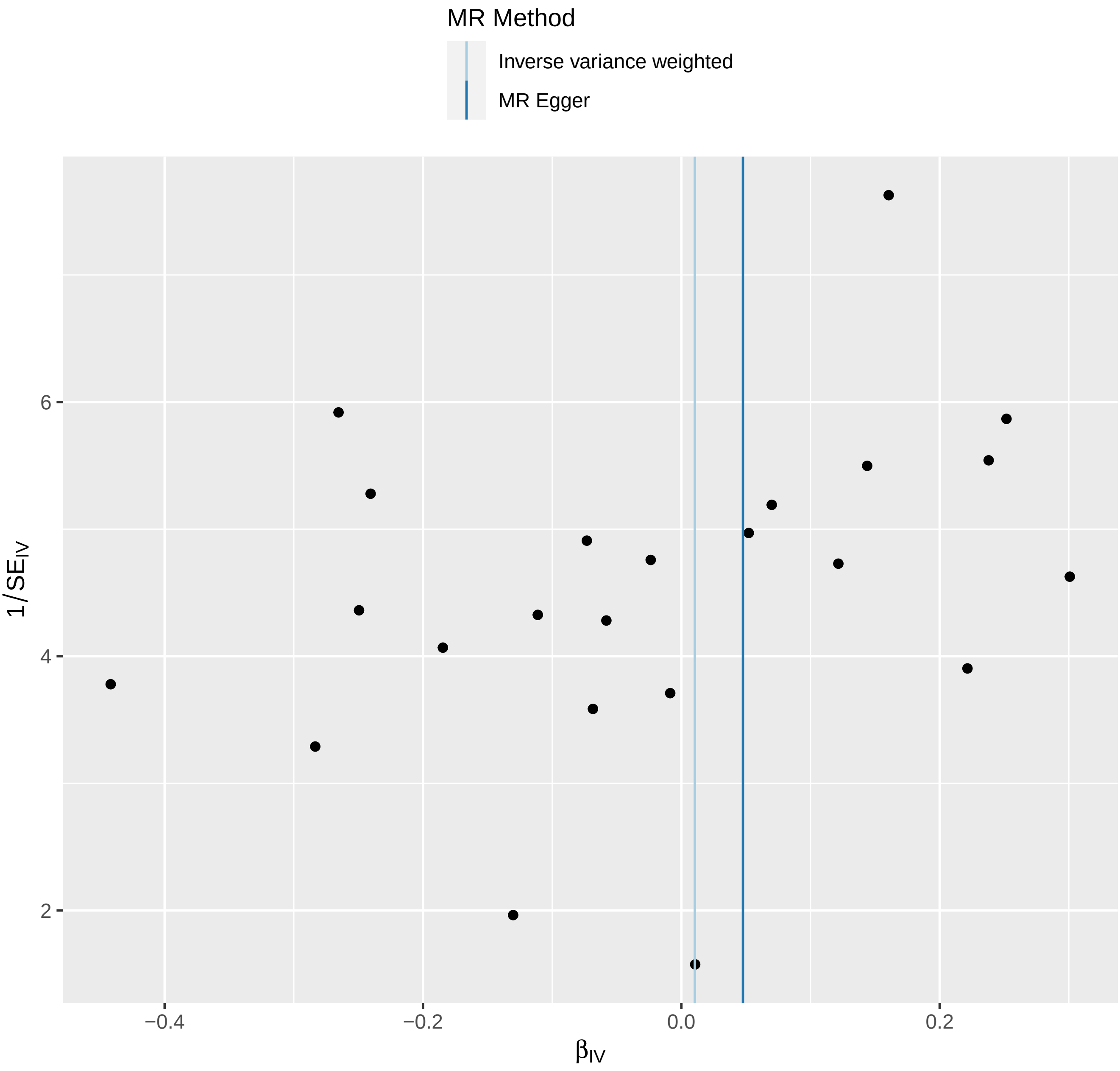

Batch 3−639 : Cytochrome c oxidase subunit 6C on Diabetic nephropathy (more control exclusions) with a threshold of 1e−05

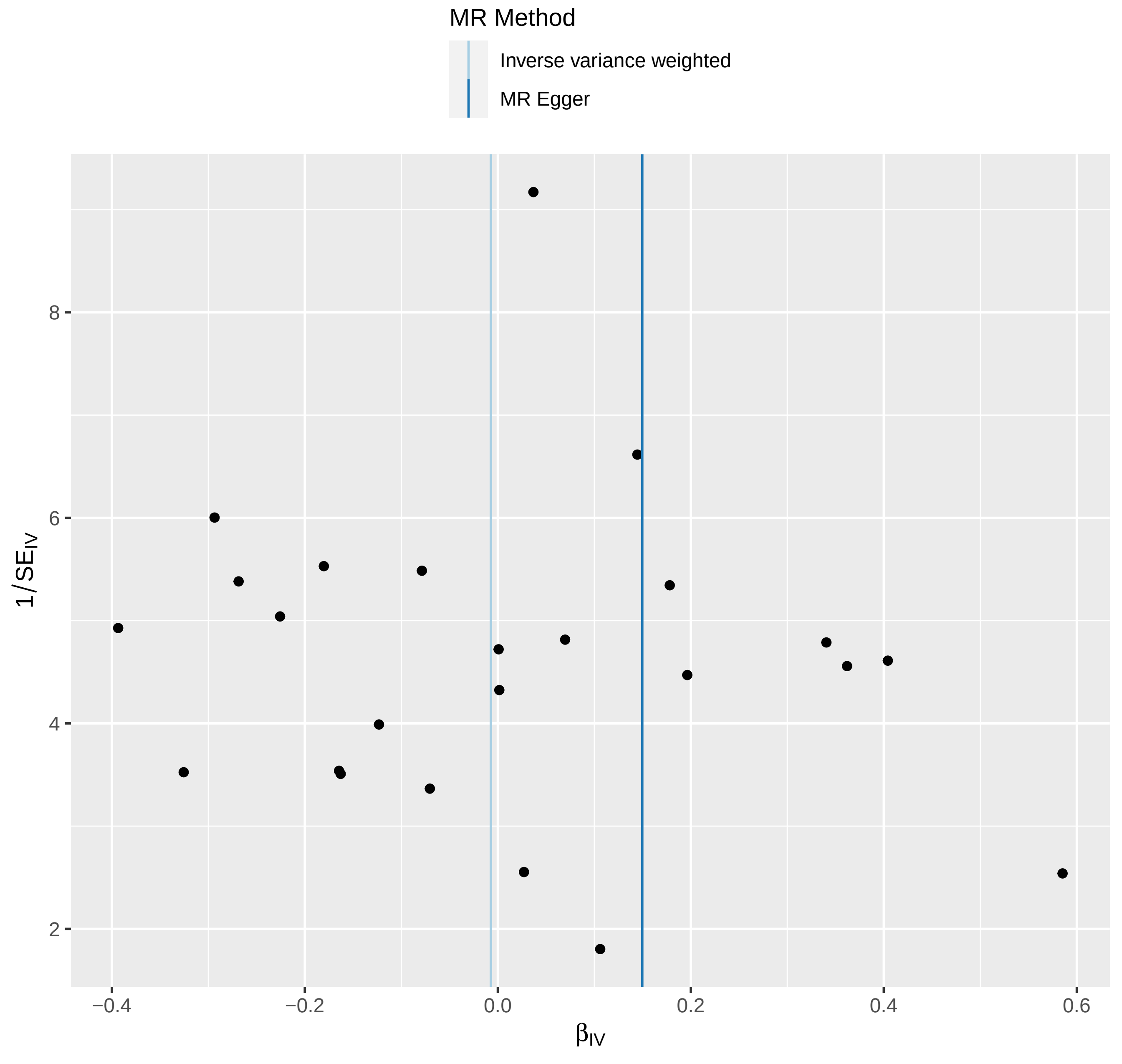

Batch 3−640 : Cytochrome c oxidase subunit 7A1, mitochondrial on Diabetic nephropathy (more control exclusions) with a threshold of 1e−05

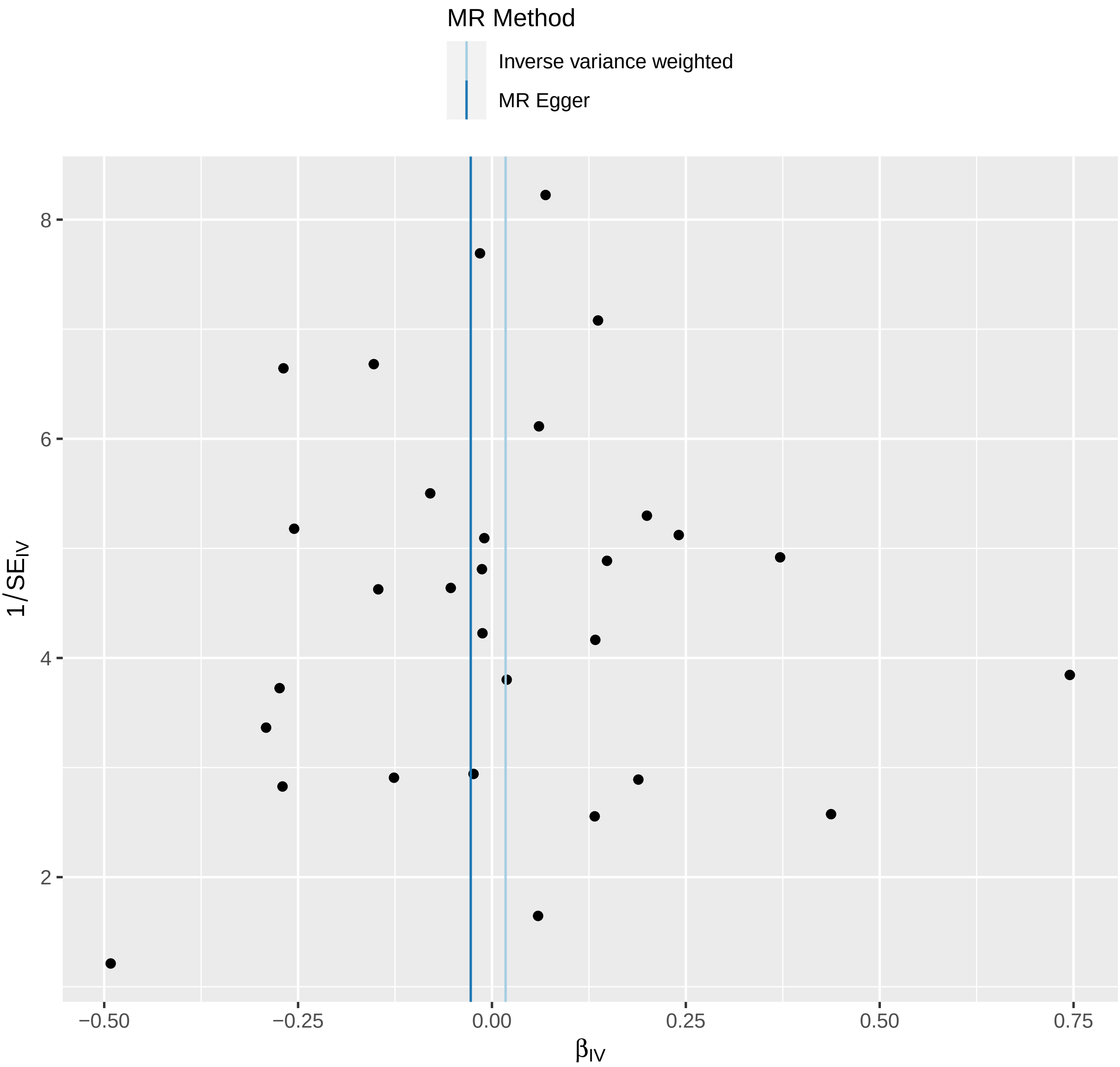

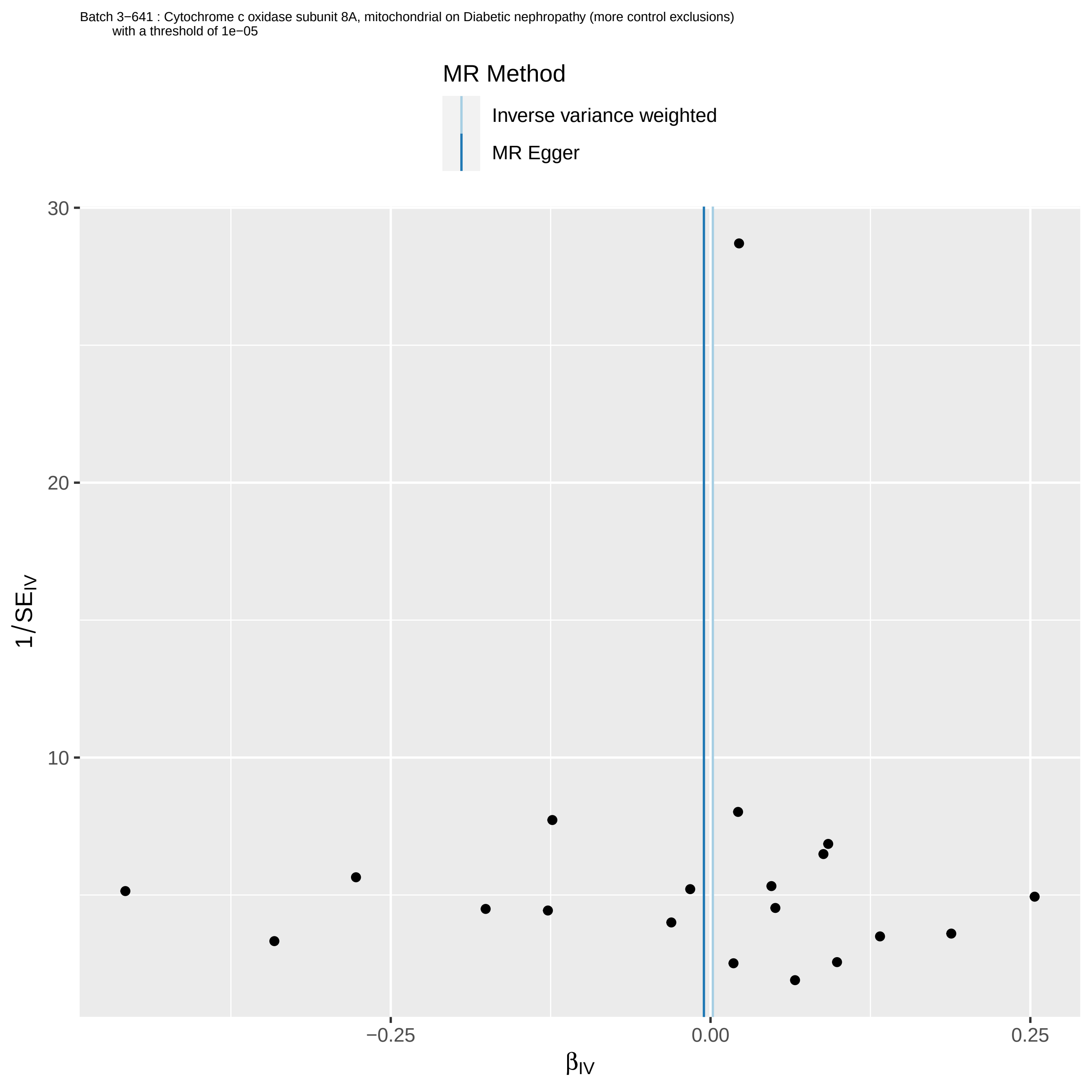

Batch 3−641 : Cytochrome c oxidase subunit 8A, mitochondrial on Diabetic nephropathy (more control exclusions)
with a threshold of 1e−05
MR Method
Inverse variance weighted
MR Egger
30
20
10
1/SE_IV
−0.25
0.00
0.25
β_IV

Batch 3−642 : Carboxypeptidase A2 on Diabetic nephropathy (more control exclusions)
with a threshold of 1e−05

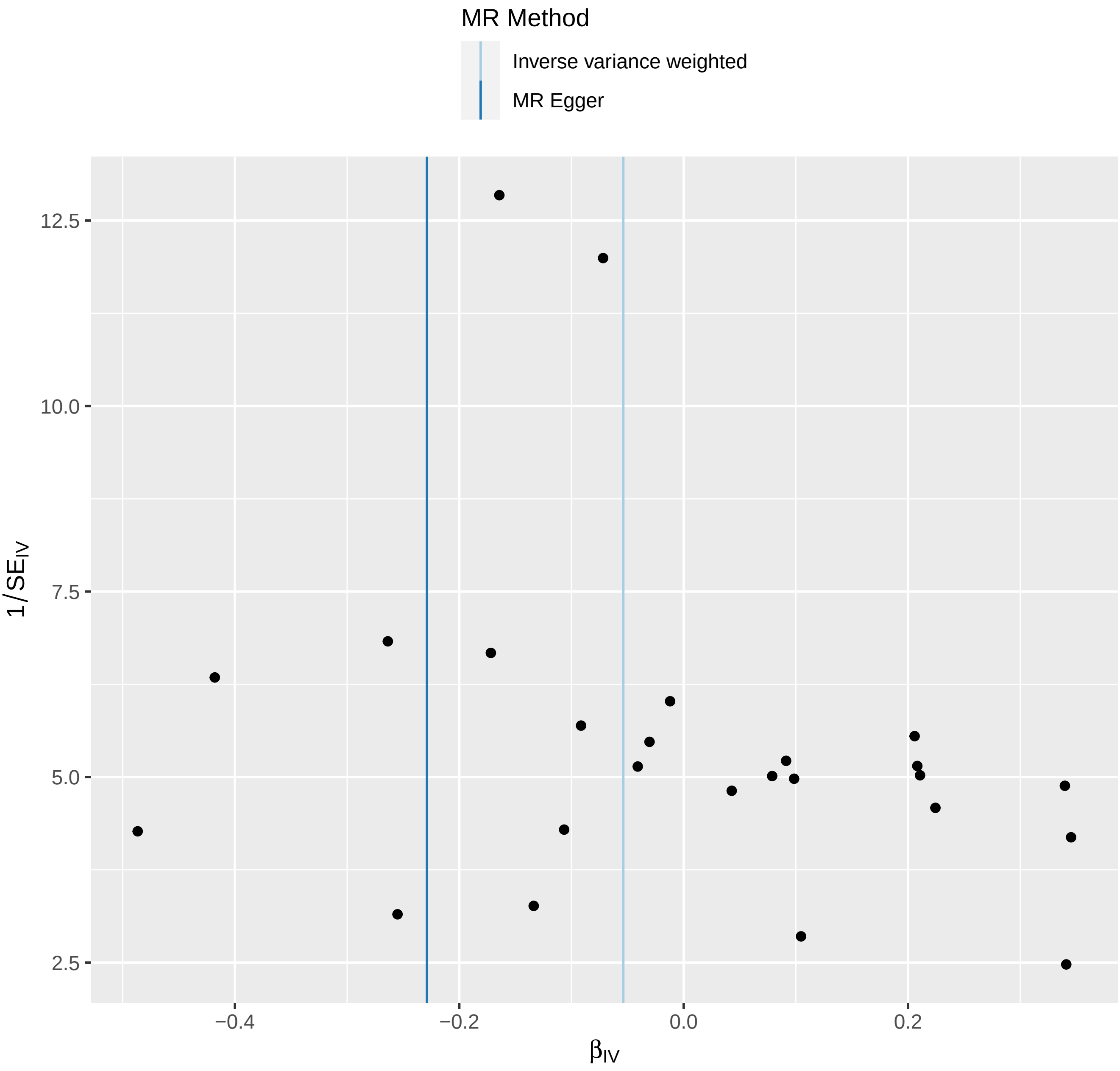

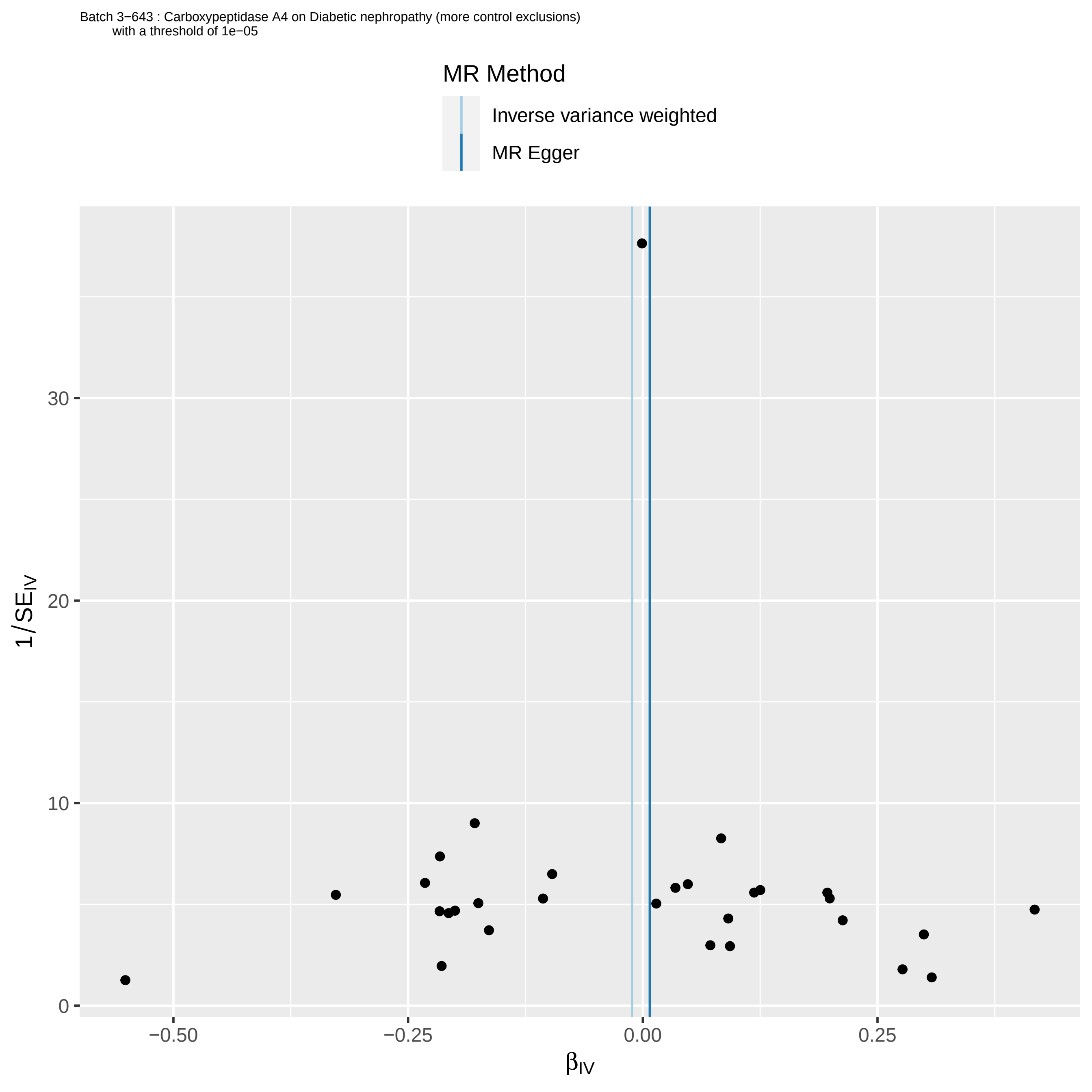

Batch 3−643 : Carboxypeptidase A4 on Diabetic nephropathy (more control exclusions)
with a threshold of 1e−05
MR Method
Inverse variance weighted
MR Egger
30
20
10
0
1/SE_IV
−0.50
−0.25
0.00
0.25
β_IV

Batch 3−644 : Carboxypeptidase B on Diabetic nephropathy (more control exclusions)
with a threshold of 1e−05

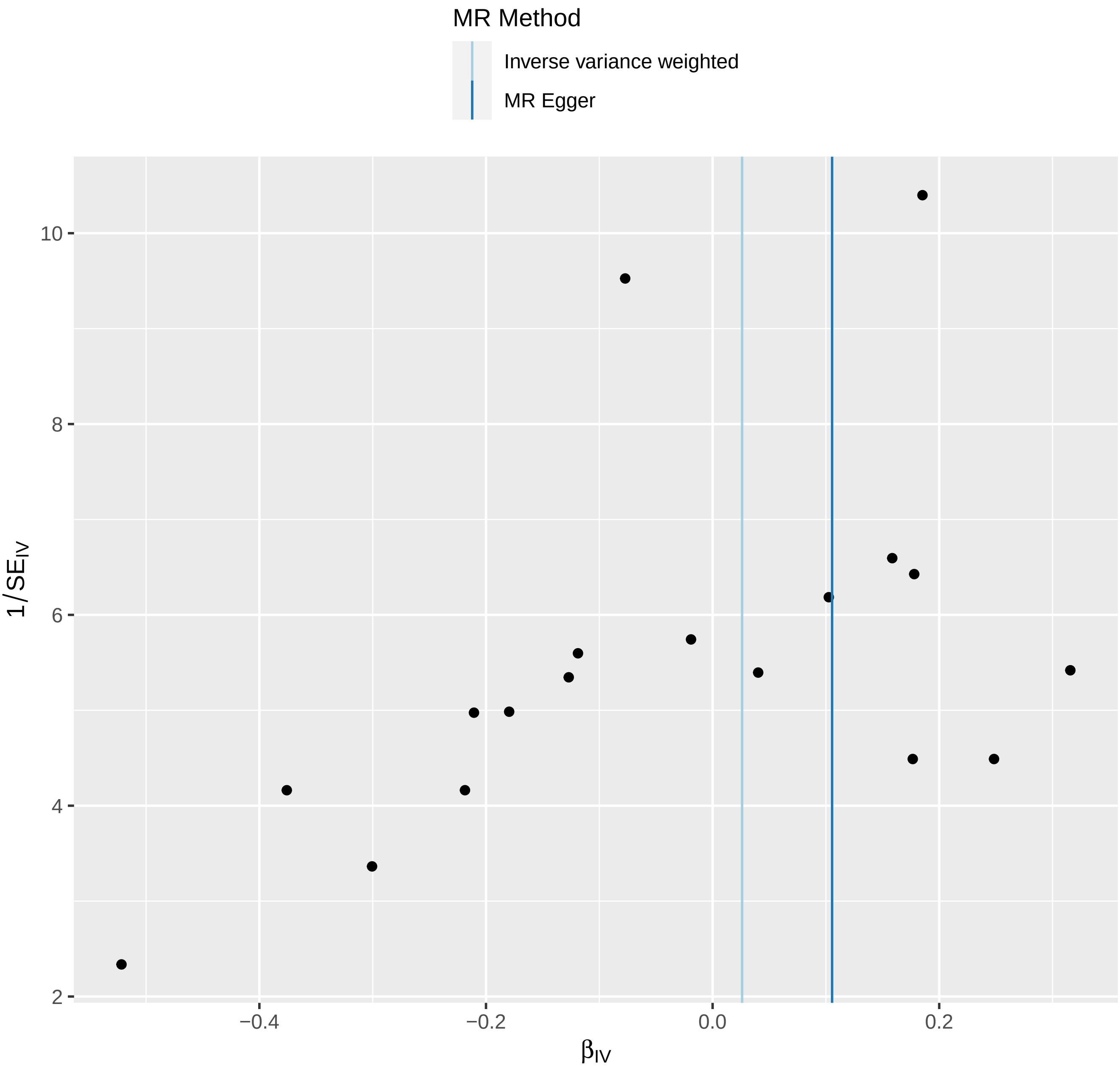

Batch 3−645 : Carboxypeptidase M on Diabetic nephropathy (more control exclusions)
with a threshold of 1e−05

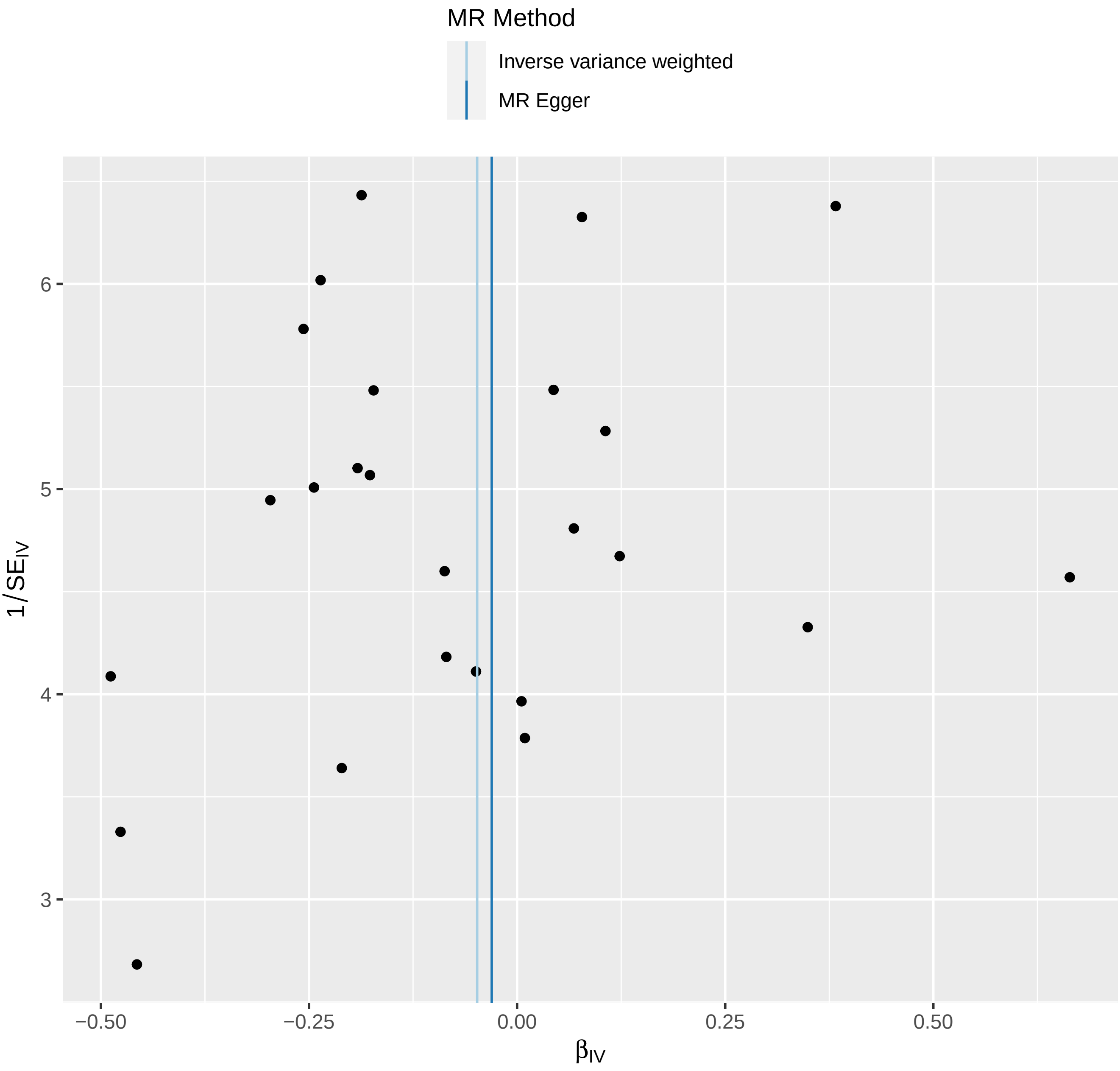

Batch 3−646 : Copine−1 on Diabetic nephropathy (more control exclusions)
with a threshold of 1e−05

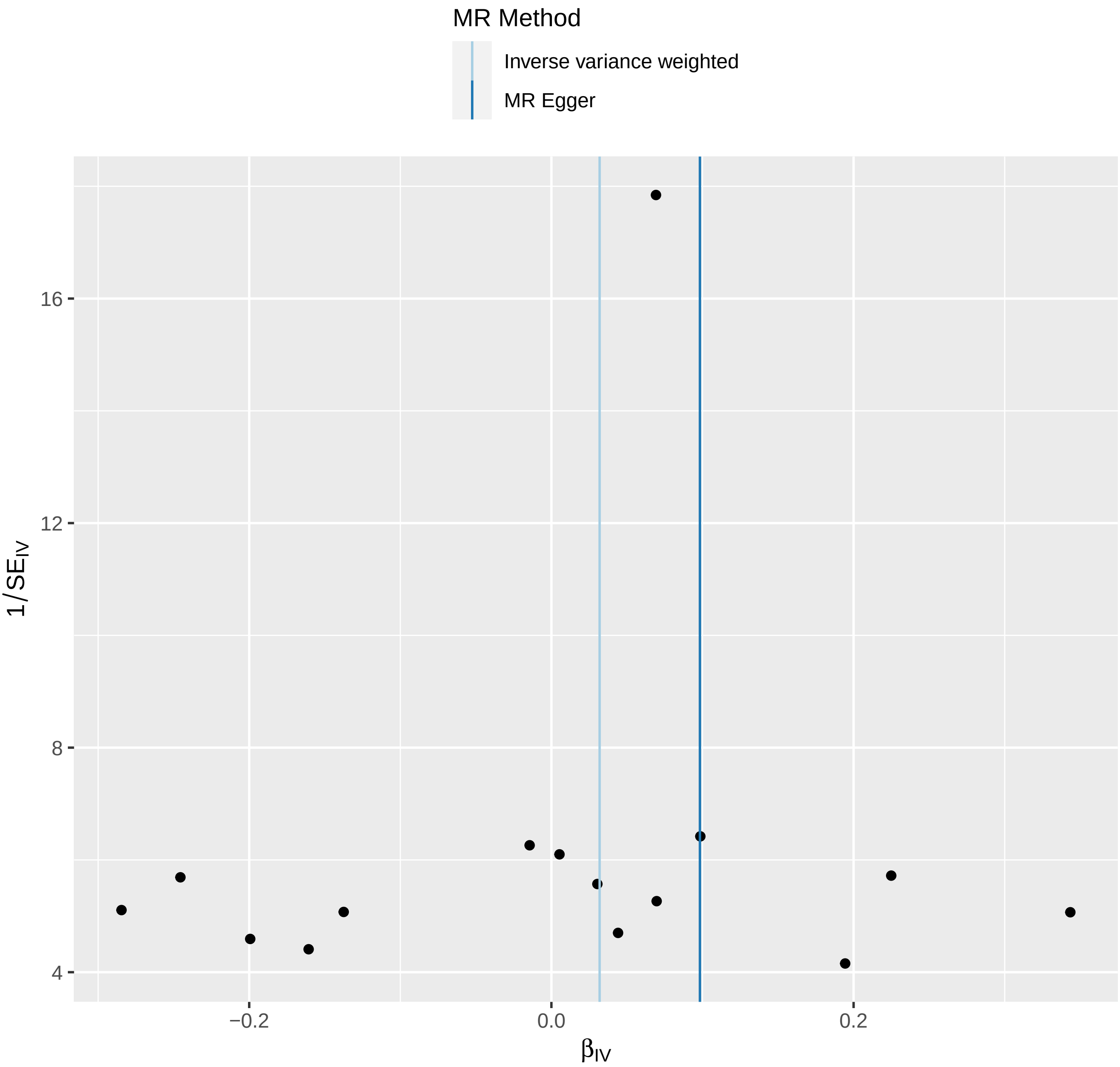

Batch 3−647 : Probable serine carboxypeptidase CPVL on Diabetic nephropathy (more control exclusions)
with a threshold of 1e−05

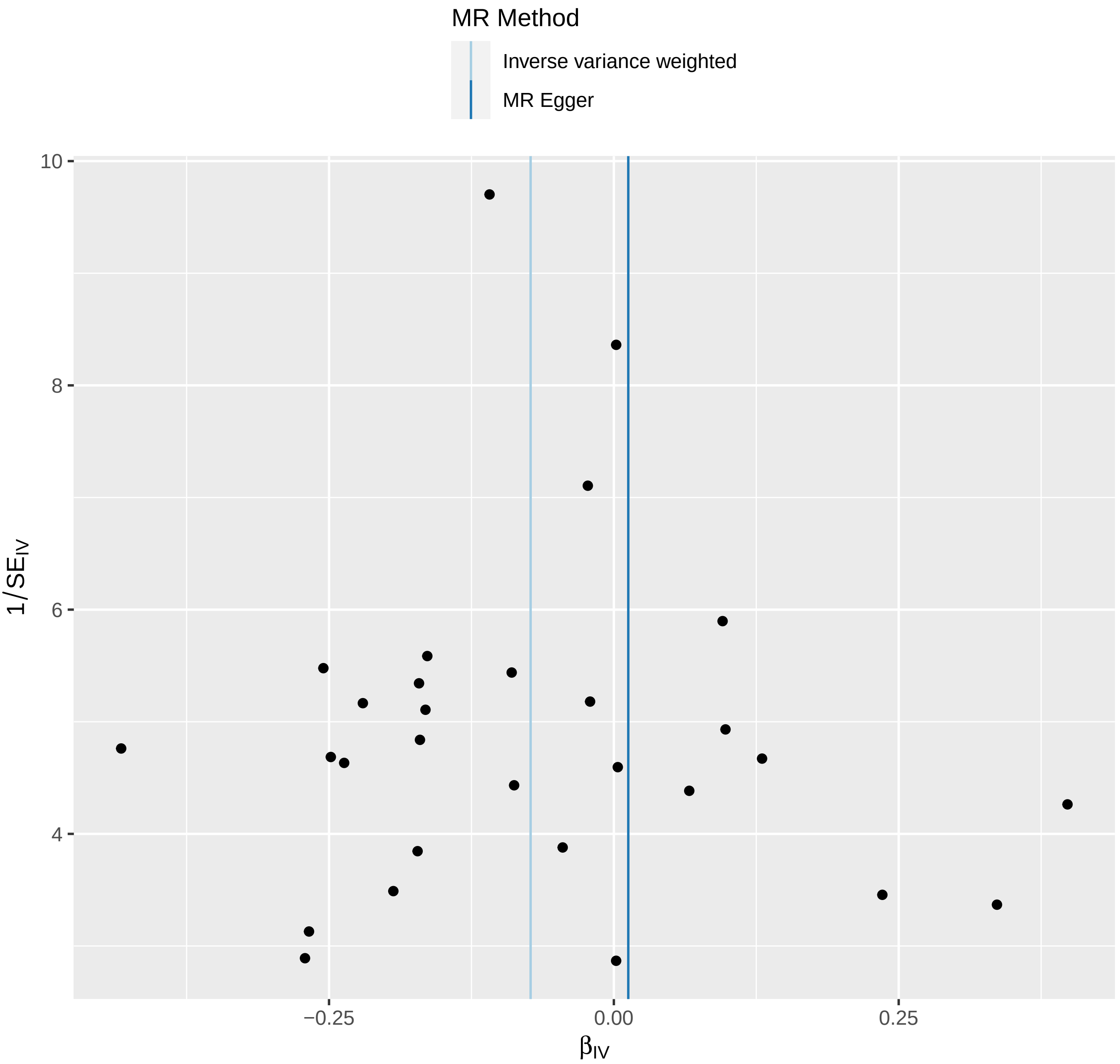

Batch 3−648 : Probable carboxypeptidase X1 on Diabetic nephropathy (more control exclusions)
with a threshold of 1e−05

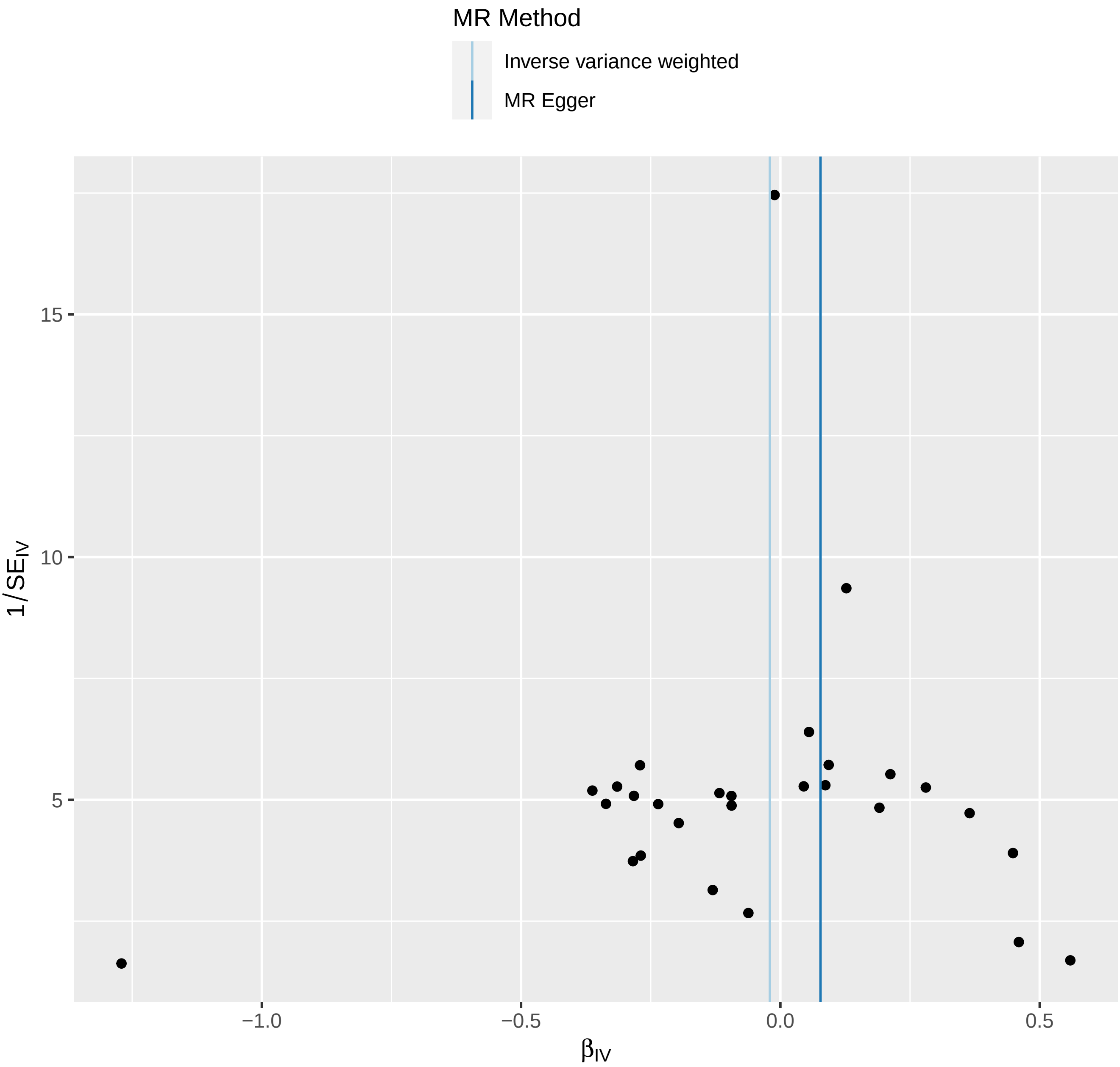

Batch 3−649 : Carboxypeptidase Z on Diabetic nephropathy (more control exclusions)
with a threshold of 1e−05

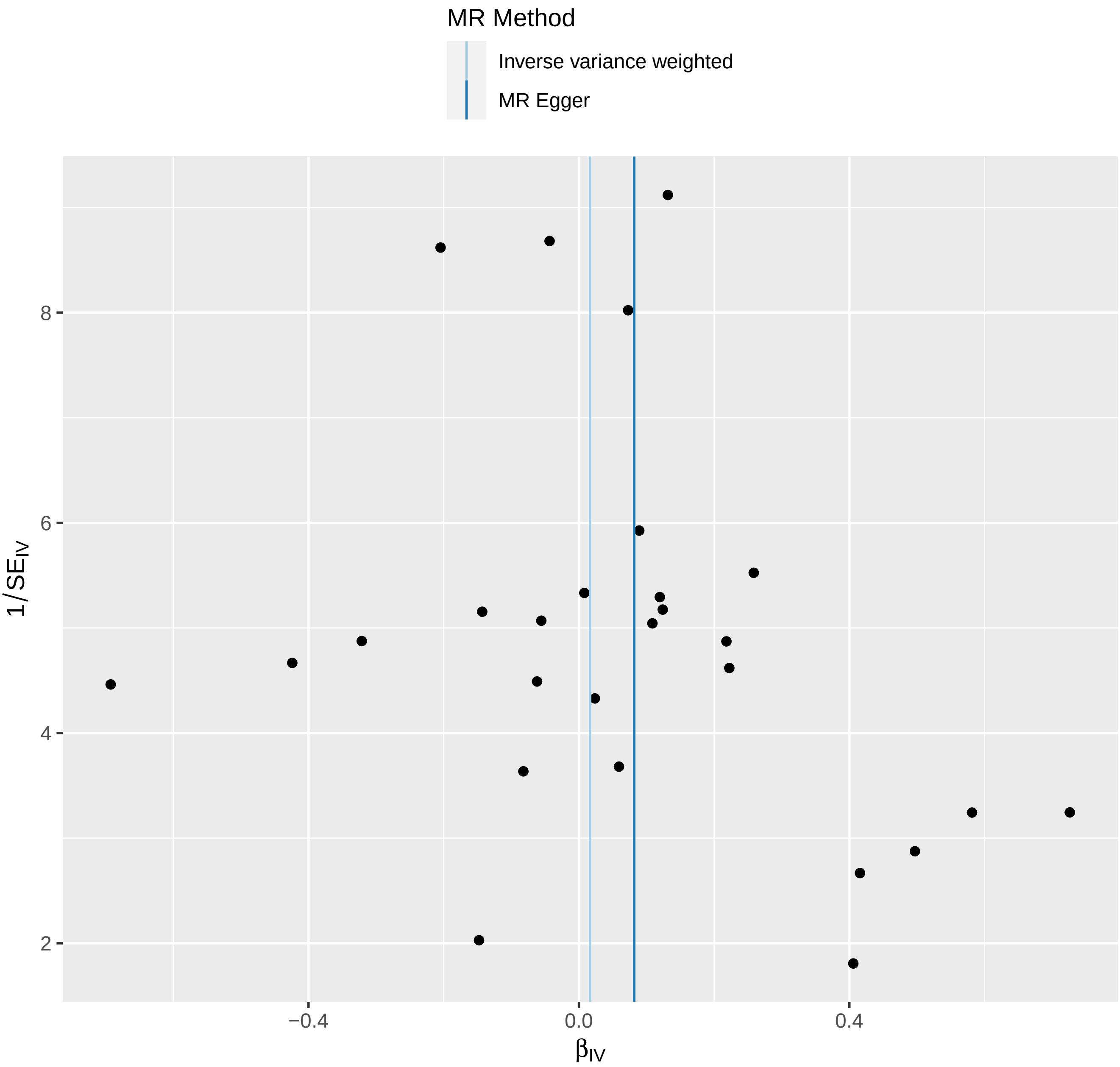

Batch 3−650 : Cellular retinoic acid−binding protein 1 on Diabetic nephropathy (more control exclusions)
with a threshold of 1e−05

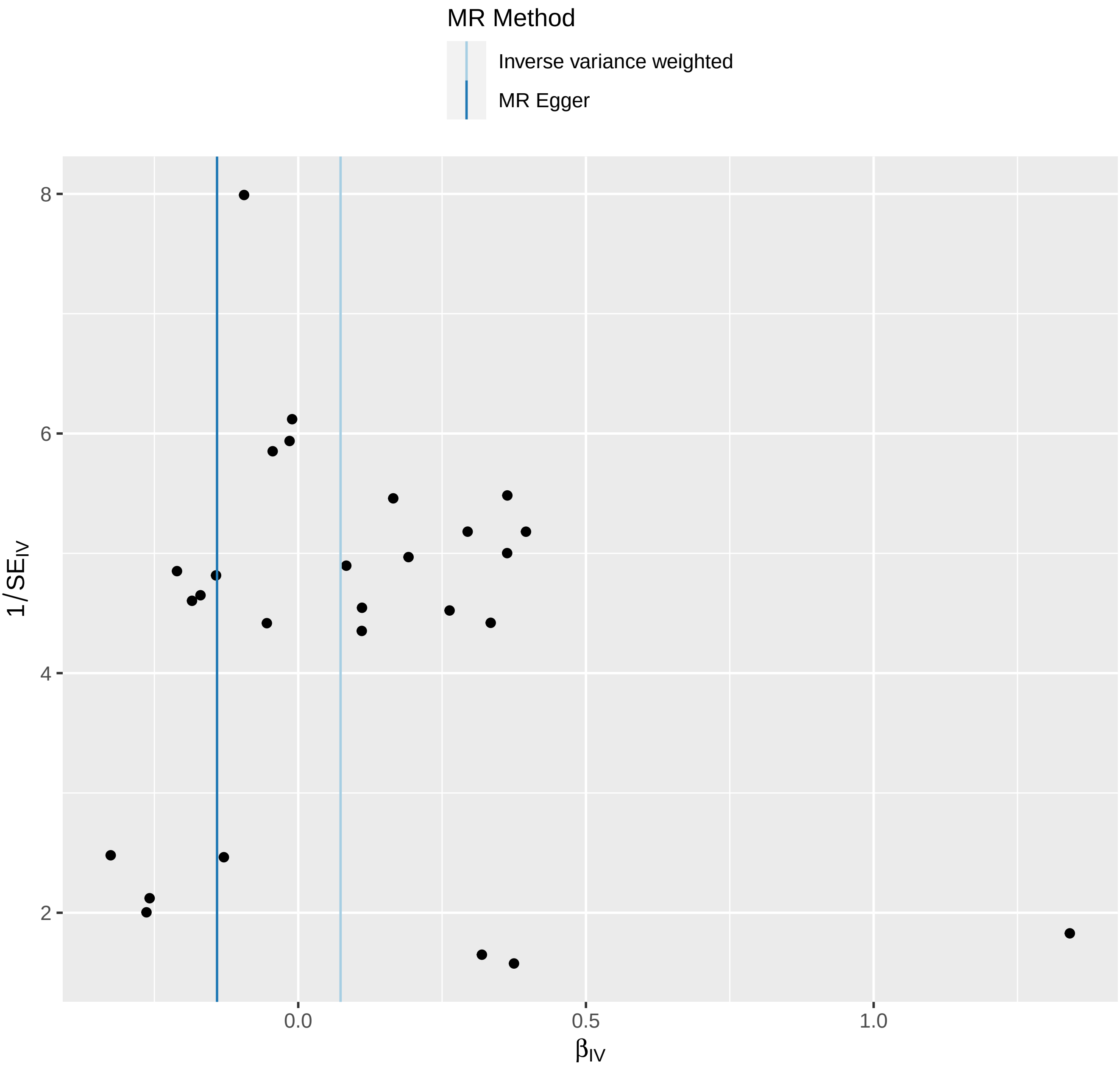

Batch 3−651 : Cellular retinoic acid−binding protein 2 on Diabetic nephropathy (more control exclusions)
with a threshold of 1e−05

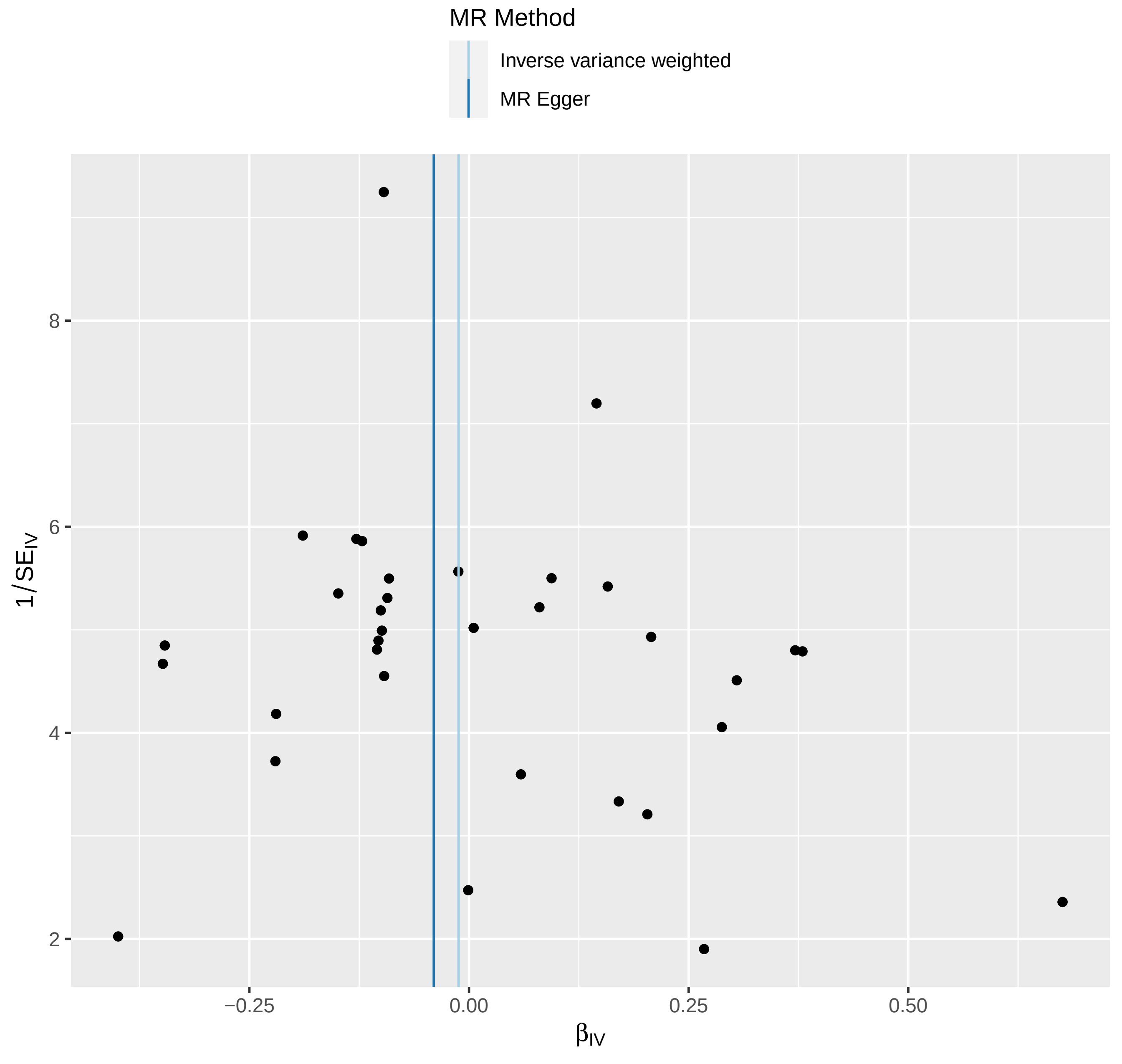

Batch 3−652 : Protein crumbs homolog 1 on Diabetic nephropathy (more control exclusions)
with a threshold of 1e−05

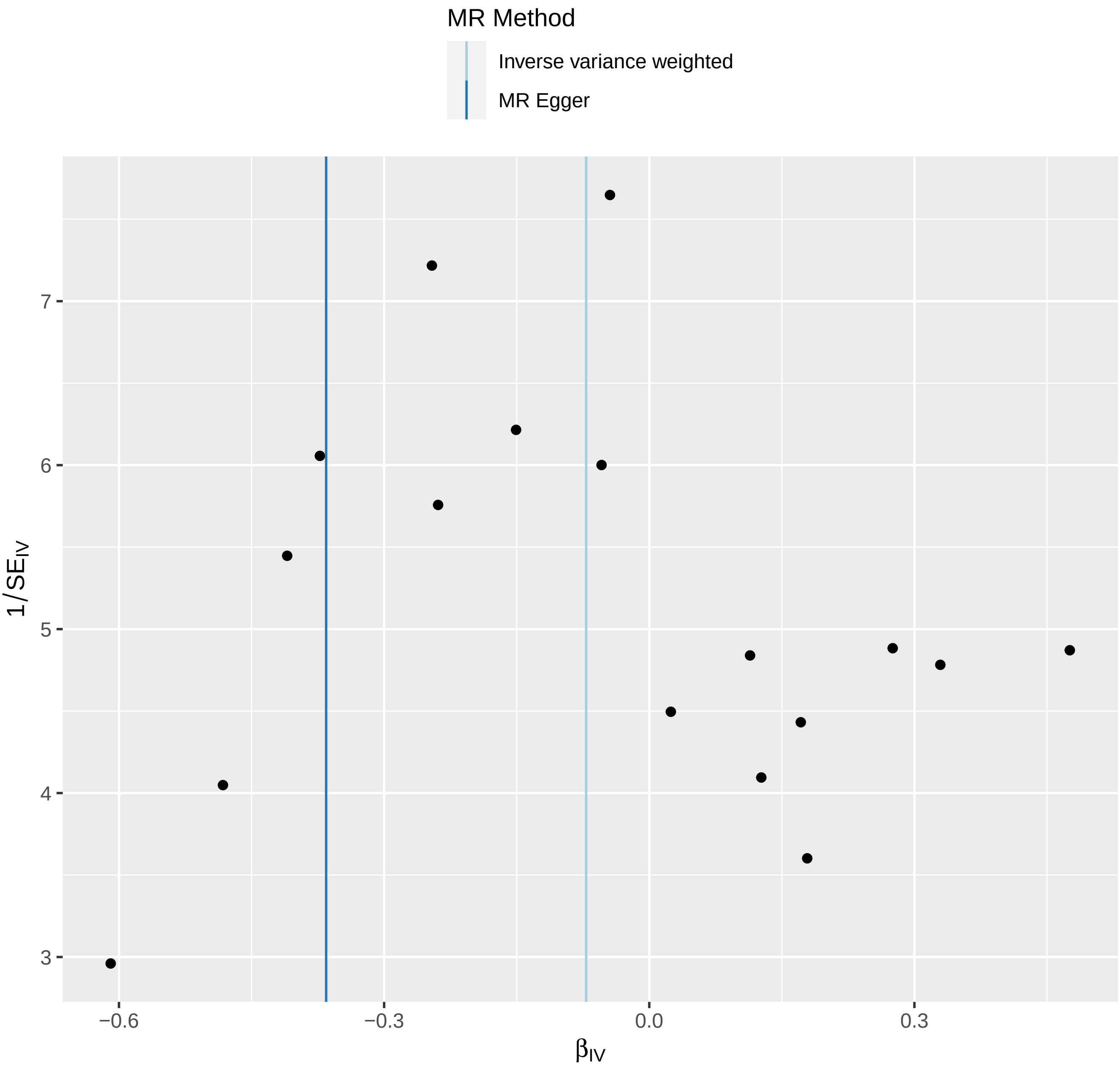

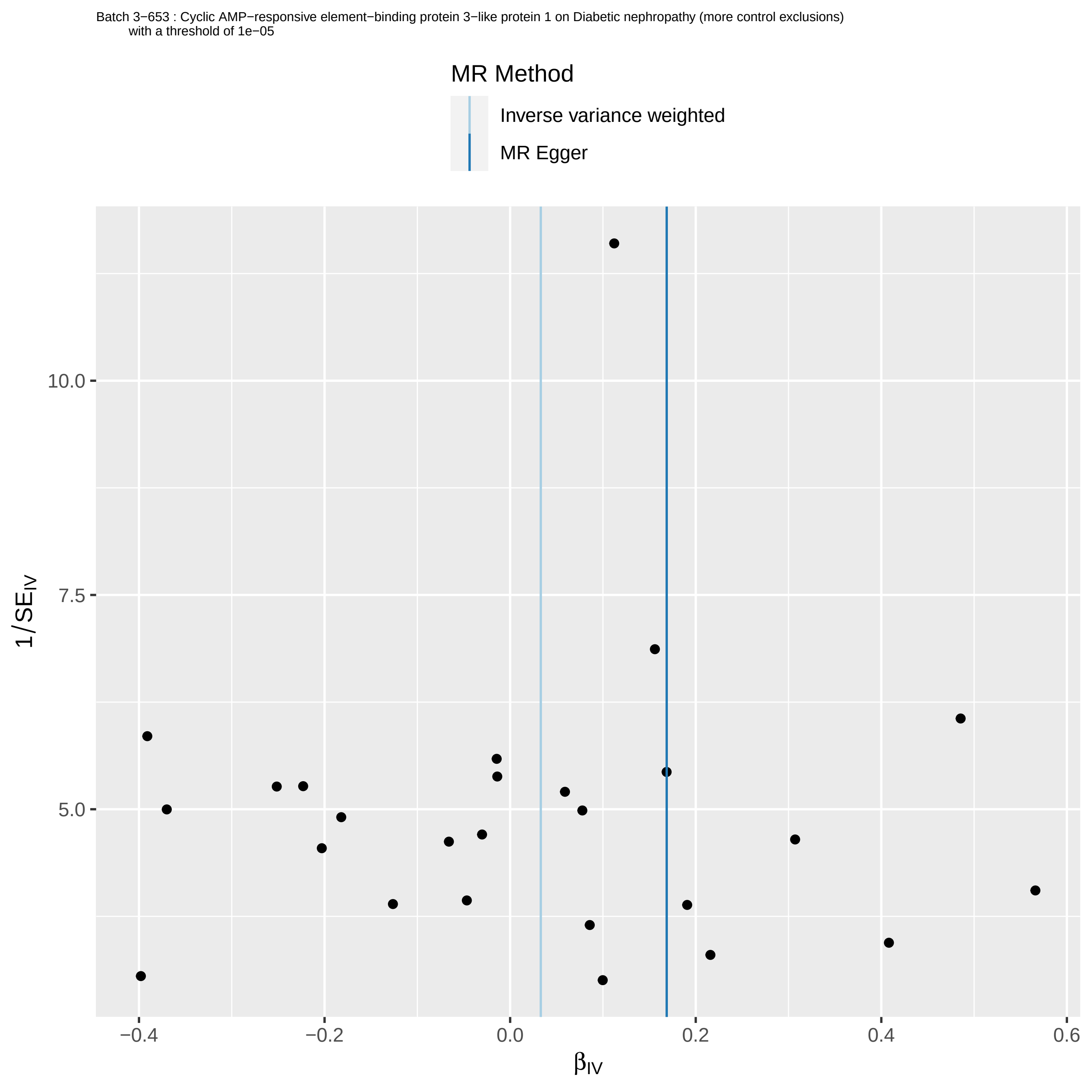
Batch 3−653 : Cyclic AMP−responsive element−binding protein 3−like protein 1 on Diabetic nephropathy (more control exclusions)
with a threshold of 1e−05
MR Method
Inverse variance weighted
MR Egger
10.0
7.5
5.0
1/SE_IV
−0.4
−0.2
0.0
0.2
0.4
0.6
β_IV

Batch 3−654 : Cyclic AMP−responsive element−binding protein 3−like protein 4 on Diabetic nephropathy (more control exclusions) with a threshold of 1e−05

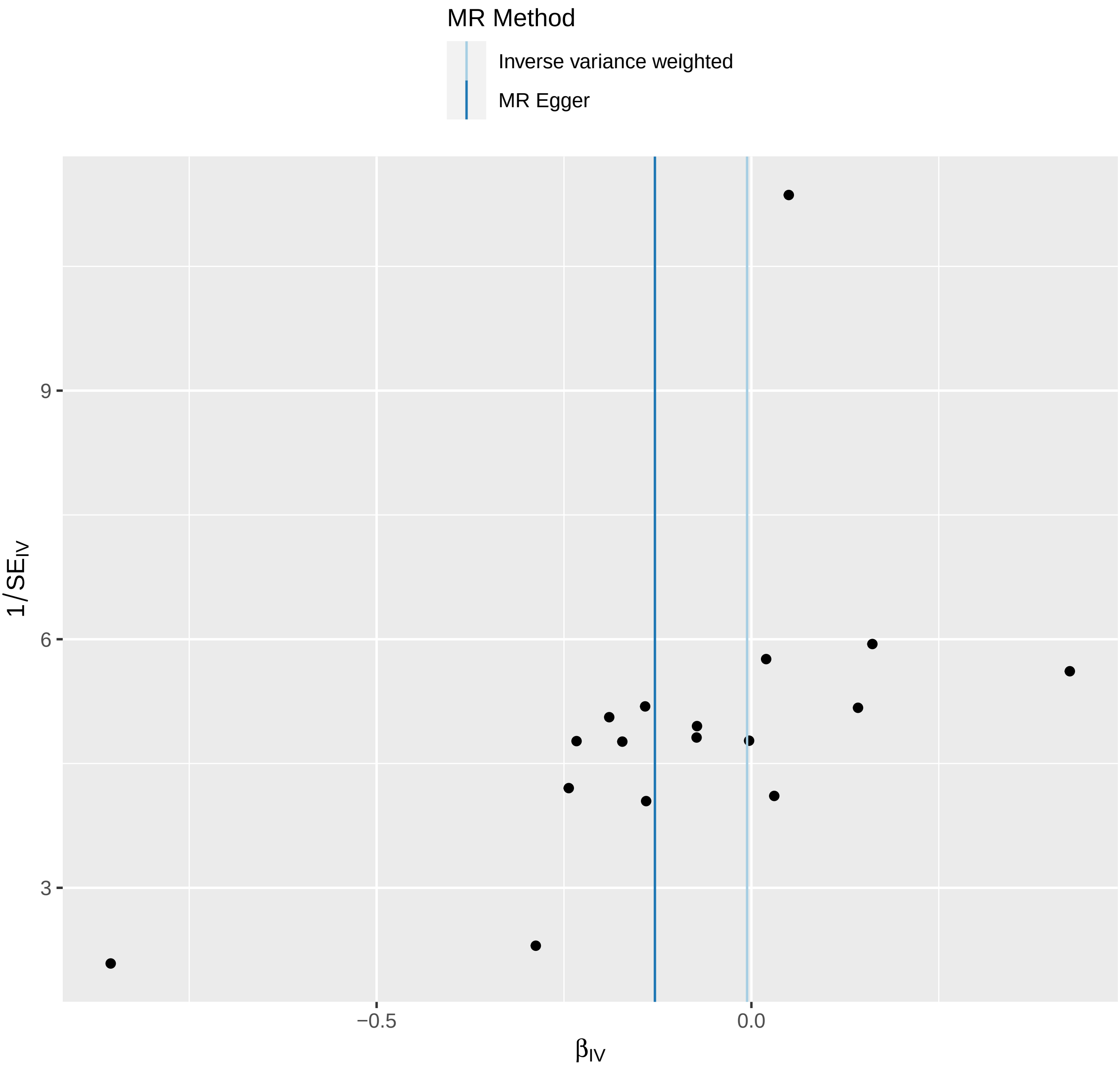

Batch 3−655 : CREB−binding protein on Diabetic nephropathy (more control exclusions)
with a threshold of 1e−05

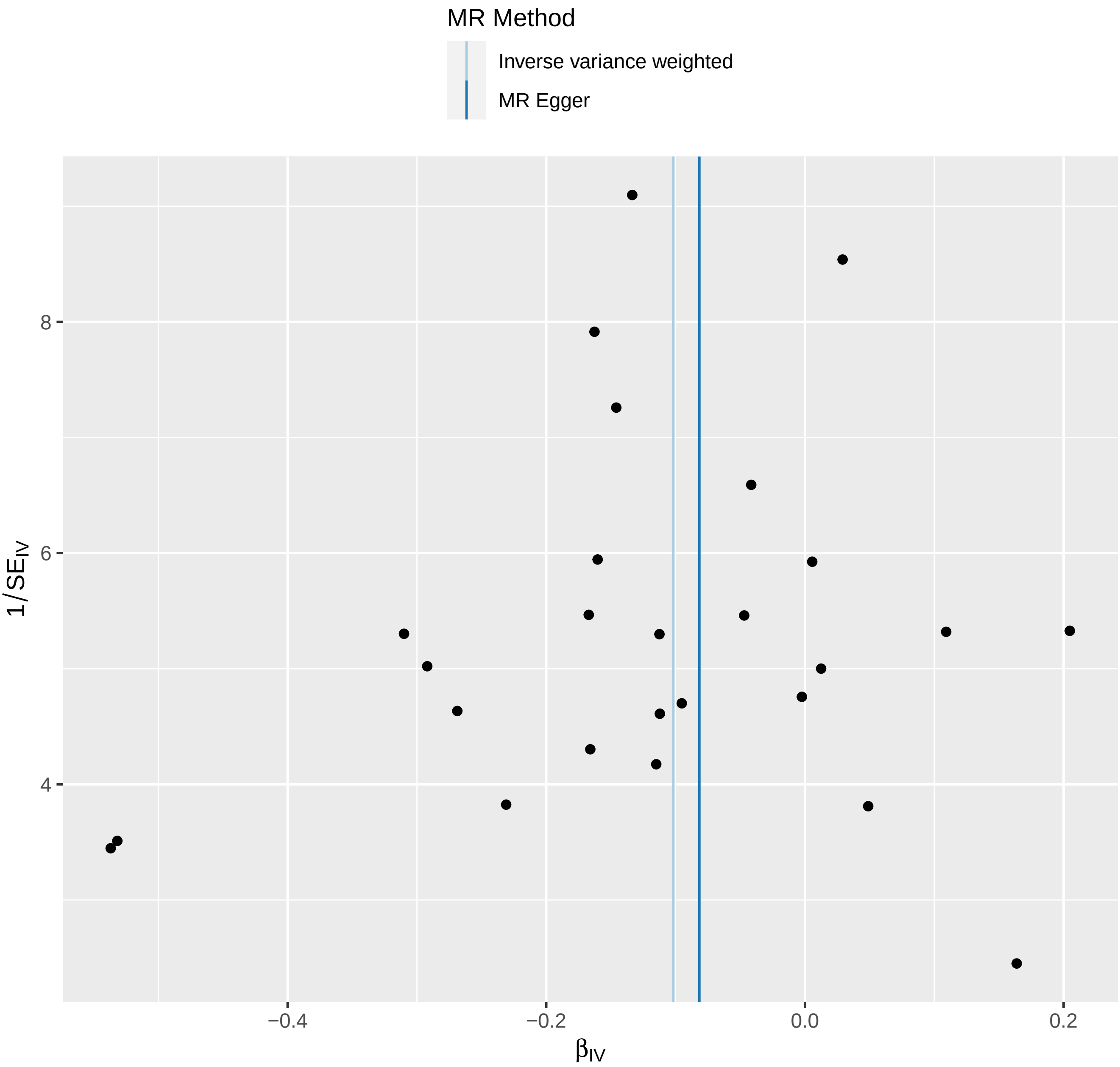

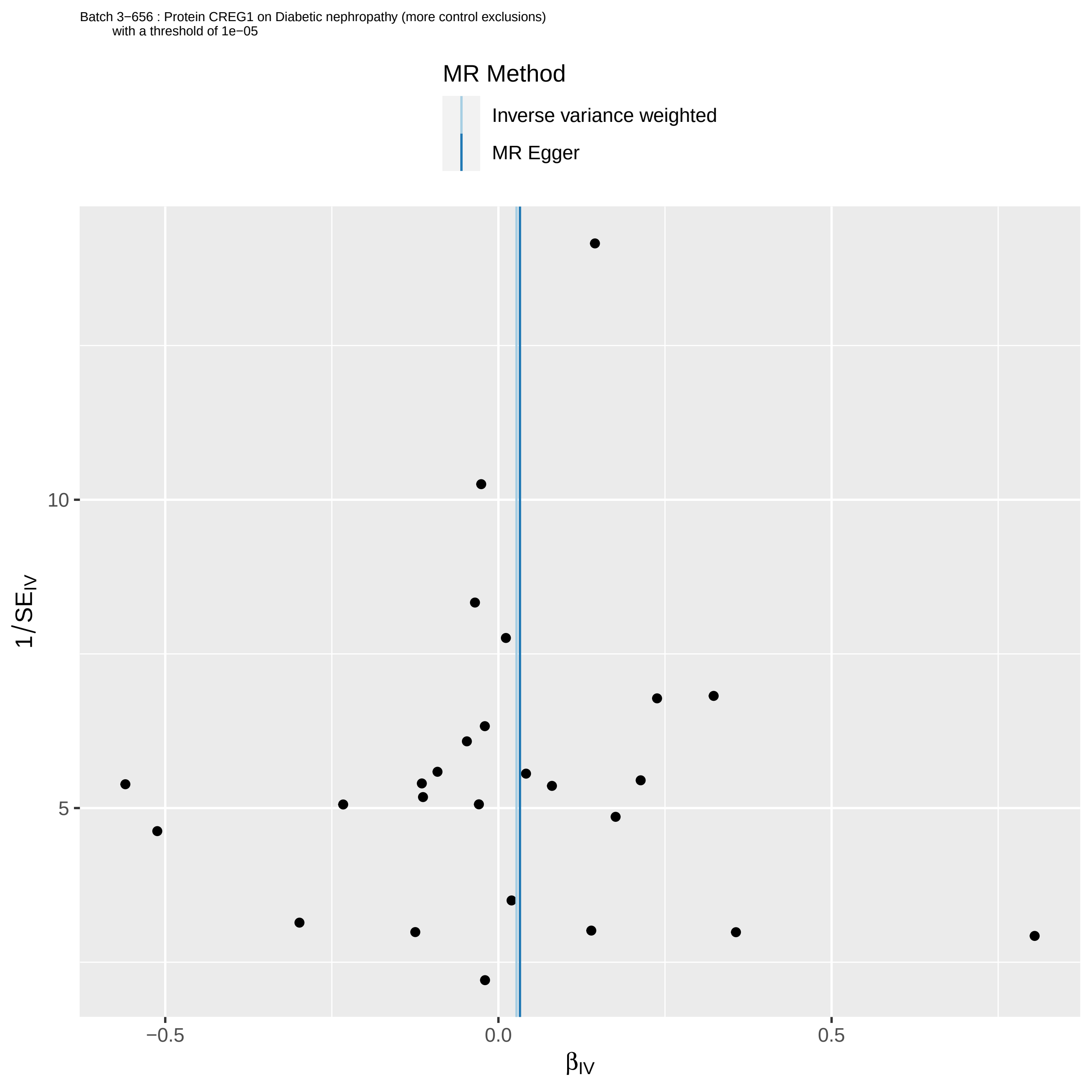

Batch 3−656 : Protein CREG1 on Diabetic nephropathy (more control exclusions)
with a threshold of 1e−05
MR Method
Inverse variance weighted
MR Egger
10
5
1/SE_IV
−0.5
0.0
0.5
β_IV

Batch 3−657 : Cysteine−rich with EGF−like domain protein 1 on Diabetic nephropathy (more control exclusions)
with a threshold of 1e−05

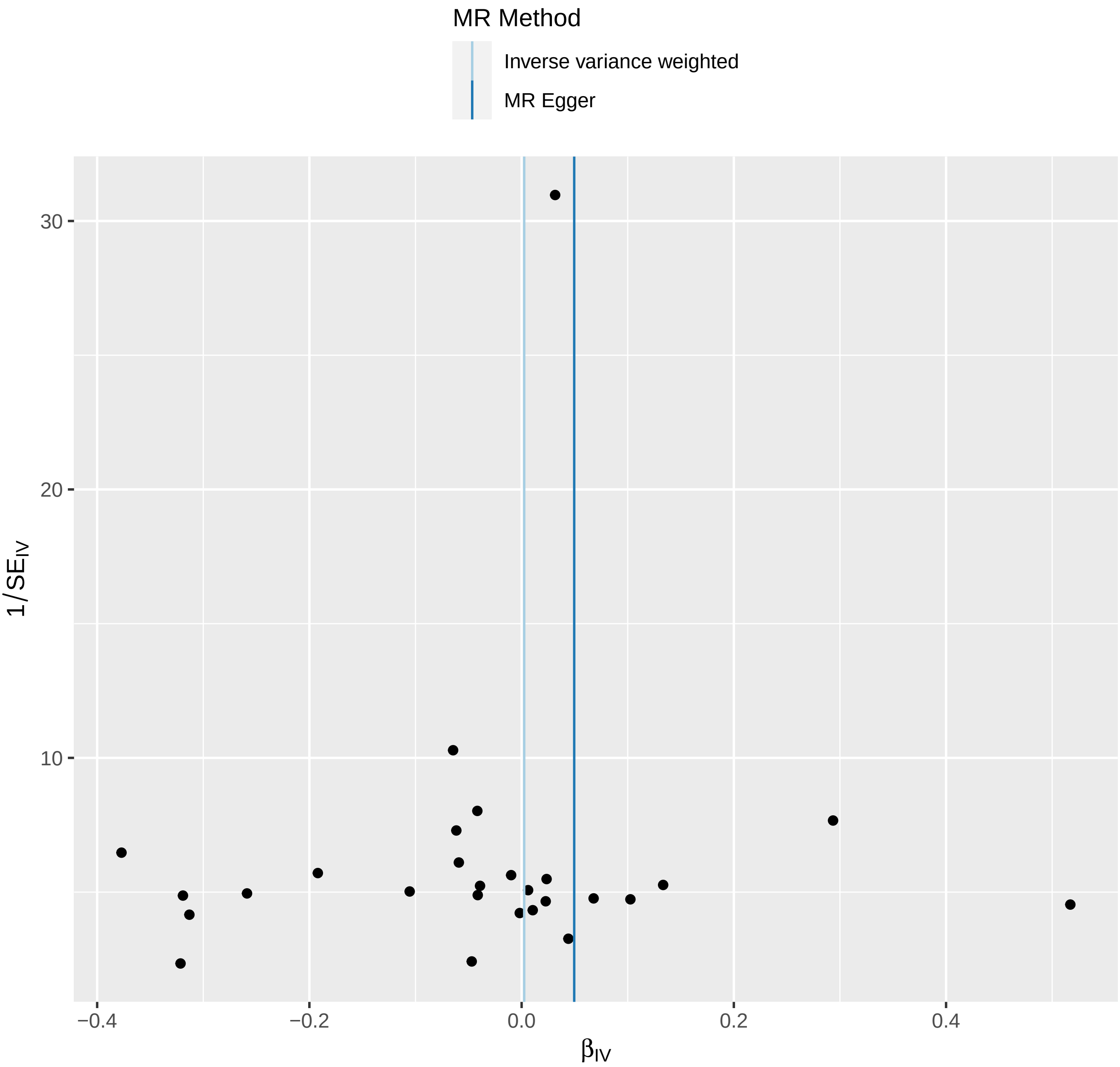

Batch 3−658 : Corticoliberin on Diabetic nephropathy (more control exclusions)
with a threshold of 1e−05

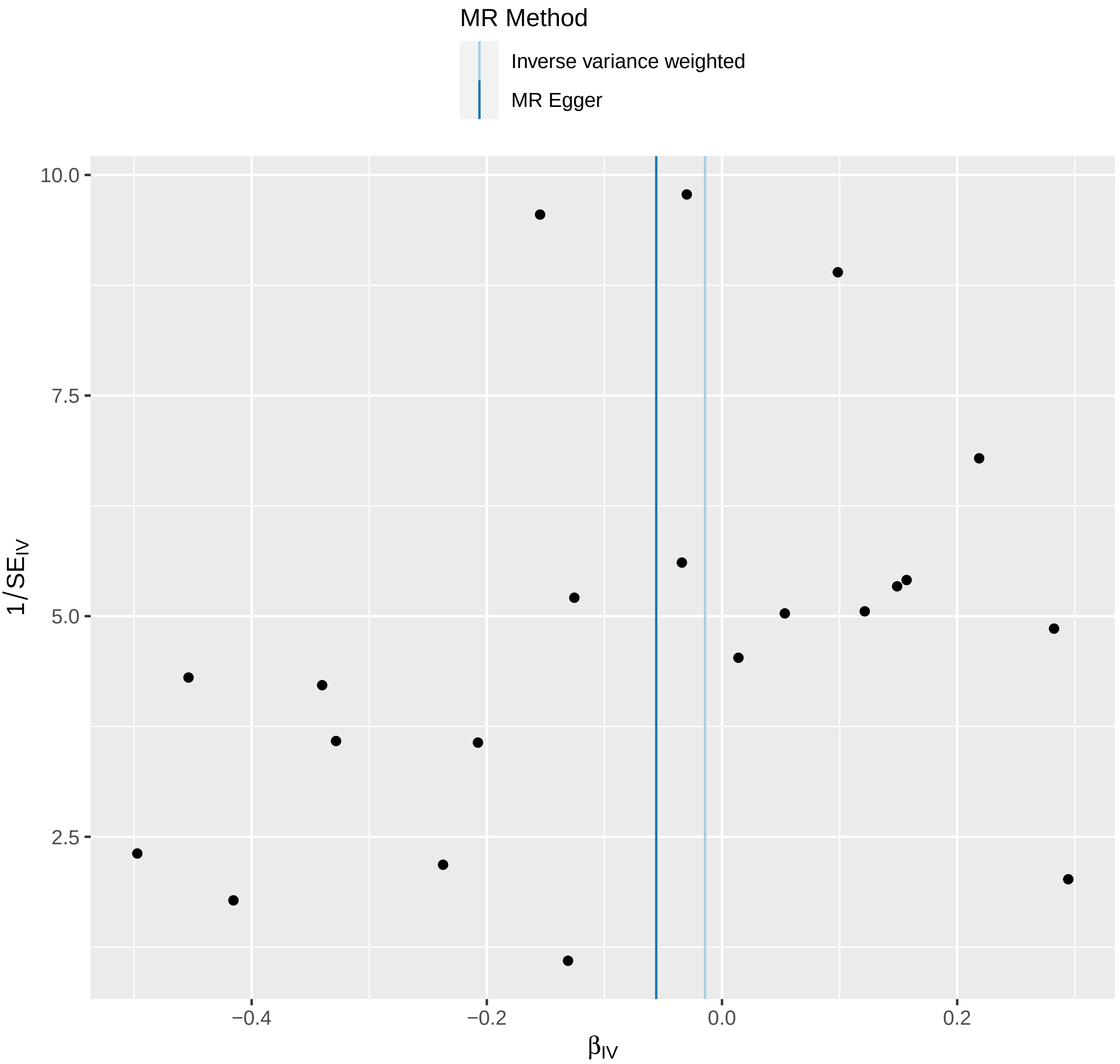

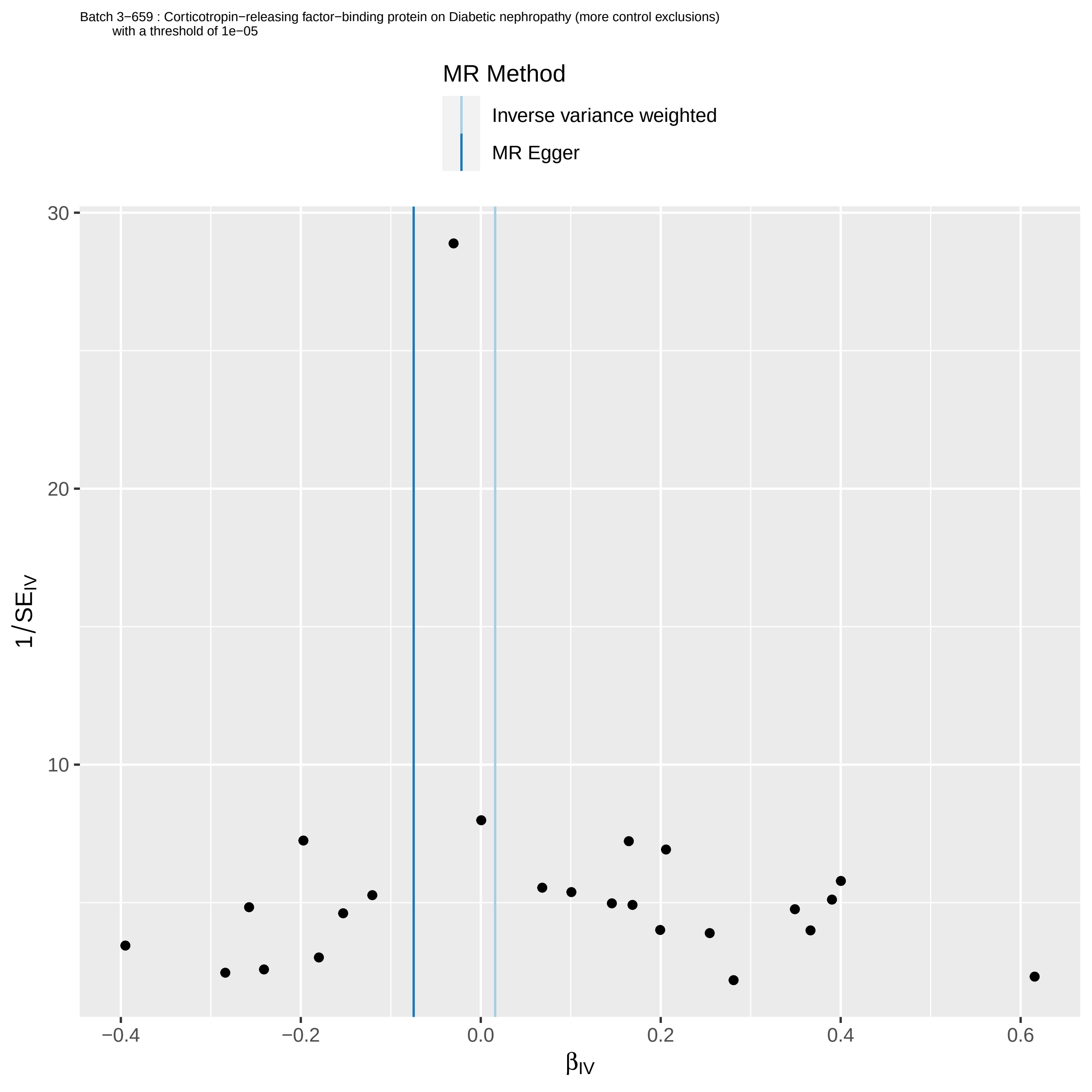
Batch 3−659 : Corticotropin−releasing factor−binding protein on Diabetic nephropathy (more control exclusions)
with a threshold of 1e−05
MR Method
Inverse variance weighted
MR Egger
30
20
10
1/SE$_{IV}$
−0.4
−0.2
0.0
0.2
0.4
0.6
β$_{IV}$

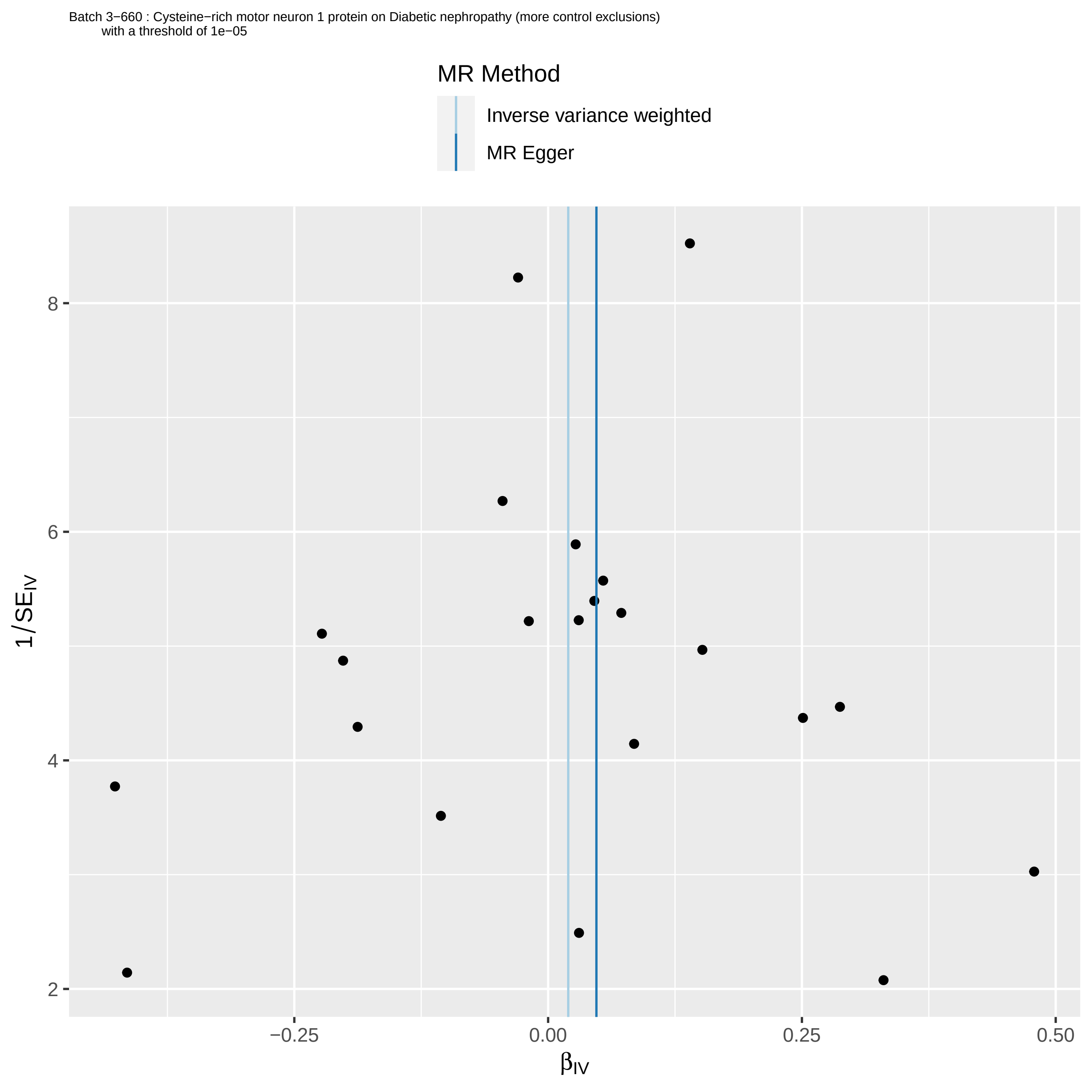
Batch 3−660 : Cysteine−rich motor neuron 1 protein on Diabetic nephropathy (more control exclusions)
with a threshold of 1e−05
MR Method
Inverse variance weighted
MR Egger
8
6
4
2
1/SE_IV
−0.25
0.00
0.25
0.50
β_IV

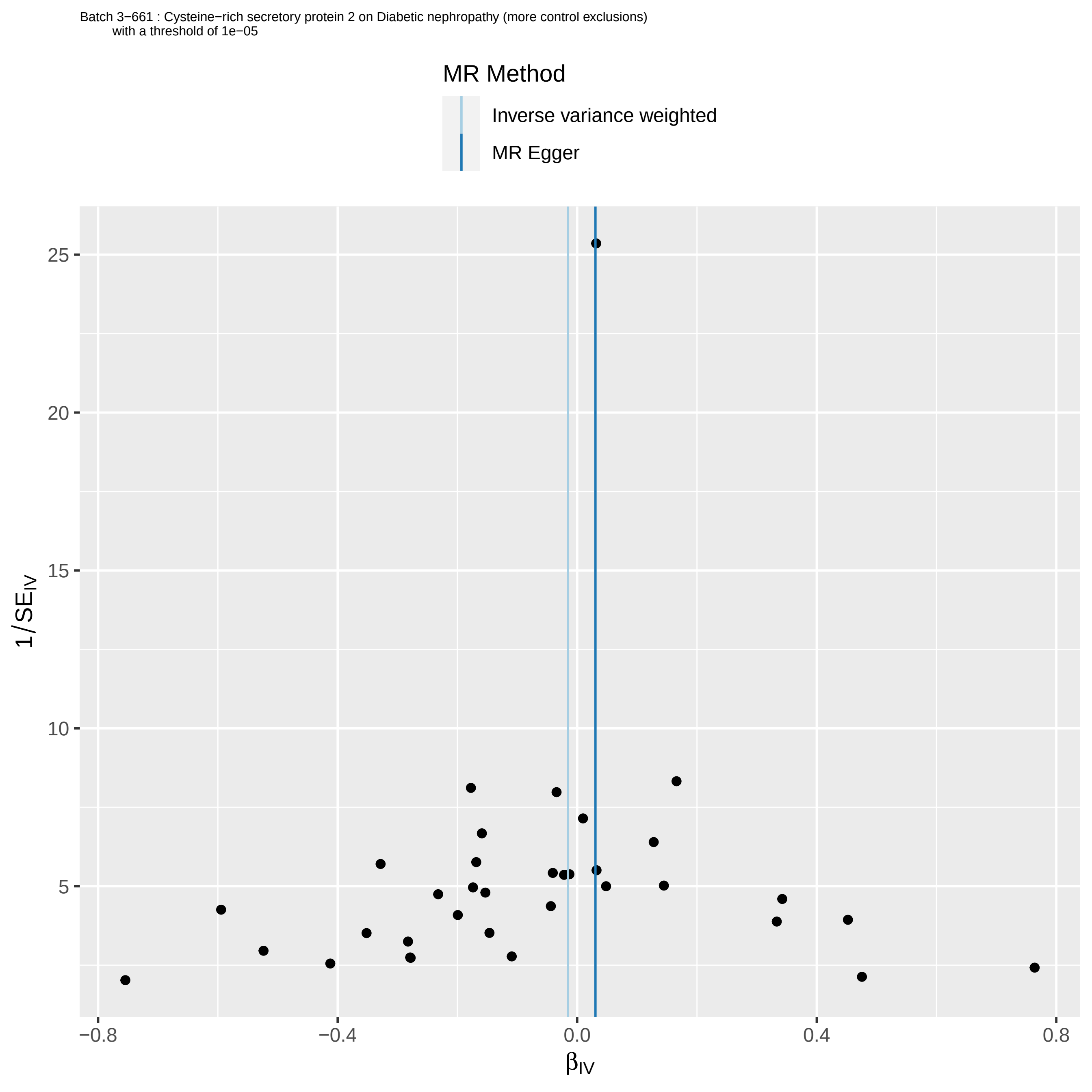
Batch 3−661 : Cysteine−rich secretory protein 2 on Diabetic nephropathy (more control exclusions)
with a threshold of 1e−05
MR Method
Inverse variance weighted
MR Egger
25
20
15
10
5
1/SE_IV
−0.8
−0.4
0.0
0.4
0.8
β_IV

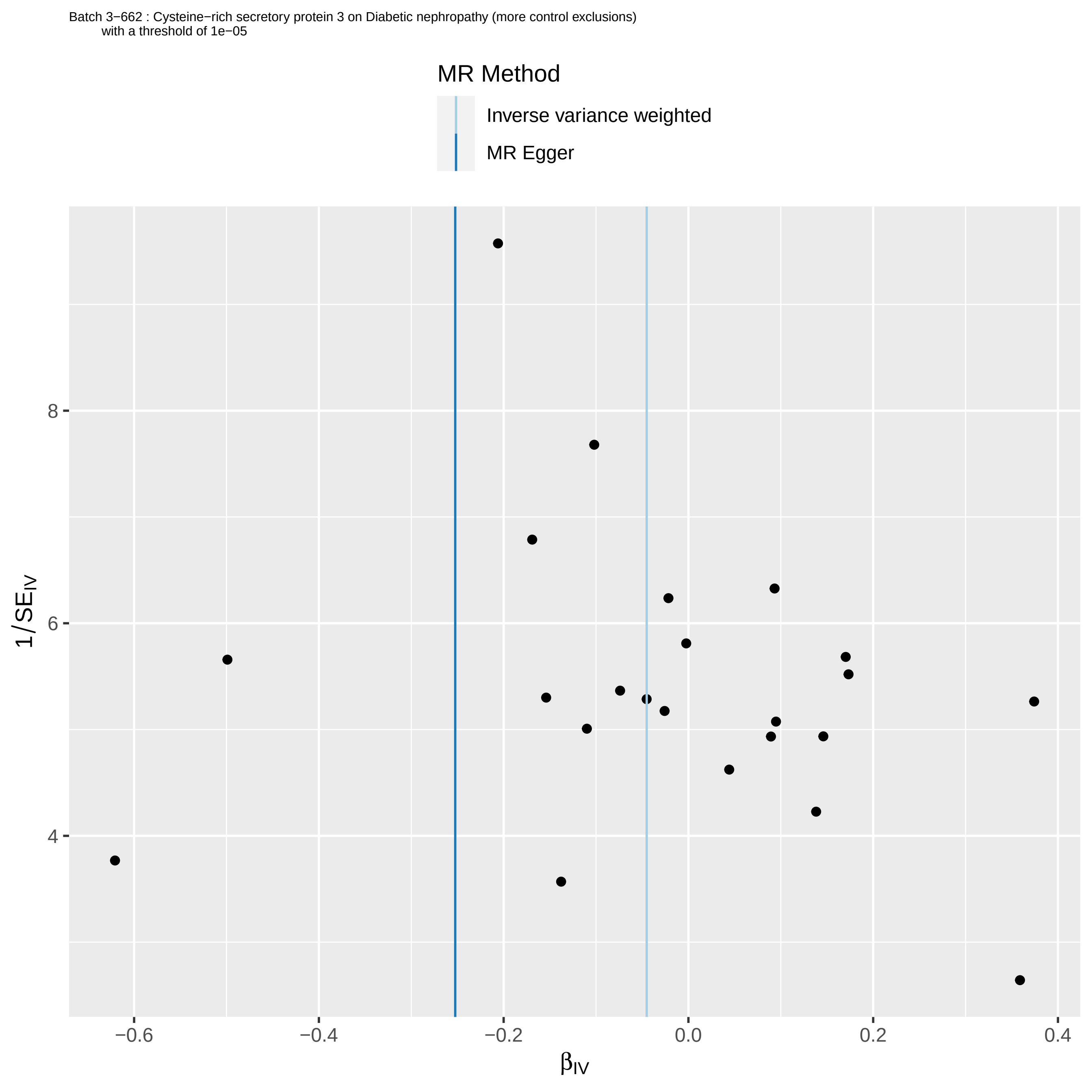

Batch 3−662 : Cysteine−rich secretory protein 3 on Diabetic nephropathy (more control exclusions)
with a threshold of 1e−05
MR Method
Inverse variance weighted
MR Egger
8
6
4
−0.6
−0.4
−0.2
0.0
0.2
0.4
$1/SE_{IV}$
β_{IV}

Batch 3−663 : Cysteine−rich secretory protein LCCL domain−containing 2 on Diabetic nephropathy (more control exclusions)
with a threshold of 1e−05

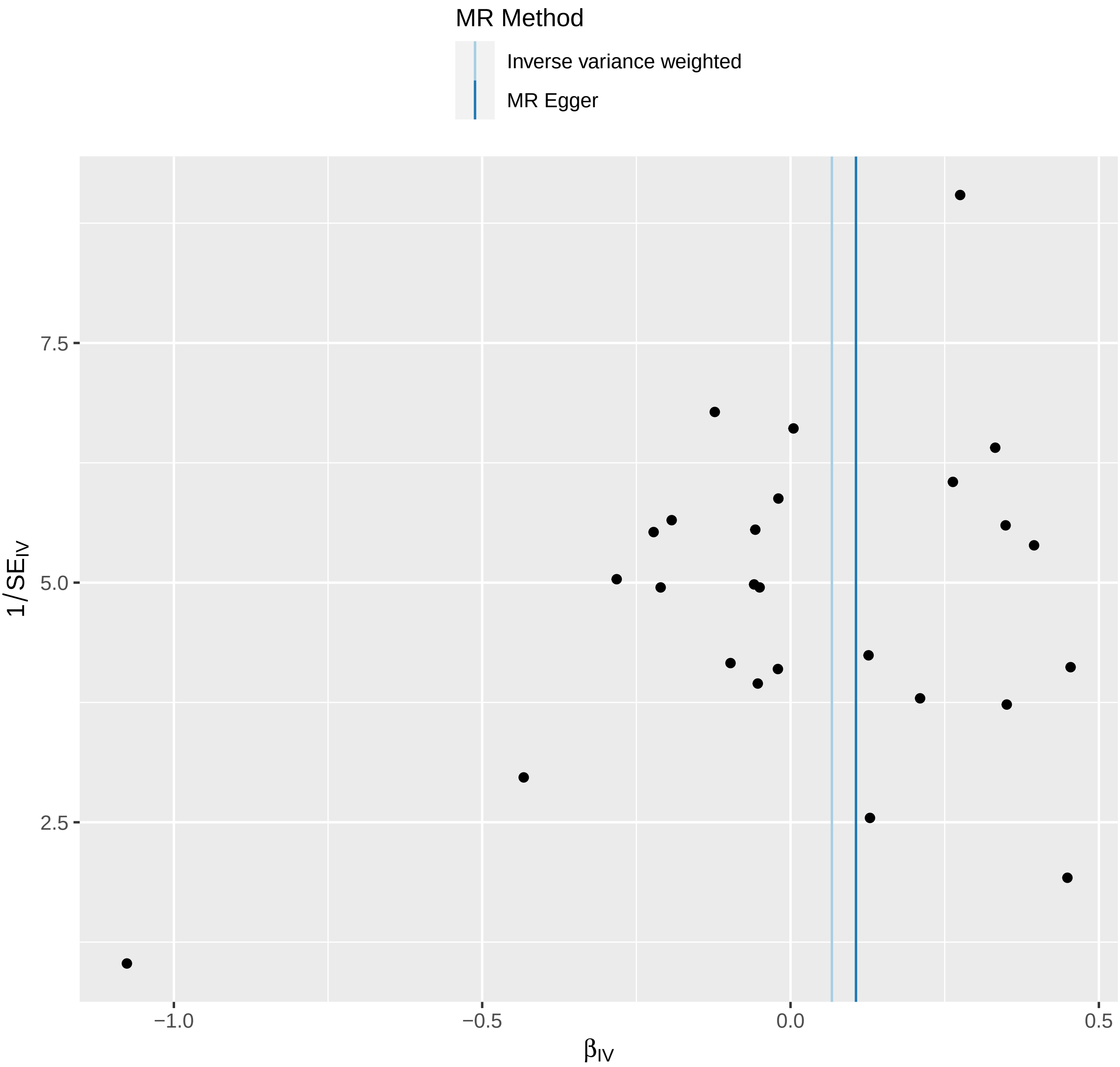

Batch 3−664 : Adapter molecule crk on Diabetic nephropathy (more control exclusions)
with a threshold of 1e−05

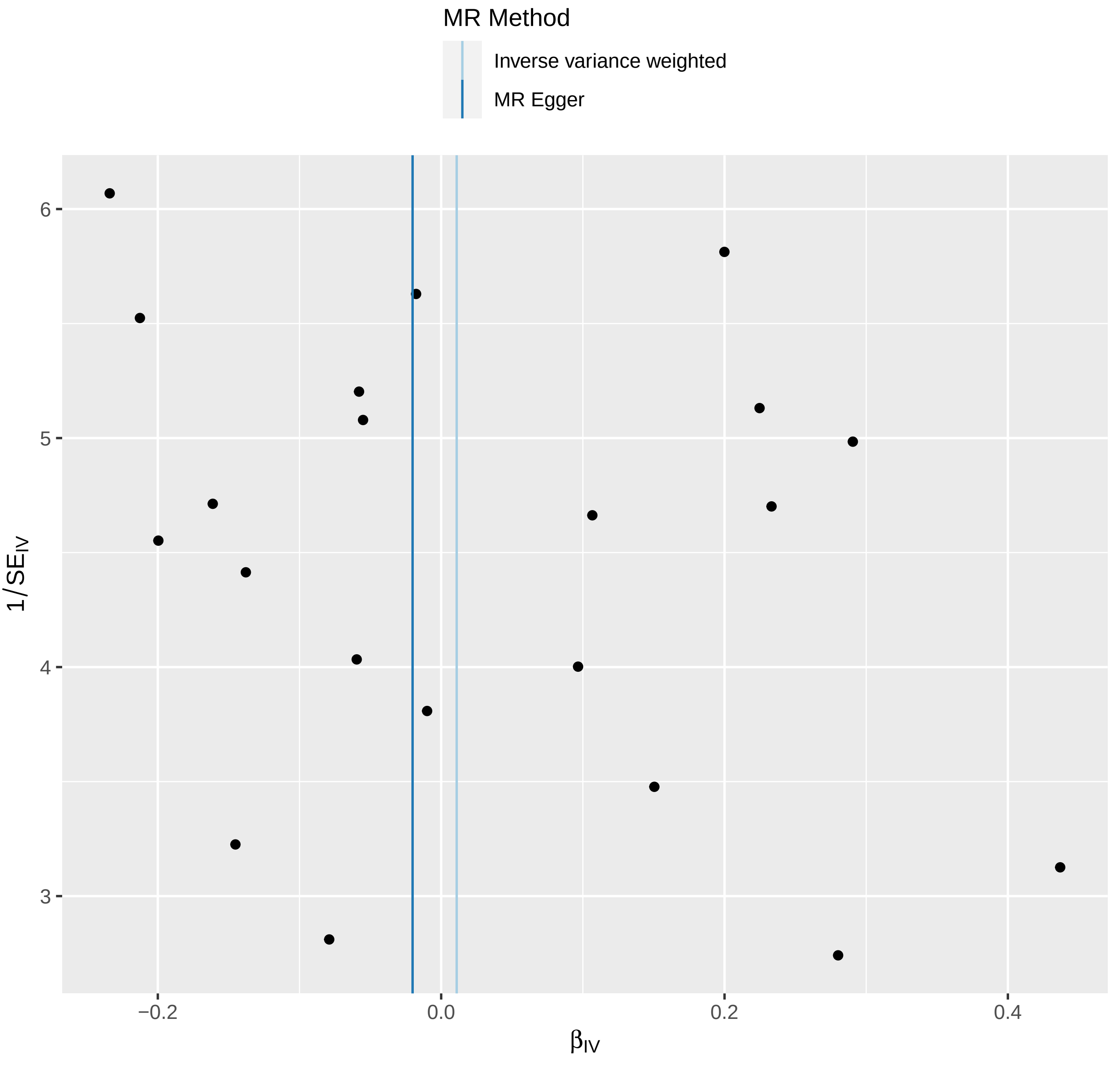

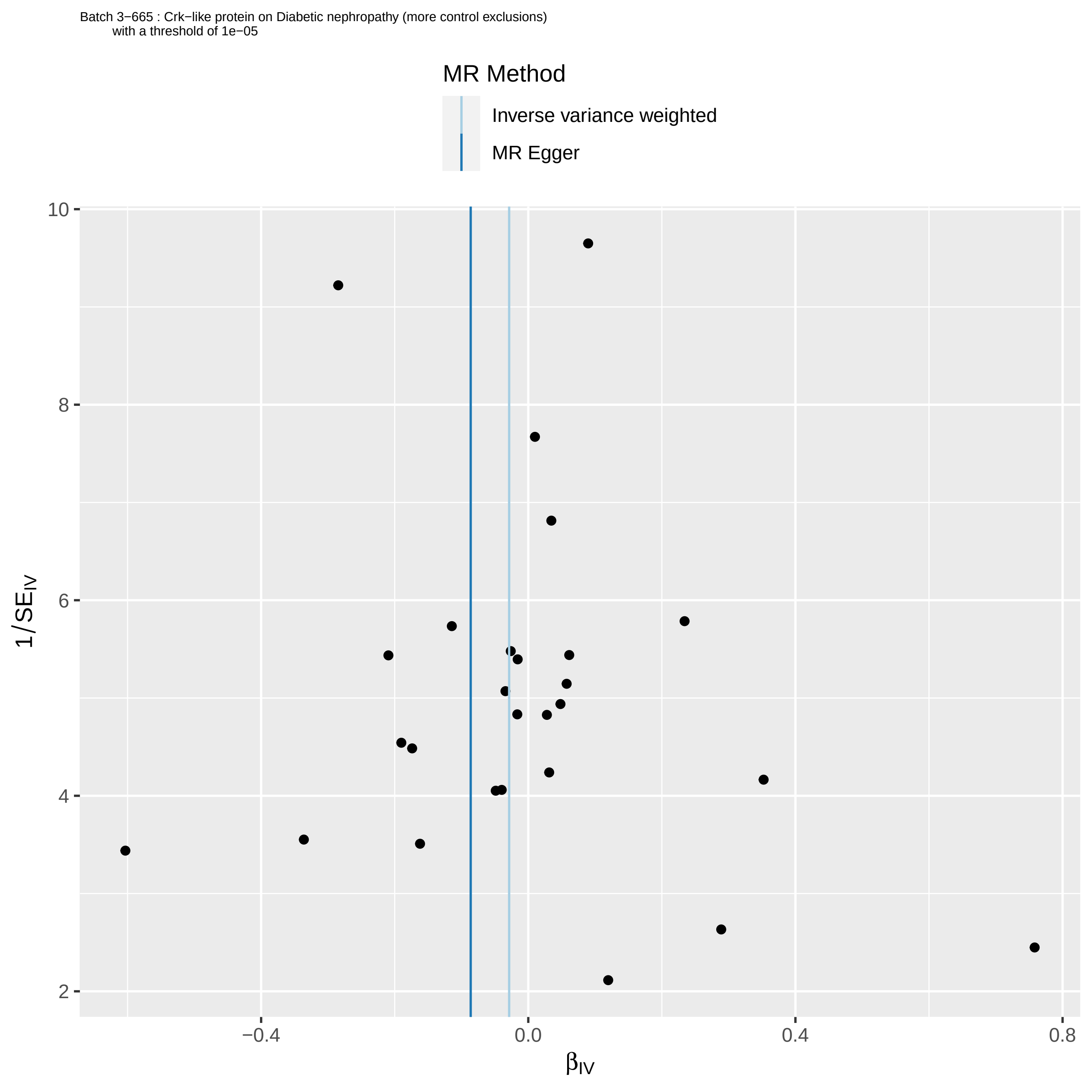

Batch 3−665 : Crk−like protein on Diabetic nephropathy (more control exclusions)
with a threshold of 1e−05
MR Method
Inverse variance weighted
MR Egger
10
8
6
4
2
1/SE_IV
−0.4
0.0
0.4
0.8
β_IV

Batch 3−666 : Cytokine receptor−like factor 1:Cardiotrophin−like cytokine factor 1 Complex on Diabetic nephropathy (more control exclusions) with a threshold of 1e−05

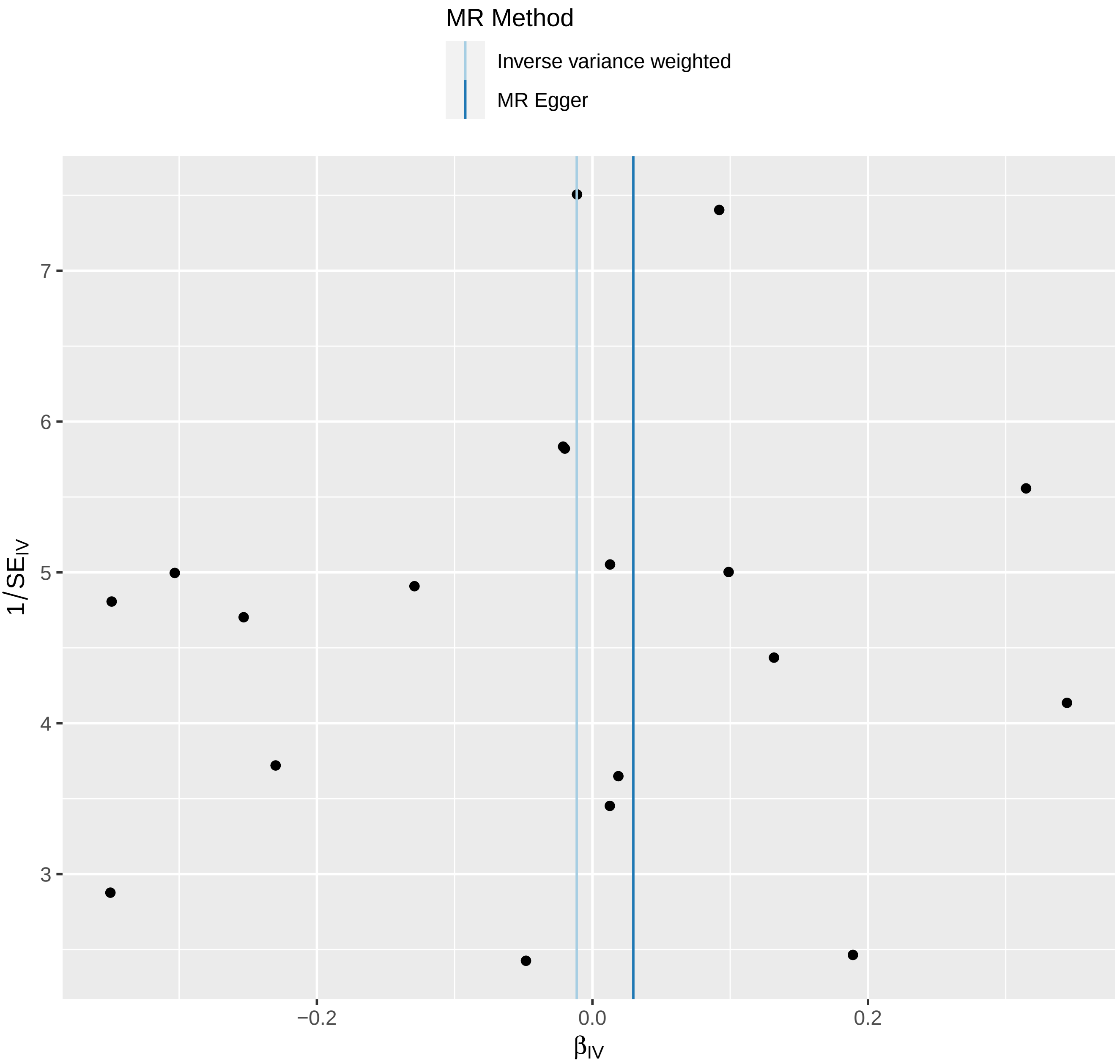

Batch 3−667 : Cytokine receptor−like factor 2 on Diabetic nephropathy (more control exclusions)
with a threshold of 1e−05

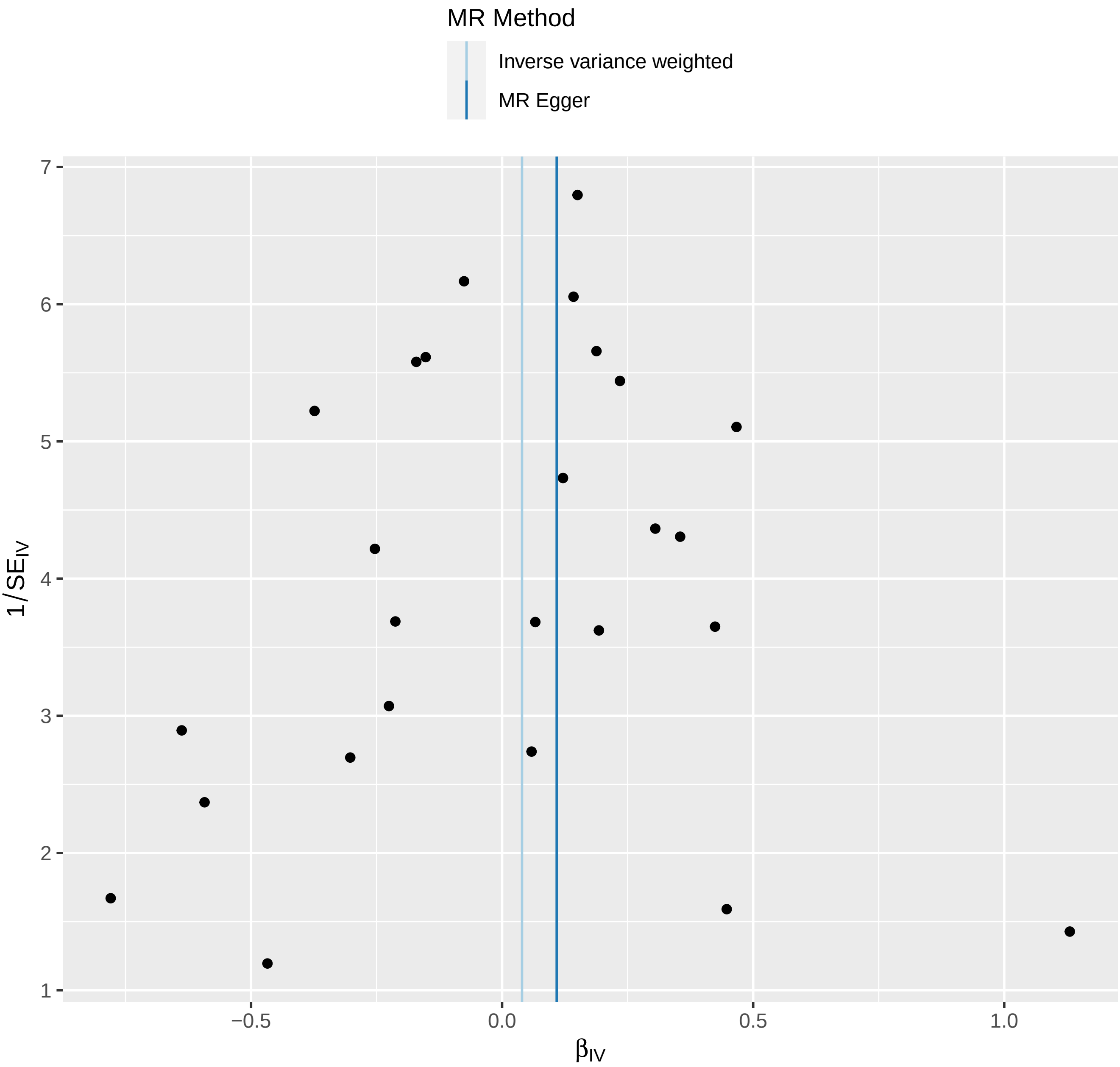

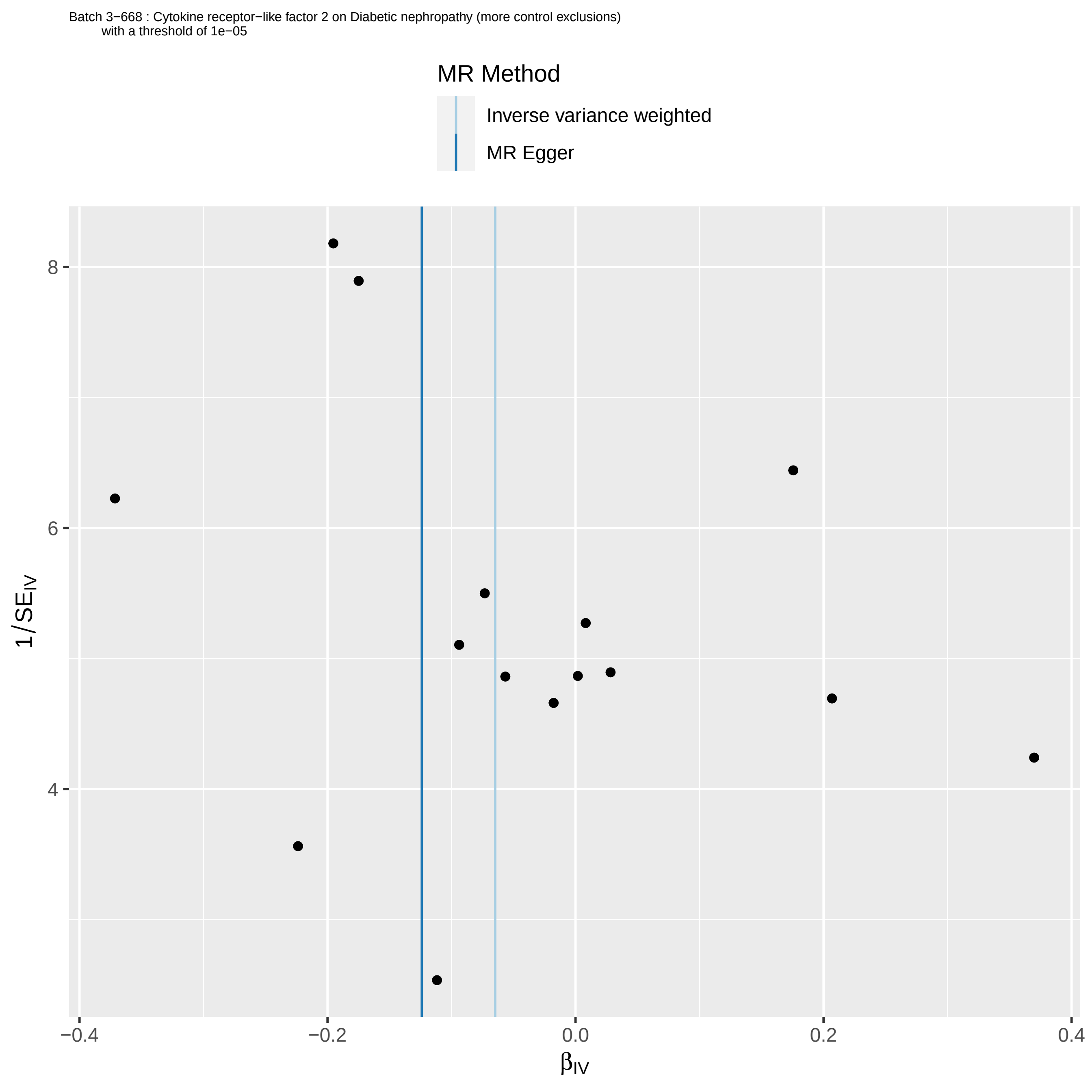

Batch 3−668 : Cytokine receptor−like factor 2 on Diabetic nephropathy (more control exclusions)
with a threshold of 1e−05
MR Method
Inverse variance weighted
MR Egger
8
6
4
1/SE$_{IV}$
−0.4
−0.2
0.0
0.2
0.4
β$_{IV}$

Batch 3−669 : Peroxisomal carnitine O−octanoyltransferase on Diabetic nephropathy (more control exclusions)
with a threshold of 1e−05

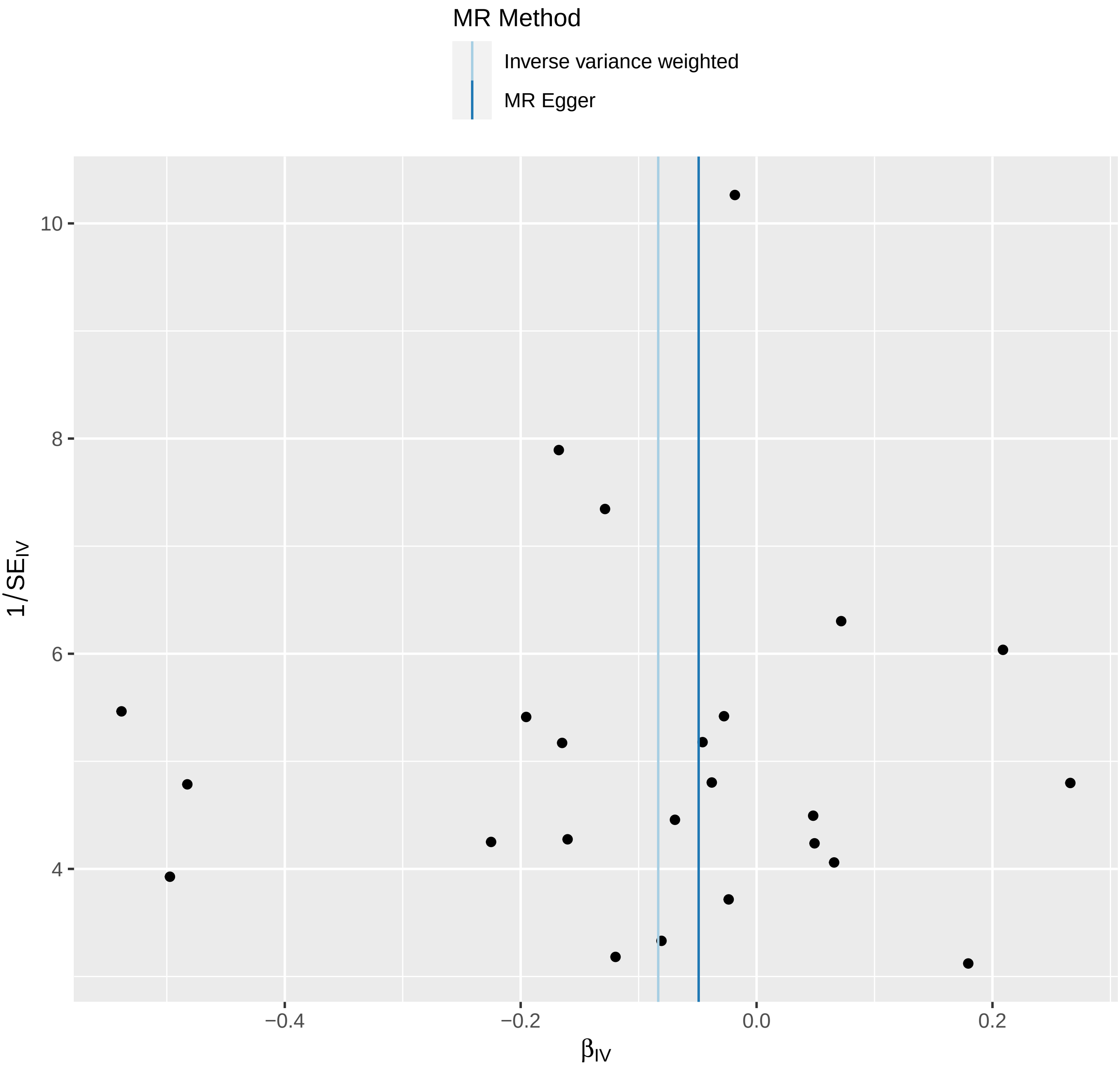

Batch 3−670 : C−reactive protein on Diabetic nephropathy (more control exclusions)
with a threshold of 1e−05

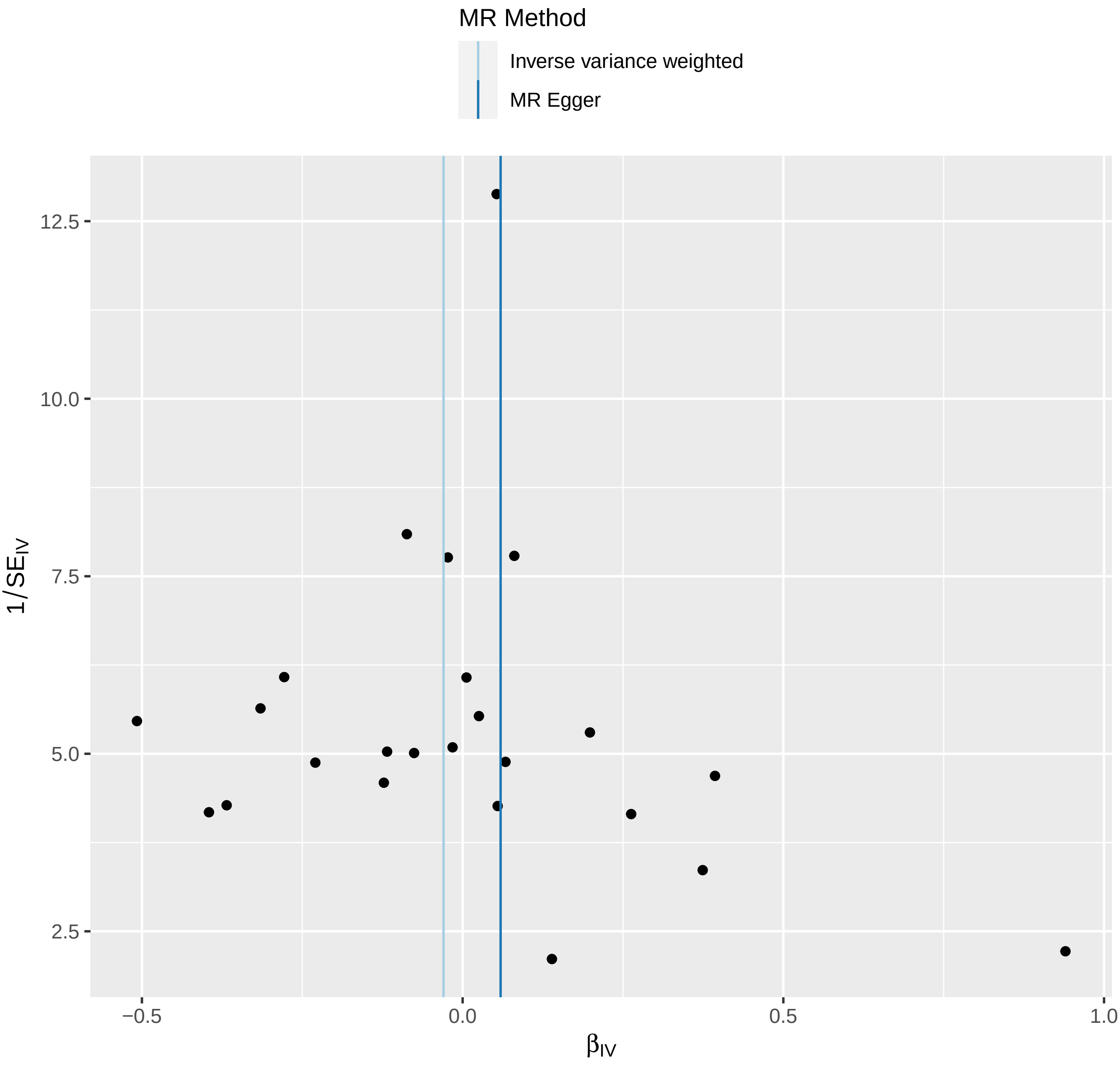

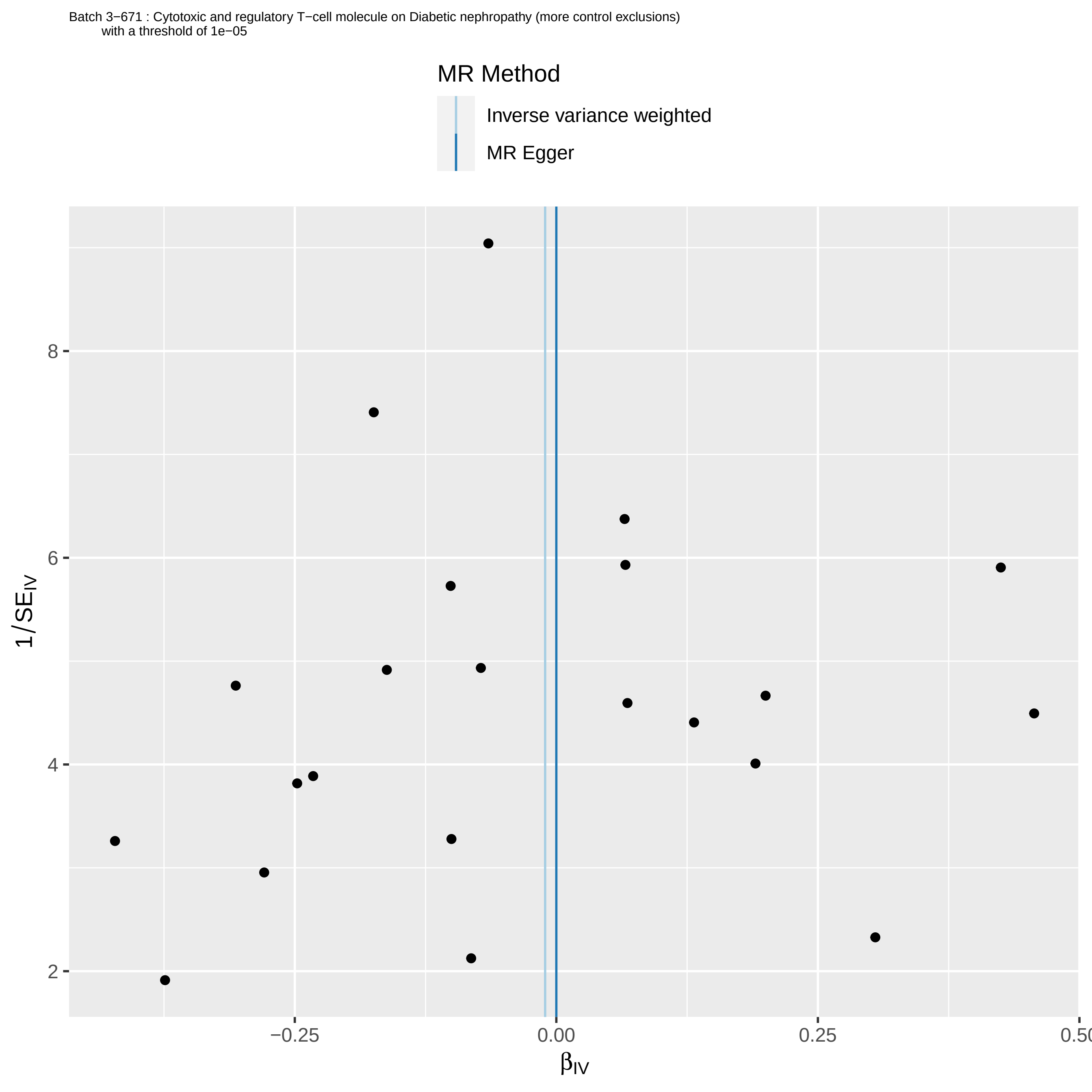

Batch 3−671 : Cytotoxic and regulatory T−cell molecule on Diabetic nephropathy (more control exclusions)
with a threshold of 1e−05
MR Method
Inverse variance weighted
MR Egger
1/SE_IV
8
6
4
2
−0.25
0.00
0.25
0.50
β_IV

Batch 3−672 : Cytotoxic and regulatory T−cell molecule on Diabetic nephropathy (more control exclusions)
with a threshold of 1e−05

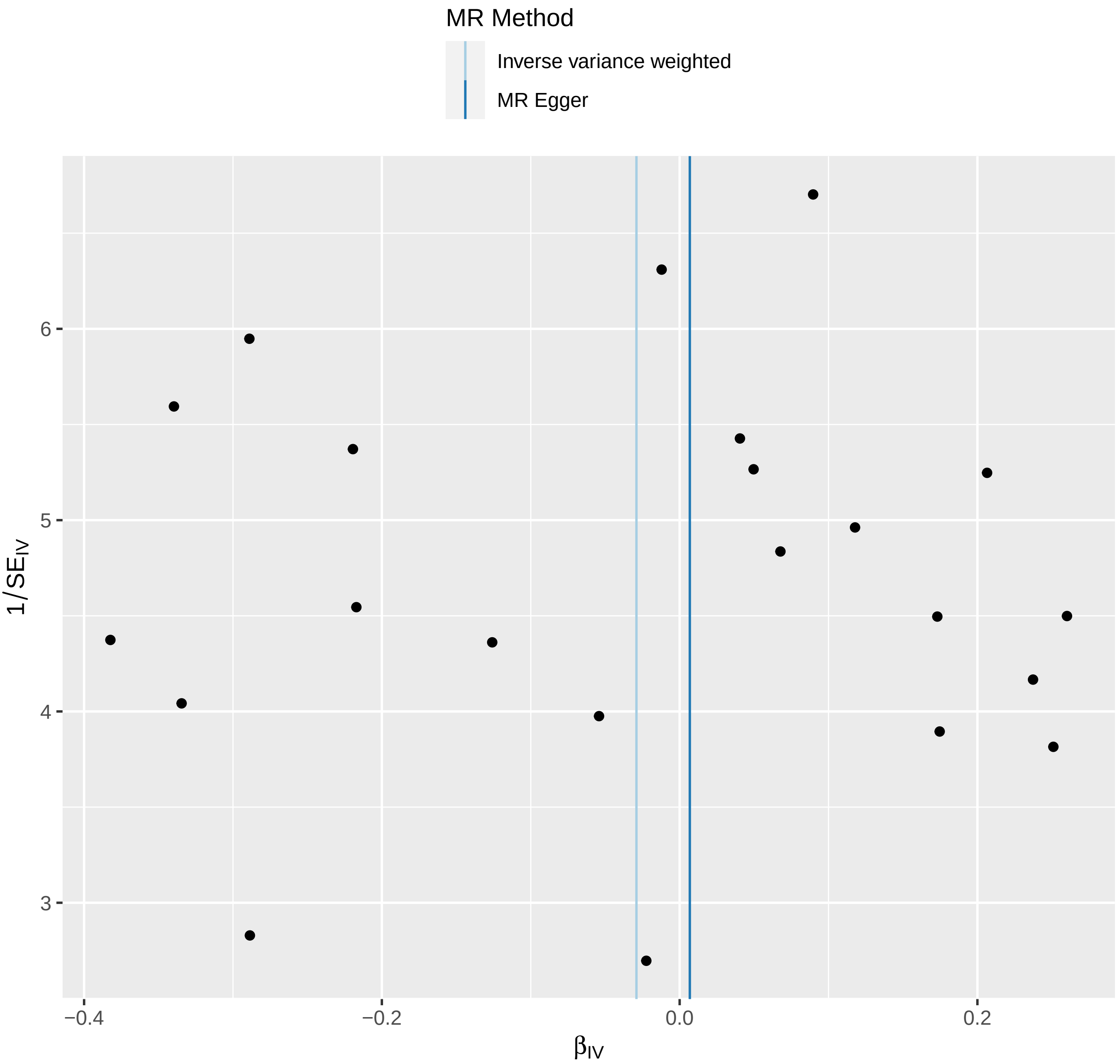

Batch 3−673 : Alpha−crystallin A chain on Diabetic nephropathy (more control exclusions)
with a threshold of 1e−05

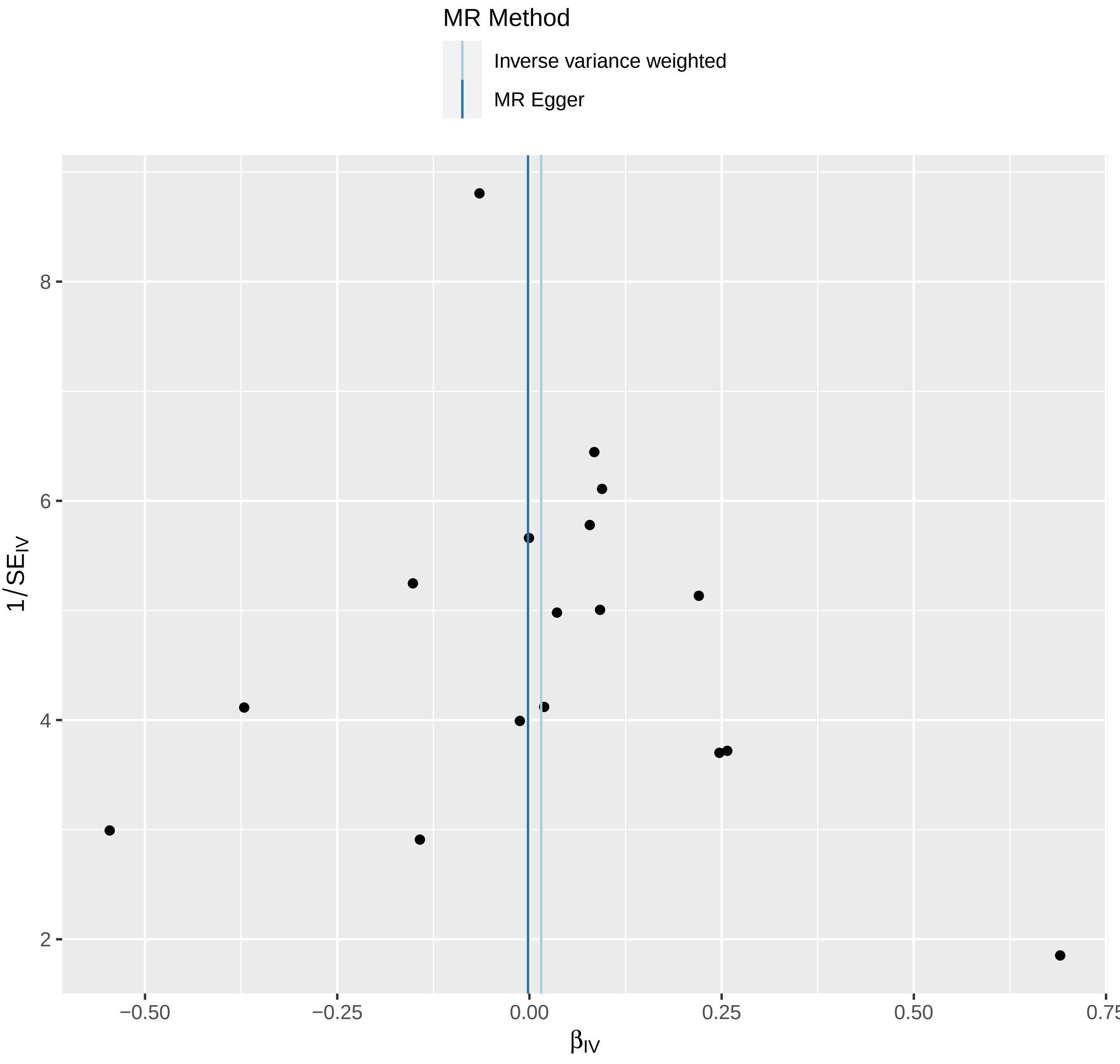

Batch 3−674 : Beta−crystallin B2 on Diabetic nephropathy (more control exclusions)
with a threshold of 1e−05

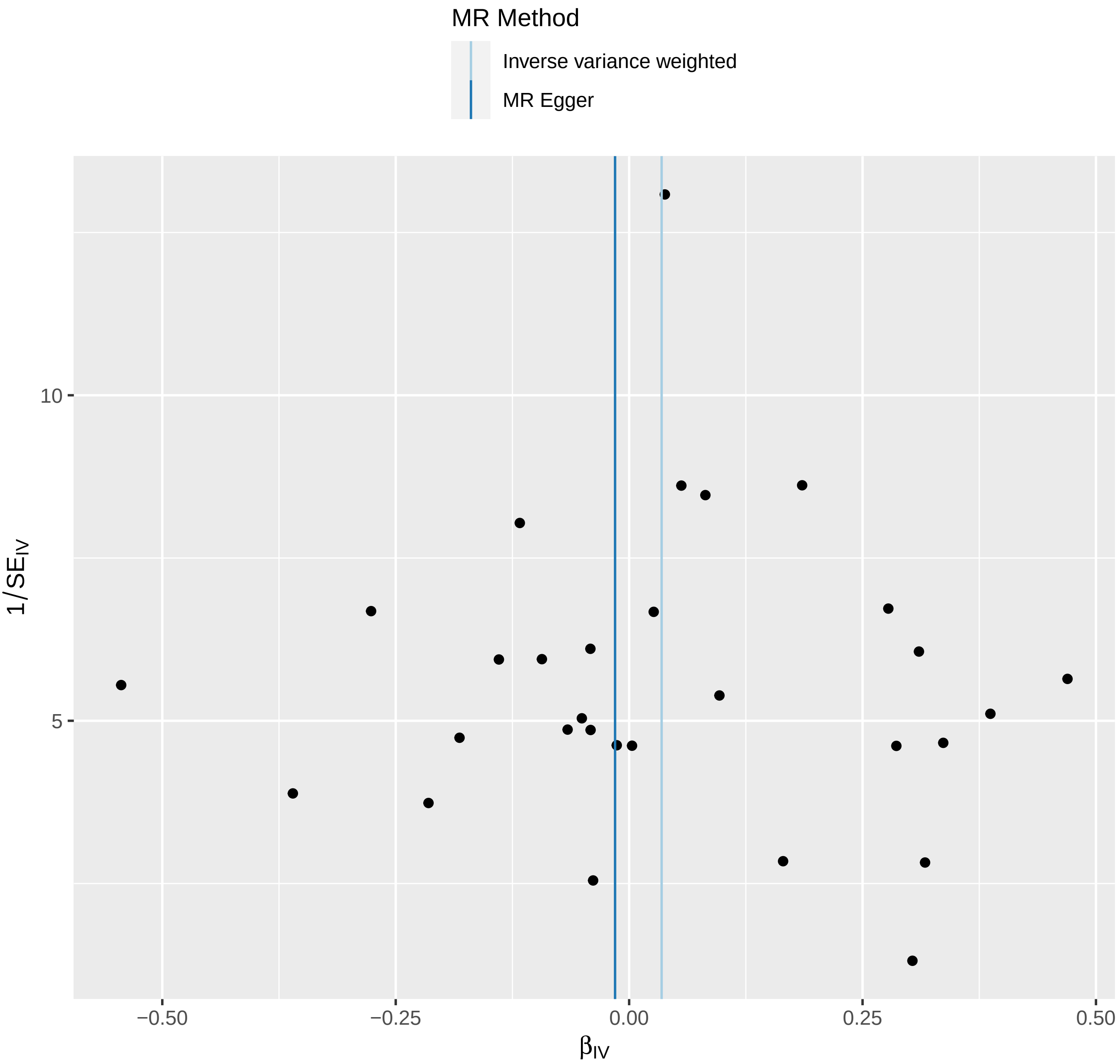

Batch 3−675 : Gamma−crystallin D on Diabetic nephropathy (more control exclusions)
with a threshold of 1e−05

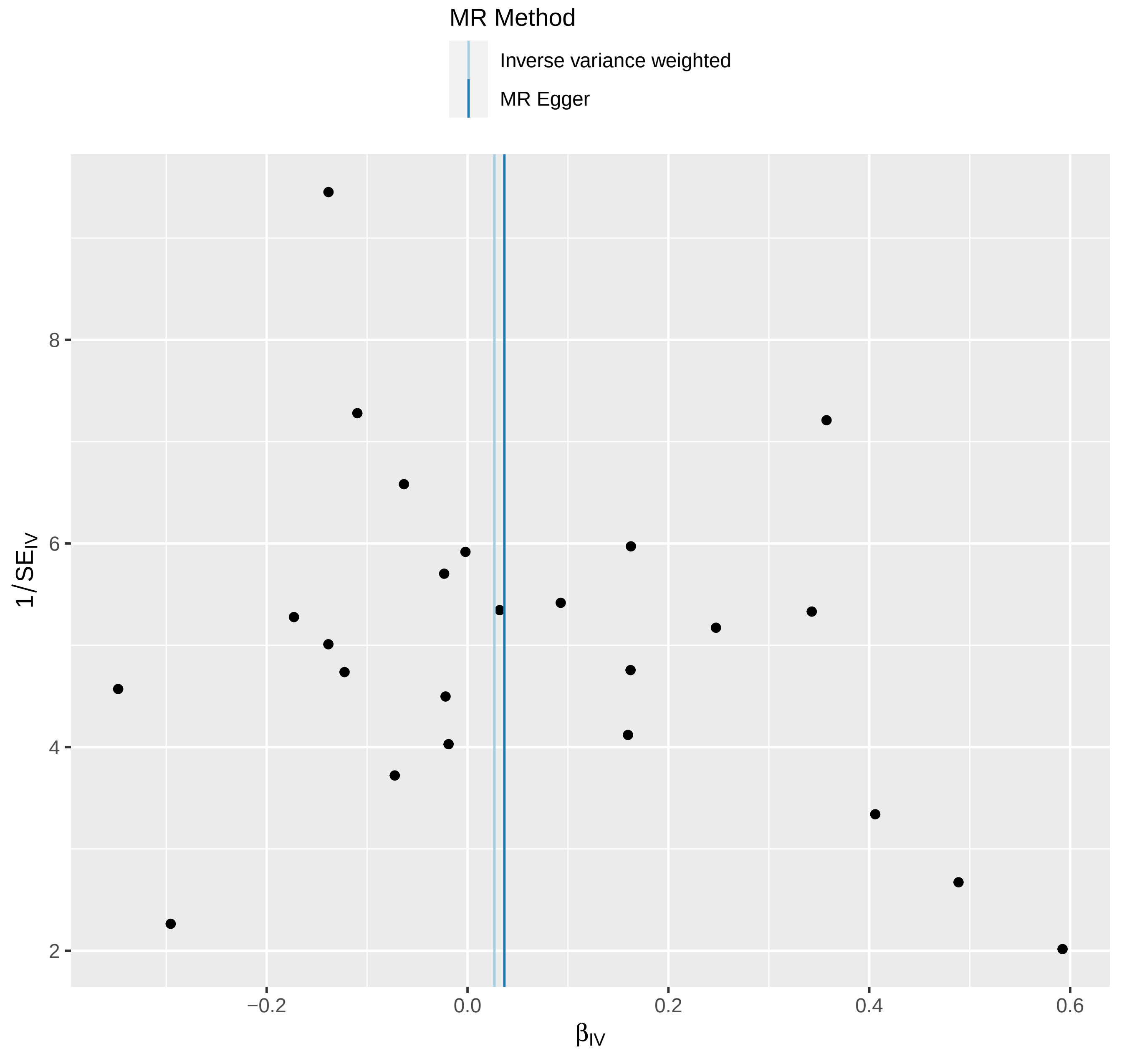

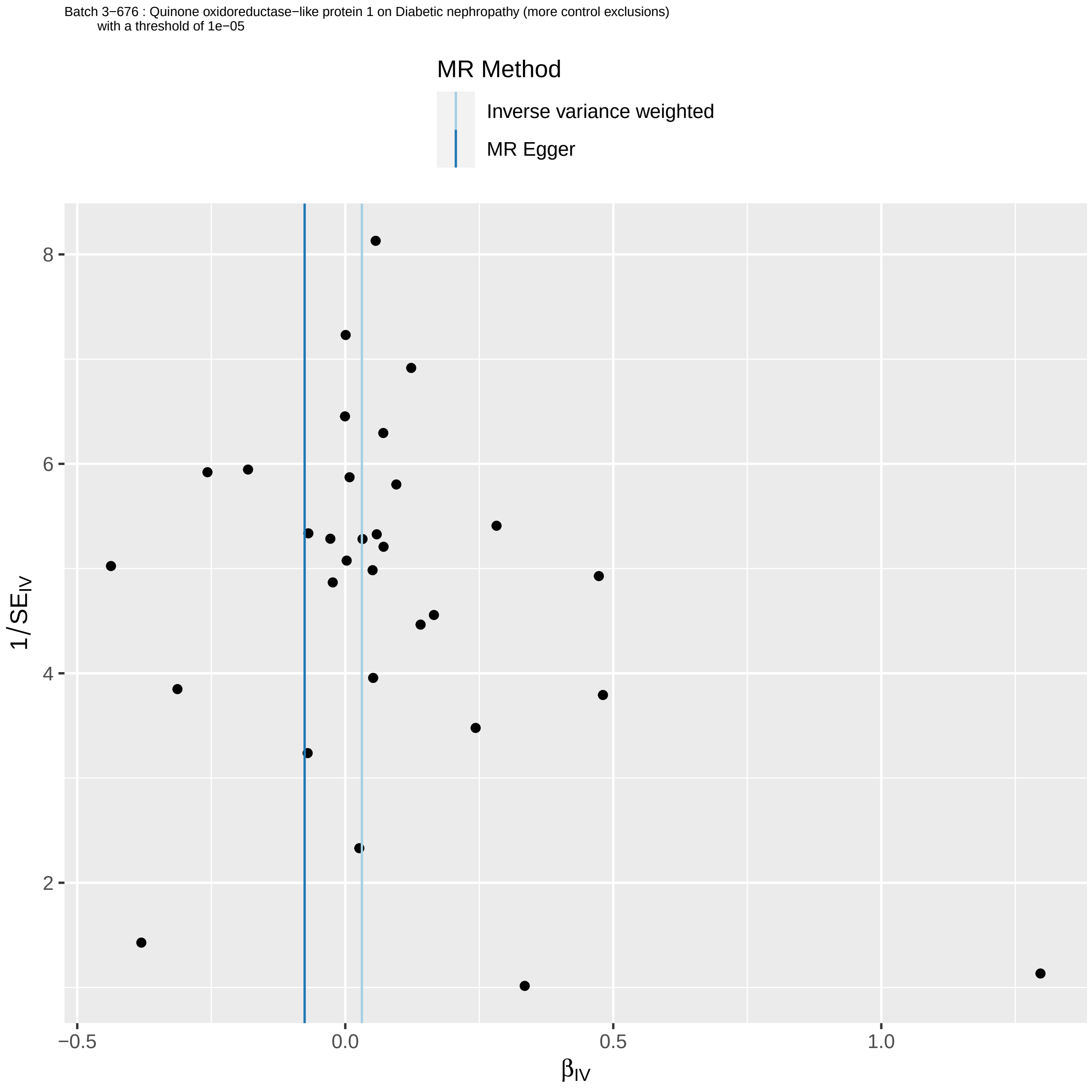

Batch 3−676 : Quinone oxidoreductase−like protein 1 on Diabetic nephropathy (more control exclusions)
with a threshold of 1e−05
MR Method
Inverse variance weighted
MR Egger
8
6
4
2
1/SE_IV
−0.5
0.0
0.5
1.0
β_IV

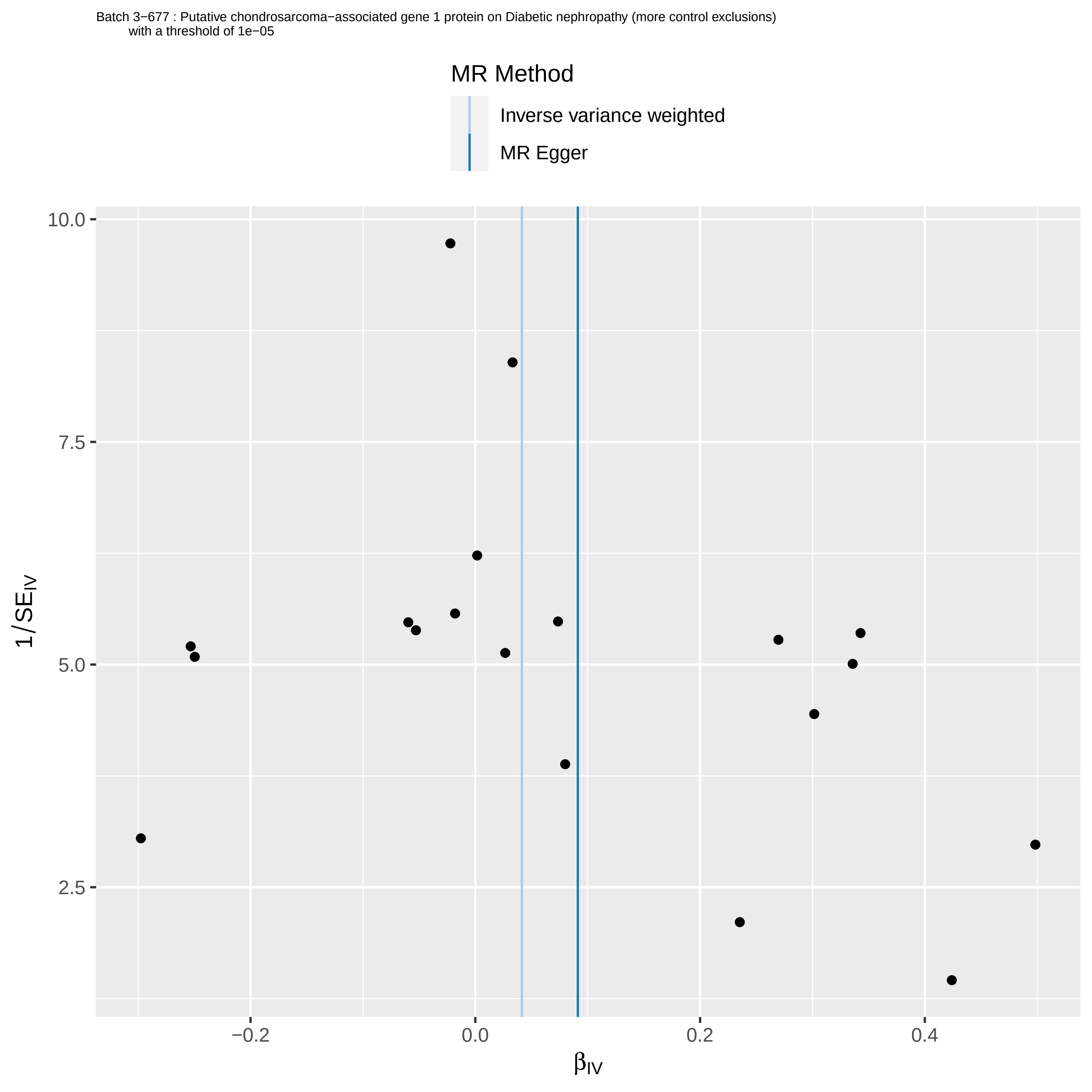

Batch 3−677 : Putative chondrosarcoma−associated gene 1 protein on Diabetic nephropathy (more control exclusions)
with a threshold of 1e−05
MR Method
Inverse variance weighted
MR Egger
10.0
7.5
5.0
2.5
1/SE_IV
−0.2
0.0
0.2
0.4
β_IV

Batch 3−678 : Cold shock domain−containing protein C2 on Diabetic nephropathy (more control exclusions)
with a threshold of 1e−05

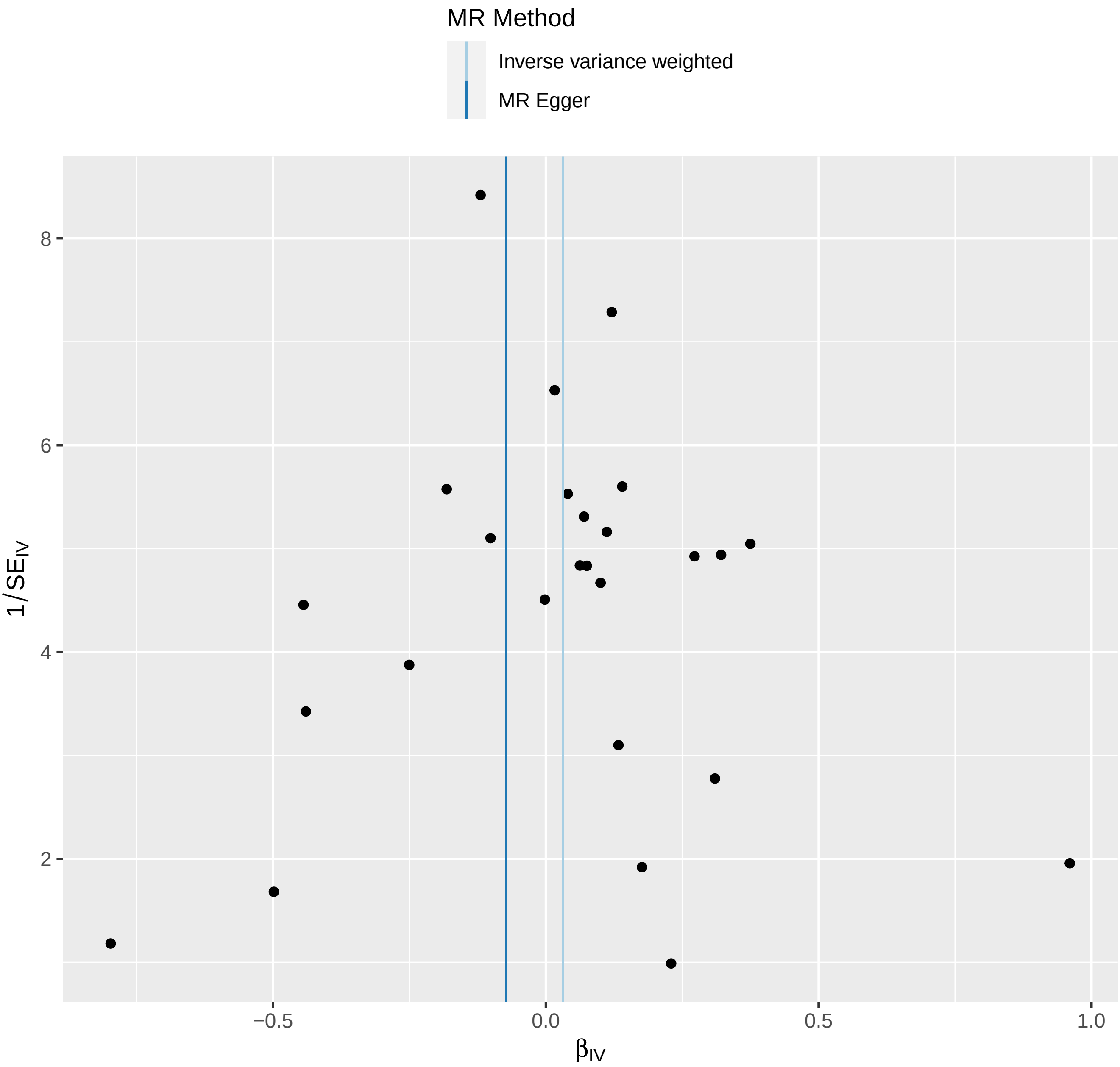

Batch 3−679 : Cold shock domain−containing protein E1 on Diabetic nephropathy (more control exclusions)
with a threshold of 1e−05

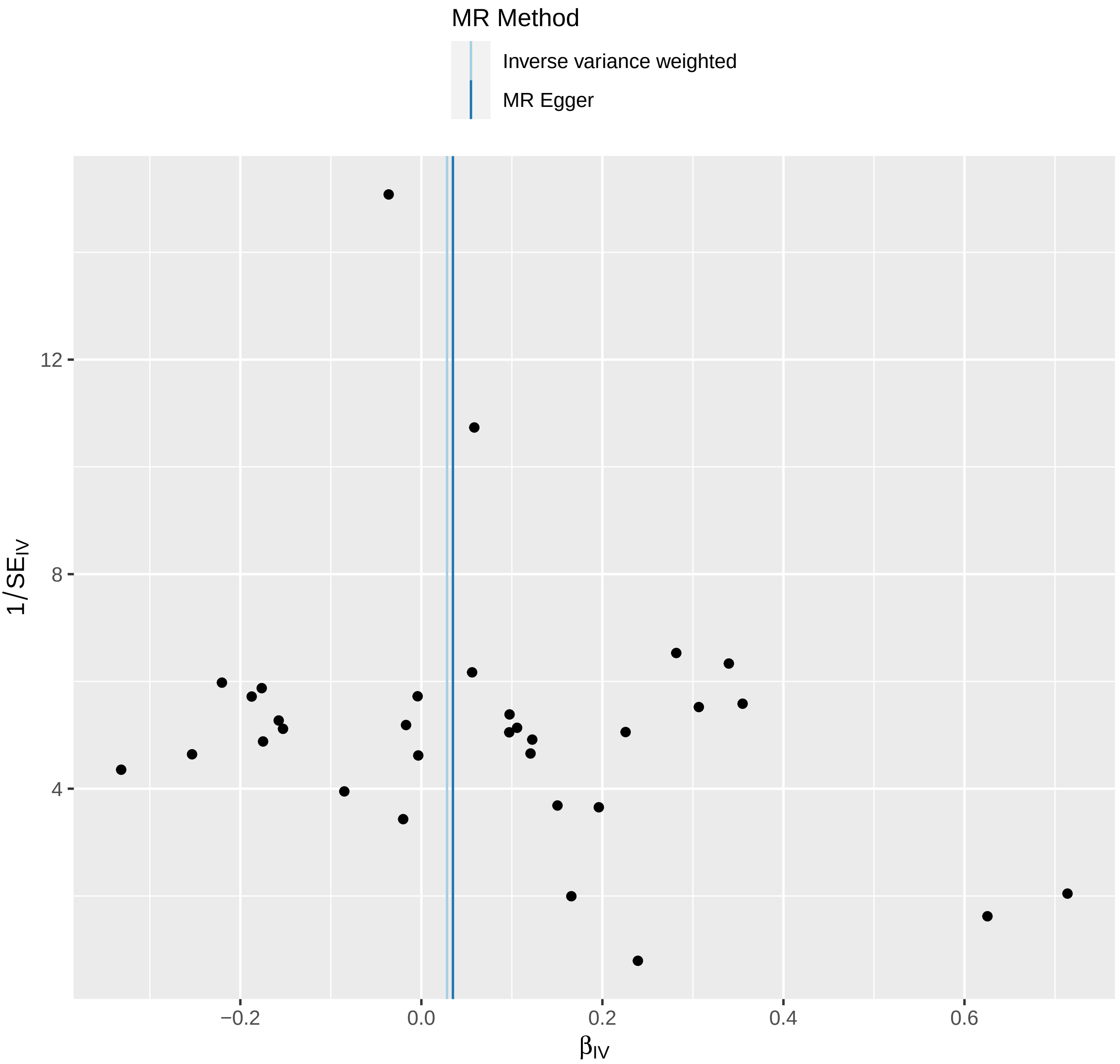

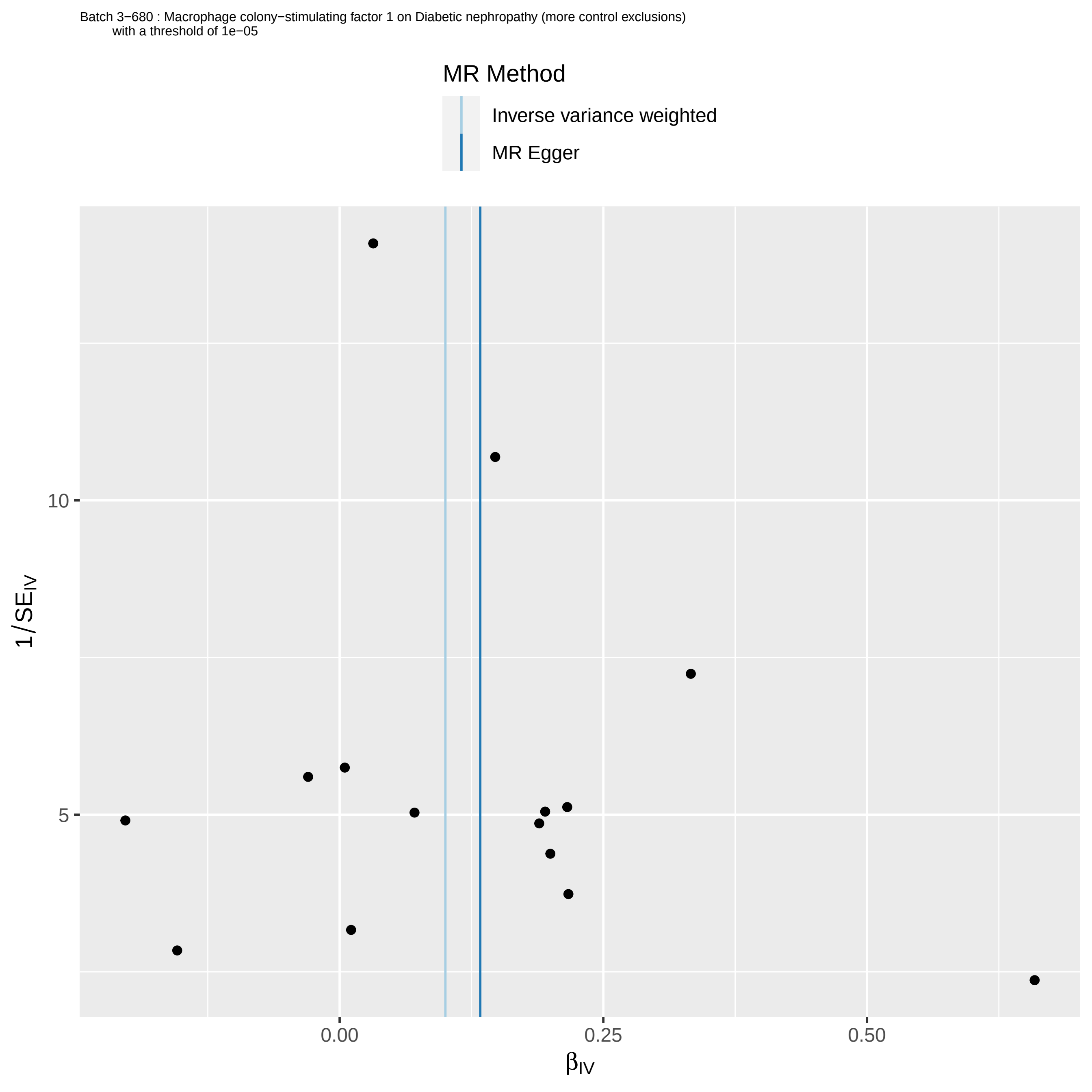
Batch 3−680 : Macrophage colony−stimulating factor 1 on Diabetic nephropathy (more control exclusions)
with a threshold of 1e−05
MR Method
Inverse variance weighted
MR Egger
10
5
1/SE_IV
0.00
0.25
0.50
β_IV

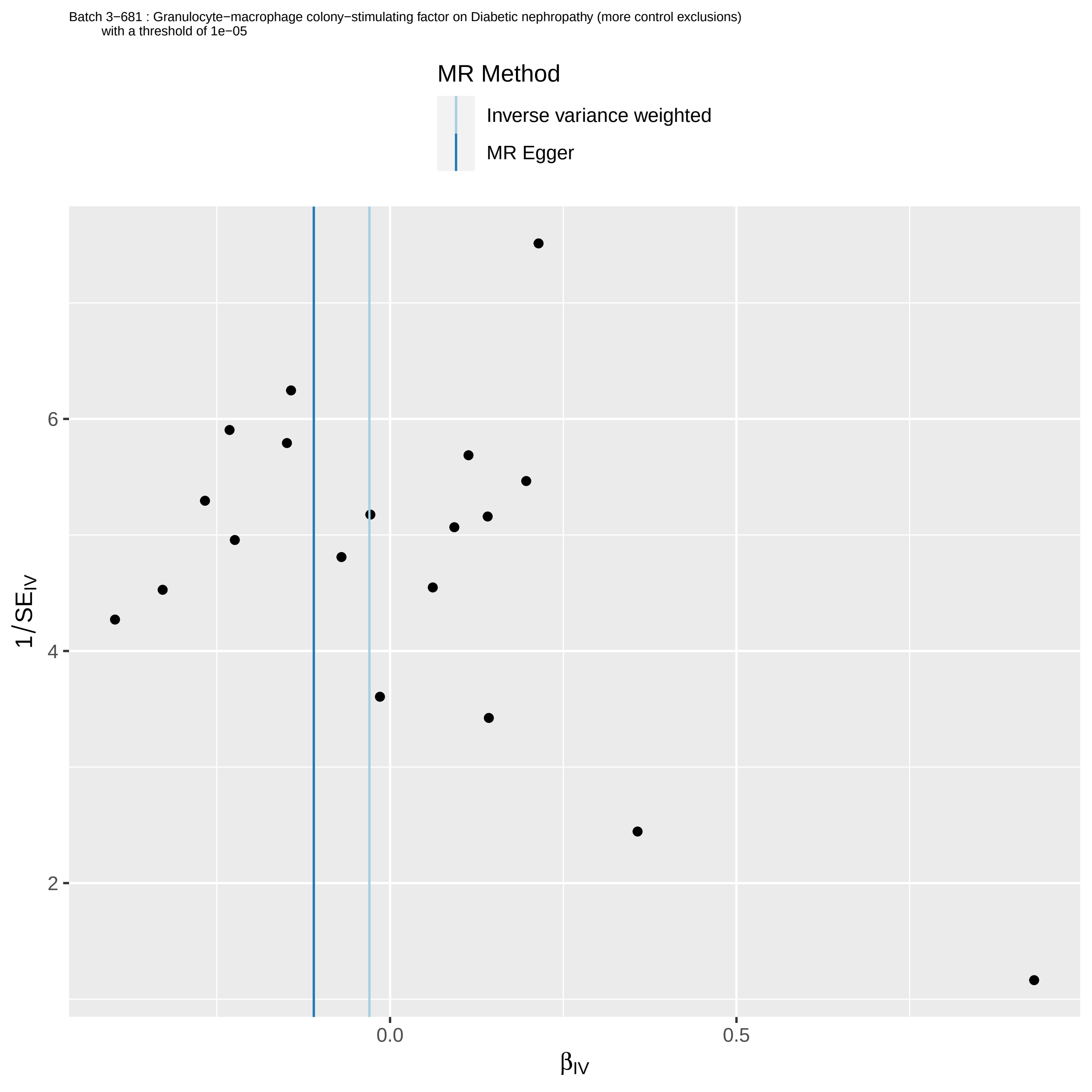

Batch 3−681 : Granulocyte−macrophage colony−stimulating factor on Diabetic nephropathy (more control exclusions)
with a threshold of 1e−05
MR Method
Inverse variance weighted
MR Egger
6
4
2
$1/SE_{IV}$
0.0
0.5
β_{IV}

Batch 3−682 : Granulocyte−macrophage colony−stimulating factor receptor subunit alpha on Diabetic nephropathy (more control exclusions) with a threshold of 1e−05

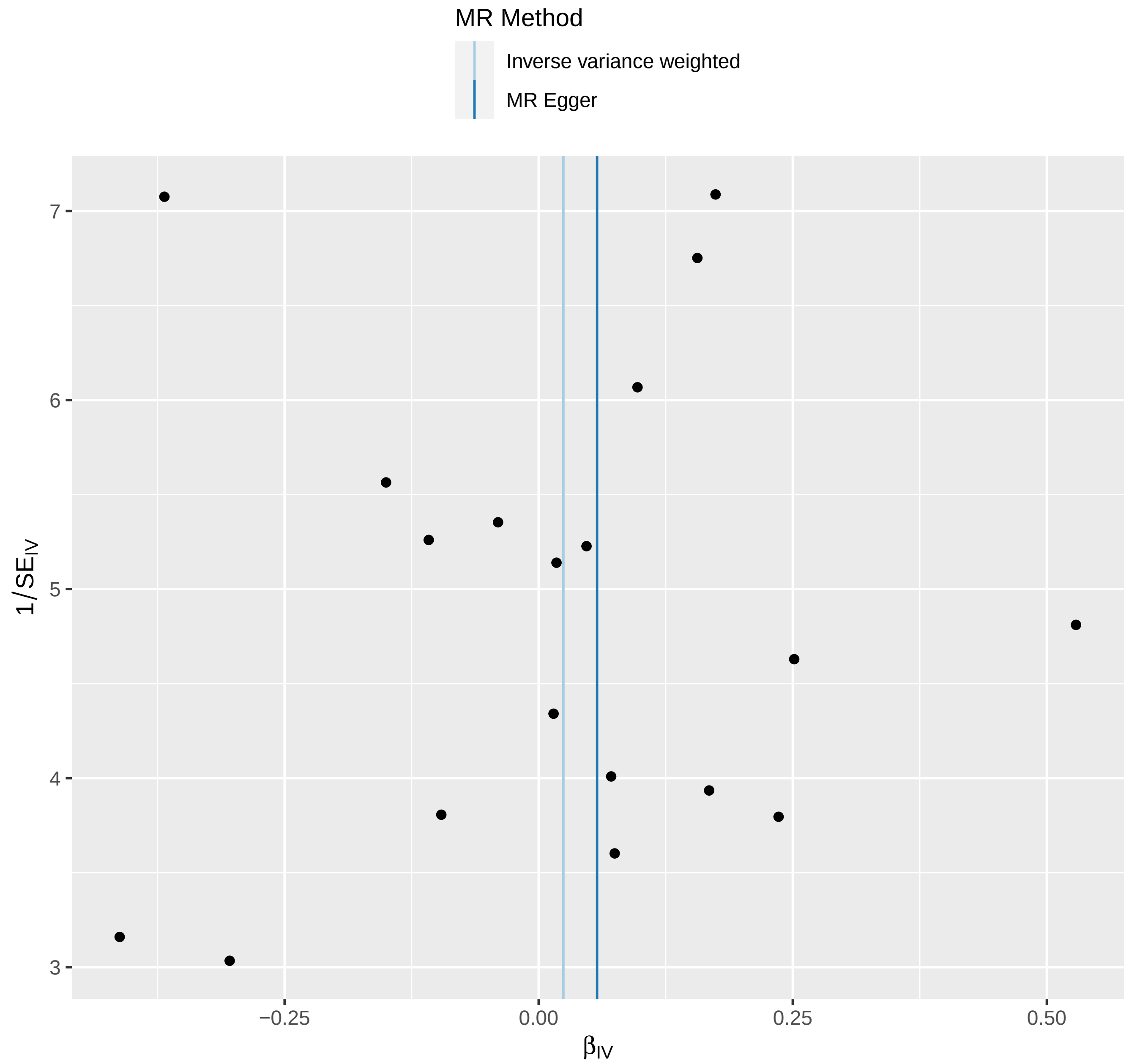

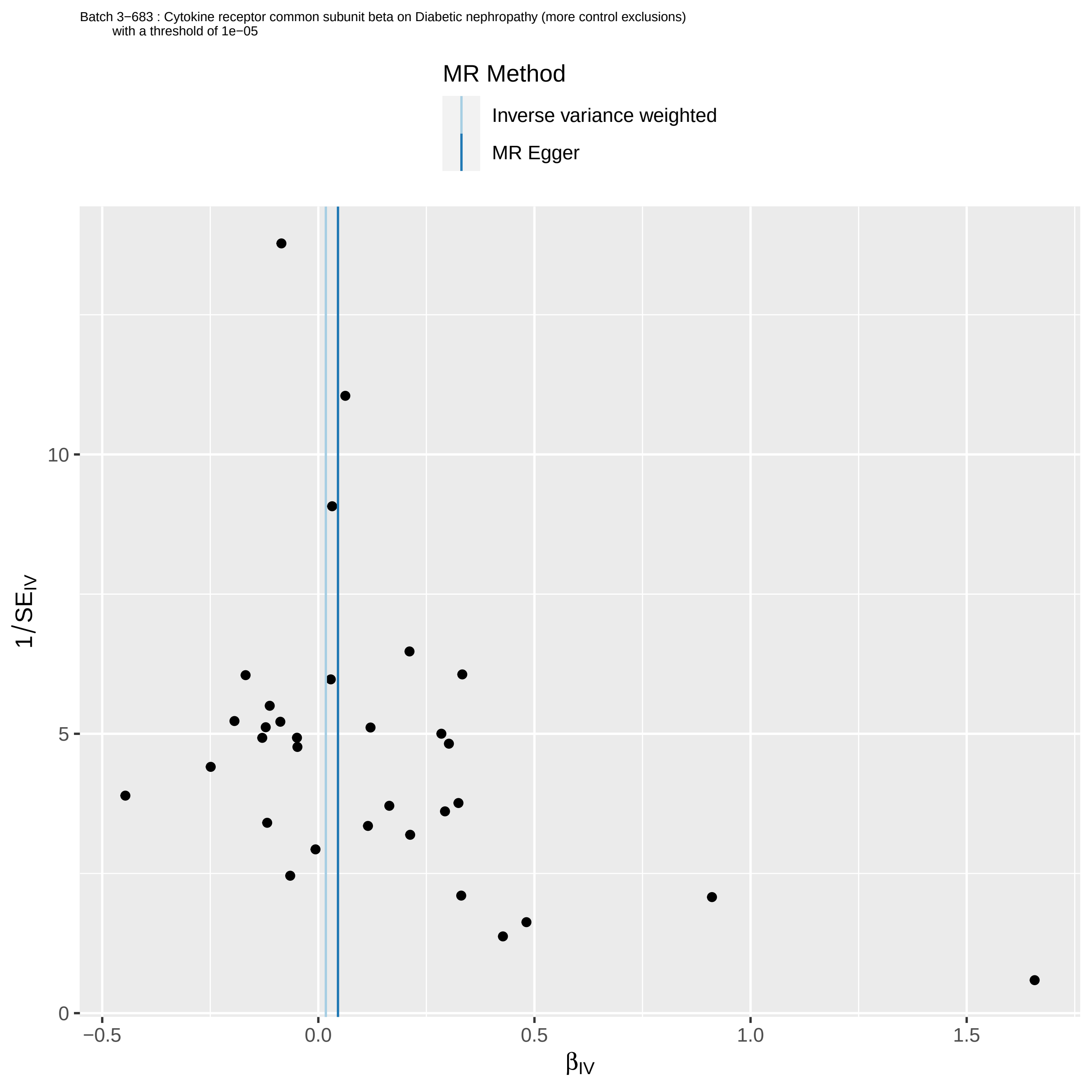

Batch 3−683 : Cytokine receptor common subunit beta on Diabetic nephropathy (more control exclusions)
with a threshold of 1e−05
MR Method
Inverse variance weighted
MR Egger
1/SE_IV
10
5
0
−0.5
0.0
0.5
1.0
1.5
β_IV

Batch 3−684 : Cytokine receptor common subunit beta on Diabetic nephropathy (more control exclusions) with a threshold of 1e−05

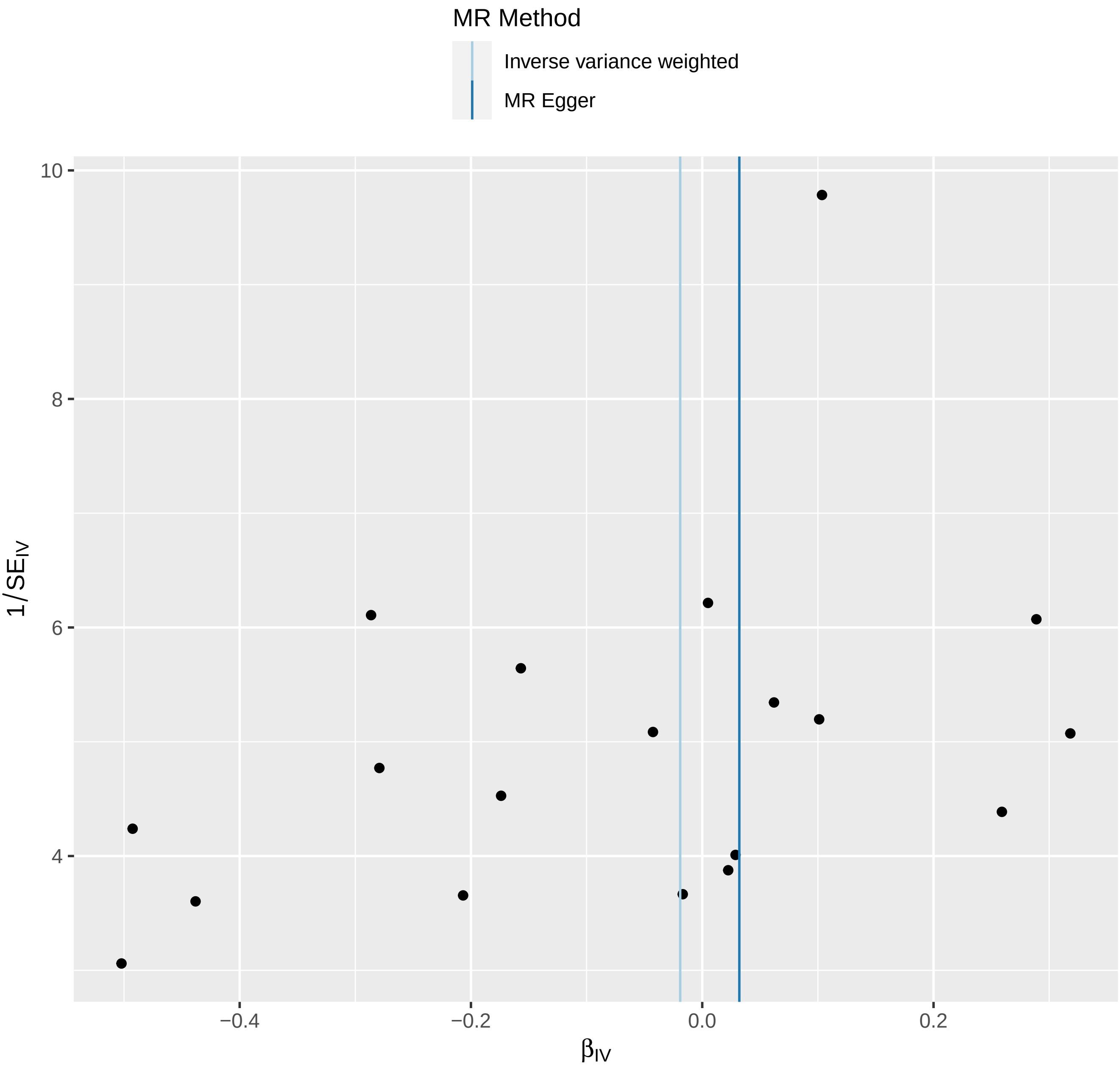

Batch 3−685 : Granulocyte colony−stimulating factor on Diabetic nephropathy (more control exclusions)
with a threshold of 1e−05

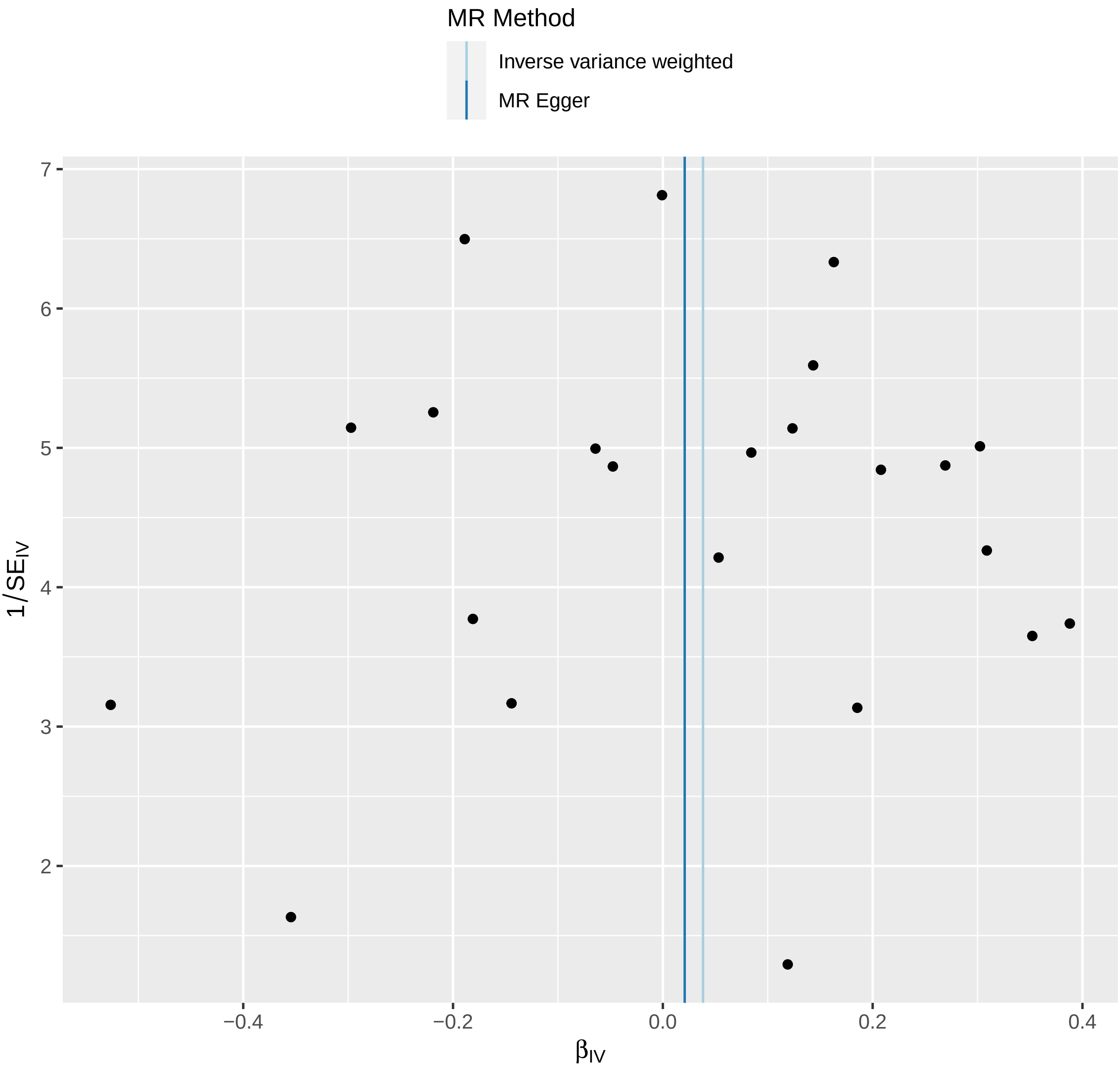

Batch 3−686 : Granulocyte colony−stimulating factor receptor on Diabetic nephropathy (more control exclusions)
with a threshold of 1e−05

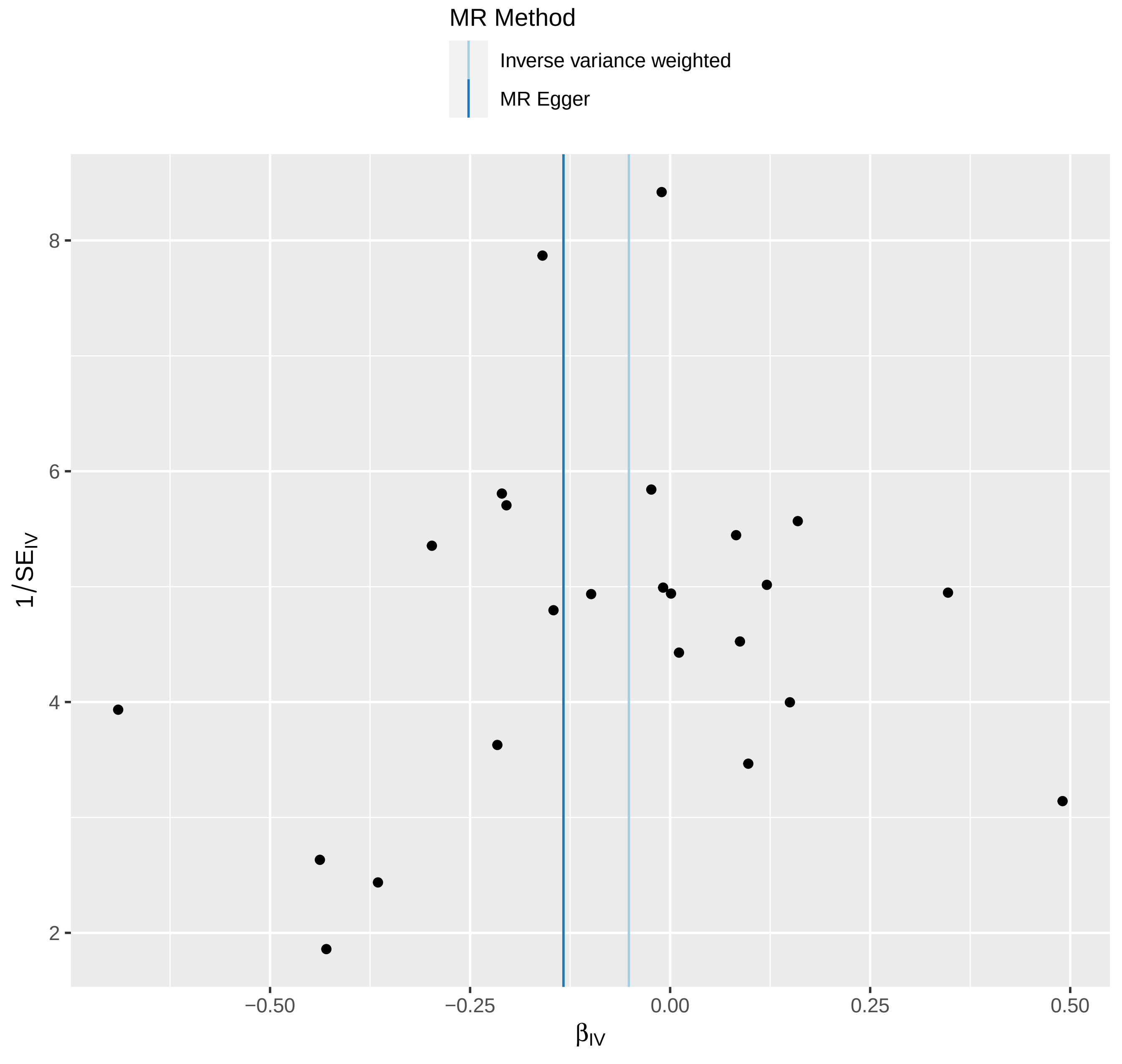

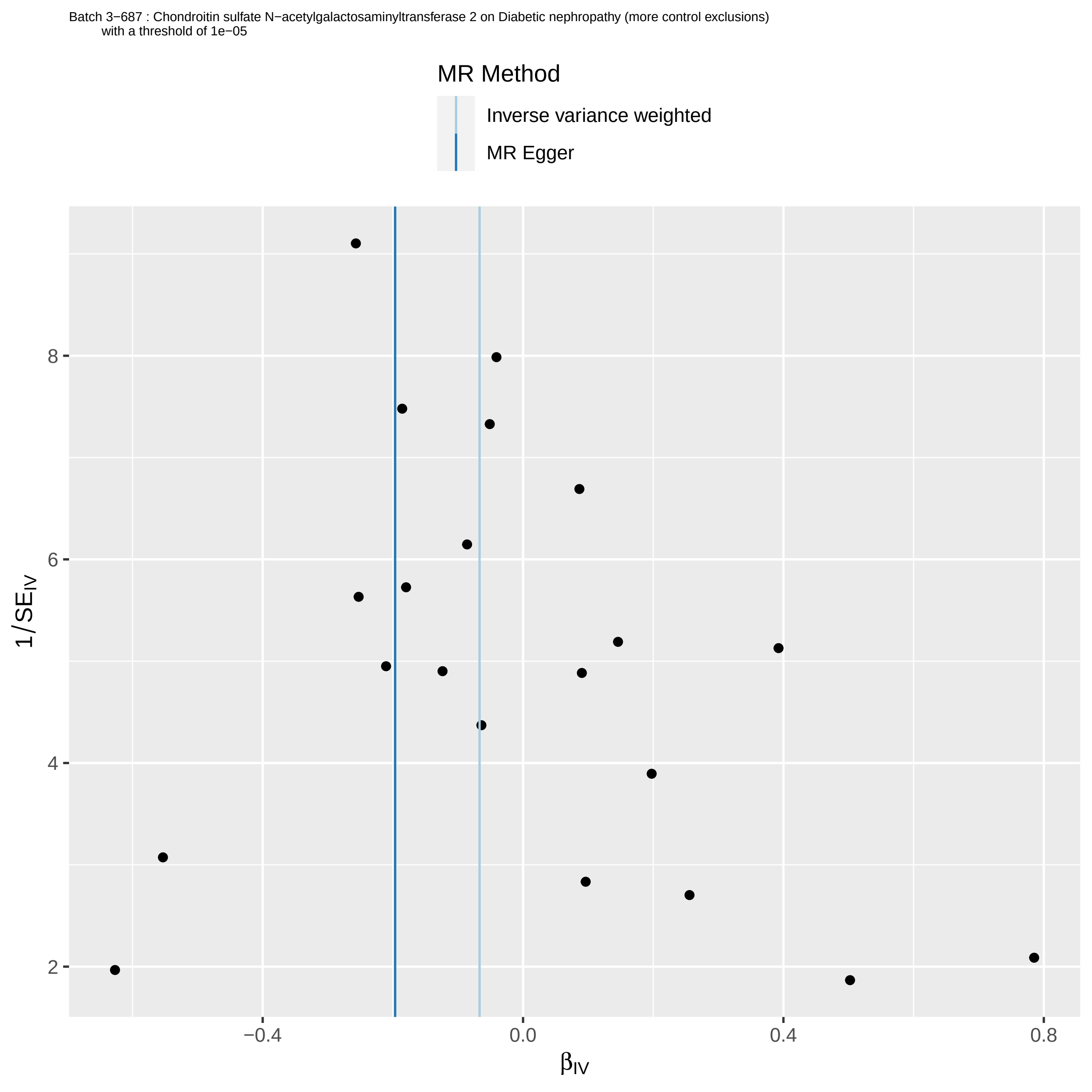
Batch 3−687 : Chondroitin sulfate N−acetylgalactosaminyltransferase 2 on Diabetic nephropathy (more control exclusions)
with a threshold of 1e−05
MR Method
Inverse variance weighted
MR Egger
8
6
4
2
1/SE_IV
−0.4
0.0
0.4
0.8
β_IV

Batch 3−688 : Chorionic somatomammotropin hormone on Diabetic nephropathy (more control exclusions)
with a threshold of 1e−05

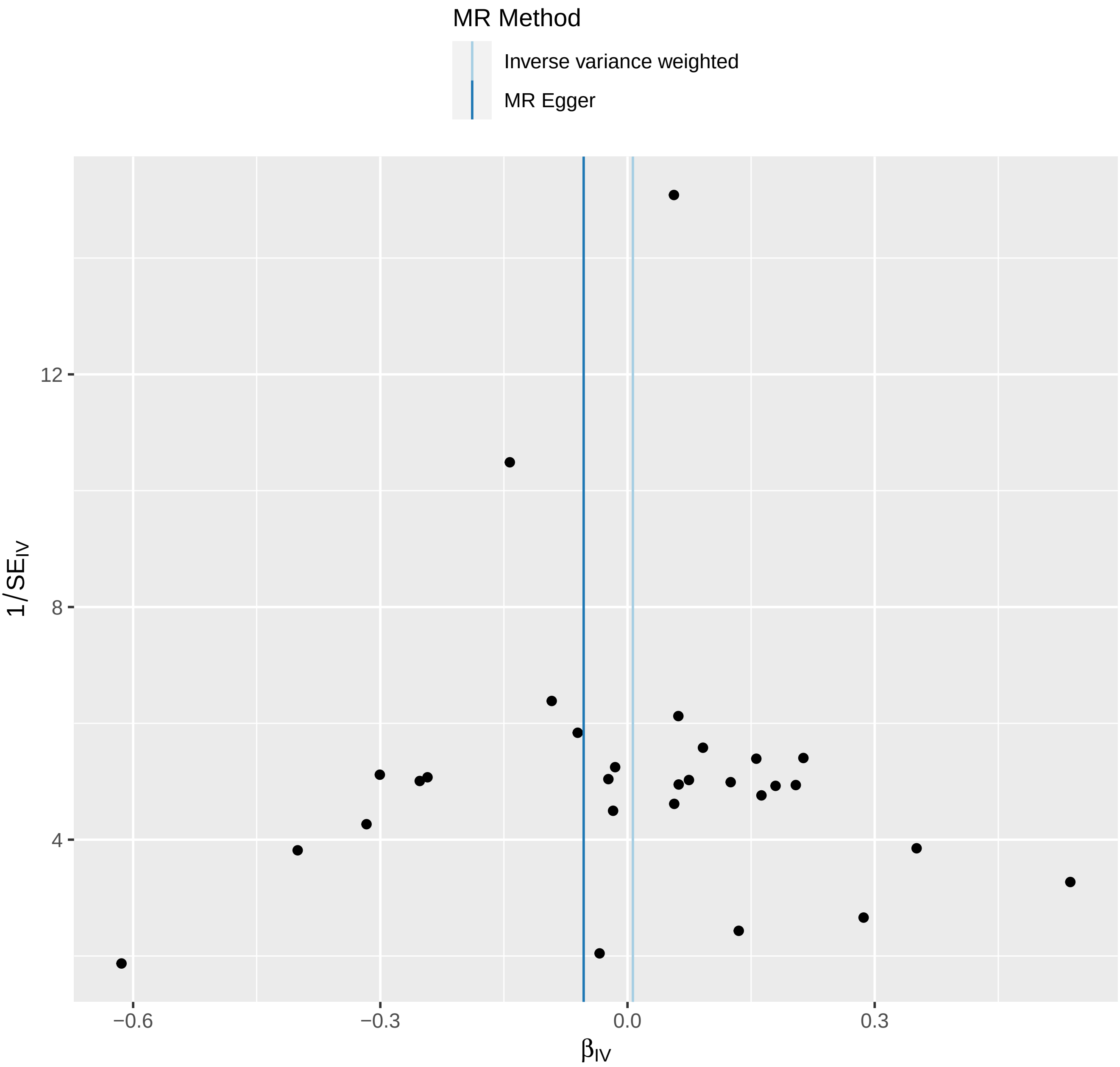

Batch 3−689 : Tyrosine−protein kinase CSK on Diabetic nephropathy (more control exclusions)
with a threshold of 1e−05

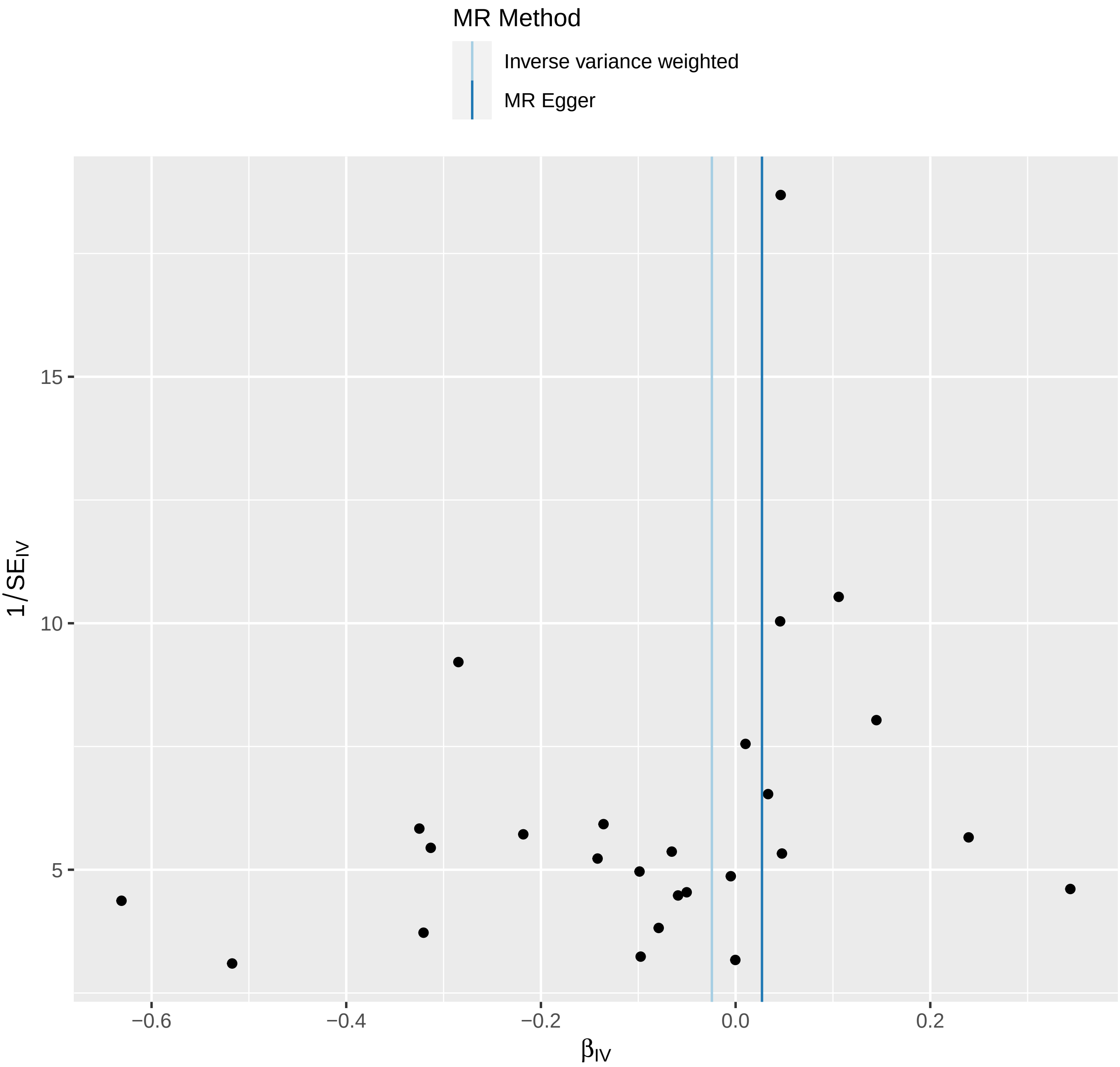

Batch 3−690 : CUB and sushi domain−containing protein 1 on Diabetic nephropathy (more control exclusions)
with a threshold of 1e−05

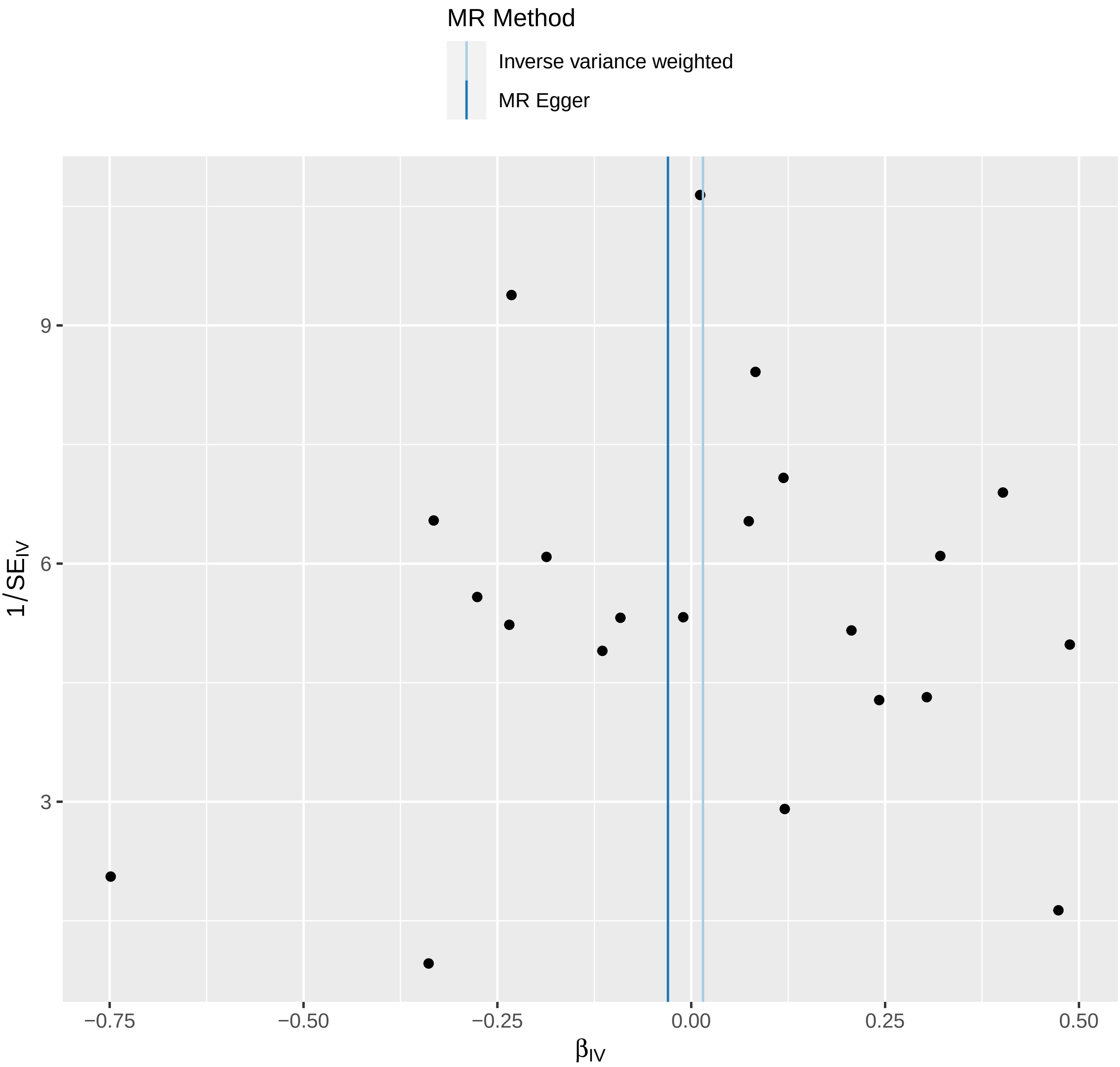

Batch 3−691 : CUB and sushi domain−containing protein 2 on Diabetic nephropathy (more control exclusions)
with a threshold of 1e−05

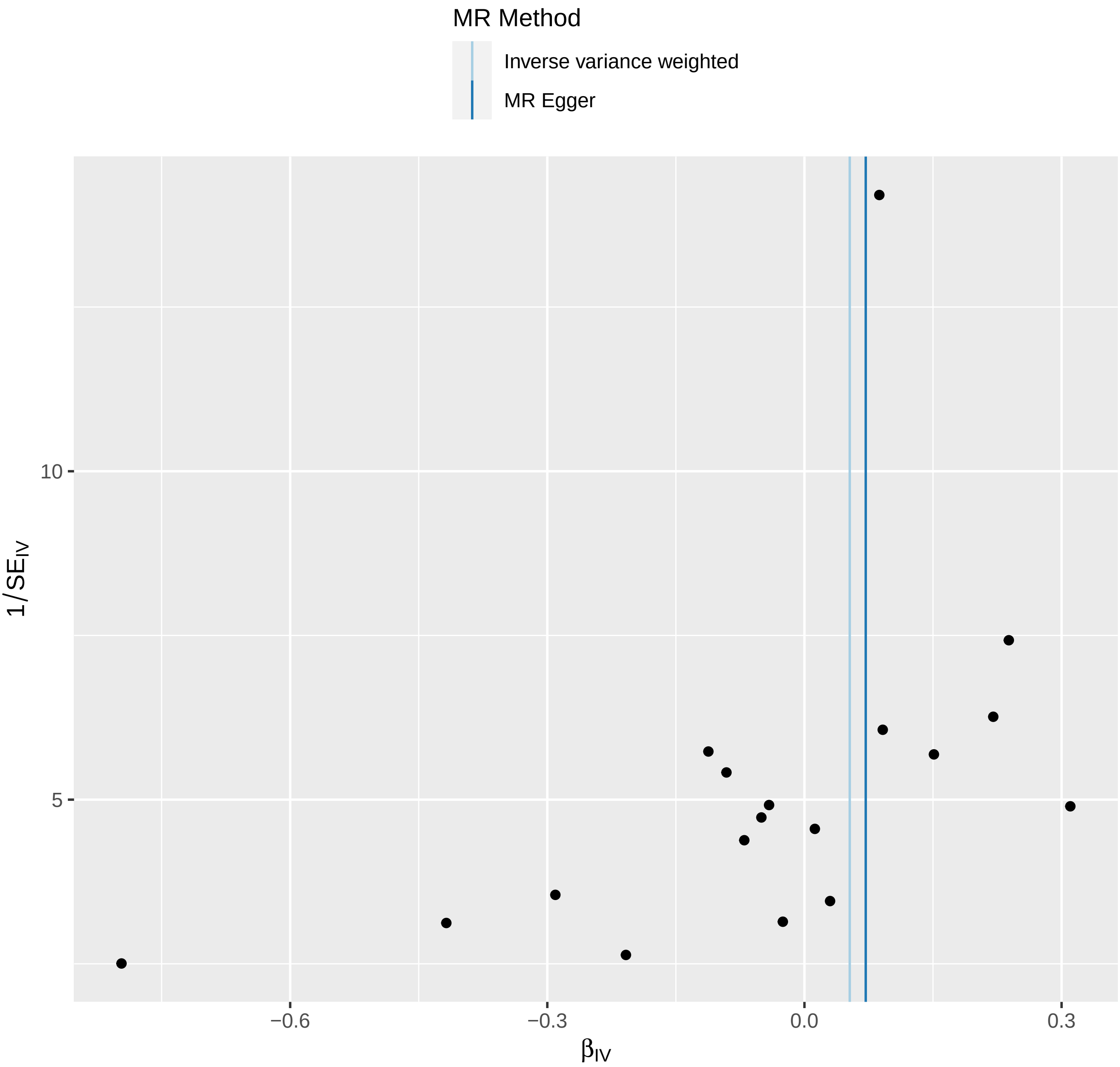

Batch 3−692 : alpha−S1−casein on Diabetic nephropathy (more control exclusions)
with a threshold of 1e−05

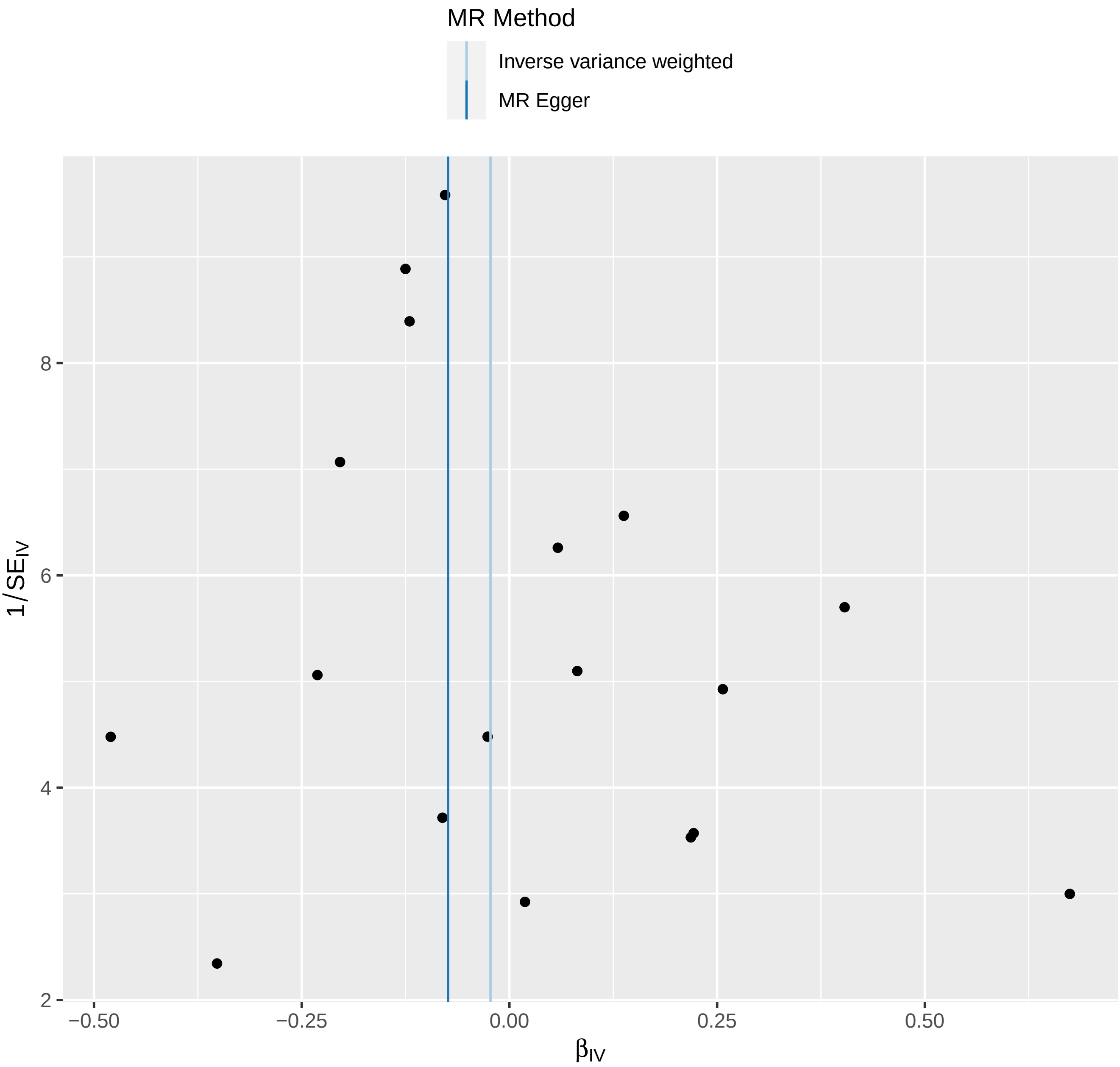

Batch 3−693 : Casein kinase I isoform delta on Diabetic nephropathy (more control exclusions)
with a threshold of 1e−05

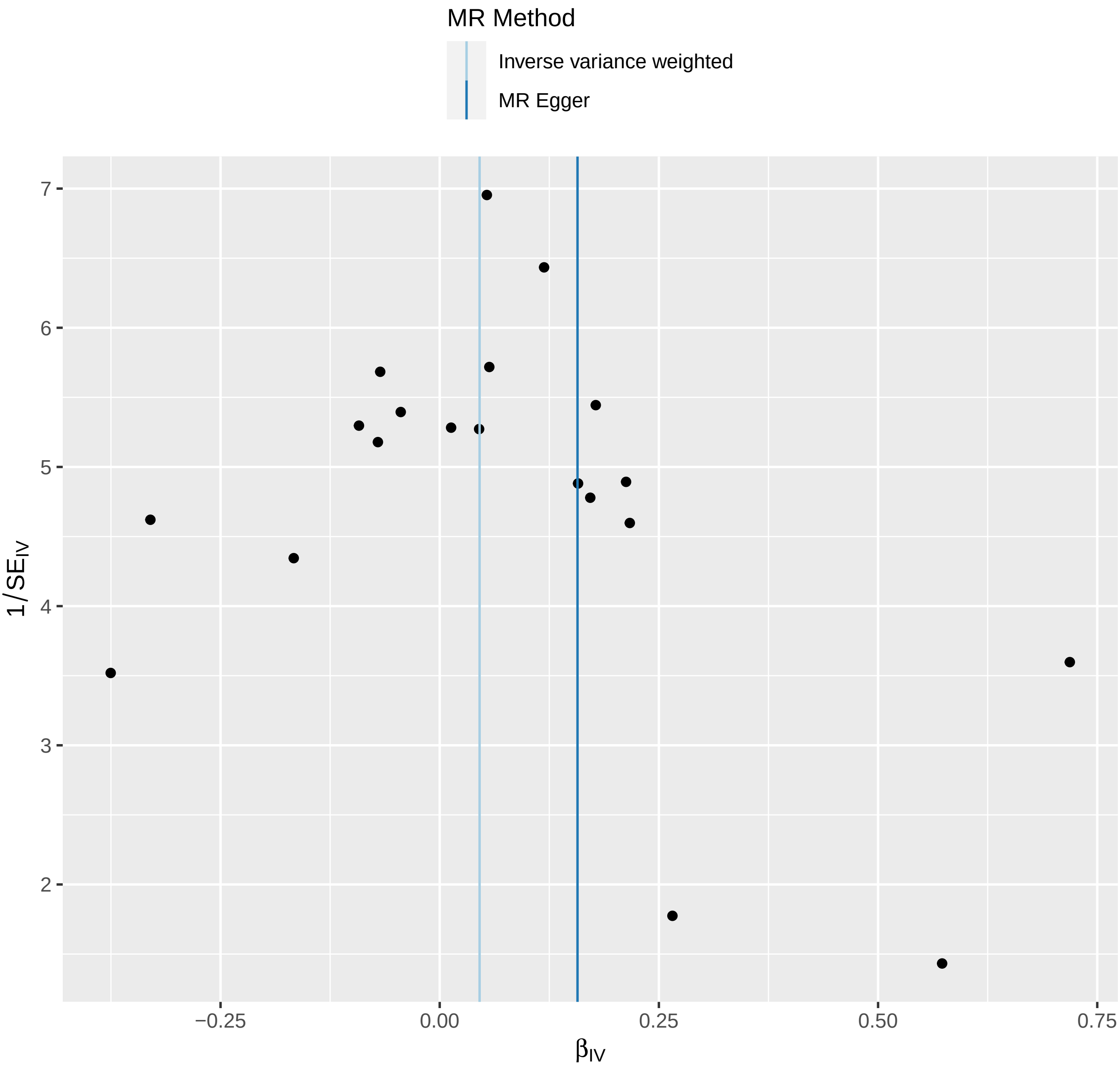

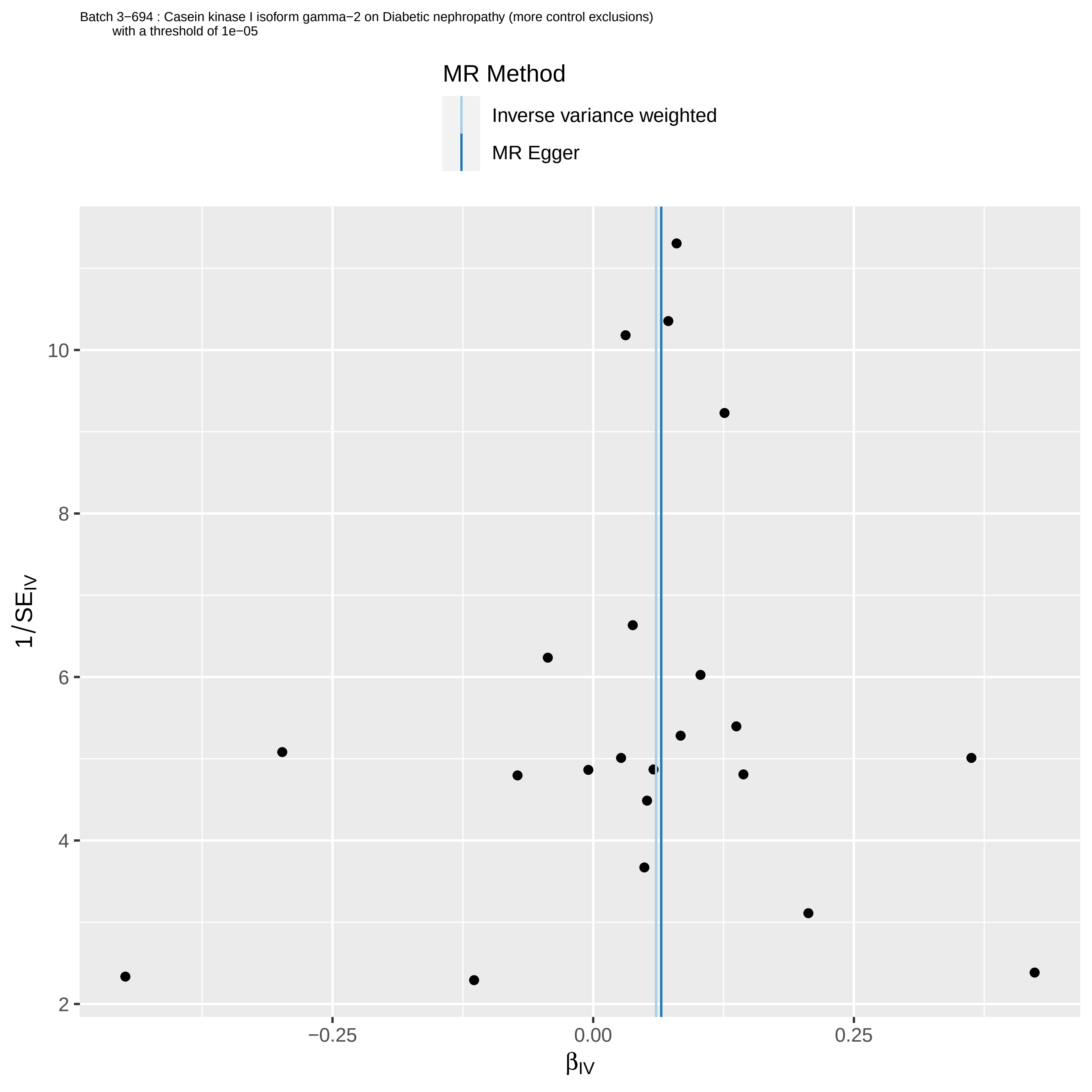
Batch 3−694 : Casein kinase I isoform gamma−2 on Diabetic nephropathy (more control exclusions)
with a threshold of 1e−05
MR Method
Inverse variance weighted
MR Egger
10
8
6
4
2
1/SE_IV
−0.25
0.00
0.25
β_IV

Batch 3−695 : Casein kinase II subunit alpha on Diabetic nephropathy (more control exclusions)
with a threshold of 1e−05

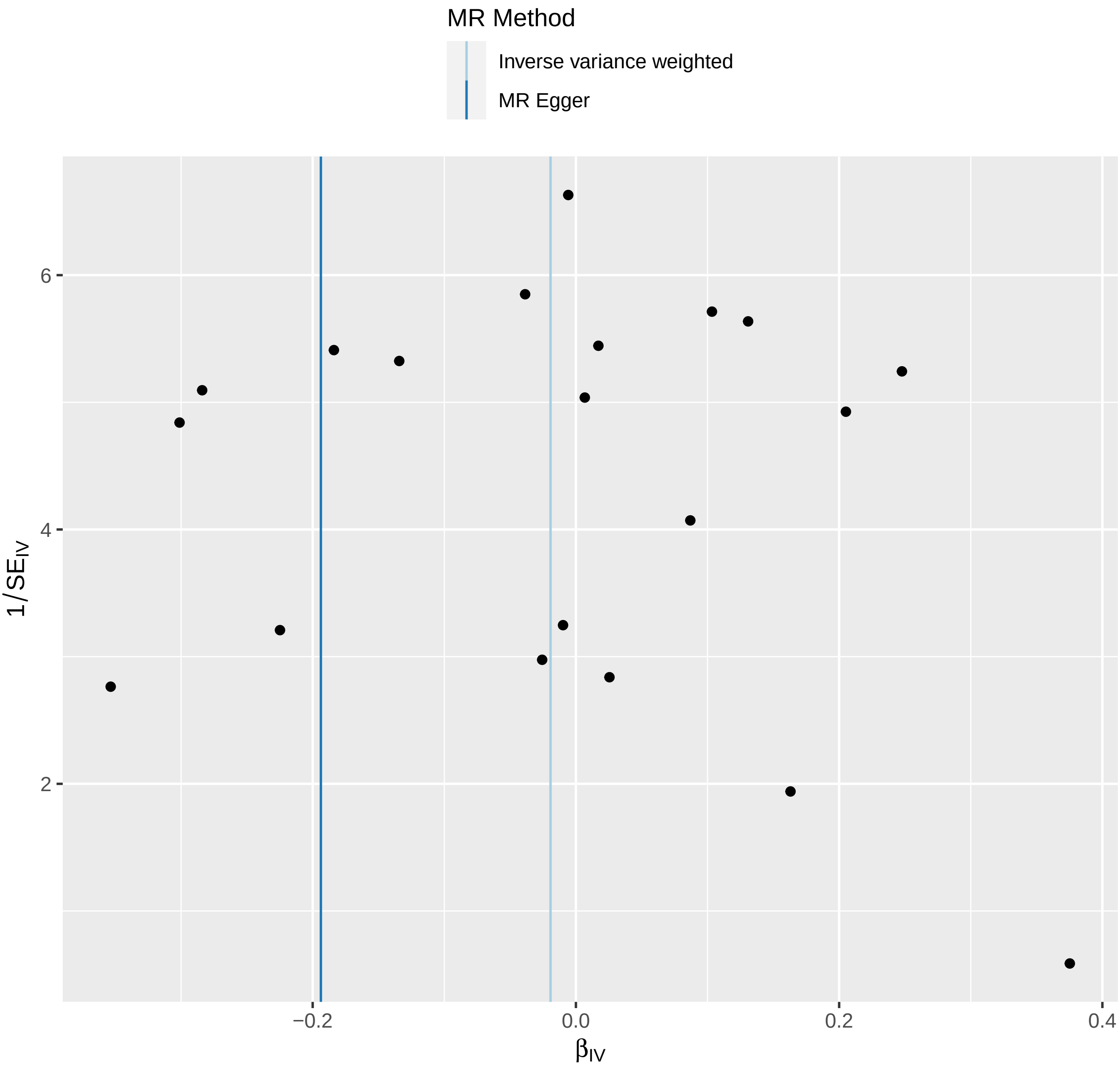

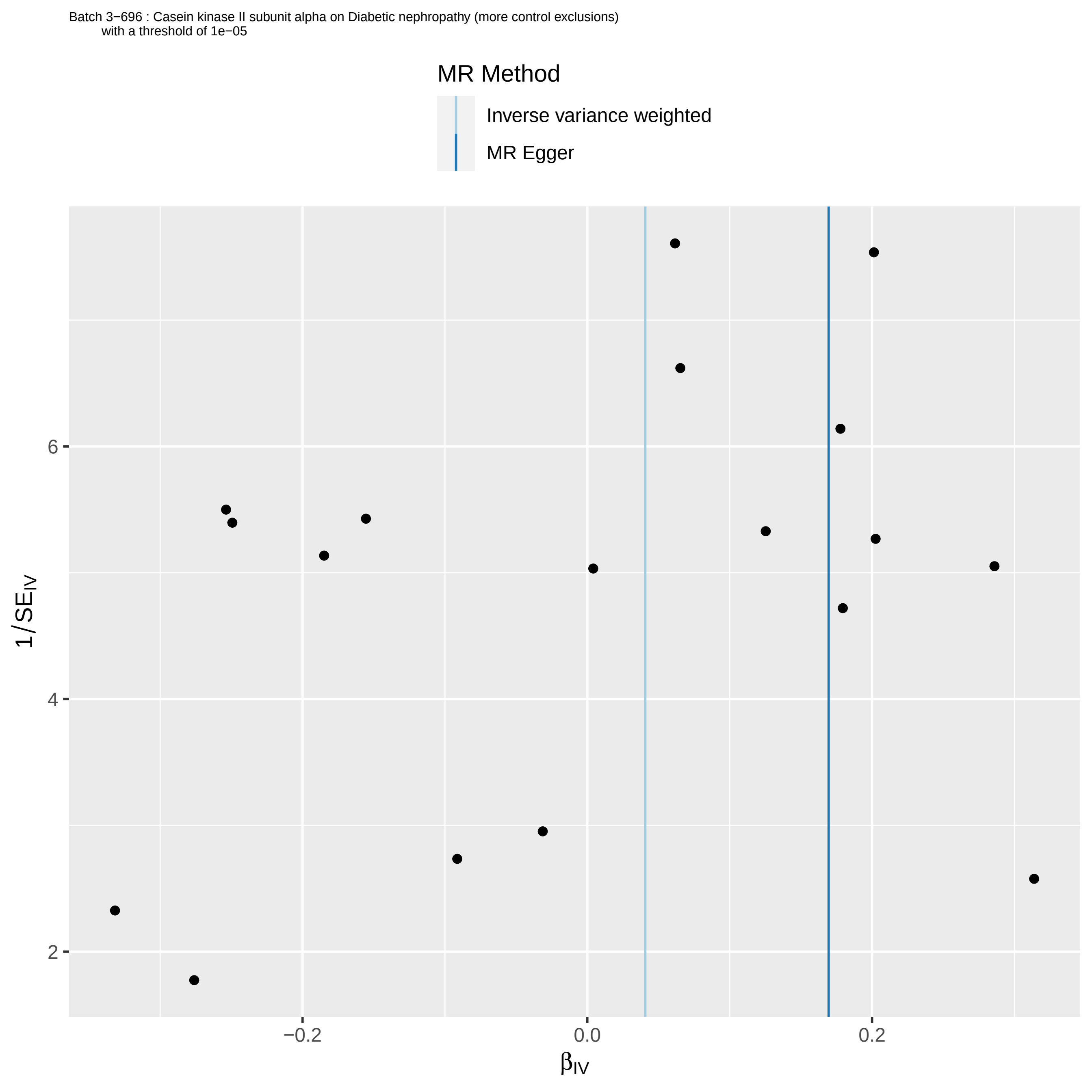
Batch 3−696 : Casein kinase II subunit alpha on Diabetic nephropathy (more control exclusions)
with a threshold of 1e−05
MR Method
Inverse variance weighted
MR Egger
1/SE_IV
β_IV
6
4
2
−0.2
0.0
0.2

Batch 3−697 : Casein kinase II 2−alpha:2−beta heterotetramer on Diabetic nephropathy (more control exclusions) with a threshold of 1e−05

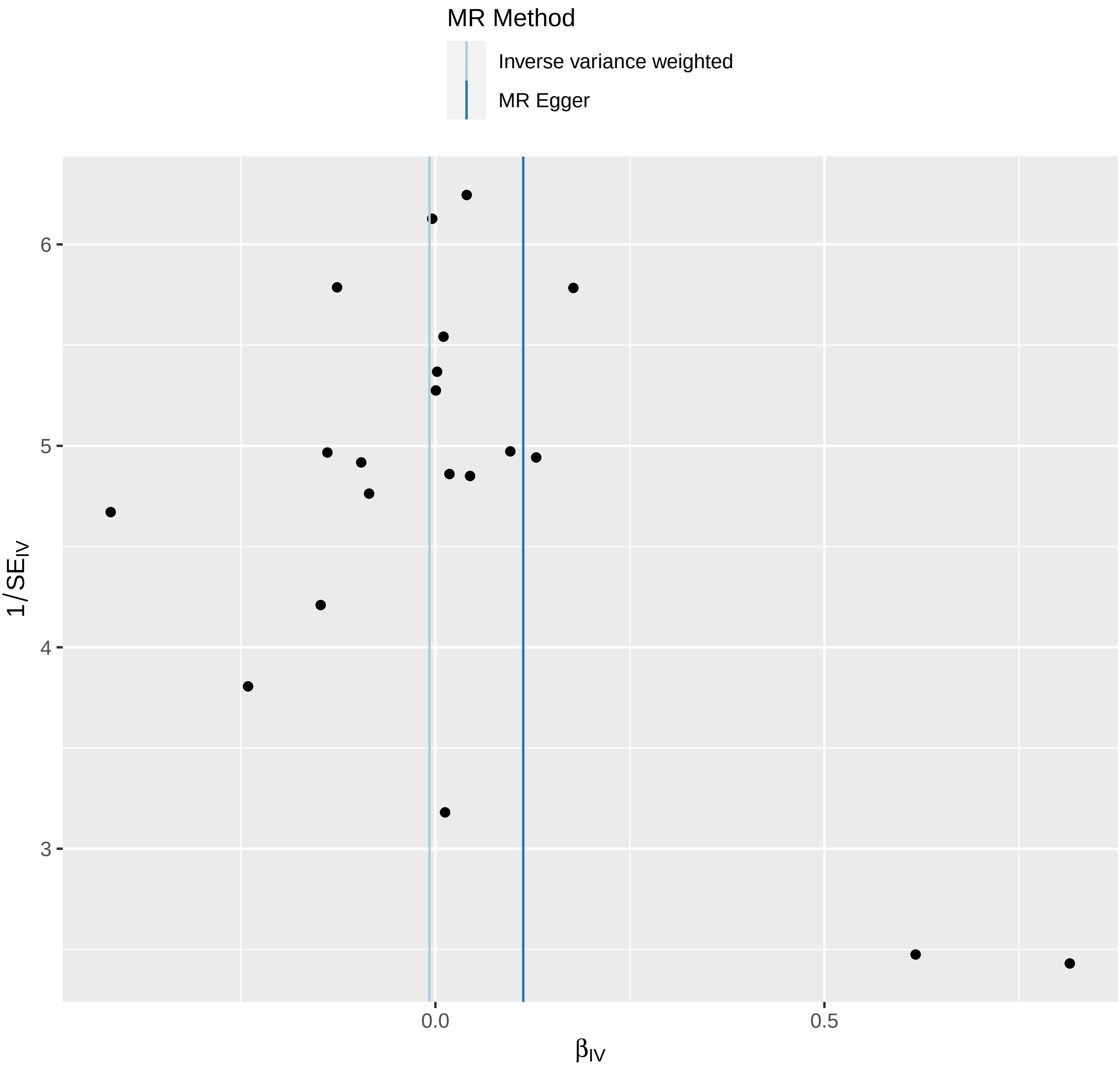

Batch 3−698 : Casein kinase II 2−alpha':2−beta heterotetramer on Diabetic nephropathy (more control exclusions)
with a threshold of 1e−05

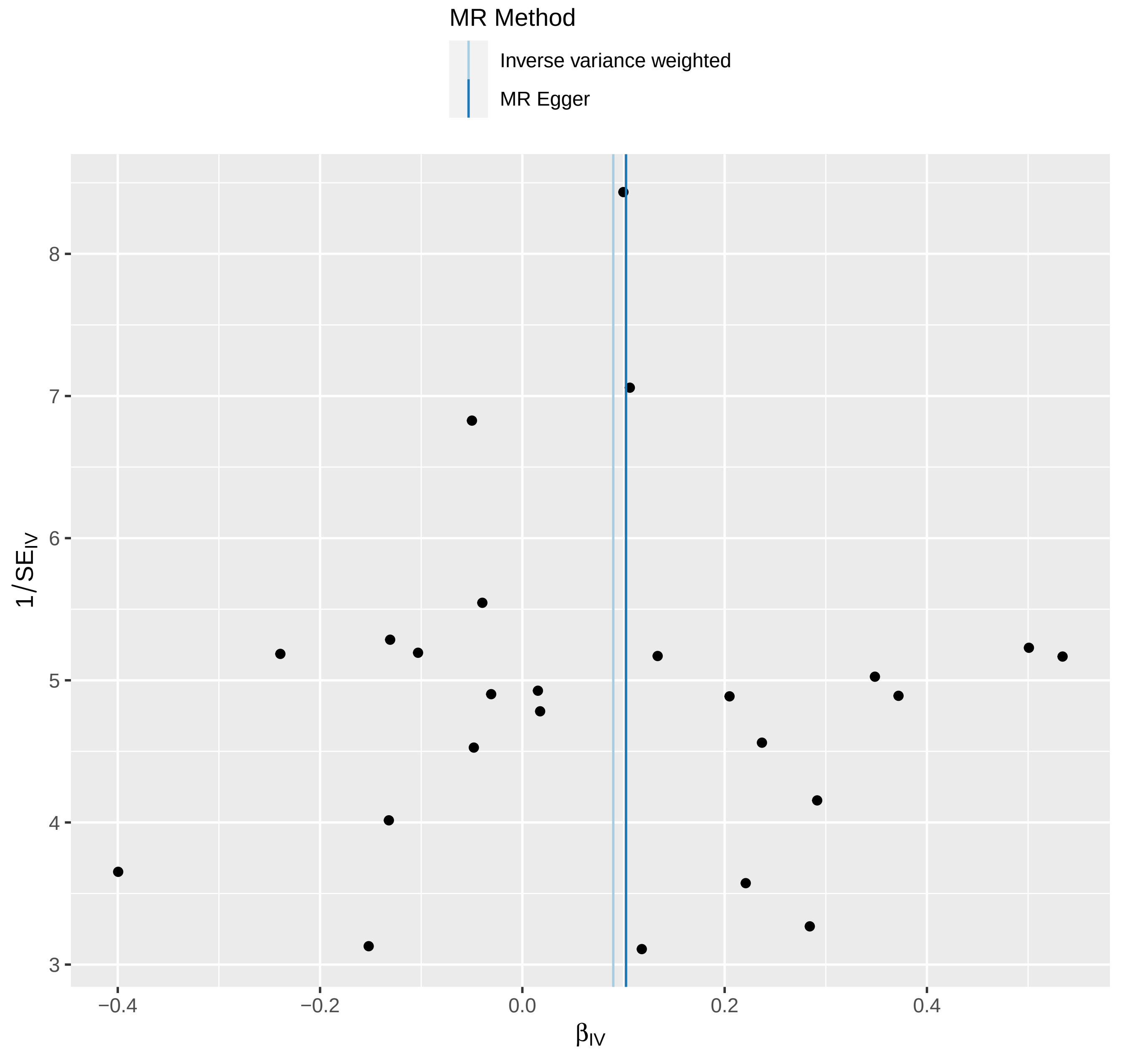

Batch 3−699 : Chondroitin sulfate proteoglycan 4 on Diabetic nephropathy (more control exclusions)
with a threshold of 1e−05

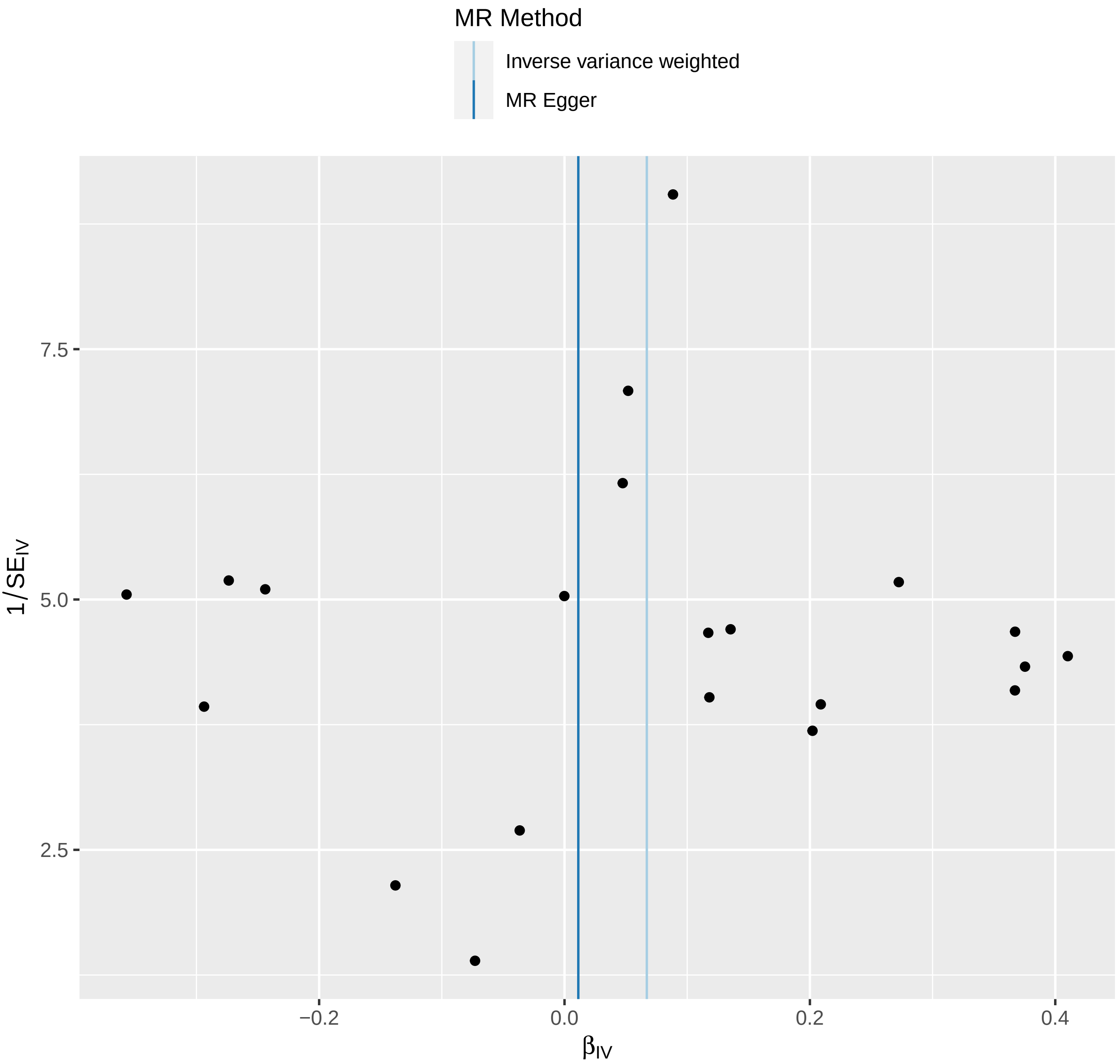

Batch 3−700 : Cysteine and glycine−rich protein 2 on Diabetic nephropathy (more control exclusions)
with a threshold of 1e−05

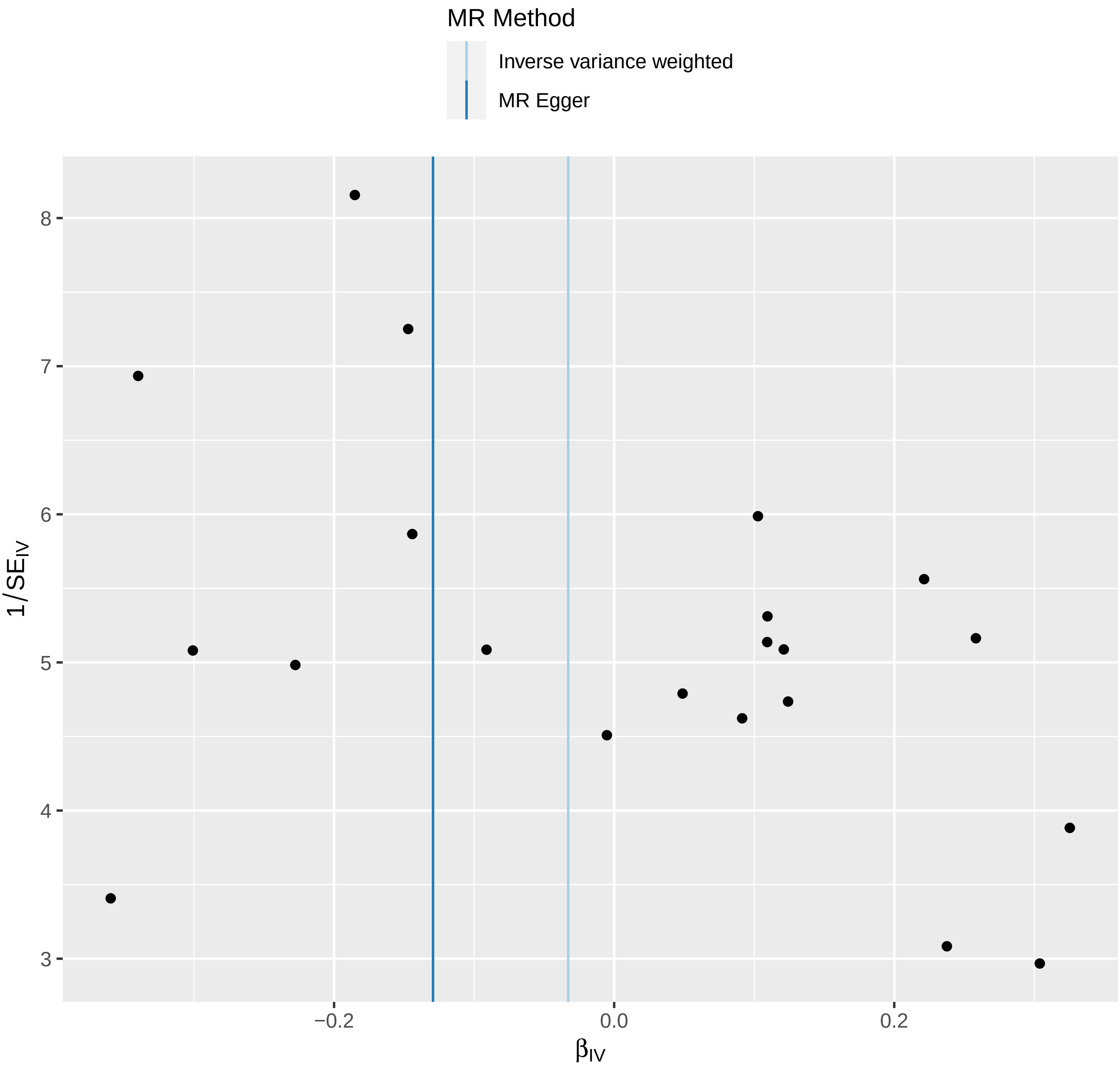

Batch 3−701 : Cysteine and glycine−rich protein 3 on Diabetic nephropathy (more control exclusions)
with a threshold of 1e−05

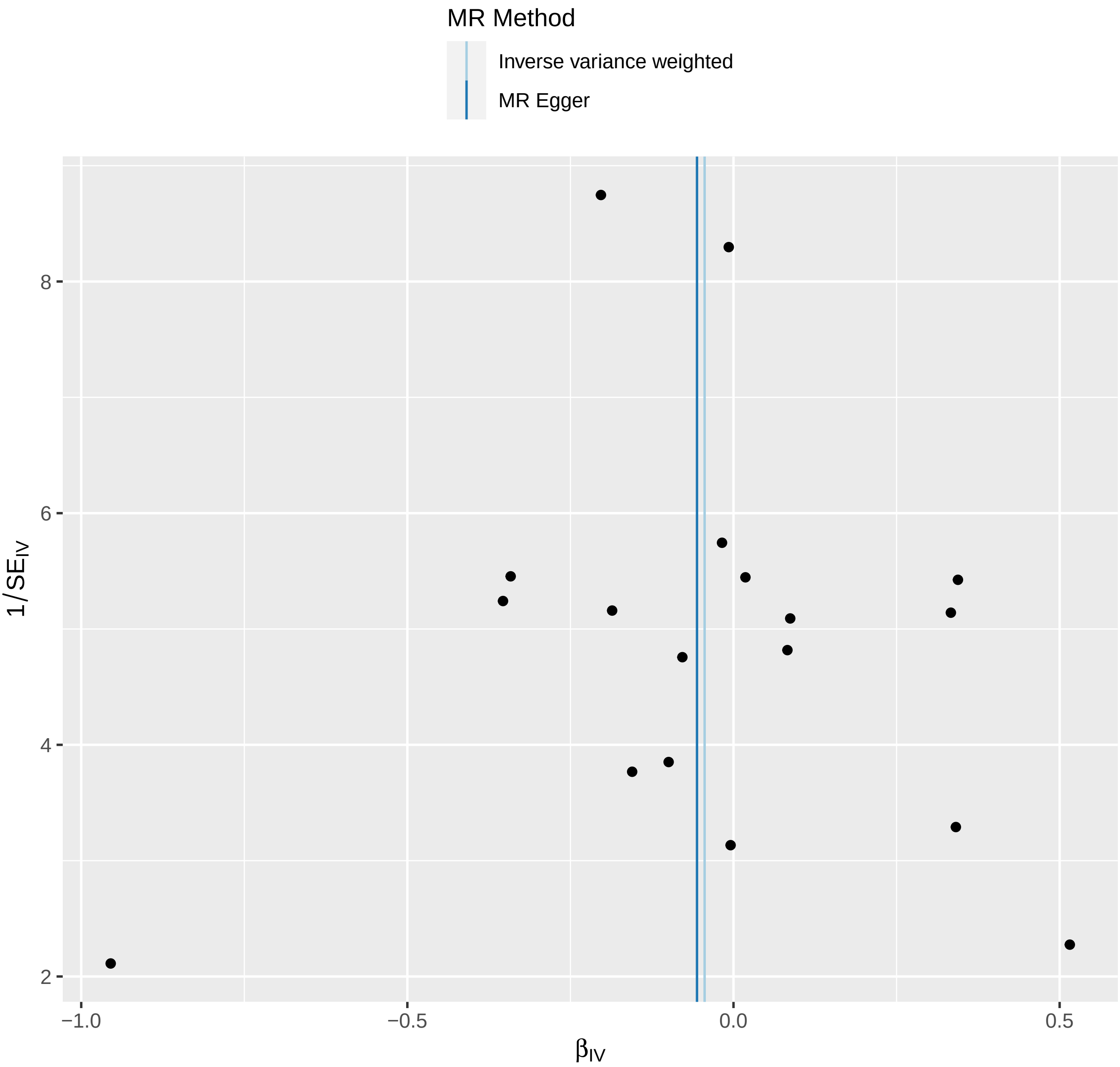

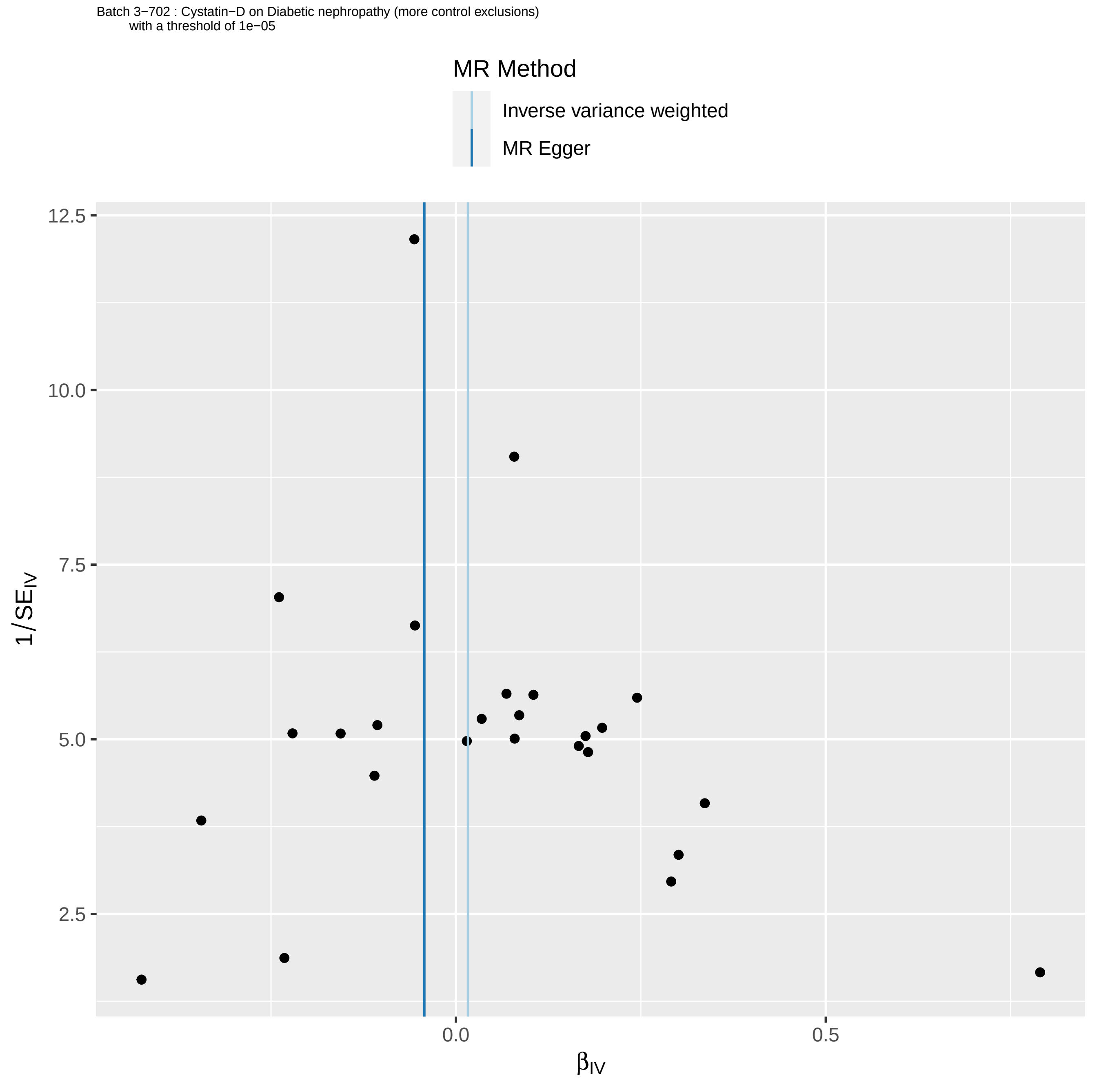
Batch 3−702 : Cystatin−D on Diabetic nephropathy (more control exclusions)
with a threshold of 1e−05
MR Method
Inverse variance weighted
MR Egger
12.5
10.0
7.5
5.0
2.5
$1/SE_{IV}$
0.0
0.5
β_{IV}

Batch 3−703 : Cystatin−M on Diabetic nephropathy (more control exclusions)
with a threshold of 1e−05

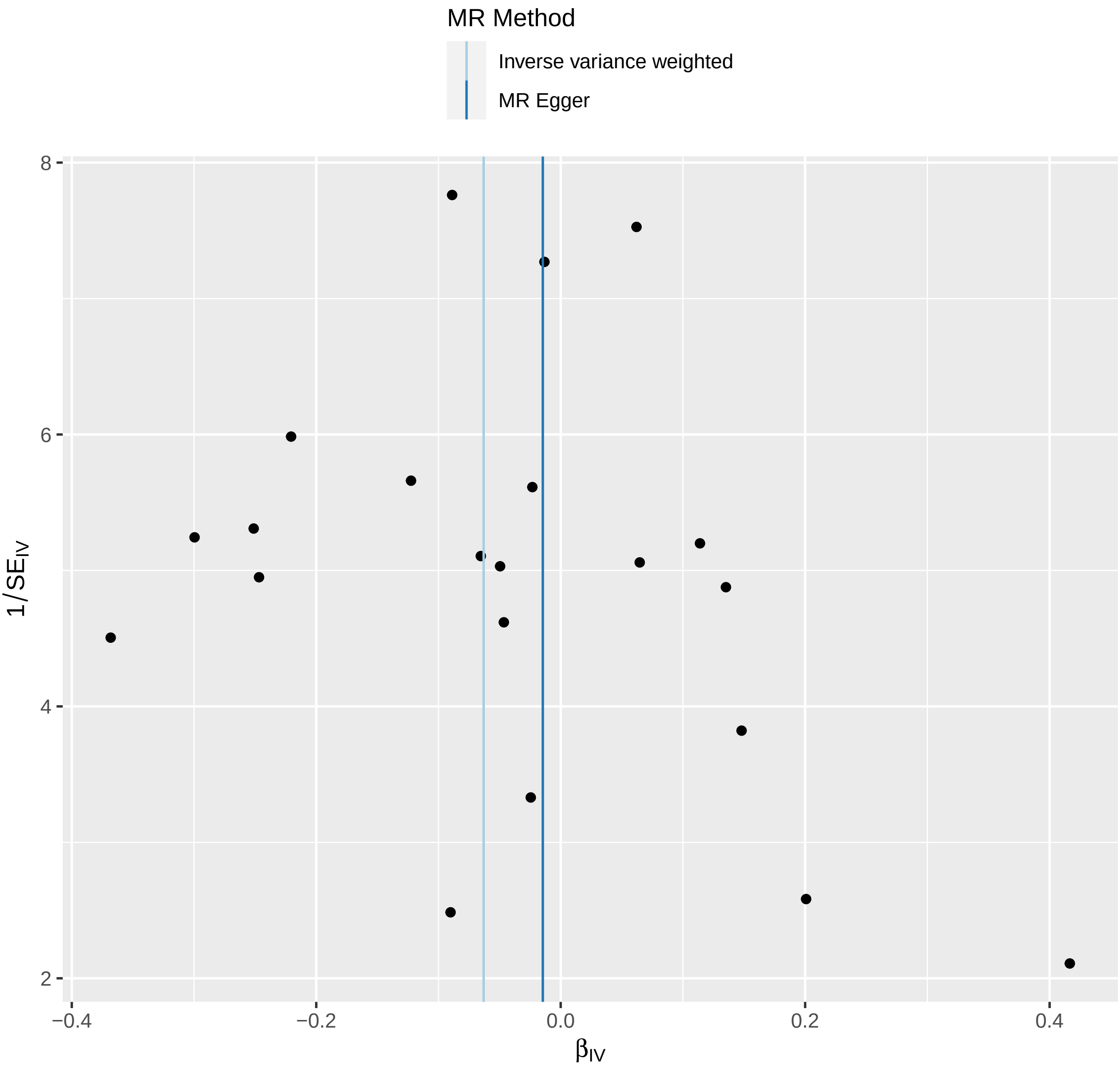

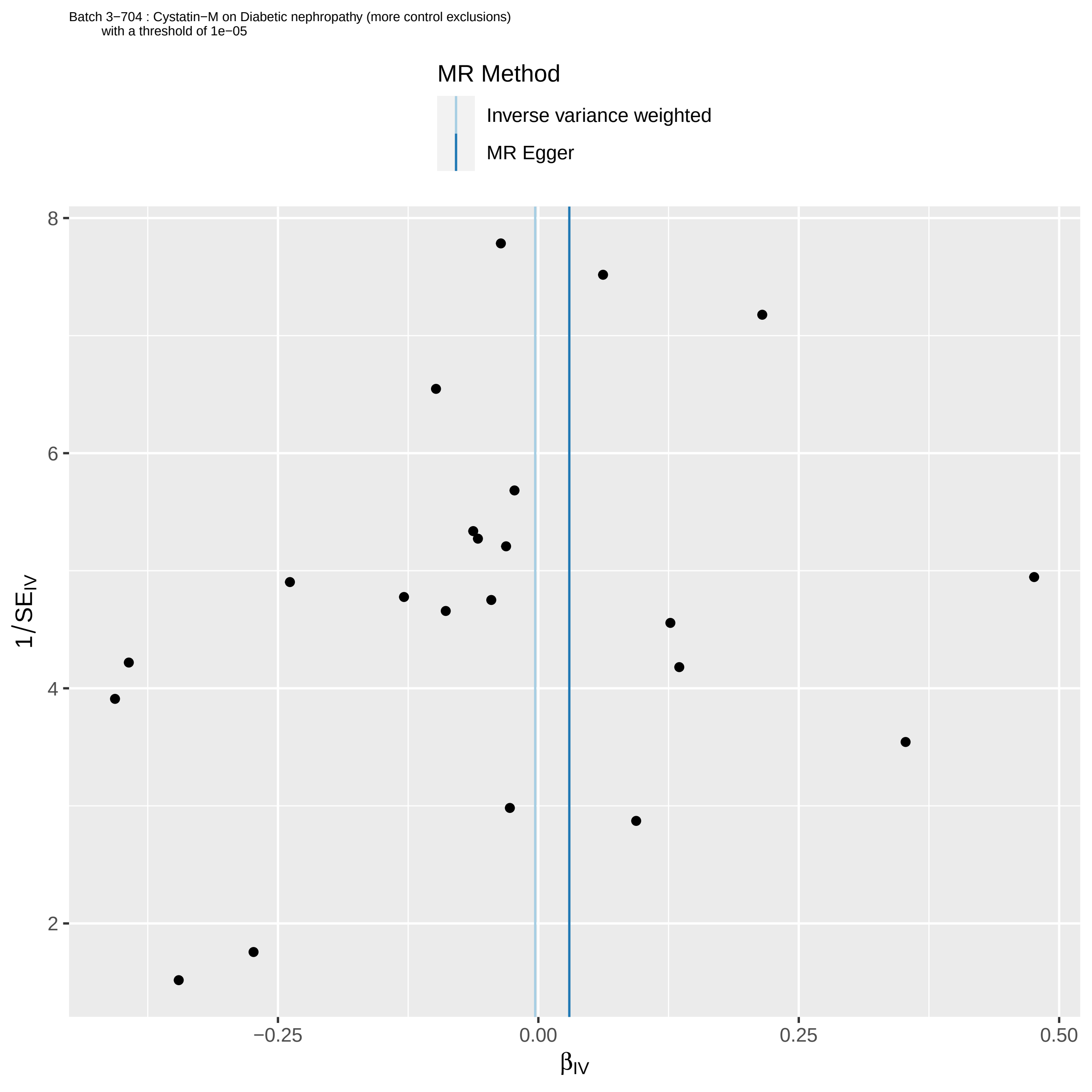
Batch 3−704 : Cystatin−M on Diabetic nephropathy (more control exclusions)
with a threshold of 1e−05
MR Method
Inverse variance weighted
MR Egger
8
6
4
2
1/SE$_{IV}$
−0.25
0.00
0.25
0.50
β$_{IV}$

Batch 3−705 : Cystatin−F on Diabetic nephropathy (more control exclusions)
with a threshold of 1e−05

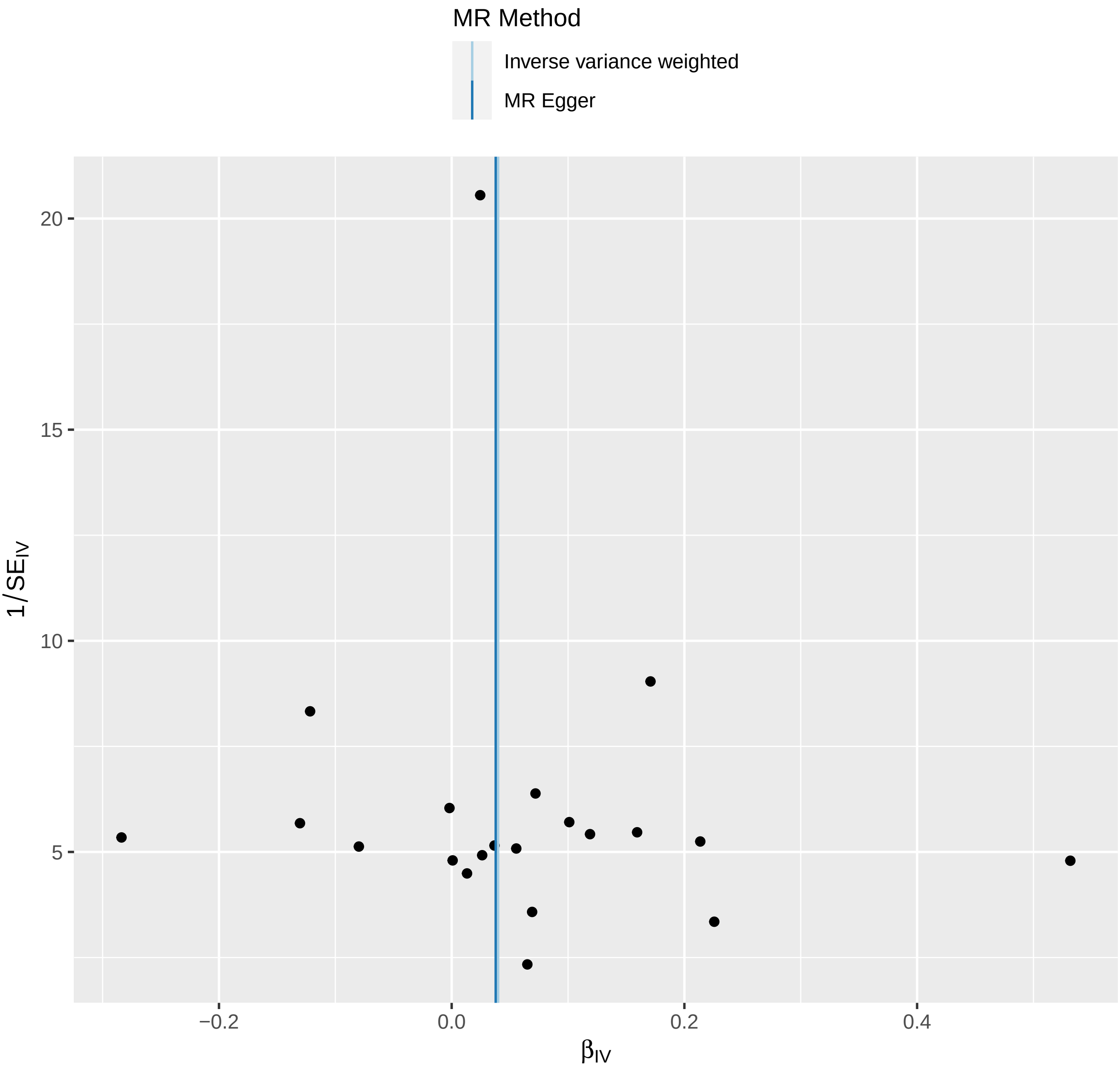

Batch 3−706 : Cystatin−8 on Diabetic nephropathy (more control exclusions)
with a threshold of 1e−05

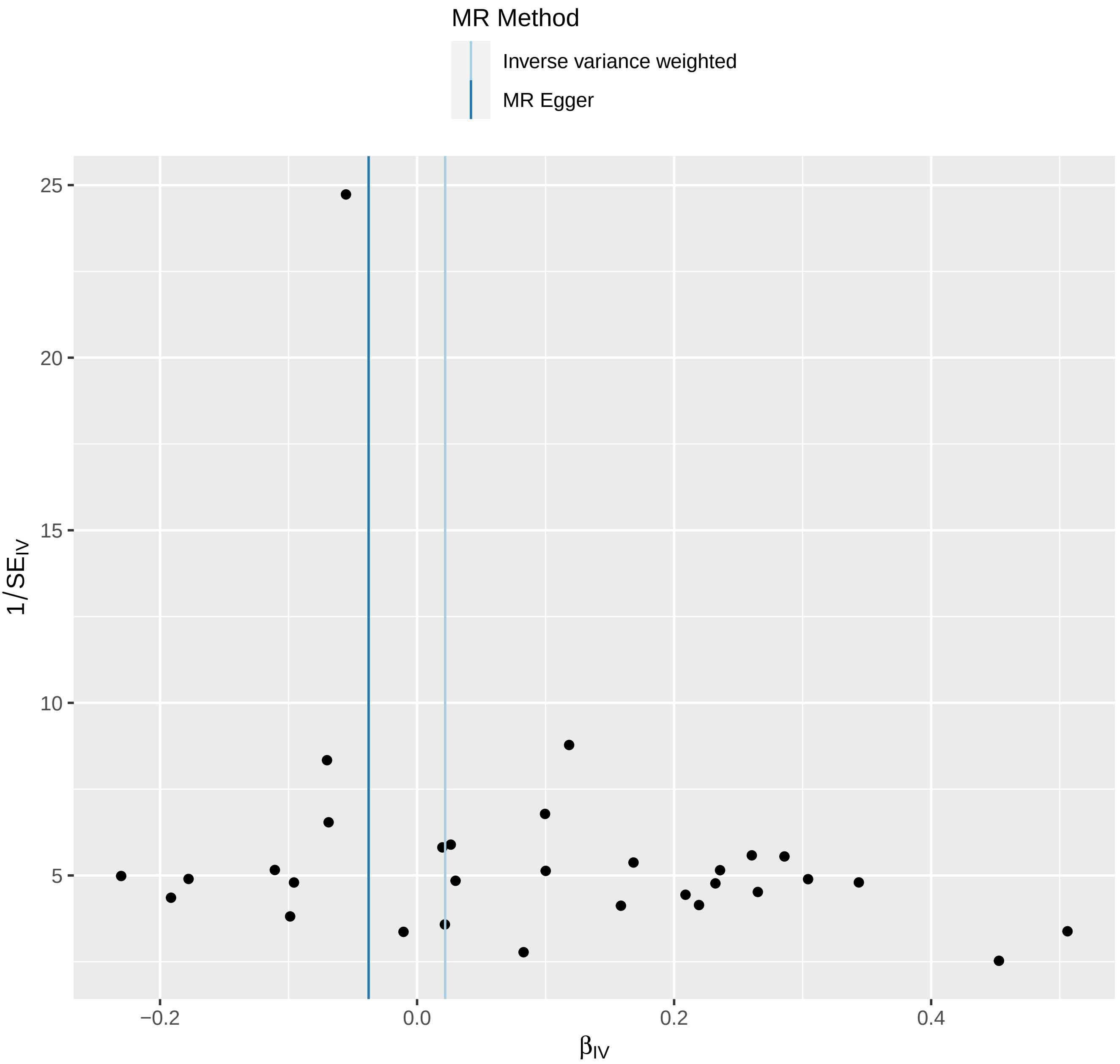

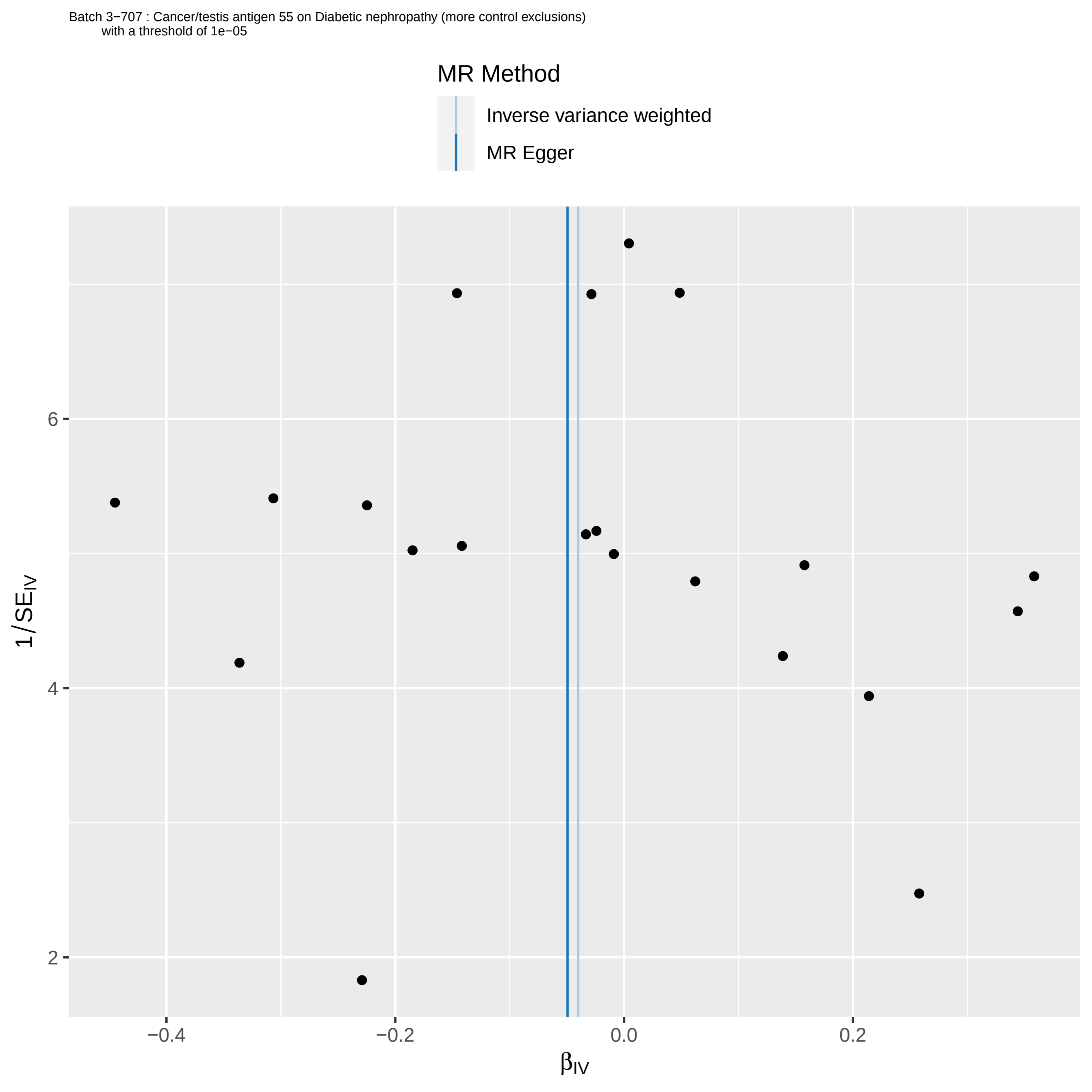

Batch 3−707 : Cancer/testis antigen 55 on Diabetic nephropathy (more control exclusions)
with a threshold of 1e−05
MR Method
Inverse variance weighted
MR Egger
6
4
2
1/SE_IV
−0.4
−0.2
0.0
0.2
β_IV

Batch 3−708 : Di−N−acetylchitobiase on Diabetic nephropathy (more control exclusions)
with a threshold of 1e−05

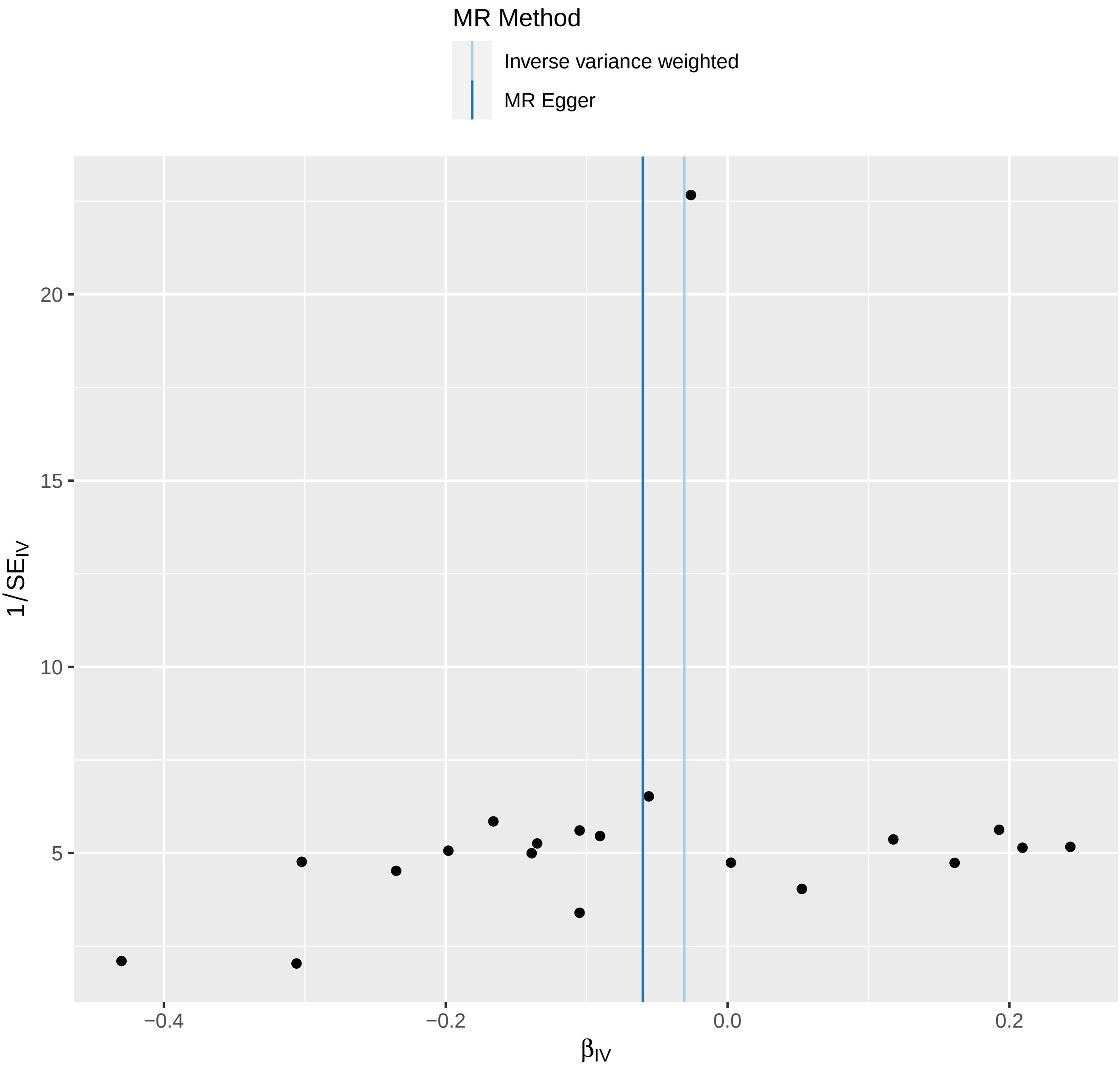

Batch 3−709 : Transcriptional repressor CTCF on Diabetic nephropathy (more control exclusions)
with a threshold of 1e−05

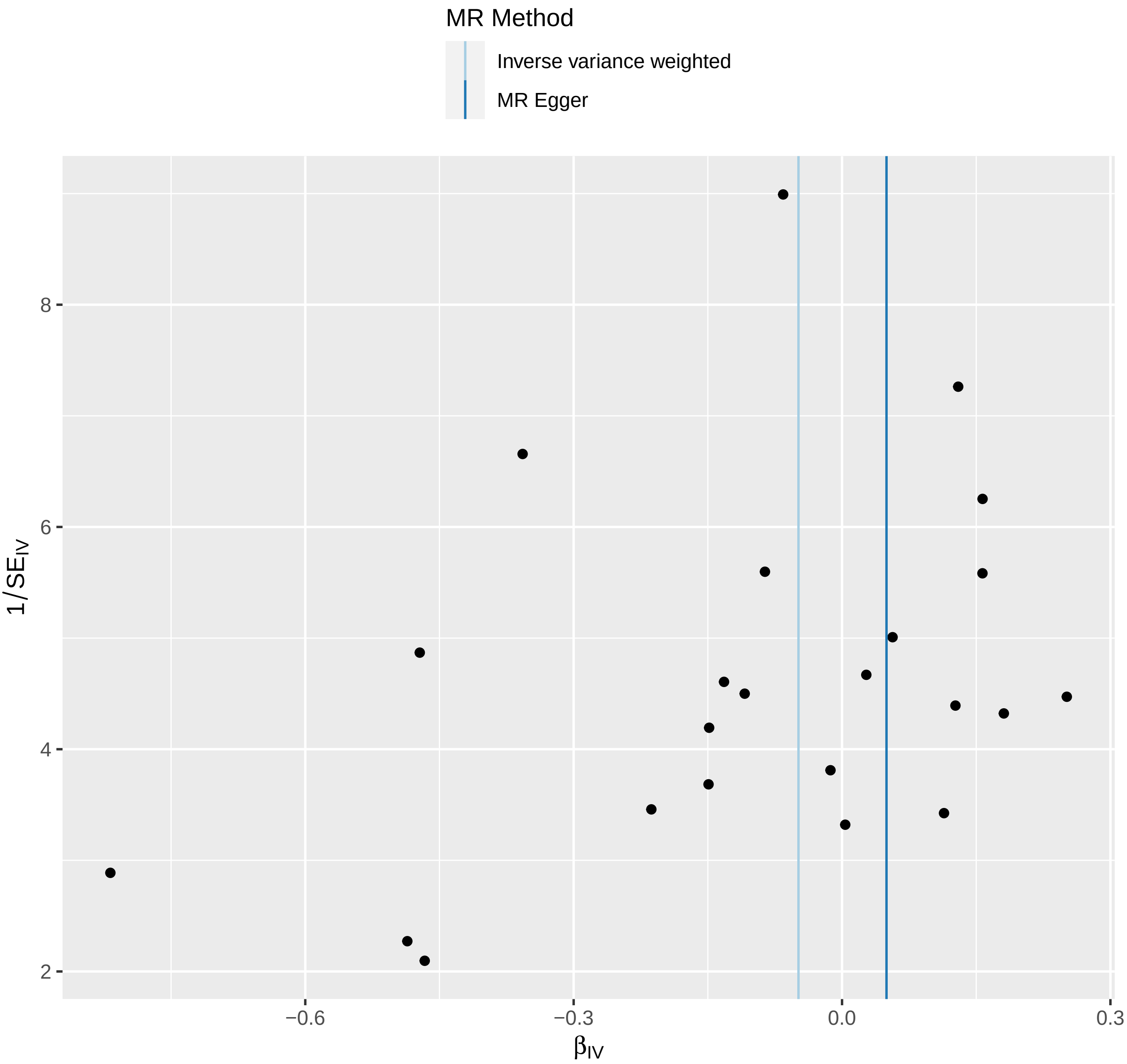

Batch 3−710 : Cardiotrophin−1 on Diabetic nephropathy (more control exclusions)
with a threshold of 1e−05

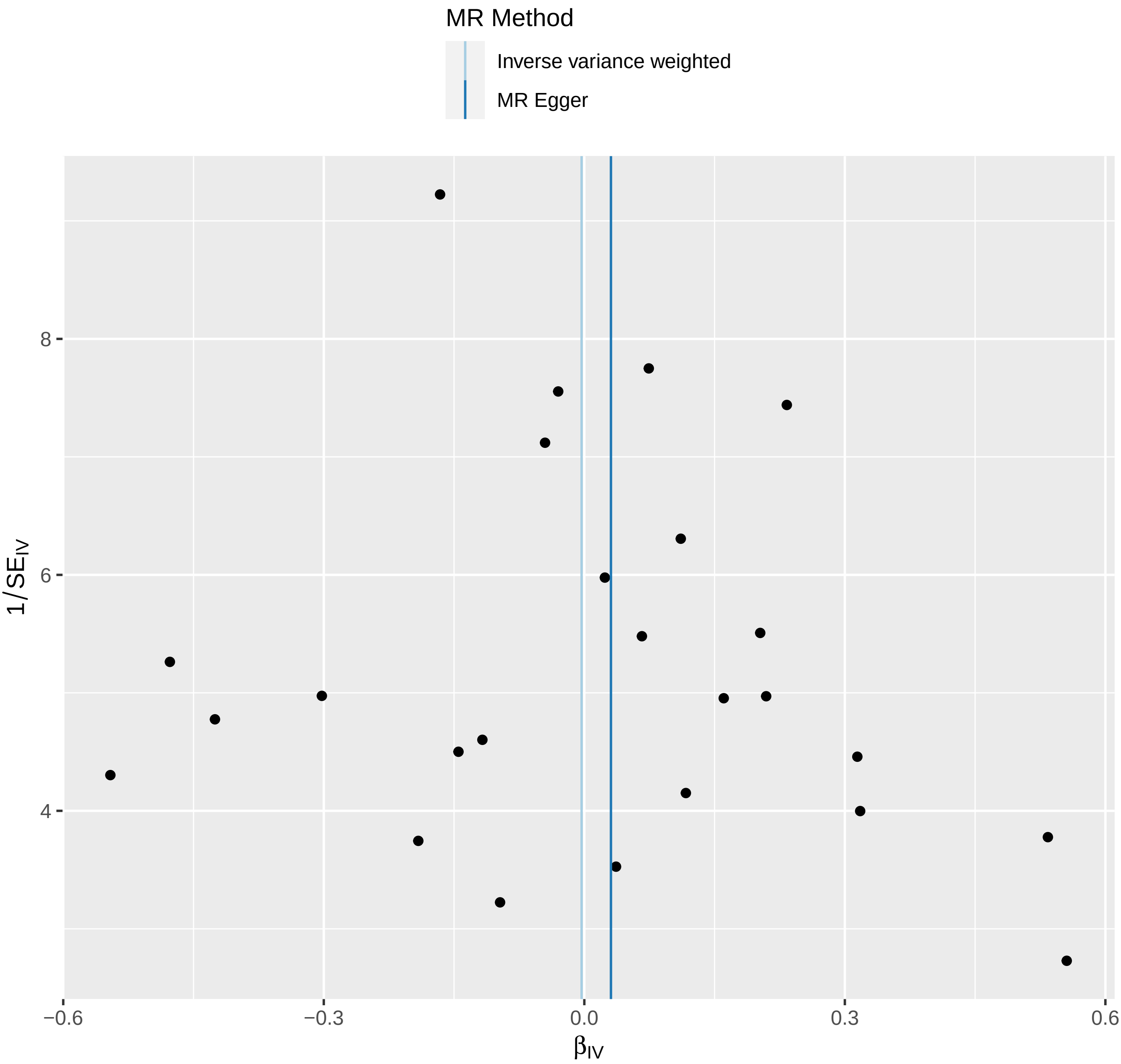

Batch 3−711 : Cardiotrophin−1 on Diabetic nephropathy (more control exclusions)
with a threshold of 1e−05

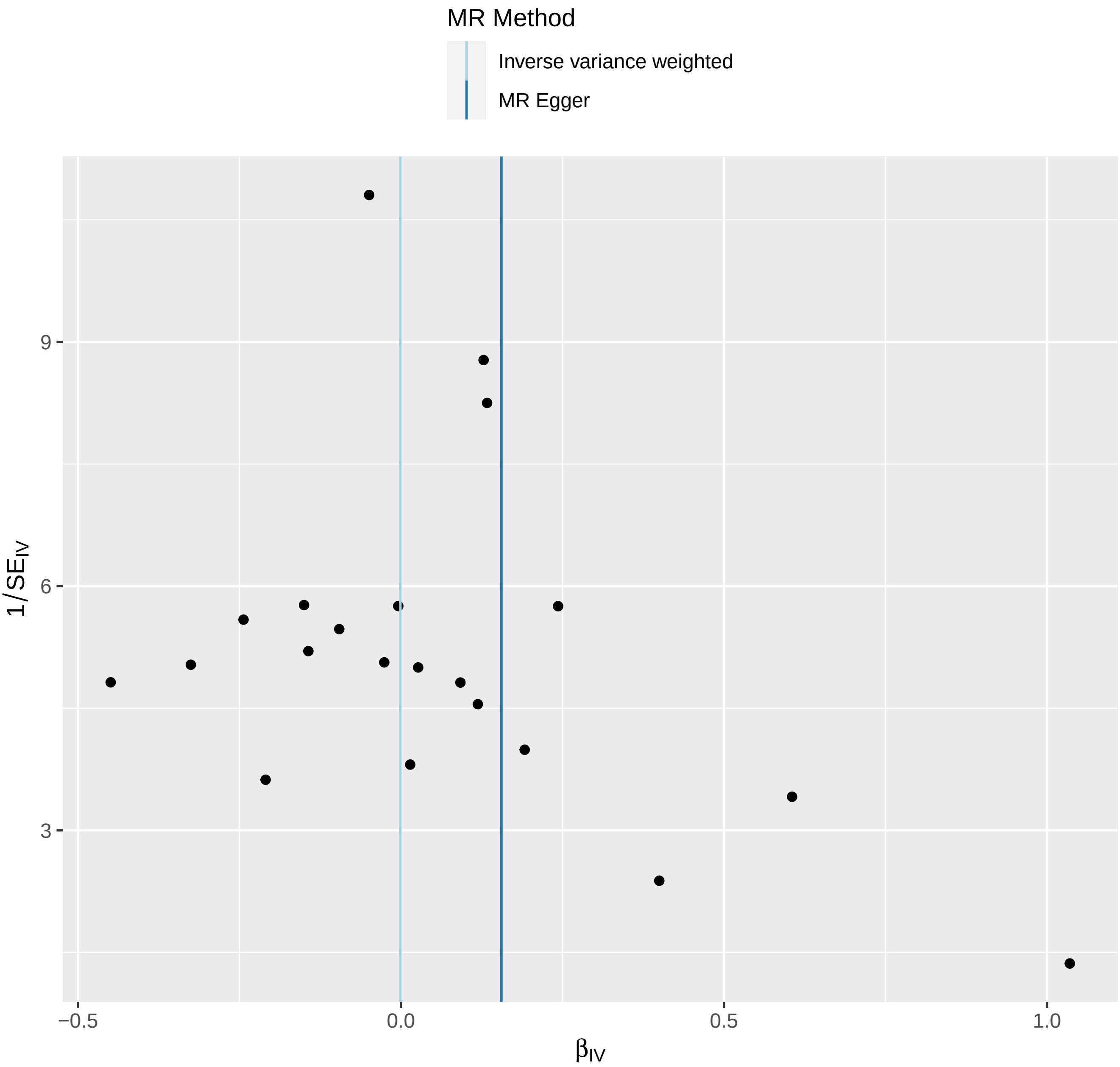

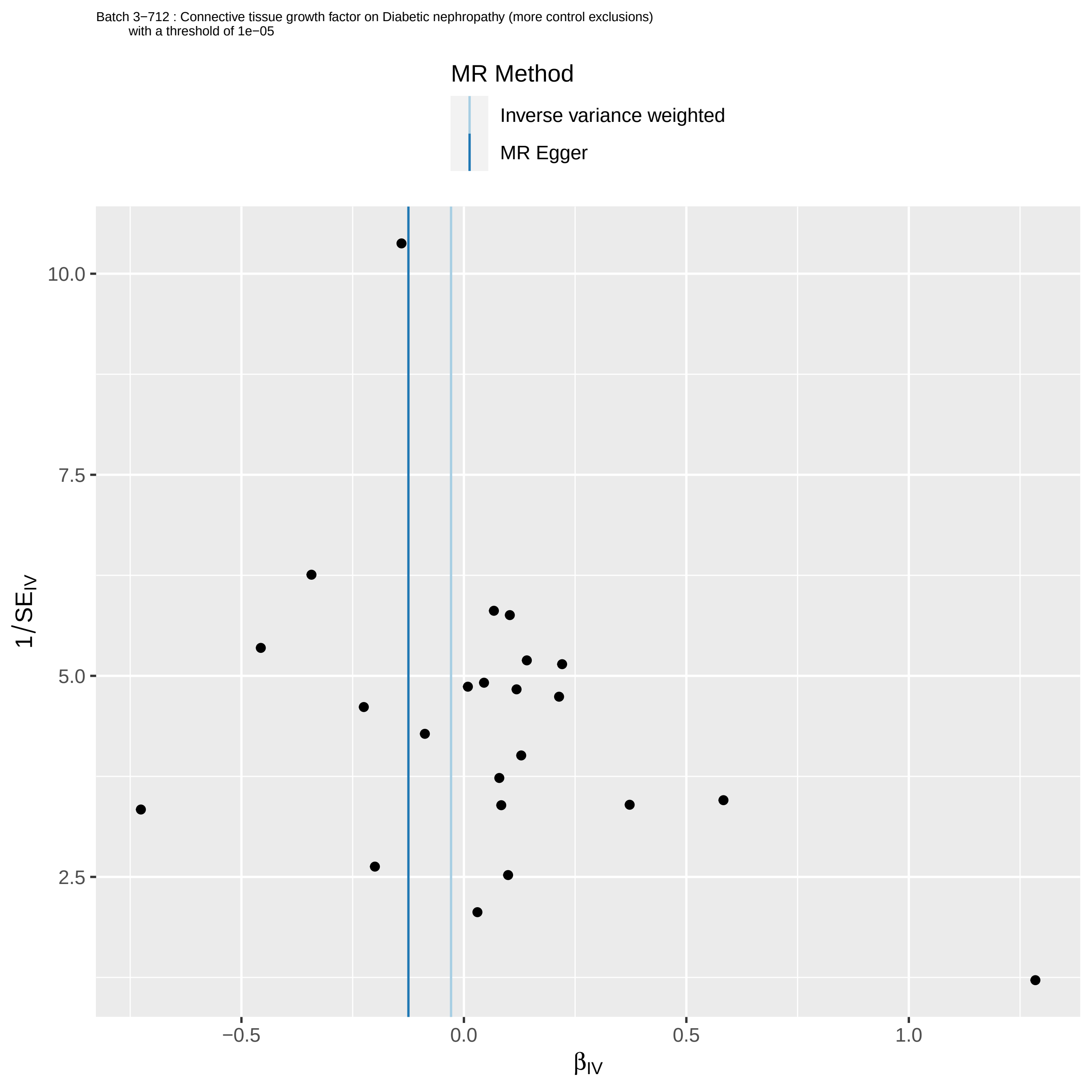

Batch 3−712 : Connective tissue growth factor on Diabetic nephropathy (more control exclusions)
with a threshold of 1e−05
MR Method
Inverse variance weighted
MR Egger
10.0
7.5
5.0
2.5
1/SE_IV
−0.5
0.0
0.5
1.0
β_IV

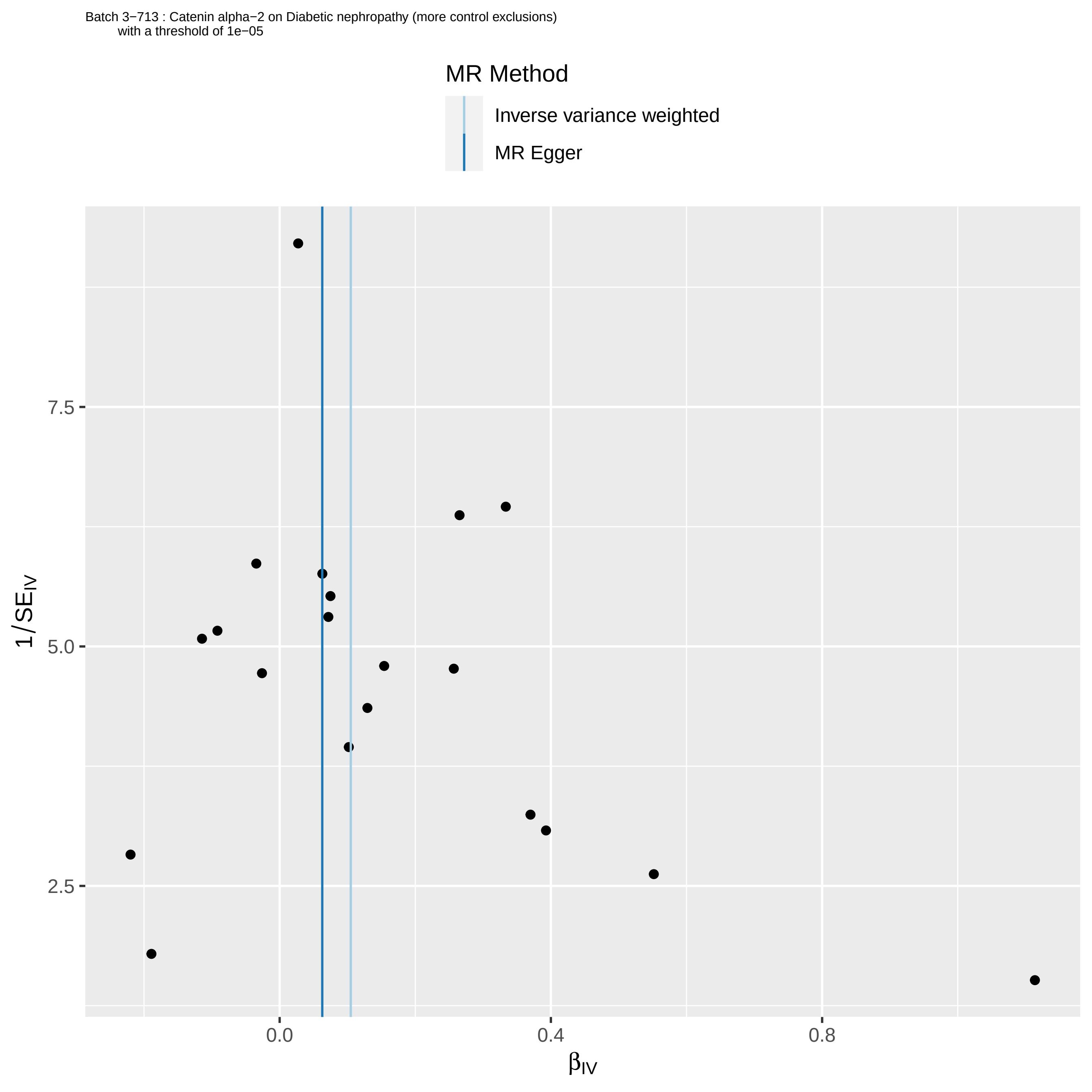
Batch 3−713 : Catenin alpha−2 on Diabetic nephropathy (more control exclusions)
with a threshold of 1e−05
MR Method
Inverse variance weighted
MR Egger
7.5
5.0
2.5
1/SE_IV
0.0
0.4
0.8
β_IV

Batch 3−714 : Chymotrypsinogen B on Diabetic nephropathy (more control exclusions)
with a threshold of 1e−05

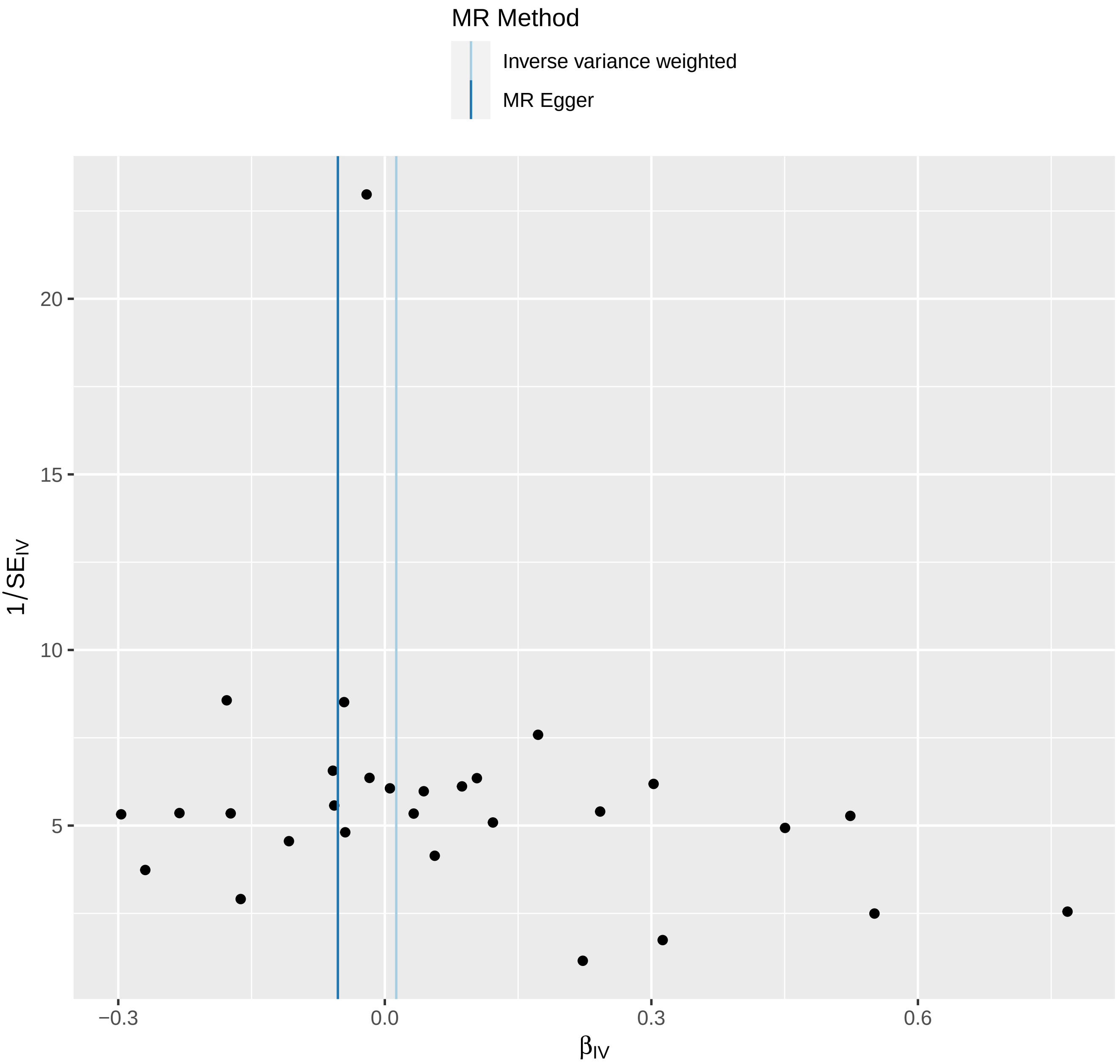

Batch 3−715 : Chymotrypsin−C on Diabetic nephropathy (more control exclusions)
with a threshold of 1e−05

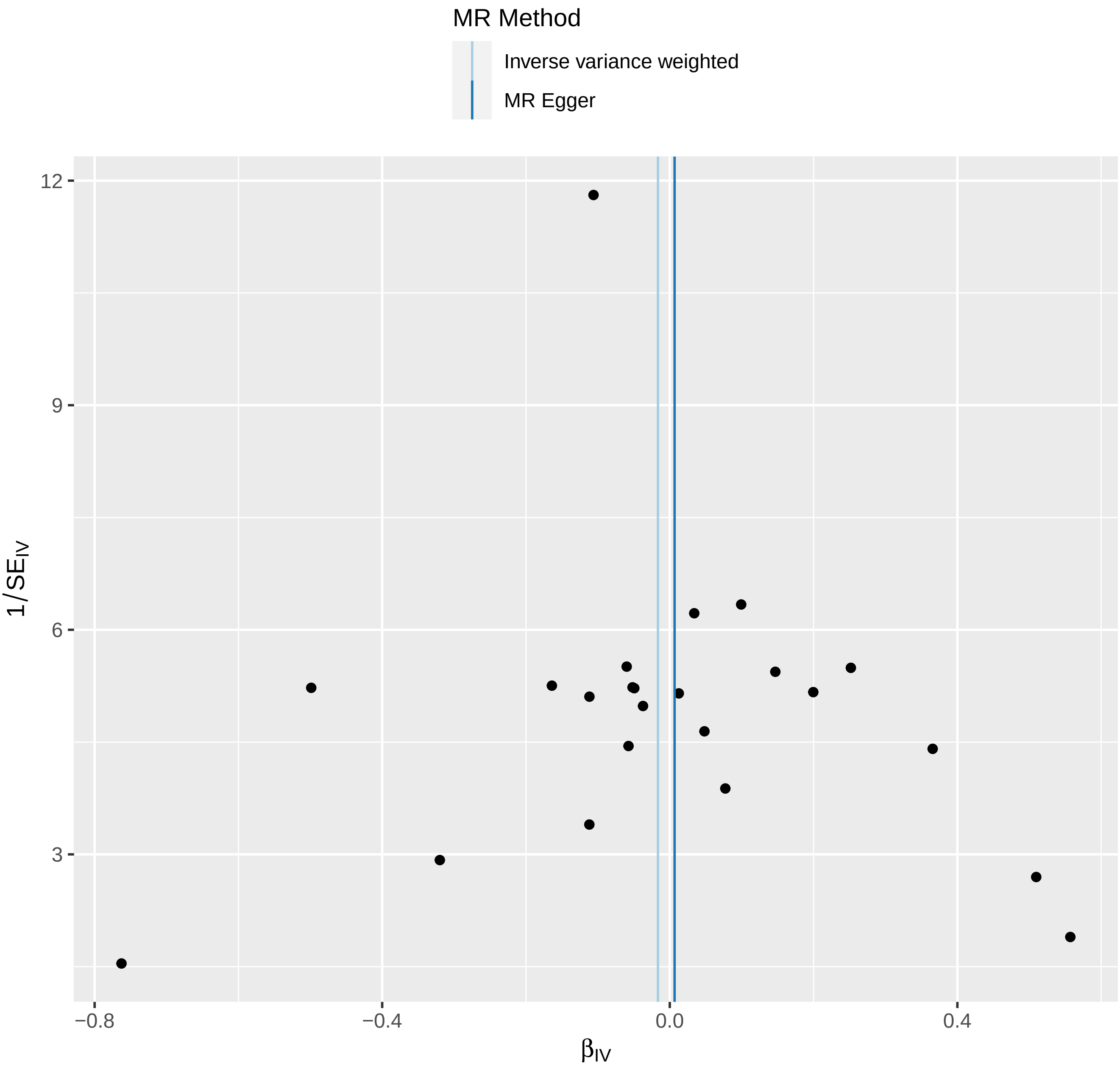

Batch 3−716 : Chymotrypsin−like protease CTRL−1 on Diabetic nephropathy (more control exclusions)
with a threshold of 1e−05

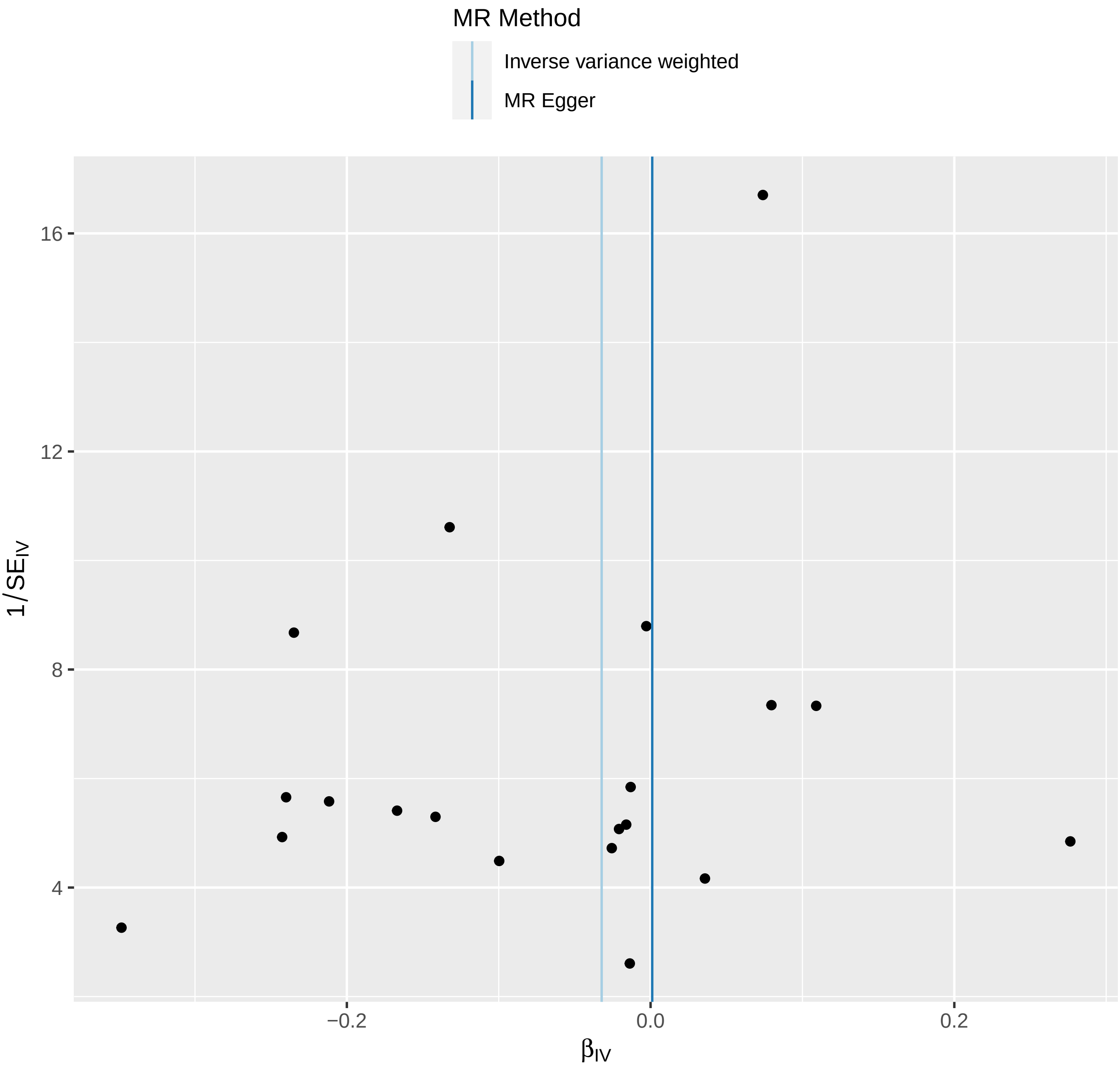

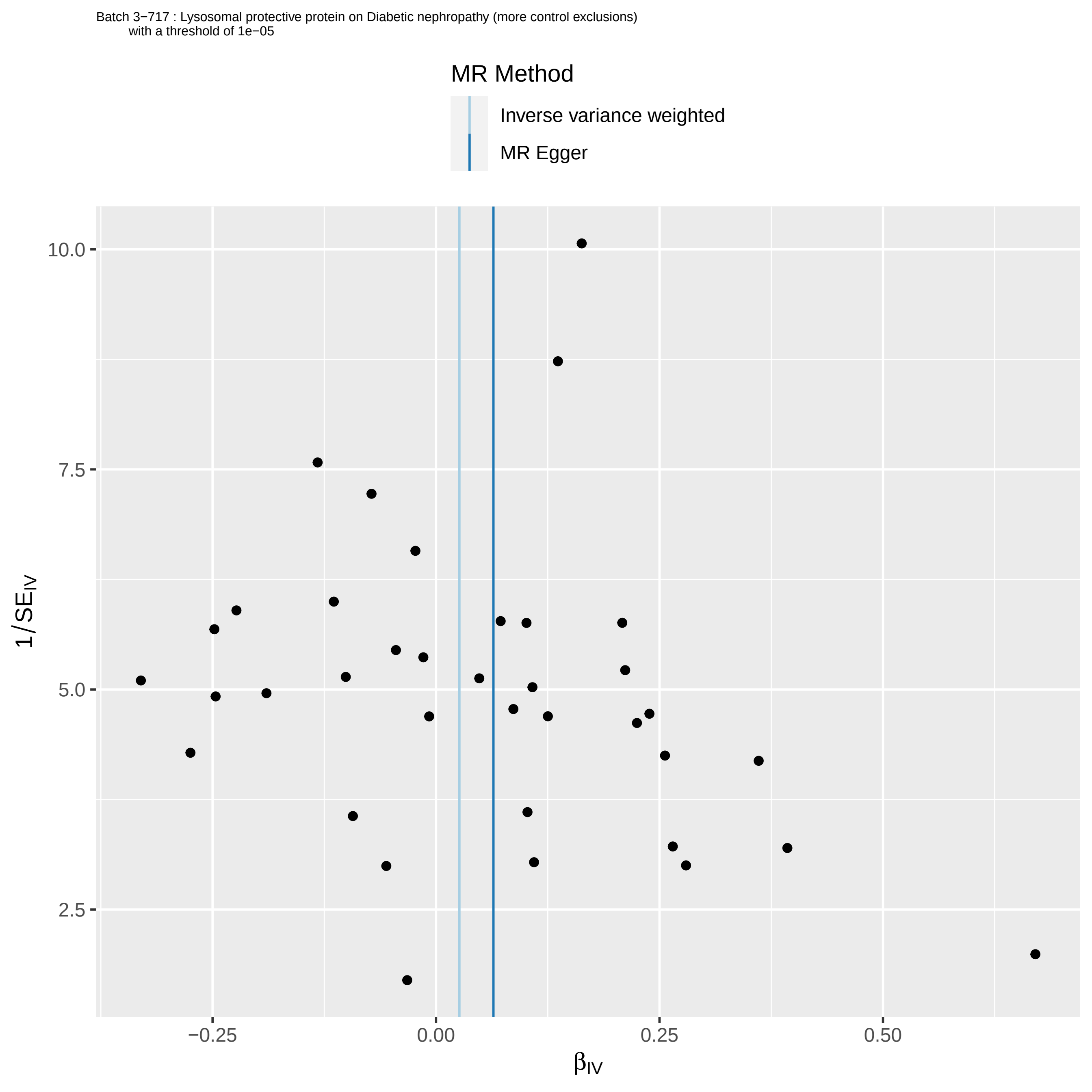

Batch 3−717 : Lysosomal protective protein on Diabetic nephropathy (more control exclusions)
with a threshold of 1e−05
MR Method
Inverse variance weighted
MR Egger
10.0
7.5
5.0
2.5
1/SE_IV
−0.25
0.00
0.25
0.50
β_IV

Batch 3−718 : Cathepsin B on Diabetic nephropathy (more control exclusions)
with a threshold of 1e−05

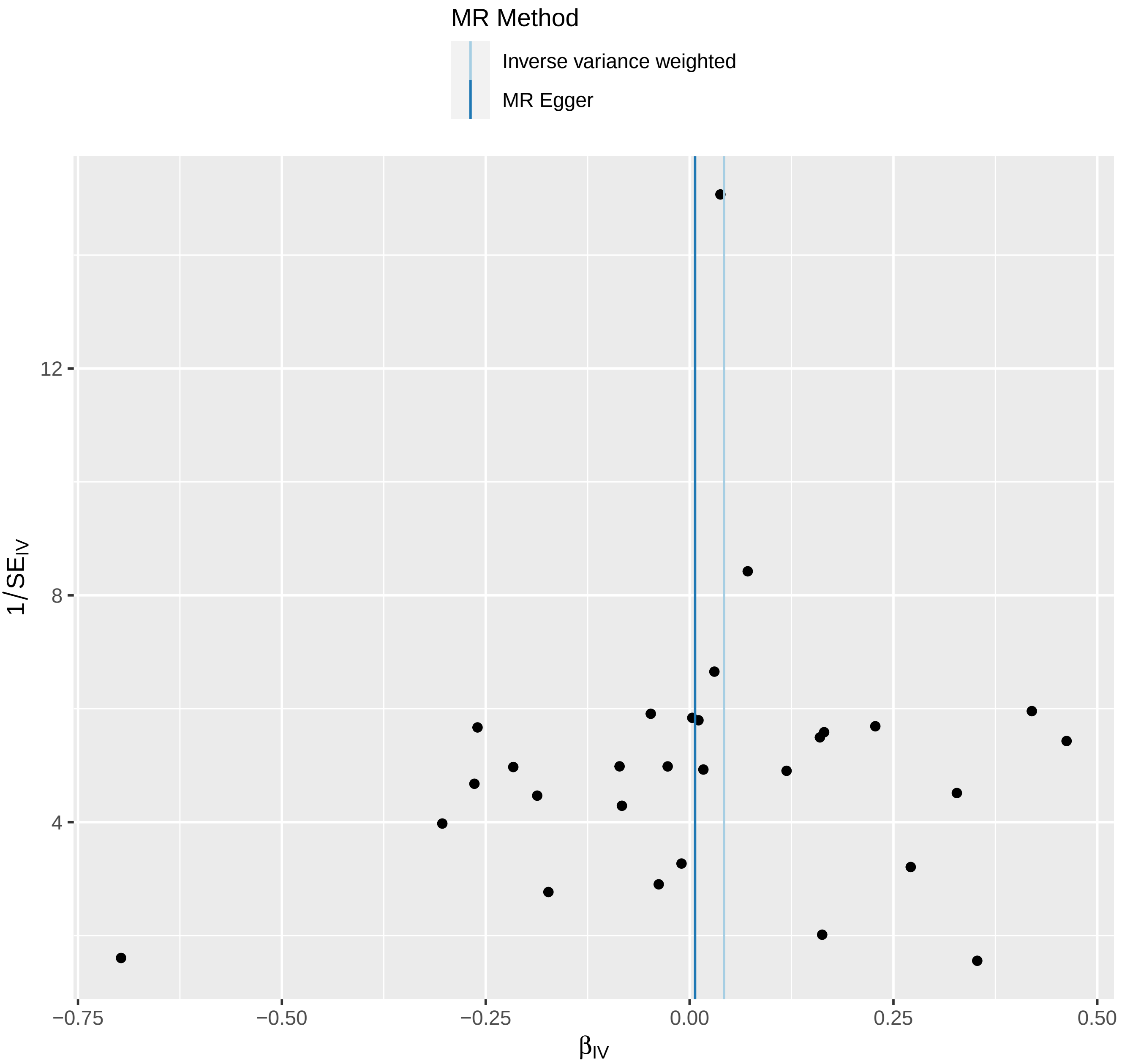

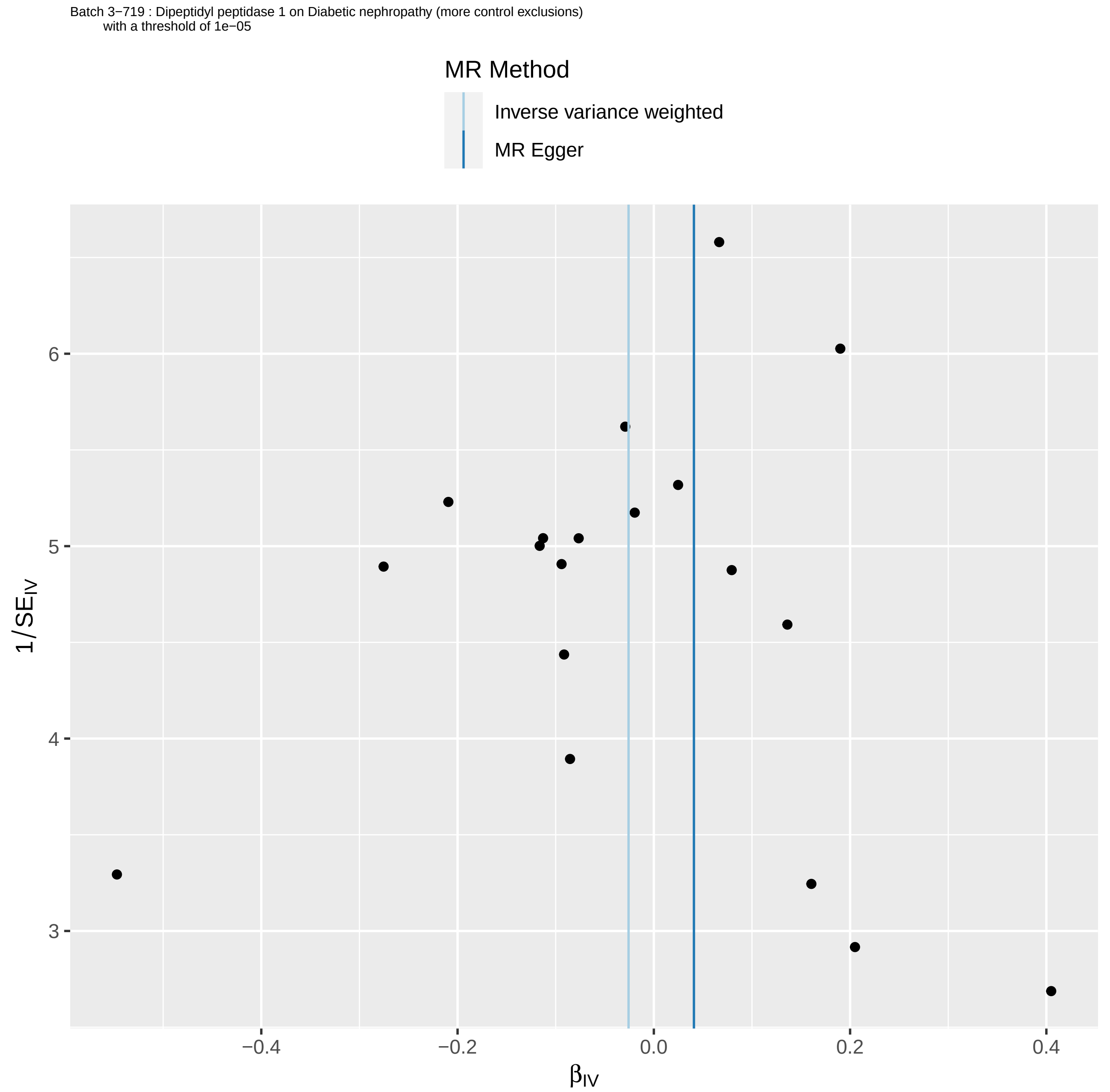
Batch 3−719 : Dipeptidyl peptidase 1 on Diabetic nephropathy (more control exclusions)
with a threshold of 1e−05
MR Method
Inverse variance weighted
MR Egger
6
5
4
3
−0.4
−0.2
0.0
0.2
0.4
1/SE_IV
β_IV

Batch 3−720 : Cathepsin E on Diabetic nephropathy (more control exclusions)
with a threshold of 1e−05

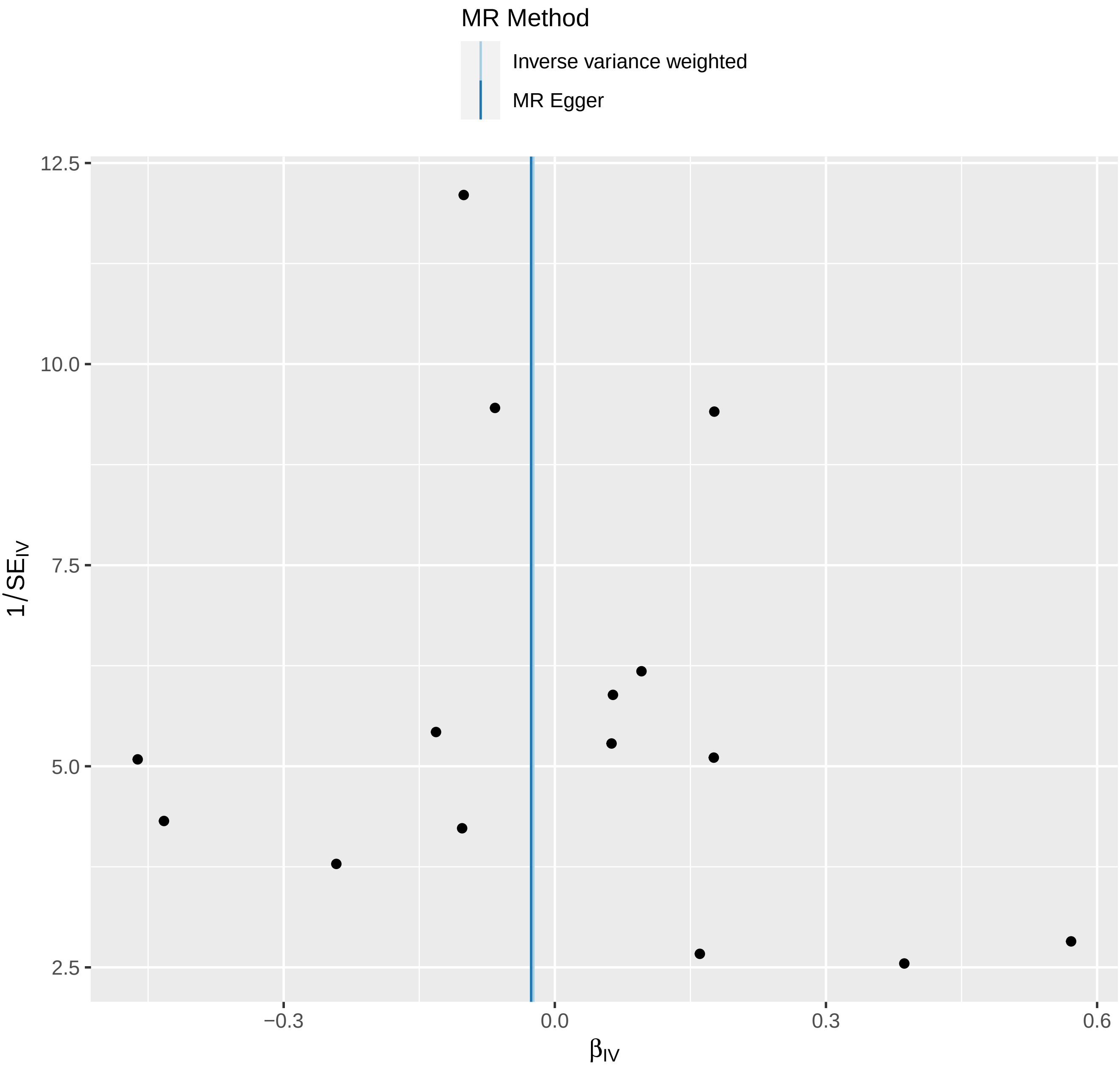

Batch 3−721 : Cathepsin F on Diabetic nephropathy (more control exclusions)
with a threshold of 1e−05

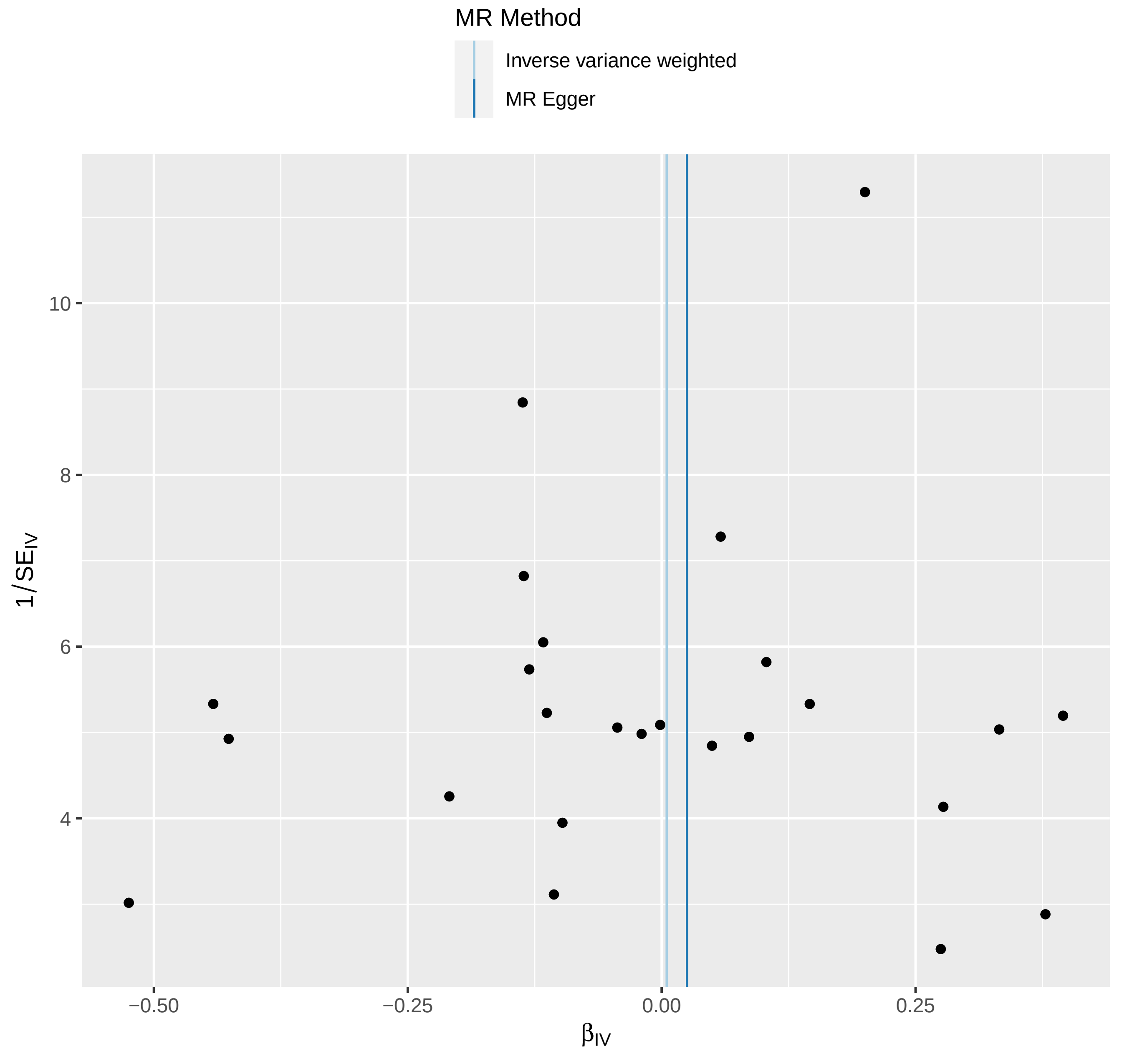

Batch 3−722 : Cathepsin F on Diabetic nephropathy (more control exclusions)
with a threshold of 1e−05

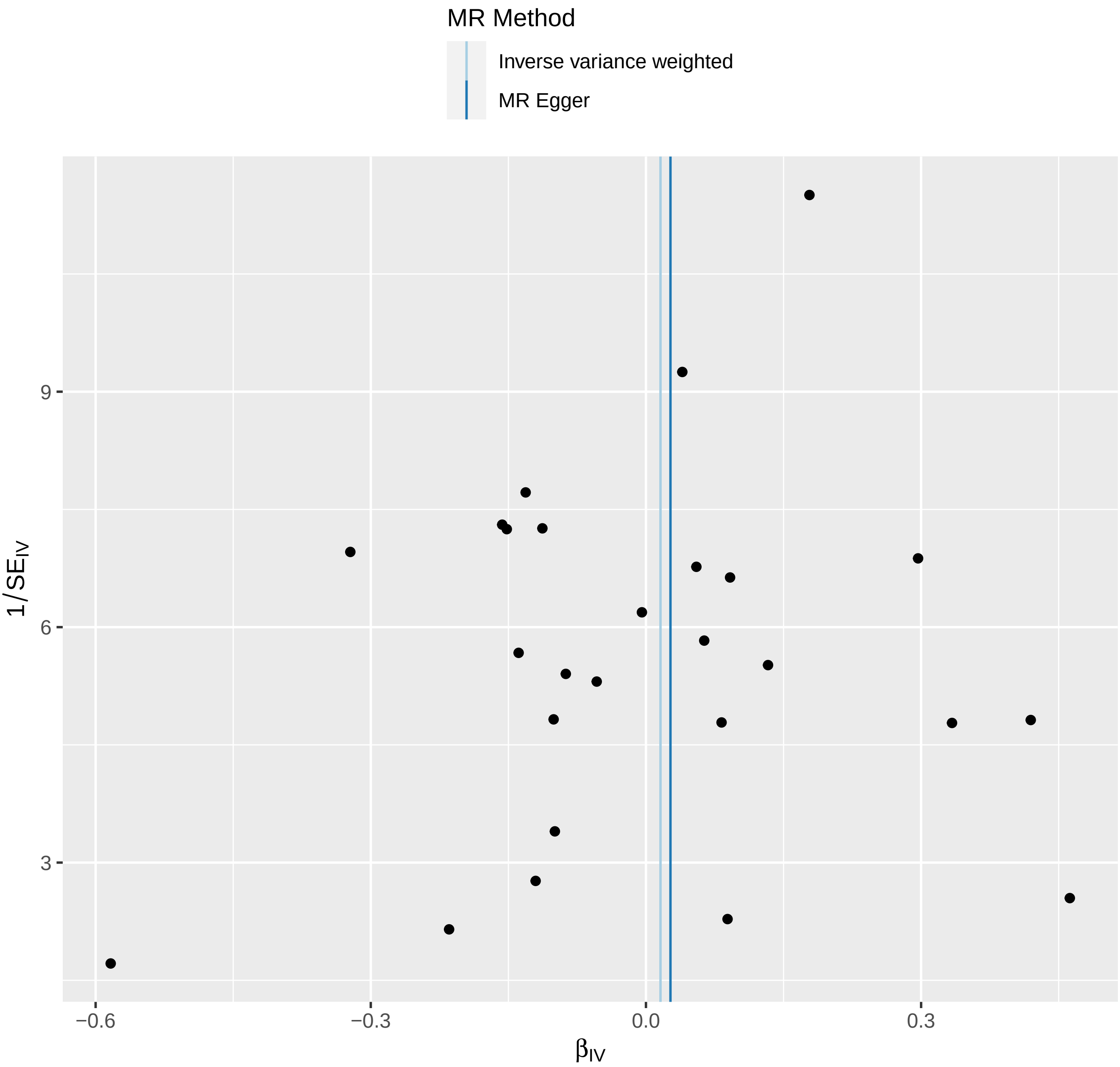

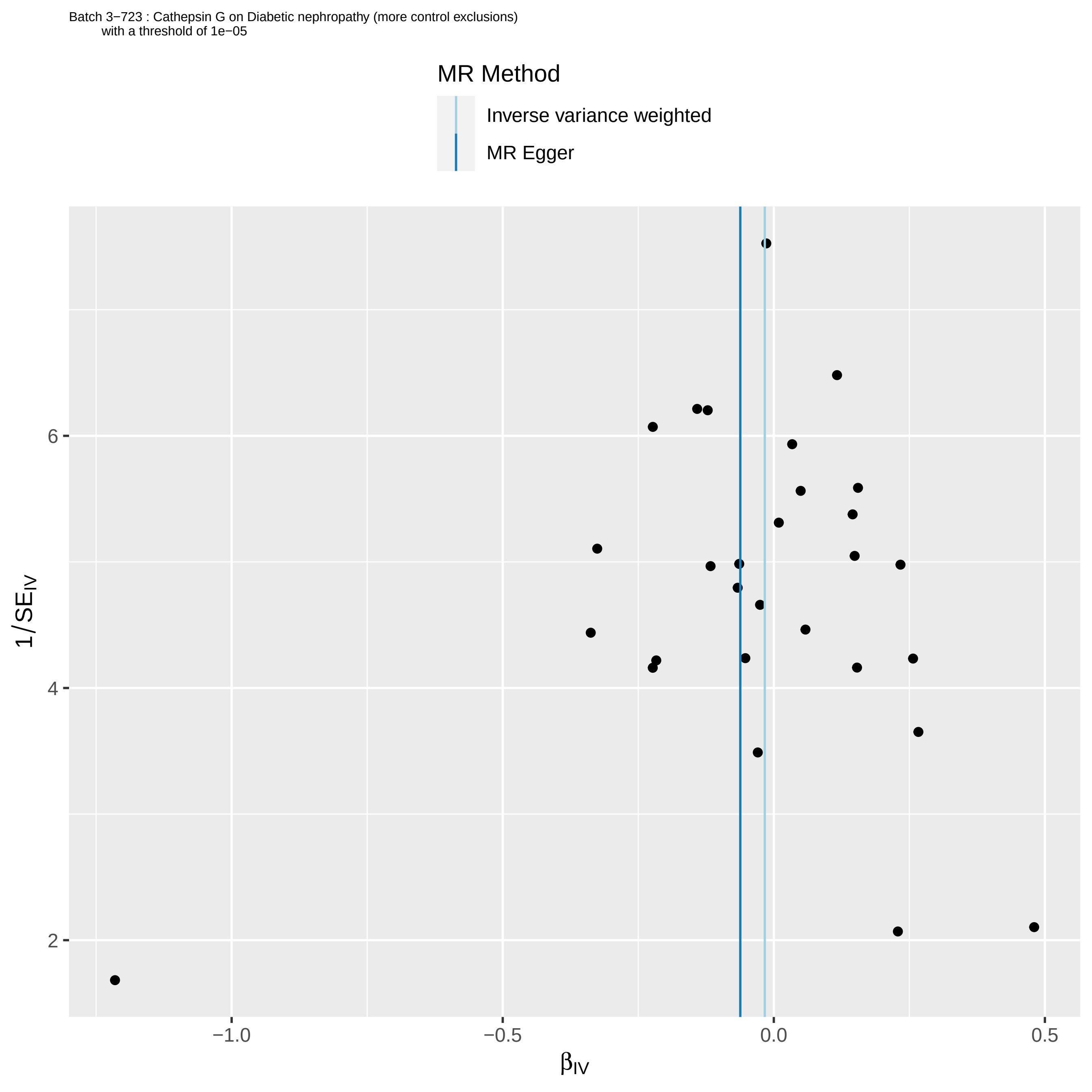
Batch 3−723 : Cathepsin G on Diabetic nephropathy (more control exclusions)
with a threshold of 1e−05
MR Method
Inverse variance weighted
MR Egger
1/SE$_{IV}$
6
4
2
−1.0
−0.5
0.0
0.5
β_{IV}

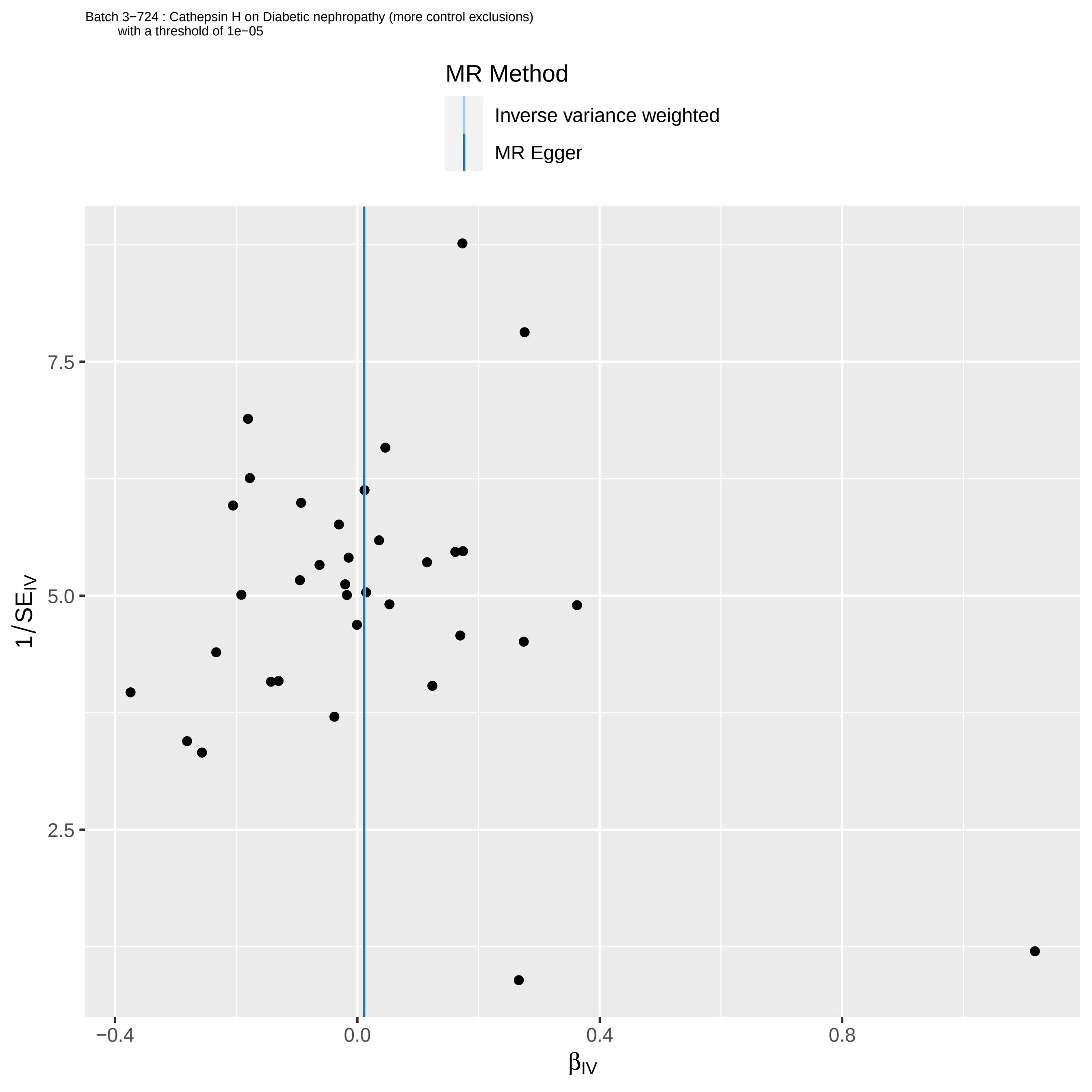

Batch 3−724 : Cathepsin H on Diabetic nephropathy (more control exclusions)
with a threshold of 1e−05
MR Method
Inverse variance weighted
MR Egger
7.5
5.0
2.5
1/SE_IV
−0.4
0.0
0.4
0.8
β_IV

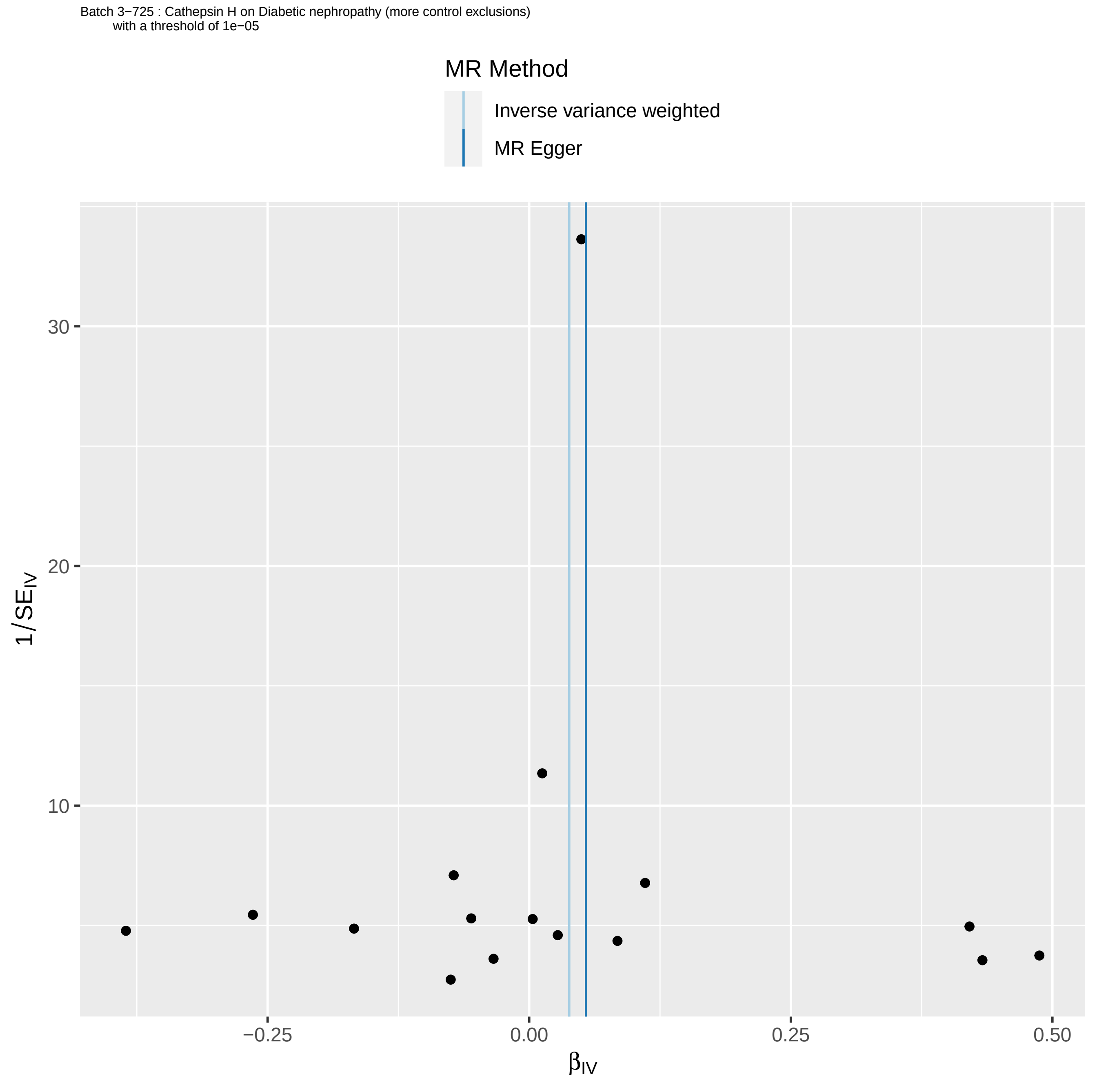

Batch 3−725 : Cathepsin H on Diabetic nephropathy (more control exclusions)
with a threshold of 1e−05
MR Method
Inverse variance weighted
MR Egger
30
20
10
1/SE_IV
−0.25
0.00
0.25
0.50
β_IV

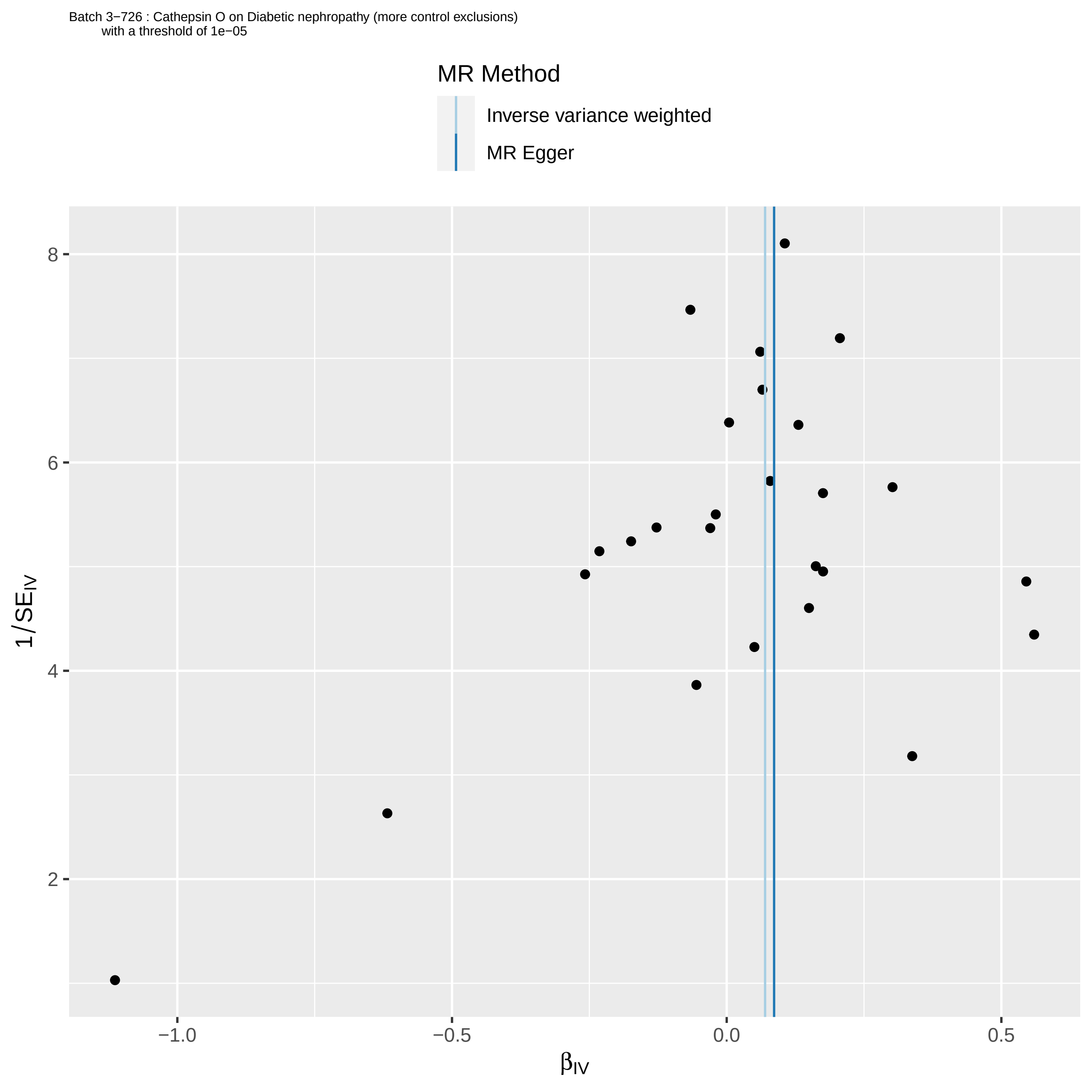
Batch 3−726 : Cathepsin O on Diabetic nephropathy (more control exclusions)
with a threshold of 1e−05
MR Method
Inverse variance weighted
MR Egger
8
6
4
2
$1/SE_{IV}$
−1.0
−0.5
0.0
0.5
β_{IV}

Batch 3−727 : Cathepsin S on Diabetic nephropathy (more control exclusions)
with a threshold of 1e−05

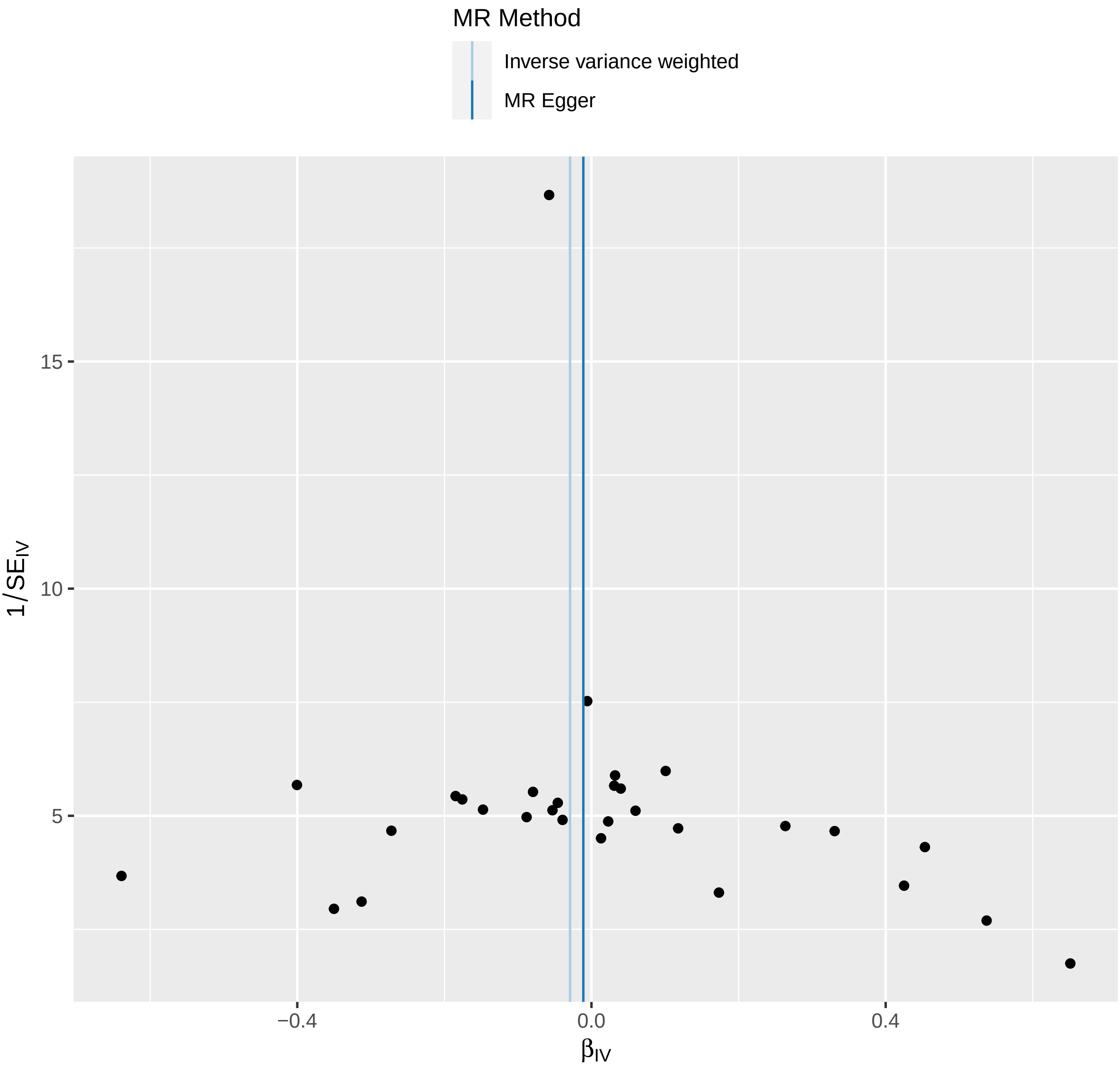

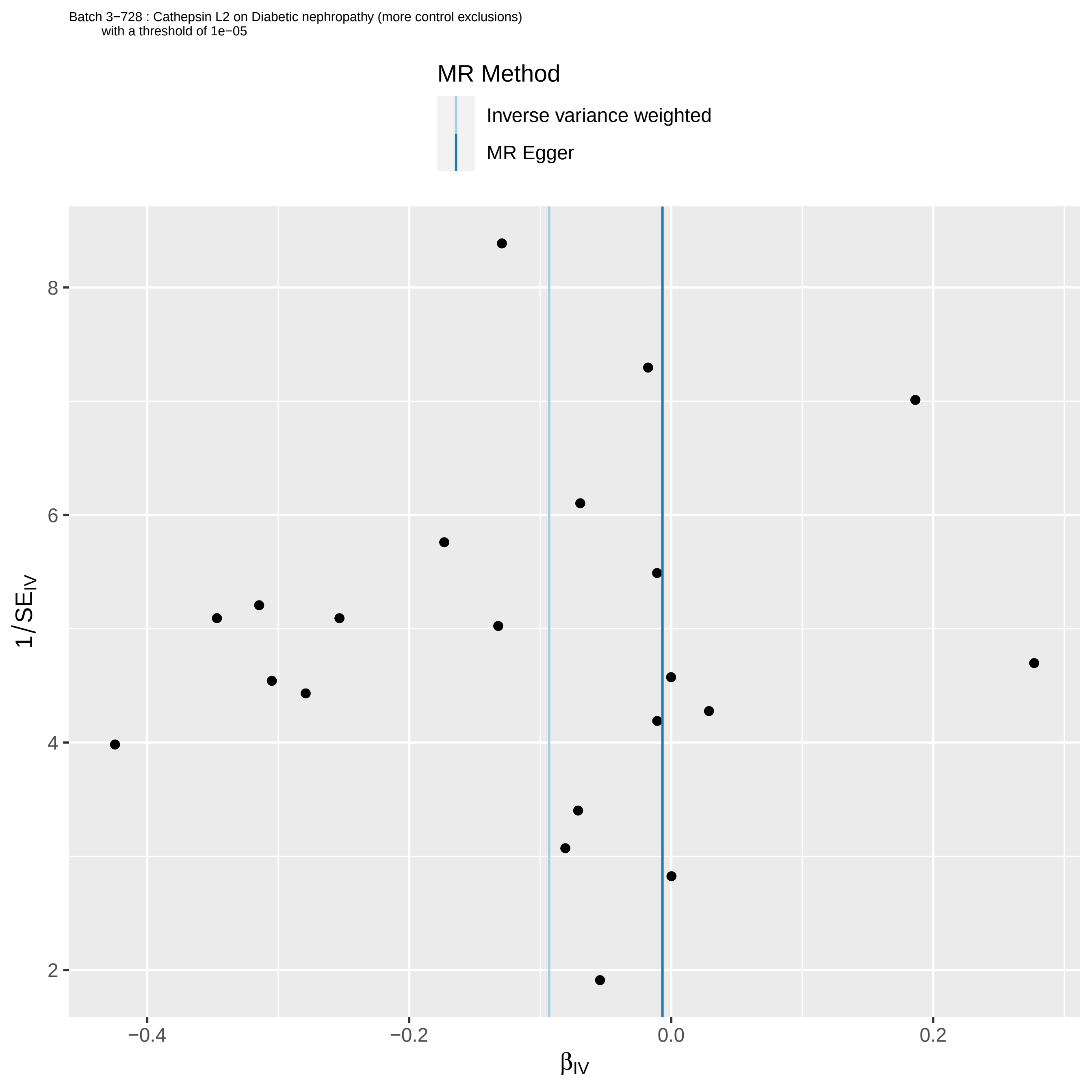

Batch 3−728 : Cathepsin L2 on Diabetic nephropathy (more control exclusions)
with a threshold of 1e−05
MR Method
Inverse variance weighted
MR Egger
8
6
4
2
−0.4
−0.2
0.0
0.2
$1/SE_{IV}$
β_{IV}

Batch 3−729 : Cathepsin Z on Diabetic nephropathy (more control exclusions)
with a threshold of 1e−05

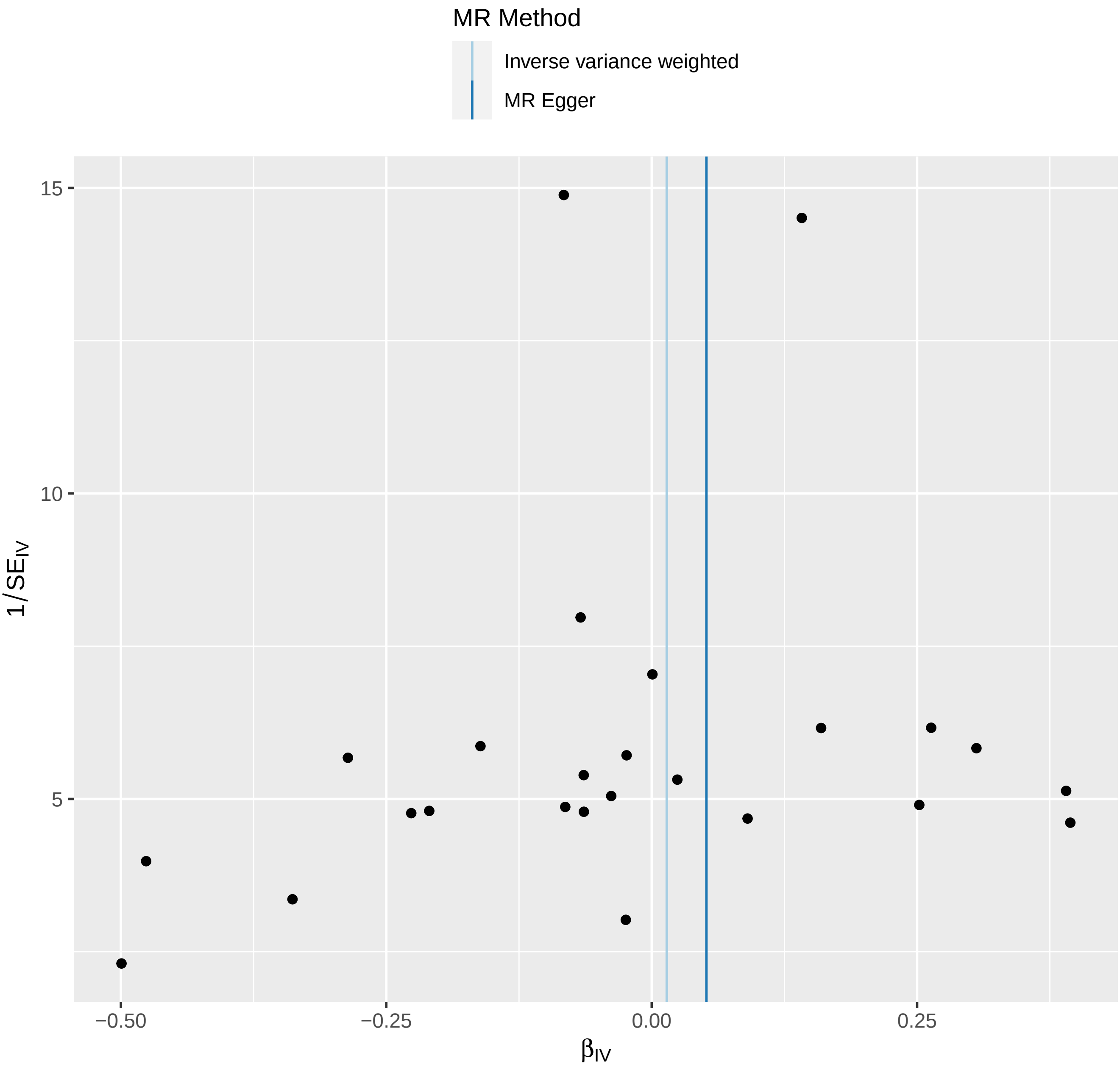

Batch 3−730 : Cortexin−3 on Diabetic nephropathy (more control exclusions)
with a threshold of 1e−05

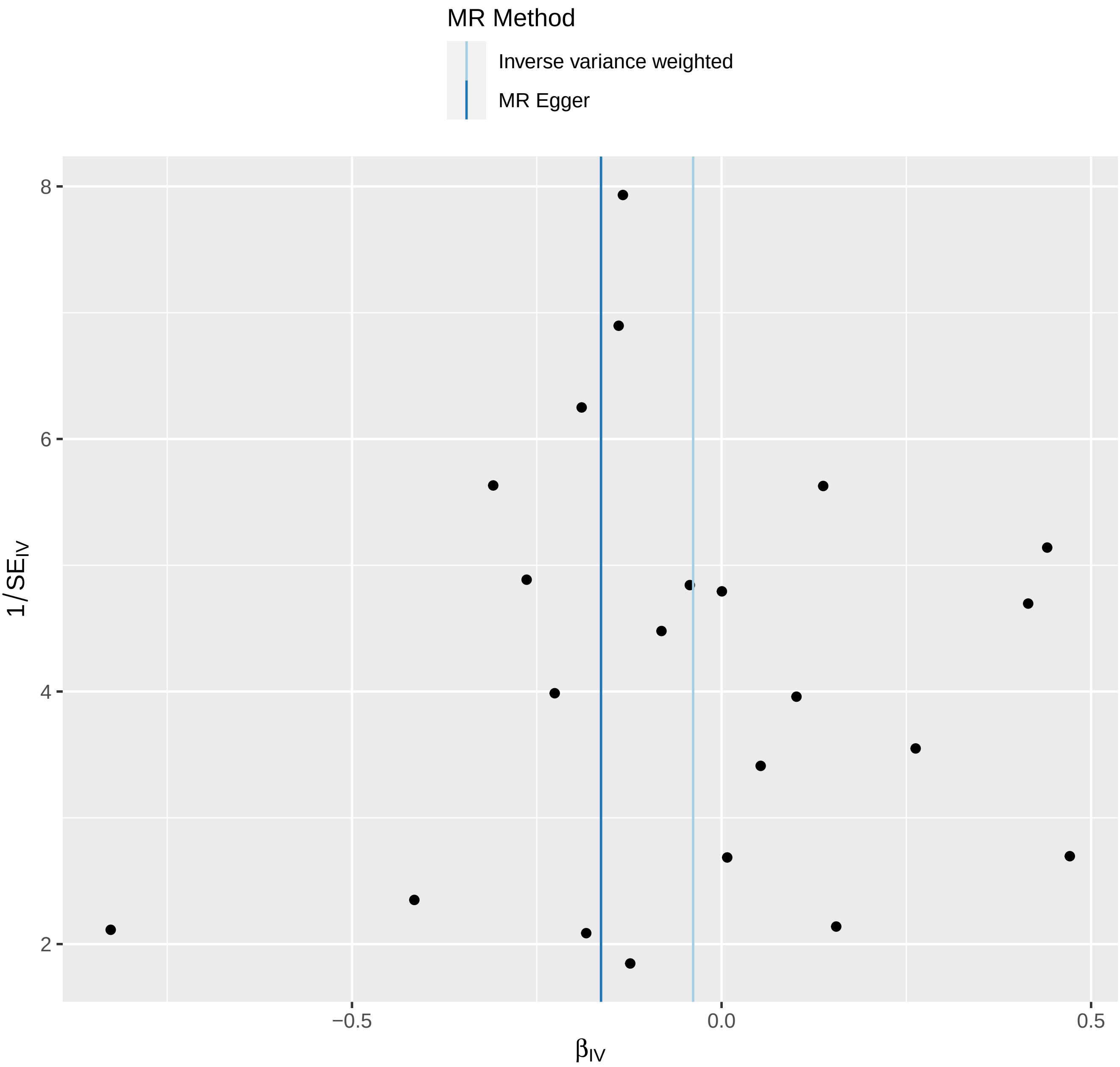

Batch 3−731 : cubilin on Diabetic nephropathy (more control exclusions)
with a threshold of 1e−05

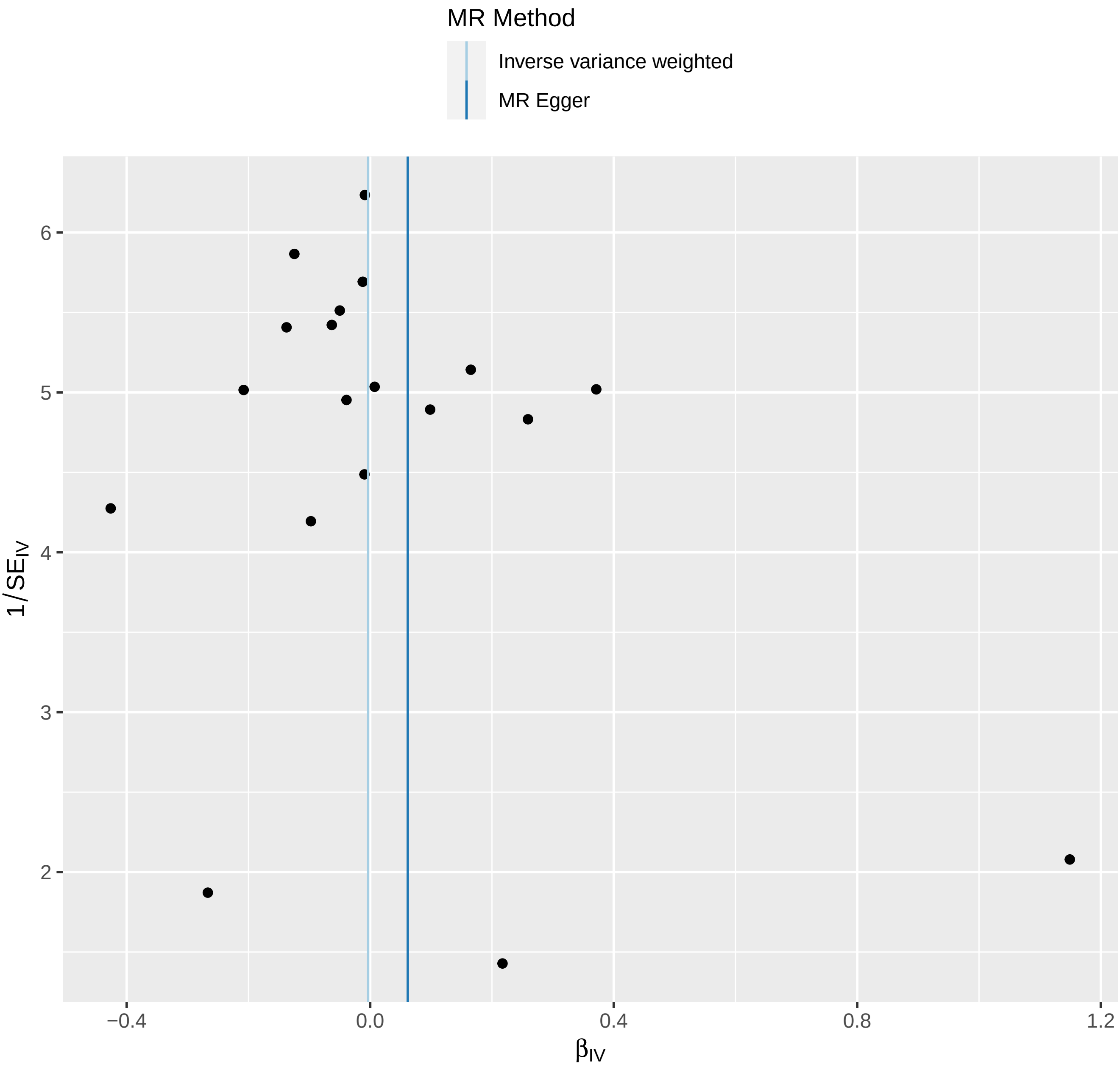

Batch 3−732 : Cullin−3 on Diabetic nephropathy (more control exclusions)
with a threshold of 1e−05

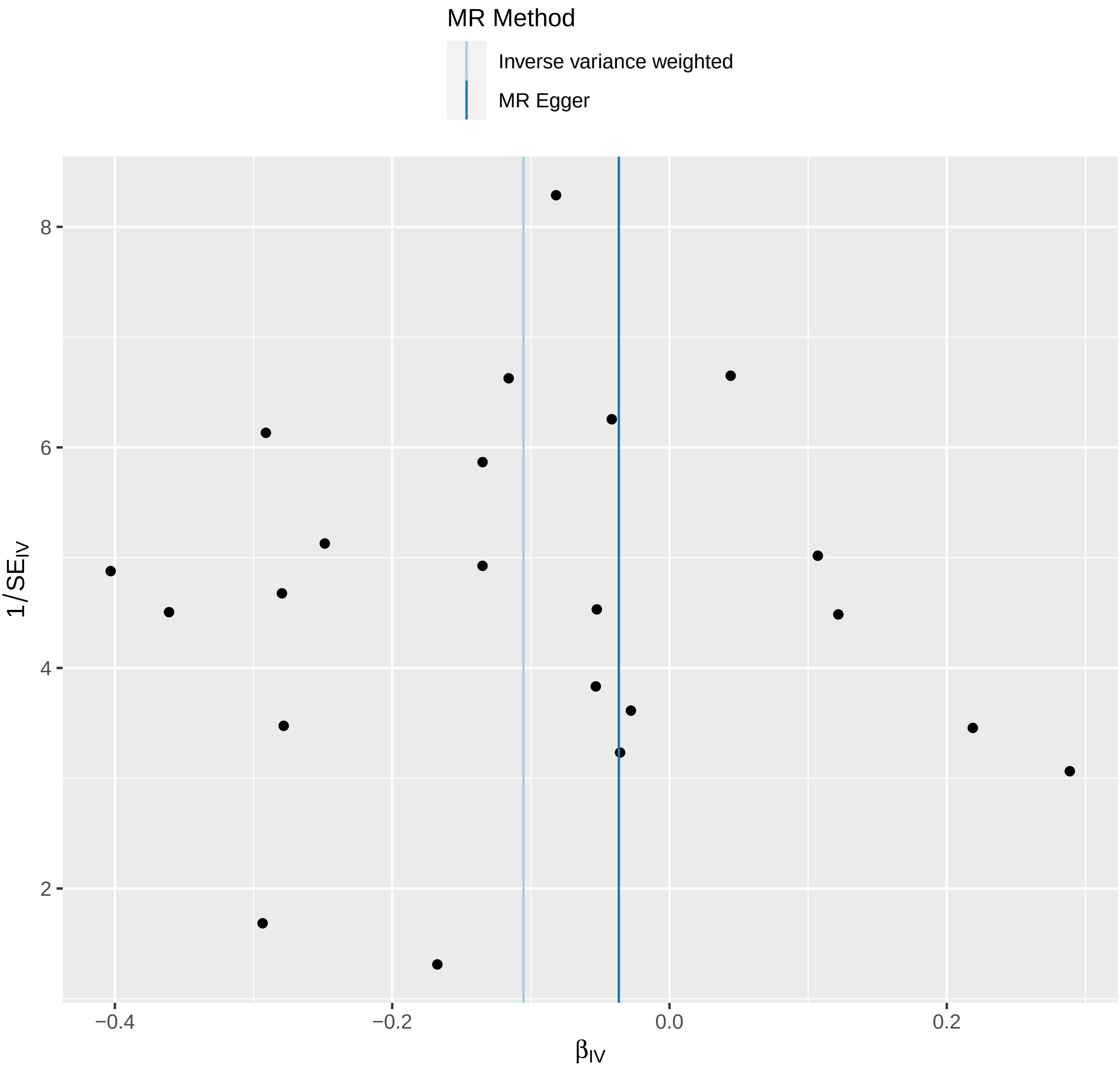

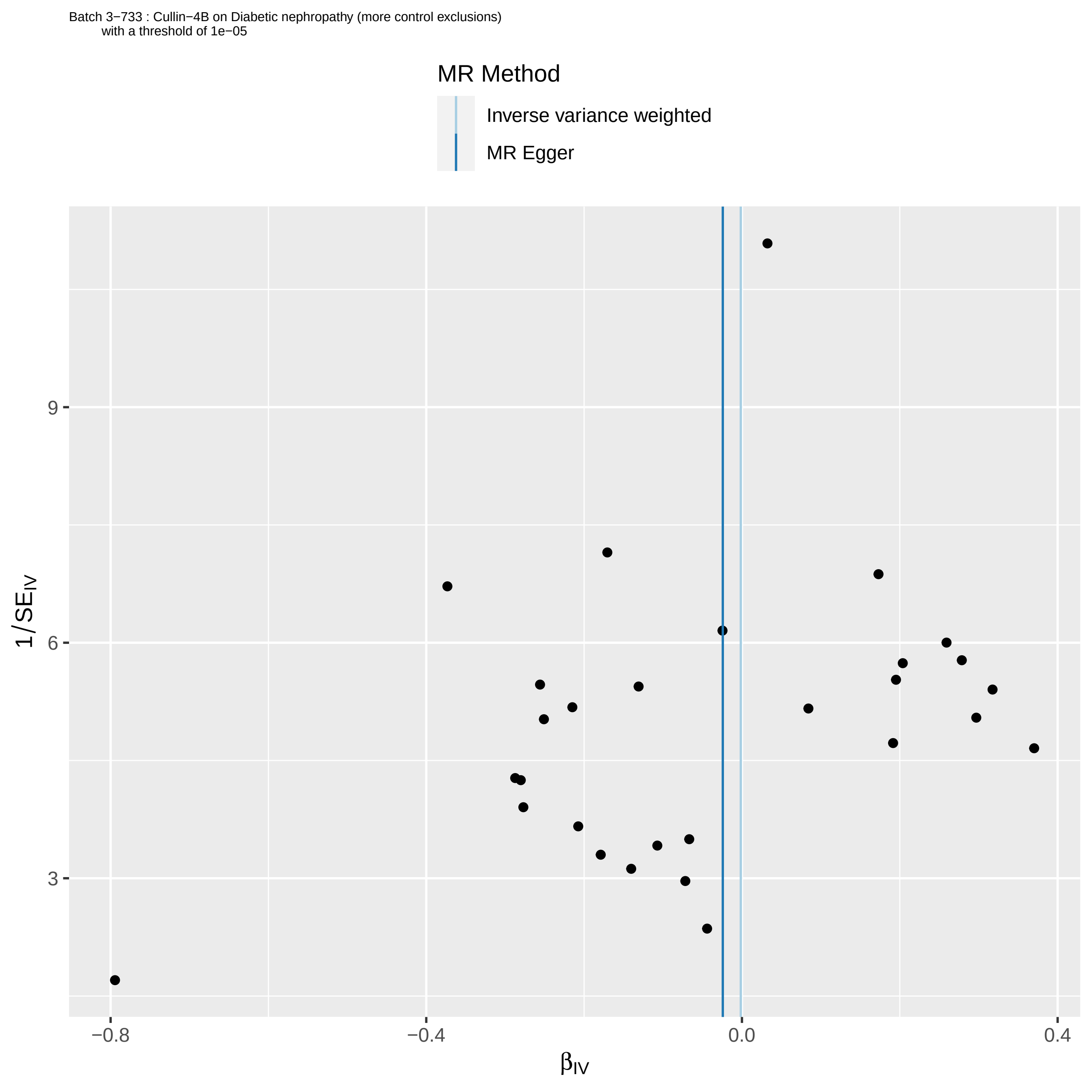

Batch 3−733 : Cullin−4B on Diabetic nephropathy (more control exclusions)
with a threshold of 1e−05
MR Method
Inverse variance weighted
MR Egger
1/SE_IV
9
6
3
−0.8
−0.4
0.0
0.4
β_IV

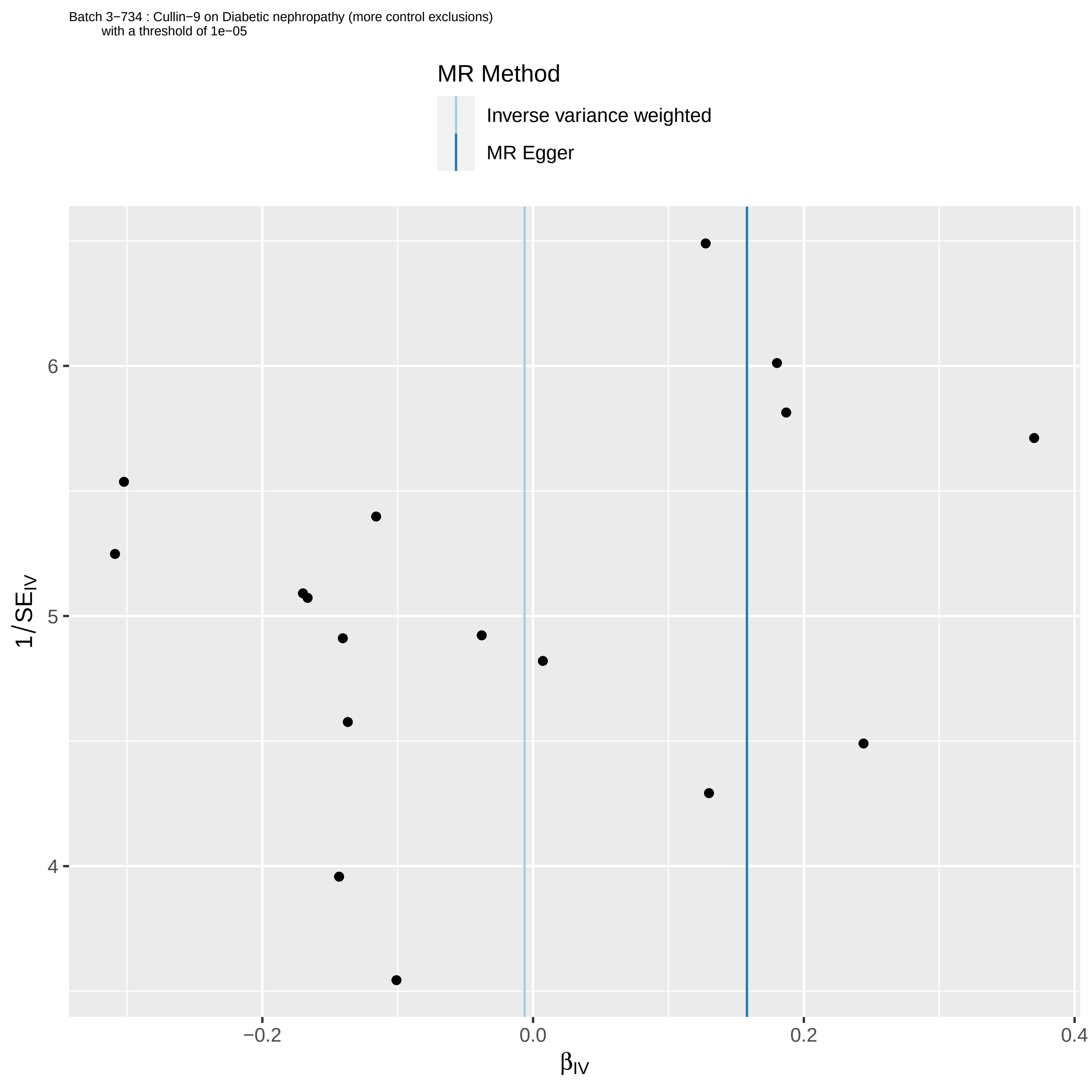
Batch 3−734 : Cullin−9 on Diabetic nephropathy (more control exclusions)
with a threshold of 1e−05
MR Method
Inverse variance weighted
MR Egger
1/SE$_{IV}$
6
5
4
−0.2
0.0
0.2
0.4
β$_{IV}$

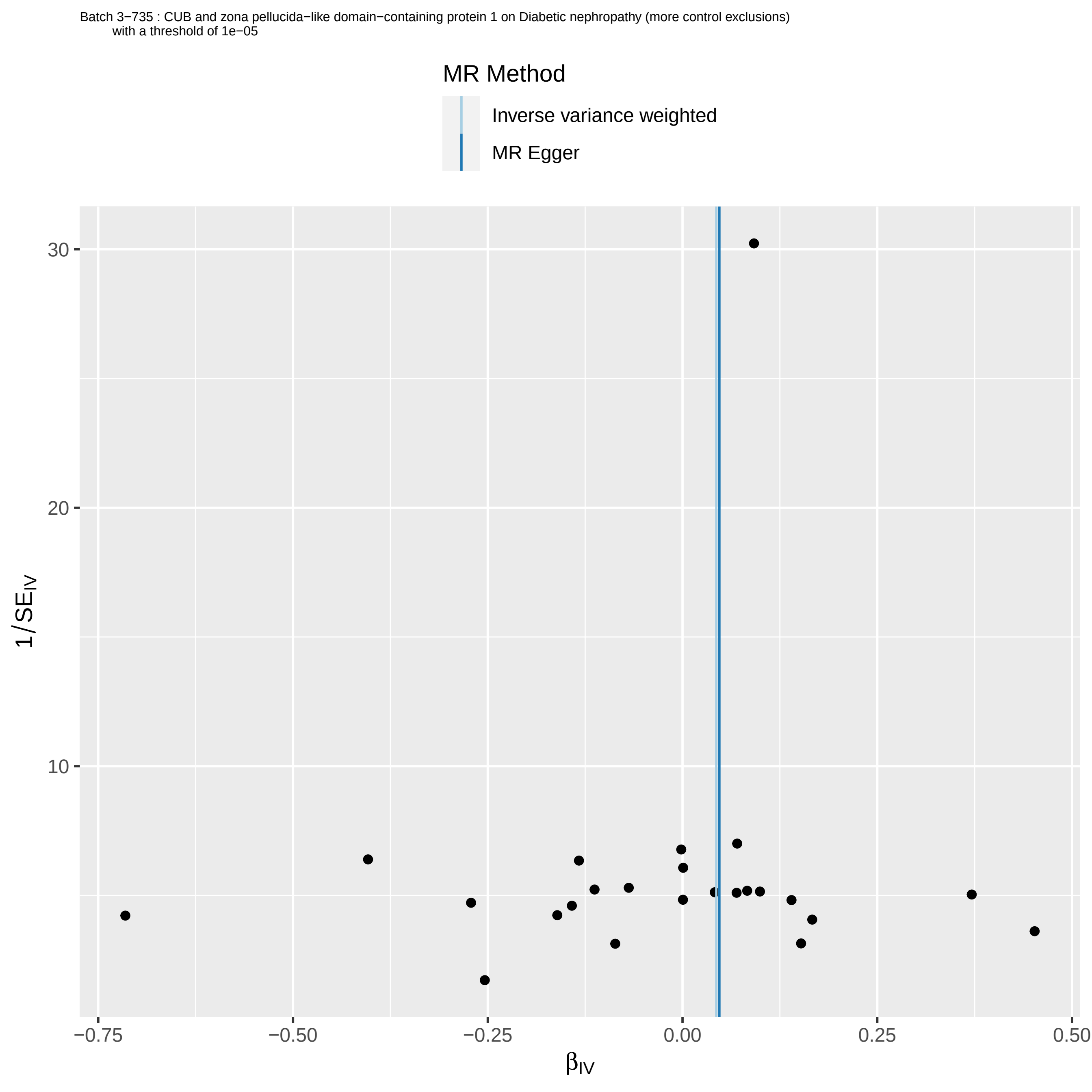
Batch 3−735 : CUB and zona pellucida−like domain−containing protein 1 on Diabetic nephropathy (more control exclusions)
with a threshold of 1e−05
MR Method
Inverse variance weighted
MR Egger
30
20
10
1/SE_IV
−0.75
−0.50
−0.25
0.00
0.25
0.50
β_IV

Batch 3−736 : Fractalkine on Diabetic nephropathy (more control exclusions)
with a threshold of 1e−05

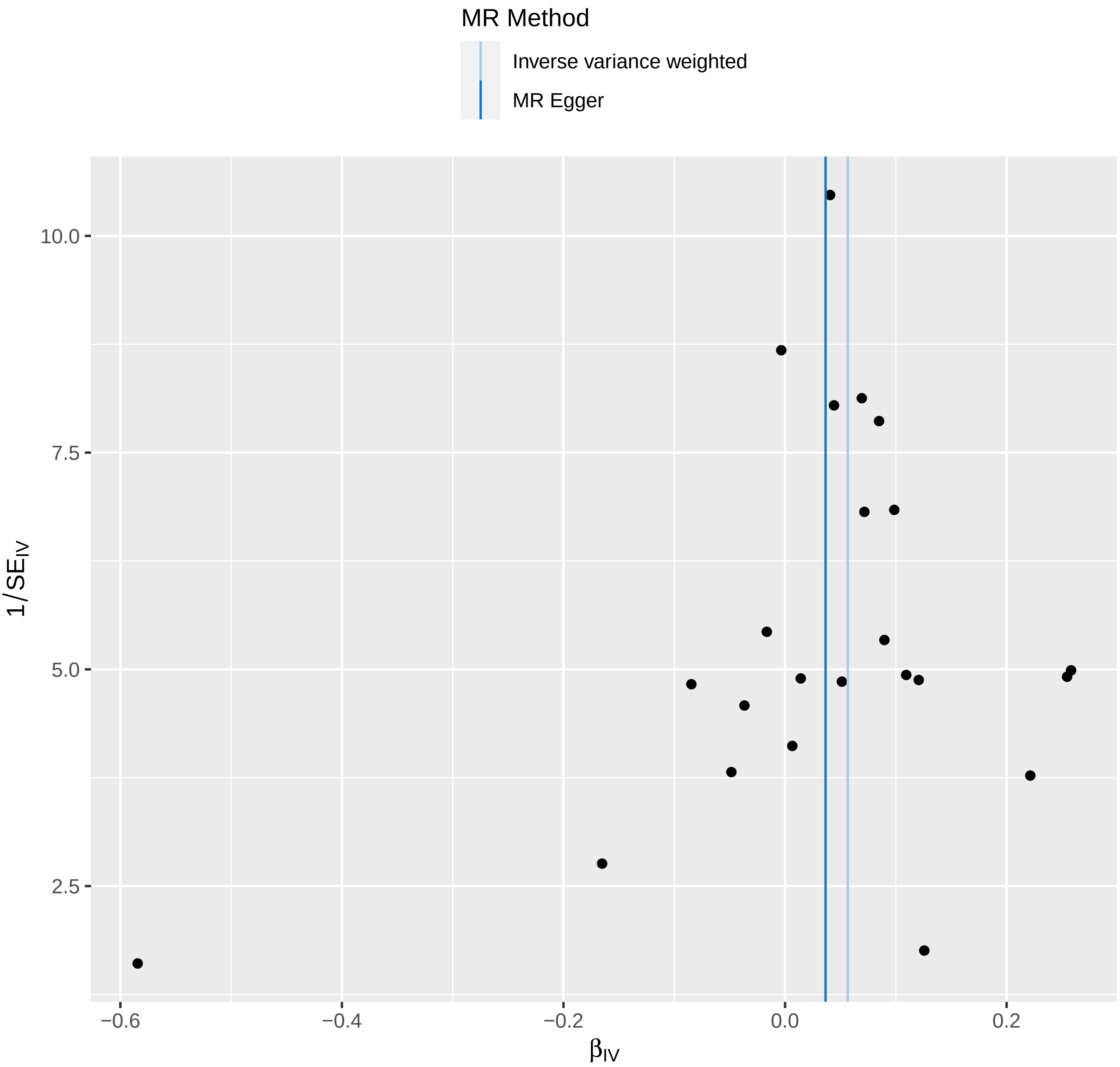

Batch 3−737 : Coxsackievirus and adenovirus receptor on Diabetic nephropathy (more control exclusions)
with a threshold of 1e−05

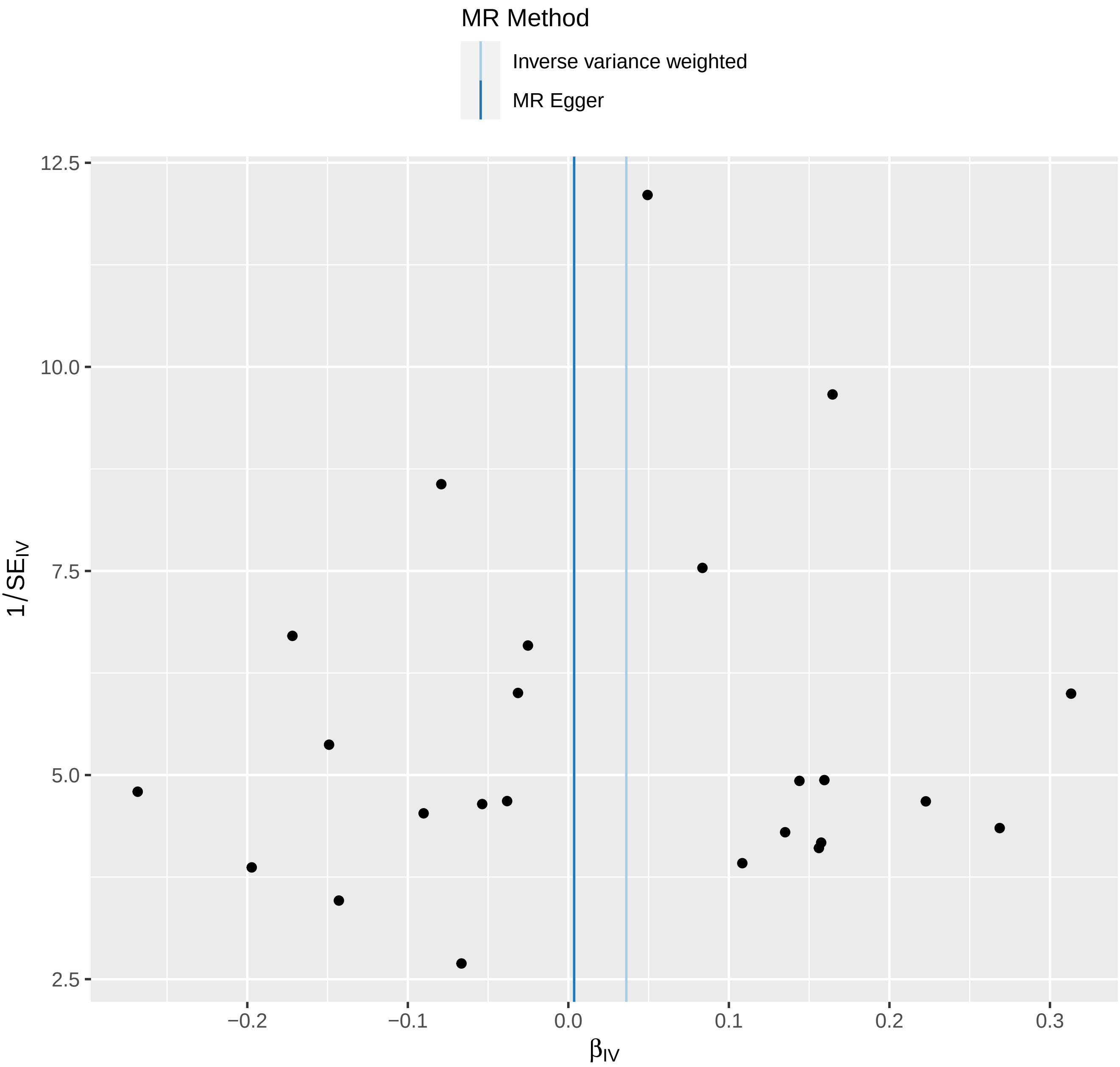

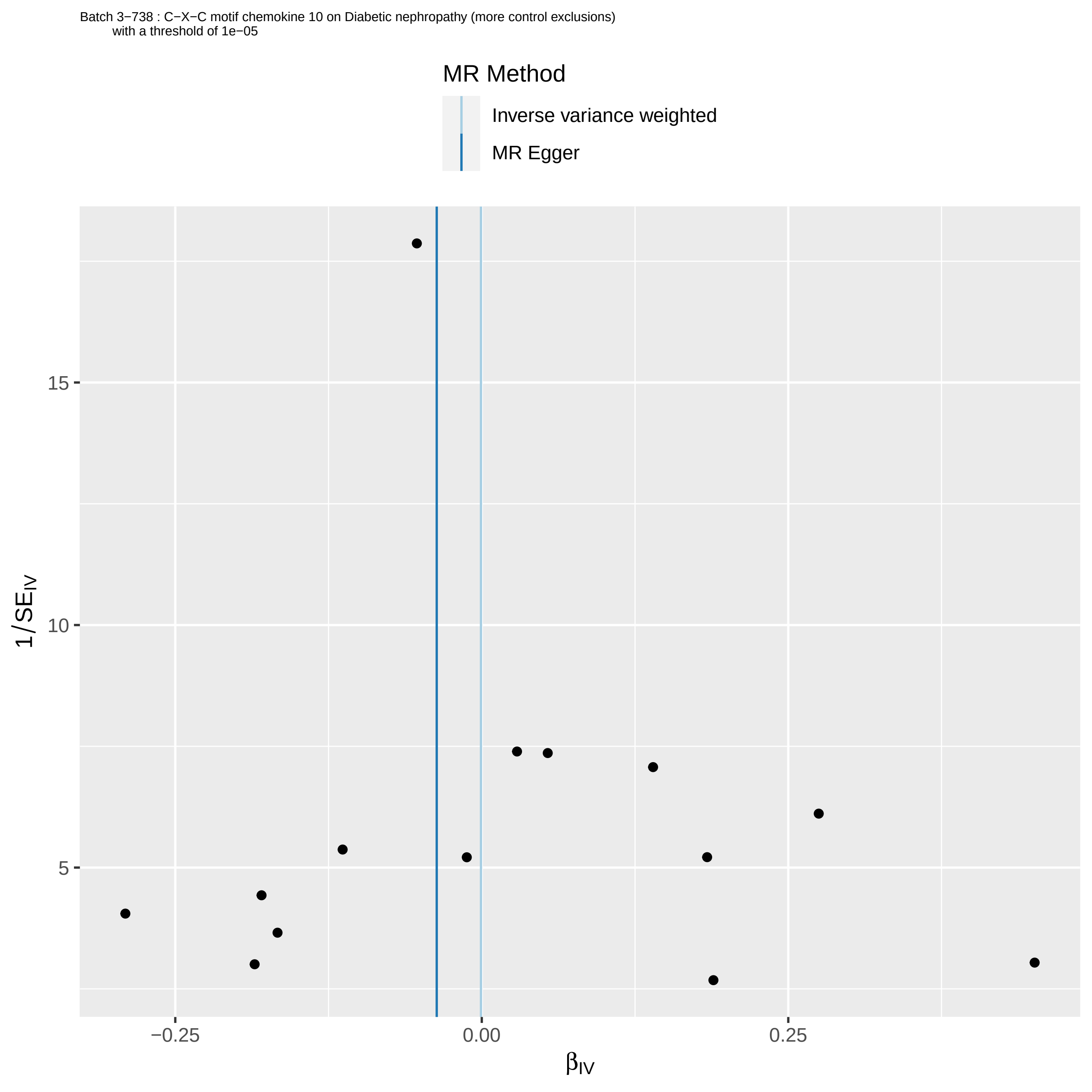
Batch 3−738 : C−X−C motif chemokine 10 on Diabetic nephropathy (more control exclusions)
with a threshold of 1e−05
MR Method
Inverse variance weighted
MR Egger
15
10
5
1/SE_IV
−0.25
0.00
0.25
β_IV

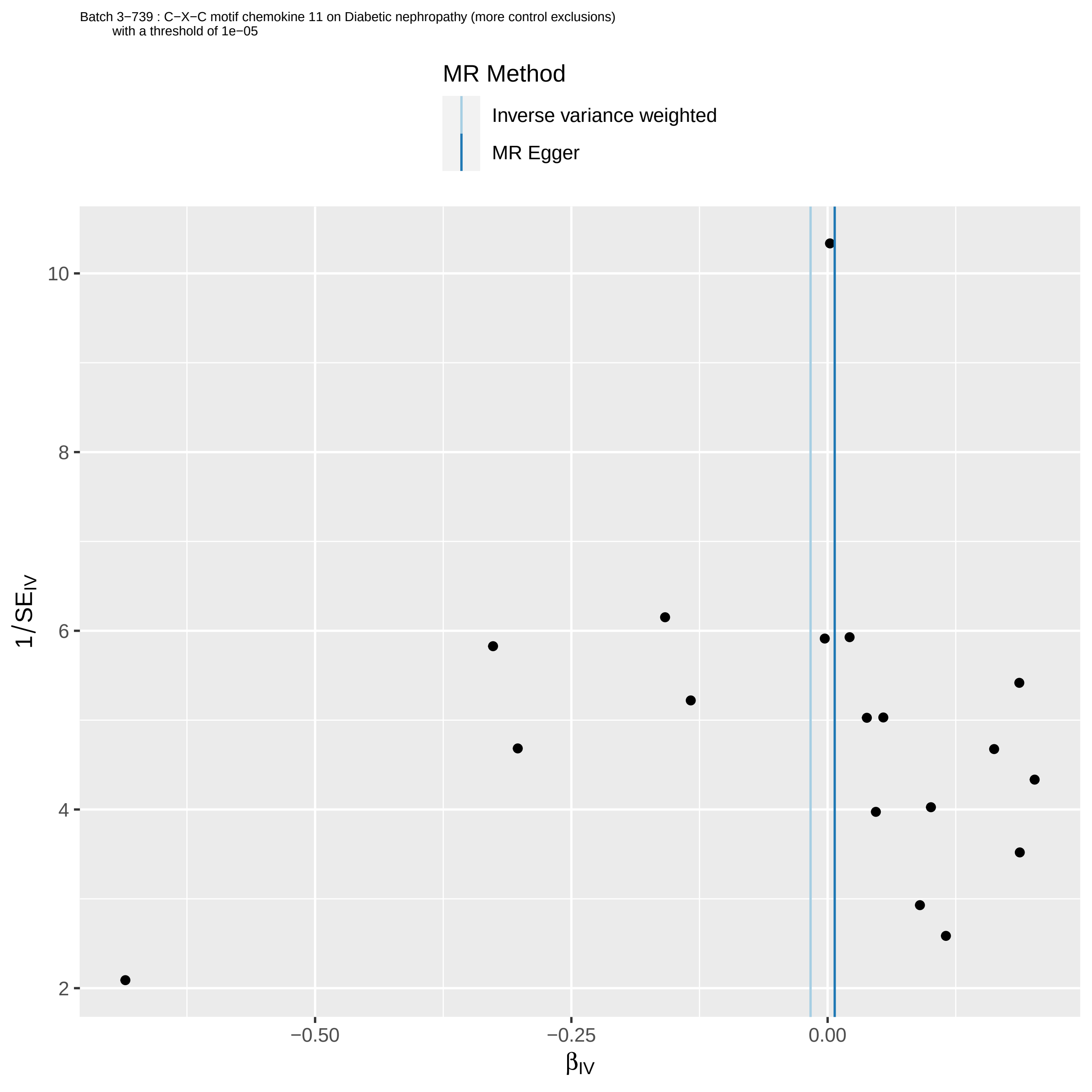
Batch 3−739 : C−X−C motif chemokine 11 on Diabetic nephropathy (more control exclusions)
with a threshold of 1e−05
MR Method
Inverse variance weighted
MR Egger
10
8
6
4
2
1/SE$_{IV}$
−0.50
−0.25
0.00
β$_{IV}$

Batch 3−740 : Stromal cell−derived factor 1 on Diabetic nephropathy (more control exclusions)
with a threshold of 1e−05

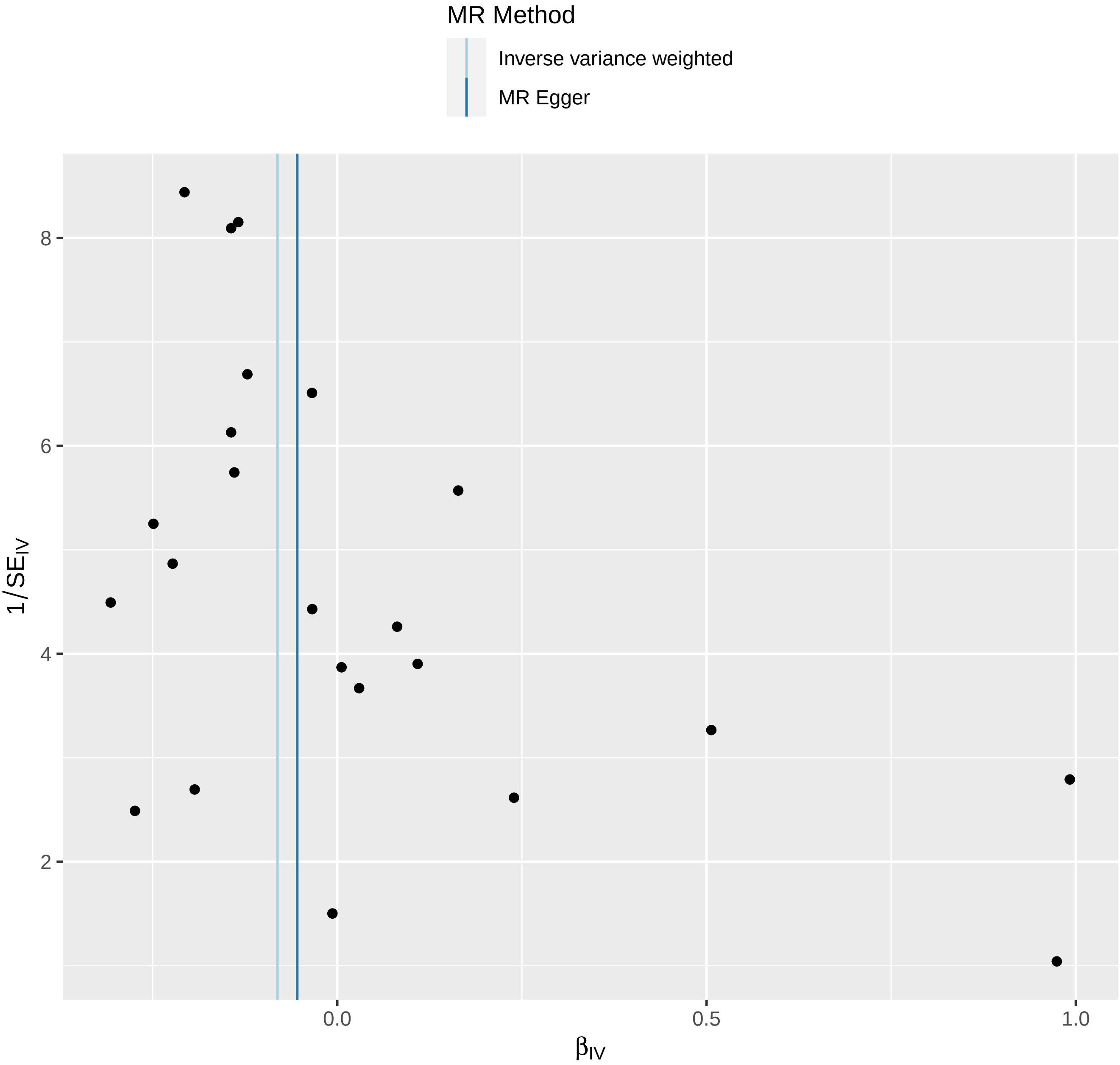

Batch 3−741 : Stromal cell−derived factor 1 on Diabetic nephropathy (more control exclusions)
with a threshold of 1e−05

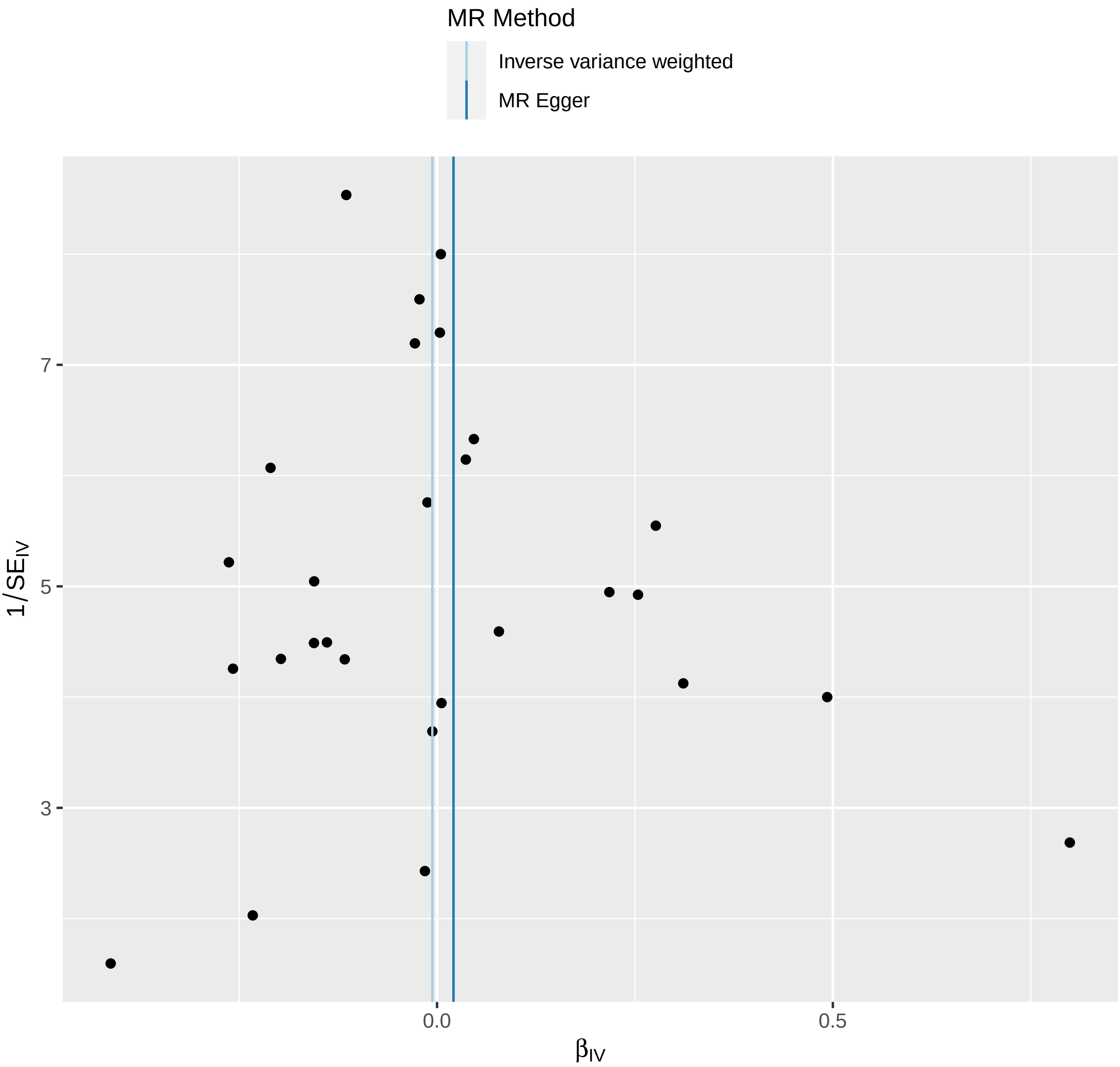

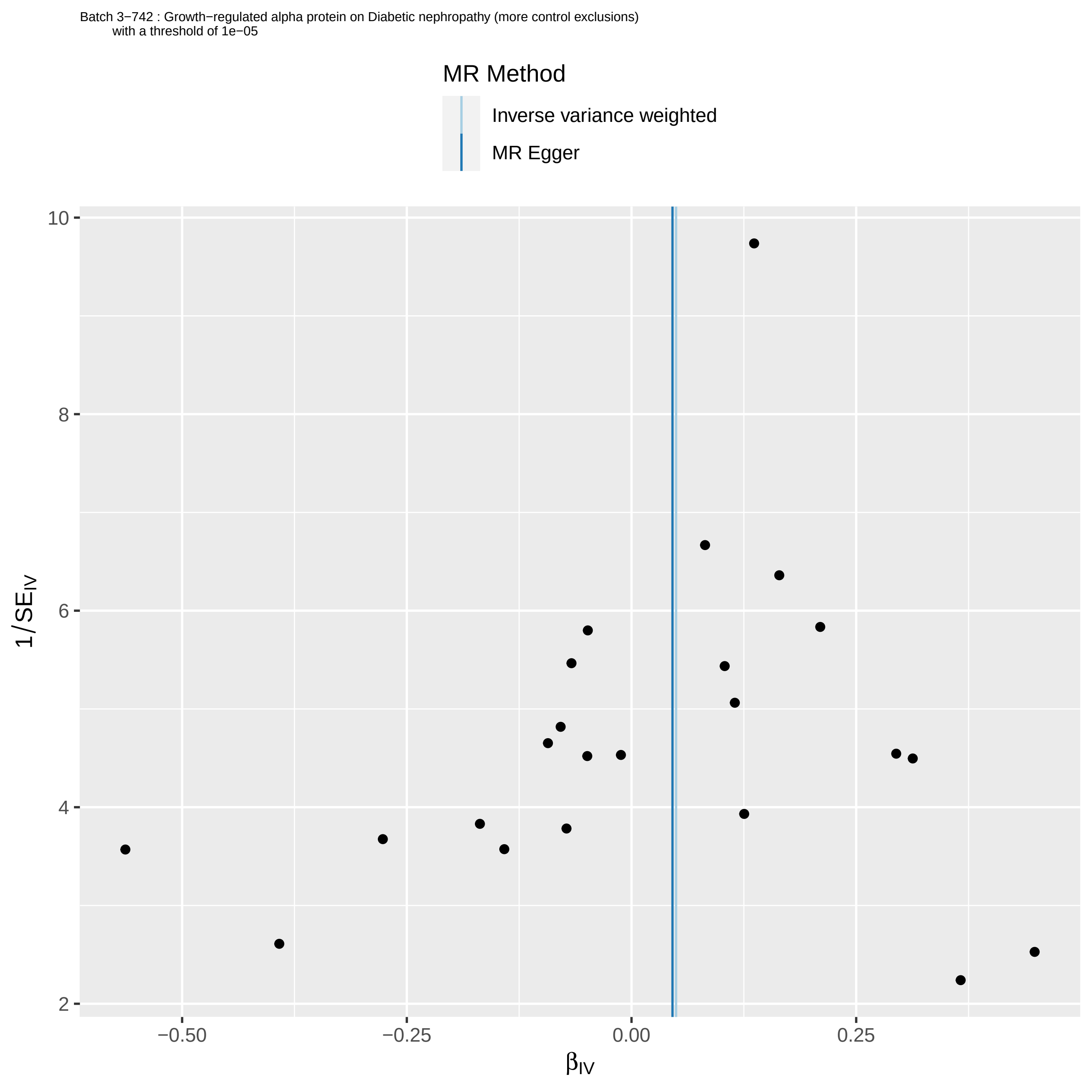

Batch 3−742 : Growth−regulated alpha protein on Diabetic nephropathy (more control exclusions)
with a threshold of 1e−05
MR Method
Inverse variance weighted
MR Egger
10
8
6
4
2
1/SE_IV
−0.50
−0.25
0.00
0.25
β_IV

Batch 3−743 : C−X−C motif chemokine 13 on Diabetic nephropathy (more control exclusions)
with a threshold of 1e−05

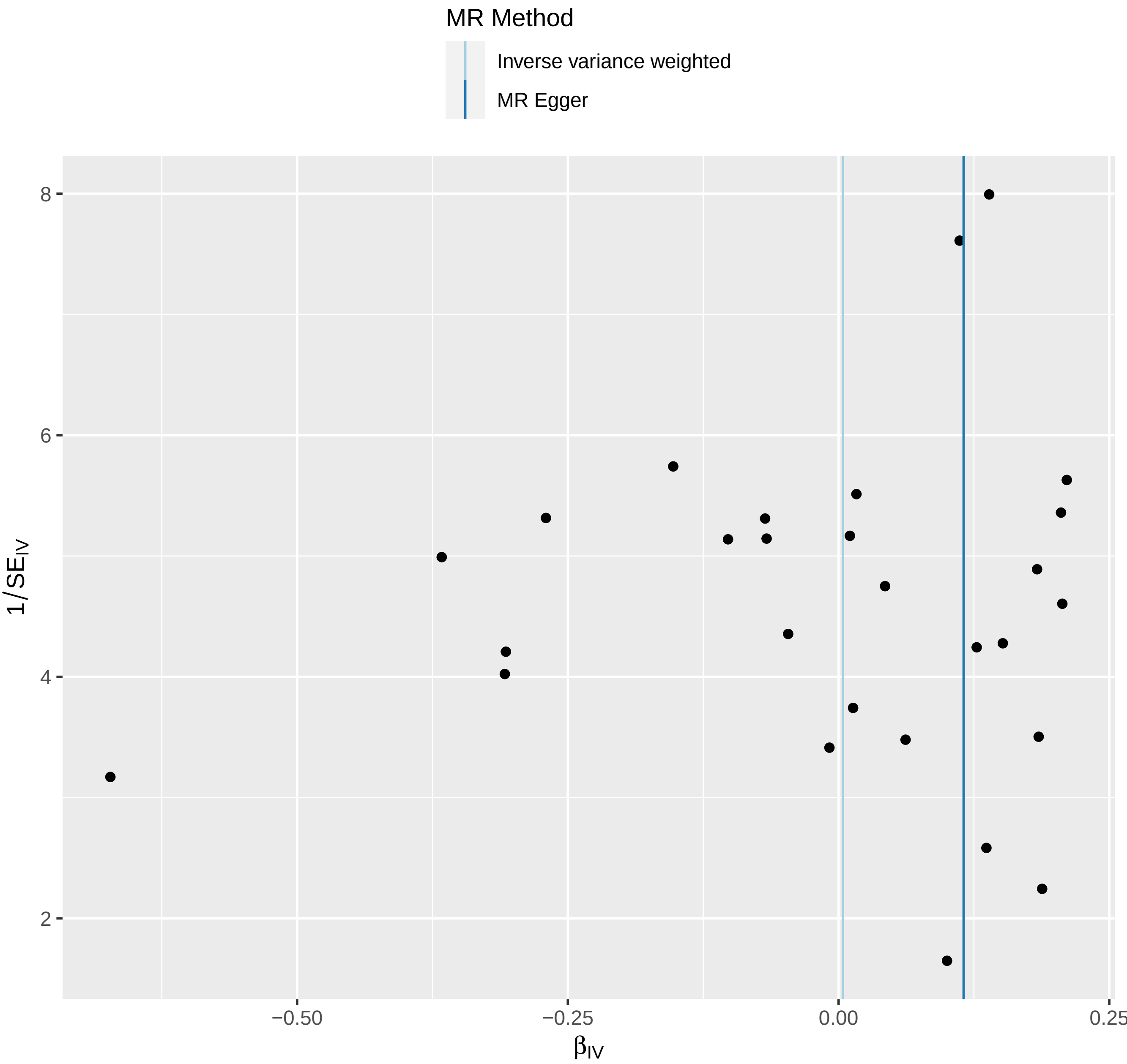

Batch 3−744 : C−X−C motif chemokine 14 on Diabetic nephropathy (more control exclusions)
with a threshold of 1e−05

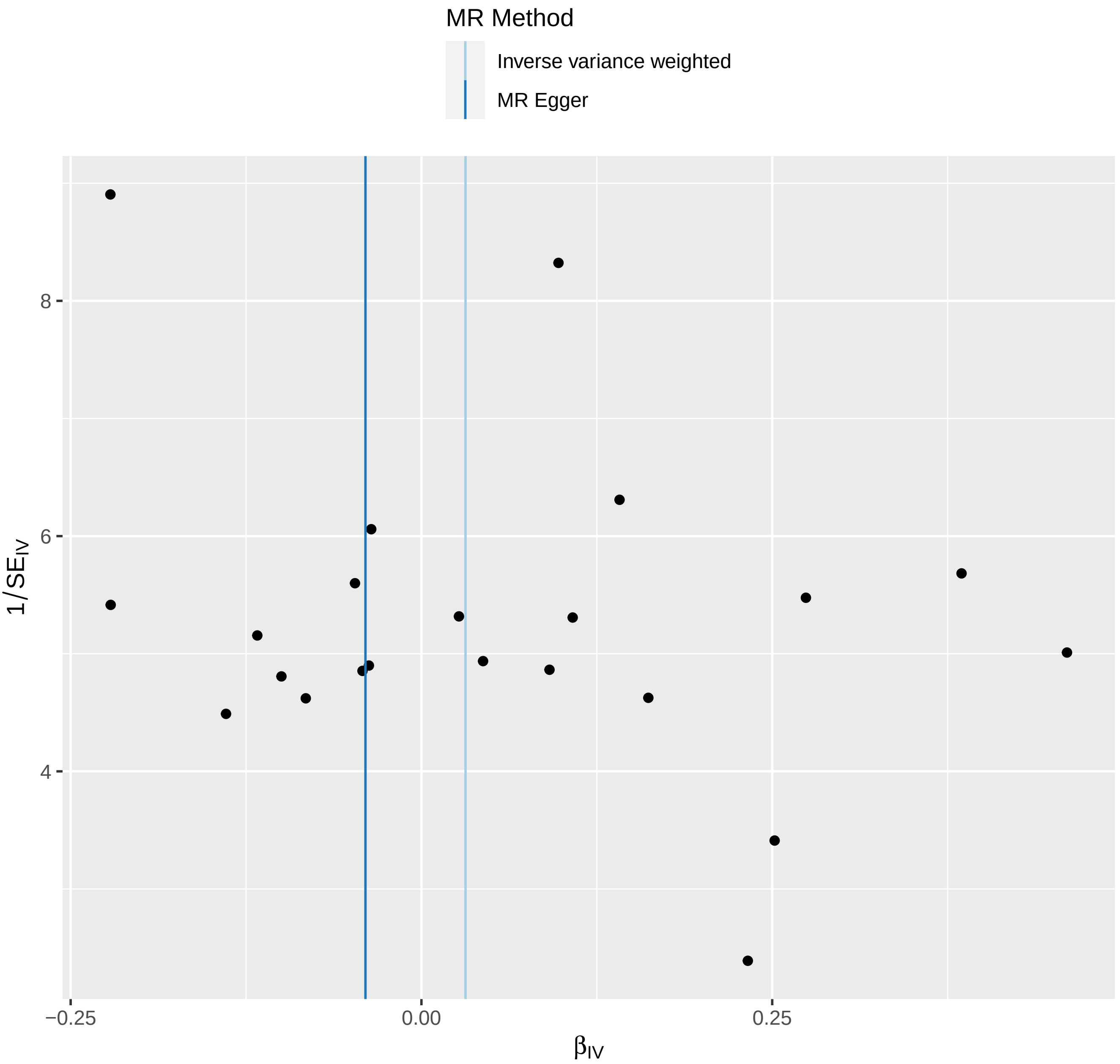

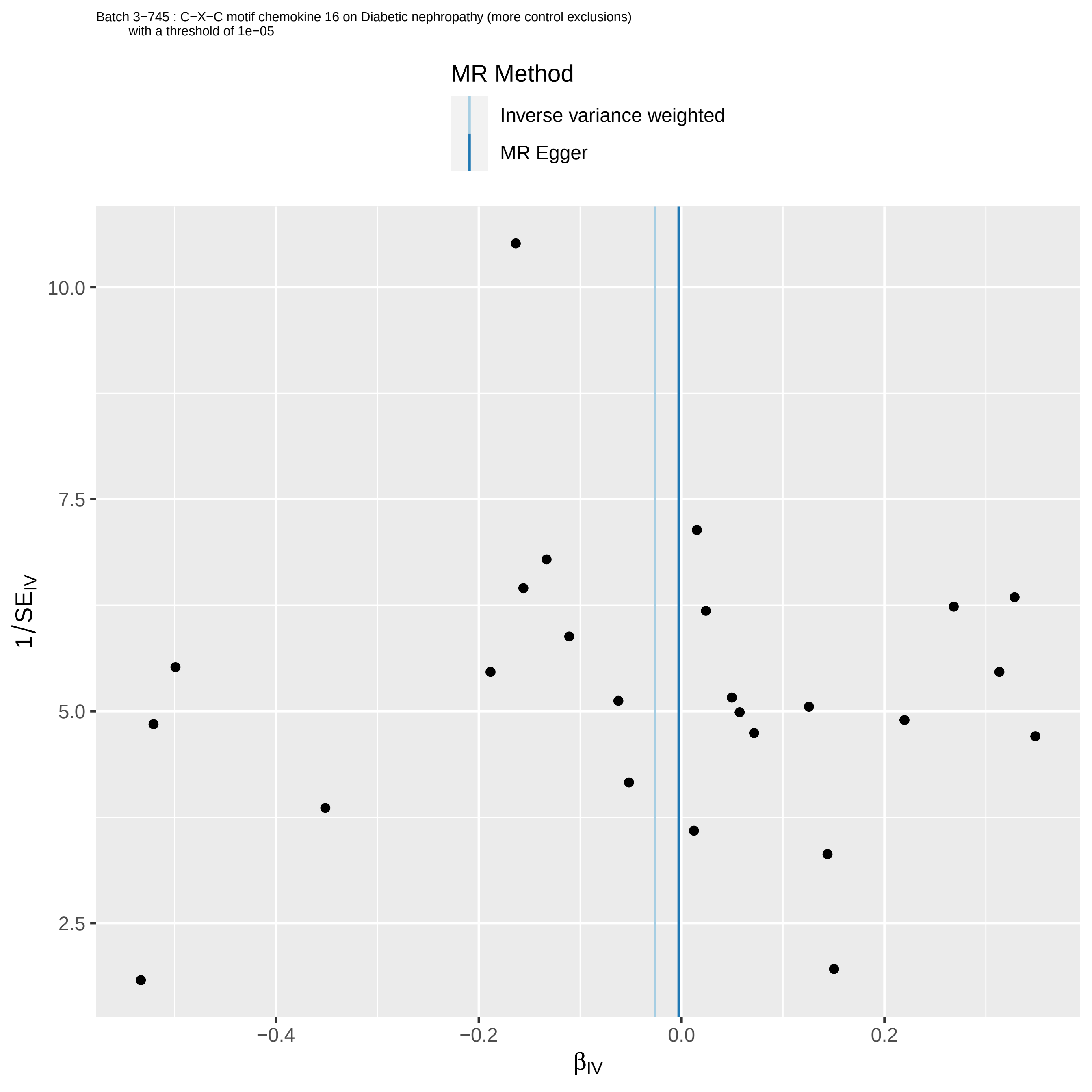
Batch 3−745 : C−X−C motif chemokine 16 on Diabetic nephropathy (more control exclusions)
with a threshold of 1e−05
MR Method
Inverse variance weighted
MR Egger
10.0
7.5
5.0
2.5
−0.4
−0.2
0.0
0.2
1/SE_IV
β_IV

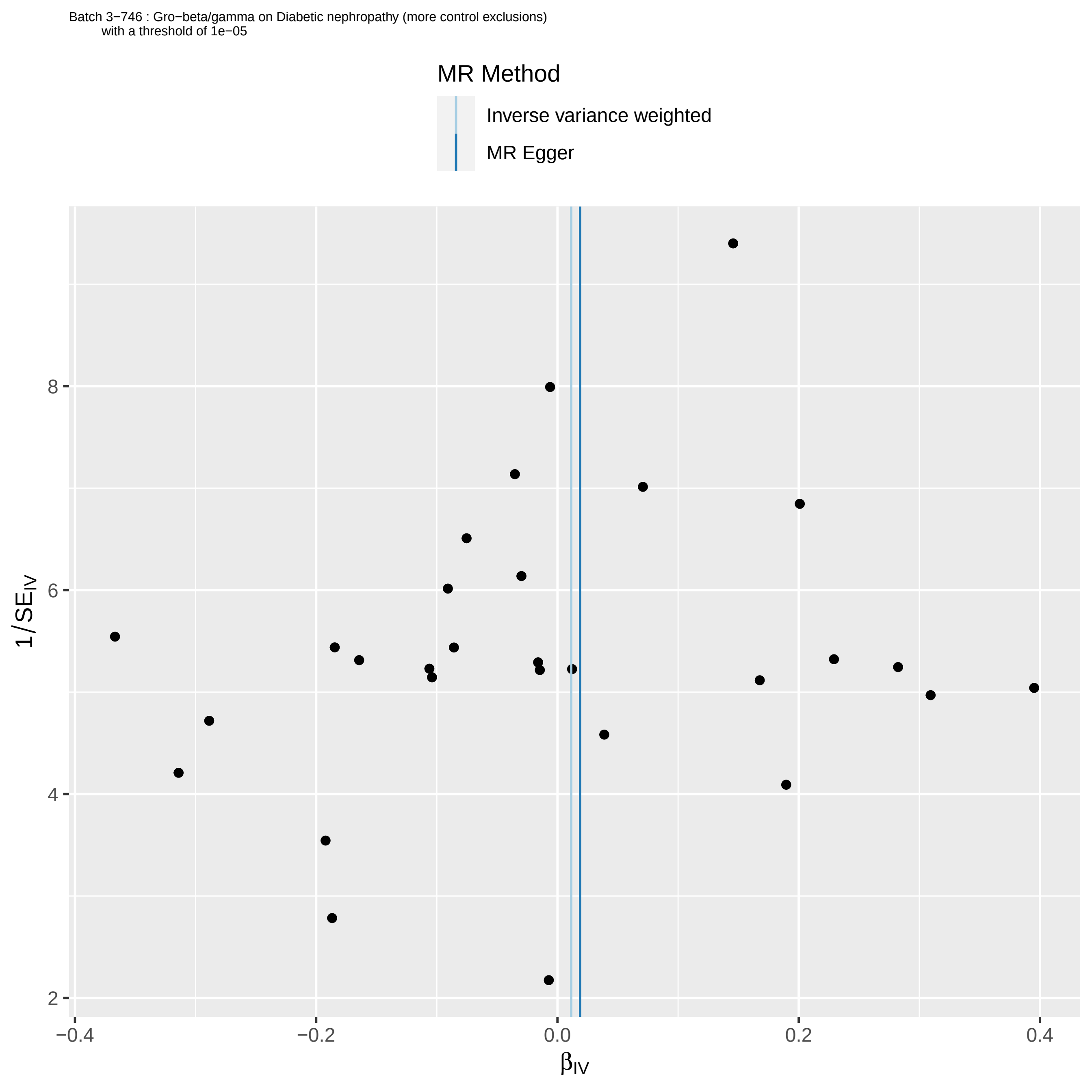
Batch 3−746 : Gro−beta/gamma on Diabetic nephropathy (more control exclusions)
with a threshold of 1e−05
MR Method
Inverse variance weighted
MR Egger
8
6
4
2
1/SE_IV
−0.4
−0.2
0.0
0.2
0.4
β_IV

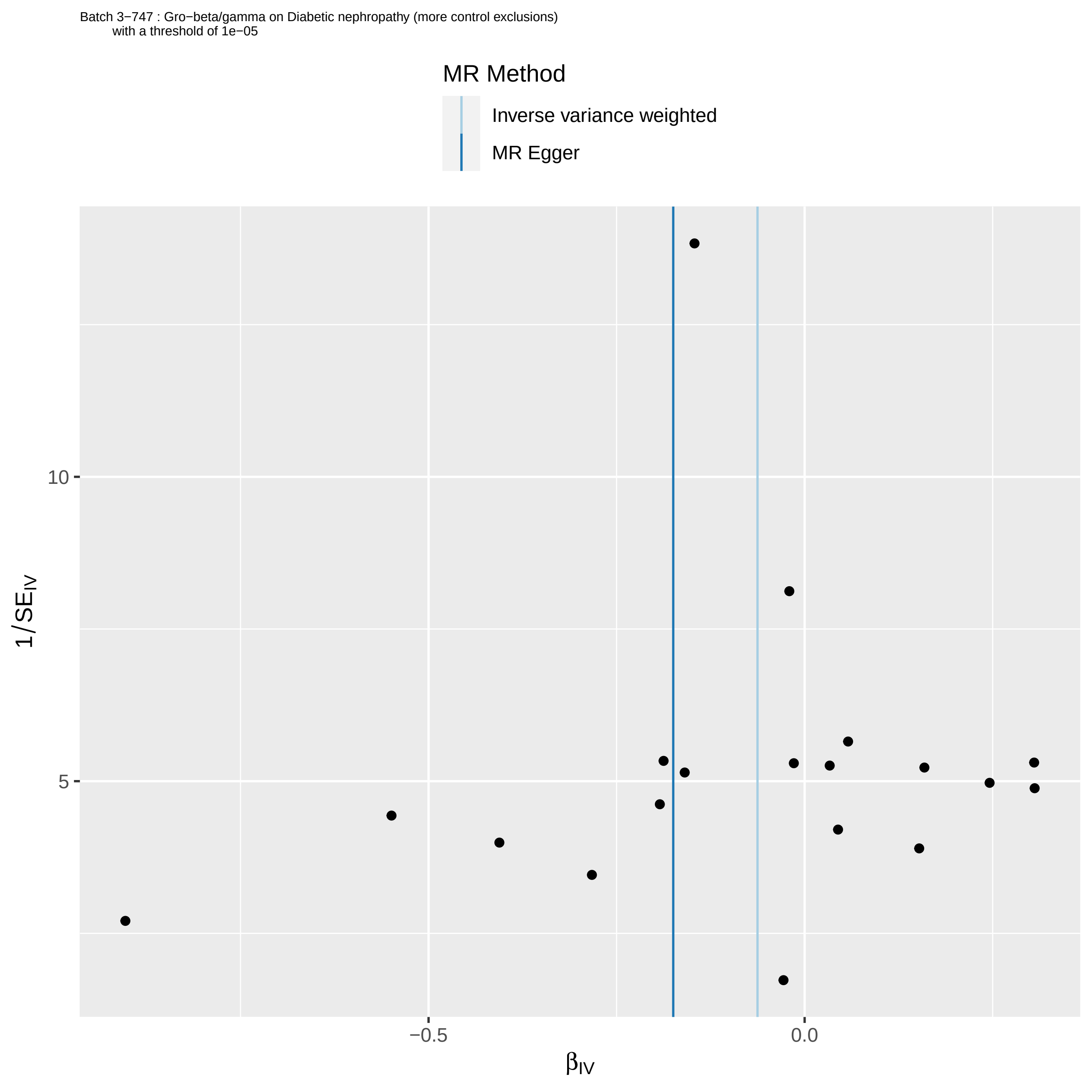

Batch 3−747 : Gro−beta/gamma on Diabetic nephropathy (more control exclusions)
with a threshold of 1e−05
MR Method
Inverse variance weighted
MR Egger
10
5
1/SE_IV
−0.5
0.0
β_IV

Batch 3−748 : C−X−C motif chemokine 5 on Diabetic nephropathy (more control exclusions)
with a threshold of 1e−05

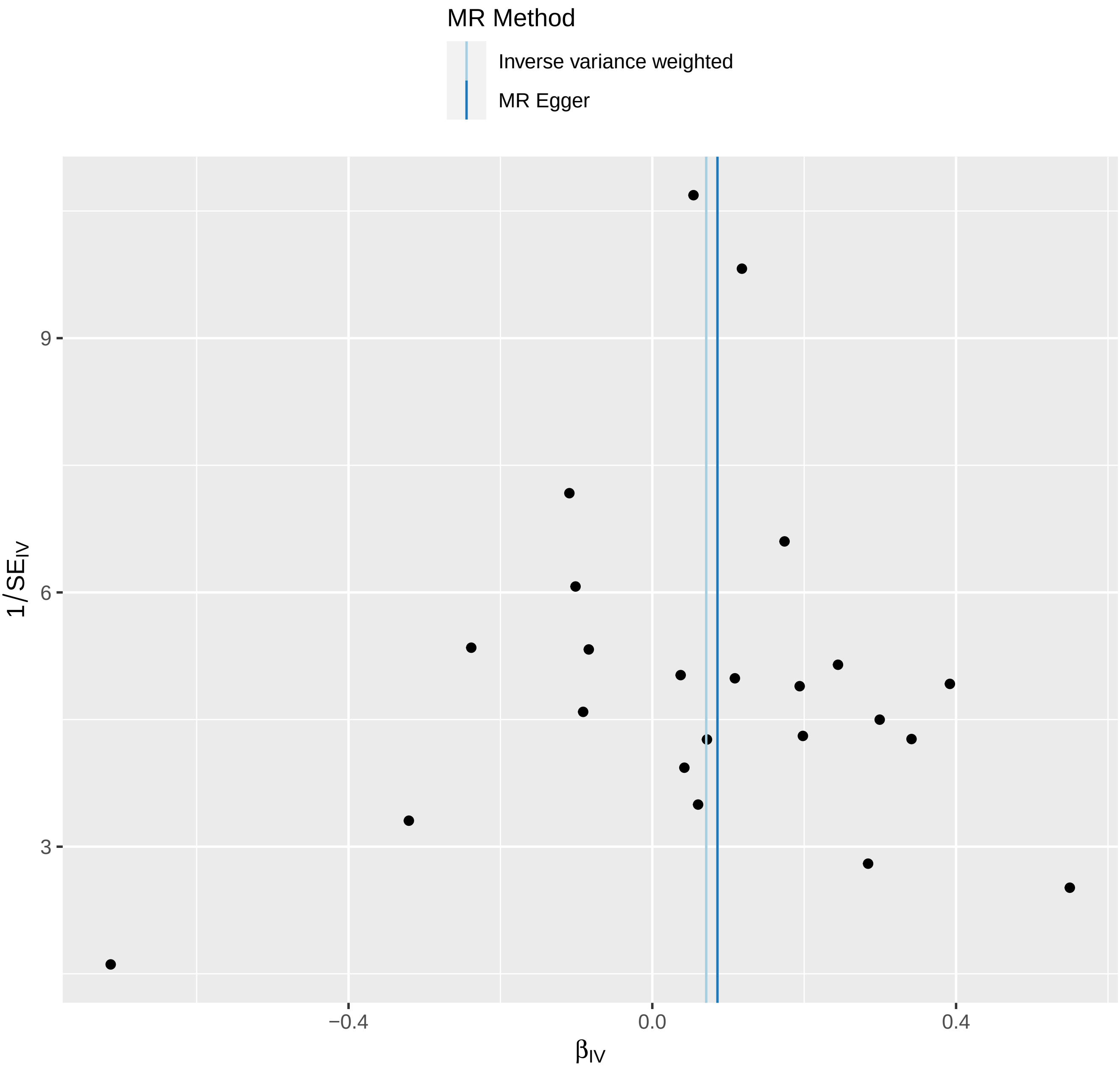

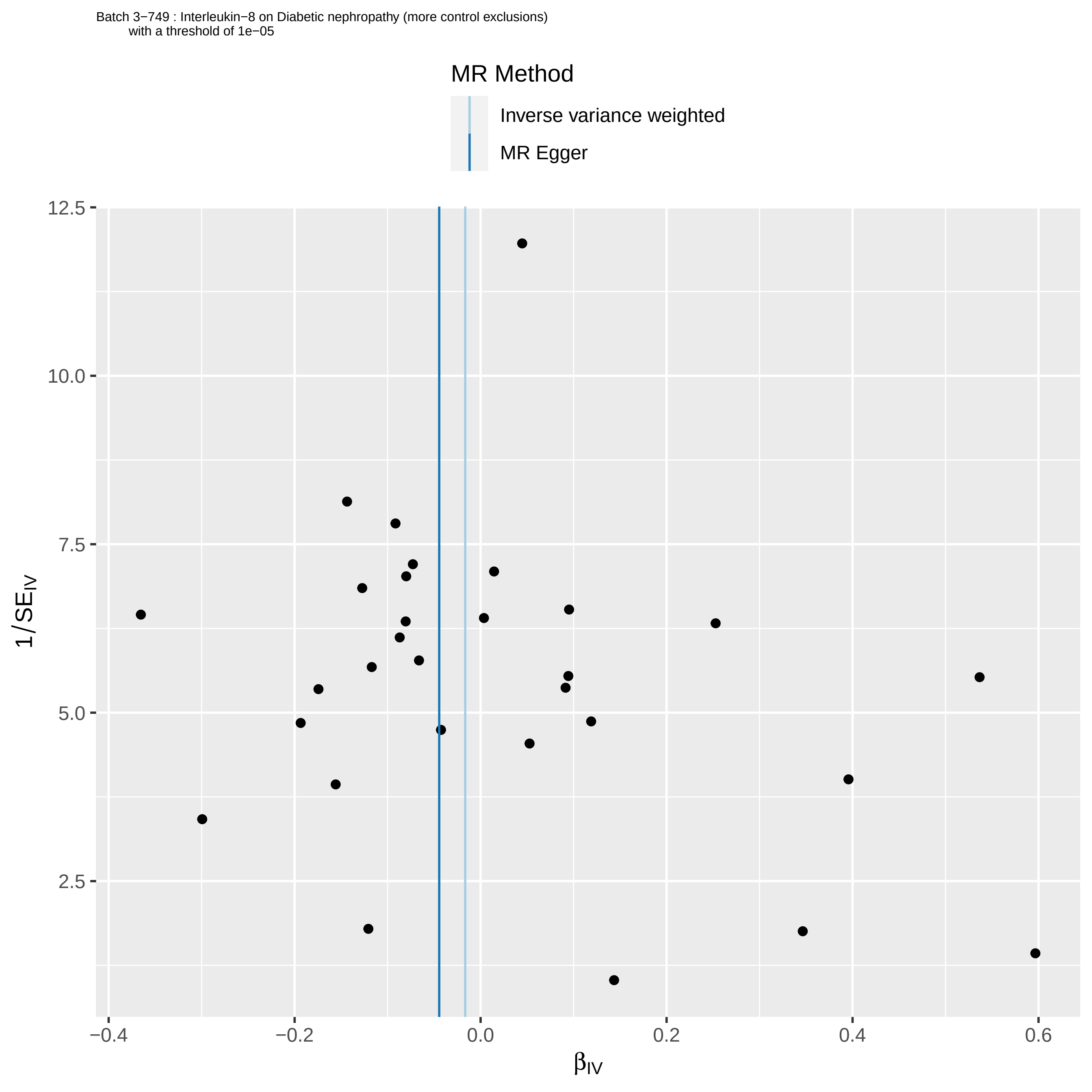

Batch 3−749 : Interleukin−8 on Diabetic nephropathy (more control exclusions)
with a threshold of 1e−05
MR Method
Inverse variance weighted
MR Egger
12.5
10.0
7.5
5.0
2.5
1/SE_IV
−0.4
−0.2
0.0
0.2
0.4
0.6
β_IV

Batch 3−750 : C−X−C motif chemokine 9 on Diabetic nephropathy (more control exclusions)
with a threshold of 1e−05

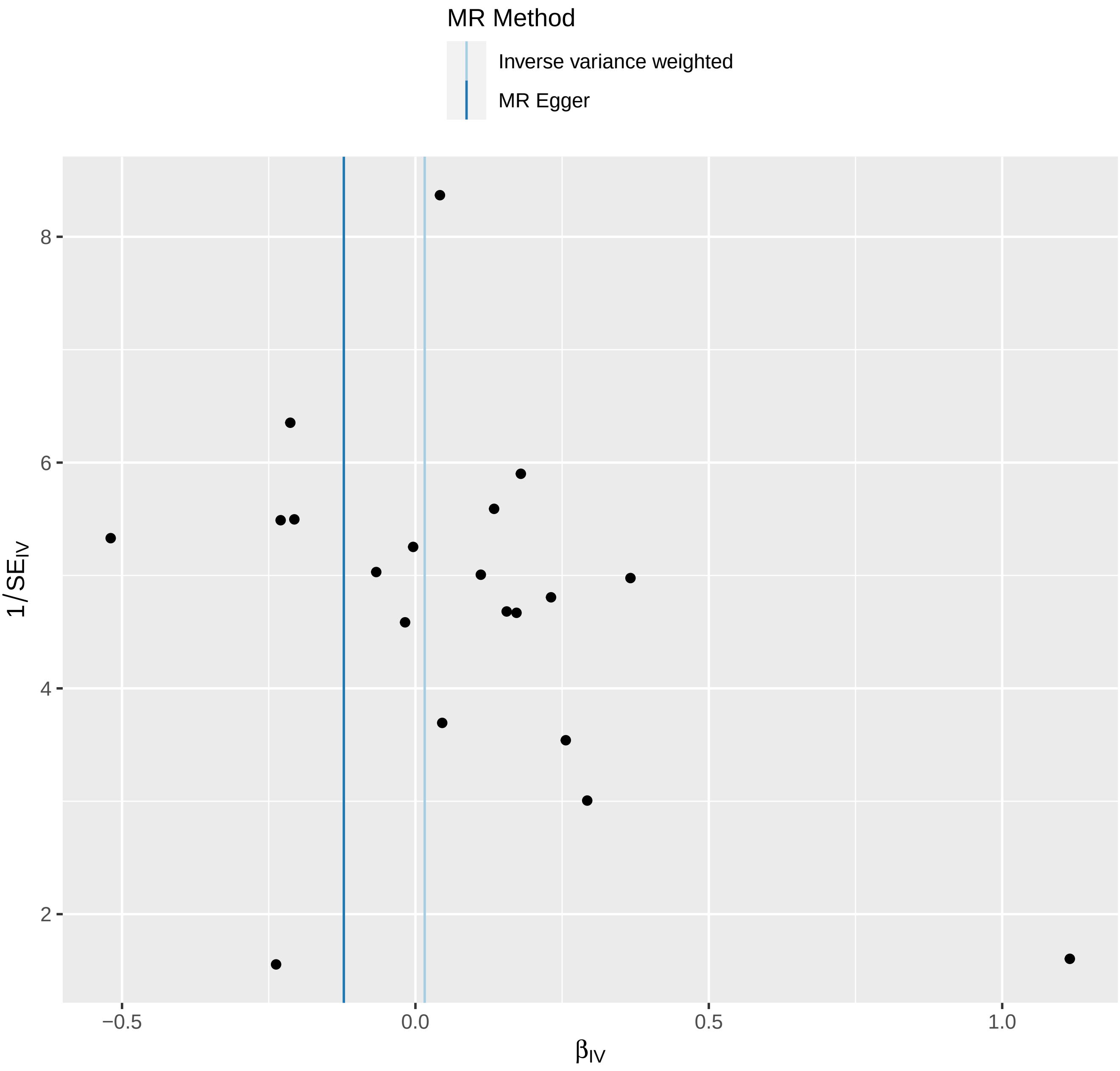

Batch 3−751 : C−X−C motif chemokine 9 on Diabetic nephropathy (more control exclusions)
with a threshold of 1e−05

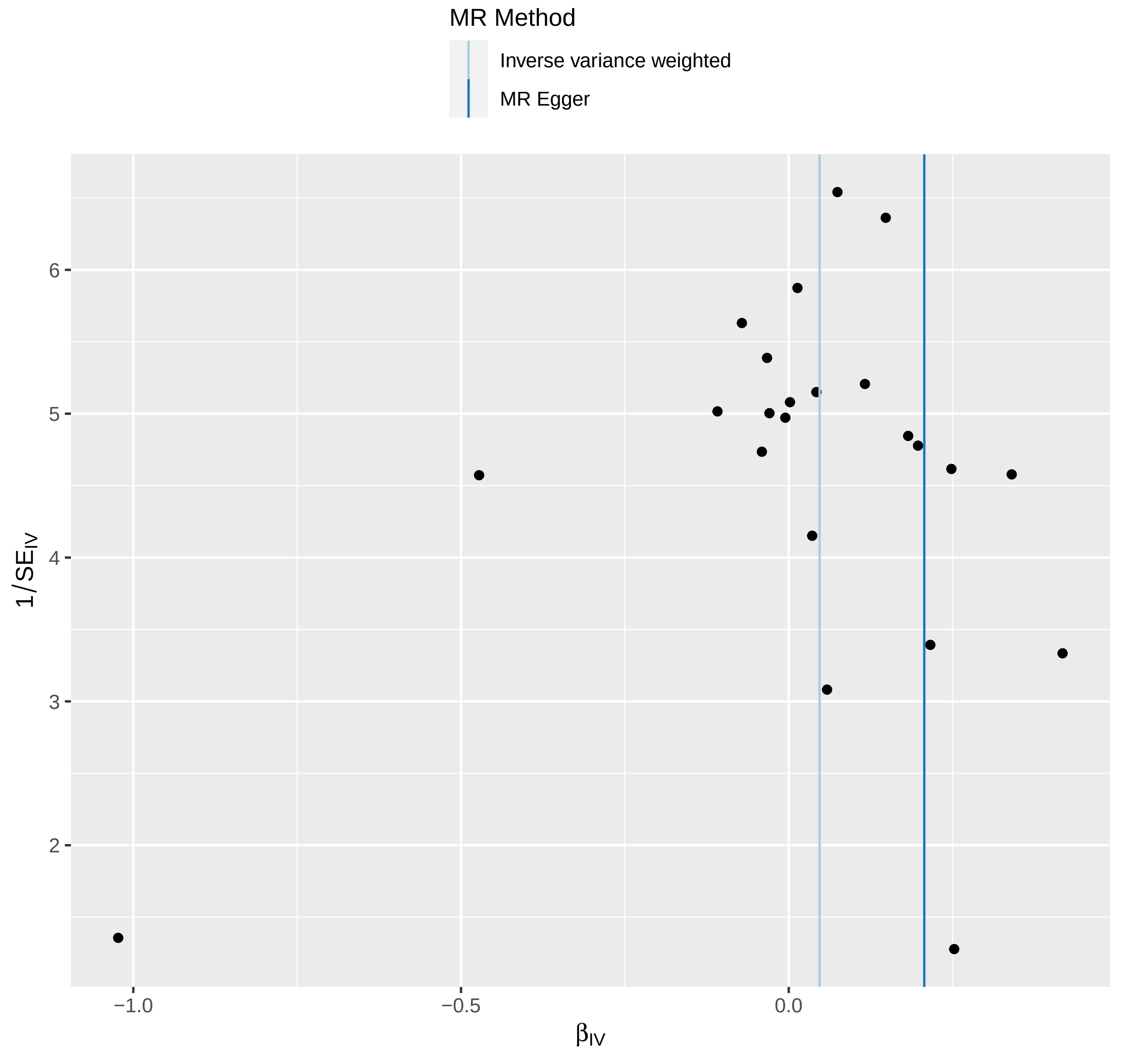

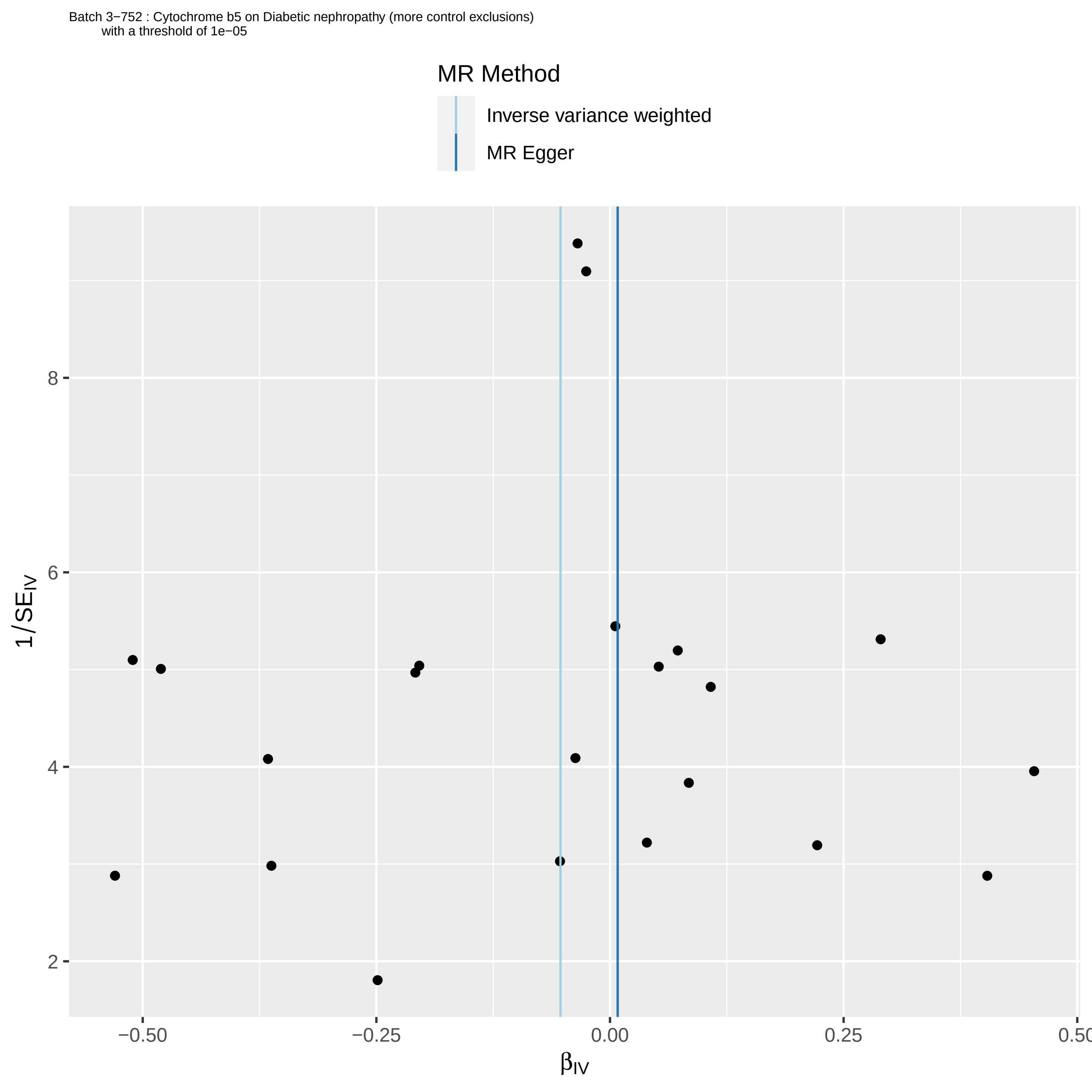

Batch 3−752 : Cytochrome b5 on Diabetic nephropathy (more control exclusions)
with a threshold of 1e−05
MR Method
Inverse variance weighted
MR Egger
8
6
4
2
1/SE$_{IV}$
−0.50
−0.25
0.00
0.25
0.50
β_{IV}

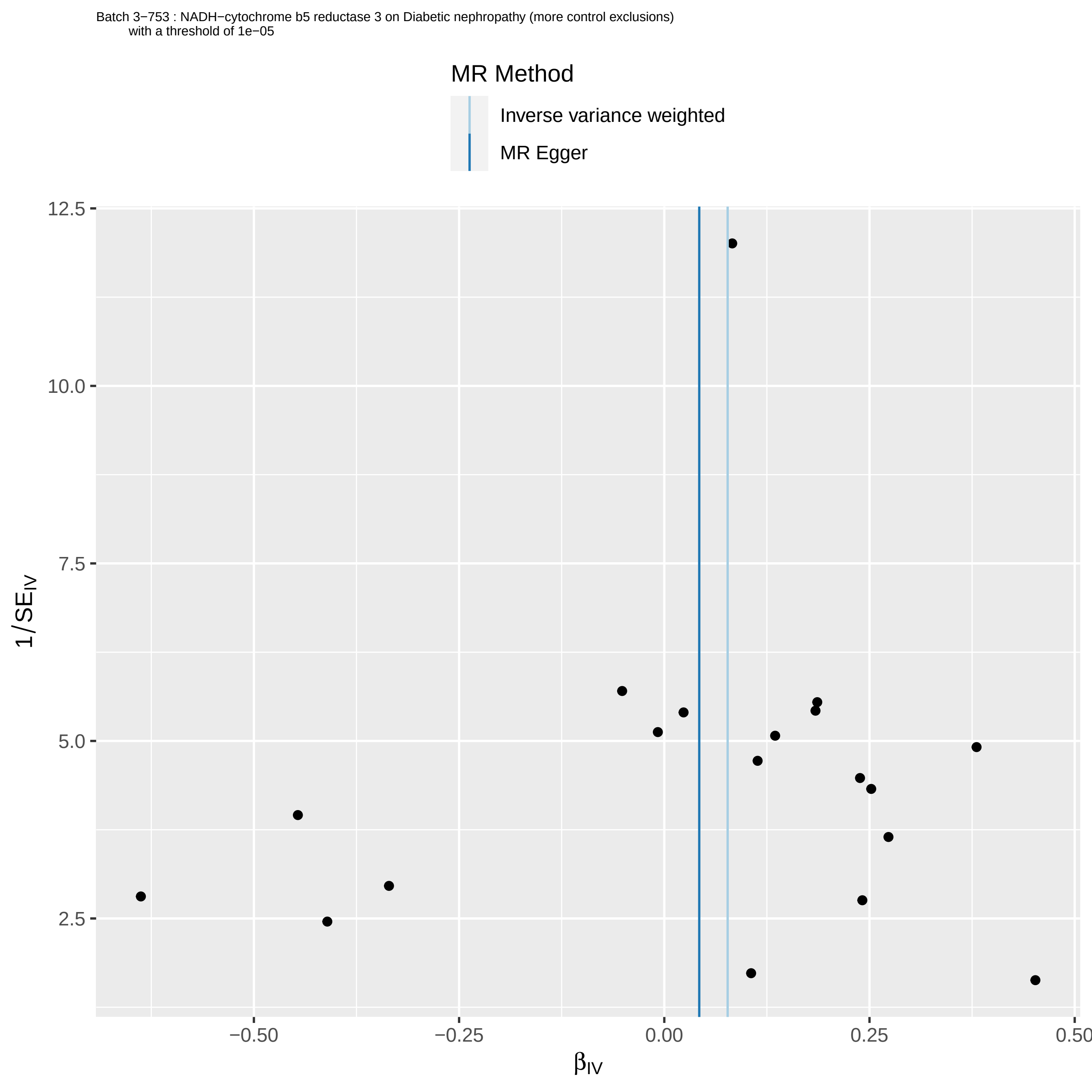

Batch 3−753 : NADH−cytochrome b5 reductase 3 on Diabetic nephropathy (more control exclusions)
with a threshold of 1e−05
MR Method
Inverse variance weighted
MR Egger
12.5
10.0
7.5
5.0
2.5
1/SE_IV
−0.50
−0.25
0.00
0.25
0.50
β_IV

Batch 3−754 : Cytochrome c on Diabetic nephropathy (more control exclusions)
with a threshold of 1e−05

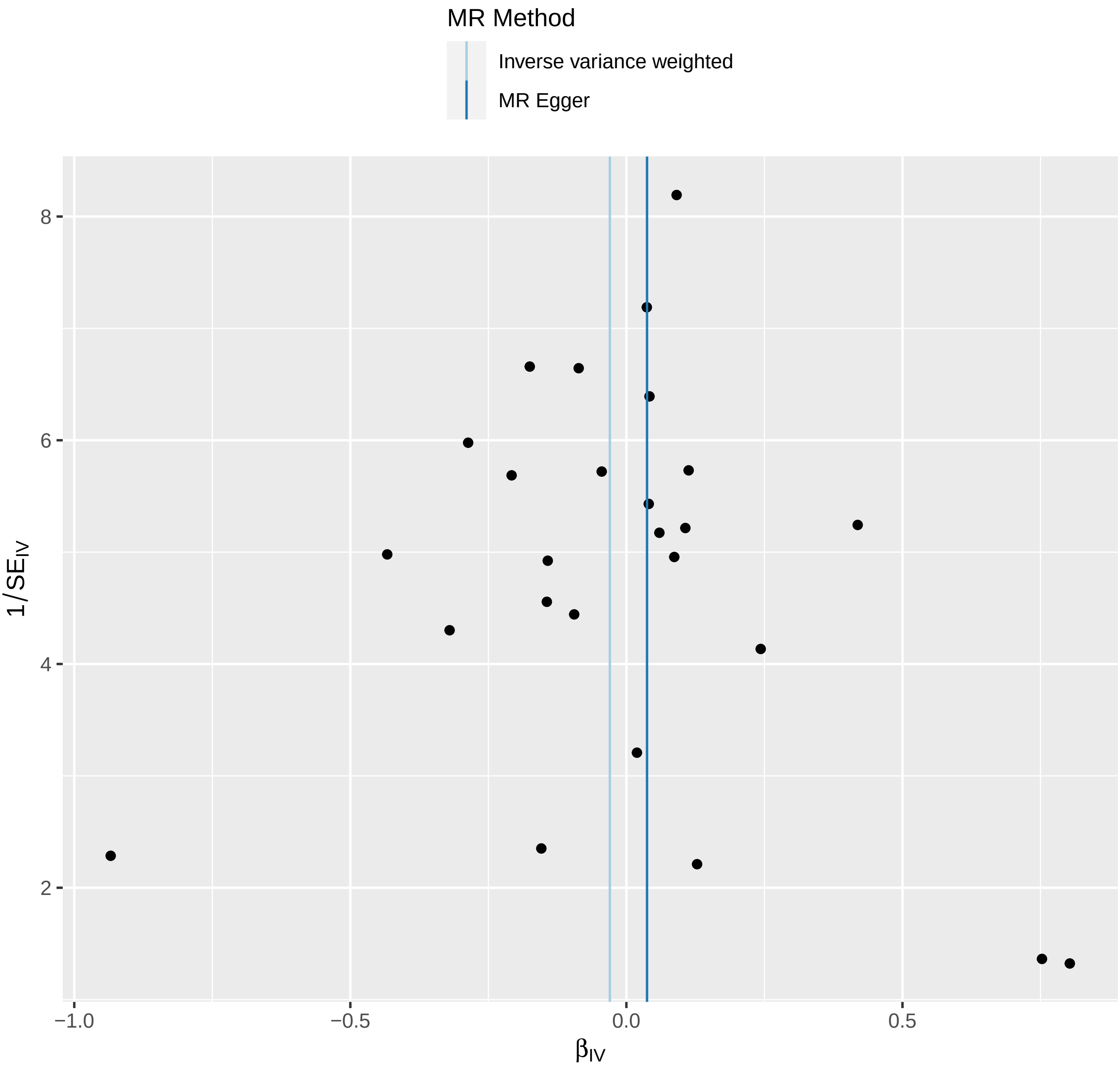

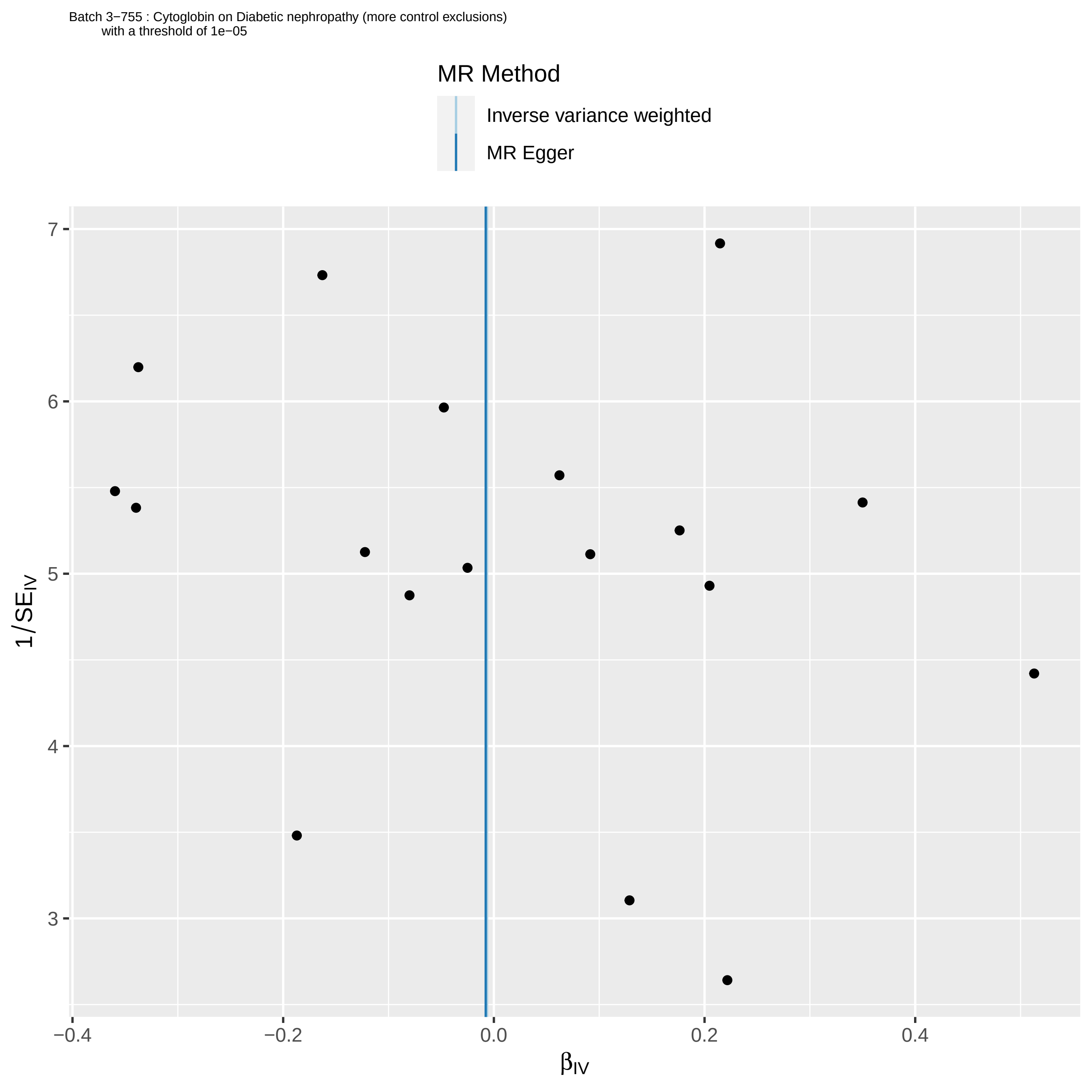
Batch 3−755 : Cytoglobin on Diabetic nephropathy (more control exclusions)
with a threshold of 1e−05
MR Method
Inverse variance weighted
MR Egger
7
6
5
4
3
1/SE_IV
−0.4
−0.2
0.0
0.2
0.4
β_IV

Batch 3−756 : Cytochrome P450 3A4 on Diabetic nephropathy (more control exclusions)
with a threshold of 1e−05

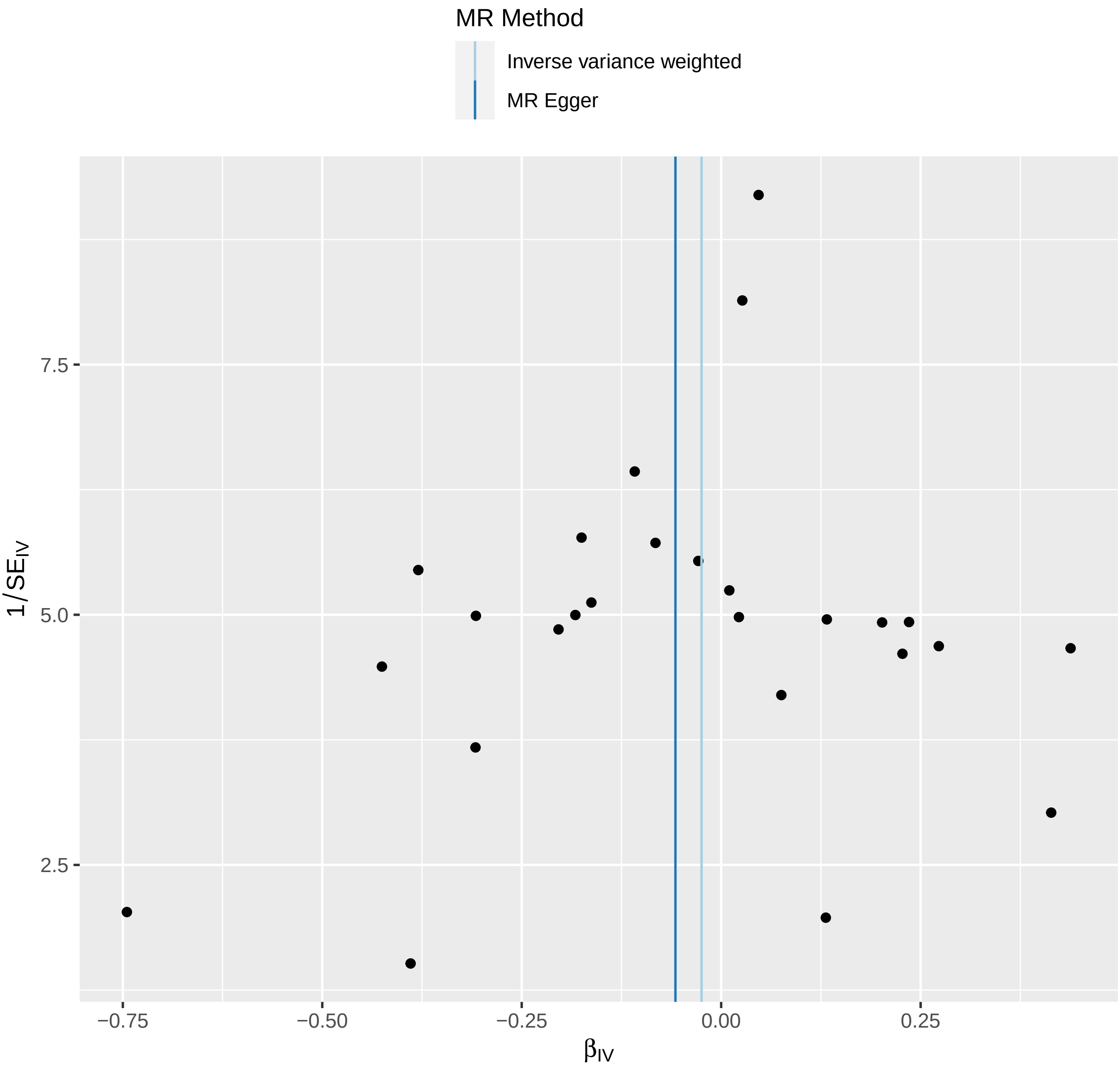

Batch 3−757 : Cytochrome P450 3A4 on Diabetic nephropathy (more control exclusions)
with a threshold of 1e−05

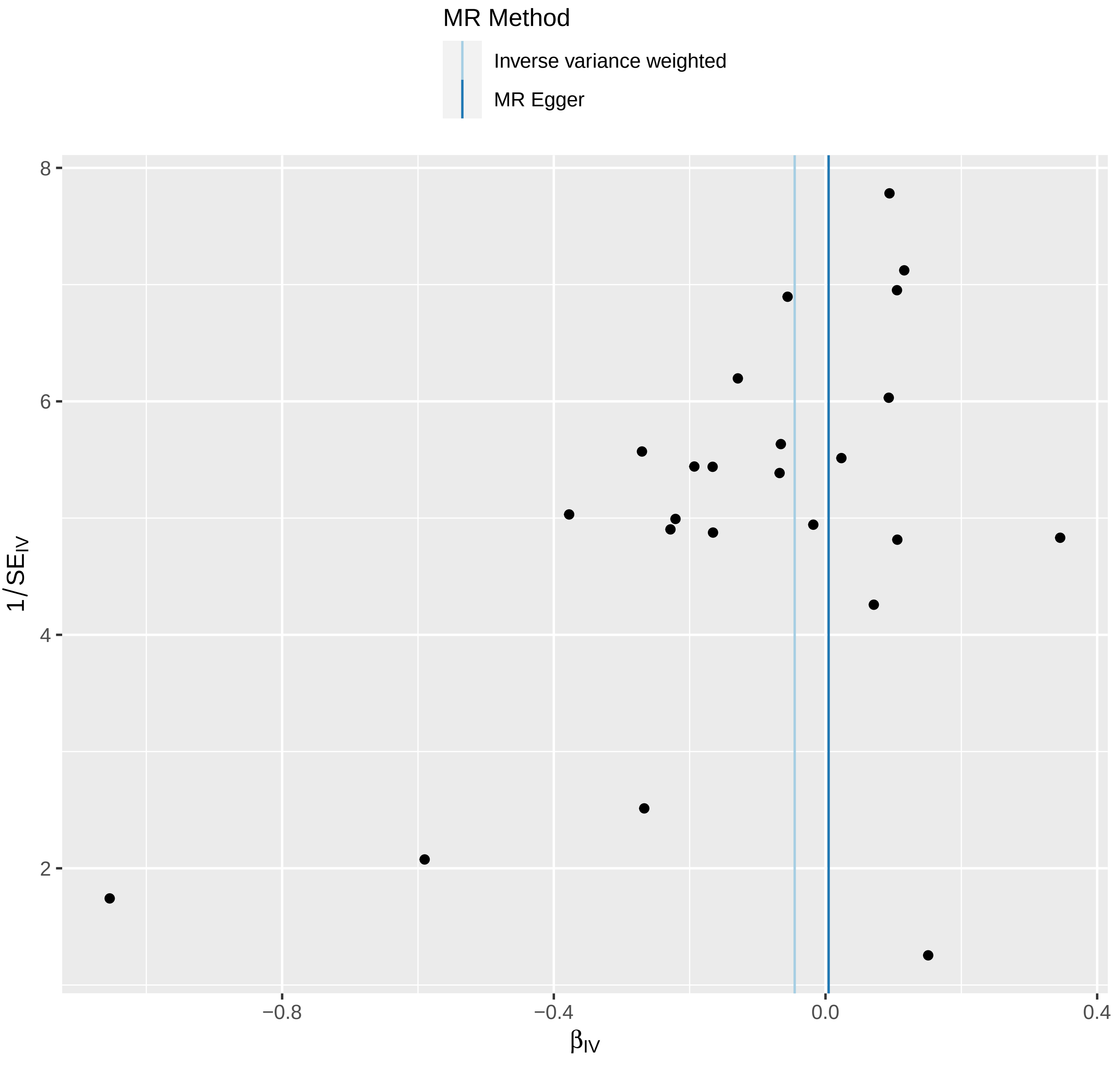

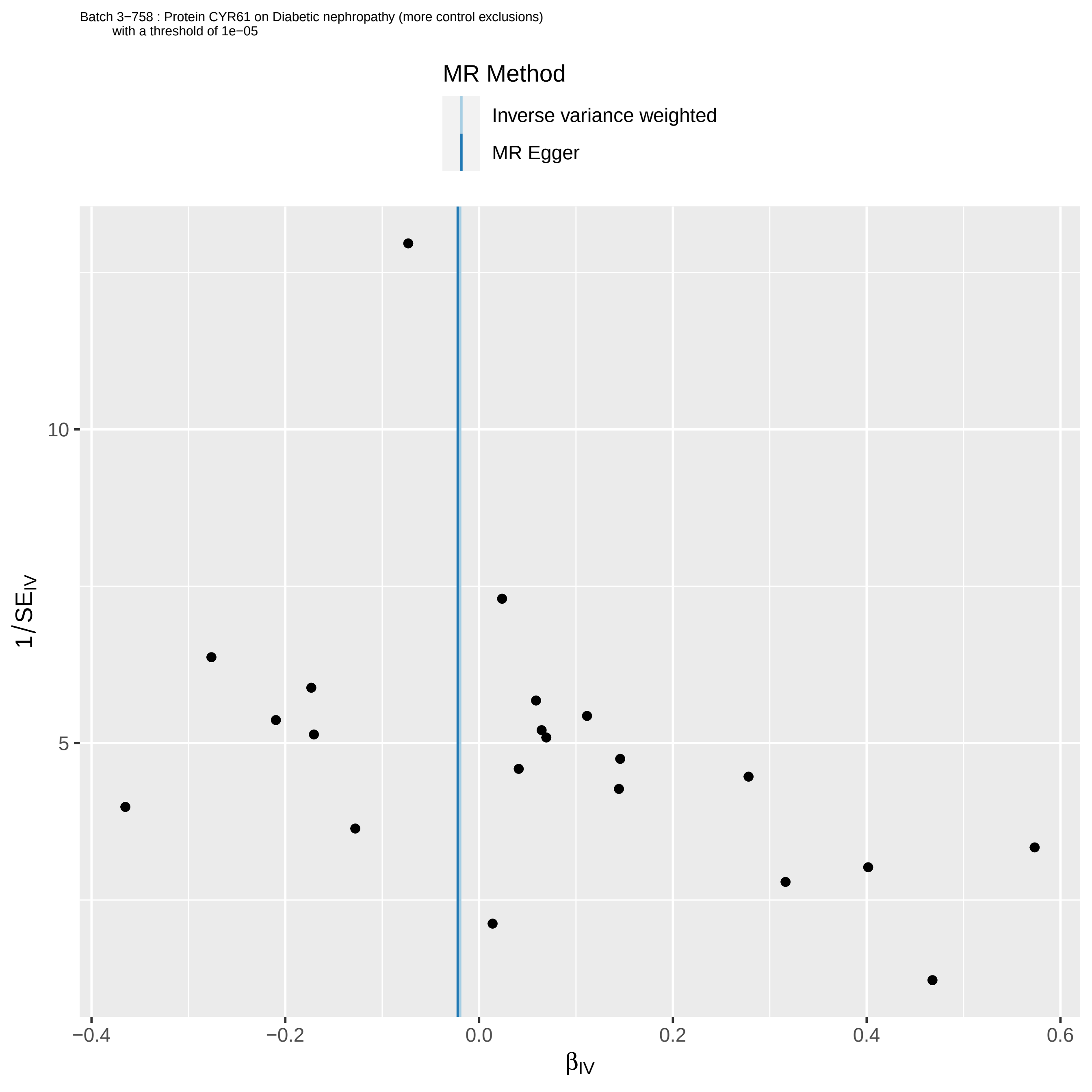

Batch 3−758 : Protein CYR61 on Diabetic nephropathy (more control exclusions)
with a threshold of 1e−05
MR Method
Inverse variance weighted
MR Egger
10
5
1/SE_IV
−0.4
−0.2
0.0
0.2
0.4
0.6
β_IV

Batch 3−759 : Cytohesin−2 on Diabetic nephropathy (more control exclusions)
with a threshold of 1e−05

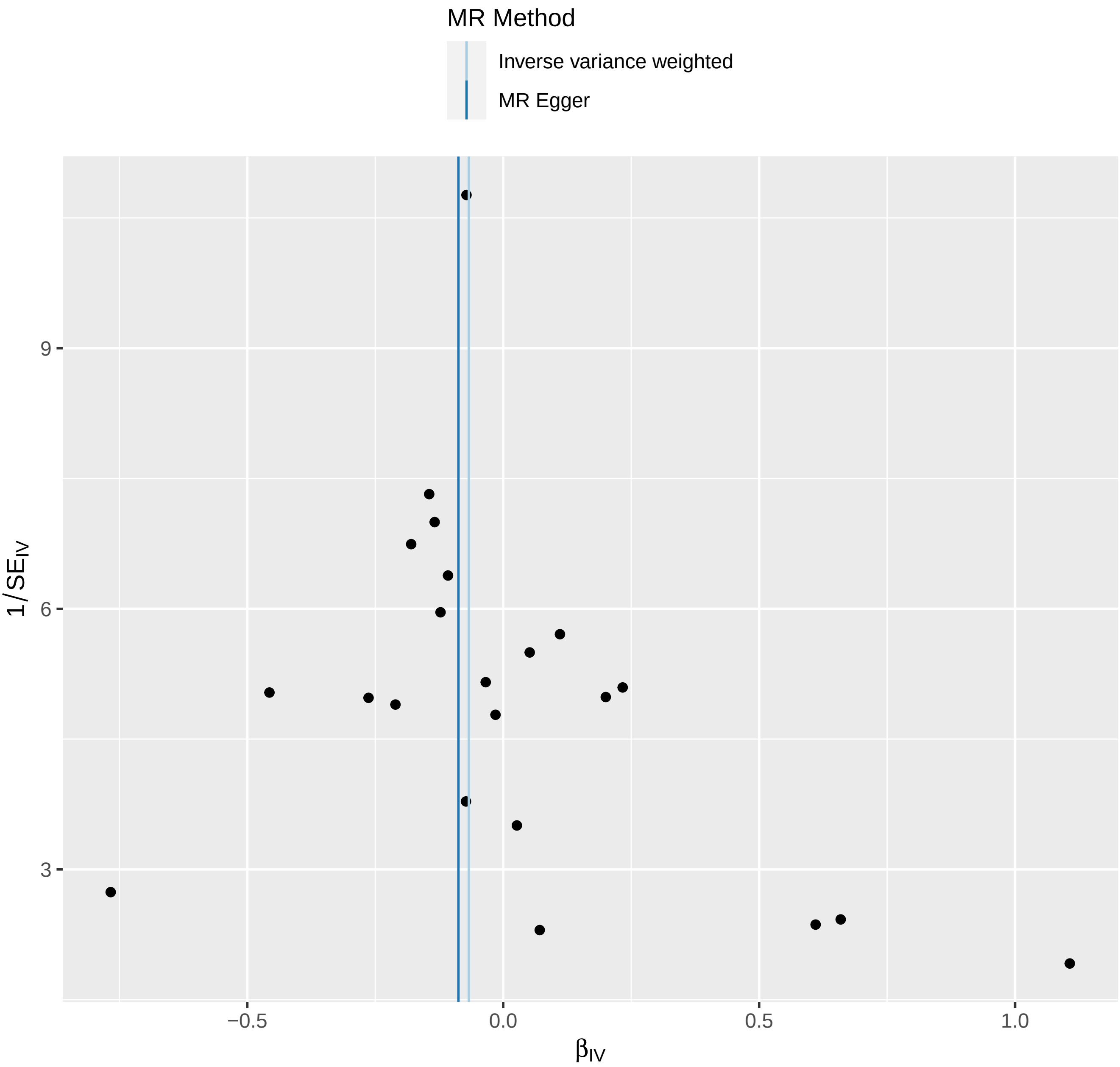

Batch 3−760 : Cytohesin−4 on Diabetic nephropathy (more control exclusions)
with a threshold of 1e−05

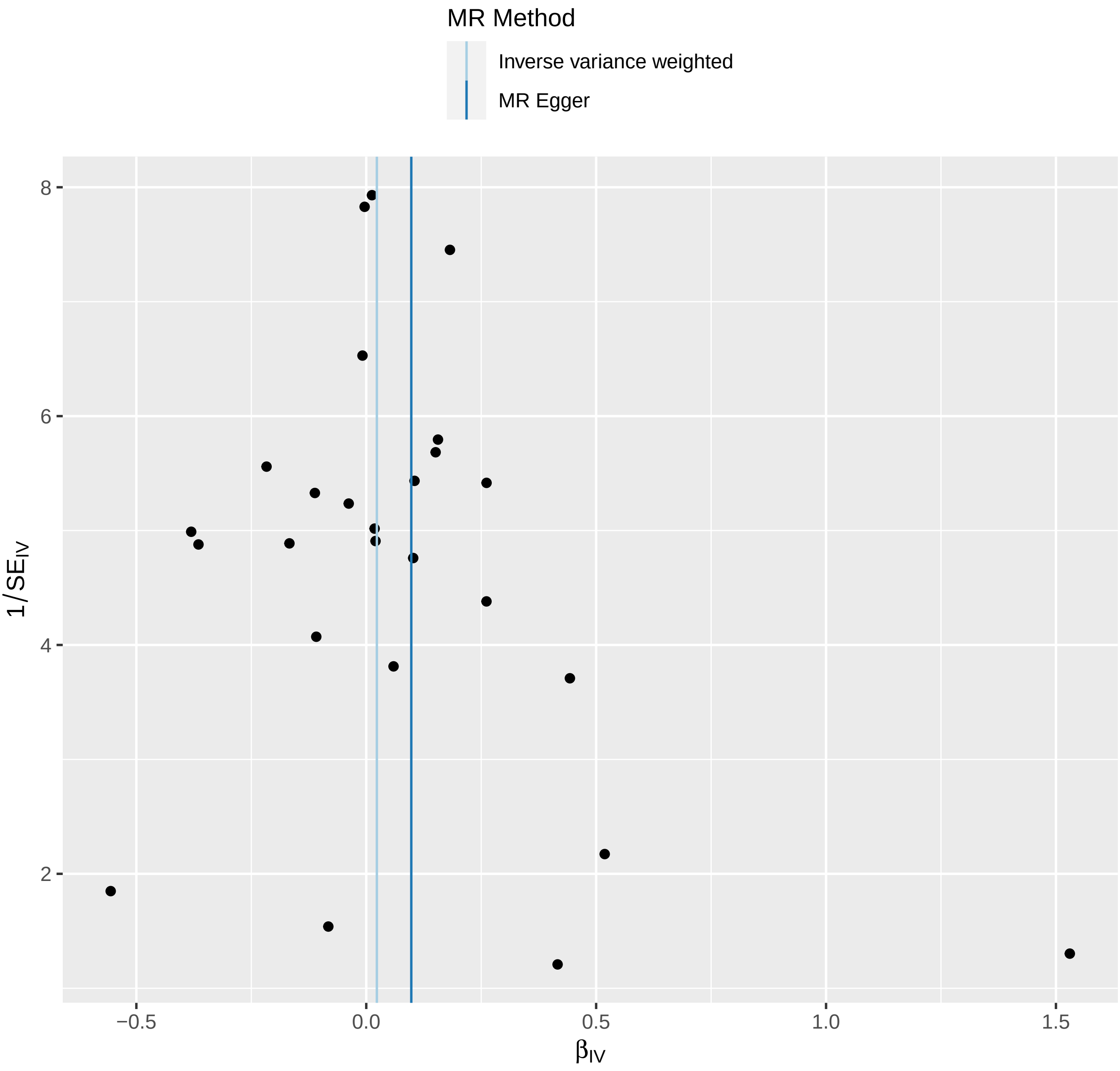

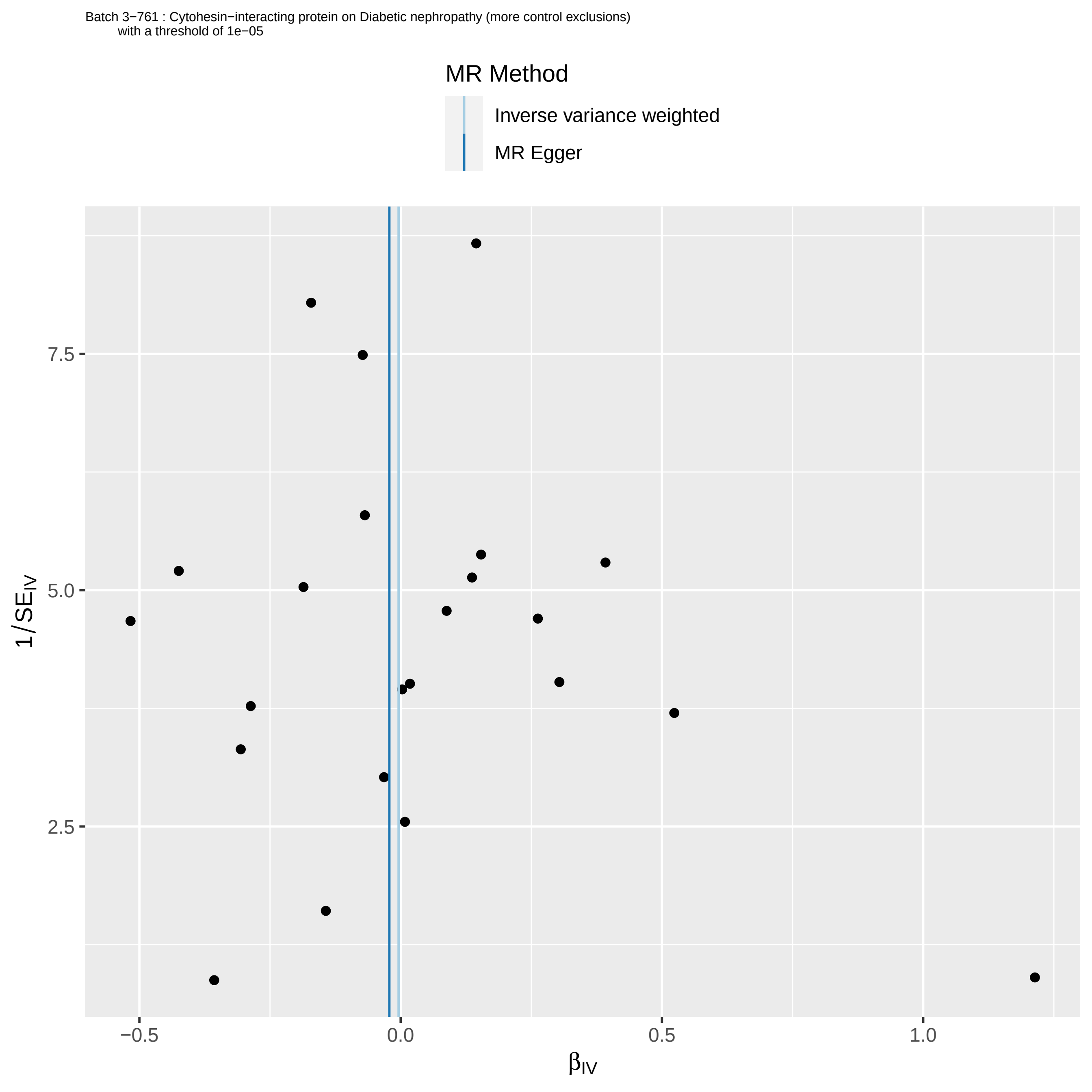
Batch 3−761 : Cytohesin−interacting protein on Diabetic nephropathy (more control exclusions)
with a threshold of 1e−05
MR Method
Inverse variance weighted
MR Egger
7.5
5.0
2.5
$1/SE_{IV}$
−0.5
0.0
0.5
1.0
β_{IV}

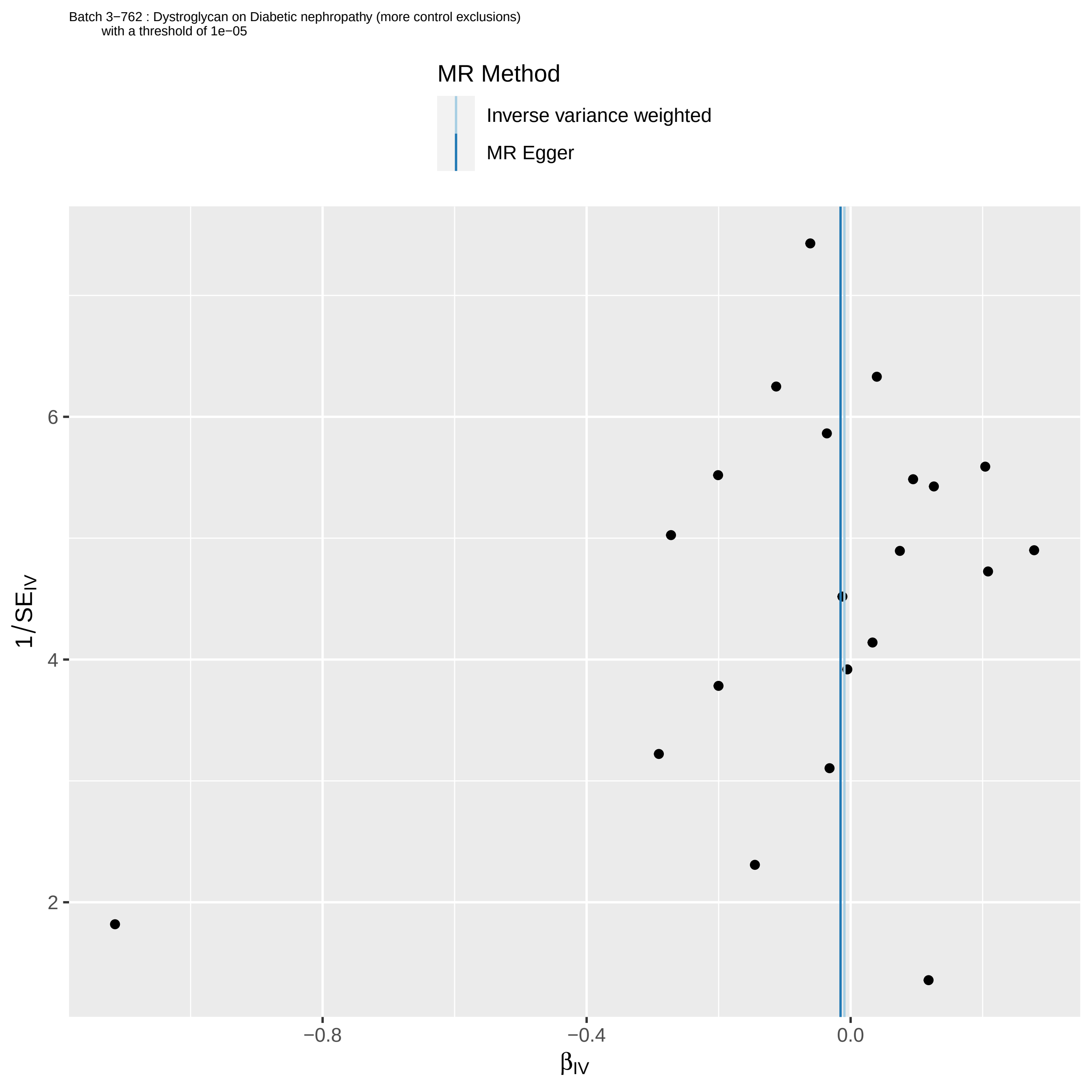

Batch 3−762 : Dystroglycan on Diabetic nephropathy (more control exclusions)
with a threshold of 1e−05
MR Method
Inverse variance weighted
MR Egger
6
4
2
1/SE$_{IV}$
−0.8
−0.4
0.0
β$_{IV}$

Batch 3−763 : Death−associated protein kinase 2 on Diabetic nephropathy (more control exclusions)
with a threshold of 1e−05

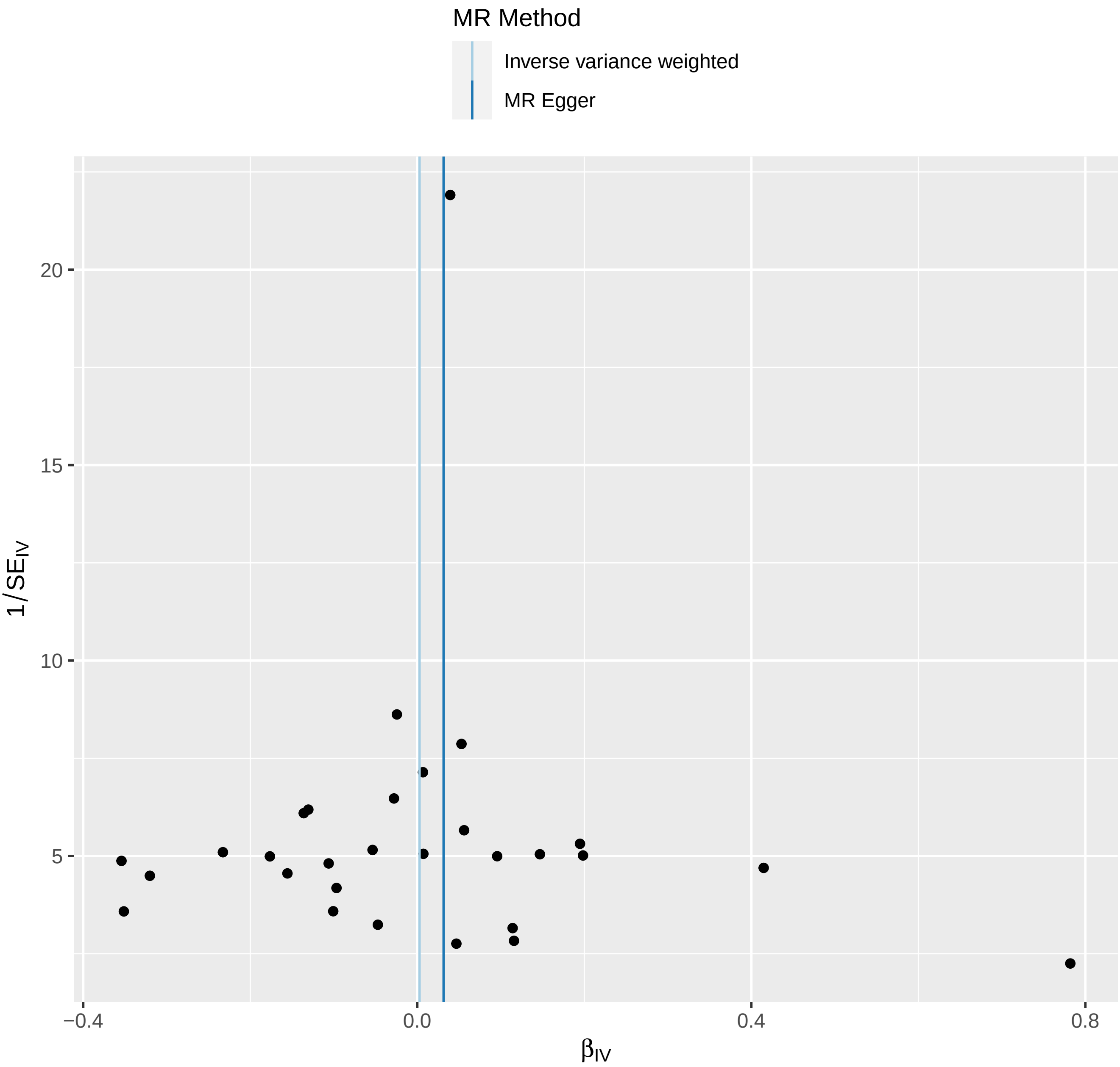

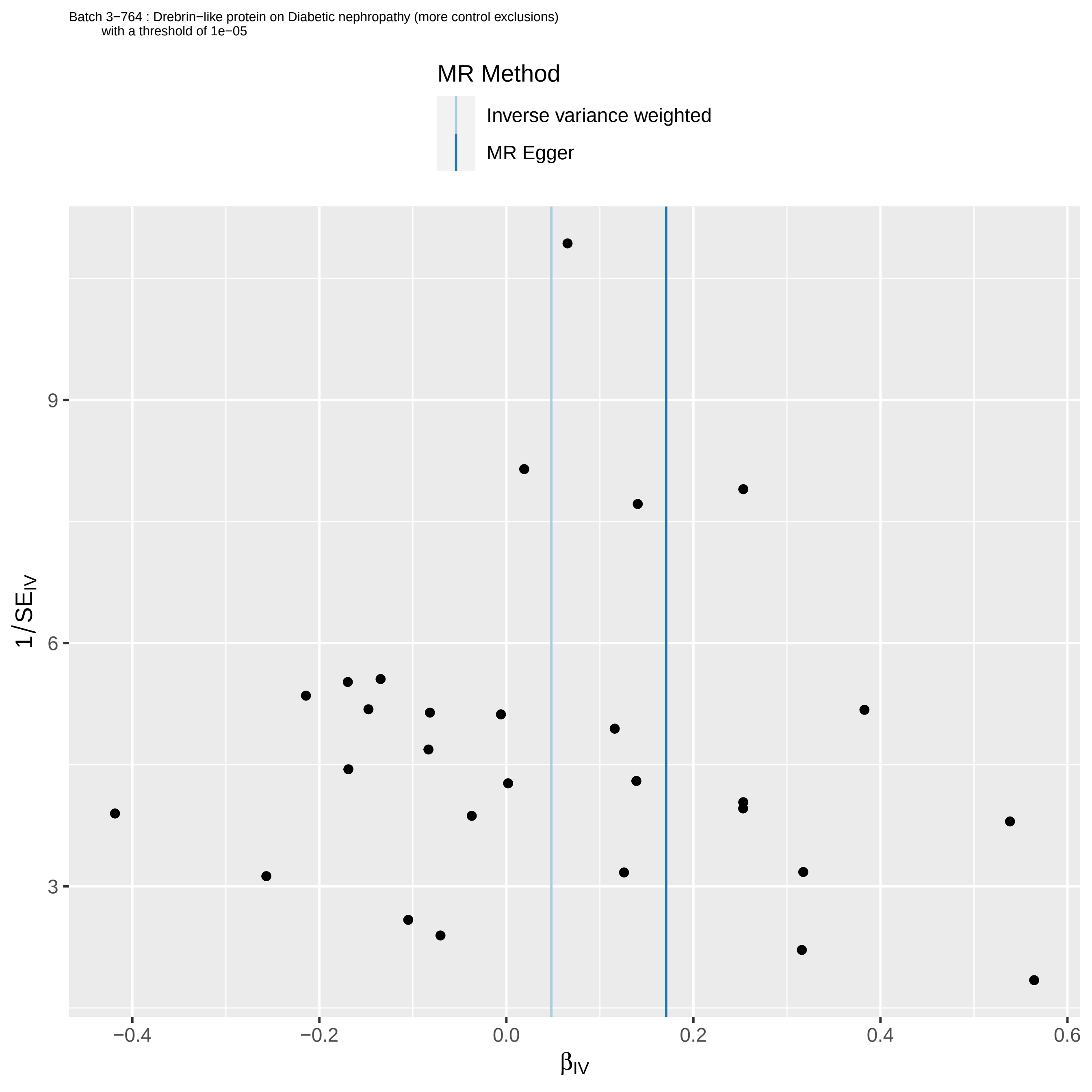

Batch 3−764 : Drebrin−like protein on Diabetic nephropathy (more control exclusions)
with a threshold of 1e−05
MR Method
Inverse variance weighted
MR Egger
1/SE_IV
9
6
3
−0.4
−0.2
0.0
0.2
0.4
0.6
β_IV

Batch 3−765 : DDB1− and CUL4−associated factor 5 on Diabetic nephropathy (more control exclusions)
with a threshold of 1e−05

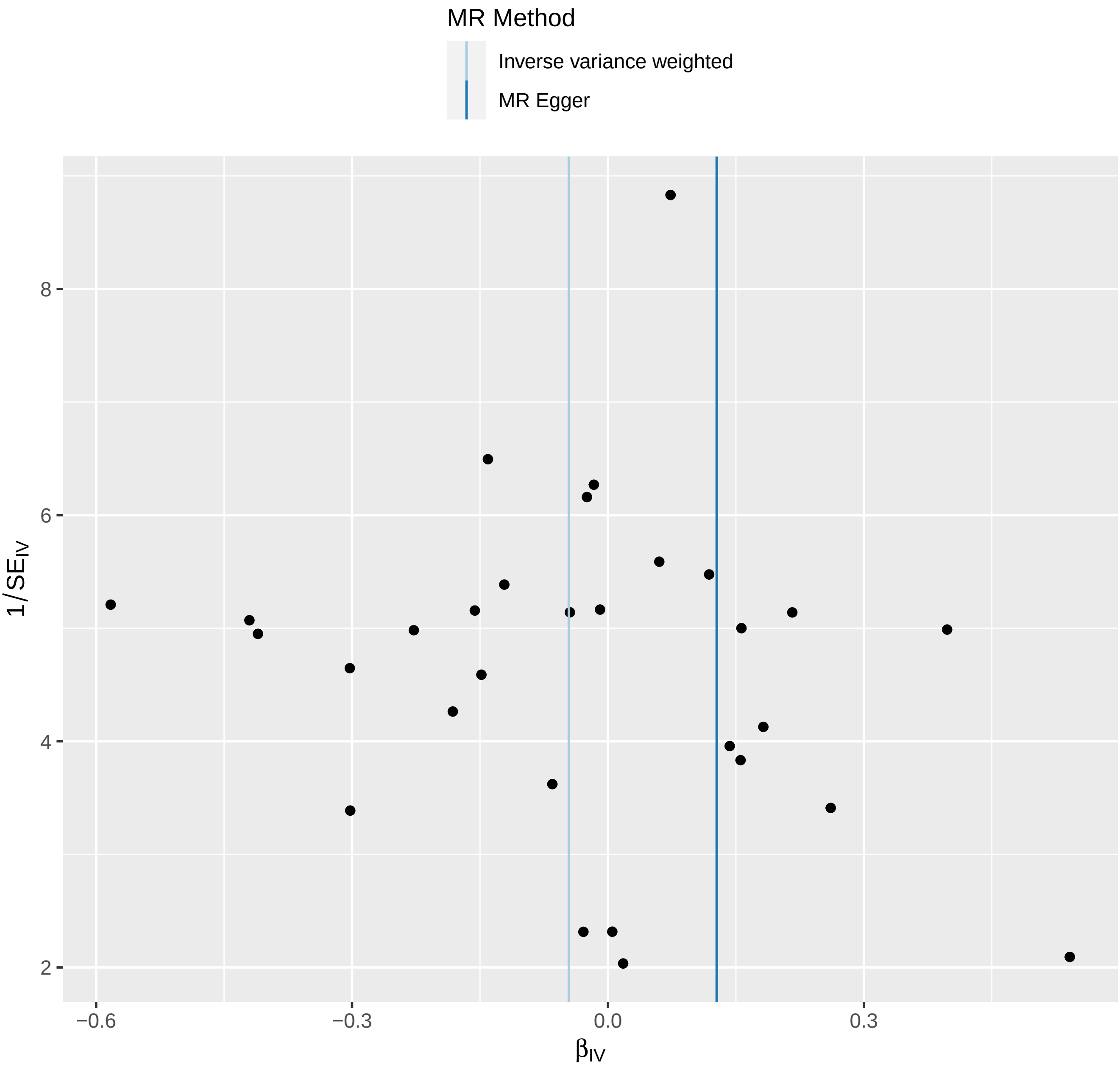

Batch 3−766 : Discoidin, CUB and LCCL domain−containing protein 2 on Diabetic nephropathy (more control exclusions) with a threshold of 1e−05

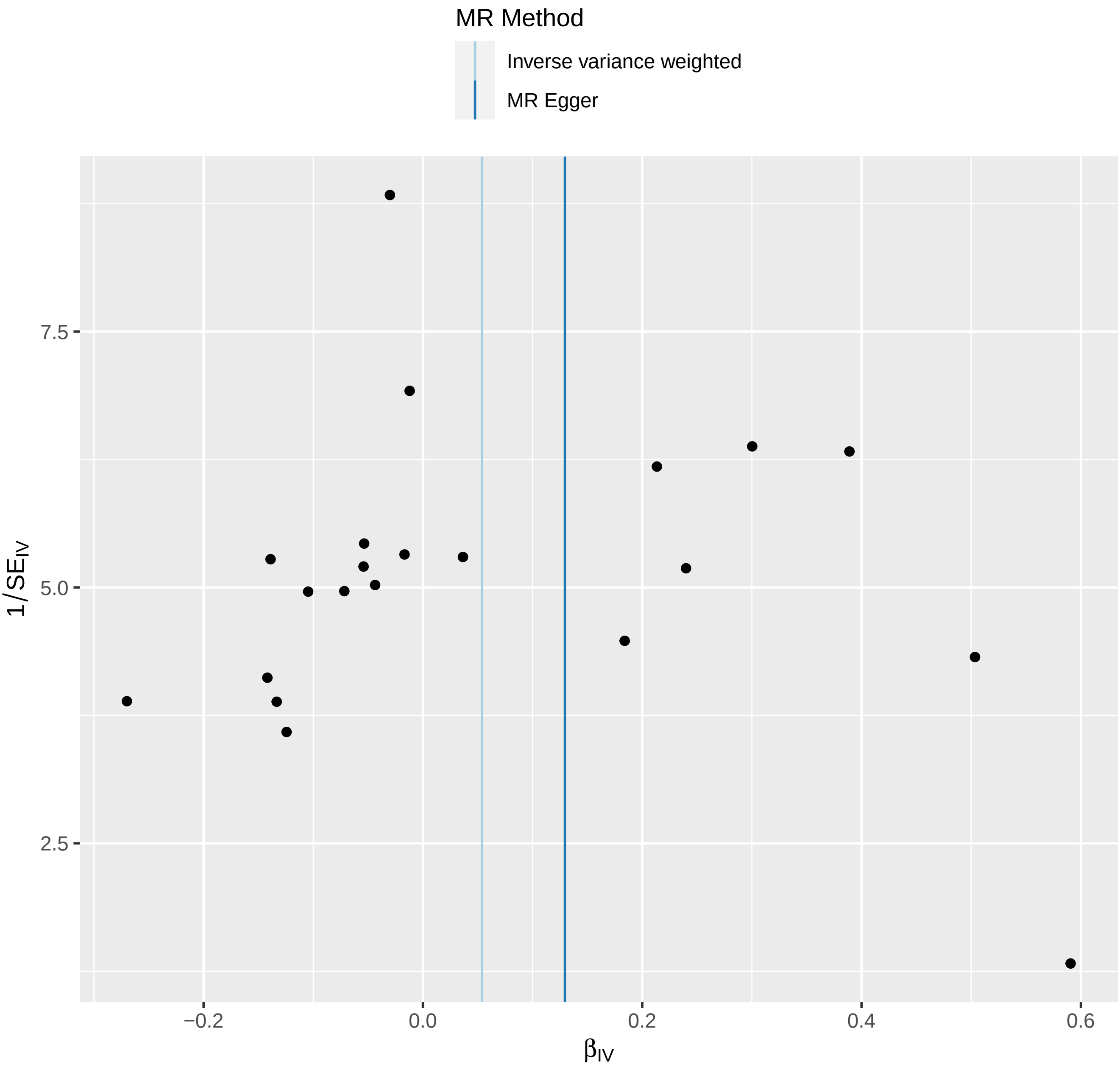

Batch 3−767 : Deoxycytidine kinase on Diabetic nephropathy (more control exclusions)
with a threshold of 1e−05

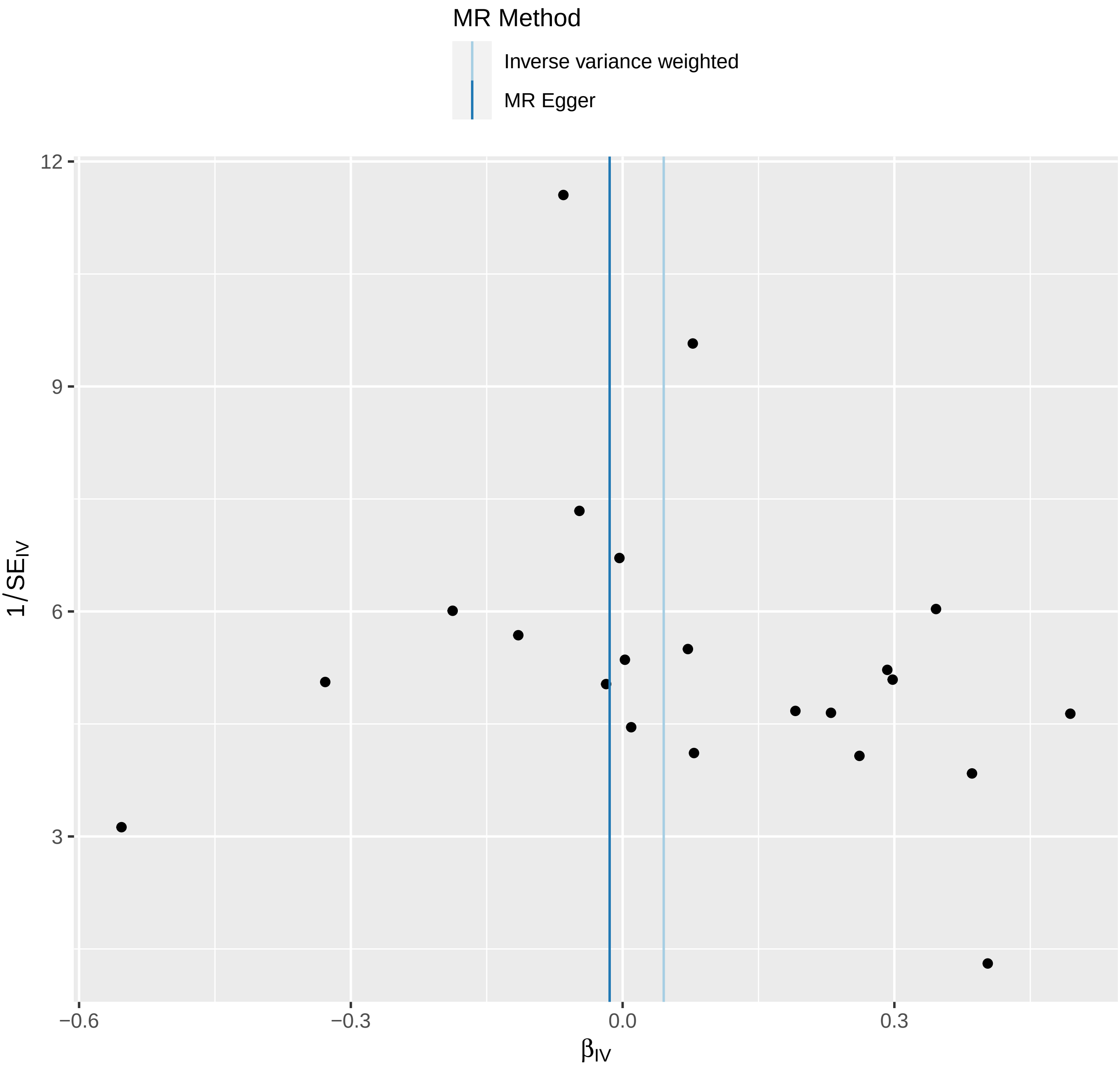

Batch 3−768 : Serine/threonine−protein kinase DCLK3 on Diabetic nephropathy (more control exclusions) with a threshold of 1e−05

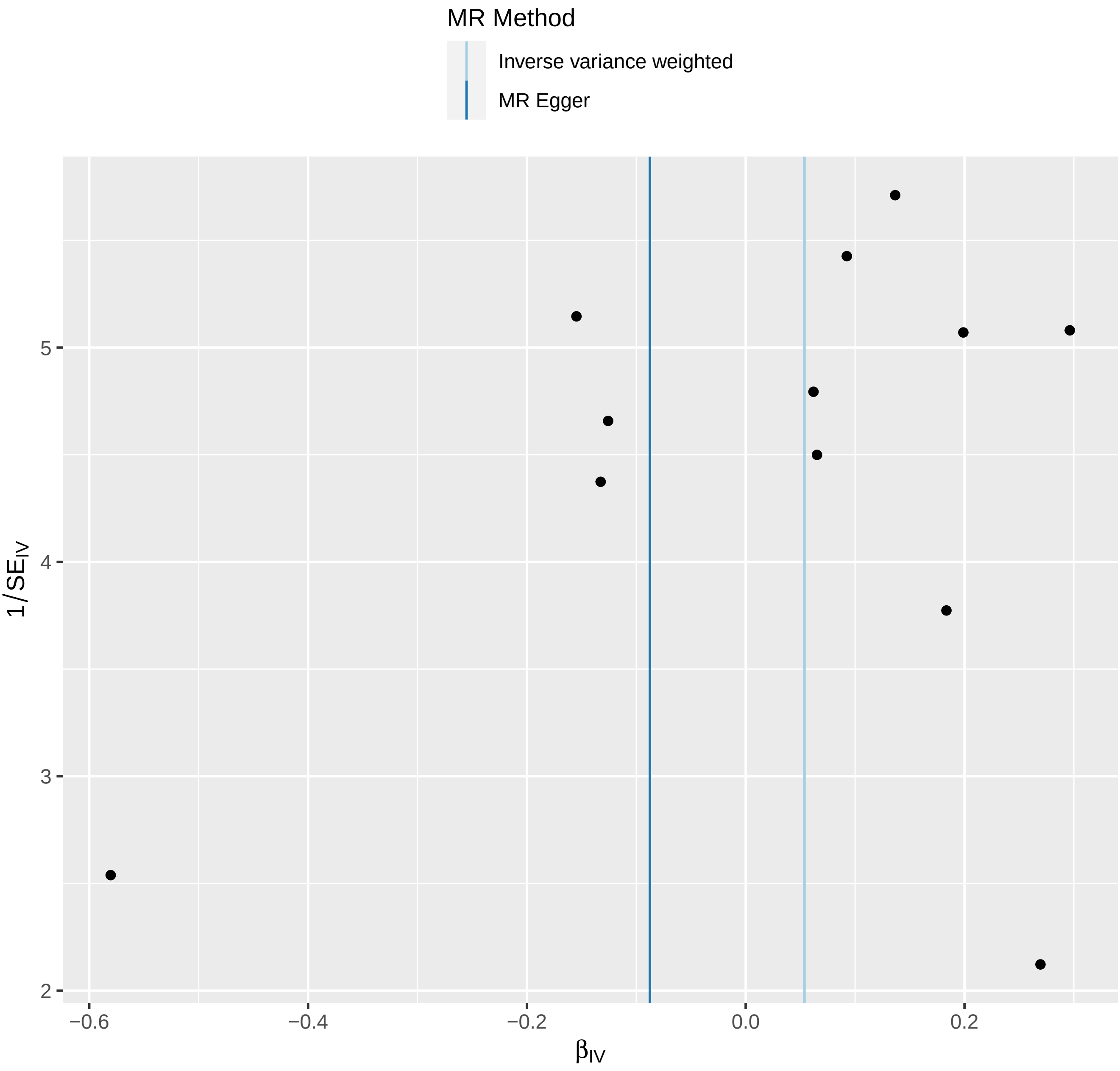

Batch 3−769 : Decorin on Diabetic nephropathy (more control exclusions)
with a threshold of 1e−05

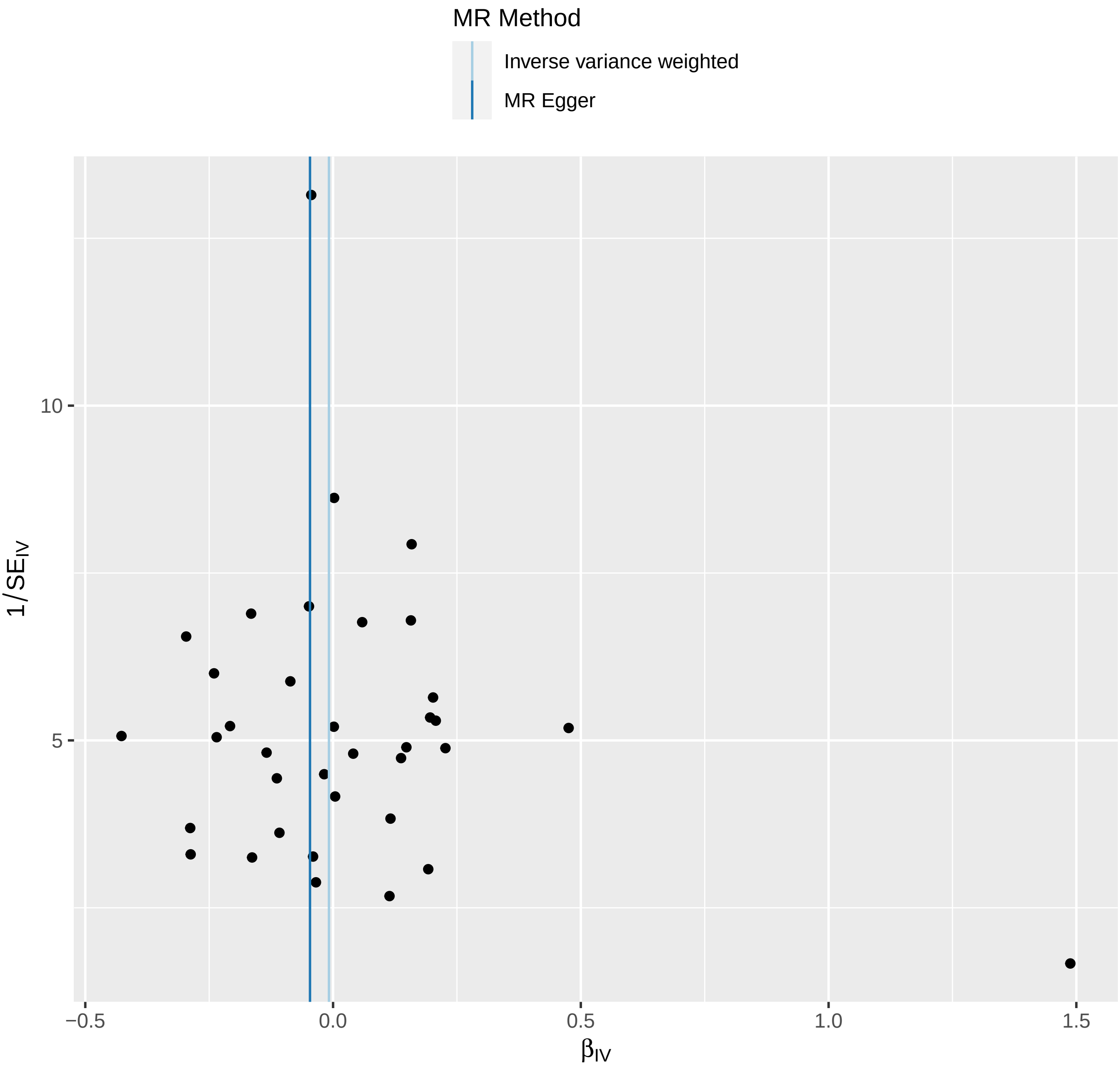

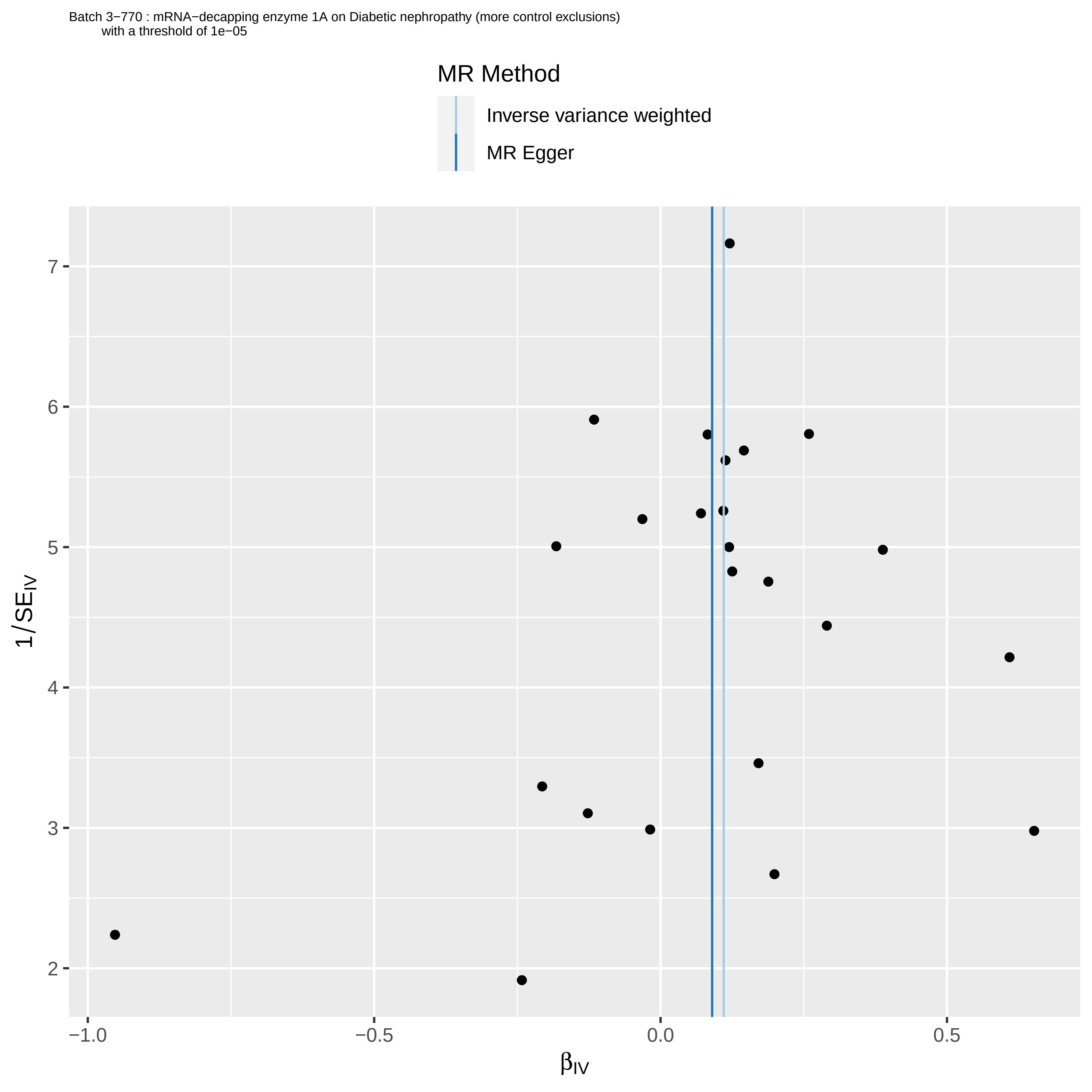

Batch 3−770 : mRNA−decapping enzyme 1A on Diabetic nephropathy (more control exclusions)
with a threshold of 1e−05
MR Method
Inverse variance weighted
MR Egger
1/SE_IV
β_IV
7
6
5
4
3
2
−1.0
−0.5
0.0
0.5

Batch 3−771 : Dynactin subunit 2 on Diabetic nephropathy (more control exclusions)
with a threshold of 1e−05

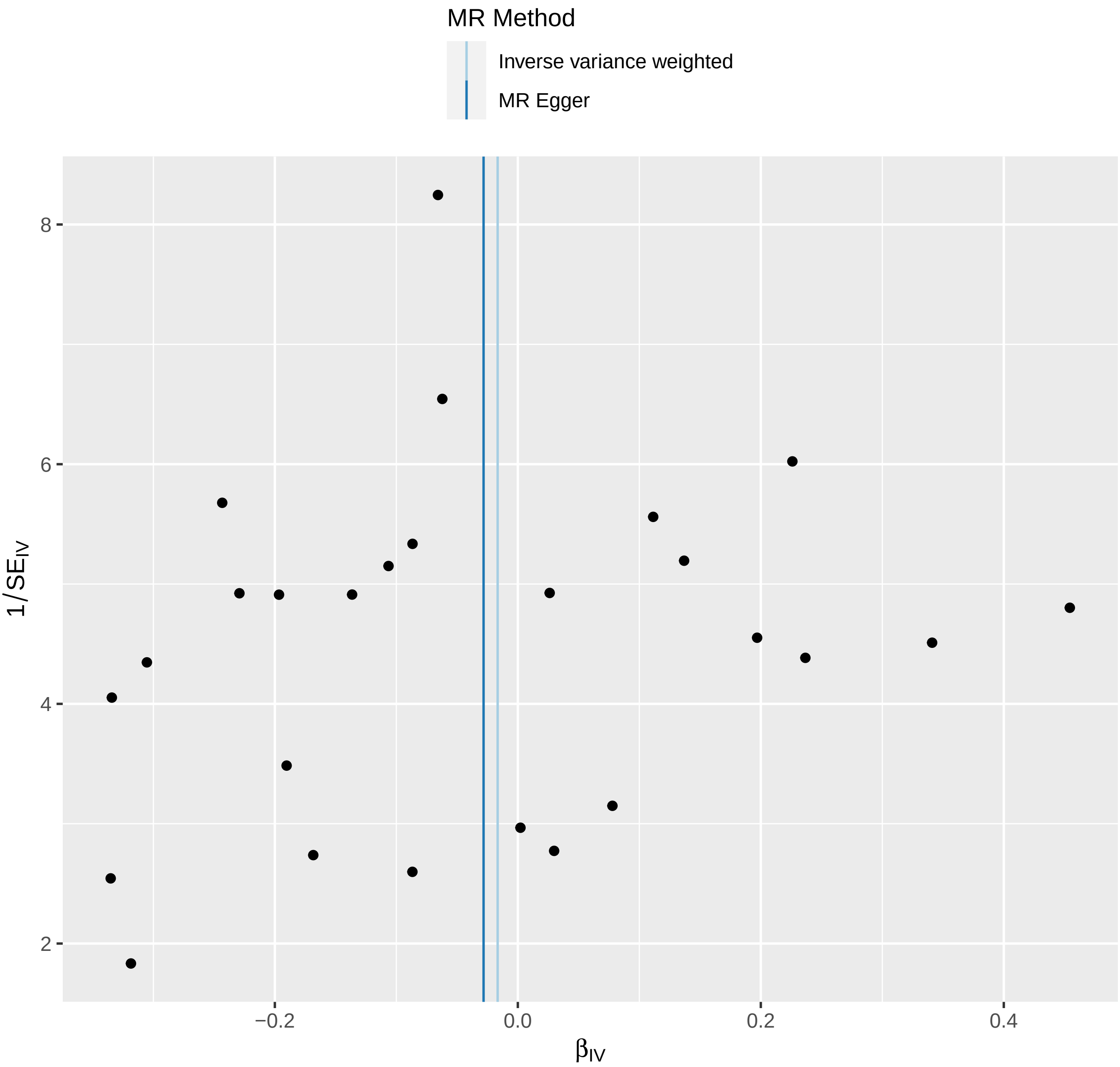

Batch 3−772 : dCTP pyrophosphatase 1 on Diabetic nephropathy (more control exclusions)
with a threshold of 1e−05

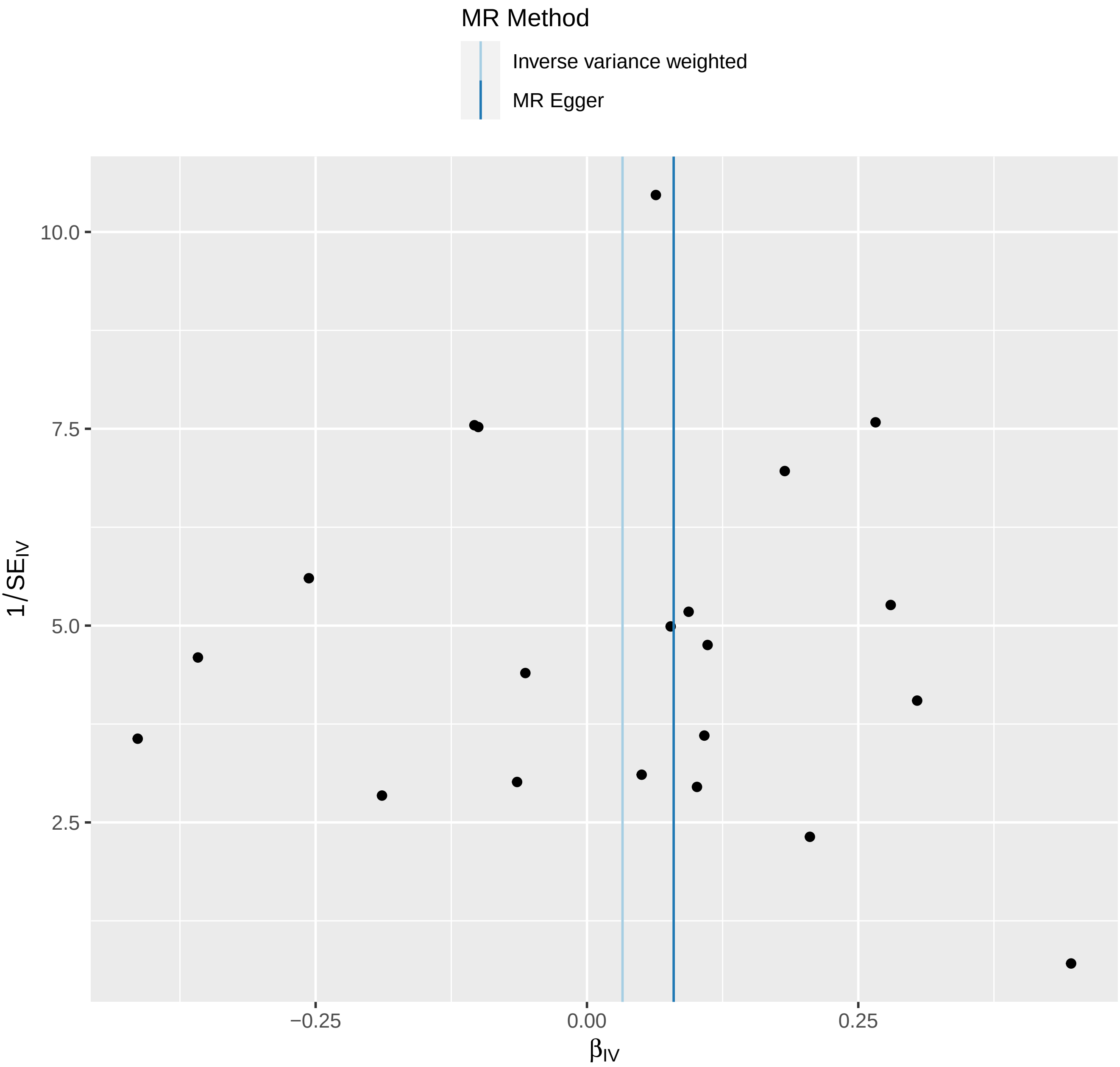

Batch 3−773 : DCN1−like protein 3 on Diabetic nephropathy (more control exclusions)
with a threshold of 1e−05

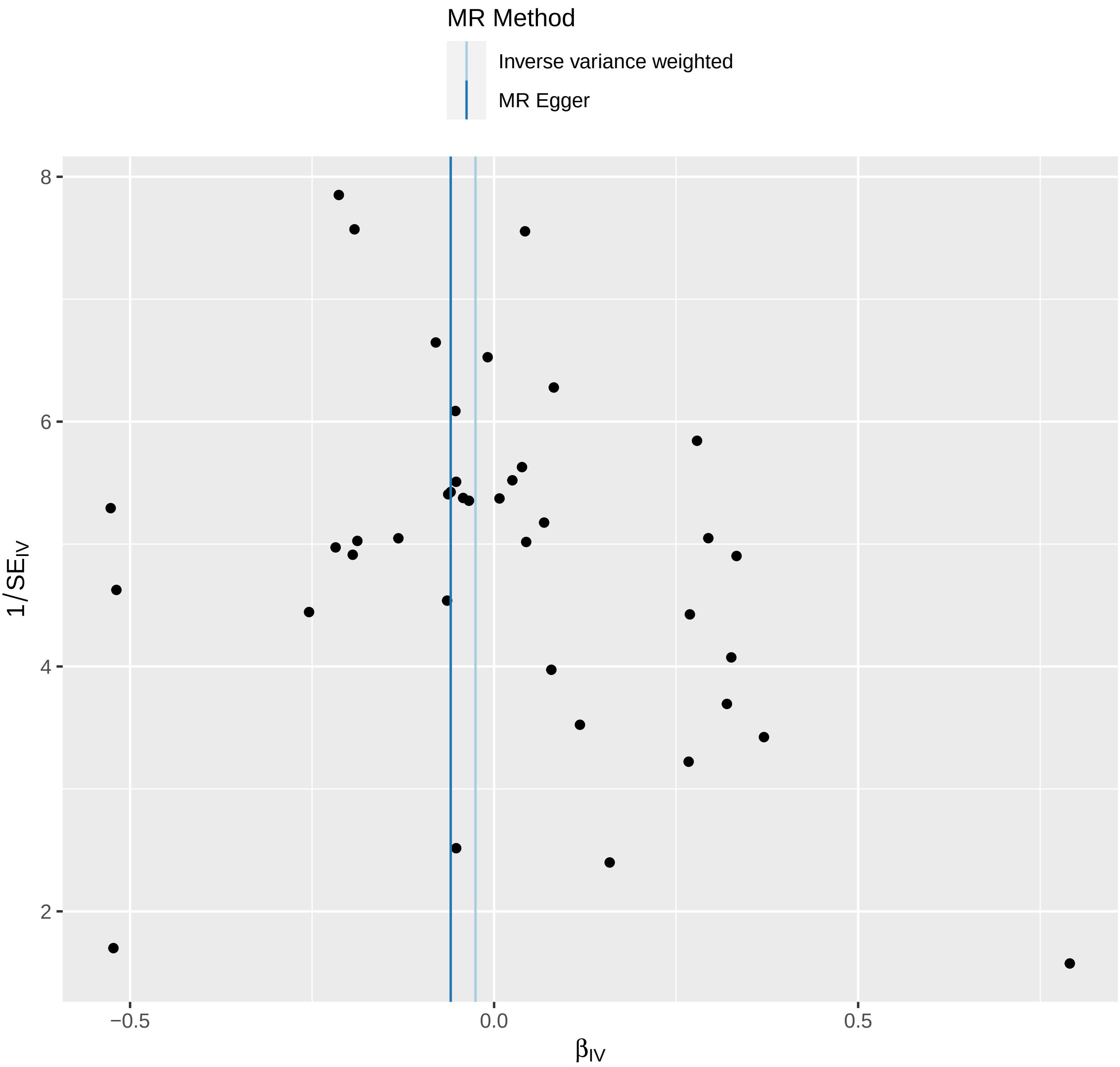

Batch 3−774 : DCN1−like protein 5 on Diabetic nephropathy (more control exclusions)
with a threshold of 1e−05

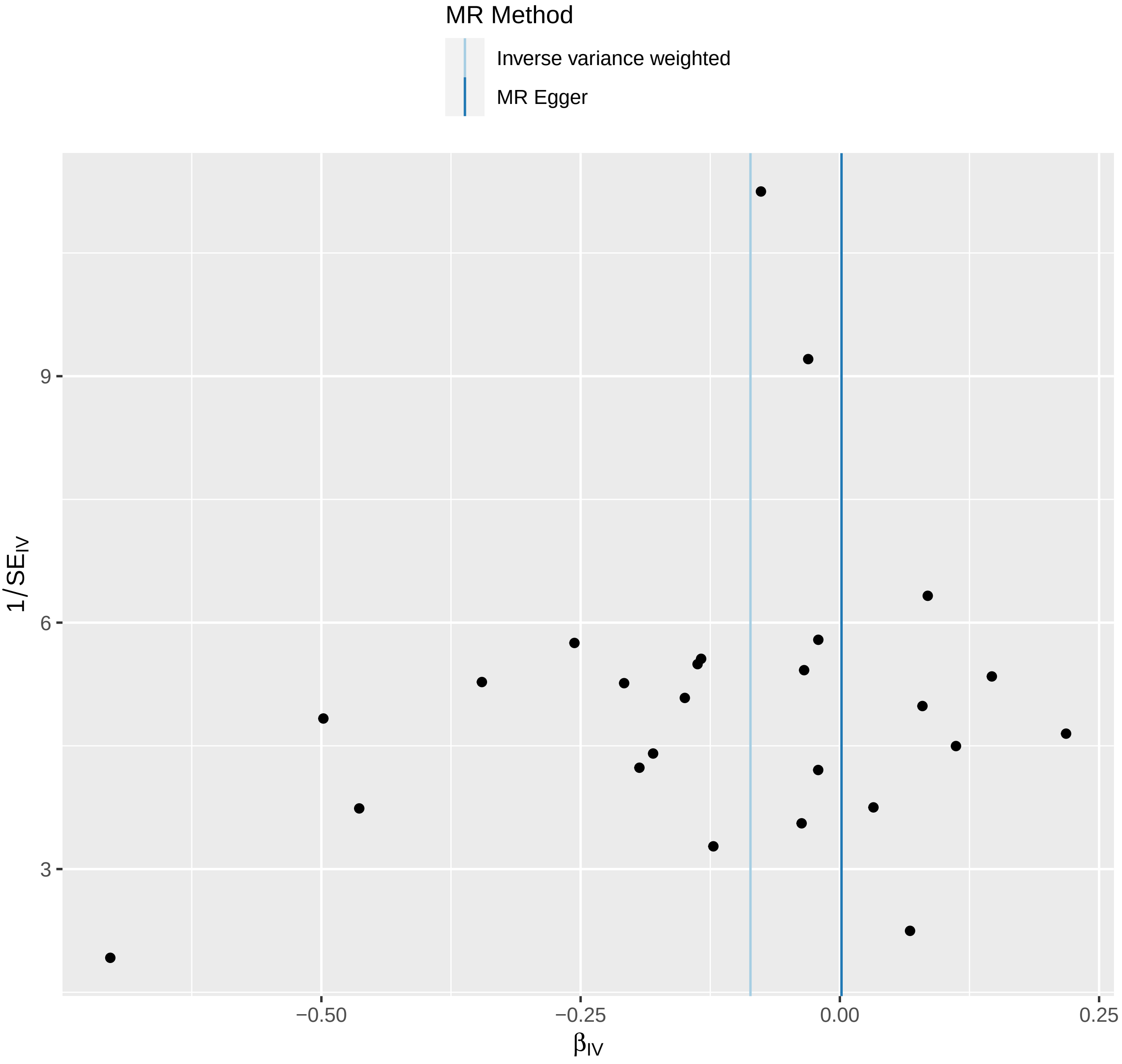

Batch 3−775 : Aromatic−L−amino−acid decarboxylase on Diabetic nephropathy (more control exclusions)
with a threshold of 1e−05

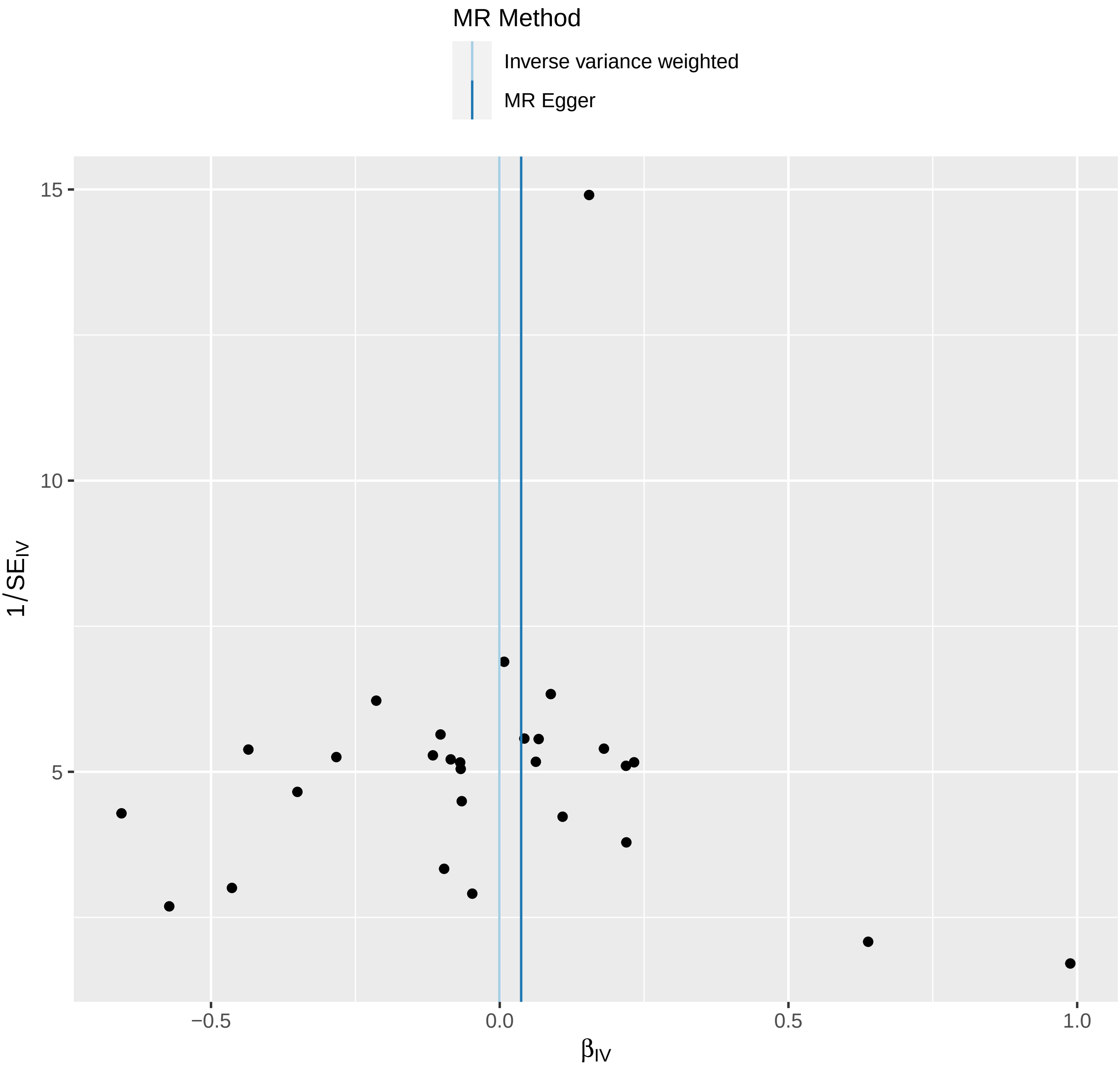

Batch 3−776 : Discoidin domain−containing receptor 2 on Diabetic nephropathy (more control exclusions)
with a threshold of 1e−05

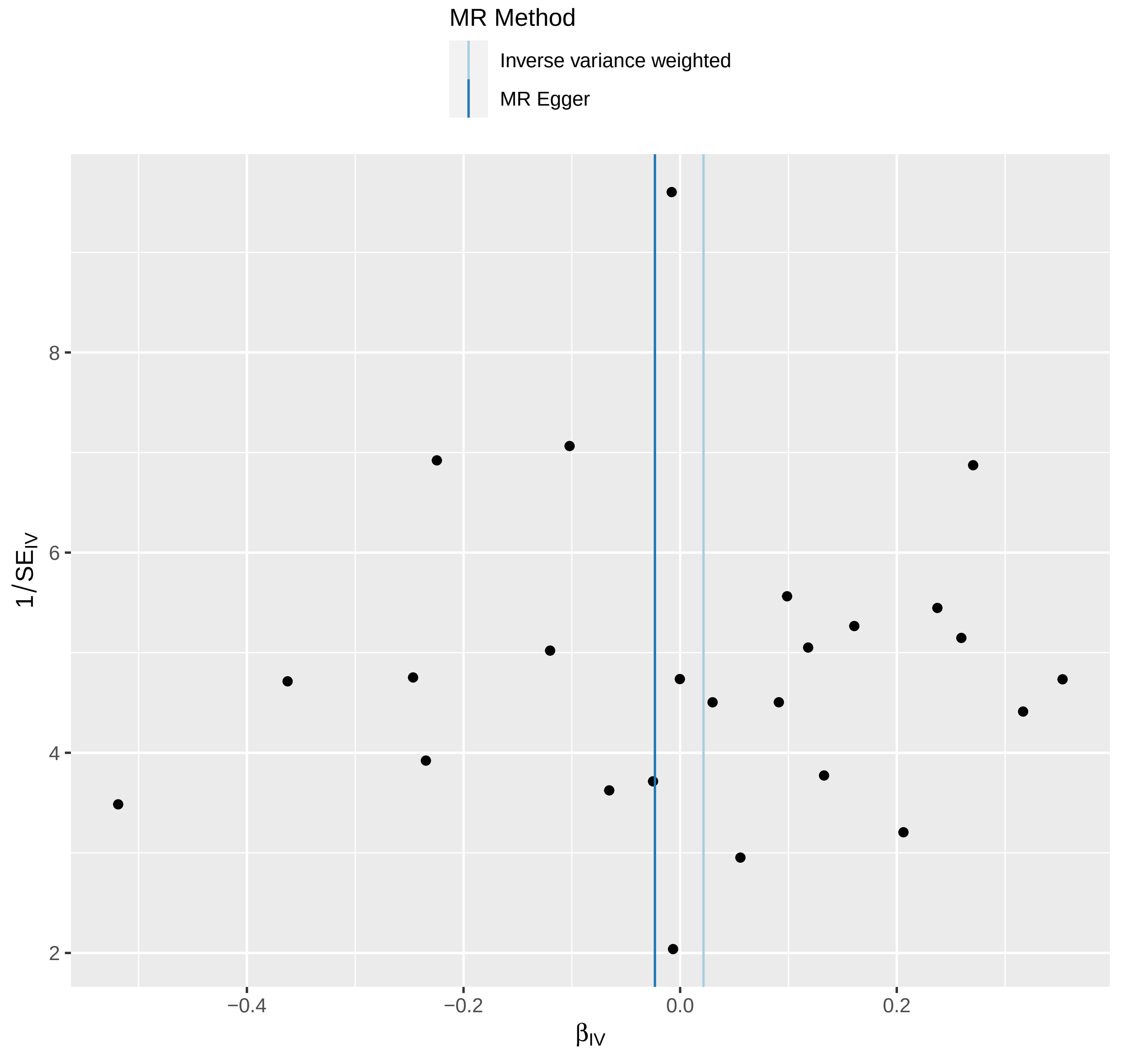

Batch 3−777 : ATP−dependent RNA helicase DDX25 on Diabetic nephropathy (more control exclusions)
with a threshold of 1e−05

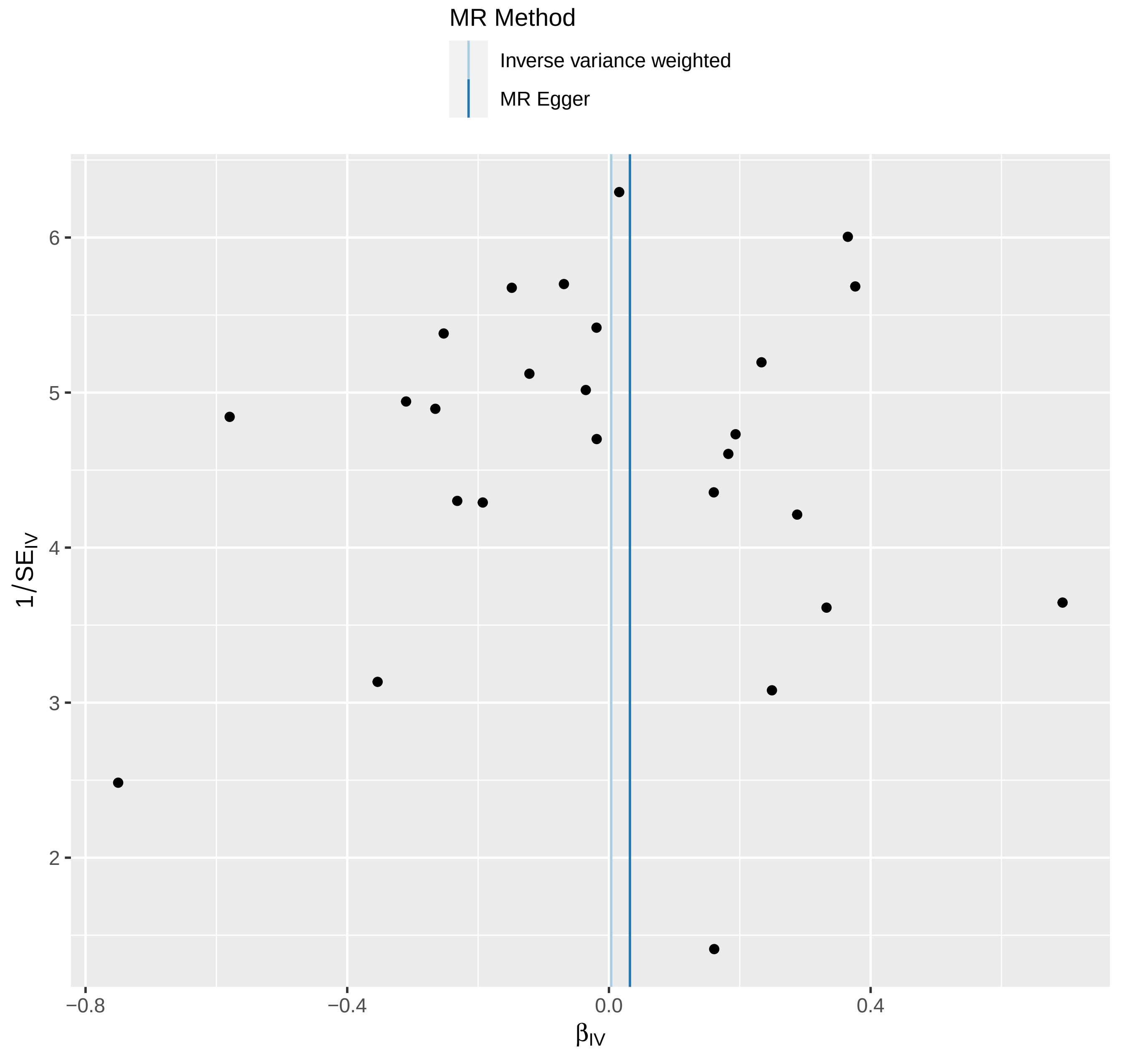

Batch 3−778 : Spliceosome RNA helicase DDX39B on Diabetic nephropathy (more control exclusions)
with a threshold of 1e−05

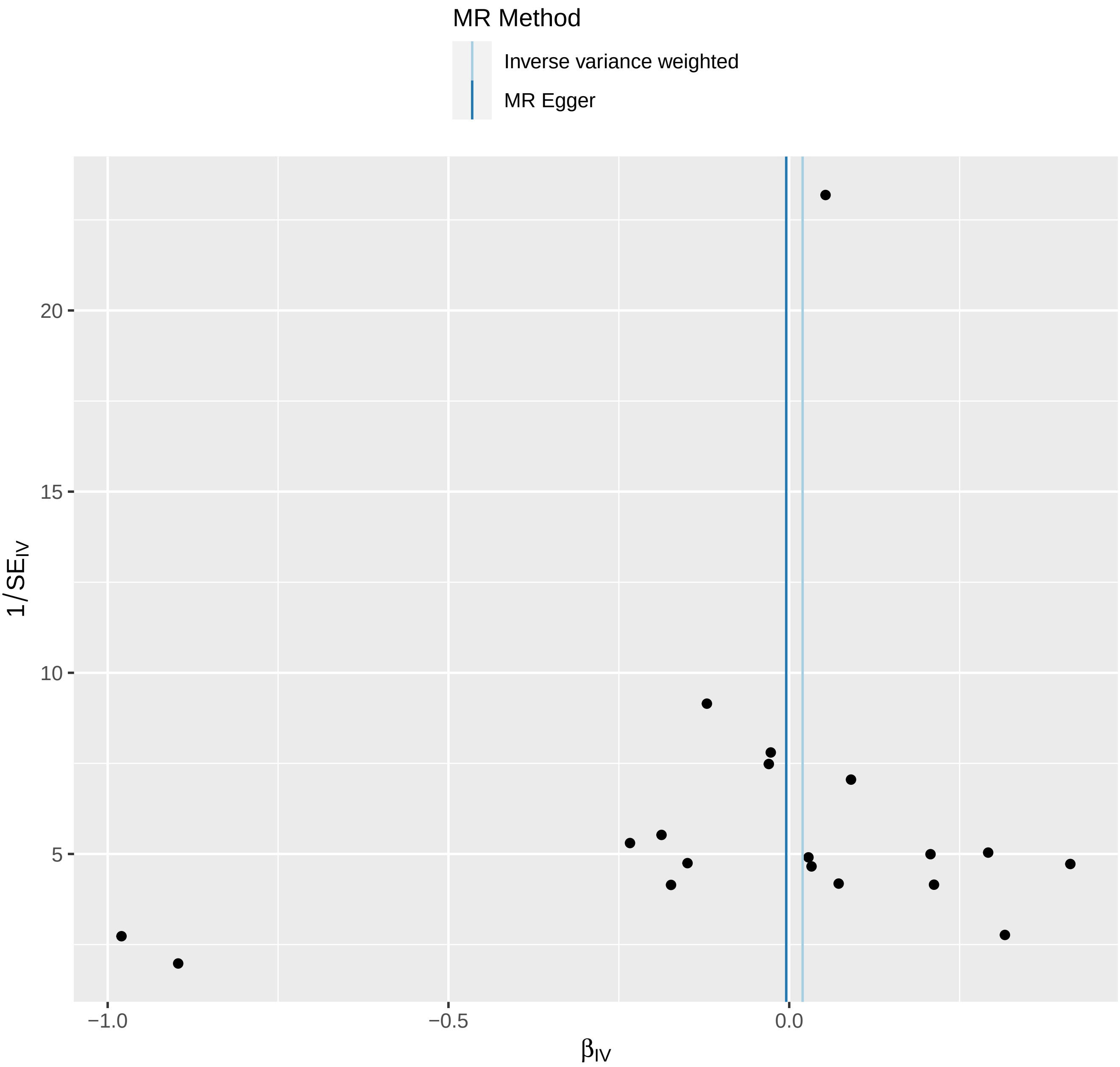

Batch 3−779 : Probable ATP−dependent RNA helicase DDX58 on Diabetic nephropathy (more control exclusions)
with a threshold of 1e−05

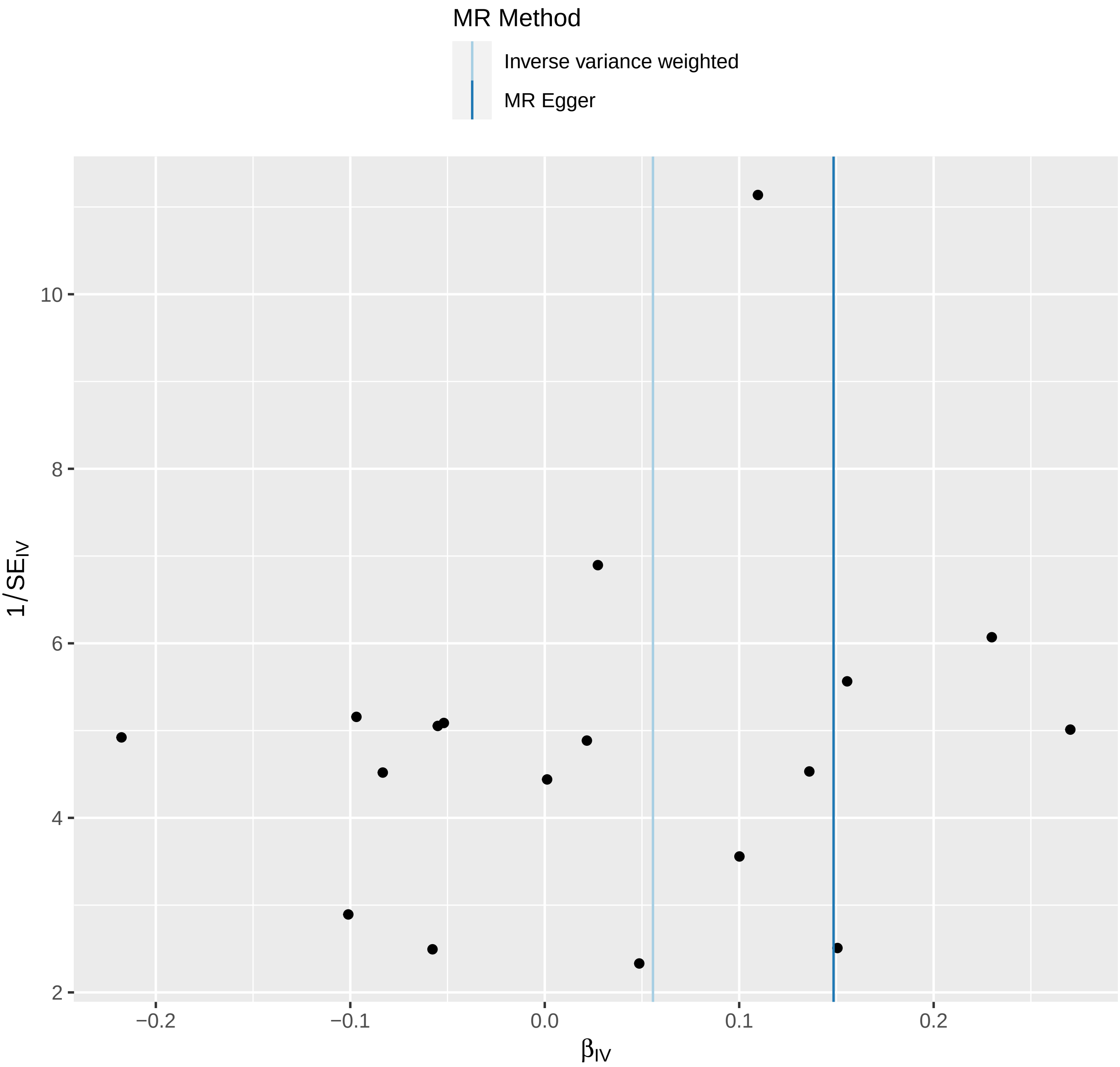

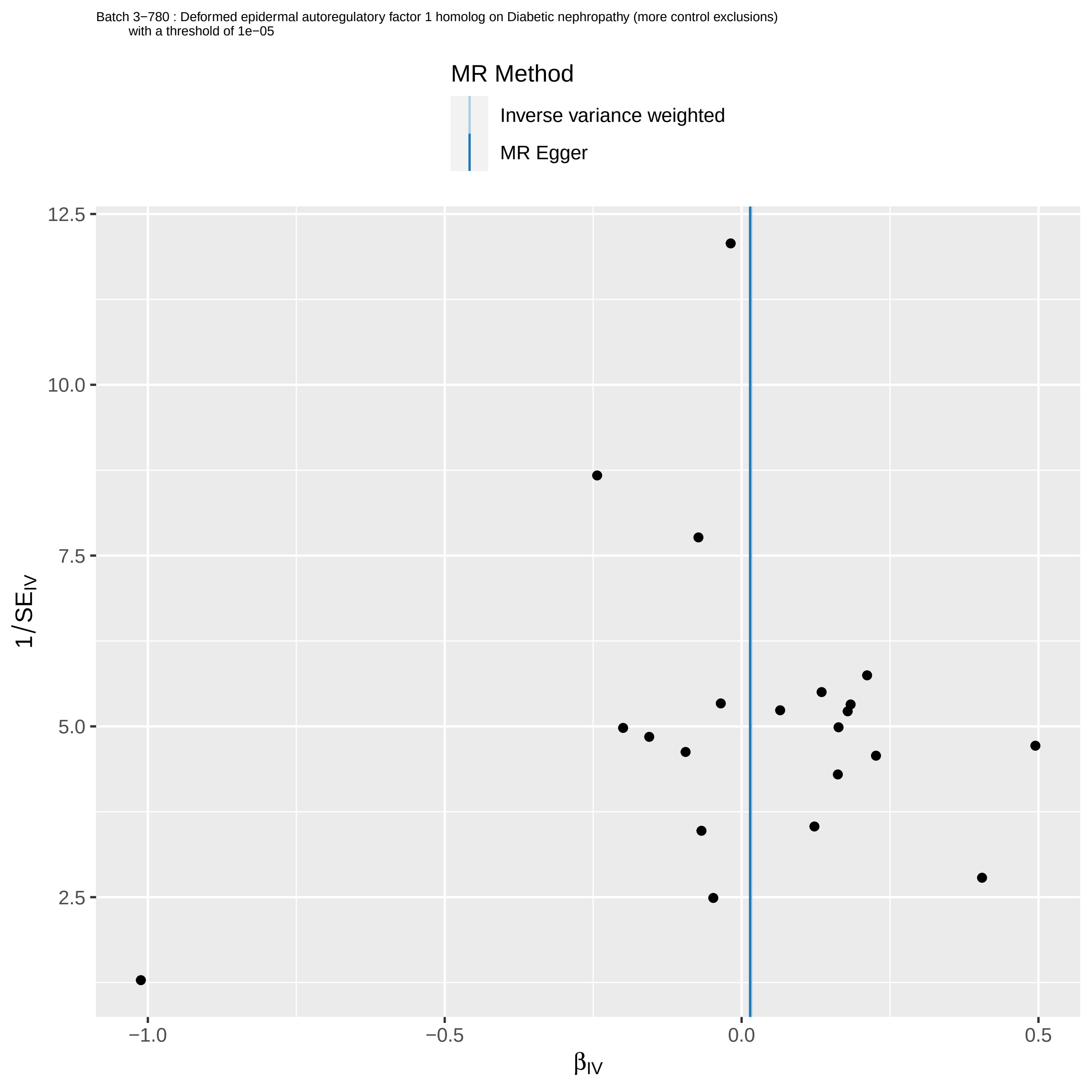
Batch 3−780 : Deformed epidermal autoregulatory factor 1 homolog on Diabetic nephropathy (more control exclusions)
with a threshold of 1e−05
MR Method
Inverse variance weighted
MR Egger
12.5
10.0
7.5
5.0
2.5
1/SE_IV
−1.0
−0.5
0.0
0.5
β_IV

Batch 3−781 : Beta−defensin 103 on Diabetic nephropathy (more control exclusions)
with a threshold of 1e−05

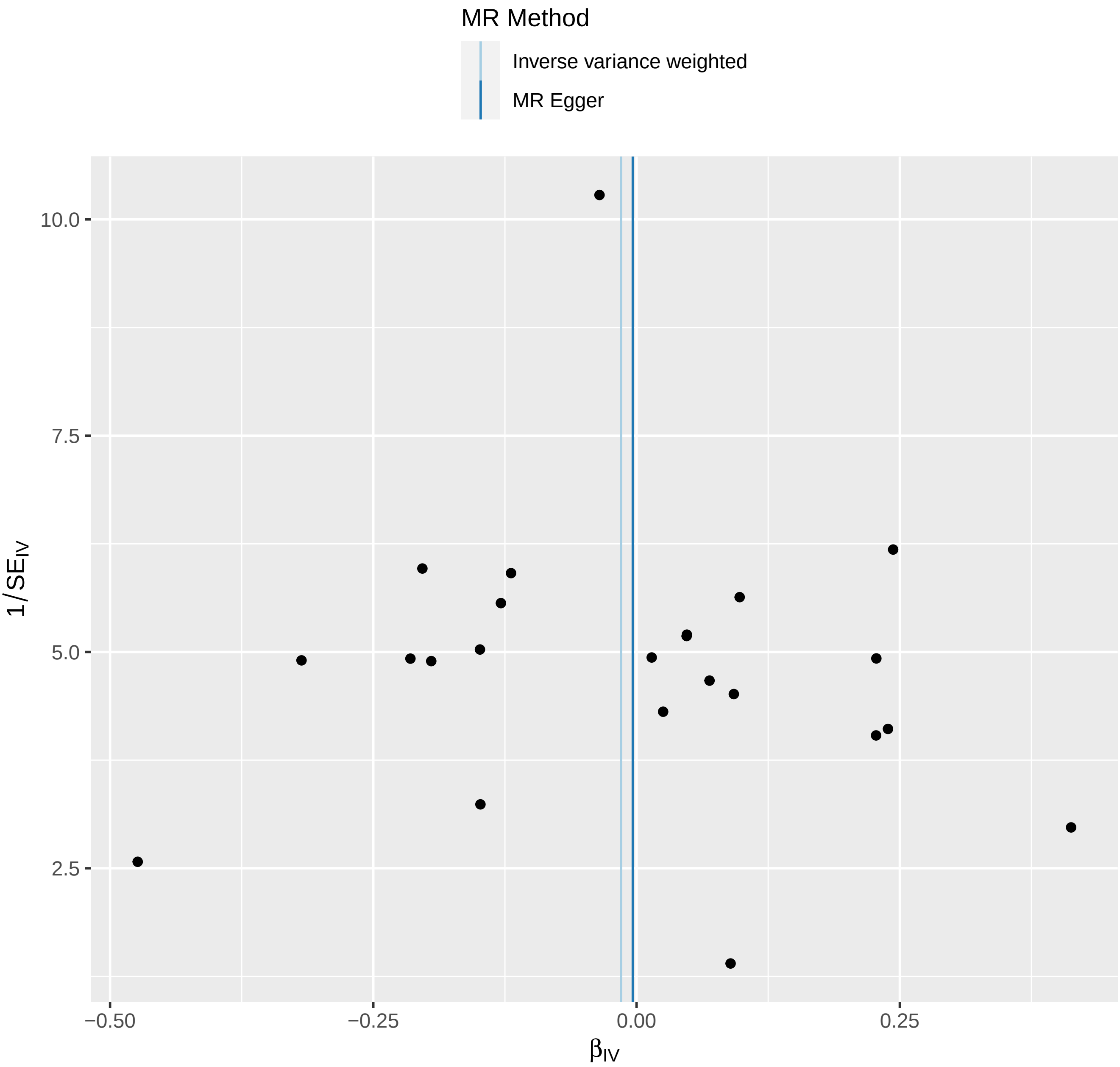

Batch 3−782 : Beta−defensin 104 on Diabetic nephropathy (more control exclusions)
with a threshold of 1e−05

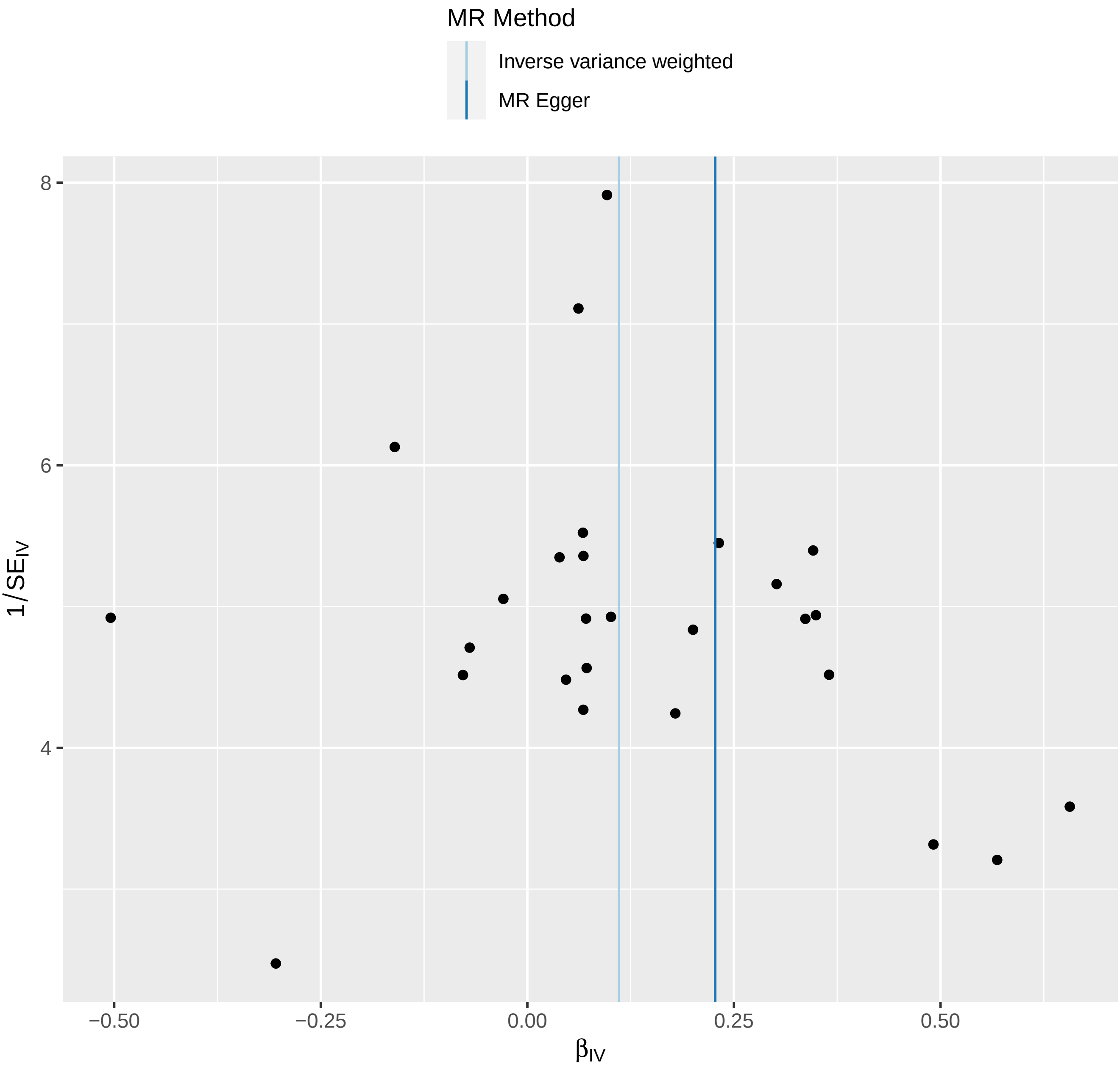

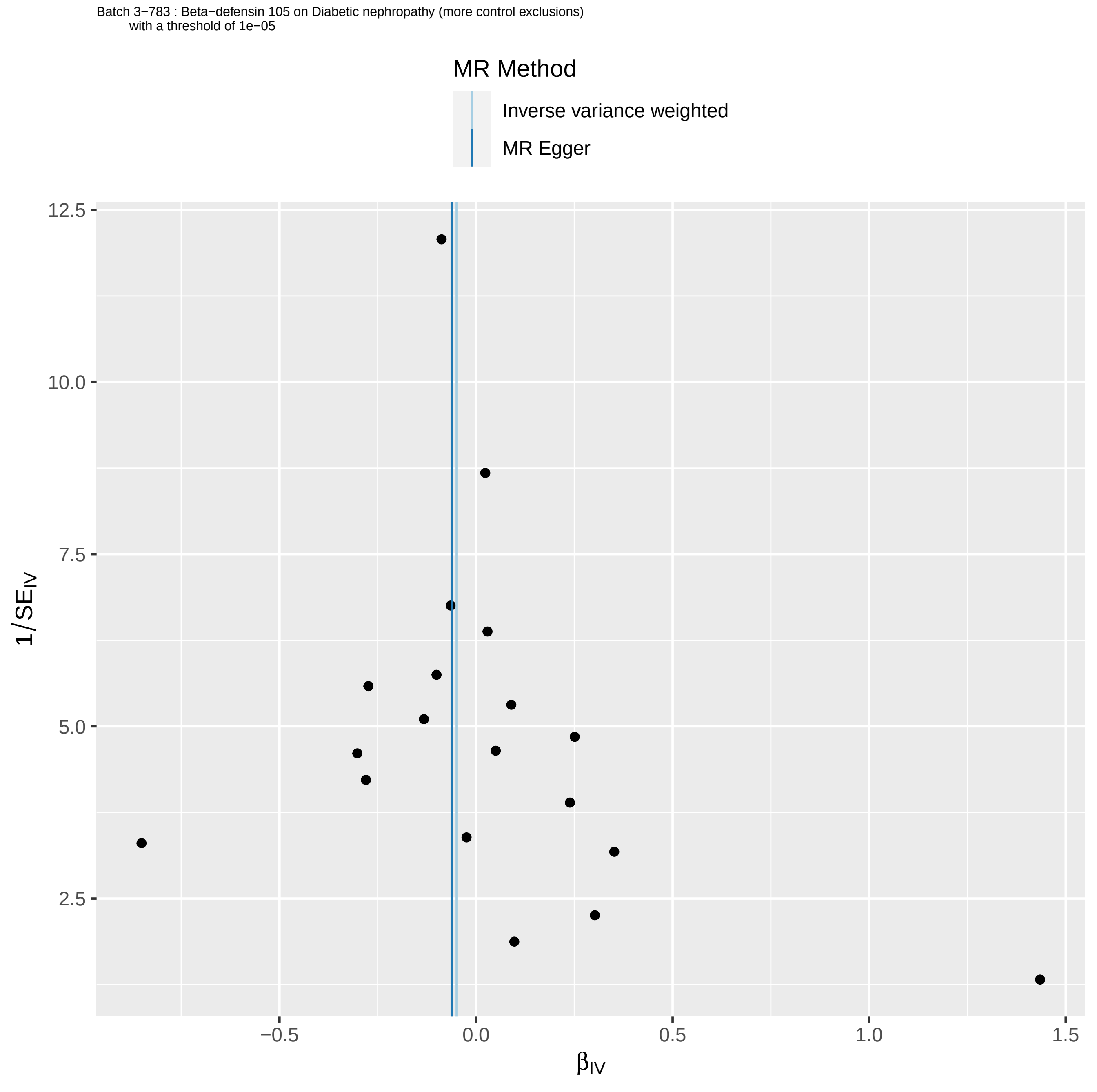
Batch 3−783 : Beta−defensin 105 on Diabetic nephropathy (more control exclusions)
with a threshold of 1e−05
MR Method
Inverse variance weighted
MR Egger
12.5
10.0
7.5
5.0
2.5
1/SE$_{IV}$
−0.5
0.0
0.5
1.0
1.5
β$_{IV}$

Batch 3−784 : Beta−defensin 106 on Diabetic nephropathy (more control exclusions)
with a threshold of 1e−05

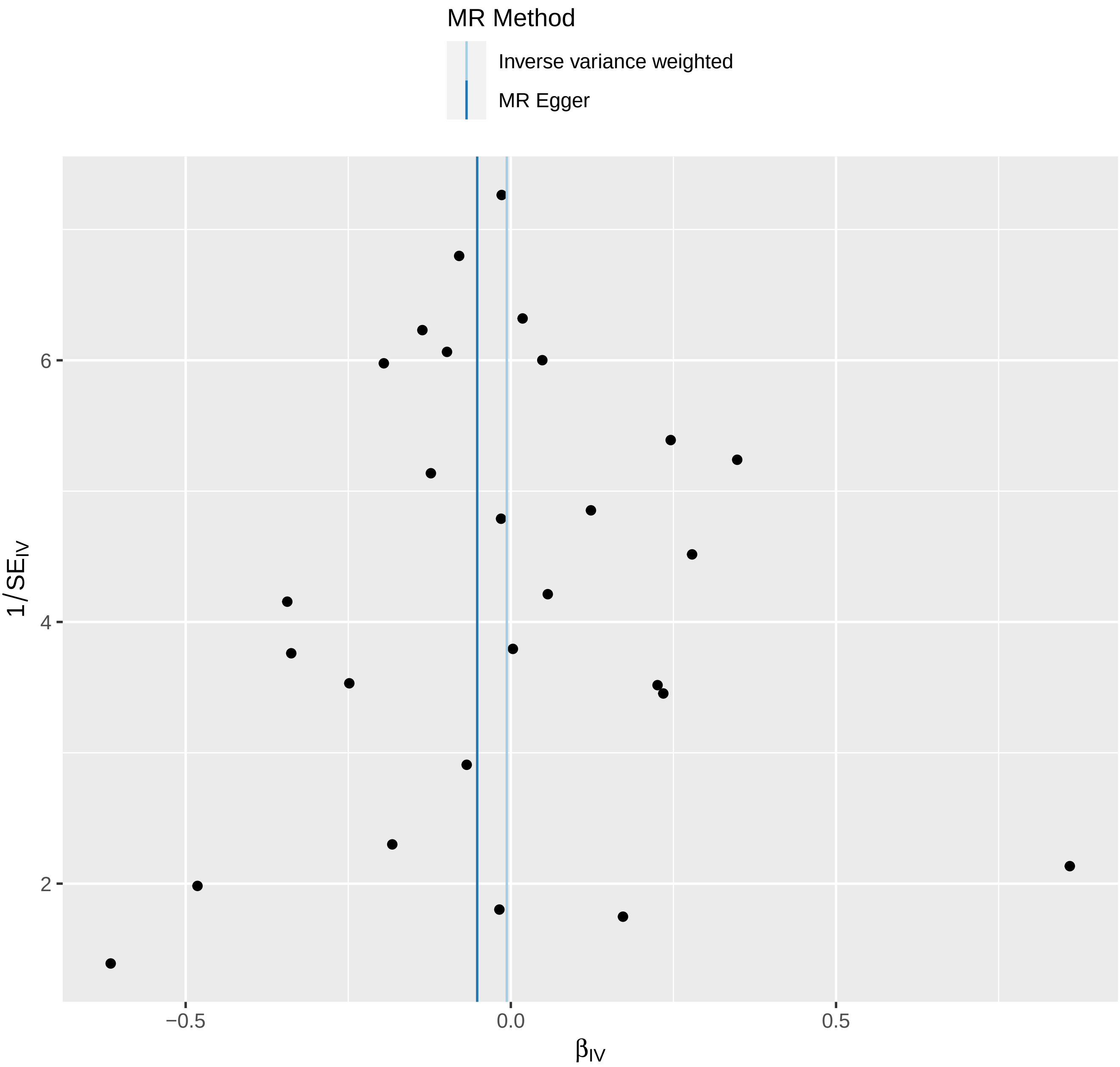

Batch 3−785 : Beta−defensin 107 on Diabetic nephropathy (more control exclusions)
with a threshold of 1e−05

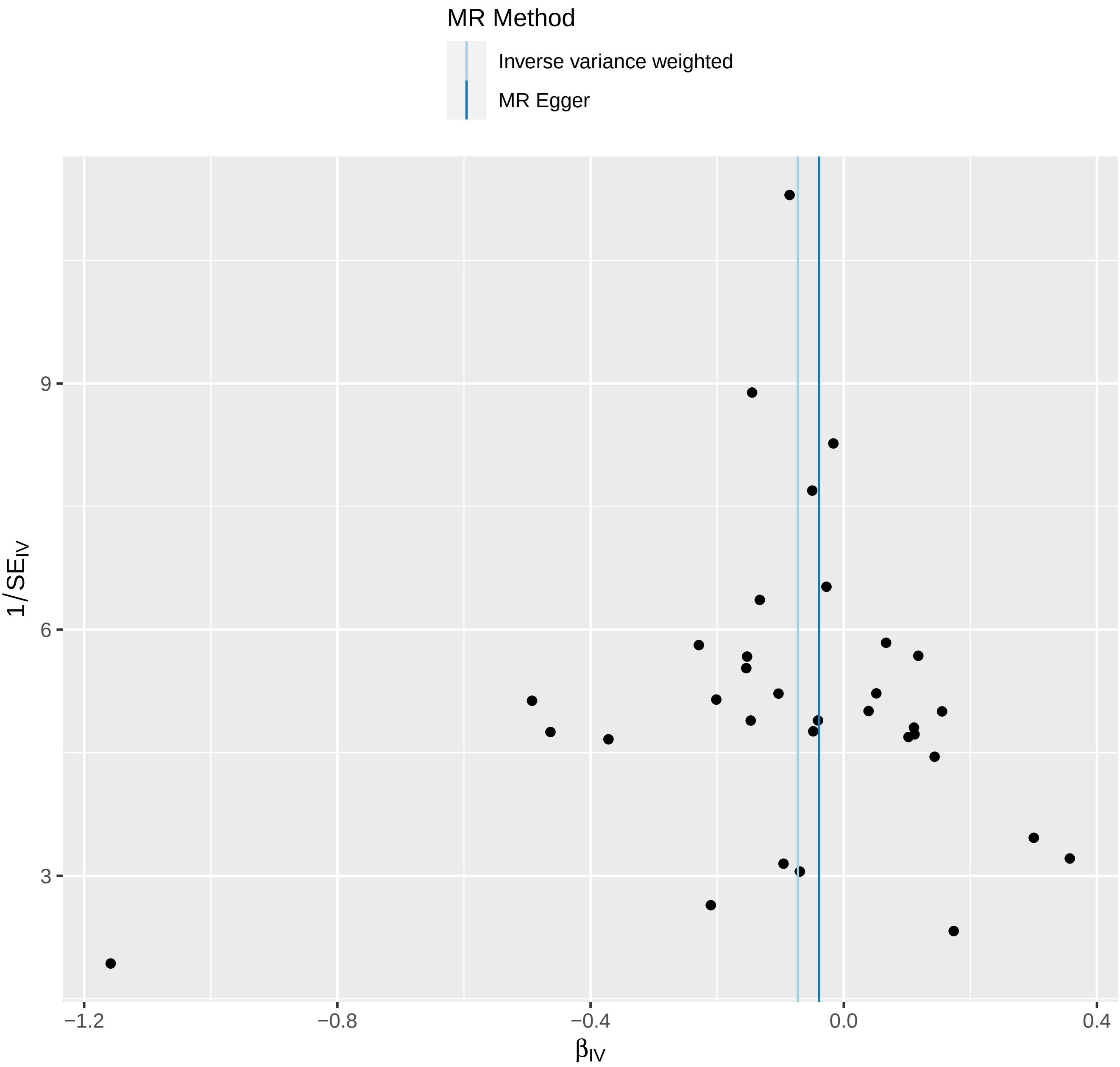

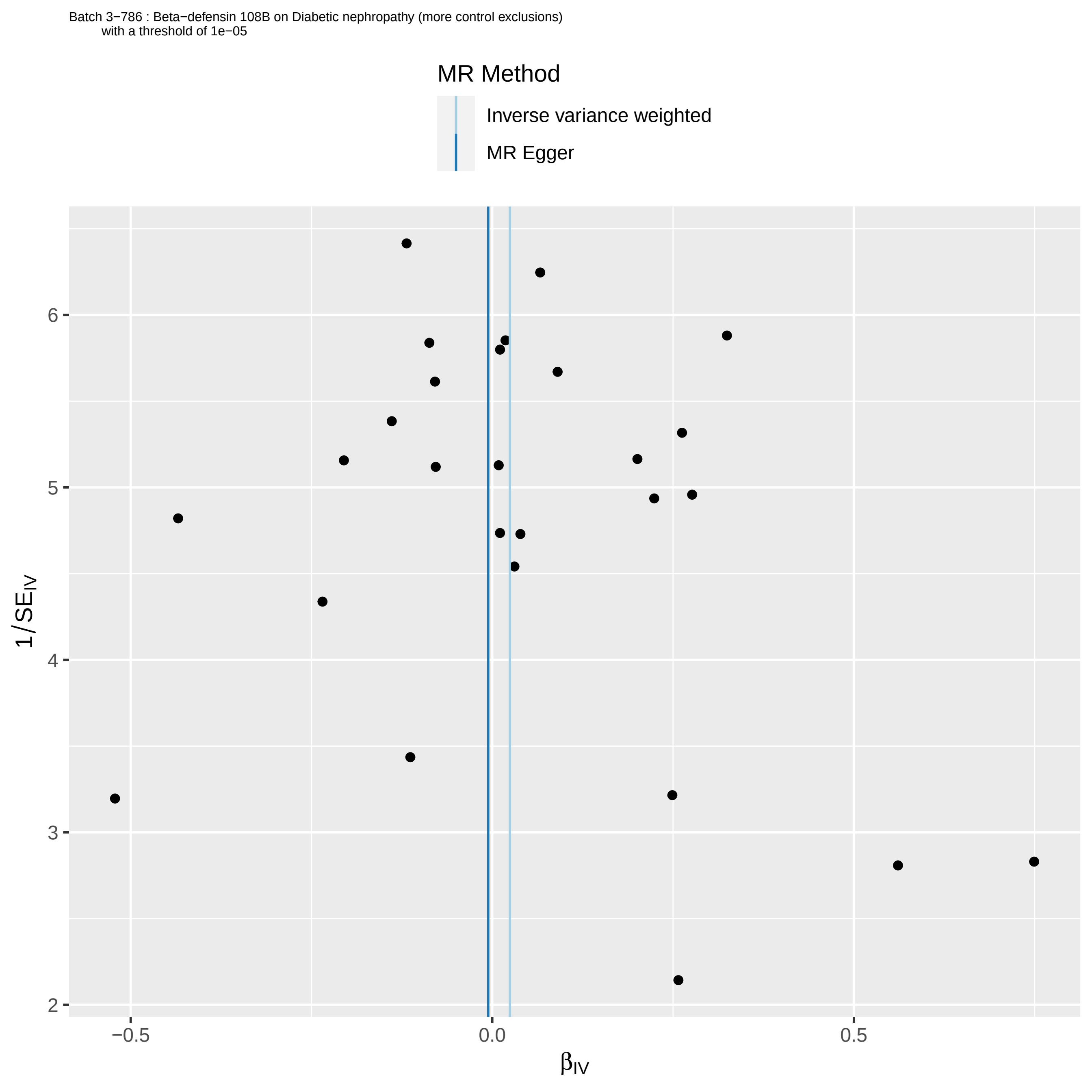

Batch 3−786 : Beta−defensin 108B on Diabetic nephropathy (more control exclusions)
with a threshold of 1e−05
MR Method
Inverse variance weighted
MR Egger
1/SE_IV
6
5
4
3
2
−0.5
0.0
0.5
β_IV

Batch 3−787 : Beta−defensin 110 on Diabetic nephropathy (more control exclusions)
with a threshold of 1e−05

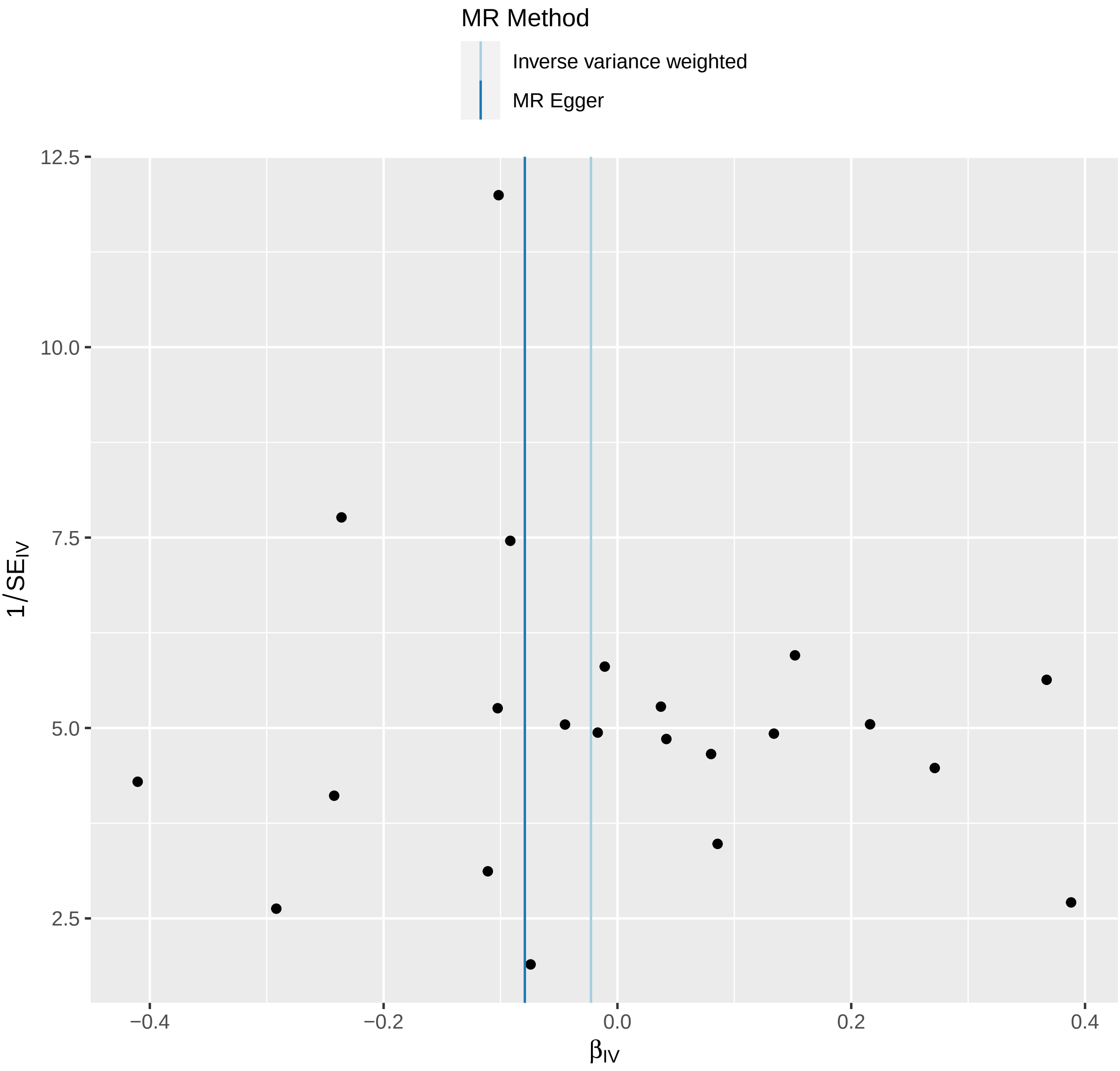

Batch 3−788 : Beta−defensin 112 on Diabetic nephropathy (more control exclusions)
with a threshold of 1e−05

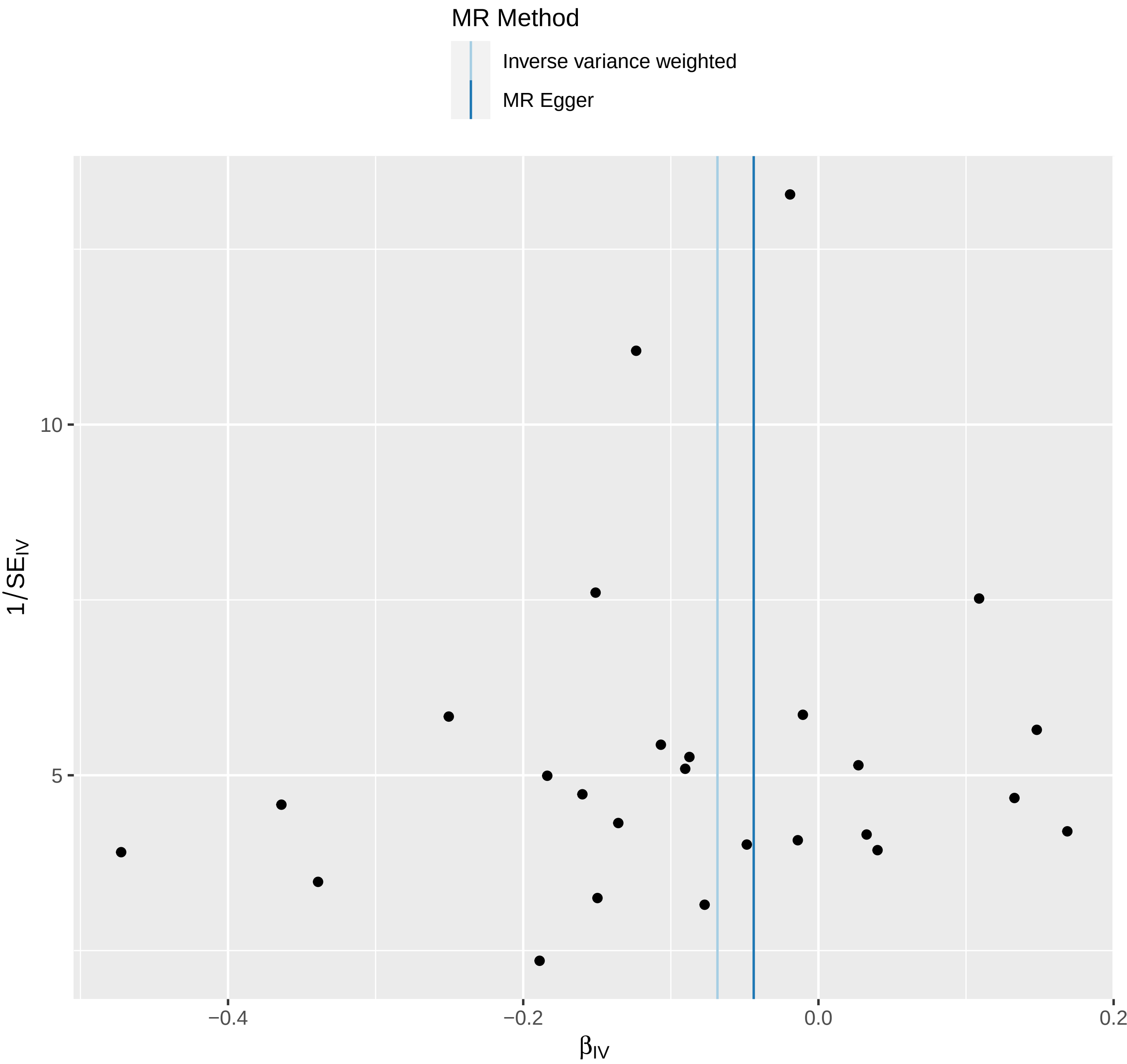

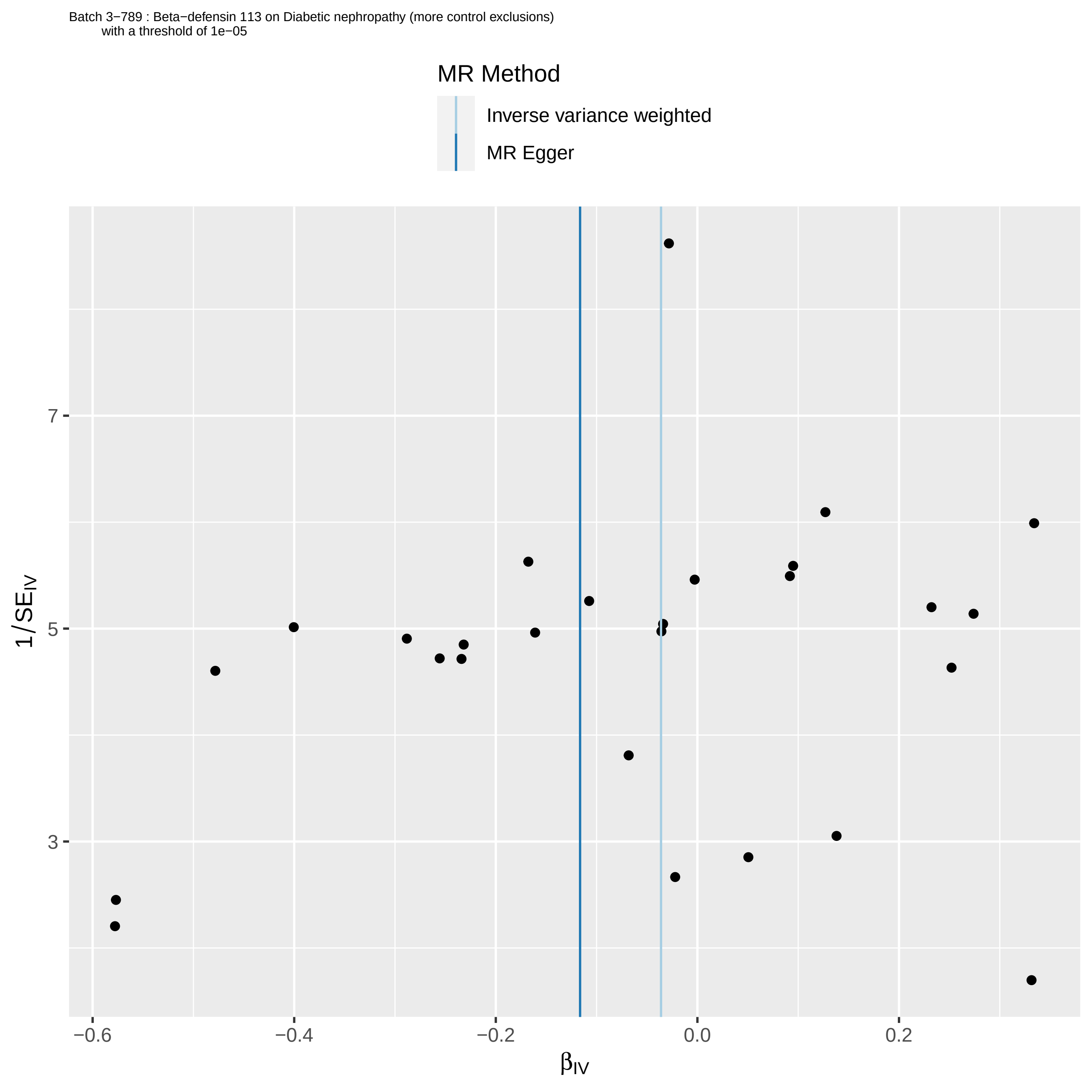
Batch 3−789 : Beta−defensin 113 on Diabetic nephropathy (more control exclusions)
with a threshold of 1e−05
MR Method
Inverse variance weighted
MR Egger
7
5
3
$1/SE_{IV}$
−0.6
−0.4
−0.2
0.0
0.2
β_{IV}

Batch 3−790 : Beta−defensin 115 on Diabetic nephropathy (more control exclusions)
with a threshold of 1e−05

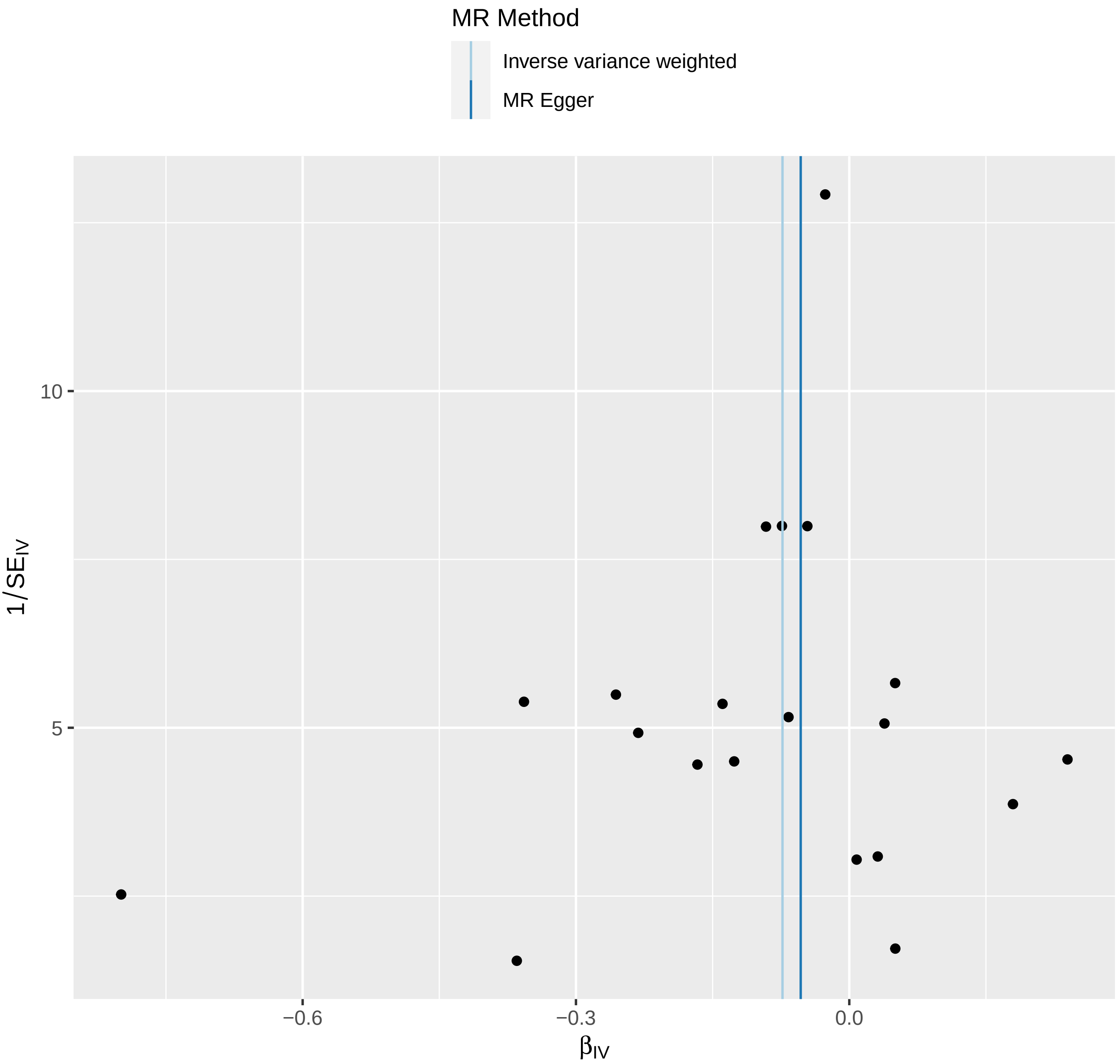

Batch 3−791 : Beta−defensin 116 on Diabetic nephropathy (more control exclusions)
with a threshold of 1e−05

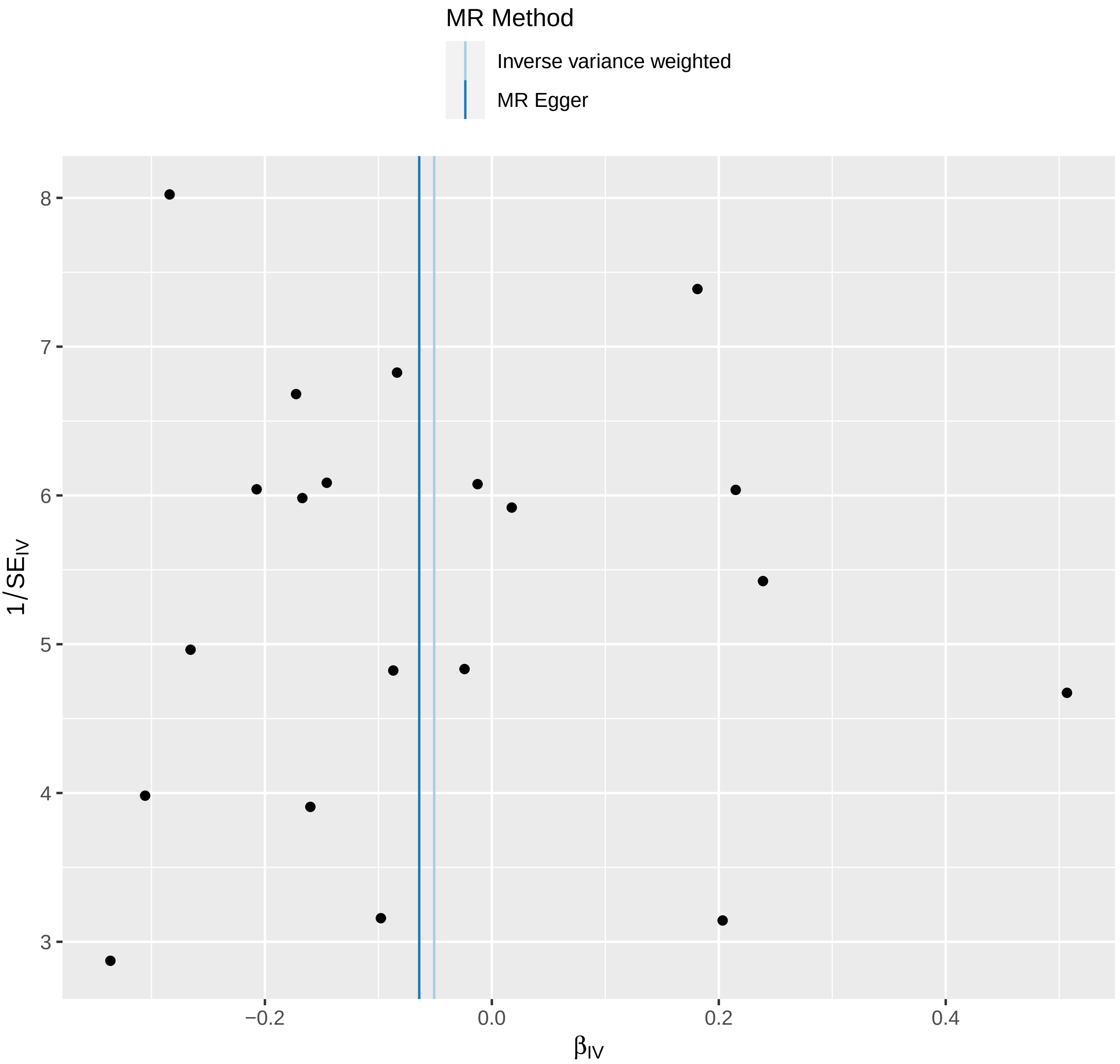

Batch 3−792 : Beta−defensin 118 on Diabetic nephropathy (more control exclusions)
with a threshold of 1e−05

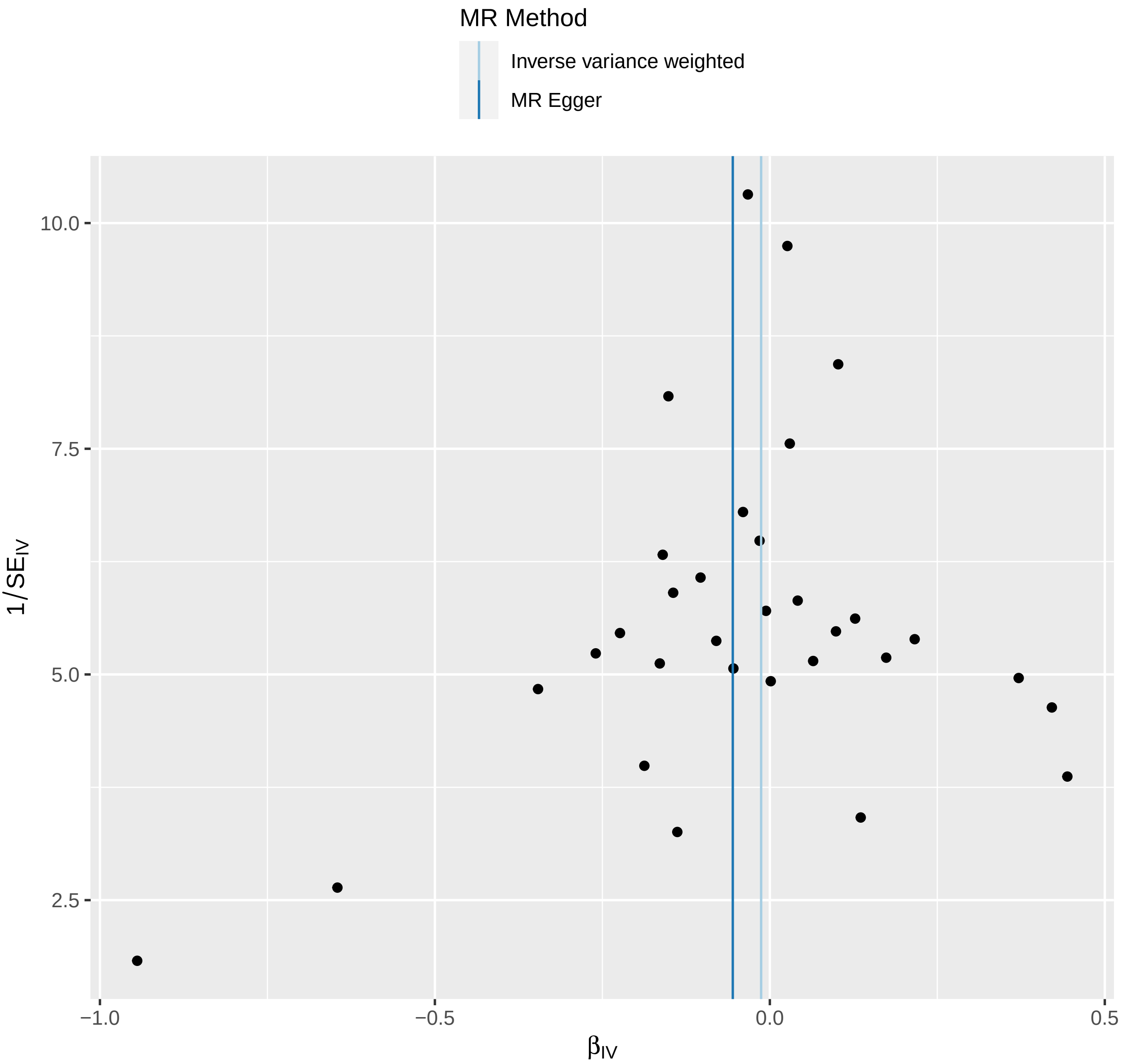

Batch 3−793 : Beta−defensin 119 on Diabetic nephropathy (more control exclusions)
with a threshold of 1e−05

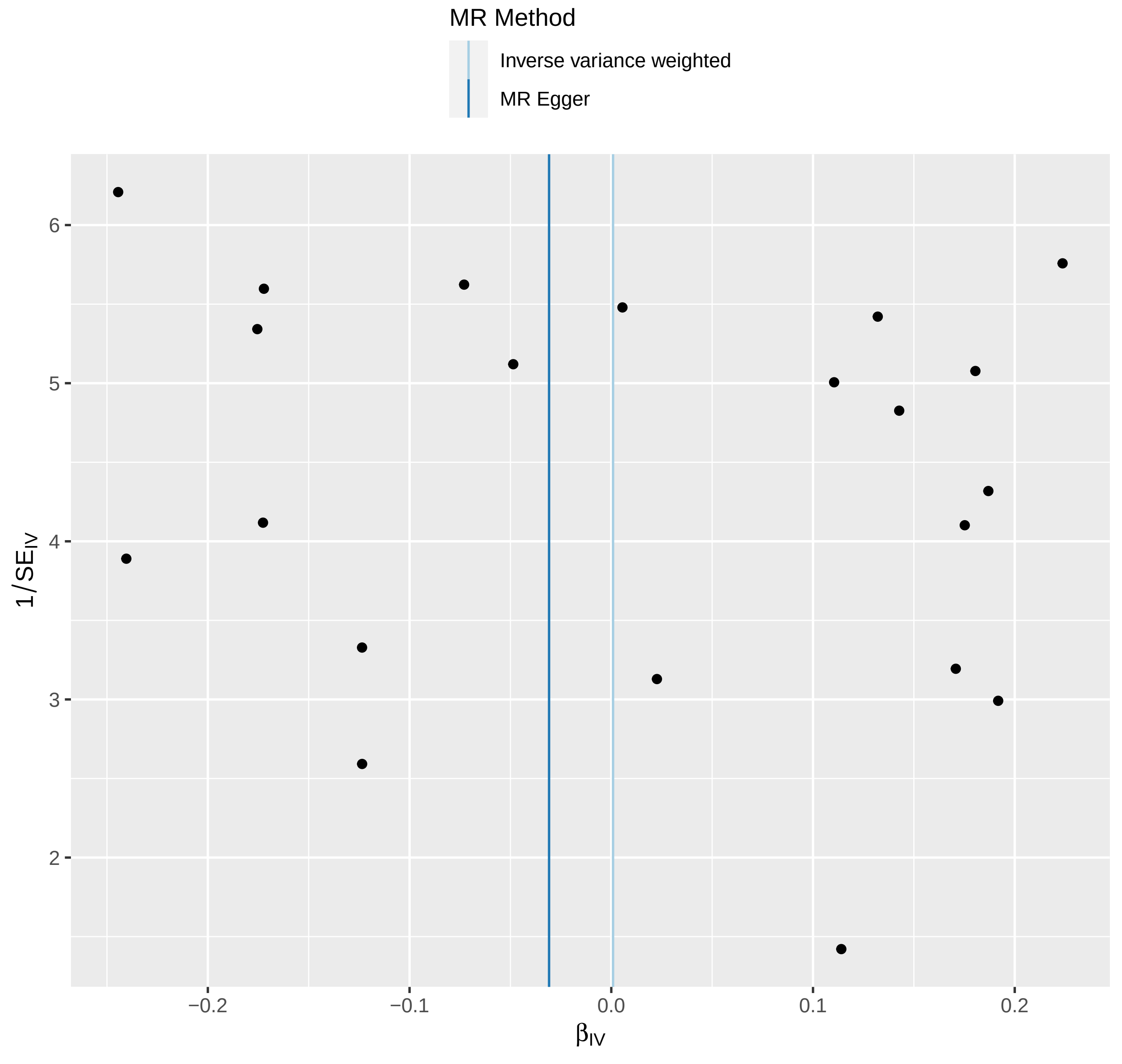

Batch 3−794 : Beta−defensin 119 on Diabetic nephropathy (more control exclusions)
with a threshold of 1e−05

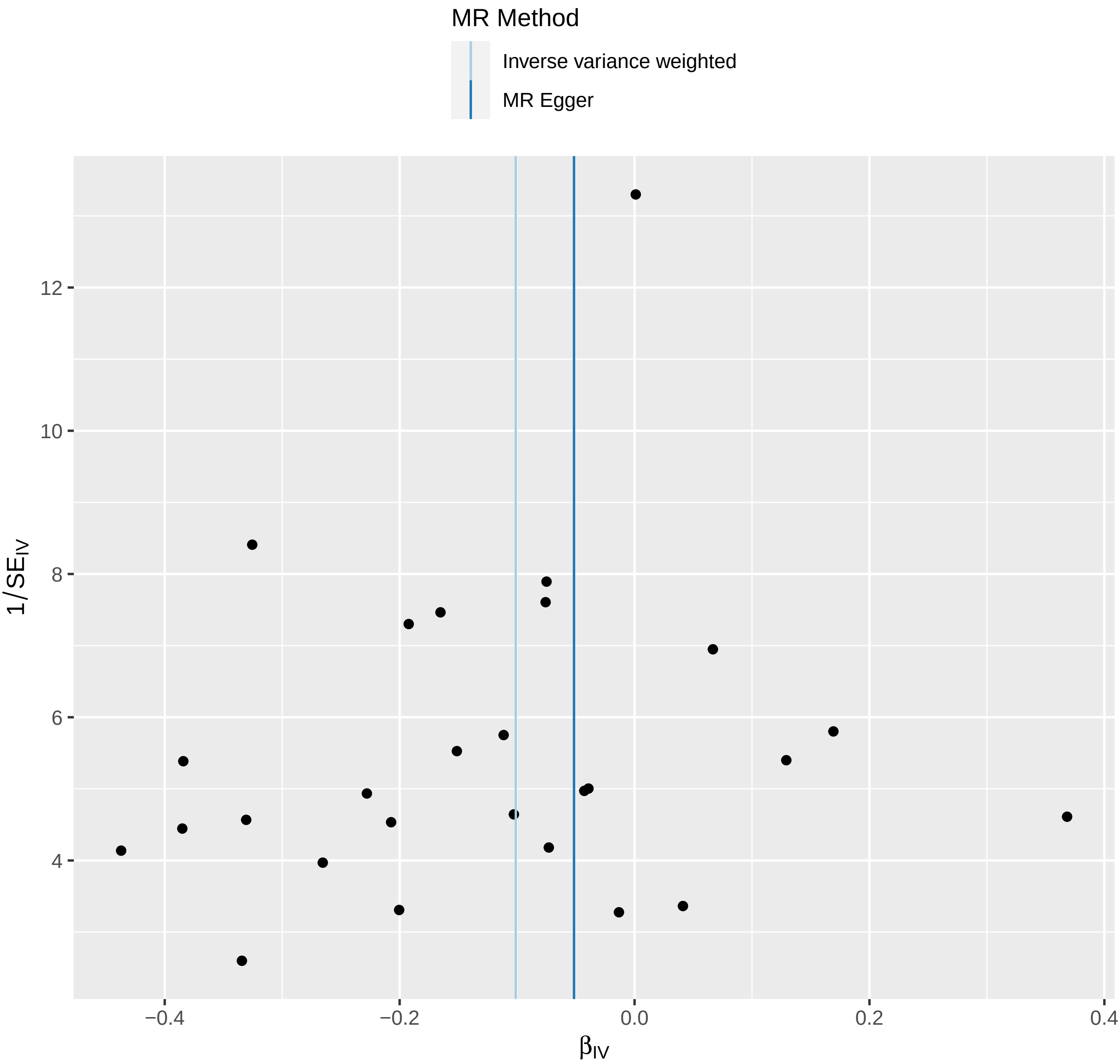

Batch 3−795 : Beta−defensin 119 on Diabetic nephropathy (more control exclusions)
with a threshold of 1e−05

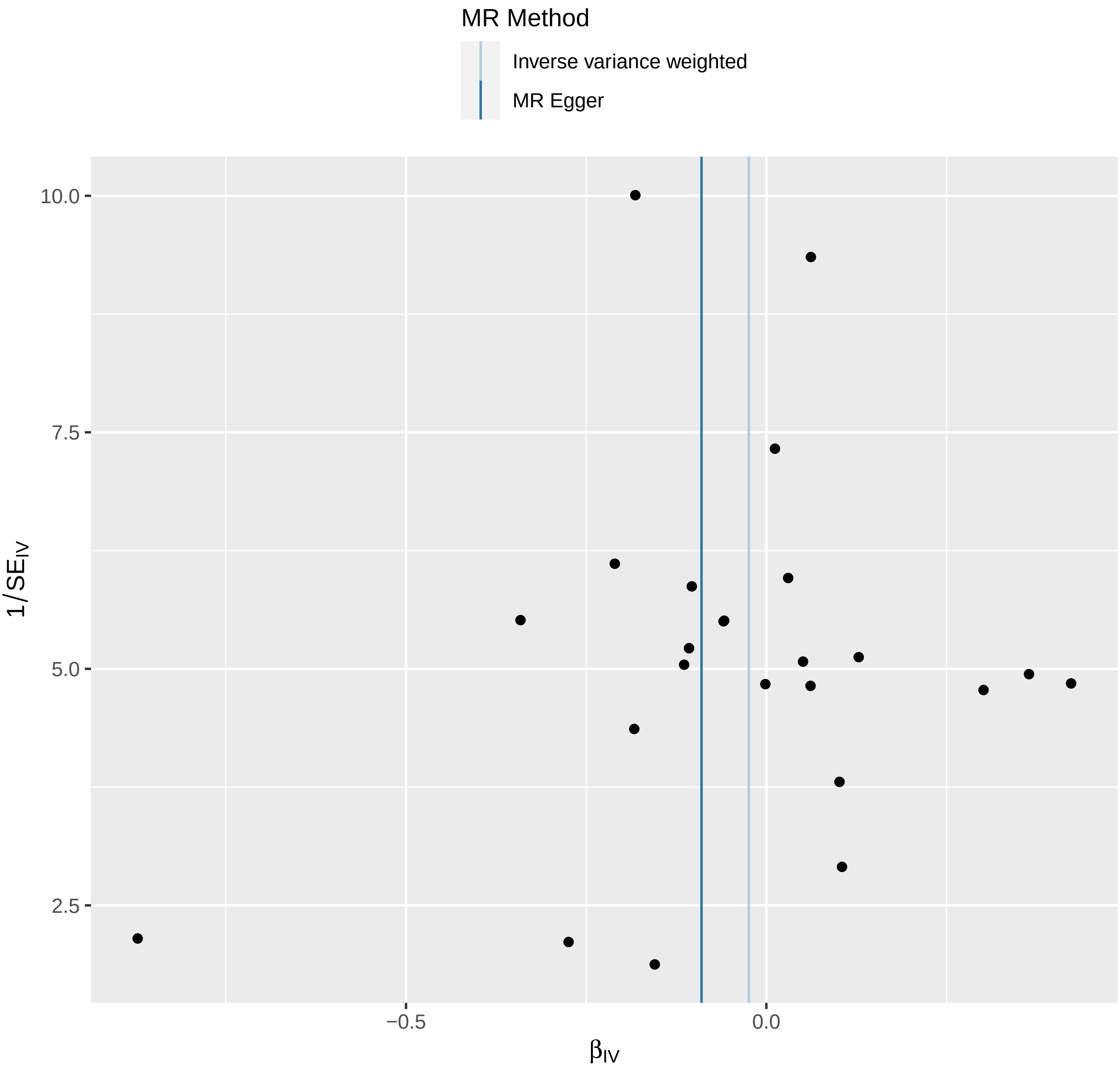

Batch 3−796 : Beta−defensin 119 on Diabetic nephropathy (more control exclusions)
with a threshold of 1e−05

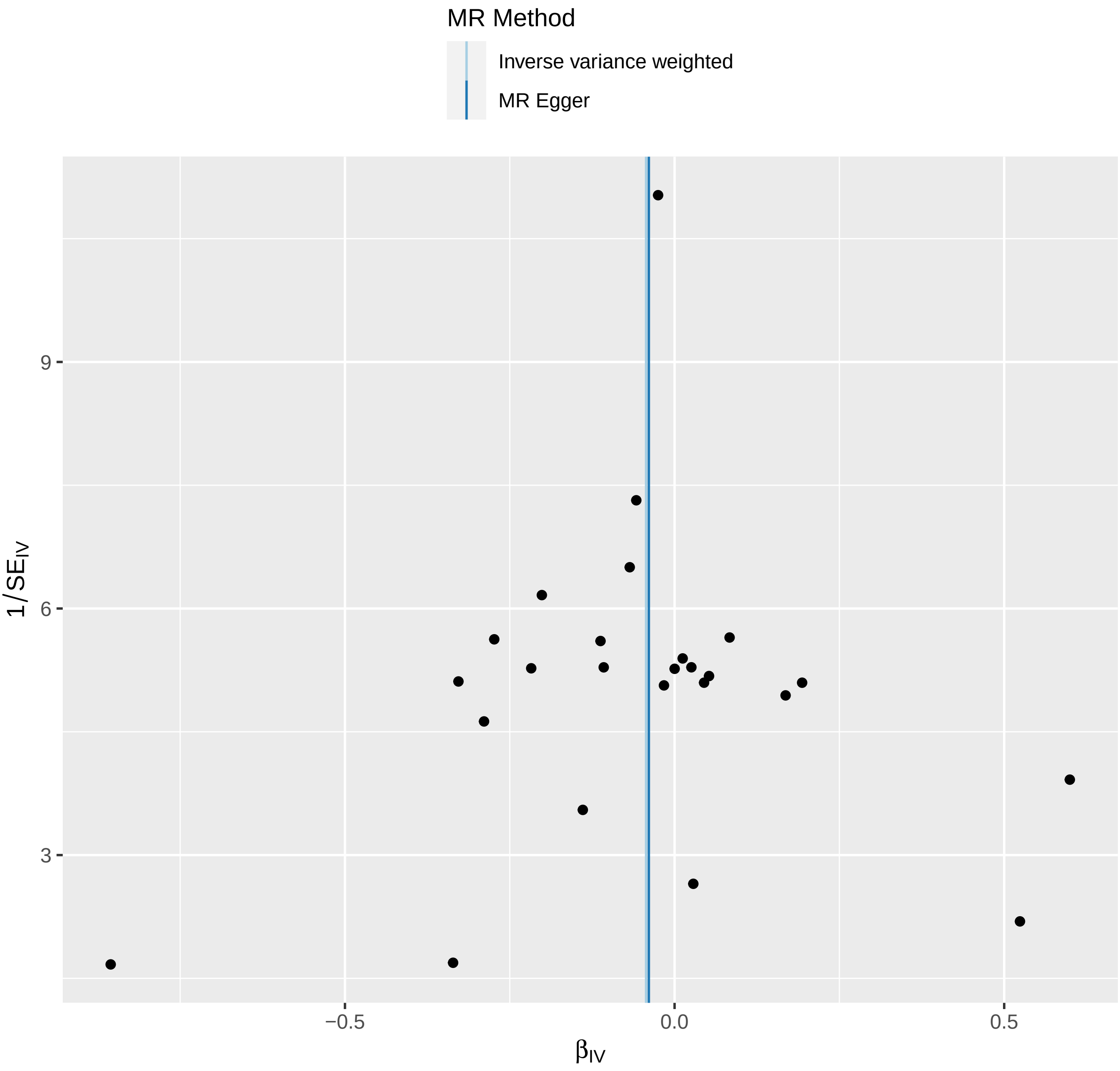

Batch 3−797 : Beta−defensin 121 on Diabetic nephropathy (more control exclusions)
with a threshold of 1e−05

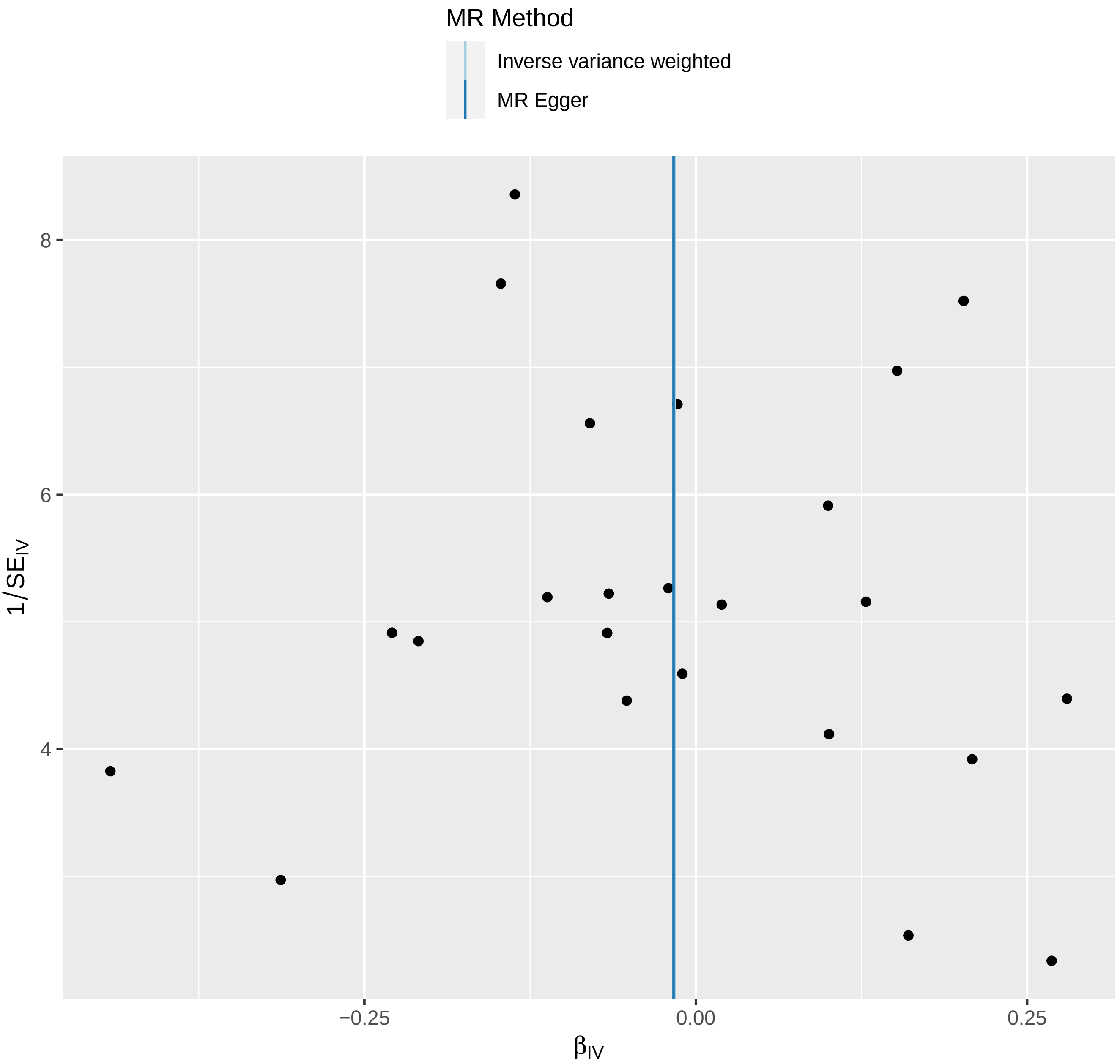

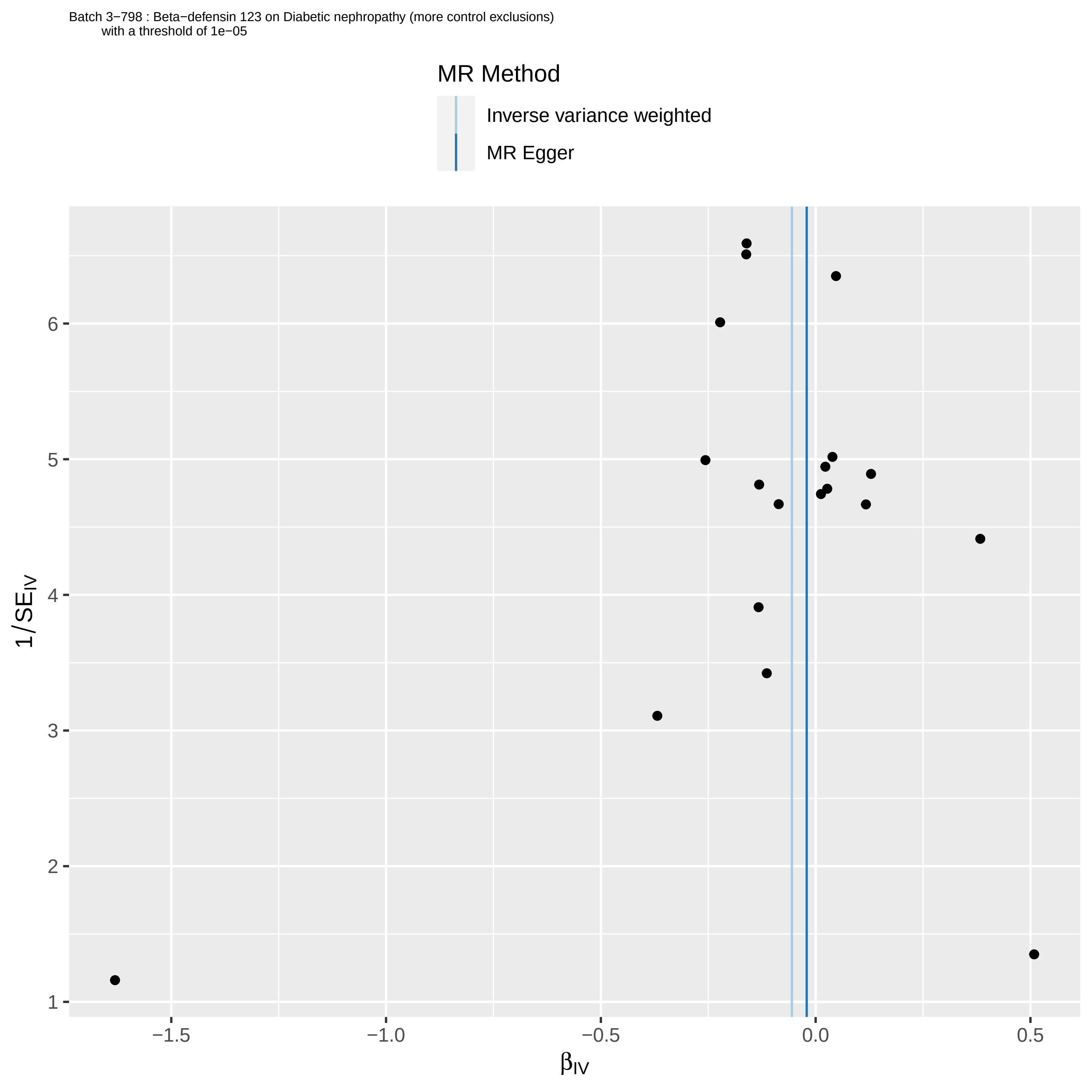

Batch 3−798 : Beta−defensin 123 on Diabetic nephropathy (more control exclusions)
with a threshold of 1e−05
MR Method
Inverse variance weighted
MR Egger
1/SE$_{IV}$
6
5
4
3
2
1
−1.5
−1.0
−0.5
0.0
0.5
β$_{IV}$

Batch 3−799 : Beta−defensin 125 on Diabetic nephropathy (more control exclusions)
with a threshold of 1e−05

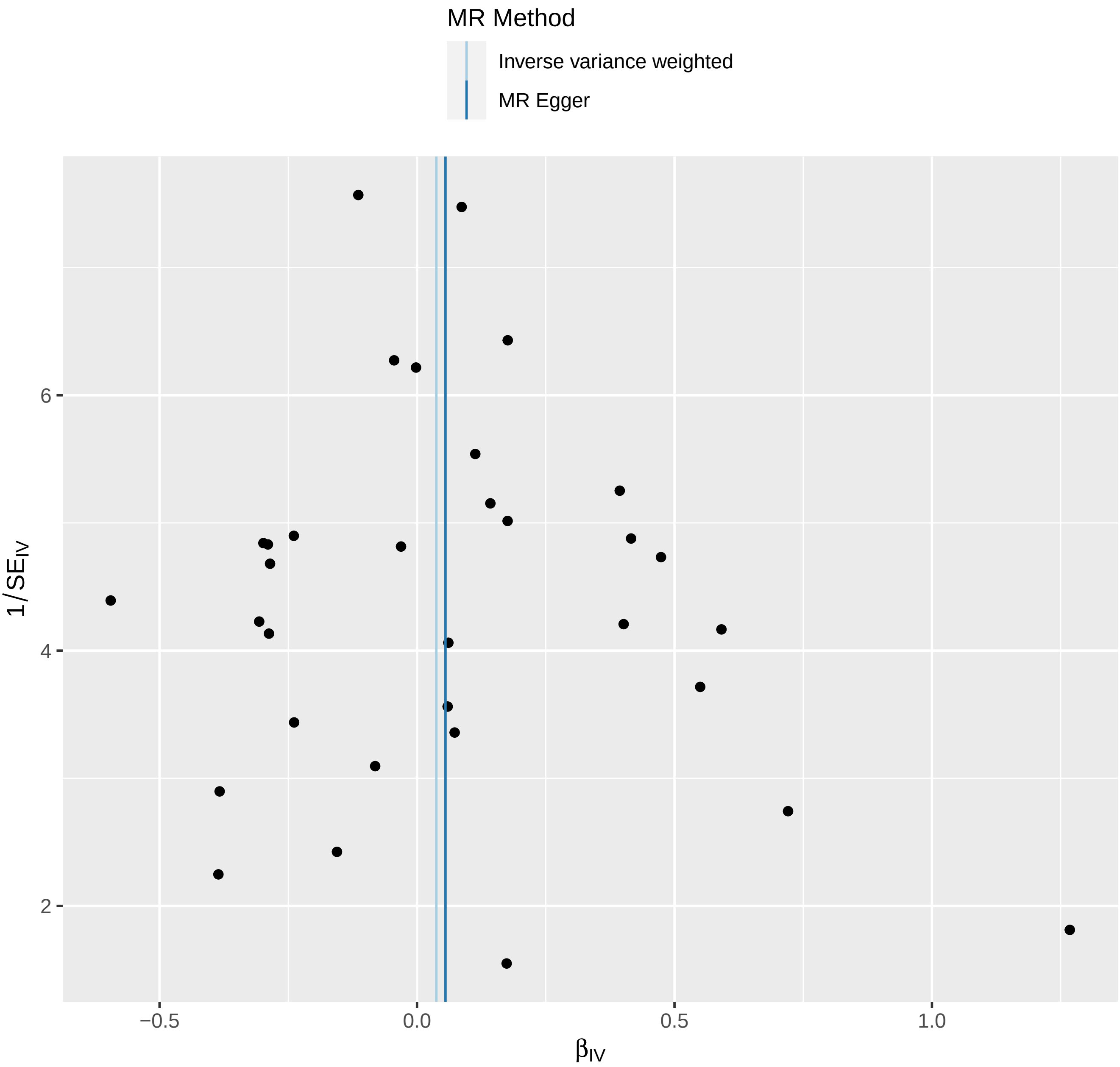

Batch 3−800 : Beta−defensin 128 on Diabetic nephropathy (more control exclusions)
with a threshold of 1e−05

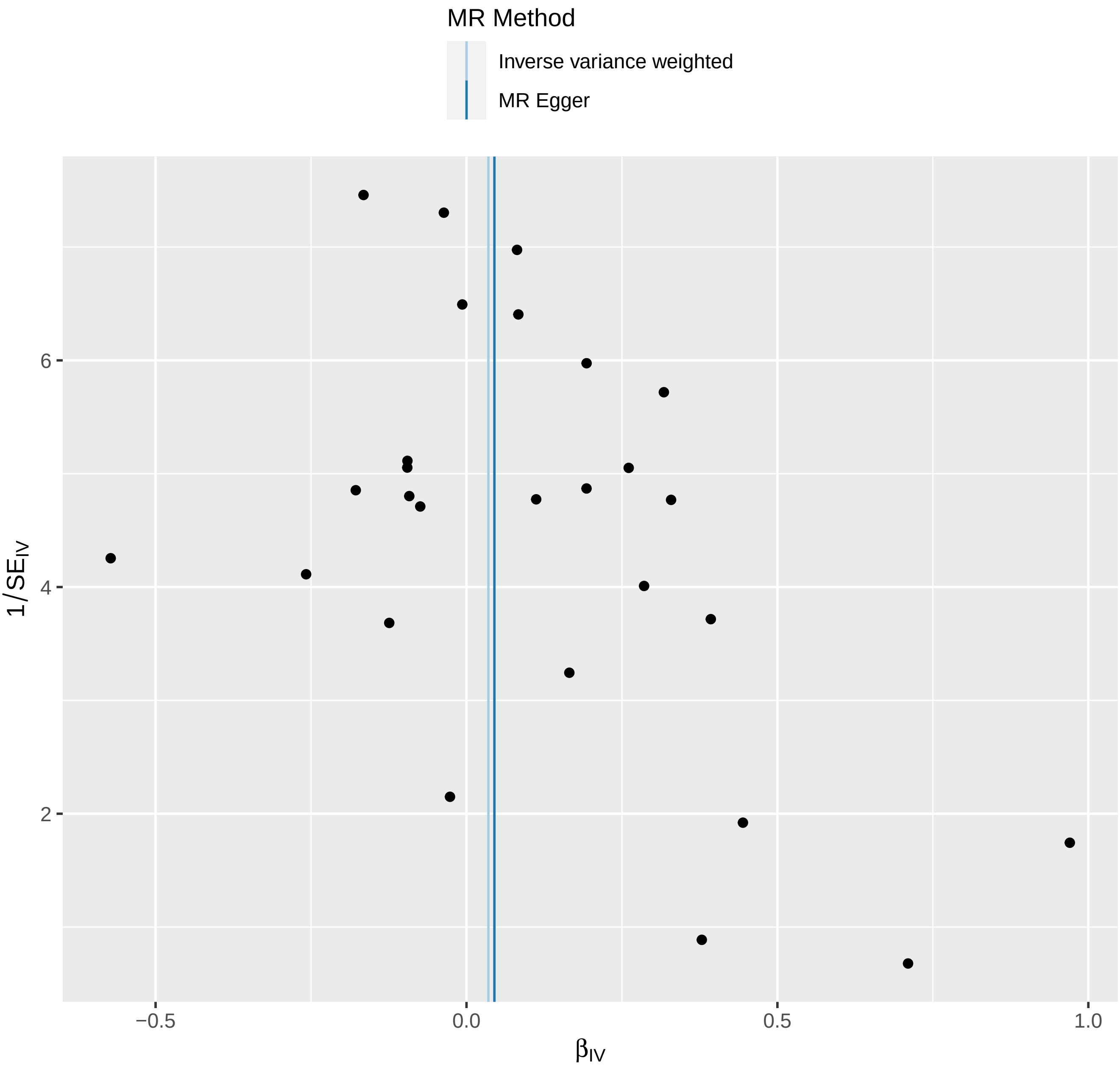

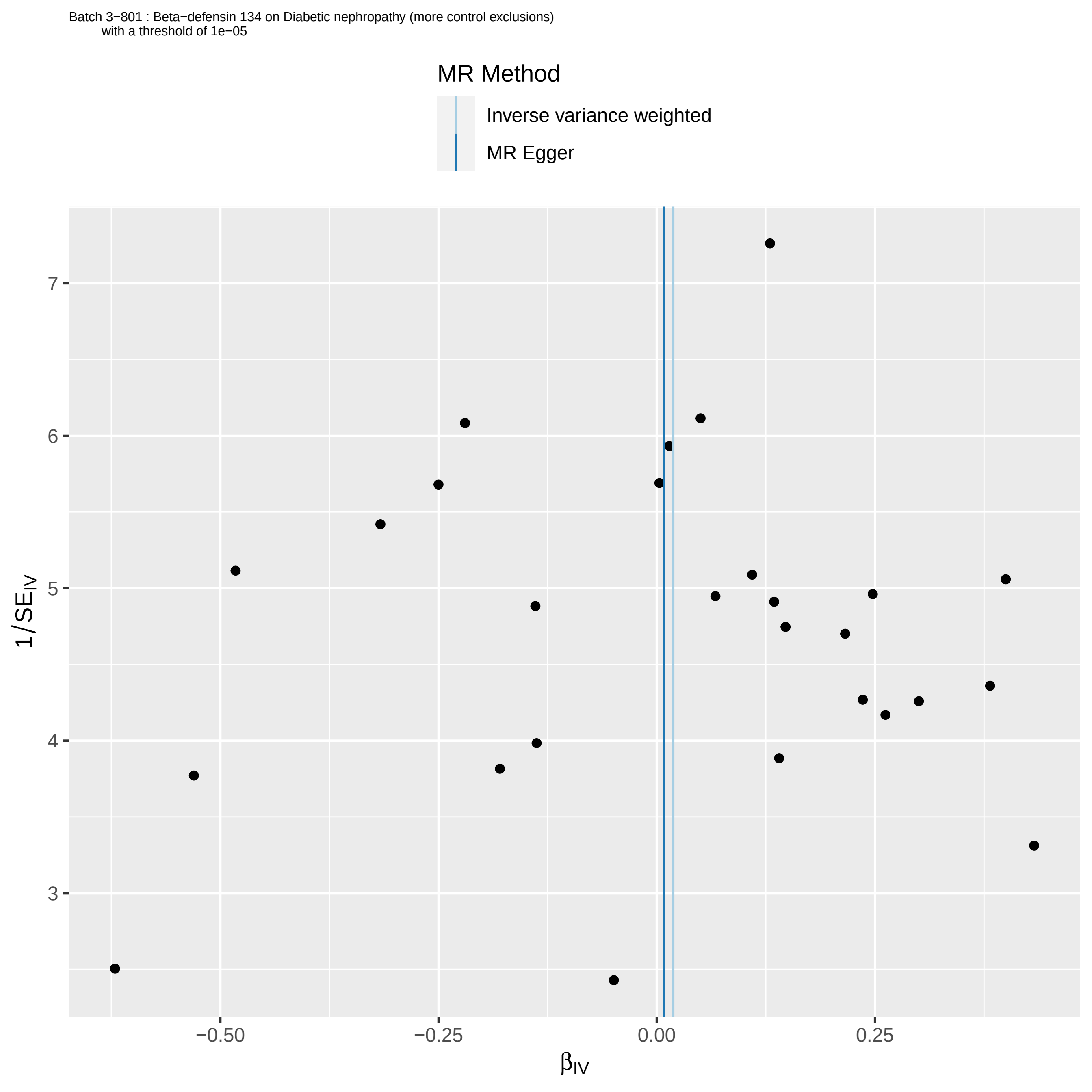

Batch 3−801 : Beta−defensin 134 on Diabetic nephropathy (more control exclusions)
with a threshold of 1e−05
MR Method
Inverse variance weighted
MR Egger
7
6
5
4
3
1/SE$_{IV}$
−0.50
−0.25
0.00
0.25
β_{IV}

Batch 3−802 : Beta−defensin 135 on Diabetic nephropathy (more control exclusions)
with a threshold of 1e−05

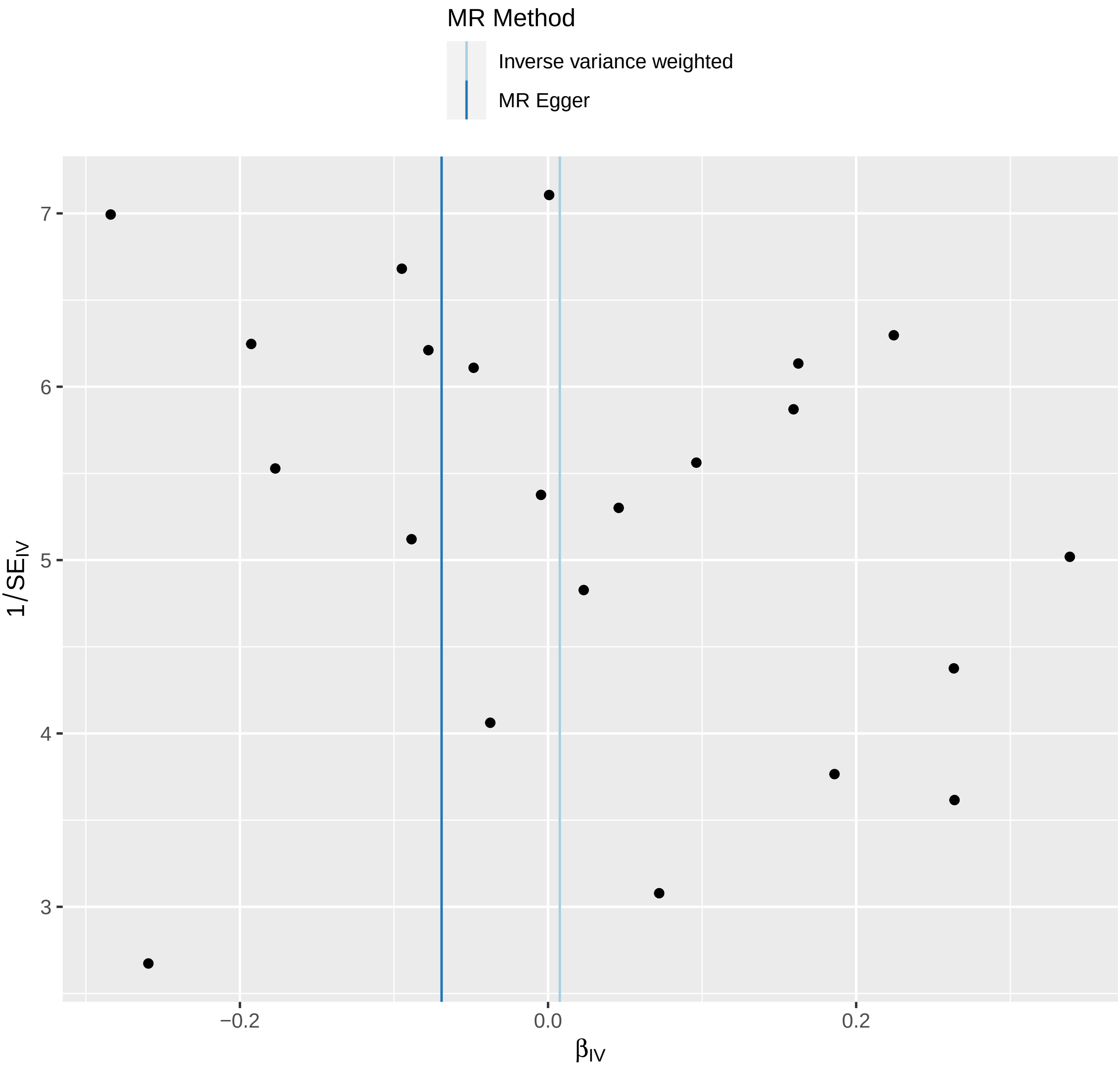

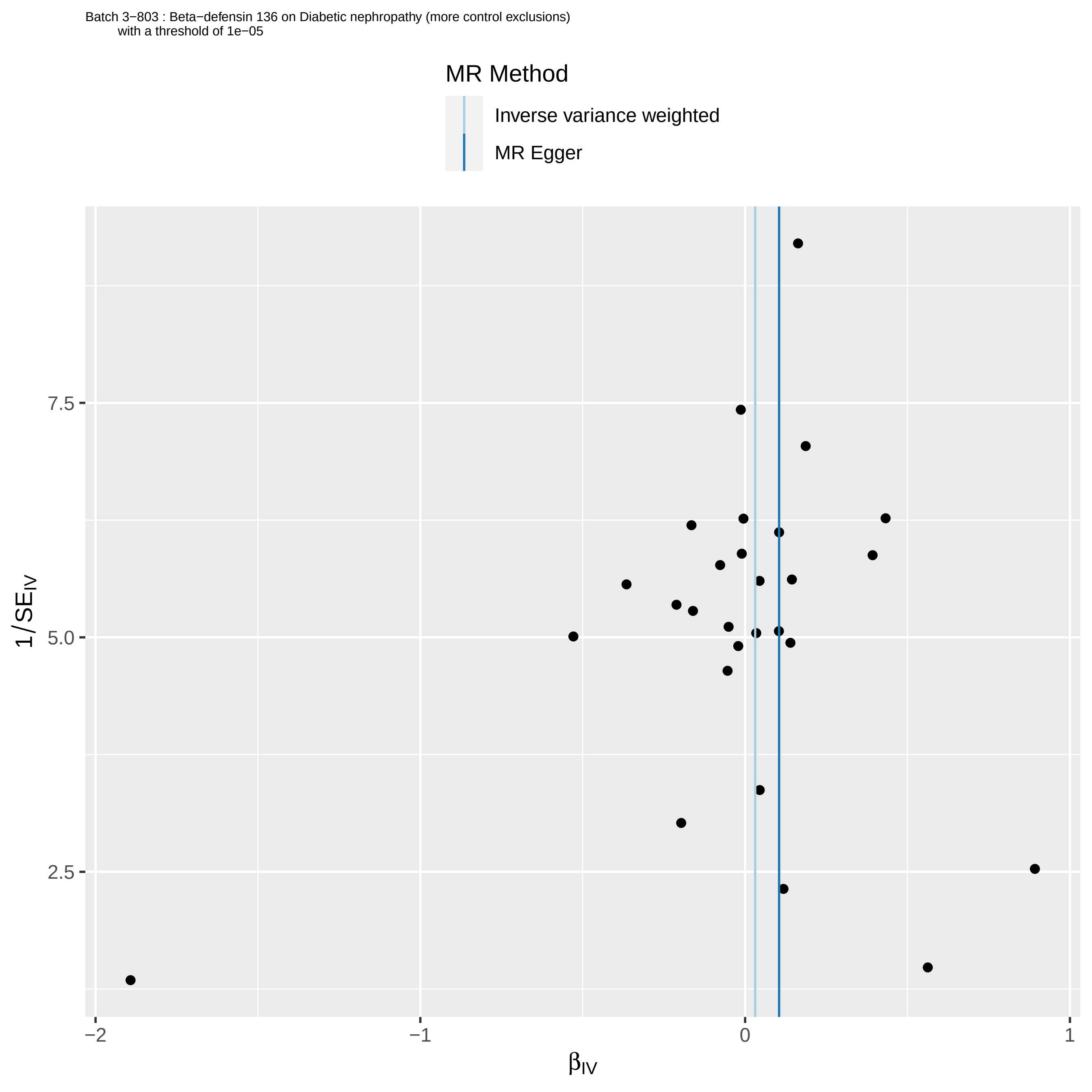

Batch 3−803 : Beta−defensin 136 on Diabetic nephropathy (more control exclusions)
with a threshold of 1e−05
MR Method
Inverse variance weighted
MR Egger
7.5
5.0
2.5
1/SE_IV
−2
−1
0
1
β_IV

Batch 3−804 : Beta−defensin 1 on Diabetic nephropathy (more control exclusions)
with a threshold of 1e−05

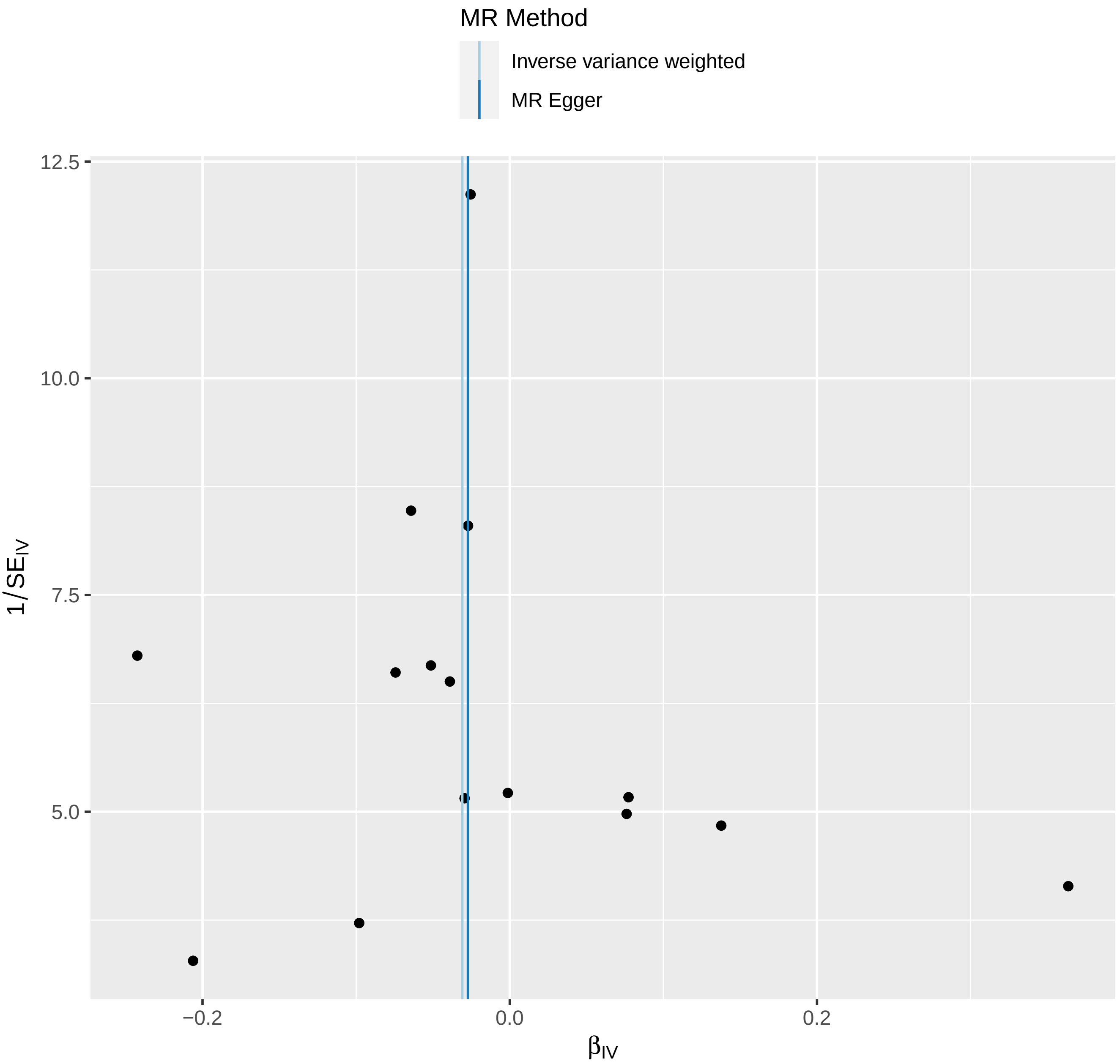

Batch 3−805 : Protein DEPP on Diabetic nephropathy (more control exclusions)
with a threshold of 1e−05

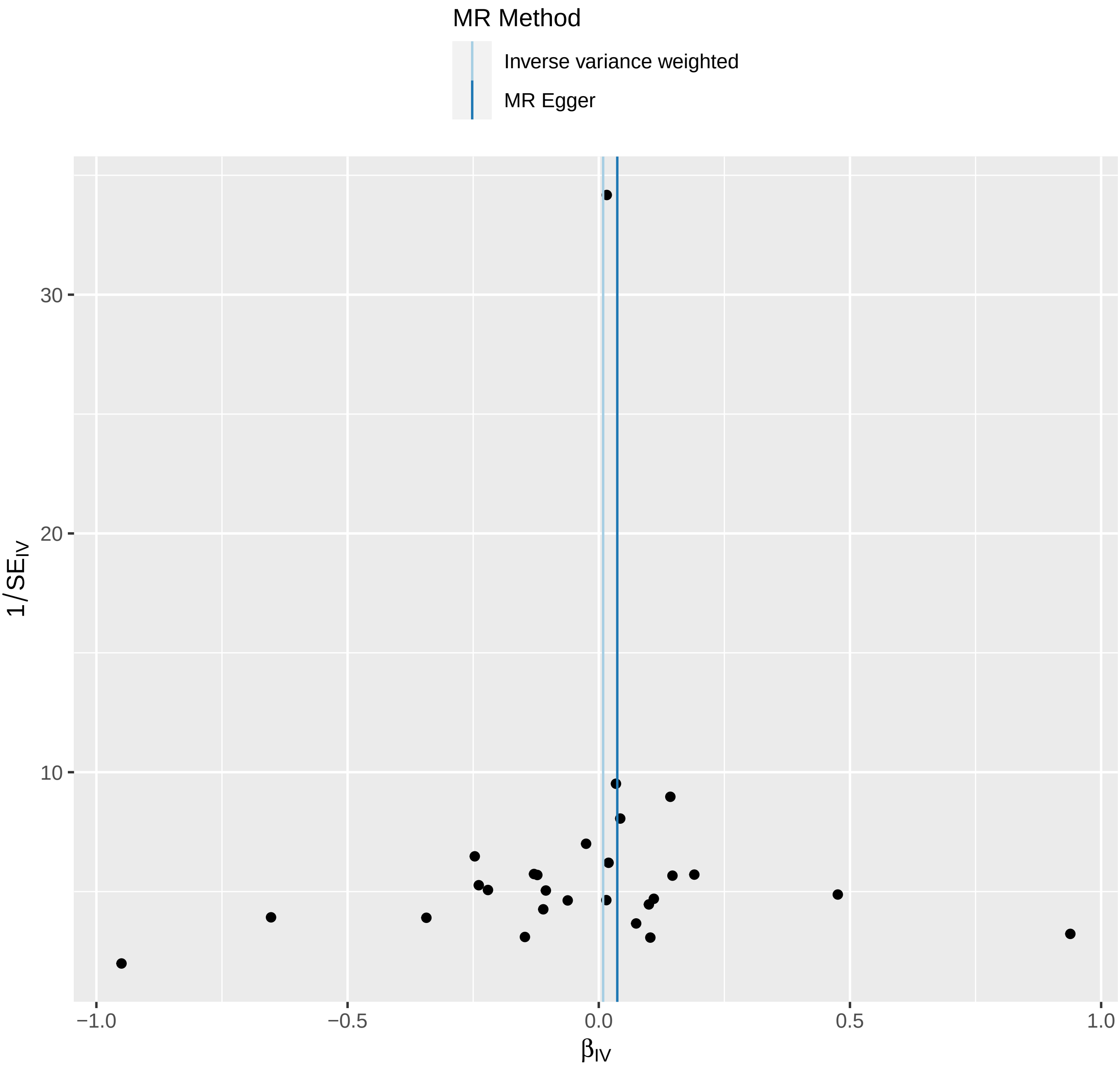

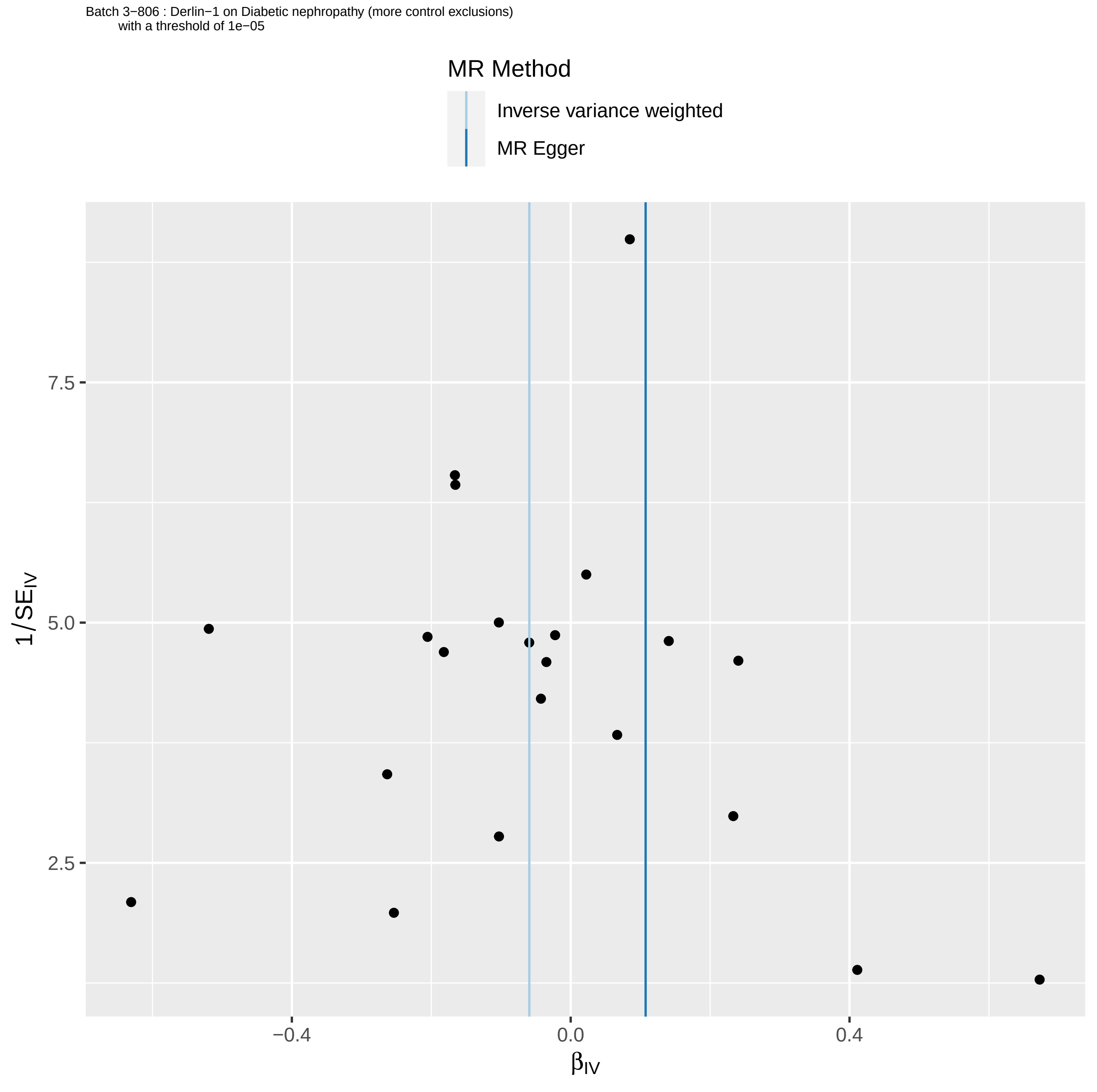

Batch 3−806 : Derlin−1 on Diabetic nephropathy (more control exclusions)
with a threshold of 1e−05
MR Method
Inverse variance weighted
MR Egger
7.5
5.0
2.5
1/SE$_{IV}$
−0.4
0.0
0.4
β$_{IV}$

Batch 3−807 : Desmin on Diabetic nephropathy (more control exclusions)
with a threshold of 1e−05

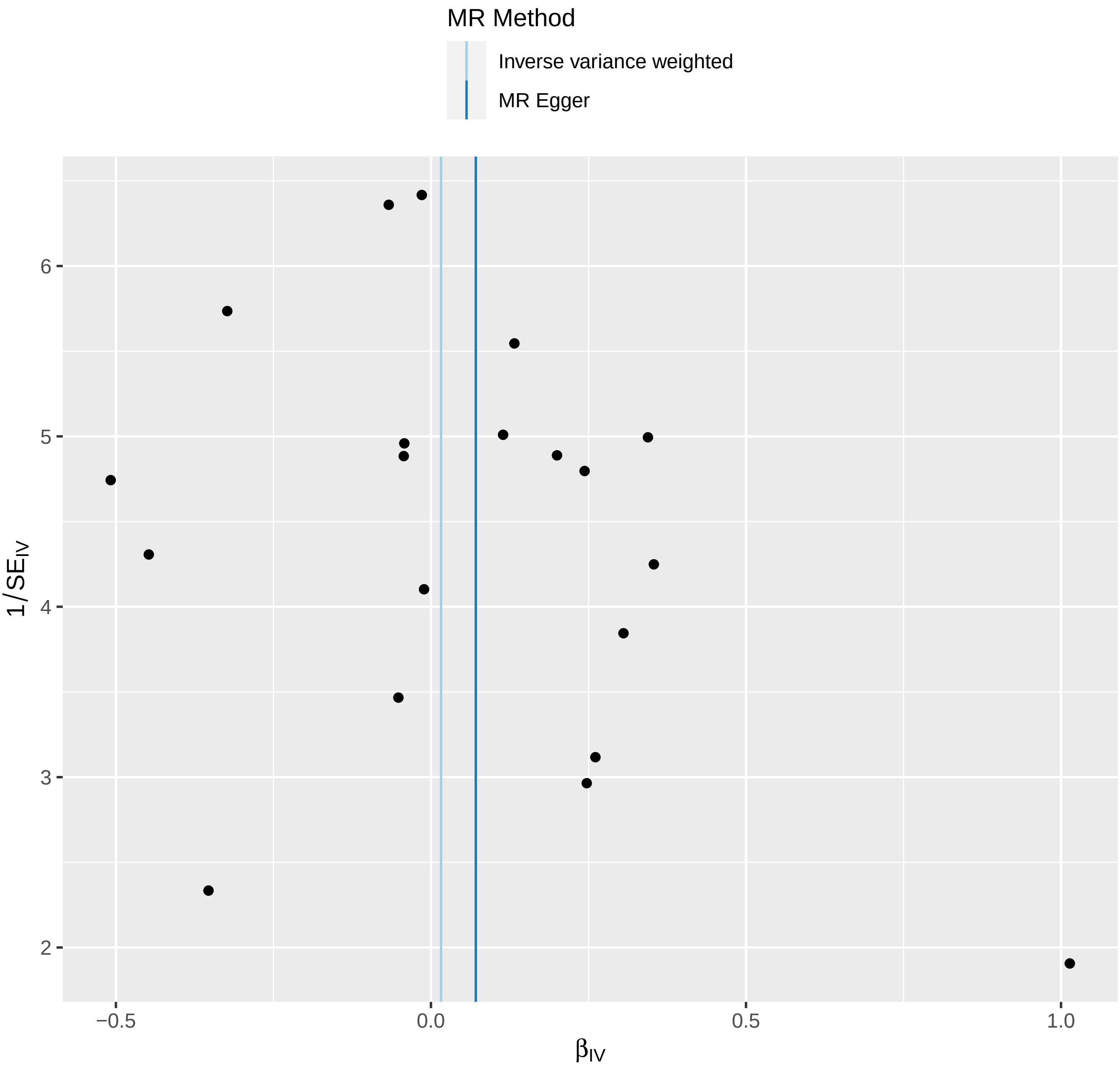

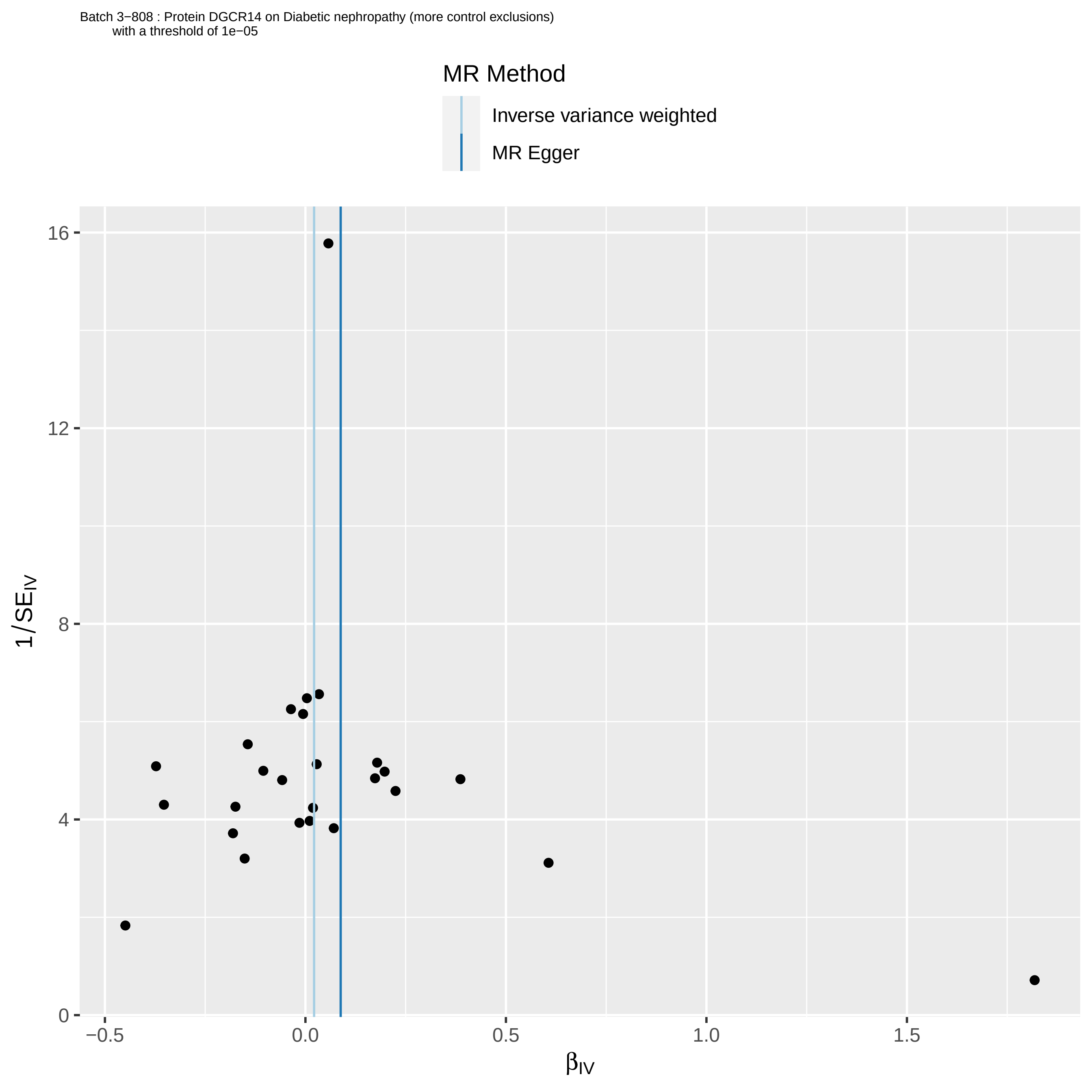

Batch 3−808 : Protein DGCR14 on Diabetic nephropathy (more control exclusions)
with a threshold of 1e−05
MR Method
Inverse variance weighted
MR Egger
16
12
8
4
0
1/SE_IV
−0.5
0.0
0.5
1.0
1.5
β_IV

Batch 3−809 : Integral membrane protein DGCR2/IDD on Diabetic nephropathy (more control exclusions)
with a threshold of 1e−05

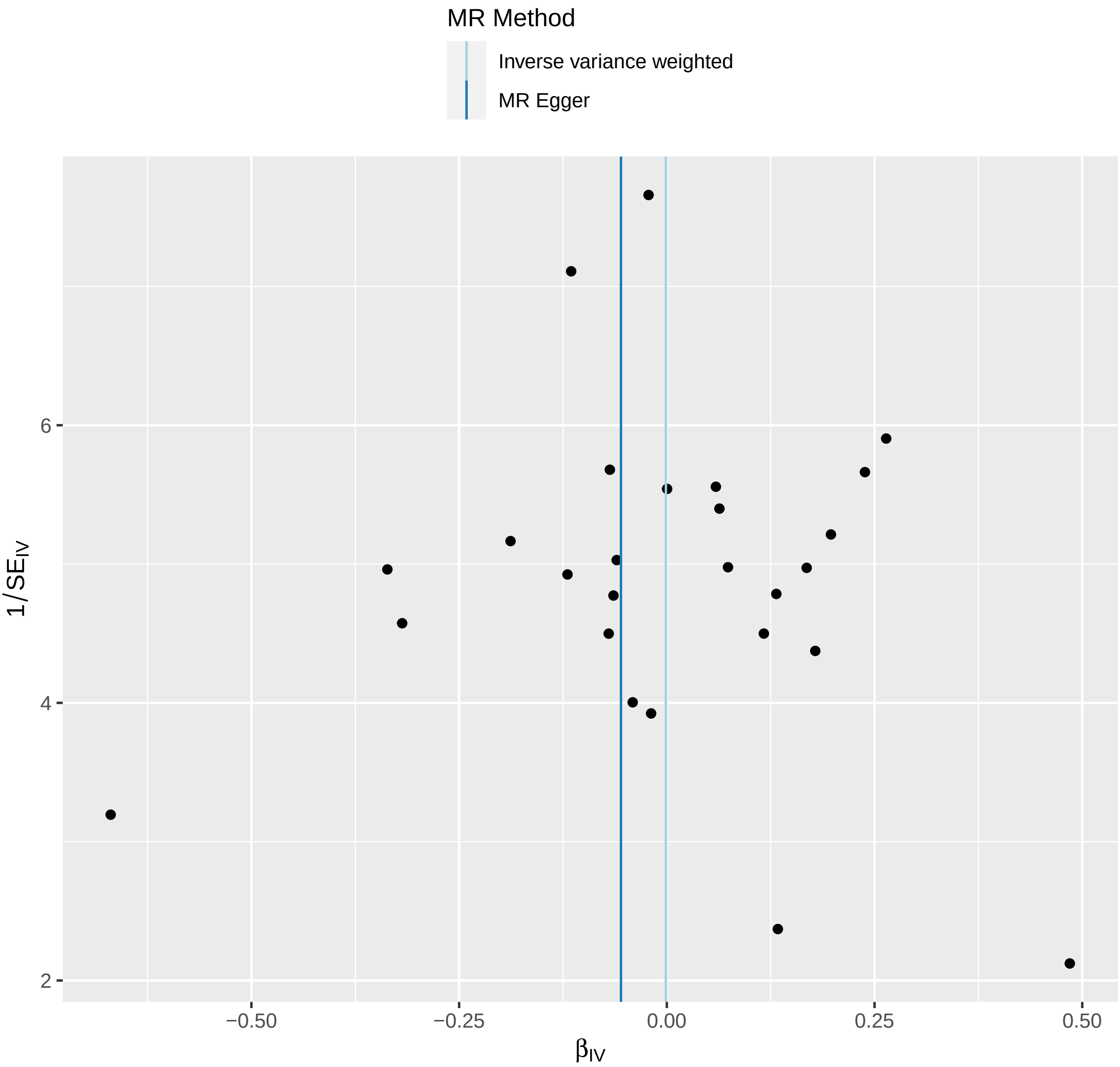

Batch 3−810 : Protein DGCR6 on Diabetic nephropathy (more control exclusions)
with a threshold of 1e−05

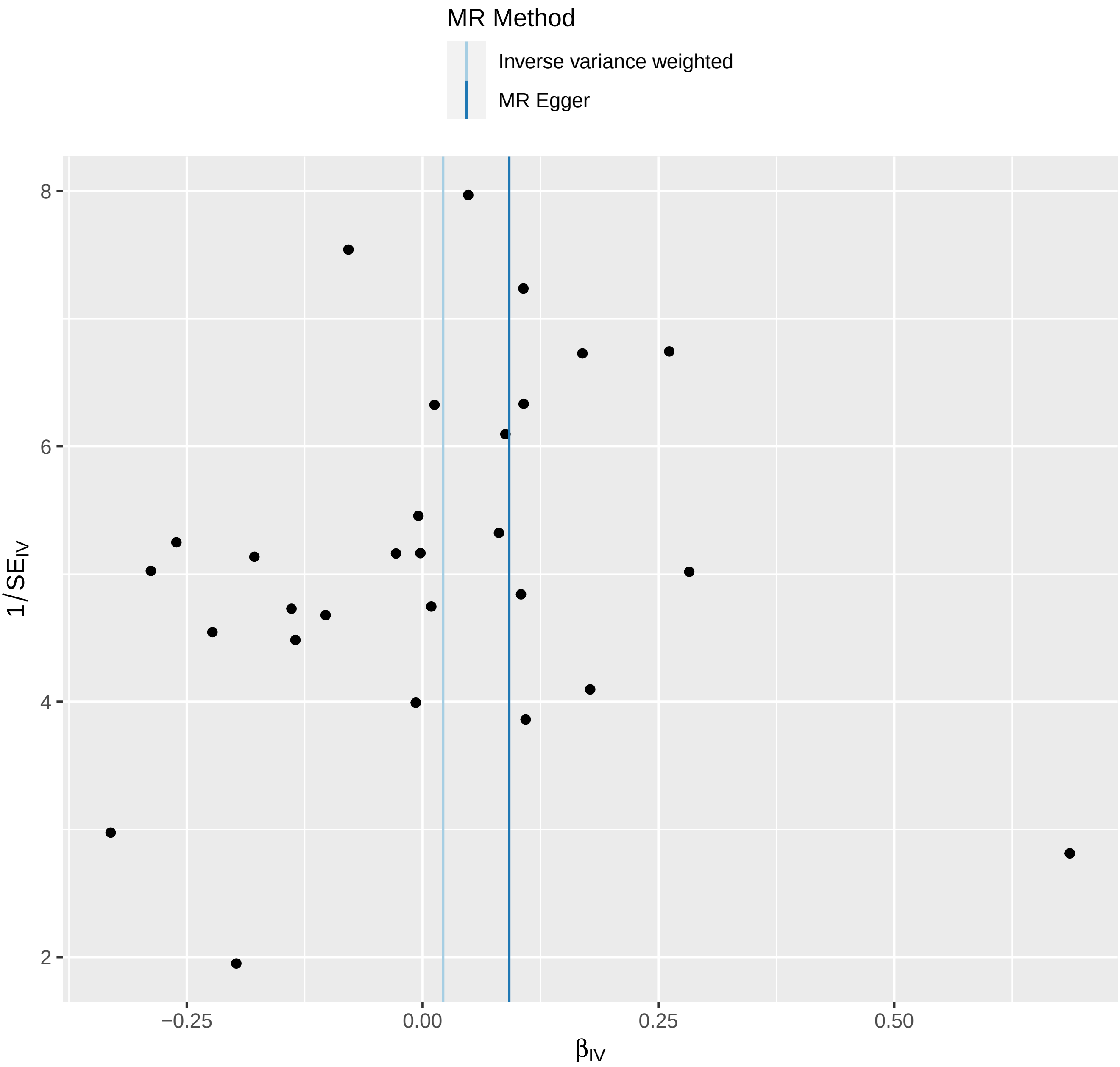

Batch 3−811 : Diacylglycerol kinase beta on Diabetic nephropathy (more control exclusions)
with a threshold of 1e−05

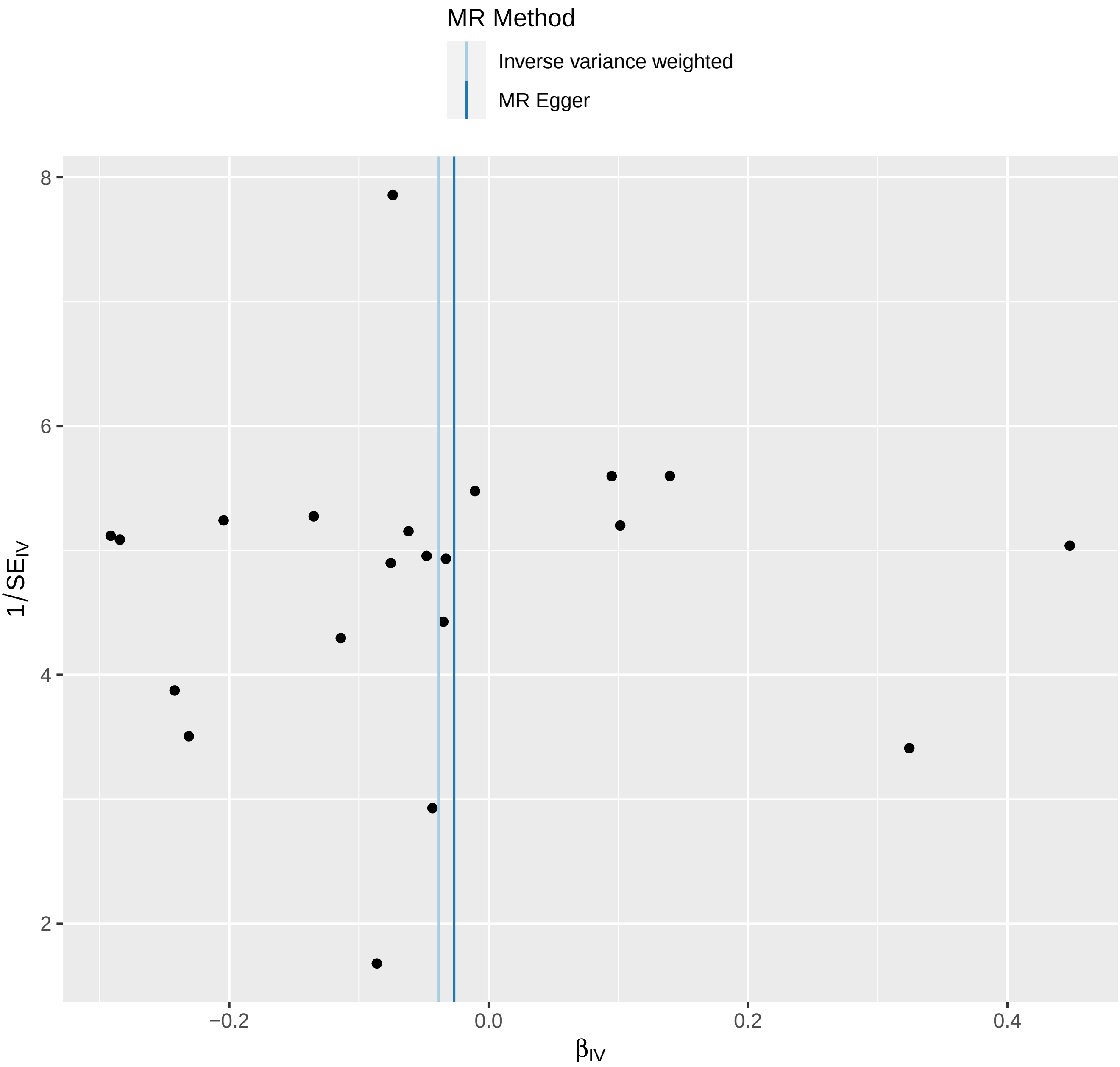

Batch 3−812 : Dihydrofolate reductase on Diabetic nephropathy (more control exclusions)
with a threshold of 1e−05

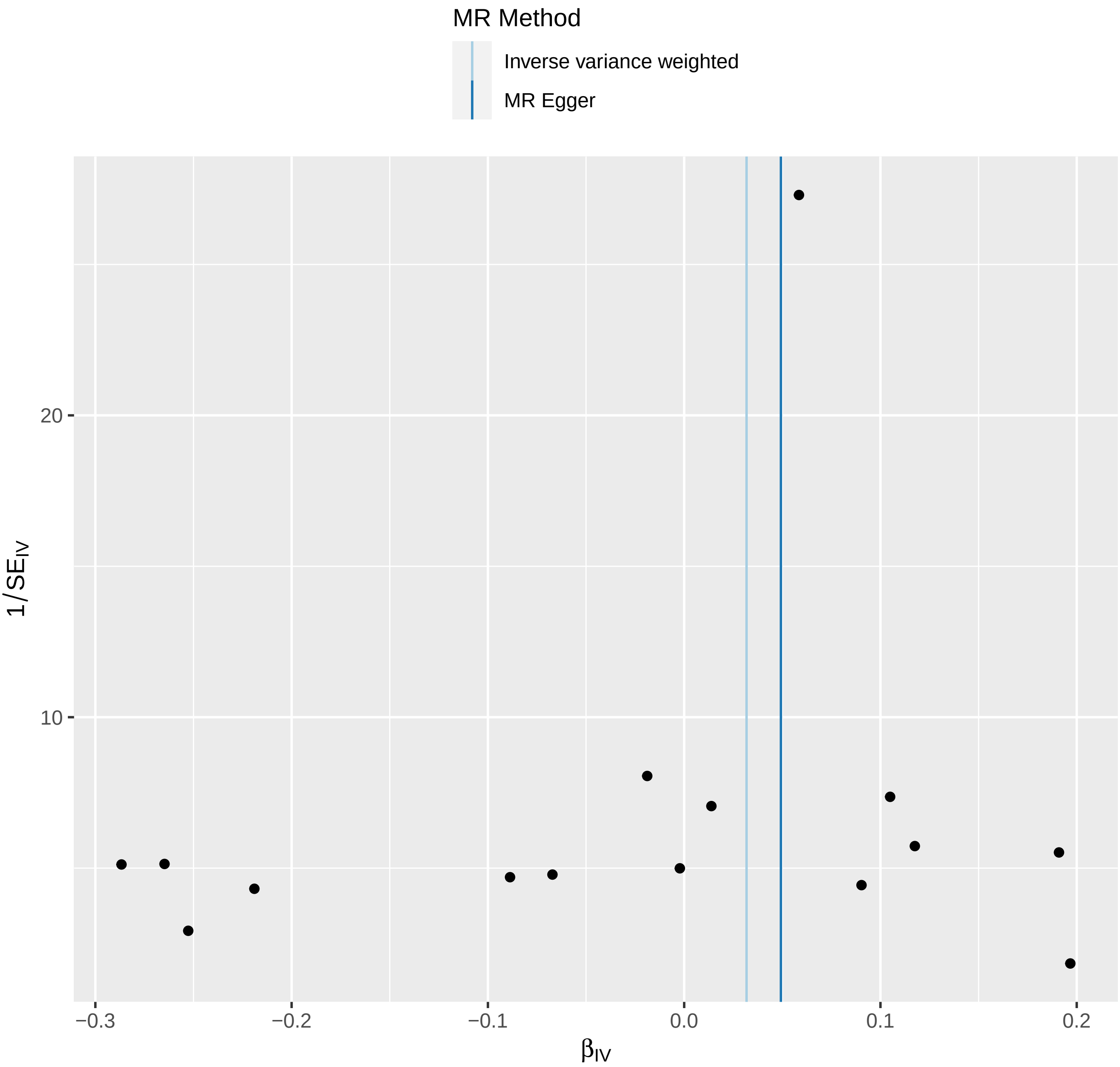

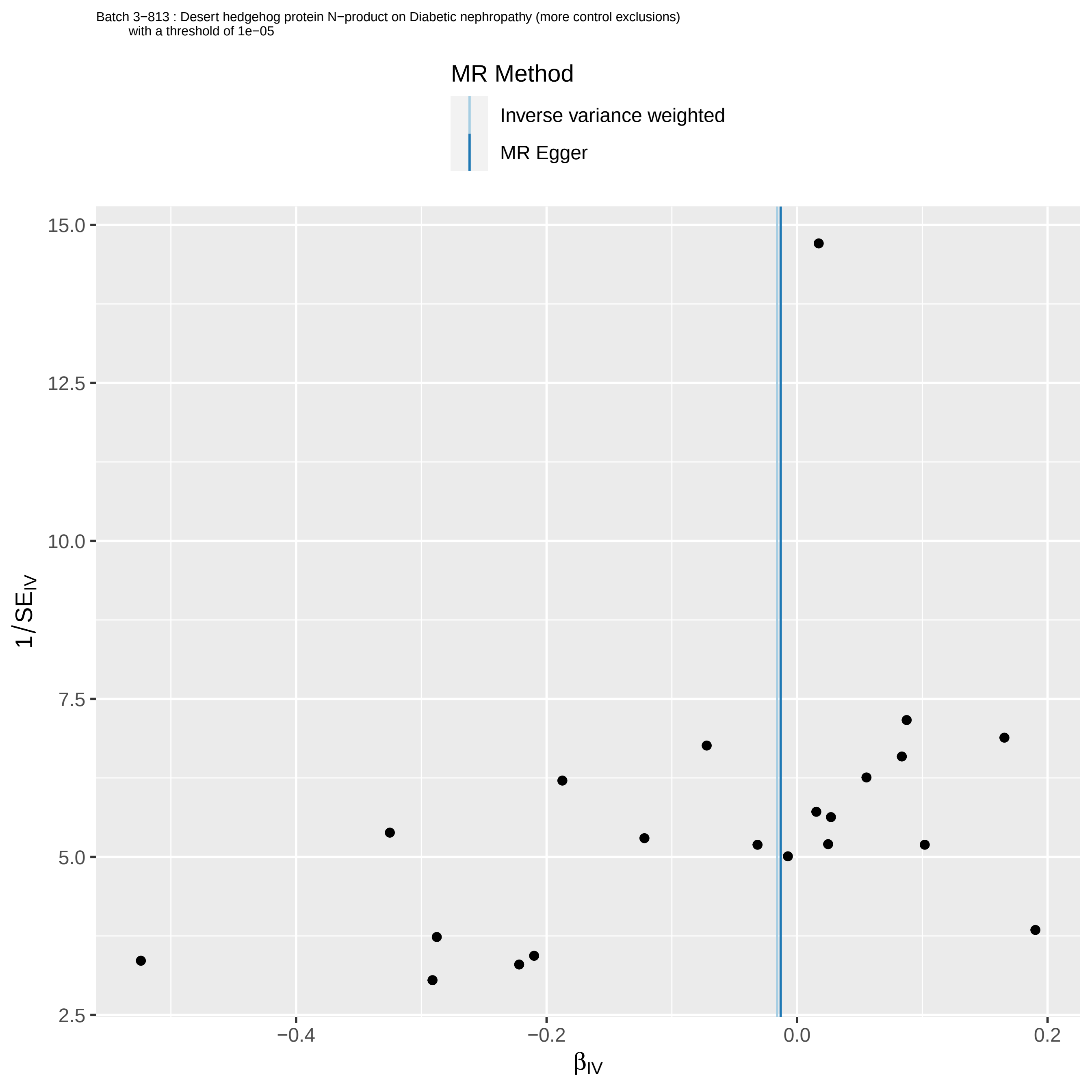
Batch 3−813 : Desert hedgehog protein N−product on Diabetic nephropathy (more control exclusions)
with a threshold of 1e−05
MR Method
Inverse variance weighted
MR Egger
15.0
12.5
10.0
7.5
5.0
2.5
1/SE_IV
−0.4
−0.2
0.0
0.2
β_IV

Batch 3−814 : Pre−mRNA−splicing factor ATP−dependent RNA helicase PRP16 on Diabetic nephropathy (more control exclusions)
with a threshold of 1e−05

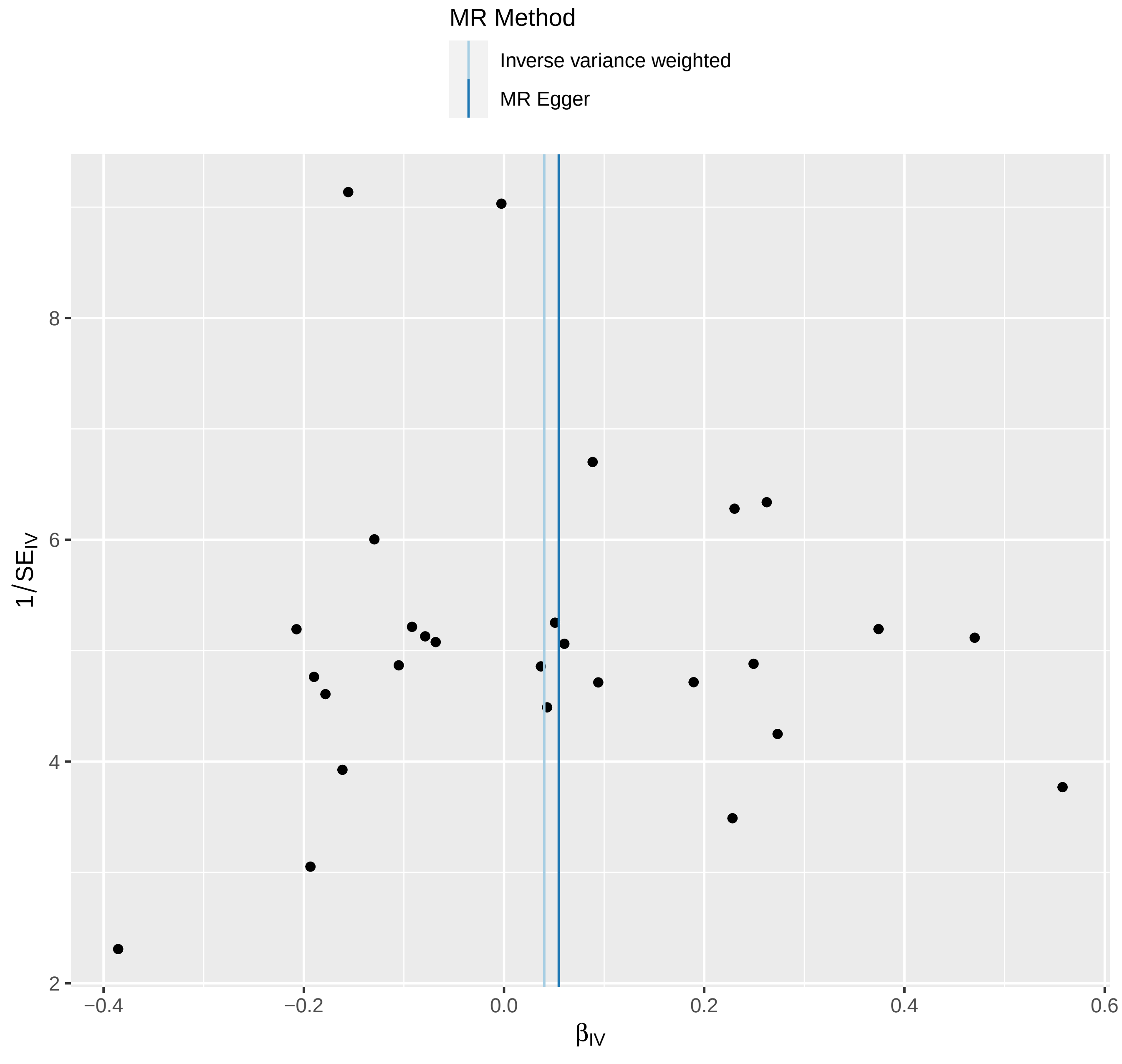

Batch 3−815 : Probable ATP−dependent RNA helicase DHX58 on Diabetic nephropathy (more control exclusions)
with a threshold of 1e−05

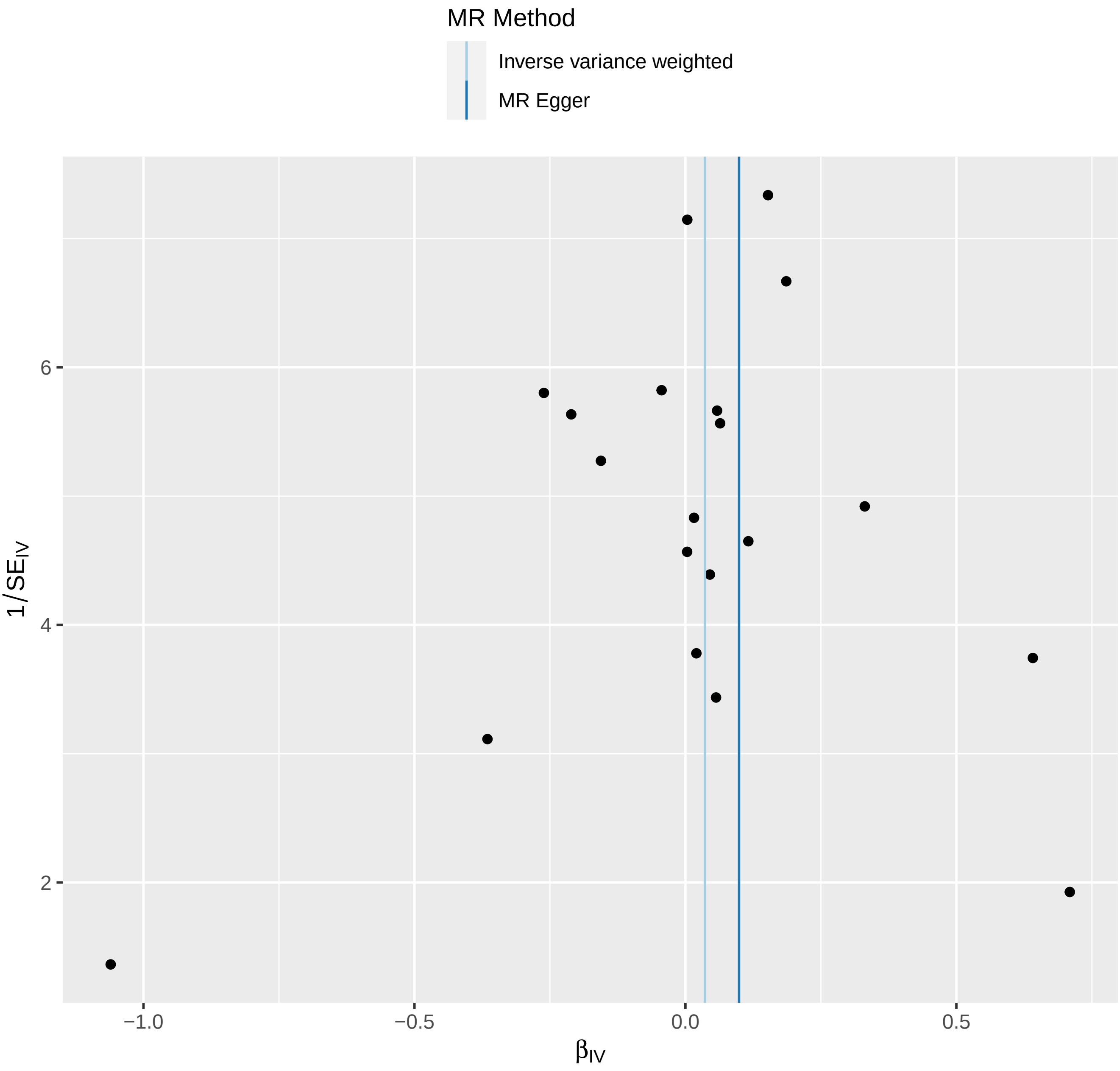

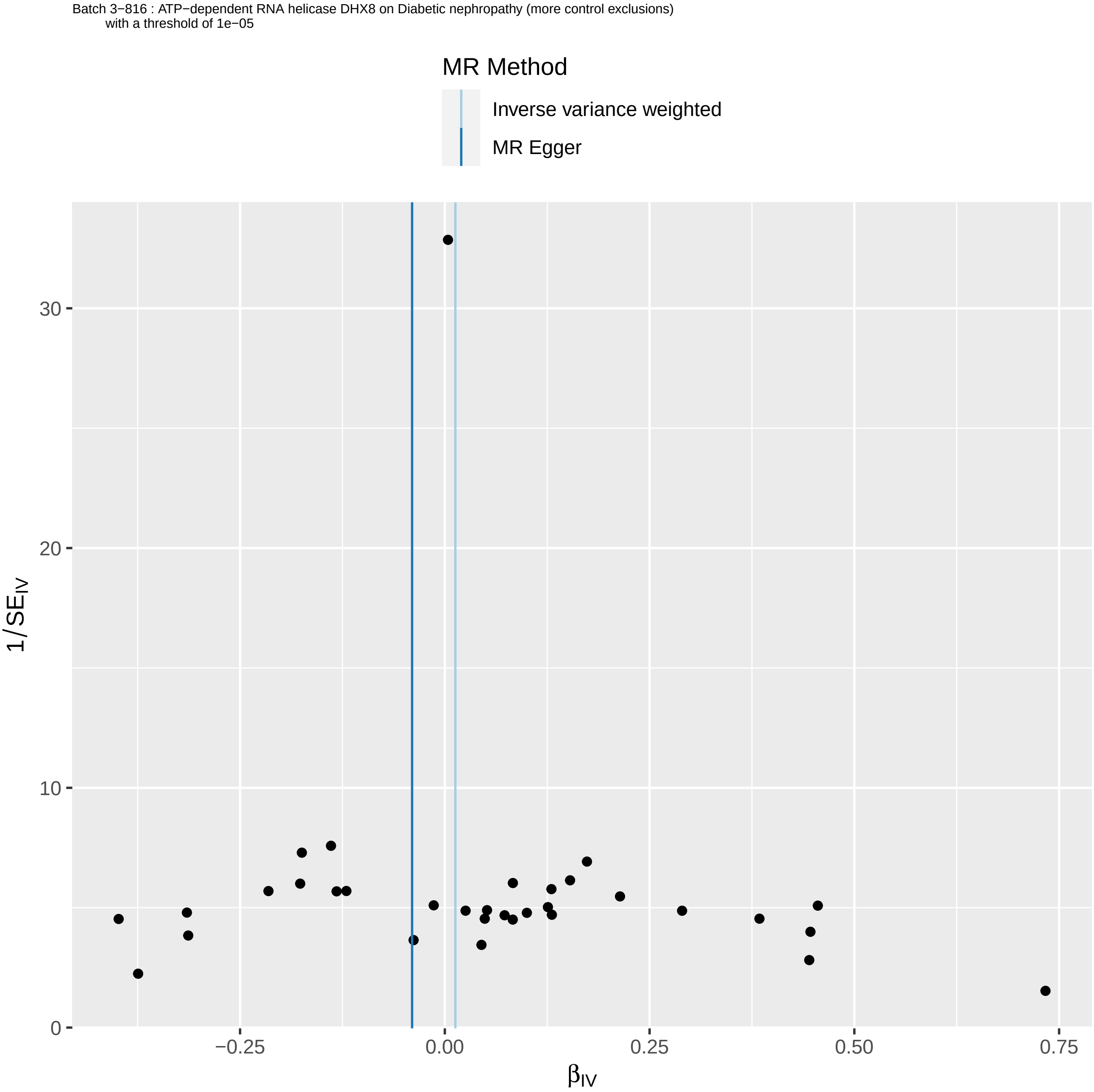

Batch 3−816 : ATP−dependent RNA helicase DHX8 on Diabetic nephropathy (more control exclusions)
with a threshold of 1e−05
MR Method
Inverse variance weighted
MR Egger
30
20
10
0
−0.25
0.00
0.25
0.50
0.75
1/SE_IV
β_IV

Batch 3−817 : ATP−dependent RNA helicase A on Diabetic nephropathy (more control exclusions)
with a threshold of 1e−05

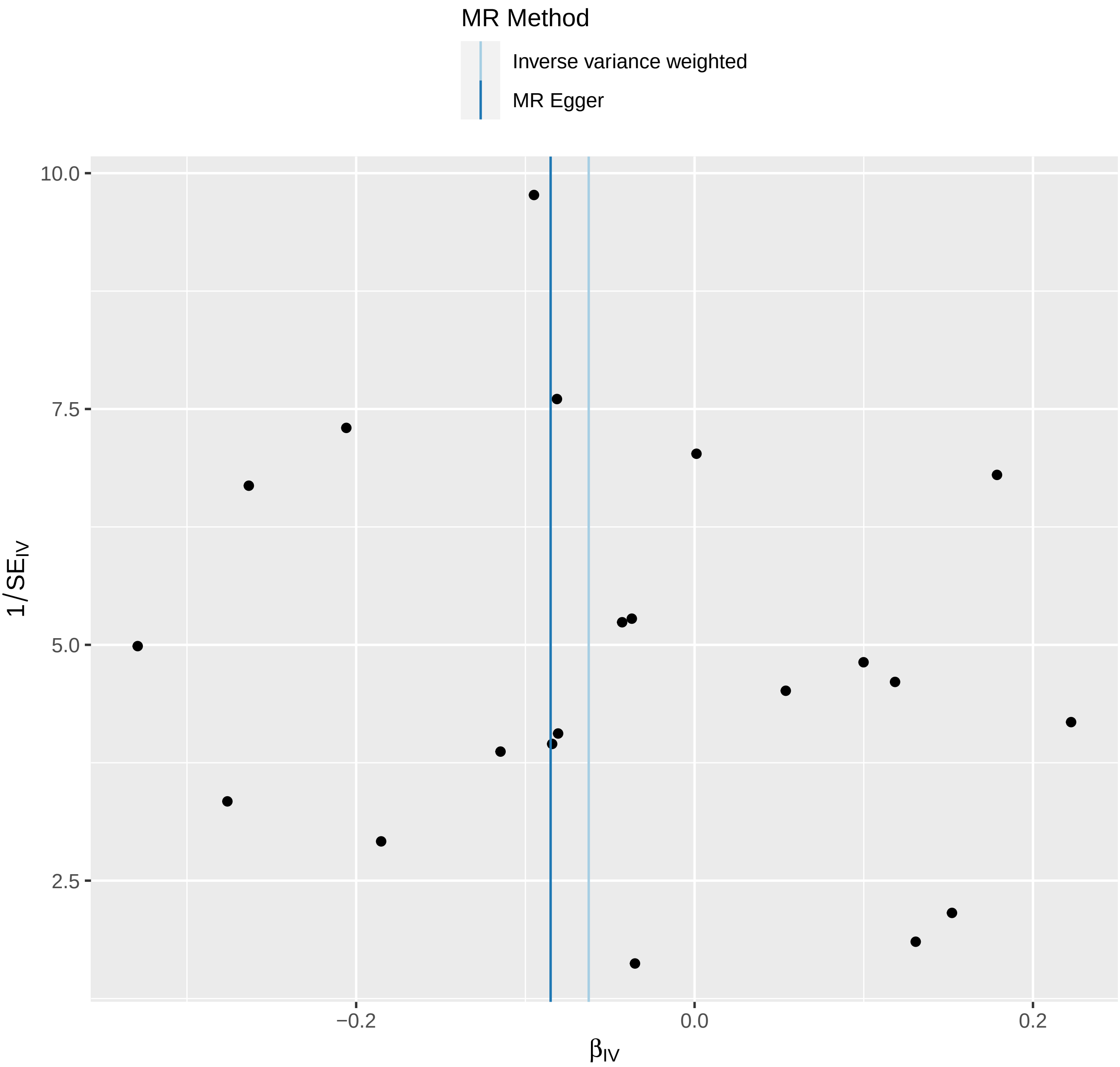

Batch 3−818 : Diablo homolog, mitochondrial on Diabetic nephropathy (more control exclusions)
with a threshold of 1e−05

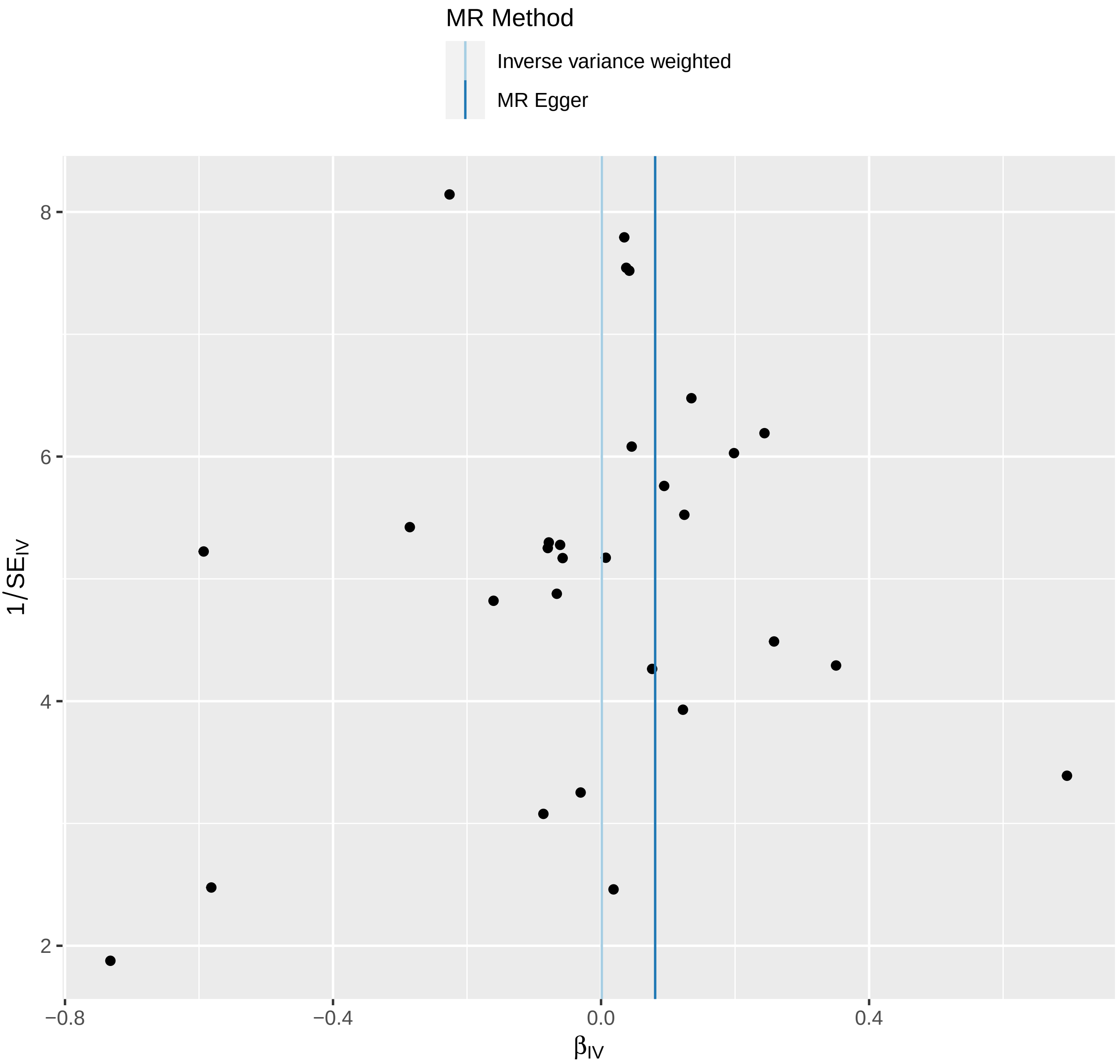

Batch 3−819 : Probable dimethyladenosine transferase on Diabetic nephropathy (more control exclusions)
with a threshold of 1e−05

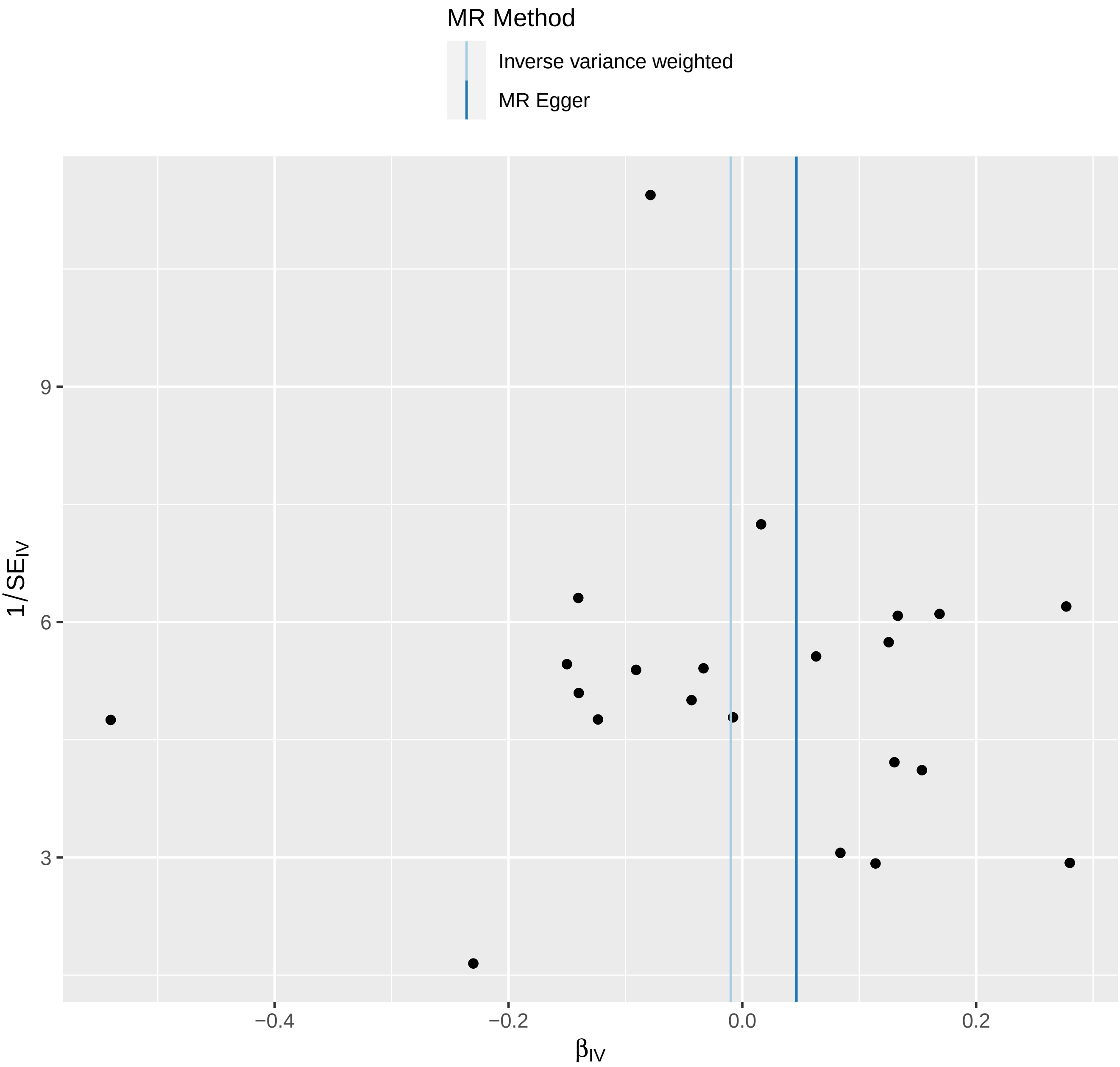

Batch 3−820 : GTP−binding protein Di−Ras3 on Diabetic nephropathy (more control exclusions) with a threshold of 1e−05

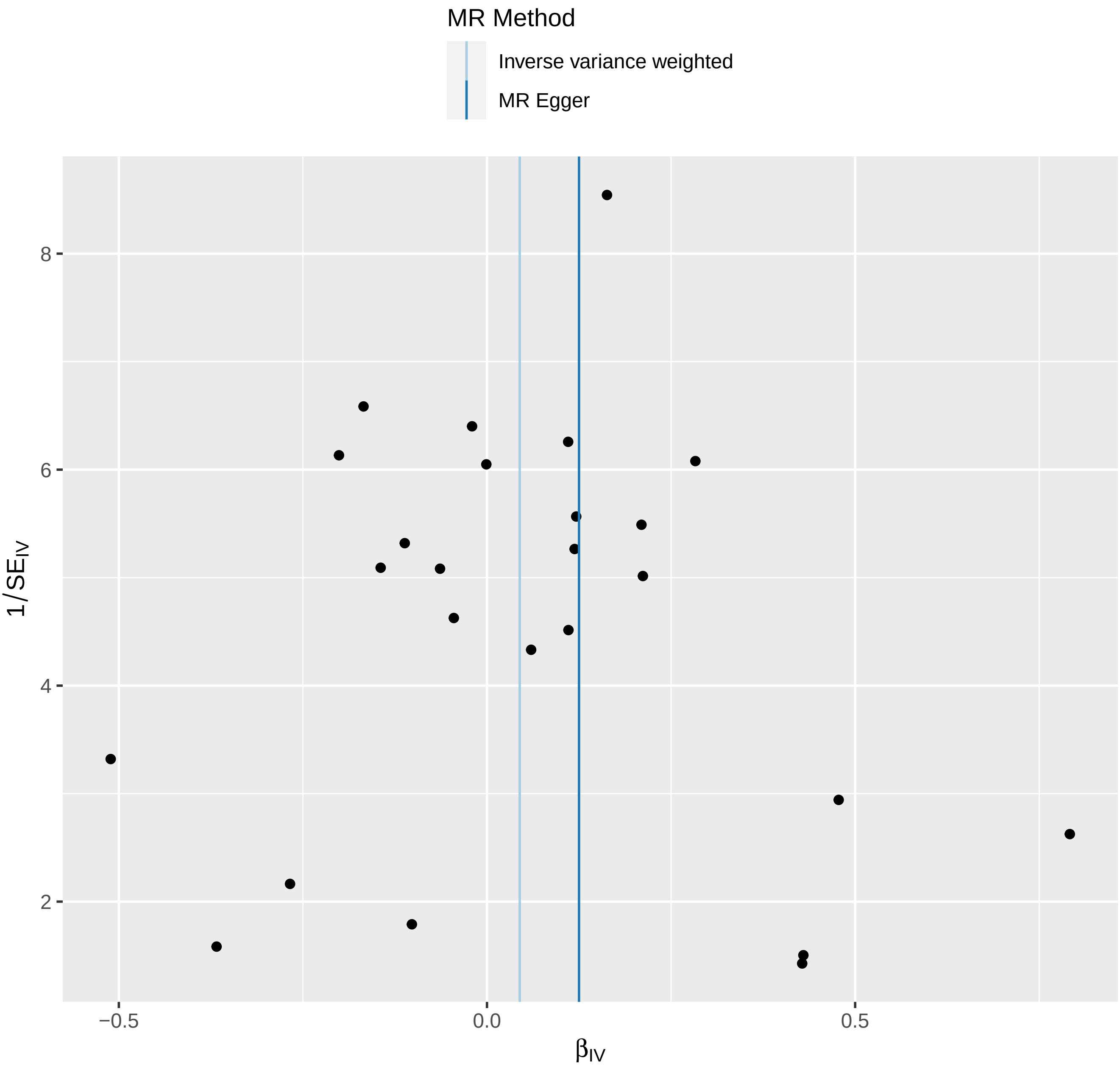

Batch 3−821 : Dickkopf−related protein 1 on Diabetic nephropathy (more control exclusions)
with a threshold of 1e−05

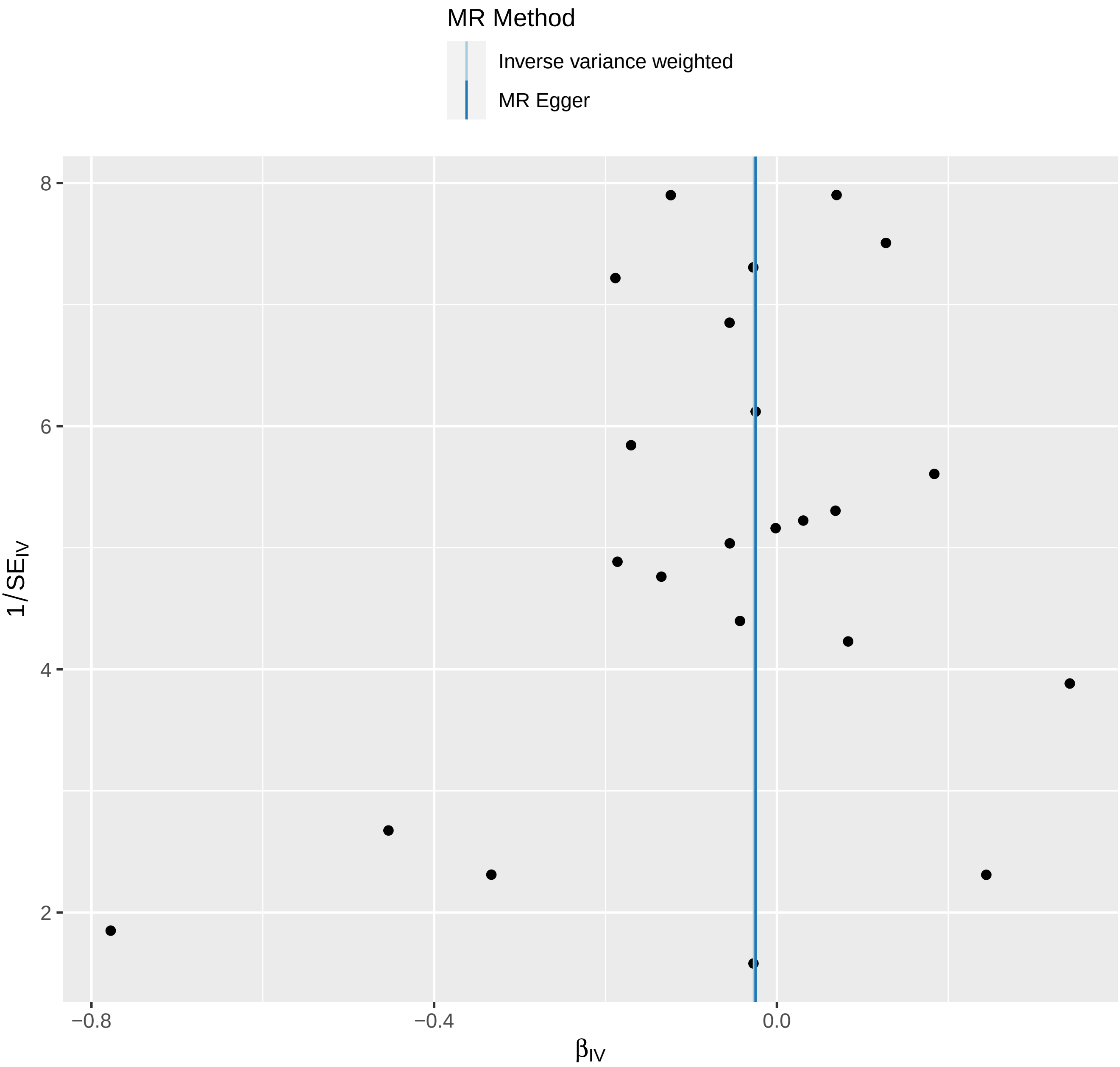

Batch 3−822 : Dickkopf−related protein 3 on Diabetic nephropathy (more control exclusions)
with a threshold of 1e−05

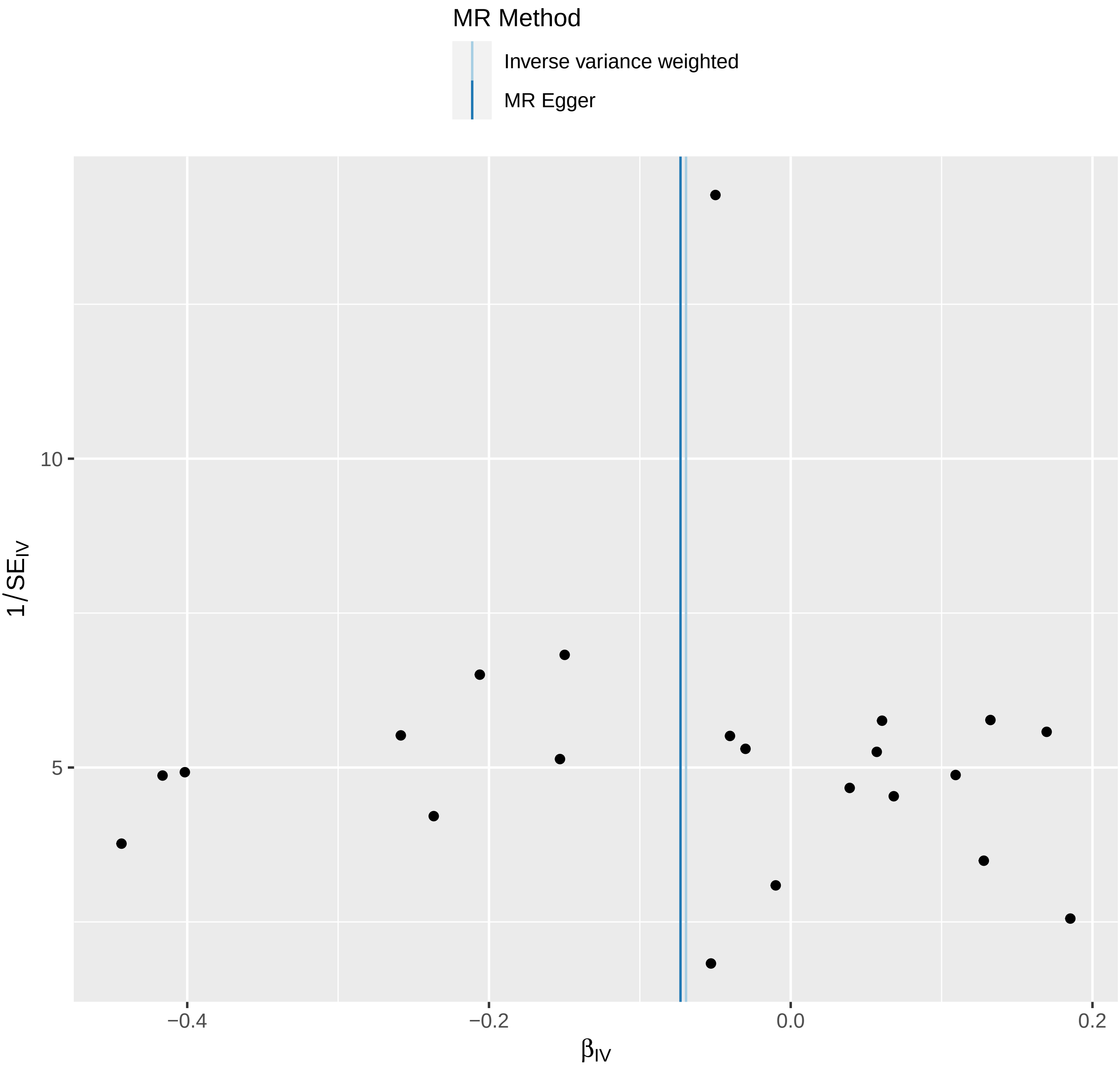

Batch 3−823 : Dickkopf−related protein 4 on Diabetic nephropathy (more control exclusions)
with a threshold of 1e−05

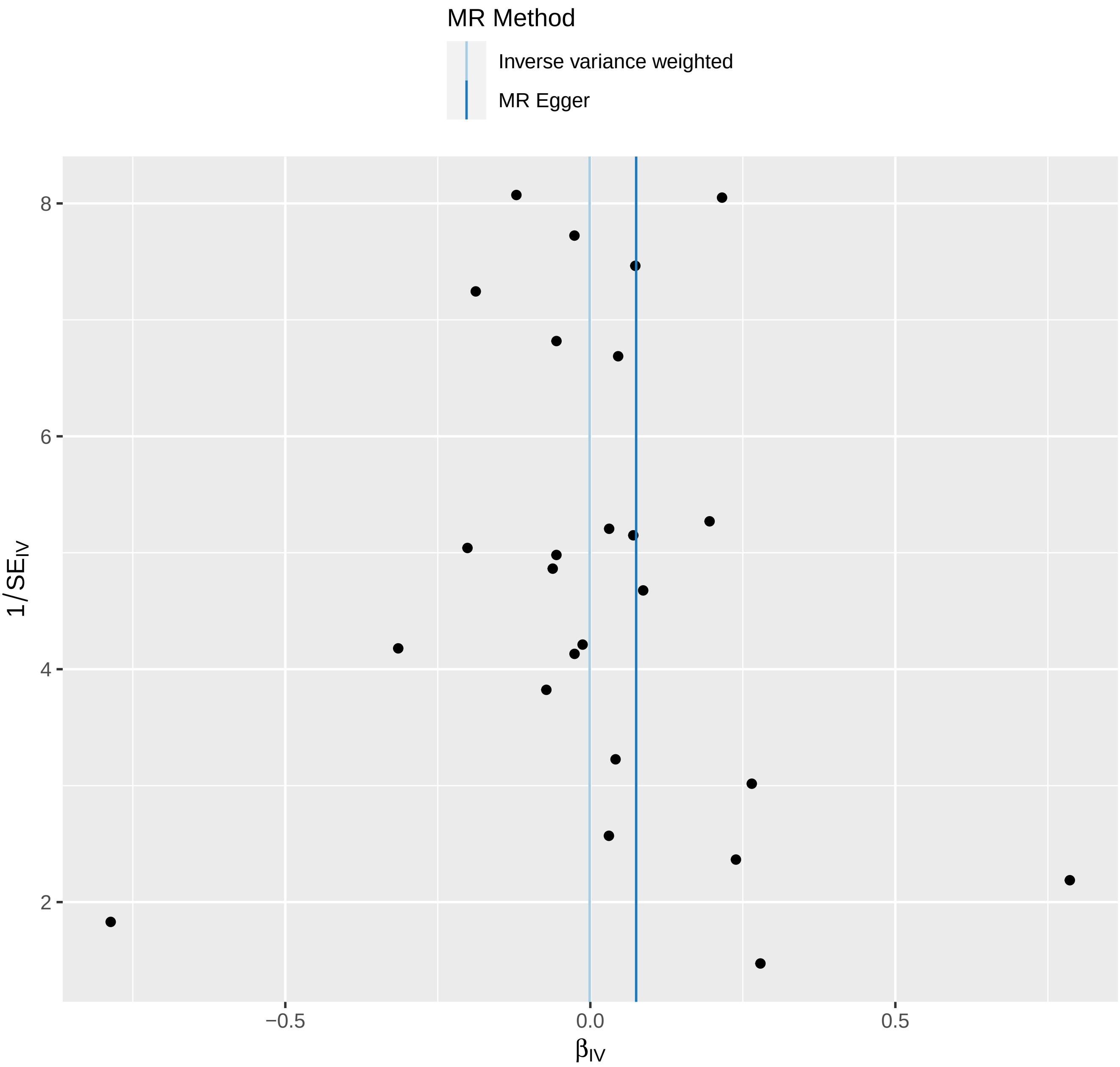

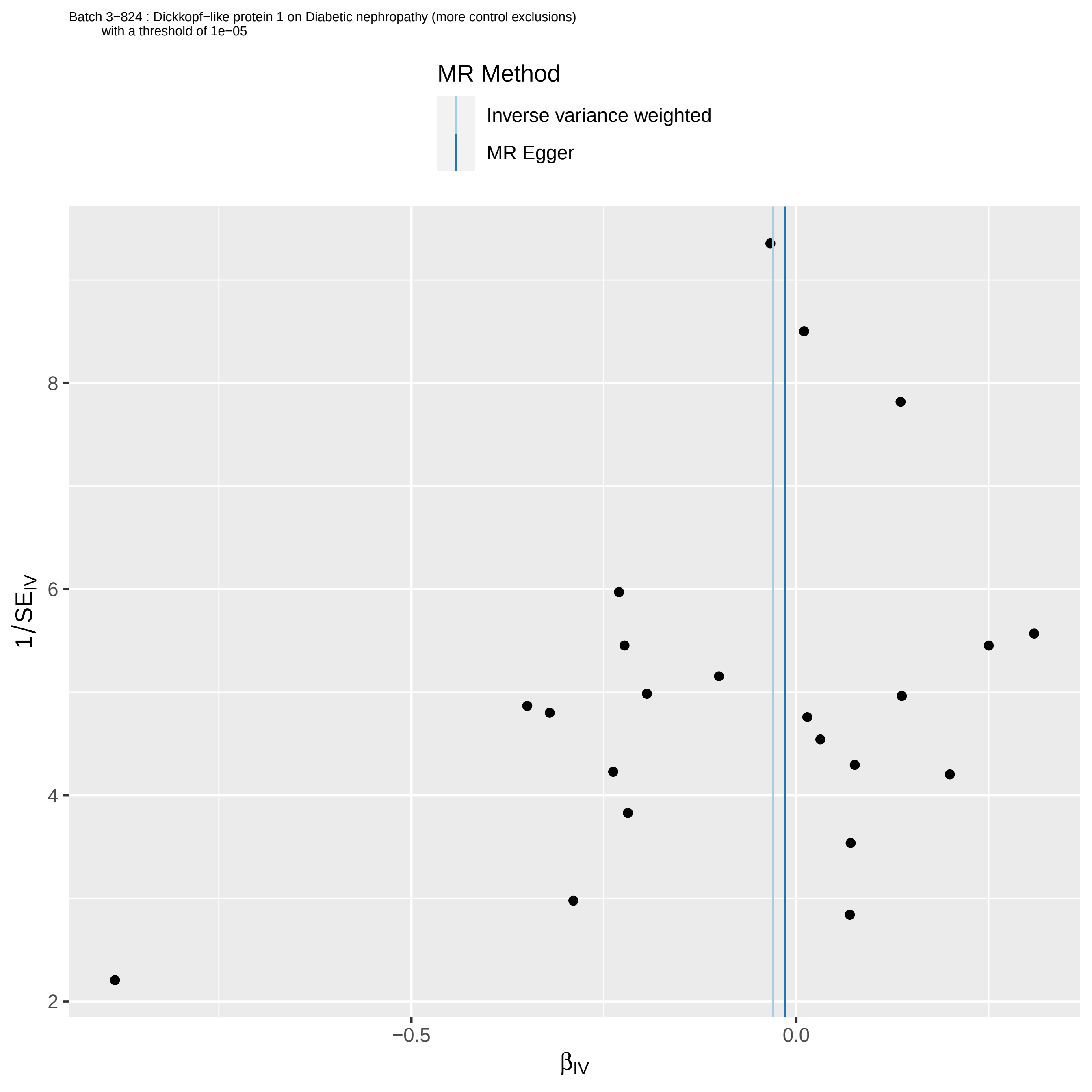
Batch 3−824 : Dickkopf−like protein 1 on Diabetic nephropathy (more control exclusions)
with a threshold of 1e−05
MR Method
Inverse variance weighted
MR Egger
8
6
4
2
1/SE_IV
−0.5
0.0
β_IV

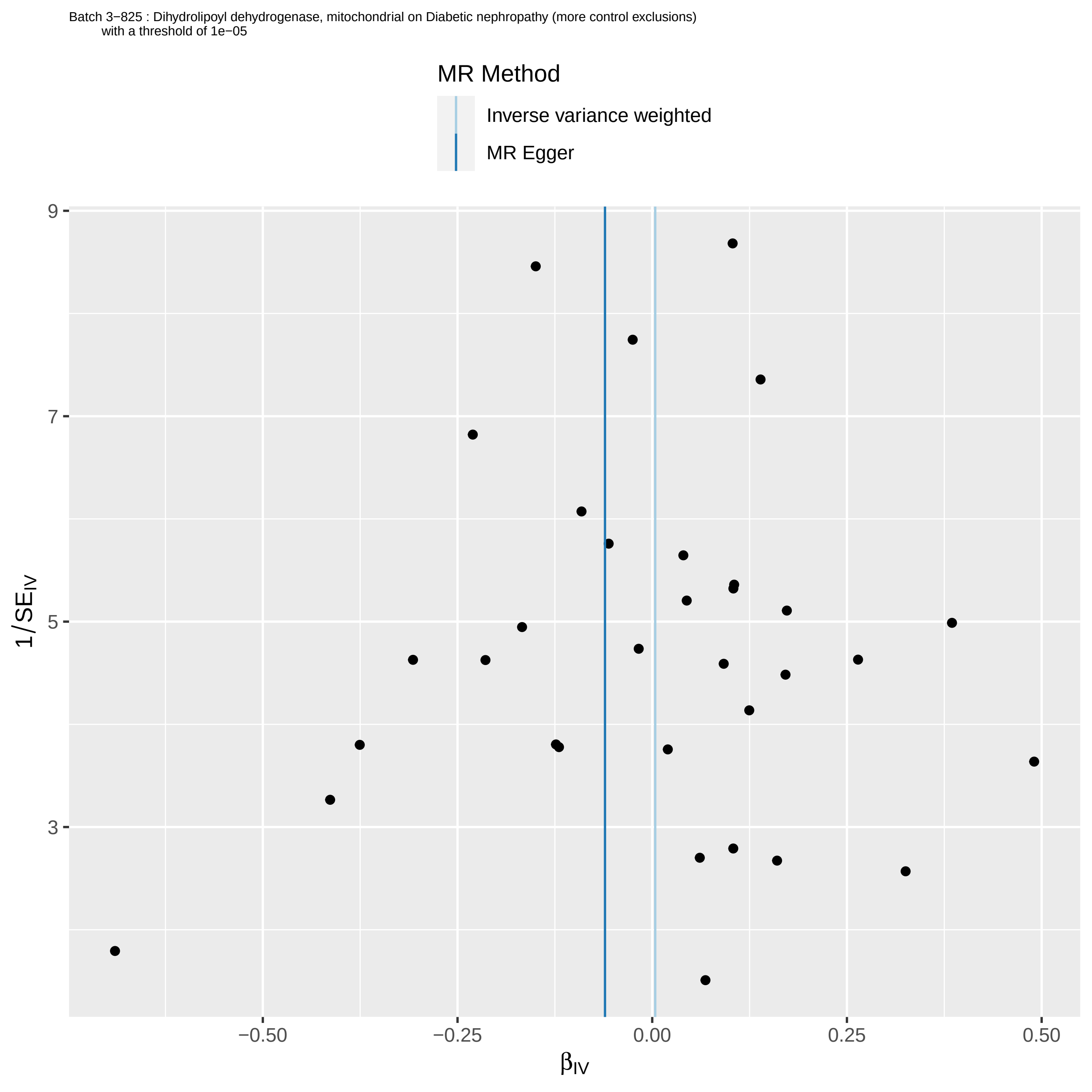

Batch 3−825 : Dihydrolipoyl dehydrogenase, mitochondrial on Diabetic nephropathy (more control exclusions)
with a threshold of 1e−05
MR Method
Inverse variance weighted
MR Egger
9
7
5
3
1/SE$_{IV}$
−0.50
−0.25
0.00
0.25
0.50
β_{IV}

Batch 3−826 : Disks large homolog 3 on Diabetic nephropathy (more control exclusions)
with a threshold of 1e−05

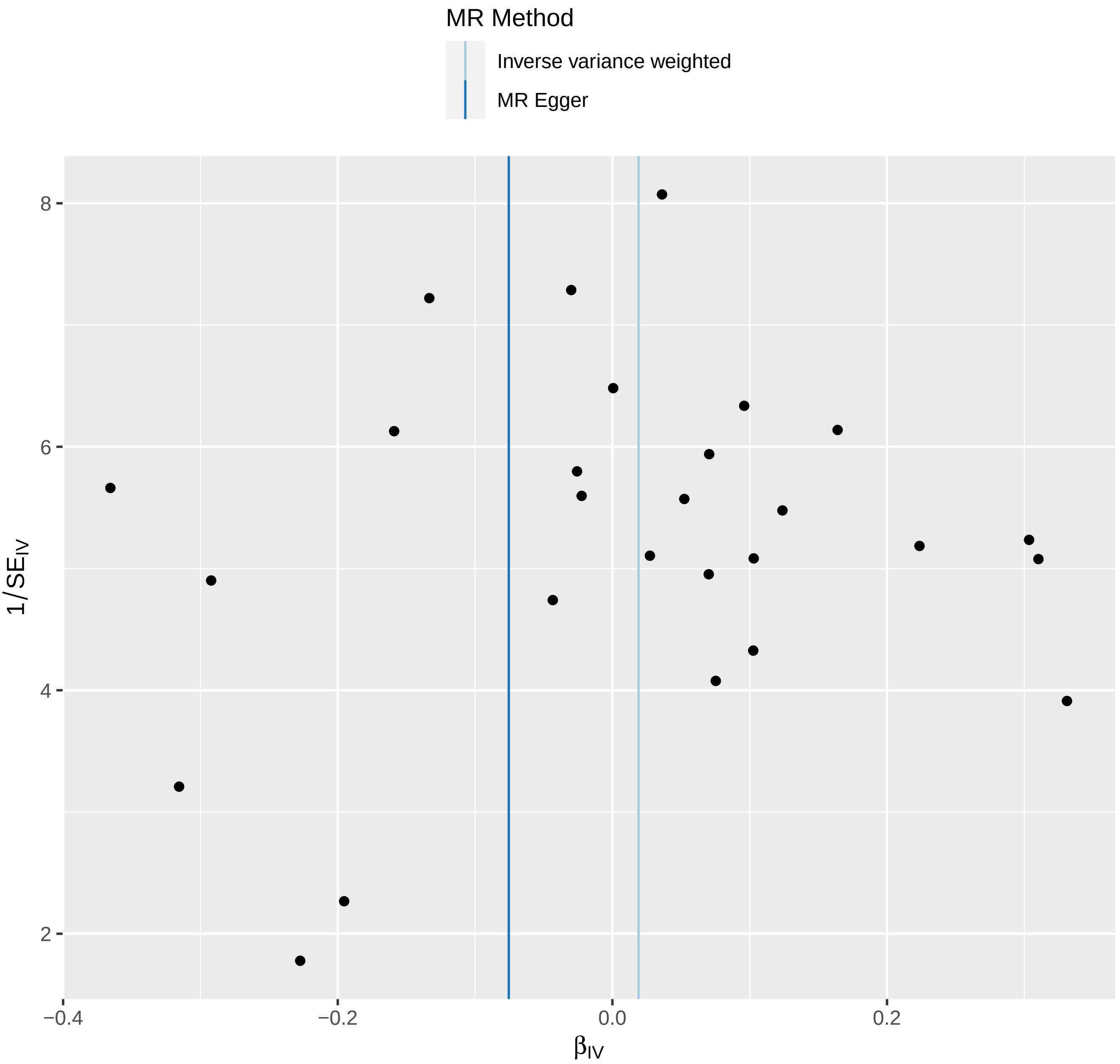

Batch 3−827 : Protein delta homolog 1 on Diabetic nephropathy (more control exclusions)
with a threshold of 1e−05

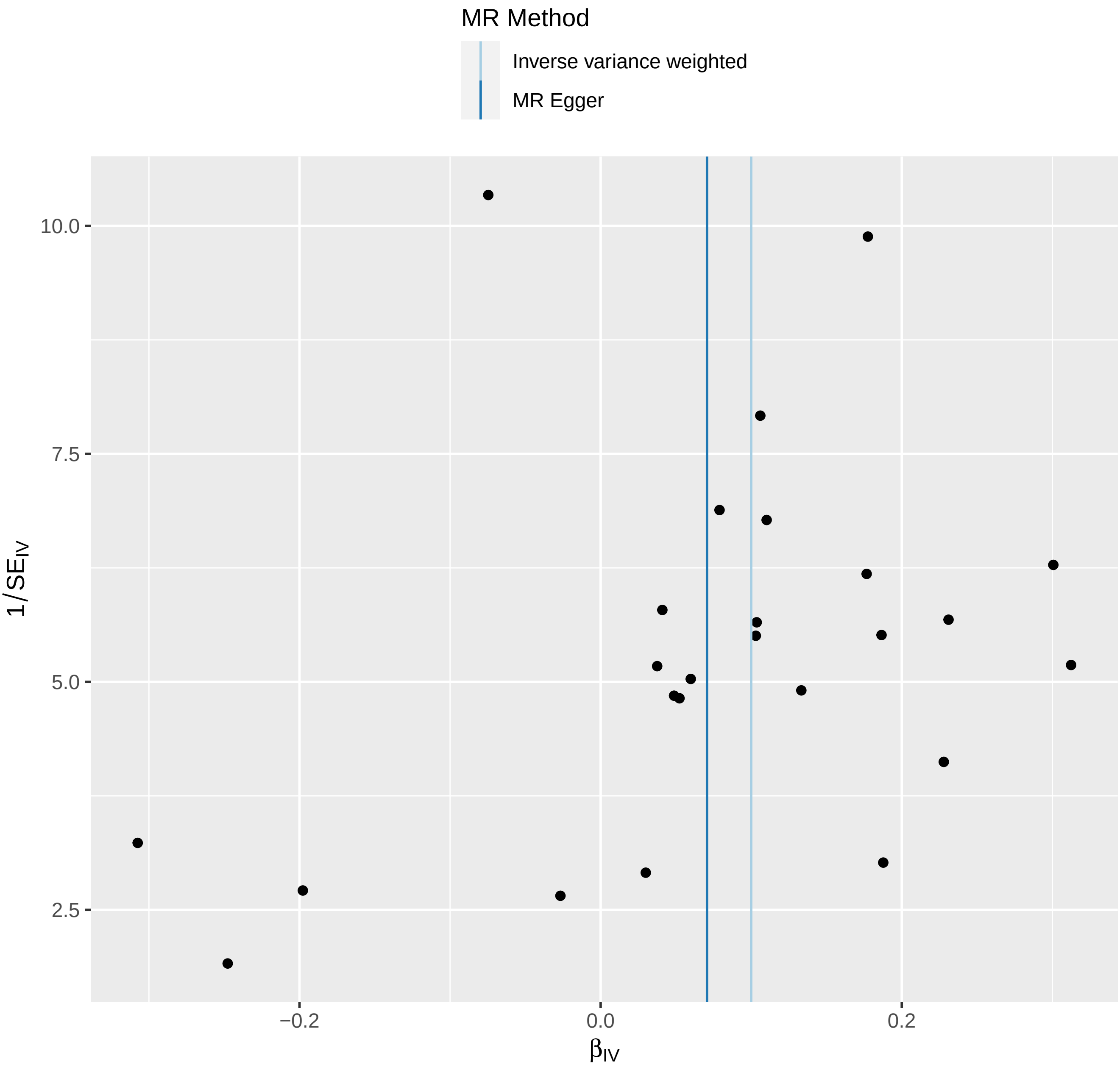

Batch 3−828 : Protein delta homolog 1 on Diabetic nephropathy (more control exclusions)
with a threshold of 1e−05

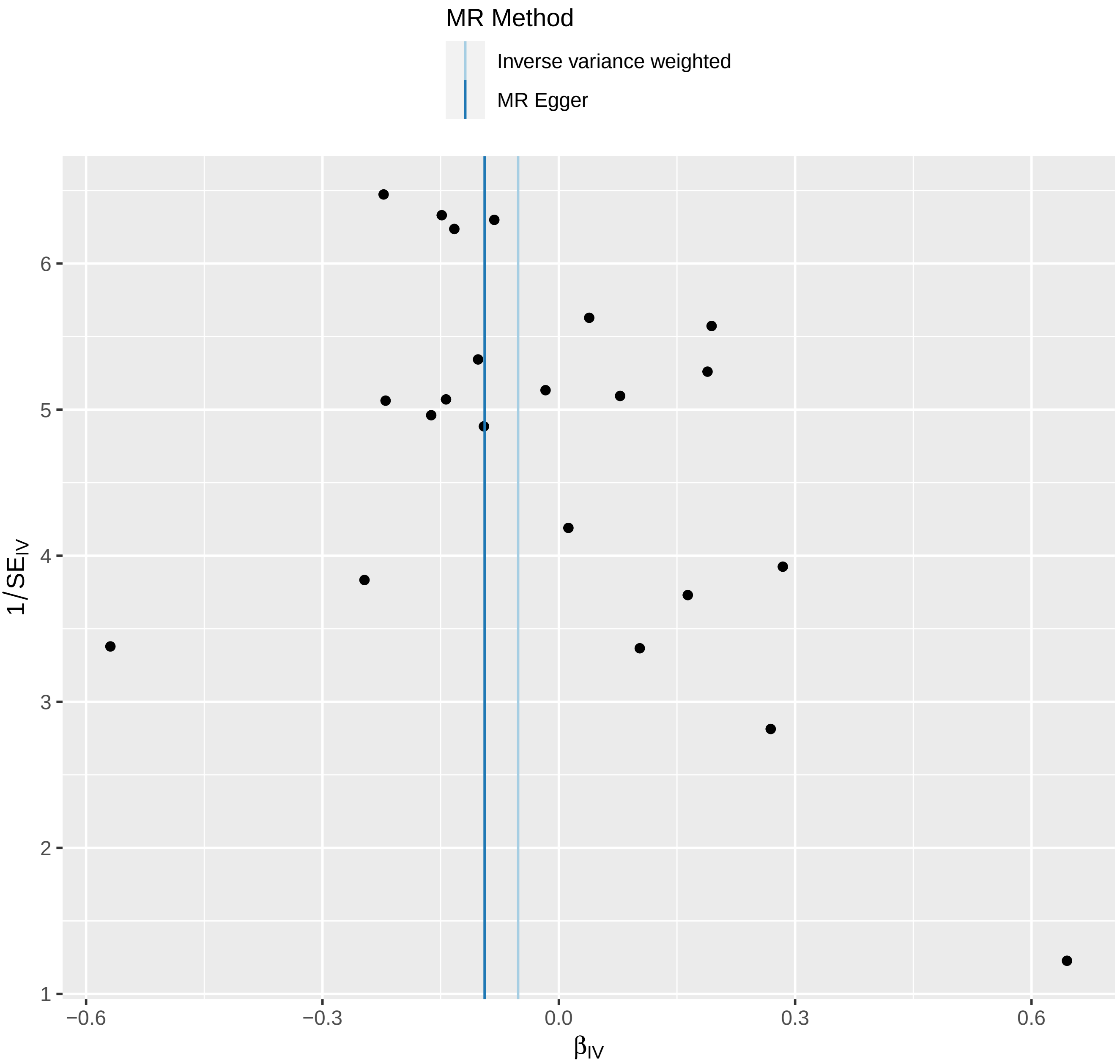

Batch 3−829 : Protein delta homolog 2 on Diabetic nephropathy (more control exclusions)
with a threshold of 1e−05

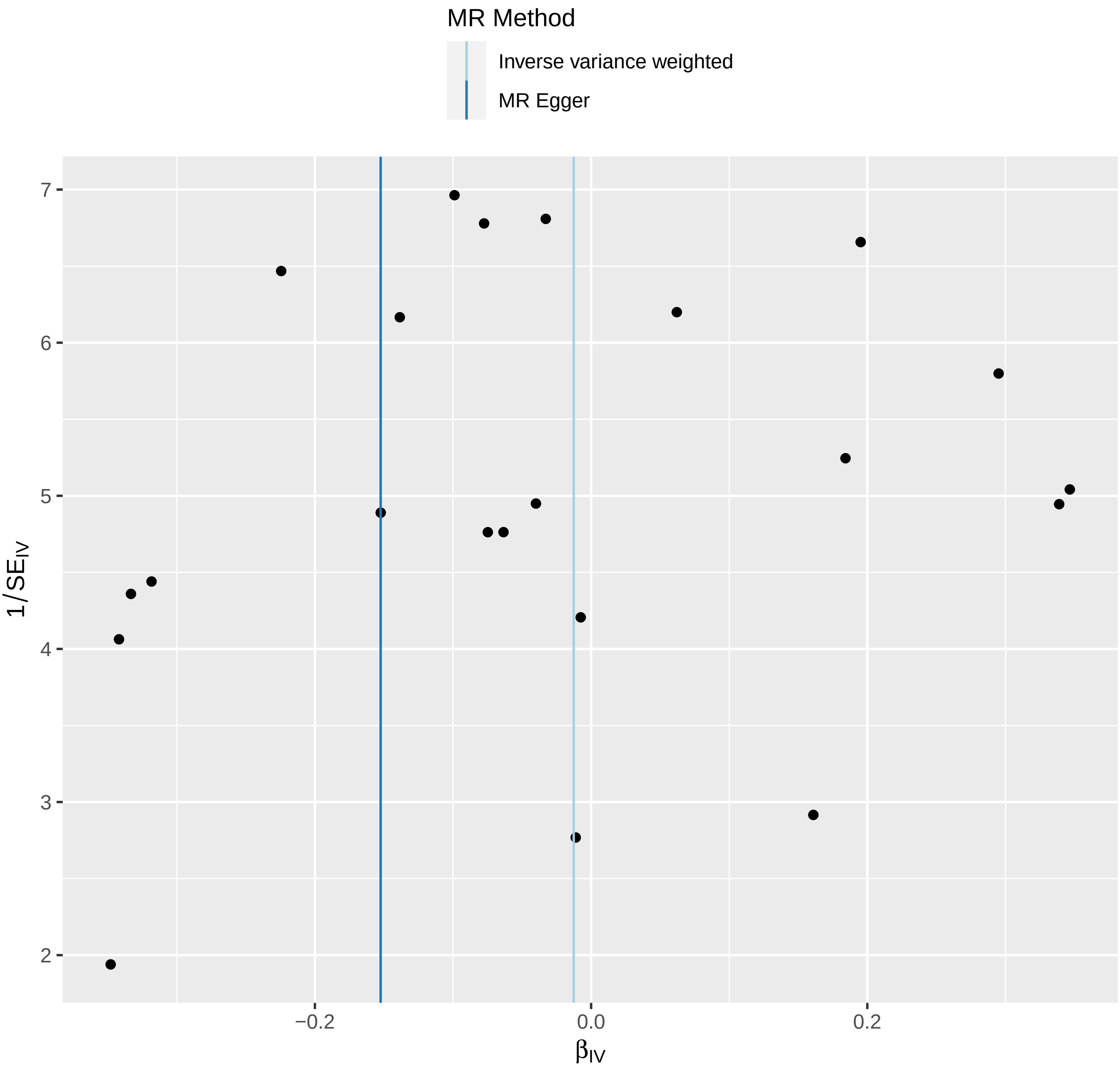

Batch 3−830 : Delta−like protein 1 on Diabetic nephropathy (more control exclusions)
with a threshold of 1e−05

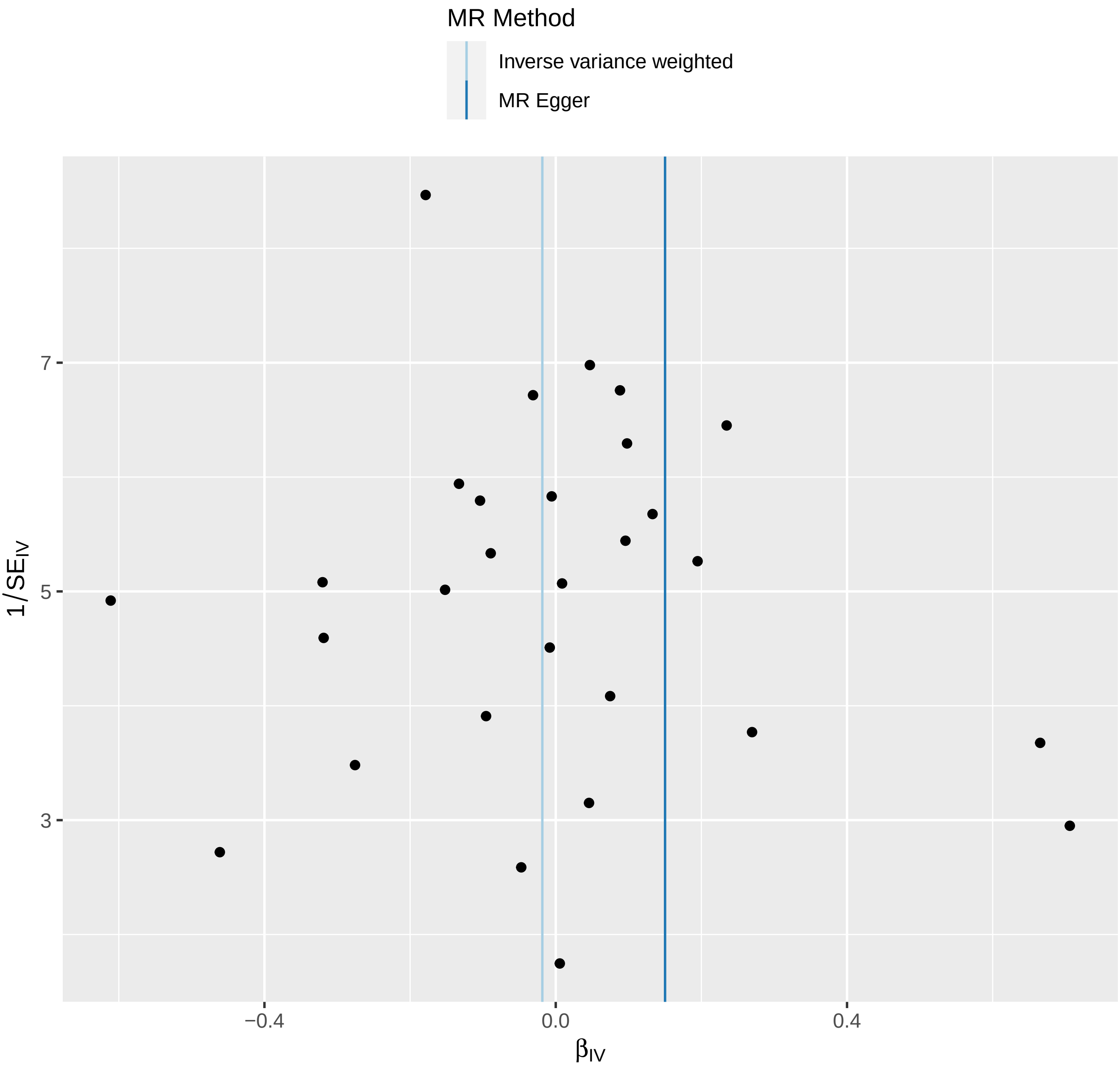

Batch 3−831 : Delta−like protein 1 on Diabetic nephropathy (more control exclusions)
with a threshold of 1e−05

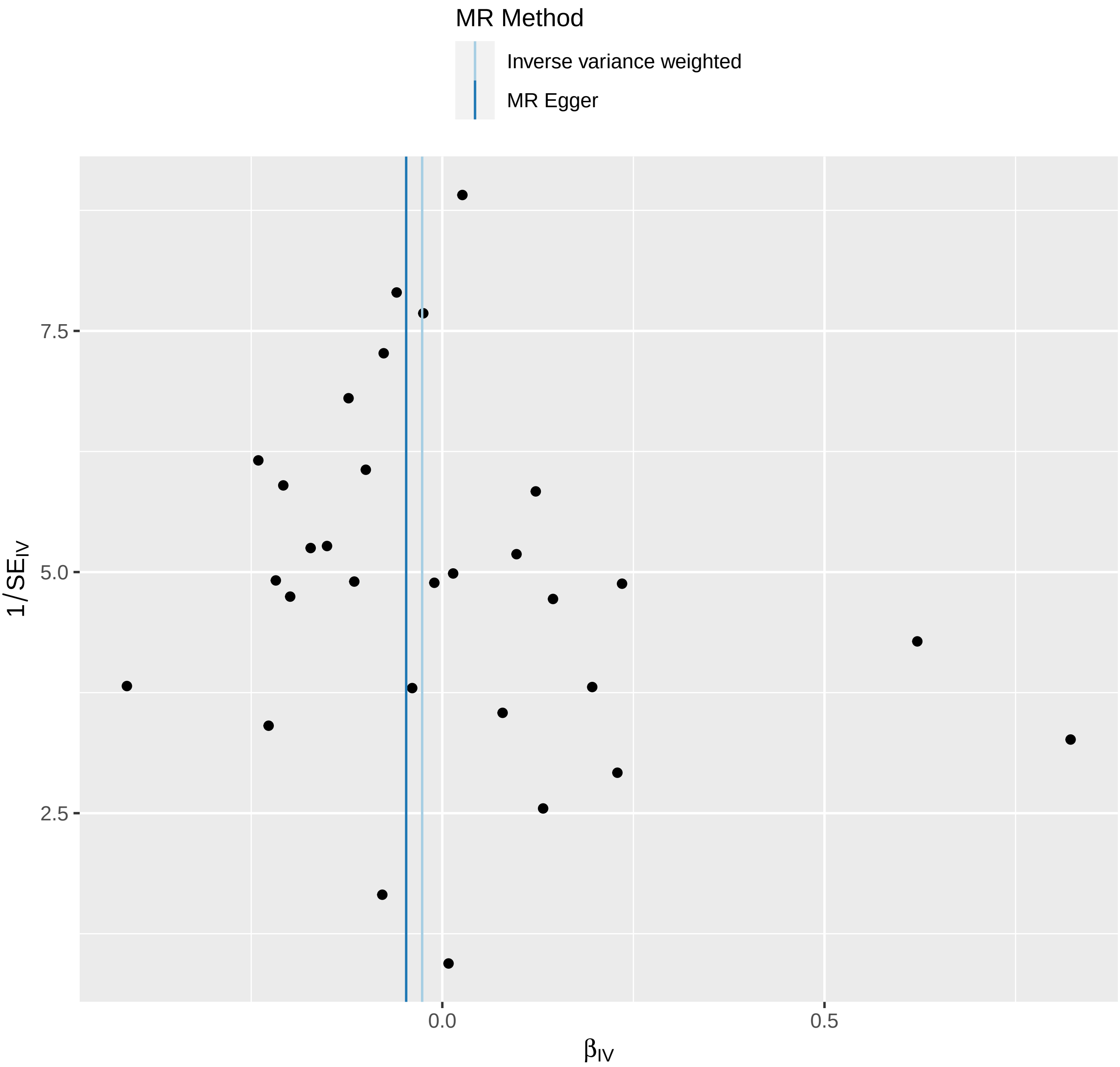

Batch 3−832 : Delta−like protein 4 on Diabetic nephropathy (more control exclusions)
with a threshold of 1e−05

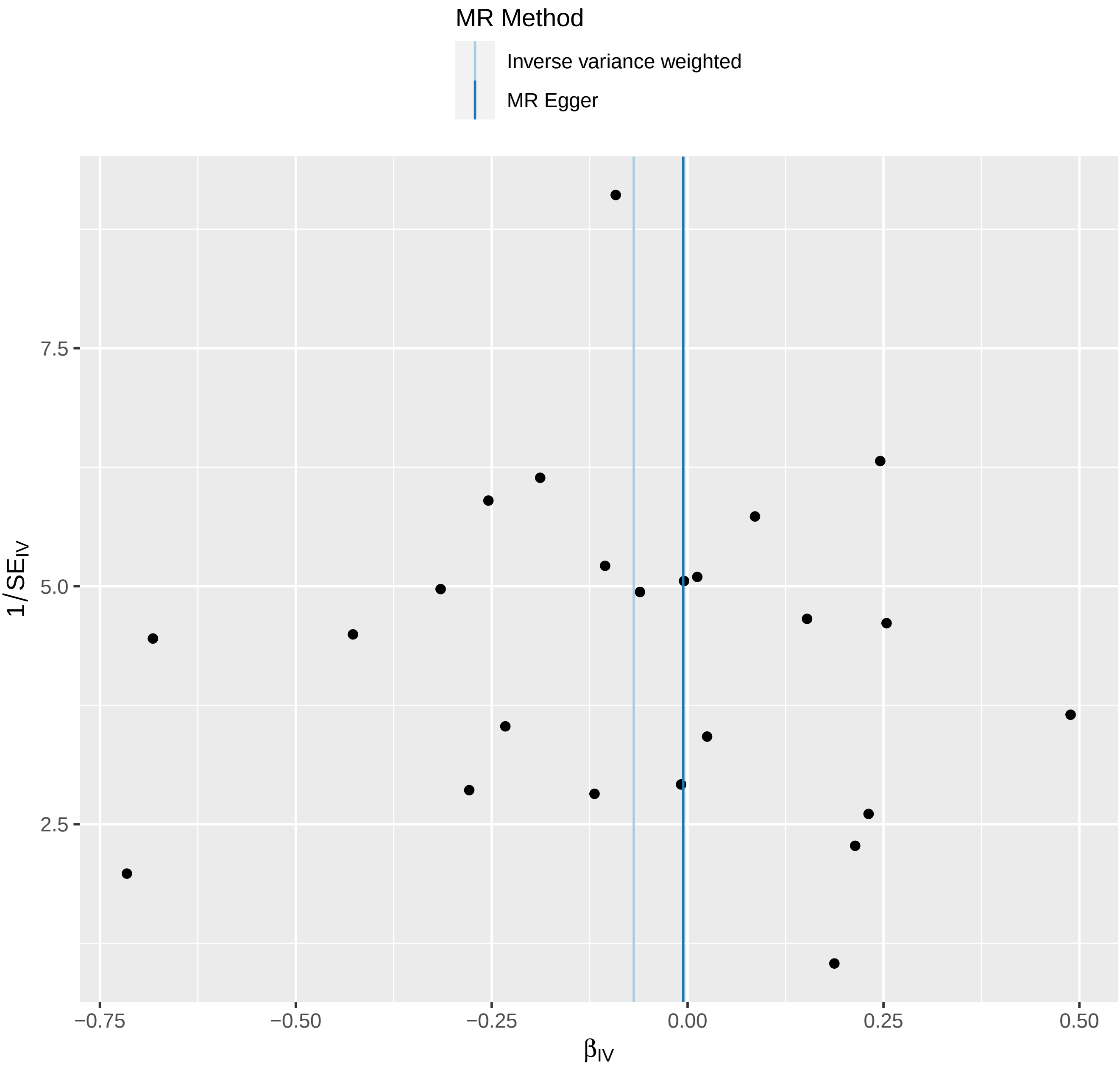

Batch 3−833 : Dermokine on Diabetic nephropathy (more control exclusions)
with a threshold of 1e−05

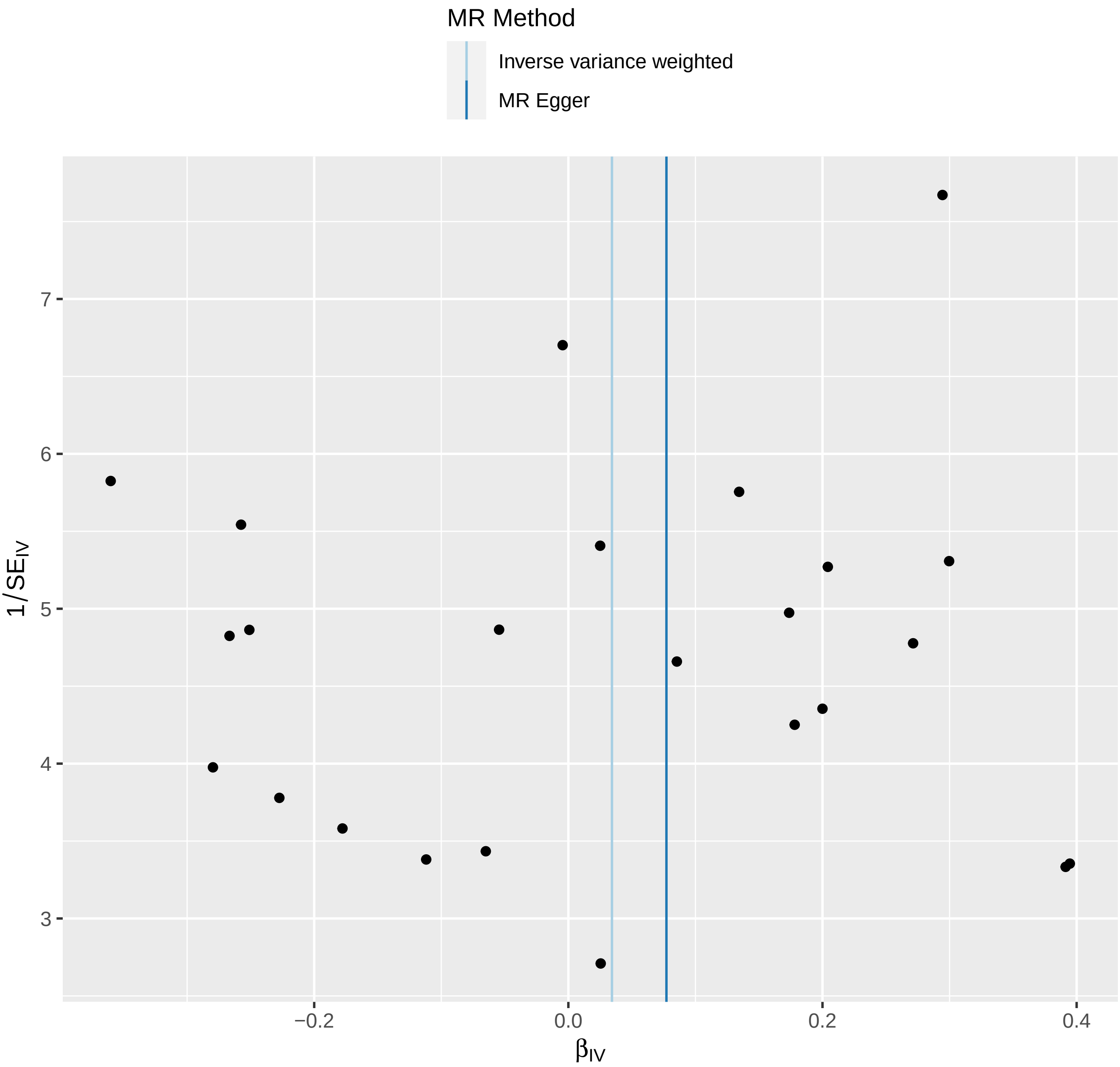

Batch 3−834 : Myotonin−protein kinase on Diabetic nephropathy (more control exclusions)
with a threshold of 1e−05

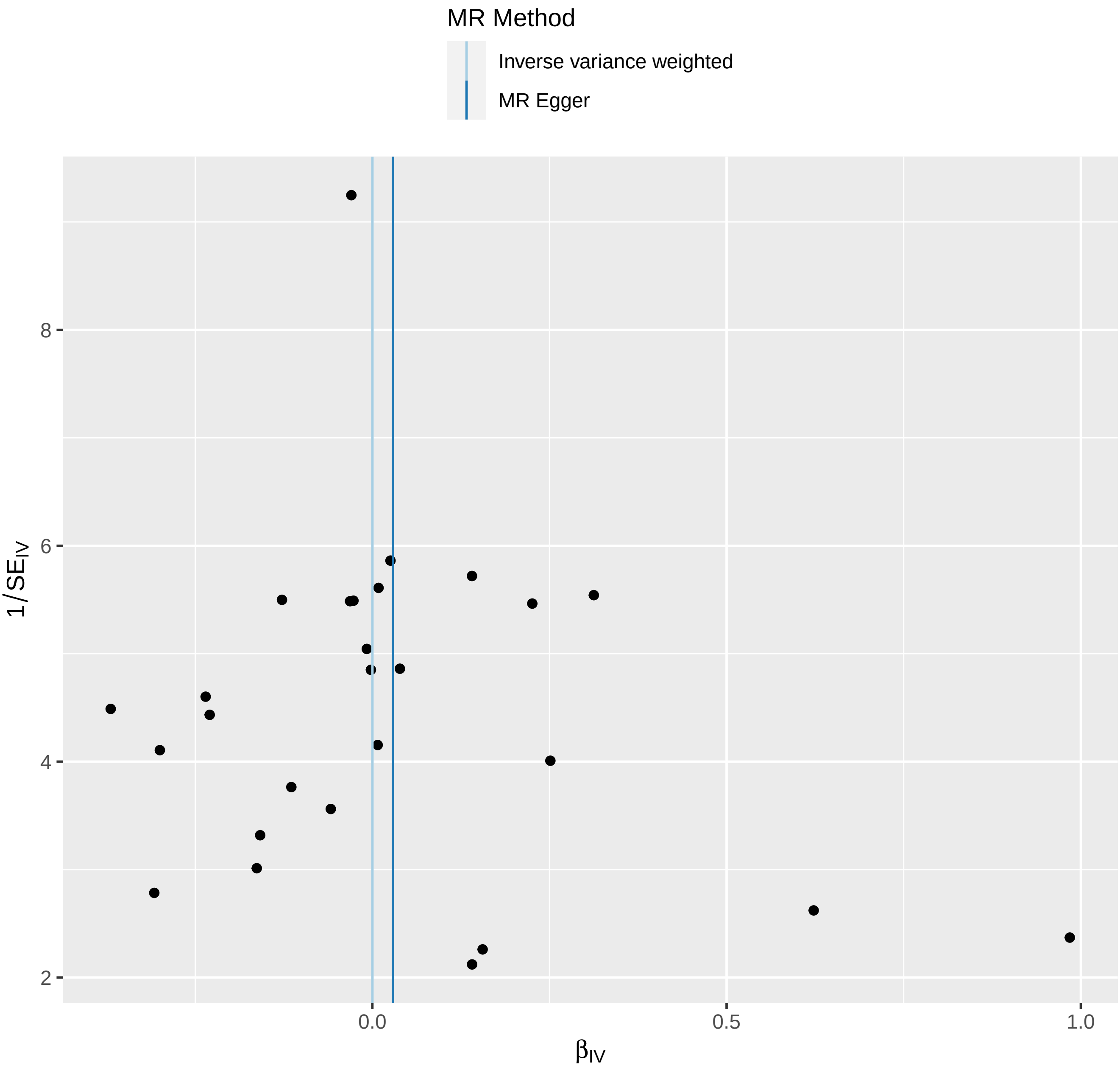

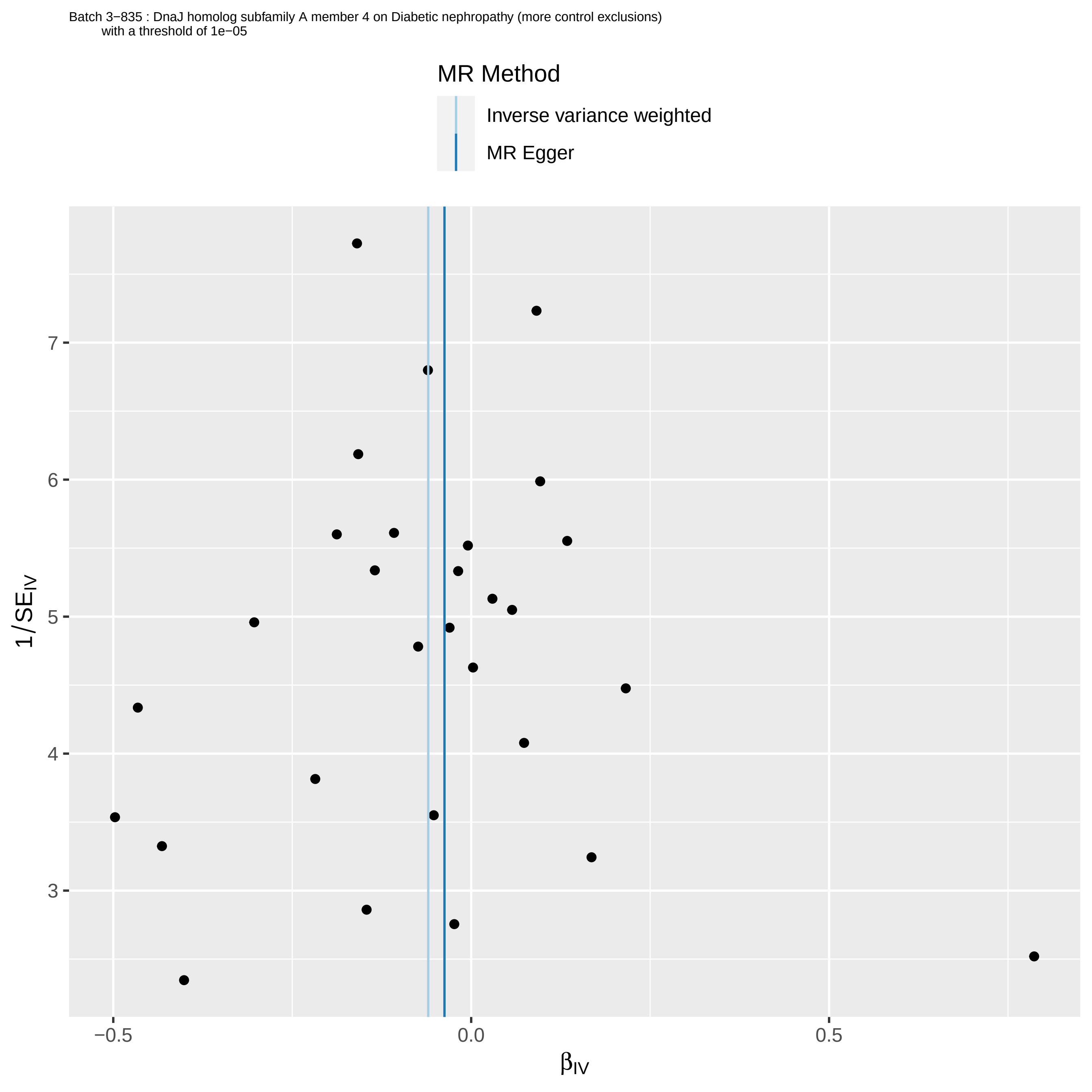
Batch 3−835 : DnaJ homolog subfamily A member 4 on Diabetic nephropathy (more control exclusions)
with a threshold of 1e−05
MR Method
Inverse variance weighted
MR Egger
7
6
5
4
3
$1/SE_{IV}$
−0.5
0.0
0.5
β_{IV}

Batch 3−836 : DnaJ homolog subfamily B member 11 on Diabetic nephropathy (more control exclusions)
with a threshold of 1e−05

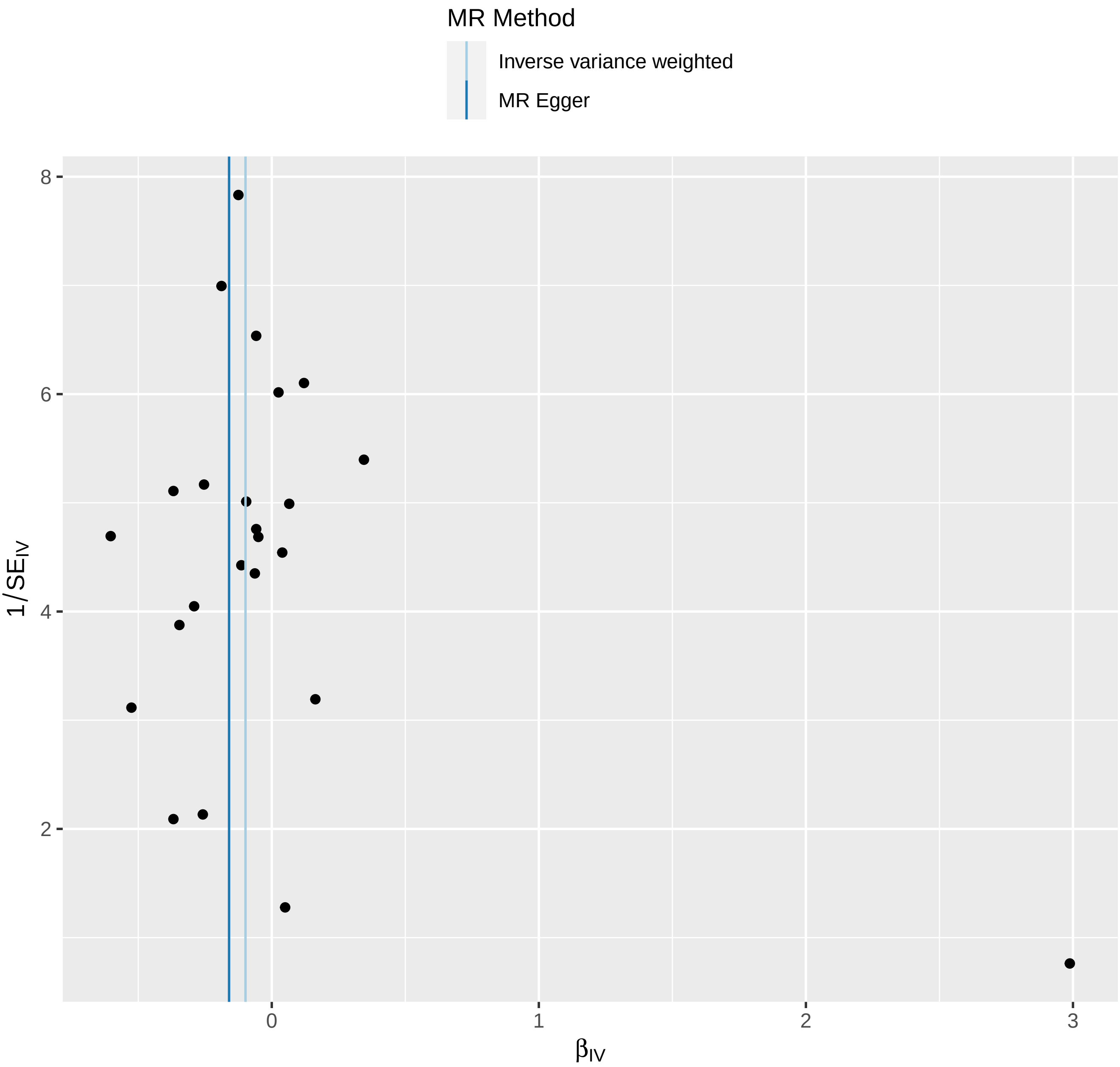

Batch 3−837 : DnaJ homolog subfamily B member 12 on Diabetic nephropathy (more control exclusions)
with a threshold of 1e−05

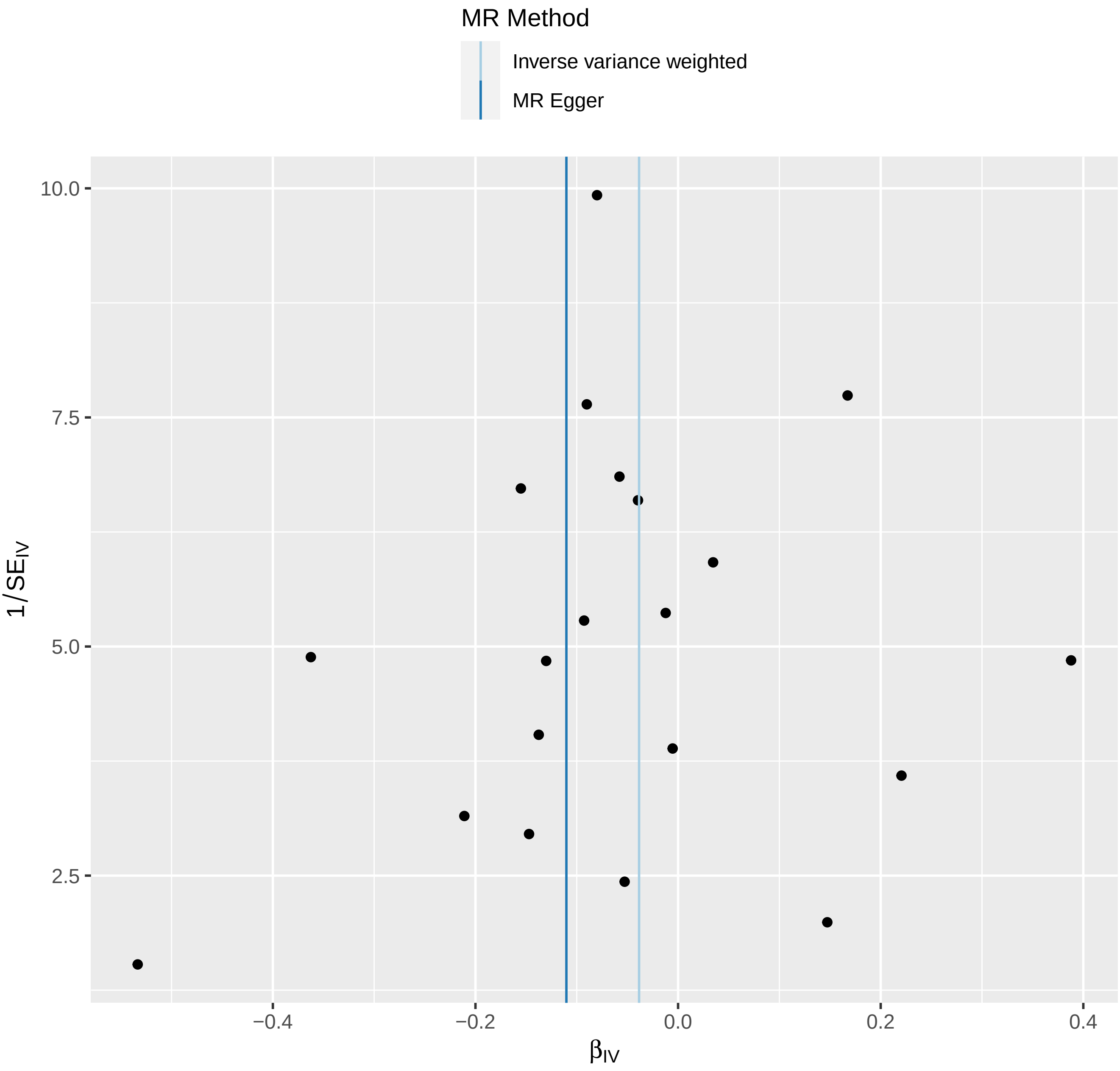

Batch 3−838 : DnaJ homolog subfamily B member 1 on Diabetic nephropathy (more control exclusions)
with a threshold of 1e−05

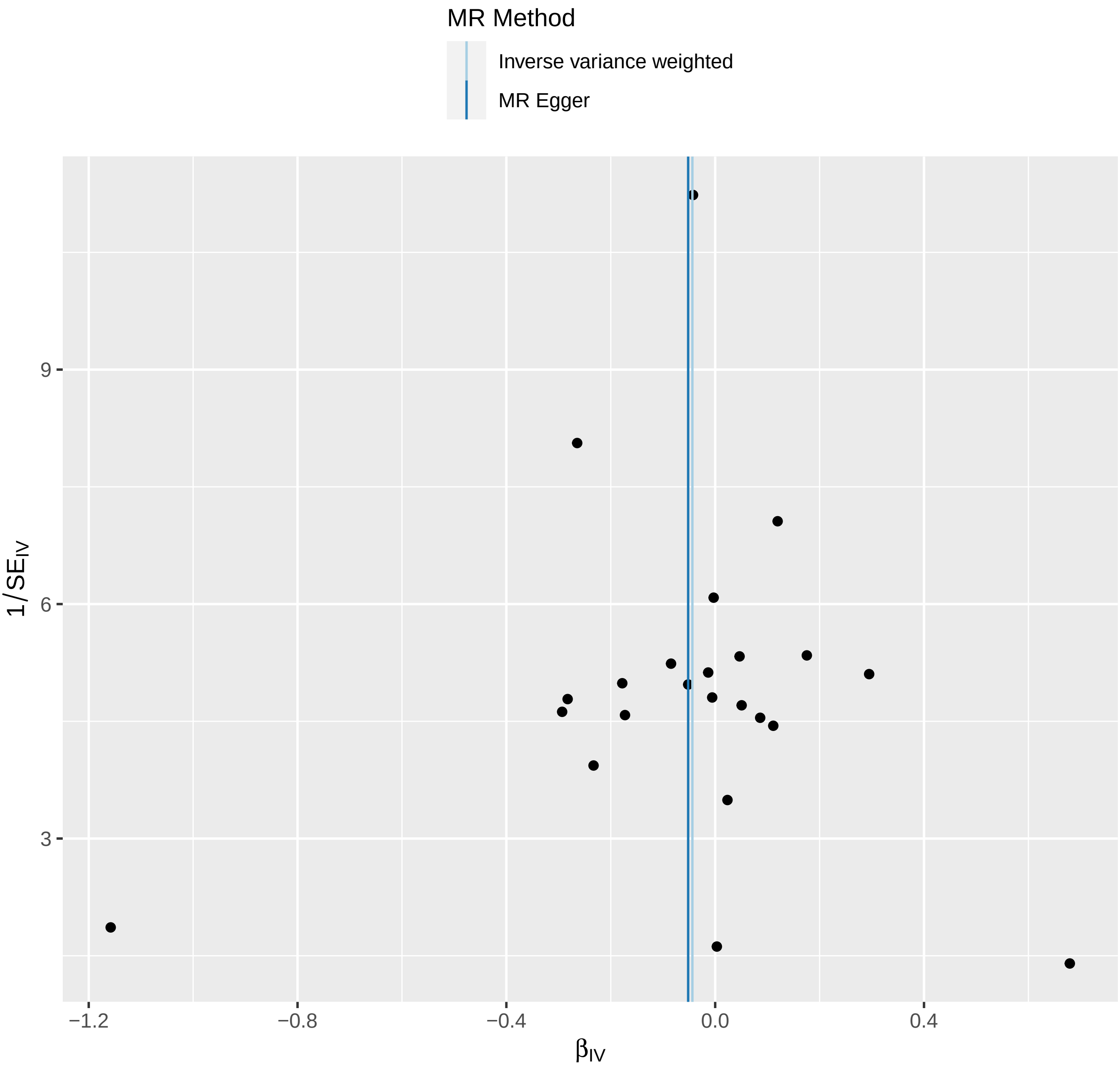

Batch 3−839 : DnaJ homolog subfamily B member 14 on Diabetic nephropathy (more control exclusions)
with a threshold of 1e−05

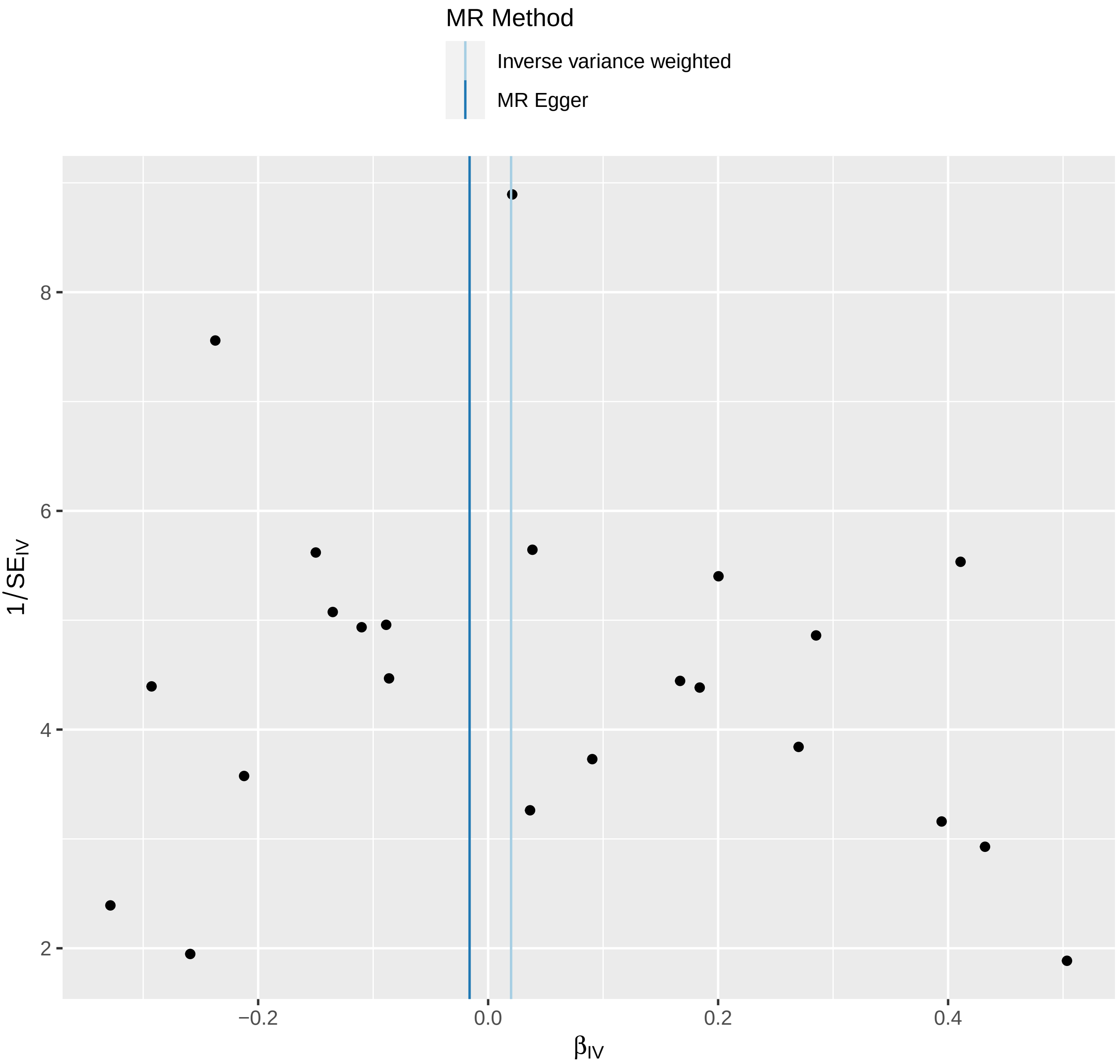

Batch 3−840 : DnaJ homolog subfamily B member 14 on Diabetic nephropathy (more control exclusions)
with a threshold of 1e−05

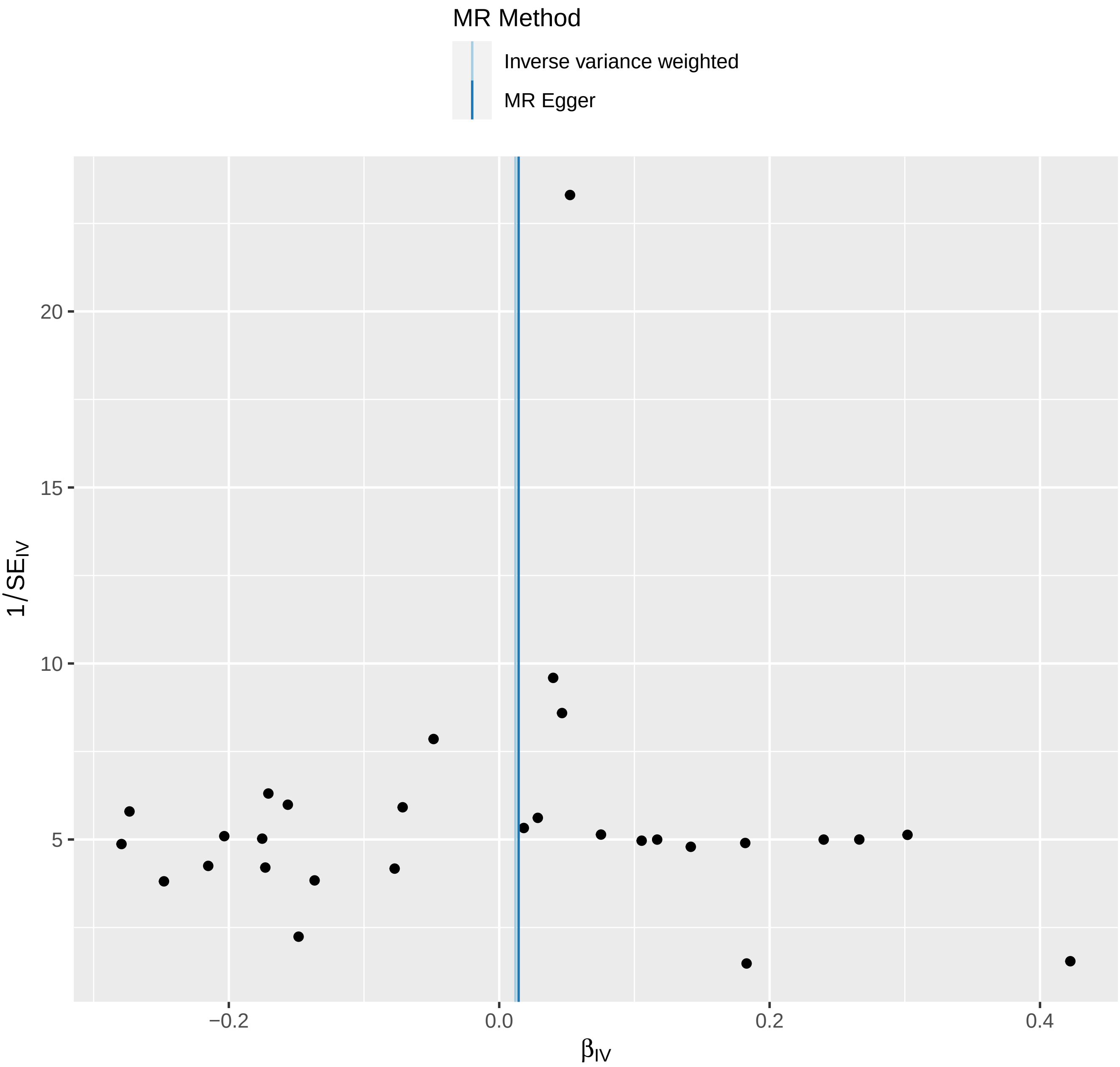

Batch 3−841 : DnaJ homolog subfamily B member 2 on Diabetic nephropathy (more control exclusions)
with a threshold of 1e−05

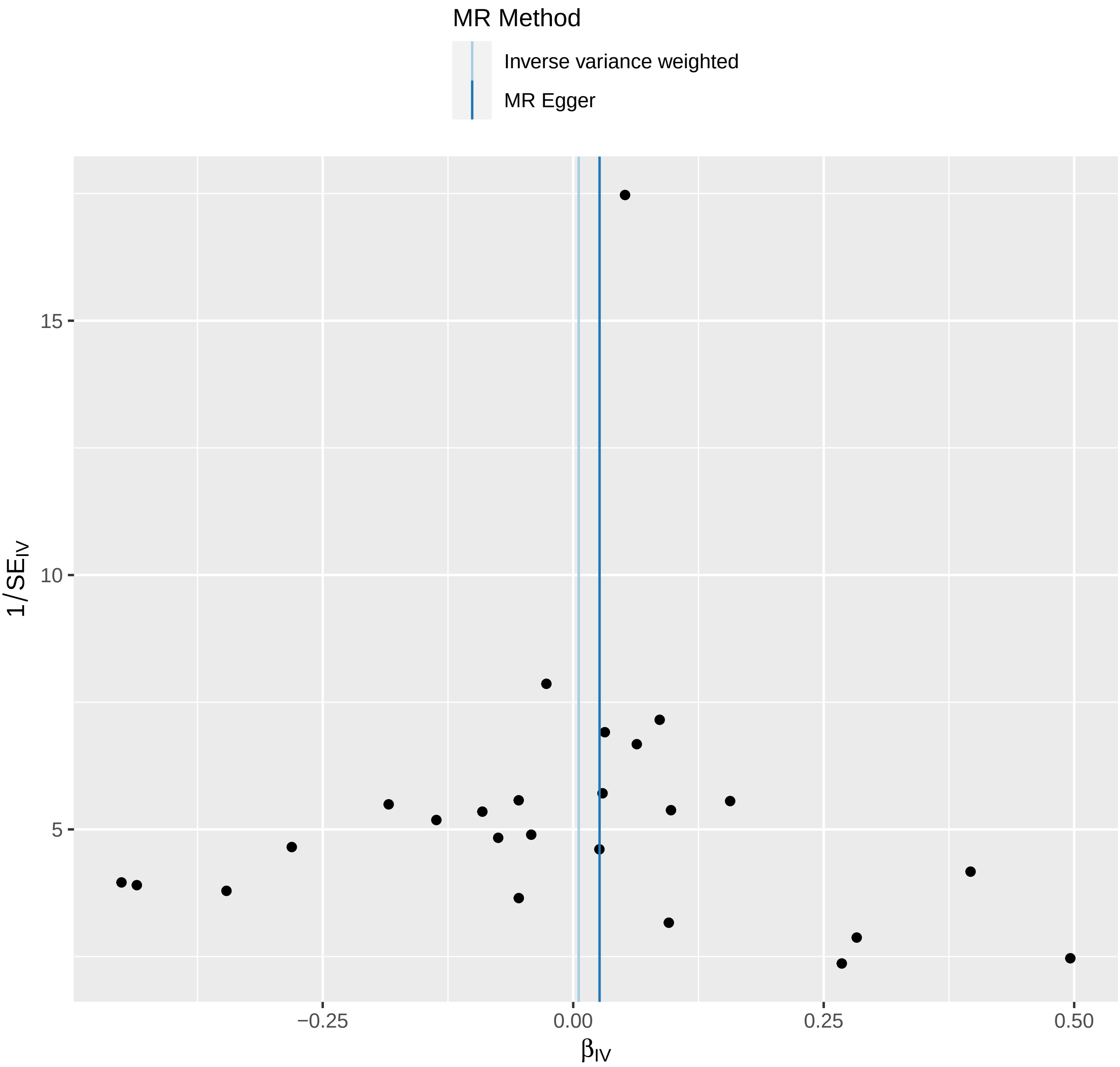

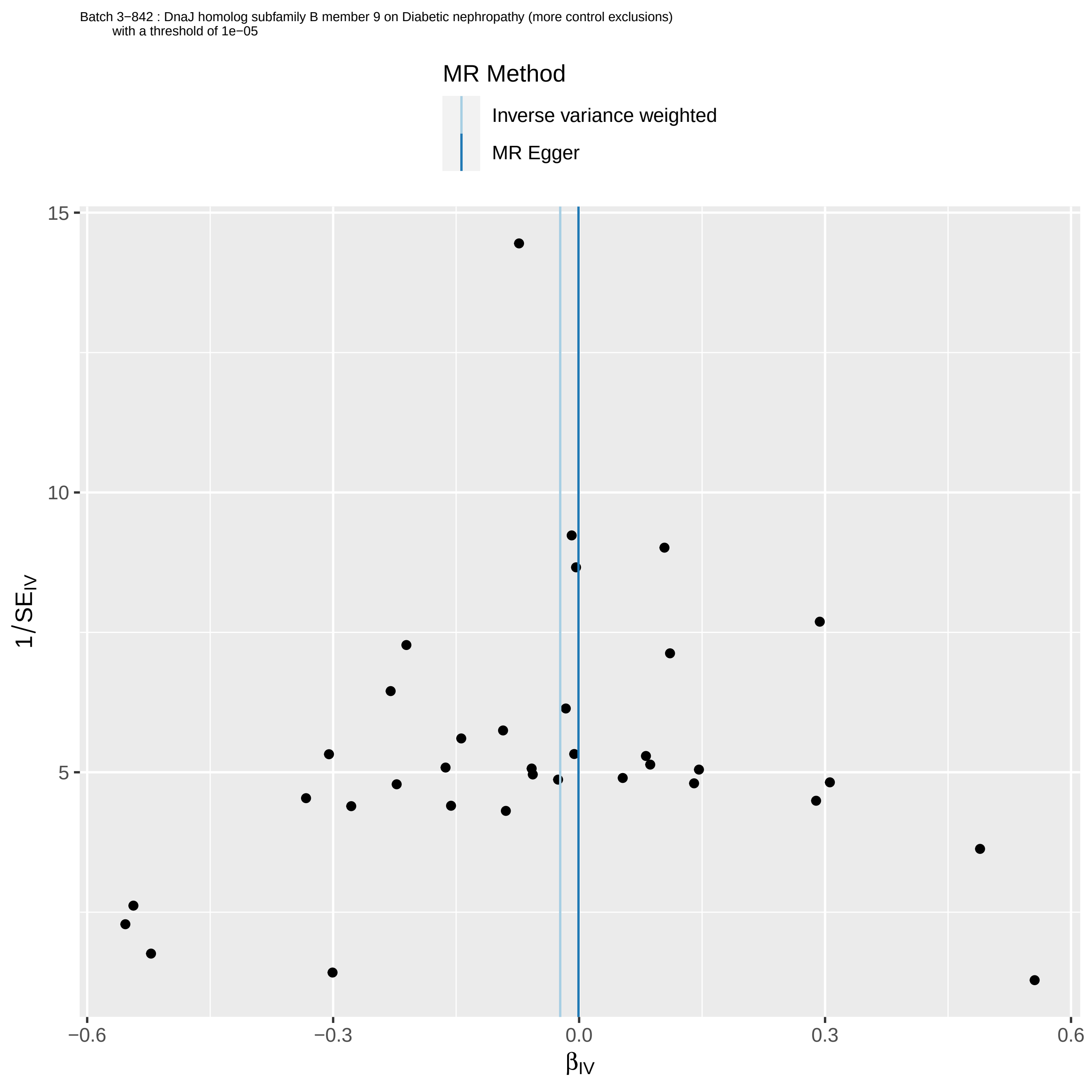
Batch 3−842 : DnaJ homolog subfamily B member 9 on Diabetic nephropathy (more control exclusions)
with a threshold of 1e−05
MR Method
Inverse variance weighted
MR Egger
15
10
5
1/SE_IV
−0.6
−0.3
0.0
0.3
0.6
β_IV

Batch 3−843 : DnaJ homolog subfamily C member 10 on Diabetic nephropathy (more control exclusions)
with a threshold of 1e−05

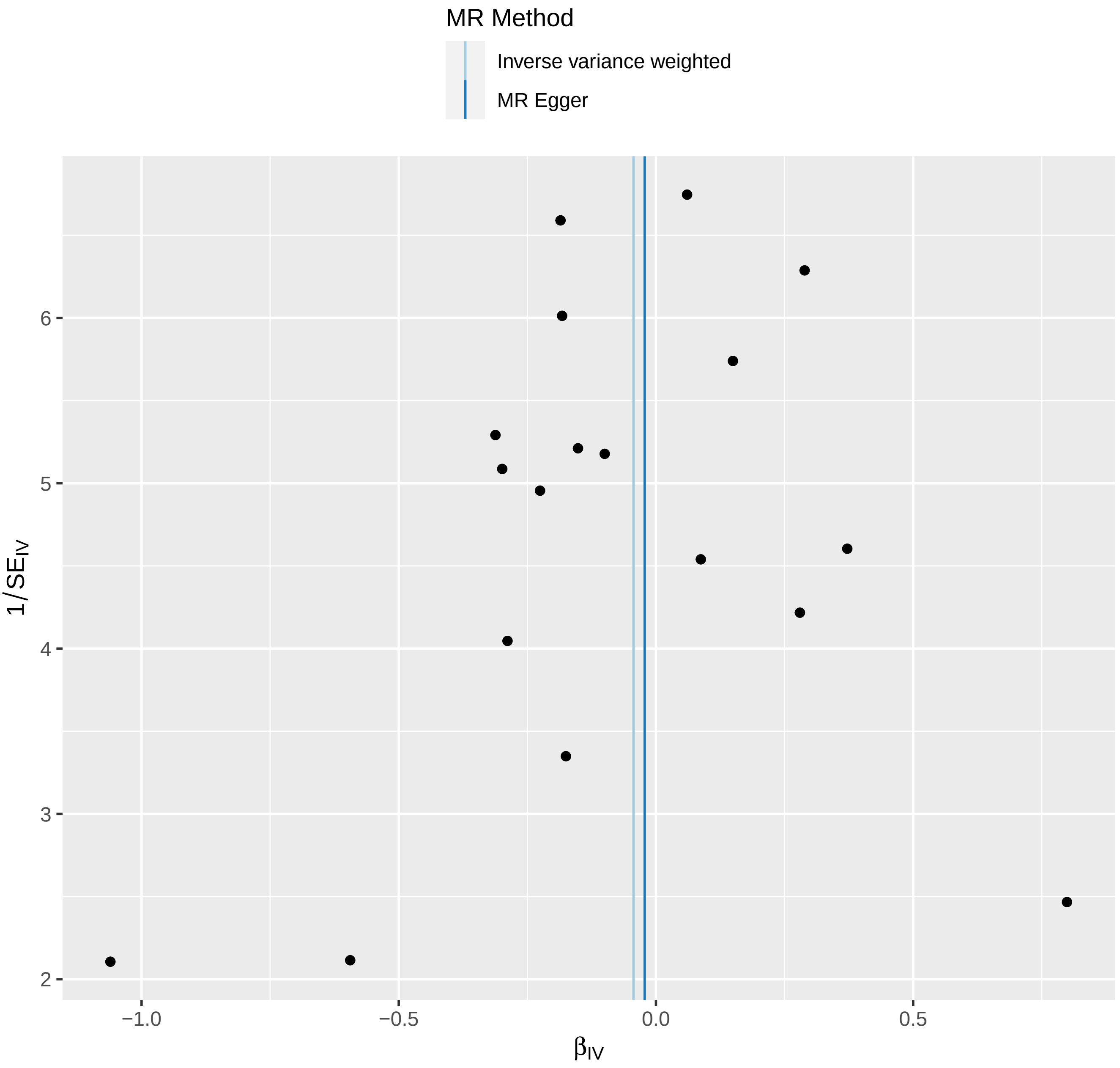

Batch 3−844 : DnaJ homolog subfamily C member 15 on Diabetic nephropathy (more control exclusions)
with a threshold of 1e−05

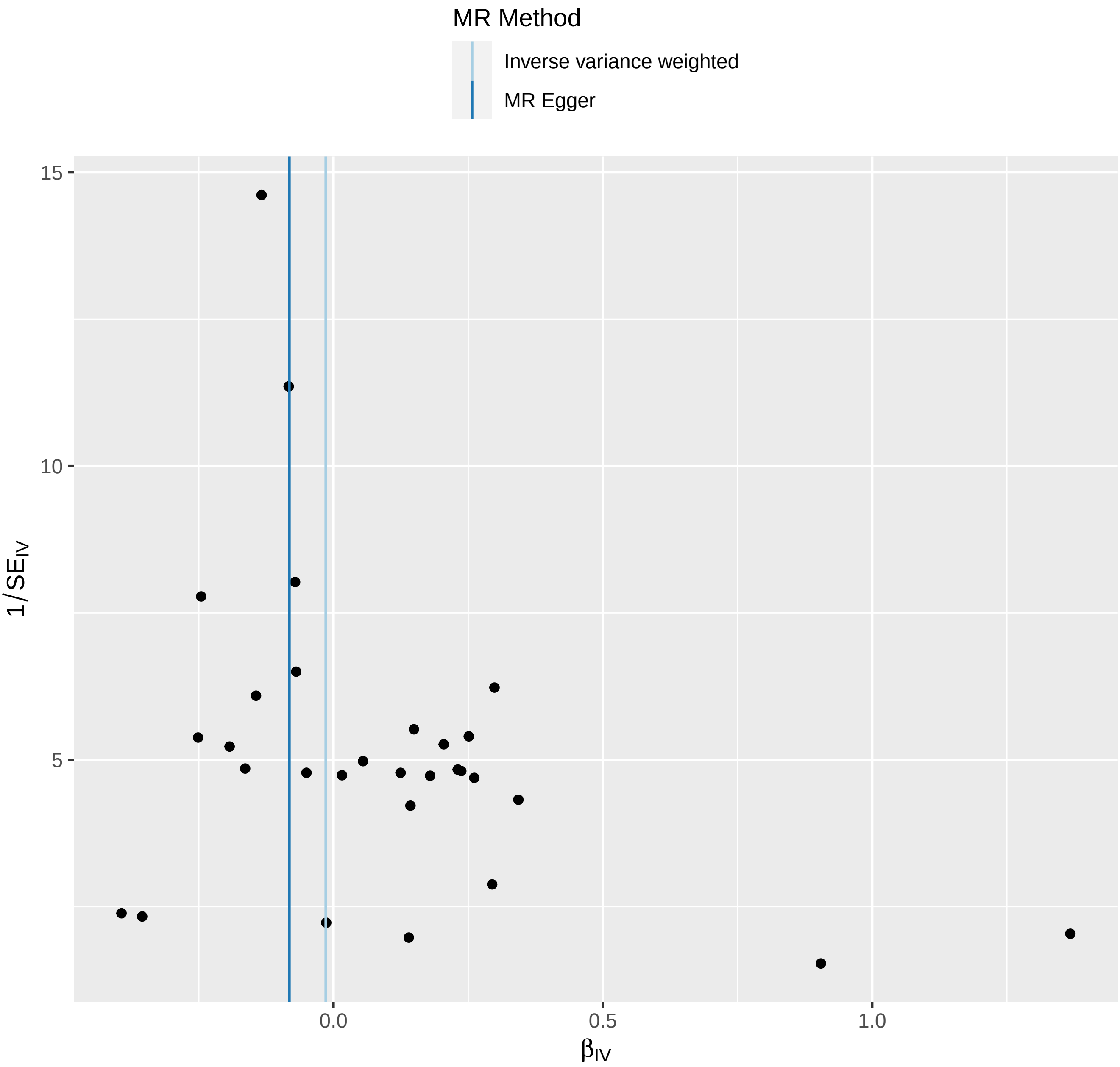

Batch 3−845 : DnaJ homolog subfamily C member 17 on Diabetic nephropathy (more control exclusions)
with a threshold of 1e−05

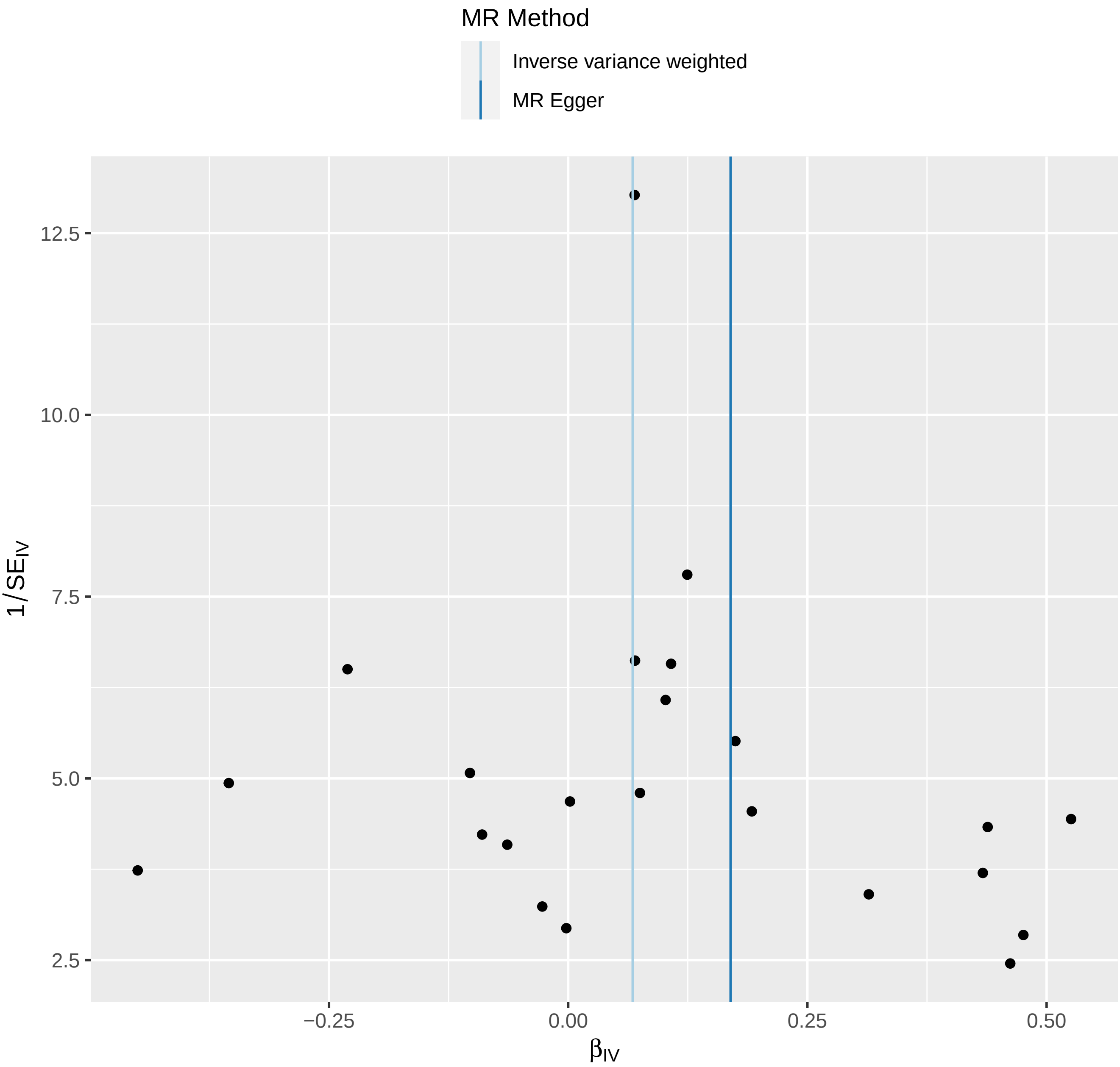

Batch 3−846 : DnaJ homolog subfamily C member 18 on Diabetic nephropathy (more control exclusions)
with a threshold of 1e−05

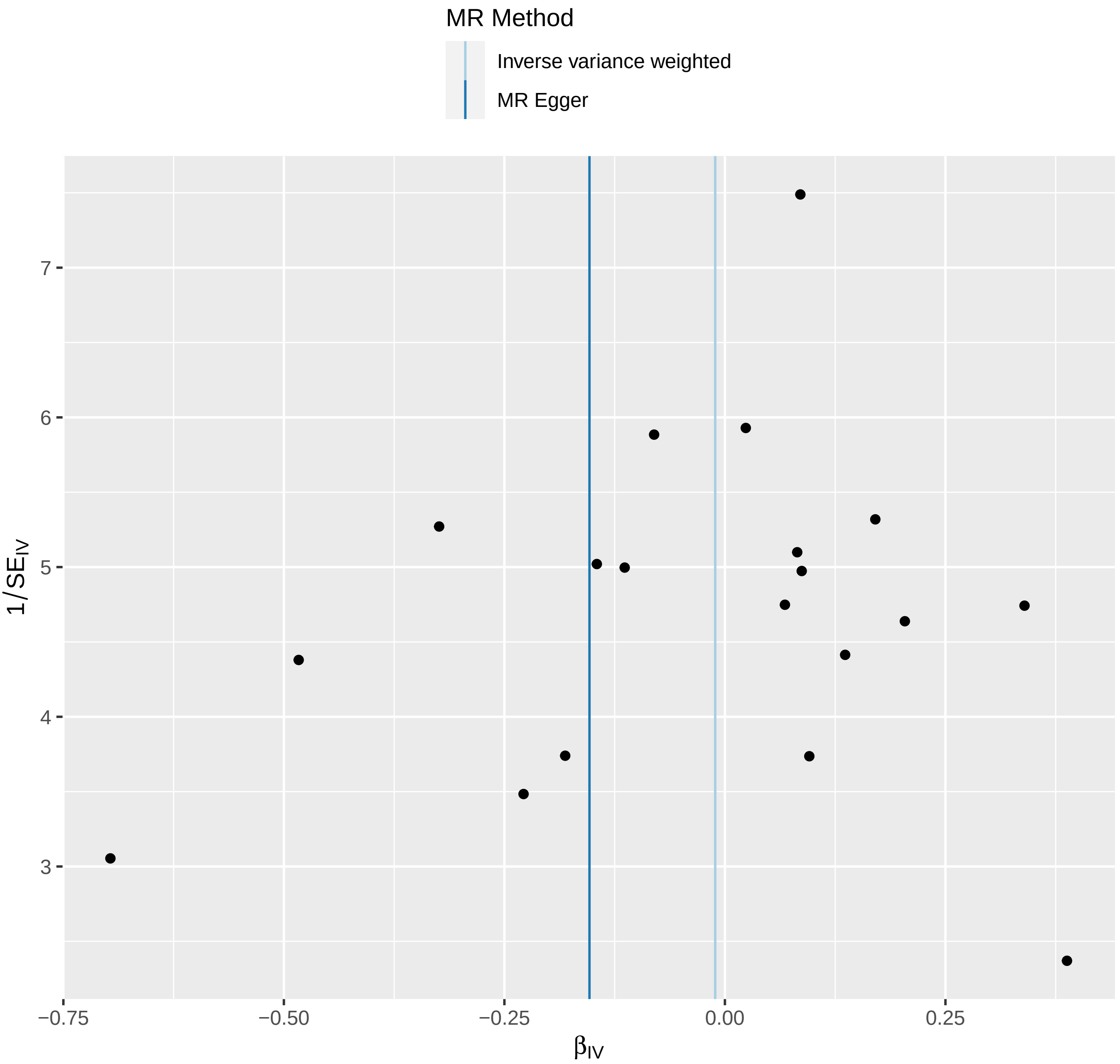

Batch 3−847 : Mitochondrial import inner membrane translocase subunit TIM14 on Diabetic nephropathy (more control exclusions) with a threshold of 1e−05

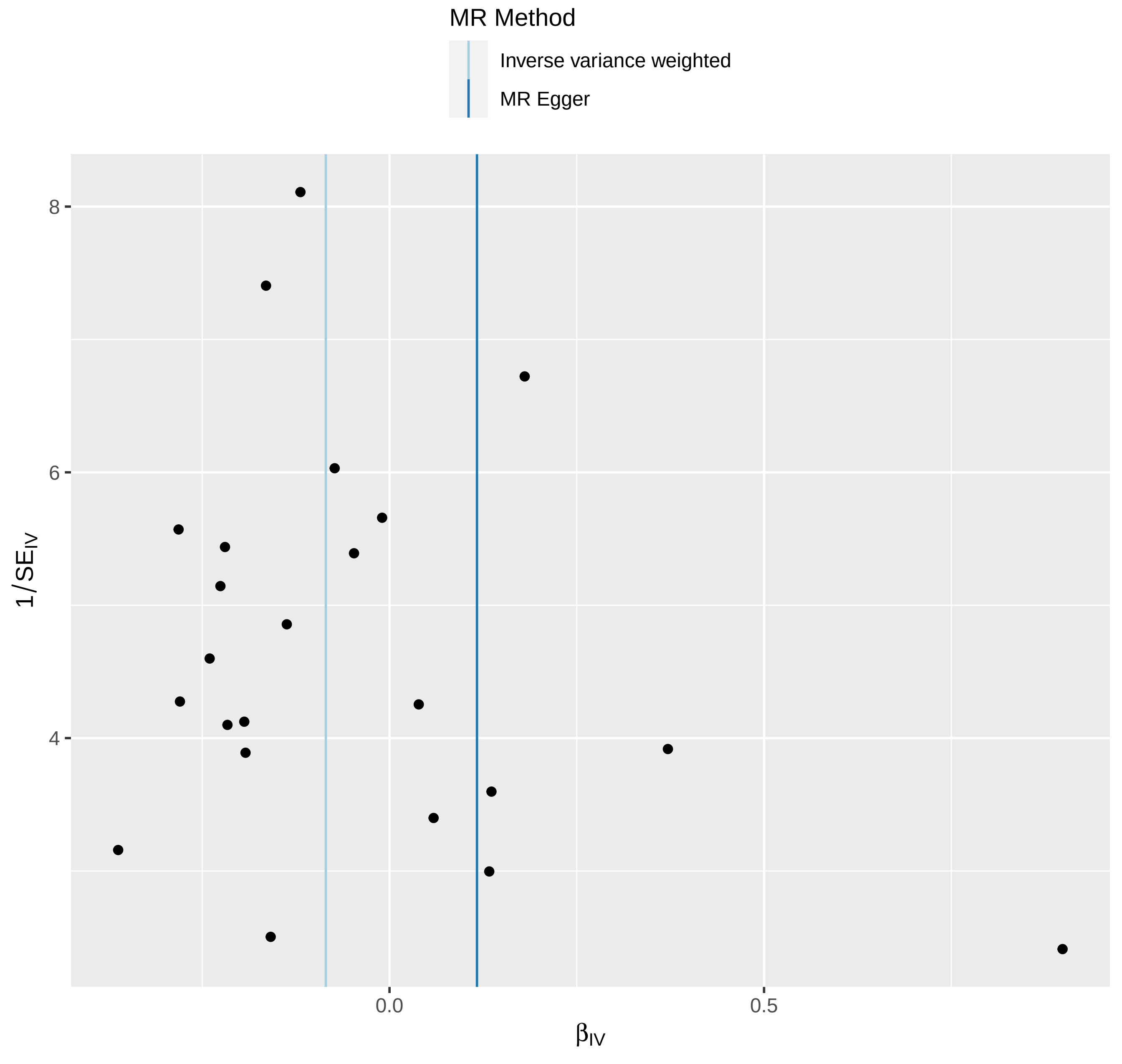

Batch 3−848 : DnaJ homolog subfamily C member 27 on Diabetic nephropathy (more control exclusions)
with a threshold of 1e−05

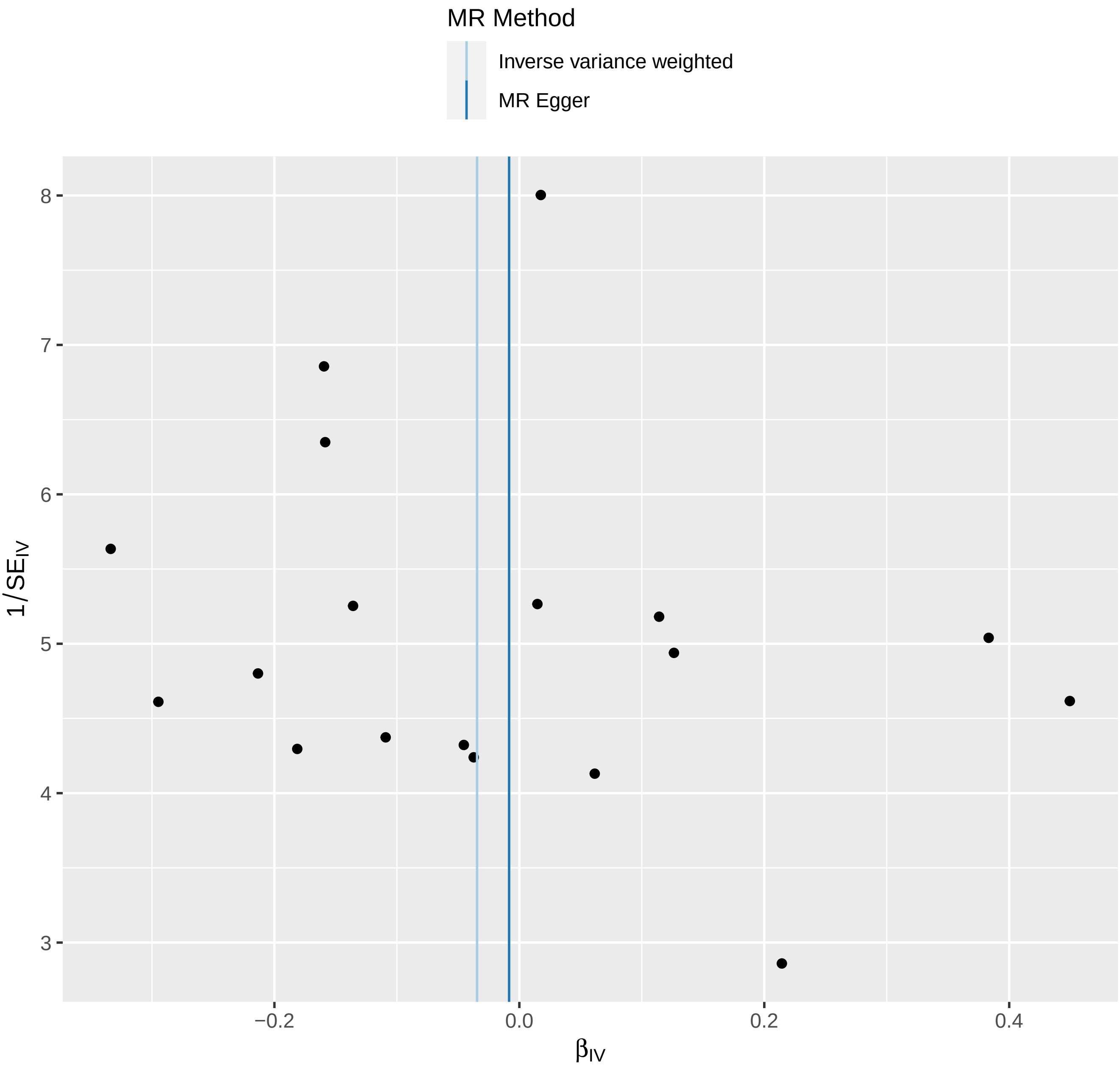

Batch 3−849 : DnaJ homolog subfamily C member 30 on Diabetic nephropathy (more control exclusions) with a threshold of 1e−05

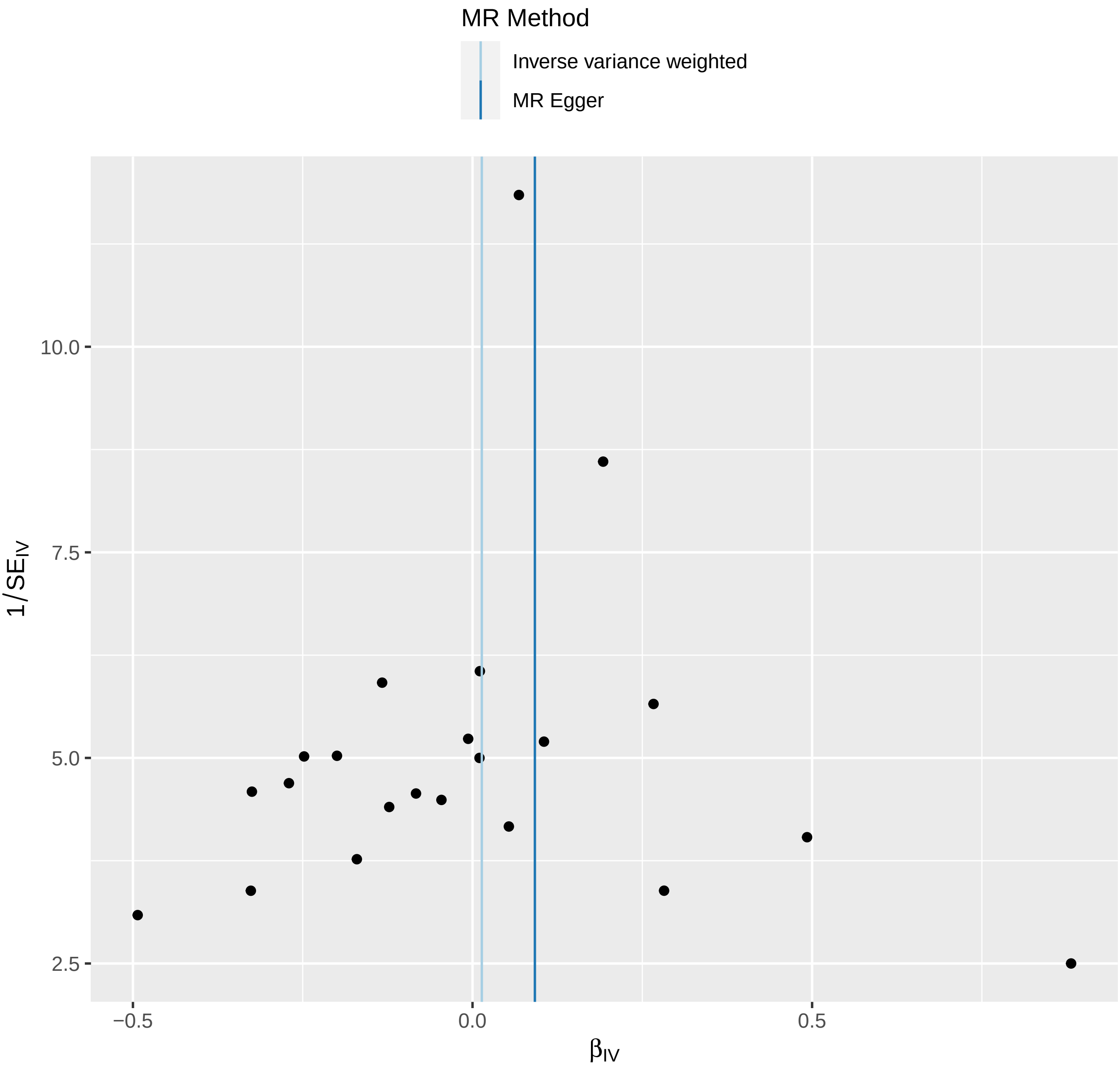

Batch 3−850 : DnaJ homolog subfamily C member 4 on Diabetic nephropathy (more control exclusions) with a threshold of 1e−05

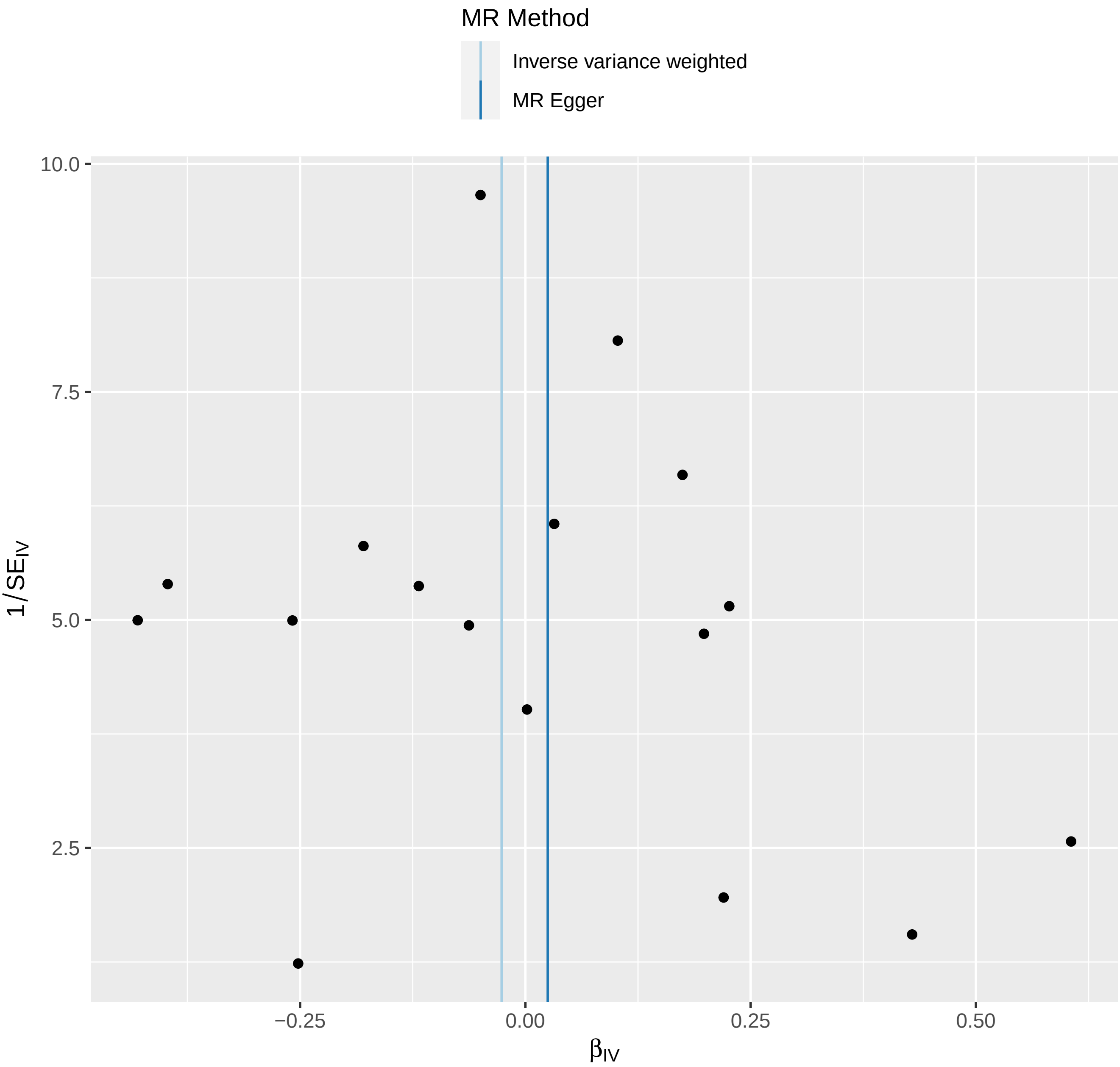

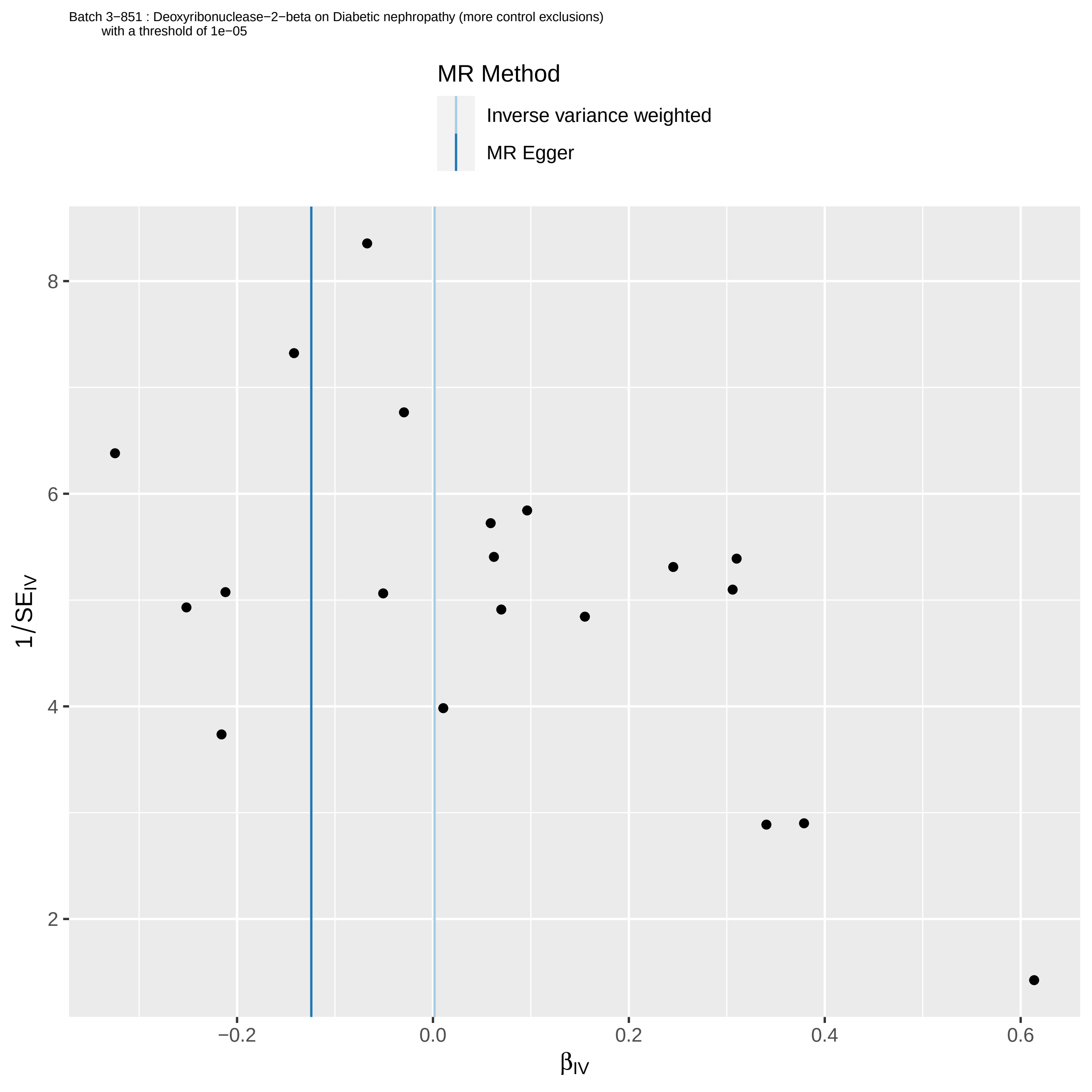
Batch 3−851 : Deoxyribonuclease−2−beta on Diabetic nephropathy (more control exclusions)
with a threshold of 1e−05
MR Method
Inverse variance weighted
MR Egger
8
6
4
2
1/SE_IV
−0.2
0.0
0.2
0.4
0.6
β_IV

Batch 3−852 : Delta and Notch−like epidermal growth factor−related receptor on Diabetic nephropathy (more control exclusions)
with a threshold of 1e−05

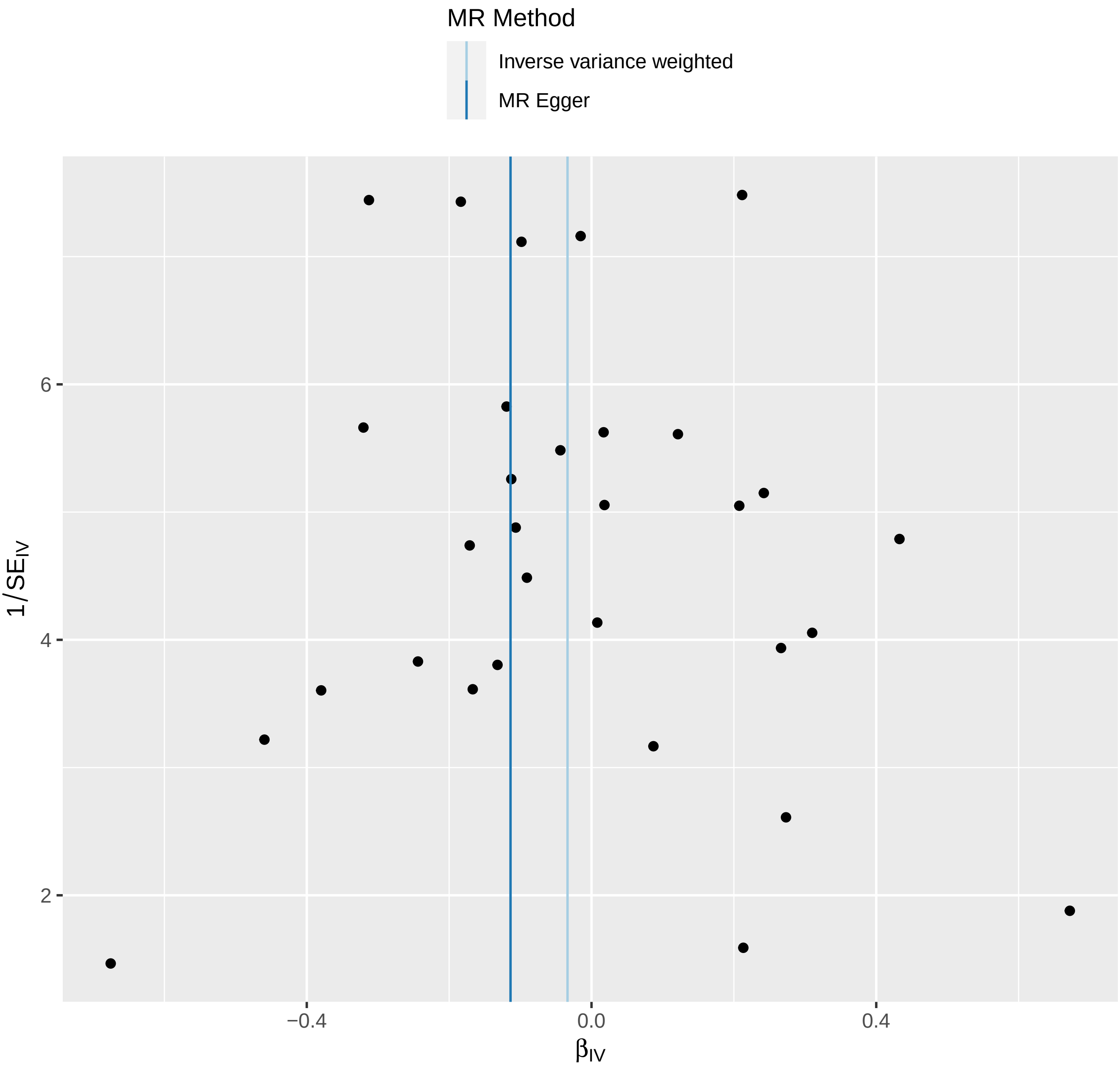

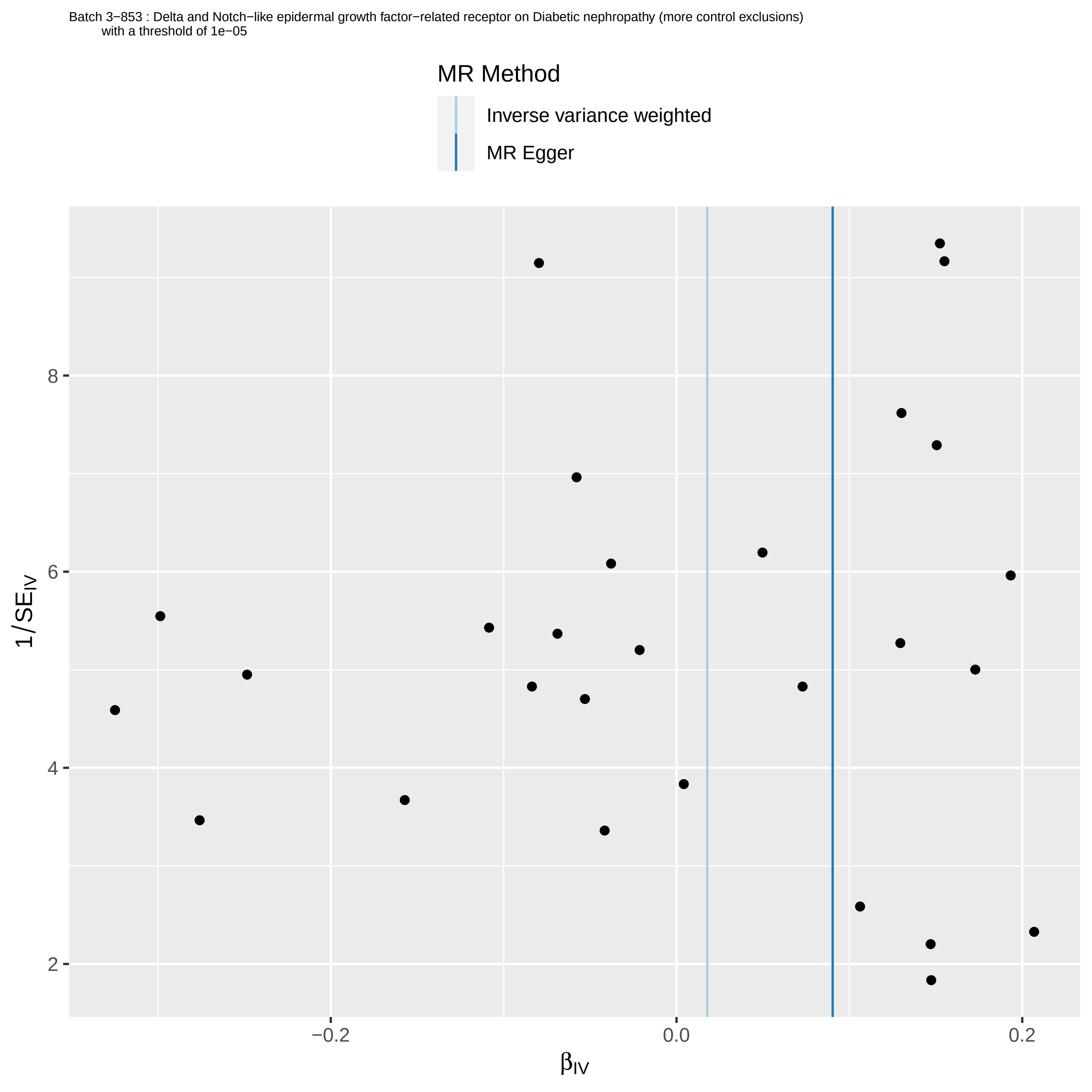
Batch 3−853 : Delta and Notch−like epidermal growth factor−related receptor on Diabetic nephropathy (more control exclusions)
with a threshold of 1e−05
MR Method
Inverse variance weighted
MR Egger
8
6
4
2
$1/SE_{IV}$
−0.2
0.0
0.2
β_{IV}

Batch 3−854 : Dedicator of cytokinesis protein 9 on Diabetic nephropathy (more control exclusions)
with a threshold of 1e−05

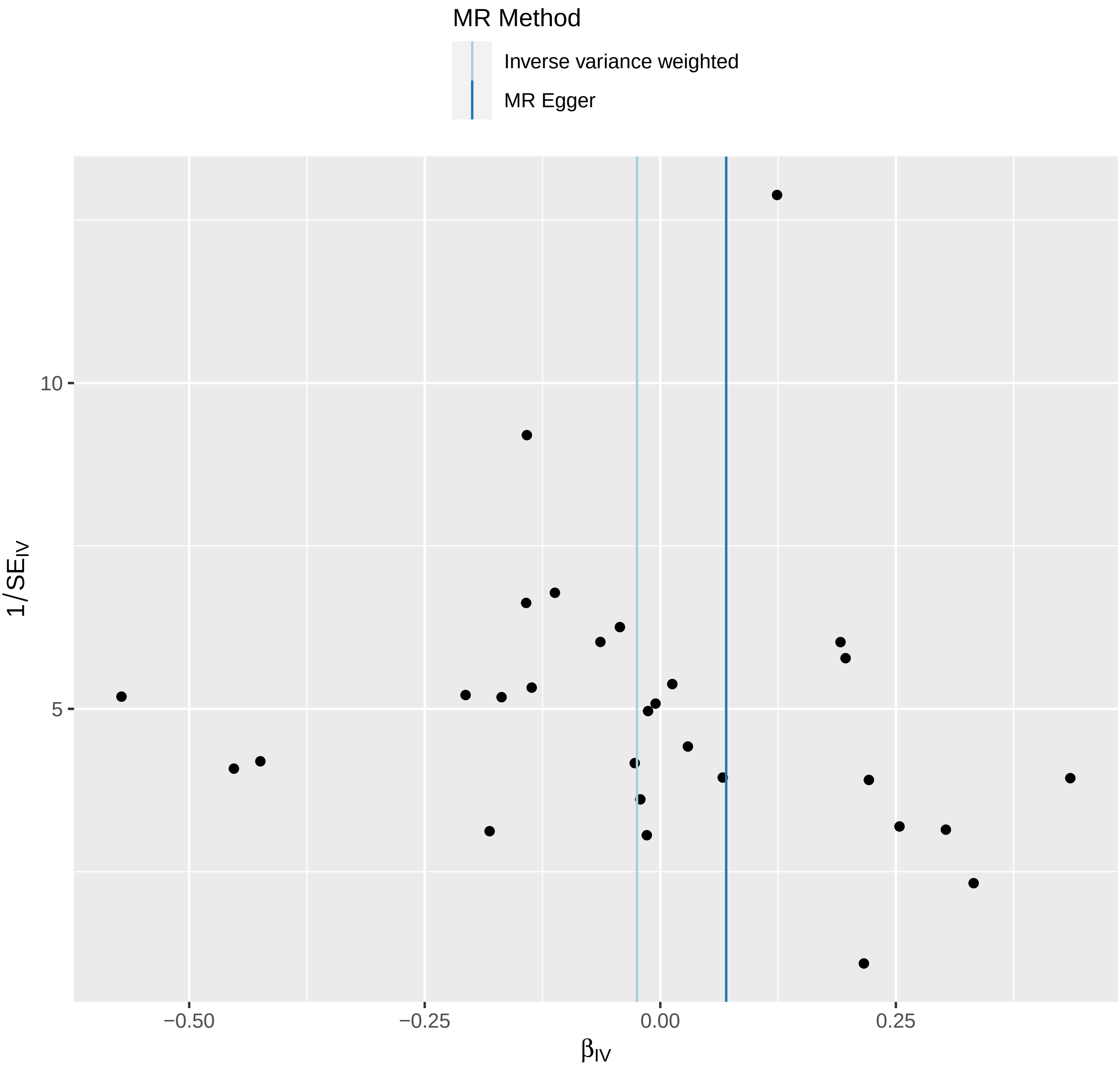

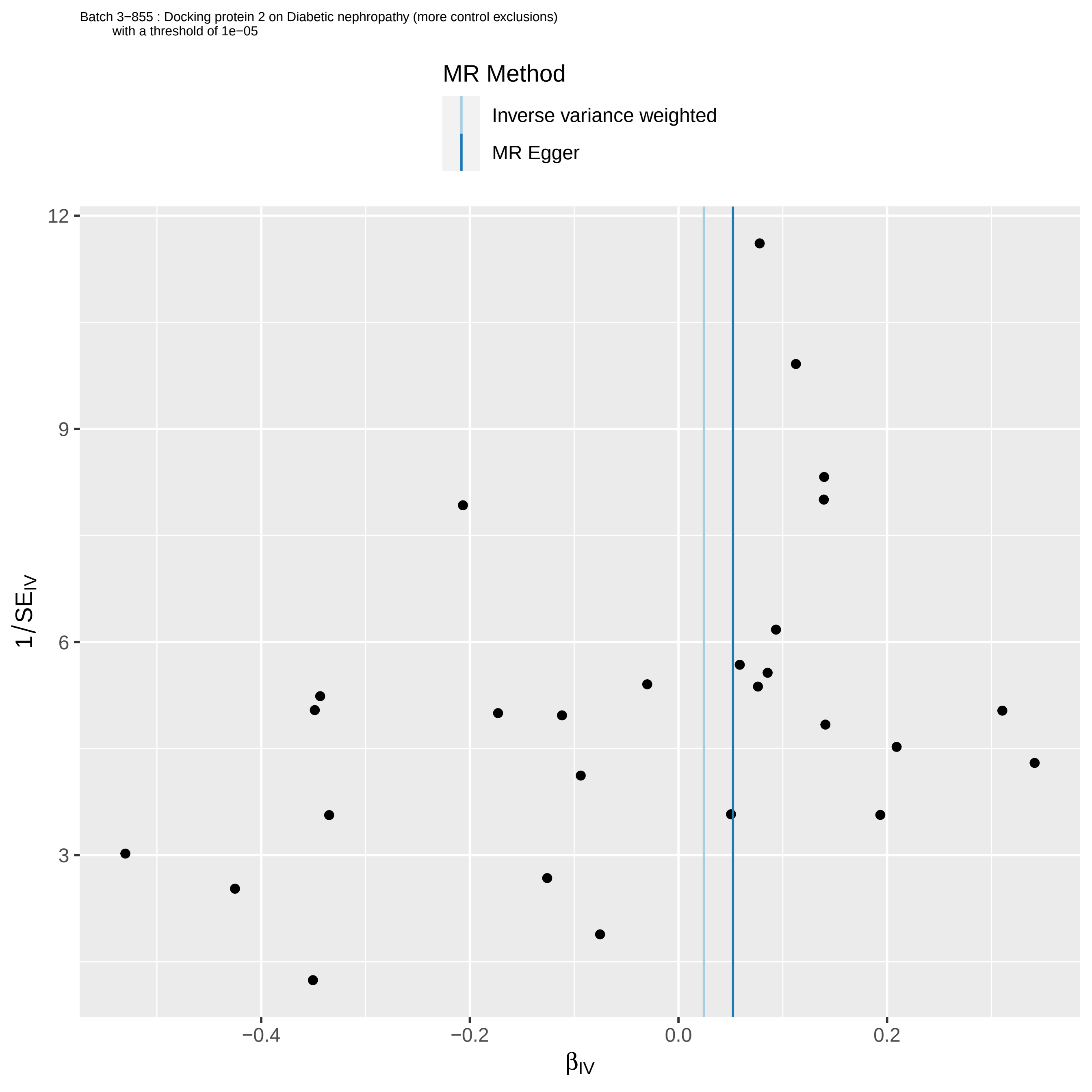

Batch 3−855 : Docking protein 2 on Diabetic nephropathy (more control exclusions)
with a threshold of 1e−05
MR Method
Inverse variance weighted
MR Egger
12
9
6
3
1/SE_IV
−0.4
−0.2
0.0
0.2
β_IV

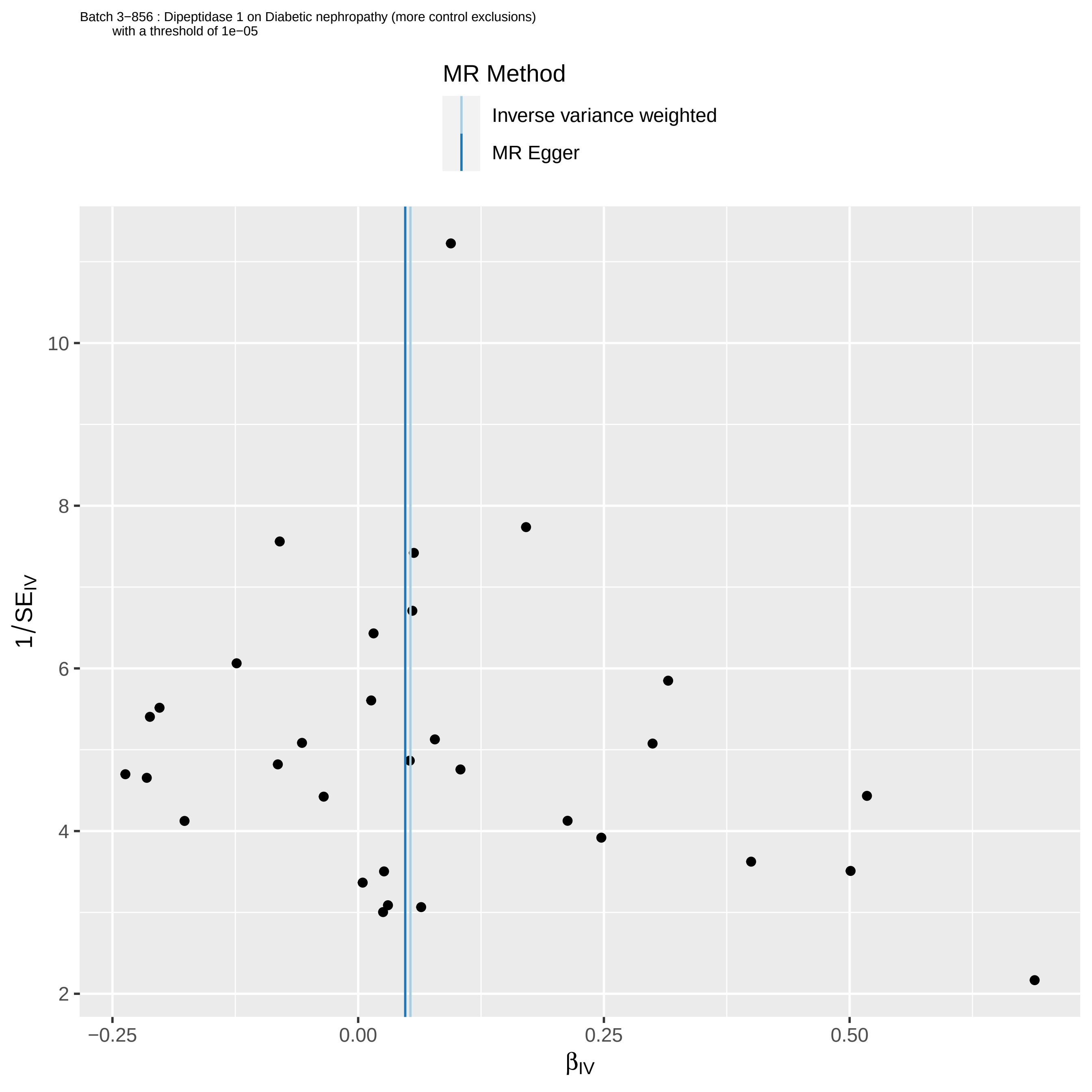
Batch 3−856 : Dipeptidase 1 on Diabetic nephropathy (more control exclusions)
with a threshold of 1e−05
MR Method
Inverse variance weighted
MR Egger
10
8
6
4
2
1/SE_IV
−0.25
0.00
0.25
0.50
β_IV

Batch 3−857 : Dipeptidase 2 on Diabetic nephropathy (more control exclusions)
with a threshold of 1e−05

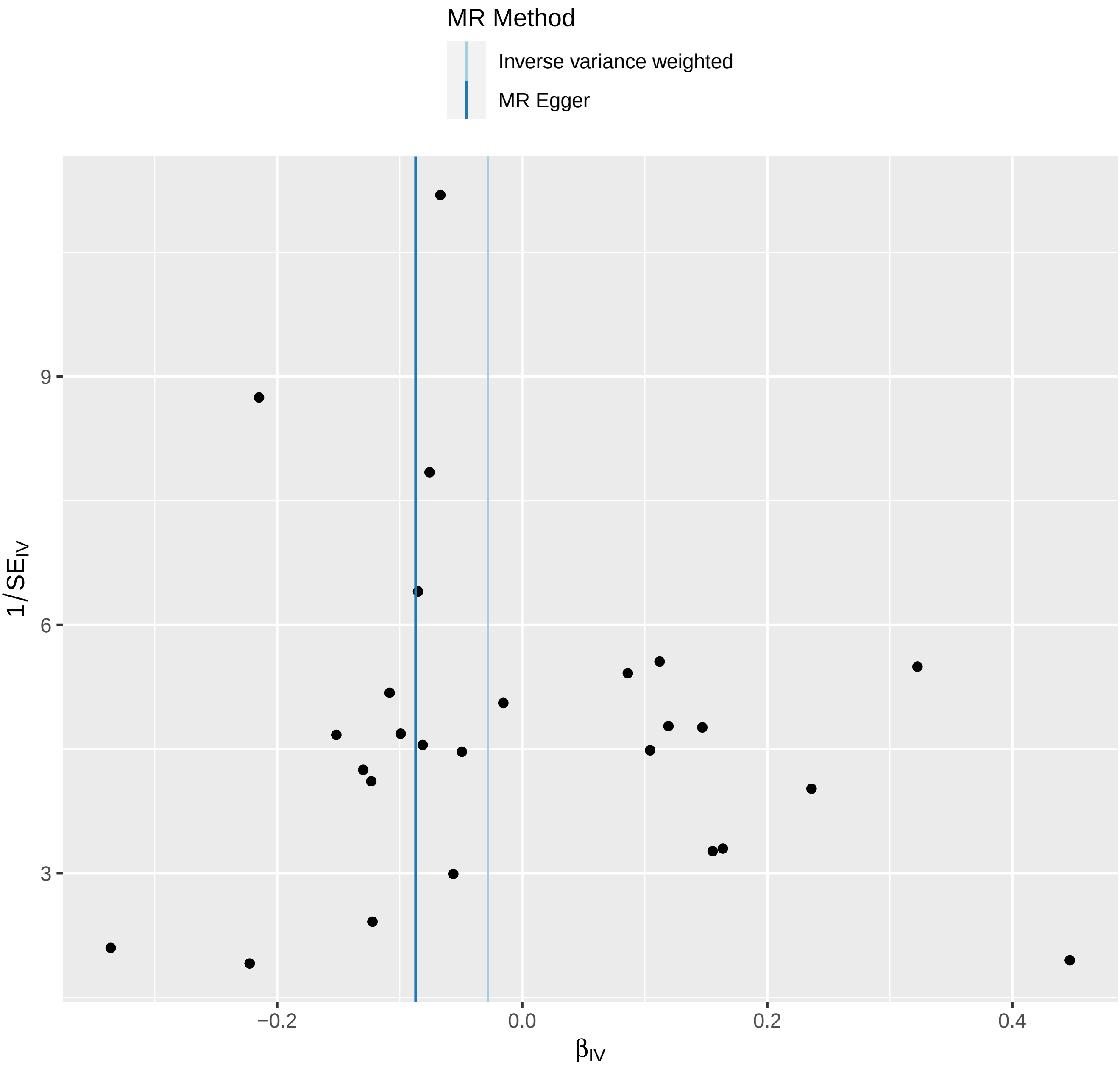

Batch 3−858 : Inactive dipeptidyl peptidase 10 on Diabetic nephropathy (more control exclusions)
with a threshold of 1e−05

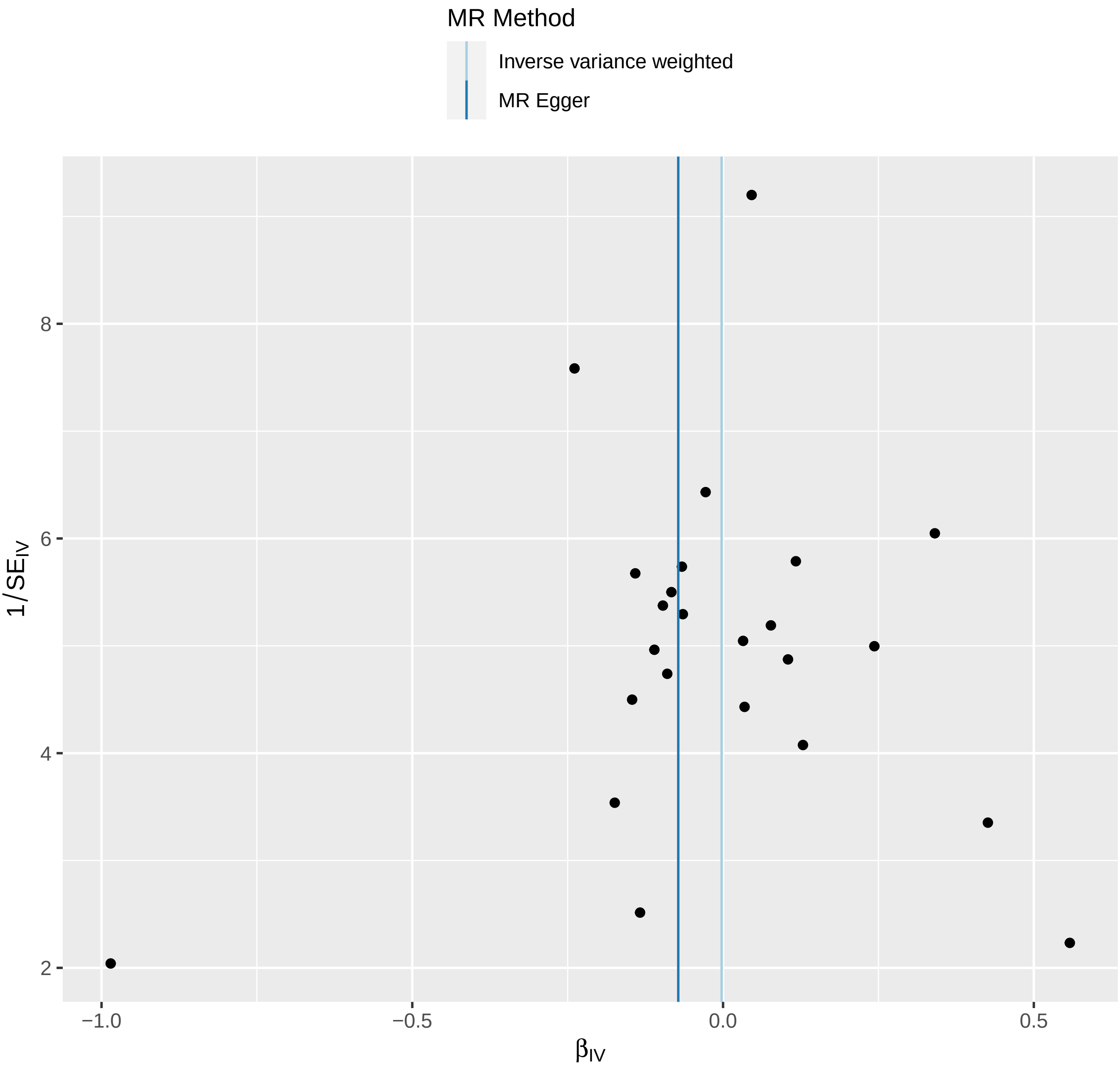

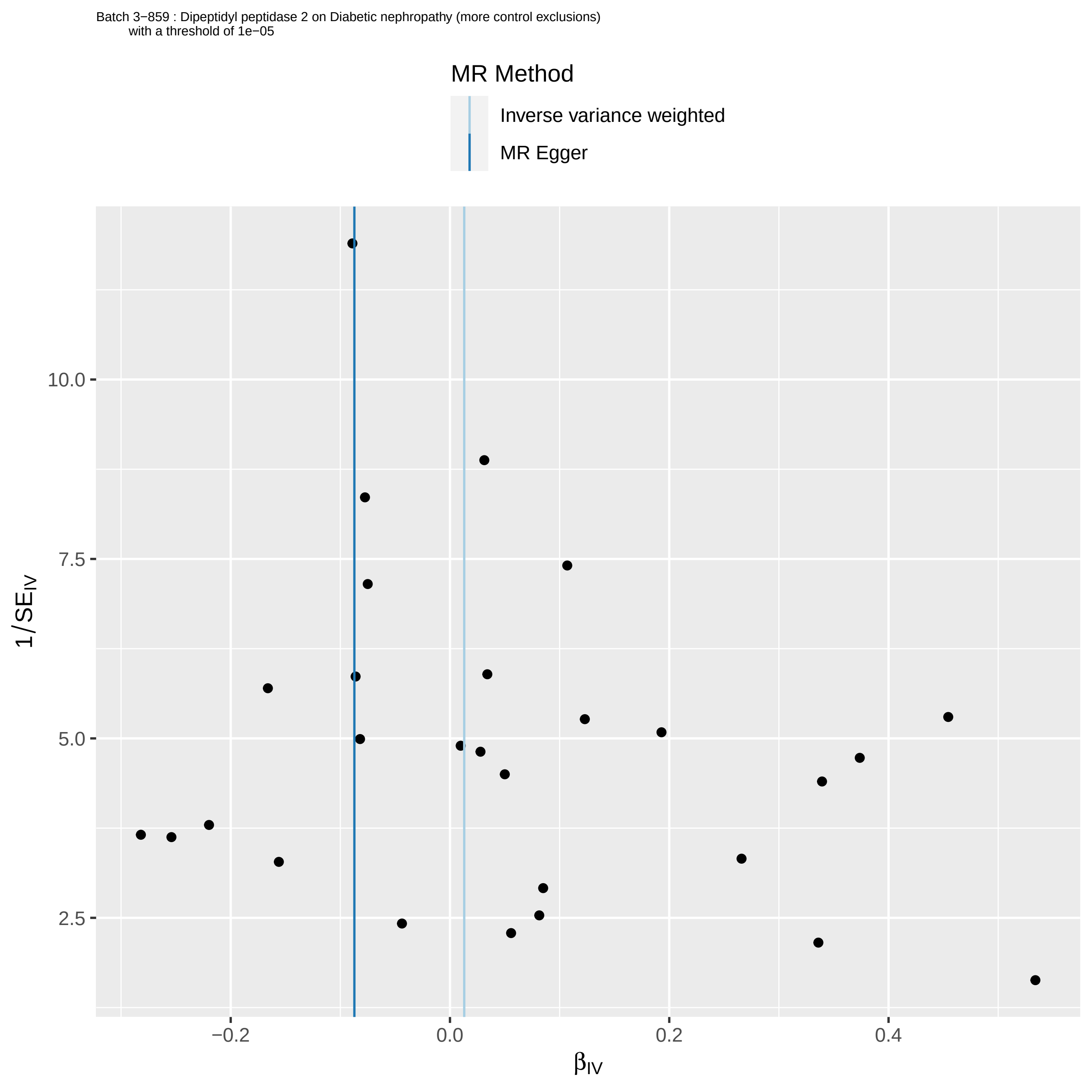
Batch 3−859 : Dipeptidyl peptidase 2 on Diabetic nephropathy (more control exclusions)
with a threshold of 1e−05
MR Method
Inverse variance weighted
MR Egger
10.0
7.5
5.0
2.5
1/SE_IV
−0.2
0.0
0.2
0.4
β_IV

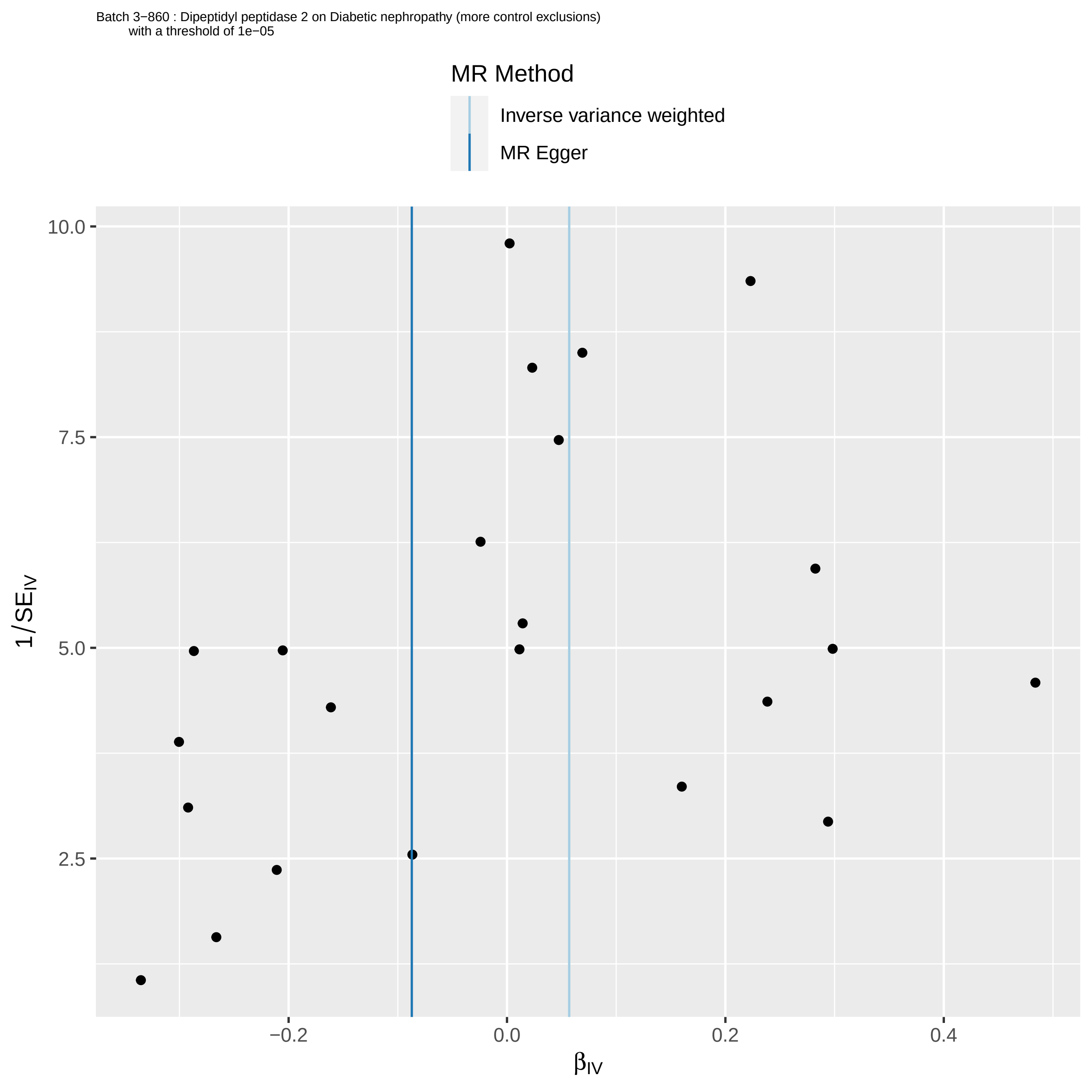

Batch 3−860 : Dipeptidyl peptidase 2 on Diabetic nephropathy (more control exclusions)
with a threshold of 1e−05
MR Method
Inverse variance weighted
MR Egger
10.0
7.5
5.0
2.5
$1/SE_{IV}$
−0.2
0.0
0.2
0.4
β_{IV}

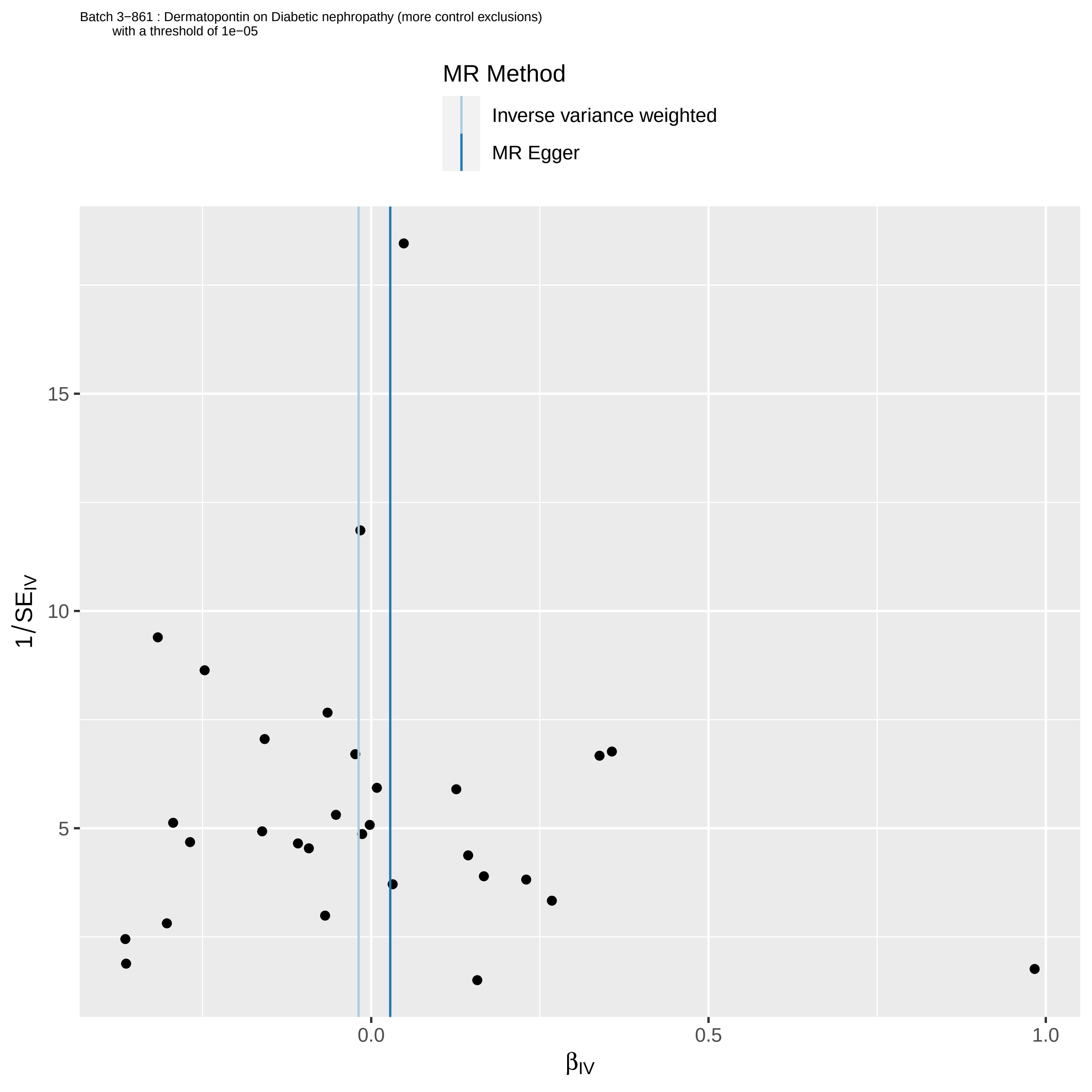
Batch 3−861 : Dermatopontin on Diabetic nephropathy (more control exclusions)
with a threshold of 1e−05
MR Method
Inverse variance weighted
MR Egger
15
10
5
0.0
0.5
1.0
1/SE_IV
β_IV

Batch 3−862 : Protein dpy−30 homolog on Diabetic nephropathy (more control exclusions)
with a threshold of 1e−05

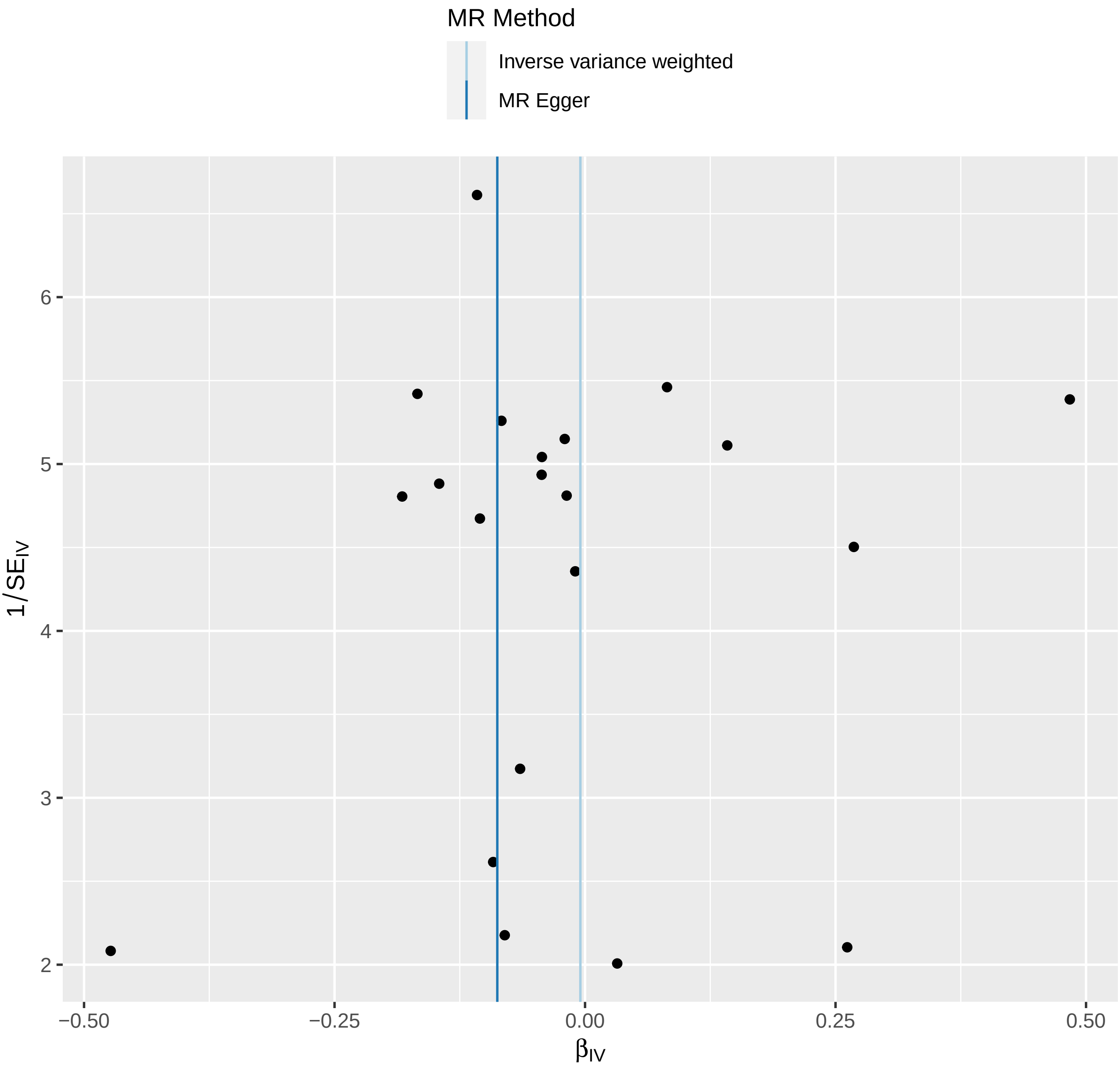

Batch 3−863 : Dihydropyrimidinase−related protein 3 on Diabetic nephropathy (more control exclusions)
with a threshold of 1e−05

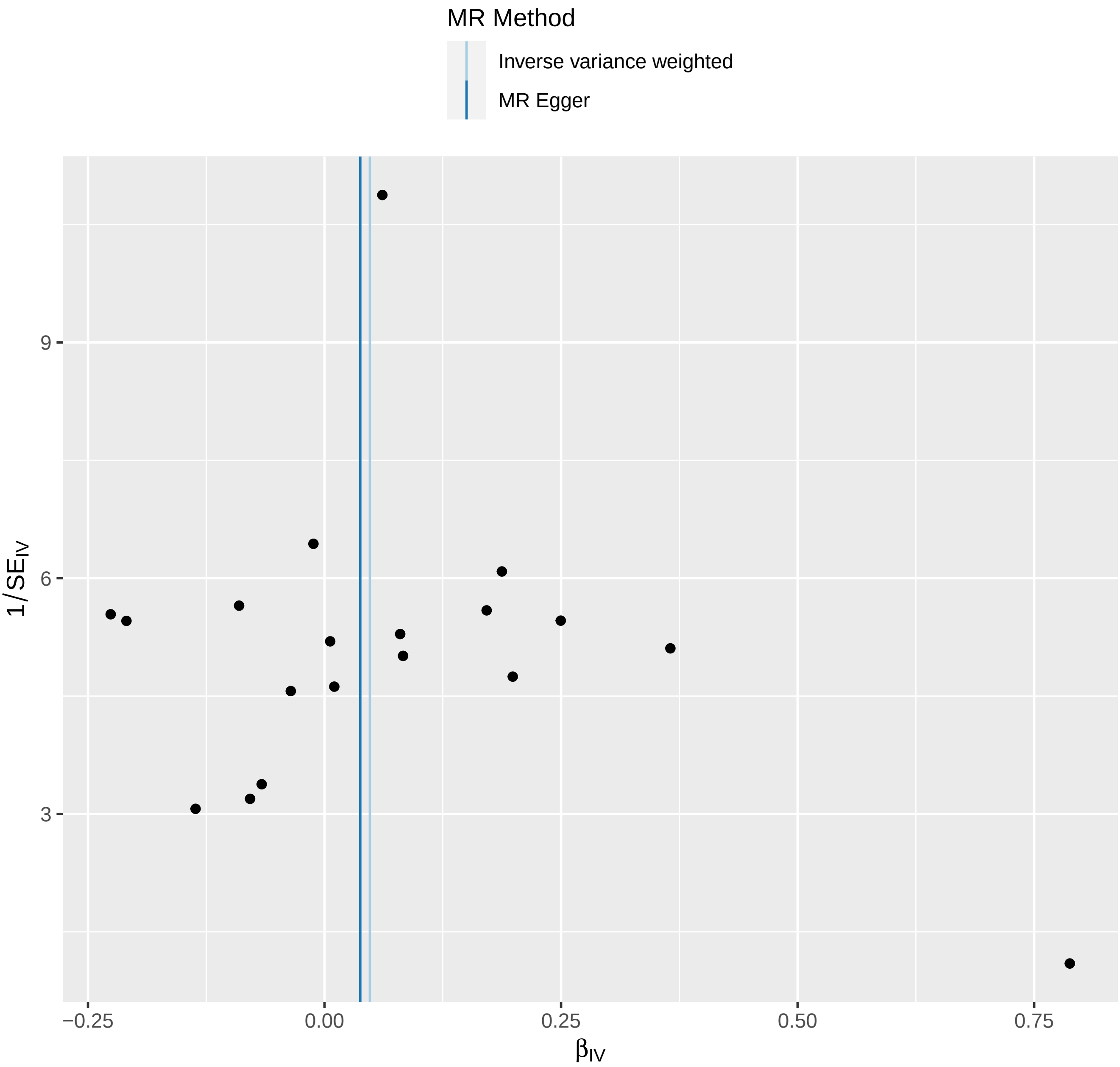

Batch 3−864 : Dihydropyrimidinase−related protein 5 on Diabetic nephropathy (more control exclusions)
with a threshold of 1e−05

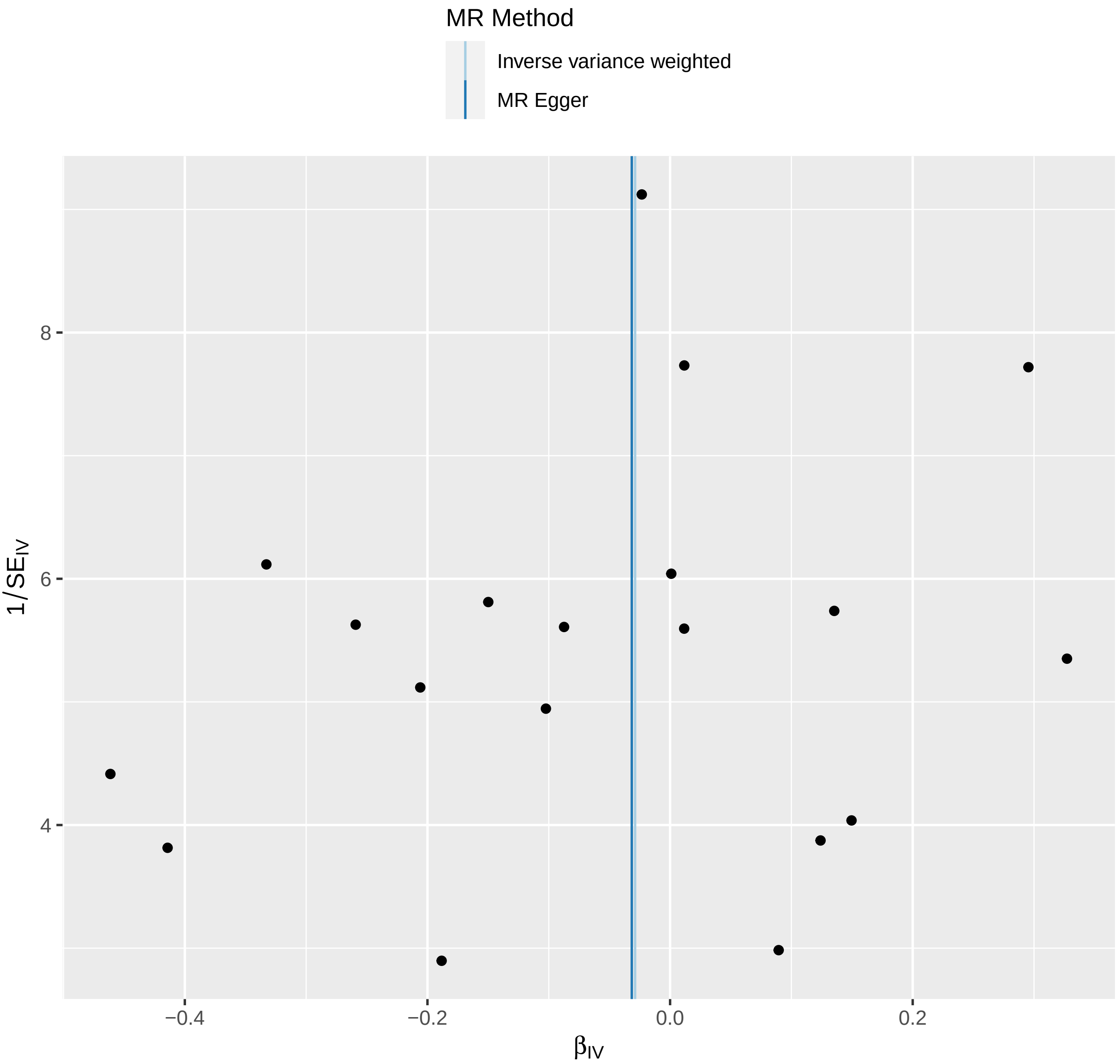

Batch 3−865 : Dorsal root ganglia homeobox protein on Diabetic nephropathy (more control exclusions)
with a threshold of 1e−05

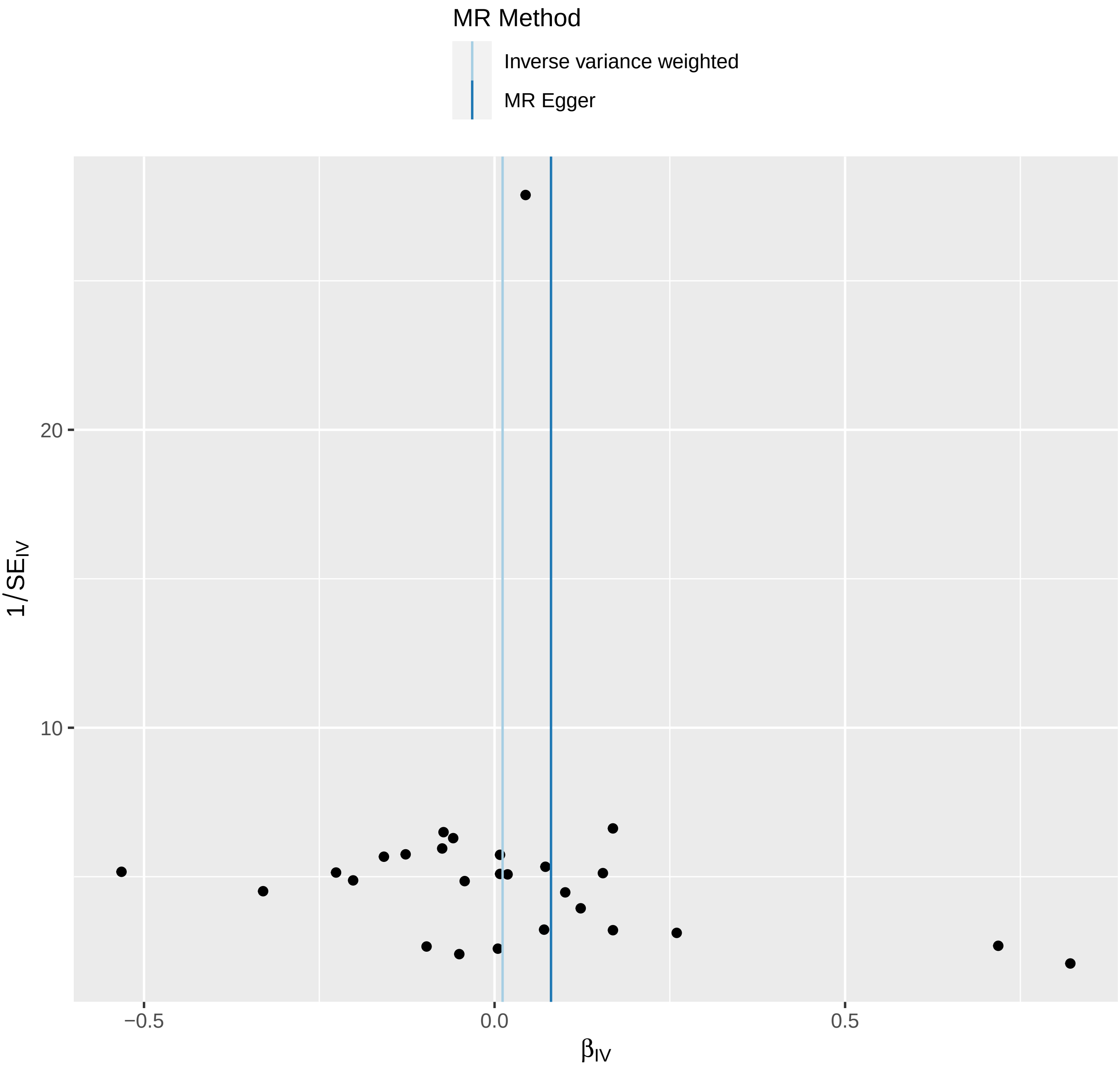

Batch 3−866 : Desmocollin−2 on Diabetic nephropathy (more control exclusions)
with a threshold of 1e−05

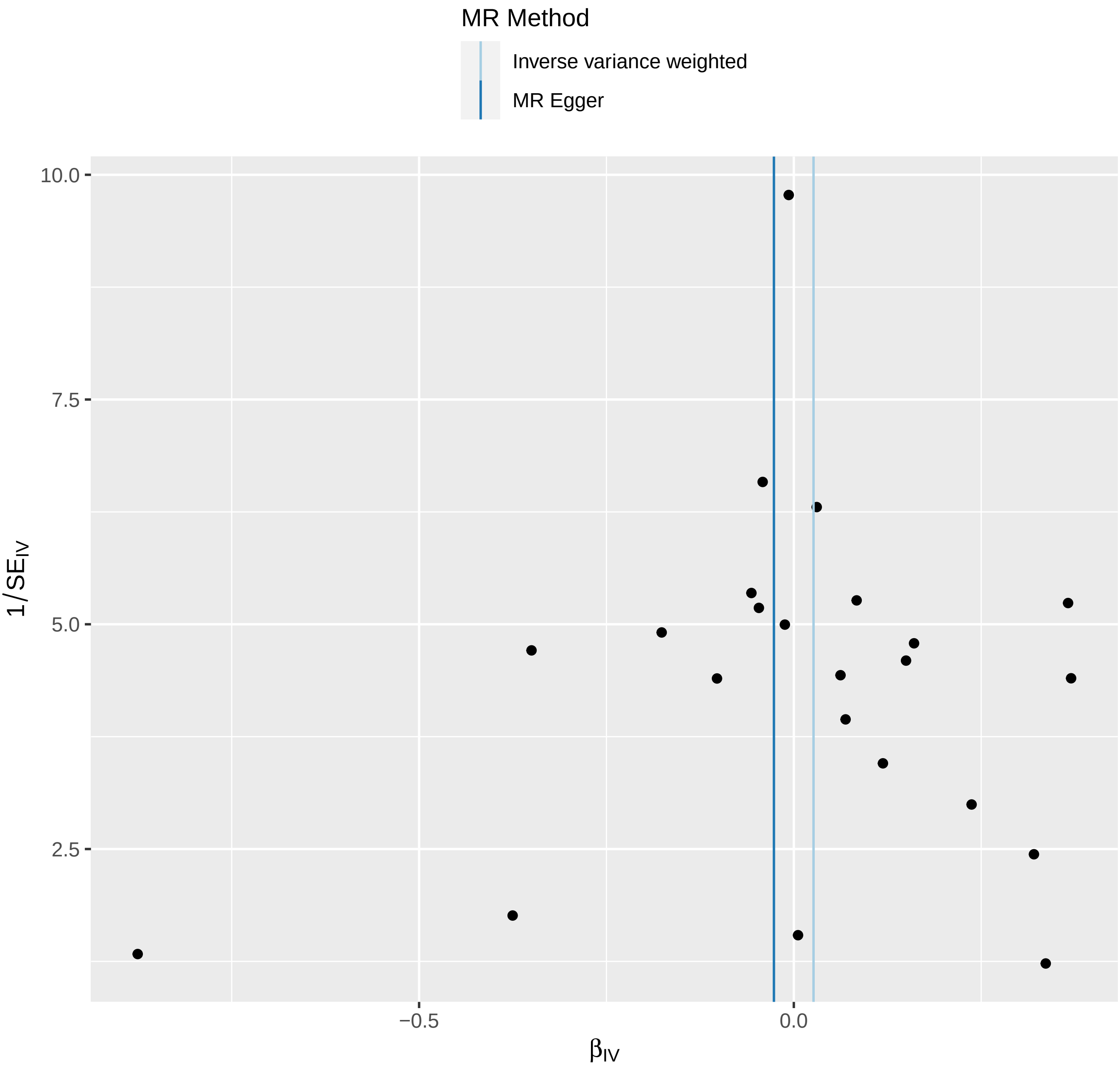

Batch 3−867 : Desmocollin−3 on Diabetic nephropathy (more control exclusions)
with a threshold of 1e−05

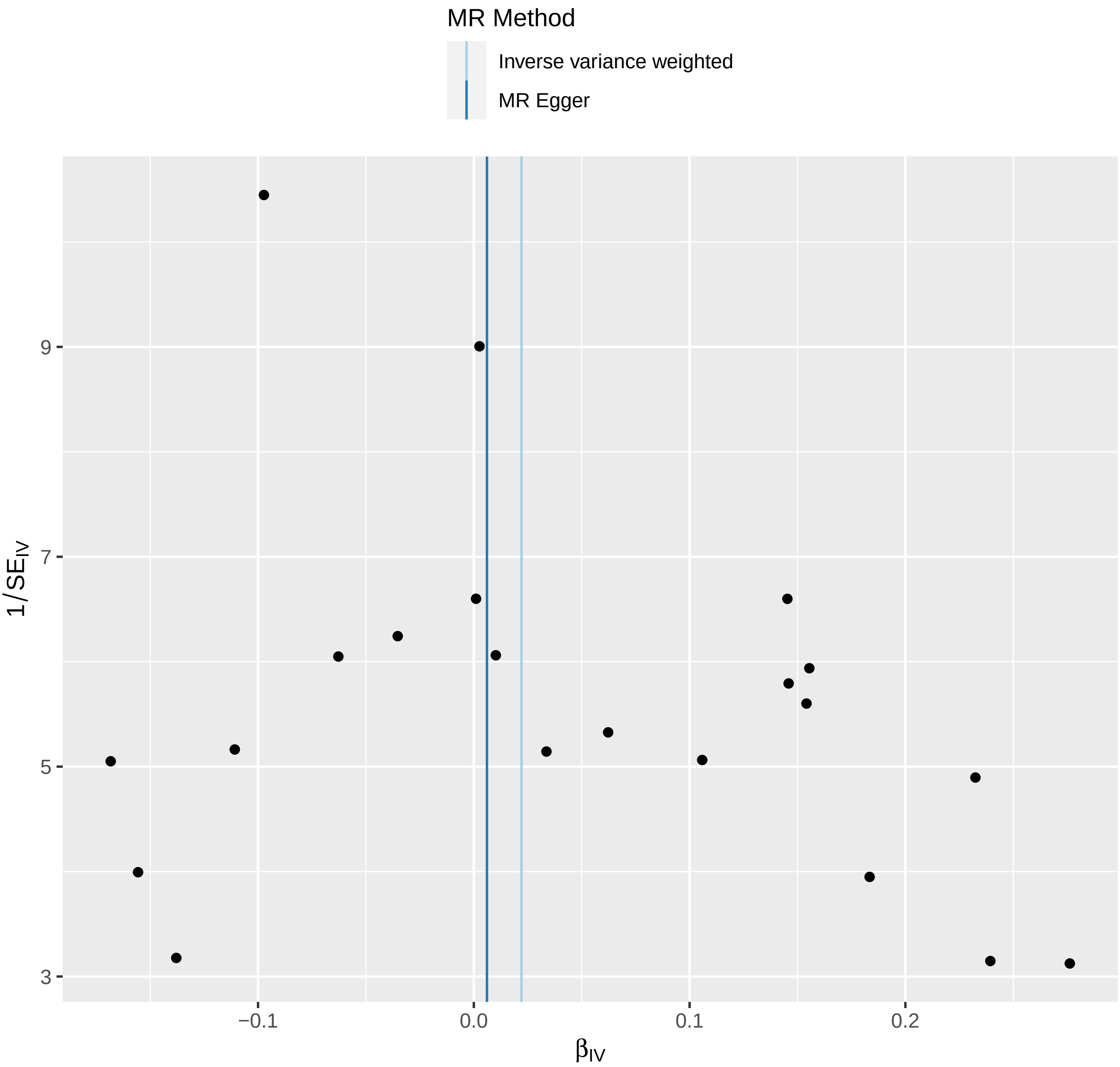

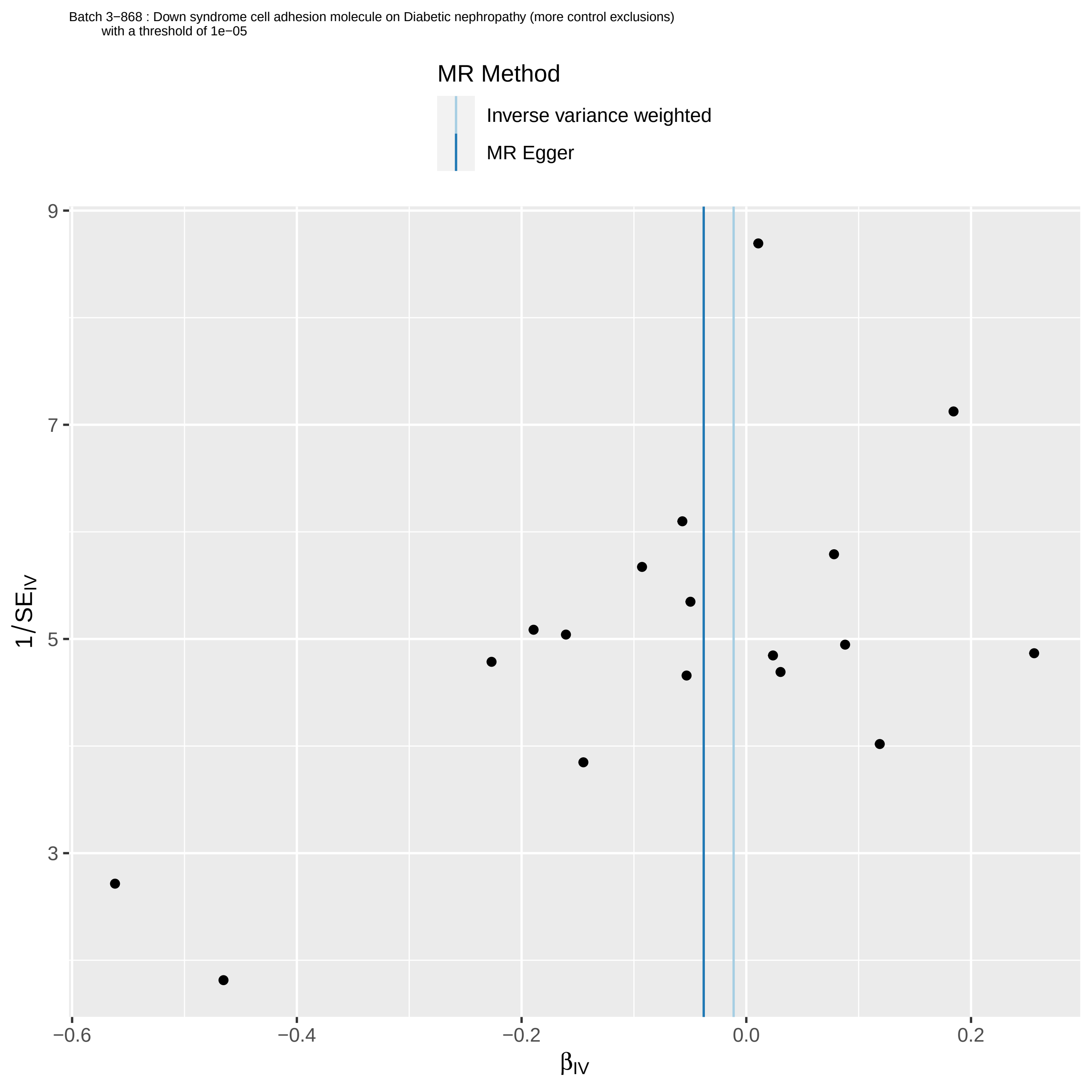
Batch 3−868 : Down syndrome cell adhesion molecule on Diabetic nephropathy (more control exclusions)
with a threshold of 1e−05
MR Method
Inverse variance weighted
MR Egger
9
7
5
3
1/SE$_{IV}$
−0.6
−0.4
−0.2
0.0
0.2
β_{IV}

Batch 3−869 : Desmoglein−1 on Diabetic nephropathy (more control exclusions)
with a threshold of 1e−05

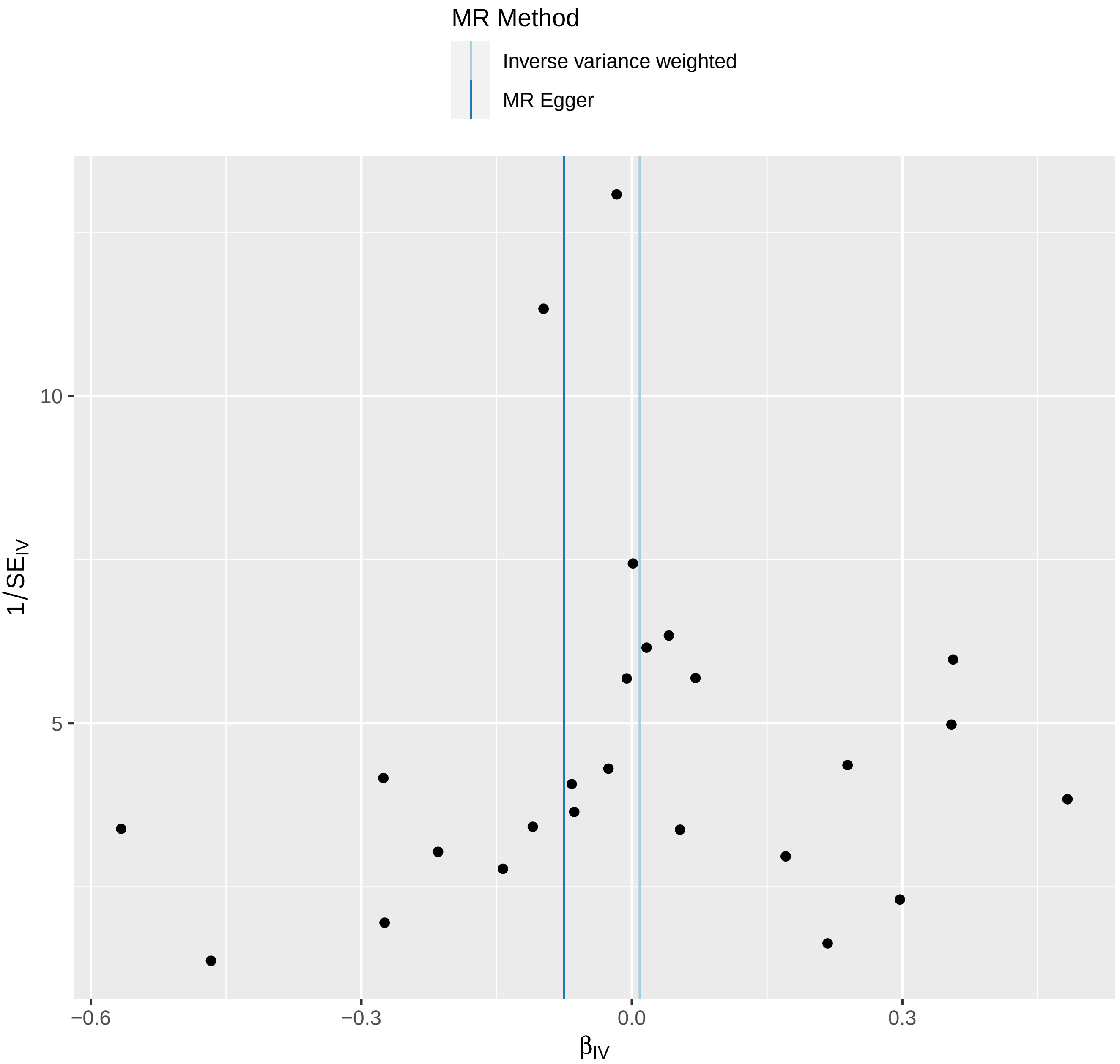

Batch 3−870 : Desmoglein−2 on Diabetic nephropathy (more control exclusions)
with a threshold of 1e−05

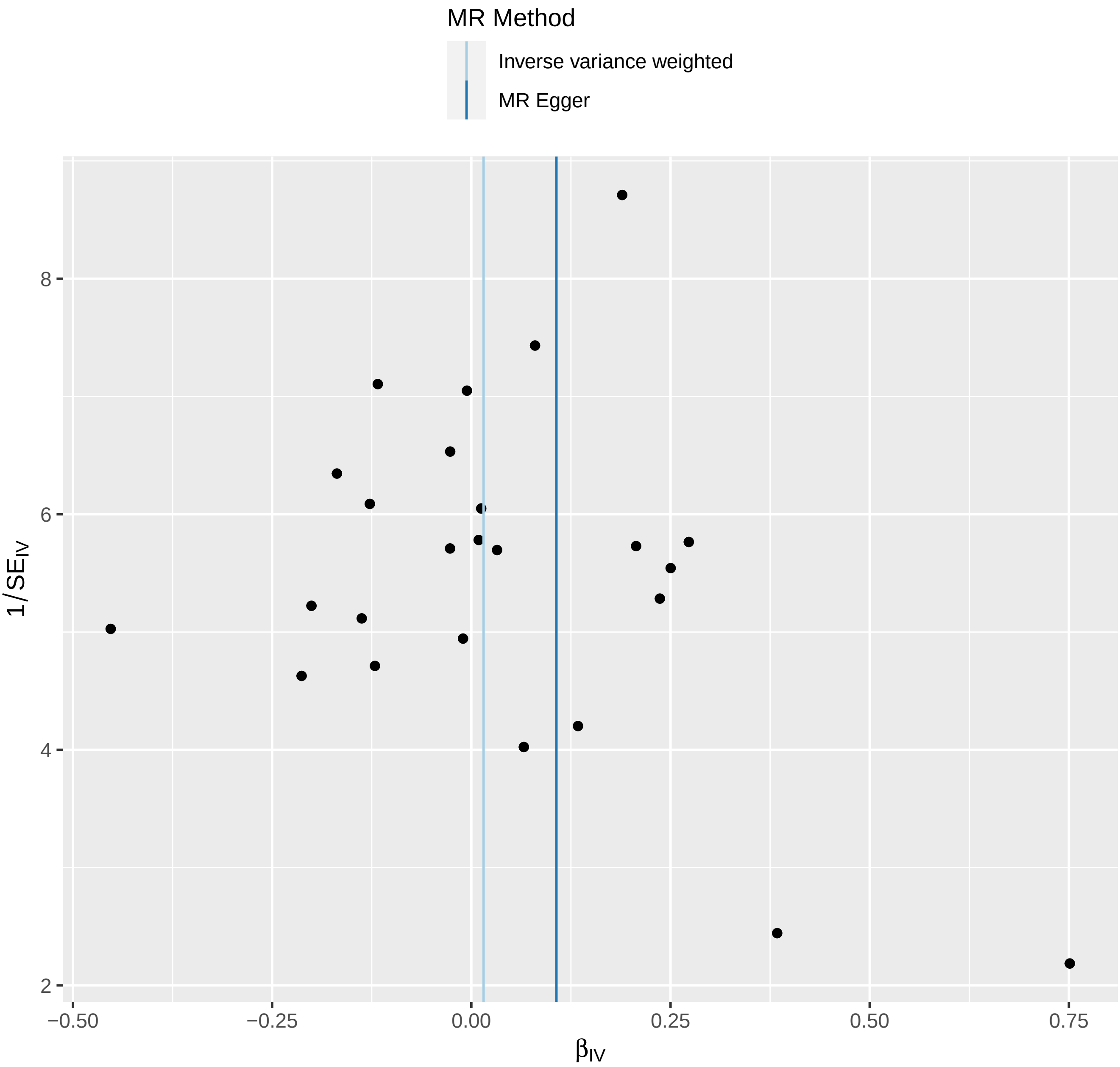

Batch 3−871 : E3 ubiquitin−protein ligase DTX1 on Diabetic nephropathy (more control exclusions)
with a threshold of 1e−05

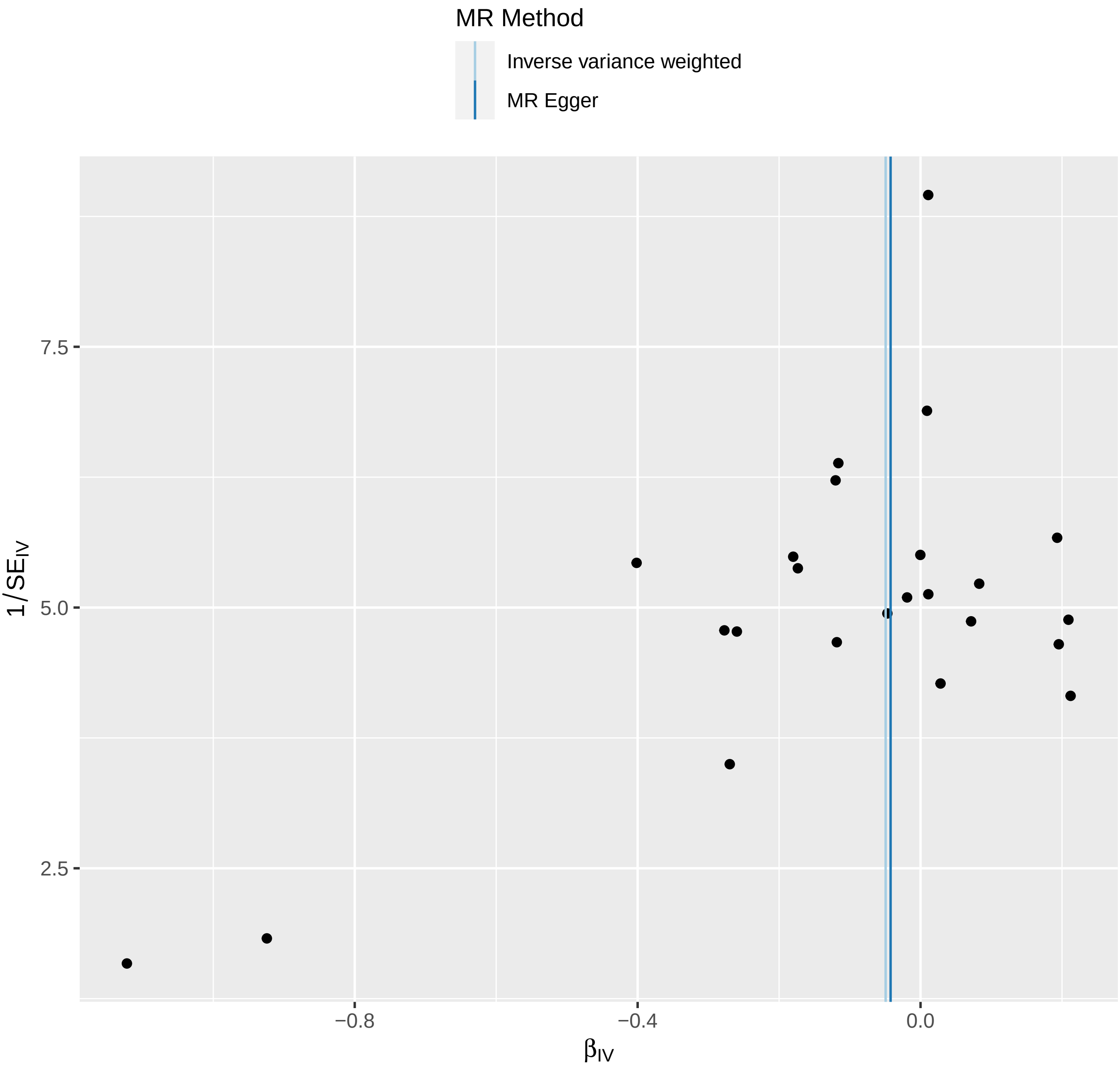

Batch 3−872 : E3 ubiquitin−protein ligase DTX3L on Diabetic nephropathy (more control exclusions) with a threshold of 1e−05

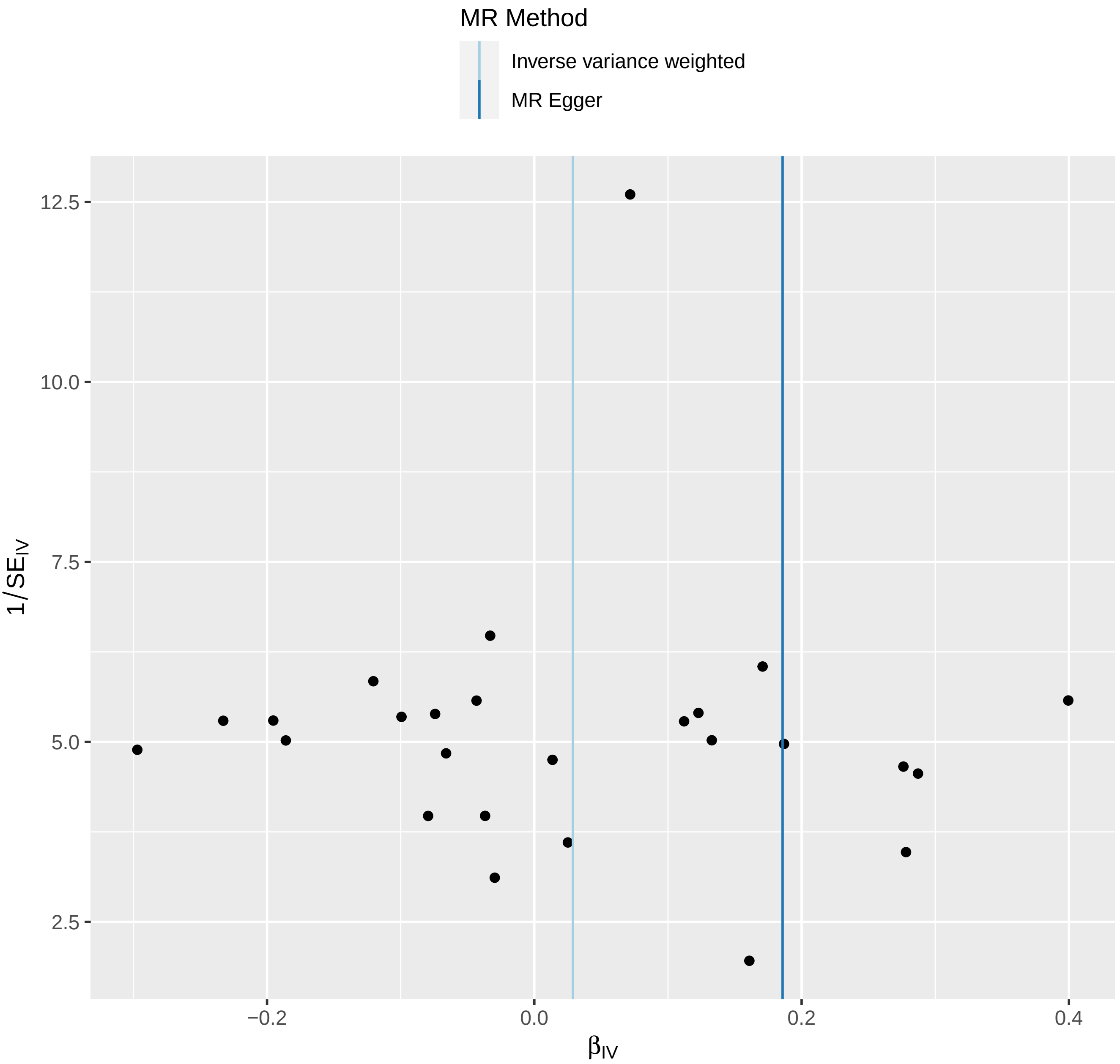

Batch 3−873 : Dual specificity protein phosphatase 13 isoform A on Diabetic nephropathy (more control exclusions)
with a threshold of 1e−05

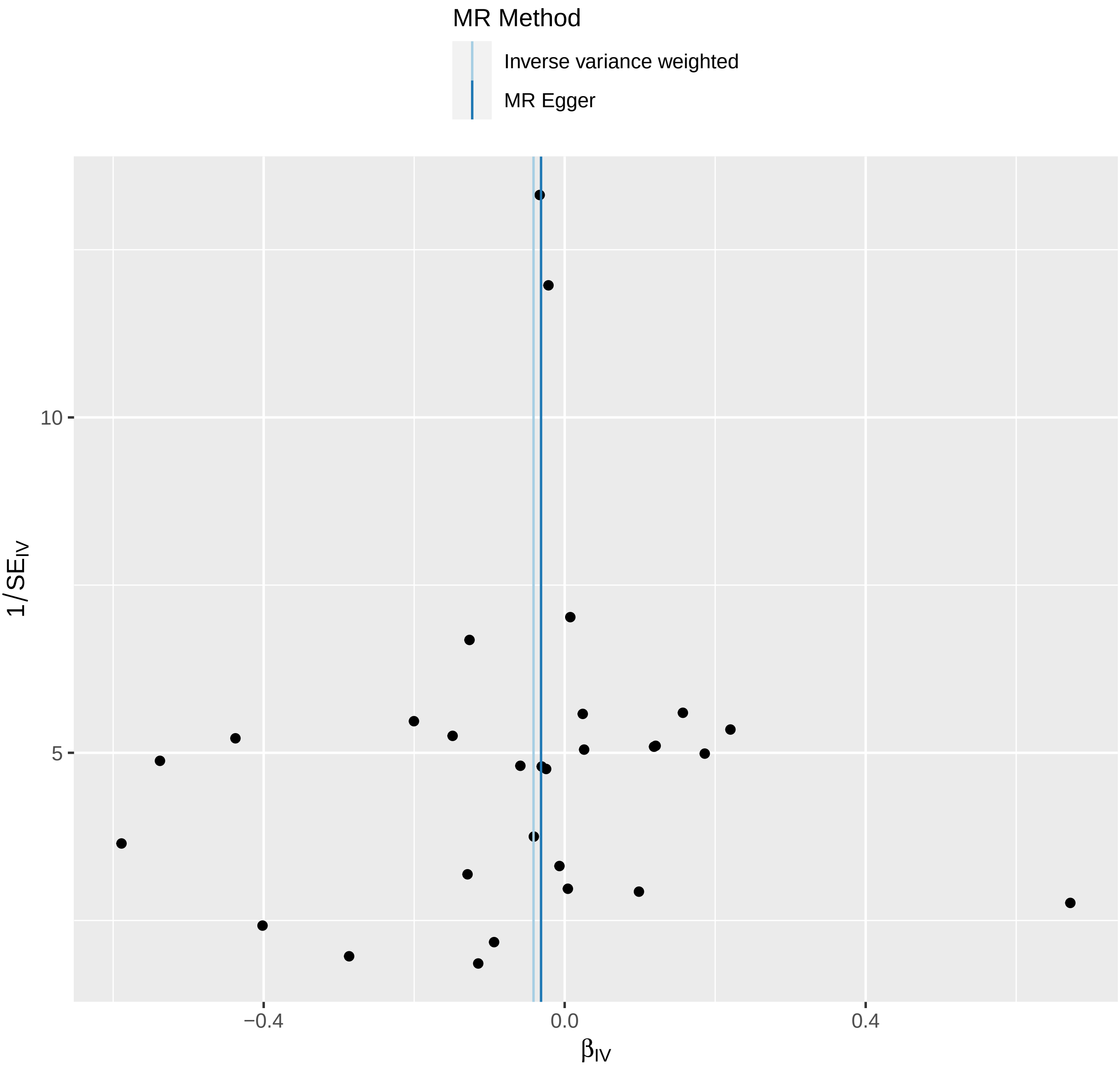

Batch 3−874 : Dual specificity protein phosphatase 15 on Diabetic nephropathy (more control exclusions) with a threshold of 1e−05

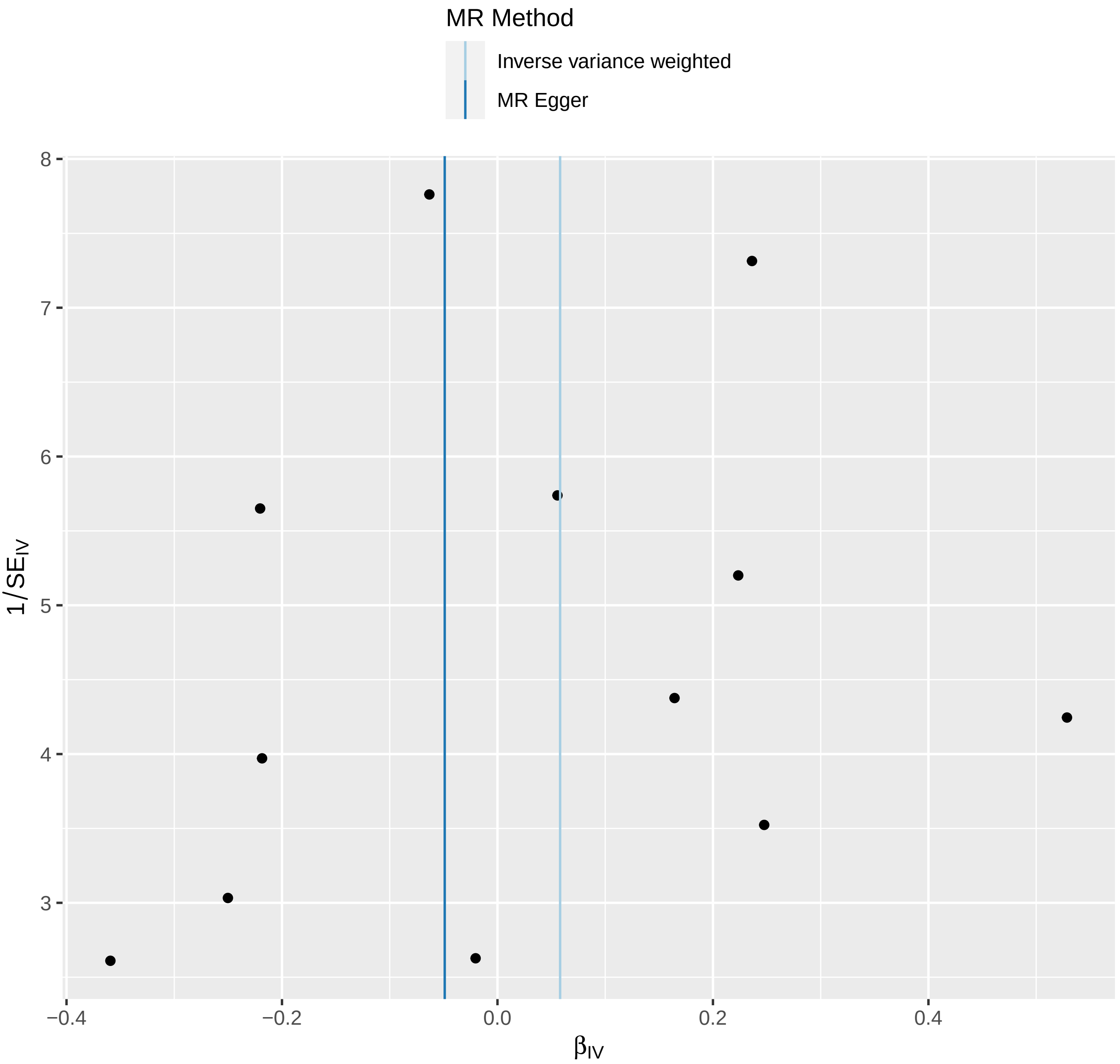

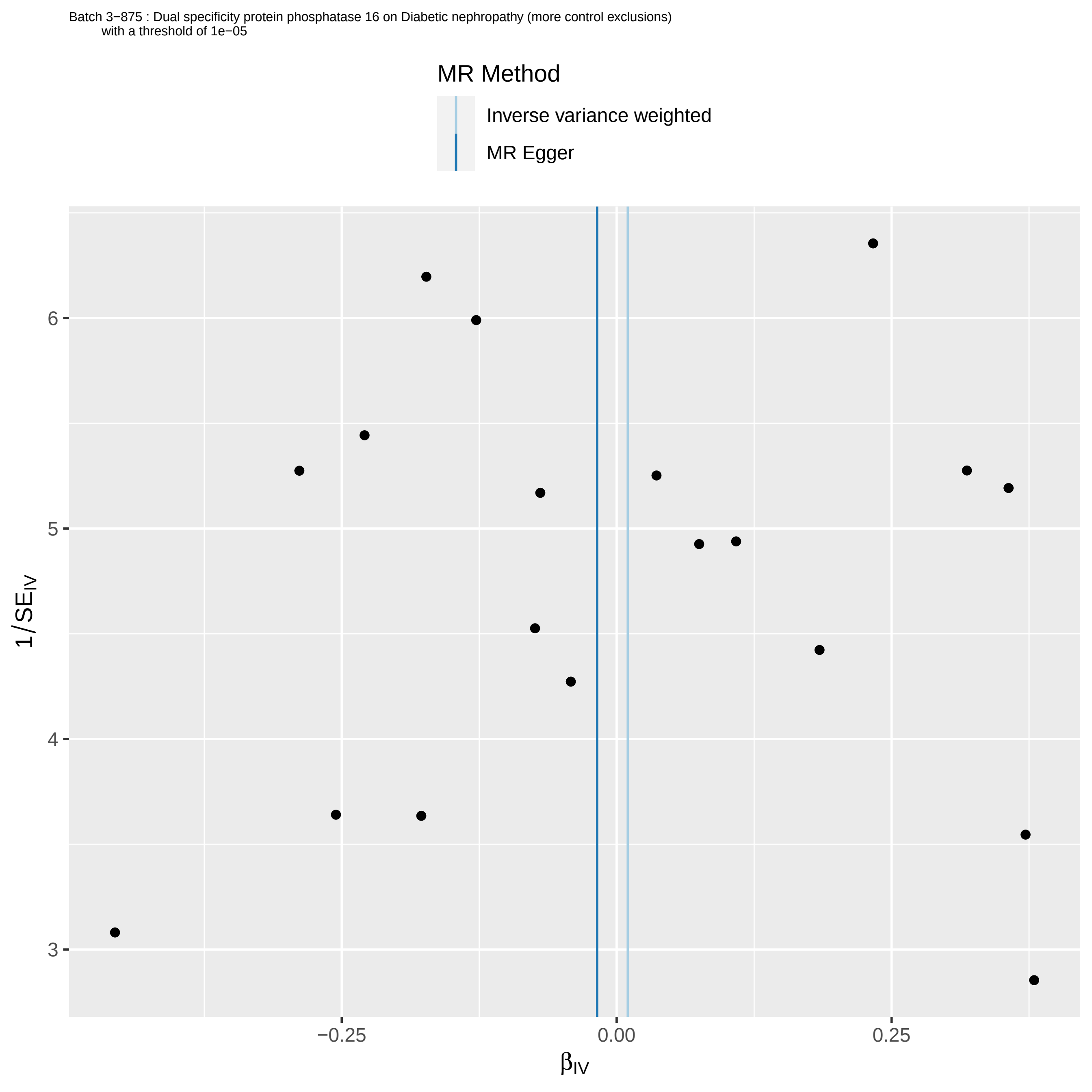

Batch 3−875 : Dual specificity protein phosphatase 16 on Diabetic nephropathy (more control exclusions)
with a threshold of 1e−05
MR Method
Inverse variance weighted
MR Egger
6
5
4
3
1/SE_IV
−0.25
0.00
0.25
β_IV

Batch 3−876 : Dual specificity protein phosphatase 4 on Diabetic nephropathy (more control exclusions)
with a threshold of 1e−05

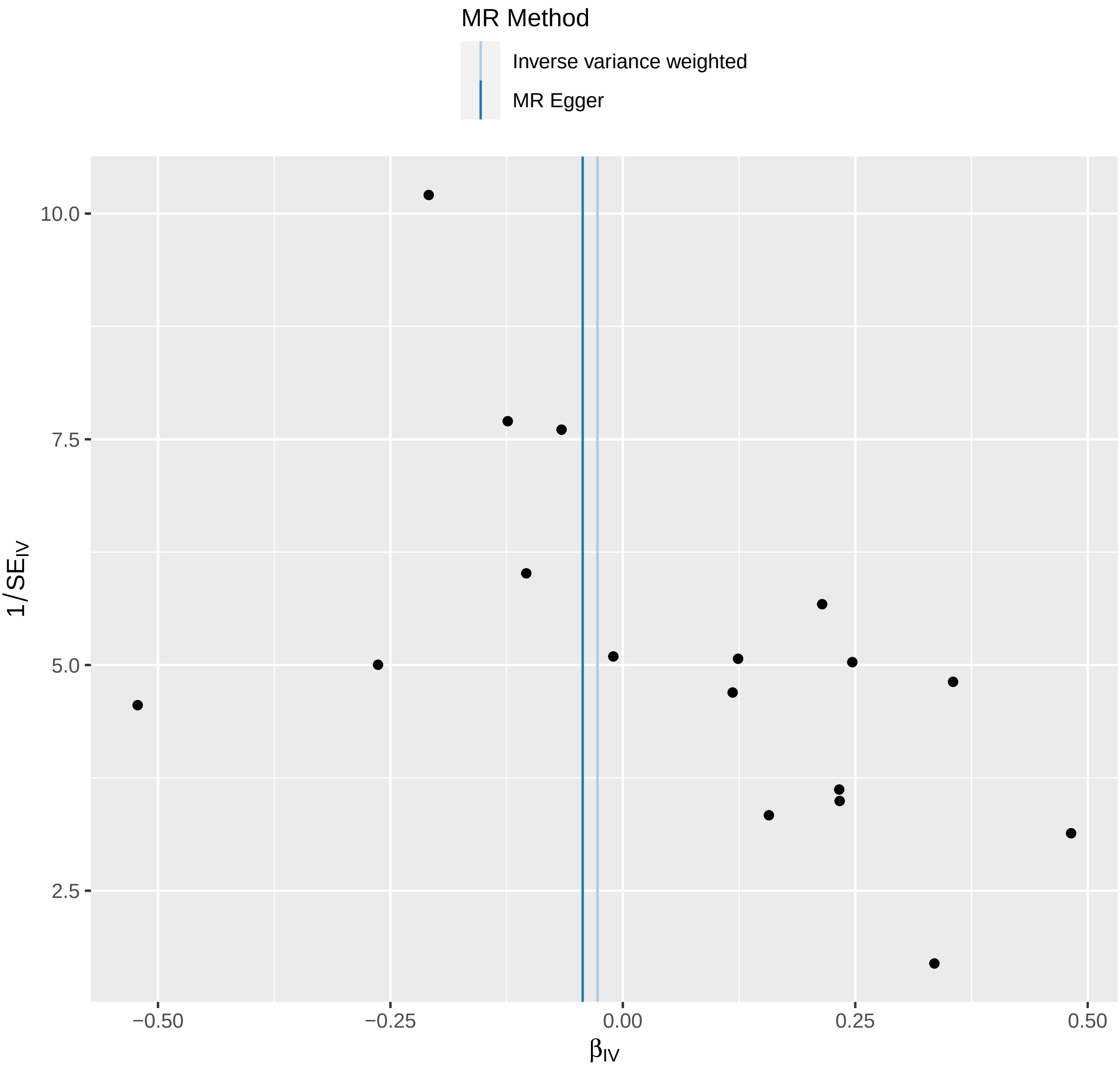

Batch 3−877 : Dual specificity protein phosphatase 6 on Diabetic nephropathy (more control exclusions)
with a threshold of 1e−05

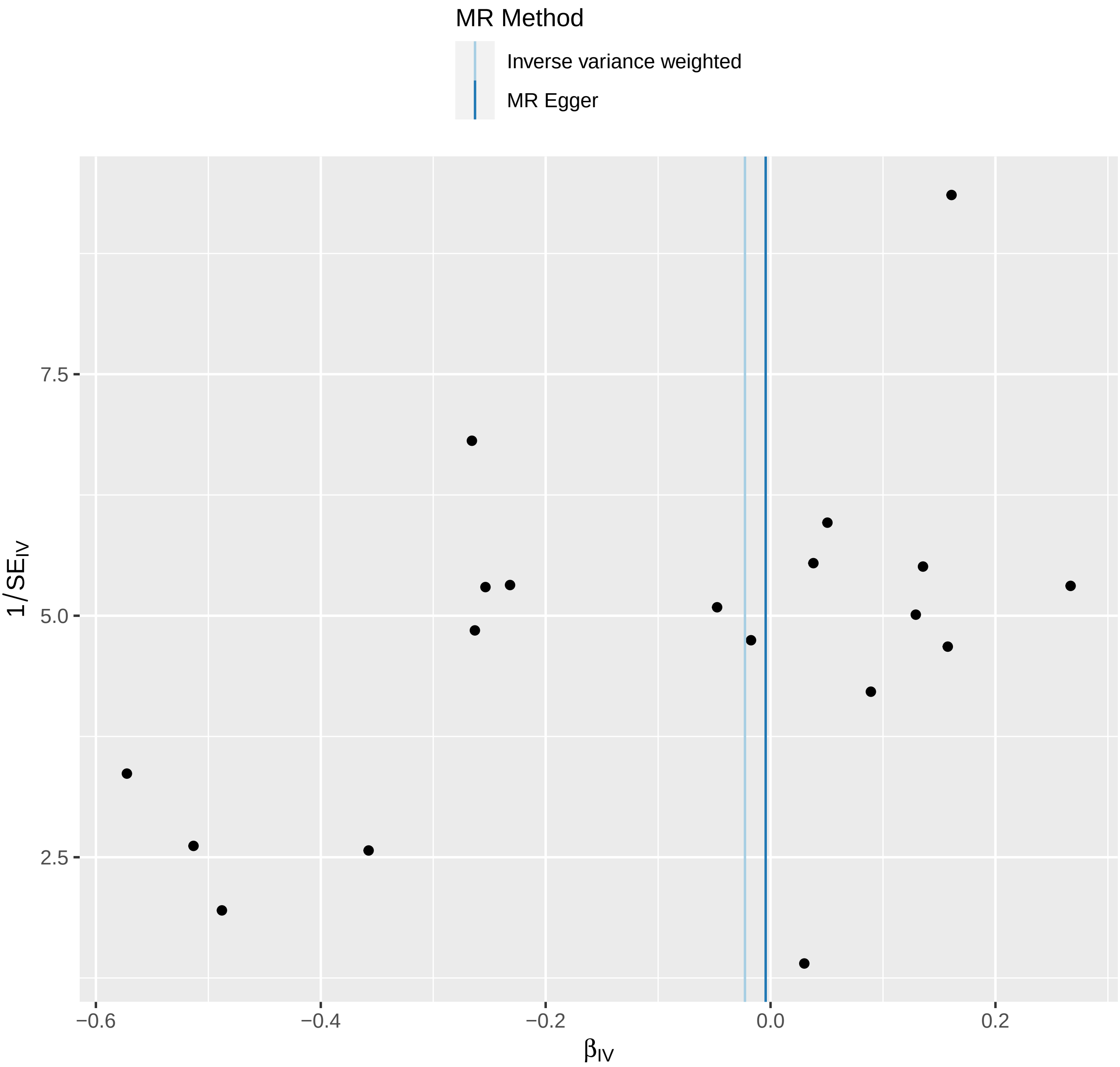

Batch 3−878 : Segment polarity protein dishevelled homolog DVL−2 on Diabetic nephropathy (more control exclusions) with a threshold of 1e−05

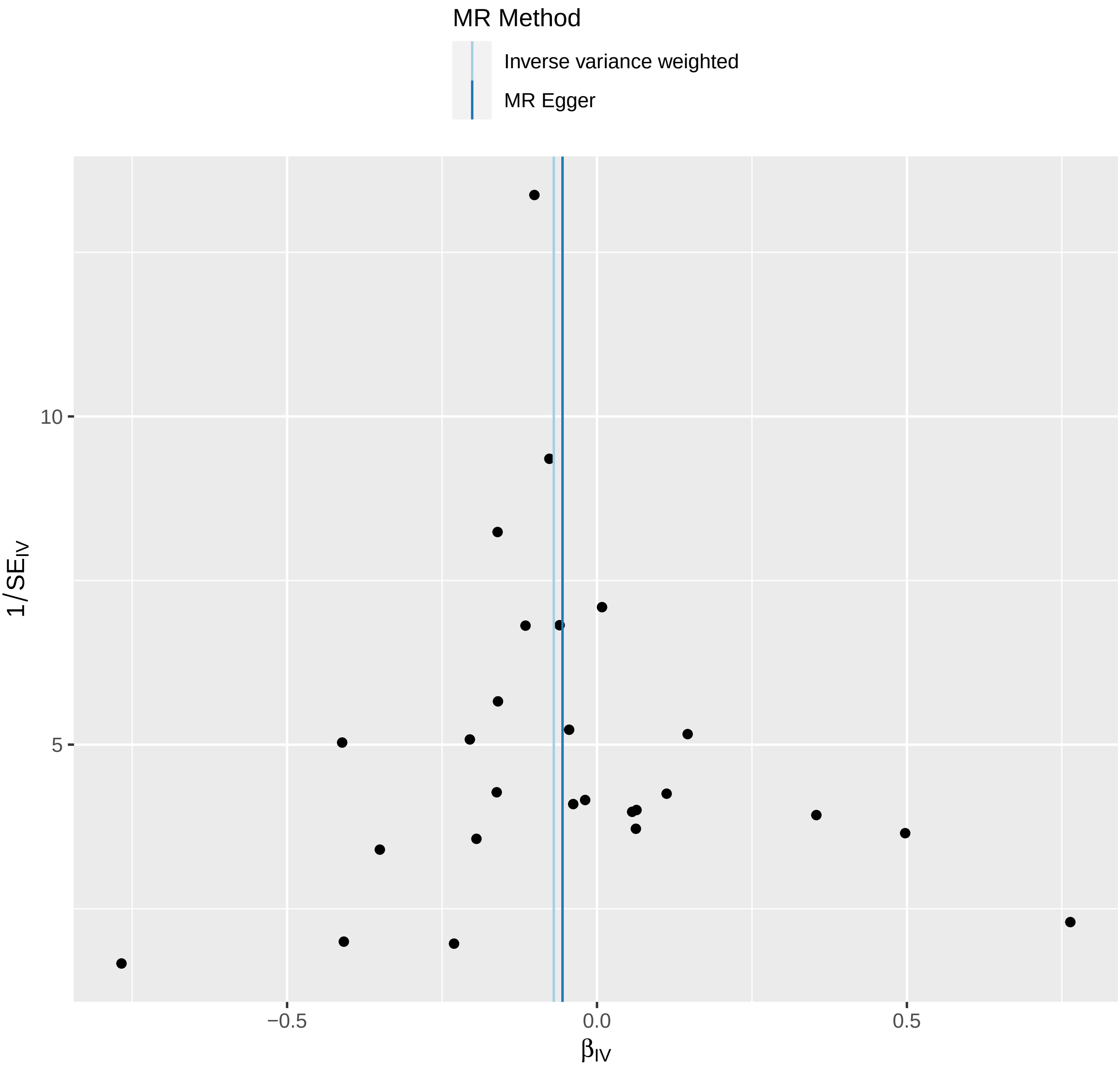

Batch 3−879 : Dynactin−associated protein on Diabetic nephropathy (more control exclusions)
with a threshold of 1e−05

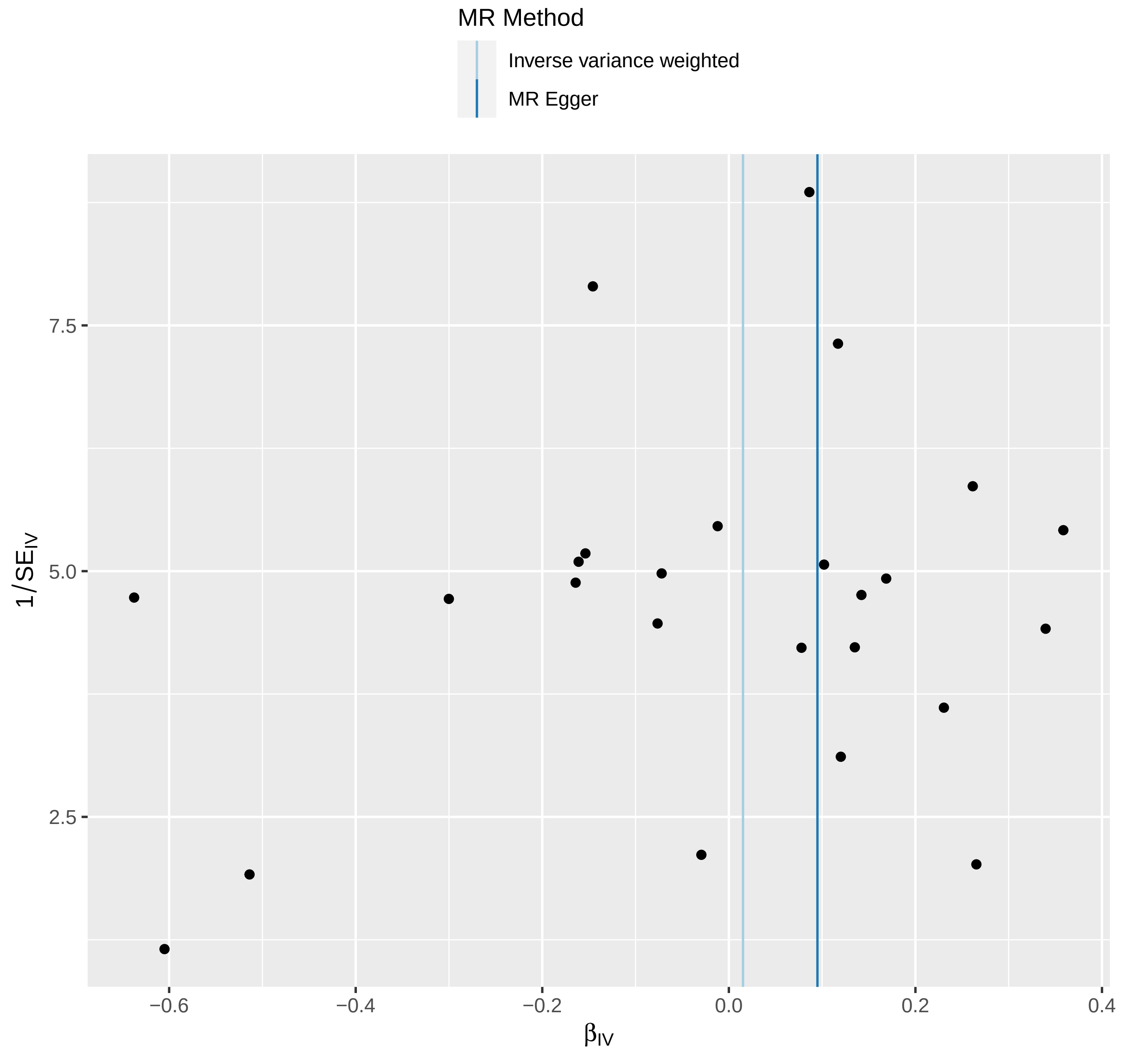

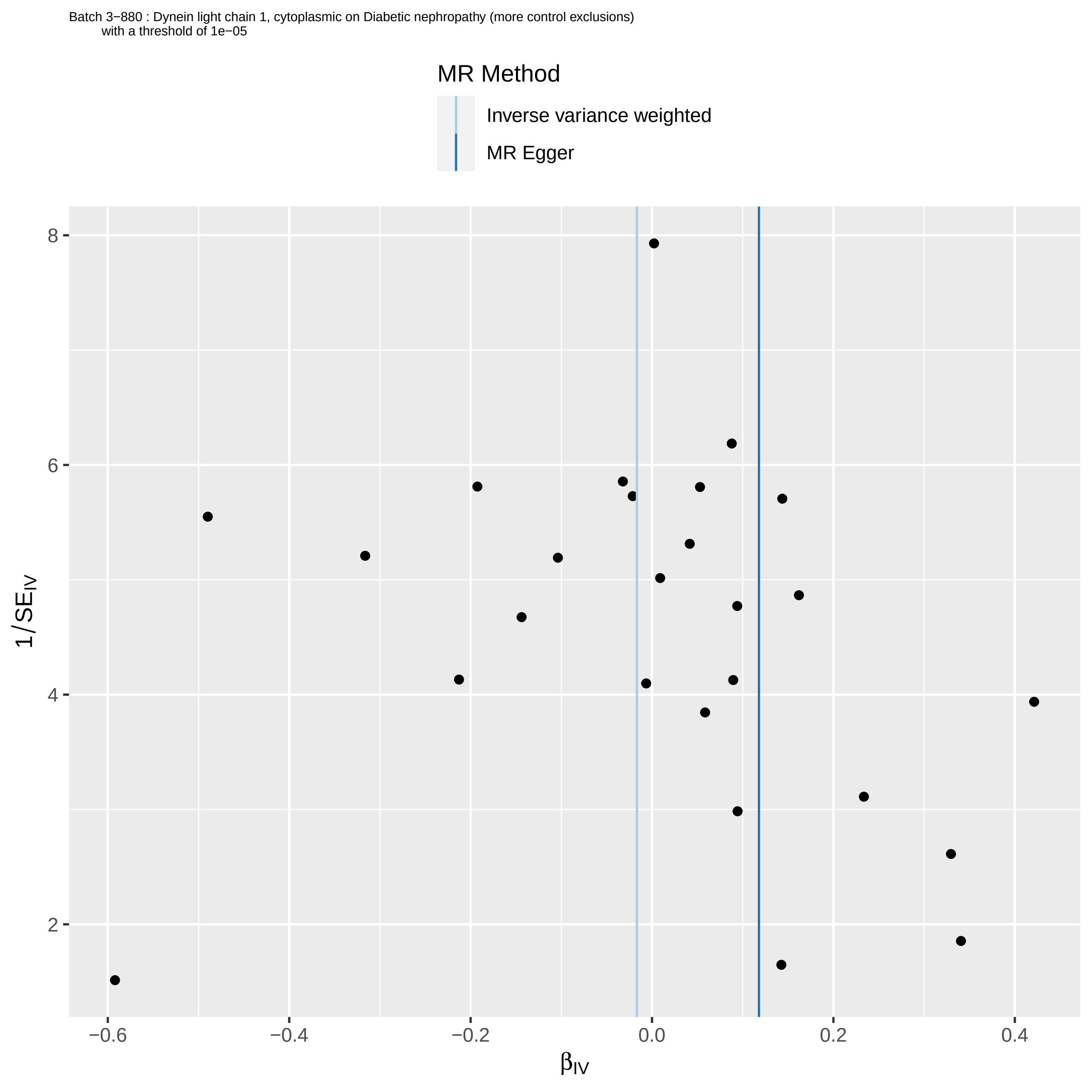
Batch 3−880 : Dynein light chain 1, cytoplasmic on Diabetic nephropathy (more control exclusions)
with a threshold of 1e−05
MR Method
Inverse variance weighted
MR Egger
8
6
4
2
1/SE_IV
−0.6
−0.4
−0.2
0.0
0.2
0.4
β_IV

Batch 3−881 : Dynein light chain Tctex−type 3 on Diabetic nephropathy (more control exclusions)
with a threshold of 1e−05

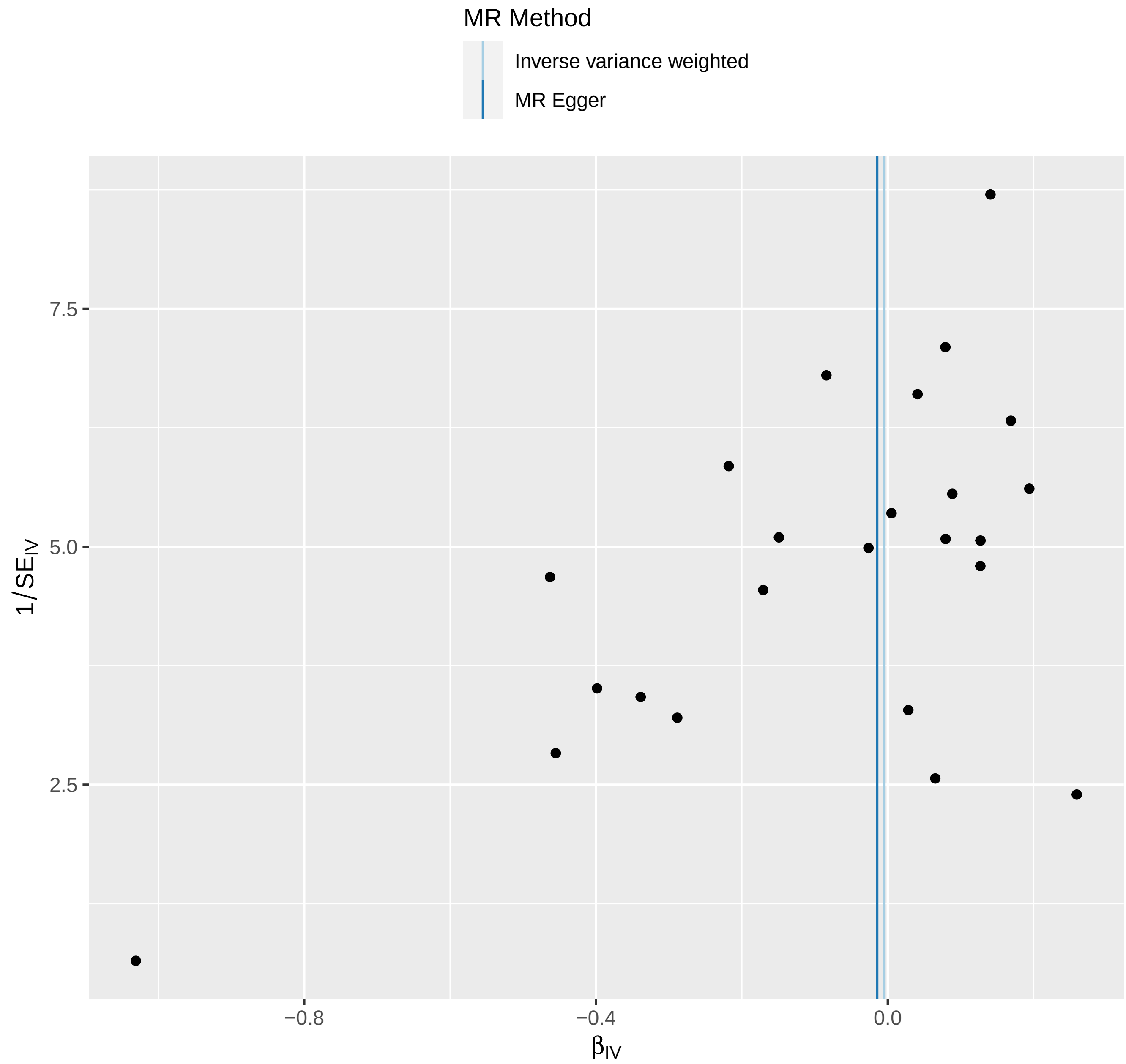

Batch 3−882 : Dual specificity tyrosine−phosphorylation−regulated kinase 3 on Diabetic nephropathy (more control exclusions)
with a threshold of 1e−05

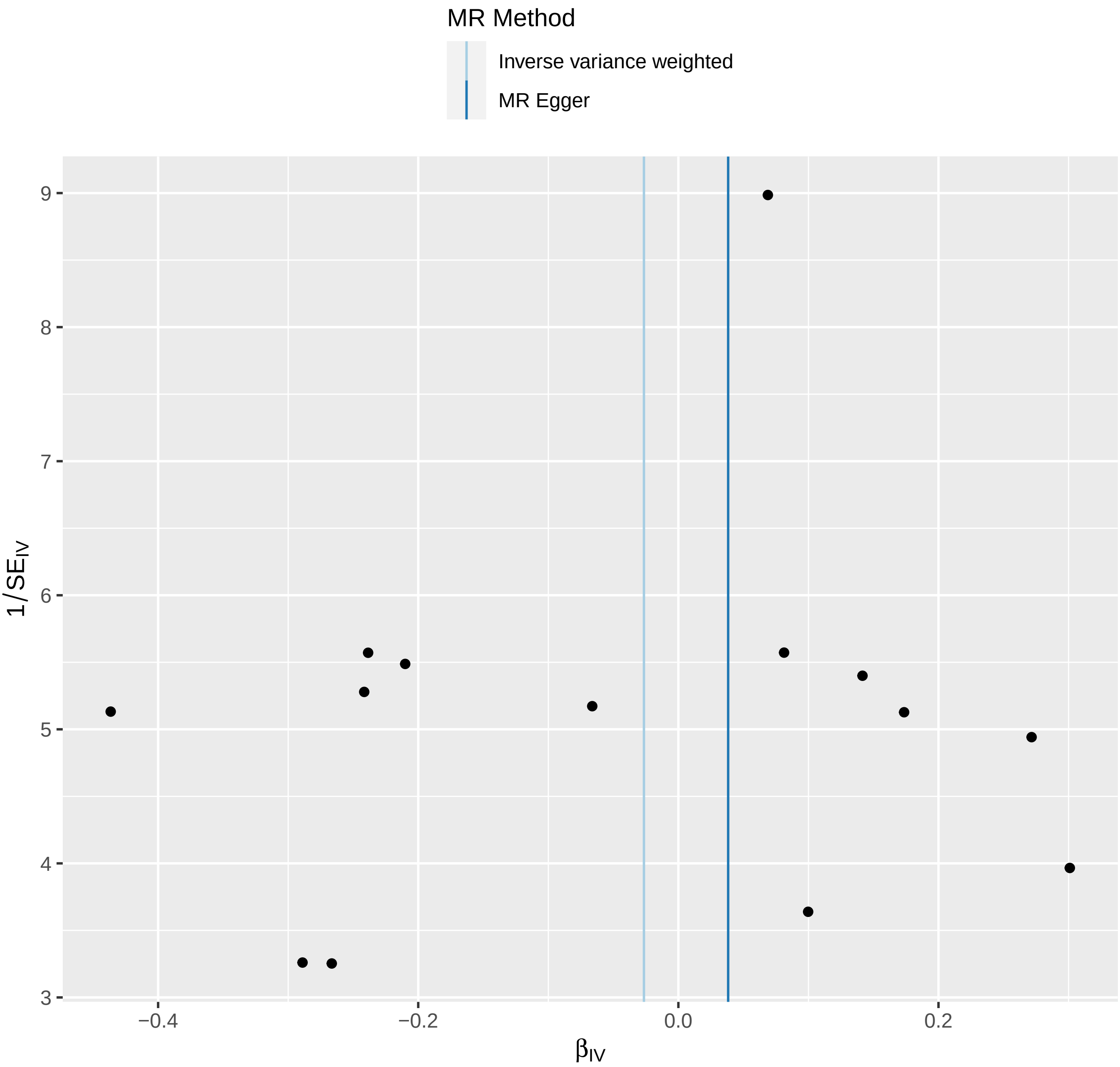

Batch 3−883 : Endothelin−converting enzyme 1 on Diabetic nephropathy (more control exclusions)
with a threshold of 1e−05

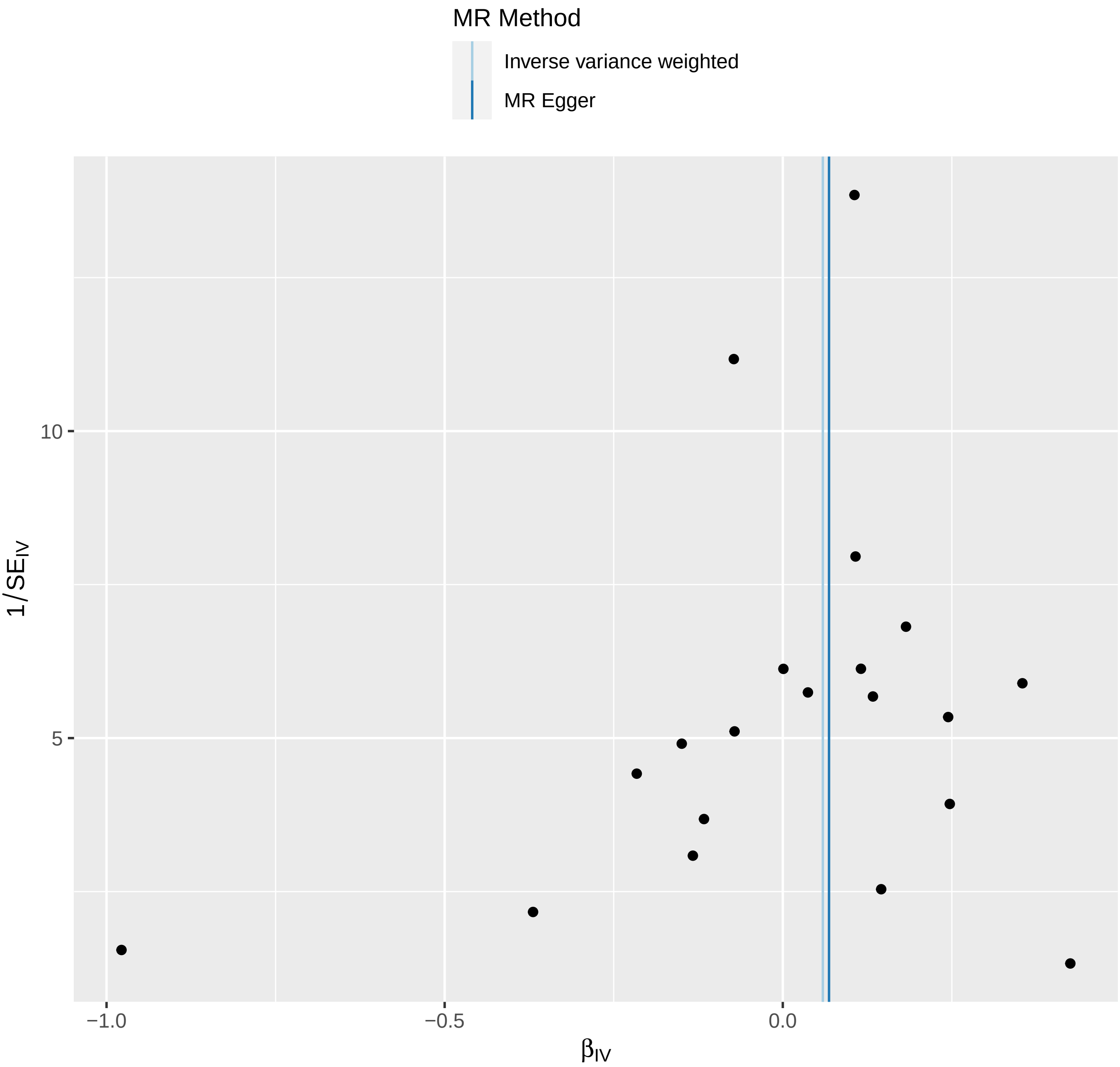

Batch 3−884 : Endothelin−converting enzyme 1 on Diabetic nephropathy (more control exclusions)
with a threshold of 1e−05

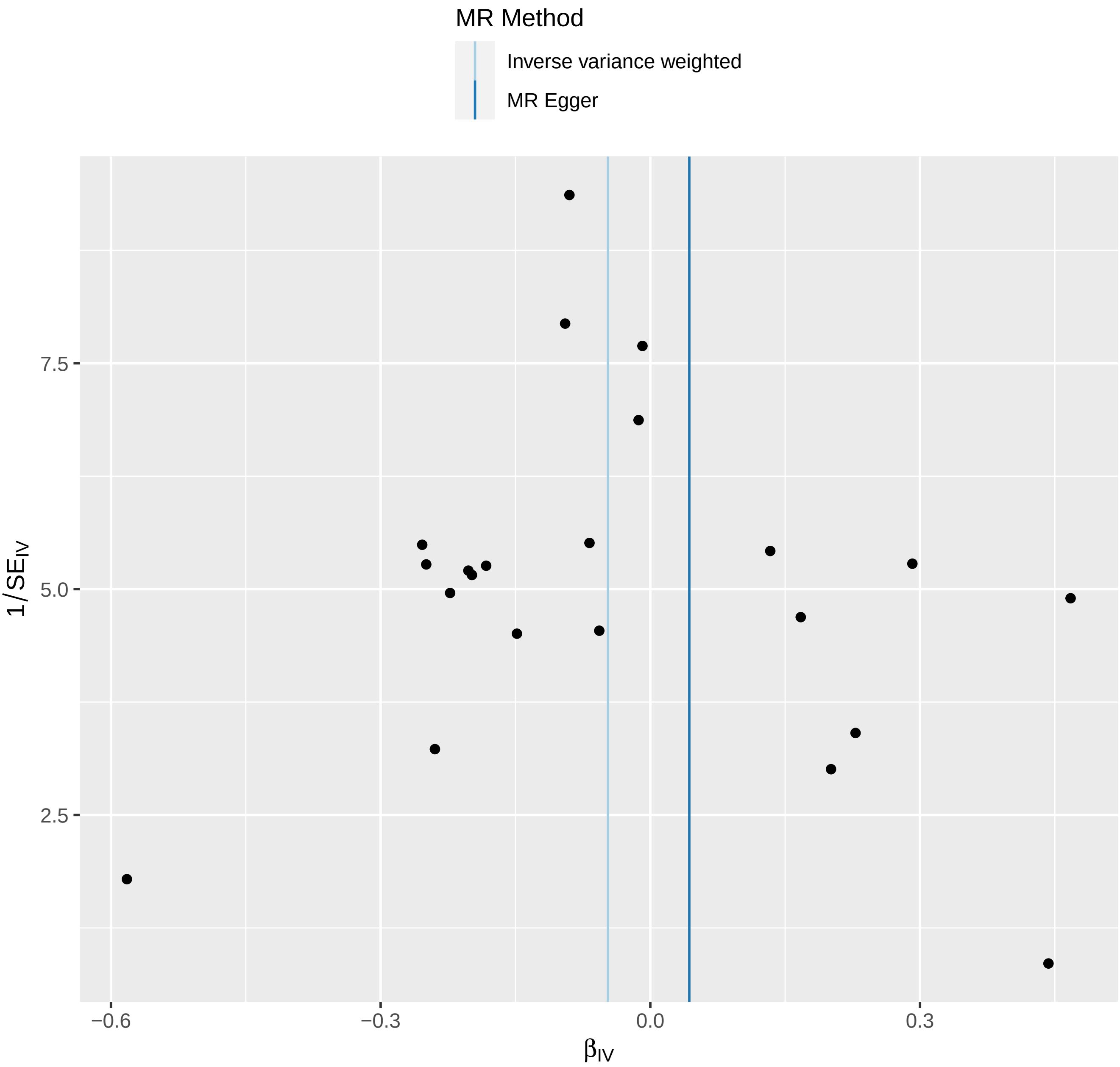

Batch 3−885 : Ectodysplasin−A, secreted form on Diabetic nephropathy (more control exclusions)
with a threshold of 1e−05

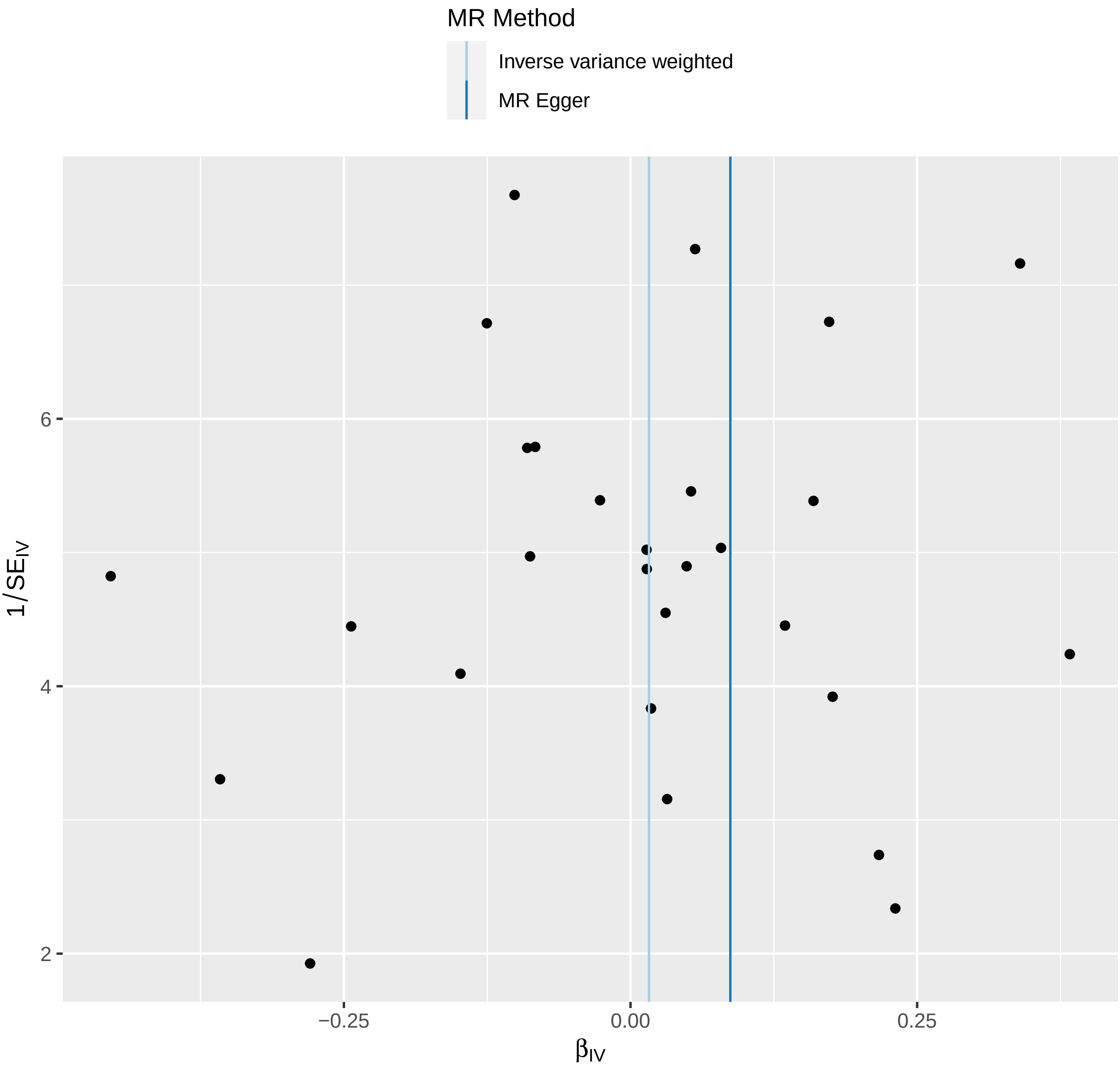

Batch 3−886 : Ectodysplasin−A, secreted form on Diabetic nephropathy (more control exclusions)
with a threshold of 1e−05

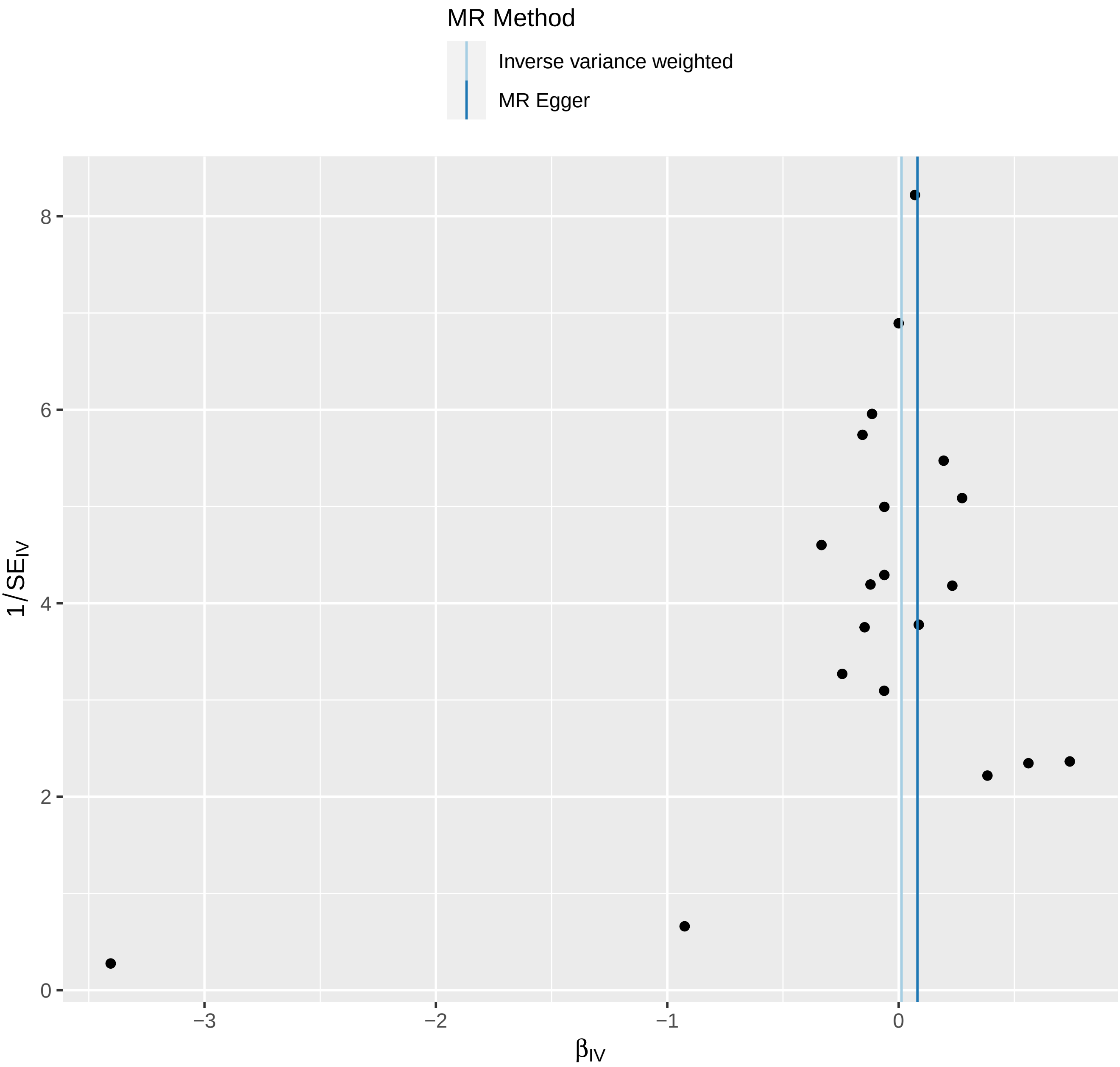

Batch 3−887 : Tumor necrosis factor receptor superfamily member 27 on Diabetic nephropathy (more control exclusions)
with a threshold of 1e−05

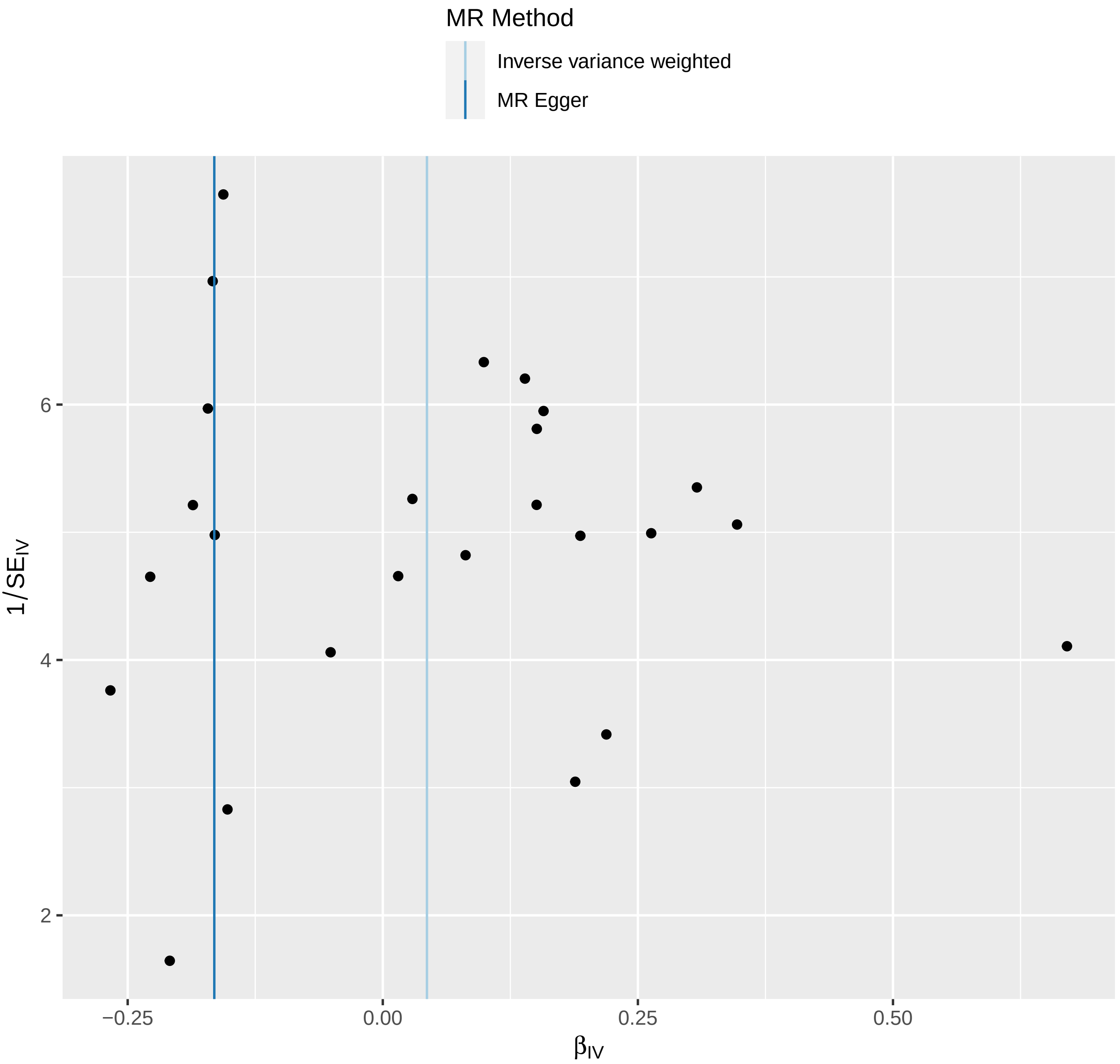

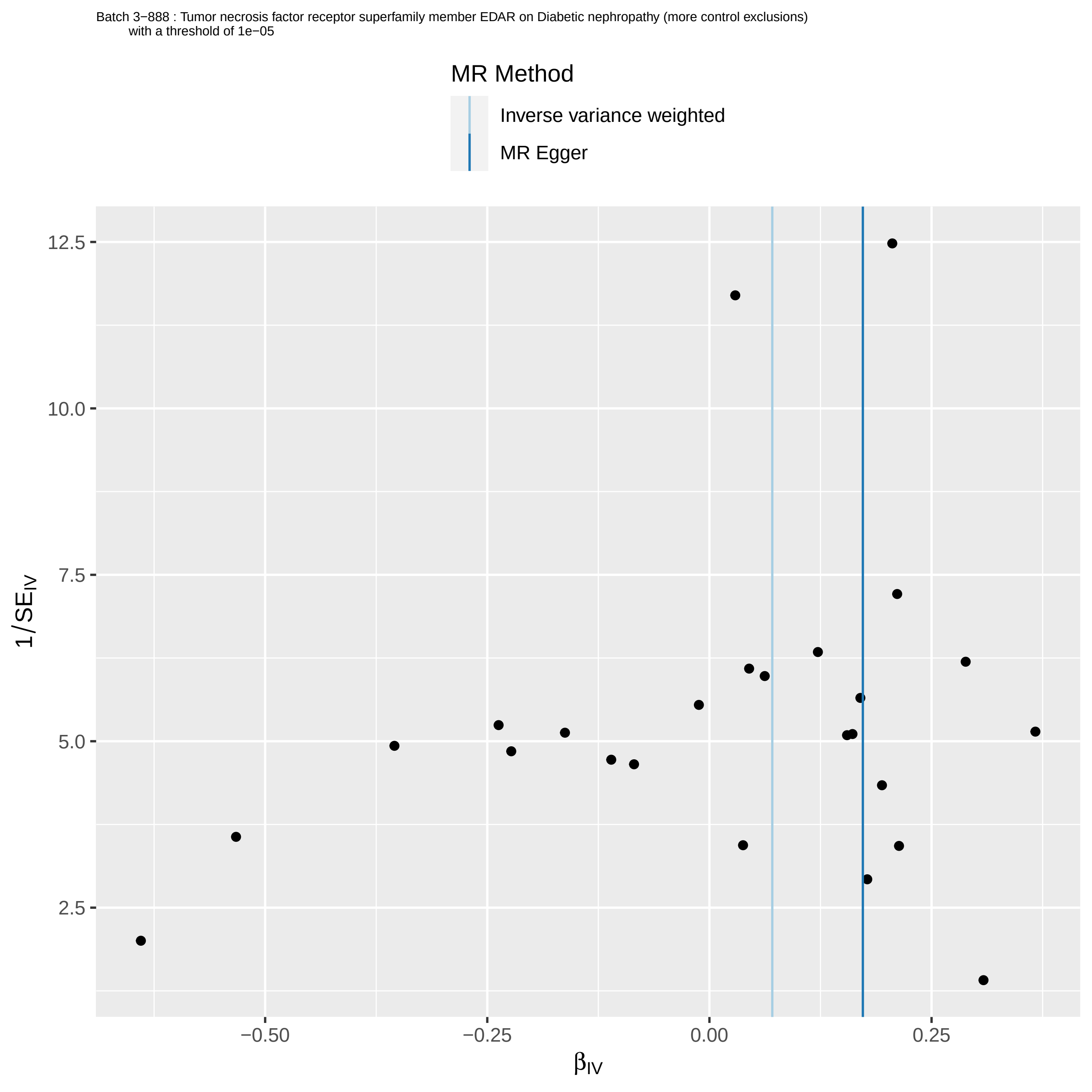

Batch 3−888 : Tumor necrosis factor receptor superfamily member EDAR on Diabetic nephropathy (more control exclusions)
with a threshold of 1e−05
MR Method
Inverse variance weighted
MR Egger
12.5
10.0
7.5
5.0
2.5
1/SE_IV
−0.50
−0.25
0.00
0.25
β_IV

Batch 3−889 : Enhancer of mRNA−decapping protein 4 on Diabetic nephropathy (more control exclusions)
with a threshold of 1e−05

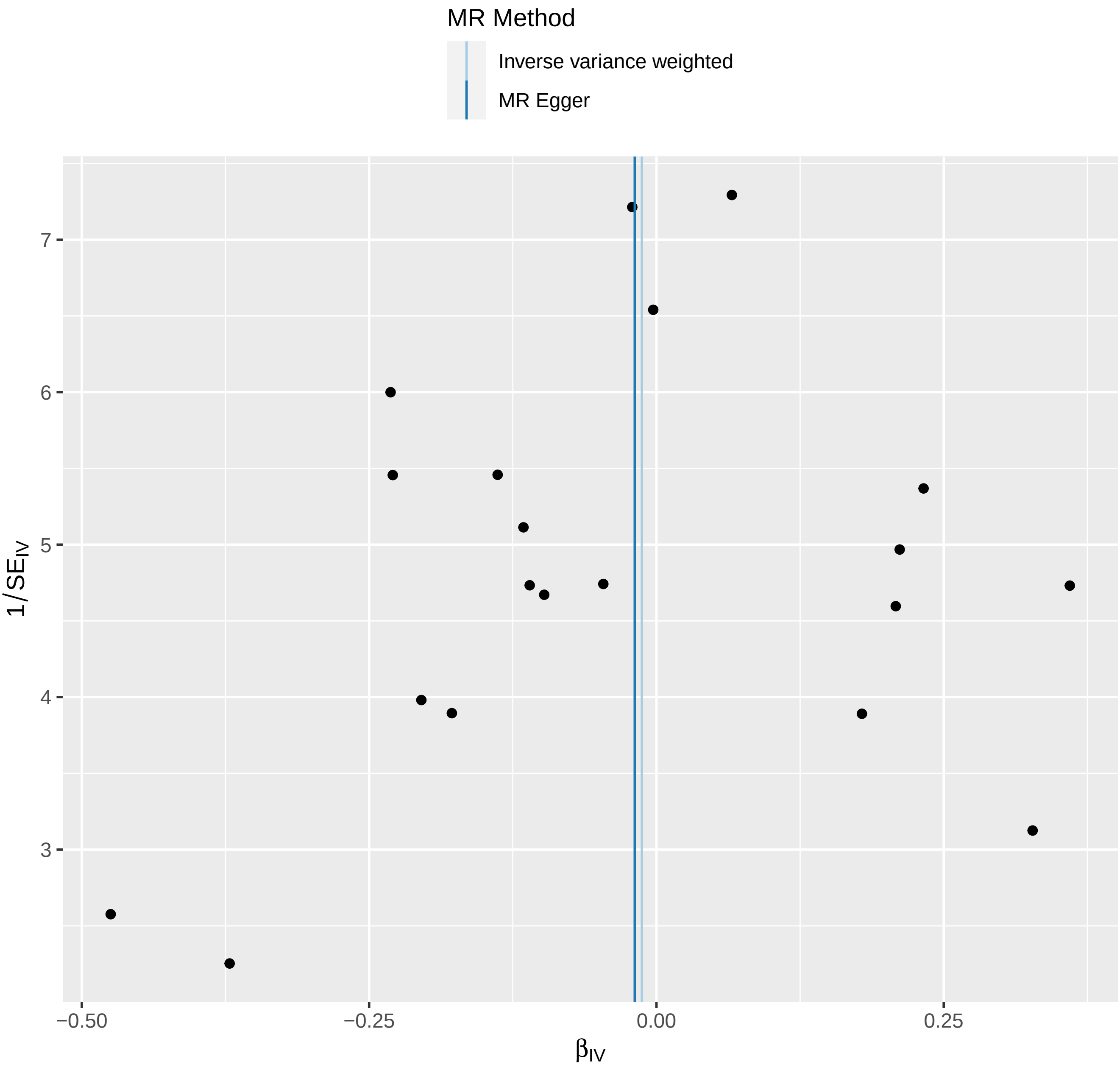

Batch 3−890 : Epididymal secretory protein E3−beta on Diabetic nephropathy (more control exclusions)
with a threshold of 1e−05

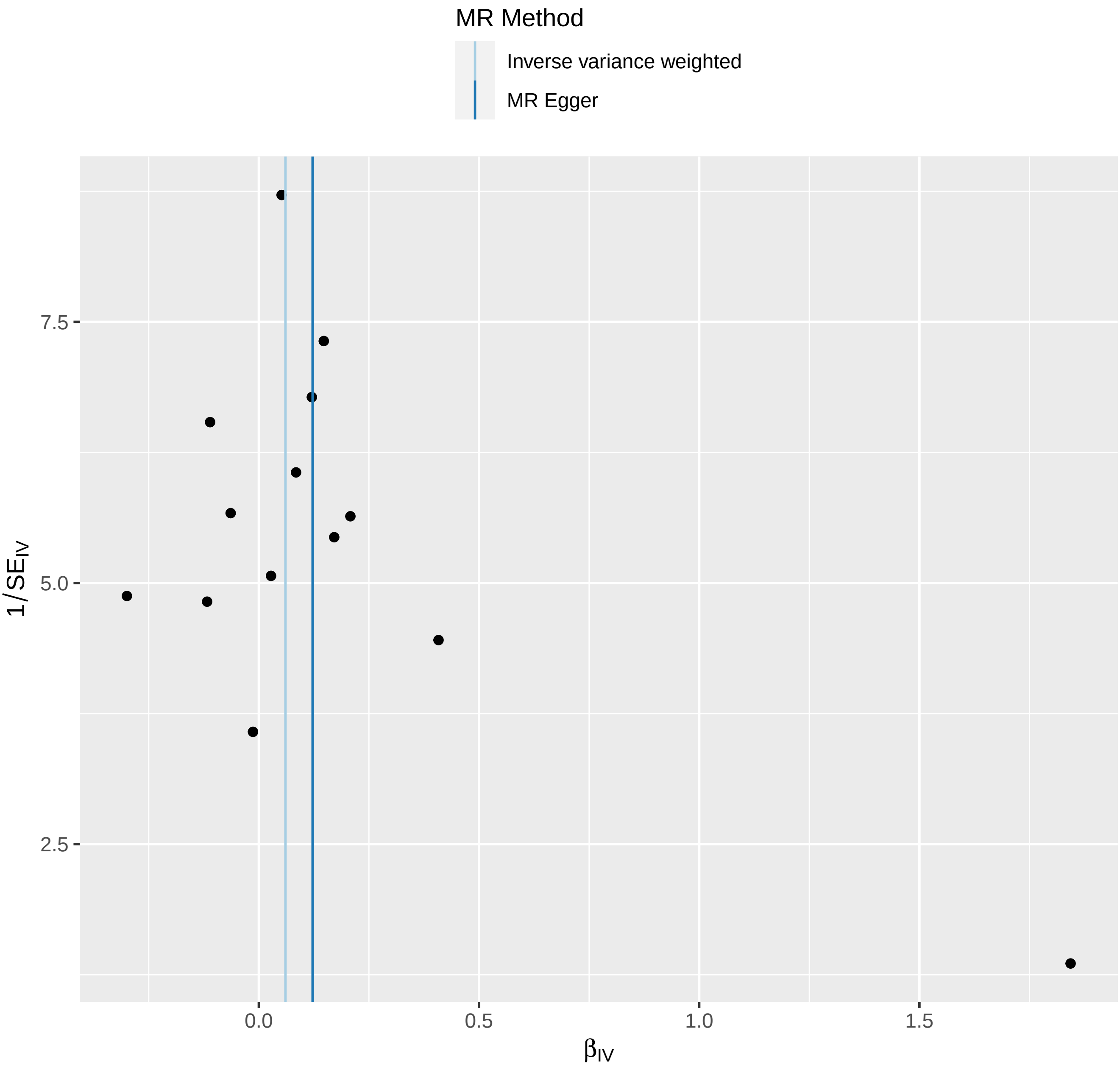

Batch 3−891 : Endothelial differentiation−related factor 1 on Diabetic nephropathy (more control exclusions)
with a threshold of 1e−05

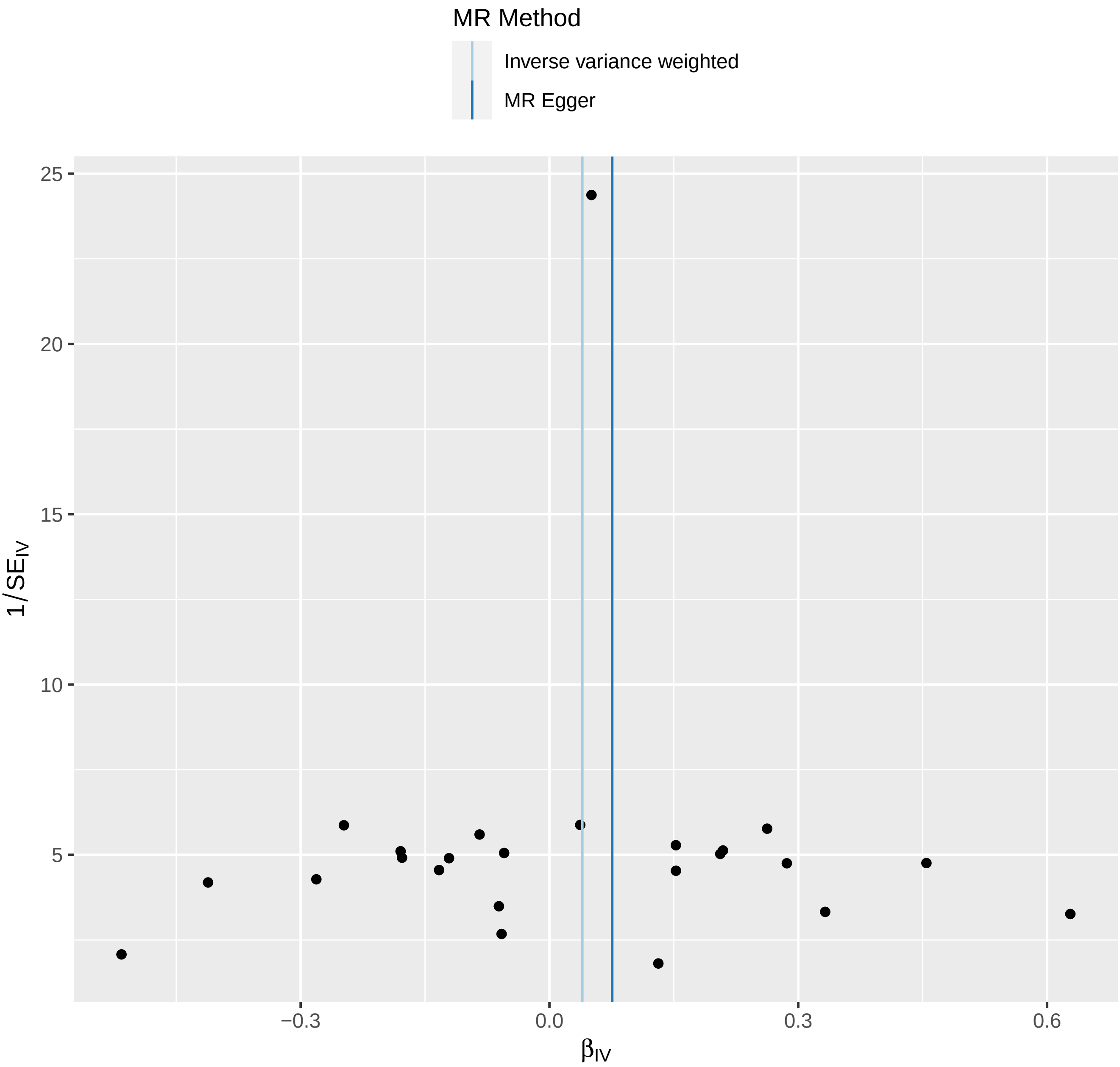

Batch 3−892 : Endothelin−2 on Diabetic nephropathy (more control exclusions)
with a threshold of 1e−05

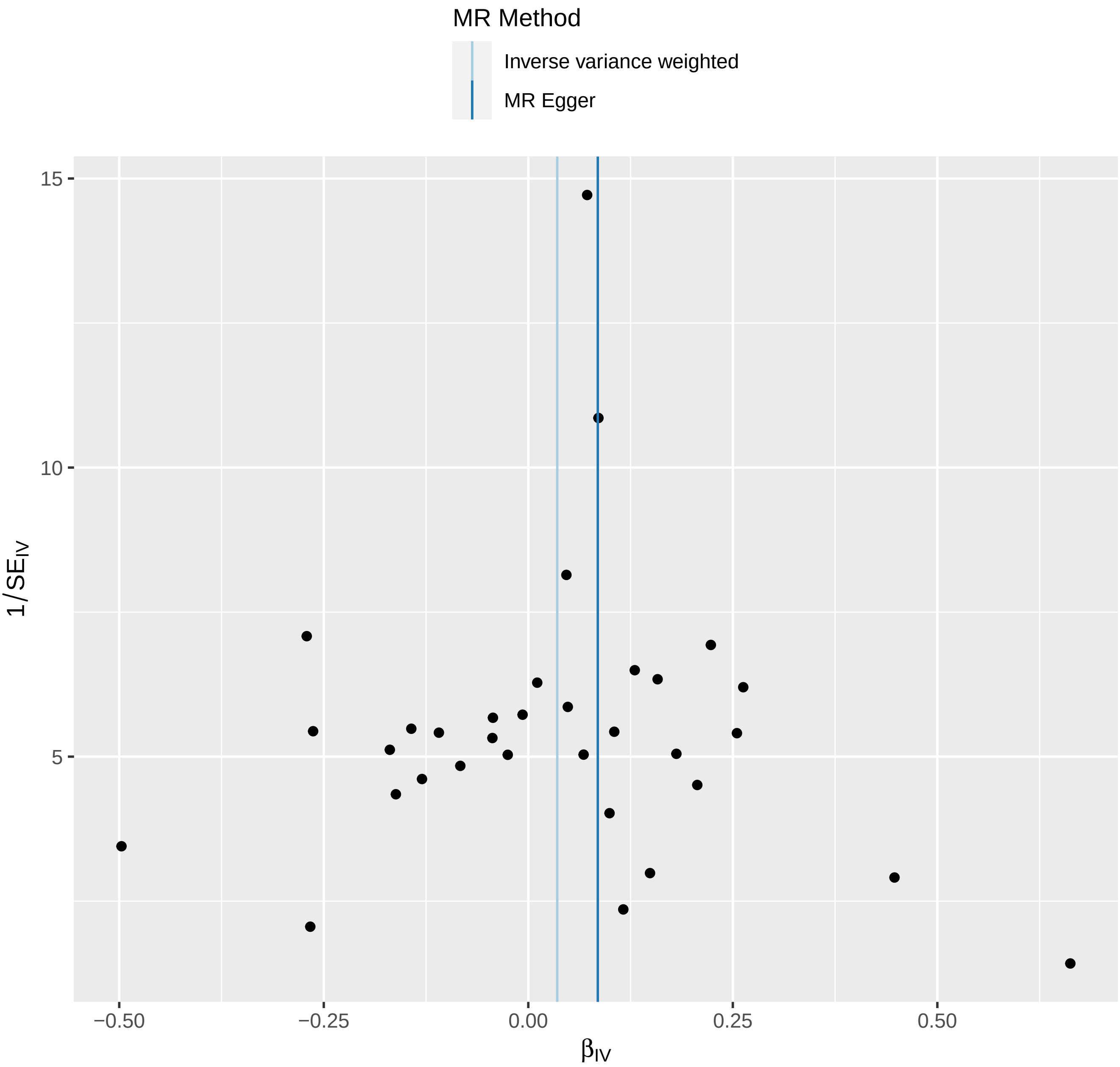

Batch 3−893 : Early endosome antigen 1 on Diabetic nephropathy (more control exclusions)
with a threshold of 1e−05

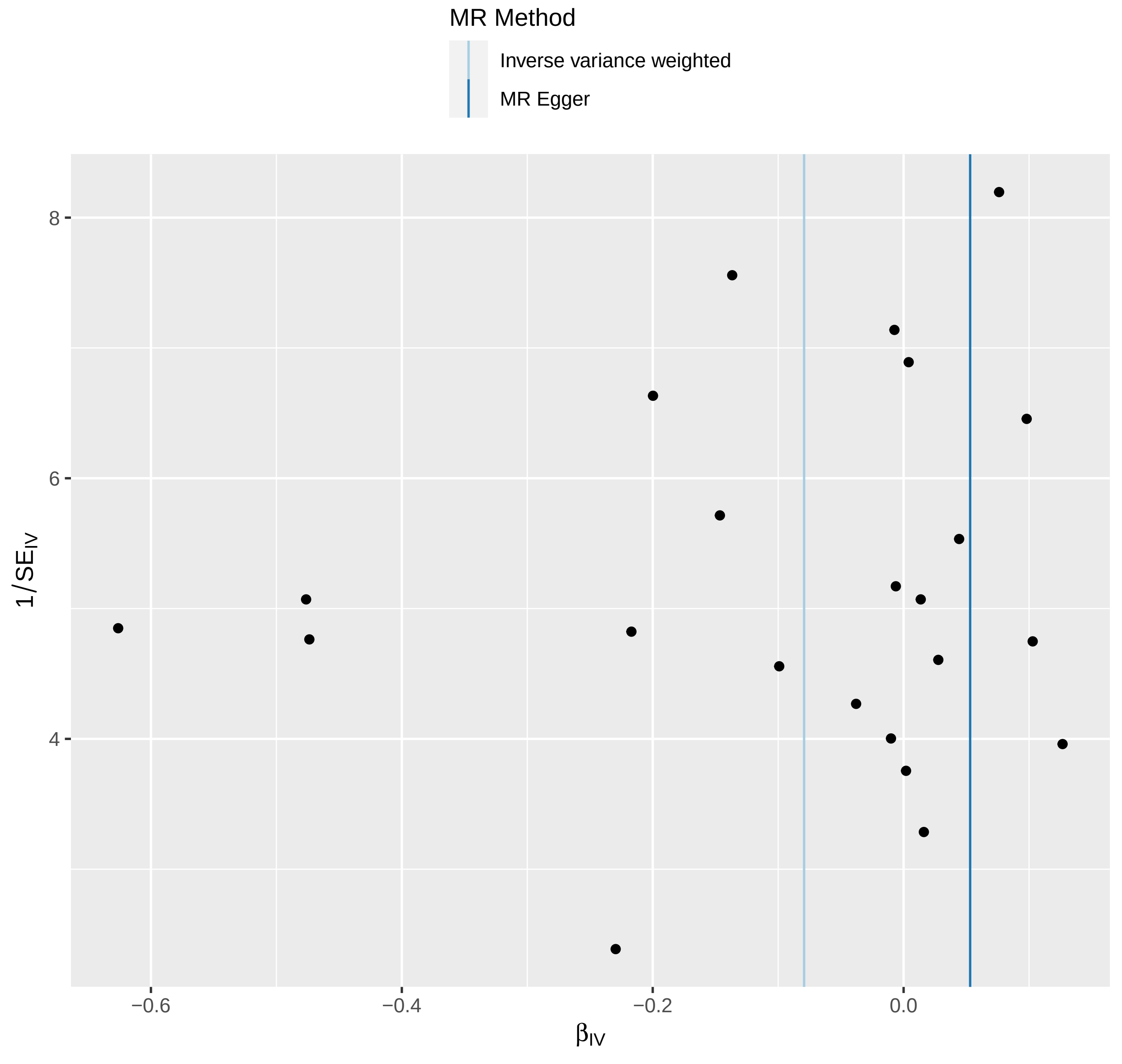

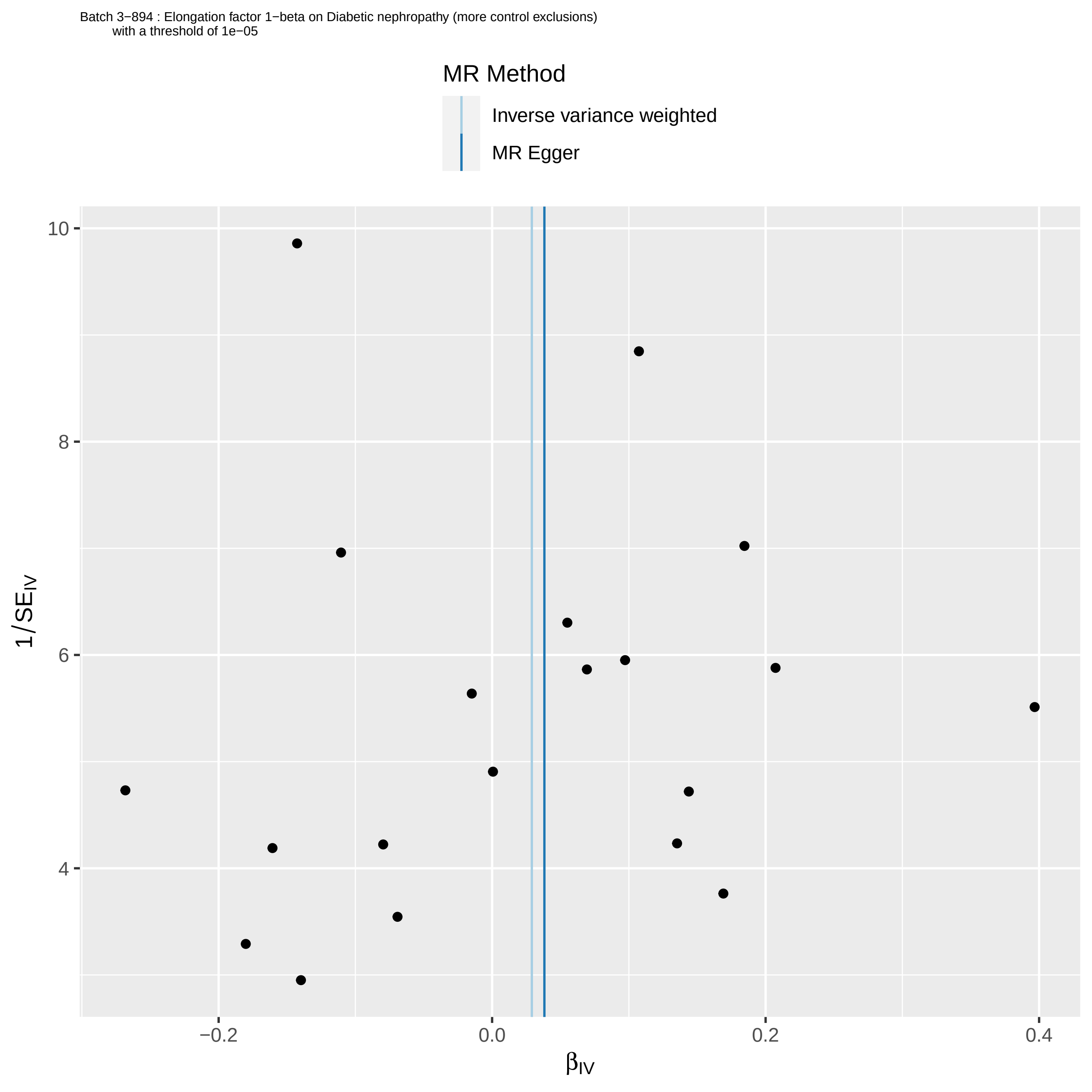

Batch 3−894 : Elongation factor 1−beta on Diabetic nephropathy (more control exclusions)
with a threshold of 1e−05
MR Method
Inverse variance weighted
MR Egger
10
8
6
4
1/SE$_{IV}$
−0.2
0.0
0.2
0.4
β$_{IV}$

Batch 3−895 : EF−hand calcium−binding domain−containing protein 14 on Diabetic nephropathy (more control exclusions)
with a threshold of 1e−05

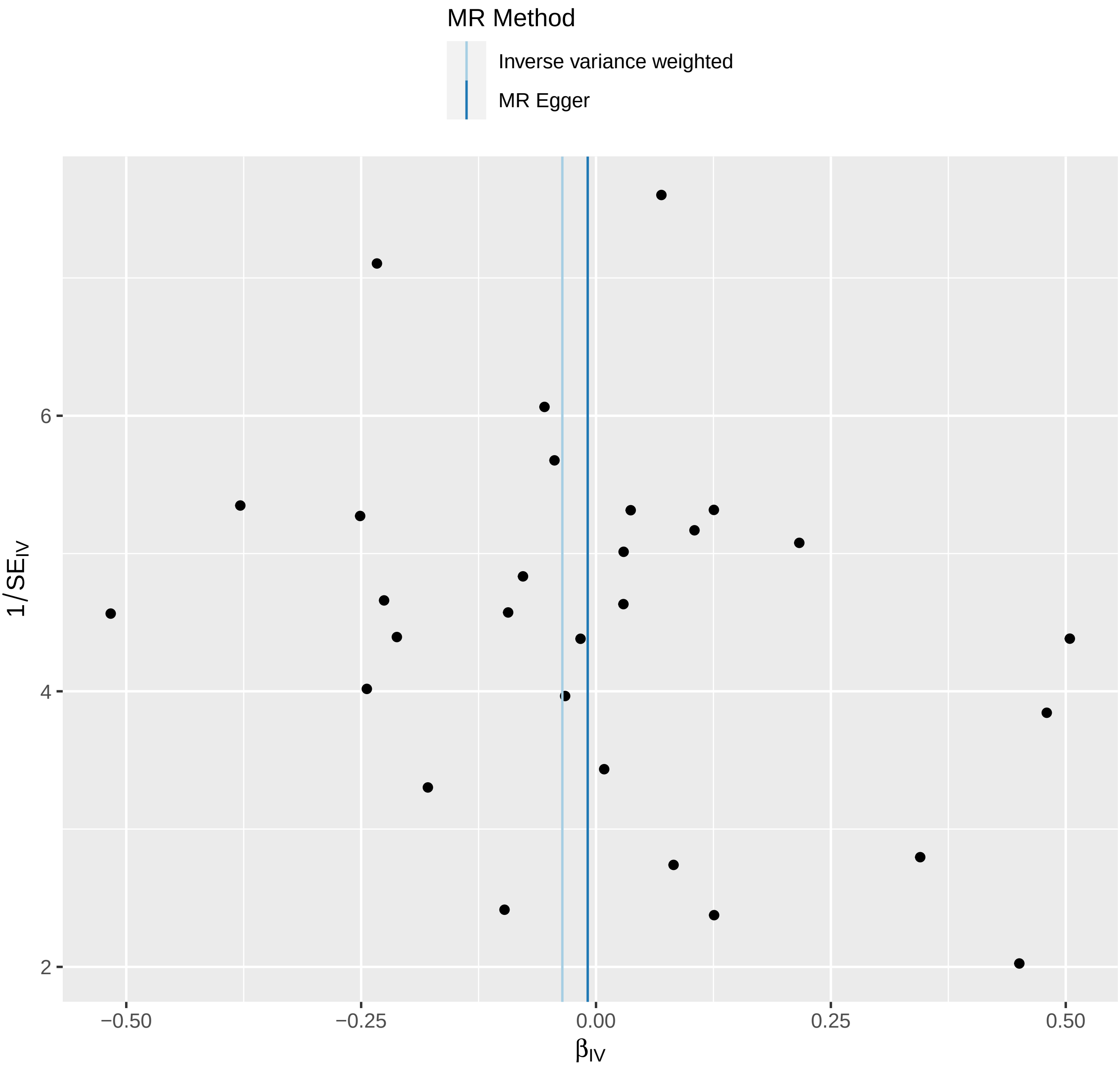

Batch 3−896 : Calcium uptake protein 3, mitochondrial on Diabetic nephropathy (more control exclusions)
with a threshold of 1e−05

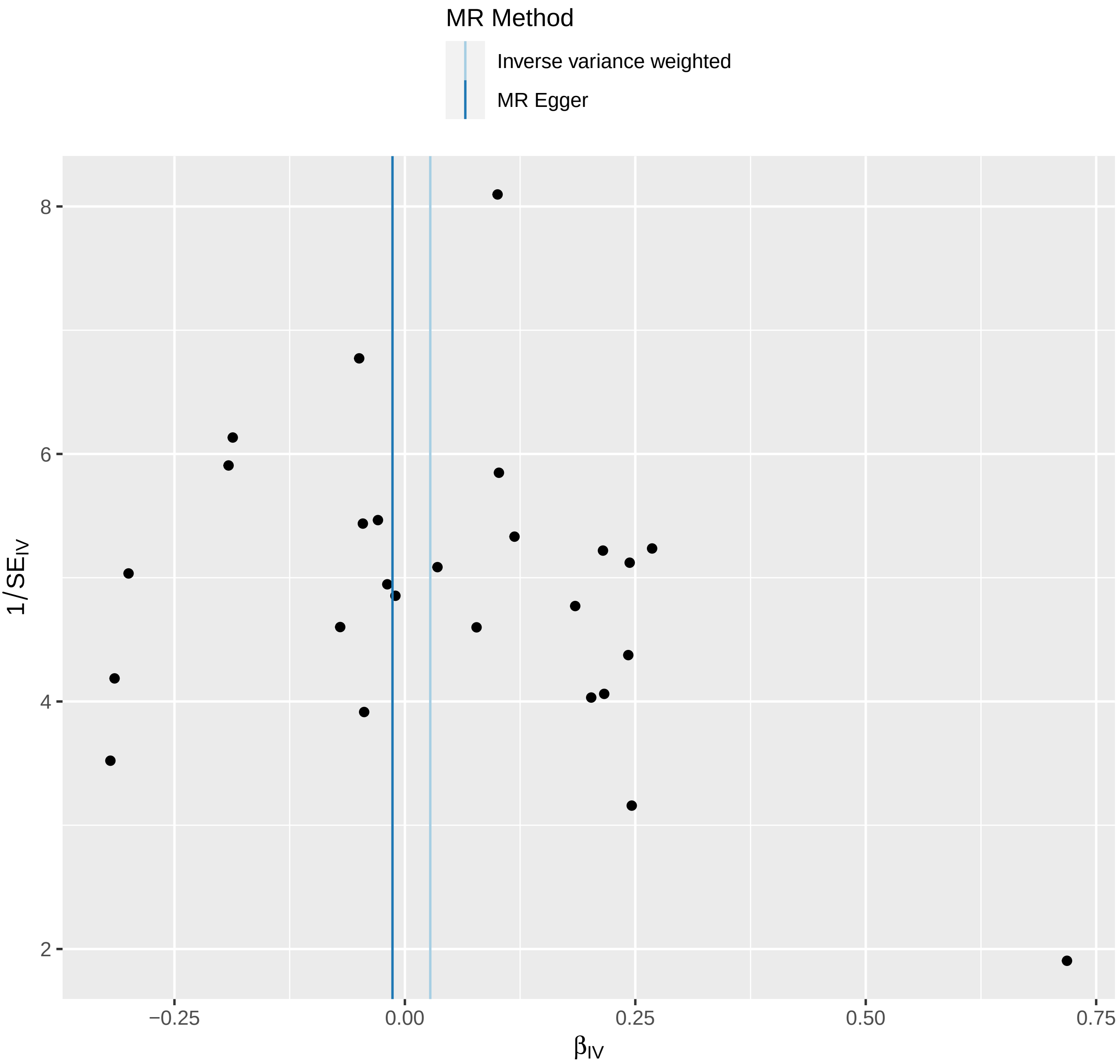

Batch 3−897 : Ephrin−A2 on Diabetic nephropathy (more control exclusions)
with a threshold of 1e−05

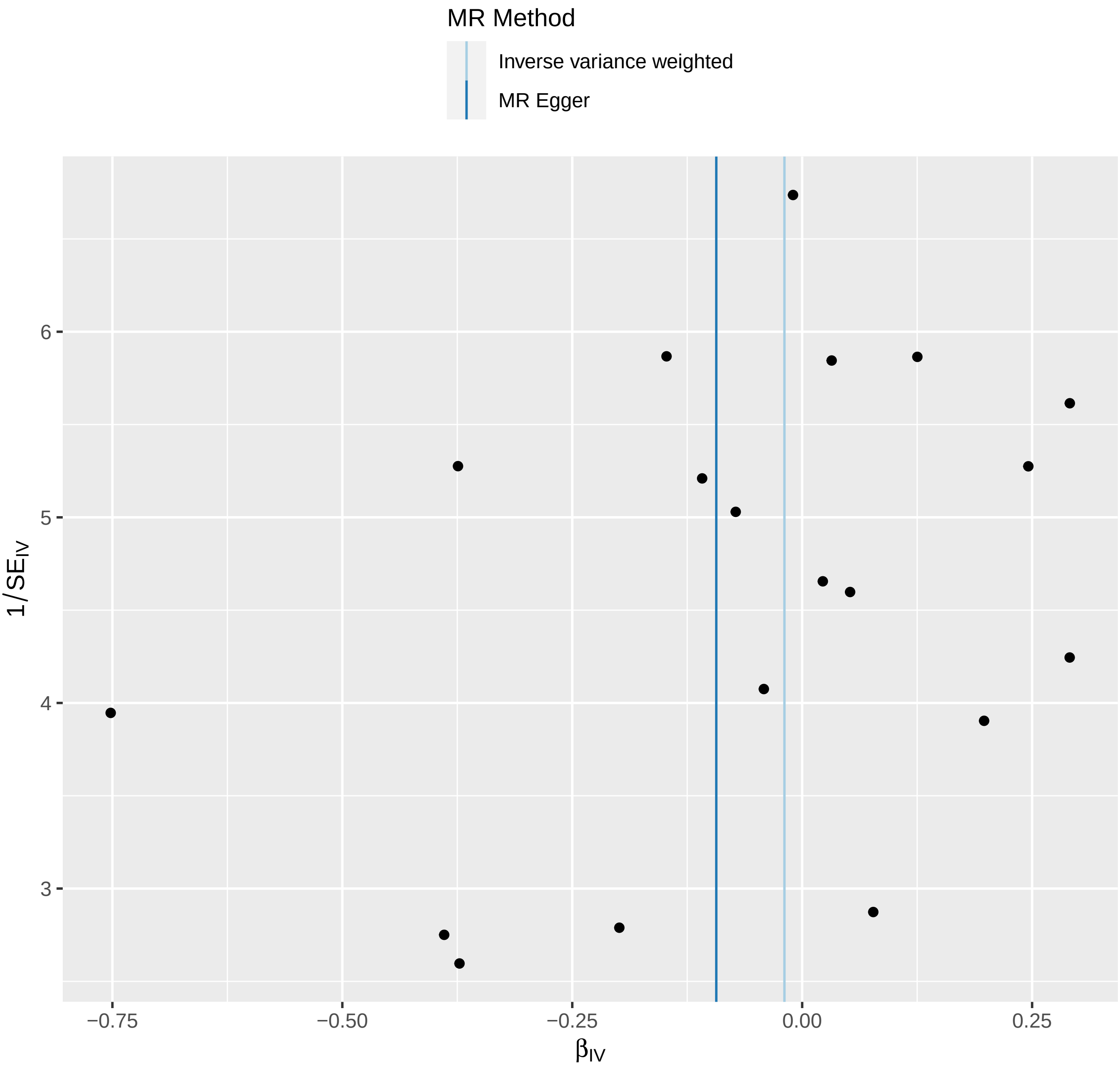

Batch 3−898 : Ephrin−A3 on Diabetic nephropathy (more control exclusions)
with a threshold of 1e−05

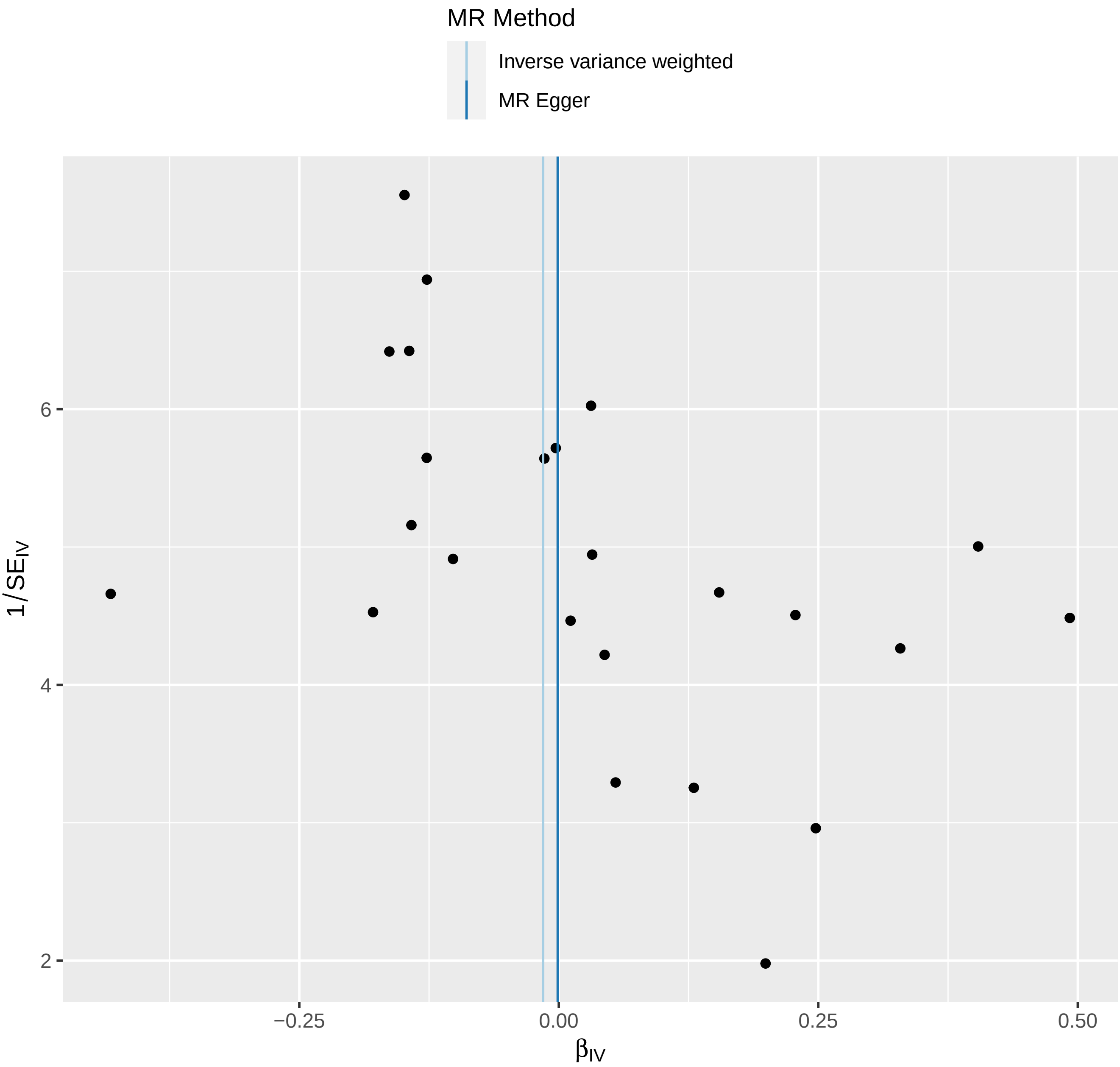

Batch 3−899 : Ephrin−A3 on Diabetic nephropathy (more control exclusions)
with a threshold of 1e−05

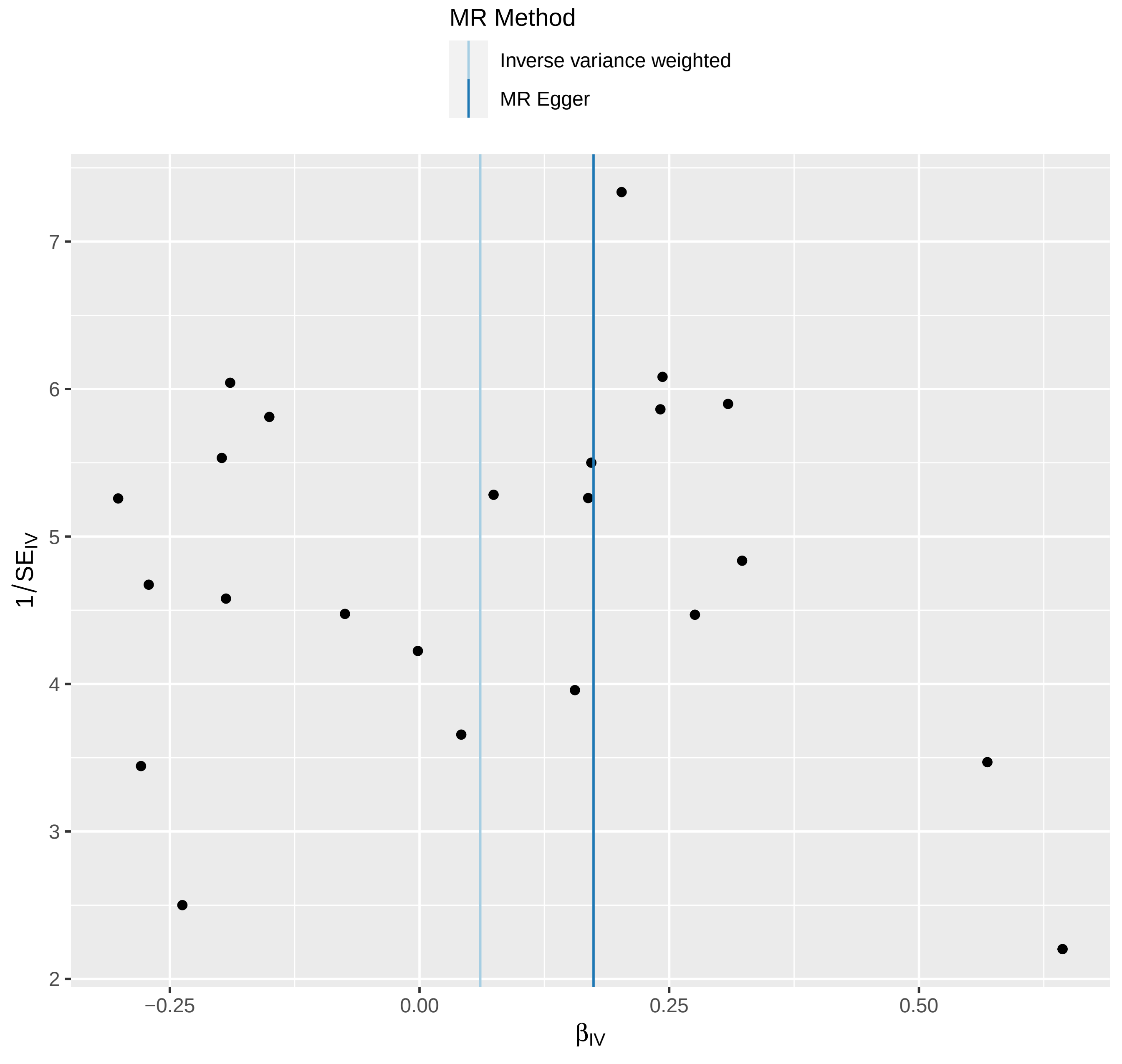

Batch 3−900 : Ephrin−A4 on Diabetic nephropathy (more control exclusions)
with a threshold of 1e−05

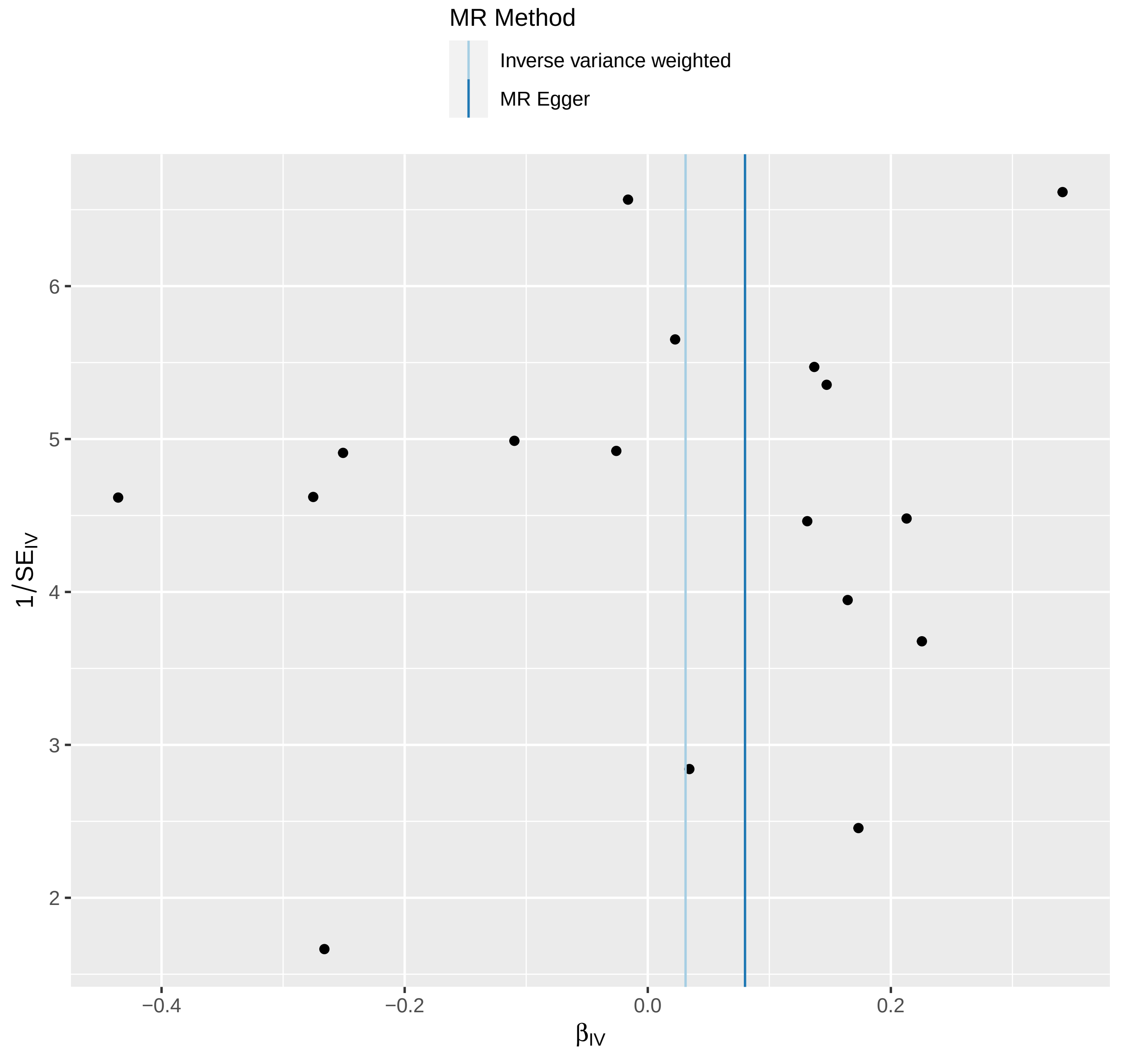

Batch 3−901 : Ephrin−A5 on Diabetic nephropathy (more control exclusions)
with a threshold of 1e−05

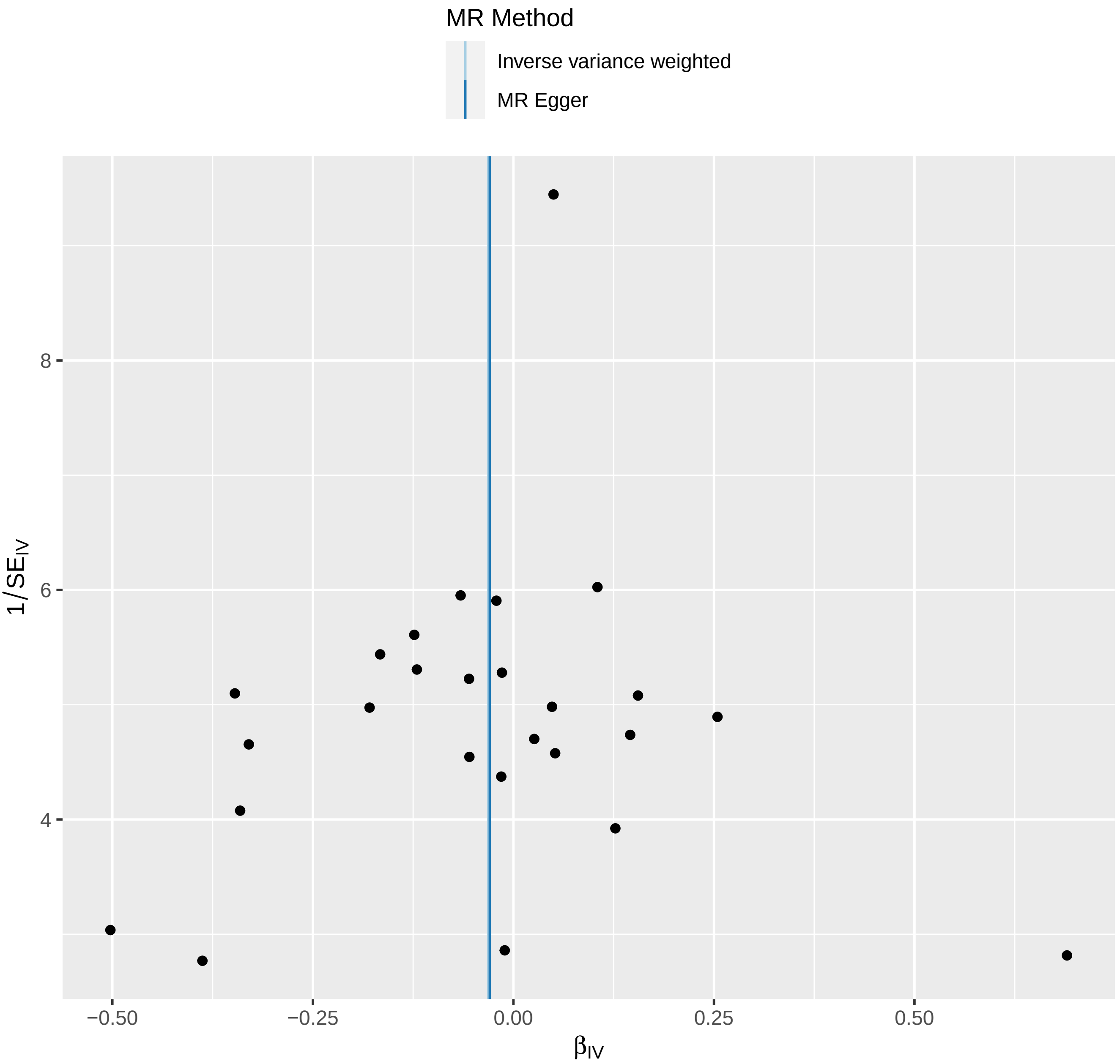

Batch 3−902 : Ephrin−B1 on Diabetic nephropathy (more control exclusions)
with a threshold of 1e−05

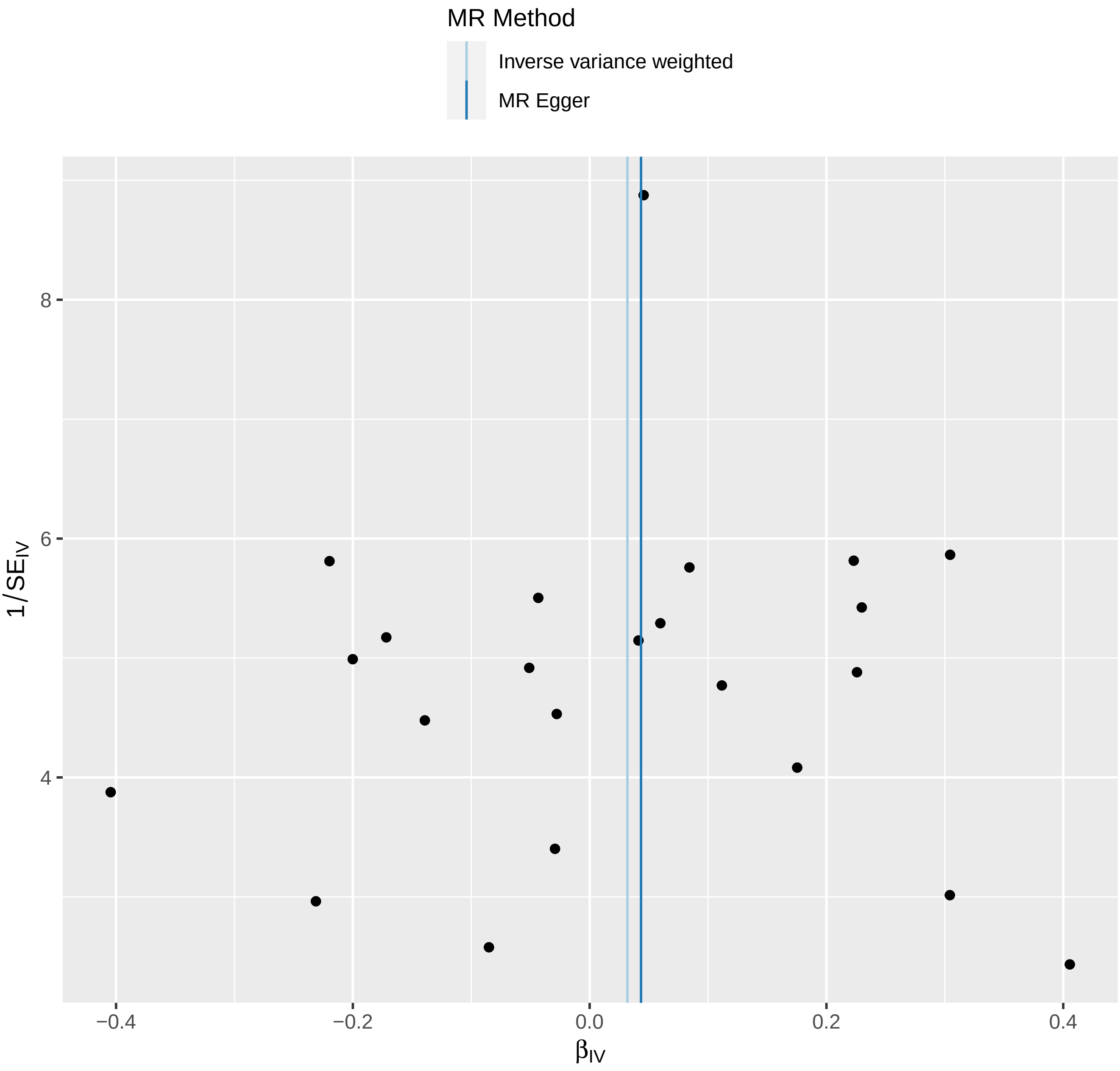

Batch 3−903 : Ephrin−B1 on Diabetic nephropathy (more control exclusions)
with a threshold of 1e−05

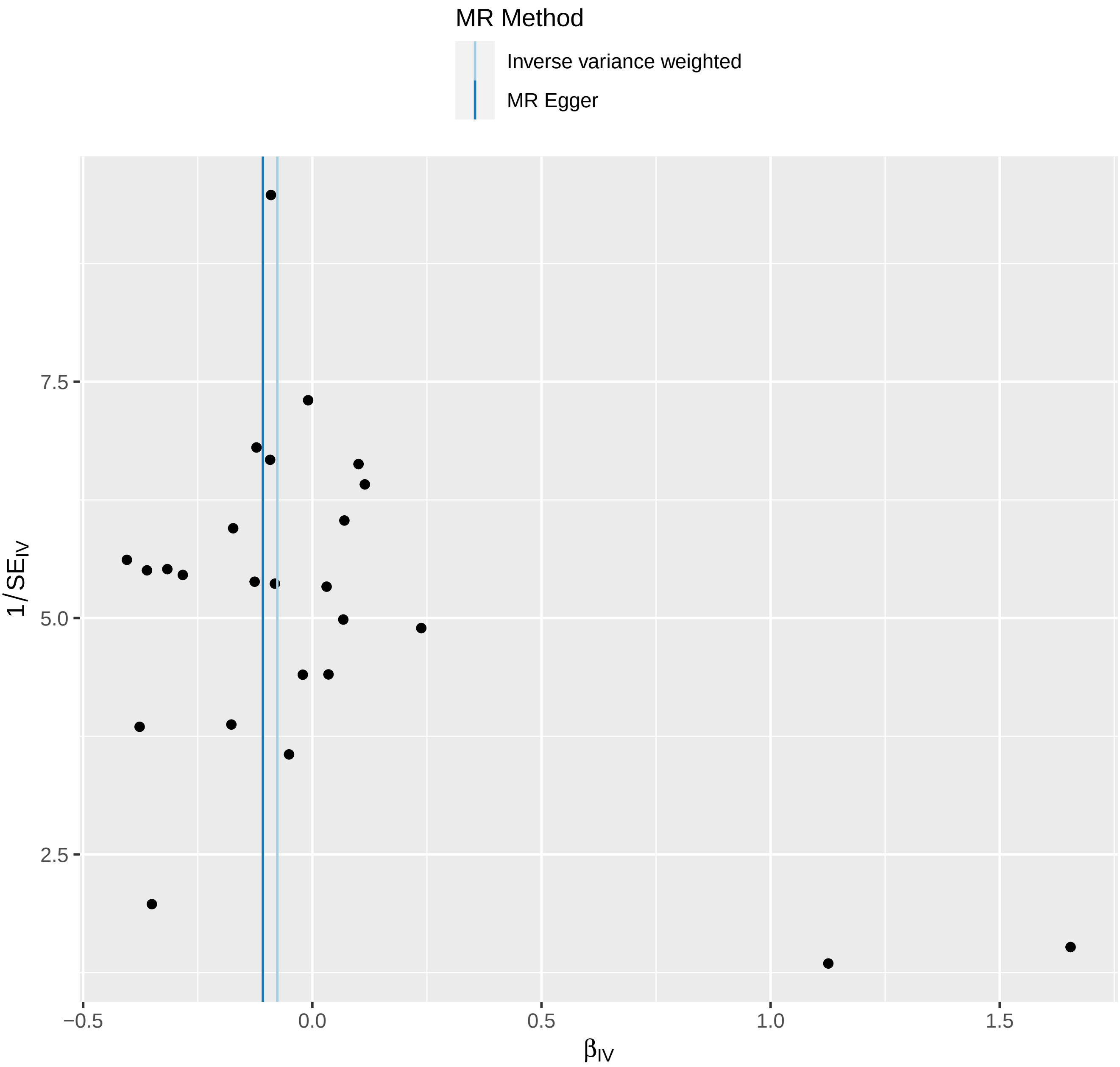

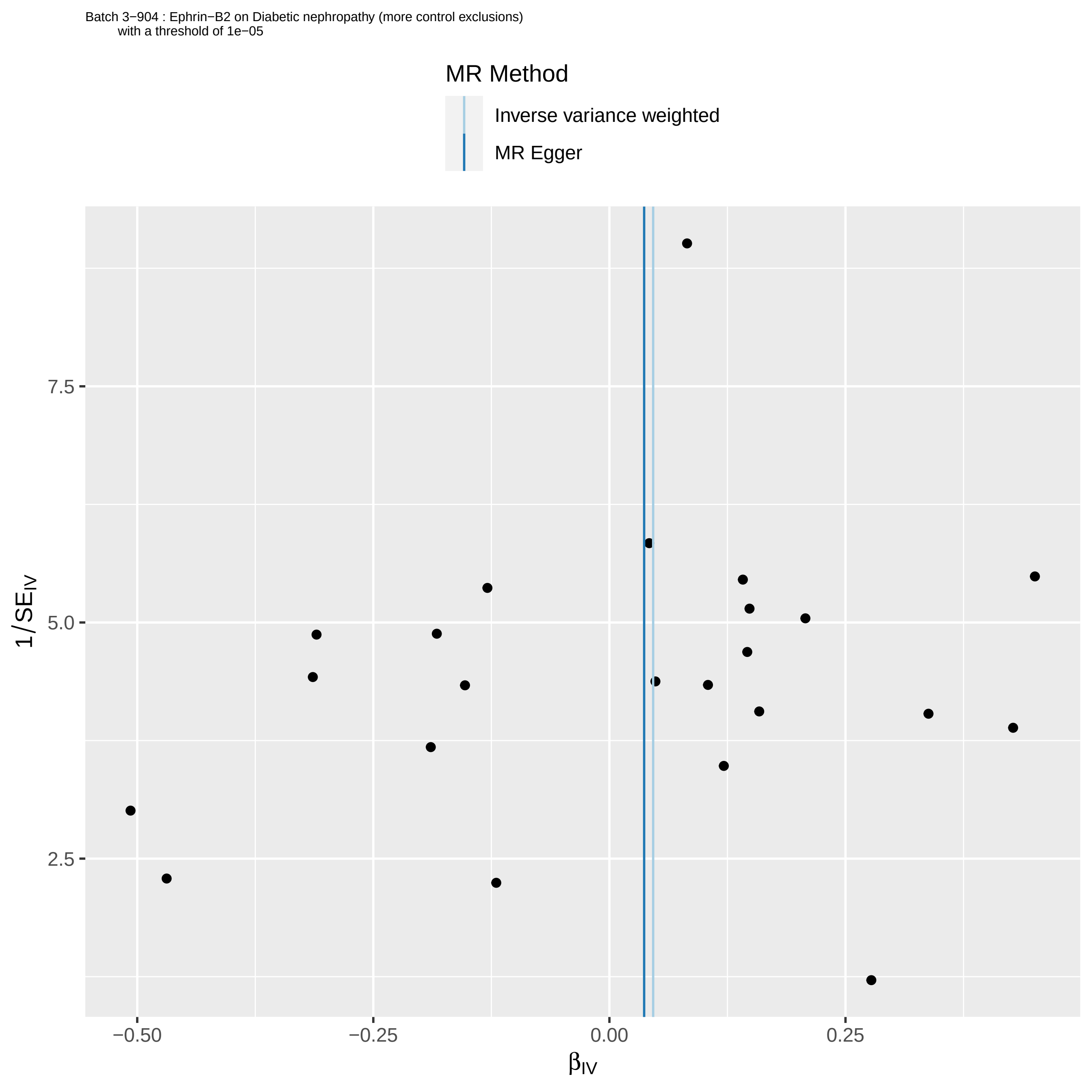
Batch 3−904 : Ephrin−B2 on Diabetic nephropathy (more control exclusions)
with a threshold of 1e−05
MR Method
Inverse variance weighted
MR Egger
7.5
5.0
2.5
1/SE$_{IV}$
−0.50
−0.25
0.00
0.25
β_{IV}

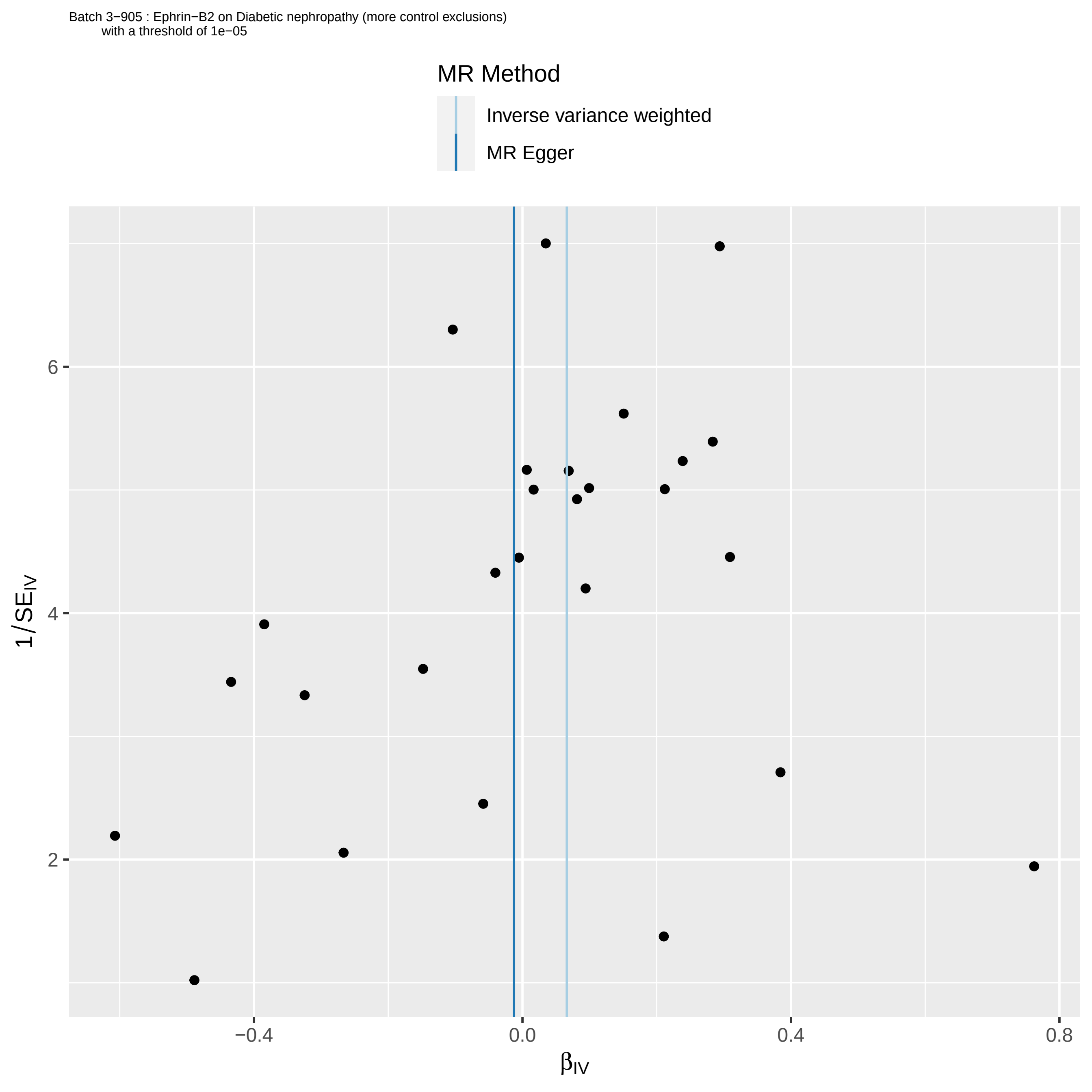
Batch 3−905 : Ephrin−B2 on Diabetic nephropathy (more control exclusions)
with a threshold of 1e−05
MR Method
Inverse variance weighted
MR Egger
6
4
2
$1/SE_{IV}$
−0.4
0.0
0.4
0.8
β_{IV}

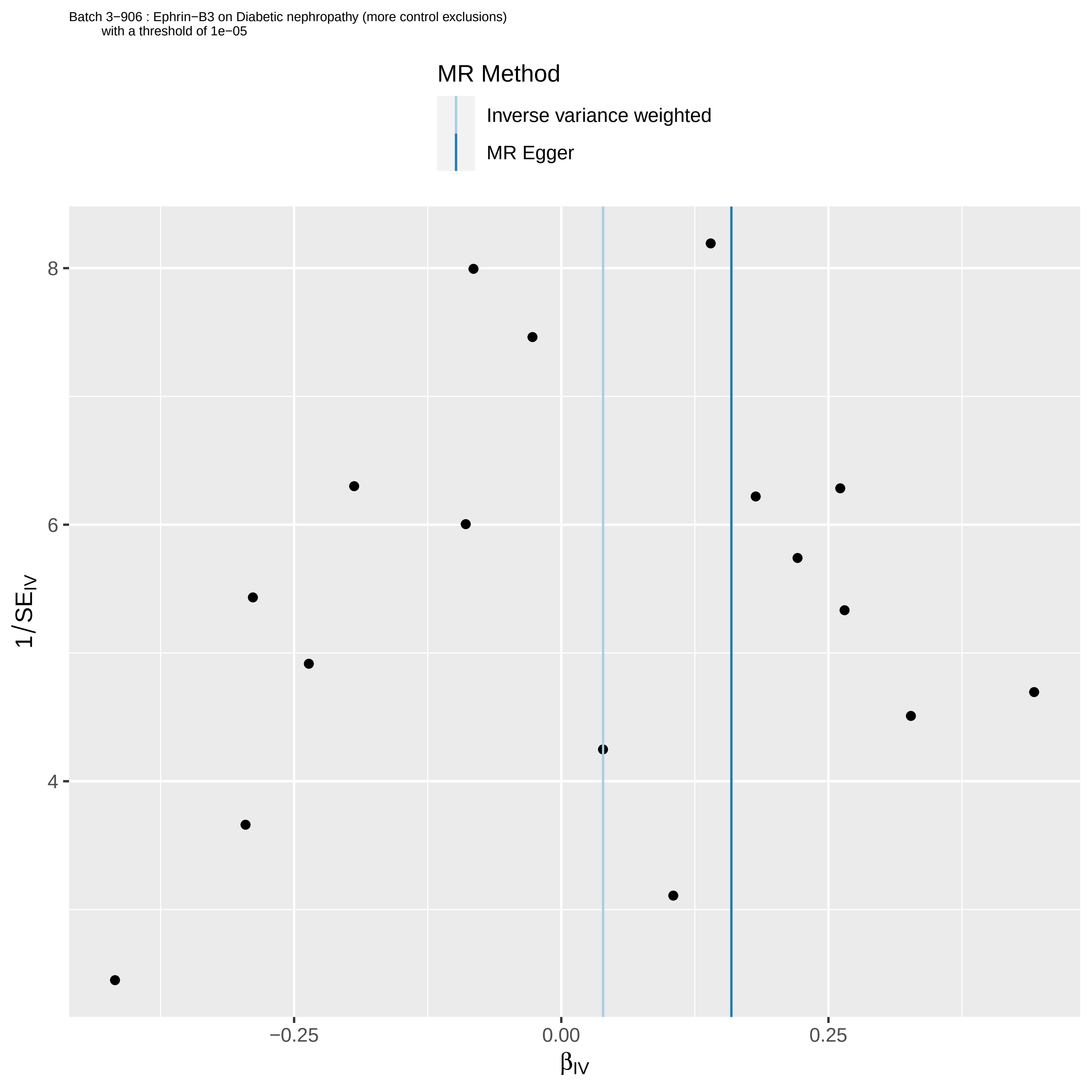

Batch 3−906 : Ephrin−B3 on Diabetic nephropathy (more control exclusions)
with a threshold of 1e−05
MR Method
Inverse variance weighted
MR Egger
8
6
4
1/SE_IV
−0.25
0.00
0.25
β_IV

Batch 3−907 : Ephrin−B3 on Diabetic nephropathy (more control exclusions)
with a threshold of 1e−05

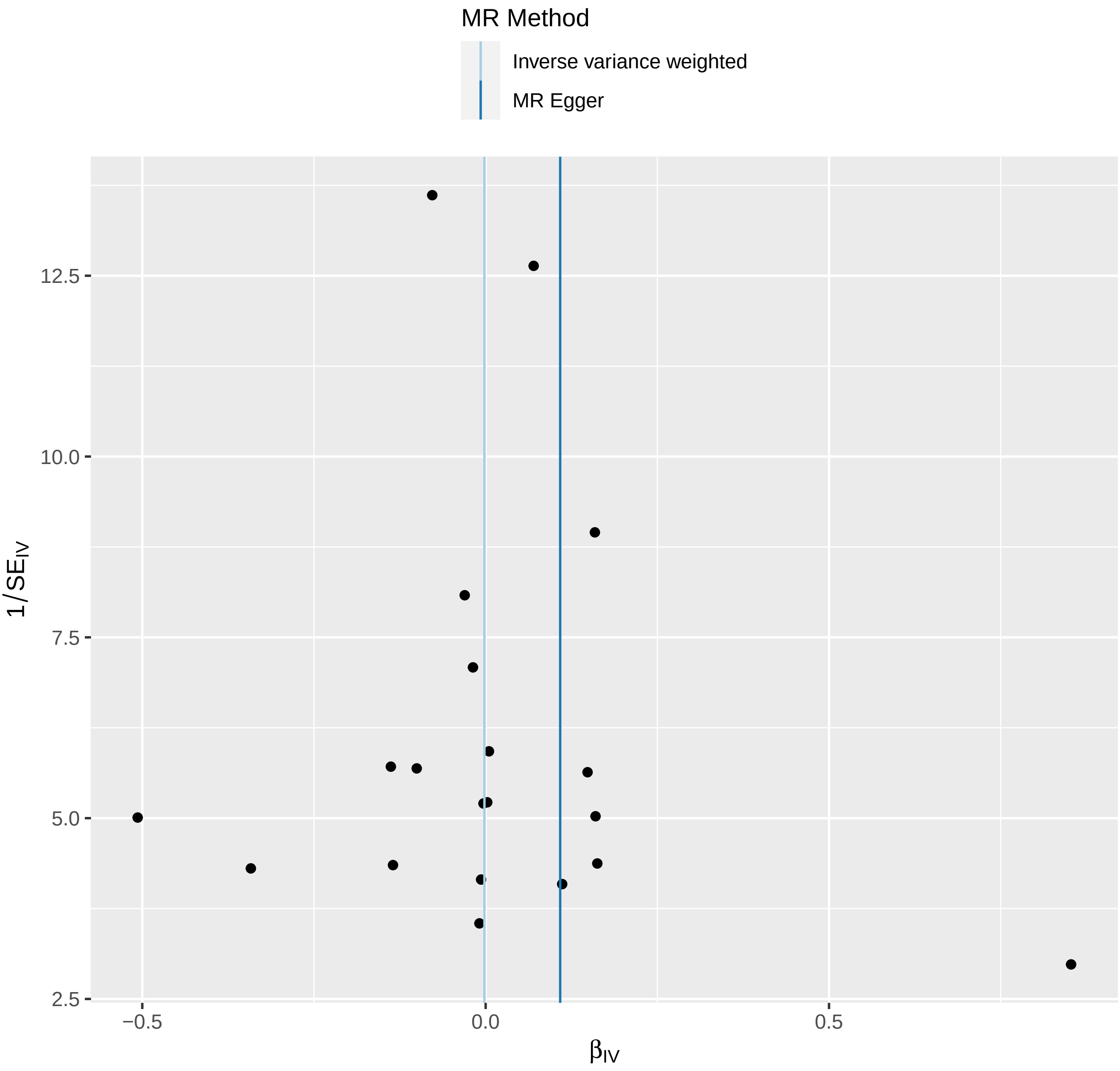

Batch 3−908 : Epidermal growth factor on Diabetic nephropathy (more control exclusions)
with a threshold of 1e−05

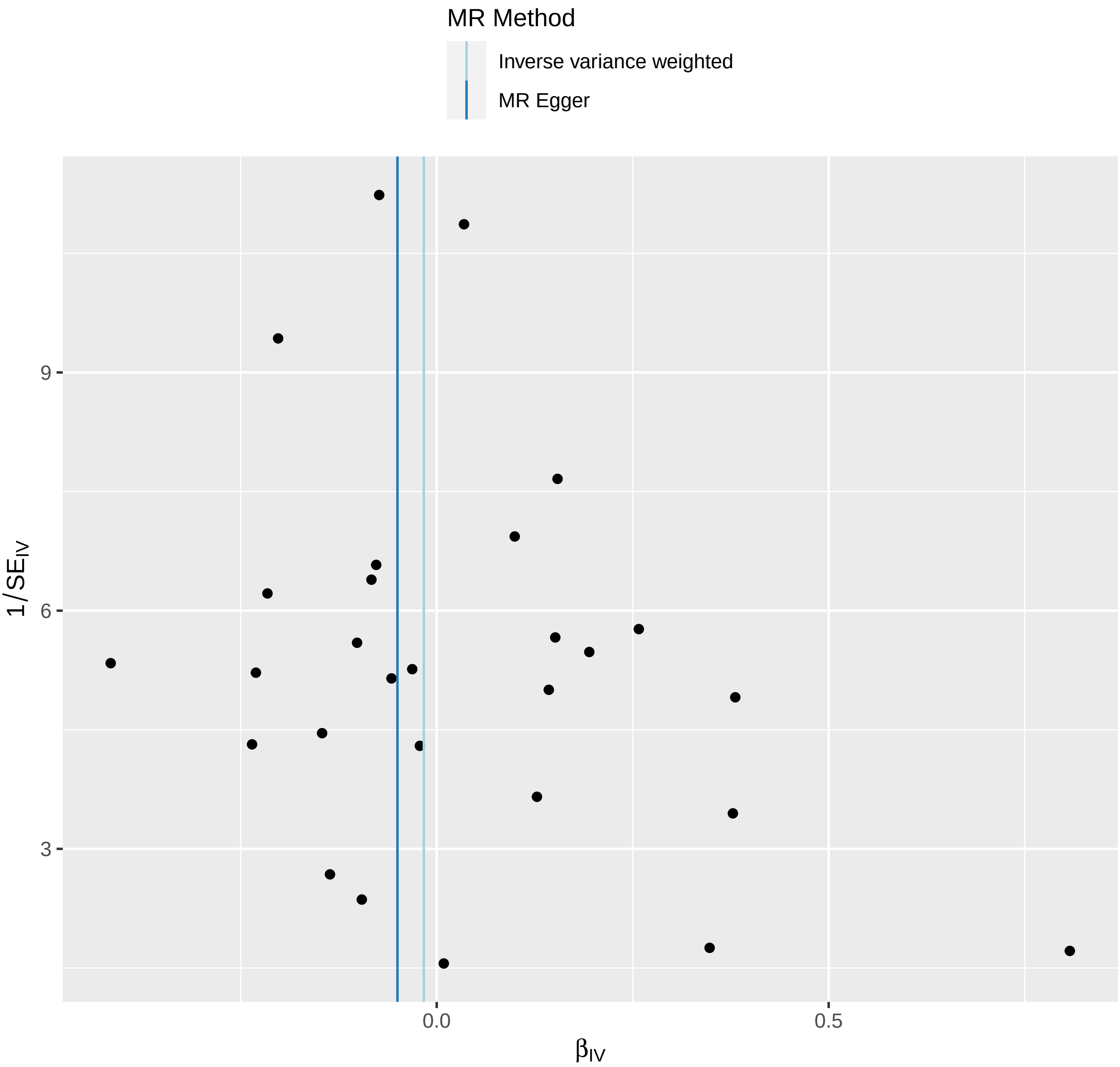

Batch 3−909 : Epidermal growth factor receptor on Diabetic nephropathy (more control exclusions)
with a threshold of 1e−05

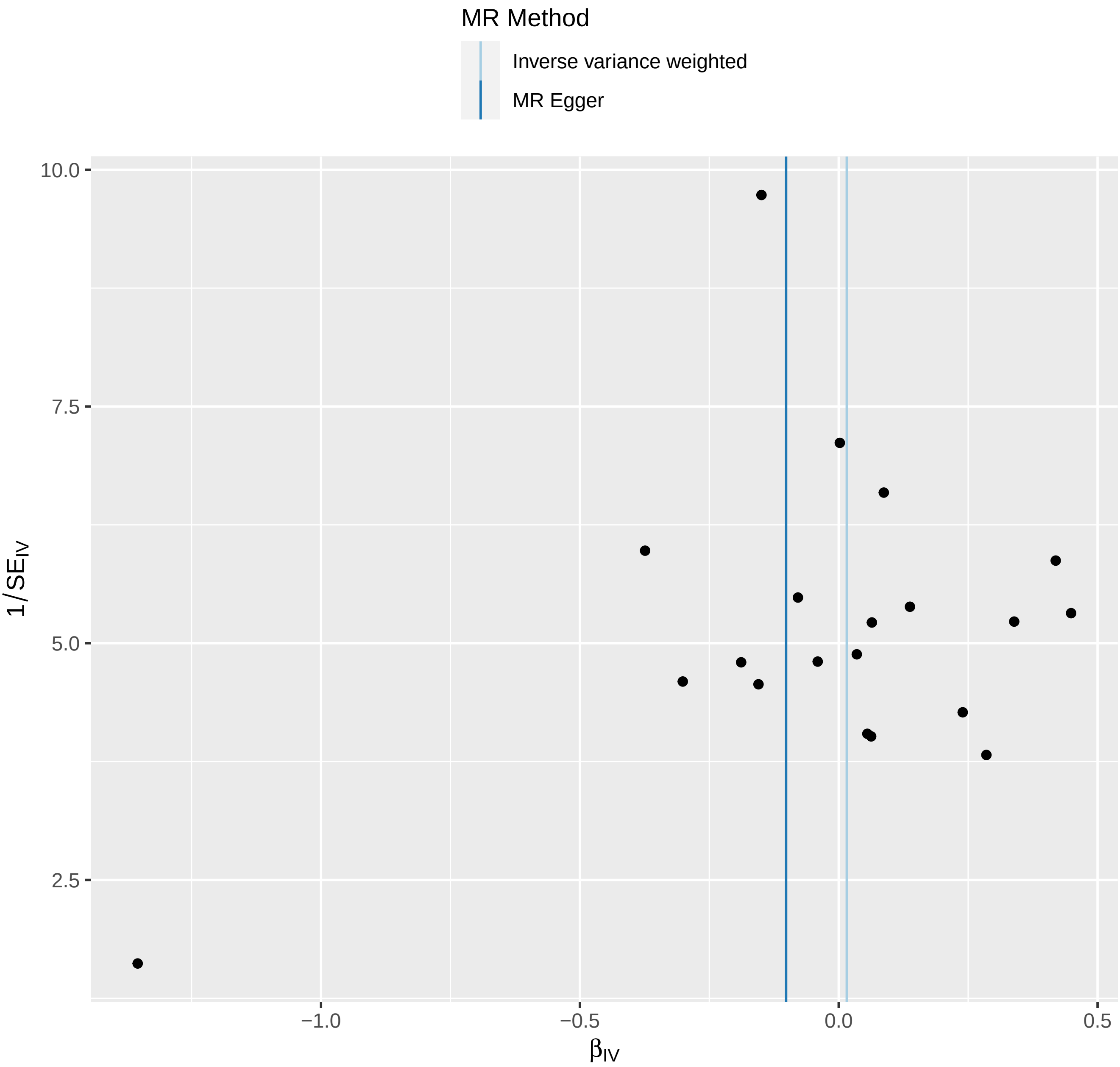

Batch 3−910 : Epidermal growth factor receptor variant III on Diabetic nephropathy (more control exclusions)
with a threshold of 1e−05

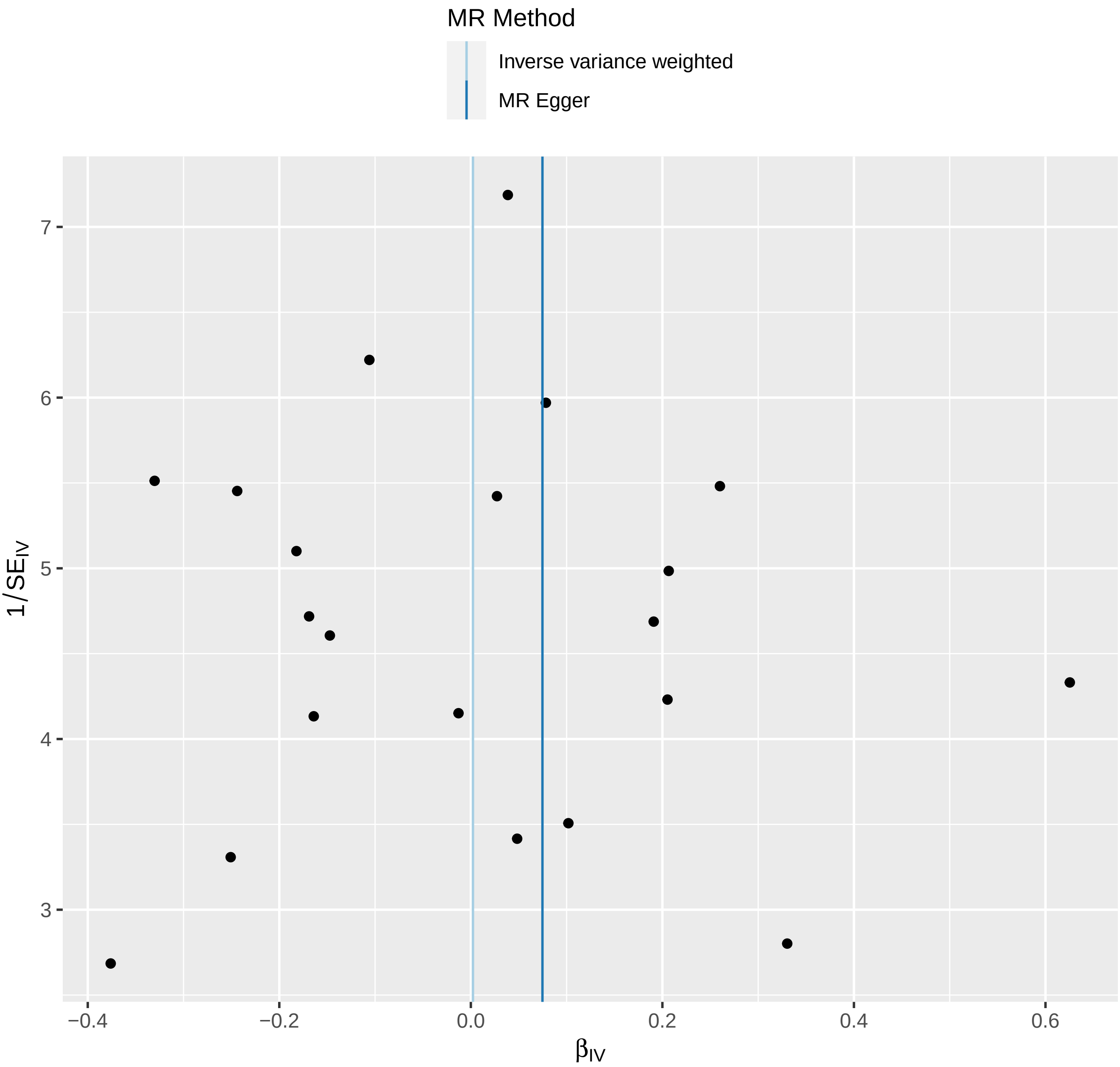

Batch 3−911 : EH domain−binding protein 1 on Diabetic nephropathy (more control exclusions)
with a threshold of 1e−05

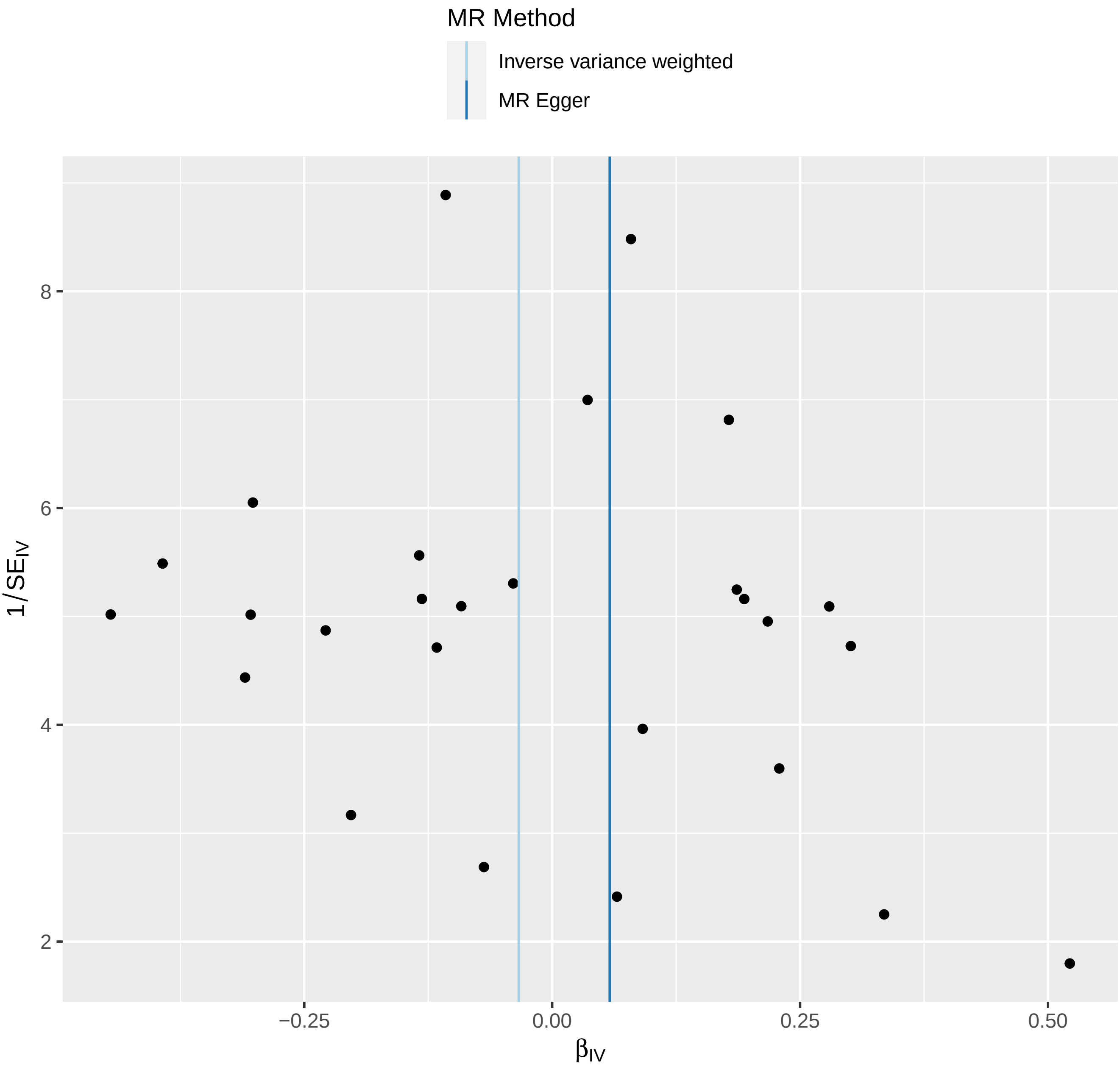

Batch 3−912 : EH domain−containing protein 4 on Diabetic nephropathy (more control exclusions)
with a threshold of 1e−05

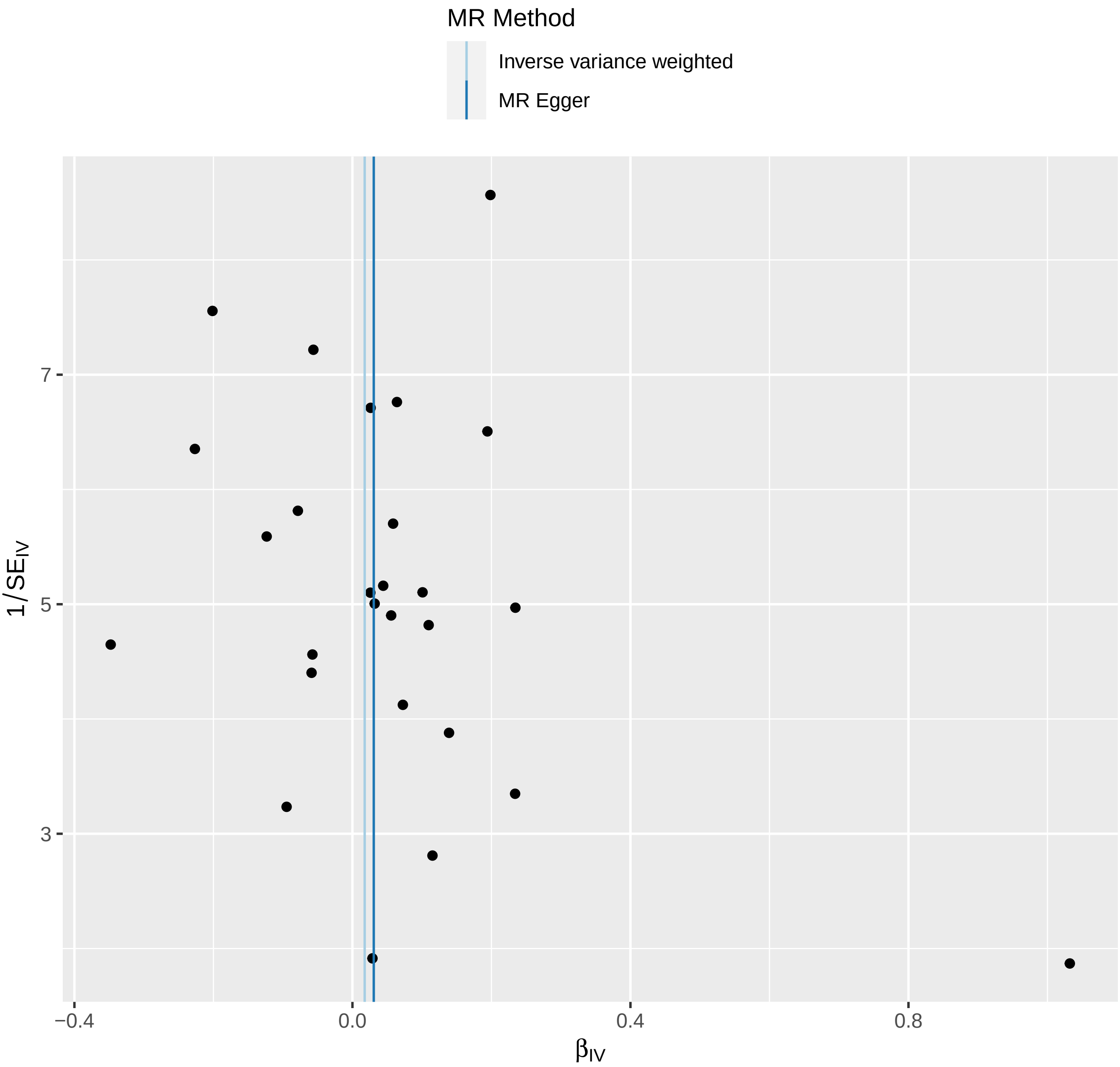

Batch 3−913 : ETS homologous factor on Diabetic nephropathy (more control exclusions)
with a threshold of 1e−05

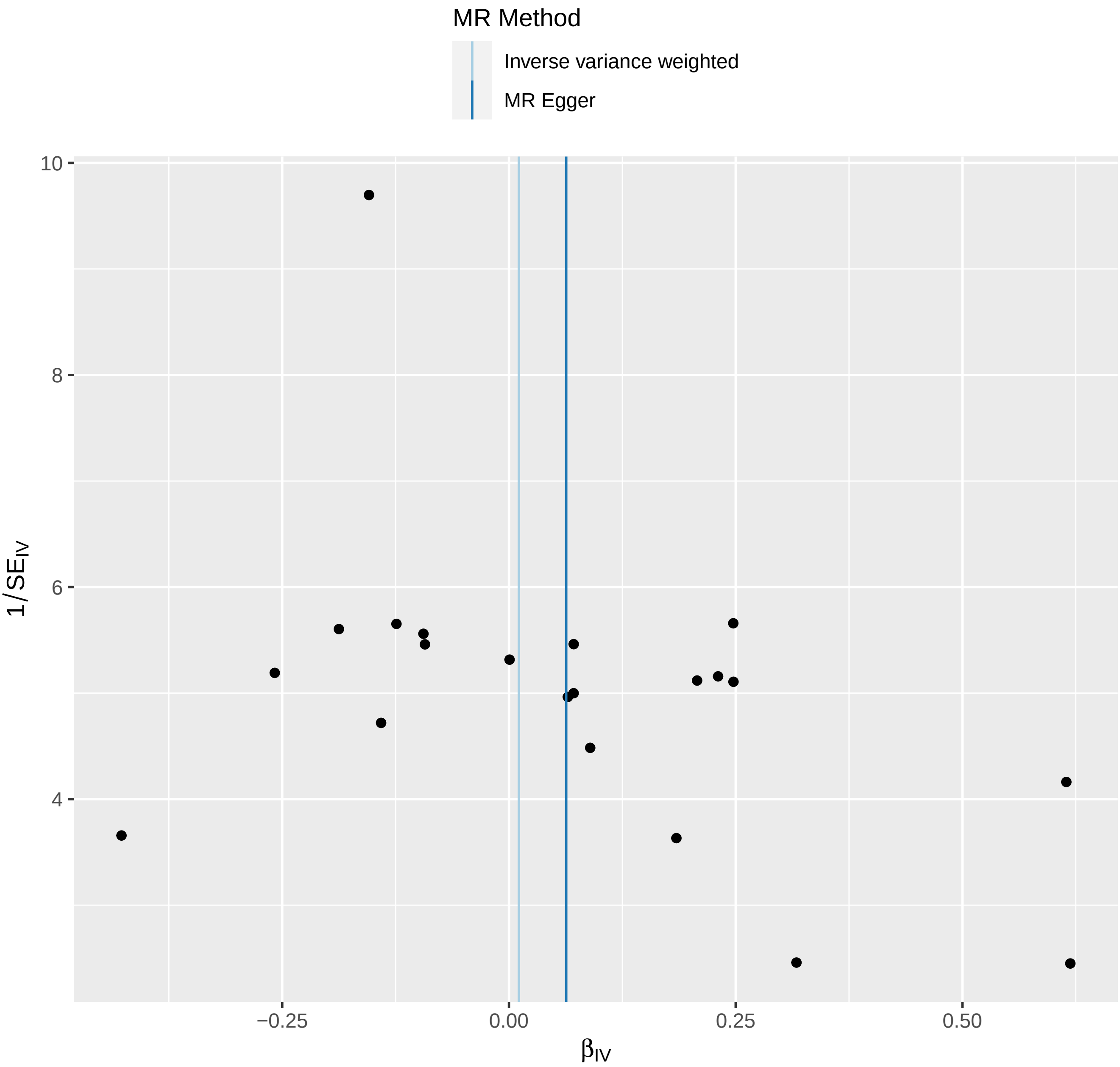

Batch 3−914 : Histone−lysine N−methyltransferase EHMT2 on Diabetic nephropathy (more control exclusions)
with a threshold of 1e−05

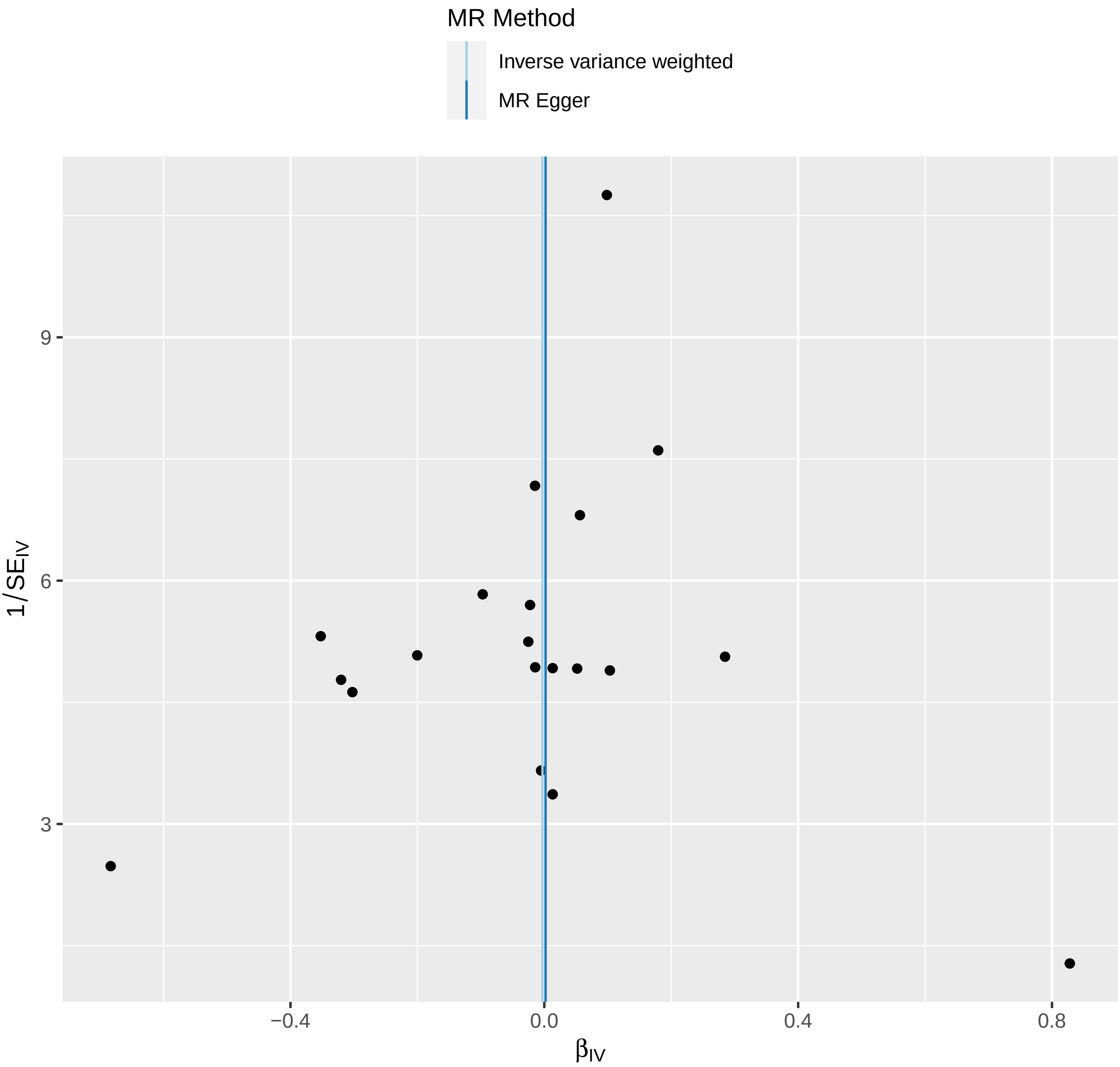

Batch 3−915 : EP300−interacting inhibitor of differentiation 3 on Diabetic nephropathy (more control exclusions)
with a threshold of 1e−05

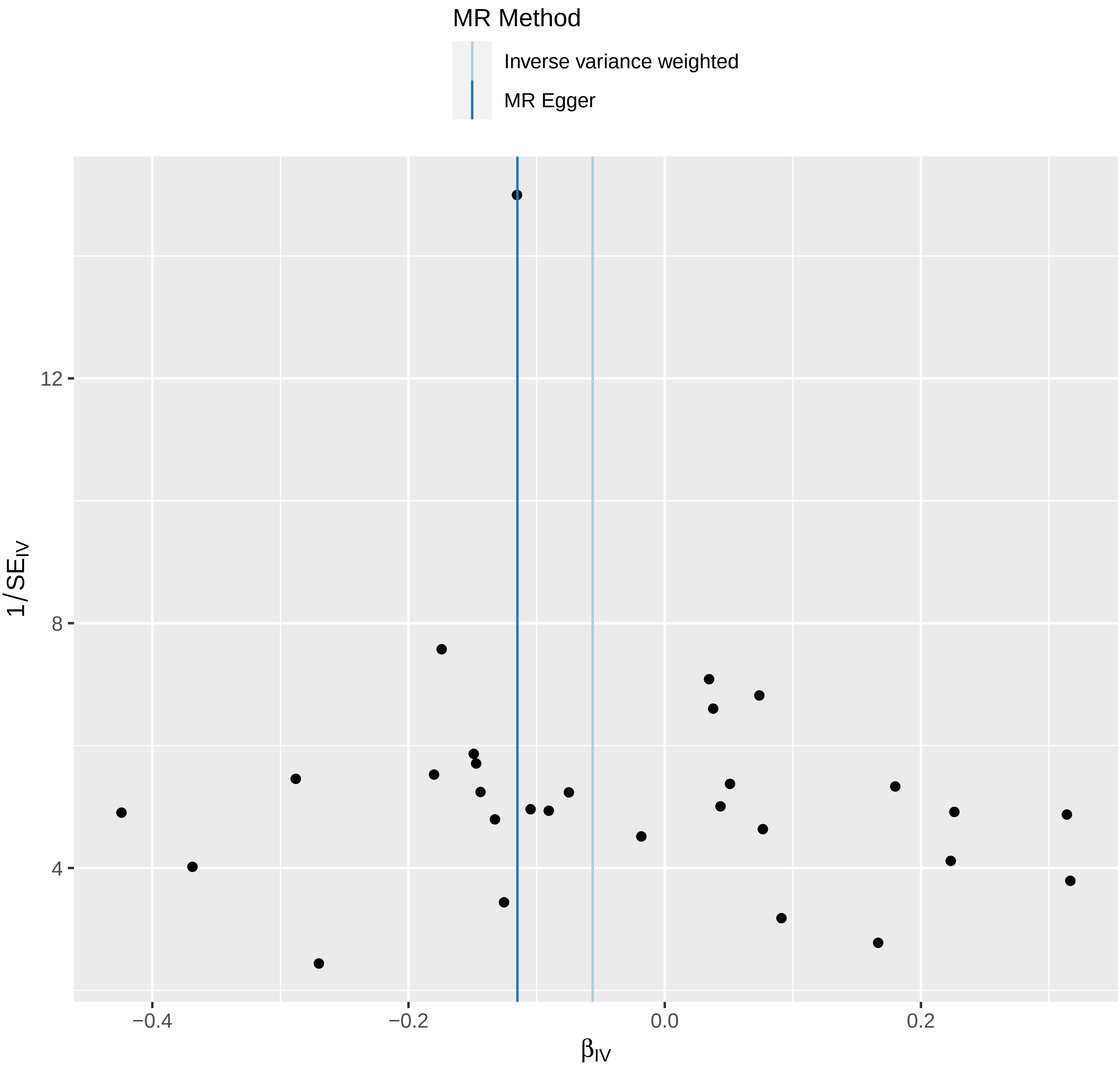

Batch 3−916 : Probable RNA−binding protein EIF1AD on Diabetic nephropathy (more control exclusions)
with a threshold of 1e−05

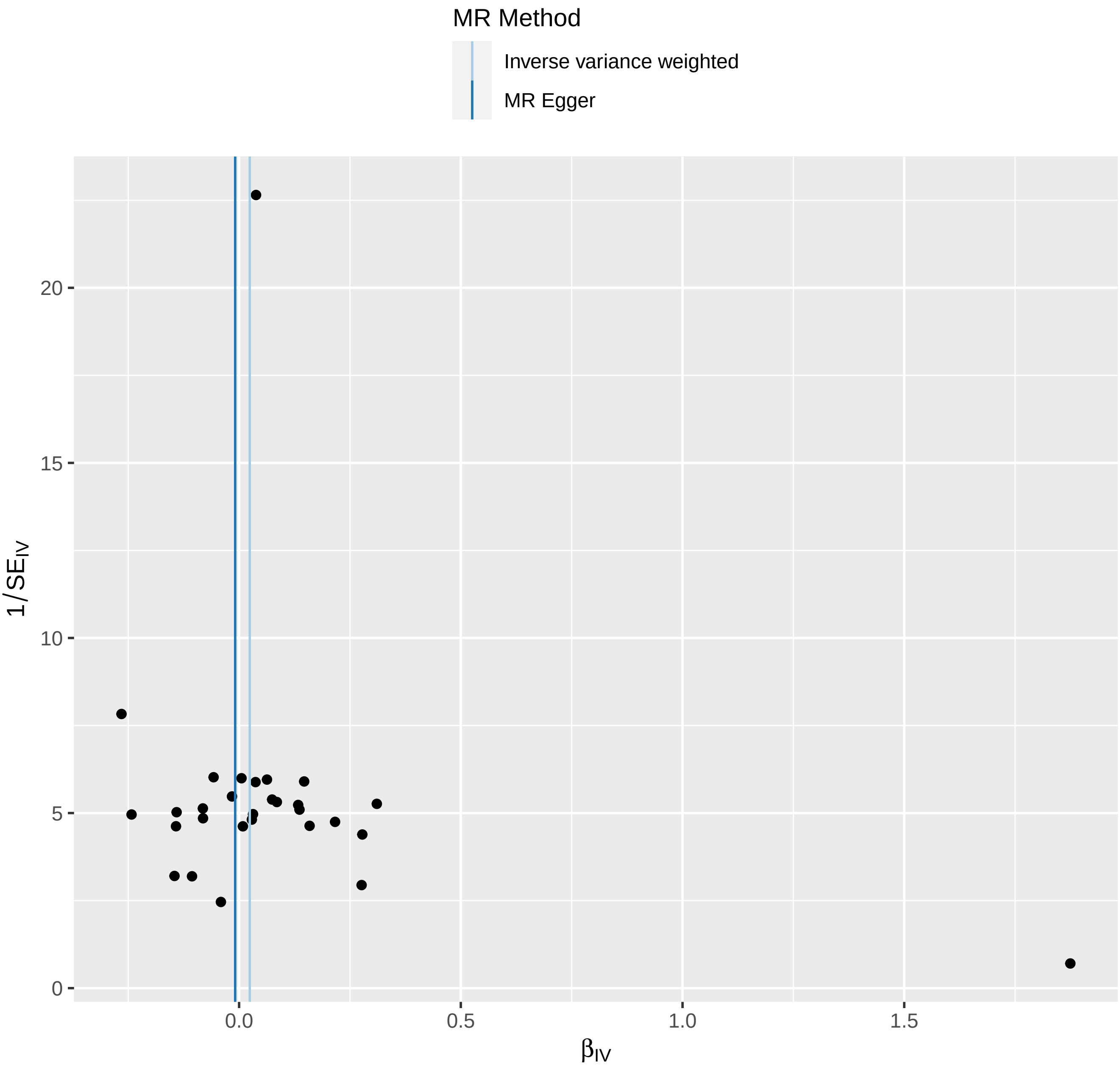

Batch 3−917 : Eukaryotic translation initiation factor 1A, X−chromosomal on Diabetic nephropathy (more control exclusions)
with a threshold of 1e−05

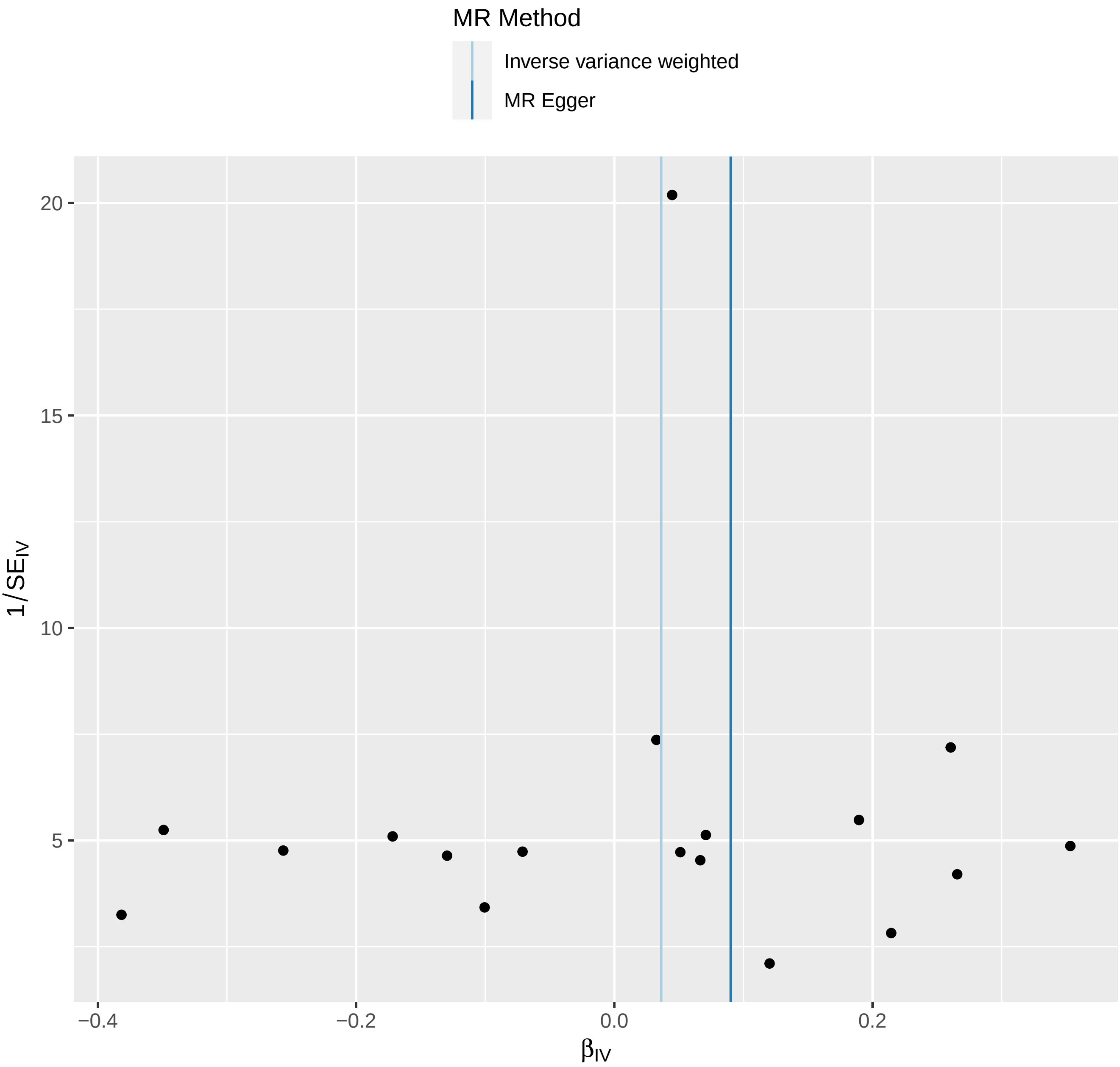

Batch 3−918 : Translation initiation factor eIF−2B subunit alpha on Diabetic nephropathy (more control exclusions) with a threshold of 1e−05

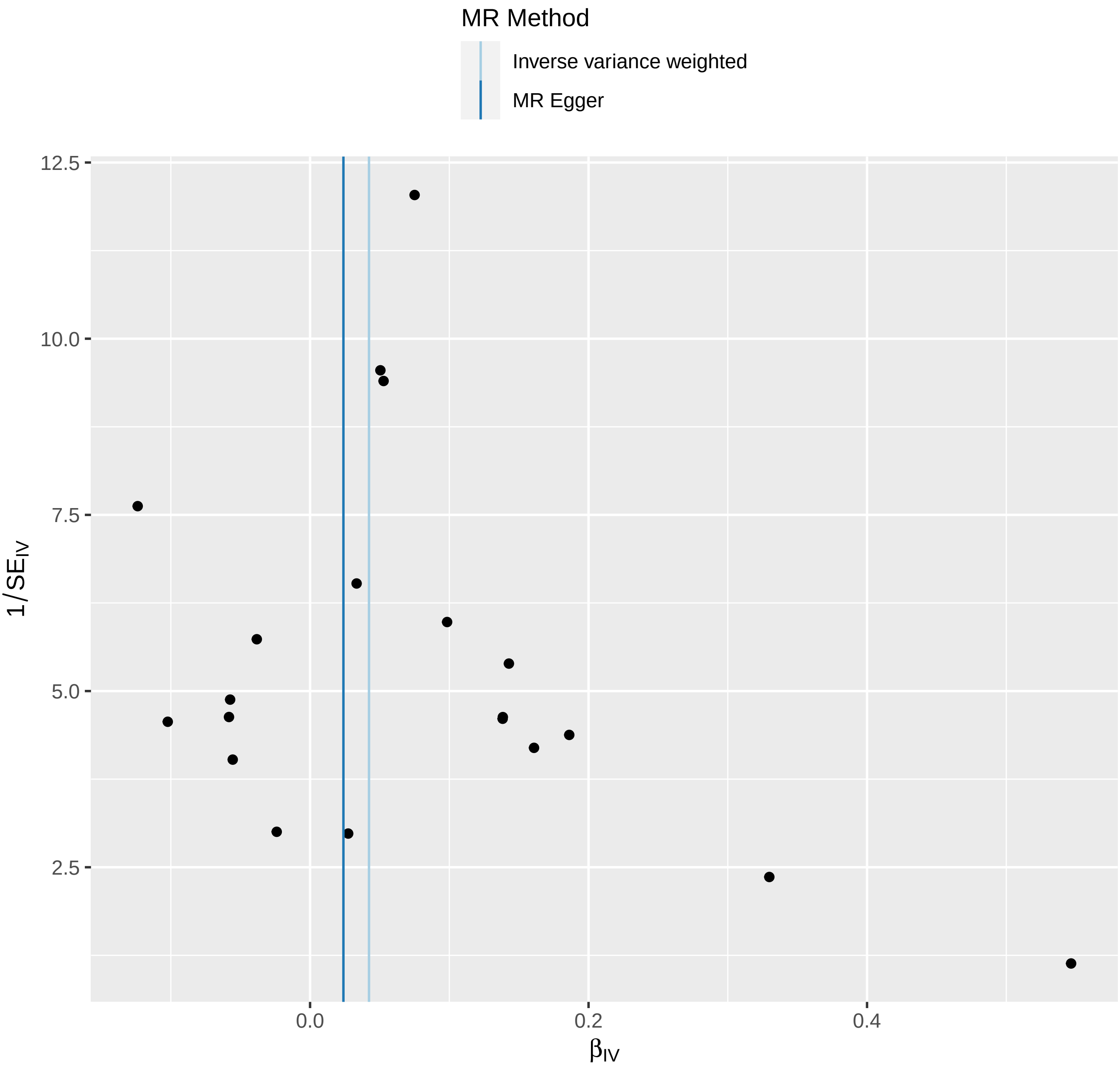

Batch 3−919 : Eukaryotic translation initiation factor 3 subunit G on Diabetic nephropathy (more control exclusions)
with a threshold of 1e−05

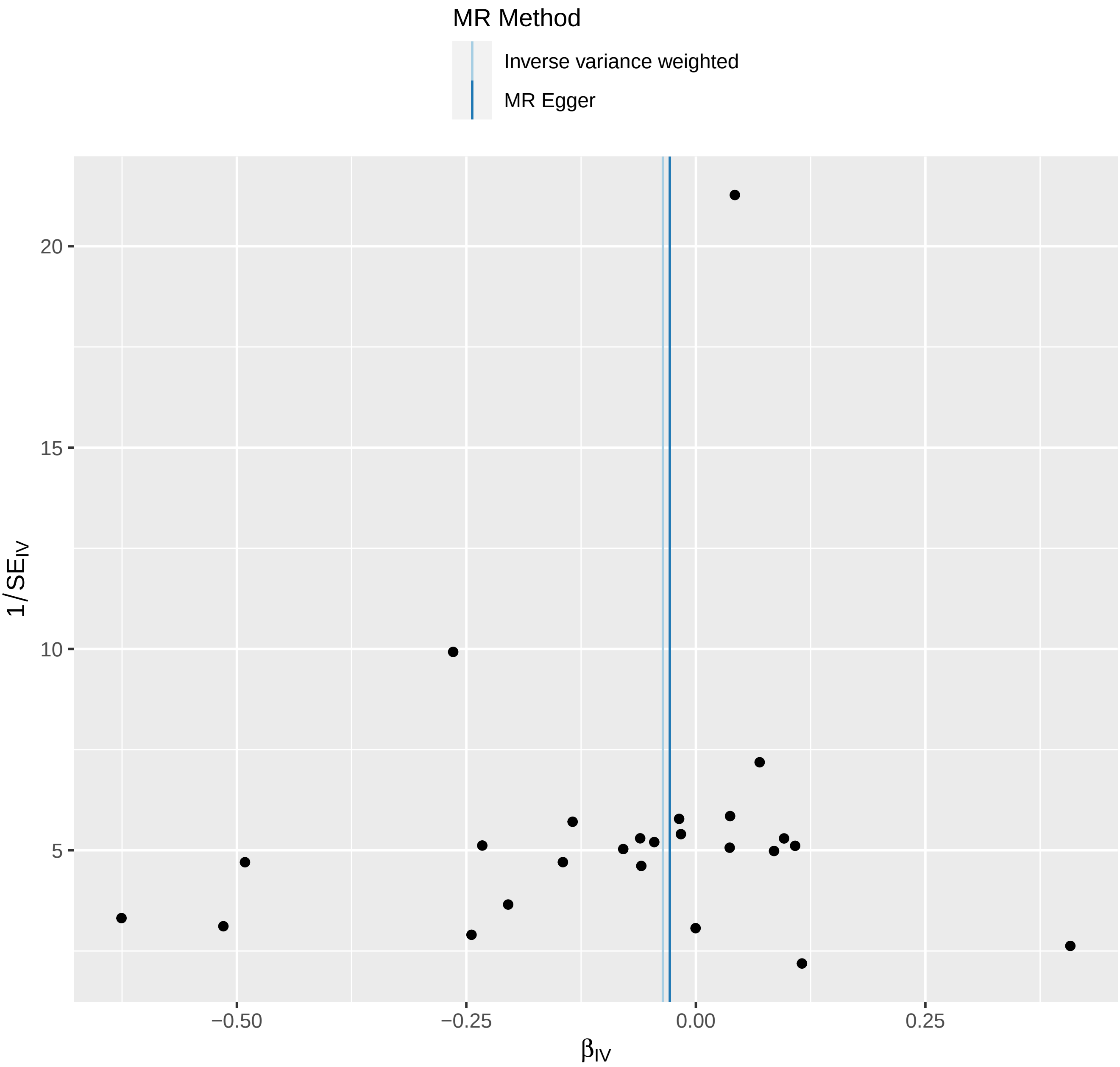

Batch 3−920 : Eukaryotic translation initiation factor 3 subunit J on Diabetic nephropathy (more control exclusions) with a threshold of 1e−05

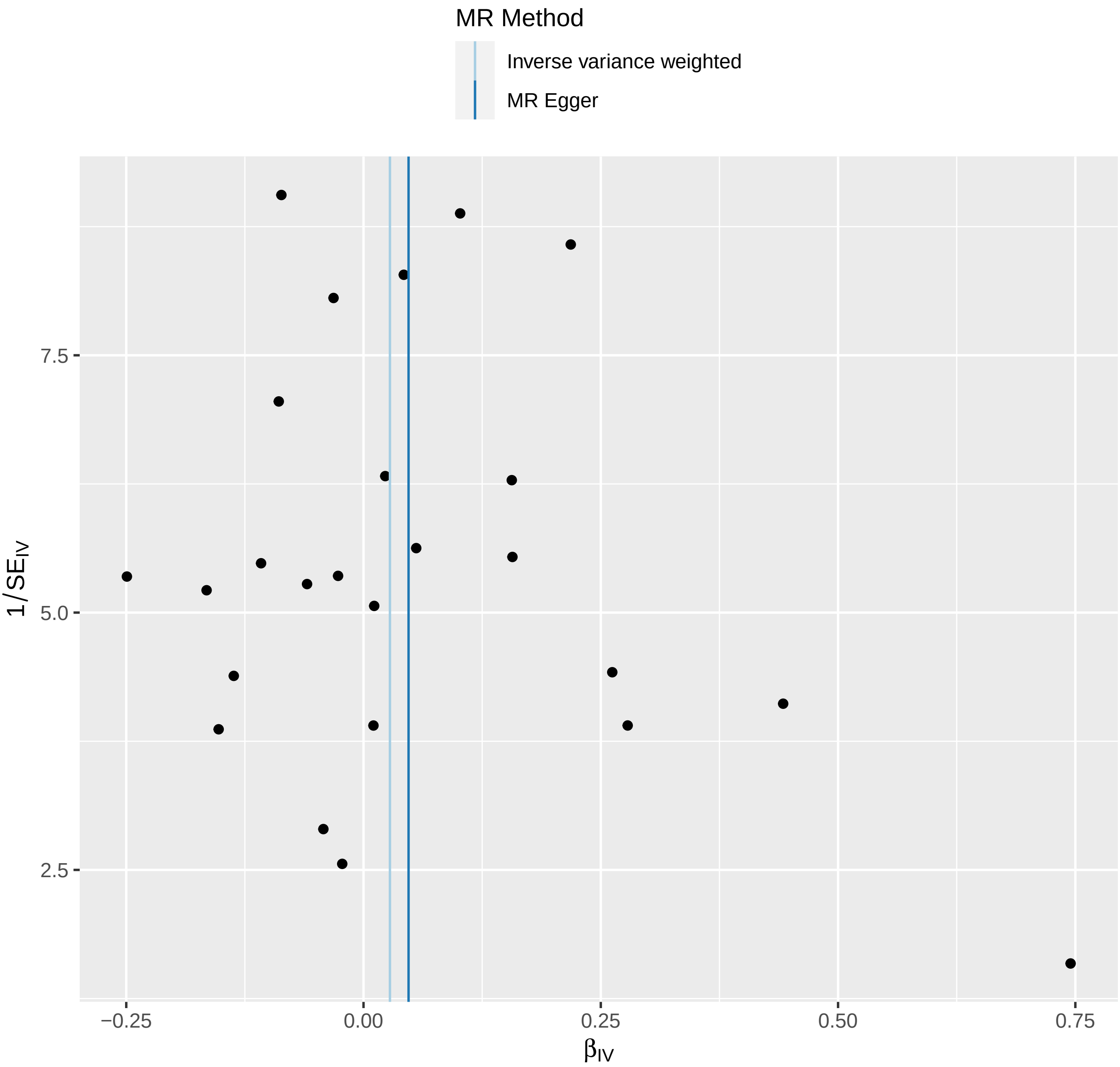

Batch 3−921 : Eukaryotic initiation factor 4A−III on Diabetic nephropathy (more control exclusions) with a threshold of 1e−05

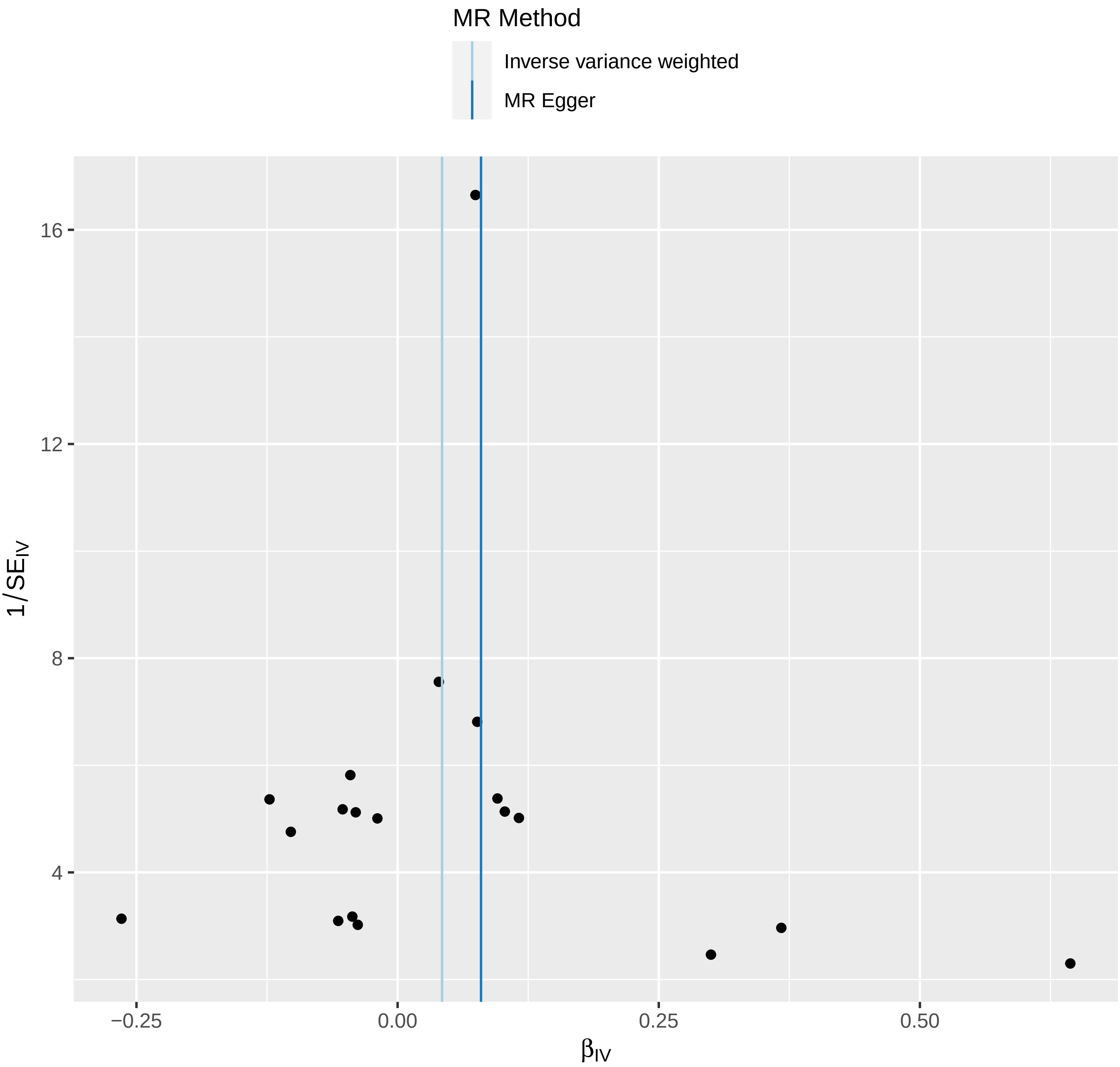

Batch 3−922 : Eukaryotic translation initiation factor 4B on Diabetic nephropathy (more control exclusions)
with a threshold of 1e−05

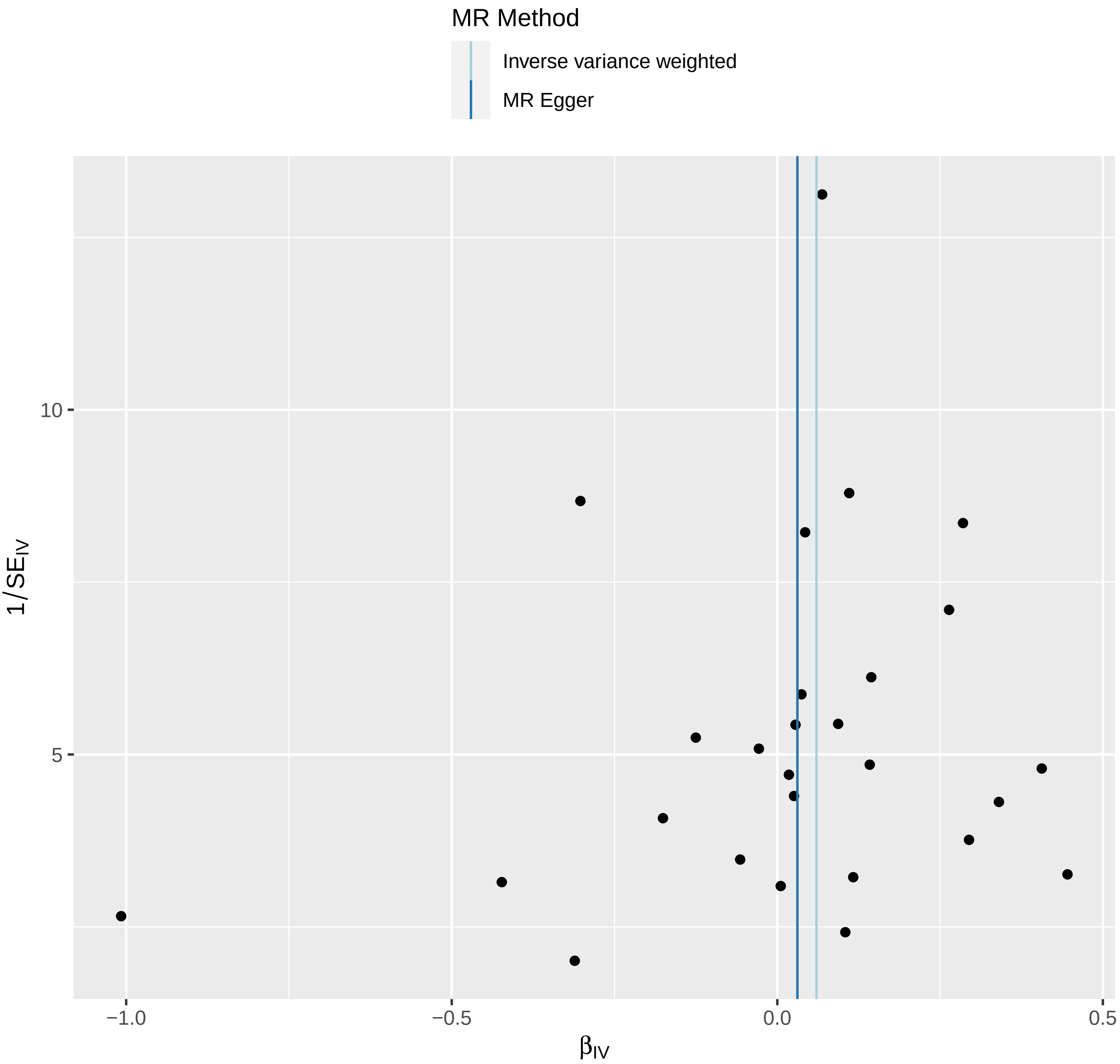

Batch 3−923 : Eukaryotic translation initiation factor 4E type 2 on Diabetic nephropathy (more control exclusions)
with a threshold of 1e−05

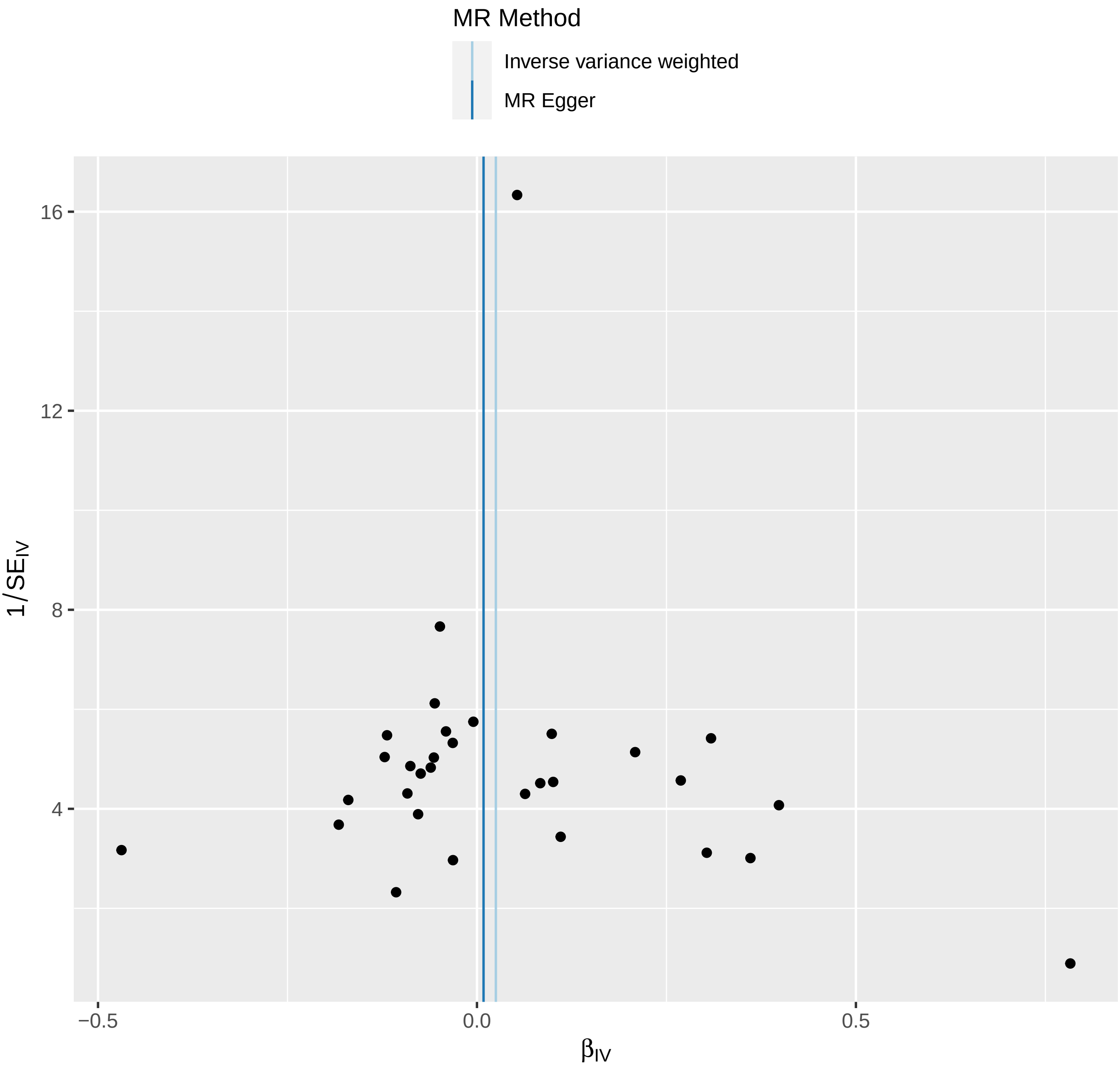

Batch 3−924 : Eukaryotic translation initiation factor 4E−binding protein 2 on Diabetic nephropathy (more control exclusions)
with a threshold of 1e−05

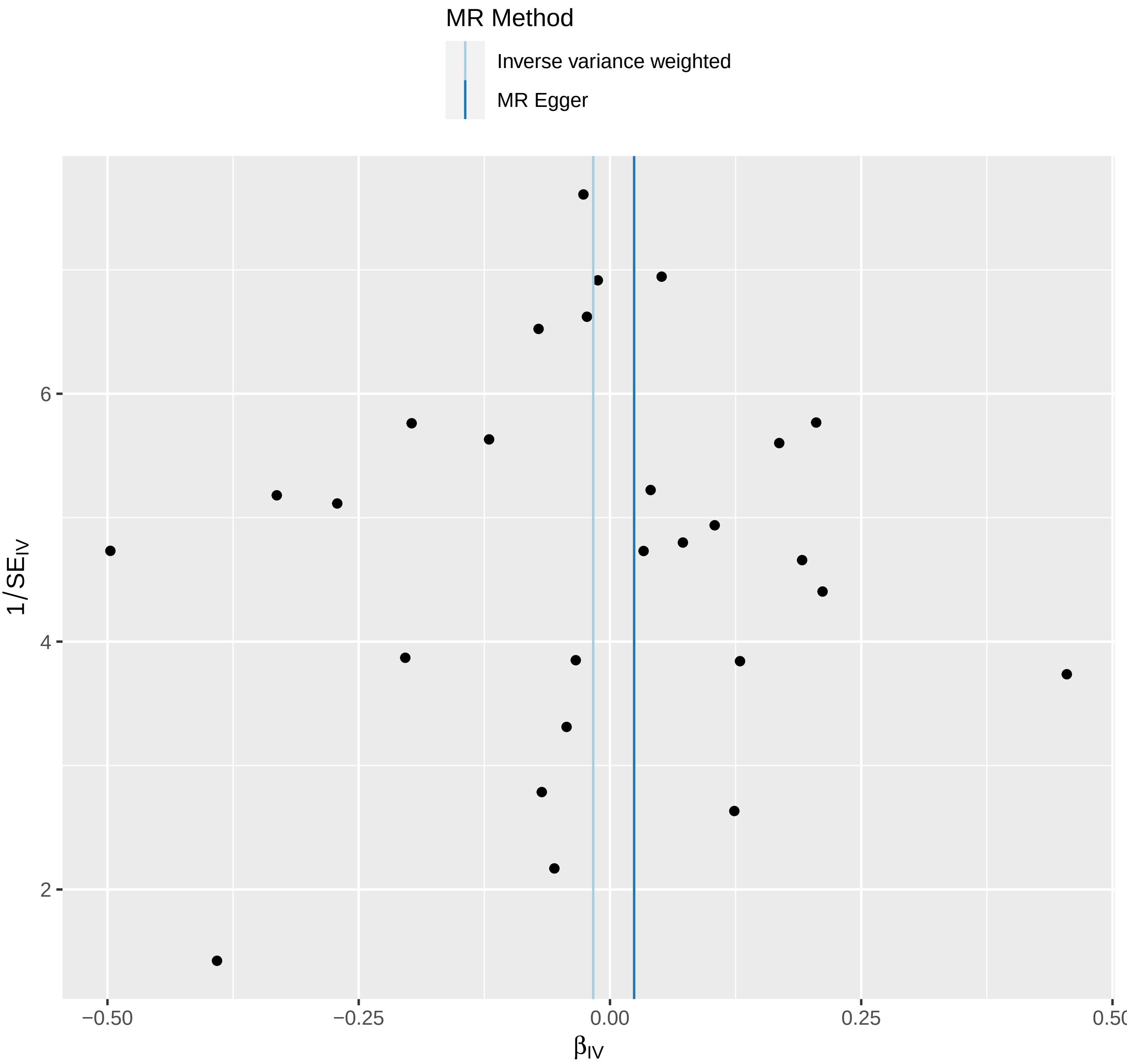

Batch 3−925 : Eukaryotic translation initiation factor 4 gamma 3 on Diabetic nephropathy (more control exclusions)
with a threshold of 1e−05

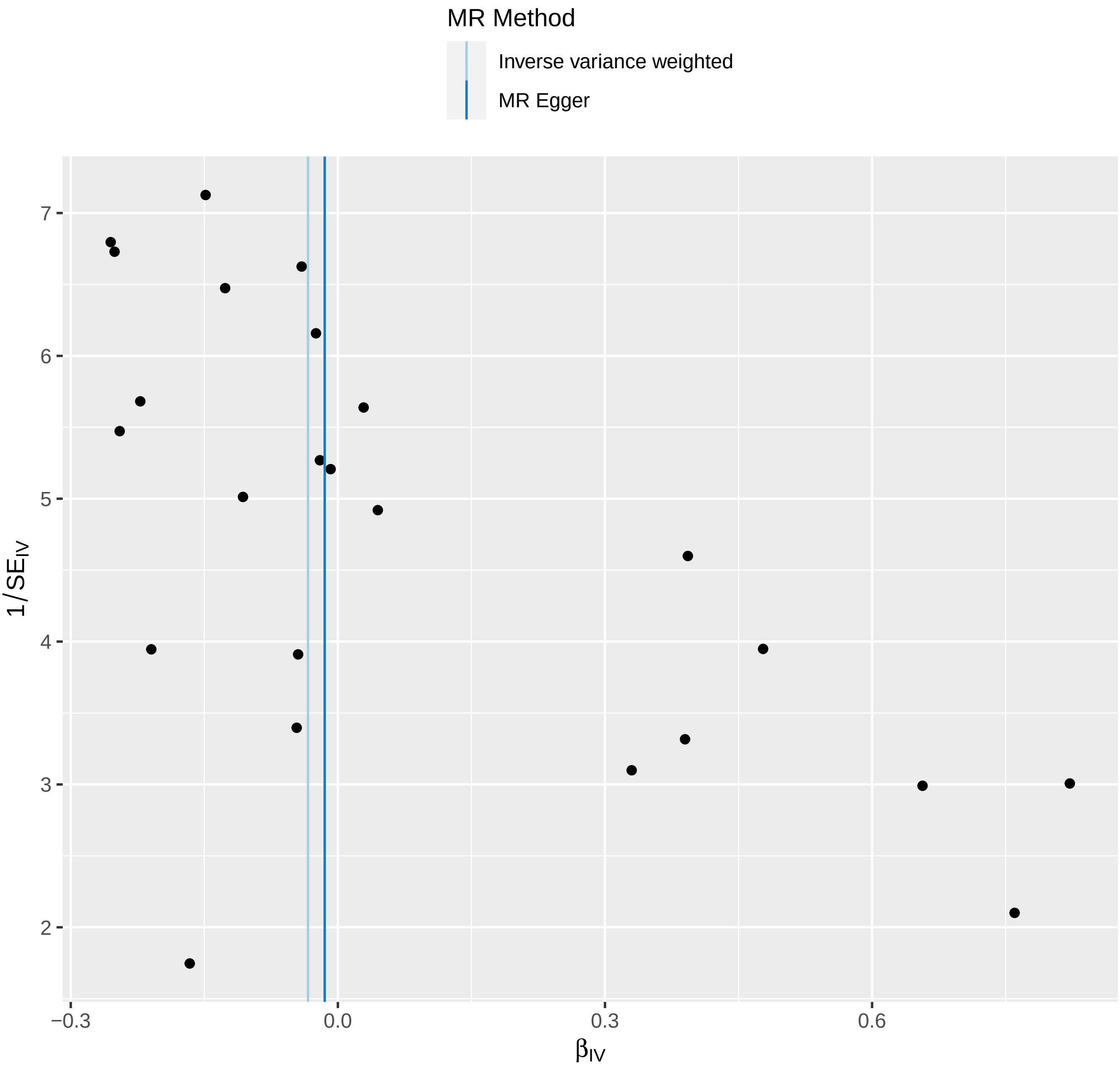

Batch 3−926 : Eukaryotic translation initiation factor 5 on Diabetic nephropathy (more control exclusions)
with a threshold of 1e−05

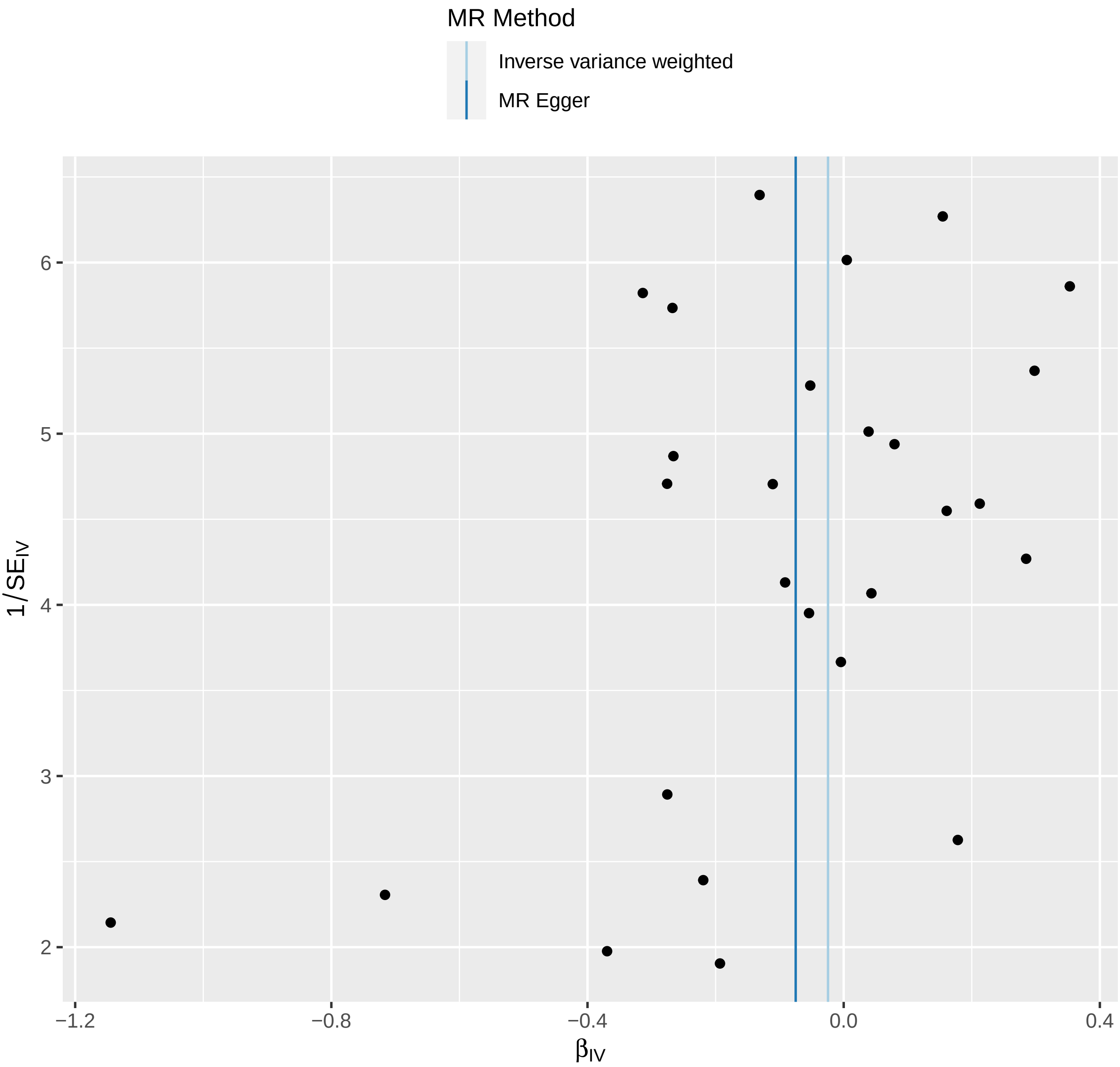

Batch 3−927 : Eukaryotic translation initiation factor 5A−1 on Diabetic nephropathy (more control exclusions)
with a threshold of 1e−05

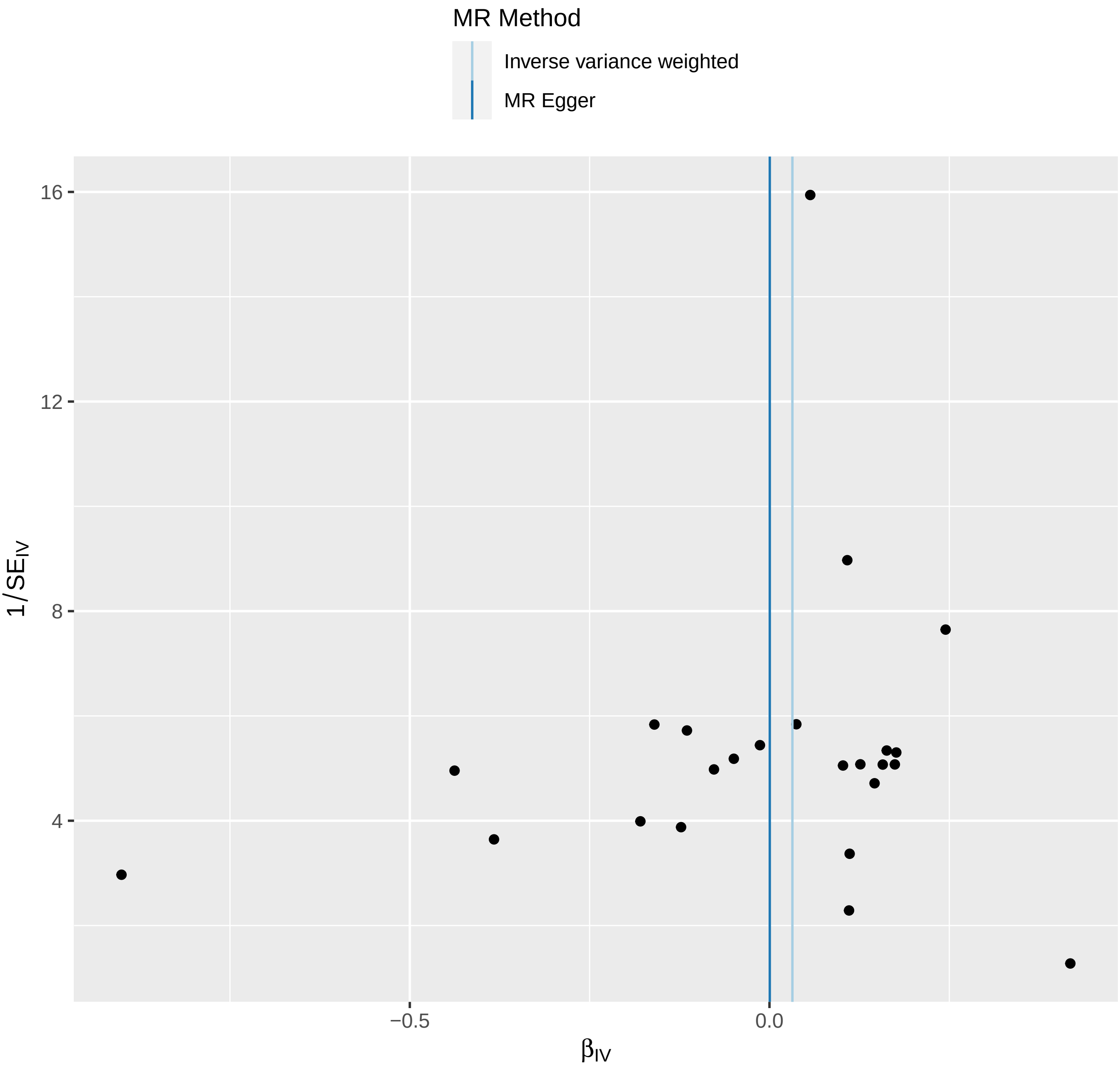

Batch 3−928 : ELAV−like protein 1 on Diabetic nephropathy (more control exclusions)
with a threshold of 1e−05

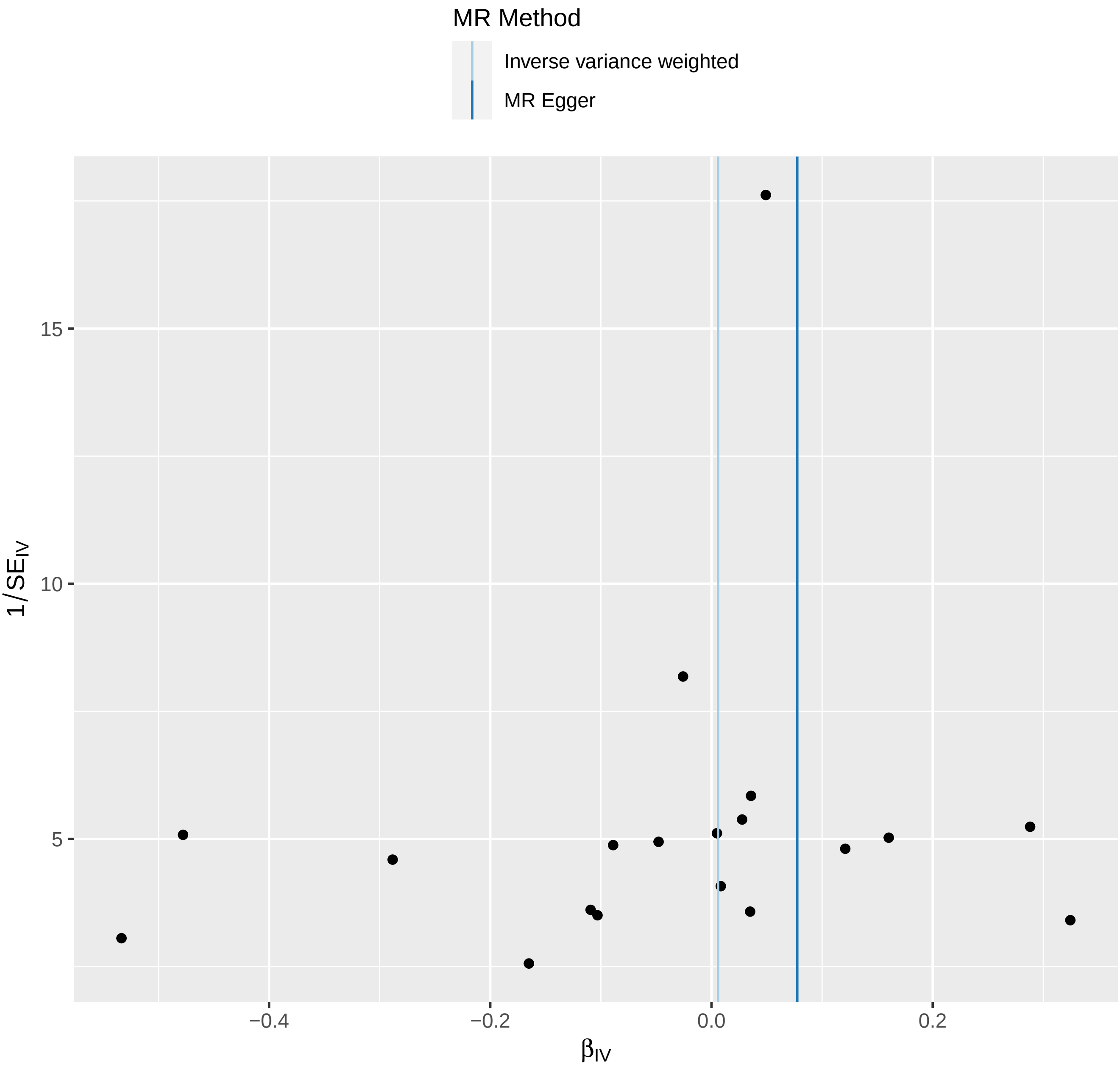

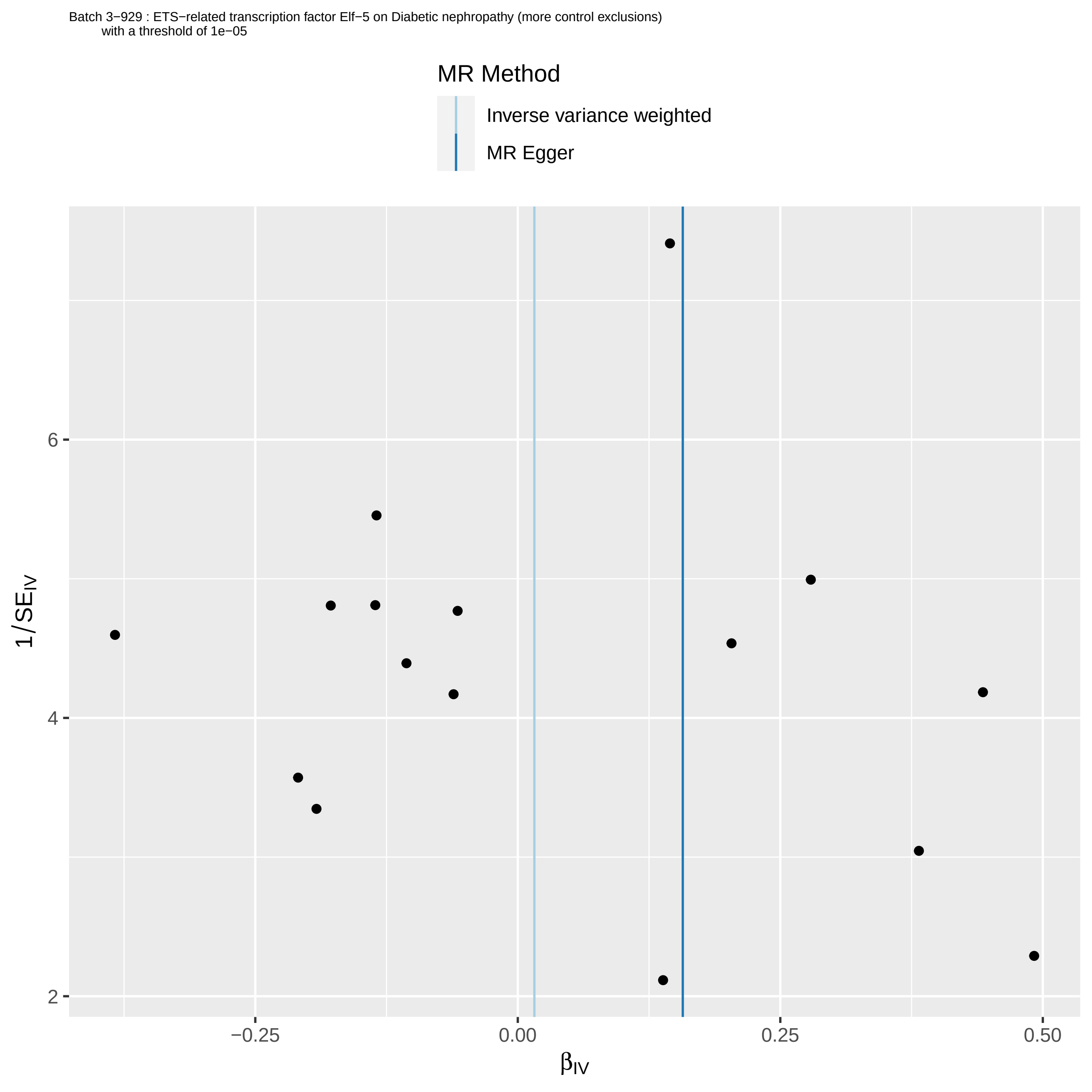
Batch 3−929 : ETS−related transcription factor Elf−5 on Diabetic nephropathy (more control exclusions)
with a threshold of 1e−05
MR Method
Inverse variance weighted
MR Egger
6
4
2
1/SE_IV
−0.25
0.00
0.25
0.50
β_IV

Batch 3−930 : ETS domain−containing protein Elk−1 on Diabetic nephropathy (more control exclusions)
with a threshold of 1e−05

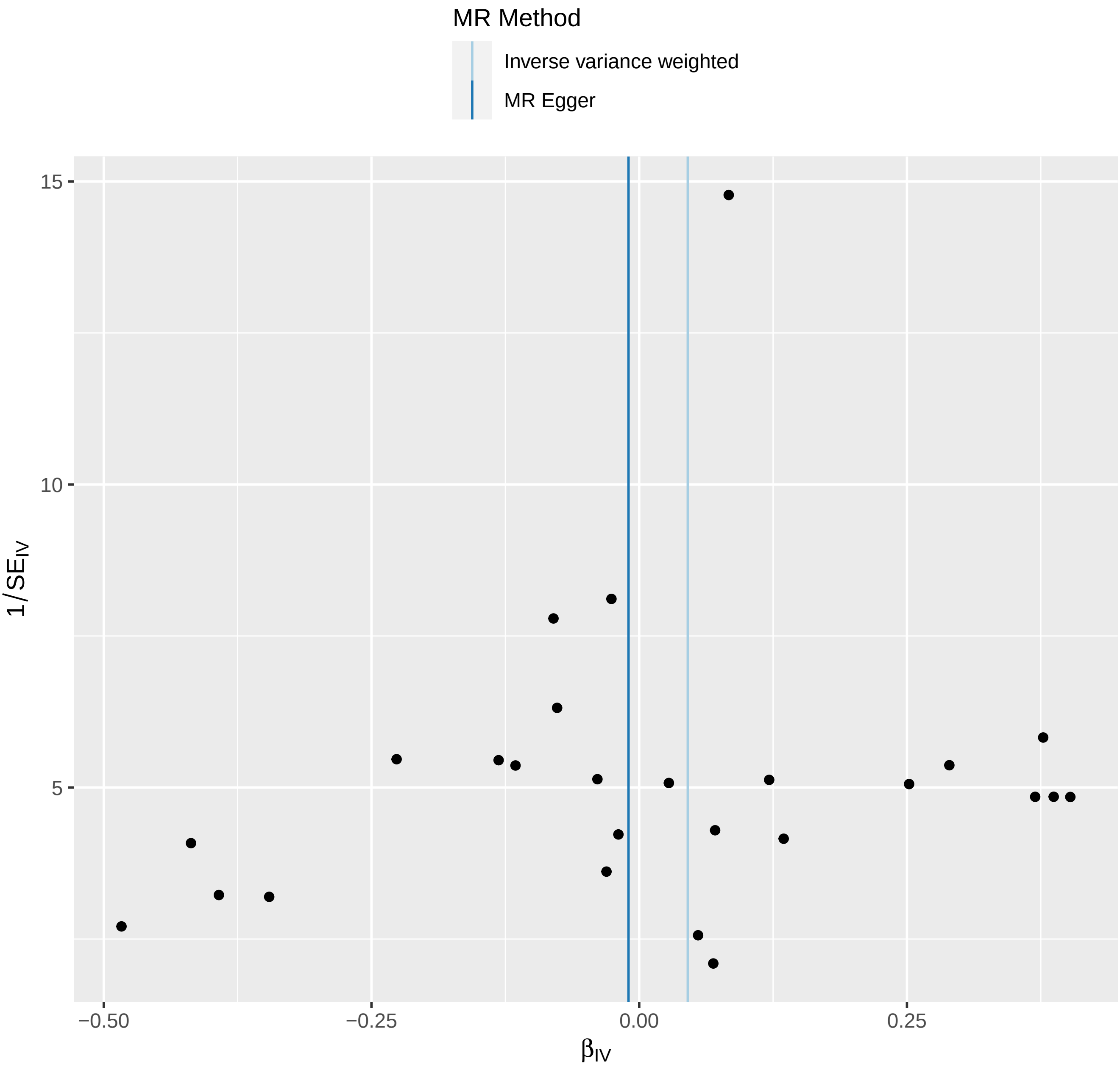

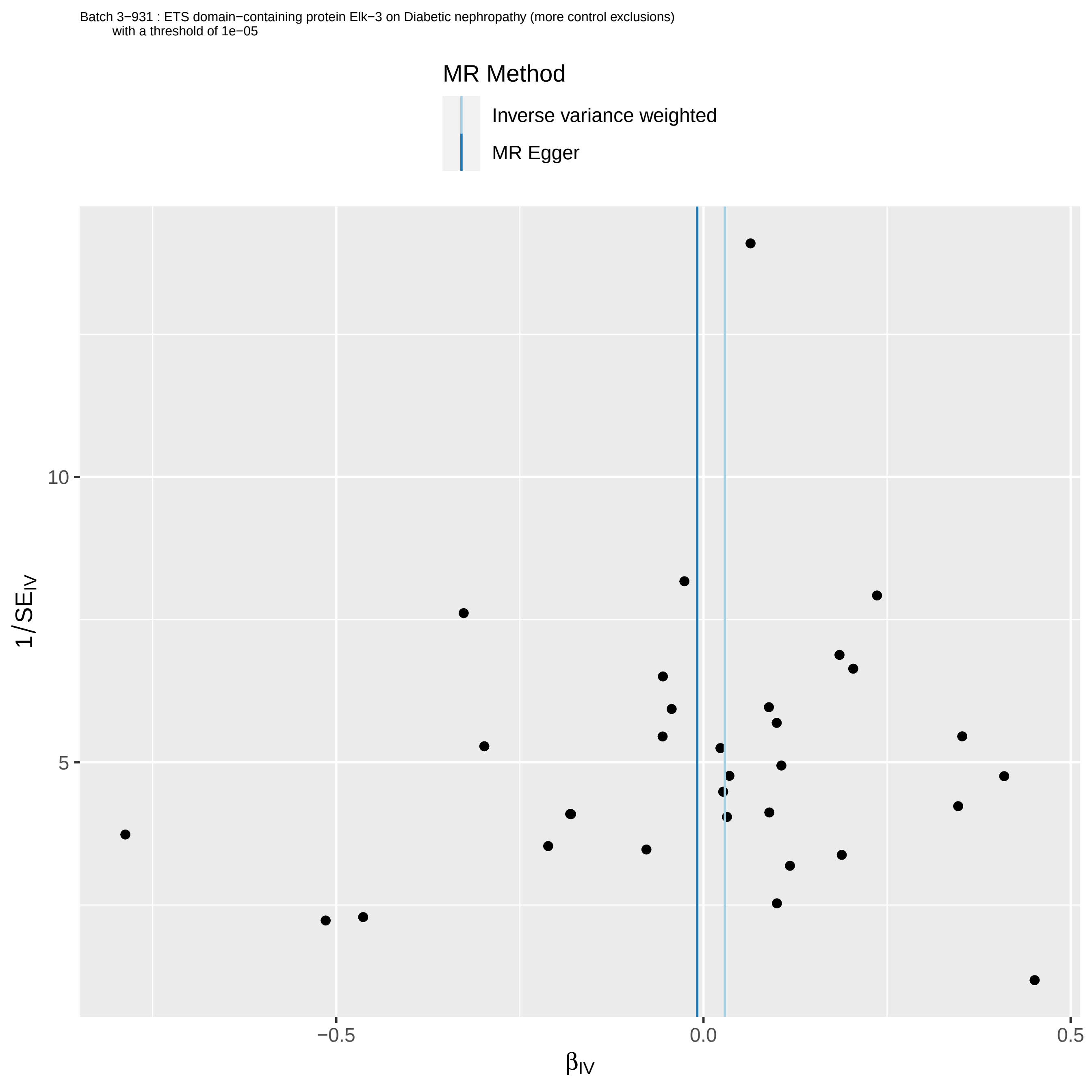
Batch 3−931 : ETS domain−containing protein Elk−3 on Diabetic nephropathy (more control exclusions)
with a threshold of 1e−05
MR Method
Inverse variance weighted
MR Egger
10
5
1/SE_IV
−0.5
0.0
0.5
β_IV

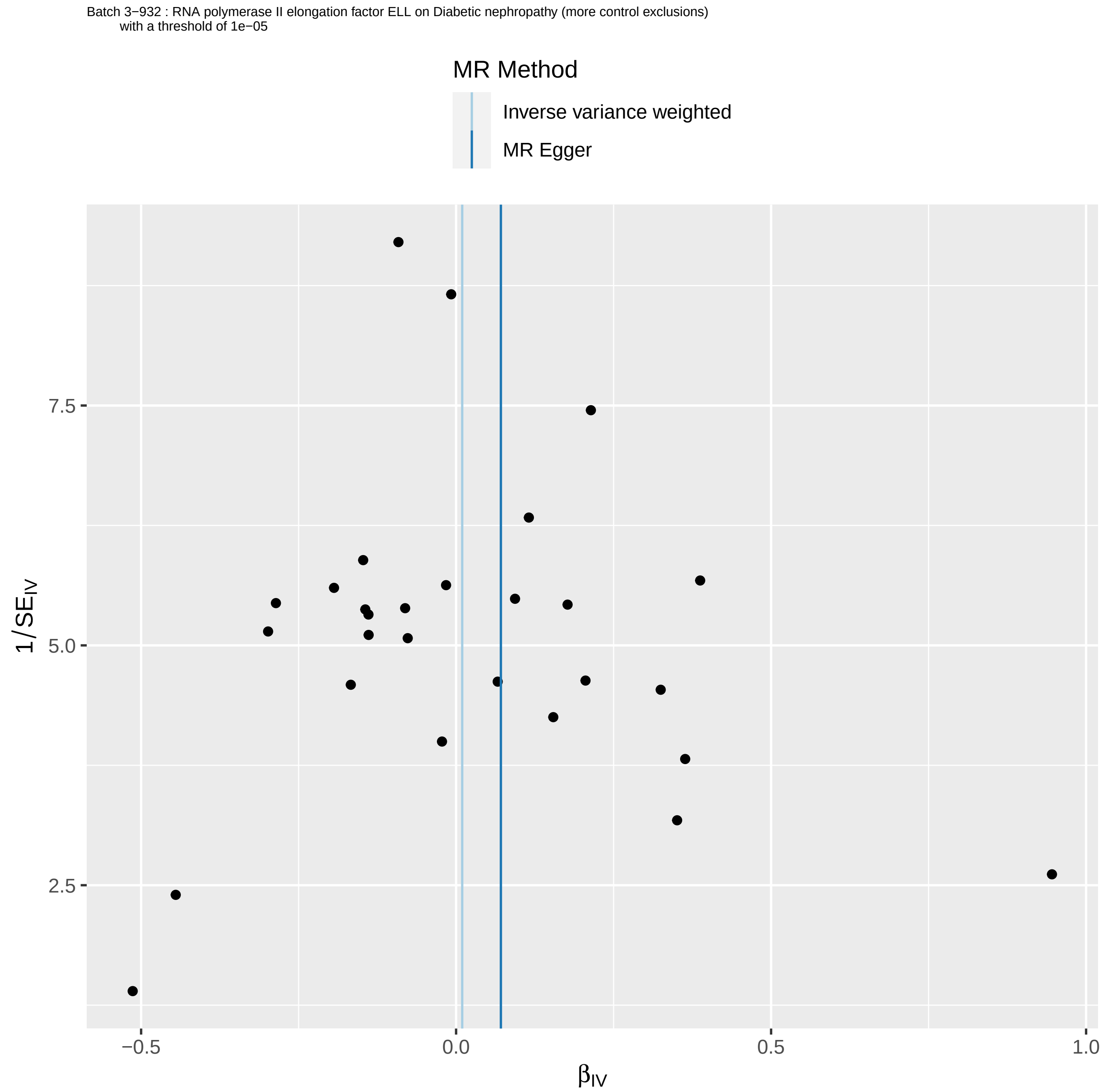
Batch 3−932 : RNA polymerase II elongation factor ELL on Diabetic nephropathy (more control exclusions)
with a threshold of 1e−05
MR Method
Inverse variance weighted
MR Egger
7.5
5.0
2.5
1/SE_IV
−0.5
0.0
0.5
1.0
β_IV

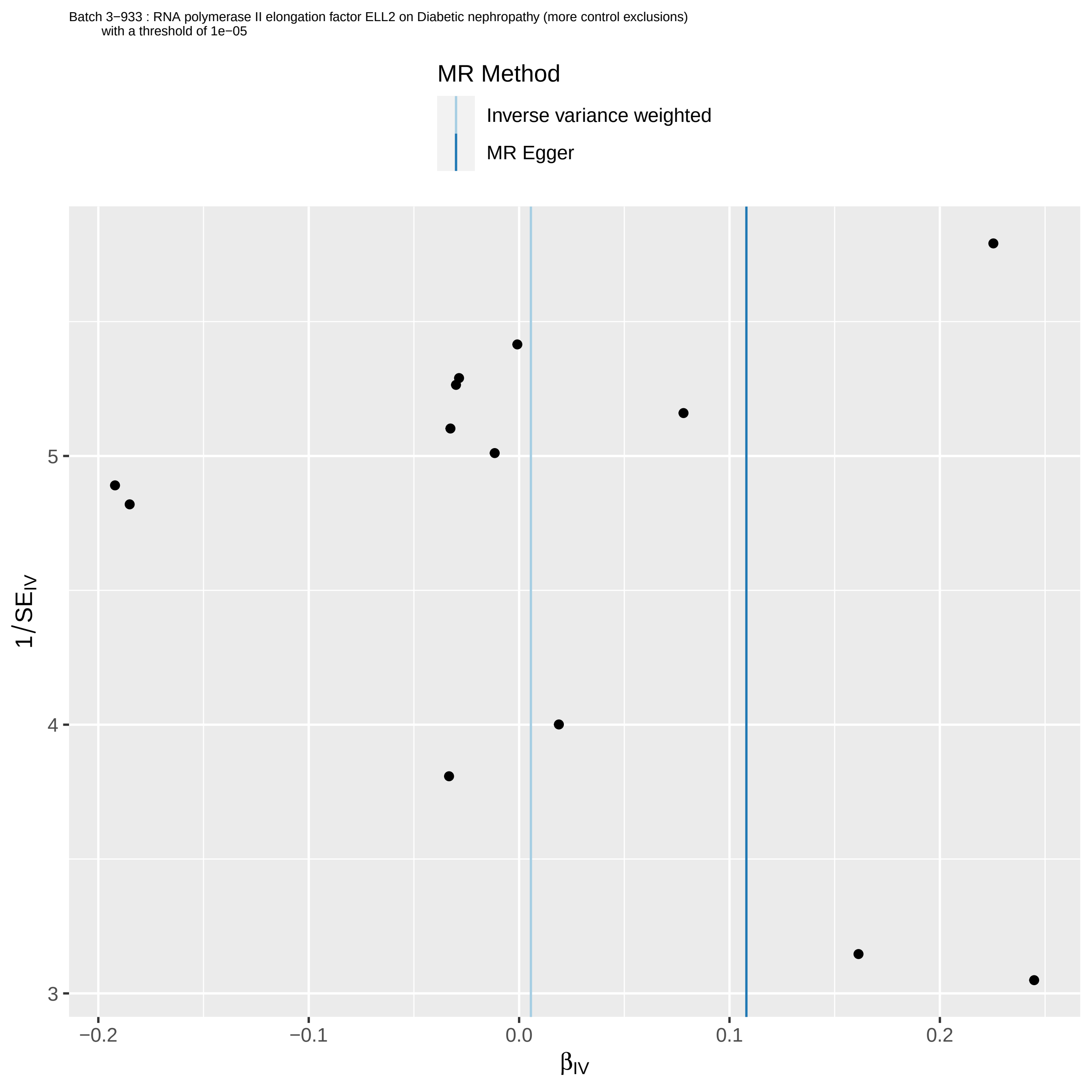

Batch 3−933 : RNA polymerase II elongation factor ELL2 on Diabetic nephropathy (more control exclusions)
with a threshold of 1e−05
MR Method
Inverse variance weighted
MR Egger
1/SE_IV
5
4
3
−0.2
−0.1
0.0
0.1
0.2
β_IV

Batch 3−934 : Engulfment and cell motility protein 1 on Diabetic nephropathy (more control exclusions)
with a threshold of 1e−05

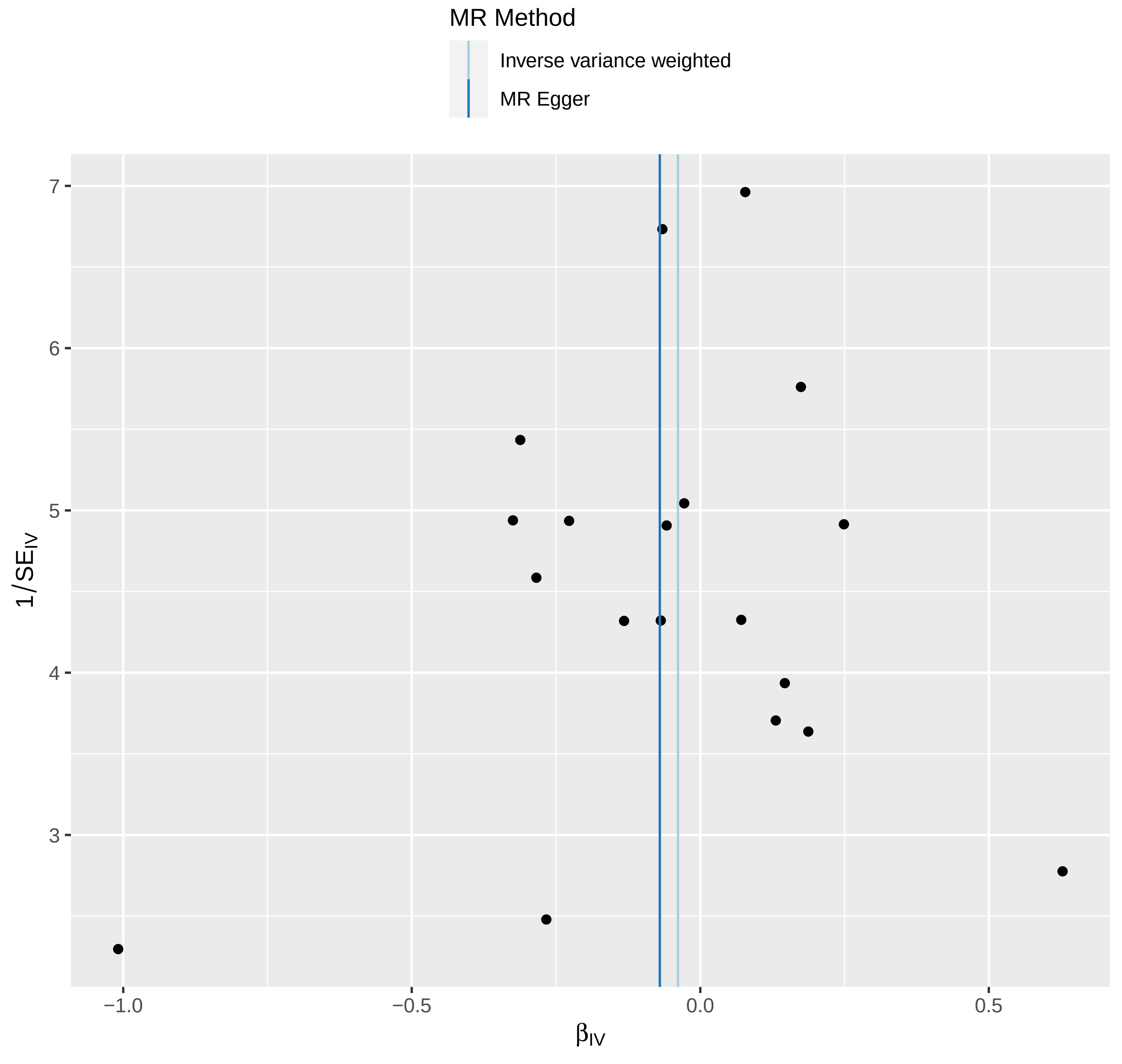

Batch 3−935 : ER membrane protein complex subunit 1 on Diabetic nephropathy (more control exclusions)
with a threshold of 1e−05

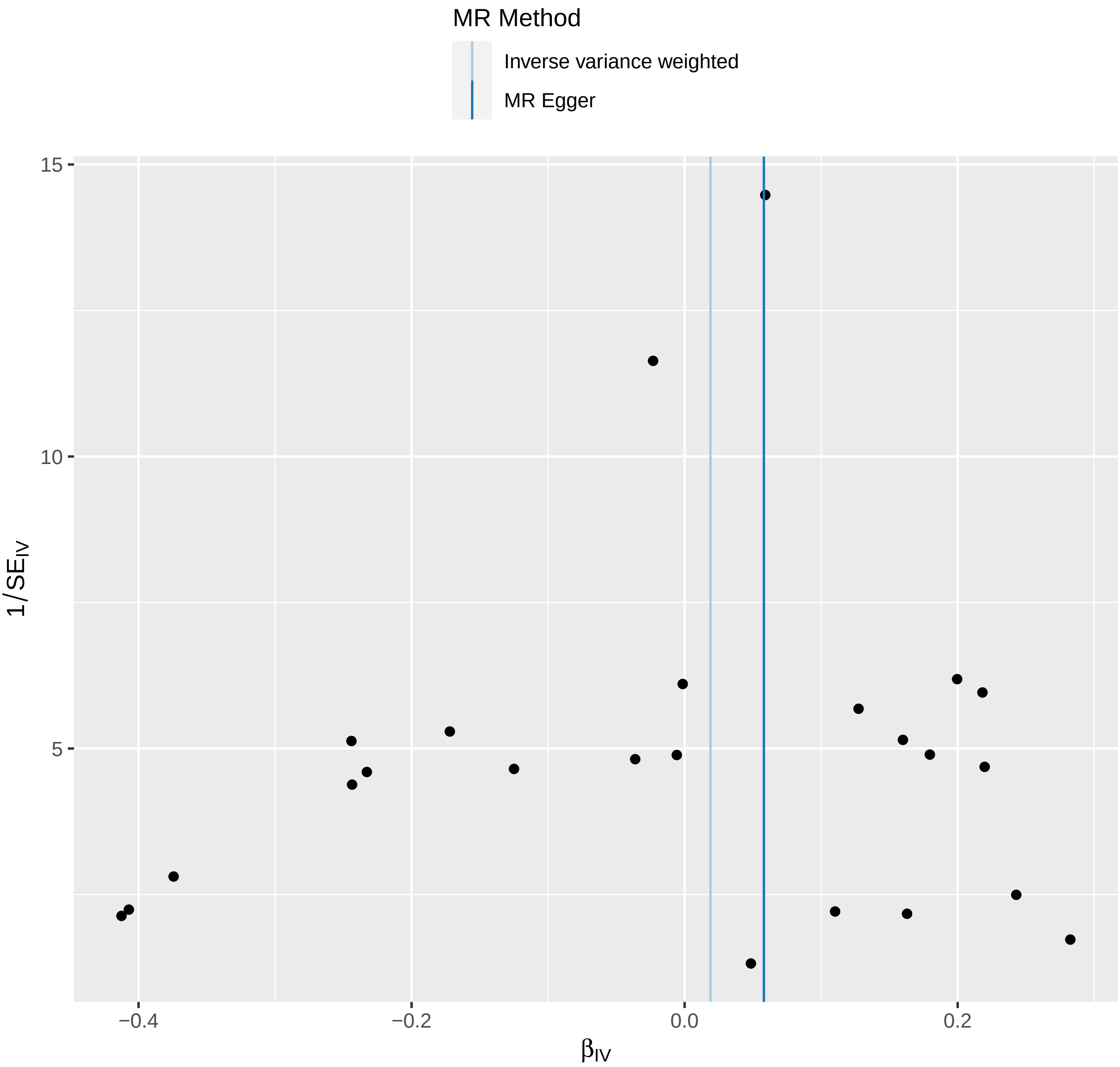

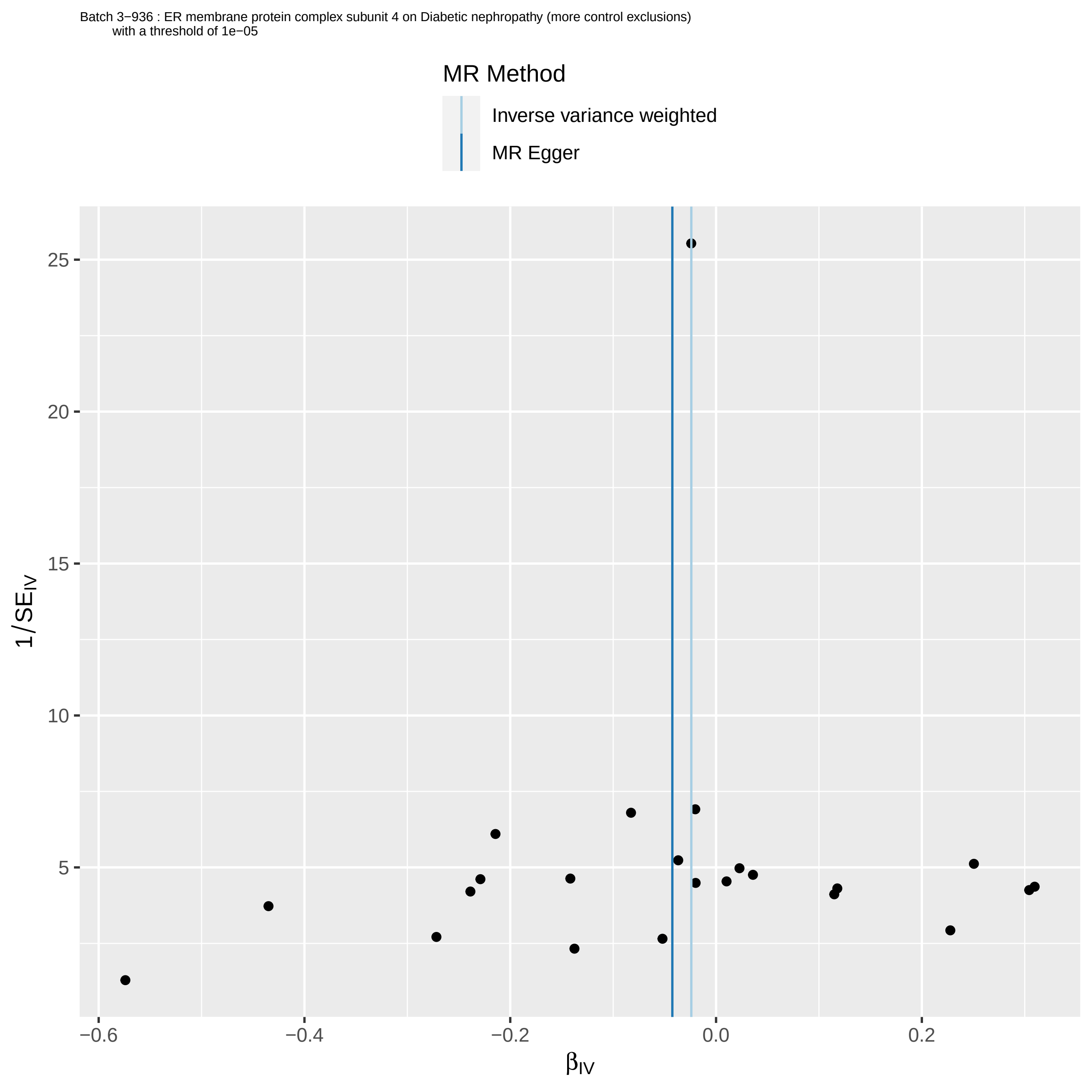

Batch 3−936 : ER membrane protein complex subunit 4 on Diabetic nephropathy (more control exclusions)
with a threshold of 1e−05
MR Method
Inverse variance weighted
MR Egger
25
20
15
10
5
1/SE_IV
−0.6
−0.4
−0.2
0.0
0.2
β_IV

Batch 3−937 : EMI domain−containing protein 1 on Diabetic nephropathy (more control exclusions)
with a threshold of 1e−05

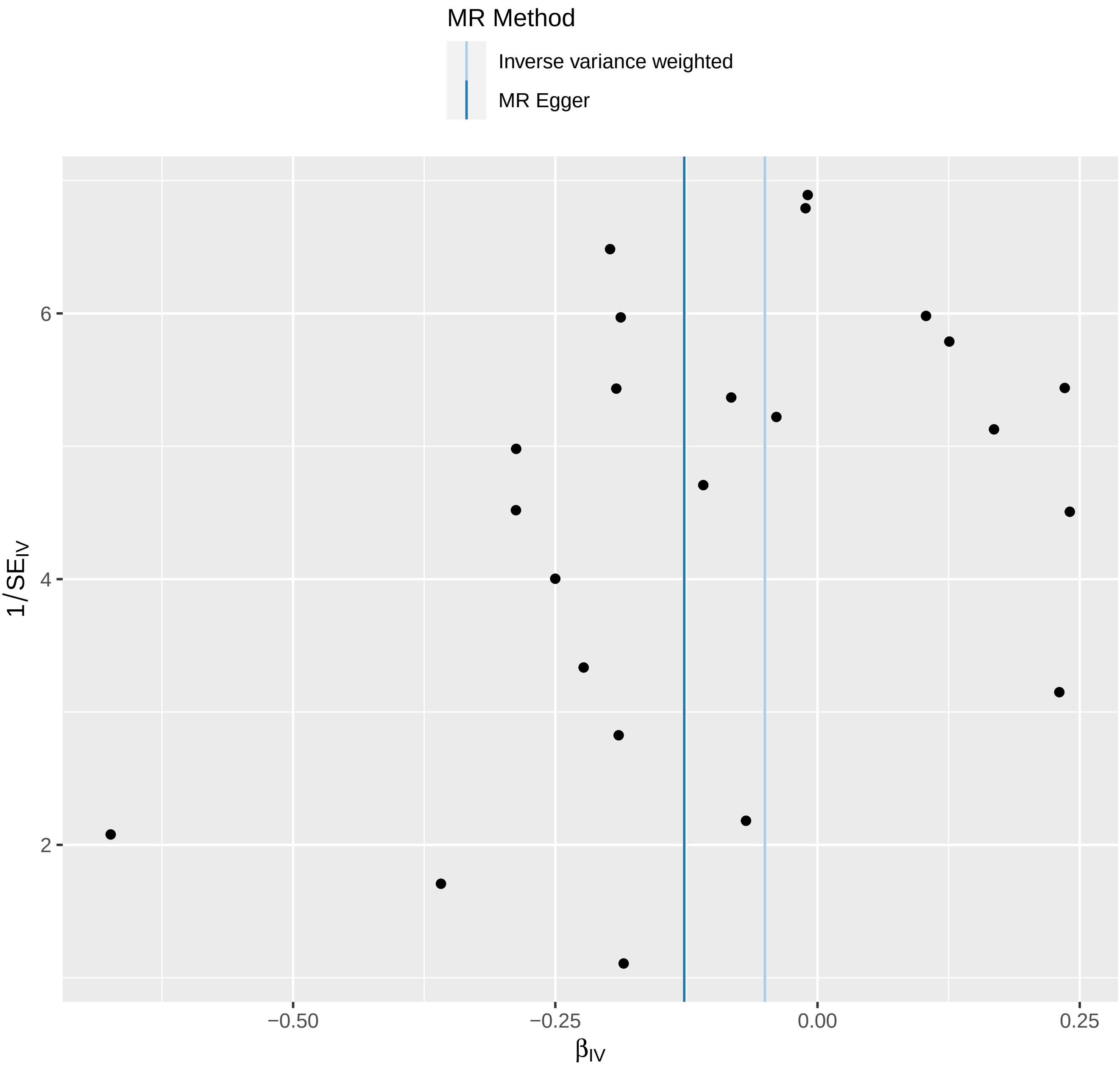

Batch 3−938 : EMILIN−3 on Diabetic nephropathy (more control exclusions)
with a threshold of 1e−05

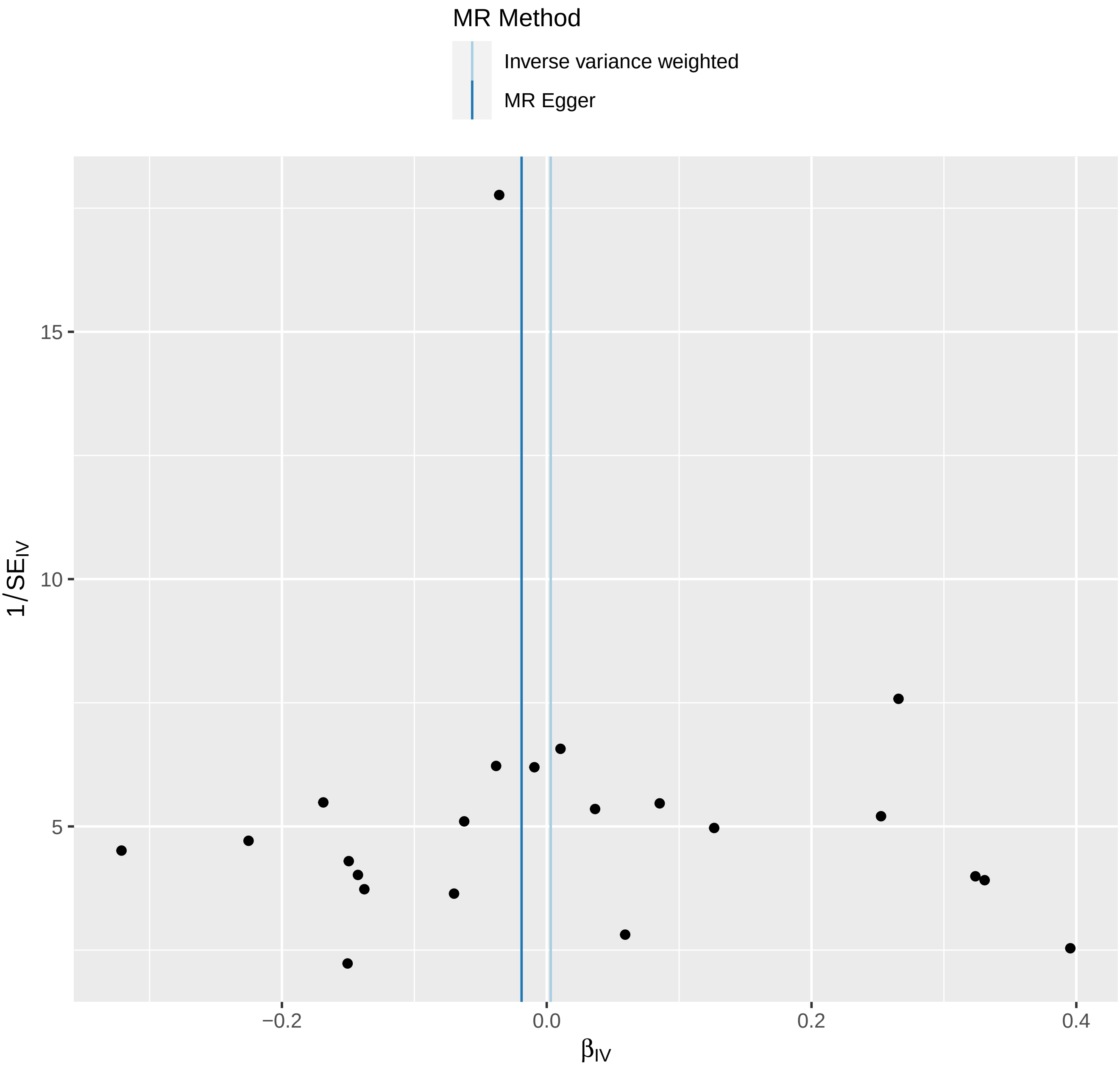

Batch 3−939 : EMILIN−3 on Diabetic nephropathy (more control exclusions)
with a threshold of 1e−05

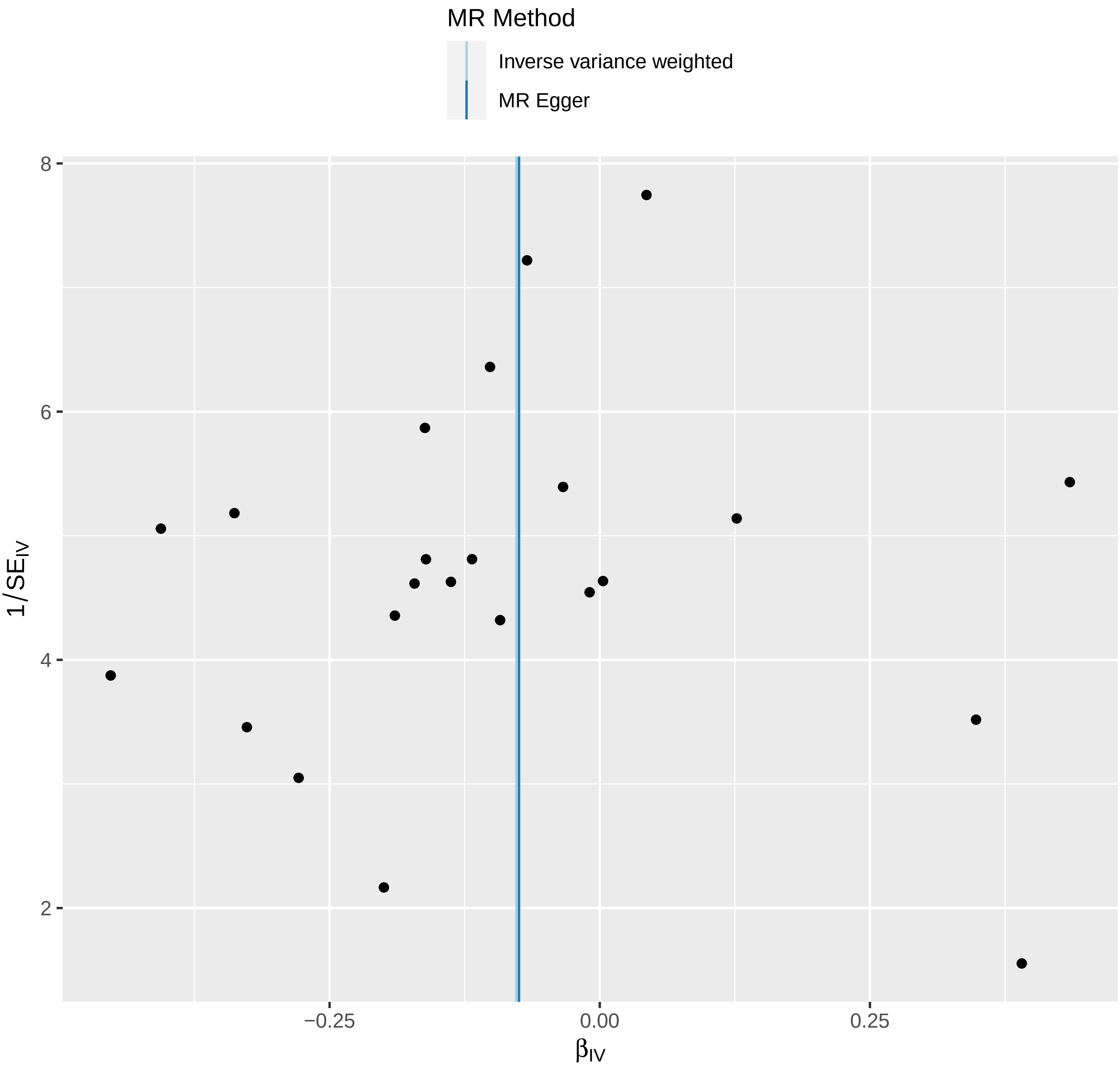

Batch 3−940 : EMILIN−3 on Diabetic nephropathy (more control exclusions)
with a threshold of 1e−05

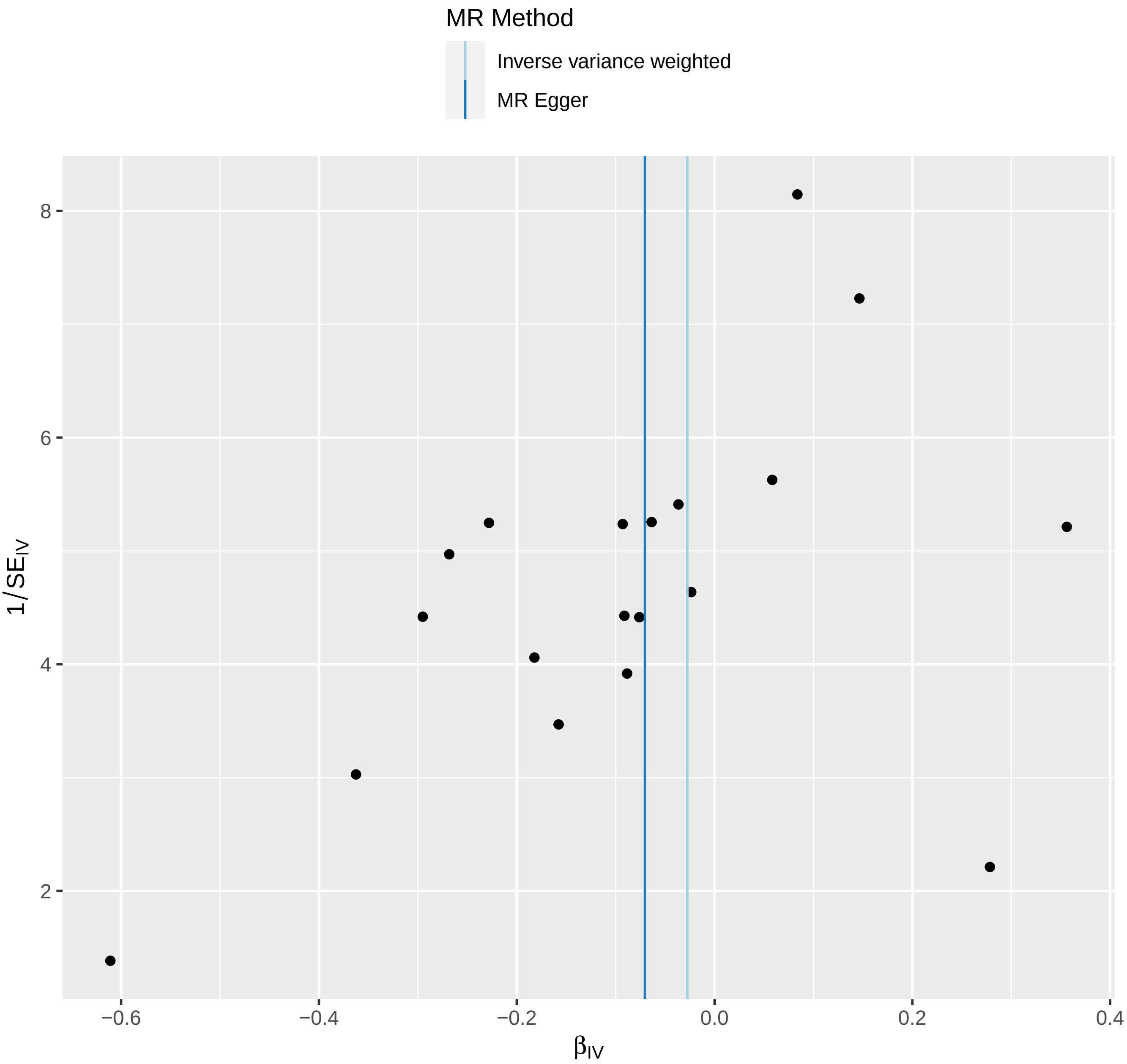

Batch 3−941 : Protein enabled homolog on Diabetic nephropathy (more control exclusions)
with a threshold of 1e−05

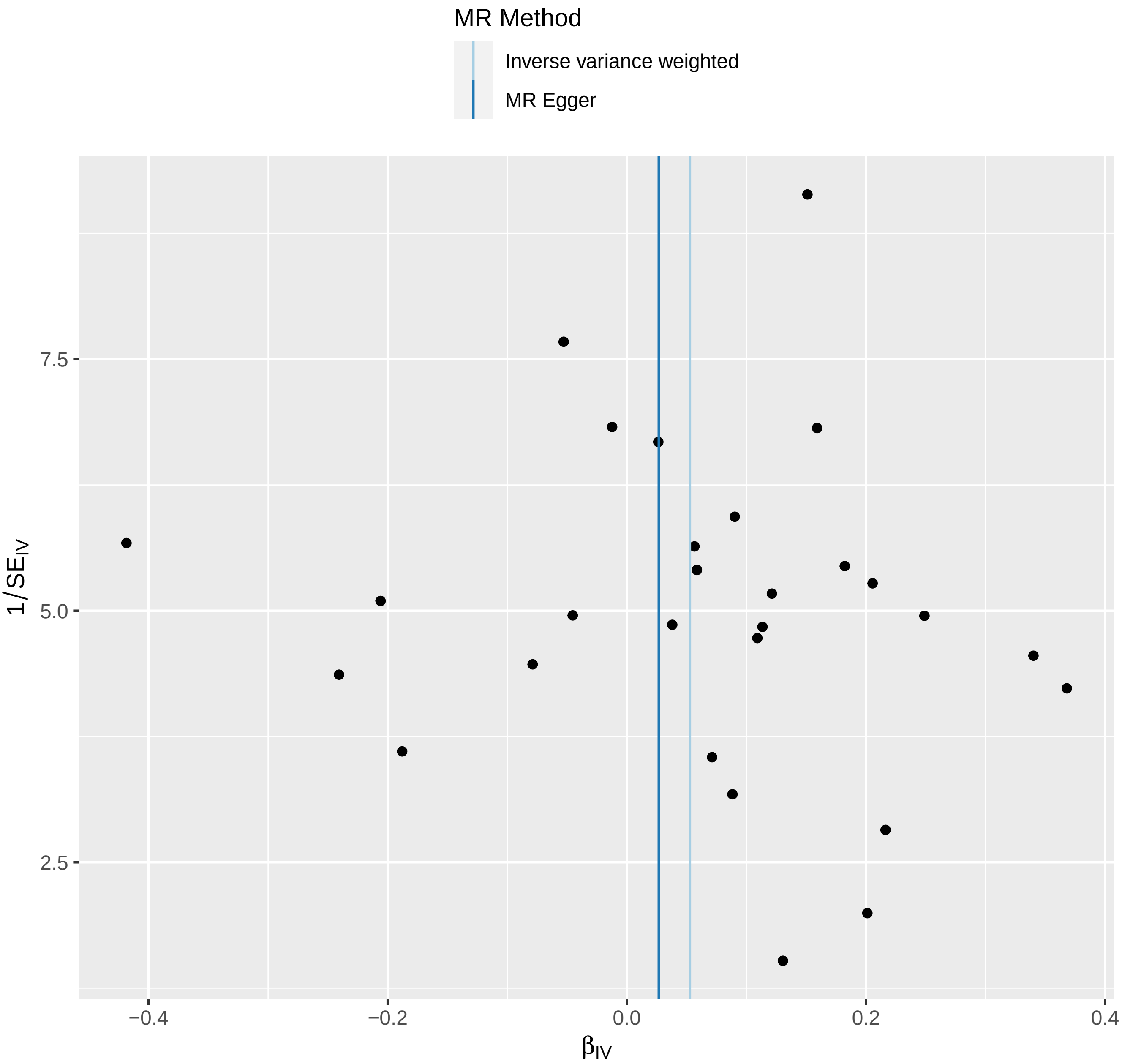

Batch 3−942 : Poly(U)−specific endoribonuclease on Diabetic nephropathy (more control exclusions) with a threshold of 1e−05

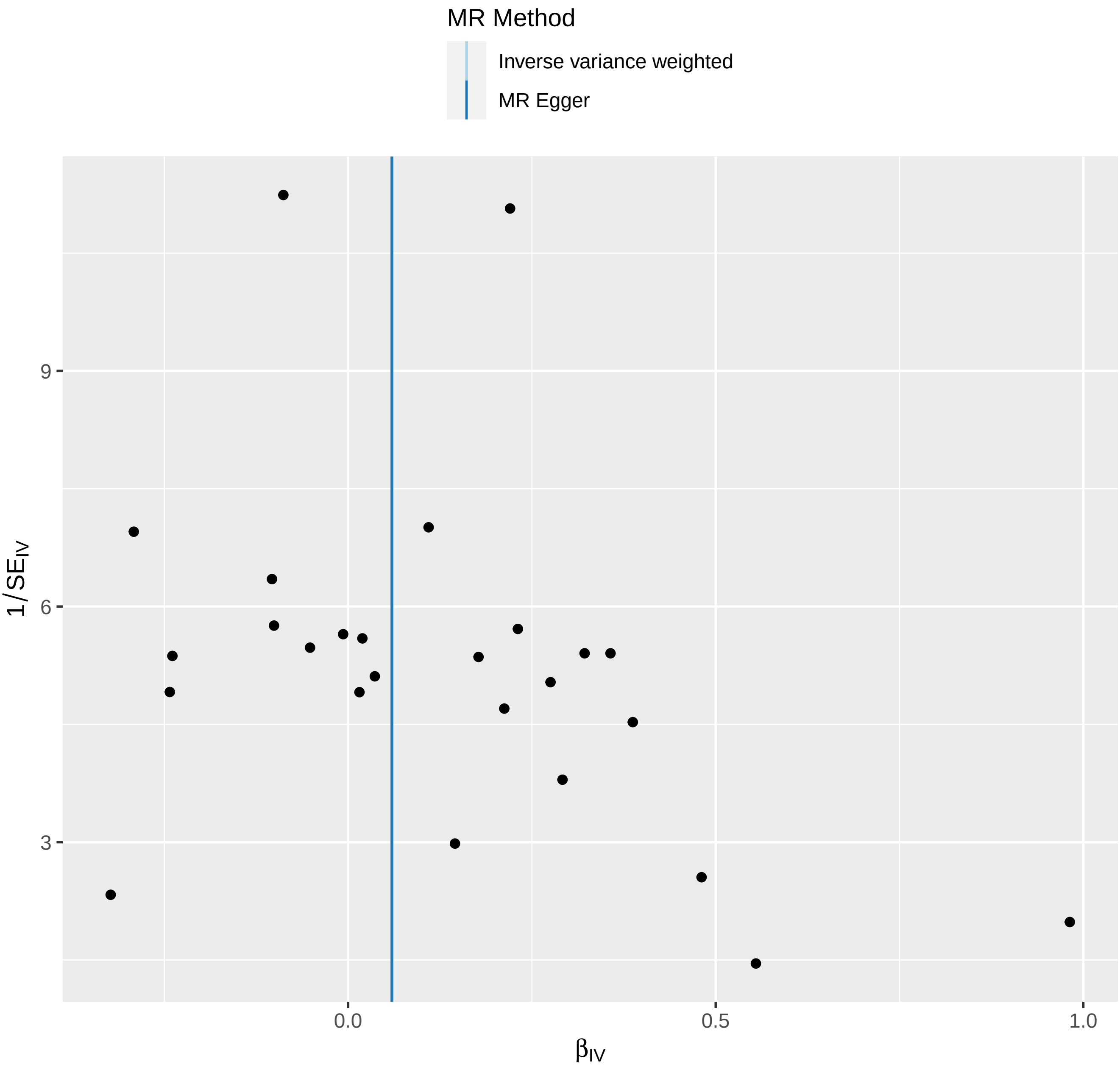

Batch 3−943 : Endoglin on Diabetic nephropathy (more control exclusions)
with a threshold of 1e−05

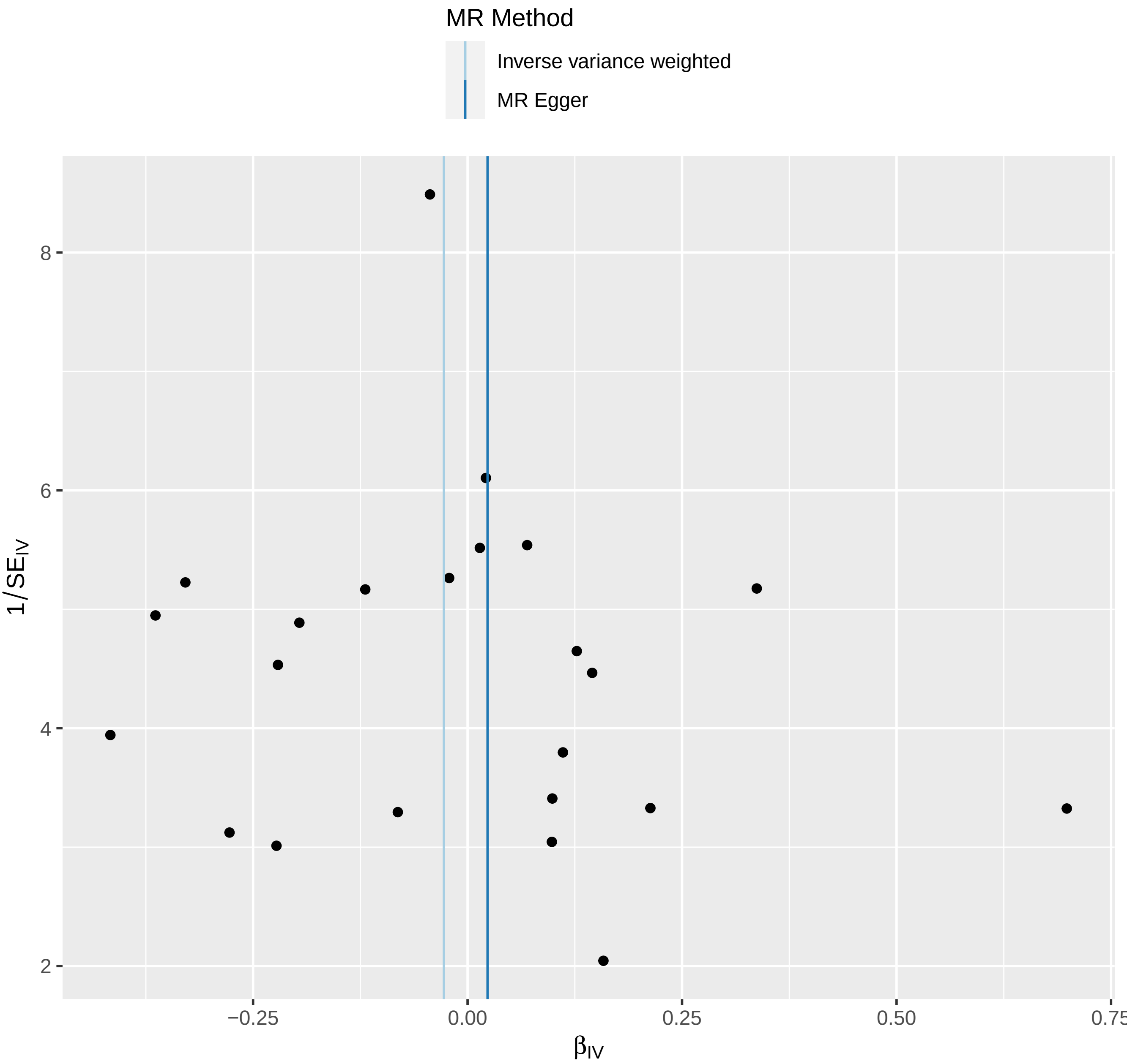

Batch 3−944 : Gamma−enolase on Diabetic nephropathy (more control exclusions)
with a threshold of 1e−05

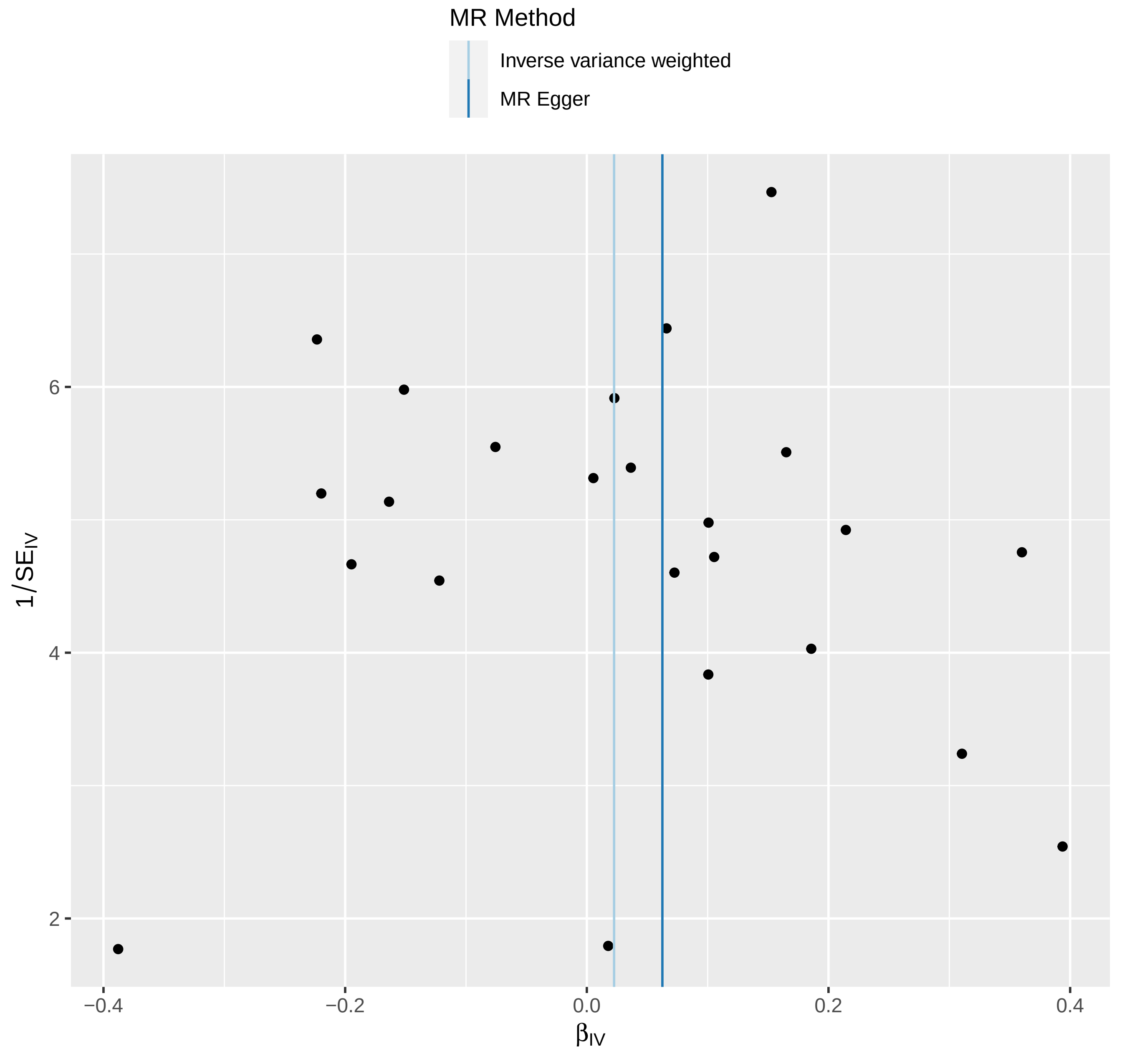

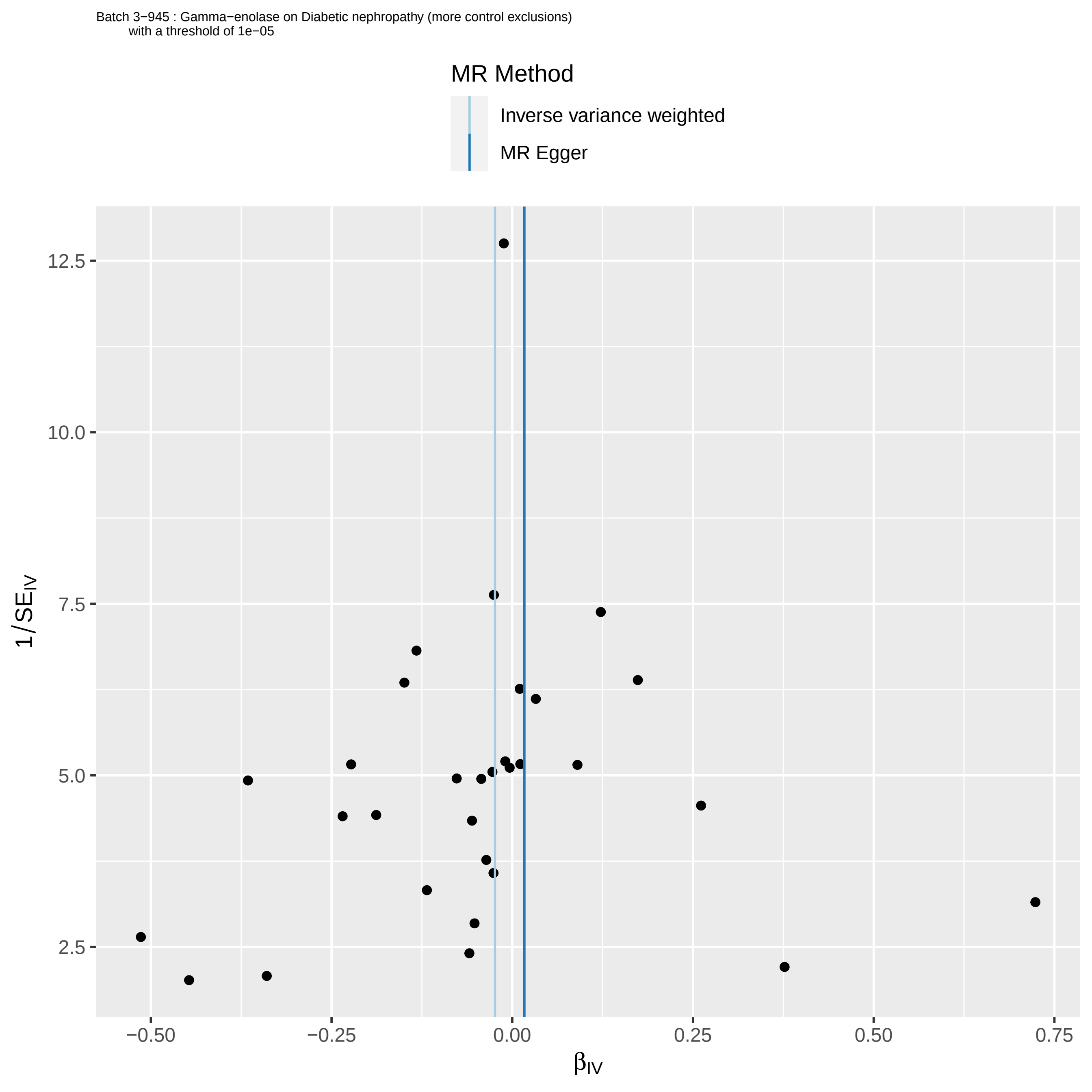
Batch 3−945 : Gamma−enolase on Diabetic nephropathy (more control exclusions)
with a threshold of 1e−05
MR Method
Inverse variance weighted
MR Egger
12.5
10.0
7.5
5.0
2.5
−0.50
−0.25
0.00
0.25
0.50
0.75
$1/SE_{IV}$
β_{IV}

Batch 3−946 : Ecto−NOX disulfide−thiol exchanger 2 on Diabetic nephropathy (more control exclusions)
with a threshold of 1e−05

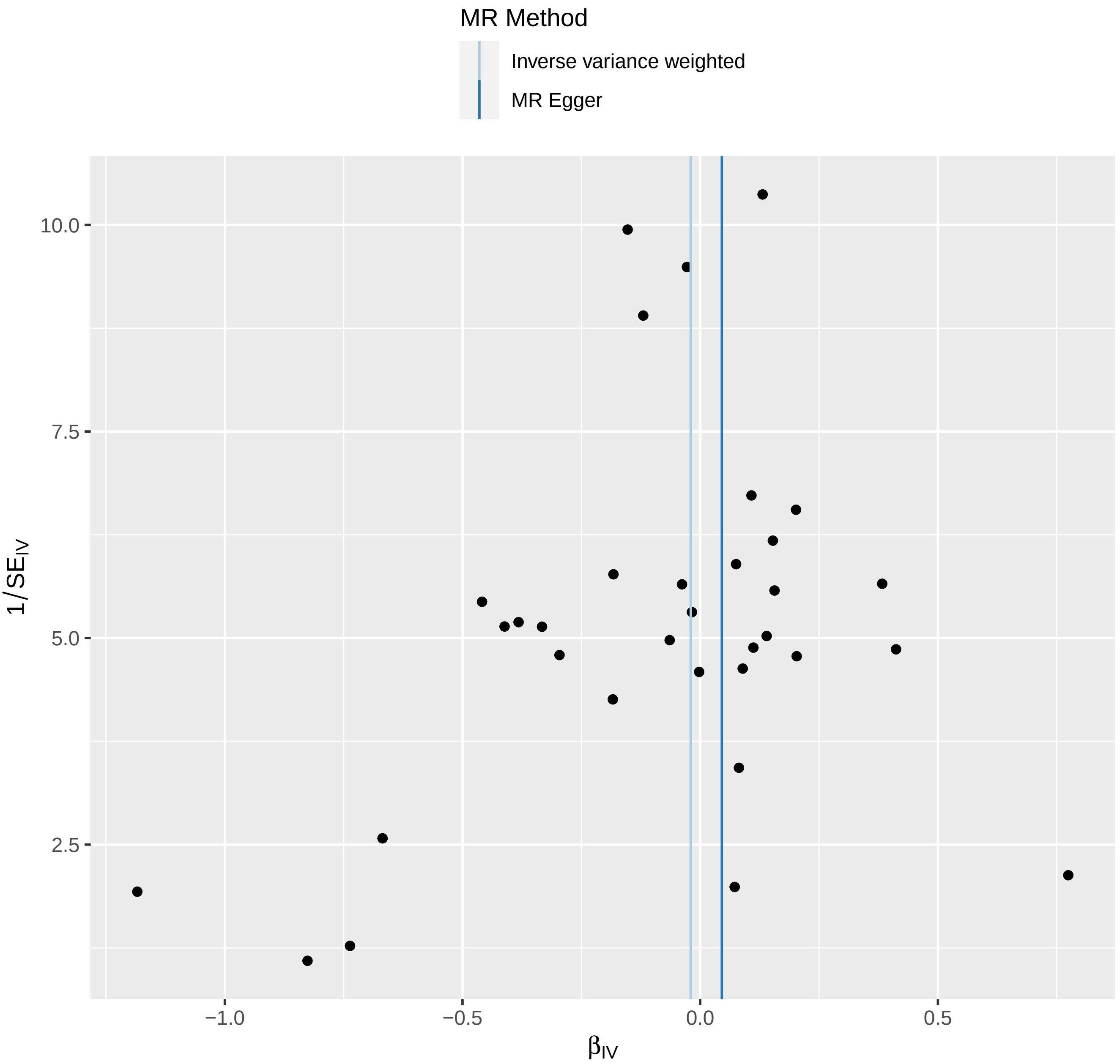

Batch 3−947 : Ectonucleotide pyrophosphatase/phosphodiesterase family member 5 on Diabetic nephropathy (more control exclusions) with a threshold of 1e−05

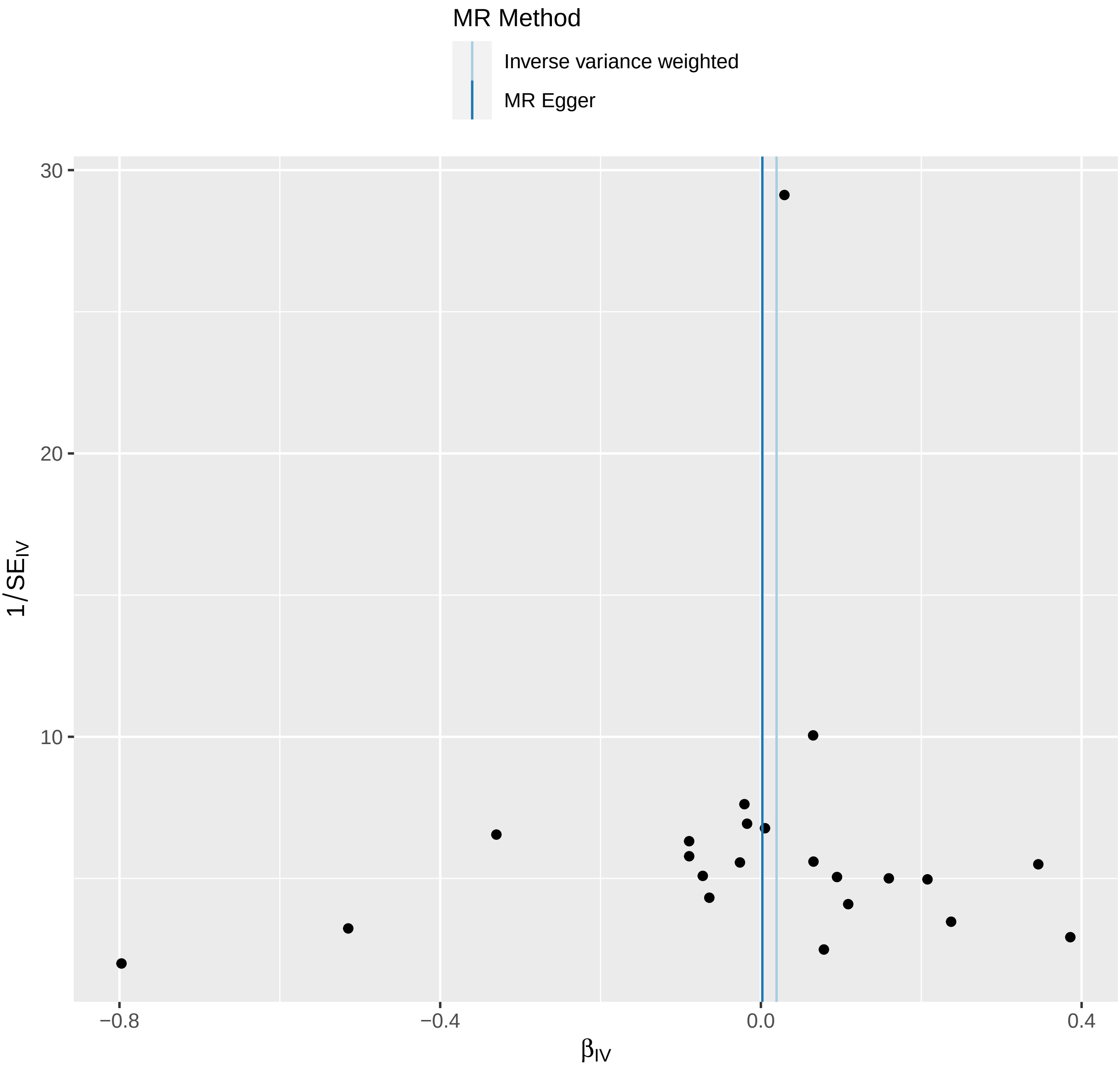

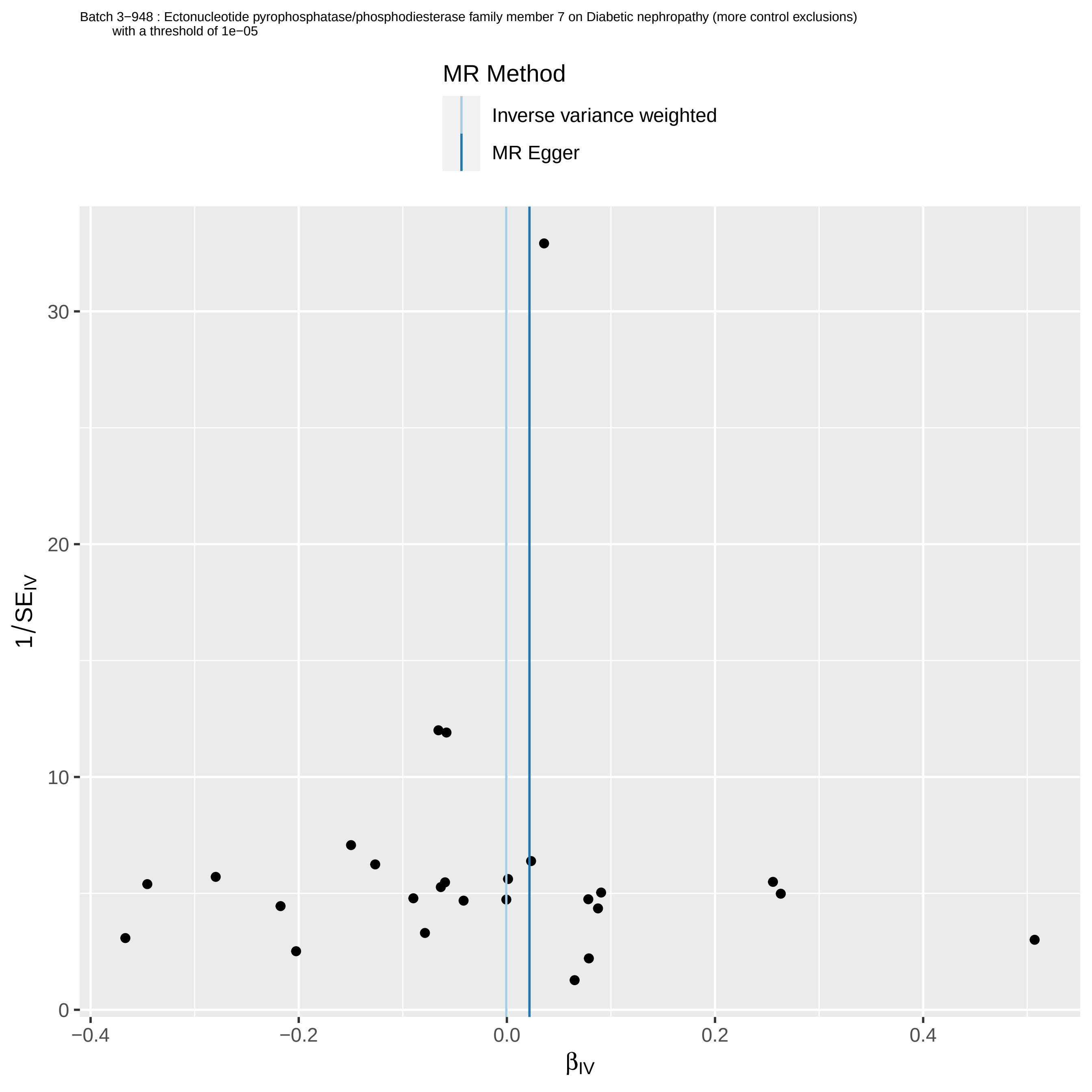
Batch 3−948 : Ectonucleotide pyrophosphatase/phosphodiesterase family member 7 on Diabetic nephropathy (more control exclusions)
with a threshold of 1e−05
MR Method
Inverse variance weighted
MR Egger
30
20
10
0
−0.4
−0.2
0.0
0.2
0.4
$1/SE_{IV}$
β_{IV}

Batch 3−949 : AP−4 complex accessory subunit tepsin on Diabetic nephropathy (more control exclusions) with a threshold of 1e−05

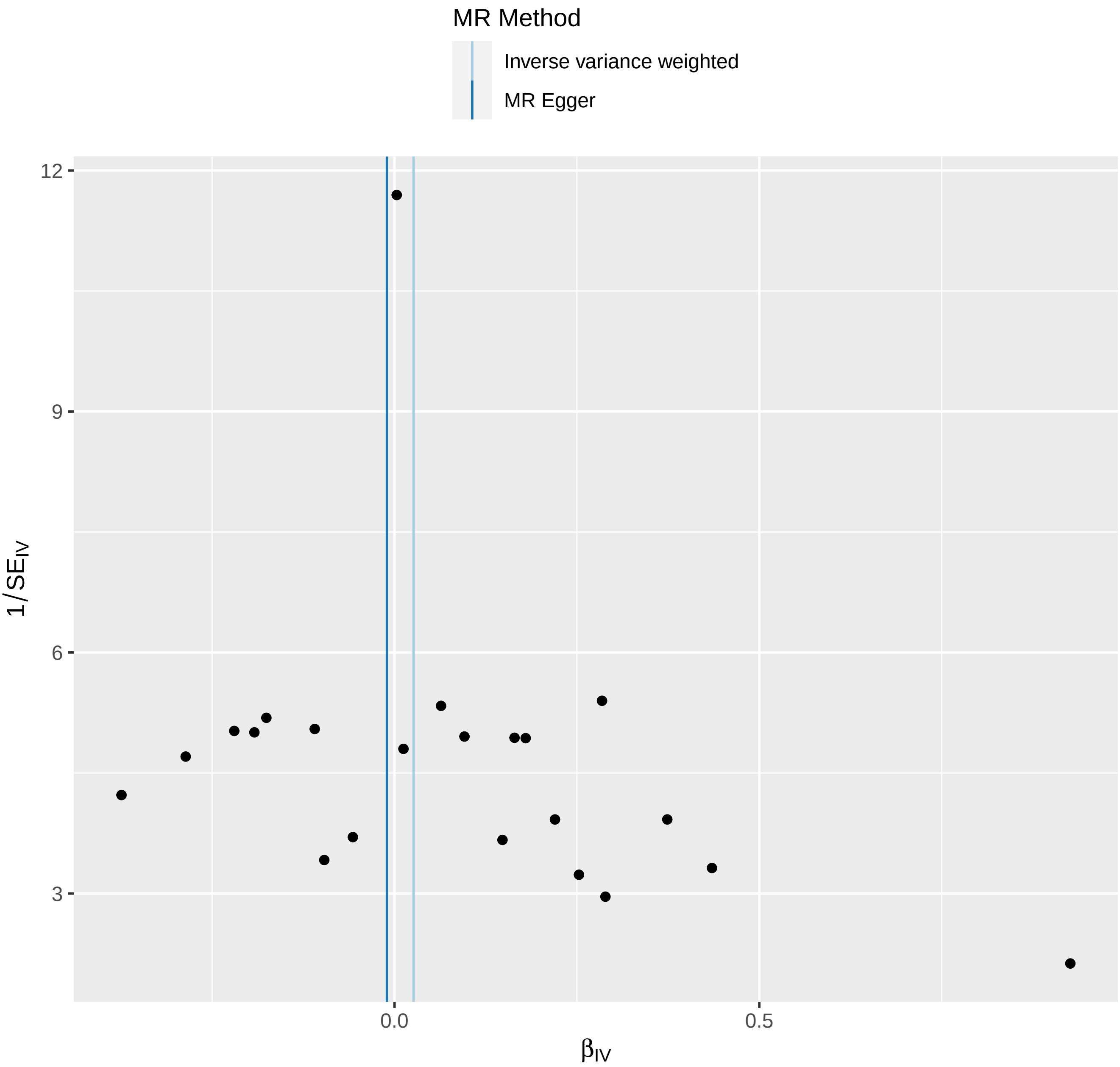

Batch 3−950 : Ectonucleoside triphosphate diphosphohydrolase 1 on Diabetic nephropathy (more control exclusions)
with a threshold of 1e−05

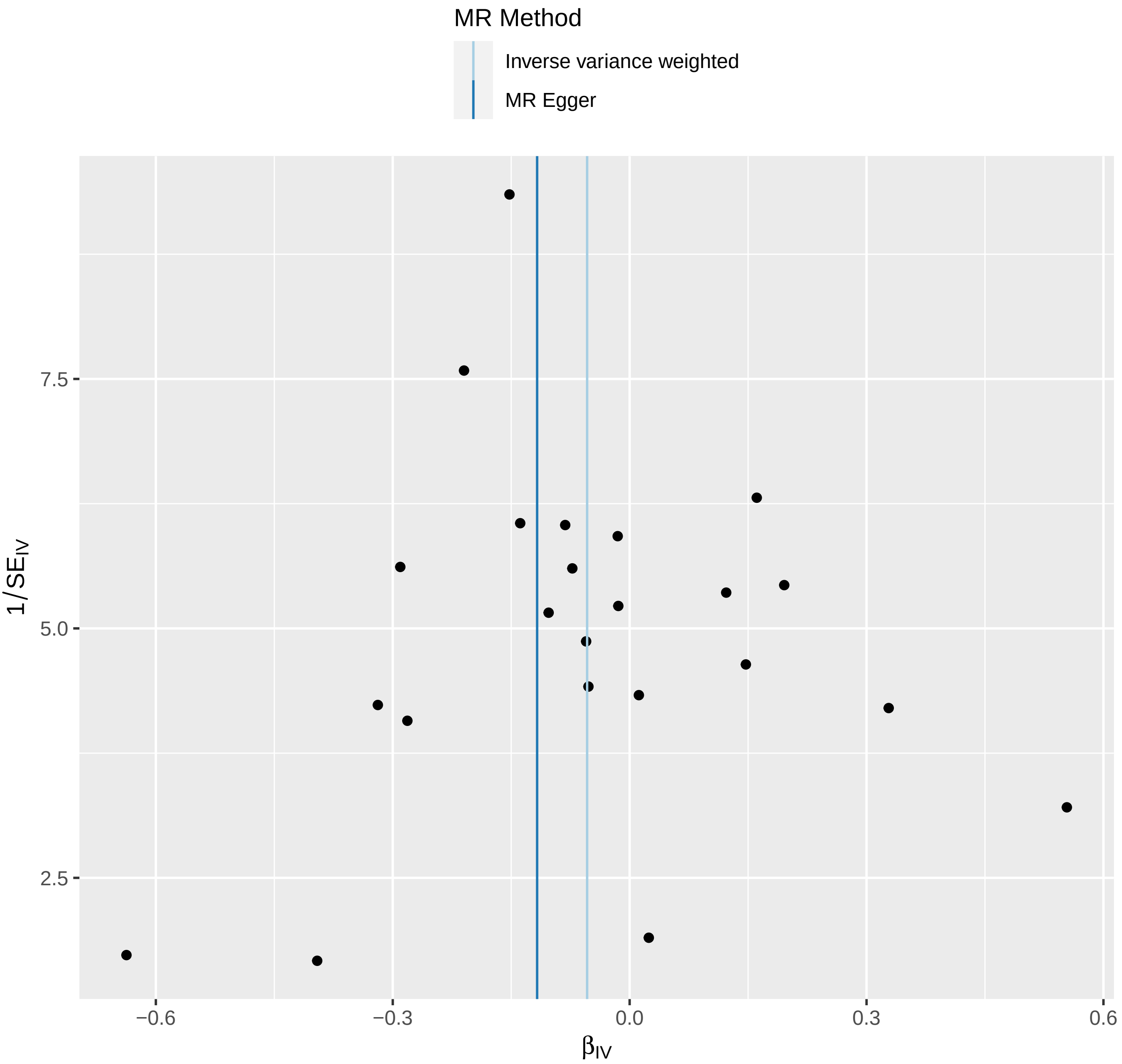

Batch 3−951 : Ectonucleoside triphosphate diphosphohydrolase 1 on Diabetic nephropathy (more control exclusions) with a threshold of 1e−05

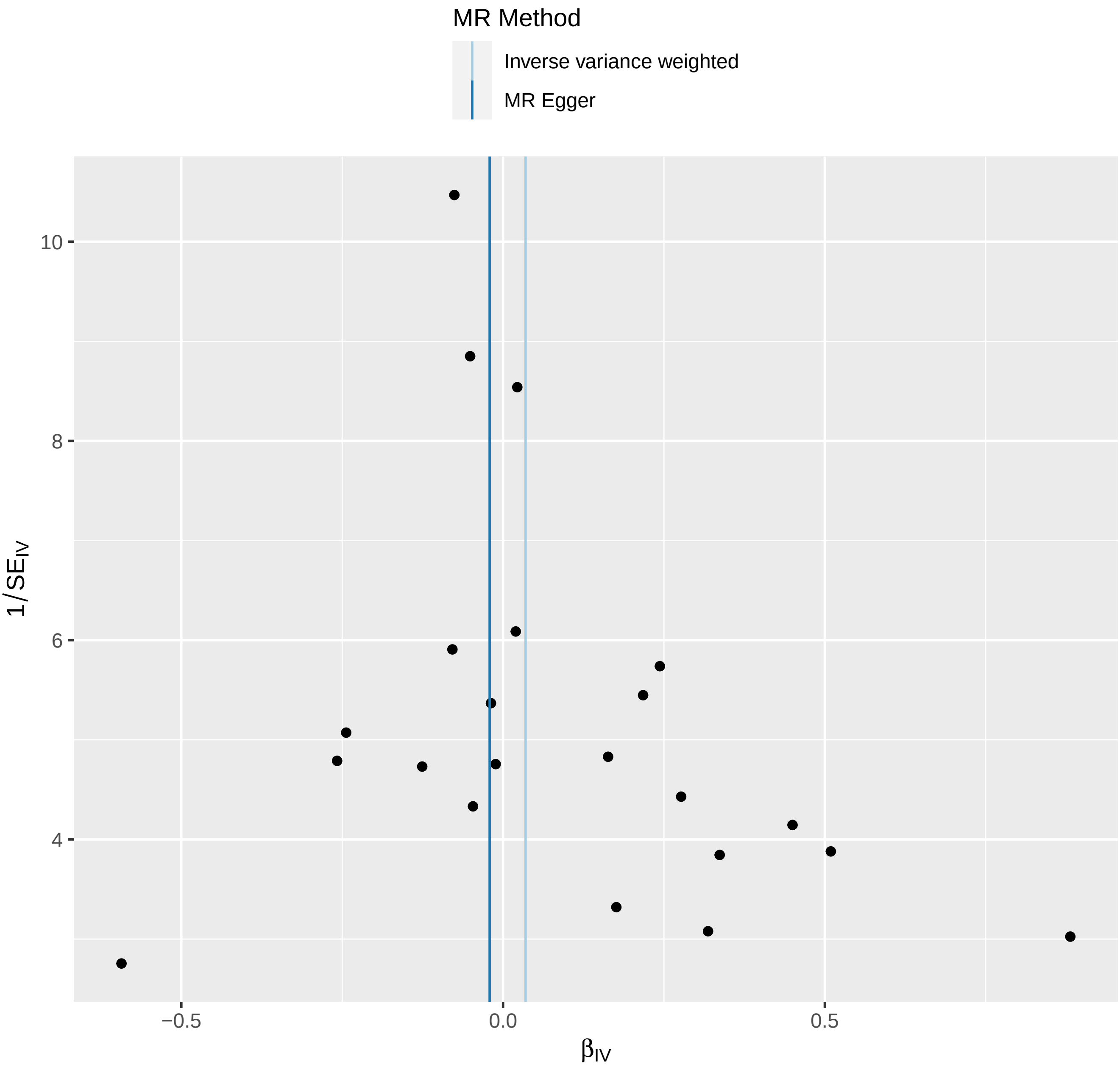

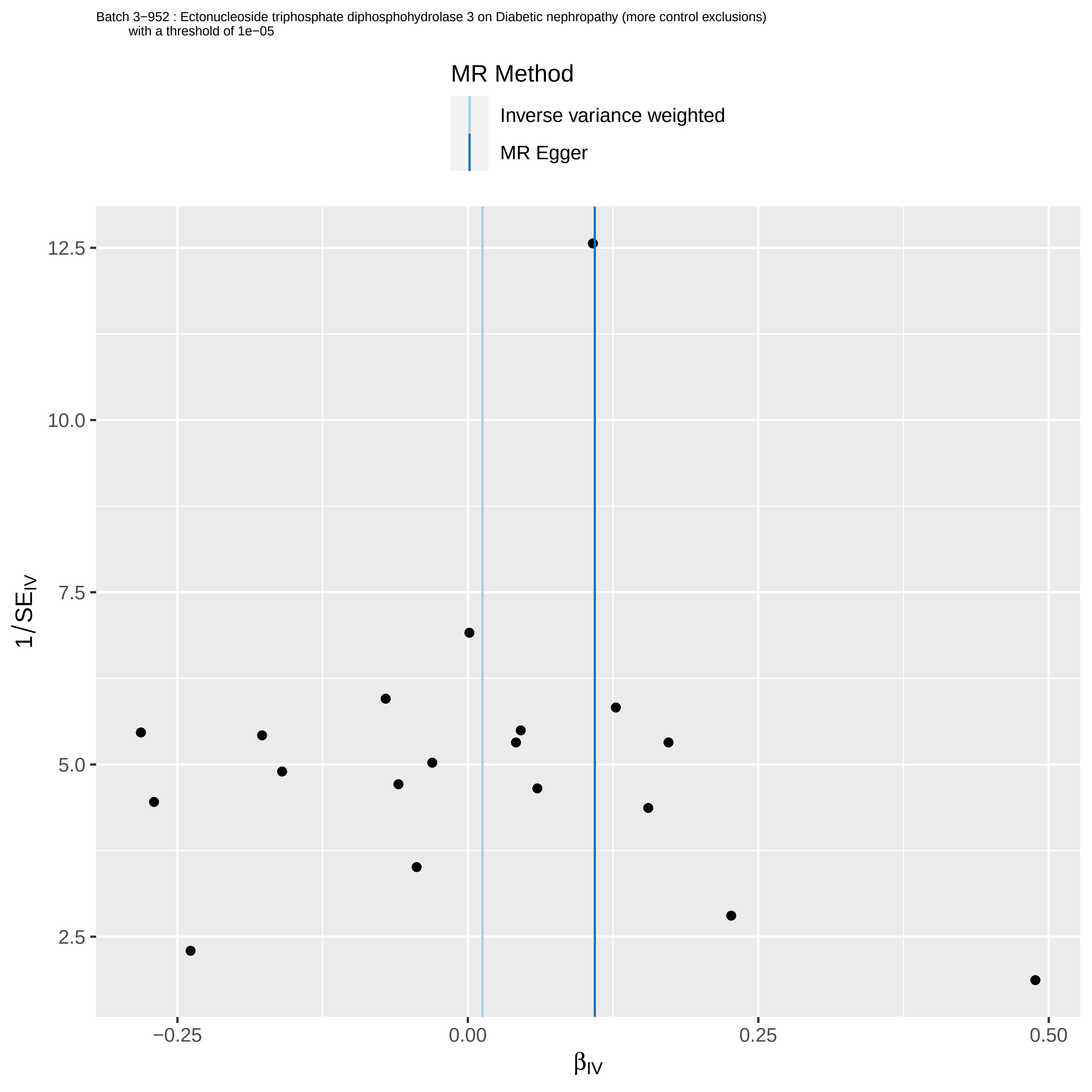

Batch 3−952 : Ectonucleoside triphosphate diphosphohydrolase 3 on Diabetic nephropathy (more control exclusions)
with a threshold of 1e−05
MR Method
Inverse variance weighted
MR Egger
12.5
10.0
7.5
5.0
2.5
$1/SE_{IV}$
−0.25
0.00
0.25
0.50
β_{IV}

Batch 3−953 : Ectonucleoside triphosphate diphosphohydrolase 5 on Diabetic nephropathy (more control exclusions)
with a threshold of 1e−05

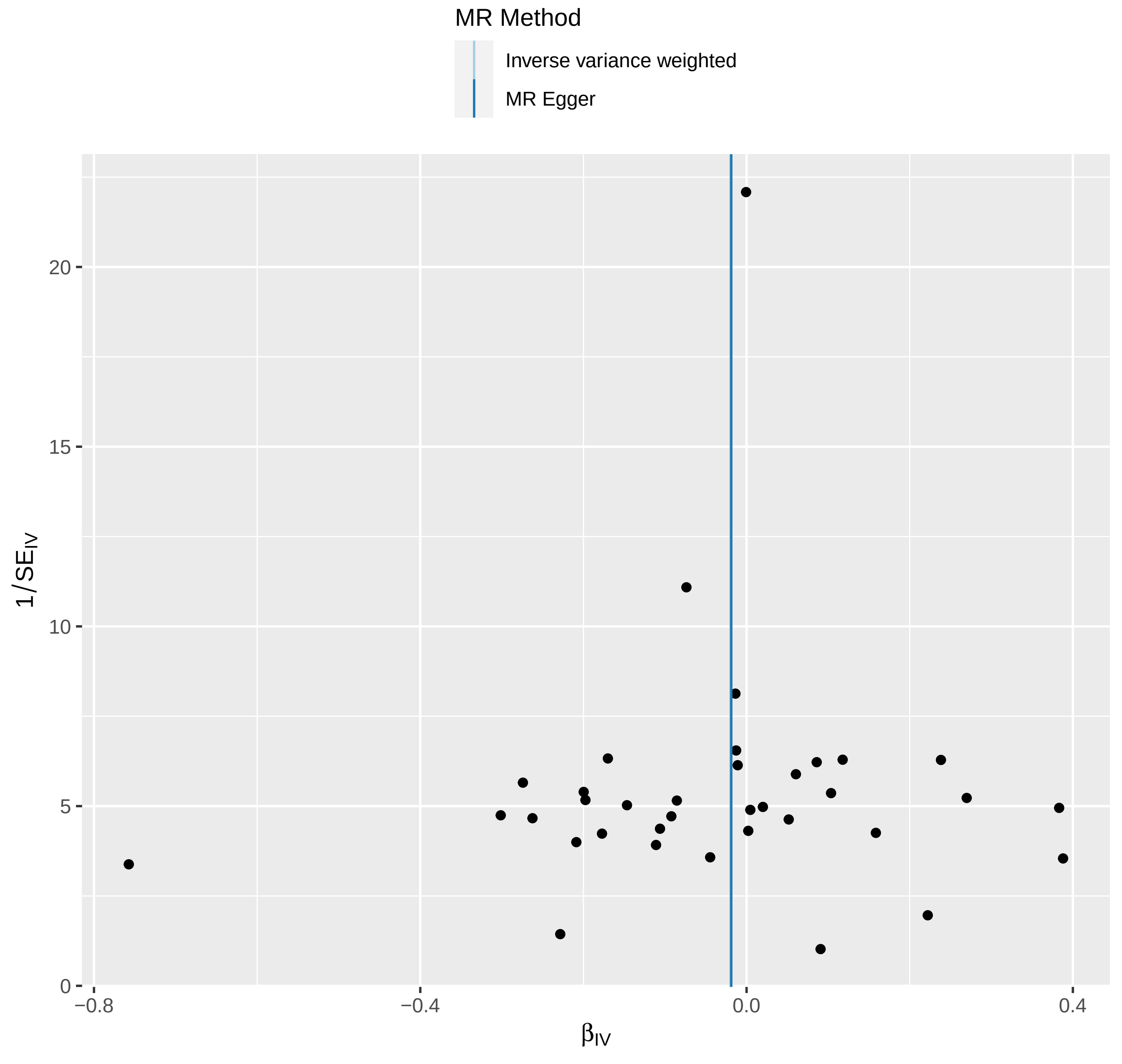

Batch 3−954 : Ectonucleoside triphosphate diphosphohydrolase 6 on Diabetic nephropathy (more control exclusions)
with a threshold of 1e−05

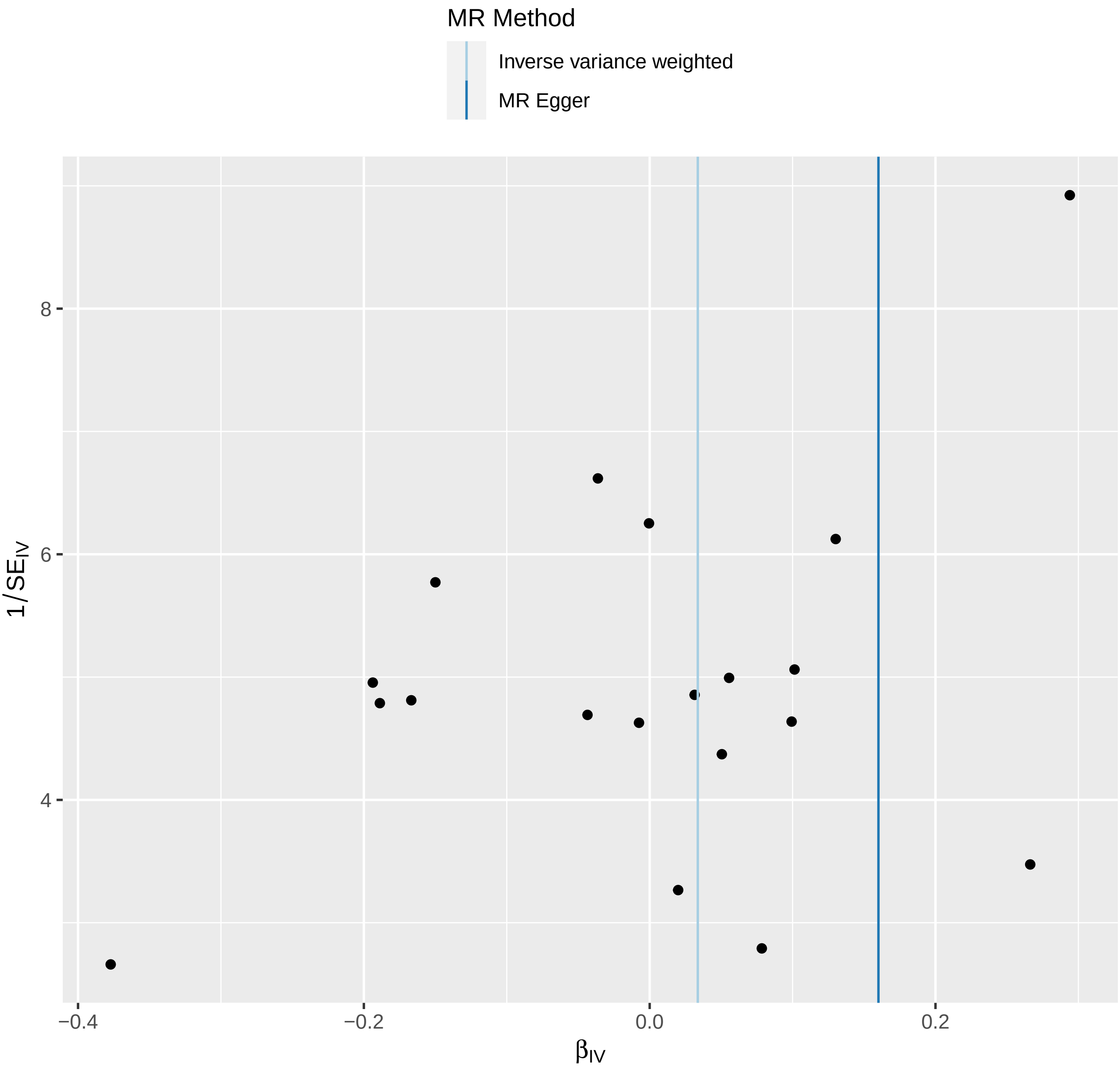

Batch 3−955 : Ephrin type−A receptor 10 on Diabetic nephropathy (more control exclusions)
with a threshold of 1e−05

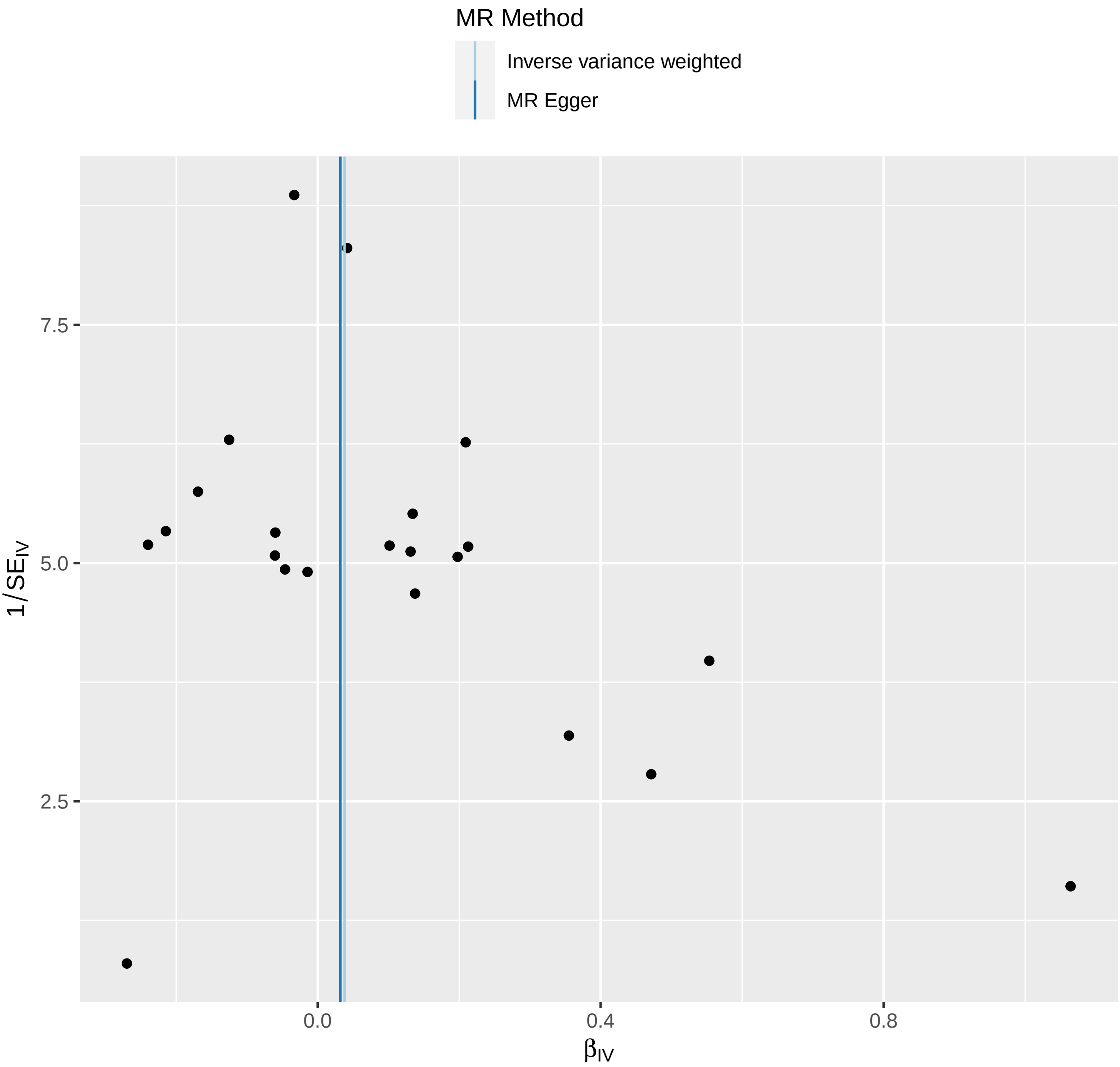

Batch 3−956 : Ephrin type−A receptor 1 on Diabetic nephropathy (more control exclusions)
with a threshold of 1e−05

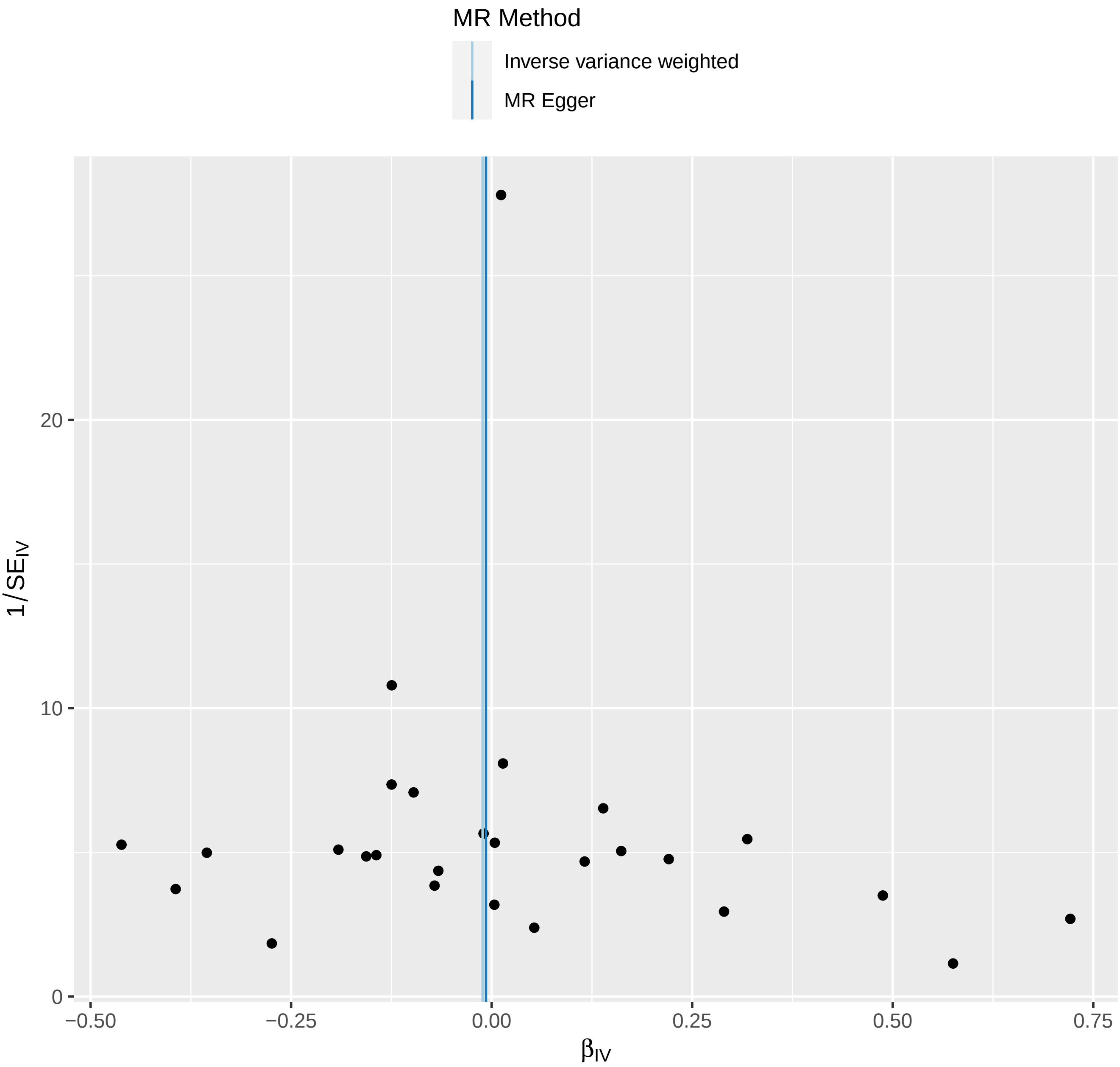

Batch 3−957 : Ephrin type−A receptor 2 on Diabetic nephropathy (more control exclusions)
with a threshold of 1e−05

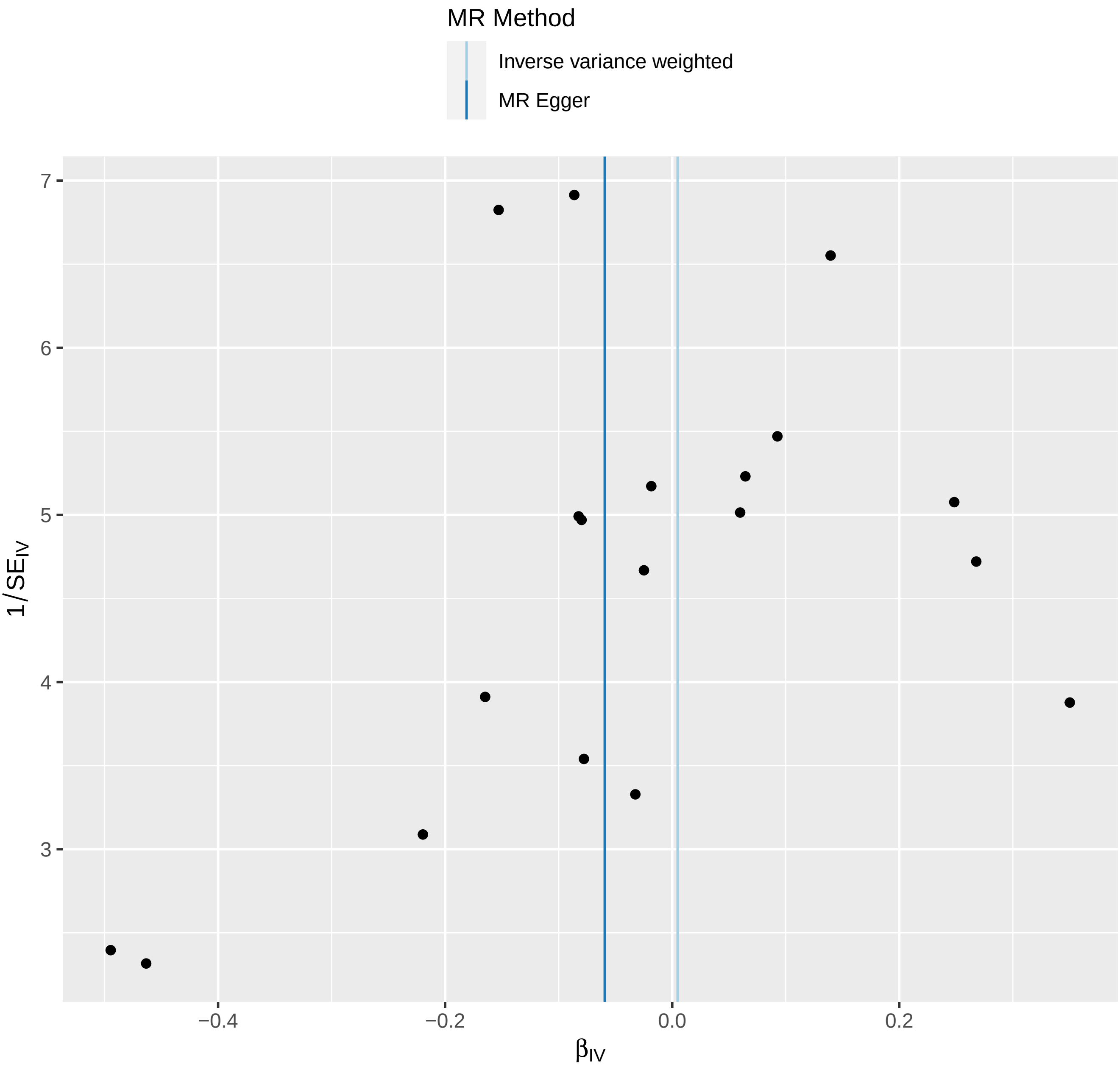

Batch 3−958 : Ephrin type−A receptor 5 on Diabetic nephropathy (more control exclusions)
with a threshold of 1e−05

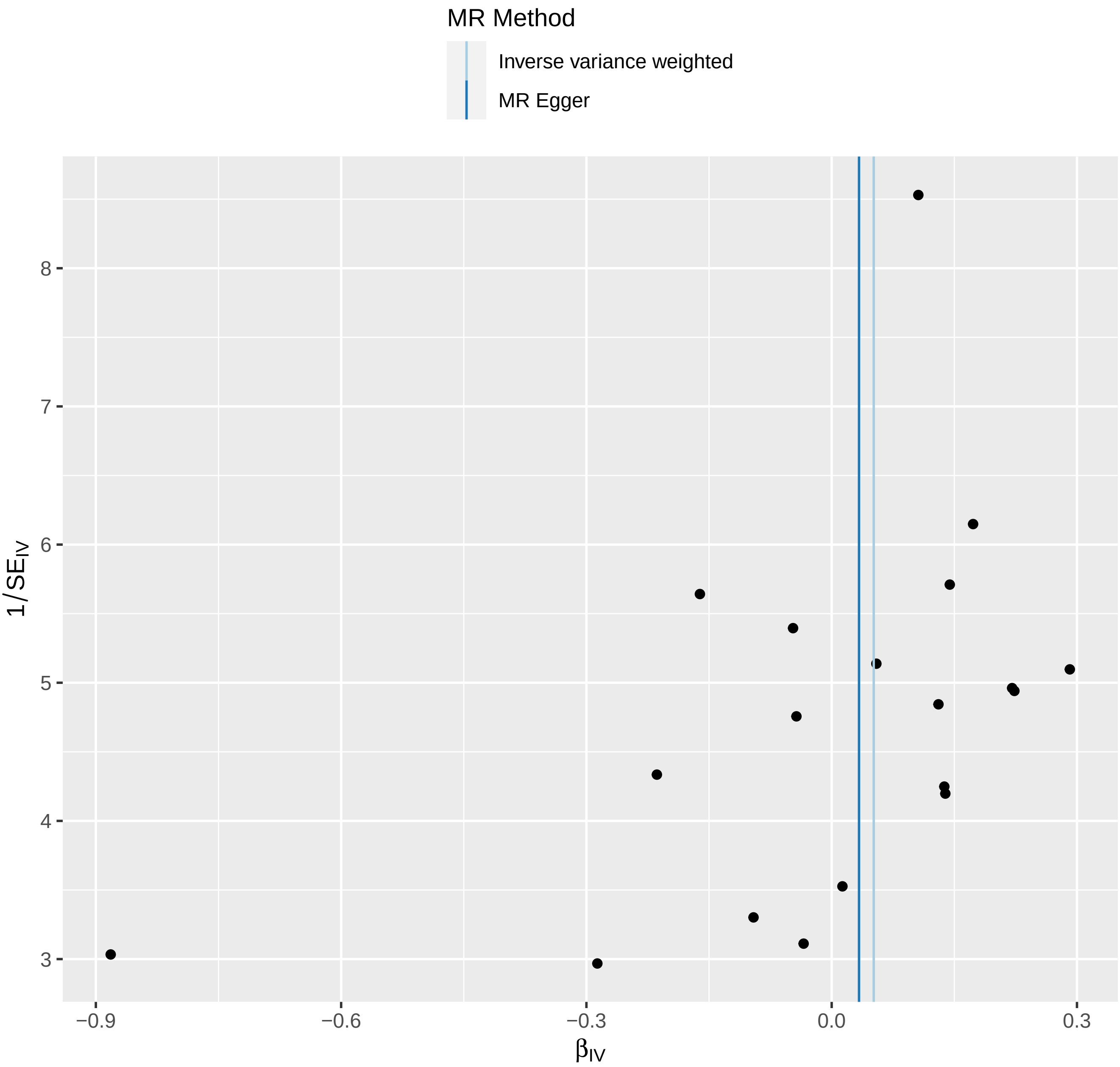

Batch 3−959 : Ephrin type−A receptor 5 on Diabetic nephropathy (more control exclusions)
with a threshold of 1e−05

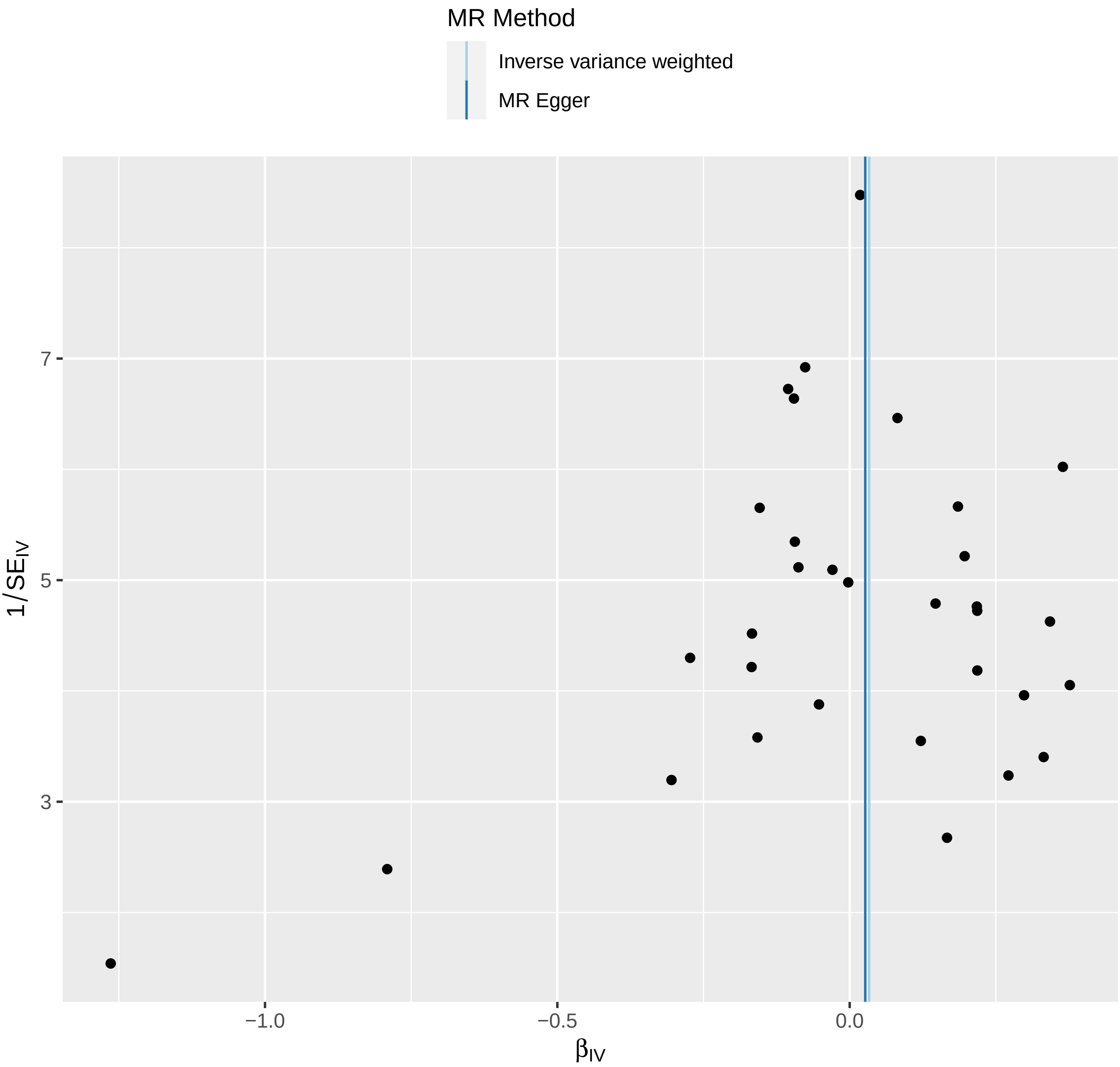

Batch 3−960 : Ephrin type−B receptor 2 on Diabetic nephropathy (more control exclusions)
with a threshold of 1e−05

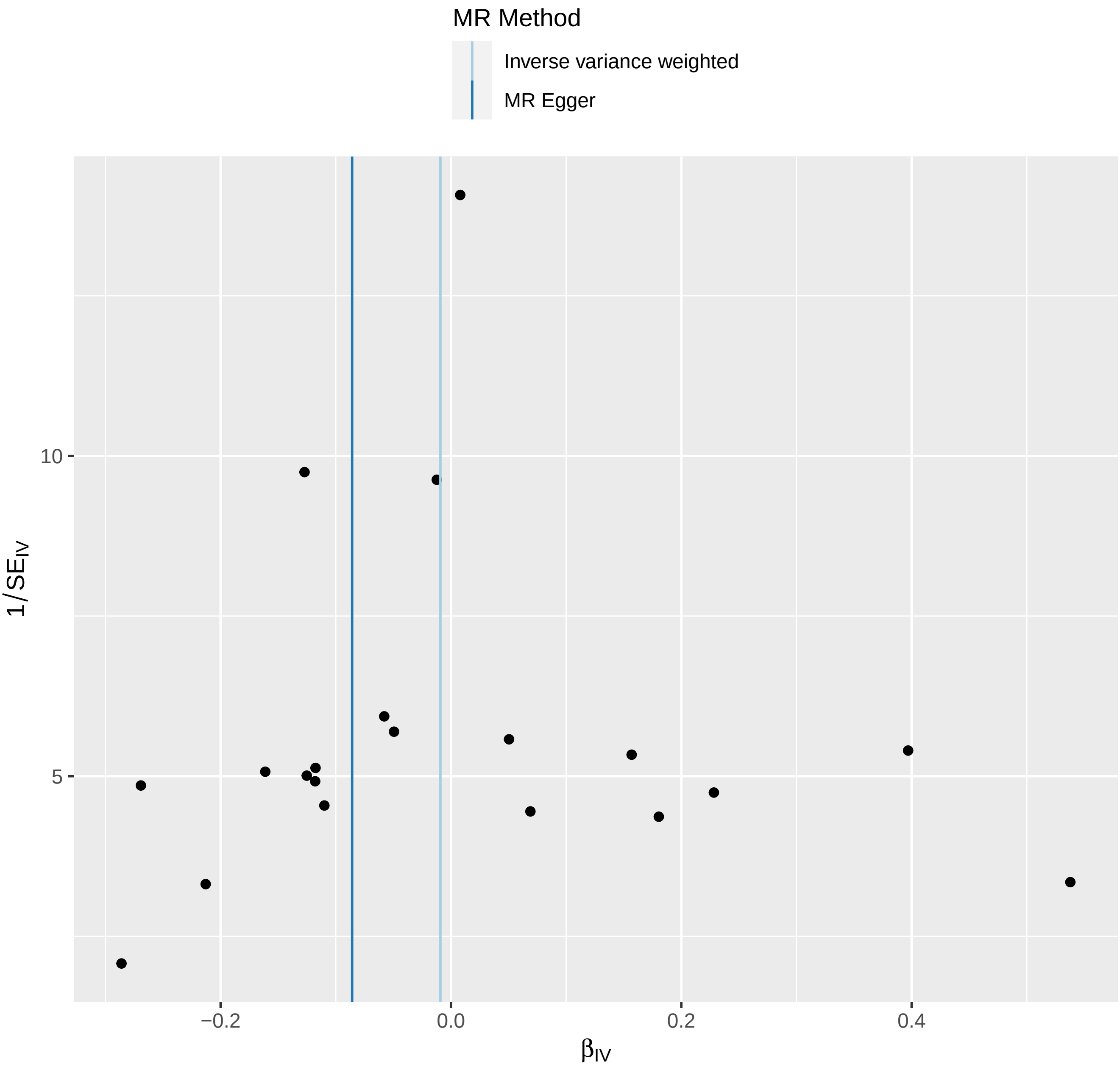

Batch 3−961 : Ephrin type−B receptor 2 on Diabetic nephropathy (more control exclusions)
with a threshold of 1e−05

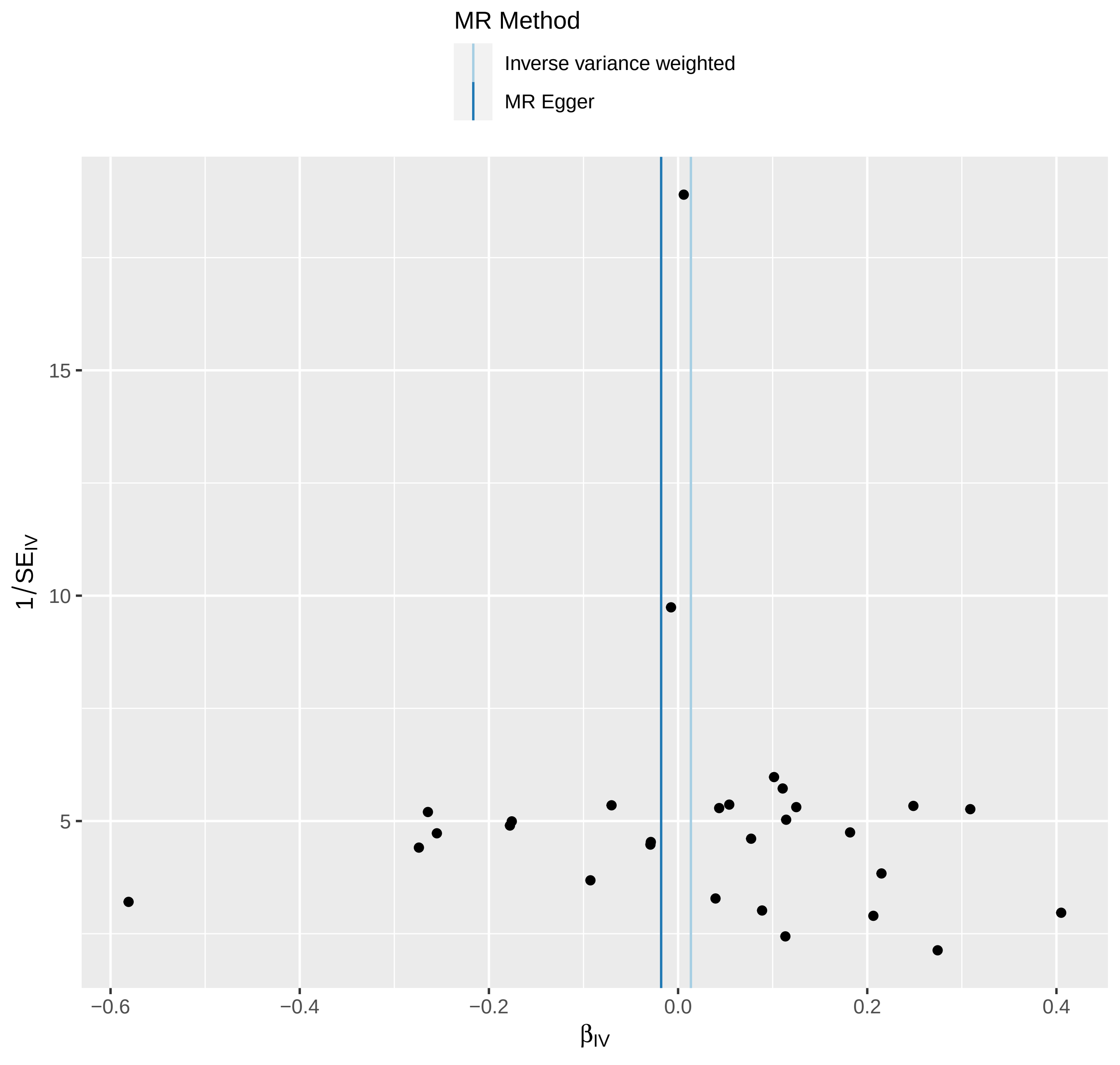

Batch 3−962 : Ephrin type−B receptor 2 on Diabetic nephropathy (more control exclusions)
with a threshold of 1e−05

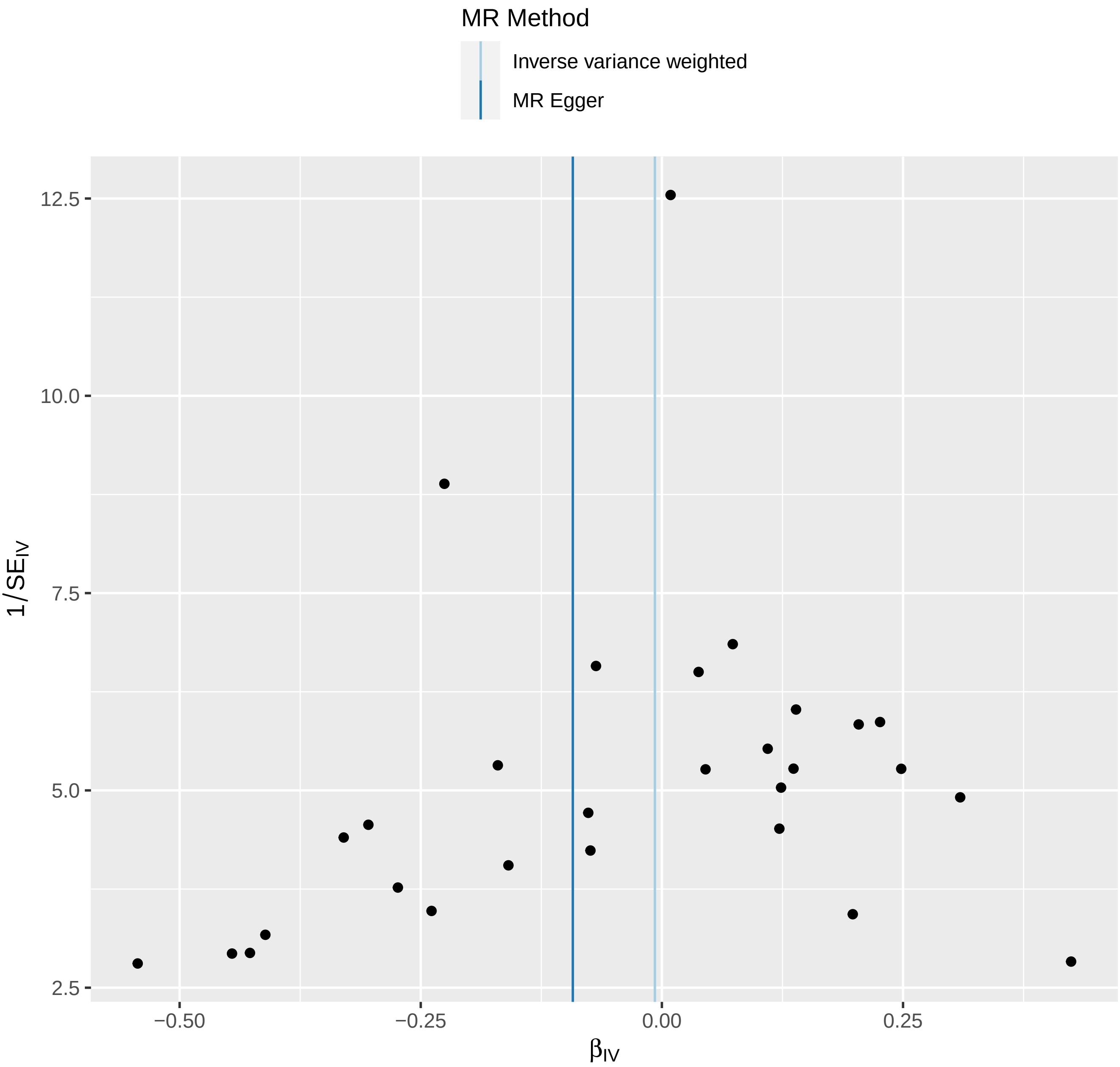

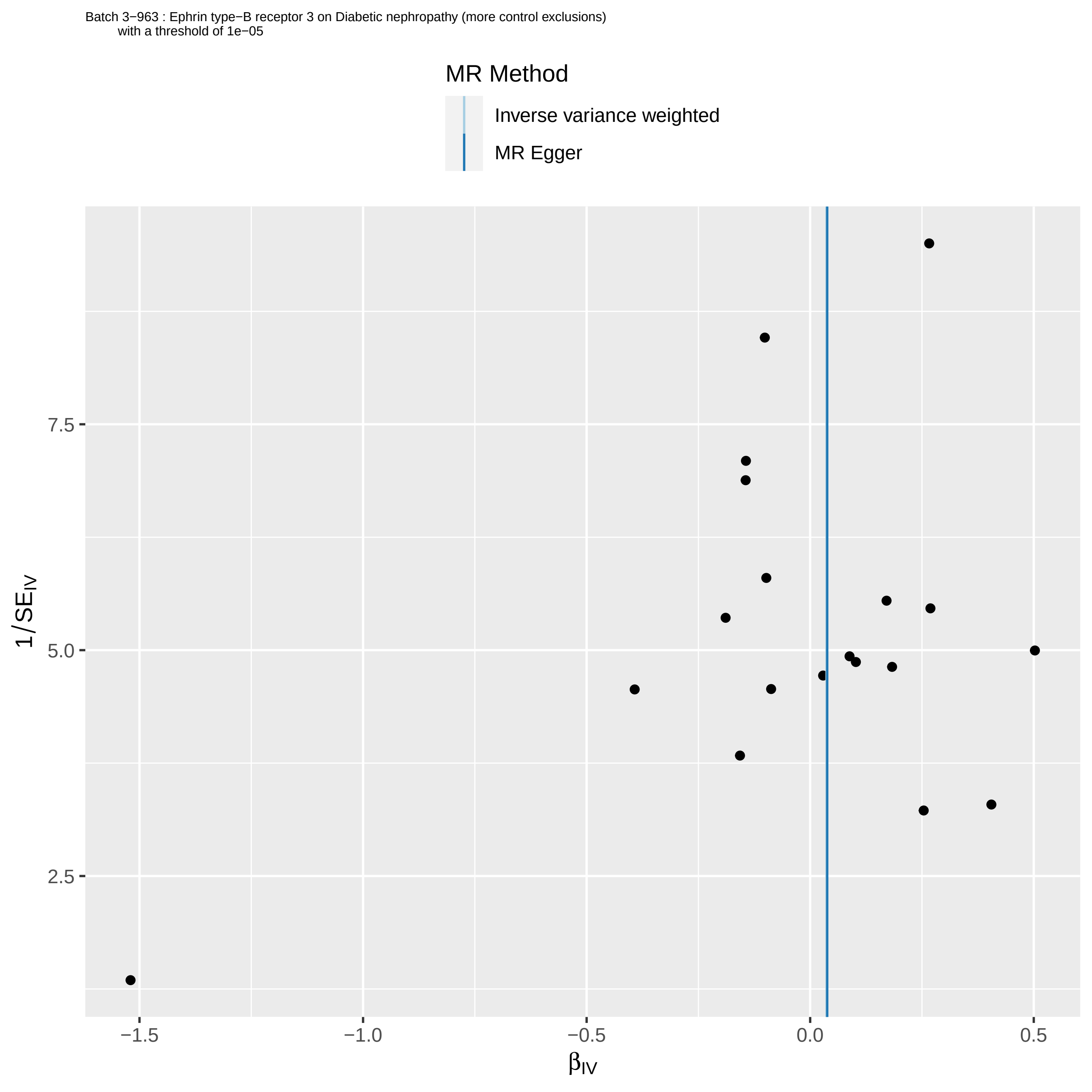
Batch 3−963 : Ephrin type−B receptor 3 on Diabetic nephropathy (more control exclusions)
with a threshold of 1e−05
MR Method
Inverse variance weighted
MR Egger
7.5
5.0
2.5
1/SE_IV
−1.5
−1.0
−0.5
0.0
0.5
β_IV

Batch 3−964 : Ephrin type−B receptor 4 on Diabetic nephropathy (more control exclusions)
with a threshold of 1e−05

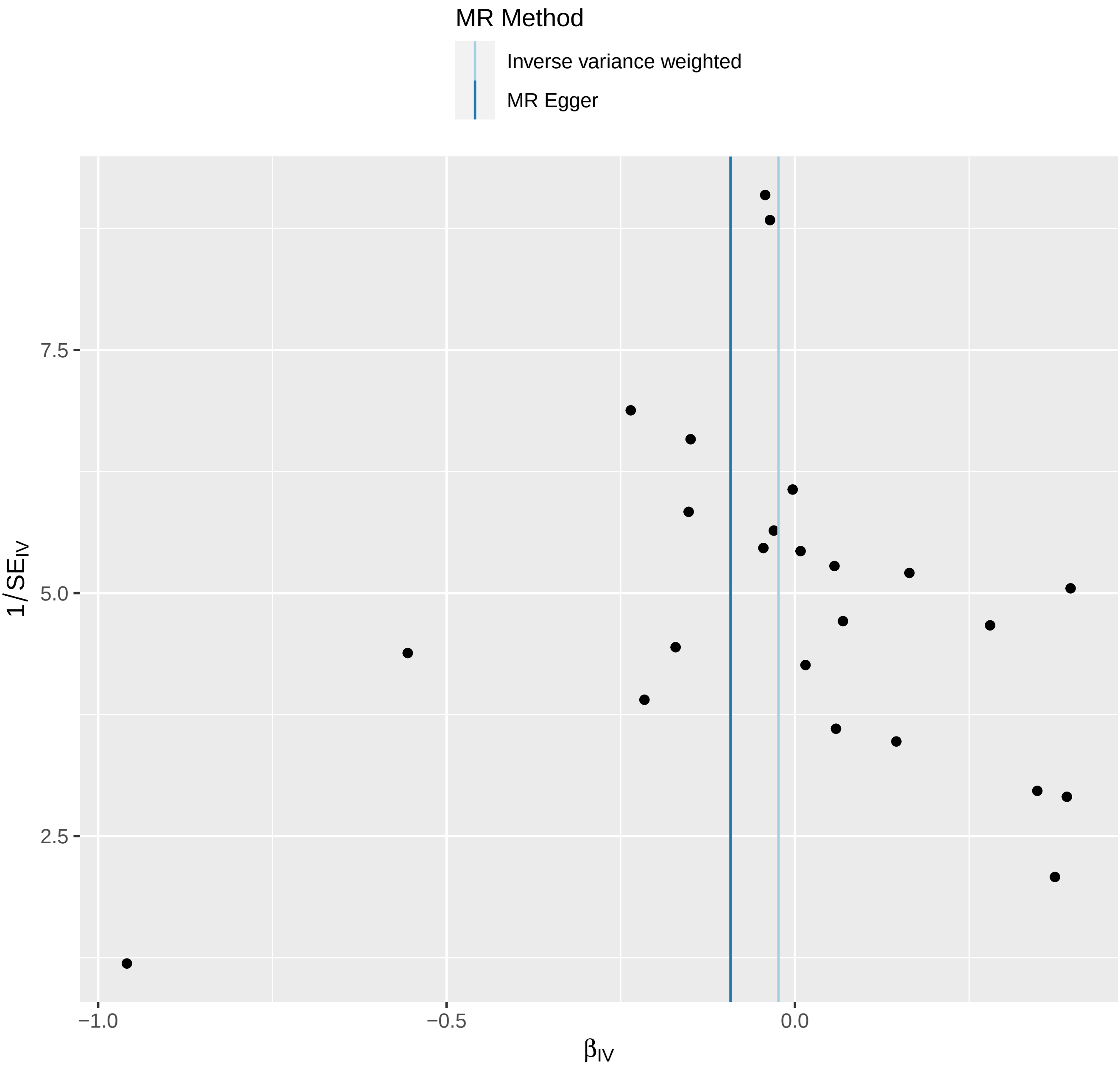

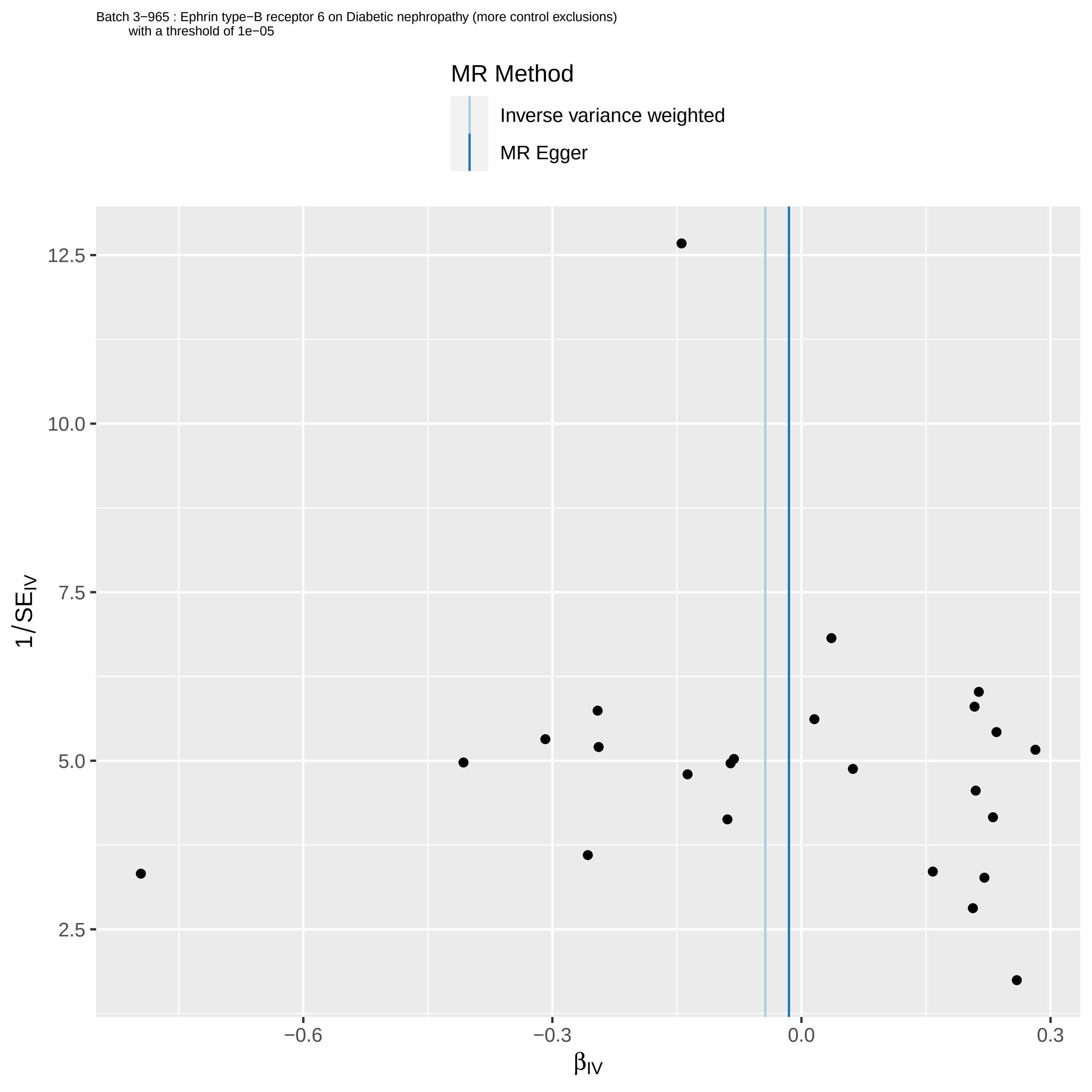
Batch 3−965 : Ephrin type−B receptor 6 on Diabetic nephropathy (more control exclusions)
with a threshold of 1e−05
MR Method
Inverse variance weighted
MR Egger
12.5
10.0
7.5
5.0
2.5
1/SE_IV
−0.6
−0.3
0.0
0.3
β_IV

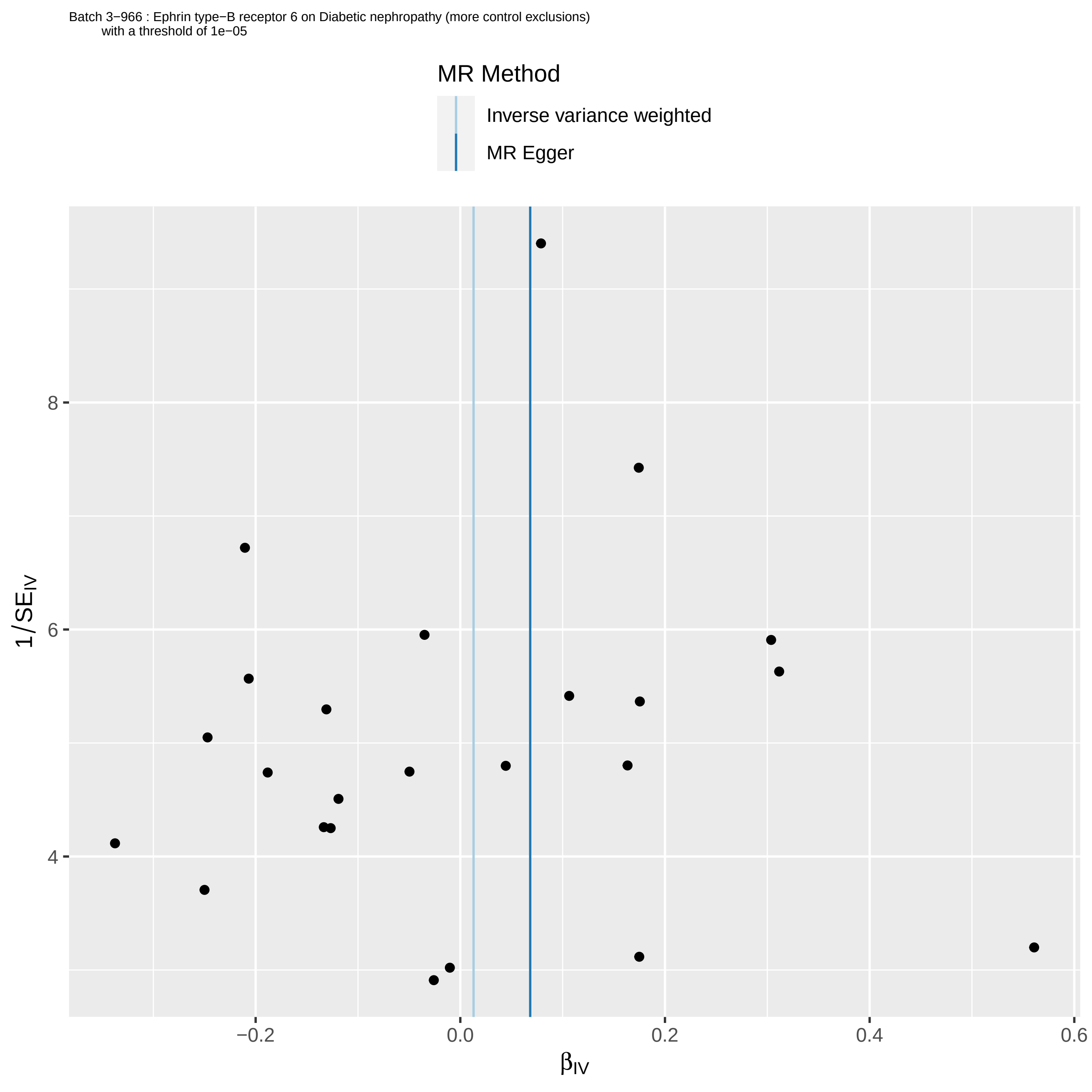

Batch 3−966 : Ephrin type−B receptor 6 on Diabetic nephropathy (more control exclusions)
with a threshold of 1e−05
MR Method
Inverse variance weighted
MR Egger
1/SE$_{IV}$
8
6
4
−0.2
0.0
0.2
0.4
0.6
β$_{IV}$

Batch 3−967 : Erythropoietin on Diabetic nephropathy (more control exclusions)
with a threshold of 1e−05

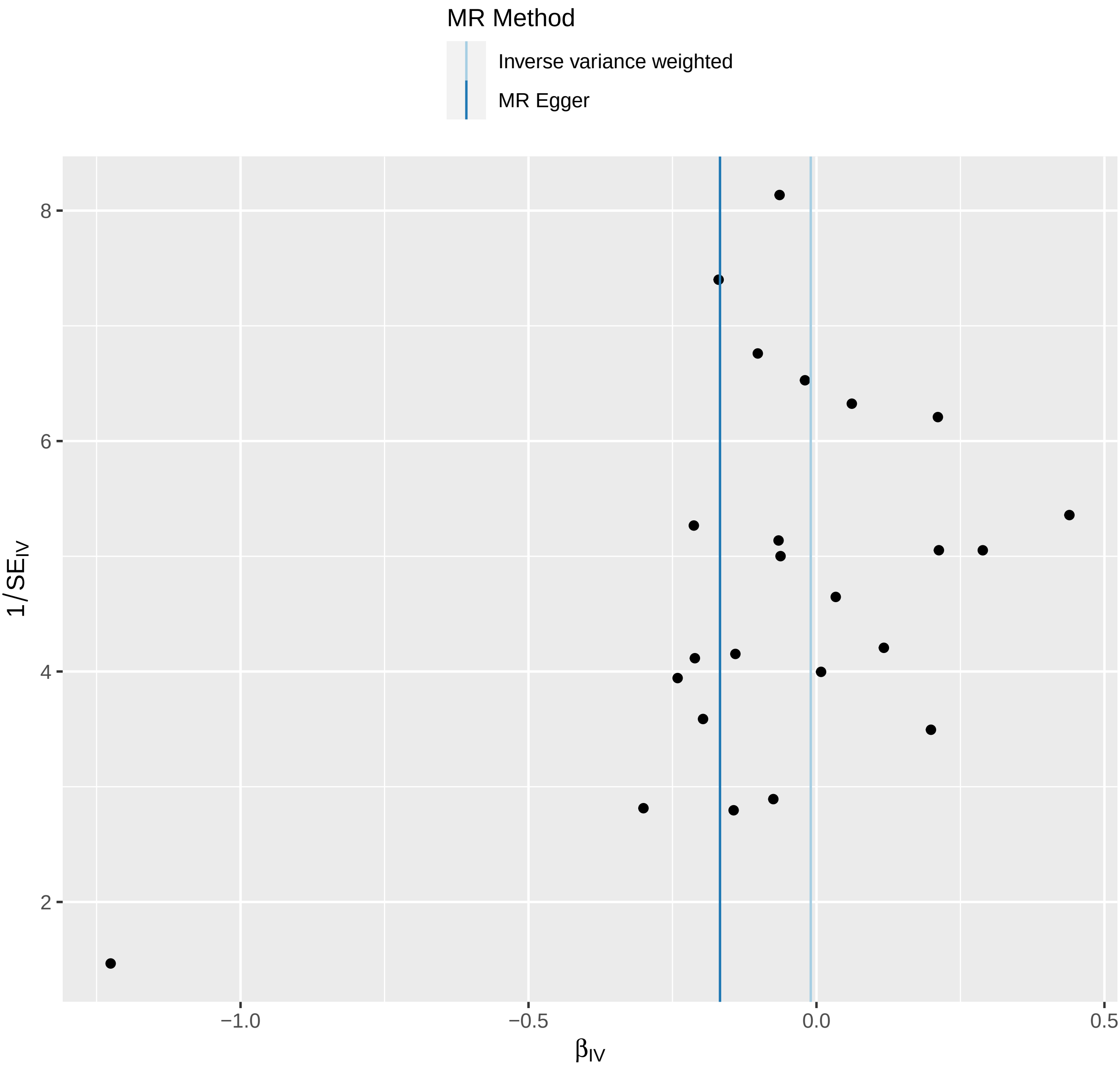

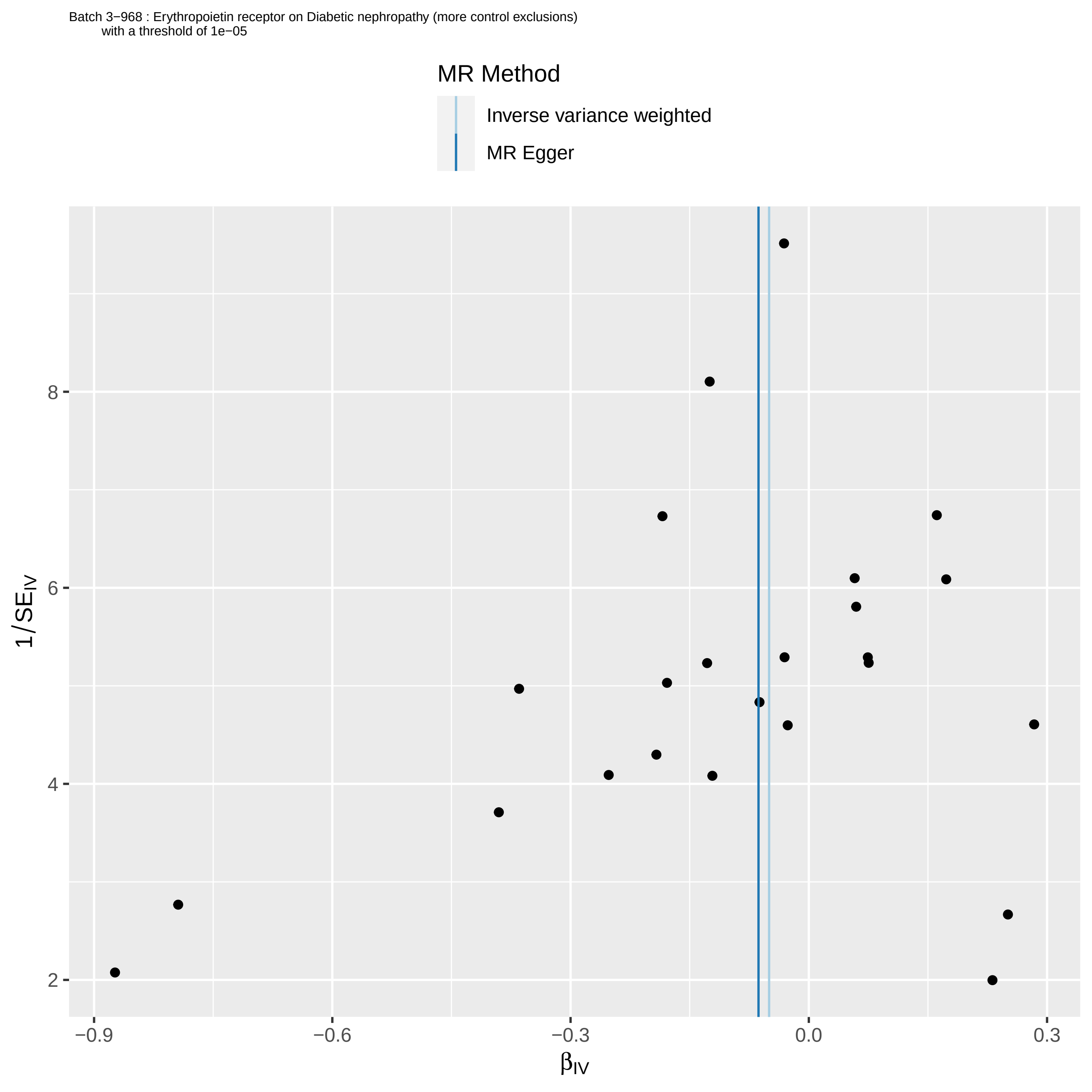

Batch 3−968 : Erythropoietin receptor on Diabetic nephropathy (more control exclusions)
with a threshold of 1e−05
MR Method
Inverse variance weighted
MR Egger
1/SE_IV
β_IV
−0.9
−0.6
−0.3
0.0
0.3
2
4
6
8

Batch 3−969 : Epidermal growth factor receptor substrate 15−like 1 on Diabetic nephropathy (more control exclusions) with a threshold of 1e−05

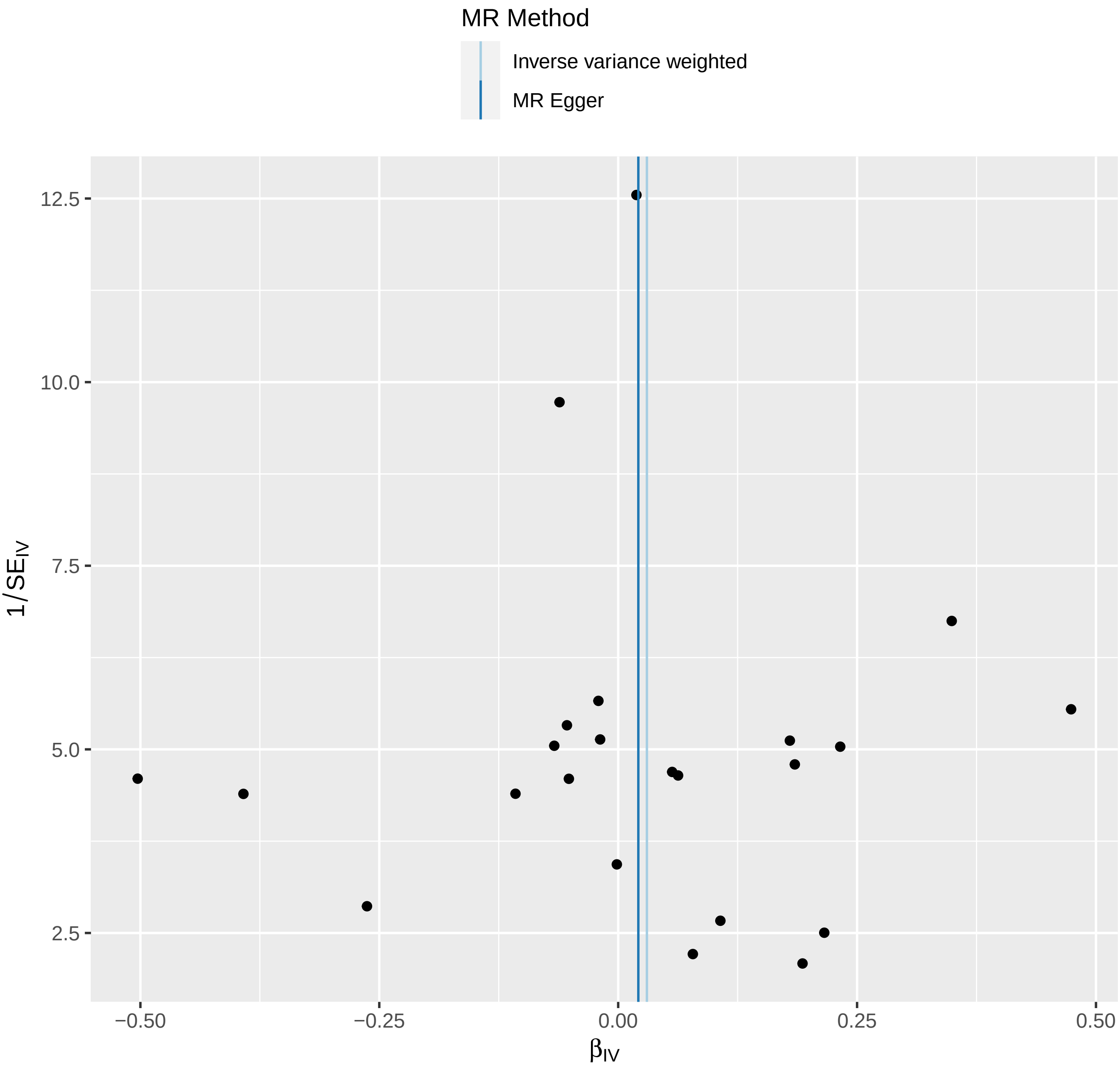

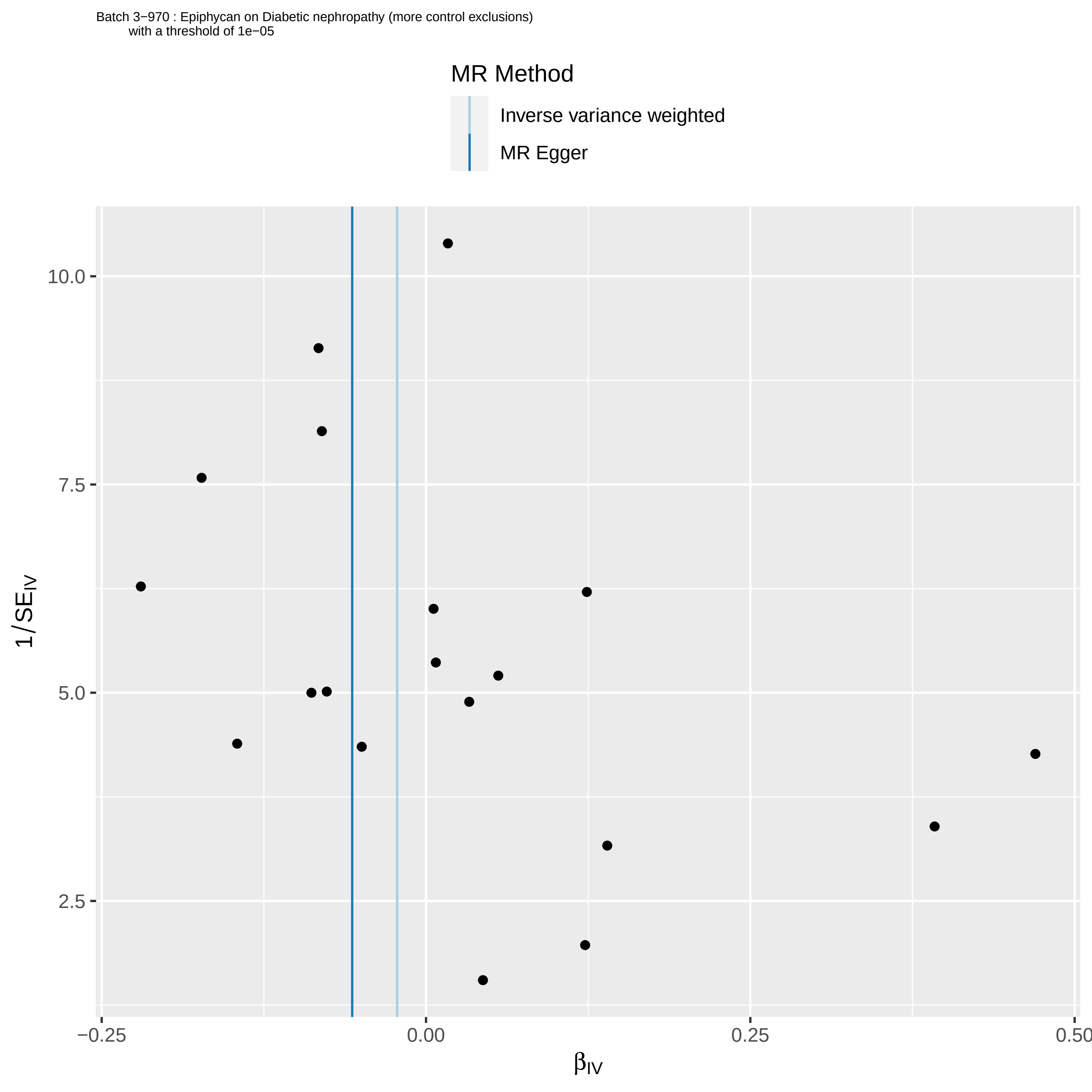

Batch 3−970 : Epiphycan on Diabetic nephropathy (more control exclusions)
with a threshold of 1e−05
MR Method
Inverse variance weighted
MR Egger
10.0
7.5
5.0
2.5
1/SE$_{IV}$
−0.25
0.00
0.25
0.50
β$_{IV}$

Batch 3−971 : Endoplasmic reticulum aminopeptidase 1 on Diabetic nephropathy (more control exclusions)
with a threshold of 1e−05

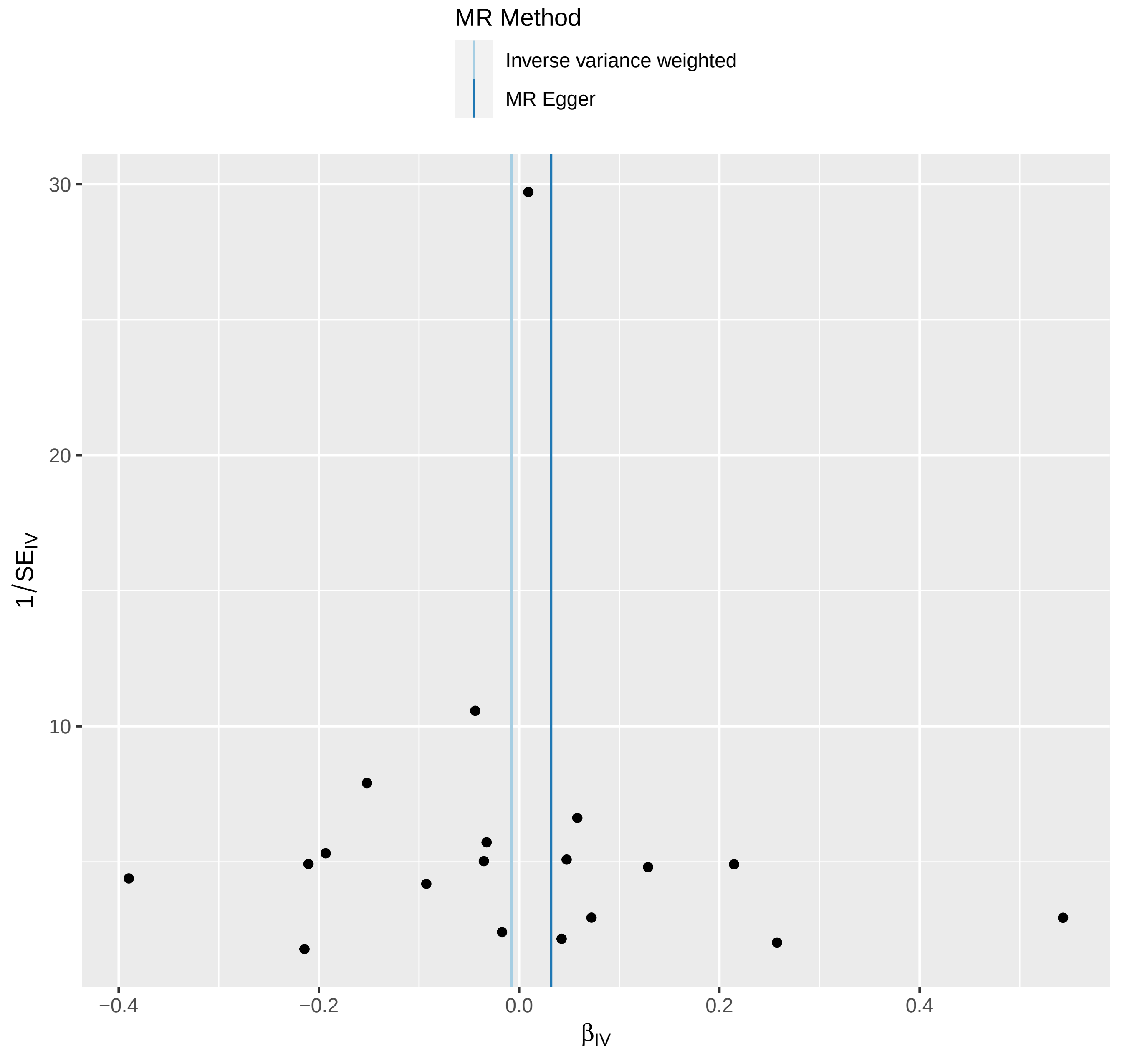

Batch 3−972 : Endoplasmic reticulum aminopeptidase 2 on Diabetic nephropathy (more control exclusions)
with a threshold of 1e−05

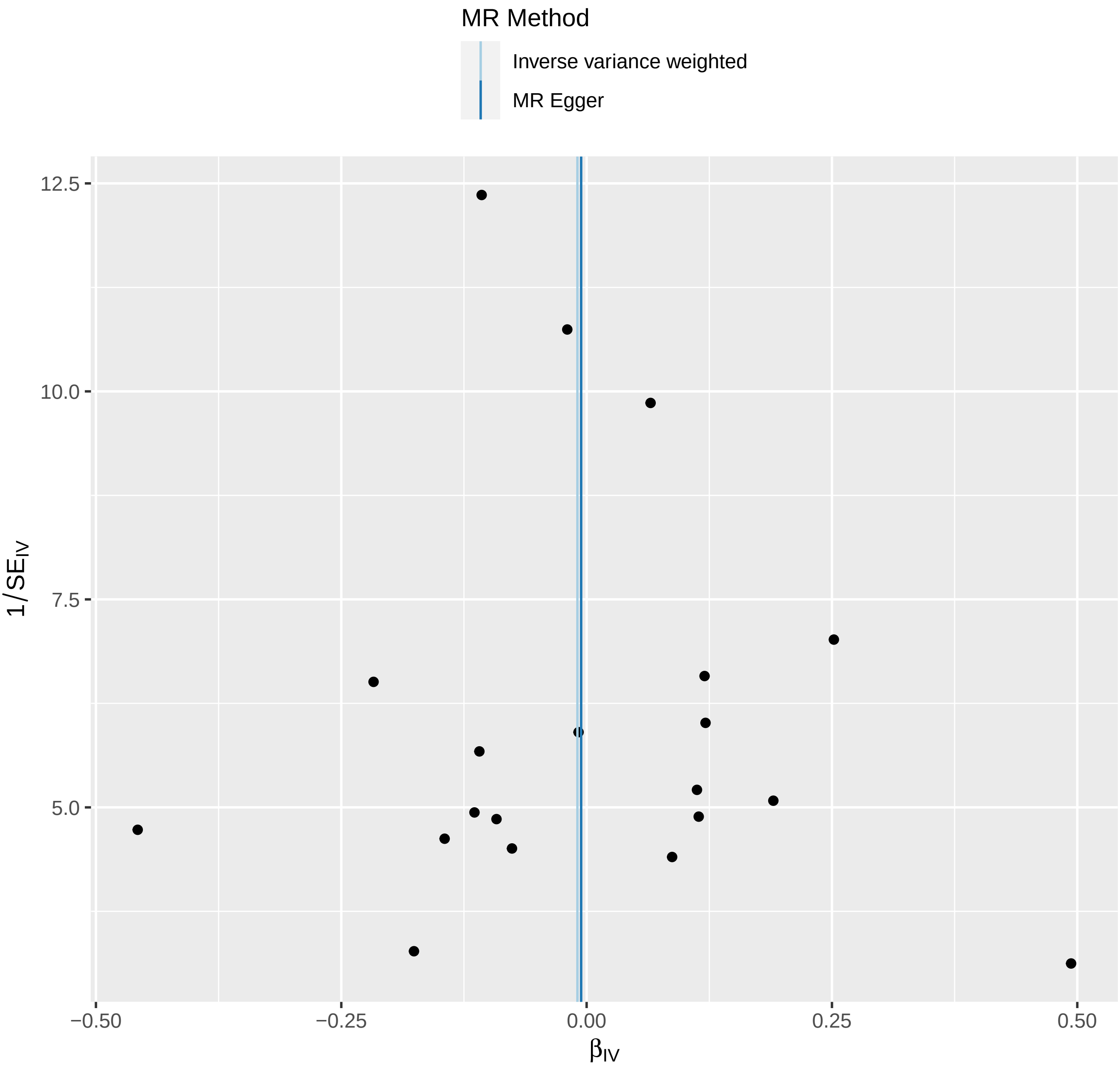

Batch 3−973 : Receptor tyrosine−protein kinase erbB−2 on Diabetic nephropathy (more control exclusions)
with a threshold of 1e−05

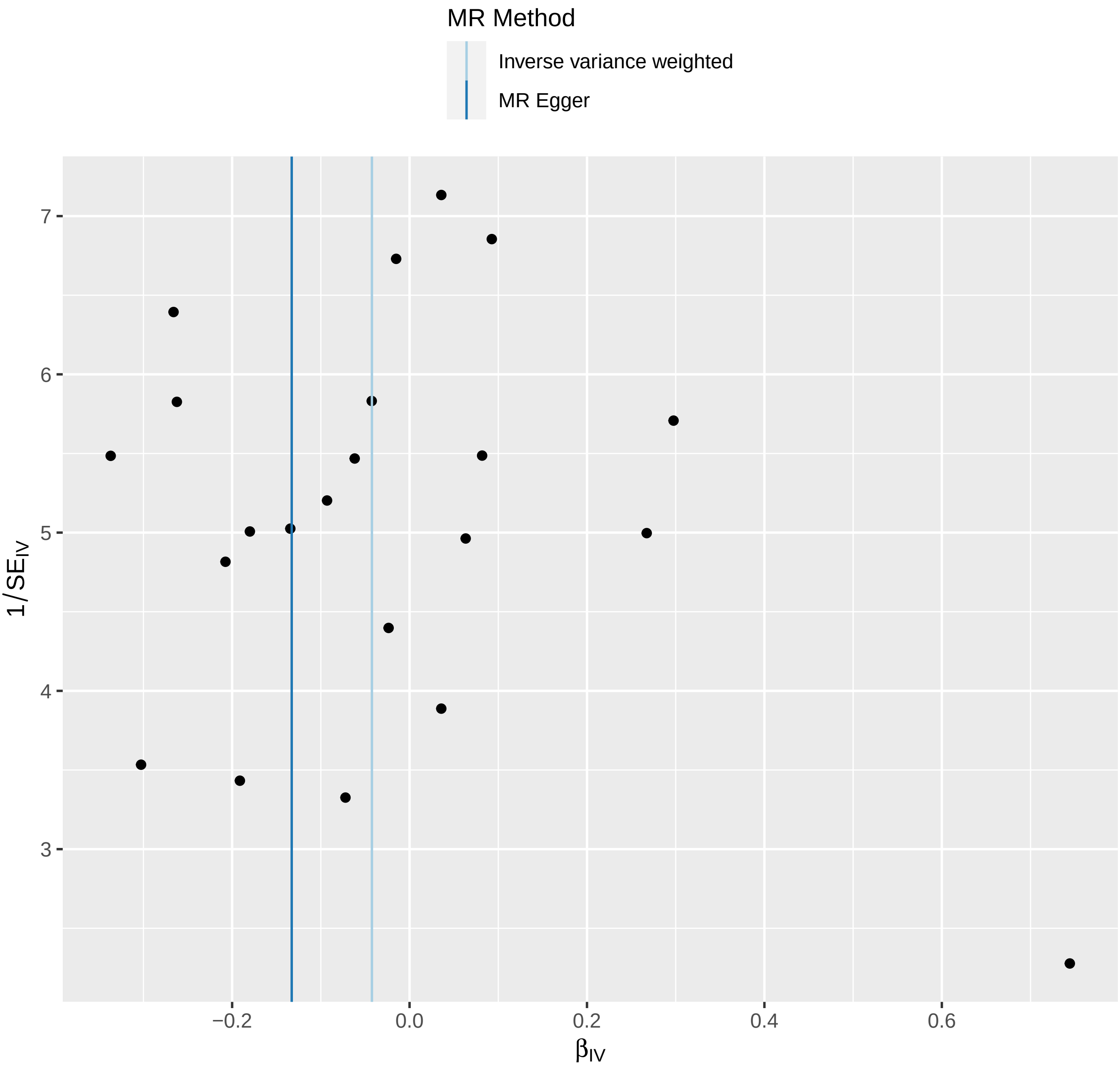

Batch 3−974 : Receptor tyrosine−protein kinase erbB−3 on Diabetic nephropathy (more control exclusions)
with a threshold of 1e−05

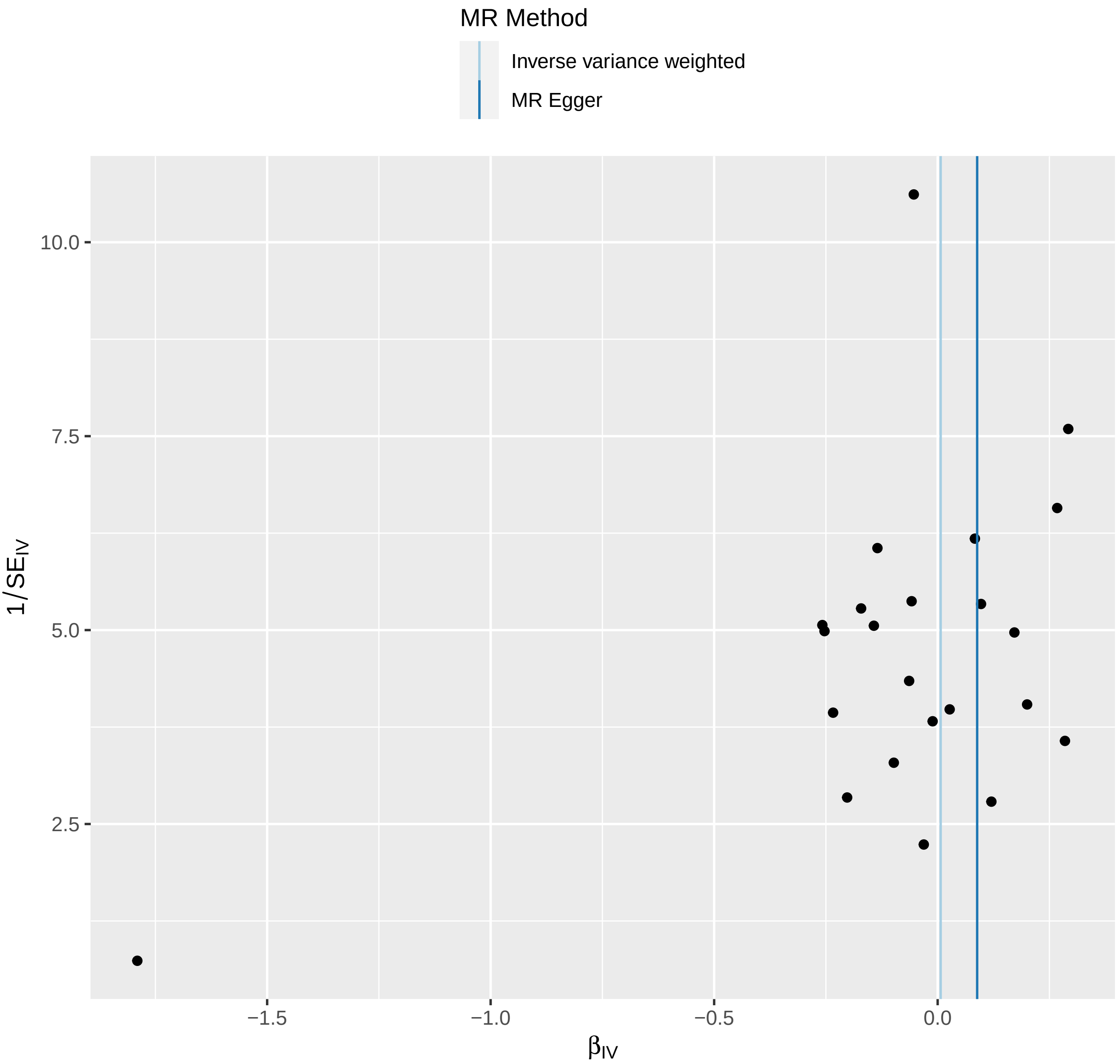

Batch 3−975 : DNA excision repair protein ERCC−1 on Diabetic nephropathy (more control exclusions)
with a threshold of 1e−05

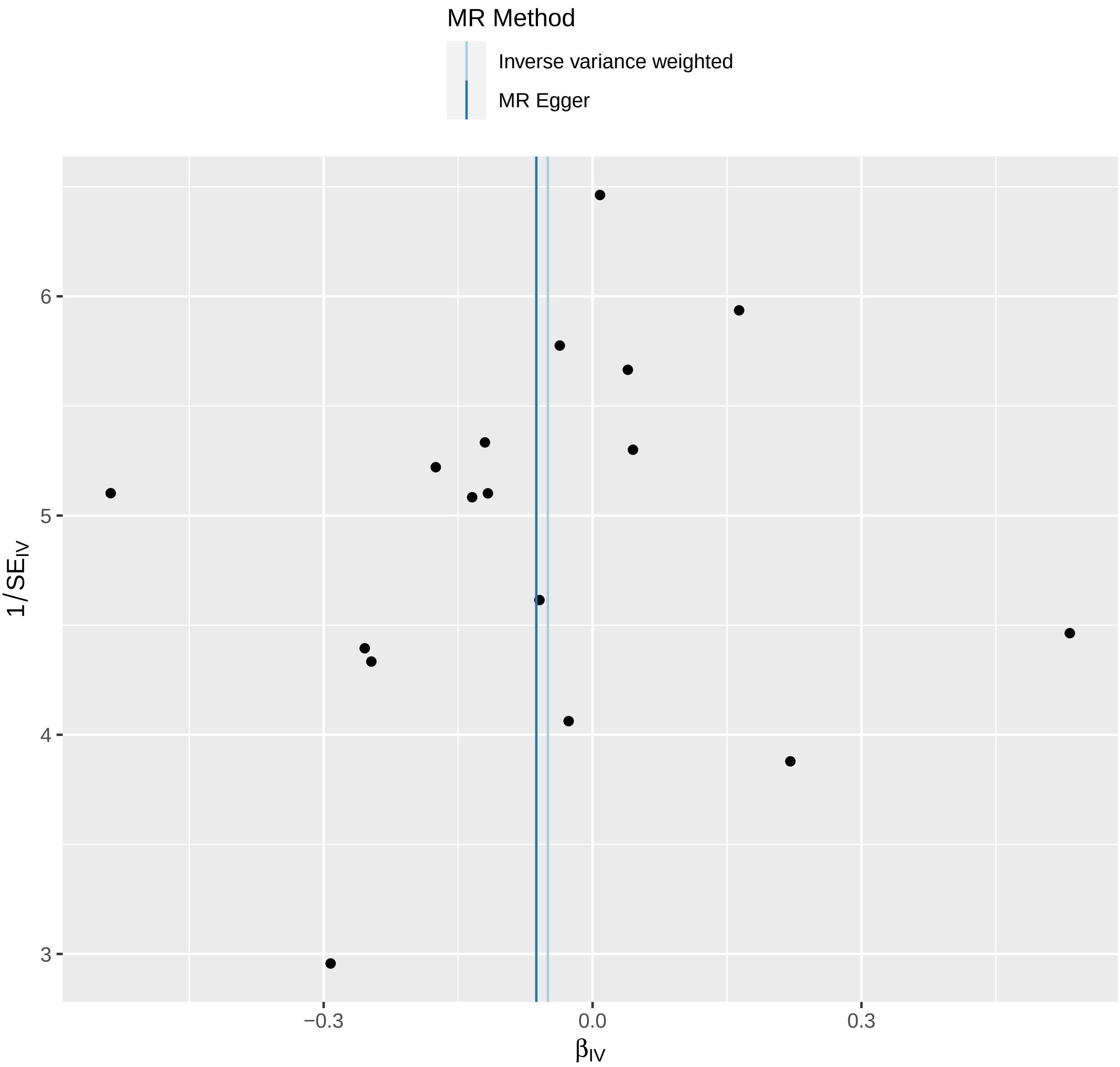

Batch 3−976 : DNA repair endonuclease XPF on Diabetic nephropathy (more control exclusions)
with a threshold of 1e−05

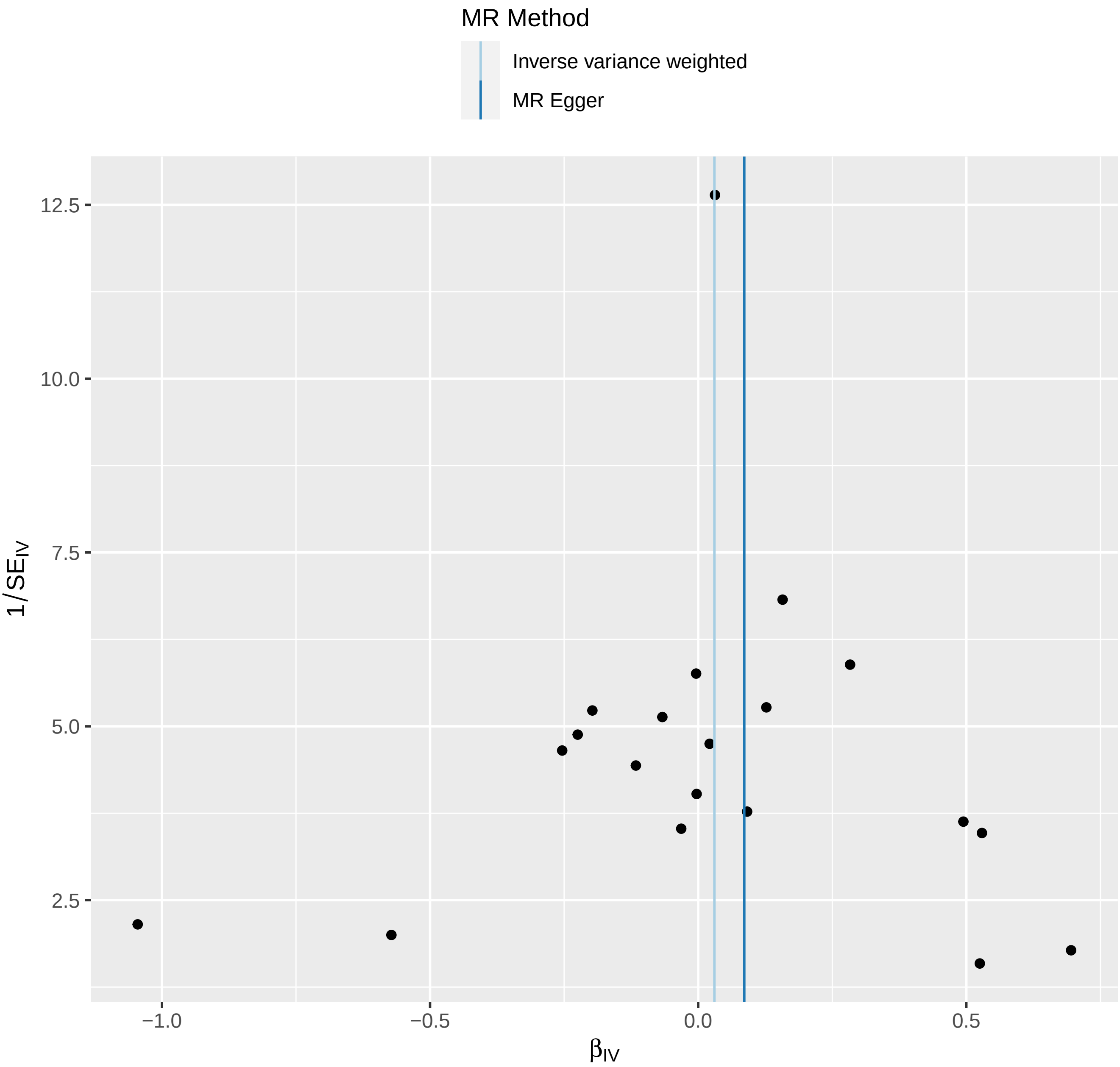

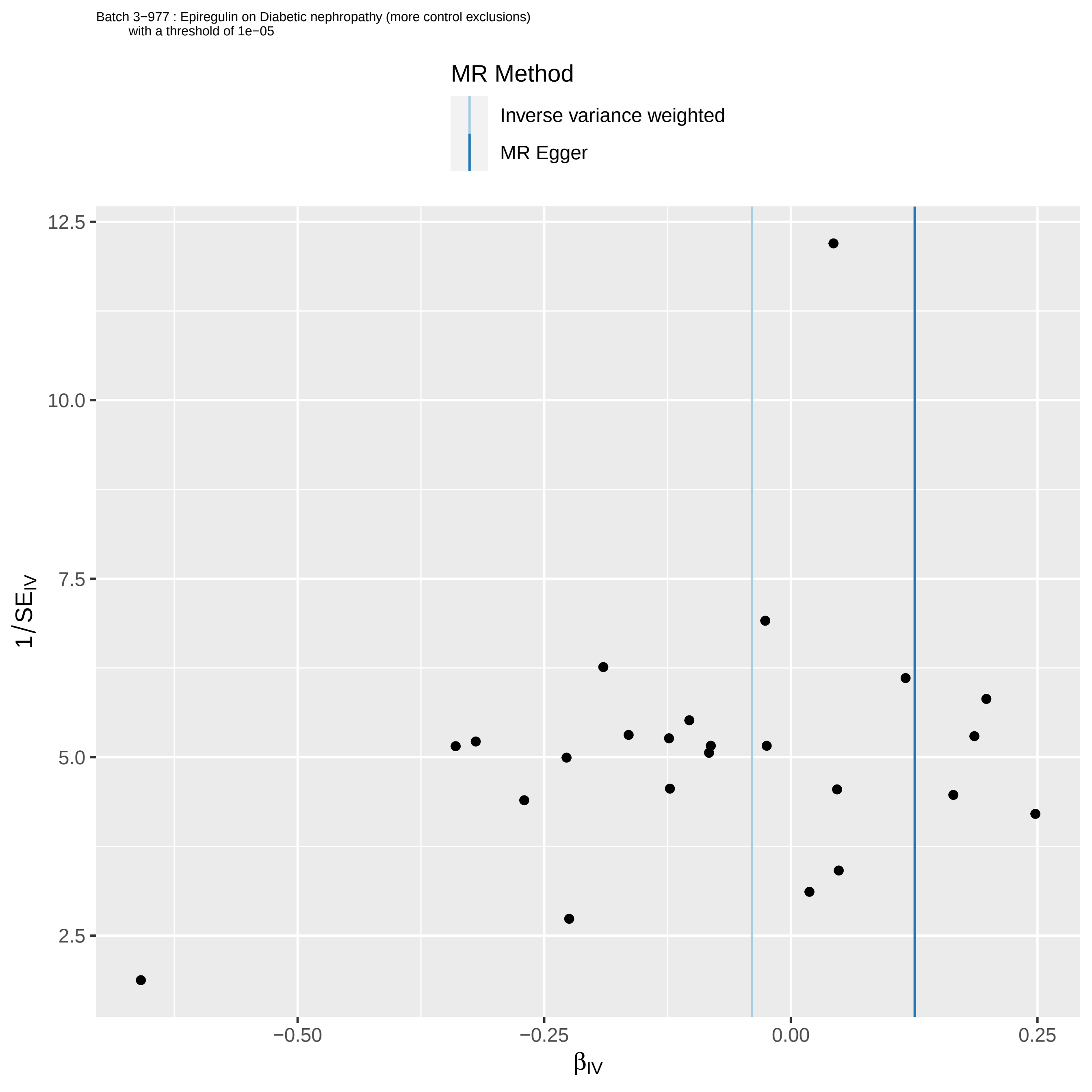
Batch 3−977 : Epiregulin on Diabetic nephropathy (more control exclusions)
with a threshold of 1e−05
MR Method
Inverse variance weighted
MR Egger
12.5
10.0
7.5
5.0
2.5
1/SE_IV
−0.50
−0.25
0.00
0.25
β_IV

Batch 3−978 : Enhancer of rudimentary homolog on Diabetic nephropathy (more control exclusions)
with a threshold of 1e−05

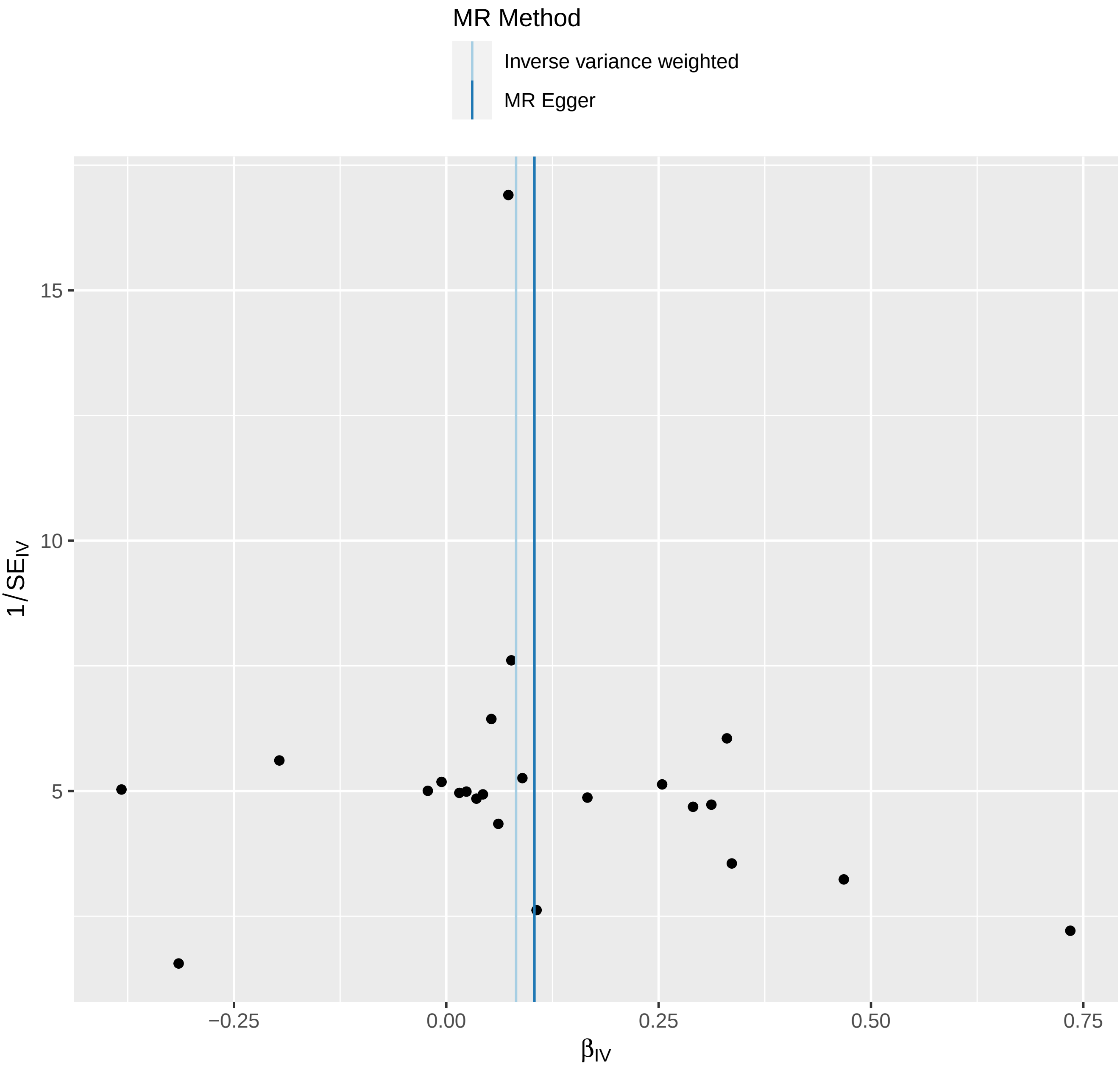

Batch 3−979 : Endoplasmic reticulum lectin 1 on Diabetic nephropathy (more control exclusions)
with a threshold of 1e−05

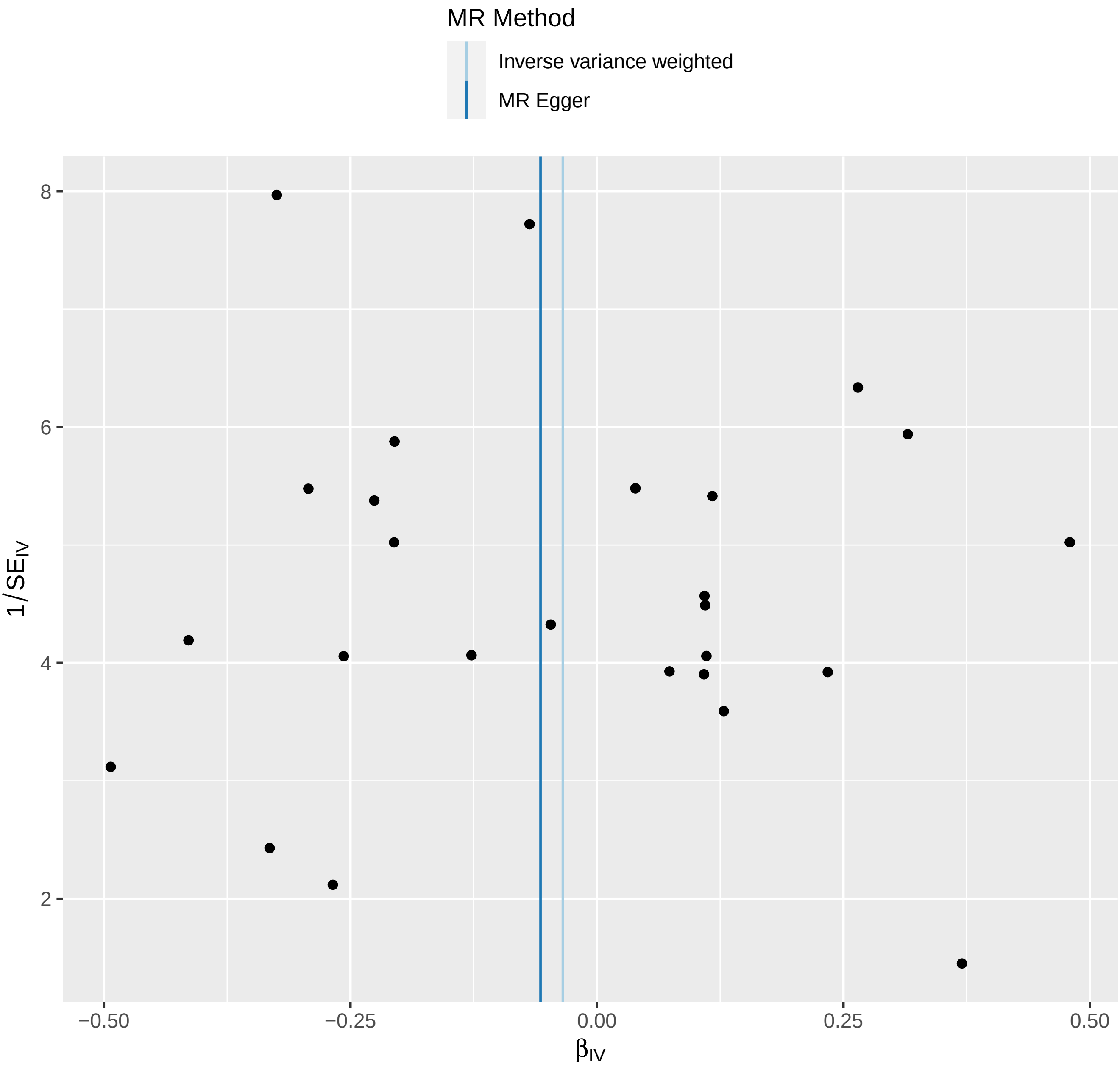

Batch 3−980 : Erlin−1 on Diabetic nephropathy (more control exclusions)
with a threshold of 1e−05

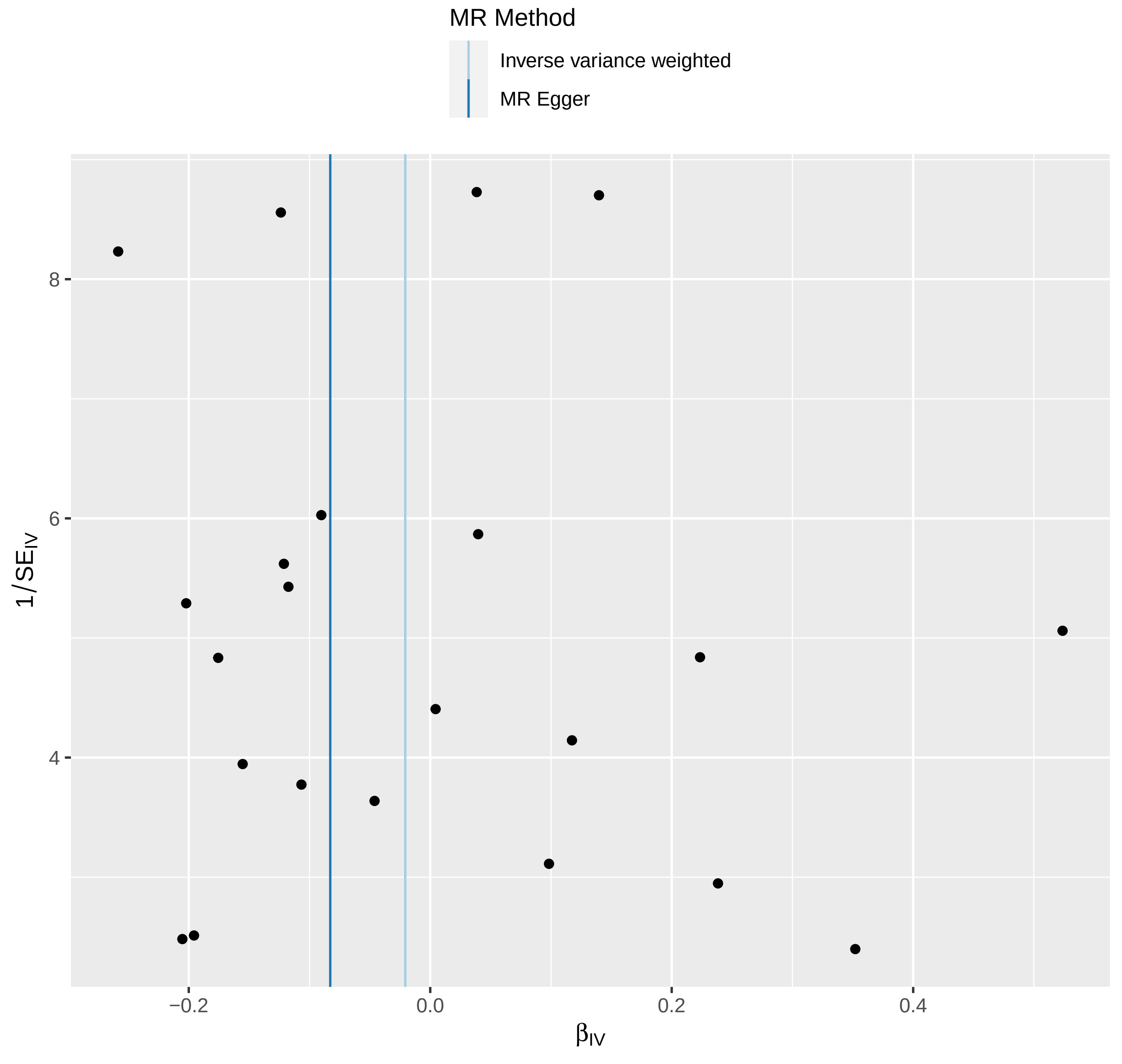

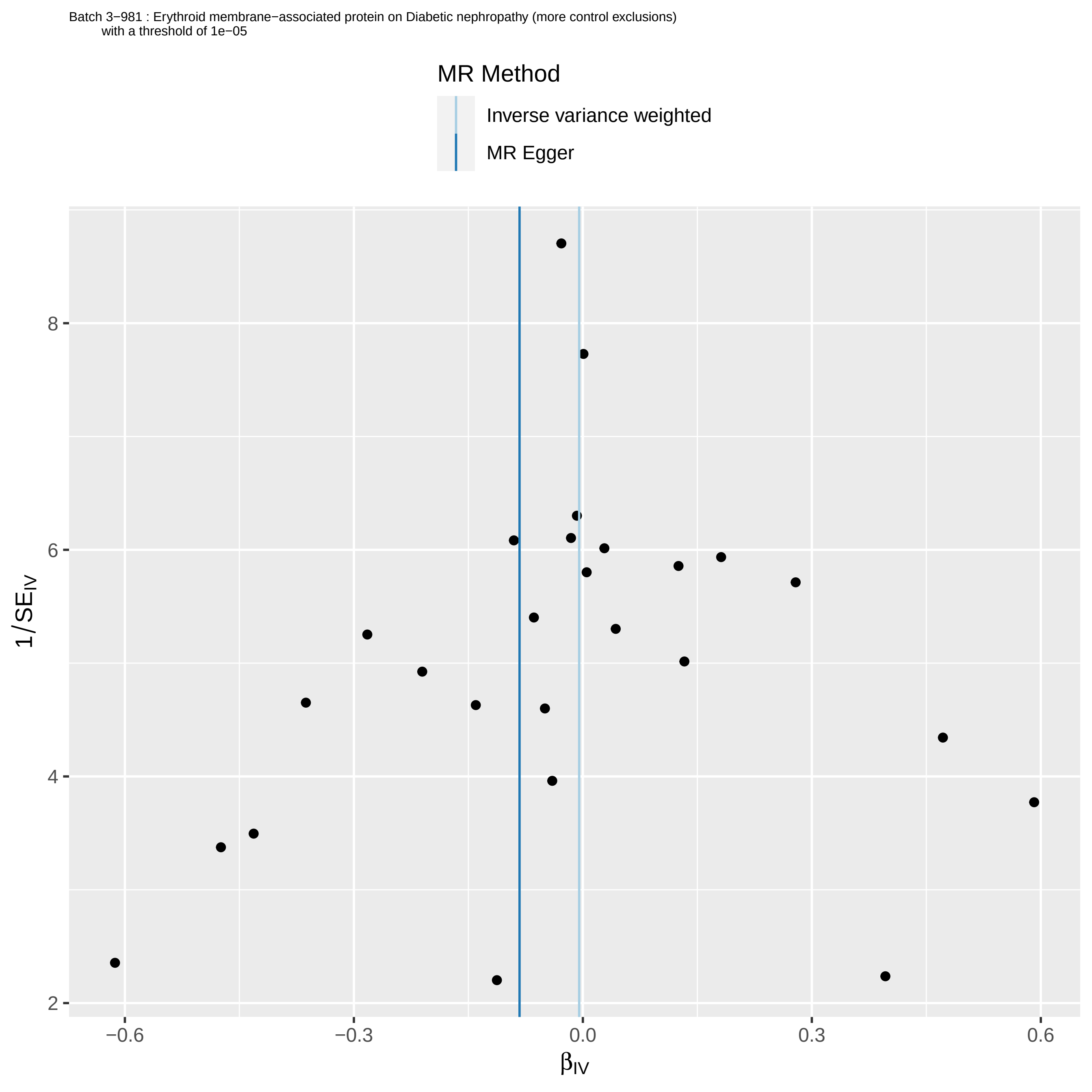

Batch 3−981 : Erythroid membrane−associated protein on Diabetic nephropathy (more control exclusions)
with a threshold of 1e−05
MR Method
Inverse variance weighted
MR Egger
8
6
4
2
1/SE_IV
−0.6
−0.3
0.0
0.3
0.6
β_IV

Batch 3−982 : ERO1−like protein alpha on Diabetic nephropathy (more control exclusions)
with a threshold of 1e−05

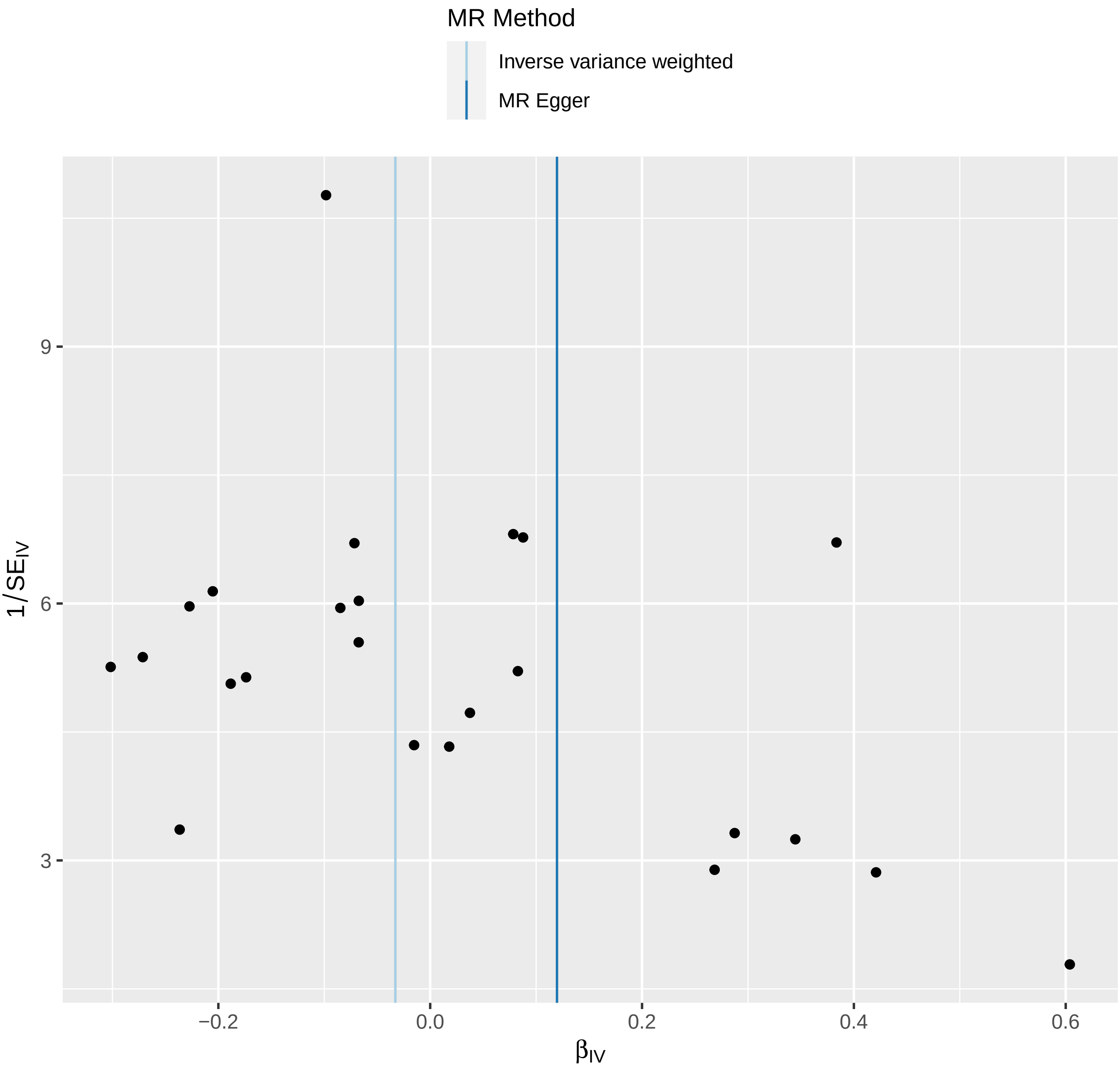

Batch 3−983 : ERO1−like protein beta on Diabetic nephropathy (more control exclusions)
with a threshold of 1e−05

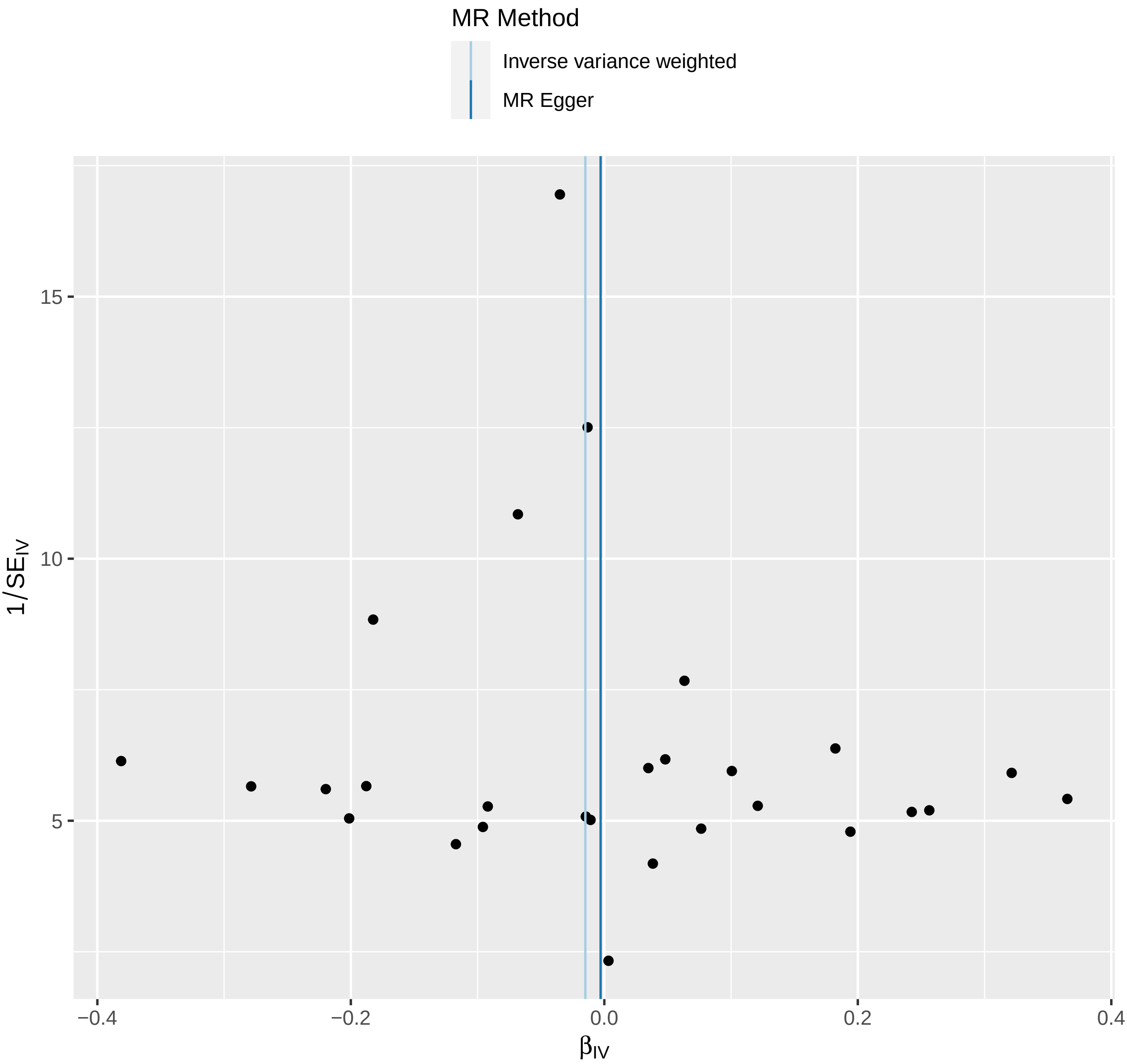

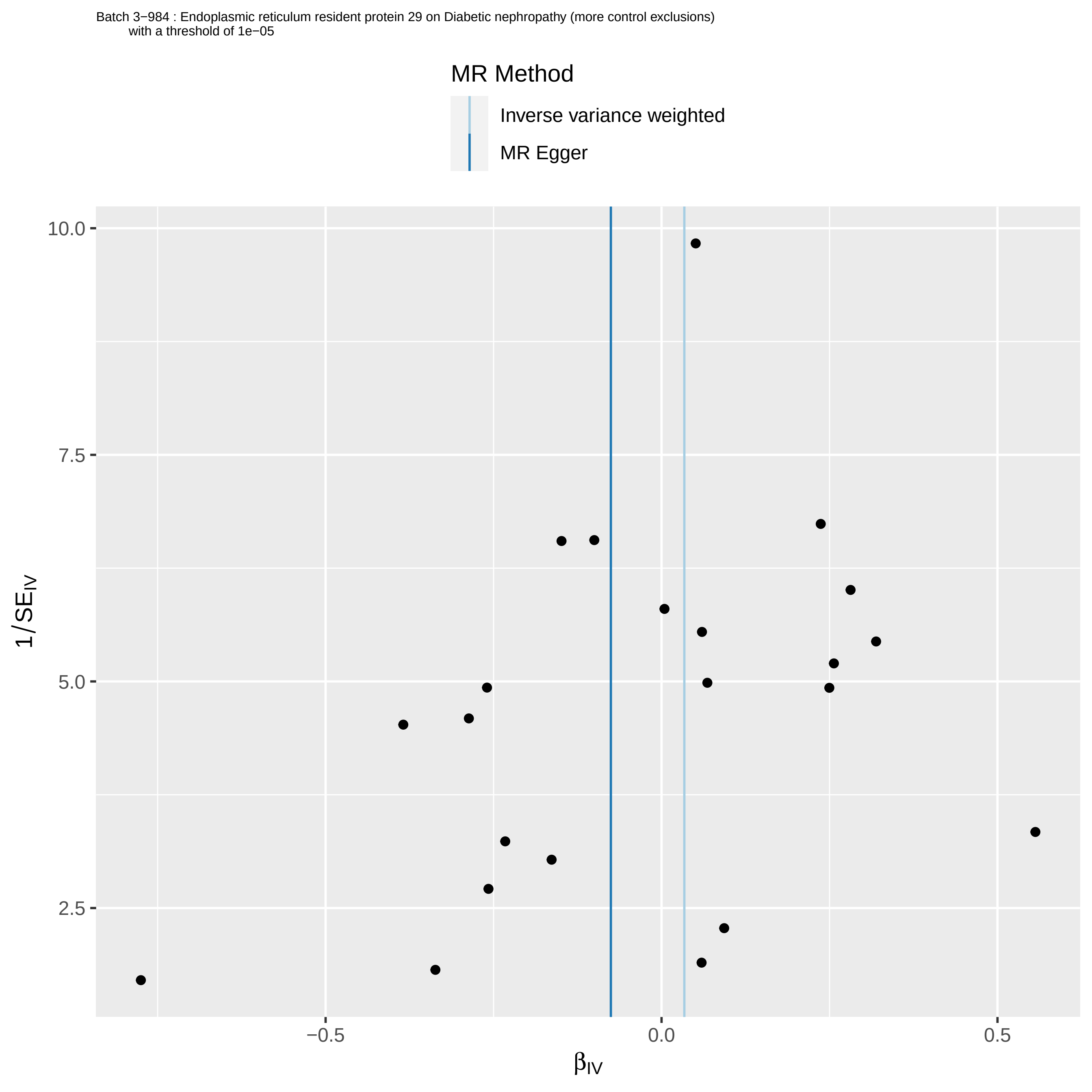
Batch 3−984 : Endoplasmic reticulum resident protein 29 on Diabetic nephropathy (more control exclusions)
with a threshold of 1e−05
MR Method
Inverse variance weighted
MR Egger
10.0
7.5
5.0
2.5
1/SE_IV
−0.5
0.0
0.5
β_IV

Batch 3−985 : Endoplasmic reticulum resident protein 44 on Diabetic nephropathy (more control exclusions)
with a threshold of 1e−05

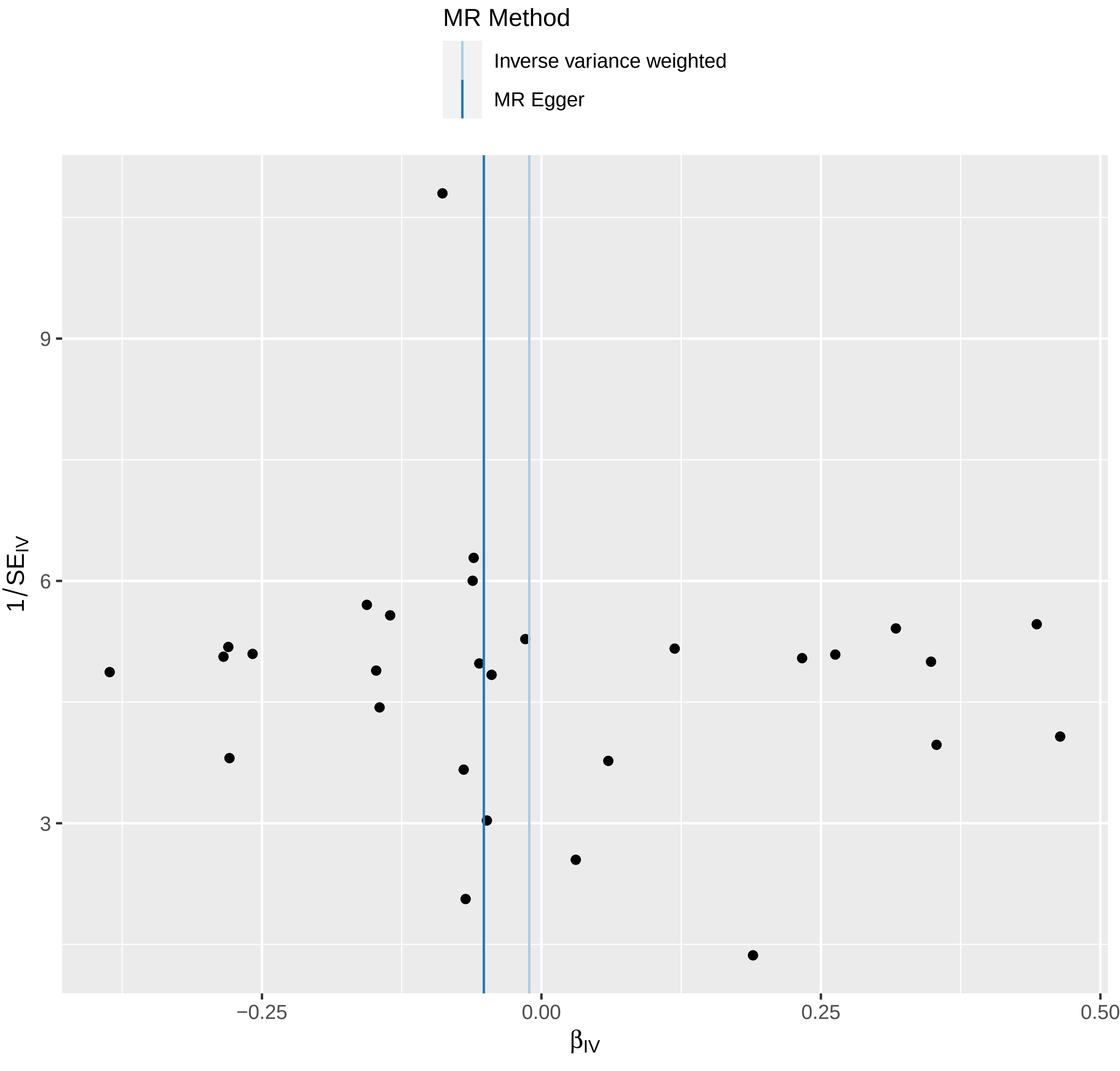

Batch 3−986 : Endogenous retrovirus group V member 1 Env polyprotein on Diabetic nephropathy (more control exclusions) with a threshold of 1e−05

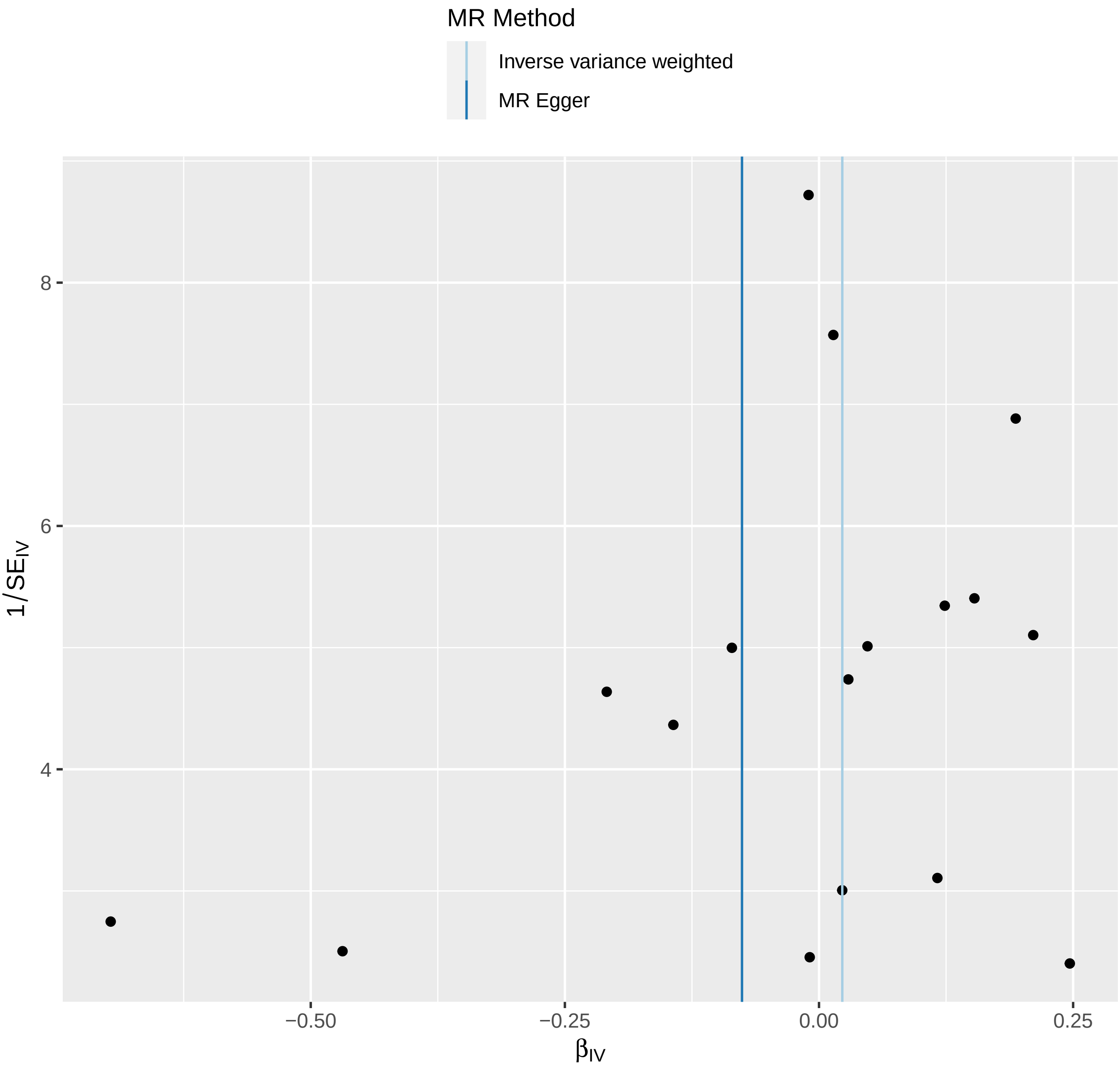

Batch 3−987 : Endothelial cell−selective adhesion molecule on Diabetic nephropathy (more control exclusions) with a threshold of 1e−05

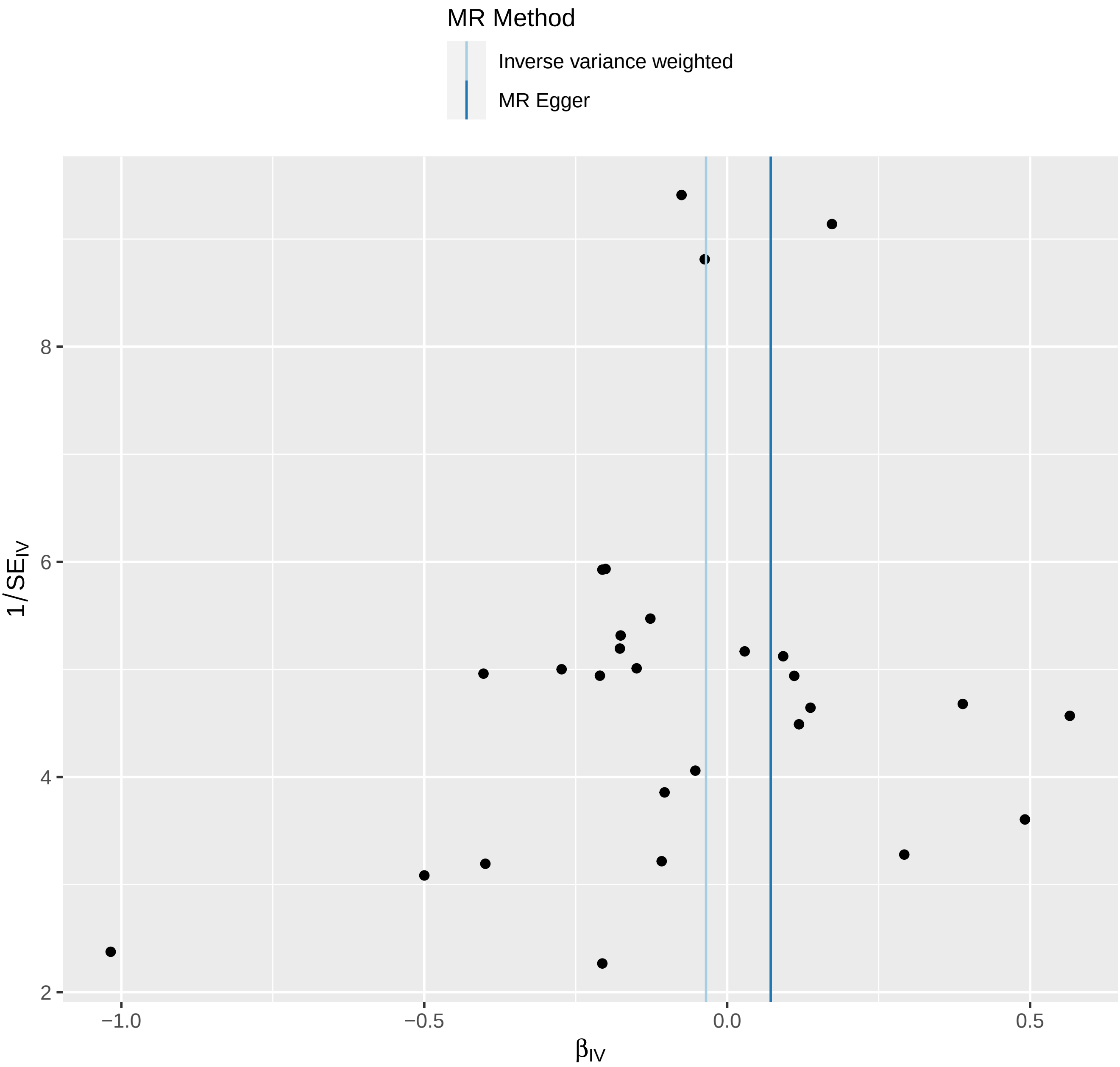

Batch 3−988 : Endothelial cell−selective adhesion molecule on Diabetic nephropathy (more control exclusions)
with a threshold of 1e−05

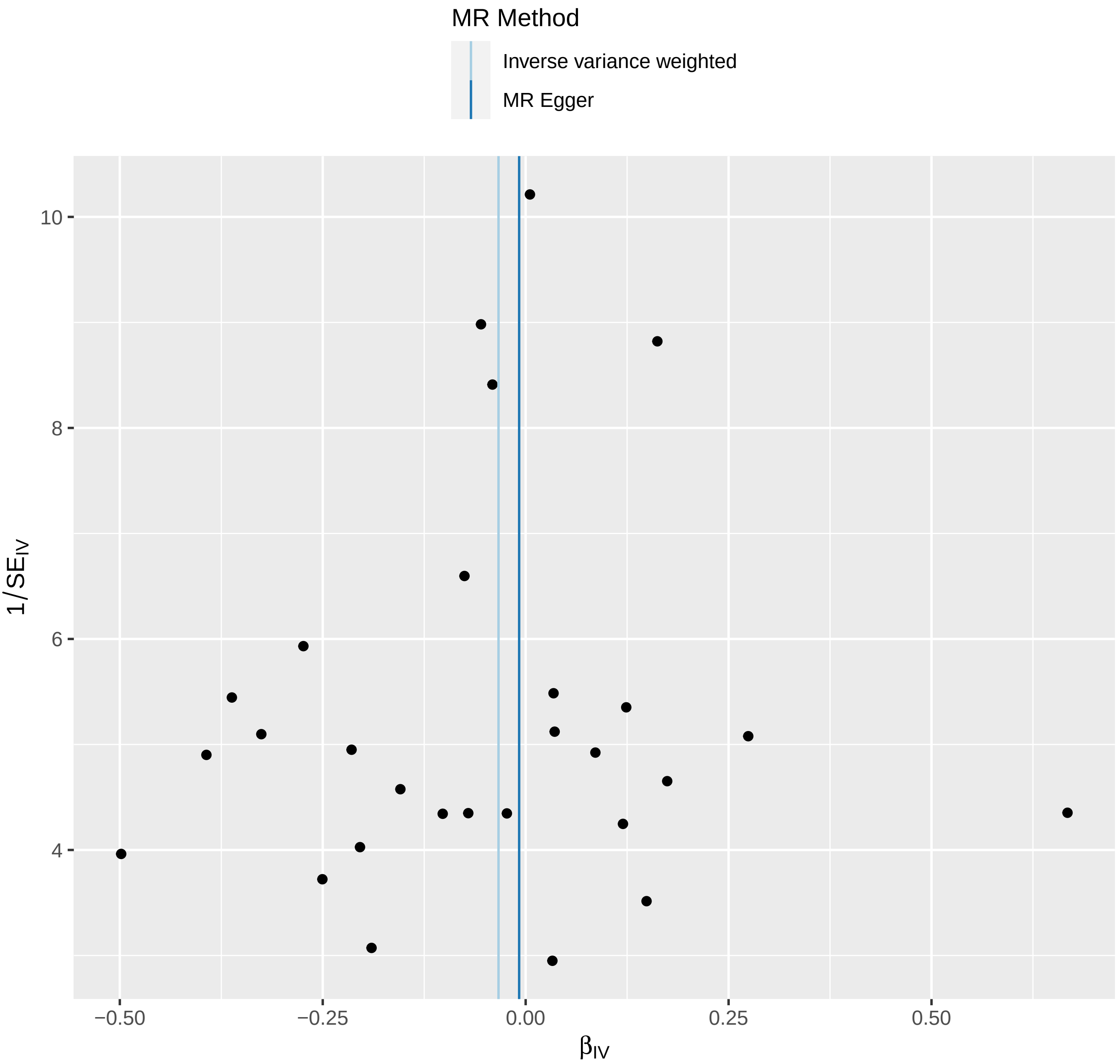

Batch 3−989 : S−formylglutathione hydrolase on Diabetic nephropathy (more control exclusions)
with a threshold of 1e−05

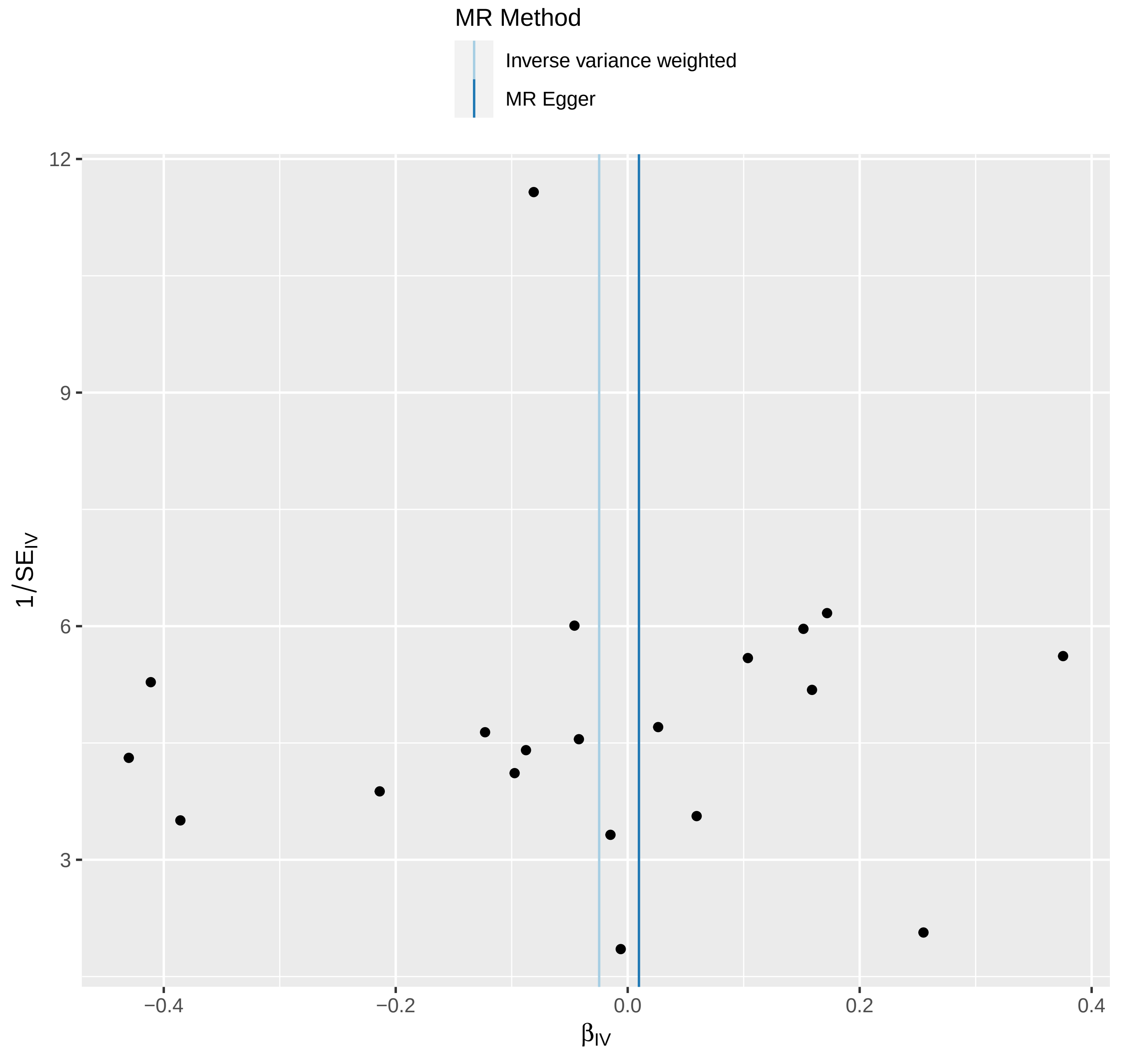

Batch 3−990 : Endothelial cell−specific molecule 1 on Diabetic nephropathy (more control exclusions)
with a threshold of 1e−05

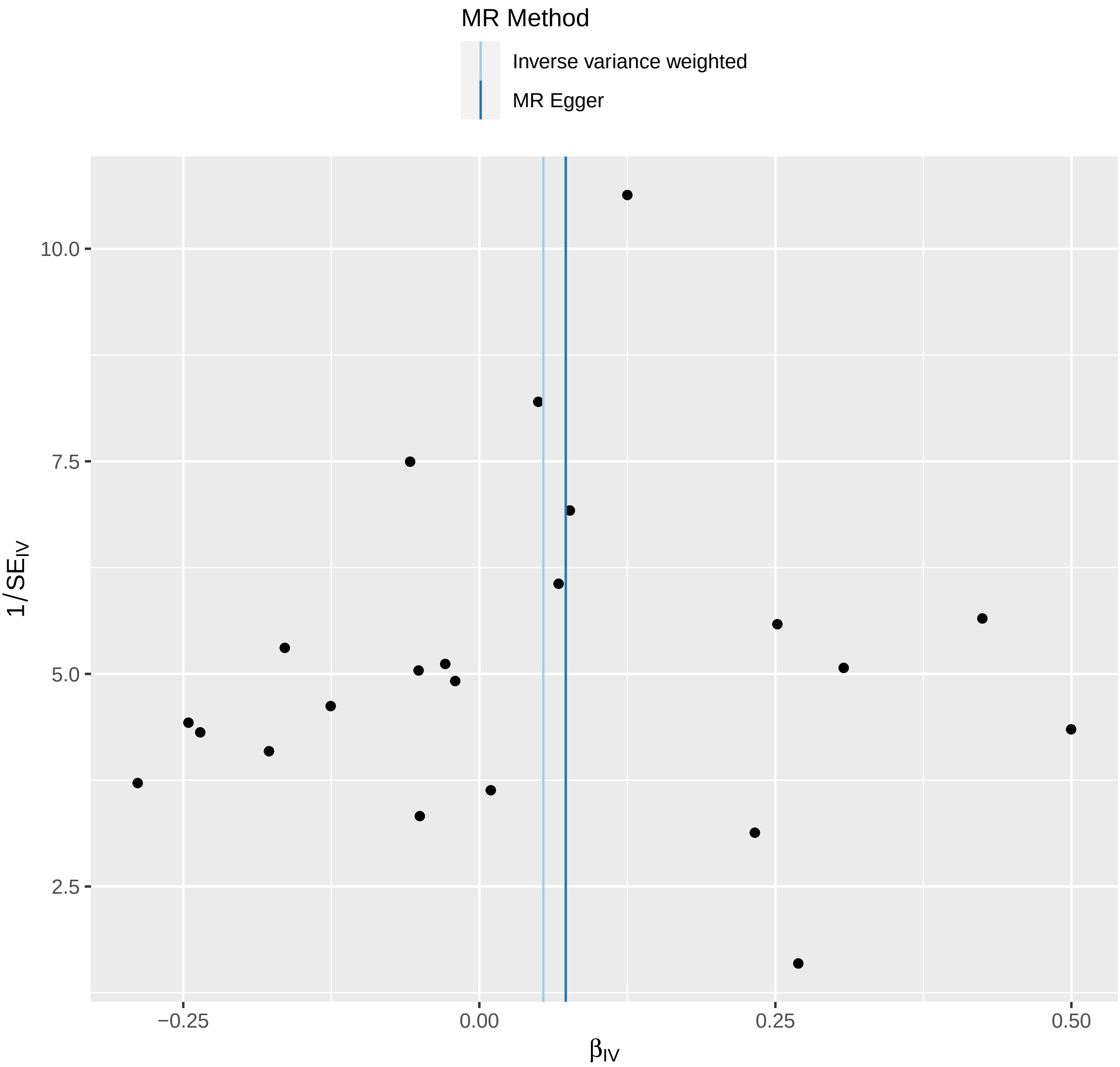

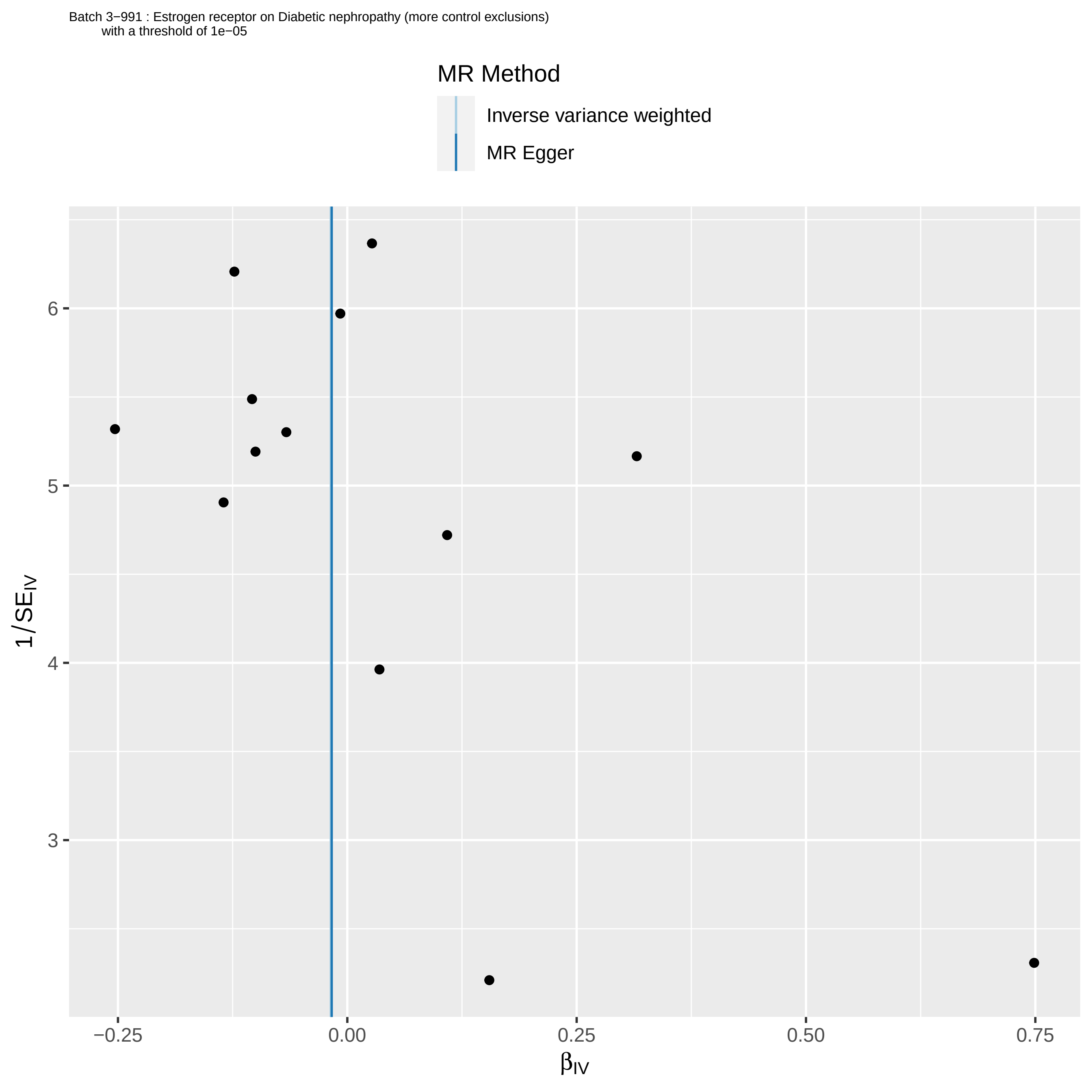
Batch 3−991 : Estrogen receptor on Diabetic nephropathy (more control exclusions)
with a threshold of 1e−05
MR Method
Inverse variance weighted
MR Egger
6
5
4
3
$1/SE_{IV}$
−0.25
0.00
0.25
0.50
0.75
β_{IV}

Batch 3−992 : Persulfide dioxygenase ETHE1, mitochondrial on Diabetic nephropathy (more control exclusions) with a threshold of 1e−05

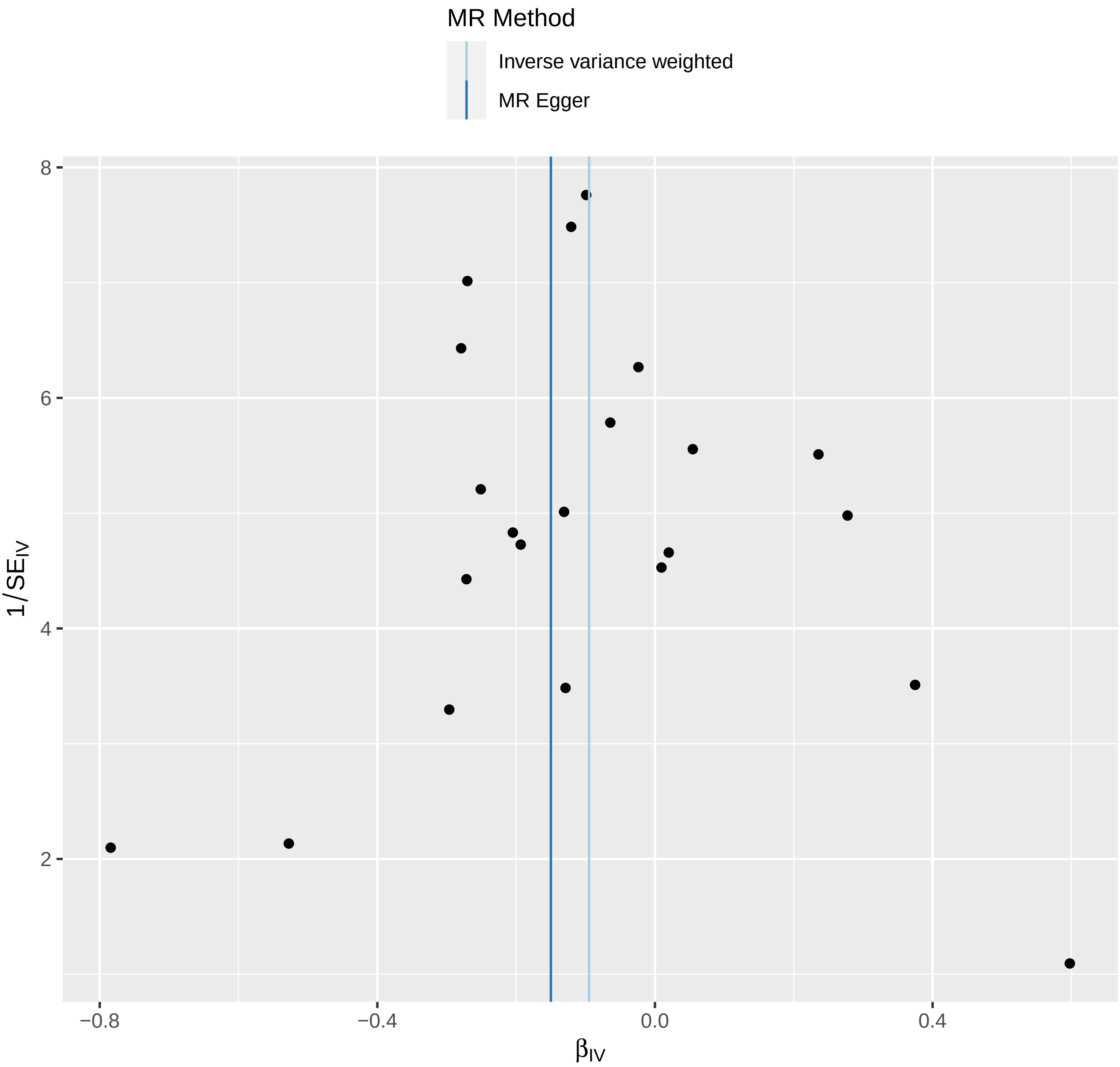

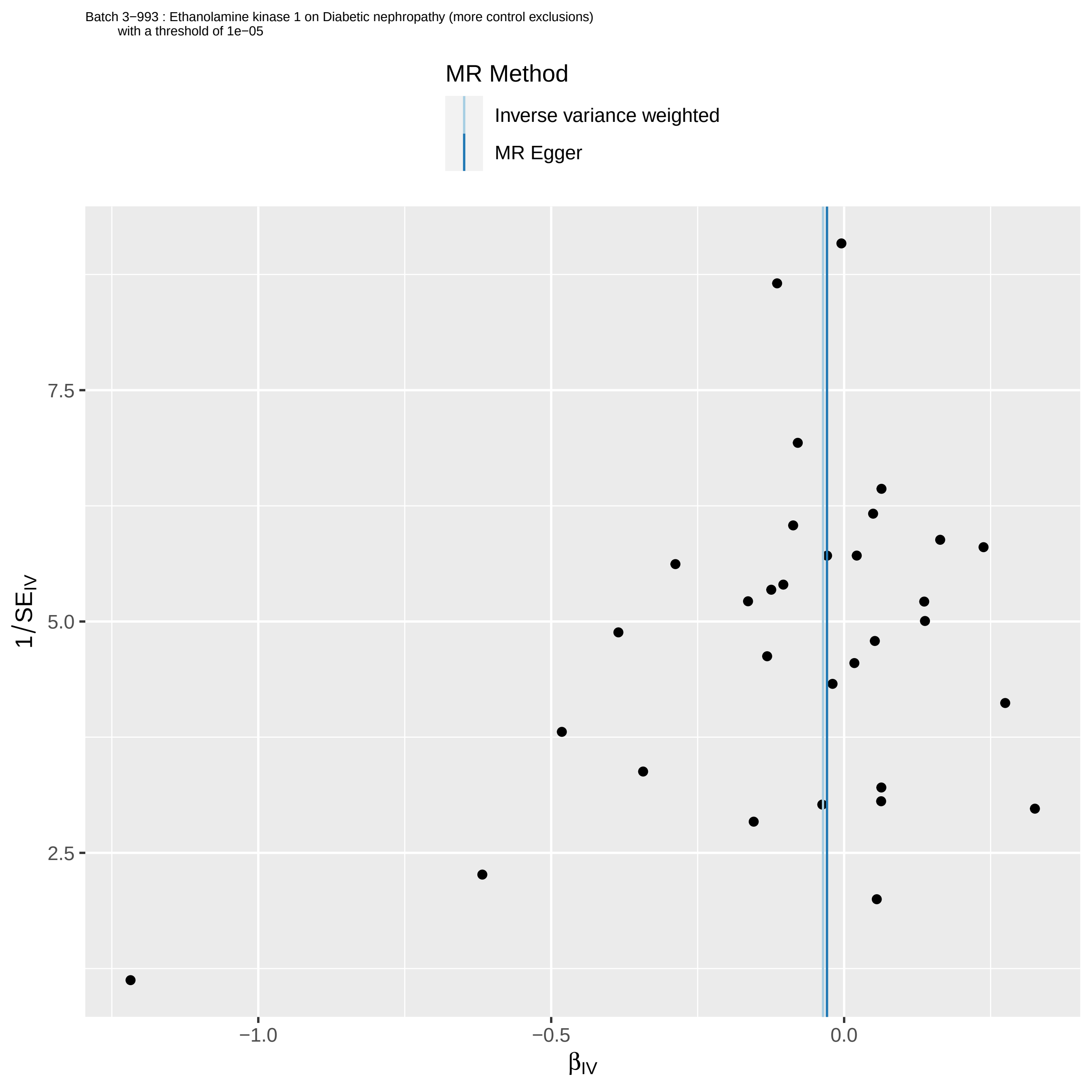
Batch 3−993 : Ethanolamine kinase 1 on Diabetic nephropathy (more control exclusions)
with a threshold of 1e−05
MR Method
Inverse variance weighted
MR Egger
7.5
5.0
2.5
$1/SE_{IV}$
−1.0
−0.5
0.0
β_{IV}

Batch 3−994 : Protein C−ets−2 on Diabetic nephropathy (more control exclusions)
with a threshold of 1e−05

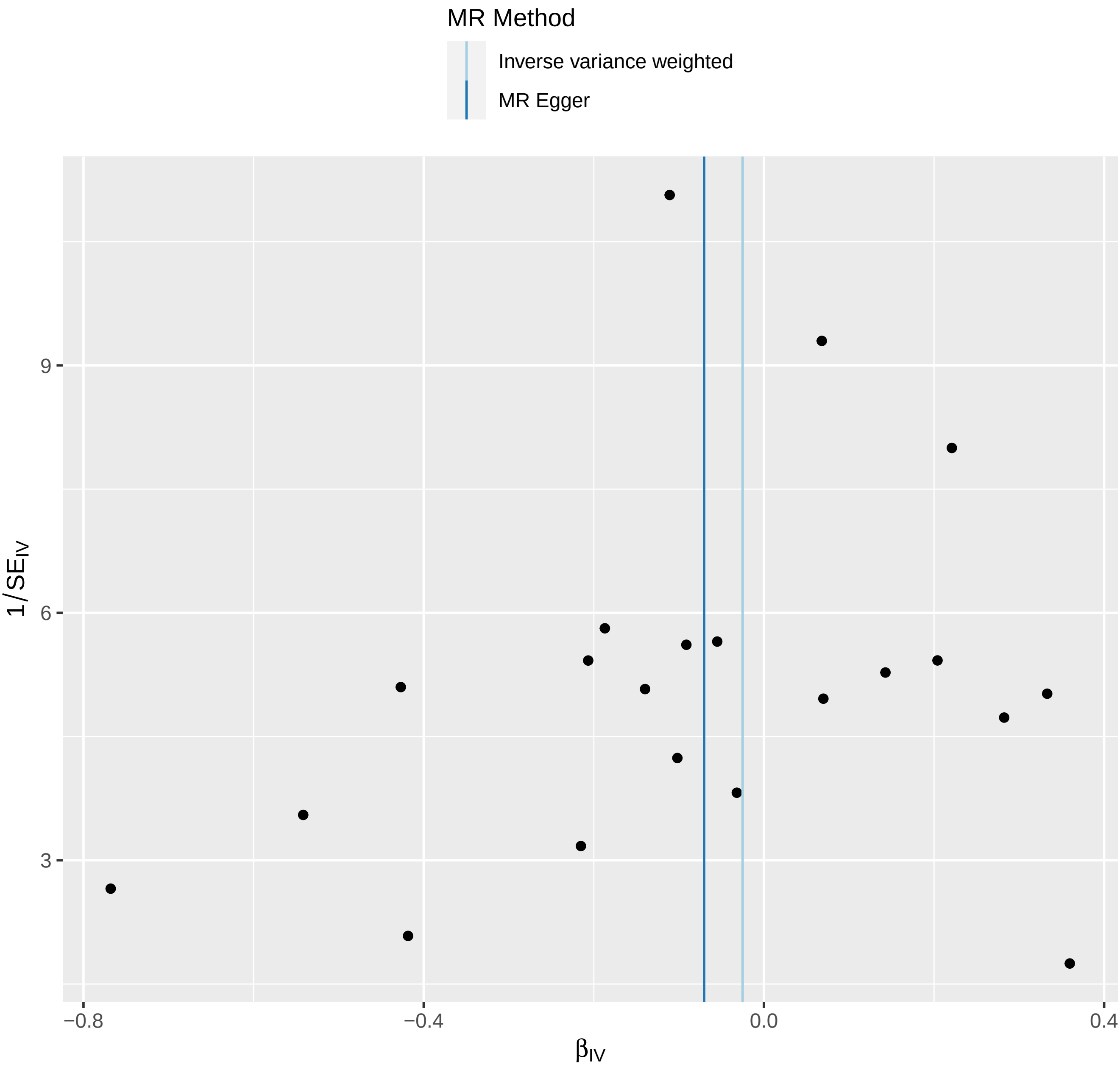

Batch 3−995 : Protein eva−1 homolog C on Diabetic nephropathy (more control exclusions)
with a threshold of 1e−05

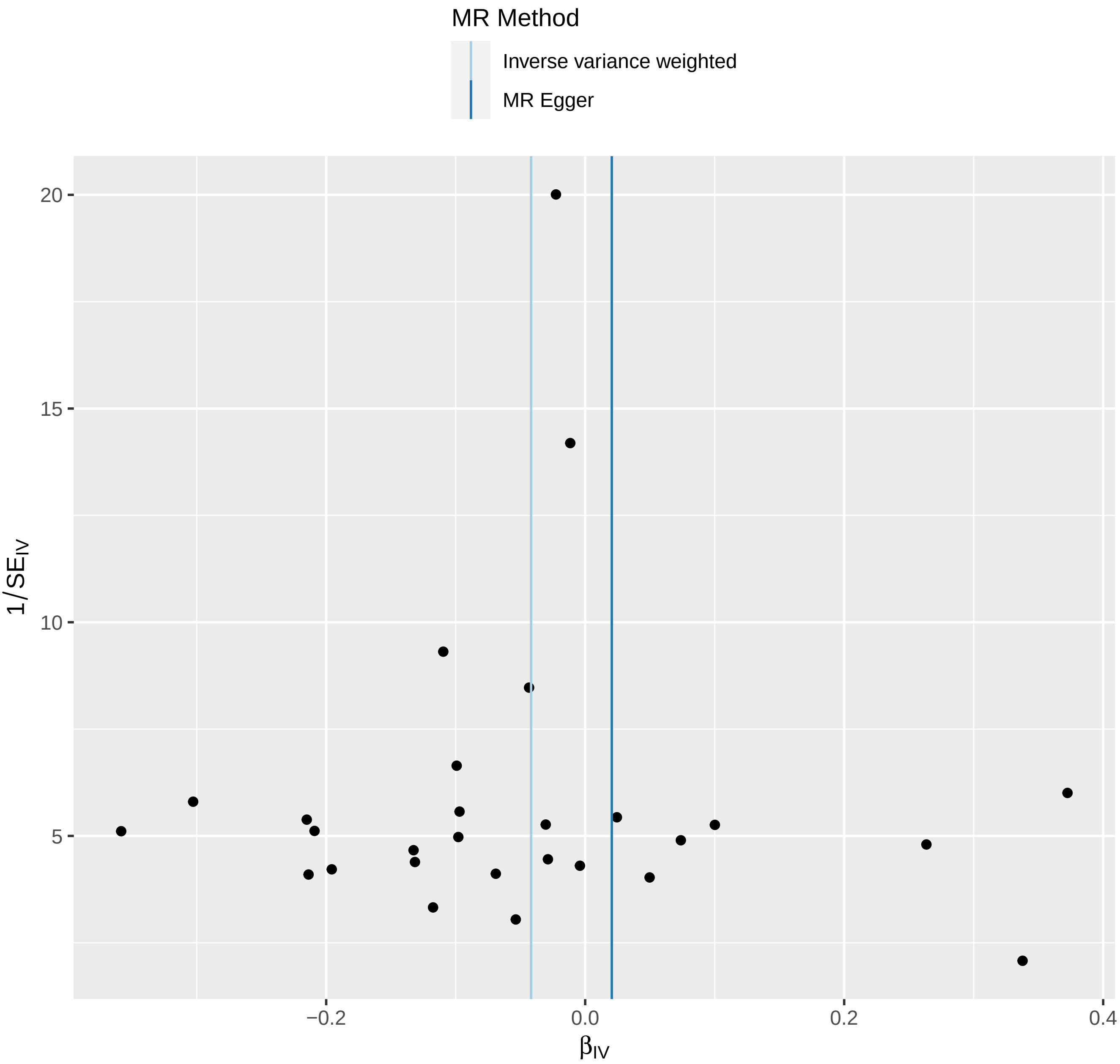

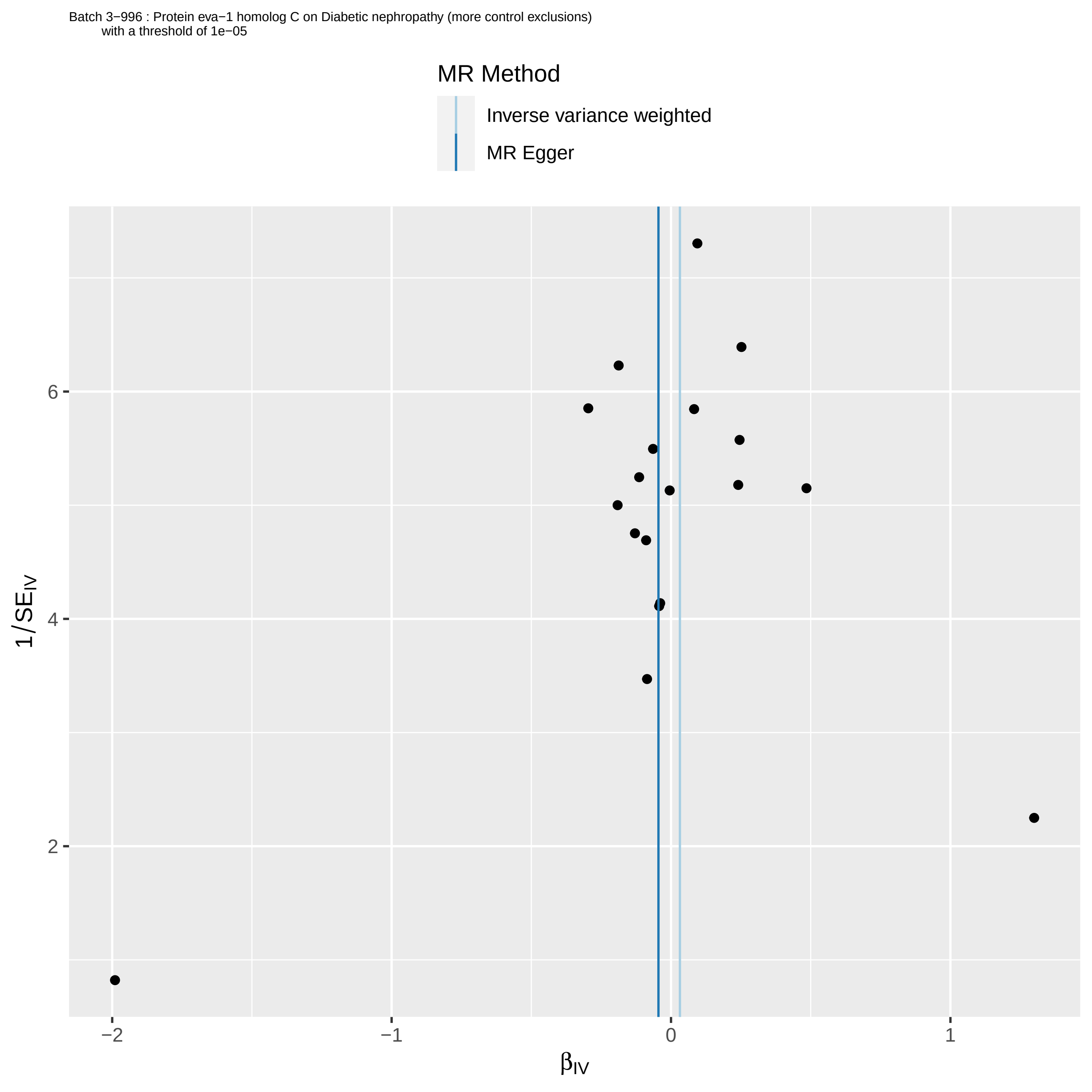

Batch 3−996 : Protein eva−1 homolog C on Diabetic nephropathy (more control exclusions)
with a threshold of 1e−05
MR Method
Inverse variance weighted
MR Egger
6
4
2
1/SE_IV
−2
−1
0
1
β_IV

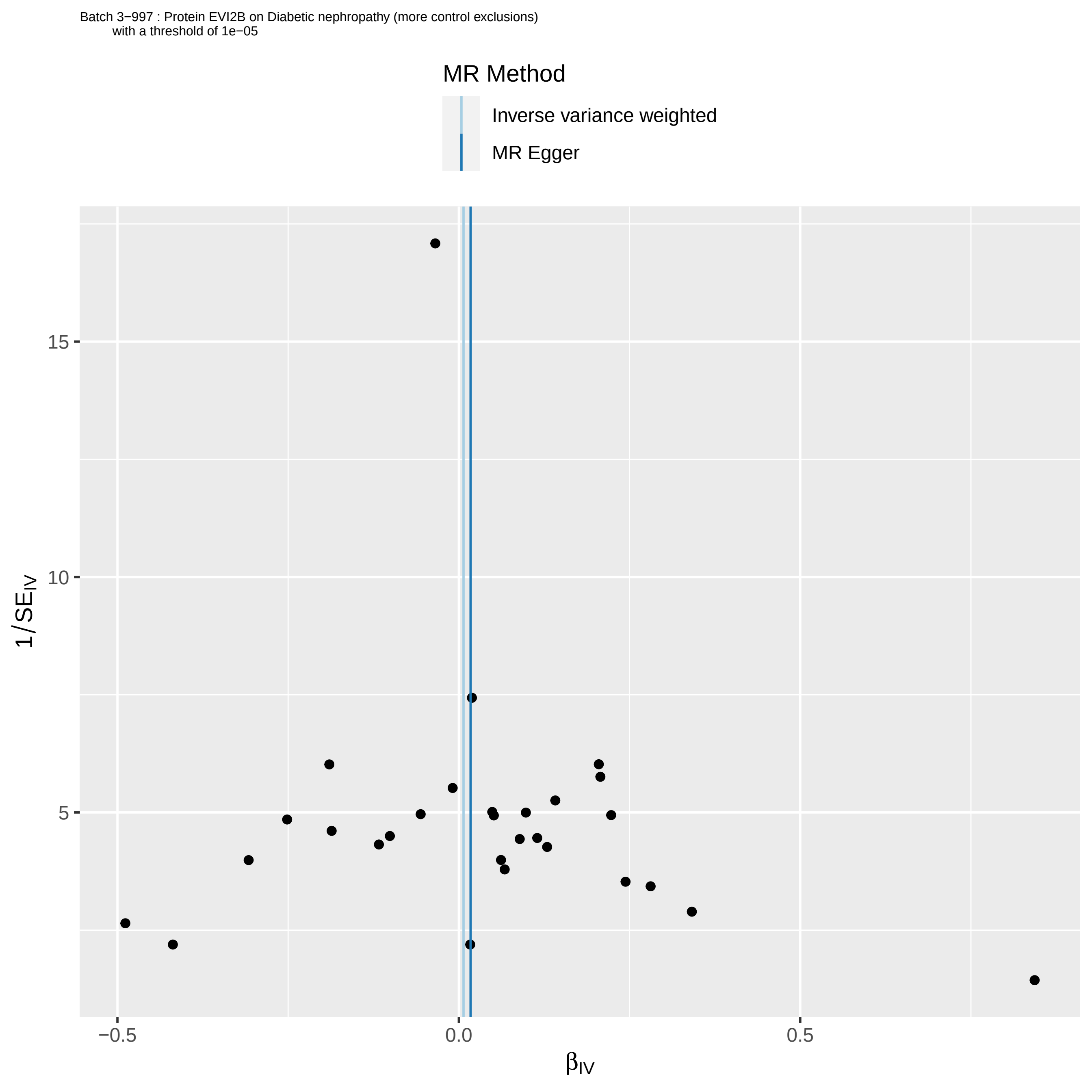

Batch 3−997 : Protein EVI2B on Diabetic nephropathy (more control exclusions)
with a threshold of 1e−05
MR Method
Inverse variance weighted
MR Egger
15
10
5
−0.5
0.0
0.5
$1/SE_{IV}$
β_{IV}

Batch 3−998 : Ena/VASP−like protein on Diabetic nephropathy (more control exclusions)
with a threshold of 1e−05

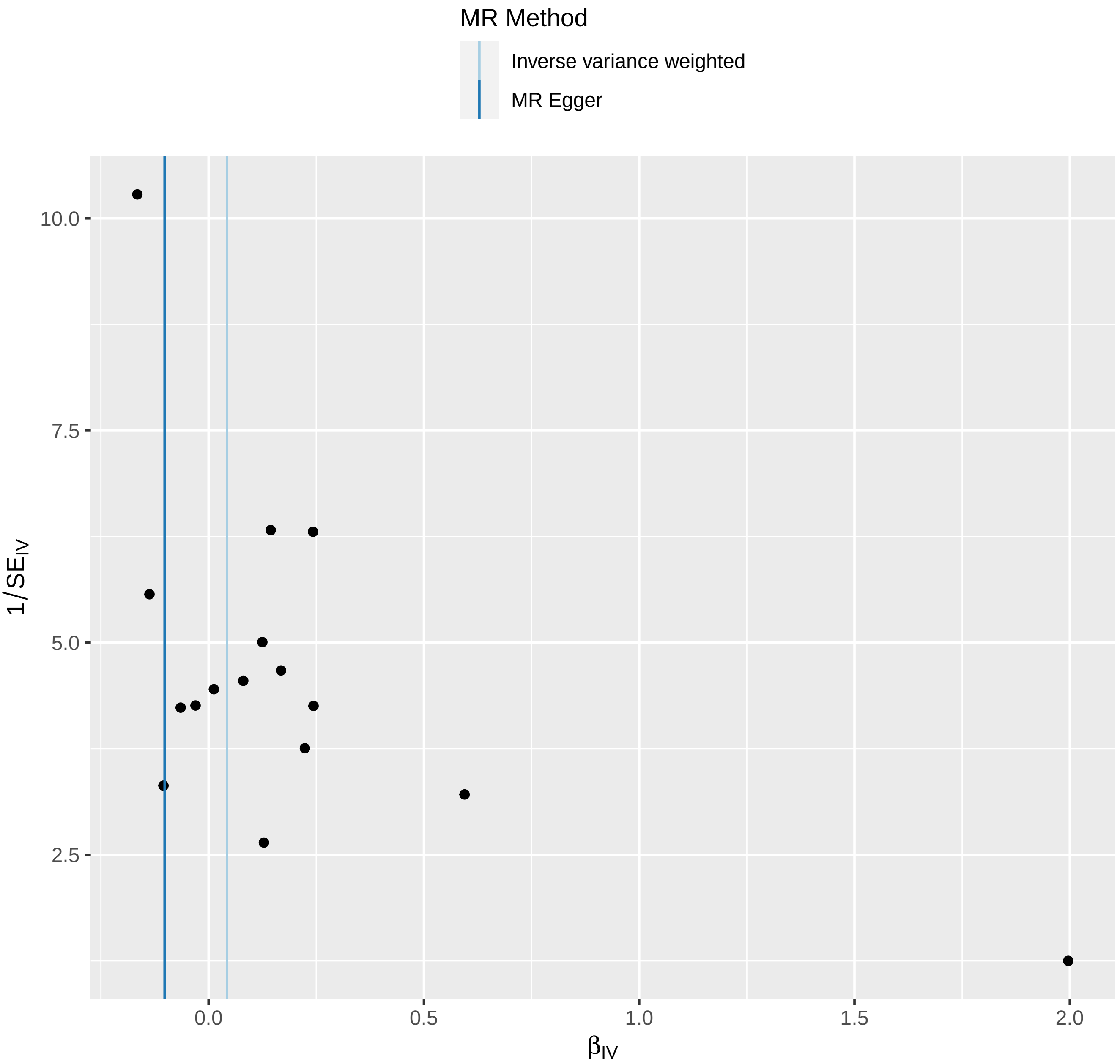

Batch 3−999 : Envoplakin on Diabetic nephropathy (more control exclusions)
with a threshold of 1e−05

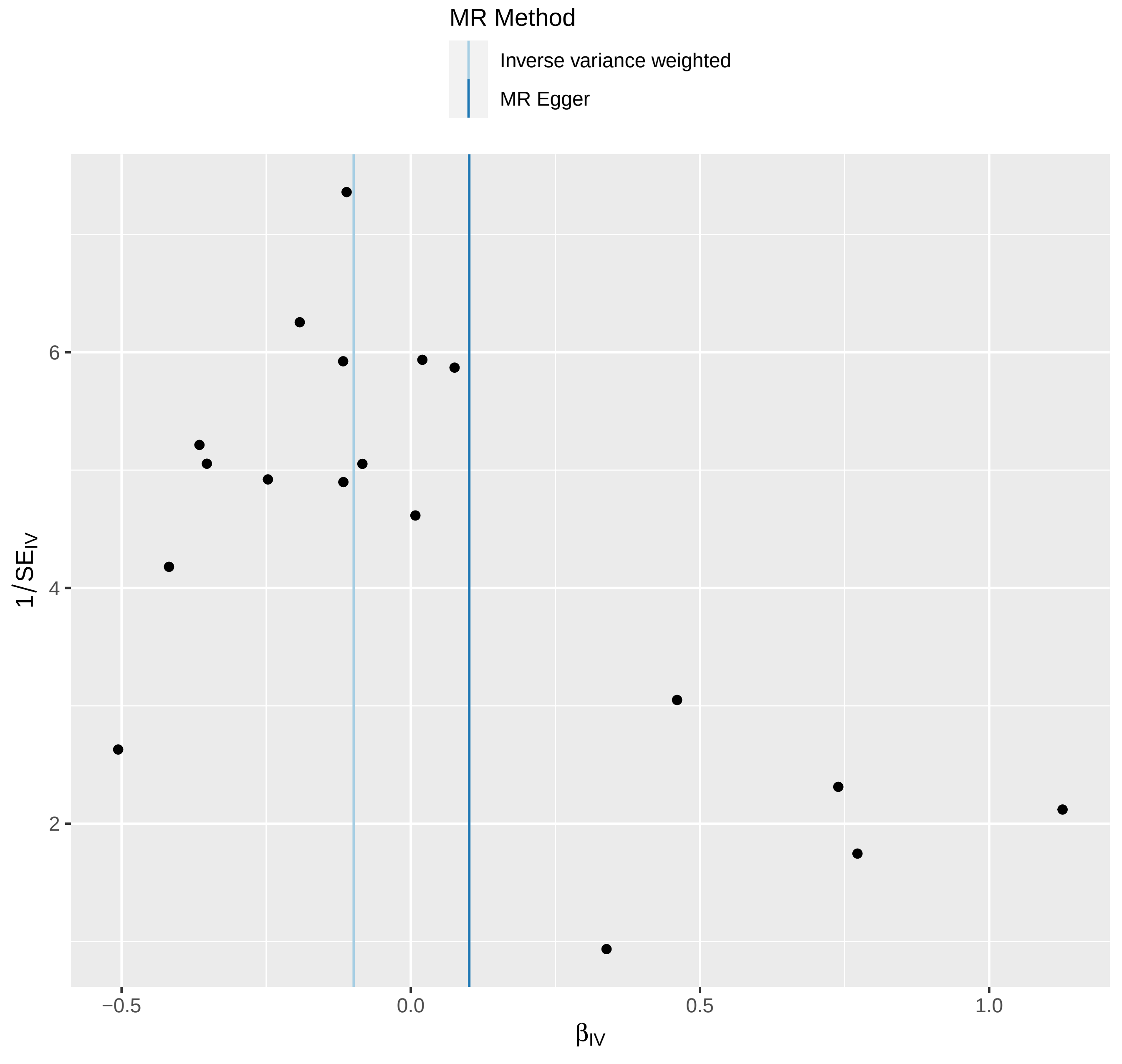

Batch 3−1000 : RNA−binding protein EWS on Diabetic nephropathy (more control exclusions)
with a threshold of 1e−05

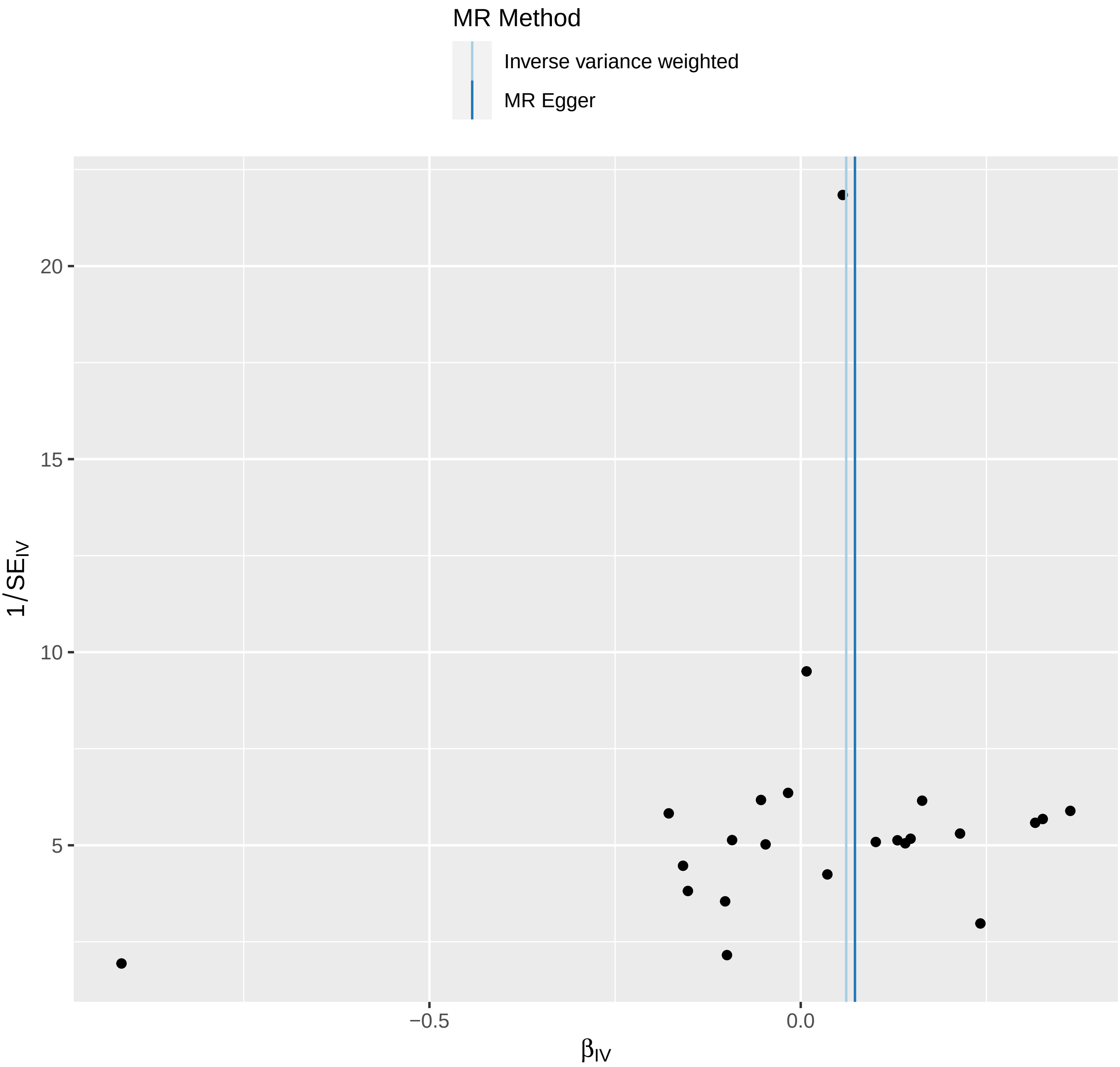

Batch 3−1001 : Exosome complex component CSL4 on Diabetic nephropathy (more control exclusions)
with a threshold of 1e−05

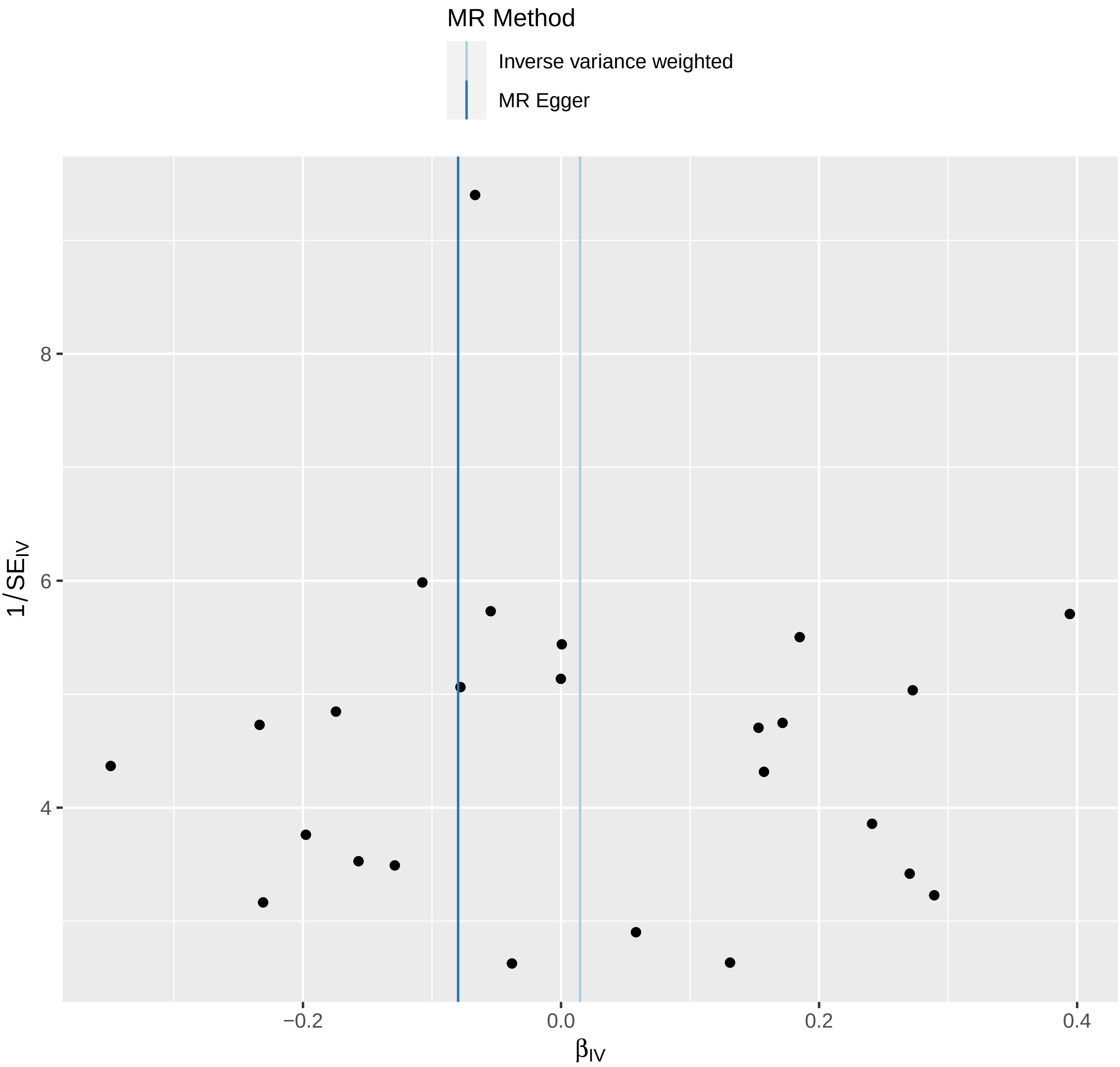

Batch 3−1002 : Exosome complex component RRP40 on Diabetic nephropathy (more control exclusions)
with a threshold of 1e−05

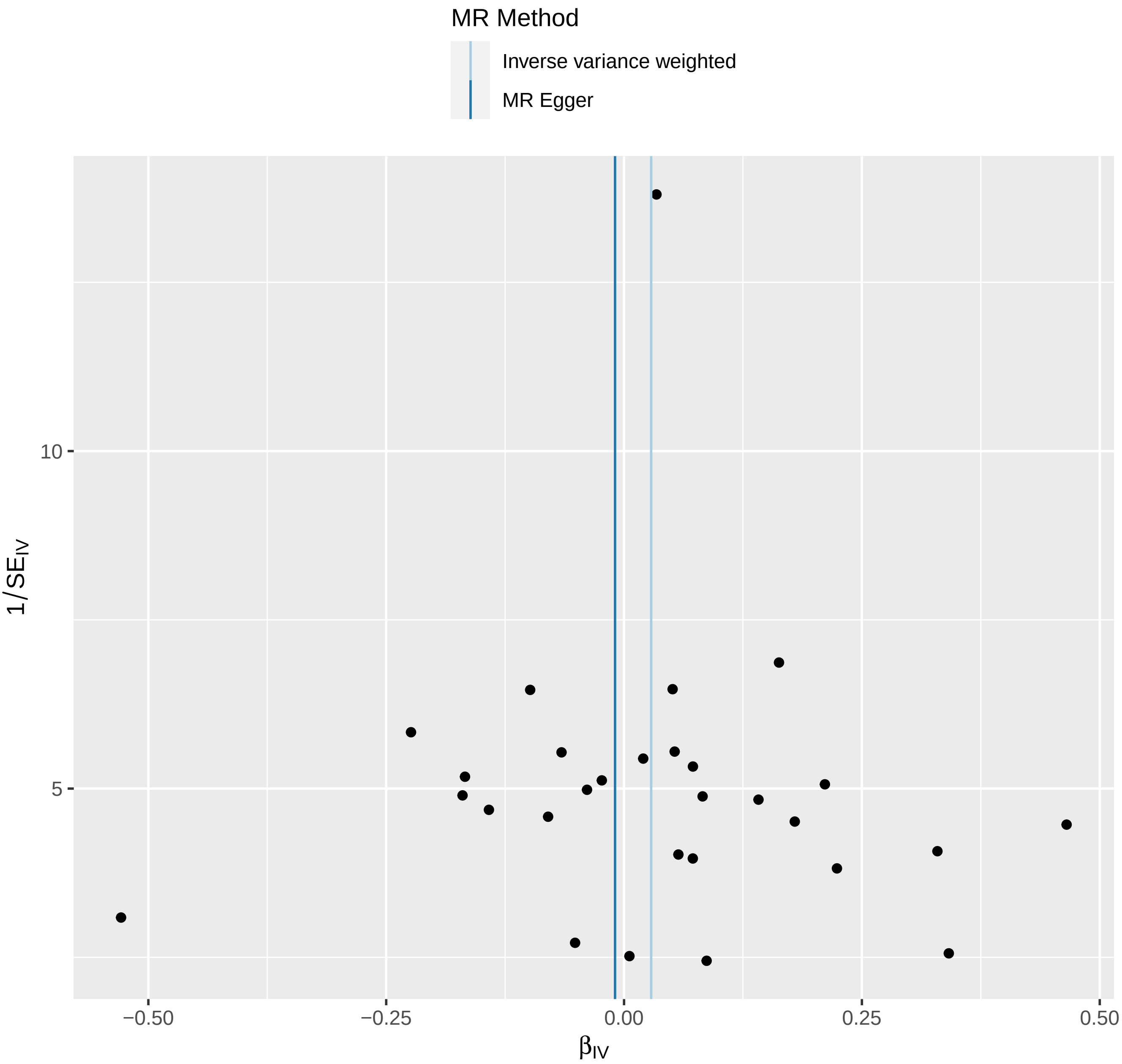

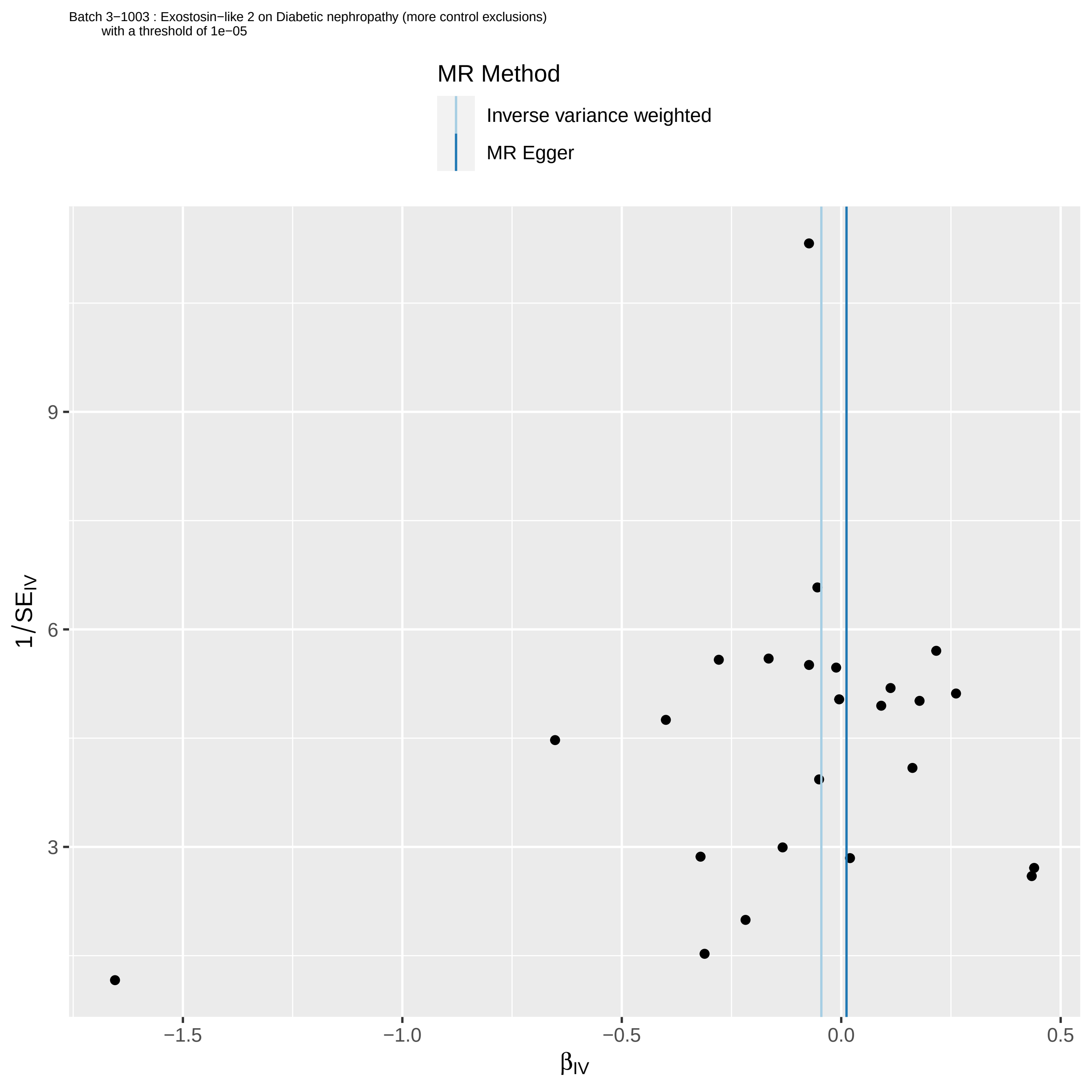

Batch 3−1003 : Exostosin−like 2 on Diabetic nephropathy (more control exclusions)
with a threshold of 1e−05
MR Method
Inverse variance weighted
MR Egger
9
6
3
1/SE_IV
−1.5
−1.0
−0.5
0.0
0.5
β_IV

Batch 3−1004 : Ezrin on Diabetic nephropathy (more control exclusions)
with a threshold of 1e−05

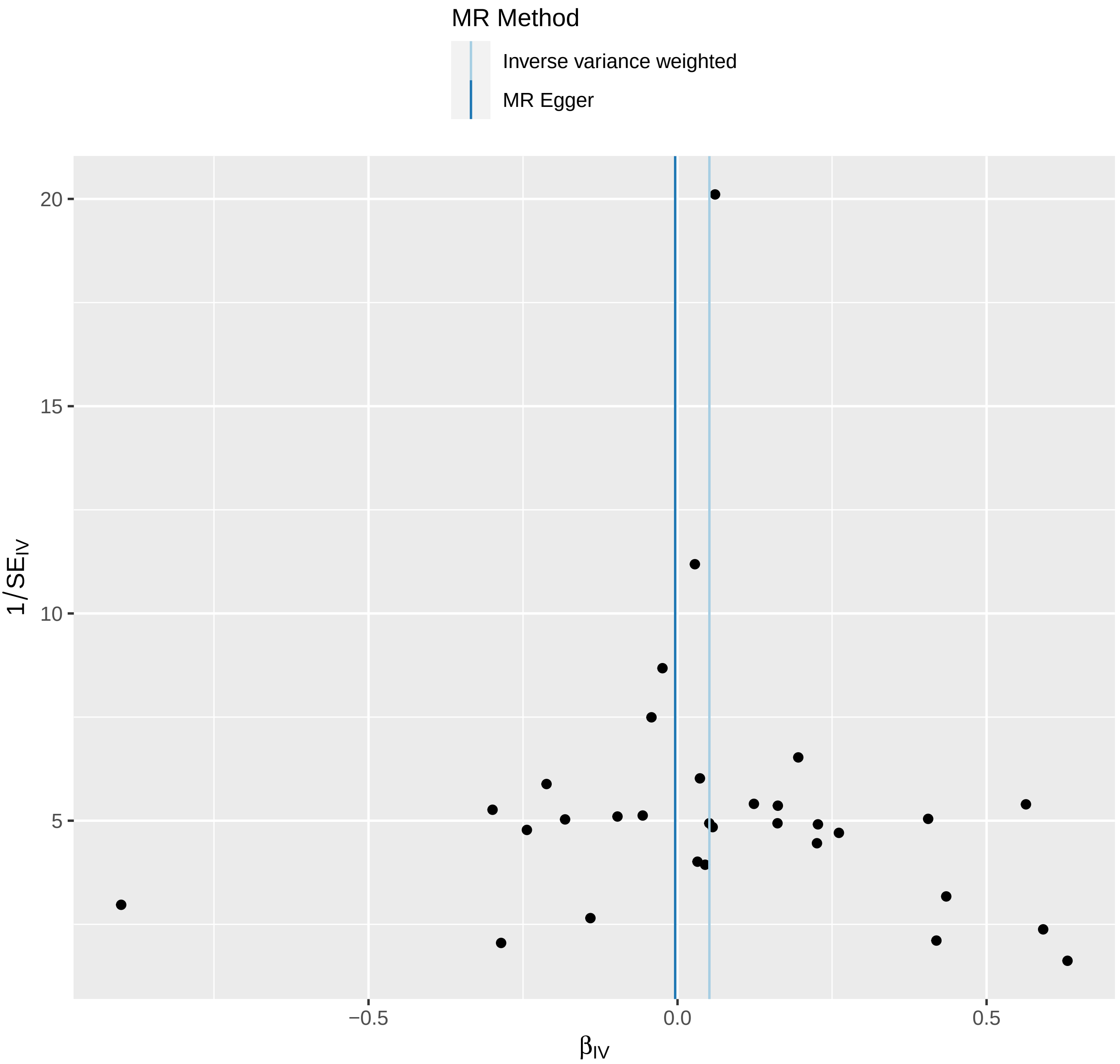

Batch 3−1005 : Coagulation factor Xa on Diabetic nephropathy (more control exclusions)
with a threshold of 1e−05

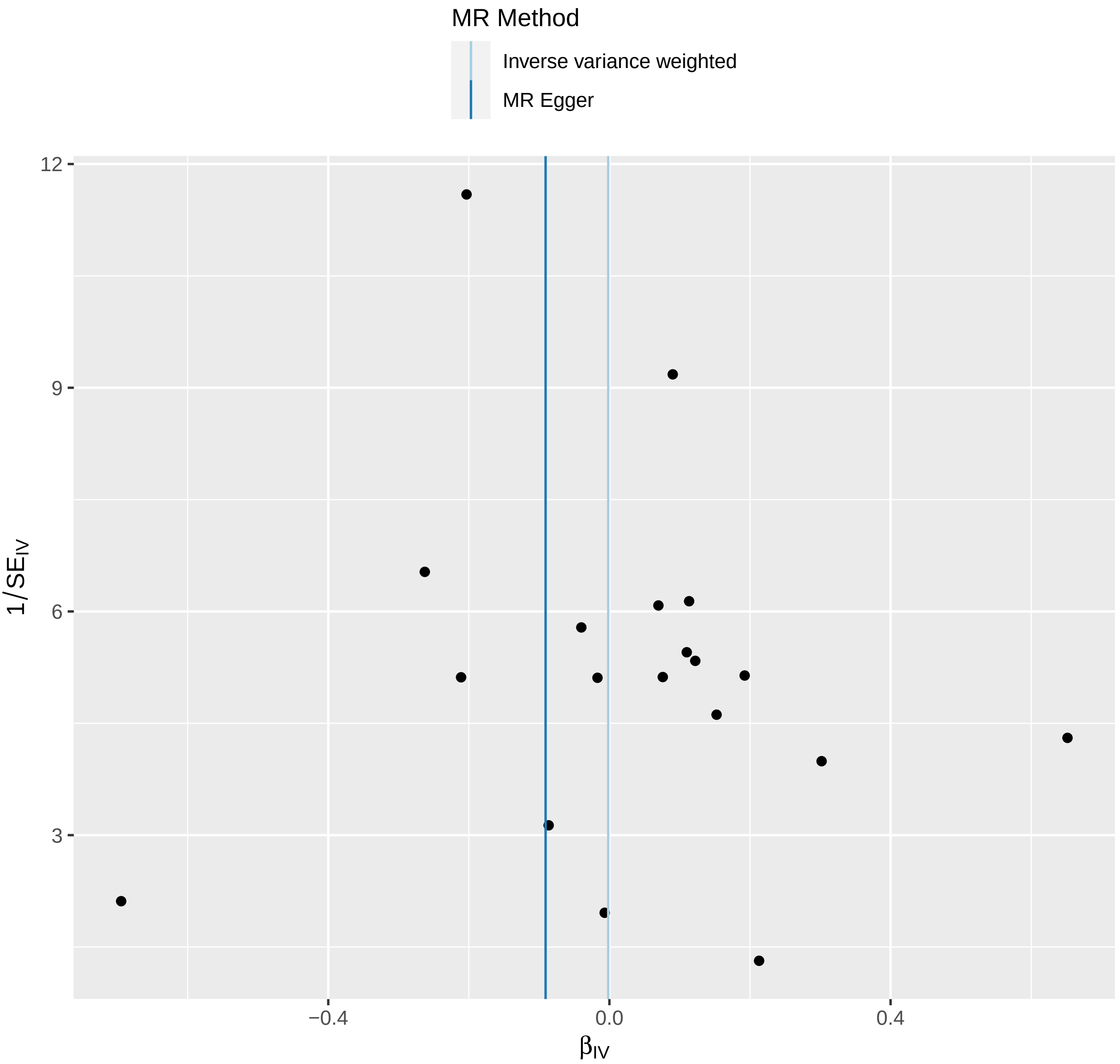

Batch 3−1006 : Coagulation Factor X on Diabetic nephropathy (more control exclusions)
with a threshold of 1e−05

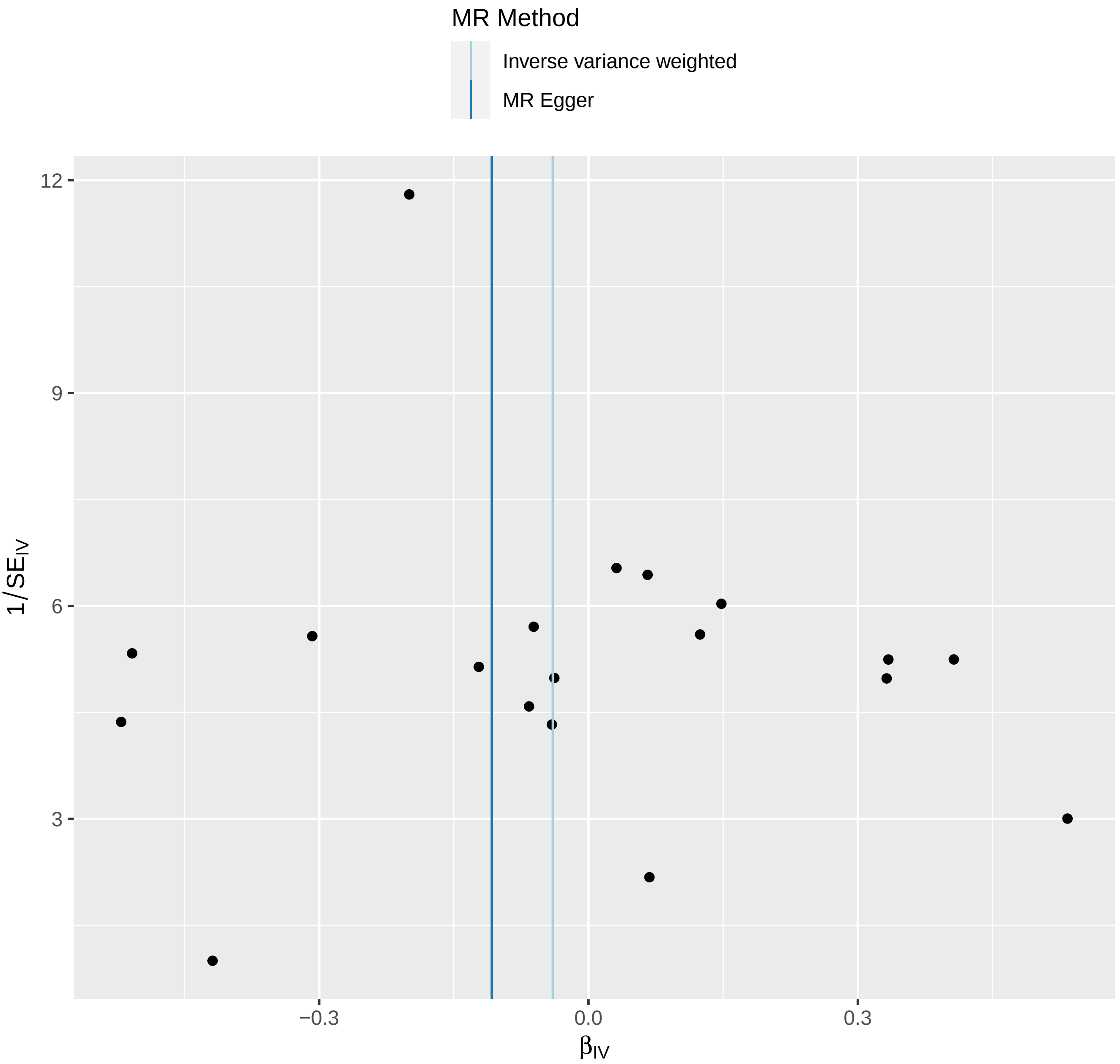

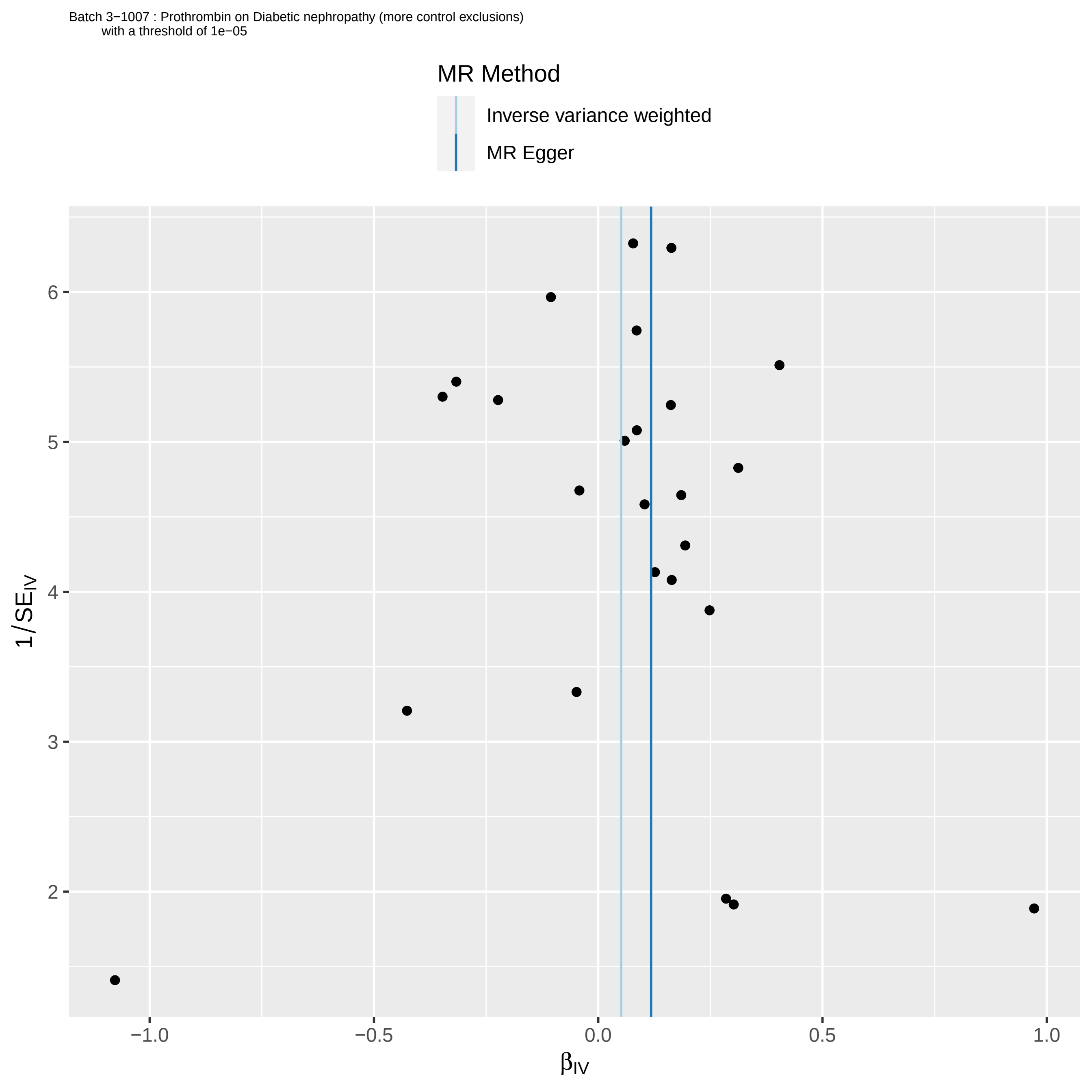
Batch 3−1007 : Prothrombin on Diabetic nephropathy (more control exclusions)
with a threshold of 1e−05
MR Method
Inverse variance weighted
MR Egger
6
5
4
3
2
1/SE_IV
−1.0
−0.5
0.0
0.5
1.0
β_IV

Batch 3−1008 : Tissue Factor on Diabetic nephropathy (more control exclusions)
with a threshold of 1e−05

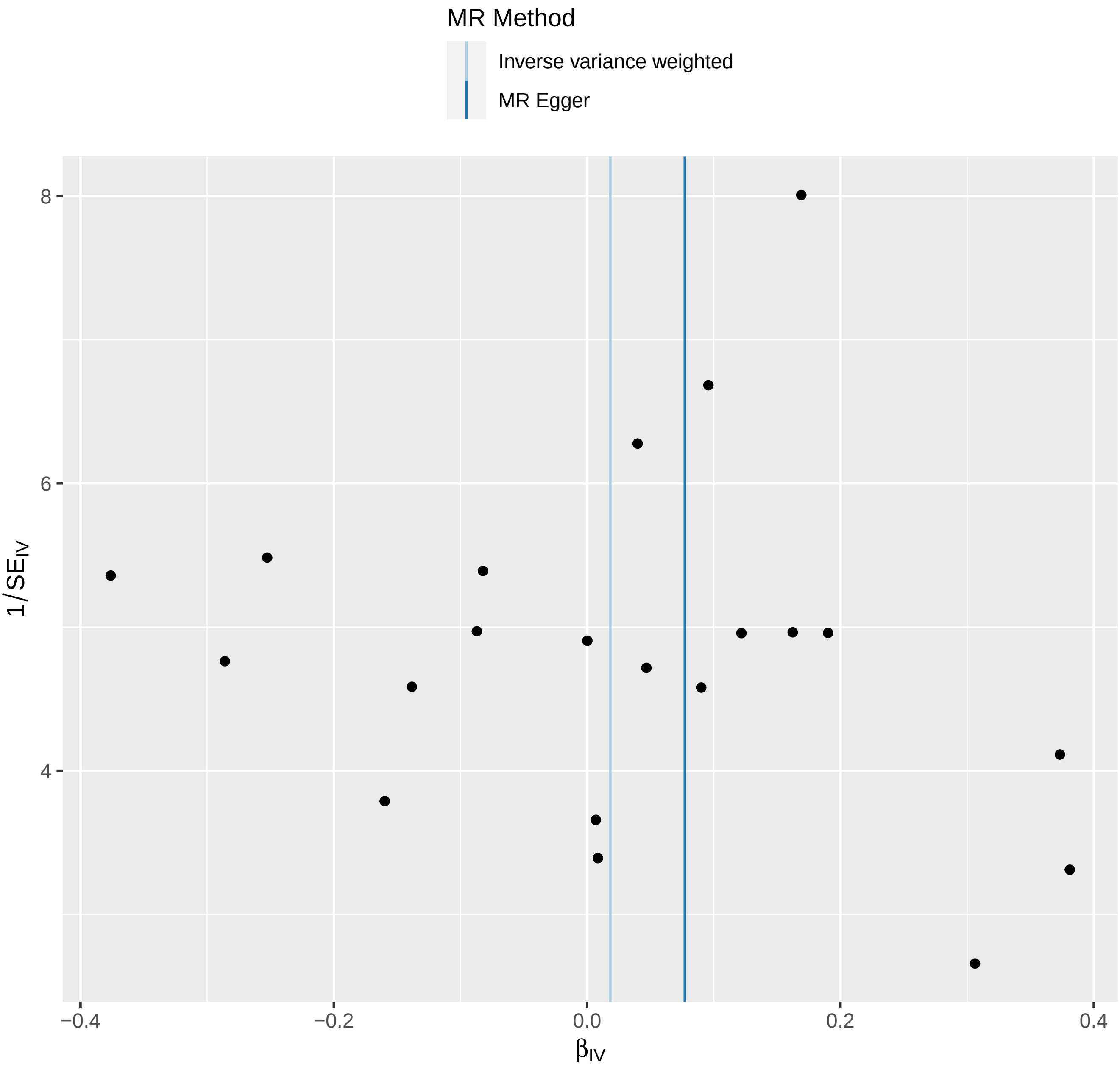

Batch 3−1009 : Coagulation Factor VIII on Diabetic nephropathy (more control exclusions)
with a threshold of 1e−05

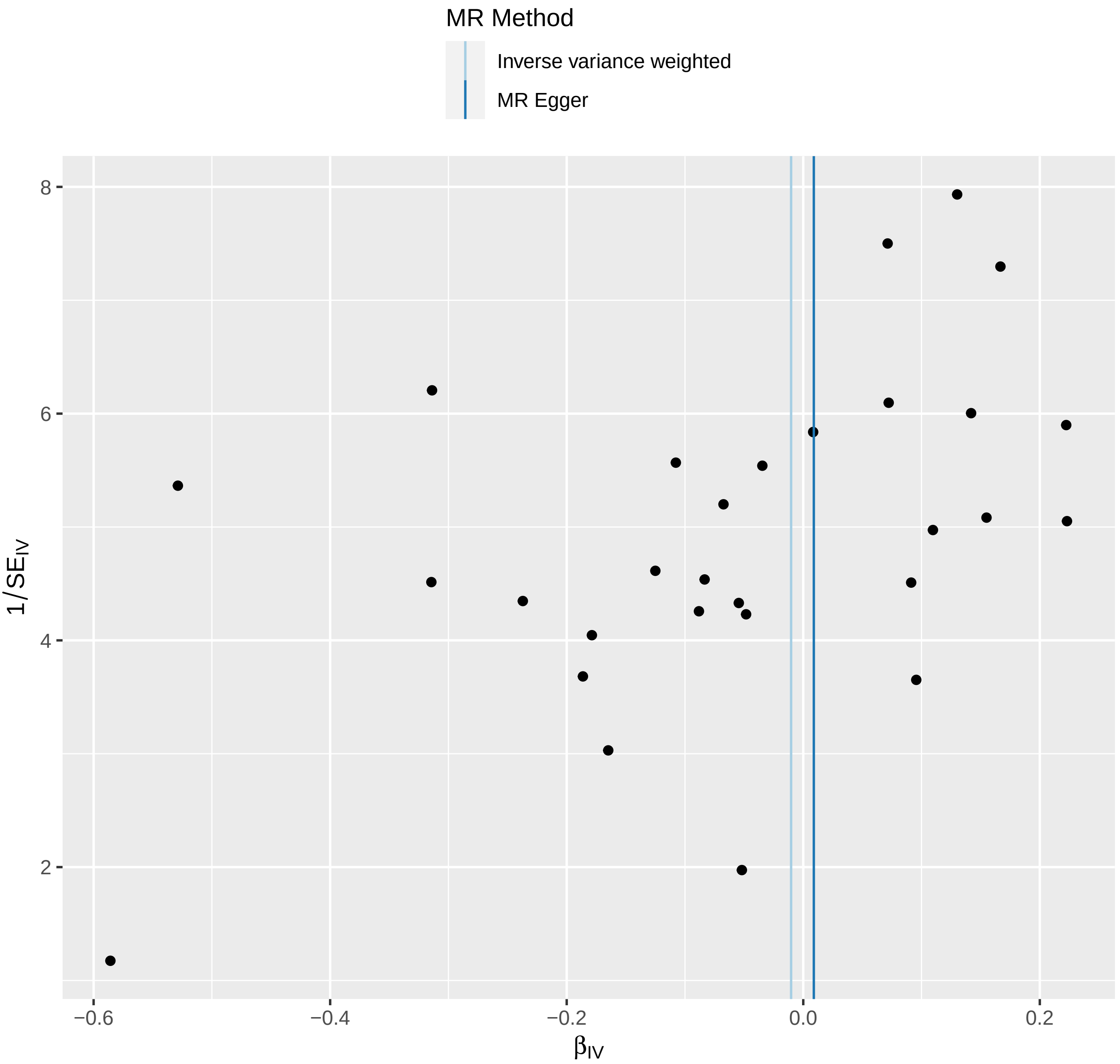

Batch 3−1010 : Fatty−acid amide hydrolase 2 on Diabetic nephropathy (more control exclusions)
with a threshold of 1e−05

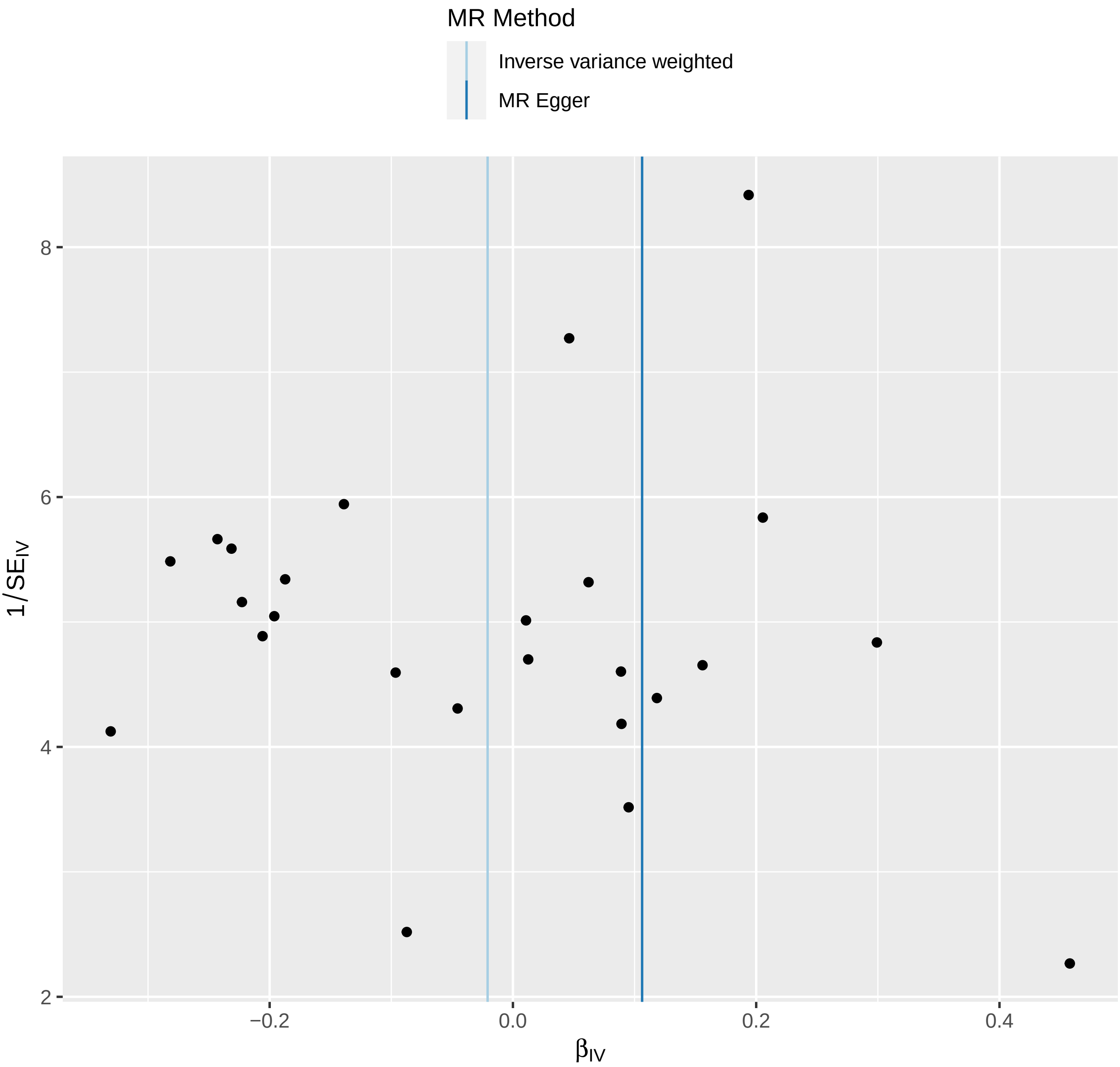

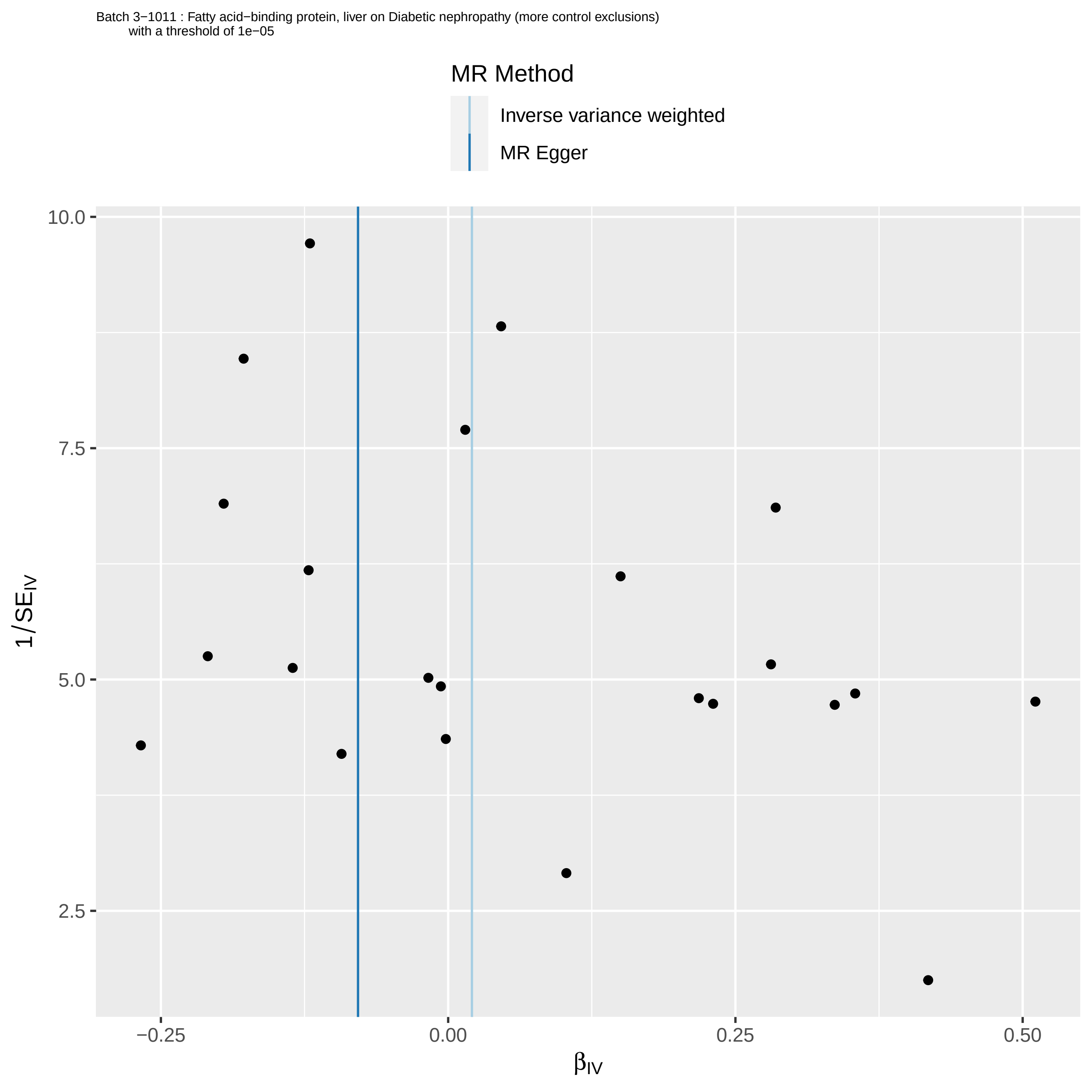

Batch 3−1011 : Fatty acid−binding protein, liver on Diabetic nephropathy (more control exclusions)
with a threshold of 1e−05
MR Method
Inverse variance weighted
MR Egger
10.0
7.5
5.0
2.5
1/SE_IV
−0.25
0.00
0.25
0.50
β_IV

Batch 3−1012 : Fatty acid−binding protein, heart on Diabetic nephropathy (more control exclusions)
with a threshold of 1e−05

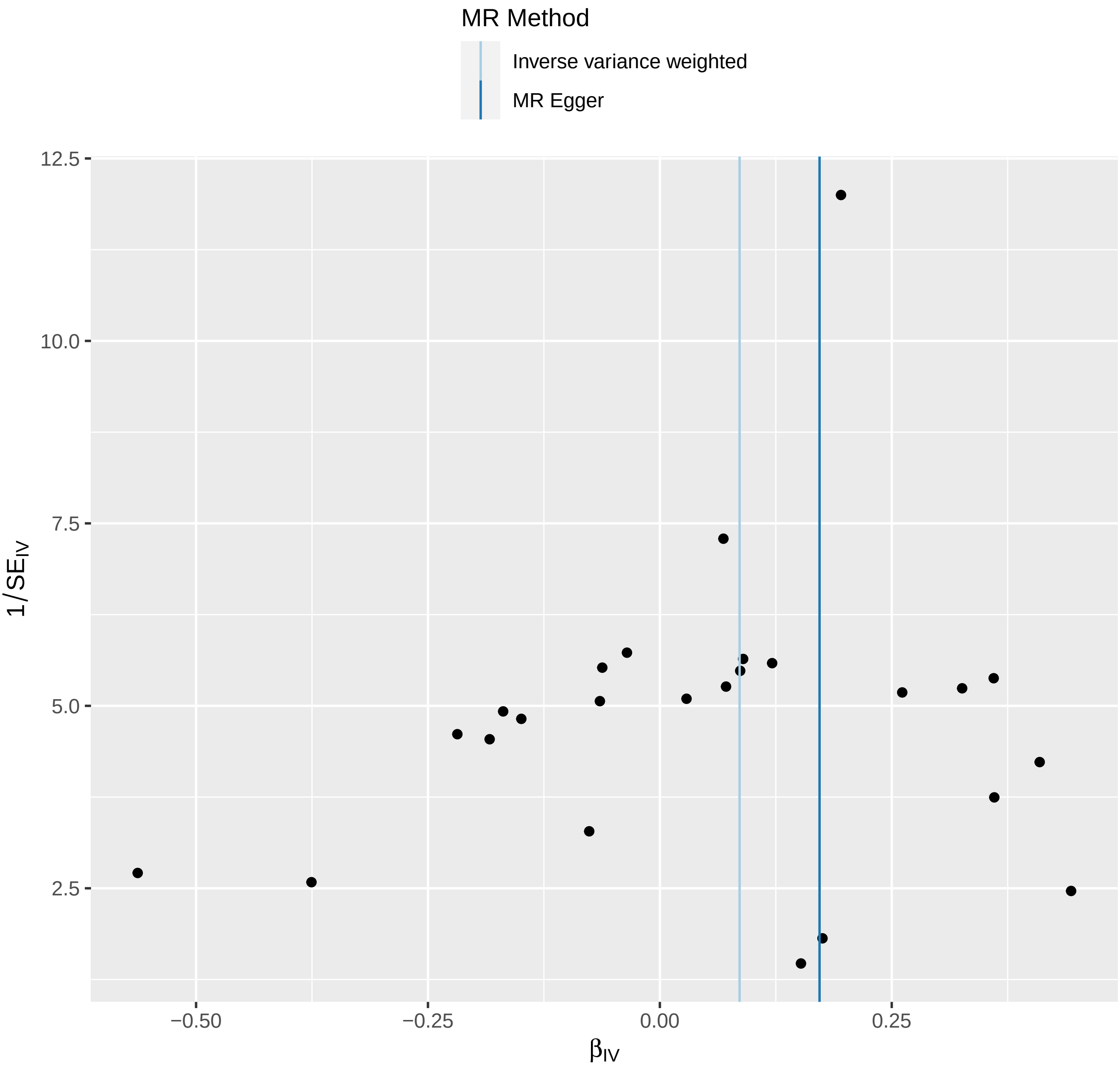

Batch 3−1013 : Fatty acid−binding protein, adipocyte on Diabetic nephropathy (more control exclusions)
with a threshold of 1e−05

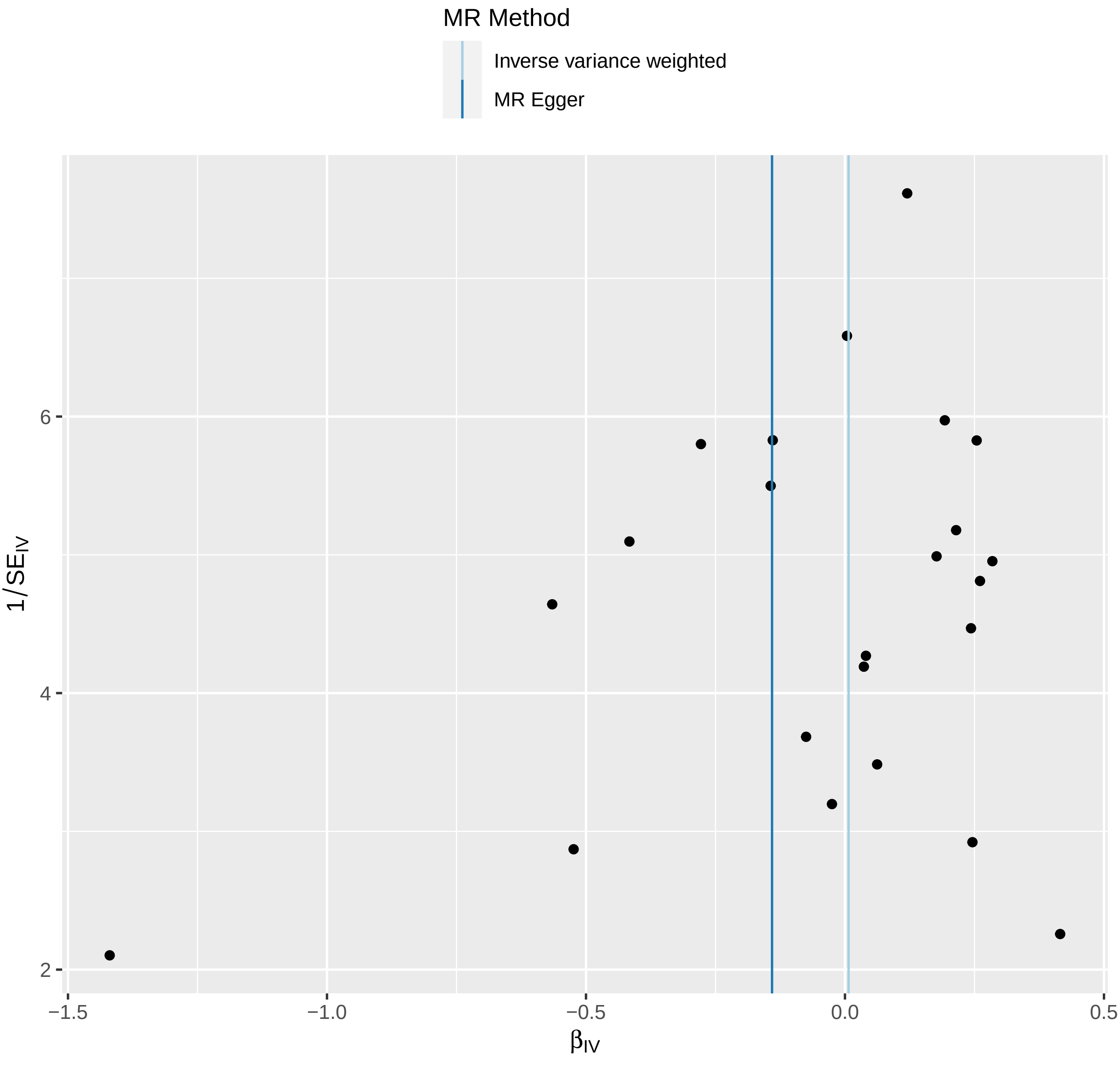

Batch 3−1014 : Fatty acid−binding protein, epidermal on Diabetic nephropathy (more control exclusions)
with a threshold of 1e−05

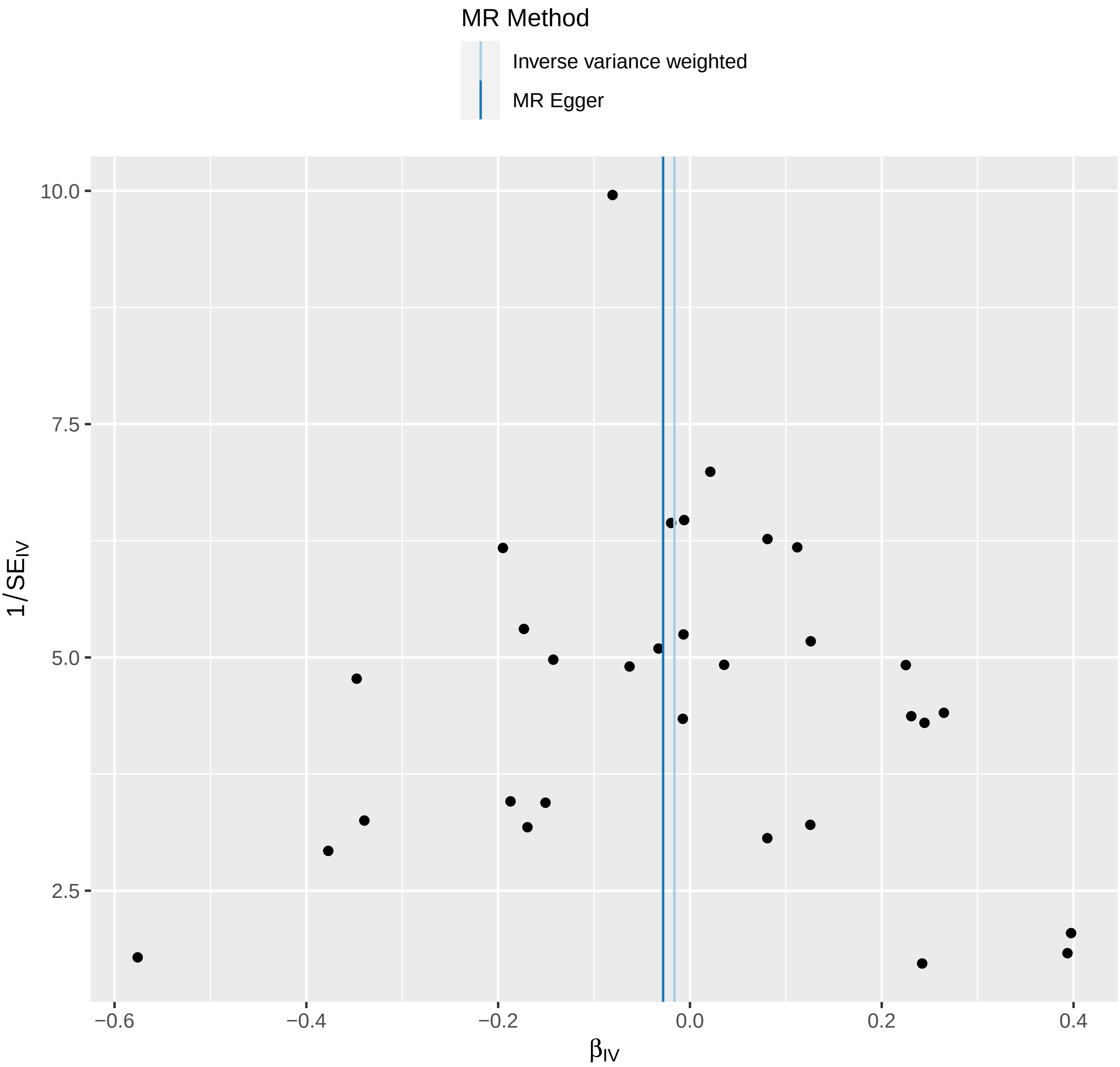

Batch 3−1015 : FAS−associated factor 2 on Diabetic nephropathy (more control exclusions)
with a threshold of 1e−05

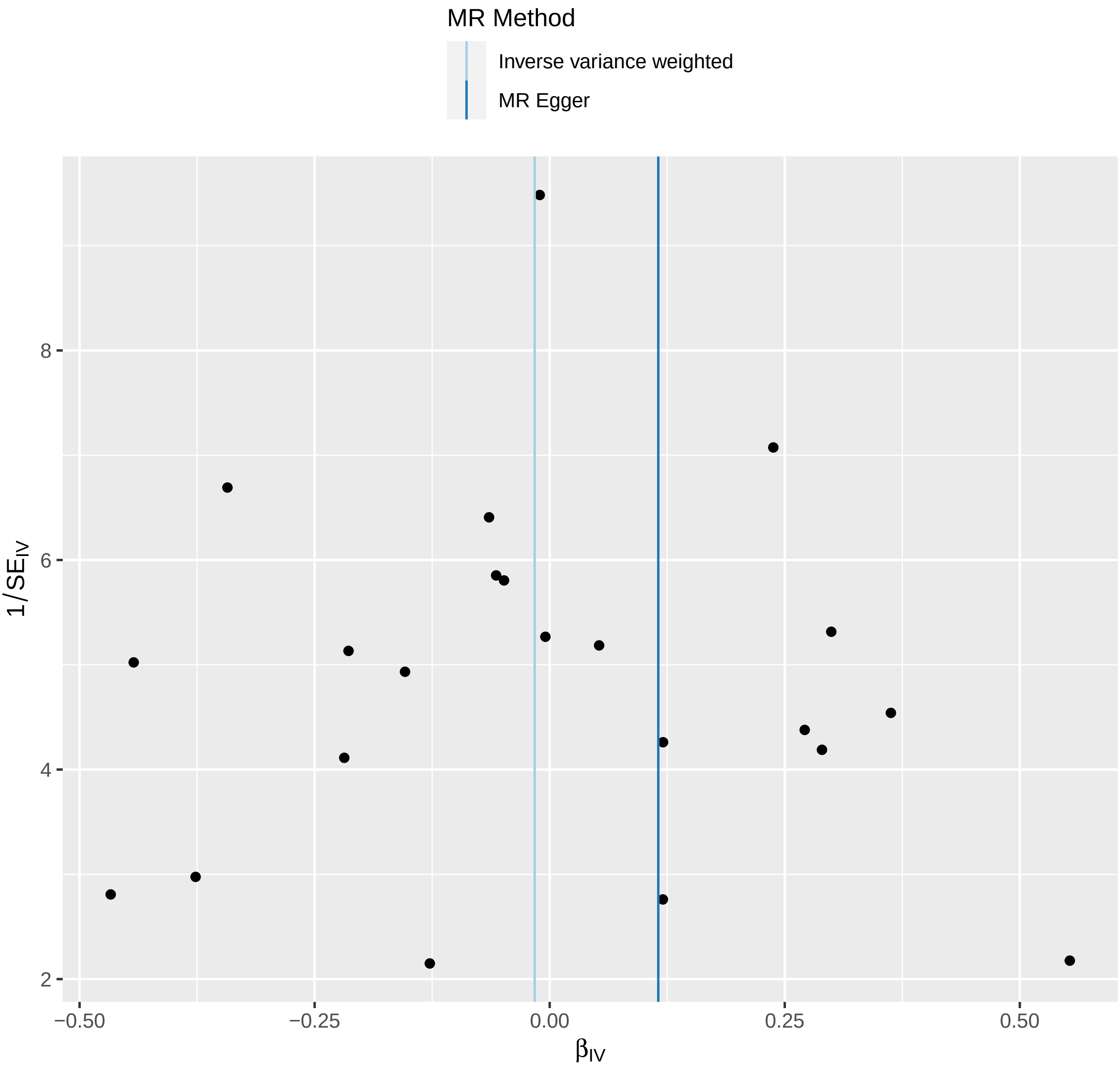

Batch 3−1016 : FAS−associated factor 2 on Diabetic nephropathy (more control exclusions)
with a threshold of 1e−05

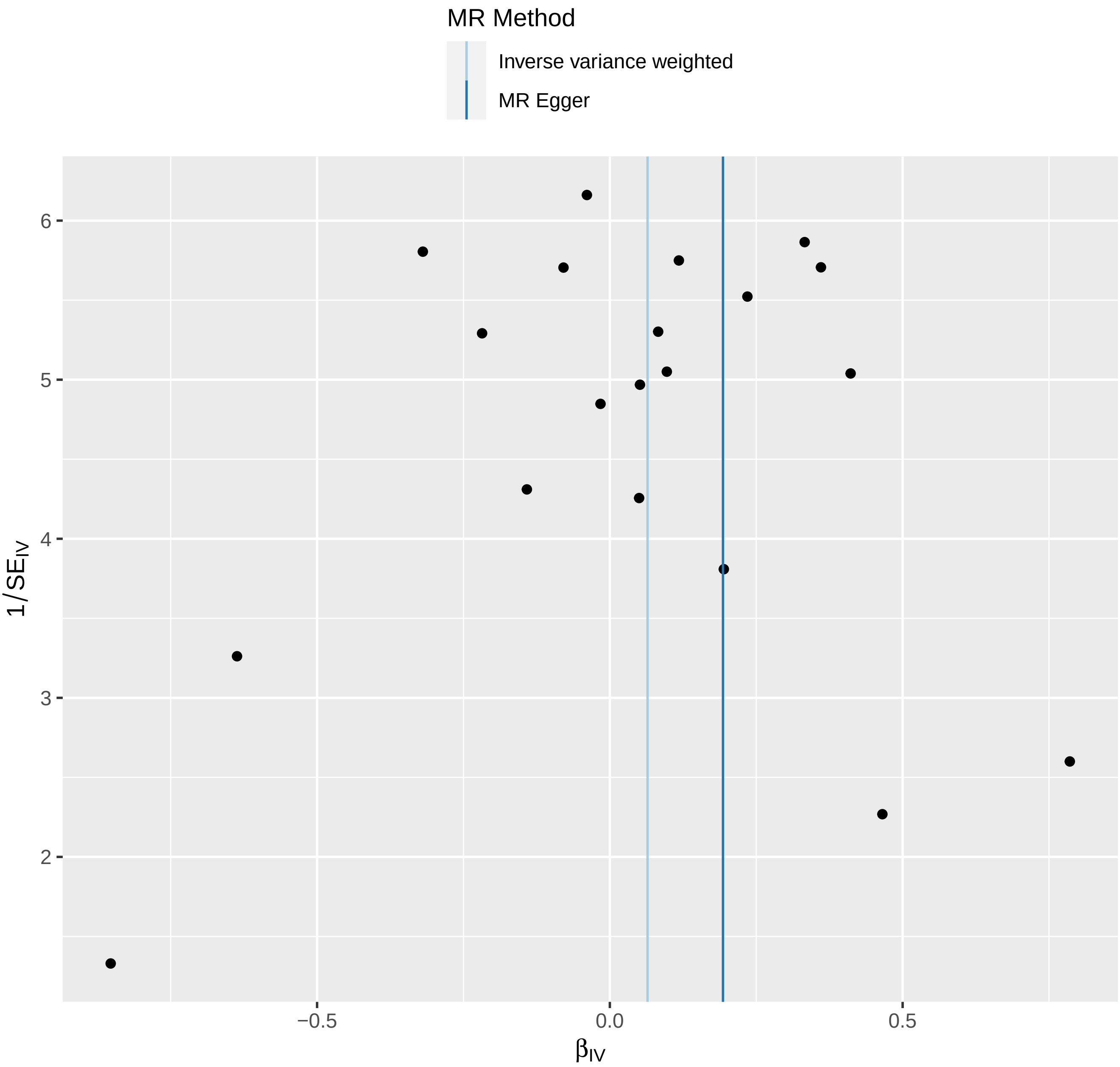

Batch 3−1017 : FAS−associated factor 2 on Diabetic nephropathy (more control exclusions)
with a threshold of 1e−05

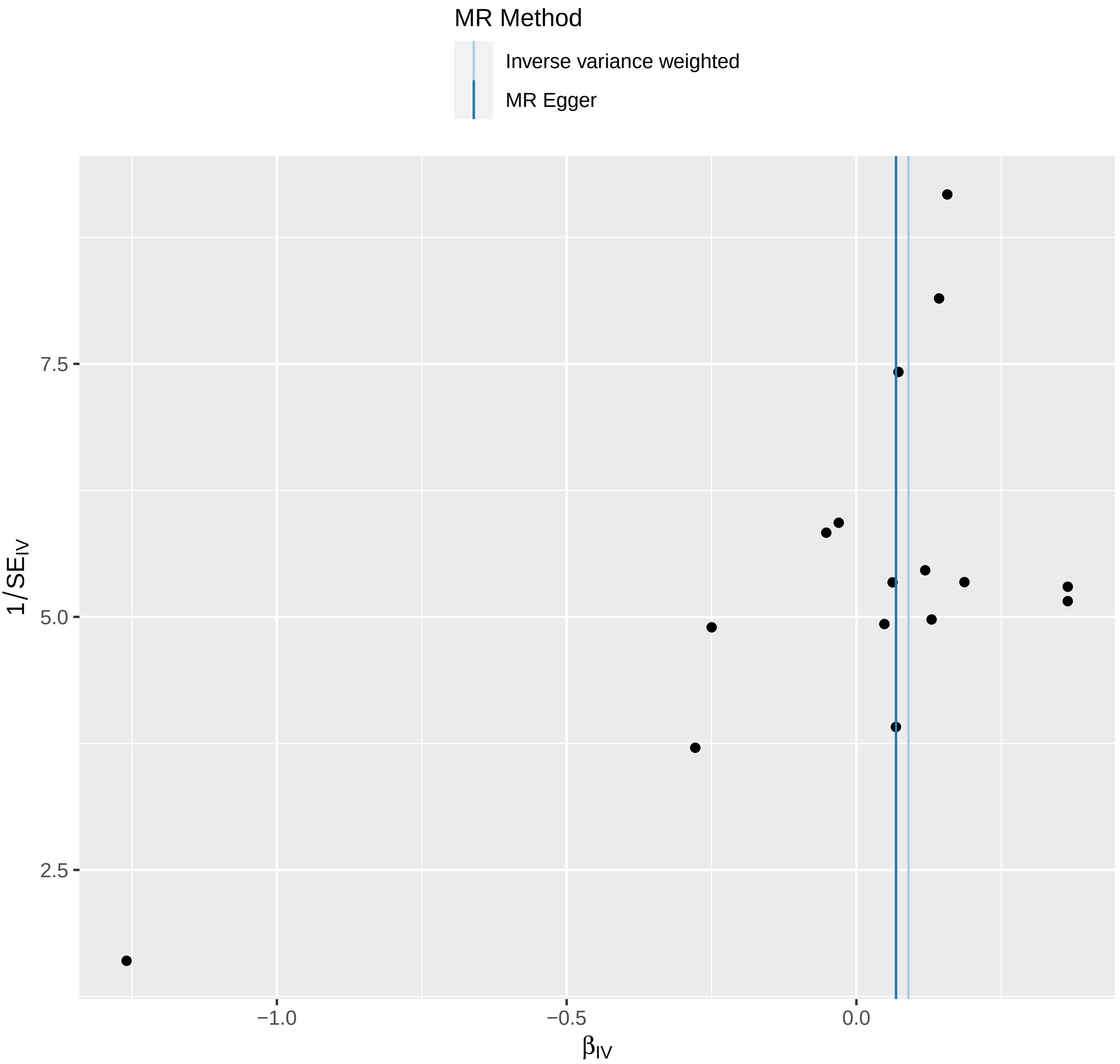

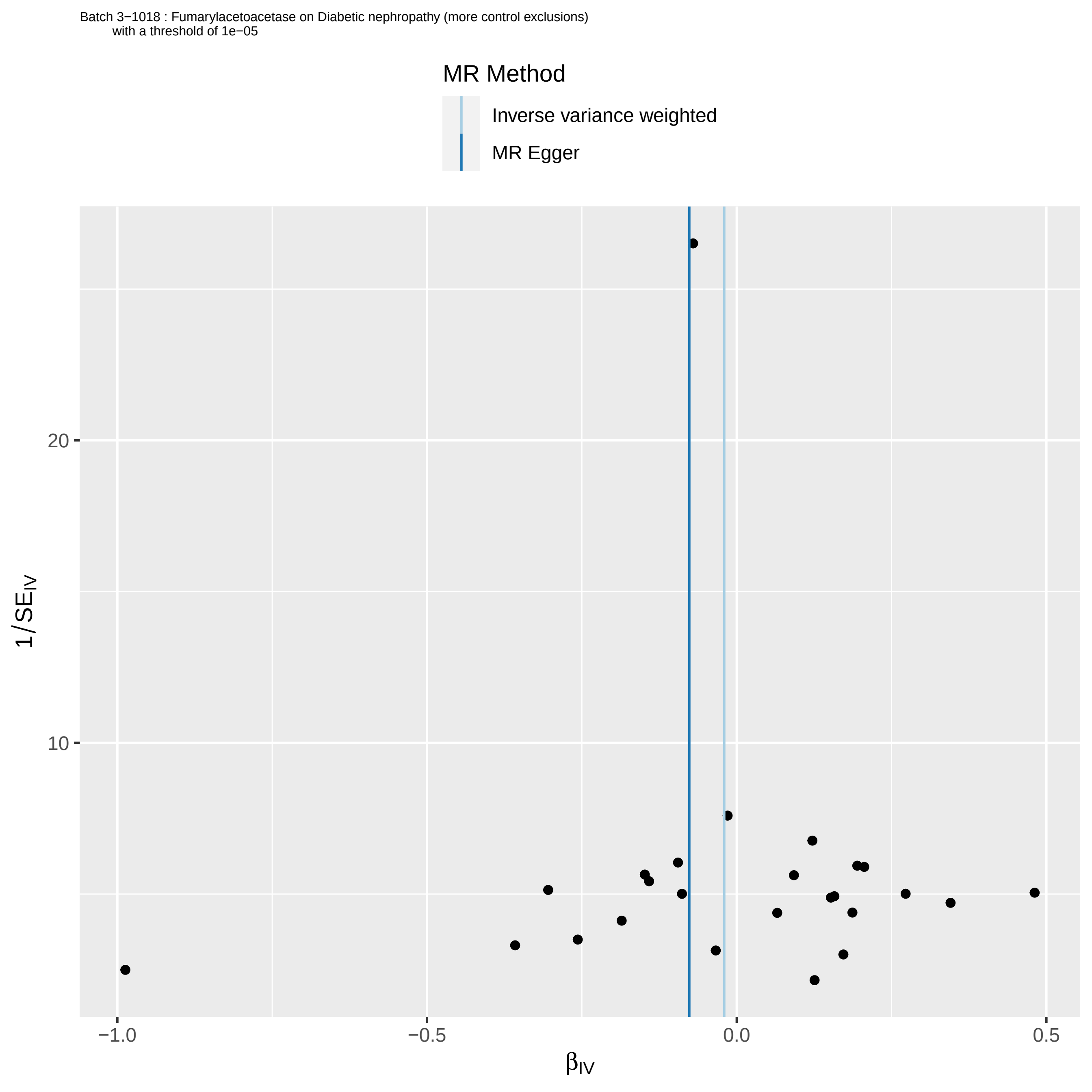

Batch 3−1018 : Fumarylacetoacetase on Diabetic nephropathy (more control exclusions)
with a threshold of 1e−05
MR Method
Inverse variance weighted
MR Egger
20
10
1/SE_IV
−1.0
−0.5
0.0
0.5
β_IV

Batch 3−1019 : Fas apoptotic inhibitory molecule 3 on Diabetic nephropathy (more control exclusions)
with a threshold of 1e−05

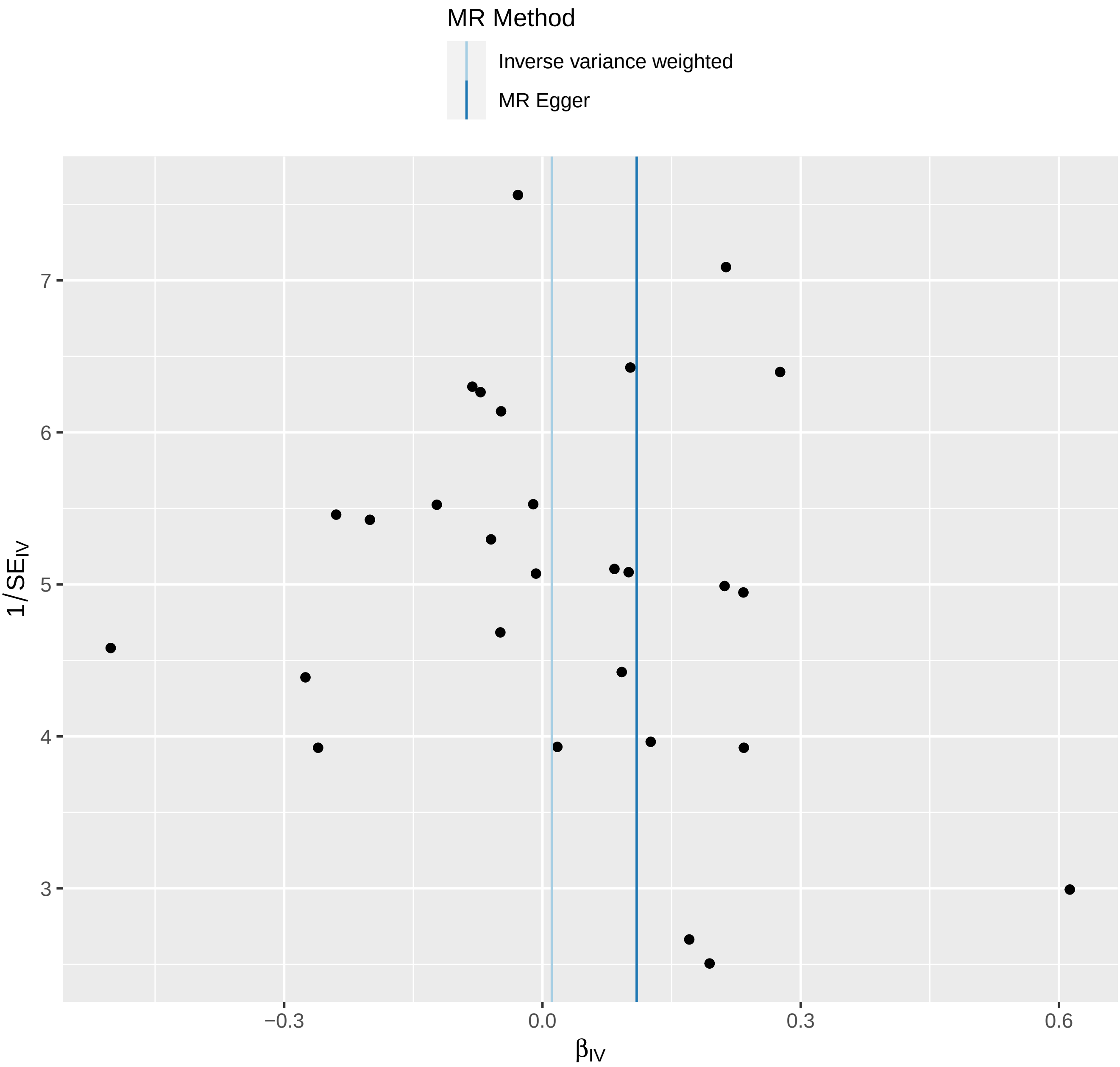

Batch 3−1020 : Protein FAM107A on Diabetic nephropathy (more control exclusions)
with a threshold of 1e−05

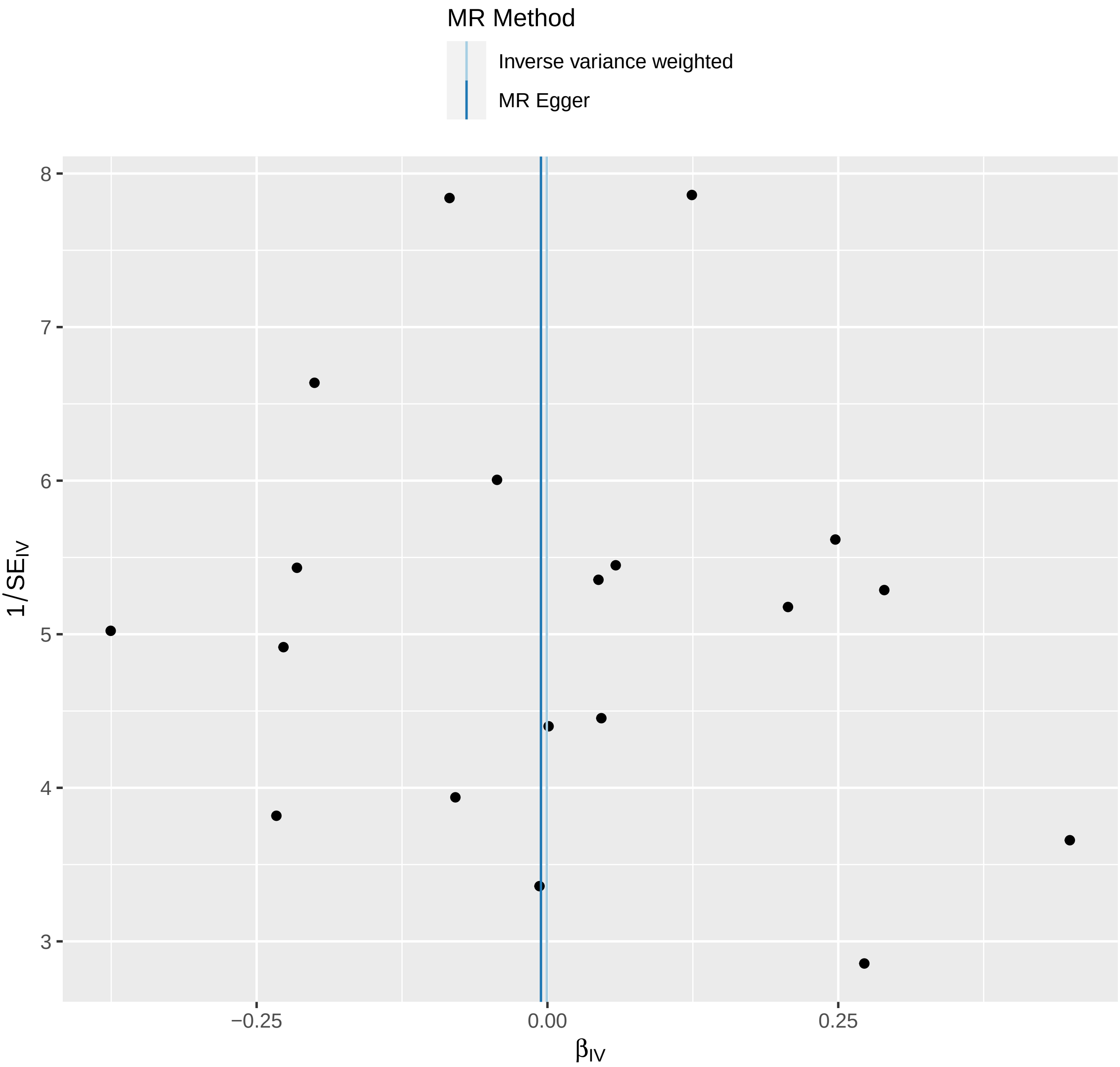

Batch 3−1021 : Protein FAM107B on Diabetic nephropathy (more control exclusions)
with a threshold of 1e−05

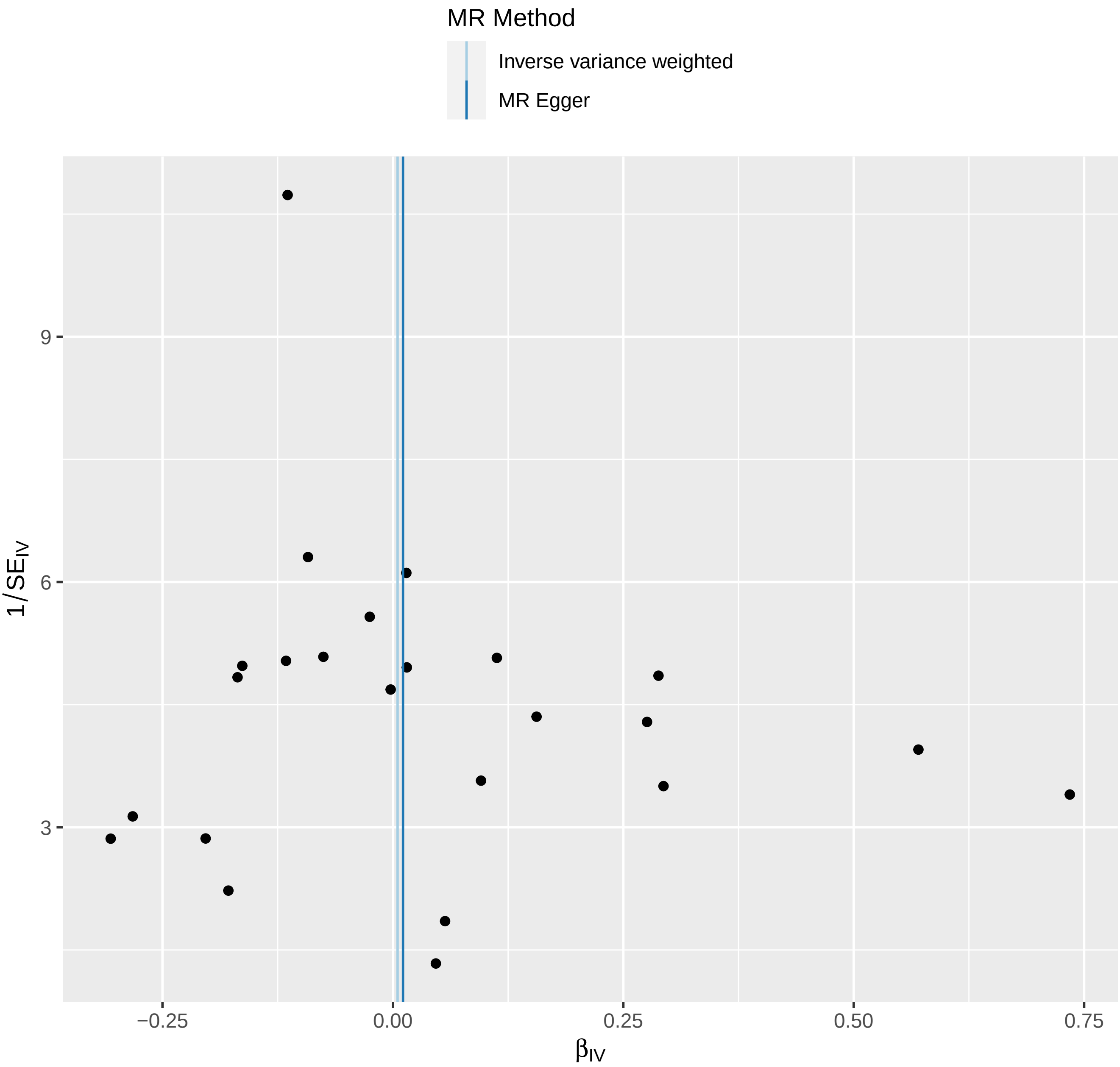

Batch 3−1022 : Protein FAM107B on Diabetic nephropathy (more control exclusions)
with a threshold of 1e−05

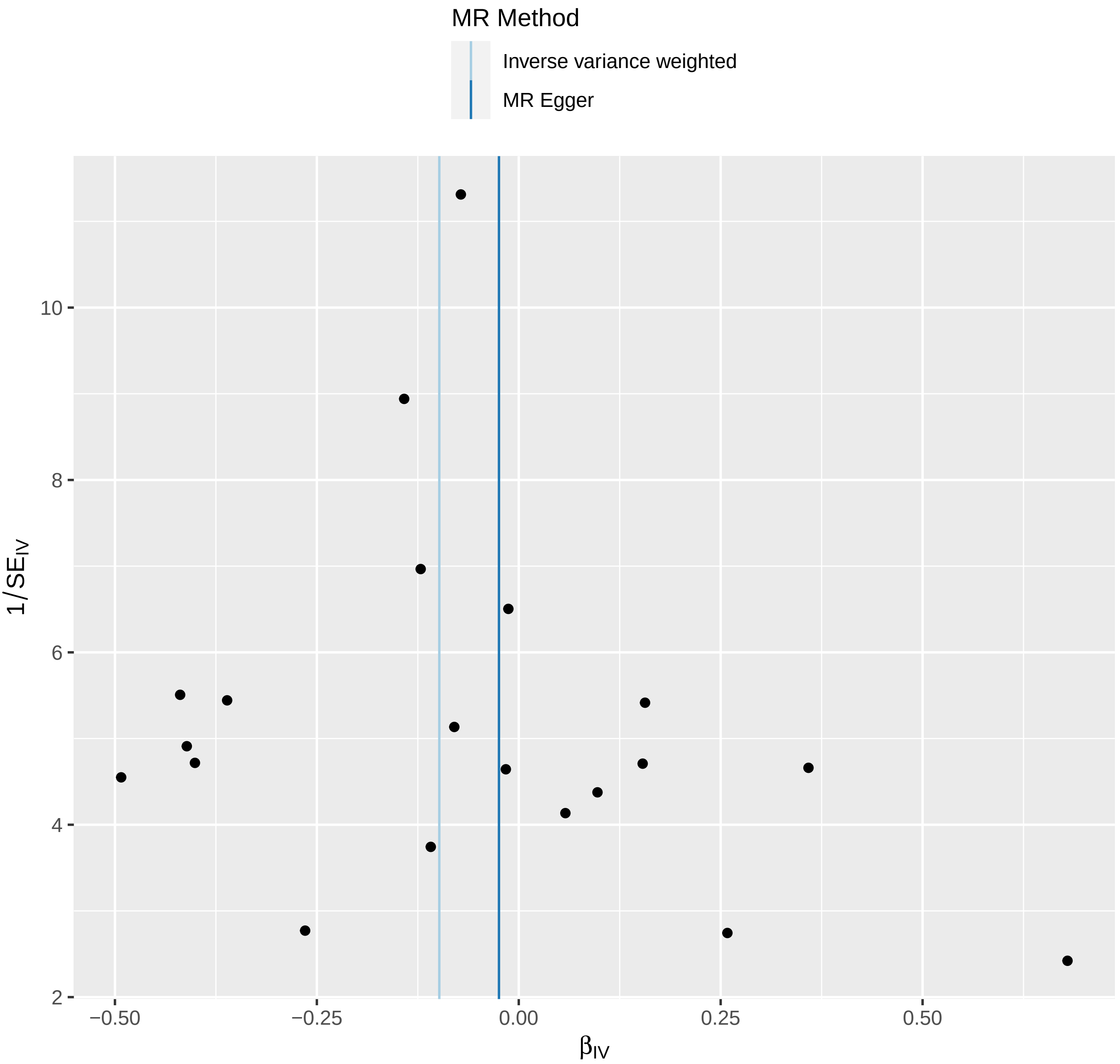

Batch 3−1023 : Protein FAM134B on Diabetic nephropathy (more control exclusions)
with a threshold of 1e−05

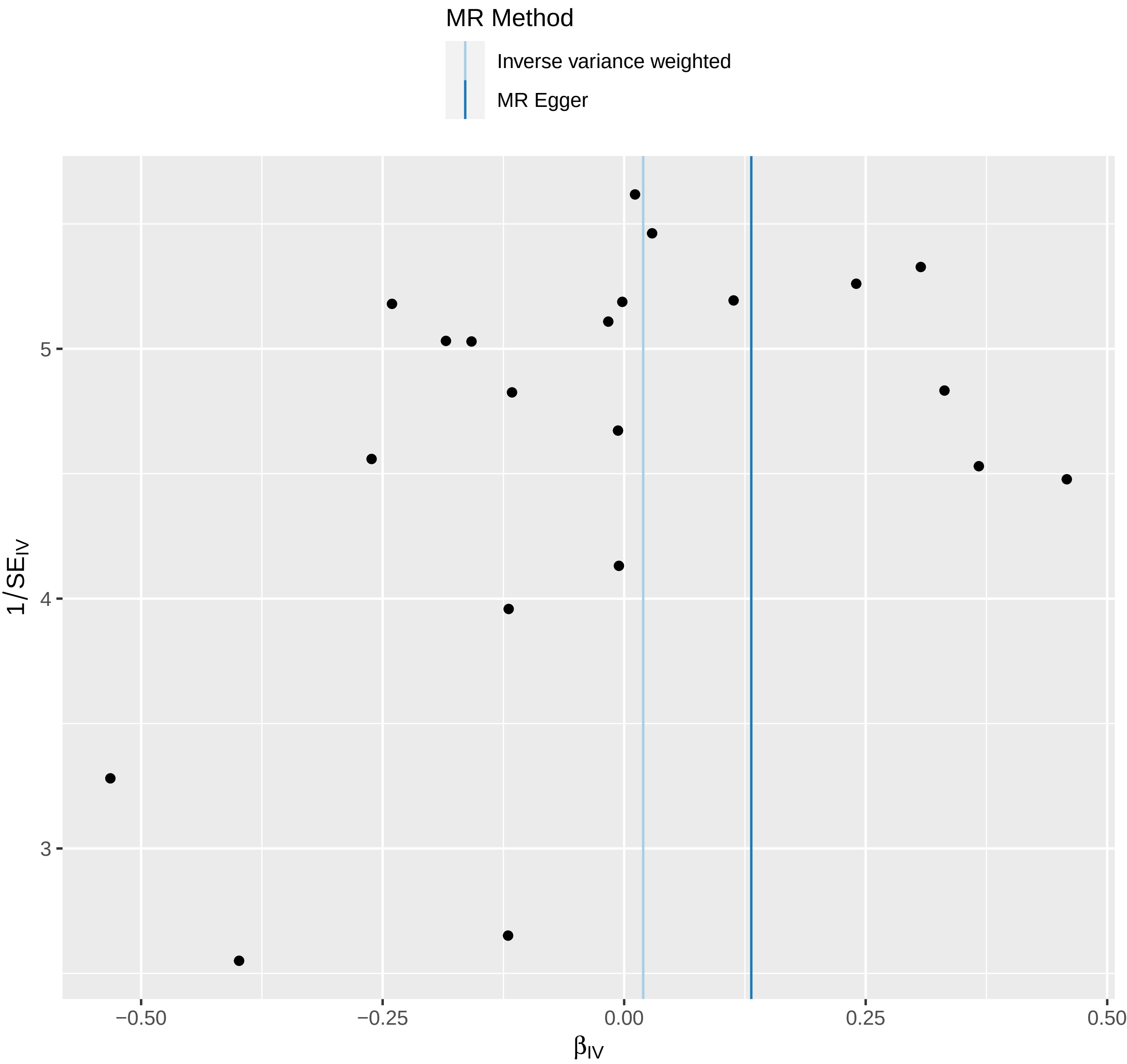

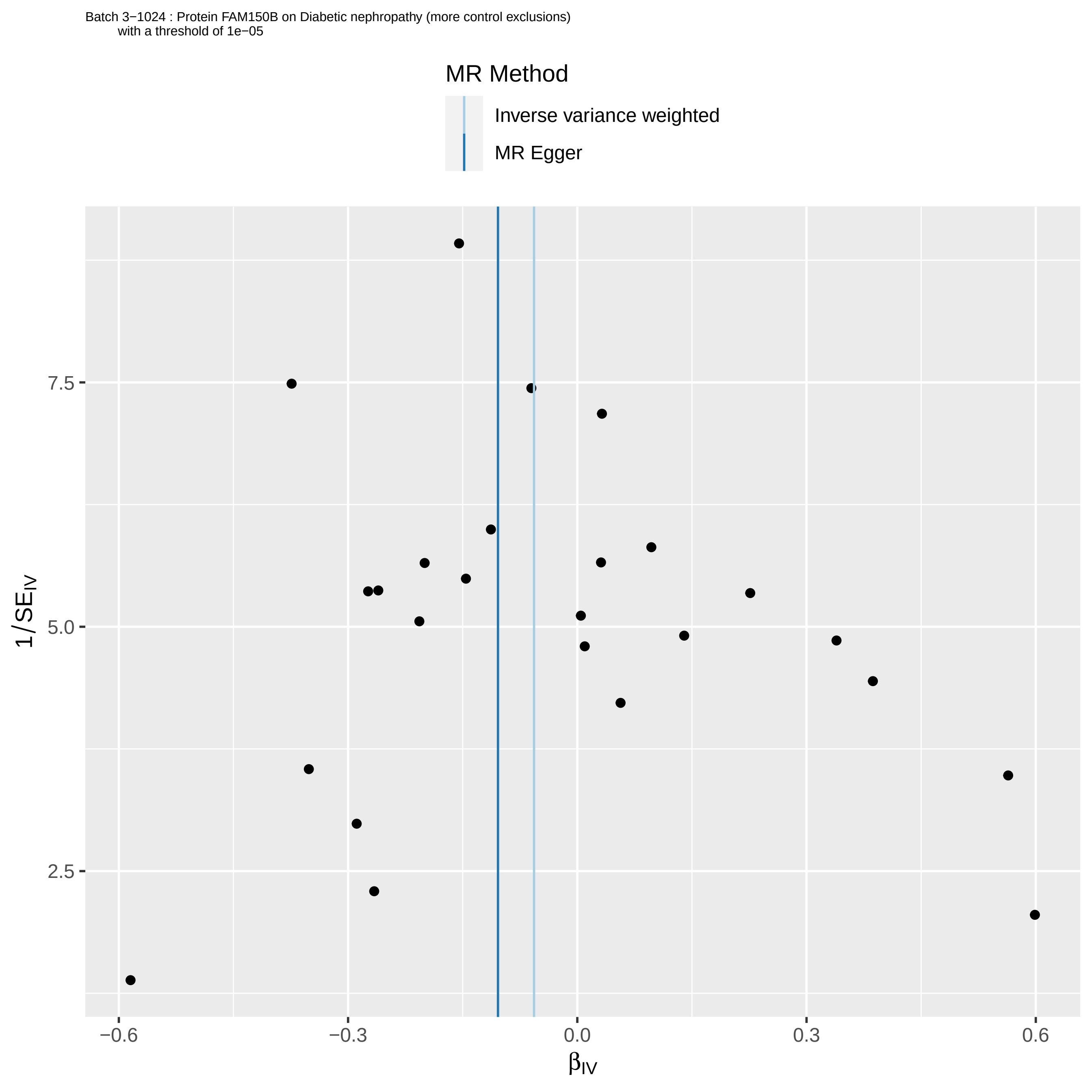

Batch 3−1024 : Protein FAM150B on Diabetic nephropathy (more control exclusions)
with a threshold of 1e−05
MR Method
Inverse variance weighted
MR Egger
7.5
5.0
2.5
1/SE$_{IV}$
−0.6
−0.3
0.0
0.3
0.6
β$_{IV}$

Batch 3−1025 : Protein FAM151A on Diabetic nephropathy (more control exclusions)
with a threshold of 1e−05

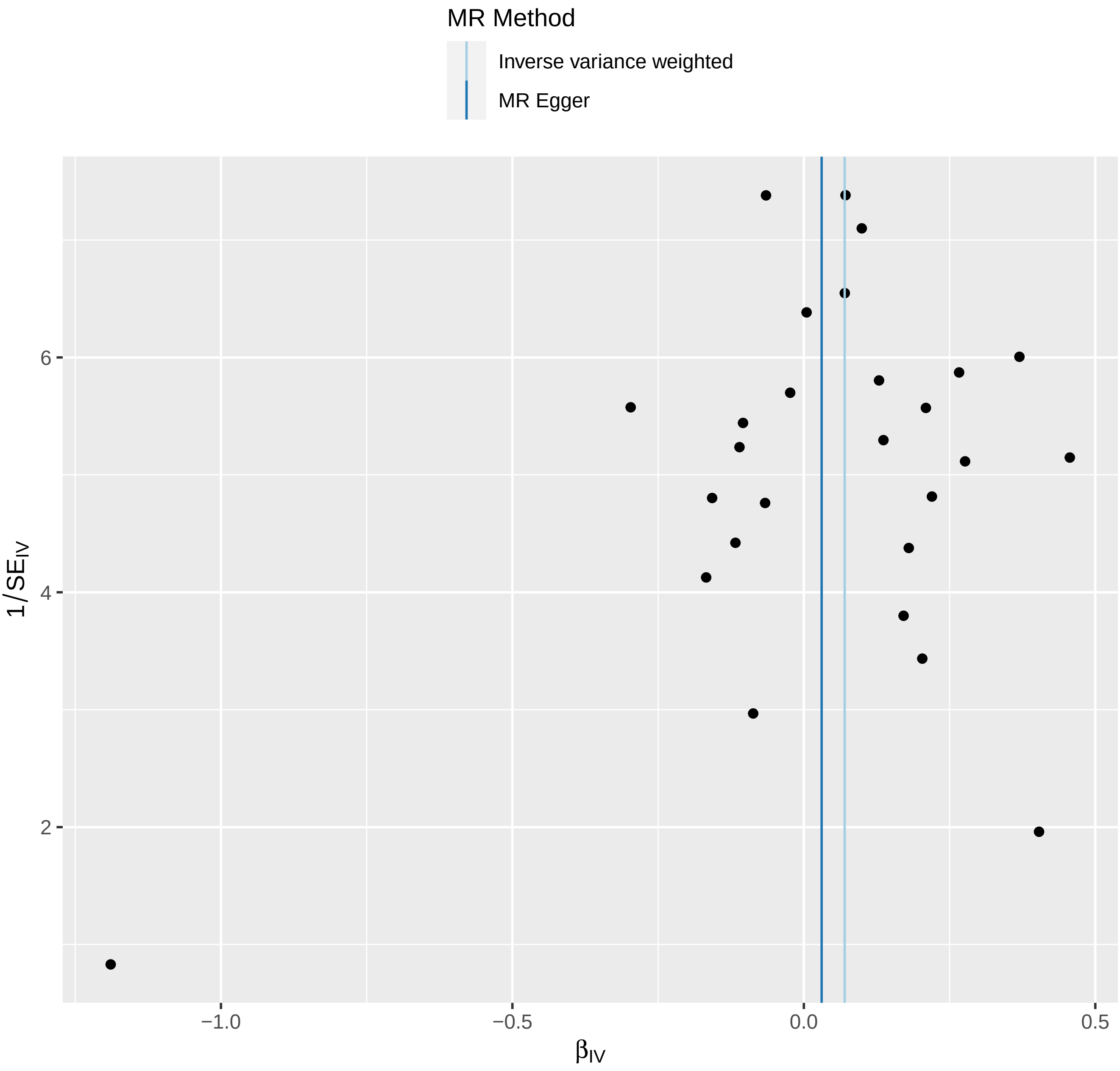

Batch 3−1026 : Membrane protein FAM159B on Diabetic nephropathy (more control exclusions)
with a threshold of 1e−05

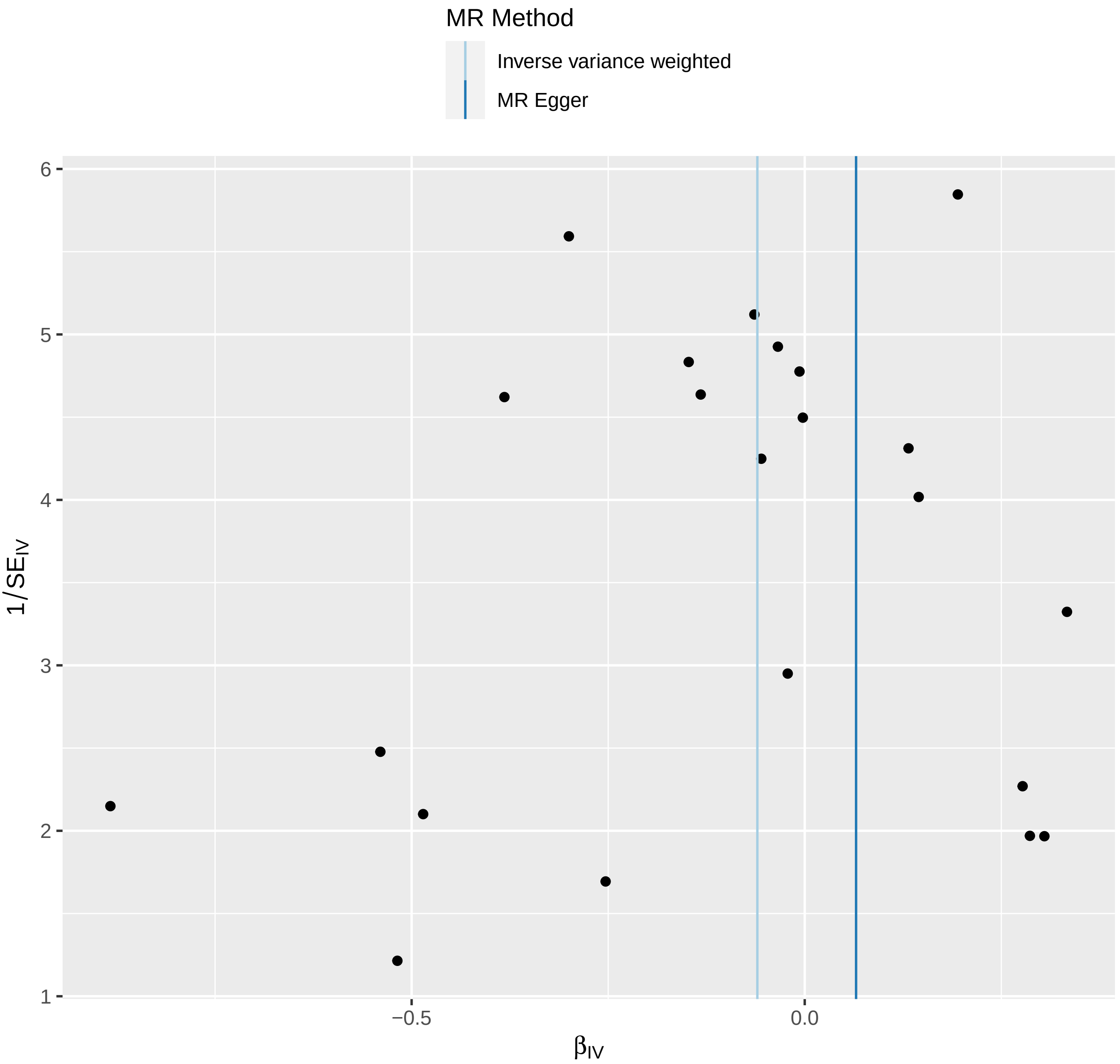

Batch 3−1027 : Protein FAM163A on Diabetic nephropathy (more control exclusions)
with a threshold of 1e−05

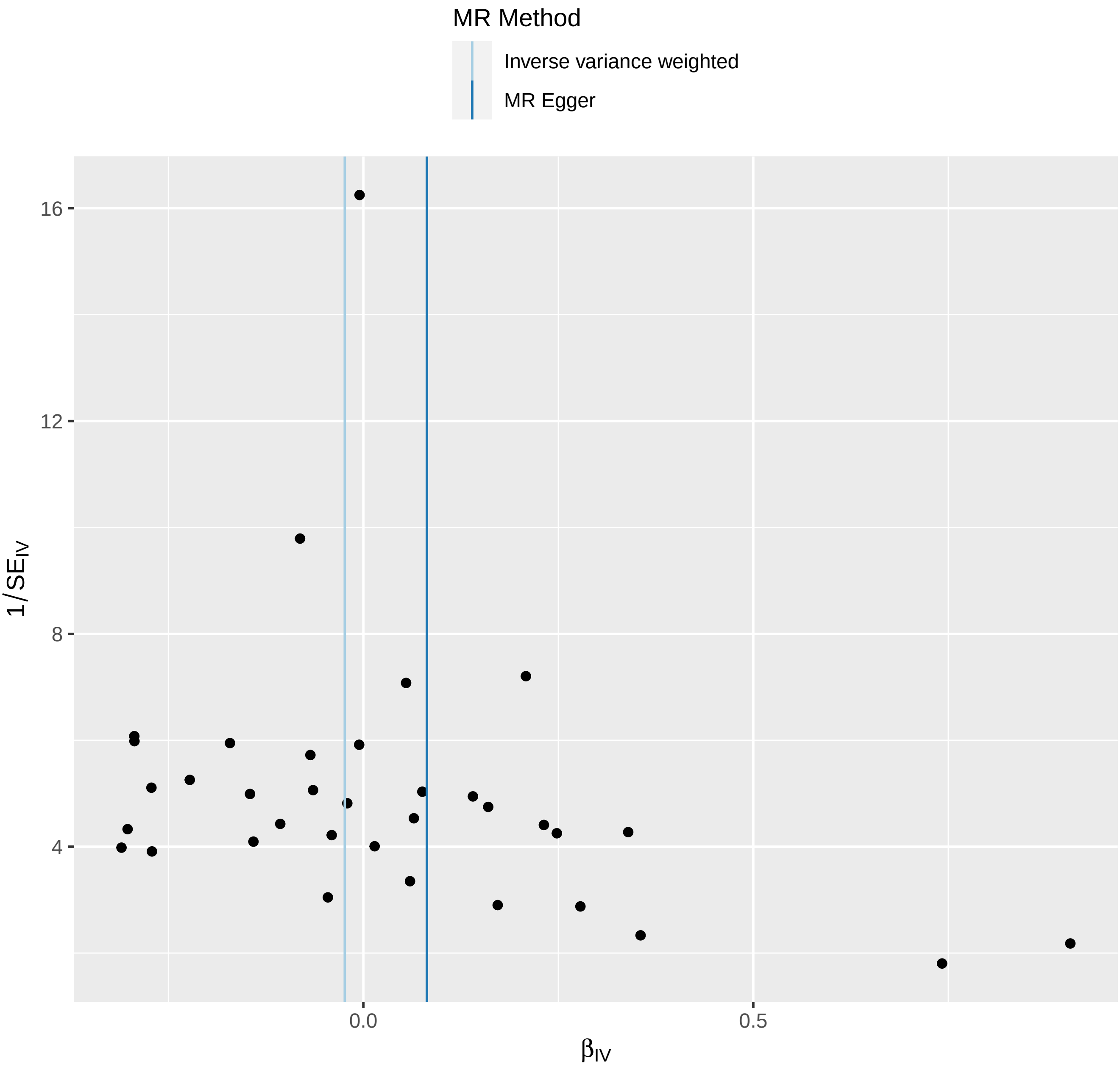

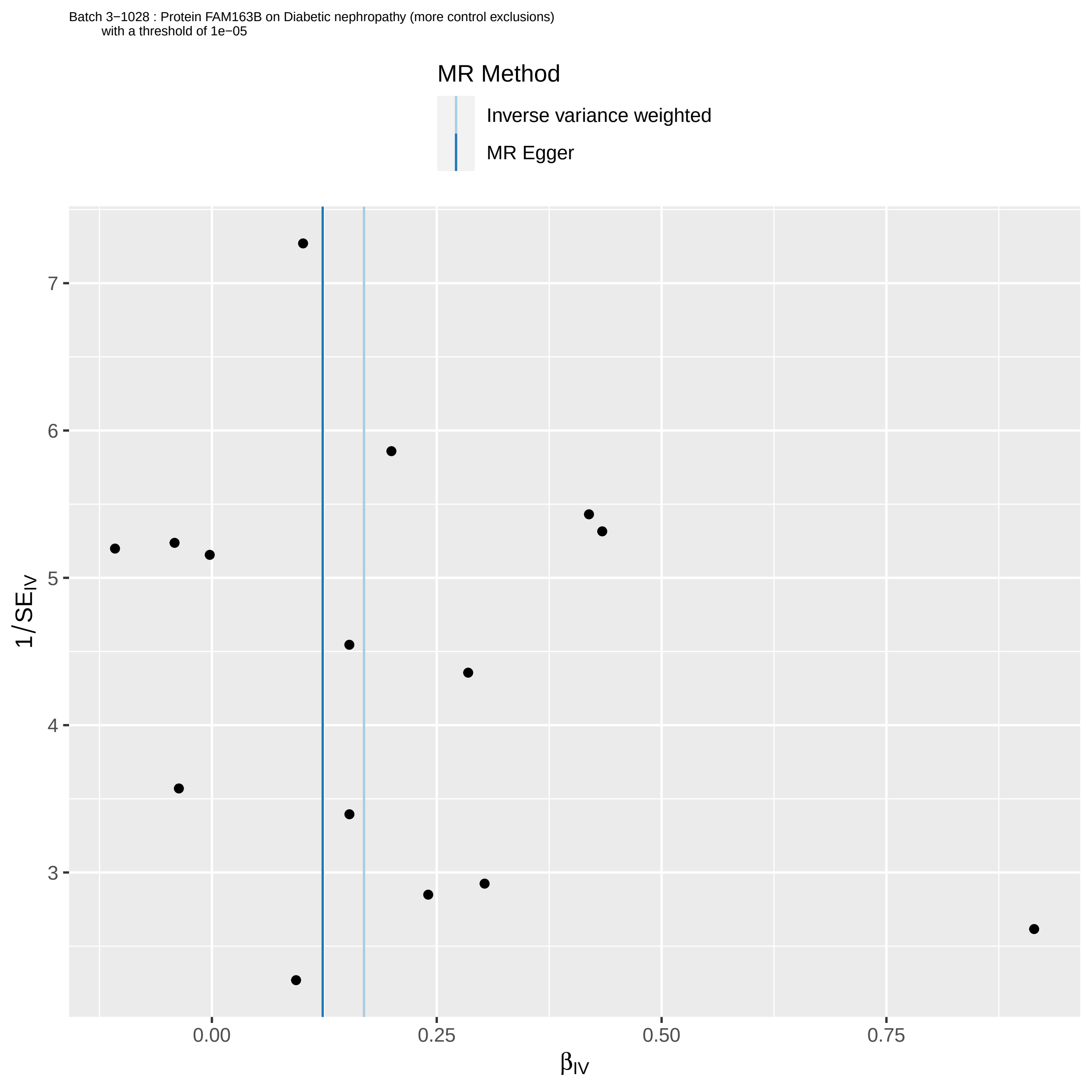

Batch 3−1028 : Protein FAM163B on Diabetic nephropathy (more control exclusions)
with a threshold of 1e−05
MR Method
Inverse variance weighted
MR Egger
7
6
5
4
3
1/SE_IV
0.00
0.25
0.50
0.75
β_IV

Batch 3−1029 : Protein FAM171B on Diabetic nephropathy (more control exclusions)
with a threshold of 1e−05

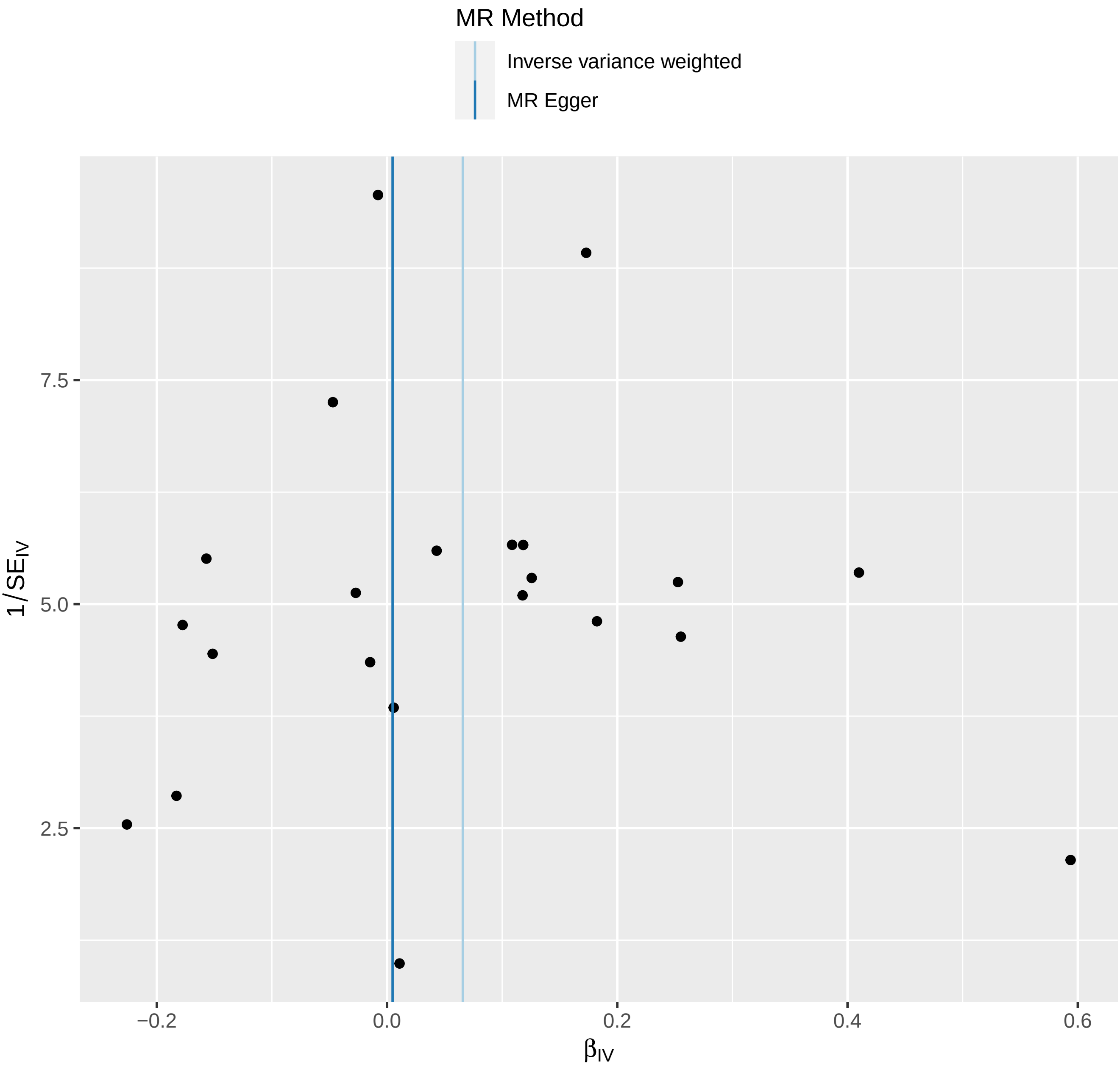

Batch 3−1030 : Protein FAM171B on Diabetic nephropathy (more control exclusions)
with a threshold of 1e−05

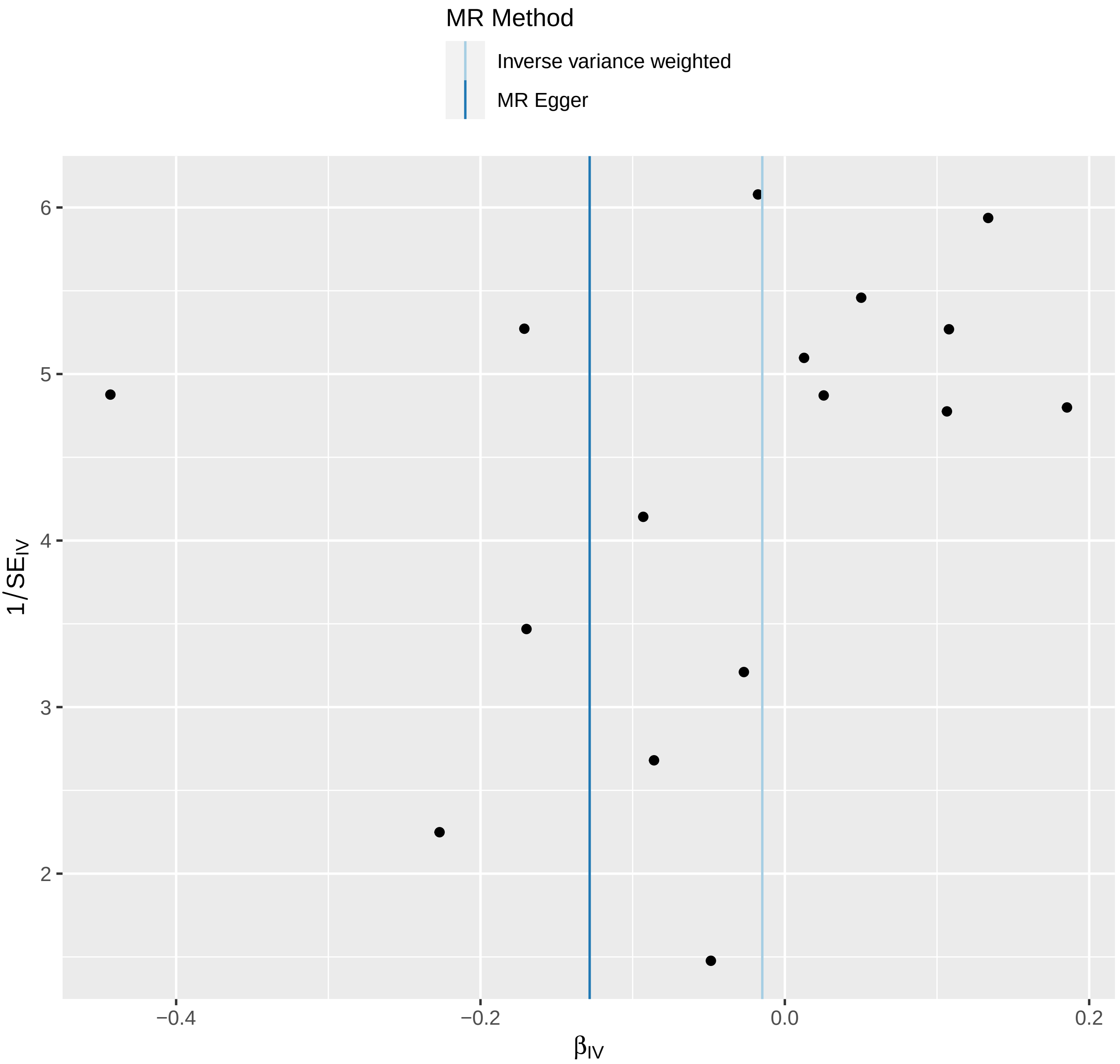

Batch 3−1031 : Protein FAM171B on Diabetic nephropathy (more control exclusions)
with a threshold of 1e−05

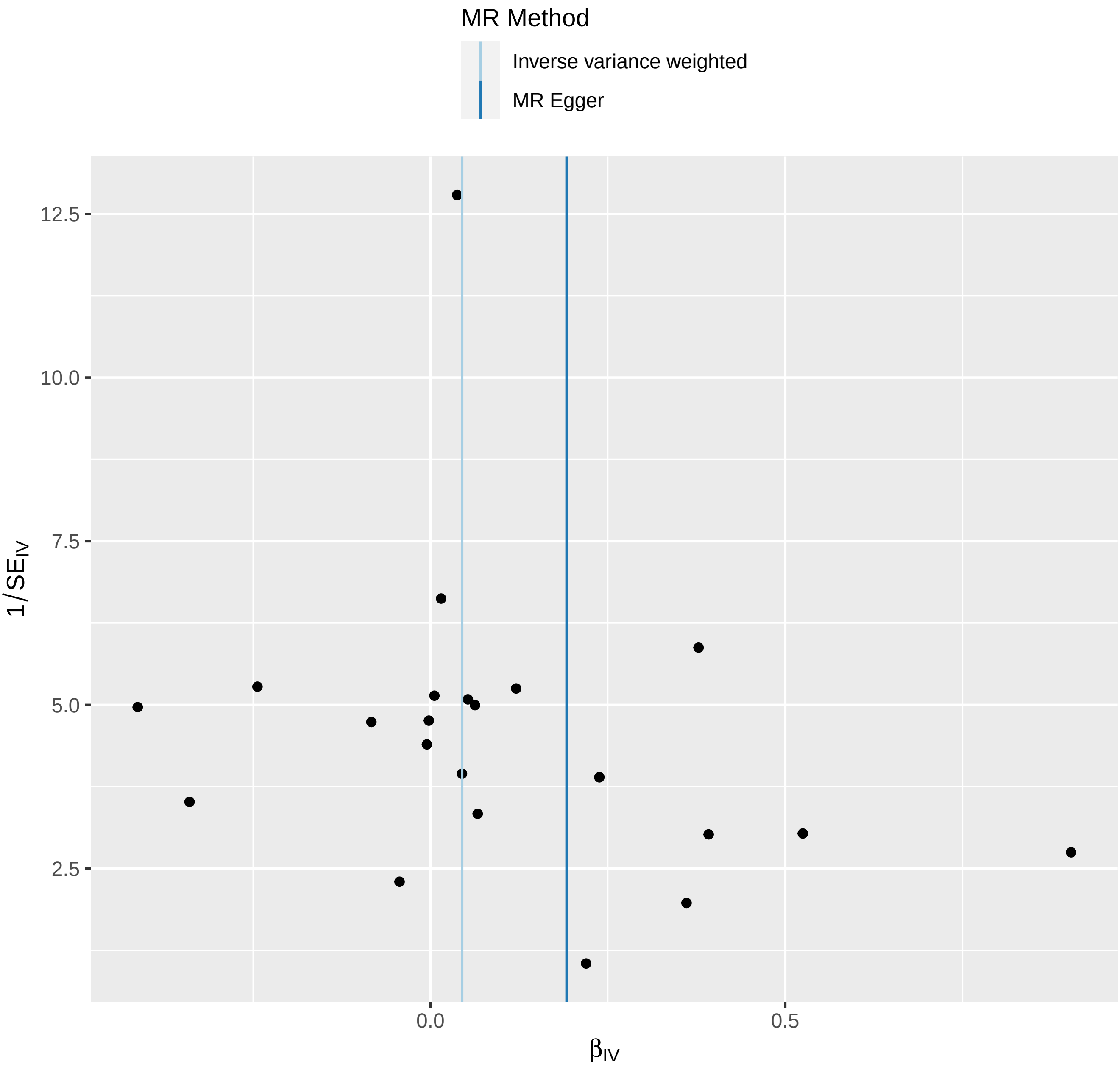

Batch 3−1032 : Protein FAM172A on Diabetic nephropathy (more control exclusions)
with a threshold of 1e−05

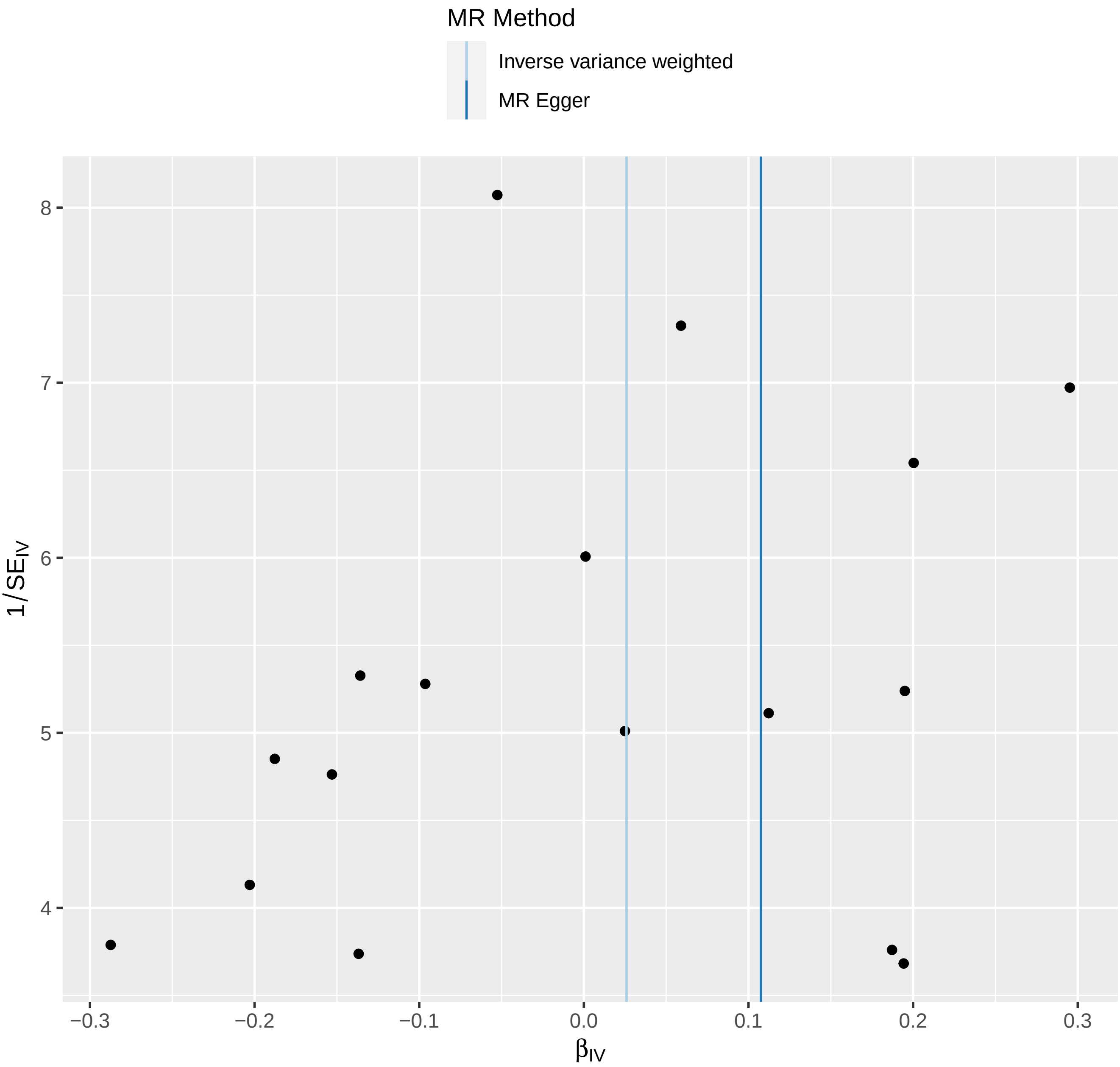

Batch 3−1033 : Protein FAM173A on Diabetic nephropathy (more control exclusions)
with a threshold of 1e−05

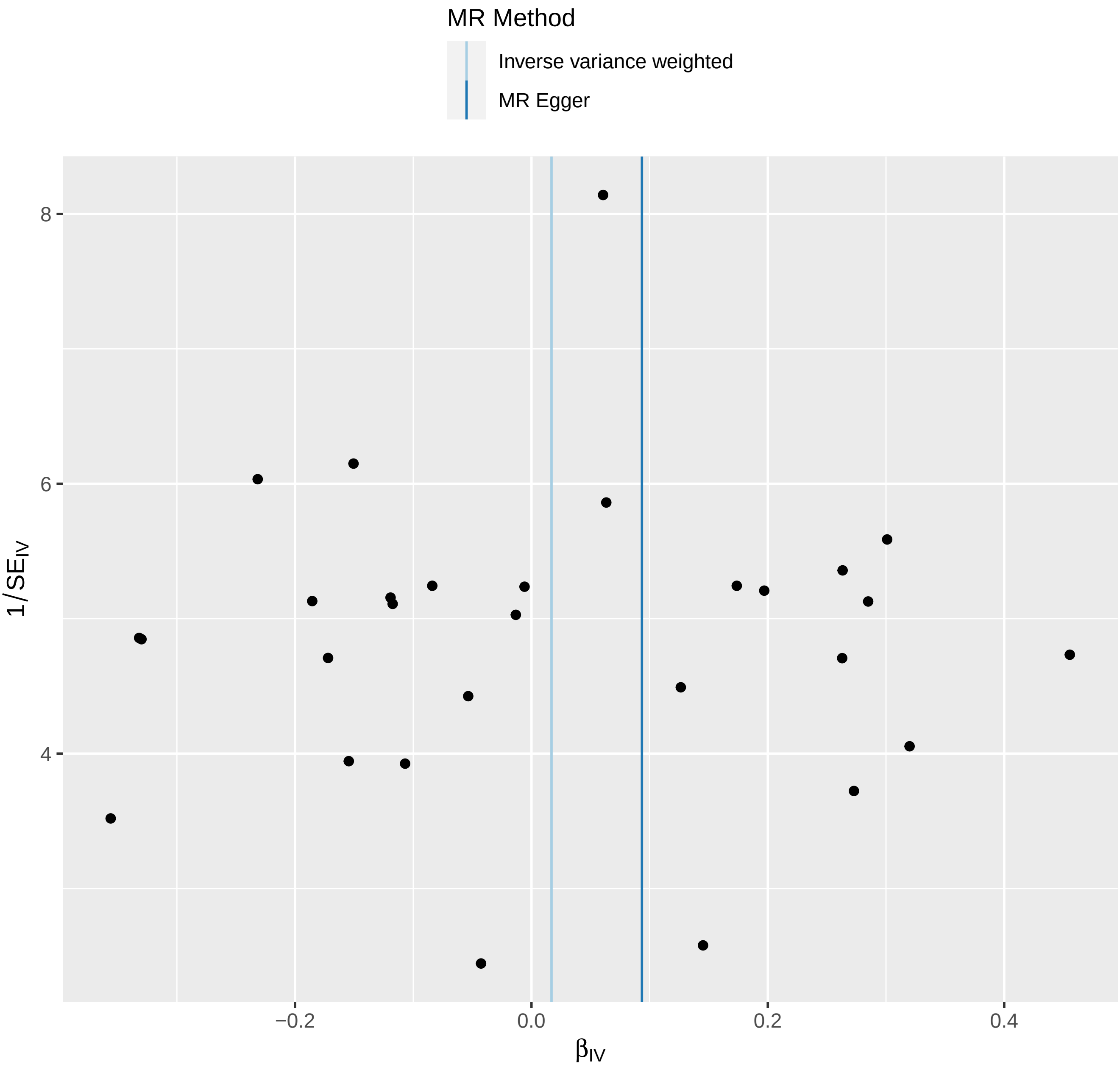

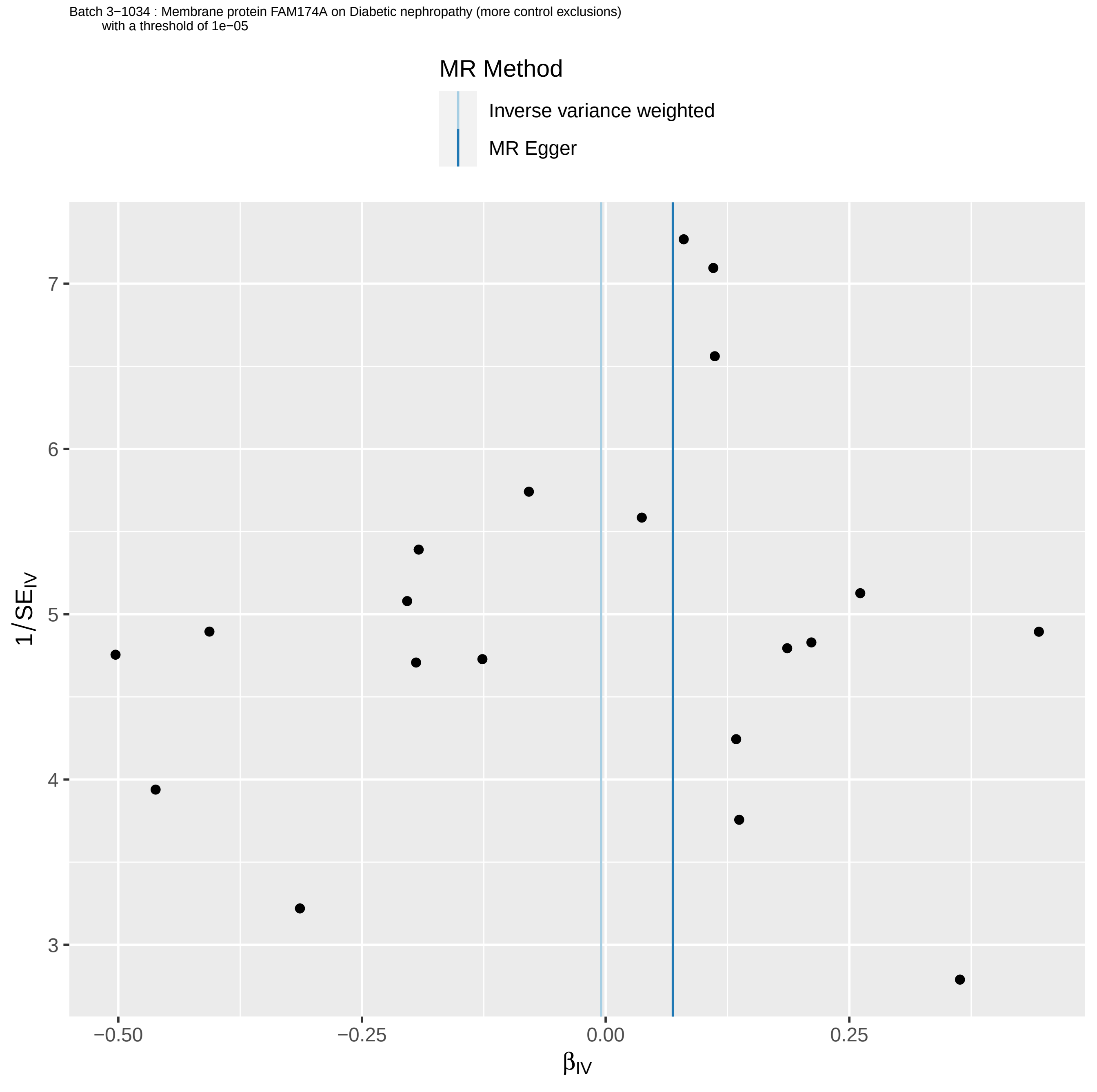

Batch 3−1034 : Membrane protein FAM174A on Diabetic nephropathy (more control exclusions)
with a threshold of 1e−05
MR Method
Inverse variance weighted
MR Egger
7
6
5
4
3
1/SE_IV
−0.50
−0.25
0.00
0.25
β_IV

Batch 3−1035 : Membrane protein FAM174B on Diabetic nephropathy (more control exclusions)
with a threshold of 1e−05

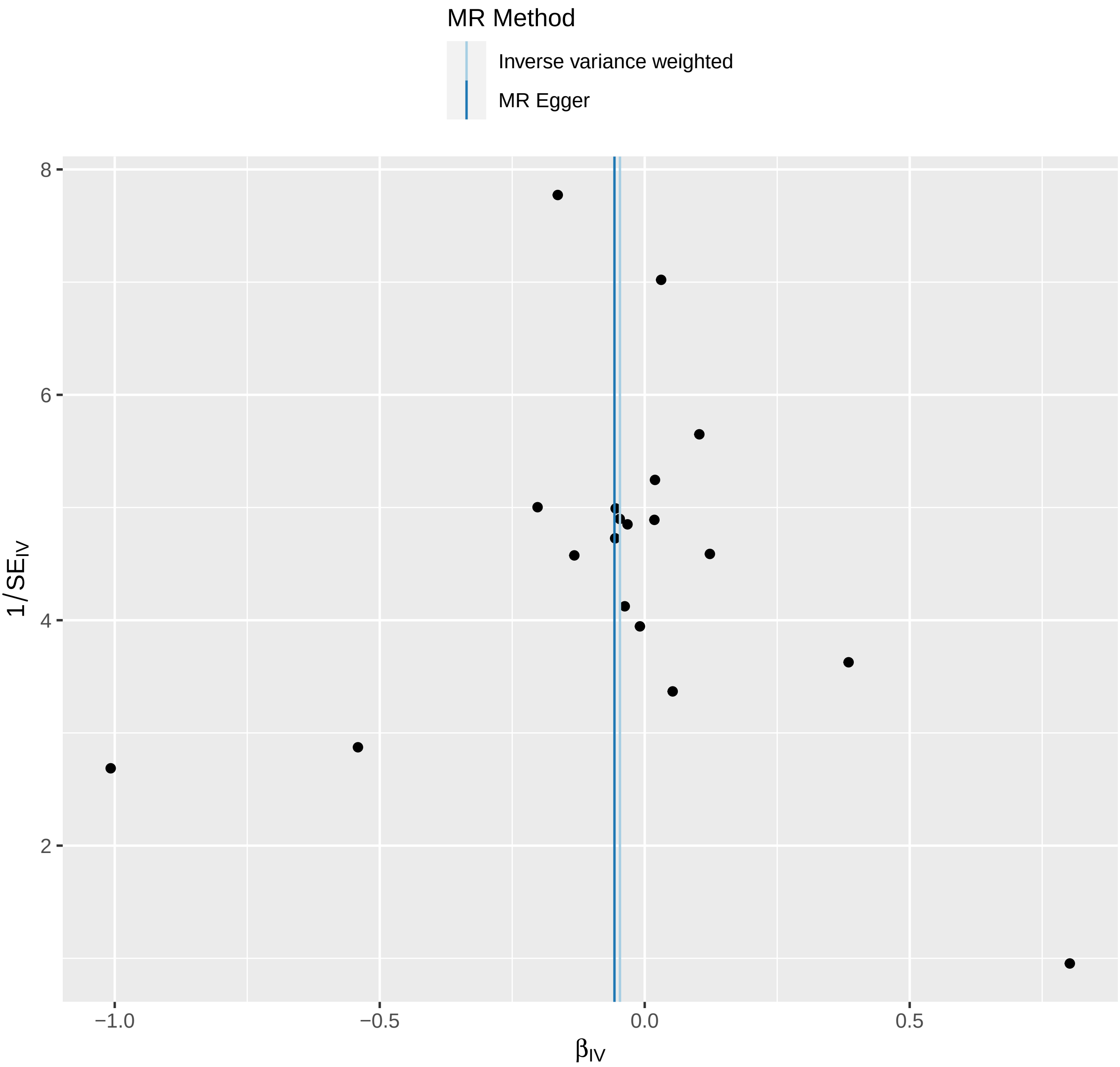

Batch 3−1036 : BRISC complex subunit Abro1 on Diabetic nephropathy (more control exclusions)
with a threshold of 1e−05

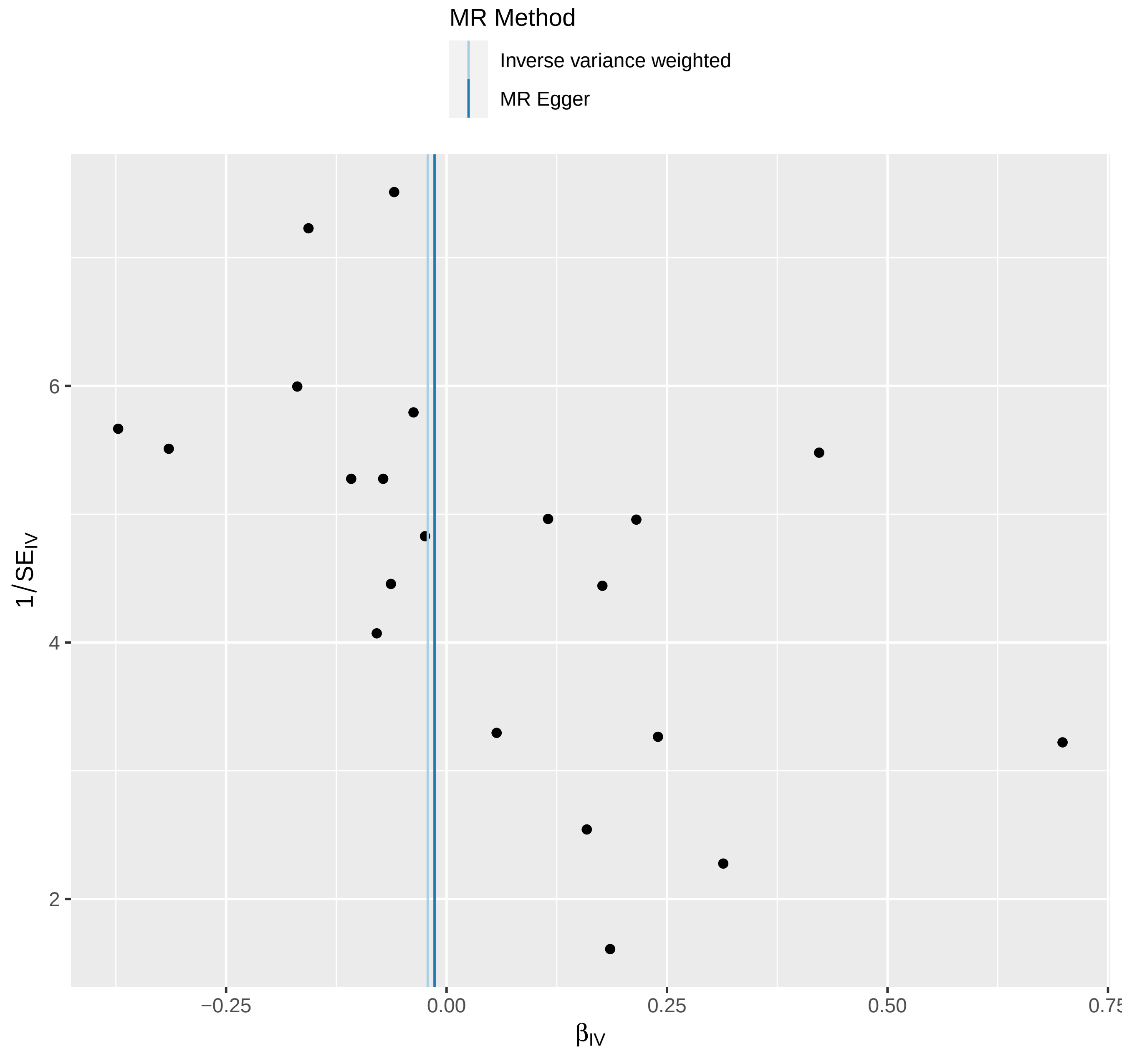

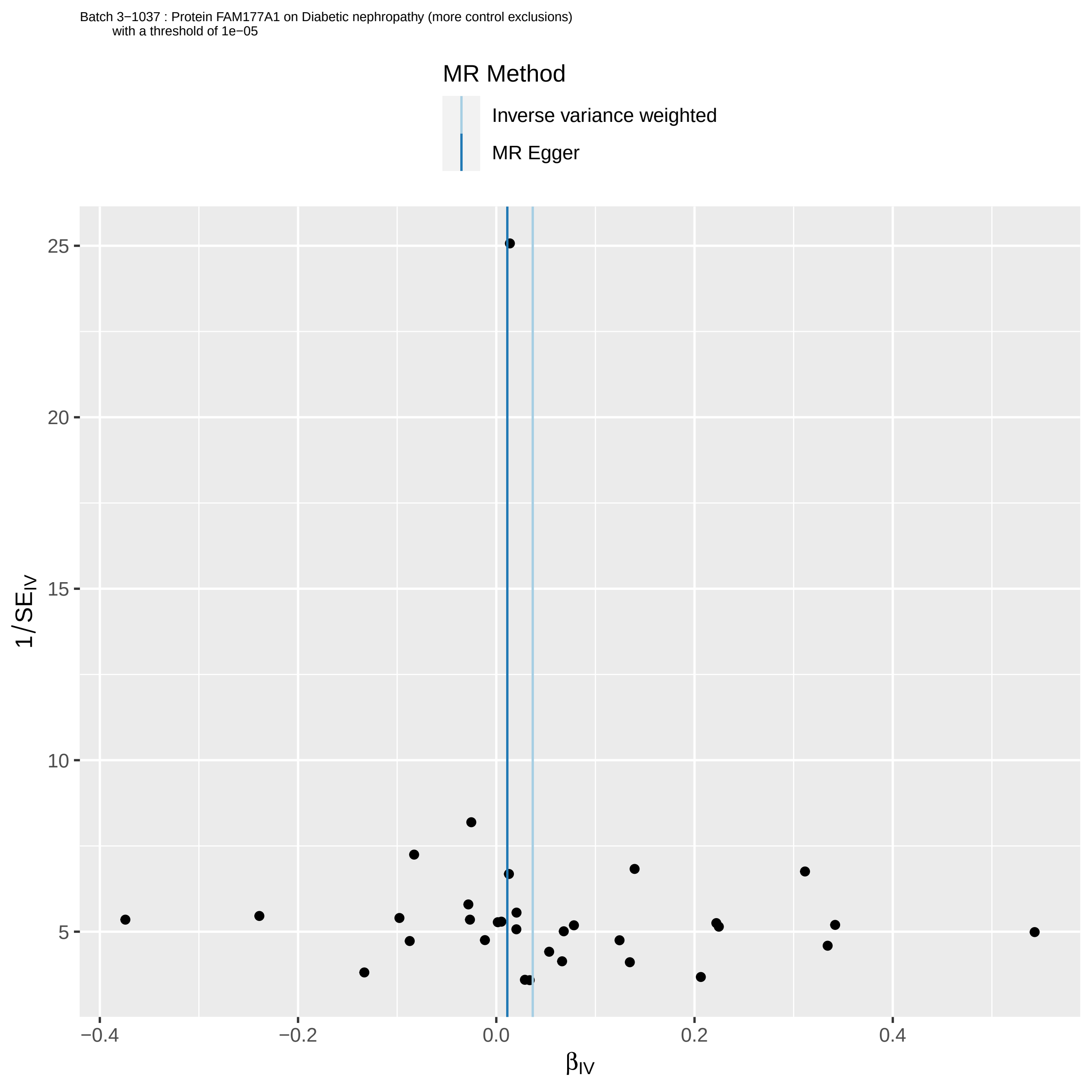
Batch 3−1037 : Protein FAM177A1 on Diabetic nephropathy (more control exclusions)
with a threshold of 1e−05
MR Method
Inverse variance weighted
MR Egger
25
20
15
10
5
1/SE_IV
−0.4
−0.2
0.0
0.2
0.4
β_IV

Batch 3−1038 : Protein FAM189A2 on Diabetic nephropathy (more control exclusions)
with a threshold of 1e−05

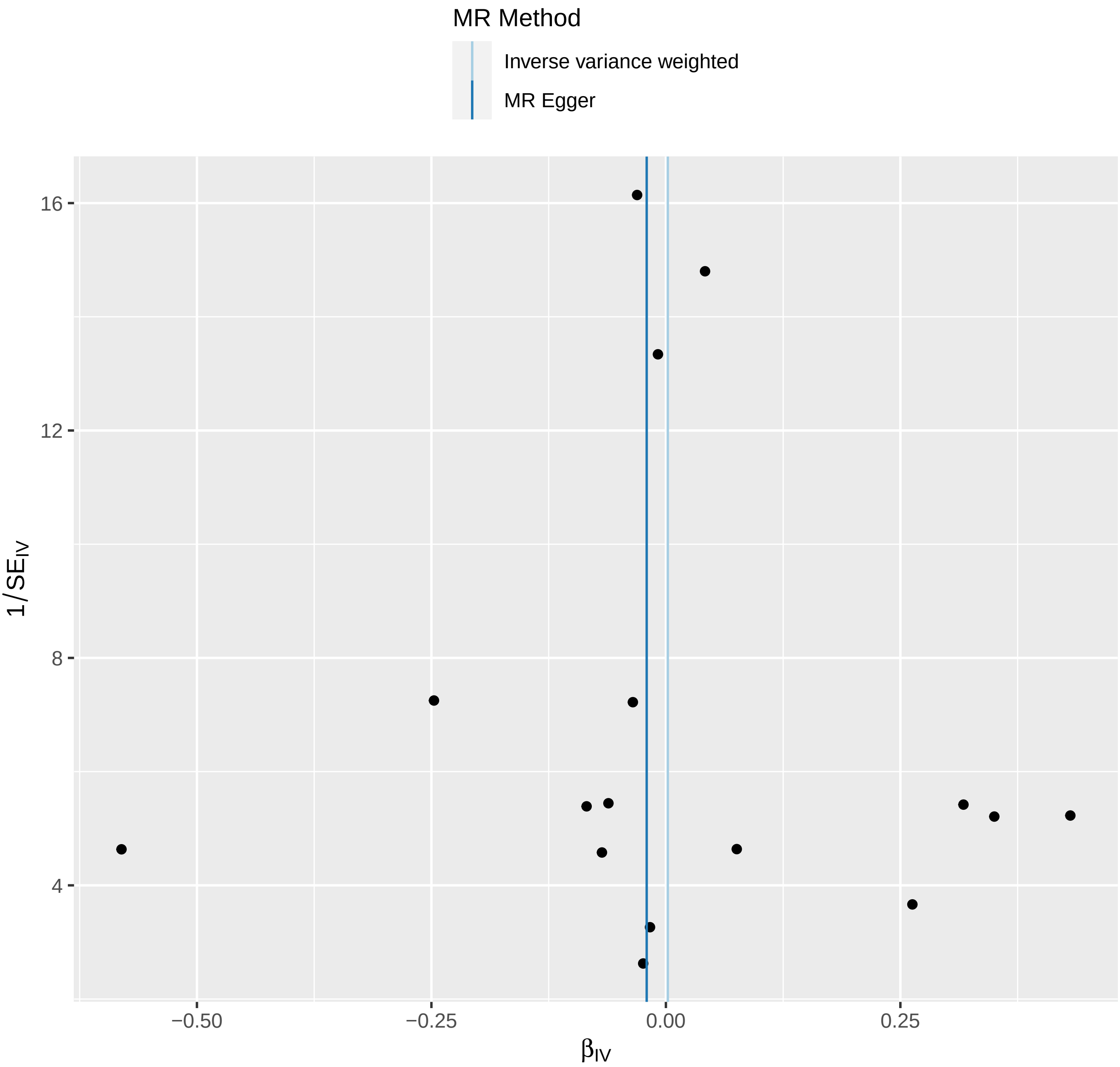

Batch 3−1039 : Protein FAM189A2 on Diabetic nephropathy (more control exclusions)
with a threshold of 1e−05

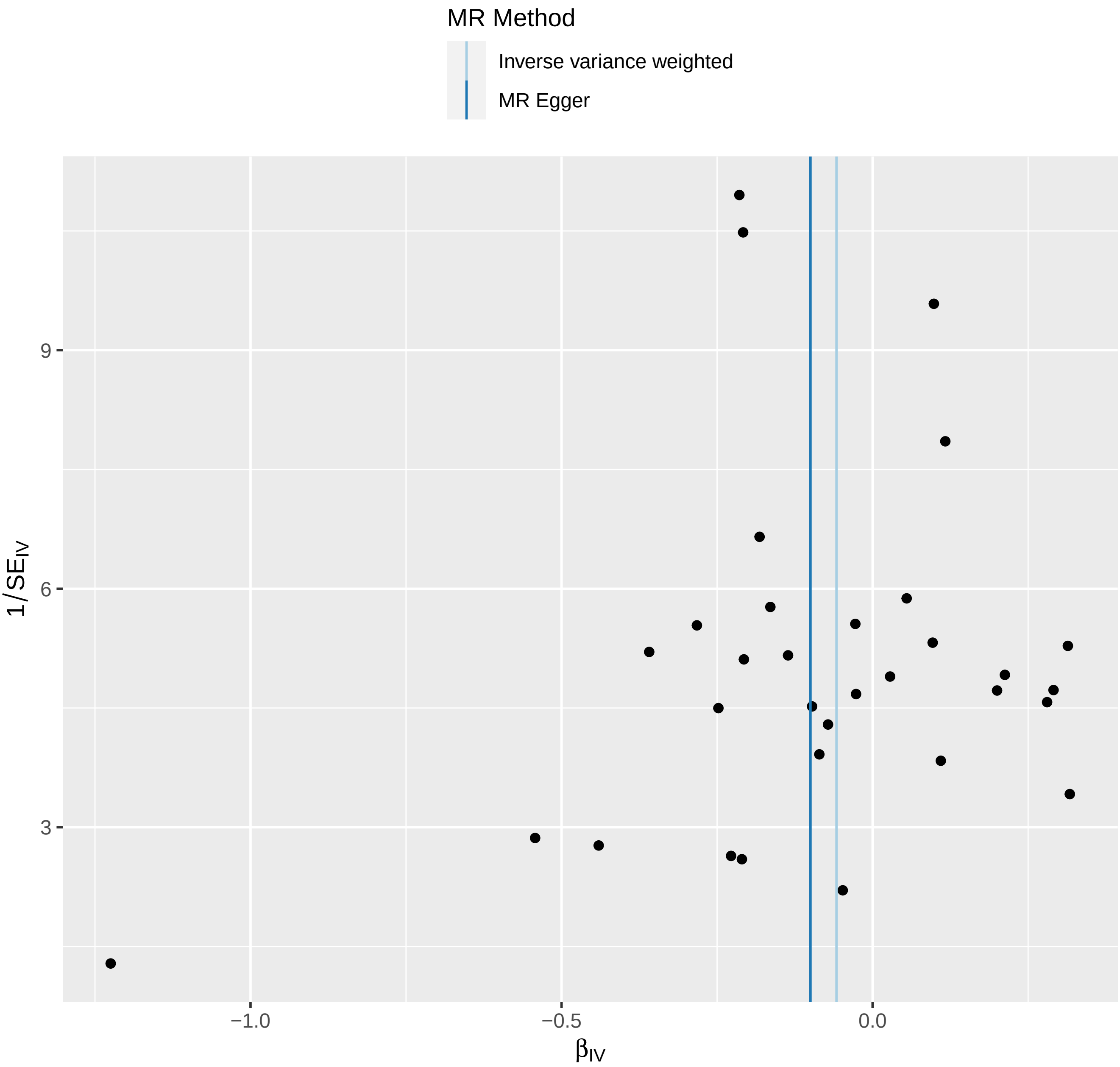

Batch 3−1040 : Protein FAM19A2 on Diabetic nephropathy (more control exclusions)
with a threshold of 1e−05

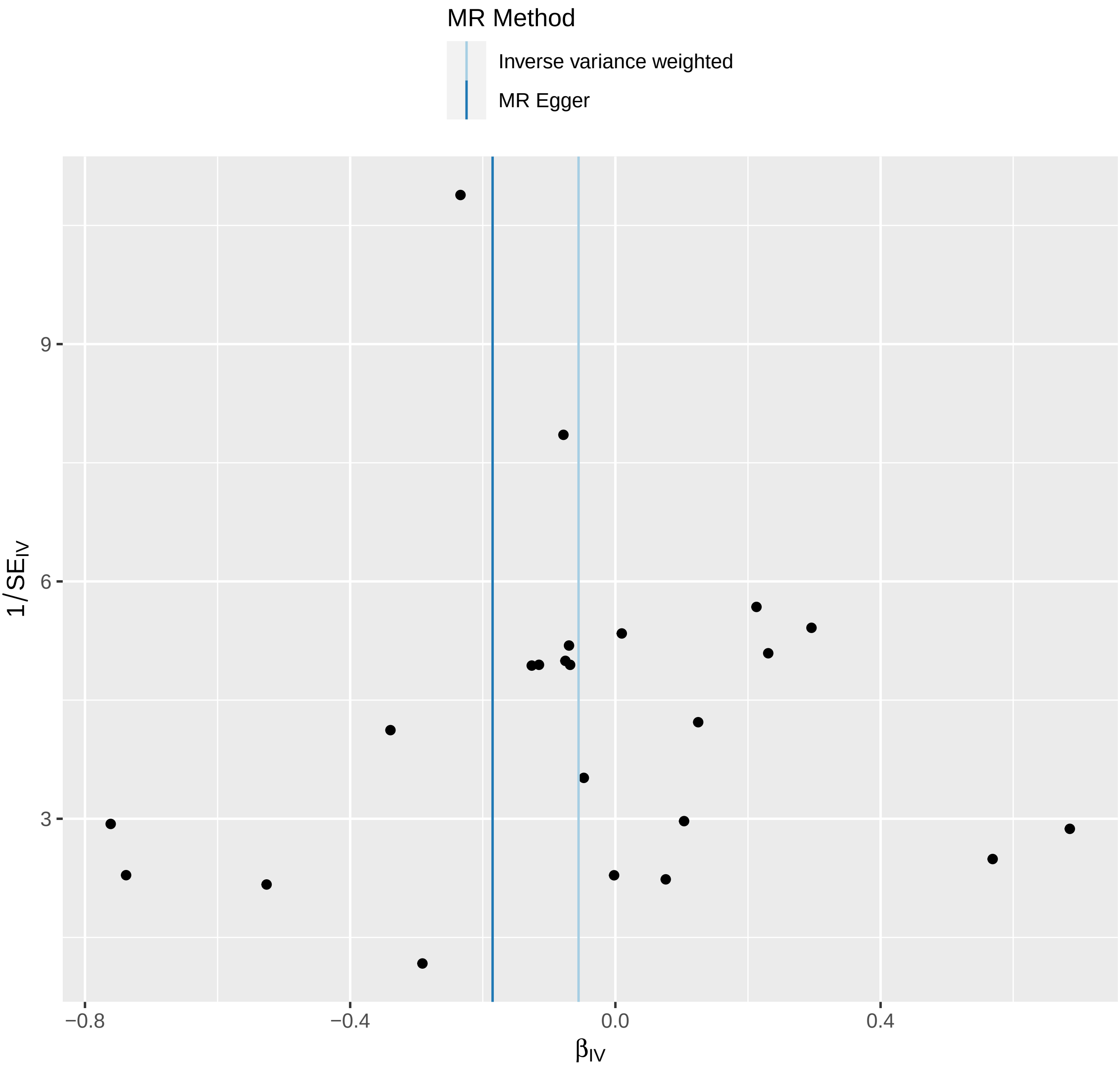

Batch 3−1041 : Protein FAM19A3 on Diabetic nephropathy (more control exclusions)
with a threshold of 1e−05

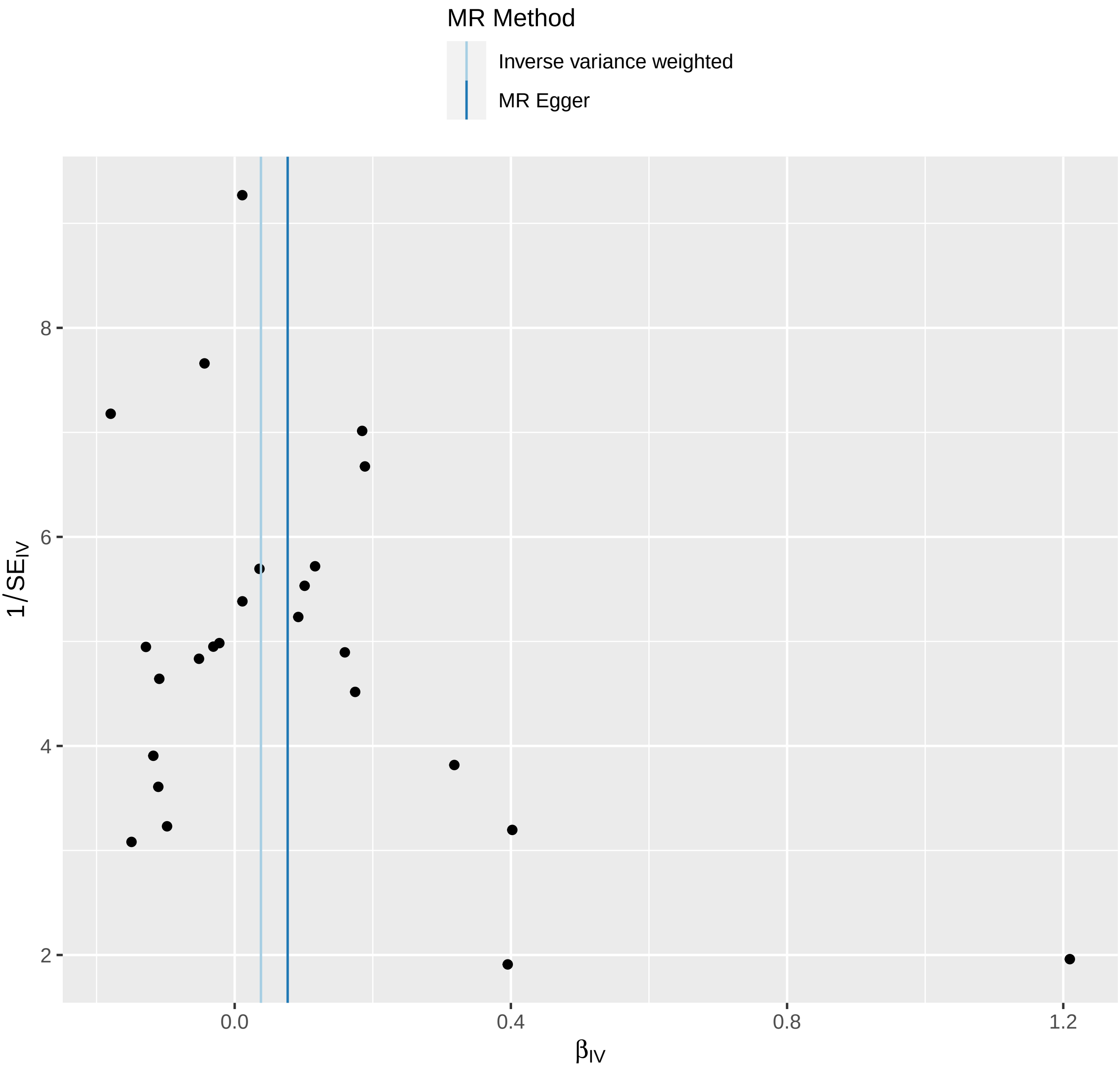

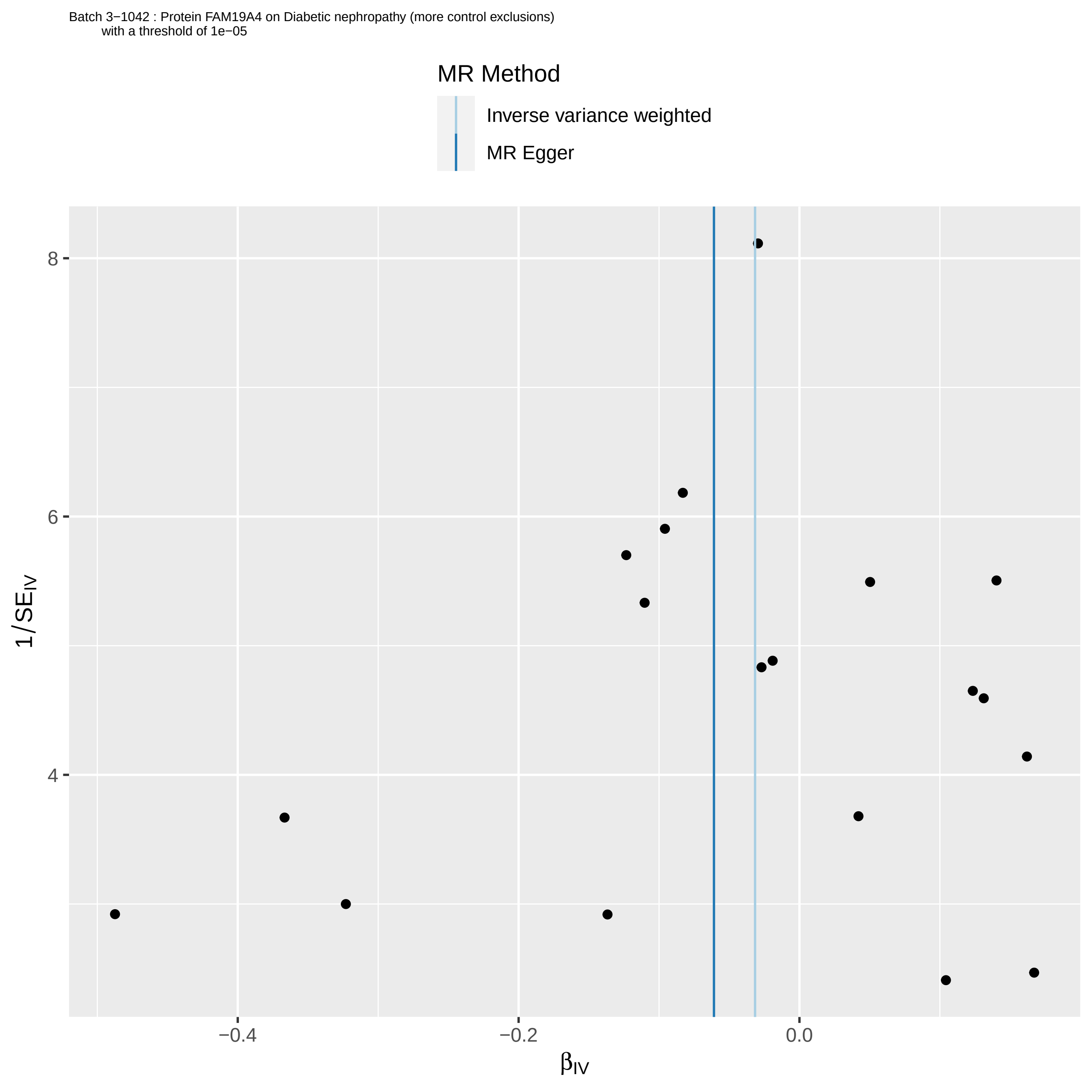

Batch 3−1042 : Protein FAM19A4 on Diabetic nephropathy (more control exclusions)
with a threshold of 1e−05
MR Method
Inverse variance weighted
MR Egger
8
6
4
1/SE_IV
−0.4
−0.2
0.0
β_IV

Batch 3−1043 : Protein FAM19A5 on Diabetic nephropathy (more control exclusions)
with a threshold of 1e−05

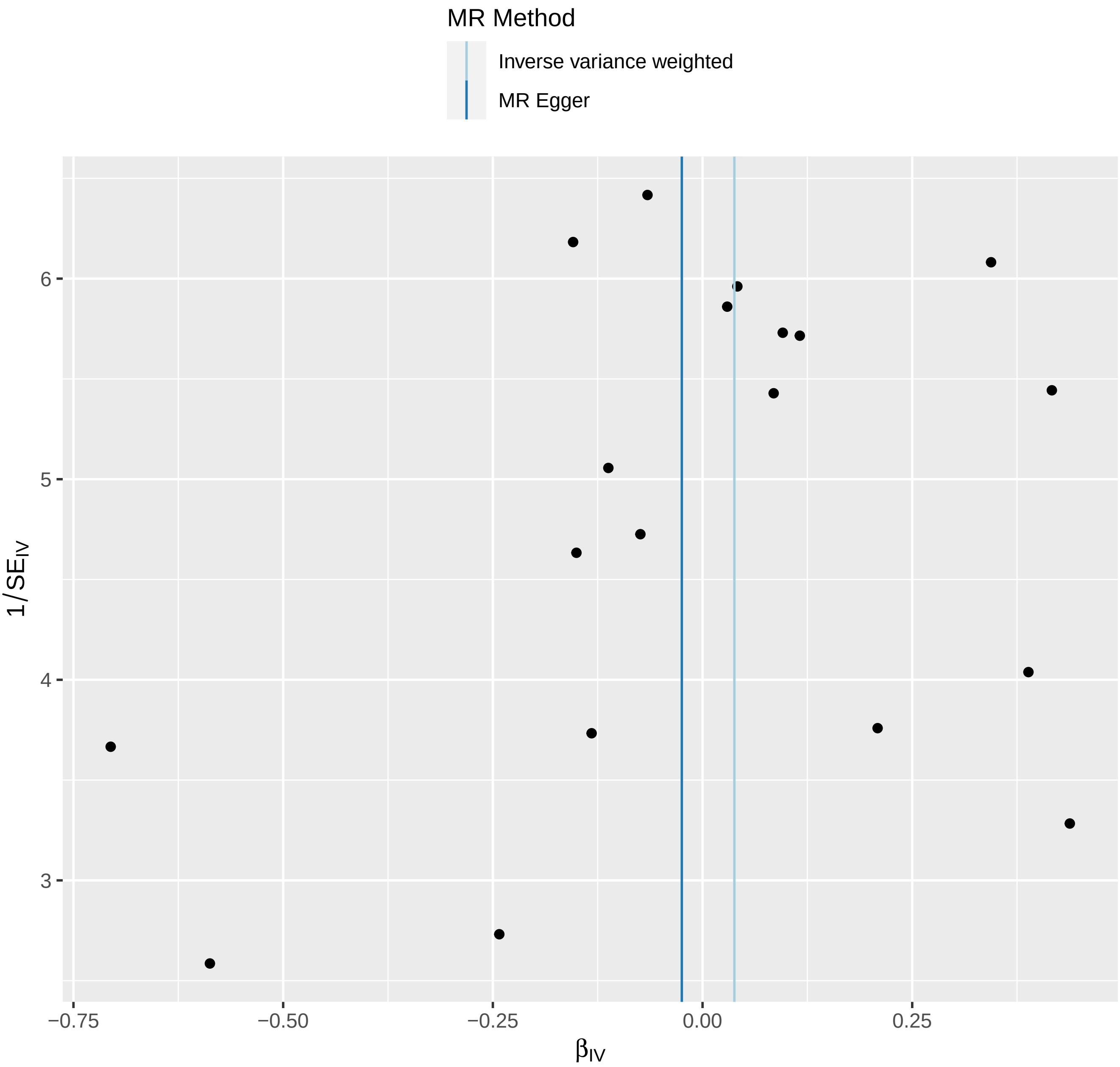

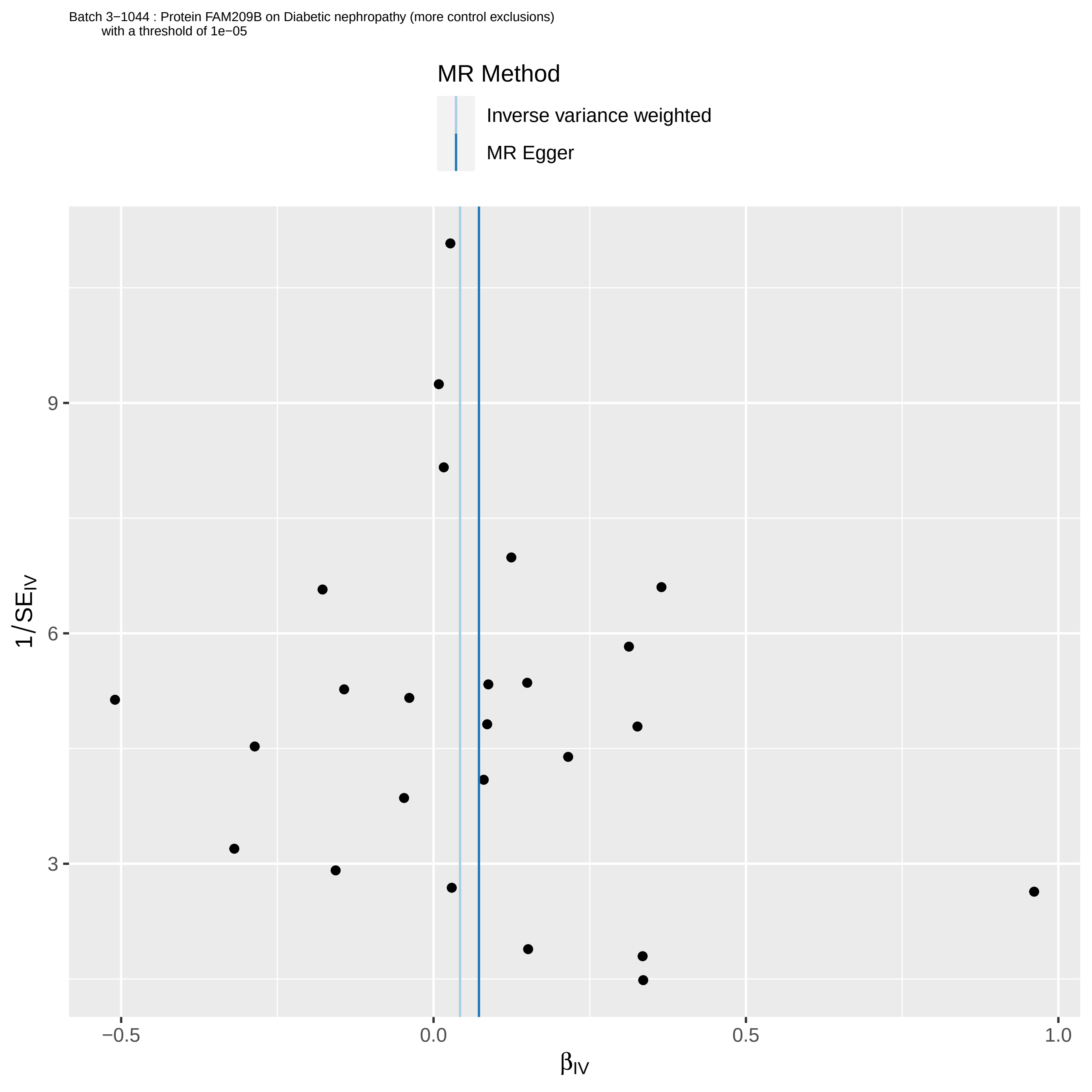
Batch 3−1044 : Protein FAM209B on Diabetic nephropathy (more control exclusions)
with a threshold of 1e−05
MR Method
Inverse variance weighted
MR Egger
9
6
3
1/SE$_{IV}$
−0.5
0.0
0.5
1.0
β$_{IV}$

Batch 3−1045 : Pseudokinase FAM20A on Diabetic nephropathy (more control exclusions)
with a threshold of 1e−05

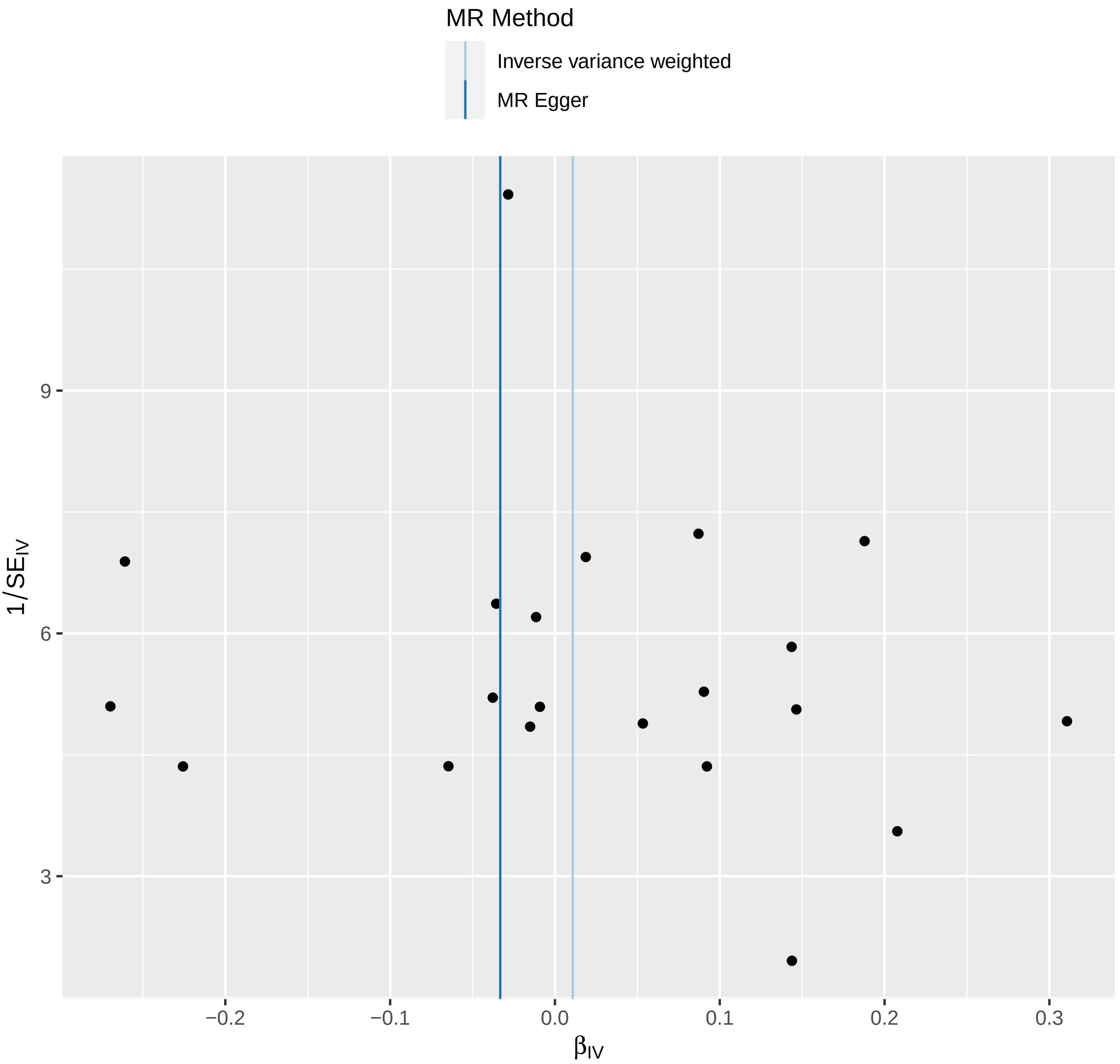

Batch 3−1046 : Glycosaminoglycan xylosylkinase on Diabetic nephropathy (more control exclusions)
with a threshold of 1e−05

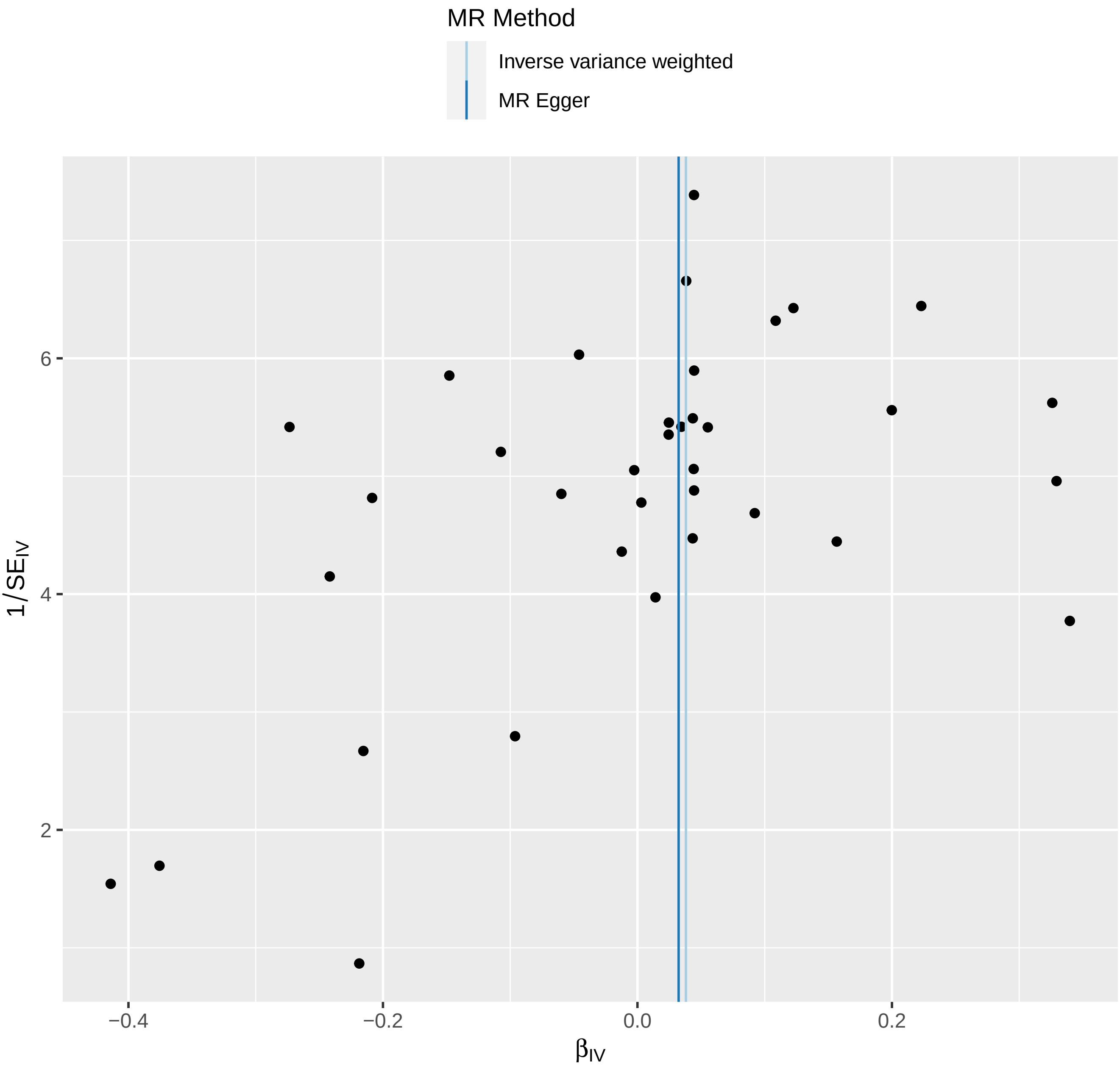

Batch 3−1047 : Redox−regulatory protein FAM213A on Diabetic nephropathy (more control exclusions)
with a threshold of 1e−05

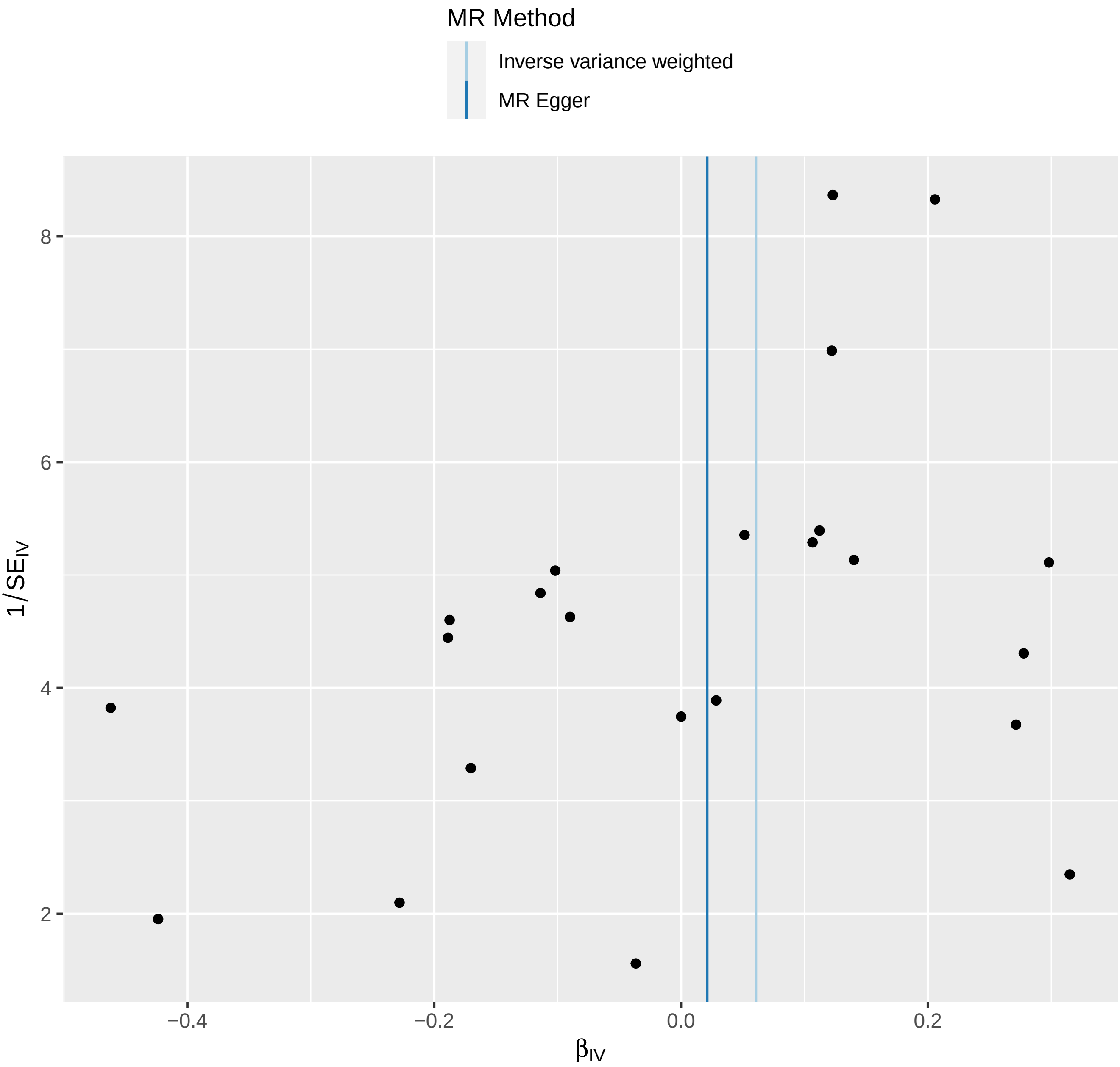

Batch 3−1048 : Protein FAM24B on Diabetic nephropathy (more control exclusions)
with a threshold of 1e−05

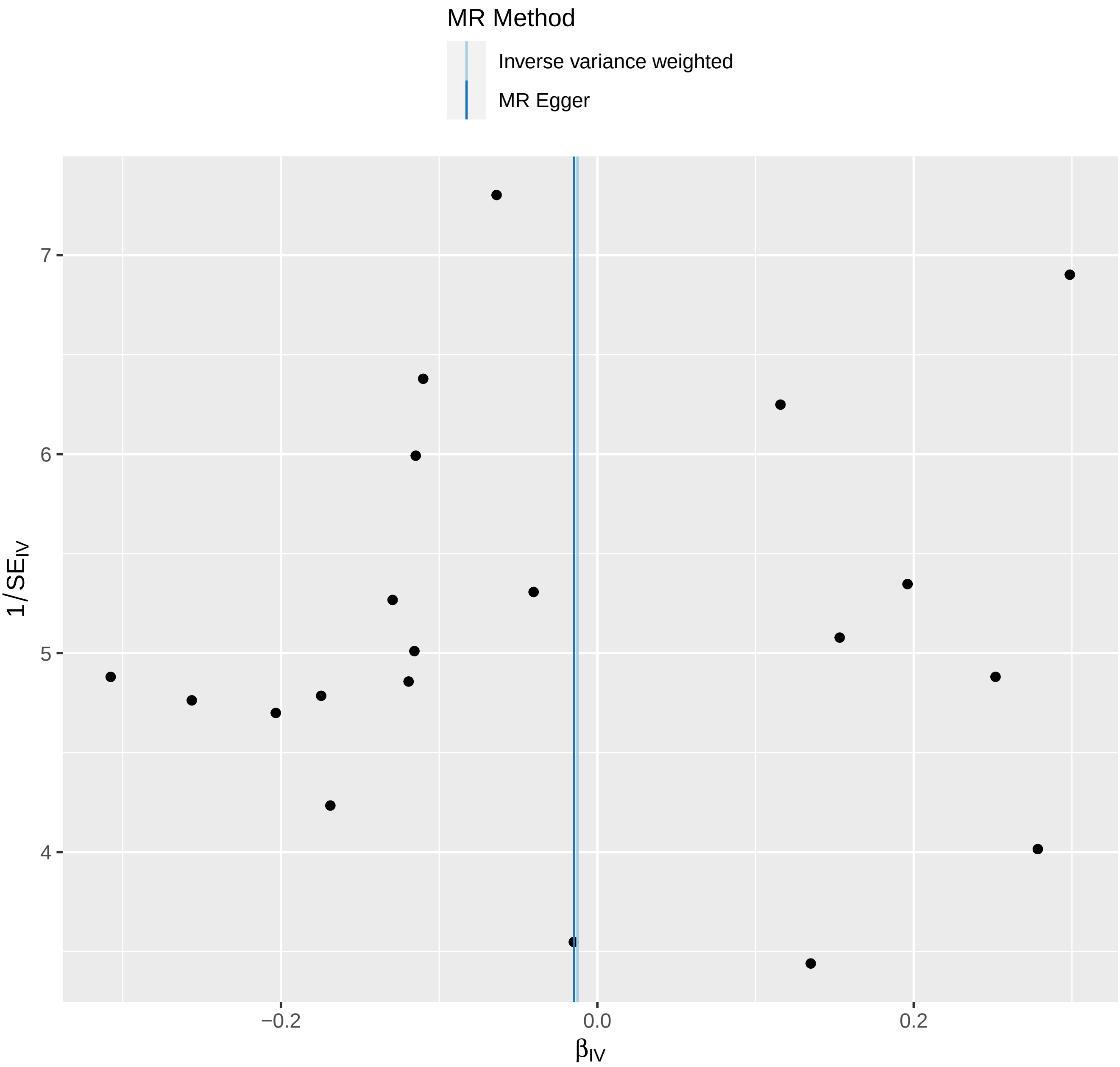

Batch 3−1049 : Protein FAM3B on Diabetic nephropathy (more control exclusions)
with a threshold of 1e−05

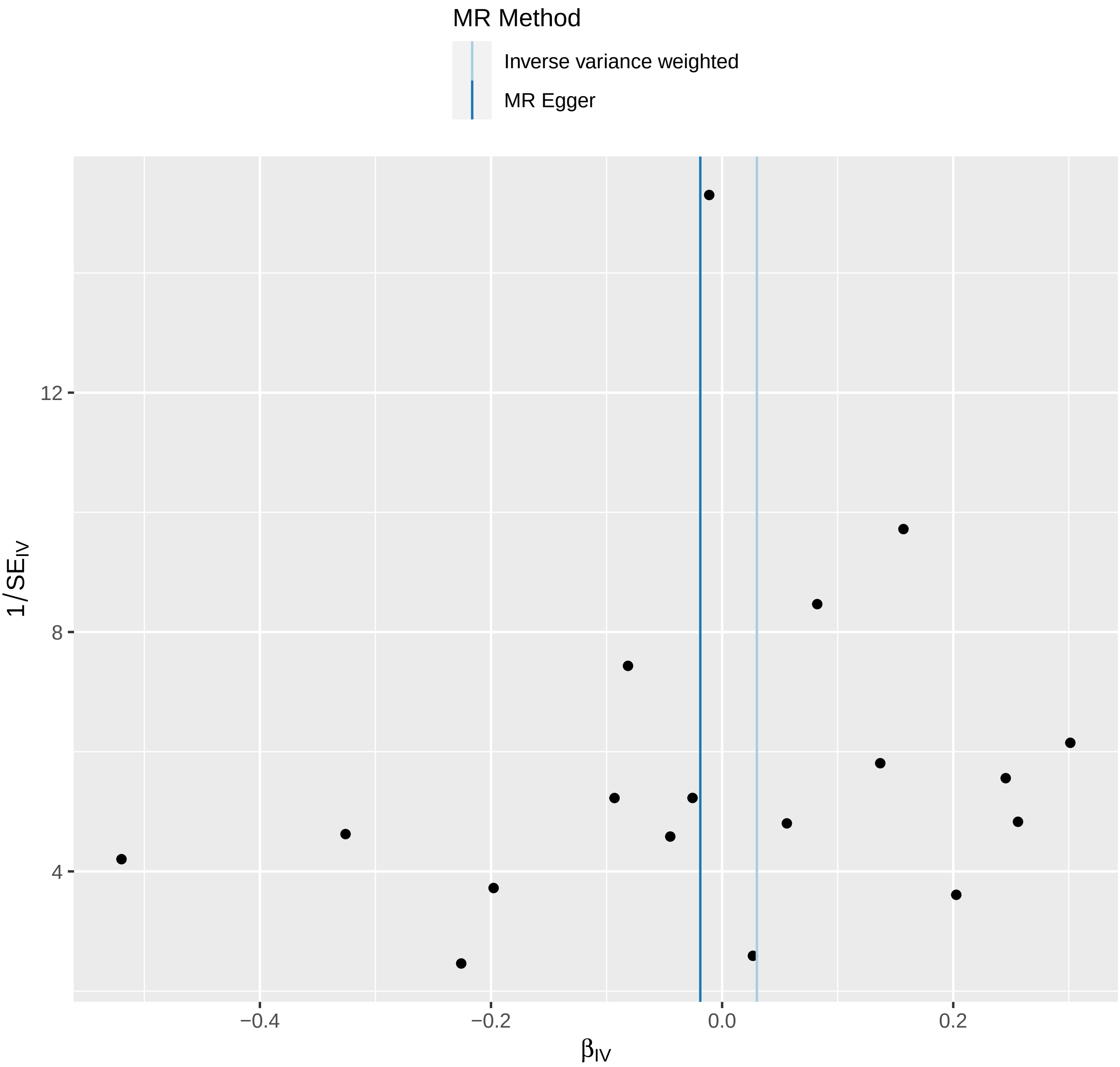

Batch 3−1050 : Protein FAM3B on Diabetic nephropathy (more control exclusions)
with a threshold of 1e−05

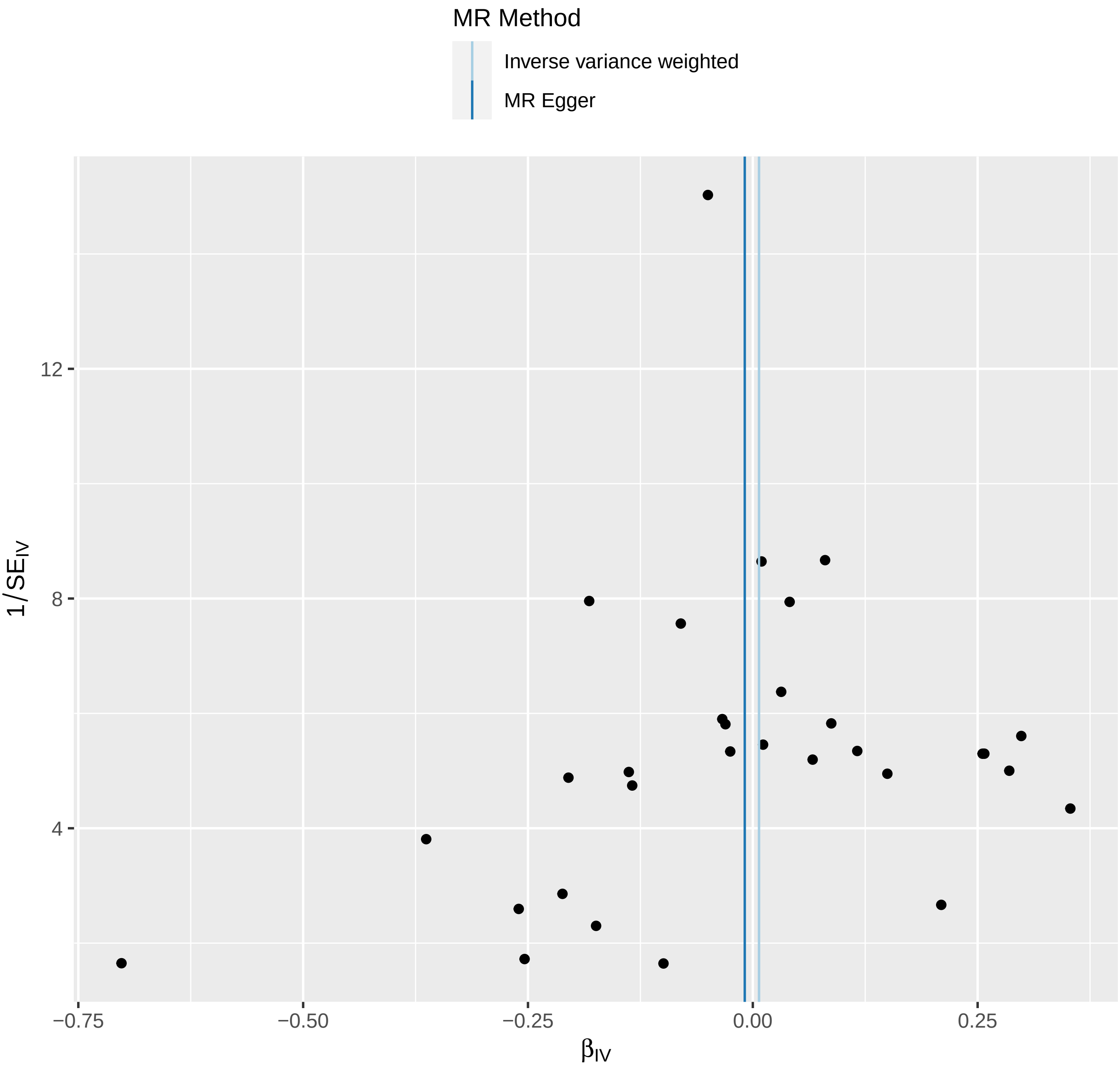

Batch 3−1051 : Protein FAM3D on Diabetic nephropathy (more control exclusions)
with a threshold of 1e−05

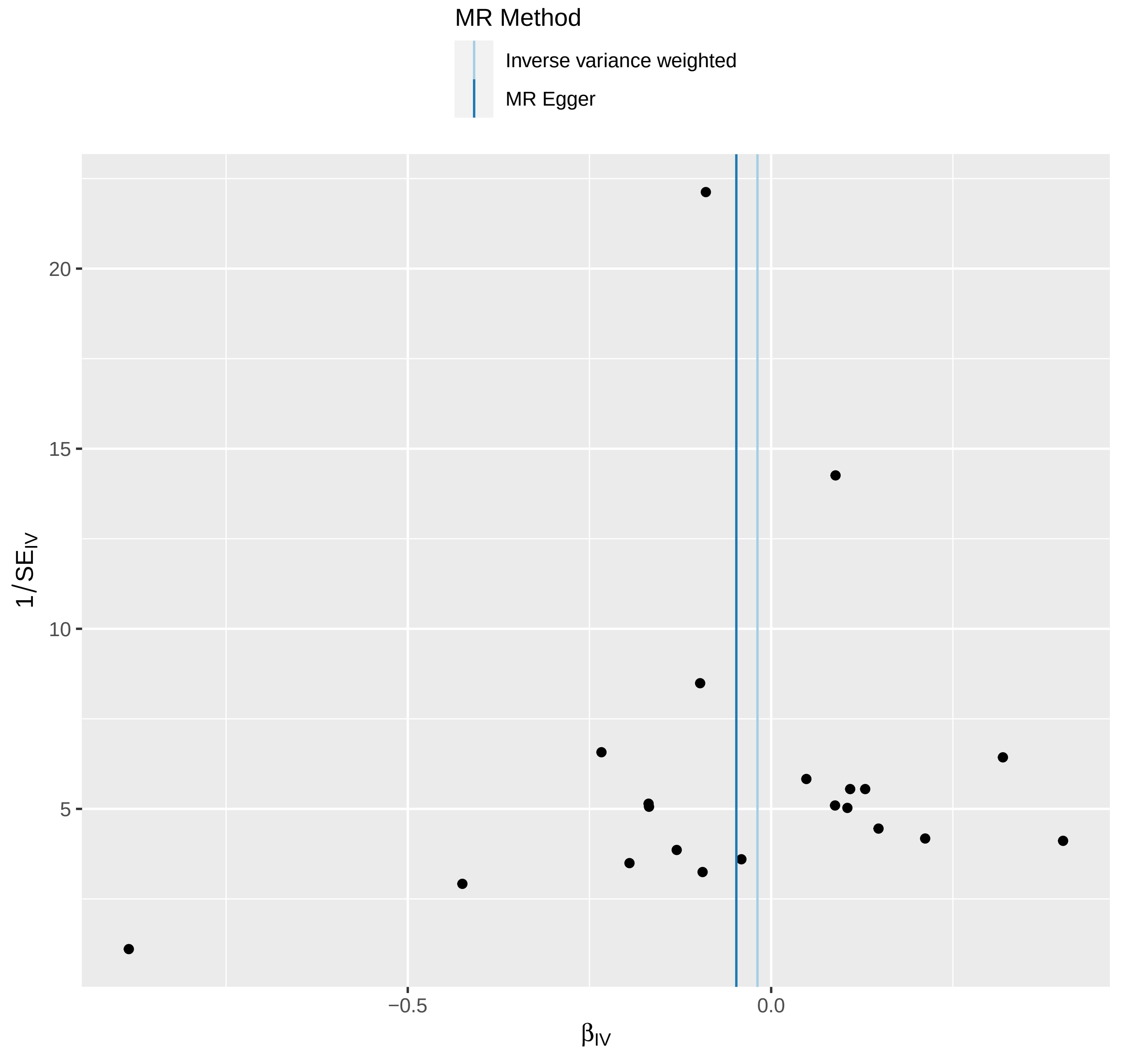

Batch 3−1052 : MIP18 family protein FAM96A on Diabetic nephropathy (more control exclusions)
with a threshold of 1e−05

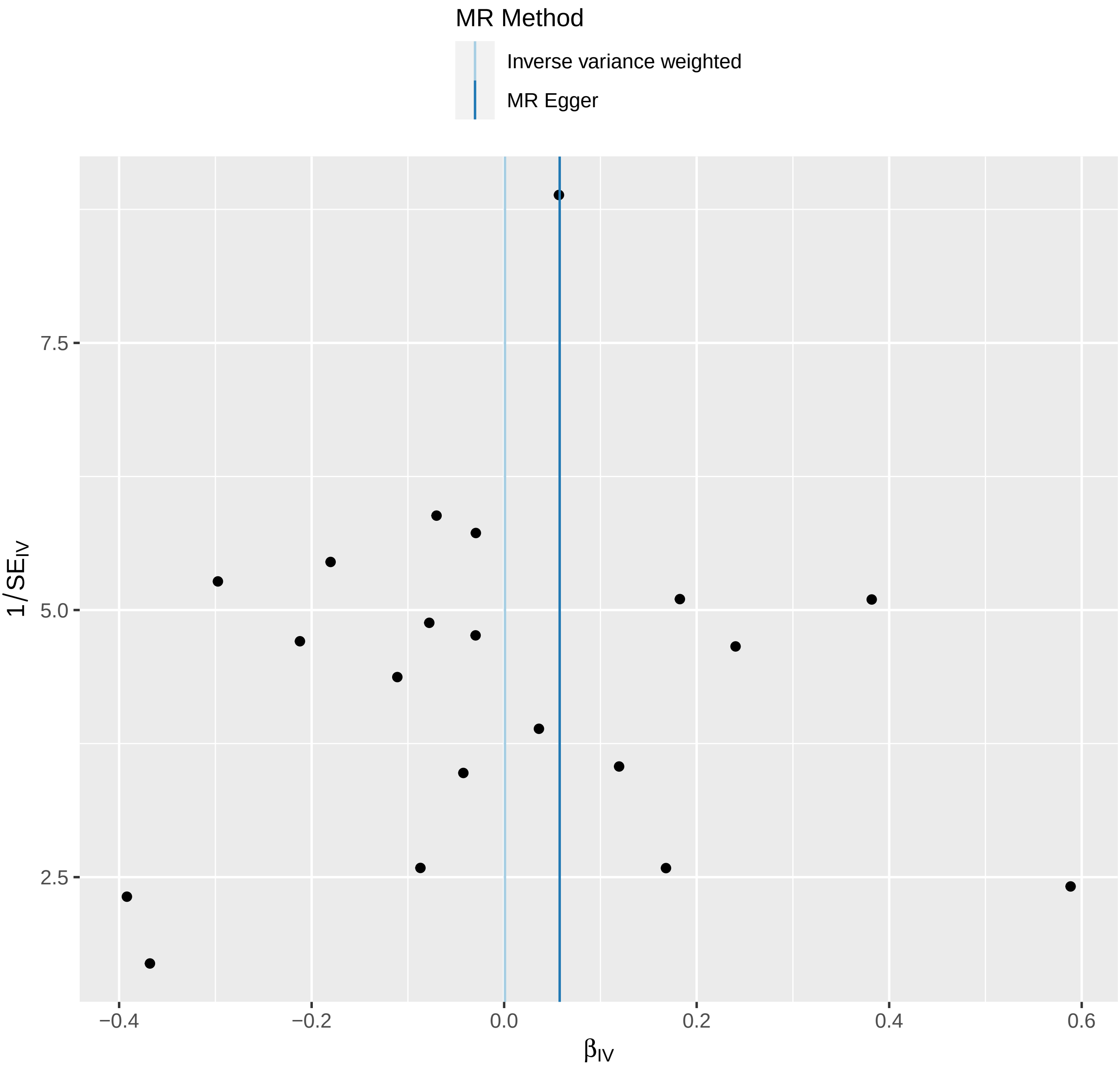

Batch 3−1053 : E3 ubiquitin−protein ligase FANCL on Diabetic nephropathy (more control exclusions)
with a threshold of 1e−05

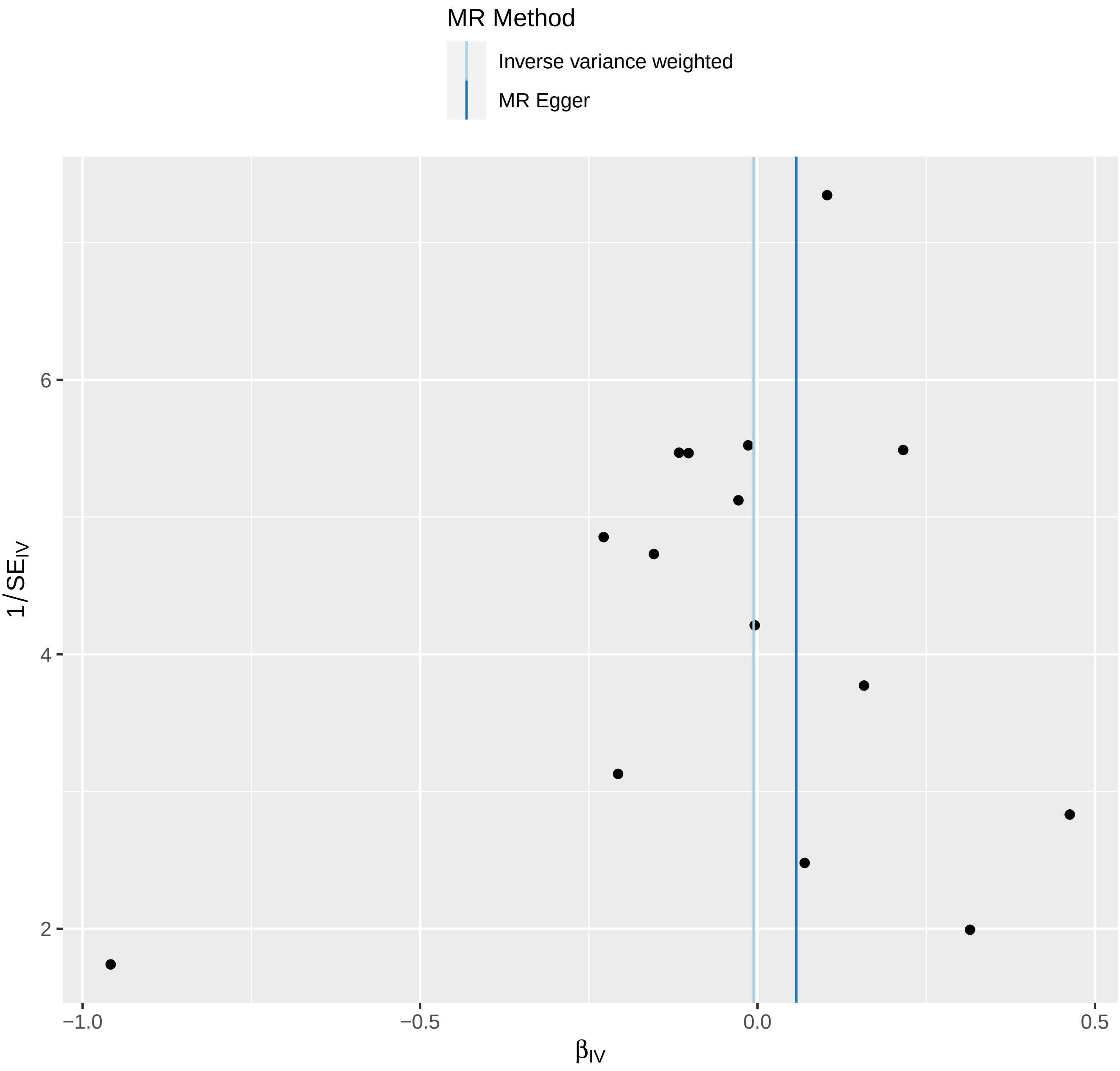

Batch 3−1054 : Prolyl endopeptidase FAP on Diabetic nephropathy (more control exclusions)
with a threshold of 1e−05

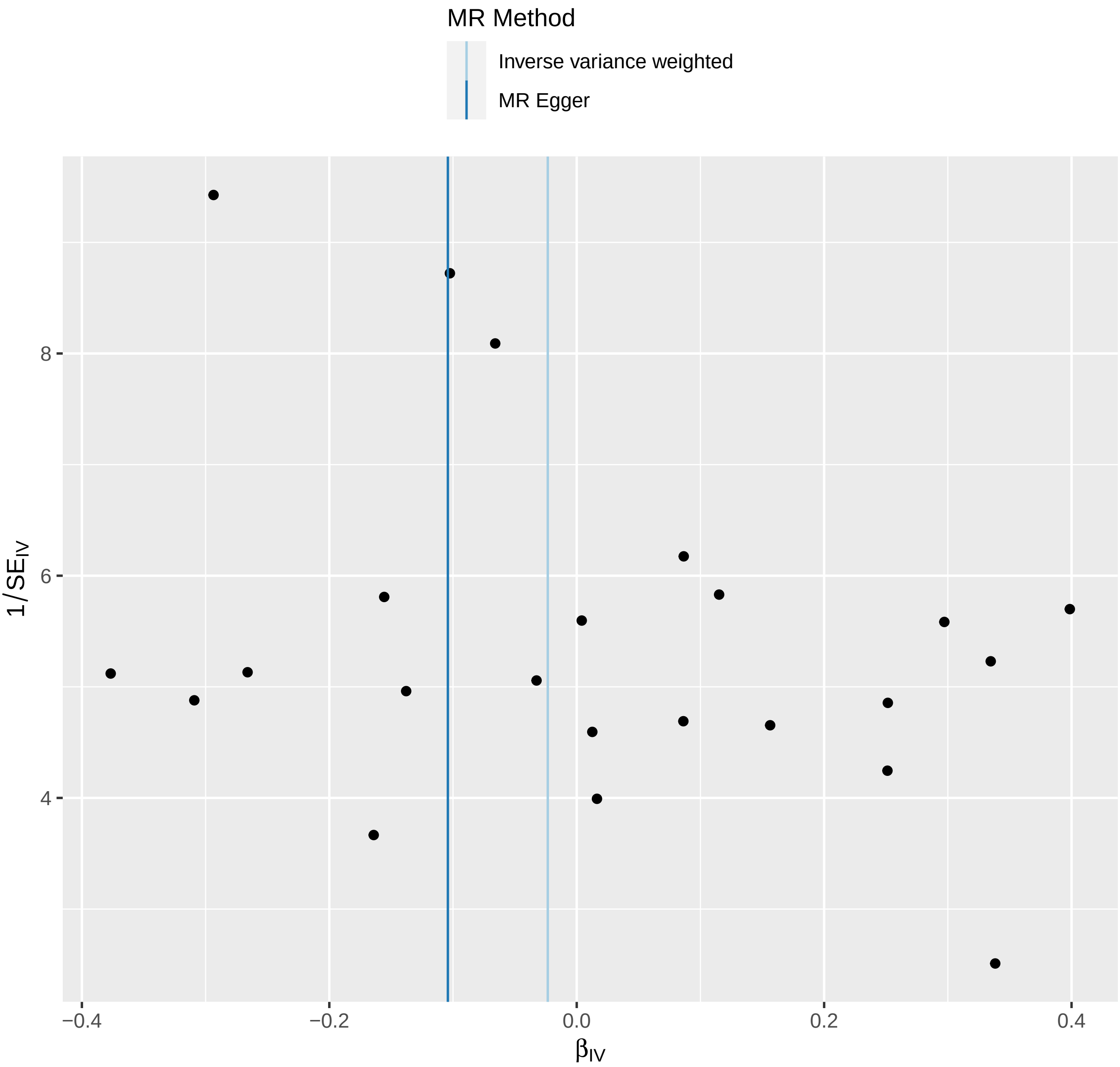

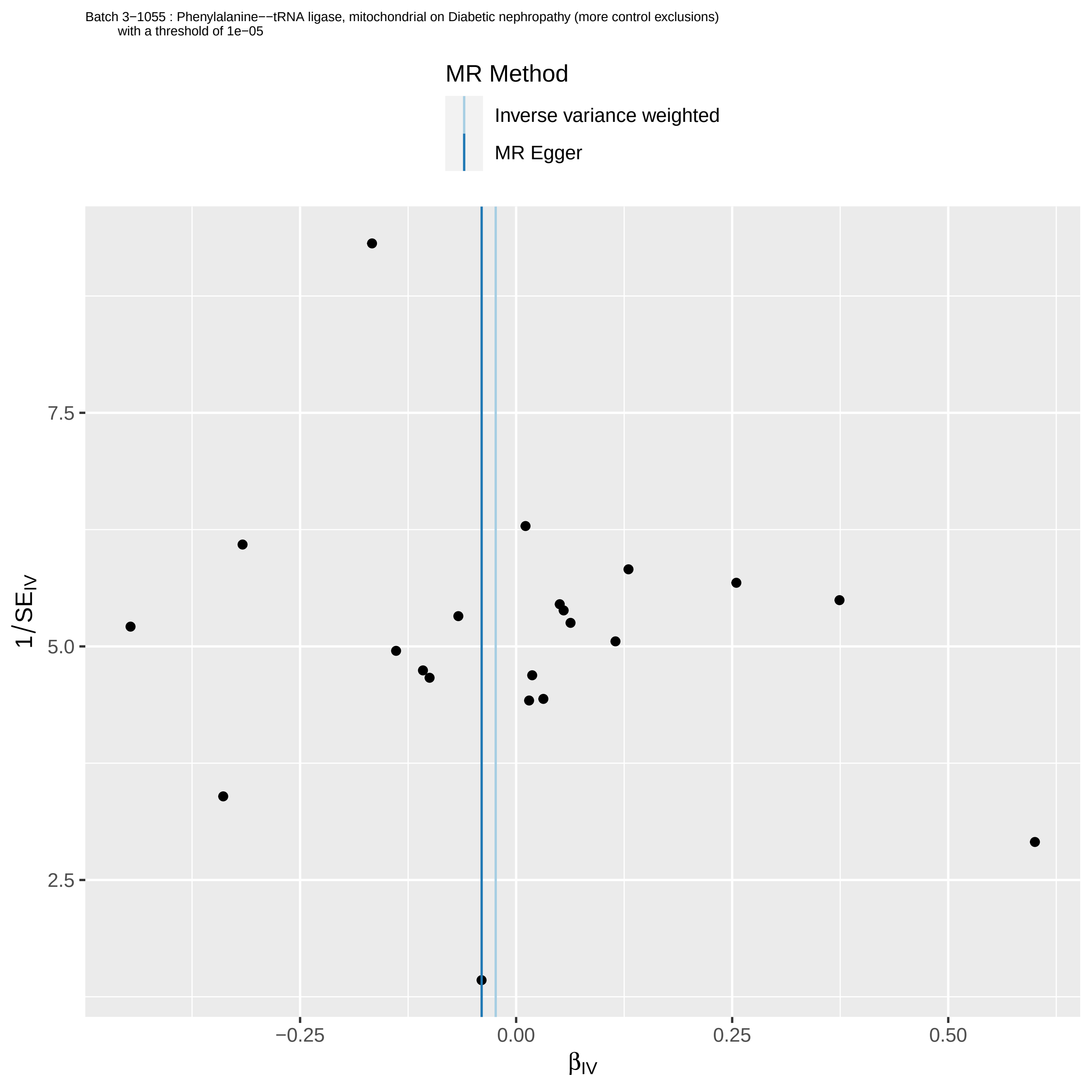

Batch 3−1055 : Phenylalanine−−tRNA ligase, mitochondrial on Diabetic nephropathy (more control exclusions)
with a threshold of 1e−05
MR Method
Inverse variance weighted
MR Egger
7.5
5.0
2.5
1/SE$_{IV}$
−0.25
0.00
0.25
0.50
β$_{IV}$

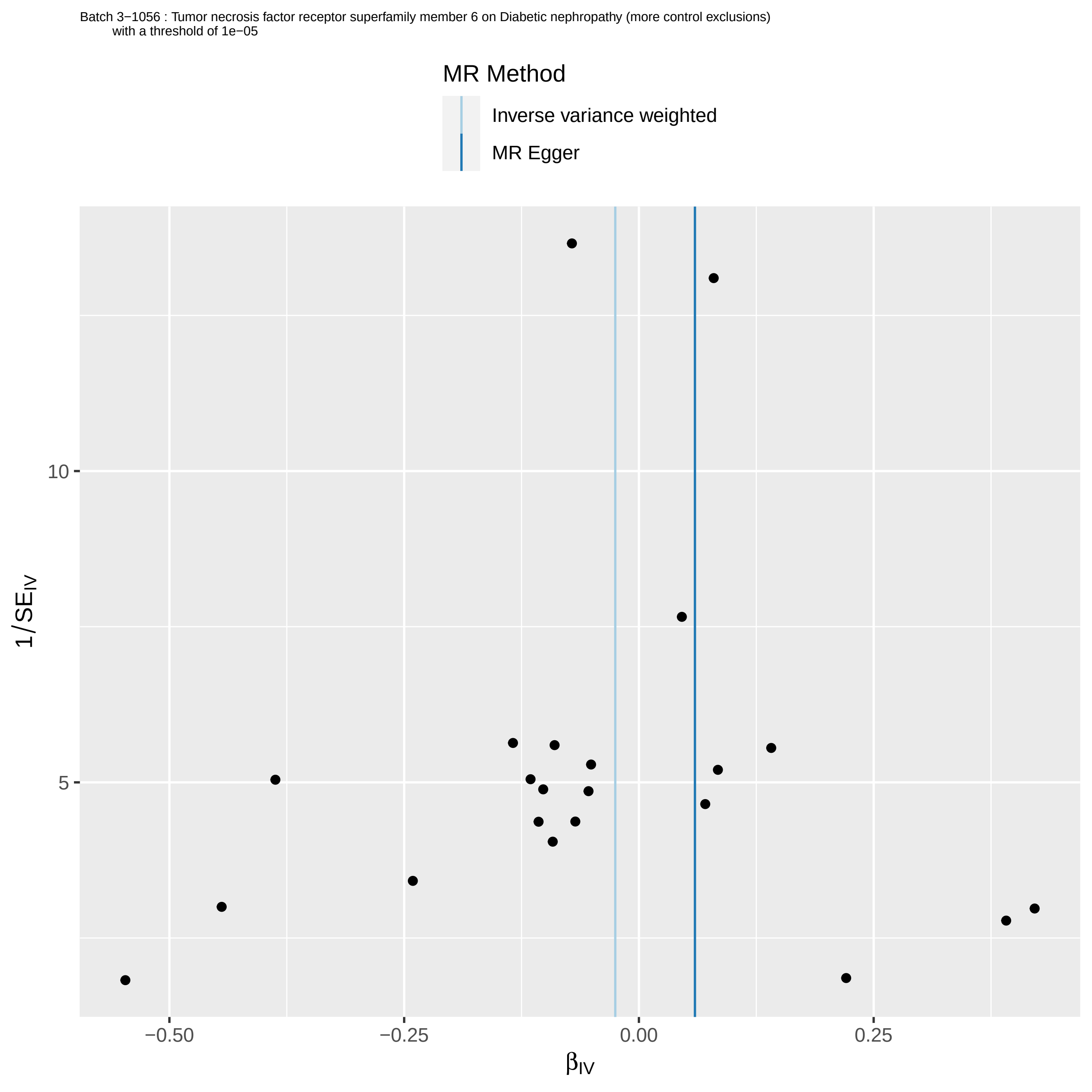

Batch 3−1056 : Tumor necrosis factor receptor superfamily member 6 on Diabetic nephropathy (more control exclusions)
with a threshold of 1e−05
MR Method
Inverse variance weighted
MR Egger
10
5
1/SE_IV
−0.50
−0.25
0.00
0.25
β_IV

Batch 3−1057 : Tumor necrosis factor receptor superfamily member 6 on Diabetic nephropathy (more control exclusions)
with a threshold of 1e−05

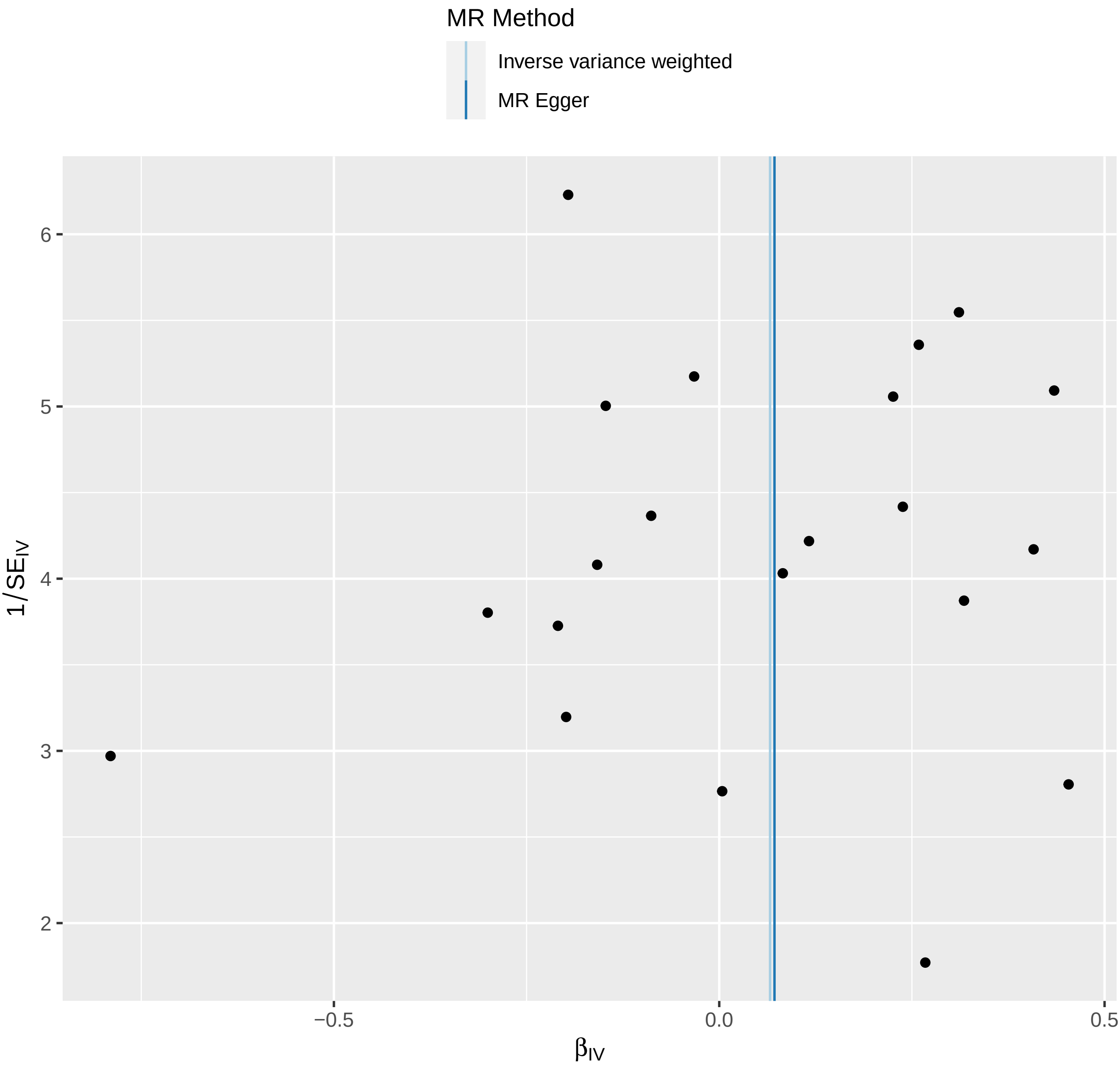

Batch 3−1058 : Tumor necrosis factor receptor superfamily member 6 on Diabetic nephropathy (more control exclusions)
with a threshold of 1e−05

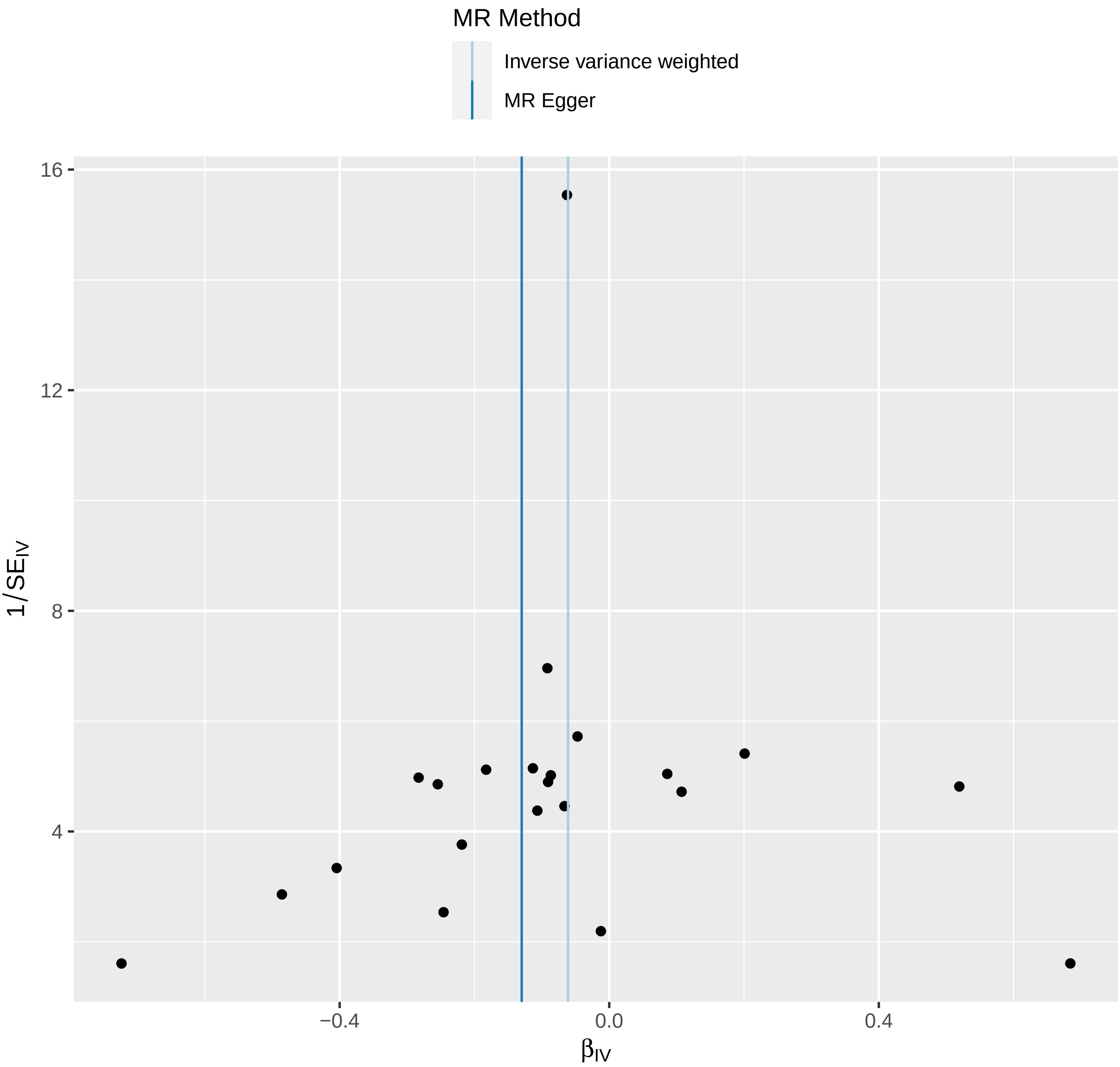

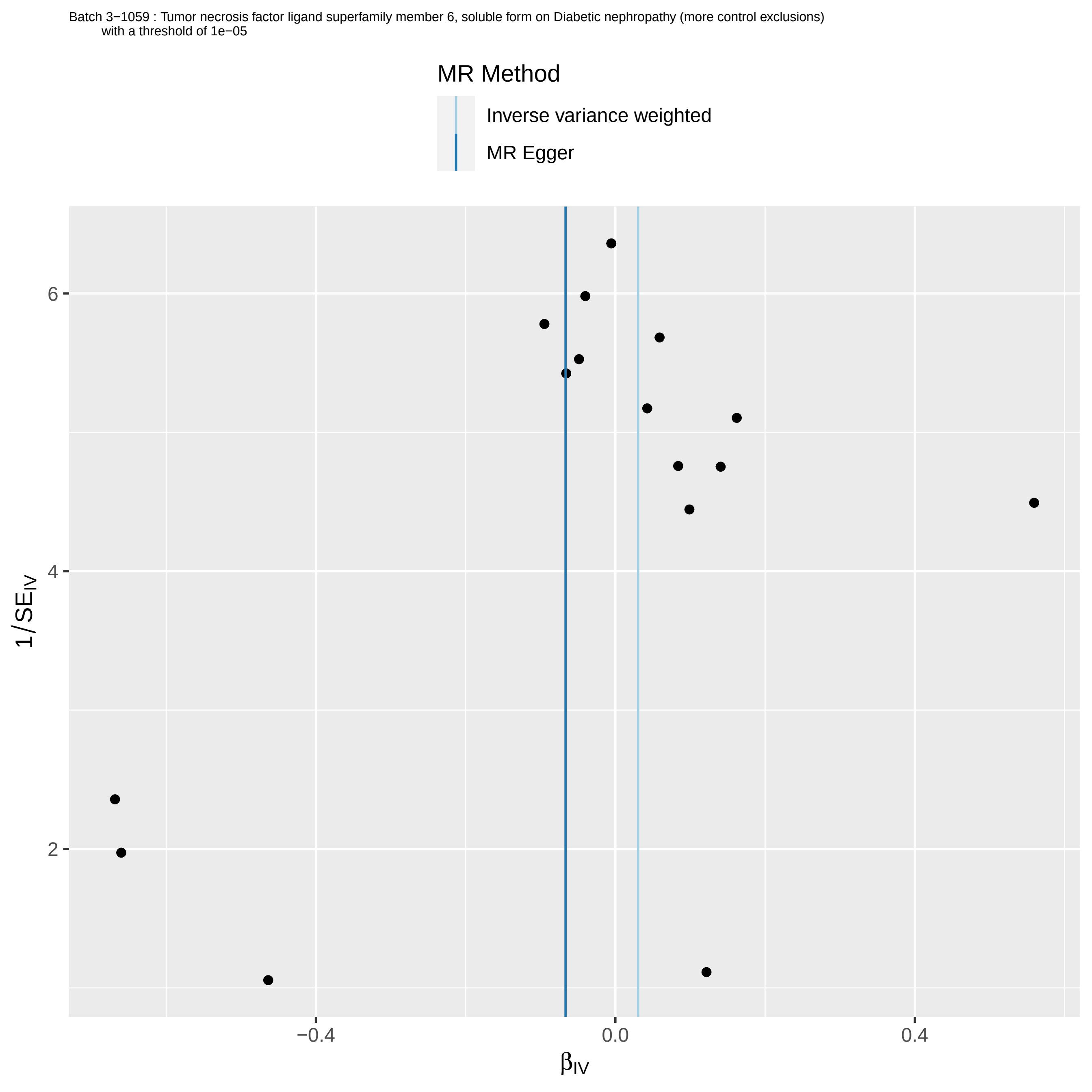

Batch 3−1059 : Tumor necrosis factor ligand superfamily member 6, soluble form on Diabetic nephropathy (more control exclusions)
with a threshold of 1e−05
MR Method
Inverse variance weighted
MR Egger
6
4
2
1/SE_IV
−0.4
0.0
0.4
β_IV

Batch 3−1060 : Fetal and adult testis−expressed transcript protein on Diabetic nephropathy (more control exclusions)
with a threshold of 1e−05

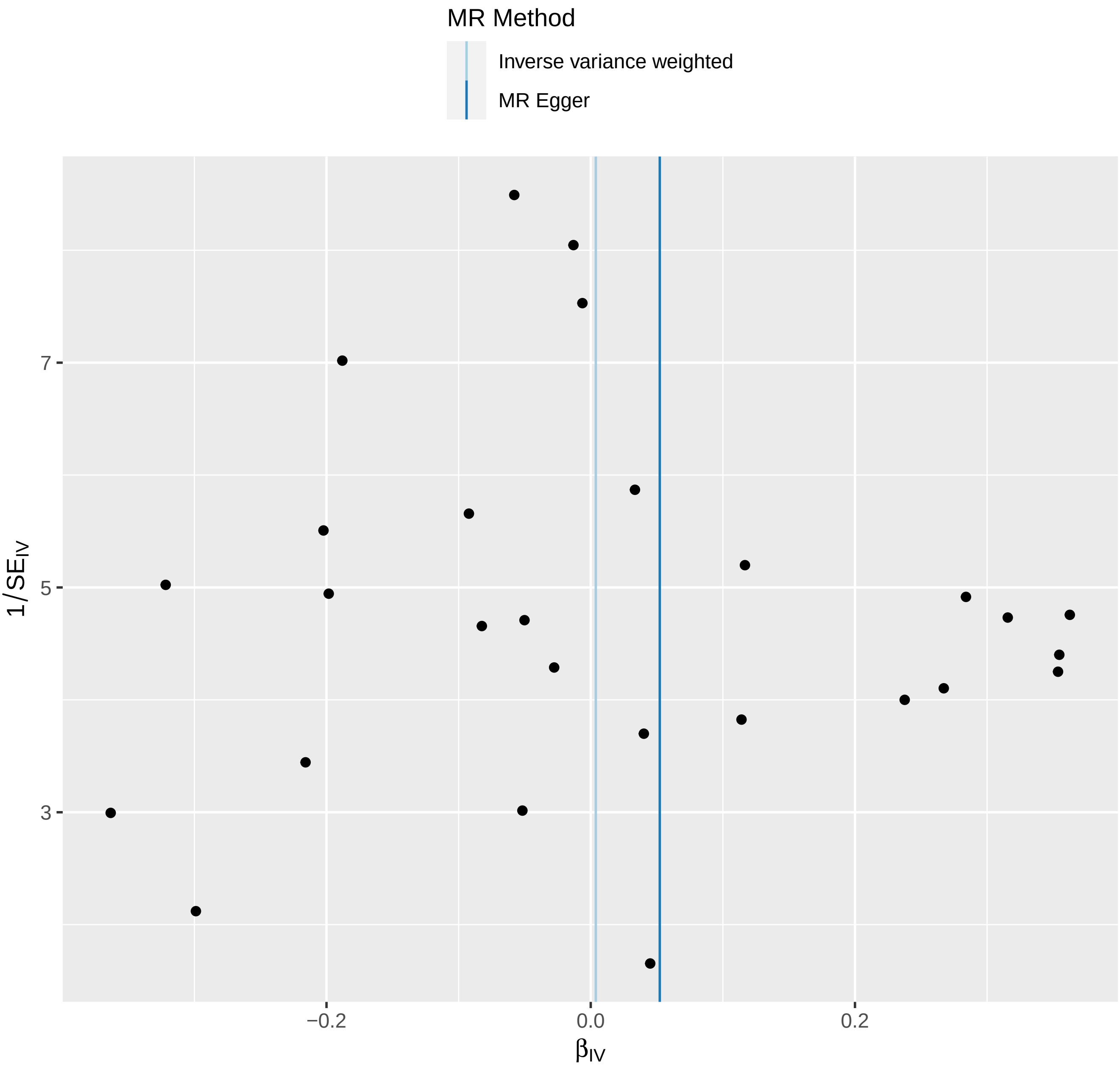

Batch 3−1061 : Fatty acid hydroxylase domain−containing protein 2 on Diabetic nephropathy (more control exclusions)
with a threshold of 1e−05

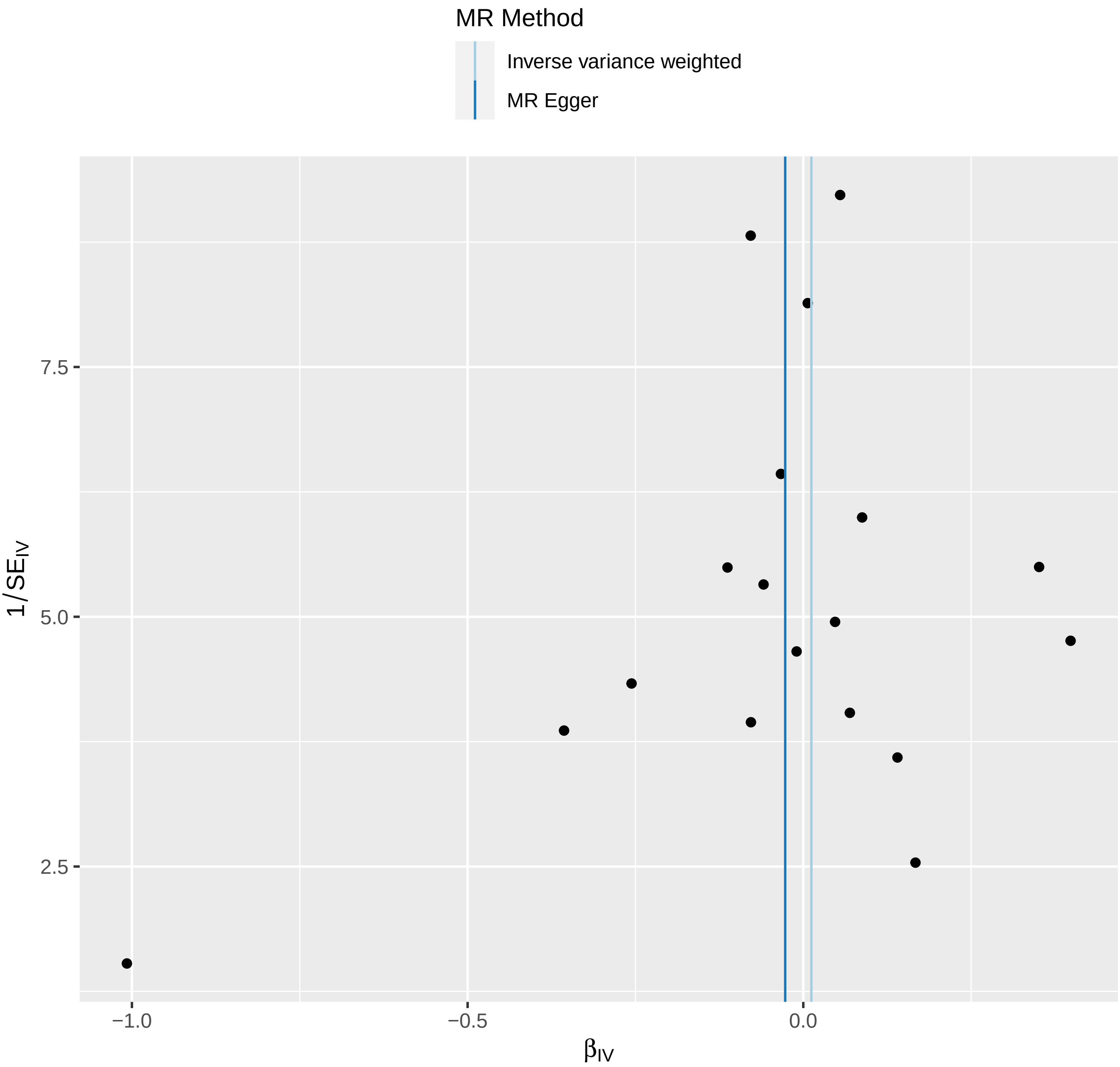

Batch 3−1062 : Fibulin−1 on Diabetic nephropathy (more control exclusions)
with a threshold of 1e−05

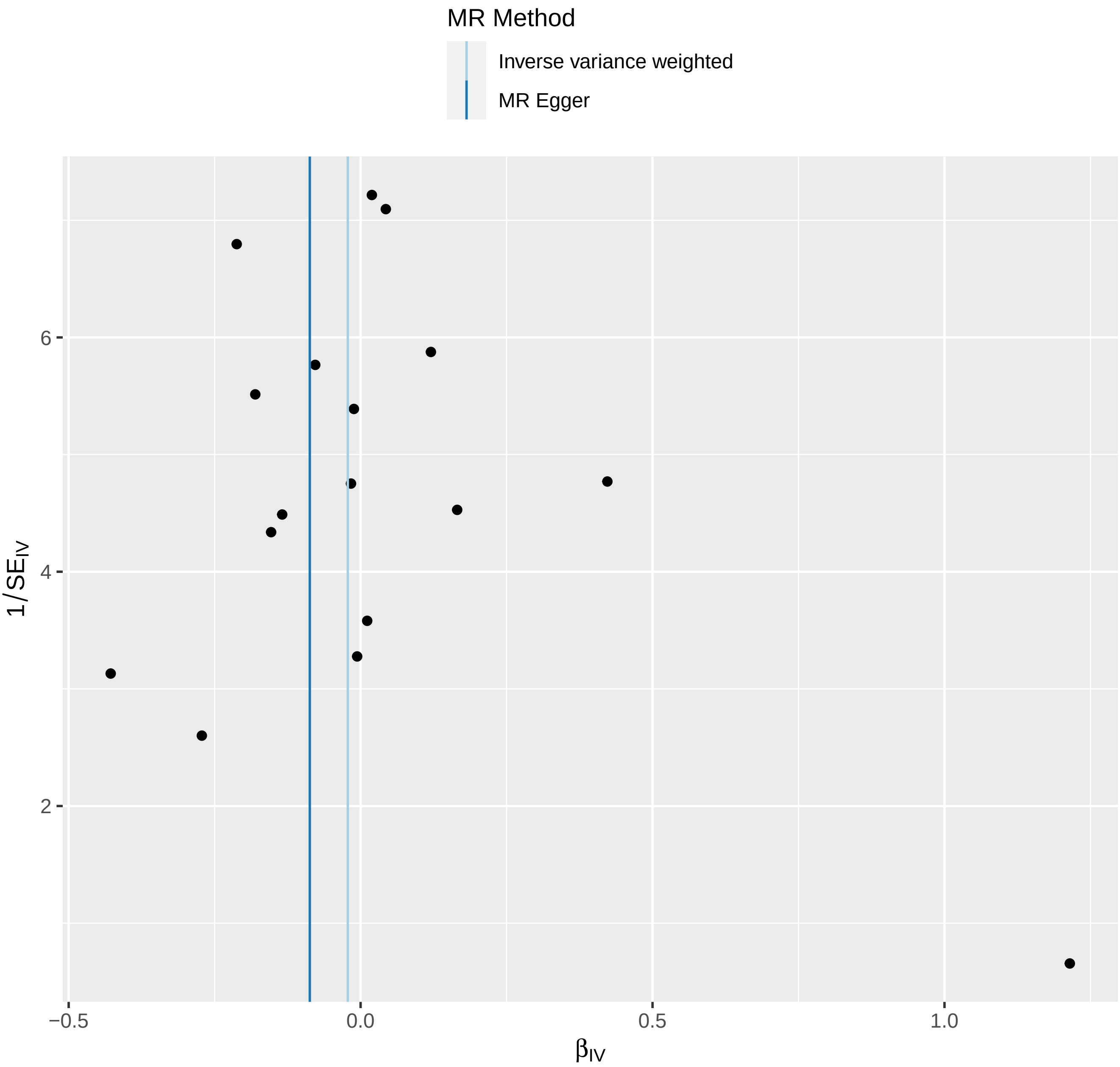

Batch 3−1063 : Fructose−1,6−bisphosphatase 1 on Diabetic nephropathy (more control exclusions) with a threshold of 1e−05

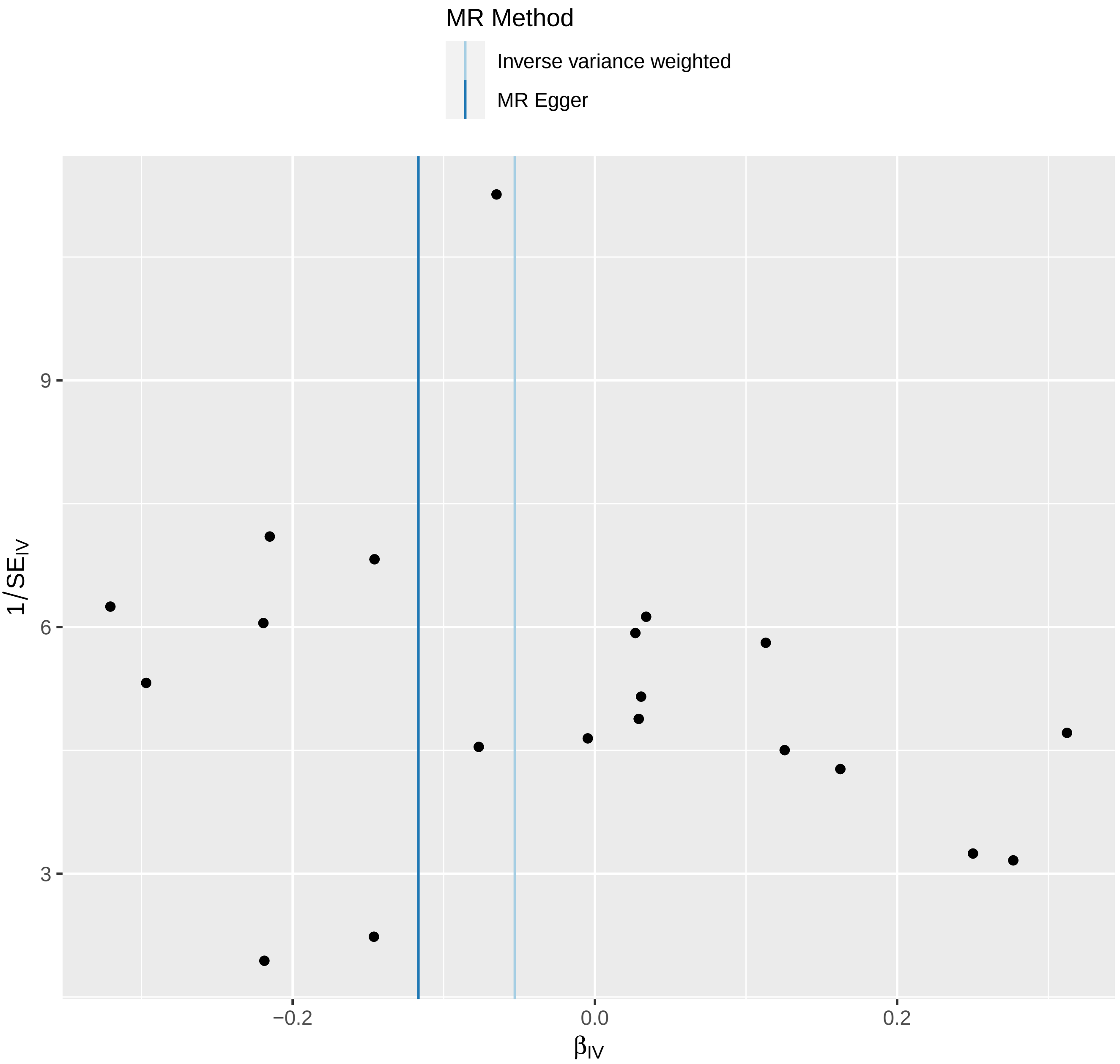

Batch 3−1064 : Fructose−1,6−bisphosphatase isozyme 2 on Diabetic nephropathy (more control exclusions)
with a threshold of 1e−05

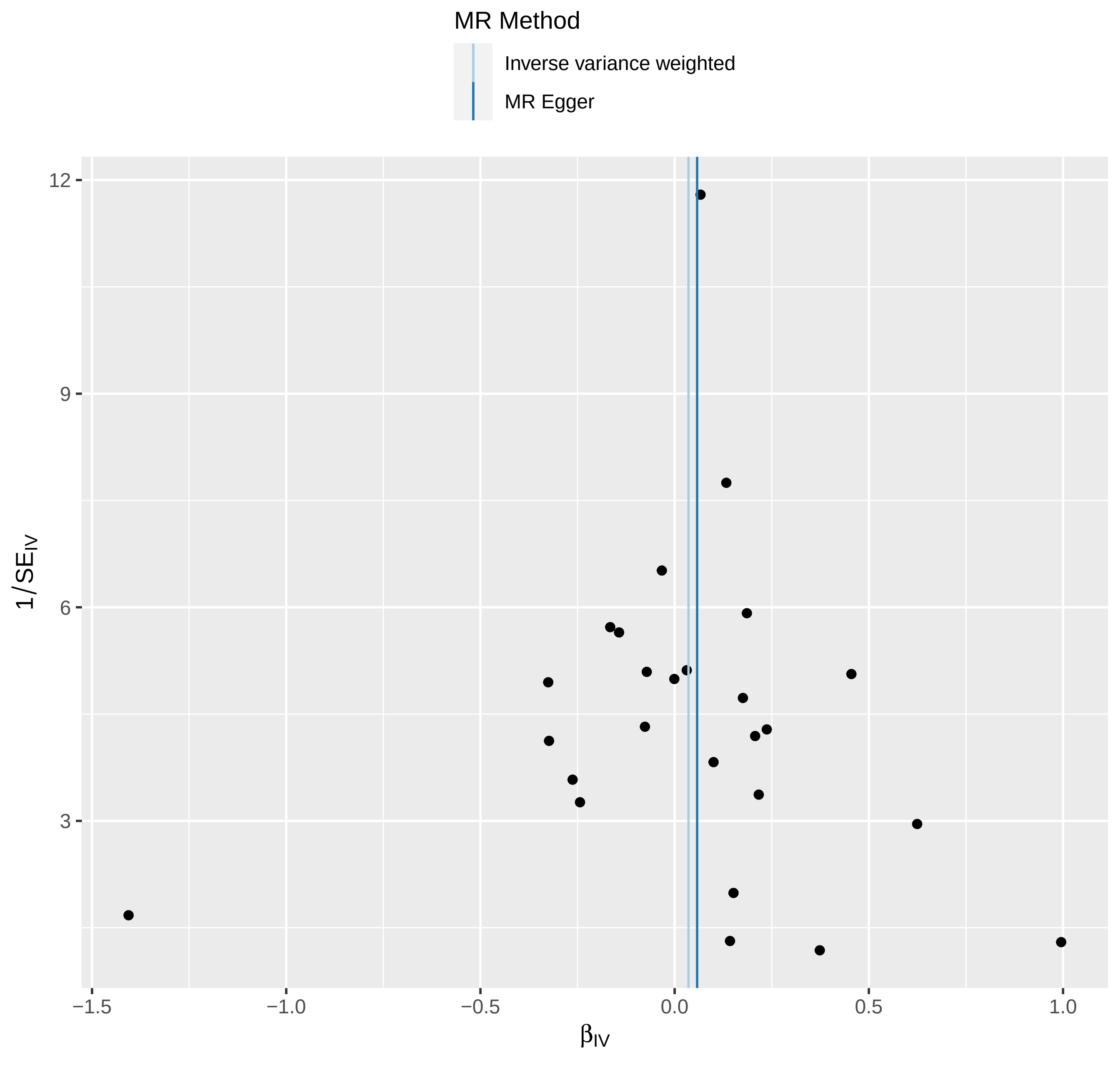

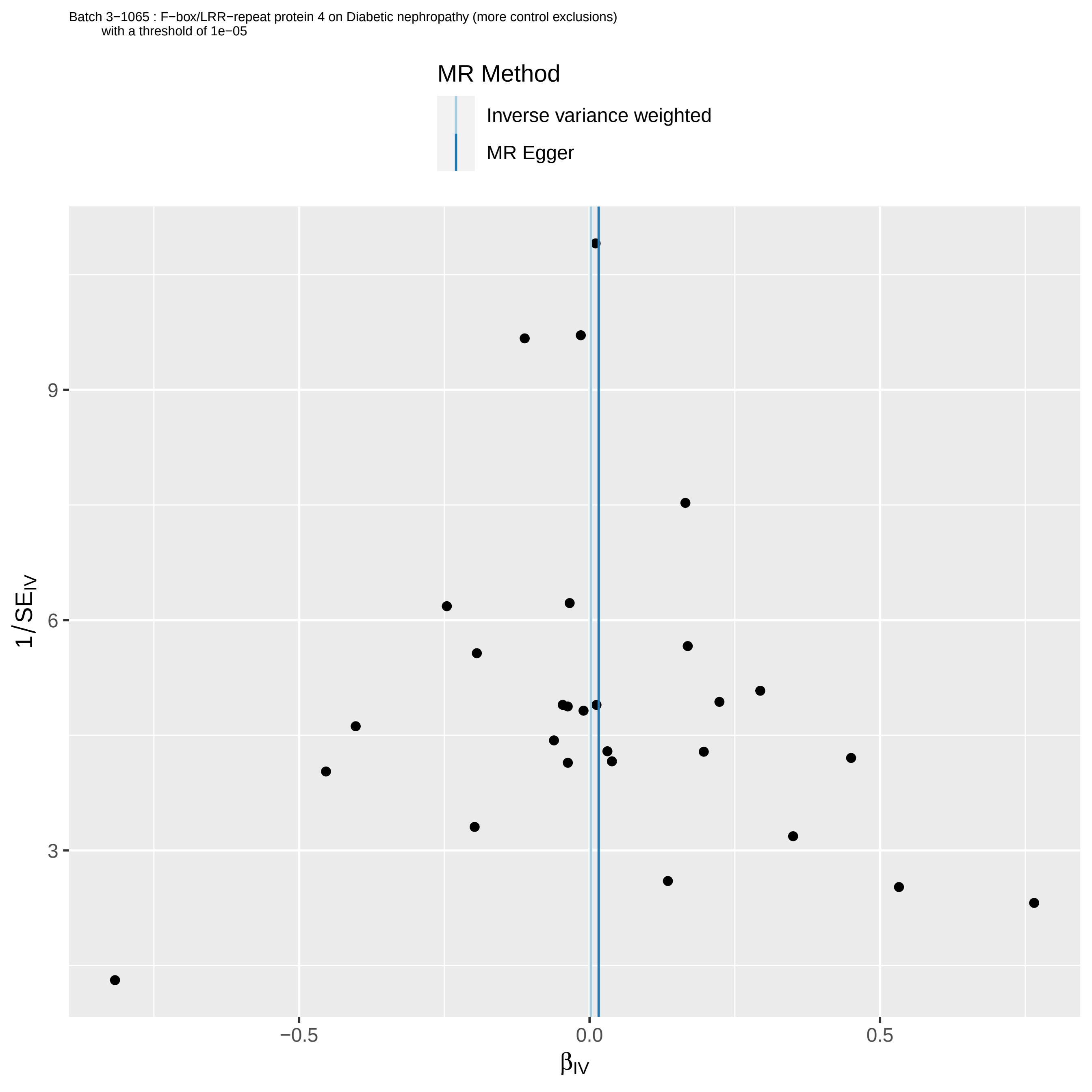

Batch 3−1065 : F−box/LRR−repeat protein 4 on Diabetic nephropathy (more control exclusions)
with a threshold of 1e−05
MR Method
Inverse variance weighted
MR Egger
9
6
3
1/SE$_{IV}$
−0.5
0.0
0.5
β_{IV}

Batch 3−1066 : F−box/LRR−repeat protein 4 on Diabetic nephropathy (more control exclusions)
with a threshold of 1e−05

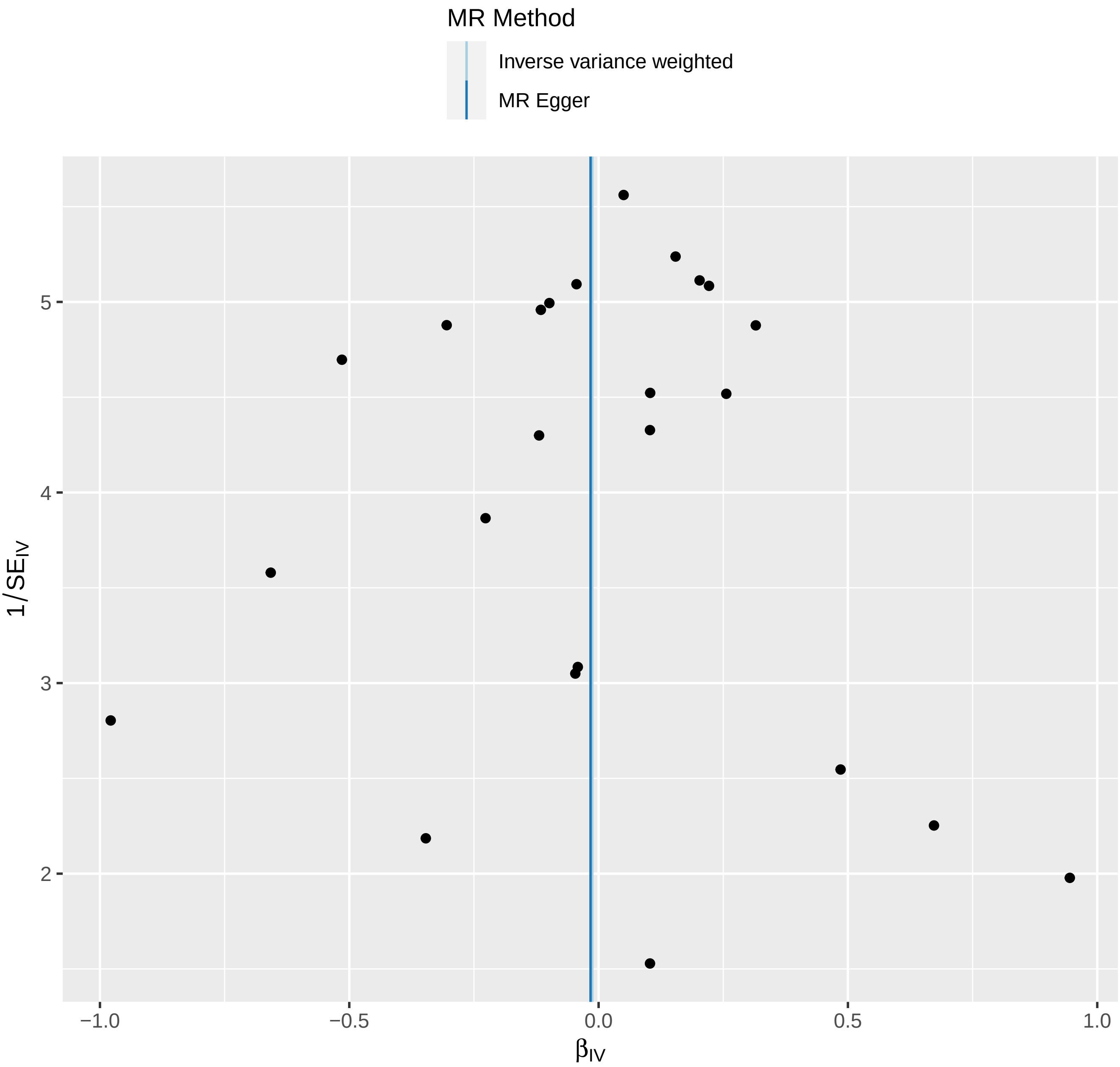

Batch 3−1067 : F−box/LRR−repeat protein 4 on Diabetic nephropathy (more control exclusions)
with a threshold of 1e−05

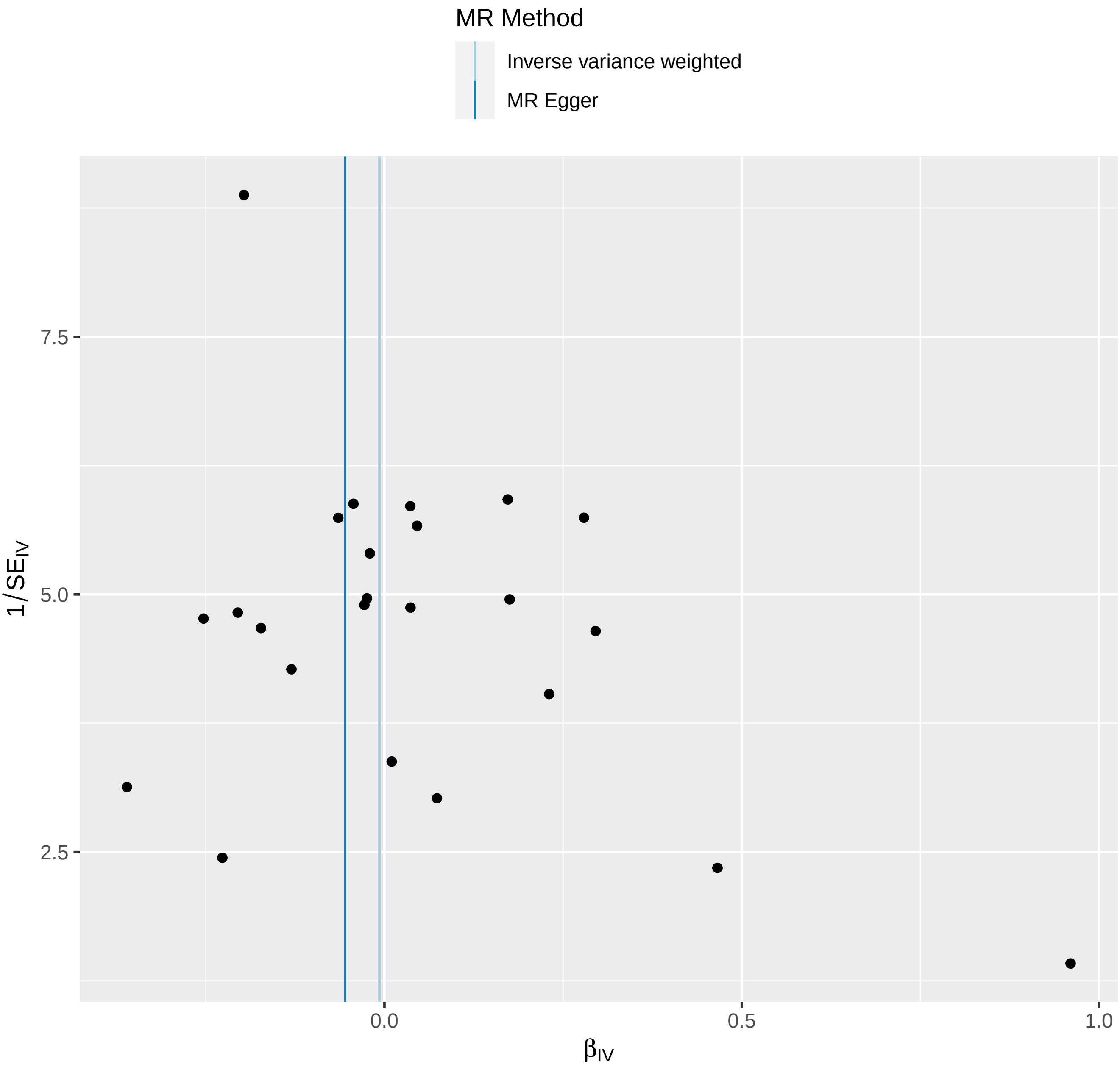

Batch 3−1068 : F−box/LRR−repeat protein 5 on Diabetic nephropathy (more control exclusions)
with a threshold of 1e−05

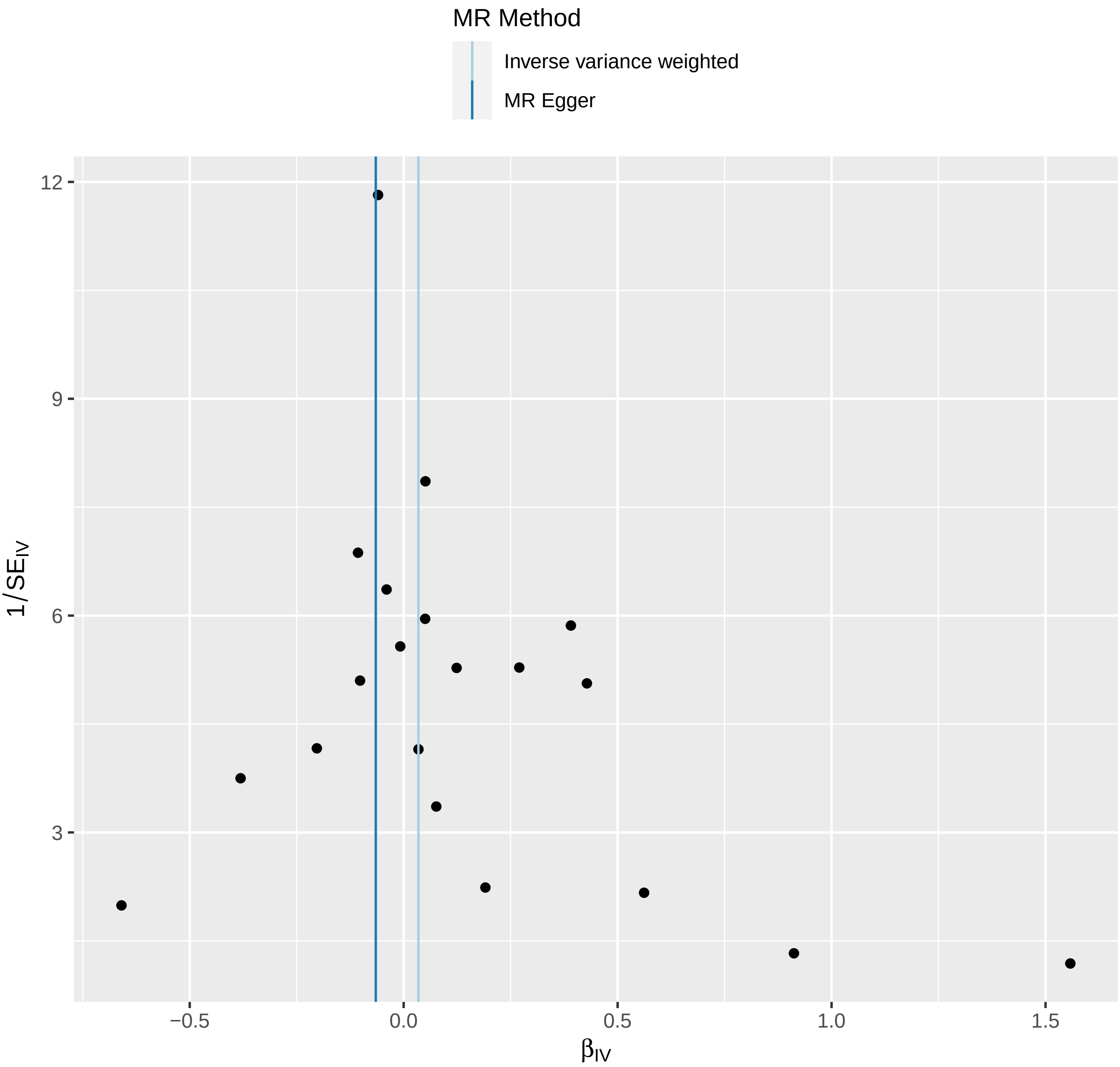

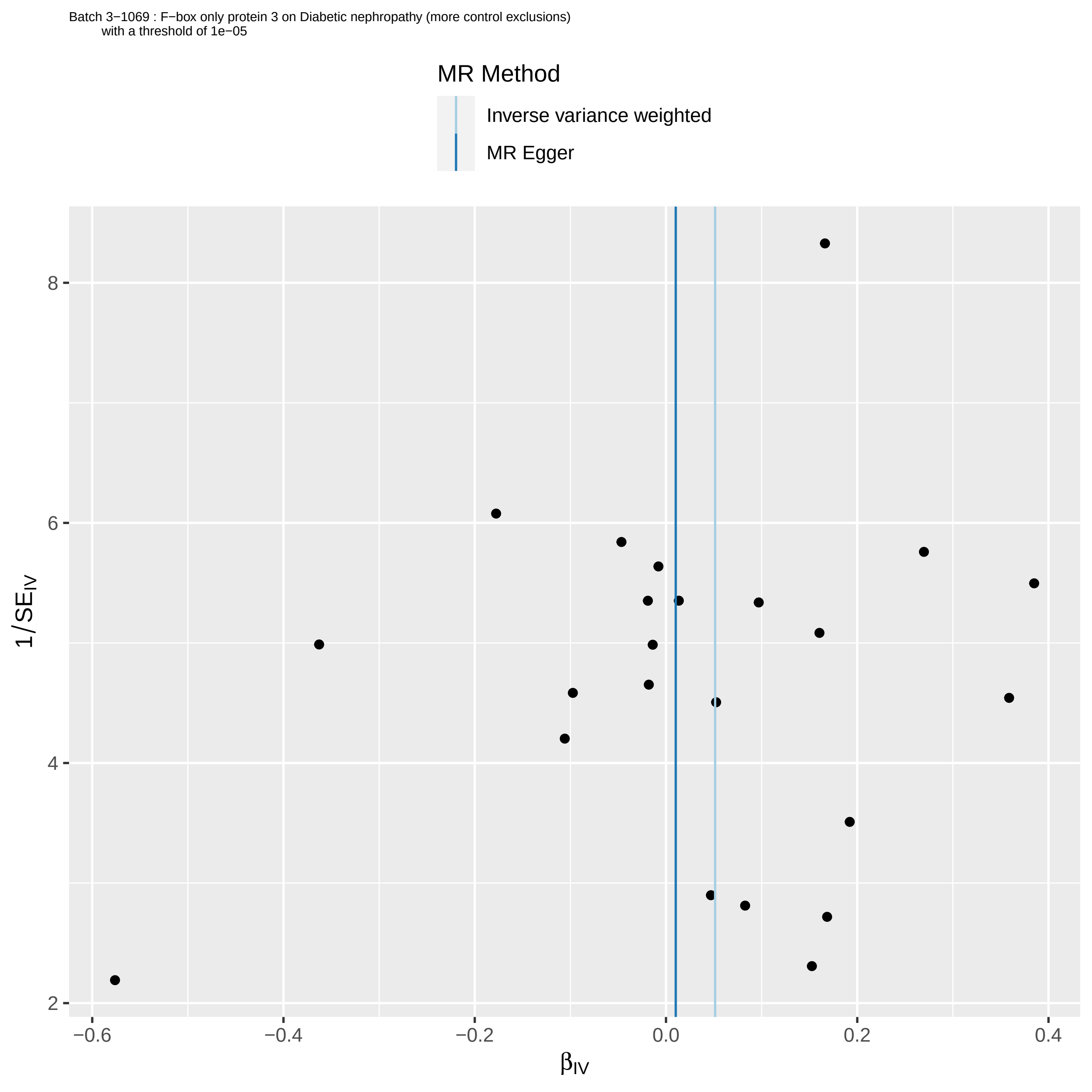
Batch 3−1069 : F−box only protein 3 on Diabetic nephropathy (more control exclusions)
with a threshold of 1e−05
MR Method
Inverse variance weighted
MR Egger
8
6
4
2
$1/SE_{IV}$
−0.6
−0.4
−0.2
0.0
0.2
0.4
β_{IV}

Batch 3−1070 : High affinity immunoglobulin alpha and immunoglobulin mu Fc receptor on Diabetic nephropathy (more control exclusions)
with a threshold of 1e−05

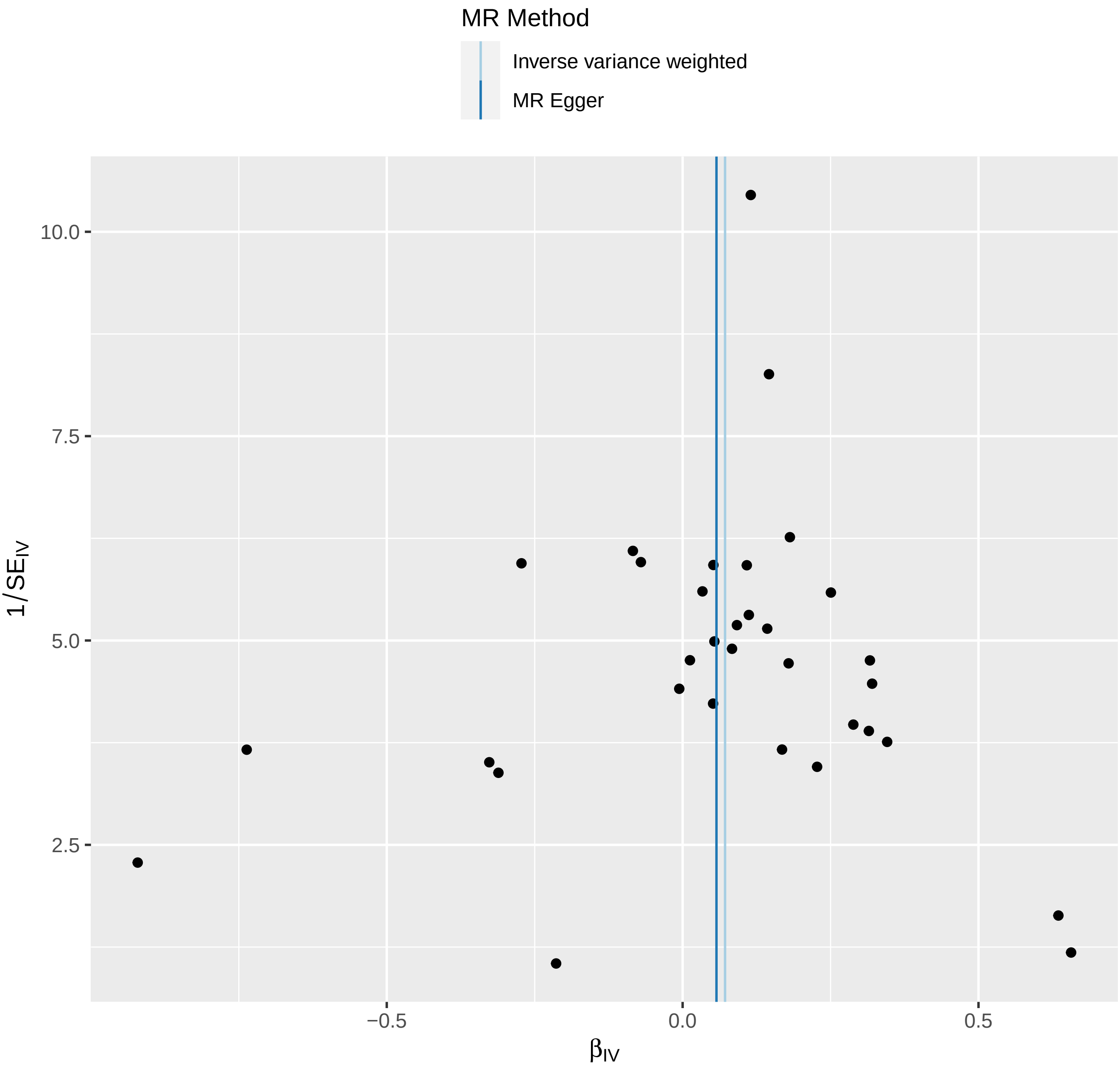

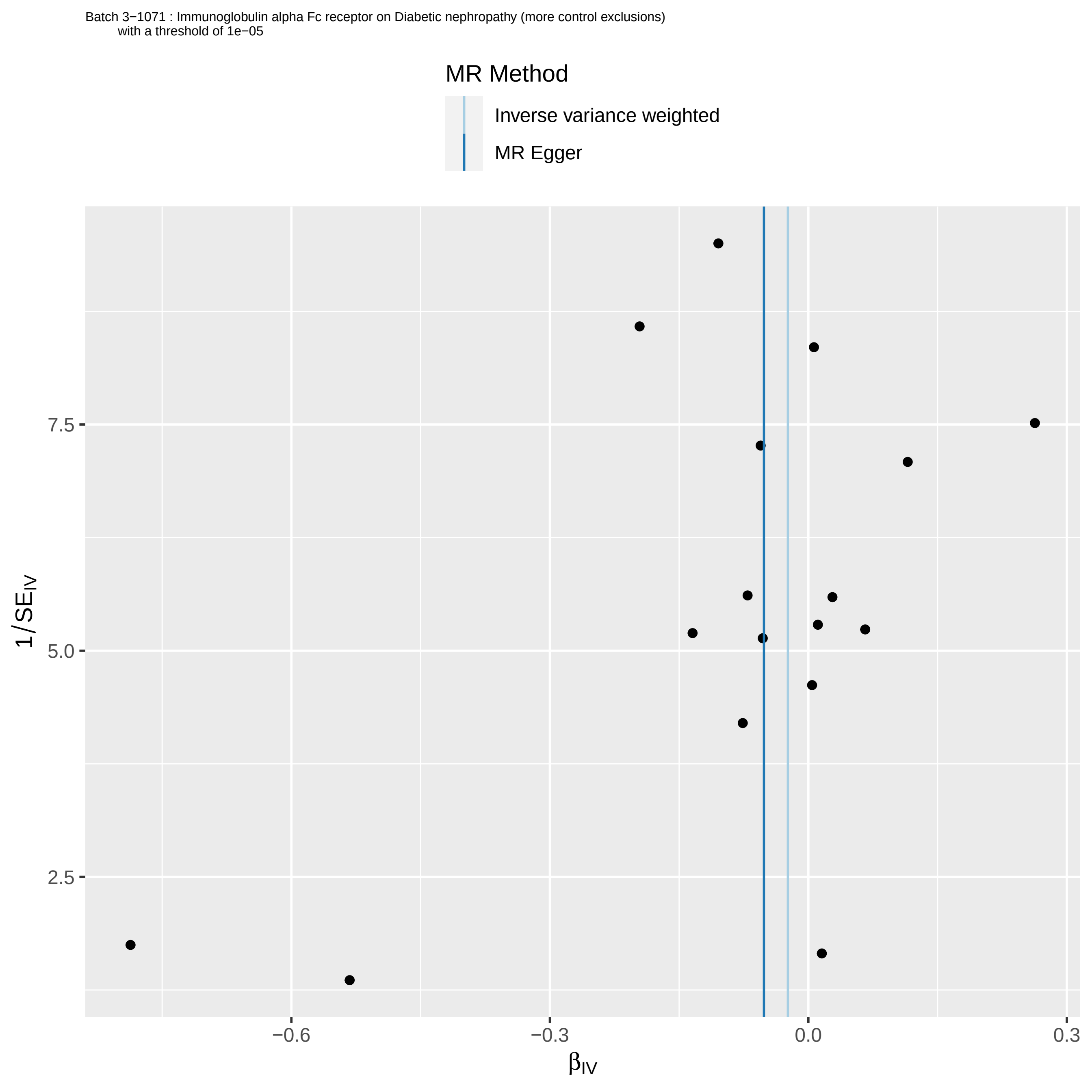

Batch 3−1071 : Immunoglobulin alpha Fc receptor on Diabetic nephropathy (more control exclusions)
with a threshold of 1e−05
MR Method
Inverse variance weighted
MR Egger
7.5
5.0
2.5
1/SE_IV
−0.6
−0.3
0.0
0.3
β_IV

Batch 3−1072 : Low affinity immunoglobulin epsilon Fc receptor on Diabetic nephropathy (more control exclusions)
with a threshold of 1e−05

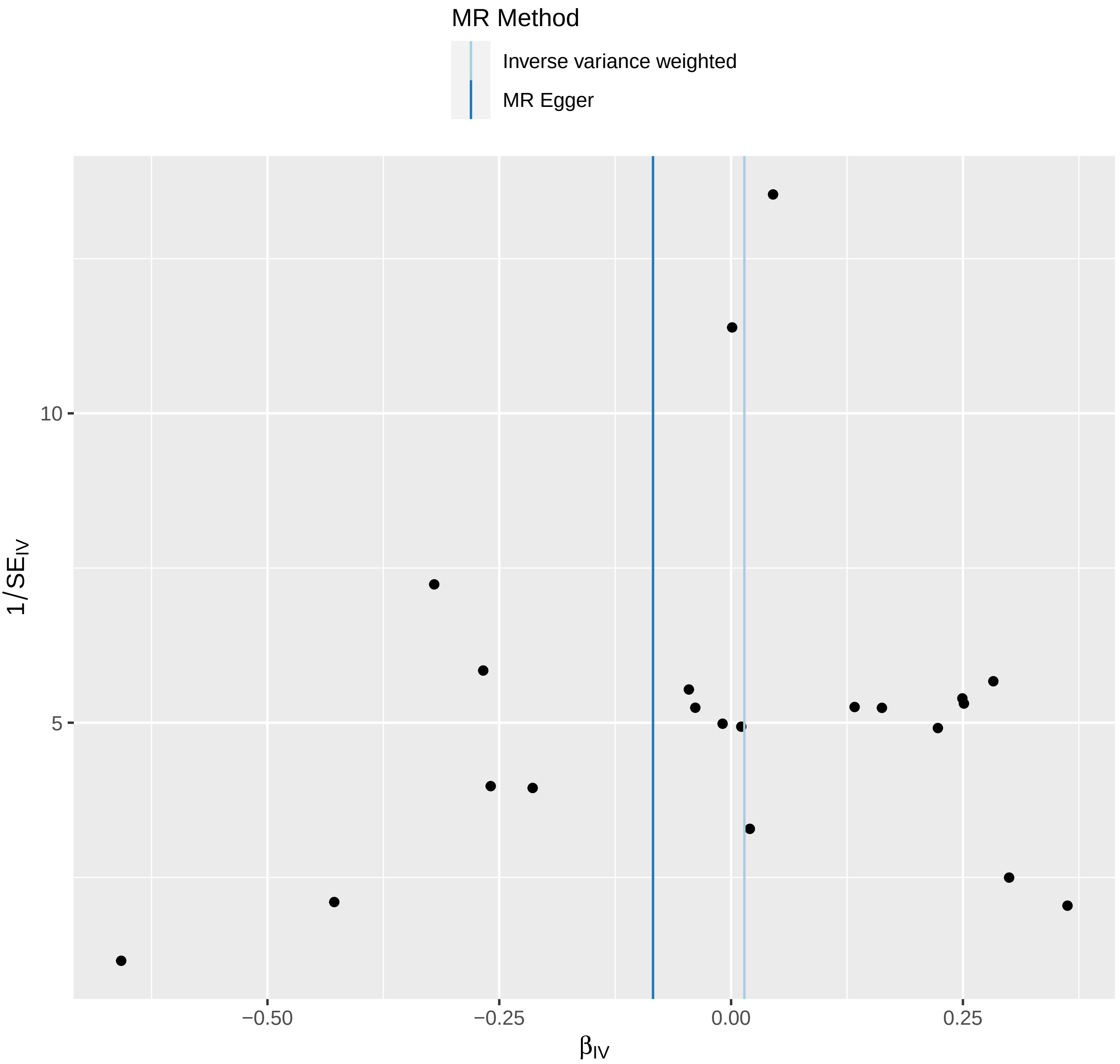

Batch 3−1073 : High affinity immunoglobulin gamma Fc receptor I on Diabetic nephropathy (more control exclusions) with a threshold of 1e−05

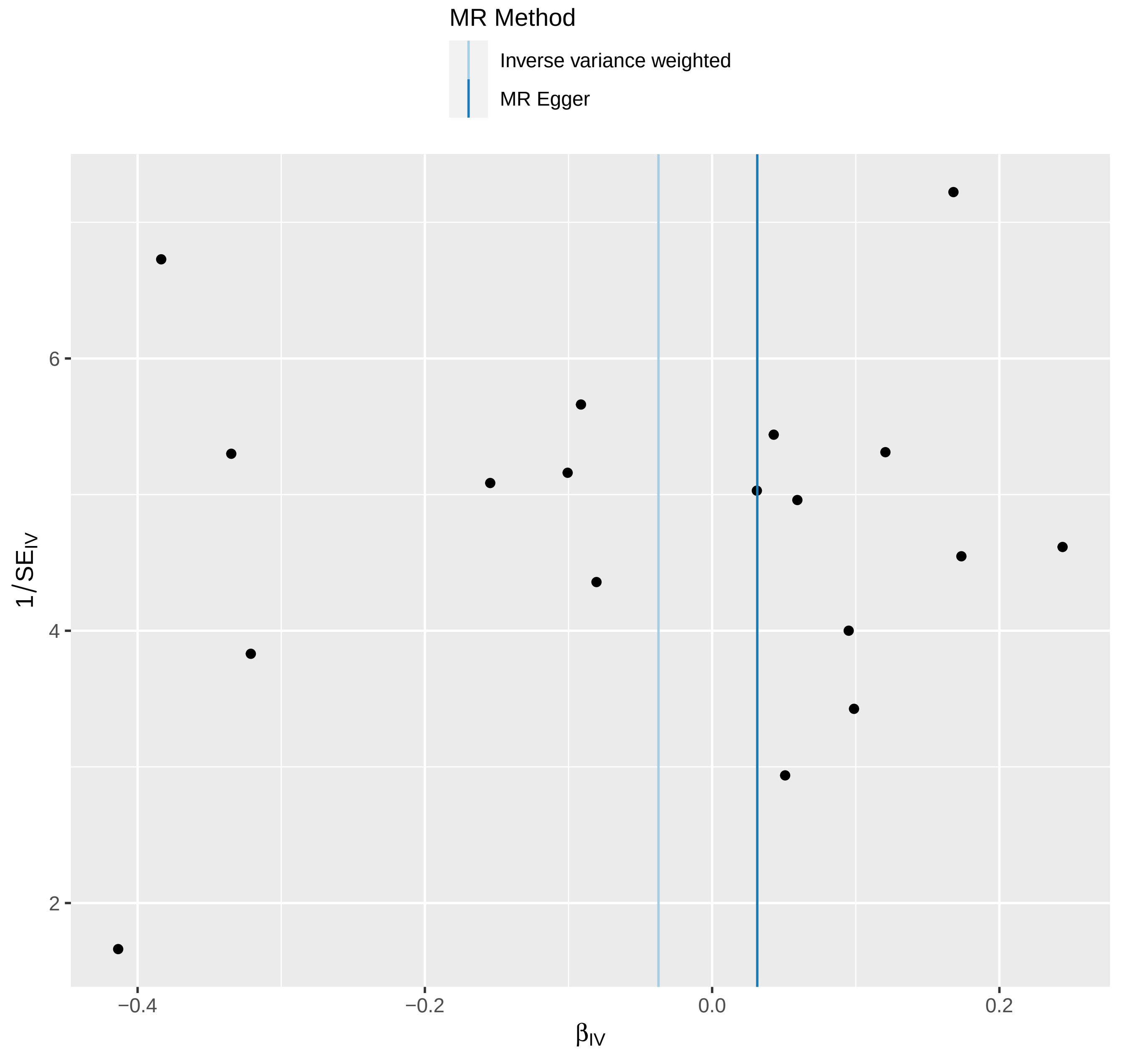

Batch 3−1074 : Low affinity immunoglobulin gamma Fc region receptor II−a on Diabetic nephropathy (more control exclusions)
with a threshold of 1e−05

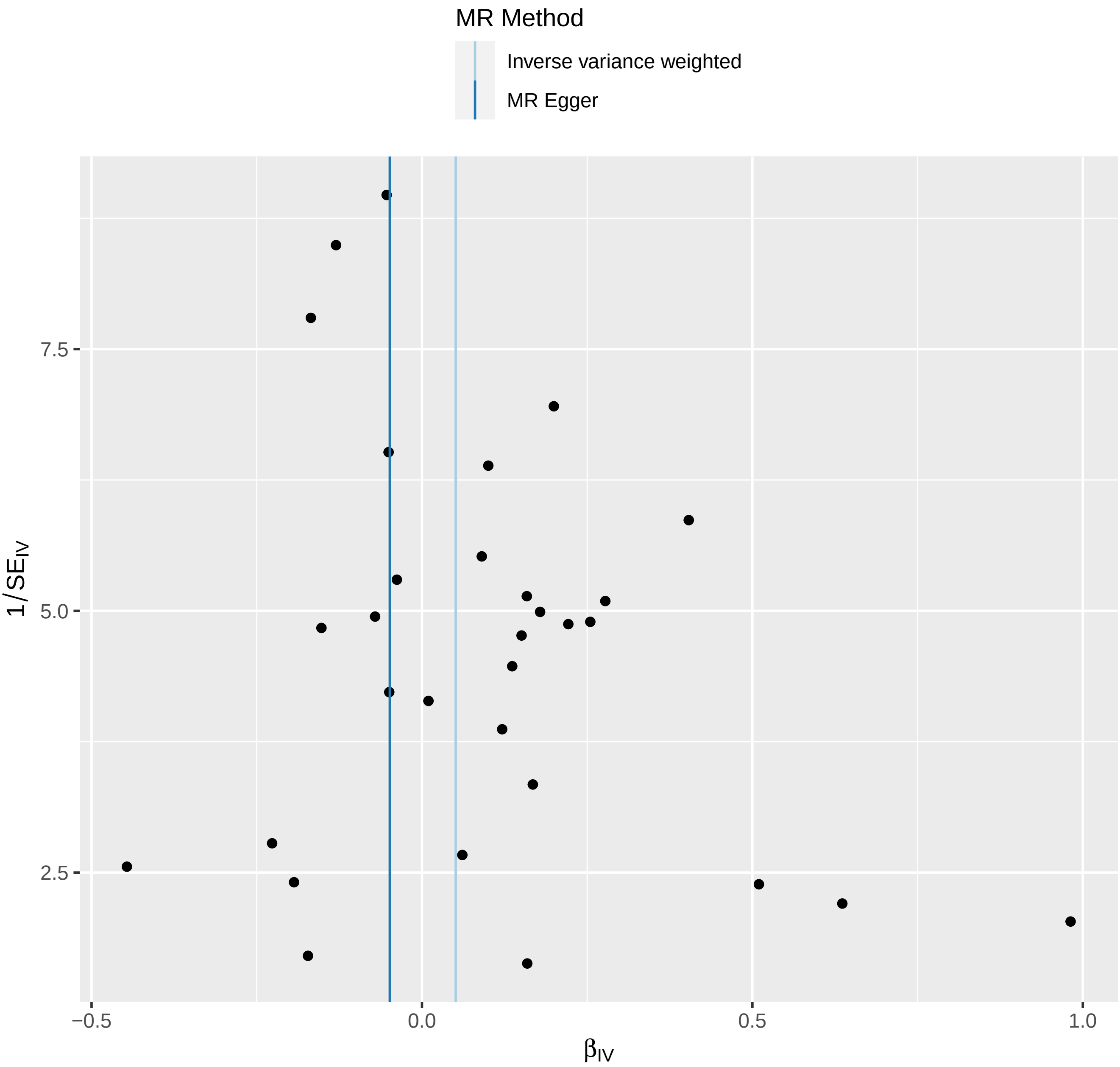

Batch 3−1075 : Low affinity immunoglobulin gamma Fc region receptor II−b on Diabetic nephropathy (more control exclusions) with a threshold of 1e−05

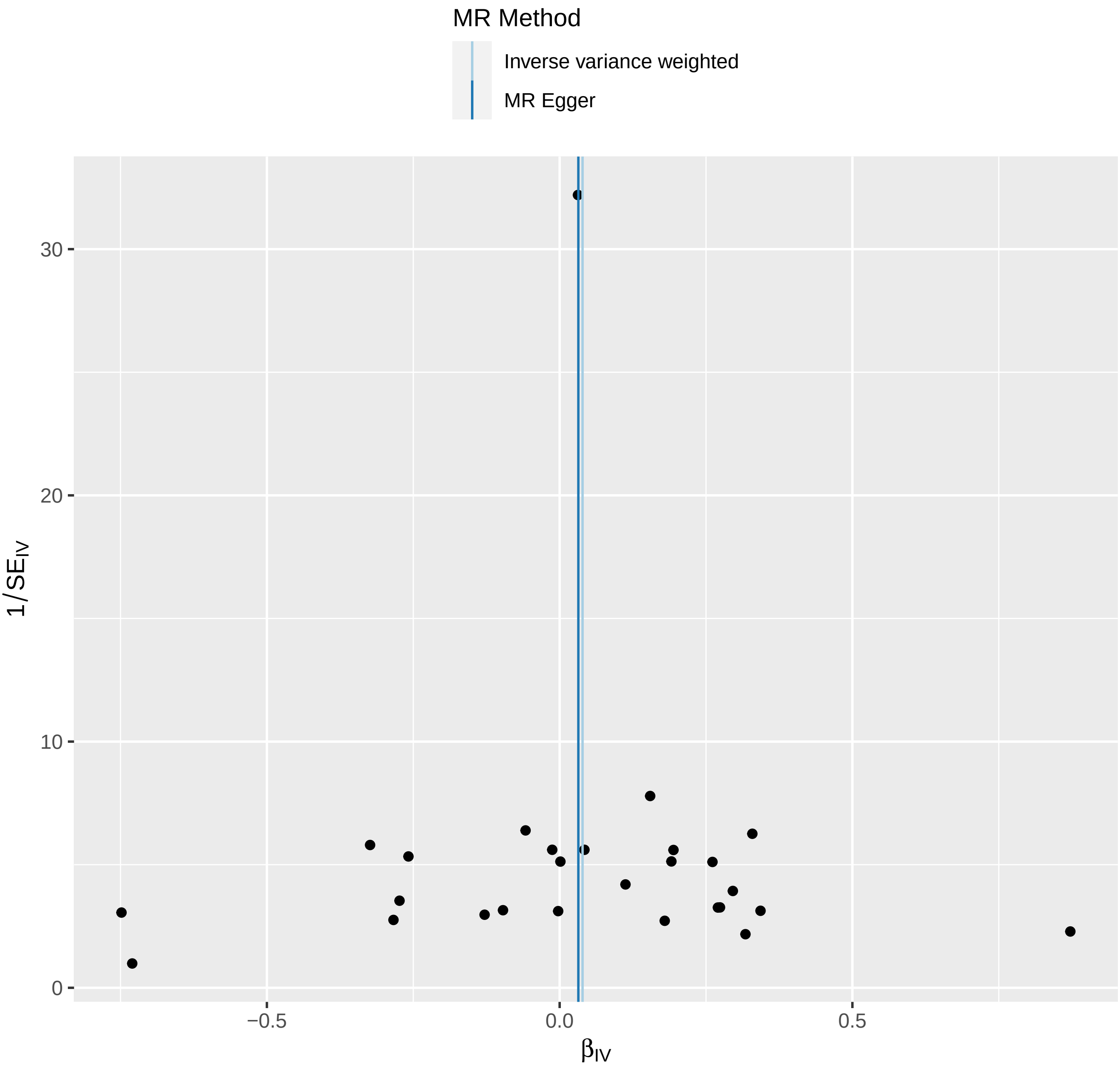

Batch 3−1076 : Low affinity immunoglobulin gamma Fc region receptor III−B on Diabetic nephropathy (more control exclusions)
with a threshold of 1e−05

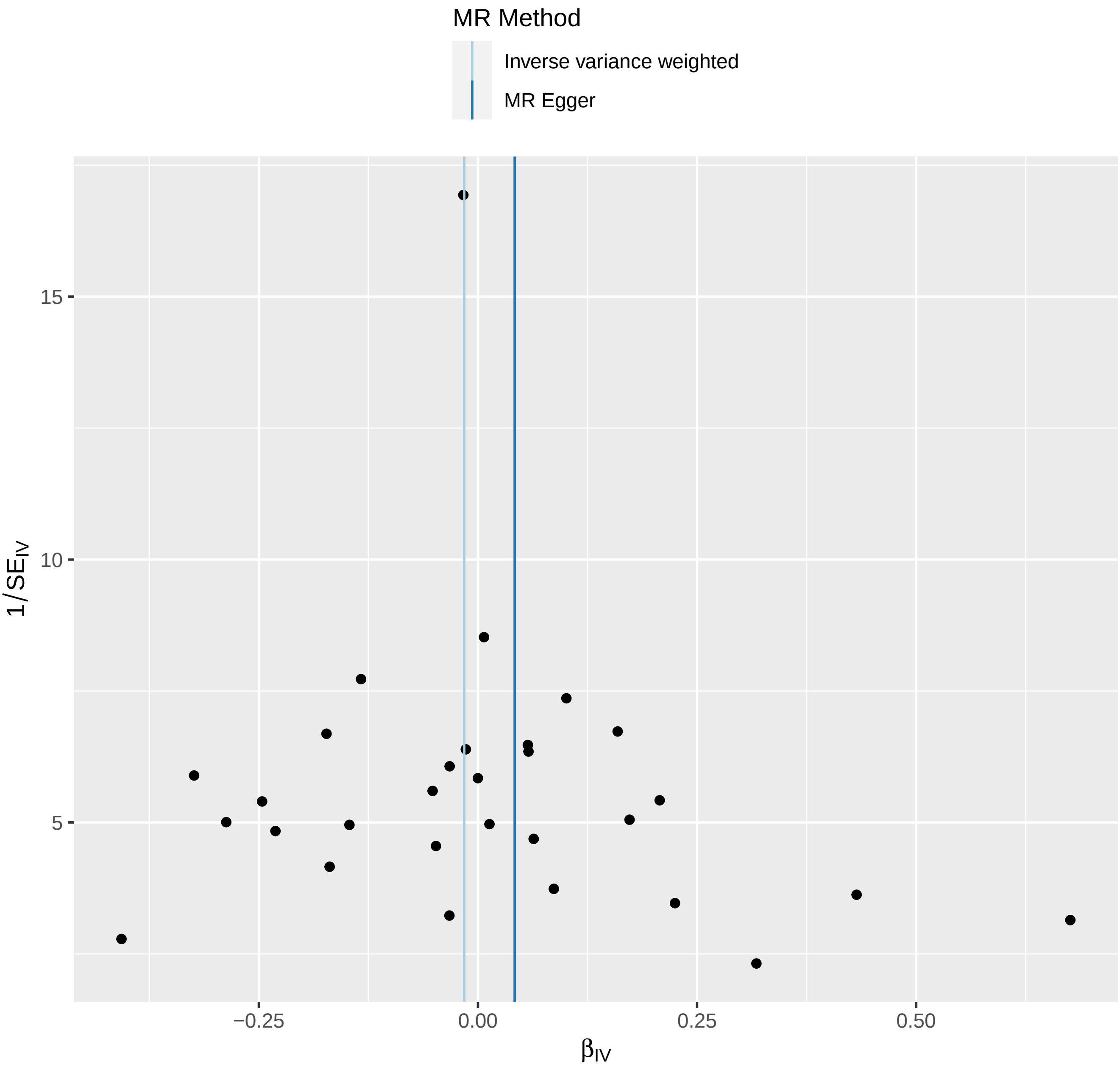

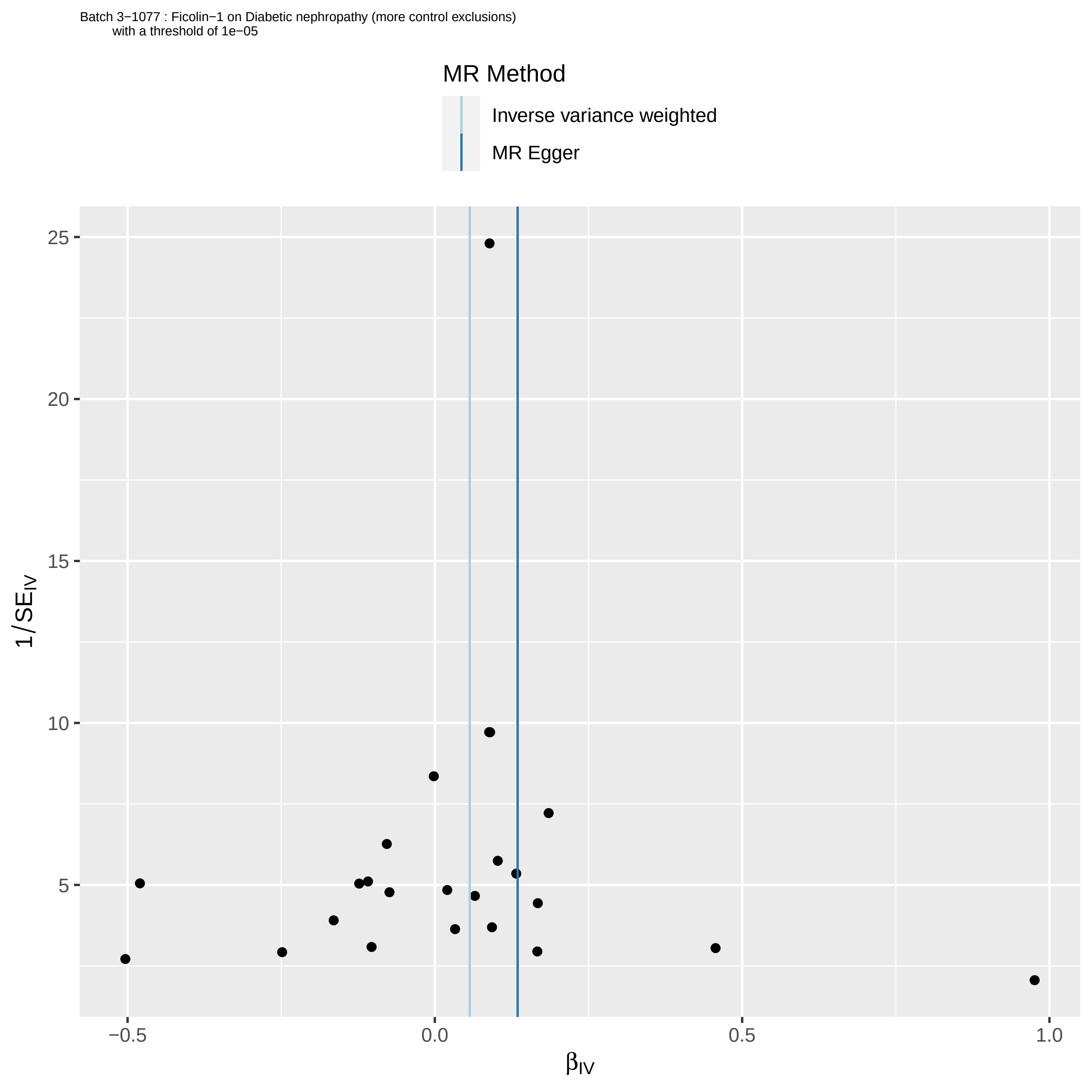
Batch 3−1077 : Ficolin−1 on Diabetic nephropathy (more control exclusions)
with a threshold of 1e−05
MR Method
Inverse variance weighted
MR Egger
25
20
15
10
5
1/SE_IV
−0.5
0.0
0.5
1.0
β_IV

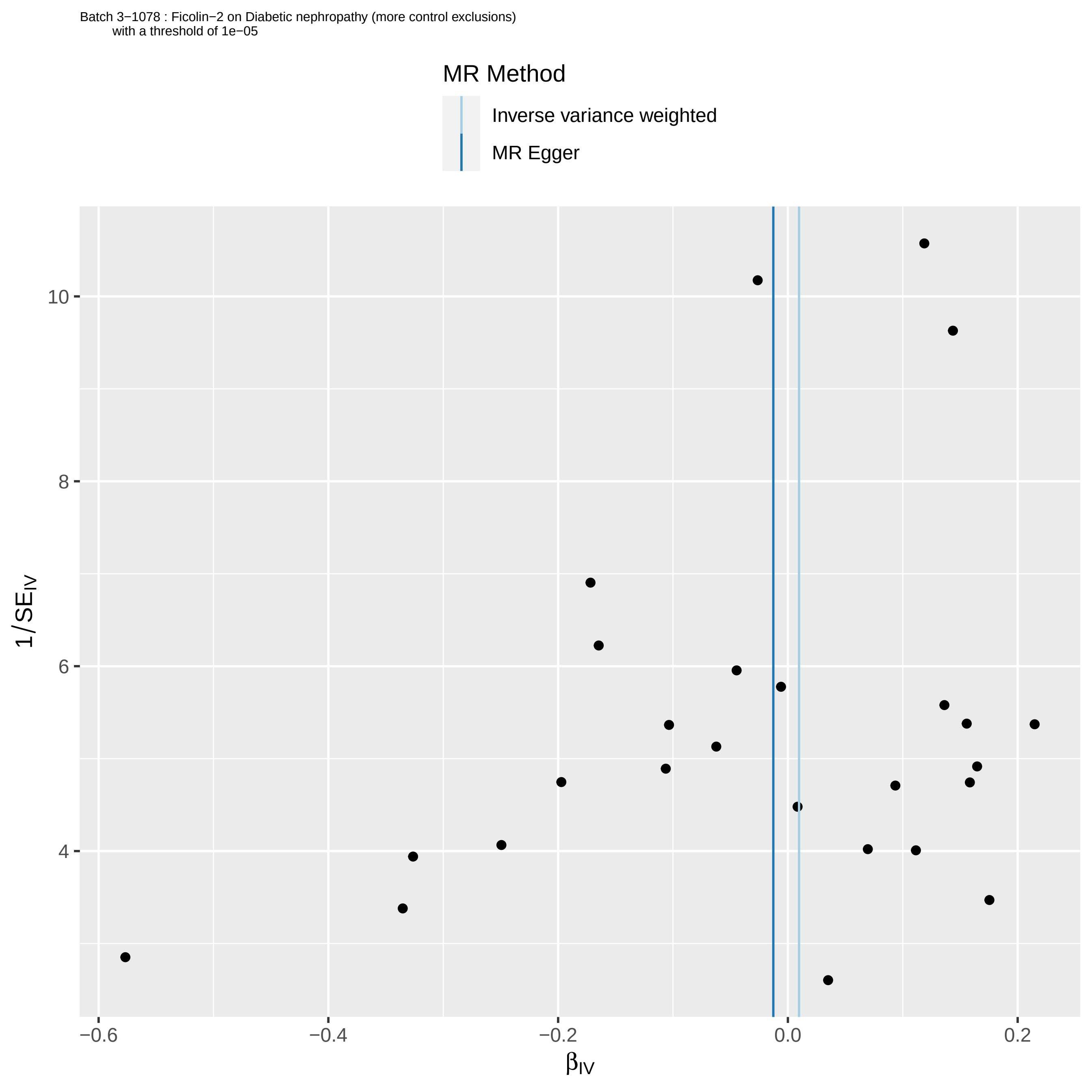

Batch 3−1078 : Ficolin−2 on Diabetic nephropathy (more control exclusions)
with a threshold of 1e−05
MR Method
Inverse variance weighted
MR Egger
10
8
6
4
−0.6
−0.4
−0.2
0.0
0.2
1/SE_IV
β_IV

Batch 3−1079 : Fc receptor−like protein 1 on Diabetic nephropathy (more control exclusions)
with a threshold of 1e−05

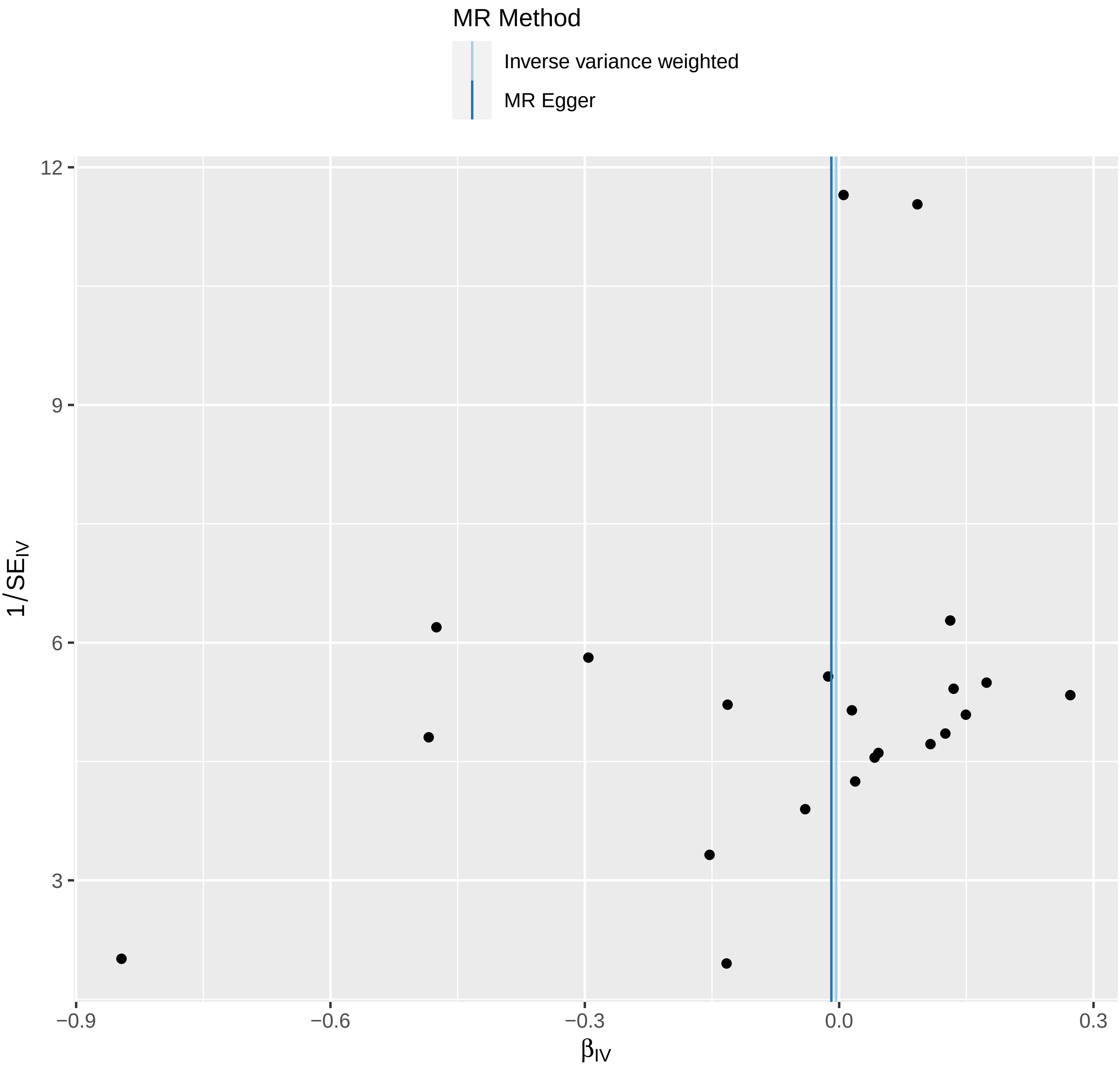

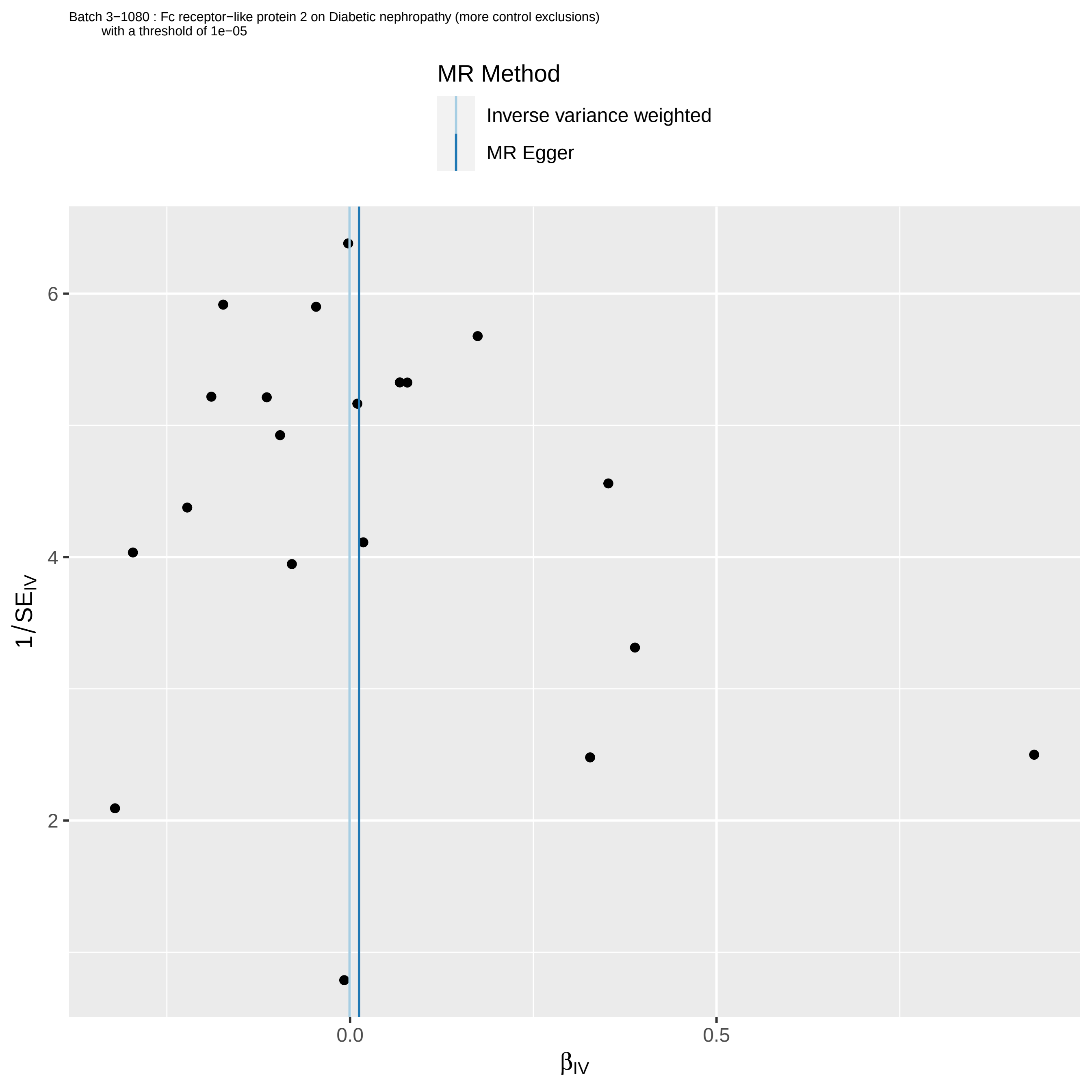

Batch 3−1080 : Fc receptor−like protein 2 on Diabetic nephropathy (more control exclusions)
with a threshold of 1e−05
MR Method
Inverse variance weighted
MR Egger
6
4
2
1/SE$_{IV}$
0.0
0.5
β_{IV}

Batch 3−1081 : Fc receptor−like protein 3 on Diabetic nephropathy (more control exclusions)
with a threshold of 1e−05

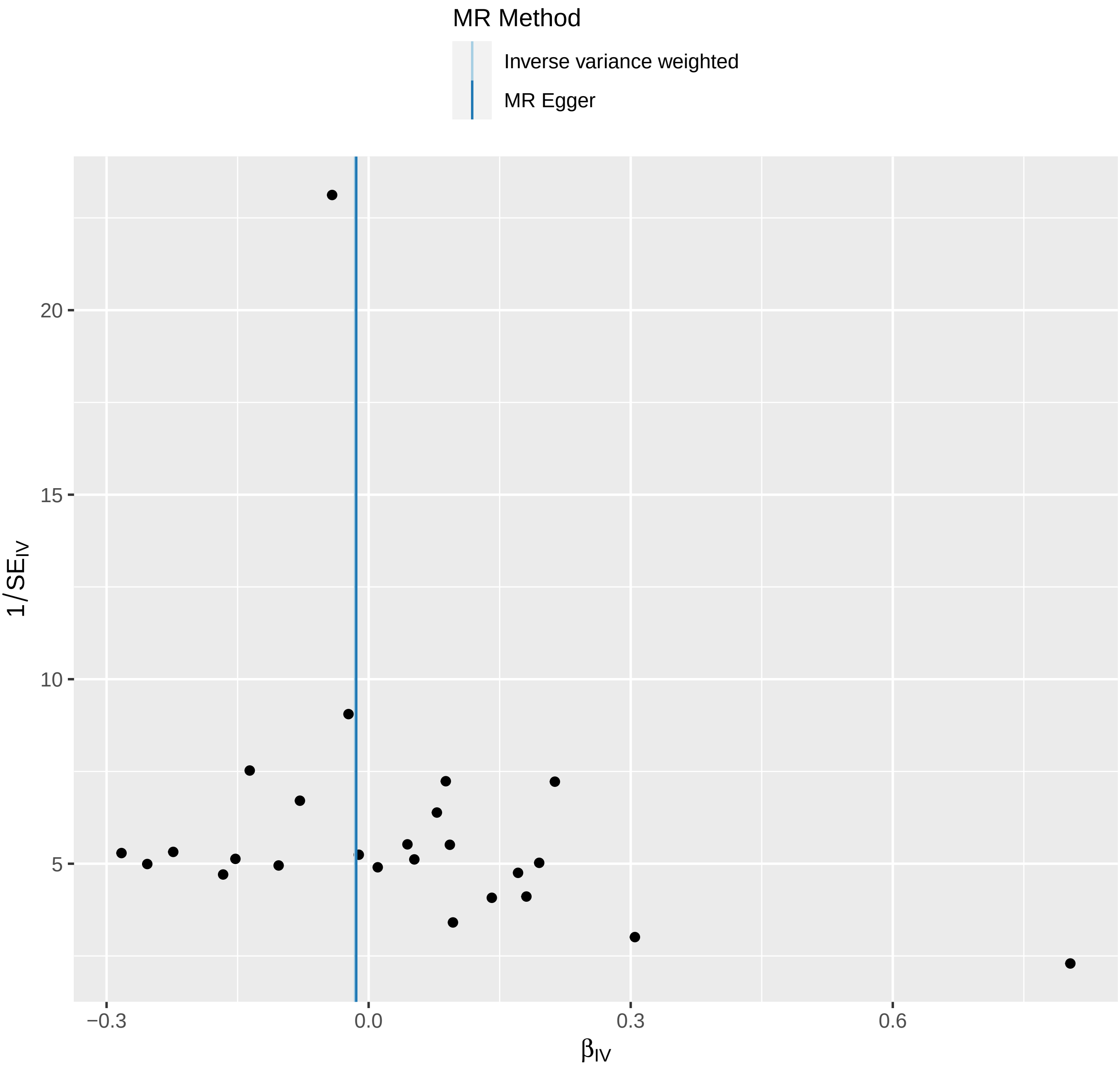

Batch 3−1082 : Fc receptor−like protein 4 on Diabetic nephropathy (more control exclusions)
with a threshold of 1e−05

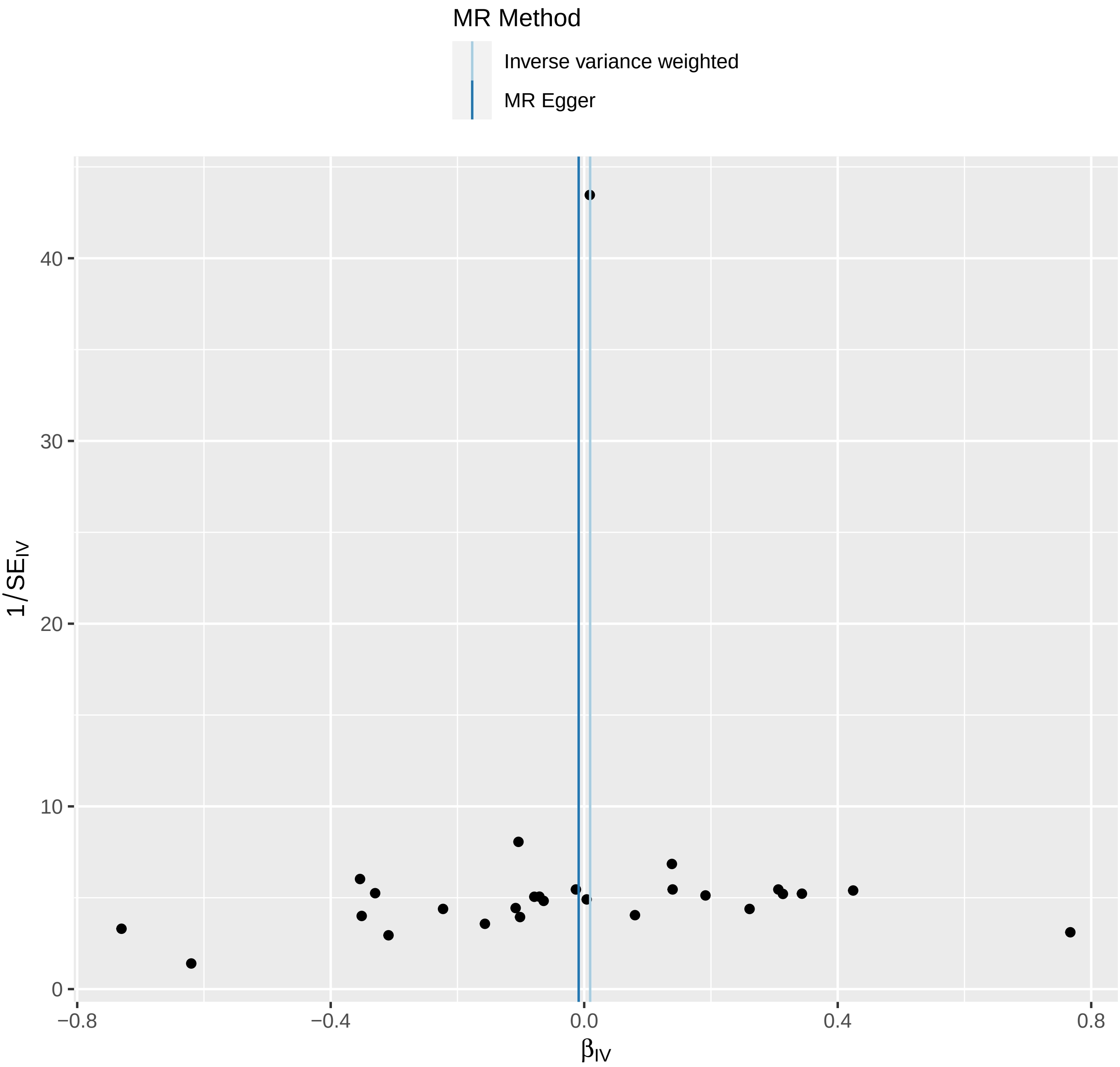

Batch 3−1083 : Fc receptor−like protein 5 on Diabetic nephropathy (more control exclusions)
with a threshold of 1e−05

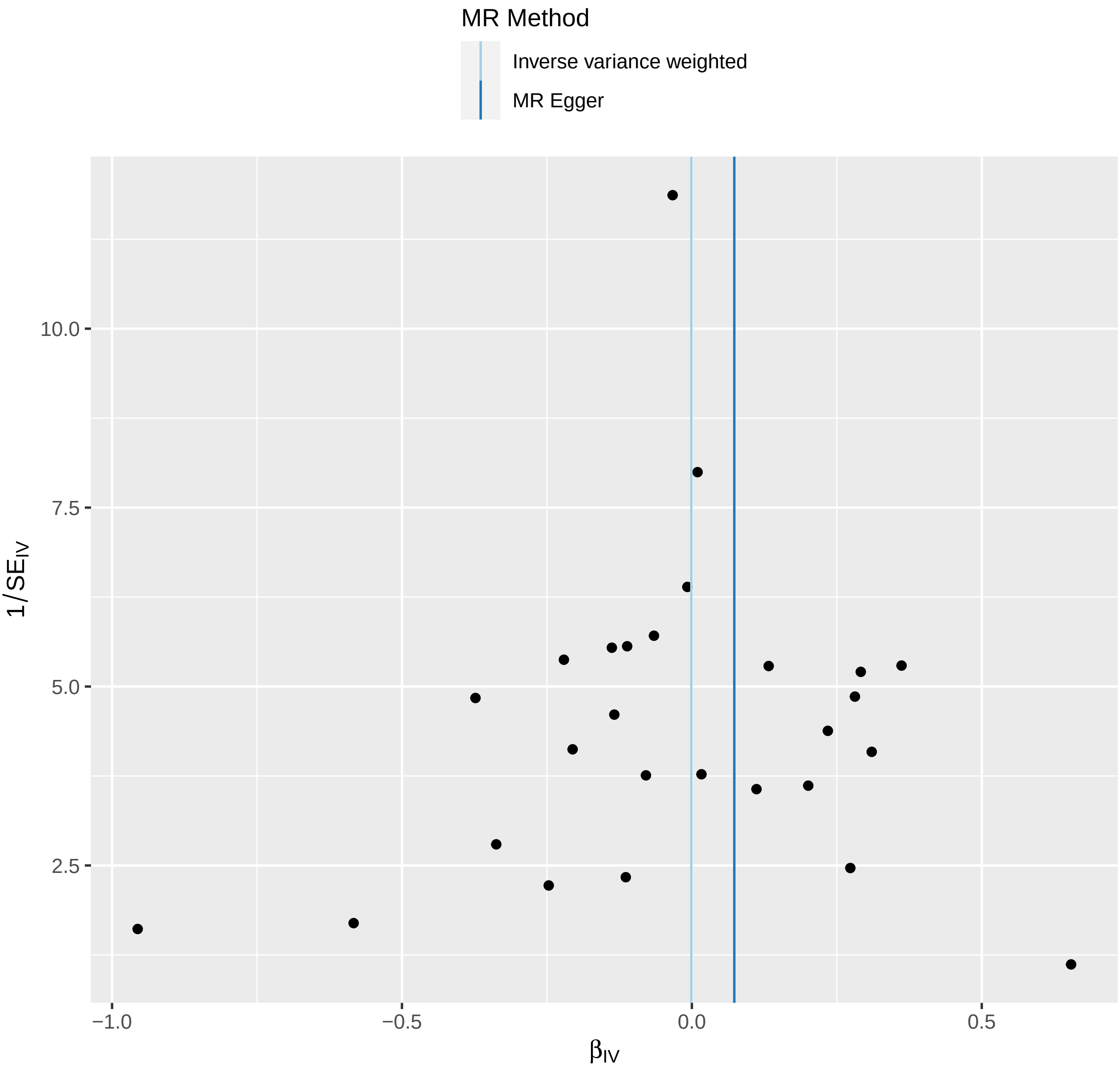

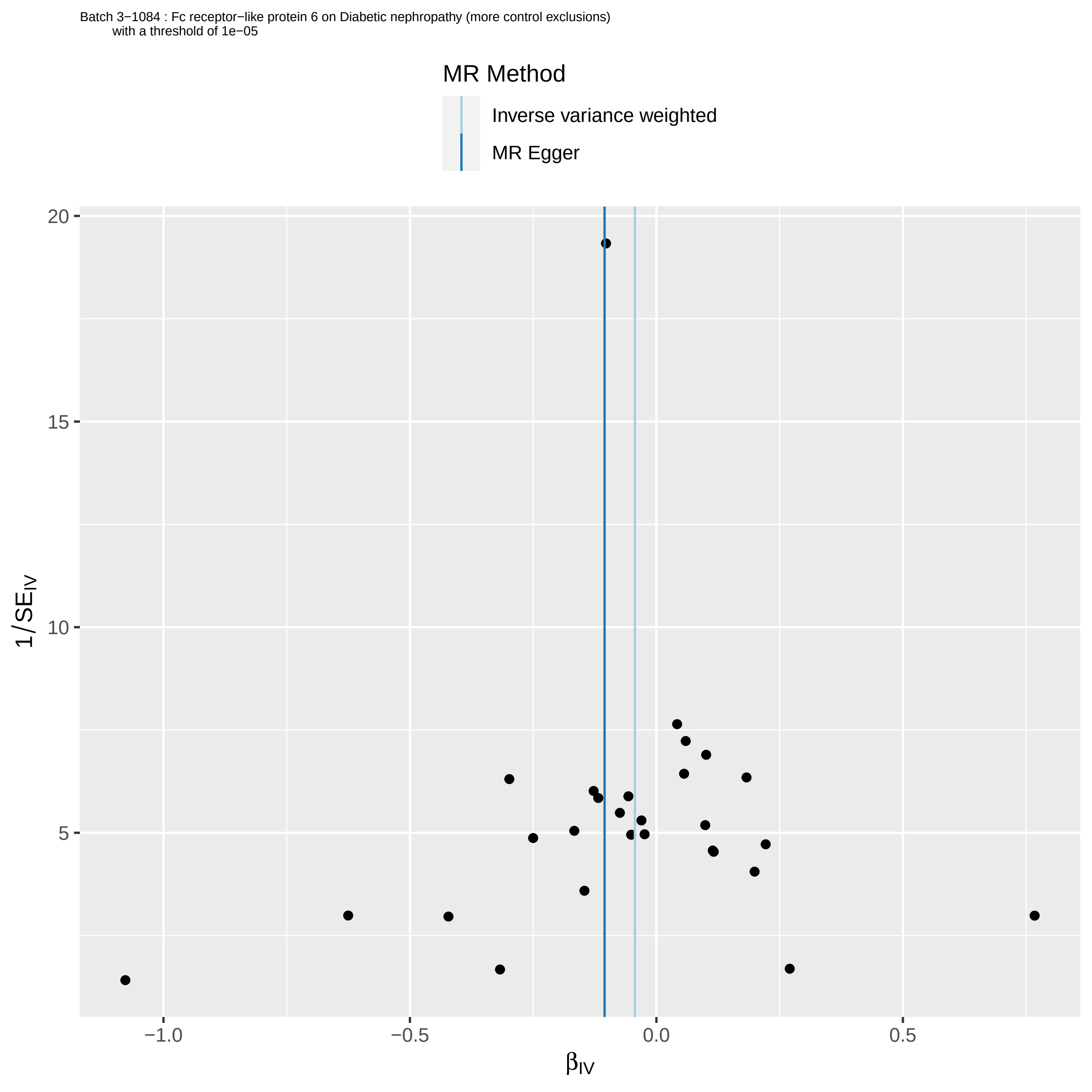

Batch 3−1084 : Fc receptor−like protein 6 on Diabetic nephropathy (more control exclusions)
with a threshold of 1e−05
MR Method
Inverse variance weighted
MR Egger
20
15
10
5
1/SE_IV
−1.0
−0.5
0.0
0.5
β_IV

Batch 3−1085 : Flap endonuclease 1 on Diabetic nephropathy (more control exclusions)
with a threshold of 1e−05

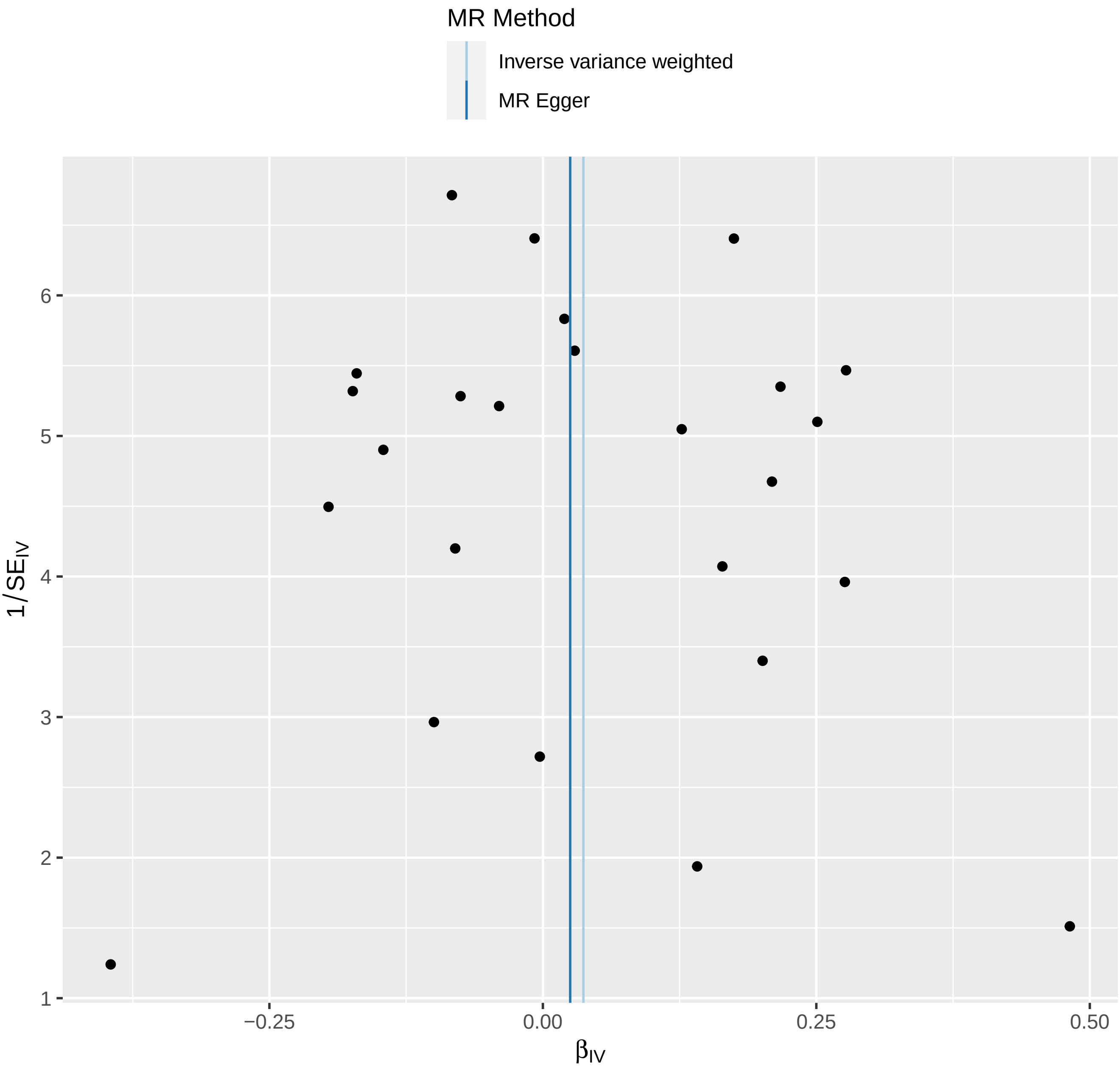

Batch 3−1086 : D−dimer on Diabetic nephropathy (more control exclusions)
with a threshold of 1e−05

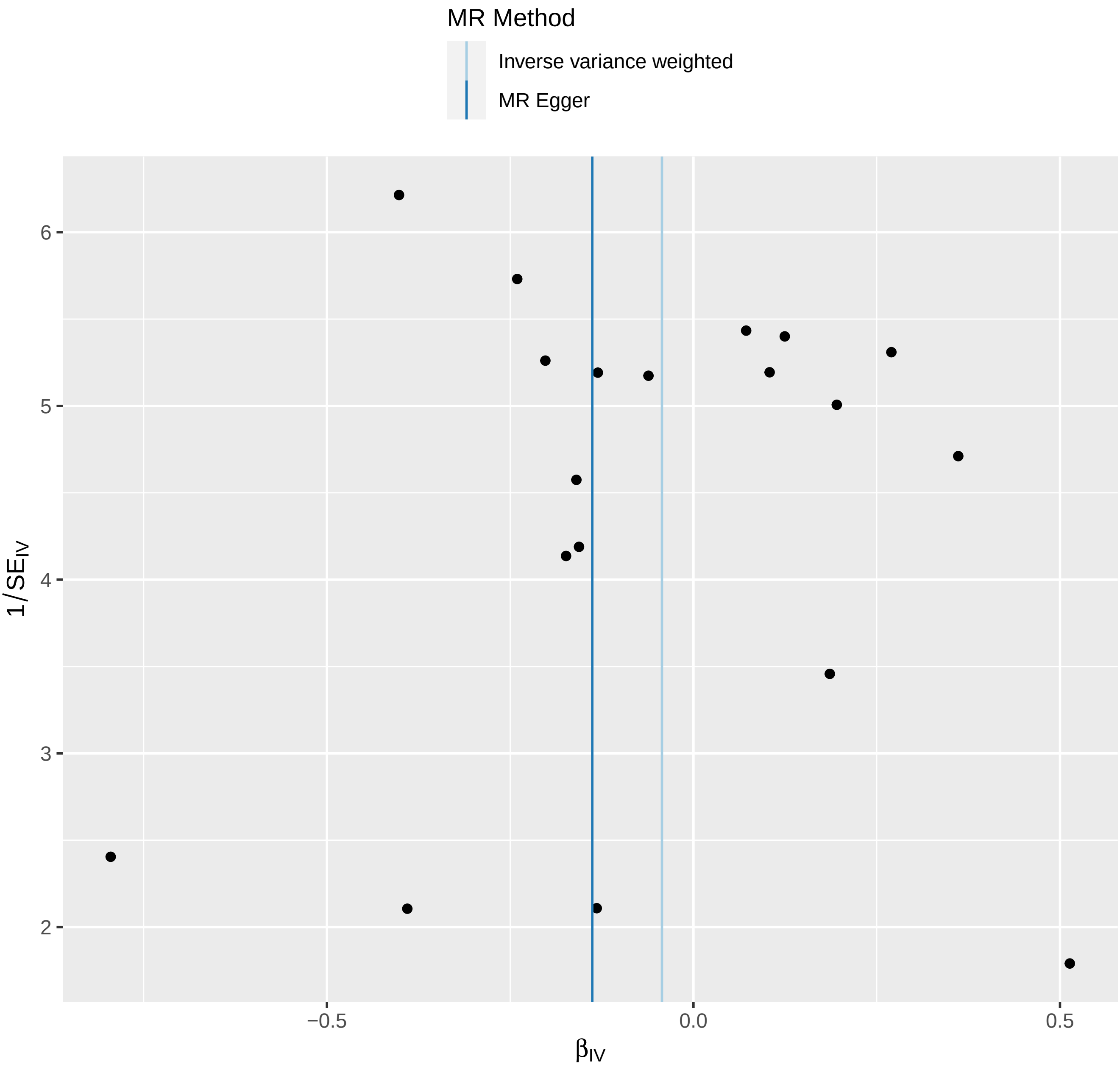

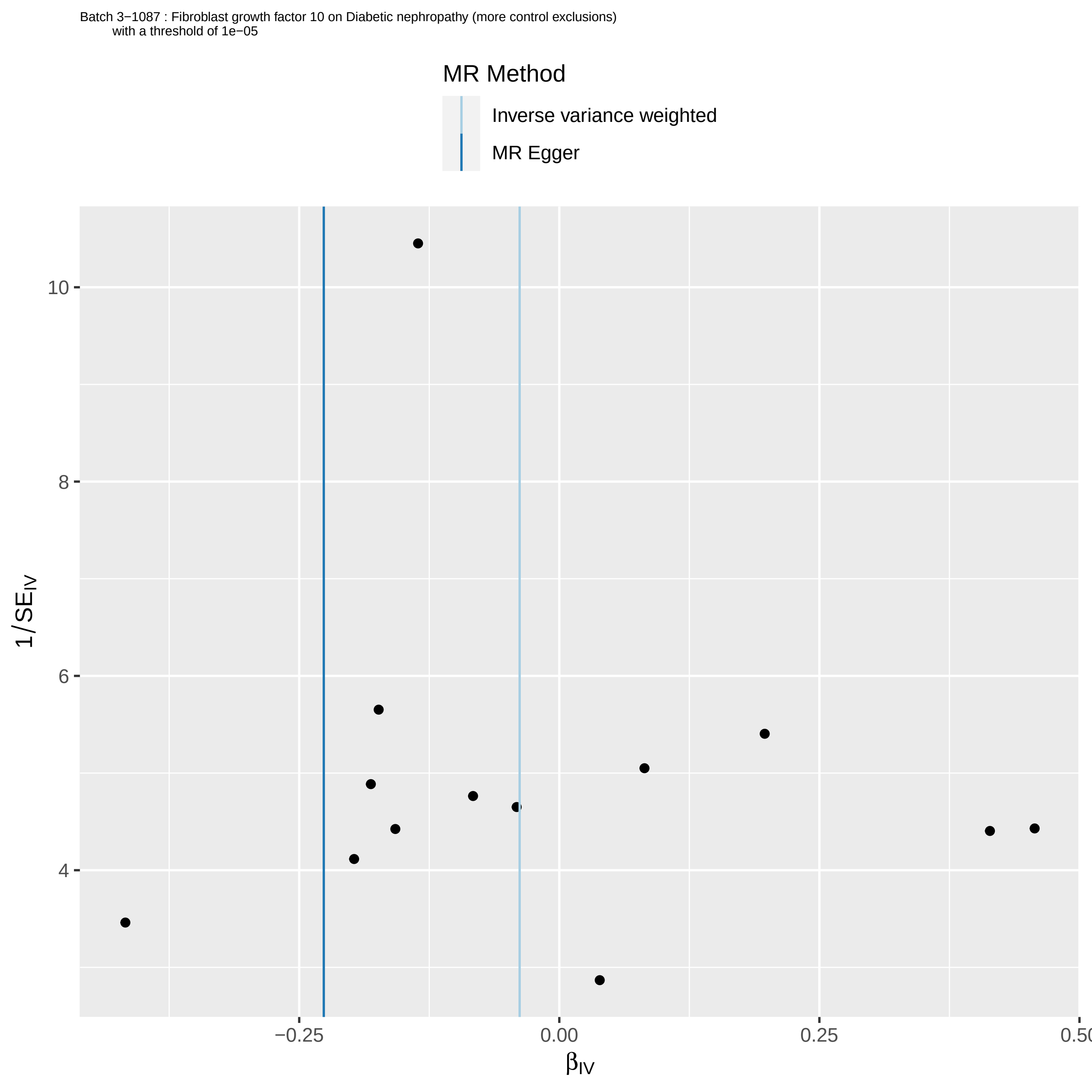
Batch 3−1087 : Fibroblast growth factor 10 on Diabetic nephropathy (more control exclusions)
with a threshold of 1e−05
MR Method
Inverse variance weighted
MR Egger
10
8
6
4
1/SE$_{IV}$
−0.25
0.00
0.25
0.50
β$_{IV}$

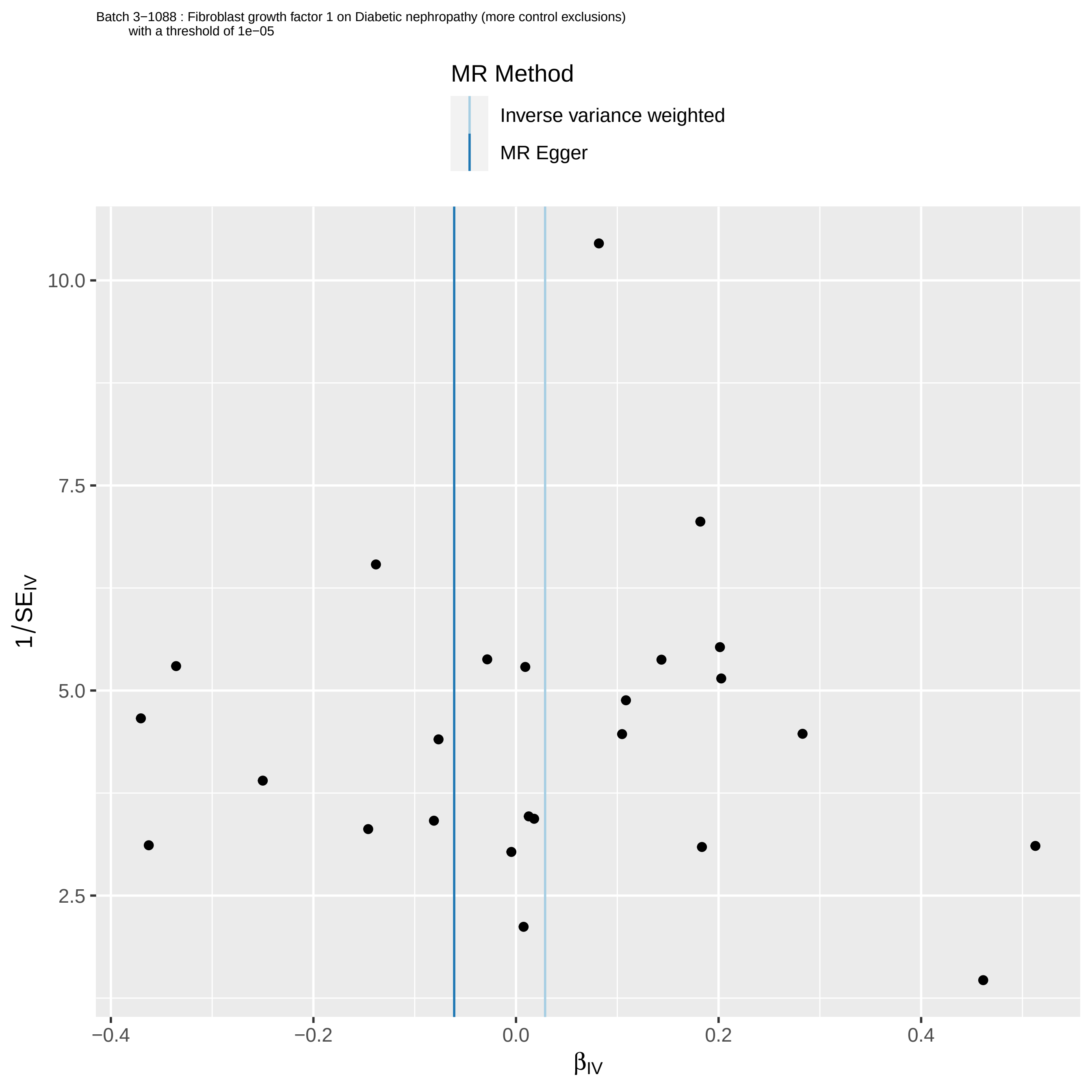
Batch 3−1088 : Fibroblast growth factor 1 on Diabetic nephropathy (more control exclusions)
with a threshold of 1e−05
MR Method
Inverse variance weighted
MR Egger
10.0
7.5
5.0
2.5
1/SE_IV
−0.4
−0.2
0.0
0.2
0.4
β_IV

Batch 3−1089 : Fibroblast growth factor 16 on Diabetic nephropathy (more control exclusions)
with a threshold of 1e−05

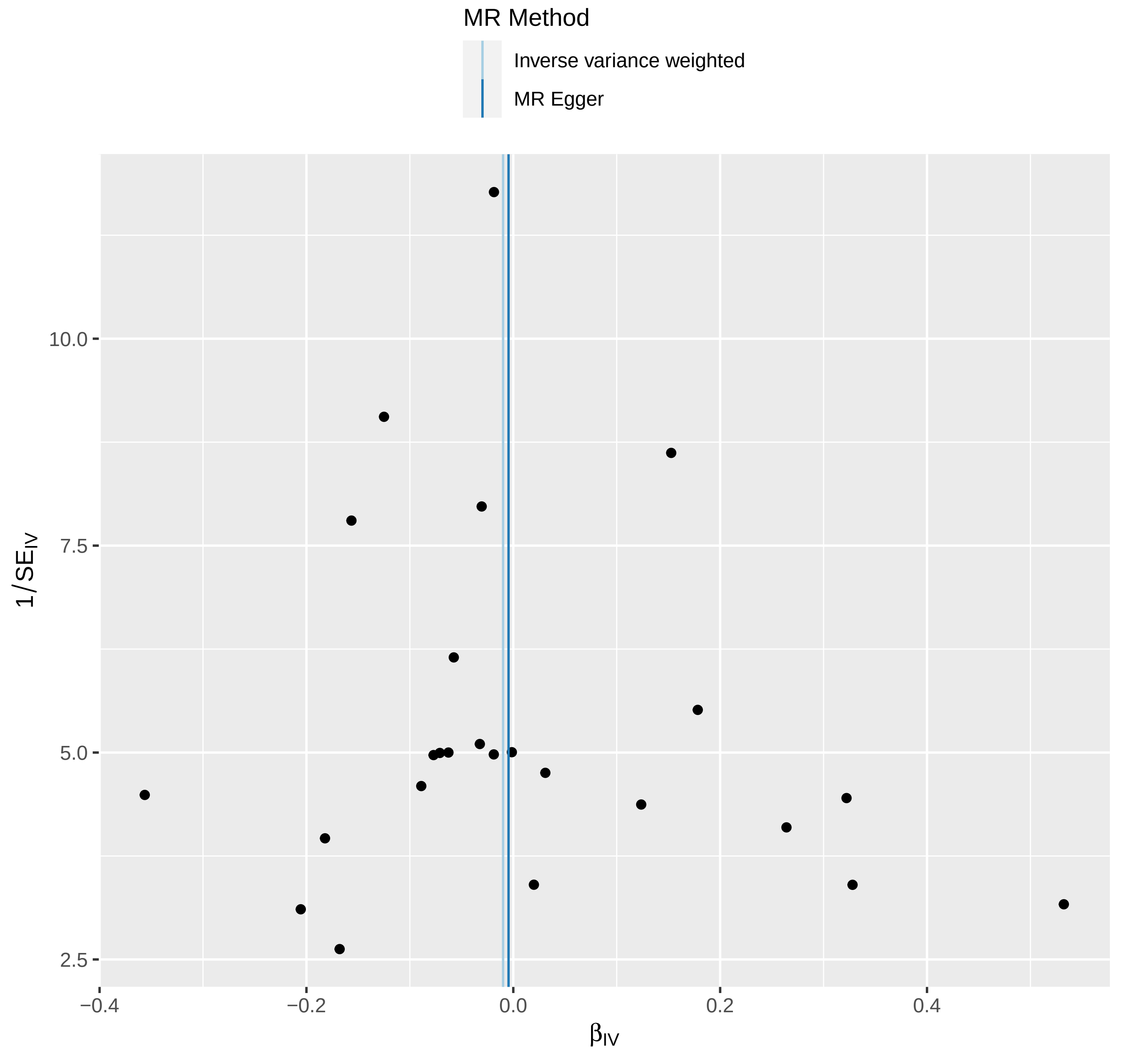

Batch 3−1090 : Fibroblast growth factor 16 on Diabetic nephropathy (more control exclusions)
with a threshold of 1e−05

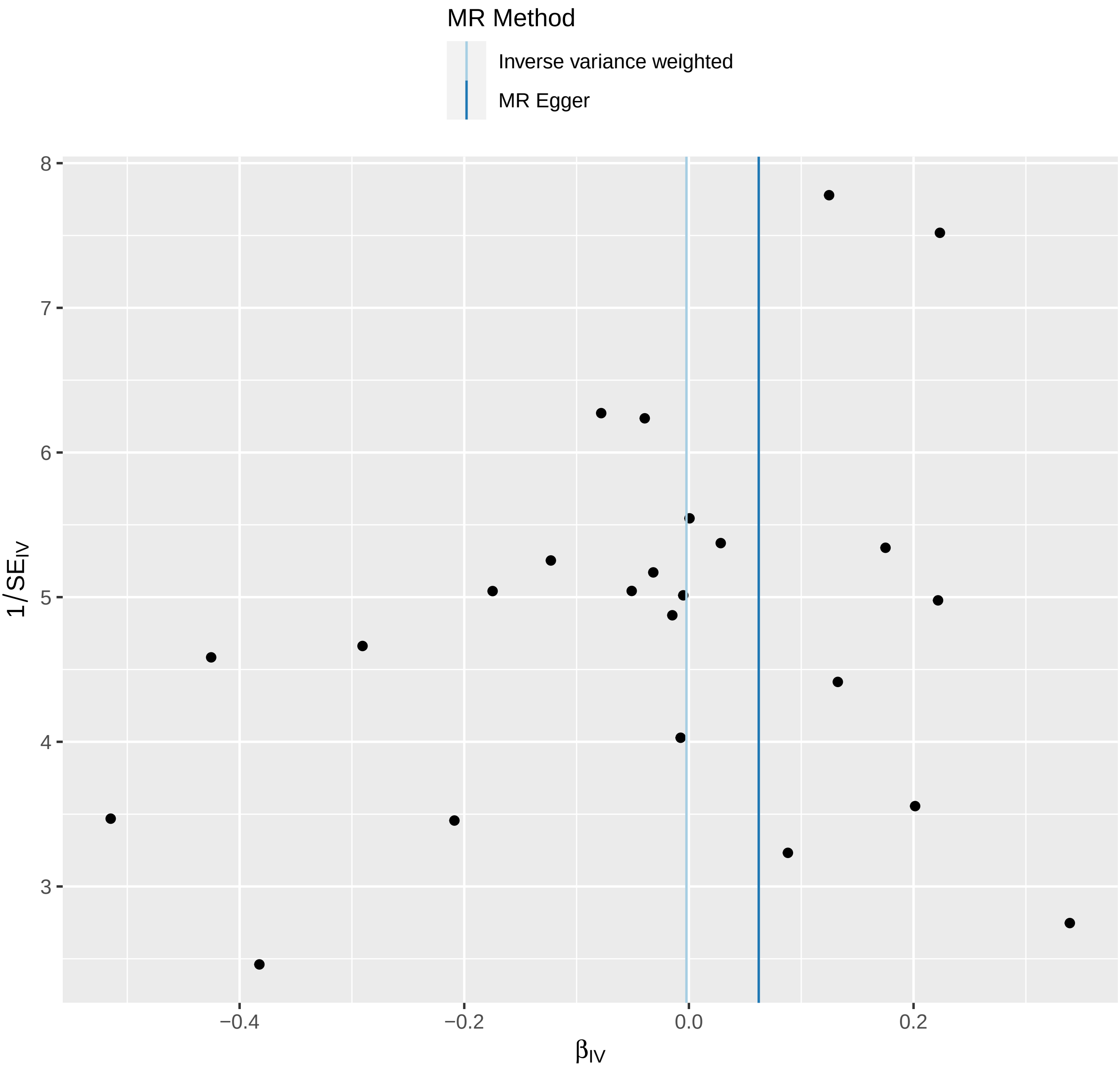

Batch 3−1091 : Fibroblast growth factor 19 on Diabetic nephropathy (more control exclusions)
with a threshold of 1e−05

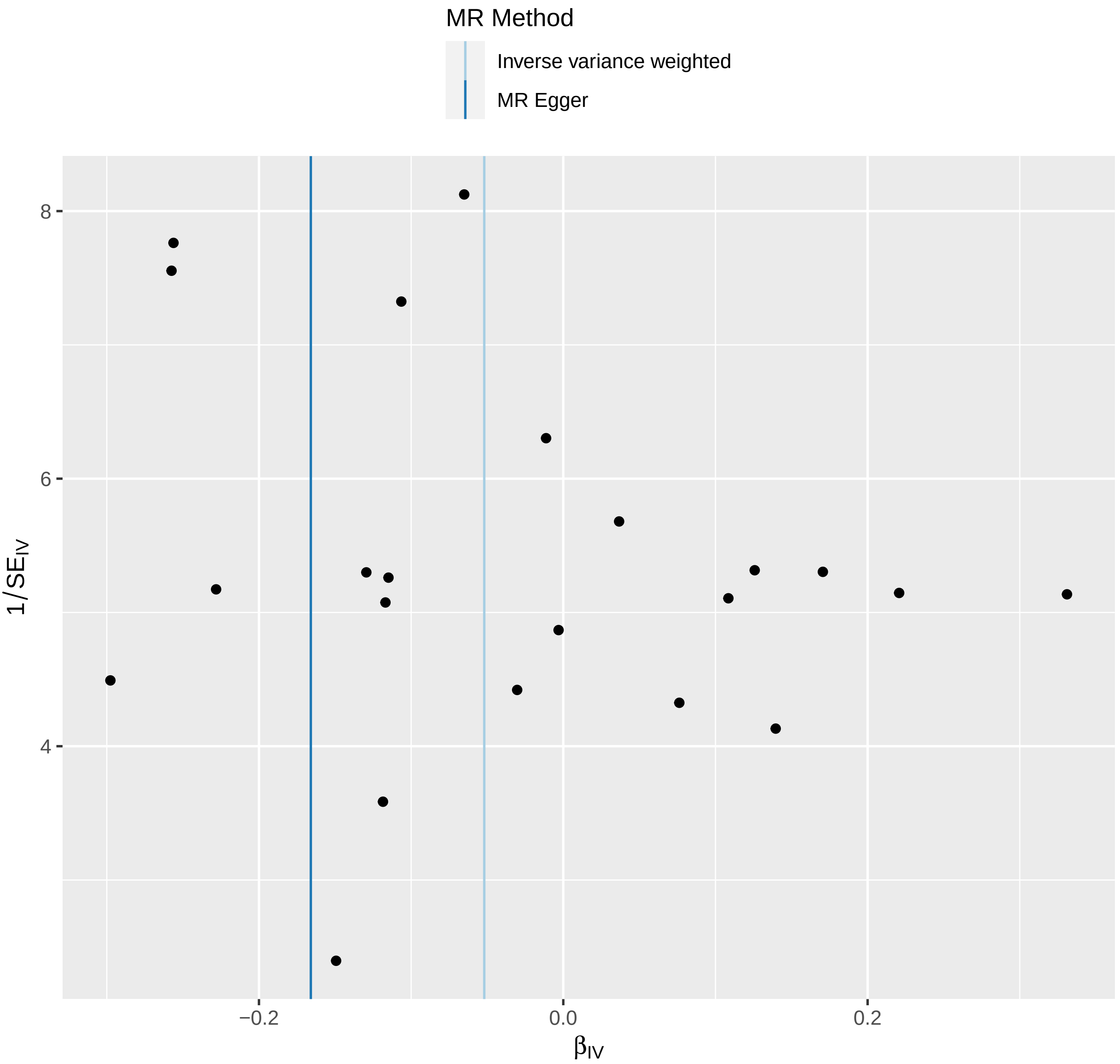

Batch 3−1092 : Fibroblast growth factor 20 on Diabetic nephropathy (more control exclusions)
with a threshold of 1e−05

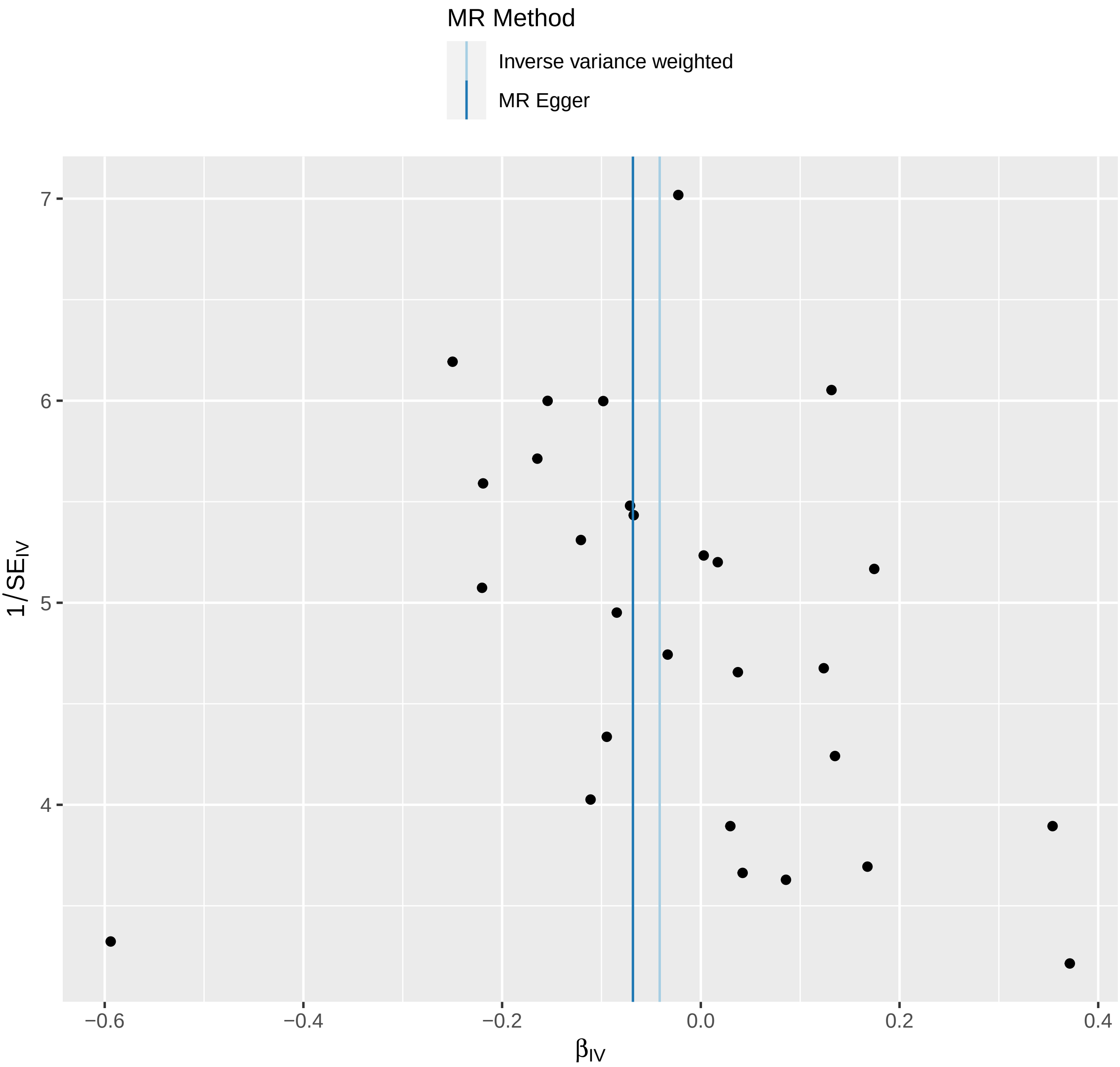

Batch 3−1093 : Fibroblast growth factor 23 on Diabetic nephropathy (more control exclusions)
with a threshold of 1e−05

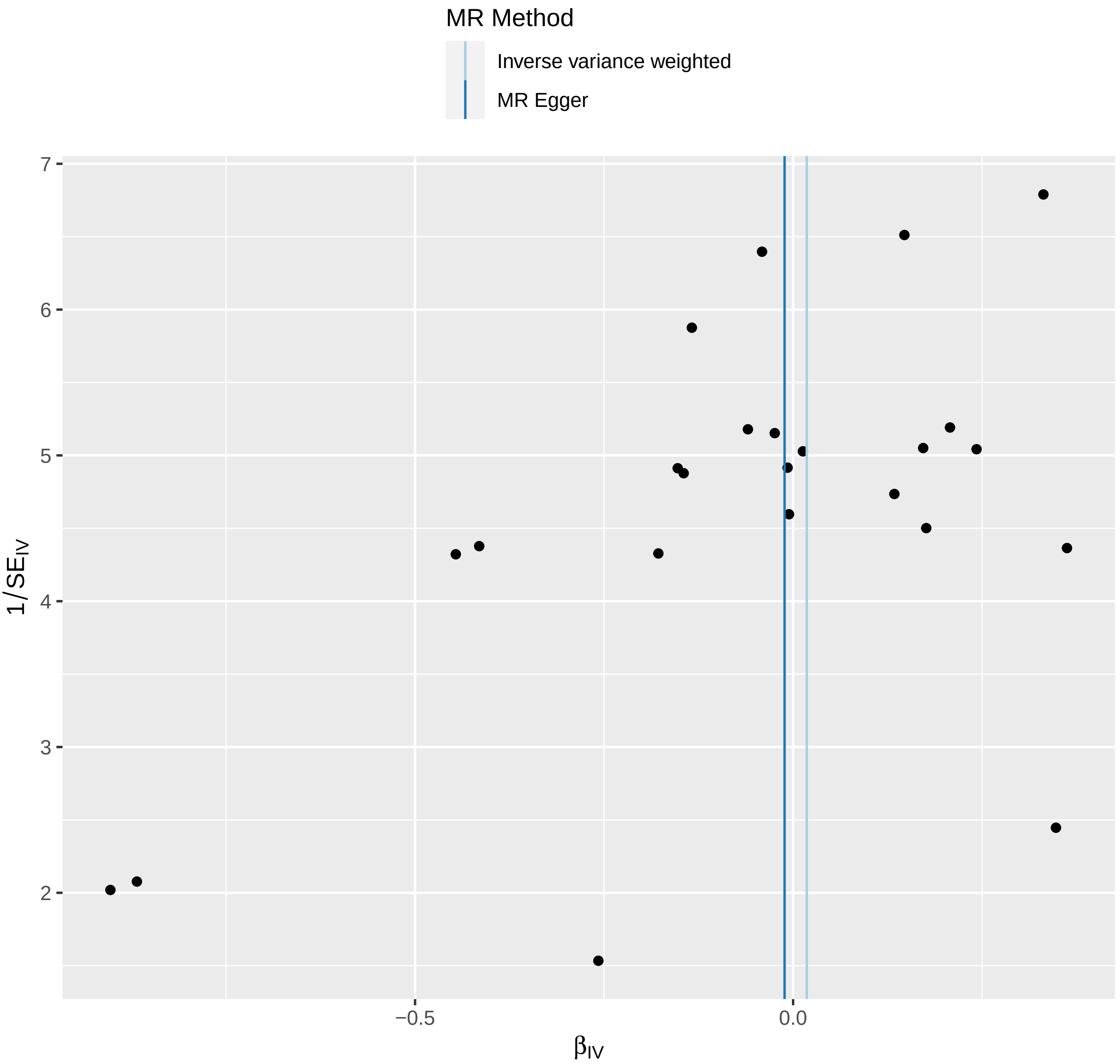

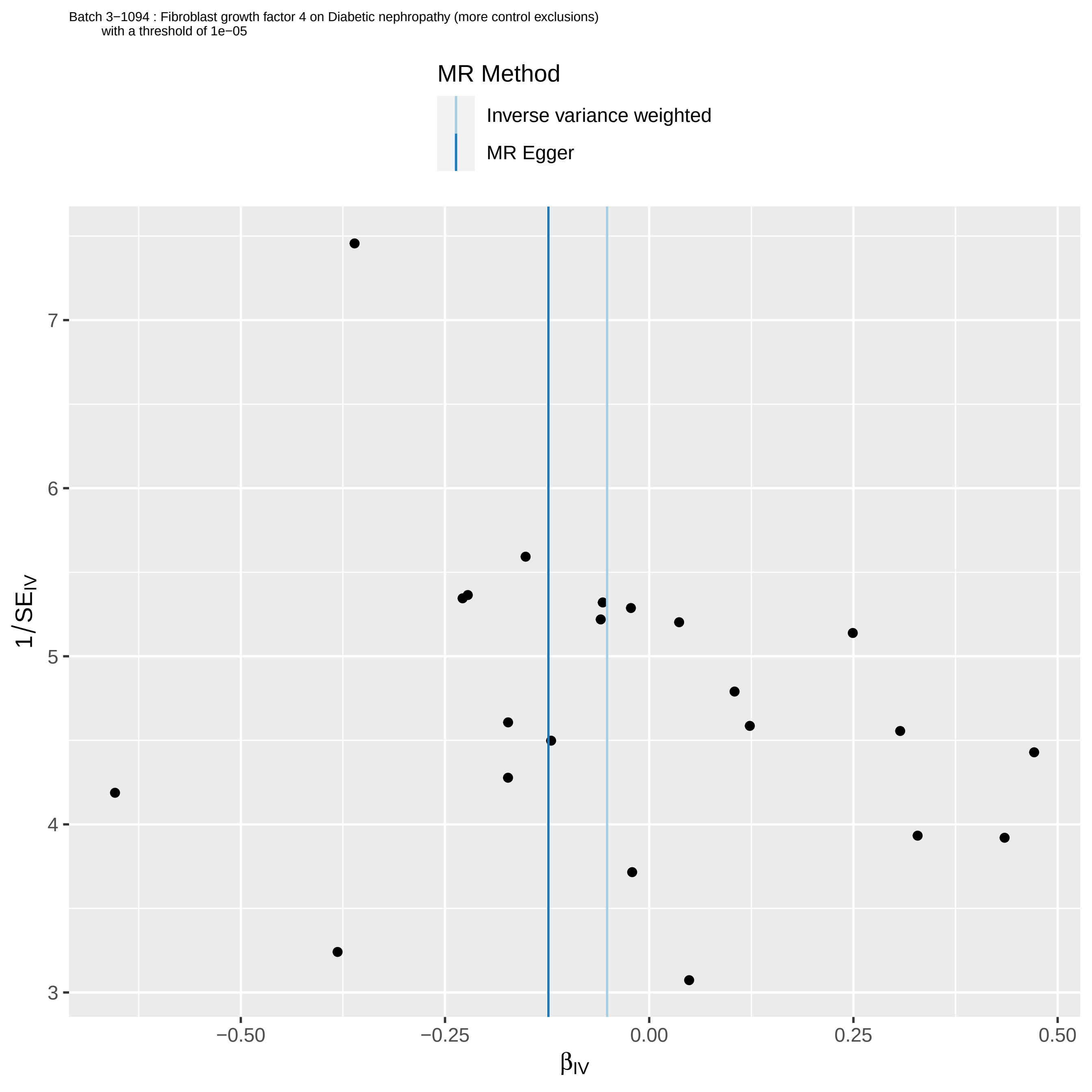

Batch 3−1094 : Fibroblast growth factor 4 on Diabetic nephropathy (more control exclusions)
with a threshold of 1e−05
MR Method
Inverse variance weighted
MR Egger
1/SE$_{IV}$
7
6
5
4
3
−0.50
−0.25
0.00
0.25
0.50
β_{IV}

Batch 3−1095 : Fibroblast growth factor 5 on Diabetic nephropathy (more control exclusions)
with a threshold of 1e−05

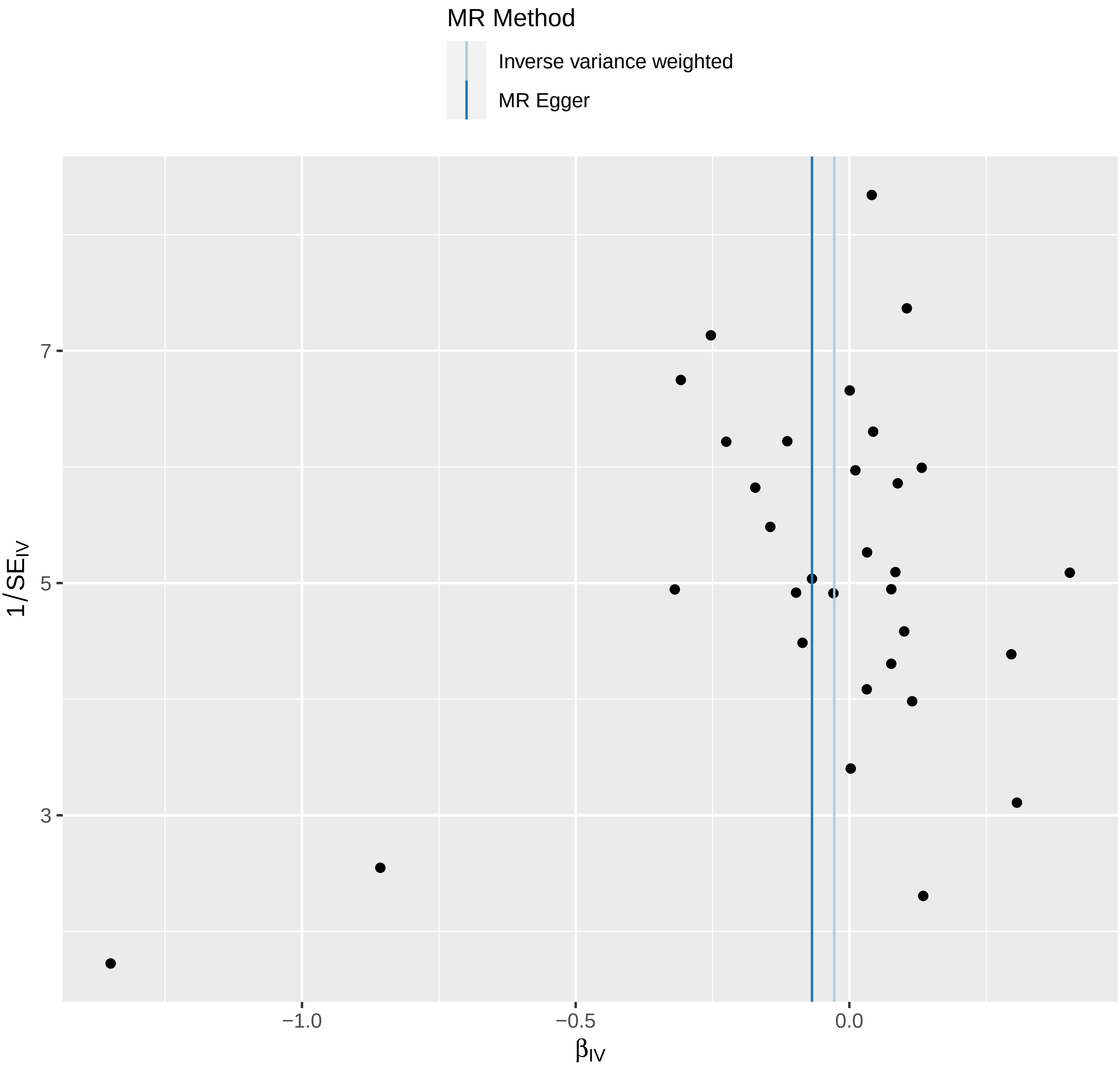

Batch 3−1096 : Fibroblast growth factor 6 on Diabetic nephropathy (more control exclusions)
with a threshold of 1e−05

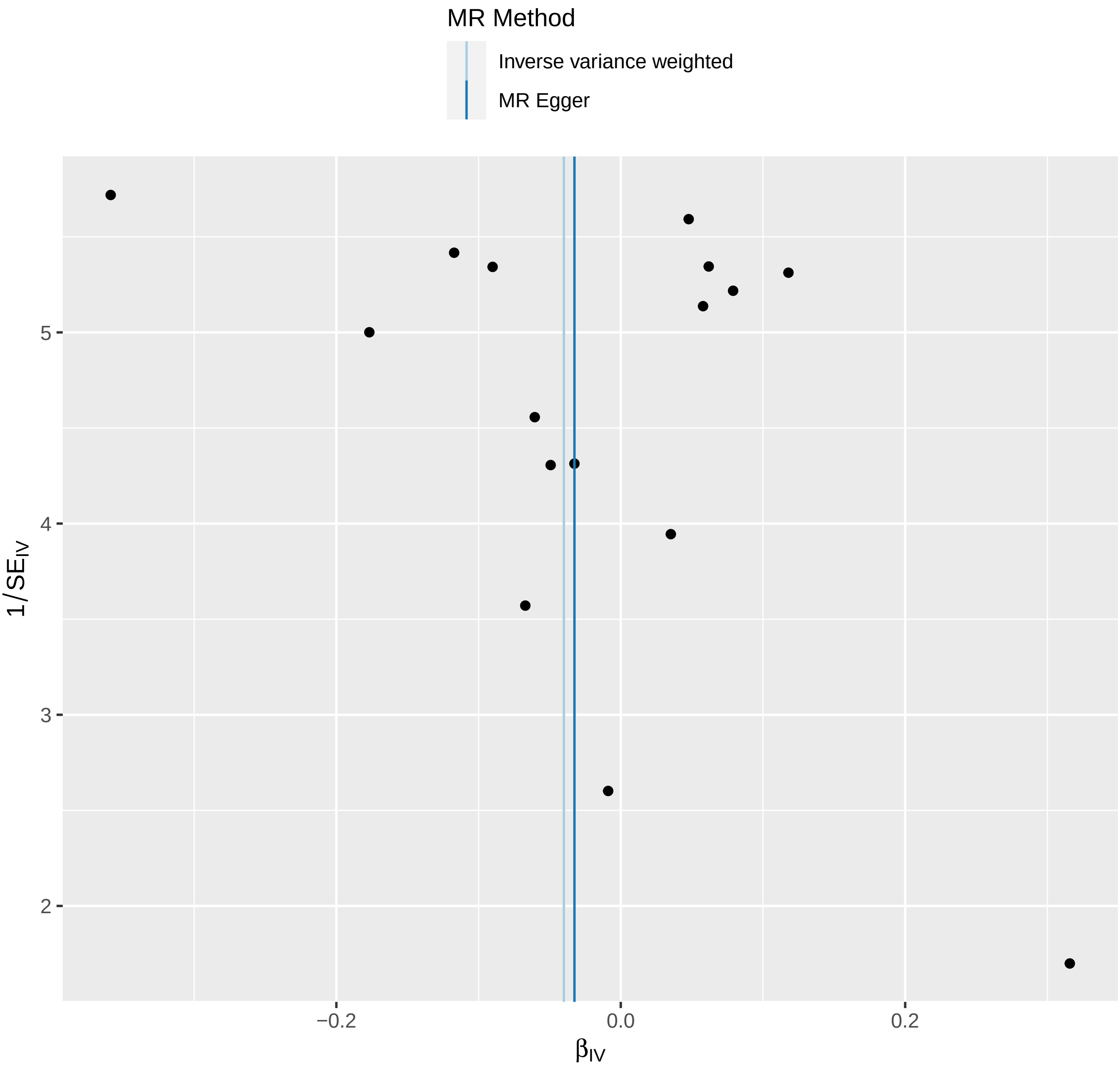

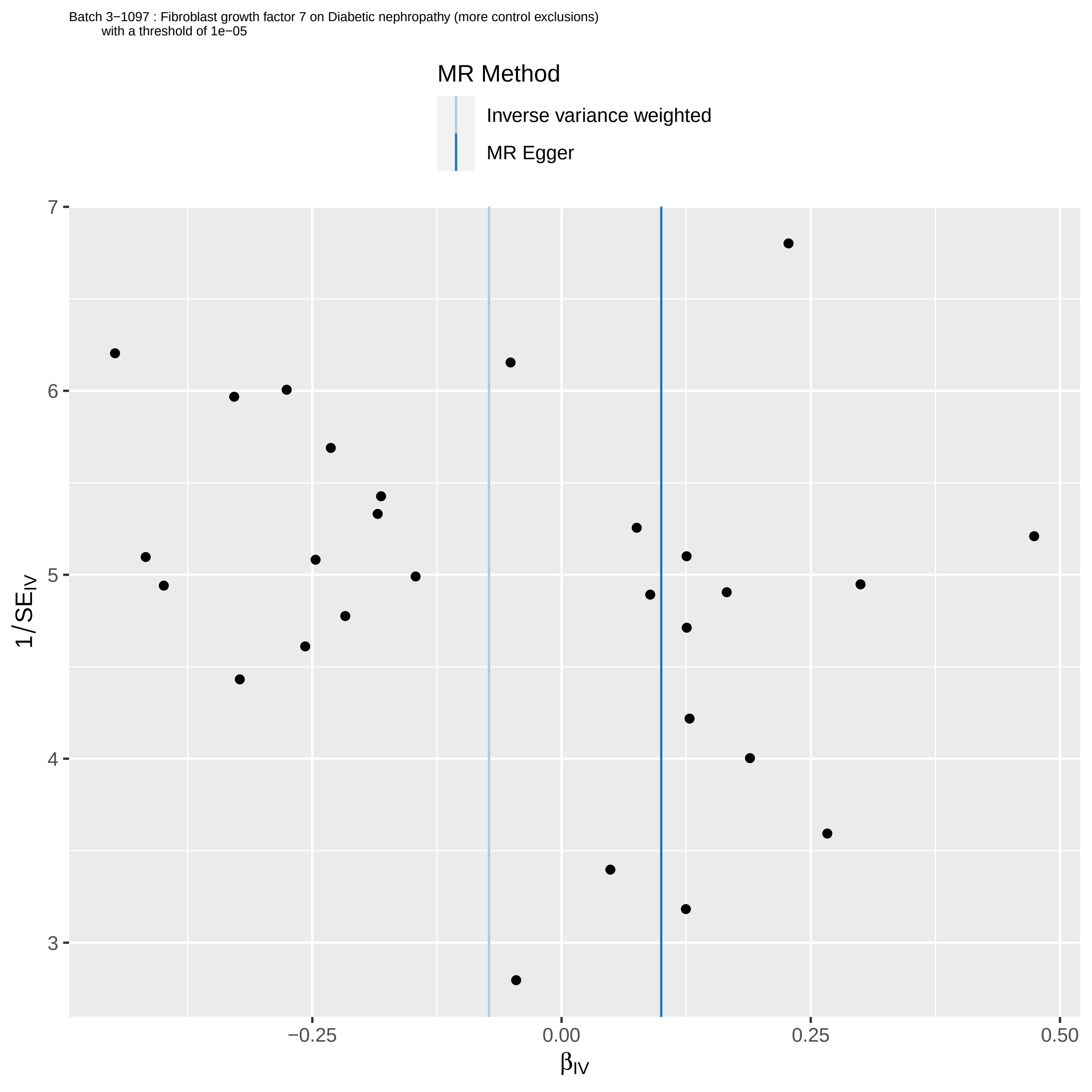

Batch 3−1097 : Fibroblast growth factor 7 on Diabetic nephropathy (more control exclusions)
with a threshold of 1e−05
MR Method
Inverse variance weighted
MR Egger
1/SE_IV
β_IV
7
6
5
4
3
−0.25
0.00
0.25
0.50

Batch 3−1098 : Fibroblast growth factor 7 on Diabetic nephropathy (more control exclusions)
with a threshold of 1e−05

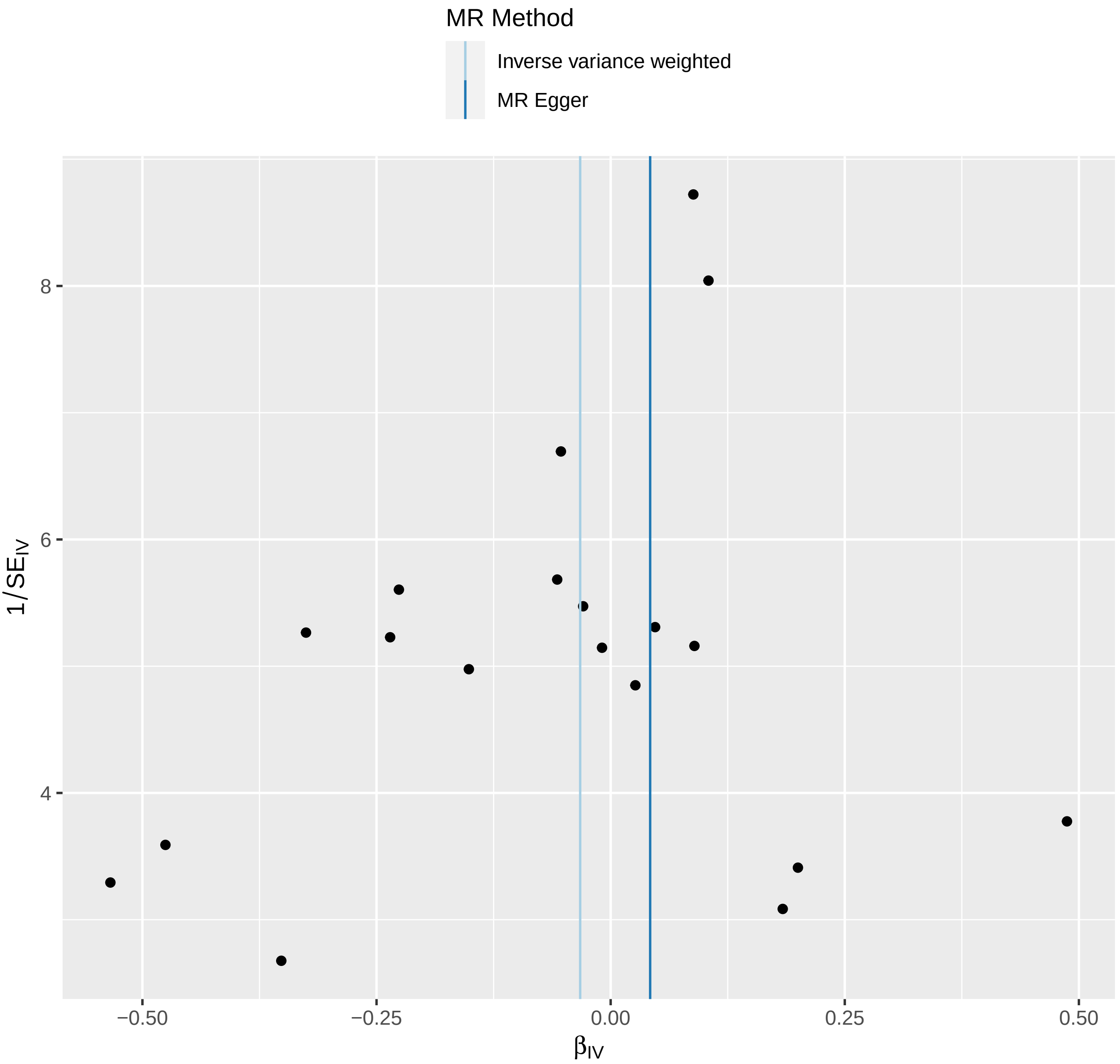

Batch 3−1099 : Fibroblast growth factor 8 isoform B on Diabetic nephropathy (more control exclusions)
with a threshold of 1e−05

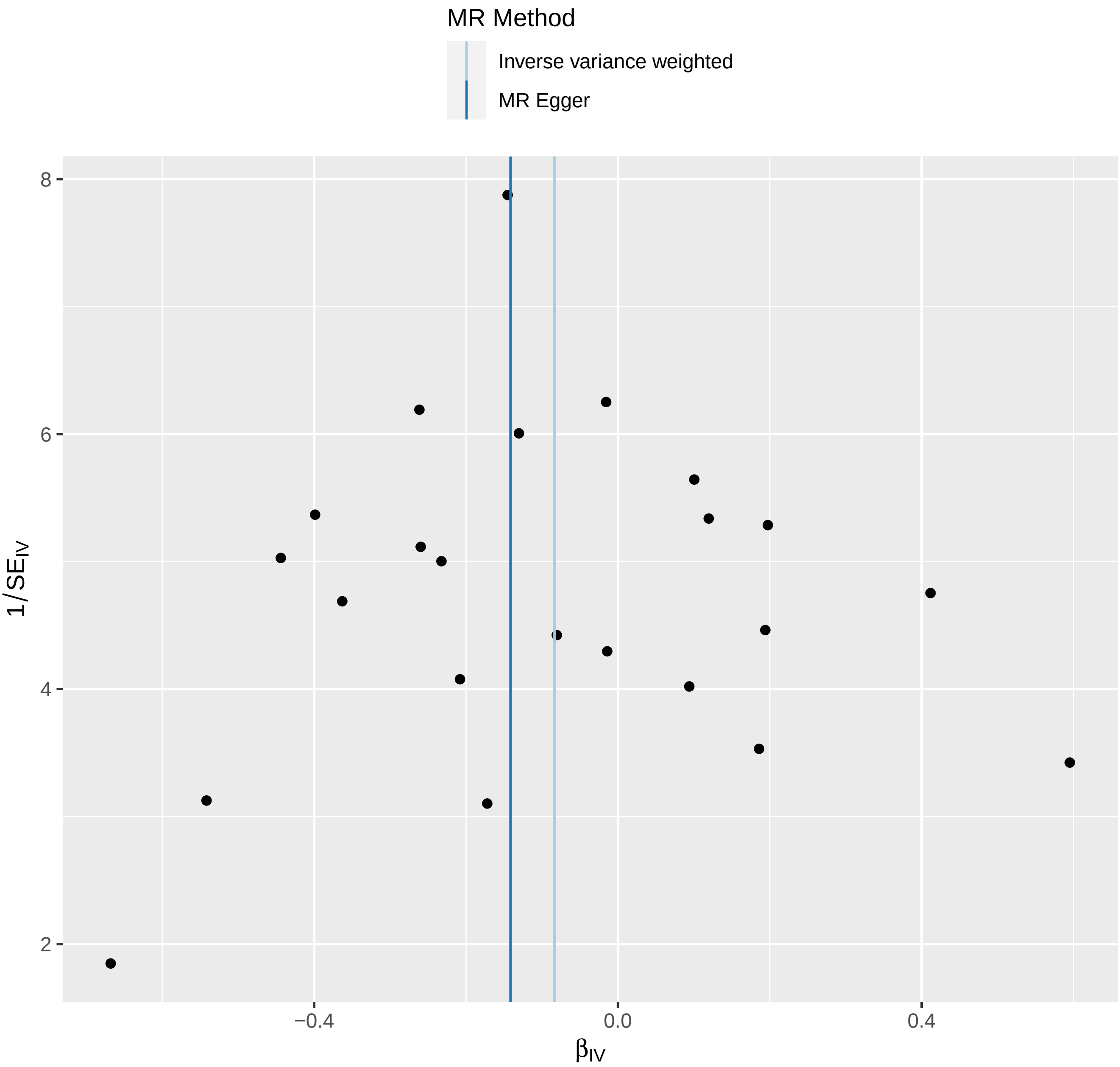

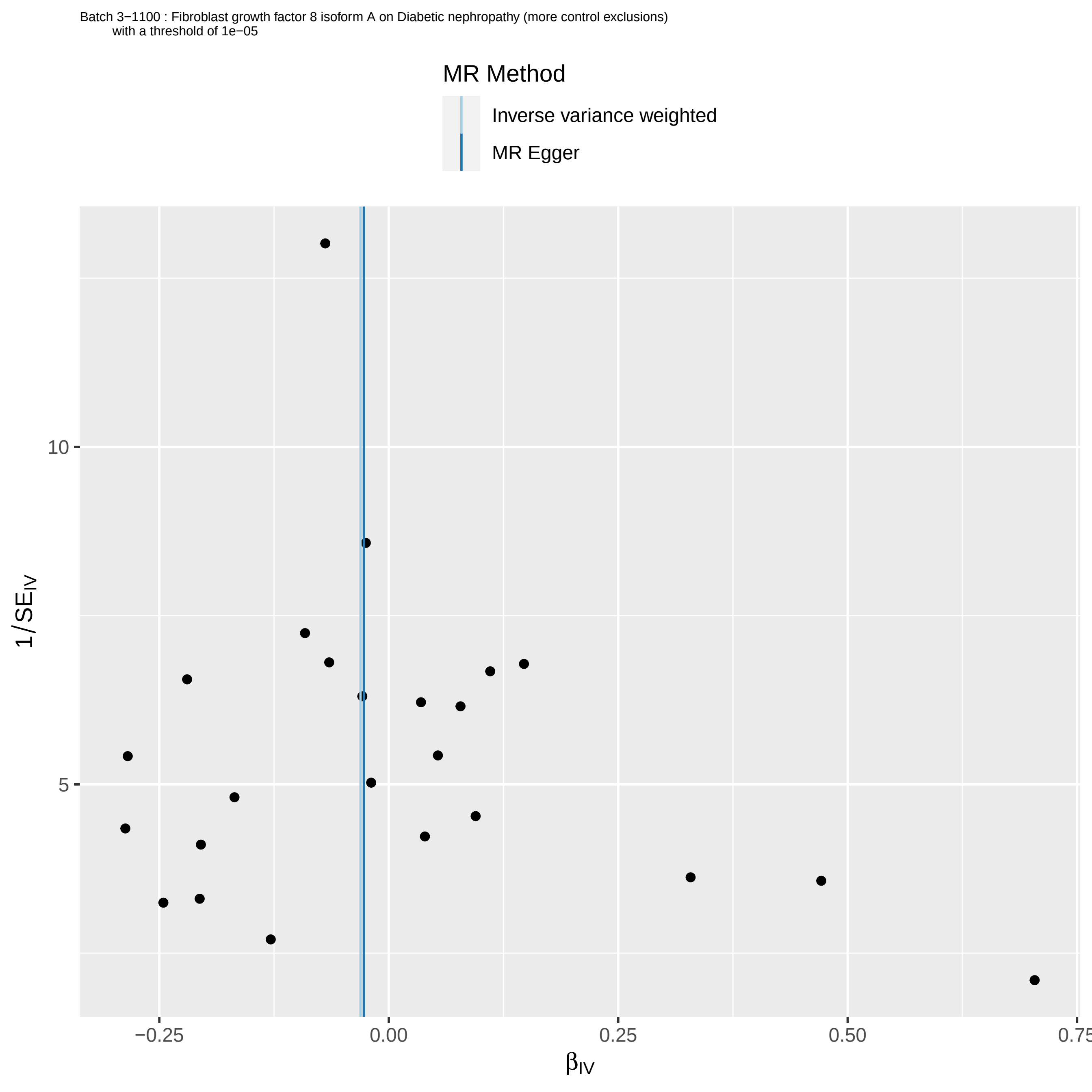

Batch 3−1100 : Fibroblast growth factor 8 isoform A on Diabetic nephropathy (more control exclusions)
with a threshold of 1e−05
MR Method
Inverse variance weighted
MR Egger
10
5
1/SE_IV
−0.25
0.00
0.25
0.50
0.75
β_IV

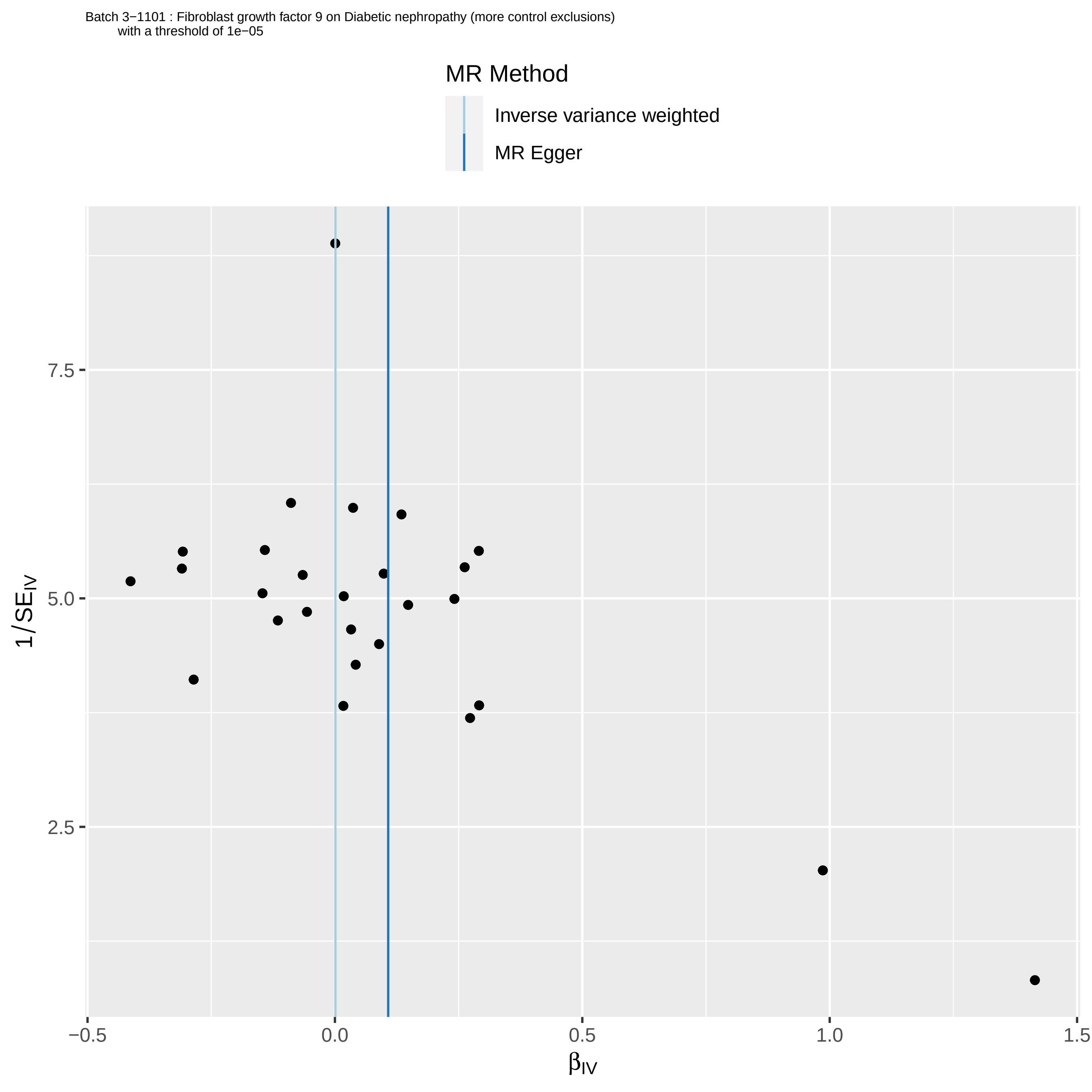

Batch 3−1101 : Fibroblast growth factor 9 on Diabetic nephropathy (more control exclusions)
with a threshold of 1e−05
MR Method
Inverse variance weighted
MR Egger
7.5
5.0
2.5
1/SE_IV
−0.5
0.0
0.5
1.0
1.5
β_IV

Batch 3−1102 : Fibroblast growth factor receptor 2 on Diabetic nephropathy (more control exclusions)
with a threshold of 1e−05

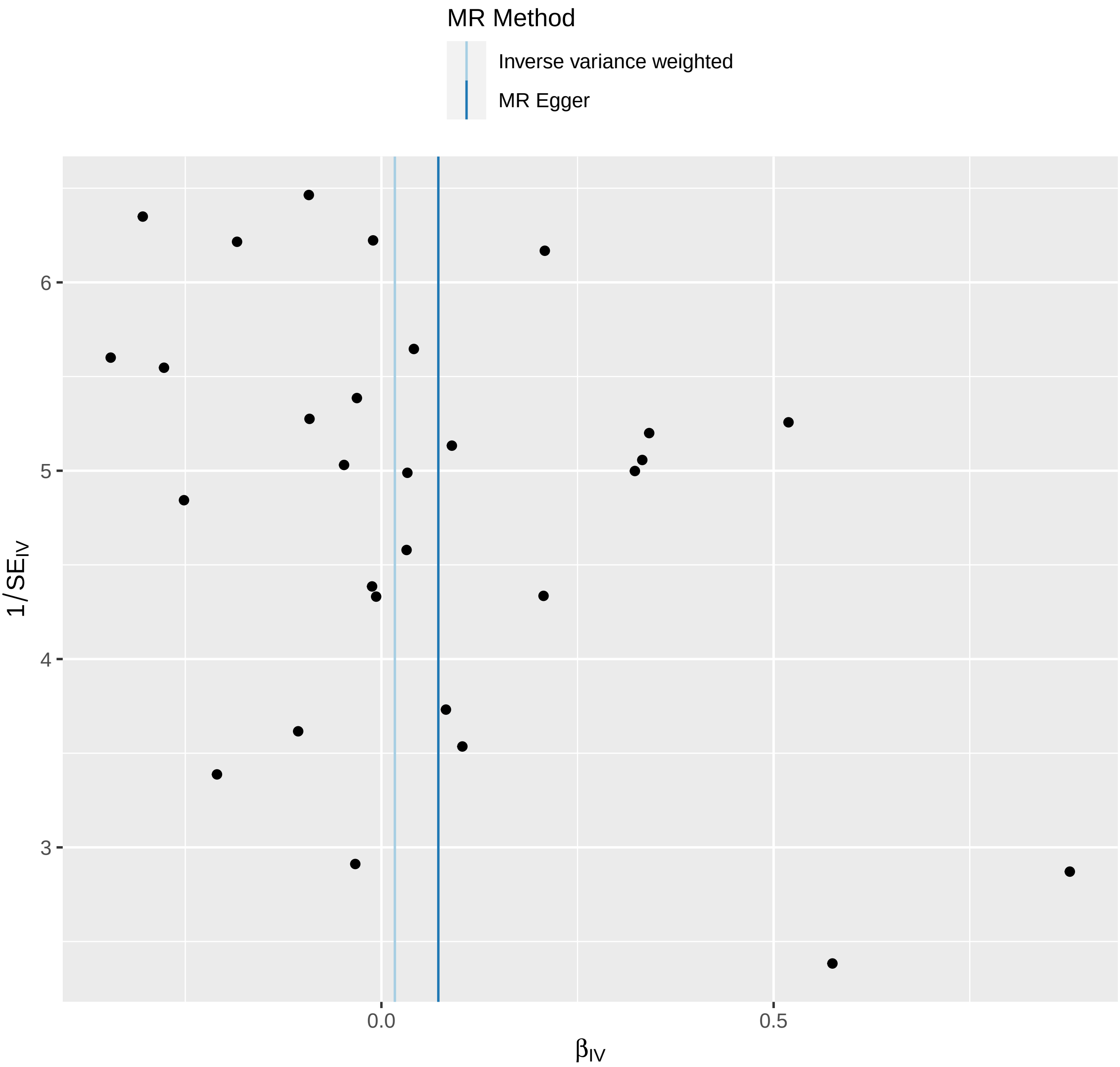

Batch 3−1103 : Fibroblast growth factor receptor 3 on Diabetic nephropathy (more control exclusions)
with a threshold of 1e−05

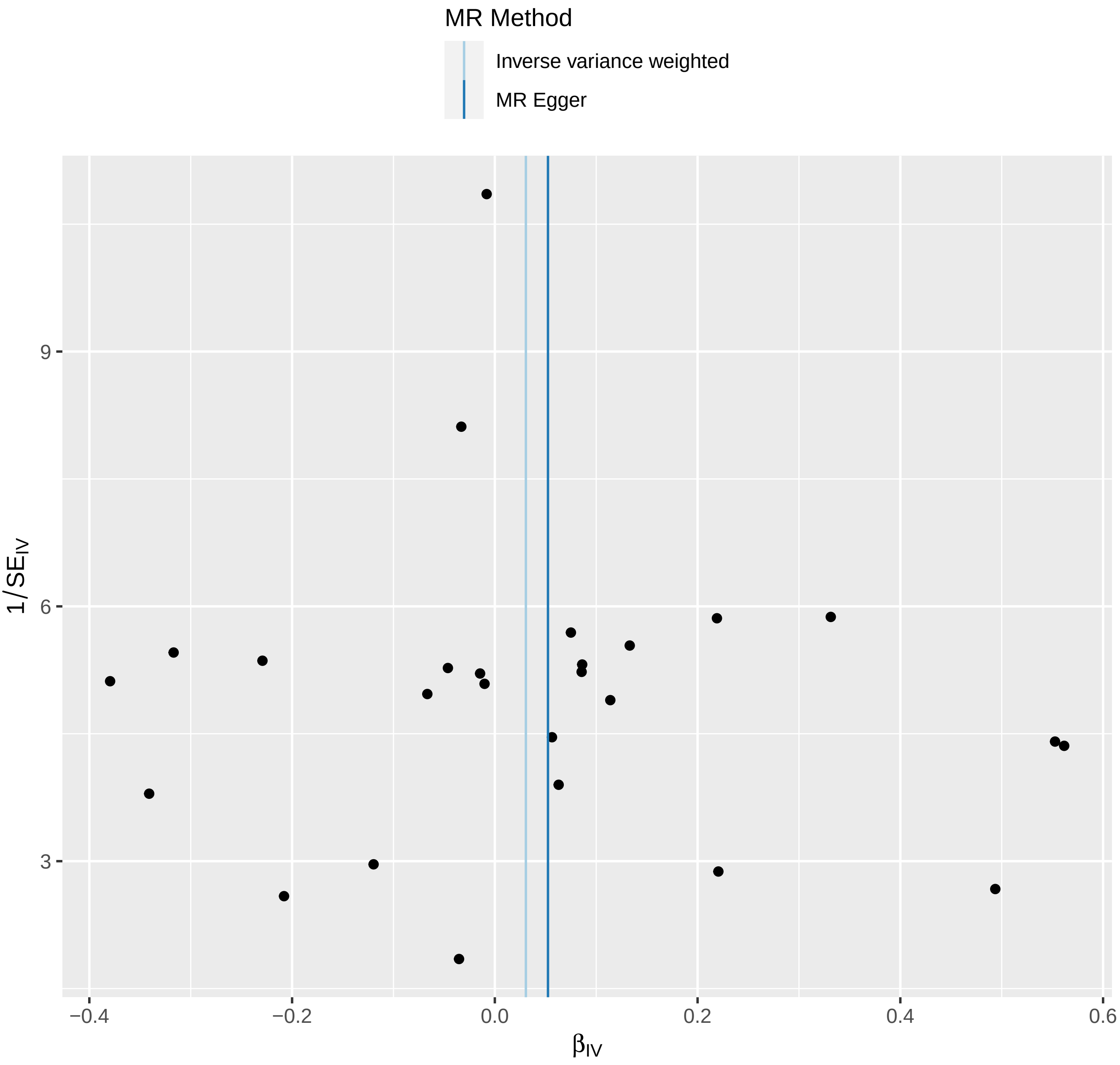

Batch 3−1104 : Fibroblast growth factor receptor 3 on Diabetic nephropathy (more control exclusions)
with a threshold of 1e−05

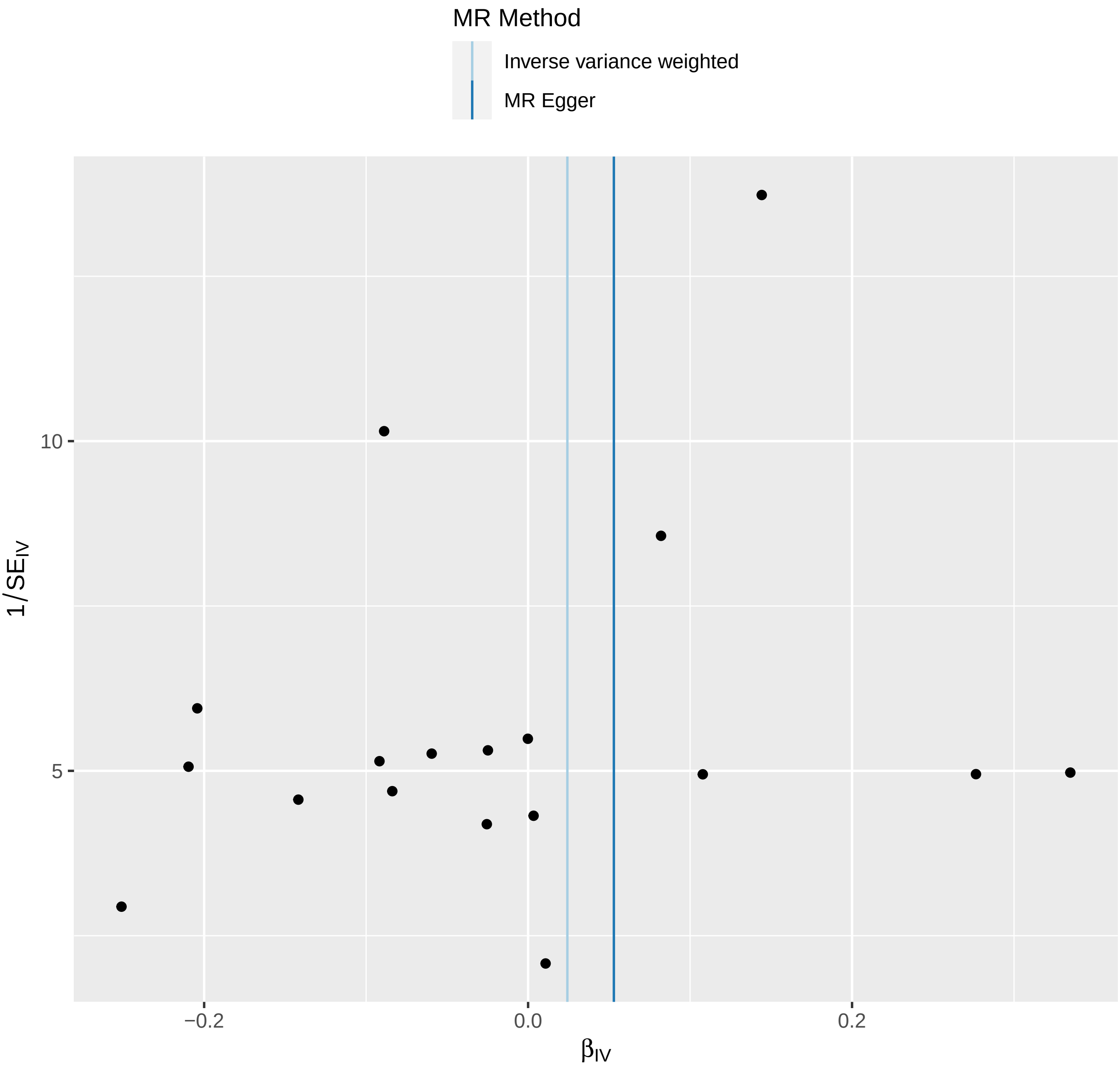

Batch 3−1105 : Fibroblast growth factor receptor 4 on Diabetic nephropathy (more control exclusions)
with a threshold of 1e−05

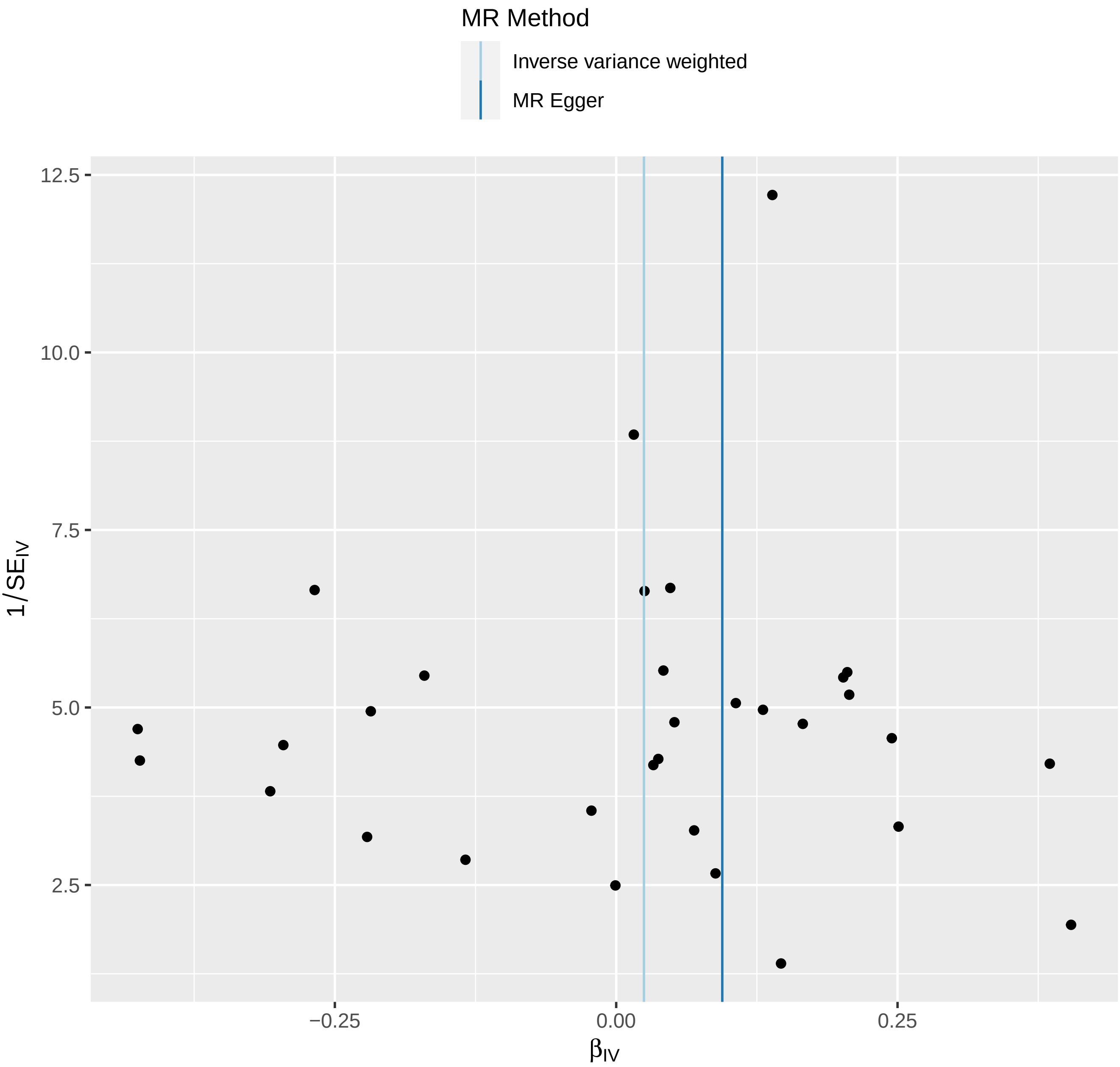

Batch 3−1106 : Tyrosine−protein kinase Fgr on Diabetic nephropathy (more control exclusions)
with a threshold of 1e−05

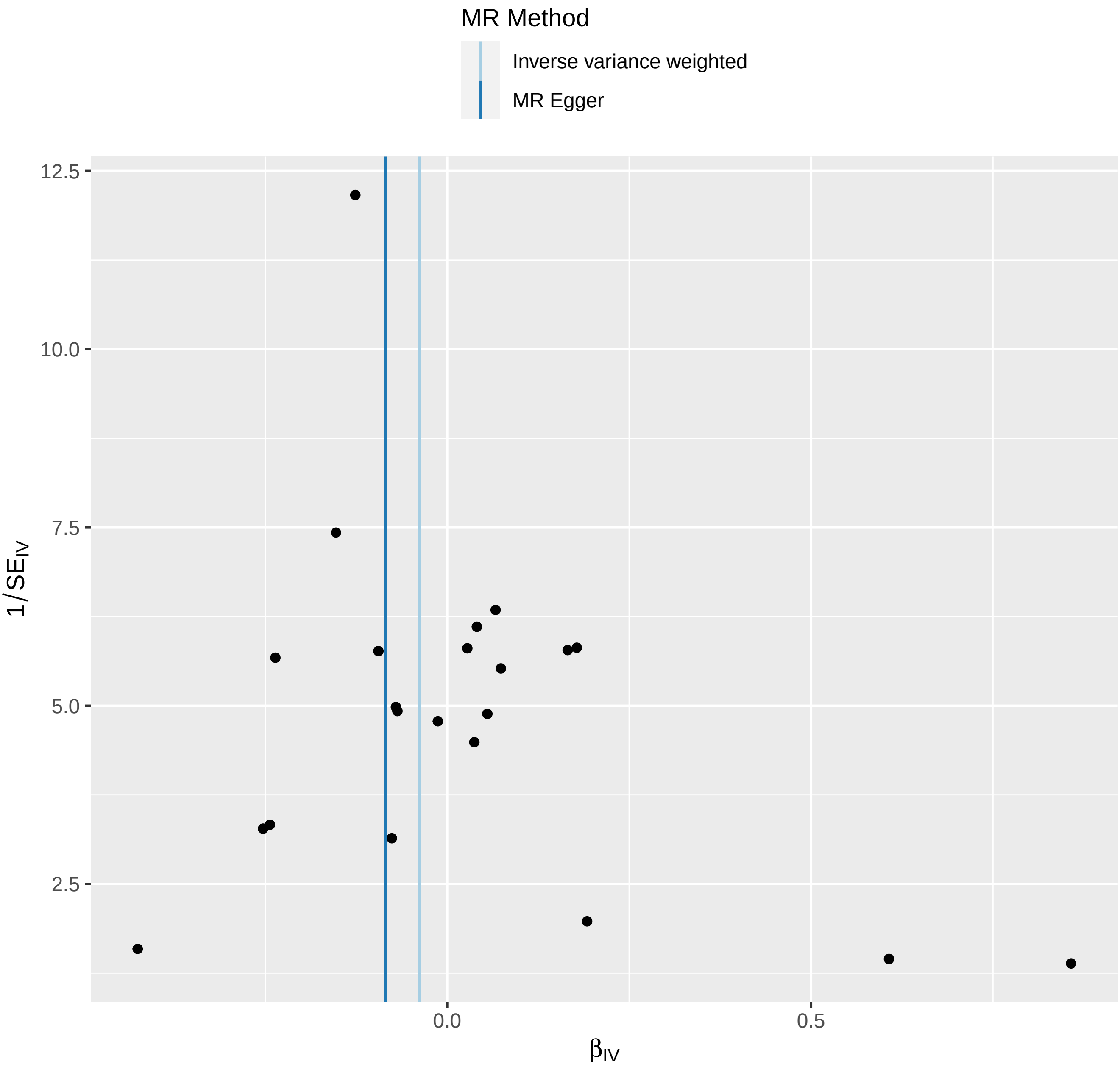

Batch 3−1107 : Bis(5'−adenosyl)−triphosphatase on Diabetic nephropathy (more control exclusions)
with a threshold of 1e−05

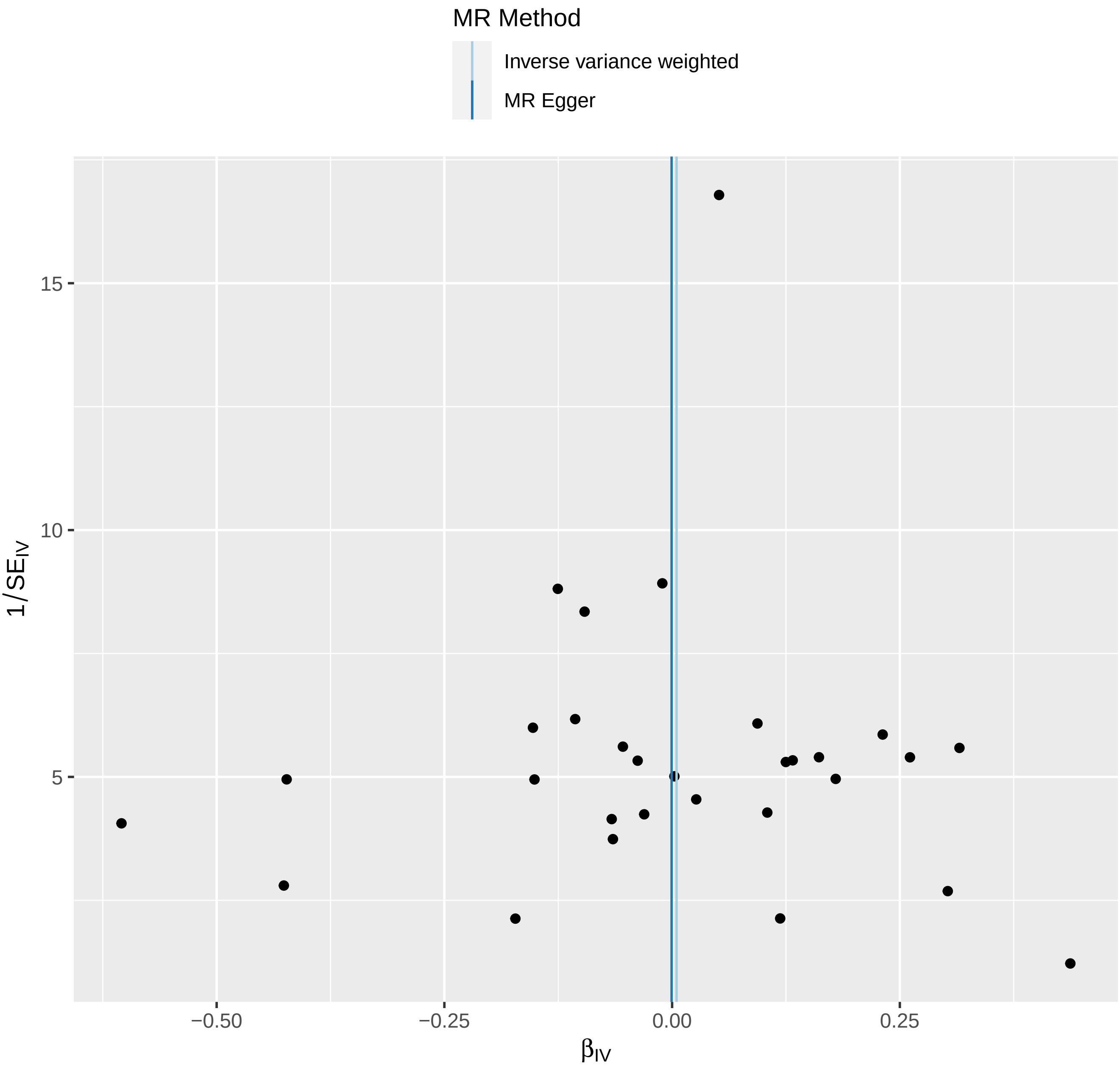

Batch 3−1108 : Fibrinogen C domain−containing protein 1 on Diabetic nephropathy (more control exclusions)
with a threshold of 1e−05

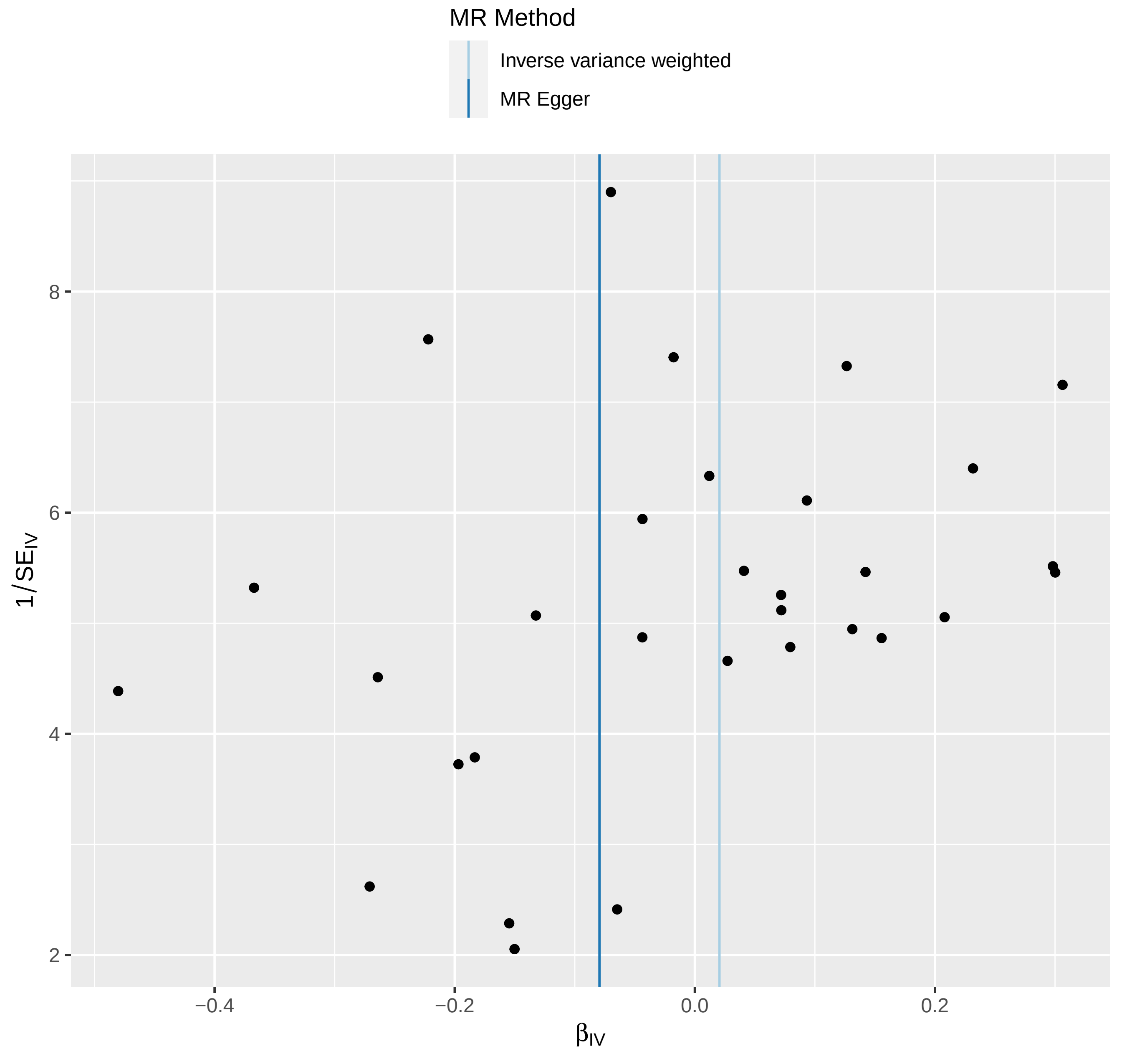

Batch 3−1109 : Polyphosphoinositide phosphatase on Diabetic nephropathy (more control exclusions)
with a threshold of 1e−05

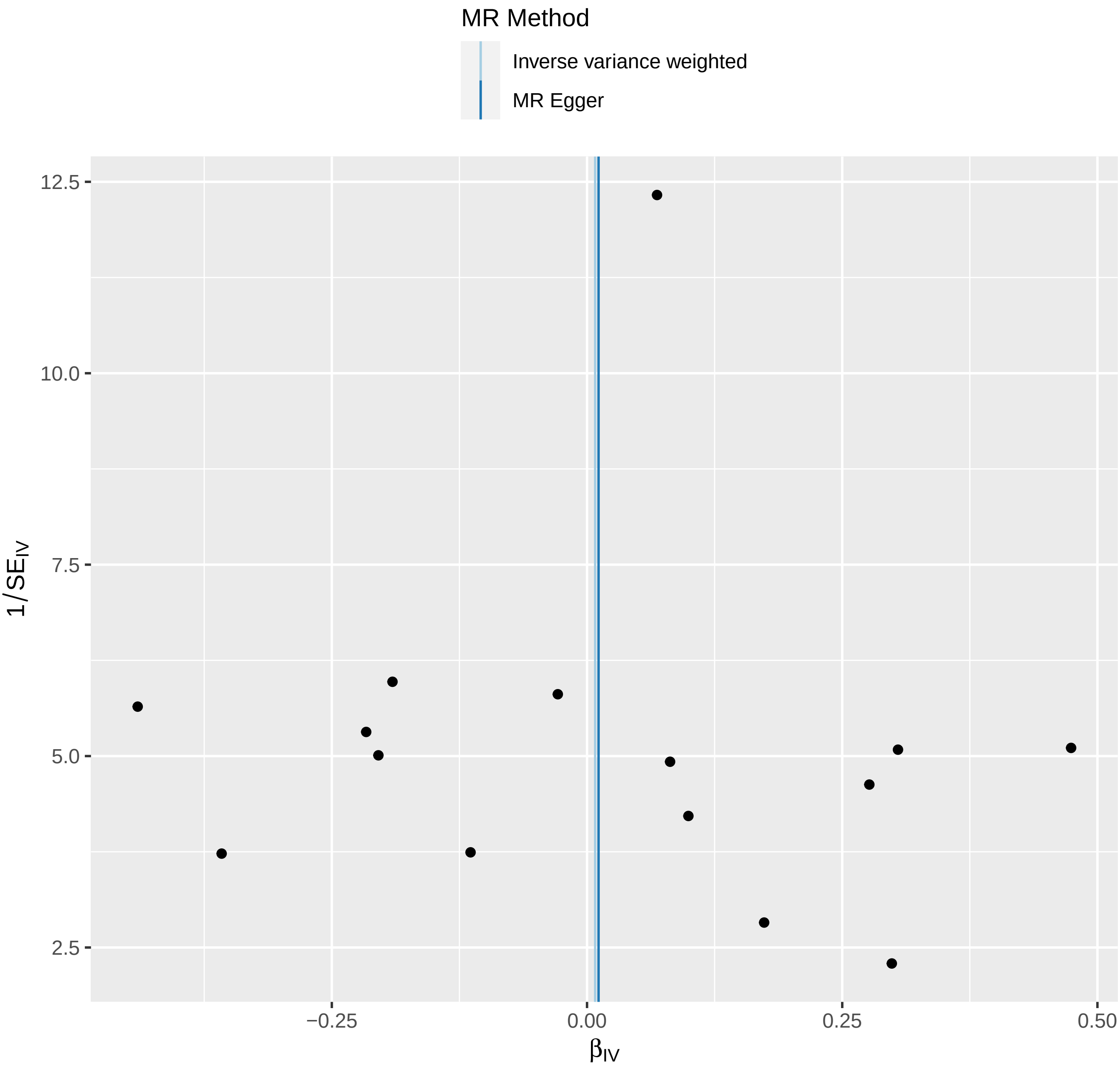

Batch 3−1110 : Vascular endothelial growth factor D on Diabetic nephropathy (more control exclusions) with a threshold of 1e−05

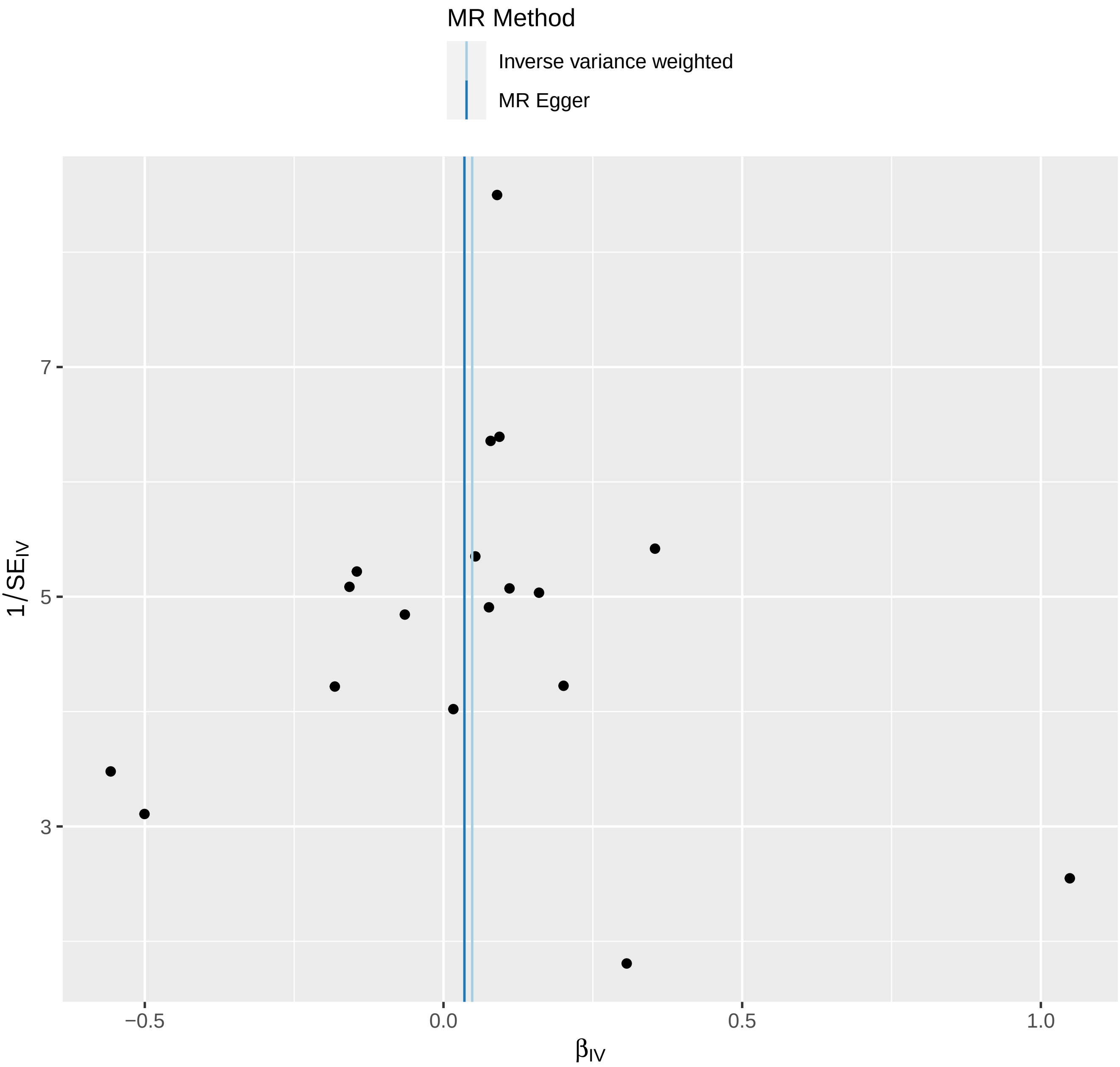

Batch 3−1111 : Vascular endothelial growth factor D on Diabetic nephropathy (more control exclusions)
with a threshold of 1e−05

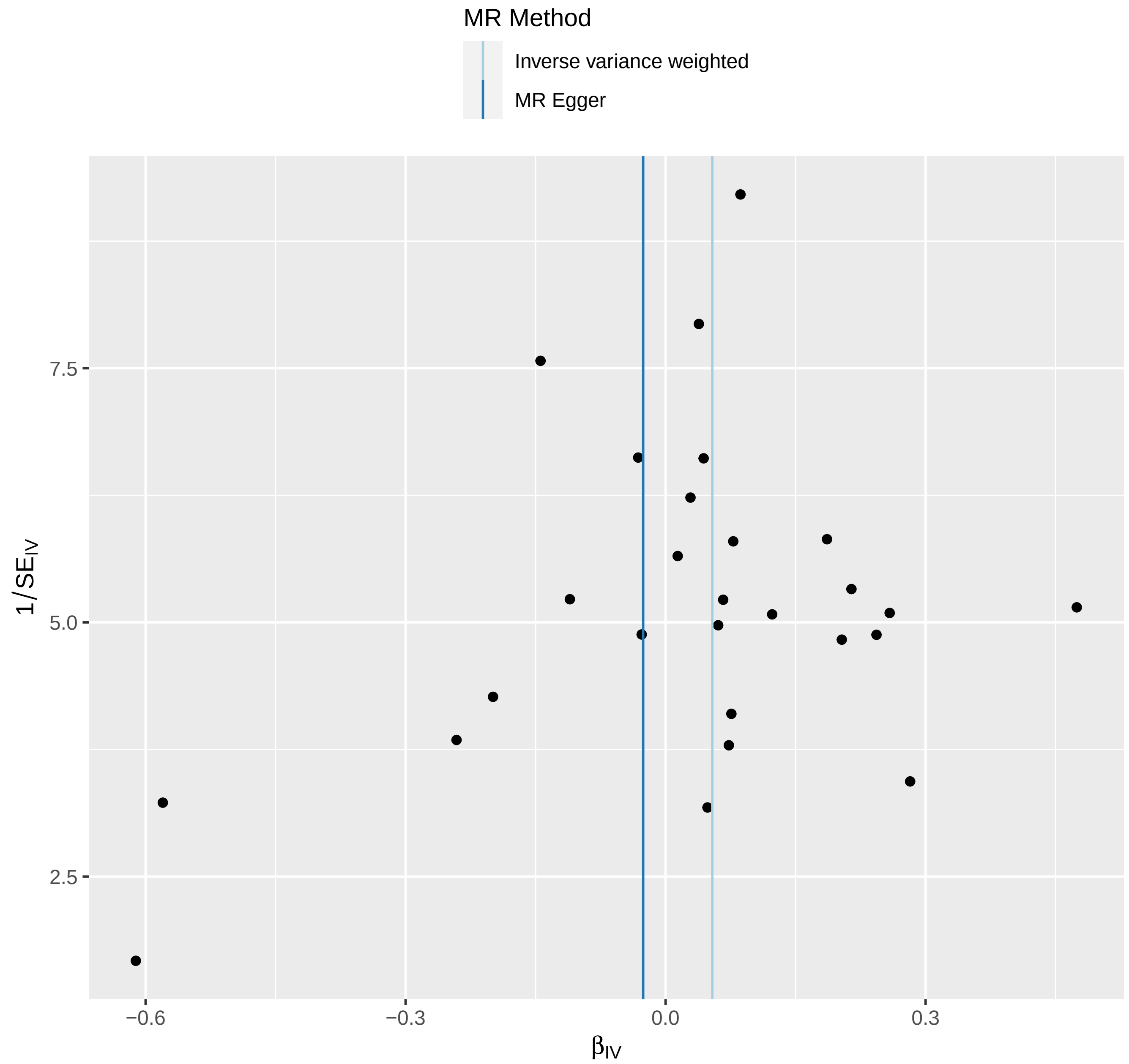

Batch 3−1112 : Four−jointed box protein 1 on Diabetic nephropathy (more control exclusions)
with a threshold of 1e−05

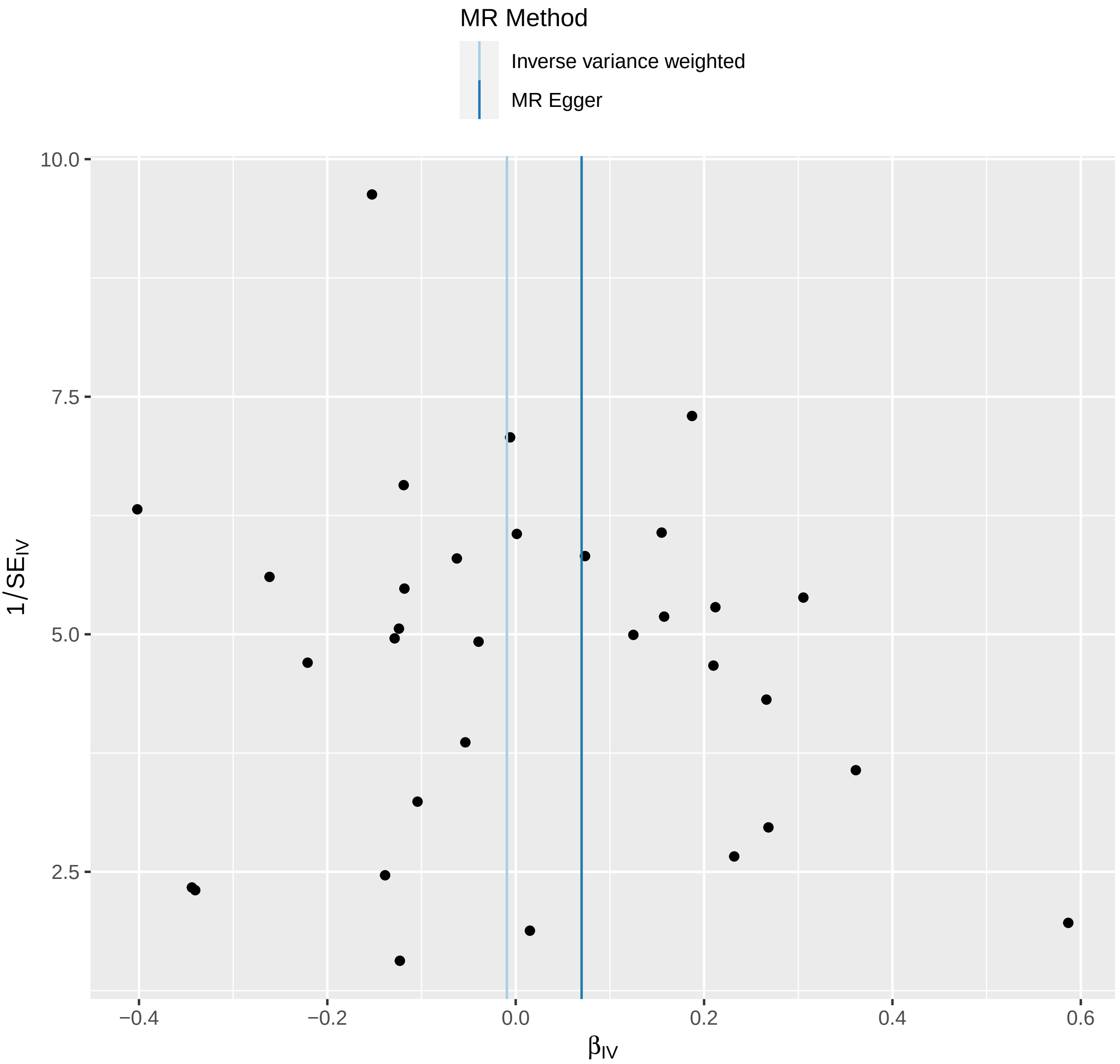

Batch 3−1113 : Peptidyl−prolyl cis−trans isomerase FKBP14 on Diabetic nephropathy (more control exclusions) with a threshold of 1e−05

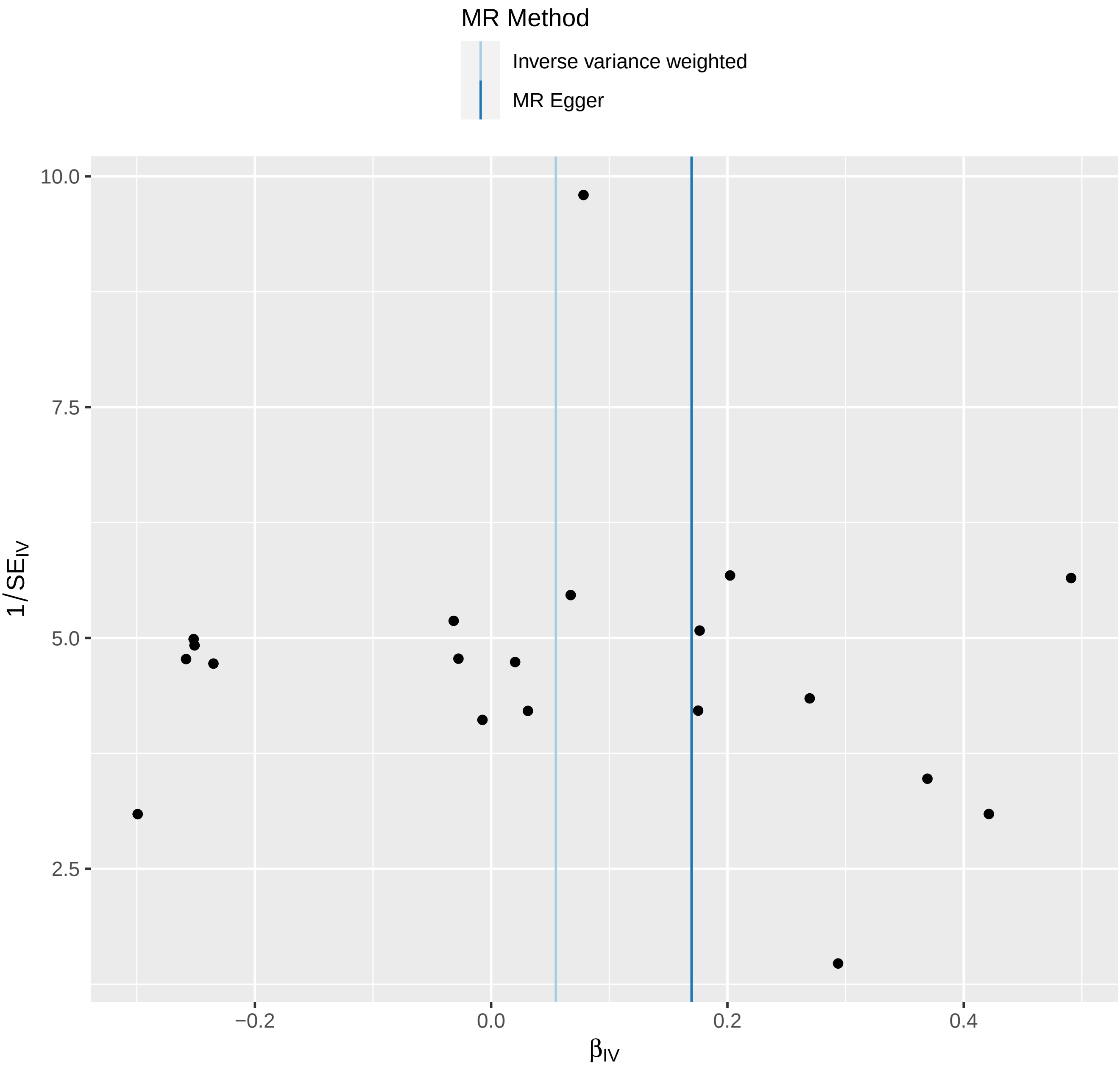

Batch 3−1114 : Peptidyl−prolyl cis−trans isomerase FKBP2 on Diabetic nephropathy (more control exclusions) with a threshold of 1e−05

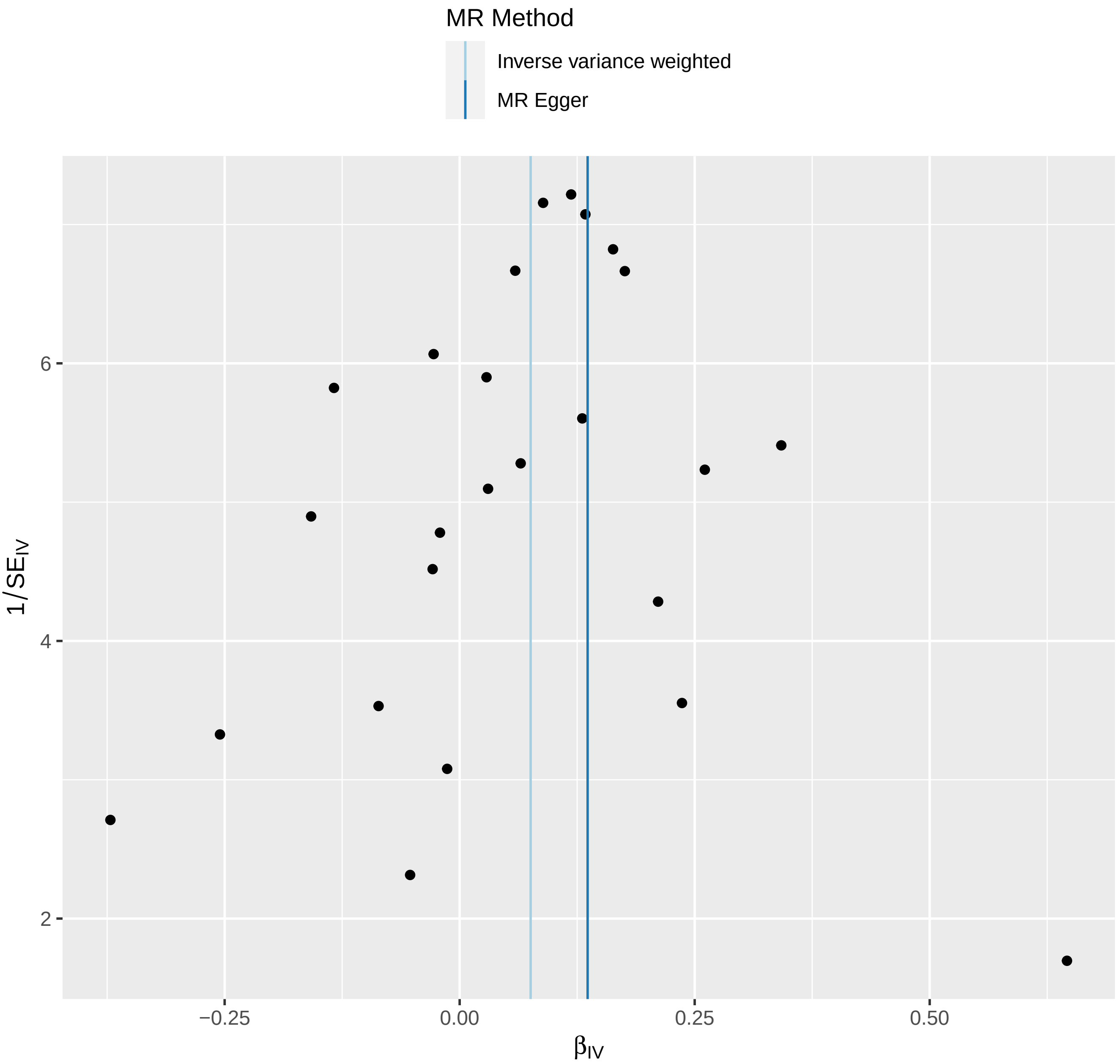

Batch 3−1115 : Inactive peptidyl−prolyl cis−trans isomerase FKBP6 on Diabetic nephropathy (more control exclusions)
with a threshold of 1e−05

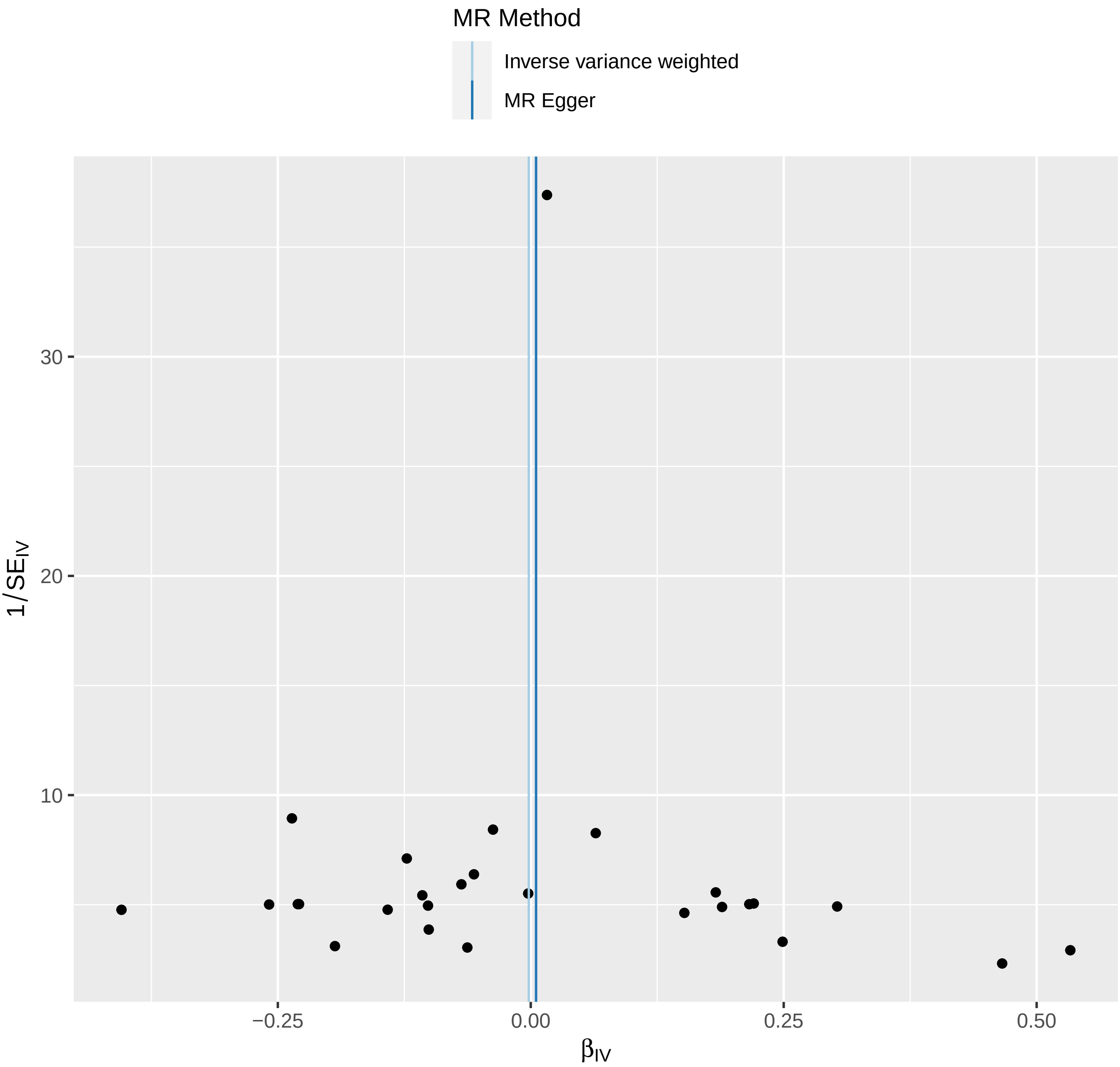

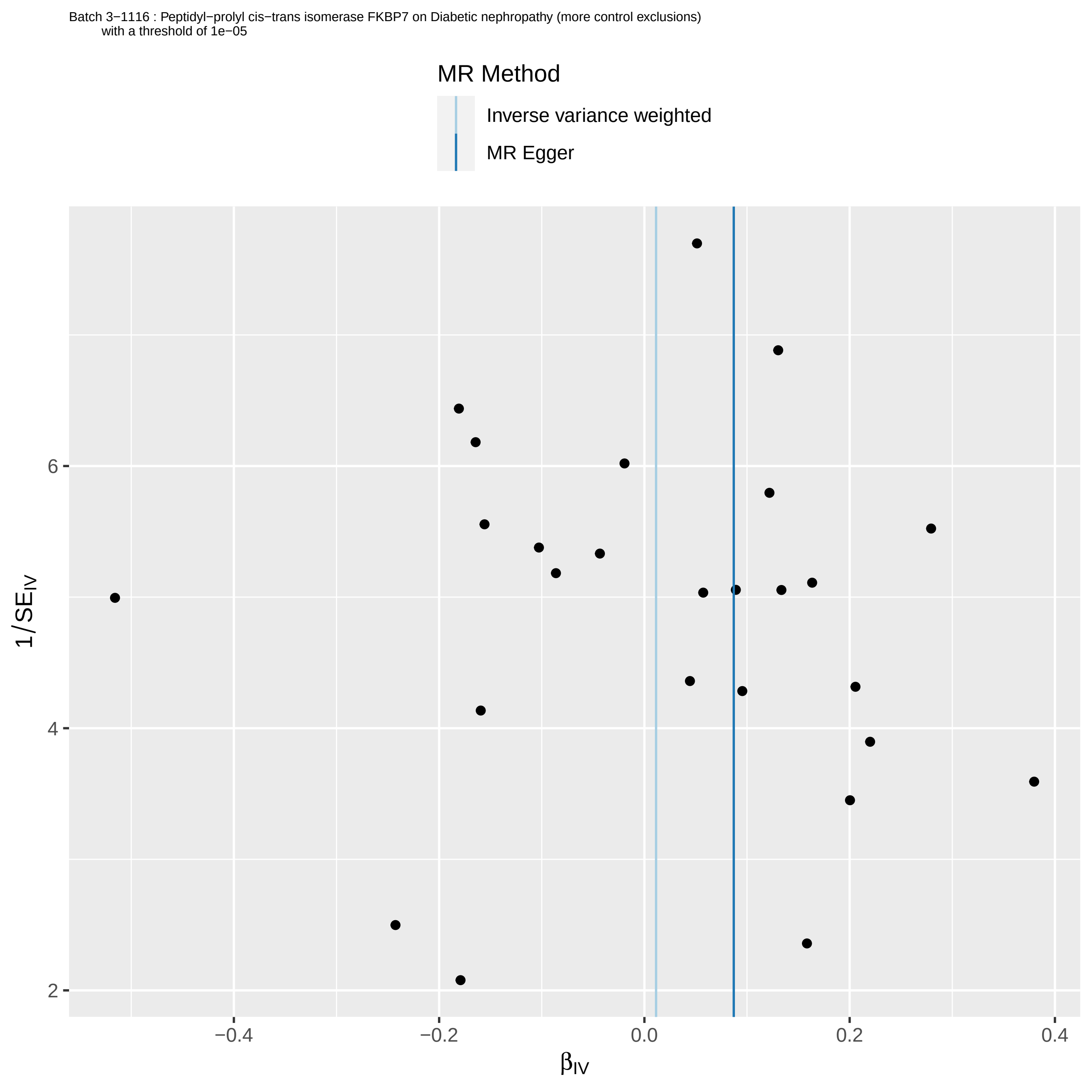
Batch 3−1116 : Peptidyl−prolyl cis−trans isomerase FKBP7 on Diabetic nephropathy (more control exclusions)
with a threshold of 1e−05
MR Method
Inverse variance weighted
MR Egger
1/SE_IV
6
4
2
−0.4
−0.2
0.0
0.2
0.4
β_IV

Batch 3−1117 : Filamin−A on Diabetic nephropathy (more control exclusions)
with a threshold of 1e−05

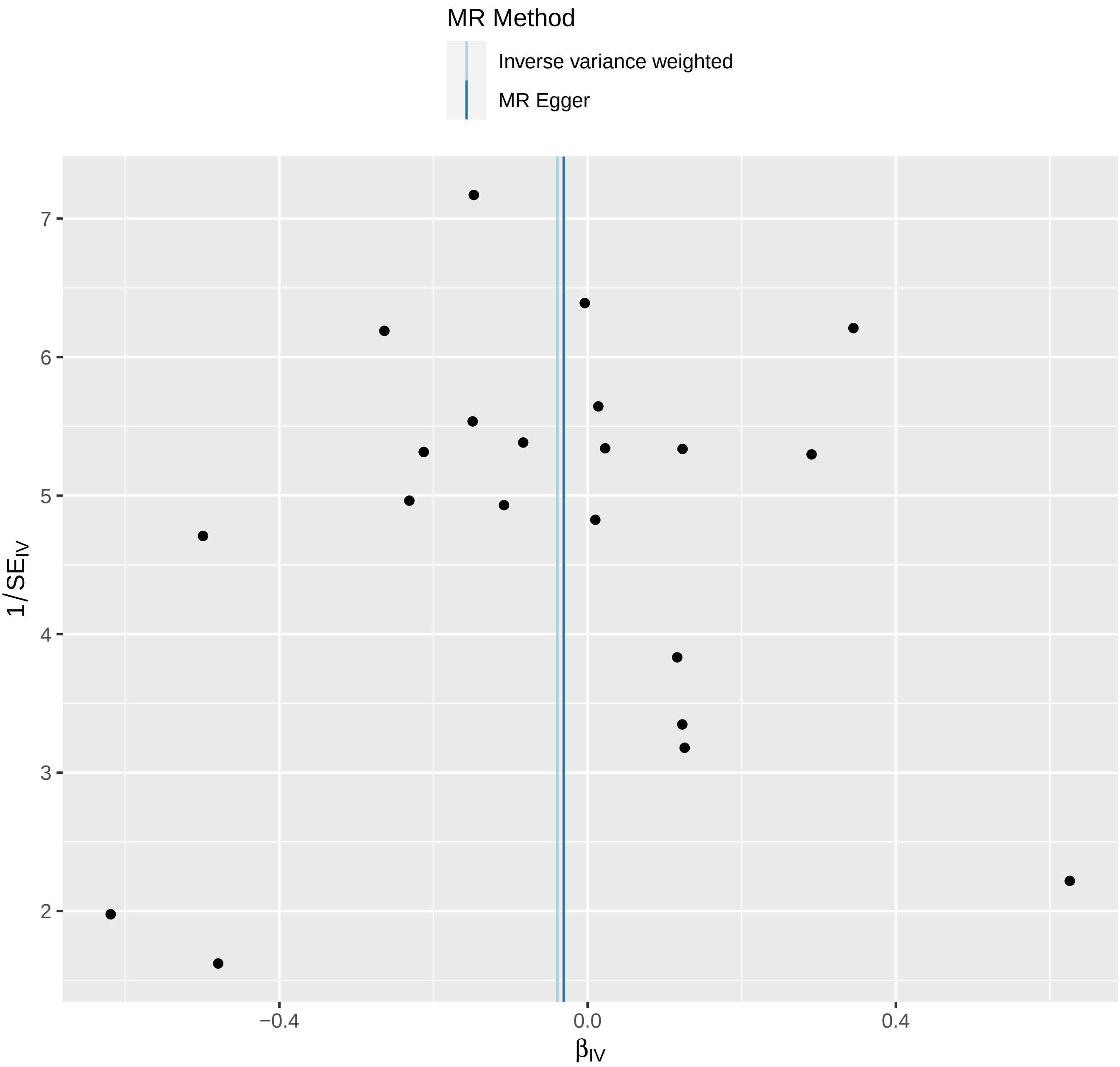

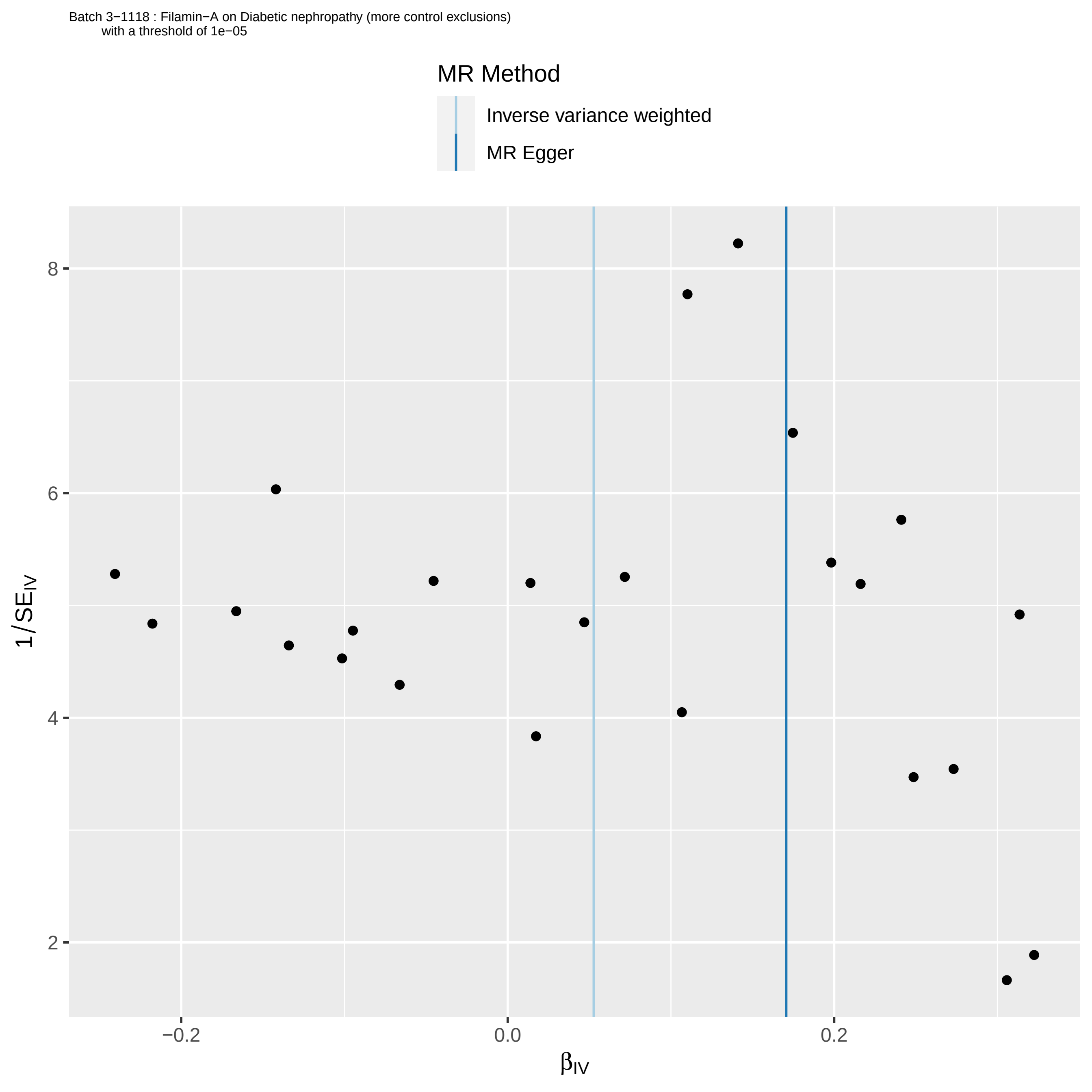

Batch 3−1118 : Filamin−A on Diabetic nephropathy (more control exclusions)
with a threshold of 1e−05
MR Method
Inverse variance weighted
MR Egger
8
6
4
2
$1/SE_{IV}$
−0.2
0.0
0.2
β_{IV}

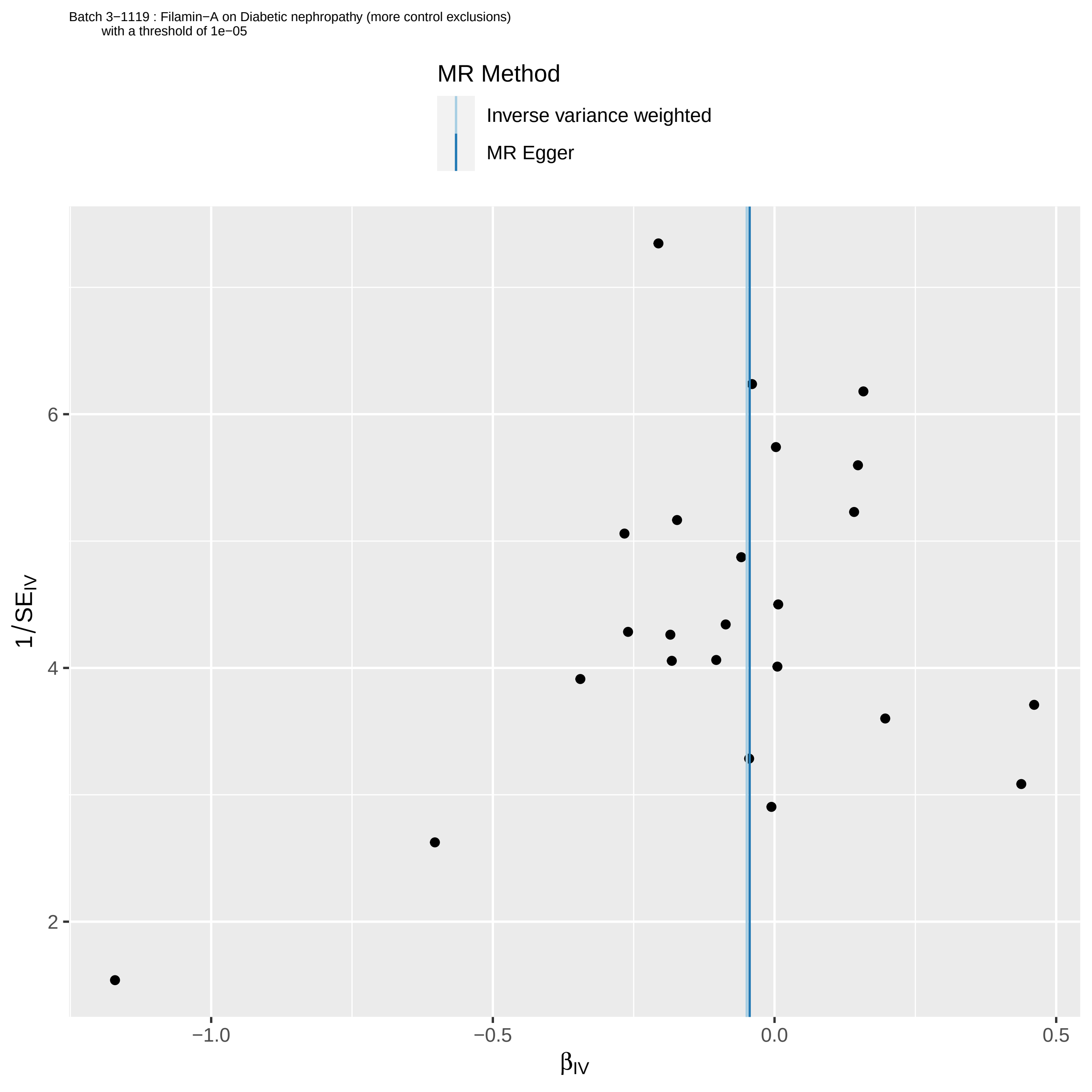
Batch 3−1119 : Filamin−A on Diabetic nephropathy (more control exclusions)
with a threshold of 1e−05
MR Method
Inverse variance weighted
MR Egger
6
4
2
1/SE$_{IV}$
−1.0
−0.5
0.0
0.5
β$_{IV}$

Batch 3−1120 : Filamin−A on Diabetic nephropathy (more control exclusions)
with a threshold of 1e−05

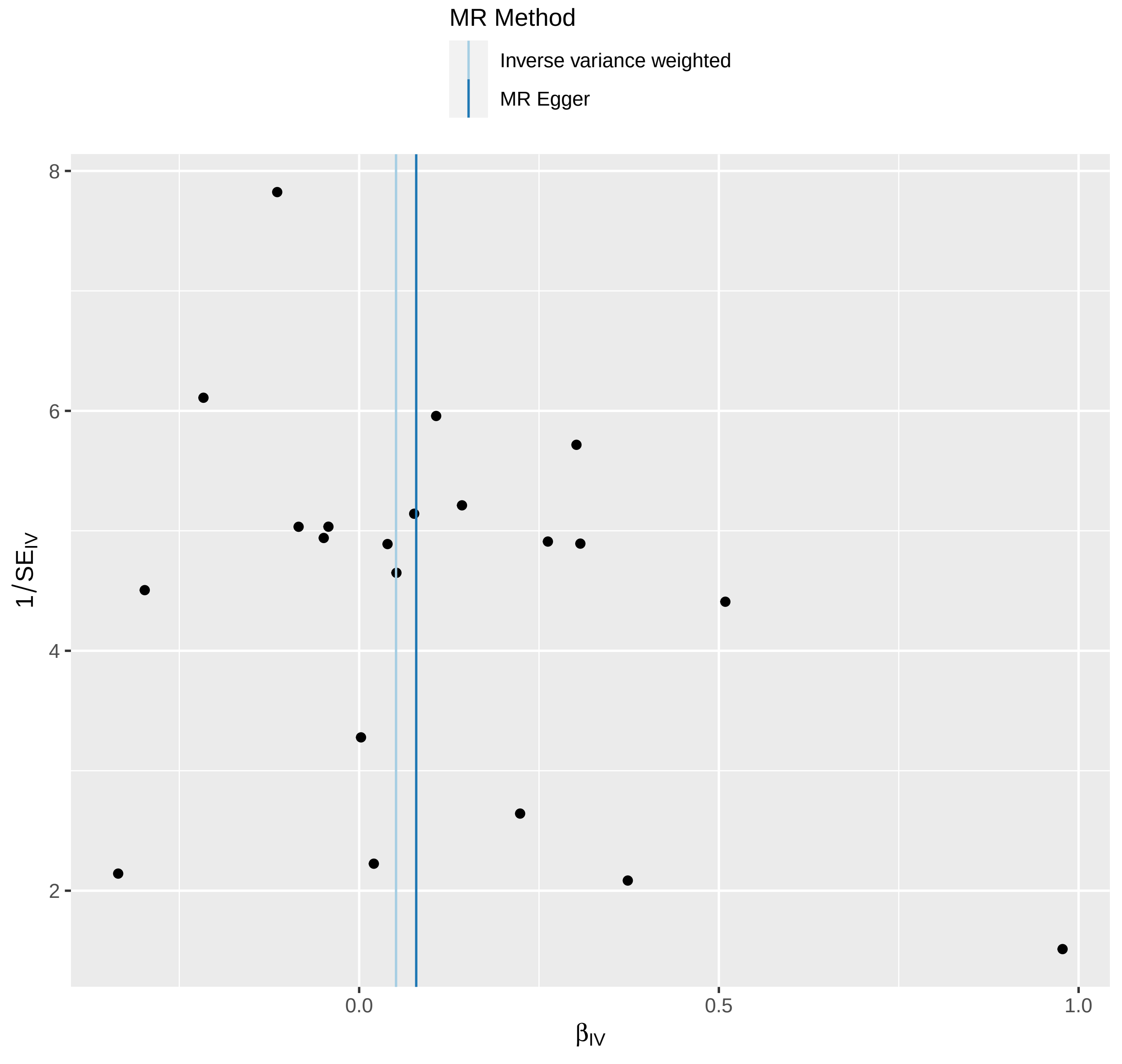

Batch 3−1121 : Leucine−rich repeat transmembrane protein FLRT1 on Diabetic nephropathy (more control exclusions) with a threshold of 1e−05

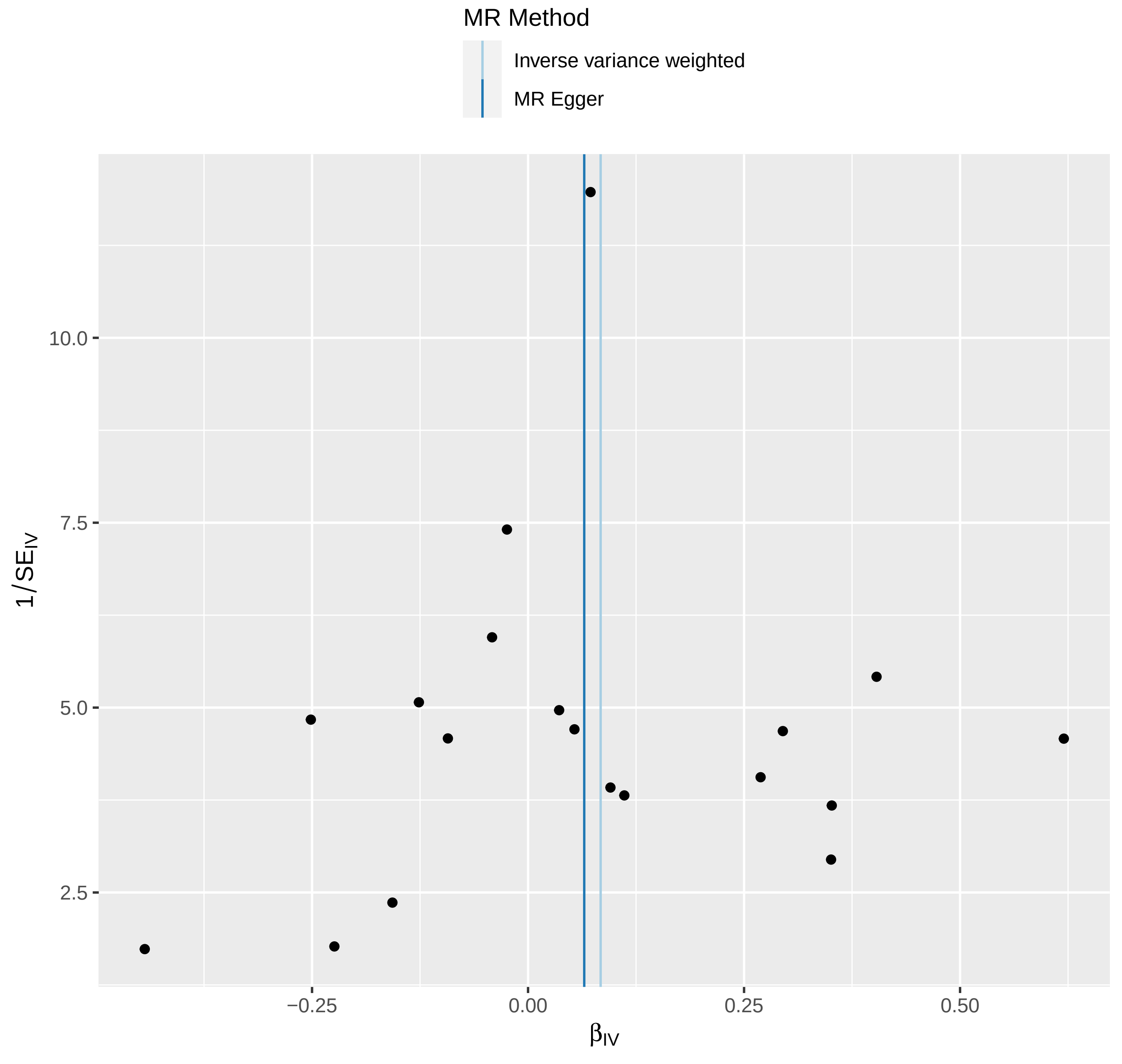

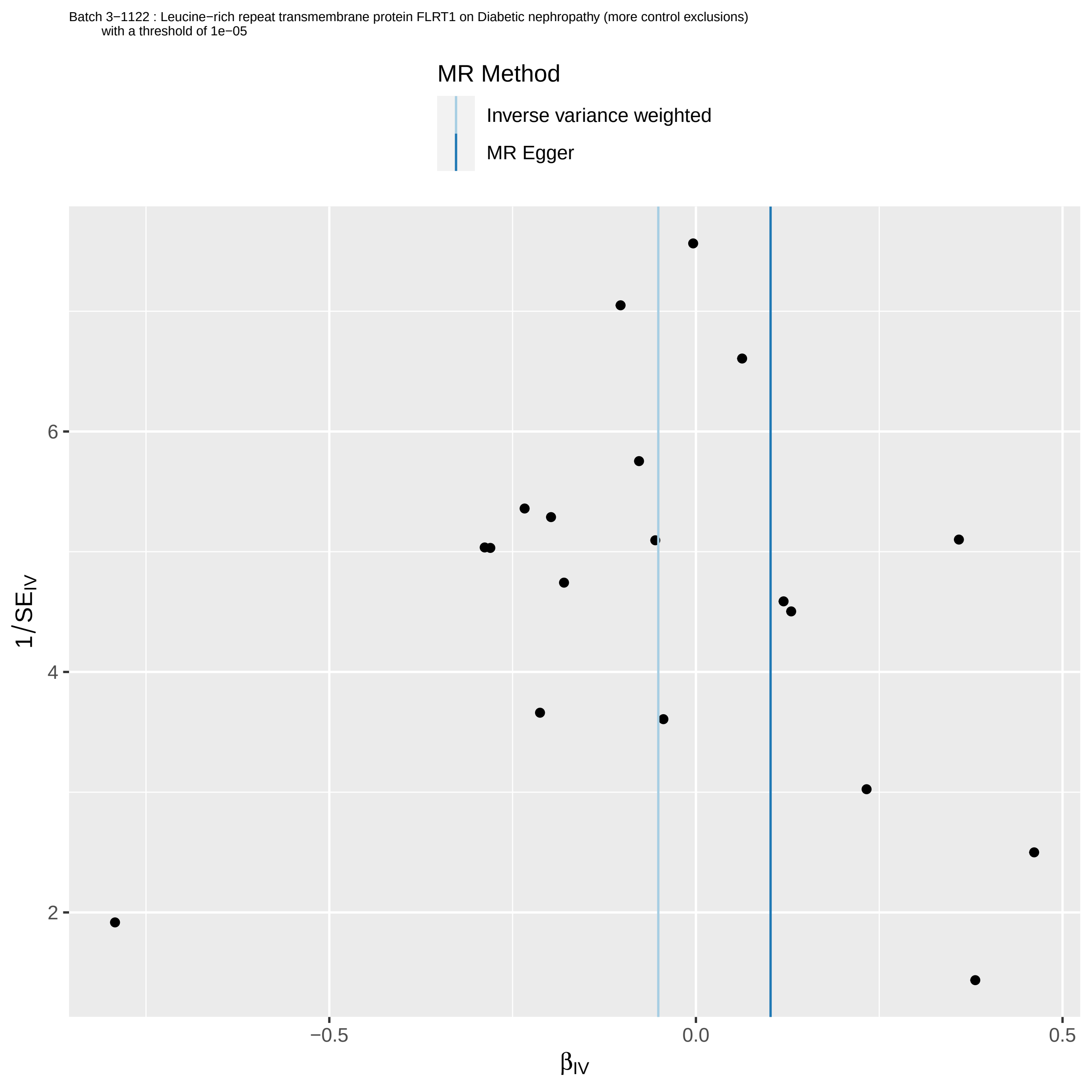
Batch 3−1122 : Leucine−rich repeat transmembrane protein FLRT1 on Diabetic nephropathy (more control exclusions)
with a threshold of 1e−05
MR Method
Inverse variance weighted
MR Egger
1/SE_IV
6
4
2
−0.5
0.0
0.5
β_IV

Batch 3−1123 : Leucine−rich repeat transmembrane protein FLRT2 on Diabetic nephropathy (more control exclusions)
with a threshold of 1e−05

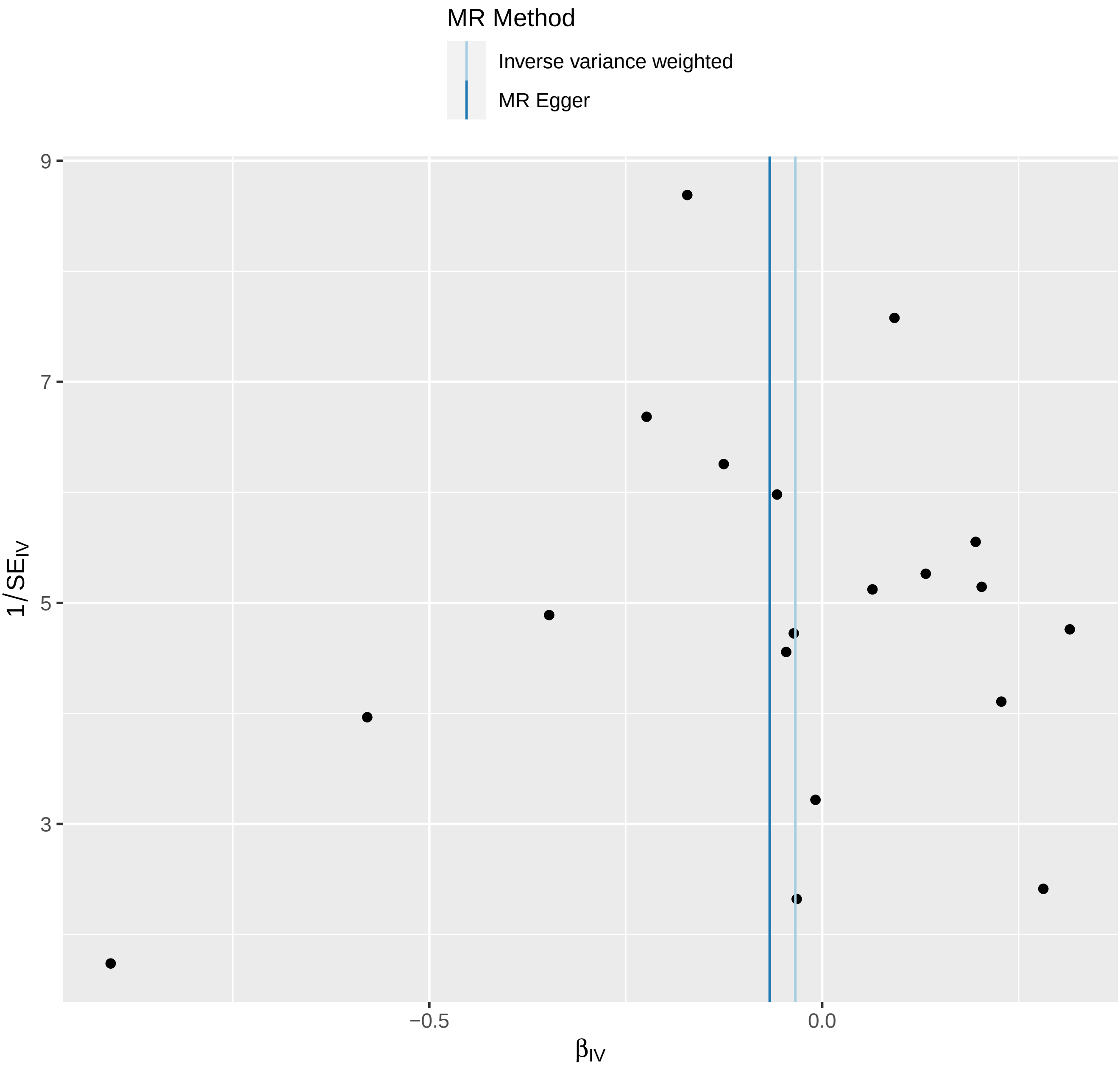

Batch 3−1124 : Leucine−rich repeat transmembrane protein FLRT3 on Diabetic nephropathy (more control exclusions) with a threshold of 1e−05

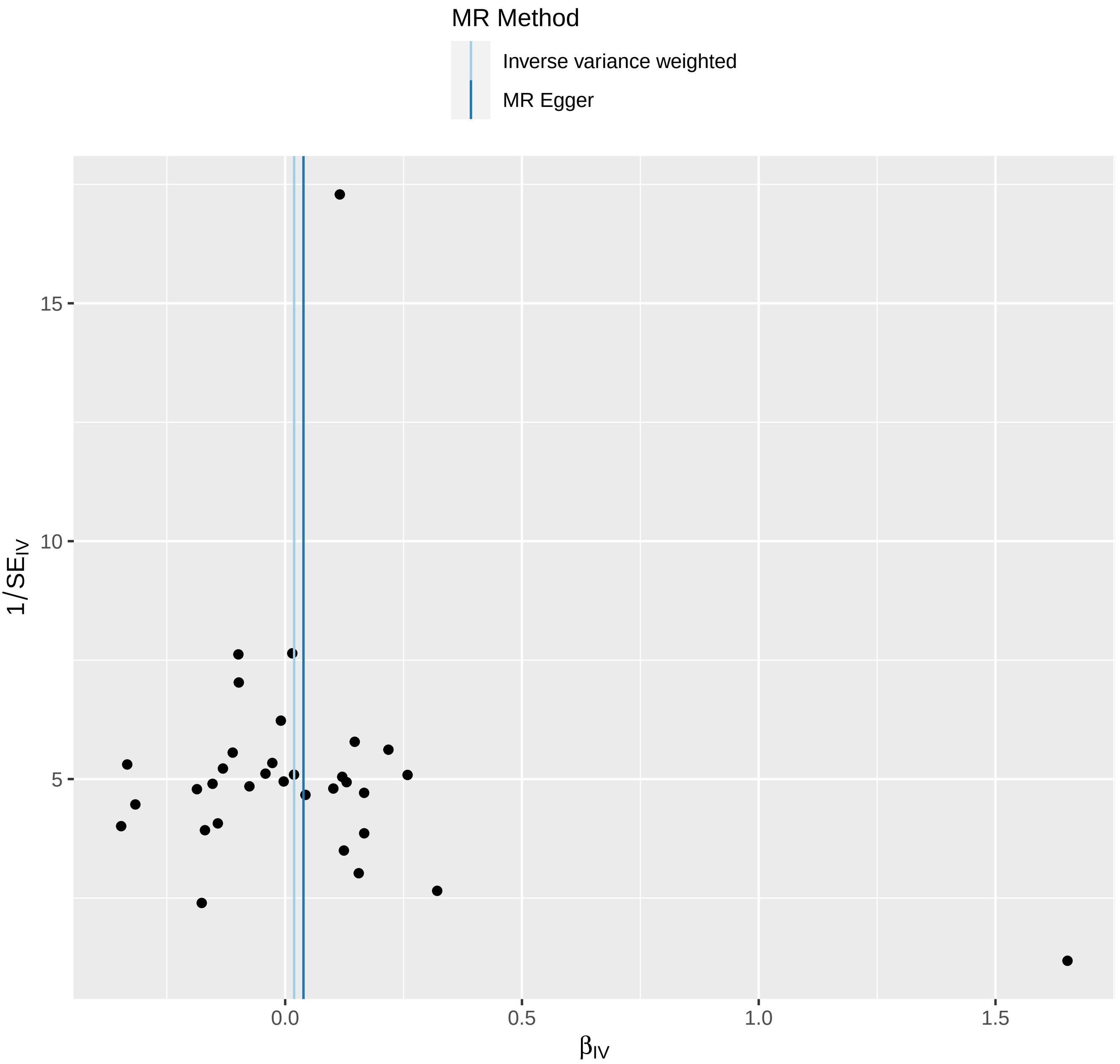

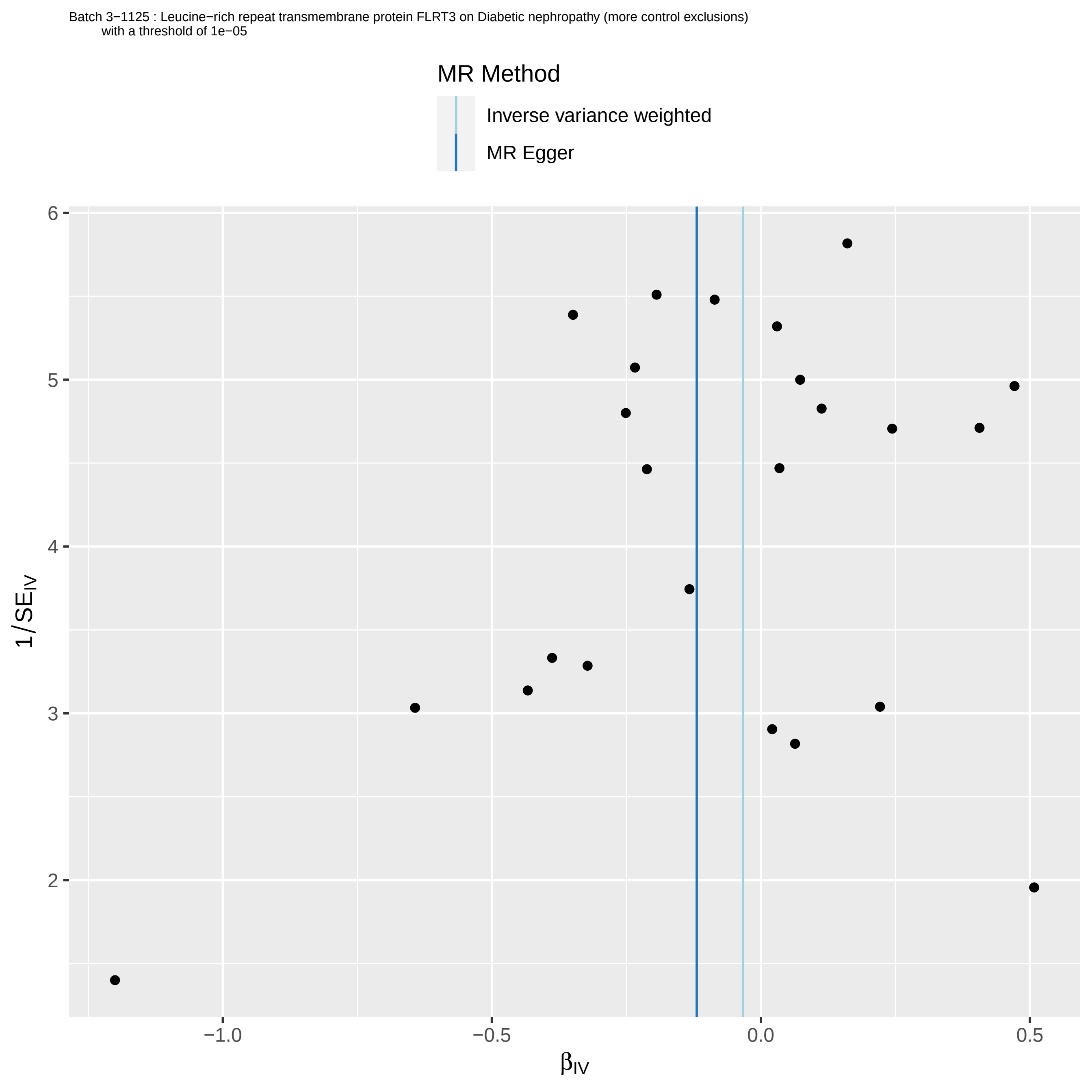

Batch 3−1125 : Leucine−rich repeat transmembrane protein FLRT3 on Diabetic nephropathy (more control exclusions)
with a threshold of 1e−05
MR Method
Inverse variance weighted
MR Egger
6
5
4
3
2
1/SE_IV
−1.0
−0.5
0.0
0.5
β_IV

Batch 3−1126 : Receptor−type tyrosine−protein kinase FLT3 on Diabetic nephropathy (more control exclusions)
with a threshold of 1e−05

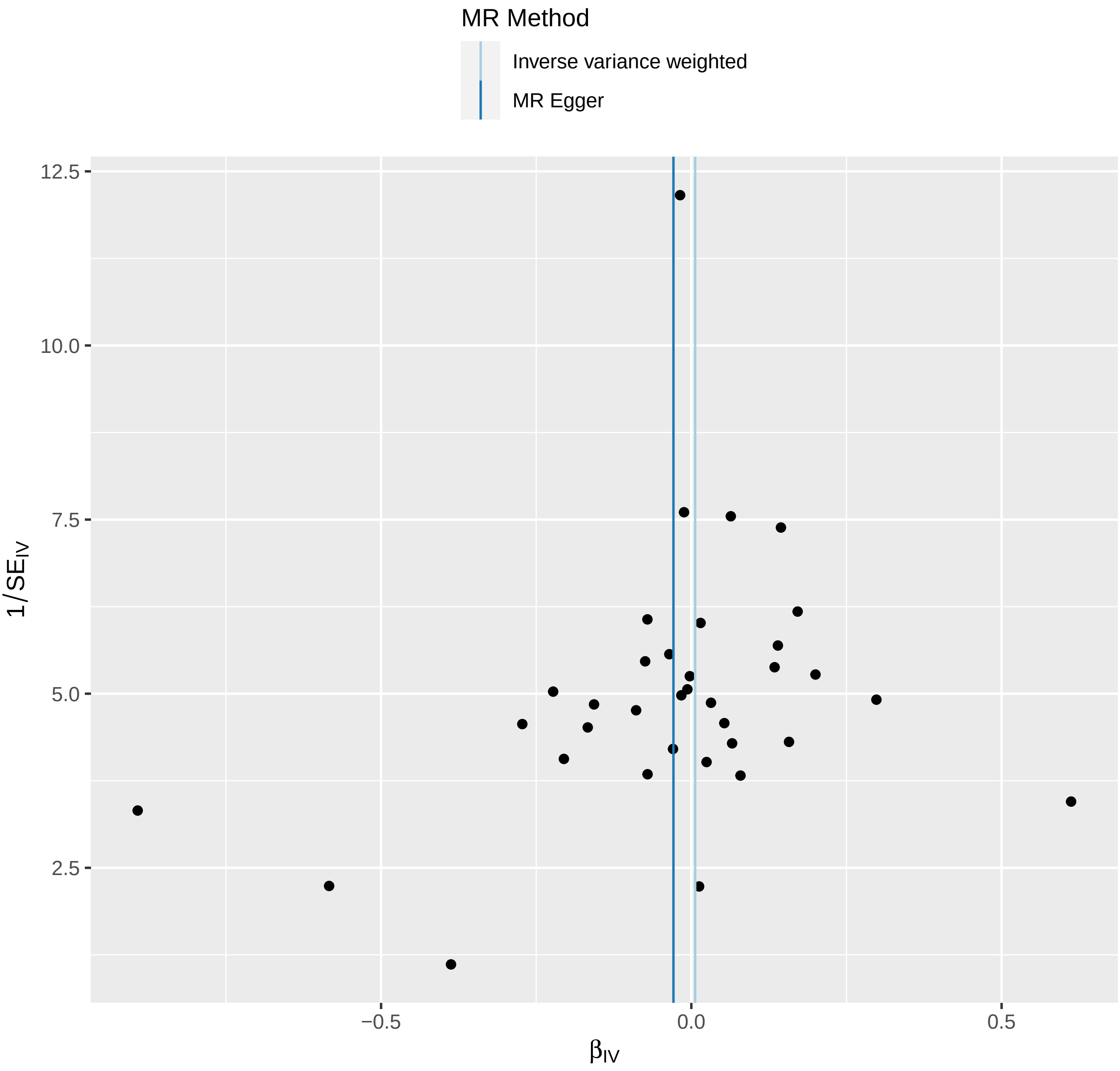

Batch 3−1127 : Fms−related tyrosine kinase 3 ligand on Diabetic nephropathy (more control exclusions)
with a threshold of 1e−05

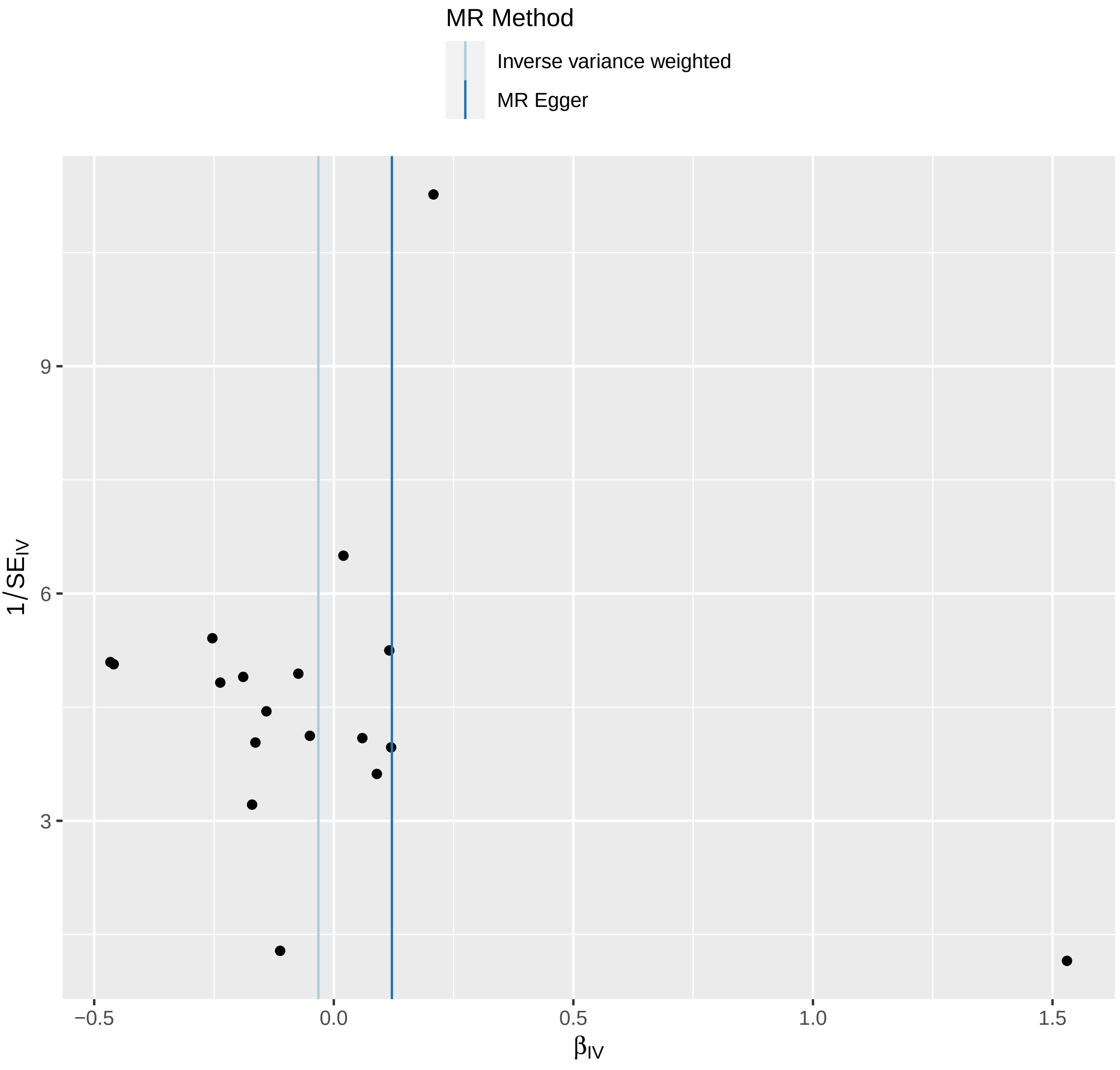

Batch 3−1128 : Fms−related tyrosine kinase 3 ligand on Diabetic nephropathy (more control exclusions) with a threshold of 1e−05

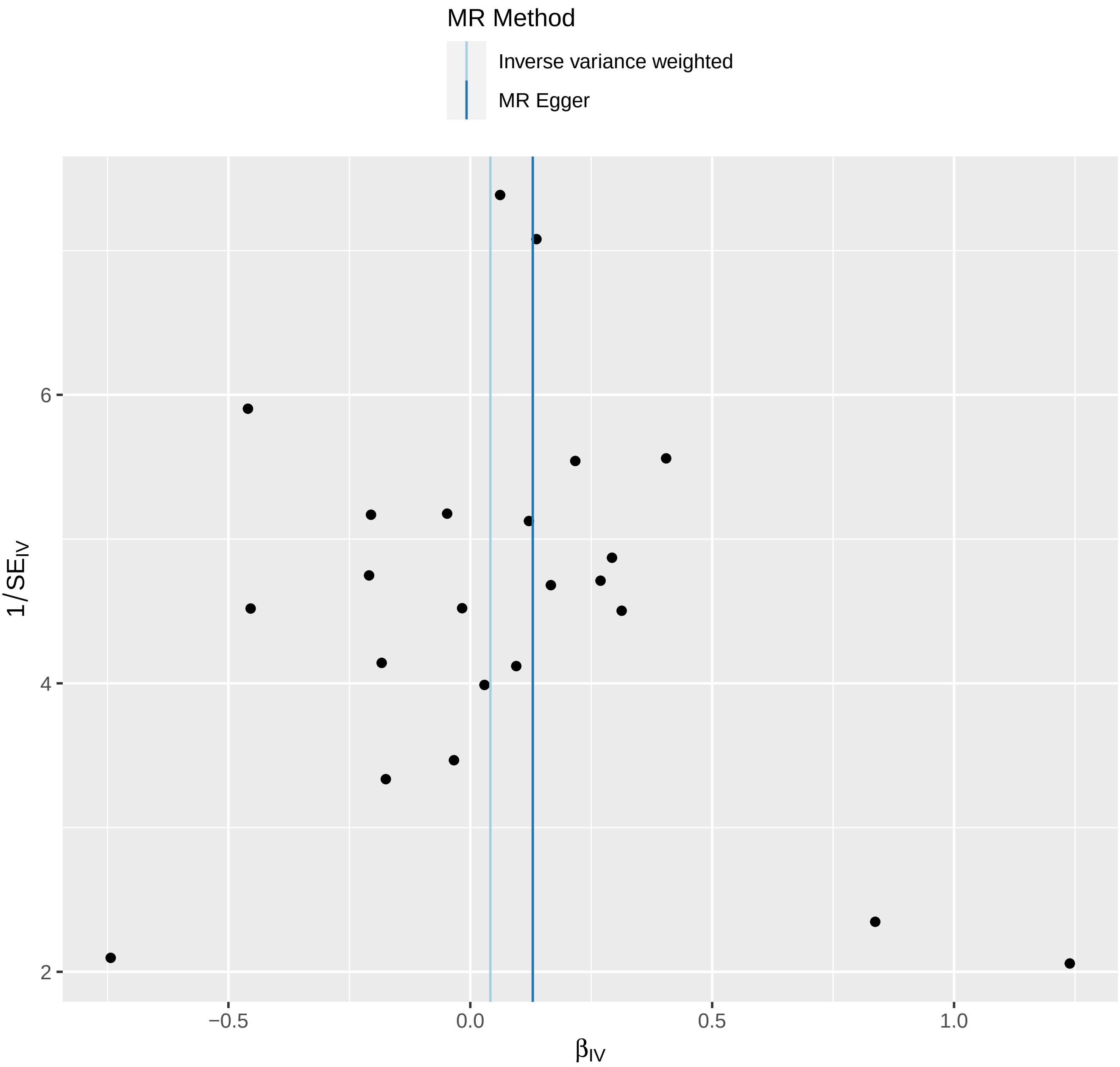

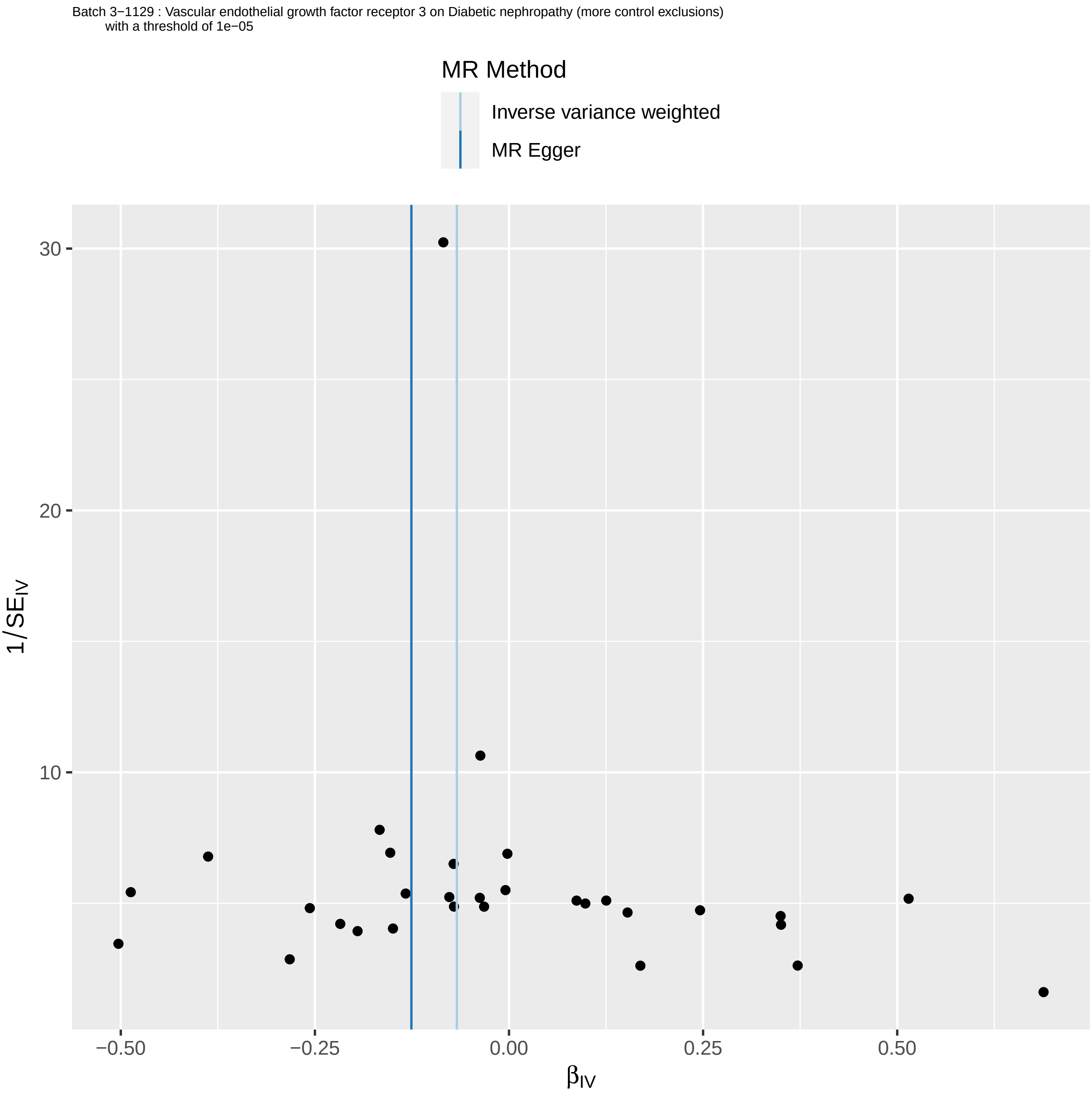
Batch 3−1129 : Vascular endothelial growth factor receptor 3 on Diabetic nephropathy (more control exclusions)
with a threshold of 1e−05
MR Method
Inverse variance weighted
MR Egger
30
20
10
1/SE_IV
−0.50
−0.25
0.00
0.25
0.50
β_IV

Batch 3−1130 : Feline leukemia virus subgroup C receptor−related protein 1 on Diabetic nephropathy (more control exclusions)
with a threshold of 1e−05

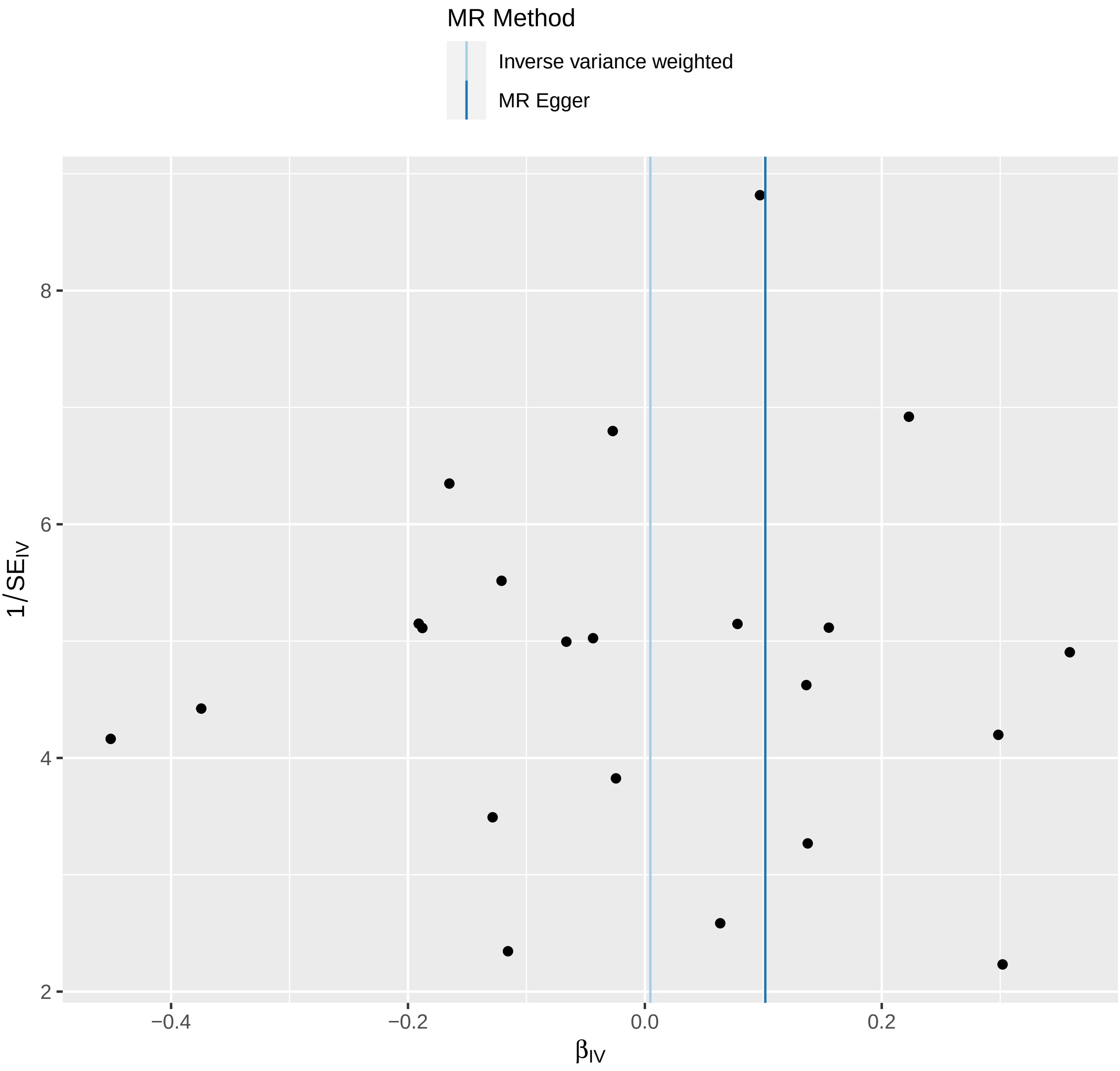

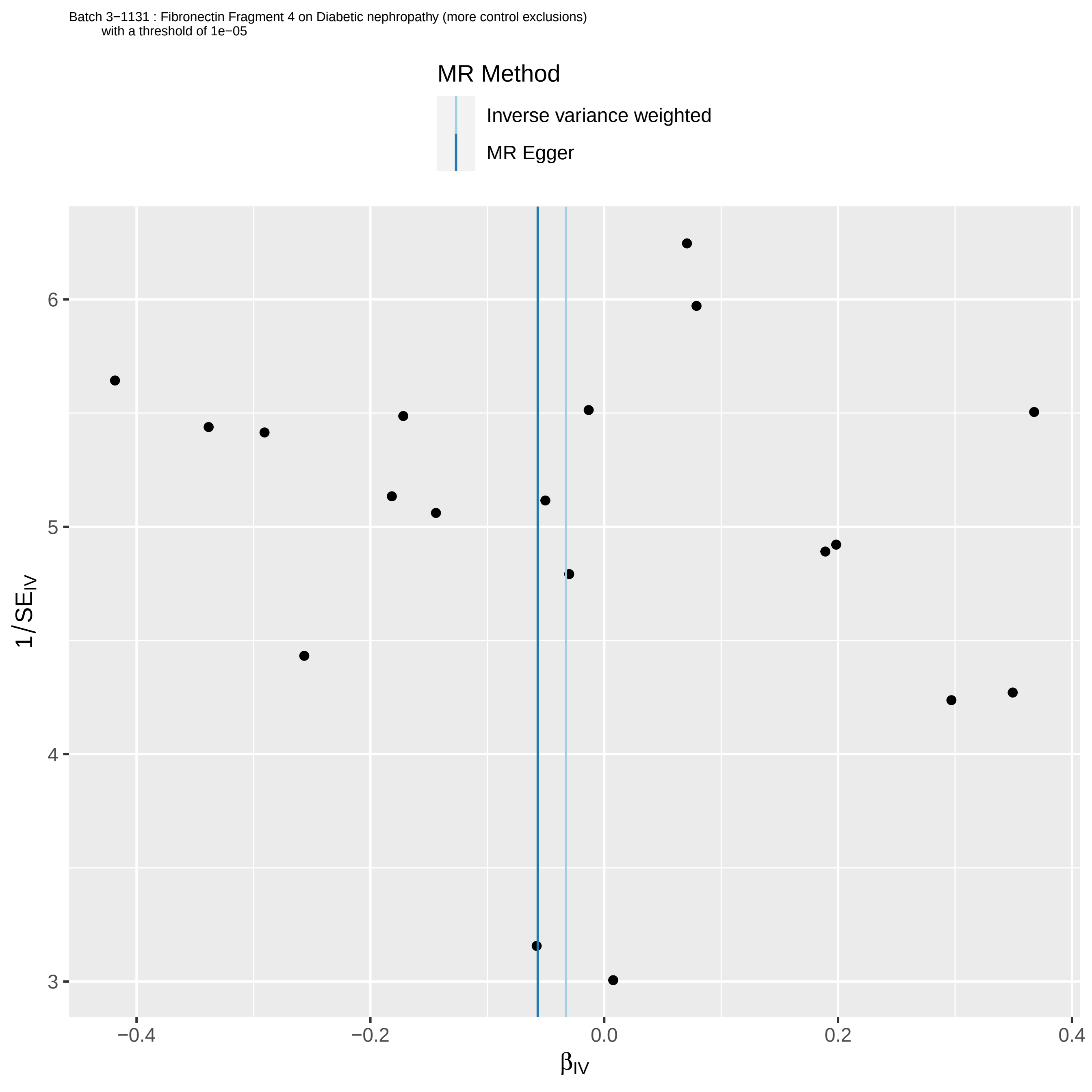

Batch 3−1131 : Fibronectin Fragment 4 on Diabetic nephropathy (more control exclusions)
with a threshold of 1e−05
MR Method
Inverse variance weighted
MR Egger
6
5
4
3
1/SE_IV
−0.4
−0.2
0.0
0.2
0.4
β_IV

Batch 3−1132 : Fibronectin type III domain−containing protein 4 on Diabetic nephropathy (more control exclusions)
with a threshold of 1e−05

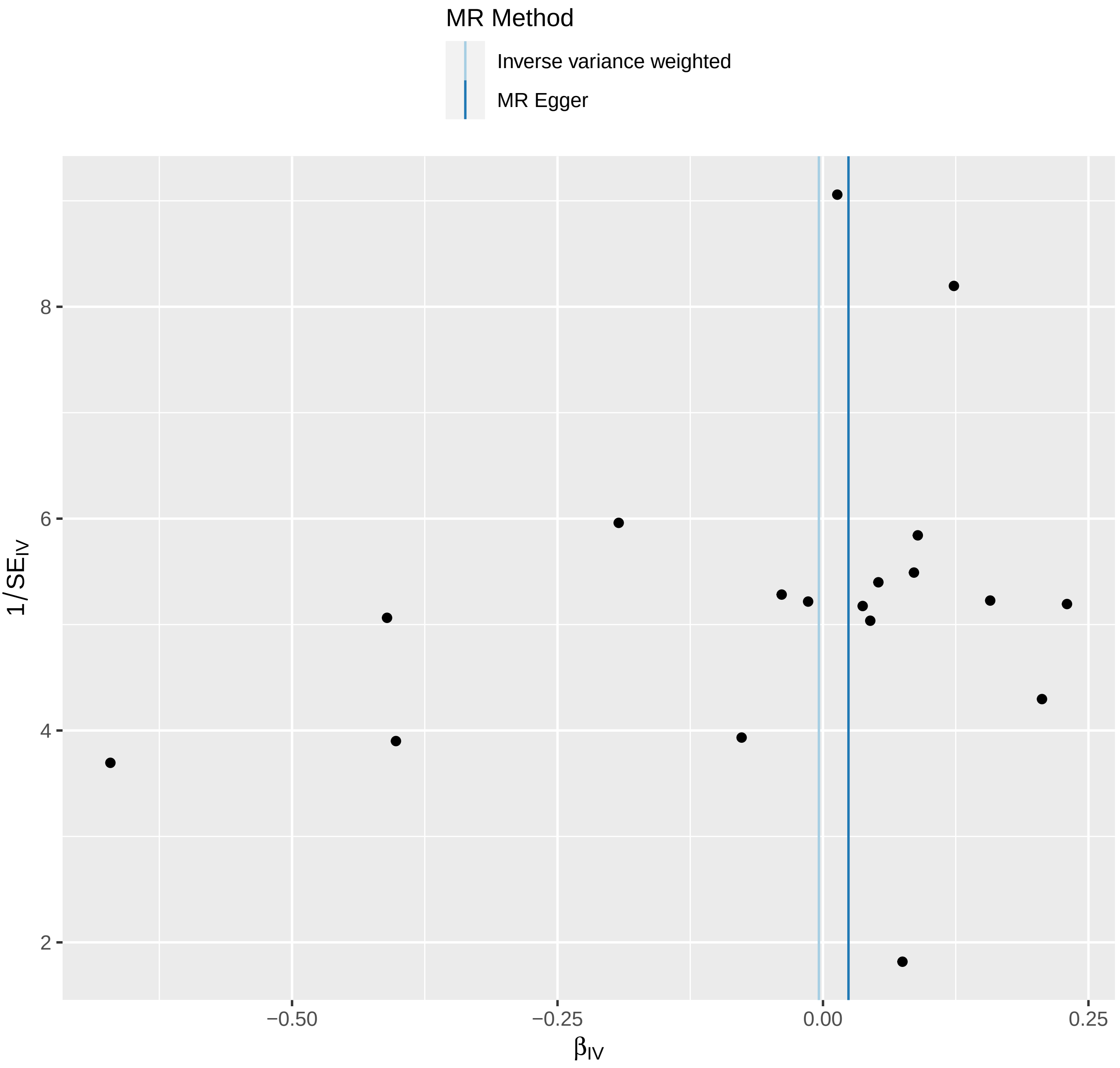

Batch 3−1133 : Fibronectin type III domain−containing protein 5 on Diabetic nephropathy (more control exclusions) with a threshold of 1e−05

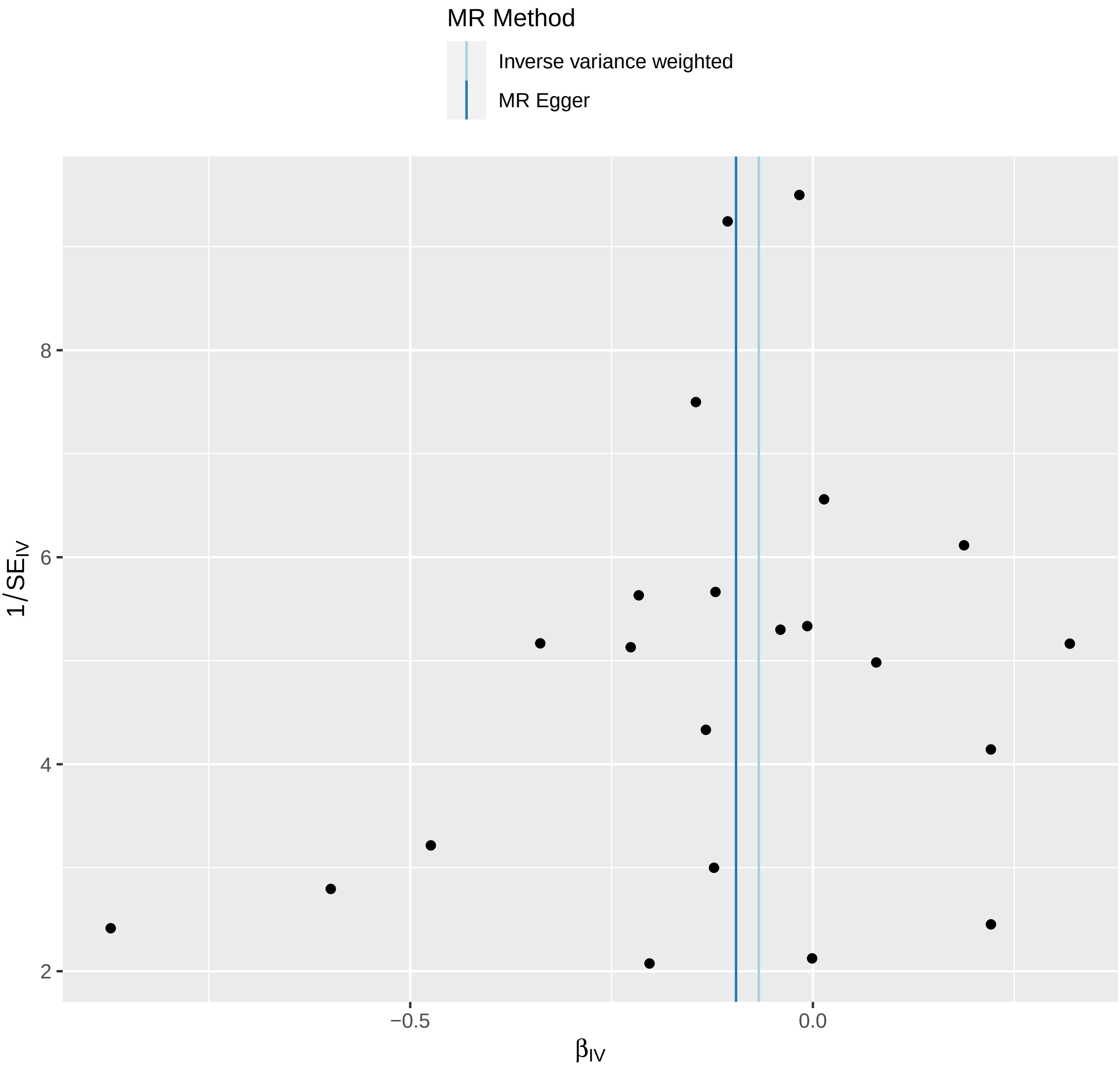

Batch 3−1134 : Glutamate carboxypeptidase 2 on Diabetic nephropathy (more control exclusions)
with a threshold of 1e−05

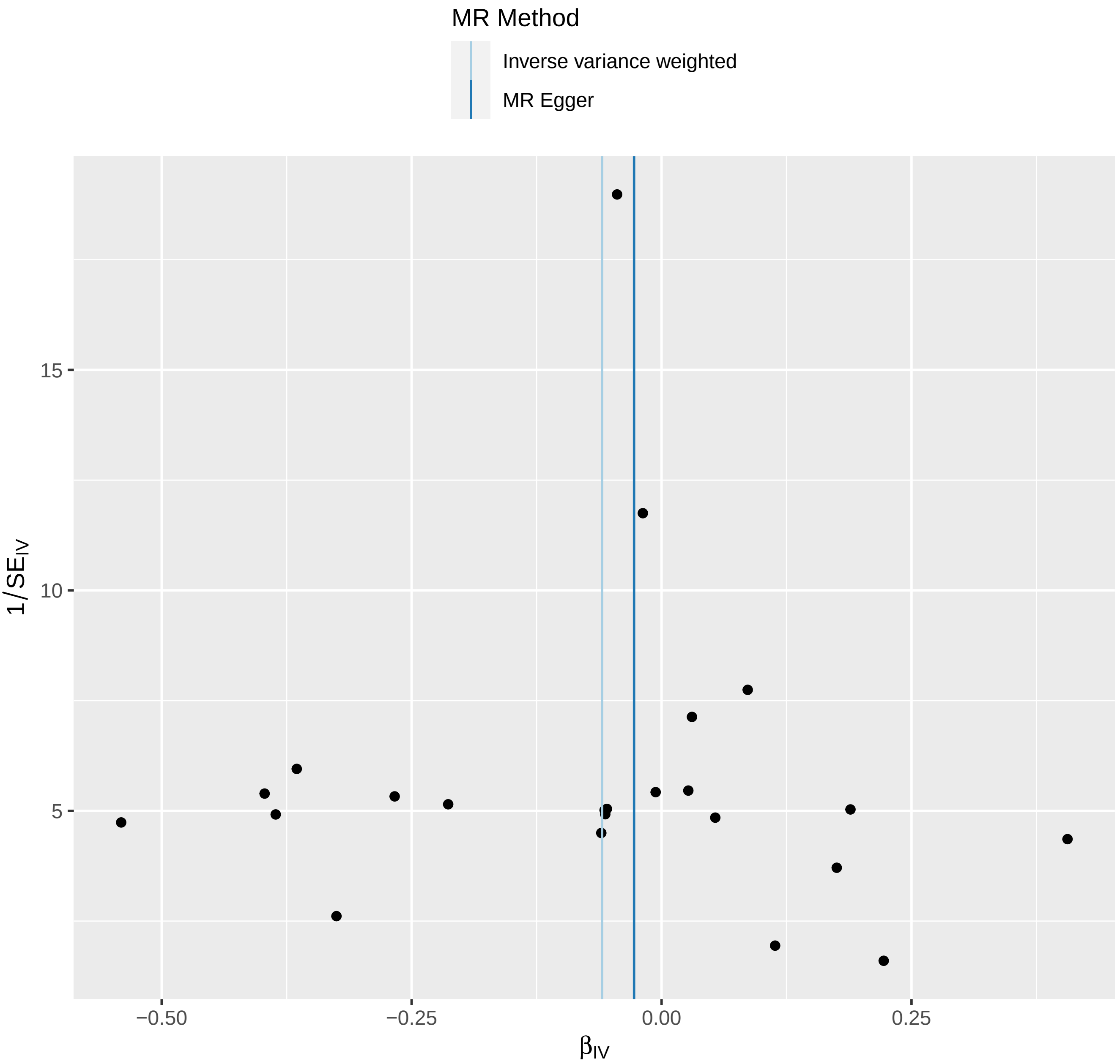

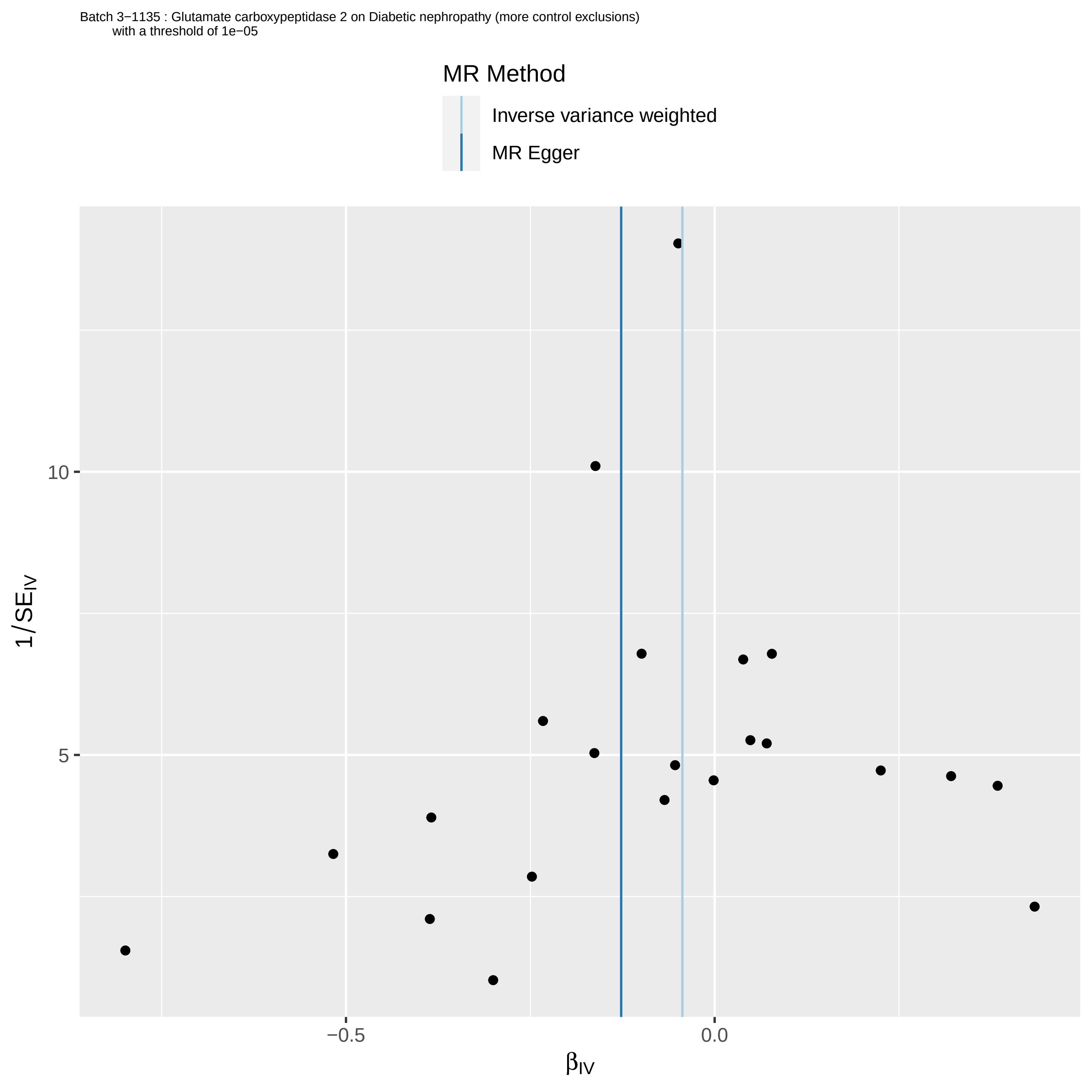

Batch 3−1135 : Glutamate carboxypeptidase 2 on Diabetic nephropathy (more control exclusions)
with a threshold of 1e−05
MR Method
Inverse variance weighted
MR Egger
10
5
1/SE_IV
−0.5
0.0
β_IV

Batch 3−1136 : Forkhead box protein C2 on Diabetic nephropathy (more control exclusions)
with a threshold of 1e−05

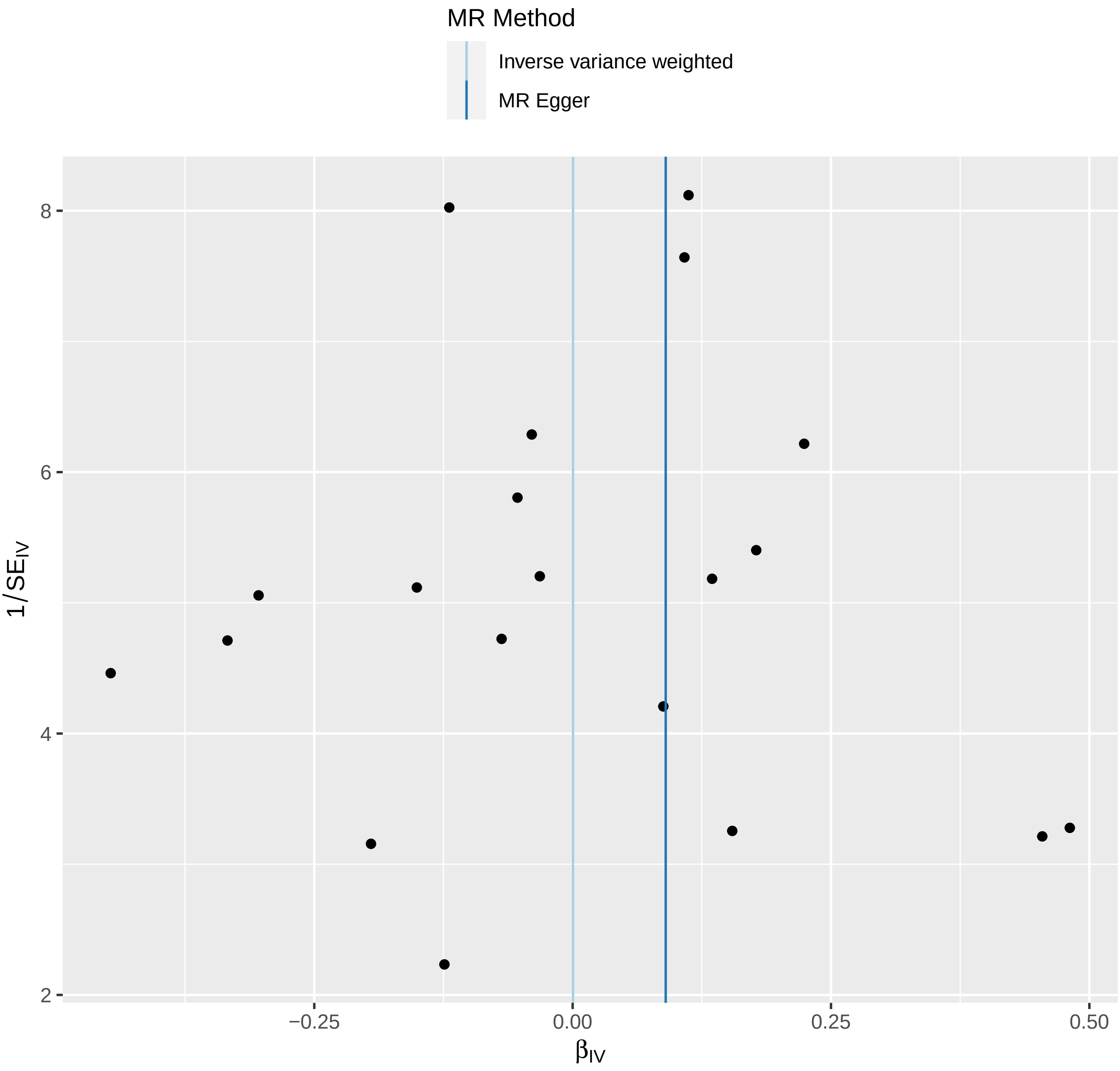

Batch 3−1137 : Forkhead box protein J2 on Diabetic nephropathy (more control exclusions)
with a threshold of 1e−05

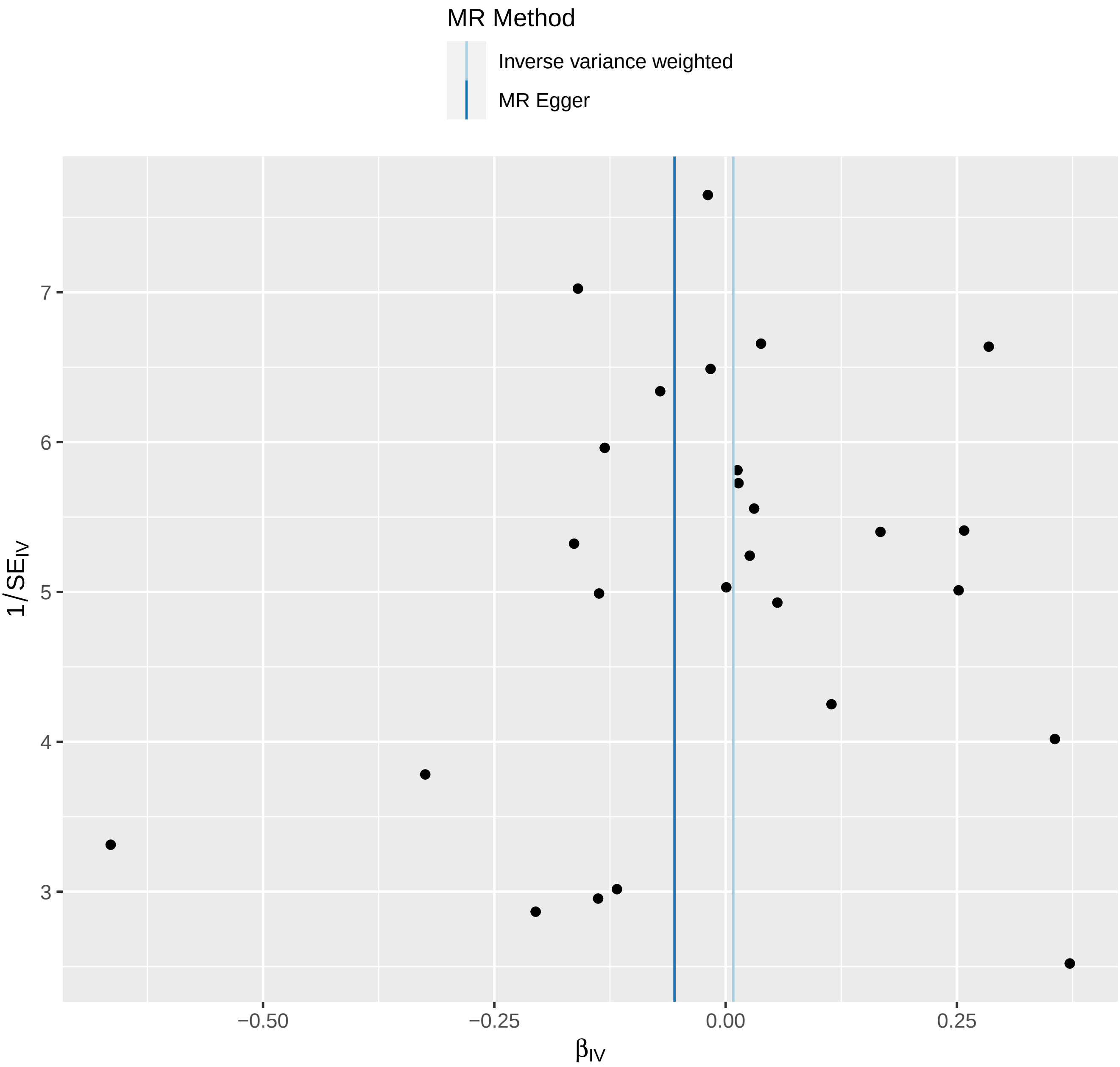

Batch 3−1138 : Forkhead box protein L2 on Diabetic nephropathy (more control exclusions)
with a threshold of 1e−05

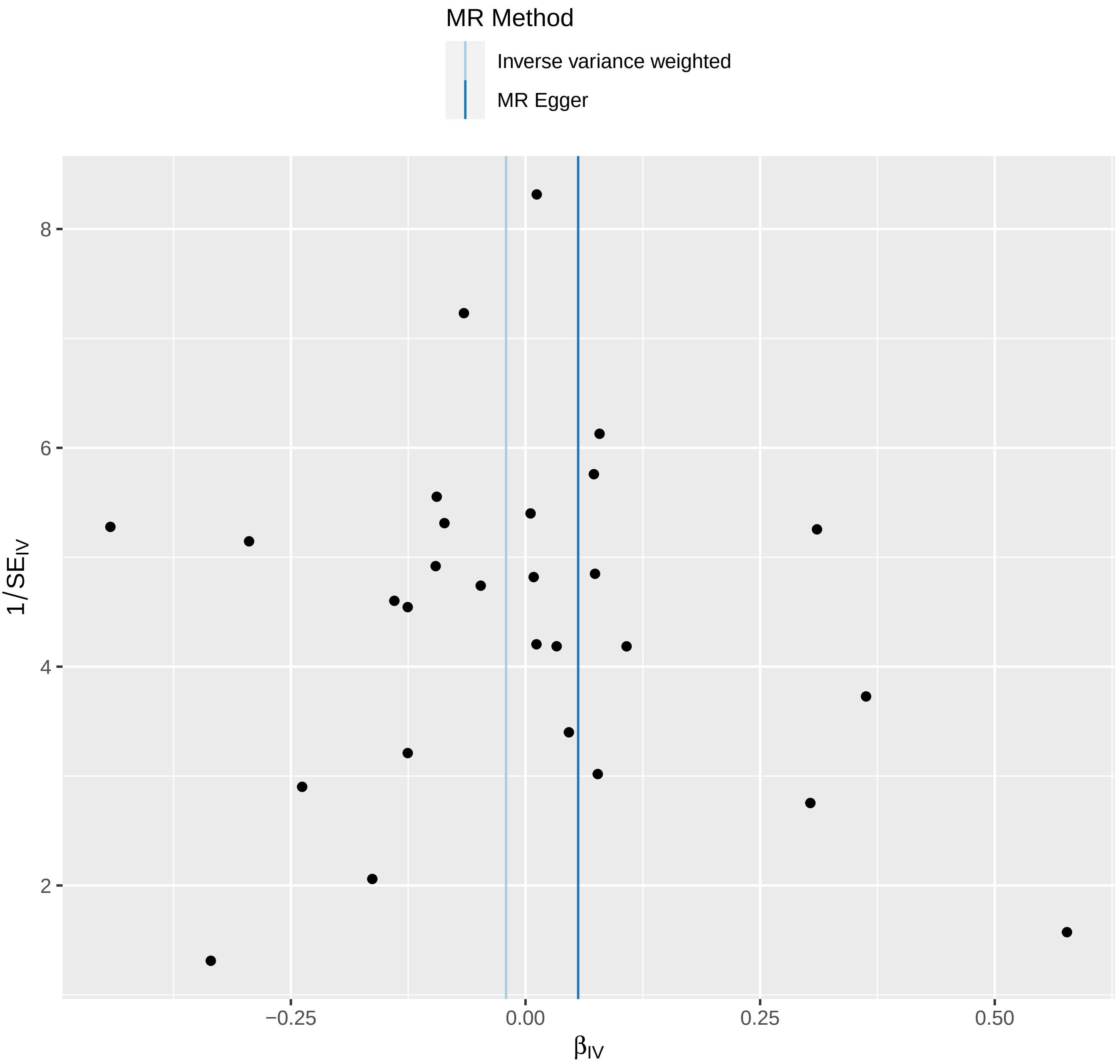

Batch 3−1139 : Tyrosine−protein kinase FRK on Diabetic nephropathy (more control exclusions)
with a threshold of 1e−05

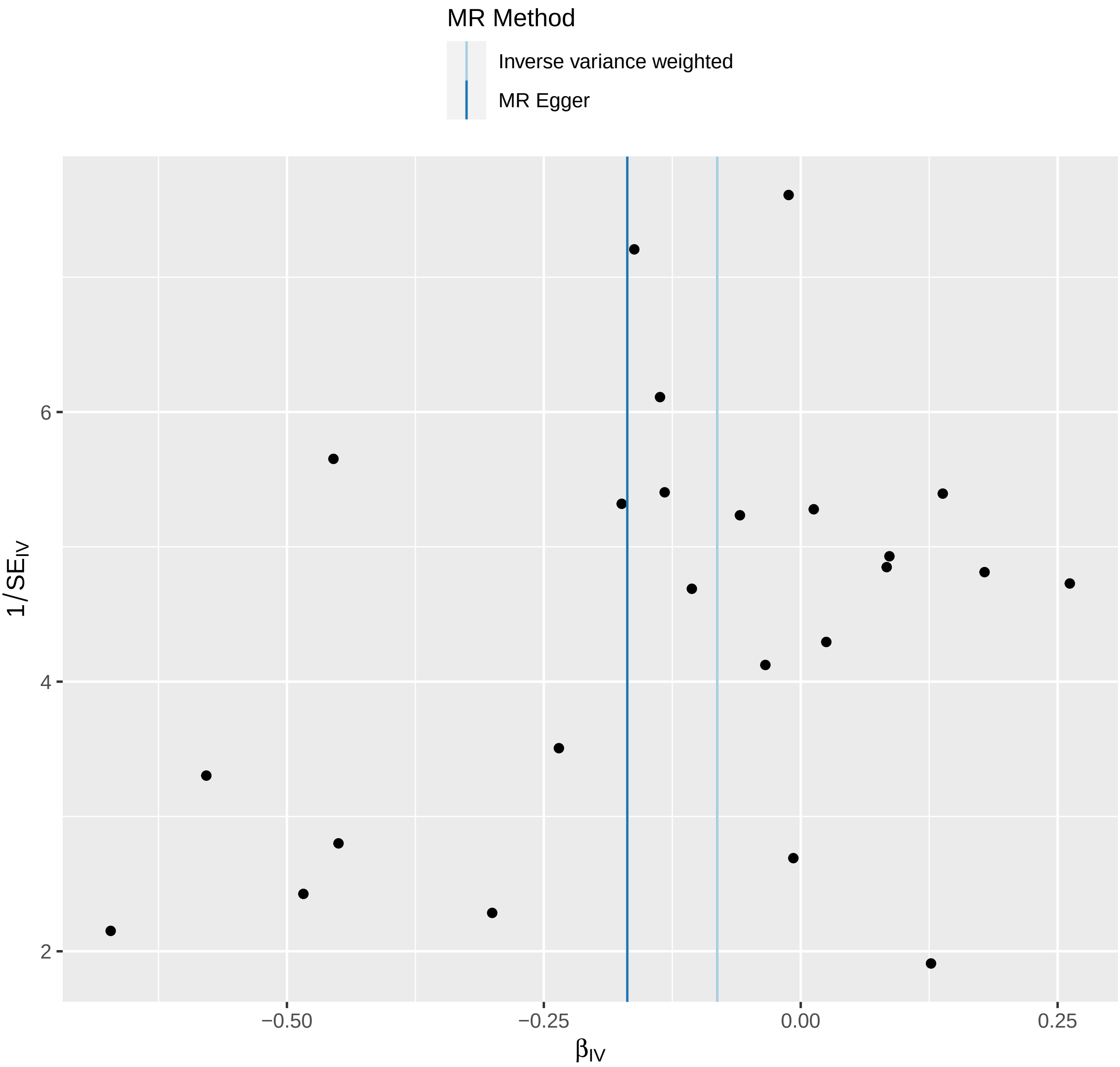

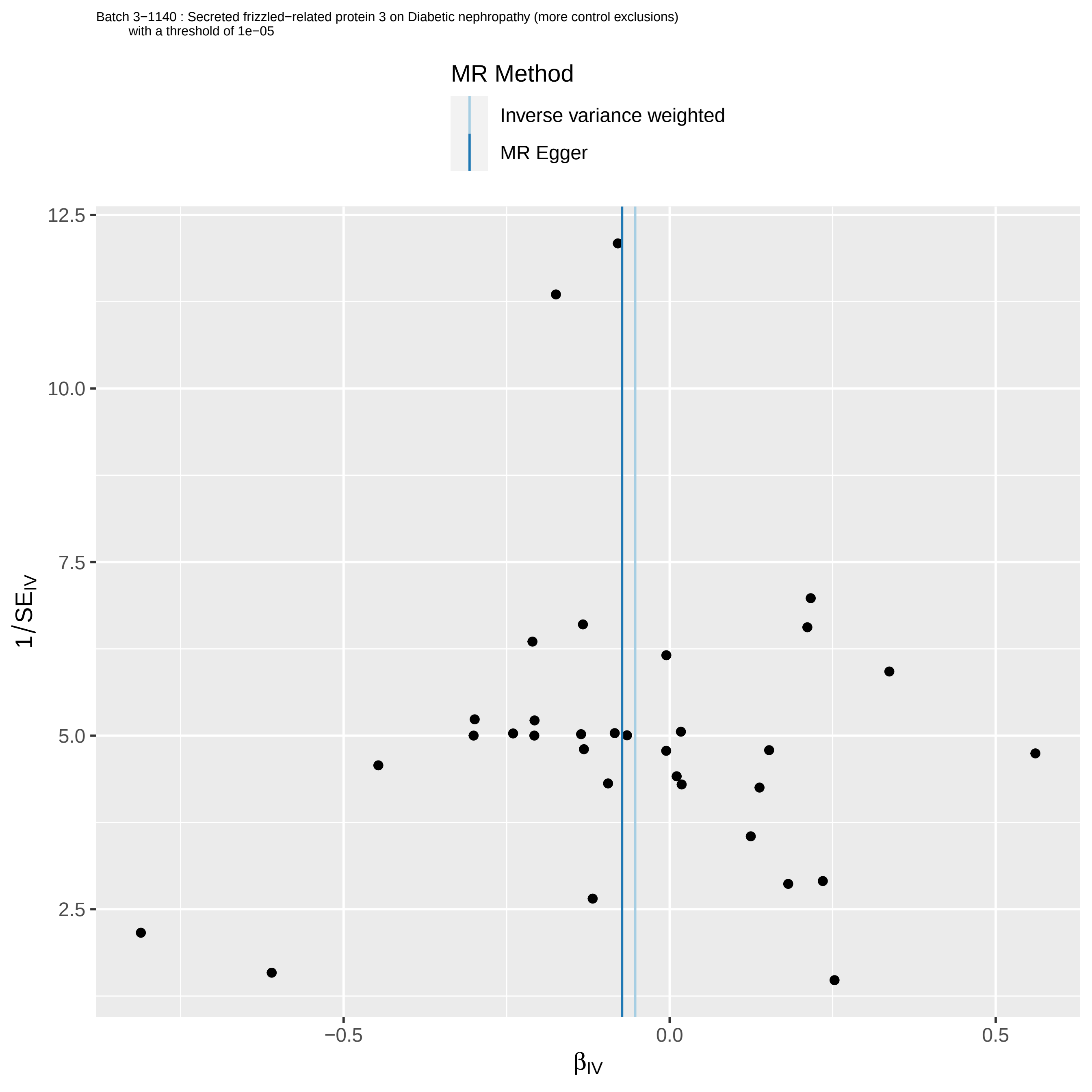
Batch 3−1140 : Secreted frizzled−related protein 3 on Diabetic nephropathy (more control exclusions)
with a threshold of 1e−05
MR Method
Inverse variance weighted
MR Egger
12.5
10.0
7.5
5.0
2.5
1/SE_IV
−0.5
0.0
0.5
β_IV

Batch 3−1141 : Secreted frizzled−related protein 3 on Diabetic nephropathy (more control exclusions)
with a threshold of 1e−05

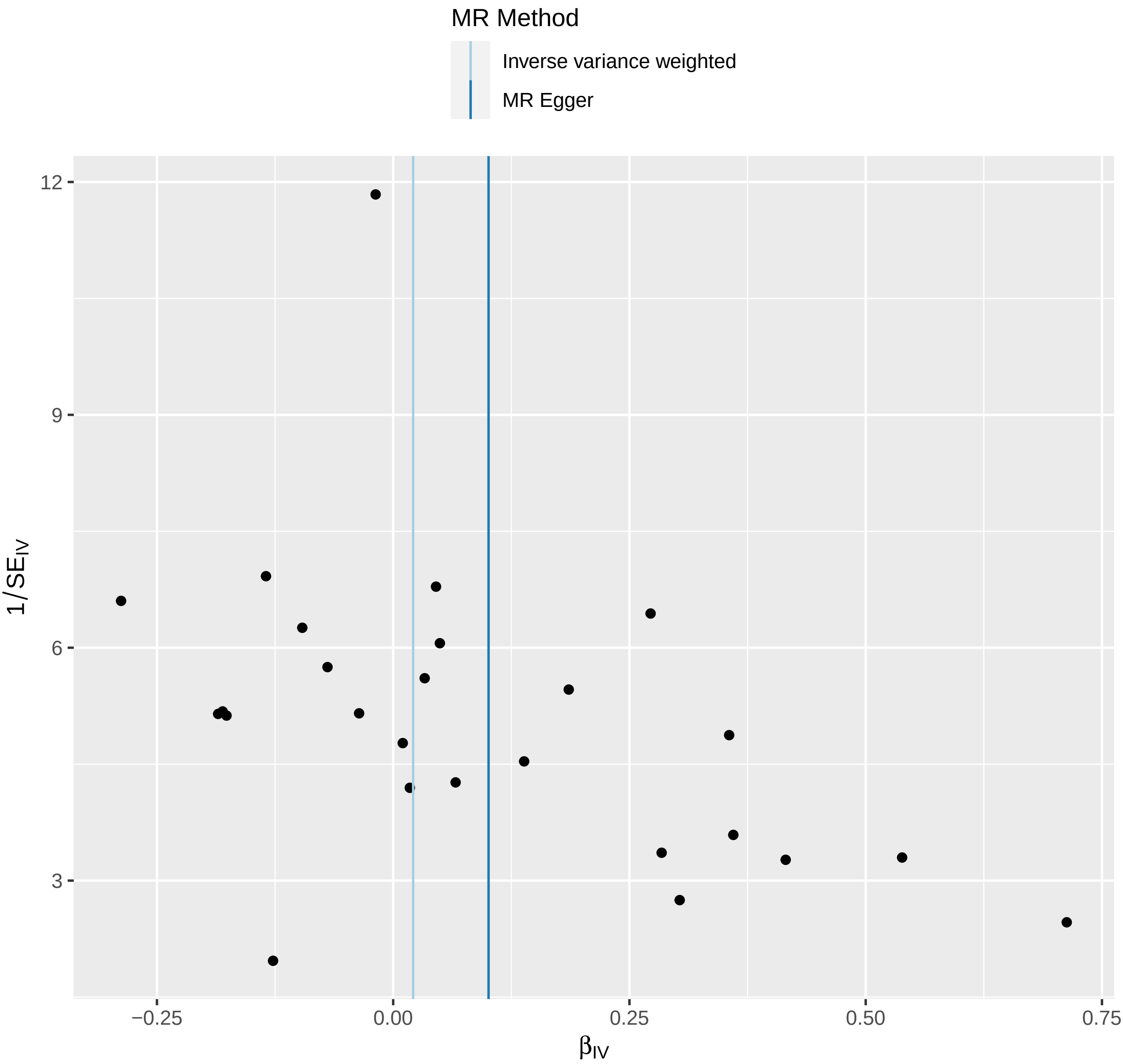

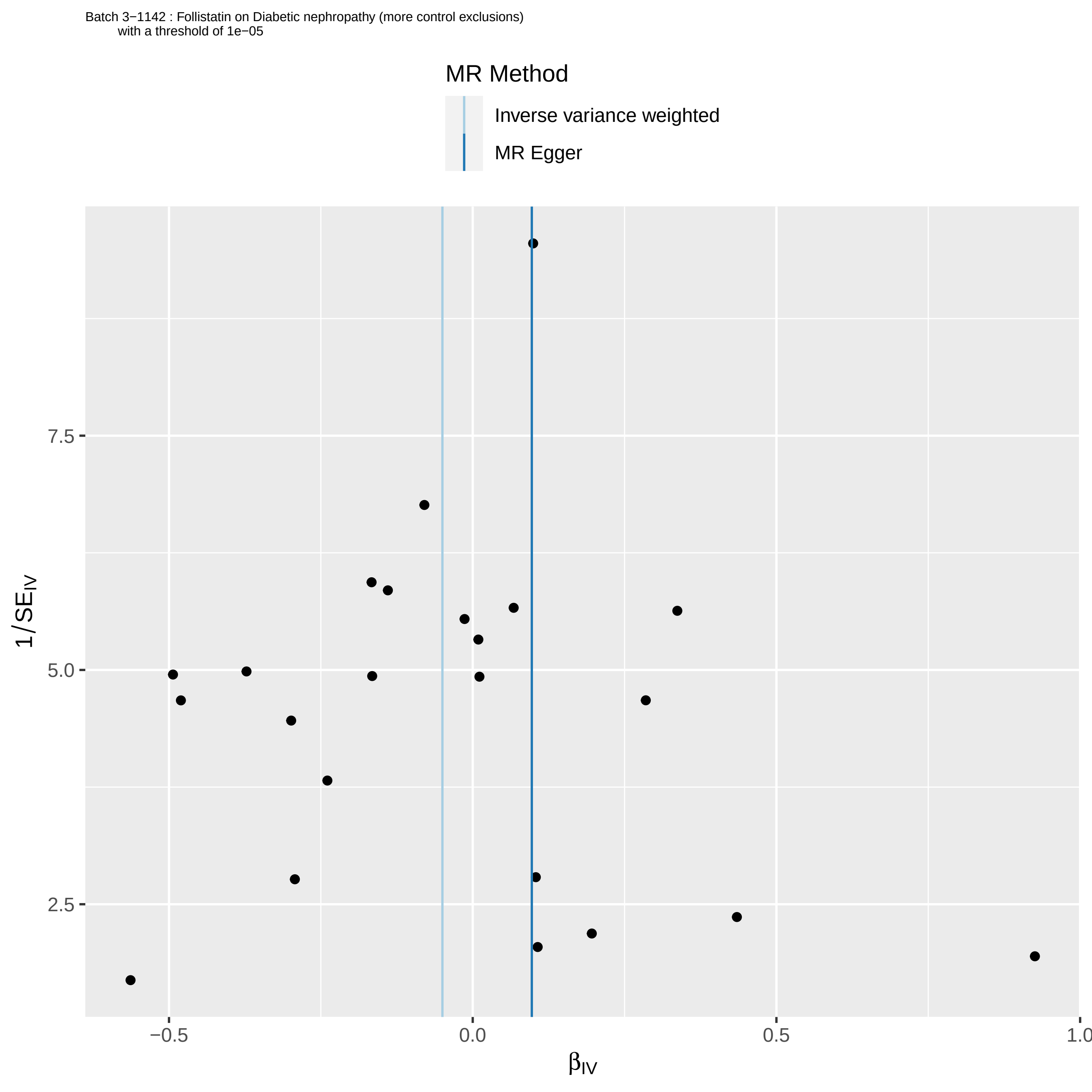

Batch 3−1142 : Follistatin on Diabetic nephropathy (more control exclusions)
with a threshold of 1e−05
MR Method
Inverse variance weighted
MR Egger
7.5
5.0
2.5
1/SE_IV
−0.5
0.0
0.5
1.0
β_IV

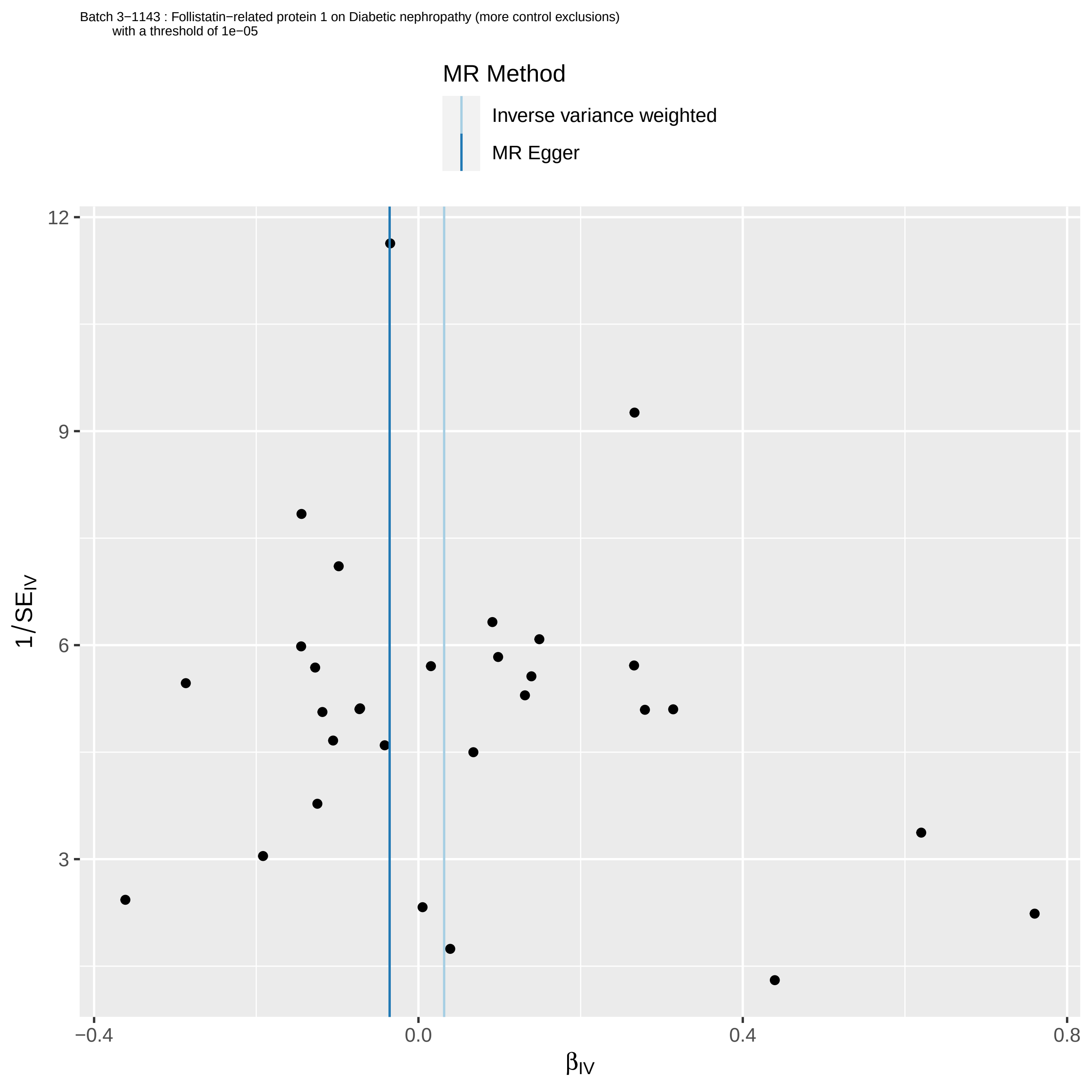

Batch 3−1143 : Follistatin−related protein 1 on Diabetic nephropathy (more control exclusions)
with a threshold of 1e−05
MR Method
Inverse variance weighted
MR Egger
12
9
6
3
1/SE_IV
−0.4
0.0
0.4
0.8
β_IV

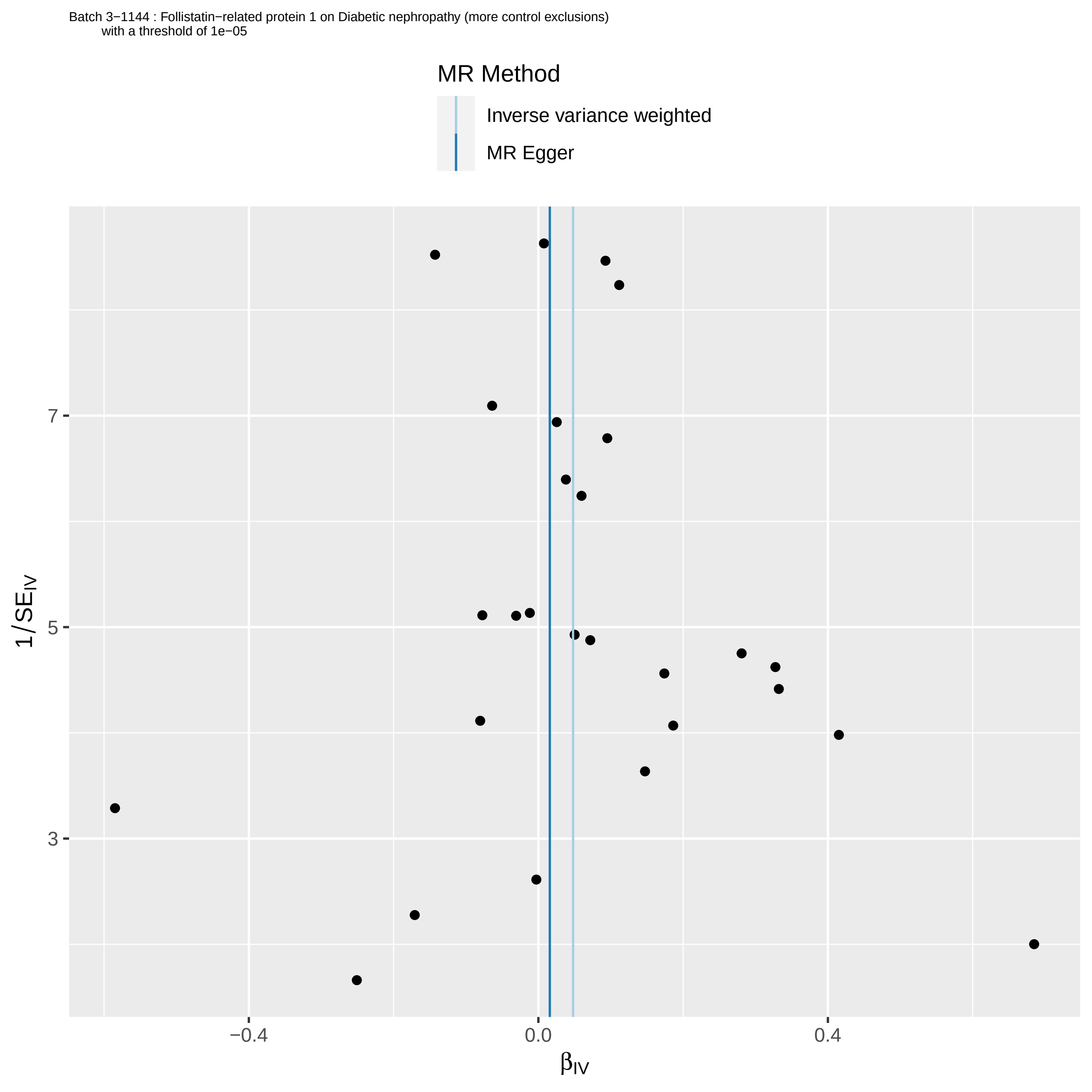

Batch 3−1144 : Follistatin−related protein 1 on Diabetic nephropathy (more control exclusions)
with a threshold of 1e−05
MR Method
Inverse variance weighted
MR Egger
7
5
3
1/SE_IV
−0.4
0.0
0.4
β_IV

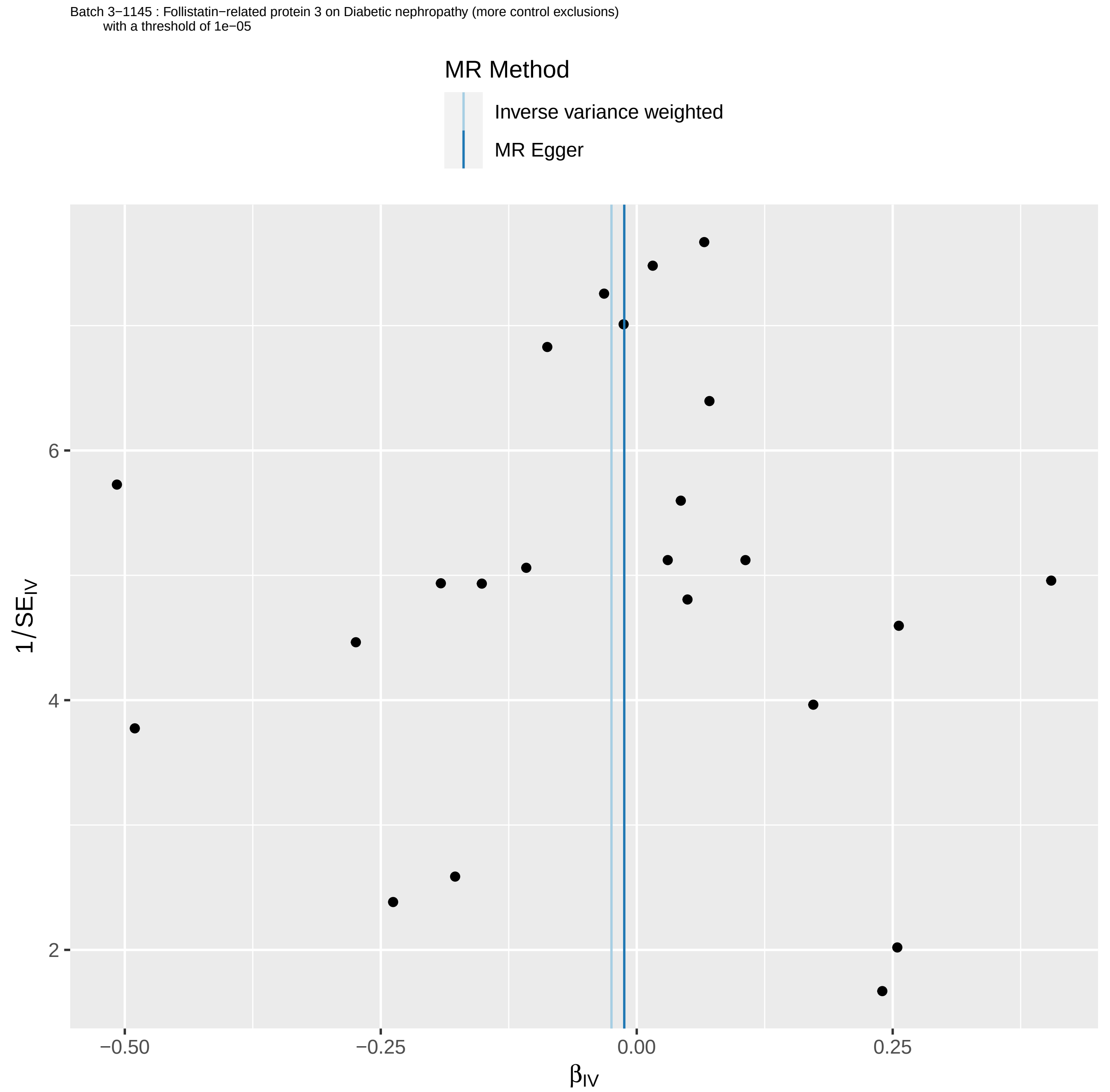
Batch 3−1145 : Follistatin−related protein 3 on Diabetic nephropathy (more control exclusions)
with a threshold of 1e−05
MR Method
Inverse variance weighted
MR Egger
6
4
2
1/SE$_{IV}$
−0.50
−0.25
0.00
0.25
β_{IV}

Batch 3−1146 : Follistatin−related protein 4 on Diabetic nephropathy (more control exclusions)
with a threshold of 1e−05

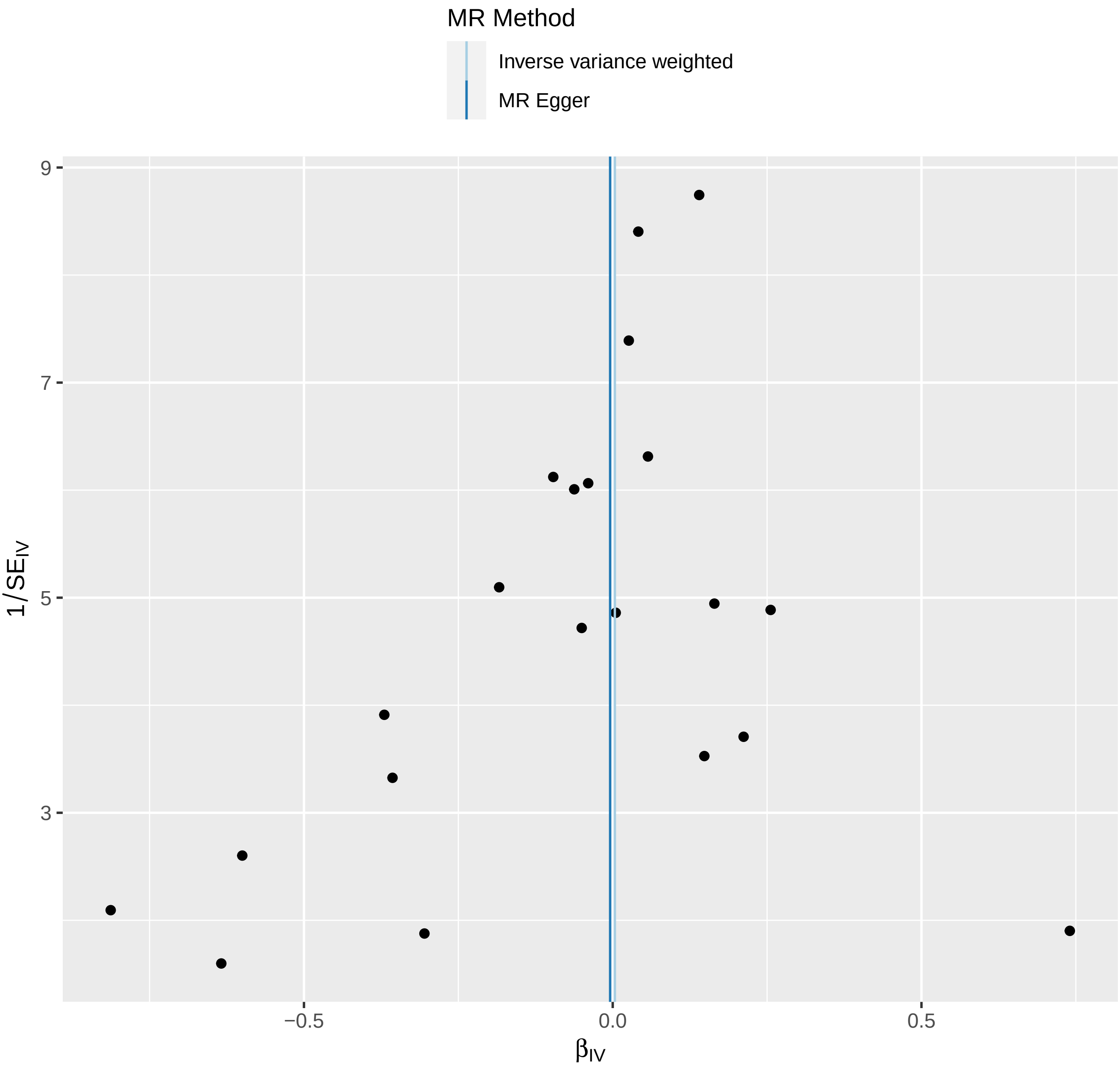

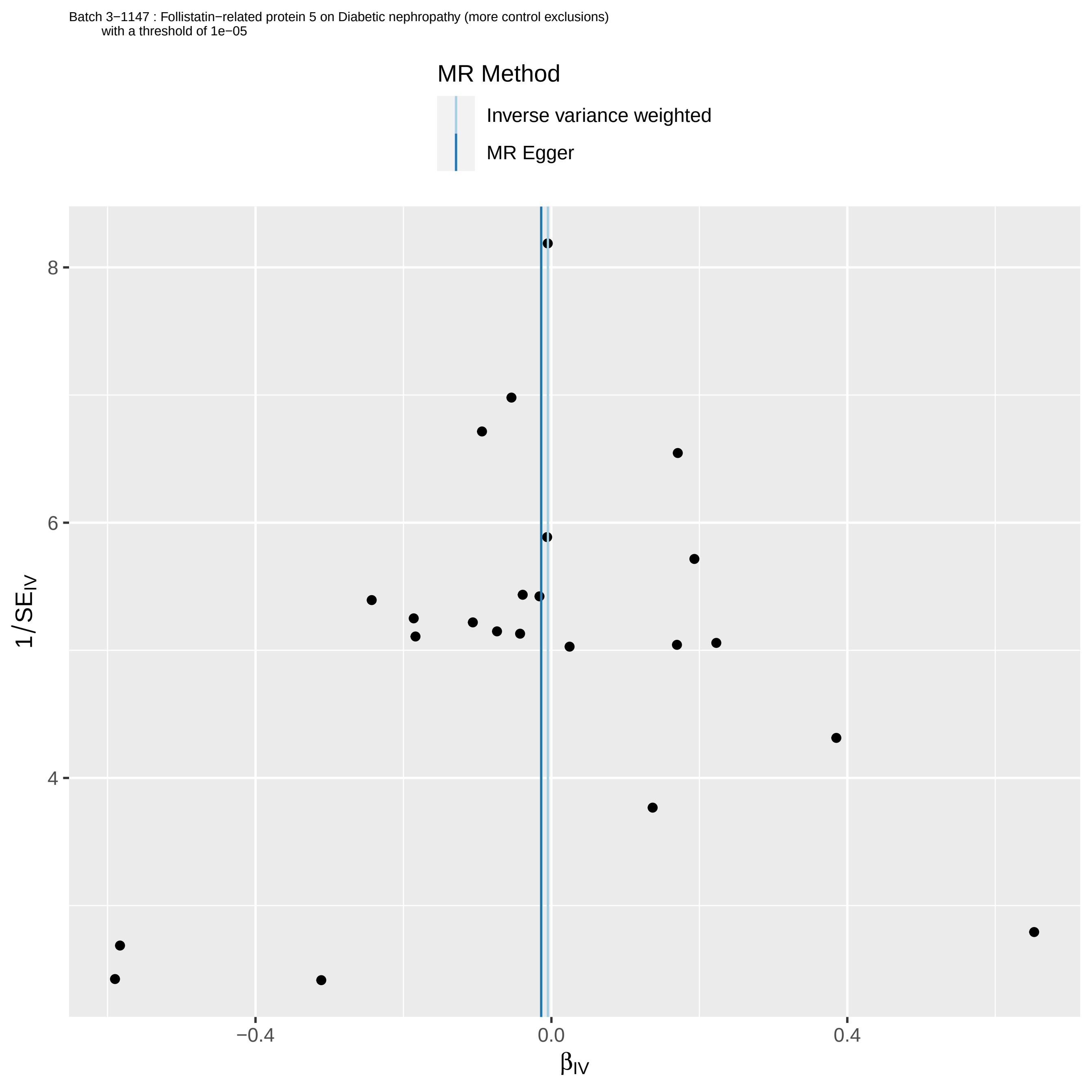

Batch 3−1147 : Follistatin−related protein 5 on Diabetic nephropathy (more control exclusions)
with a threshold of 1e−05
MR Method
Inverse variance weighted
MR Egger
8
6
4
1/SE$_{IV}$
−0.4
0.0
0.4
β$_{IV}$

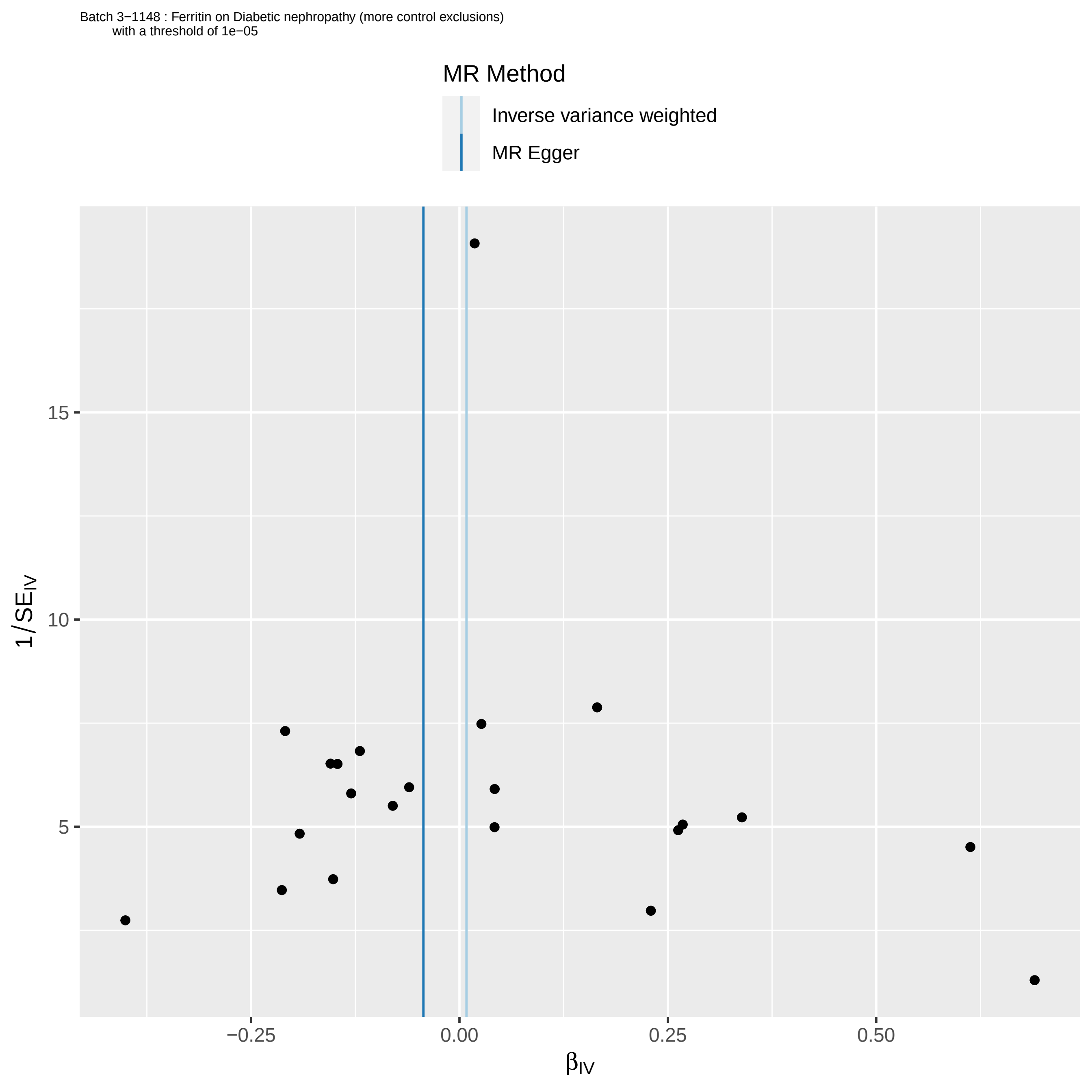

Batch 3−1148 : Ferritin on Diabetic nephropathy (more control exclusions)
with a threshold of 1e−05
MR Method
Inverse variance weighted
MR Egger
15
10
5
$1/SE_{IV}$
−0.25
0.00
0.25
0.50
β_{IV}

Batch 3−1149 : Ferritin on Diabetic nephropathy (more control exclusions)
with a threshold of 1e−05

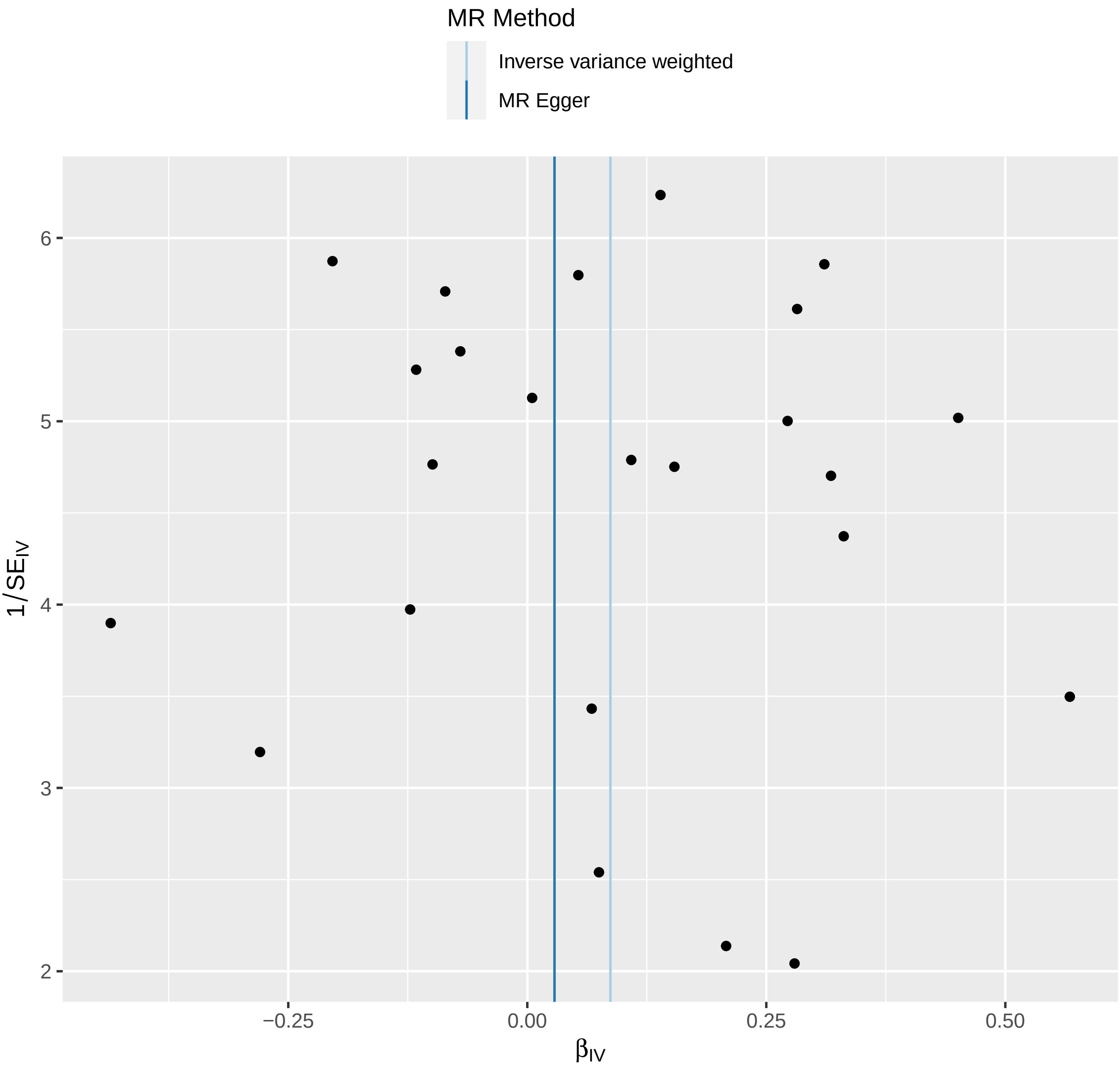

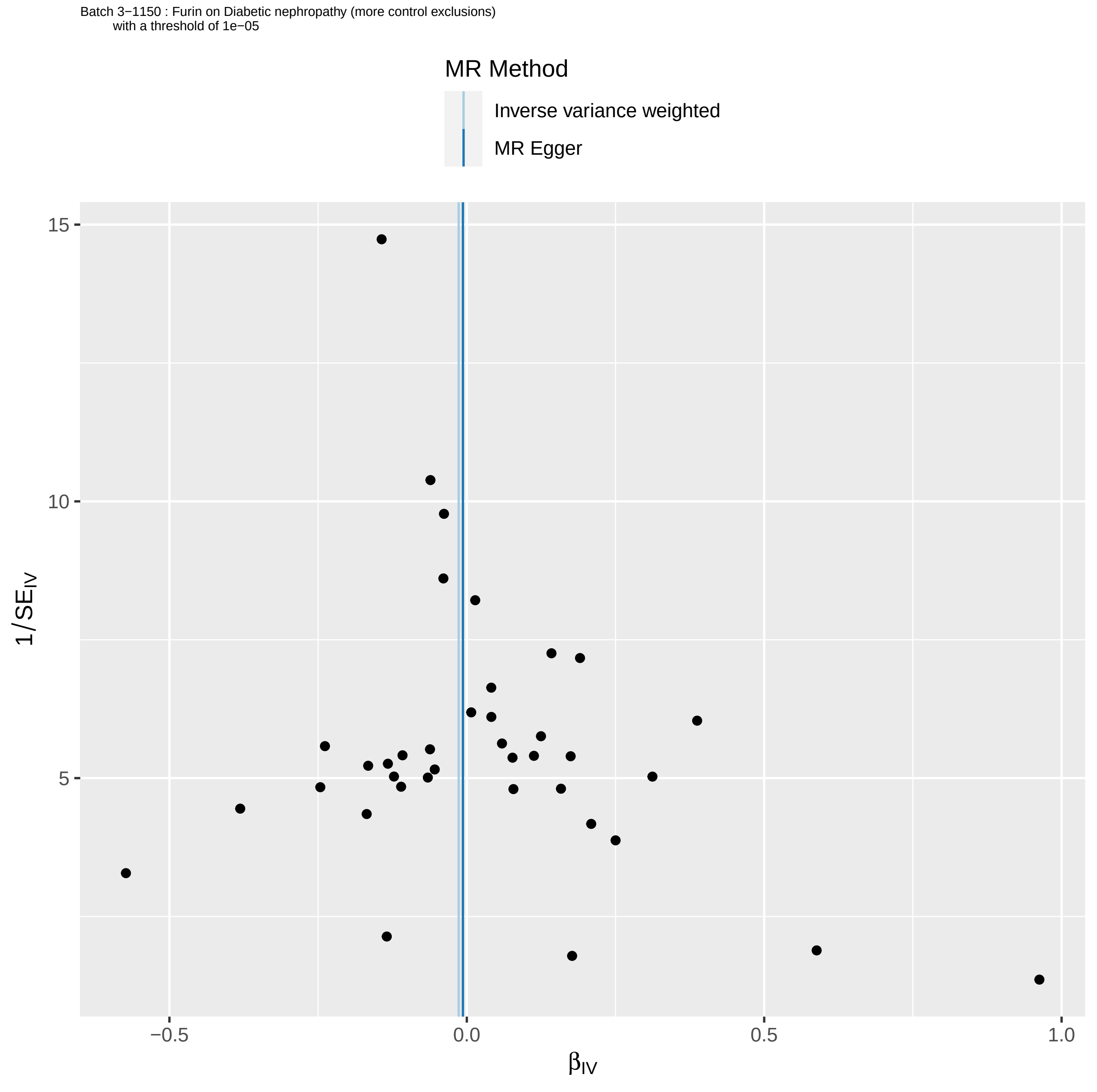

Batch 3−1150 : Furin on Diabetic nephropathy (more control exclusions)
with a threshold of 1e−05
MR Method
Inverse variance weighted
MR Egger
15
10
5
1/SE_IV
−0.5
0.0
0.5
1.0
β_IV

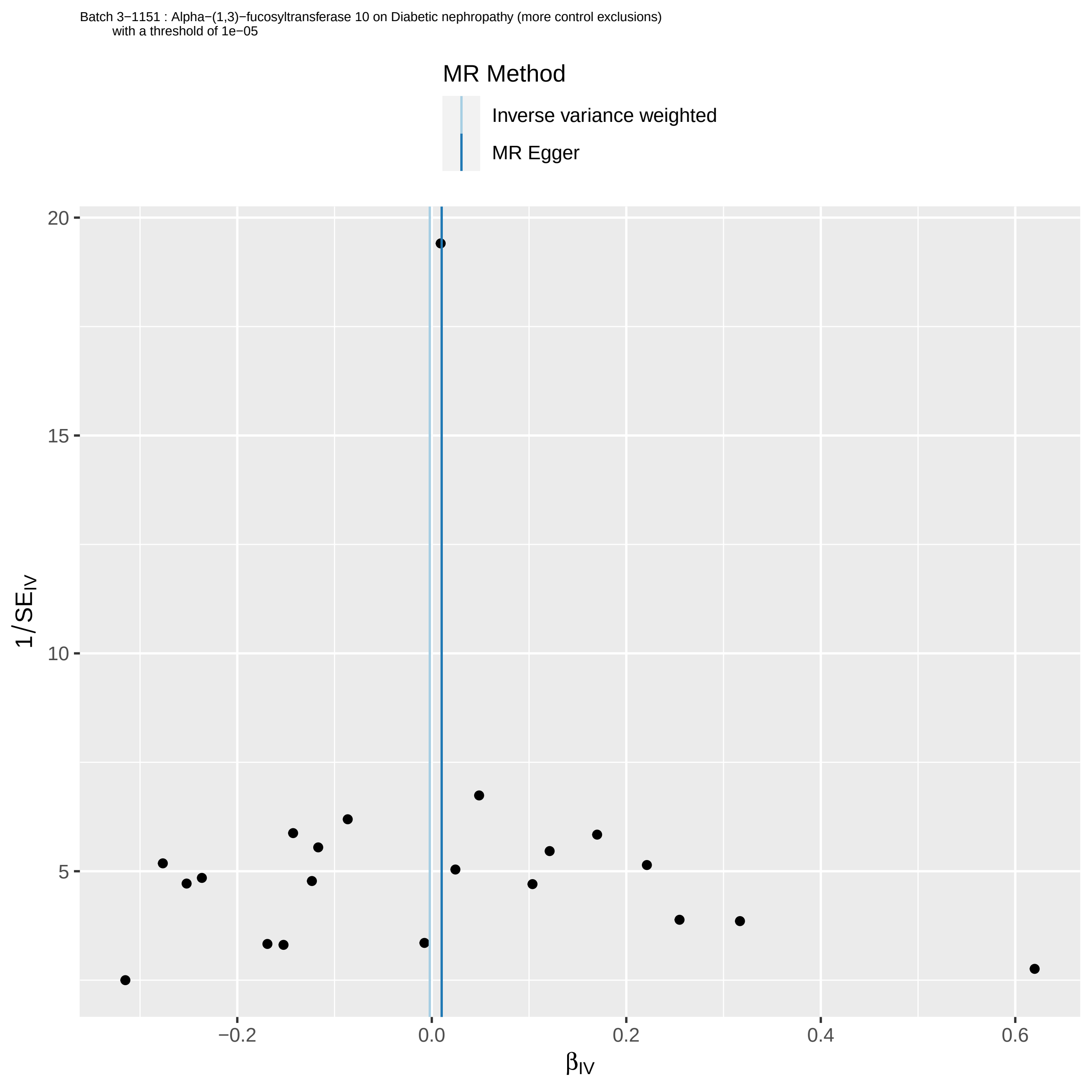
Batch 3−1151 : Alpha−(1,3)−fucosyltransferase 10 on Diabetic nephropathy (more control exclusions)
with a threshold of 1e−05
MR Method
Inverse variance weighted
MR Egger
20
15
10
5
1/SE_IV
−0.2
0.0
0.2
0.4
0.6
β_IV

Batch 3−1152 : Galactoside 3(4)−L−fucosyltransferase on Diabetic nephropathy (more control exclusions)
with a threshold of 1e−05

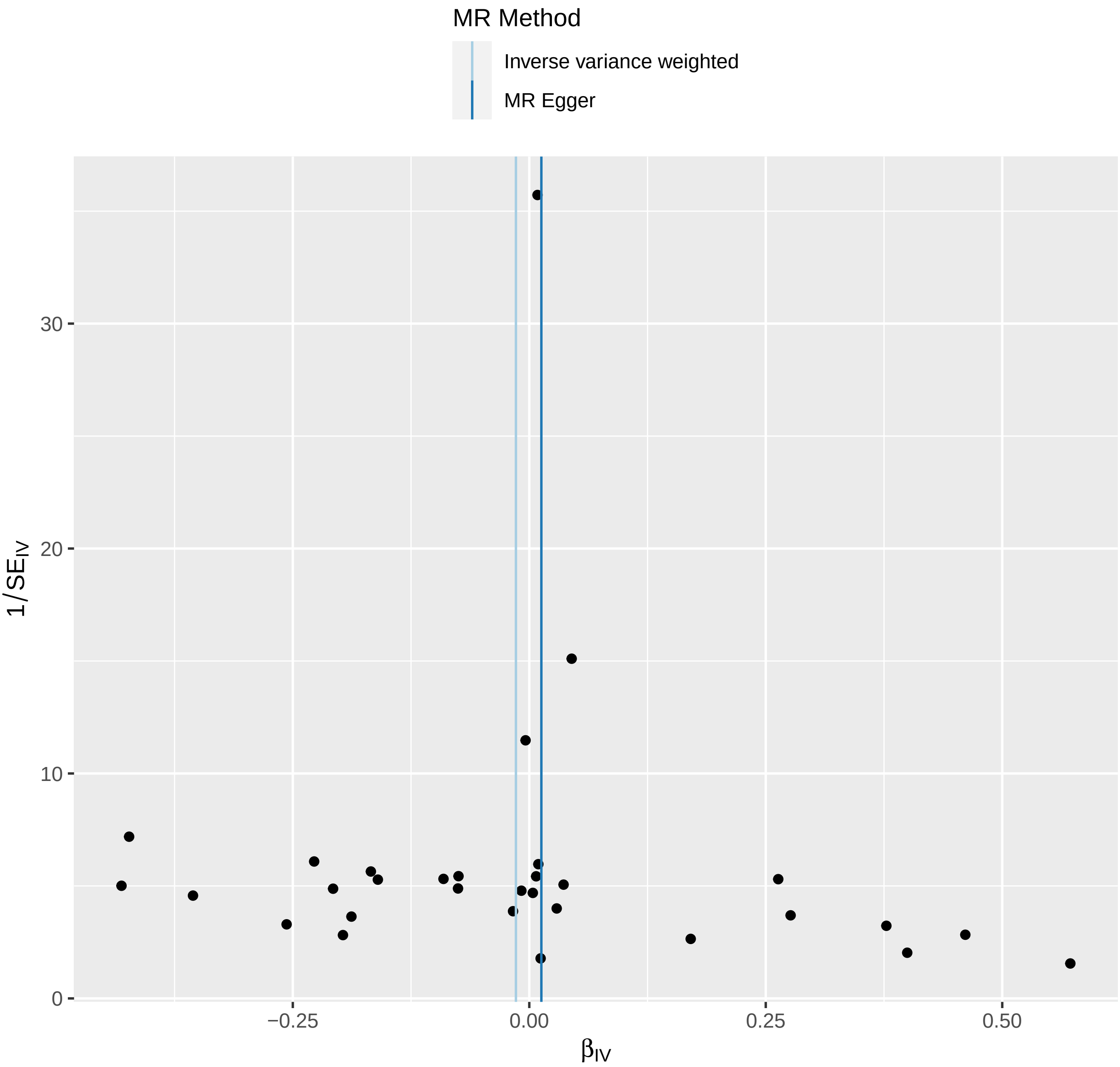

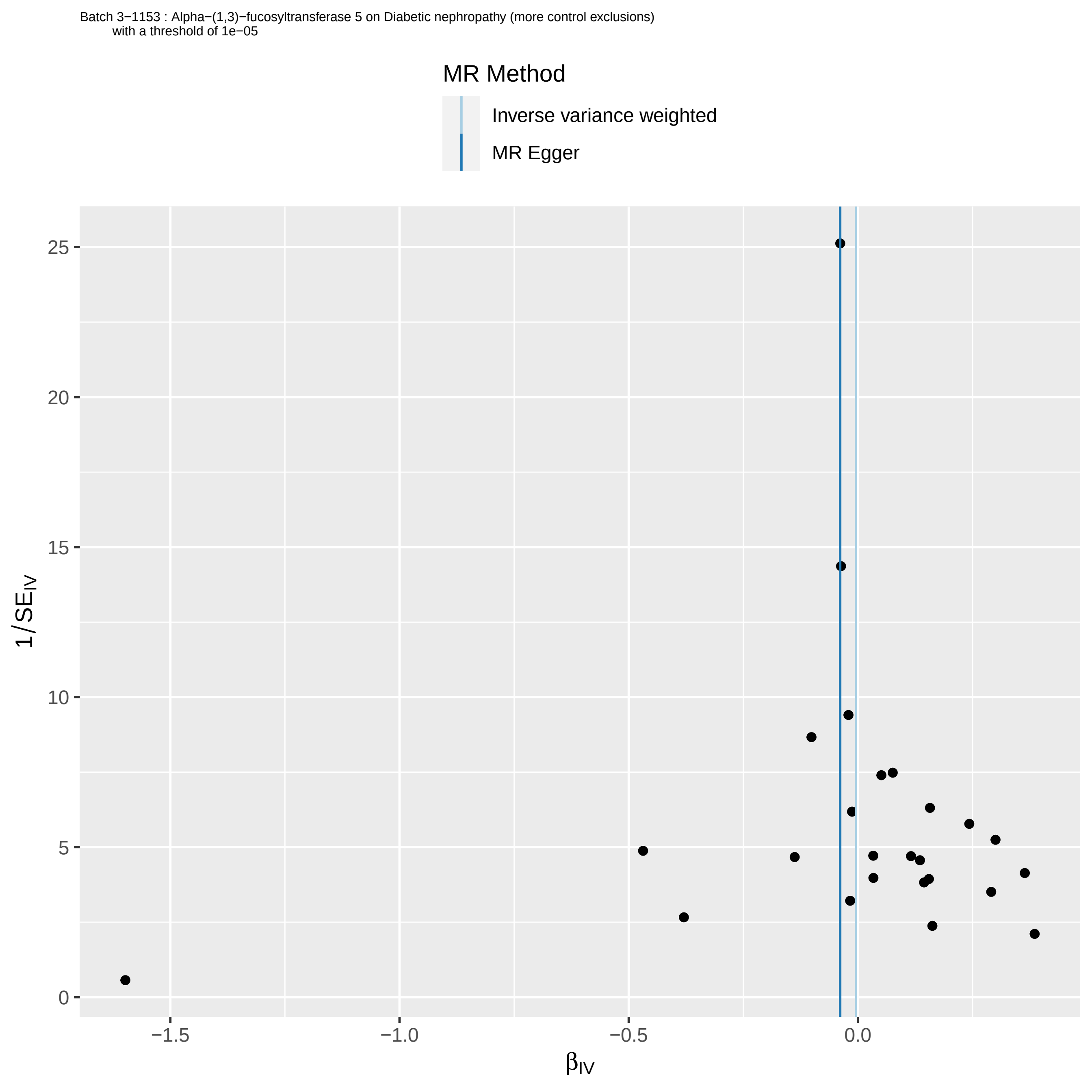

Batch 3−1153 : Alpha−(1,3)−fucosyltransferase 5 on Diabetic nephropathy (more control exclusions)
with a threshold of 1e−05
MR Method
Inverse variance weighted
MR Egger
25
20
15
10
5
0
1/SE_IV
−1.5
−1.0
−0.5
0.0
β_IV

Batch 3−1154 : Alpha−(1,6)−fucosyltransferase on Diabetic nephropathy (more control exclusions)
with a threshold of 1e−05

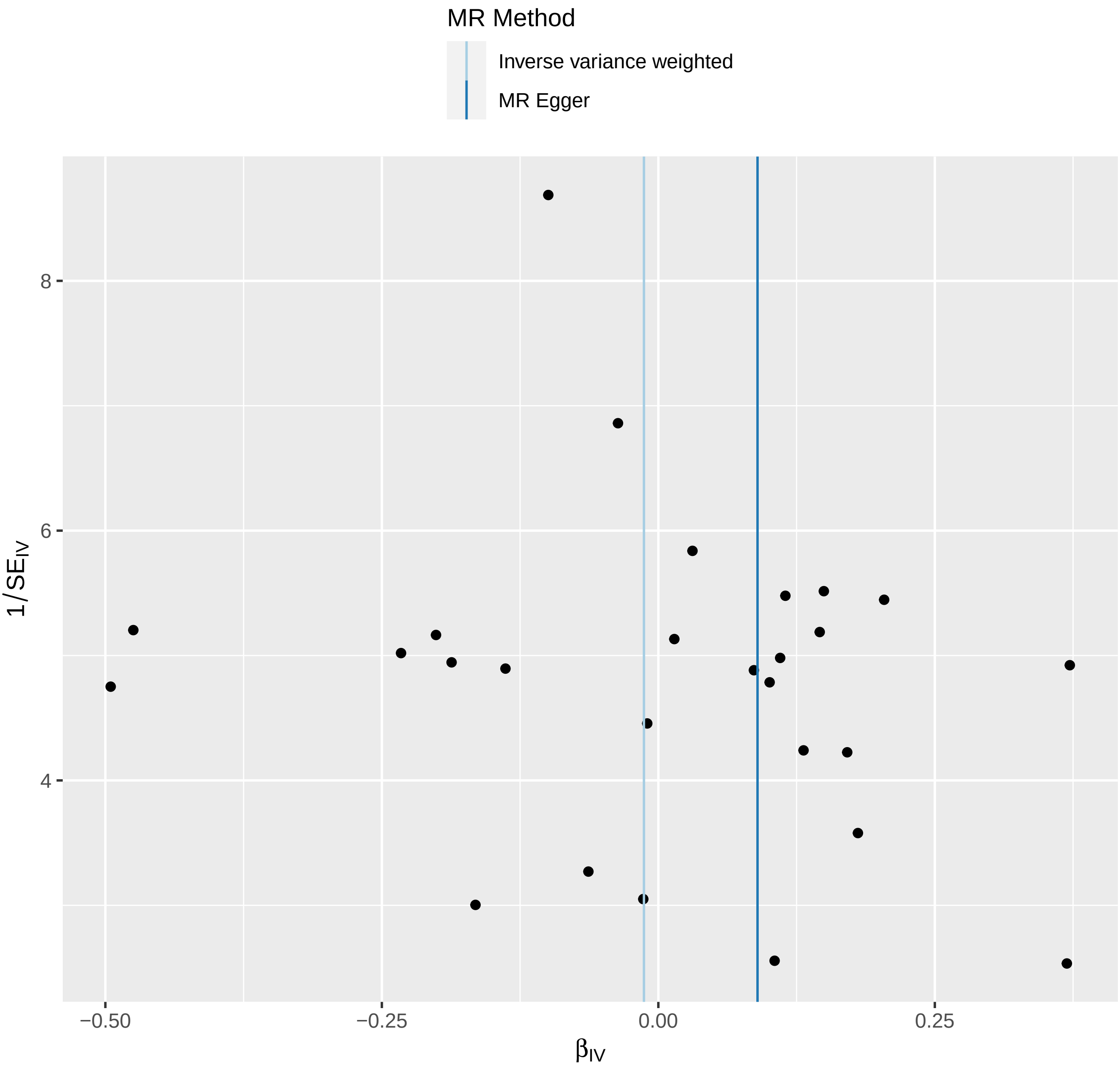

Batch 3−1155 : Alpha−(1,3)−fucosyltransferase 9 on Diabetic nephropathy (more control exclusions)
with a threshold of 1e−05

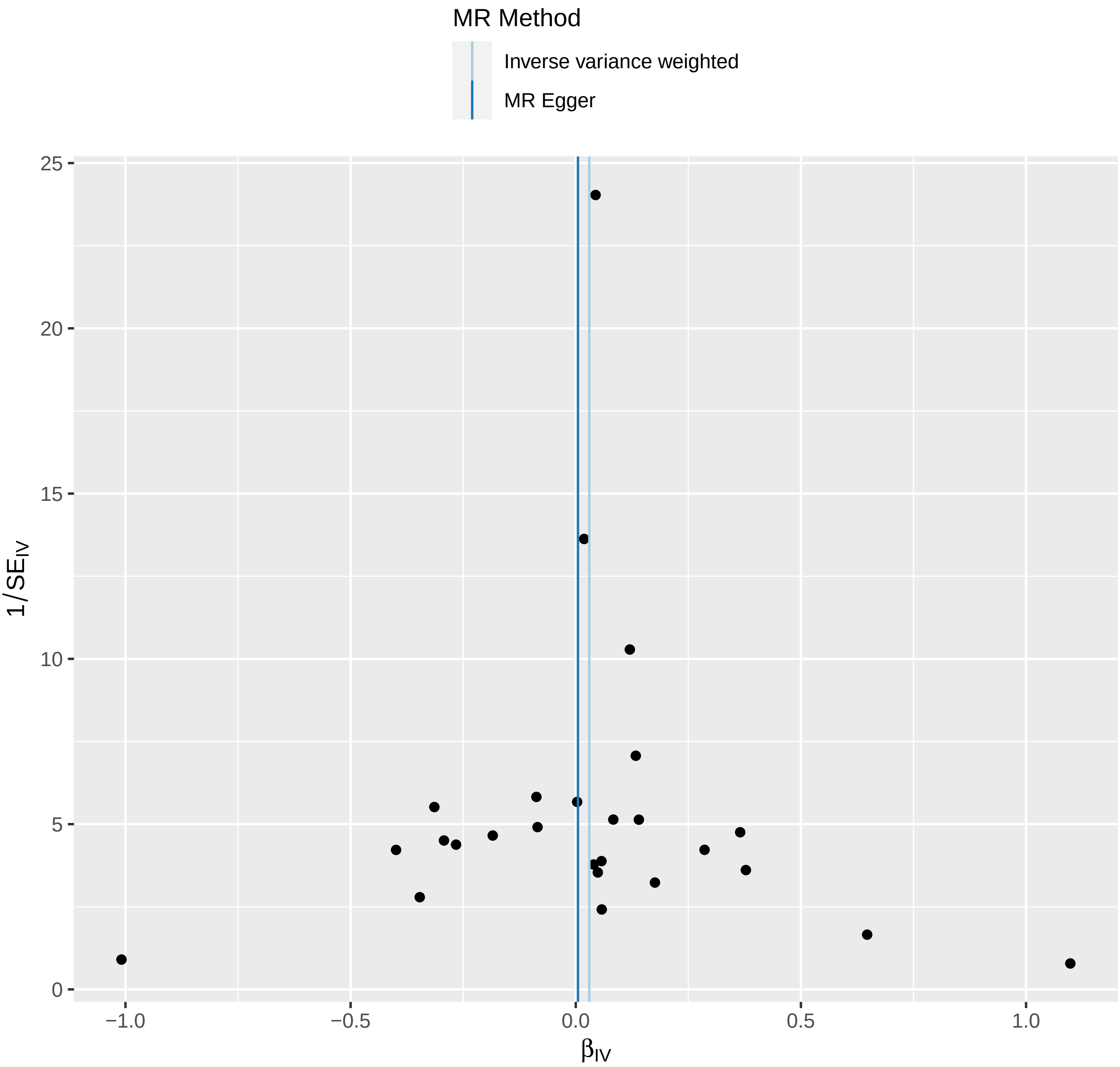

Batch 3−1156 : Fragile X mental retardation syndrome−related protein 1 on Diabetic nephropathy (more control exclusions)
with a threshold of 1e−05

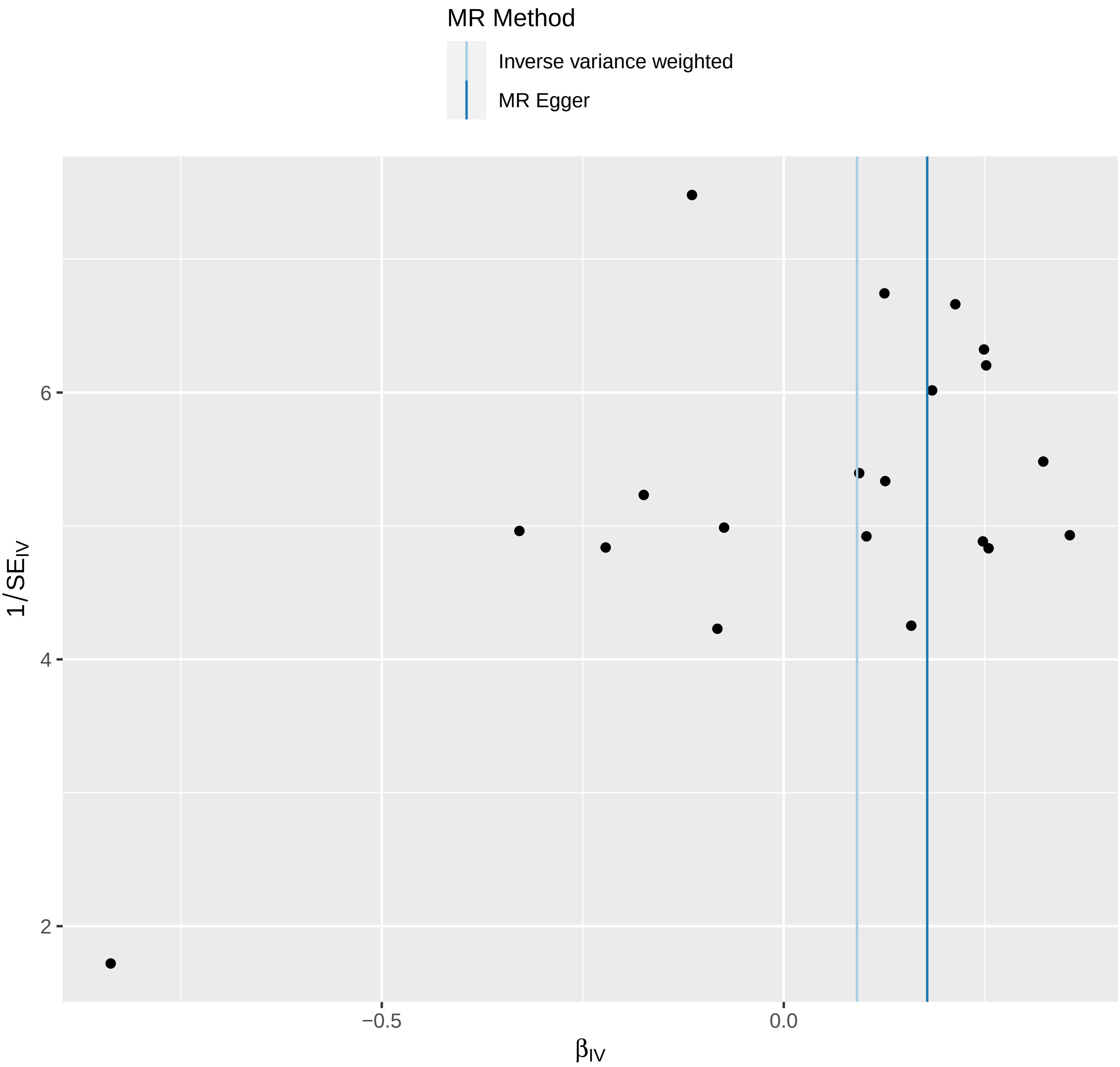

Batch 3−1157 : Tyrosine−protein kinase Fyn on Diabetic nephropathy (more control exclusions)
with a threshold of 1e−05

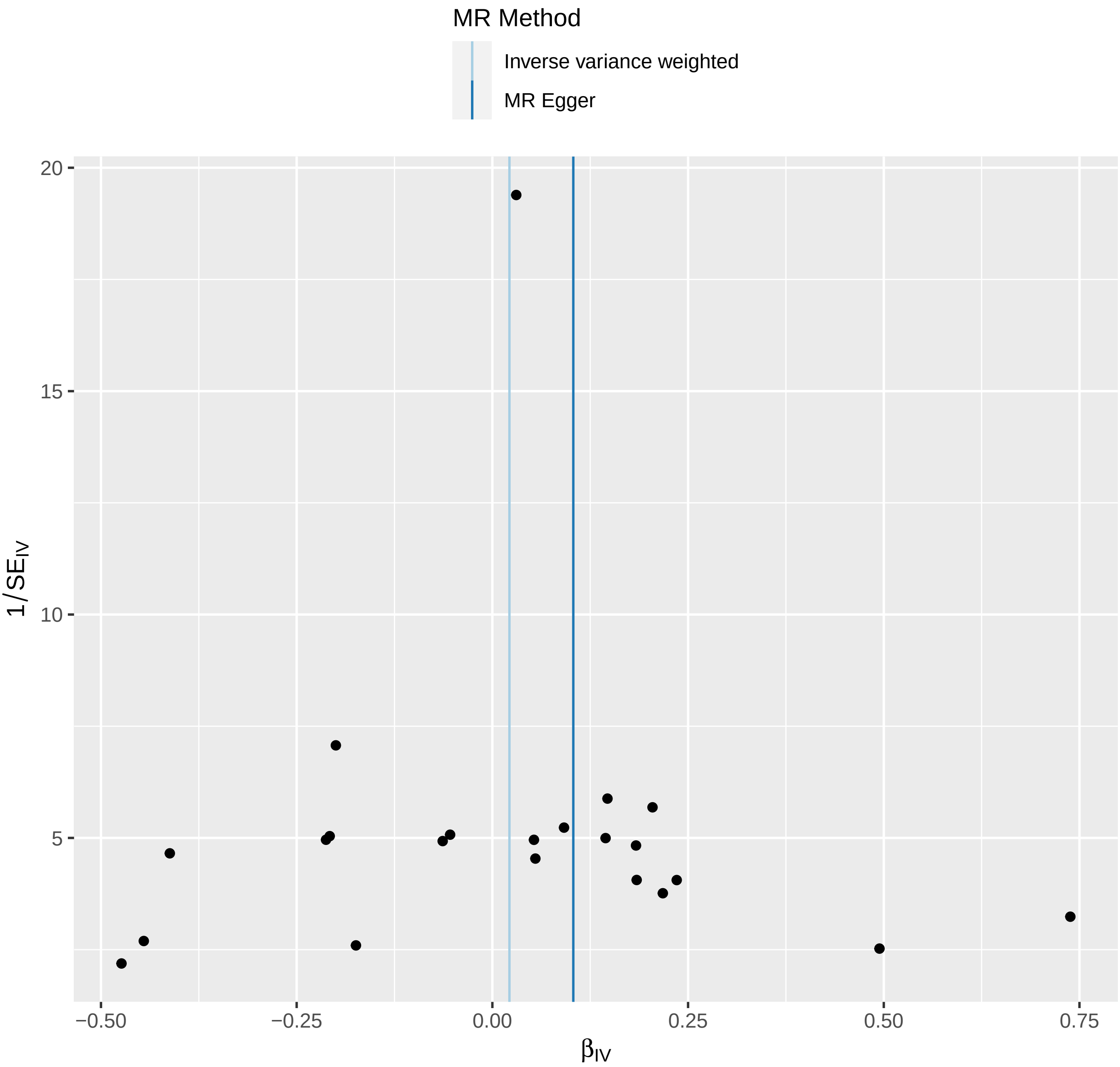

Batch 3−1158 : G0/G1 switch protein 2 on Diabetic nephropathy (more control exclusions)
with a threshold of 1e−05

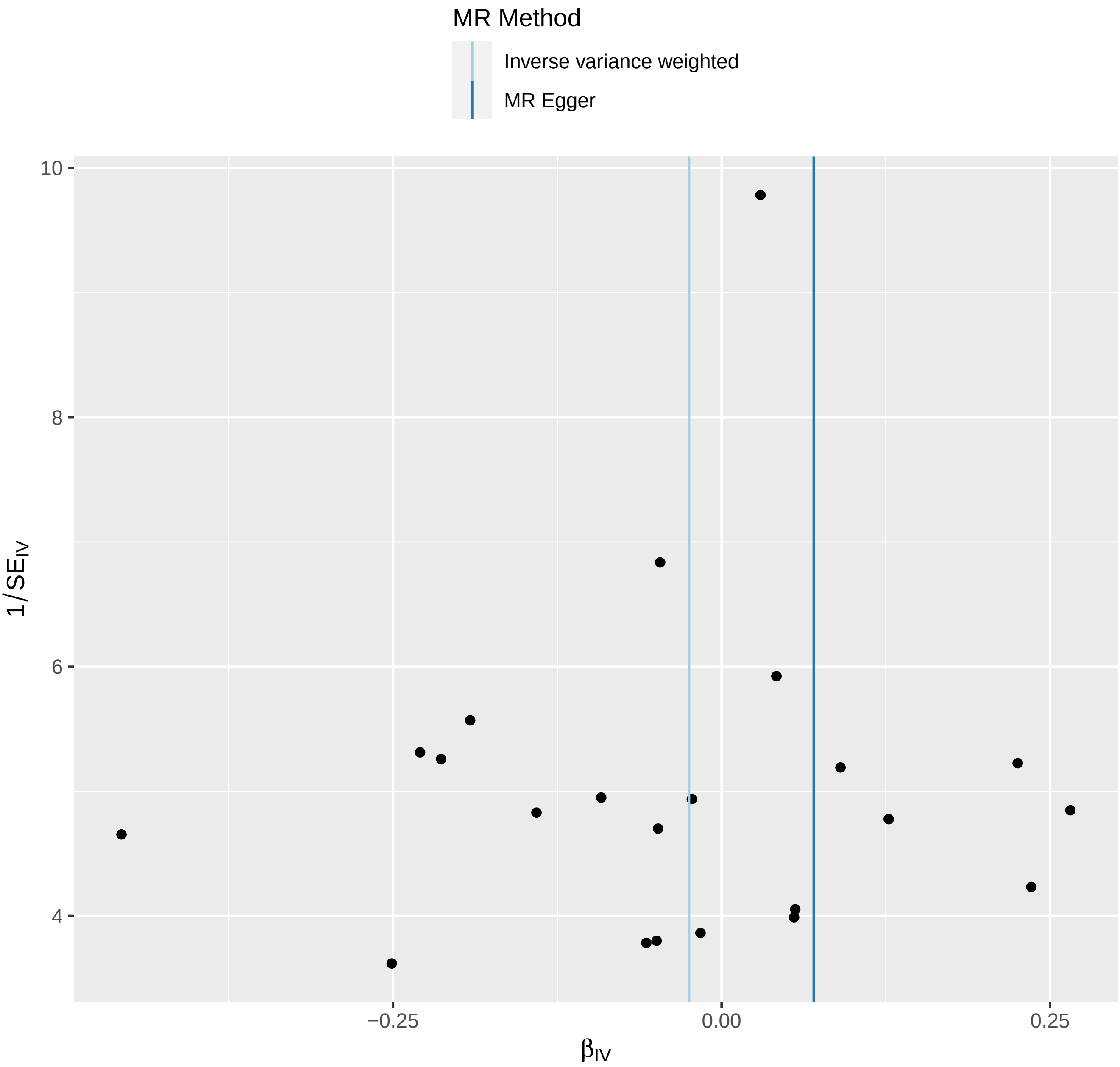

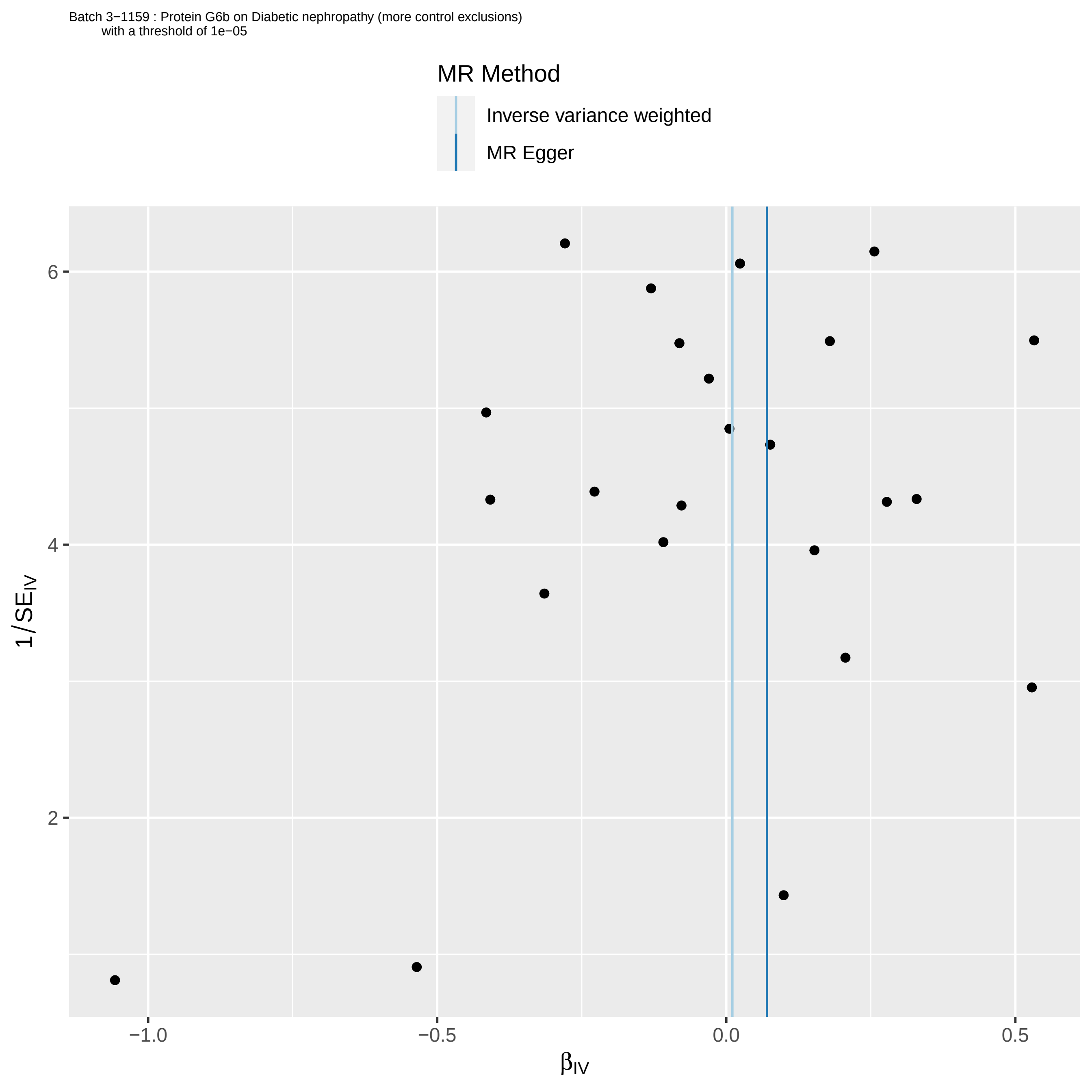

Batch 3−1159 : Protein G6b on Diabetic nephropathy (more control exclusions)
with a threshold of 1e−05
MR Method
Inverse variance weighted
MR Egger
6
4
2
1/SE_IV
−1.0
−0.5
0.0
0.5
β_IV

Batch 3−1160 : Protein G6b on Diabetic nephropathy (more control exclusions)
with a threshold of 1e−05

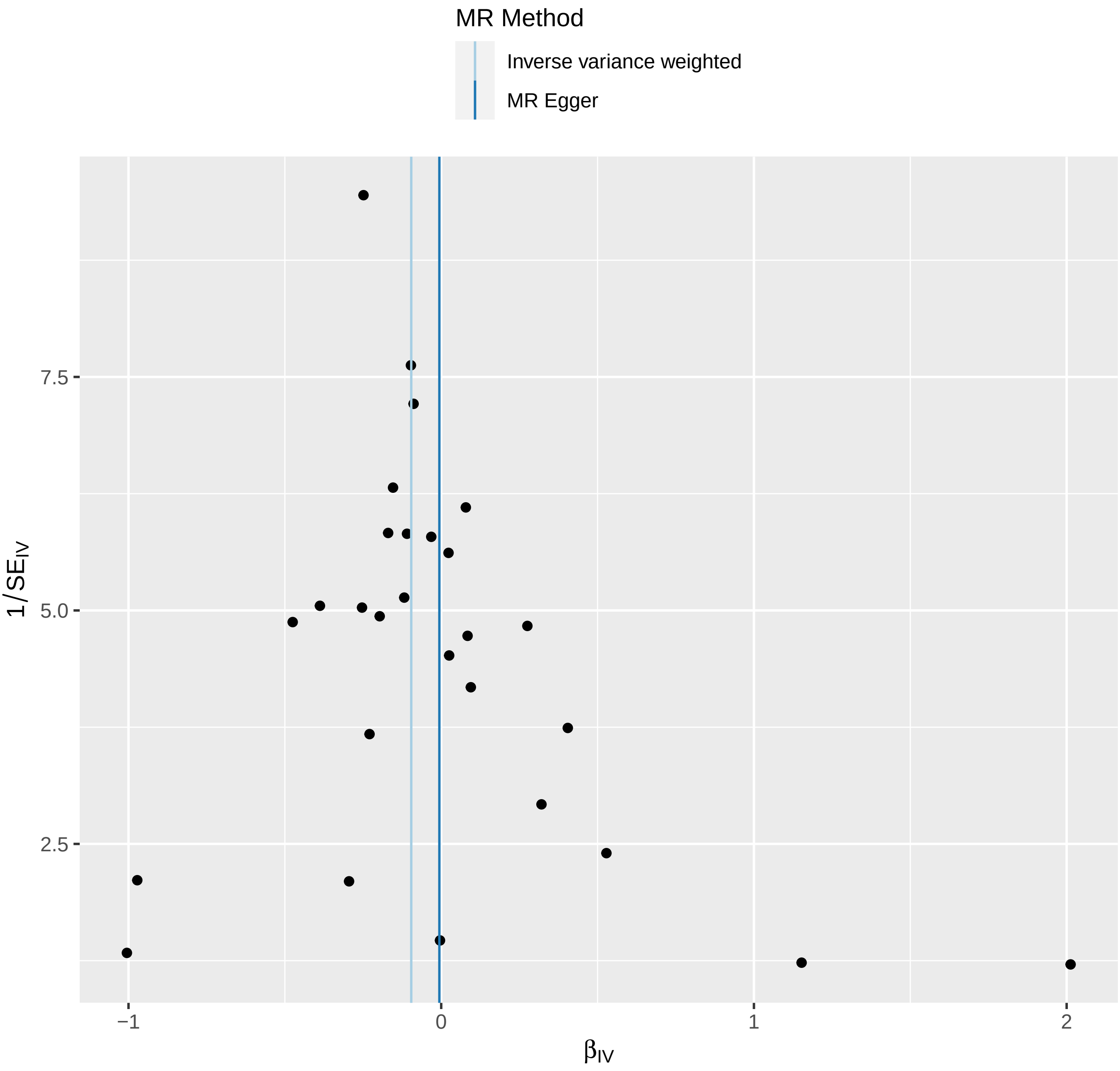

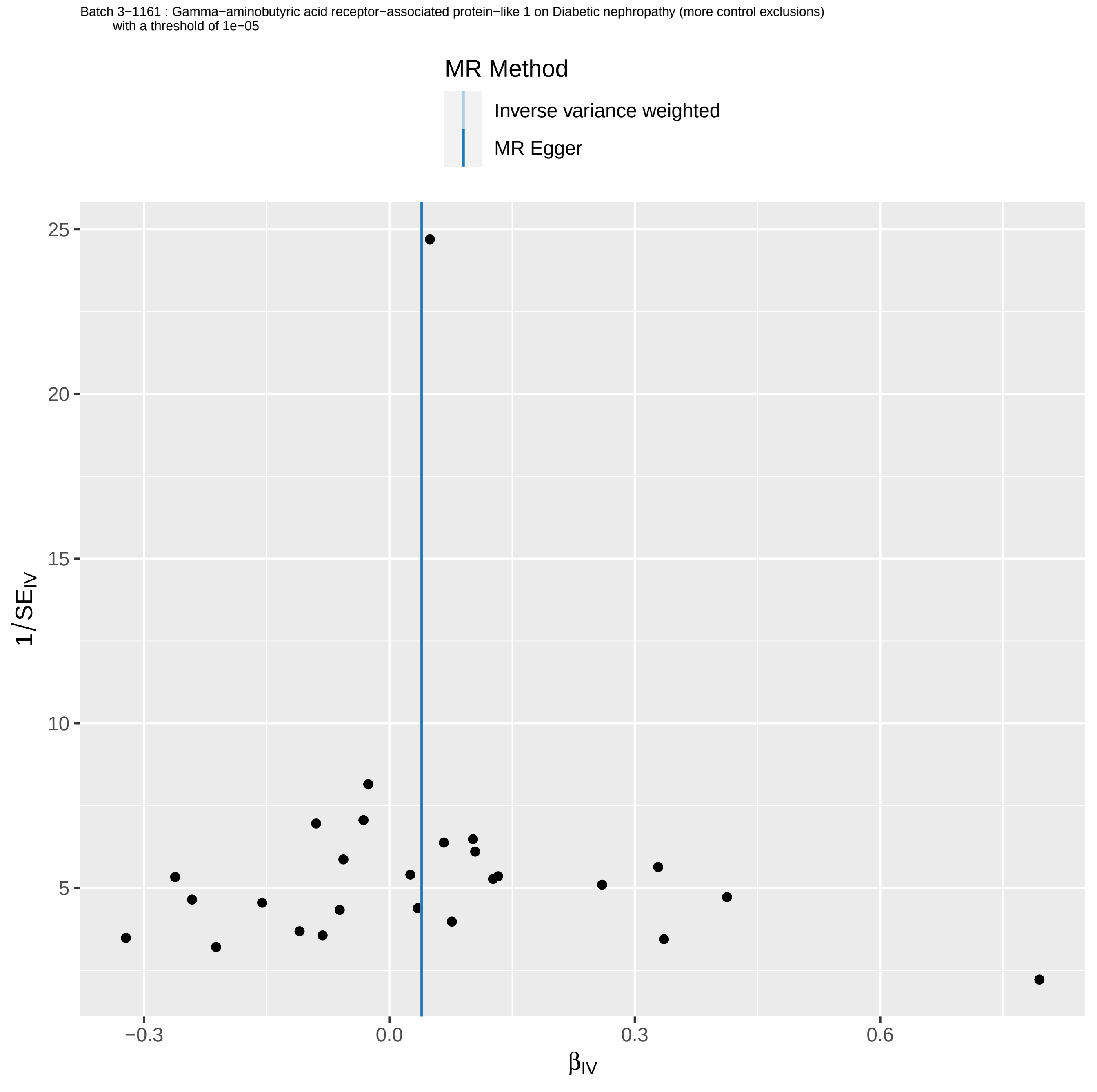

Batch 3−1161 : Gamma−aminobutyric acid receptor−associated protein−like 1 on Diabetic nephropathy (more control exclusions)
with a threshold of 1e−05
MR Method
Inverse variance weighted
MR Egger
25
20
15
10
5
1/SE_IV
−0.3
0.0
0.3
0.6
β_IV

Batch 3−1162 : Gamma−aminobutyric acid receptor−associated protein−like 2 on Diabetic nephropathy (more control exclusions) with a threshold of 1e−05

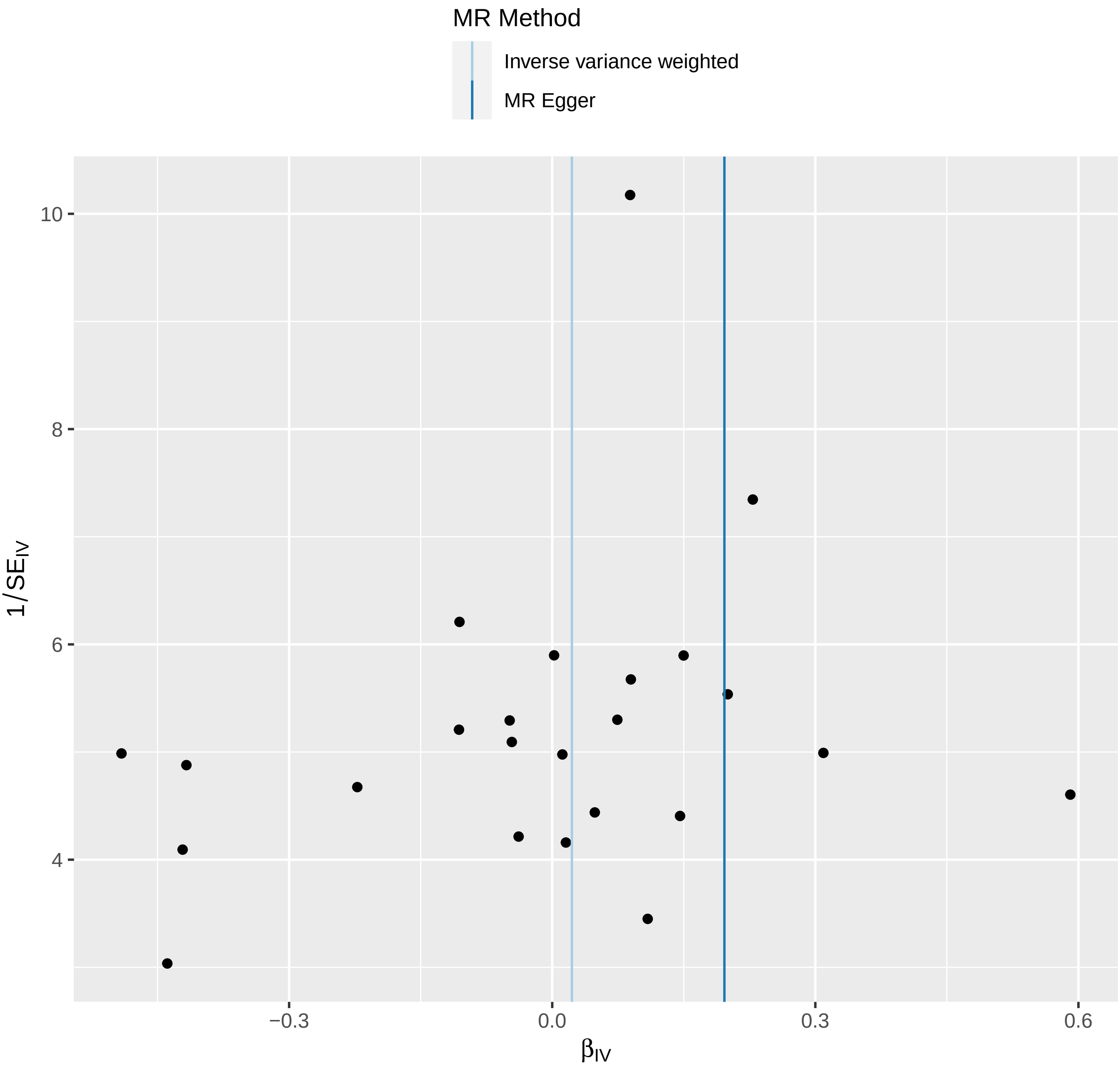

Batch 3−1163 : Gamma−aminobutyric acid type B receptor subunit 2 on Diabetic nephropathy (more control exclusions)
with a threshold of 1e−05

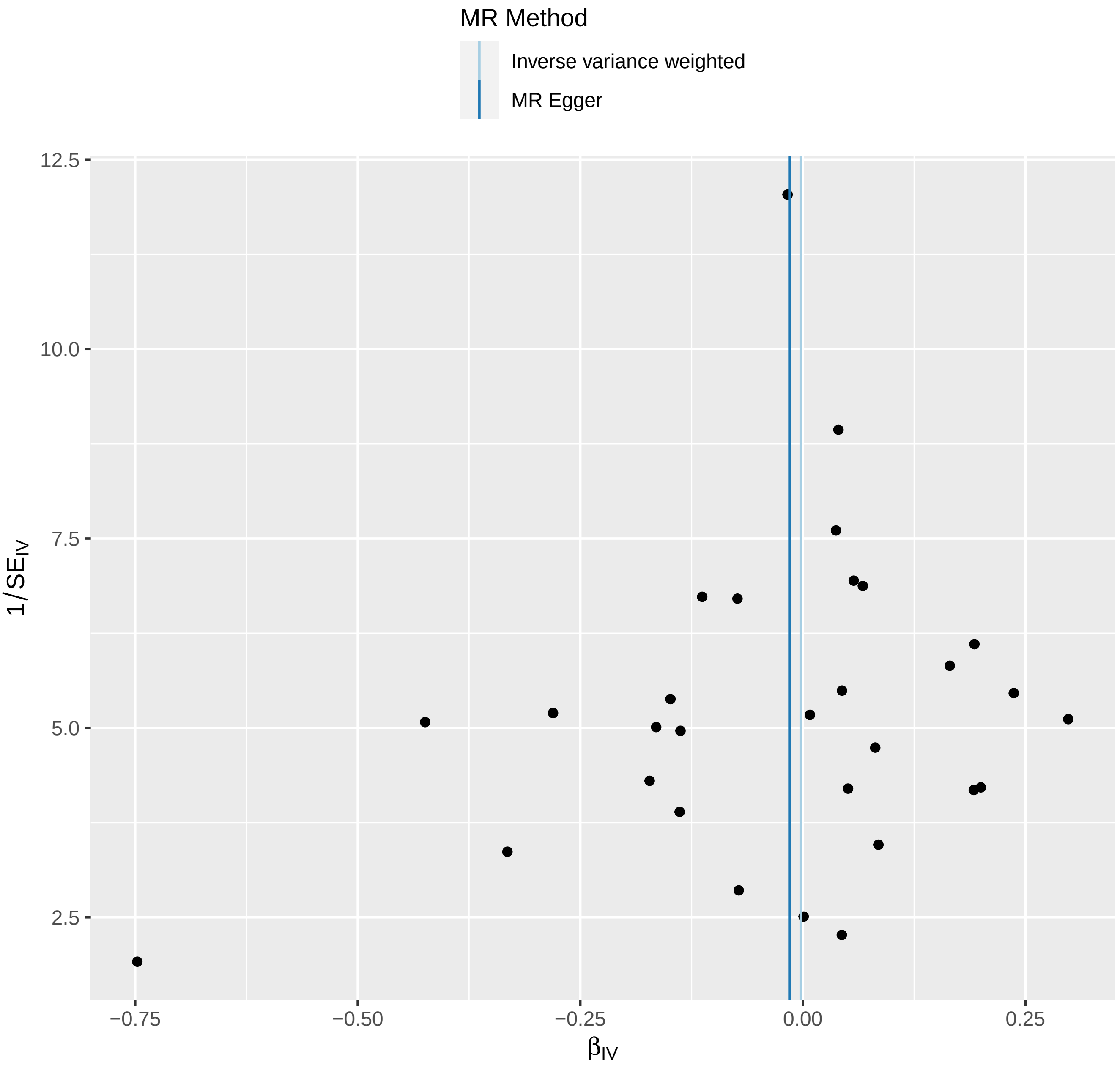

Batch 3−1164 : Gamma−aminobutyric acid type B receptor subunit 2 on Diabetic nephropathy (more control exclusions) with a threshold of 1e−05

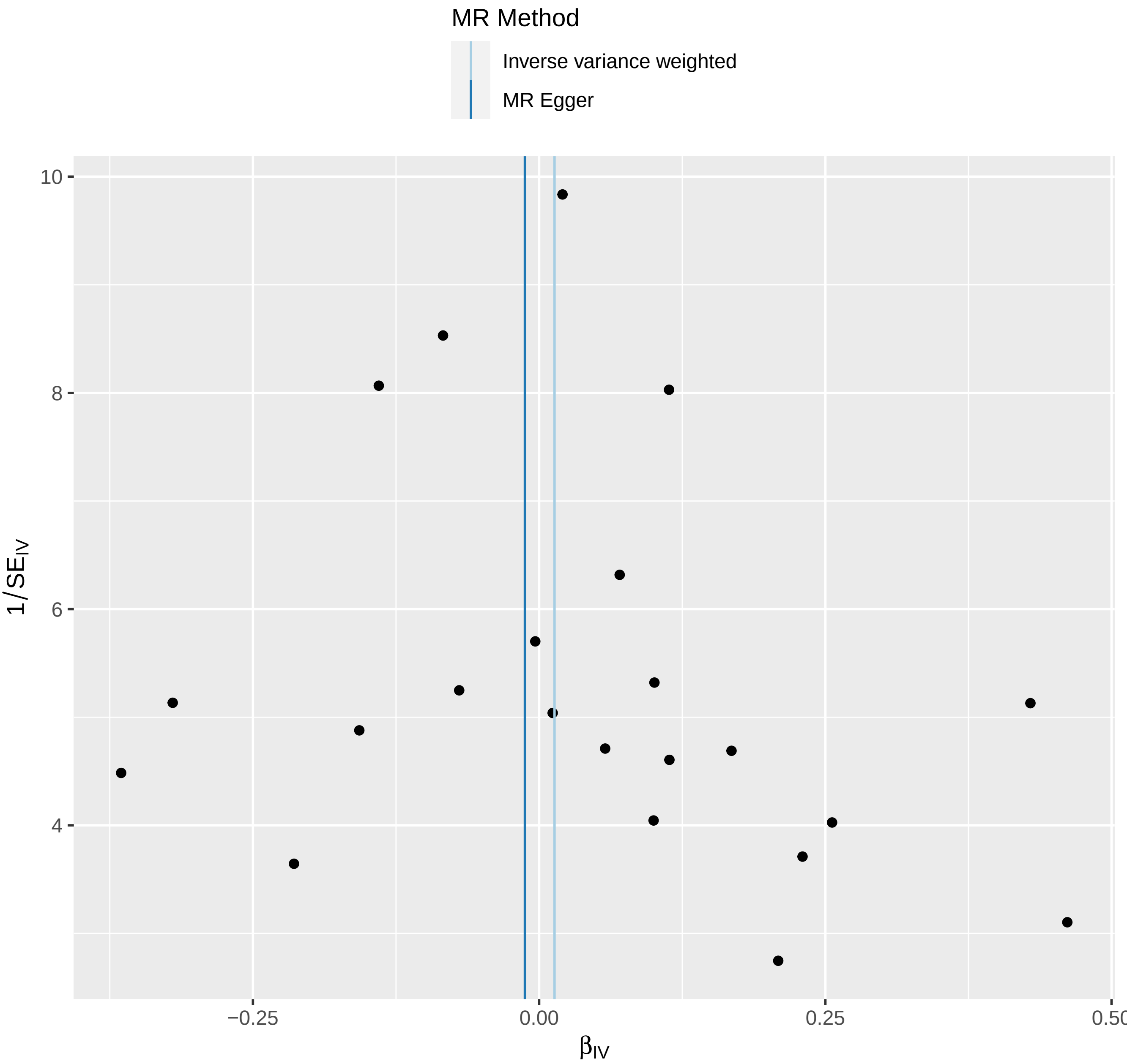

Batch 3−1165 : Growth arrest and DNA damage−inducible proteins−interacting protein 1 on Diabetic nephropathy (more control exclusions) with a threshold of 1e−05

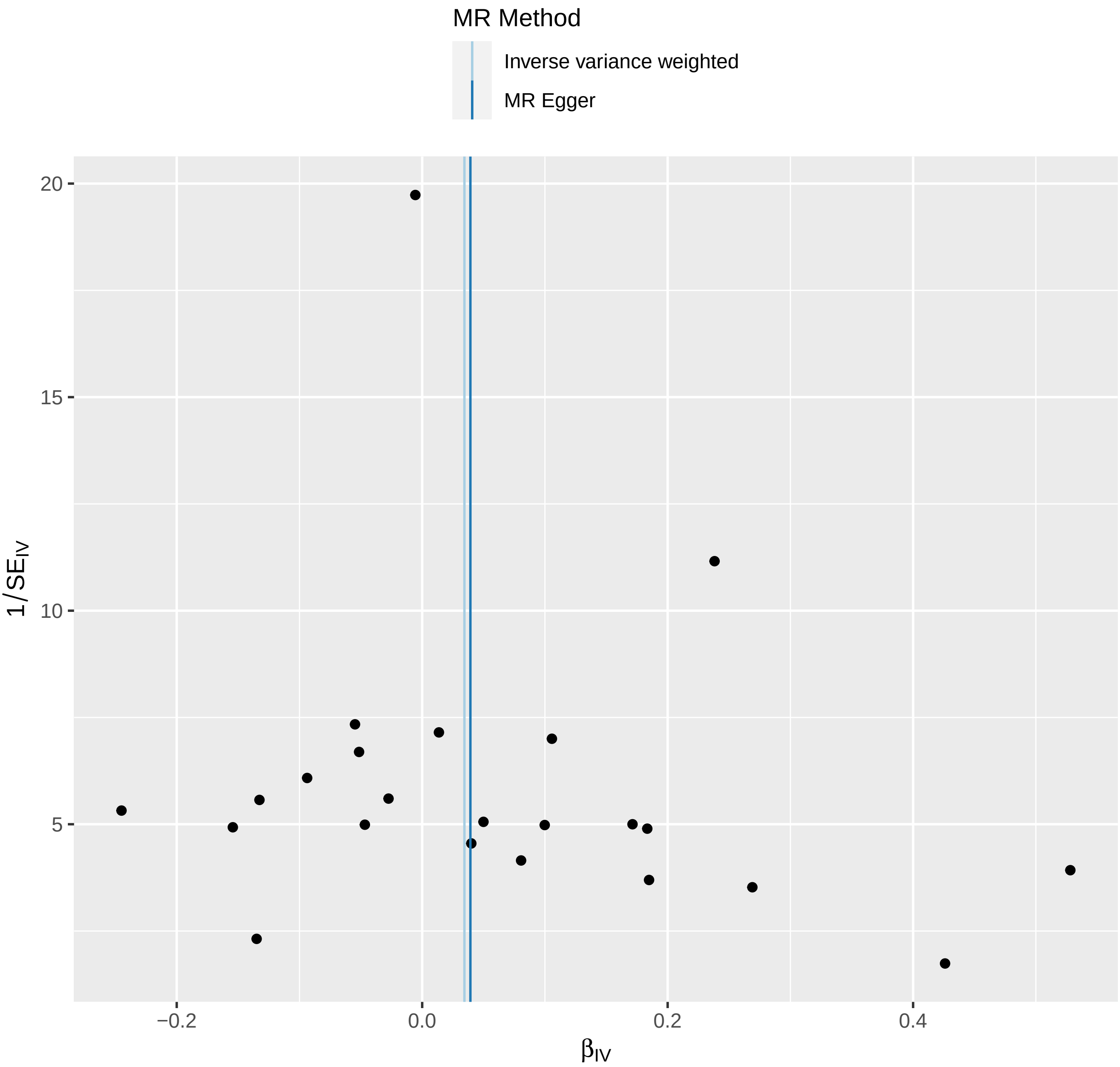

Batch 3−1166 : Galanin peptides on Diabetic nephropathy (more control exclusions)
with a threshold of 1e−05

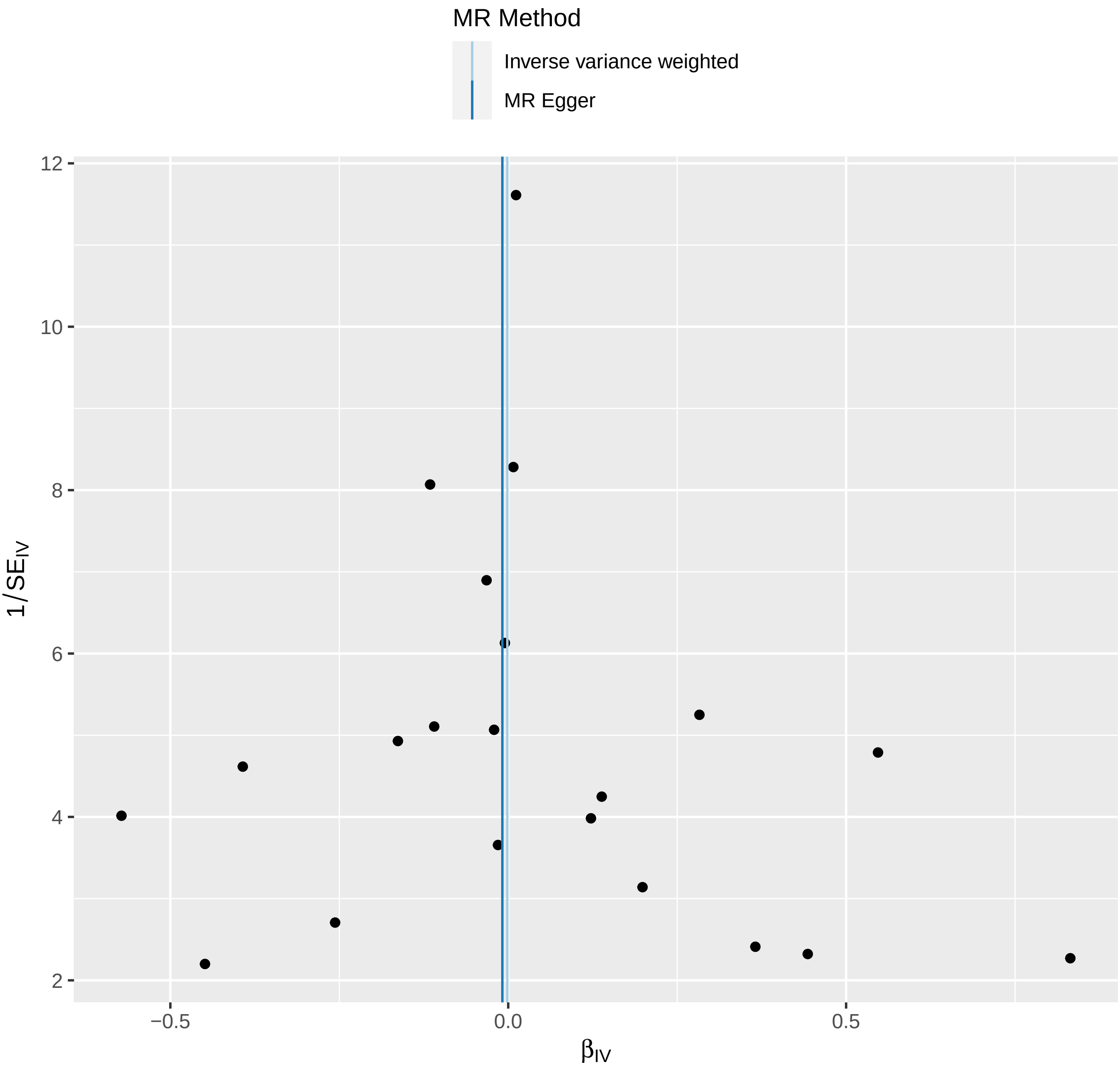

Batch 3−1167 : Polypeptide N−acetylgalactosaminyltransferase 10 on Diabetic nephropathy (more control exclusions) with a threshold of 1e−05

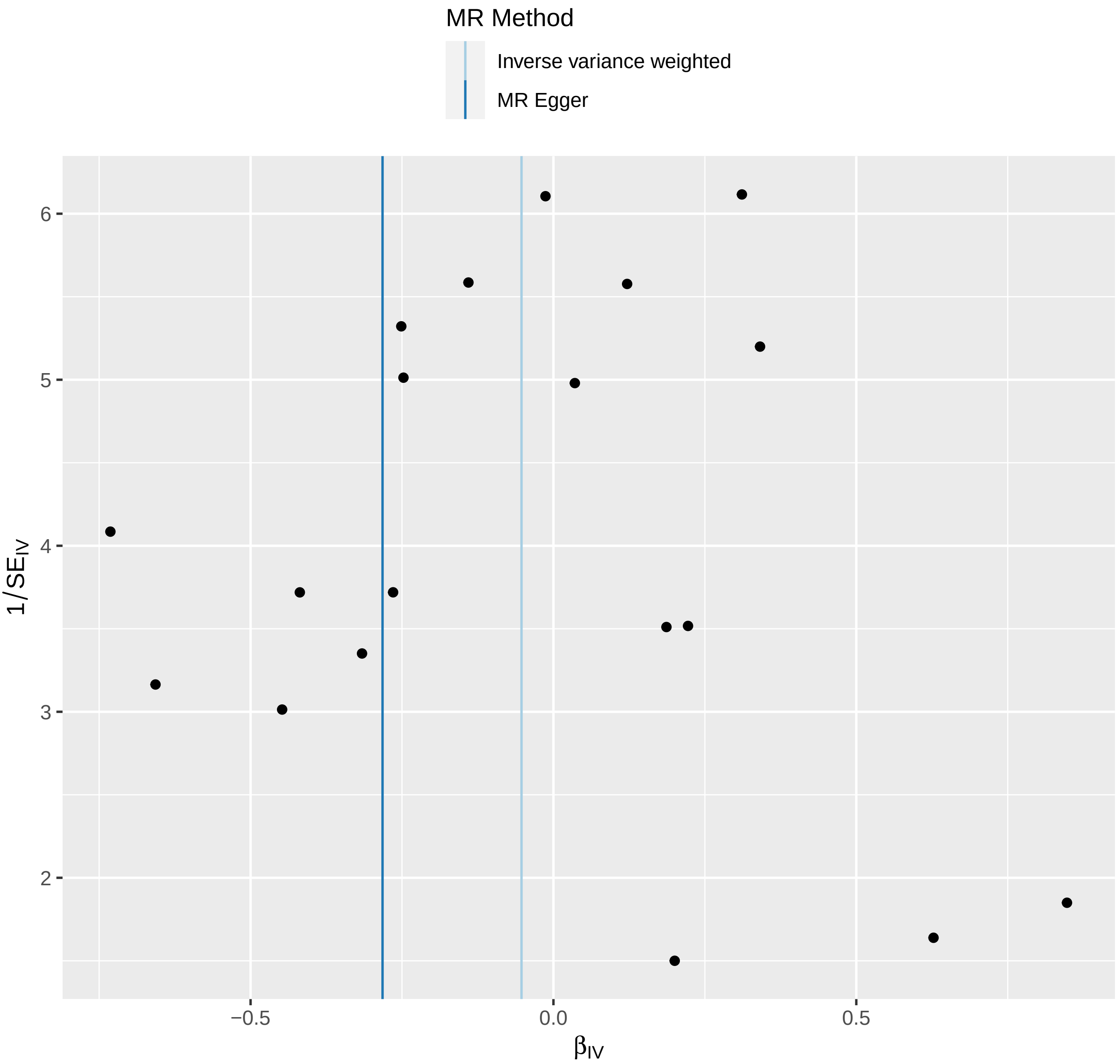

Batch 3−1168 : Polypeptide N−acetylgalactosaminyltransferase 11 on Diabetic nephropathy (more control exclusions) with a threshold of 1e−05

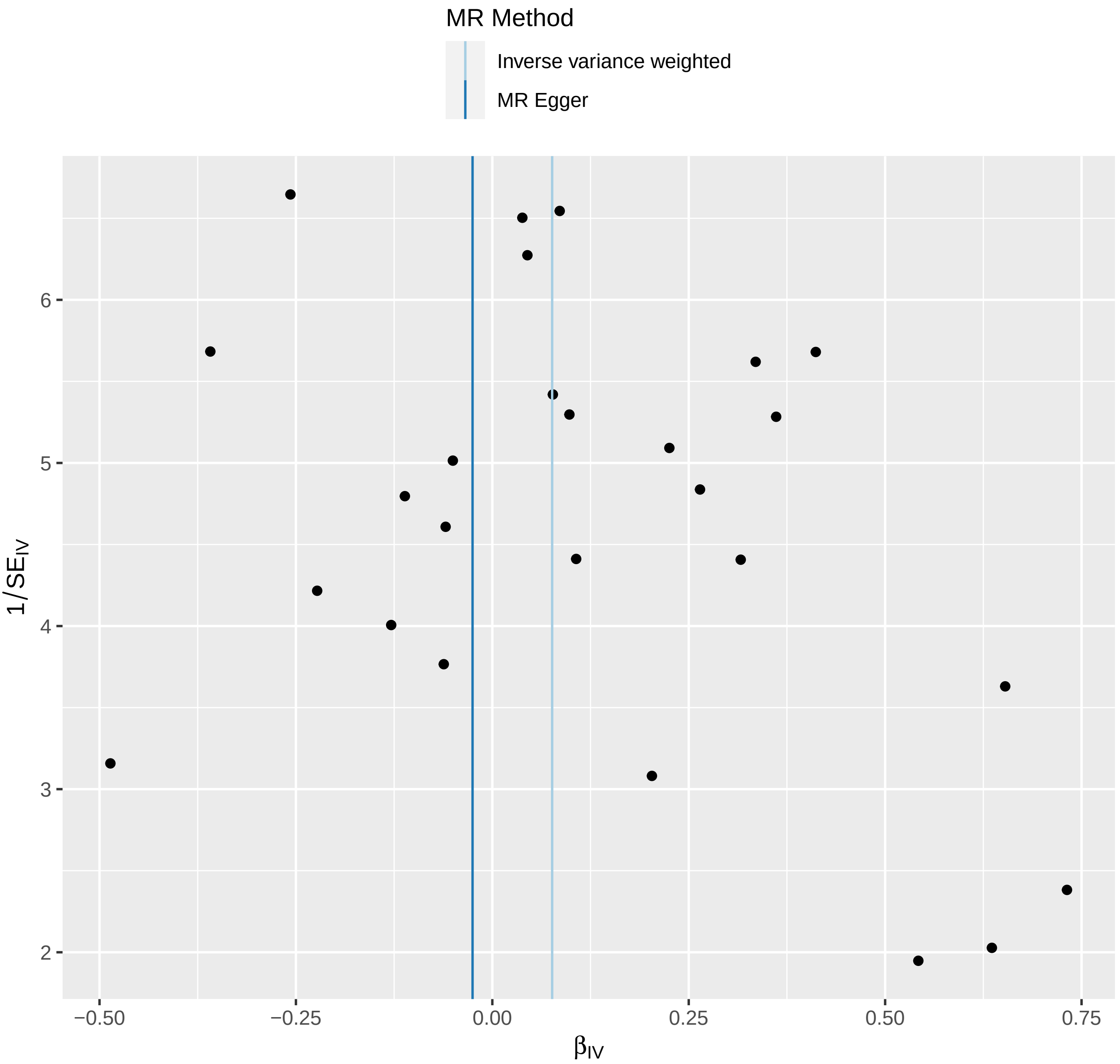

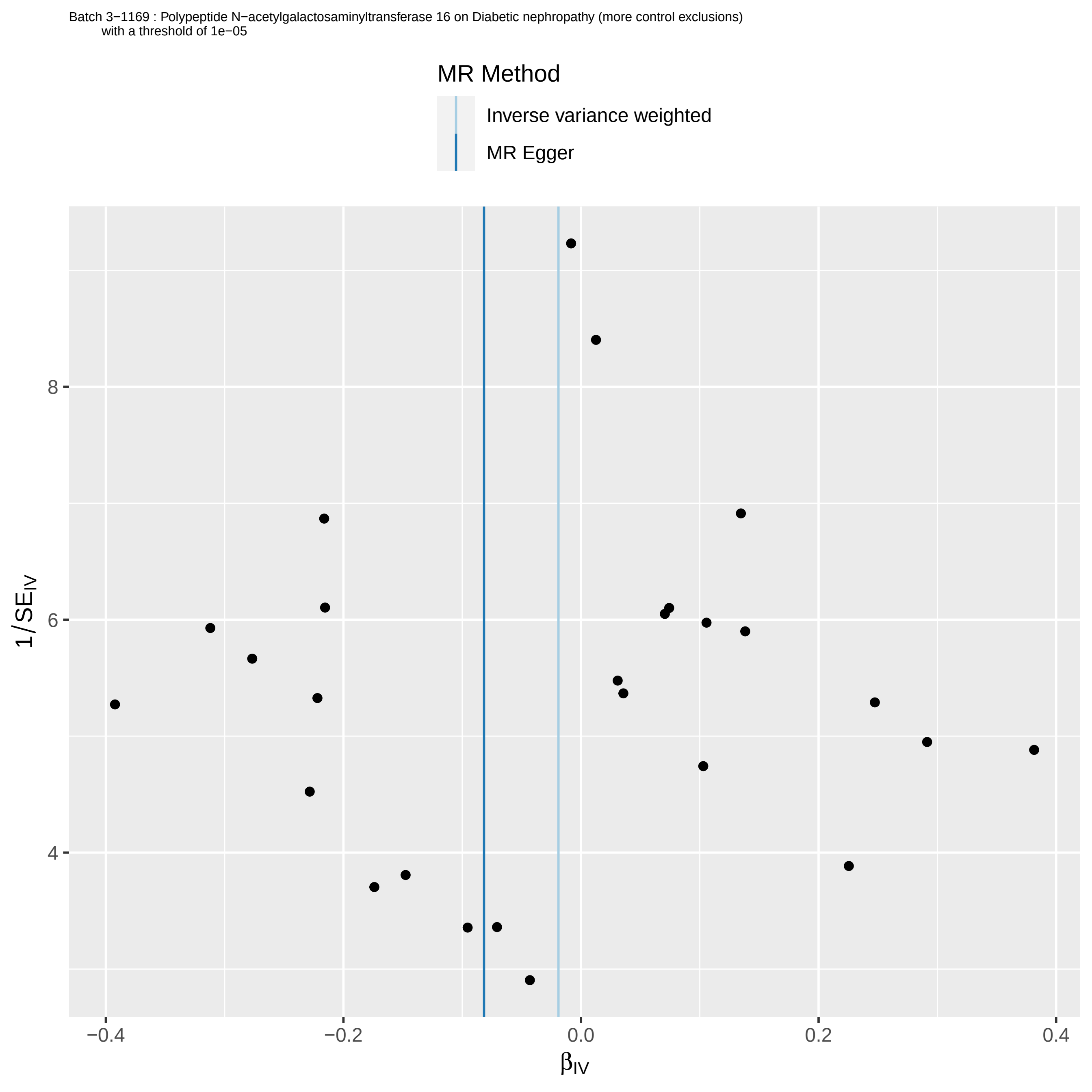
Batch 3−1169 : Polypeptide N−acetylgalactosaminyltransferase 16 on Diabetic nephropathy (more control exclusions)
with a threshold of 1e−05
MR Method
Inverse variance weighted
MR Egger
8
6
4
−0.4
−0.2
0.0
0.2
0.4
$1/SE_{IV}$
β_{IV}

Batch 3−1170 : Polypeptide N−acetylgalactosaminyltransferase 1 on Diabetic nephropathy (more control exclusions)
with a threshold of 1e−05

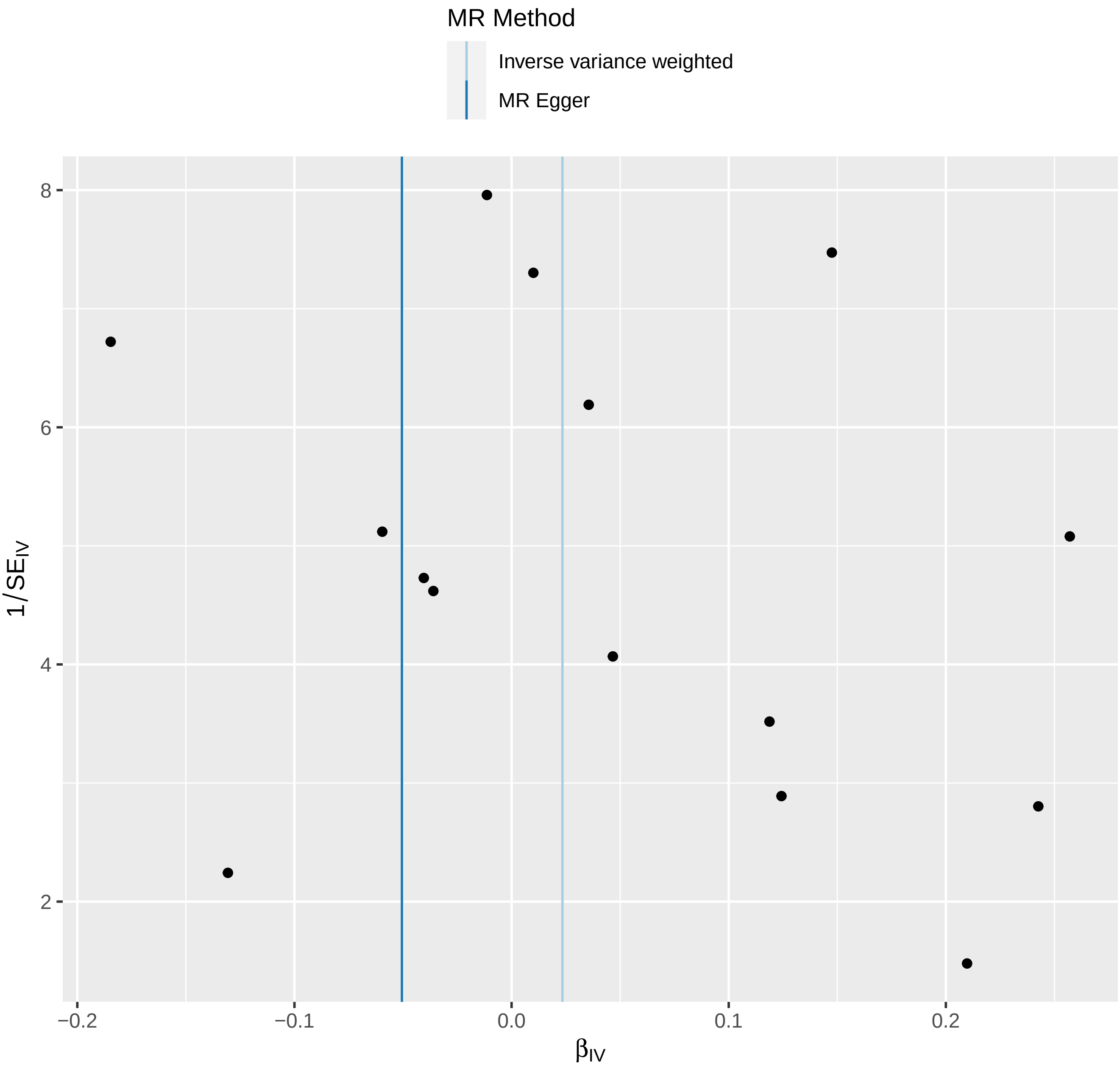

Batch 3−1171 : Polypeptide N−acetylgalactosaminyltransferase 2 on Diabetic nephropathy (more control exclusions) with a threshold of 1e−05

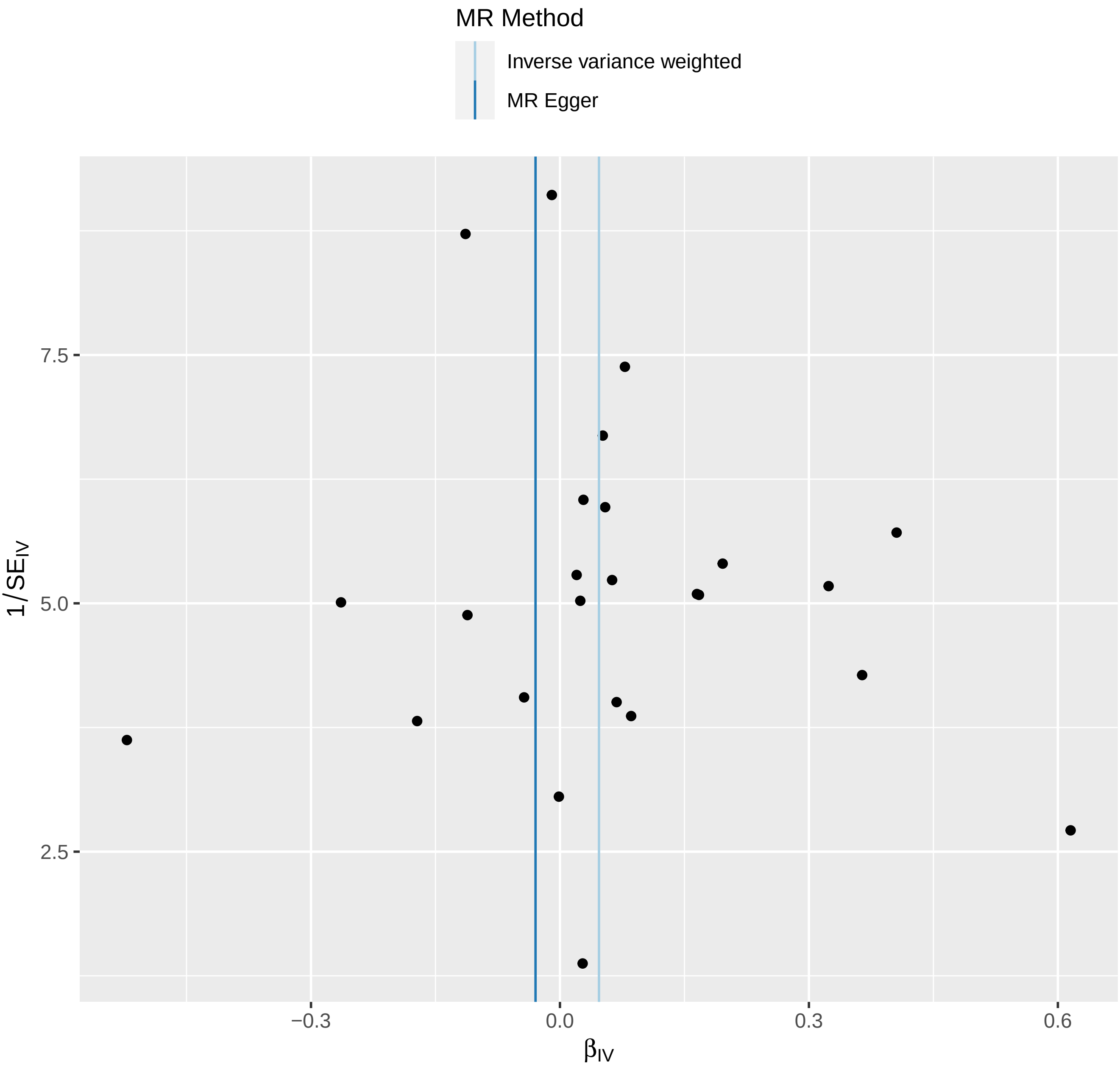

Batch 3−1172 : Polypeptide N−acetylgalactosaminyltransferase 3 on Diabetic nephropathy (more control exclusions) with a threshold of 1e−05

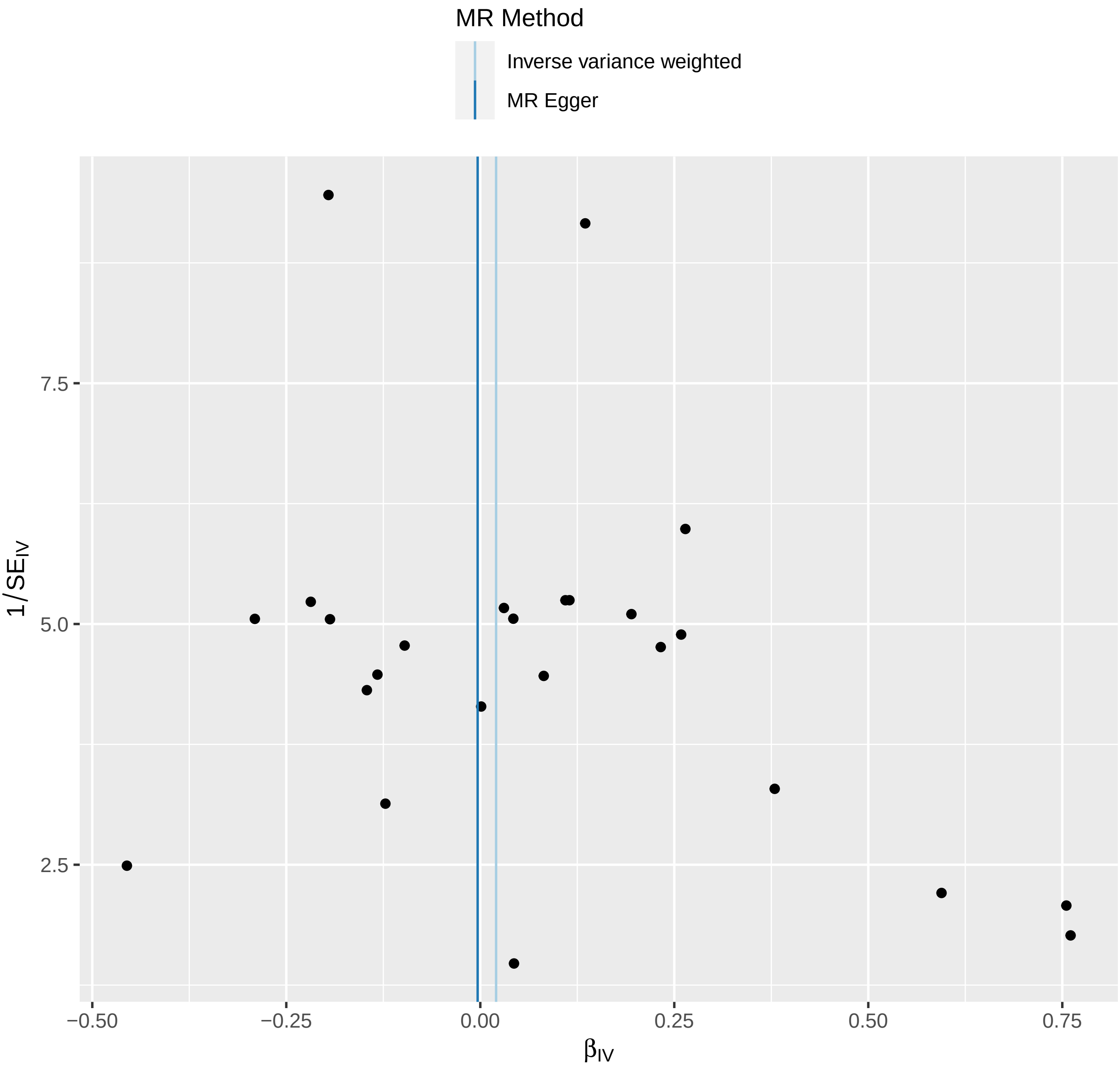

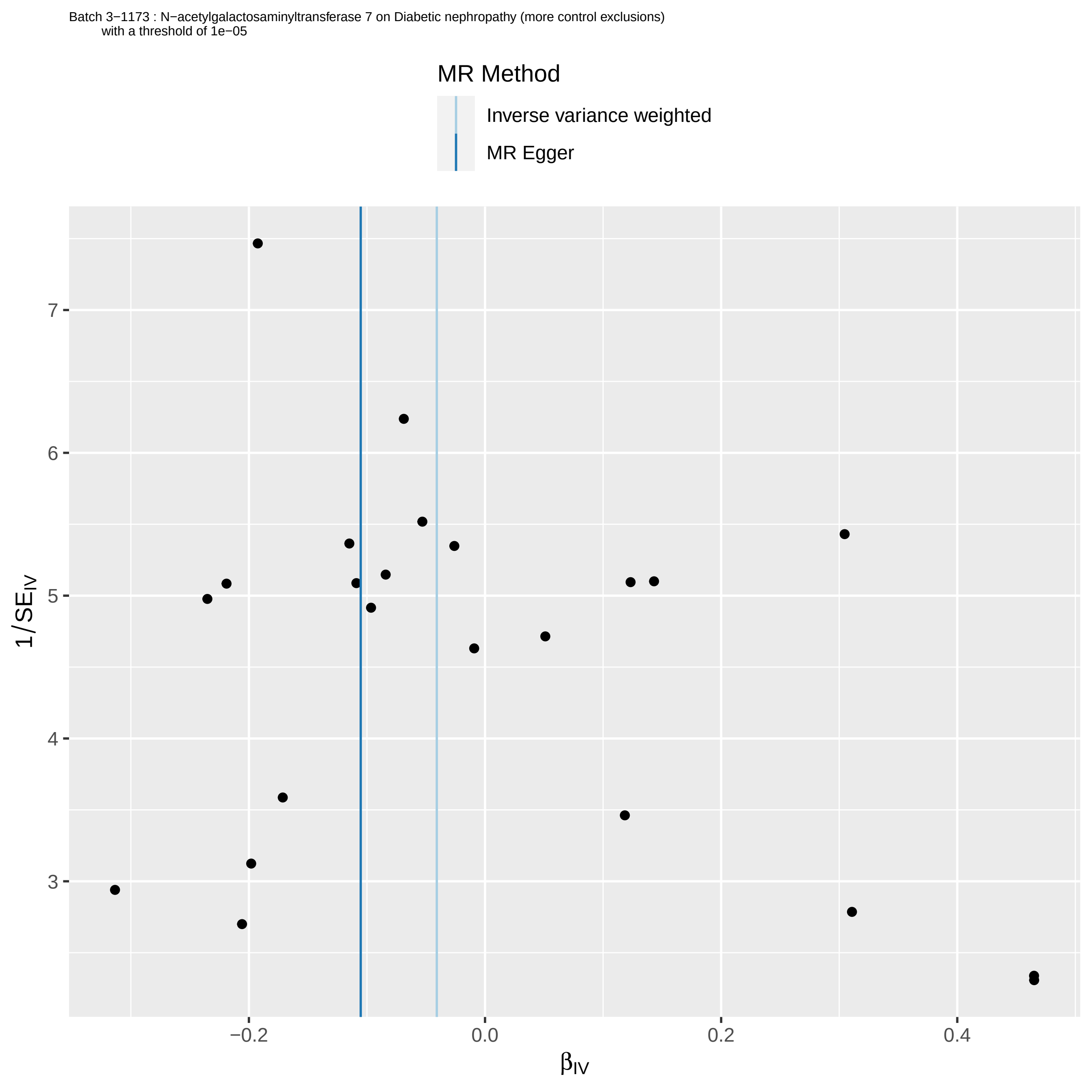

Batch 3−1173 : N−acetylgalactosaminyltransferase 7 on Diabetic nephropathy (more control exclusions)
with a threshold of 1e−05
MR Method
Inverse variance weighted
MR Egger
7
6
5
4
3
1/SE_IV
−0.2
0.0
0.2
0.4
β_IV

Batch 3−1174 : Galanin−like peptide on Diabetic nephropathy (more control exclusions)
with a threshold of 1e−05

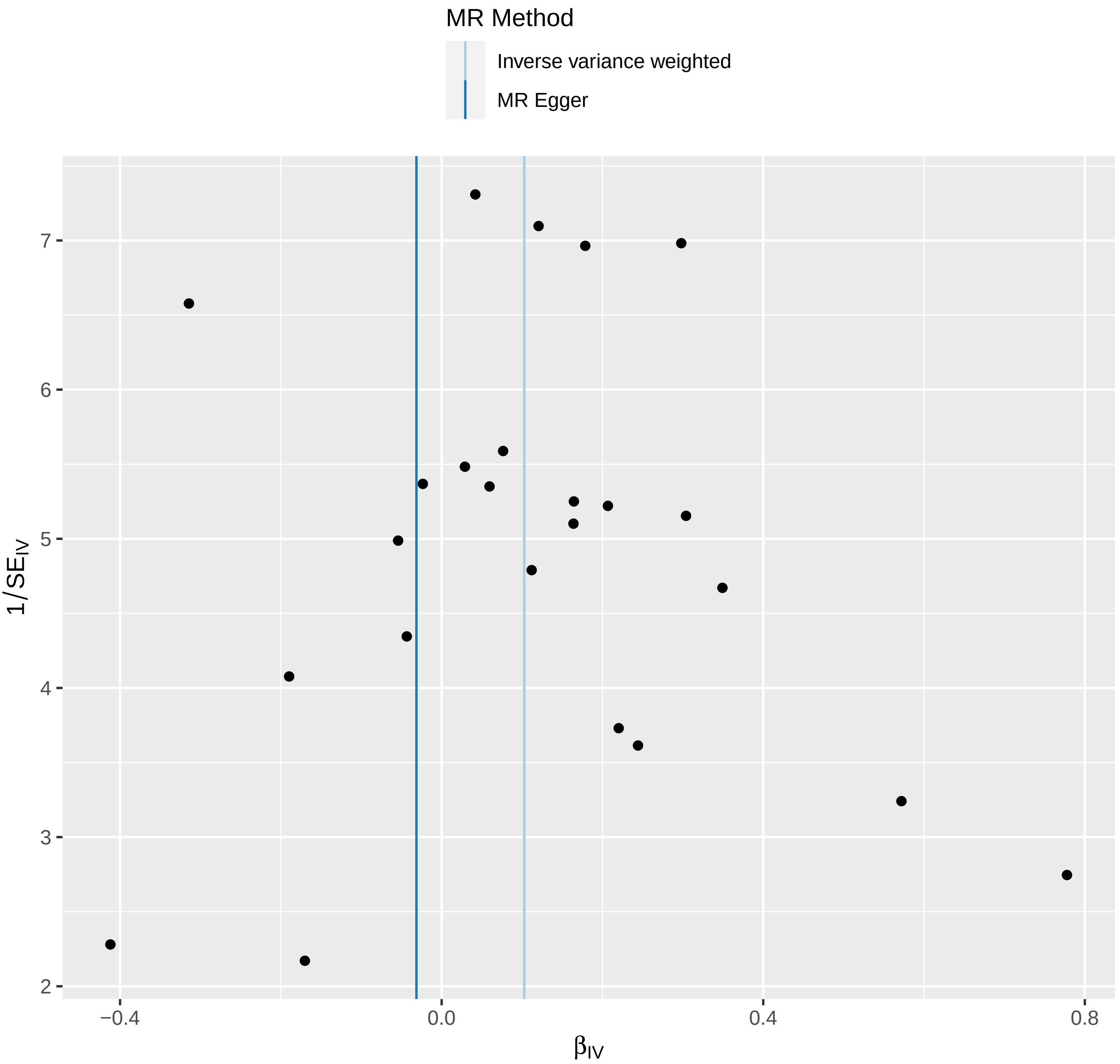

Batch 3−1175 : Gigaxonin on Diabetic nephropathy (more control exclusions)
with a threshold of 1e−05

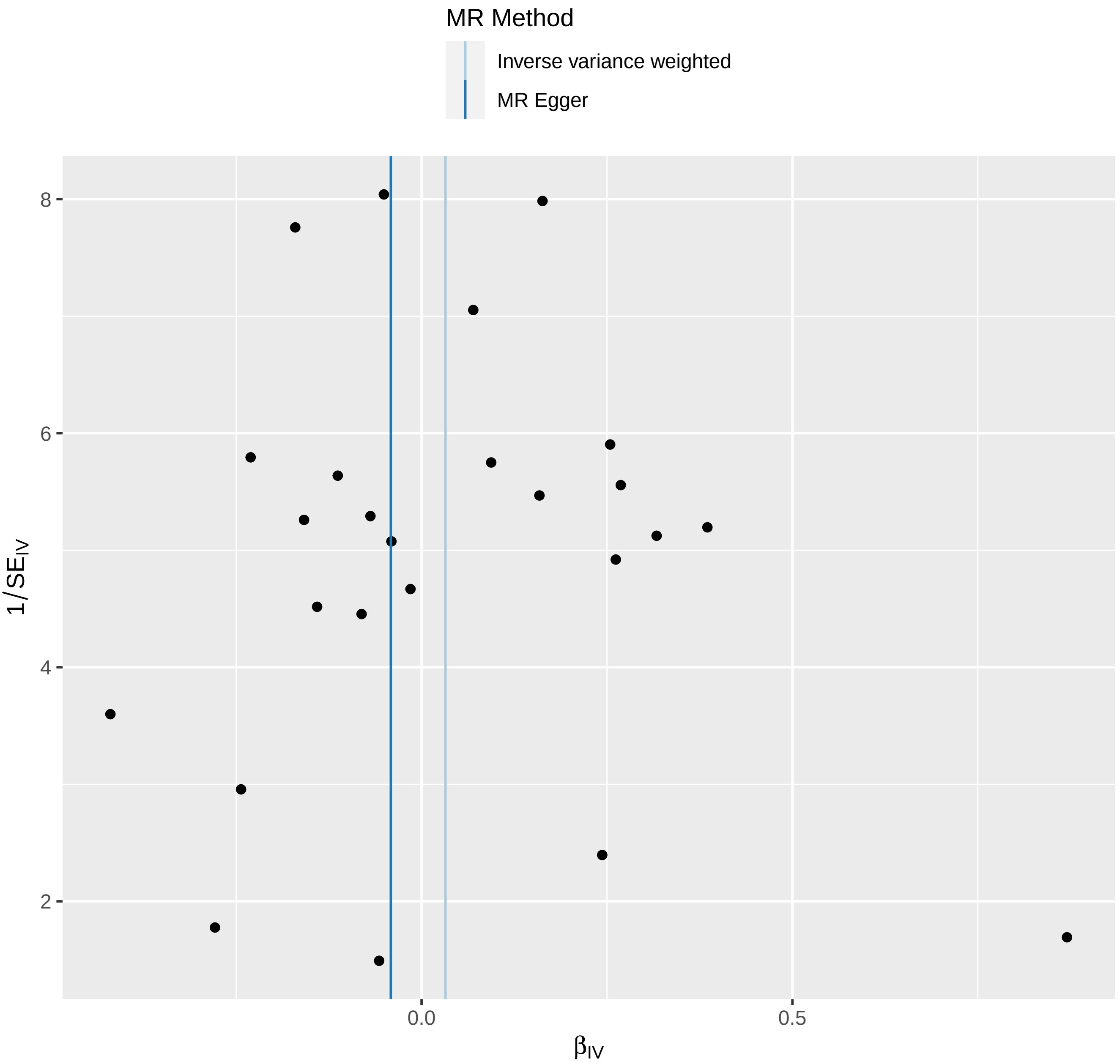

Batch 3−1176 : Glyceraldehyde−3−phosphate dehydrogenase, testis−specific on Diabetic nephropathy (more control exclusions) with a threshold of 1e−05

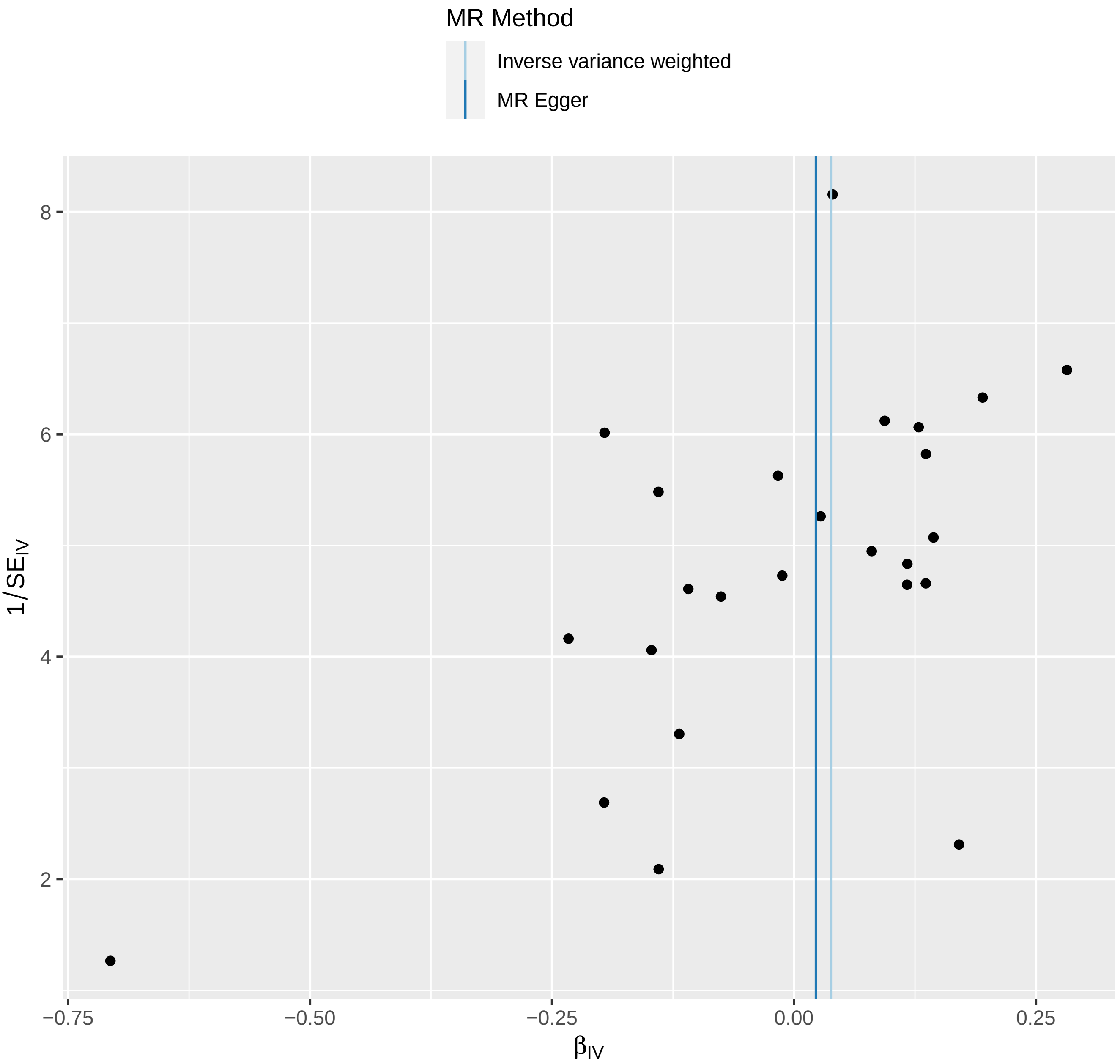

Batch 3−1177 : Growth arrest−specific protein 1 on Diabetic nephropathy (more control exclusions)
with a threshold of 1e−05

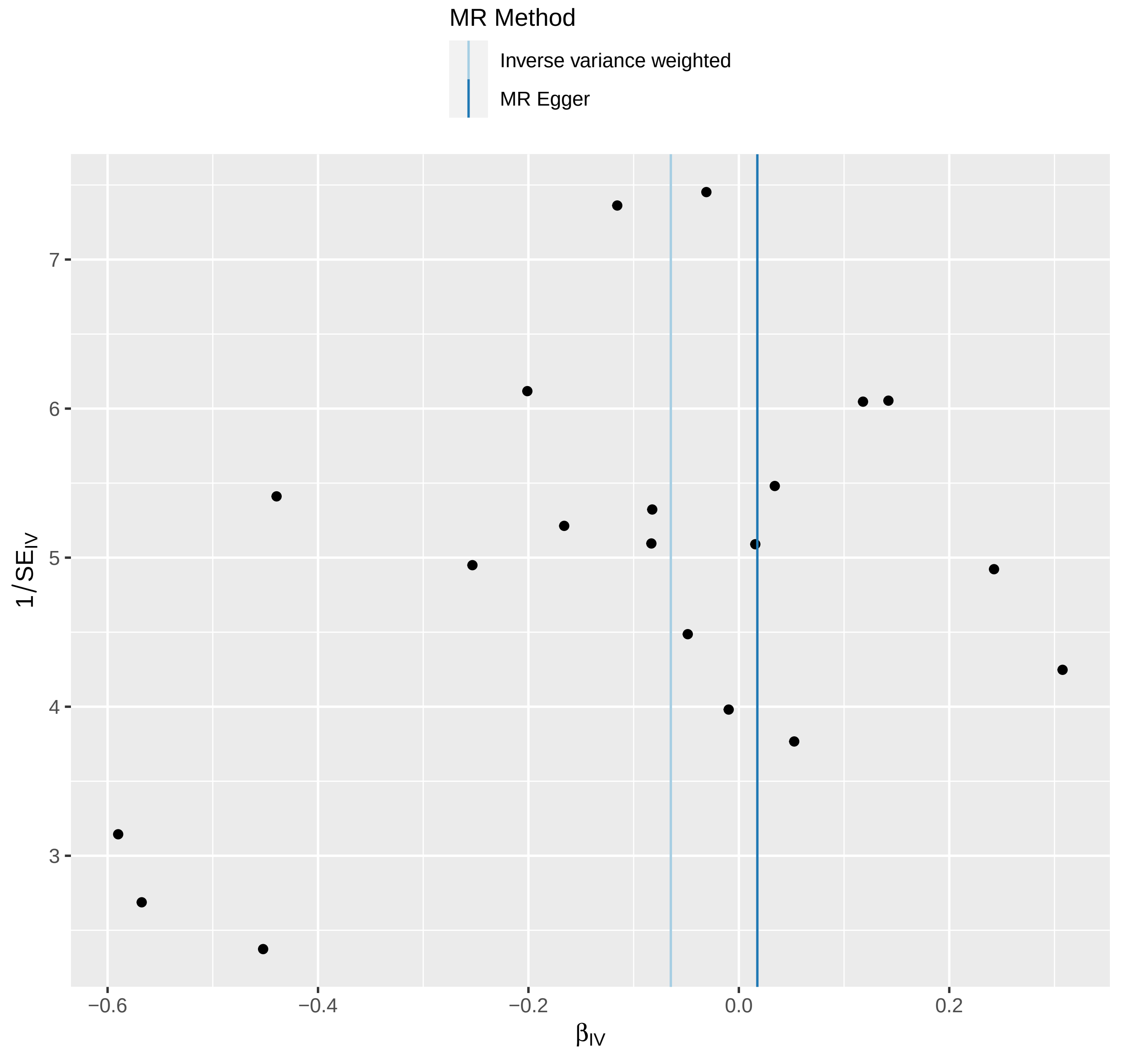

Batch 3−1178 : Guanylate−binding protein 6 on Diabetic nephropathy (more control exclusions)
with a threshold of 1e−05

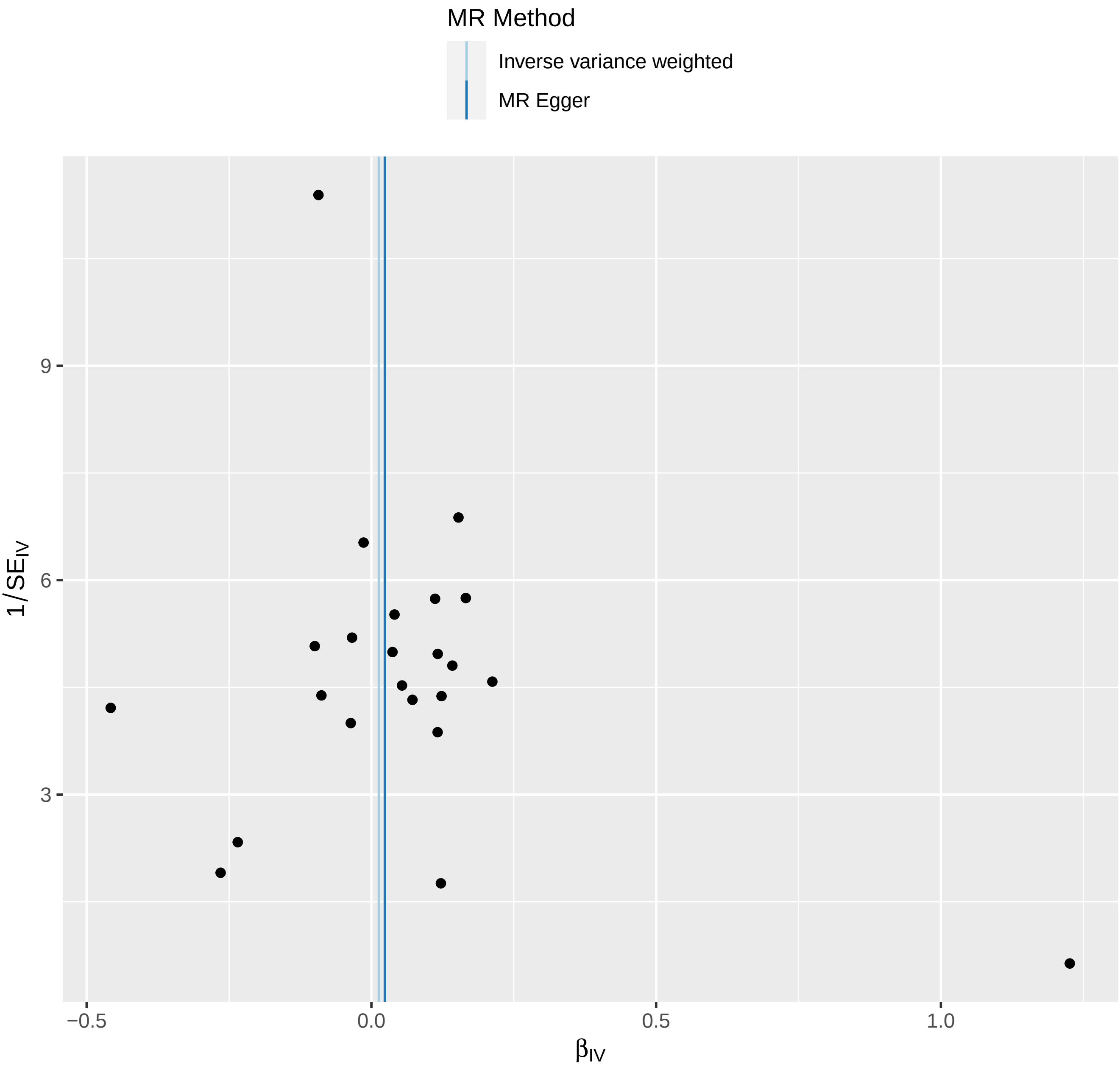

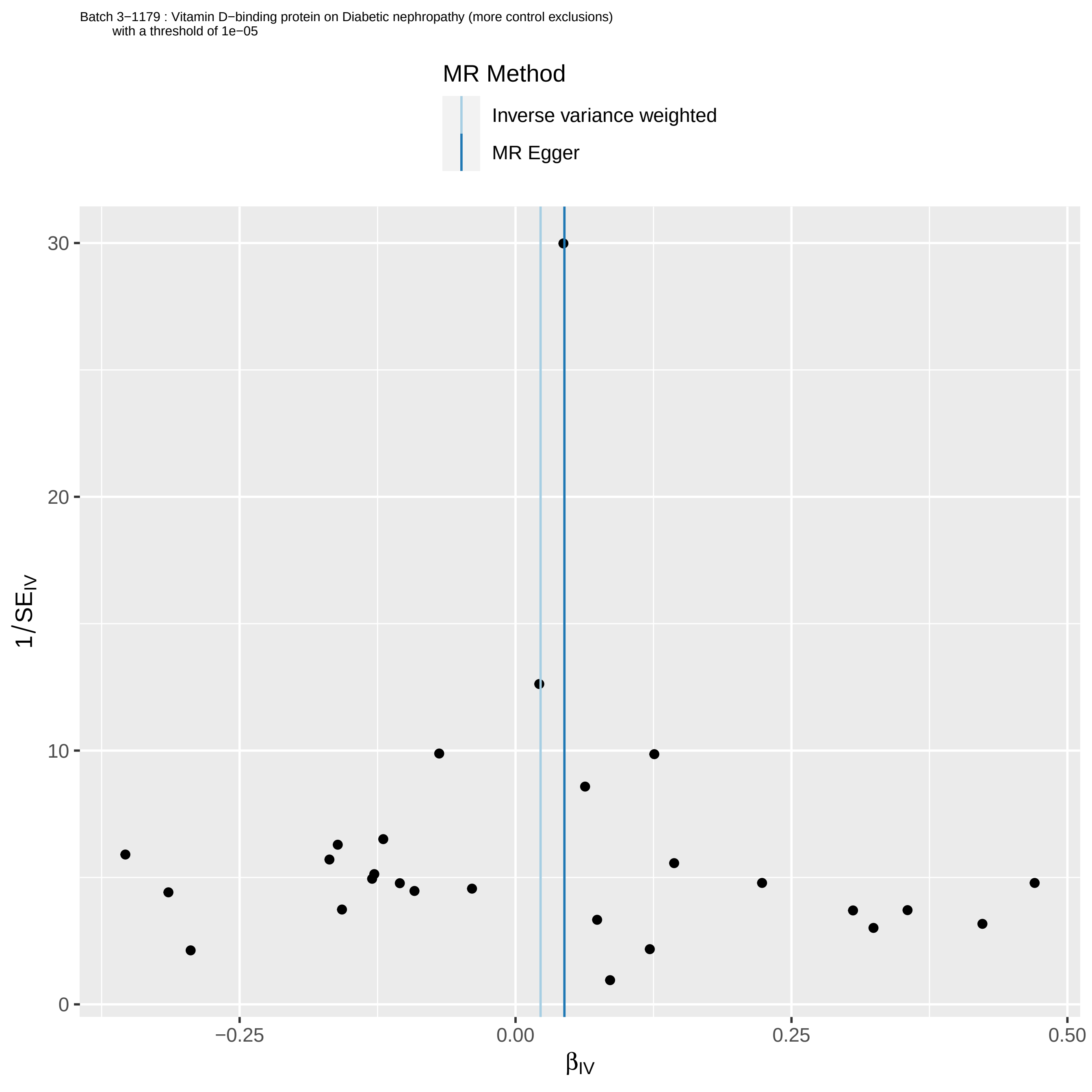

Batch 3−1179 : Vitamin D−binding protein on Diabetic nephropathy (more control exclusions)
with a threshold of 1e−05
MR Method
Inverse variance weighted
MR Egger
30
20
10
0
1/SE_IV
−0.25
0.00
0.25
0.50
β_IV

Batch 3−1180 : Grancalcin on Diabetic nephropathy (more control exclusions)
with a threshold of 1e−05

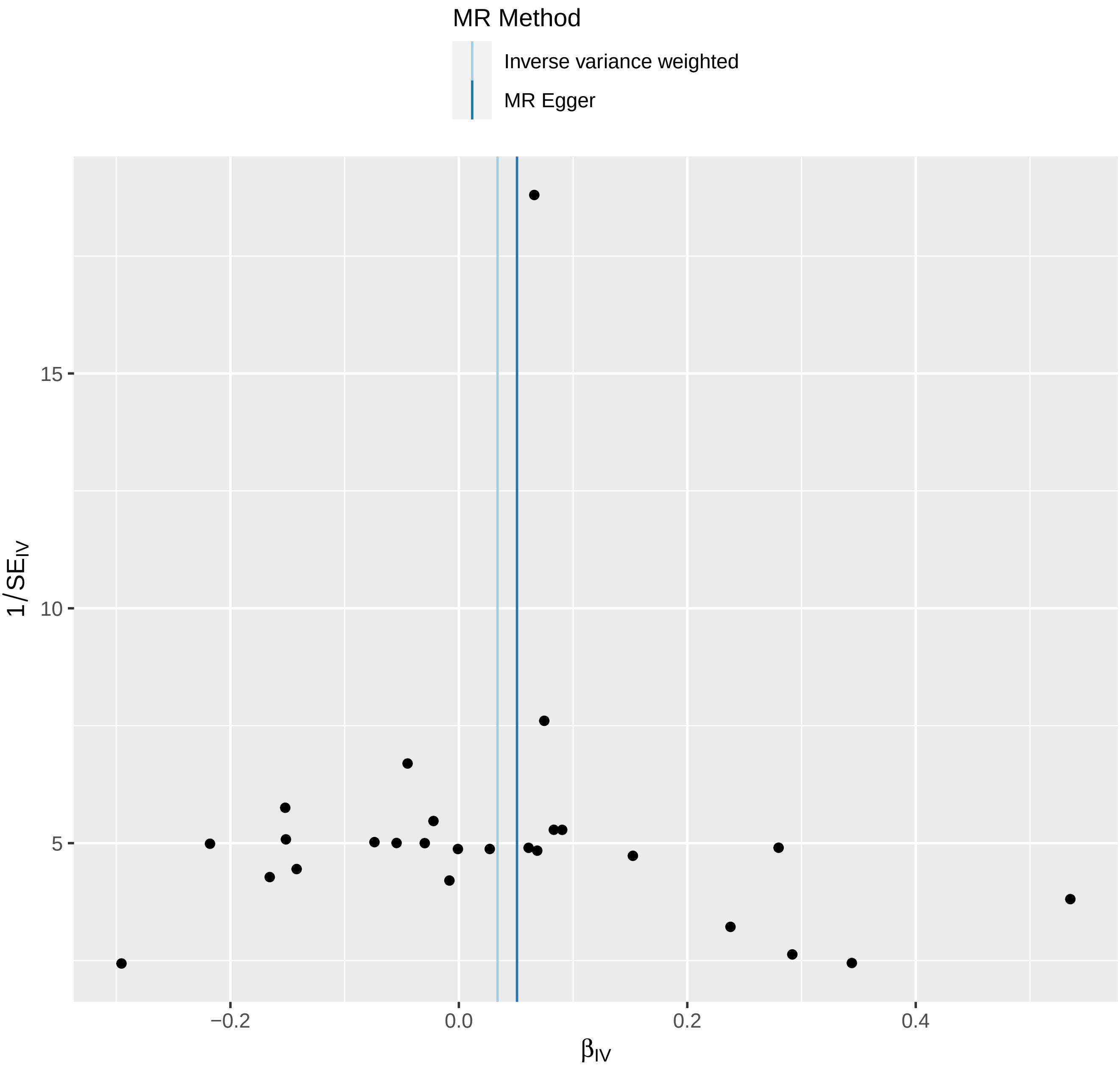

Batch 3−1181 : Glucagon on Diabetic nephropathy (more control exclusions)
with a threshold of 1e−05

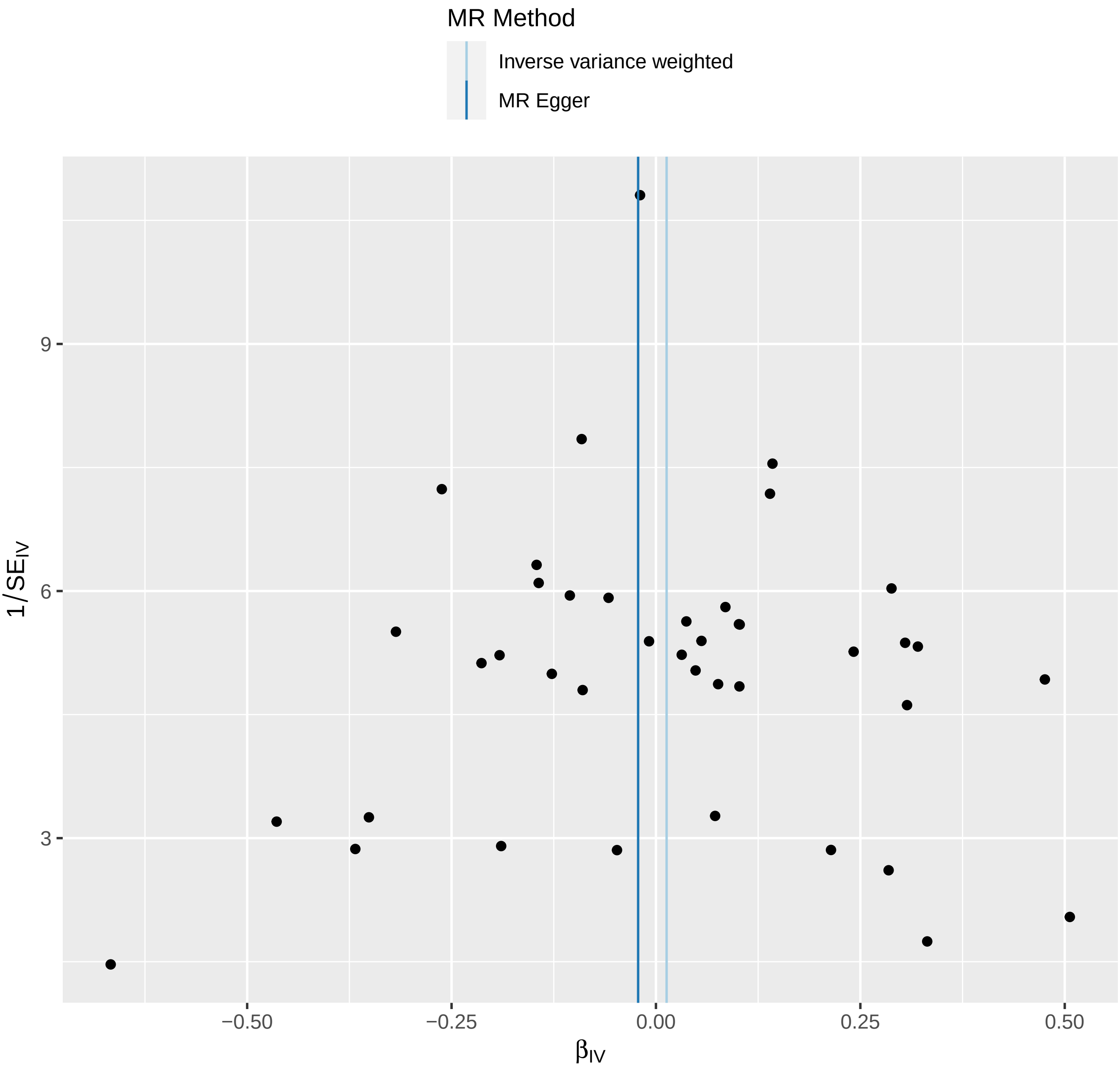

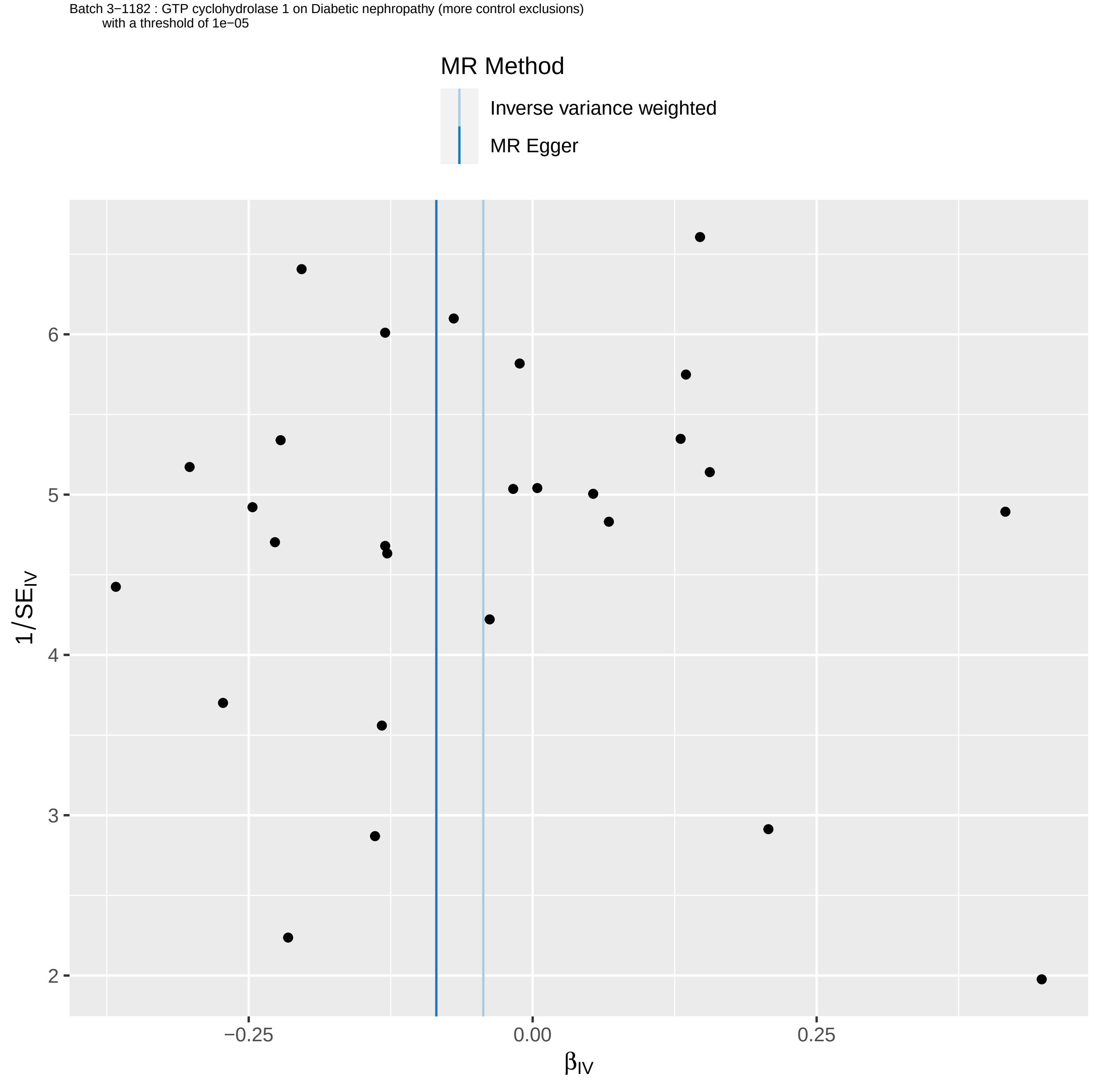
Batch 3−1182 : GTP cyclohydrolase 1 on Diabetic nephropathy (more control exclusions)
with a threshold of 1e−05
MR Method
Inverse variance weighted
MR Egger
1/SE_IV
6
5
4
3
2
−0.25
0.00
0.25
β_IV

Batch 3−1183 : Glucokinase on Diabetic nephropathy (more control exclusions)
with a threshold of 1e−05

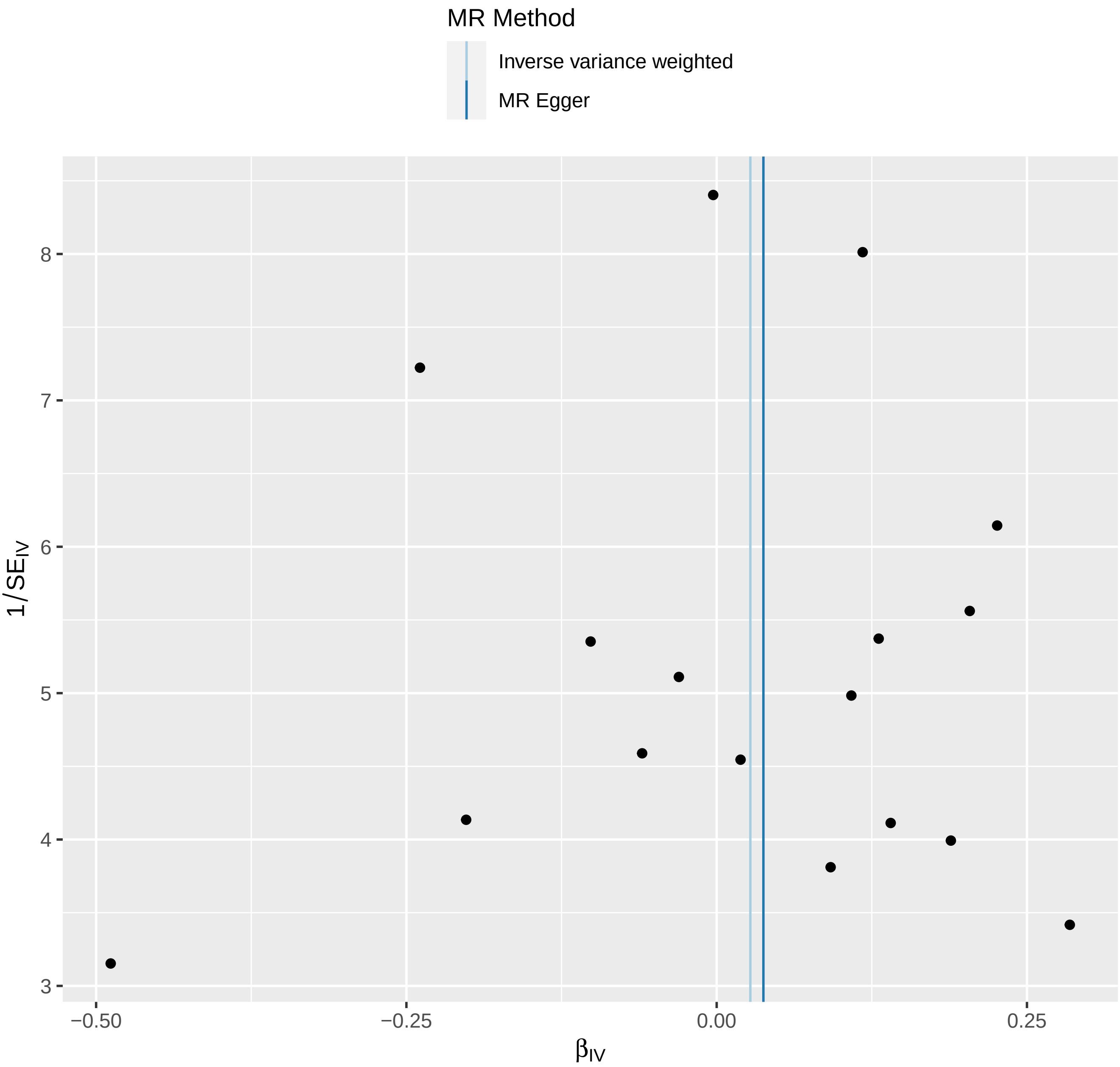

Batch 3−1184 : Glucokinase regulatory protein on Diabetic nephropathy (more control exclusions)
with a threshold of 1e−05

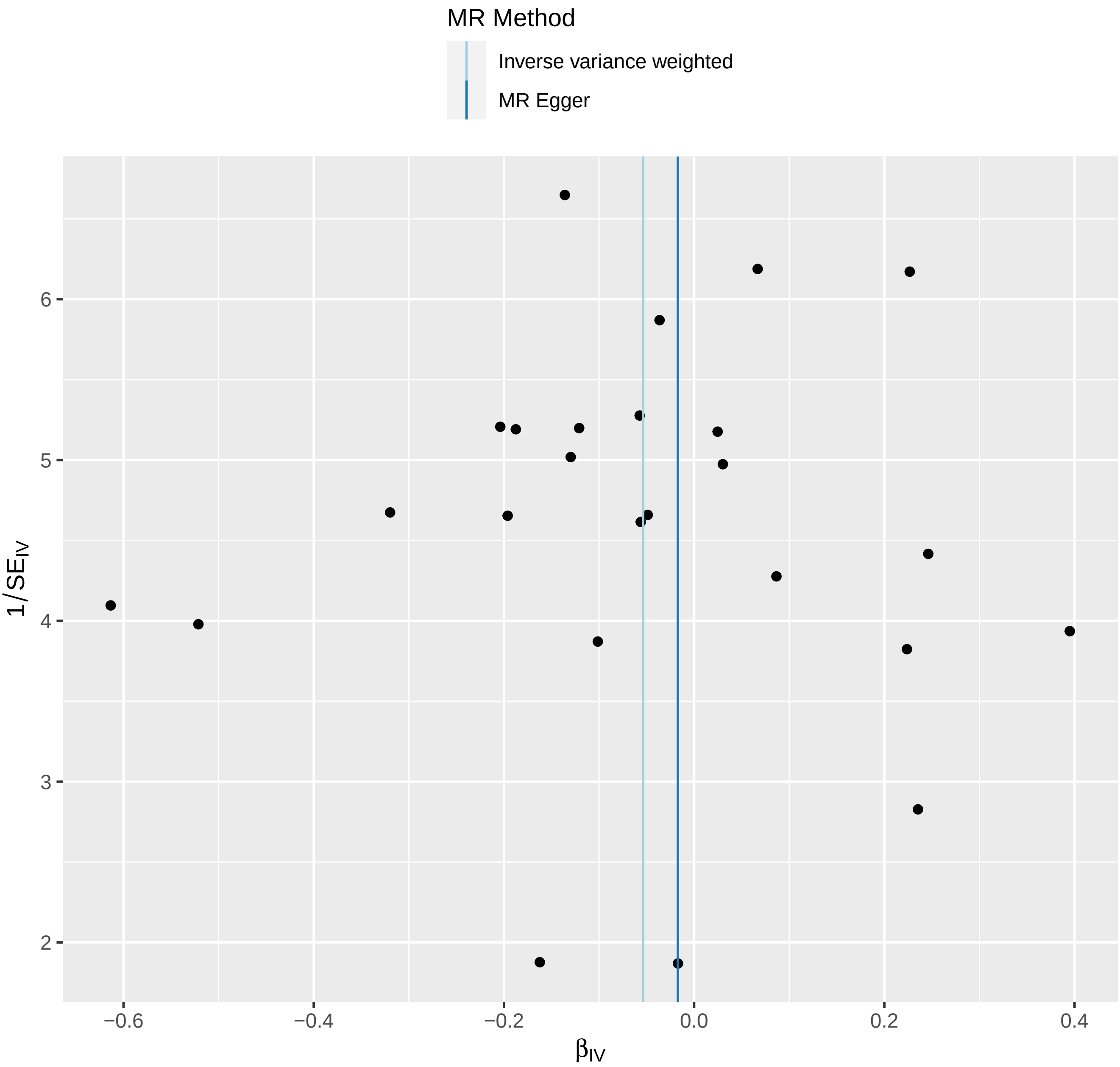

Batch 3−1185 : Beta−1,3−galactosyl−O−glycosyl−glycoprotein beta−1,6−N−acetylglucosaminyltransferase on Diabetic nephropathy (more control exclusions) with a threshold of 1e−05

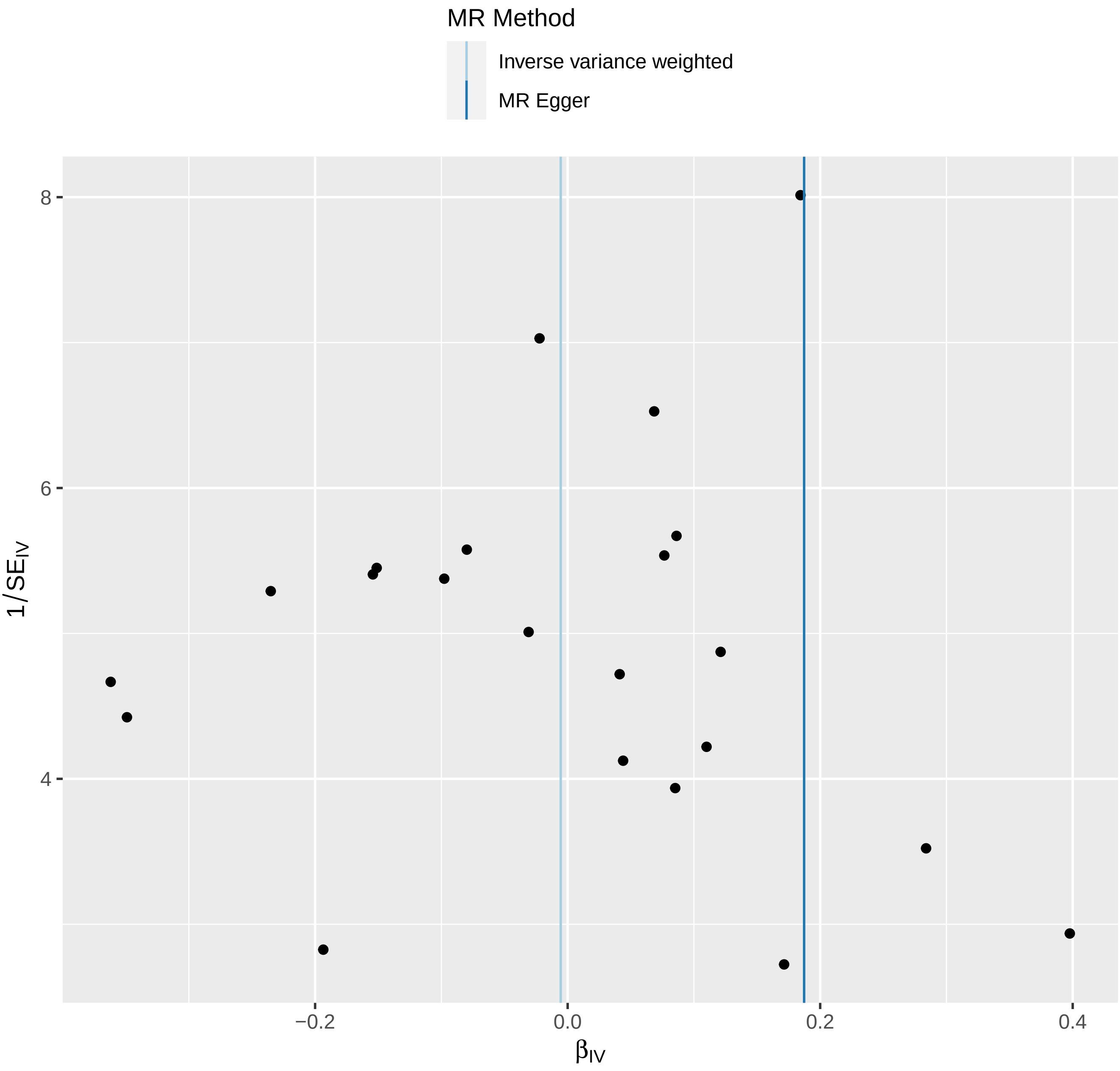

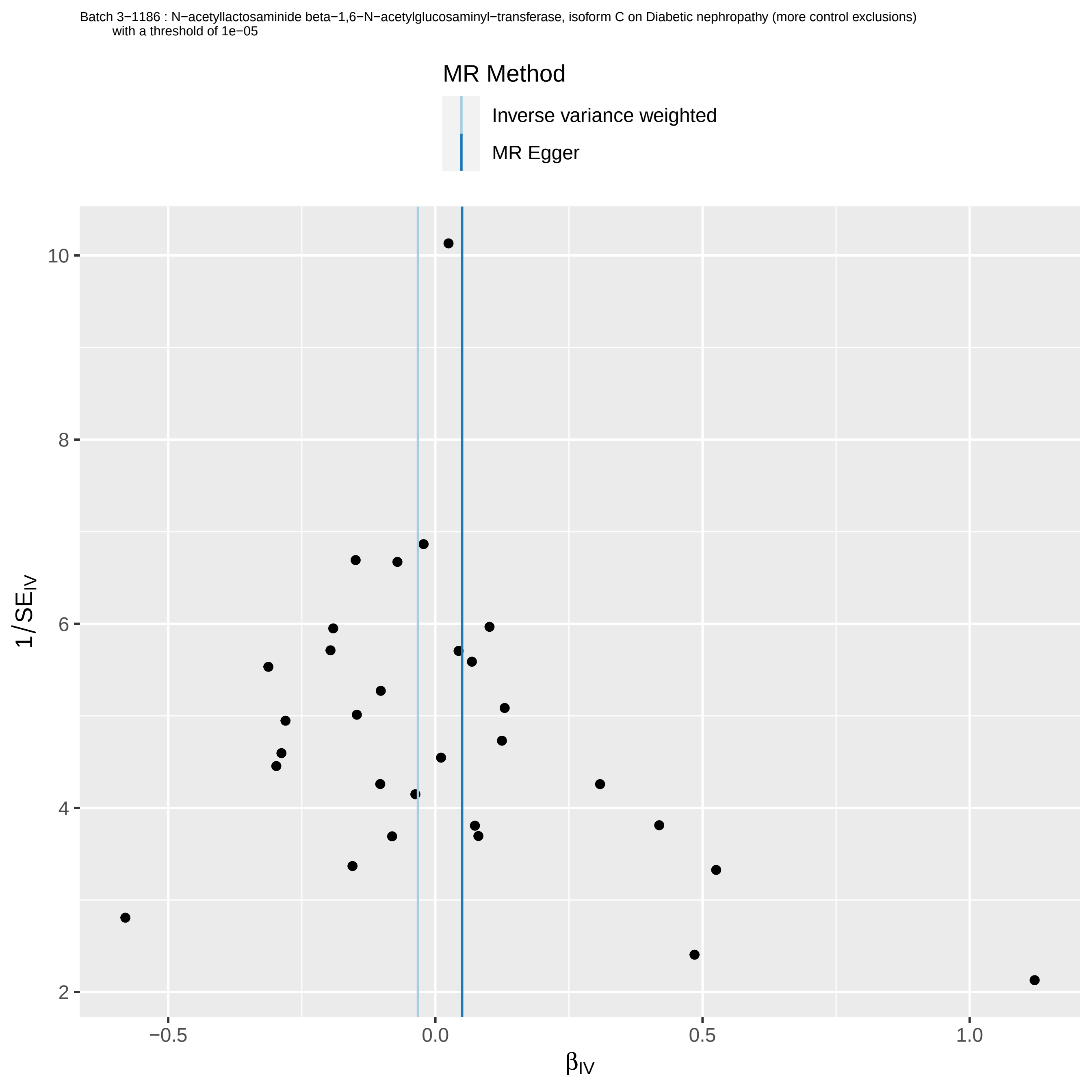

Batch 3−1186 : N−acetyllactosaminide beta−1,6−N−acetylglucosaminyl−transferase, isoform C on Diabetic nephropathy (more control exclusions) with a threshold of 1e−05
MR Method
Inverse variance weighted
MR Egger
10
8
6
4
2
−0.5
0.0
0.5
1.0
$1/SE_{IV}$
β_{IV}

Batch 3−1187 : Beta−1,3−galactosyl−O−glycosyl−glycoprotein beta−1,6−N−acetylglucosaminyltransferase 4 on Diabetic nephropathy (more control exclusions) with a threshold of 1e−05

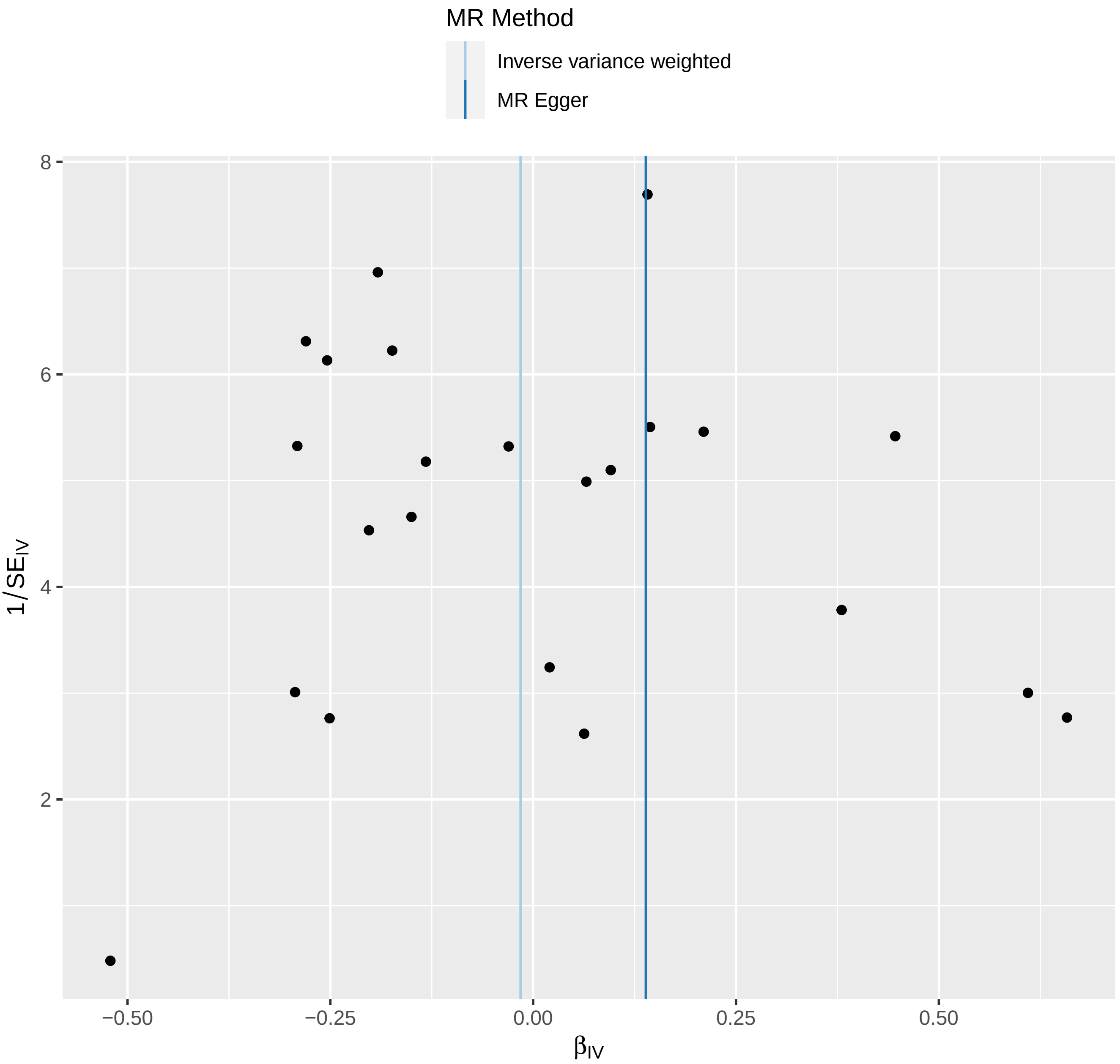

Batch 3−1188 : Beta−1,3−galactosyl−O−glycosyl−glycoprotein beta−1,6−N−acetylglucosaminyltransferase 4 on Diabetic nephropathy (more control exclusions) with a threshold of 1e−05

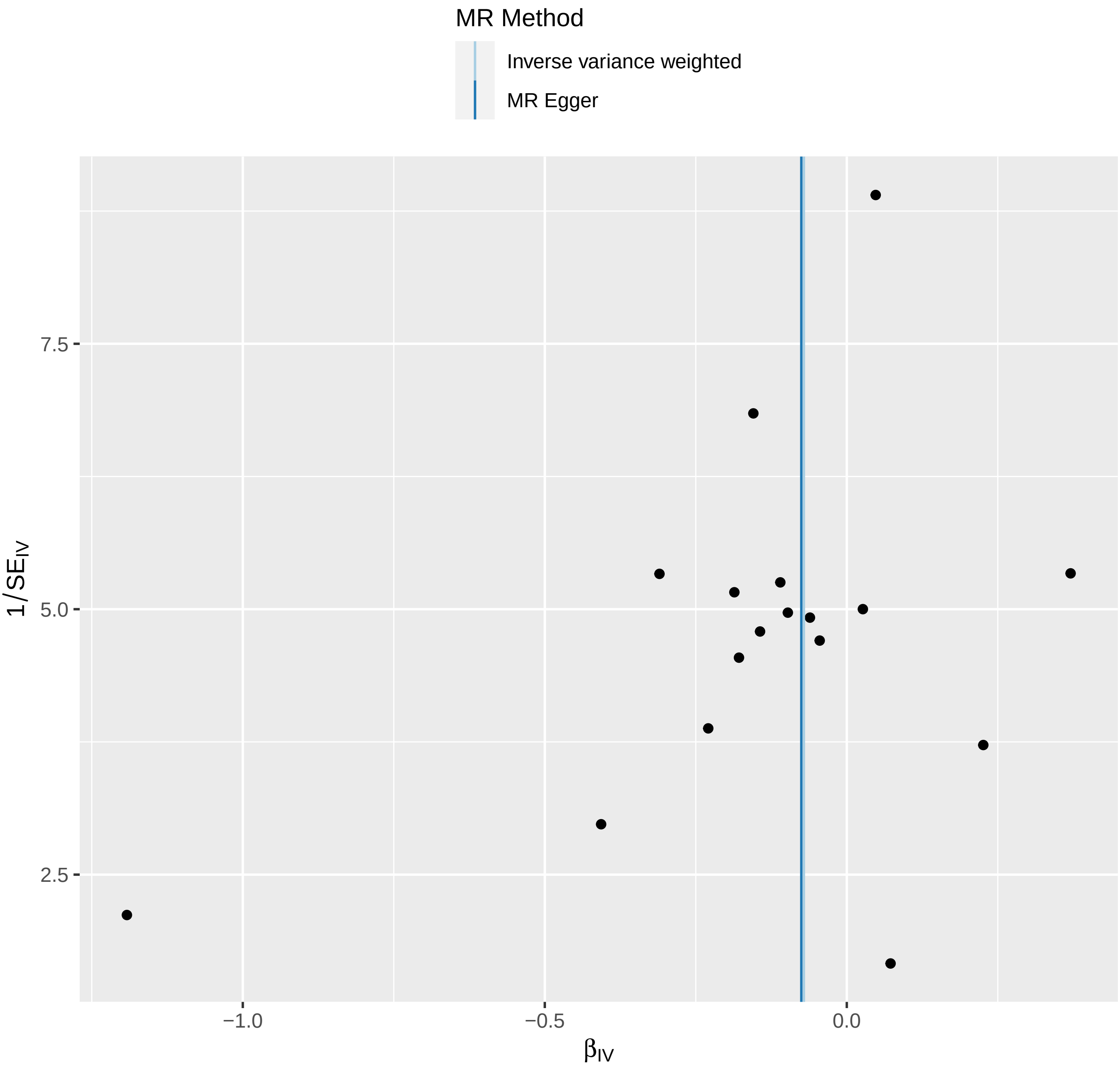

Batch 3−1189 : Guanine deaminase on Diabetic nephropathy (more control exclusions)
with a threshold of 1e−05

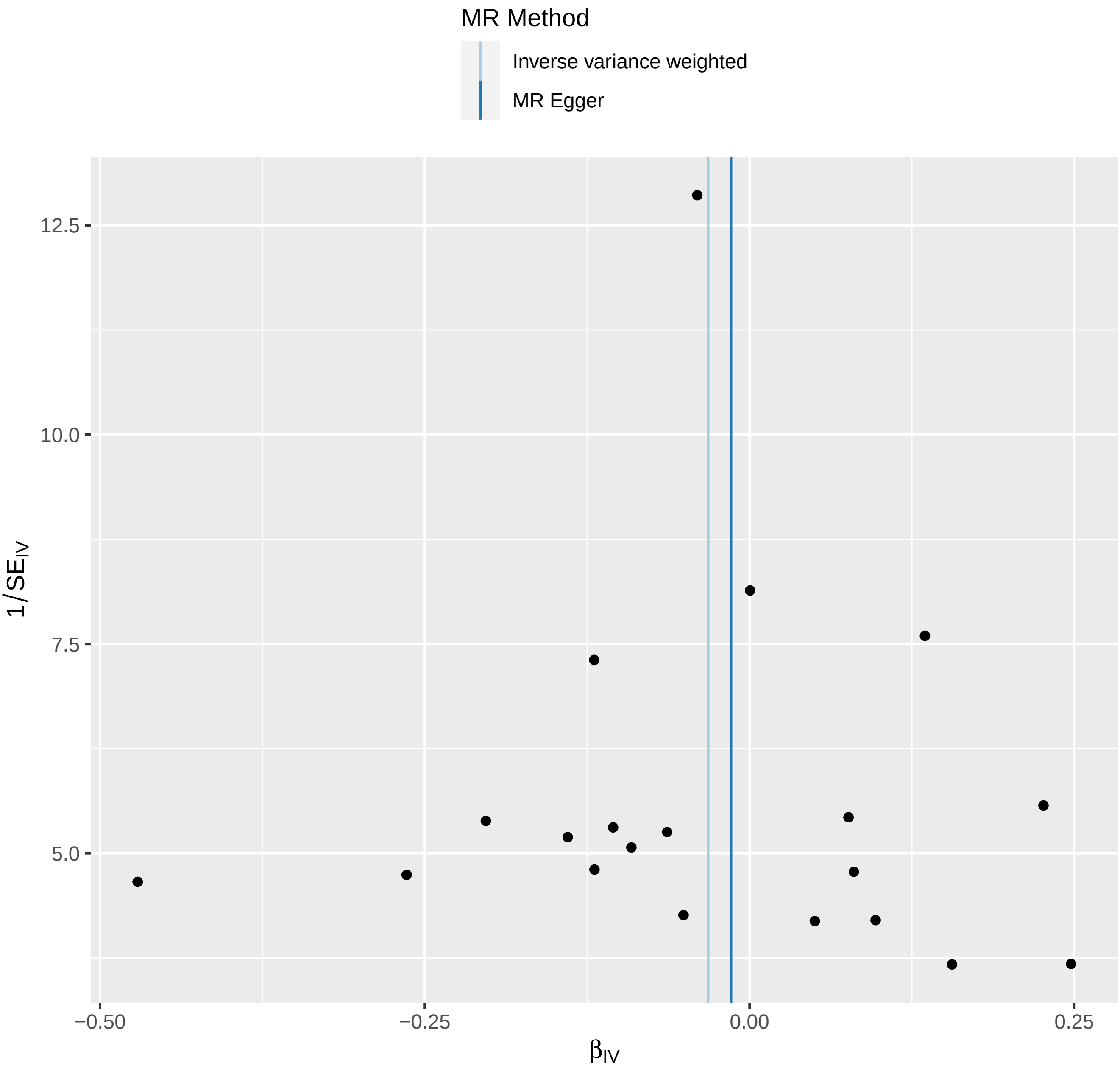

Batch 3−1190 : Growth/differentiation factor 11 on Diabetic nephropathy (more control exclusions)
with a threshold of 1e−05

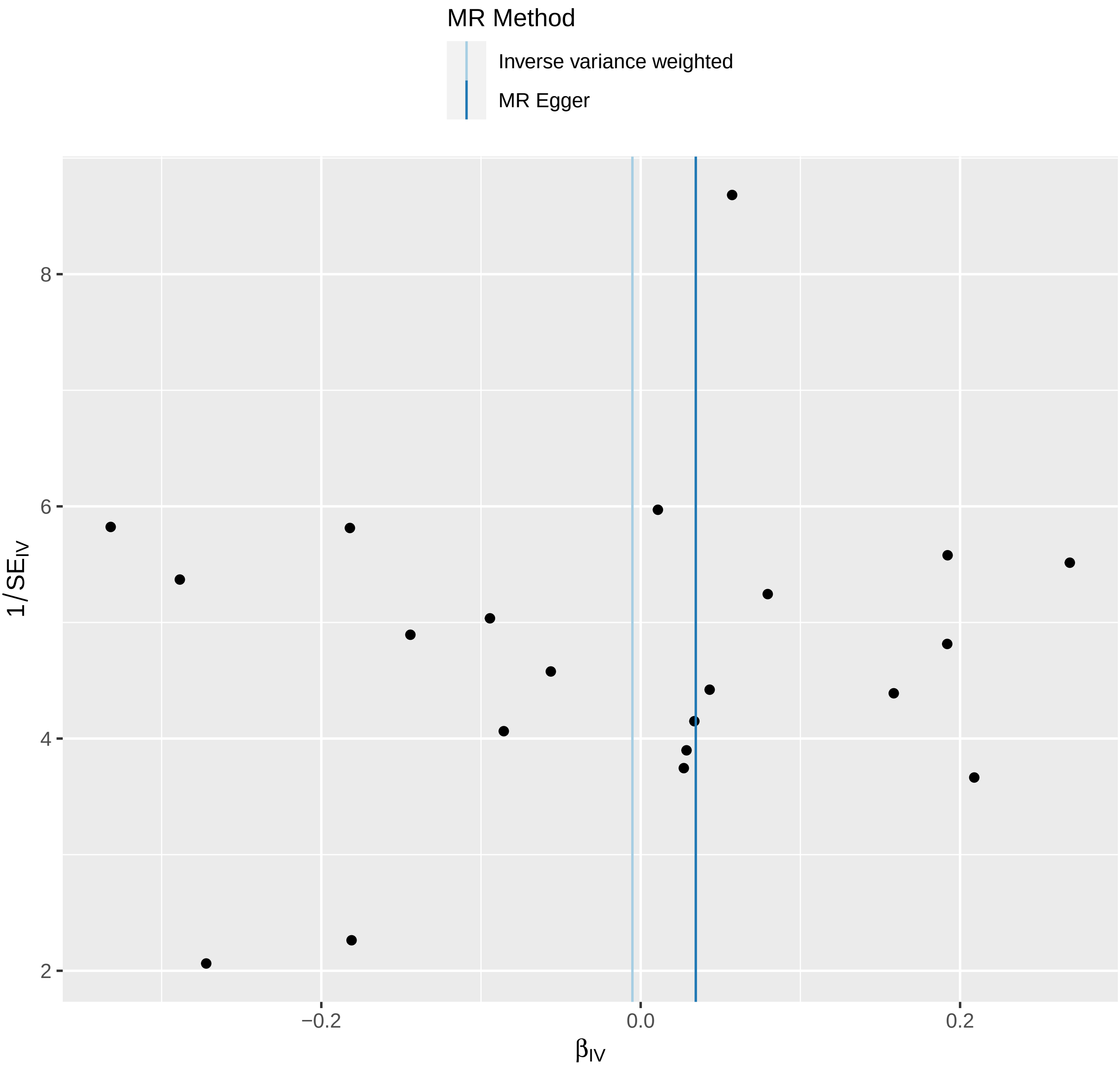

Batch 3−1191 : Growth/differentiation factor 11 on Diabetic nephropathy (more control exclusions) with a threshold of 1e−05

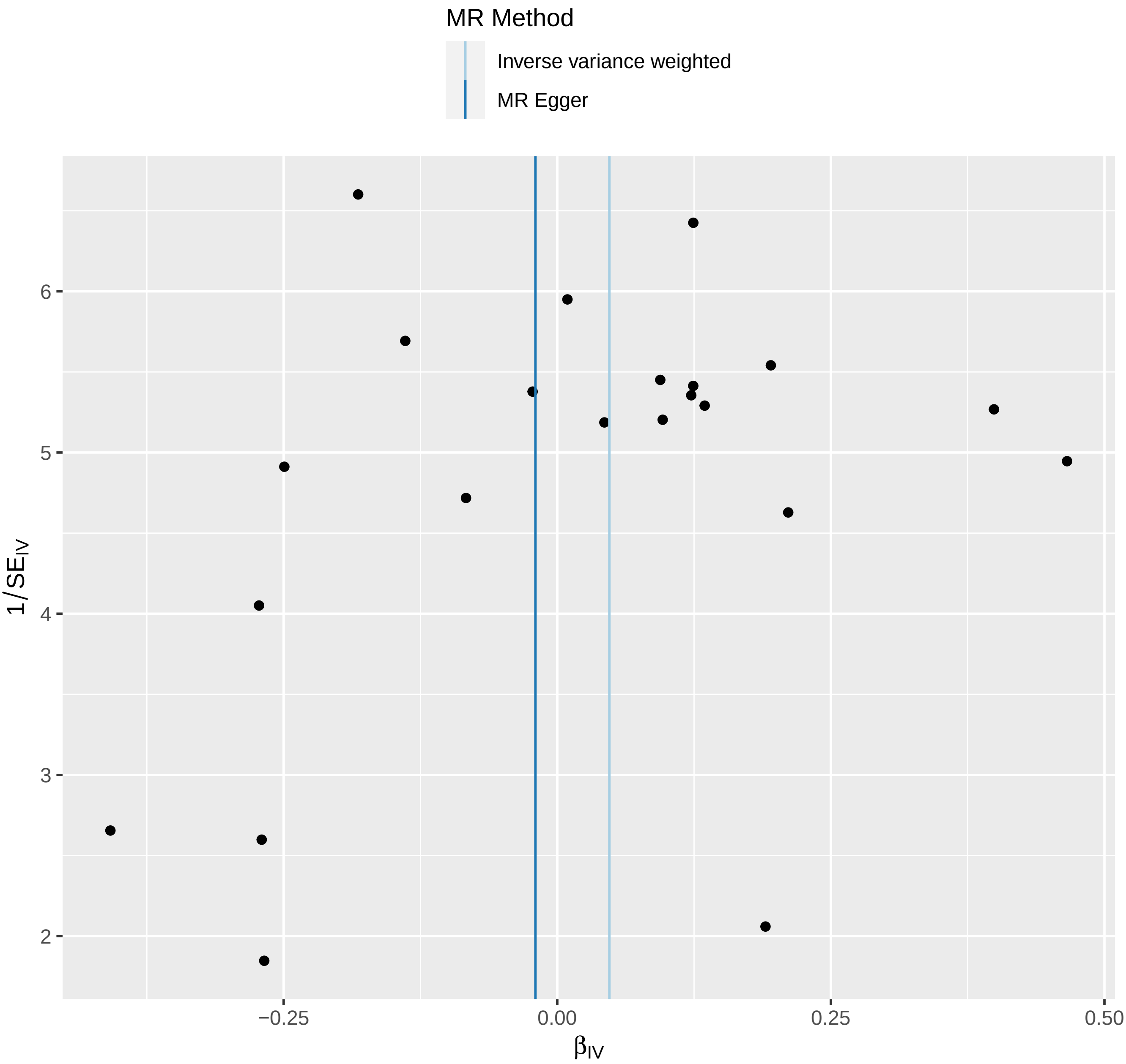

Batch 3−1192 : Growth/differentiation factor 11 on Diabetic nephropathy (more control exclusions)
with a threshold of 1e−05

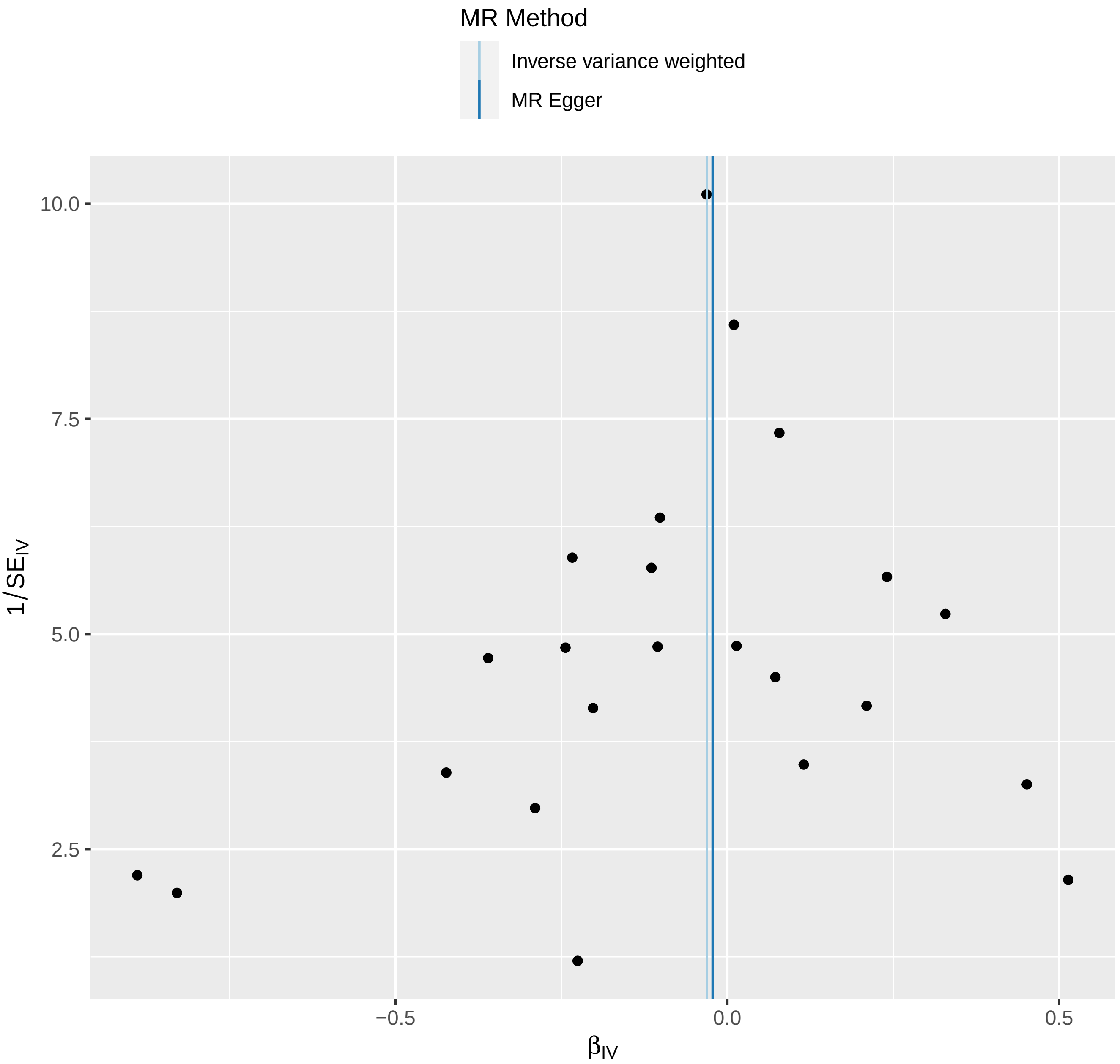

Batch 3−1193 : Growth/differentiation factor 11 on Diabetic nephropathy (more control exclusions)
with a threshold of 1e−05

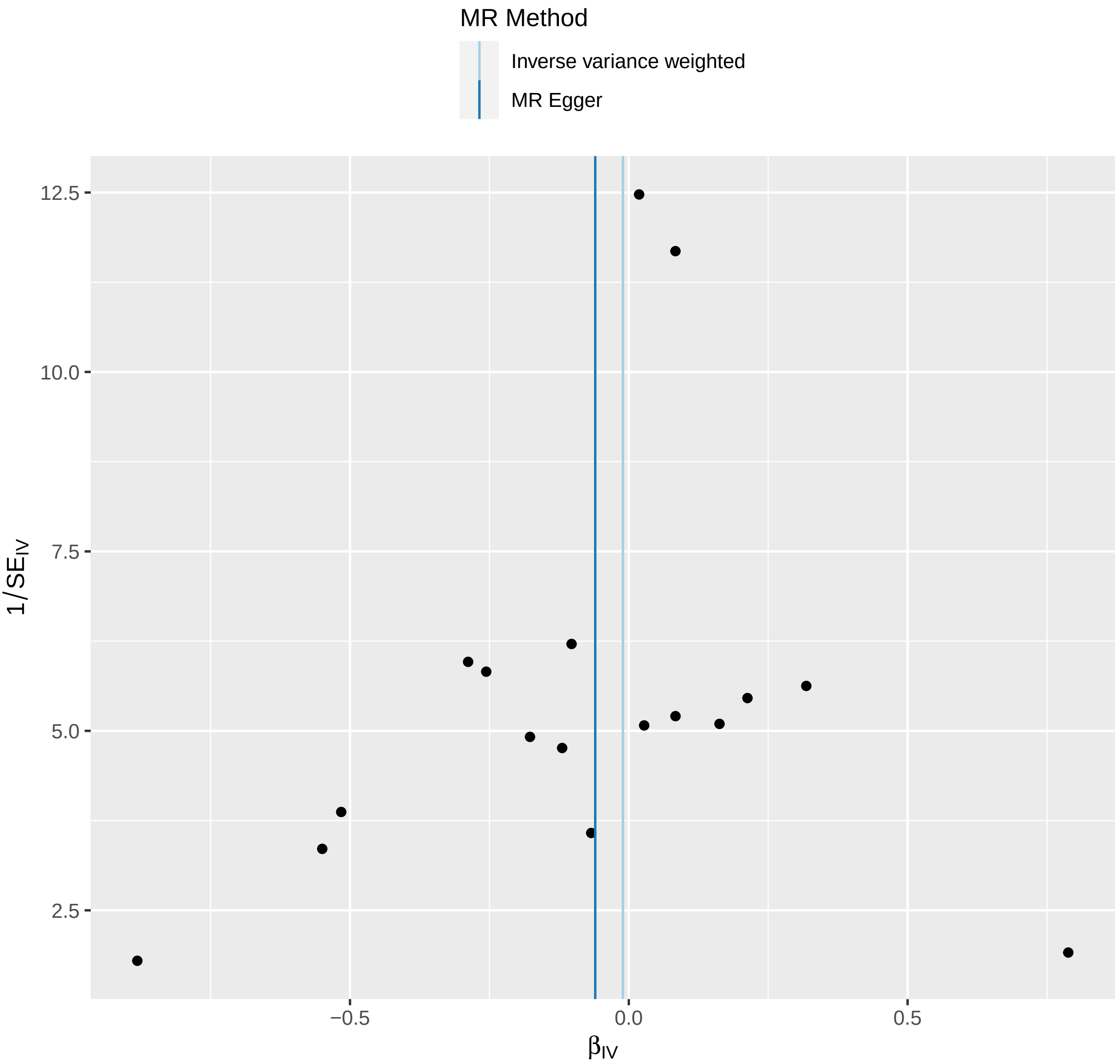

Batch 3−1194 : Growth/differentiation factor 11/8 on Diabetic nephropathy (more control exclusions)
with a threshold of 1e−05

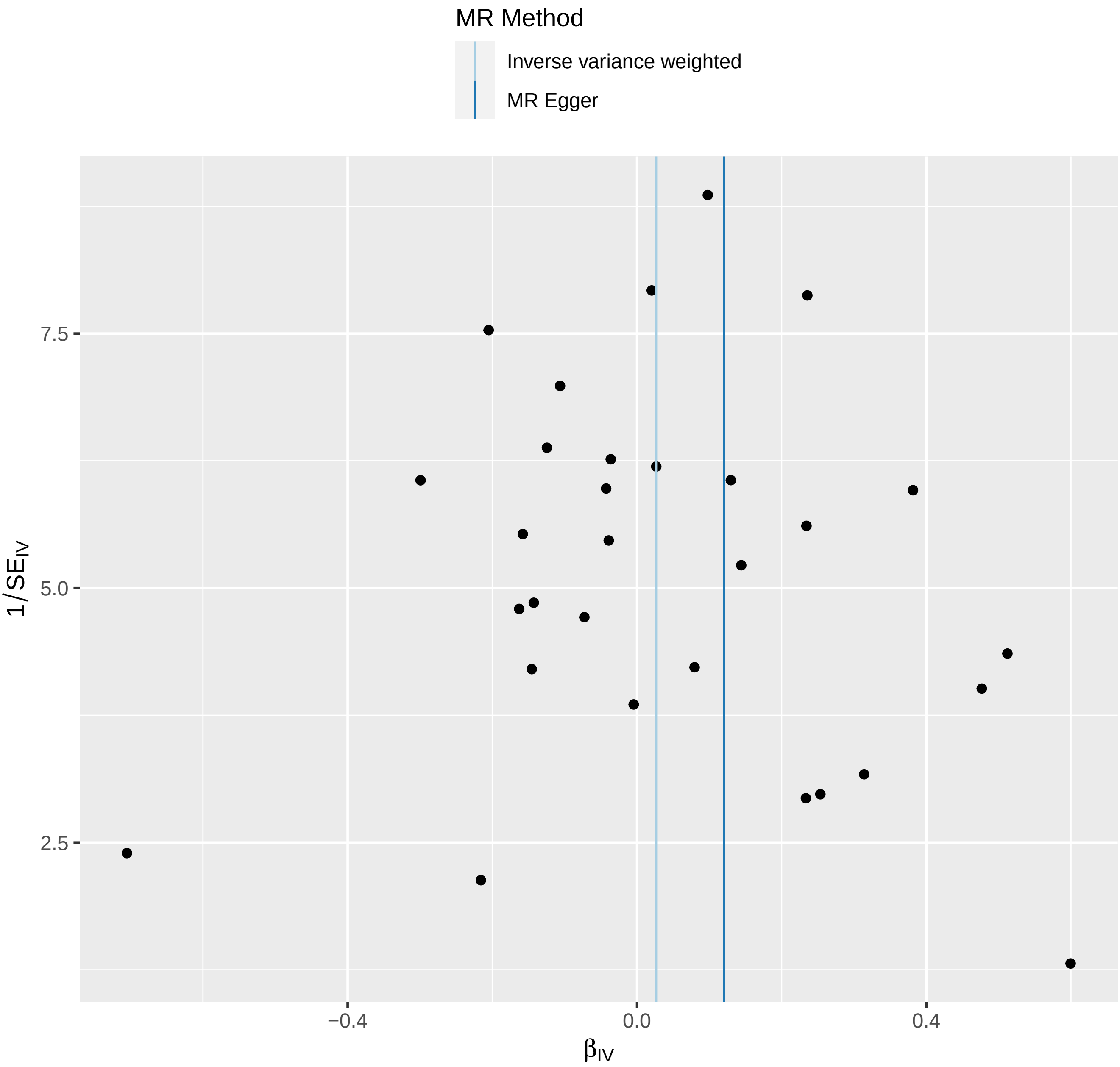

Batch 3−1195 : Growth/differentiation factor 15 on Diabetic nephropathy (more control exclusions)
with a threshold of 1e−05

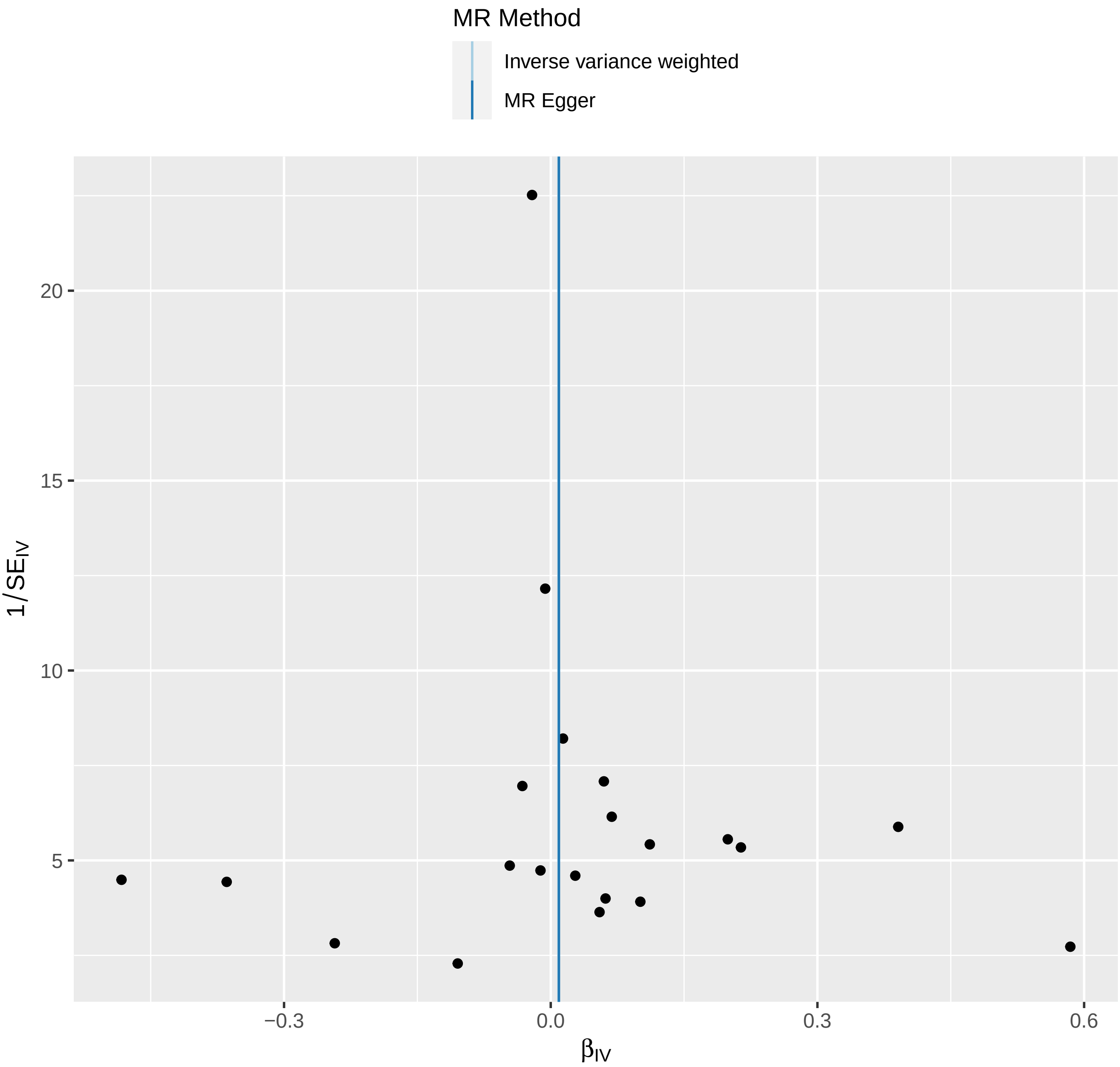

Batch 3−1196 : Growth/differentiation factor 5 on Diabetic nephropathy (more control exclusions)
with a threshold of 1e−05

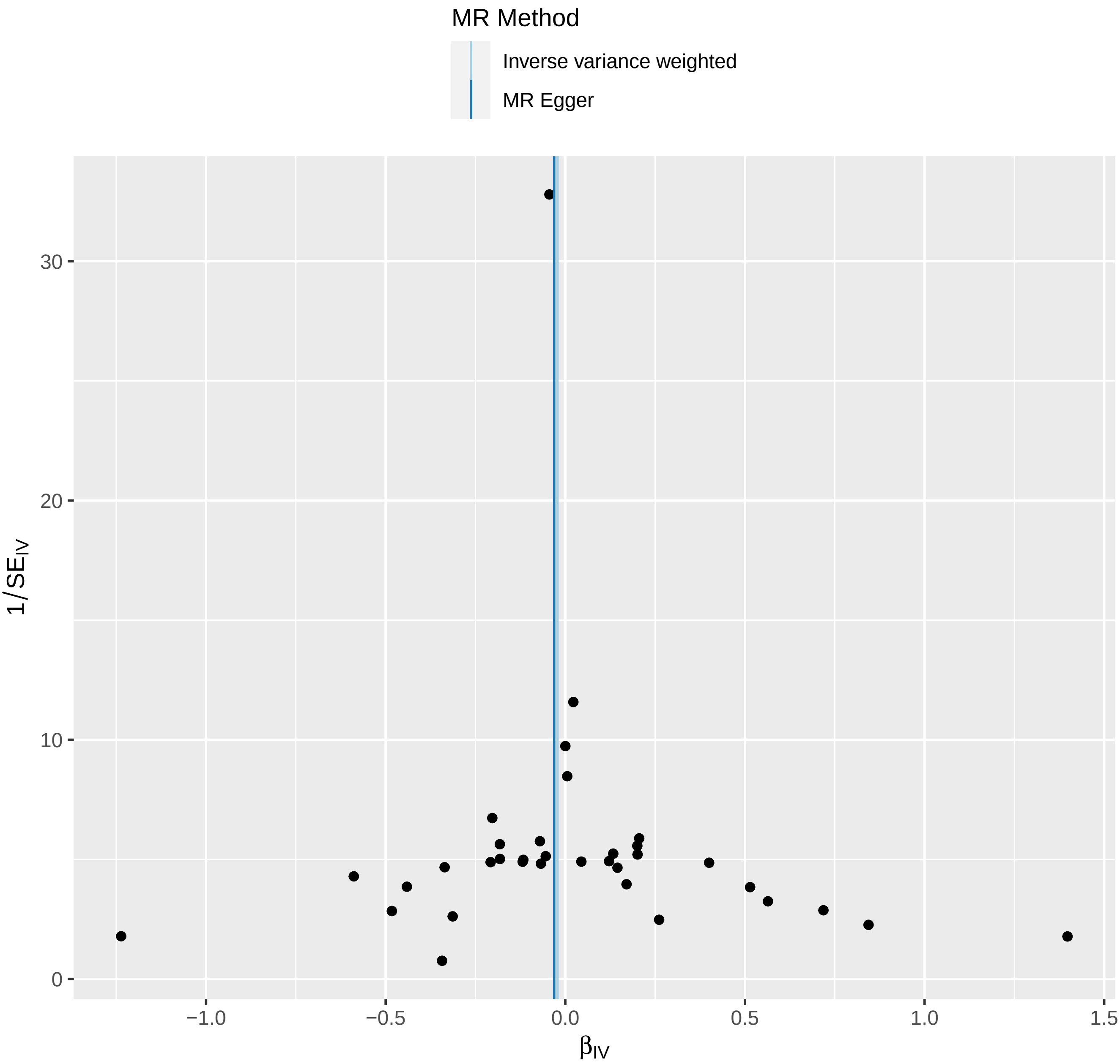

Batch 3−1197 : Growth/differentiation factor 9 on Diabetic nephropathy (more control exclusions)
with a threshold of 1e−05

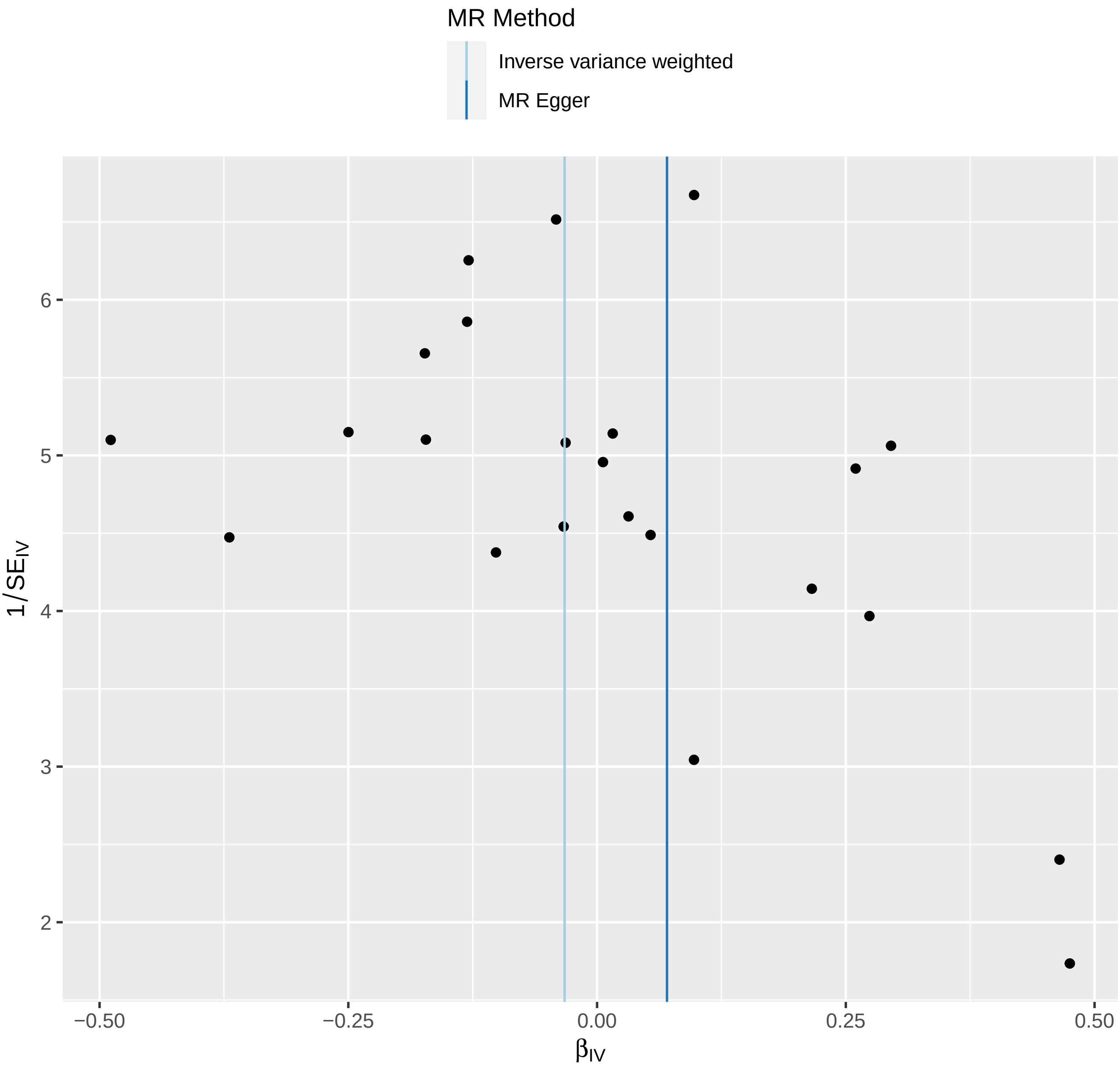

Batch 3−1198 : Glial cell line−derived neurotrophic factor on Diabetic nephropathy (more control exclusions) with a threshold of 1e−05

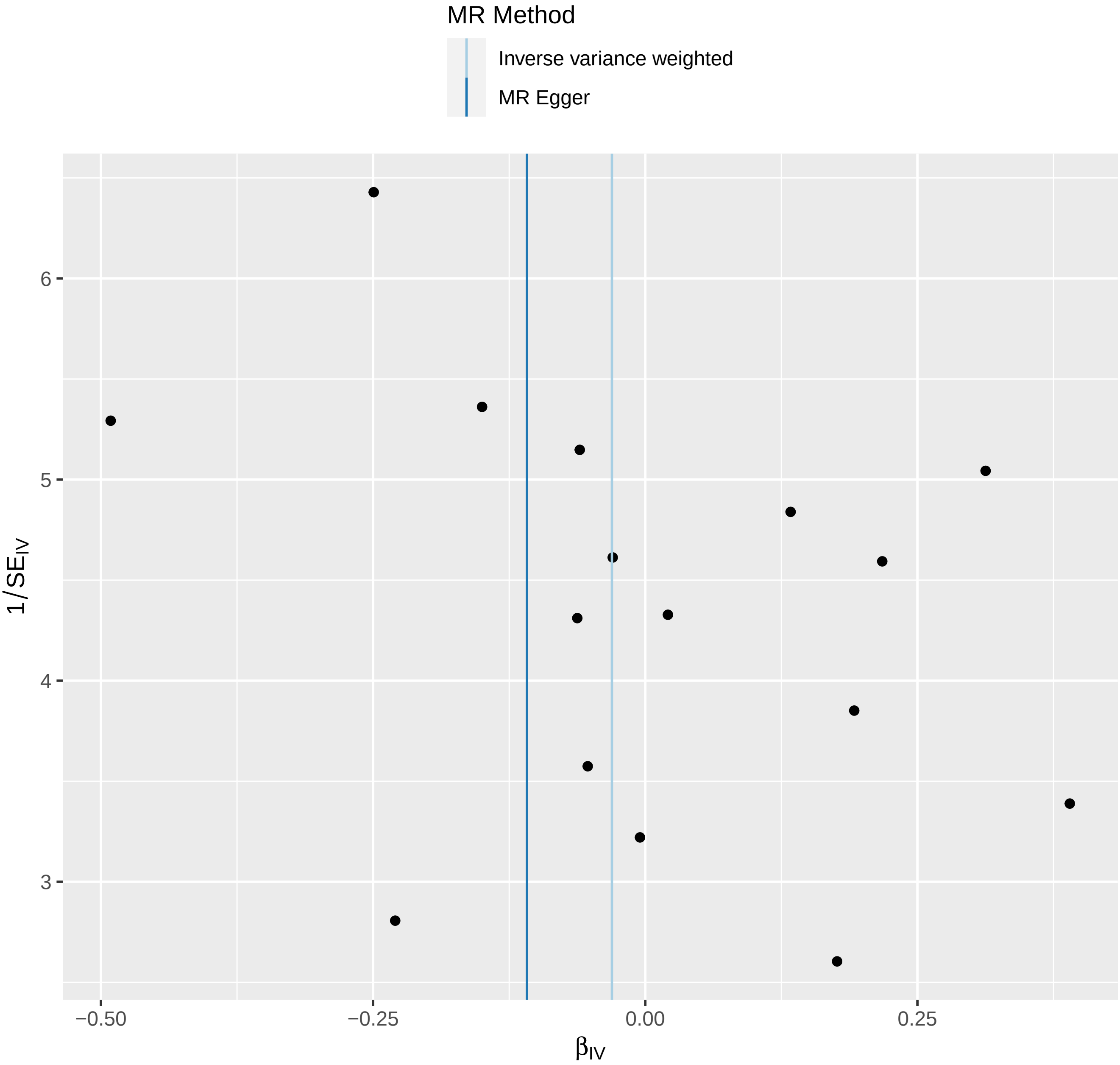

Batch 3−1199 : GTP−binding protein GEM on Diabetic nephropathy (more control exclusions)
with a threshold of 1e−05

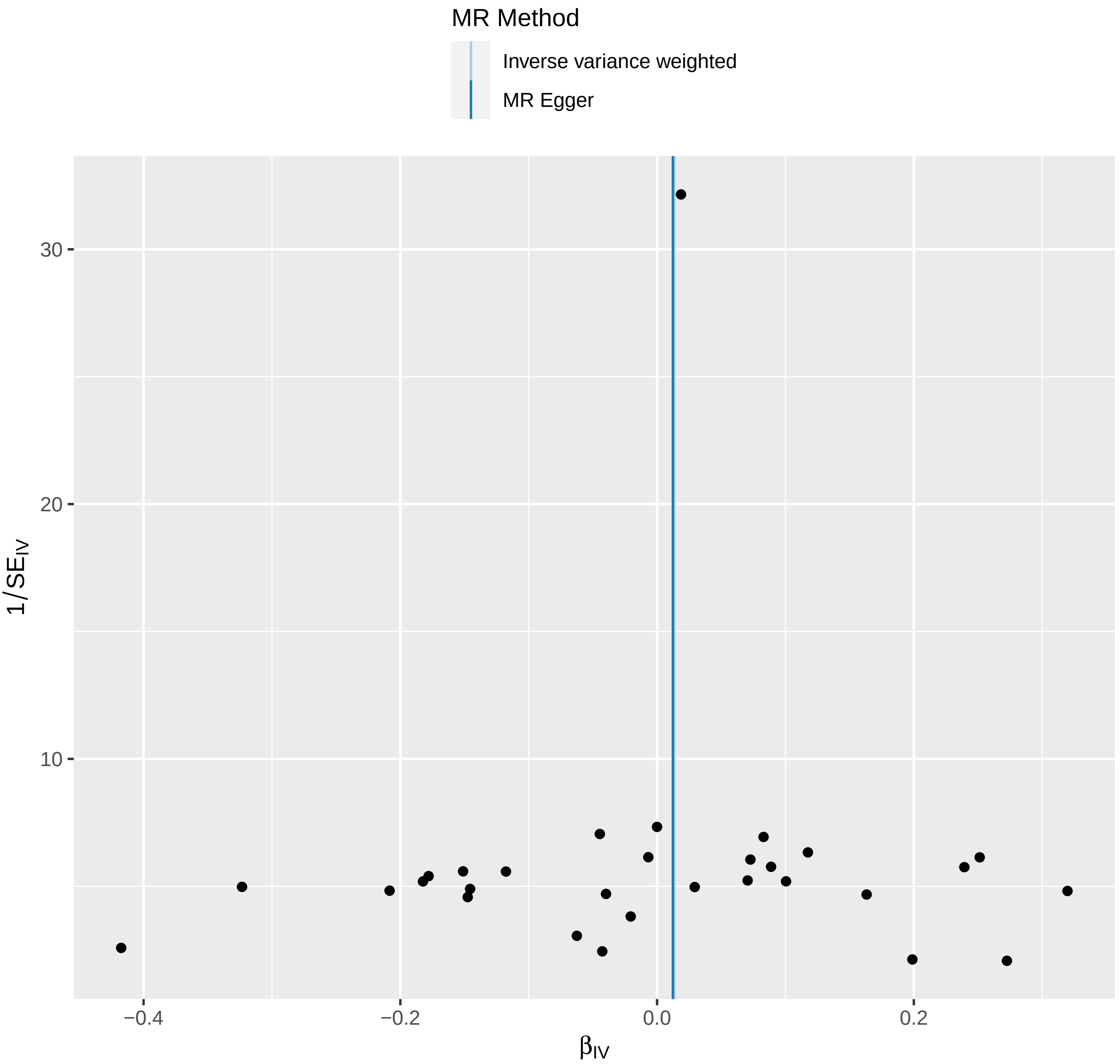

Batch 3−1200 : Glial fibrillary acidic protein on Diabetic nephropathy (more control exclusions)
with a threshold of 1e−05

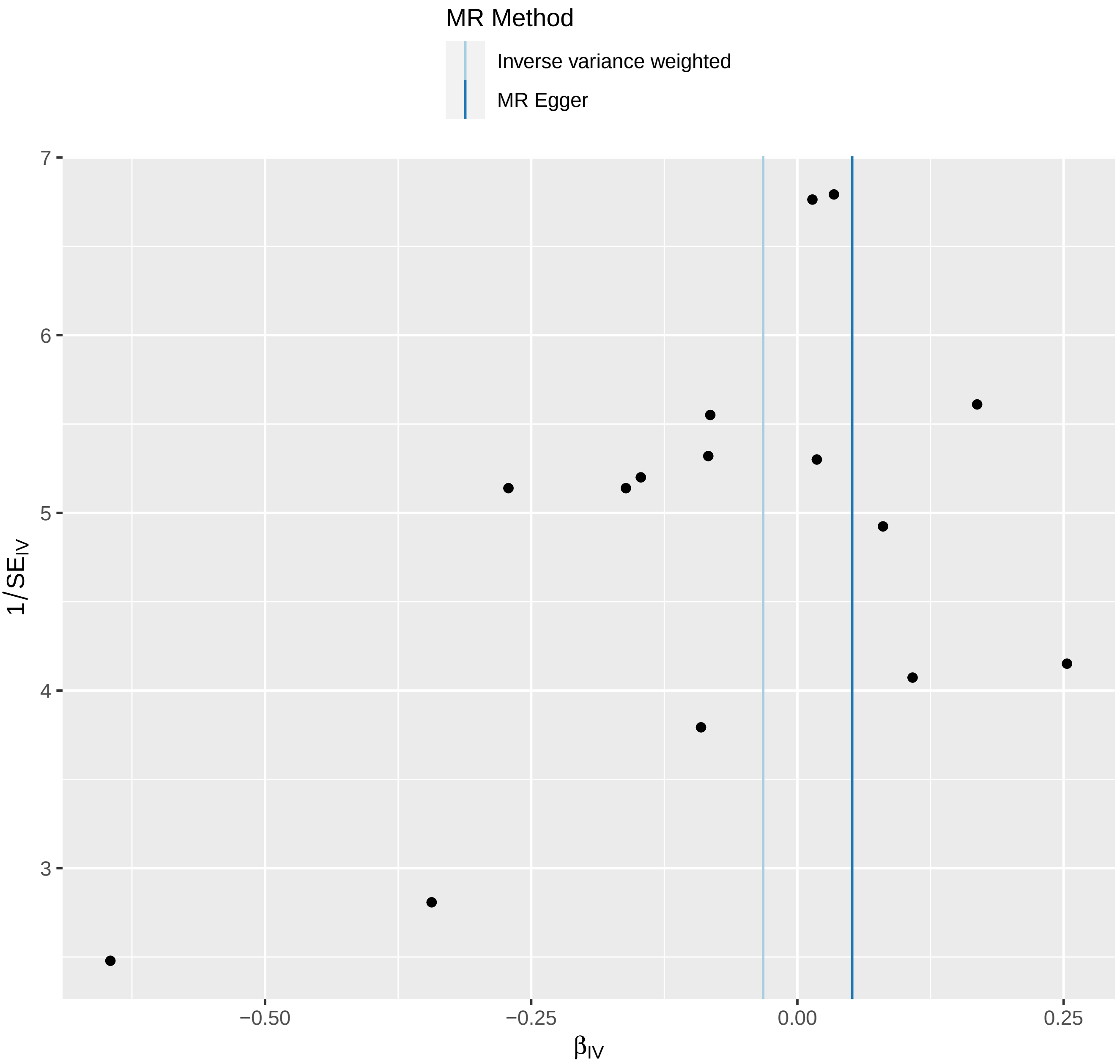

Batch 3−1201 : GDNF family receptor alpha−1 on Diabetic nephropathy (more control exclusions)
with a threshold of 1e−05

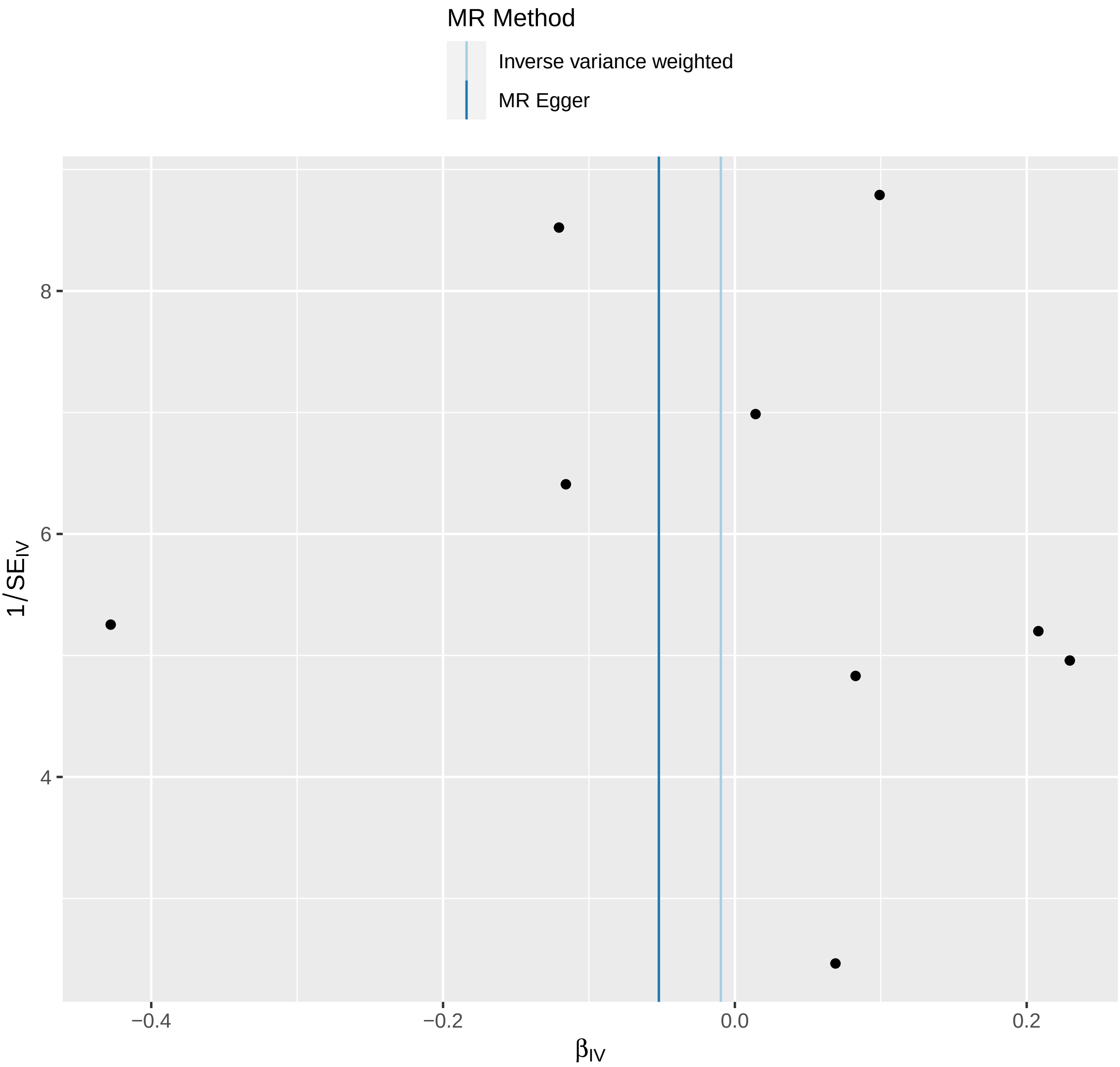

Batch 3−1202 : GDNF family receptor alpha−2 on Diabetic nephropathy (more control exclusions)
with a threshold of 1e−05

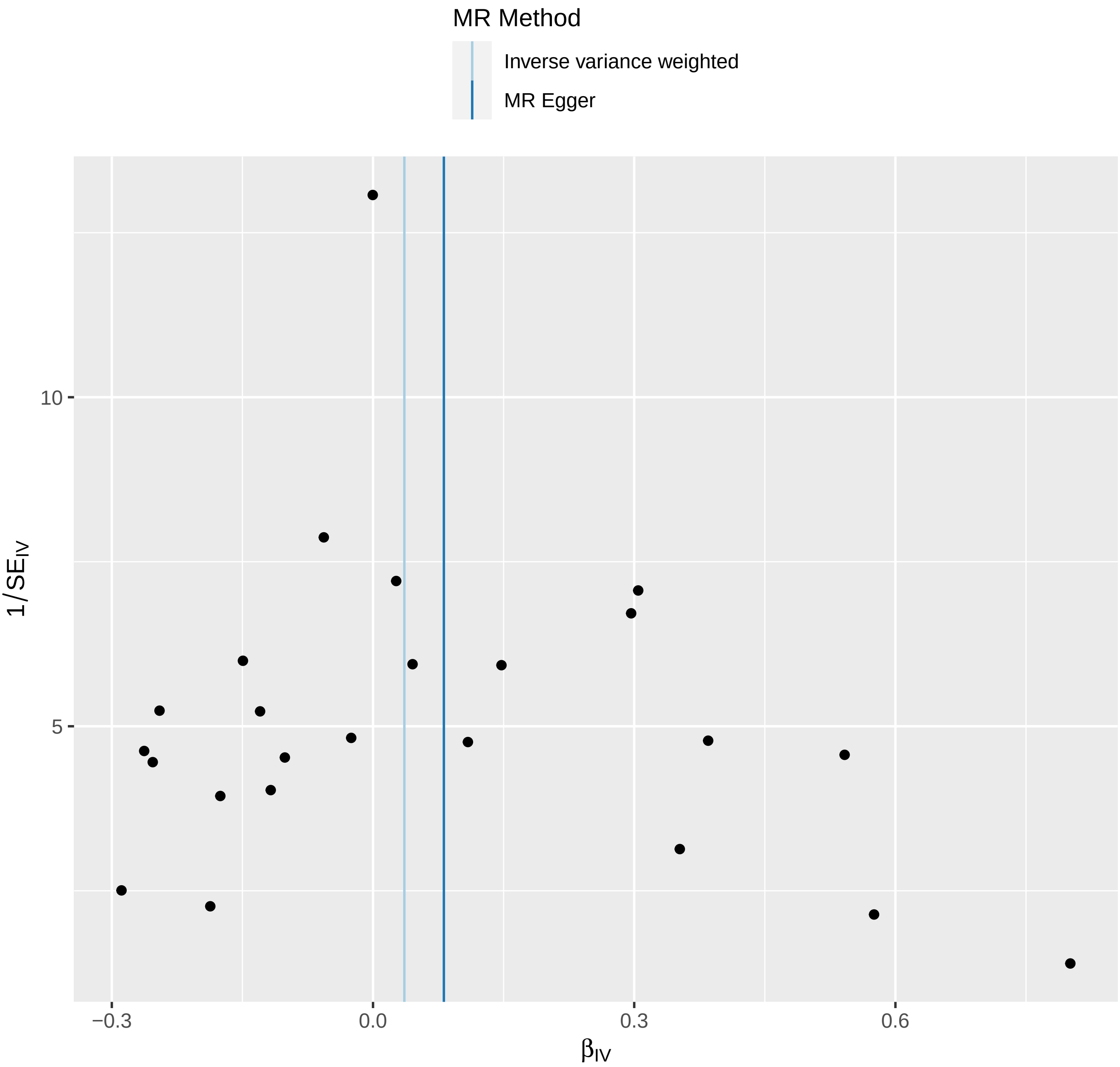

Batch 3−1203 : GDNF family receptor alpha−3 on Diabetic nephropathy (more control exclusions)
with a threshold of 1e−05

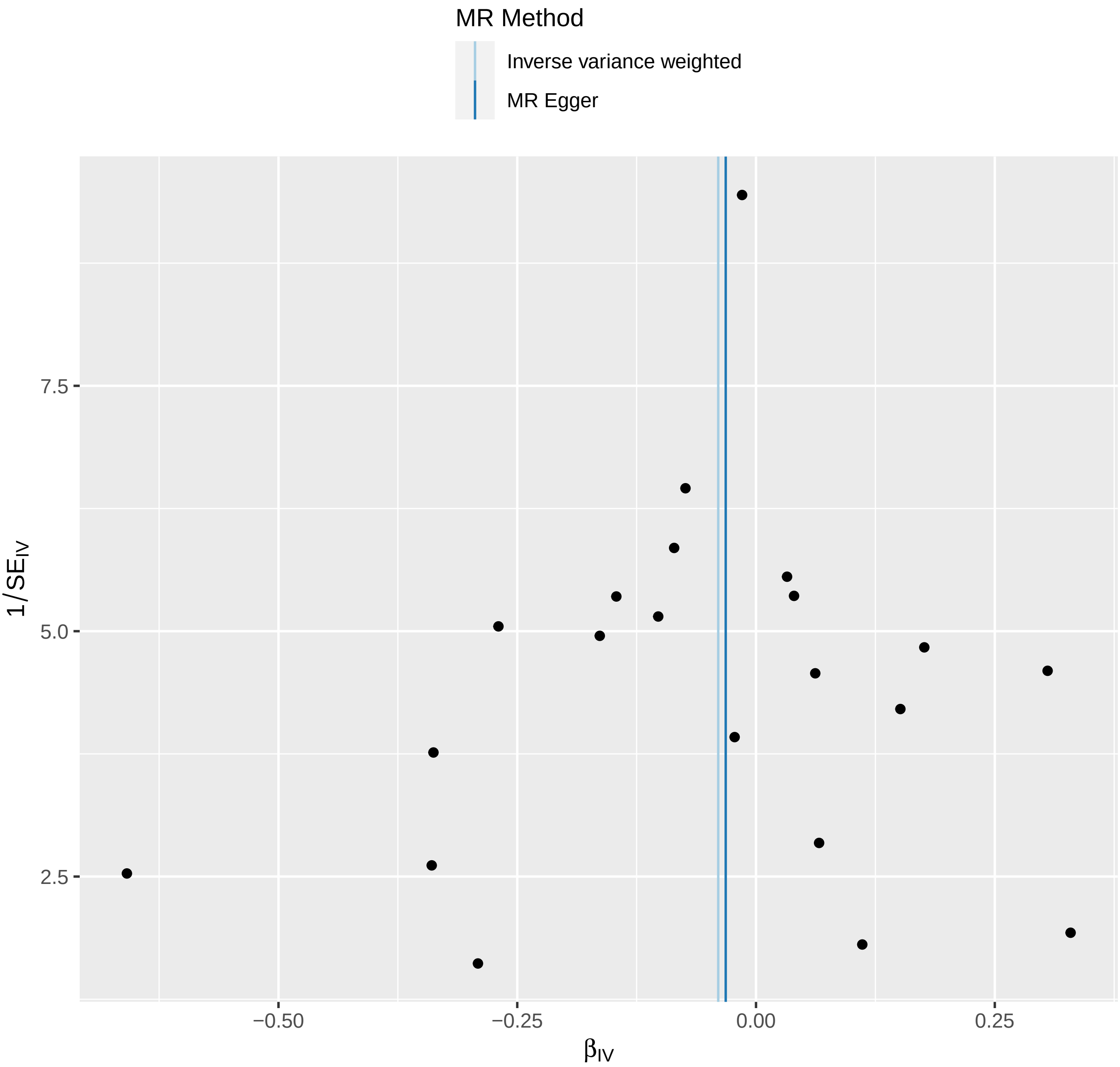

Batch 3−1204 : GDNF family receptor alpha−like on Diabetic nephropathy (more control exclusions)
with a threshold of 1e−05

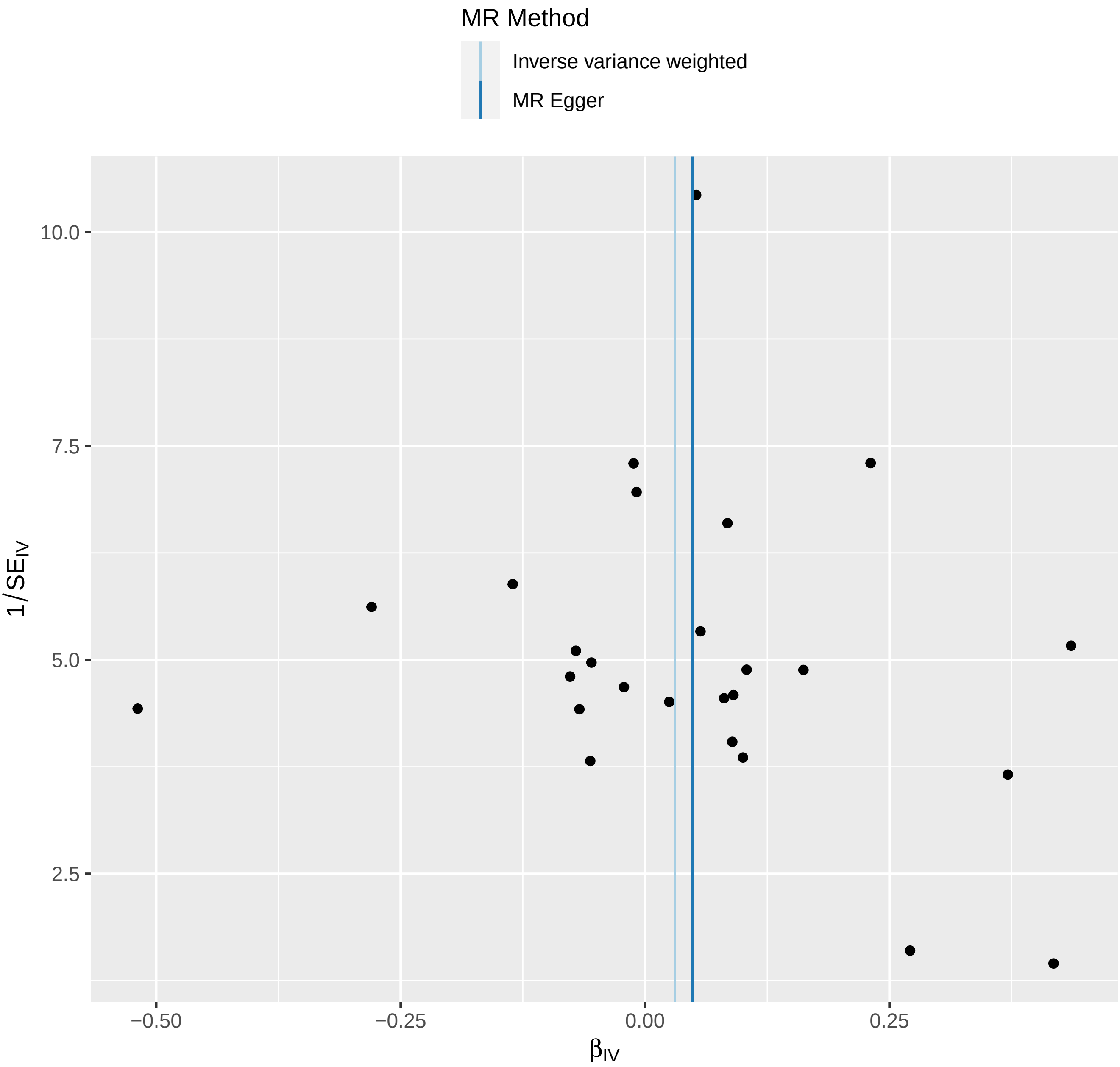

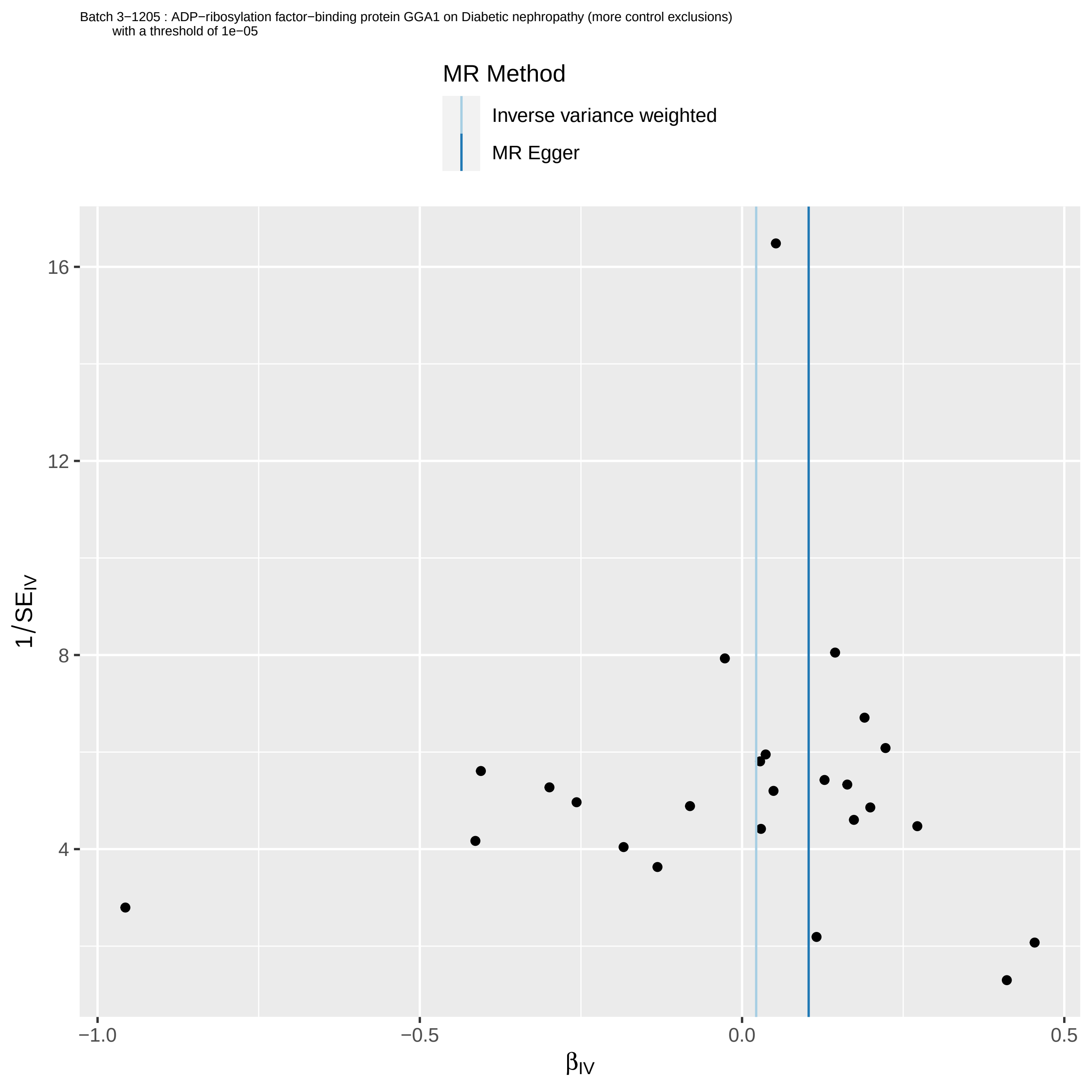
Batch 3−1205 : ADP−ribosylation factor−binding protein GGA1 on Diabetic nephropathy (more control exclusions)
with a threshold of 1e−05
MR Method
Inverse variance weighted
MR Egger
1/SE_IV
16
12
8
4
−1.0
−0.5
0.0
0.5
β_IV

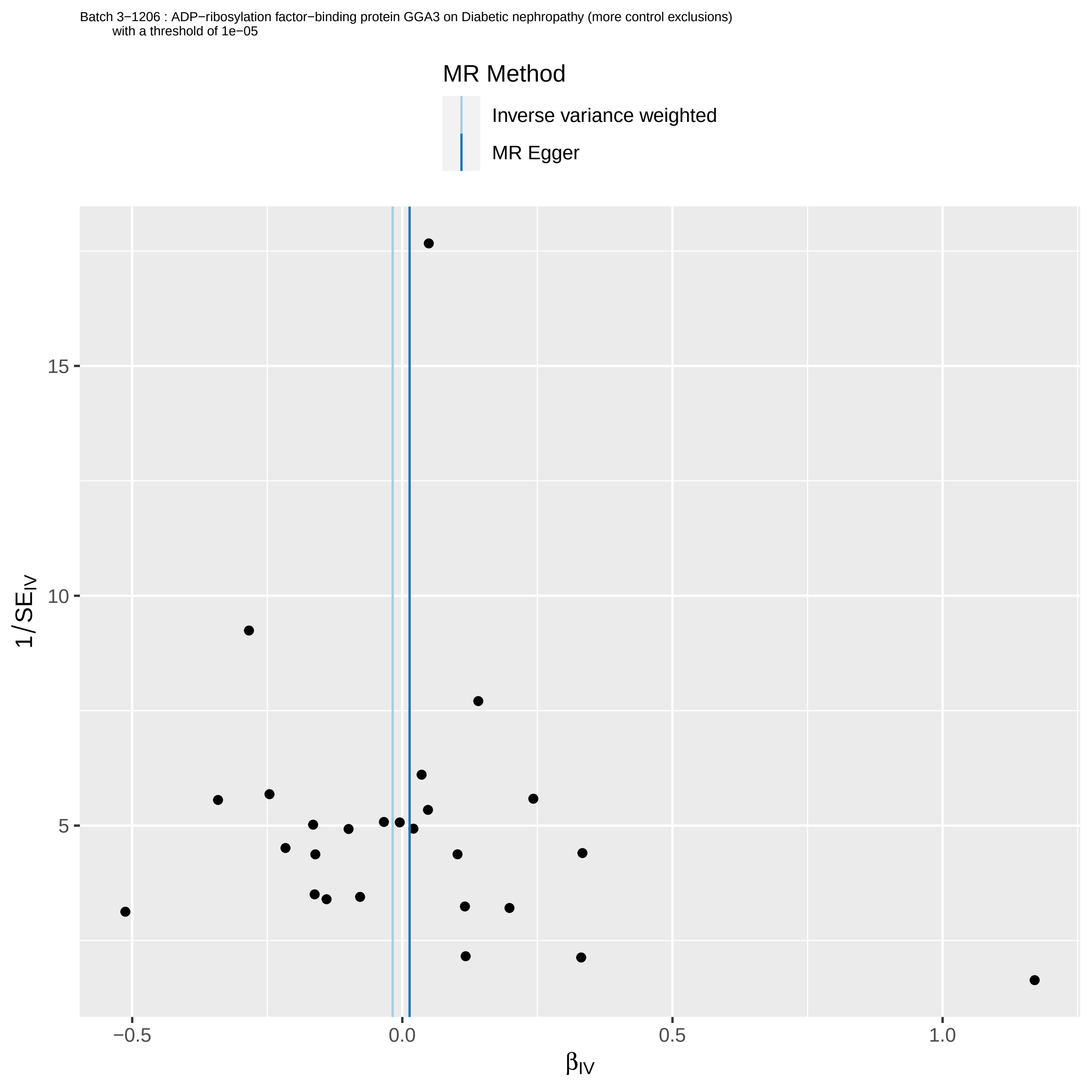
Batch 3−1206 : ADP−ribosylation factor−binding protein GGA3 on Diabetic nephropathy (more control exclusions)
with a threshold of 1e−05
MR Method
Inverse variance weighted
MR Egger
15
10
5
1/SE_IV
−0.5
0.0
0.5
1.0
β_IV

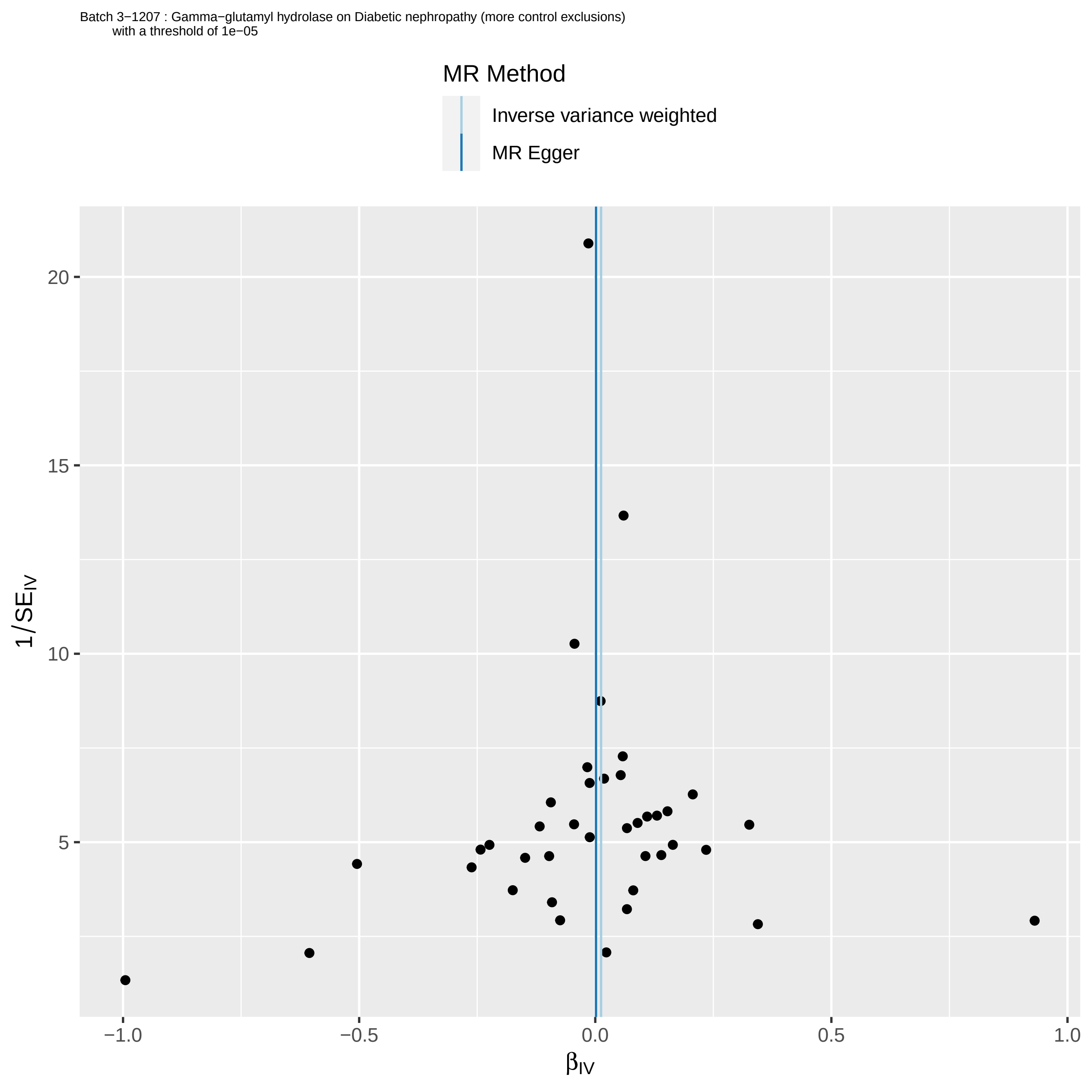
Batch 3−1207 : Gamma−glutamyl hydrolase on Diabetic nephropathy (more control exclusions)
with a threshold of 1e−05
MR Method
Inverse variance weighted
MR Egger
20
15
10
5
1/SE_IV
−1.0
−0.5
0.0
0.5
1.0
β_IV

Batch 3−1208 : Inactive gamma−glutamyltranspeptidase 2 on Diabetic nephropathy (more control exclusions)
with a threshold of 1e−05

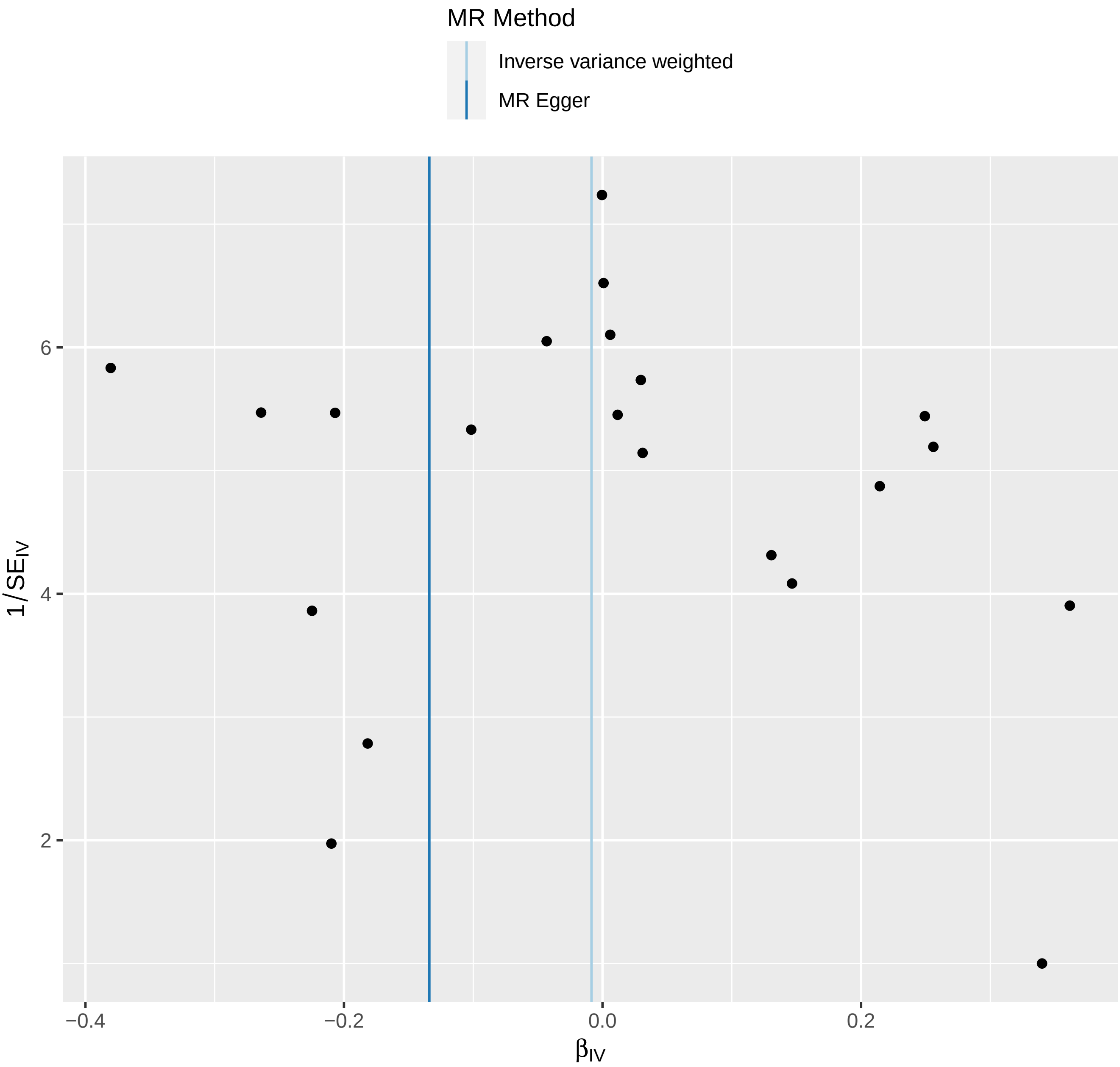

Batch 3−1209 : Somatotropin on Diabetic nephropathy (more control exclusions)
with a threshold of 1e−05

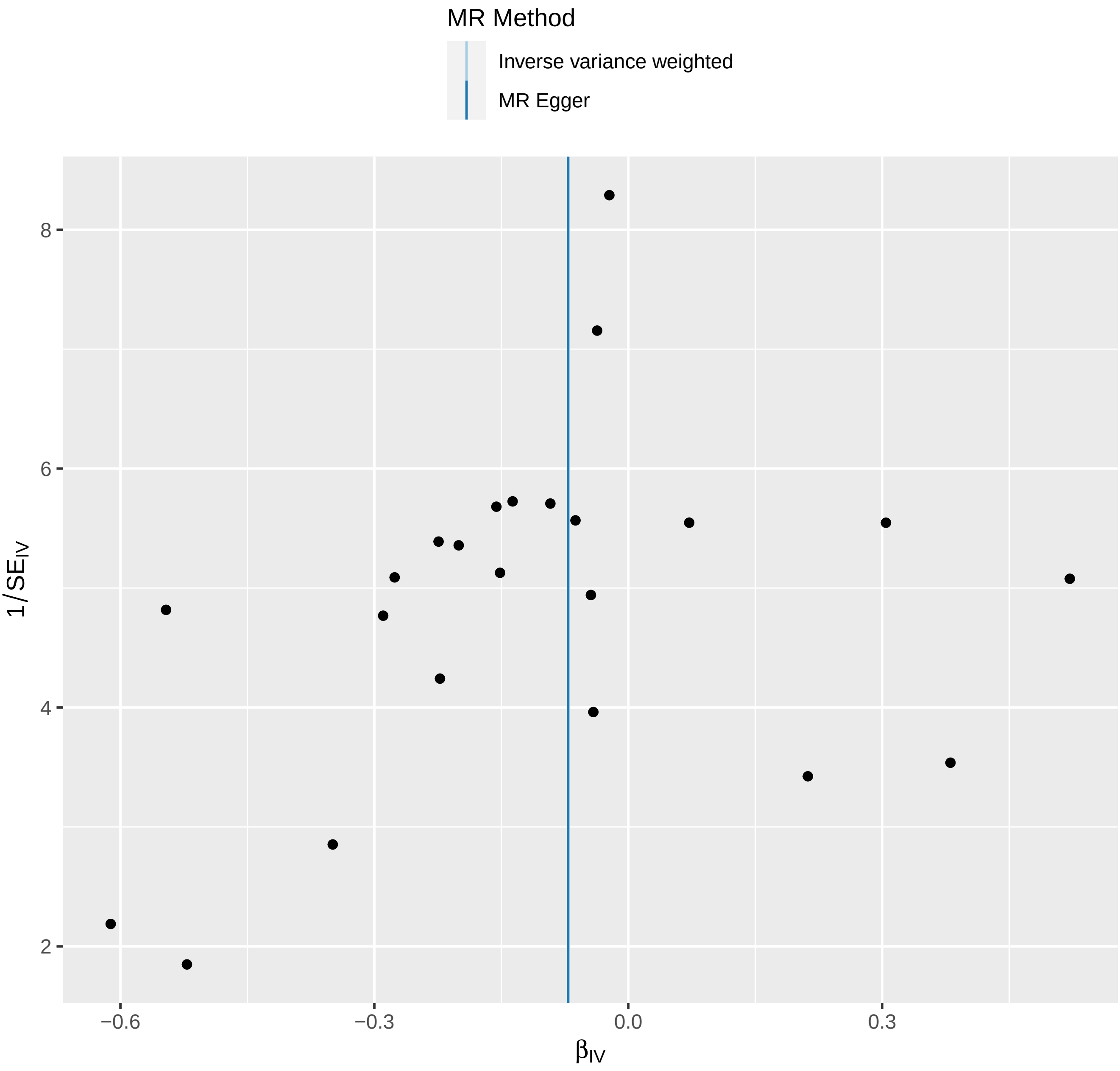

Batch 3−1210 : GH3 domain−containing protein on Diabetic nephropathy (more control exclusions)
with a threshold of 1e−05

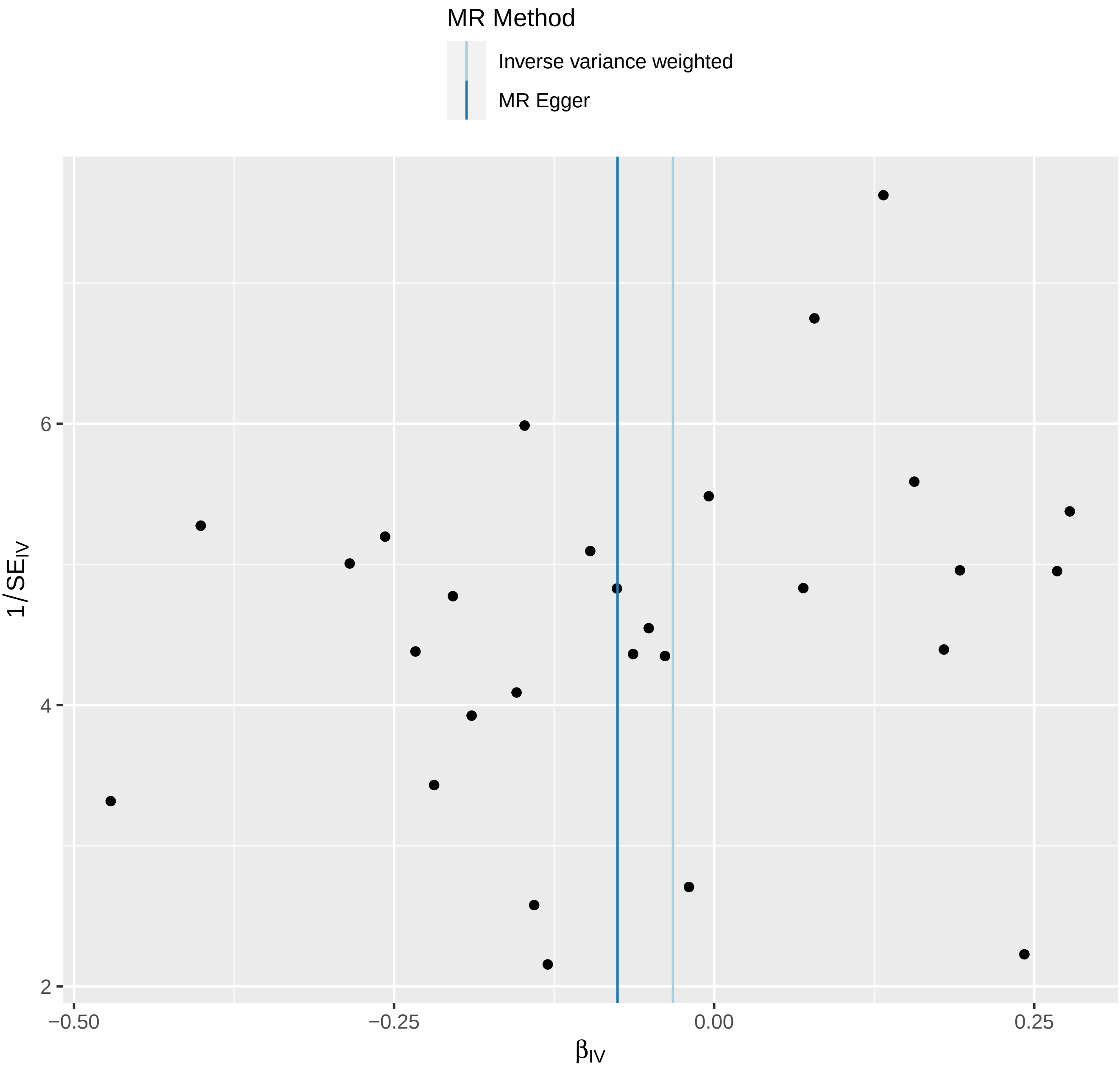

Batch 3−1211 : Growth hormone receptor on Diabetic nephropathy (more control exclusions)
with a threshold of 1e−05

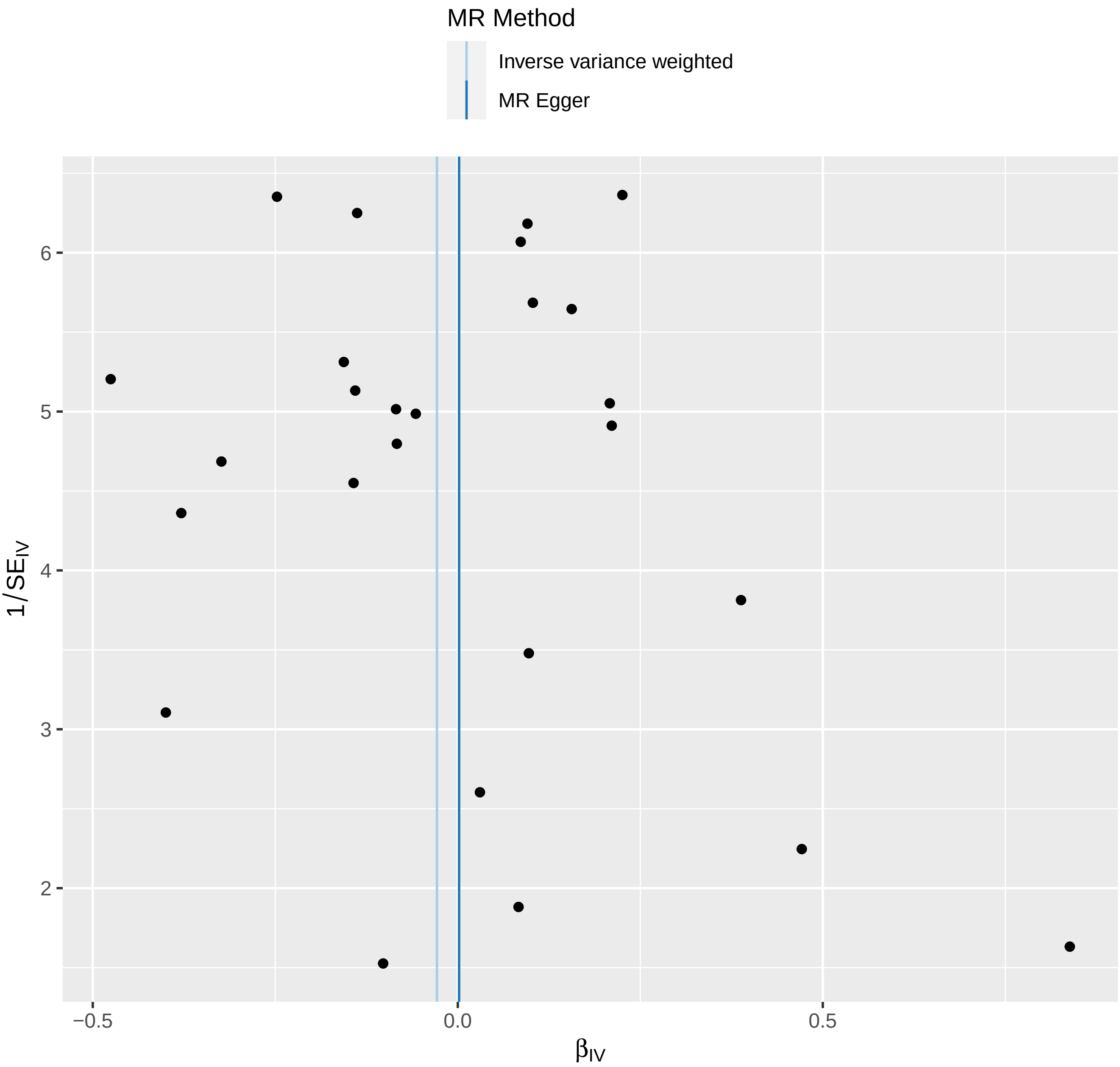

Batch 3−1212 : Appetite−regulating hormone on Diabetic nephropathy (more control exclusions)
with a threshold of 1e−05

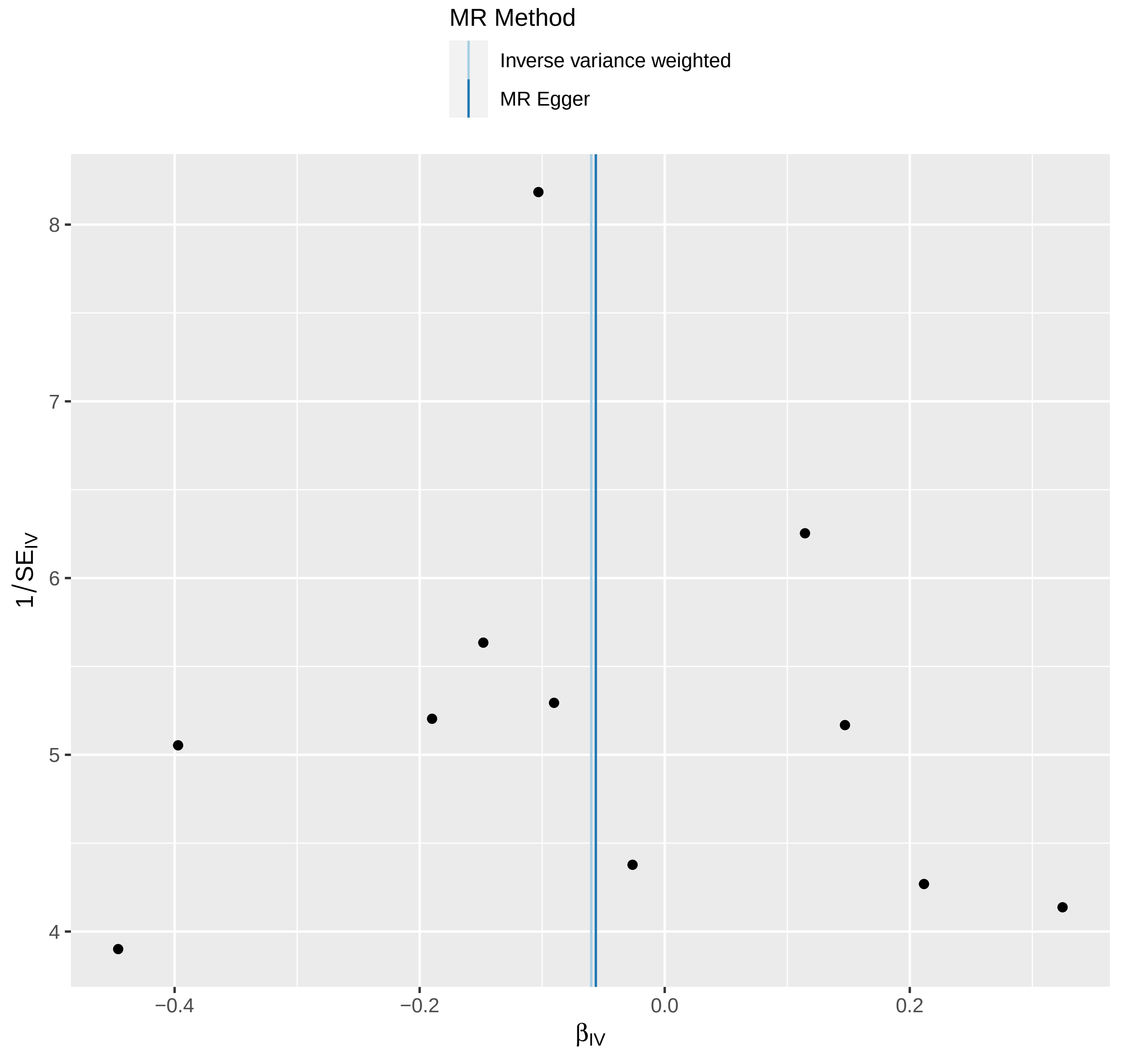

Batch 3−1213 : Gap junction alpha−1 protein on Diabetic nephropathy (more control exclusions)
with a threshold of 1e−05

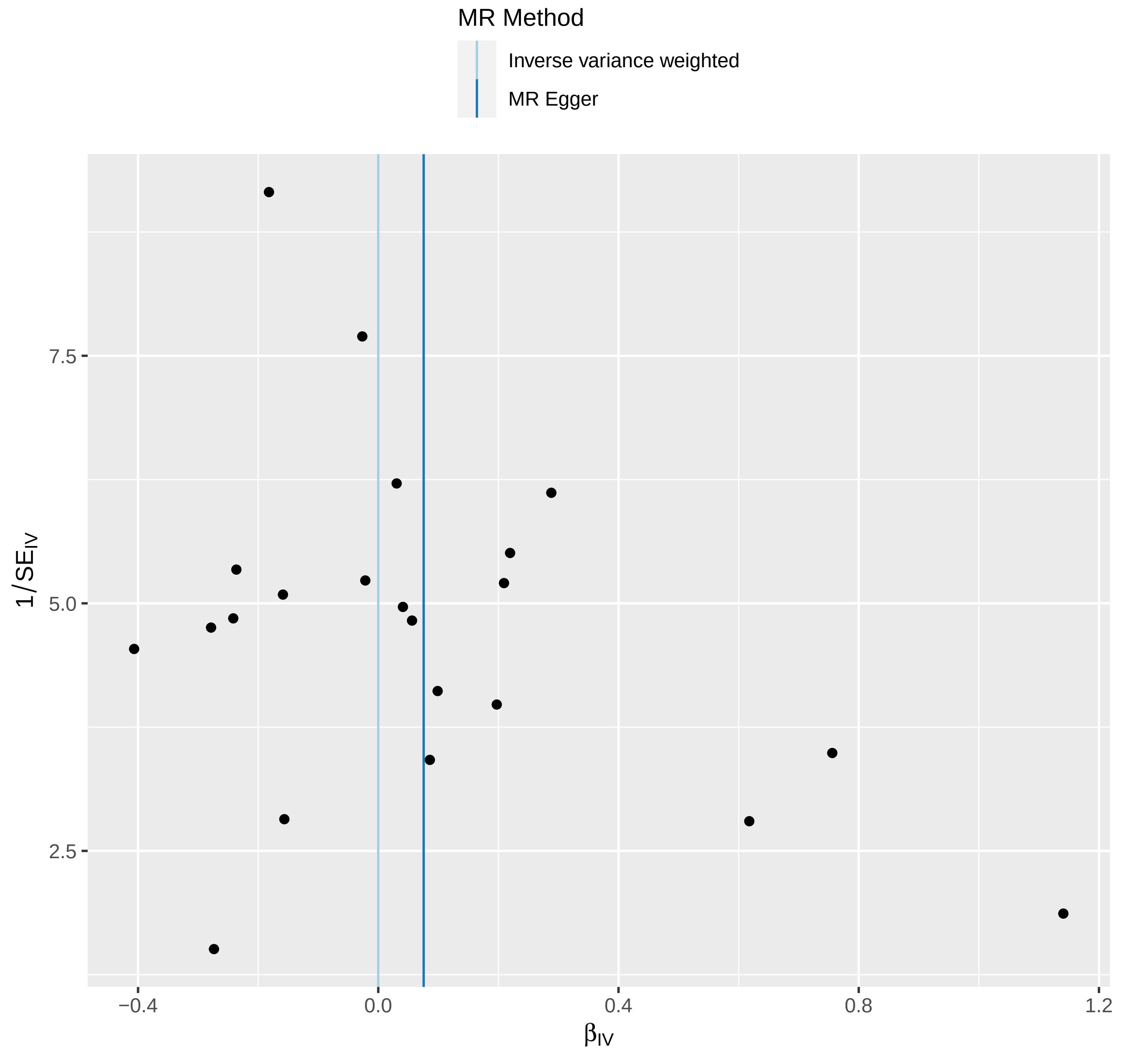

Batch 3−1214 : Gap junction alpha−8 protein on Diabetic nephropathy (more control exclusions)
with a threshold of 1e−05

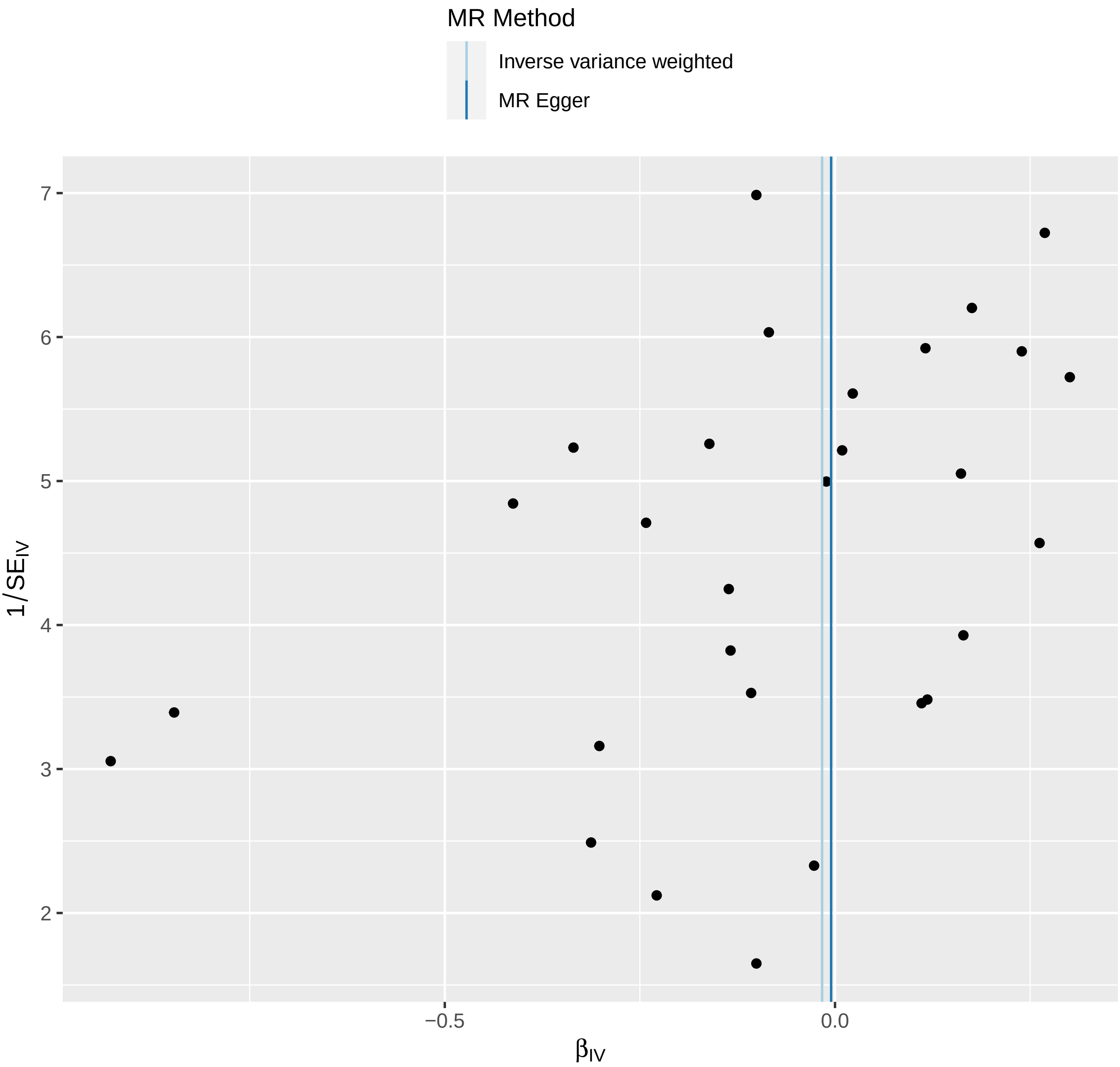

Batch 3−1215 : Gap junction delta−2 protein on Diabetic nephropathy (more control exclusions)
with a threshold of 1e−05

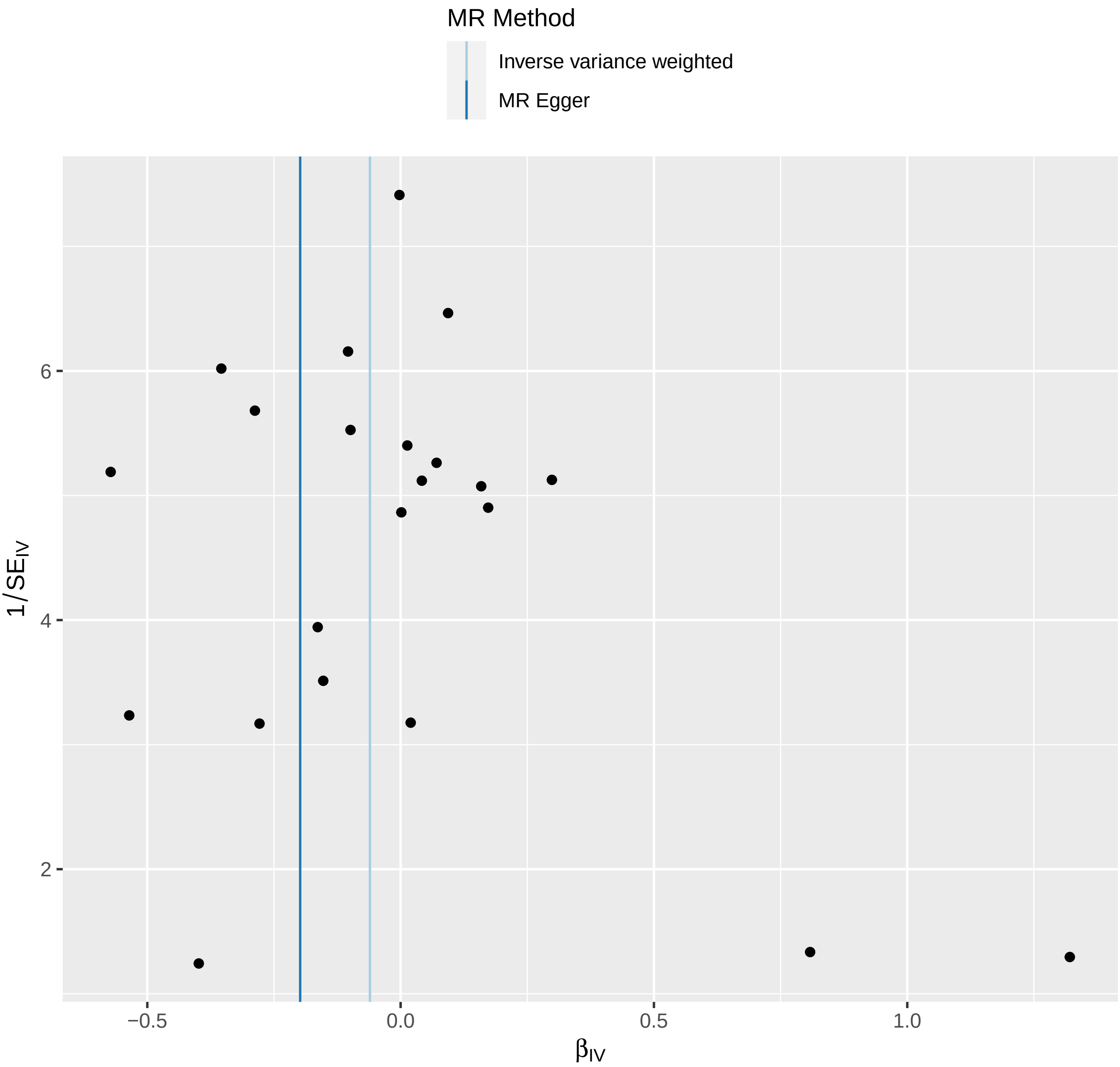

Batch 3−1216 : D−glucuronyl C5−epimerase on Diabetic nephropathy (more control exclusions)
with a threshold of 1e−05

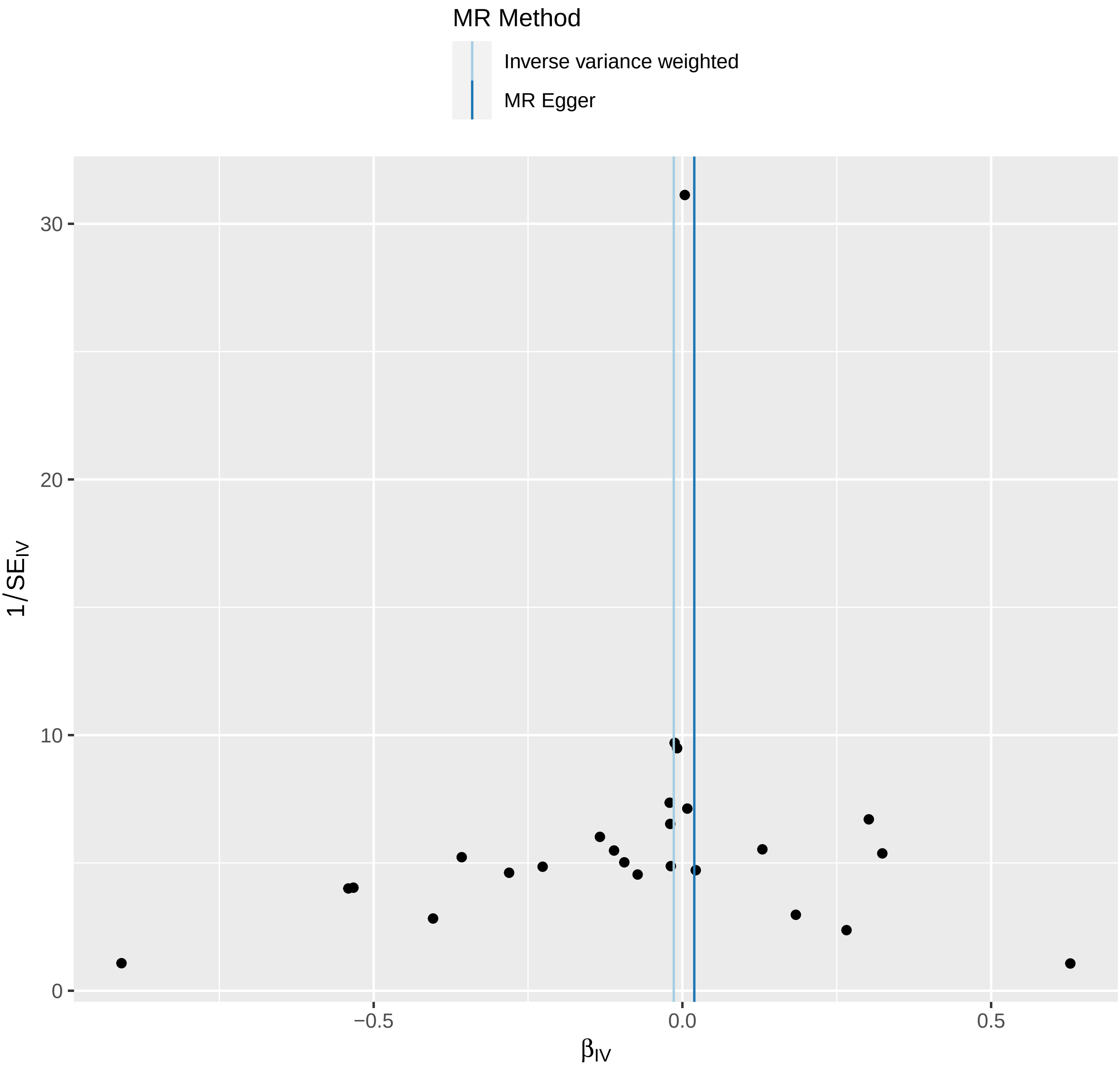

Batch 3−1217 : Glioma pathogenesis−related protein 1 on Diabetic nephropathy (more control exclusions)
with a threshold of 1e−05

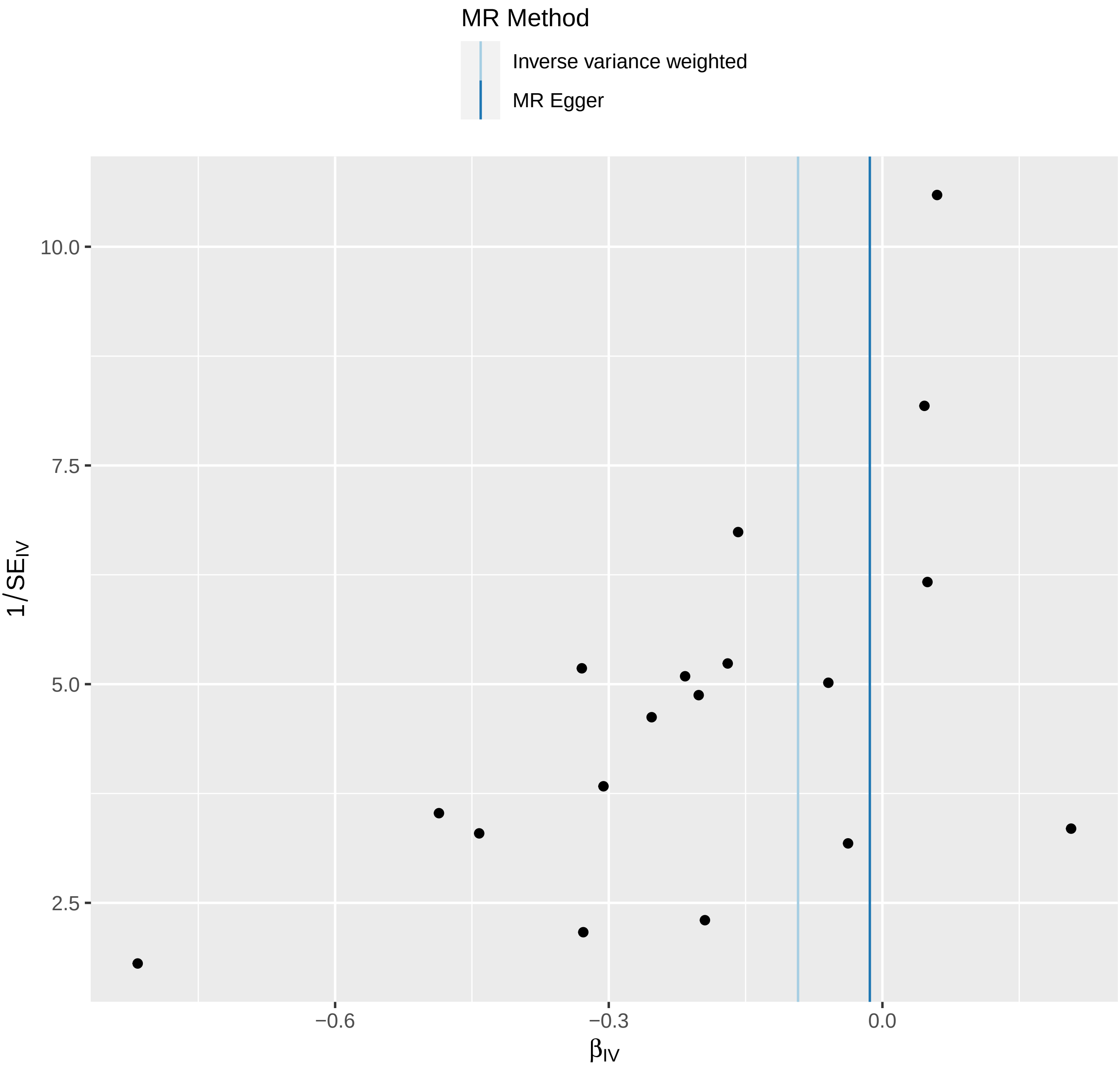

Batch 3−1218 : Lactoylglutathione lyase on Diabetic nephropathy (more control exclusions)
with a threshold of 1e−05

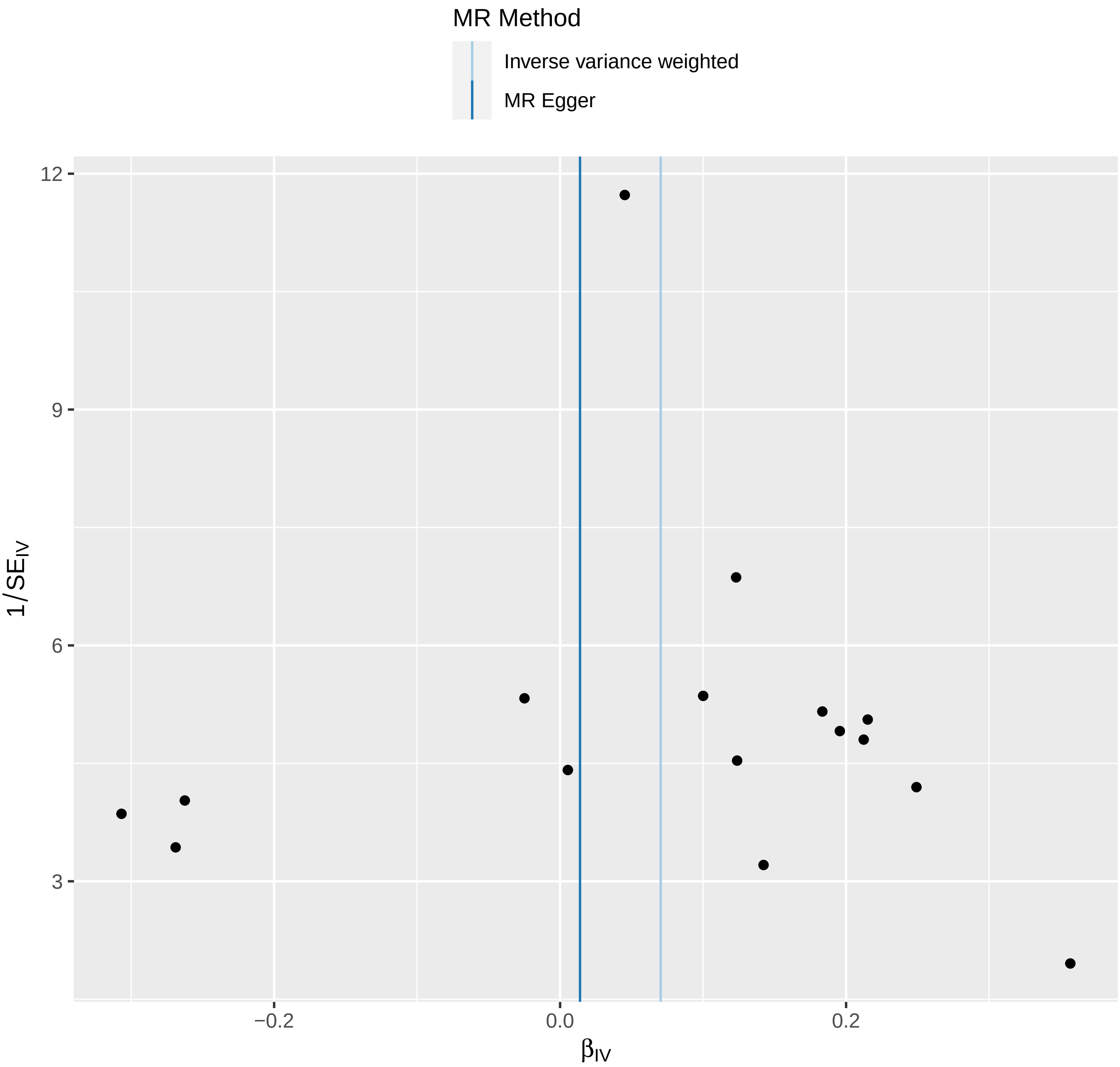

Batch 3−1219 : Glucagon−like peptide 1 receptor on Diabetic nephropathy (more control exclusions) with a threshold of 1e−05

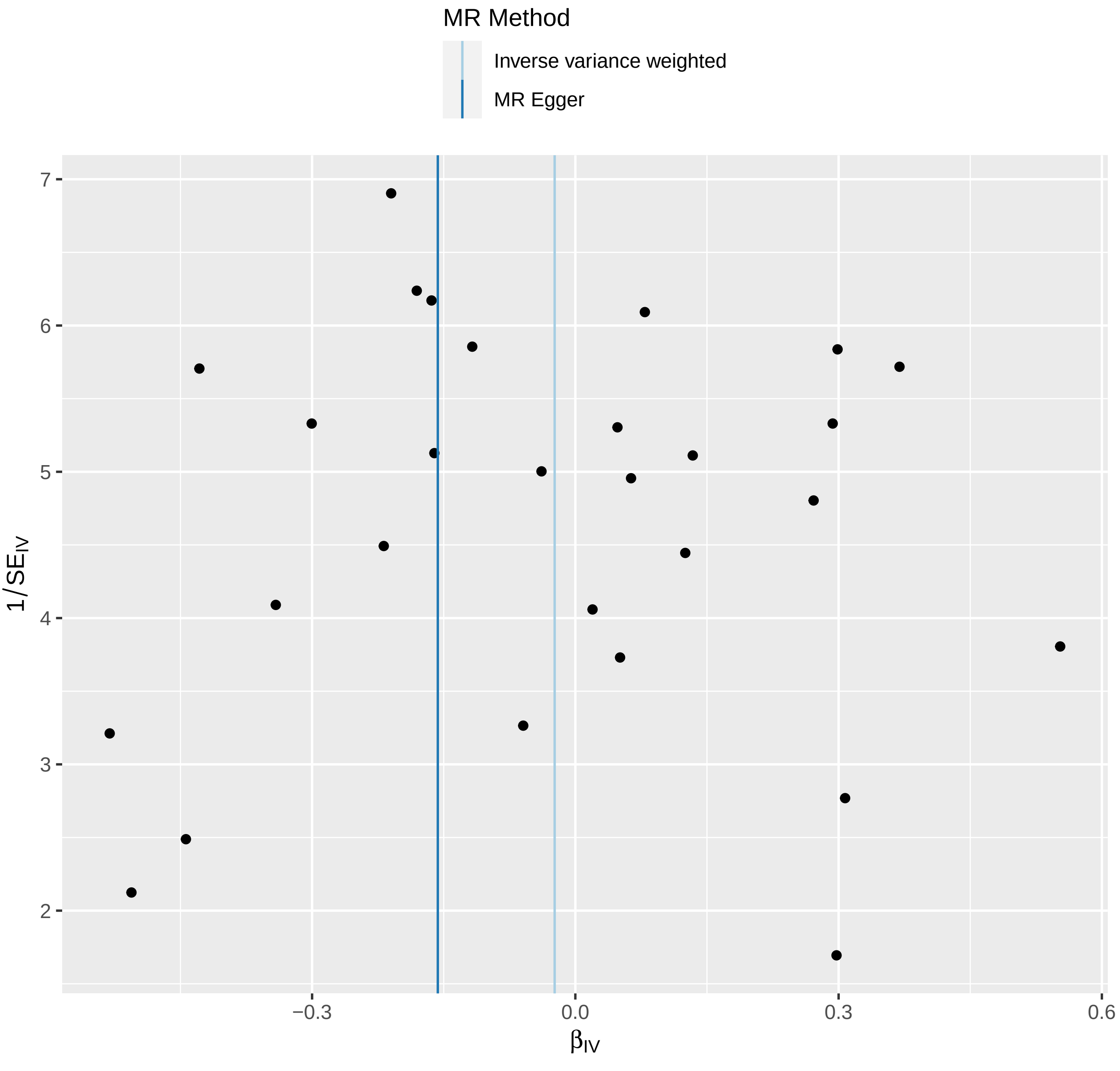

Batch 3−1220 : Glutaredoxin−2, mitochondrial on Diabetic nephropathy (more control exclusions)
with a threshold of 1e−05

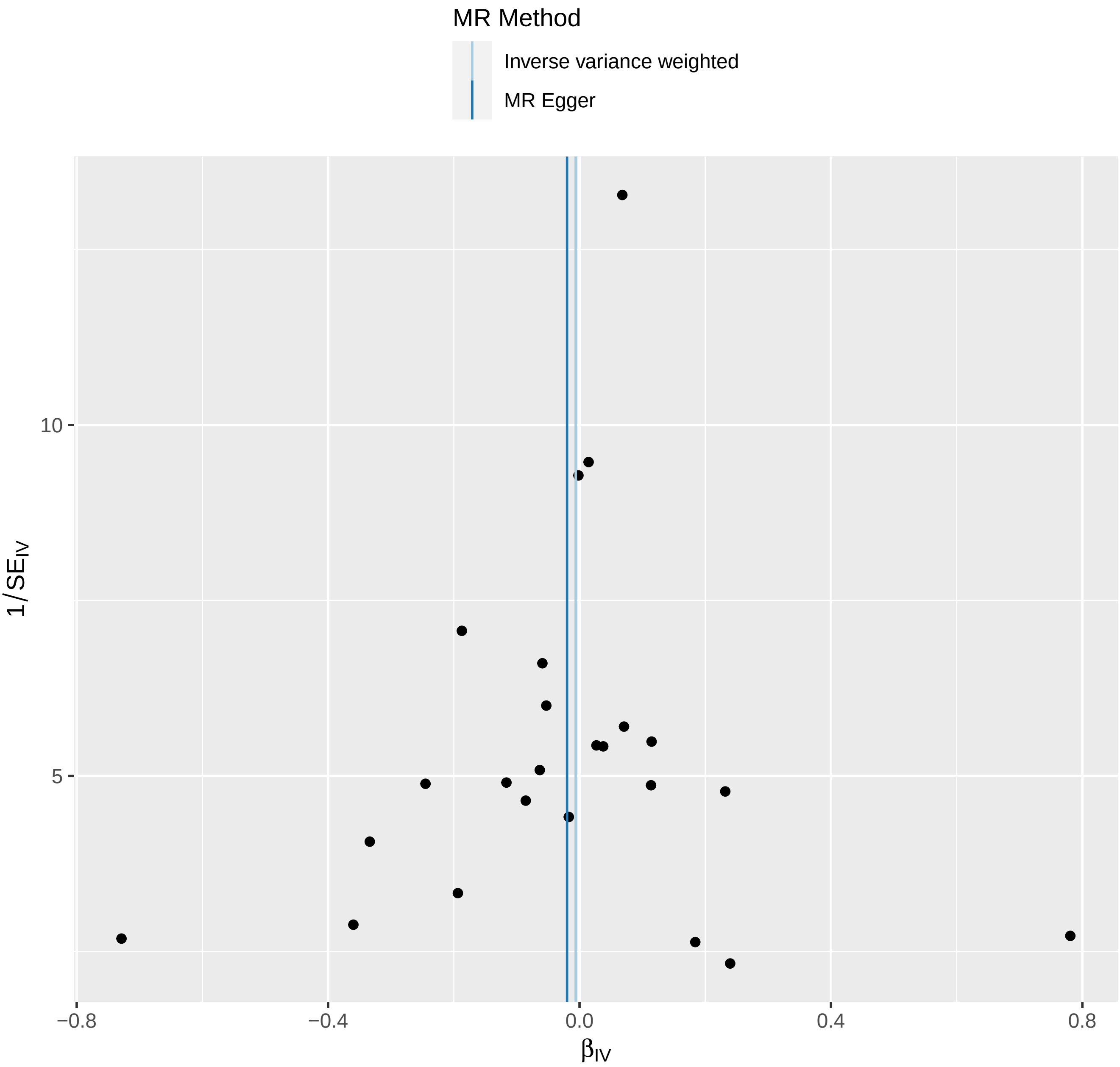

Batch 3−1221 : Glycosyltransferase 8 domain−containing protein 1 on Diabetic nephropathy (more control exclusions) with a threshold of 1e−05

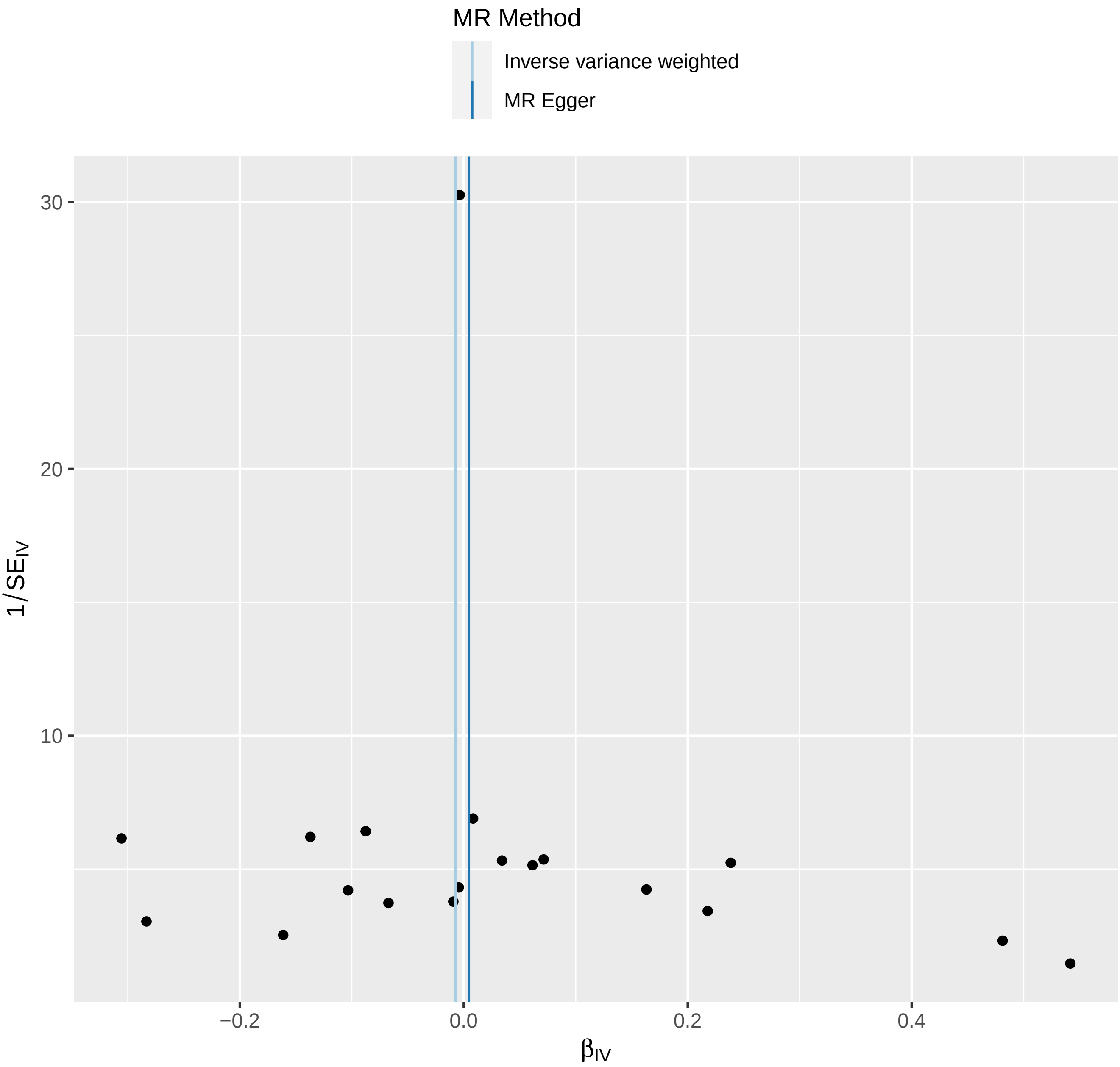

Batch 3−1222 : Glycolipid transfer protein on Diabetic nephropathy (more control exclusions)
with a threshold of 1e−05

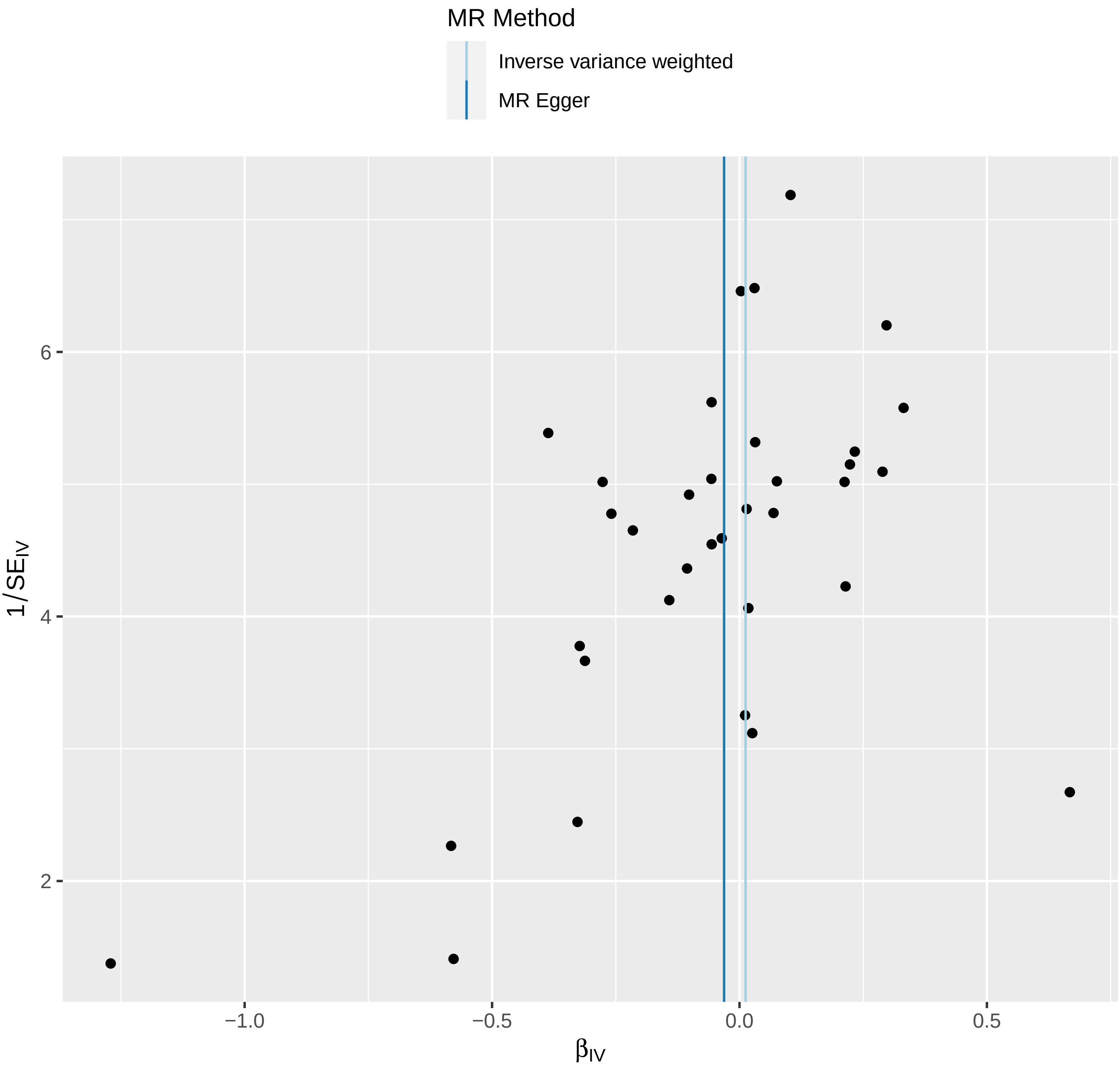

Batch 3−1223 : Glycolipid transfer protein domain−containing protein 2 on Diabetic nephropathy (more control exclusions)
with a threshold of 1e−05

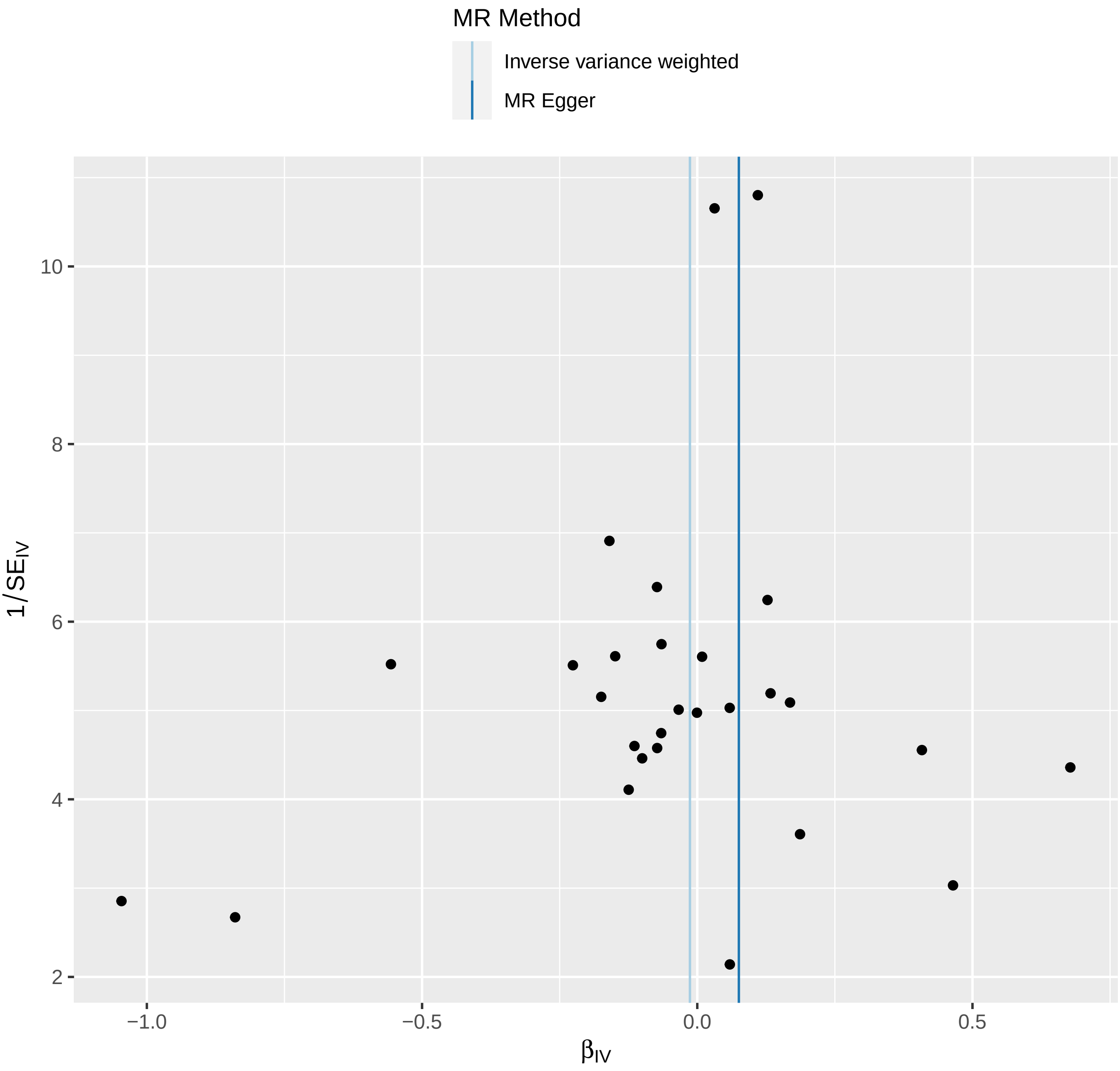

Batch 3−1224 : Glucocorticoid modulatory element−binding protein 2 on Diabetic nephropathy (more control exclusions)
with a threshold of 1e−05

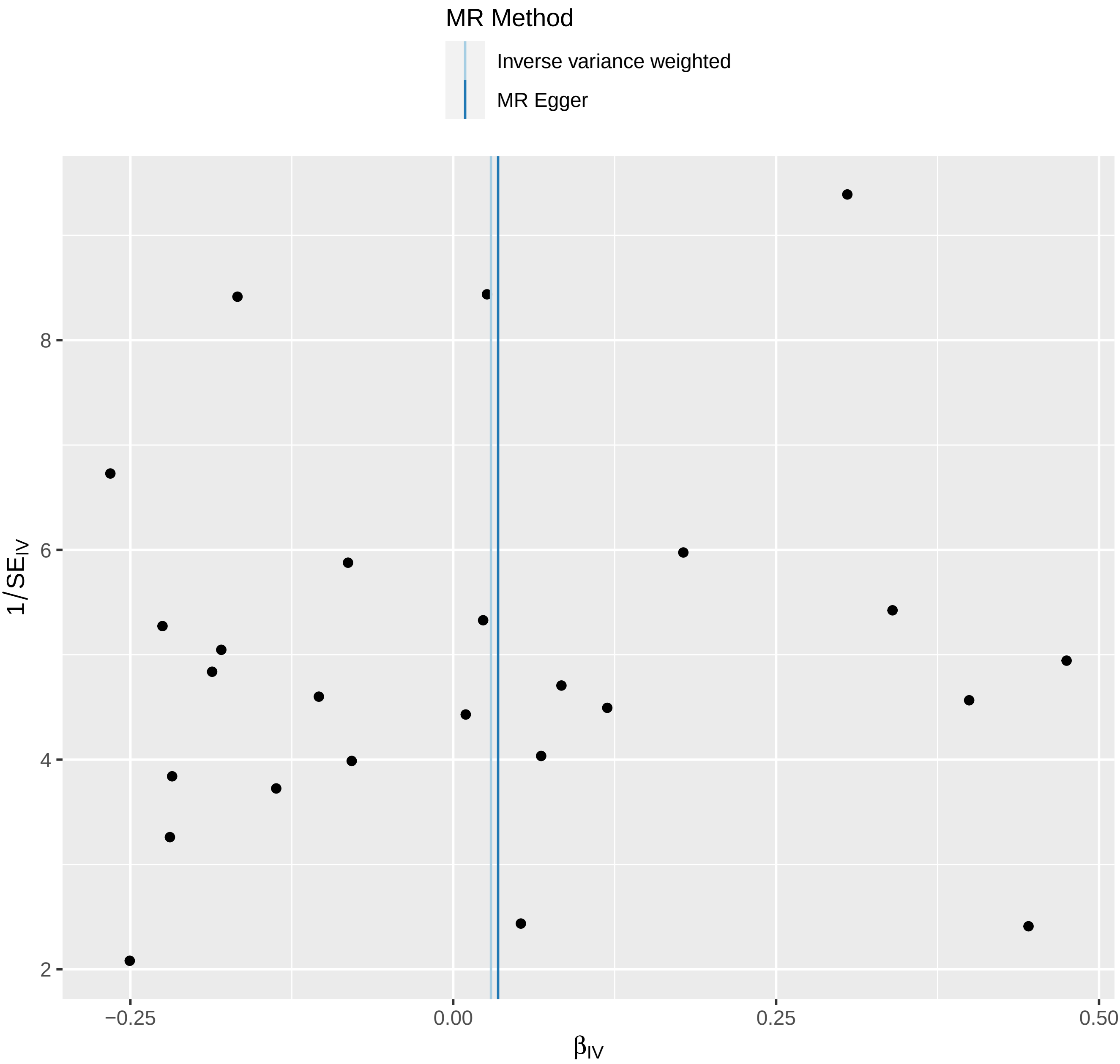

Batch 3−1225 : Glia maturation factor gamma on Diabetic nephropathy (more control exclusions) with a threshold of 1e−05

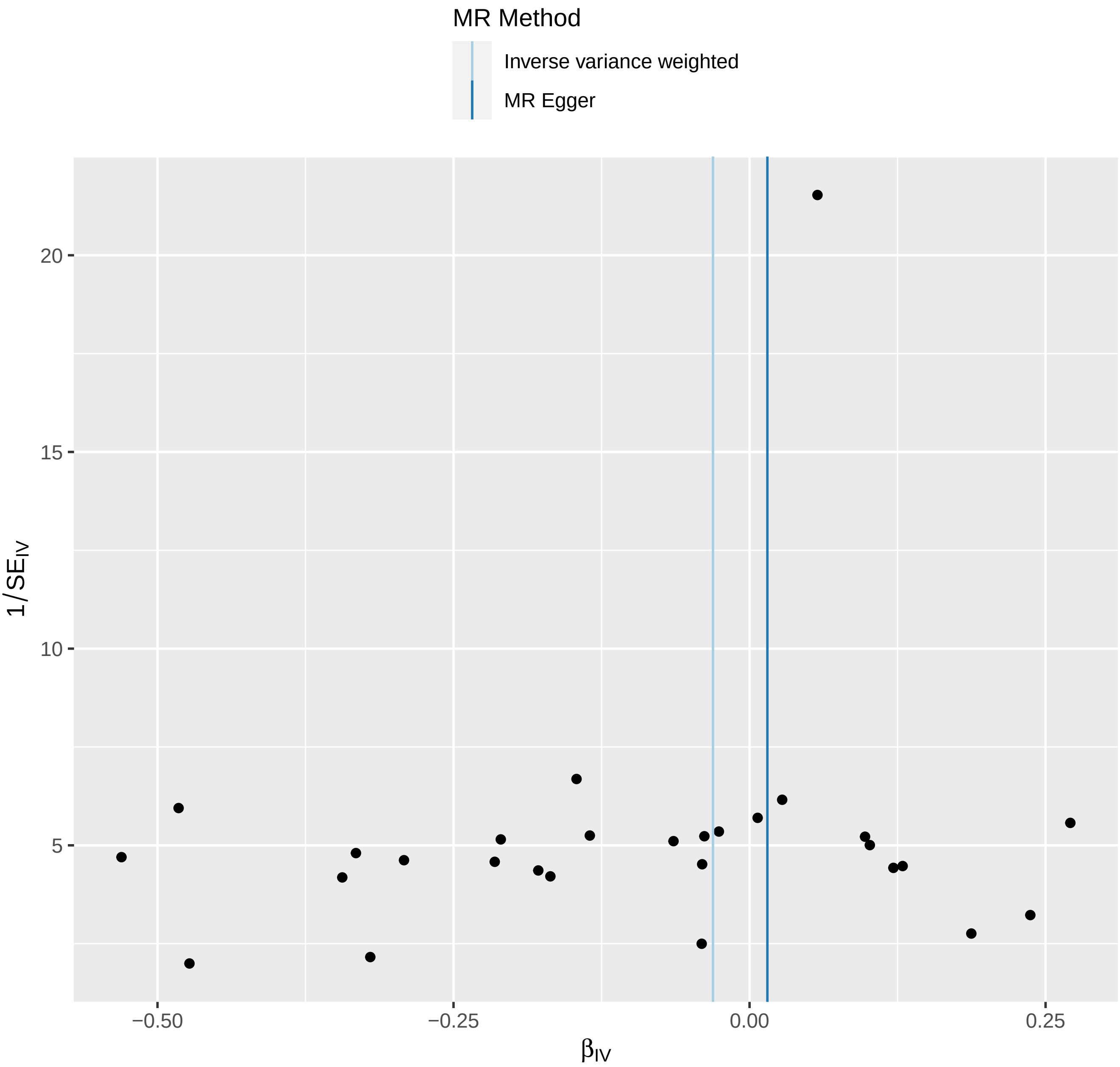

Batch 3−1226 : Guanine nucleotide−binding protein G(k) subunit alpha on Diabetic nephropathy (more control exclusions)
with a threshold of 1e−05

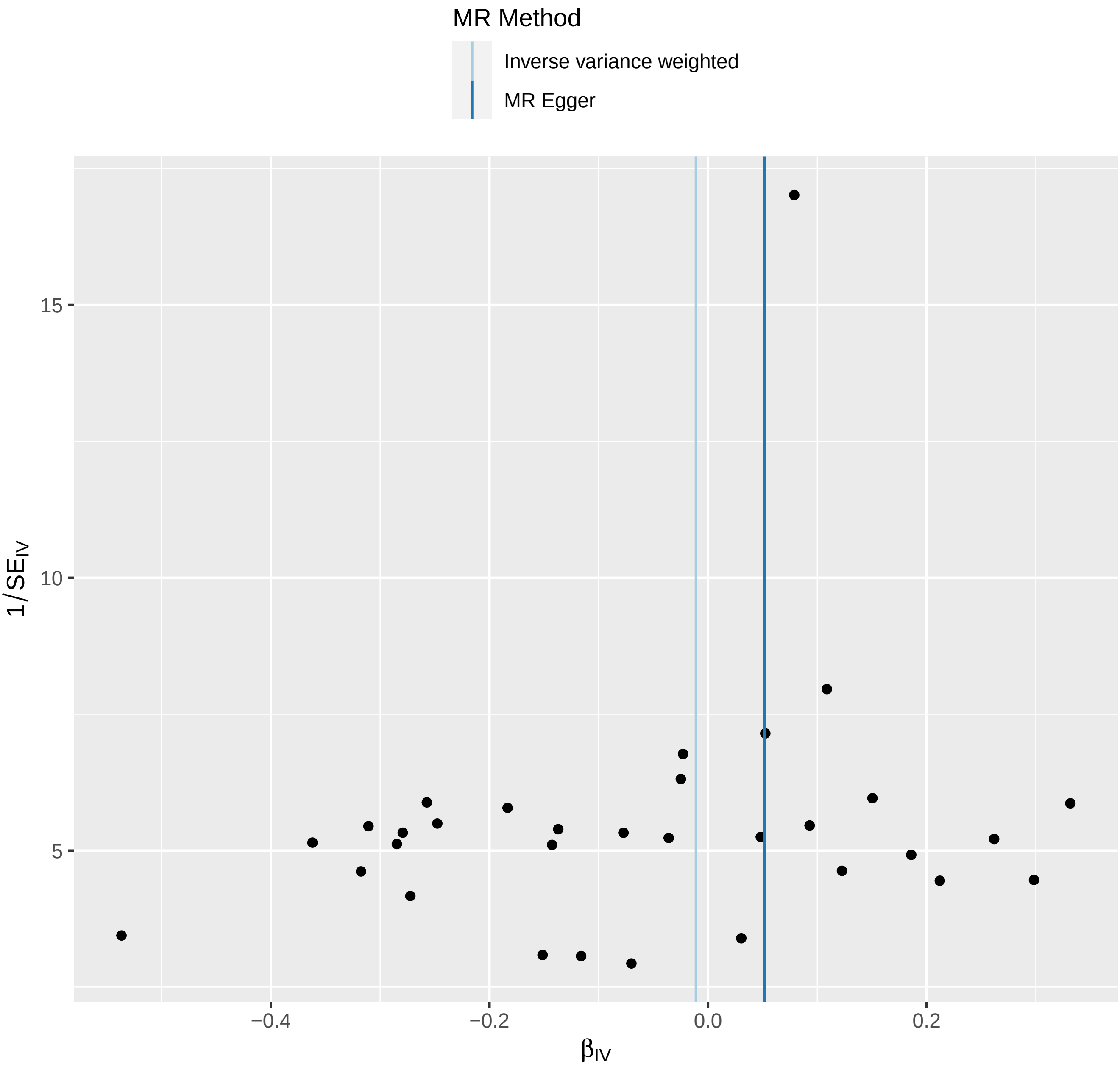

Batch 3−1227 : Guanine nucleotide−binding protein G(I)/G(S)/G(O) subunit gamma−T2 on Diabetic nephropathy (more control exclusions) with a threshold of 1e−05

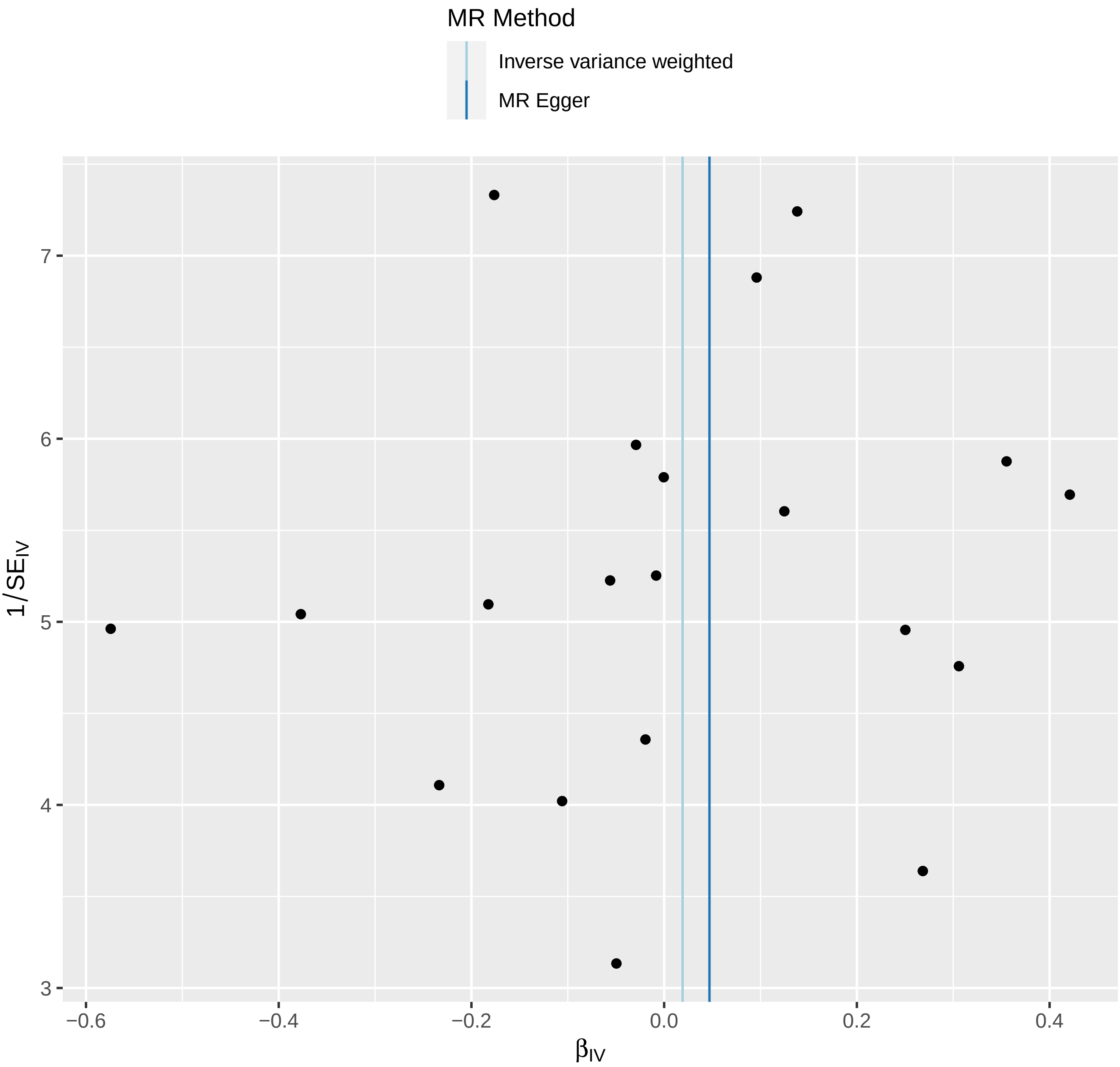

Batch 3−1228 : Granulysin on Diabetic nephropathy (more control exclusions)
with a threshold of 1e−05

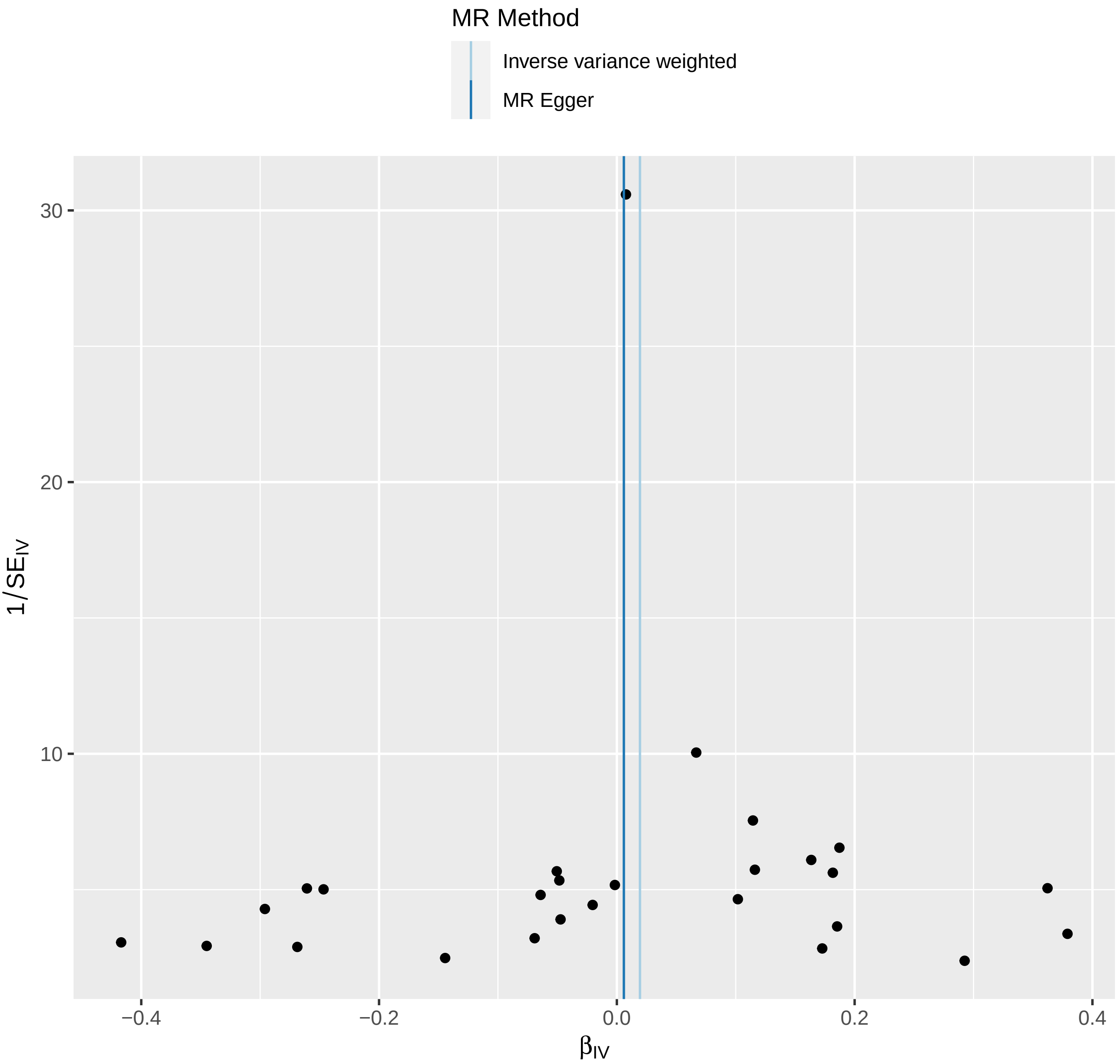

Batch 3−1229 : Glycine N−methyltransferase on Diabetic nephropathy (more control exclusions)
with a threshold of 1e−05

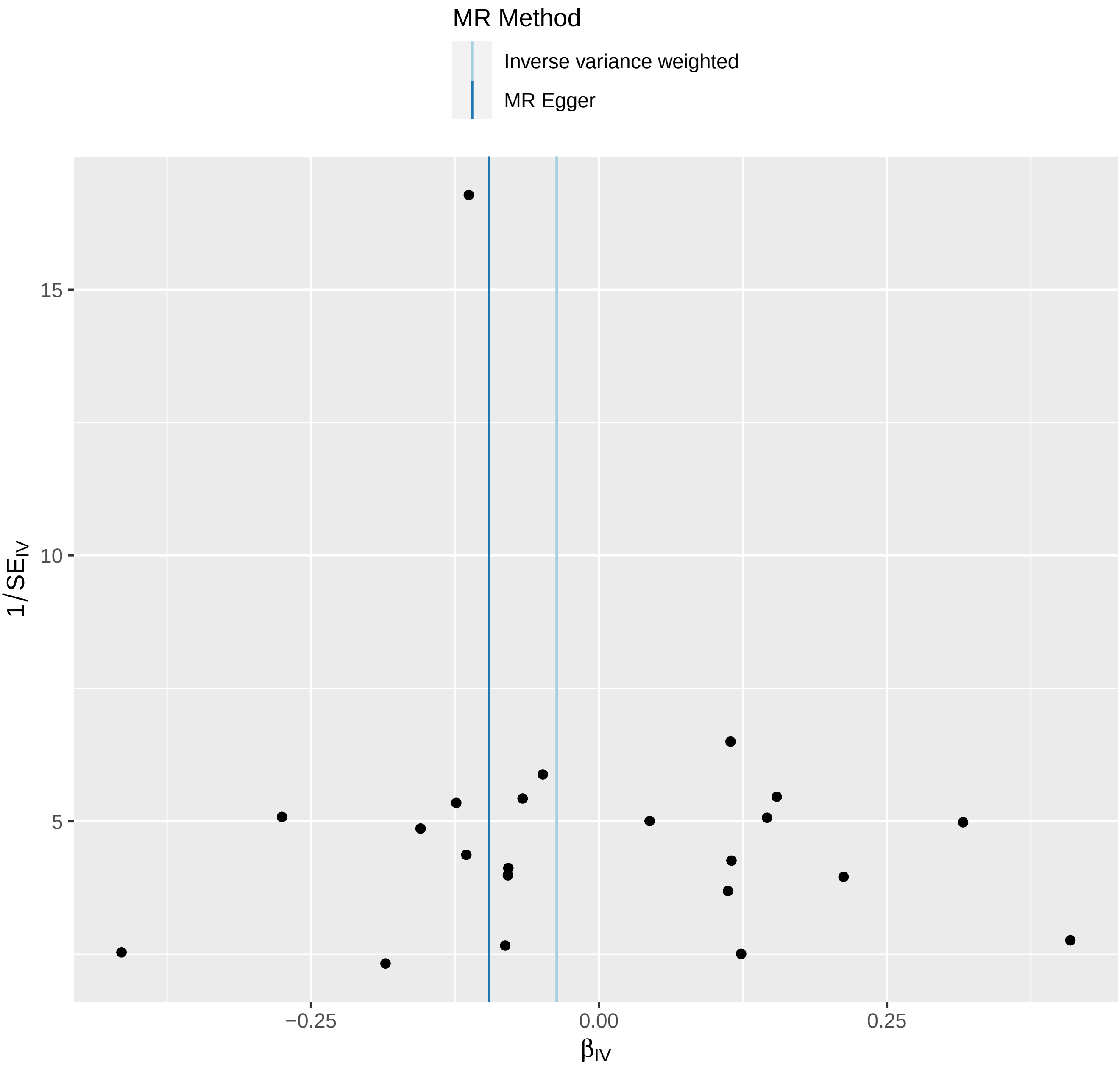

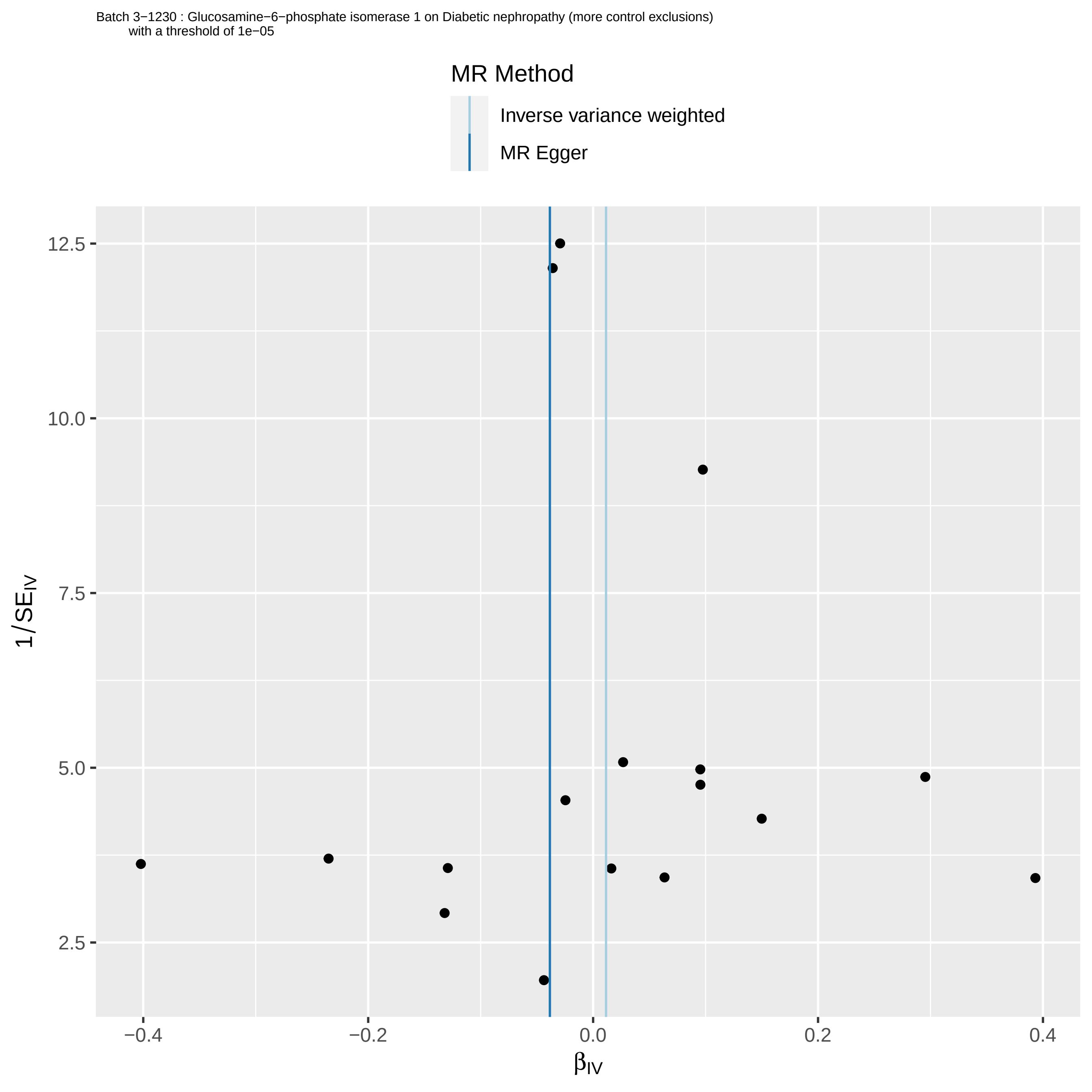

Batch 3−1230 : Glucosamine−6−phosphate isomerase 1 on Diabetic nephropathy (more control exclusions)
with a threshold of 1e−05
MR Method
Inverse variance weighted
MR Egger
12.5
10.0
7.5
5.0
2.5
$1/SE_{IV}$
−0.4
−0.2
0.0
0.2
0.4
β_{IV}

Batch 3−1231 : Glucosamine 6−phosphate N−acetyltransferase on Diabetic nephropathy (more control exclusions)
with a threshold of 1e−05

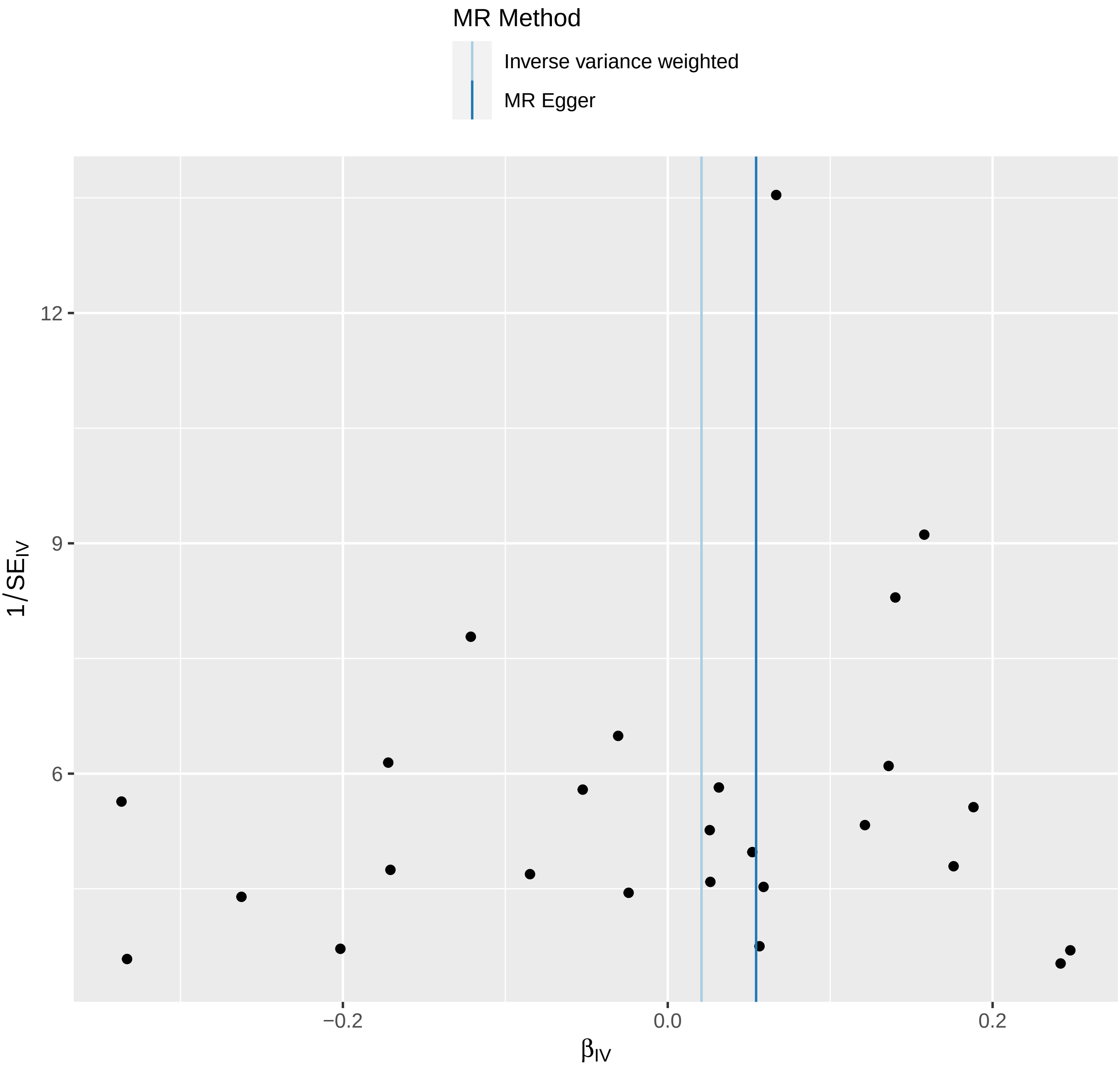

Batch 3−1232 : N−acetylglucosamine−1−phosphotransferase subunit gamma on Diabetic nephropathy (more control exclusions) with a threshold of 1e−05

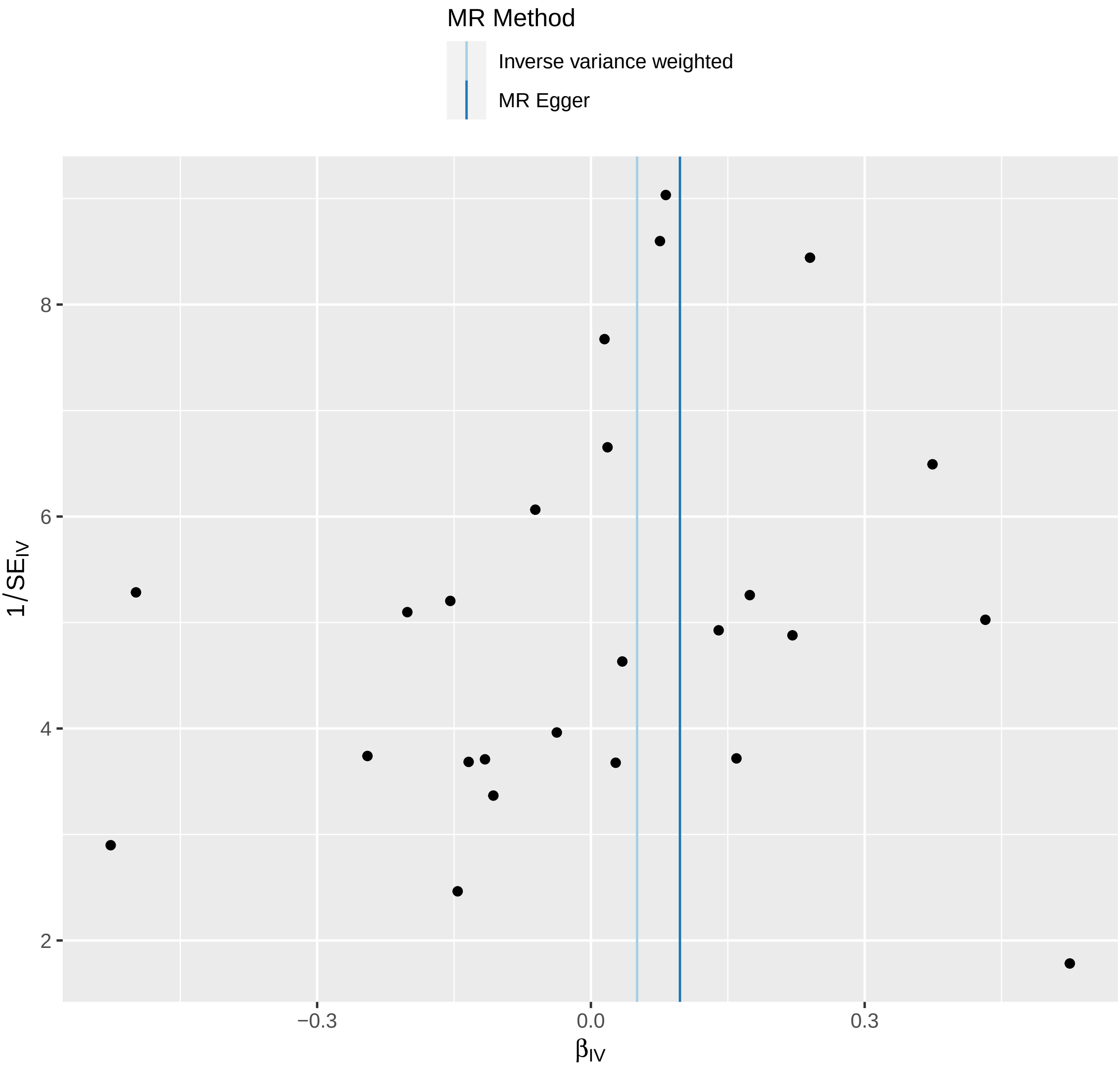

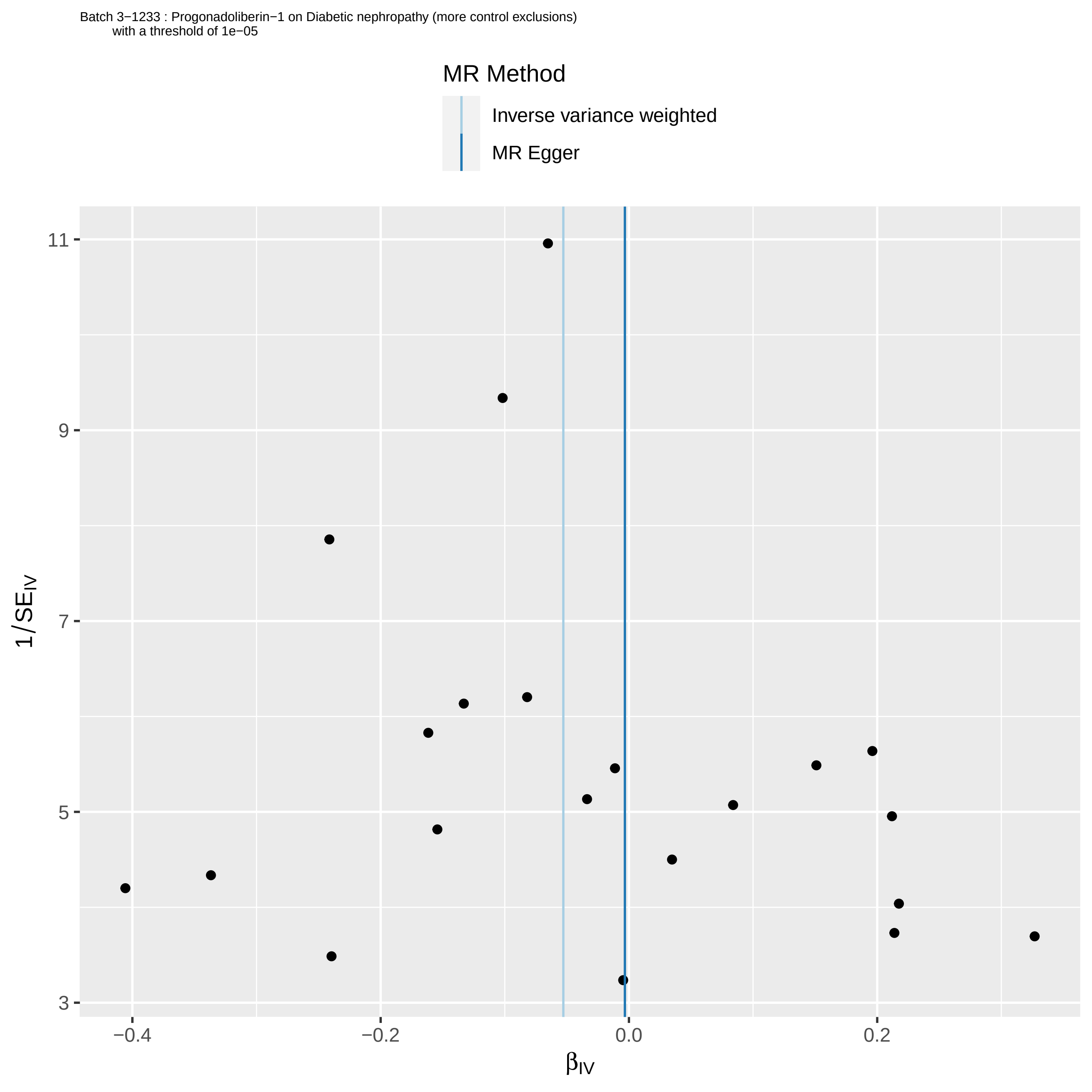

Batch 3−1233 : Progonadoliberin−1 on Diabetic nephropathy (more control exclusions)
with a threshold of 1e−05
MR Method
Inverse variance weighted
MR Egger
11
9
7
5
3
1/SE_IV
−0.4
−0.2
0.0
0.2
β_IV

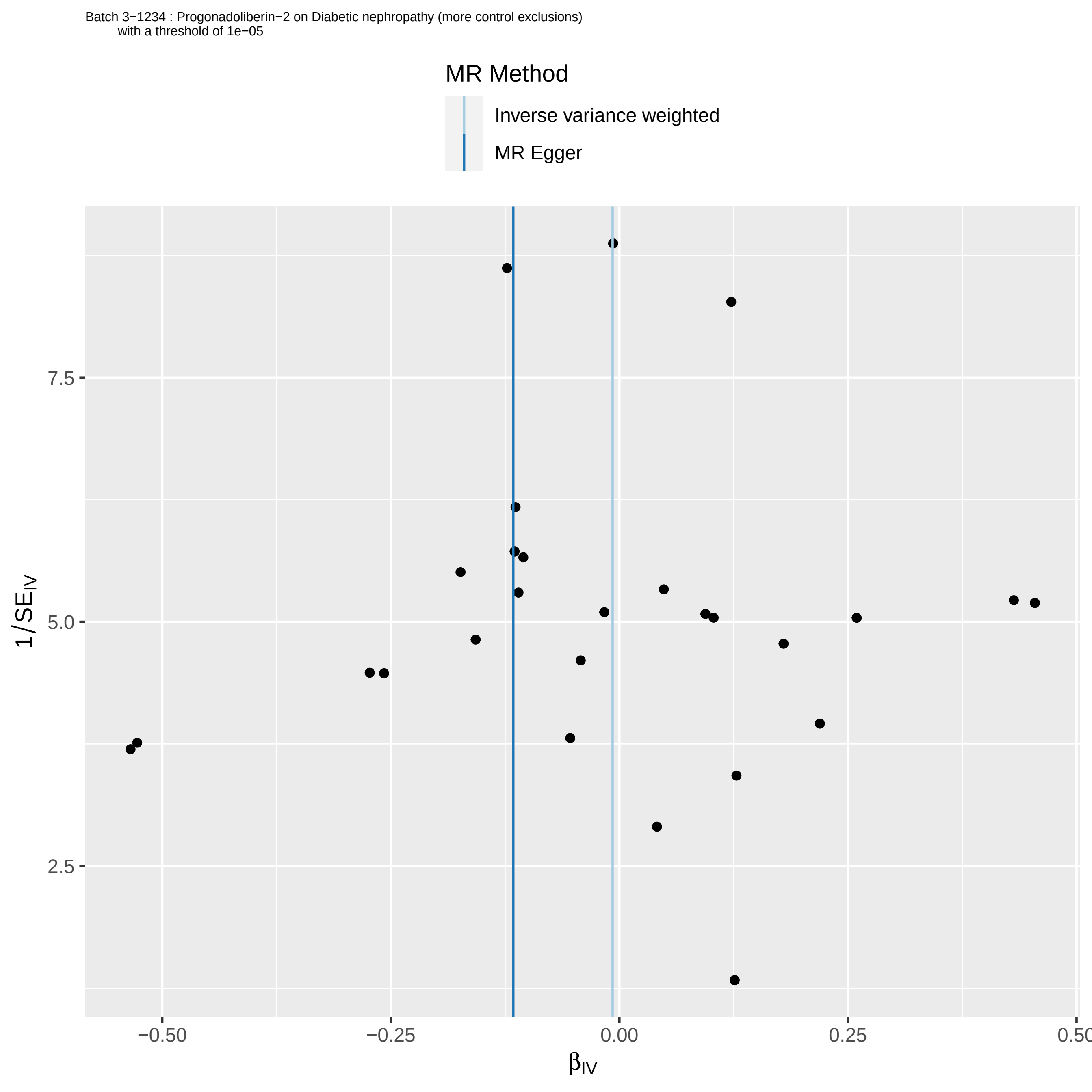
Batch 3−1234 : Progonadoliberin−2 on Diabetic nephropathy (more control exclusions)
with a threshold of 1e−05
MR Method
Inverse variance weighted
MR Egger
7.5
5.0
2.5
1/SE_IV
−0.50
−0.25
0.00
0.25
0.50
β_IV

Batch 3−1235 : N−acetylglucosamine−6−sulfatase on Diabetic nephropathy (more control exclusions)
with a threshold of 1e−05

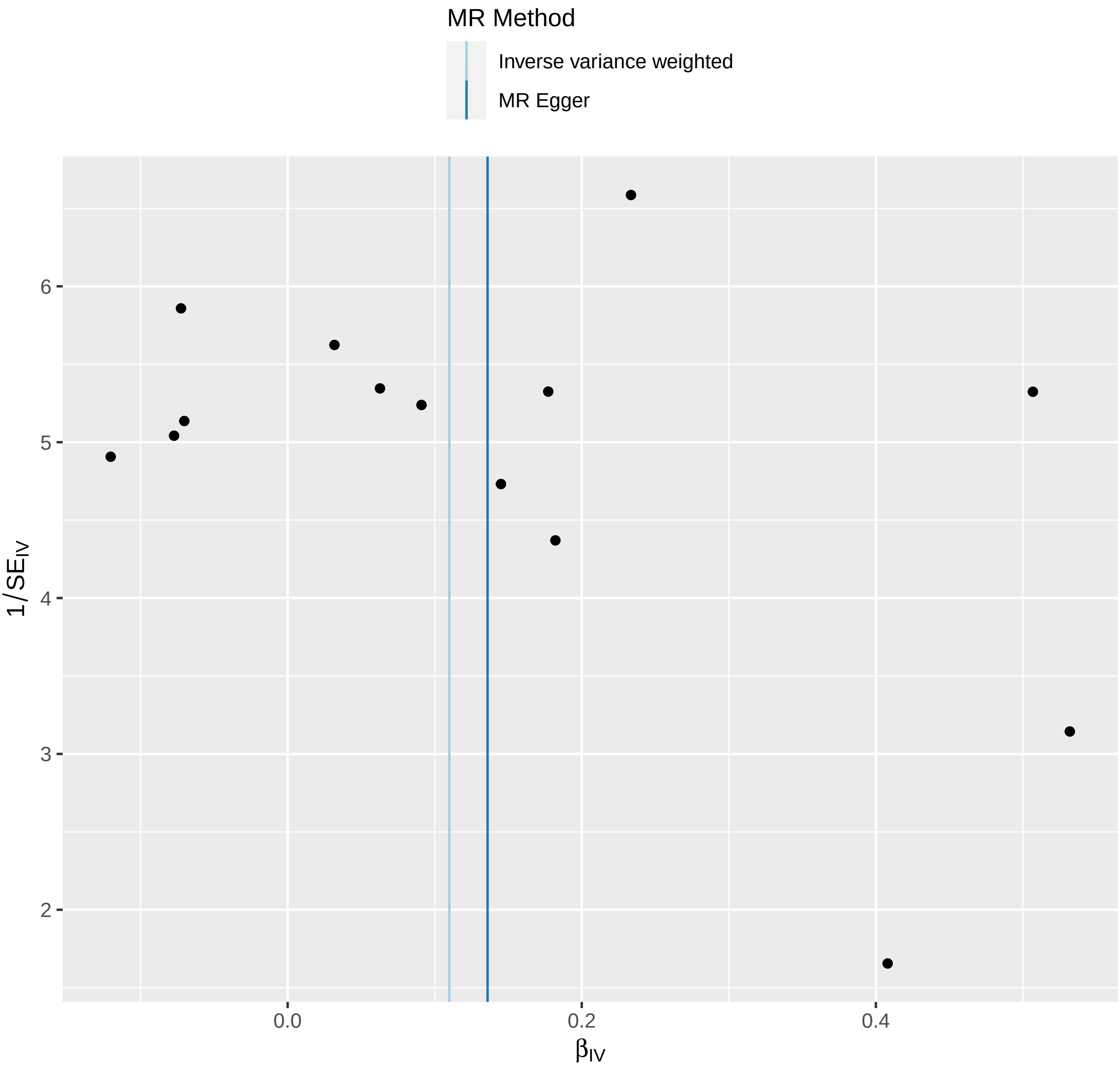

Batch 3−1236 : N−acetylglucosamine−6−sulfatase on Diabetic nephropathy (more control exclusions)
with a threshold of 1e−05

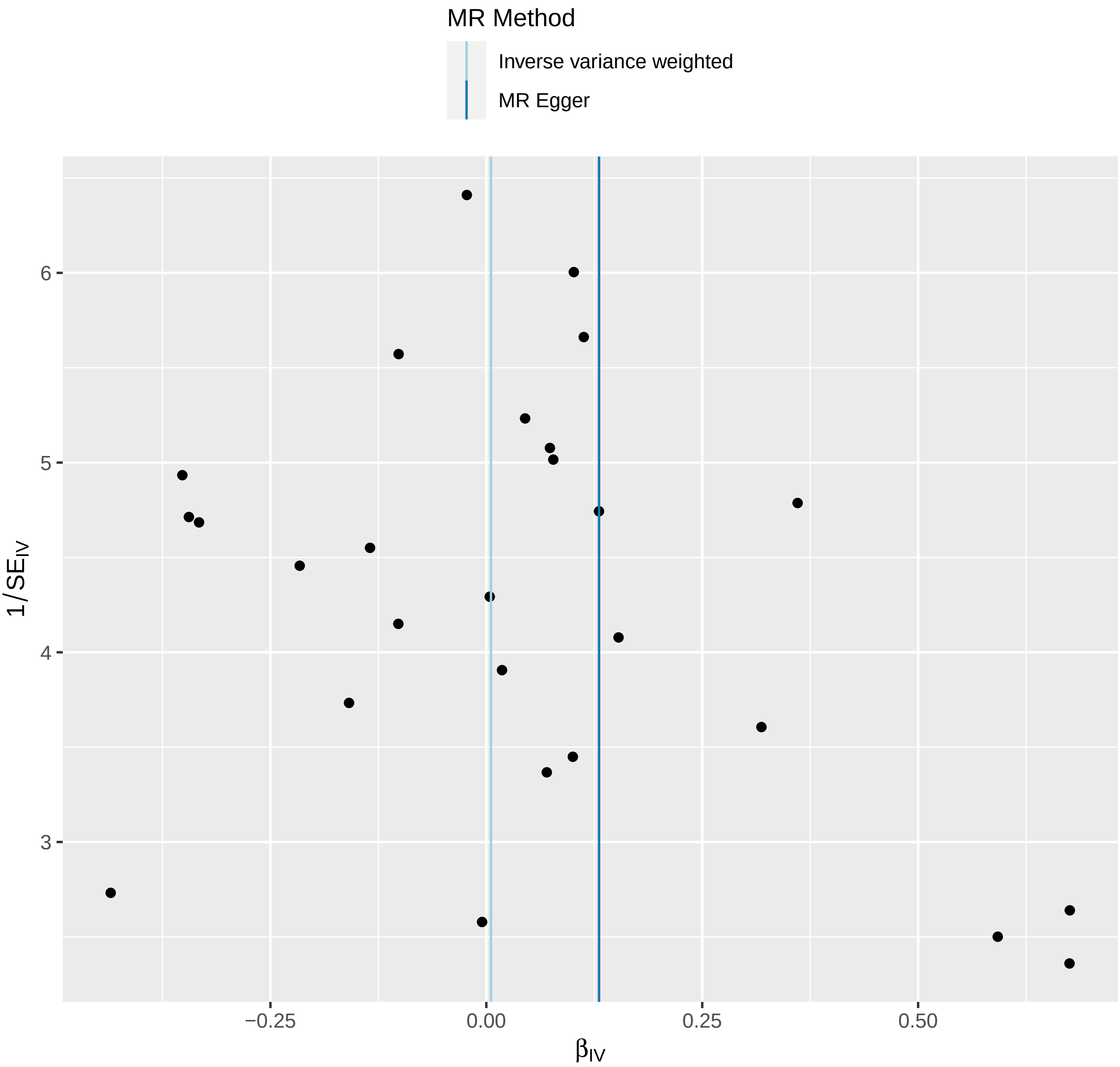

Batch 3−1237 : Golgi membrane protein 1 on Diabetic nephropathy (more control exclusions)
with a threshold of 1e−05

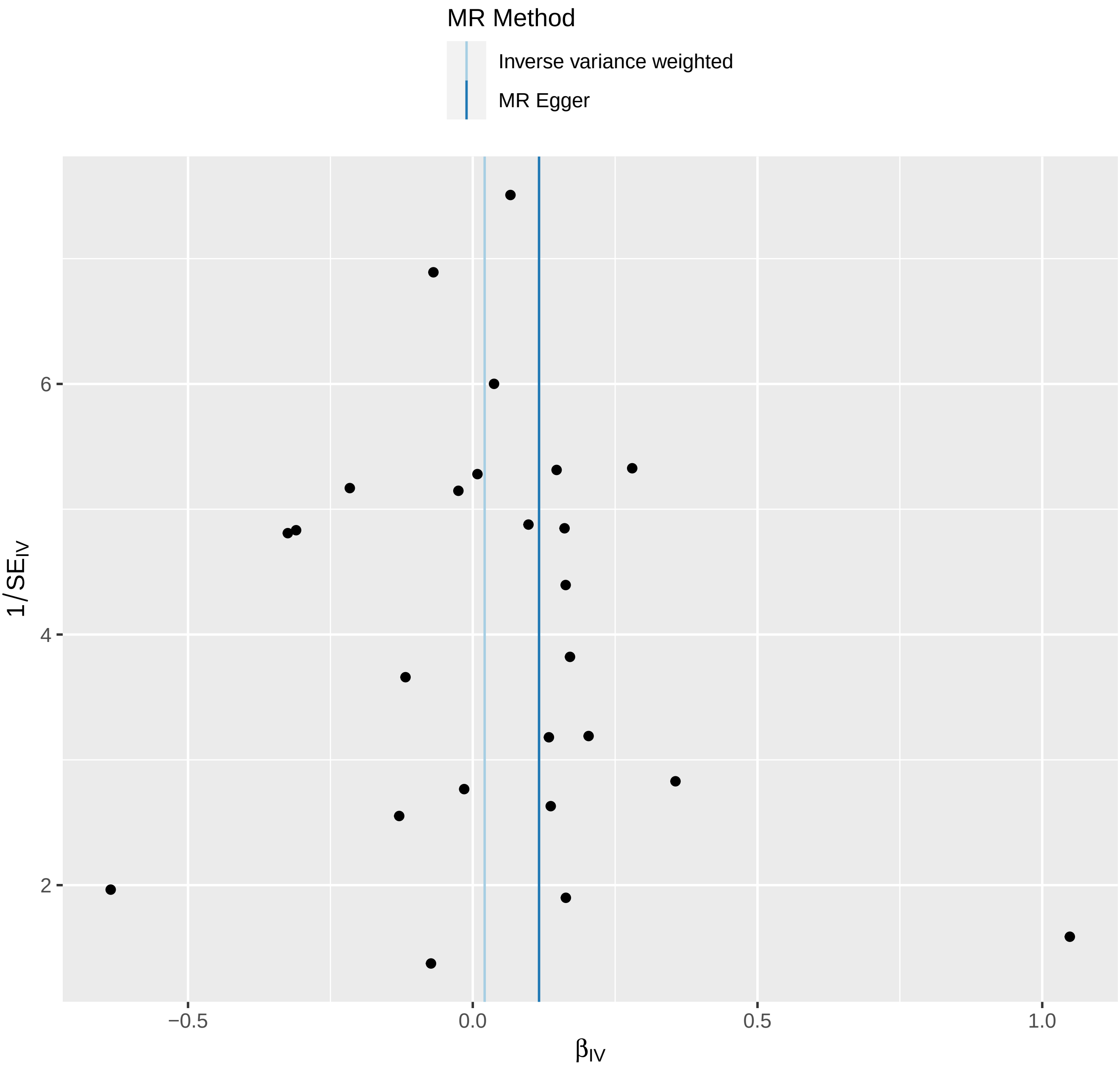

Batch 3−1238 : Golgi membrane protein 1 on Diabetic nephropathy (more control exclusions)
with a threshold of 1e−05

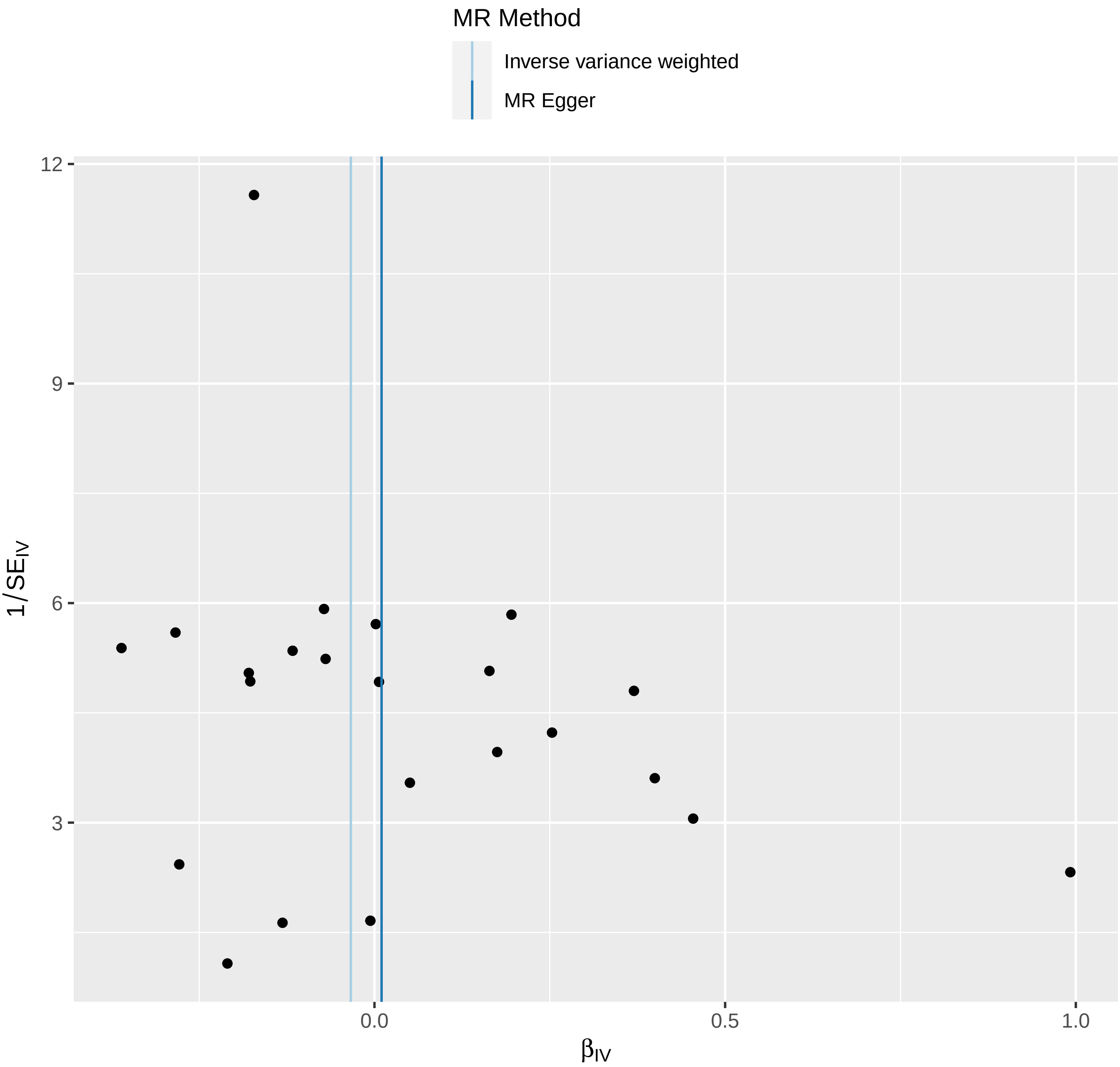

Batch 3−1239 : RAB6−interacting golgin on Diabetic nephropathy (more control exclusions)
with a threshold of 1e−05

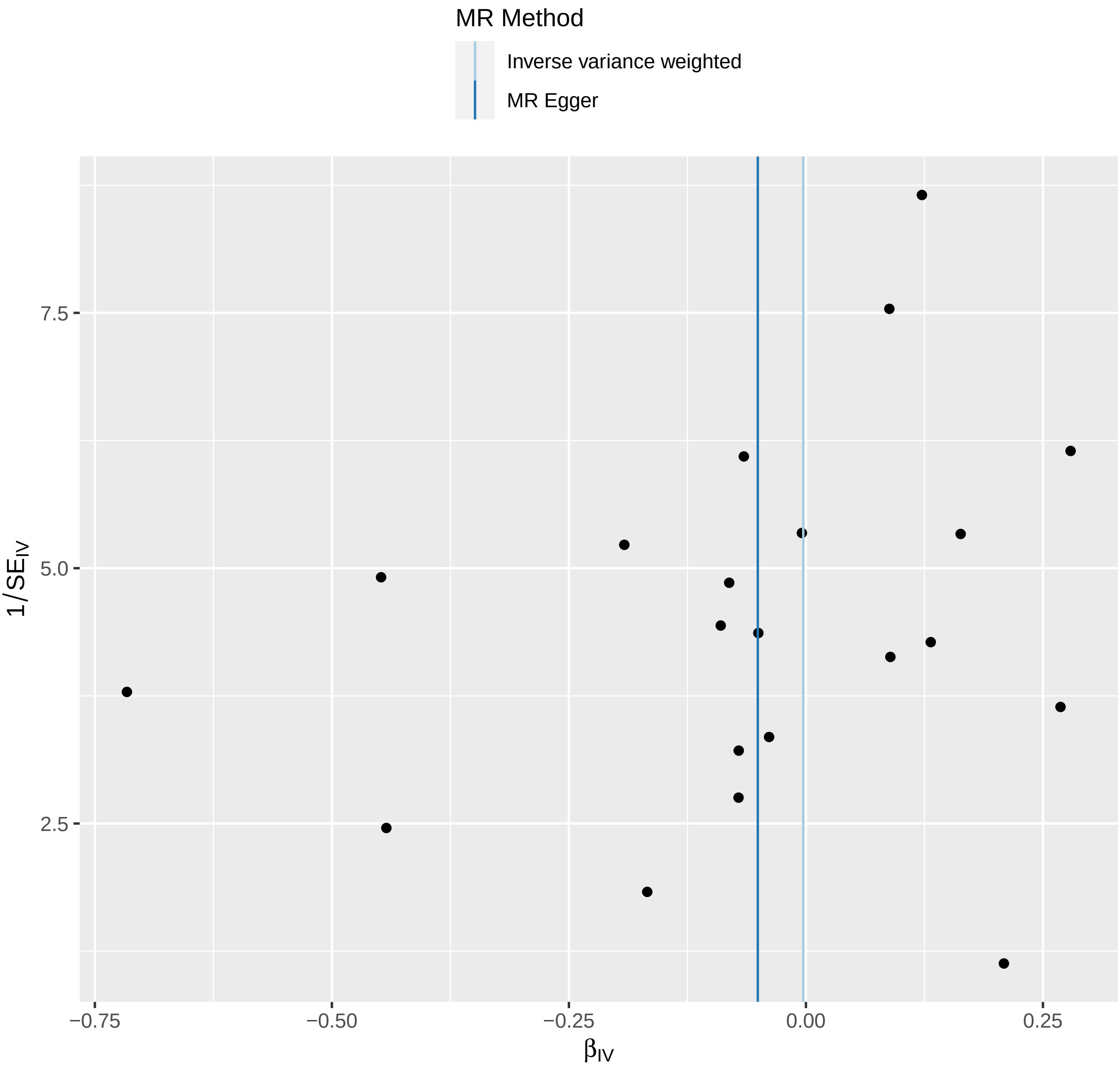

Batch 3−1240 : Golgi SNAP receptor complex member 1 on Diabetic nephropathy (more control exclusions)
with a threshold of 1e−05

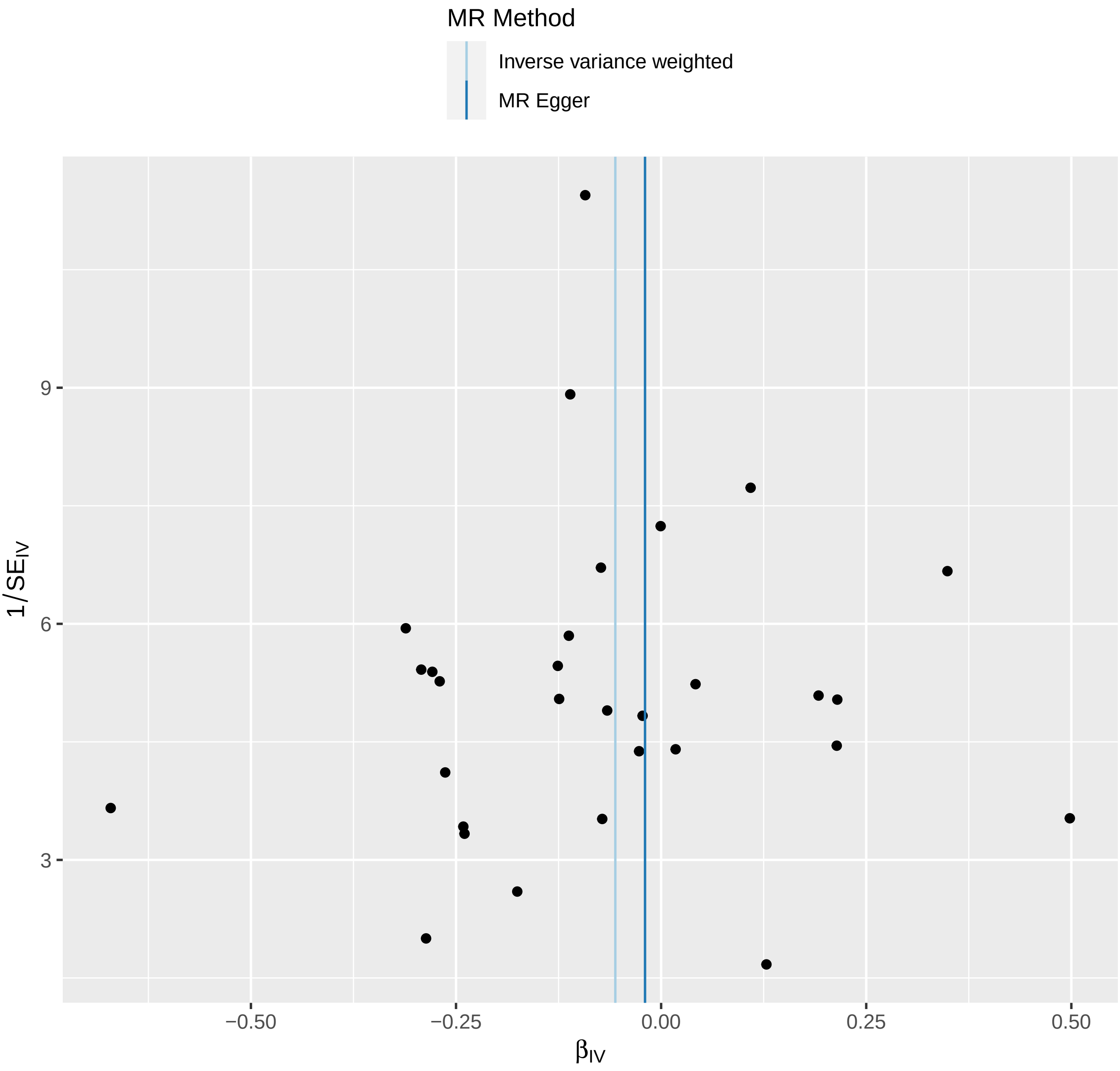

Batch 3−1241 : Aspartate aminotransferase, cytoplasmic on Diabetic nephropathy (more control exclusions) with a threshold of 1e−05

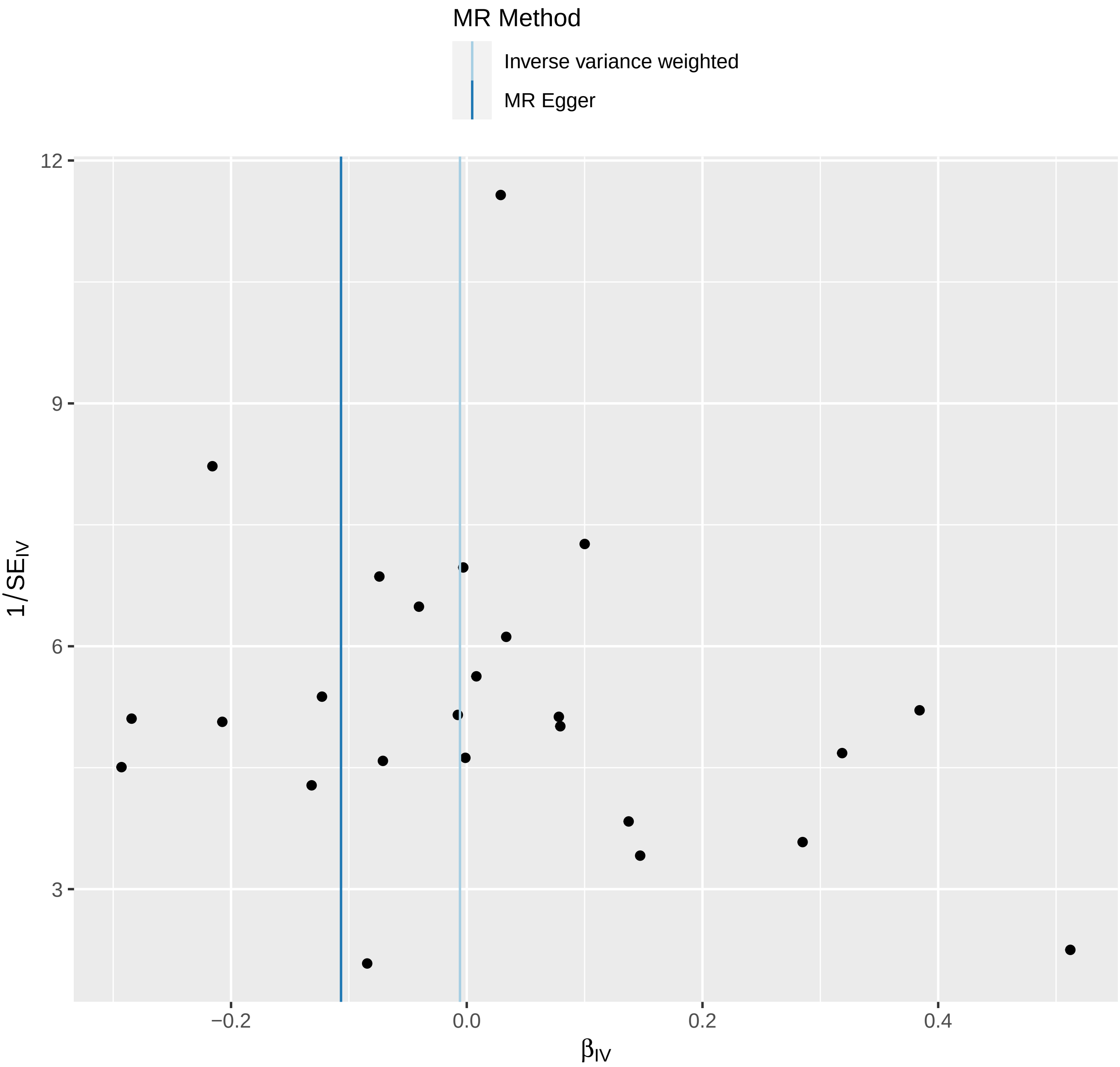

Batch 3−1242 : Platelet glycoprotein Ib alpha chain on Diabetic nephropathy (more control exclusions)
with a threshold of 1e−05

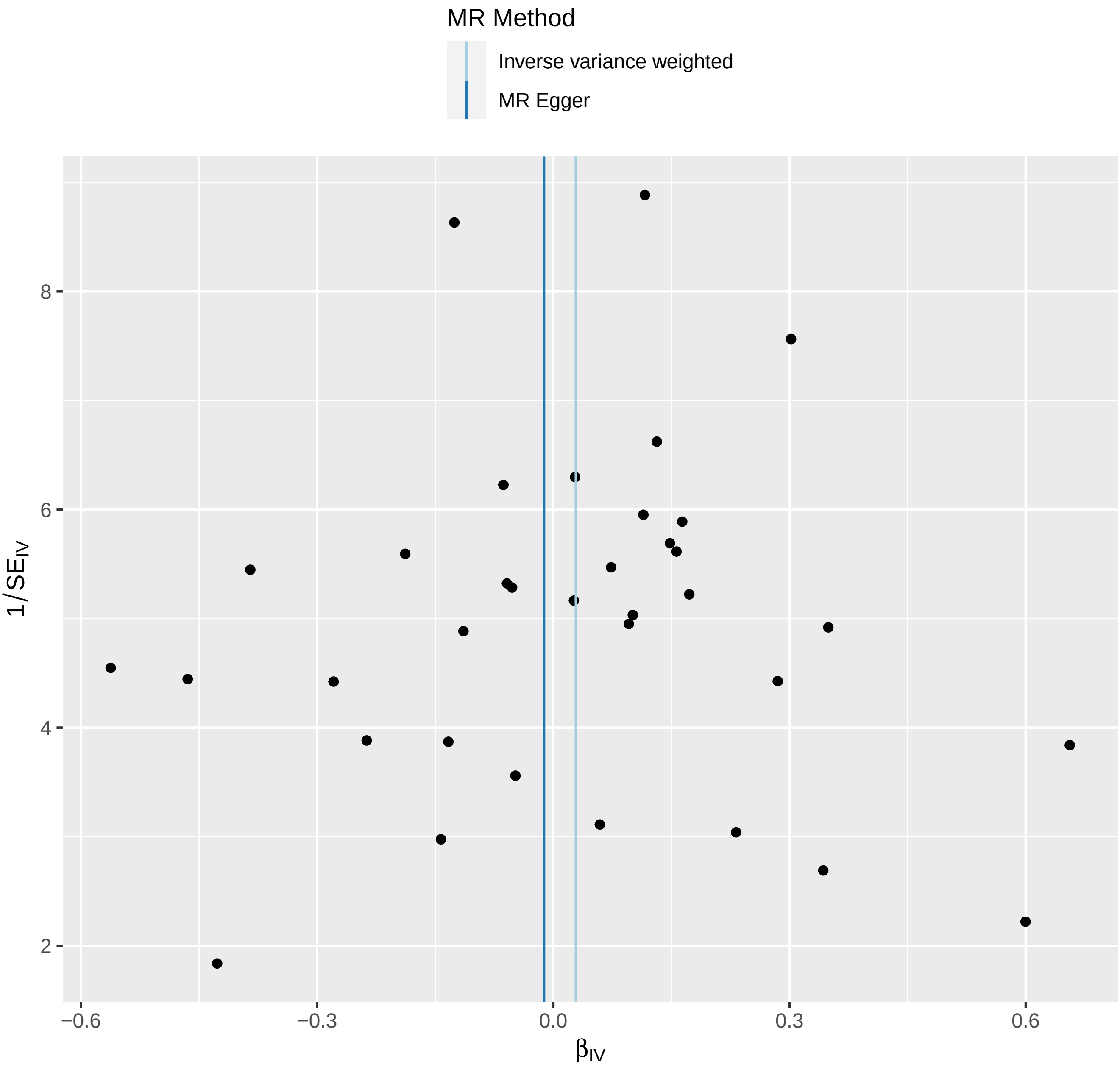

Batch 3−1243 : Platelet glycoprotein V on Diabetic nephropathy (more control exclusions)
with a threshold of 1e−05

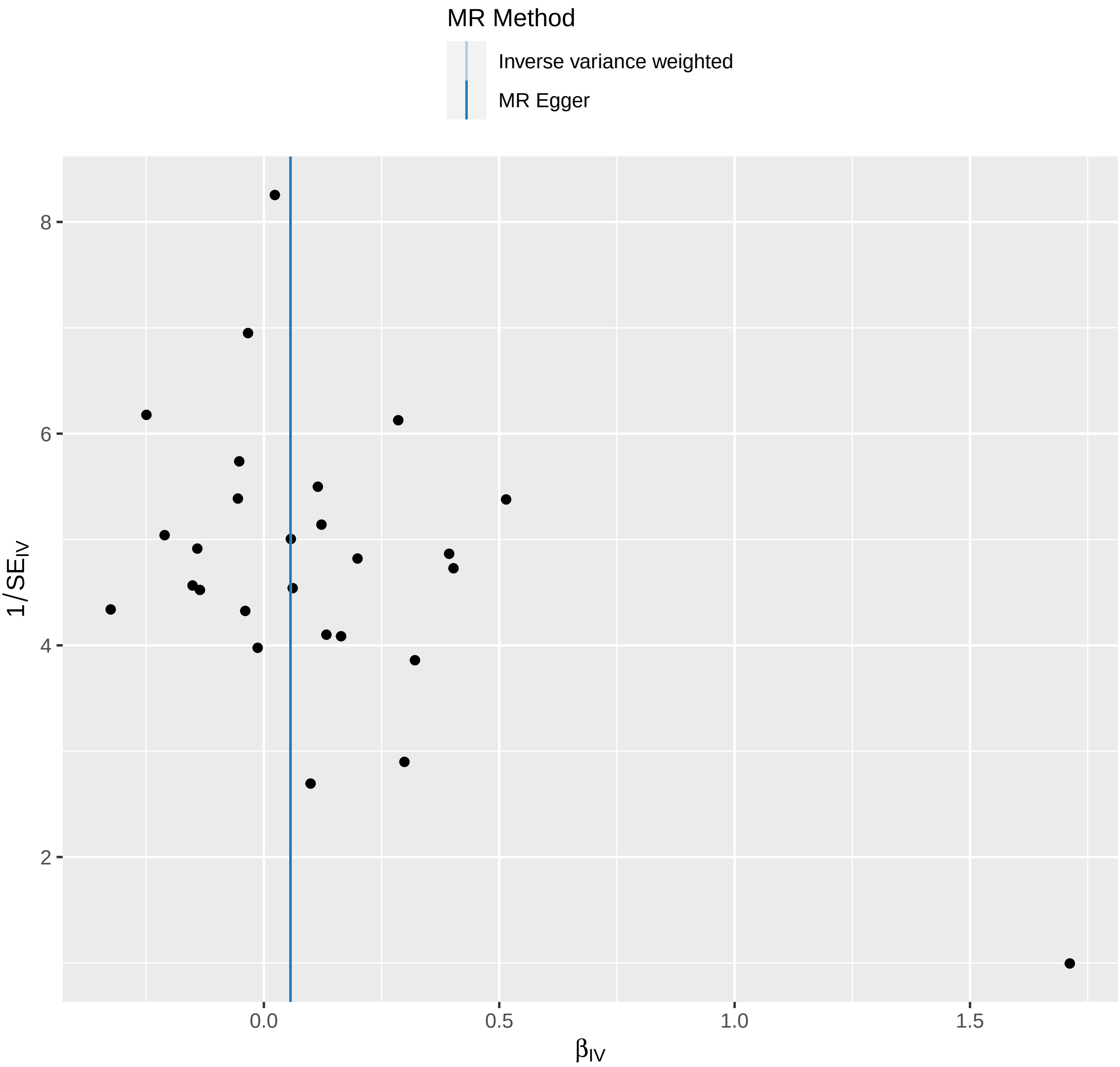

Batch 3−1244 : Cell surface A33 antigen on Diabetic nephropathy (more control exclusions)
with a threshold of 1e−05

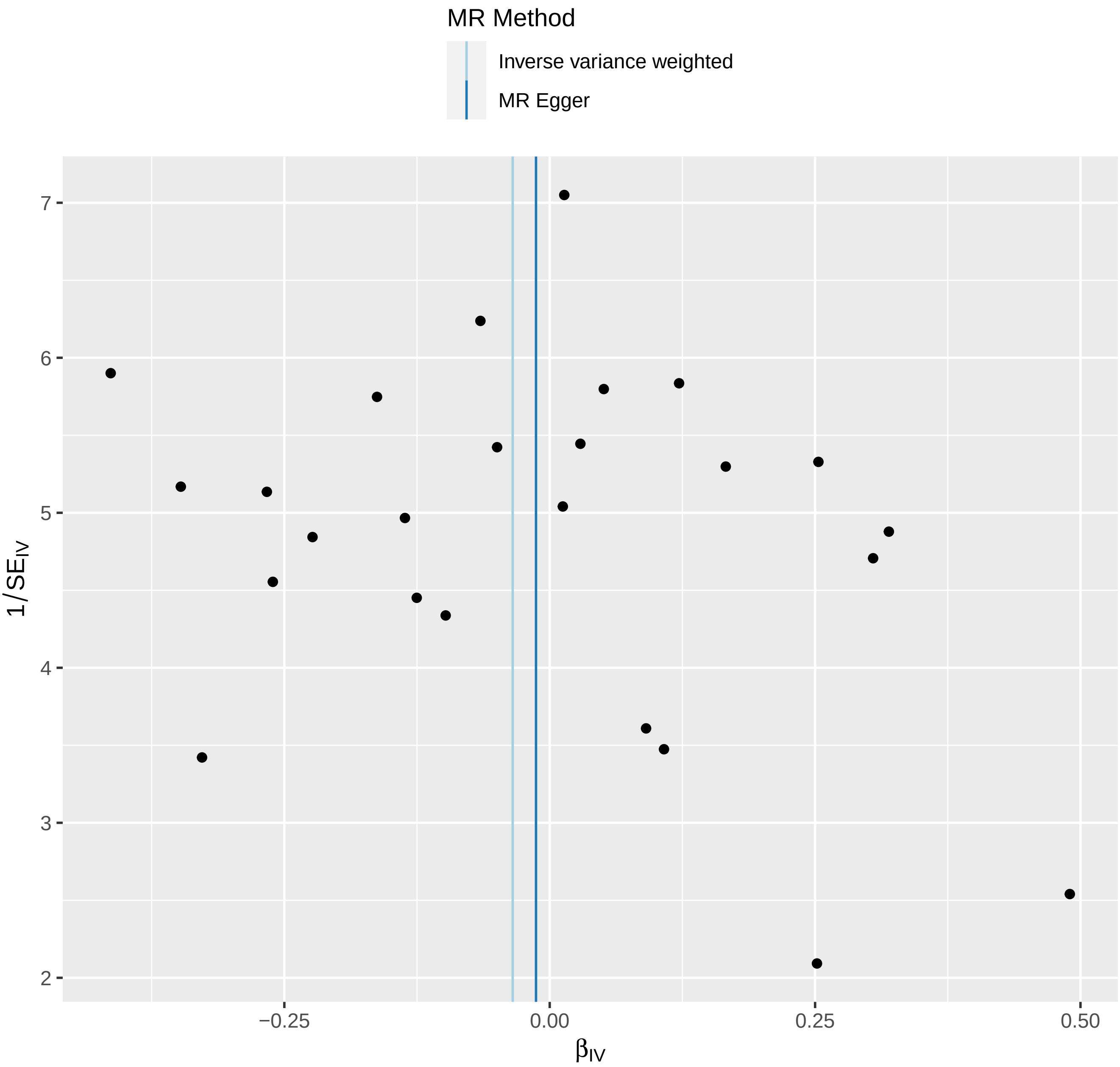

Batch 3−1245 : Glypican−1 on Diabetic nephropathy (more control exclusions)
with a threshold of 1e−05

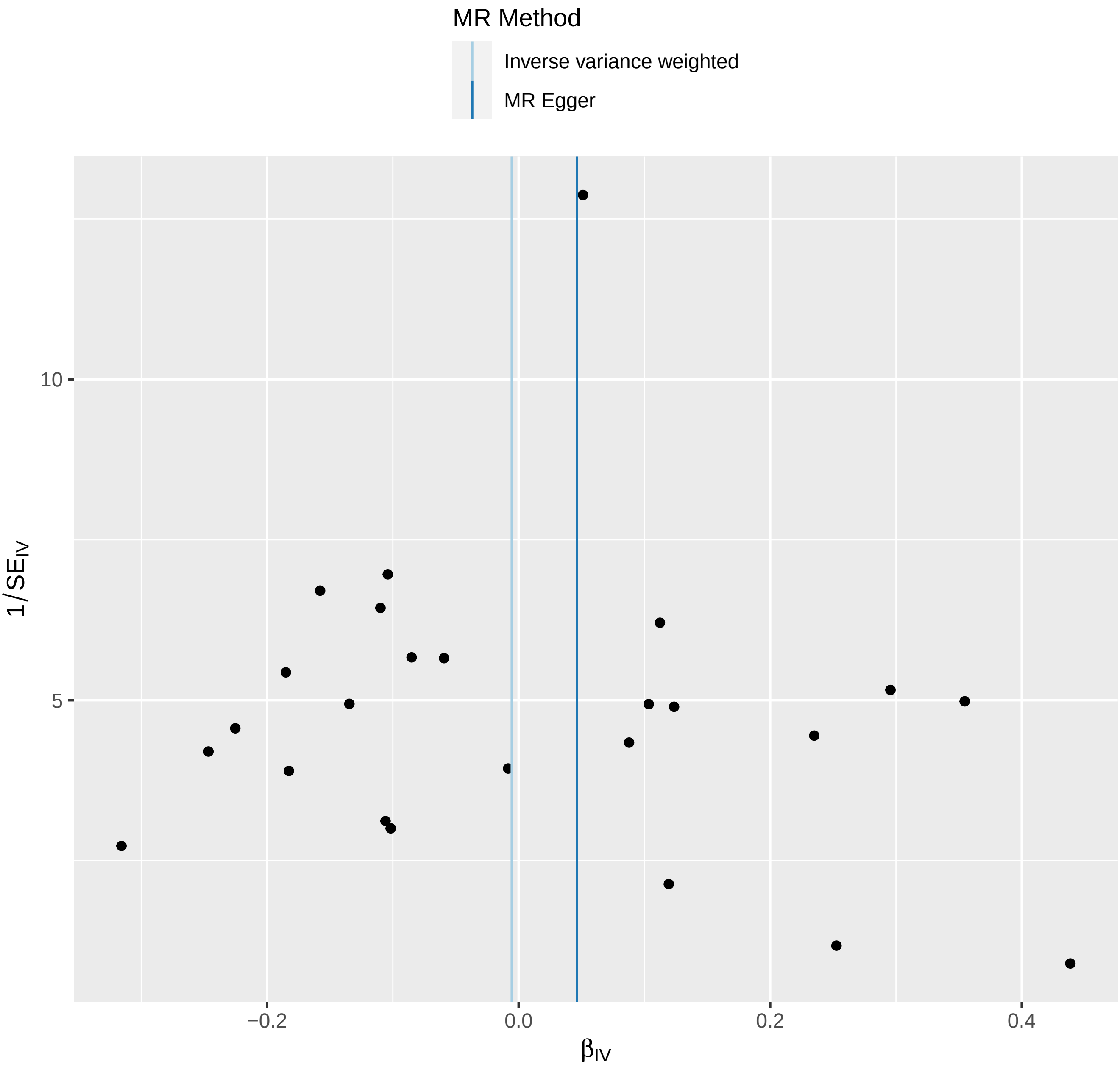

Batch 3−1246 : Glypican−3 on Diabetic nephropathy (more control exclusions)
with a threshold of 1e−05

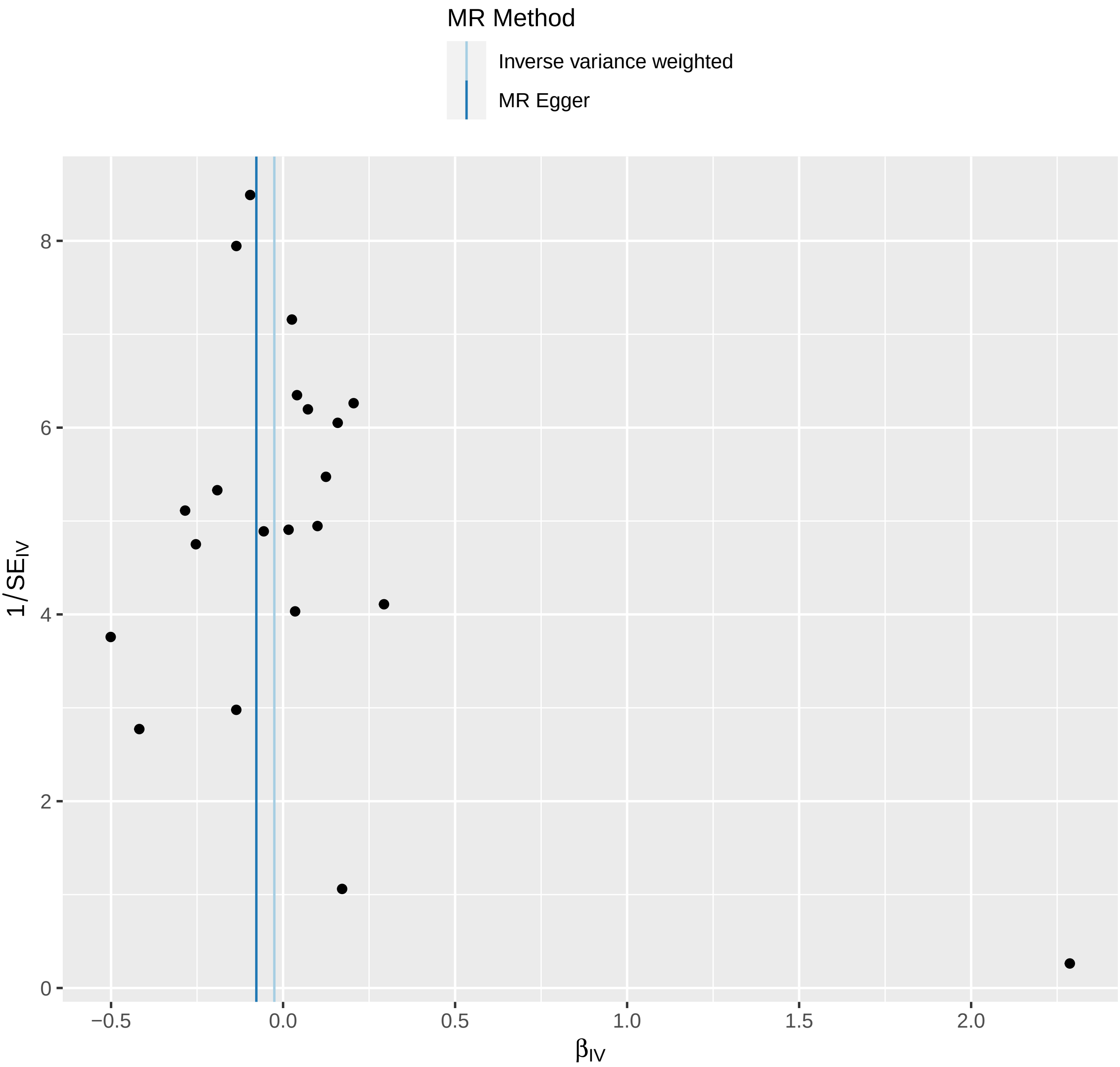

Batch 3−1247 : Glypican−5 on Diabetic nephropathy (more control exclusions)
with a threshold of 1e−05

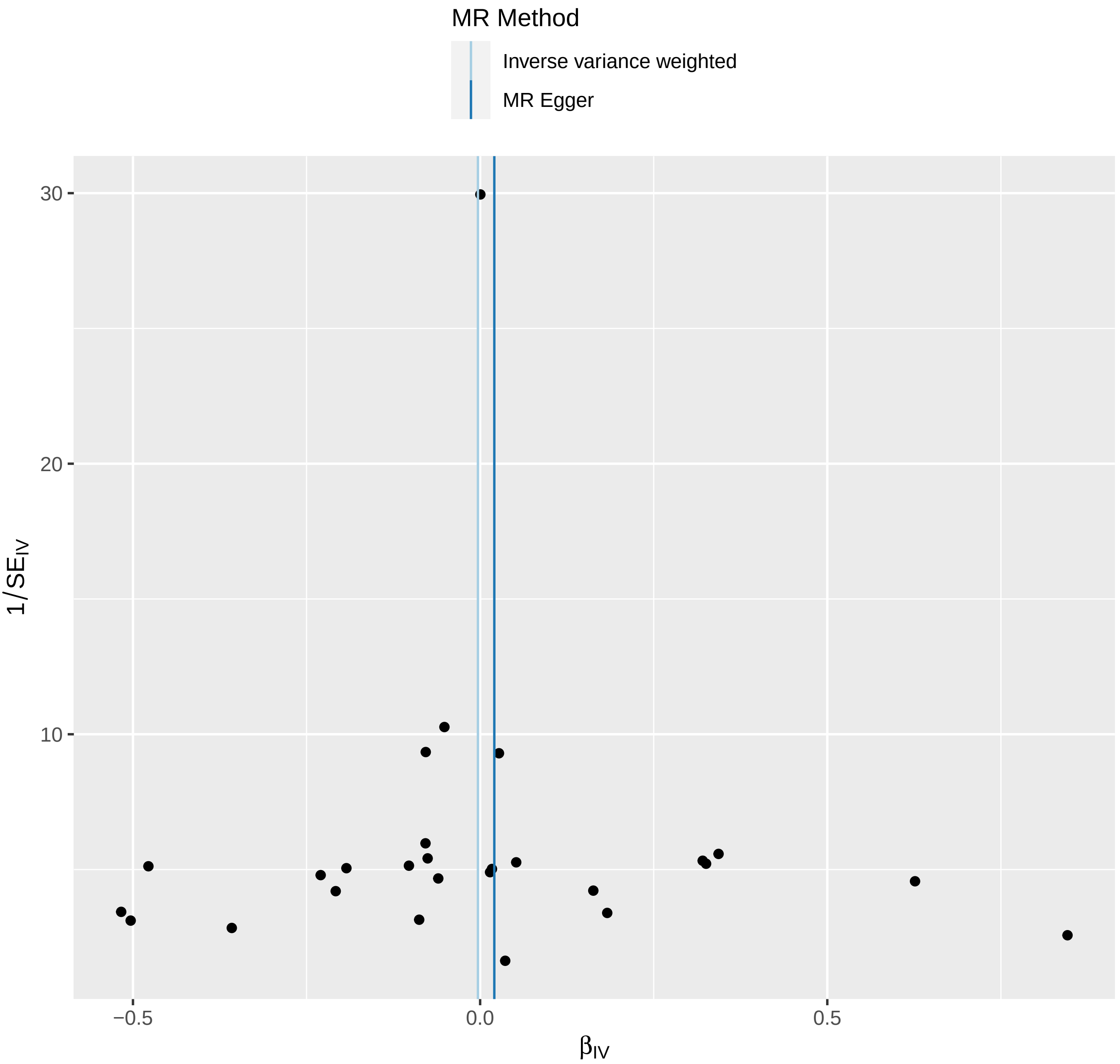

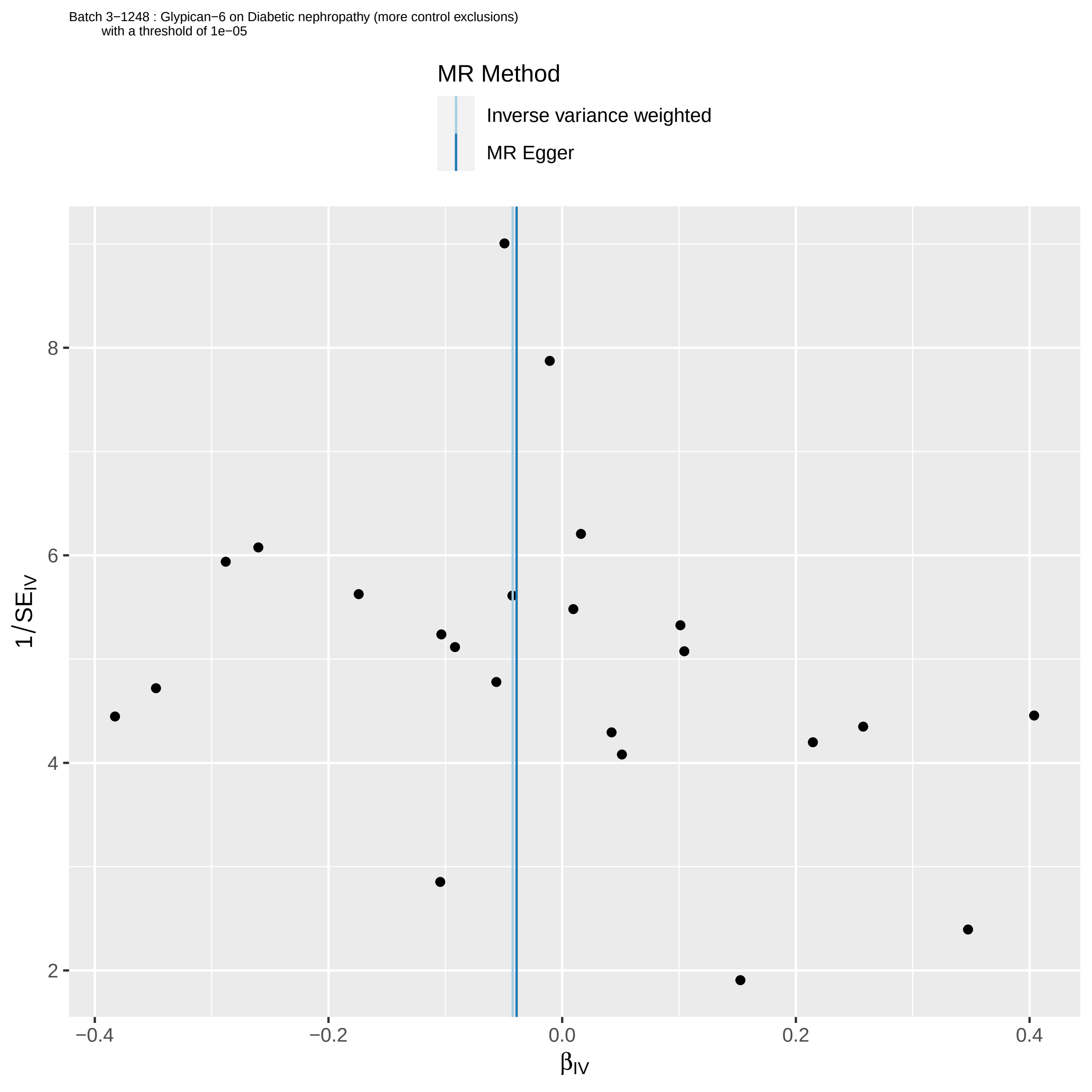

Batch 3−1248 : Glypican−6 on Diabetic nephropathy (more control exclusions)
with a threshold of 1e−05
MR Method
Inverse variance weighted
MR Egger
8
6
4
2
1/SE_IV
−0.4
−0.2
0.0
0.2
0.4
β_IV

Batch 3−1249 : Glycerophosphocholine phosphodiesterase GPCPD1 on Diabetic nephropathy (more control exclusions)
with a threshold of 1e−05

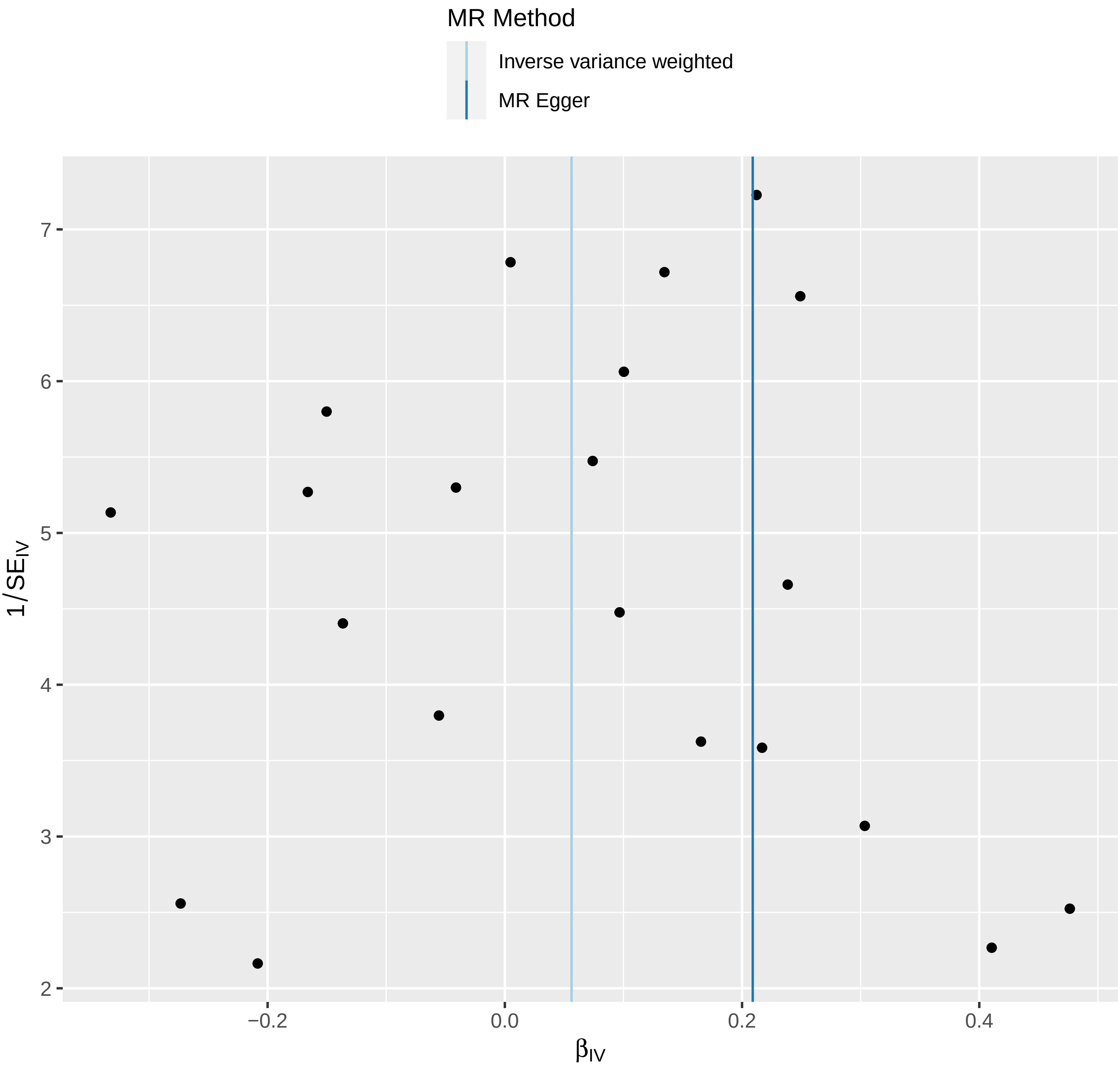

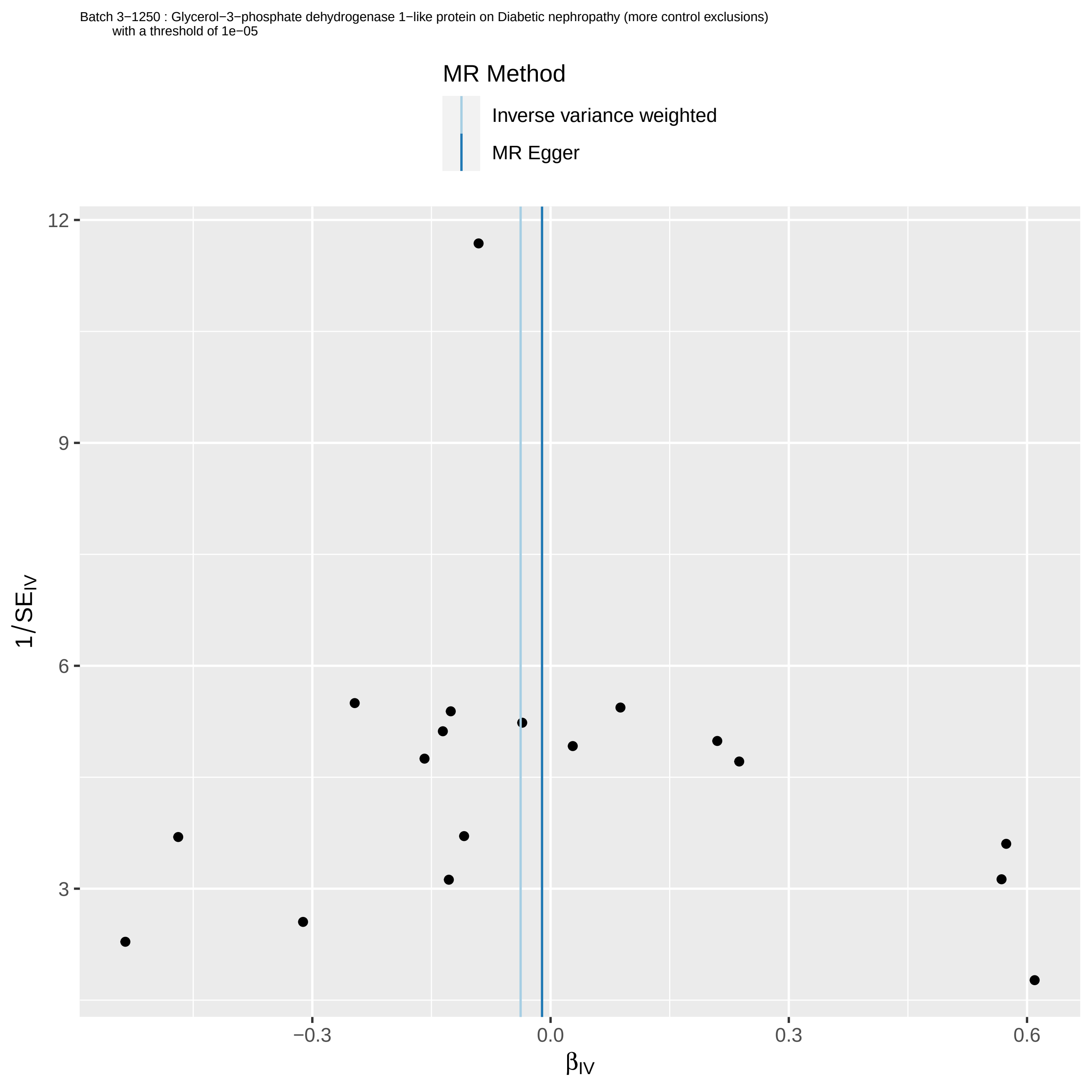
Batch 3−1250 : Glycerol−3−phosphate dehydrogenase 1−like protein on Diabetic nephropathy (more control exclusions)
with a threshold of 1e−05
MR Method
Inverse variance weighted
MR Egger
12
9
6
3
1/SE_IV
−0.3
0.0
0.3
0.6
β_IV

Batch 3−1251 : Glycoprotein hormone alpha−2 on Diabetic nephropathy (more control exclusions)
with a threshold of 1e−05

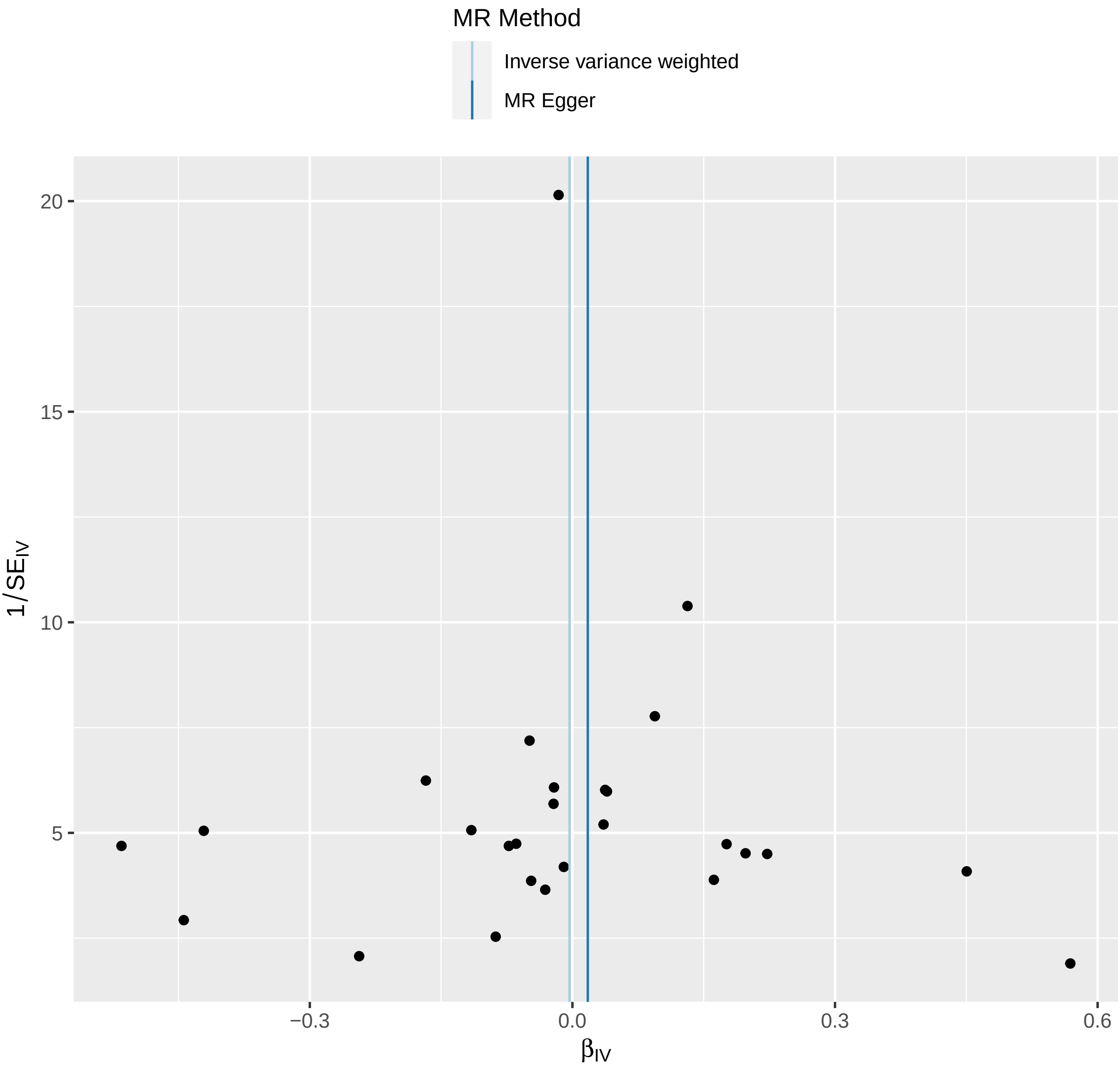

Batch 3−1252 : Glucose−6−phosphate isomerase on Diabetic nephropathy (more control exclusions)
with a threshold of 1e−05

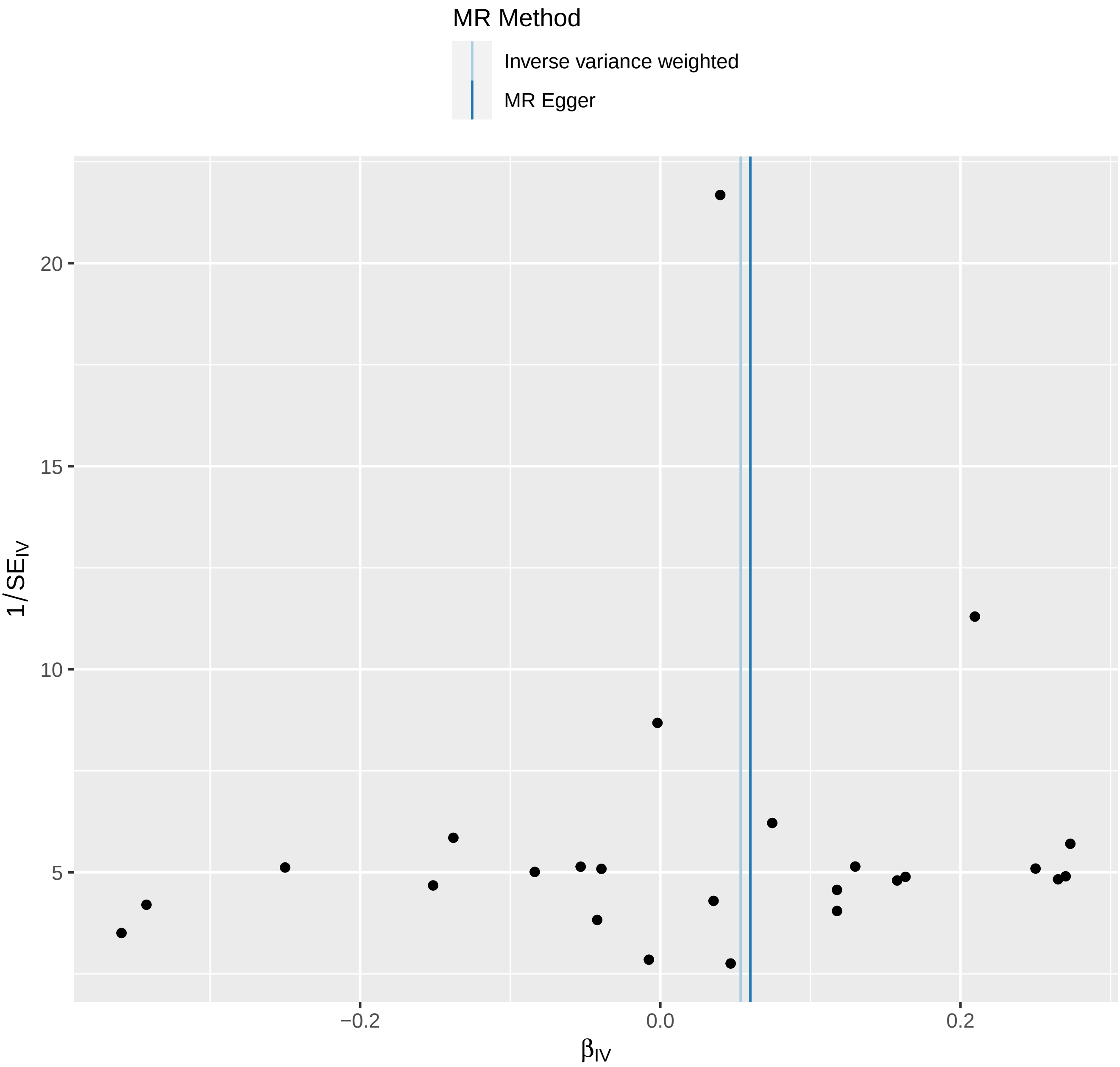

Batch 3−1253 : Phosphatidylinositol−glycan−specific phospholipase D on Diabetic nephropathy (more control exclusions)
with a threshold of 1e−05

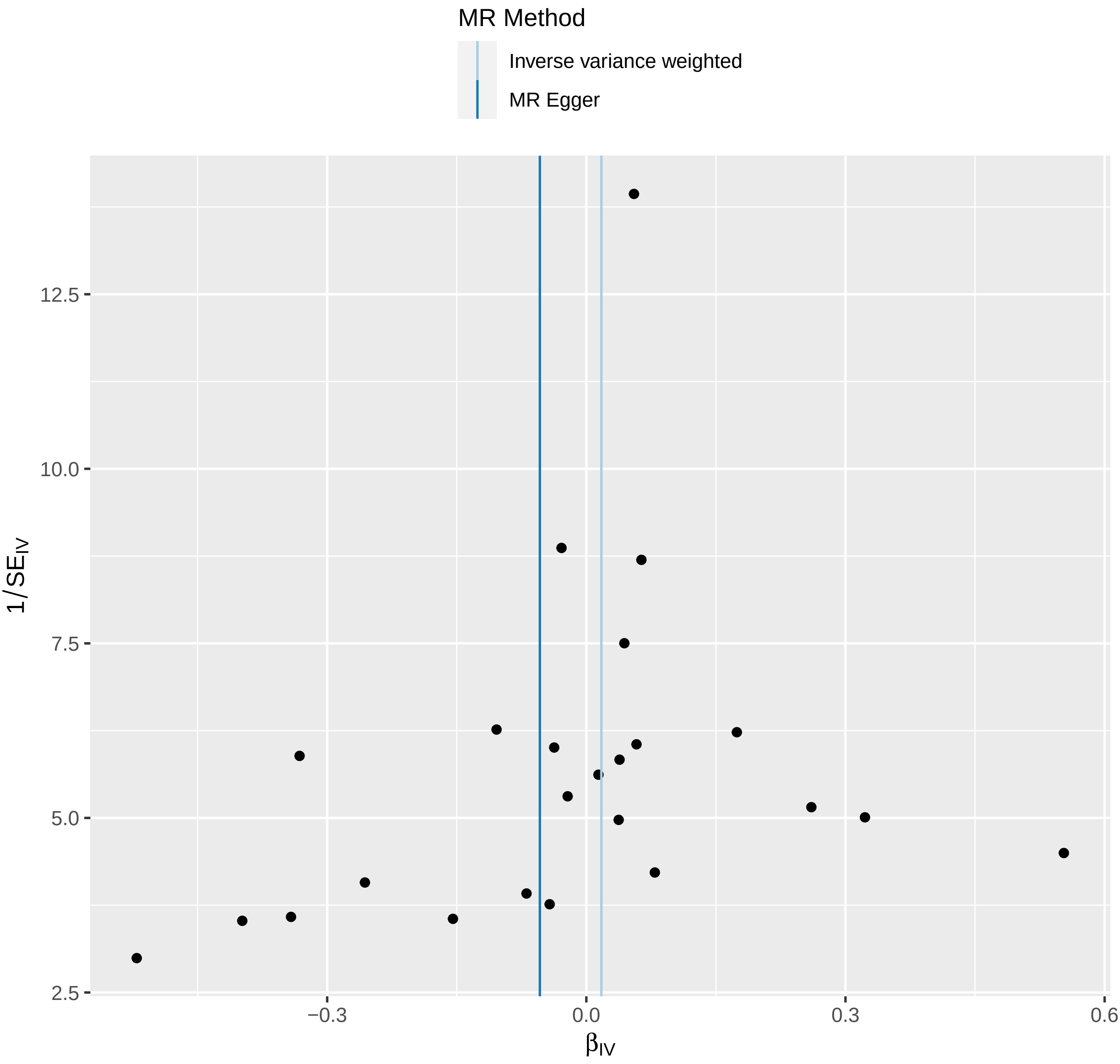

Batch 3−1254 : Phosphatidylinositol−glycan−specific phospholipase D on Diabetic nephropathy (more control exclusions)
with a threshold of 1e−05

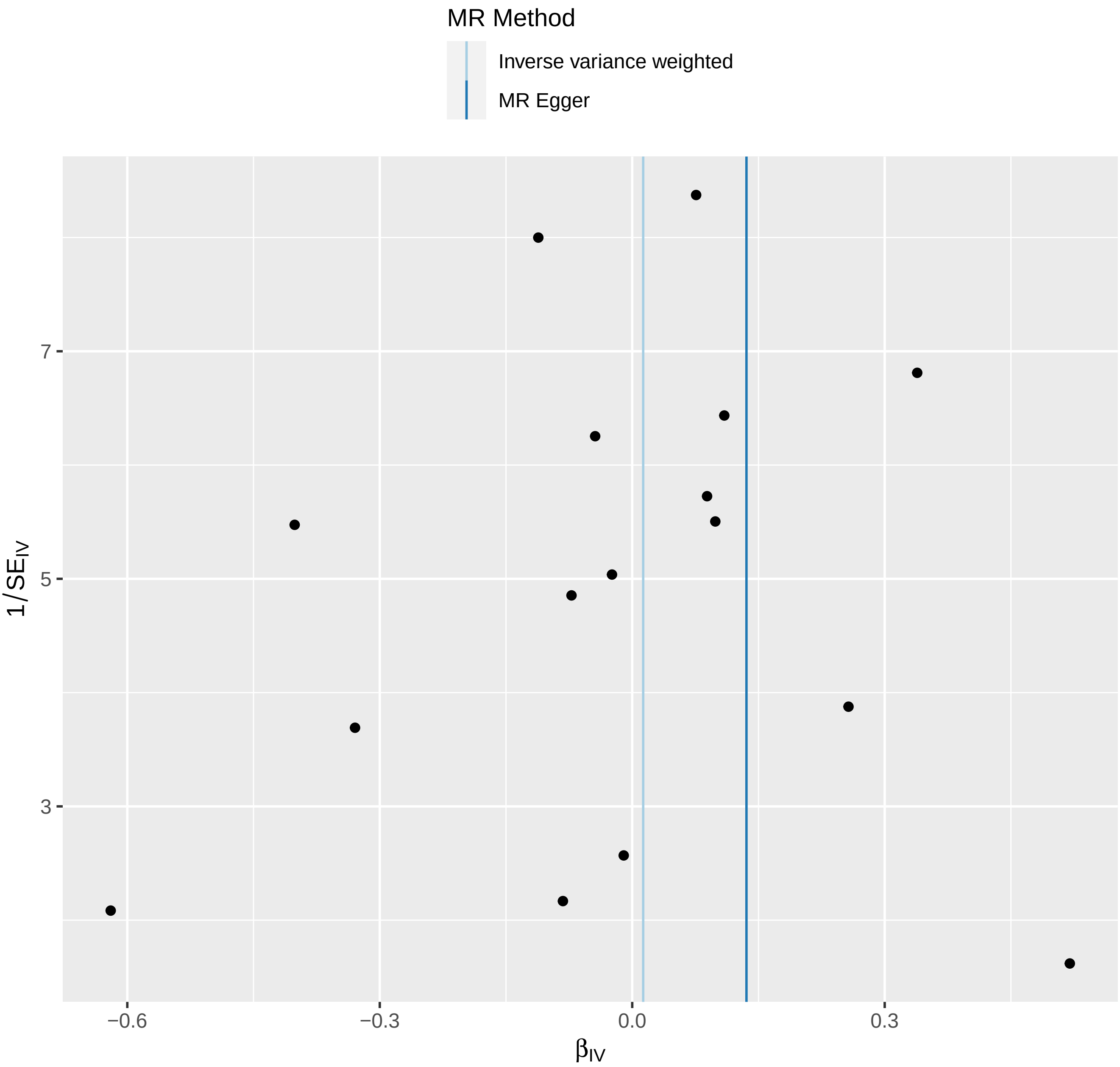

Batch 3−1255 : Transmembrane glycoprotein NMB on Diabetic nephropathy (more control exclusions)
with a threshold of 1e−05

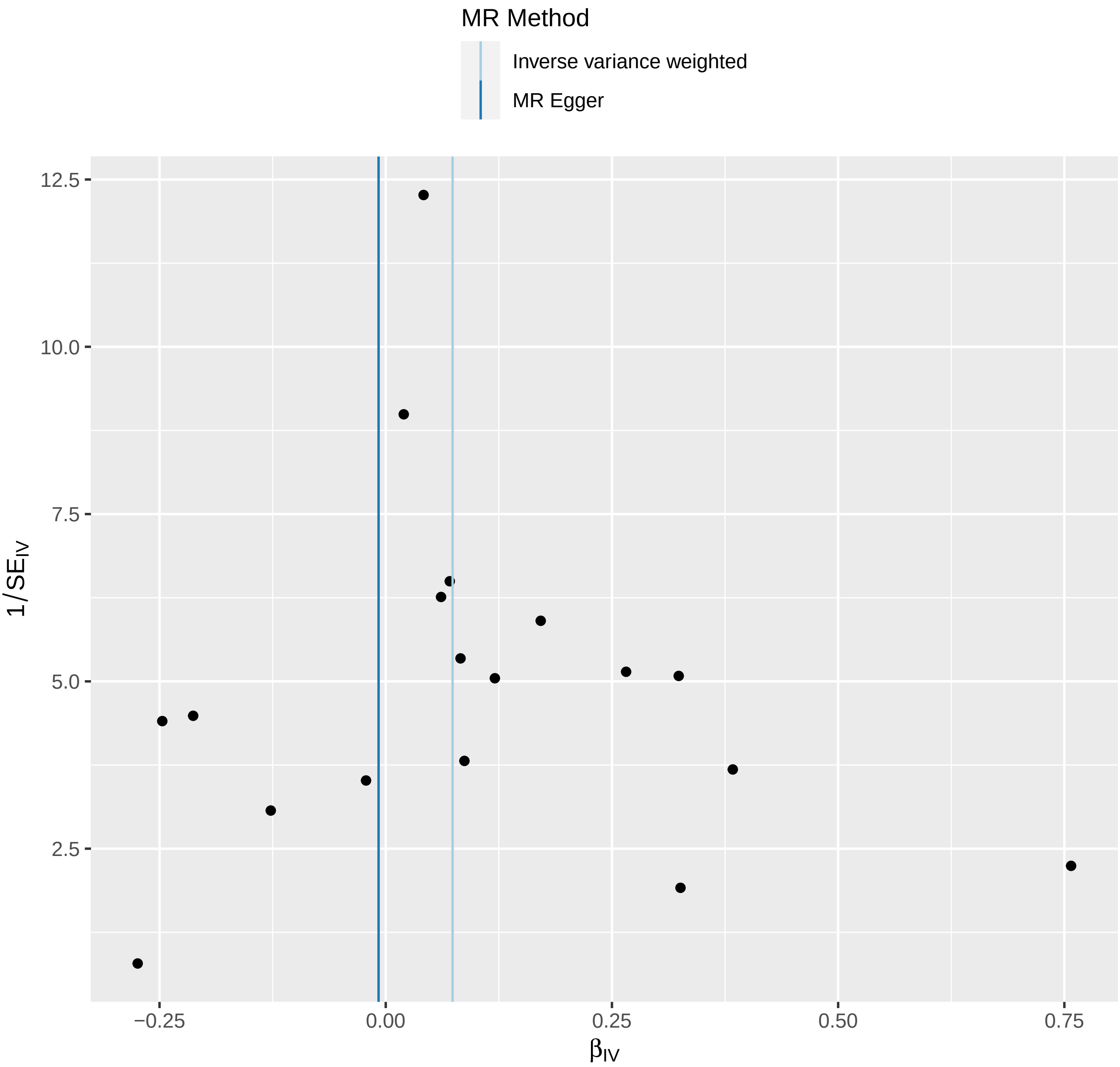

Batch 3−1256 : Transmembrane glycoprotein NMB on Diabetic nephropathy (more control exclusions)
with a threshold of 1e−05

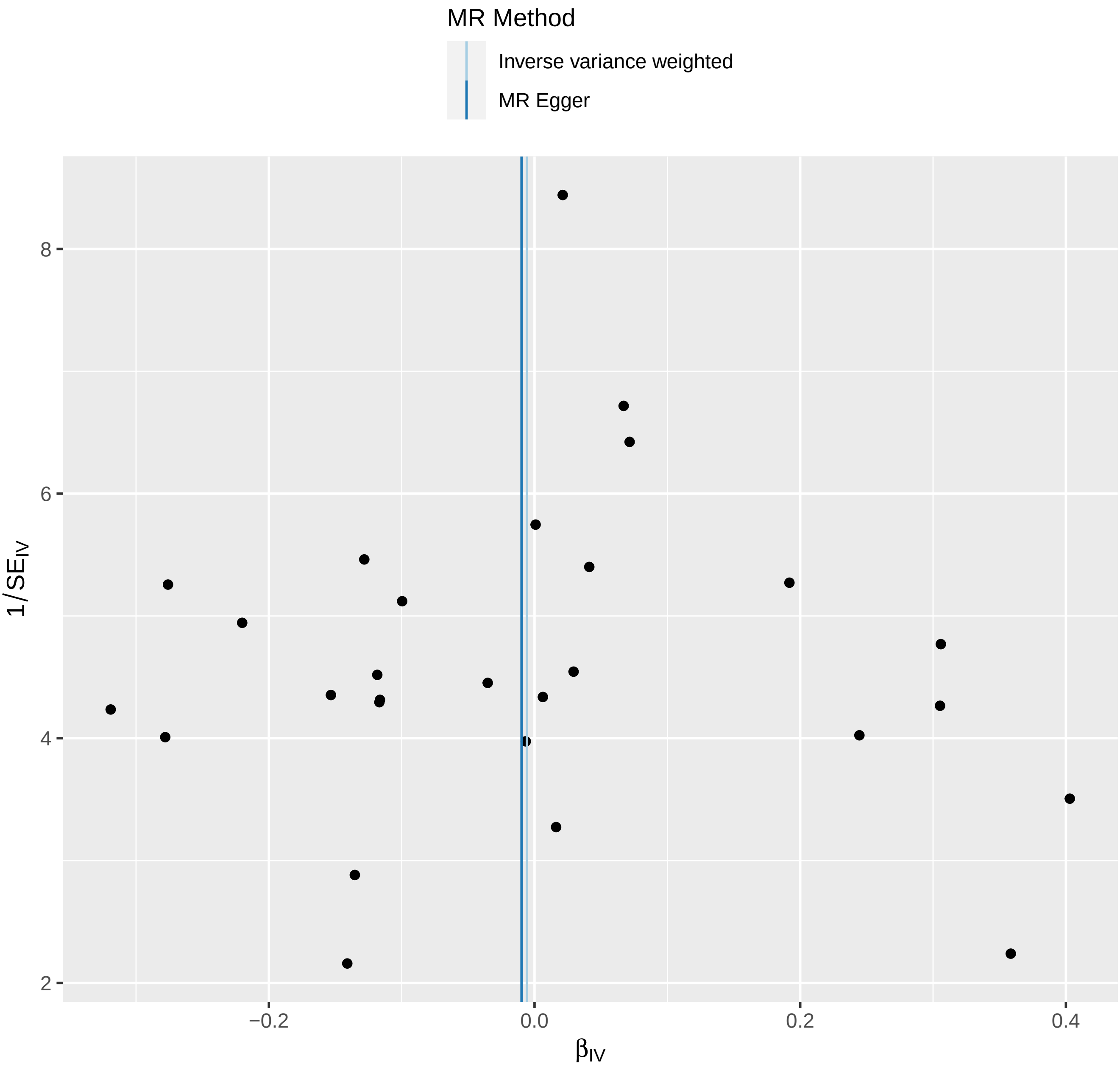

Batch 3−1257 : Transmembrane glycoprotein NMB on Diabetic nephropathy (more control exclusions)
with a threshold of 1e−05

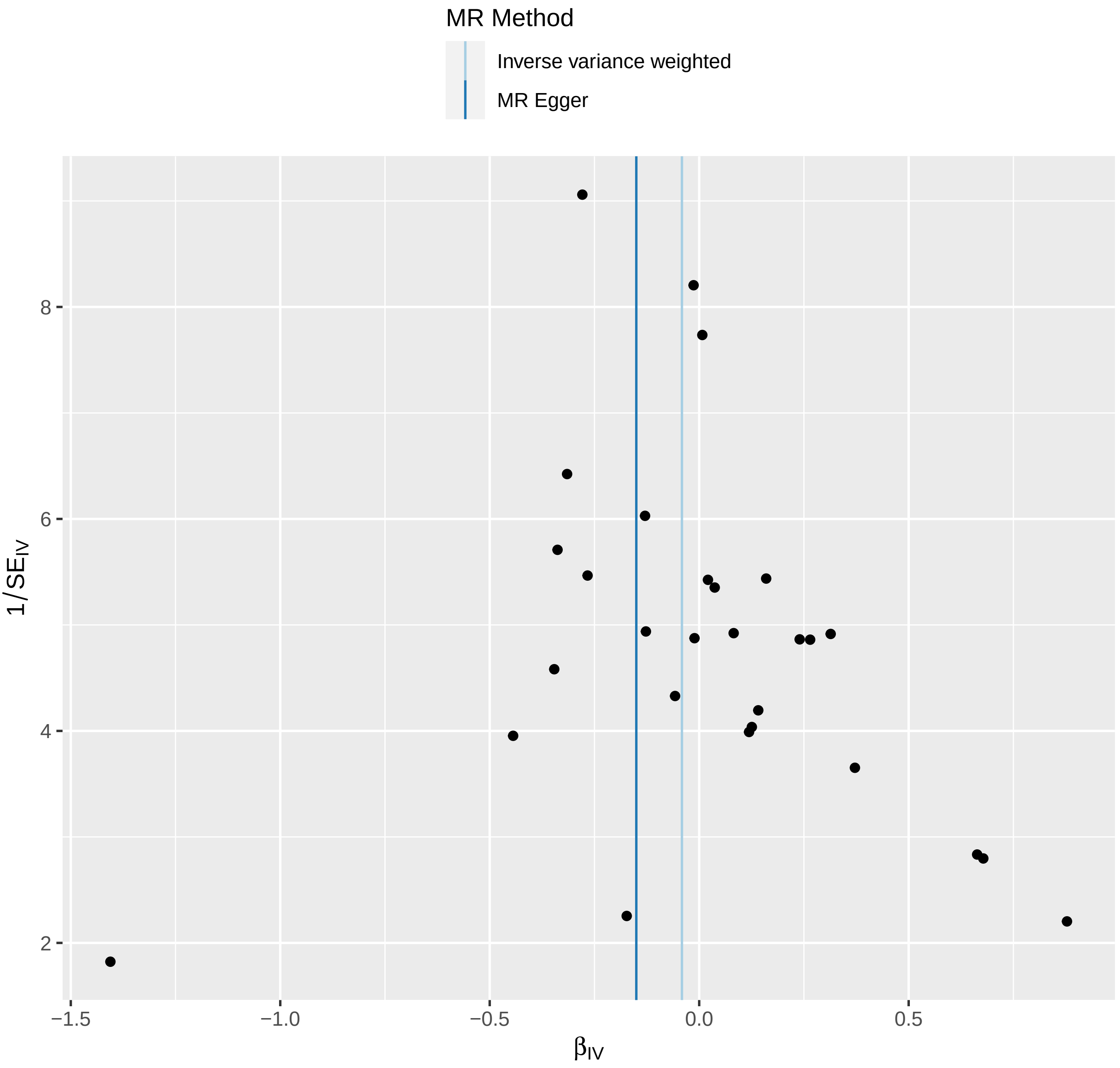

Batch 3−1258 : Transmembrane glycoprotein NMB on Diabetic nephropathy (more control exclusions)
with a threshold of 1e−05

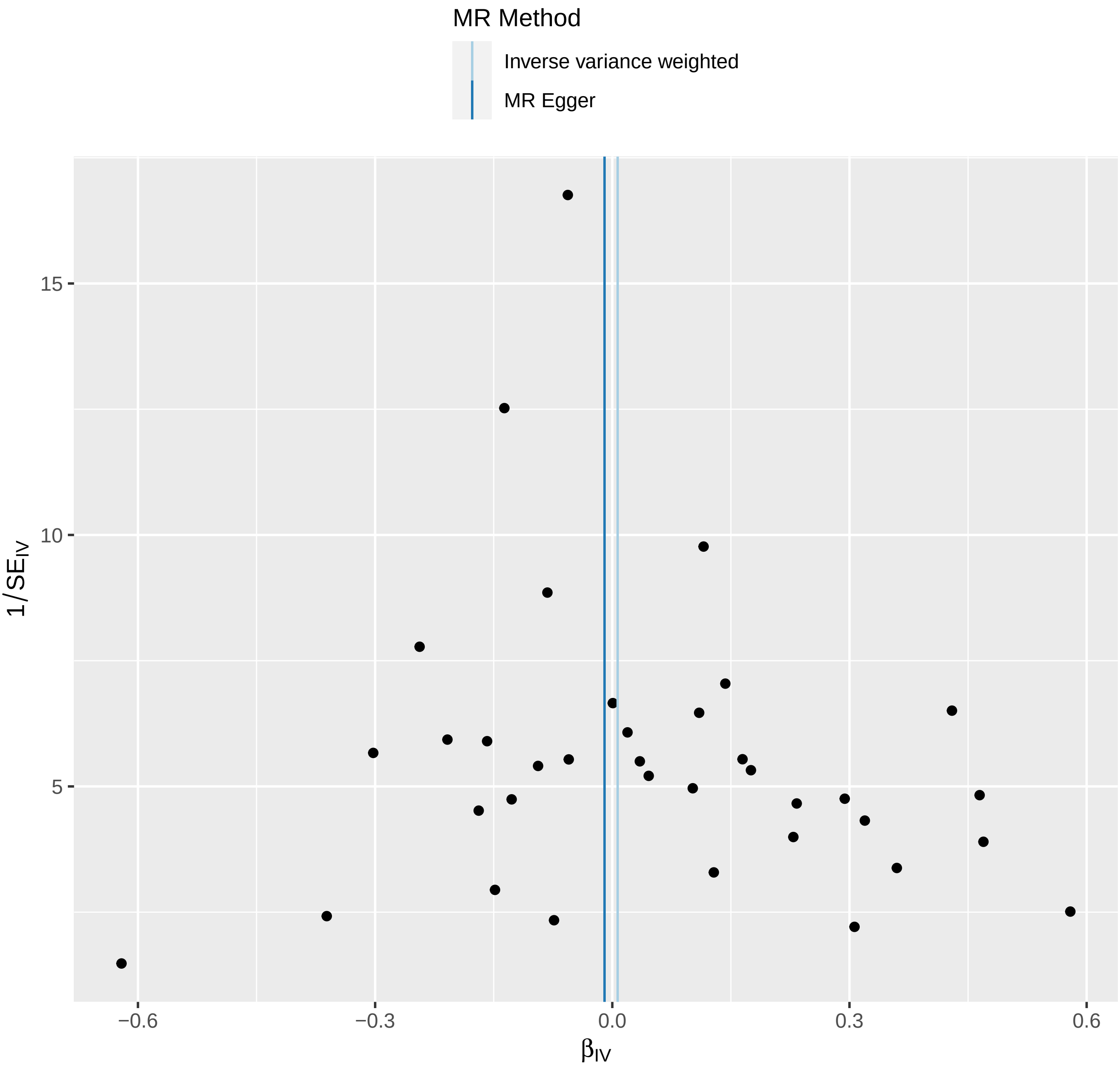

Batch 3−1259 : Probable G−protein coupled receptor 101 on Diabetic nephropathy (more control exclusions)
with a threshold of 1e−05

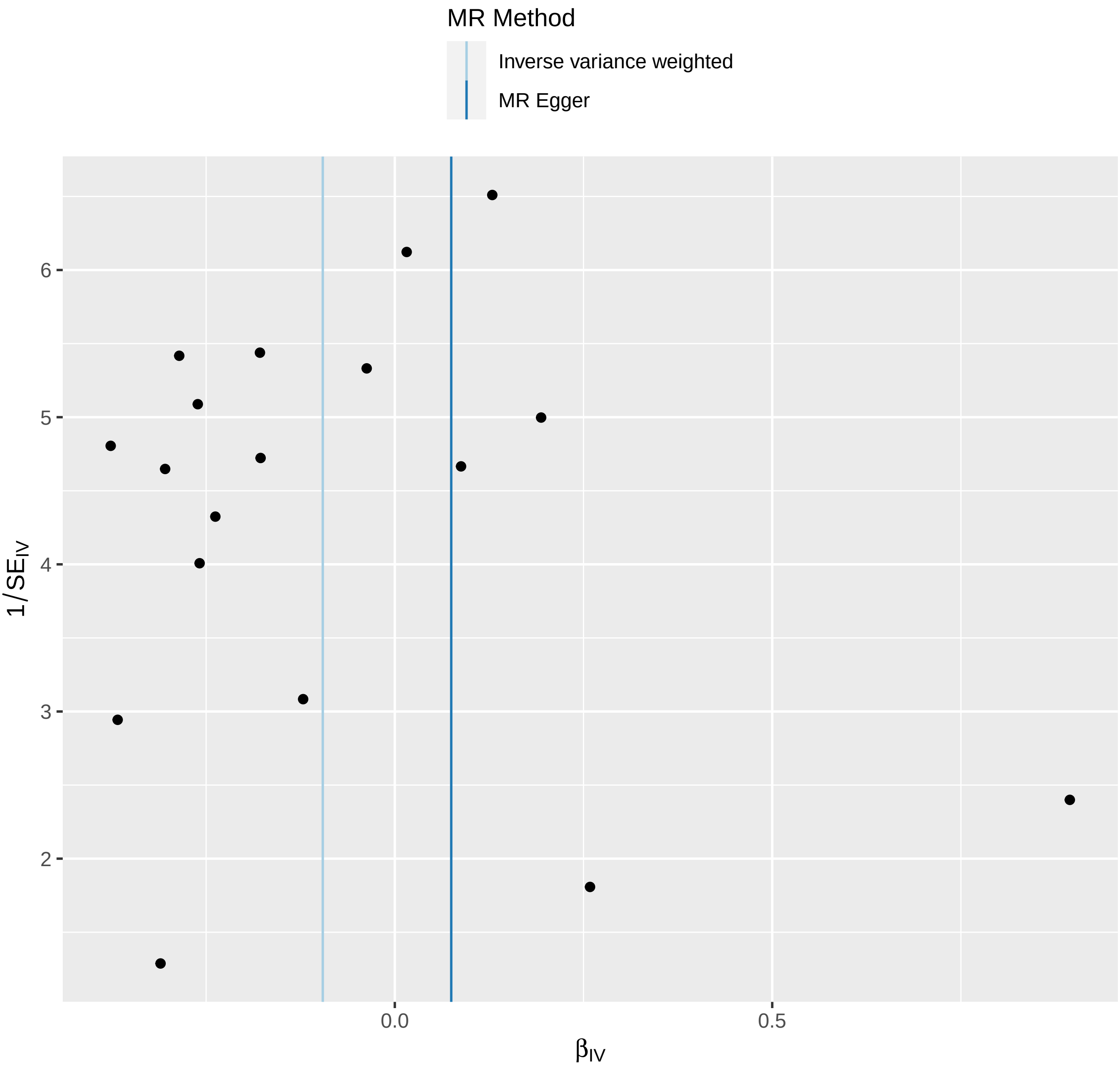

Batch 3−1260 : Probable G−protein coupled receptor 101 on Diabetic nephropathy (more control exclusions)
with a threshold of 1e−05

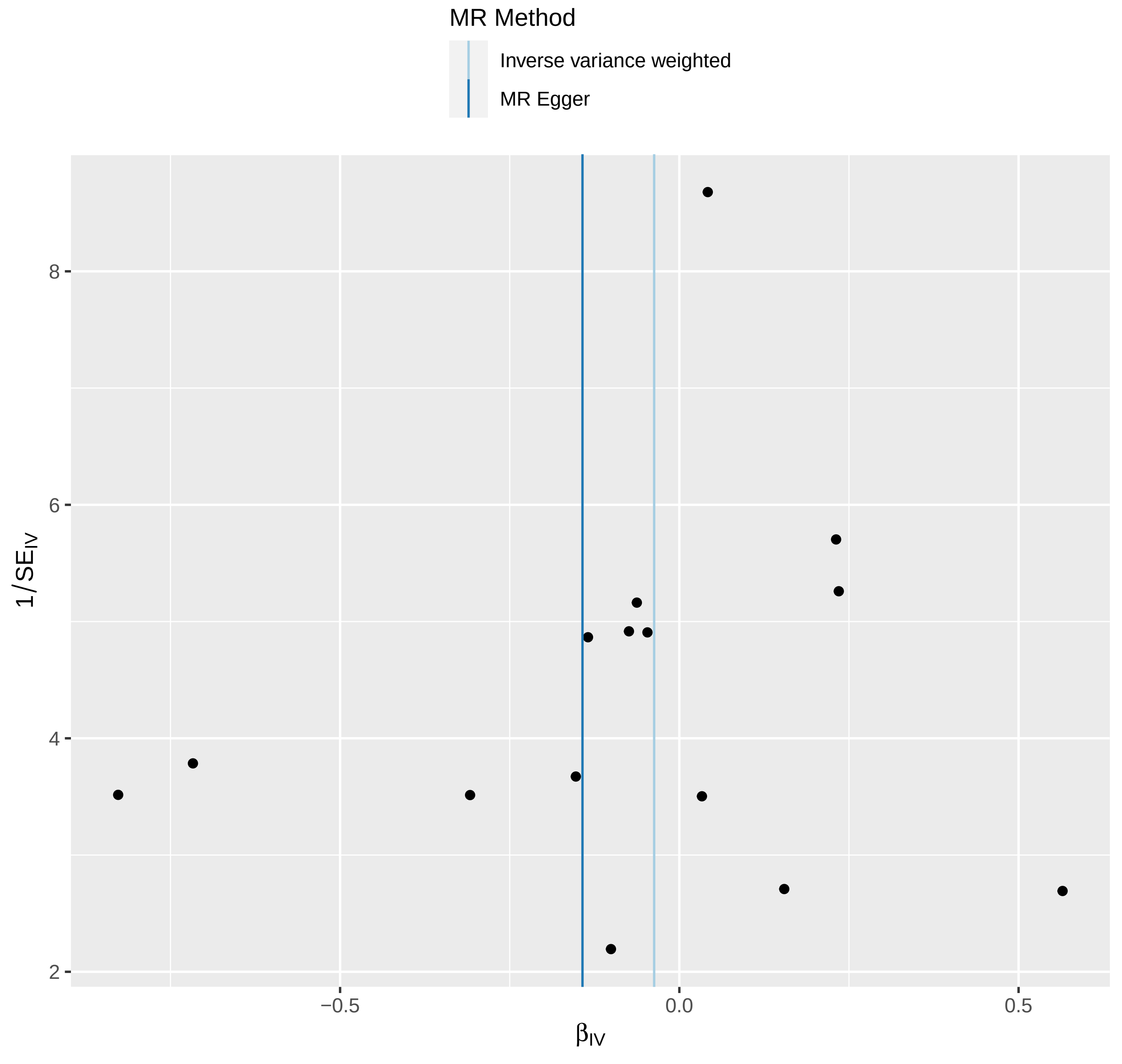

Batch 3−1261 : Protein GPR107 on Diabetic nephropathy (more control exclusions)
with a threshold of 1e−05

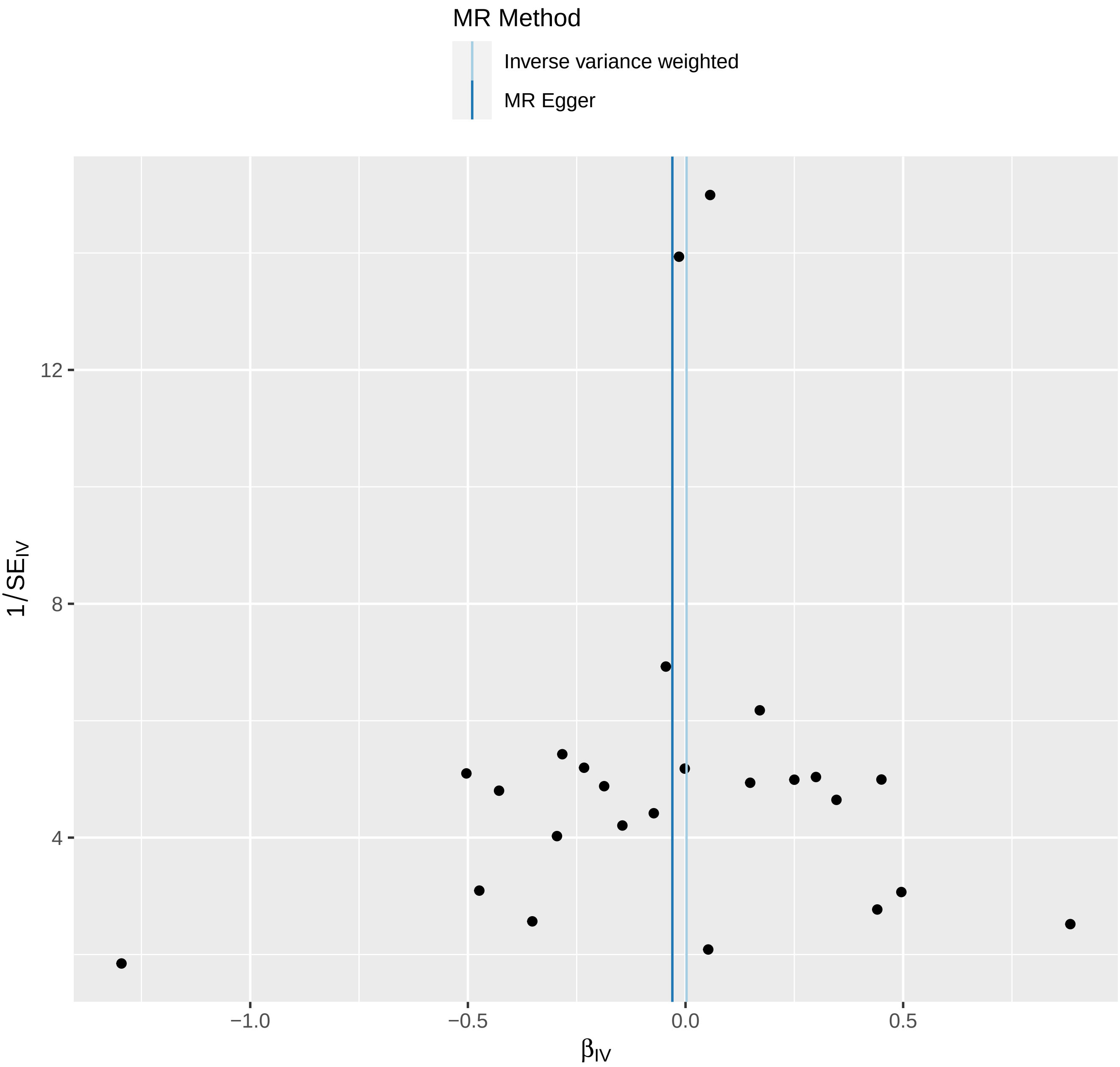

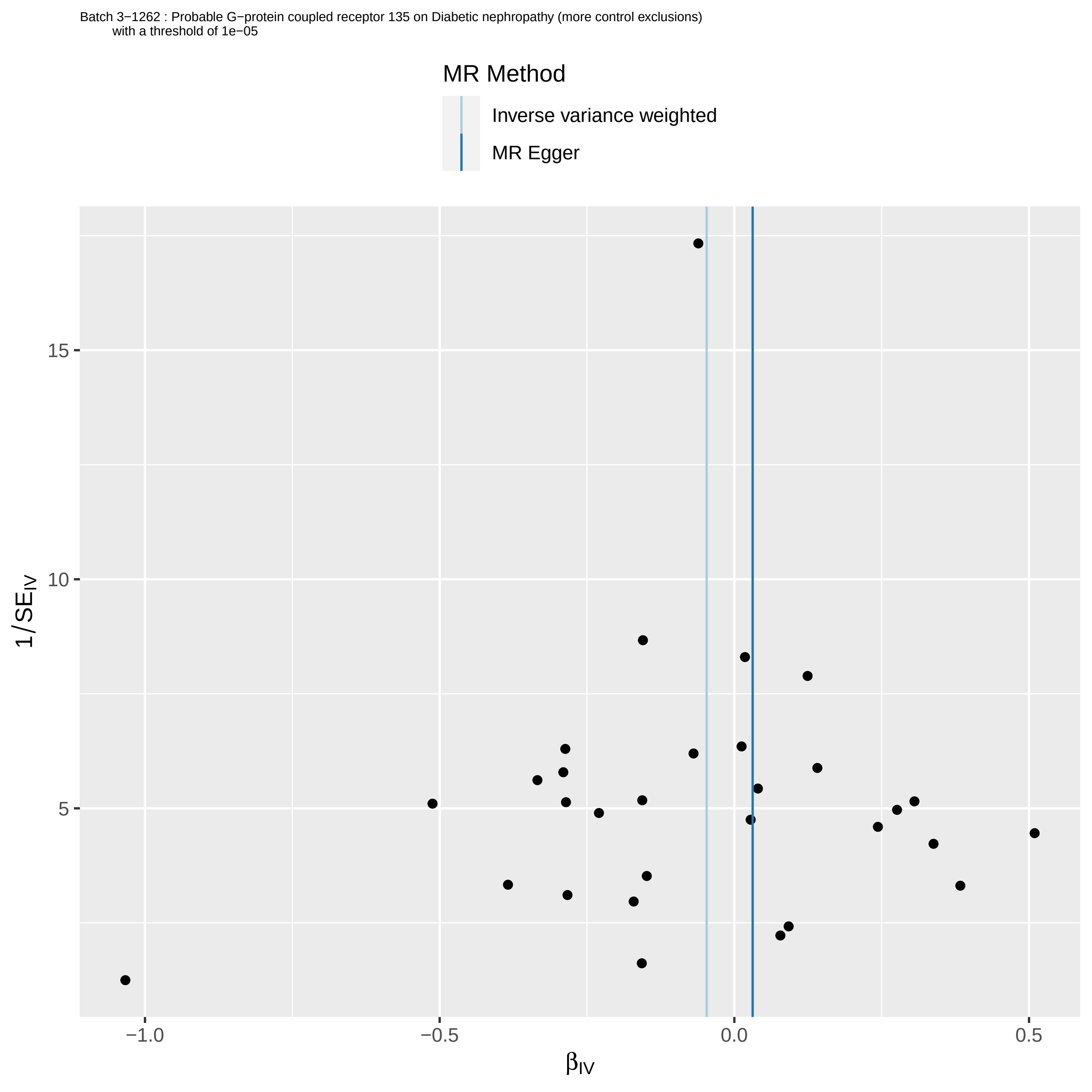
Batch 3−1262 : Probable G−protein coupled receptor 135 on Diabetic nephropathy (more control exclusions)
with a threshold of 1e−05
MR Method
Inverse variance weighted
MR Egger
15
10
5
1/SE_IV
−1.0
−0.5
0.0
0.5
β_IV

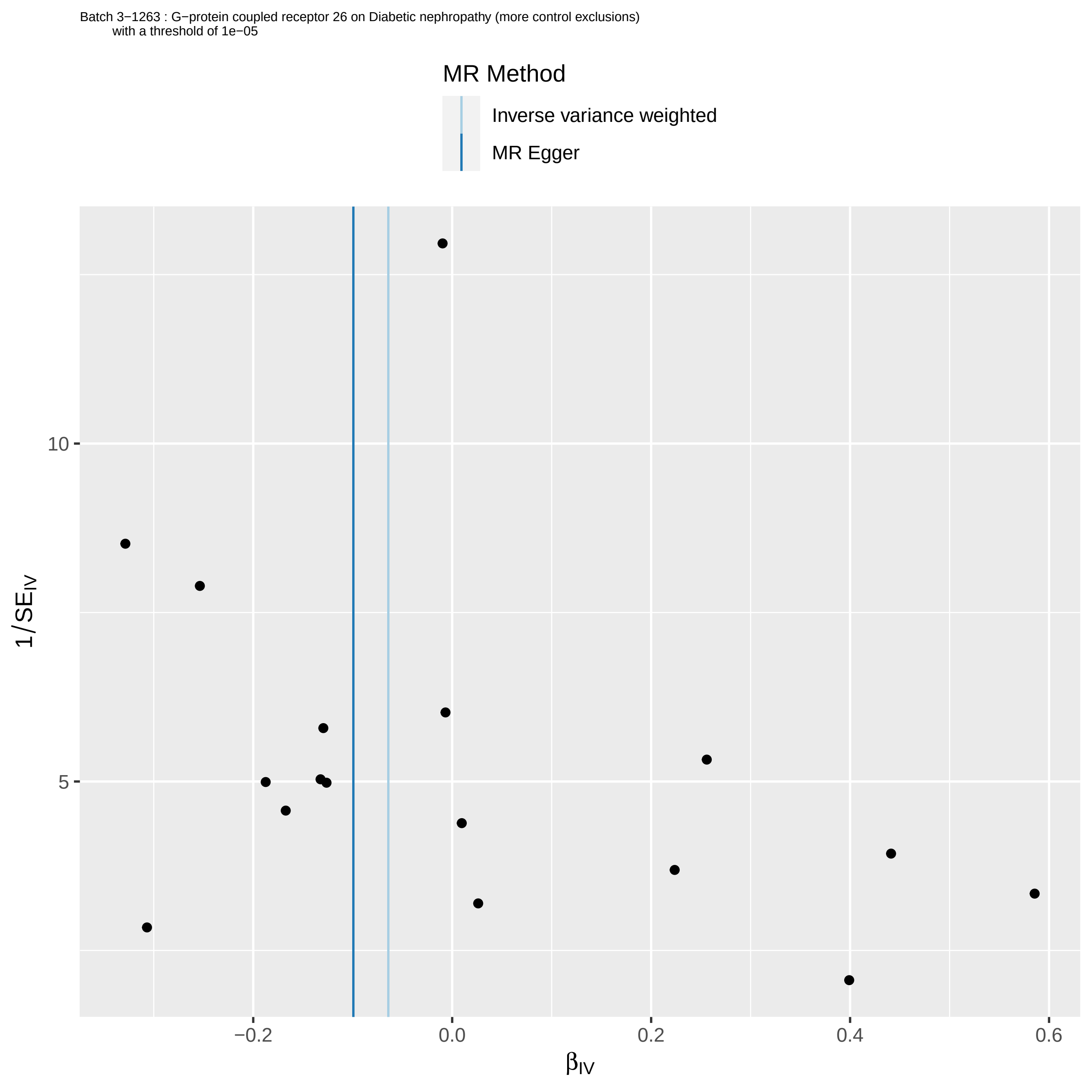
Batch 3−1263 : G−protein coupled receptor 26 on Diabetic nephropathy (more control exclusions)
with a threshold of 1e−05
MR Method
Inverse variance weighted
MR Egger
10
5
1/SE_IV
−0.2
0.0
0.2
0.4
0.6
β_IV

Batch 3−1264 : Alanine aminotransferase 1 on Diabetic nephropathy (more control exclusions)
with a threshold of 1e−05

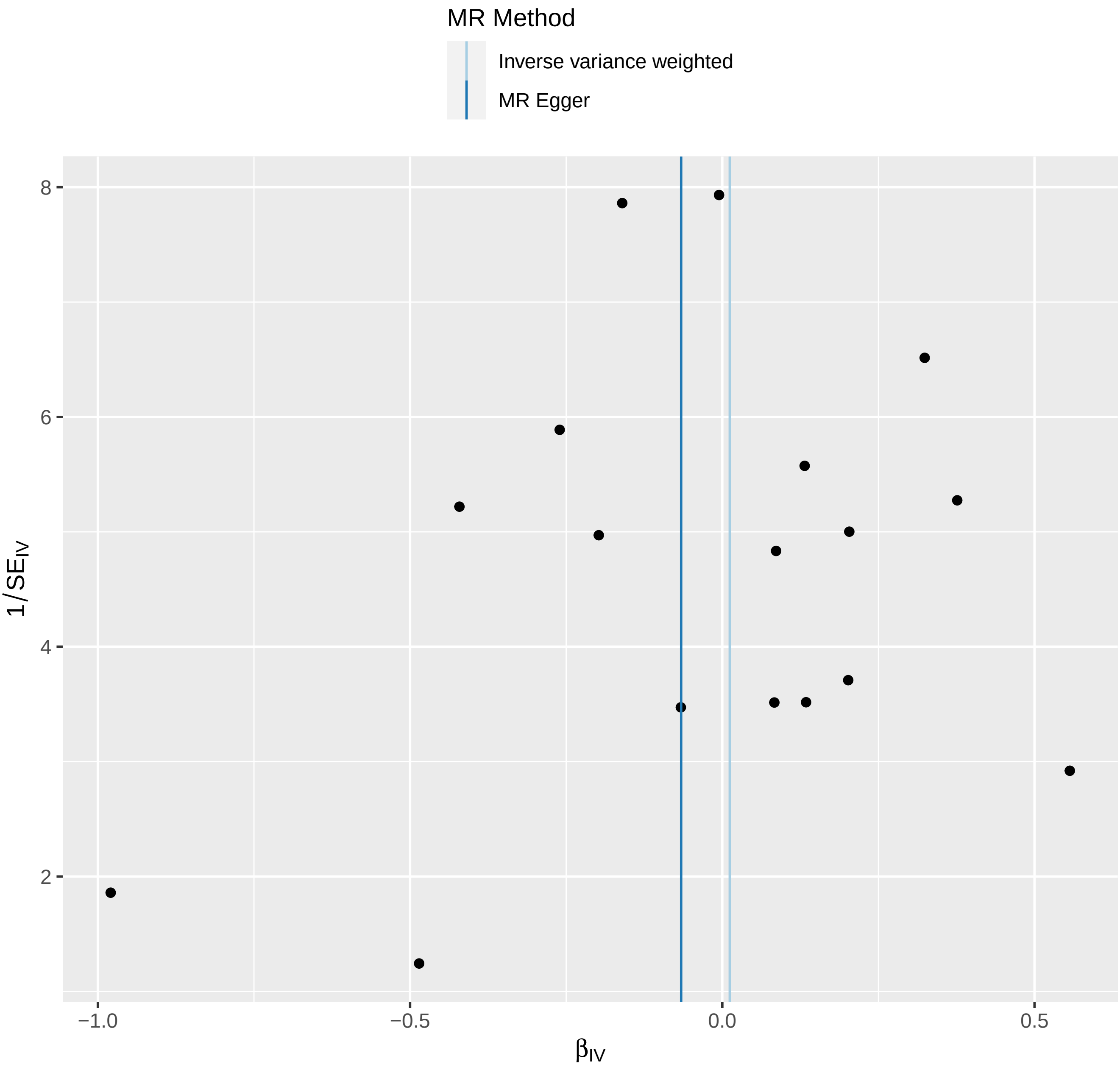

Batch 3−1265 : Glutathione peroxidase 7 on Diabetic nephropathy (more control exclusions)
with a threshold of 1e−05

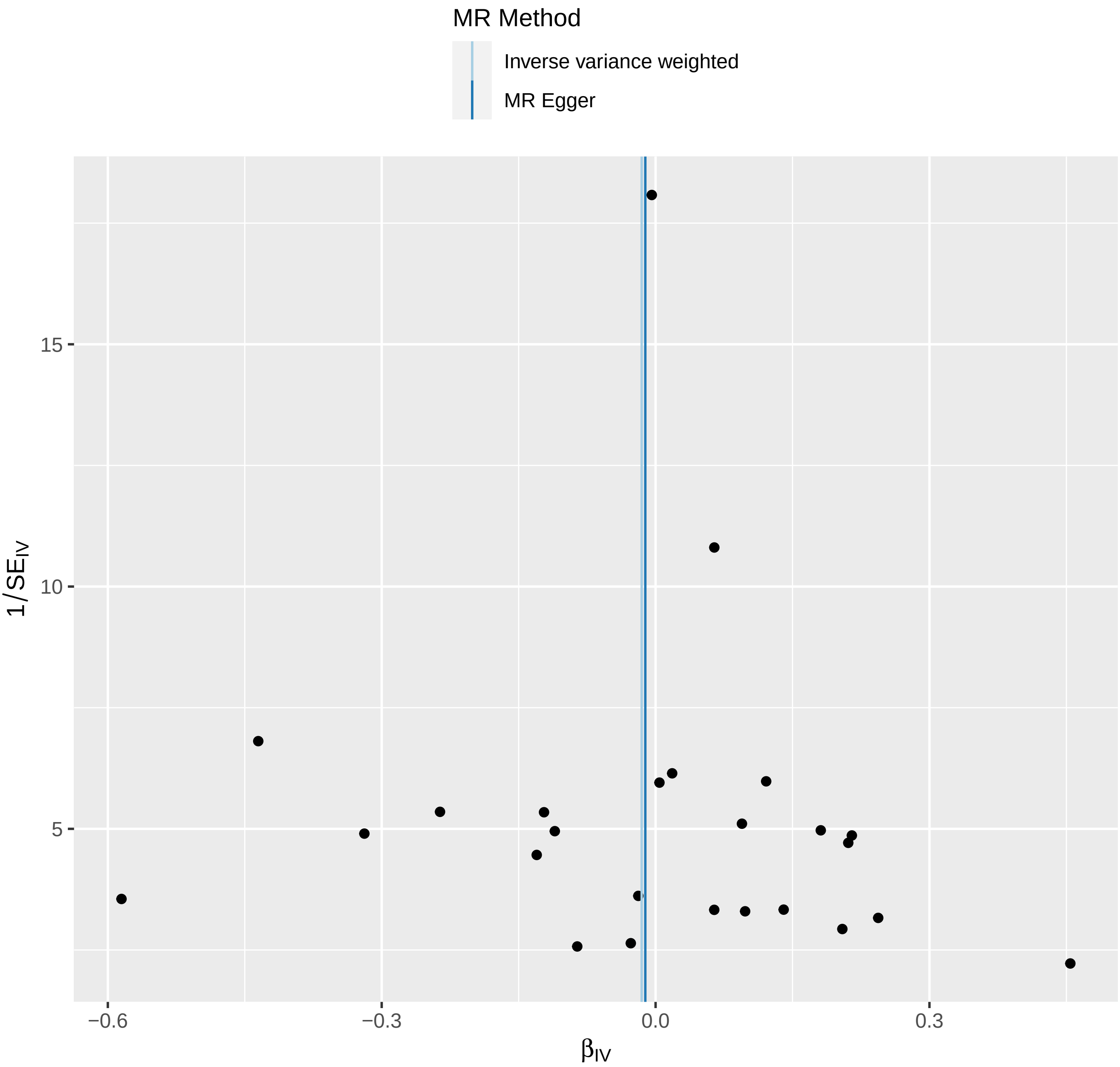

Batch 3−1266 : GRAM domain−containing protein 1C on Diabetic nephropathy (more control exclusions)
with a threshold of 1e−05

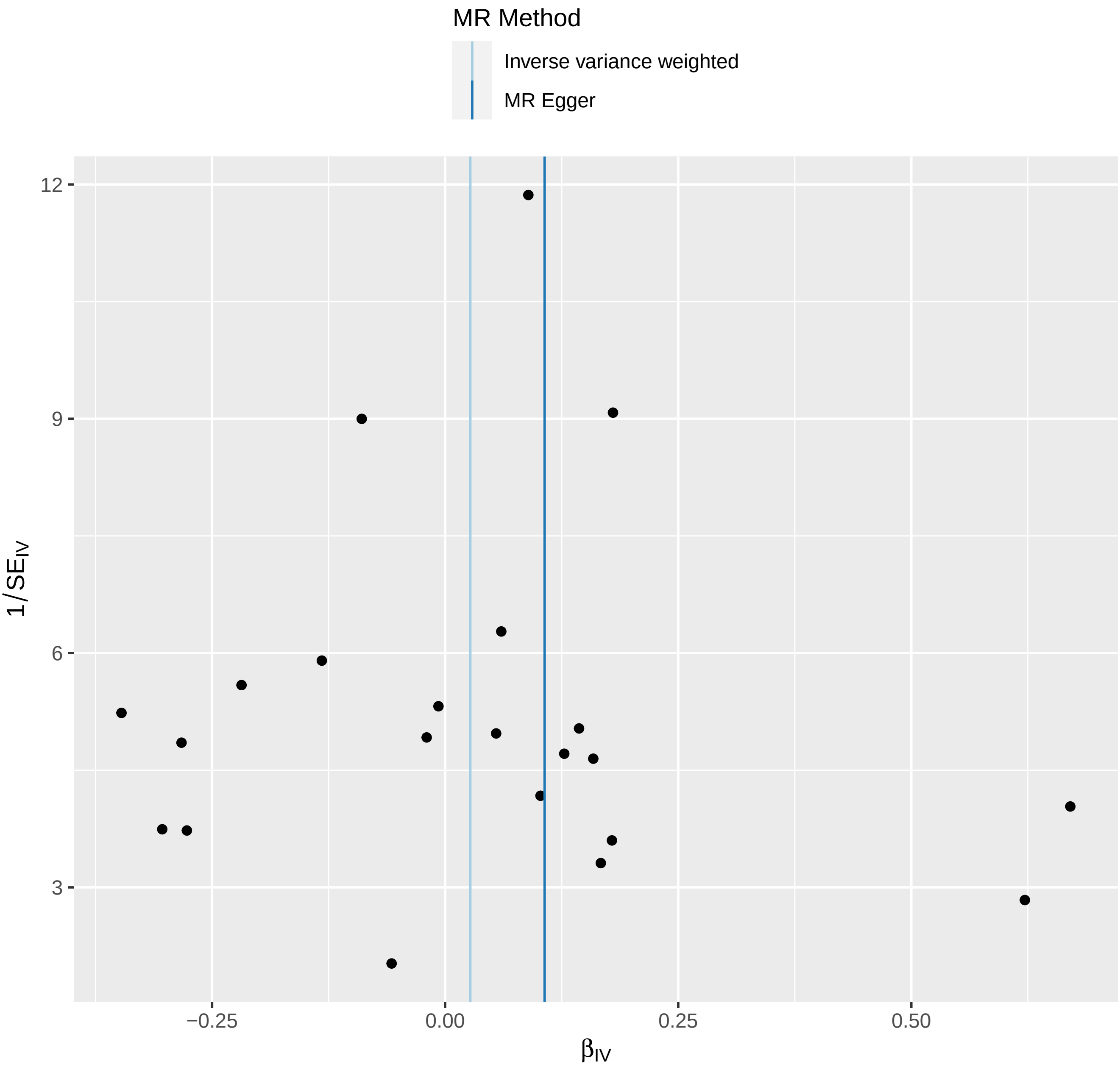

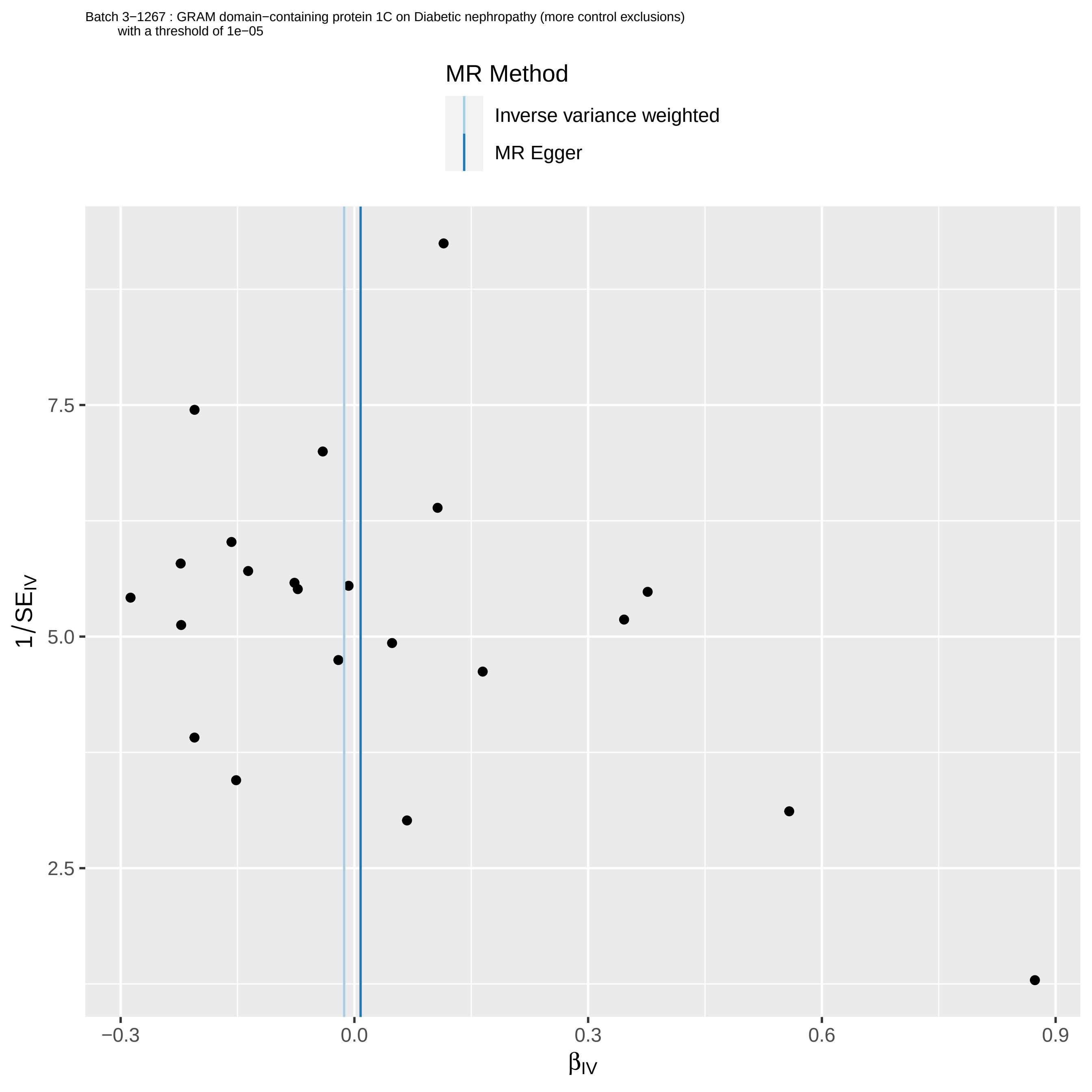

Batch 3−1267 : GRAM domain−containing protein 1C on Diabetic nephropathy (more control exclusions)
with a threshold of 1e−05
MR Method
Inverse variance weighted
MR Egger
7.5
5.0
2.5
1/SE_IV
−0.3
0.0
0.3
0.6
0.9
β_IV

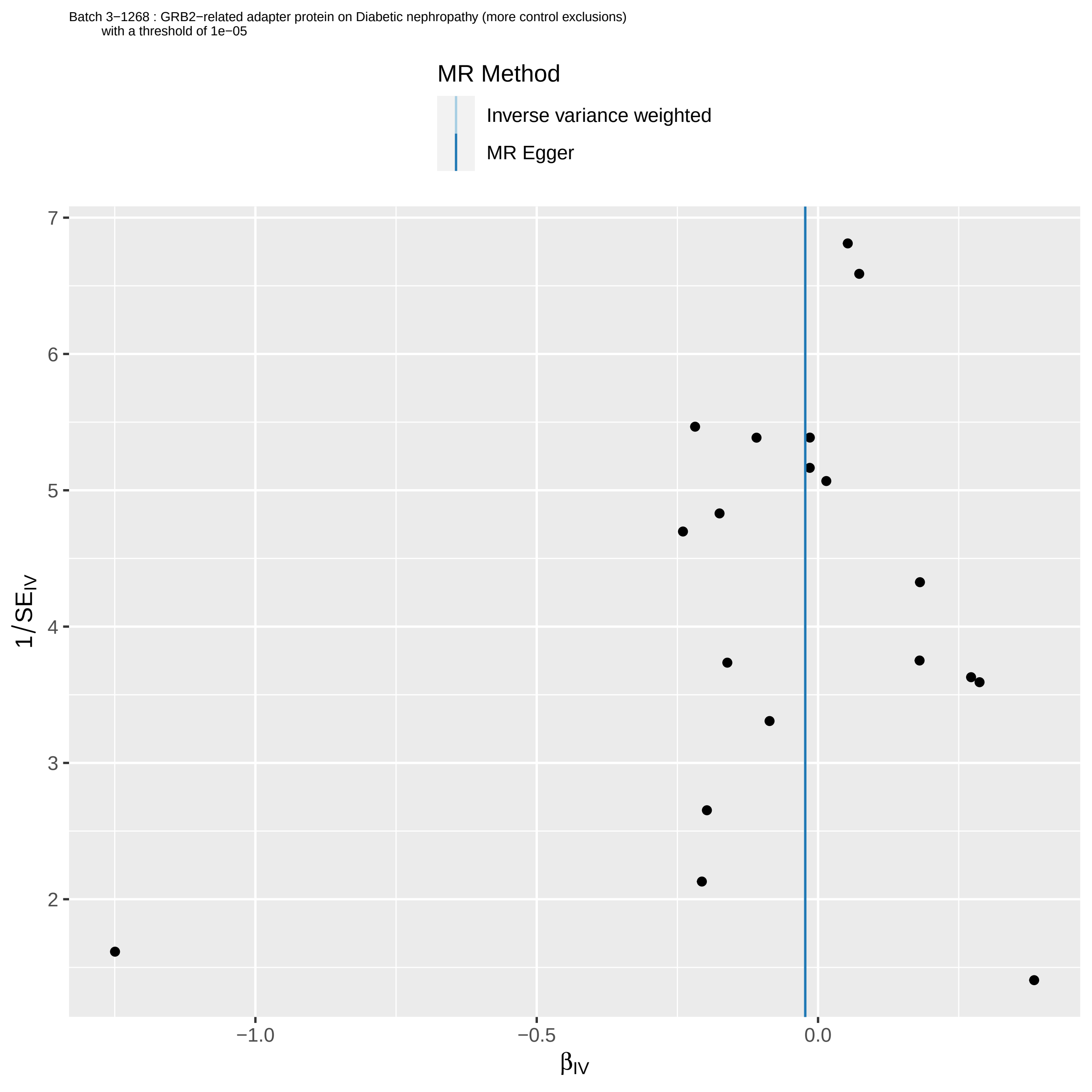
Batch 3−1268 : GRB2−related adapter protein on Diabetic nephropathy (more control exclusions) with a threshold of 1e−05
MR Method
Inverse variance weighted
MR Egger
7
6
5
4
3
2
1/SE_IV
−1.0
−0.5
0.0
β_IV

Batch 3−1269 : GRB2−related adapter protein 2 on Diabetic nephropathy (more control exclusions)
with a threshold of 1e−05

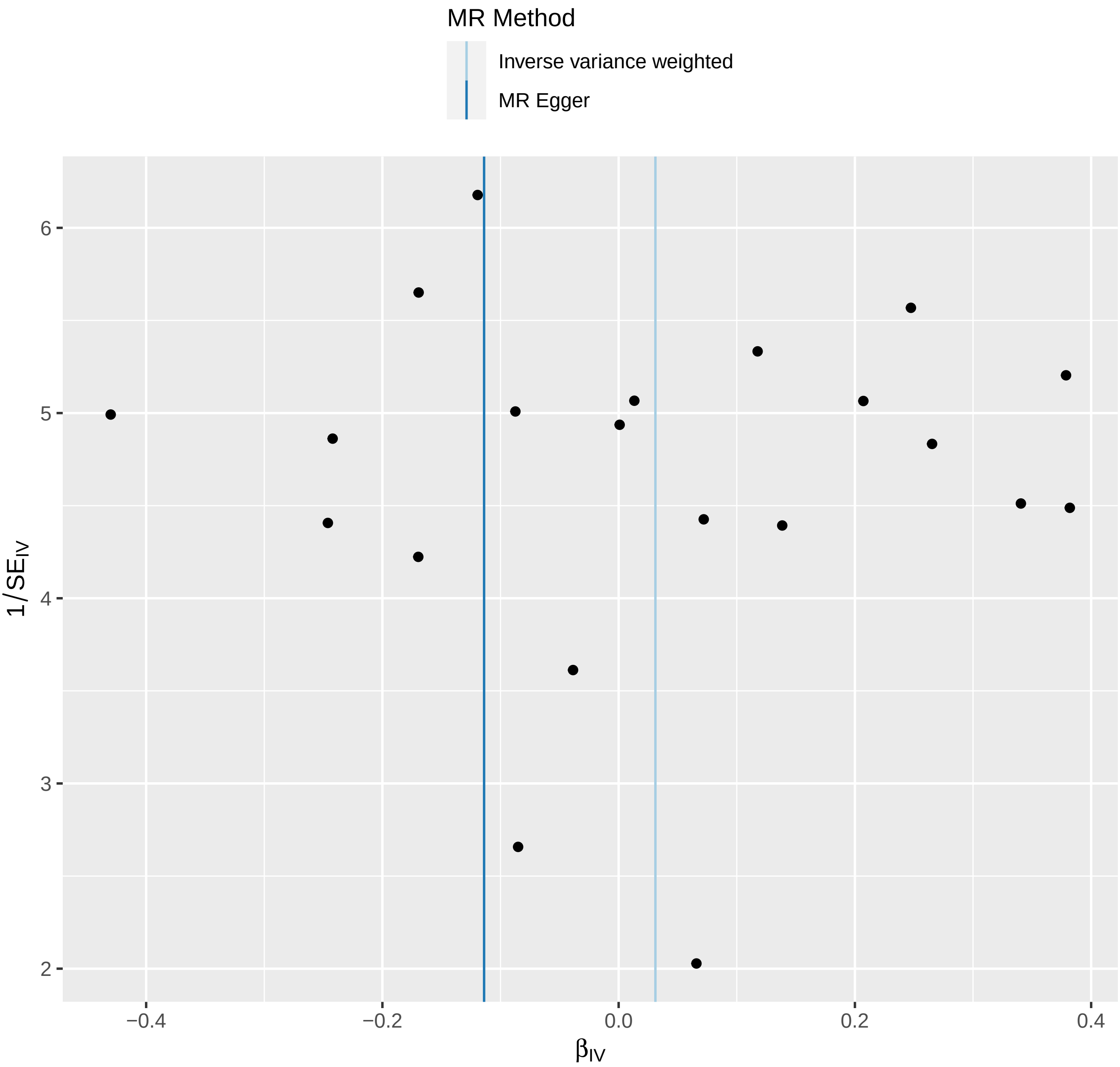

Batch 3−1270 : Growth factor receptor−bound protein 14 on Diabetic nephropathy (more control exclusions)
with a threshold of 1e−05

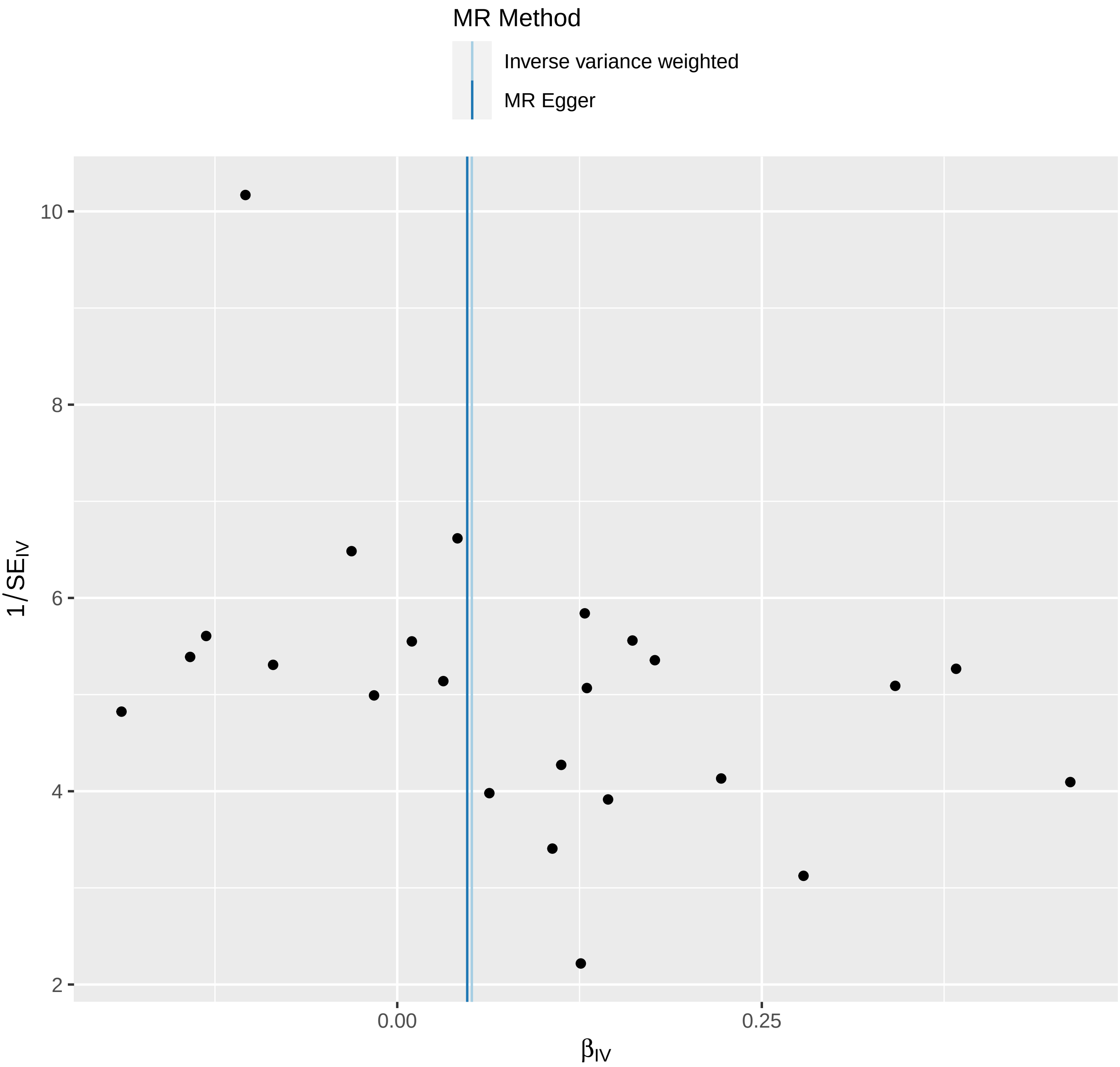

Batch 3−1271 : Growth factor receptor−bound protein 2 on Diabetic nephropathy (more control exclusions)
with a threshold of 1e−05

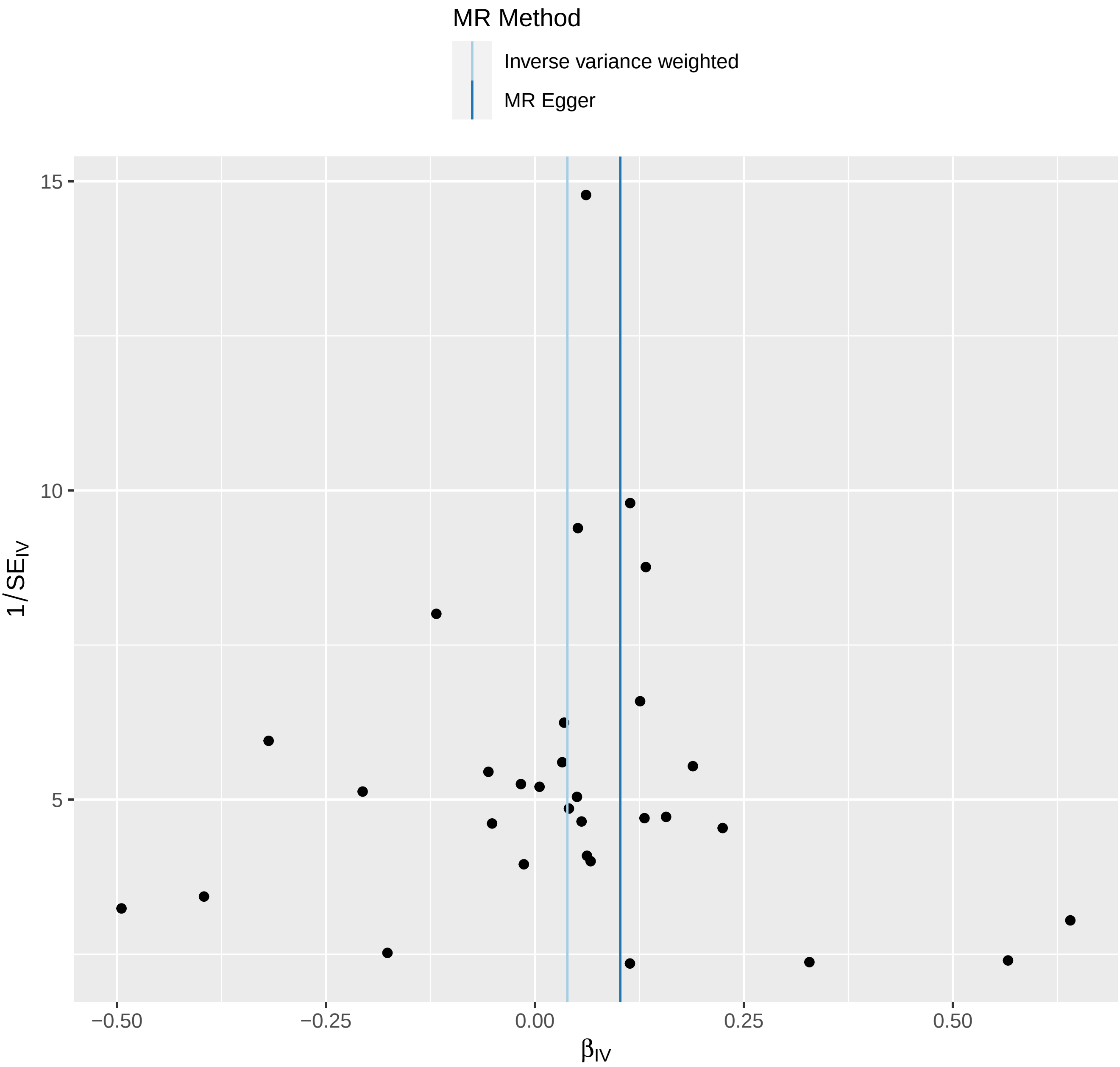

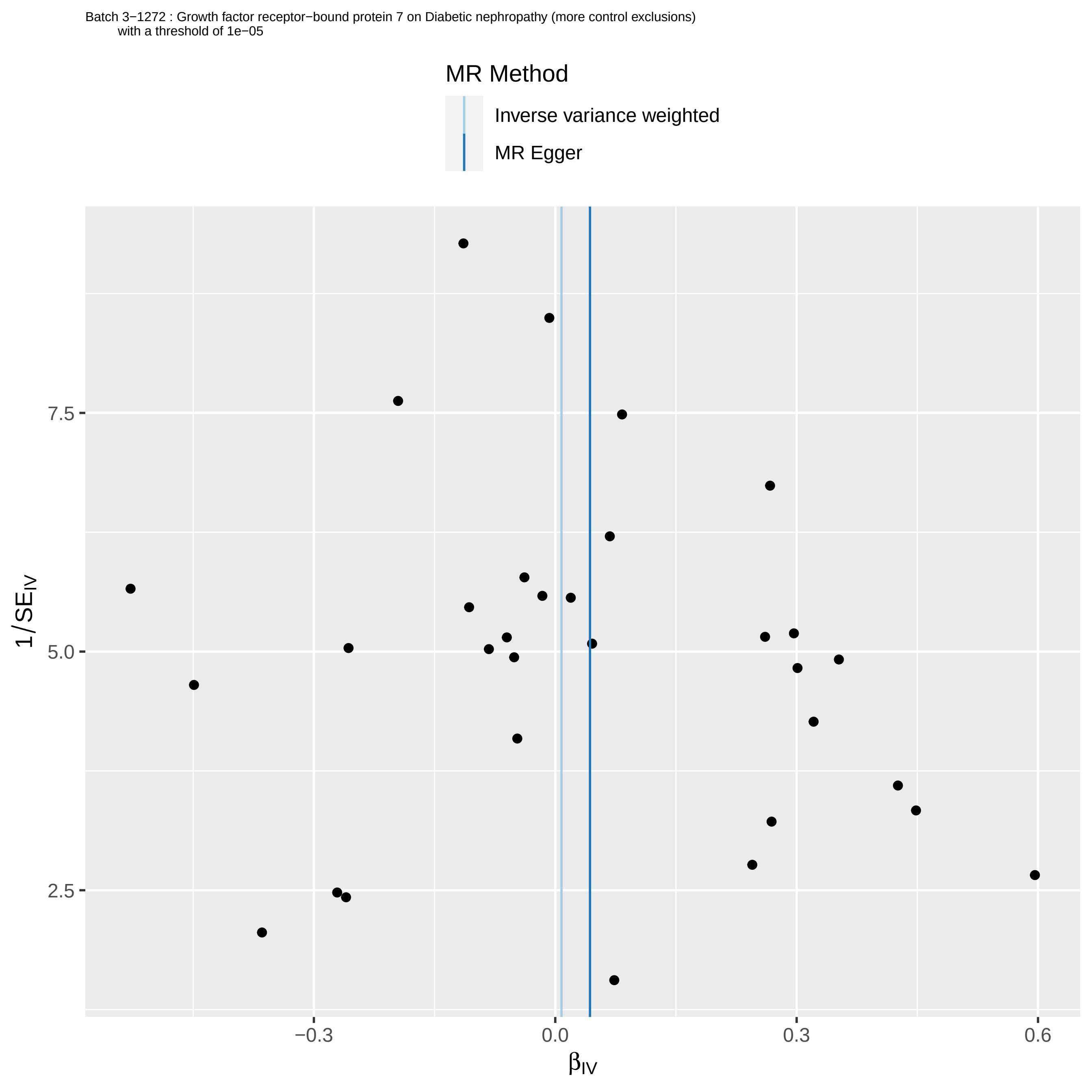

Batch 3−1272 : Growth factor receptor−bound protein 7 on Diabetic nephropathy (more control exclusions) with a threshold of 1e−05
MR Method
Inverse variance weighted
MR Egger
7.5
5.0
2.5
$1/SE_{IV}$
−0.3
0.0
0.3
0.6
β_{IV}

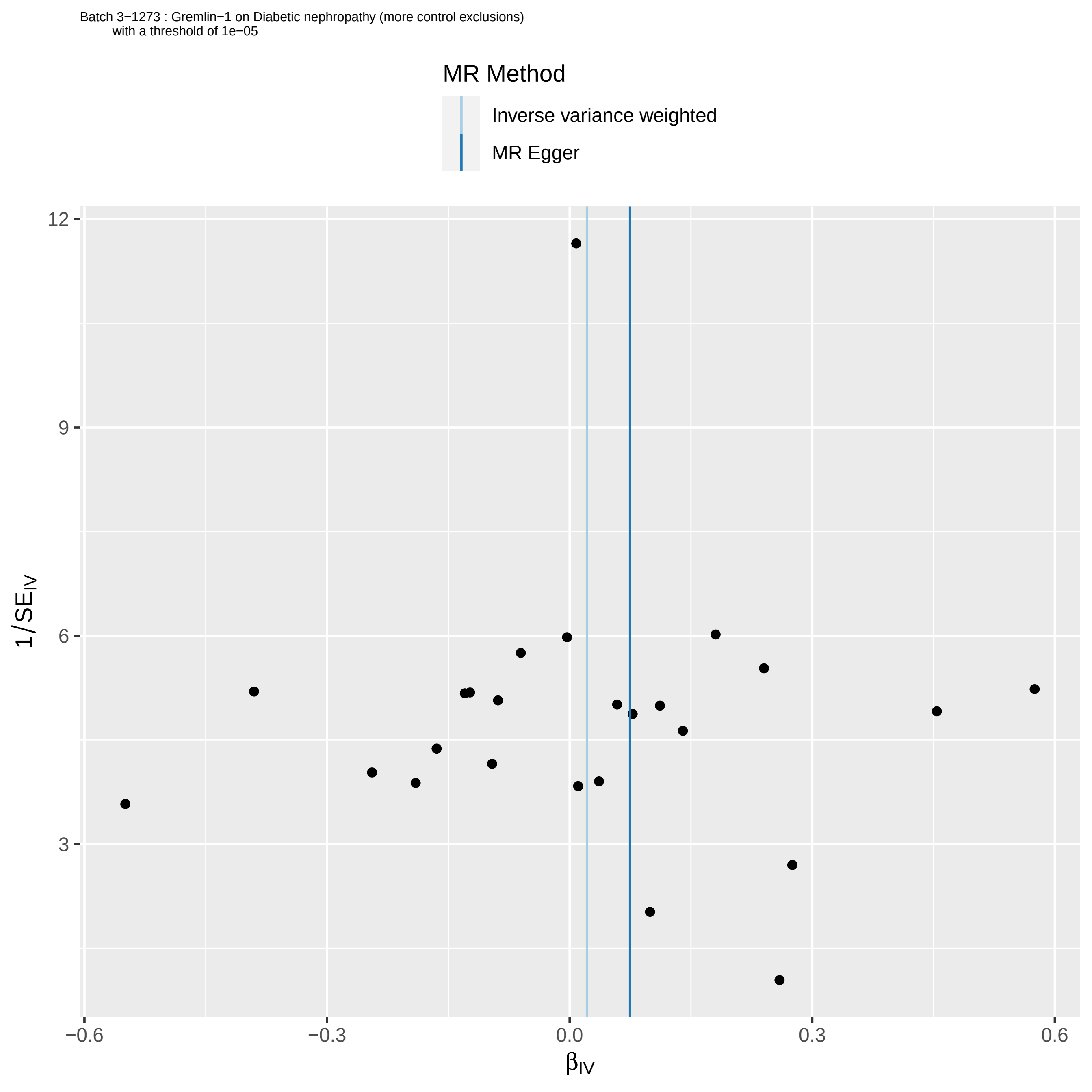

Batch 3−1273 : Gremlin−1 on Diabetic nephropathy (more control exclusions)
with a threshold of 1e−05
MR Method
Inverse variance weighted
MR Egger
12
9
6
3
1/SE$_{IV}$
−0.6
−0.3
0.0
0.3
0.6
β$_{IV}$

Batch 3−1274 : Gremlin−2 on Diabetic nephropathy (more control exclusions)
with a threshold of 1e−05

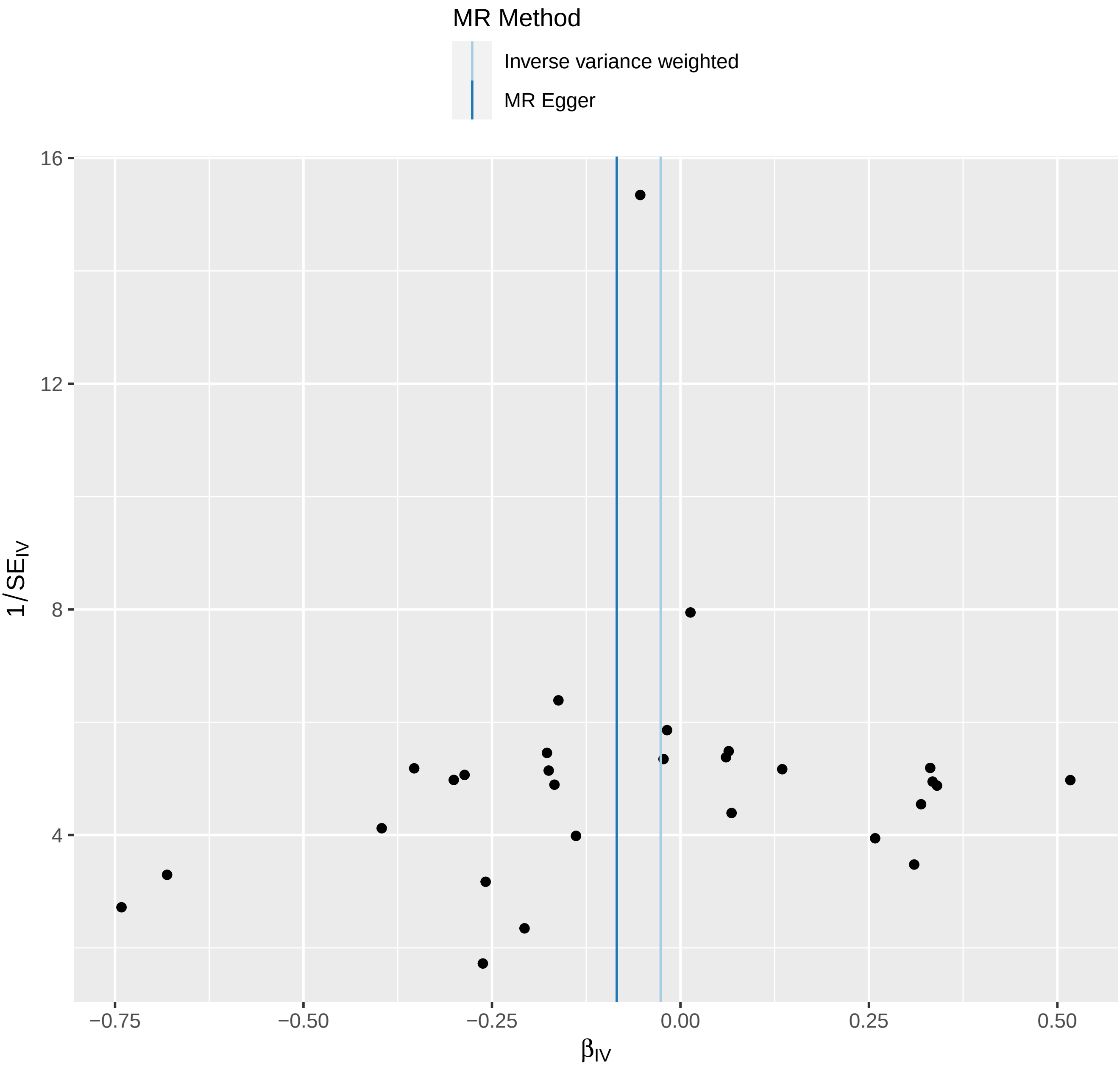

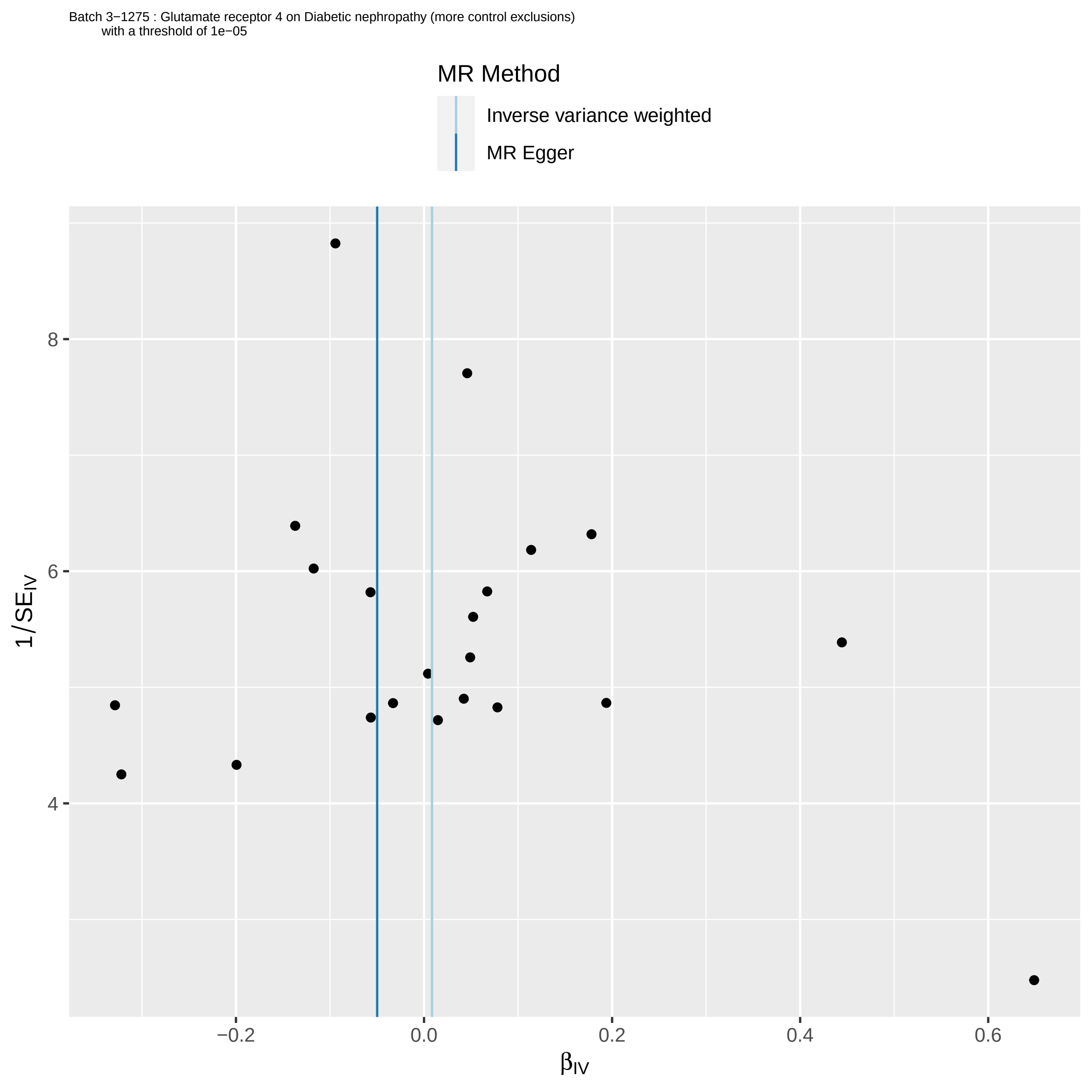

Batch 3−1275 : Glutamate receptor 4 on Diabetic nephropathy (more control exclusions)
with a threshold of 1e−05
MR Method
Inverse variance weighted
MR Egger
8
6
4
1/SE_IV
−0.2
0.0
0.2
0.4
0.6
β_IV

Batch 3−1276 : Glutamate receptor ionotropic, delta−2 on Diabetic nephropathy (more control exclusions) with a threshold of 1e−05

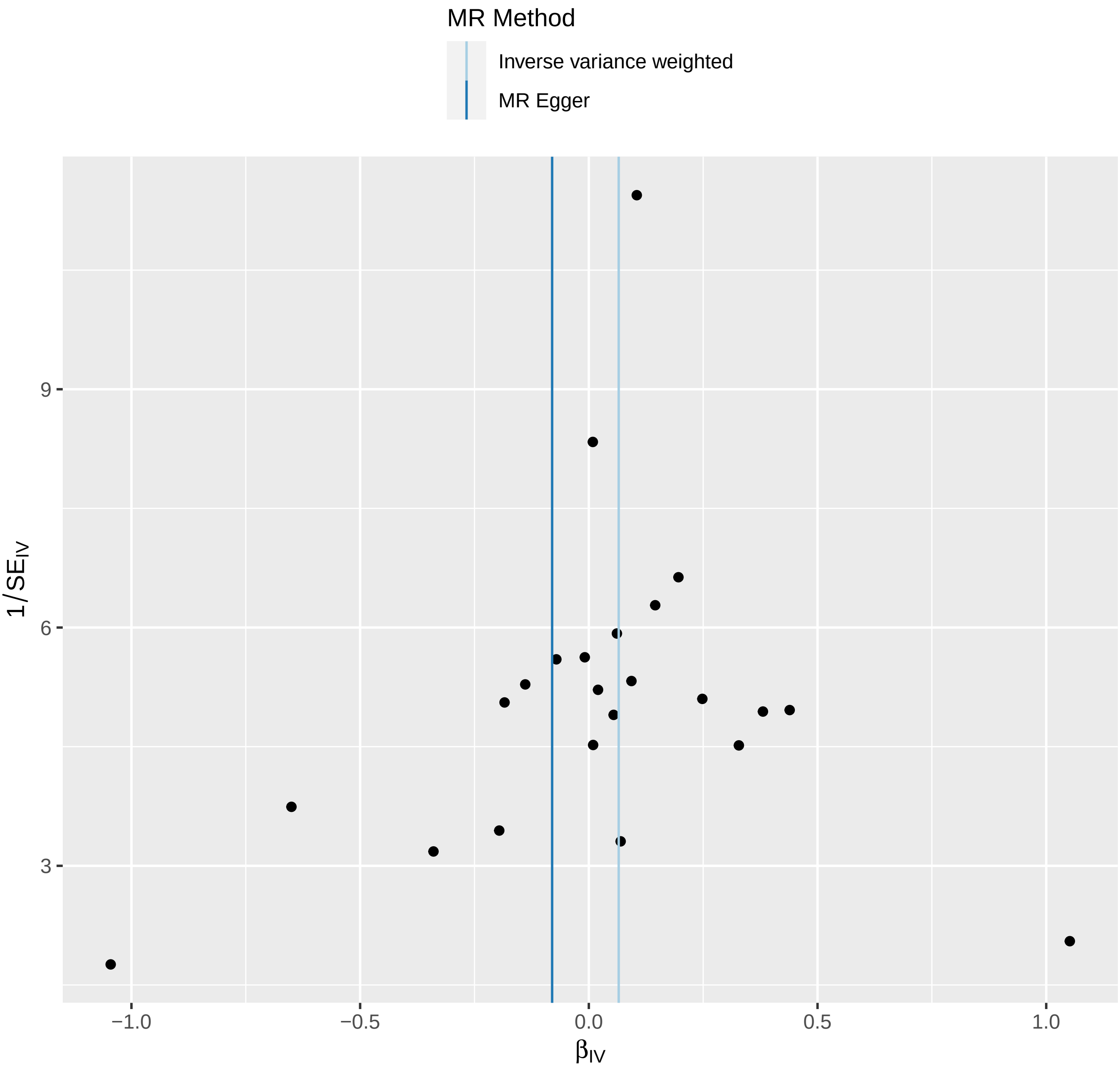

Batch 3−1277 : Granulins on Diabetic nephropathy (more control exclusions)
with a threshold of 1e−05

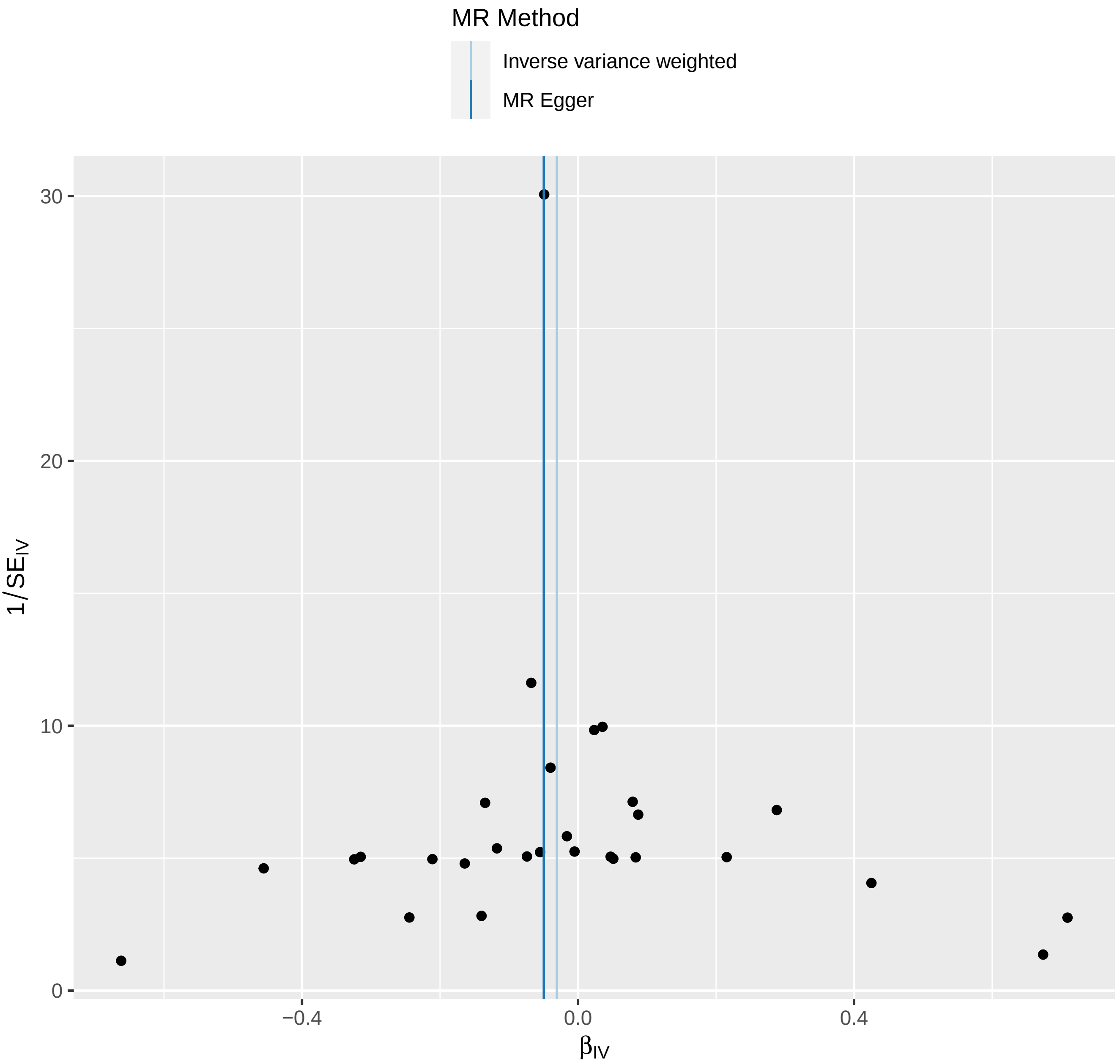

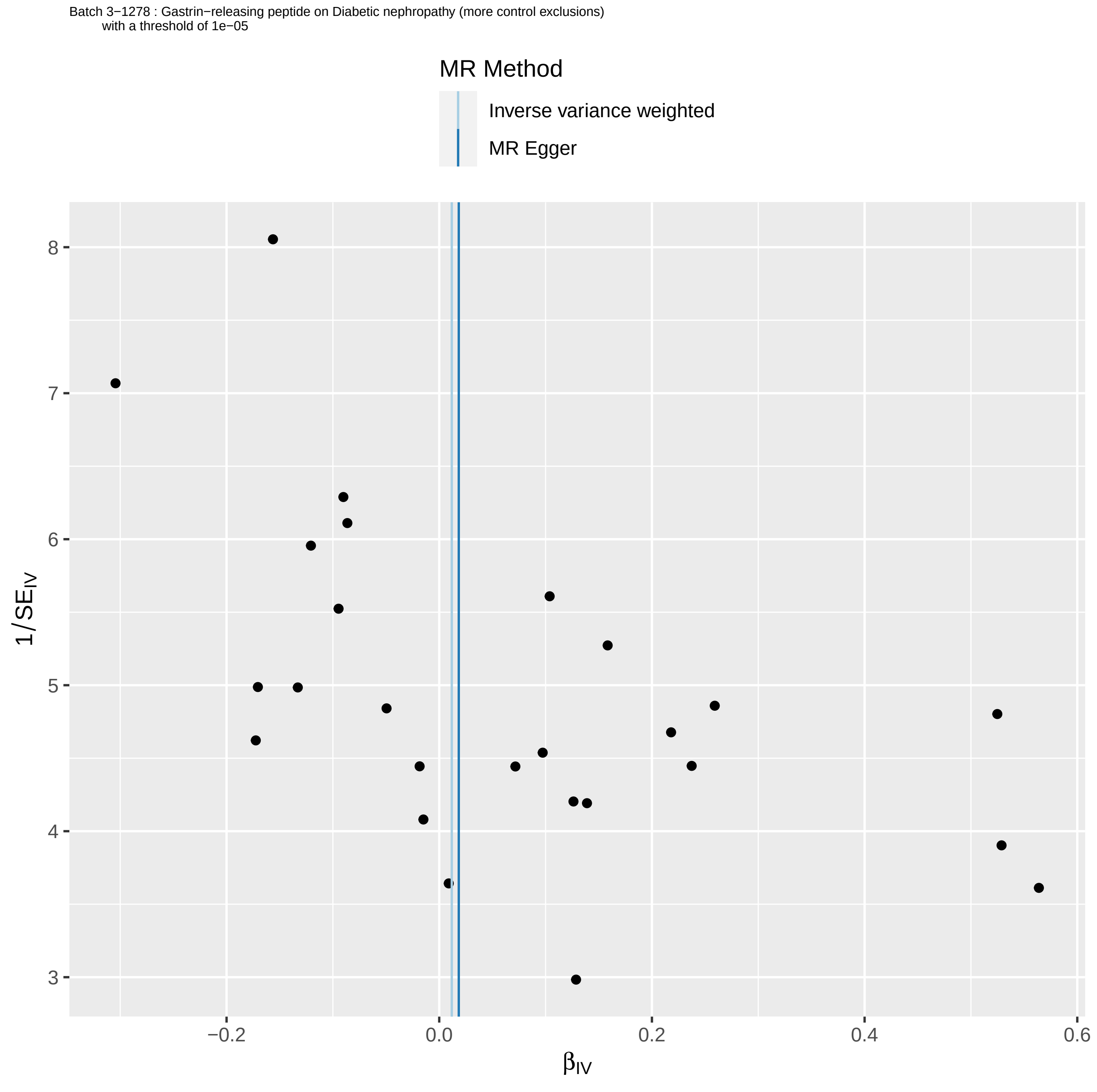

Batch 3−1278 : Gastrin−releasing peptide on Diabetic nephropathy (more control exclusions)
with a threshold of 1e−05
MR Method
Inverse variance weighted
MR Egger
1/SE_IV
8
7
6
5
4
3
−0.2
0.0
0.2
0.4
0.6
β_IV

Batch 3−1279 : Gastrin−releasing peptide on Diabetic nephropathy (more control exclusions)
with a threshold of 1e−05

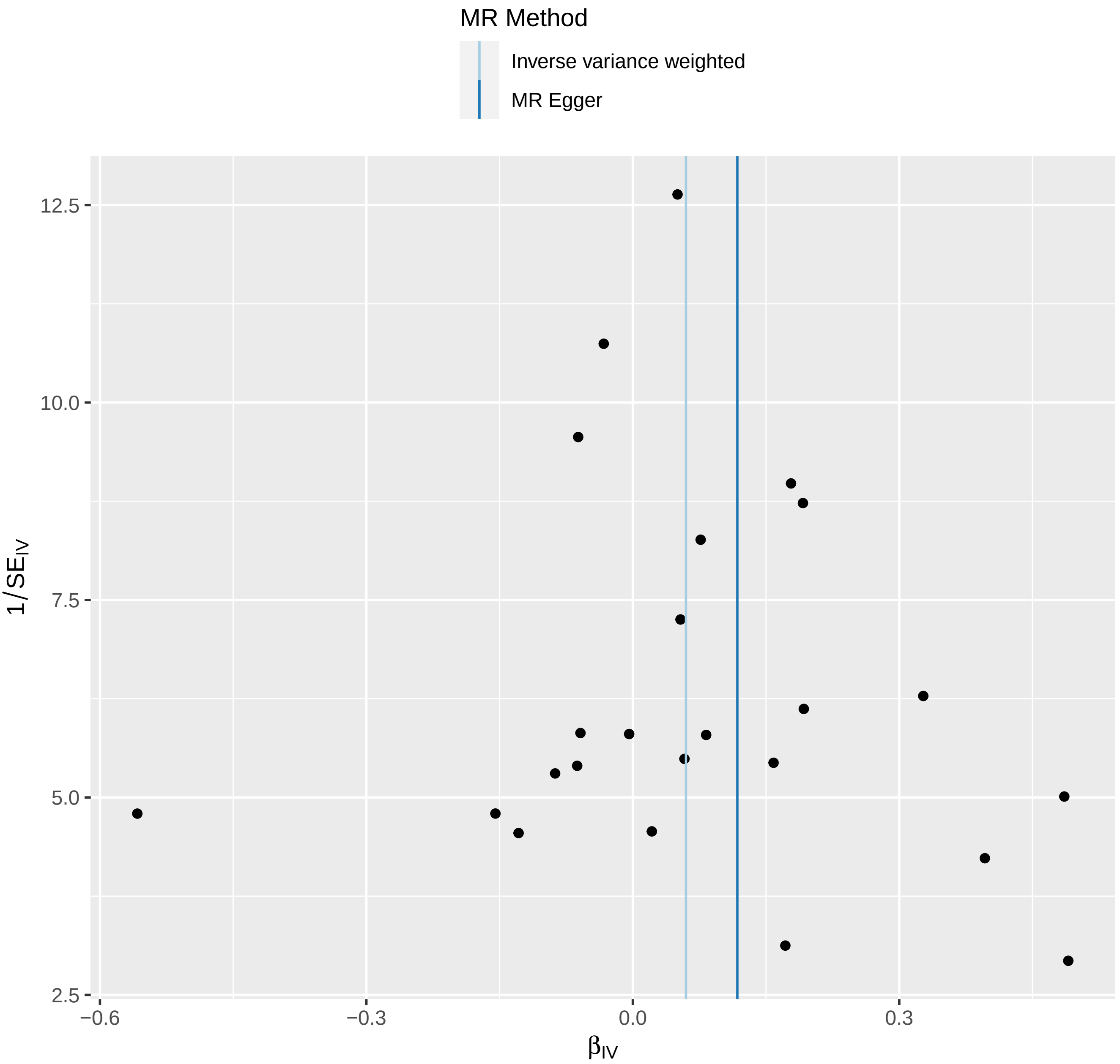

Batch 3−1280 : Gastrin−releasing peptide on Diabetic nephropathy (more control exclusions)
with a threshold of 1e−05

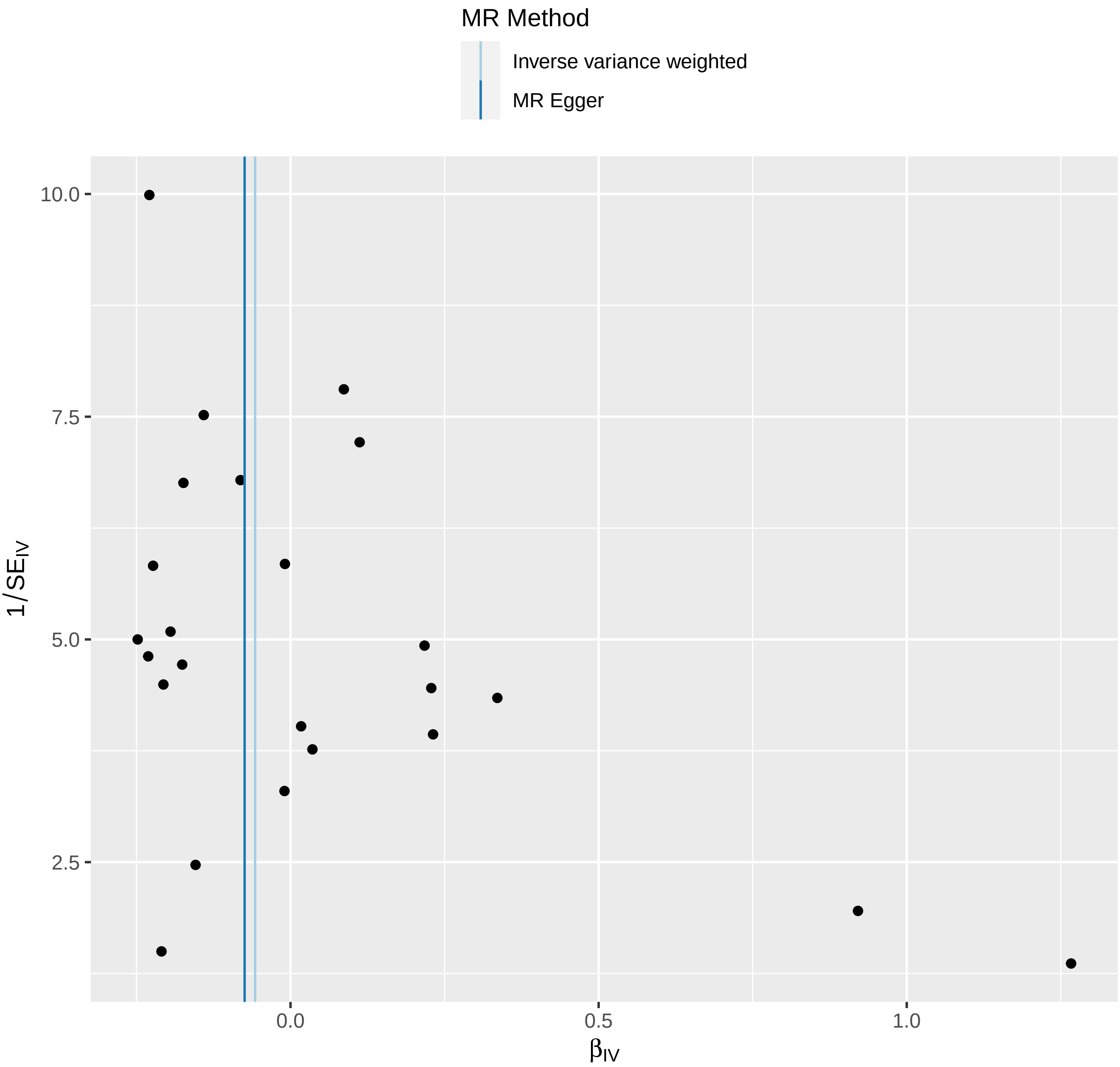

Batch 3−1281 : GrpE protein homolog 1, mitochondrial on Diabetic nephropathy (more control exclusions) with a threshold of 1e−05

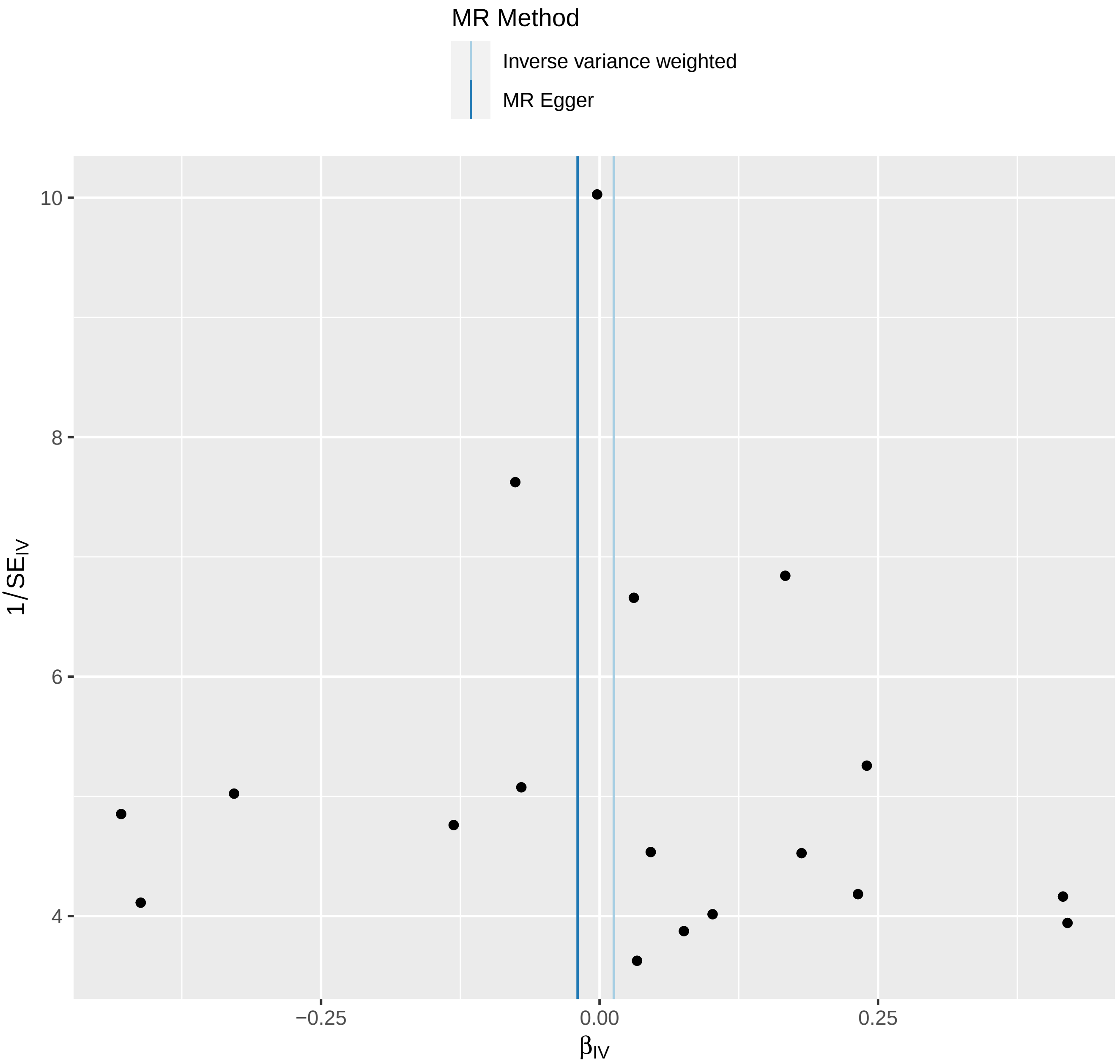

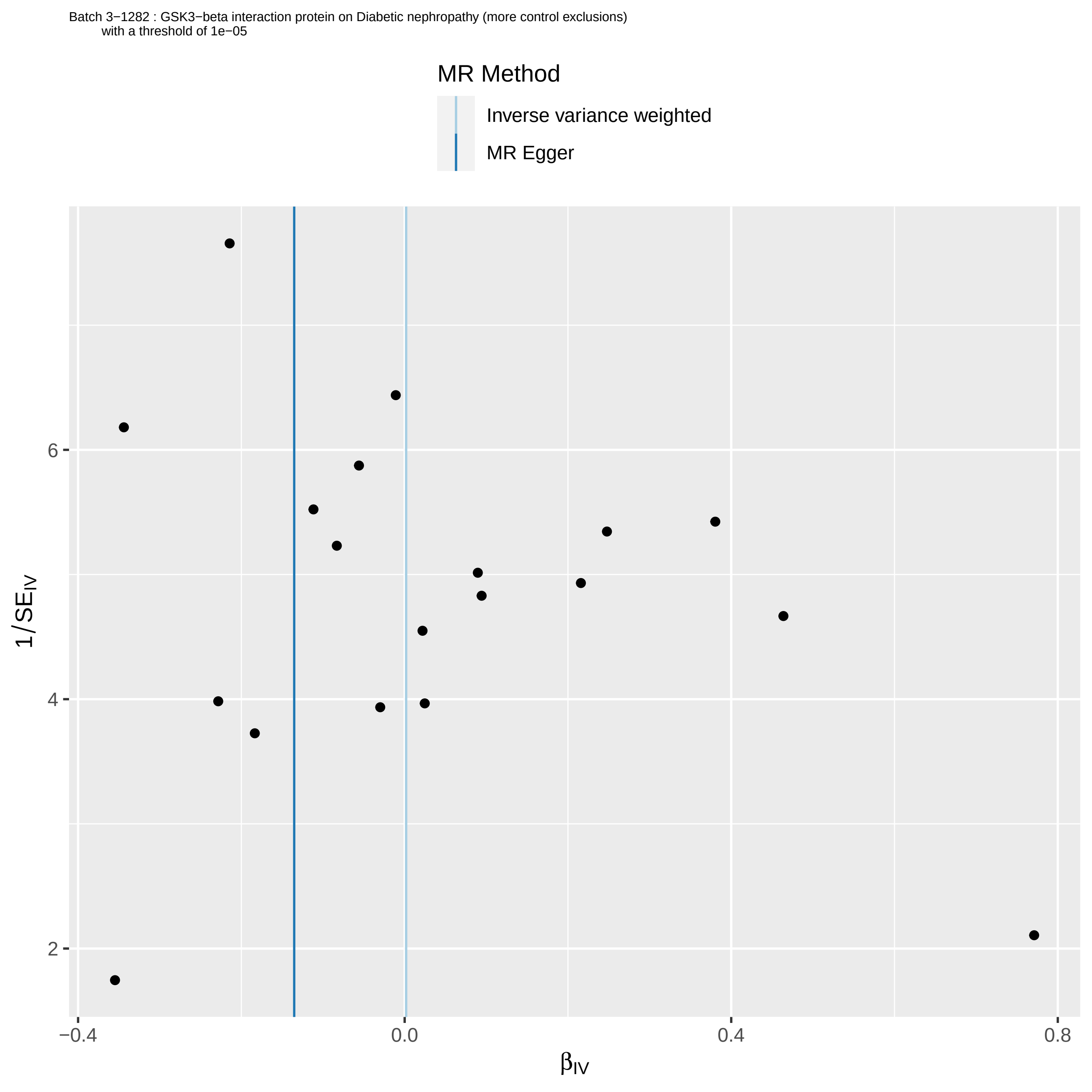
Batch 3−1282 : GSK3−beta interaction protein on Diabetic nephropathy (more control exclusions)
with a threshold of 1e−05
MR Method
Inverse variance weighted
MR Egger
1/SE_IV
β_IV
−0.4
0.0
0.4
0.8
2
4
6

Batch 3−1283 : Glutathione S−transferase A1 on Diabetic nephropathy (more control exclusions)
with a threshold of 1e−05

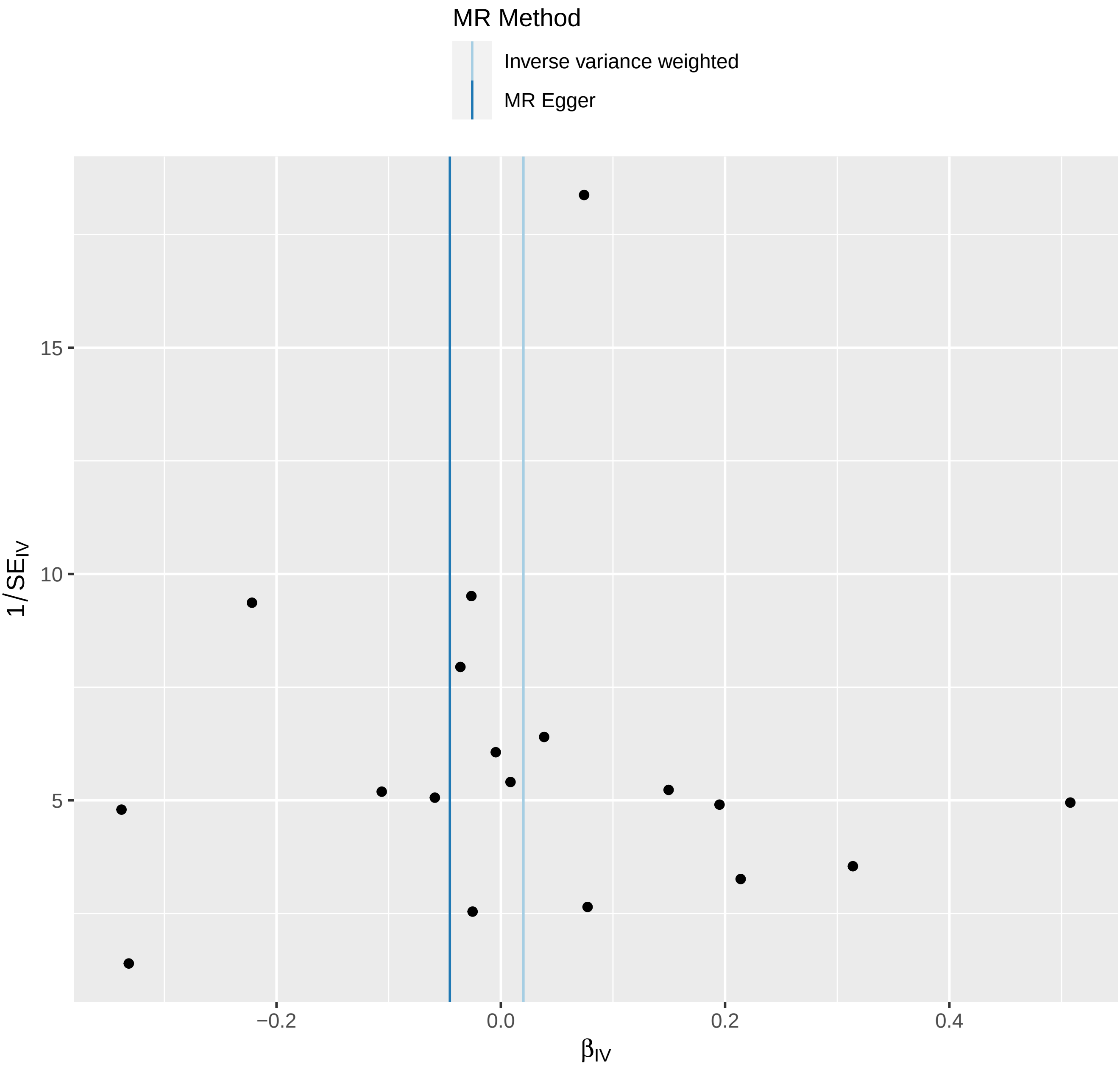

Batch 3−1284 : Glutathione S−transferase A3 on Diabetic nephropathy (more control exclusions)
with a threshold of 1e−05

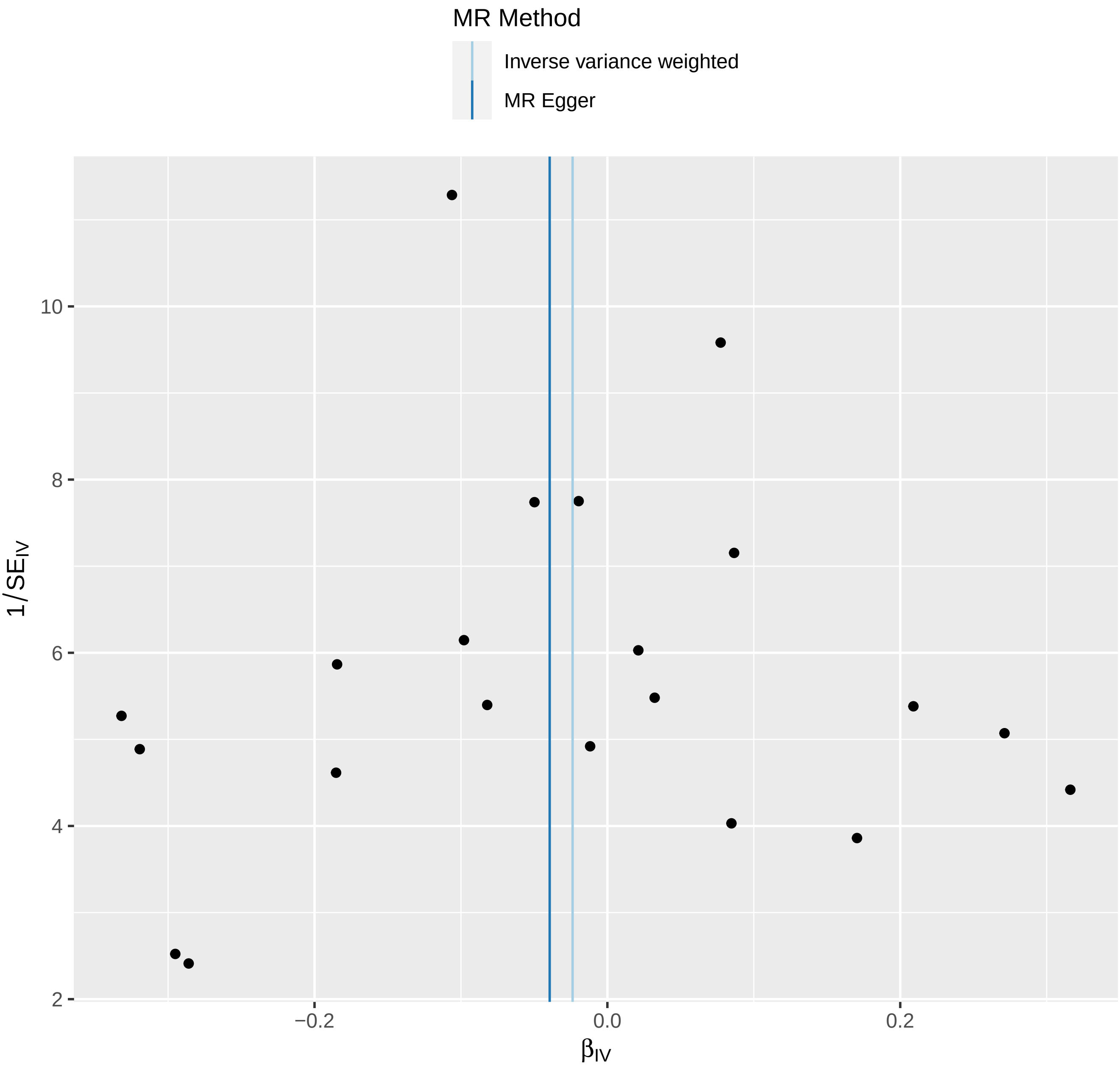

Batch 3−1285 : Glutathione S−transferase A4 on Diabetic nephropathy (more control exclusions)
with a threshold of 1e−05

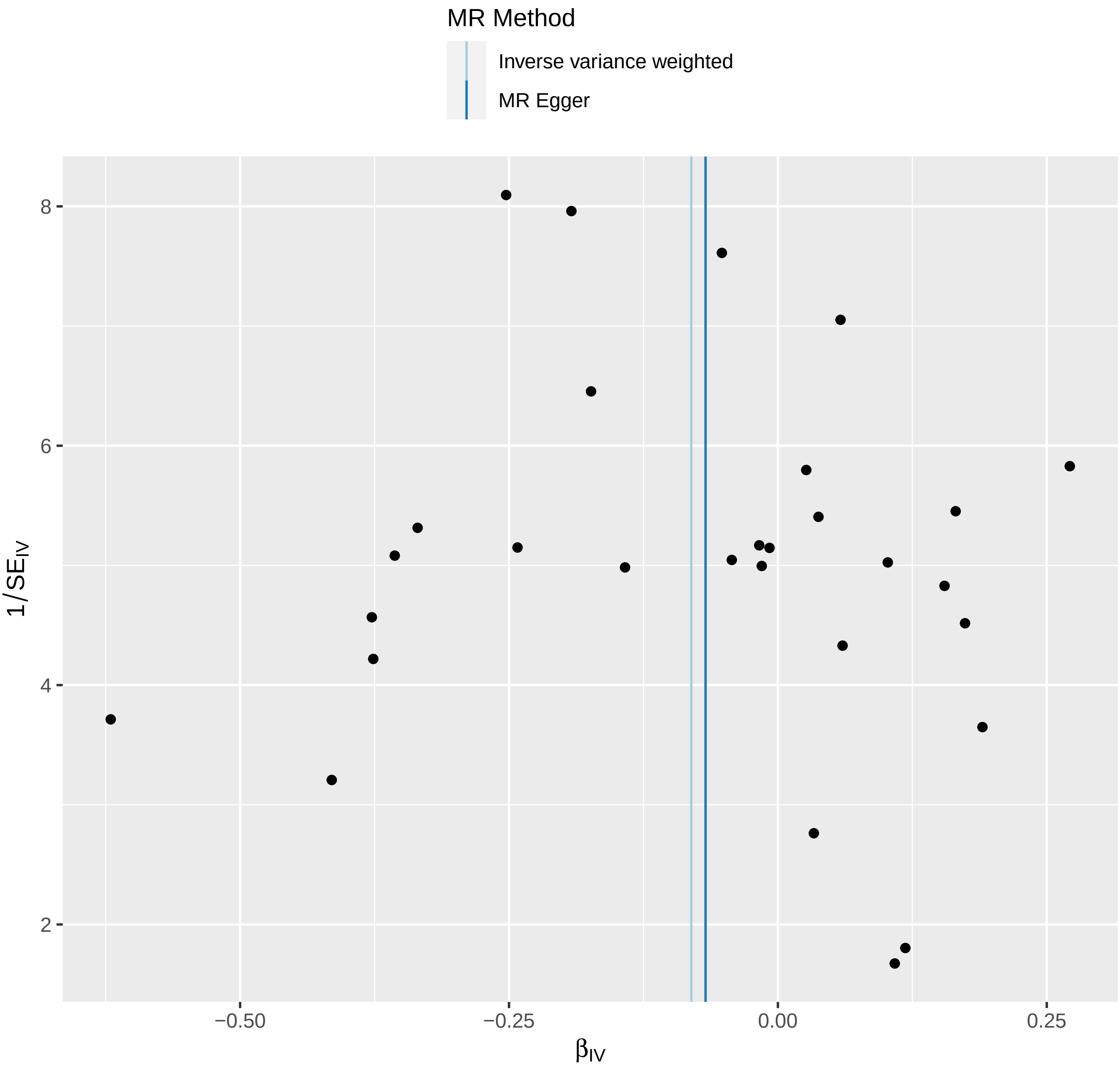

Batch 3−1286 : Glutathione S−transferase kappa 1 on Diabetic nephropathy (more control exclusions)
with a threshold of 1e−05

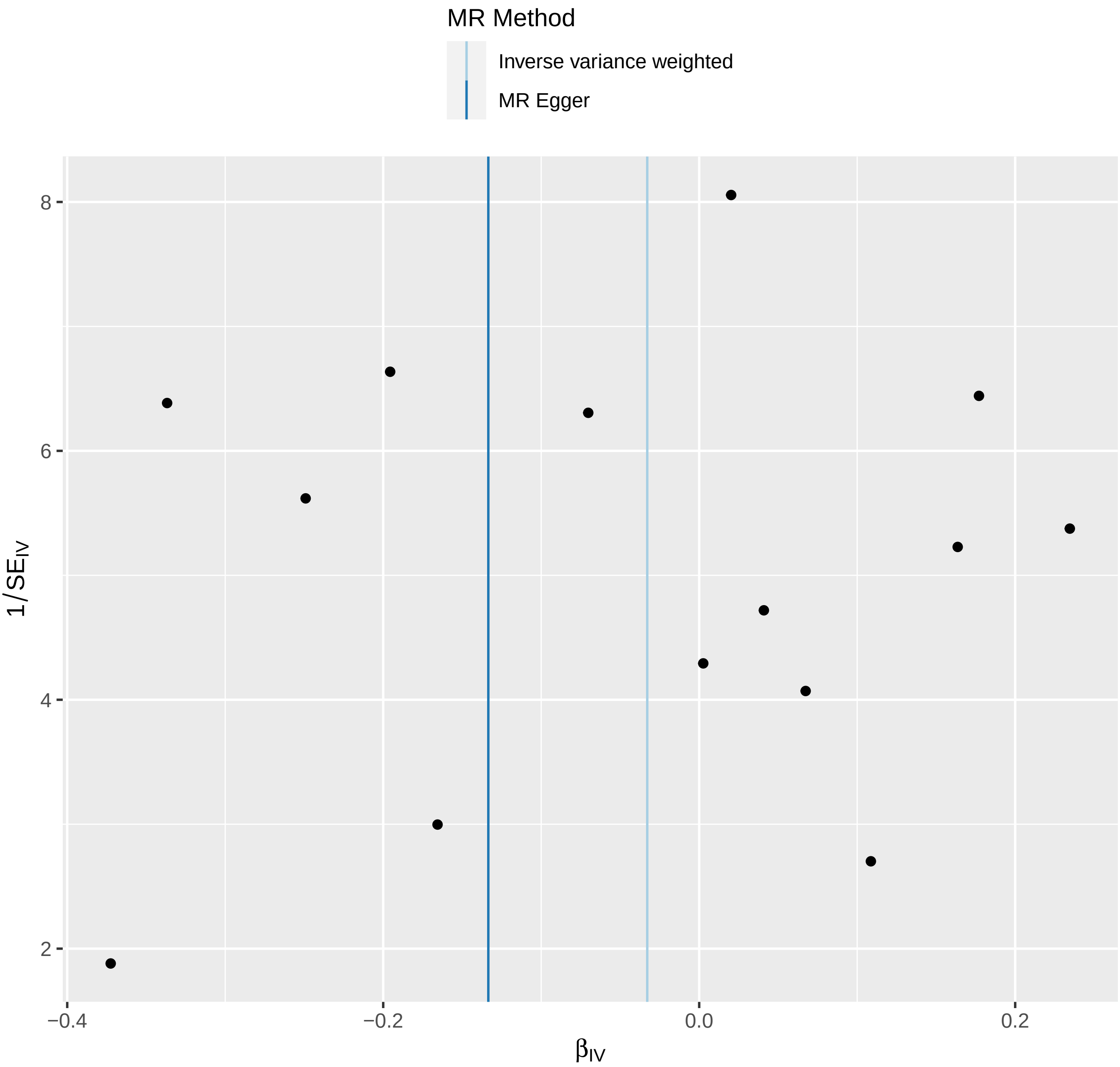

Batch 3−1287 : Glutathione S−transferase Mu 1 on Diabetic nephropathy (more control exclusions) with a threshold of 1e−05

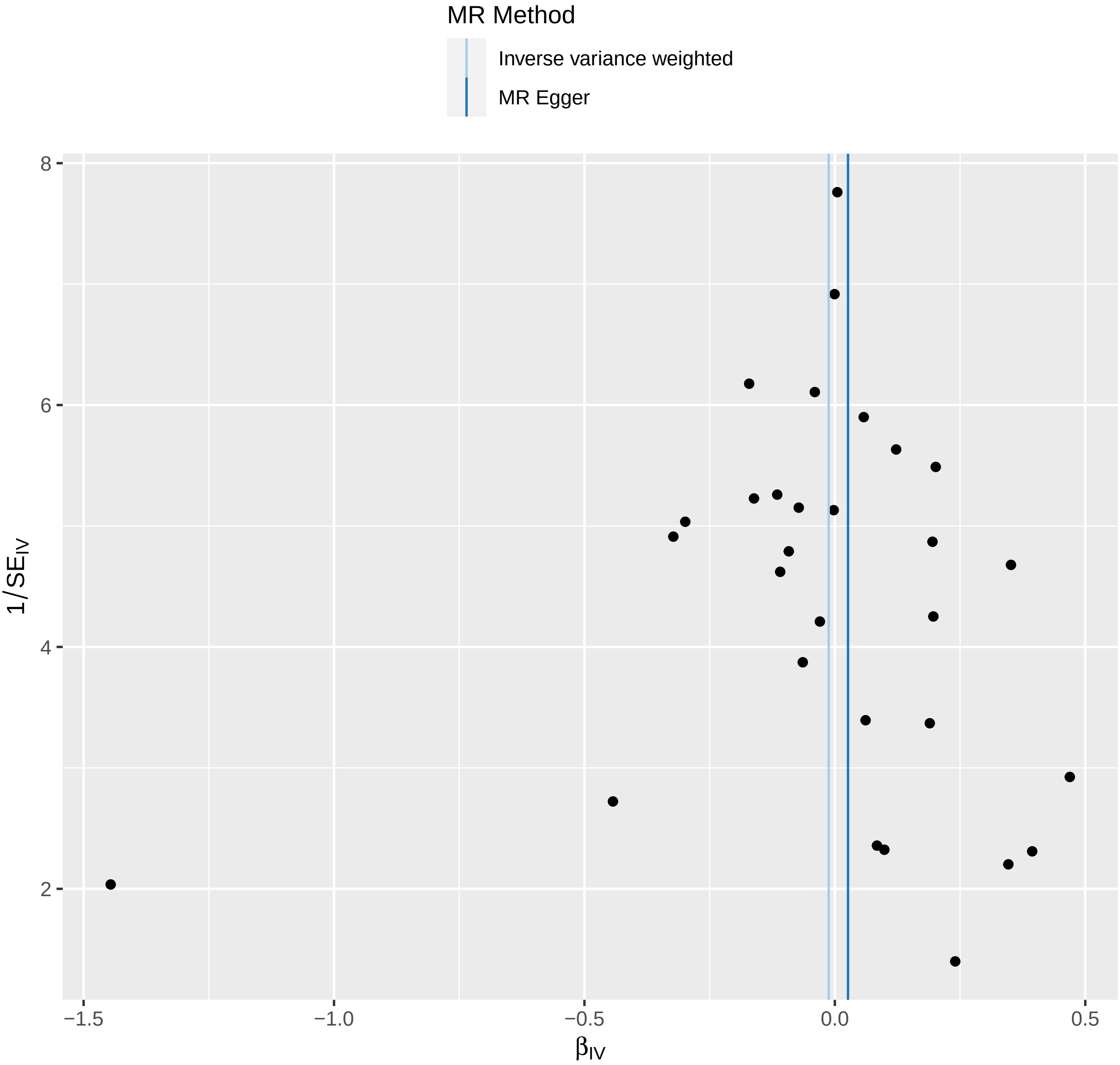

Batch 3−1288 : Glutathione S−transferase omega−1 on Diabetic nephropathy (more control exclusions)
with a threshold of 1e−05

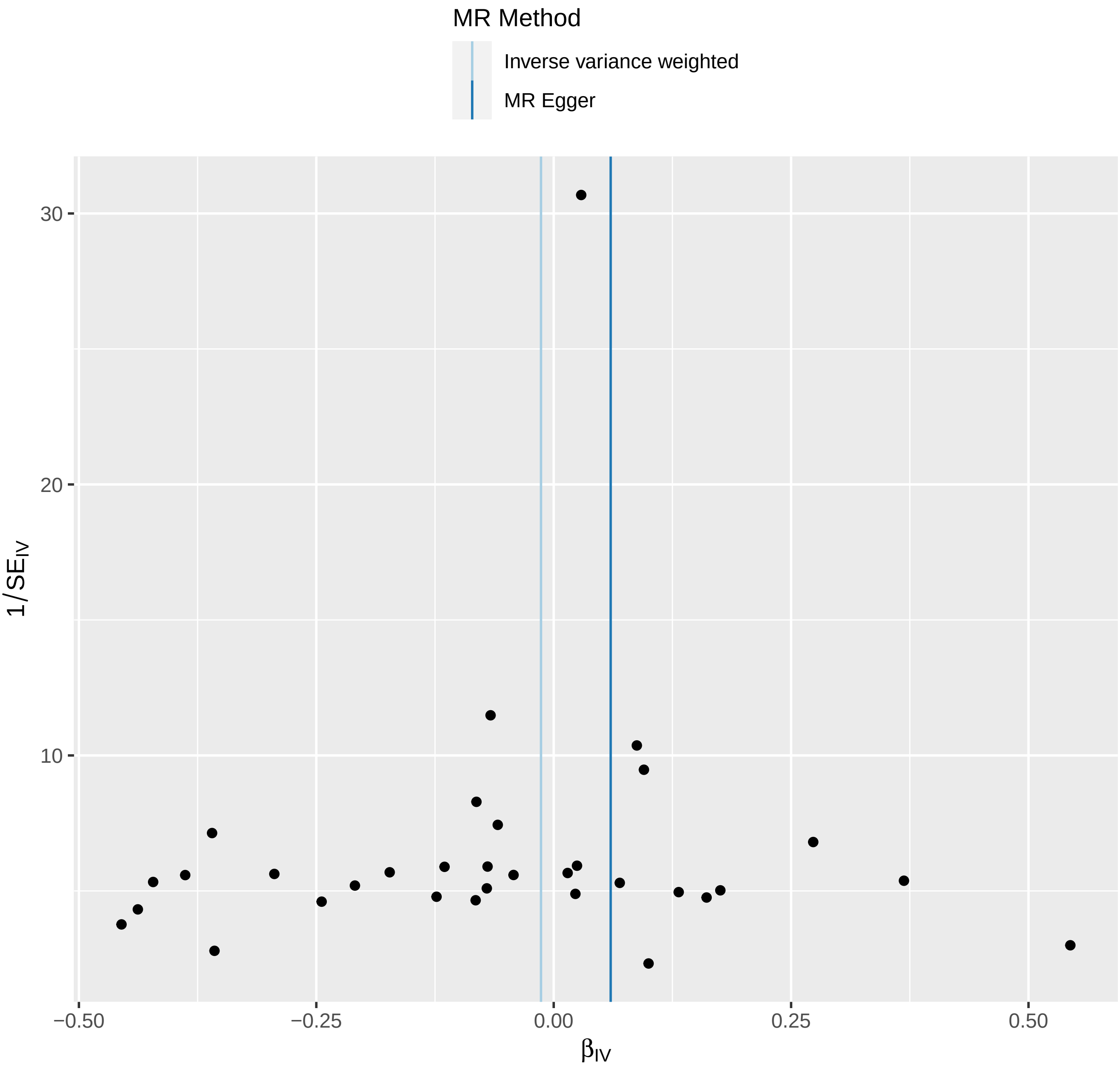

Batch 3−1289 : Glutathione S−transferase P on Diabetic nephropathy (more control exclusions)
with a threshold of 1e−05

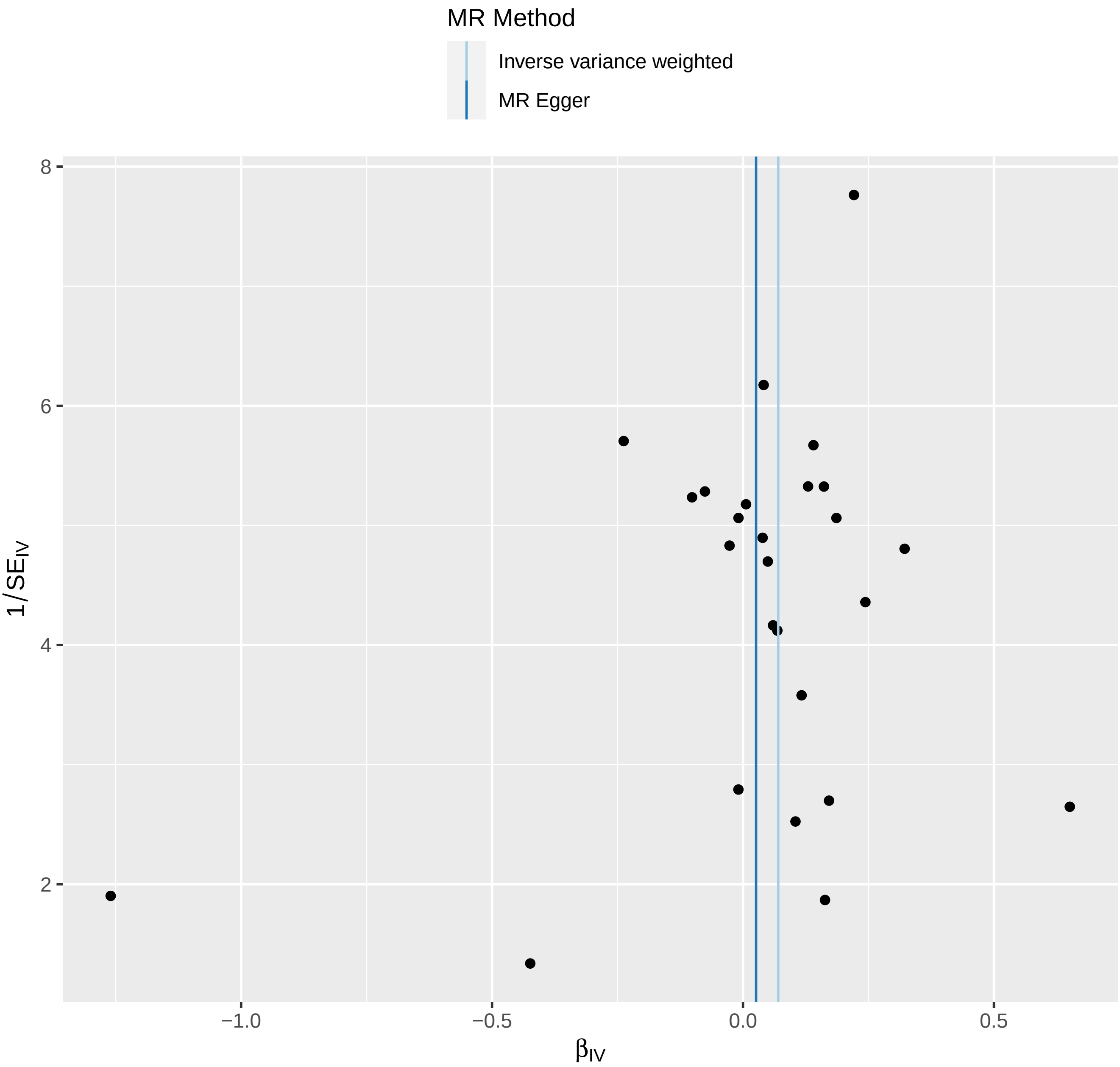

Batch 3−1290 : Glutathione S−transferase P on Diabetic nephropathy (more control exclusions)
with a threshold of 1e−05

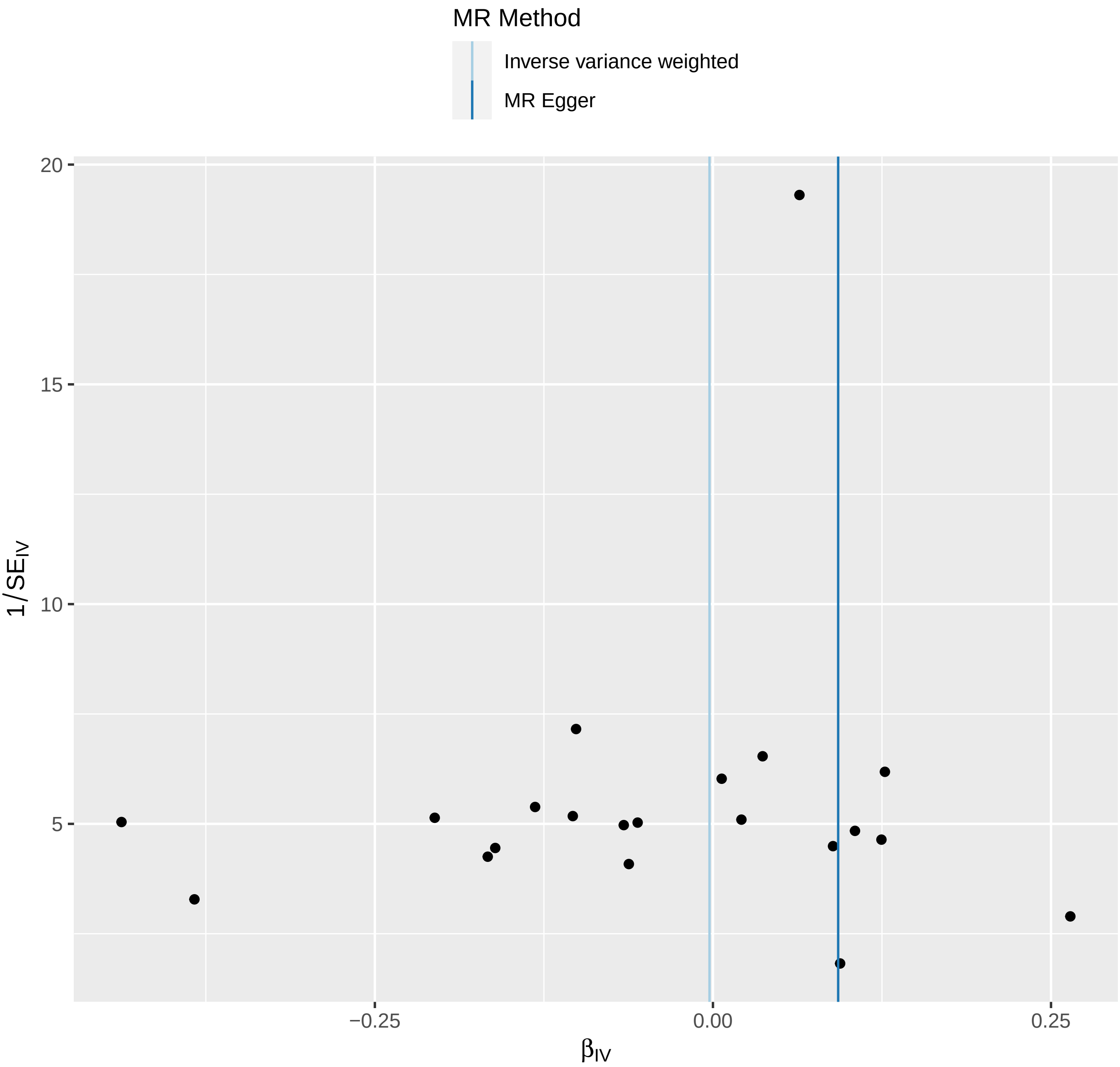

Batch 3−1291 : Glutathione S−transferase theta−2B on Diabetic nephropathy (more control exclusions)
with a threshold of 1e−05

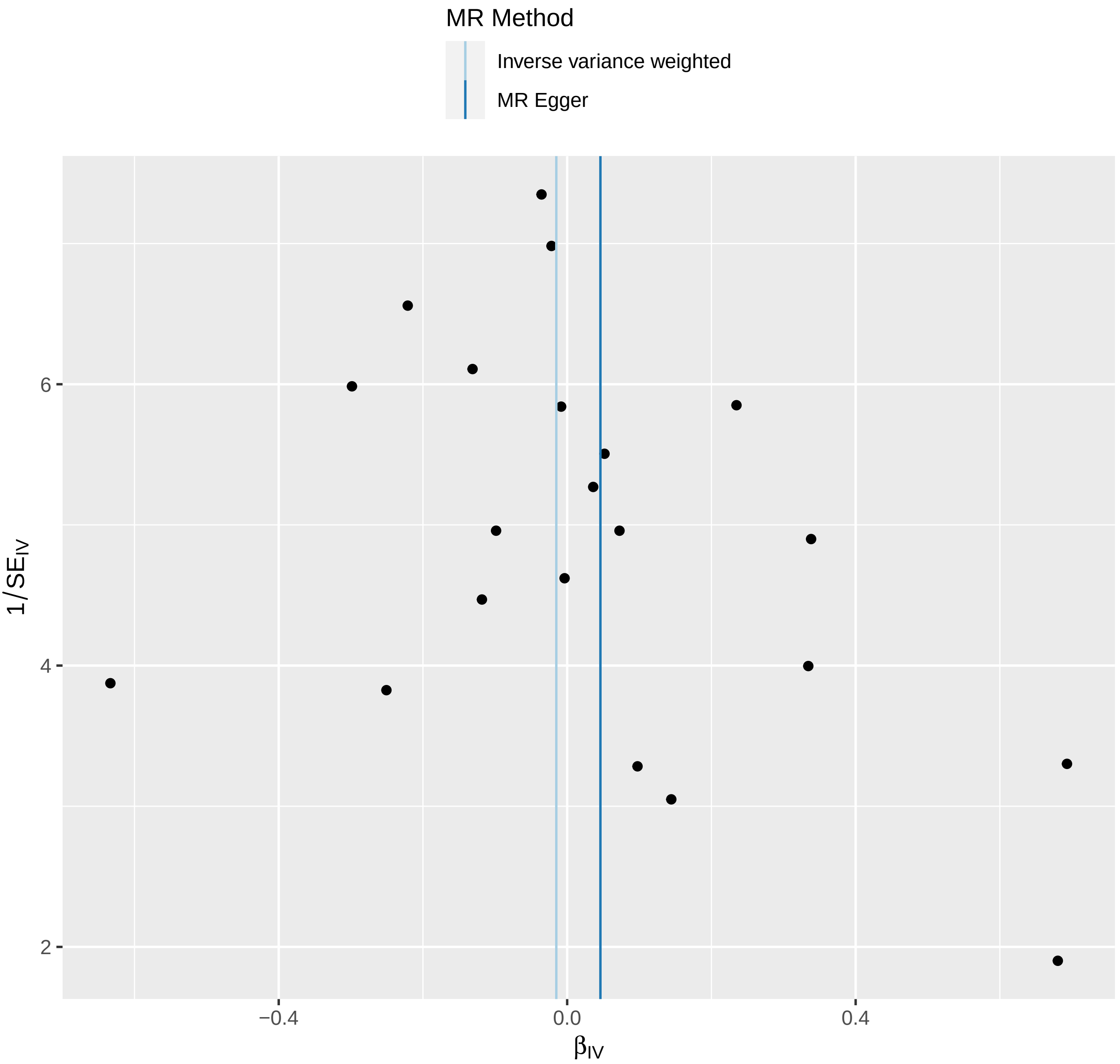

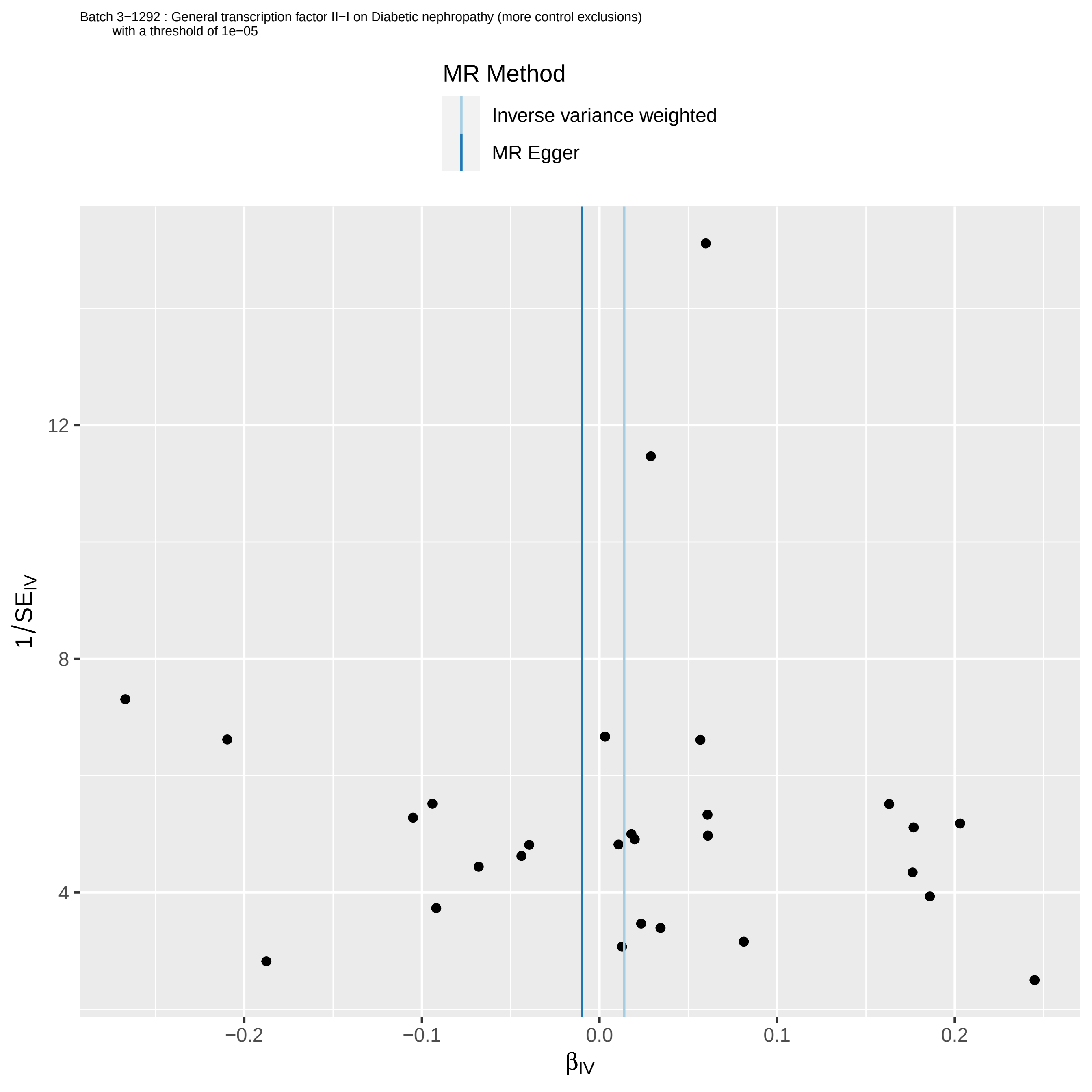

Batch 3−1292 : General transcription factor II−I on Diabetic nephropathy (more control exclusions)
with a threshold of 1e−05
MR Method
Inverse variance weighted
MR Egger
12
8
4
1/SE_IV
−0.2
−0.1
0.0
0.1
0.2
β_IV

Batch 3−1293 : Guanylyl cyclase−activating protein 1 on Diabetic nephropathy (more control exclusions)
with a threshold of 1e−05

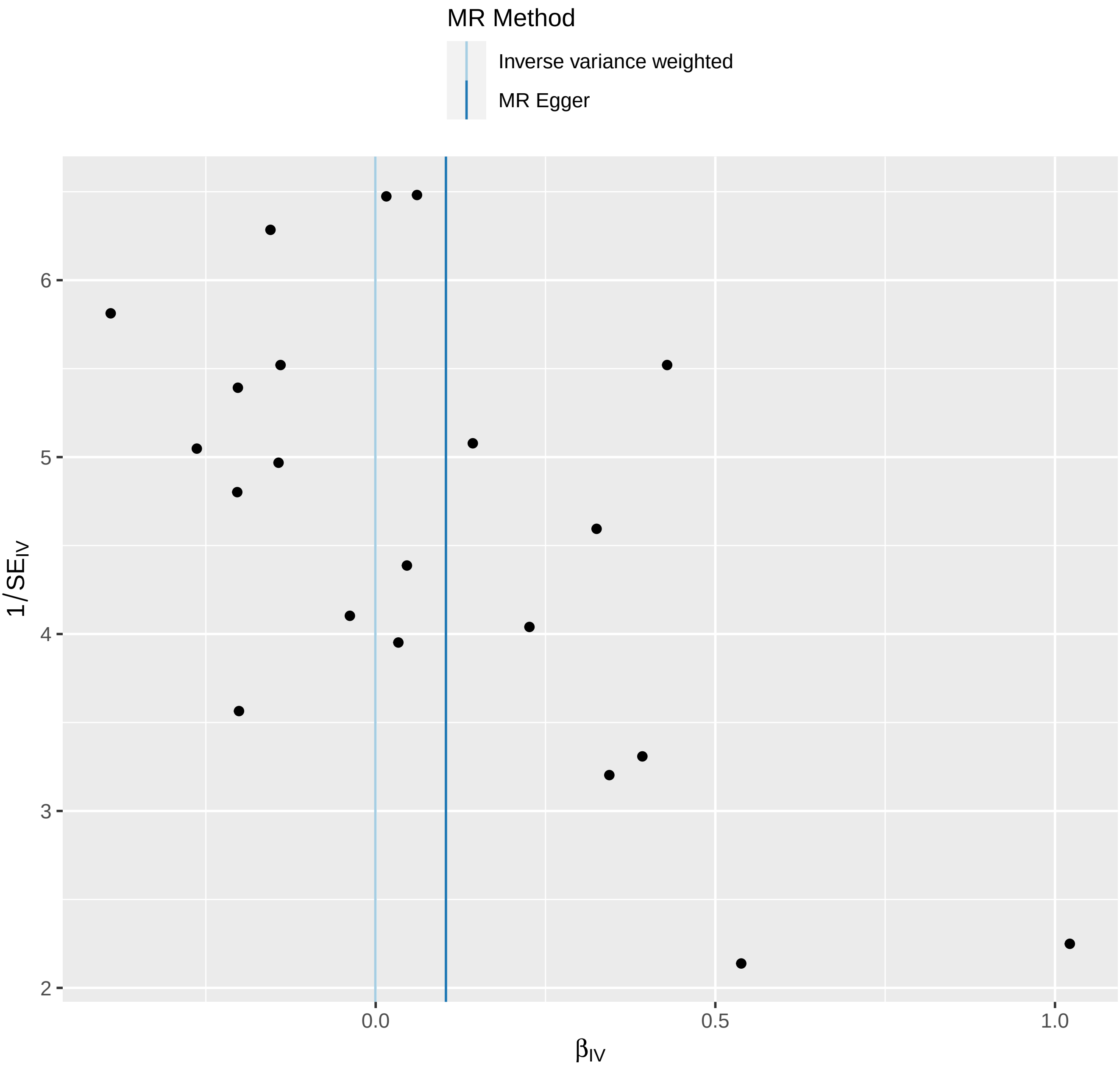

Batch 3−1294 : Guanylate cyclase activator 2B on Diabetic nephropathy (more control exclusions)
with a threshold of 1e−05

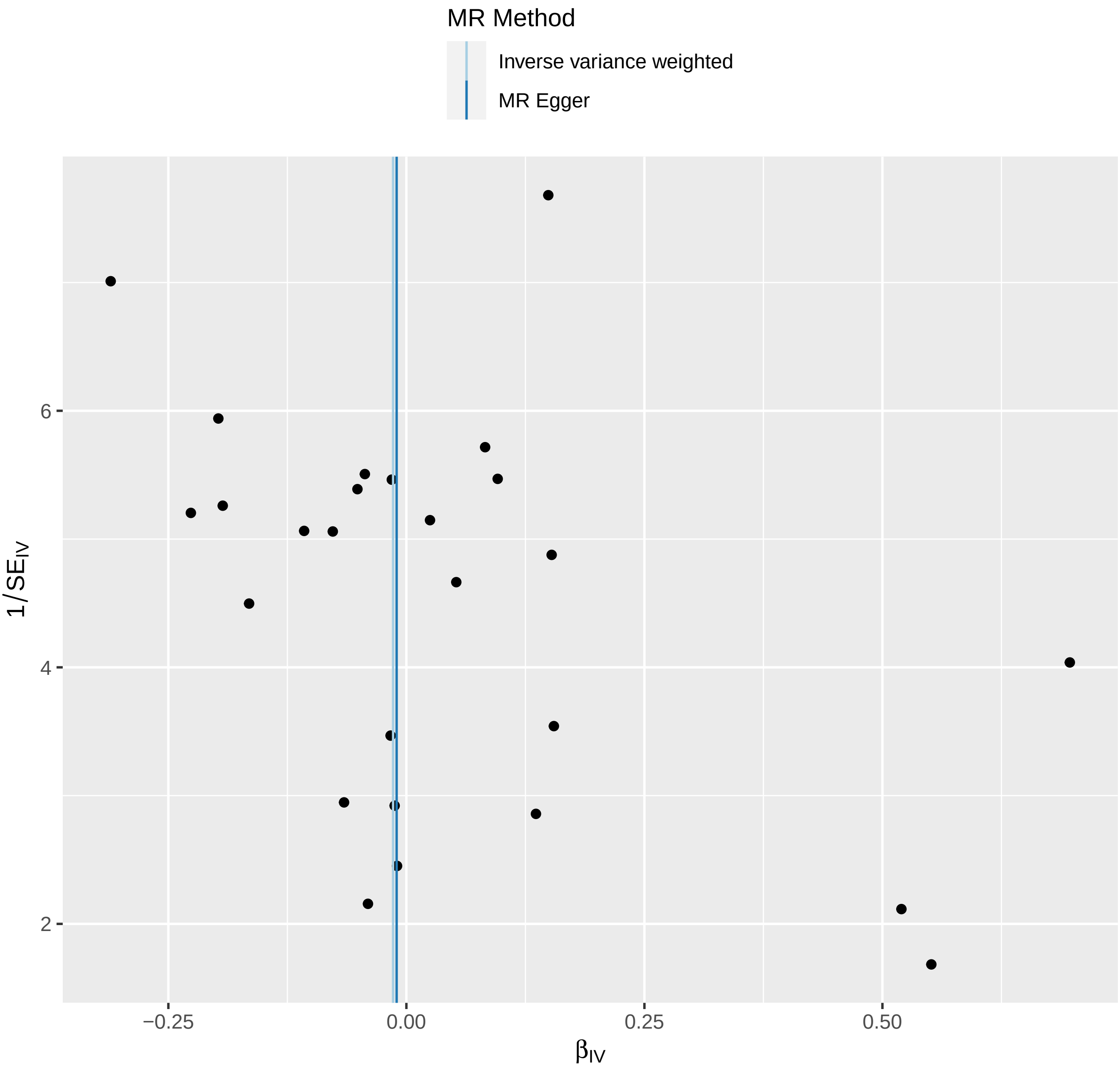

Batch 3−1295 : Glucoside xylosyltransferase 1 on Diabetic nephropathy (more control exclusions)
with a threshold of 1e−05

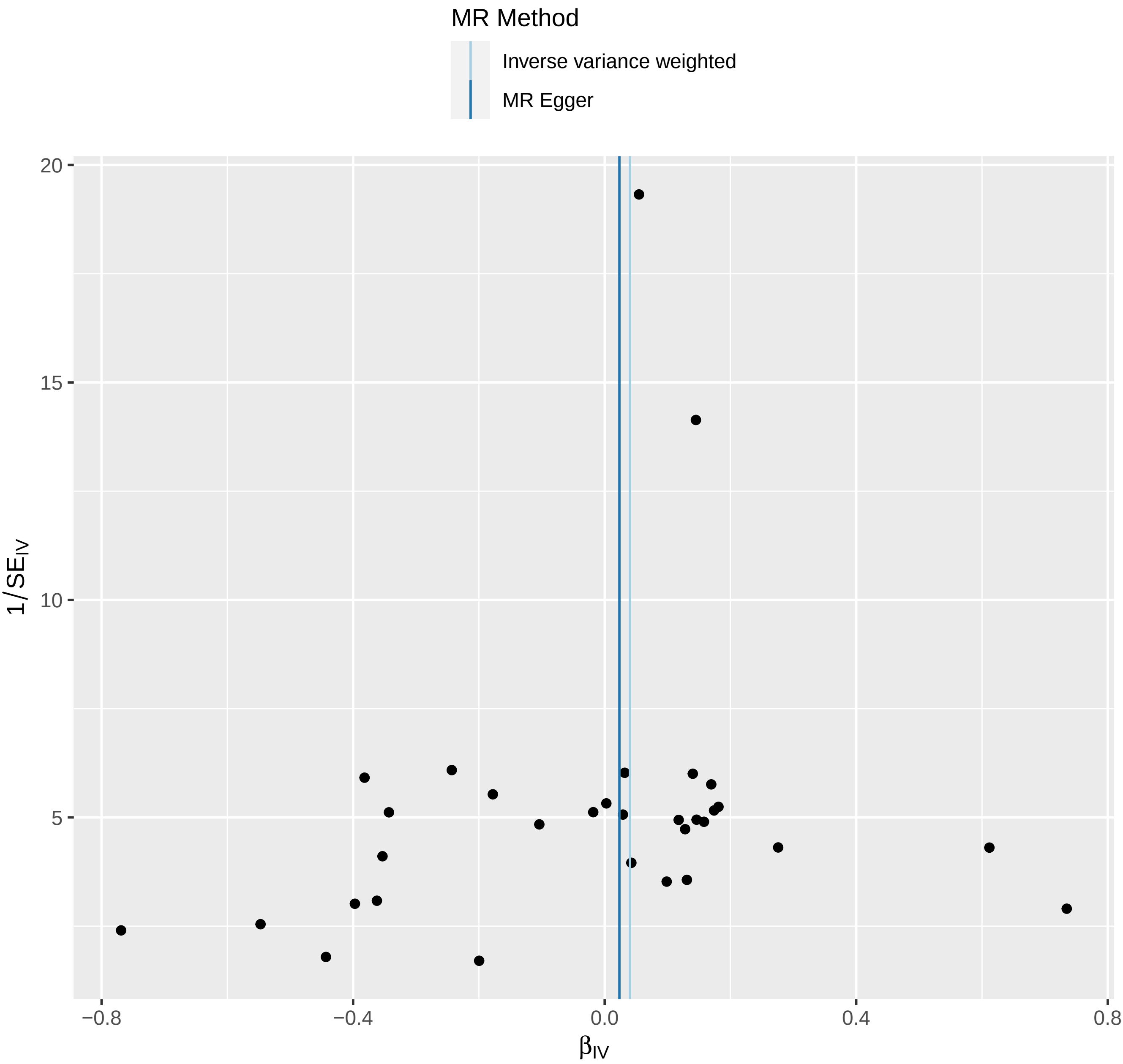

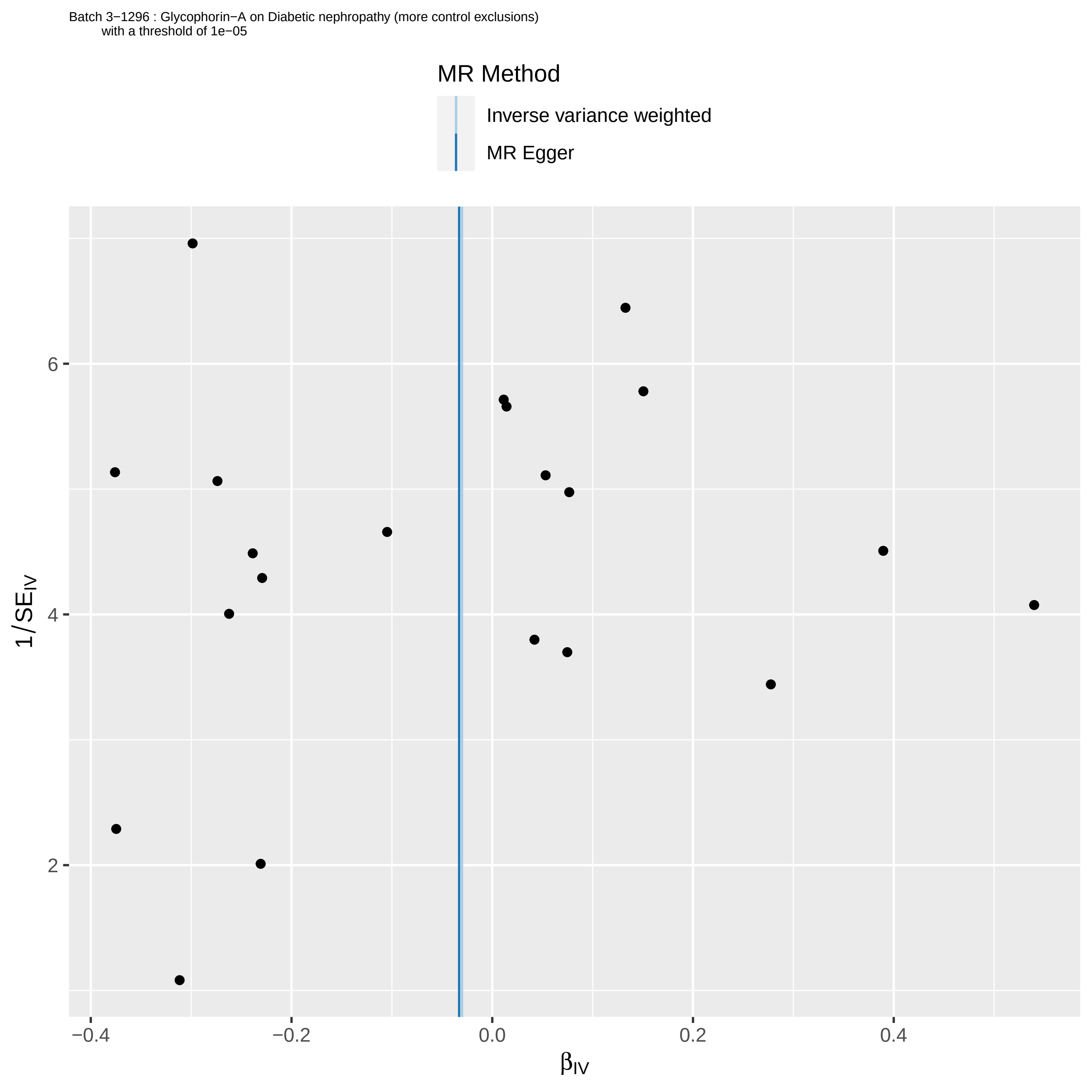

Batch 3−1296 : Glycophorin−A on Diabetic nephropathy (more control exclusions)
with a threshold of 1e−05
MR Method
Inverse variance weighted
MR Egger
6
4
2
1/SE_IV
−0.4
−0.2
0.0
0.2
0.4
β_IV

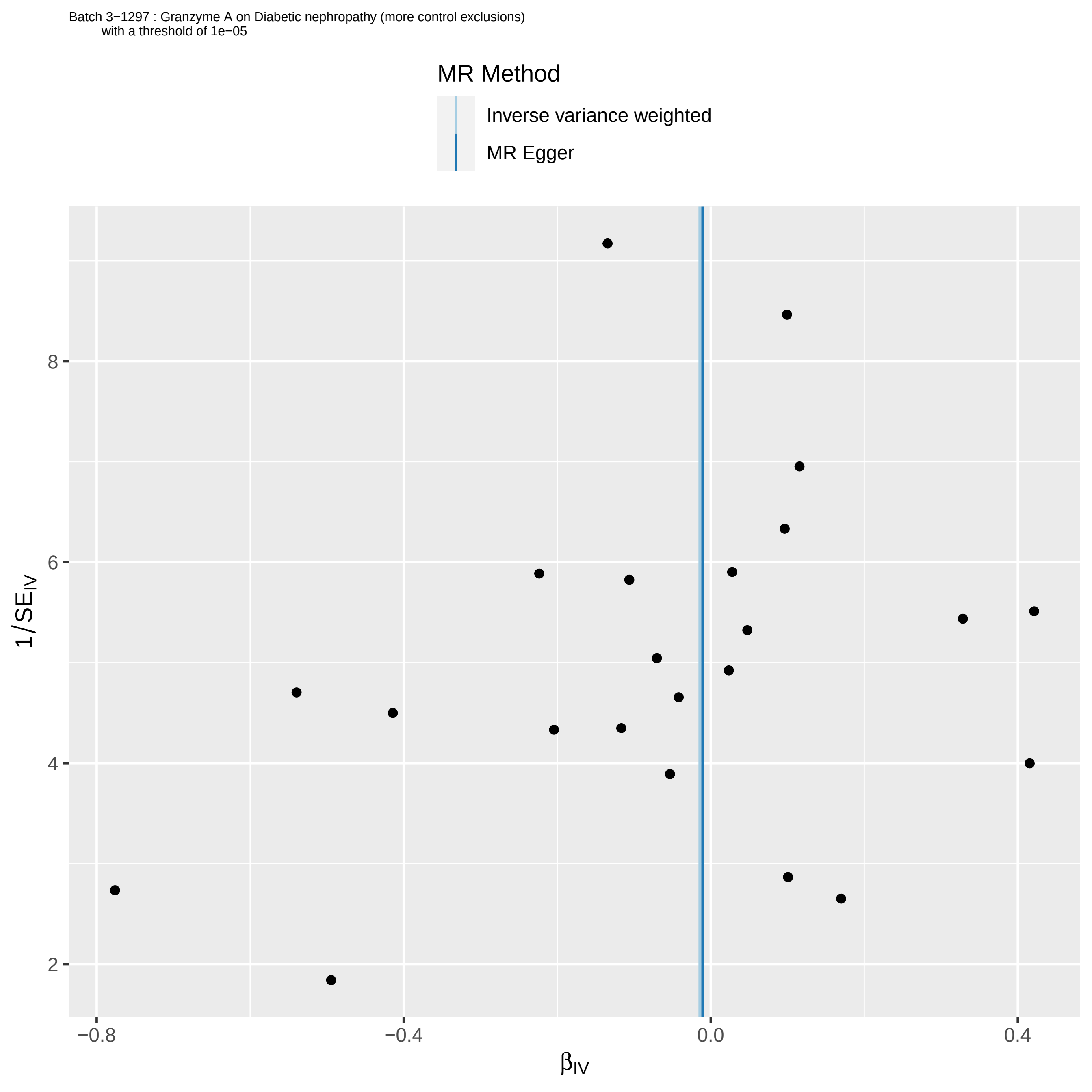

Batch 3−1297 : Granzyme A on Diabetic nephropathy (more control exclusions)
with a threshold of 1e−05
MR Method
Inverse variance weighted
MR Egger
8
6
4
2
−0.8
−0.4
0.0
0.4
$1/SE_{IV}$
β_{IV}

Batch 3−1298 : Granzyme B on Diabetic nephropathy (more control exclusions)
with a threshold of 1e−05

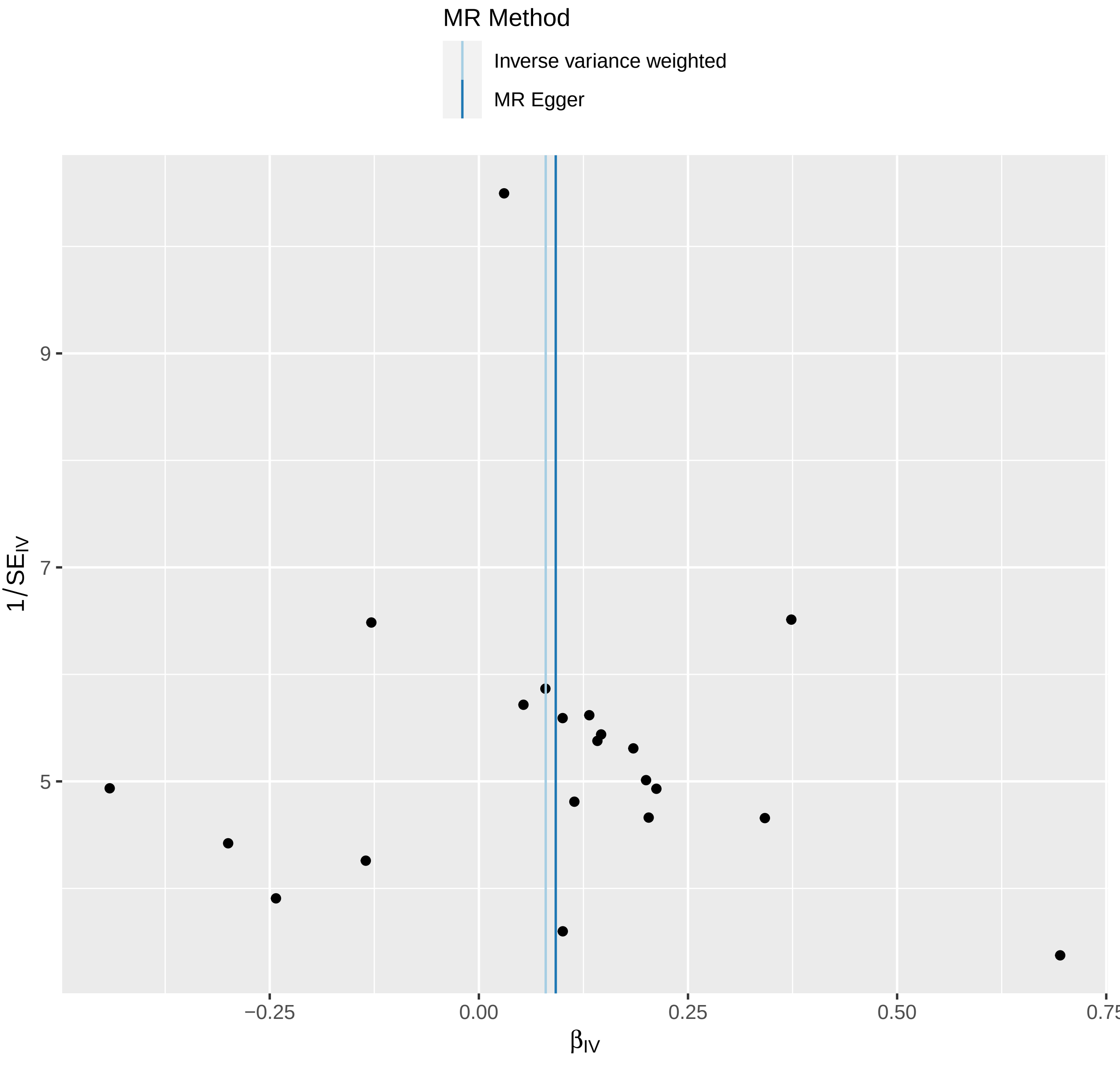

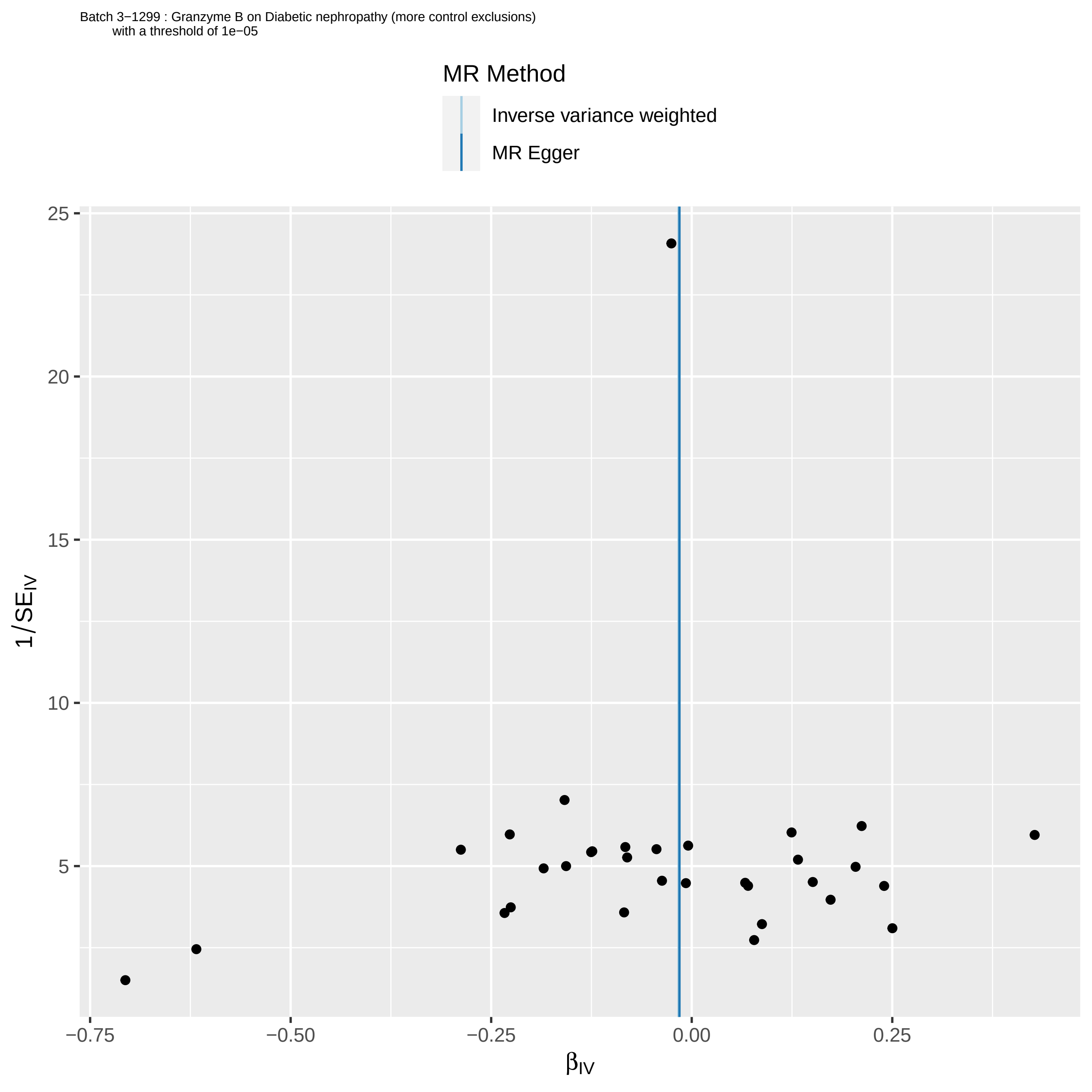
Batch 3−1299 : Granzyme B on Diabetic nephropathy (more control exclusions)
with a threshold of 1e−05
MR Method
Inverse variance weighted
MR Egger
25
20
15
10
5
1/SE_IV
−0.75
−0.50
−0.25
0.00
0.25
β_IV

Batch 3−1300 : Granzyme H on Diabetic nephropathy (more control exclusions)
with a threshold of 1e−05

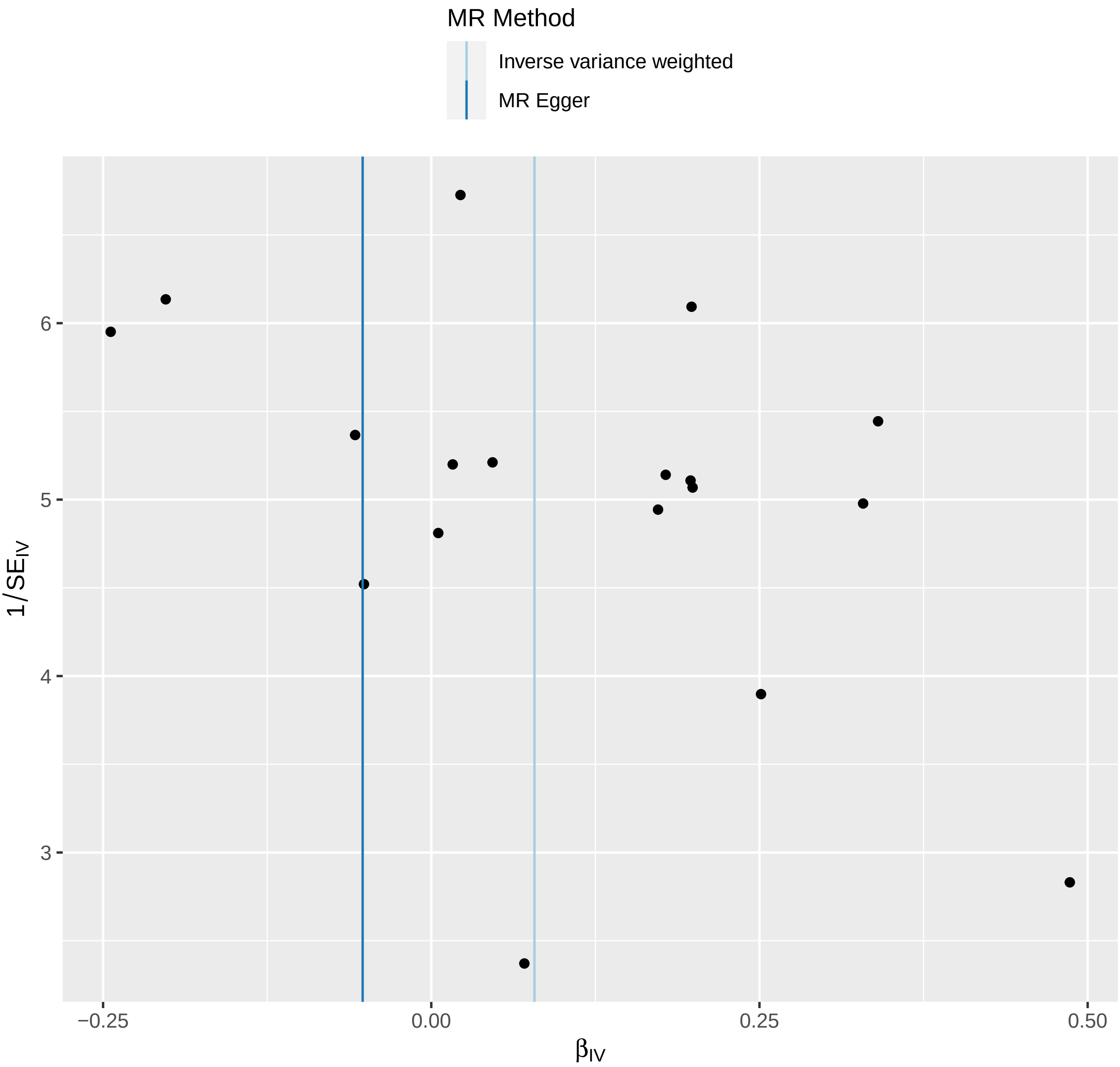

Batch 3−1301 : Granzyme K on Diabetic nephropathy (more control exclusions)
with a threshold of 1e−05

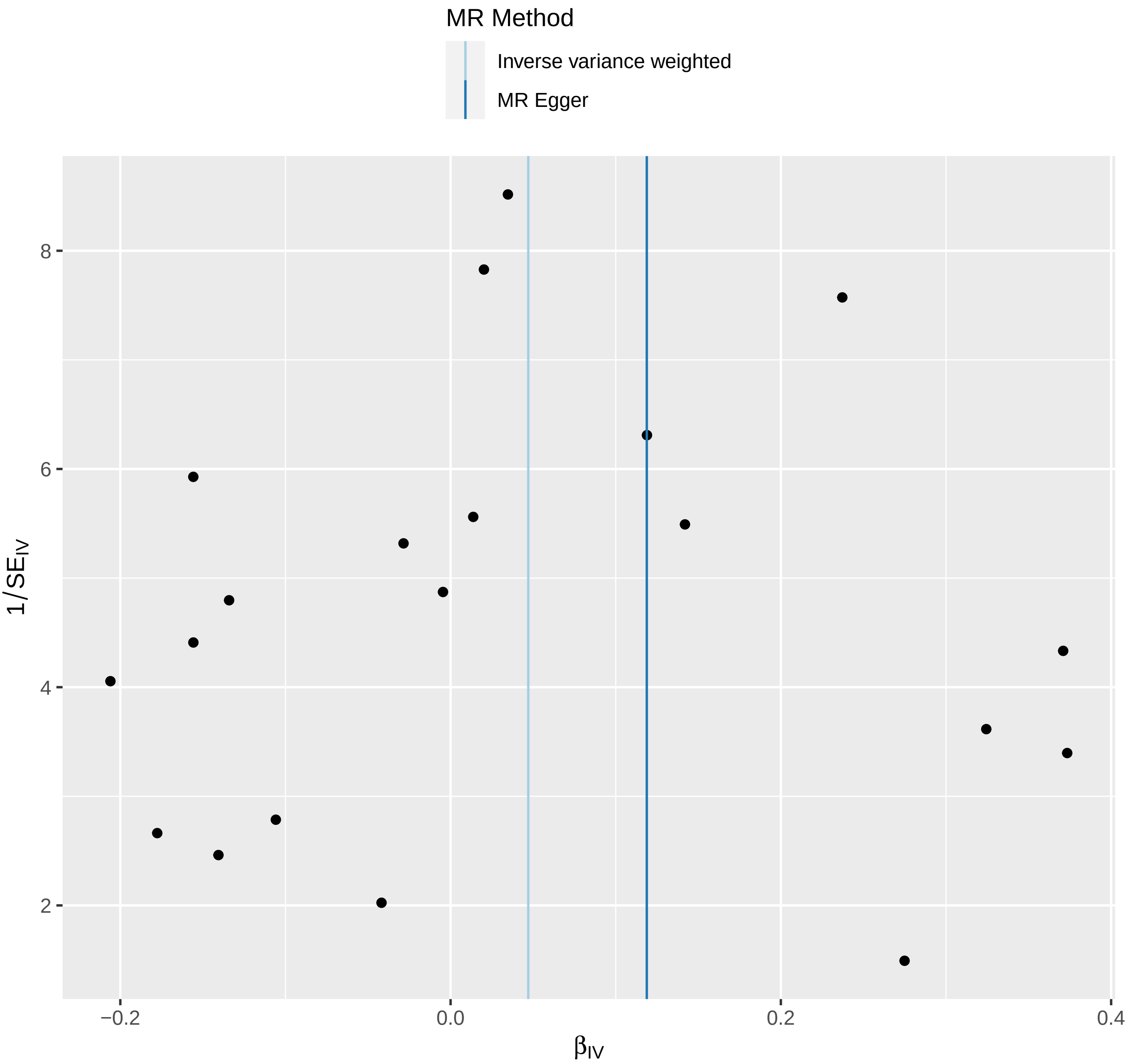

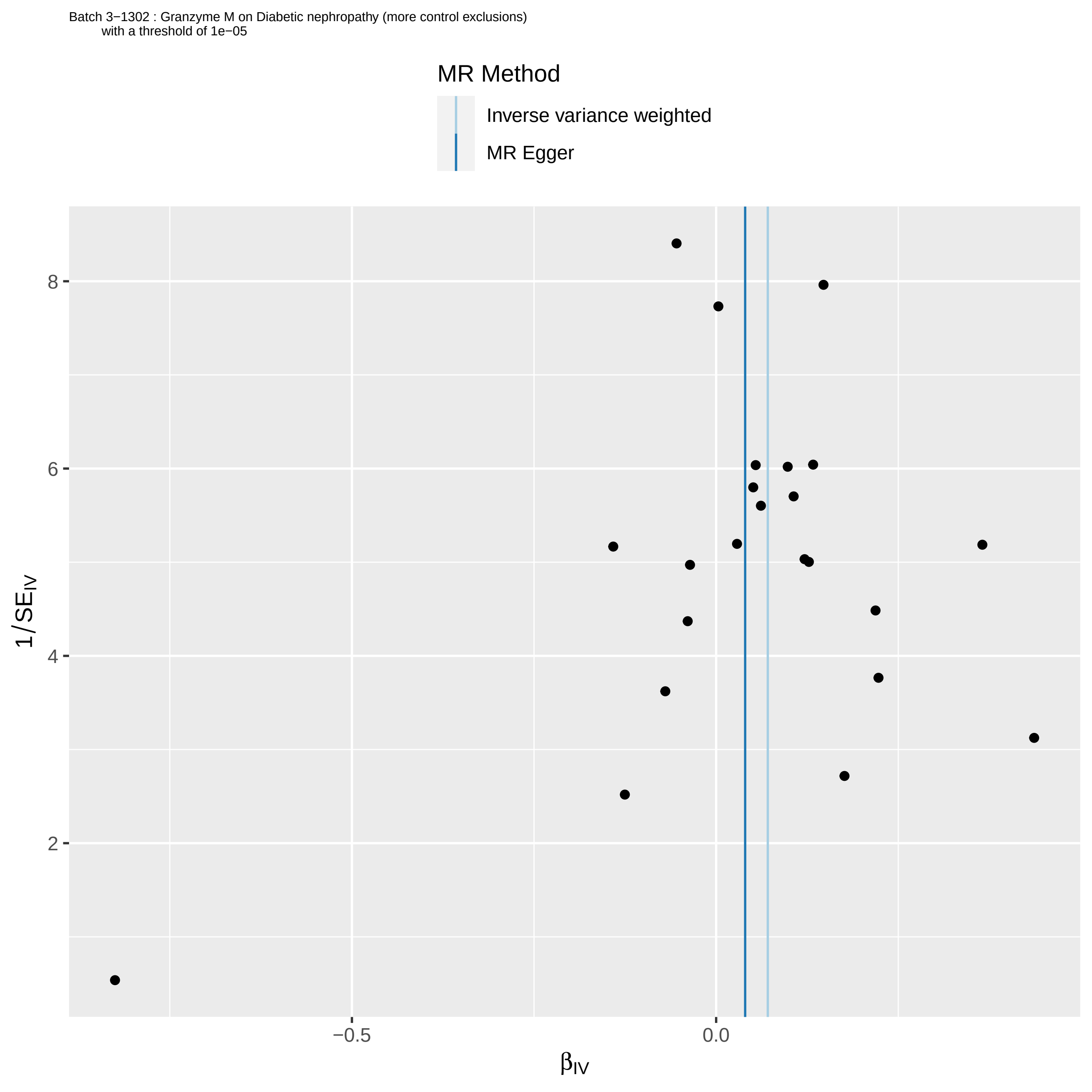

Batch 3−1302 : Granzyme M on Diabetic nephropathy (more control exclusions)
with a threshold of 1e−05
MR Method
Inverse variance weighted
MR Egger
8
6
4
2
1/SE_IV
−0.5
0.0
β_IV

Batch 3−1303 : Histone H1x on Diabetic nephropathy (more control exclusions)
with a threshold of 1e−05

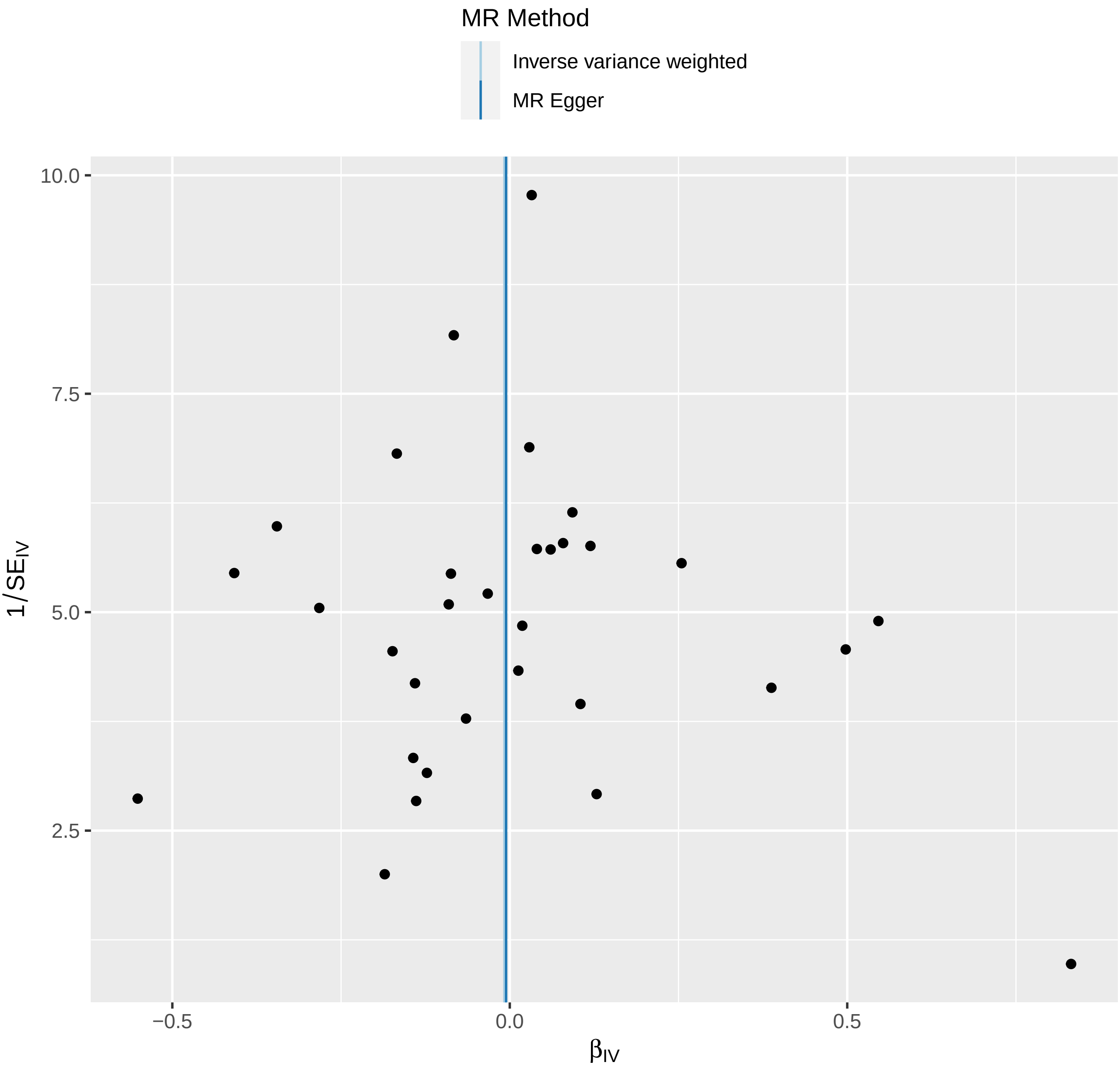

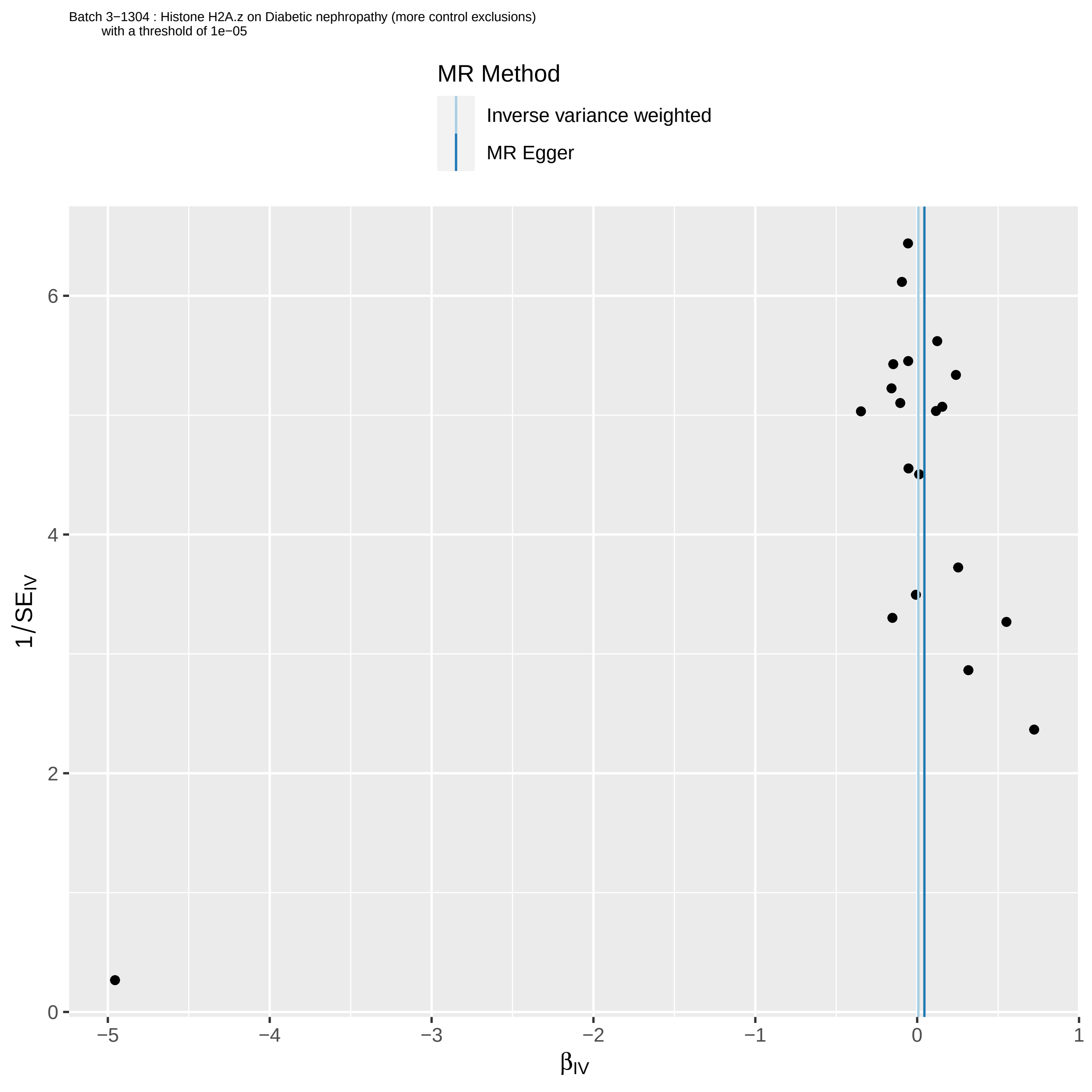
Batch 3−1304 : Histone H2A.z on Diabetic nephropathy (more control exclusions)
with a threshold of 1e−05
MR Method
Inverse variance weighted
MR Egger
6
4
2
0
−5
−4
−3
−2
−1
0
1
$1/SE_{IV}$
β_{IV}

Batch 3−1305 : GDH/6PGL endoplasmic bifunctional protein on Diabetic nephropathy (more control exclusions) with a threshold of 1e−05

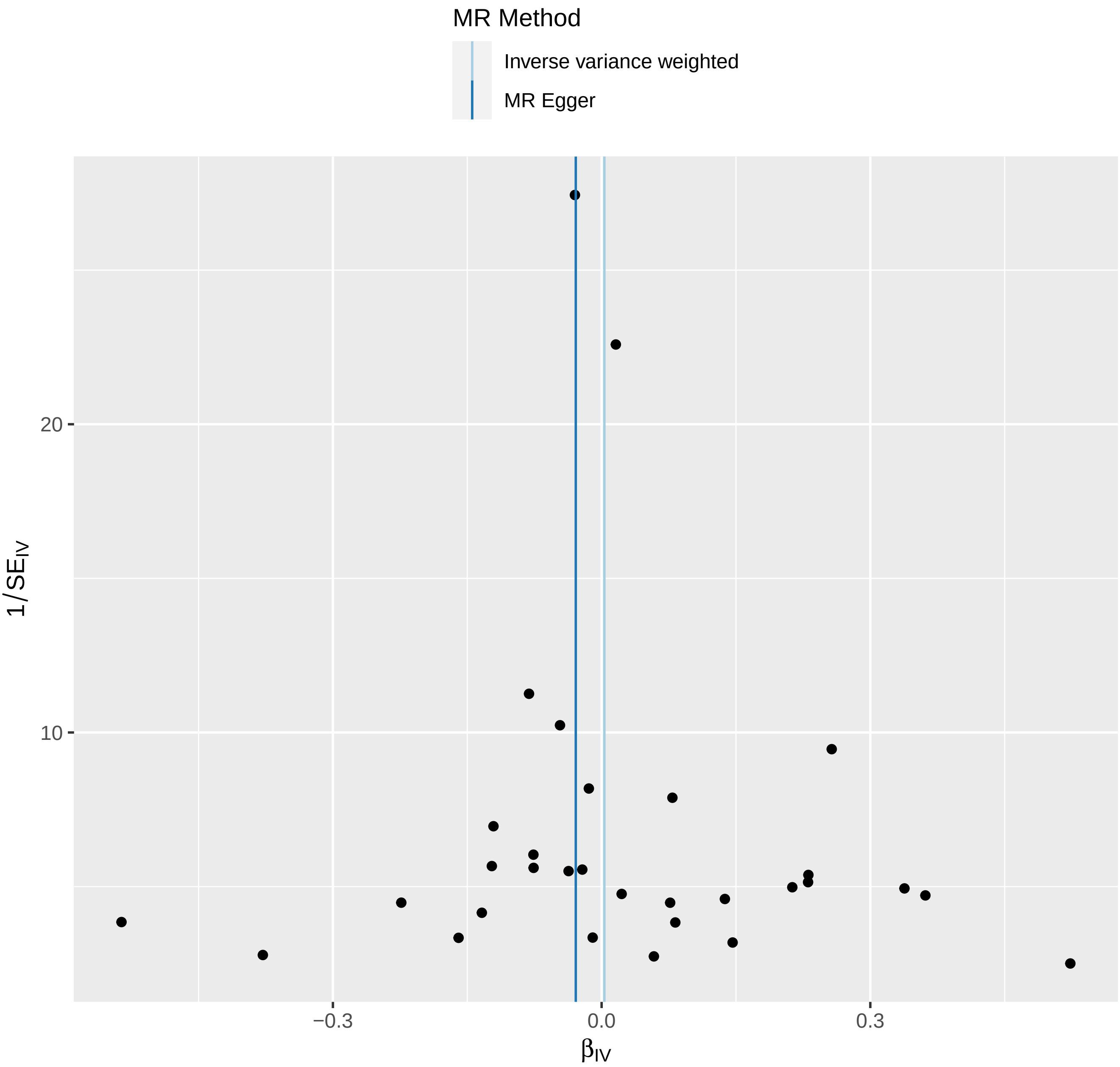

Batch 3−1306 : 3−hydroxyanthranilate 3,4−dioxygenase on Diabetic nephropathy (more control exclusions)
with a threshold of 1e−05

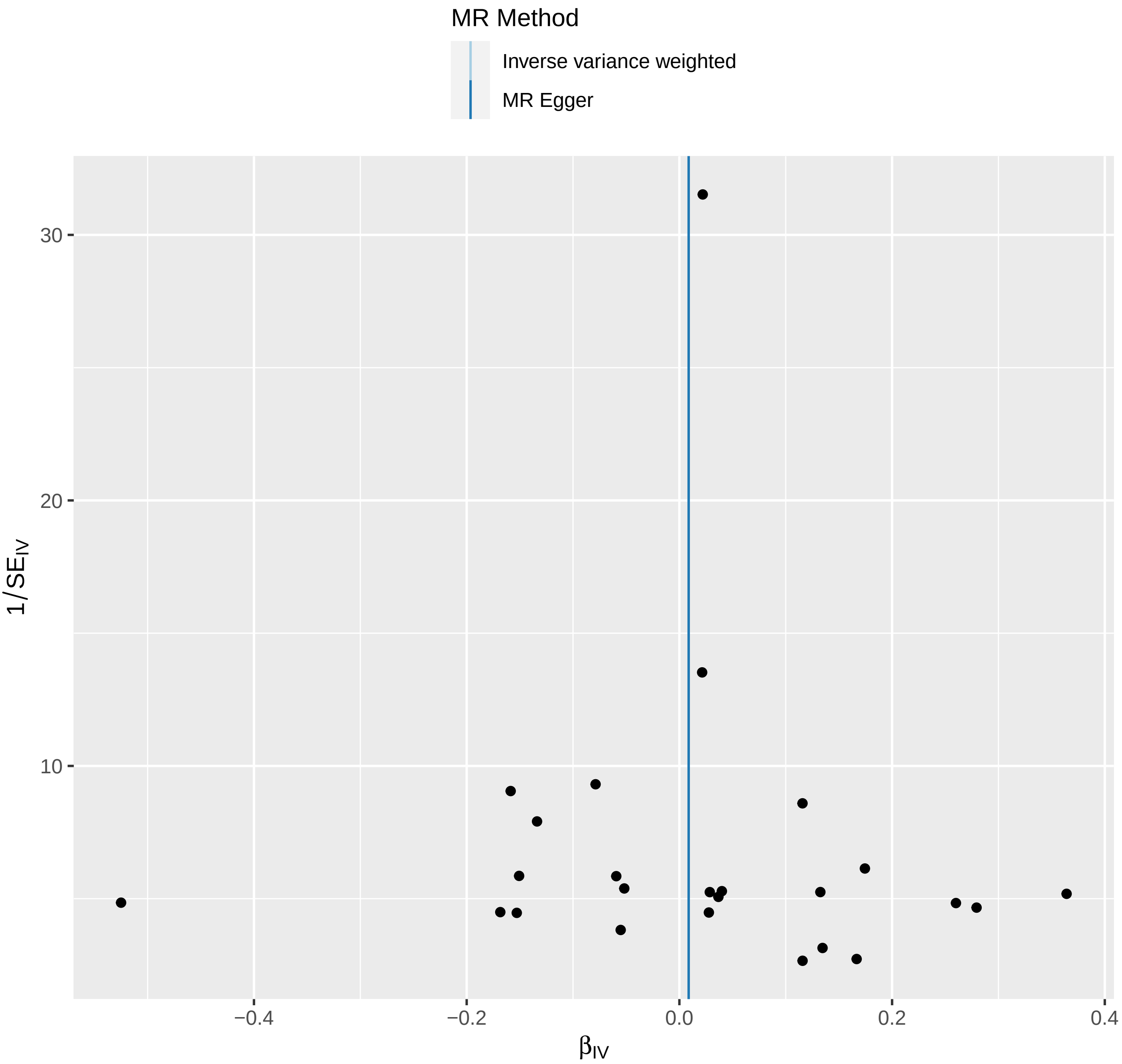

Batch 3−1307 : Hepcidin on Diabetic nephropathy (more control exclusions)
with a threshold of 1e−05

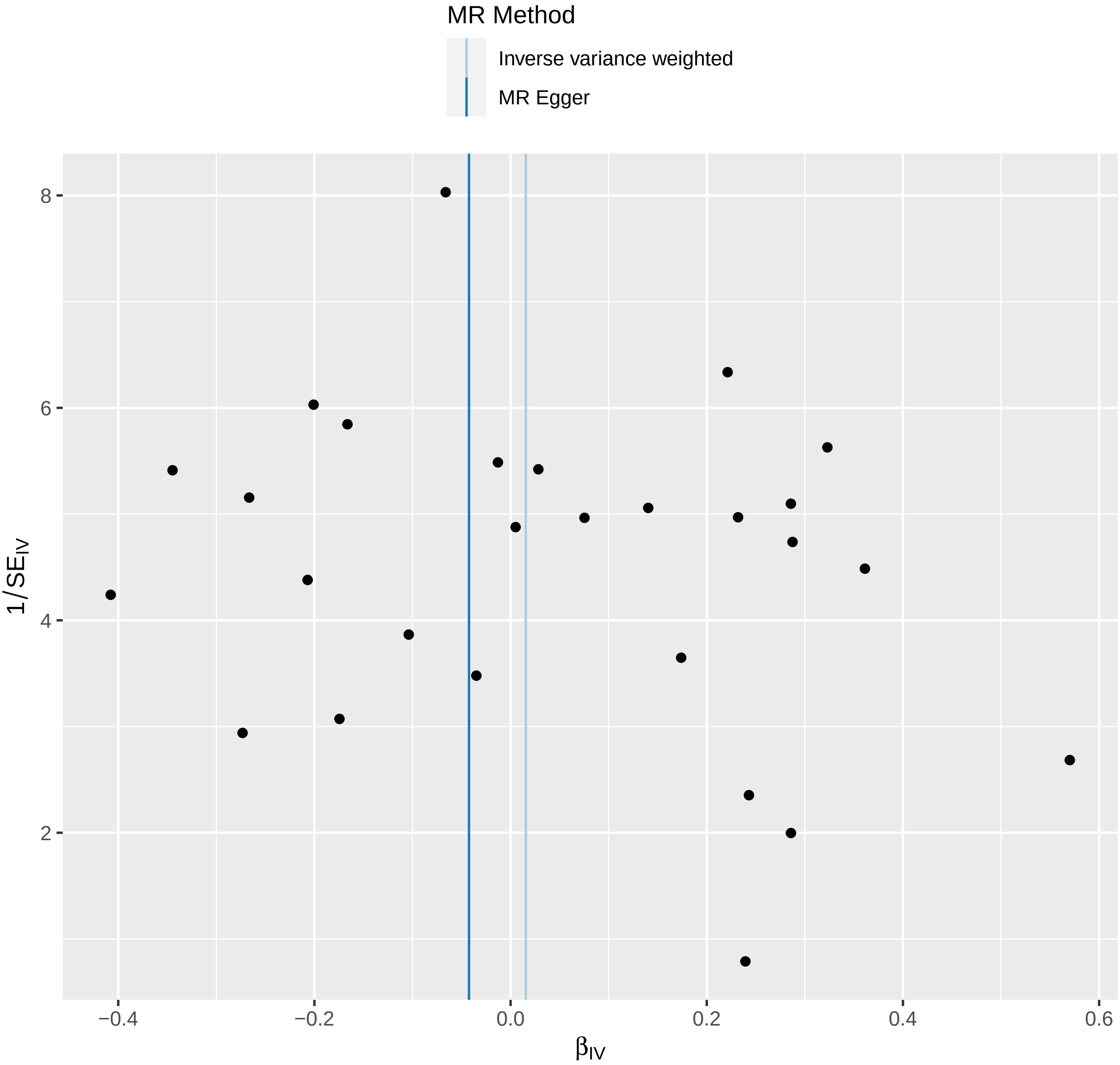

Batch 3−1308 : Hyaluronan and proteoglycan link protein 1 on Diabetic nephropathy (more control exclusions)
with a threshold of 1e−05

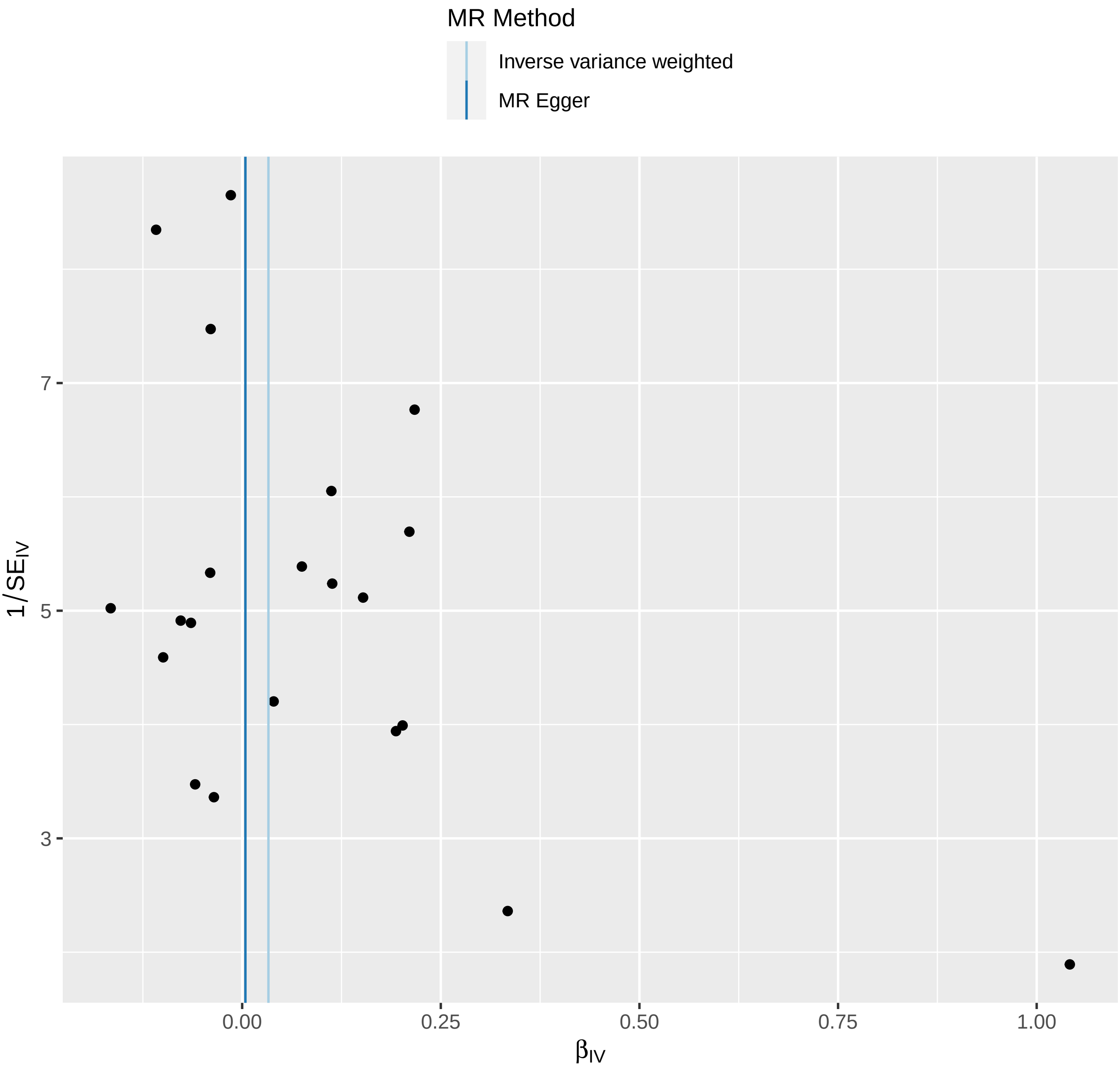

Batch 3−1309 : Histone acetyltransferase type B catalytic subunit on Diabetic nephropathy (more control exclusions)
with a threshold of 1e−05

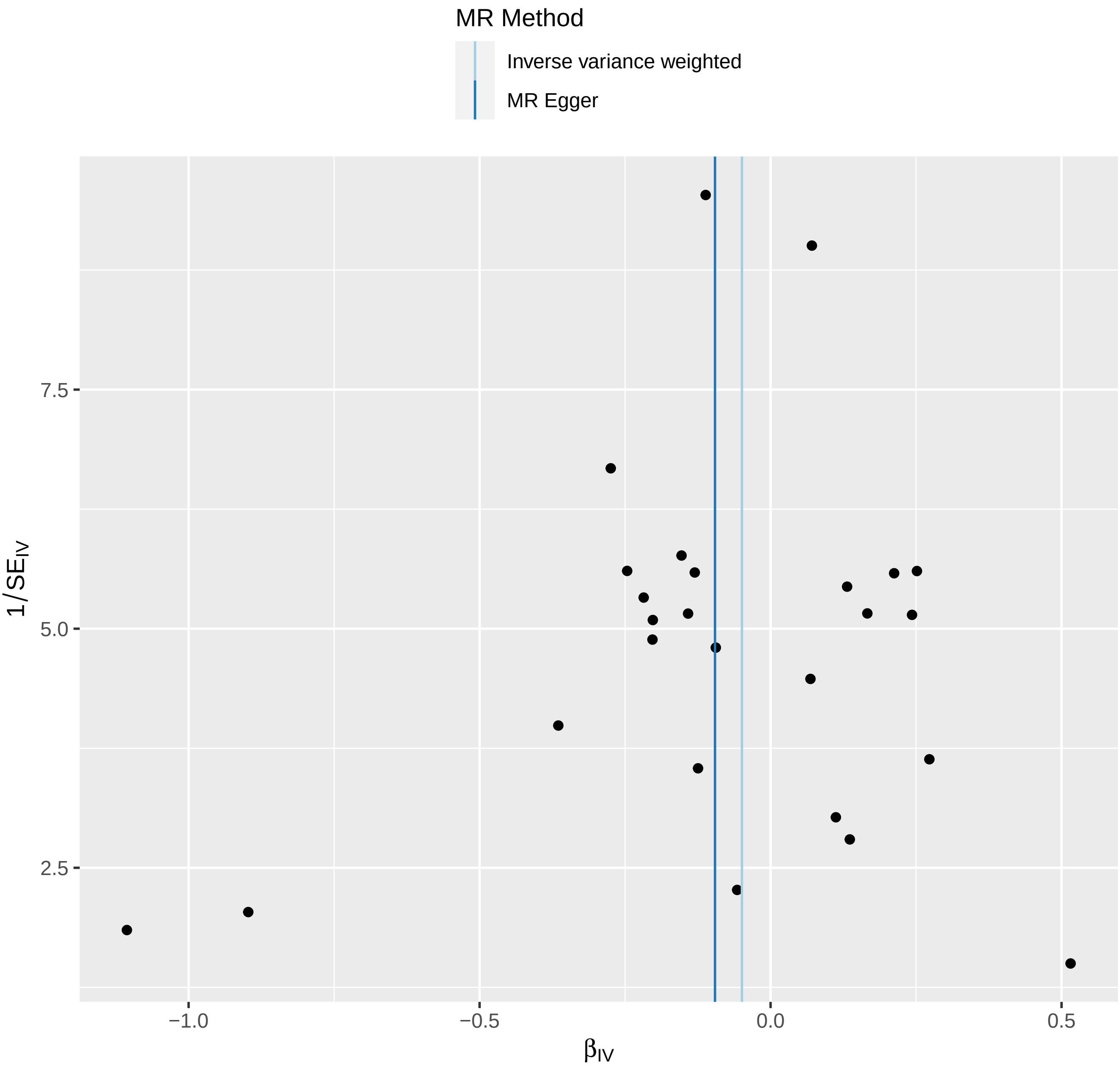

Batch 3−1310 : Hepatitis A virus cellular receptor 1 on Diabetic nephropathy (more control exclusions)
with a threshold of 1e−05

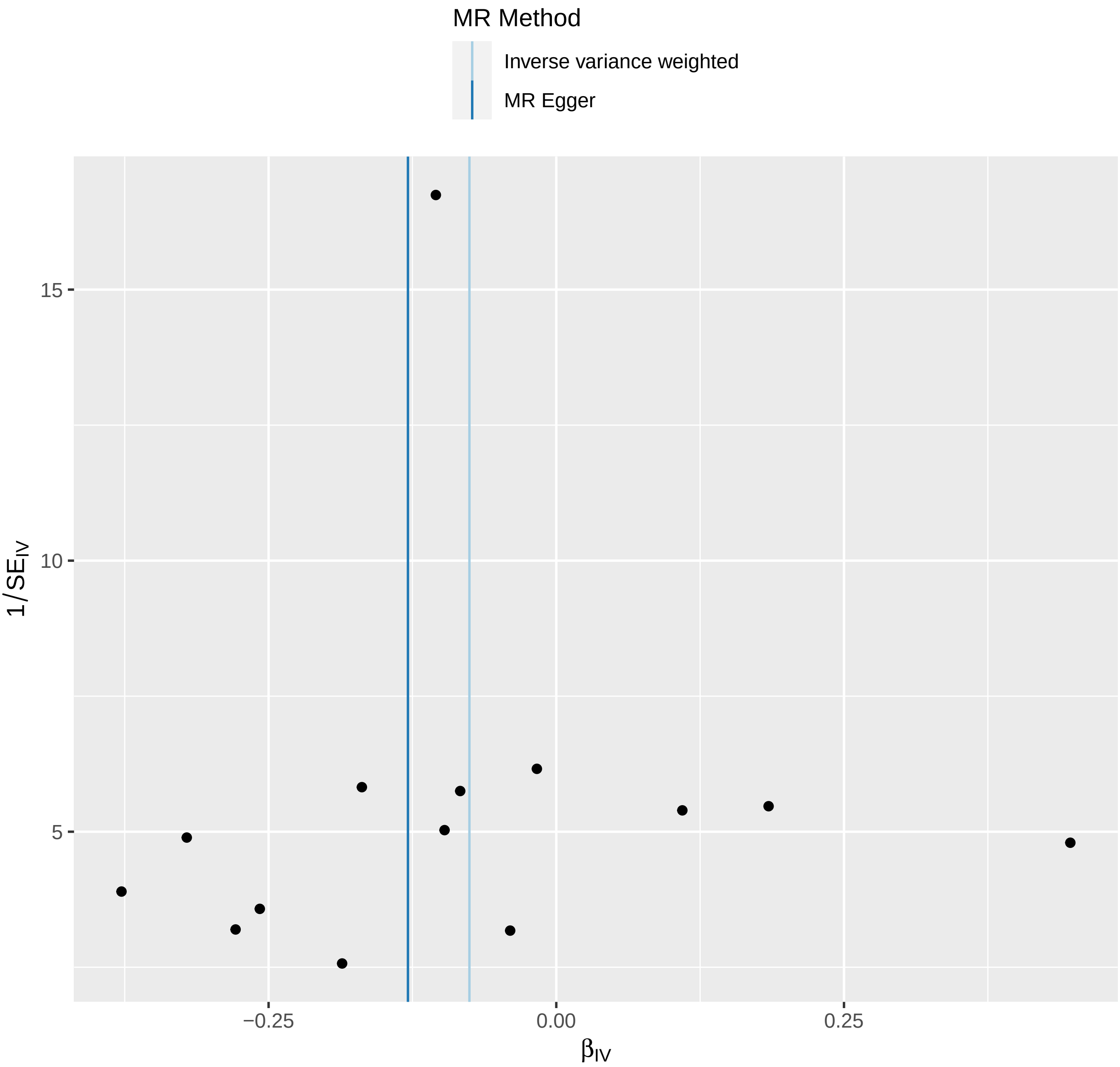

Batch 3−1311 : Hepatitis A virus cellular receptor 2 on Diabetic nephropathy (more control exclusions)
with a threshold of 1e−05

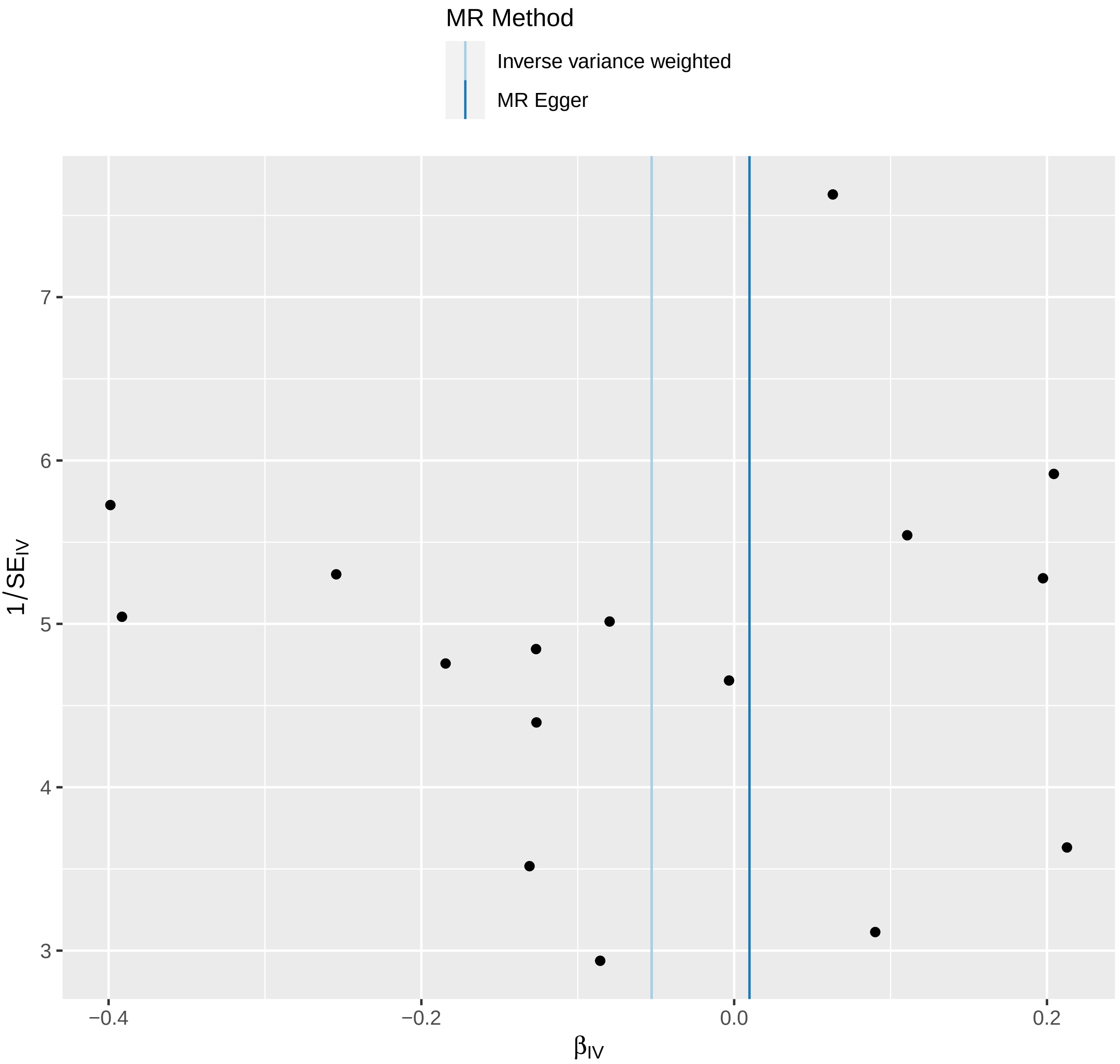

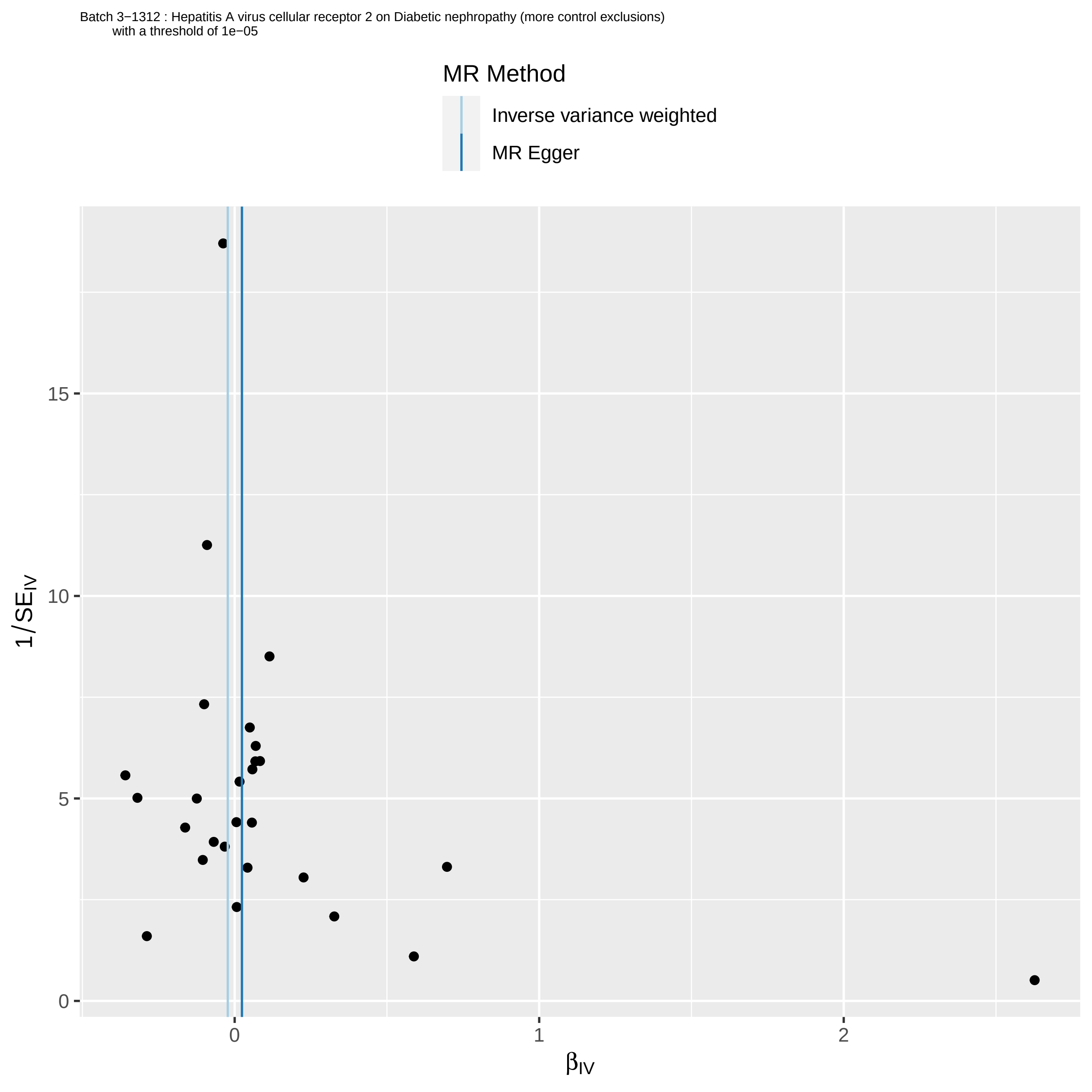

Batch 3−1312 : Hepatitis A virus cellular receptor 2 on Diabetic nephropathy (more control exclusions)
with a threshold of 1e−05
MR Method
Inverse variance weighted
MR Egger
1/SE_IV
β_IV
0
5
10
15
0
1
2

Batch 3−1313 : Heparin−binding EGF−like growth factor on Diabetic nephropathy (more control exclusions)
with a threshold of 1e−05

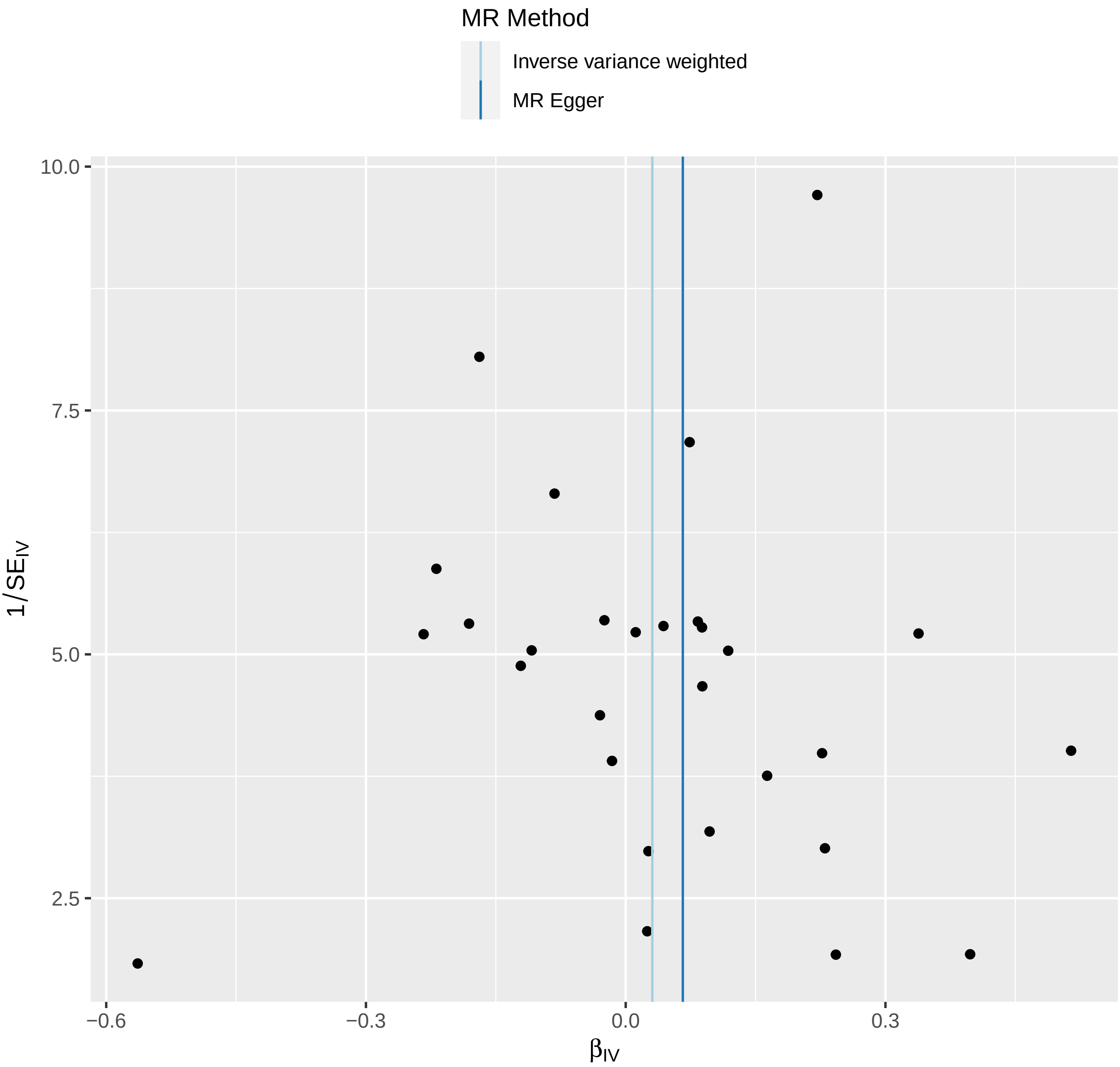

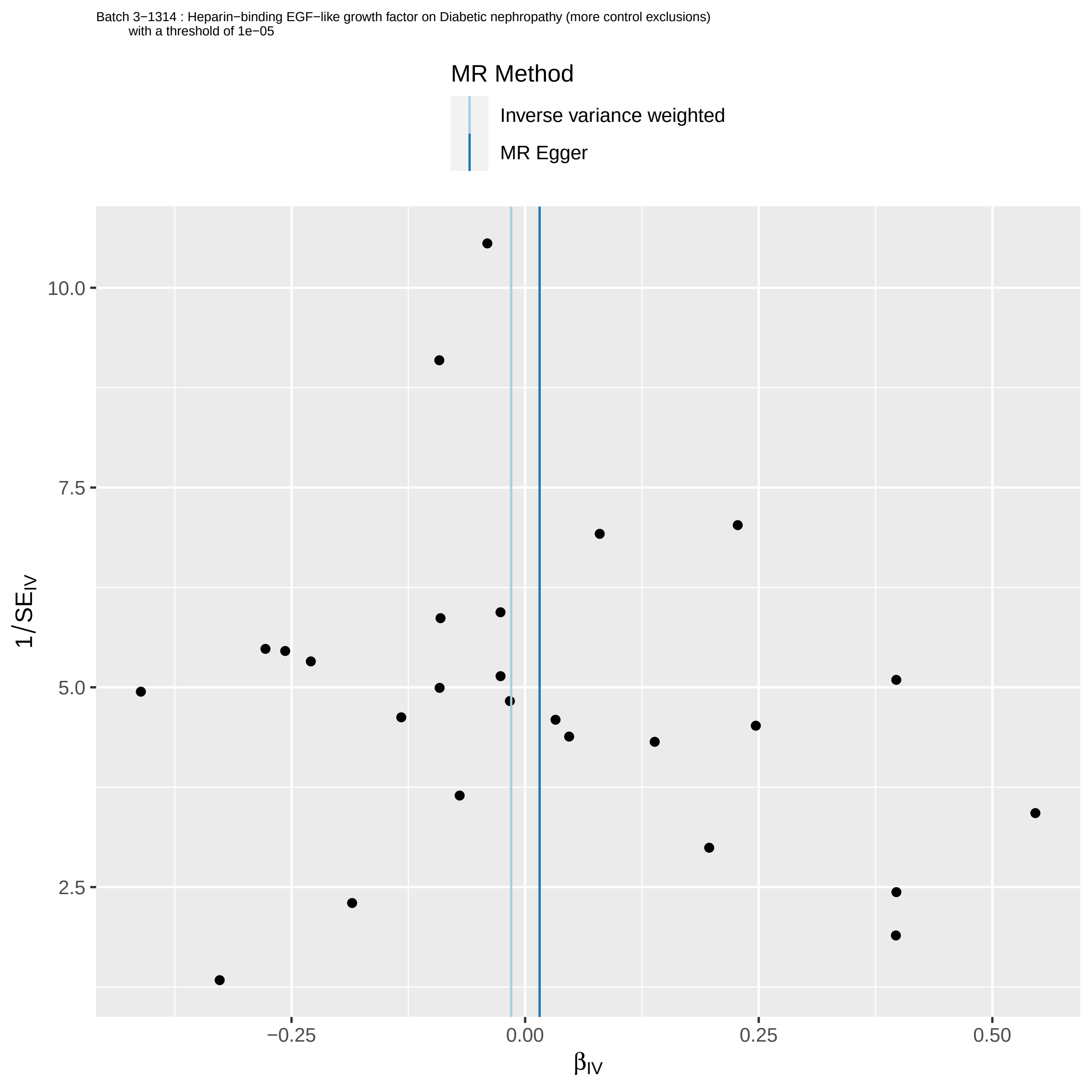
Batch 3−1314 : Heparin−binding EGF−like growth factor on Diabetic nephropathy (more control exclusions)
with a threshold of 1e−05
MR Method
Inverse variance weighted
MR Egger
10.0
7.5
5.0
2.5
−0.25
0.00
0.25
0.50
1/SE_IV
β_IV

Batch 3−1315 : Hemoglobin subunit gamma−1 on Diabetic nephropathy (more control exclusions)
with a threshold of 1e−05

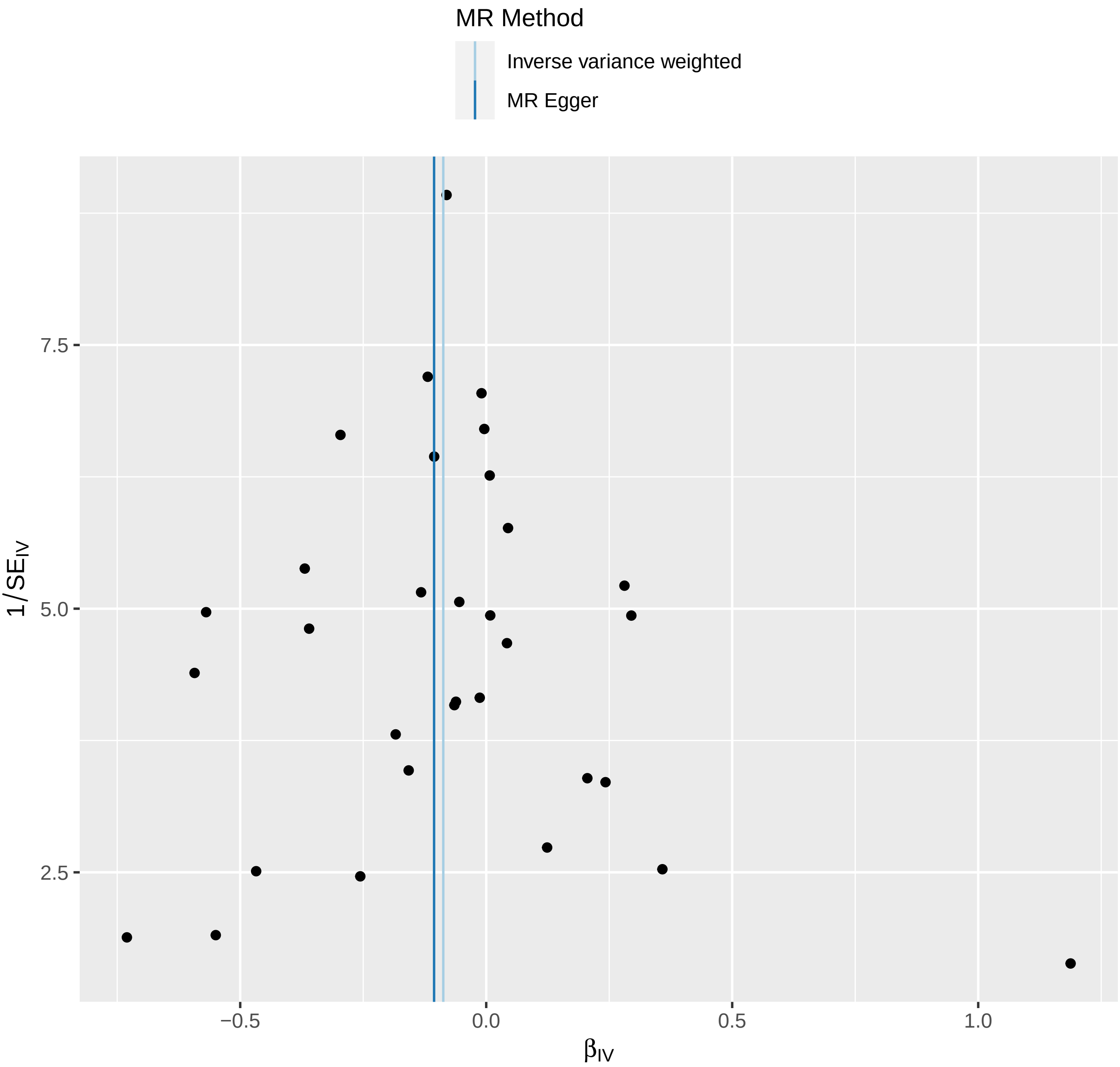

Batch 3−1316 : Hemoglobin subunit theta−1 on Diabetic nephropathy (more control exclusions)
with a threshold of 1e−05

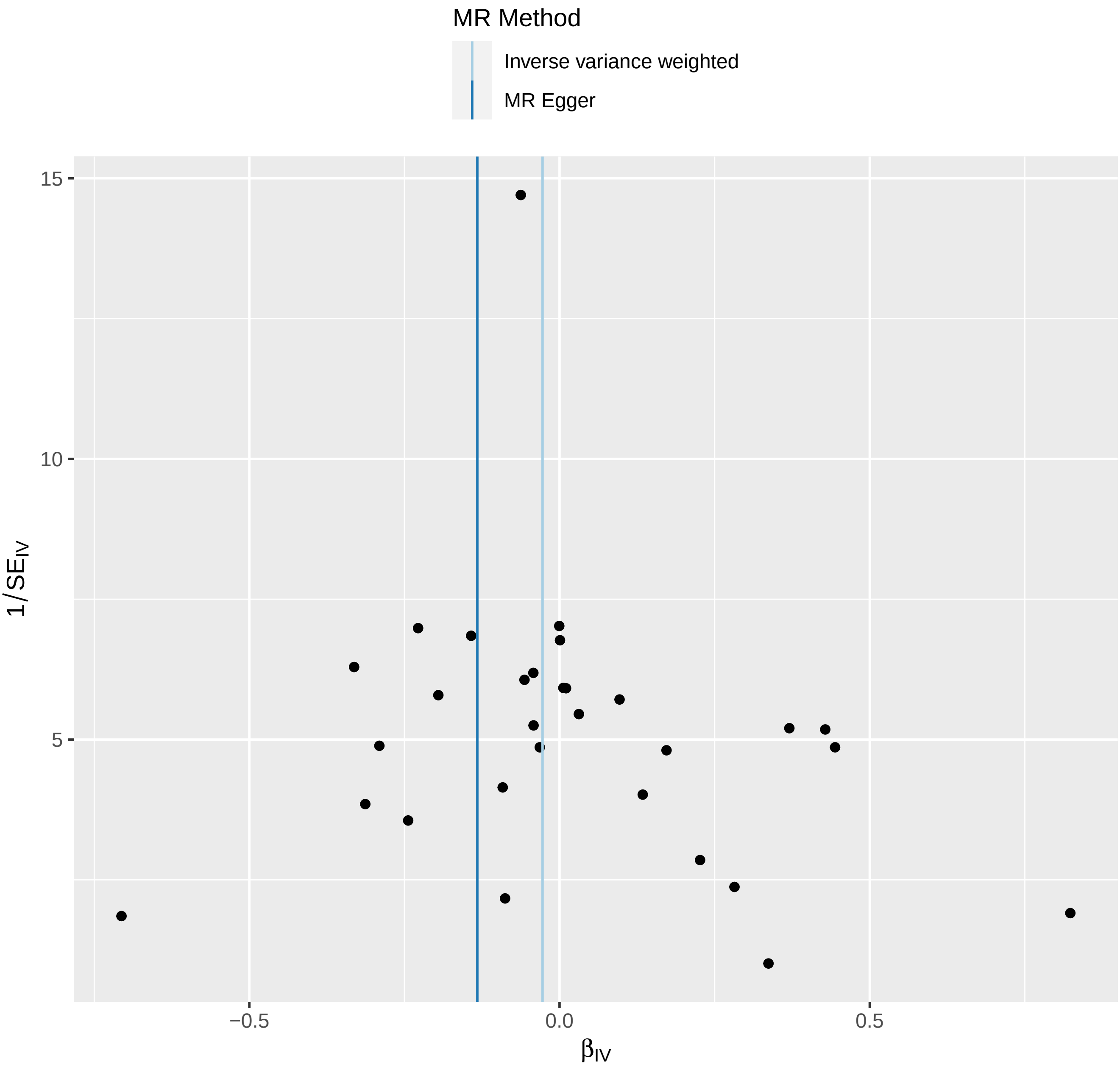

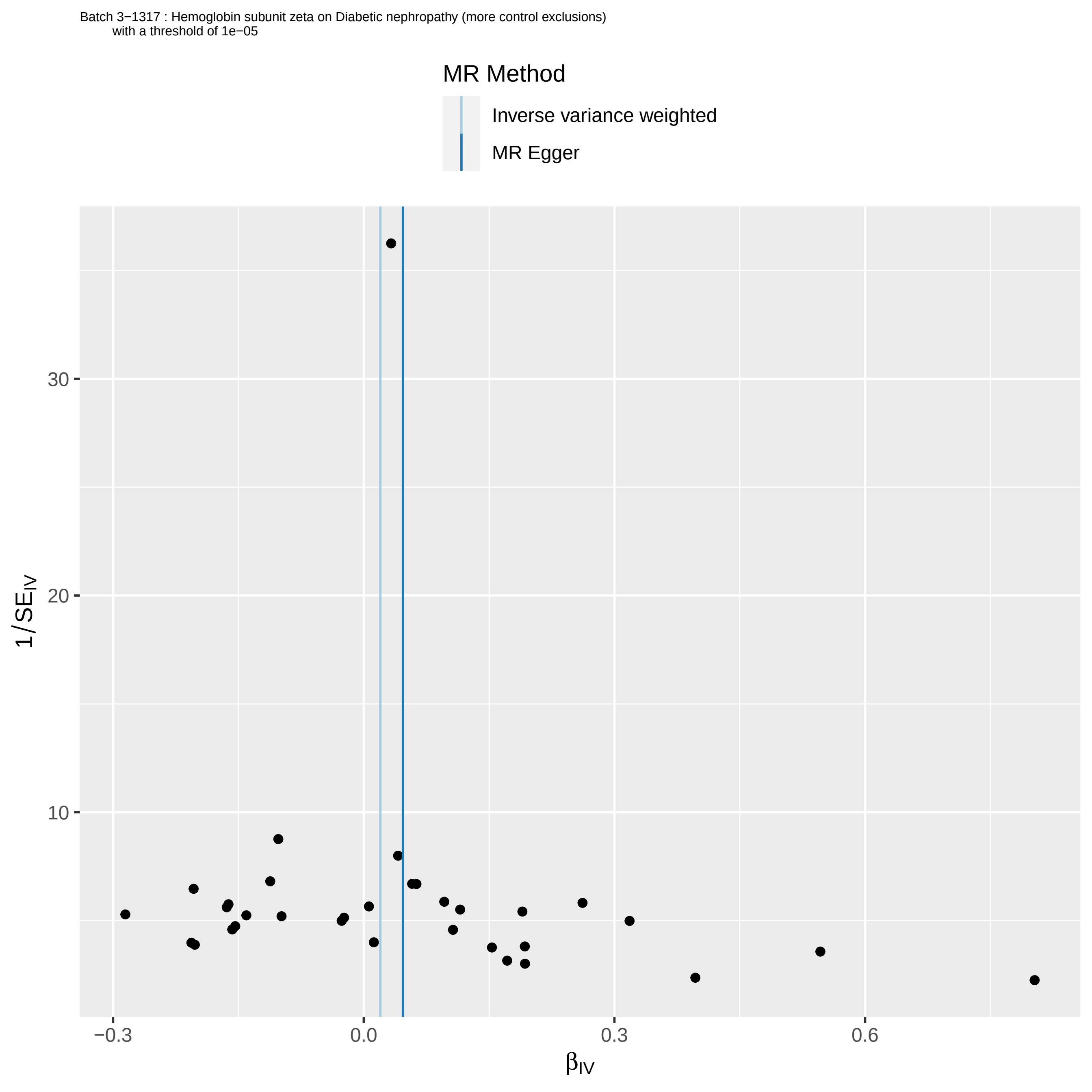

Batch 3−1317 : Hemoglobin subunit zeta on Diabetic nephropathy (more control exclusions)
with a threshold of 1e−05
MR Method
Inverse variance weighted
MR Egger
30
20
10
1/SE_IV
−0.3
0.0
0.3
0.6
β_IV

Batch 3−1318 : Hydroxycarboxylic acid receptor 2 on Diabetic nephropathy (more control exclusions)
with a threshold of 1e−05

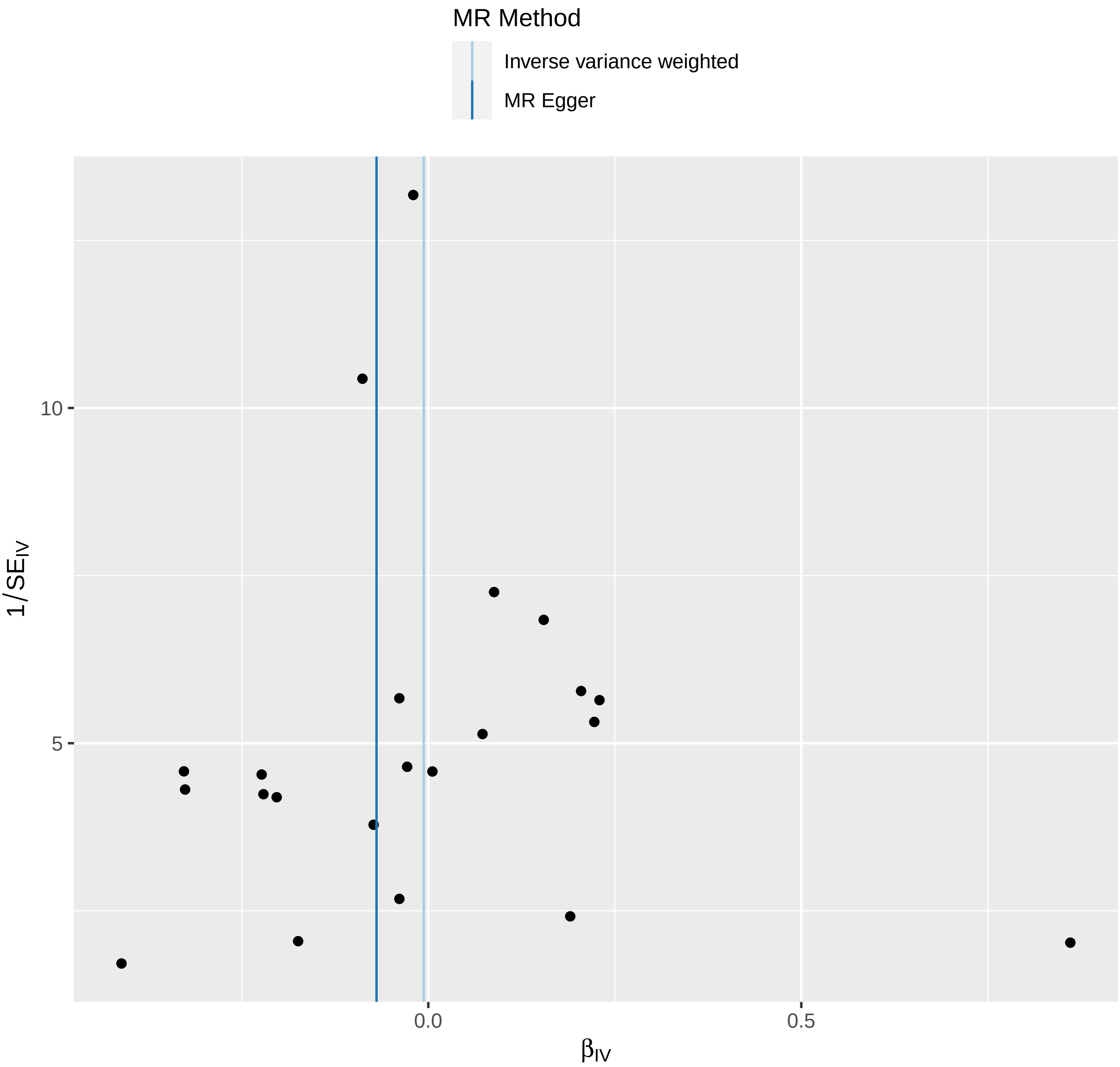

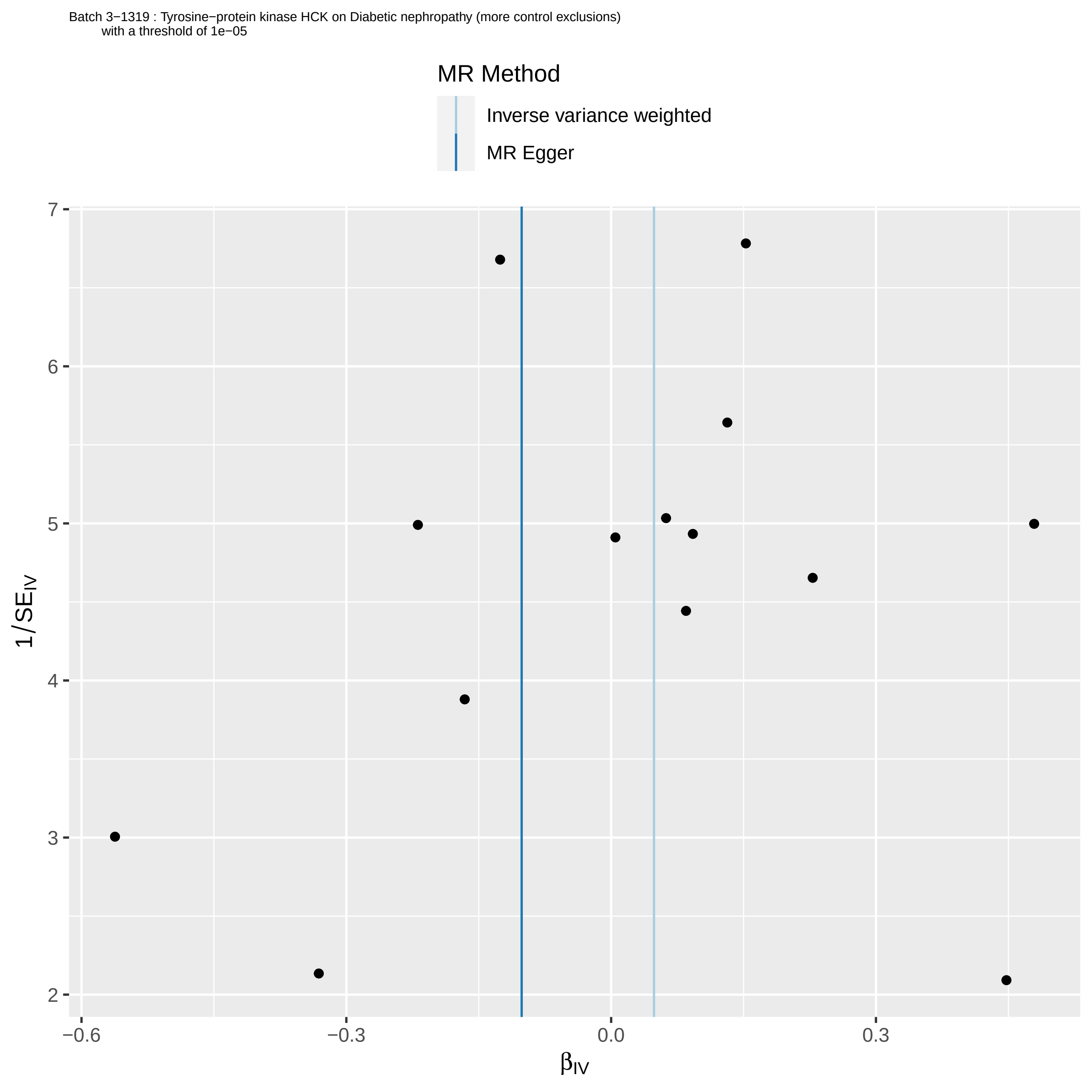
Batch 3−1319 : Tyrosine−protein kinase HCK on Diabetic nephropathy (more control exclusions)
with a threshold of 1e−05
MR Method
Inverse variance weighted
MR Egger
1/SE_IV
β_IV
7
6
5
4
3
2
−0.6
−0.3
0.0
0.3

Batch 3−1320 : Histone deacetylase 8 on Diabetic nephropathy (more control exclusions)
with a threshold of 1e−05

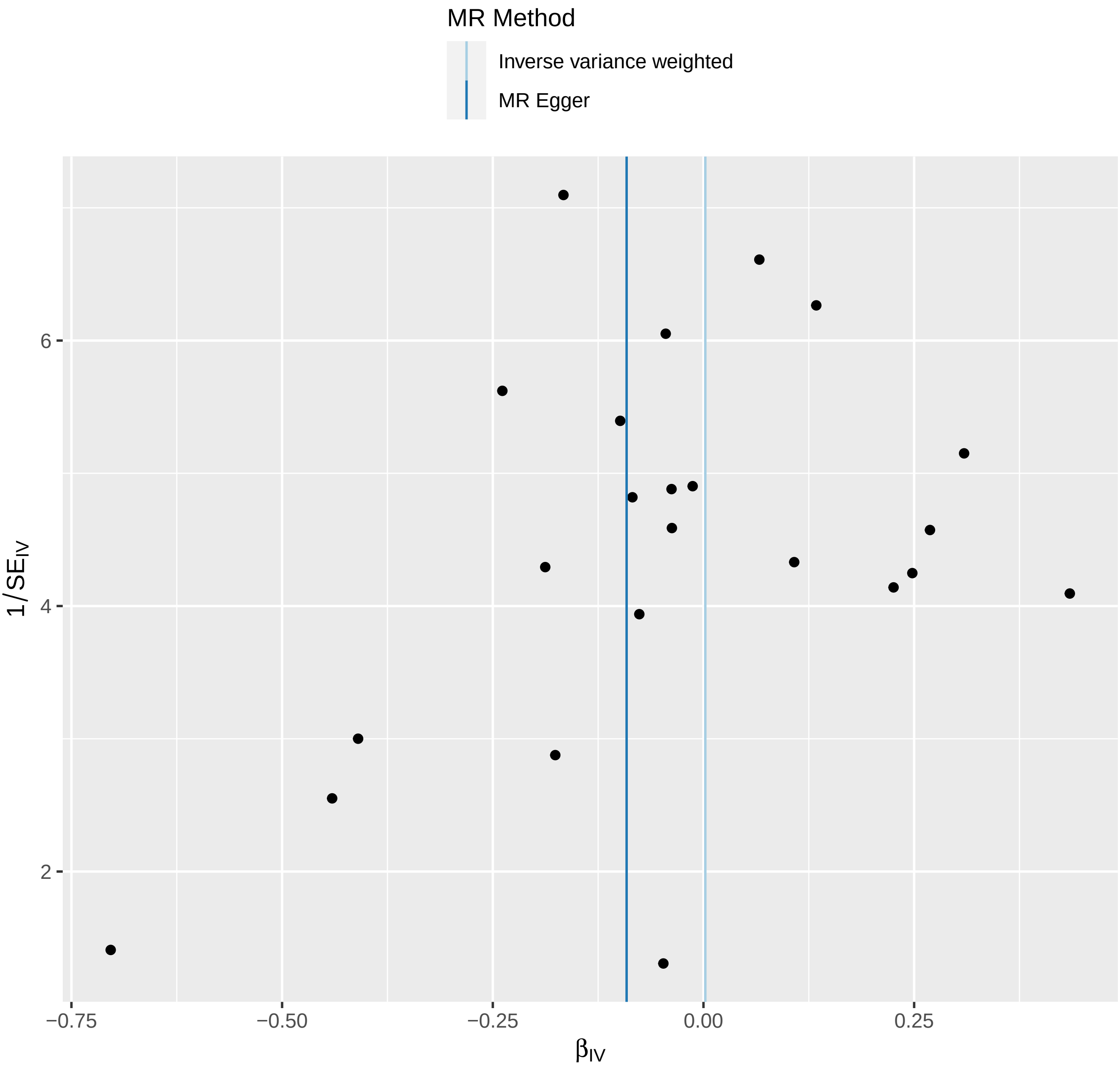

Batch 3−1321 : Hepatoma−derived growth factor−related protein 2 on Diabetic nephropathy (more control exclusions)
with a threshold of 1e−05

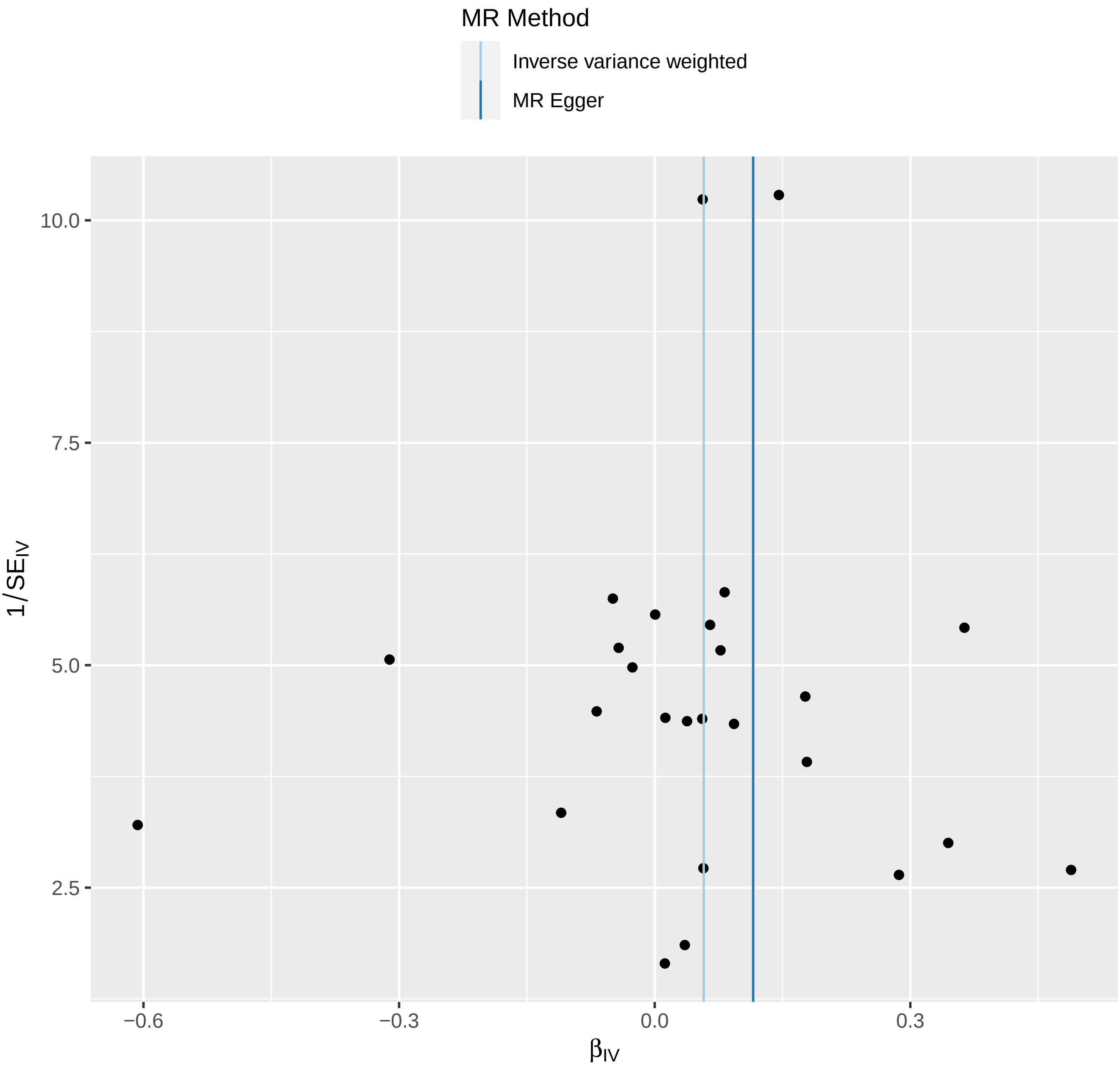

Batch 3−1322 : Haloacid dehalogenase−like hydrolase domain−containing protein 2 on Diabetic nephropathy (more control exclusions) with a threshold of 1e−05

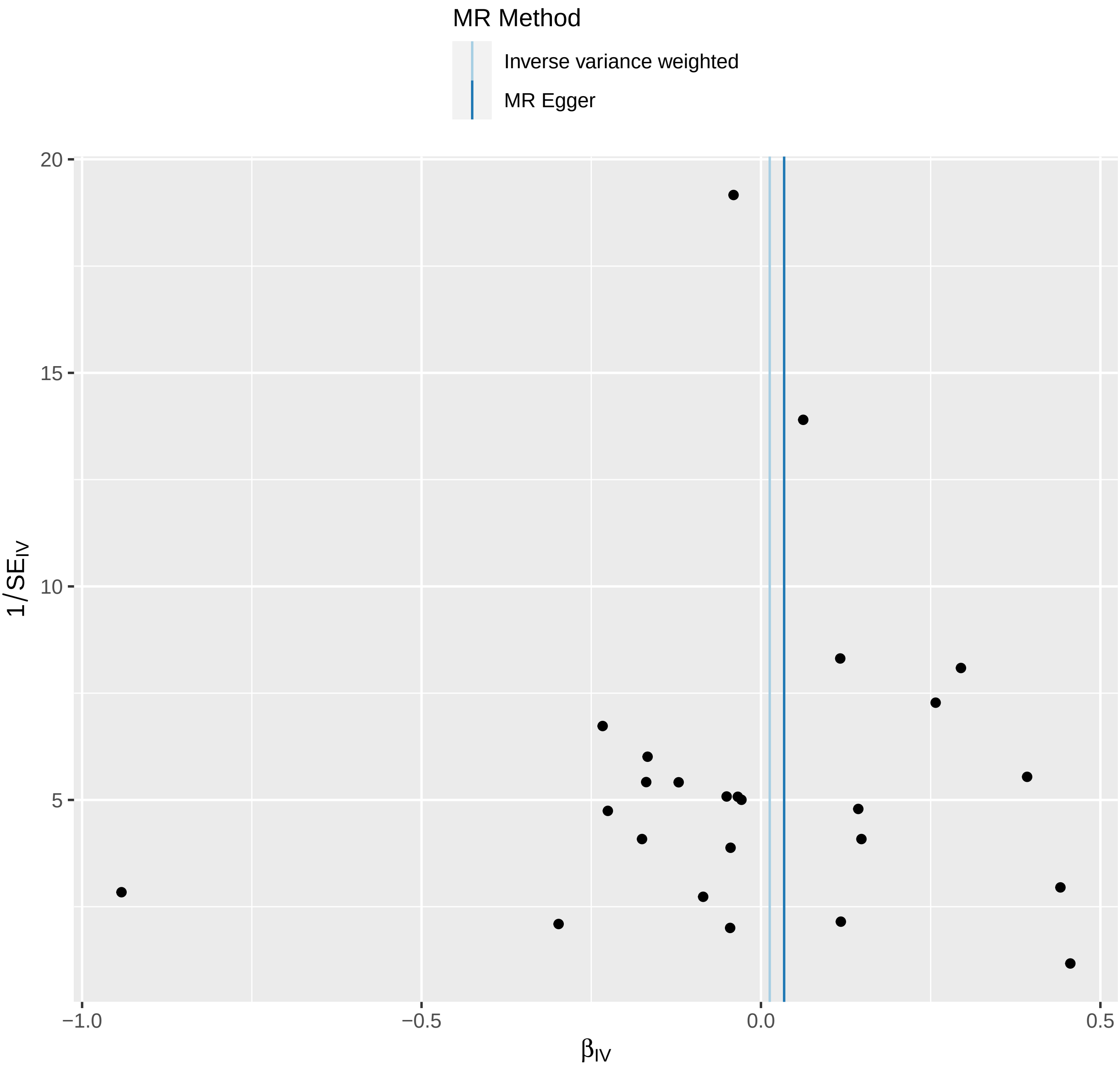

Batch 3−1323 : Vigilin on Diabetic nephropathy (more control exclusions)
with a threshold of 1e−05

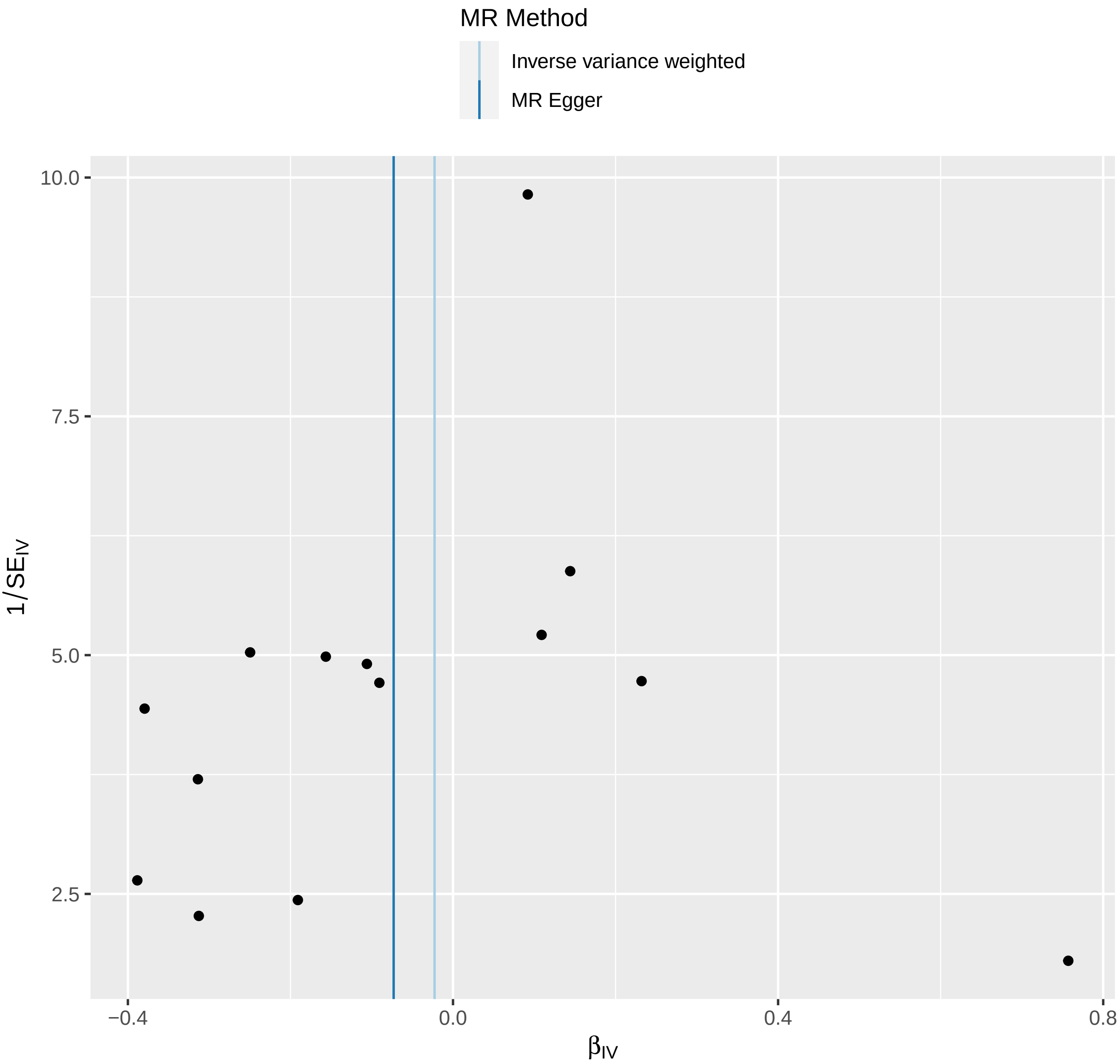

Batch 3−1324 : E3 ubiquitin−protein ligase HECW1 on Diabetic nephropathy (more control exclusions)
with a threshold of 1e−05

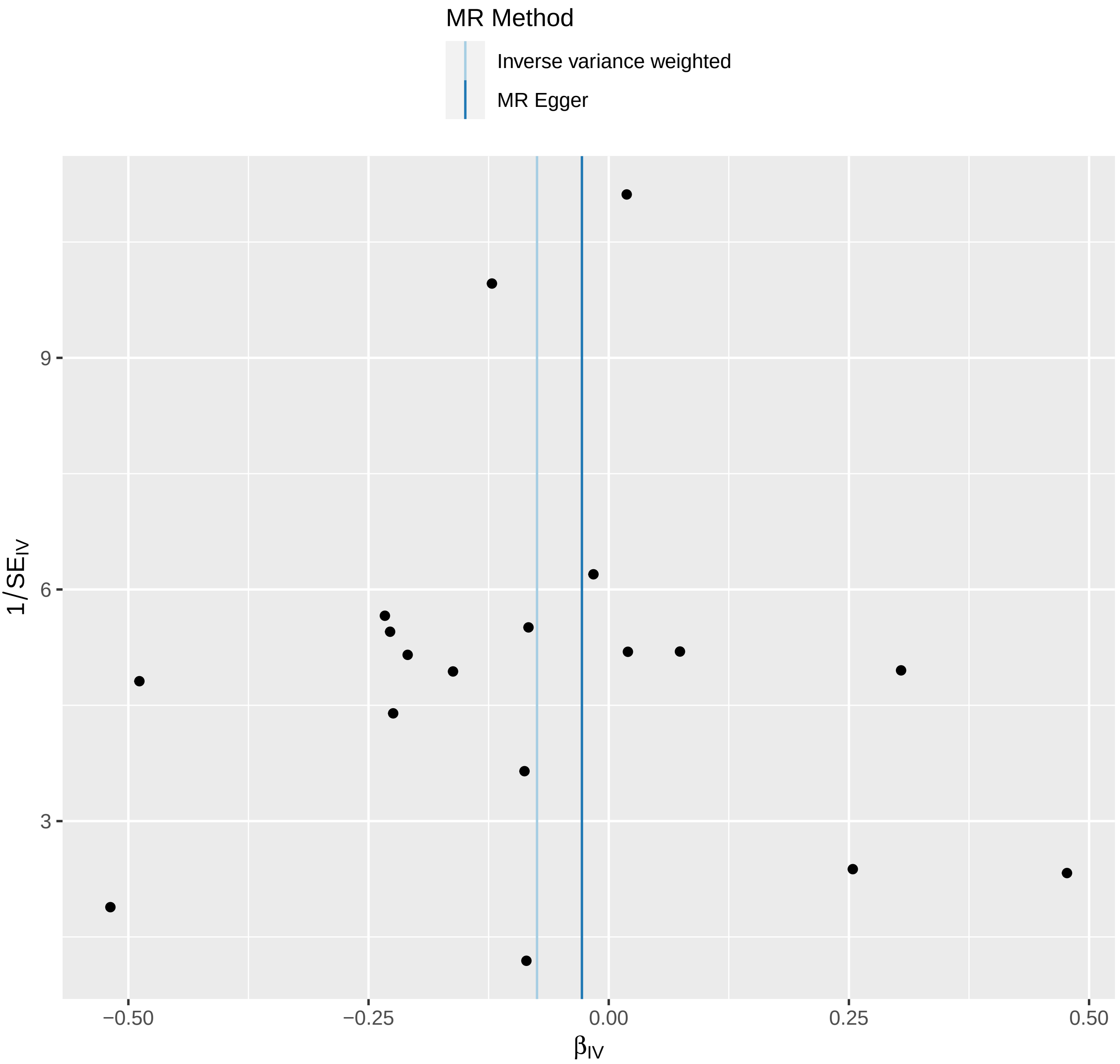

Batch 3−1325 : Protein HEG homolog 1 on Diabetic nephropathy (more control exclusions)
with a threshold of 1e−05

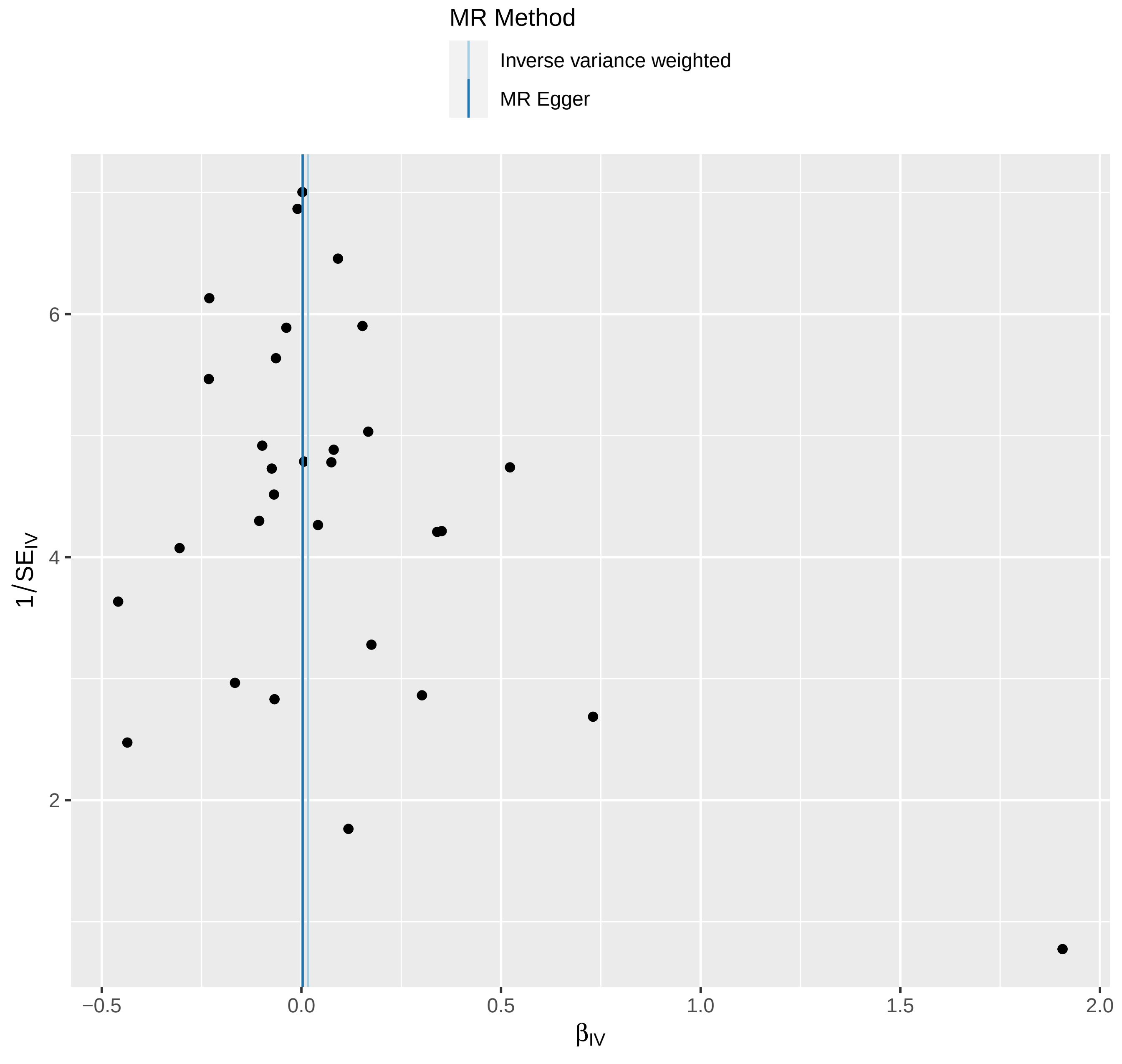

Batch 3−1326 : HEPACAM family member 2 on Diabetic nephropathy (more control exclusions)
with a threshold of 1e−05

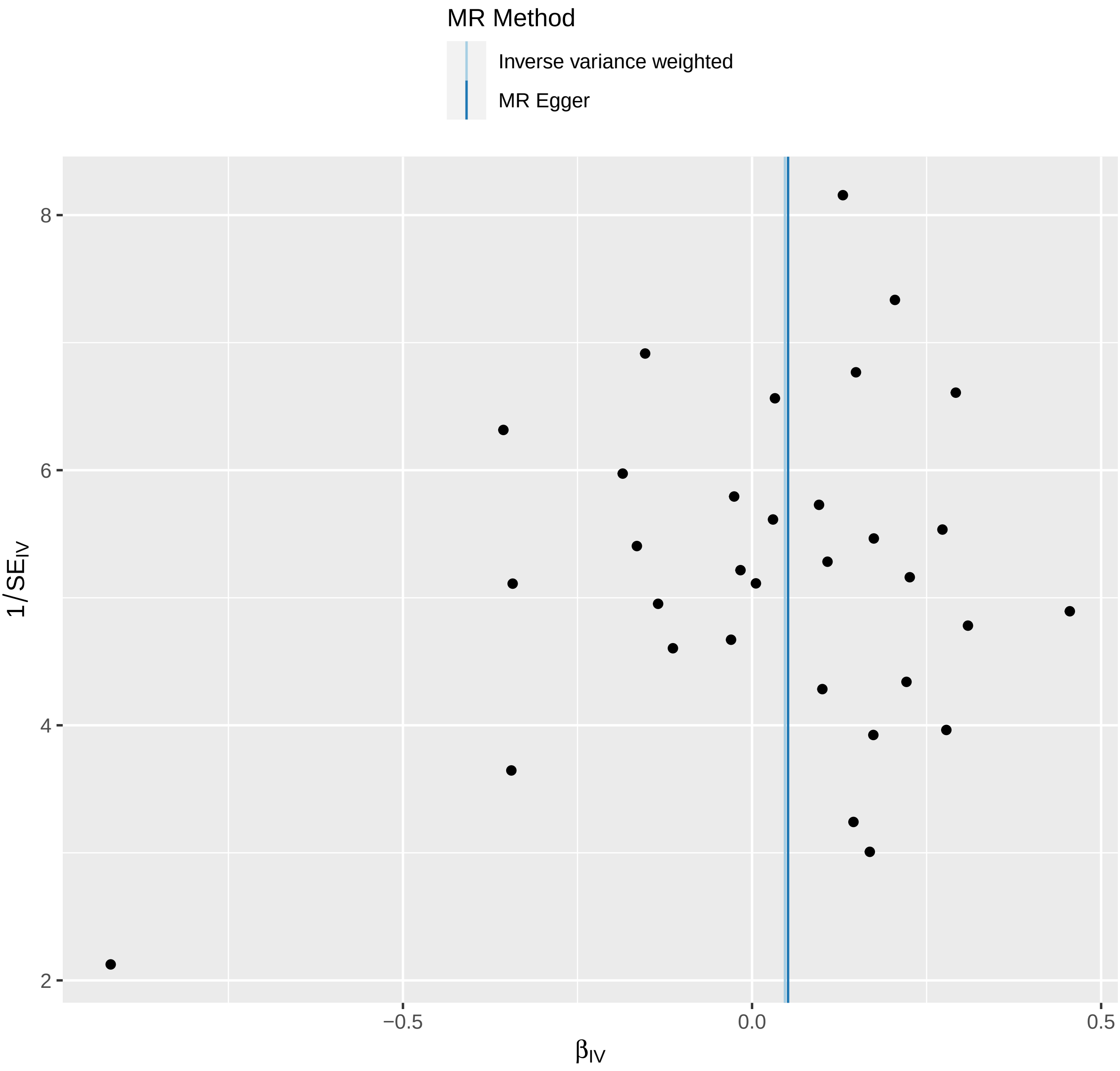

Batch 3−1327 : HEPACAM family member 2 on Diabetic nephropathy (more control exclusions)
with a threshold of 1e−05

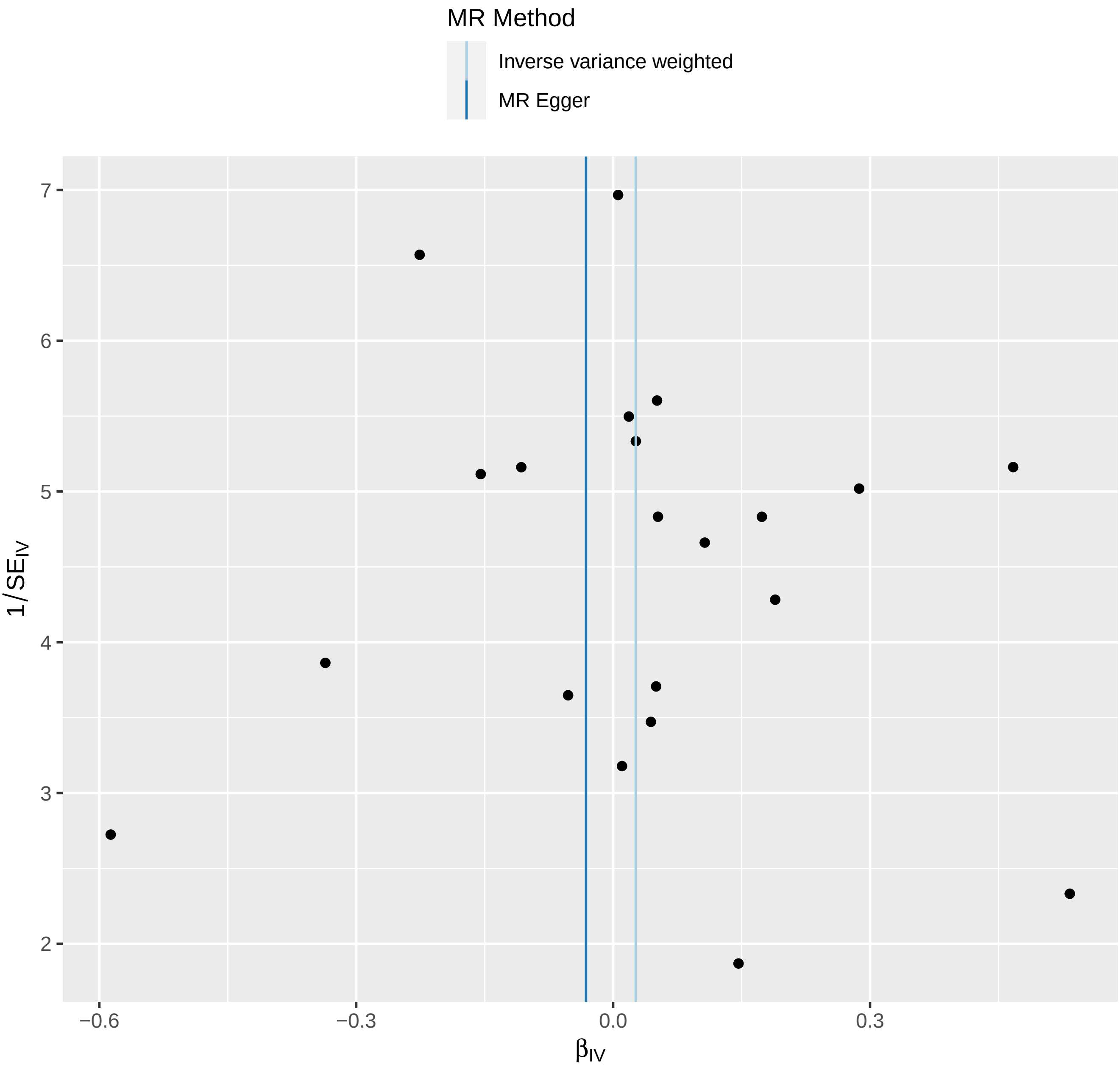

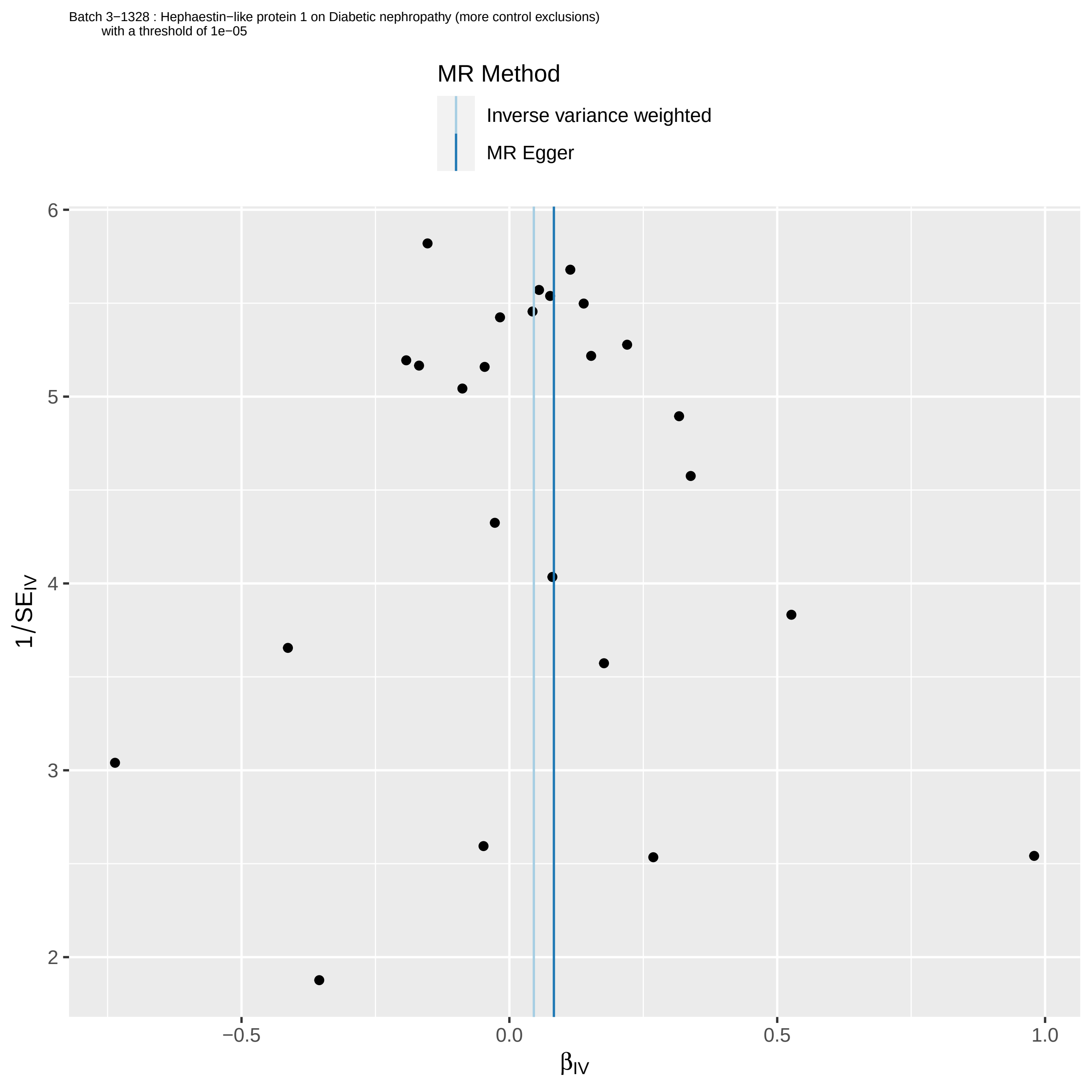
Batch 3−1328 : Hephaestin−like protein 1 on Diabetic nephropathy (more control exclusions)
with a threshold of 1e−05
MR Method
Inverse variance weighted
MR Egger
6
5
4
3
2
1/SE_IV
−0.5
0.0
0.5
1.0
β_IV

Batch 3−1329 : Probable E3 ubiquitin−protein ligase HERC1 on Diabetic nephropathy (more control exclusions)
with a threshold of 1e−05

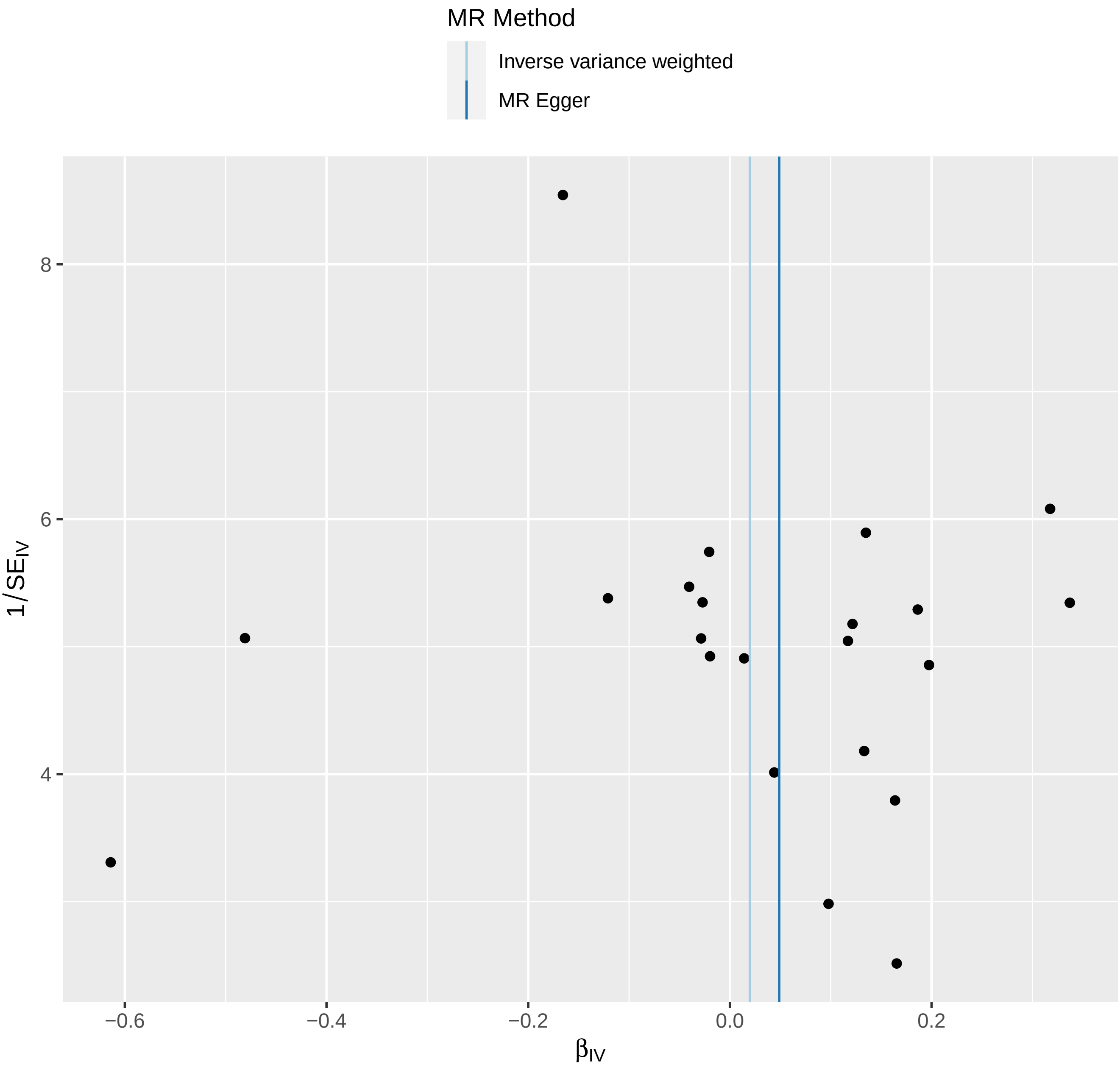

Batch 3−1330 : Probable E3 ubiquitin−protein ligase HERC4 on Diabetic nephropathy (more control exclusions) with a threshold of 1e−05

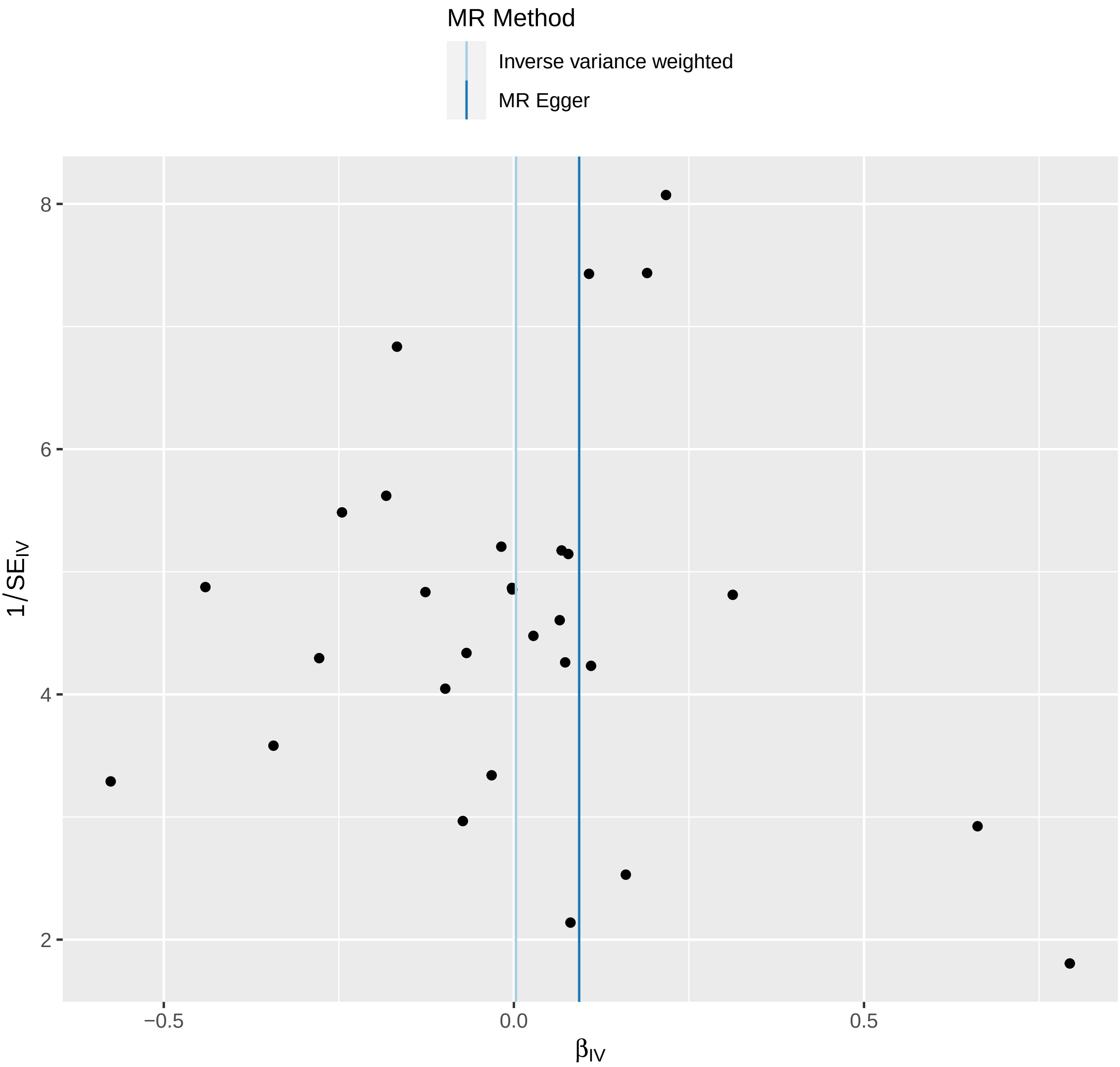

Batch 3−1331 : E3 ISG15−−protein ligase HERC5 on Diabetic nephropathy (more control exclusions)
with a threshold of 1e−05

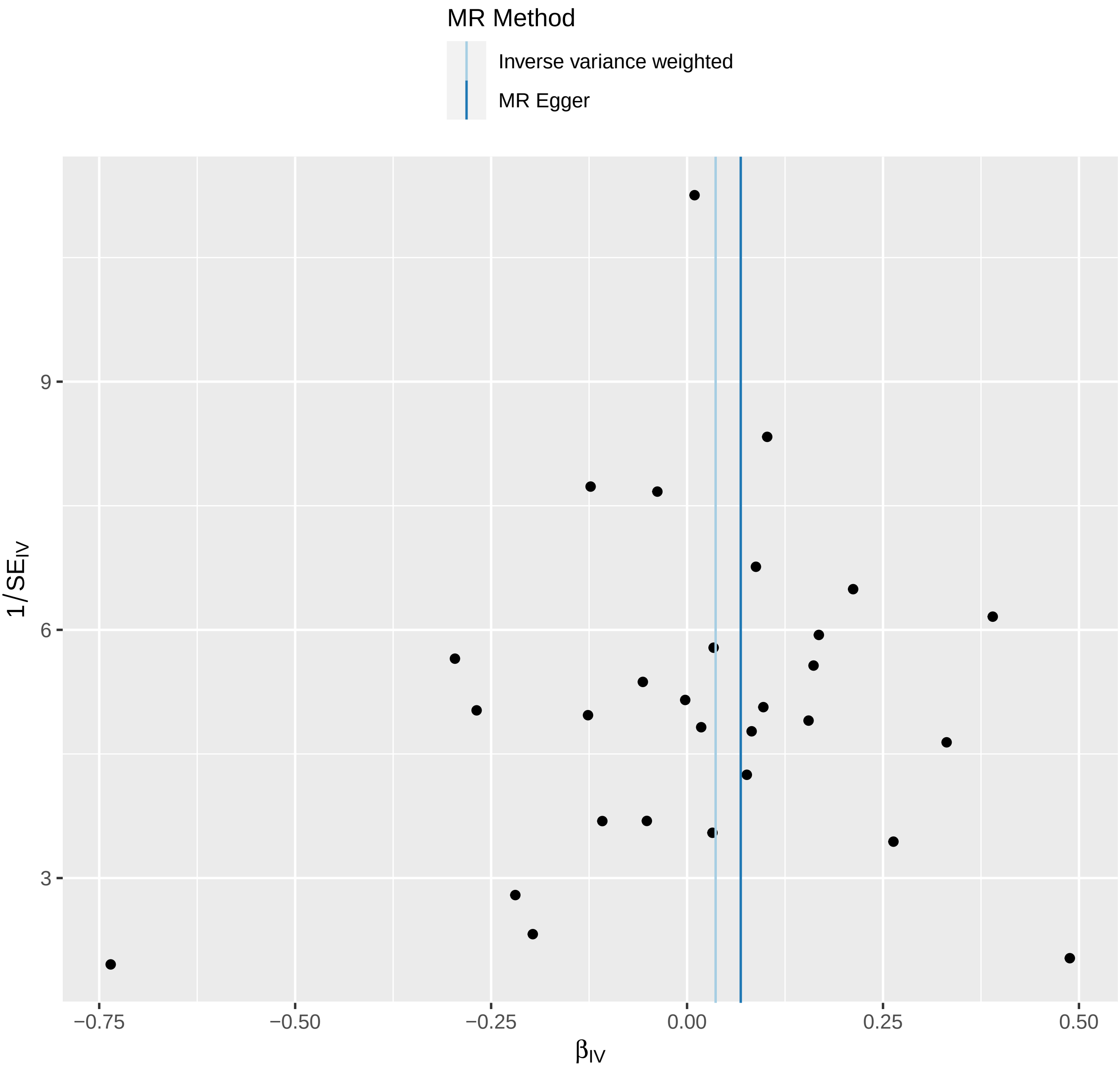

Batch 3−1332 : Hemojuvelin on Diabetic nephropathy (more control exclusions)
with a threshold of 1e−05

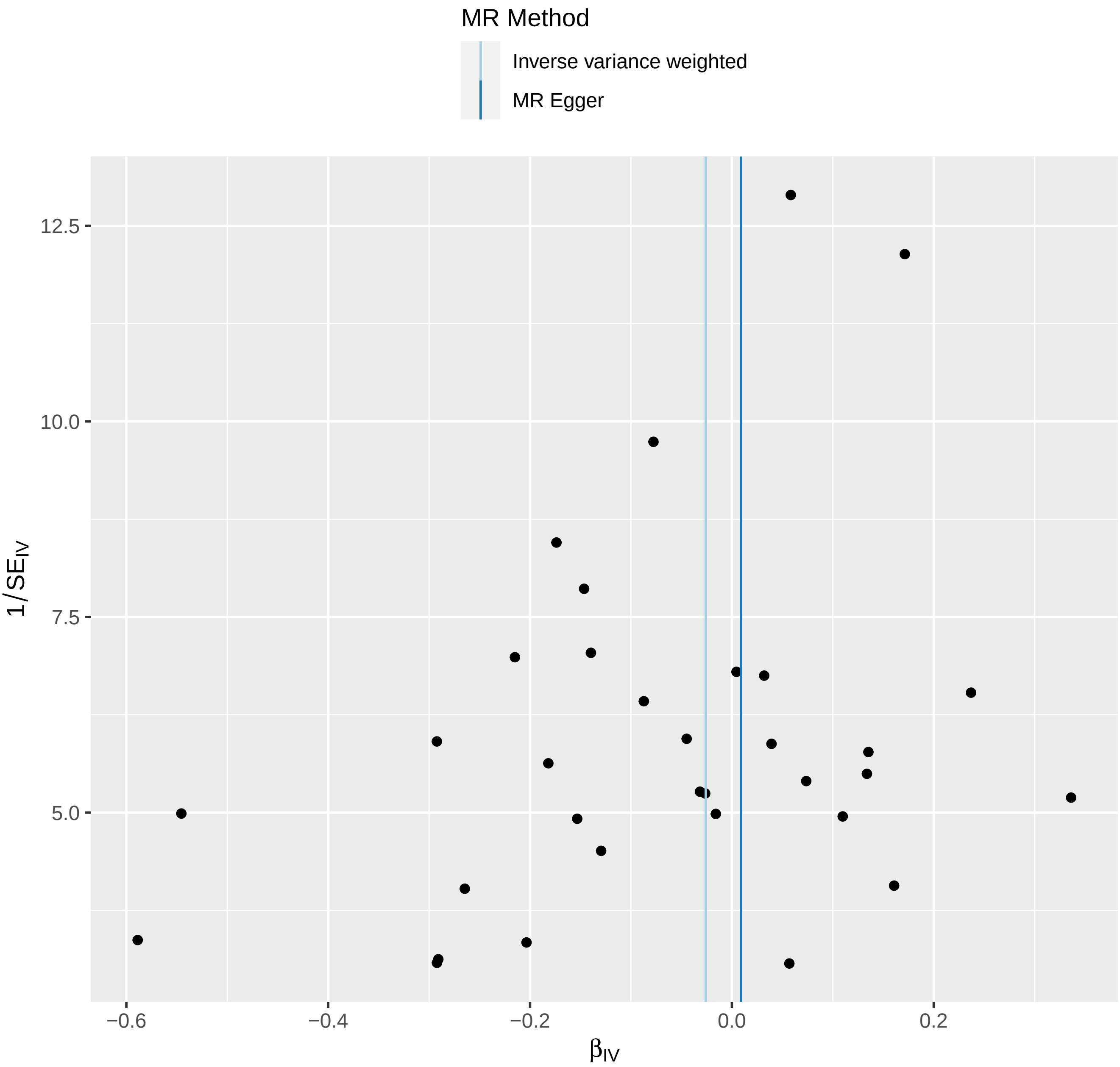

Batch 3−1333 : Homogentisate 1,2−dioxygenase on Diabetic nephropathy (more control exclusions)
with a threshold of 1e−05

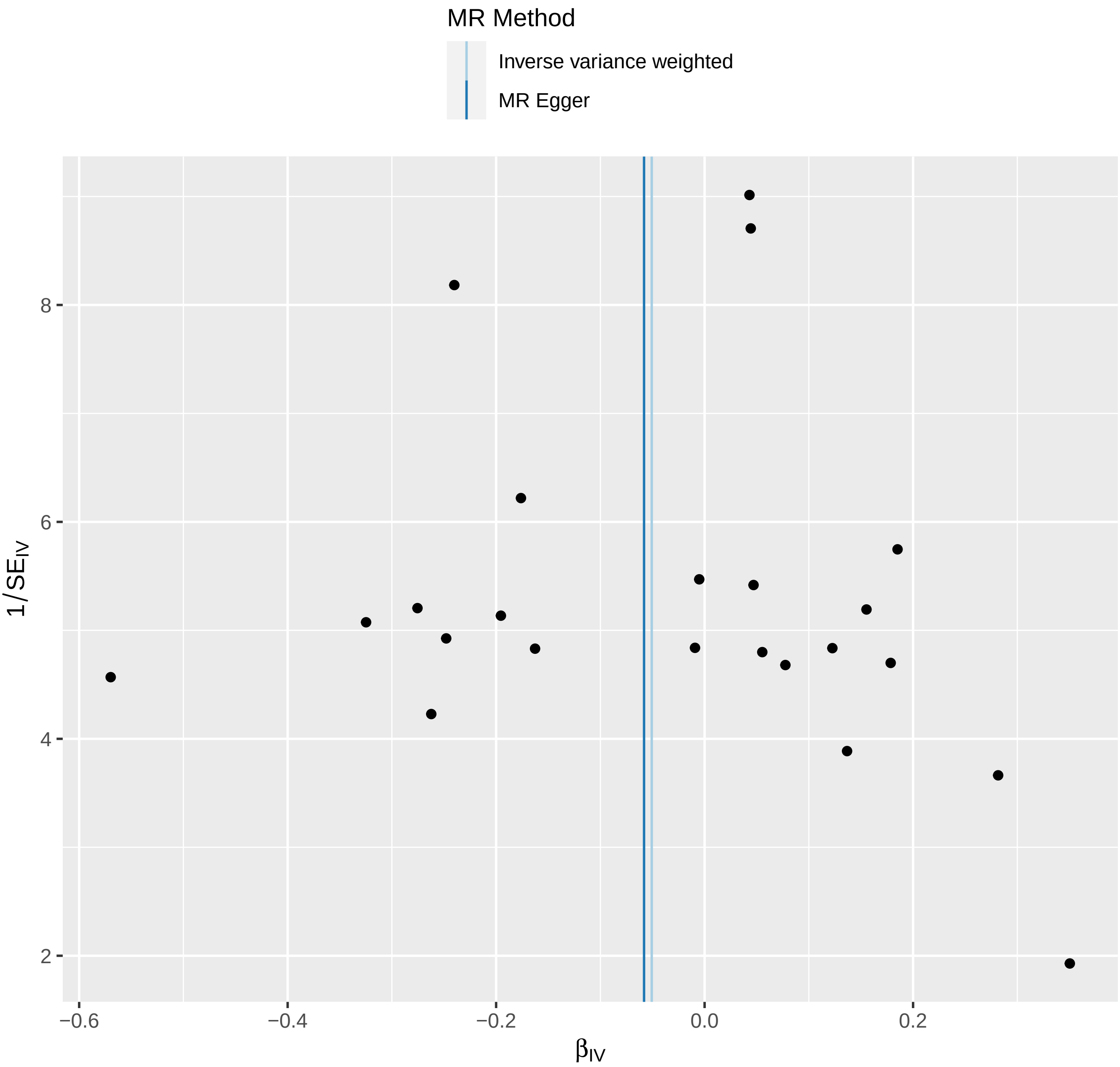

Batch 3−1334 : Hepatocyte growth factor on Diabetic nephropathy (more control exclusions)
with a threshold of 1e−05

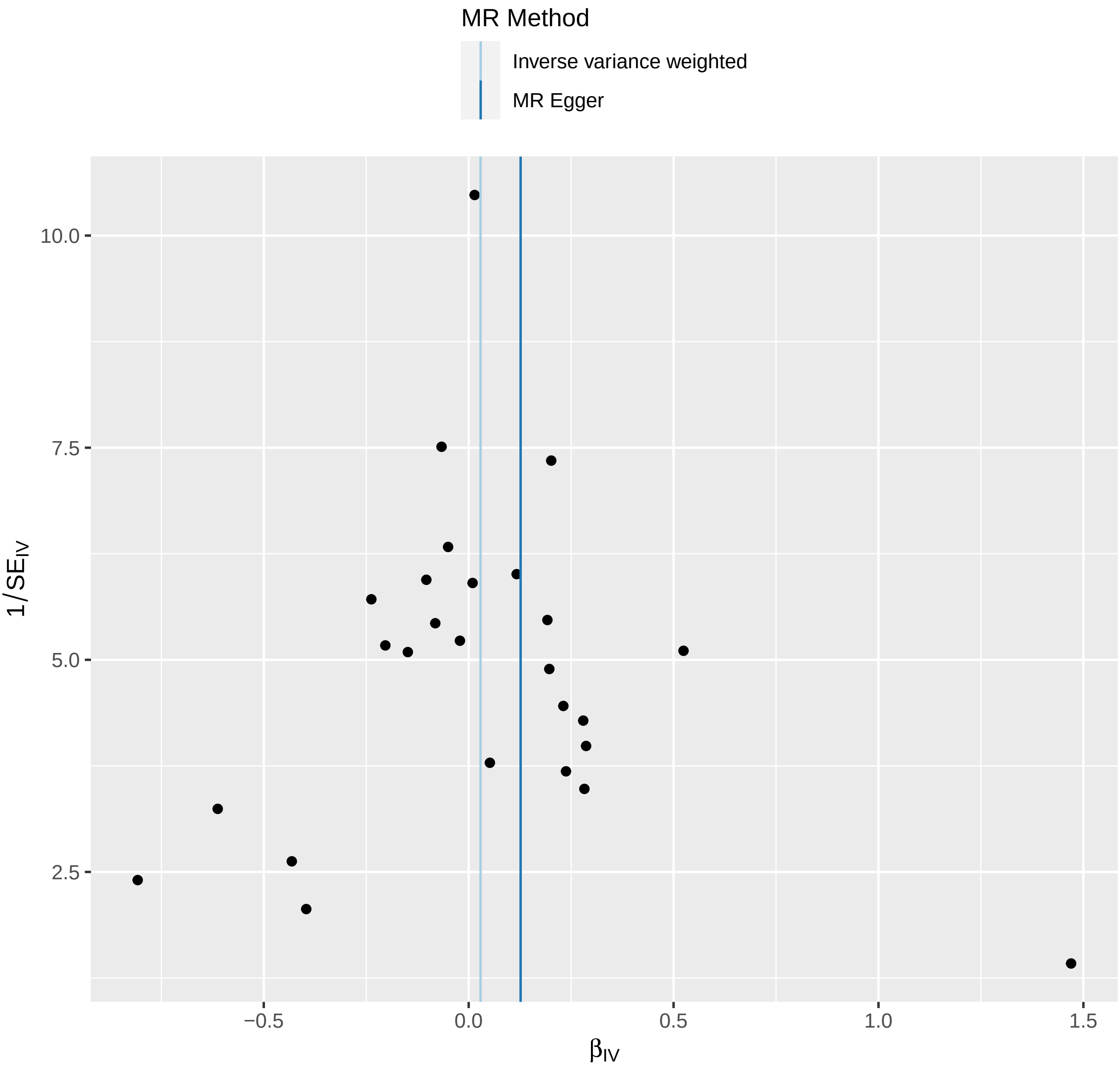

Batch 3−1335 : Hepatocyte growth factor−regulated tyrosine kinase substrate on Diabetic nephropathy (more control exclusions)
with a threshold of 1e−05

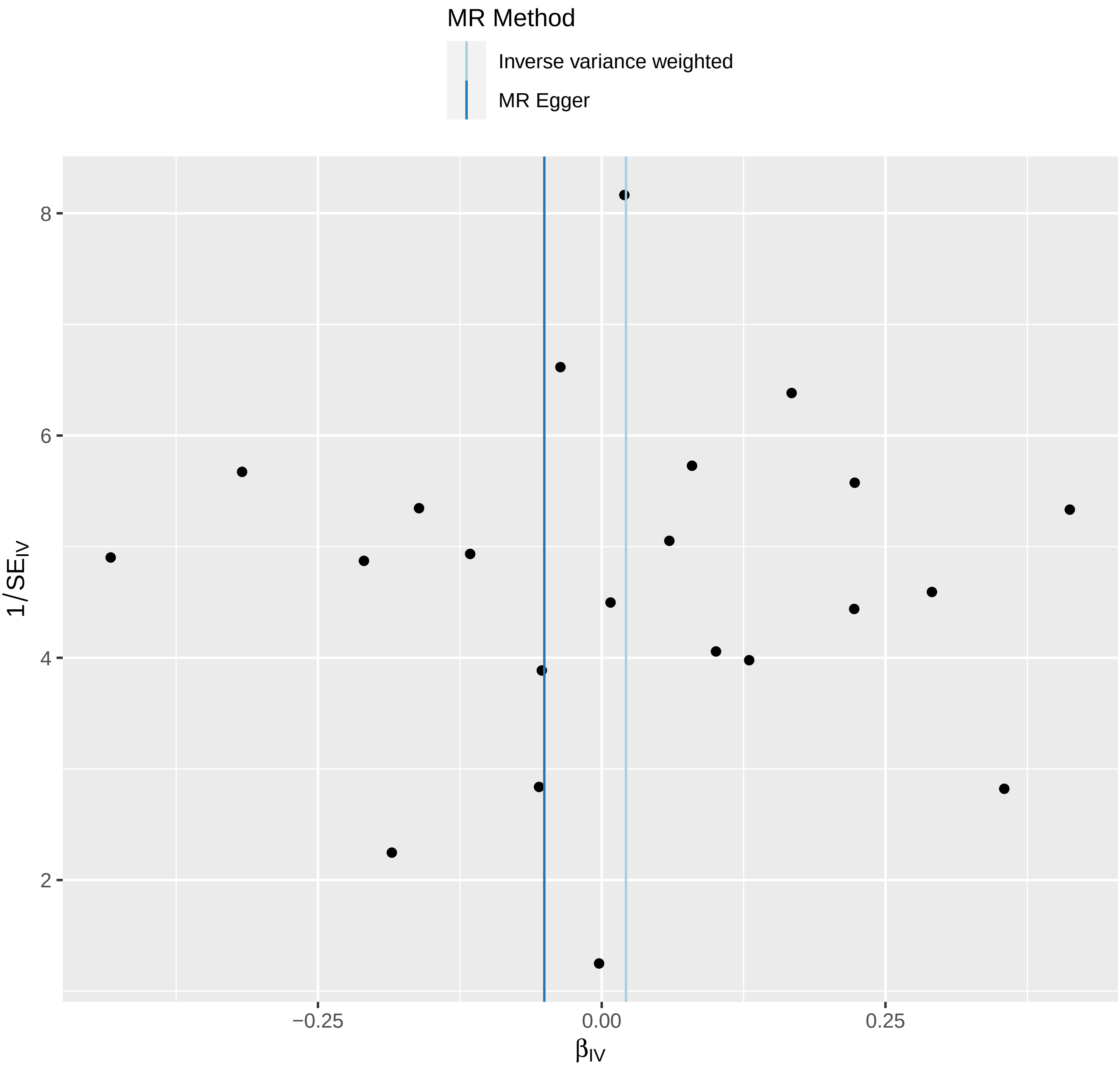

Batch 3−1336 : HERV−H LTR−associating protein 2 on Diabetic nephropathy (more control exclusions)
with a threshold of 1e−05

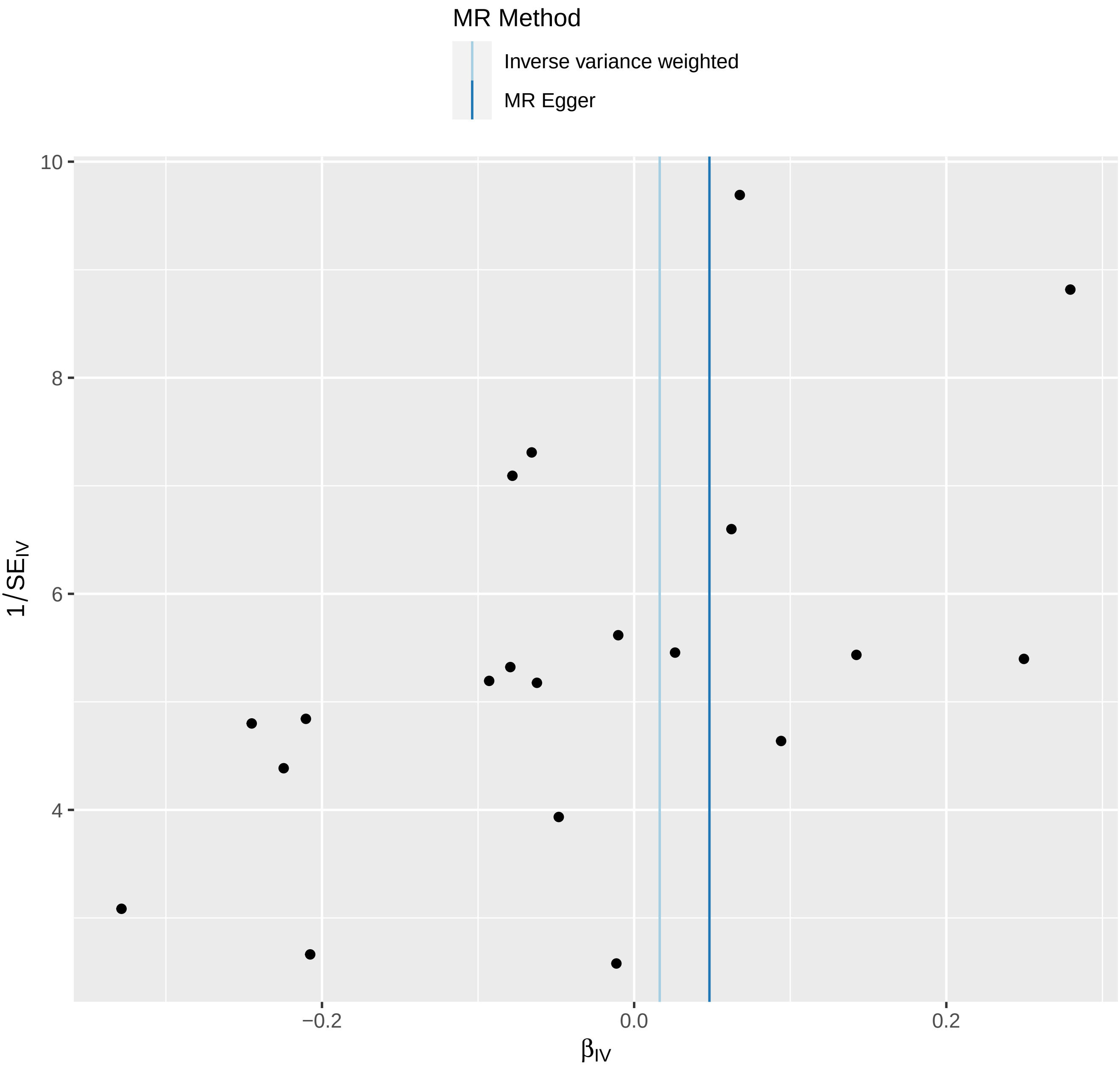

Batch 3−1337 : Hypoxia−inducible factor 1−alpha on Diabetic nephropathy (more control exclusions)
with a threshold of 1e−05

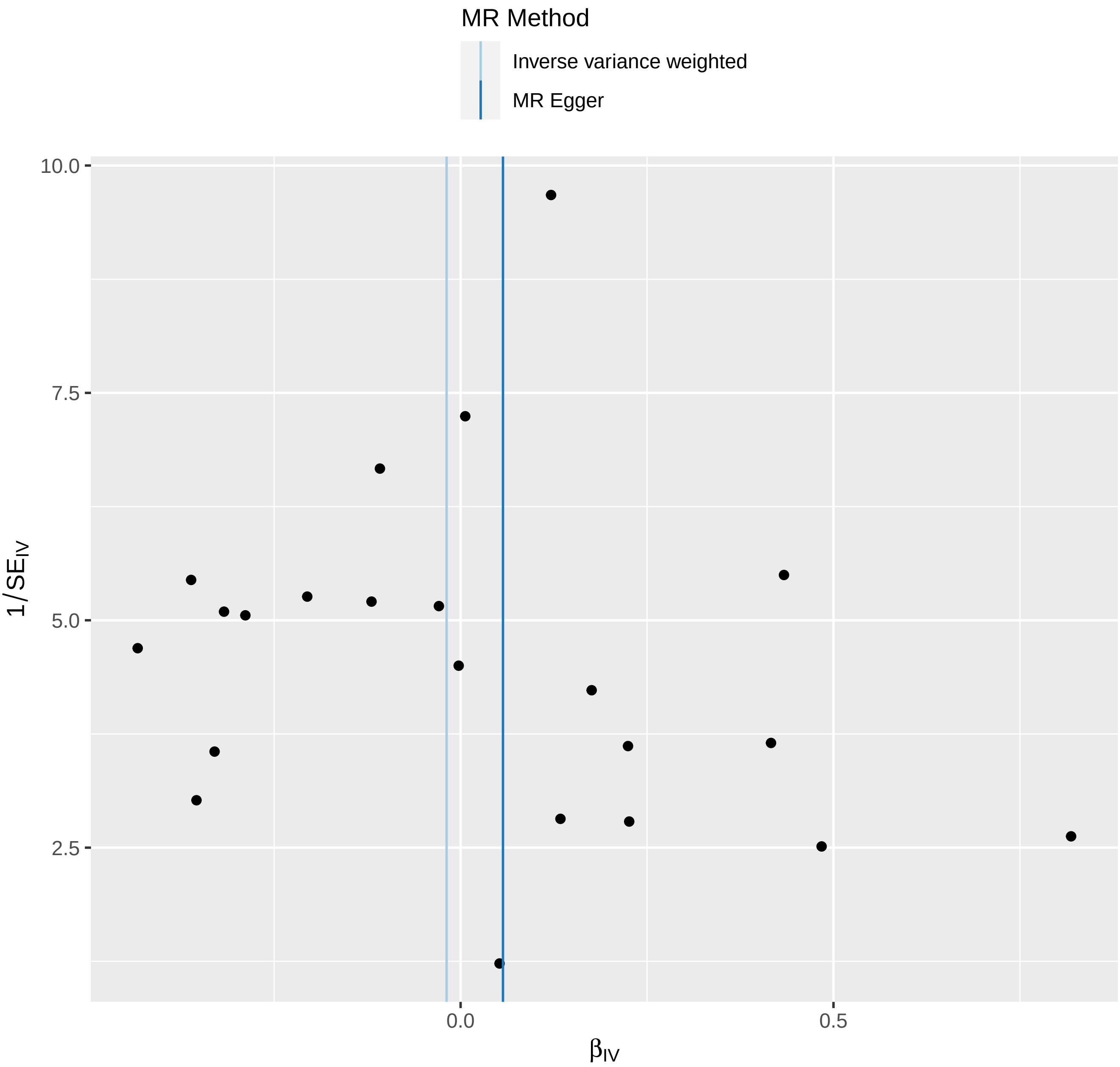

Batch 3−1338 : Histidine triad nucleotide−binding protein 1 on Diabetic nephropathy (more control exclusions)
with a threshold of 1e−05

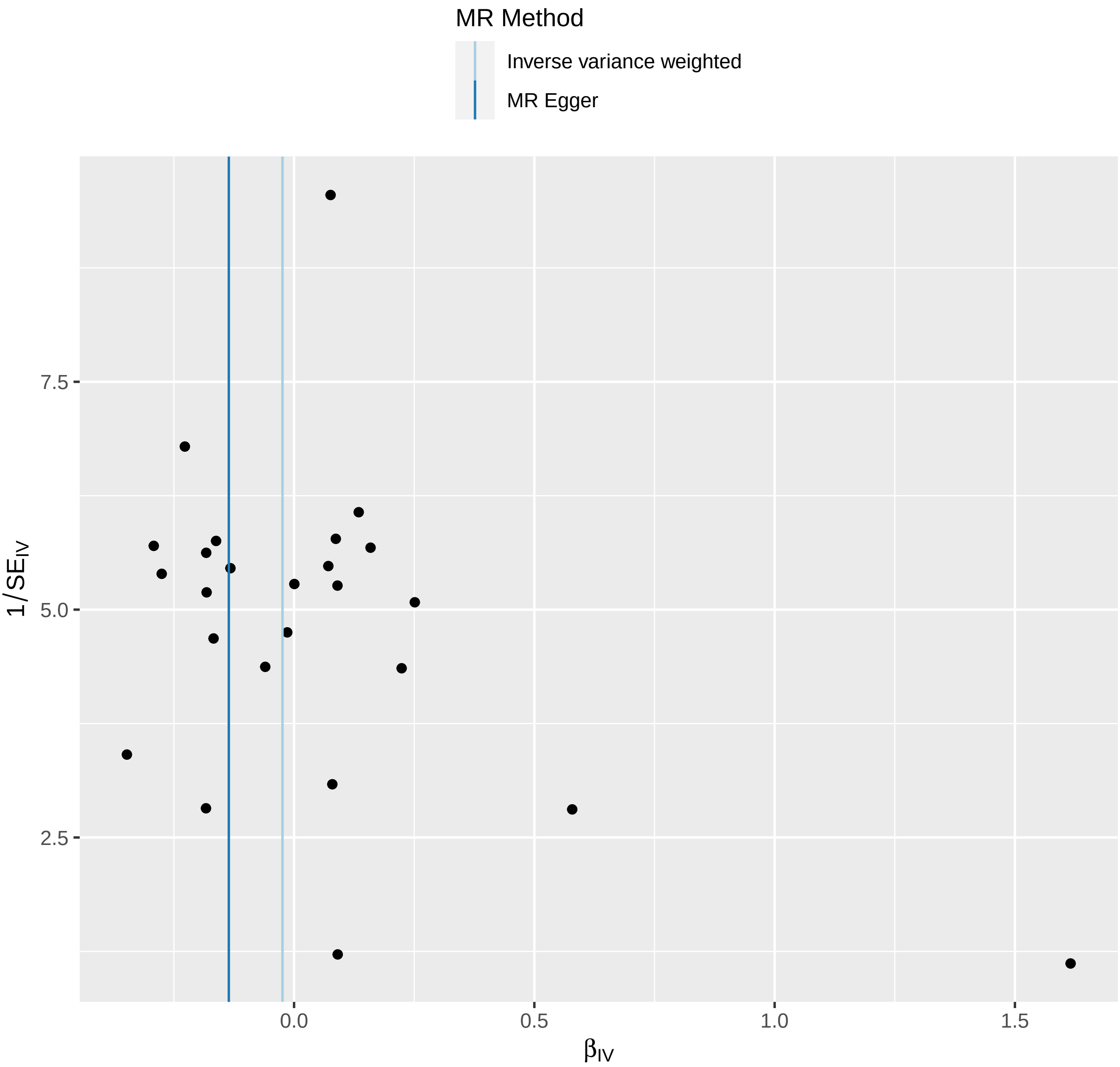

Batch 3−1339 : Histidine triad nucleotide−binding protein 2, mitochondrial on Diabetic nephropathy (more control exclusions) with a threshold of 1e−05

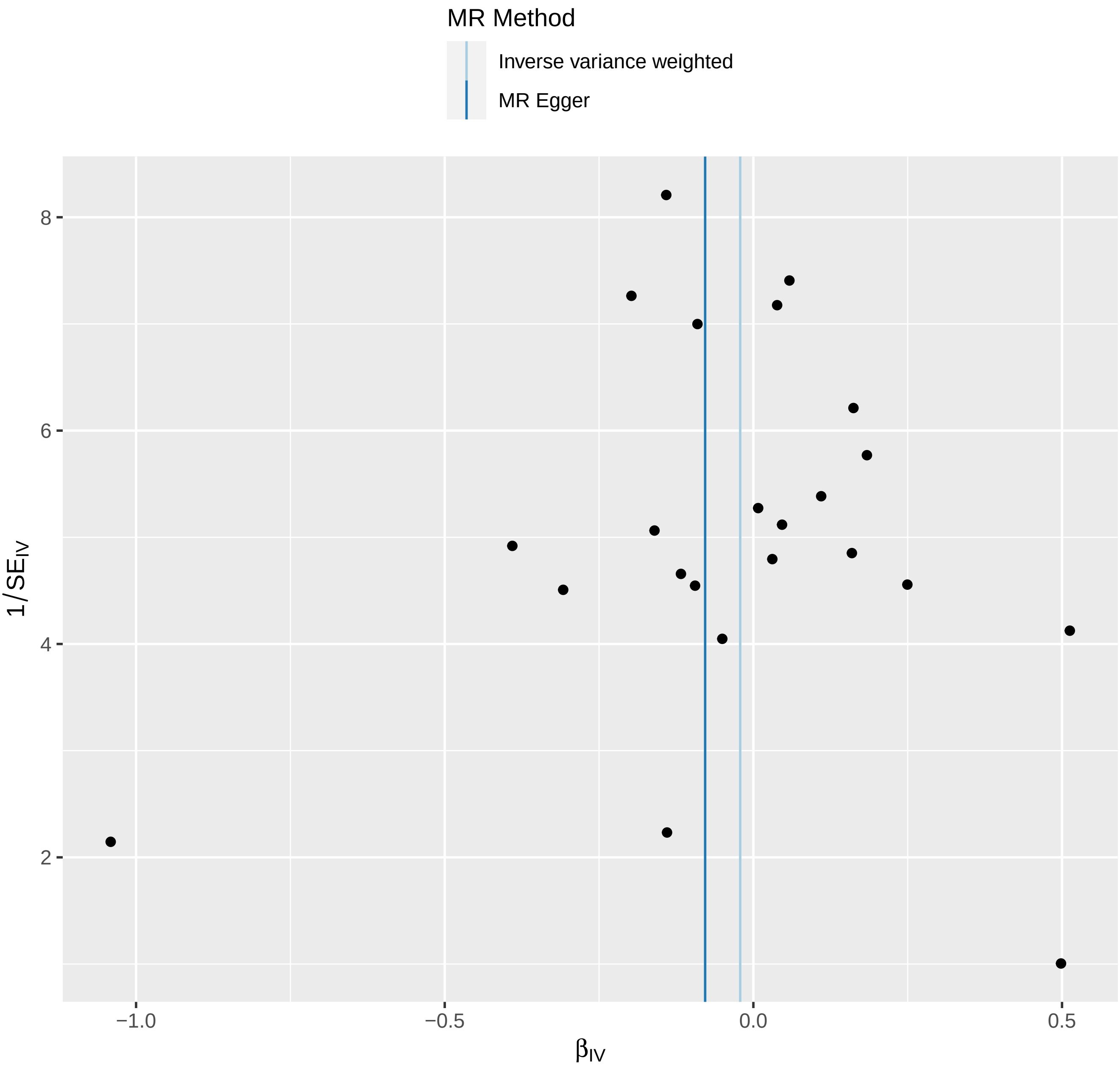

Batch 3−1340 : Homeodomain−interacting protein kinase 3 on Diabetic nephropathy (more control exclusions) with a threshold of 1e−05

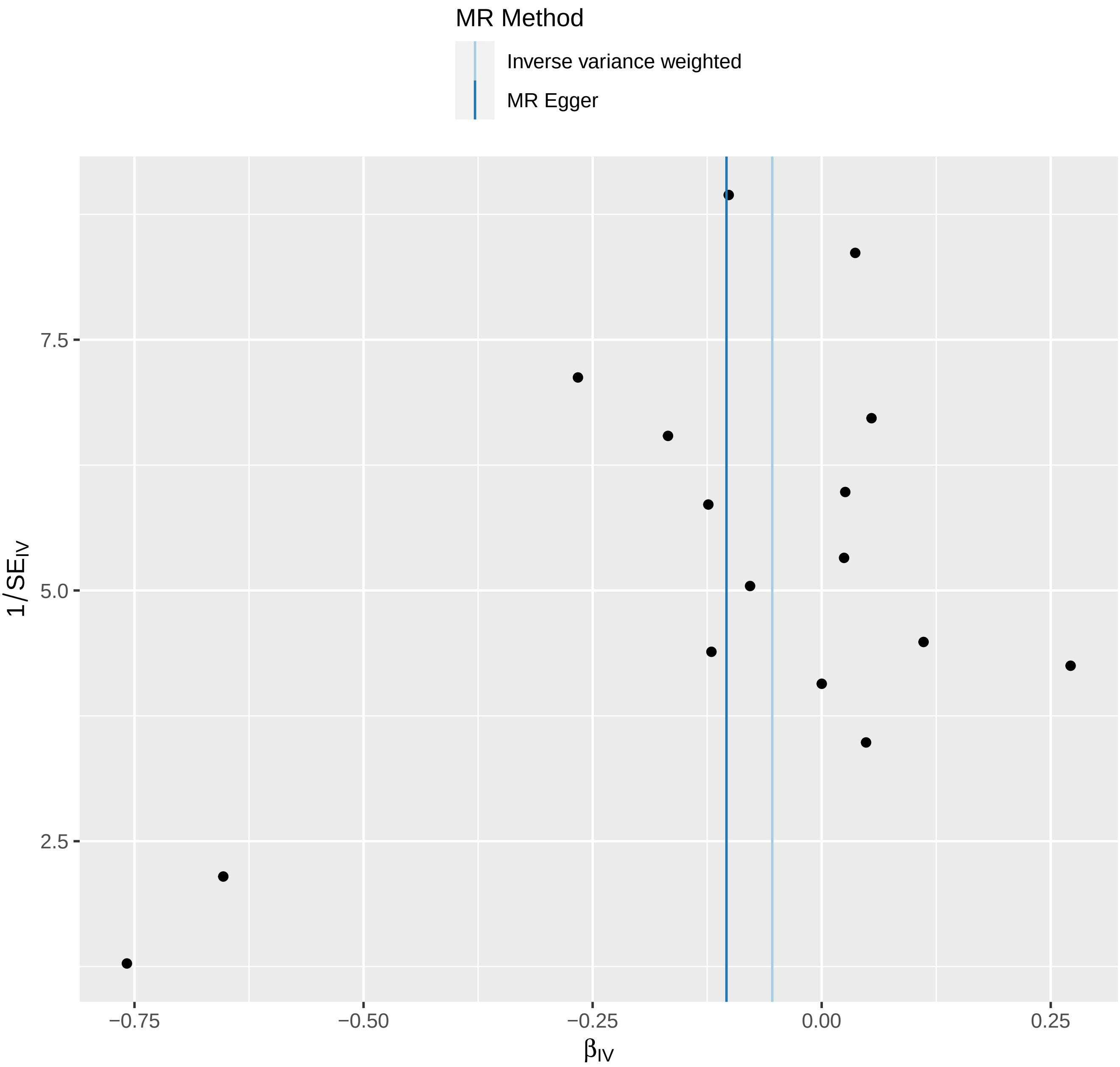

Batch 3−1341 : Histone H1.2 on Diabetic nephropathy (more control exclusions)
with a threshold of 1e−05

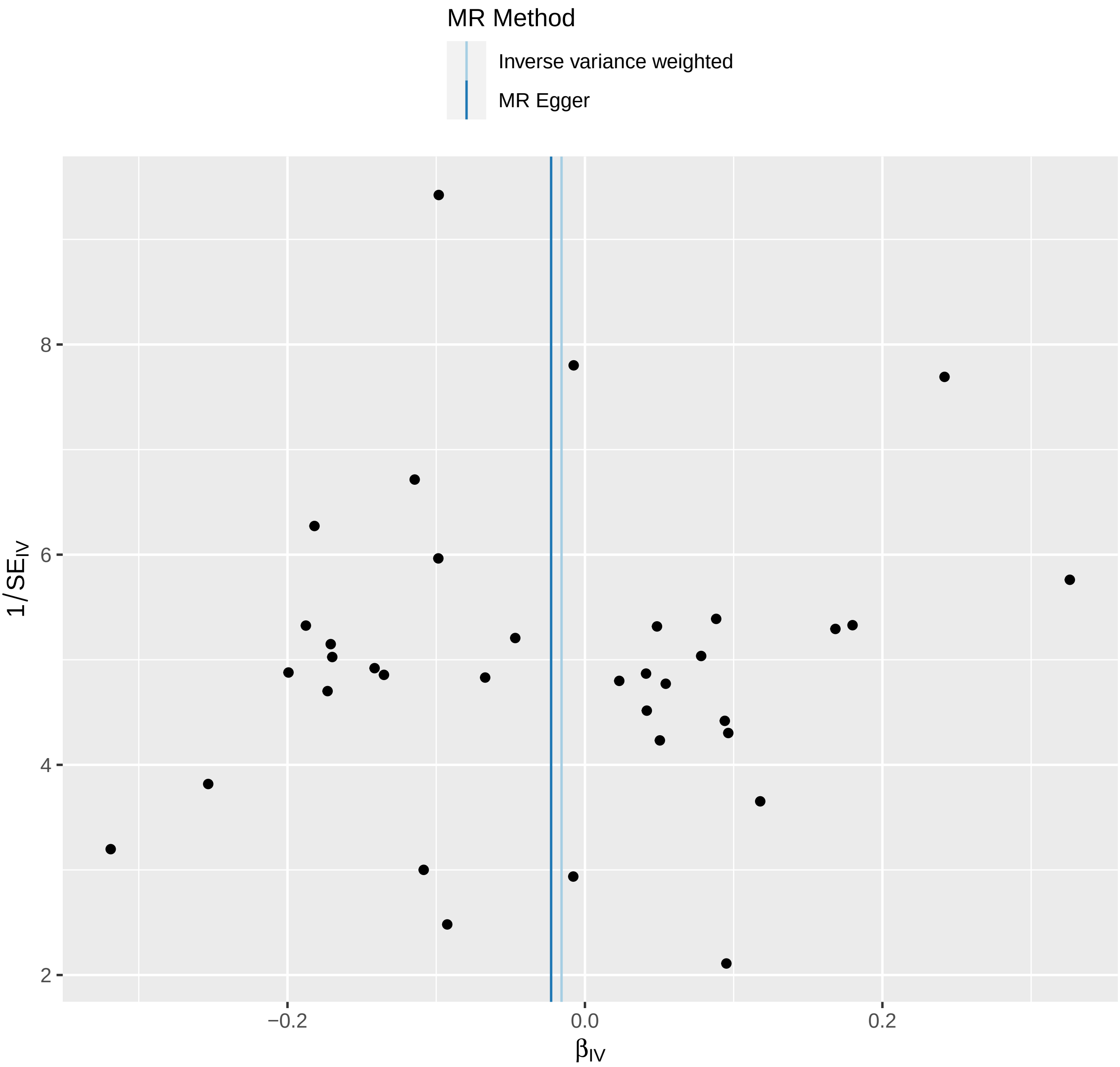

Batch 3−1342 : Histone H2A type 3 on Diabetic nephropathy (more control exclusions)
with a threshold of 1e−05

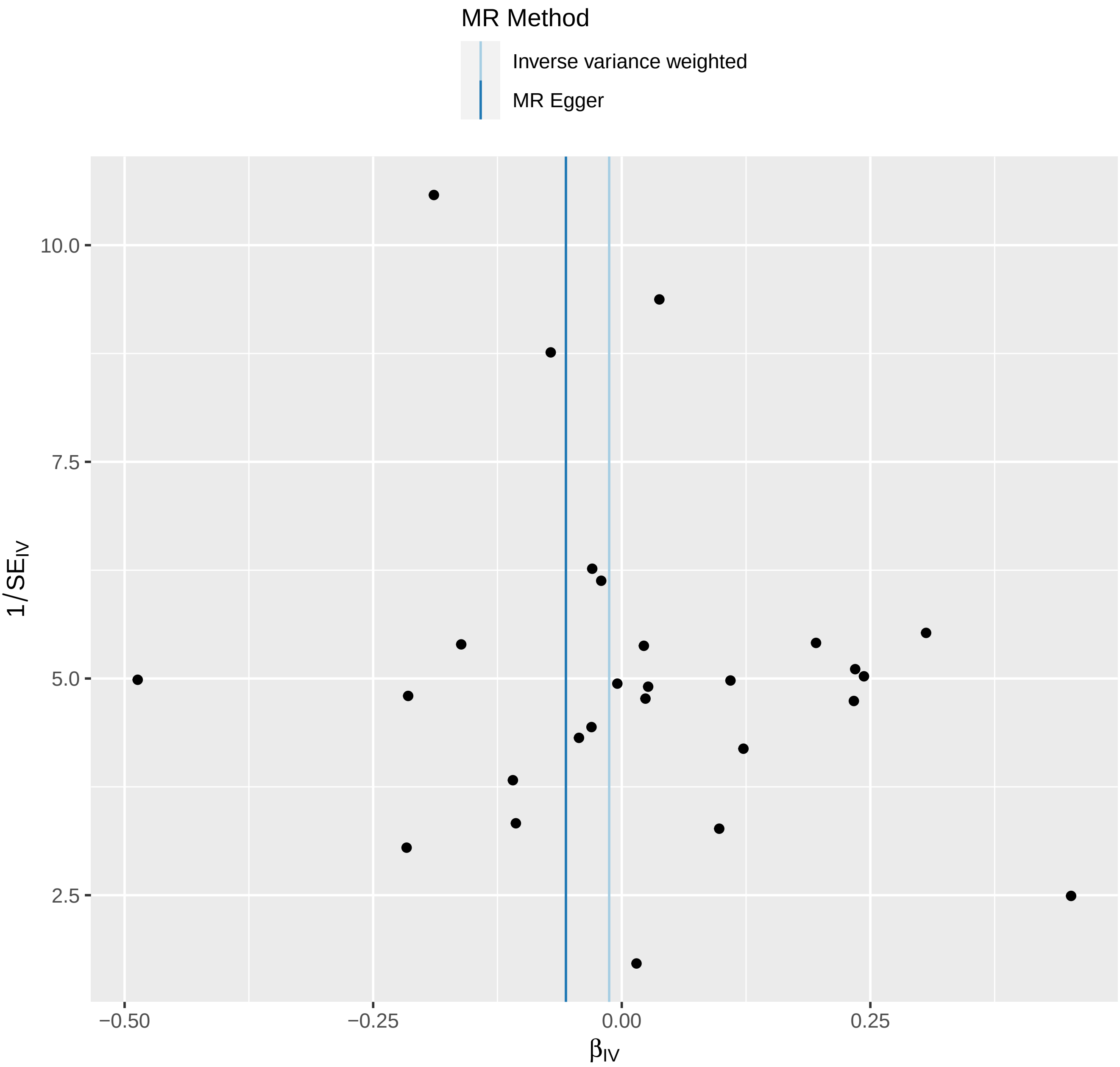

Batch 3−1343 : Hexokinase−1 on Diabetic nephropathy (more control exclusions)
with a threshold of 1e−05

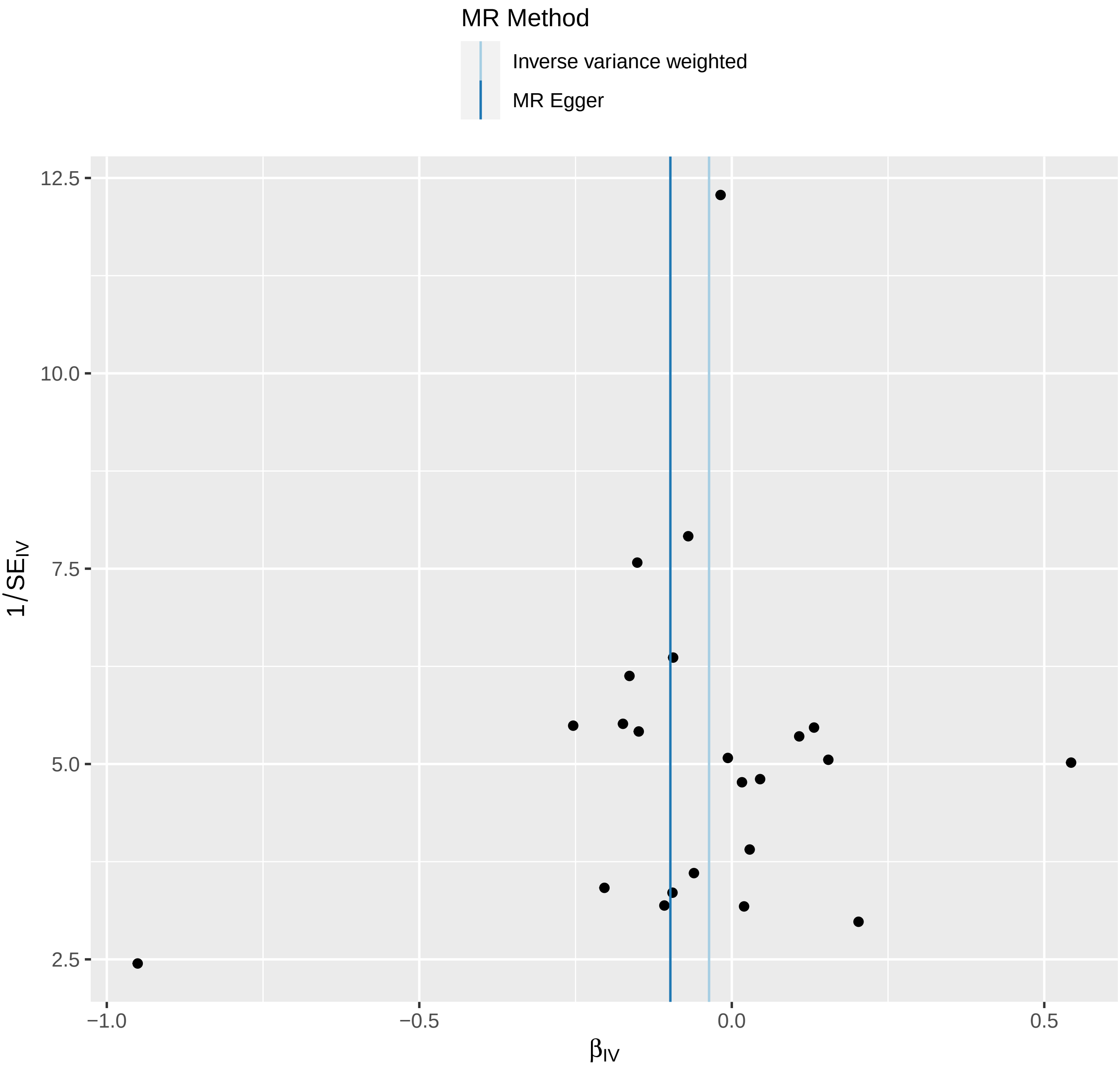

Batch 3−1344 : Hexokinase−2 on Diabetic nephropathy (more control exclusions)
with a threshold of 1e−05

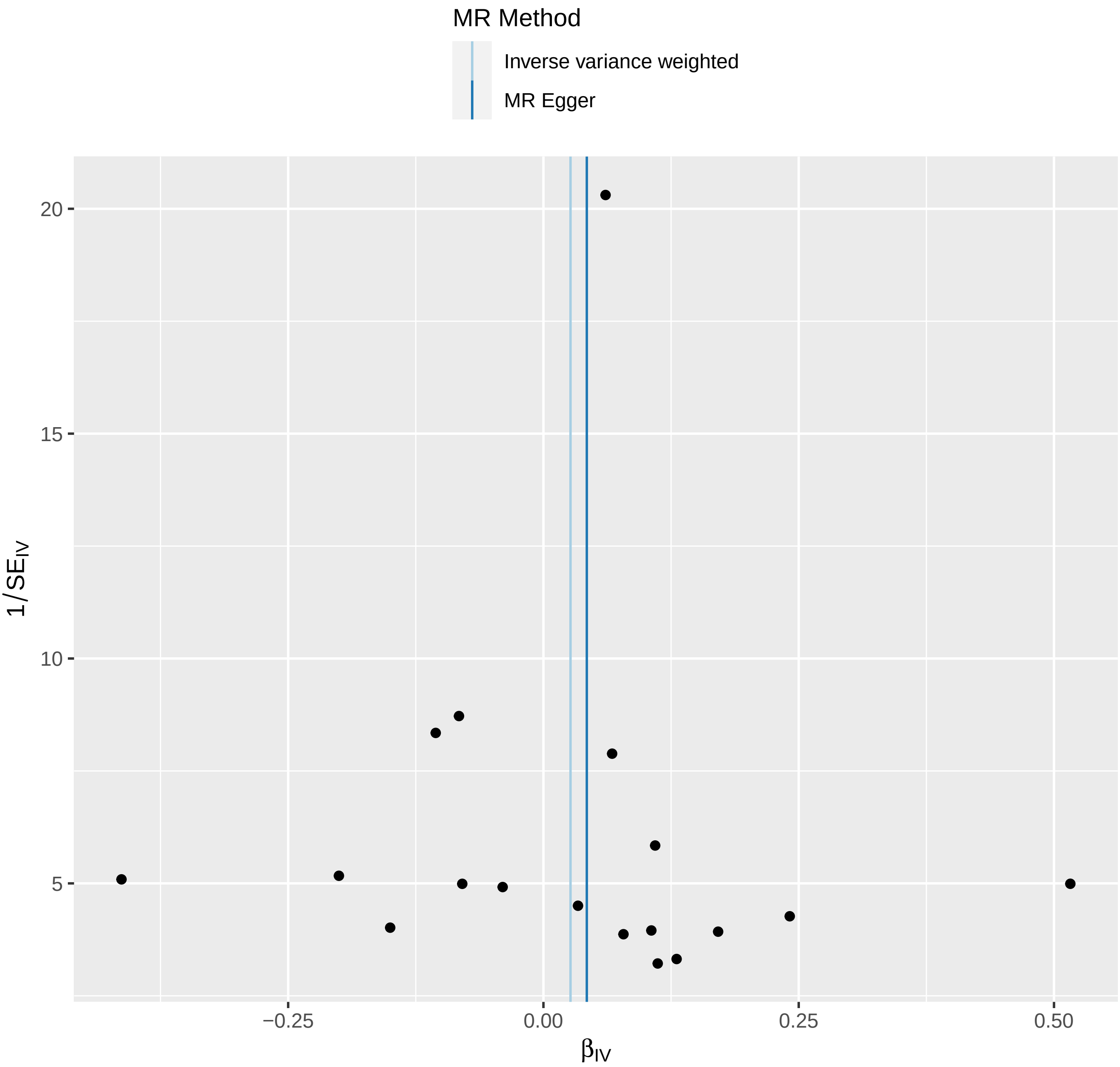

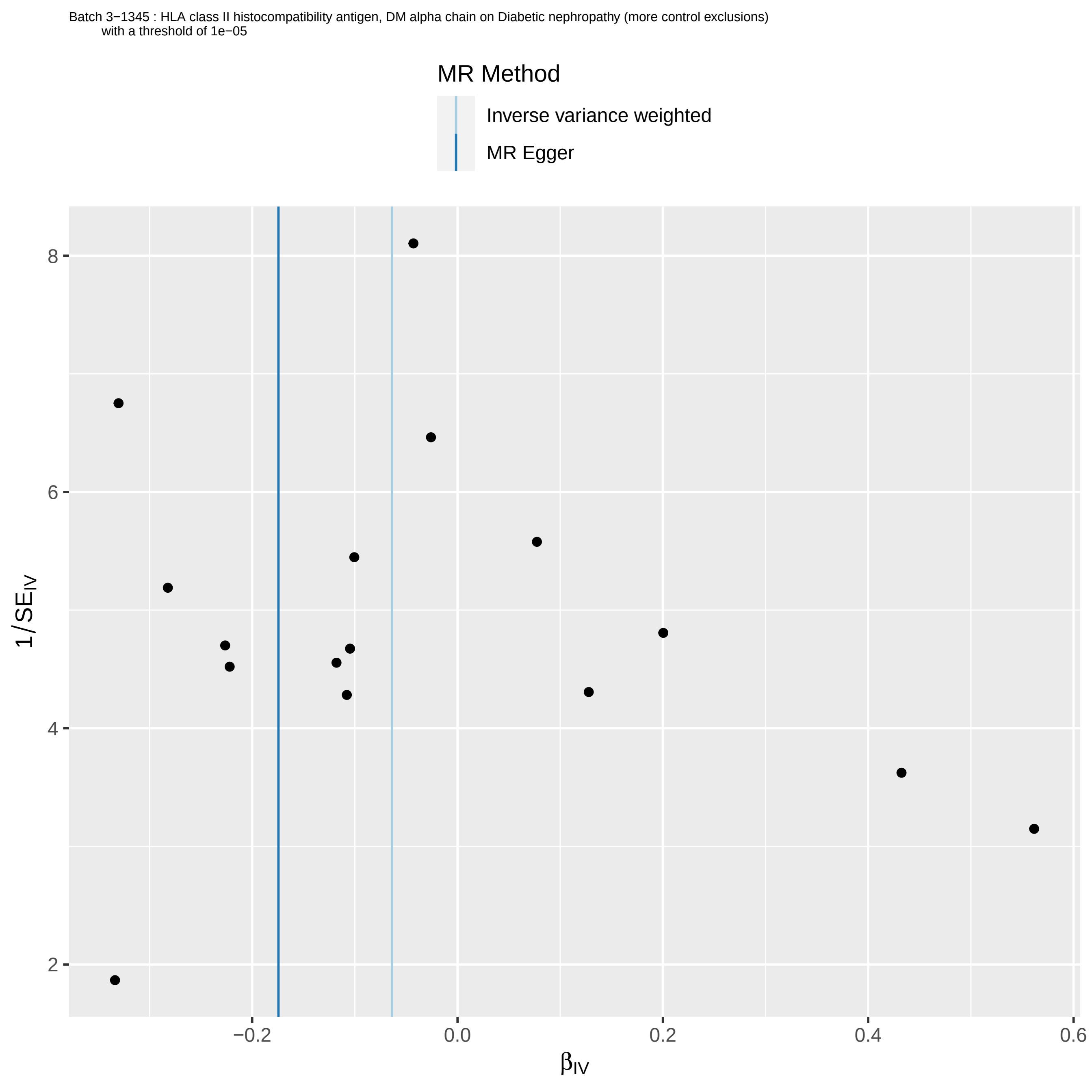
Batch 3−1345 : HLA class II histocompatibility antigen, DM alpha chain on Diabetic nephropathy (more control exclusions)
with a threshold of 1e−05
MR Method
Inverse variance weighted
MR Egger
8
6
4
2
$1/SE_{IV}$
−0.2
0.0
0.2
0.4
0.6
β_{IV}

Batch 3−1346 : HLA class II histocompatibility antigen, DP beta 1 chain on Diabetic nephropathy (more control exclusions)
with a threshold of 1e−05

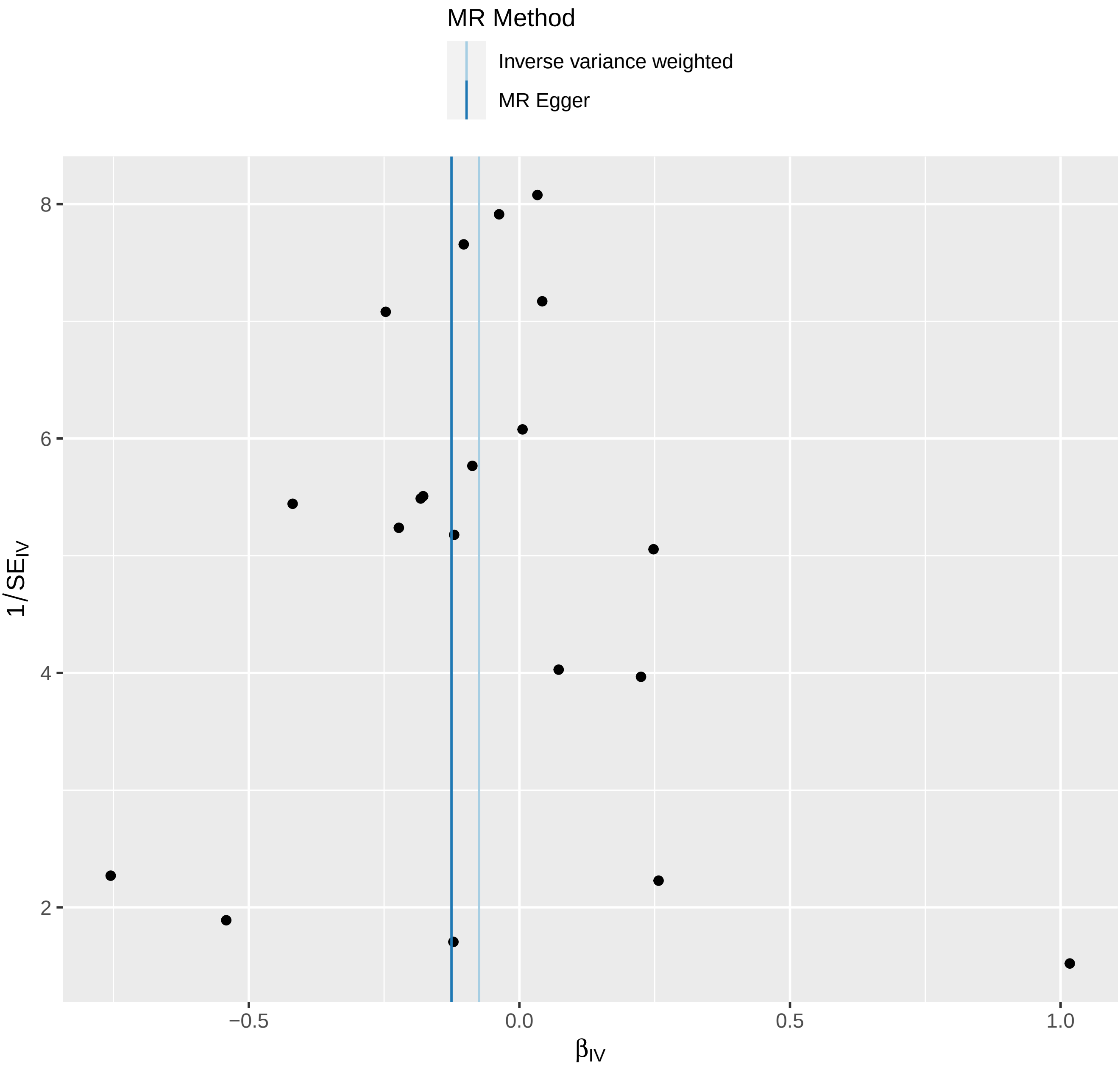

Batch 3−1347 : HLA class II histocompatibility antigen, DQ alpha 2 chain on Diabetic nephropathy (more control exclusions) with a threshold of 1e−05

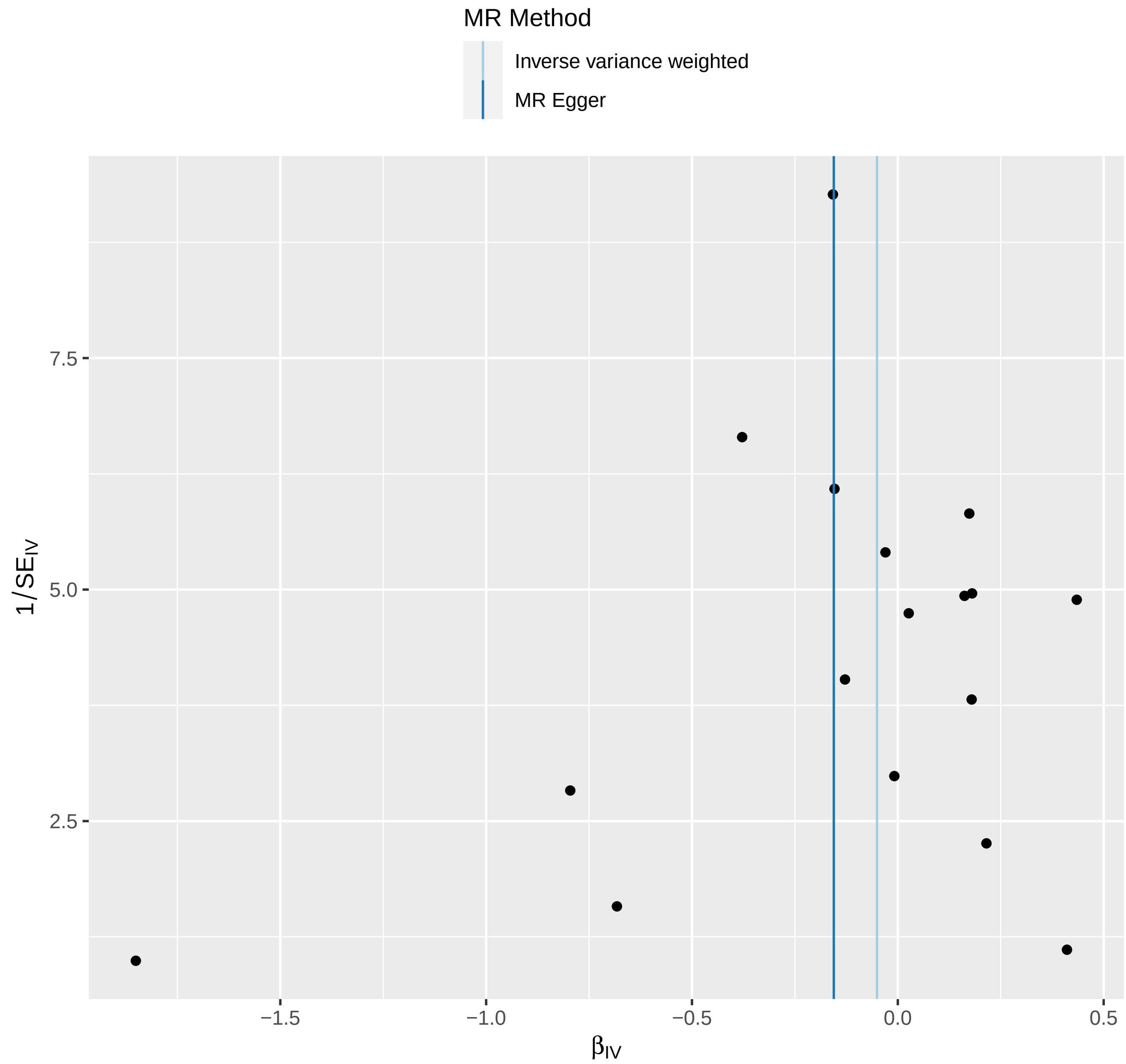

Batch 3−1348 : Porphobilinogen deaminase on Diabetic nephropathy (more control exclusions)
with a threshold of 1e−05

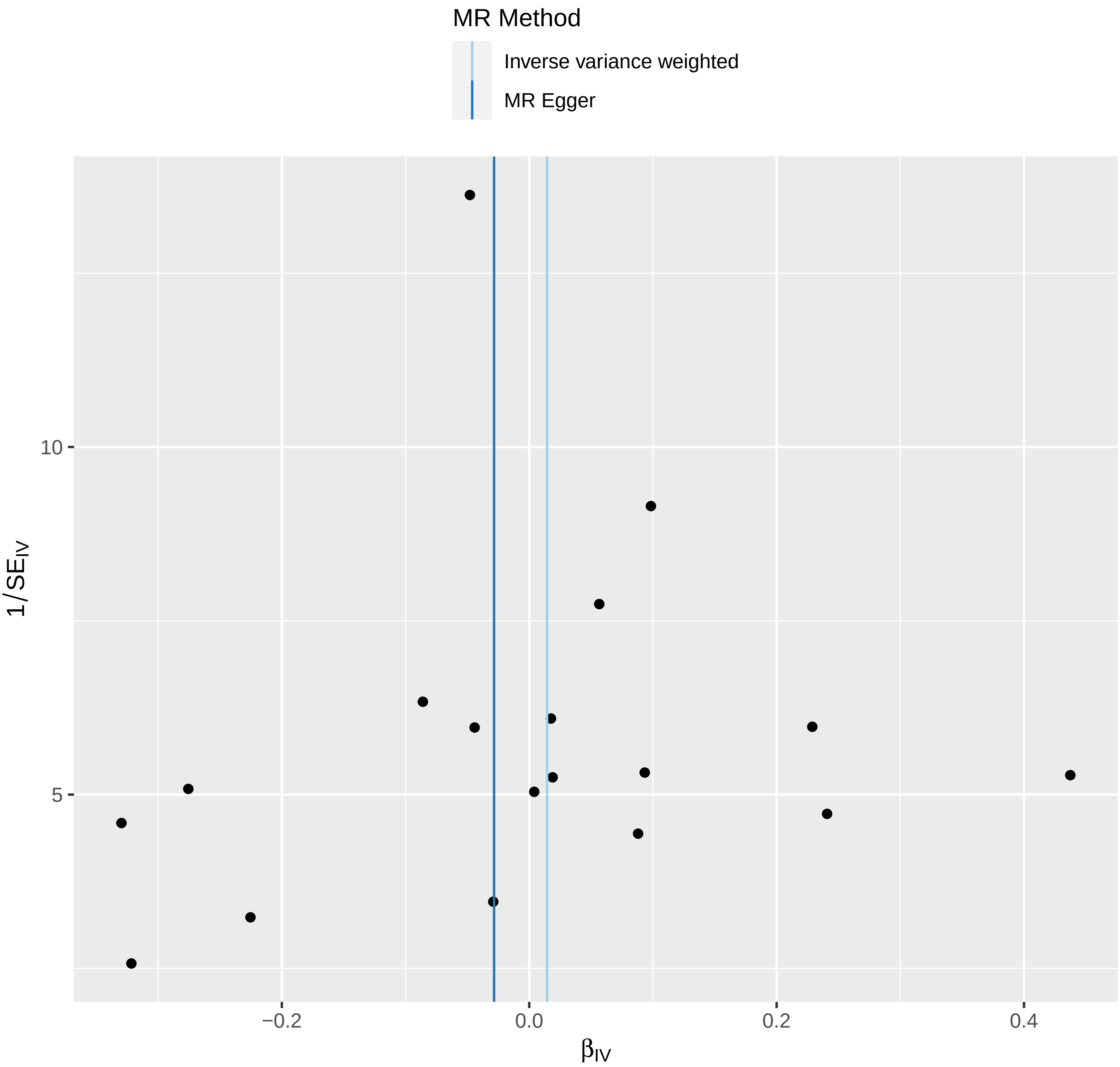

Batch 3−1349 : High mobility group protein 20A on Diabetic nephropathy (more control exclusions)
with a threshold of 1e−05

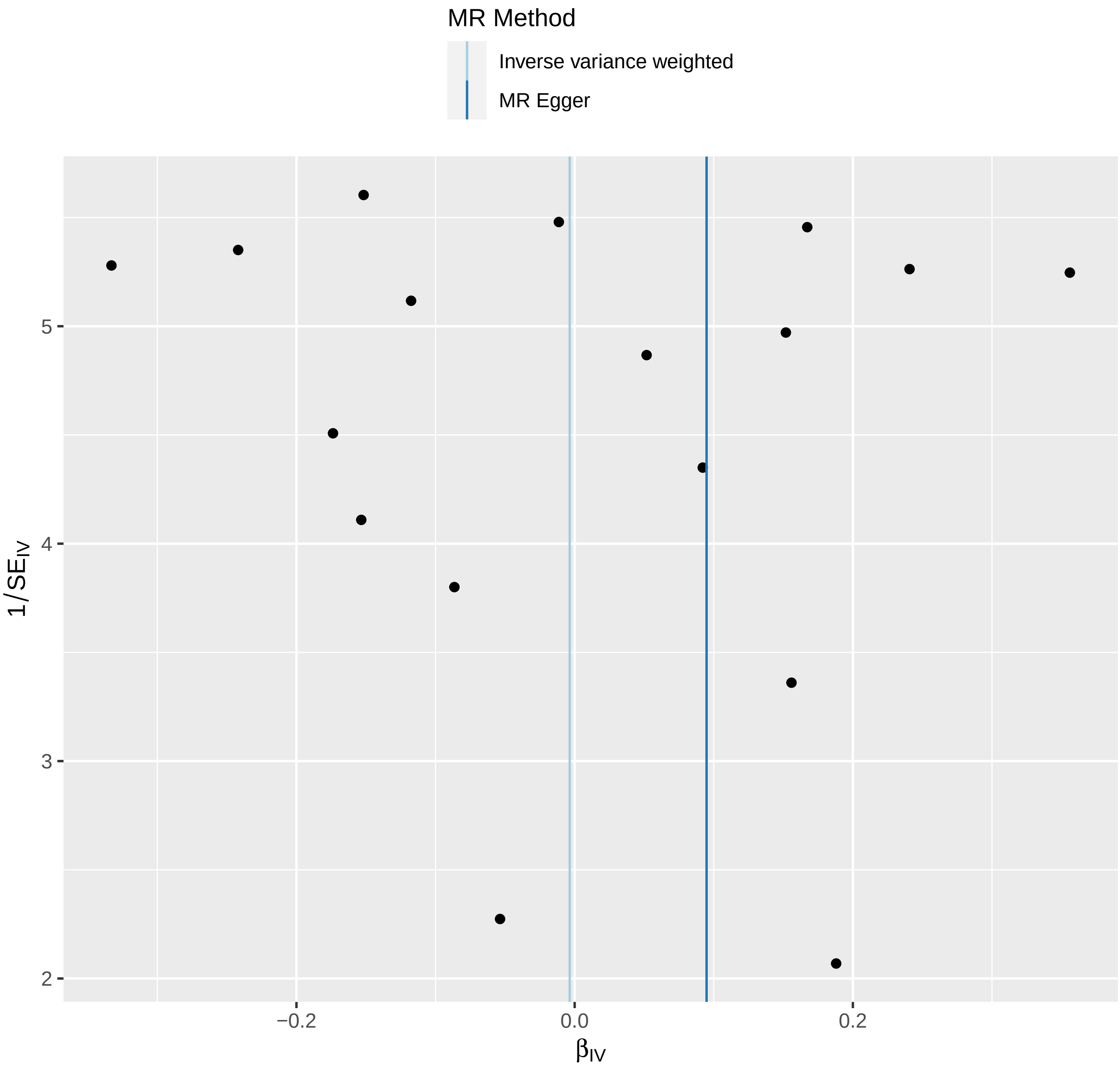

Batch 3−1350 : SWI/SNF−related matrix−associated actin−dependent regulator of chromatin subfamily E member 1−related on Diabetic nephropathy (more control exclusions) with a threshold of 1e−05

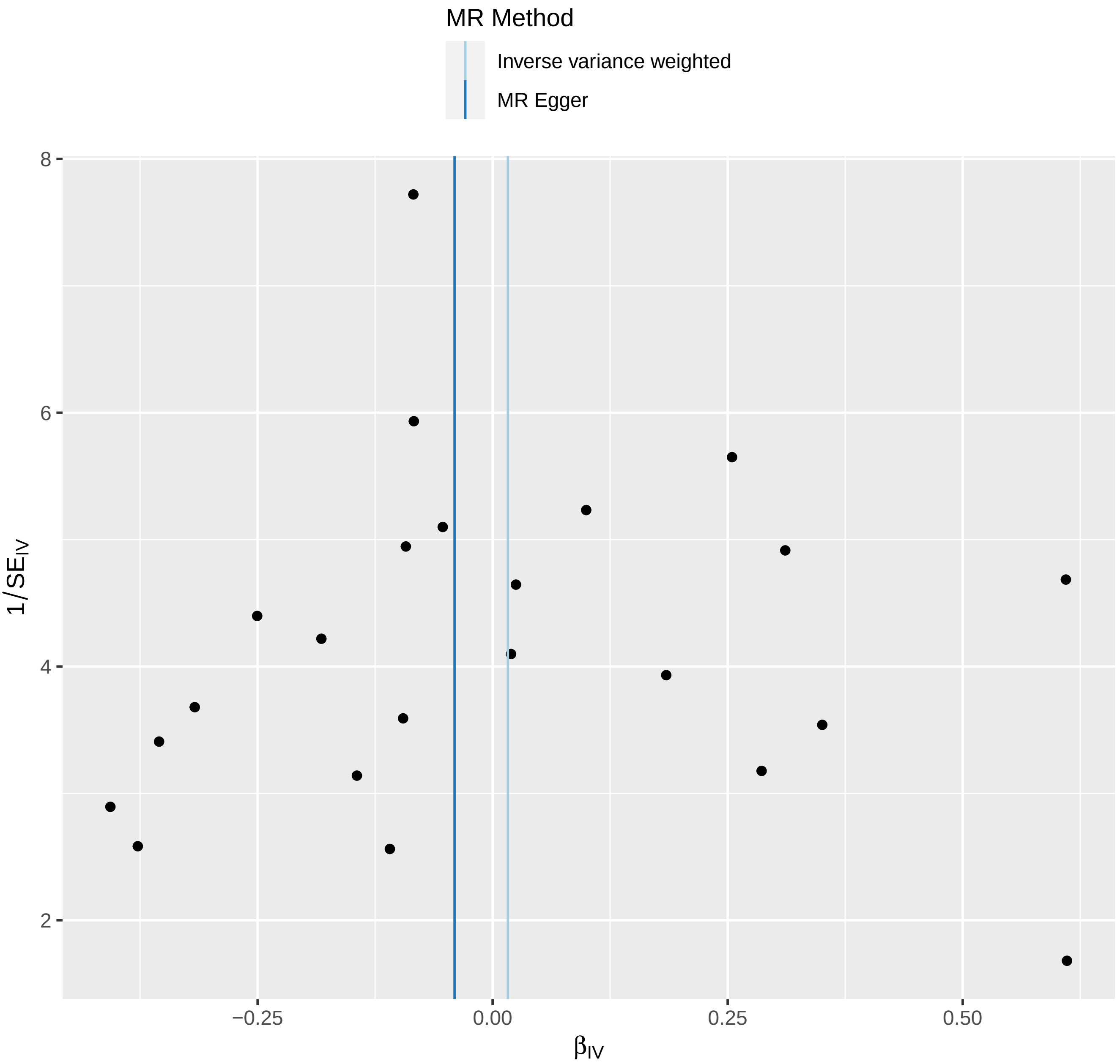

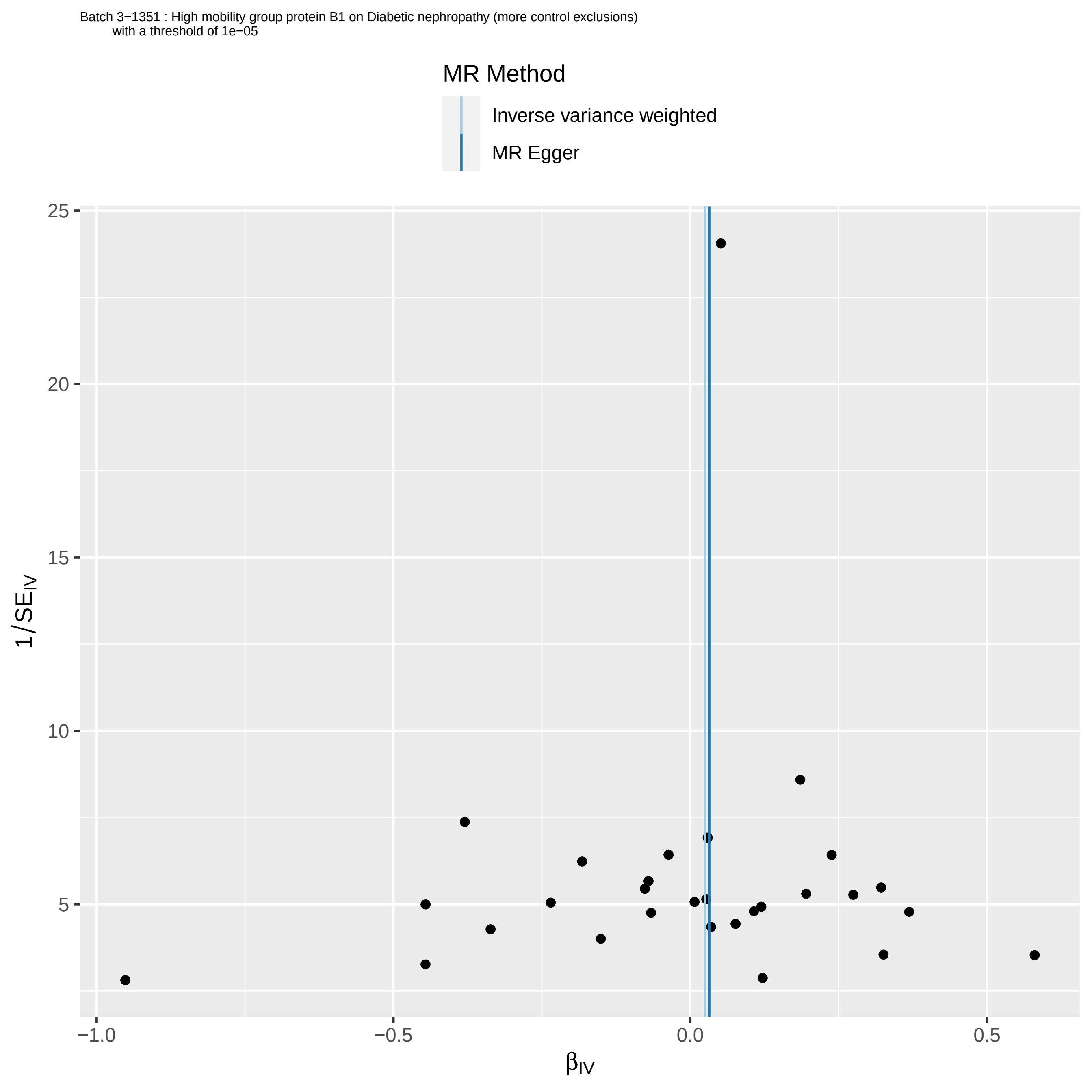
Batch 3−1351 : High mobility group protein B1 on Diabetic nephropathy (more control exclusions)
with a threshold of 1e−05
MR Method
Inverse variance weighted
MR Egger
25
20
15
10
5
1/SE_IV
−1.0
−0.5
0.0
0.5
β_IV

Batch 3−1352 : High mobility group protein B2 on Diabetic nephropathy (more control exclusions)
with a threshold of 1e−05

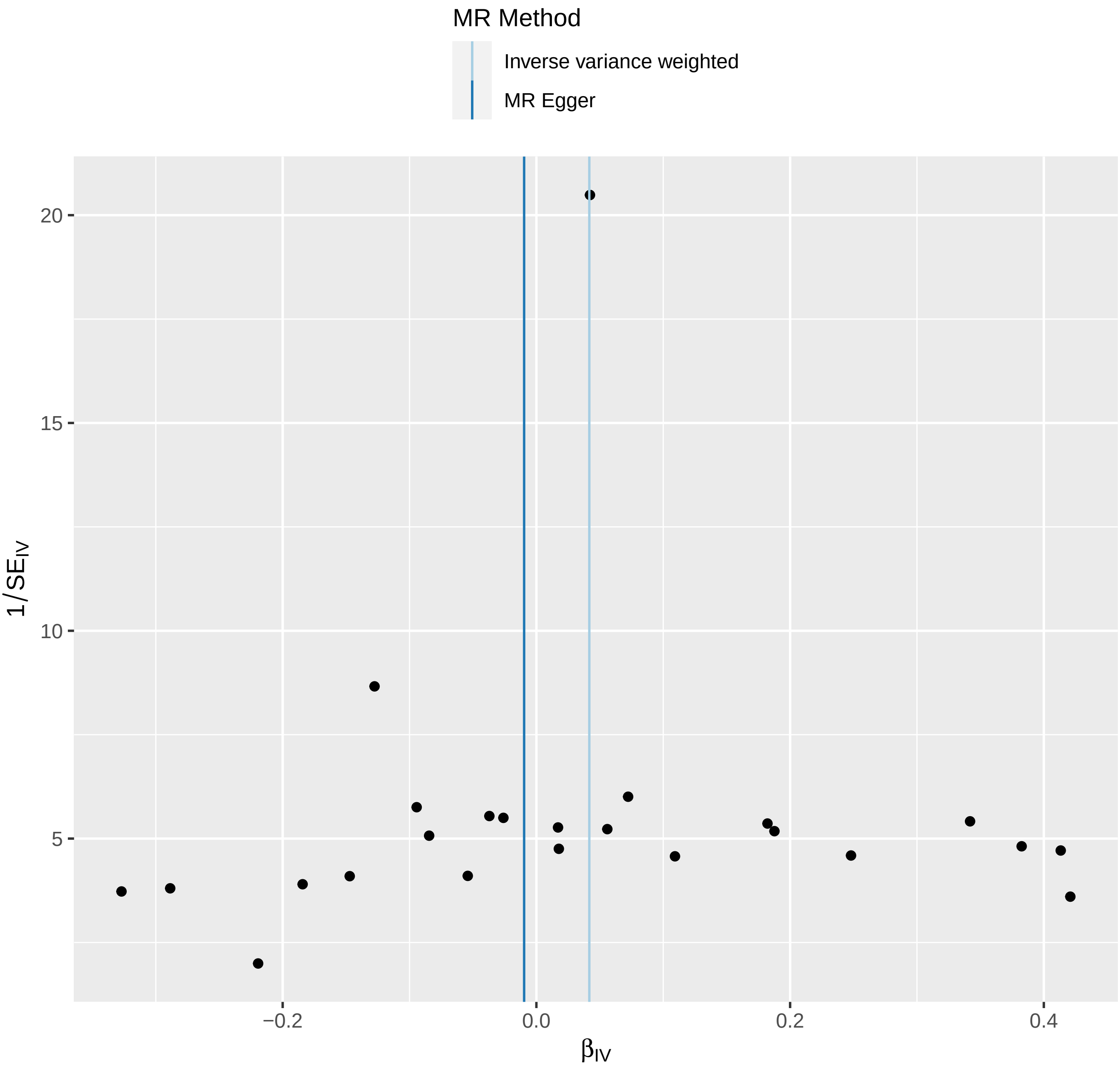

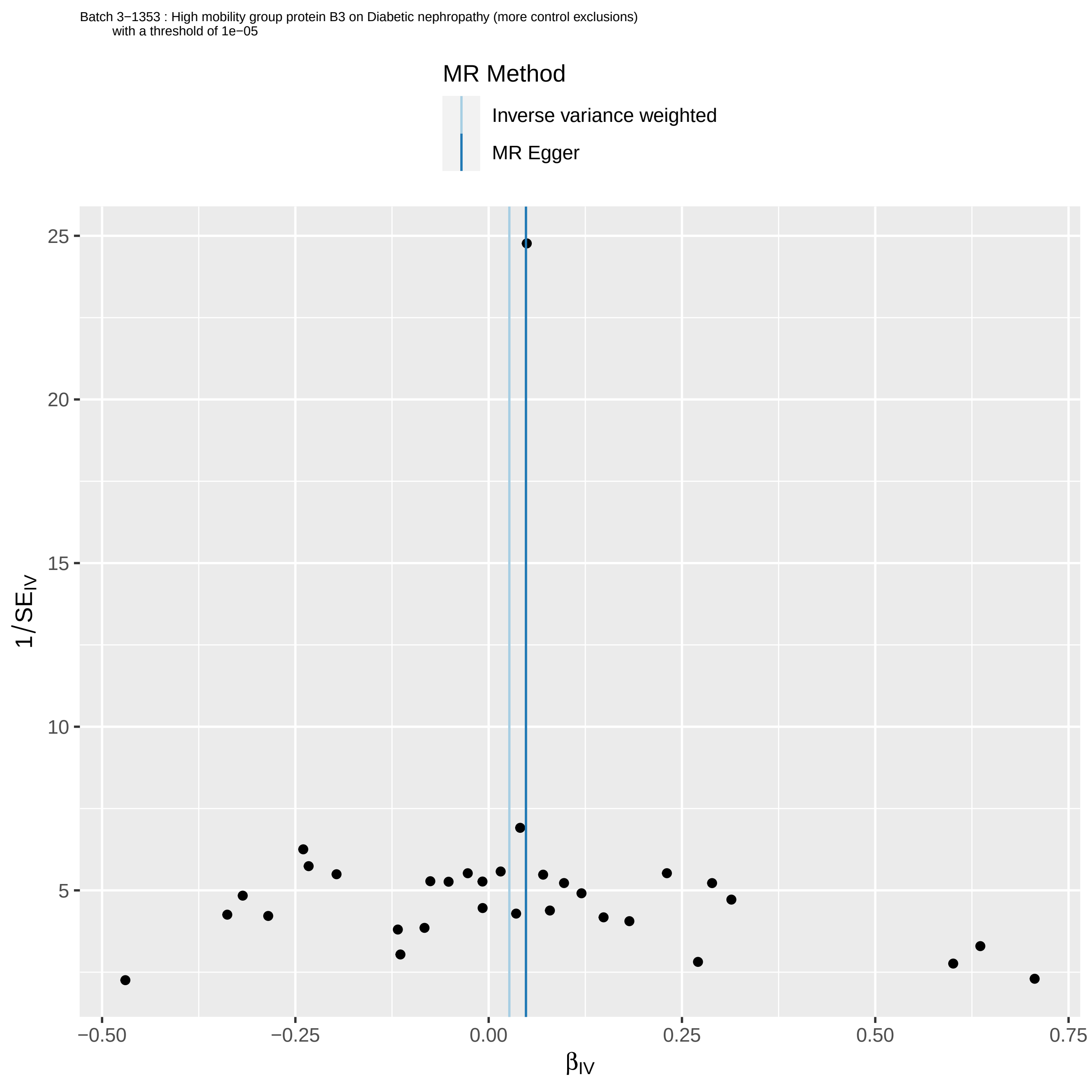
Batch 3−1353 : High mobility group protein B3 on Diabetic nephropathy (more control exclusions)
with a threshold of 1e−05
MR Method
Inverse variance weighted
MR Egger
25
20
15
10
5
1/SE_IV
−0.50
−0.25
0.00
0.25
0.50
0.75
β_IV

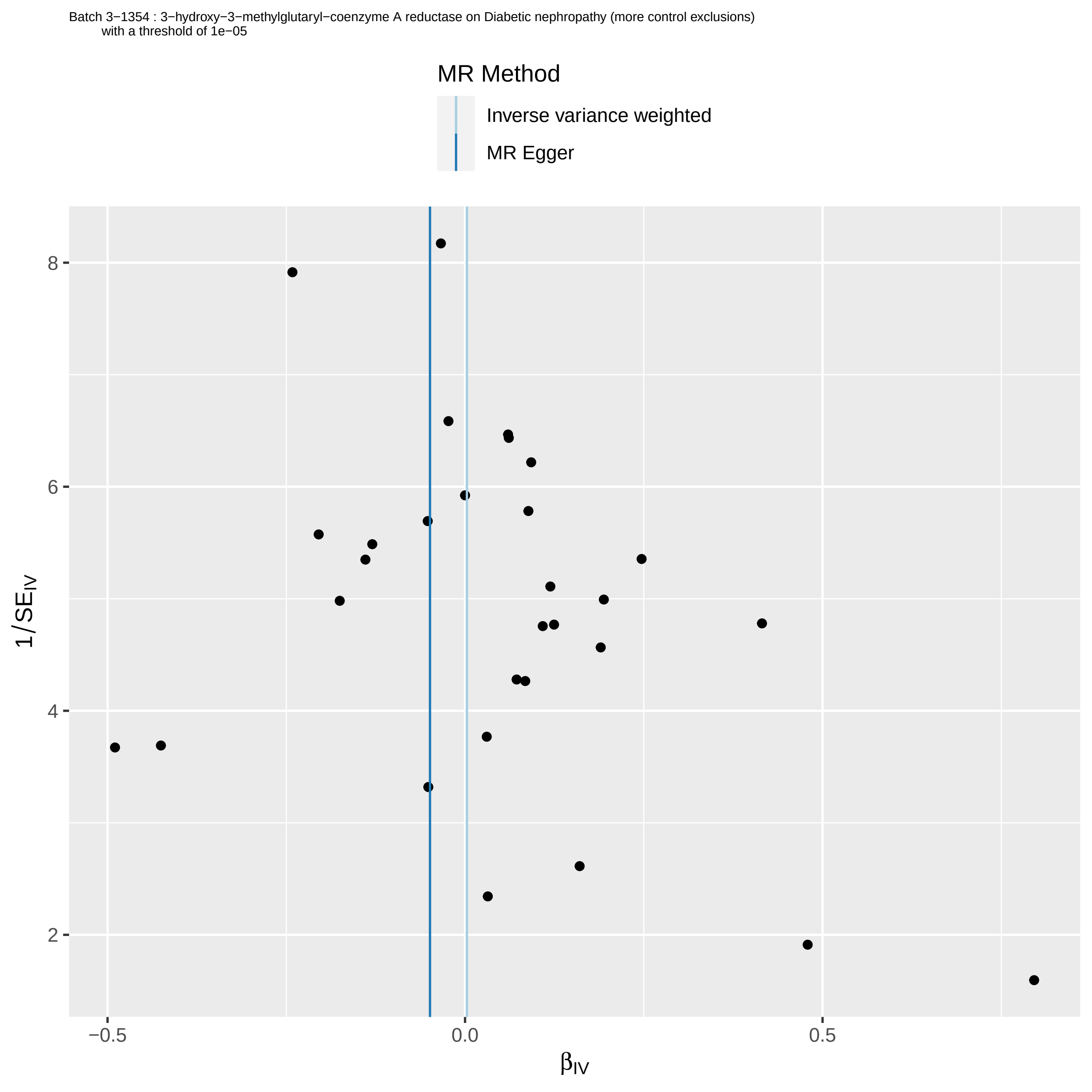

Batch 3−1354 : 3−hydroxy−3−methylglutaryl−coenzyme A reductase on Diabetic nephropathy (more control exclusions)
with a threshold of 1e−05
MR Method
Inverse variance weighted
MR Egger
8
6
4
2
1/SE$_{IV}$
−0.5
0.0
0.5
β_{IV}

Batch 3−1355 : Hydroxymethylglutaryl−CoA synthase, cytoplasmic on Diabetic nephropathy (more control exclusions)
with a threshold of 1e−05

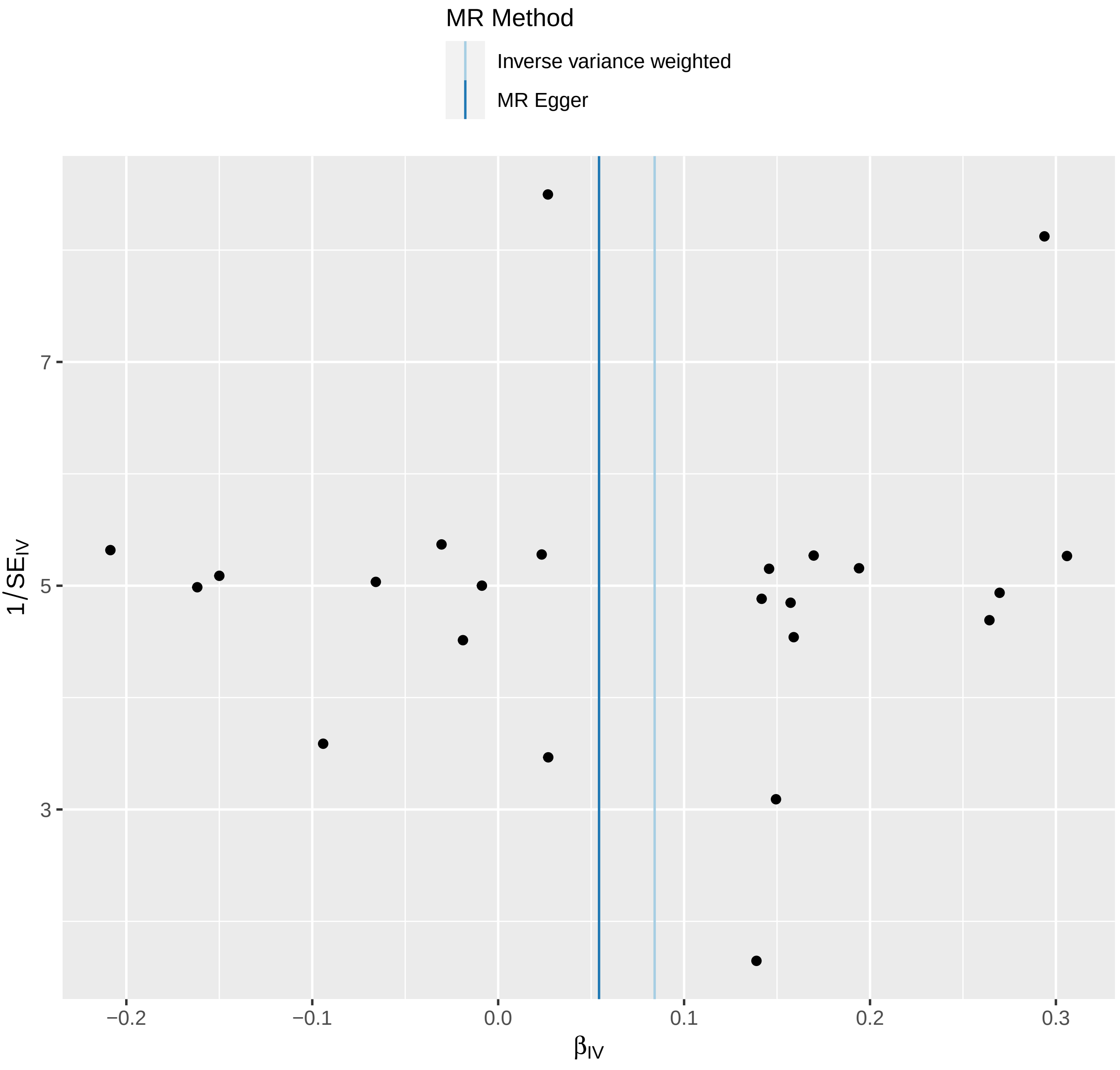

Batch 3−1356 : Hydroxymethylglutaryl−CoA synthase, mitochondrial on Diabetic nephropathy (more control exclusions)
with a threshold of 1e−05

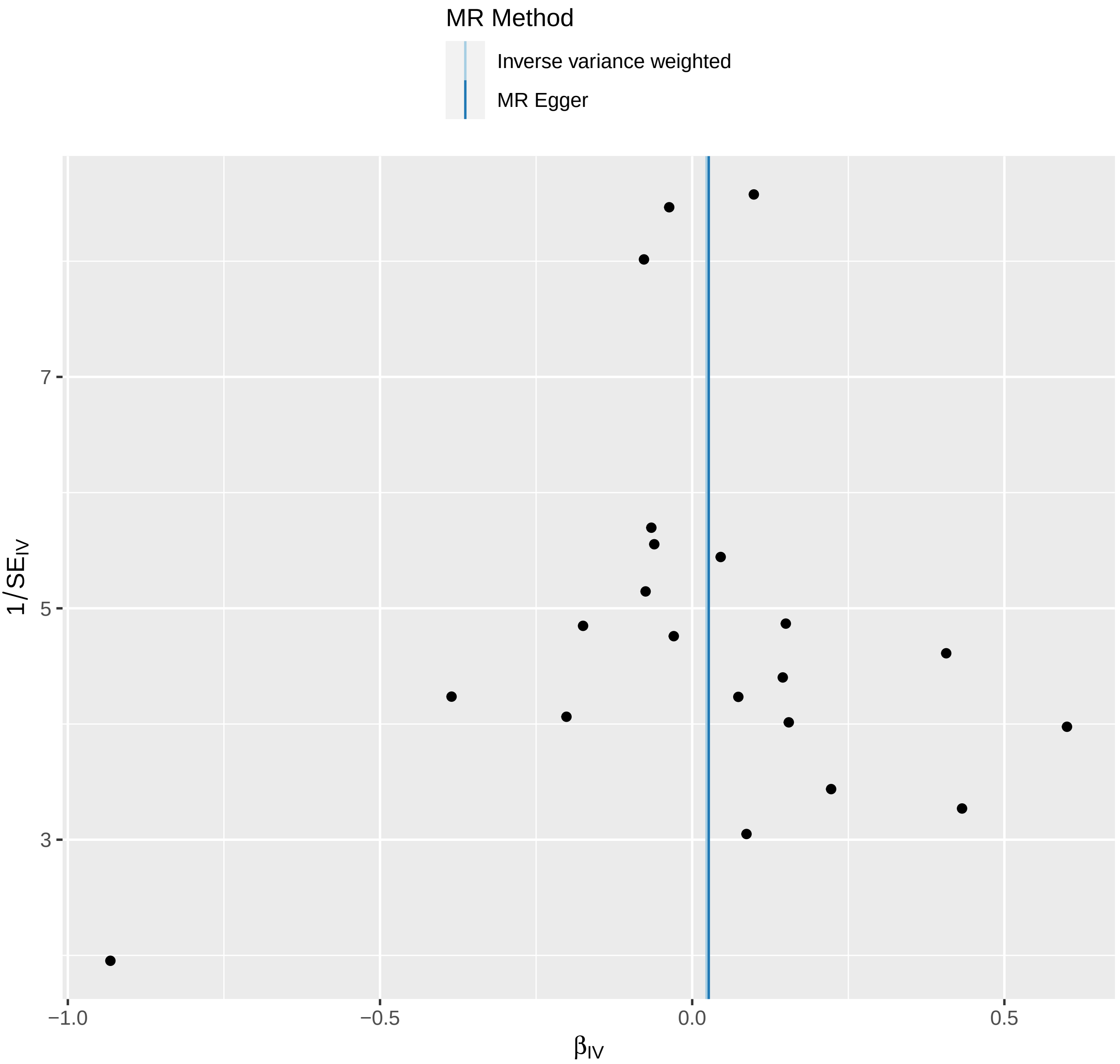

Batch 3−1357 : Non−histone chromosomal protein HMG−14 on Diabetic nephropathy (more control exclusions)
with a threshold of 1e−05

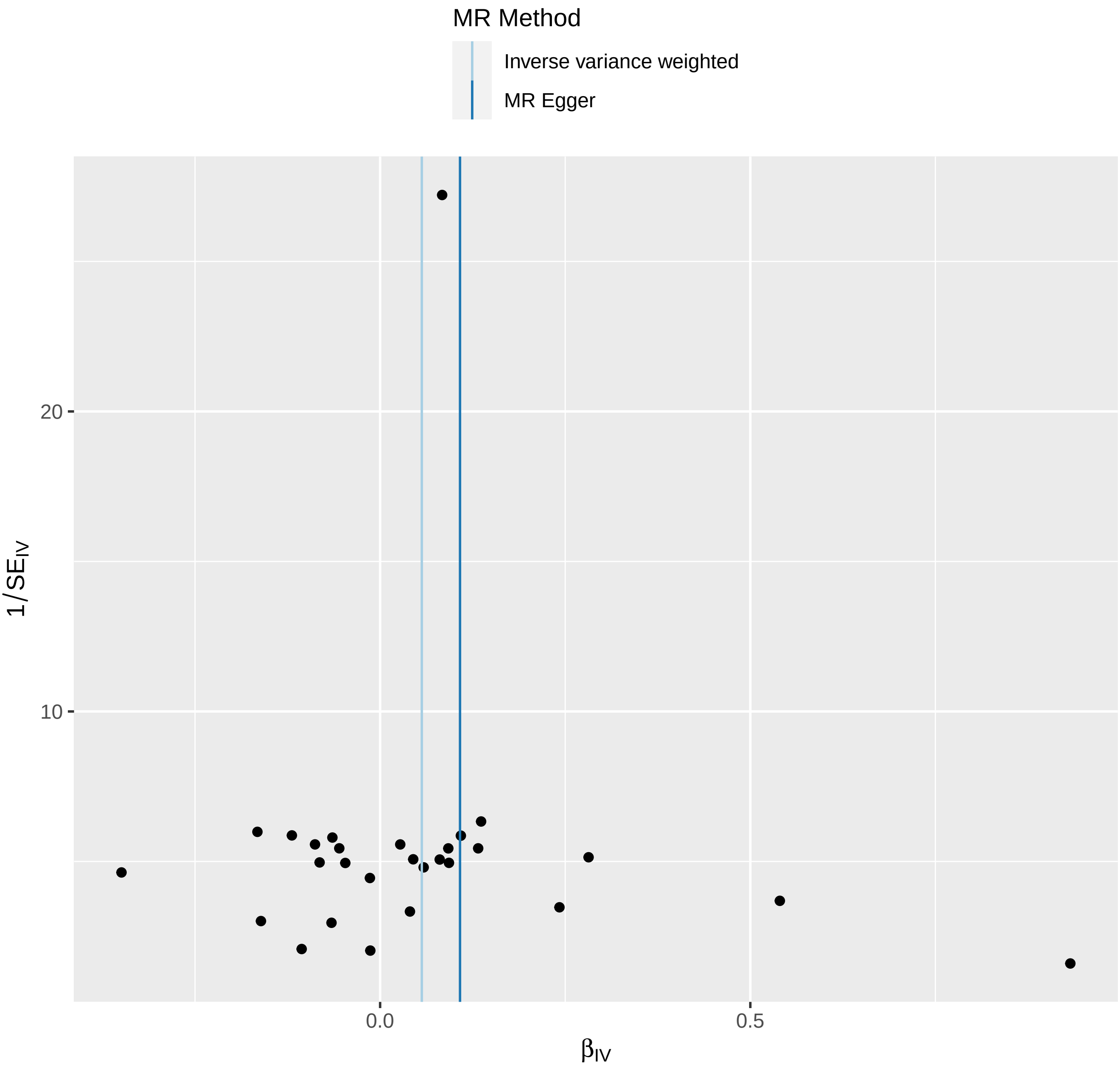

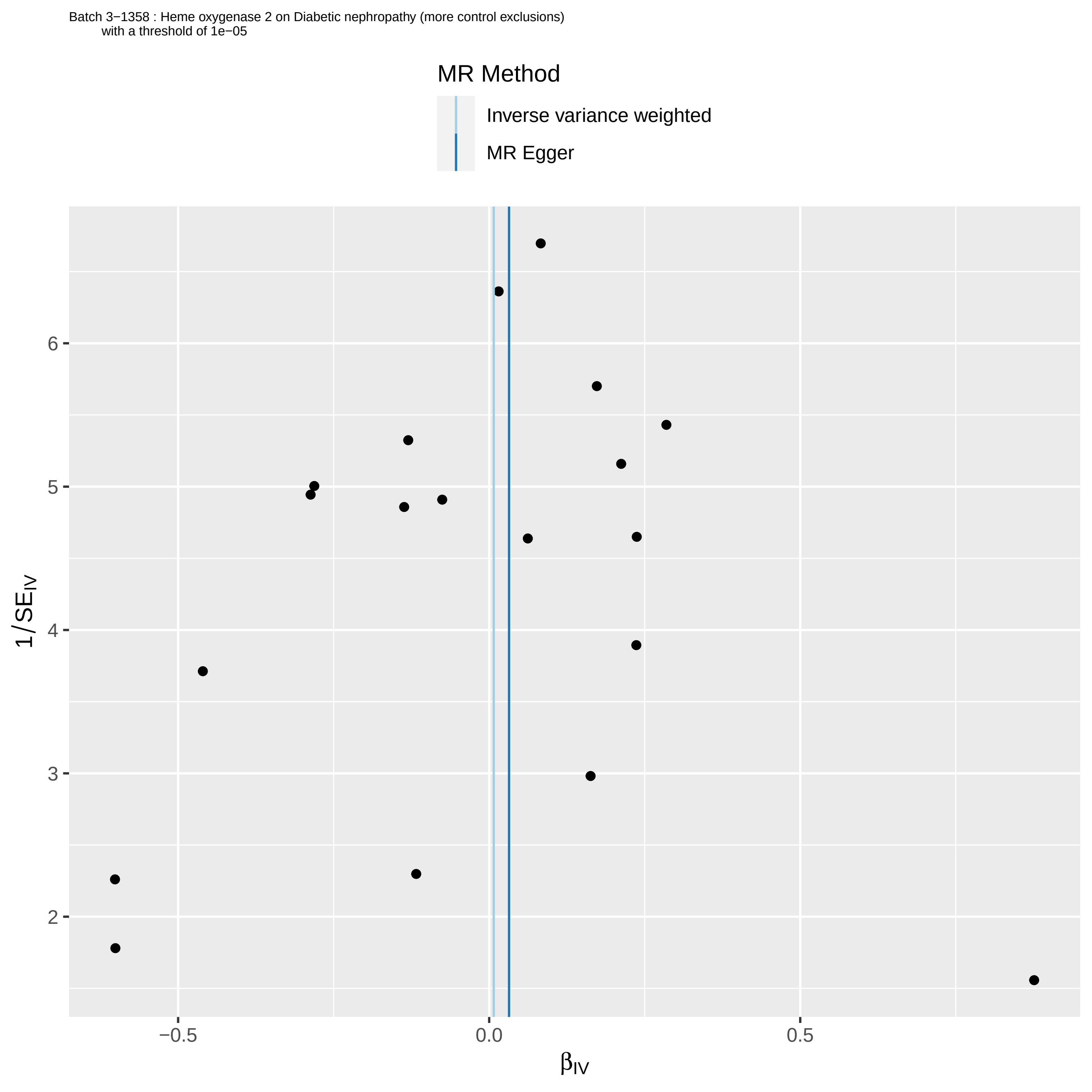

Batch 3−1358 : Heme oxygenase 2 on Diabetic nephropathy (more control exclusions)
with a threshold of 1e−05
MR Method
Inverse variance weighted
MR Egger
6
5
4
3
2
$1/SE_{IV}$
−0.5
0.0
0.5
β_{IV}

Batch 3−1359 : Hepatocyte nuclear factor 1−alpha on Diabetic nephropathy (more control exclusions)
with a threshold of 1e−05

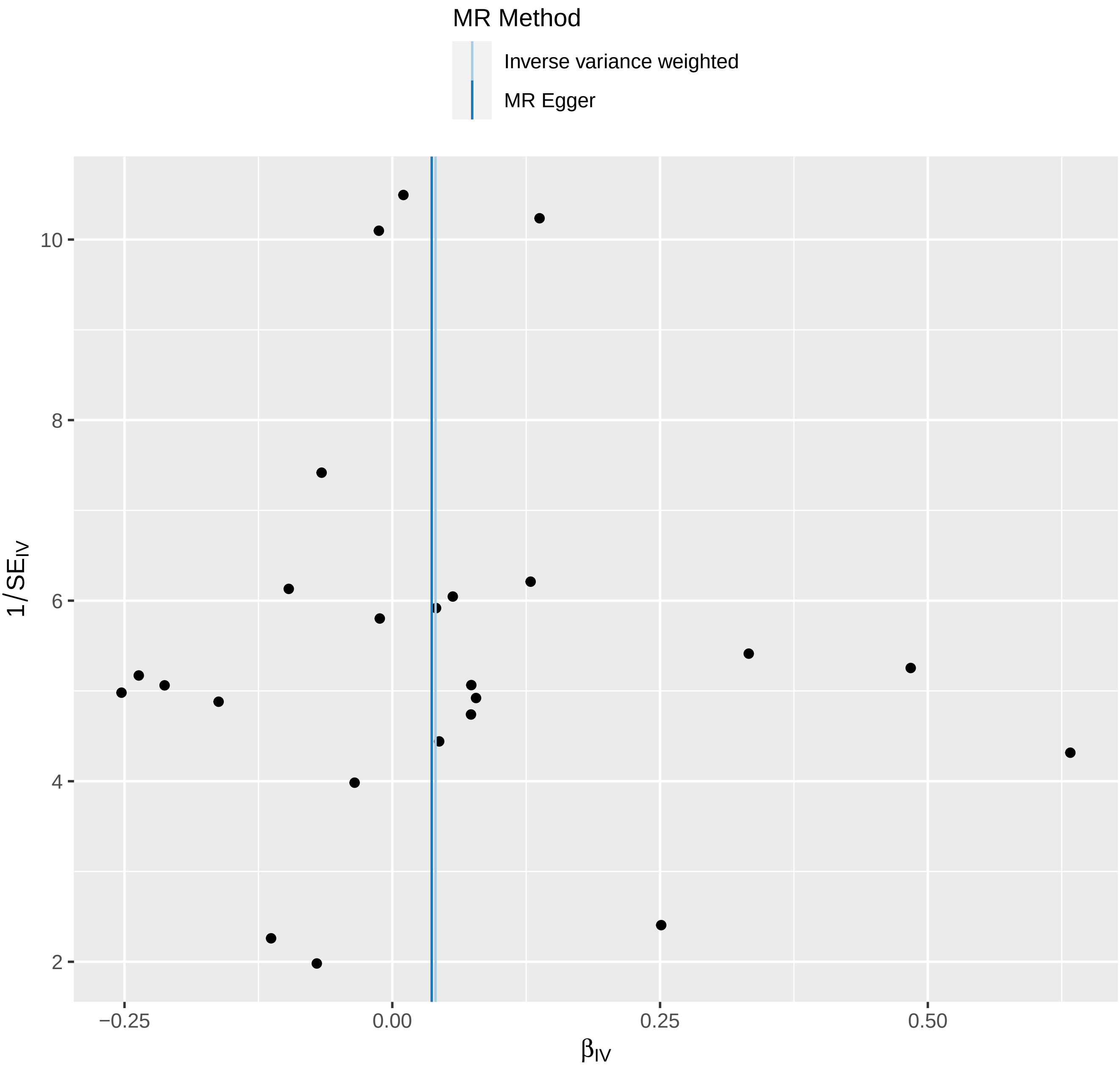

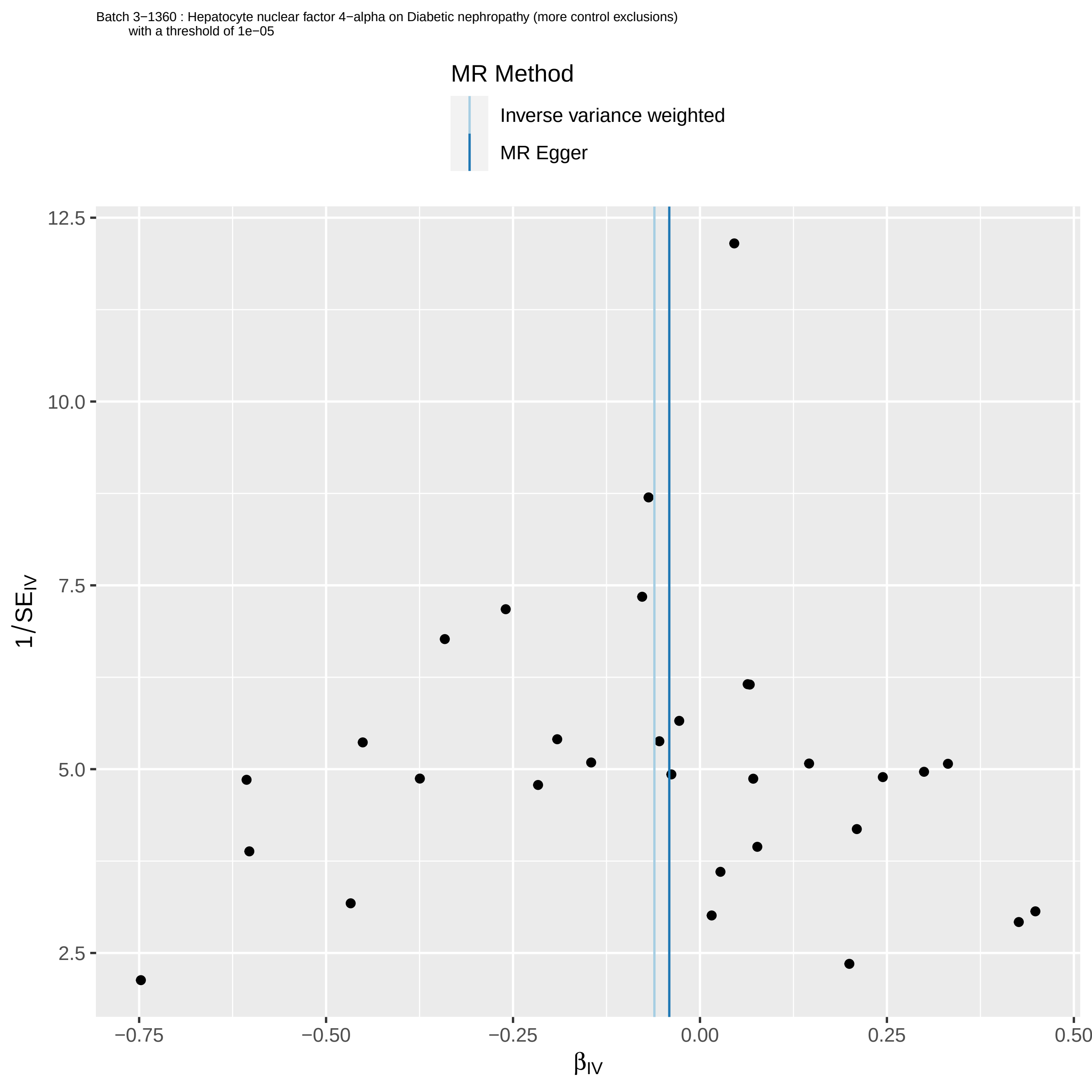

Batch 3−1360 : Hepatocyte nuclear factor 4−alpha on Diabetic nephropathy (more control exclusions)
with a threshold of 1e−05
MR Method
Inverse variance weighted
MR Egger
12.5
10.0
7.5
5.0
2.5
1/SE_IV
−0.75
−0.50
−0.25
0.00
0.25
0.50
β_IV

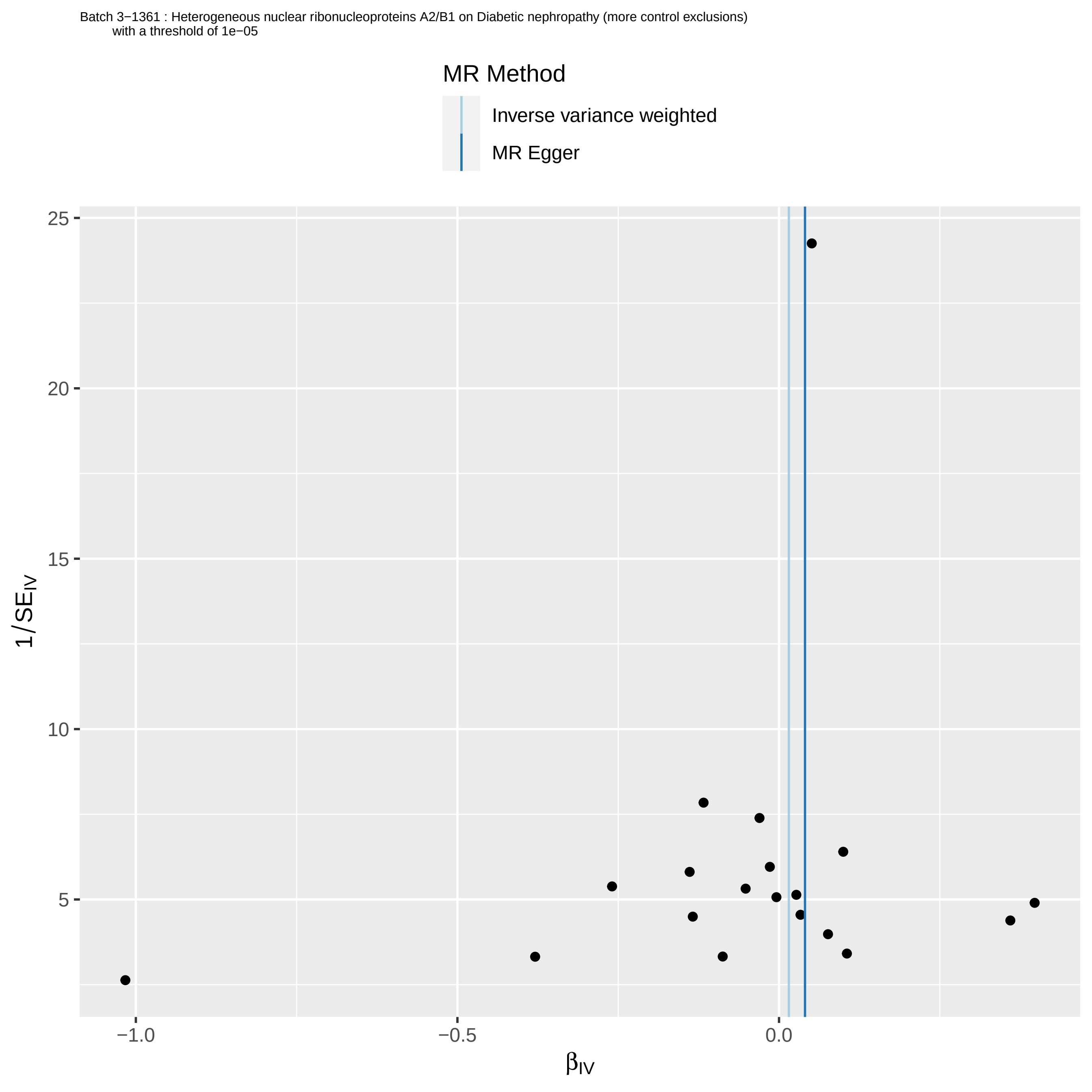
Batch 3−1361 : Heterogeneous nuclear ribonucleoproteins A2/B1 on Diabetic nephropathy (more control exclusions)
with a threshold of 1e−05
MR Method
Inverse variance weighted
MR Egger
25
20
15
10
5
1/SE_IV
−1.0
−0.5
0.0
β_IV

Batch 3−1362 : Heterogeneous nuclear ribonucleoprotein A/B on Diabetic nephropathy (more control exclusions)
with a threshold of 1e−05

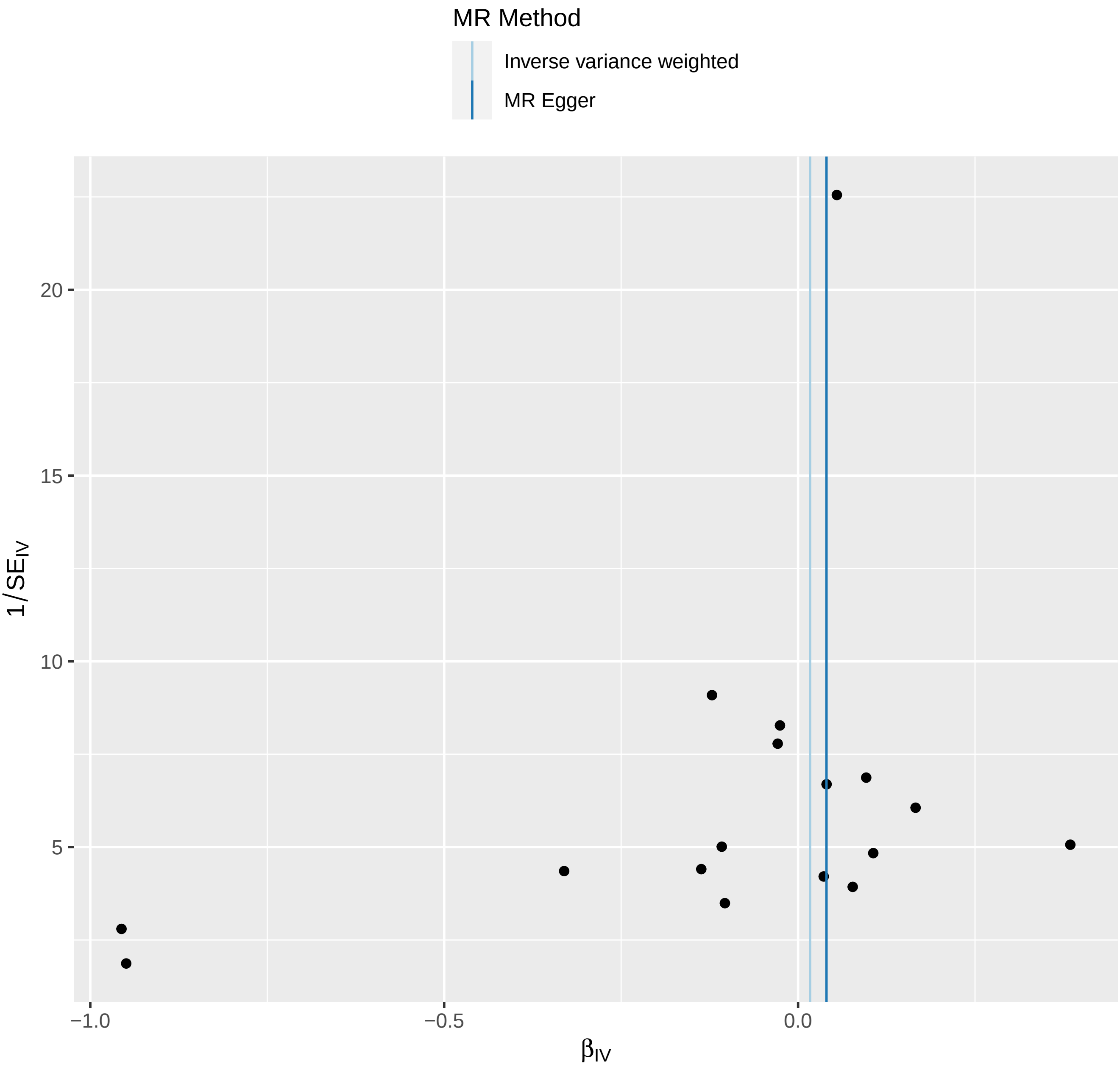

Batch 3−1363 : Heterogeneous nuclear ribonucleoprotein A/B on Diabetic nephropathy (more control exclusions)
with a threshold of 1e−05

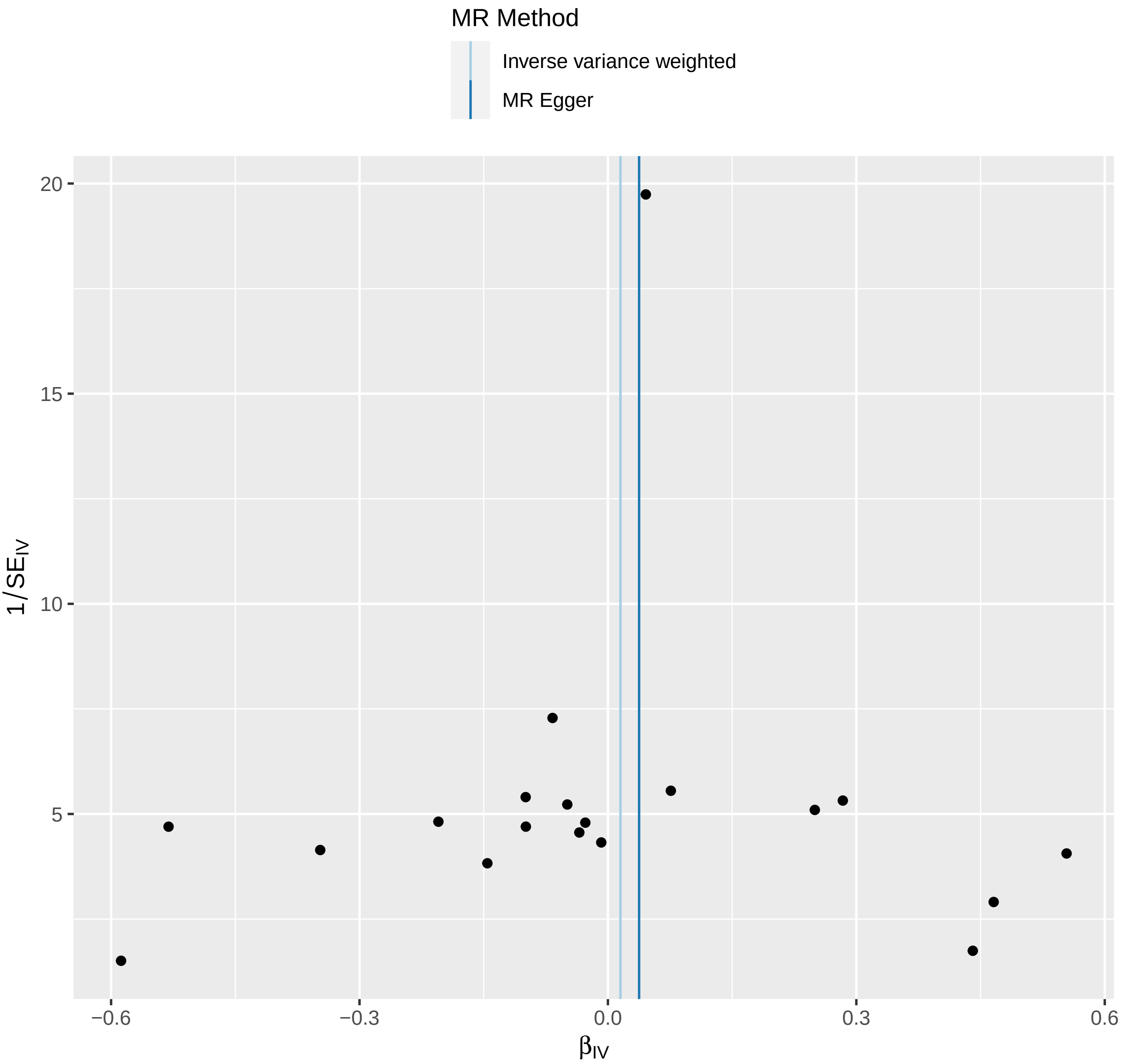

Batch 3−1364 : Heterogeneous nuclear ribonucleoproteins C1/C2 on Diabetic nephropathy (more control exclusions) with a threshold of 1e−05

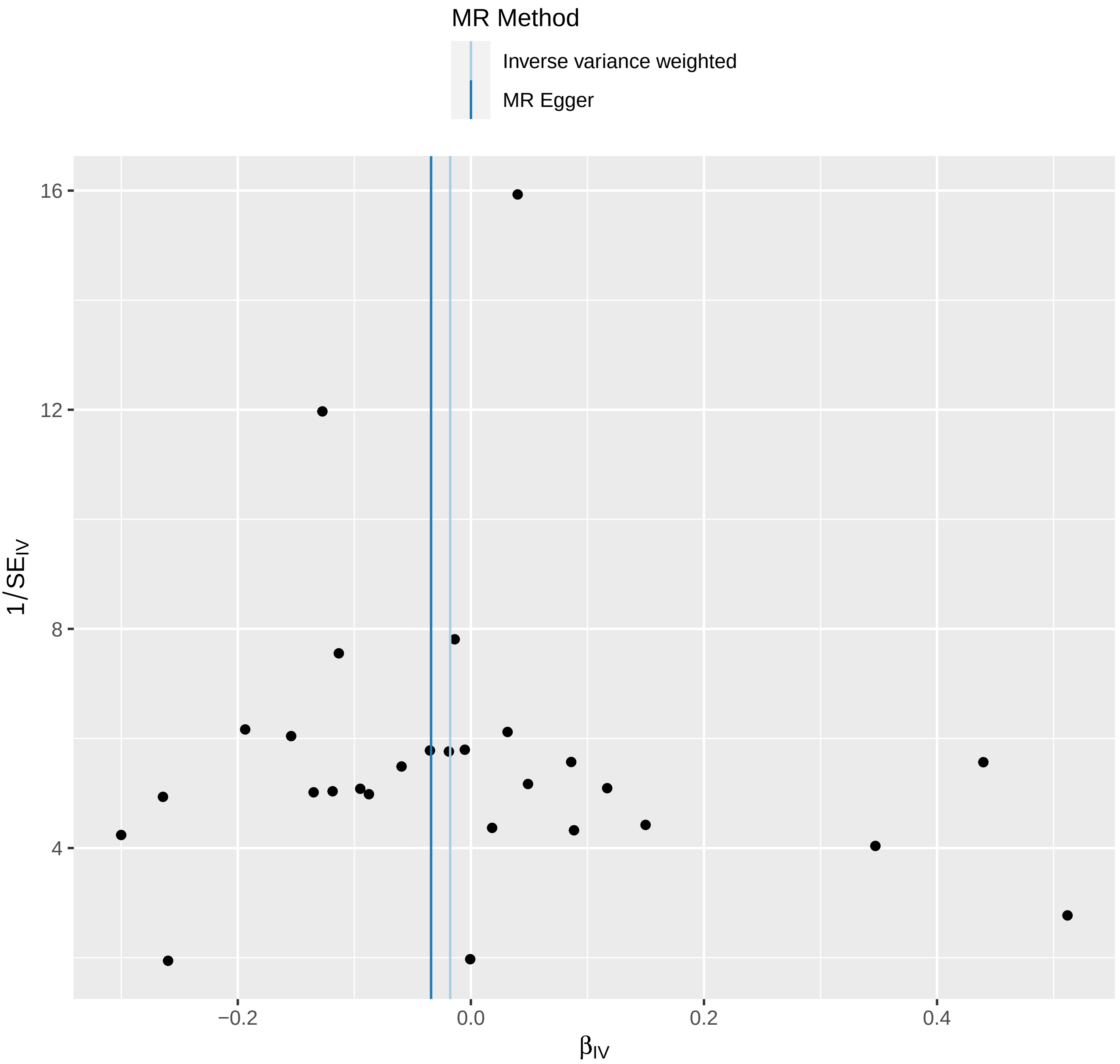

Batch 3−1365 : Heterogeneous nuclear ribonucleoprotein D−like on Diabetic nephropathy (more control exclusions)
with a threshold of 1e−05

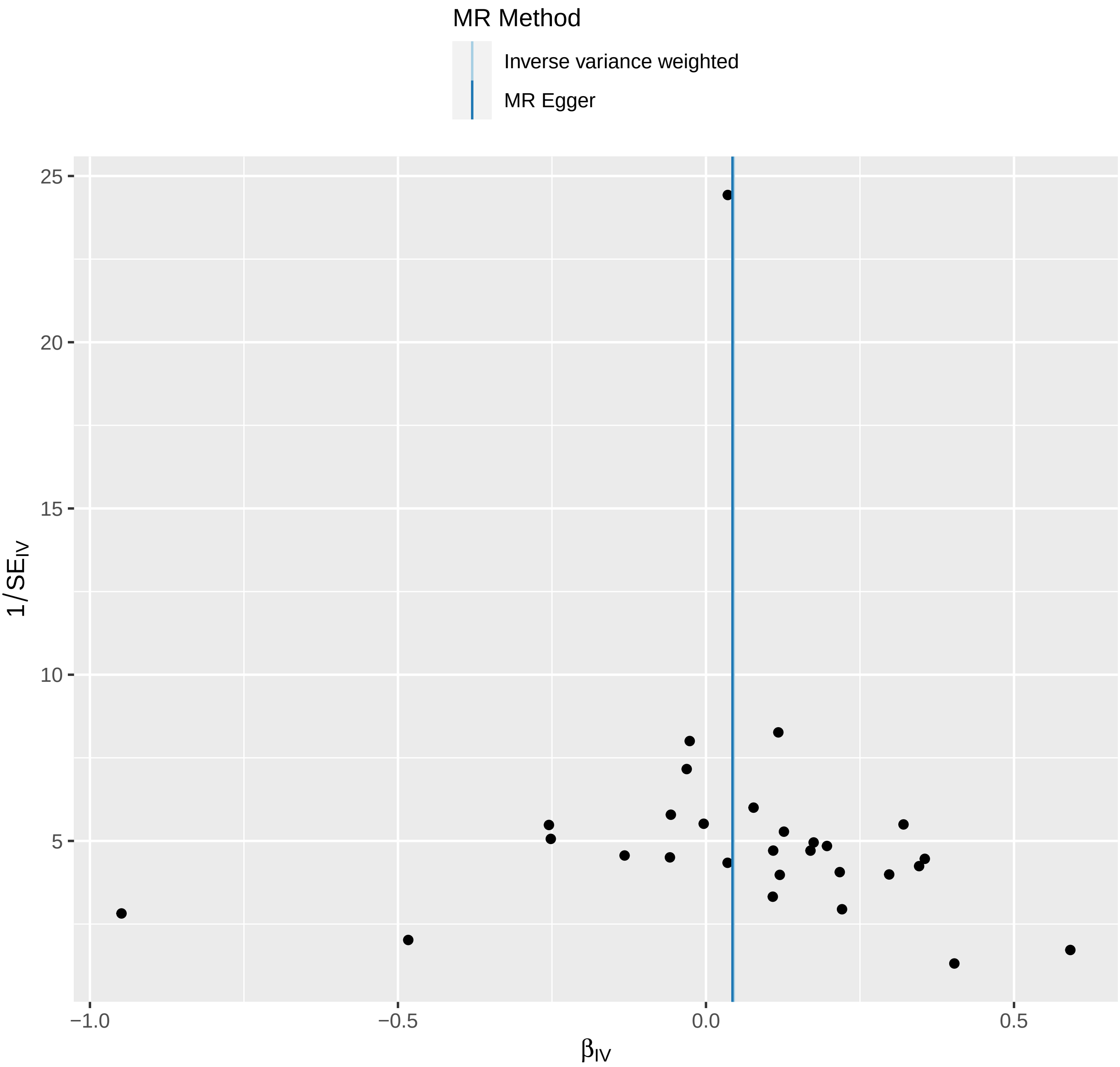

Batch 3−1366 : Heterogeneous nuclear ribonucleoprotein K on Diabetic nephropathy (more control exclusions)
with a threshold of 1e−05

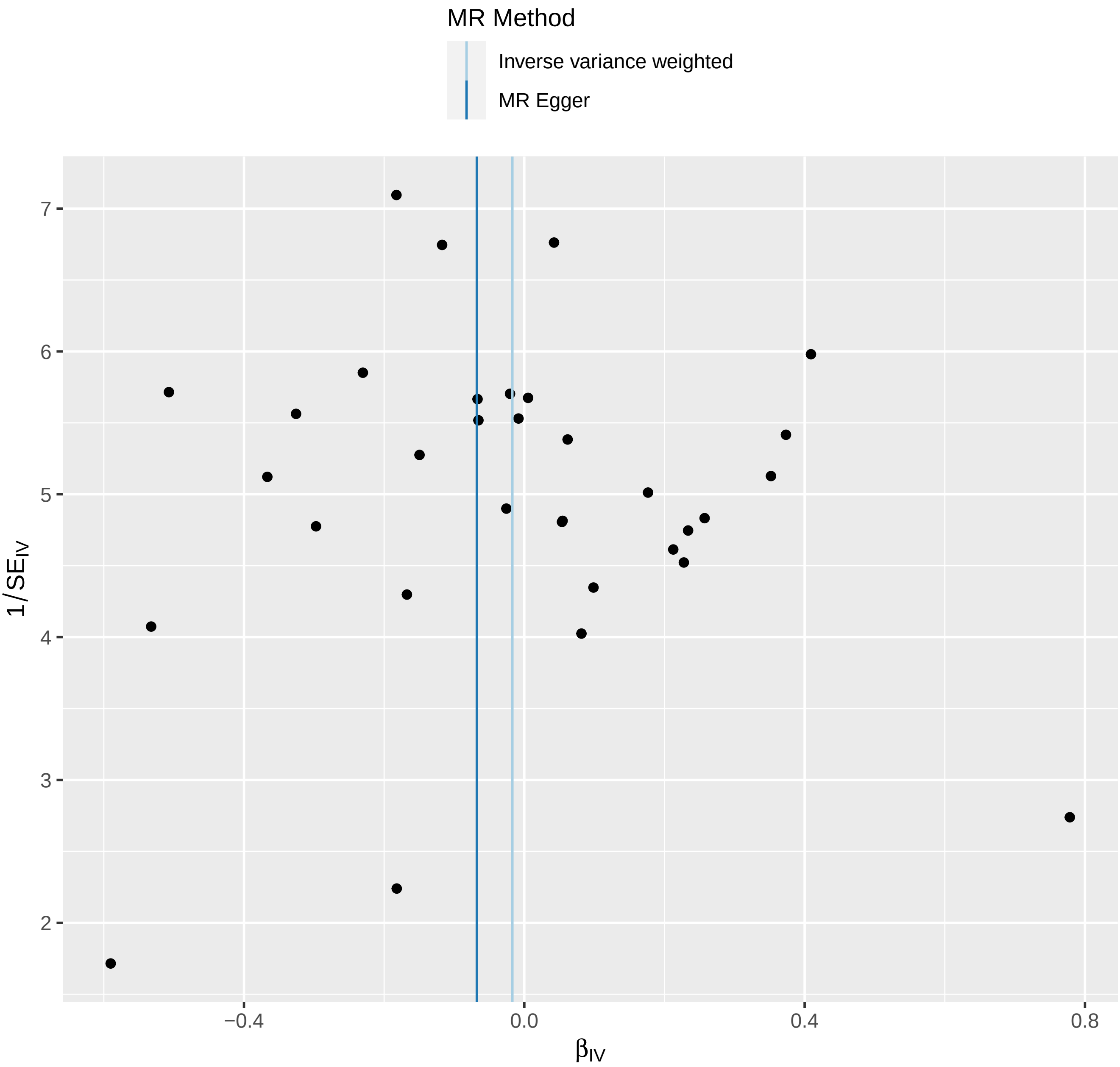

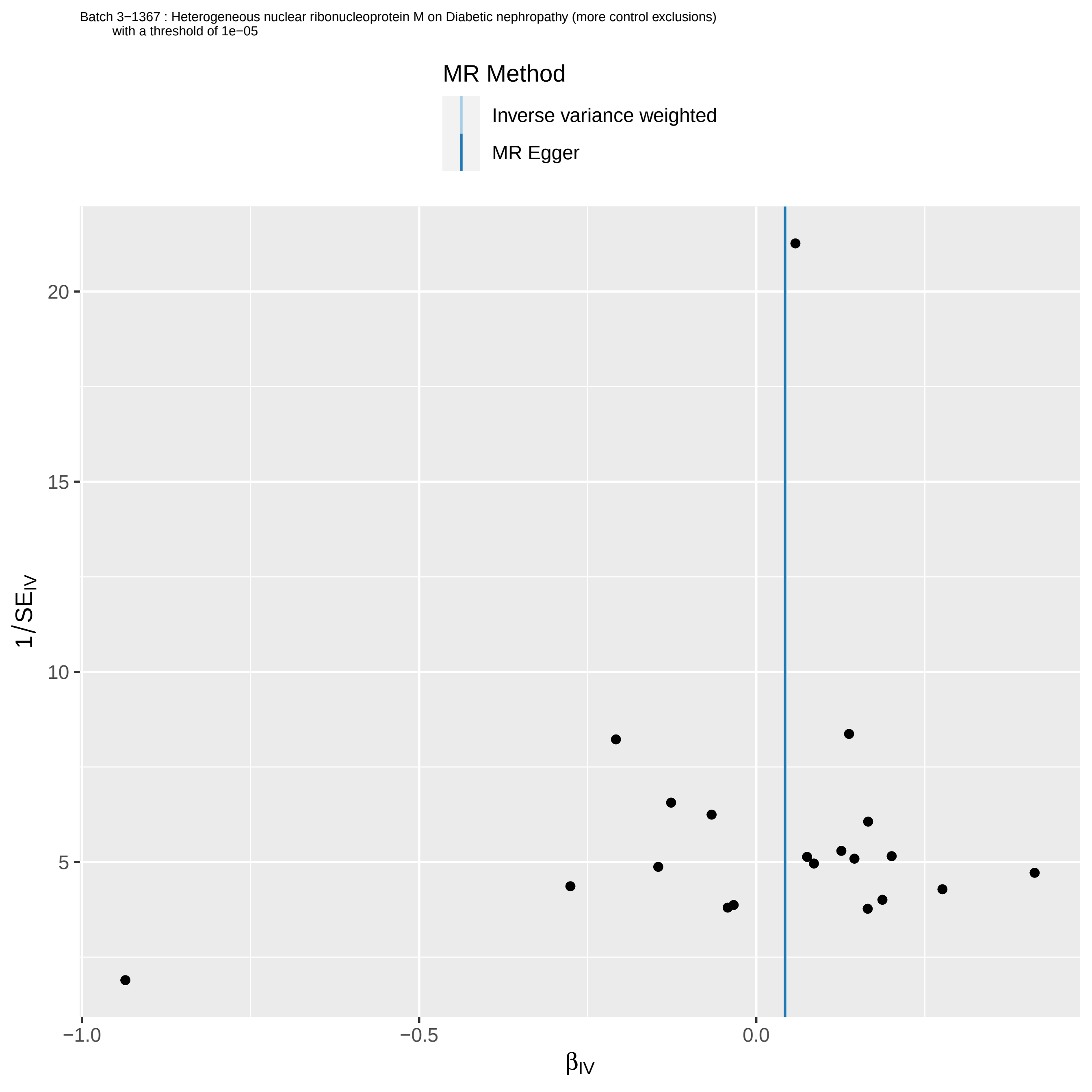
Batch 3−1367 : Heterogeneous nuclear ribonucleoprotein M on Diabetic nephropathy (more control exclusions)
with a threshold of 1e−05
MR Method
Inverse variance weighted
MR Egger
20
15
10
5
1/SE_IV
−1.0
−0.5
0.0
β_IV

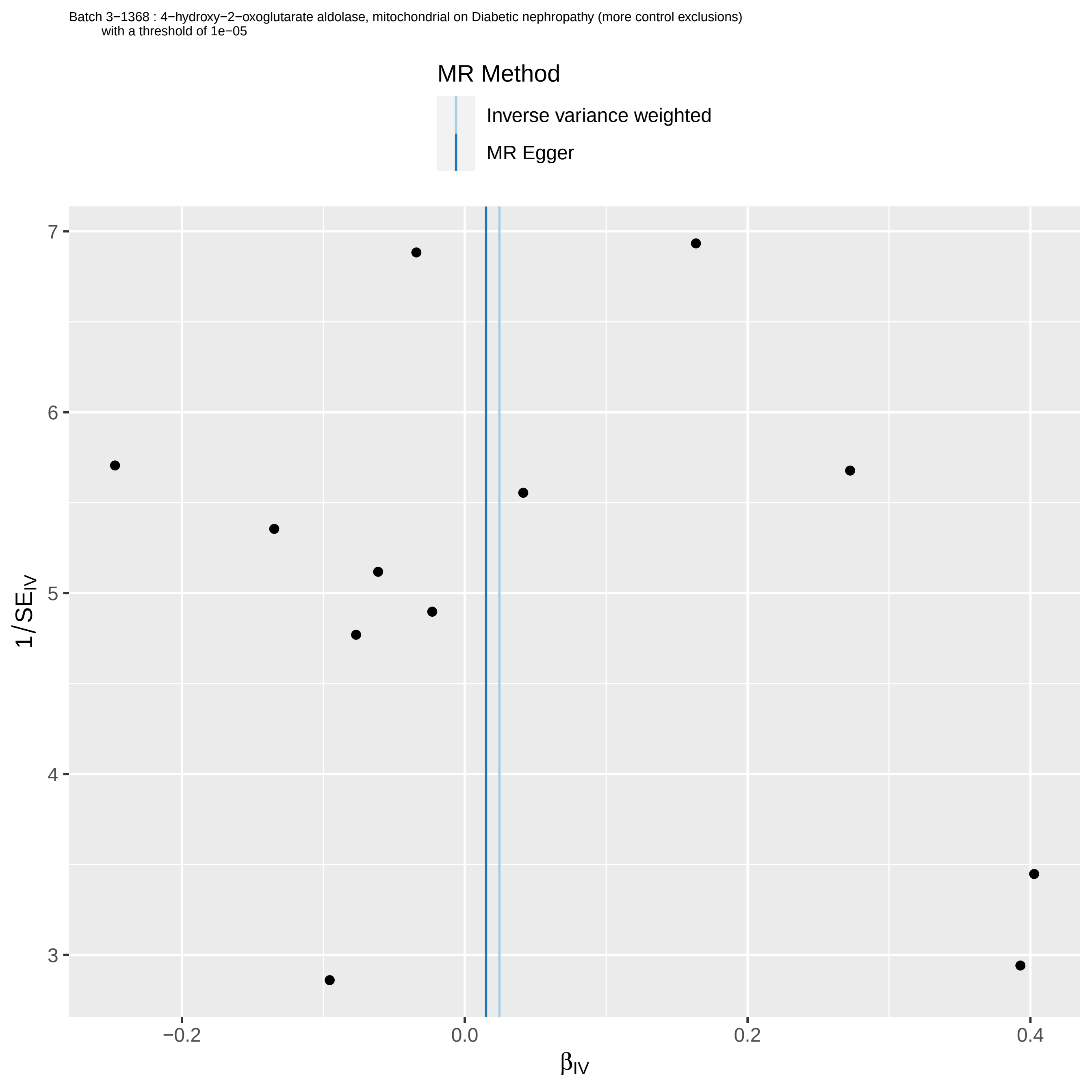

Batch 3−1368 : 4−hydroxy−2−oxoglutarate aldolase, mitochondrial on Diabetic nephropathy (more control exclusions)
with a threshold of 1e−05
MR Method
Inverse variance weighted
MR Egger
7
6
5
4
3
1/SE_IV
−0.2
0.0
0.2
0.4
β_IV

Batch 3−1369 : Haptoglobin on Diabetic nephropathy (more control exclusions)
with a threshold of 1e−05

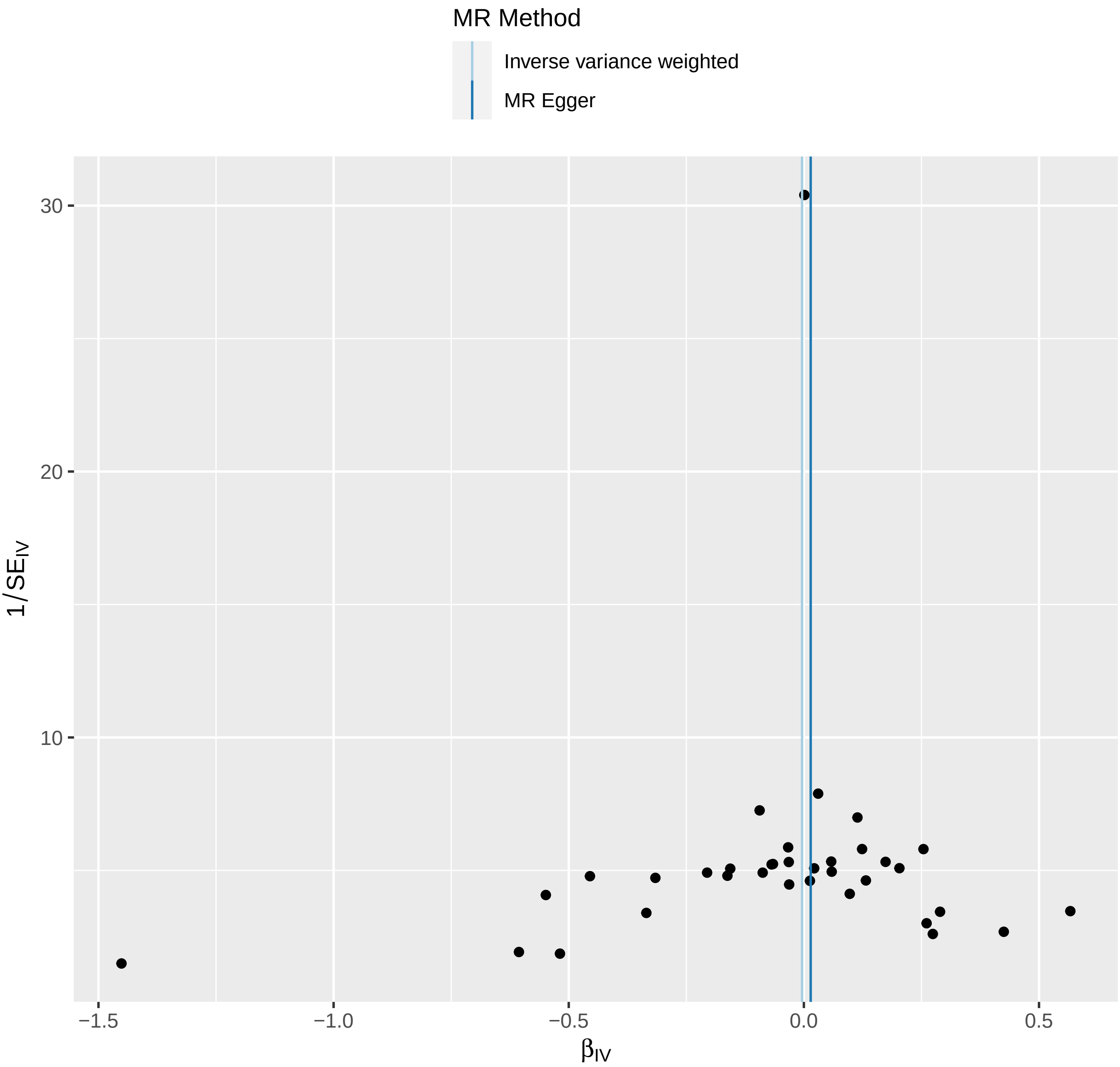

Batch 3−1370 : 15−hydroxyprostaglandin dehydrogenase [NAD(+)] on Diabetic nephropathy (more control exclusions) with a threshold of 1e−05

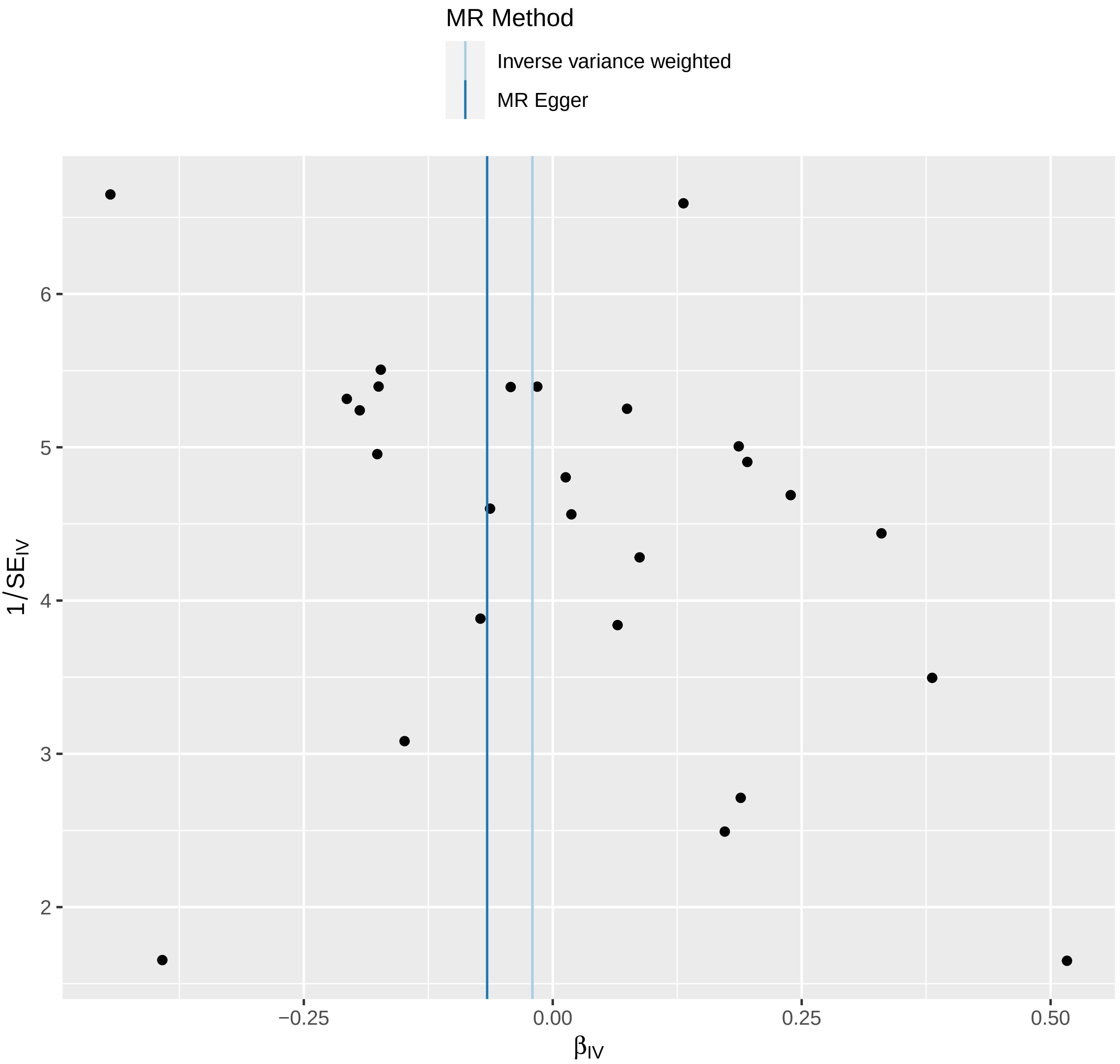

Batch 3−1371 : Hematopoietic prostaglandin D synthase on Diabetic nephropathy (more control exclusions)
with a threshold of 1e−05

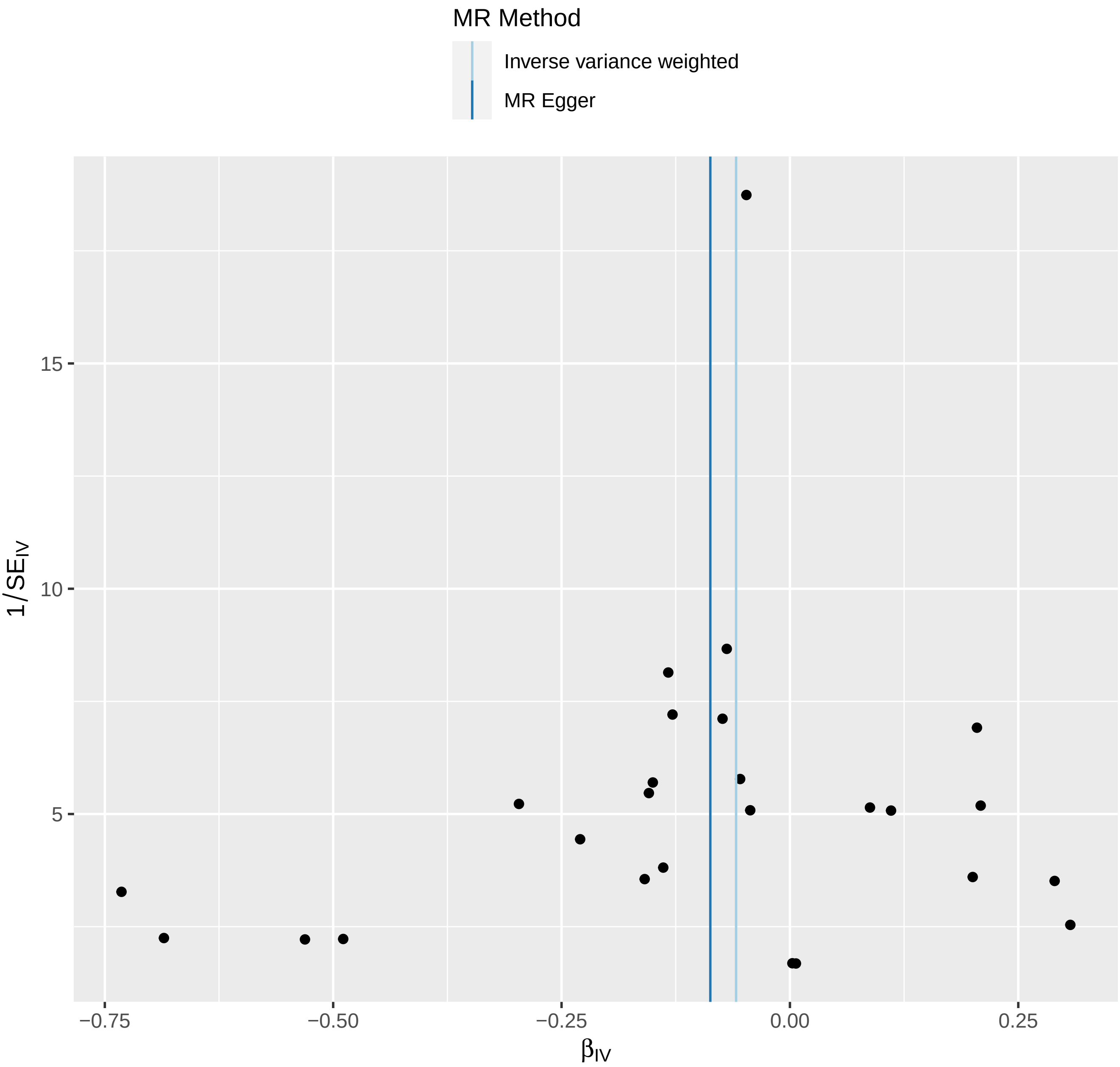

Batch 3−1372 : HRAS−like suppressor 2 on Diabetic nephropathy (more control exclusions)
with a threshold of 1e−05

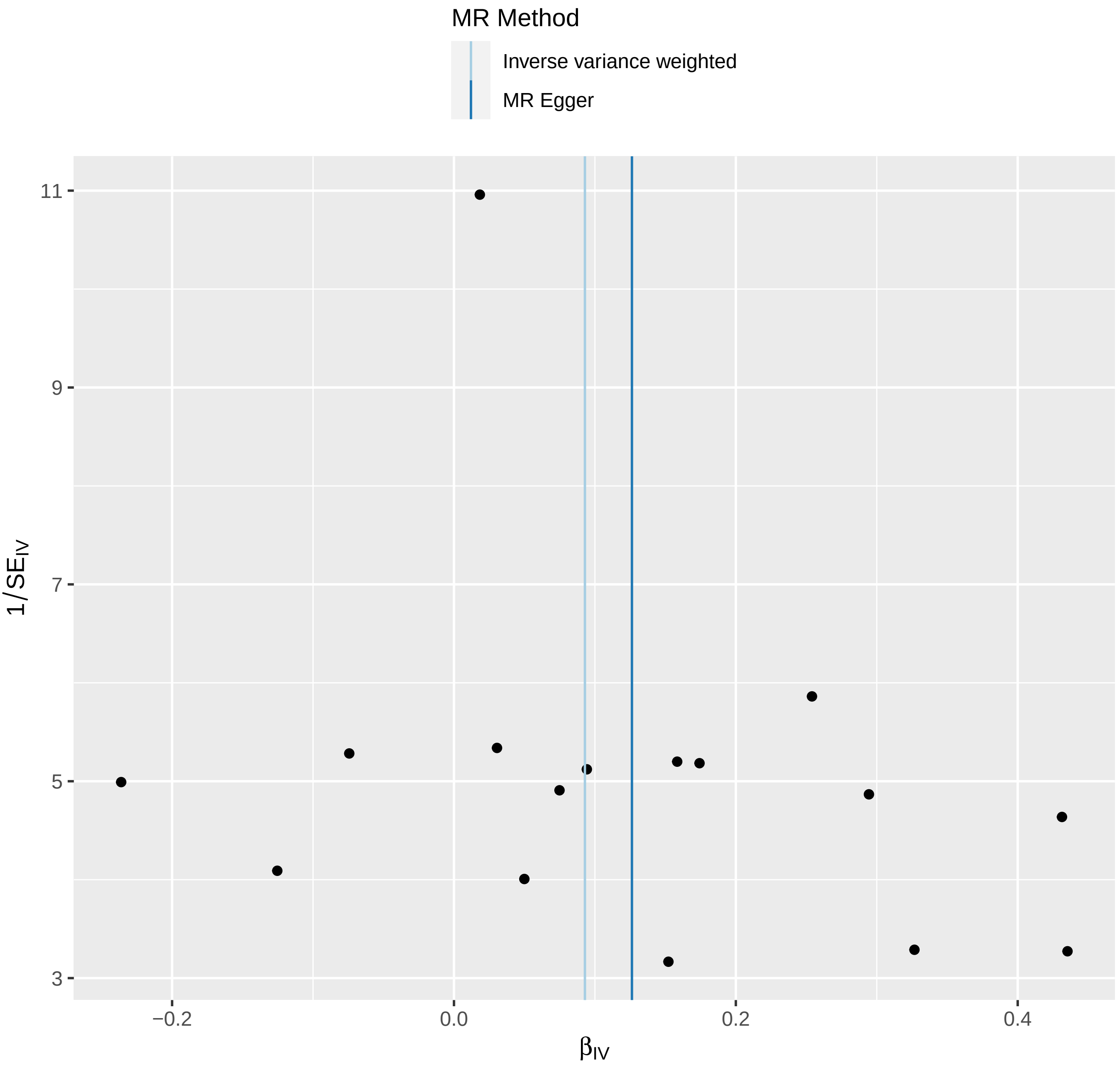

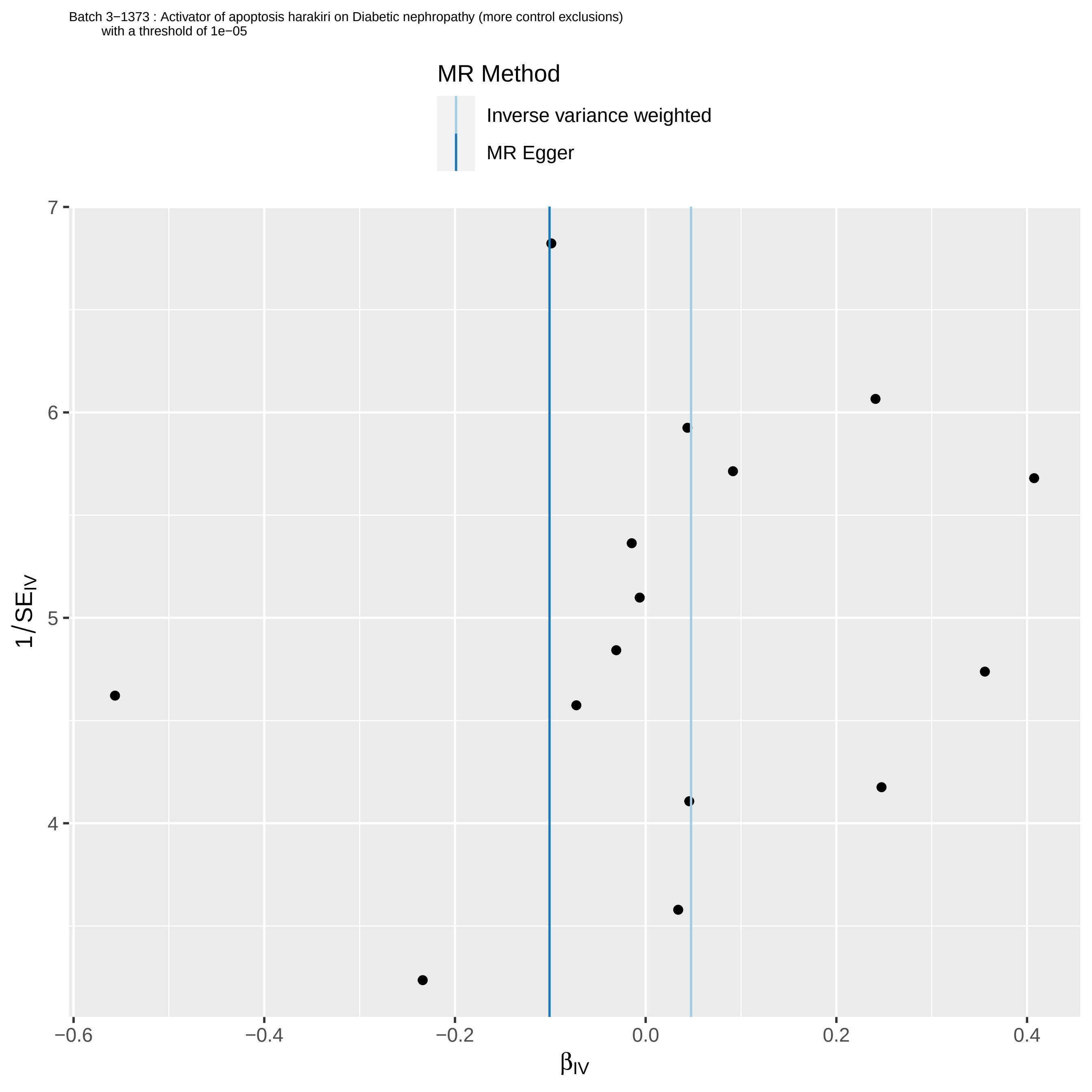
Batch 3−1373 : Activator of apoptosis harakiri on Diabetic nephropathy (more control exclusions)
with a threshold of 1e−05
MR Method
Inverse variance weighted
MR Egger
1/SE_IV
β_IV
7
6
5
4
−0.6
−0.4
−0.2
0.0
0.2
0.4

Batch 3−1374 : Ribonuclease UK114 on Diabetic nephropathy (more control exclusions)
with a threshold of 1e−05

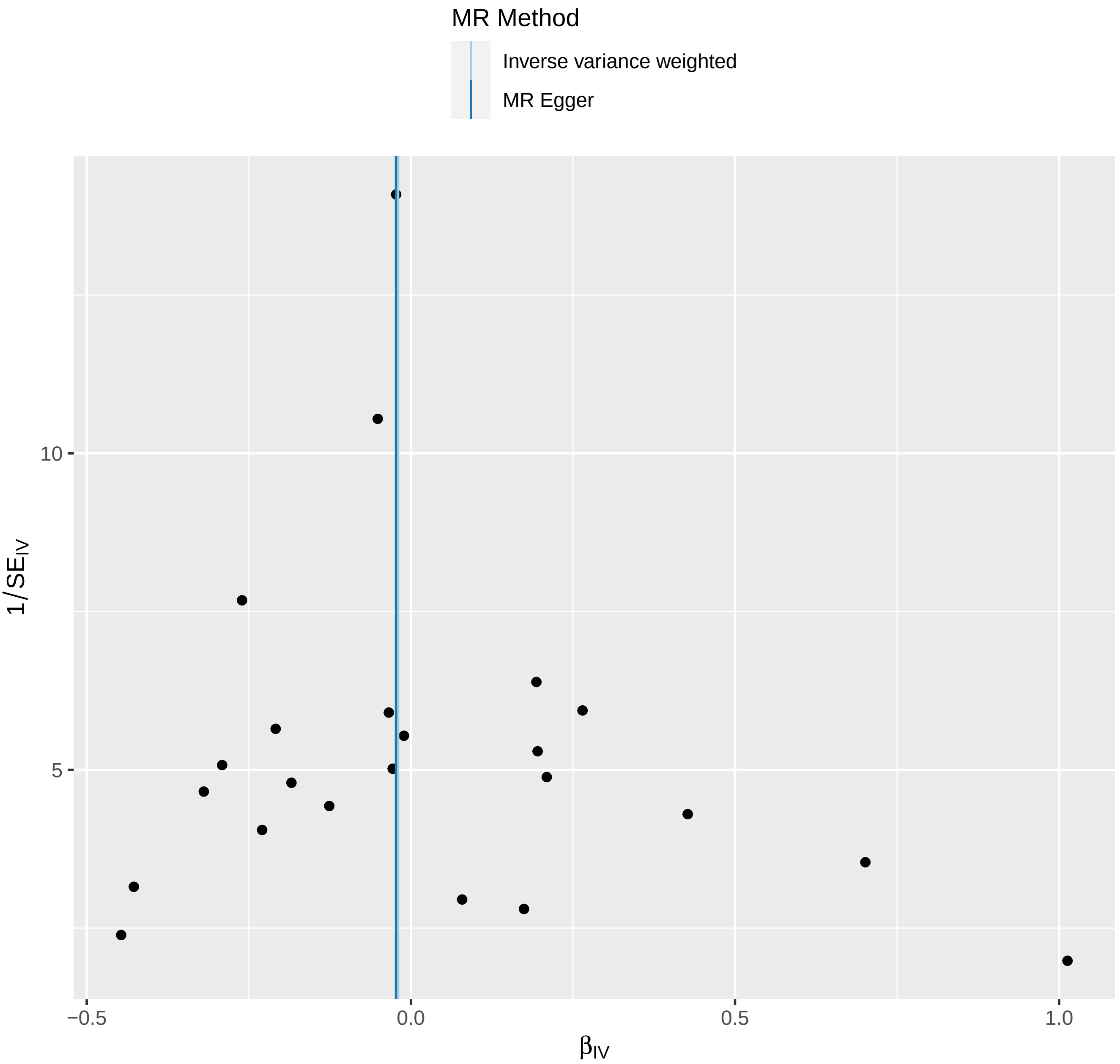

Batch 3−1375 : Heparan sulfate glucosamine 3−O−sulfotransferase 3A1 on Diabetic nephropathy (more control exclusions)
with a threshold of 1e−05

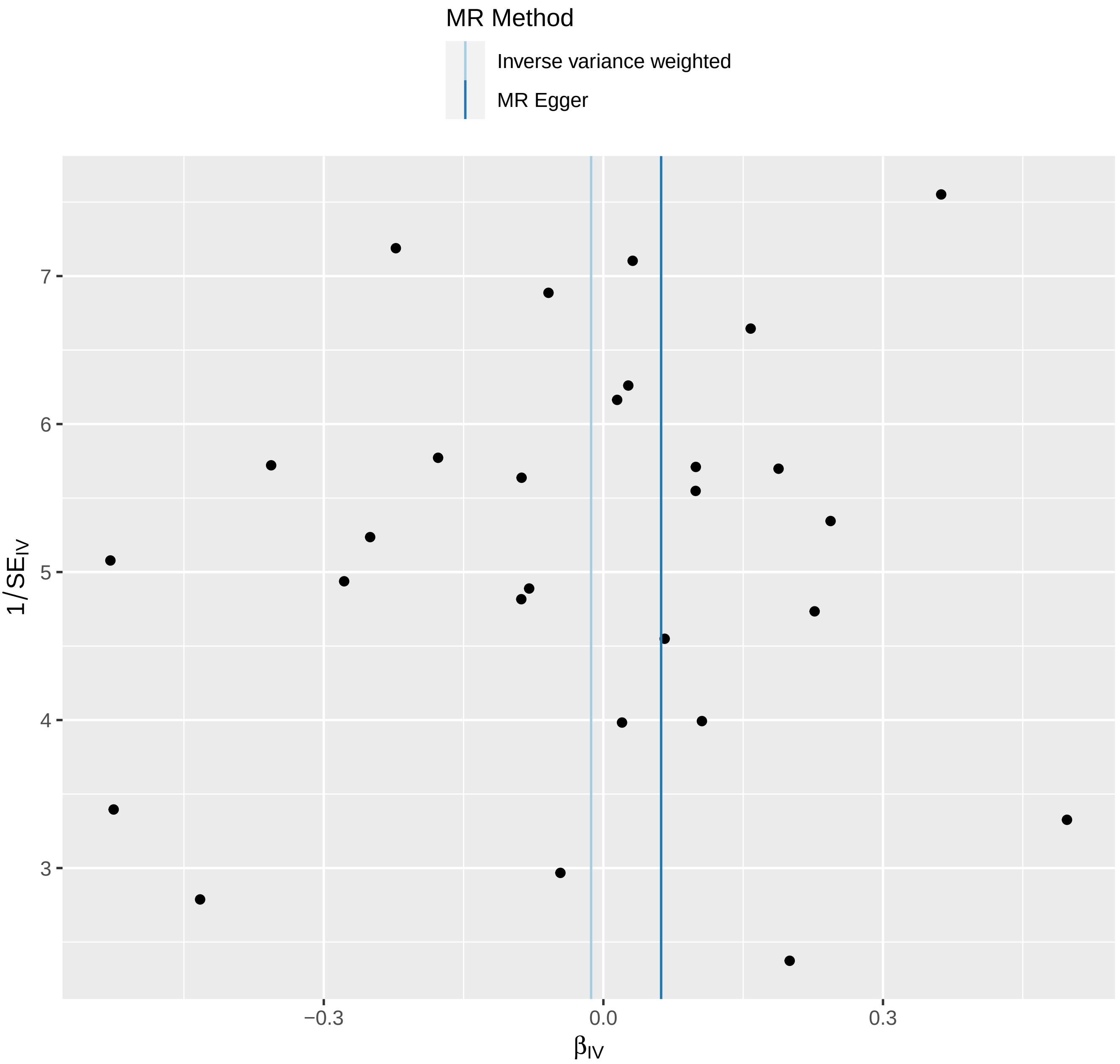

Batch 3−1376 : Heparan sulfate glucosamine 3−O−sulfotransferase 3B1 on Diabetic nephropathy (more control exclusions)
with a threshold of 1e−05

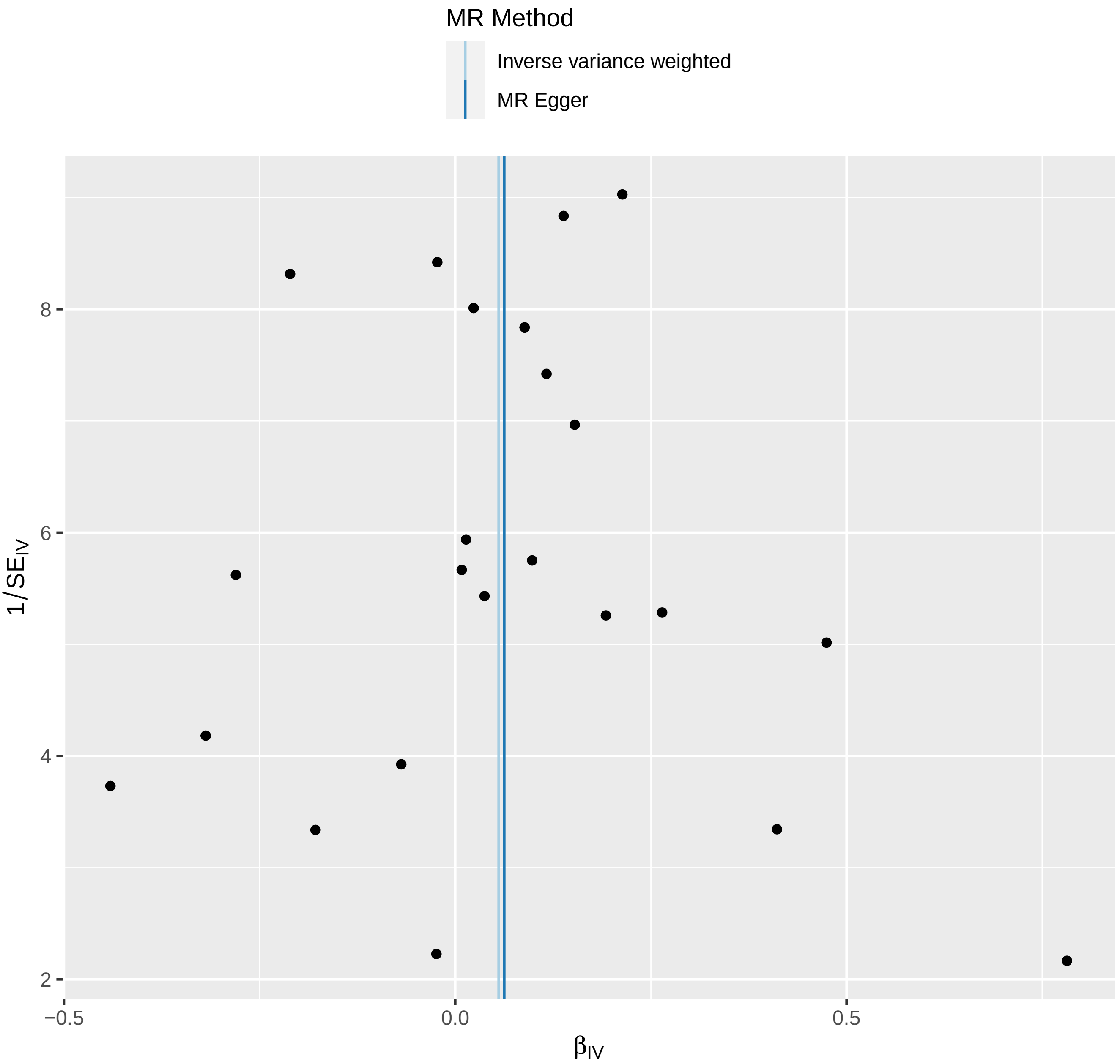

Batch 3−1377 : Heparan sulfate glucosamine 3−O−sulfotransferase 4 on Diabetic nephropathy (more control exclusions) with a threshold of 1e−05

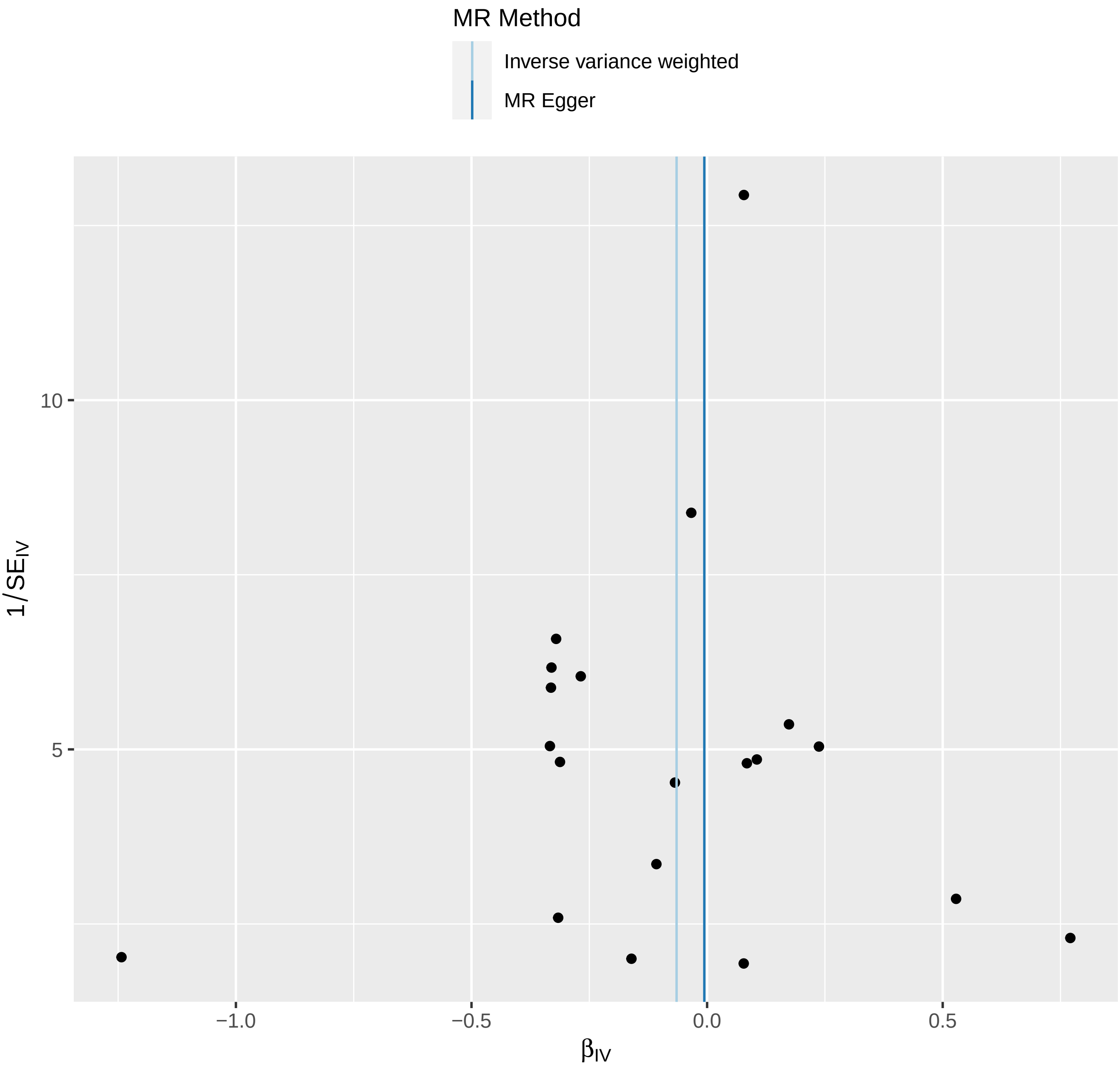

Batch 3−1378 : Heparan−sulfate 6−O−sulfotransferase 1 on Diabetic nephropathy (more control exclusions)
with a threshold of 1e−05

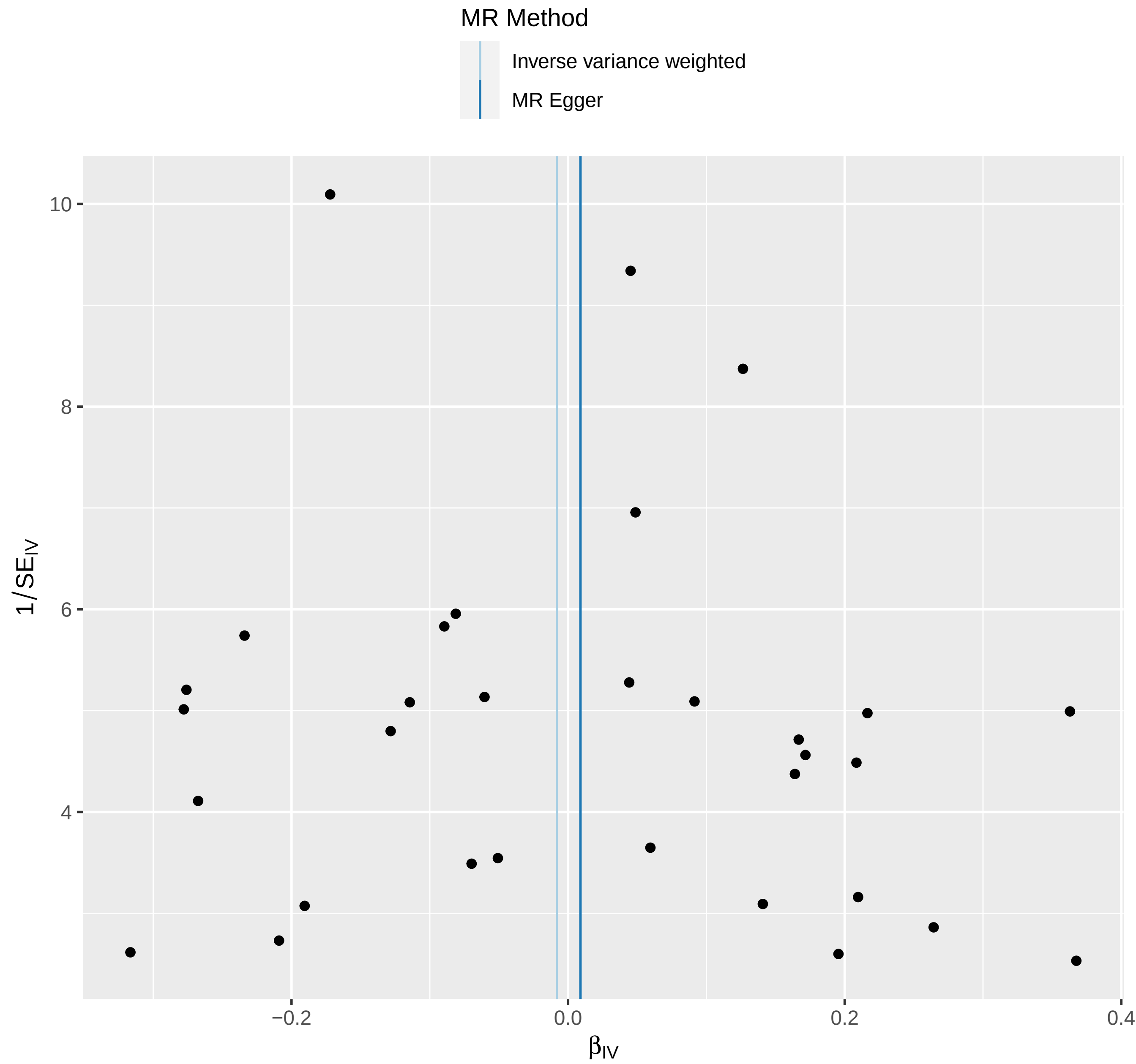

Batch 3−1379 : Heparan−sulfate 6−O−sulfotransferase 2 on Diabetic nephropathy (more control exclusions)
with a threshold of 1e−05

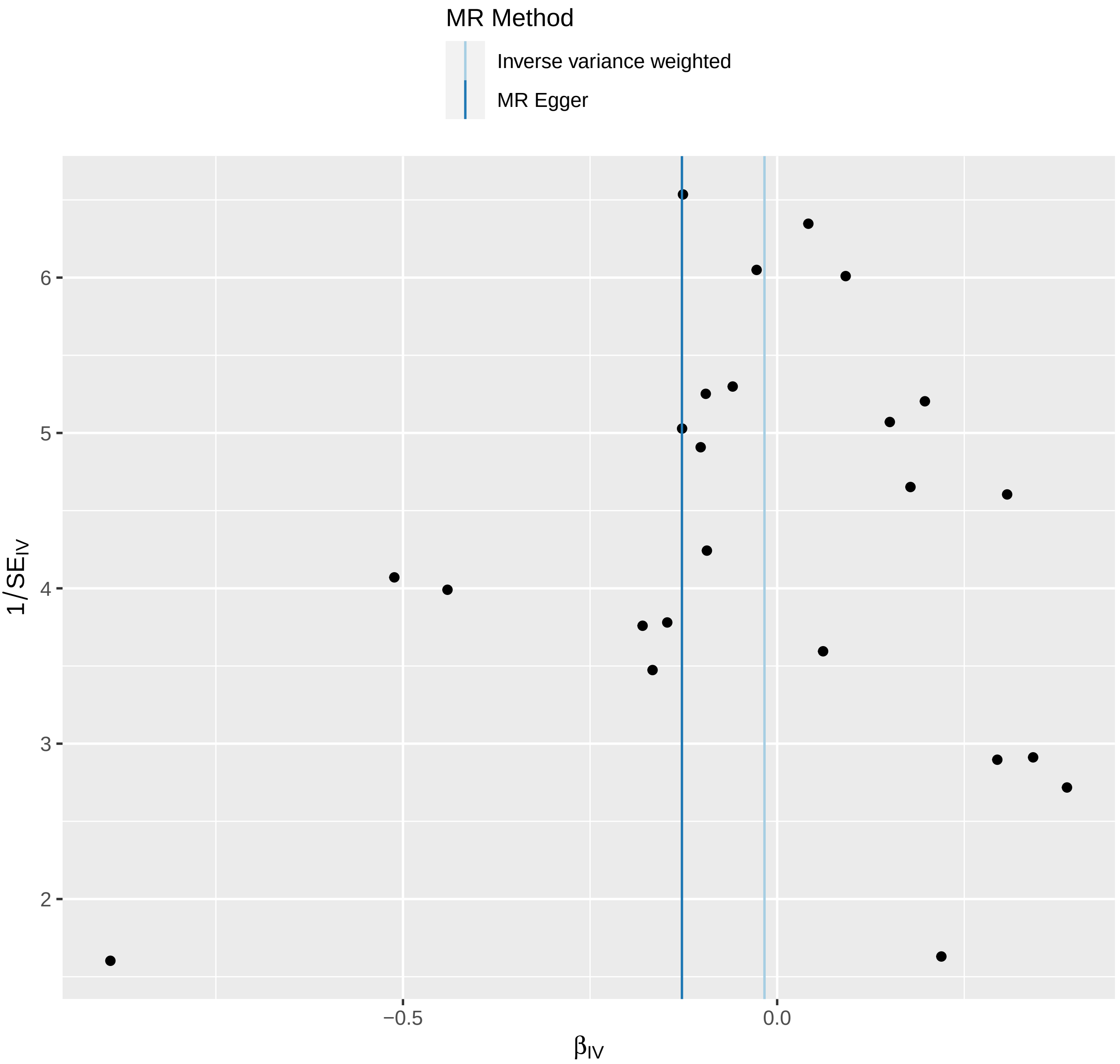

Batch 3−1380 : 3−hydroxyacyl−CoA dehydrogenase type−2 on Diabetic nephropathy (more control exclusions)
with a threshold of 1e−05

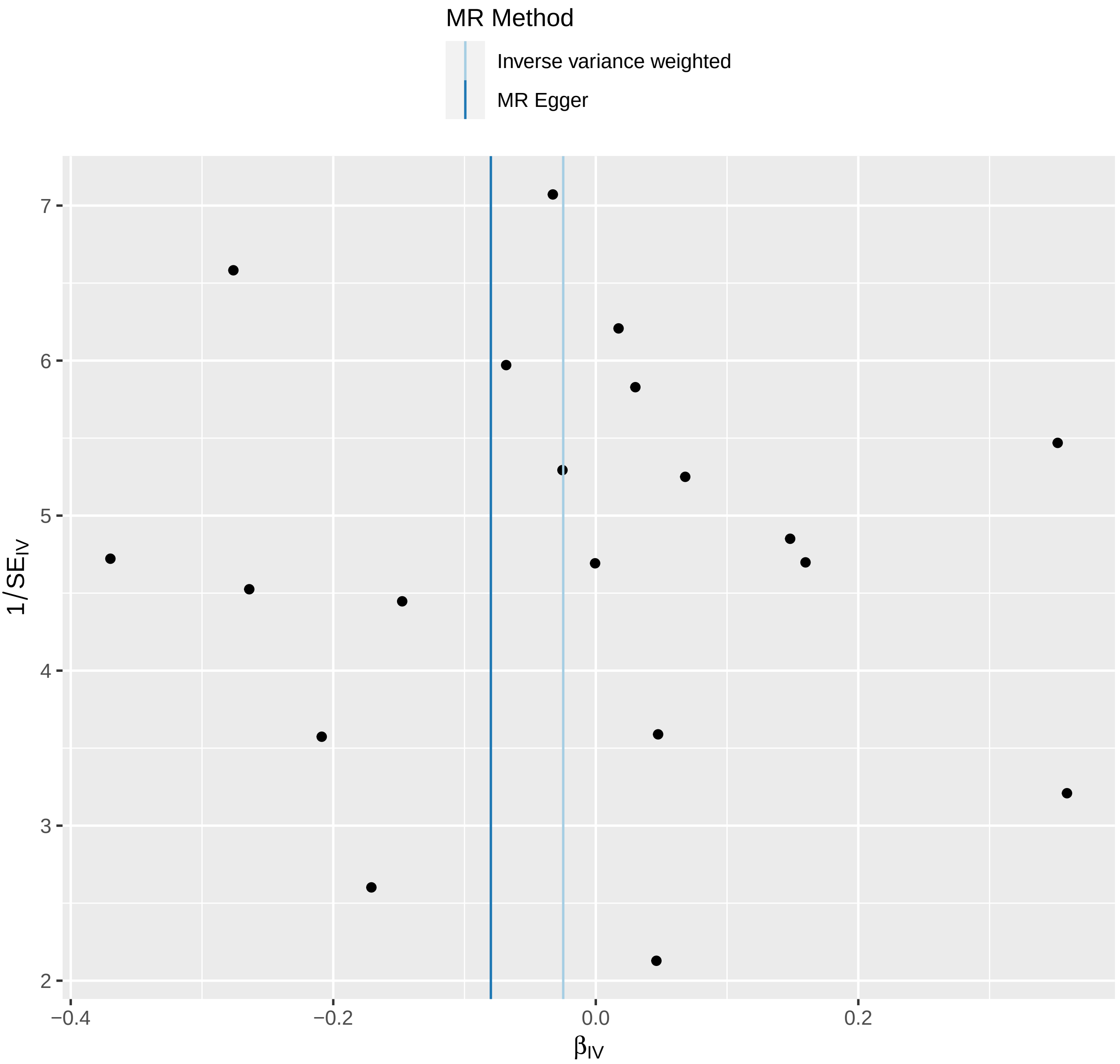

Batch 3−1381 : 17−beta−hydroxysteroid dehydrogenase 14 on Diabetic nephropathy (more control exclusions)
with a threshold of 1e−05

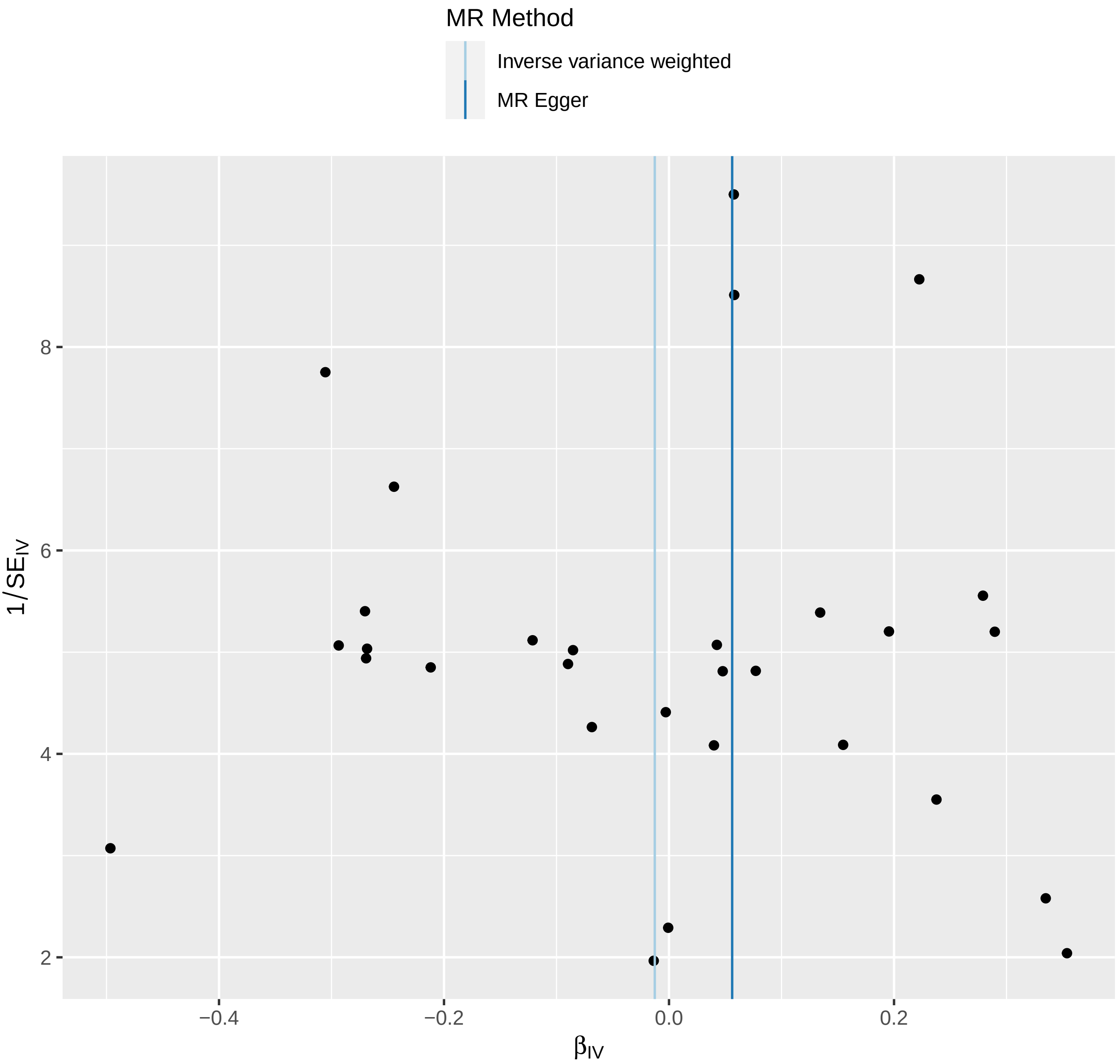

Batch 3−1382 : Estradiol 17−beta−dehydrogenase 1 on Diabetic nephropathy (more control exclusions)
with a threshold of 1e−05

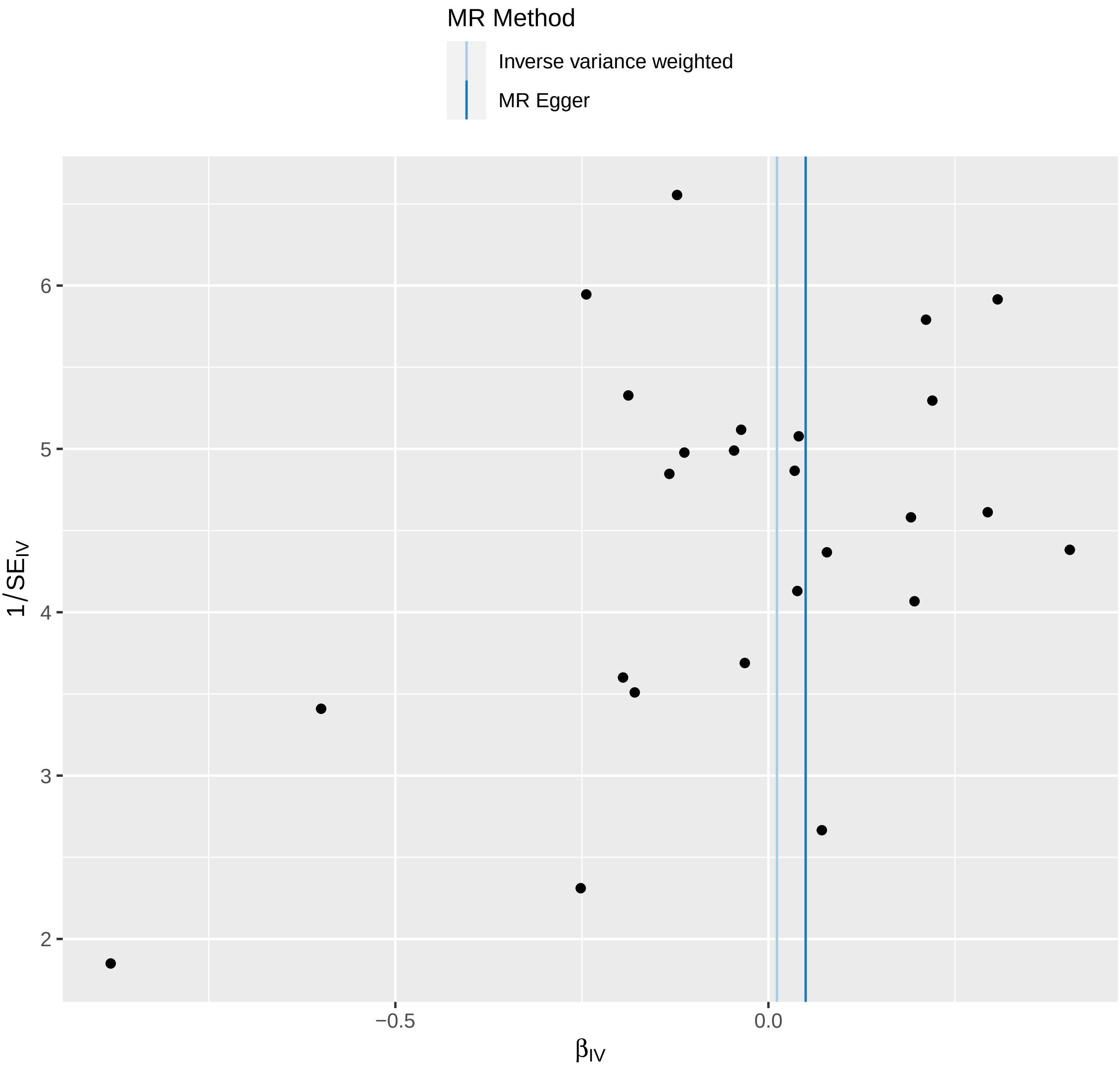

Batch 3−1383 : Estradiol 17−beta−dehydrogenase 2 on Diabetic nephropathy (more control exclusions)
with a threshold of 1e−05

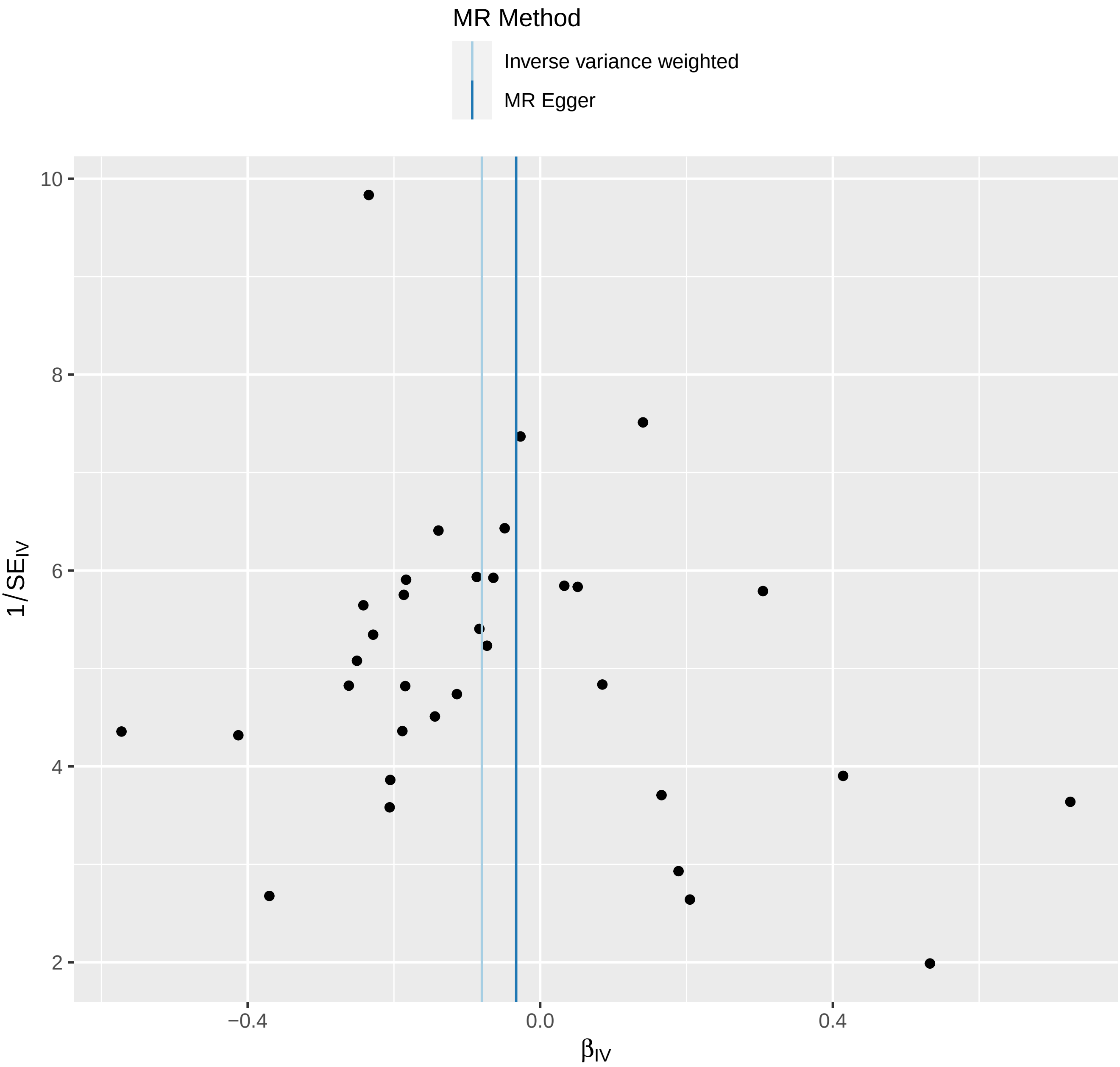

Batch 3−1384 : Heat shock protein HSP 90−alpha/beta on Diabetic nephropathy (more control exclusions)
with a threshold of 1e−05

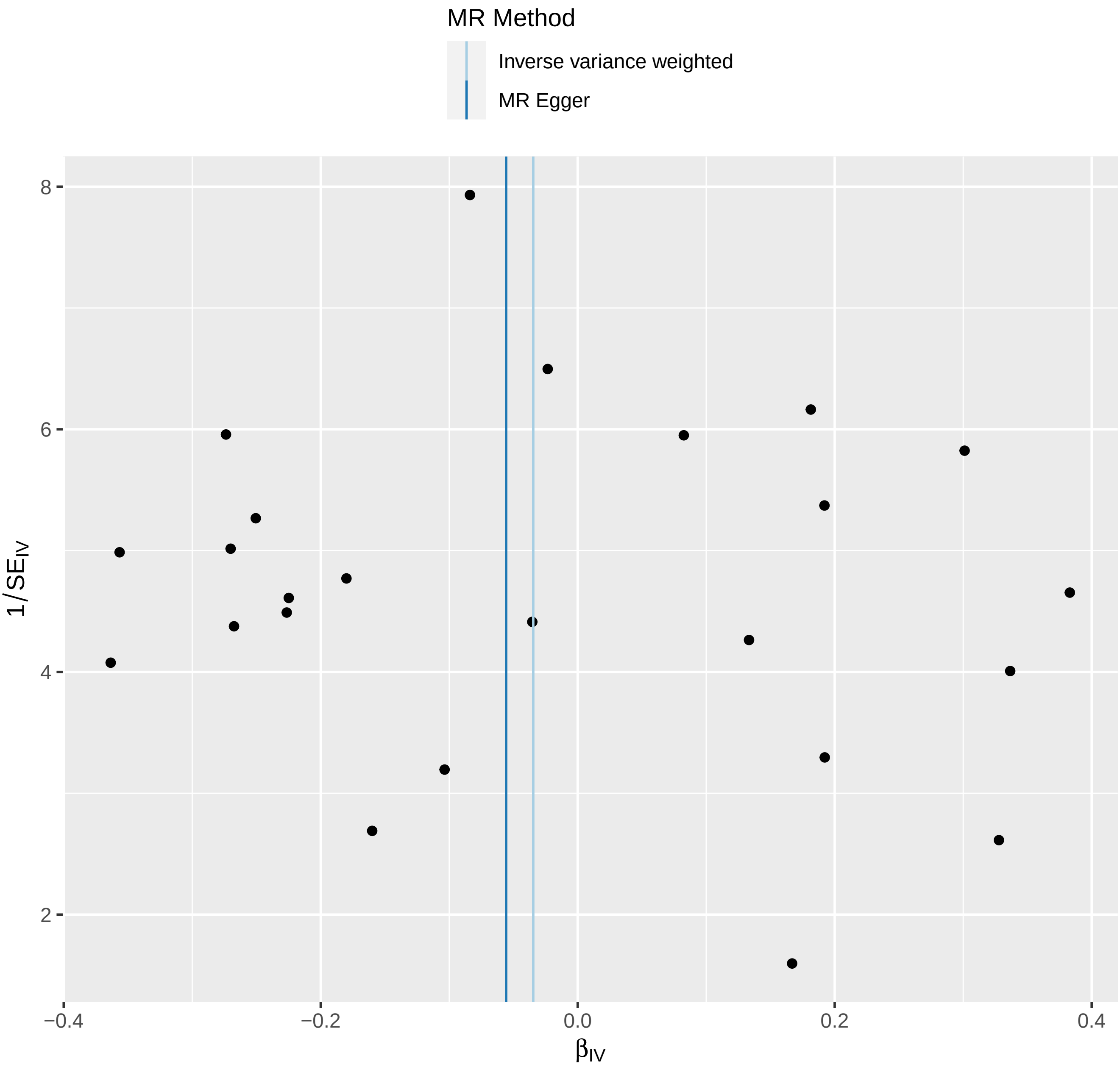

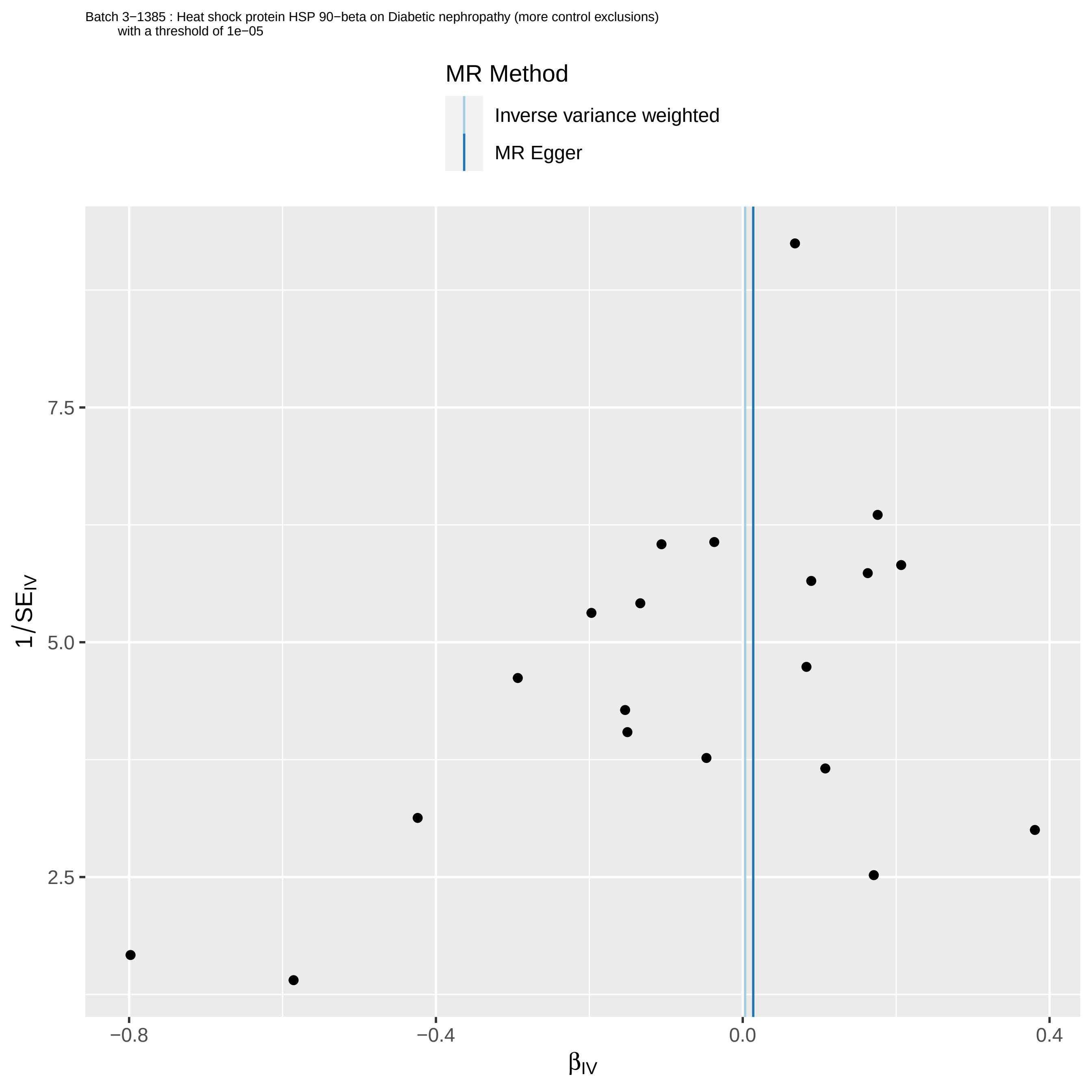

Batch 3−1385 : Heat shock protein HSP 90−beta on Diabetic nephropathy (more control exclusions)
with a threshold of 1e−05
MR Method
Inverse variance weighted
MR Egger
7.5
5.0
2.5
1/SE_IV
−0.8
−0.4
0.0
0.4
β_IV

Batch 3−1386 : Endoplasmin on Diabetic nephropathy (more control exclusions)
with a threshold of 1e−05

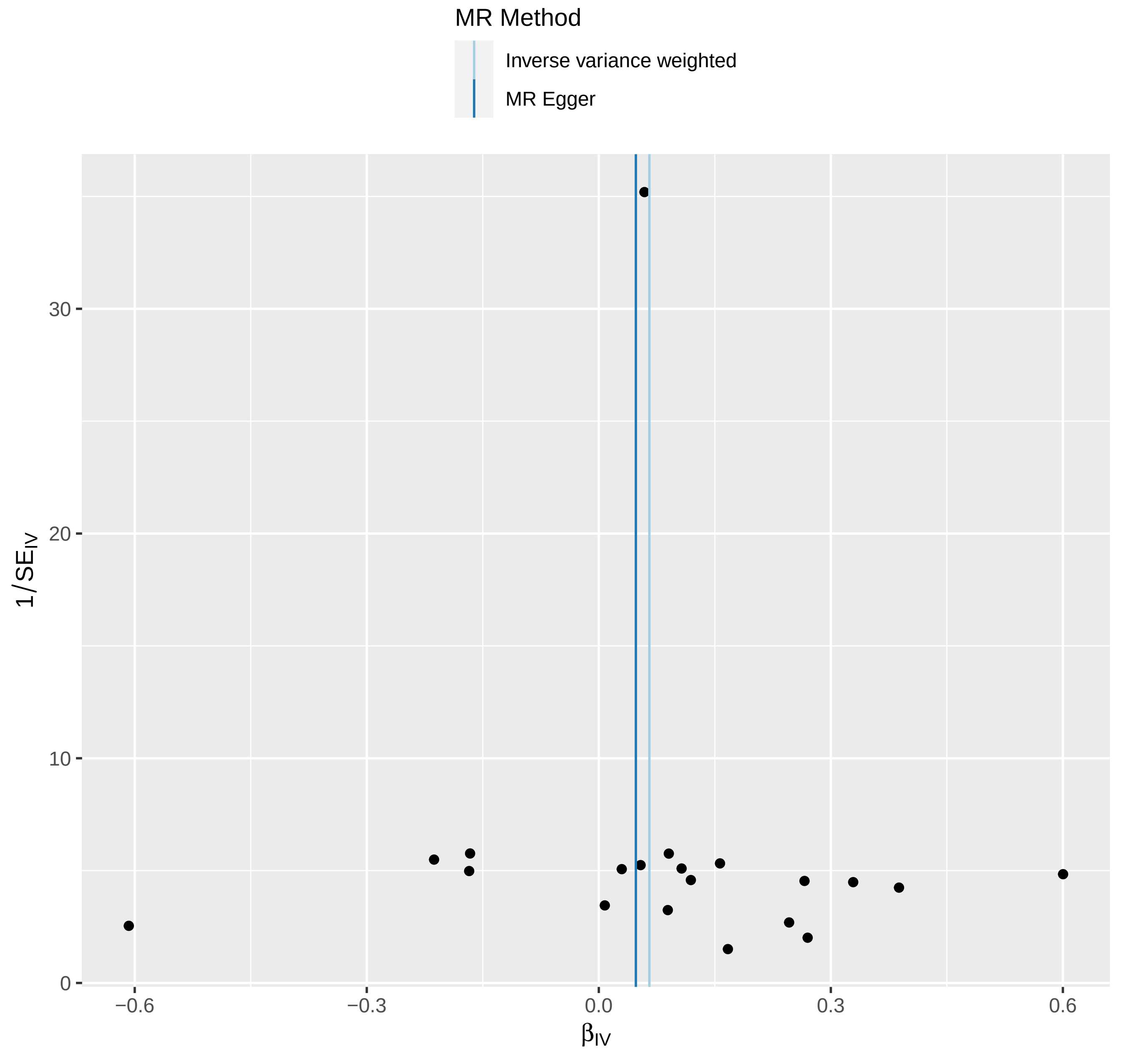

Batch 3−1387 : Heat shock 70 kDa protein 1−like on Diabetic nephropathy (more control exclusions)
with a threshold of 1e−05

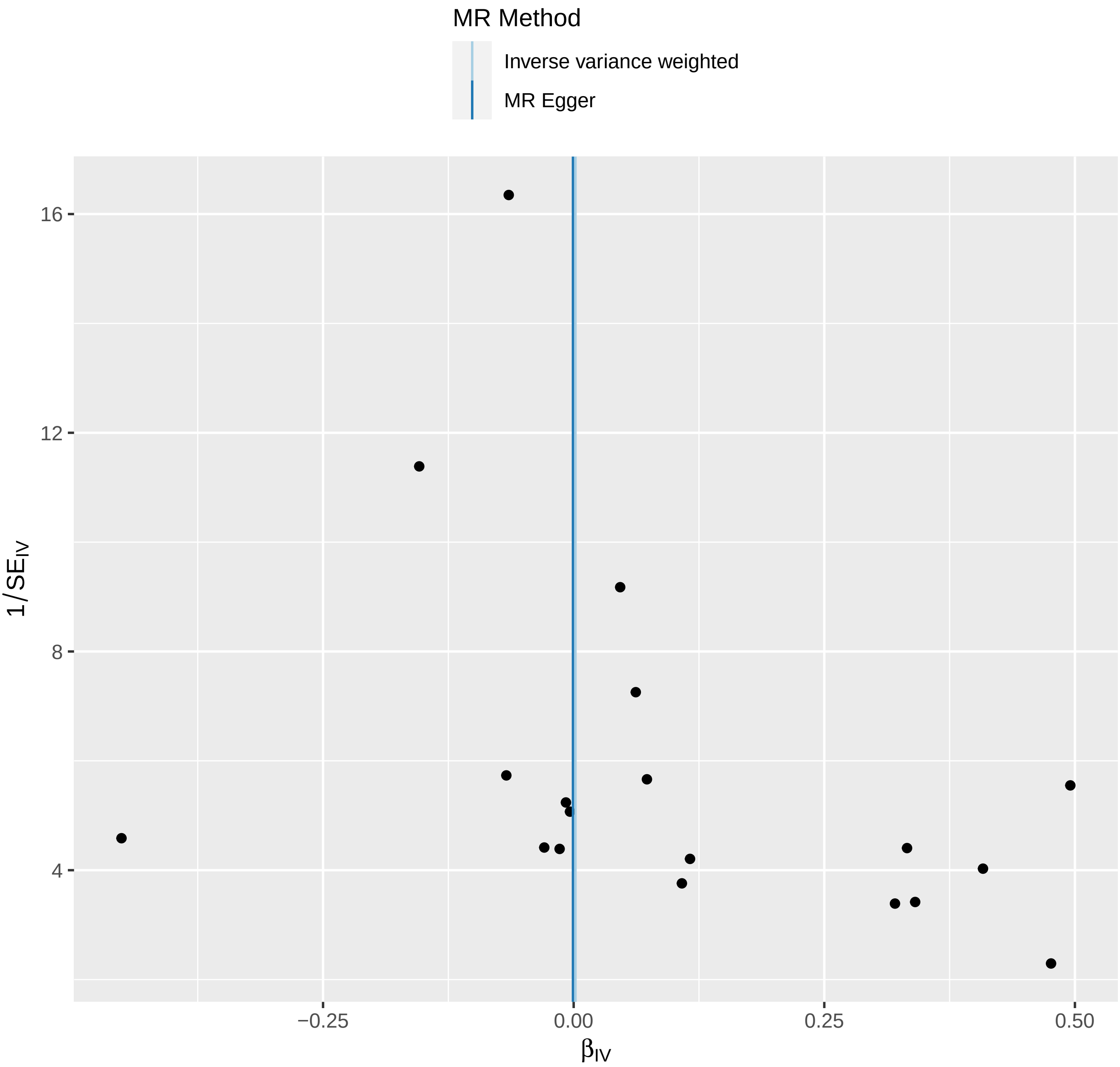

Batch 3−1388 : Heat shock 70 kDa protein 6 on Diabetic nephropathy (more control exclusions)
with a threshold of 1e−05

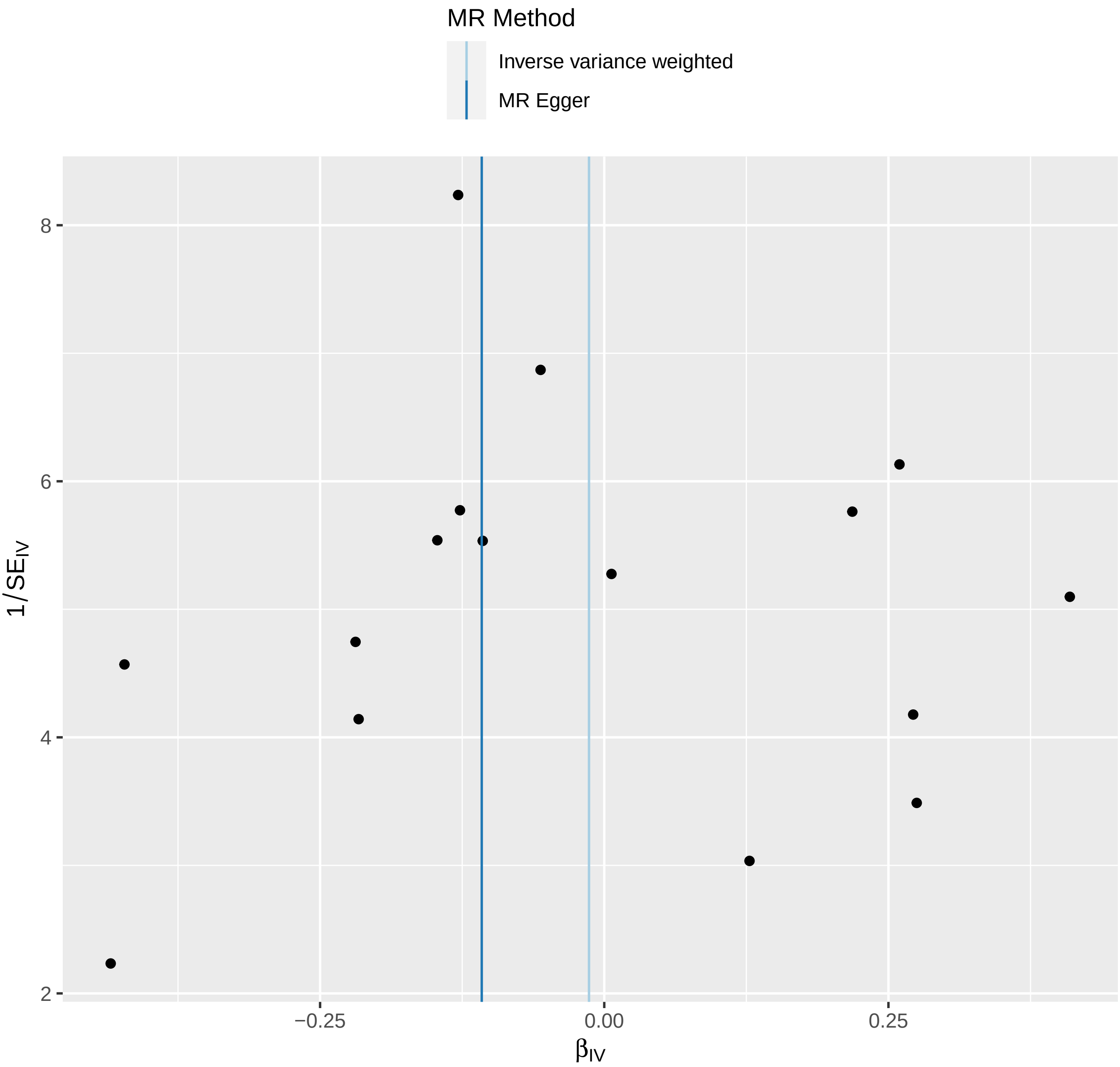

Batch 3−1389 : Oxidoreductase HTATIP2 on Diabetic nephropathy (more control exclusions)
with a threshold of 1e−05

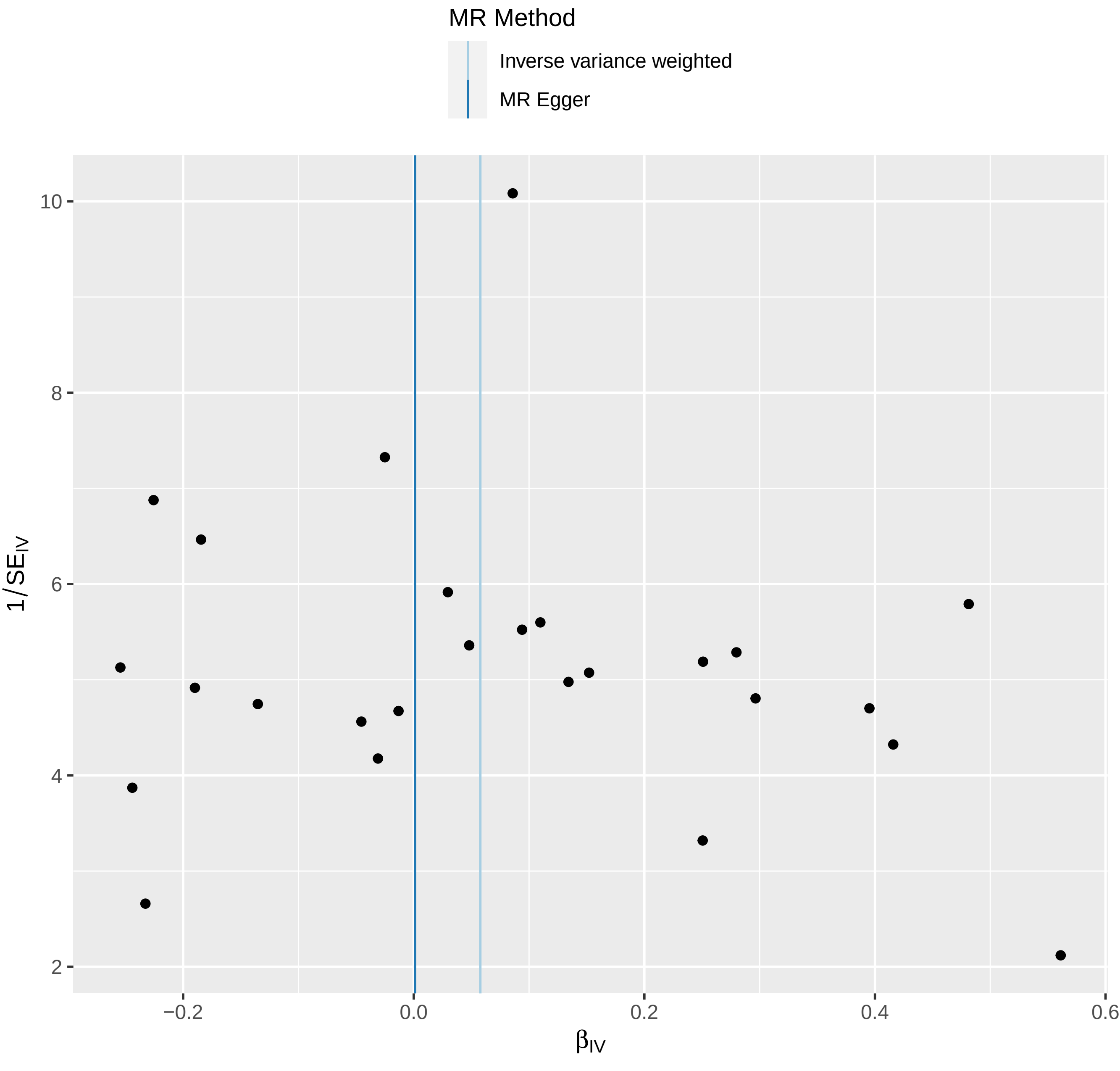

Batch 3−1390 : Oxidoreductase HTATIP2 on Diabetic nephropathy (more control exclusions)
with a threshold of 1e−05

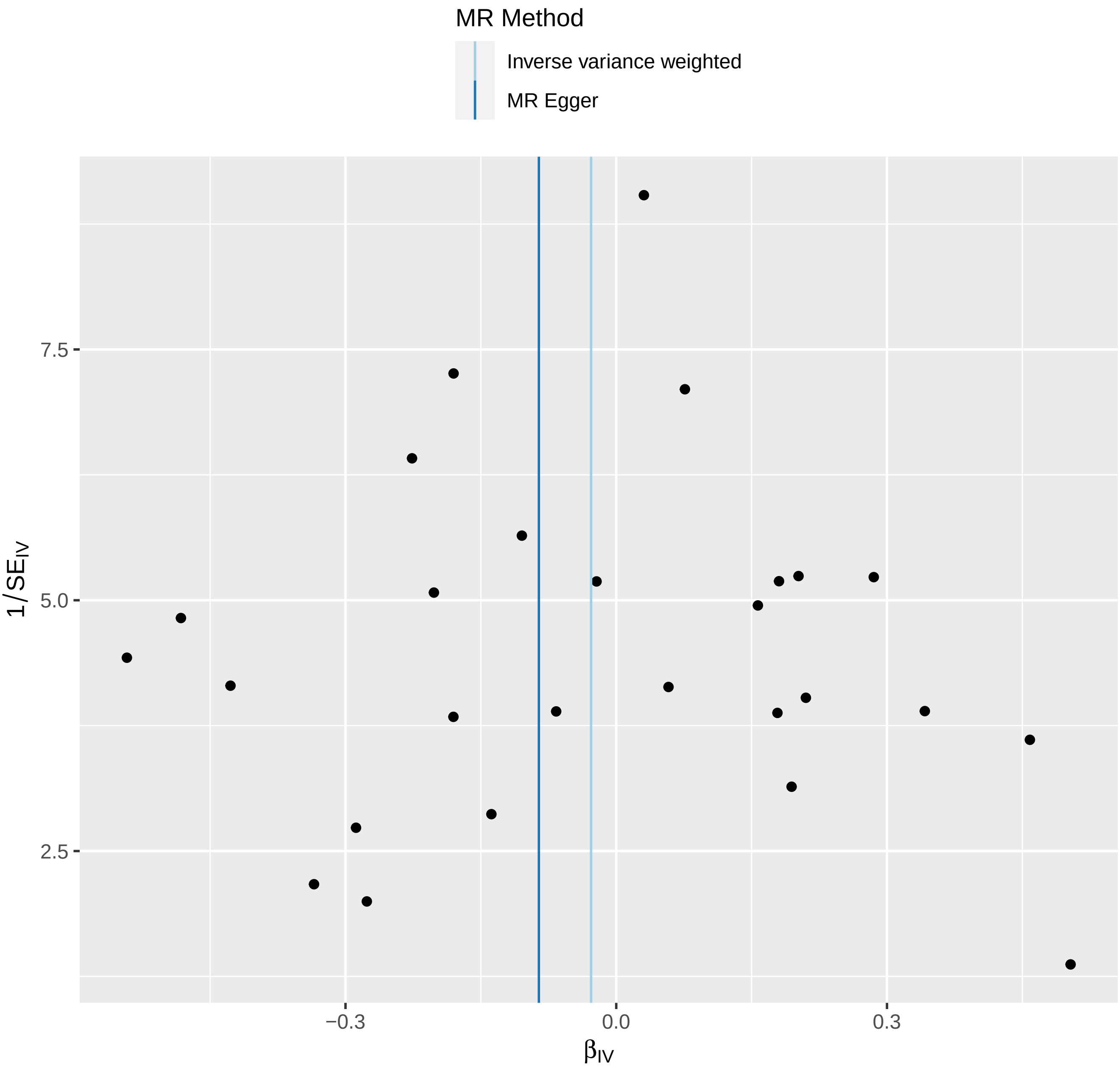

Batch 3−1391 : Histatin−1 on Diabetic nephropathy (more control exclusions)
with a threshold of 1e−05

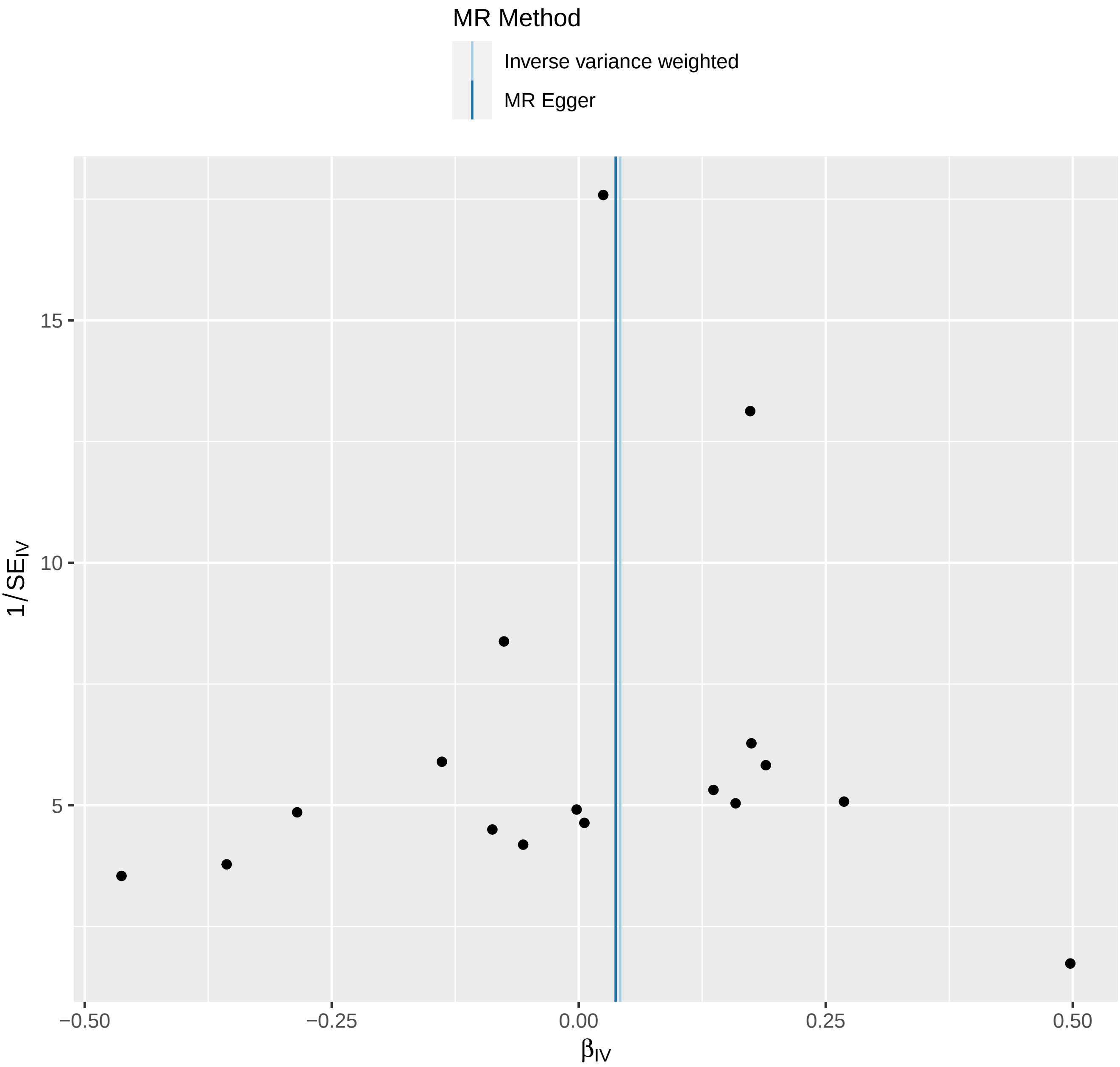

Batch 3−1392 : Serine protease HTRA2, mitochondrial on Diabetic nephropathy (more control exclusions)
with a threshold of 1e−05

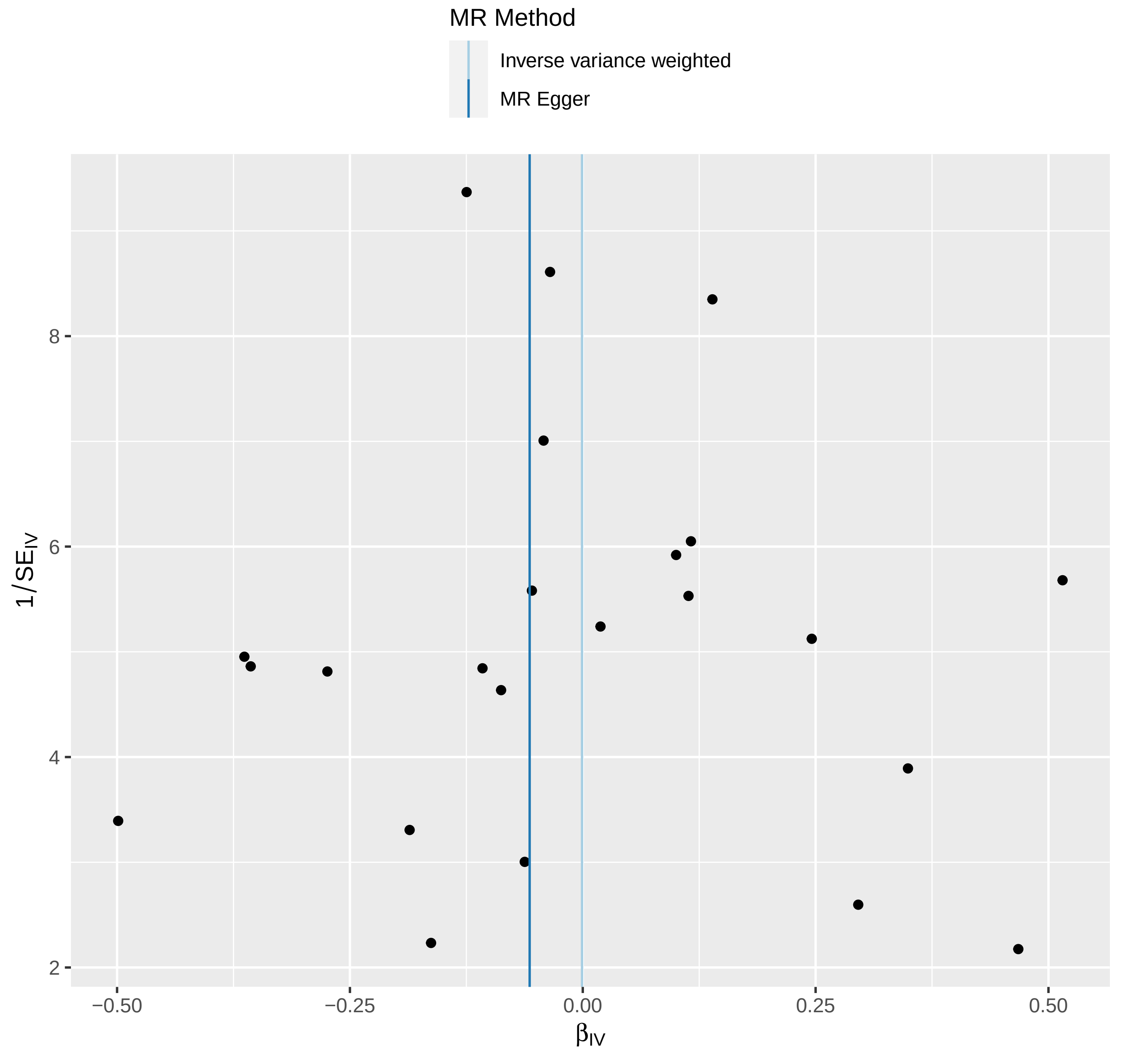

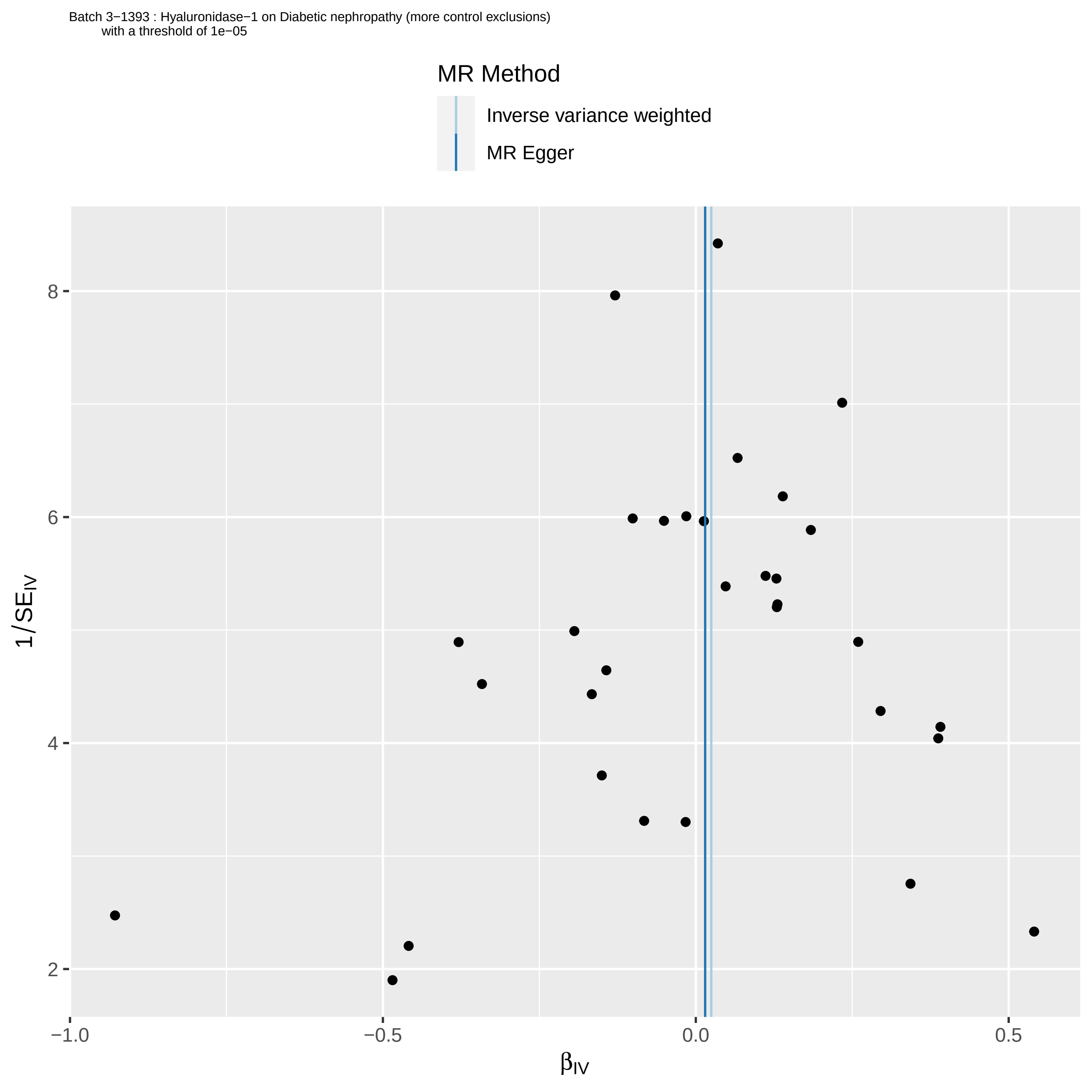
Batch 3−1393 : Hyaluronidase−1 on Diabetic nephropathy (more control exclusions)
with a threshold of 1e−05
MR Method
Inverse variance weighted
MR Egger
8
6
4
2
−1.0
−0.5
0.0
0.5
$1/SE_{IV}$
β_{IV}

Batch 3−1394 : Isoleucine−−tRNA ligase, cytoplasmic on Diabetic nephropathy (more control exclusions) with a threshold of 1e−05

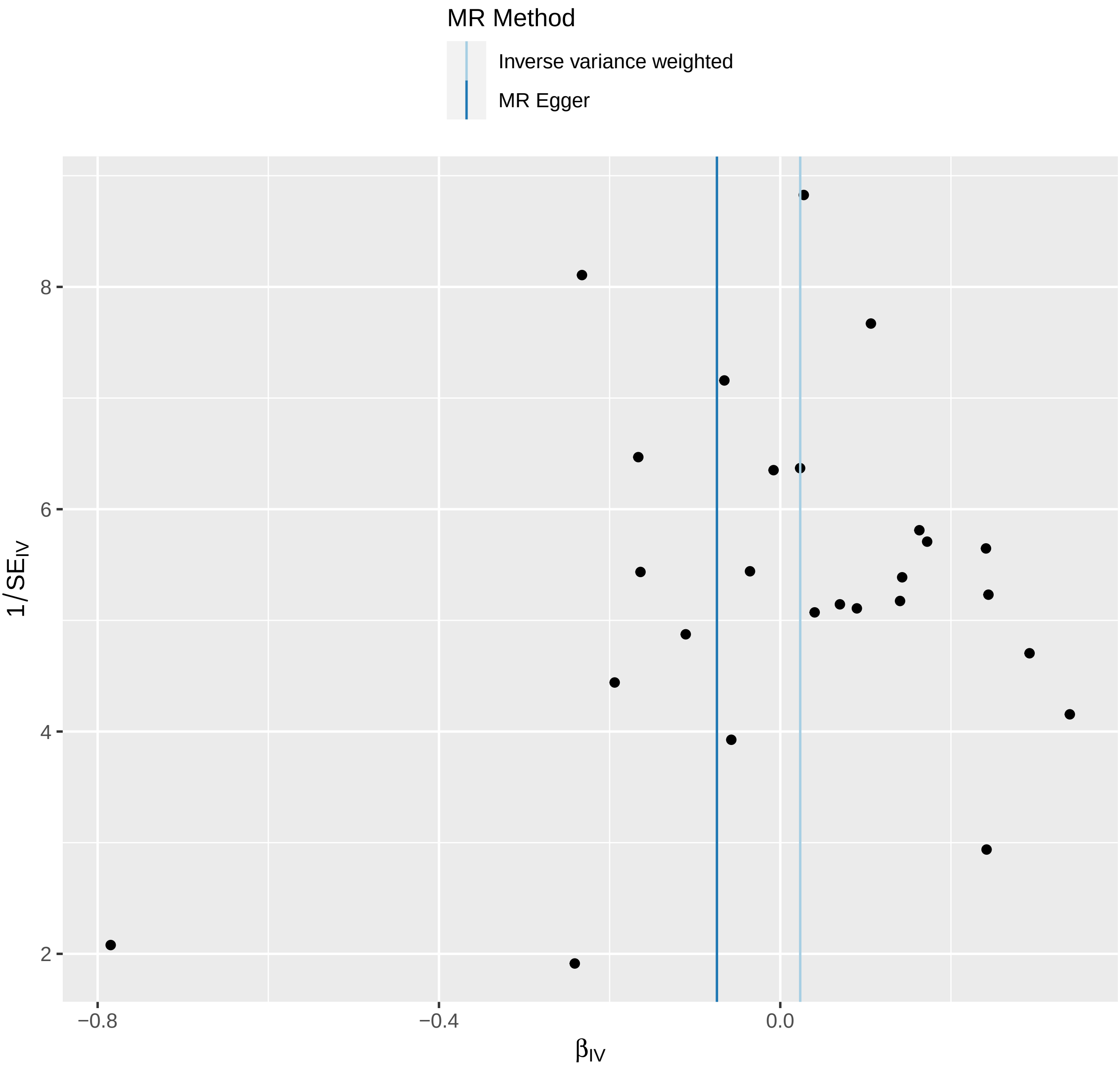

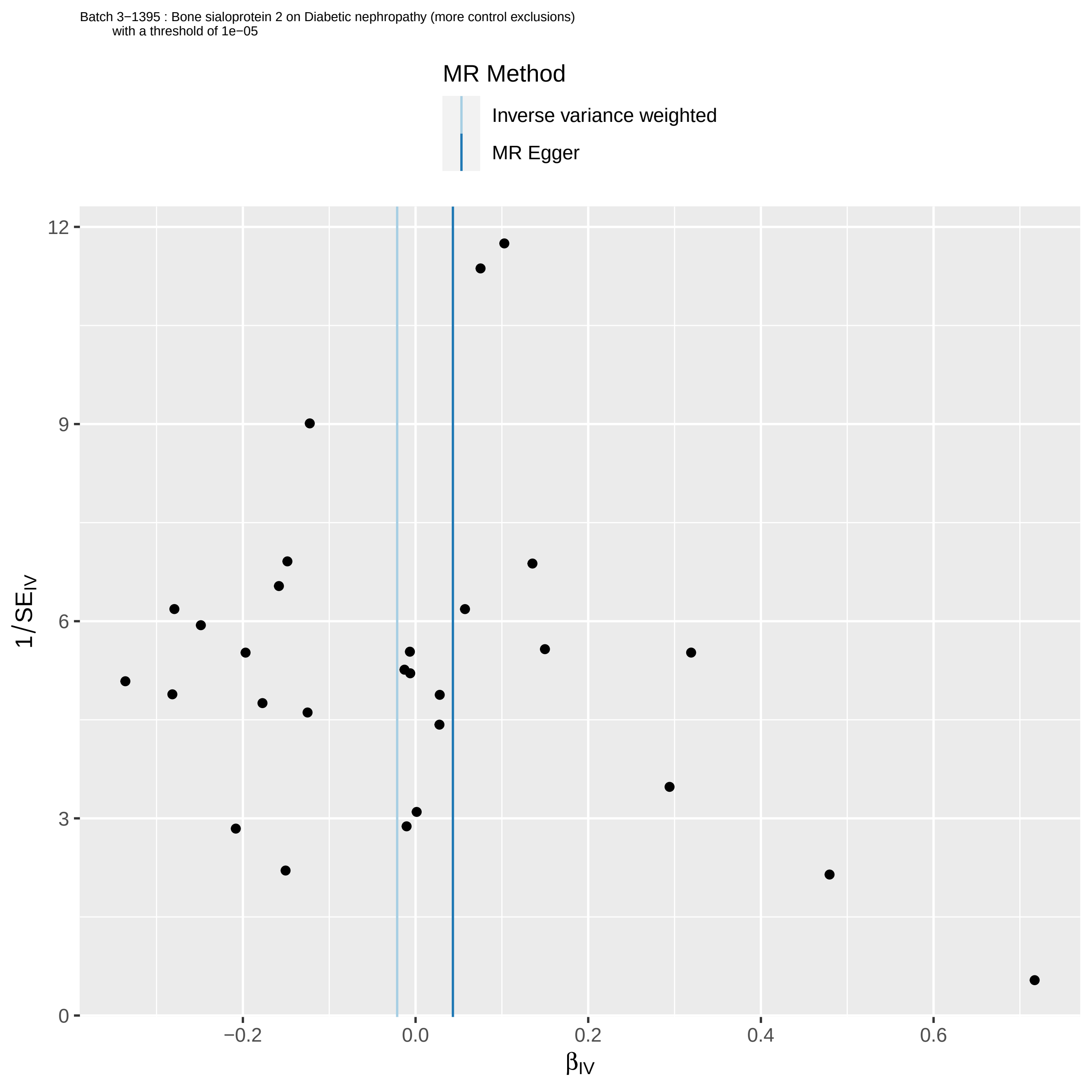

Batch 3−1395 : Bone sialoprotein 2 on Diabetic nephropathy (more control exclusions)
with a threshold of 1e−05
MR Method
Inverse variance weighted
MR Egger
12
9
6
3
0
1/SE_IV
−0.2
0.0
0.2
0.4
0.6
β_IV

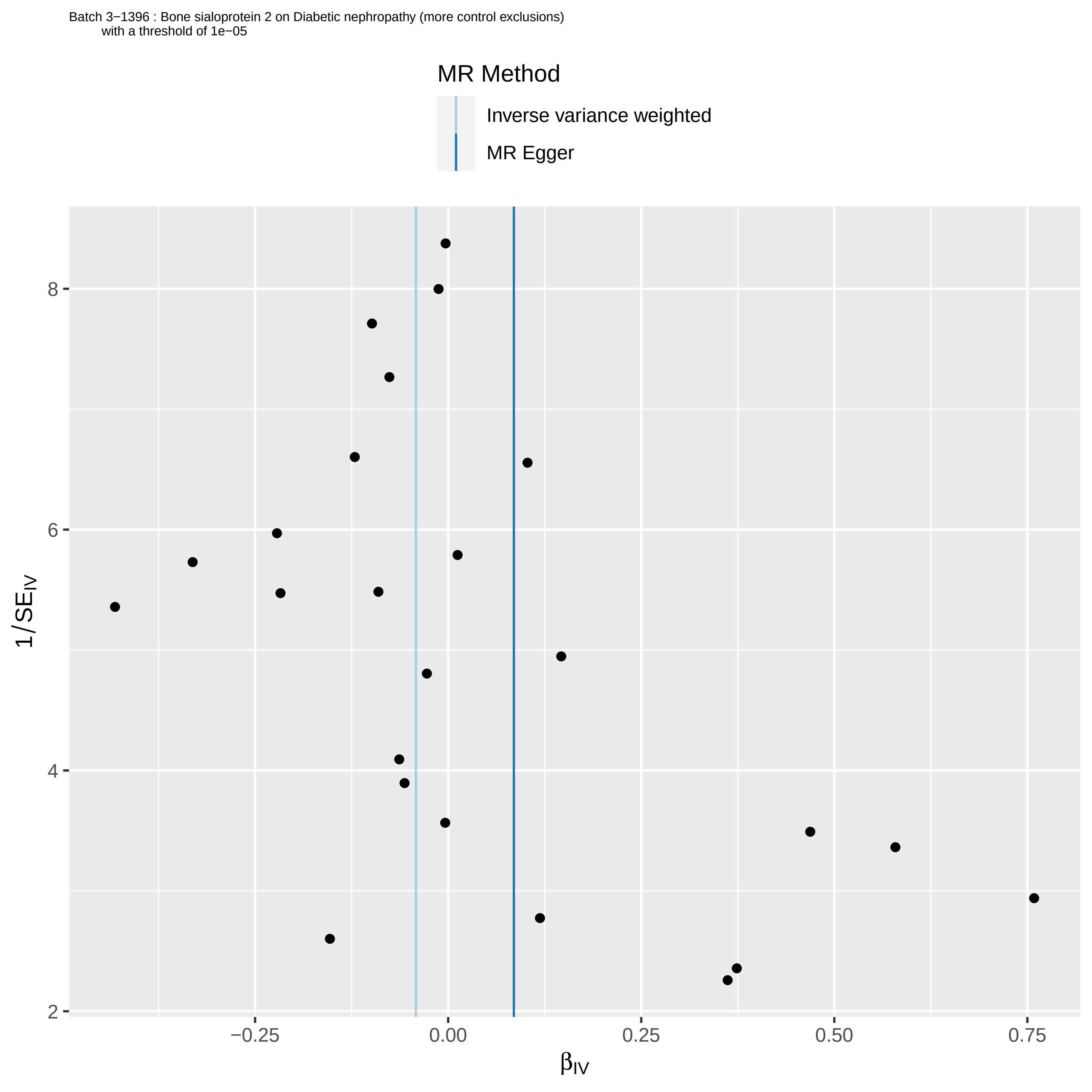

Batch 3−1396 : Bone sialoprotein 2 on Diabetic nephropathy (more control exclusions)
with a threshold of 1e−05
MR Method
Inverse variance weighted
MR Egger
8
6
4
2
1/SE_IV
−0.25
0.00
0.25
0.50
0.75
β_IV

Batch 3−1397 : Intercellular adhesion molecule 1 on Diabetic nephropathy (more control exclusions)
with a threshold of 1e−05

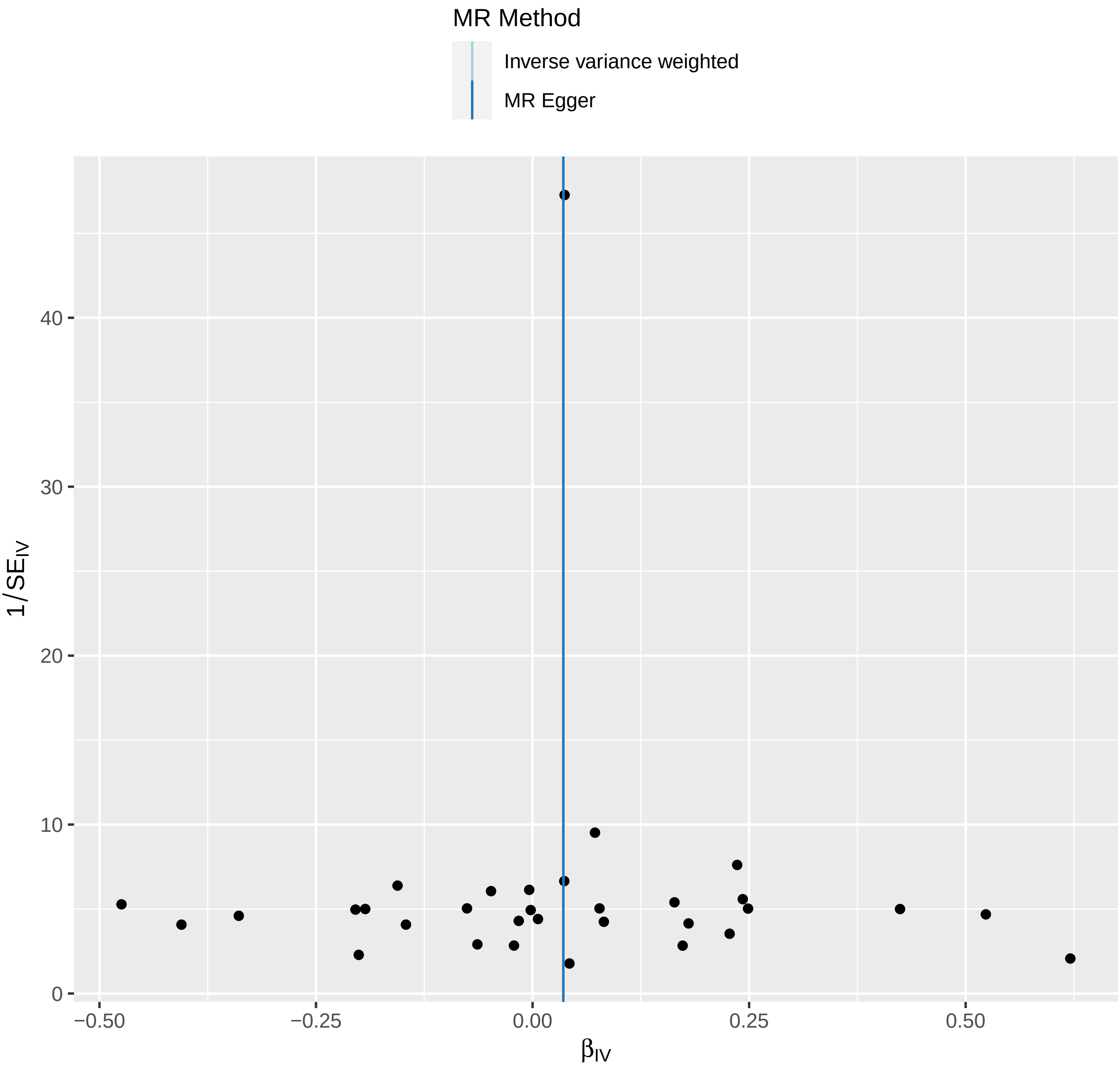

Batch 3−1398 : Intercellular adhesion molecule 2 on Diabetic nephropathy (more control exclusions)
with a threshold of 1e−05

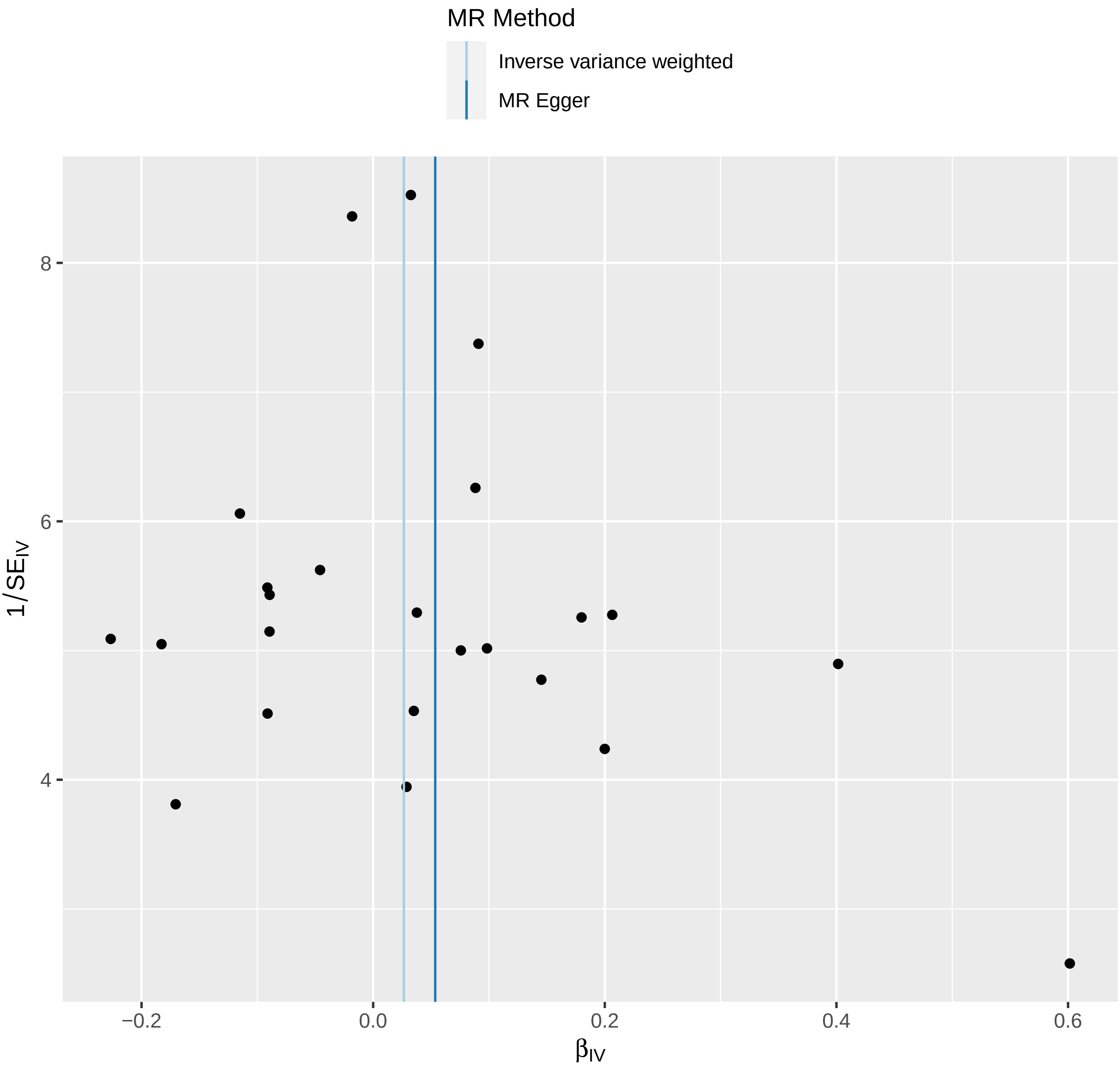

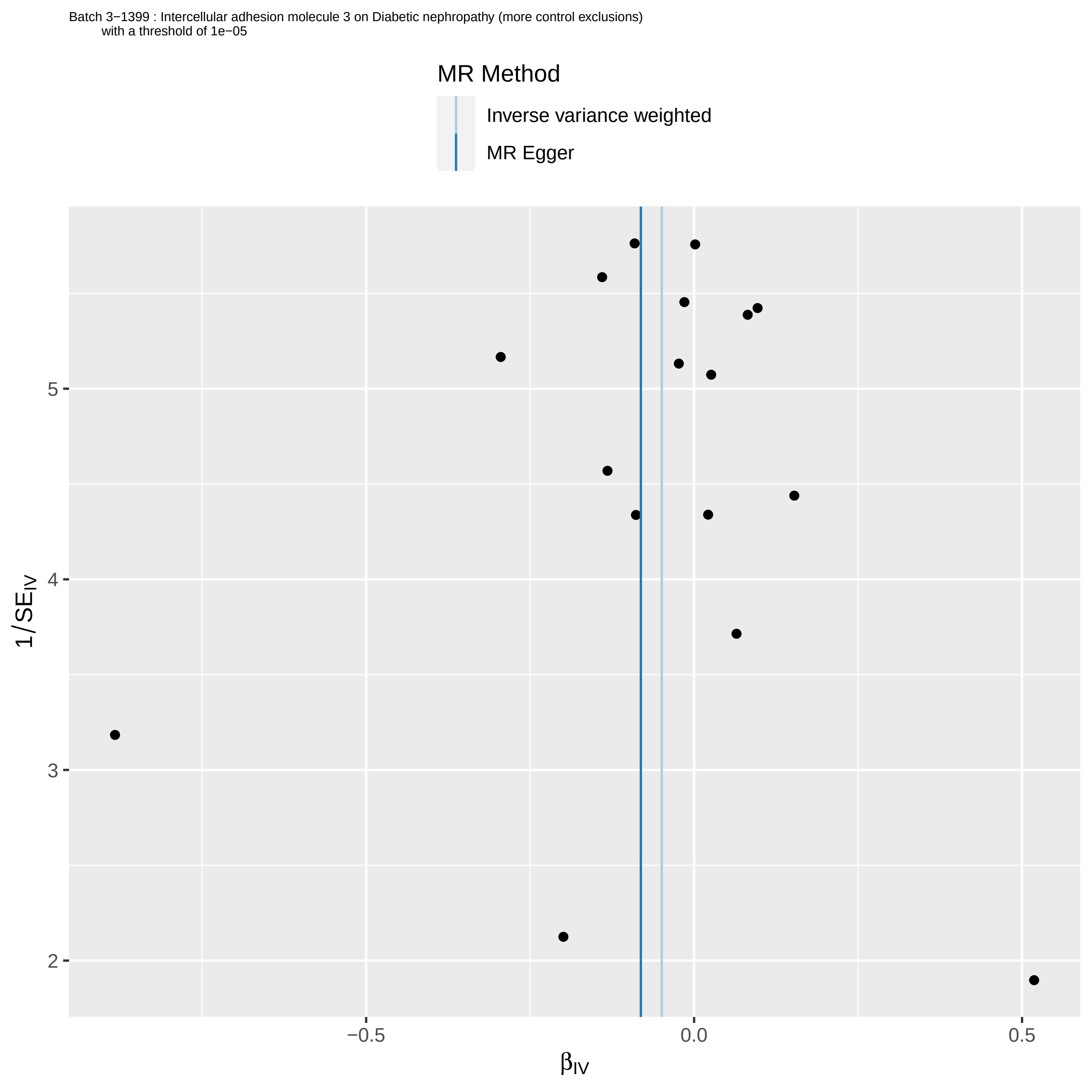

Batch 3−1399 : Intercellular adhesion molecule 3 on Diabetic nephropathy (more control exclusions)
with a threshold of 1e−05
MR Method
Inverse variance weighted
MR Egger
1/SE_IV
5
4
3
2
−0.5
0.0
0.5
β_IV

Batch 3−1400 : Intercellular adhesion molecule 4 on Diabetic nephropathy (more control exclusions)
with a threshold of 1e−05

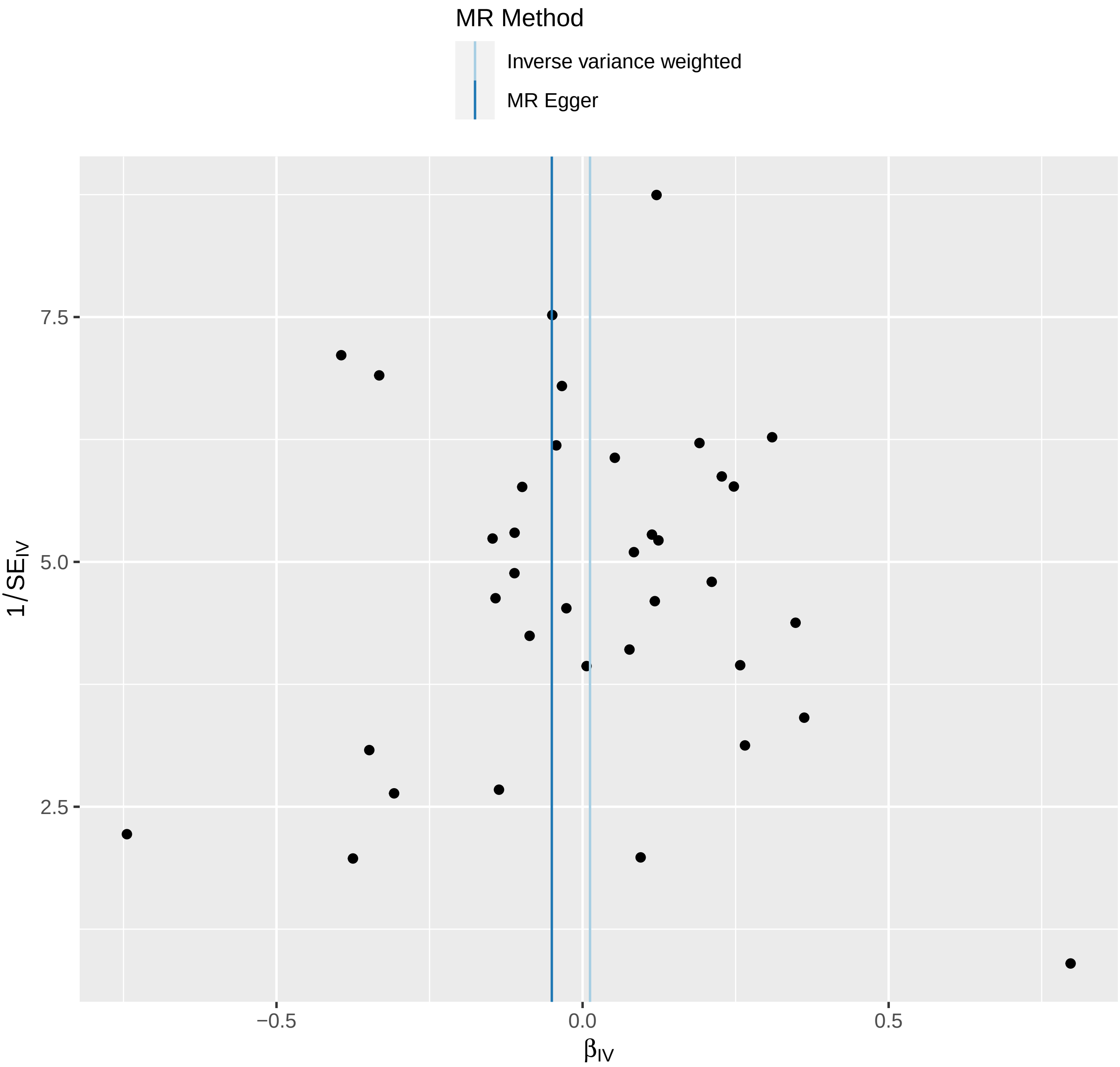

Batch 3−1401 : Intercellular adhesion molecule 5 on Diabetic nephropathy (more control exclusions)
with a threshold of 1e−05

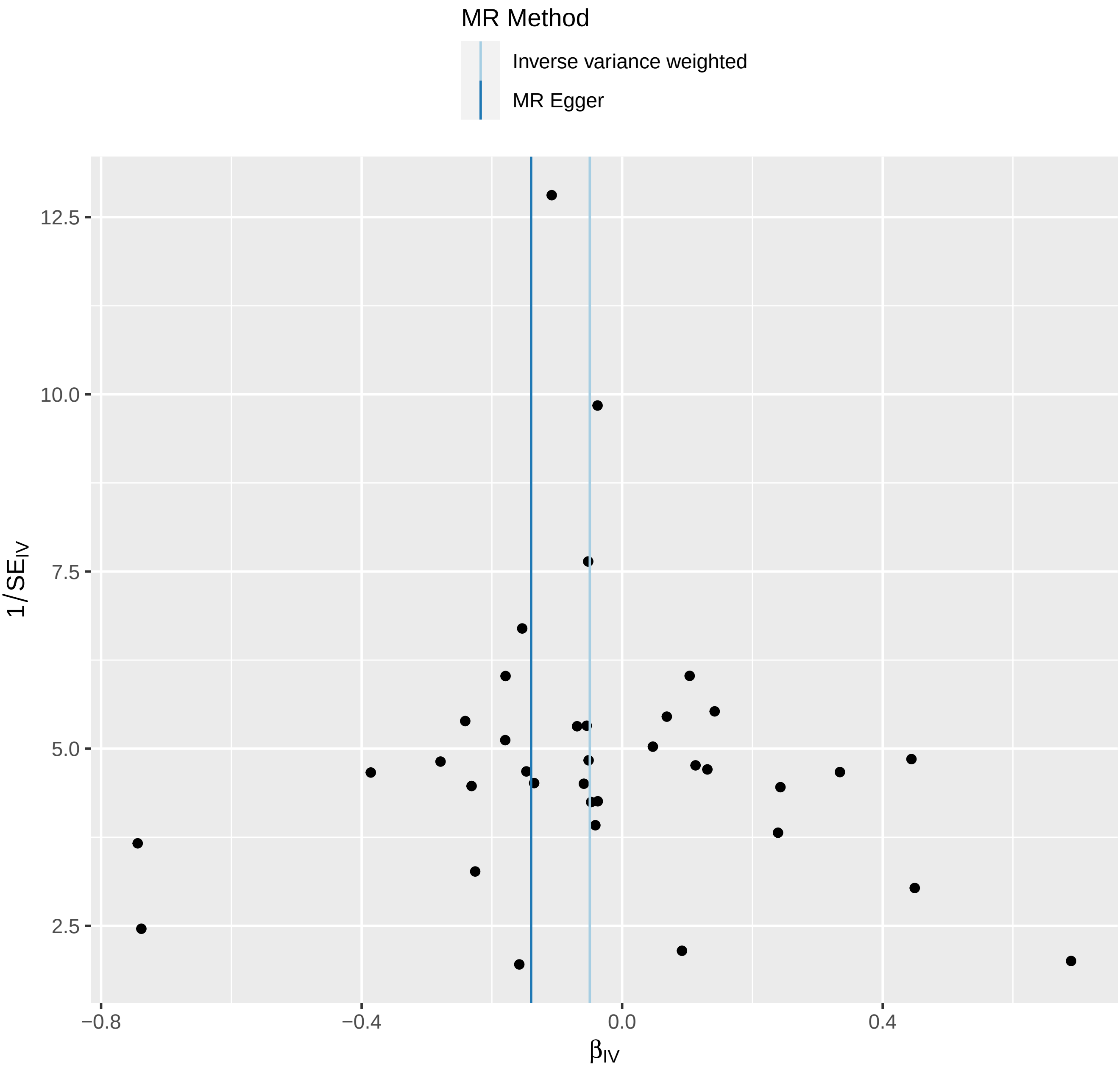

Batch 3−1402 : Intercellular adhesion molecule 5 on Diabetic nephropathy (more control exclusions)
with a threshold of 1e−05

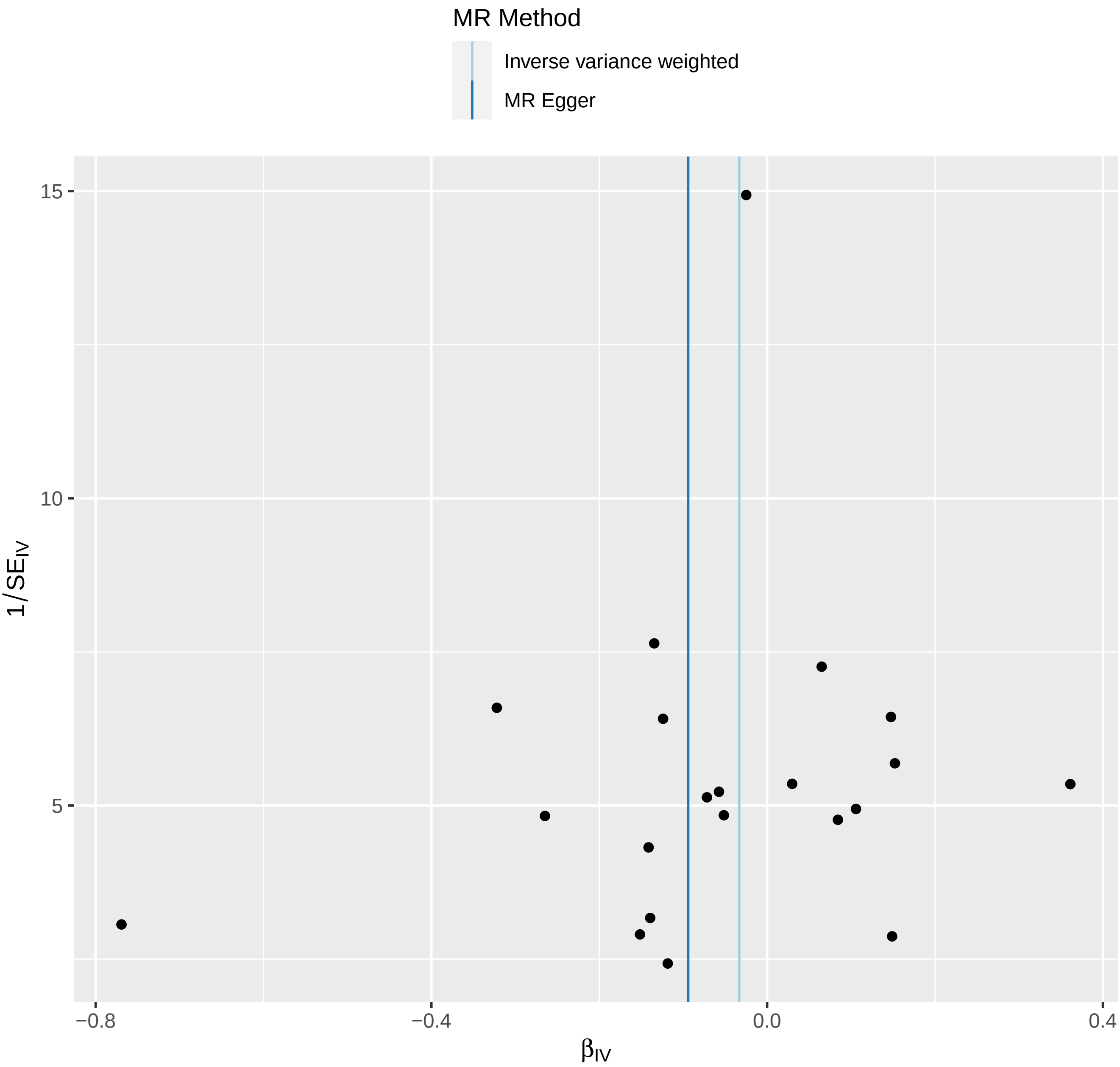

Batch 3−1403 : Inducible T−cell costimulator on Diabetic nephropathy (more control exclusions)
with a threshold of 1e−05

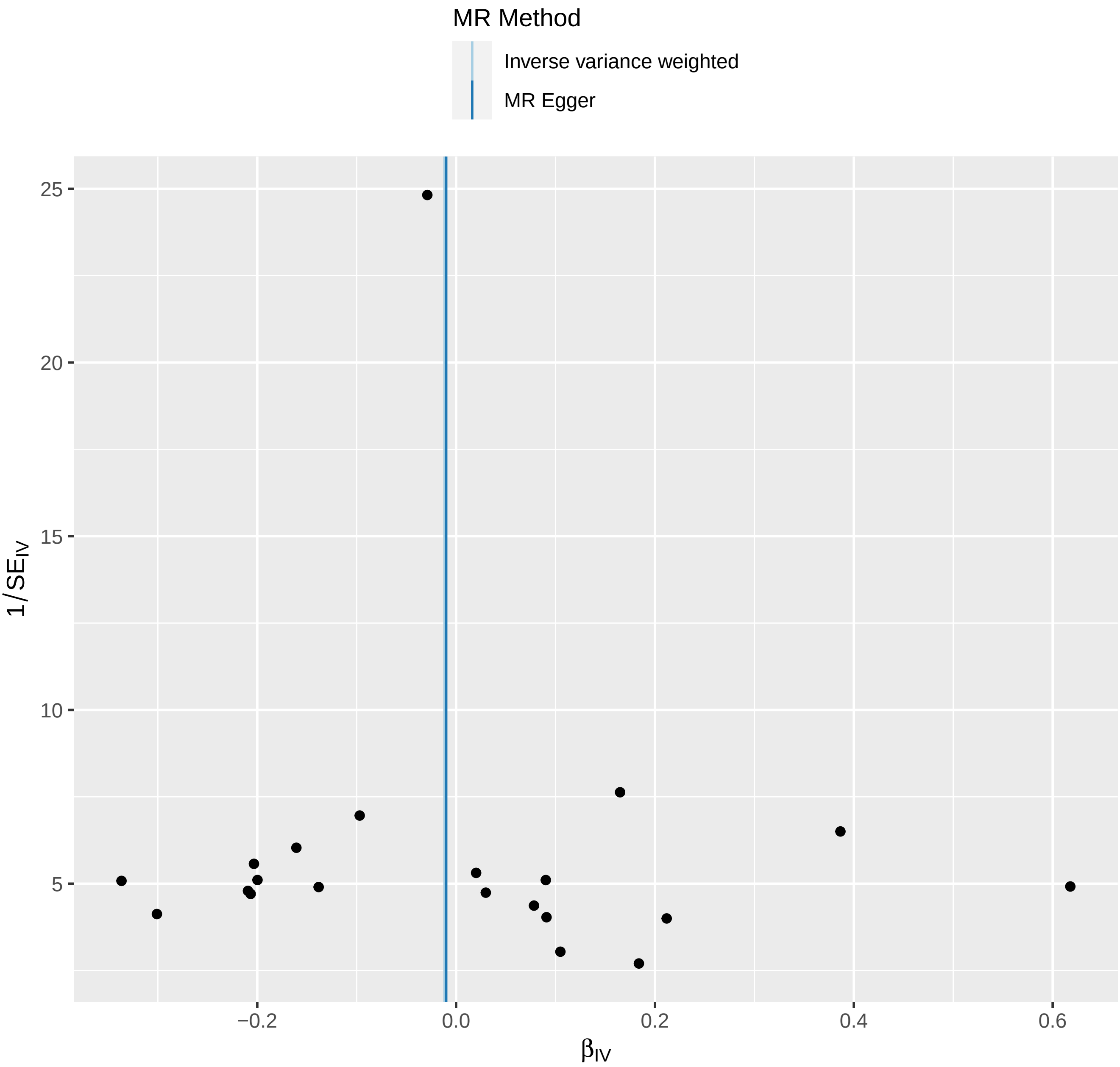

Batch 3−1404 : Inducible T−cell costimulator on Diabetic nephropathy (more control exclusions)
with a threshold of 1e−05

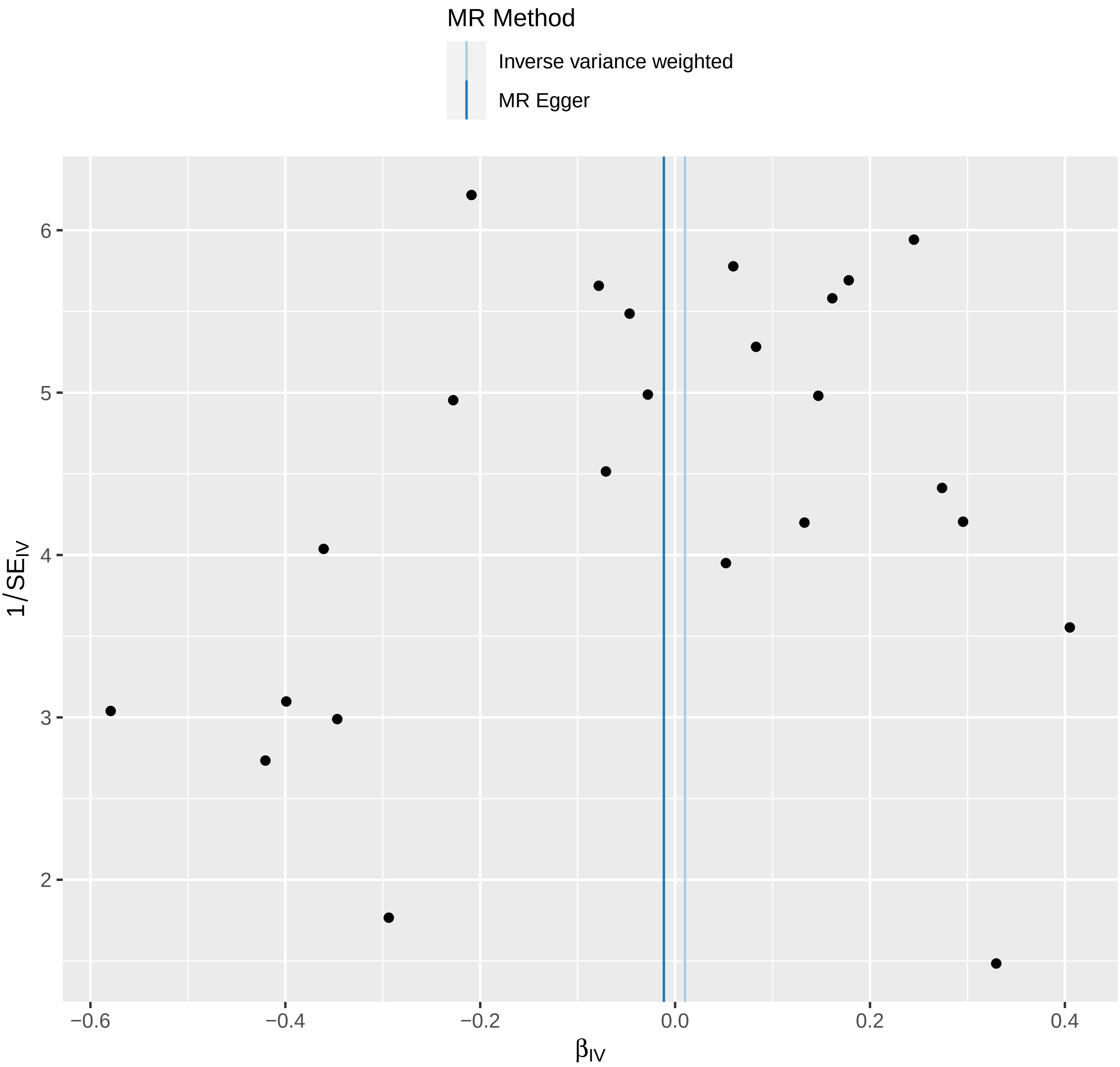

Batch 3−1405 : ICOS ligand on Diabetic nephropathy (more control exclusions)
with a threshold of 1e−05

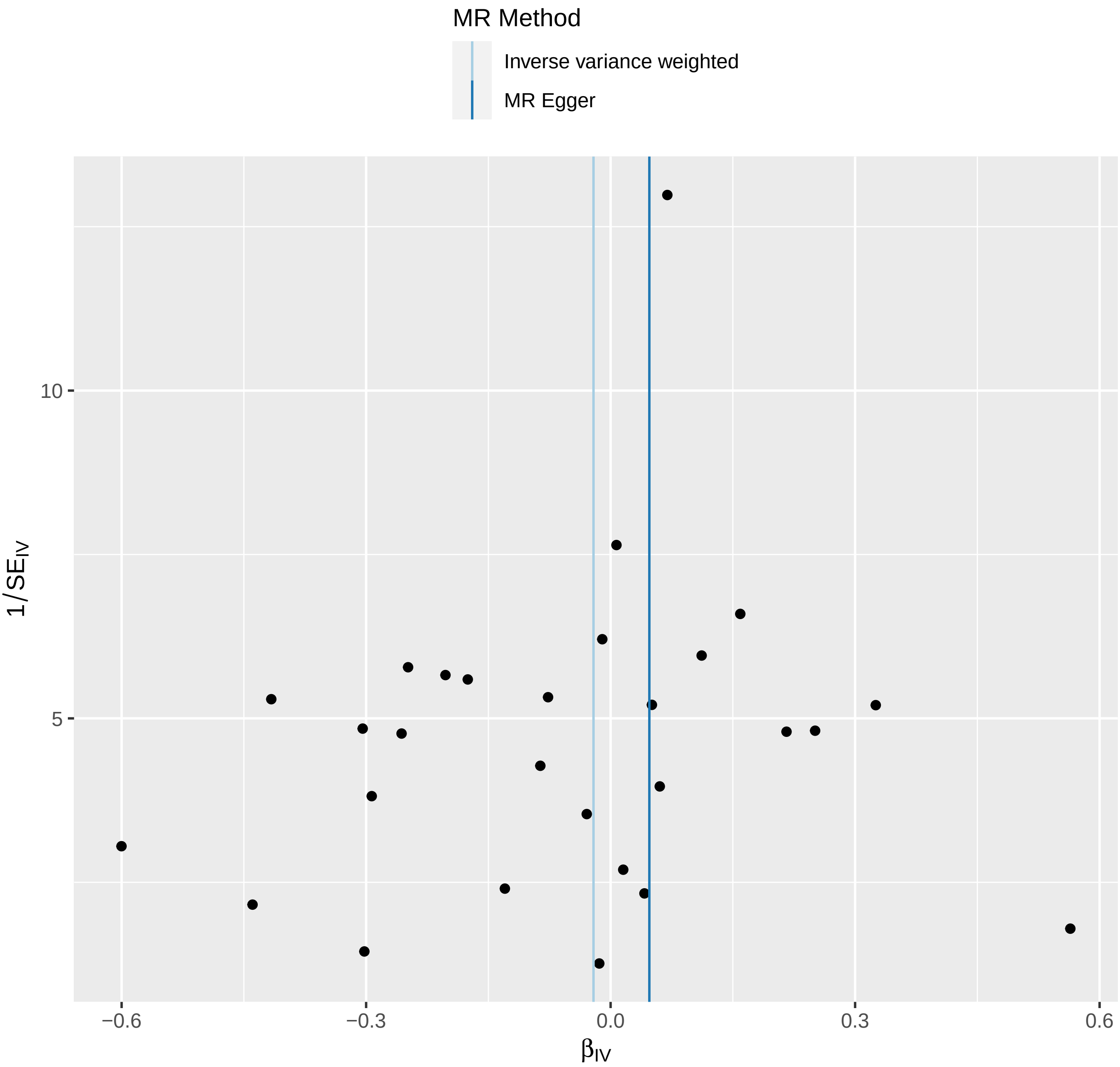

Batch 3−1406 : ICOS ligand on Diabetic nephropathy (more control exclusions)
with a threshold of 1e−05

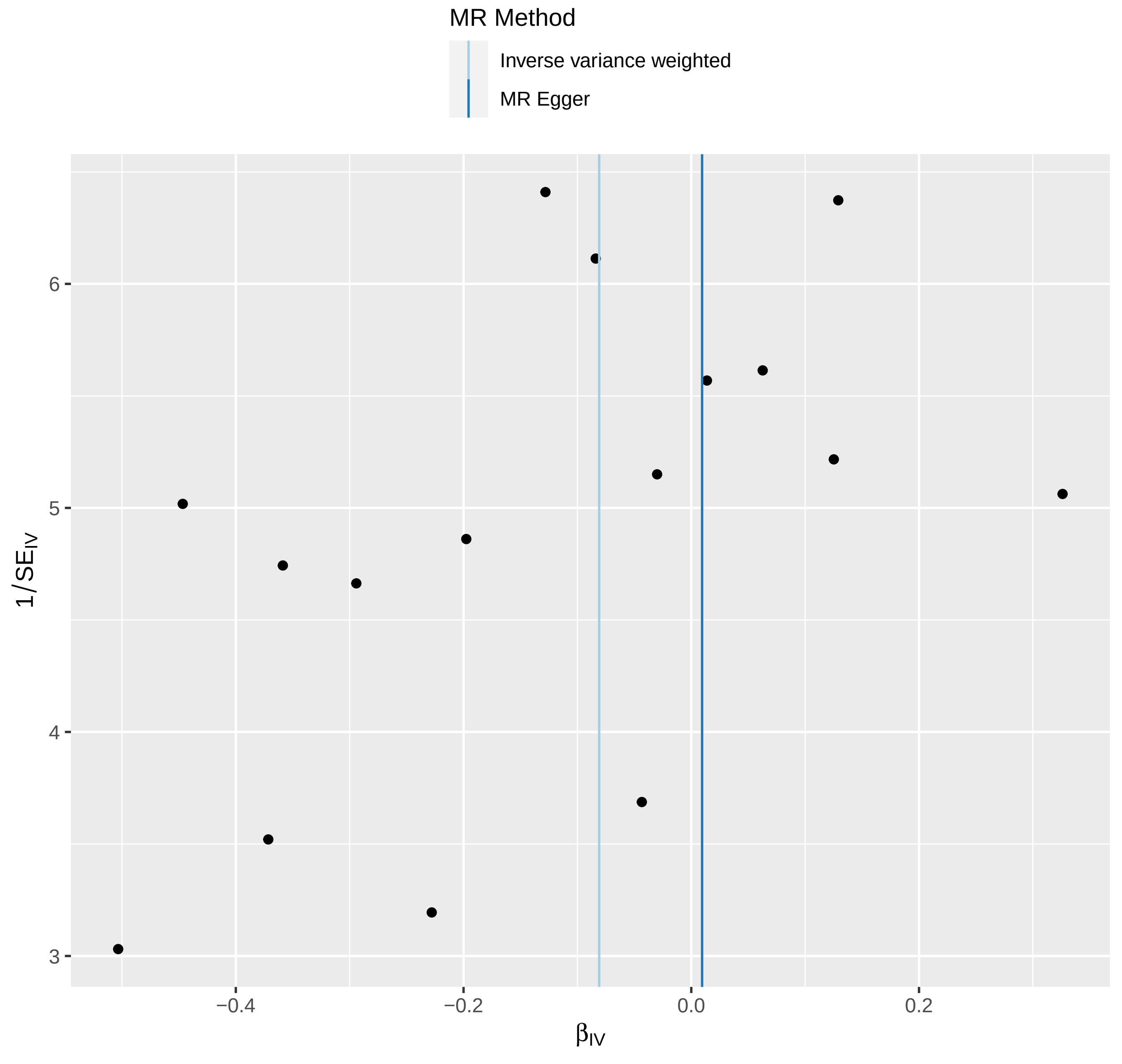

Batch 3−1407 : DNA−binding protein inhibitor ID−1 on Diabetic nephropathy (more control exclusions)
with a threshold of 1e−05

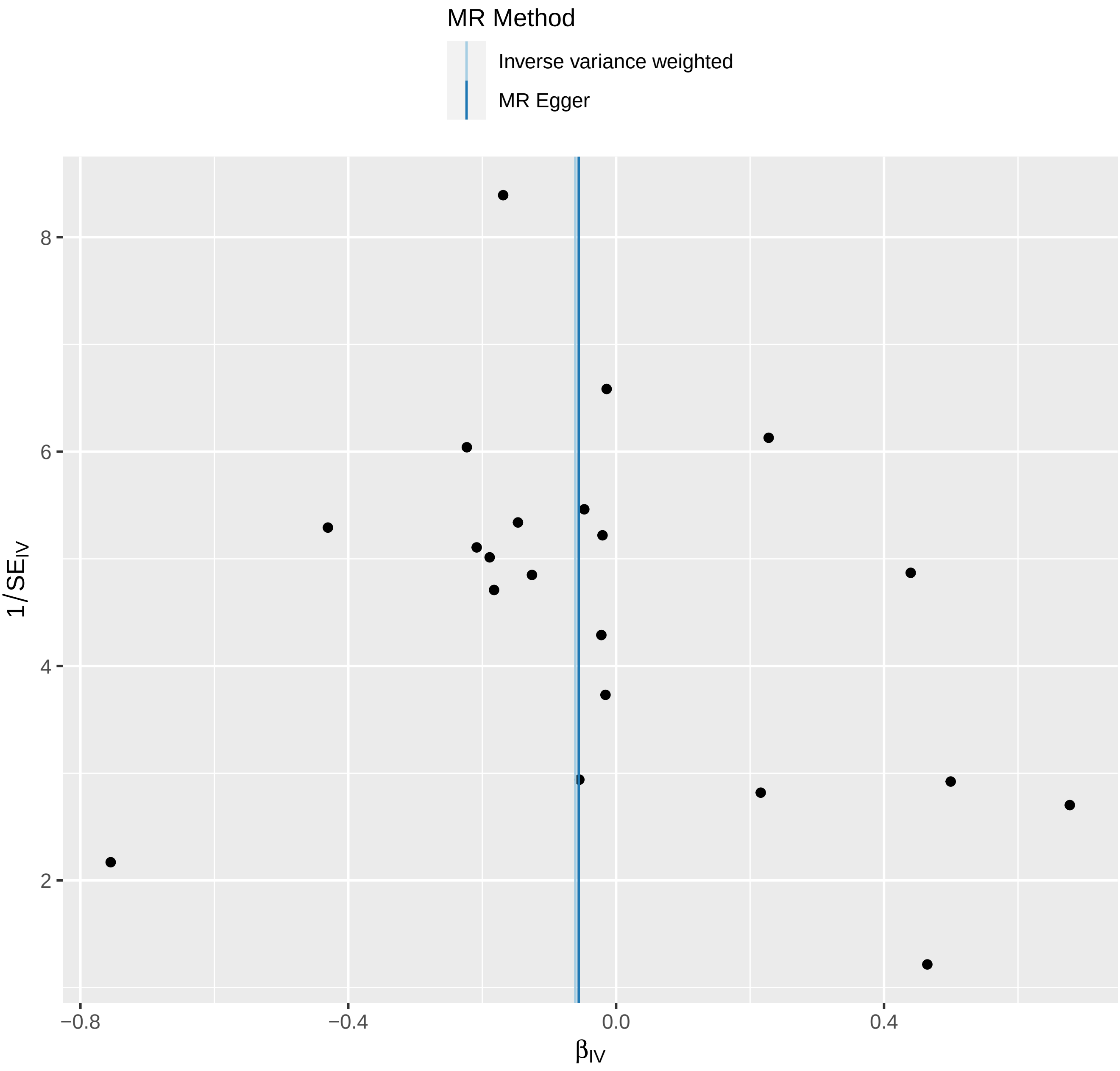

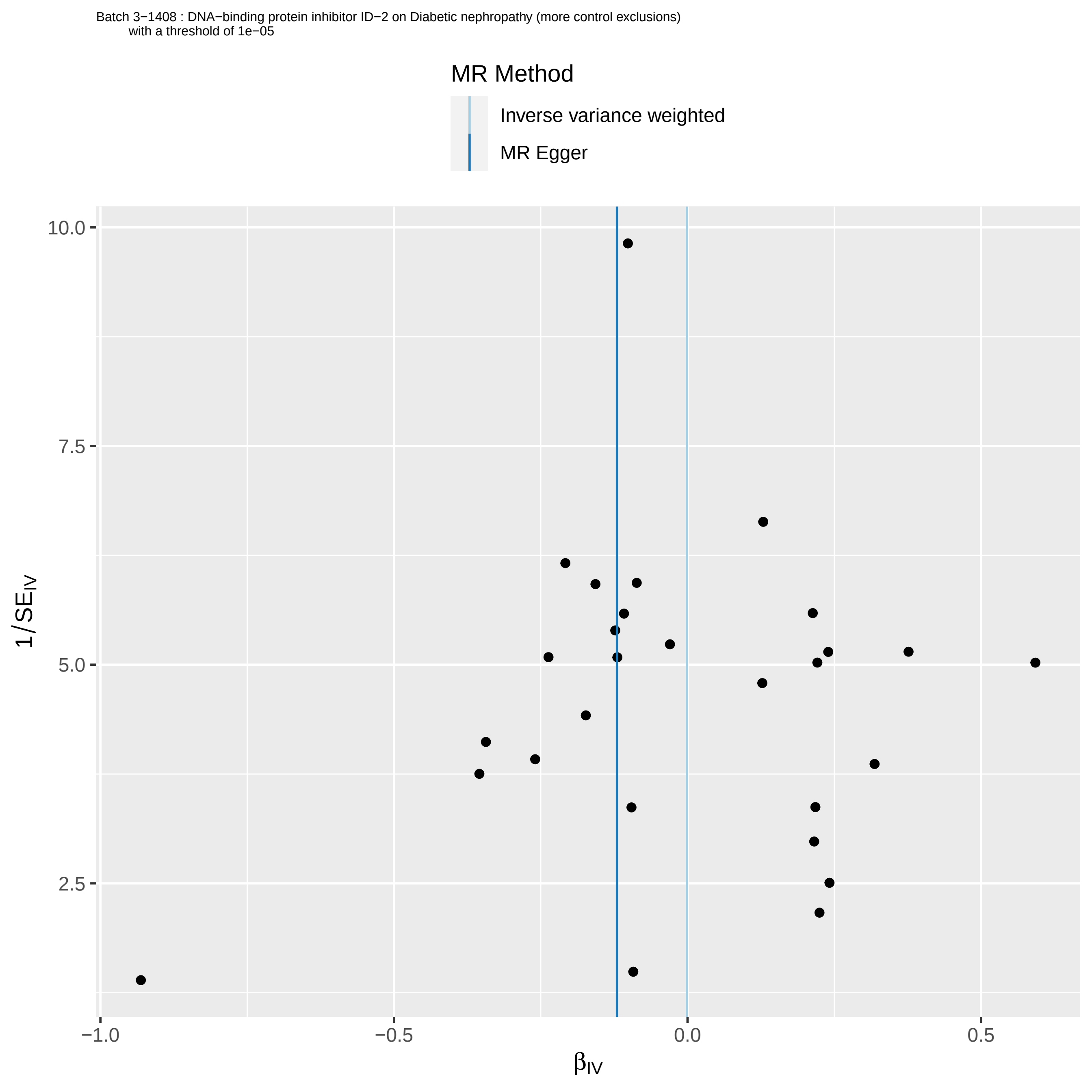
Batch 3−1408 : DNA−binding protein inhibitor ID−2 on Diabetic nephropathy (more control exclusions)
with a threshold of 1e−05
MR Method
Inverse variance weighted
MR Egger
10.0
7.5
5.0
2.5
1/SE_IV
−1.0
−0.5
0.0
0.5
β_IV

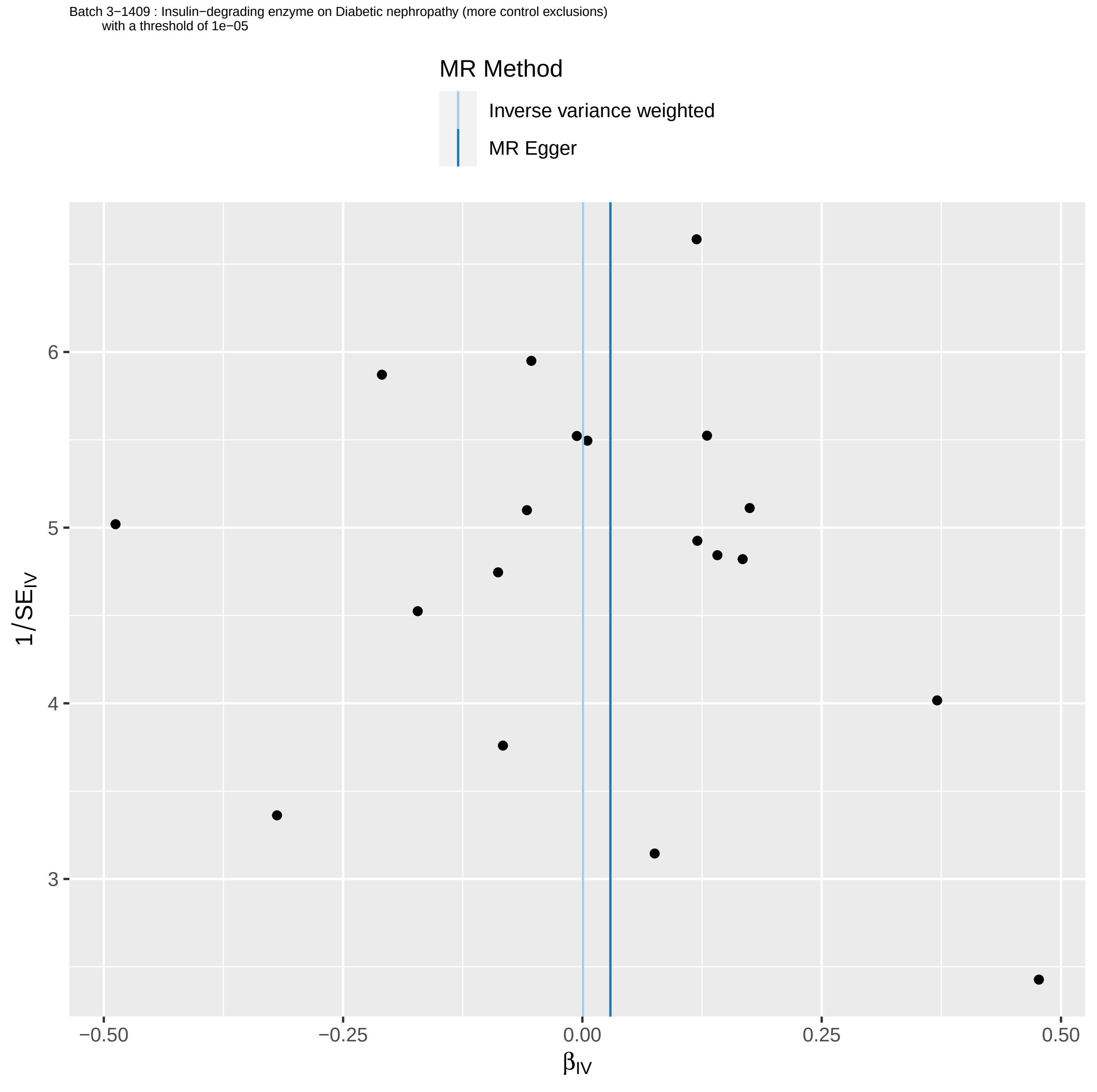
Batch 3−1409 : Insulin−degrading enzyme on Diabetic nephropathy (more control exclusions)
with a threshold of 1e−05
MR Method
Inverse variance weighted
MR Egger
6
5
4
3
1/SE_IV
−0.50
−0.25
0.00
0.25
0.50
β_IV

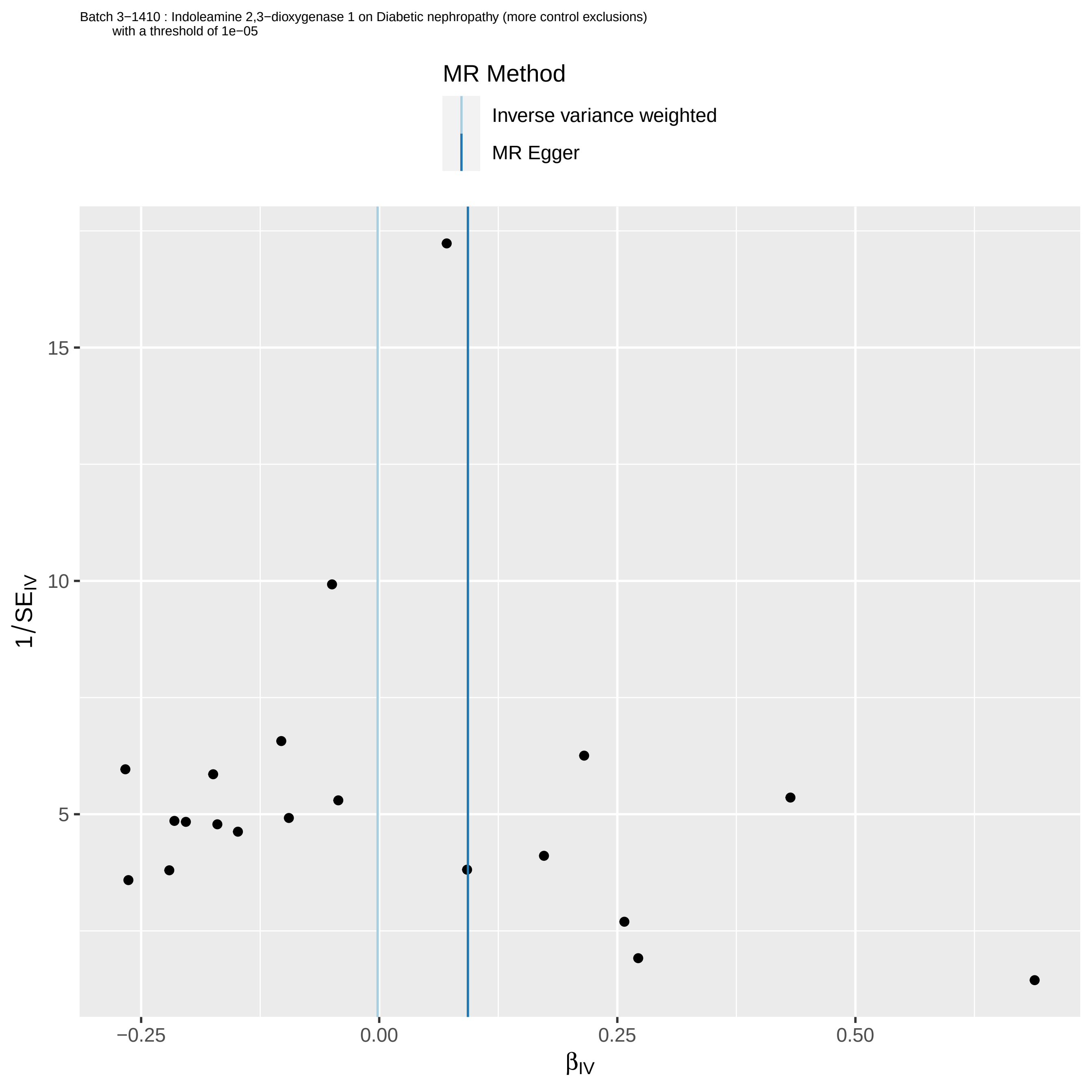
Batch 3−1410 : Indoleamine 2,3−dioxygenase 1 on Diabetic nephropathy (more control exclusions)
with a threshold of 1e−05
MR Method
Inverse variance weighted
MR Egger
15
10
5
1/SE_IV
−0.25
0.00
0.25
0.50
β_IV

Batch 3−1411 : Alpha−L−iduronidase on Diabetic nephropathy (more control exclusions)
with a threshold of 1e−05

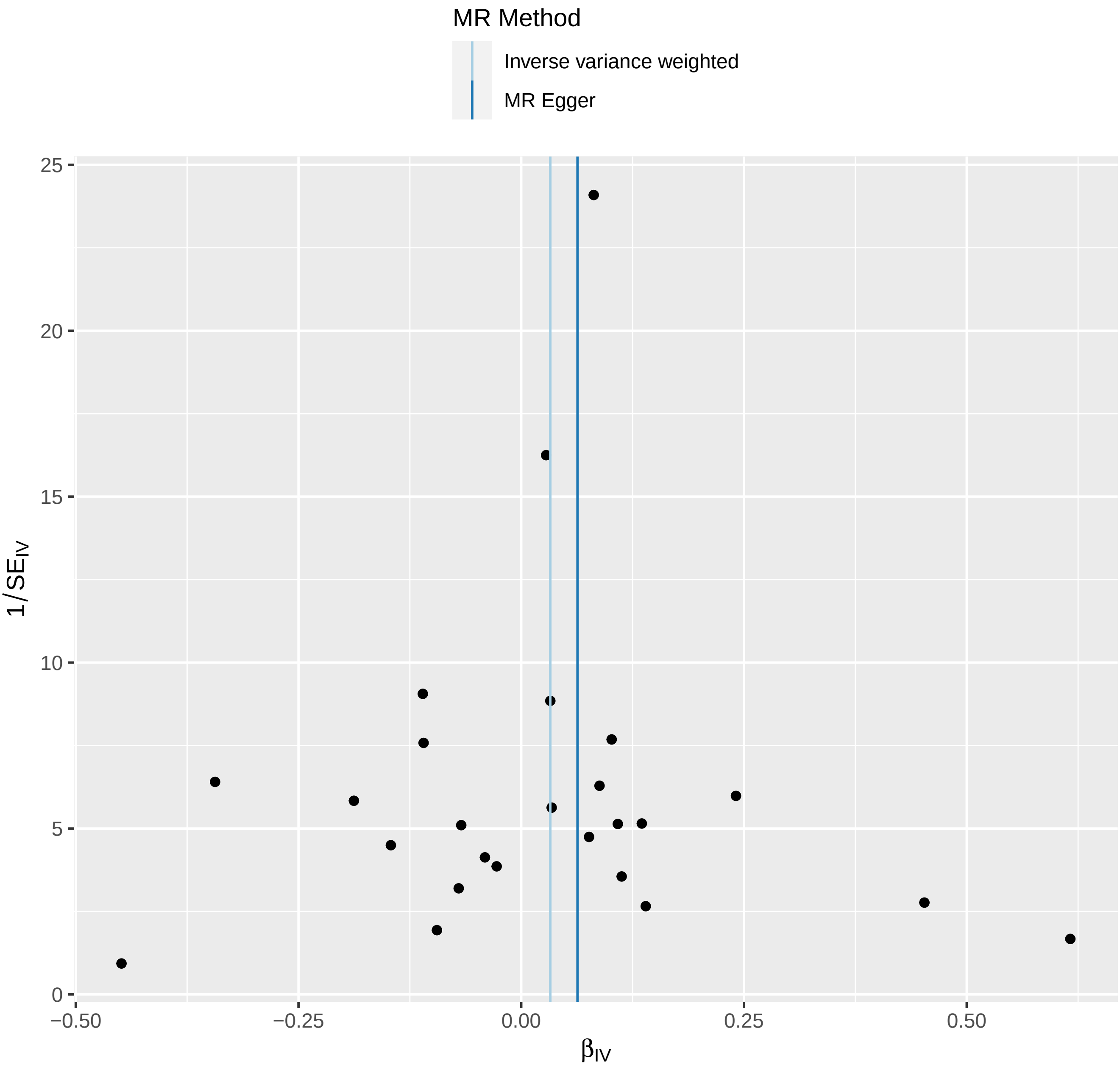

Batch 3−1412 : Radiation−inducible immediate−early gene IEX−1 on Diabetic nephropathy (more control exclusions)
with a threshold of 1e−05

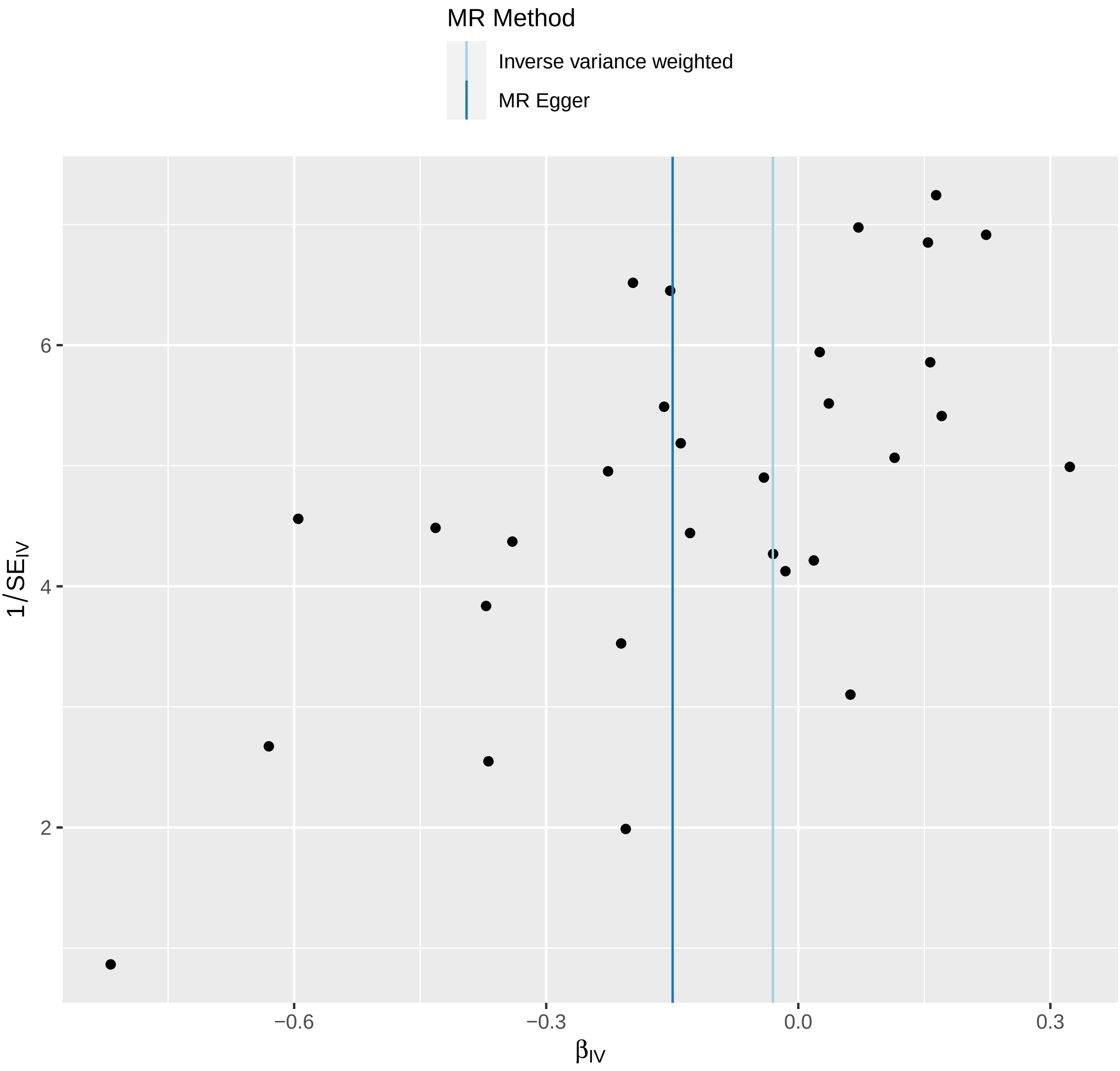

Batch 3−1413 : Immediate early response 3−interacting protein 1 on Diabetic nephropathy (more control exclusions)
with a threshold of 1e−05

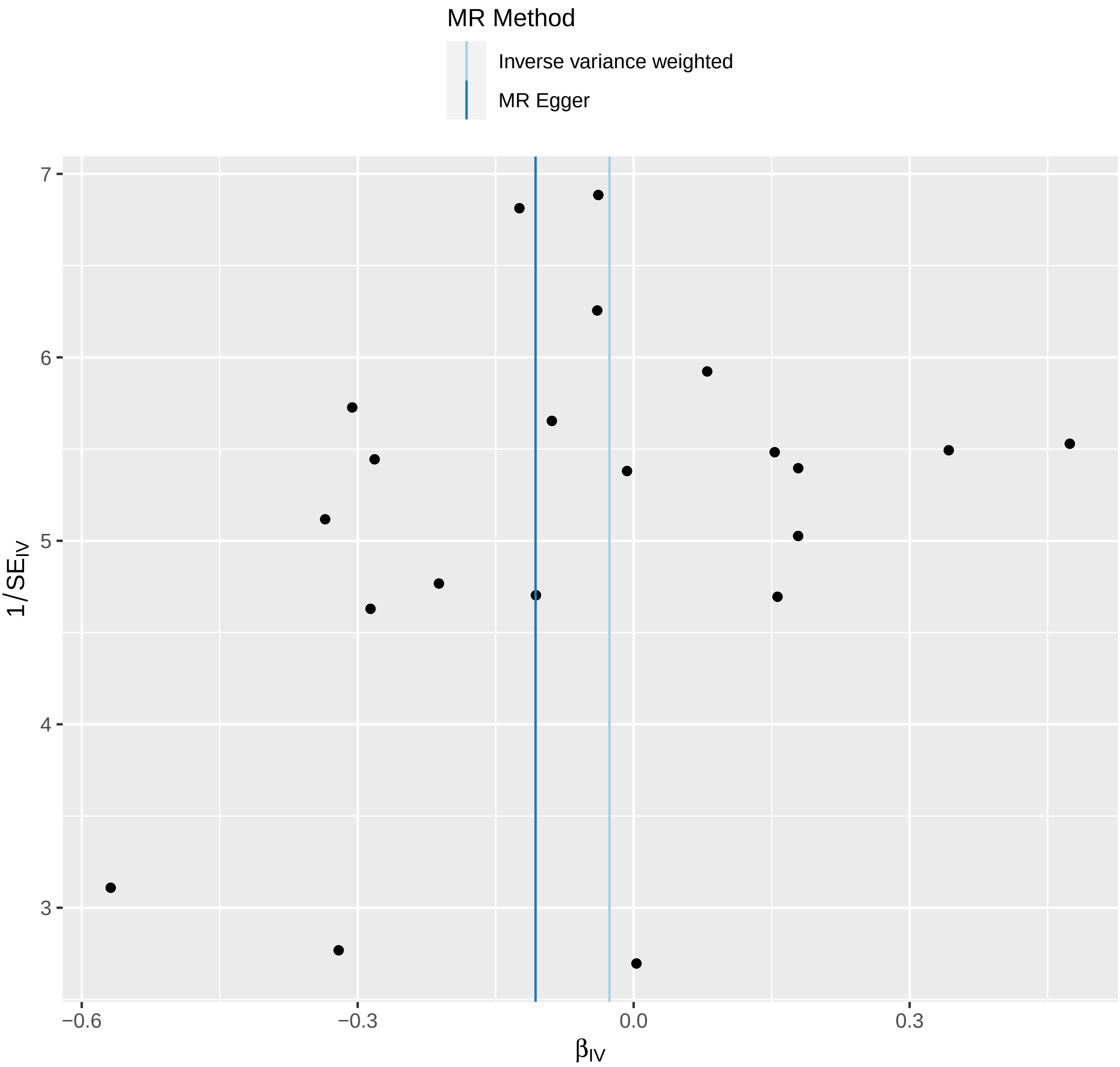

Batch 3−1414 : Gamma−interferon−inducible protein 16 on Diabetic nephropathy (more control exclusions) with a threshold of 1e−05

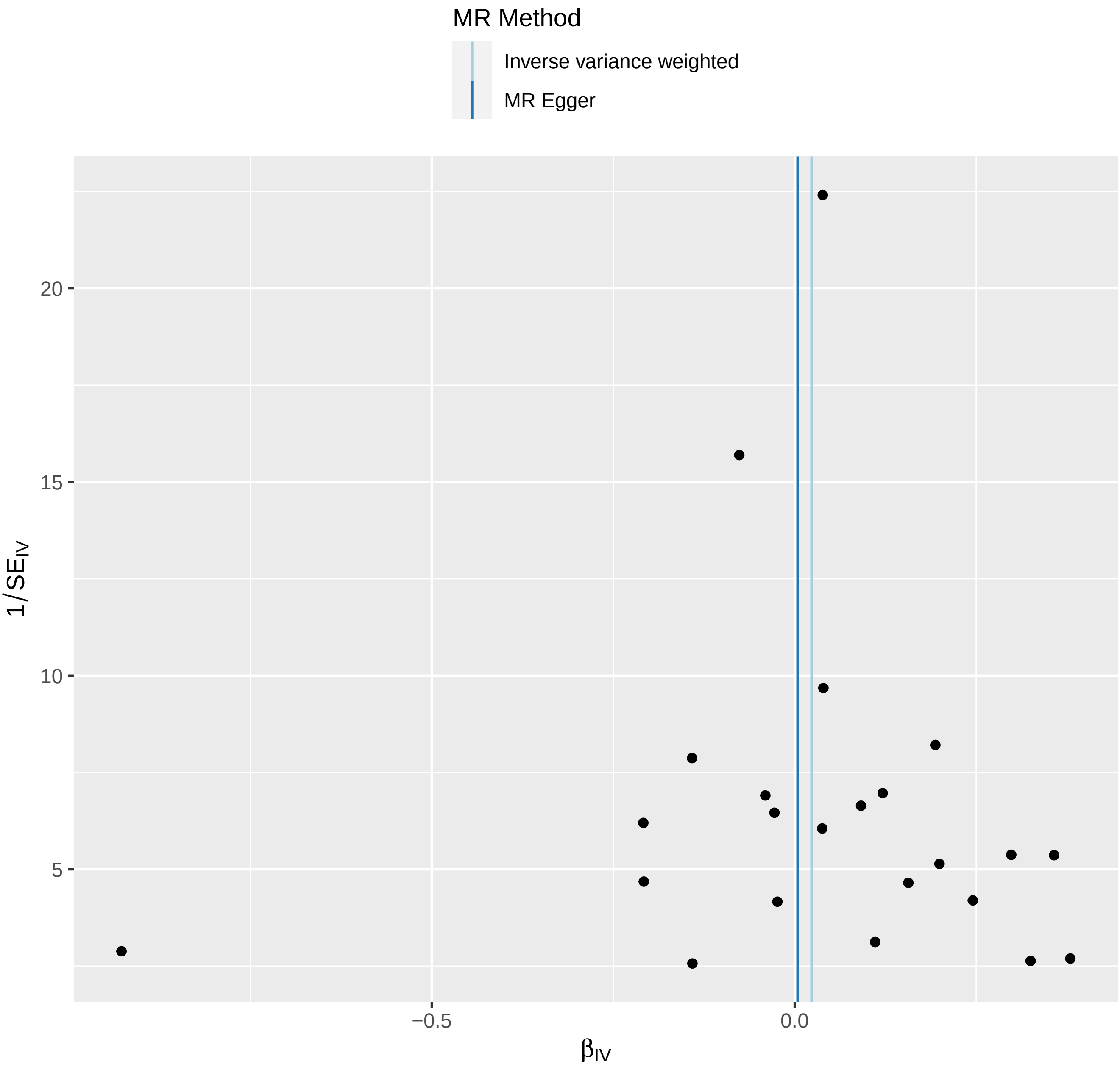

Batch 3−1415 : Gamma−interferon−inducible protein 16 on Diabetic nephropathy (more control exclusions)
with a threshold of 1e−05

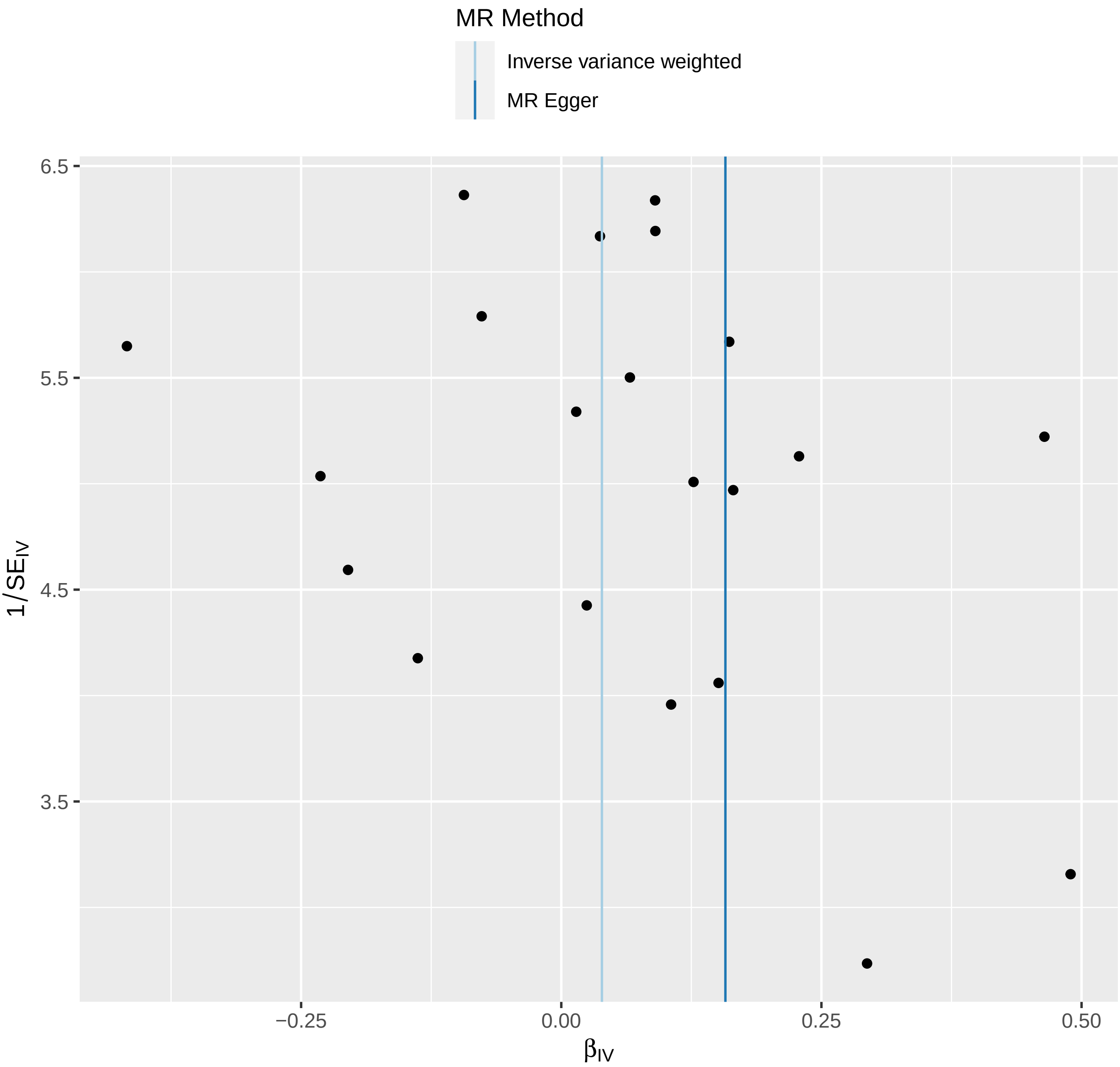

Batch 3−1416 : Interferon−induced protein with tetratricopeptide repeats 2 on Diabetic nephropathy (more control exclusions)
with a threshold of 1e−05

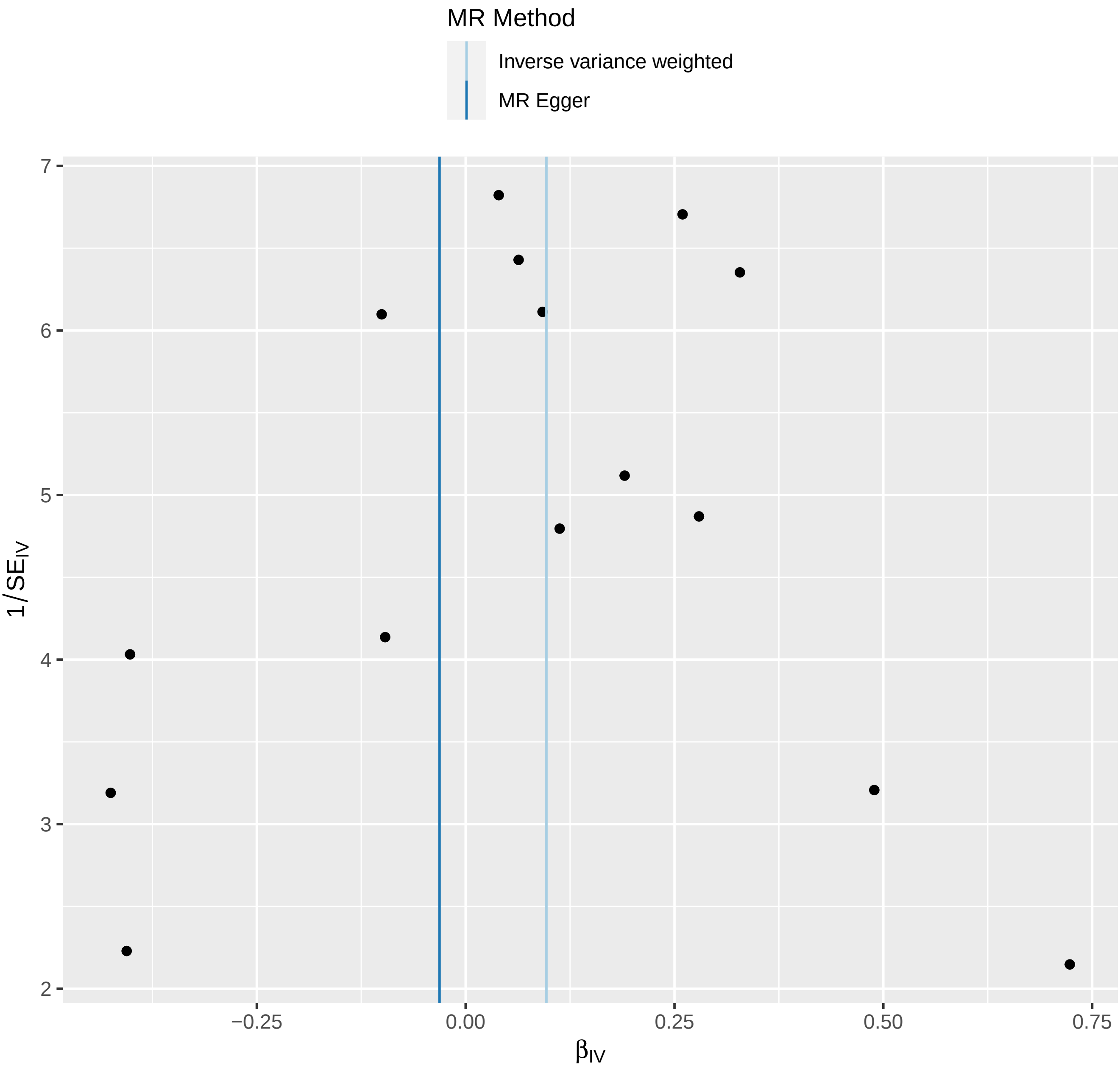

Batch 3−1417 : Interferon−induced protein with tetratricopeptide repeats 3 on Diabetic nephropathy (more control exclusions) with a threshold of 1e−05

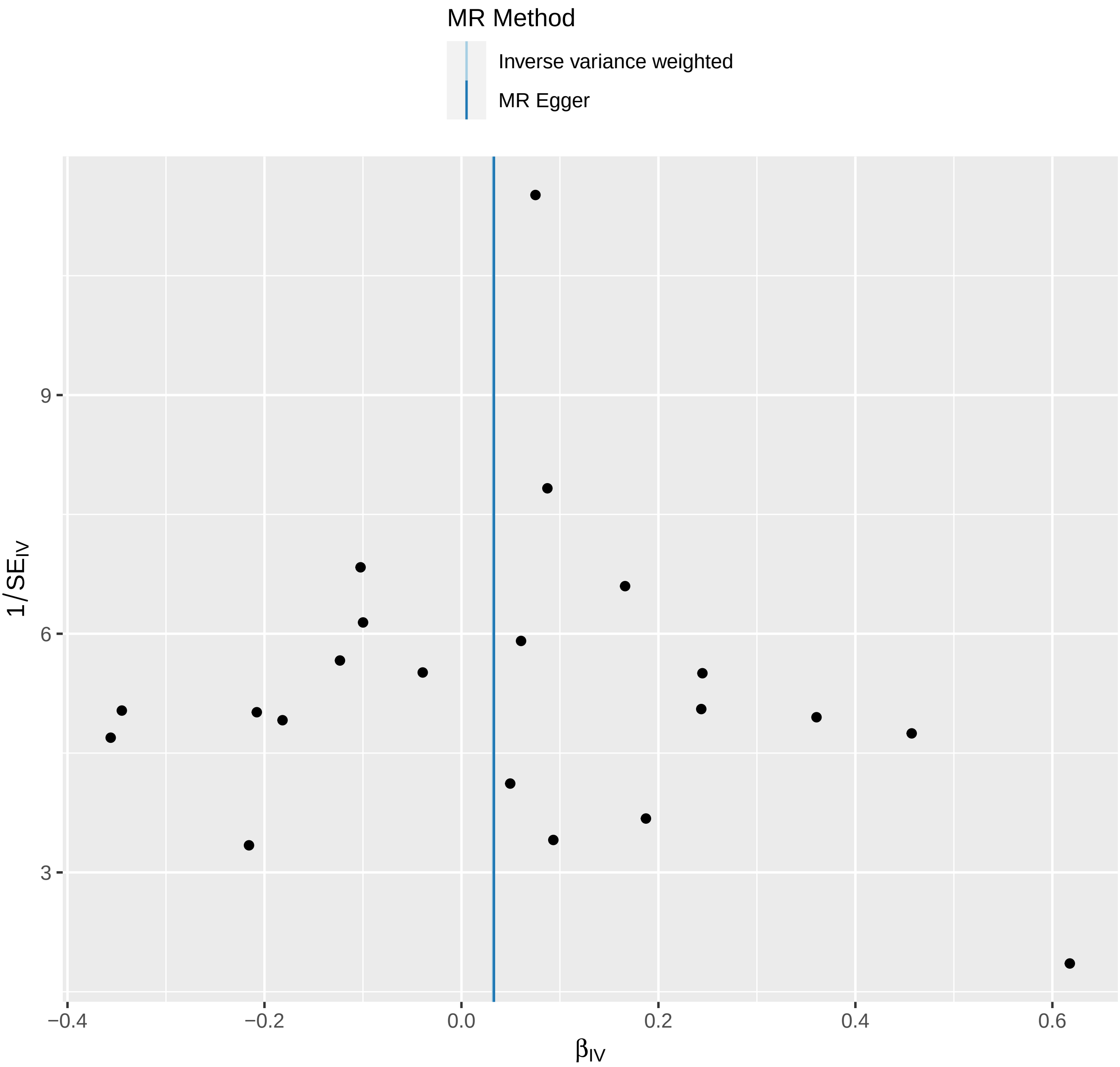

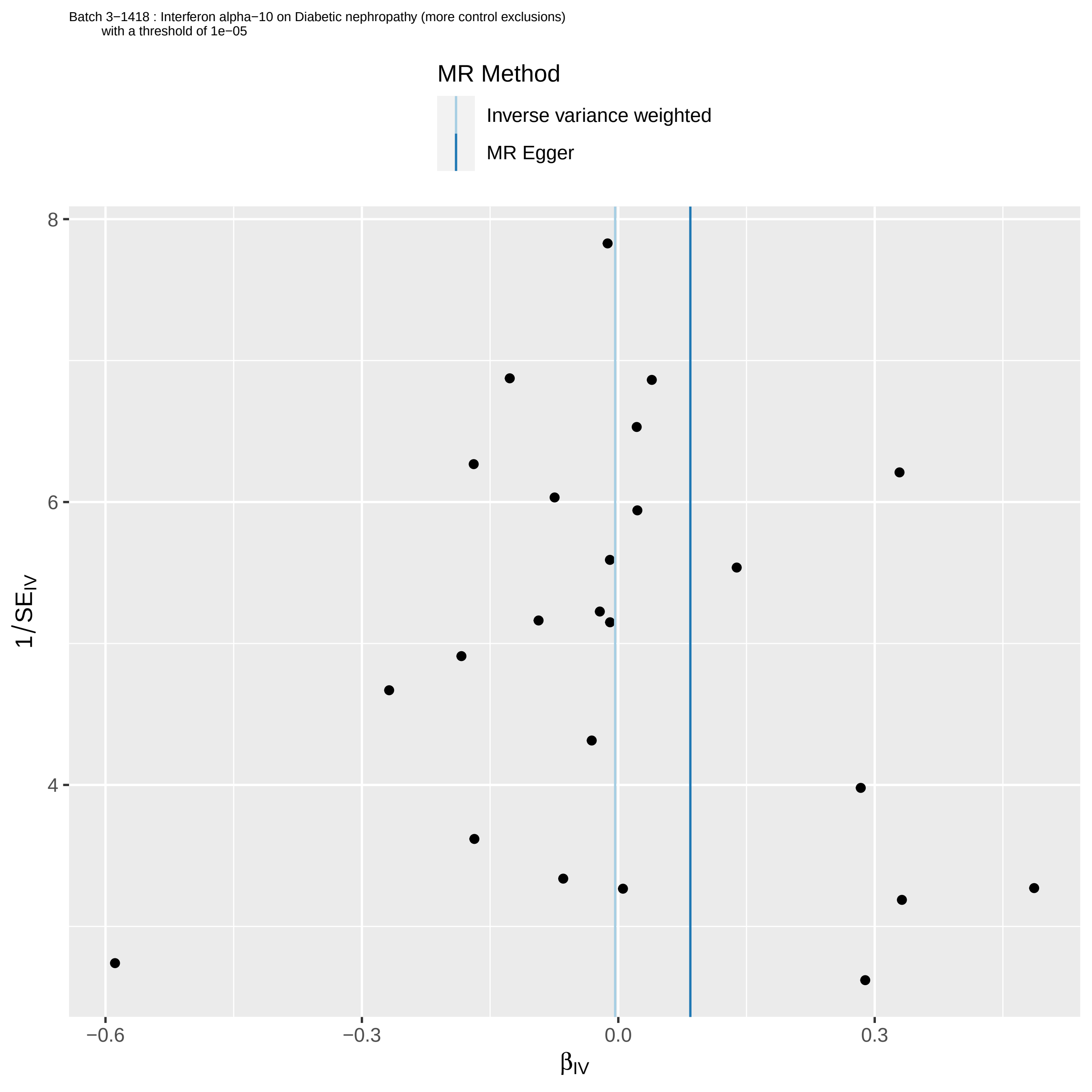

Batch 3−1418 : Interferon alpha−10 on Diabetic nephropathy (more control exclusions)
with a threshold of 1e−05
MR Method
Inverse variance weighted
MR Egger
8
6
4
1/SE_IV
−0.6
−0.3
0.0
0.3
β_IV

Batch 3−1419 : Interferon alpha−10 on Diabetic nephropathy (more control exclusions)
with a threshold of 1e−05

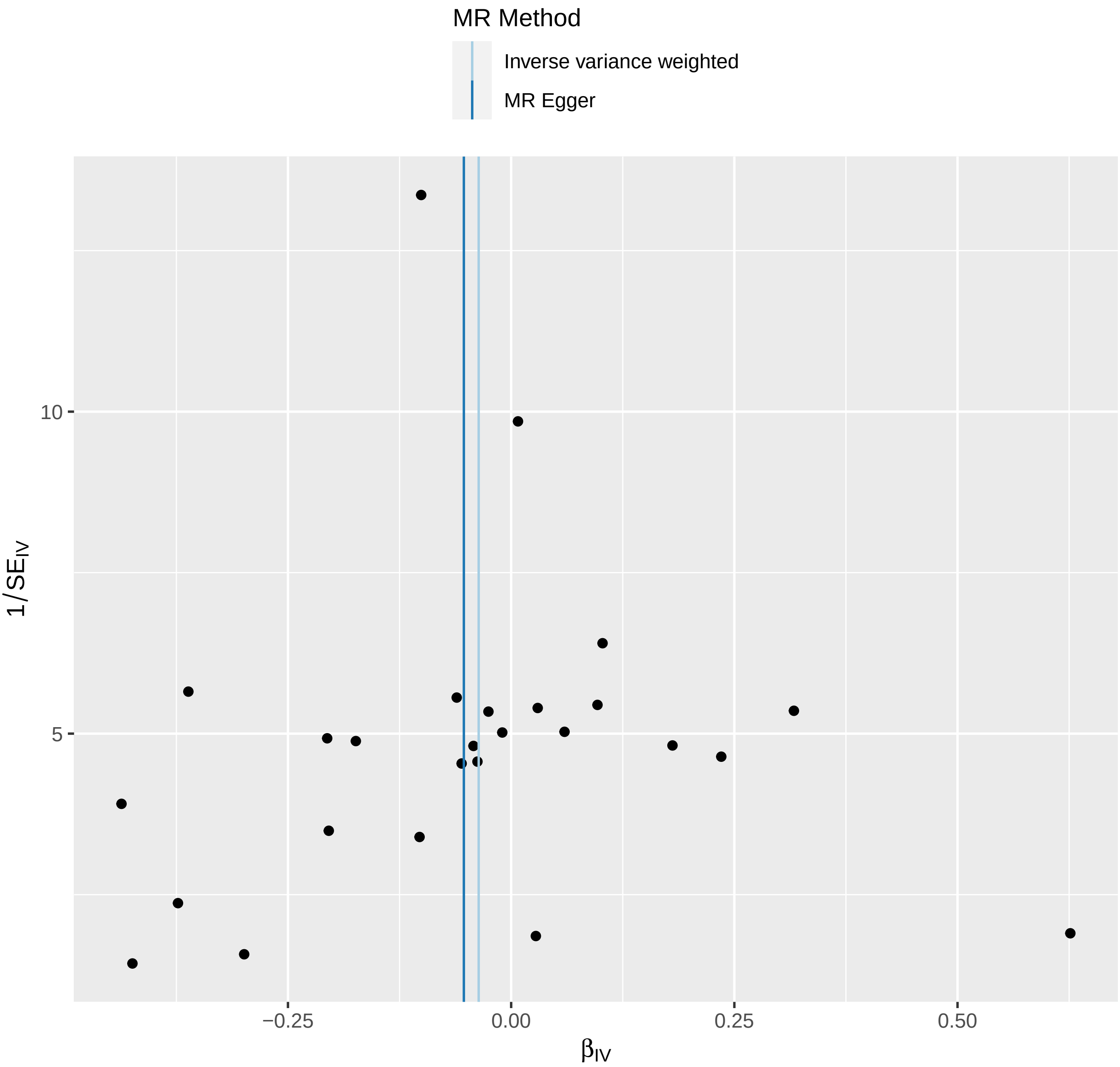

Batch 3−1420 : Interferon alpha−14 on Diabetic nephropathy (more control exclusions)
with a threshold of 1e−05

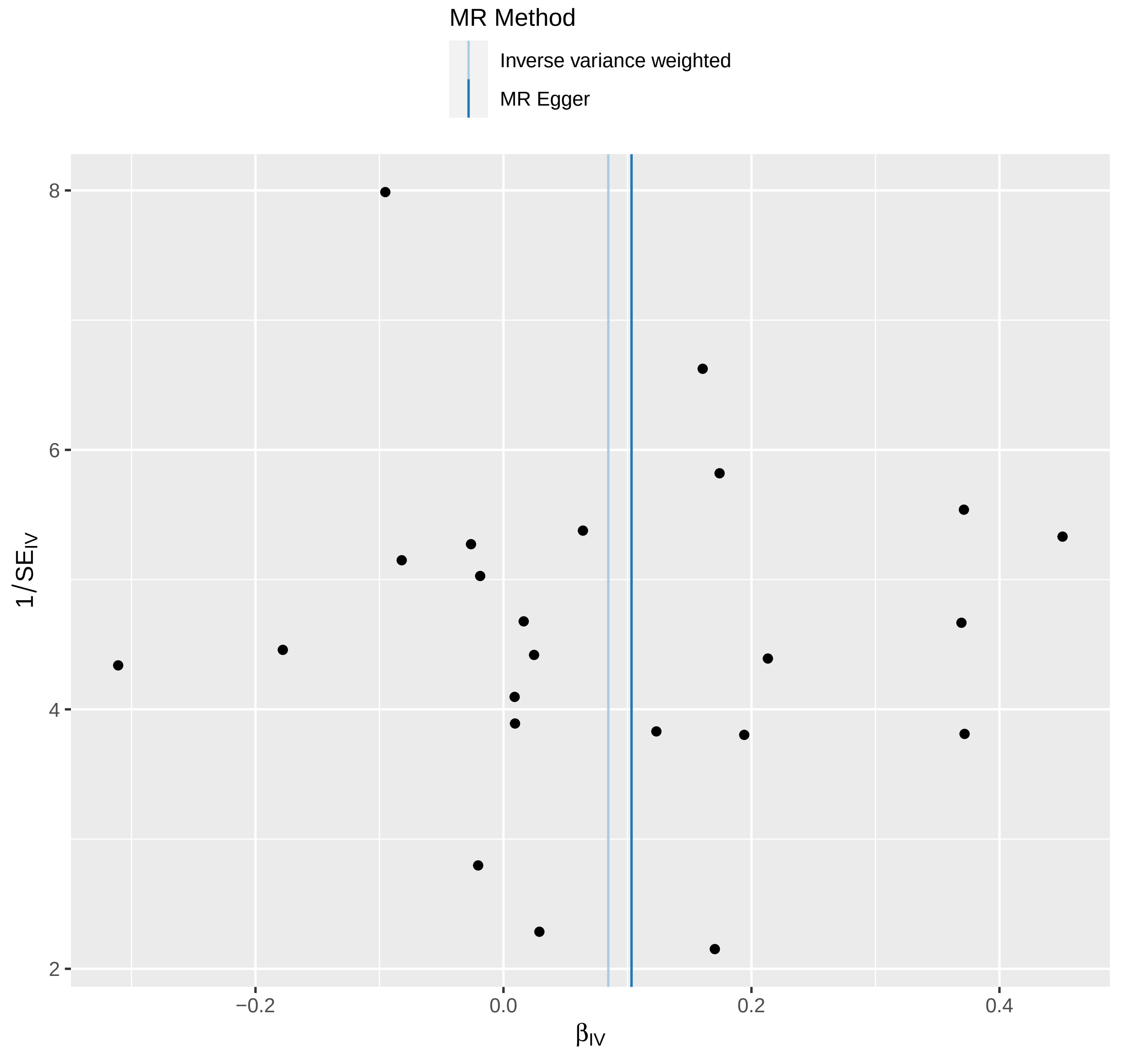

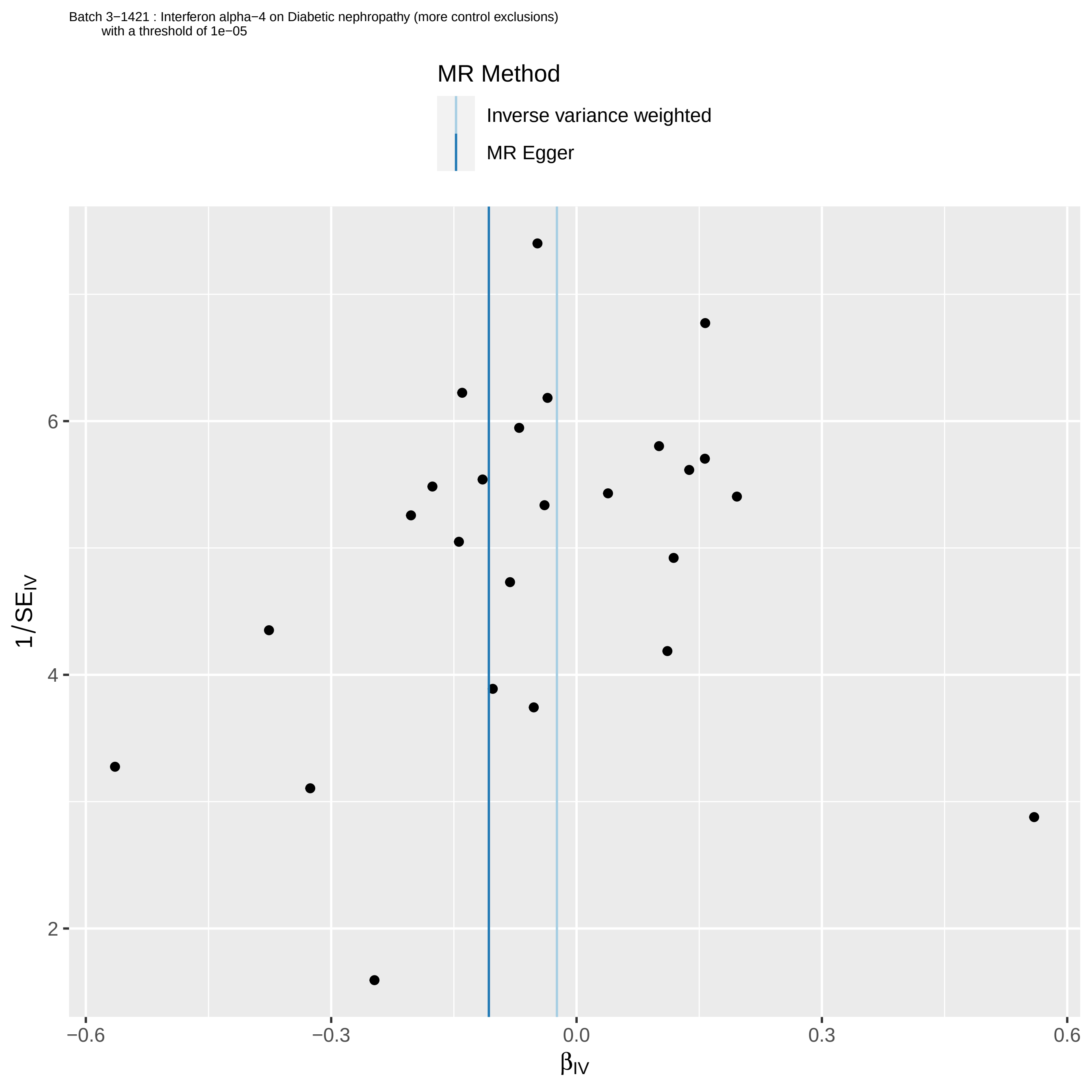
Batch 3−1421 : Interferon alpha−4 on Diabetic nephropathy (more control exclusions)
with a threshold of 1e−05
MR Method
Inverse variance weighted
MR Egger
6
4
2
1/SE$_{IV}$
−0.6
−0.3
0.0
0.3
0.6
β$_{IV}$

Batch 3−1422 : Interferon alpha−5 on Diabetic nephropathy (more control exclusions)
with a threshold of 1e−05

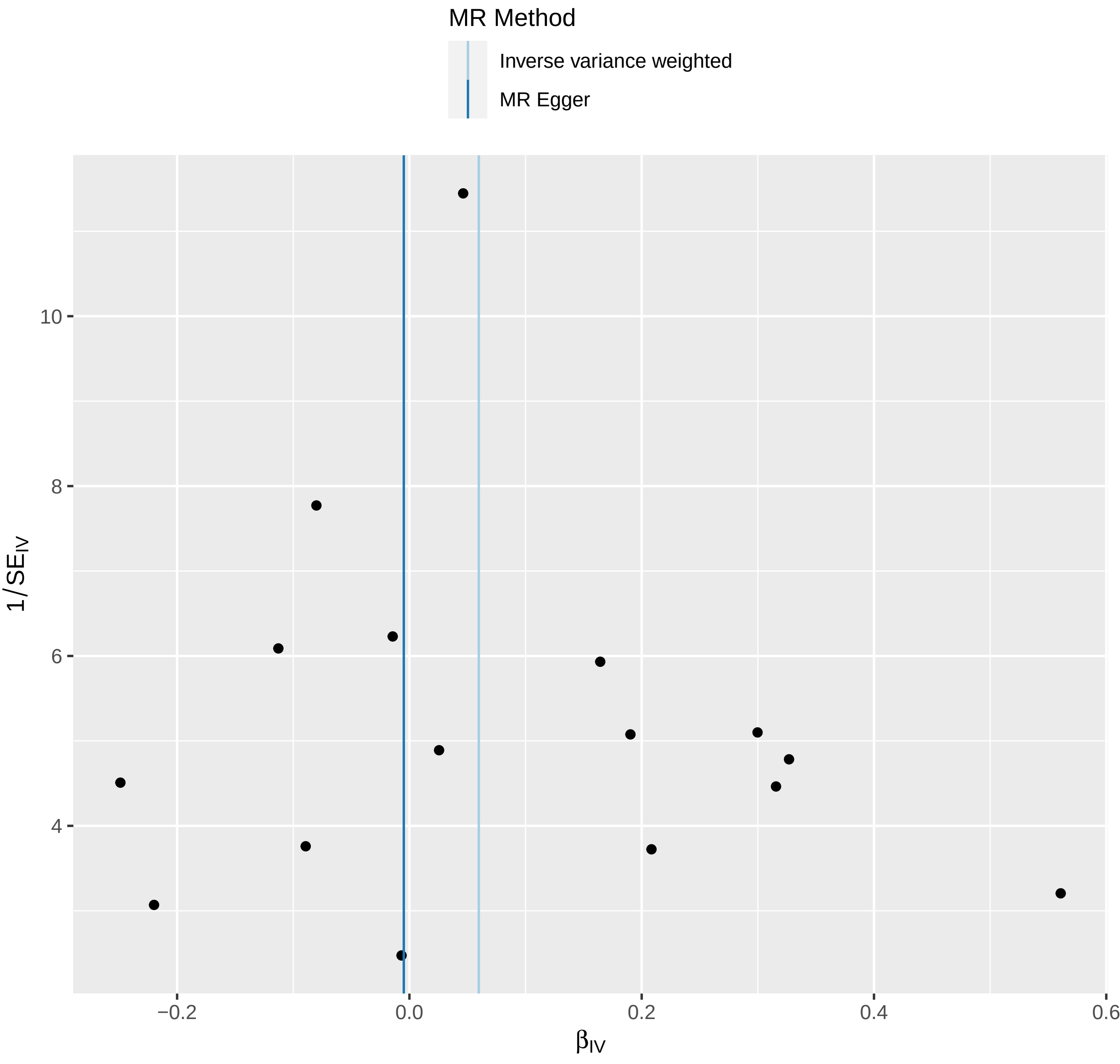

Batch 3−1423 : Interferon alpha−6 on Diabetic nephropathy (more control exclusions)
with a threshold of 1e−05

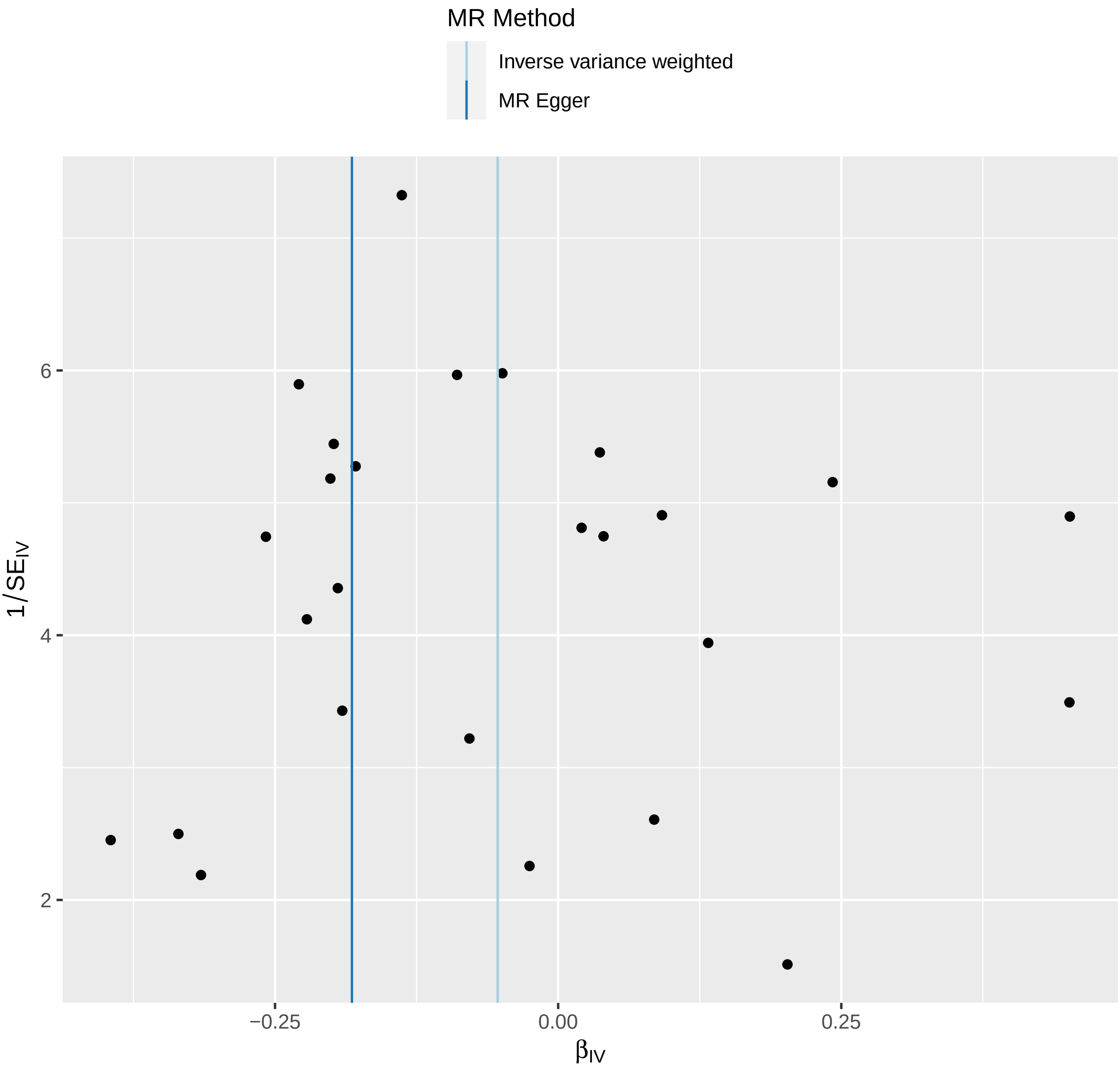

Batch 3−1424 : Interferon alpha−7 on Diabetic nephropathy (more control exclusions)
with a threshold of 1e−05

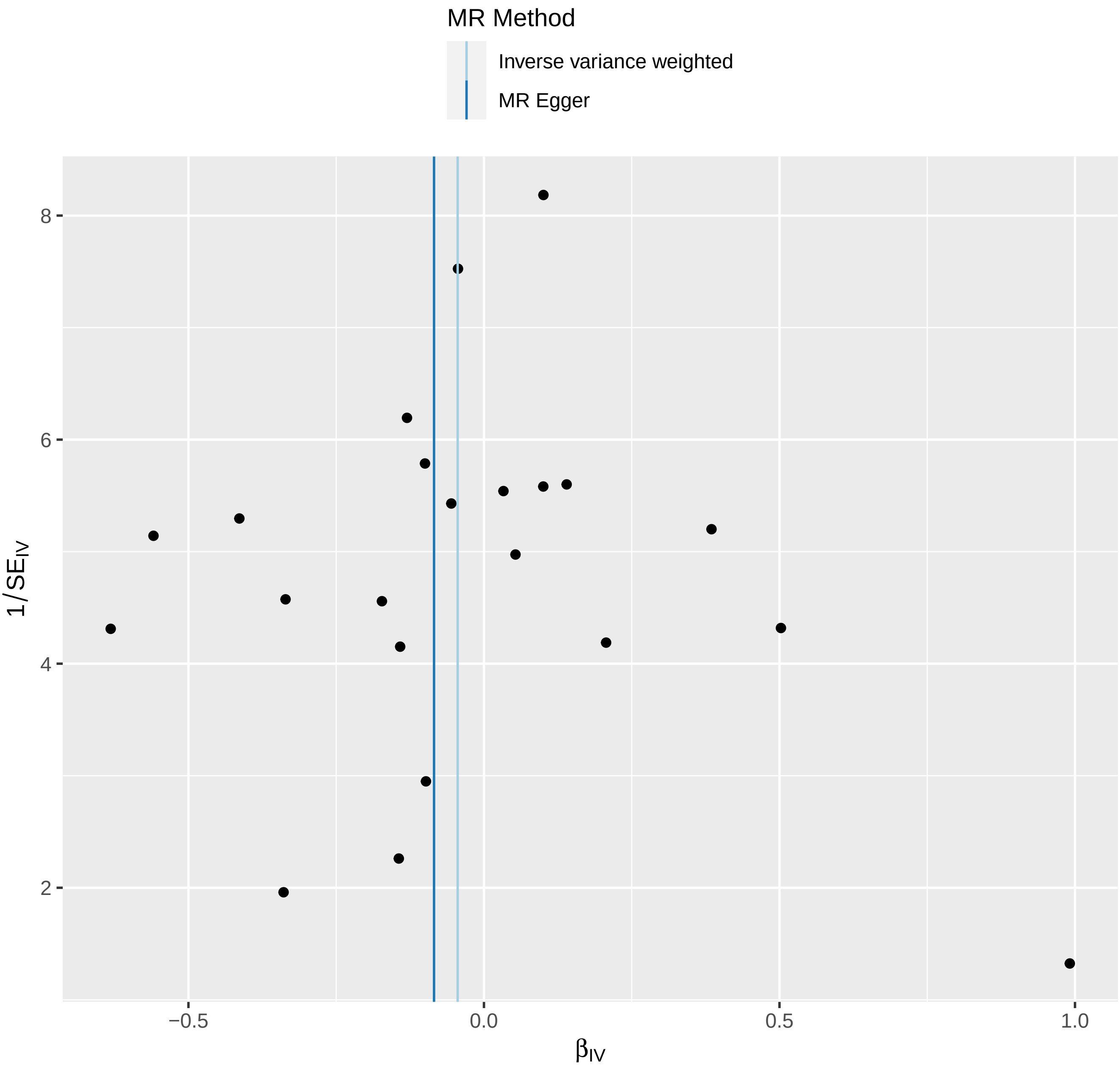

Batch 3−1425 : Interferon alpha−8 on Diabetic nephropathy (more control exclusions)
with a threshold of 1e−05

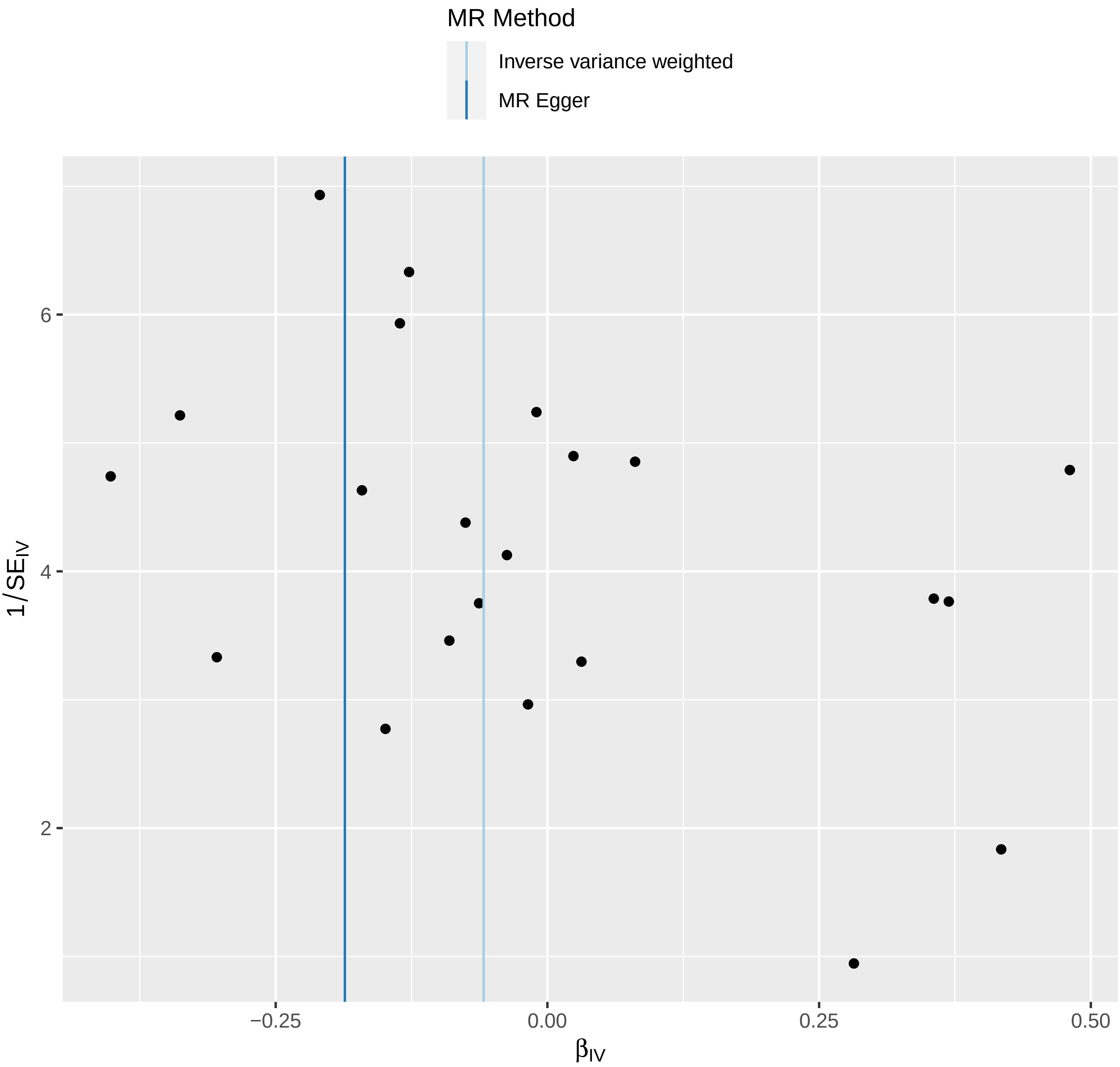

Batch 3−1426 : Interferon alpha/beta receptor 1 on Diabetic nephropathy (more control exclusions) with a threshold of 1e−05

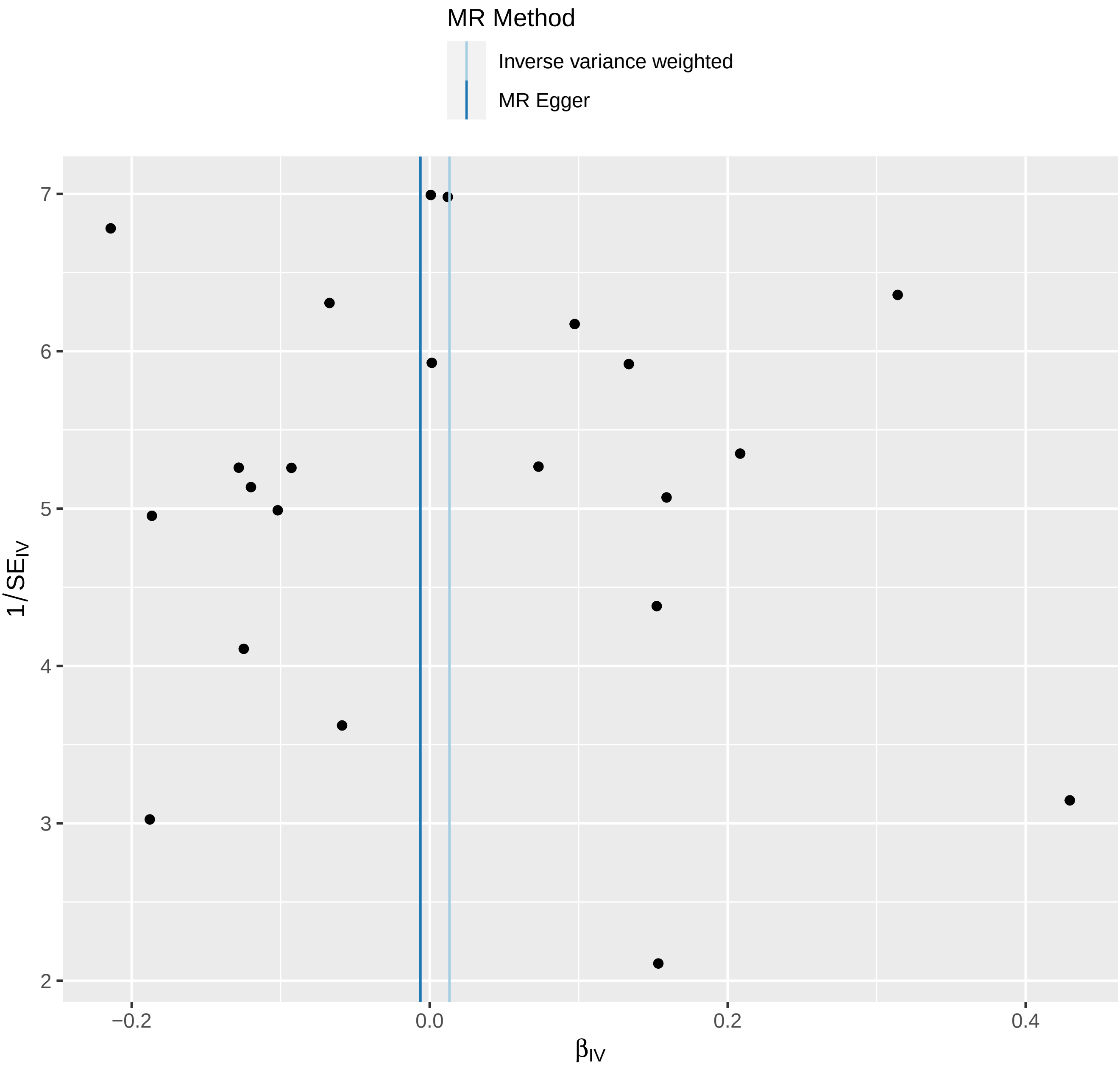

Batch 3−1427 : Interferon beta on Diabetic nephropathy (more control exclusions)
with a threshold of 1e−05

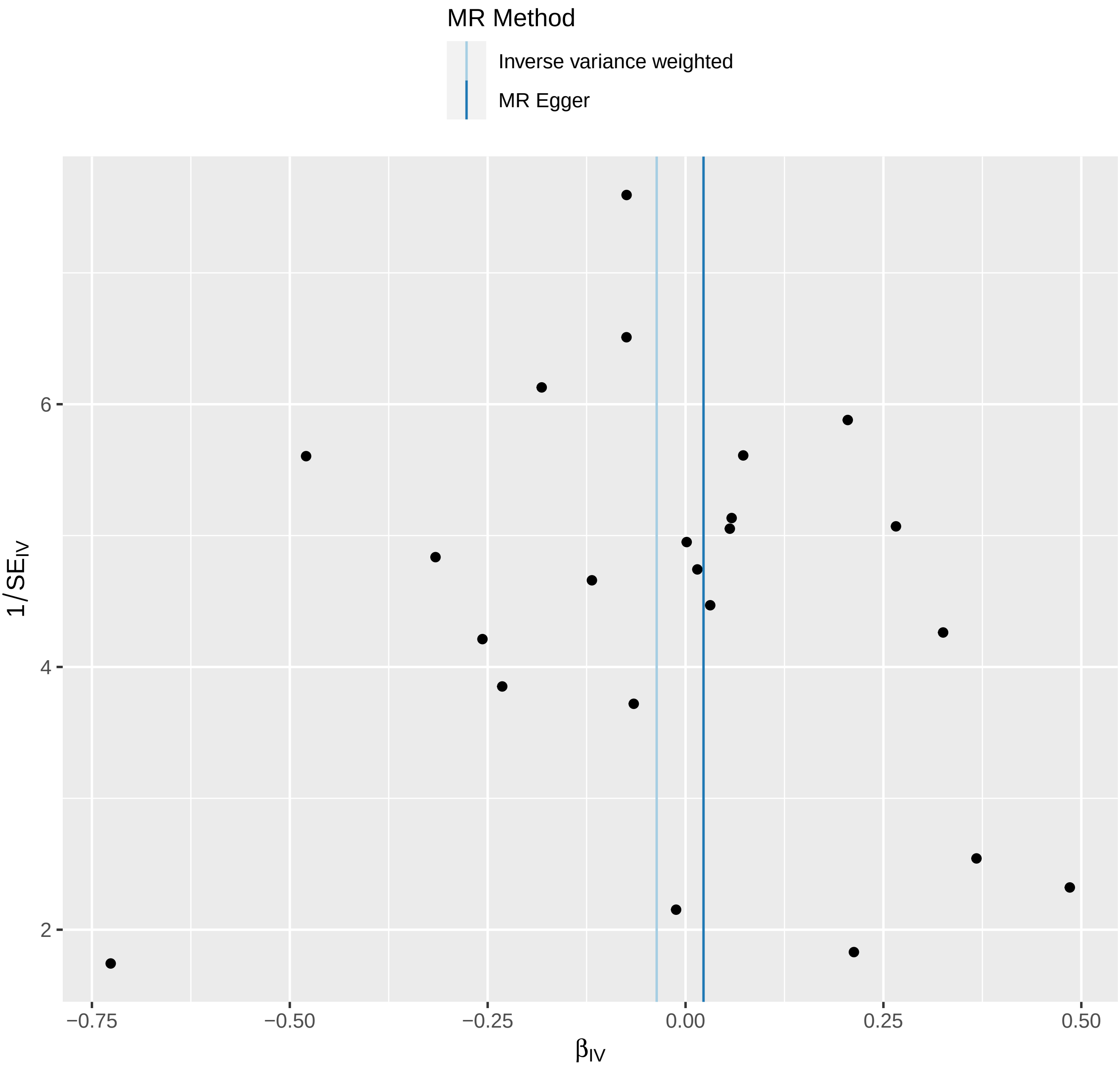

Batch 3−1428 : Interferon gamma on Diabetic nephropathy (more control exclusions)
with a threshold of 1e−05

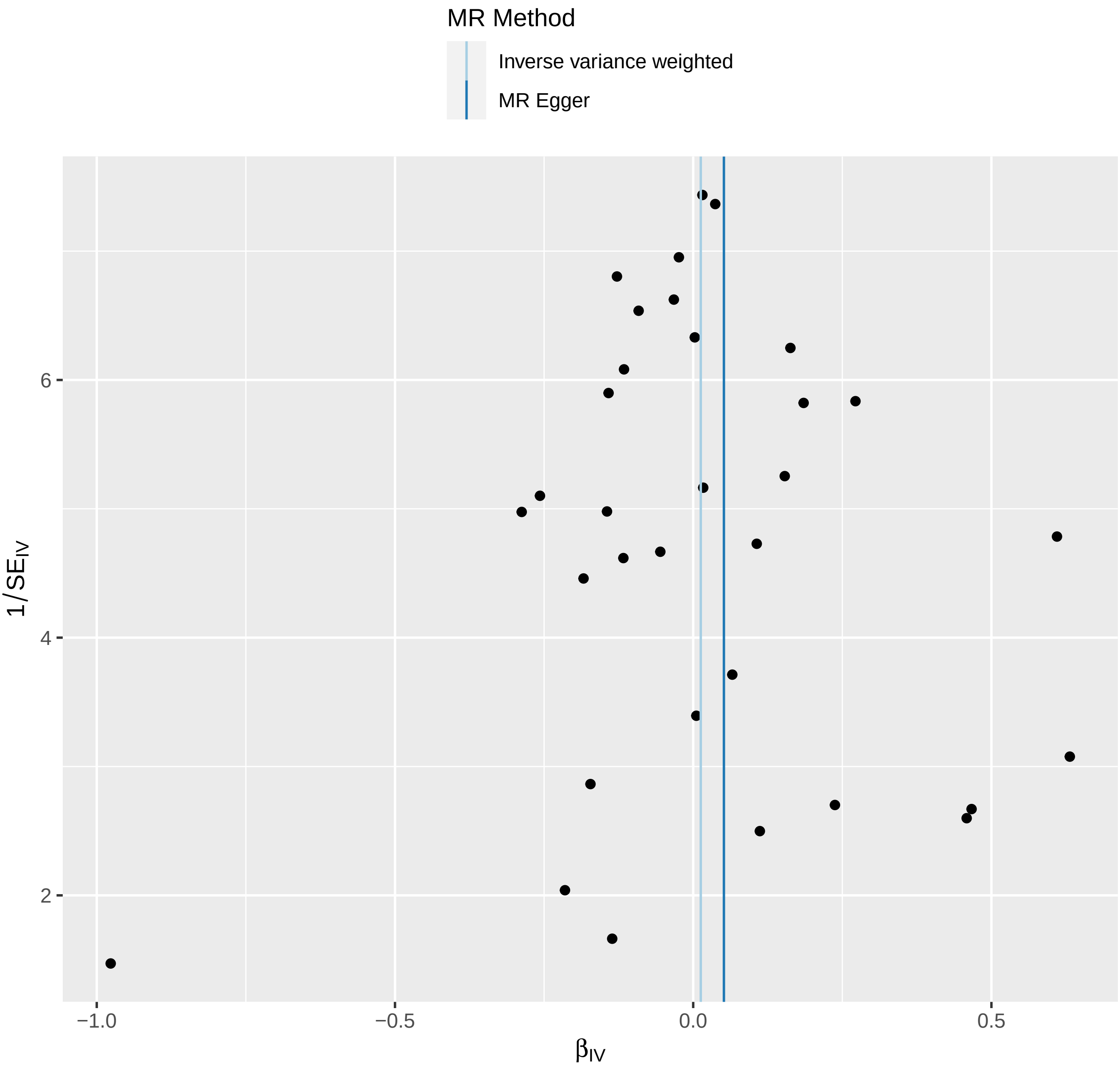

Batch 3−1429 : Interferon gamma on Diabetic nephropathy (more control exclusions)
with a threshold of 1e−05

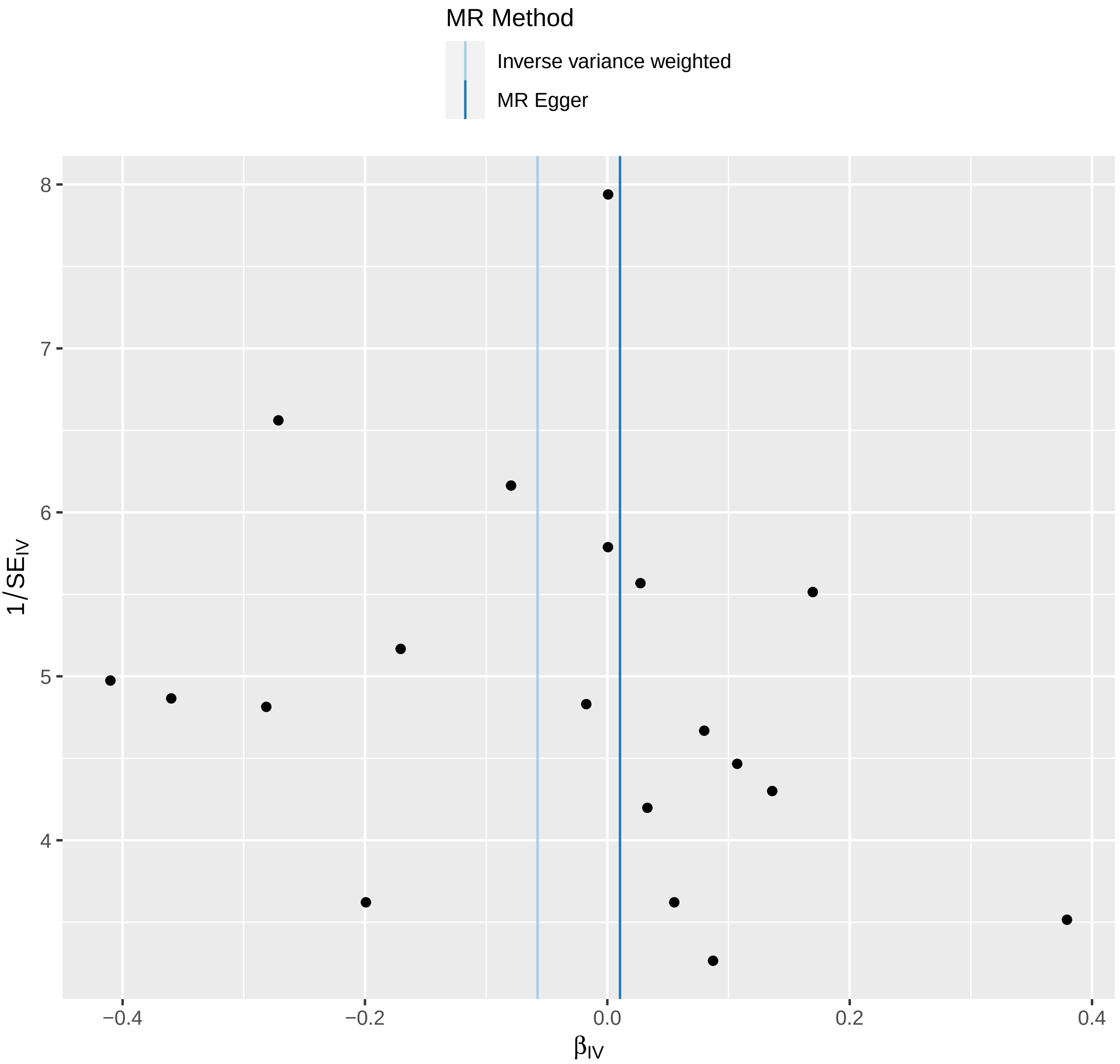

Batch 3−1430 : Interferon gamma receptor 1 on Diabetic nephropathy (more control exclusions) with a threshold of 1e−05

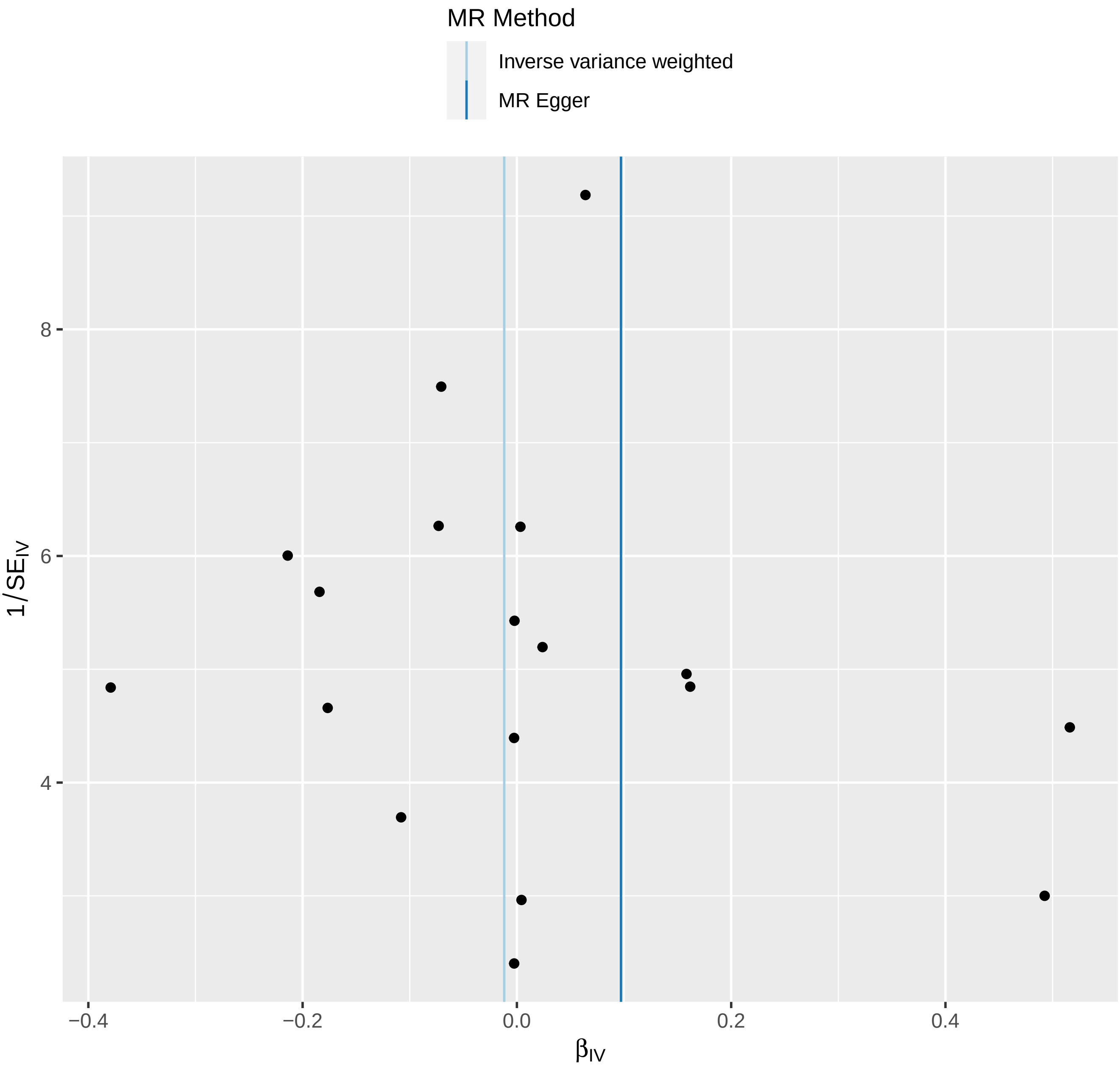

Batch 3−1431 : Interferon gamma receptor 2 on Diabetic nephropathy (more control exclusions)
with a threshold of 1e−05

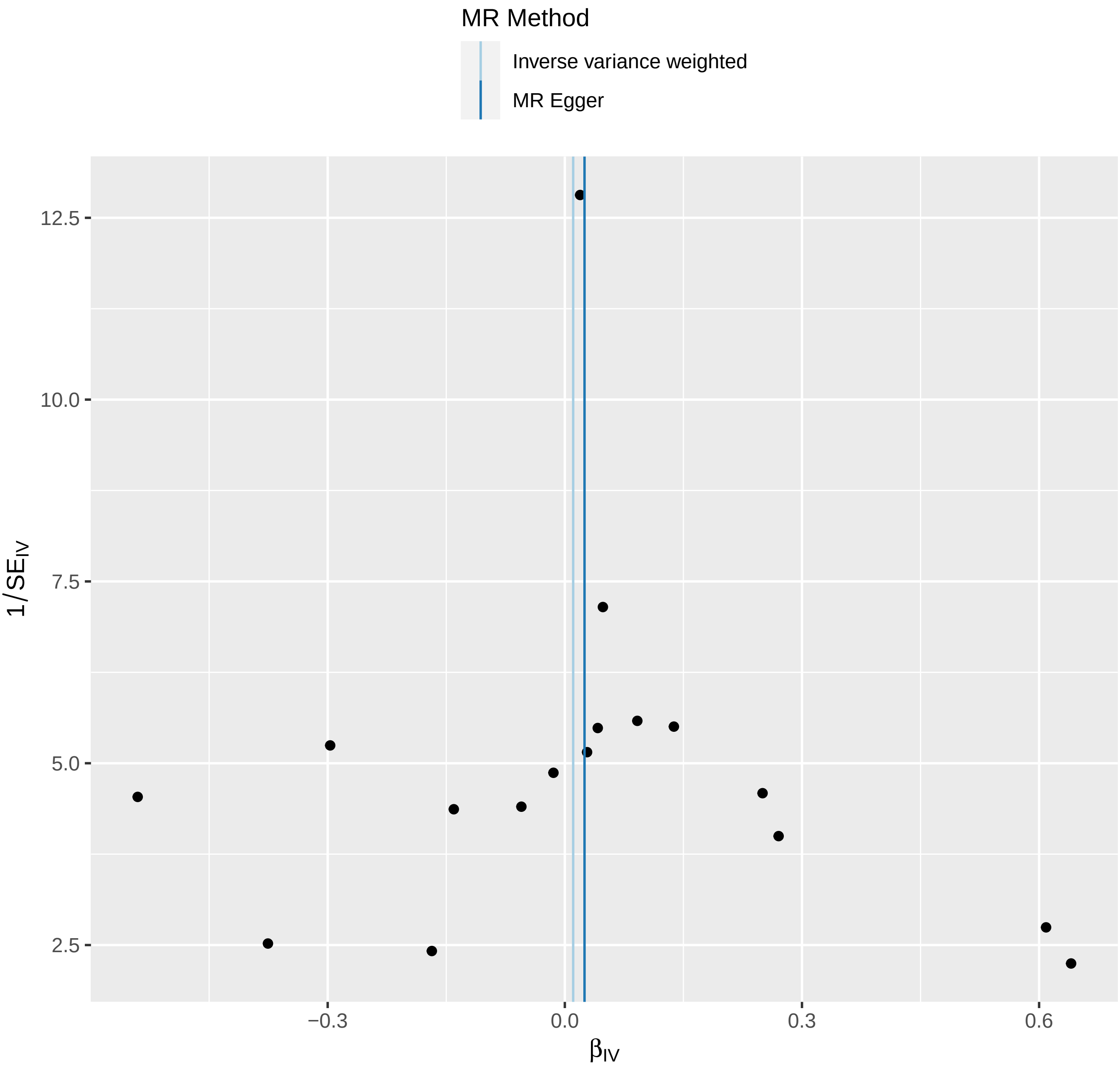

Batch 3−1432 : Interferon gamma receptor 2 on Diabetic nephropathy (more control exclusions) with a threshold of 1e−05

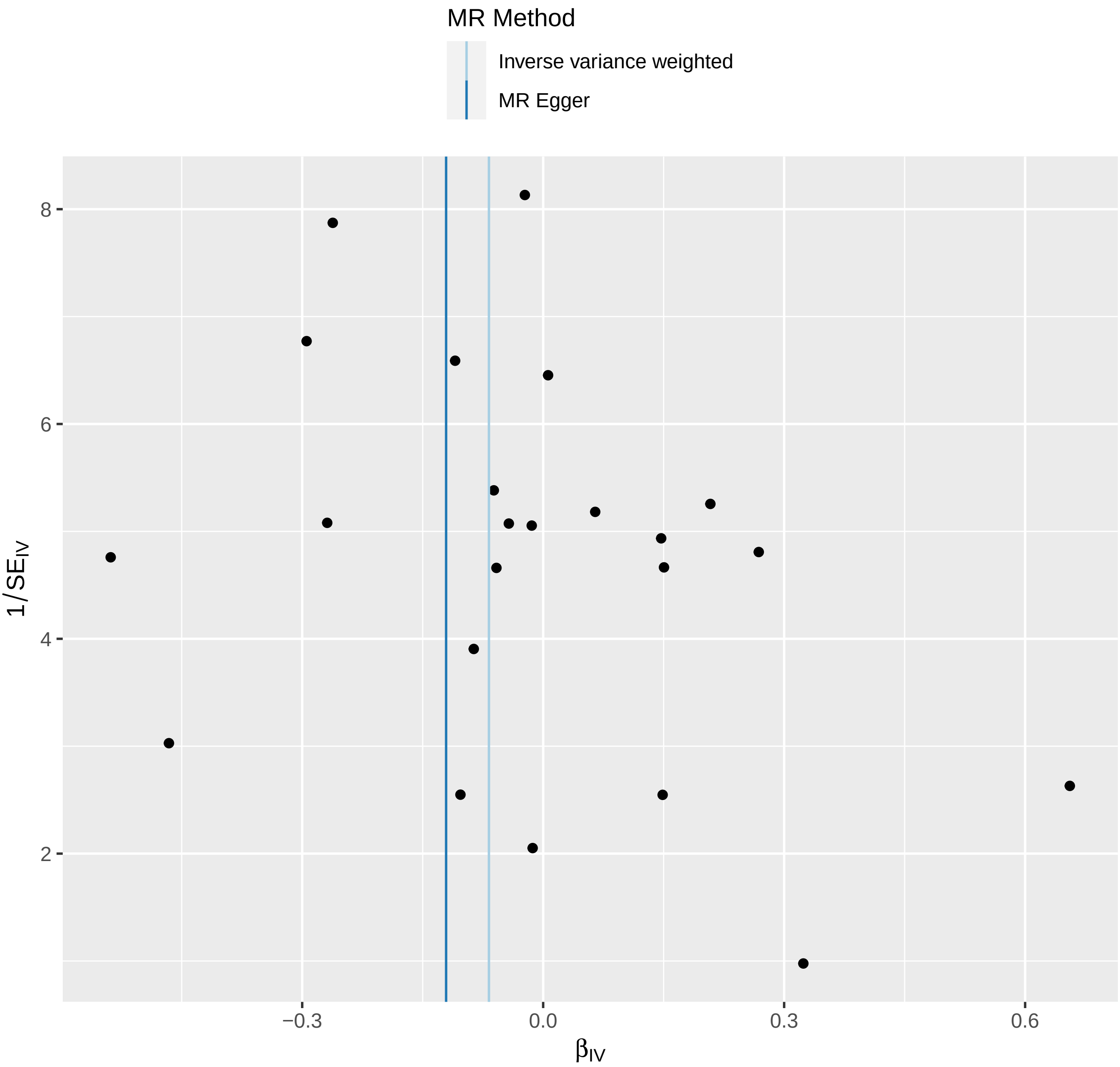

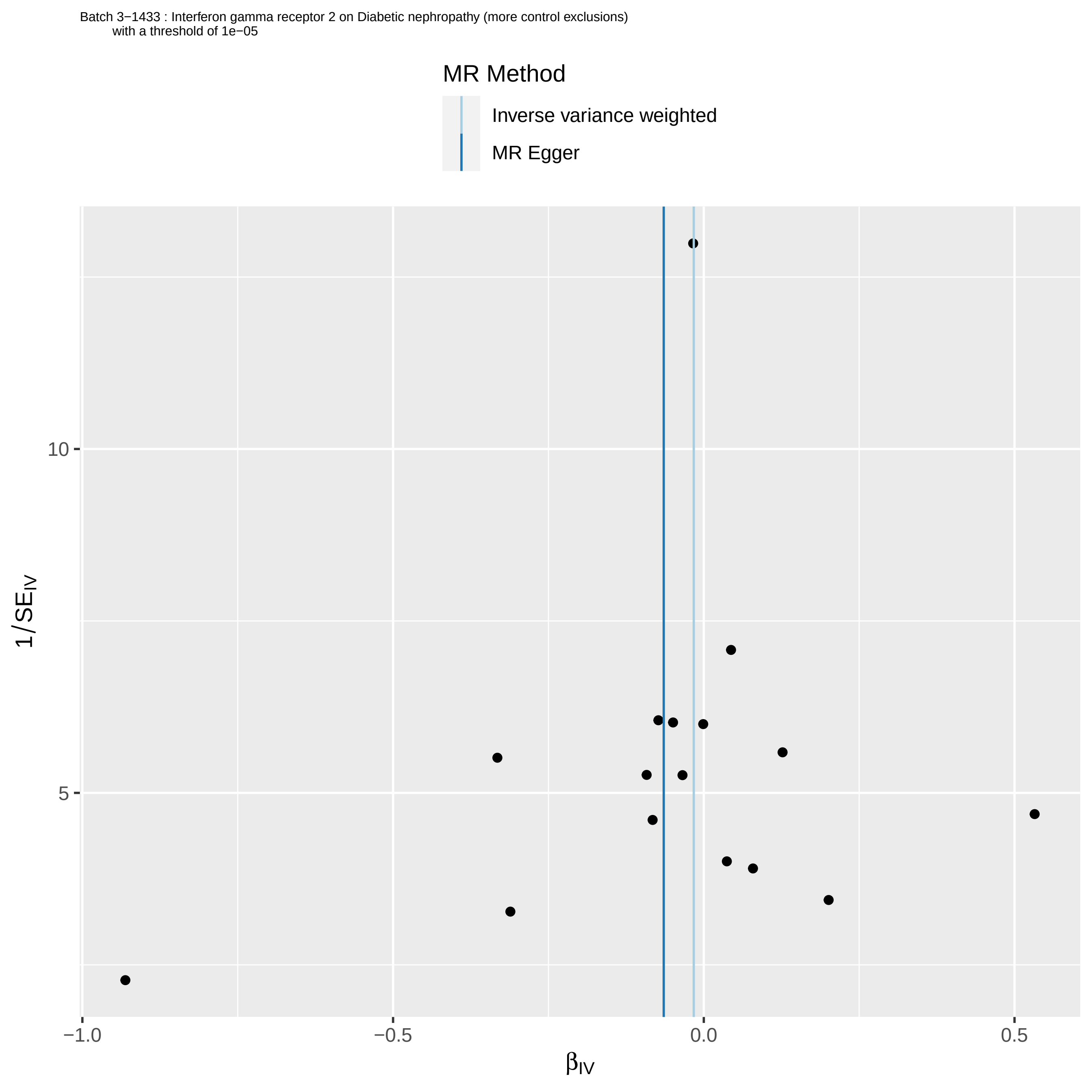

Batch 3−1433 : Interferon gamma receptor 2 on Diabetic nephropathy (more control exclusions)
with a threshold of 1e−05
MR Method
Inverse variance weighted
MR Egger
10
5
1/SE_IV
−1.0
−0.5
0.0
0.5
β_IV

Batch 3−1434 : Interferon lambda−1 on Diabetic nephropathy (more control exclusions)
with a threshold of 1e−05

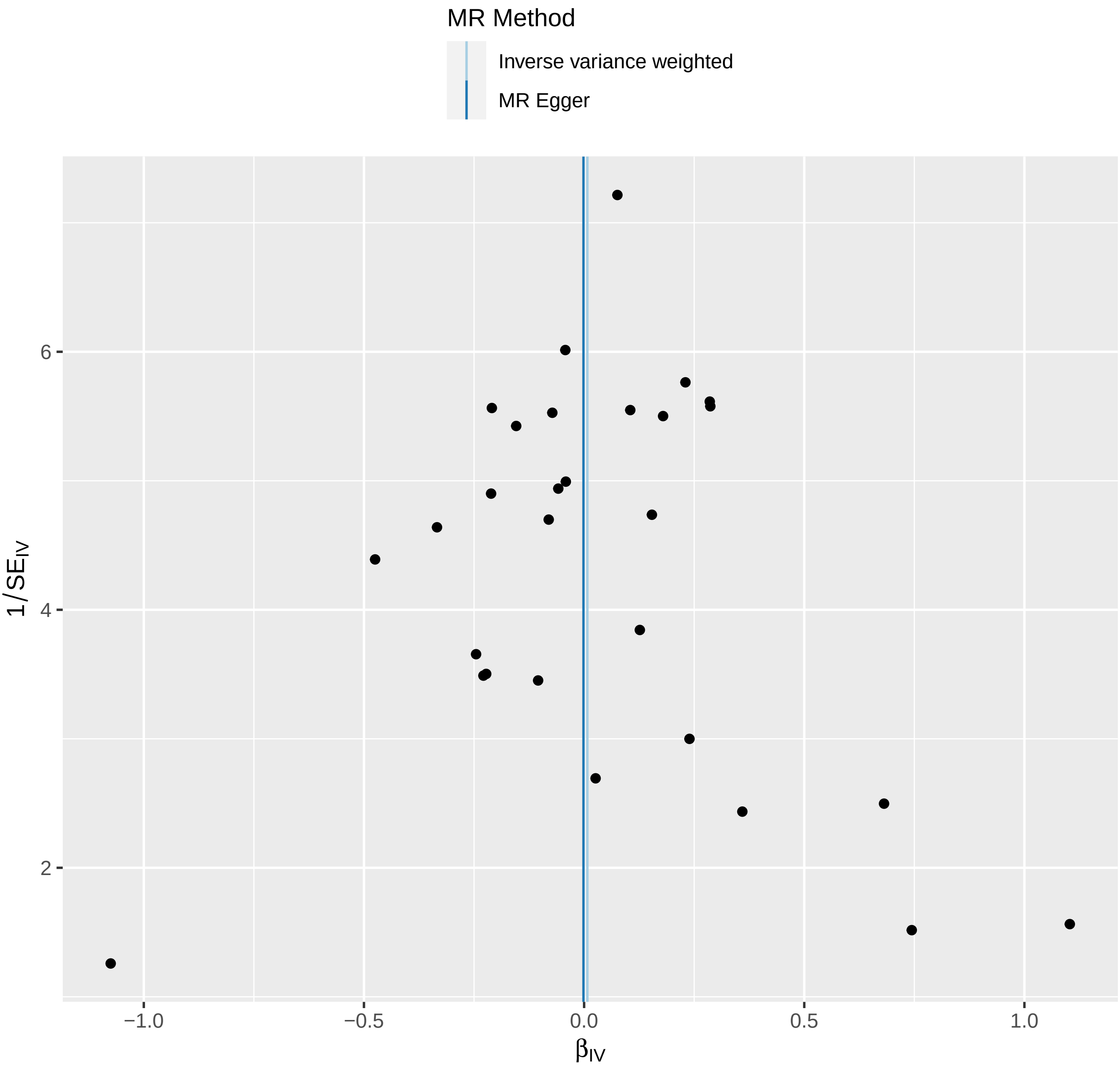

Batch 3−1435 : Interferon lambda−1 on Diabetic nephropathy (more control exclusions)
with a threshold of 1e−05

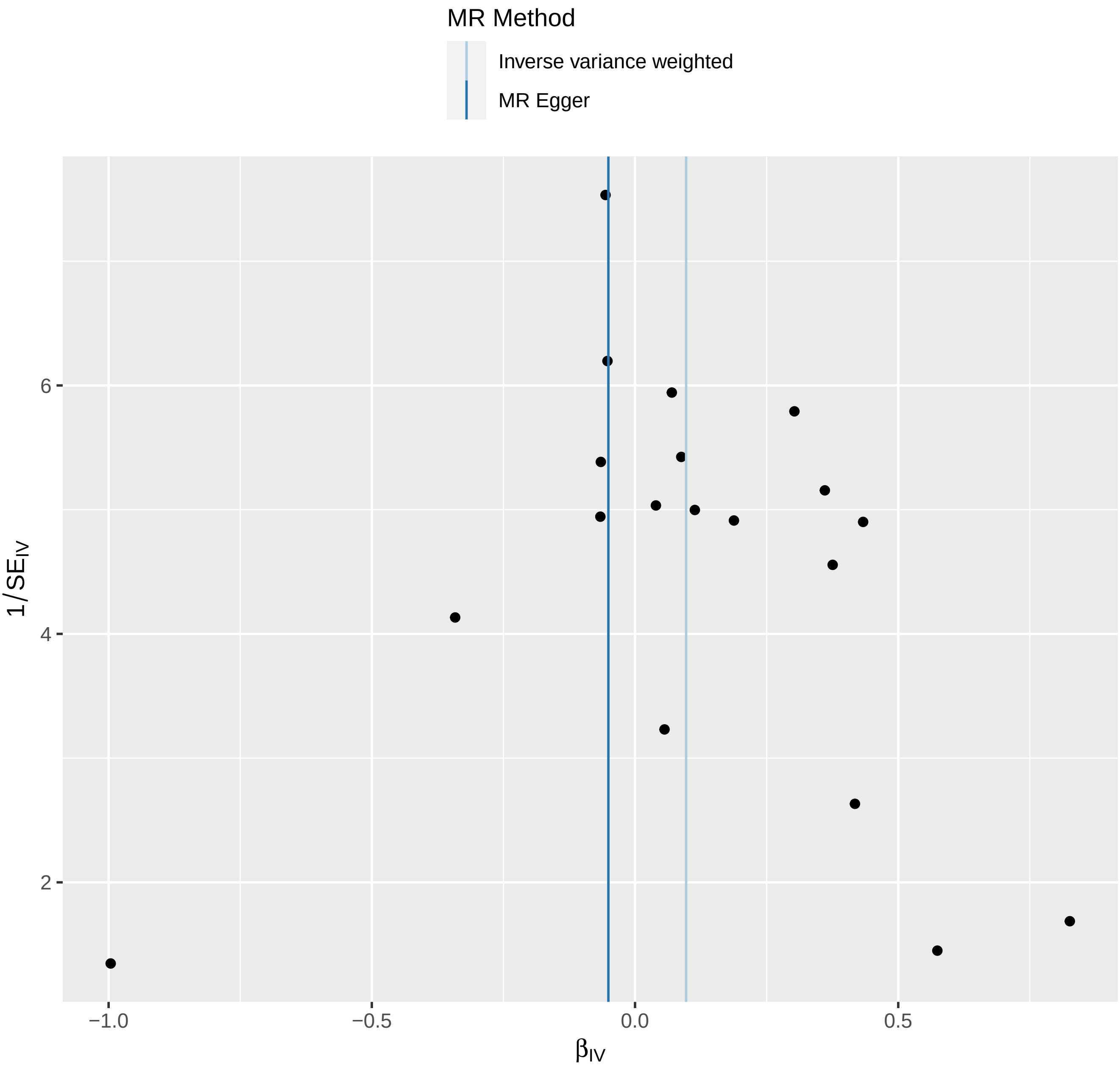

Batch 3−1436 : Interferon lambda−2 on Diabetic nephropathy (more control exclusions)
with a threshold of 1e−05

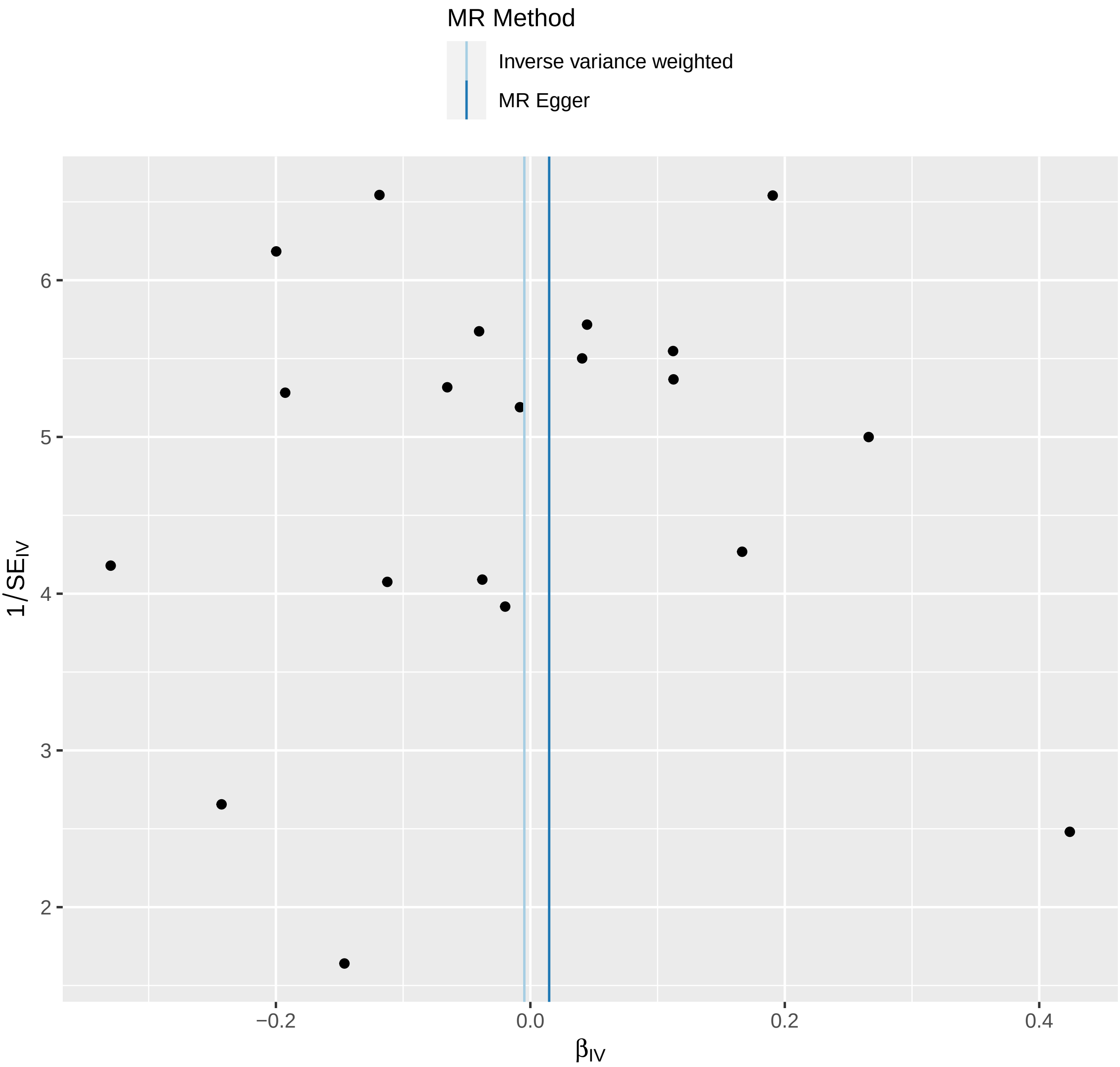

Batch 3−1437 : Interferon lambda receptor 1 on Diabetic nephropathy (more control exclusions)
with a threshold of 1e−05

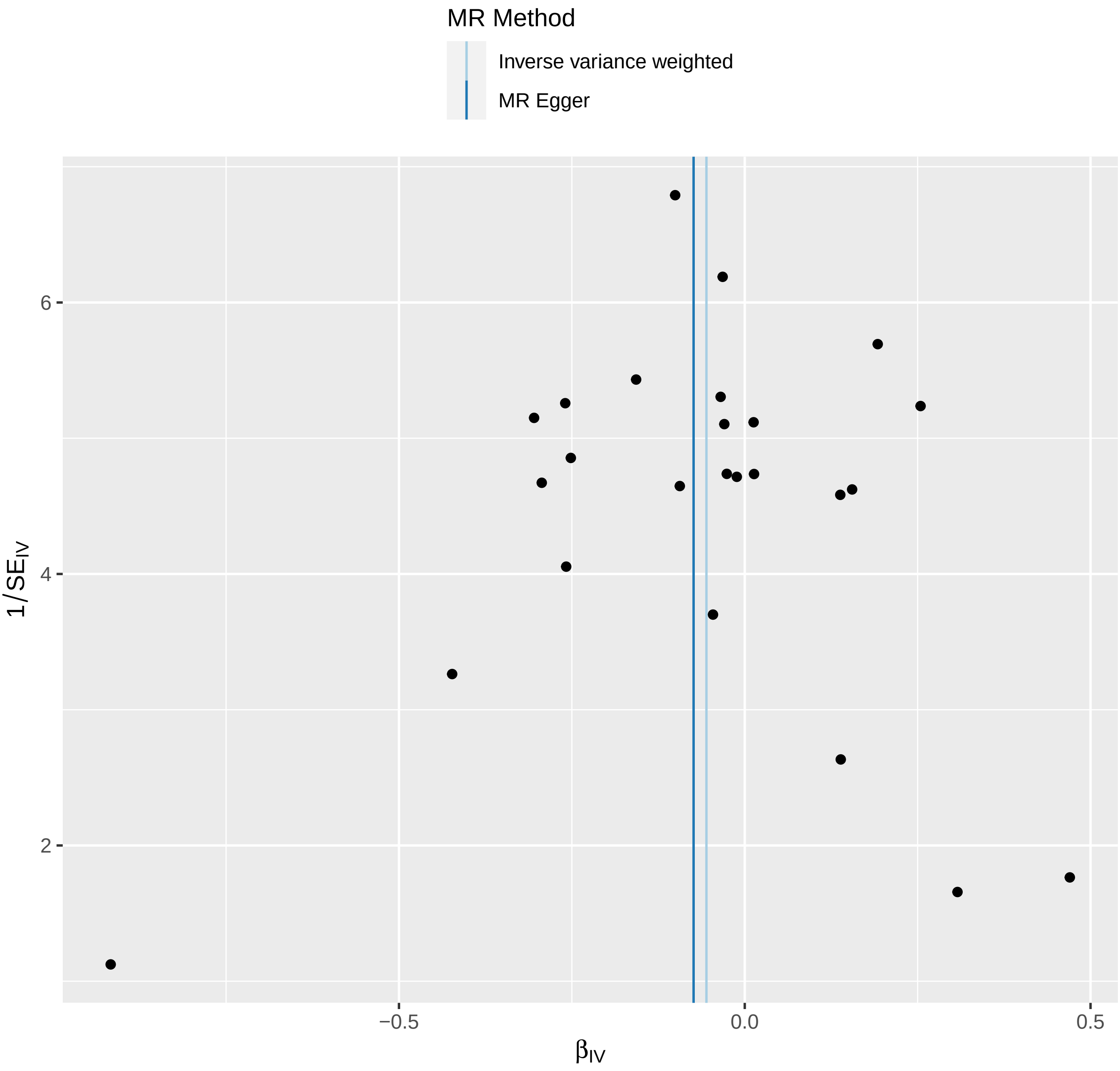

Batch 3−1438 : Interferon omega−1 on Diabetic nephropathy (more control exclusions)
with a threshold of 1e−05

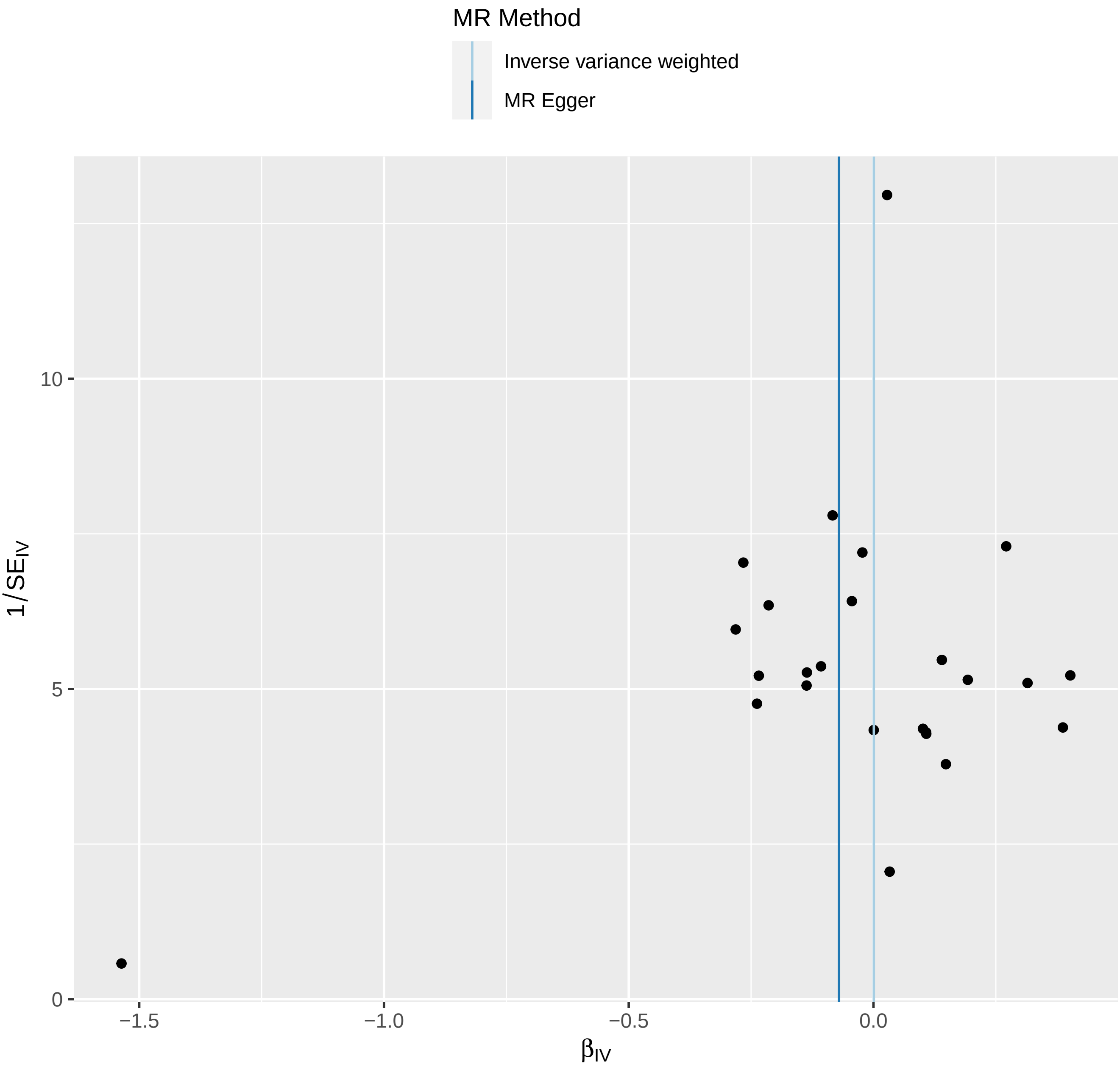

Batch 3−1439 : Immunoglobulin−binding protein 1 on Diabetic nephropathy (more control exclusions)
with a threshold of 1e−05

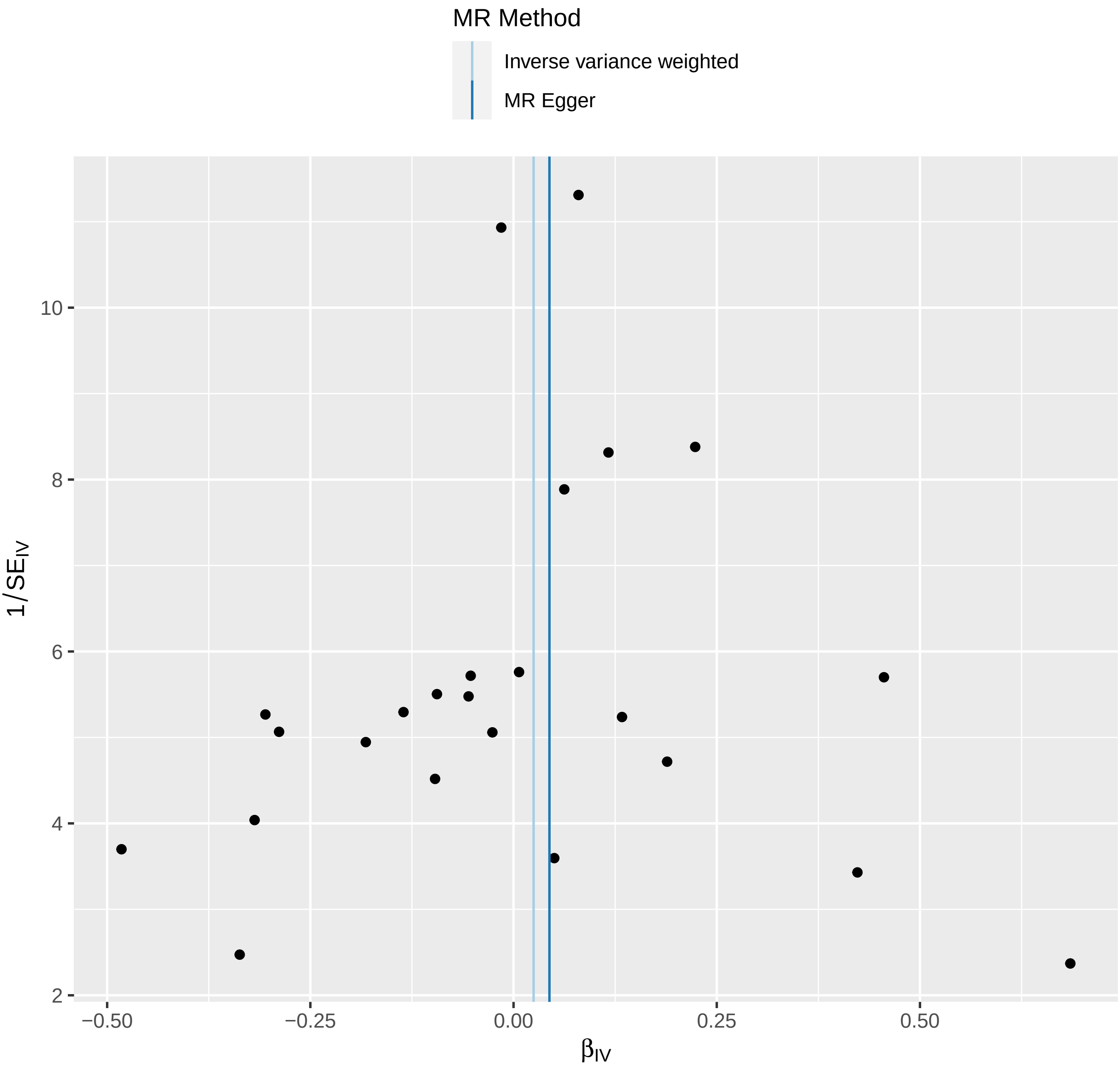

Batch 3−1440 : Immunoglobulin superfamily DCC subclass member 3 on Diabetic nephropathy (more control exclusions)
with a threshold of 1e−05

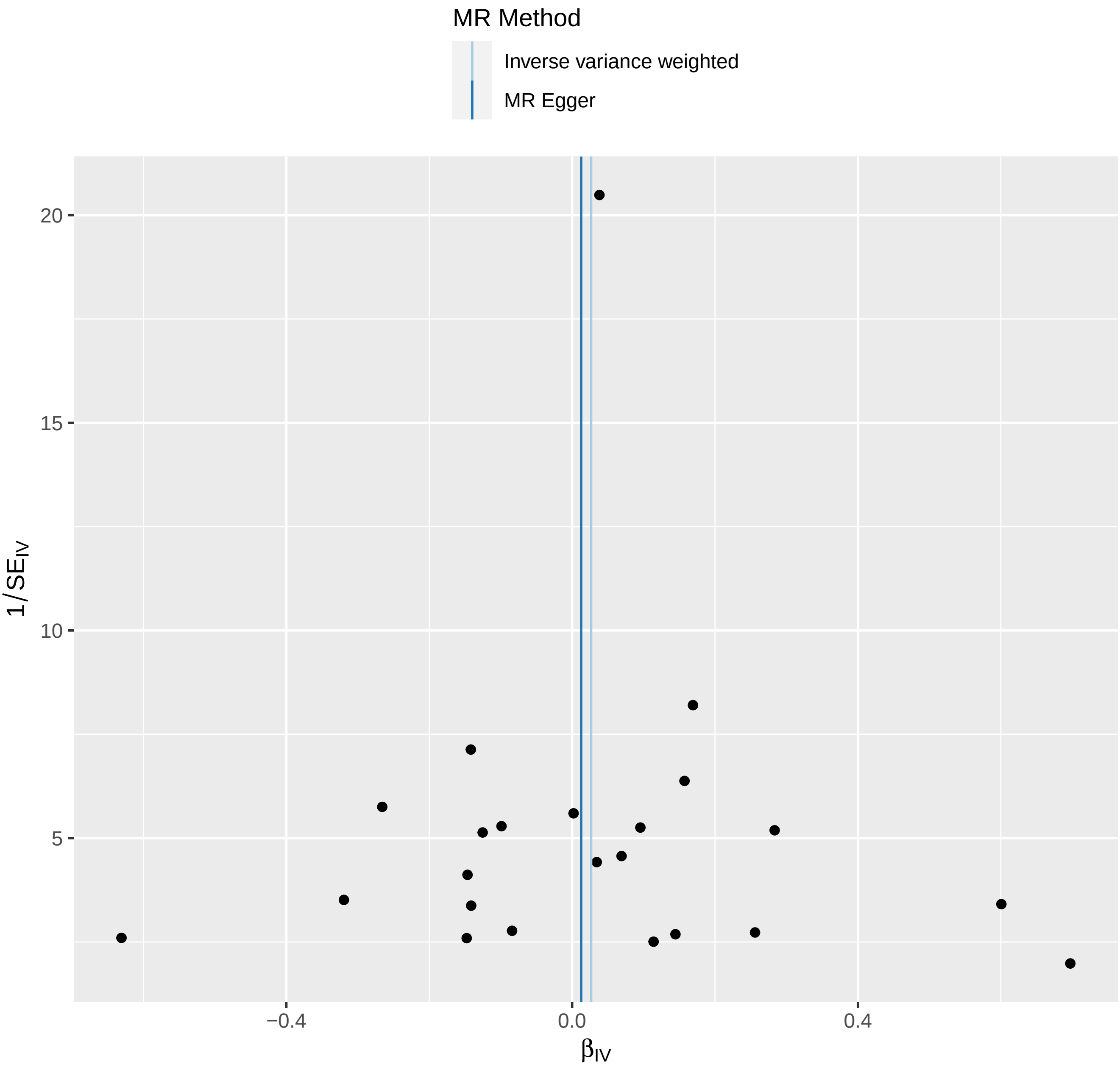

Batch 3−1441 : Immunoglobulin superfamily DCC subclass member 3 on Diabetic nephropathy (more control exclusions)
with a threshold of 1e−05

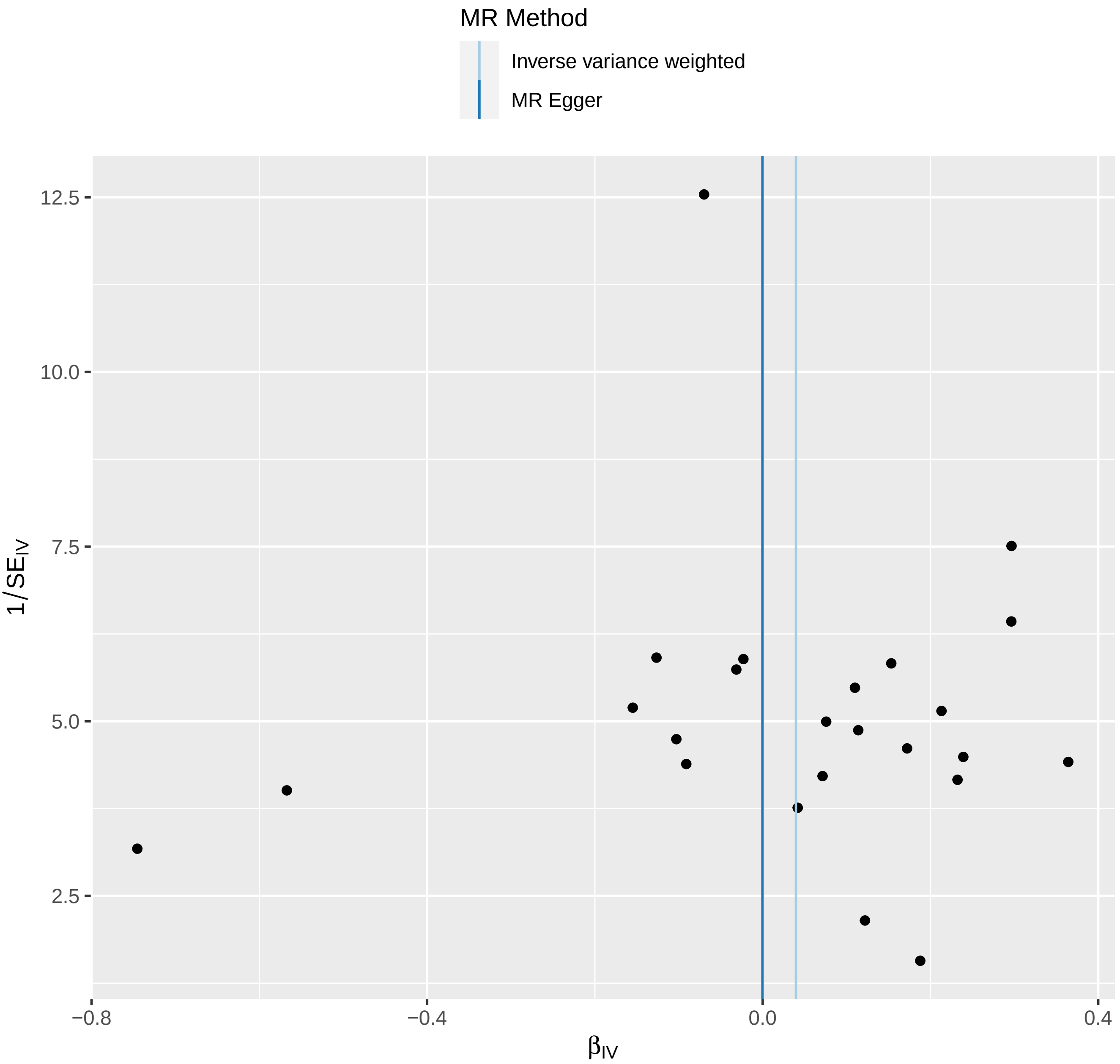

Batch 3−1442 : Immunoglobulin superfamily DCC subclass member 4 on Diabetic nephropathy (more control exclusions) with a threshold of 1e−05

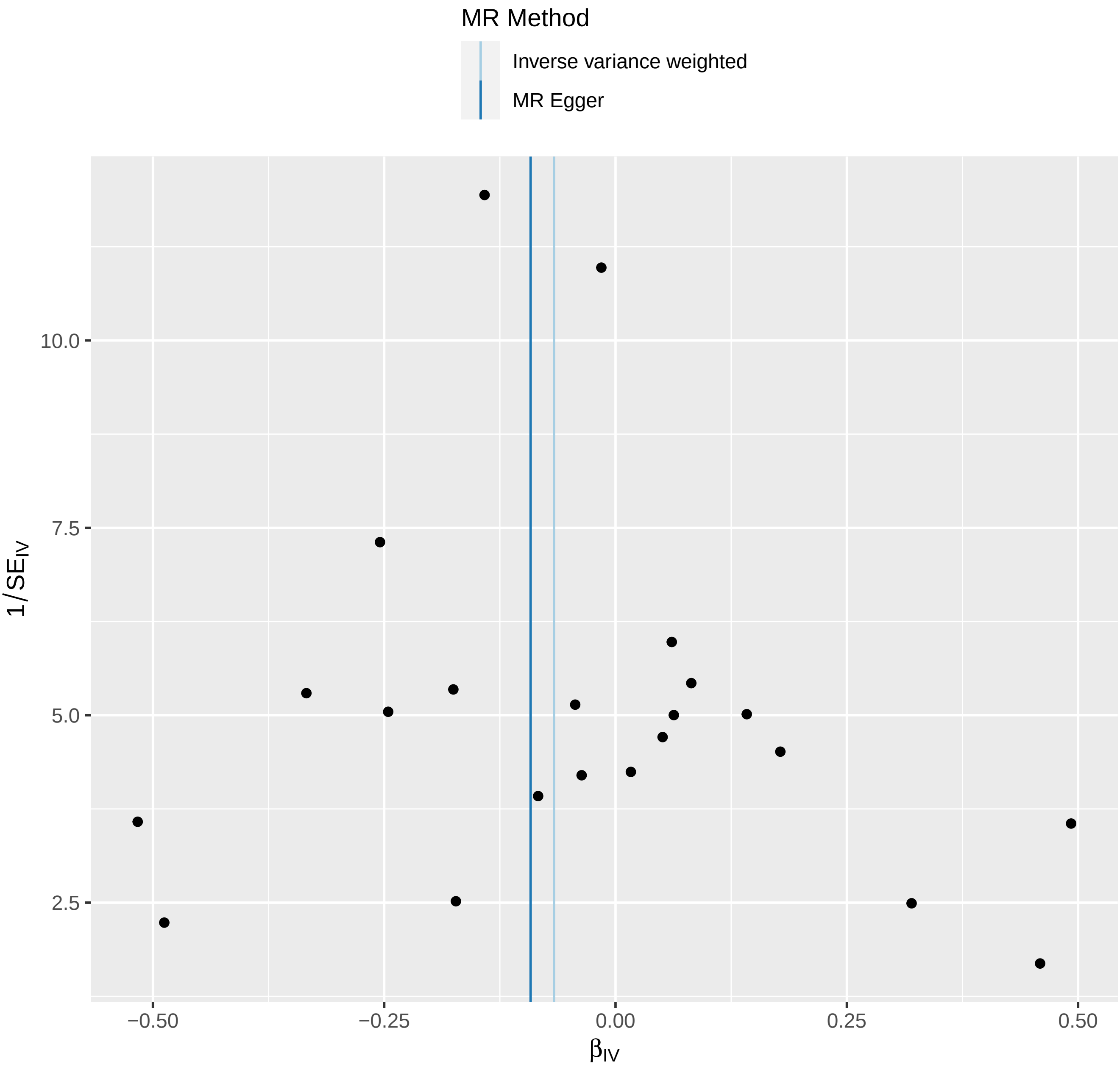

Batch 3−1443 : Insulin−like growth factor I on Diabetic nephropathy (more control exclusions)
with a threshold of 1e−05

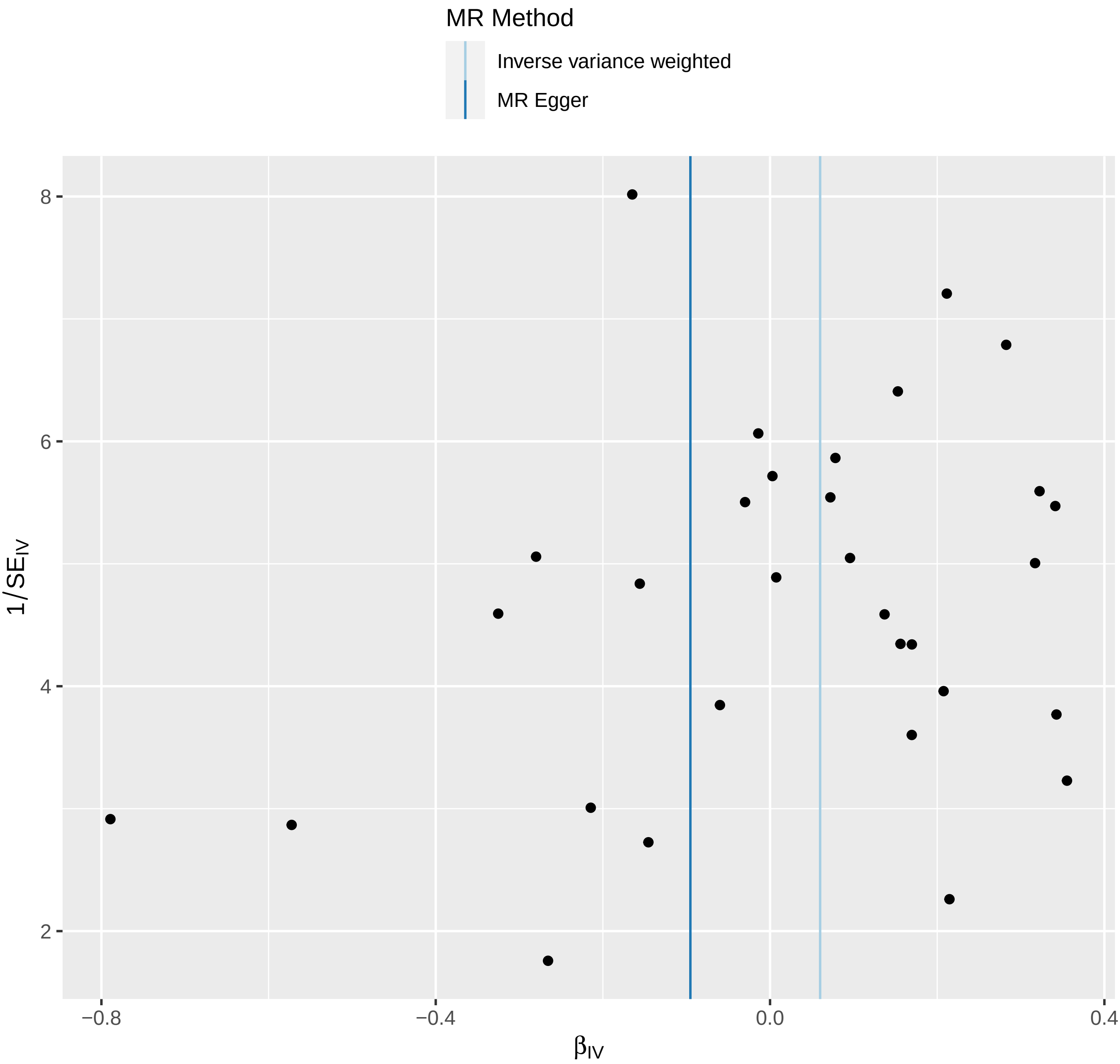

Batch 3−1444 : Insulin−like growth factor 1 receptor on Diabetic nephropathy (more control exclusions) with a threshold of 1e−05

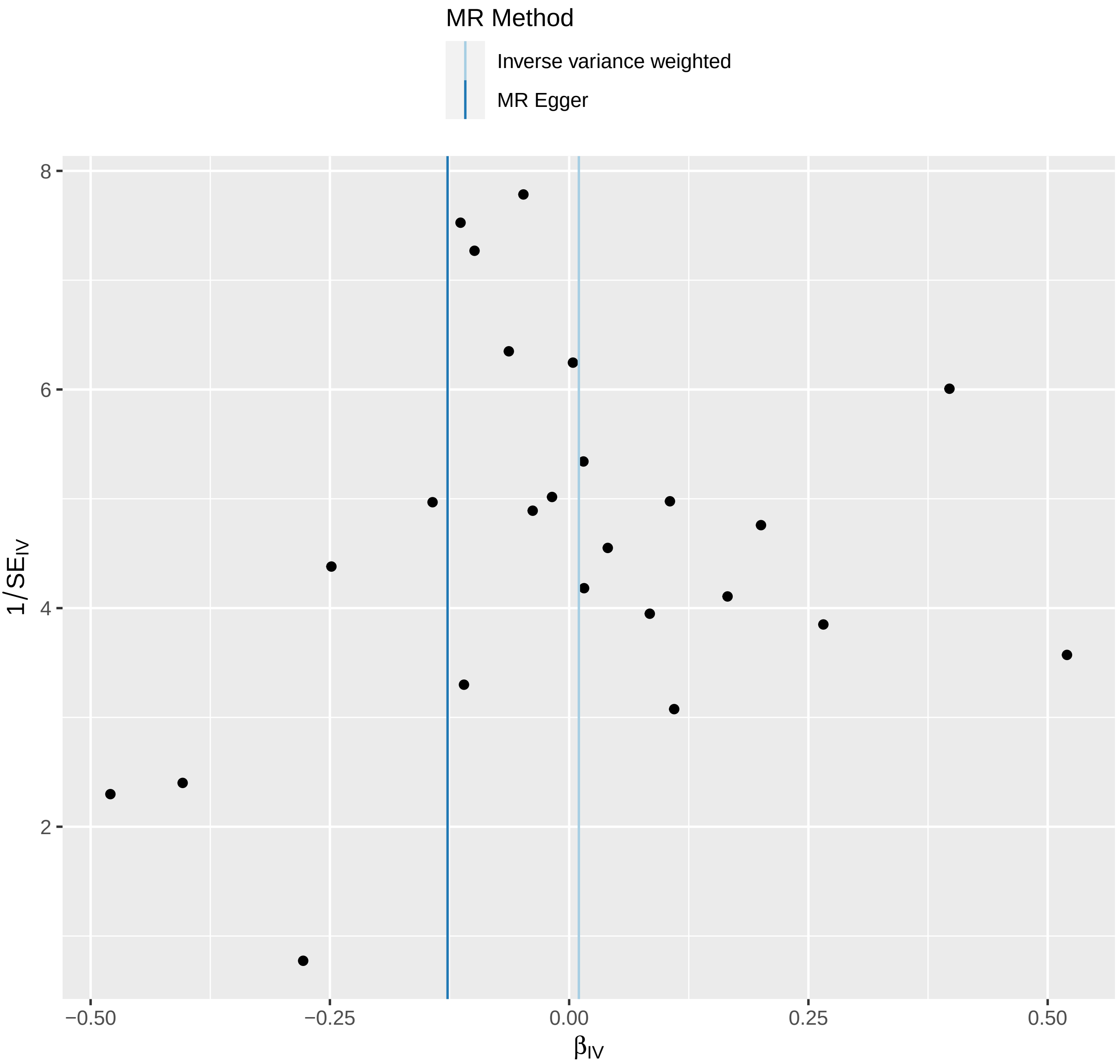

Batch 3−1445 : Cation−independent mannose−6−phosphate receptor on Diabetic nephropathy (more control exclusions)
with a threshold of 1e−05

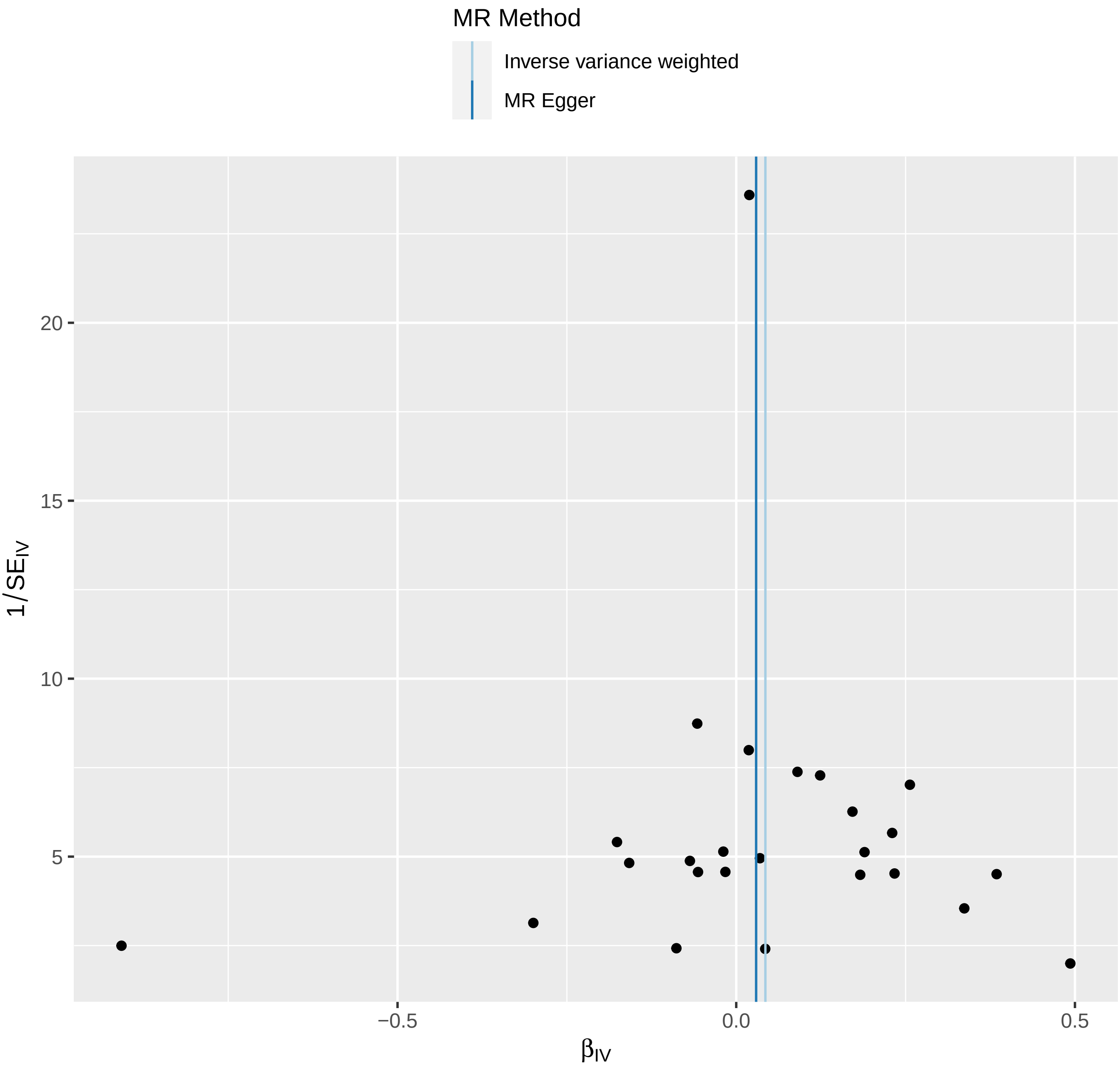

Batch 3−1446 : Insulin−like growth factor−binding protein 1 on Diabetic nephropathy (more control exclusions)
with a threshold of 1e−05

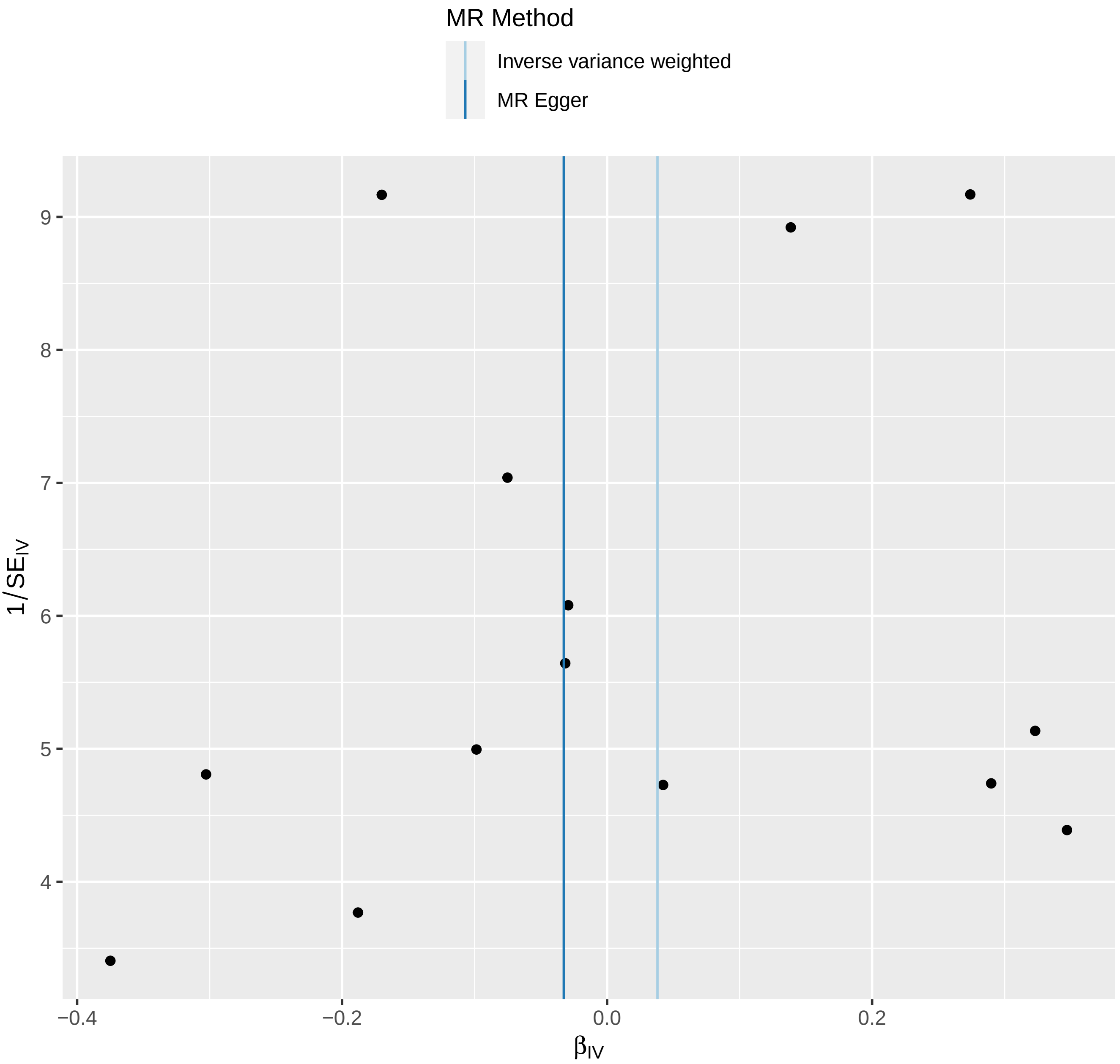

Batch 3−1447 : Insulin−like growth factor−binding protein 1 on Diabetic nephropathy (more control exclusions) with a threshold of 1e−05

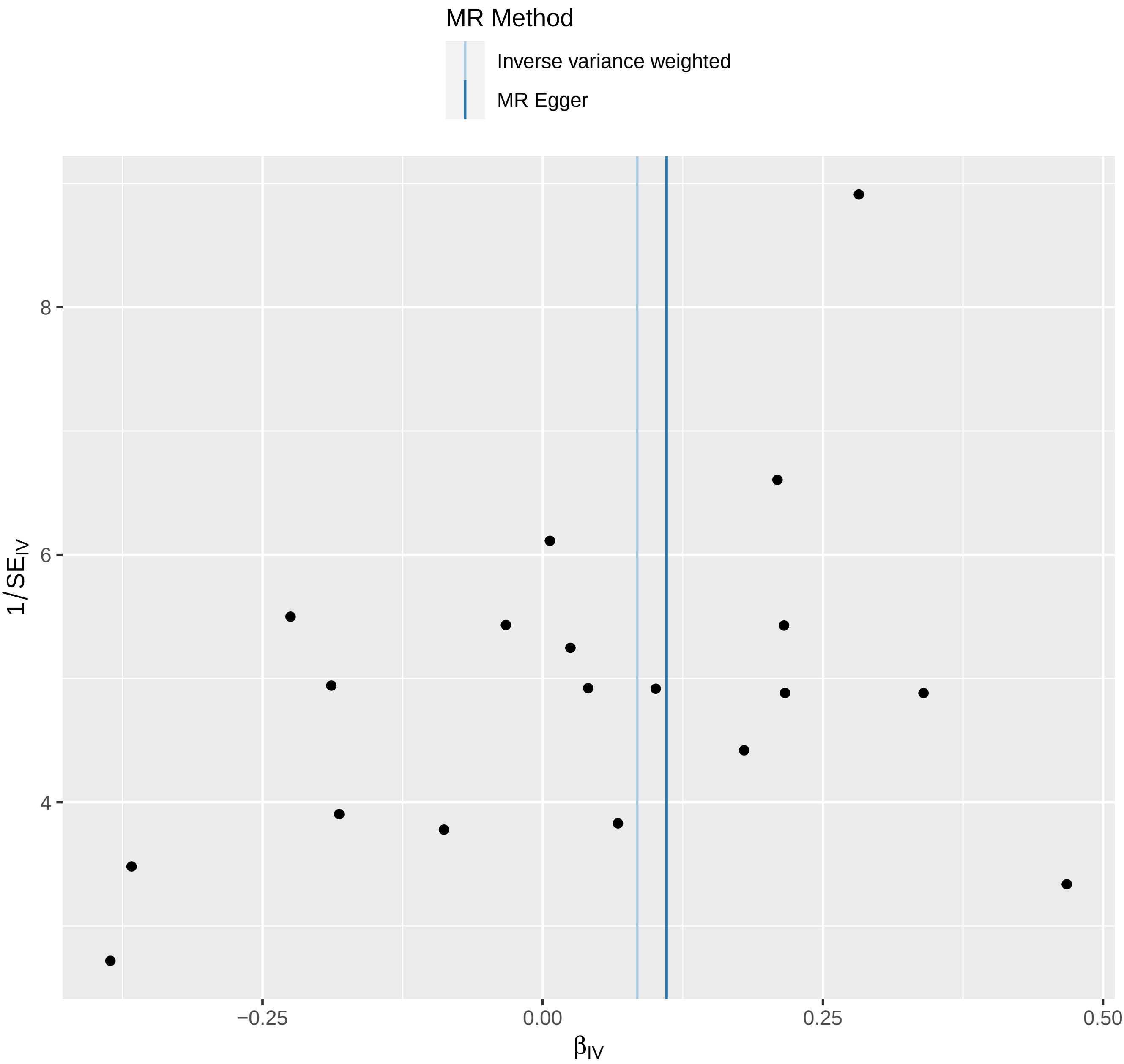

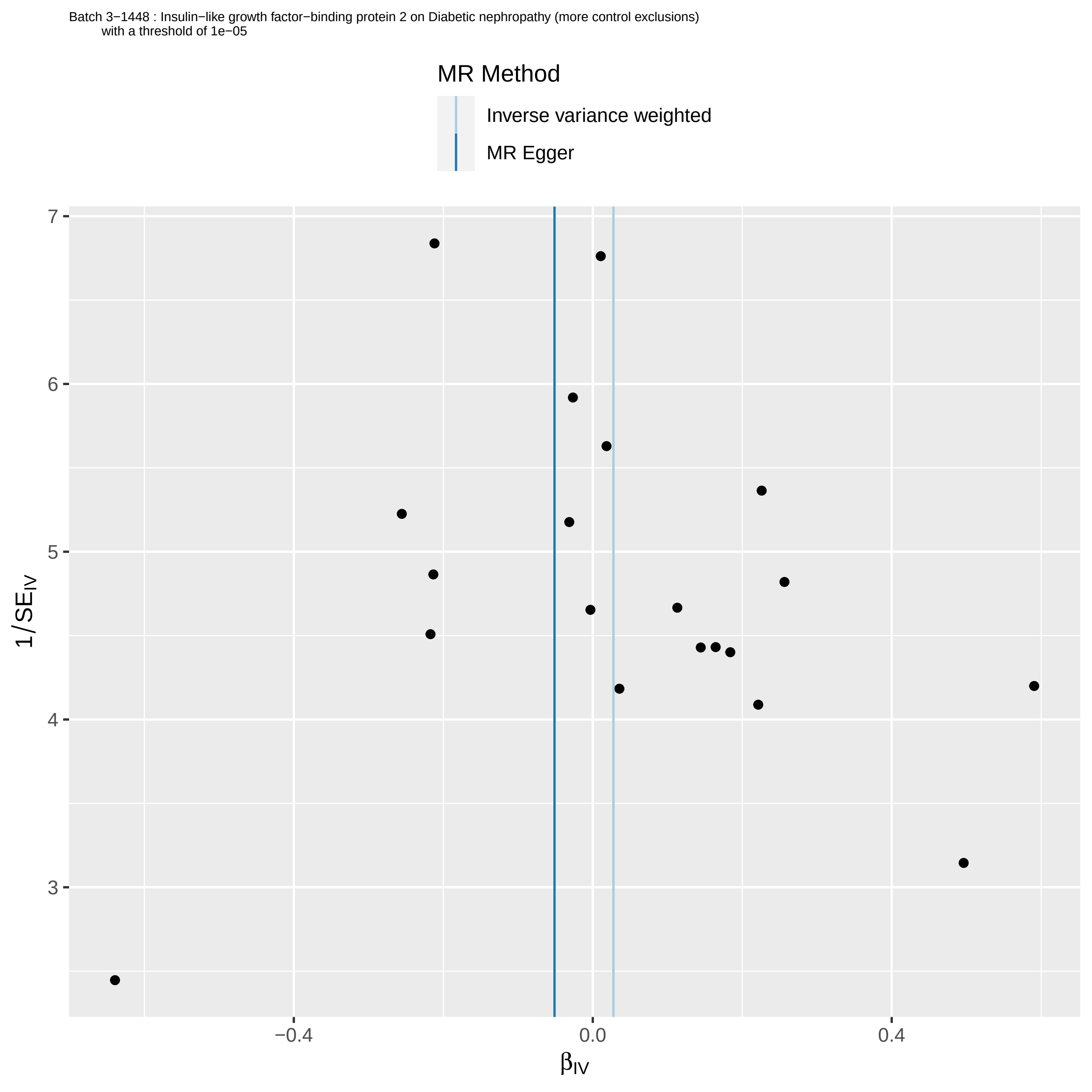

Batch 3−1448 : Insulin−like growth factor−binding protein 2 on Diabetic nephropathy (more control exclusions)
with a threshold of 1e−05
MR Method
Inverse variance weighted
MR Egger
7
6
5
4
3
1/SE_IV
−0.4
0.0
0.4
β_IV

Batch 3−1449 : Insulin−like growth factor−binding protein 3 on Diabetic nephropathy (more control exclusions) with a threshold of 1e−05

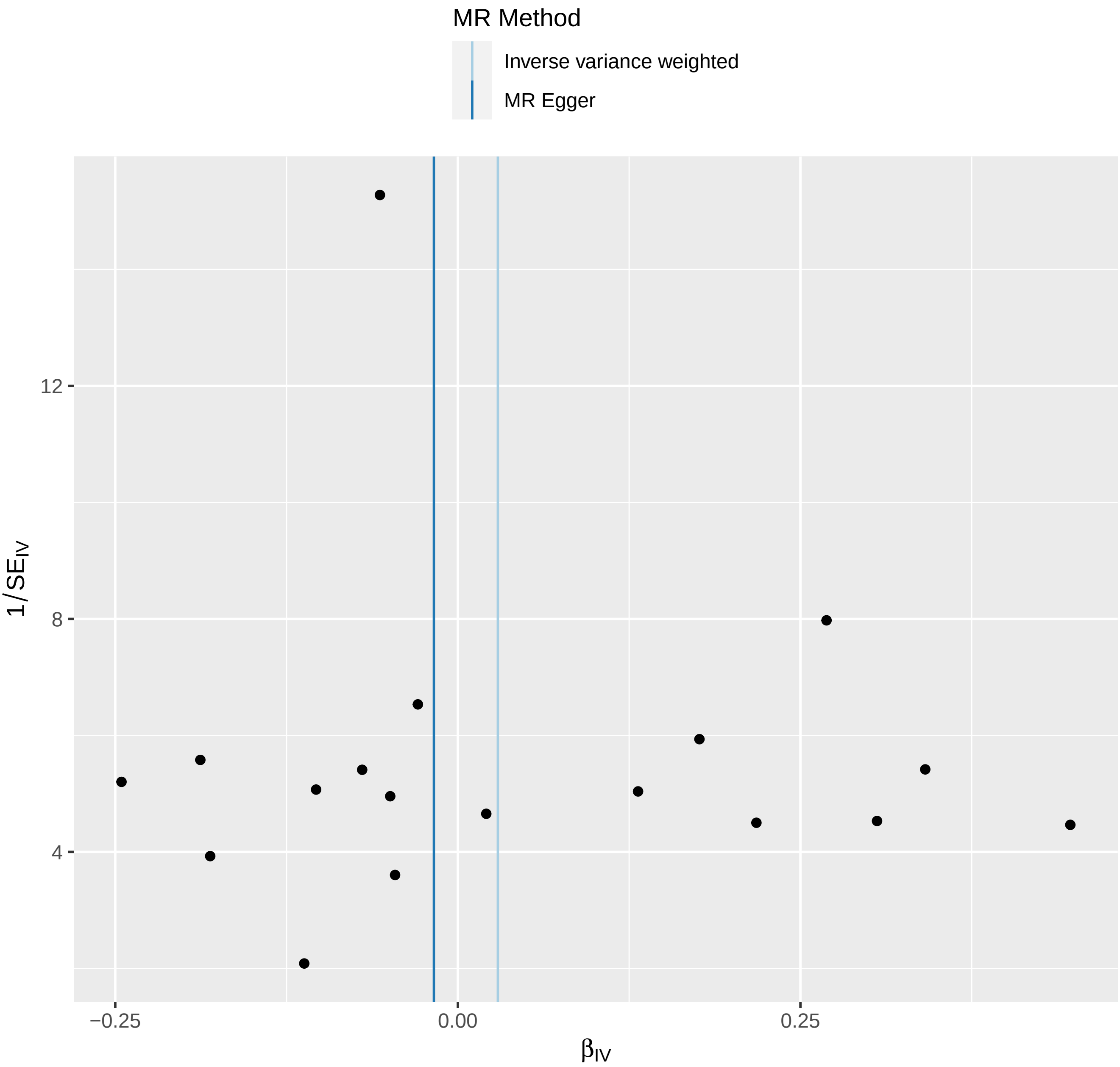

Batch 3−1450 : Insulin−like growth factor−binding protein 6 on Diabetic nephropathy (more control exclusions)
with a threshold of 1e−05

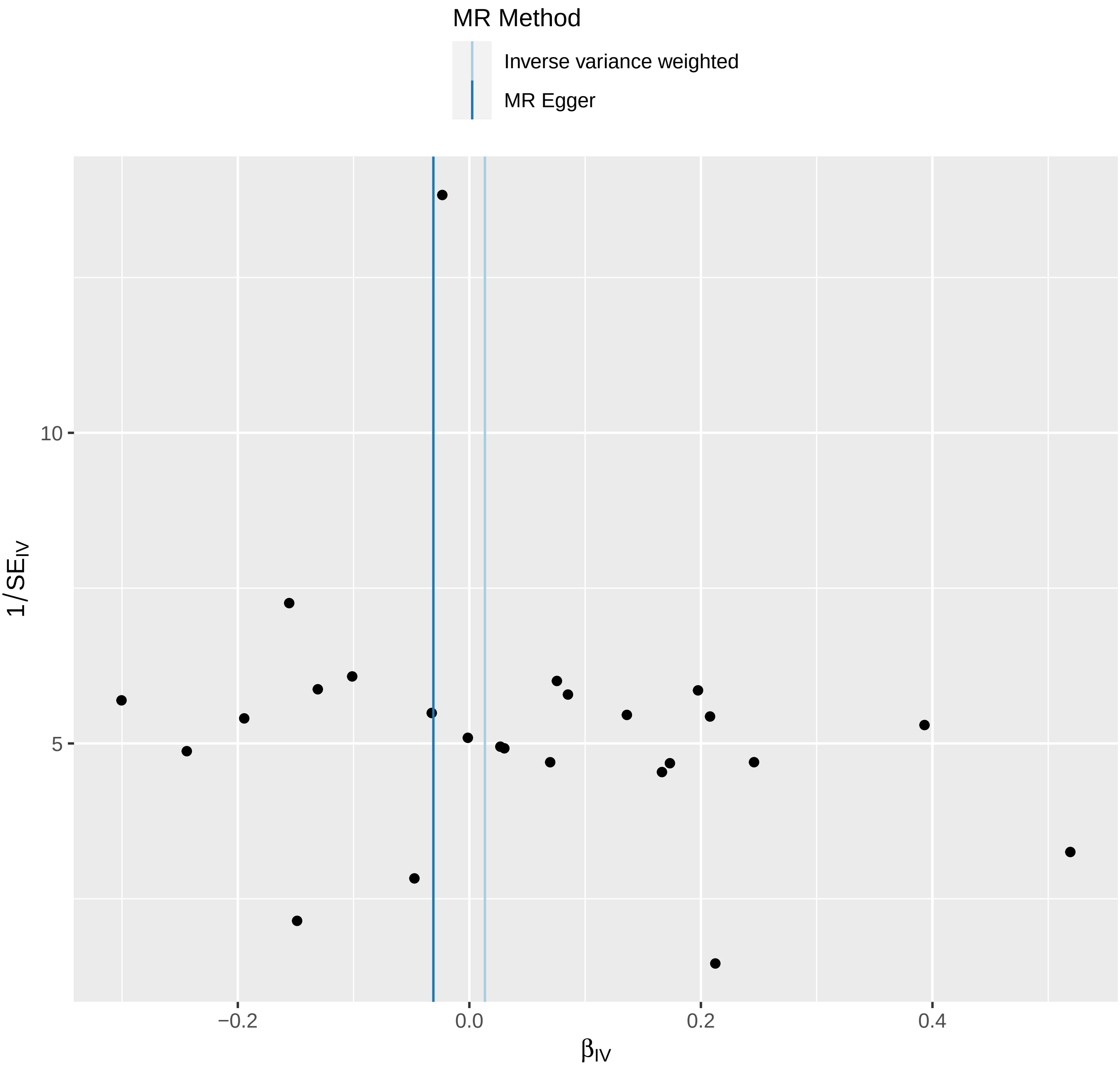

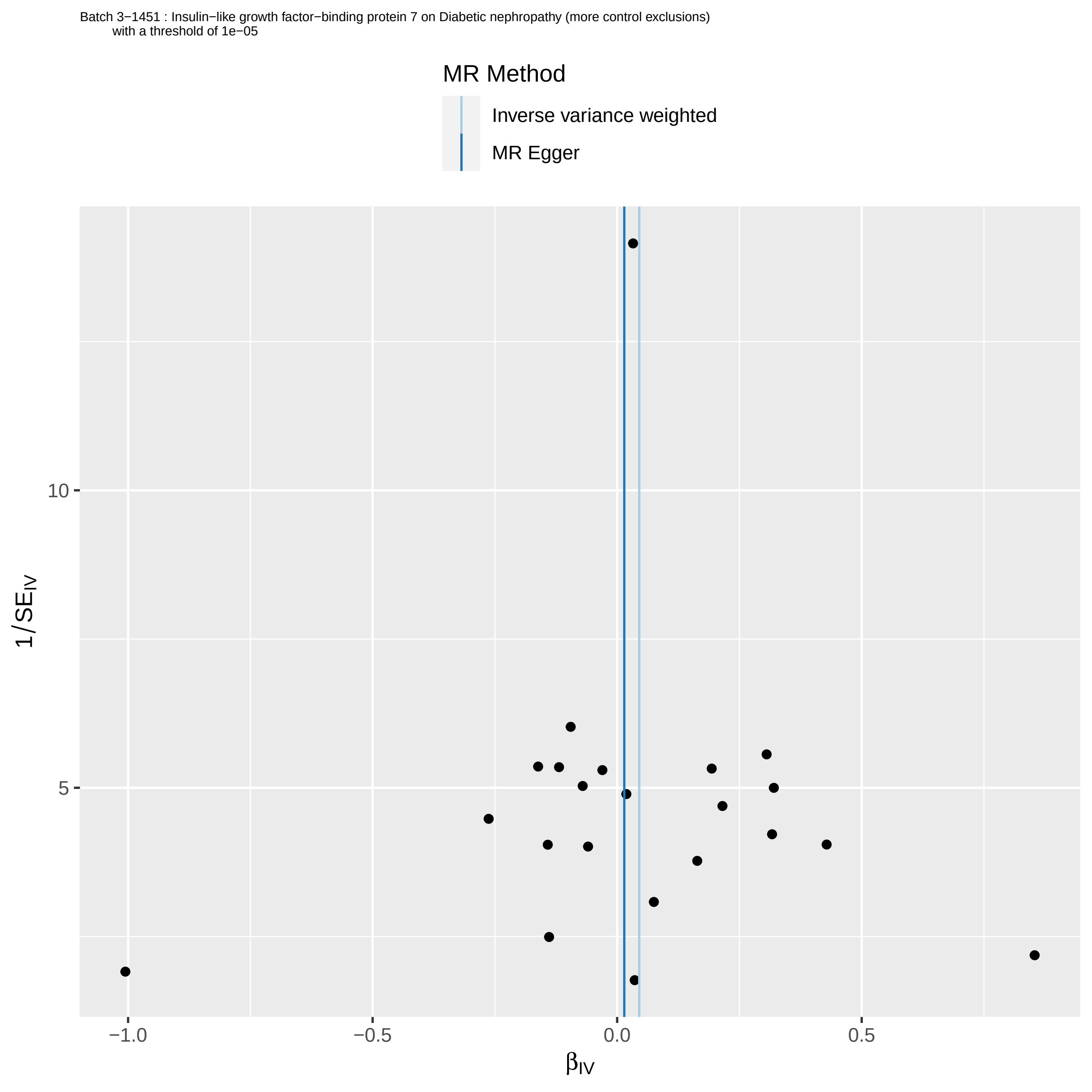

Batch 3−1451 : Insulin−like growth factor−binding protein 7 on Diabetic nephropathy (more control exclusions)
with a threshold of 1e−05
MR Method
Inverse variance weighted
MR Egger
10
5
1/SE_IV
−1.0
−0.5
0.0
0.5
β_IV

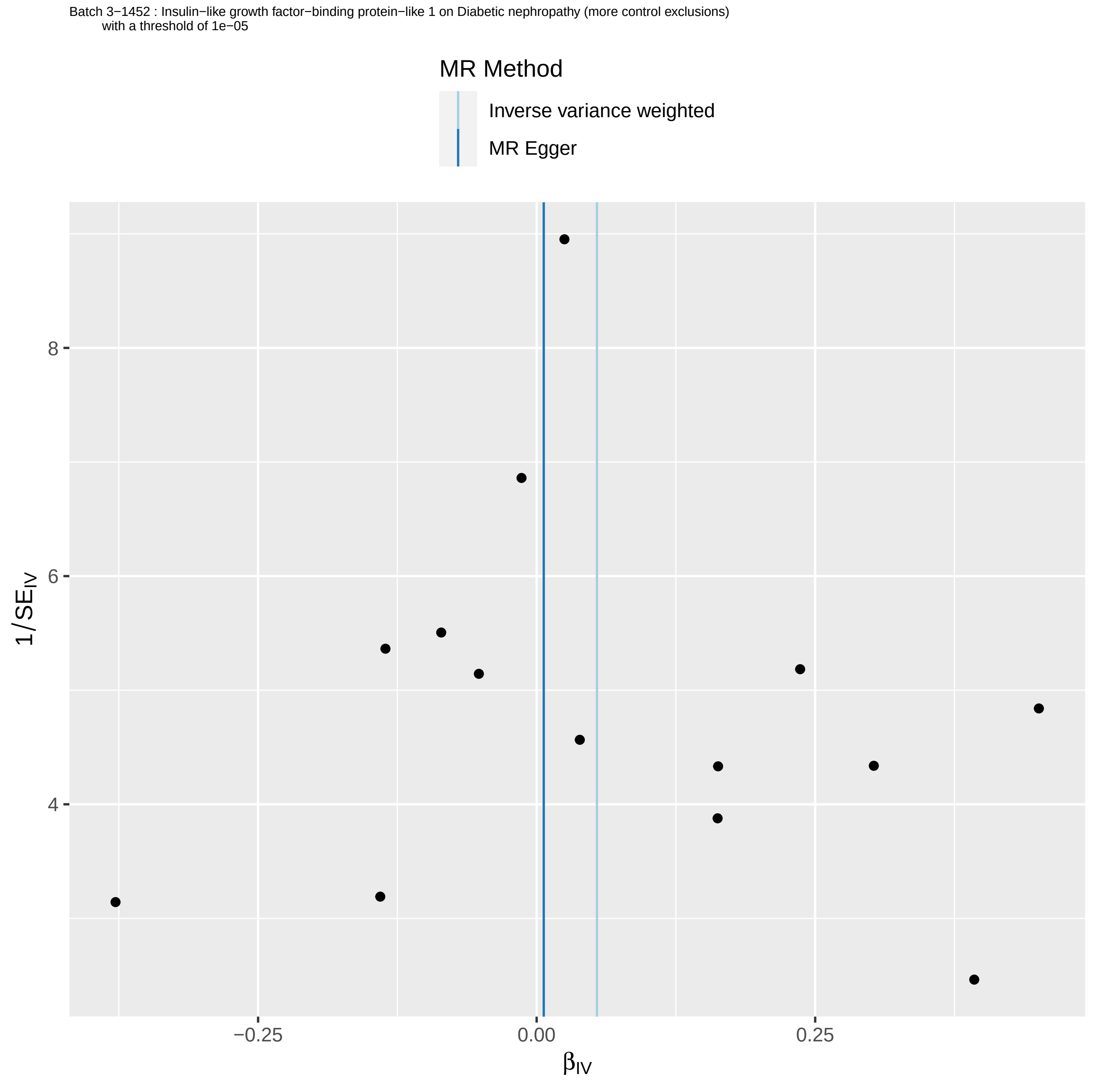

Batch 3−1452 : Insulin−like growth factor−binding protein−like 1 on Diabetic nephropathy (more control exclusions)
with a threshold of 1e−05
MR Method
Inverse variance weighted
MR Egger
8
6
4
1/SE$_{IV}$
−0.25
0.00
0.25
β_{IV}

Batch 3−1453 : Insulin growth factor−like family member 3 on Diabetic nephropathy (more control exclusions) with a threshold of 1e−05

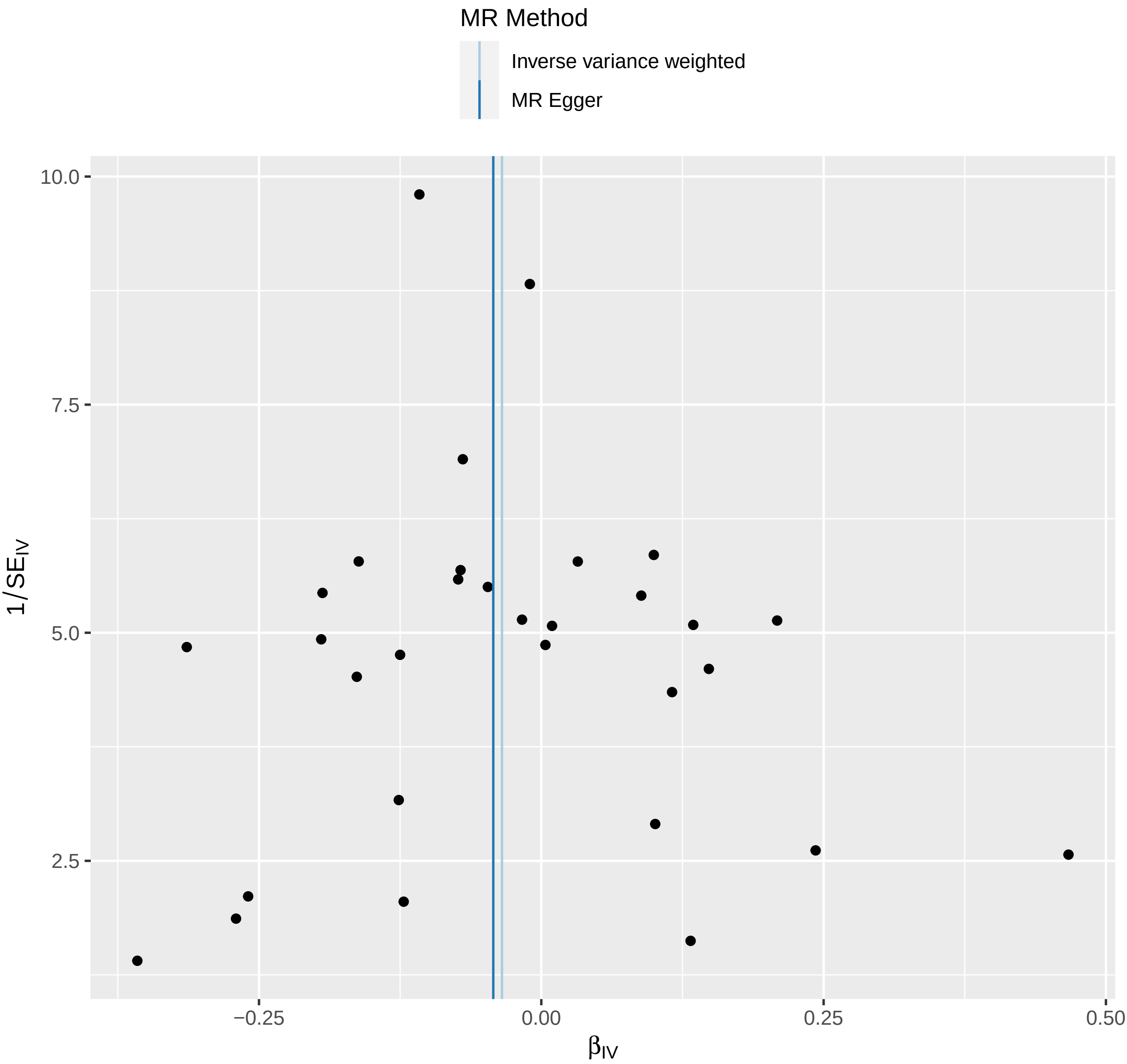

Batch 3−1454 : Insulin growth factor−like family member 4 on Diabetic nephropathy (more control exclusions)
with a threshold of 1e−05

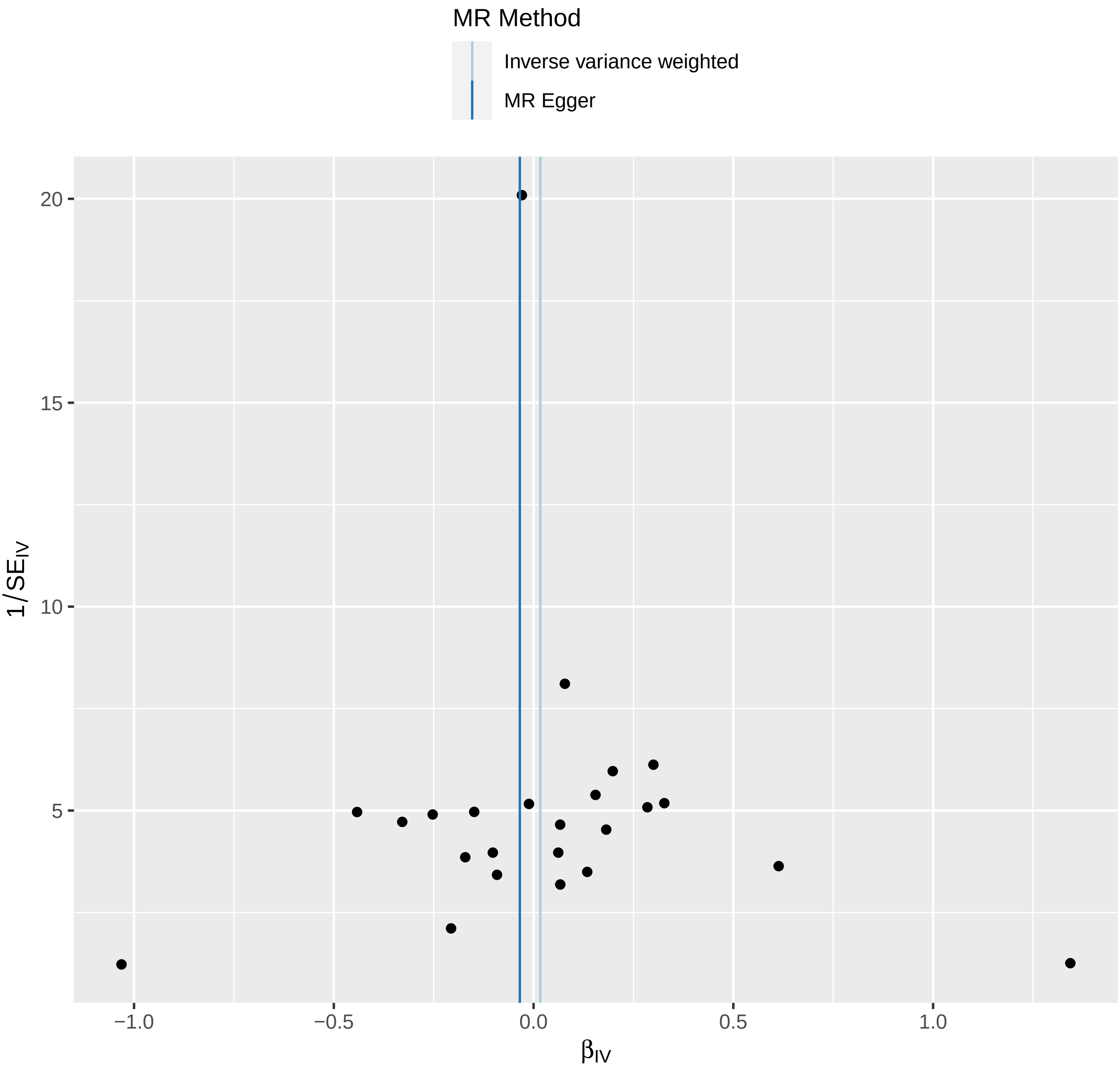

Batch 3−1455 : IGF−like family receptor 1 on Diabetic nephropathy (more control exclusions)
with a threshold of 1e−05

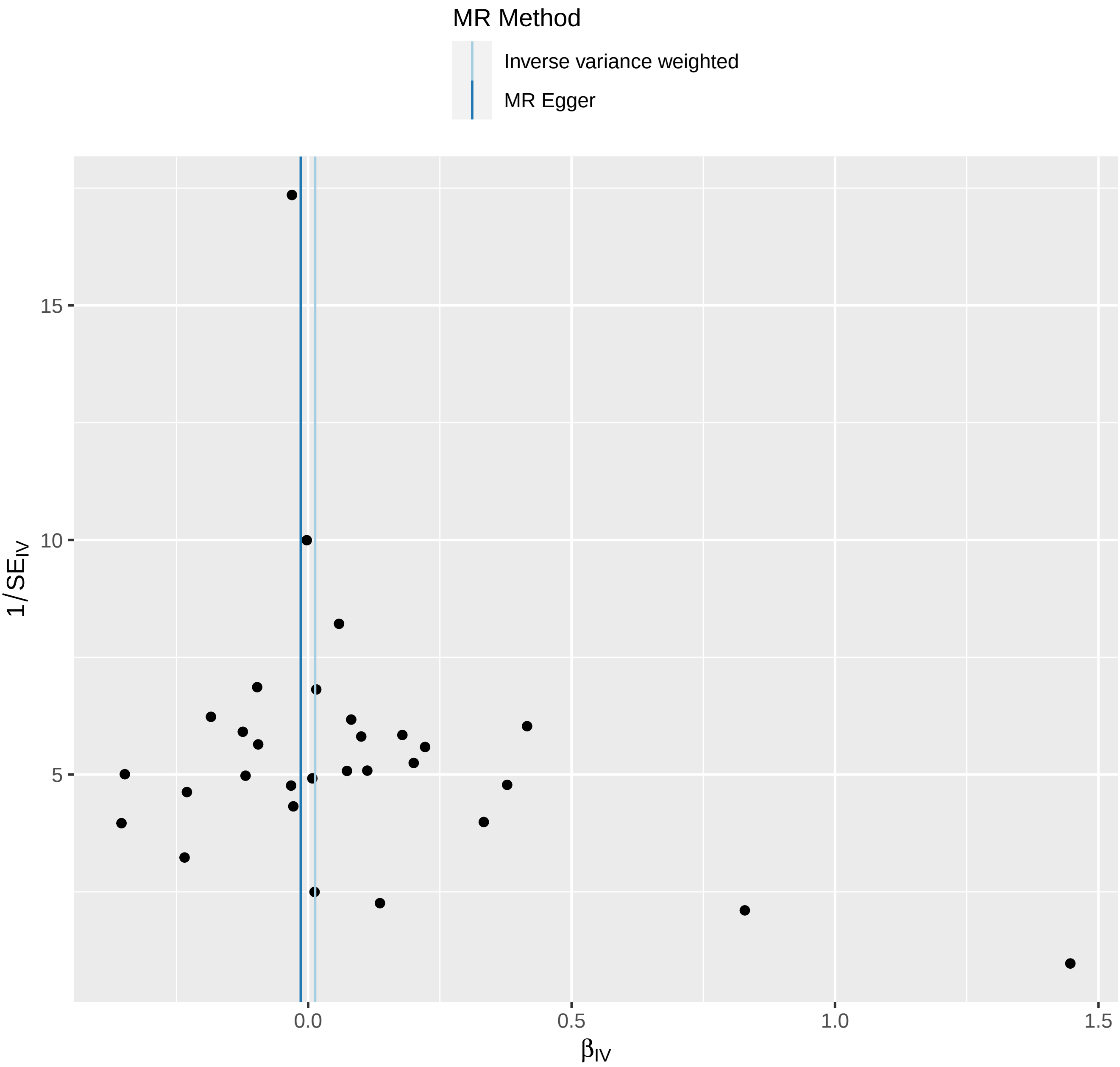

Batch 3−1456 : Immunoglobulin E on Diabetic nephropathy (more control exclusions)
with a threshold of 1e−05

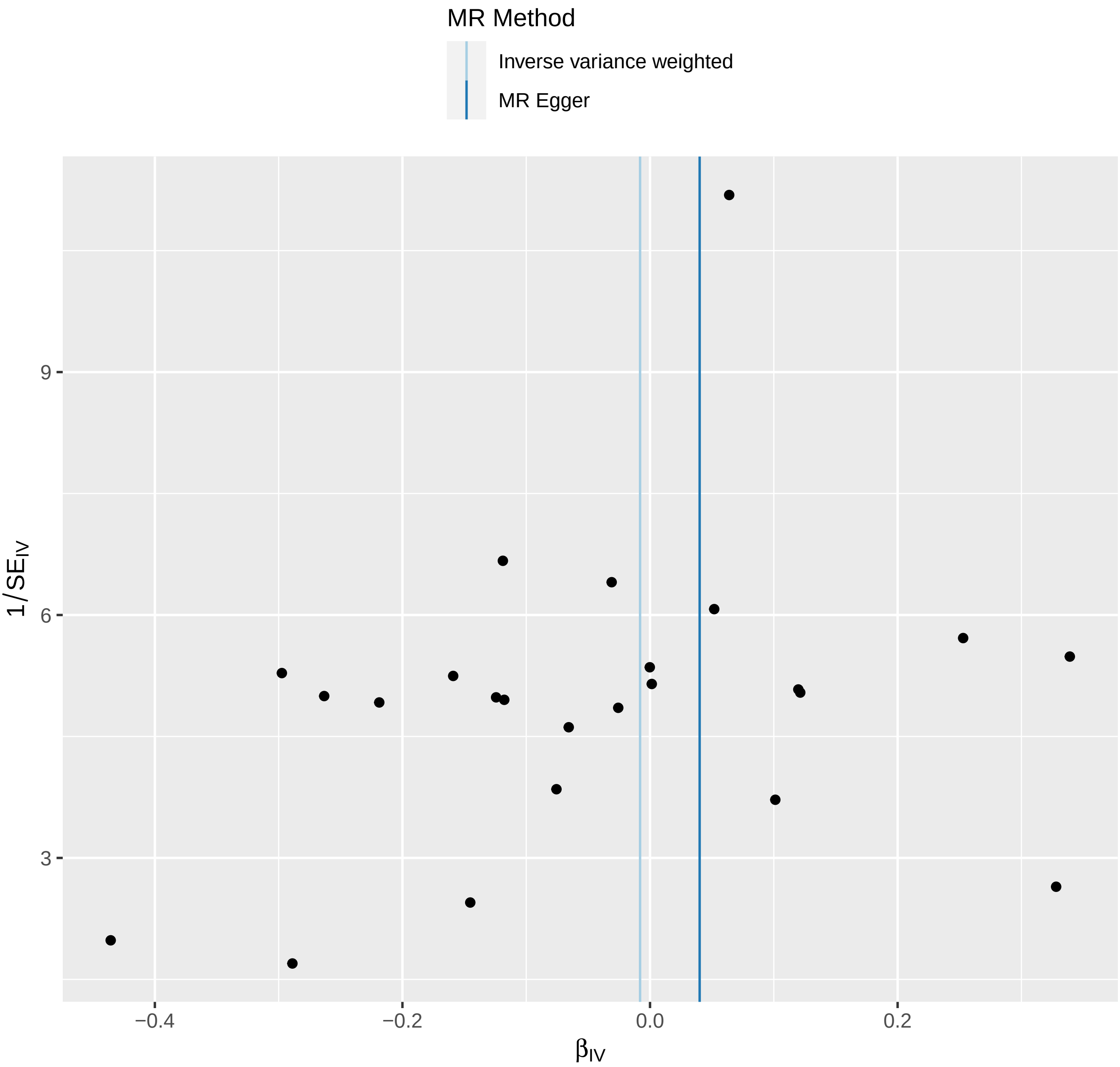

Batch 3−1457 : Immunoglobulin J chain on Diabetic nephropathy (more control exclusions)
with a threshold of 1e−05

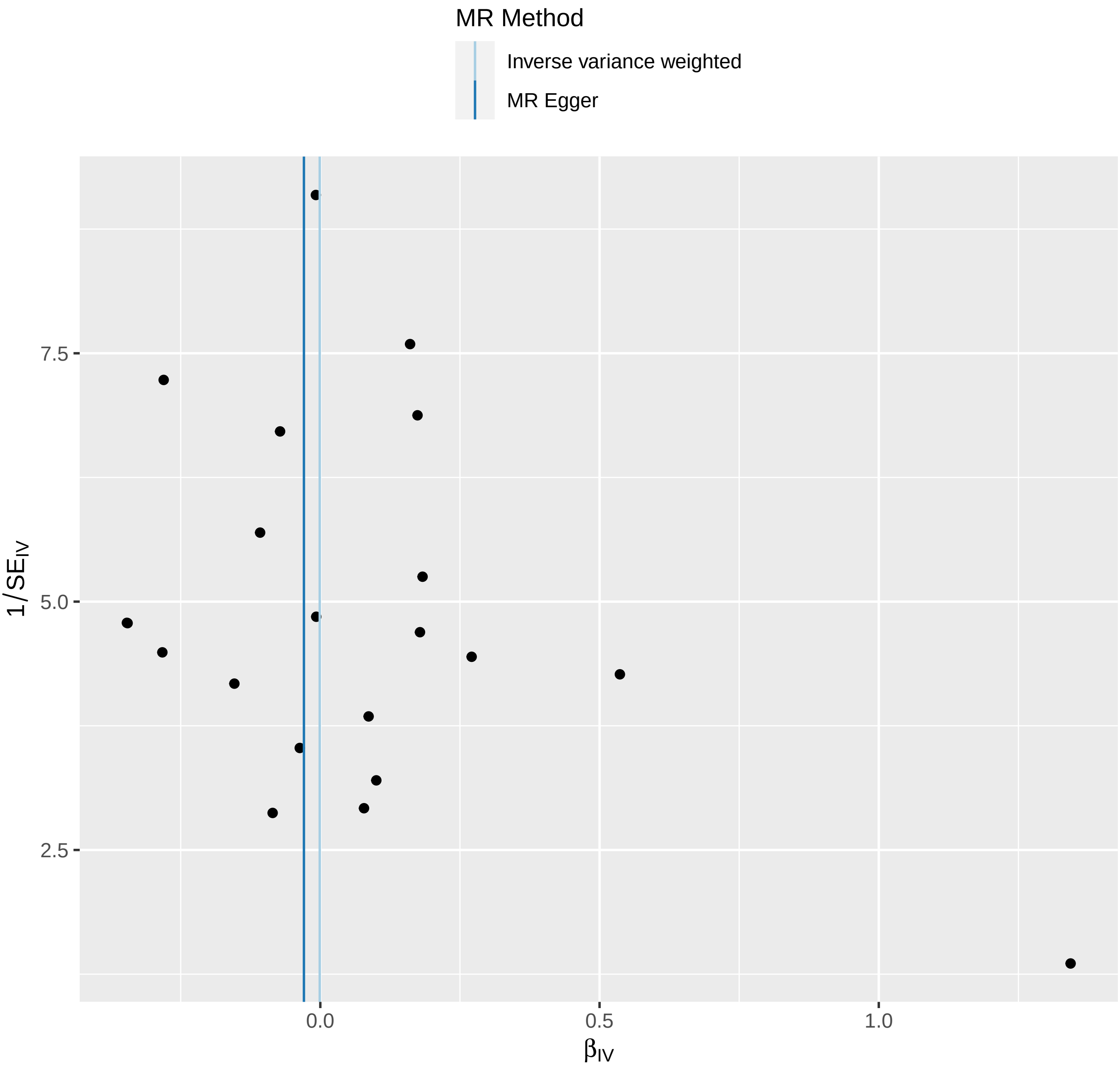

Batch 3−1458 : Immunoglobulin lambda−like polypeptide 1 on Diabetic nephropathy (more control exclusions) with a threshold of 1e−05

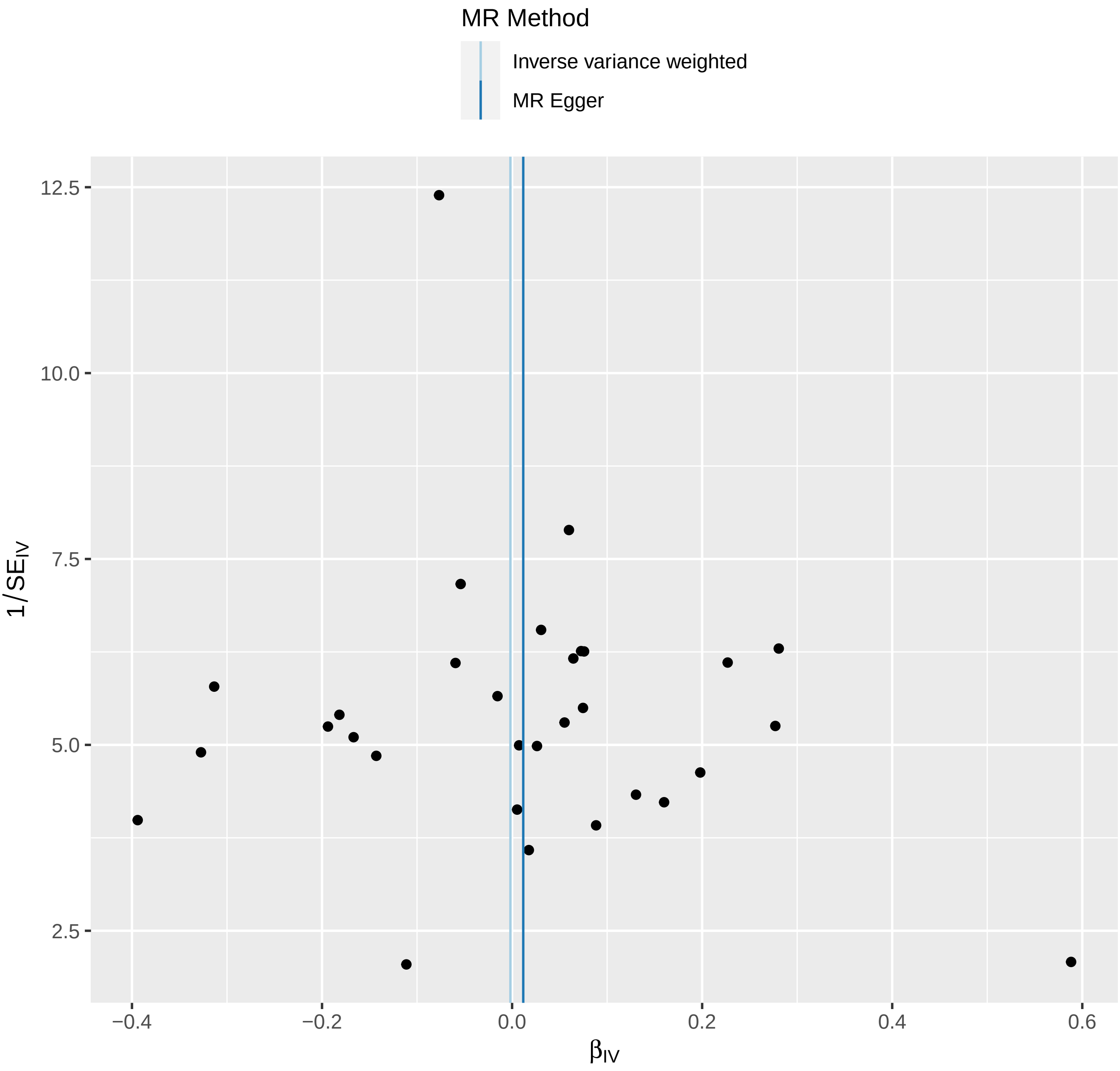

Batch 3−1459 : IgLON family member 5 on Diabetic nephropathy (more control exclusions)
with a threshold of 1e−05

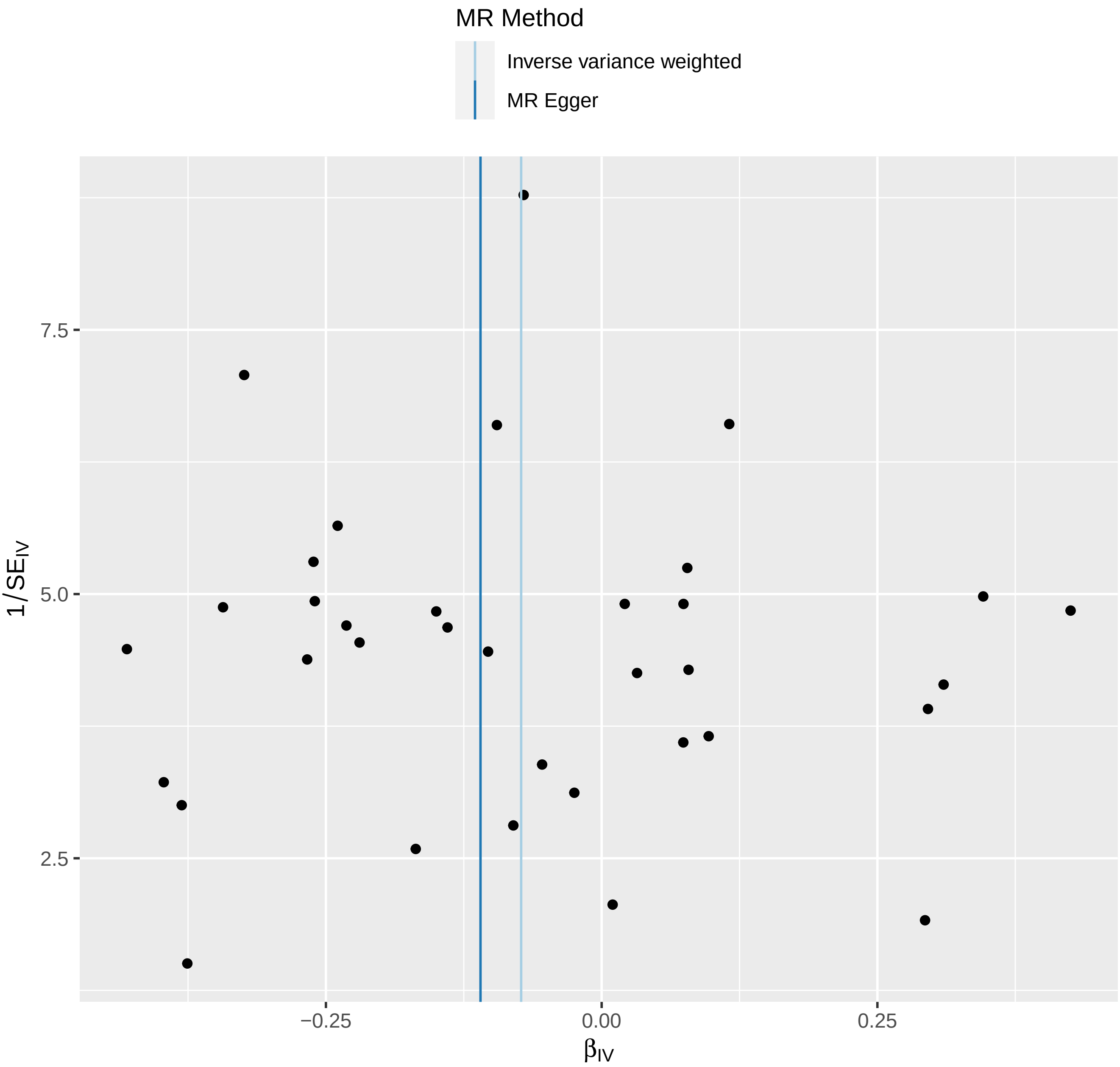

Batch 3−1460 : Immunoglobulin superfamily member 11 on Diabetic nephropathy (more control exclusions)
with a threshold of 1e−05

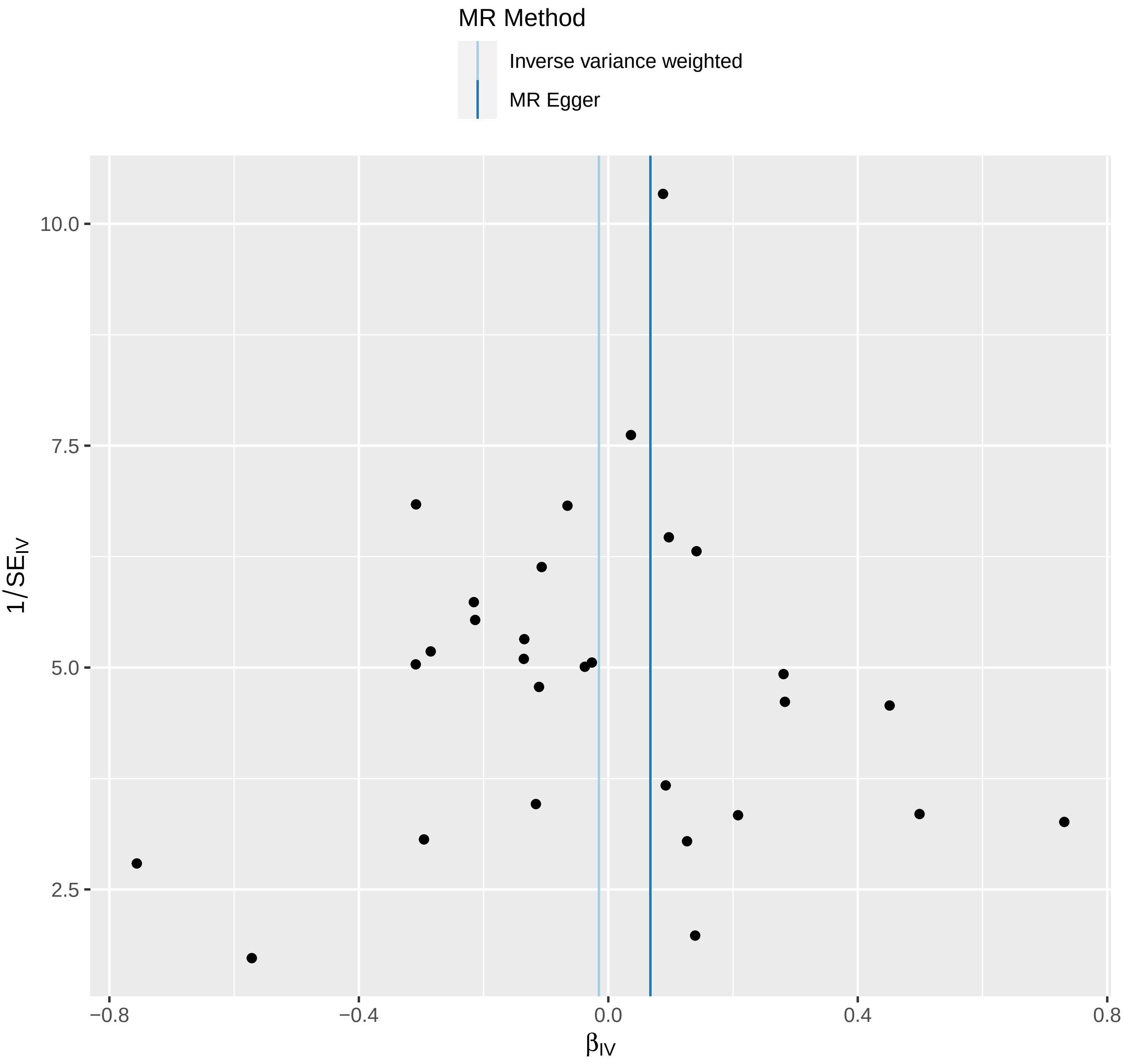

Batch 3−1461 : Immunoglobulin superfamily member 11 on Diabetic nephropathy (more control exclusions)
with a threshold of 1e−05

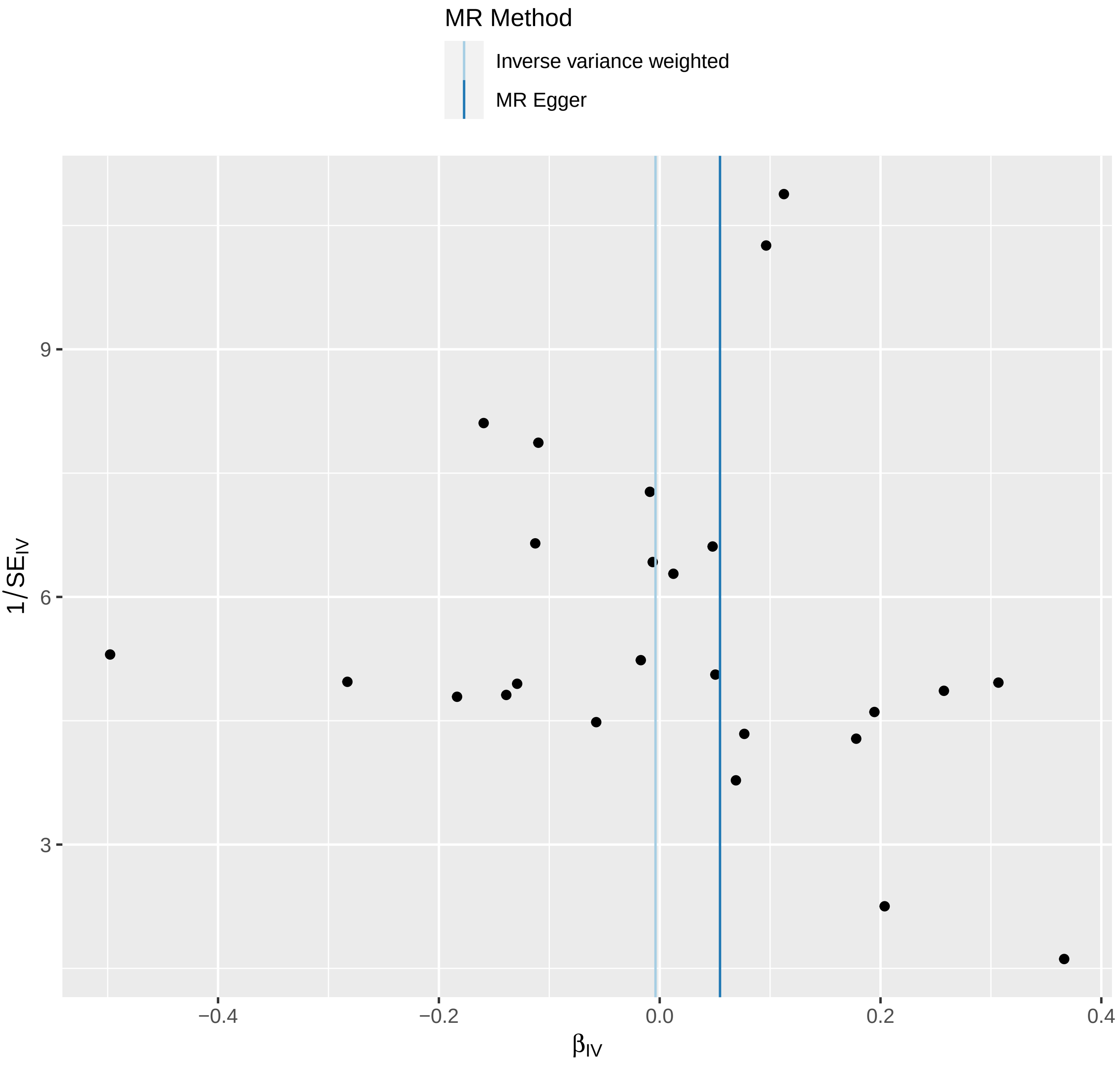

Batch 3−1462 : Immunoglobulin superfamily member 8 on Diabetic nephropathy (more control exclusions)
with a threshold of 1e−05

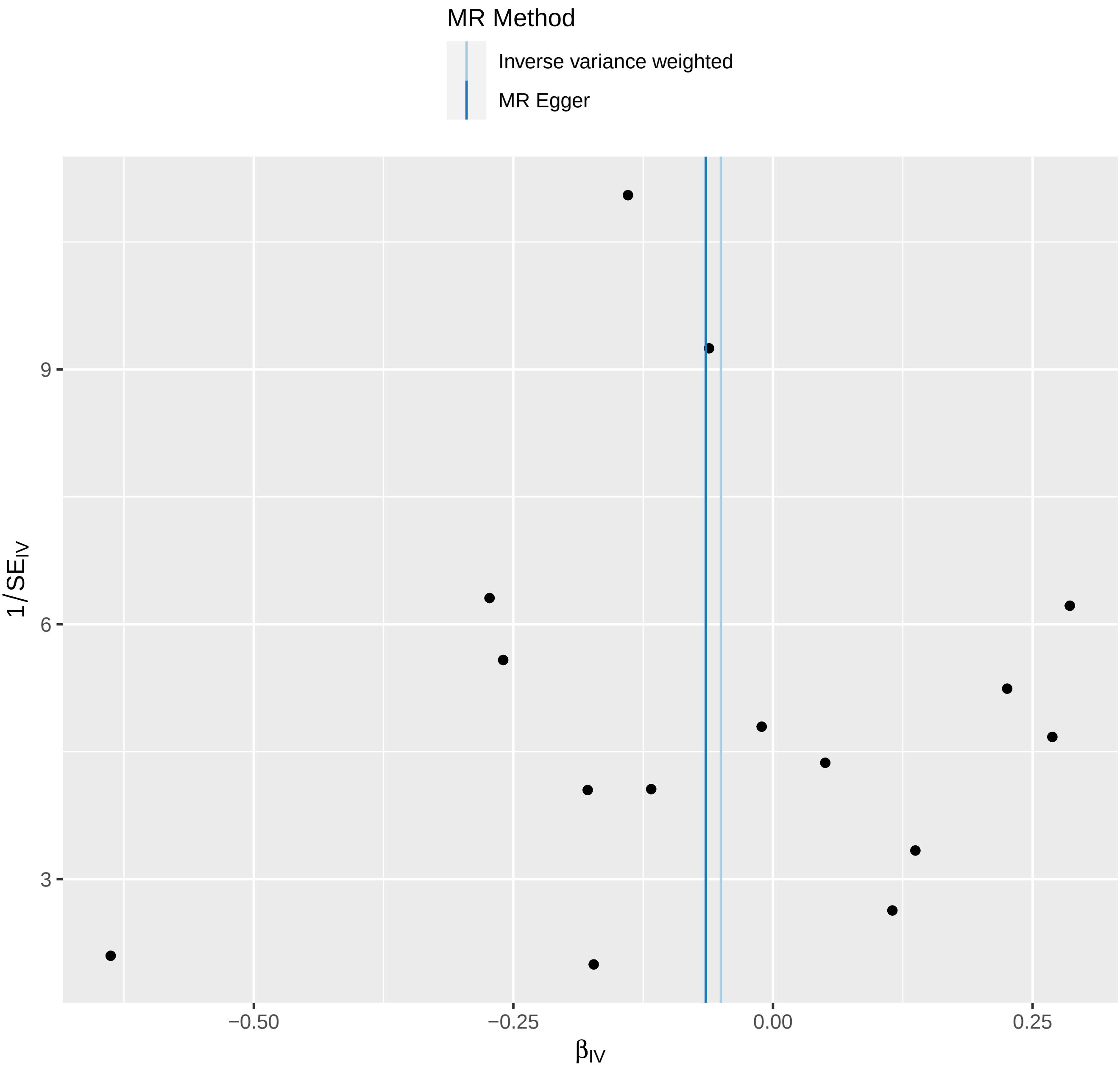

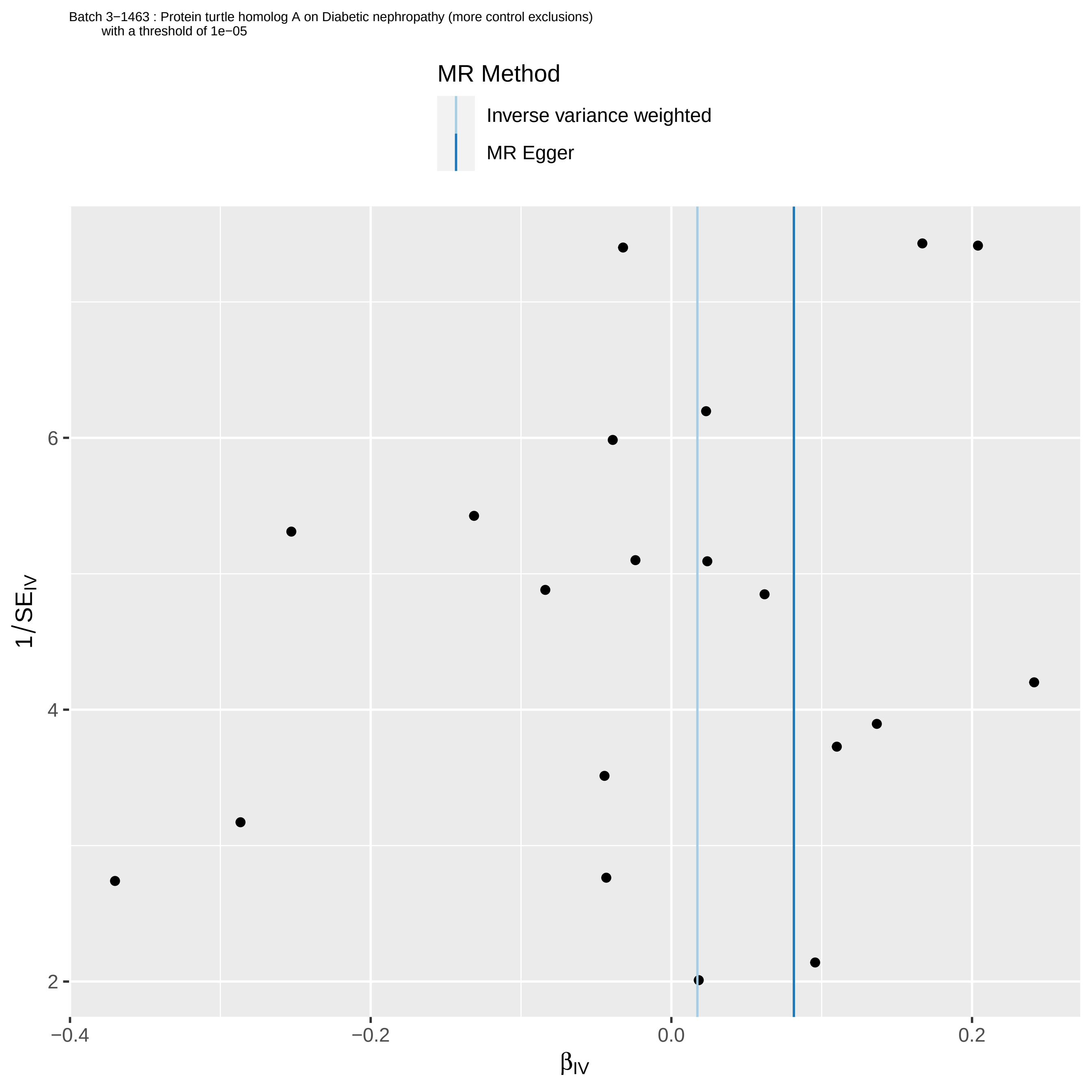

Batch 3−1463 : Protein turtle homolog A on Diabetic nephropathy (more control exclusions)
with a threshold of 1e−05
MR Method
Inverse variance weighted
MR Egger
6
4
2
1/SE_IV
−0.4
−0.2
0.0
0.2
β_IV

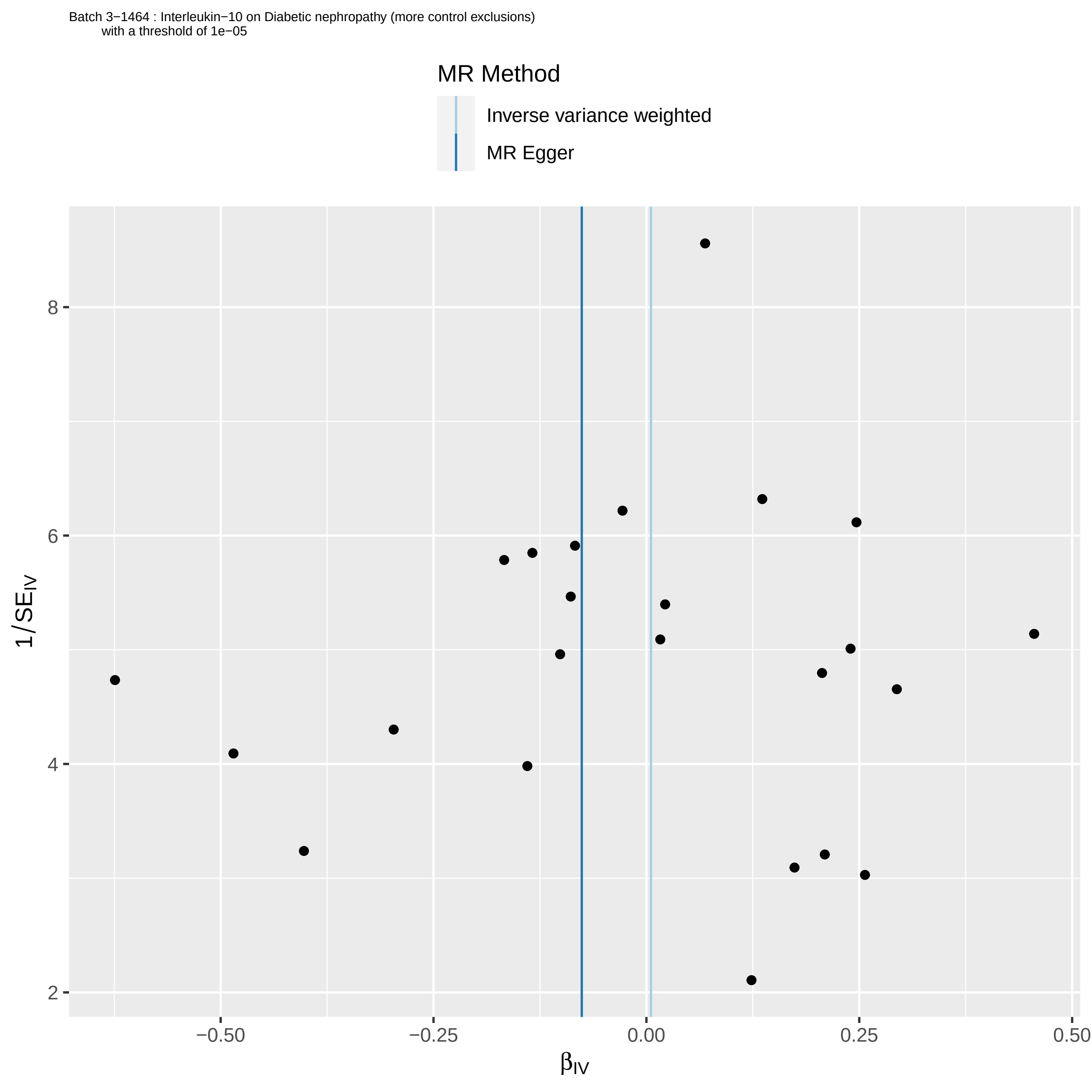
Batch 3−1464 : Interleukin−10 on Diabetic nephropathy (more control exclusions)
with a threshold of 1e−05
MR Method
Inverse variance weighted
MR Egger
8
6
4
2
−0.50
−0.25
0.00
0.25
0.50
$1/SE_{IV}$
β_{IV}

Batch 3−1465 : Interleukin−10 receptor subunit beta on Diabetic nephropathy (more control exclusions)
with a threshold of 1e−05

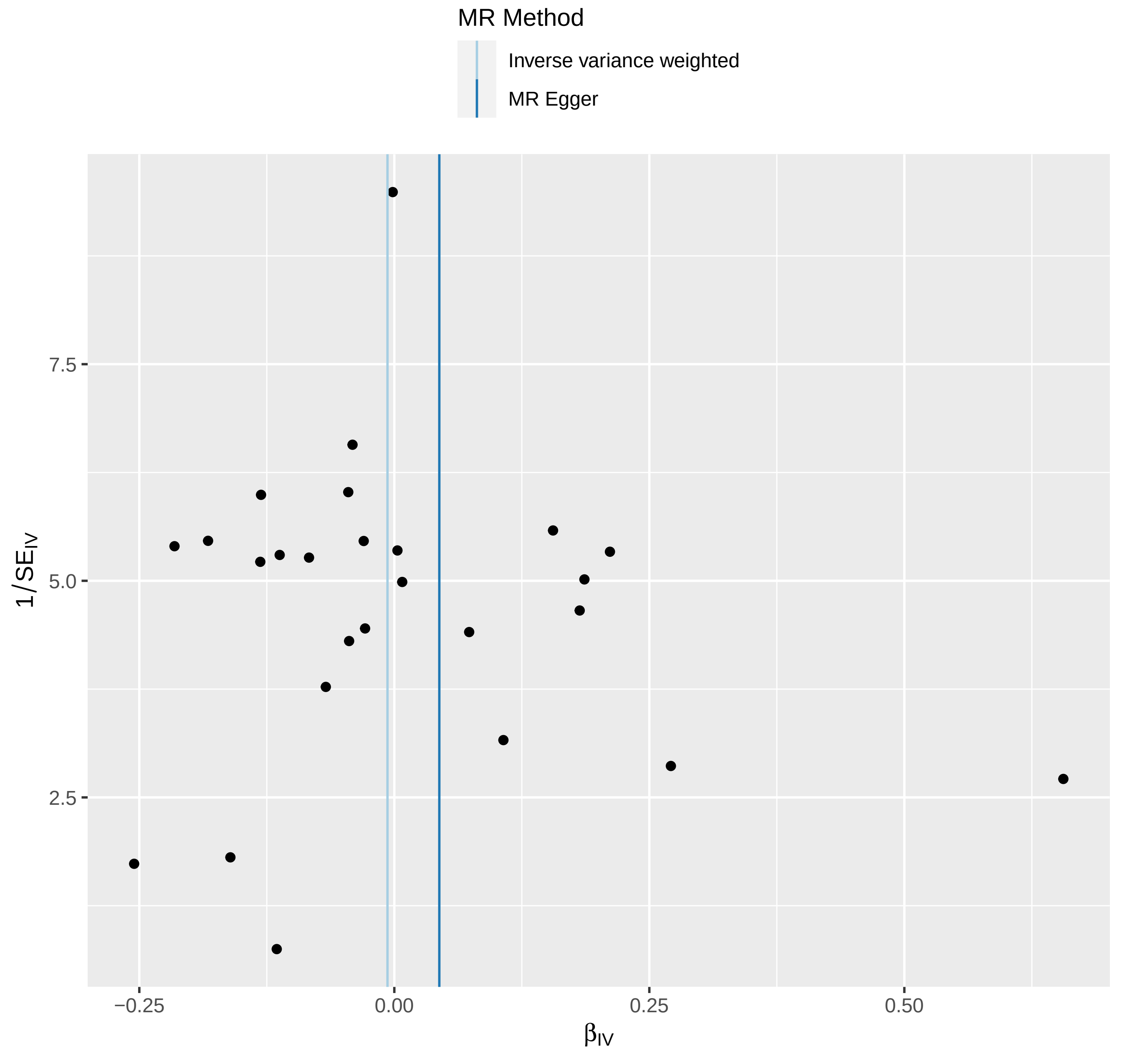

Batch 3−1466 : Interleukin−11 on Diabetic nephropathy (more control exclusions)
with a threshold of 1e−05

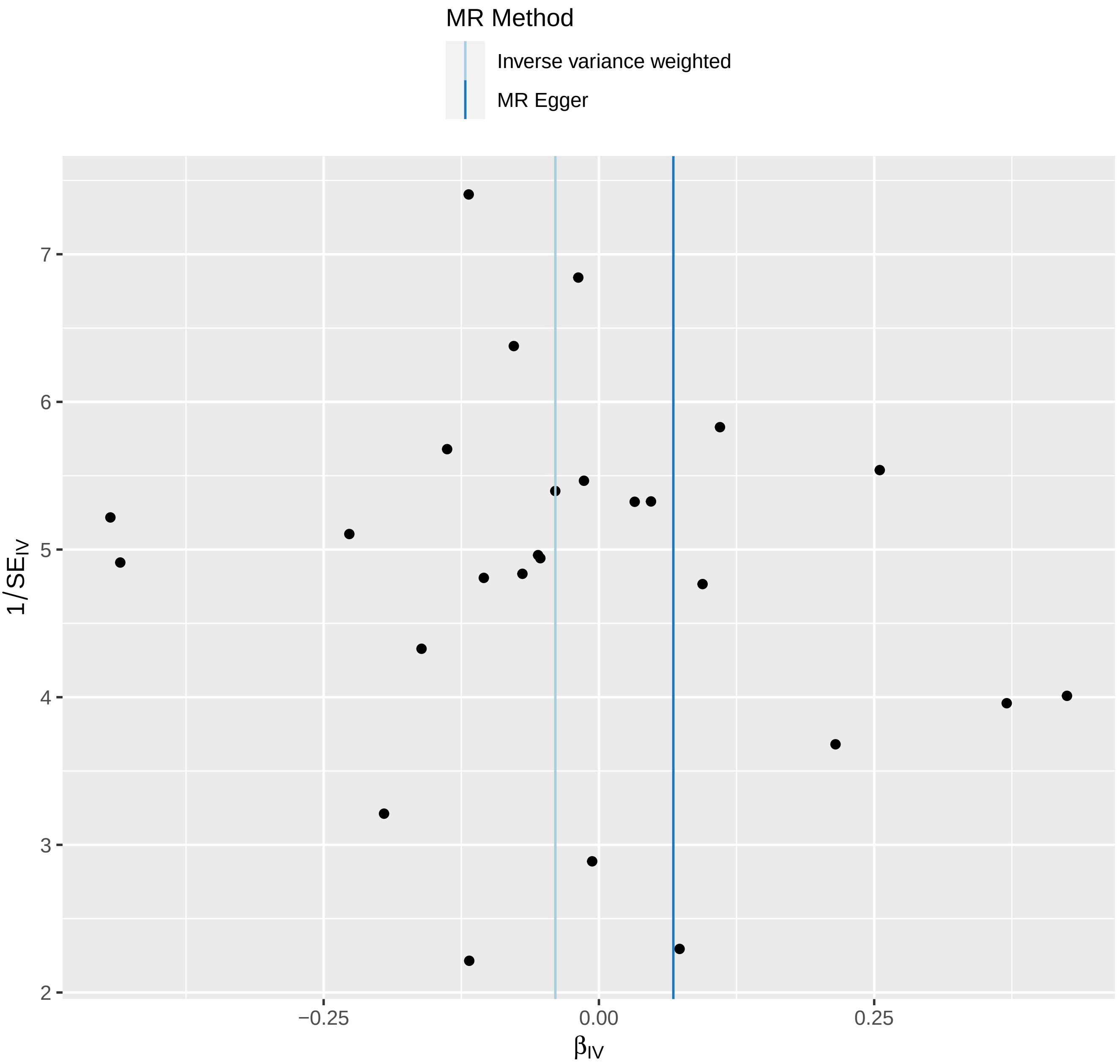

Batch 3−1467 : Interleukin−11 receptor subunit alpha on Diabetic nephropathy (more control exclusions)
with a threshold of 1e−05

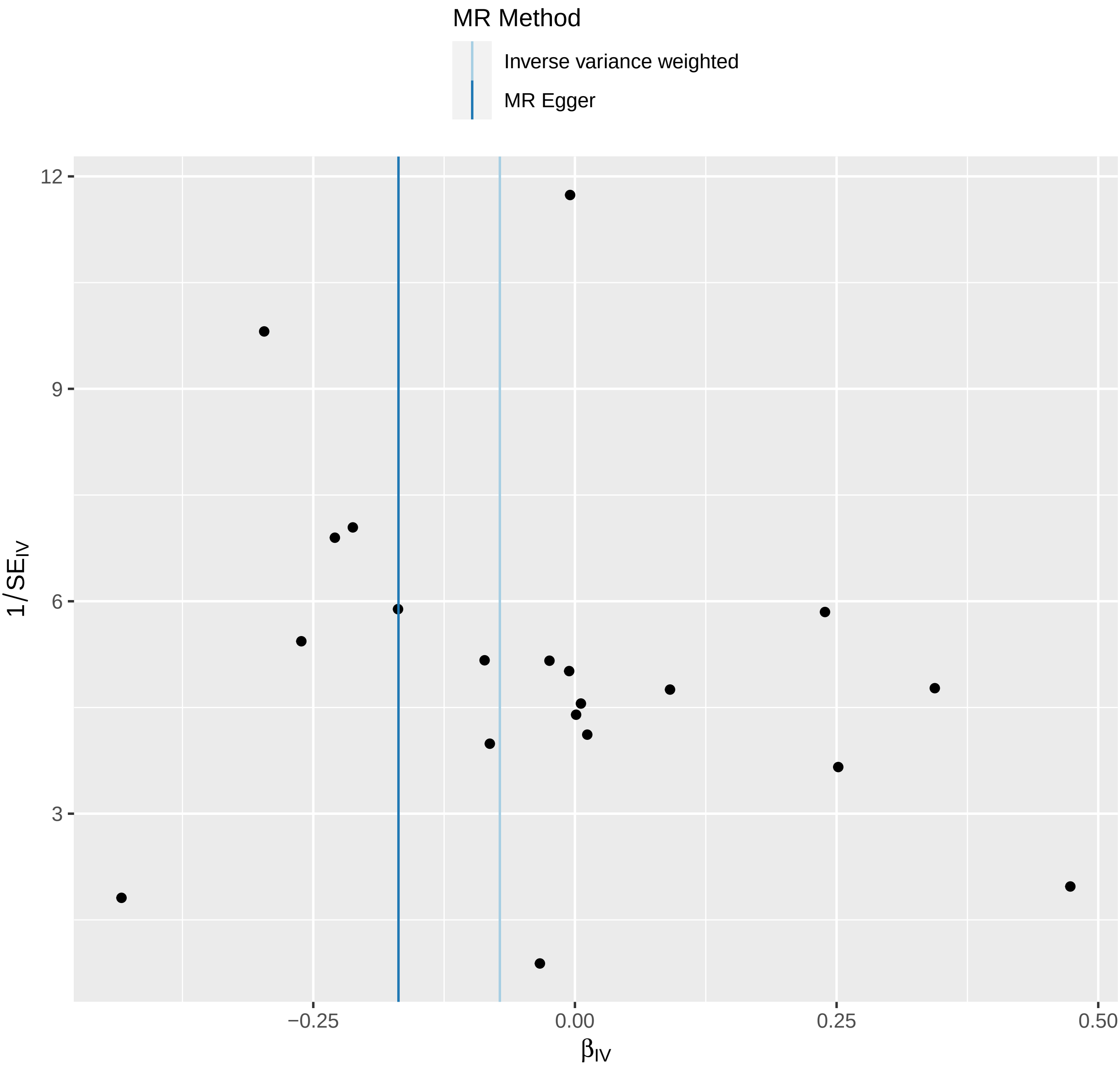

Batch 3−1468 : Interleukin−12 on Diabetic nephropathy (more control exclusions)
with a threshold of 1e−05

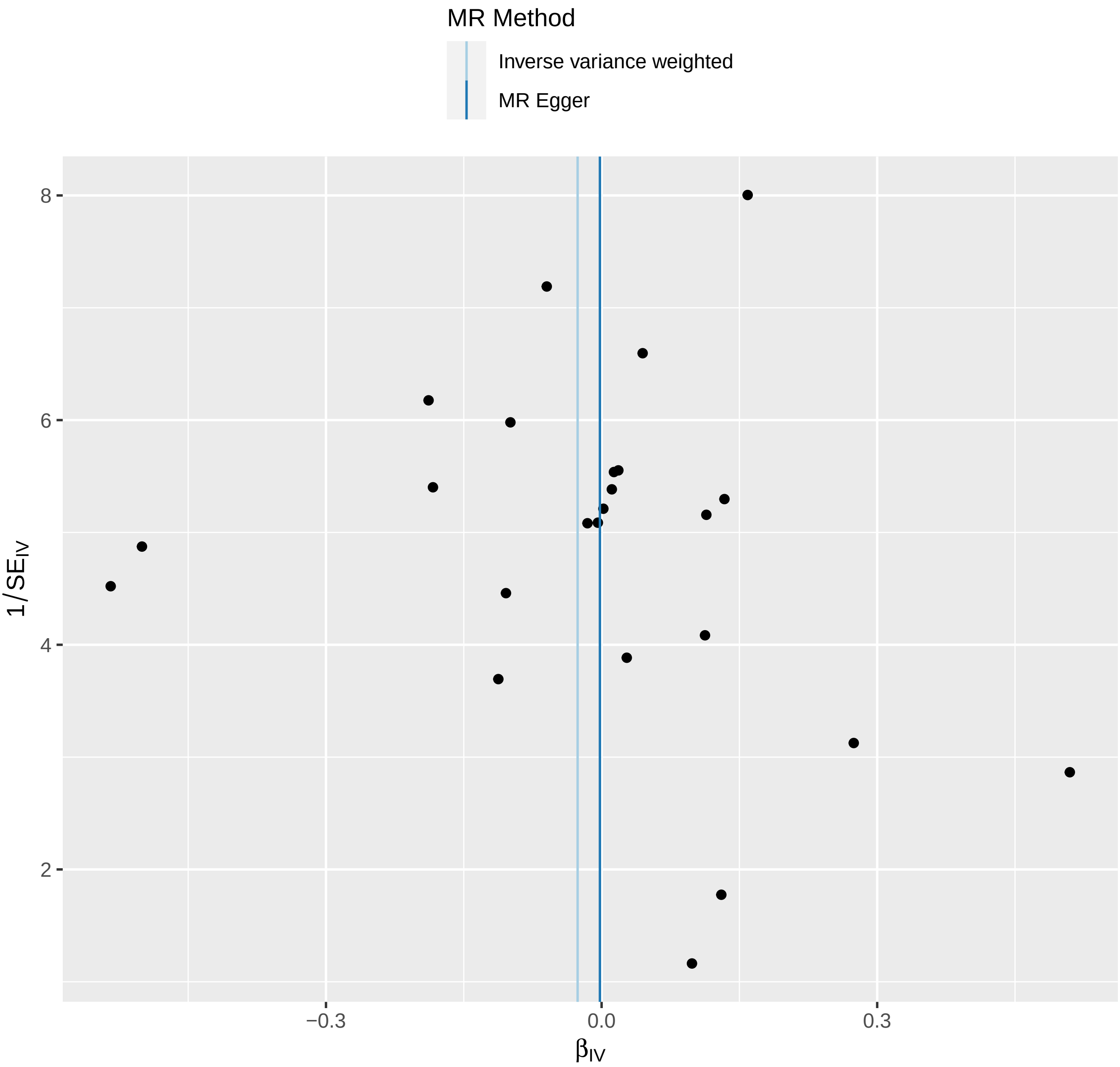

Batch 3−1469 : Interleukin−12 on Diabetic nephropathy (more control exclusions)
with a threshold of 1e−05

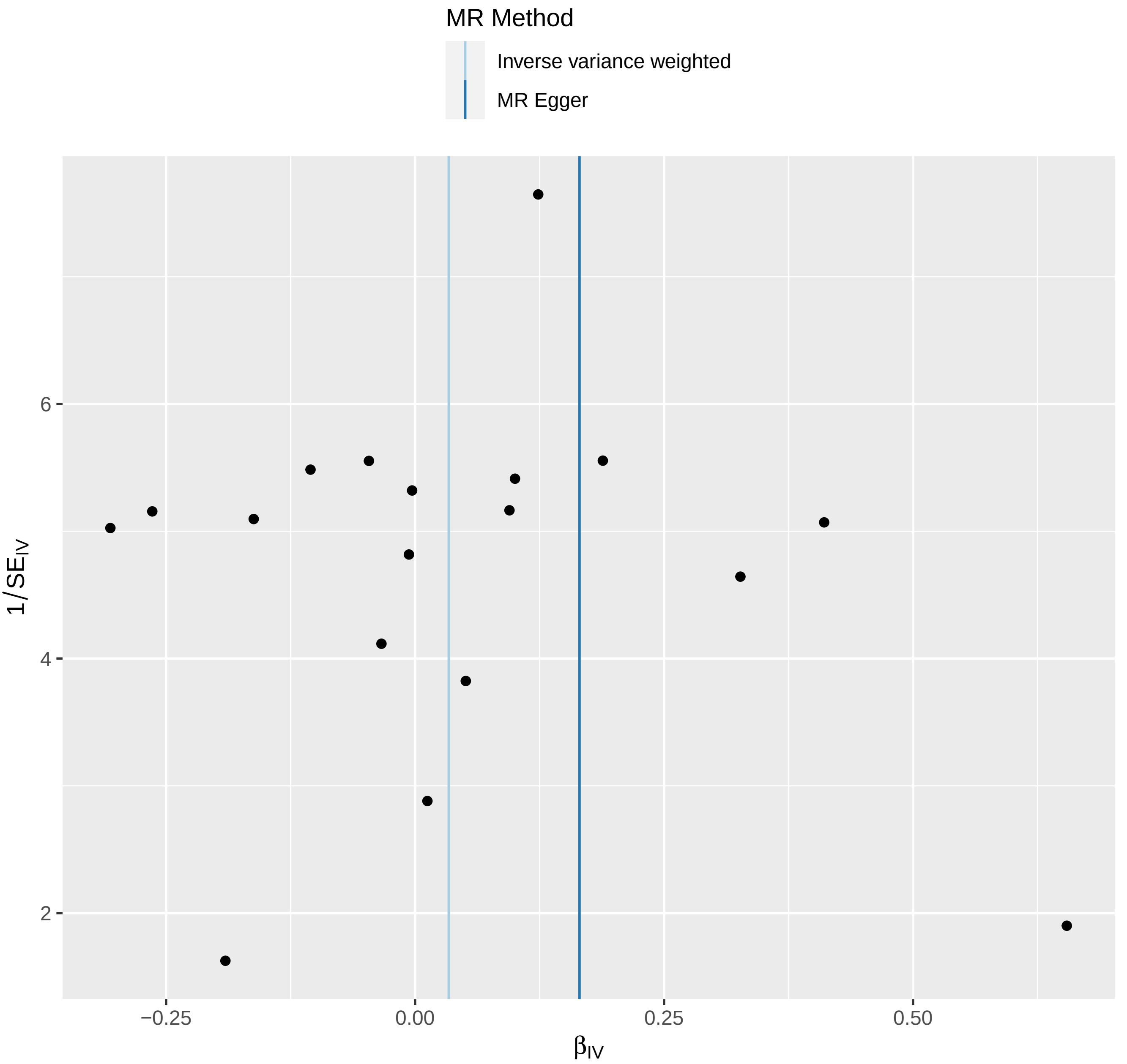

Batch 3−1470 : Interleukin−12 on Diabetic nephropathy (more control exclusions)
with a threshold of 1e−05

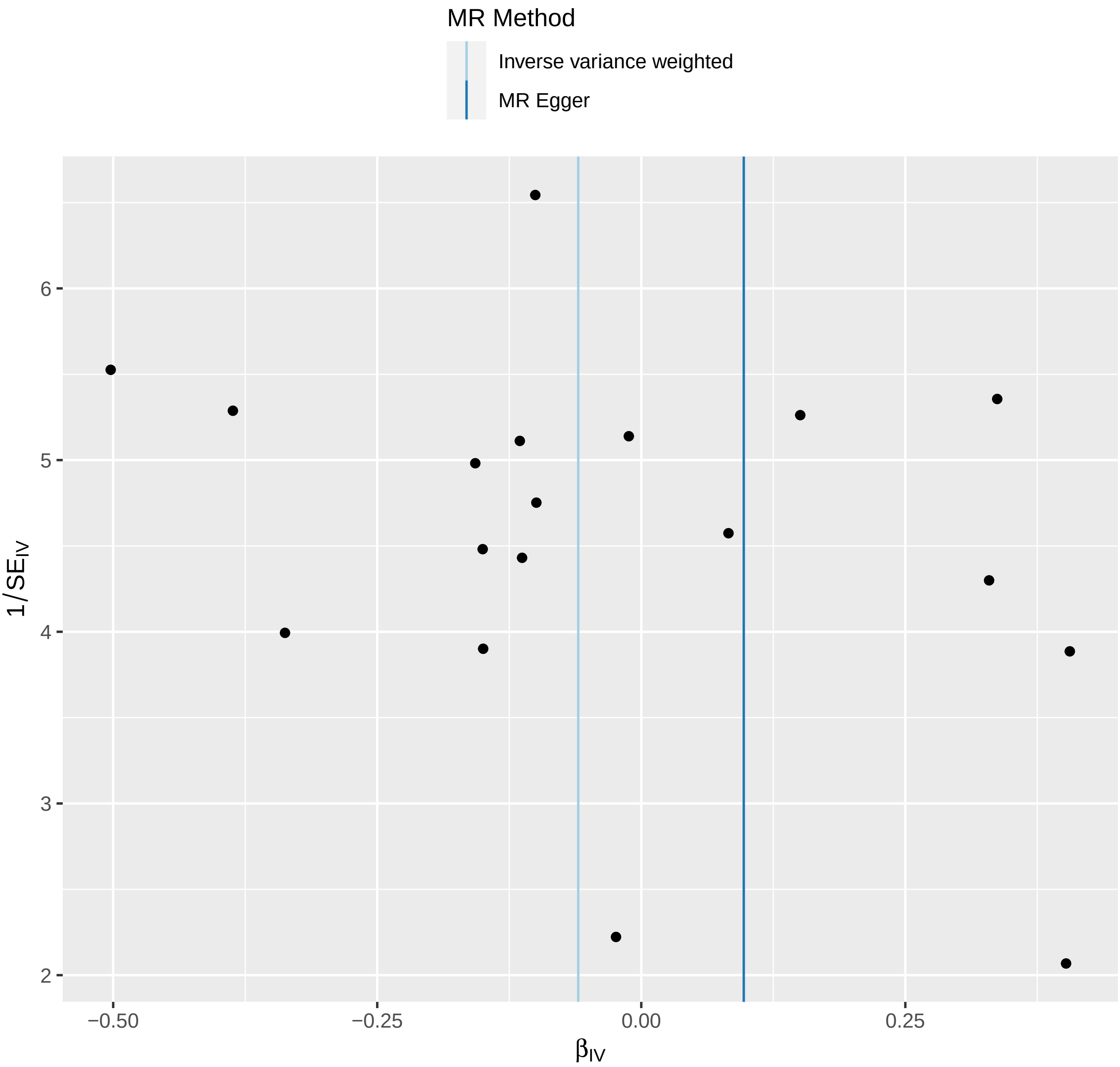

Batch 3−1471 : Interleukin−23 on Diabetic nephropathy (more control exclusions)
with a threshold of 1e−05

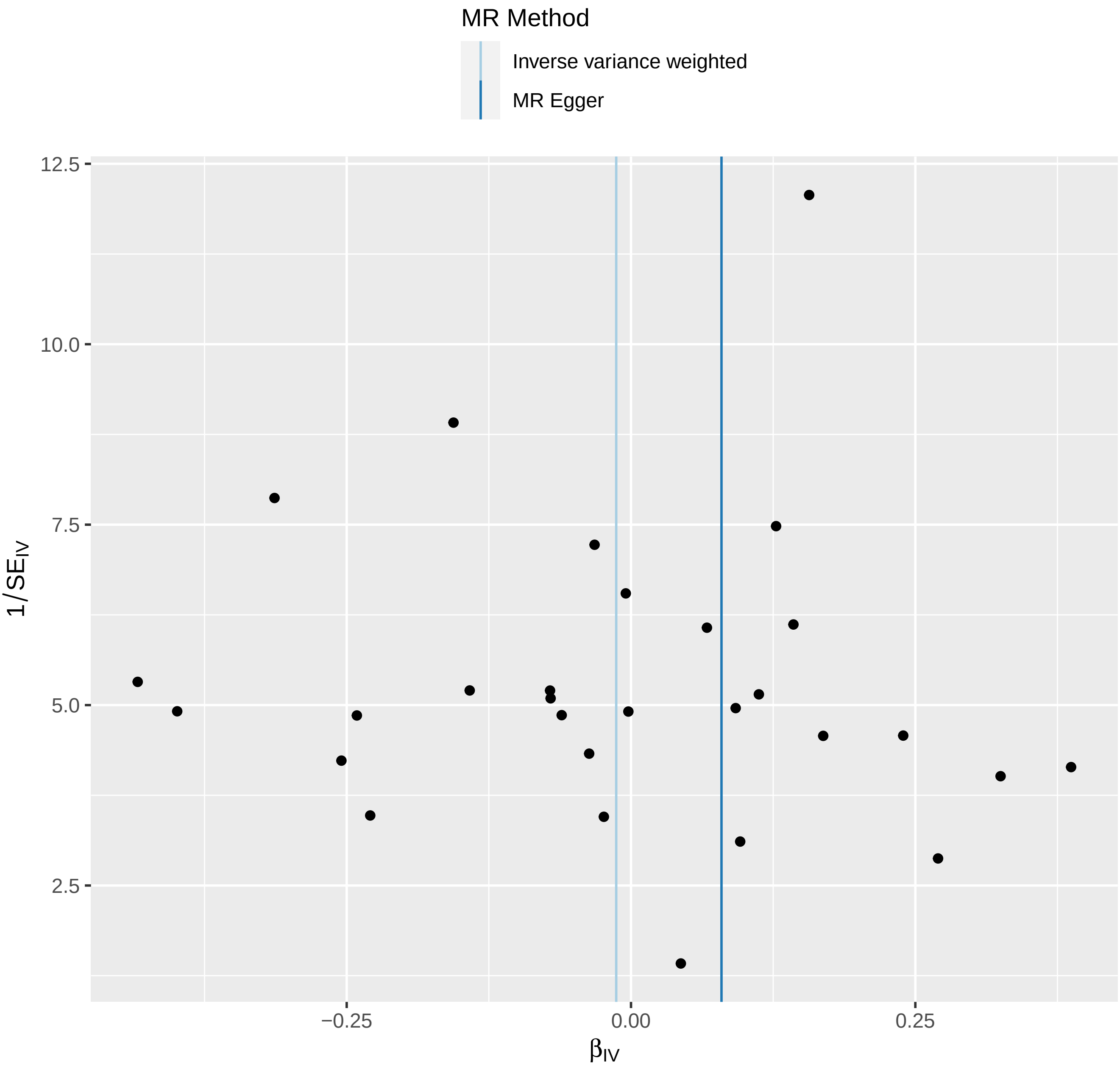

Batch 3−1472 : Interleukin−23 on Diabetic nephropathy (more control exclusions)
with a threshold of 1e−05

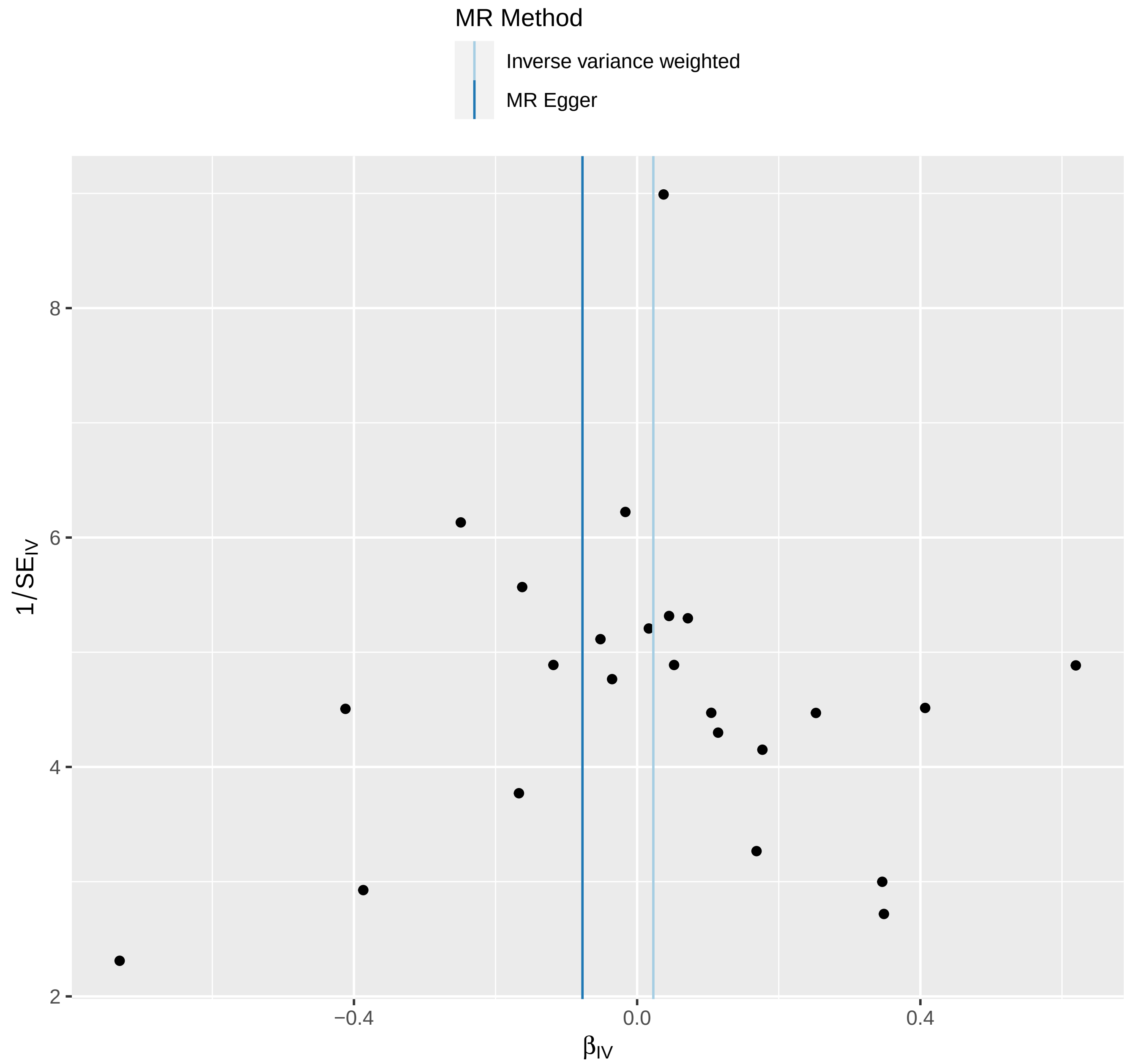

Batch 3−1473 : Interleukin−12 receptor subunit beta−1 on Diabetic nephropathy (more control exclusions)
with a threshold of 1e−05

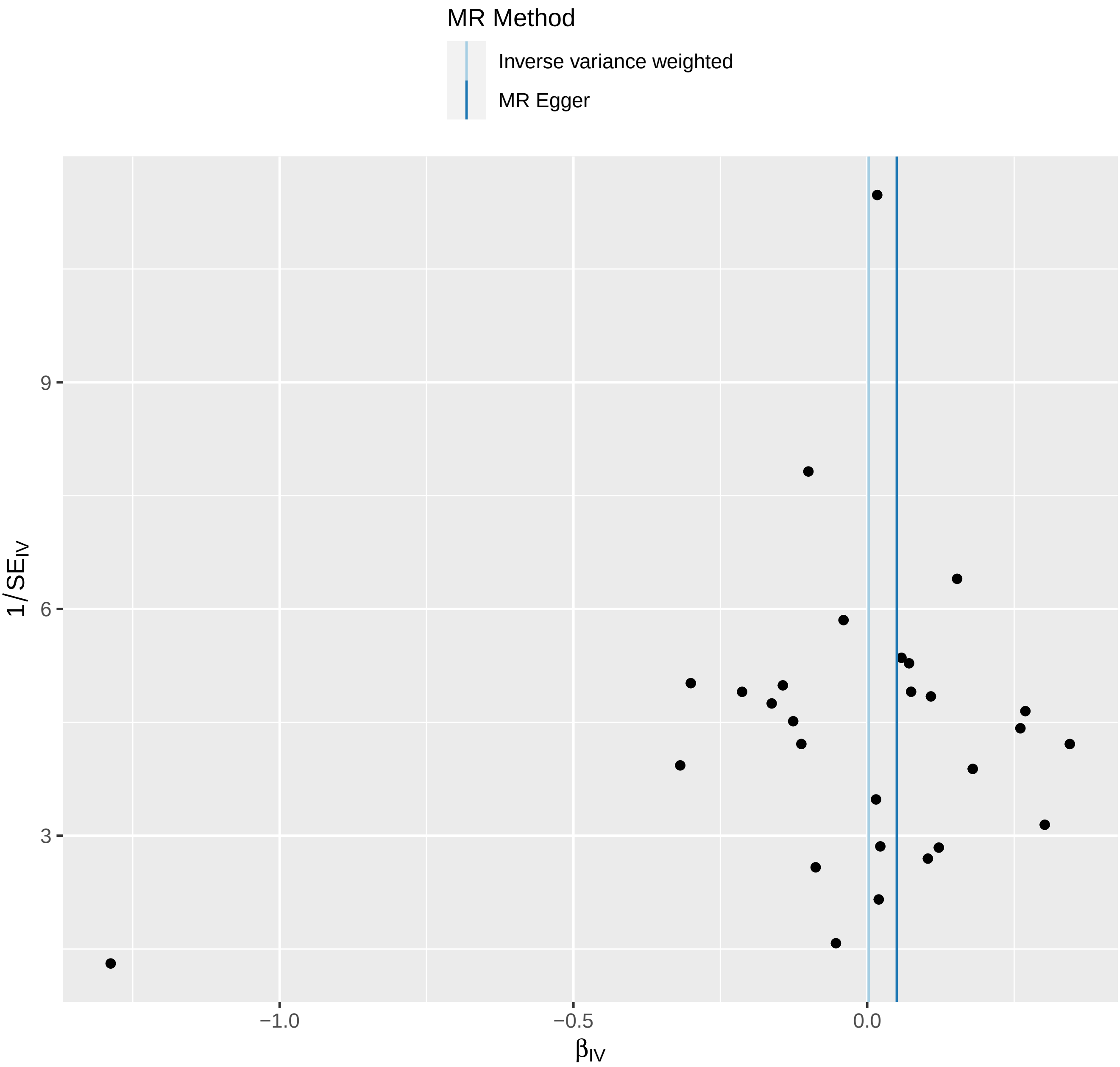

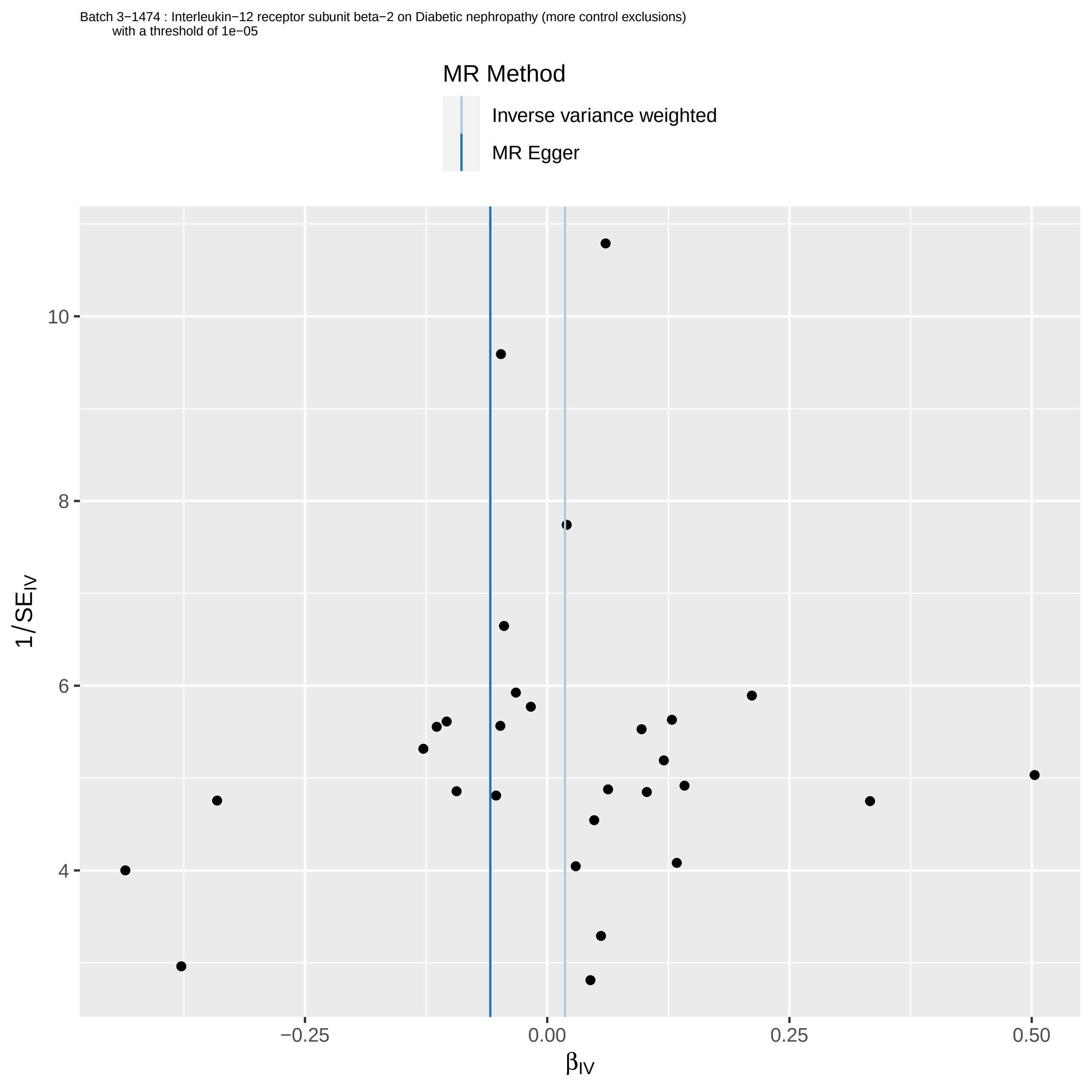
Batch 3−1474 : Interleukin−12 receptor subunit beta−2 on Diabetic nephropathy (more control exclusions)
with a threshold of 1e−05
MR Method
Inverse variance weighted
MR Egger
1/SE_IV
10
8
6
4
−0.25
0.00
0.25
0.50
β_IV

Batch 3−1475 : Interleukin−13 on Diabetic nephropathy (more control exclusions)
with a threshold of 1e−05

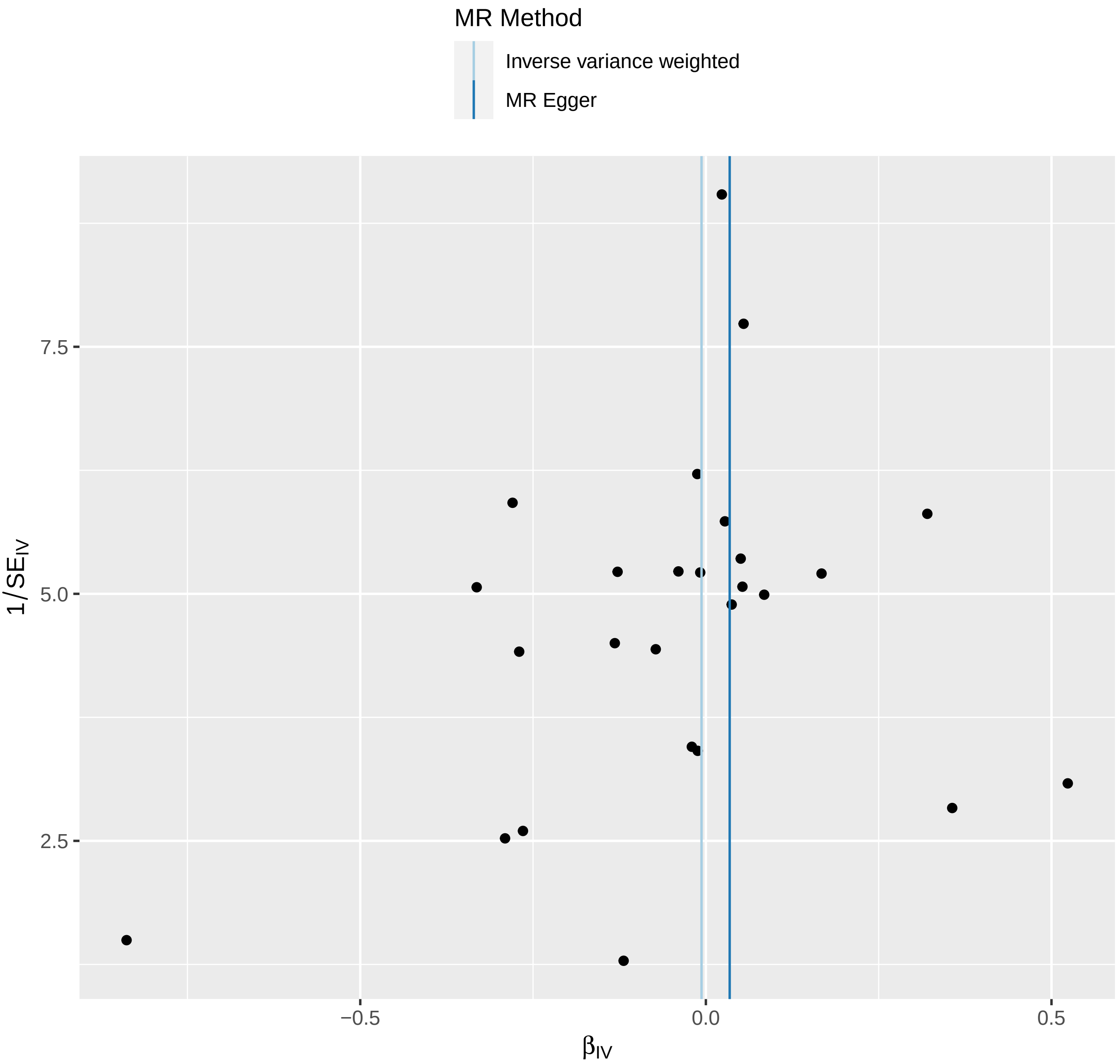

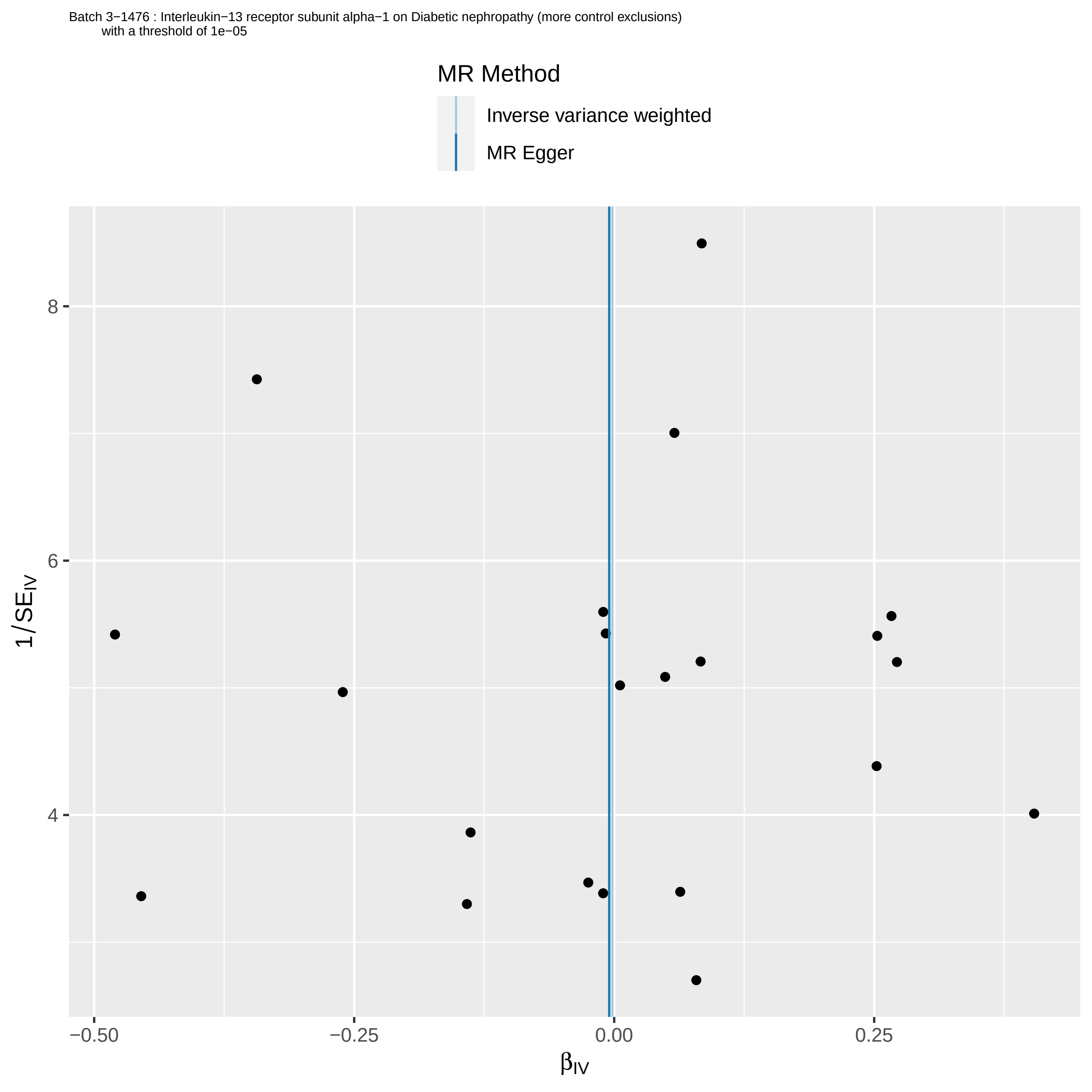
Batch 3−1476 : Interleukin−13 receptor subunit alpha−1 on Diabetic nephropathy (more control exclusions)
with a threshold of 1e−05
MR Method
Inverse variance weighted
MR Egger
8
6
4
1/SE_IV
−0.50
−0.25
0.00
0.25
β_IV

Batch 3−1477 : Interleukin−15 receptor subunit alpha on Diabetic nephropathy (more control exclusions)
with a threshold of 1e−05

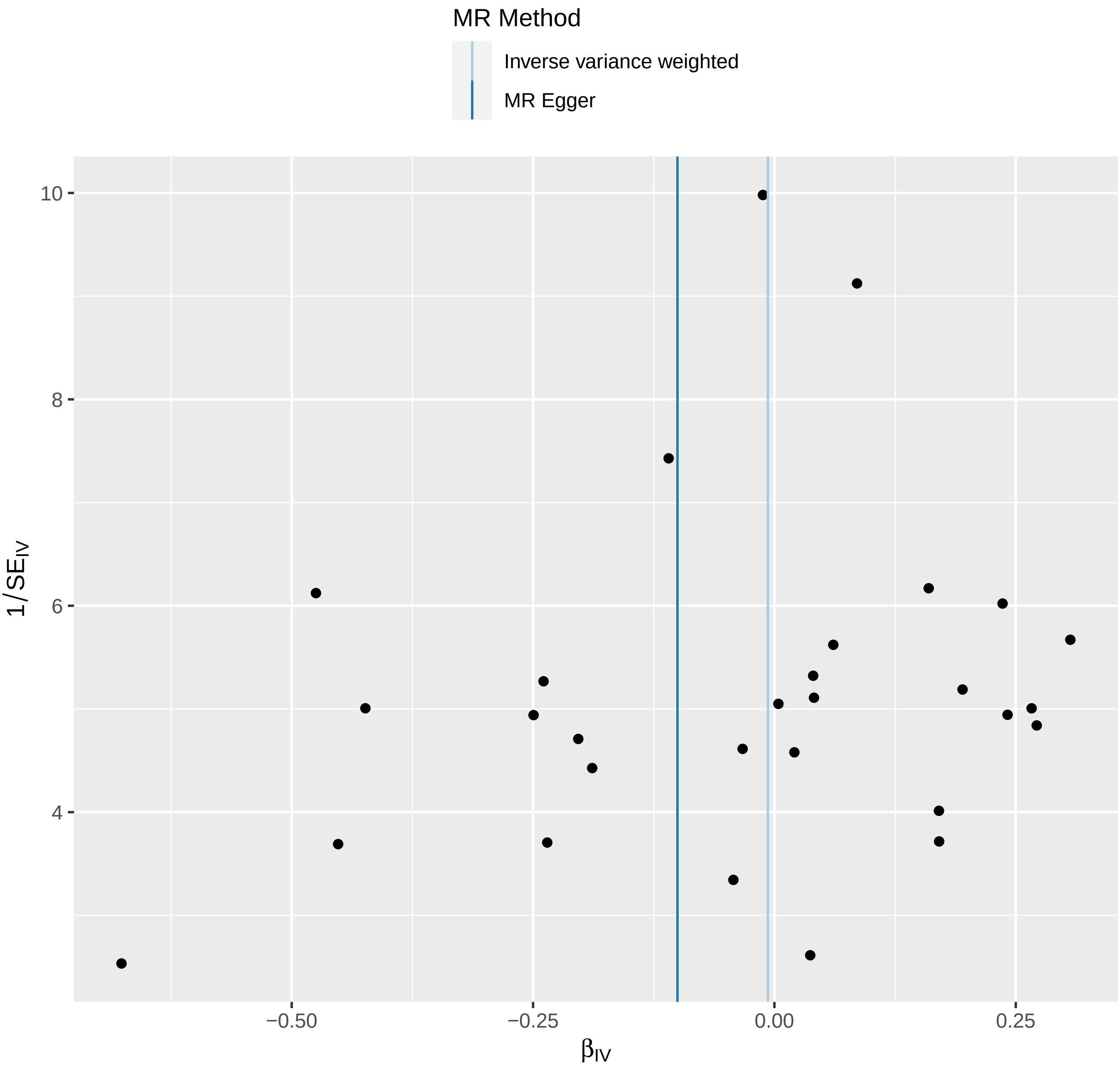

Batch 3−1478 : Interleukin−15 receptor subunit alpha on Diabetic nephropathy (more control exclusions)
with a threshold of 1e−05

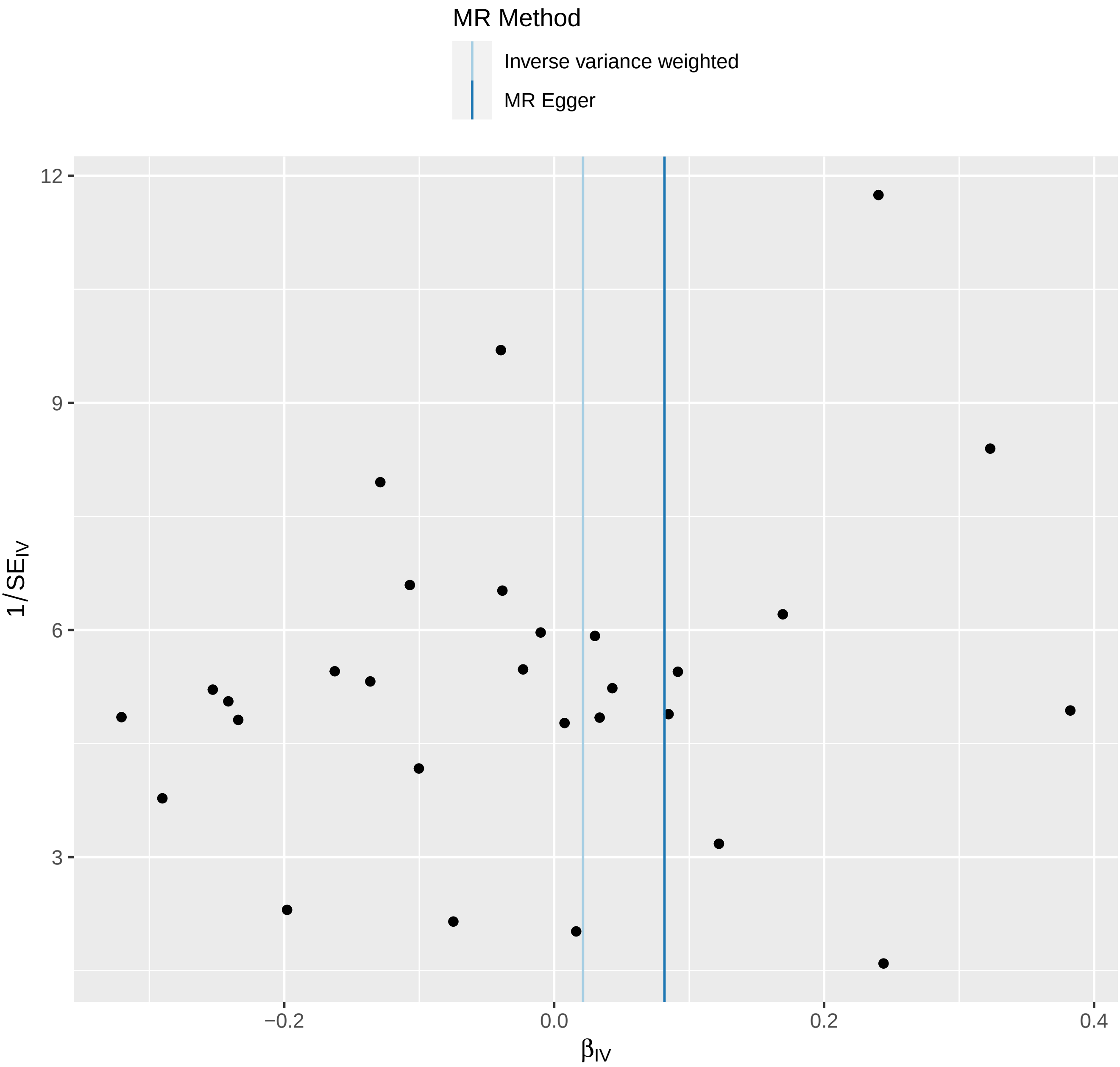

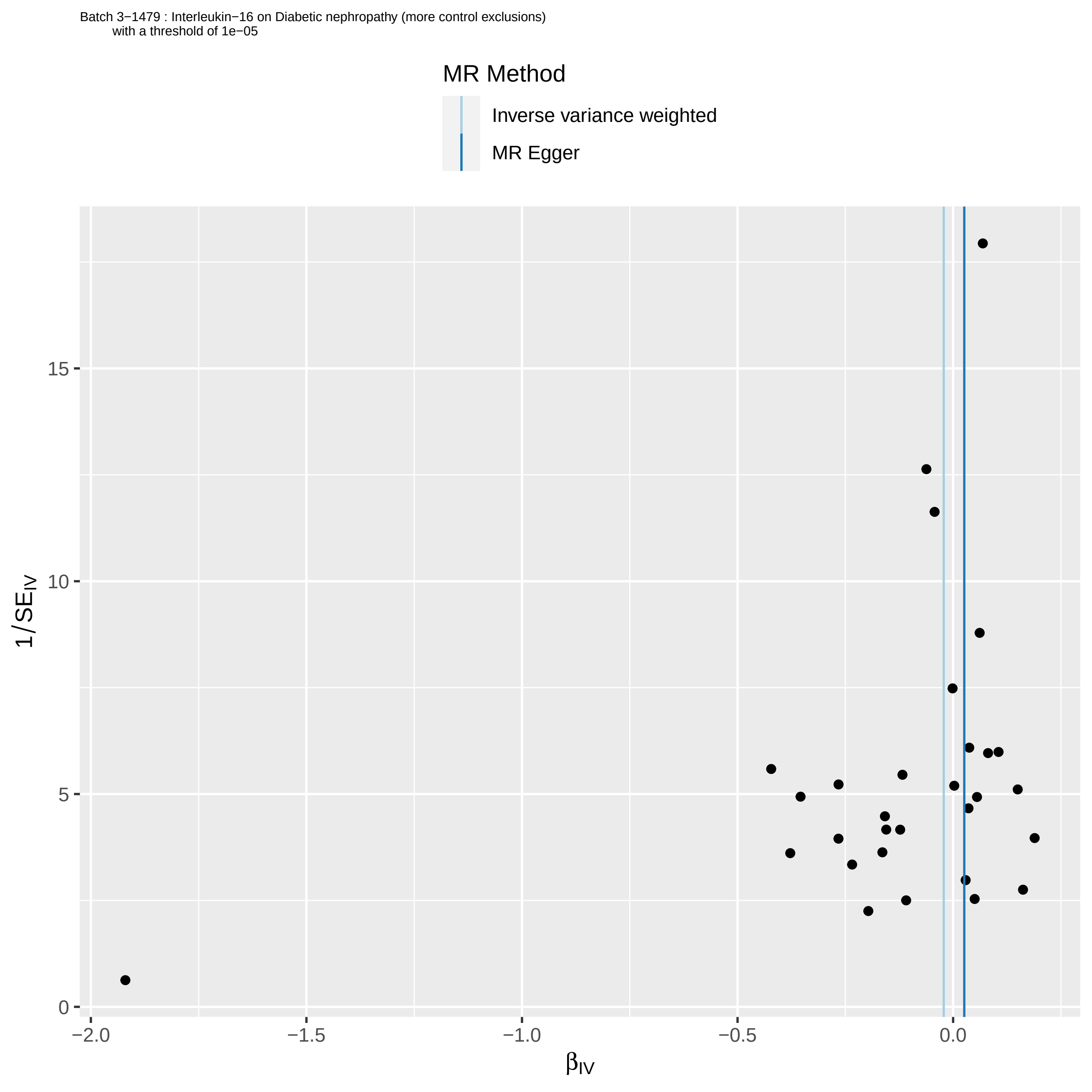
Batch 3−1479 : Interleukin−16 on Diabetic nephropathy (more control exclusions)
with a threshold of 1e−05
MR Method
Inverse variance weighted
MR Egger
15
10
5
0
1/SE_IV
−2.0
−1.5
−1.0
−0.5
0.0
β_IV

Batch 3−1480 : Interleukin−17A on Diabetic nephropathy (more control exclusions)
with a threshold of 1e−05

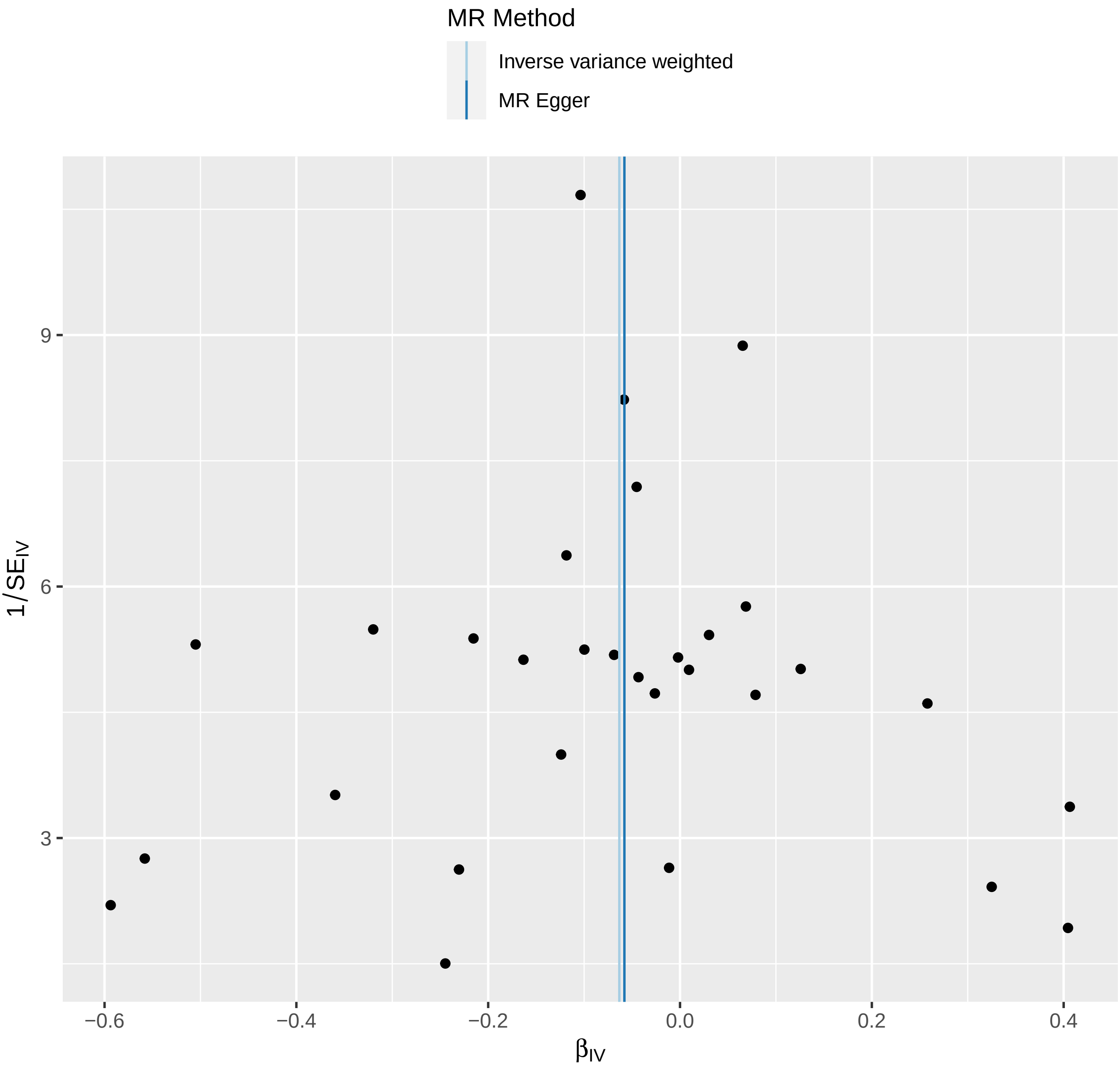

Batch 3−1481 : Interleukin−17A on Diabetic nephropathy (more control exclusions)
with a threshold of 1e−05

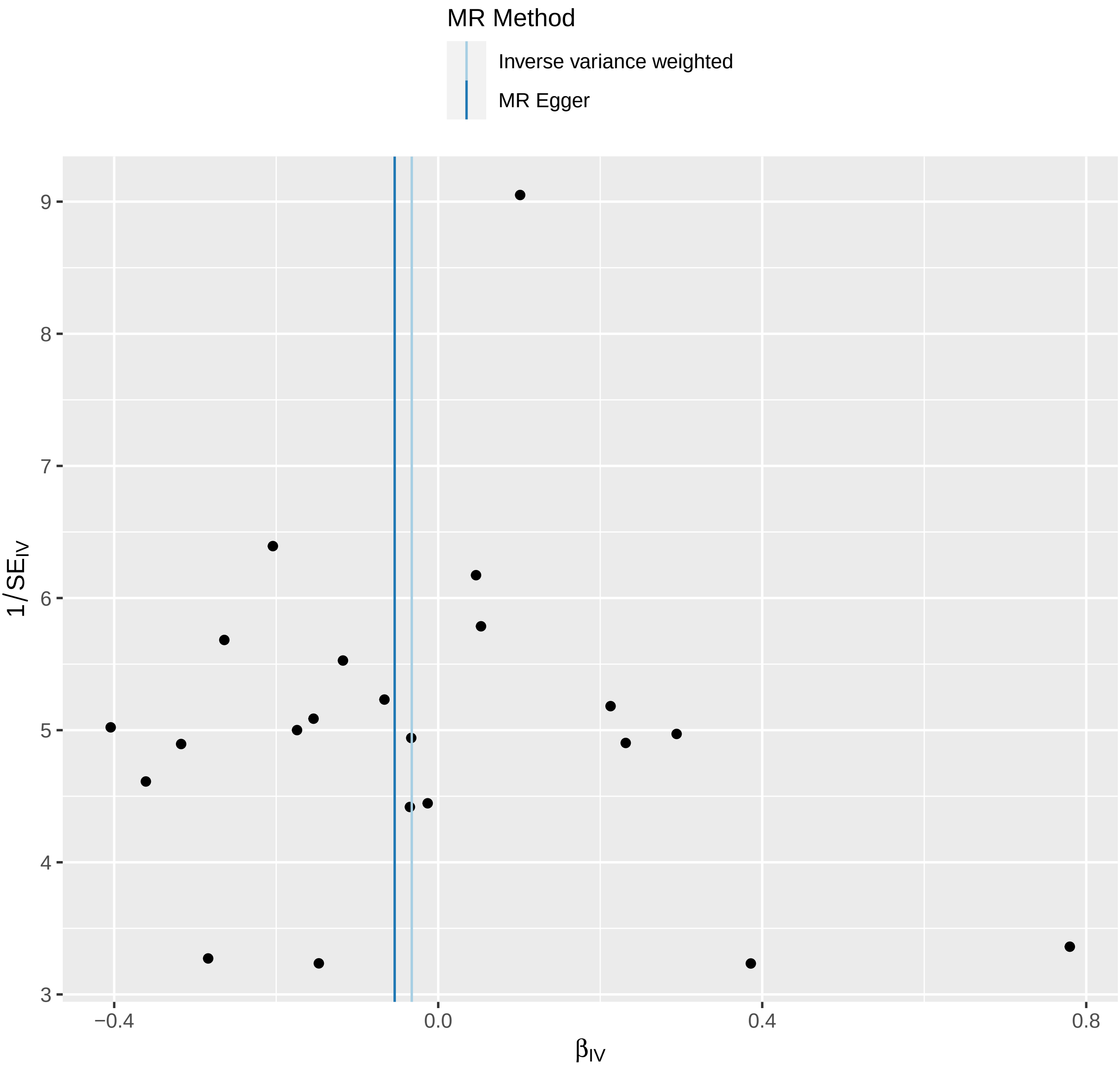

Batch 3−1482 : Interleukin−17A on Diabetic nephropathy (more control exclusions)
with a threshold of 1e−05

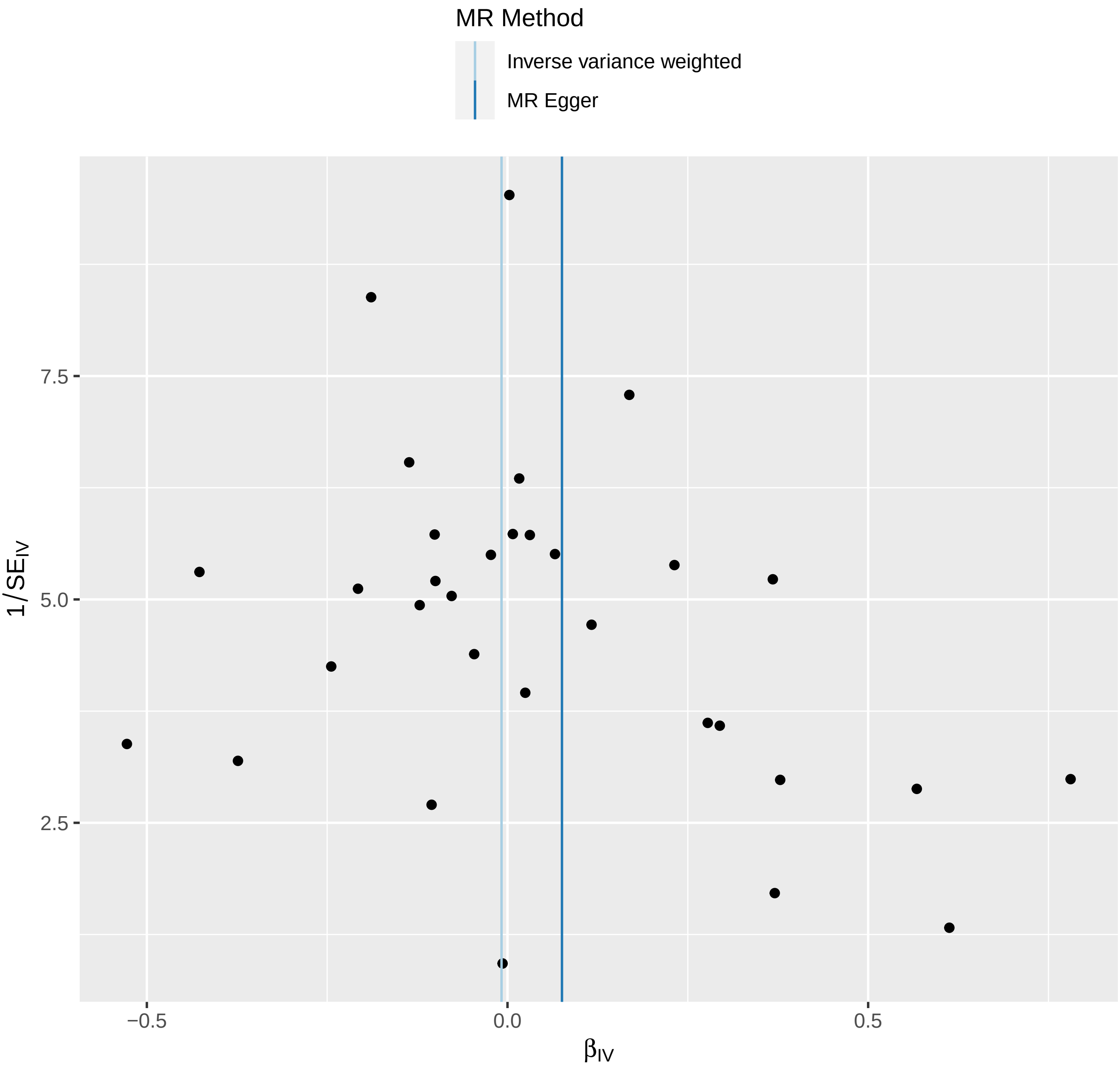

Batch 3−1483 : Interleukin−17C on Diabetic nephropathy (more control exclusions)
with a threshold of 1e−05

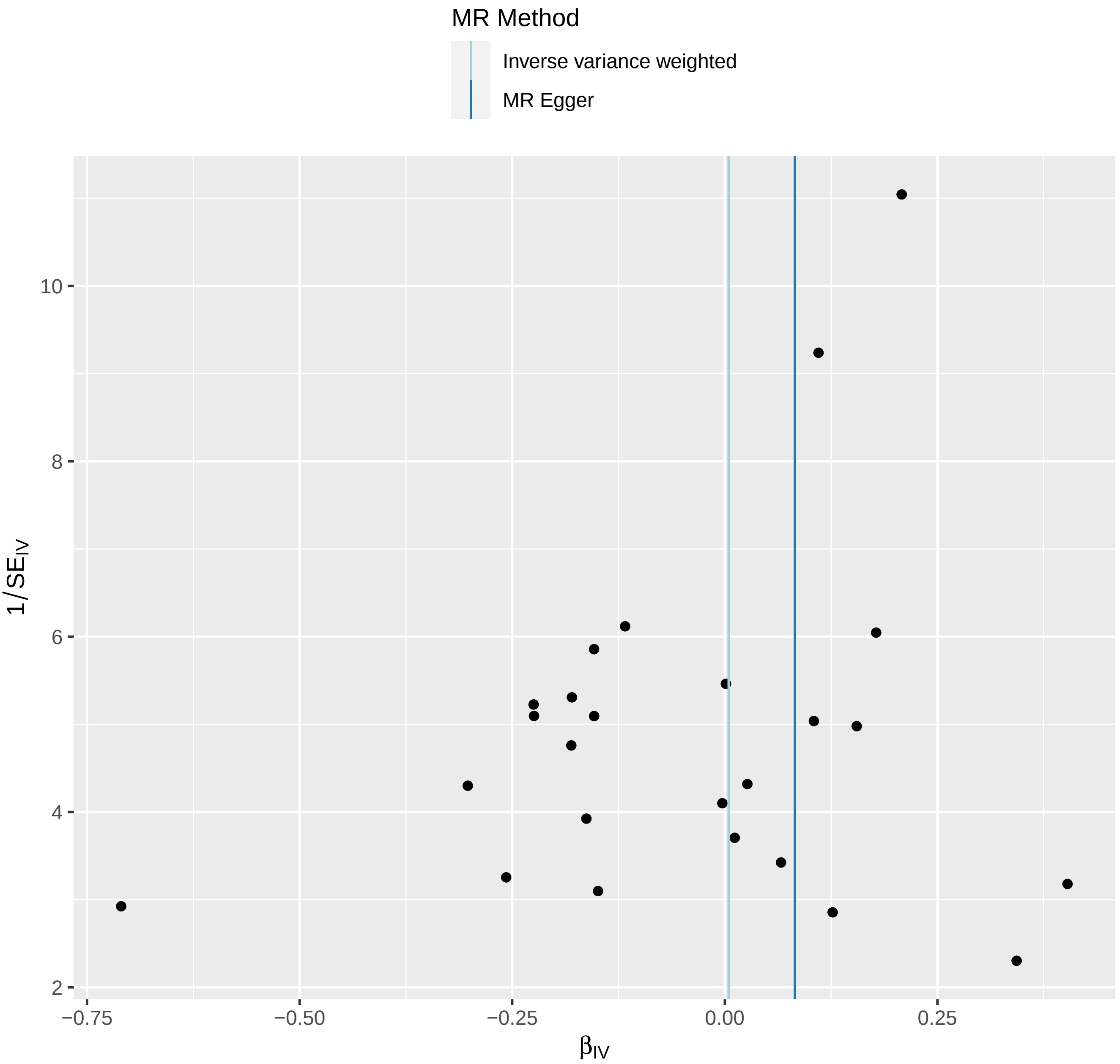

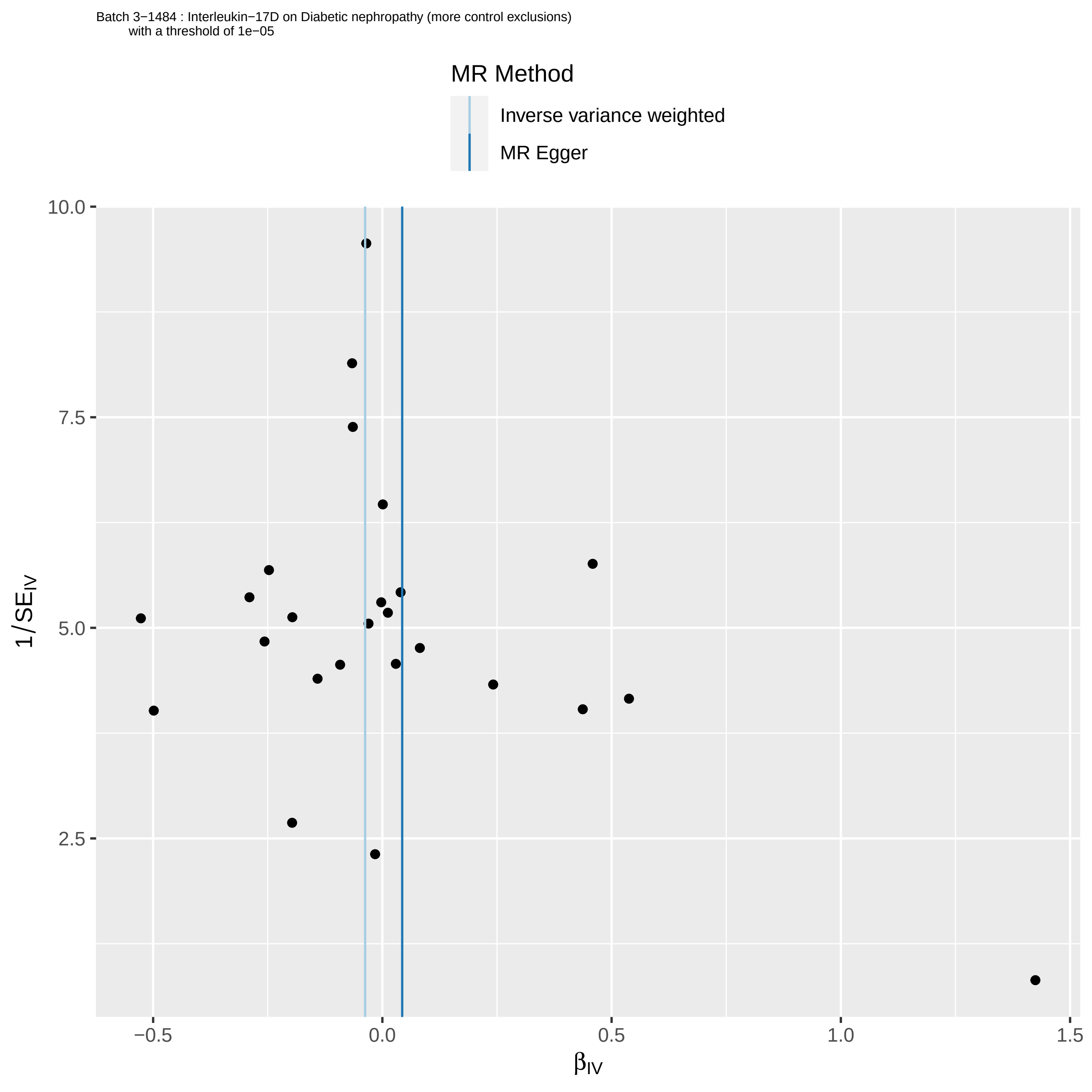

Batch 3−1484 : Interleukin−17D on Diabetic nephropathy (more control exclusions)
with a threshold of 1e−05
MR Method
Inverse variance weighted
MR Egger
10.0
7.5
5.0
2.5
1/SE_IV
−0.5
0.0
0.5
1.0
1.5
β_IV

Batch 3−1485 : Interleukin−17F on Diabetic nephropathy (more control exclusions)
with a threshold of 1e−05

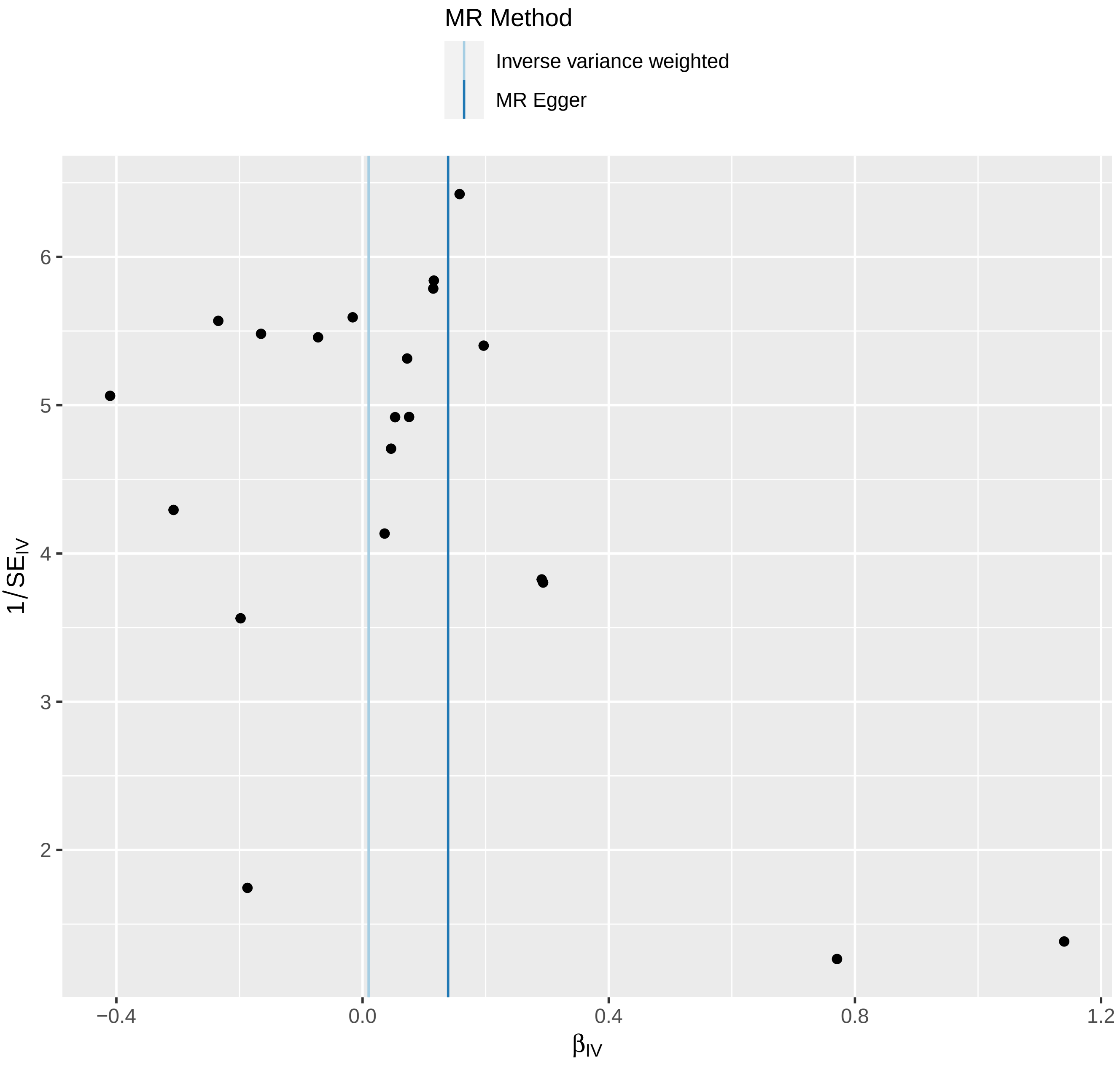

Batch 3−1486 : Interleukin−17 receptor A on Diabetic nephropathy (more control exclusions)
with a threshold of 1e−05

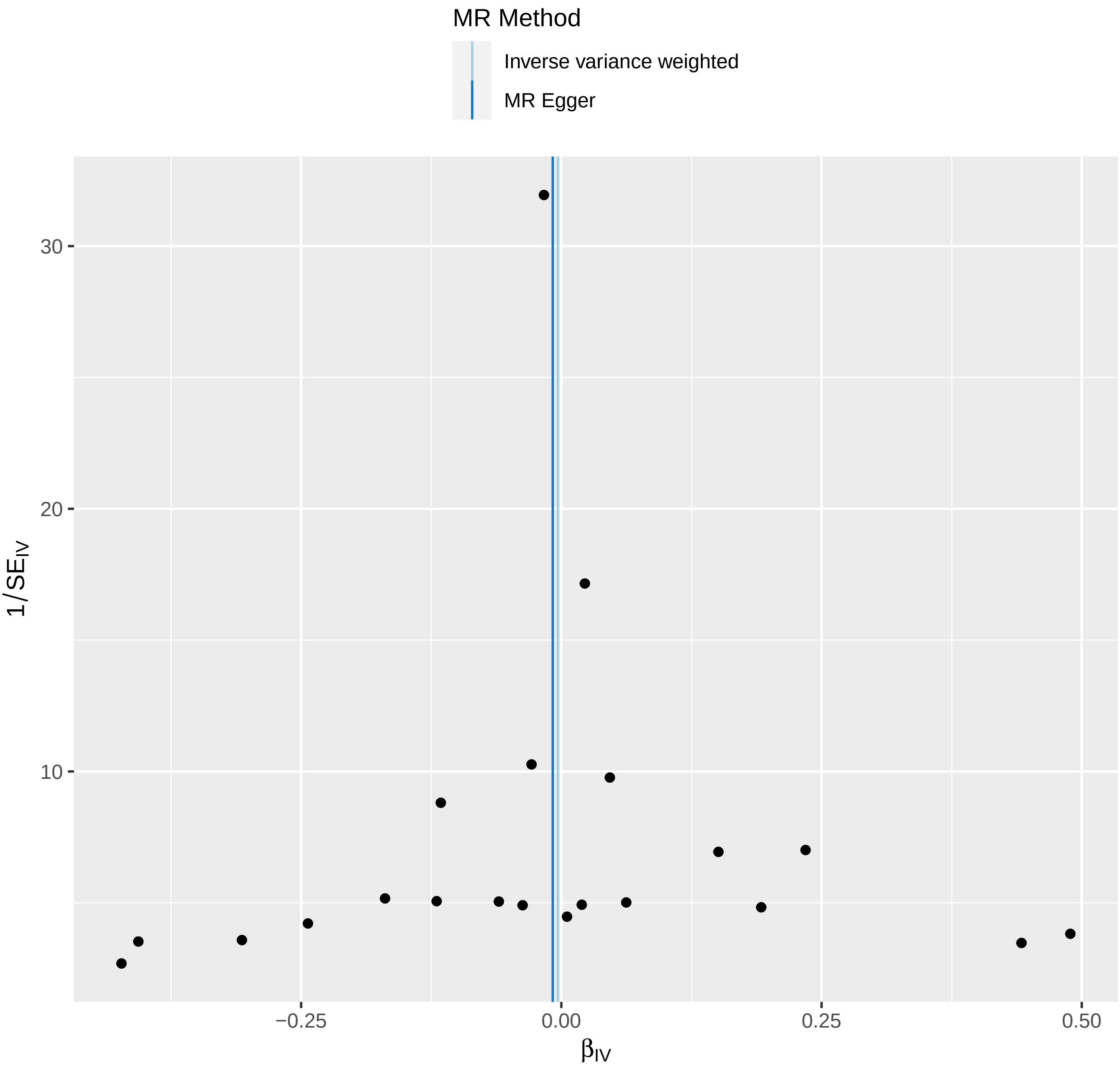

Batch 3−1487 : Interleukin−17 receptor B on Diabetic nephropathy (more control exclusions)
with a threshold of 1e−05

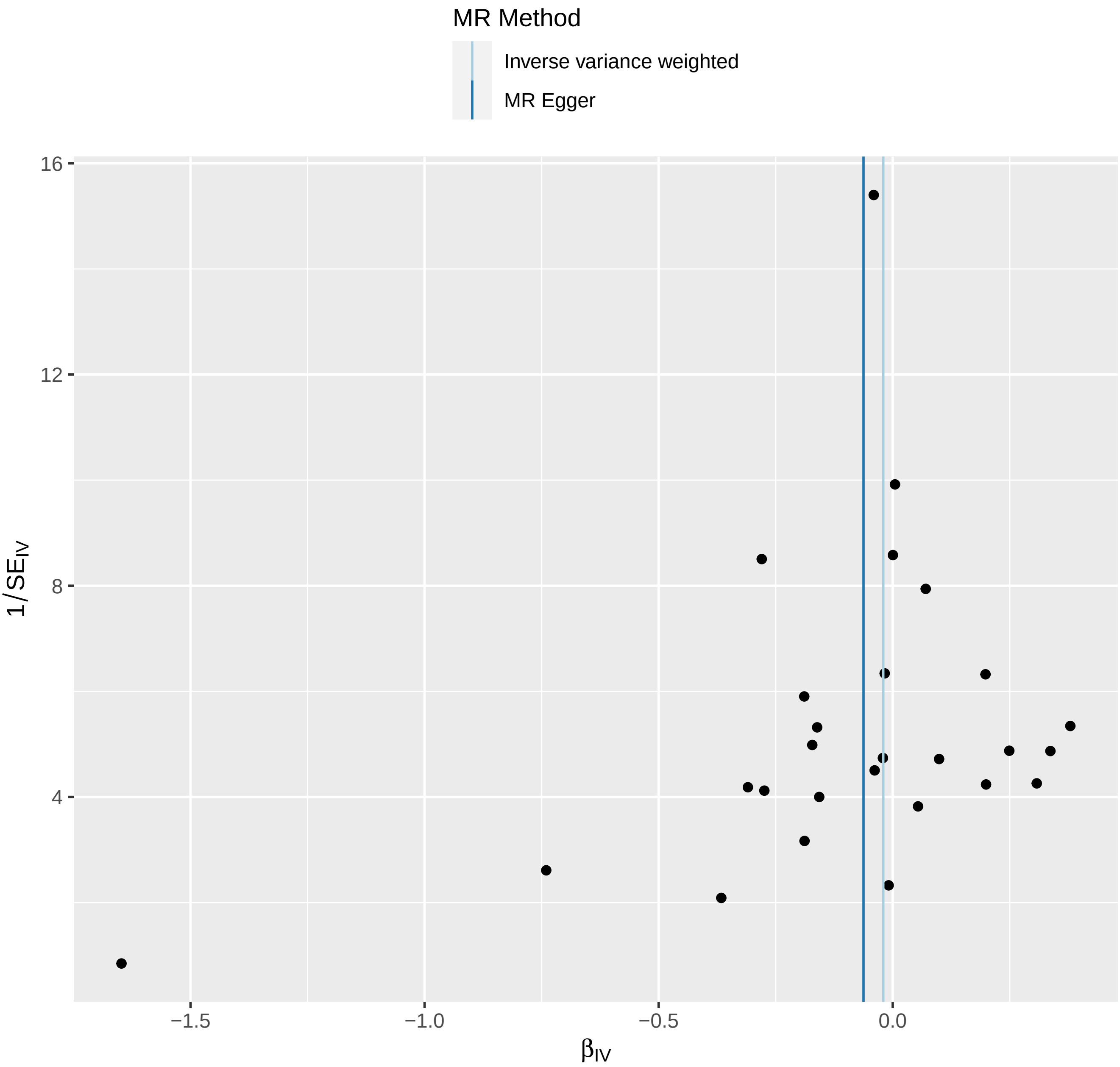

Batch 3−1488 : Interleukin−17 receptor C on Diabetic nephropathy (more control exclusions)
with a threshold of 1e−05

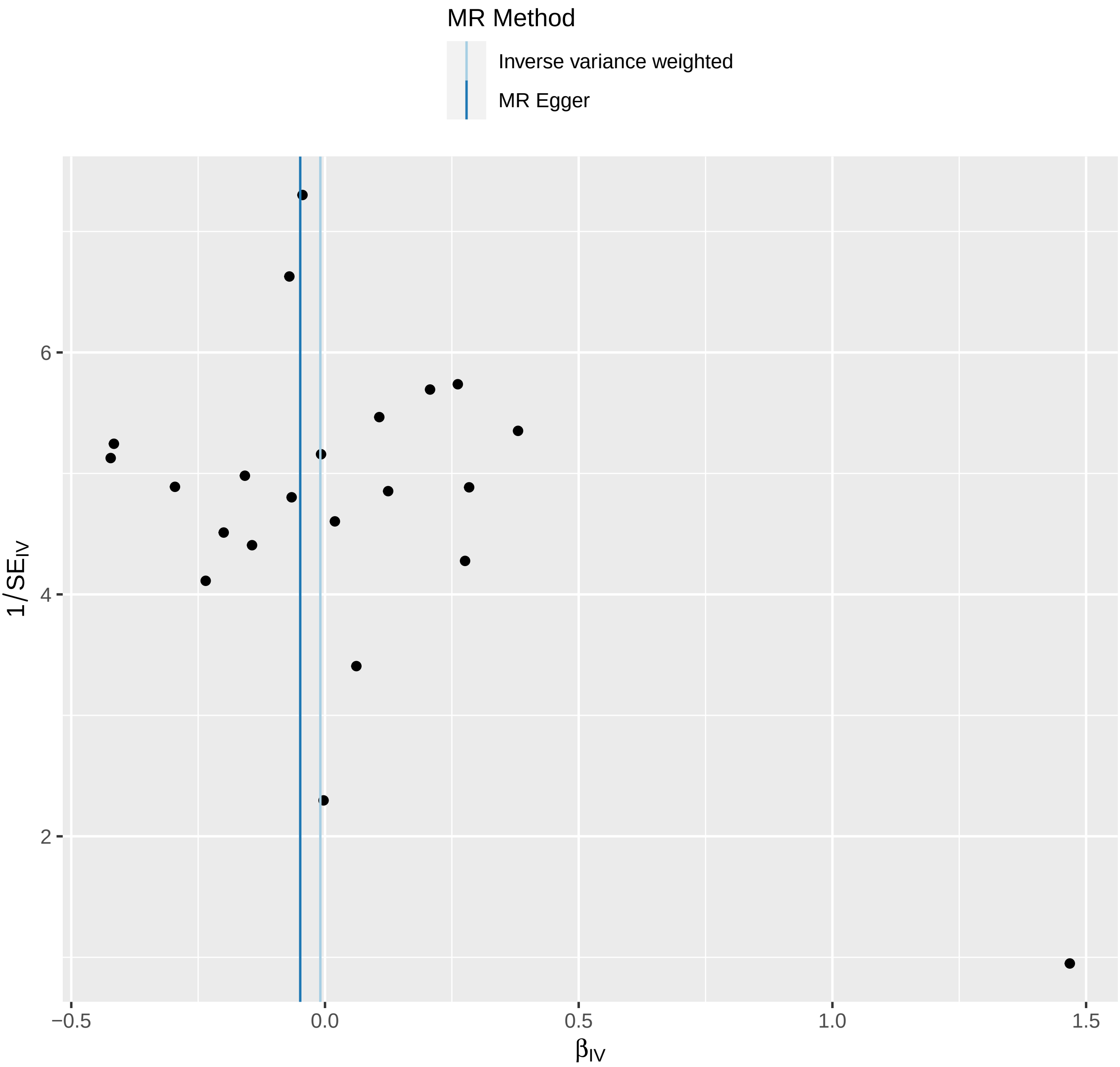

Batch 3−1489 : Interleukin−17 receptor D on Diabetic nephropathy (more control exclusions)
with a threshold of 1e−05

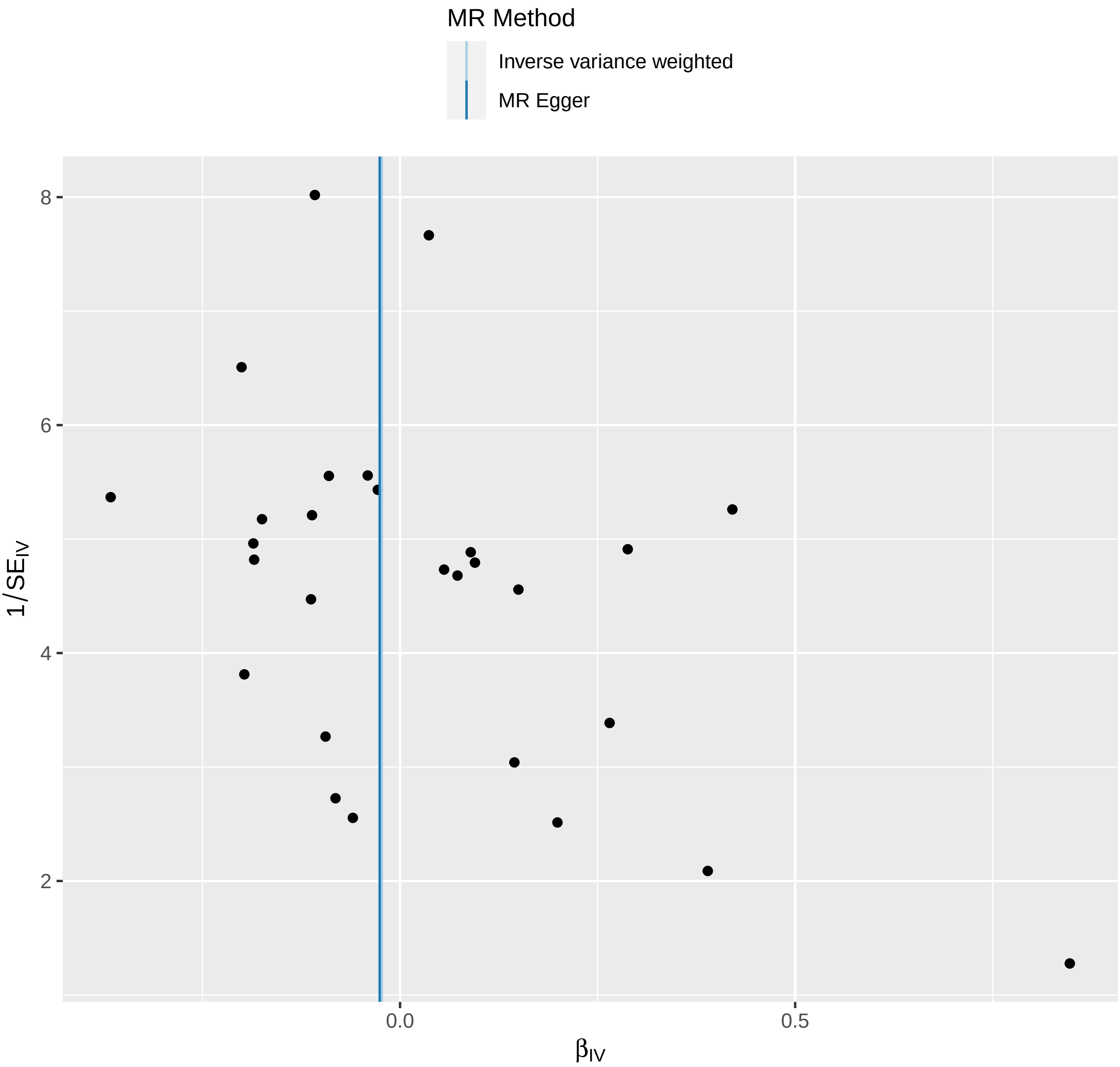

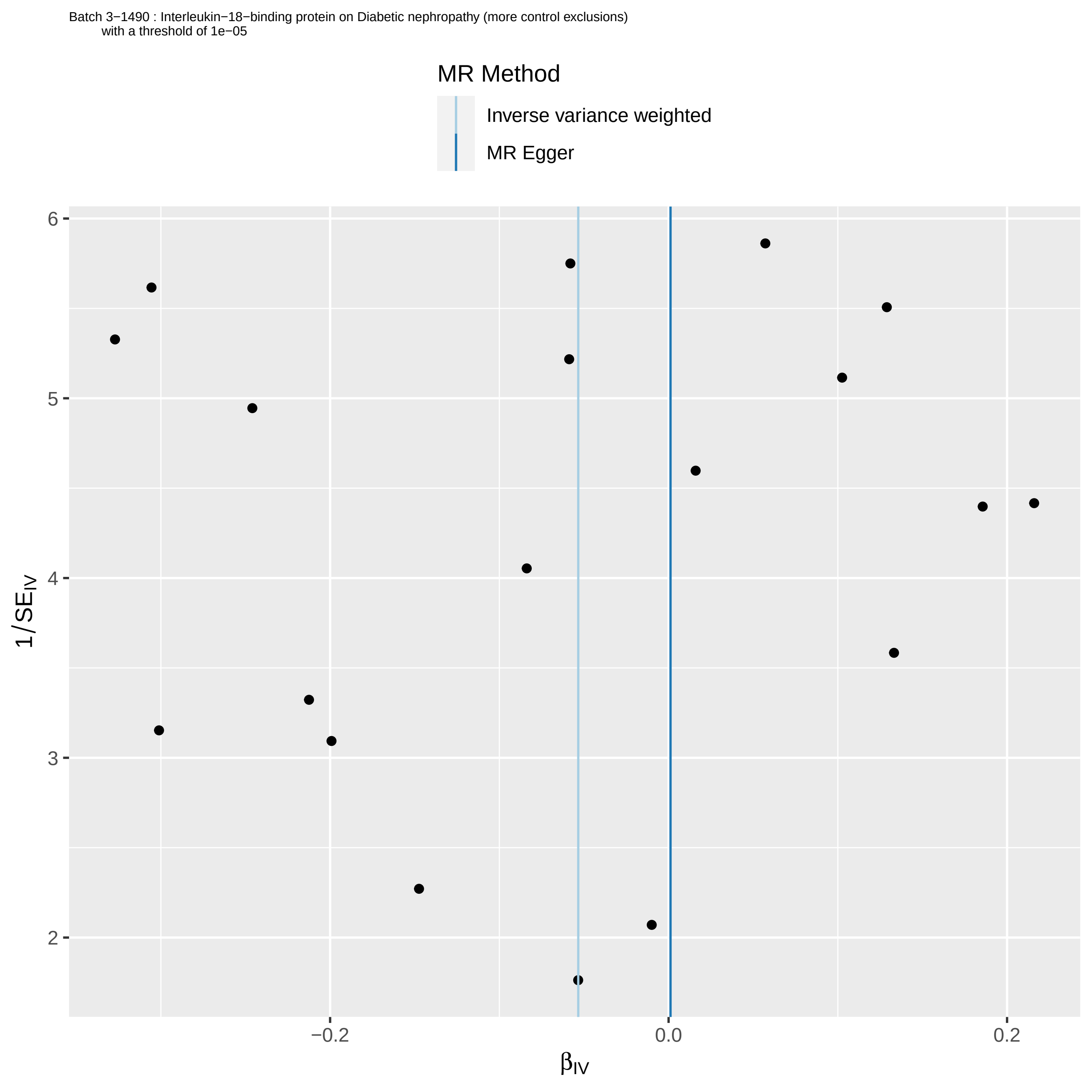

Batch 3−1490 : Interleukin−18−binding protein on Diabetic nephropathy (more control exclusions)
with a threshold of 1e−05
MR Method
Inverse variance weighted
MR Egger
6
5
4
3
2
1/SE$_{IV}$
−0.2
0.0
0.2
β$_{IV}$

Batch 3−1491 : Interleukin−18 receptor 1 on Diabetic nephropathy (more control exclusions)
with a threshold of 1e−05

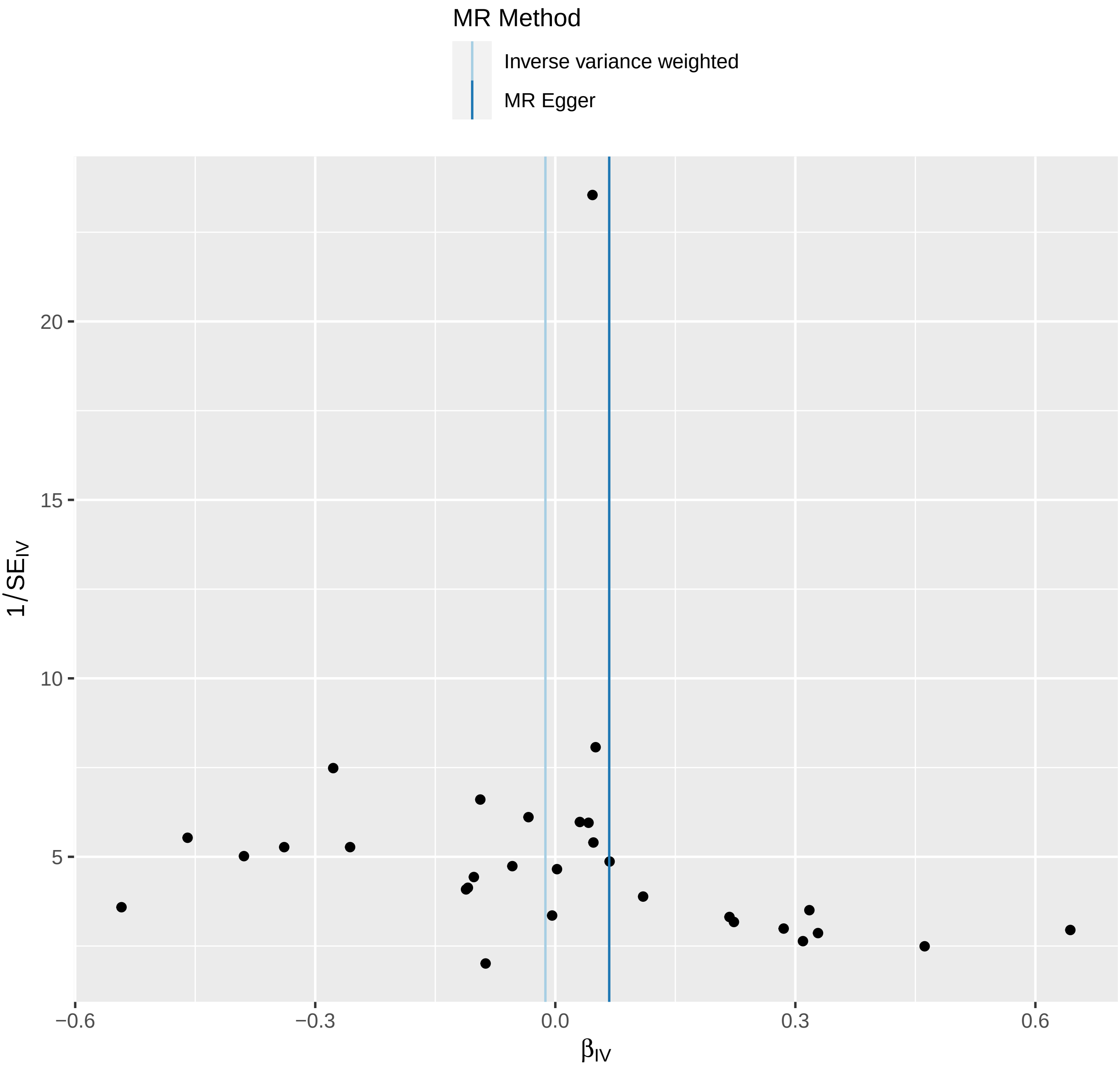

Batch 3−1492 : Interleukin−18 receptor 1 on Diabetic nephropathy (more control exclusions)
with a threshold of 1e−05

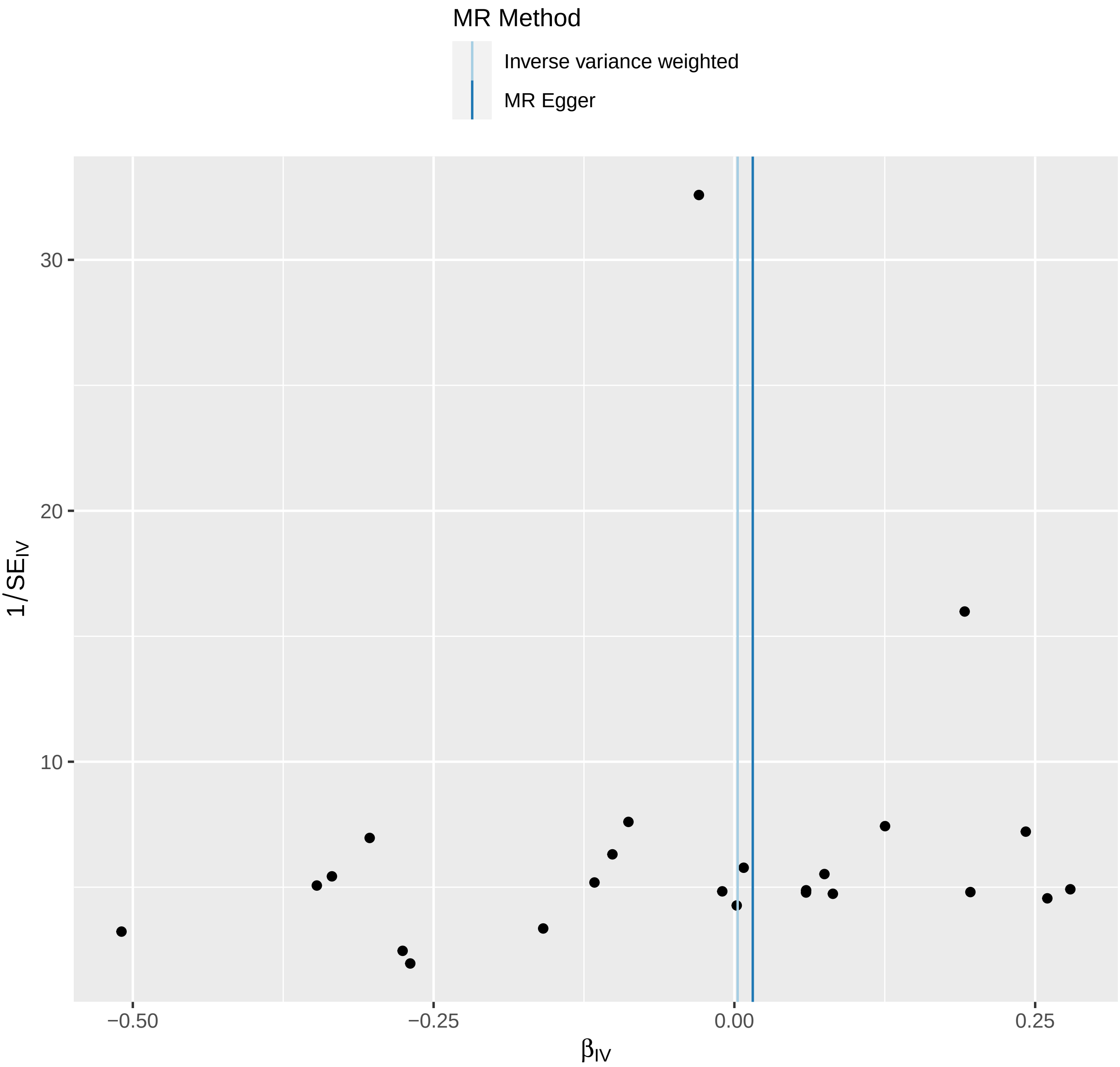

Batch 3−1493 : Interleukin−19 on Diabetic nephropathy (more control exclusions)
with a threshold of 1e−05

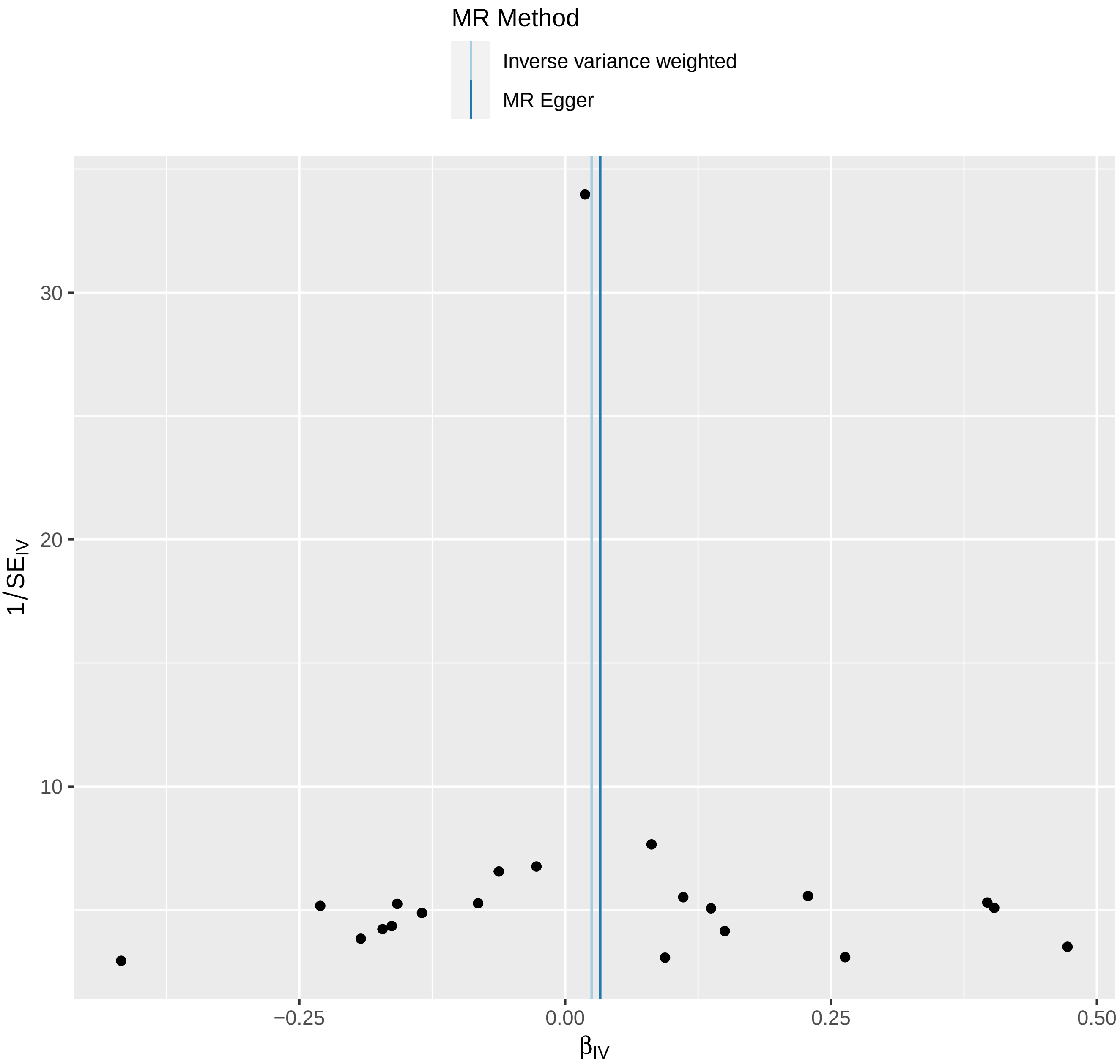

Batch 3−1494 : Interleukin−1 alpha on Diabetic nephropathy (more control exclusions)
with a threshold of 1e−05

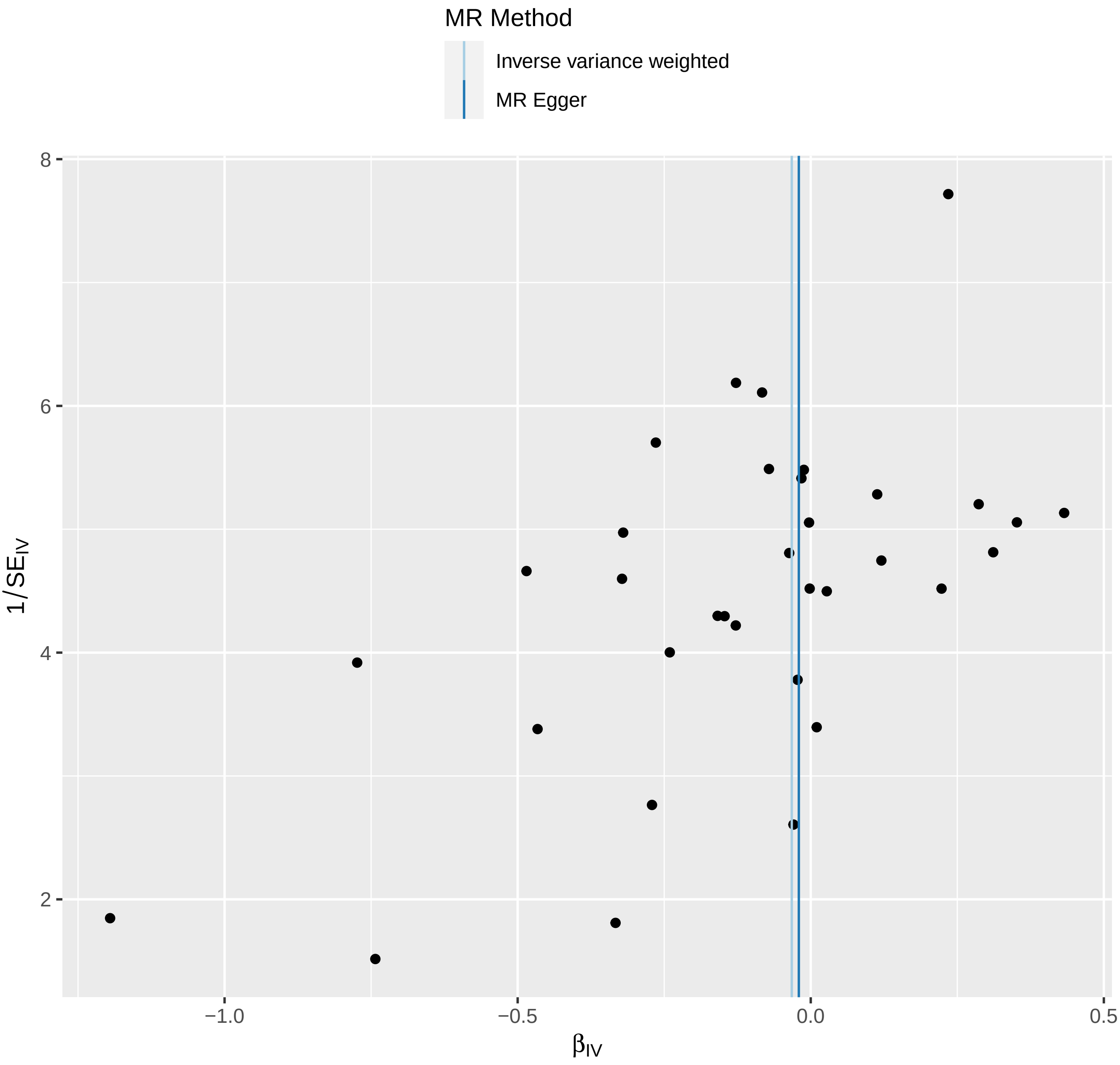

Batch 3−1495 : Interleukin−1 beta on Diabetic nephropathy (more control exclusions)
with a threshold of 1e−05

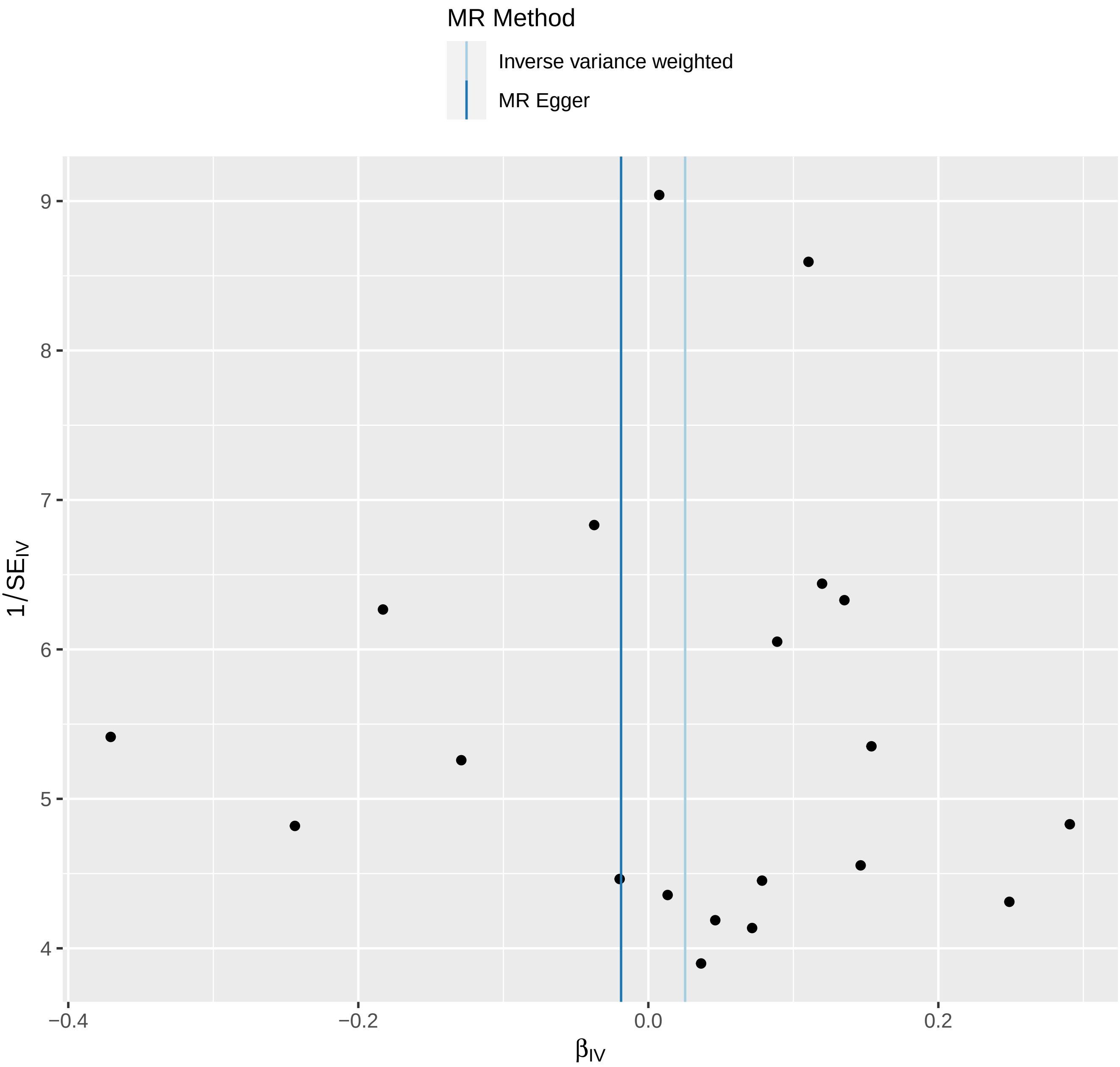

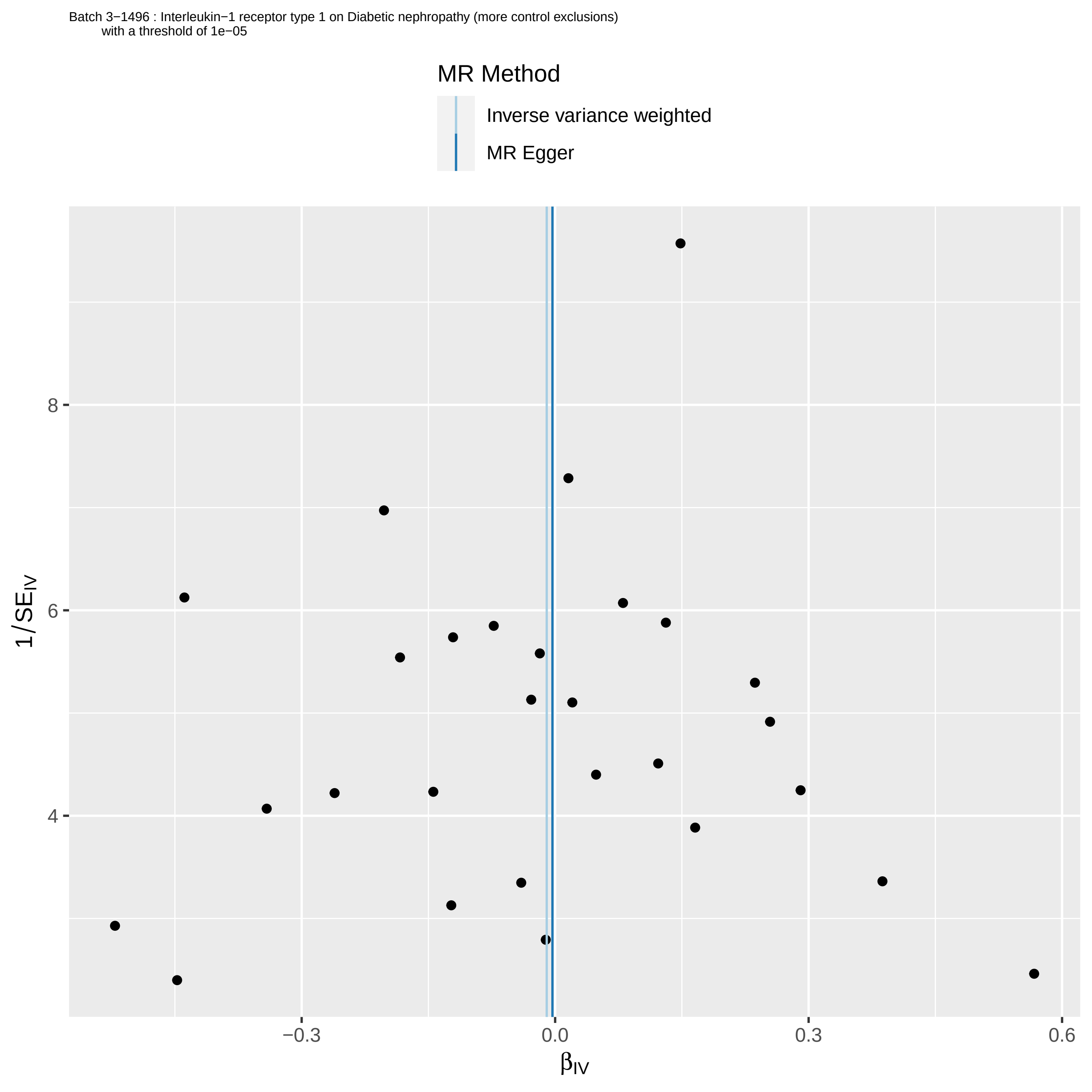
Batch 3−1496 : Interleukin−1 receptor type 1 on Diabetic nephropathy (more control exclusions)
with a threshold of 1e−05
MR Method
Inverse variance weighted
MR Egger
1/SE_IV
8
6
4
−0.3
0.0
0.3
0.6
β_IV

Batch 3−1497 : Interleukin−1 receptor type 2 on Diabetic nephropathy (more control exclusions)
with a threshold of 1e−05

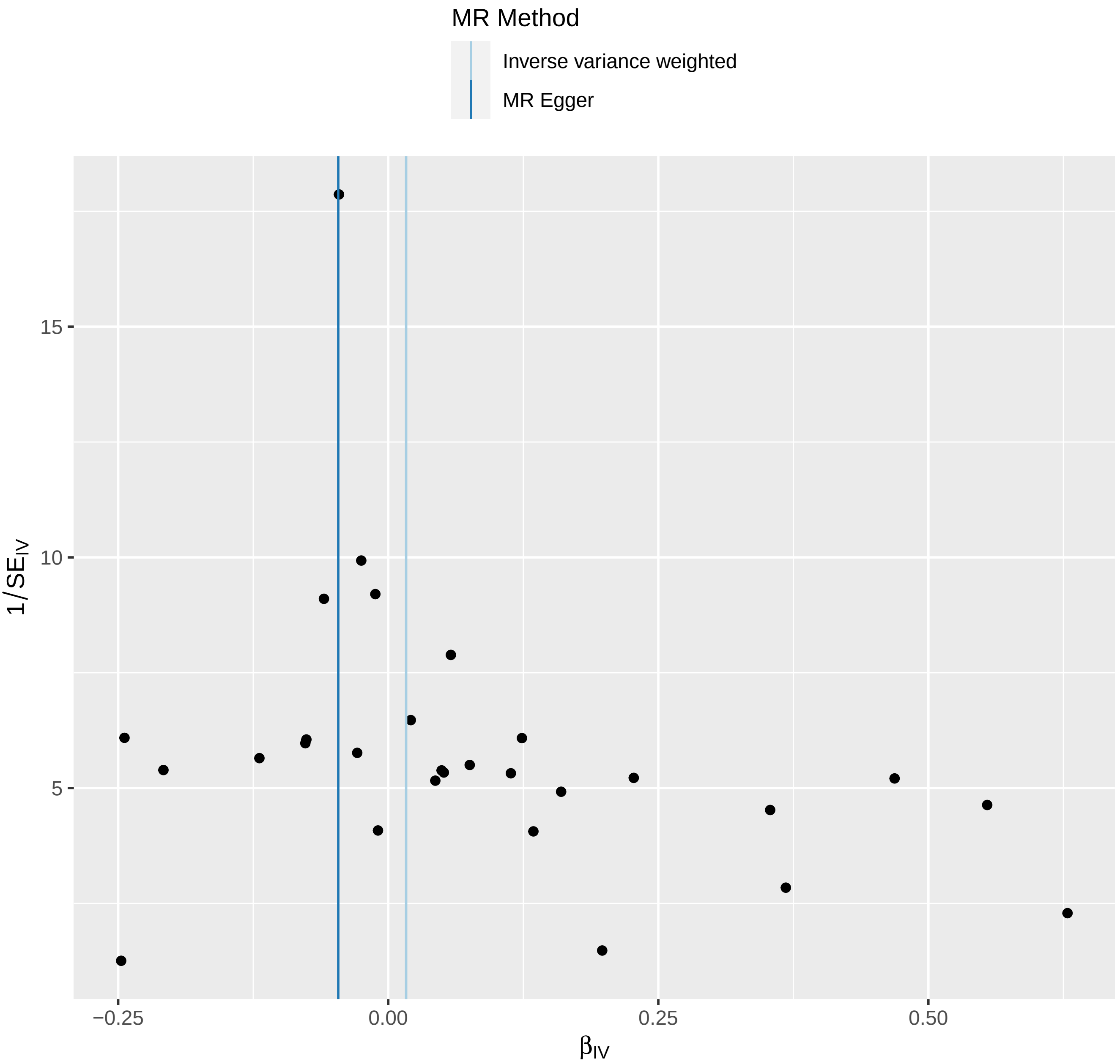

Batch 3−1498 : Interleukin−1 receptor type 2 on Diabetic nephropathy (more control exclusions)
with a threshold of 1e−05

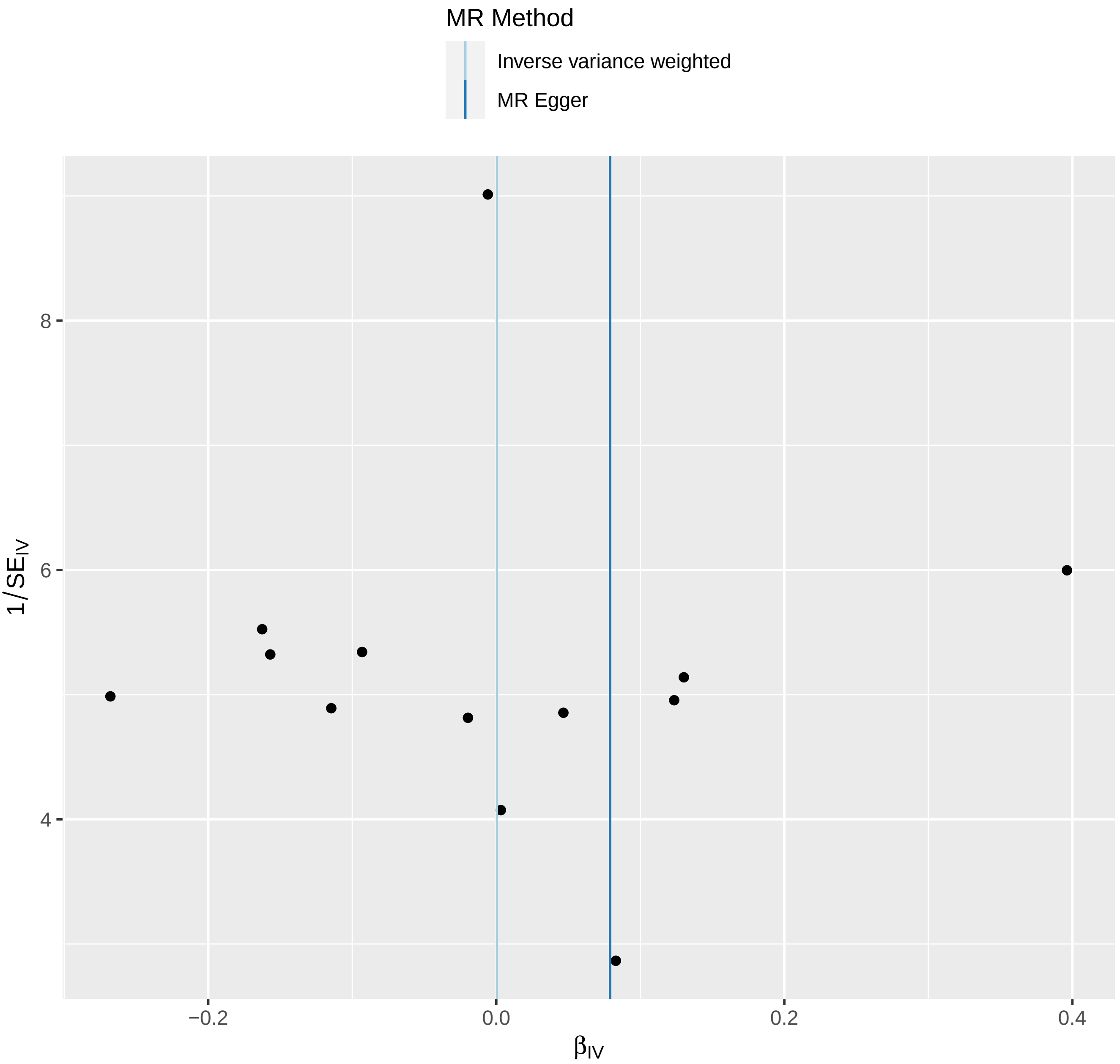

Batch 3−1499 : Interleukin−1 Receptor accessory protein on Diabetic nephropathy (more control exclusions)
with a threshold of 1e−05

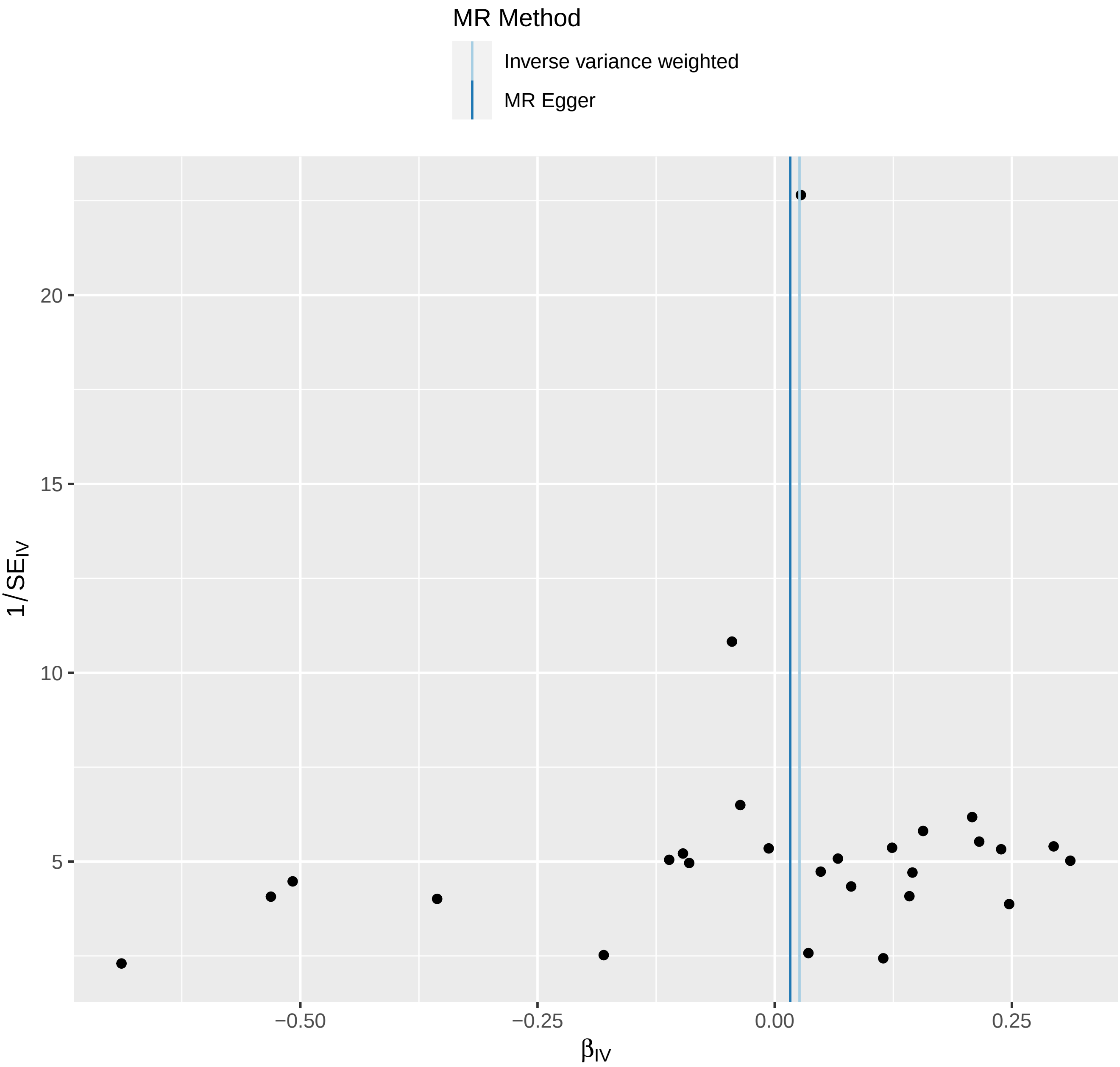

Batch 3−1500 : Interleukin−1 Receptor accessory protein on Diabetic nephropathy (more control exclusions)
with a threshold of 1e−05

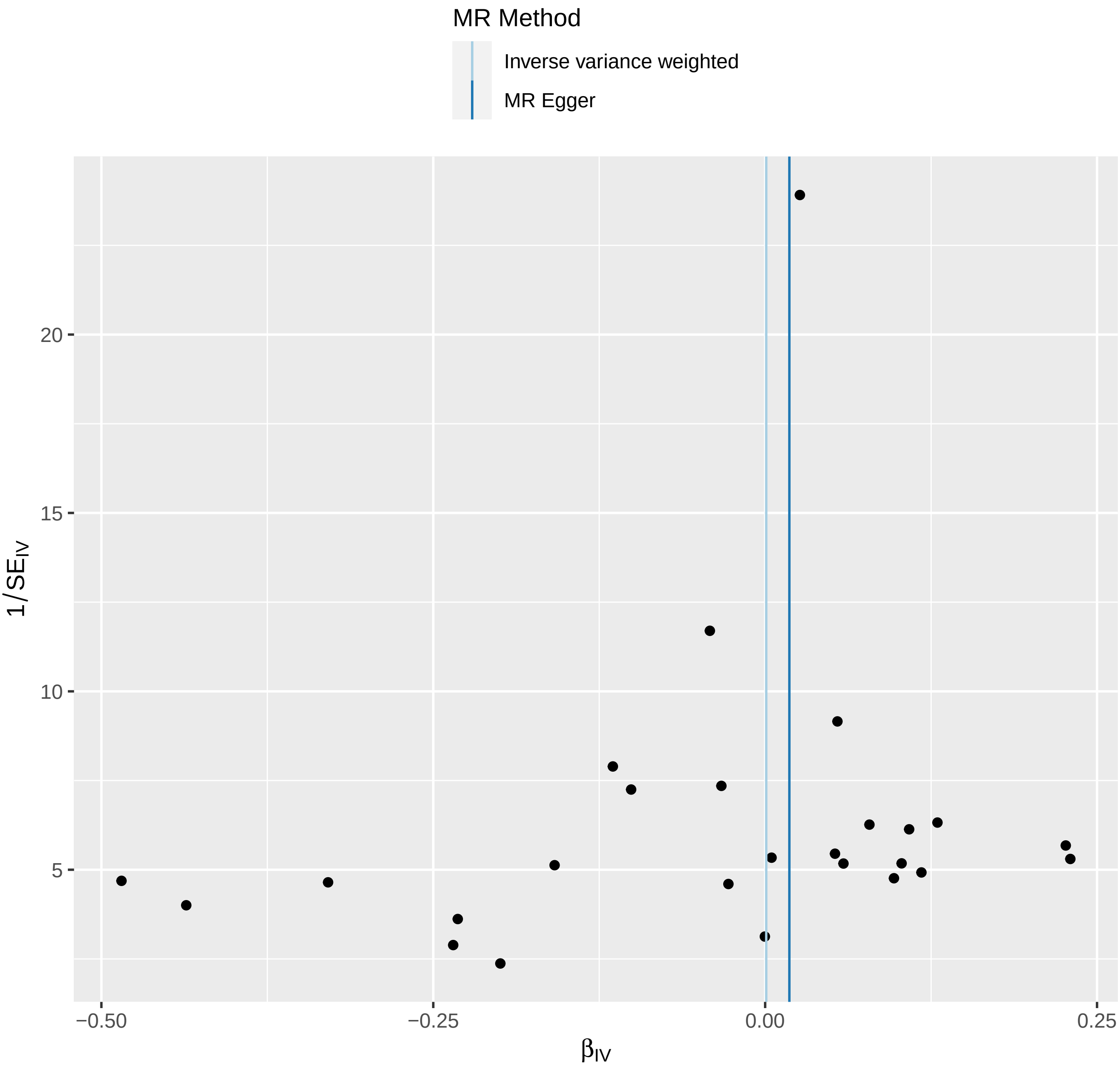

Batch 3−1501 : X−linked interleukin−1 receptor accessory protein−like 2 on Diabetic nephropathy (more control exclusions)
with a threshold of 1e−05

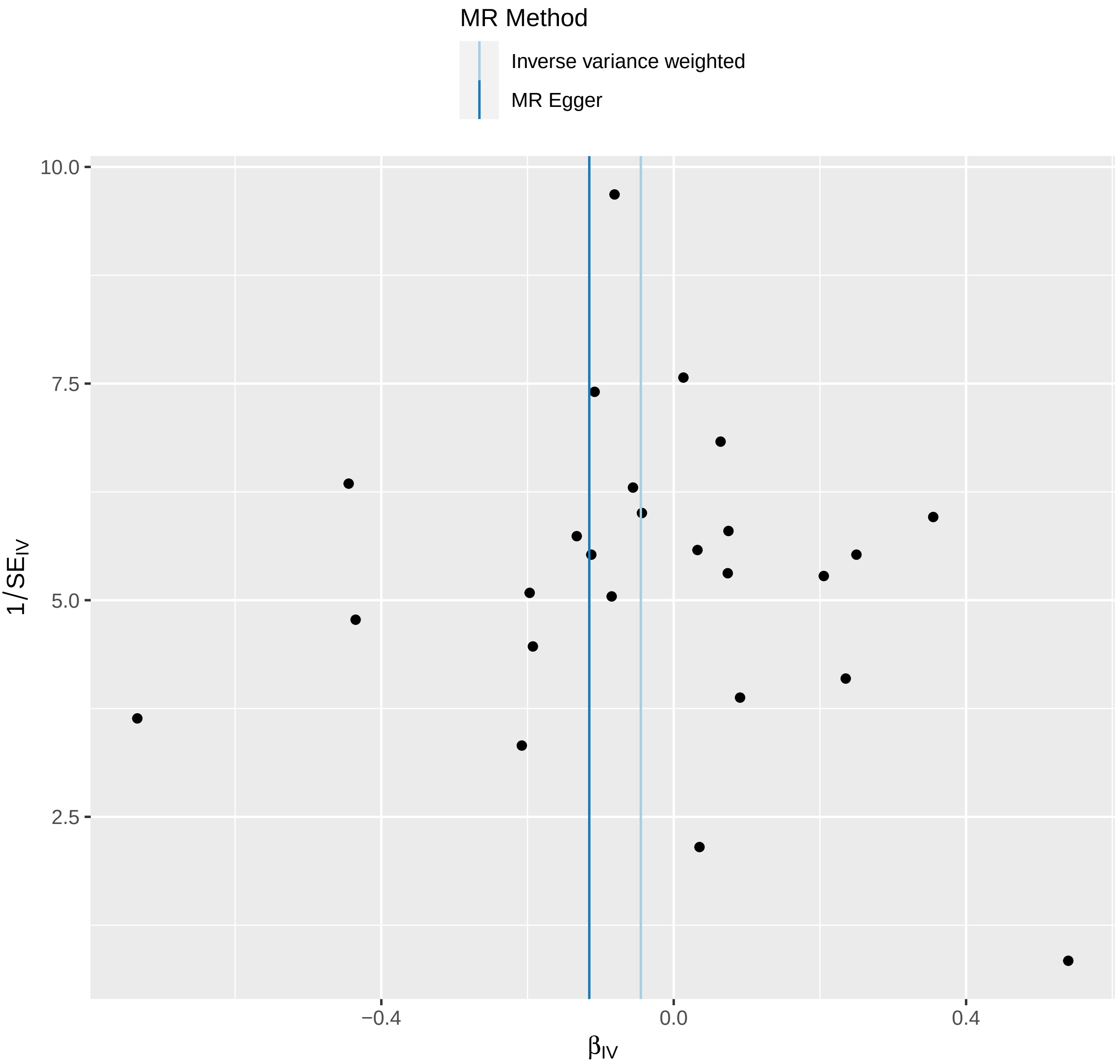

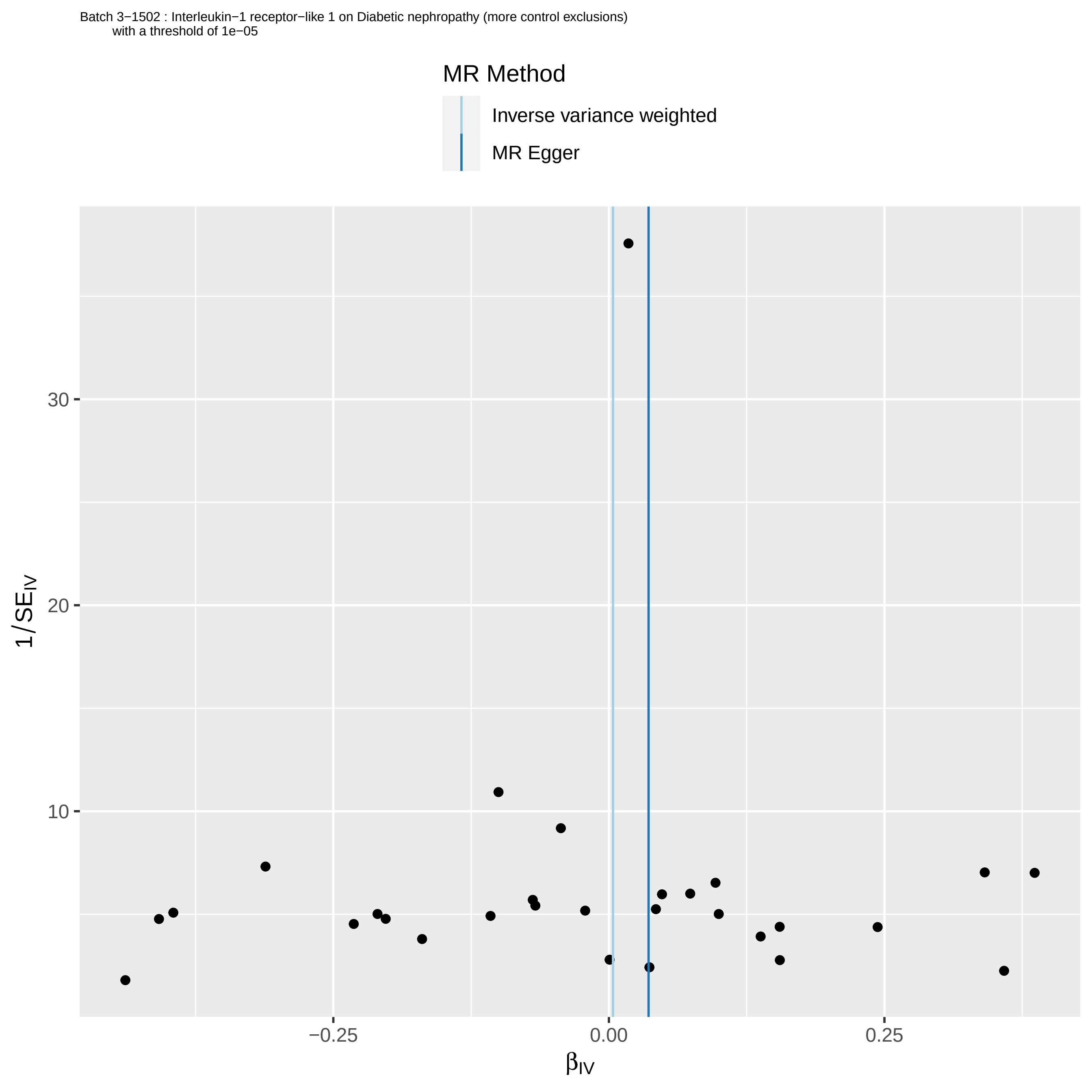

Batch 3−1502 : Interleukin−1 receptor−like 1 on Diabetic nephropathy (more control exclusions)
with a threshold of 1e−05
MR Method
Inverse variance weighted
MR Egger
30
20
10
1/SE_IV
−0.25
0.00
0.25
β_IV

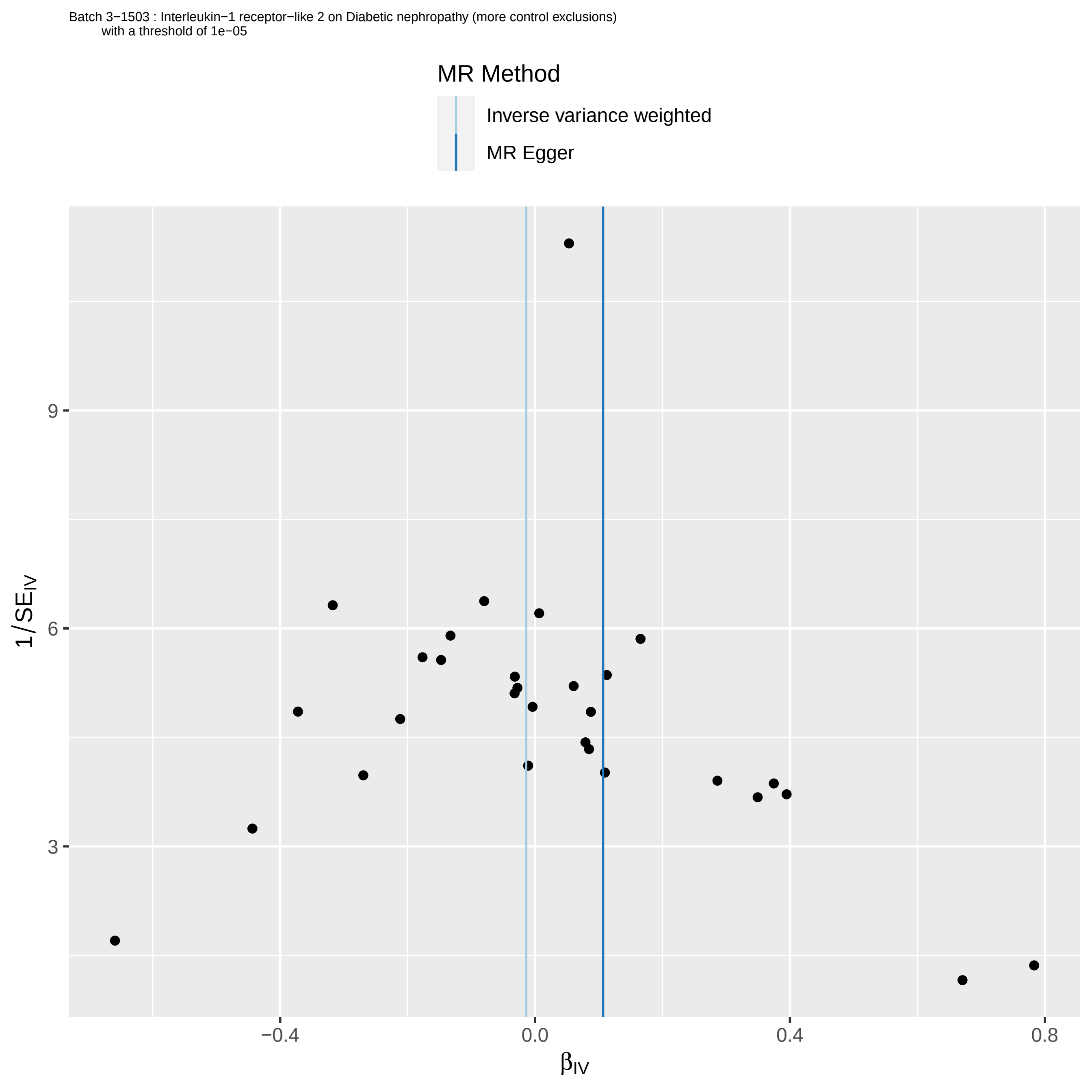
Batch 3−1503 : Interleukin−1 receptor−like 2 on Diabetic nephropathy (more control exclusions)
with a threshold of 1e−05
MR Method
Inverse variance weighted
MR Egger
9
6
3
$1/SE_{IV}$
−0.4
0.0
0.4
0.8
β_{IV}

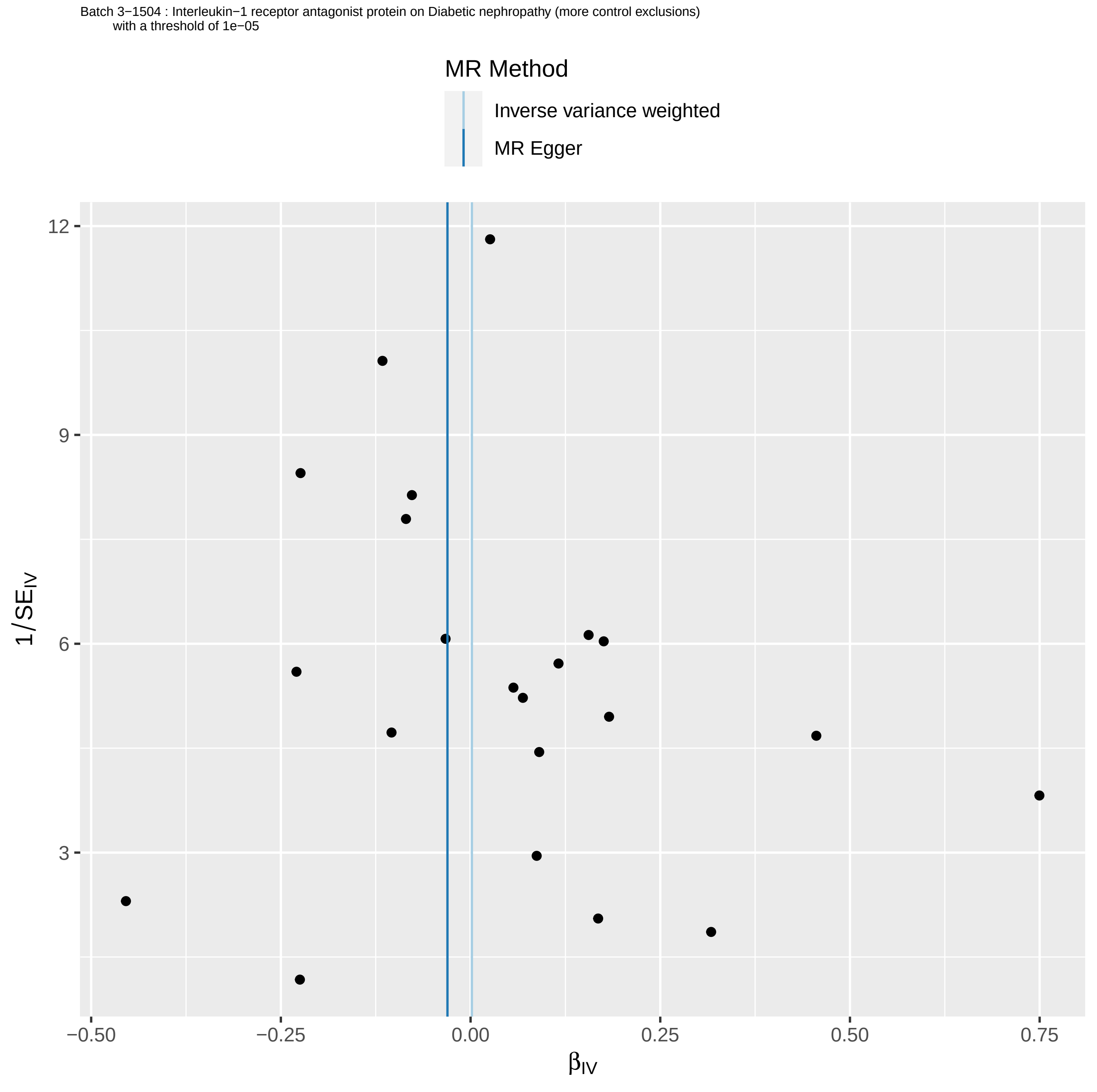
Batch 3−1504 : Interleukin−1 receptor antagonist protein on Diabetic nephropathy (more control exclusions)
with a threshold of 1e−05
MR Method
Inverse variance weighted
MR Egger
12
9
6
3
1/SEIV
−0.50
−0.25
0.00
0.25
0.50
0.75
βIV

Batch 3−1505 : Interleukin−20 on Diabetic nephropathy (more control exclusions)
with a threshold of 1e−05

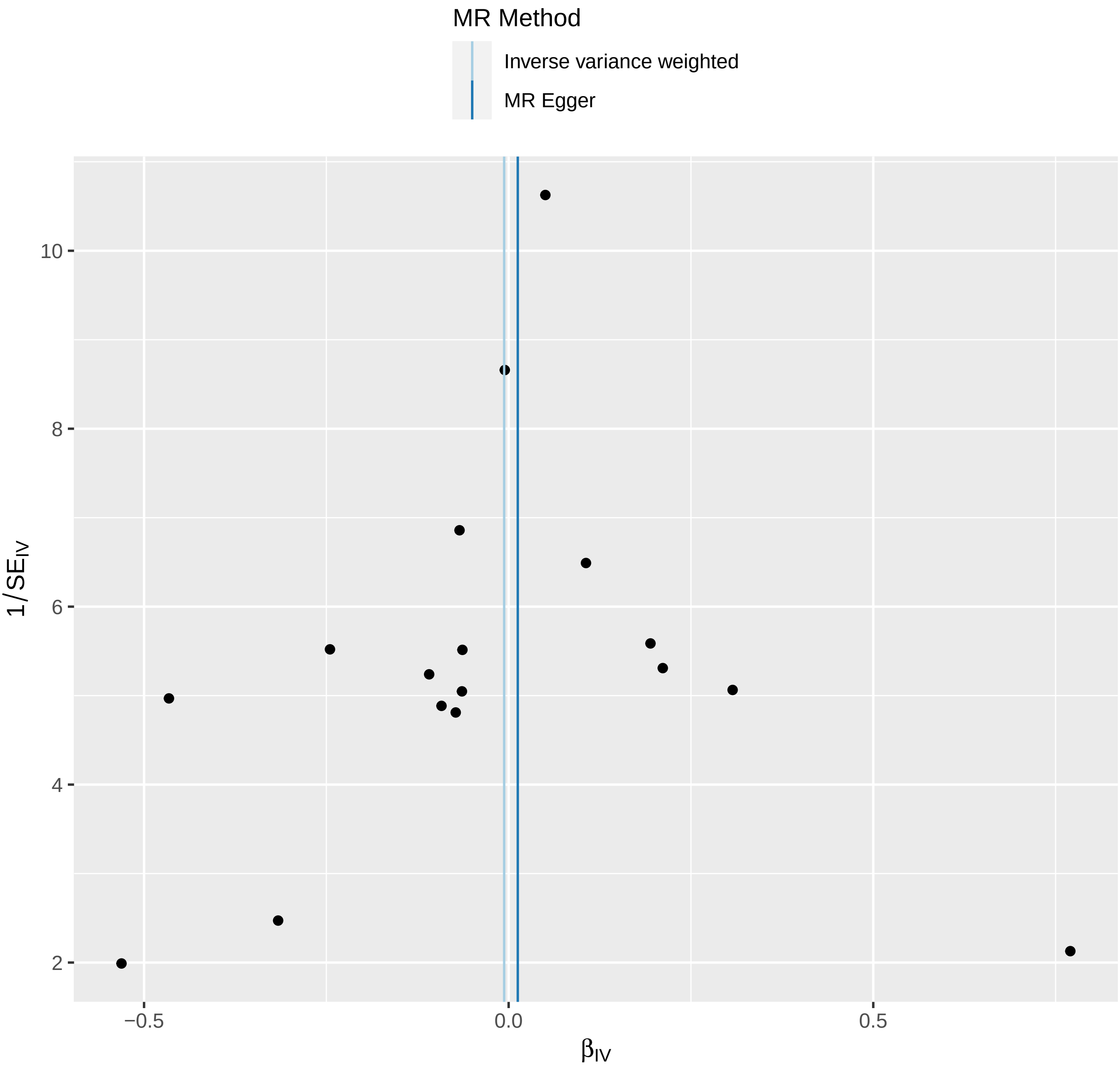

Batch 3−1506 : Interleukin−21 on Diabetic nephropathy (more control exclusions)
with a threshold of 1e−05

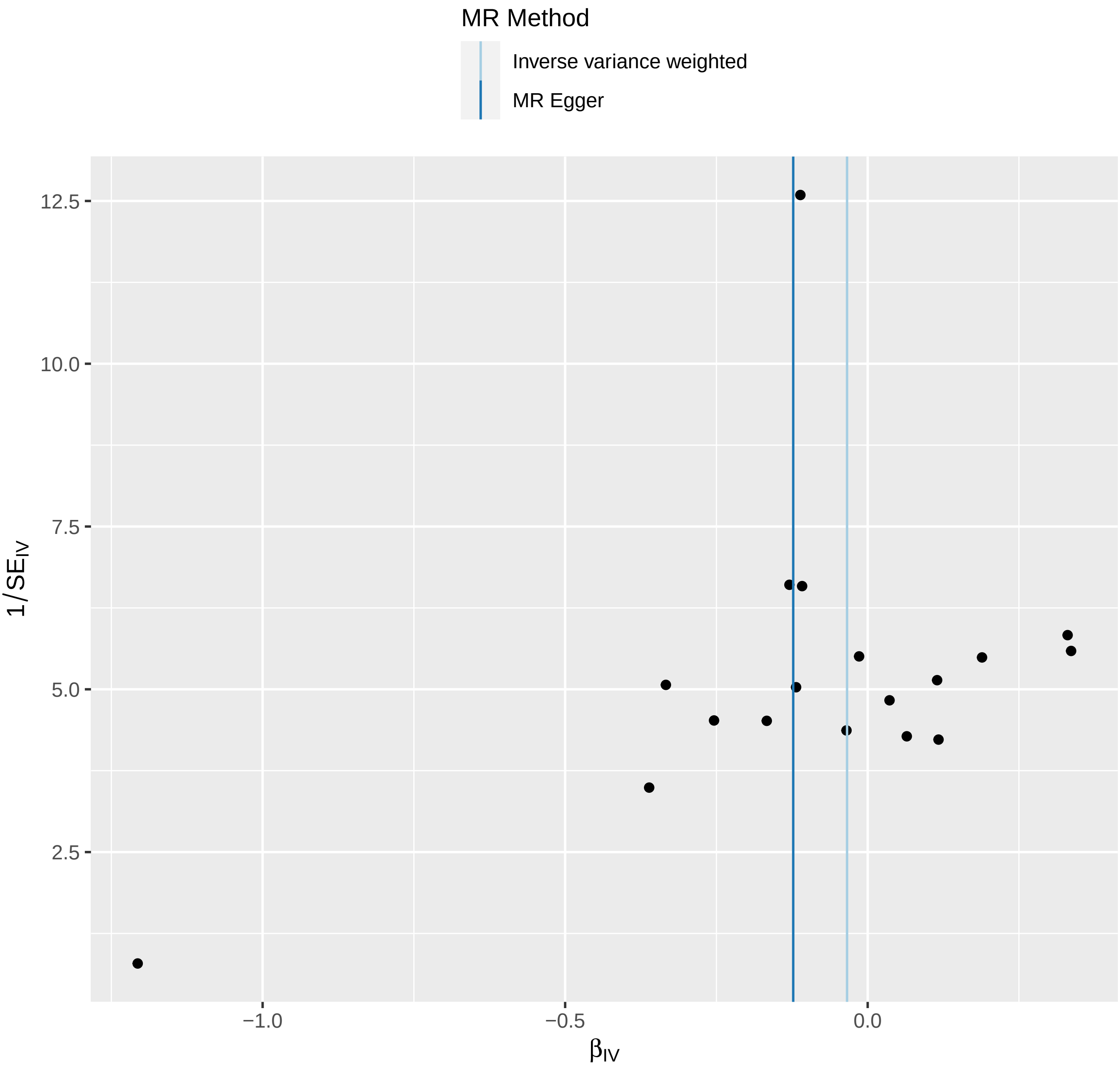

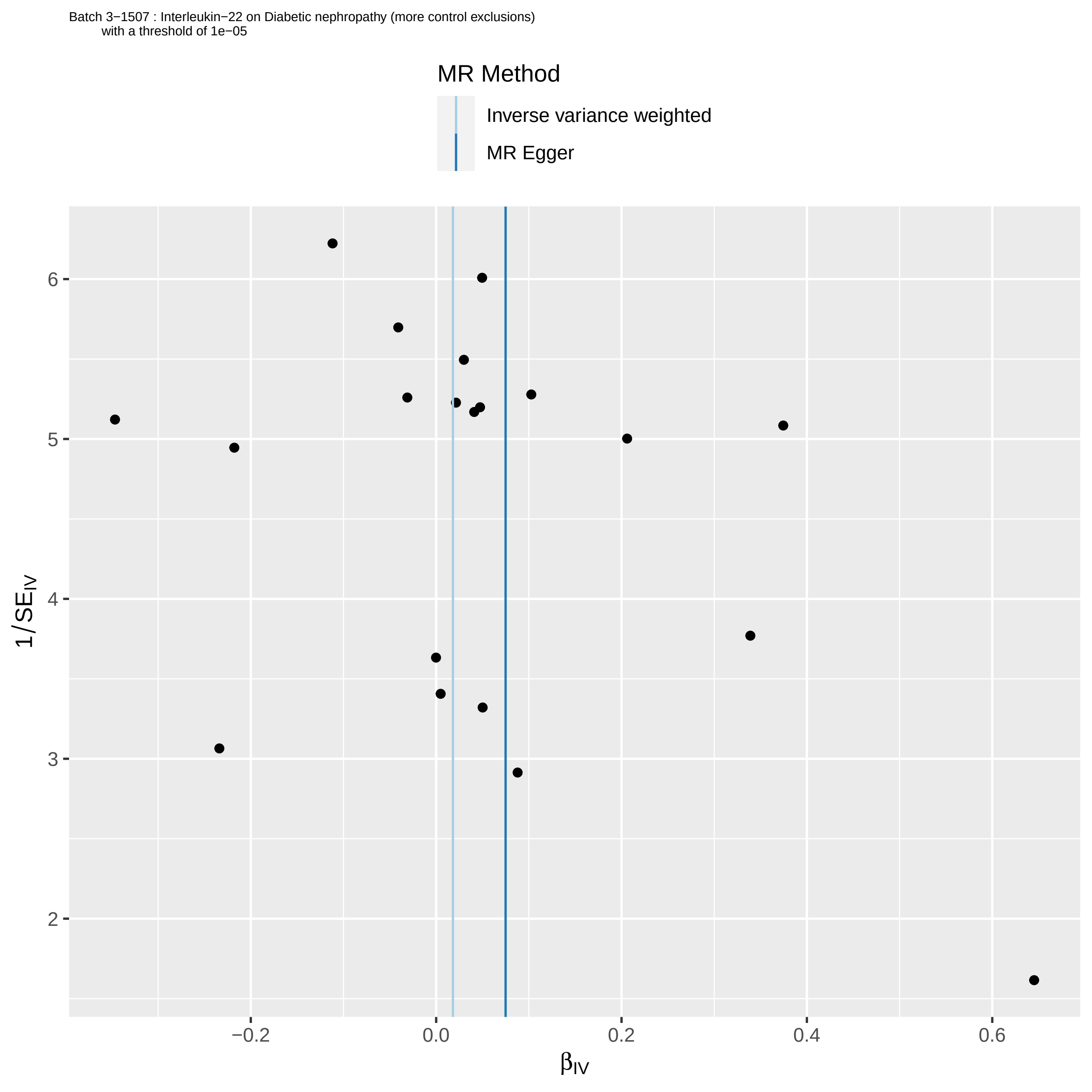

Batch 3−1507 : Interleukin−22 on Diabetic nephropathy (more control exclusions)
with a threshold of 1e−05
MR Method
Inverse variance weighted
MR Egger
6
5
4
3
2
$1/SE_{IV}$
−0.2
0.0
0.2
0.4
0.6
β_{IV}

Batch 3−1508 : Interleukin−22 receptor subunit alpha−1 on Diabetic nephropathy (more control exclusions) with a threshold of 1e−05

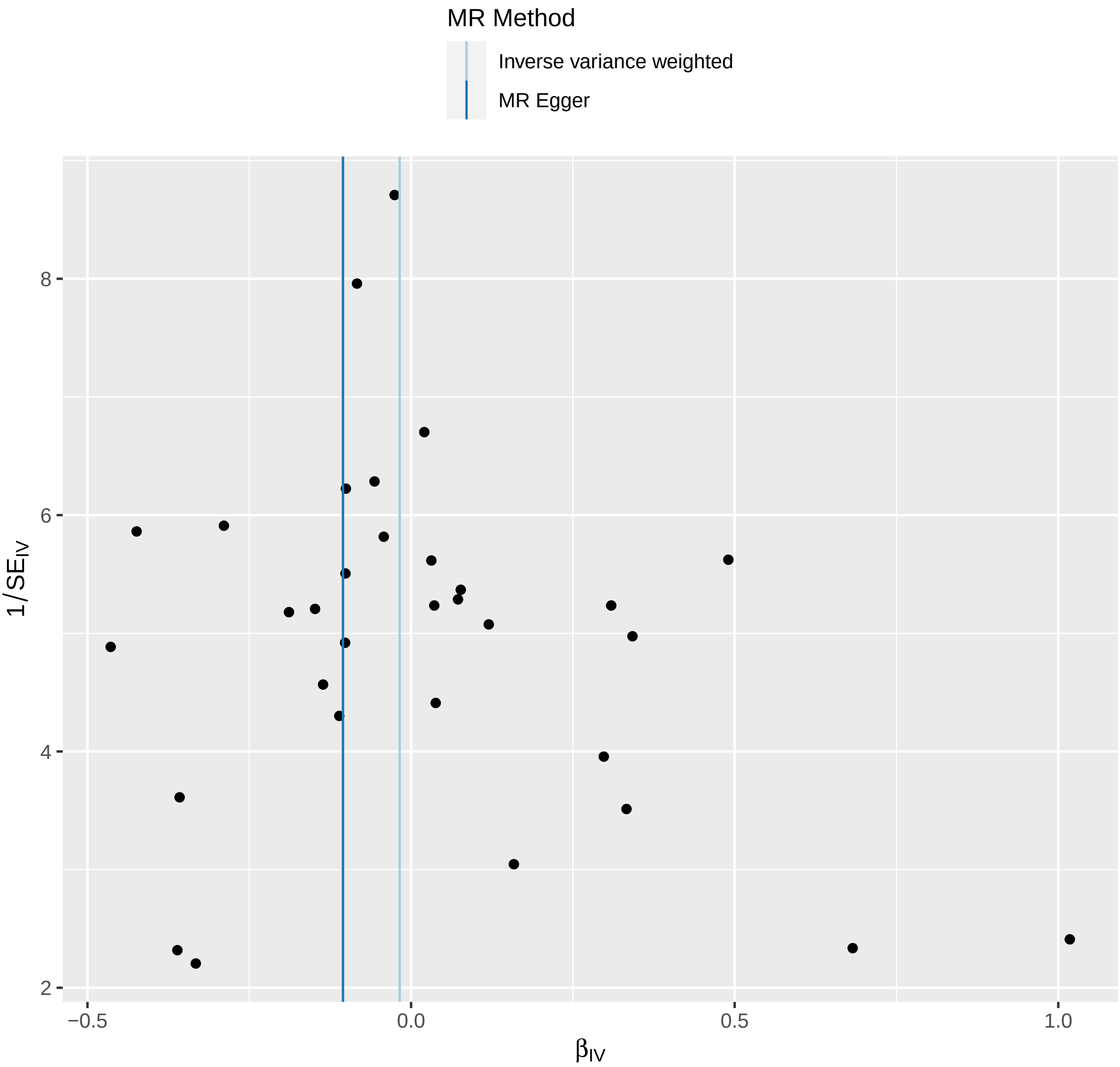

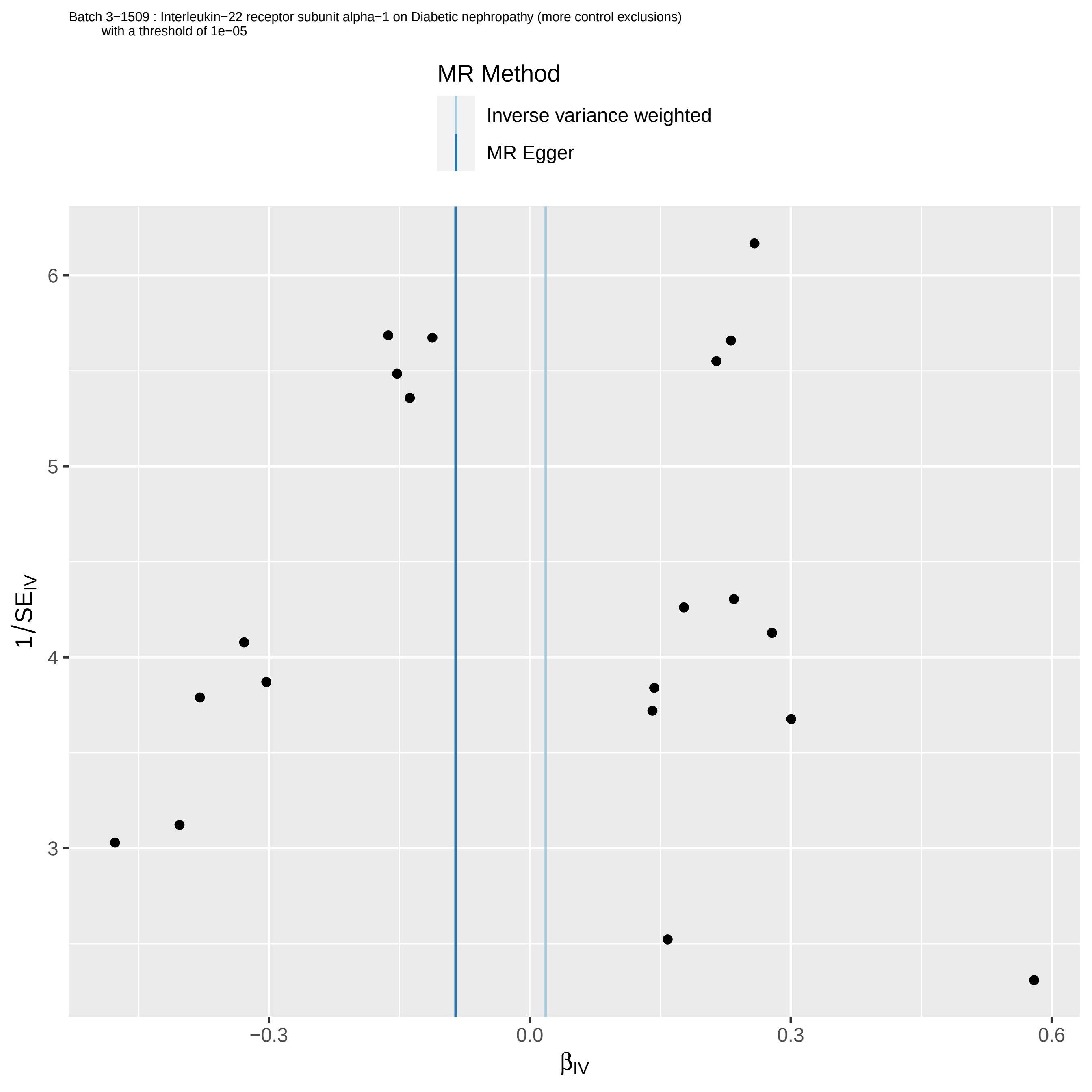
Batch 3−1509 : Interleukin−22 receptor subunit alpha−1 on Diabetic nephropathy (more control exclusions)
with a threshold of 1e−05
MR Method
Inverse variance weighted
MR Egger
6
5
4
3
1/SE$_{IV}$
−0.3
0.0
0.3
0.6
β_{IV}

Batch 3−1510 : Interleukin−22 receptor subunit alpha−2 on Diabetic nephropathy (more control exclusions)
with a threshold of 1e−05

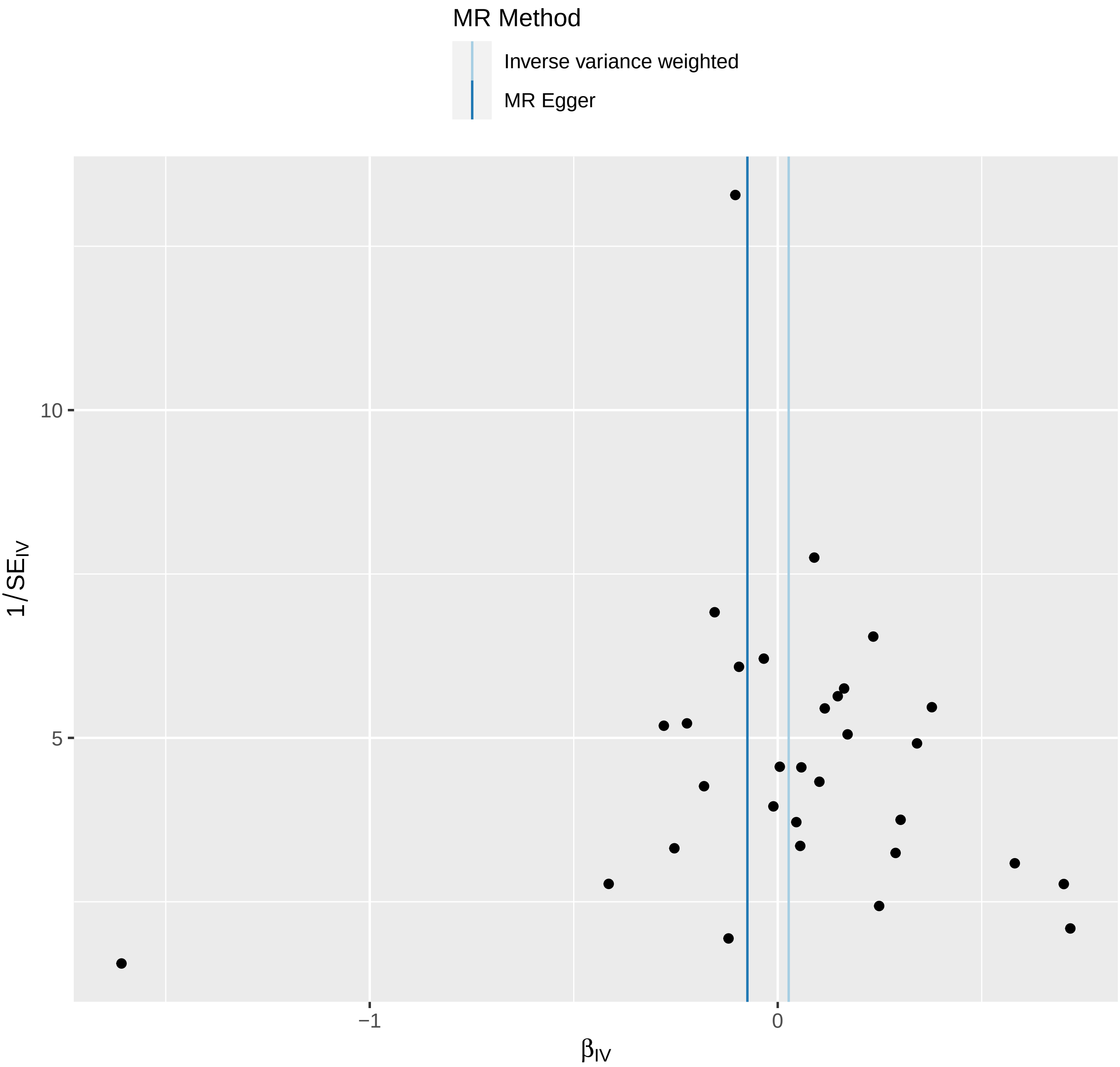

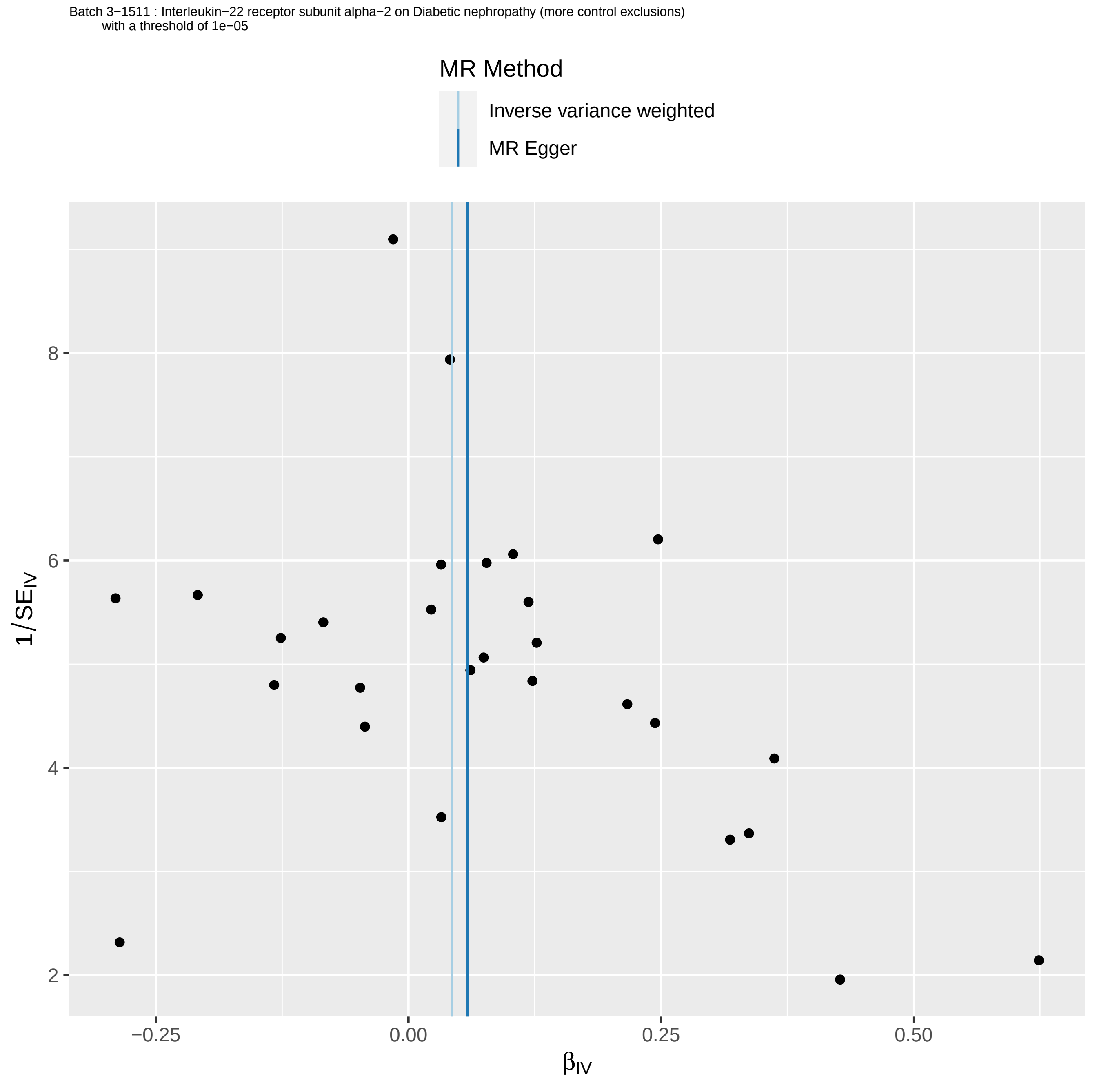

Batch 3−1511 : Interleukin−22 receptor subunit alpha−2 on Diabetic nephropathy (more control exclusions)
with a threshold of 1e−05
MR Method
Inverse variance weighted
MR Egger
8
6
4
2
1/SE_IV
−0.25
0.00
0.25
0.50
β_IV

Batch 3−1512 : Interleukin−2 on Diabetic nephropathy (more control exclusions)
with a threshold of 1e−05

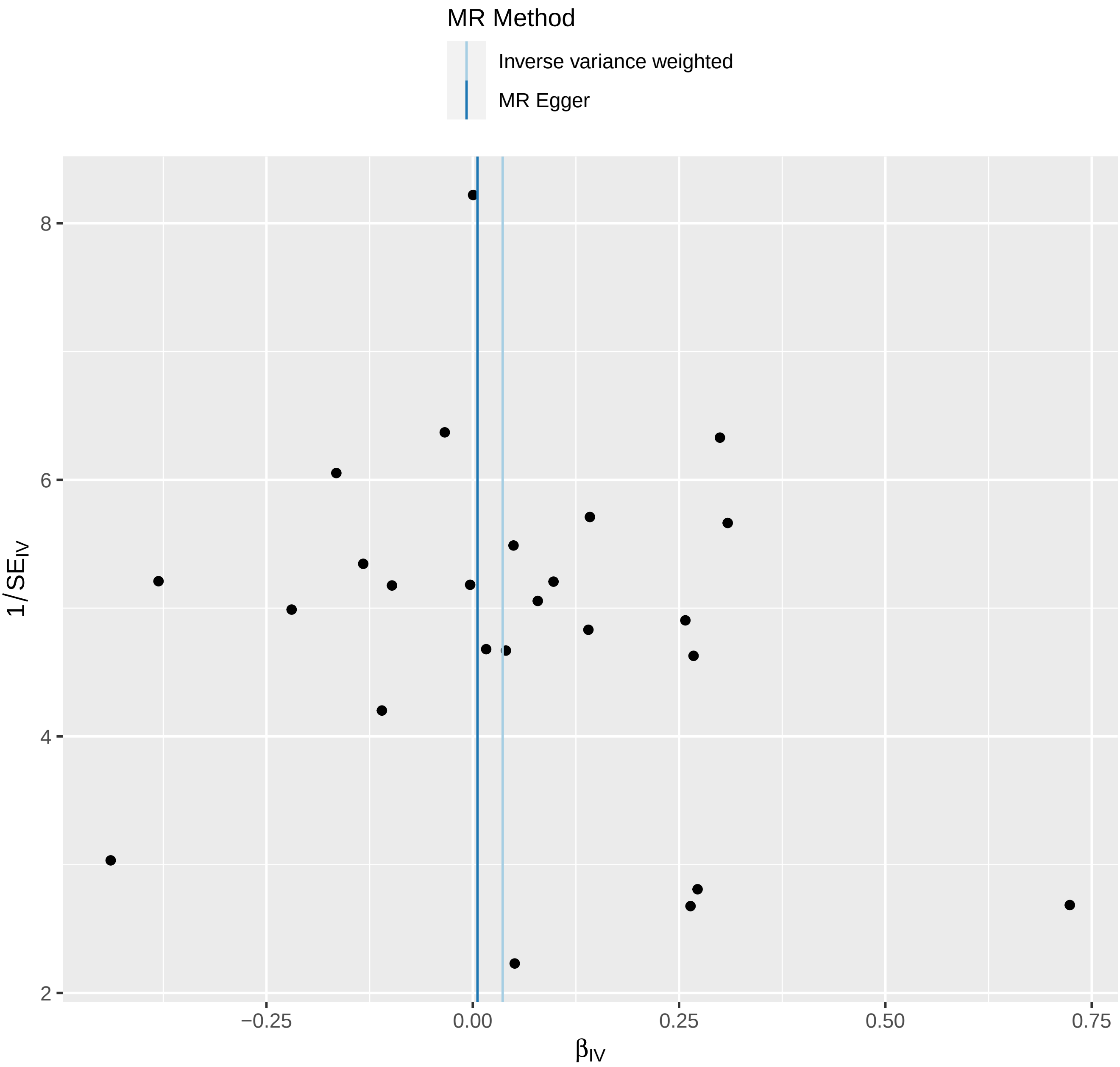

Batch 3−1513 : Interleukin−23 receptor on Diabetic nephropathy (more control exclusions)
with a threshold of 1e−05

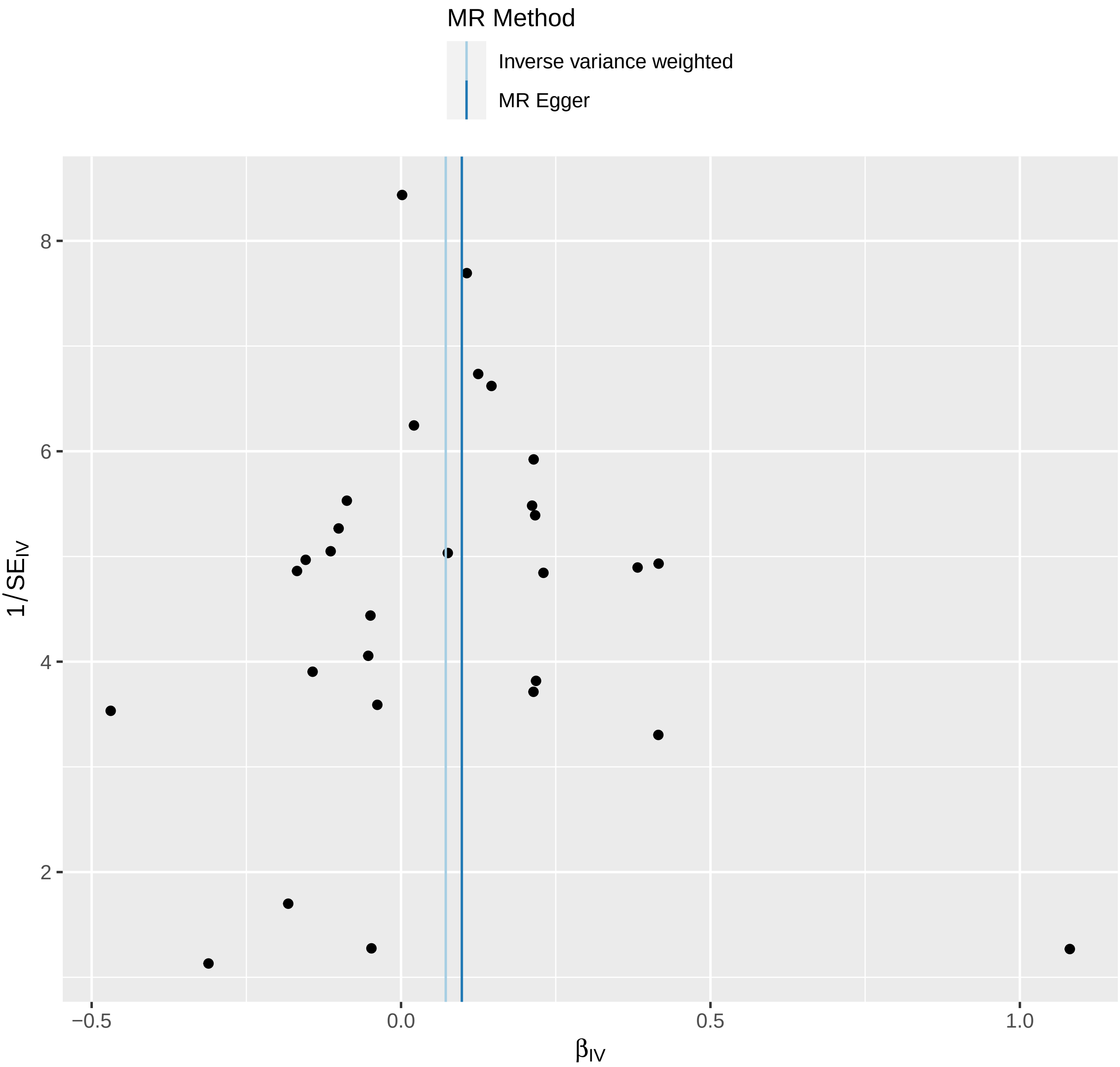

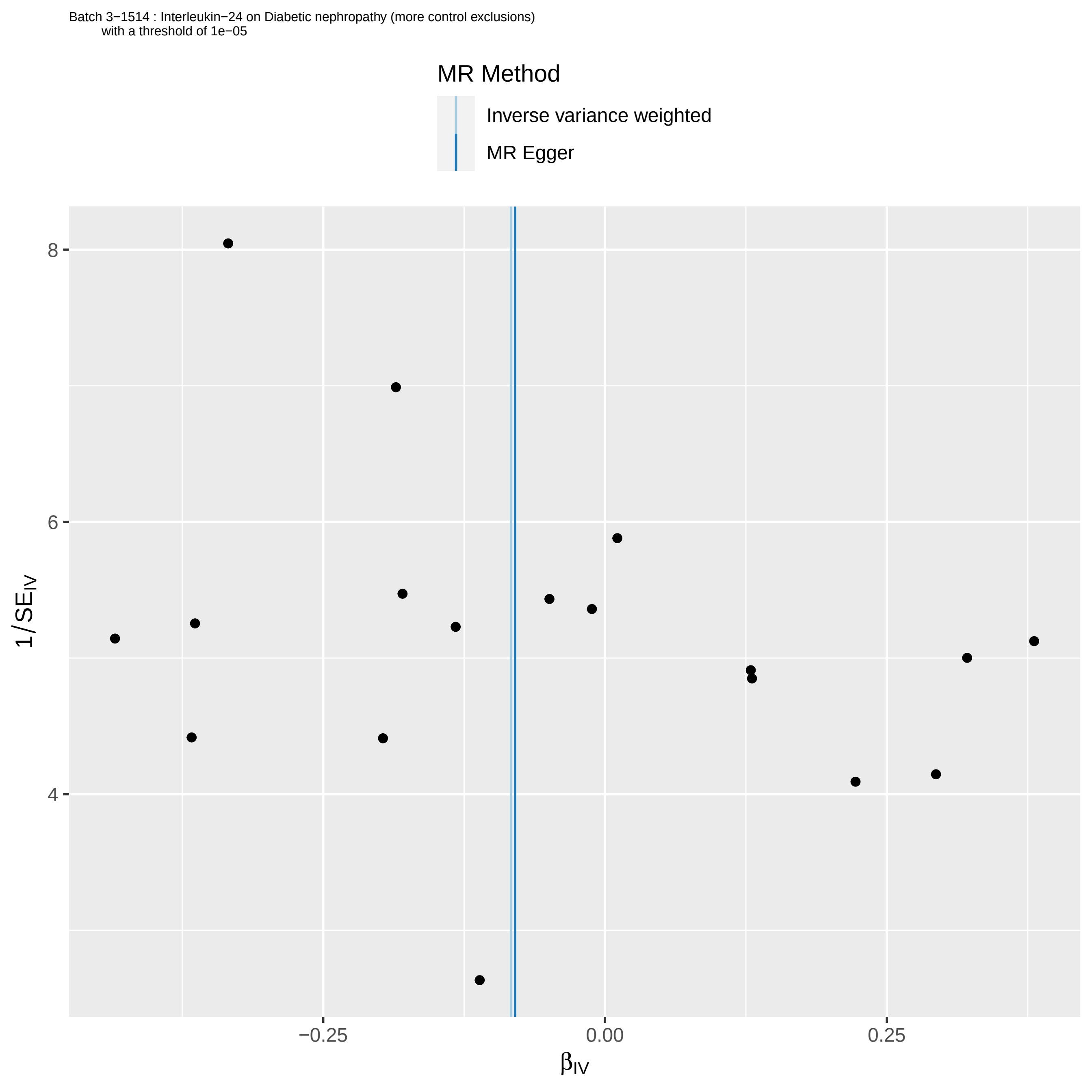
Batch 3−1514 : Interleukin−24 on Diabetic nephropathy (more control exclusions)
with a threshold of 1e−05
MR Method
Inverse variance weighted
MR Egger
8
6
4
1/SE$_{IV}$
−0.25
0.00
0.25
β_{IV}

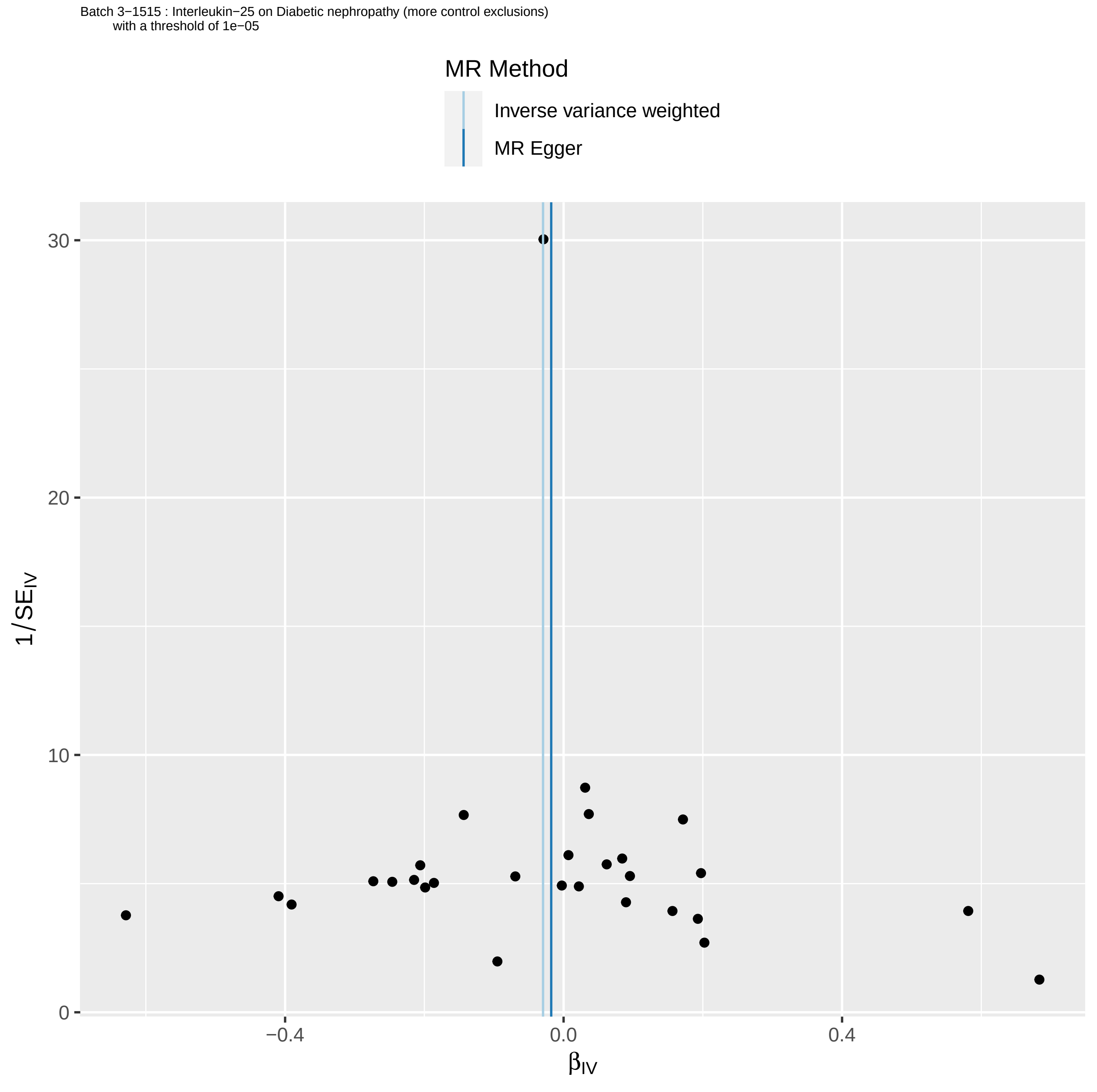
Batch 3−1515 : Interleukin−25 on Diabetic nephropathy (more control exclusions)
with a threshold of 1e−05
MR Method
Inverse variance weighted
MR Egger
30
20
10
0
1/SE_IV
−0.4
0.0
0.4
β_IV

Batch 3−1516 : Interleukin−27 on Diabetic nephropathy (more control exclusions)
with a threshold of 1e−05

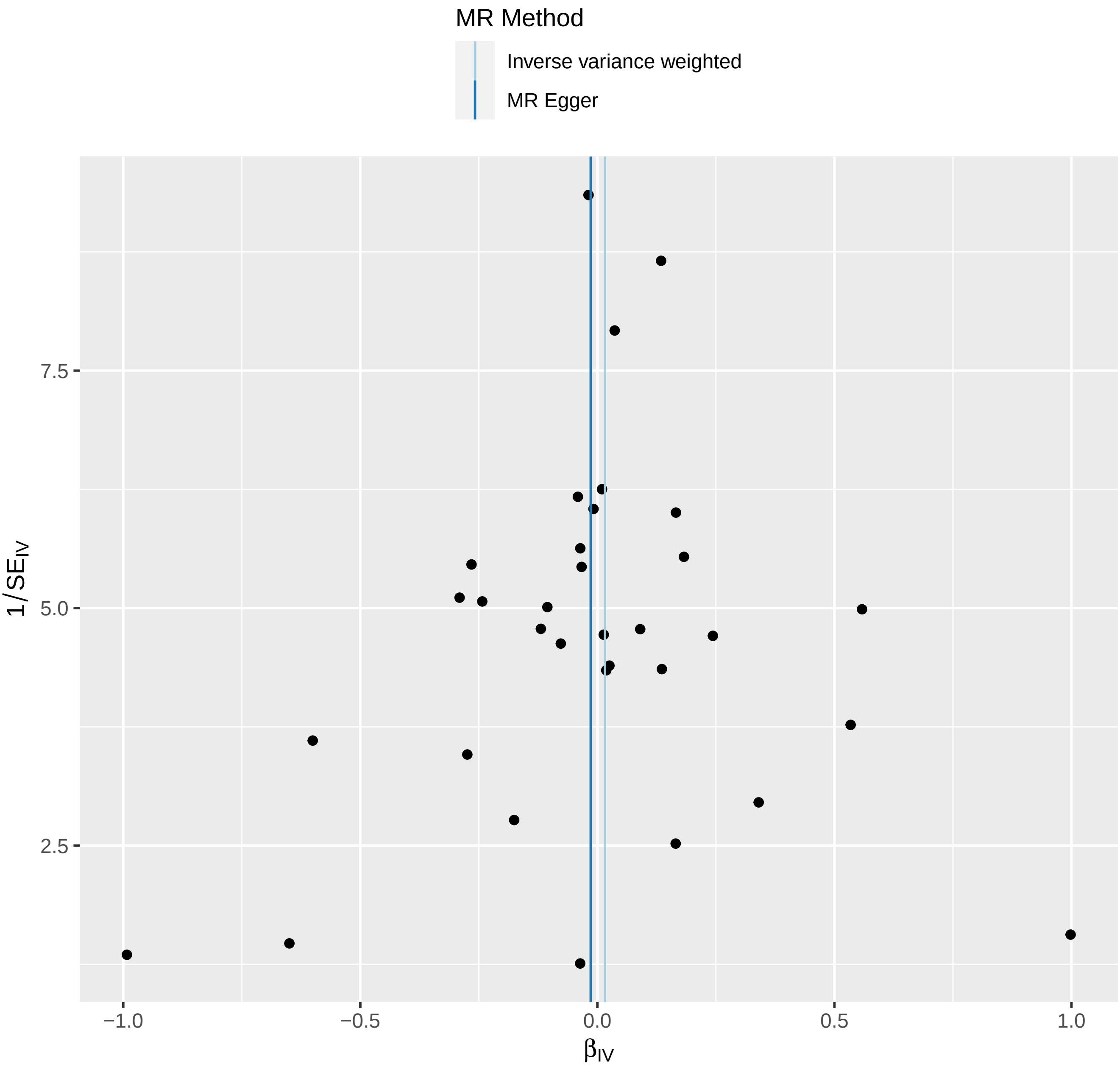

Batch 3−1517 : Interleukin−27 receptor subunit alpha on Diabetic nephropathy (more control exclusions)
with a threshold of 1e−05

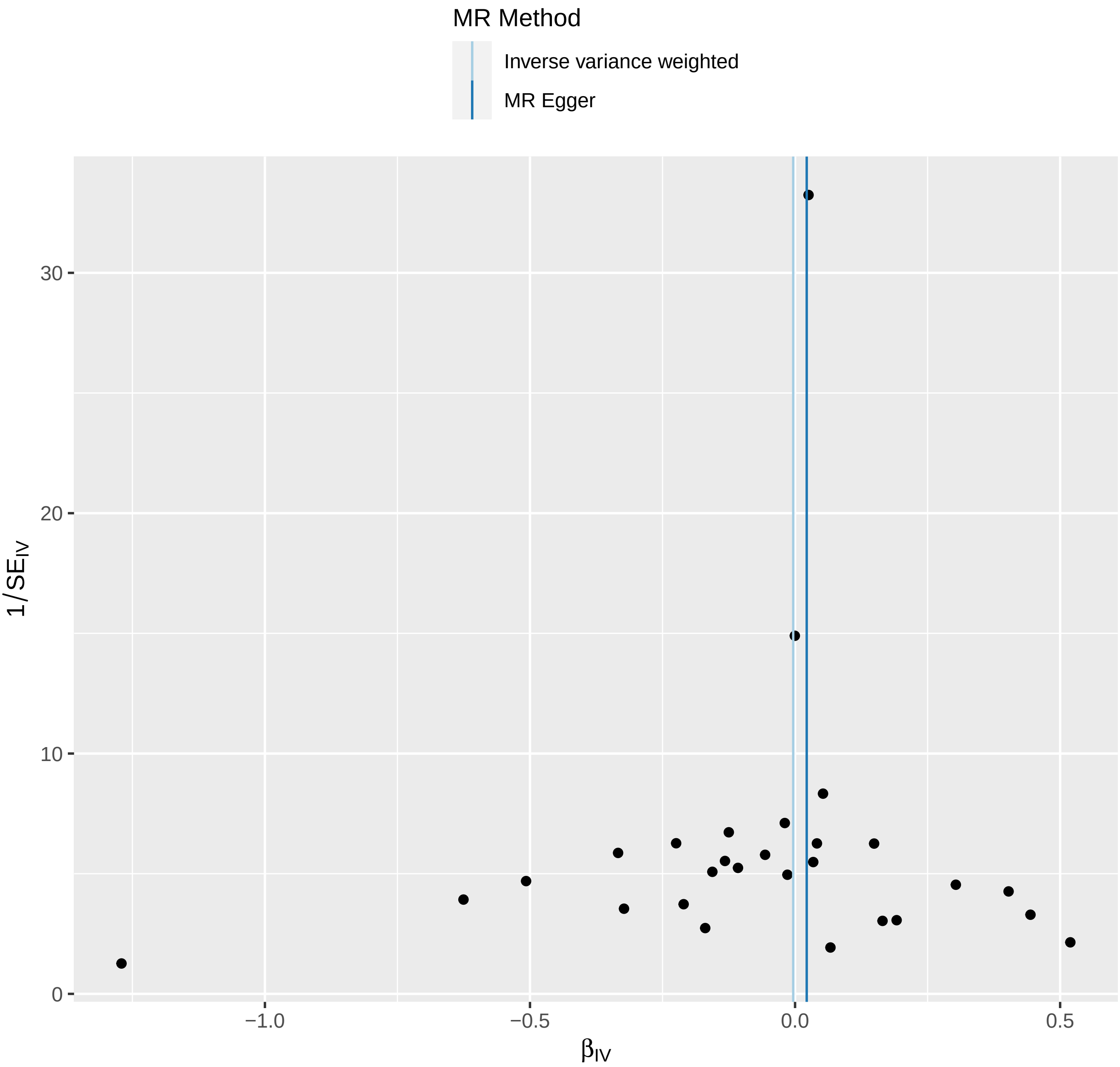

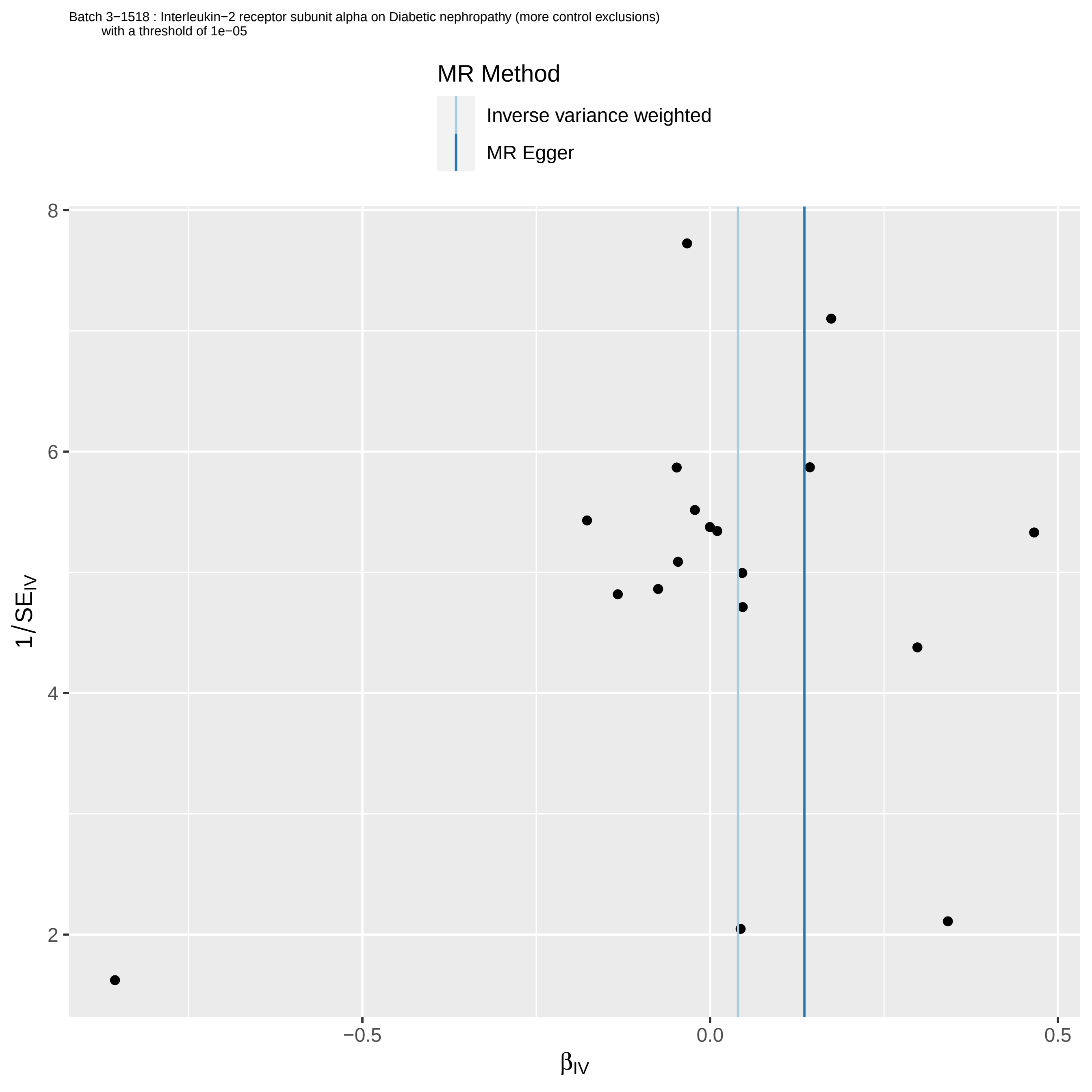
Batch 3−1518 : Interleukin−2 receptor subunit alpha on Diabetic nephropathy (more control exclusions)
with a threshold of 1e−05
MR Method
Inverse variance weighted
MR Egger
8
6
4
2
1/SE_IV
−0.5
0.0
0.5
β_IV

Batch 3−1519 : Interleukin−2 receptor subunit beta on Diabetic nephropathy (more control exclusions)
with a threshold of 1e−05

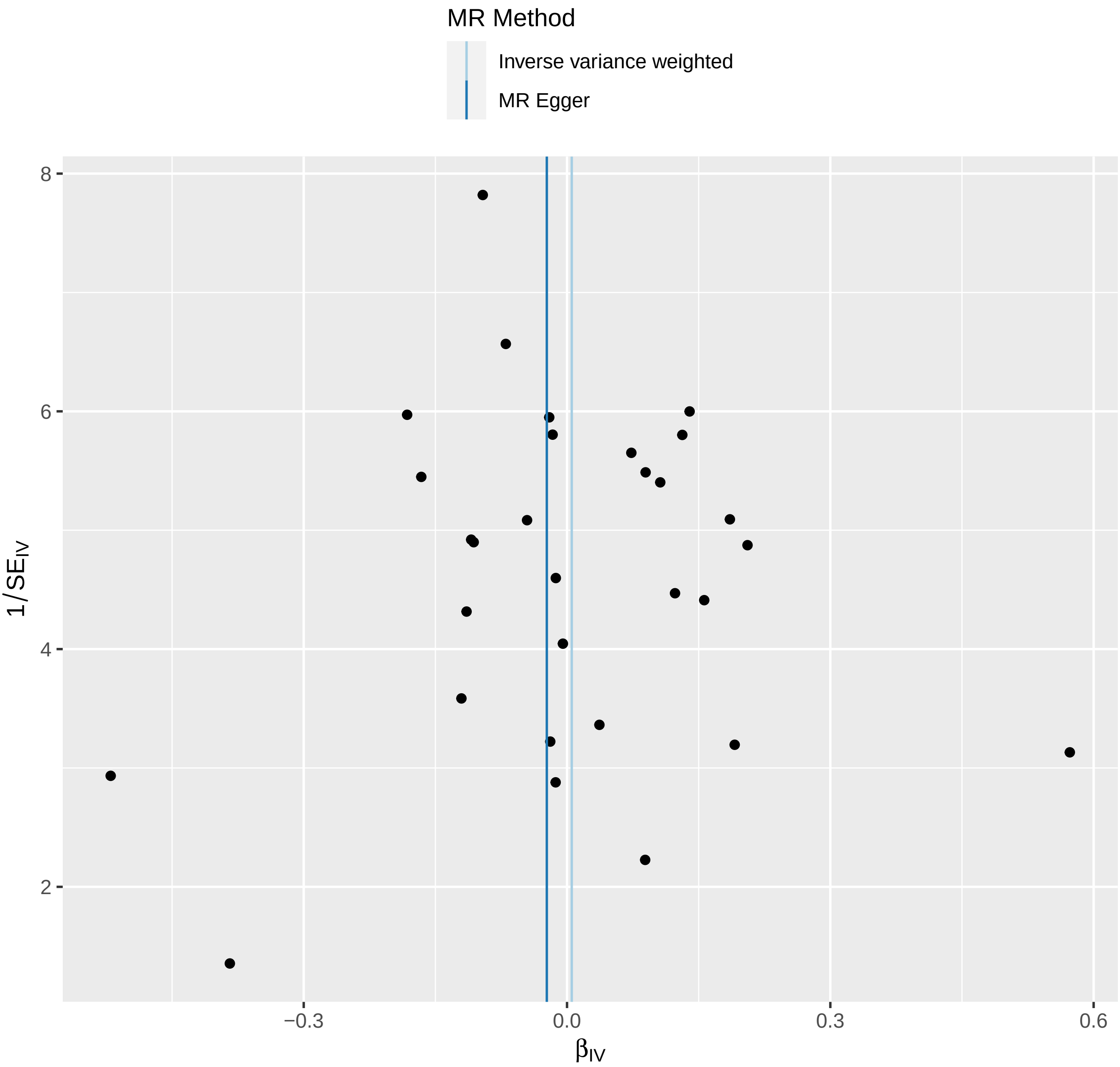

Batch 3−1520 : Cytokine receptor common subunit gamma on Diabetic nephropathy (more control exclusions)
with a threshold of 1e−05

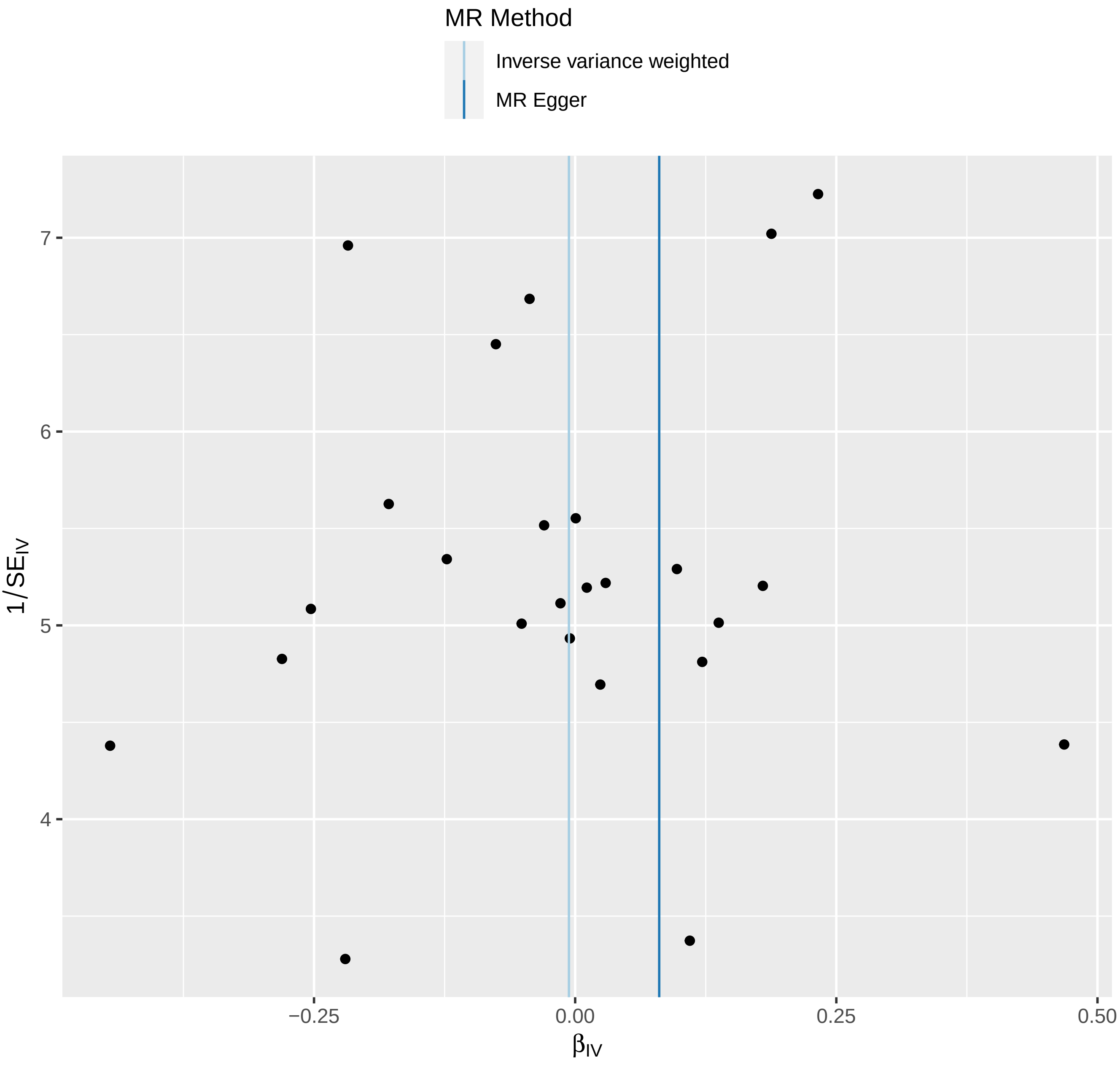

Batch 3−1521 : Interleukin−31 on Diabetic nephropathy (more control exclusions)
with a threshold of 1e−05

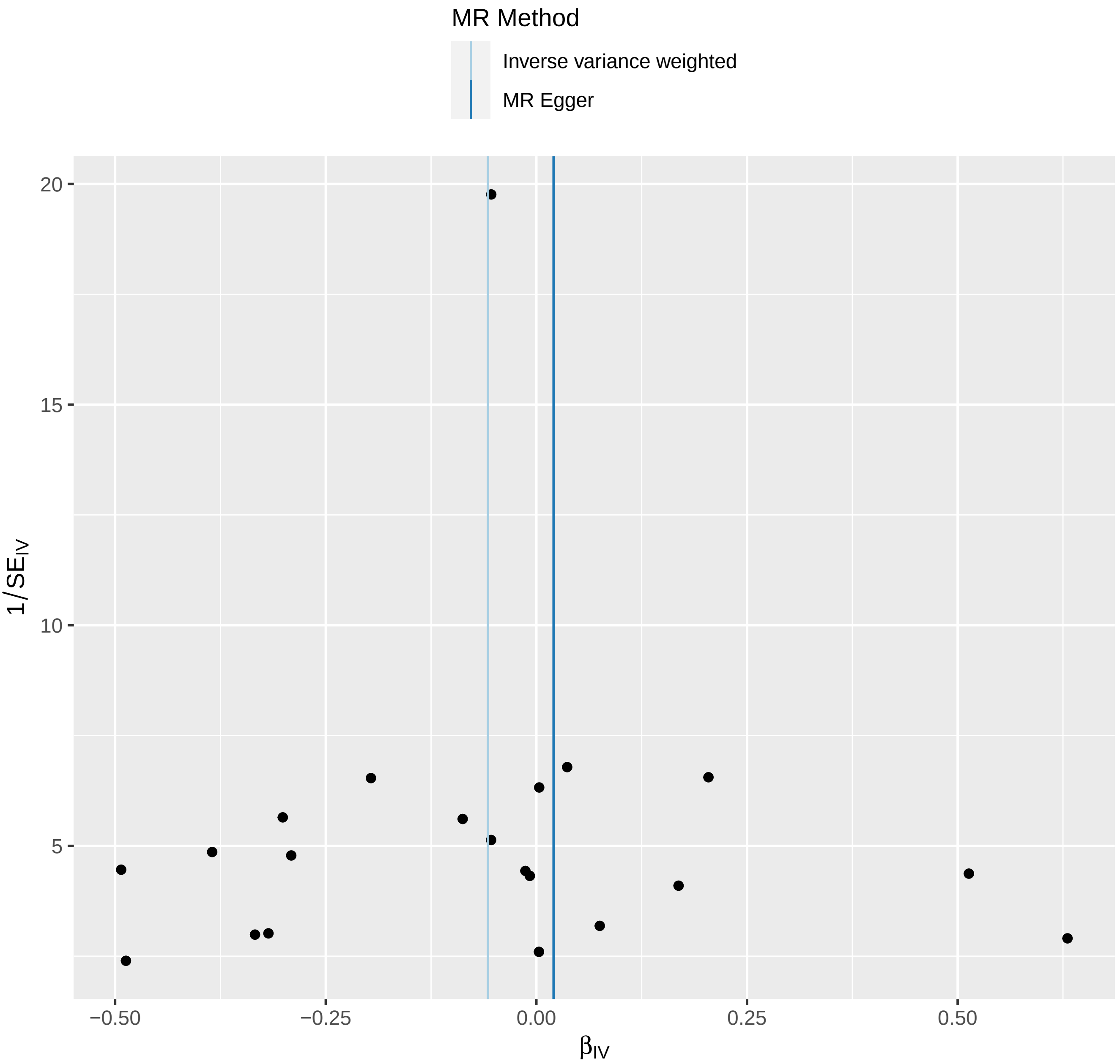

Batch 3−1522 : Interleukin−31 receptor subunit alpha on Diabetic nephropathy (more control exclusions) with a threshold of 1e−05

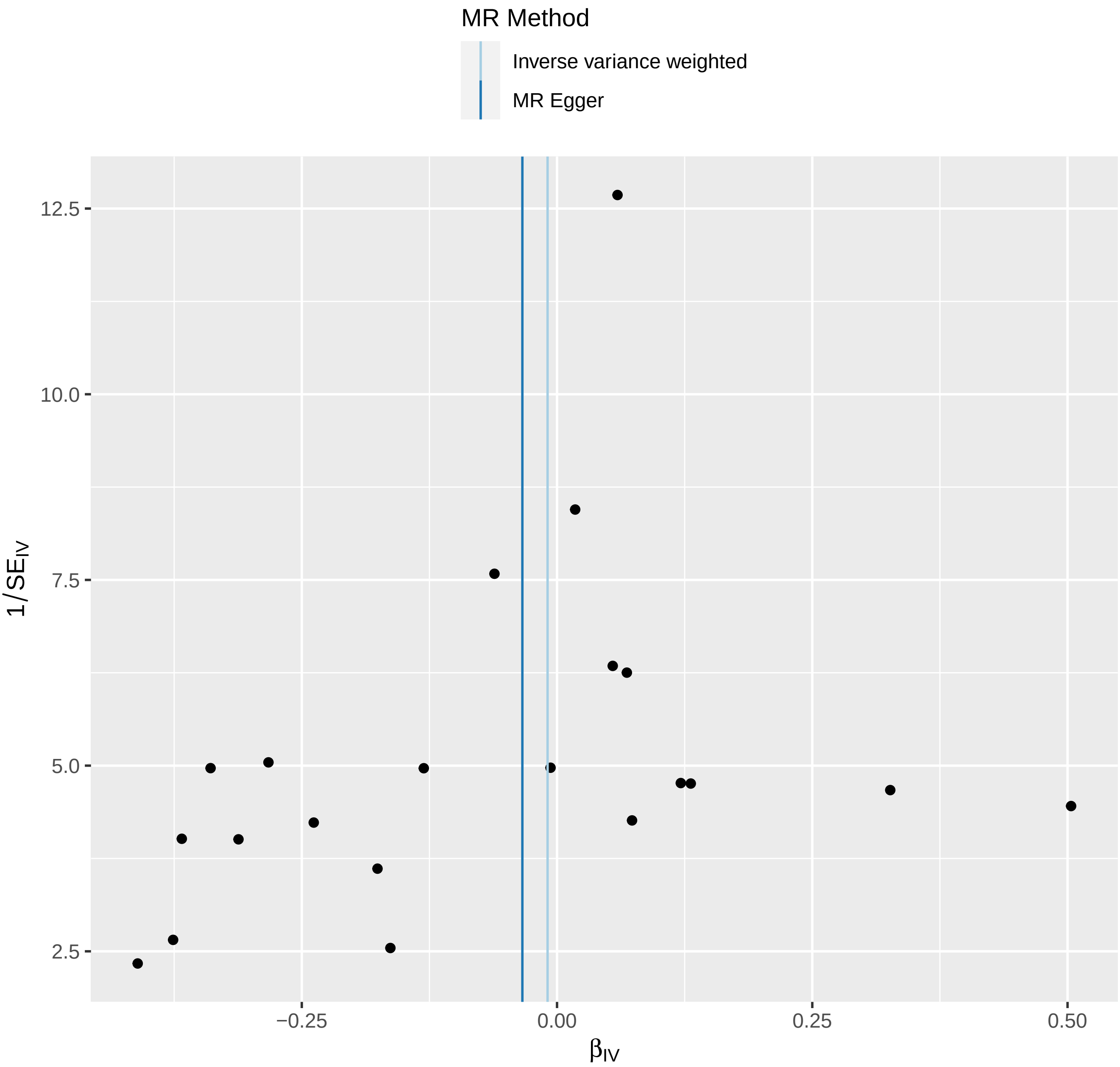

Batch 3−1523 : Interleukin−32 on Diabetic nephropathy (more control exclusions)
with a threshold of 1e−05

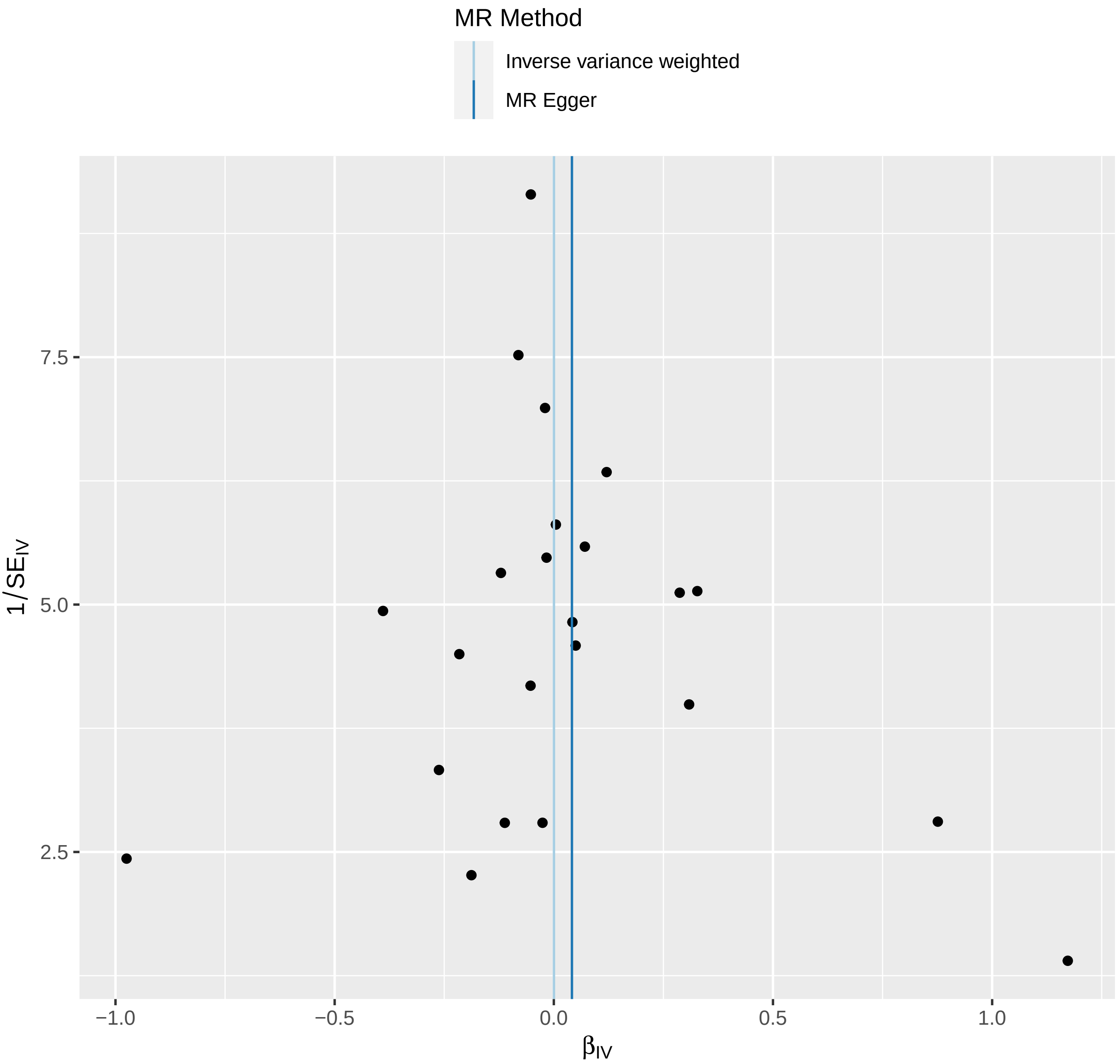

Batch 3−1524 : Interleukin−34 on Diabetic nephropathy (more control exclusions)
with a threshold of 1e−05

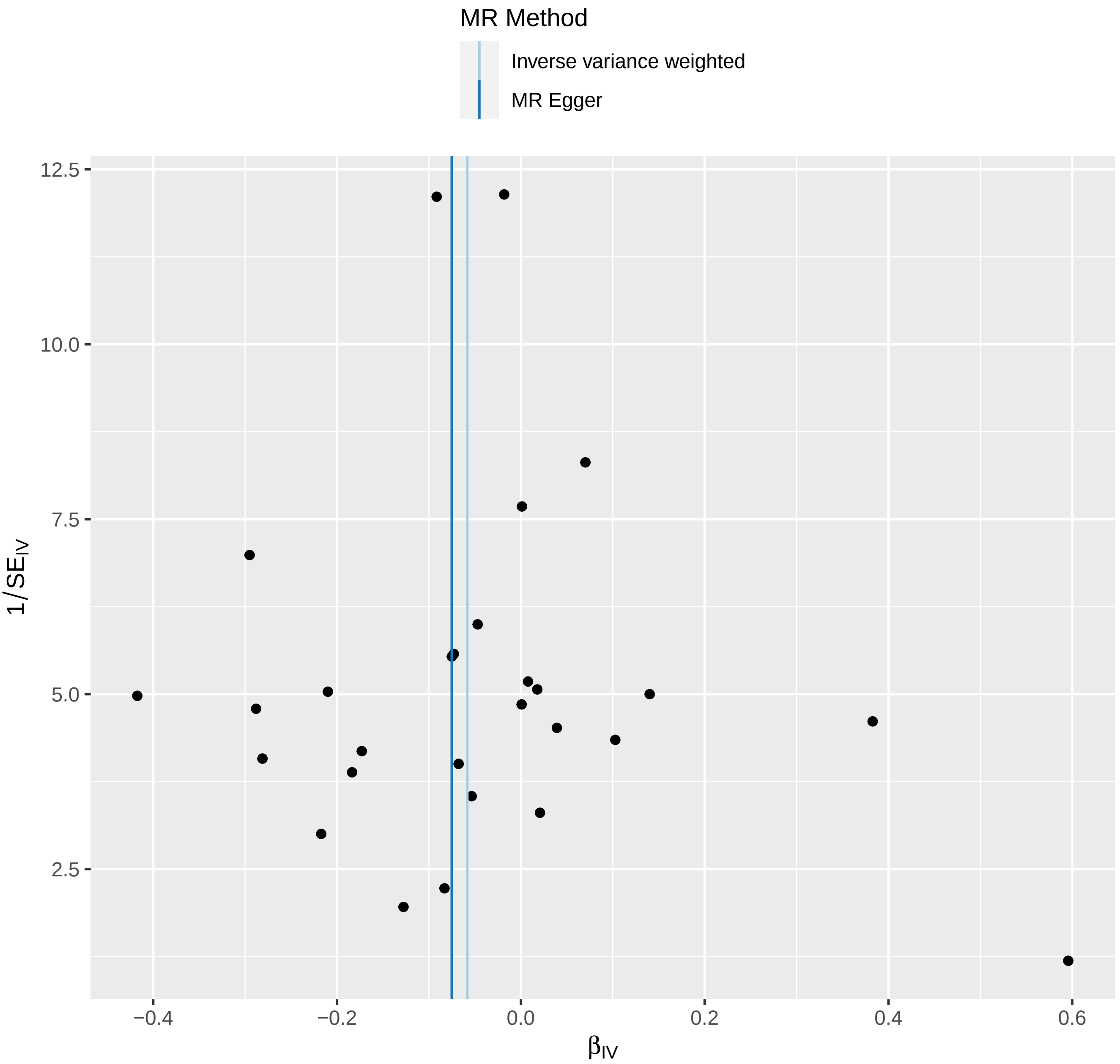

Batch 3−1525 : Interleukin−3 on Diabetic nephropathy (more control exclusions)
with a threshold of 1e−05

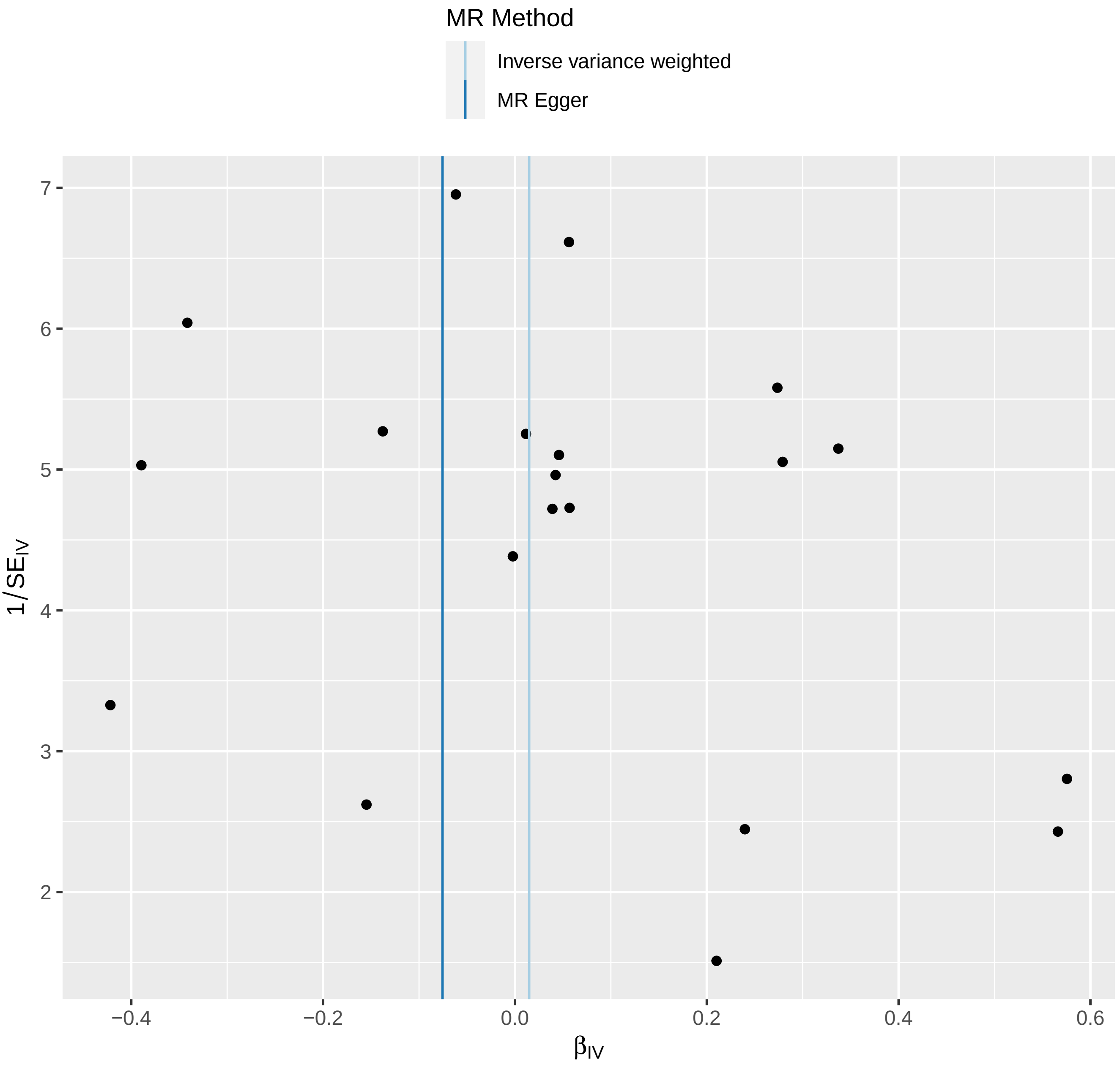

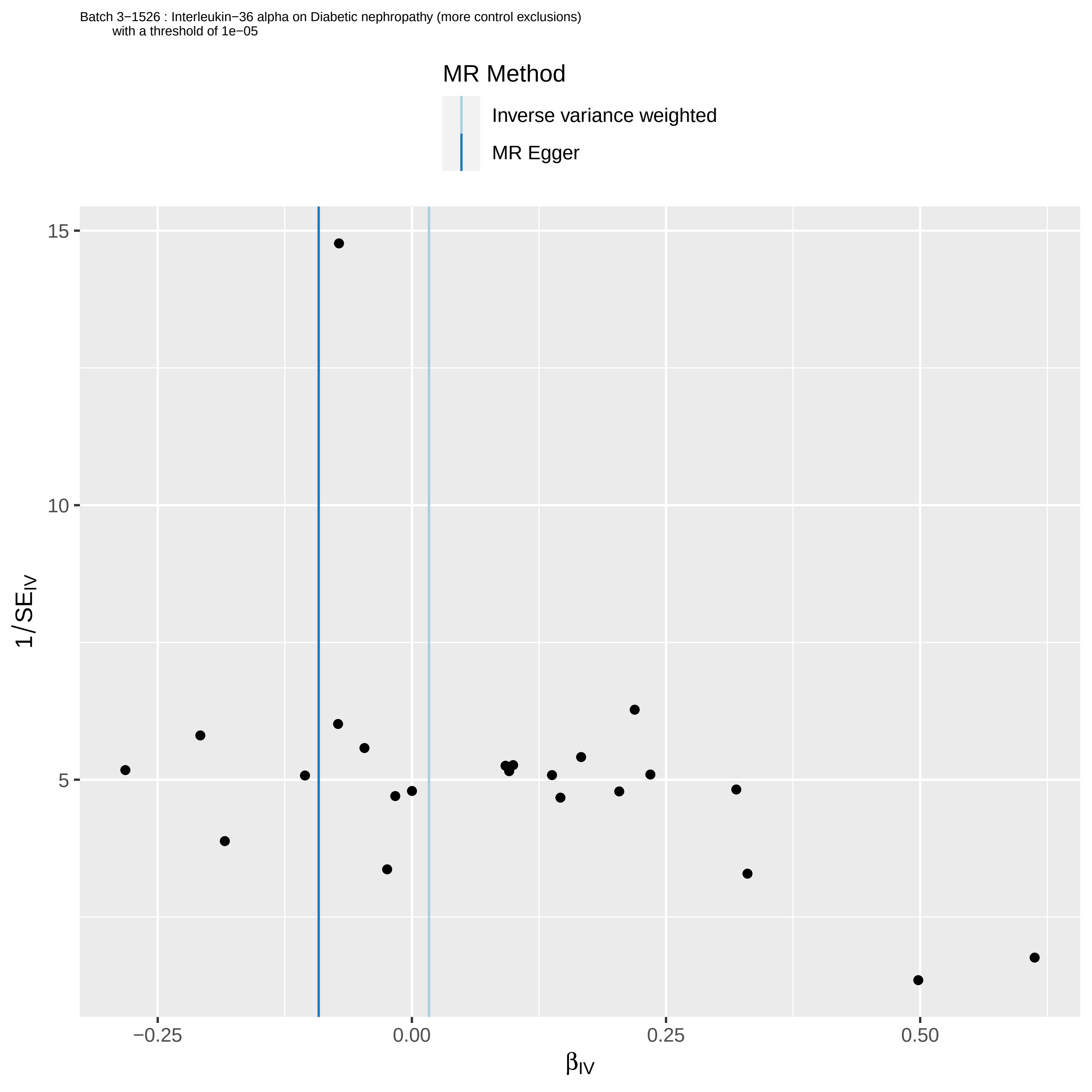

Batch 3−1526 : Interleukin−36 alpha on Diabetic nephropathy (more control exclusions)
with a threshold of 1e−05
MR Method
Inverse variance weighted
MR Egger
15
10
5
1/SE_IV
−0.25
0.00
0.25
0.50
β_IV

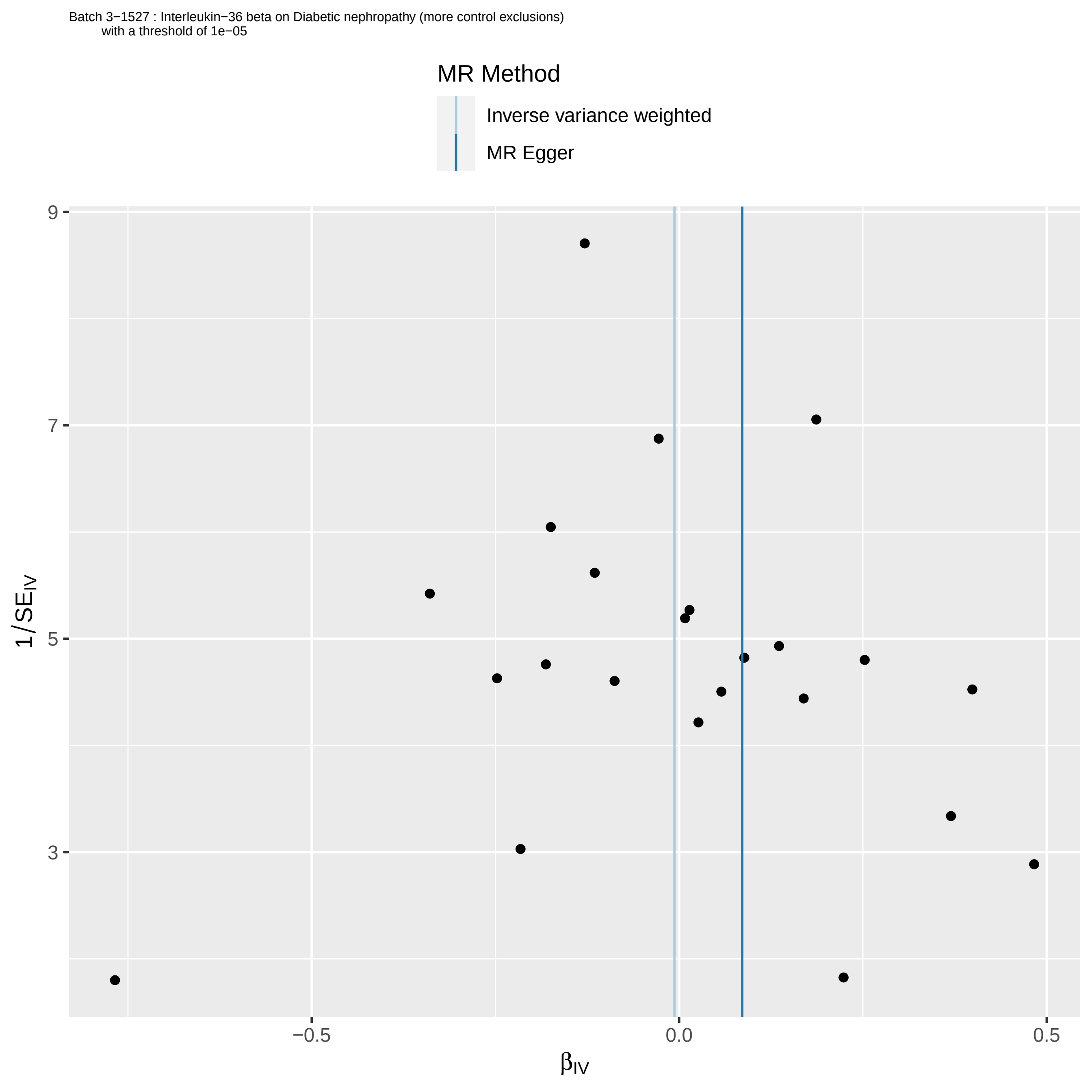
Batch 3−1527 : Interleukin−36 beta on Diabetic nephropathy (more control exclusions)
with a threshold of 1e−05
MR Method
Inverse variance weighted
MR Egger
9
7
5
3
1/SE$_{IV}$
−0.5
0.0
0.5
β_{IV}

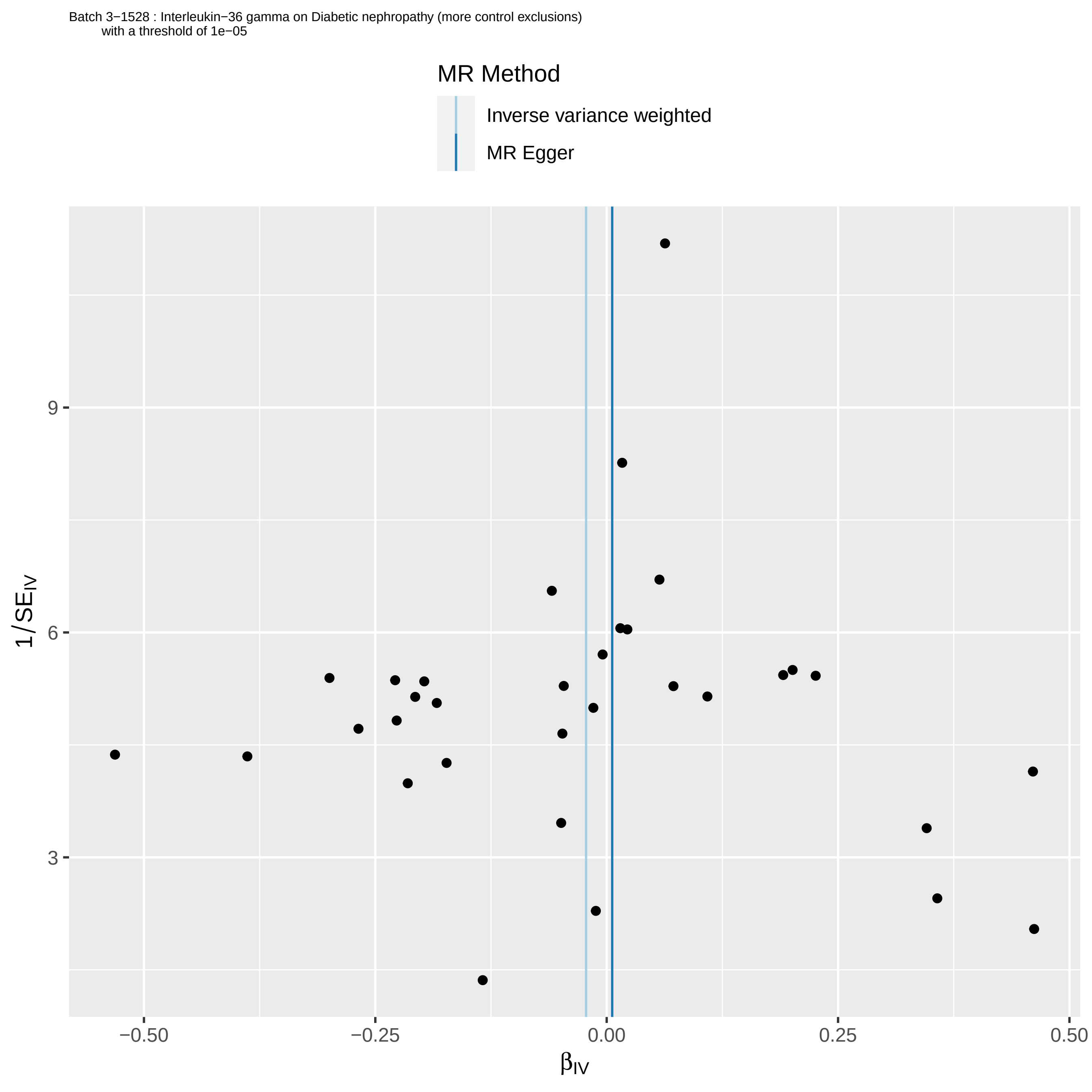
Batch 3−1528 : Interleukin−36 gamma on Diabetic nephropathy (more control exclusions)
with a threshold of 1e−05
MR Method
Inverse variance weighted
MR Egger
9
6
3
1/SE$_{IV}$
−0.50
−0.25
0.00
0.25
0.50
β_{IV}

Batch 3−1529 : Interleukin−37 on Diabetic nephropathy (more control exclusions)
with a threshold of 1e−05

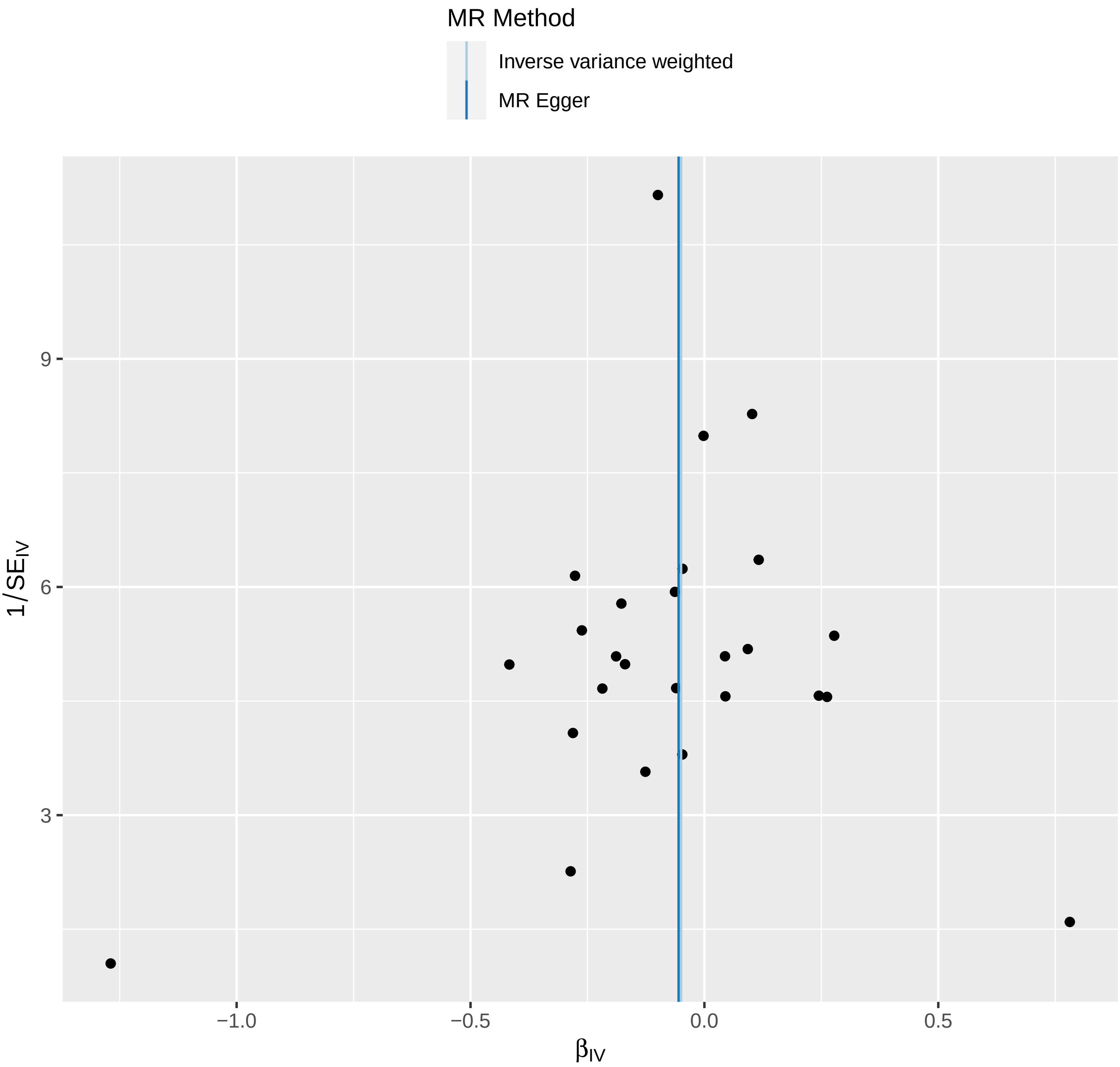

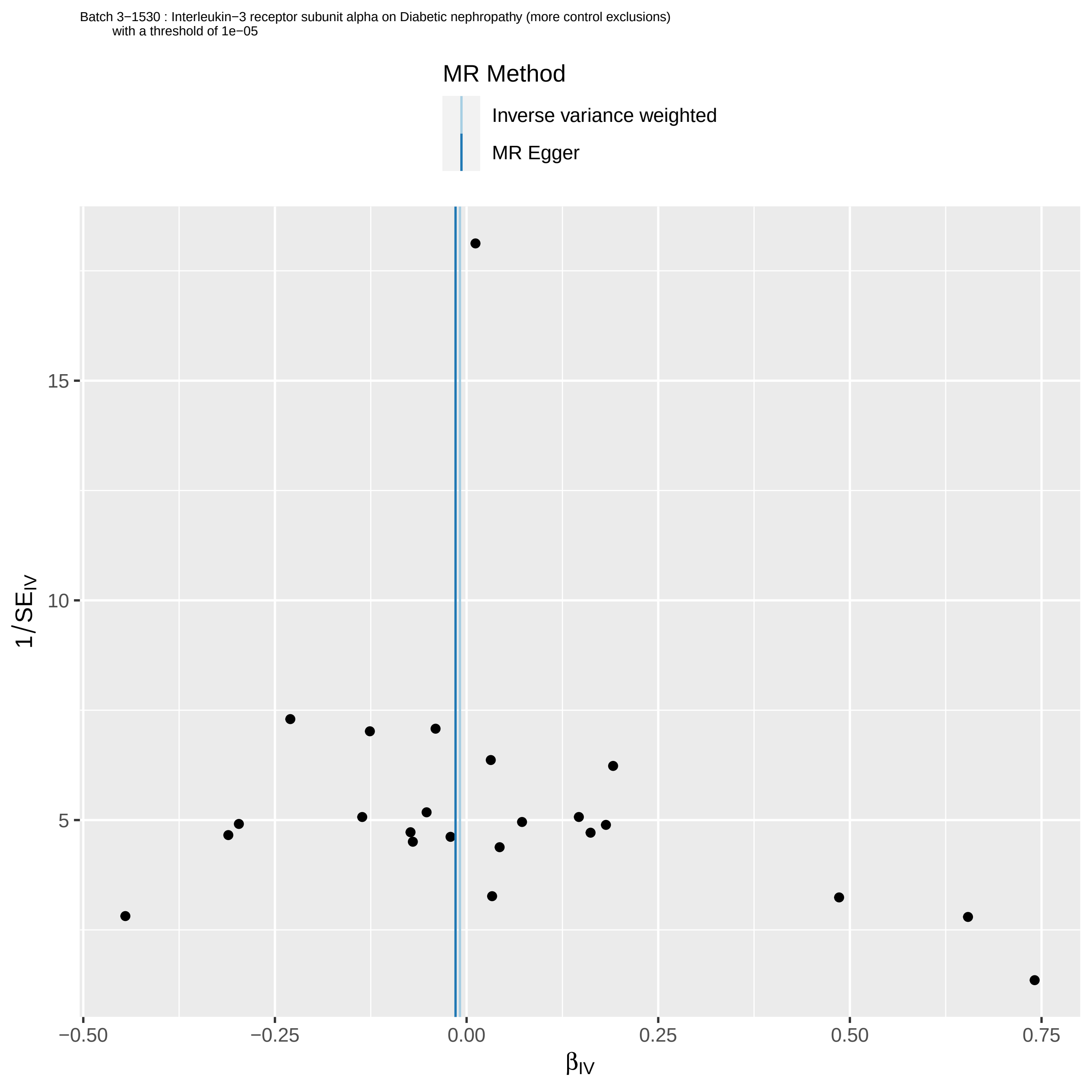

Batch 3−1530 : Interleukin−3 receptor subunit alpha on Diabetic nephropathy (more control exclusions)
with a threshold of 1e−05
MR Method
Inverse variance weighted
MR Egger
15
10
5
1/SE_IV
−0.50
−0.25
0.00
0.25
0.50
0.75
β_IV

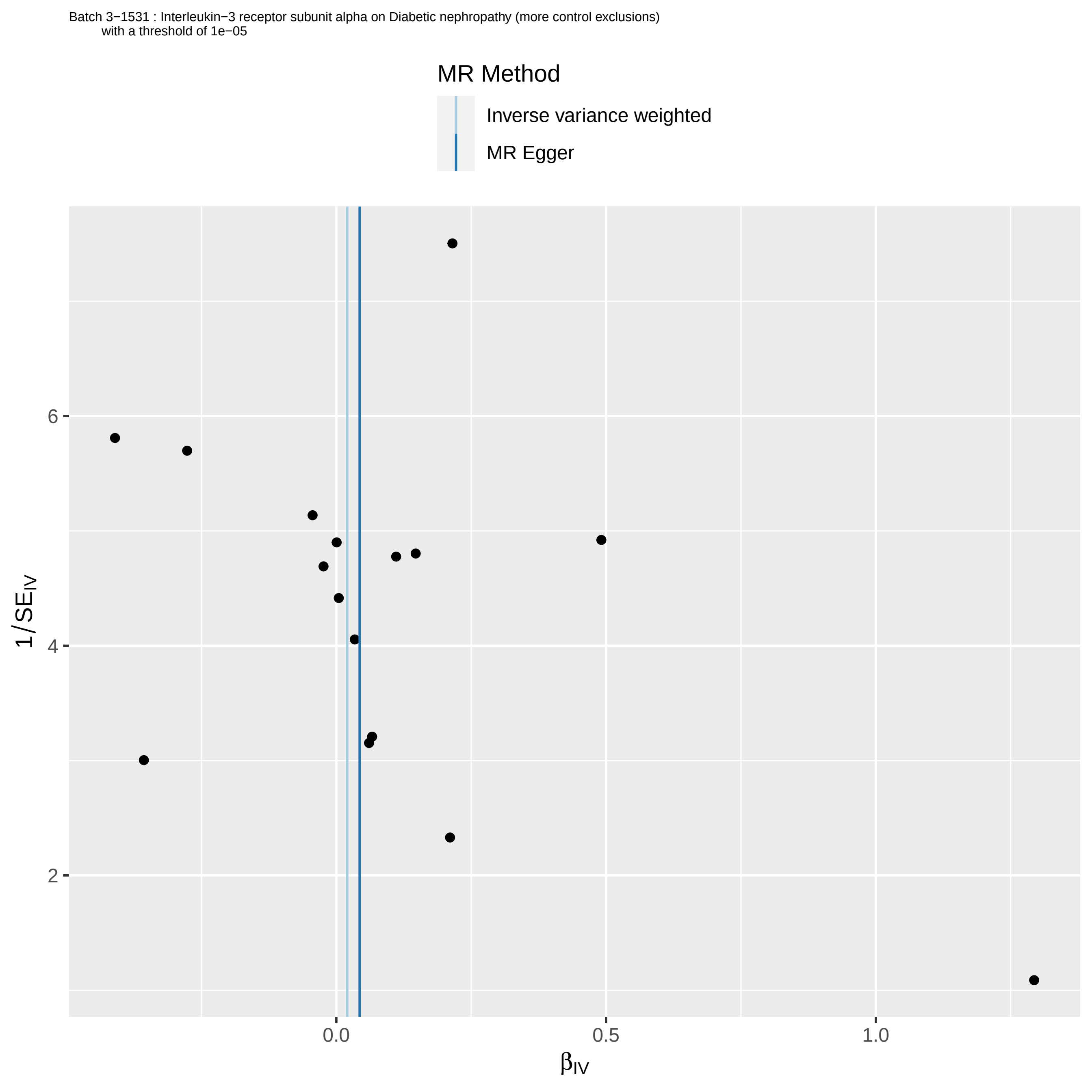

Batch 3−1531 : Interleukin−3 receptor subunit alpha on Diabetic nephropathy (more control exclusions)
with a threshold of 1e−05
MR Method
Inverse variance weighted
MR Egger
6
4
2
$1/SE_{IV}$
0.0
0.5
1.0
β_{IV}

Batch 3−1532 : Interleukin−4 on Diabetic nephropathy (more control exclusions)
with a threshold of 1e−05

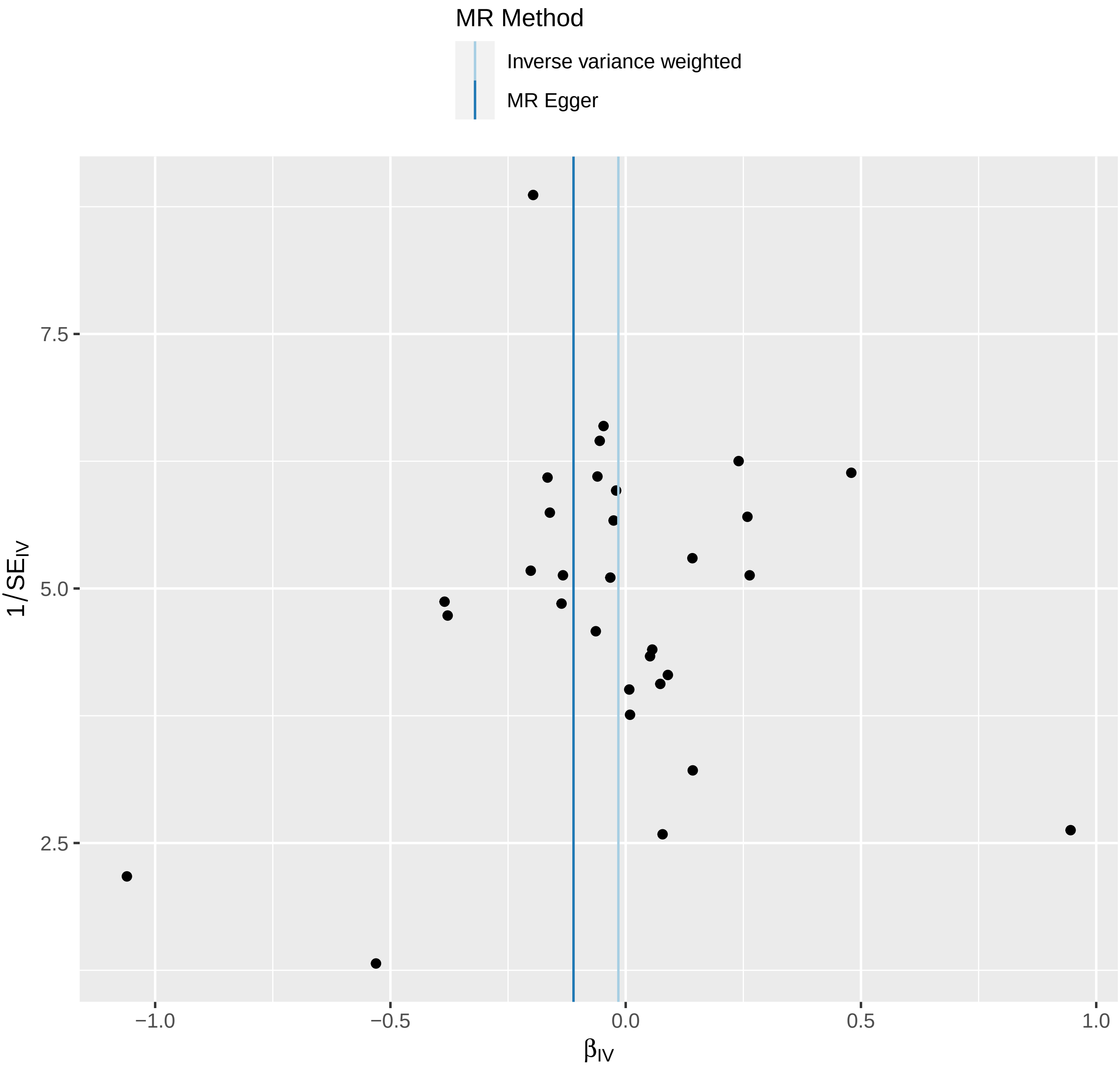

Batch 3−1533 : Interleukin−4 receptor subunit alpha on Diabetic nephropathy (more control exclusions) with a threshold of 1e−05

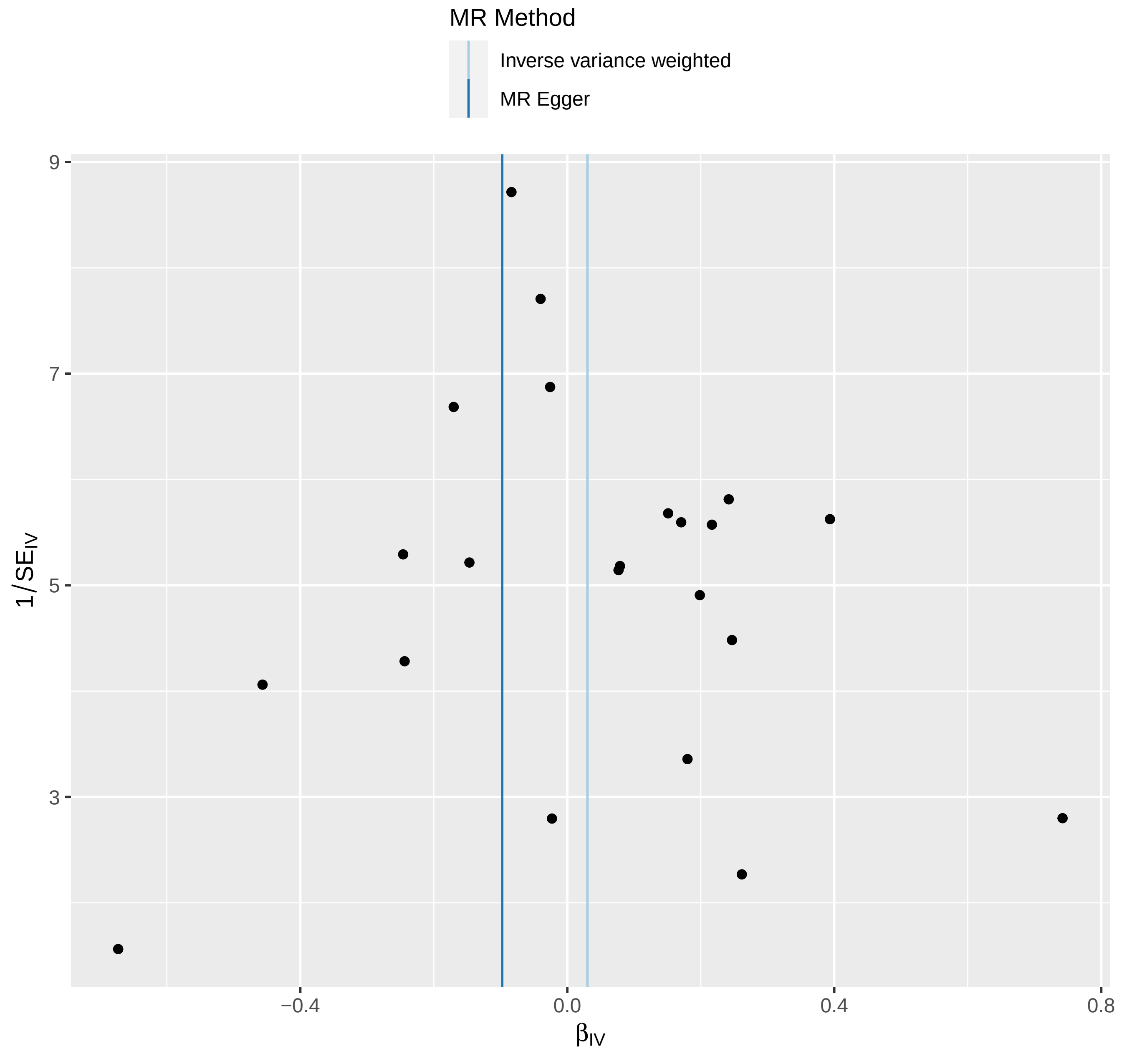

Batch 3−1534 : Interleukin−5 on Diabetic nephropathy (more control exclusions)
with a threshold of 1e−05

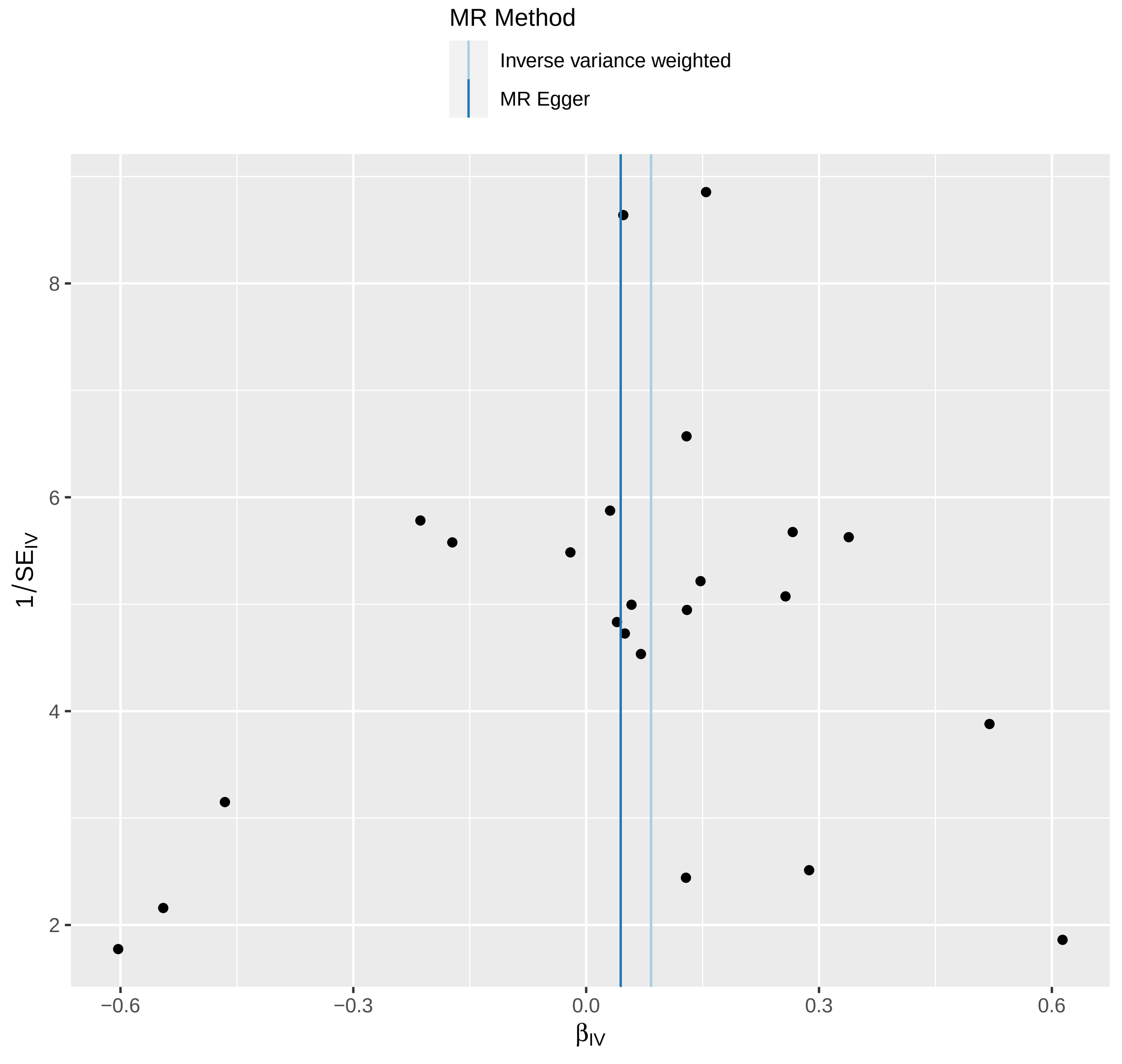

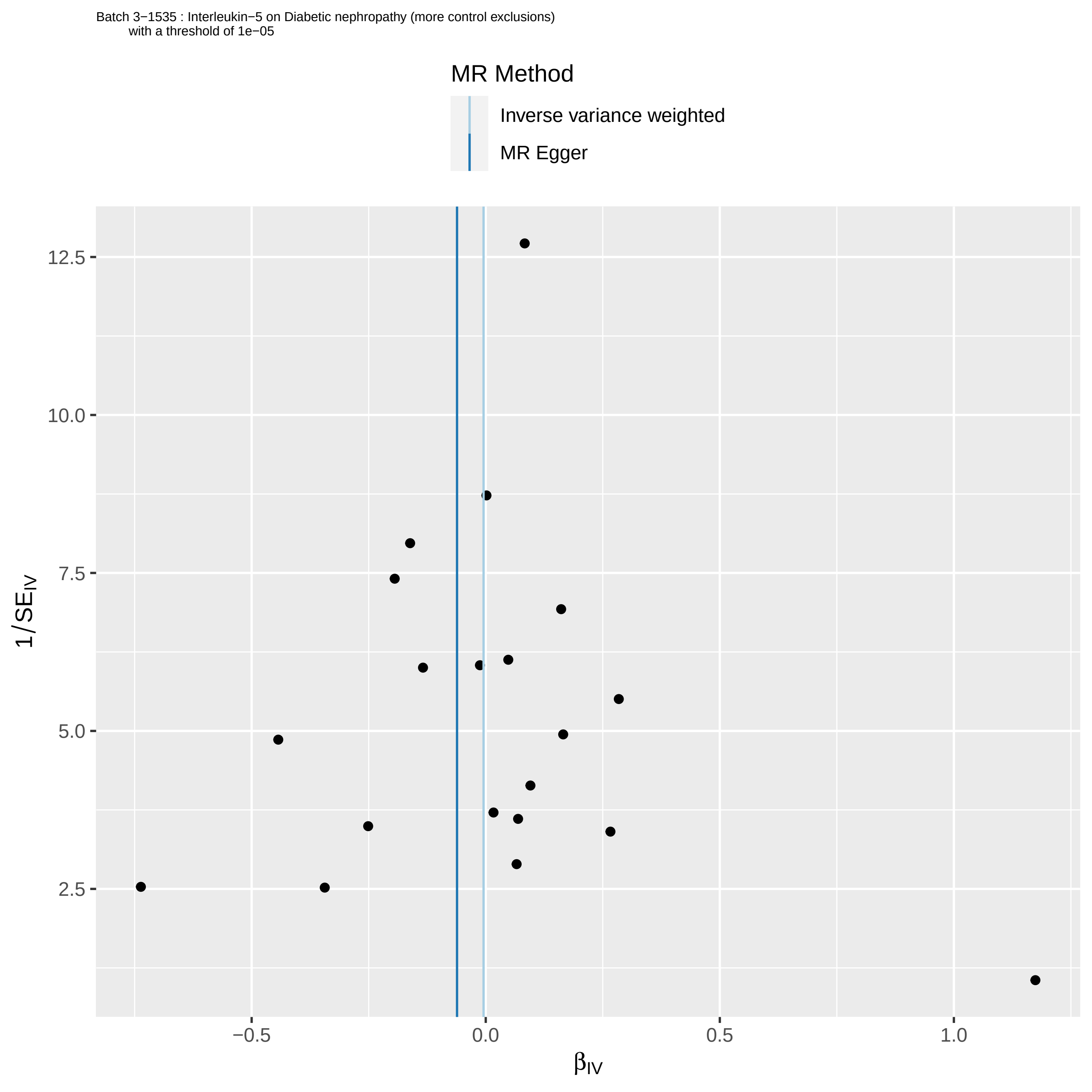

Batch 3−1535 : Interleukin−5 on Diabetic nephropathy (more control exclusions)
with a threshold of 1e−05
MR Method
Inverse variance weighted
MR Egger
12.5
10.0
7.5
5.0
2.5
1/SE_IV
−0.5
0.0
0.5
1.0
β_IV

Batch 3−1536 : Interleukin−5 receptor subunit alpha on Diabetic nephropathy (more control exclusions) with a threshold of 1e−05

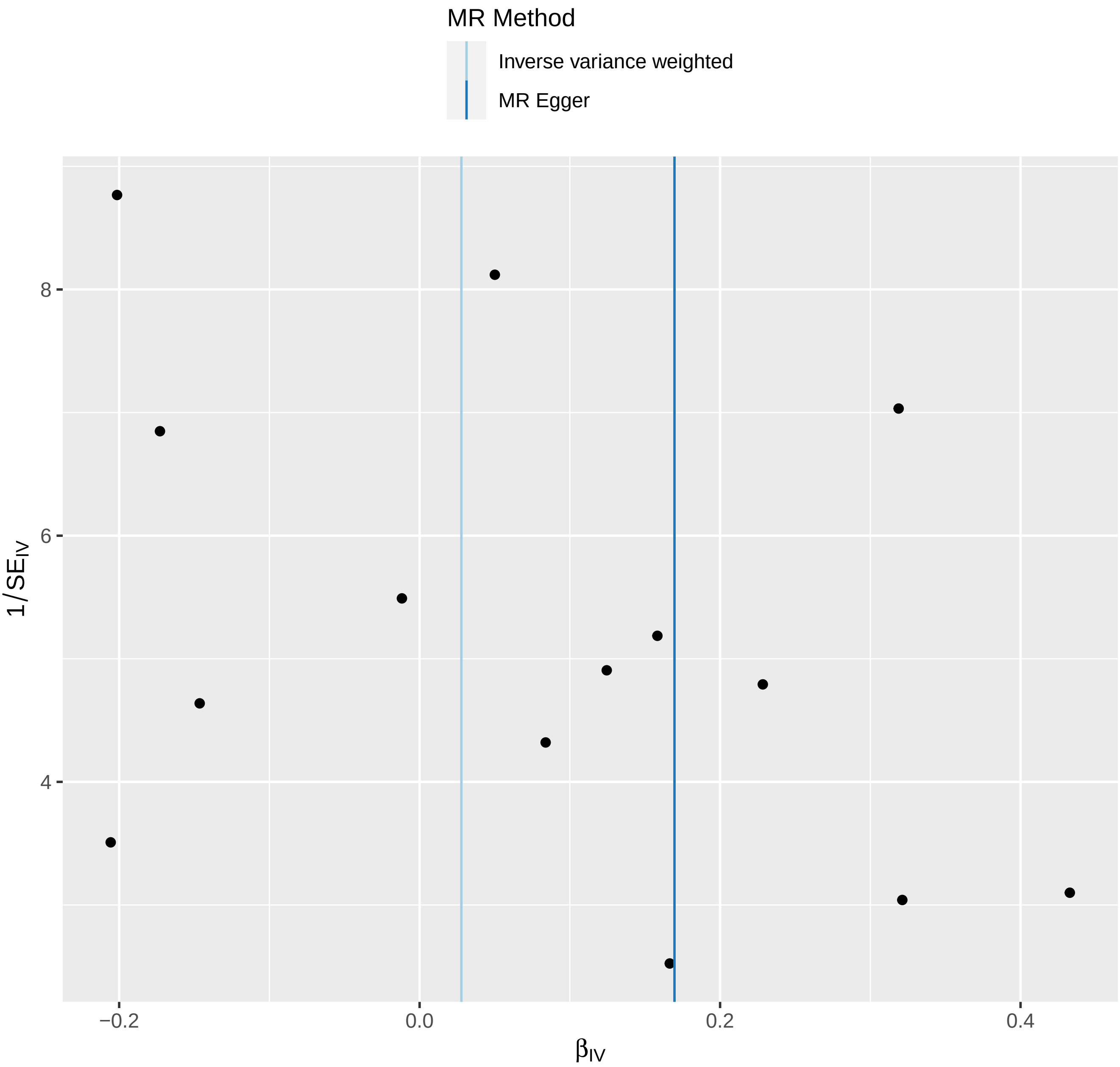

Batch 3−1537 : Interleukin−5 receptor subunit alpha on Diabetic nephropathy (more control exclusions)
with a threshold of 1e−05

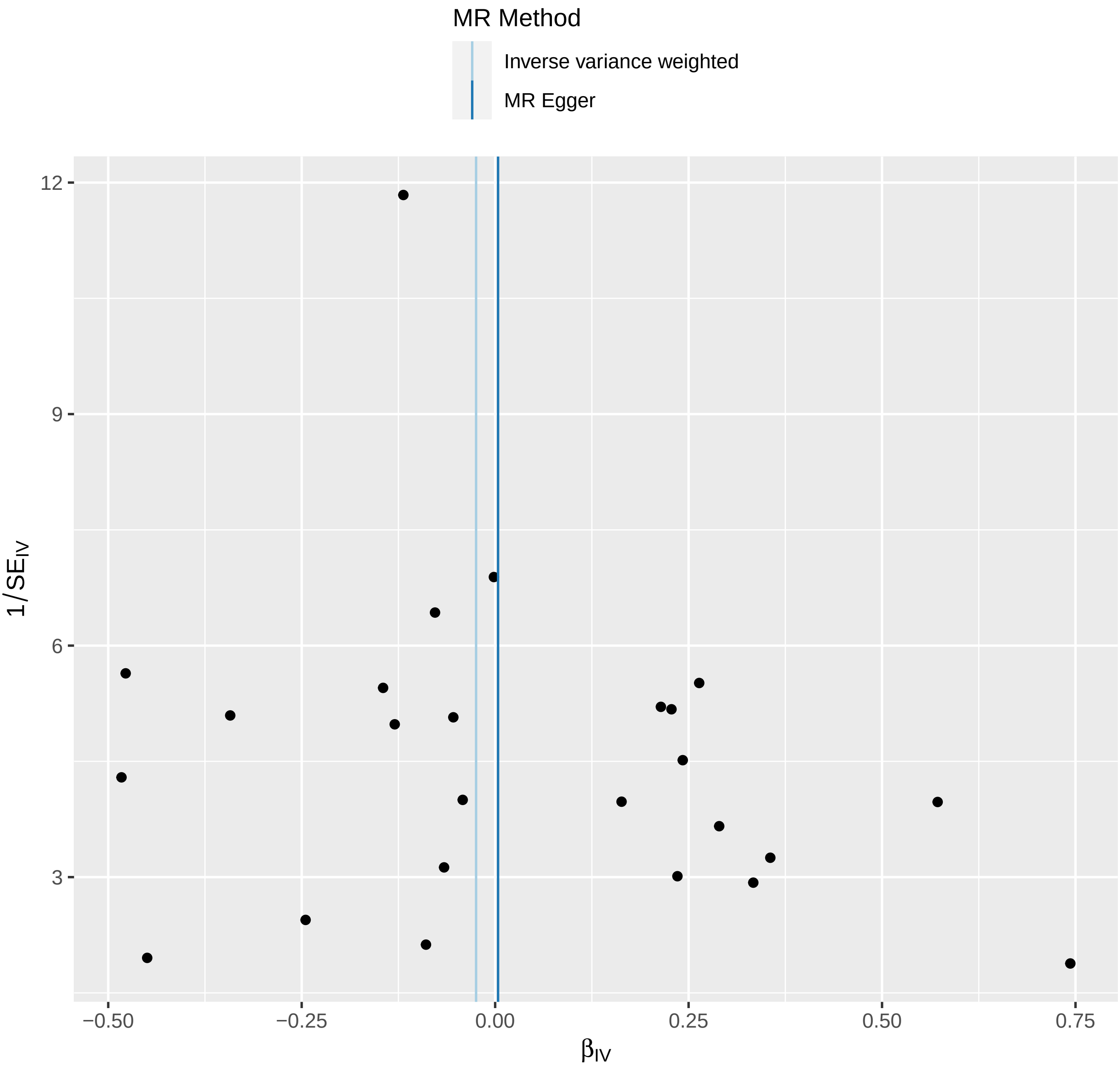

Batch 3−1538 : Interleukin−6 on Diabetic nephropathy (more control exclusions)
with a threshold of 1e−05

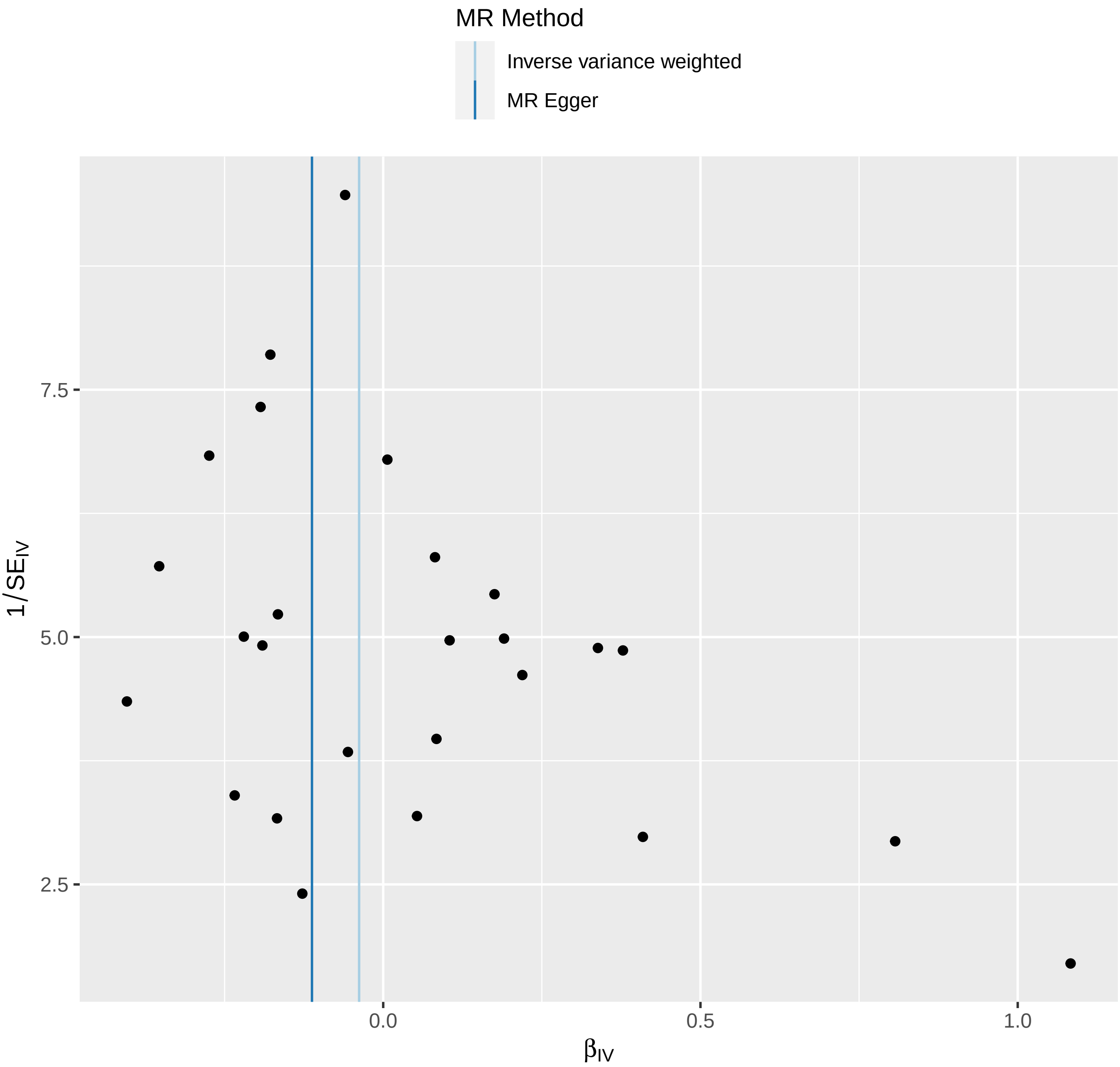

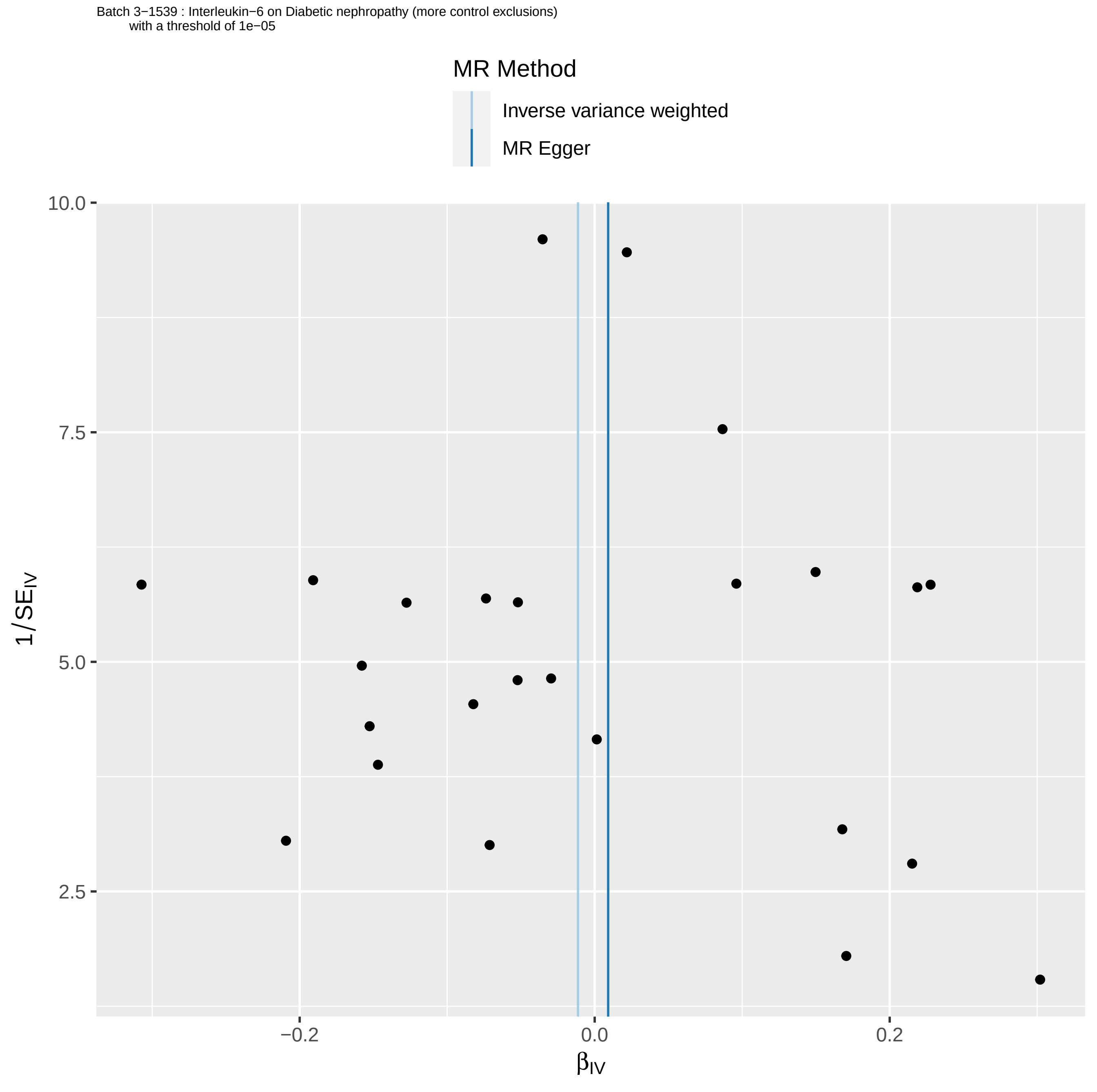
Batch 3−1539 : Interleukin−6 on Diabetic nephropathy (more control exclusions)
with a threshold of 1e−05
MR Method
Inverse variance weighted
MR Egger
10.0
7.5
5.0
2.5
1/SE_IV
−0.2
0.0
0.2
β_IV

Batch 3−1540 : Interleukin−6 receptor subunit alpha on Diabetic nephropathy (more control exclusions)
with a threshold of 1e−05

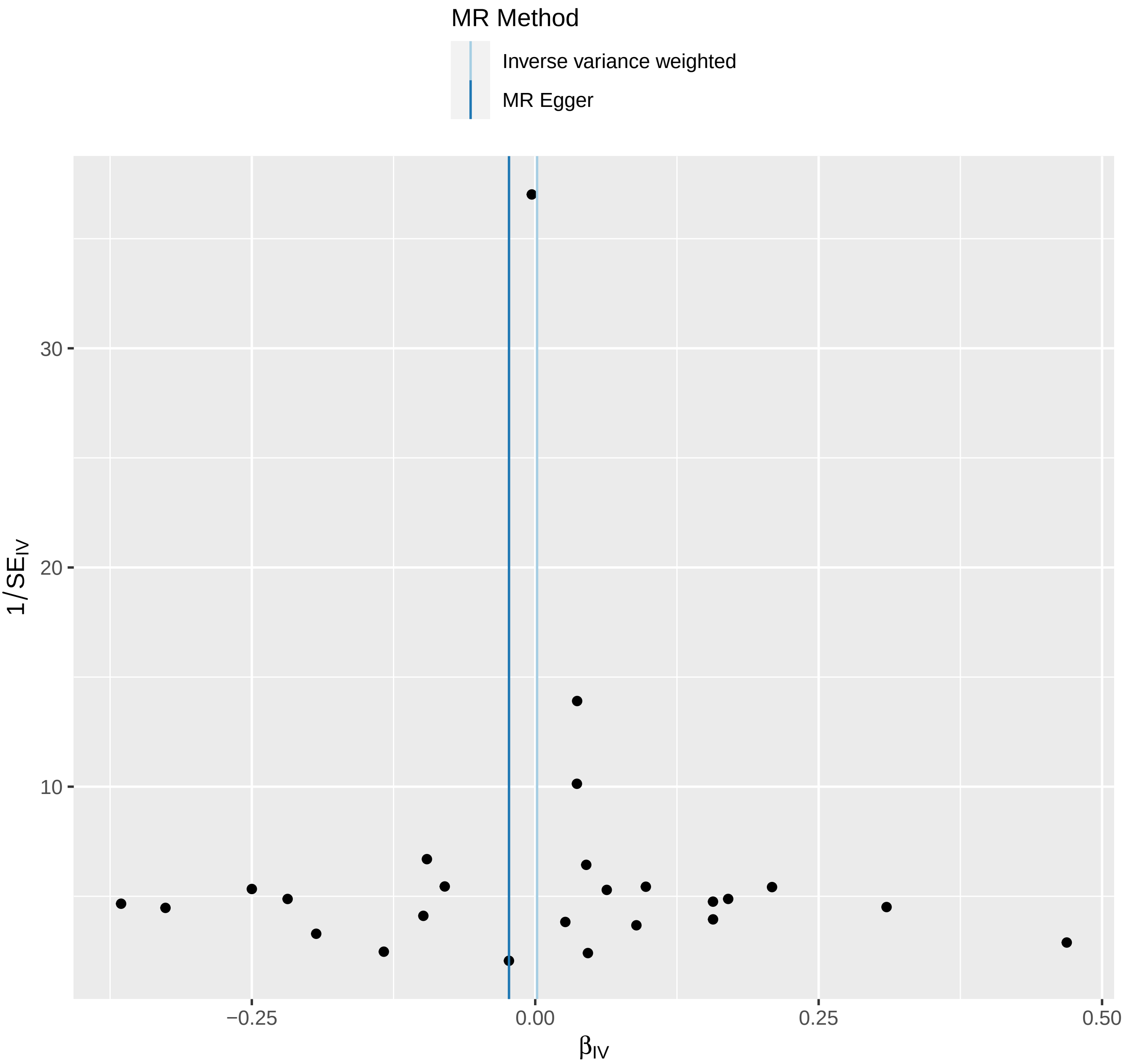

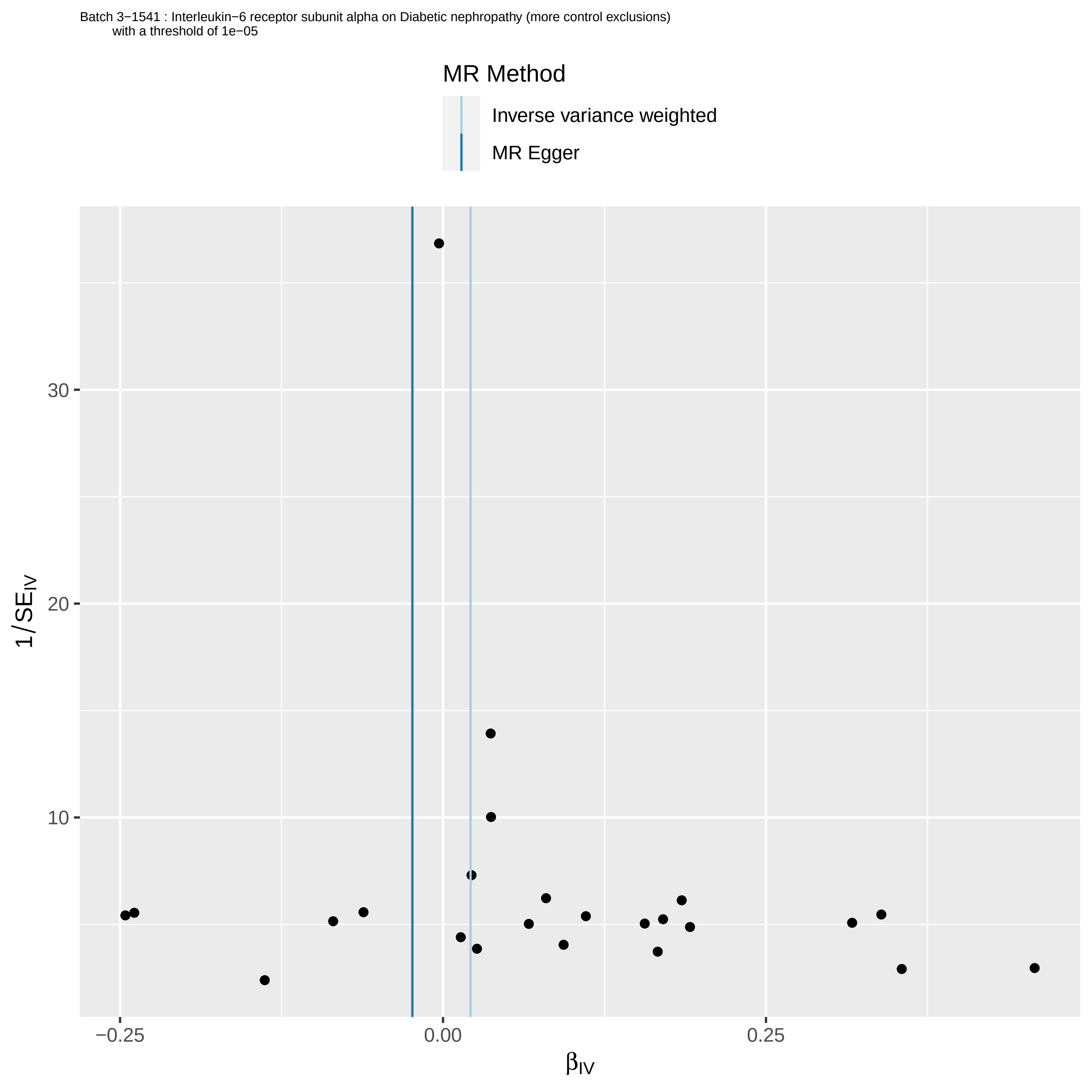
Batch 3−1541 : Interleukin−6 receptor subunit alpha on Diabetic nephropathy (more control exclusions)
with a threshold of 1e−05
MR Method
Inverse variance weighted
MR Egger
30
20
10
1/SE$_{IV}$
−0.25
0.00
0.25
β_{IV}

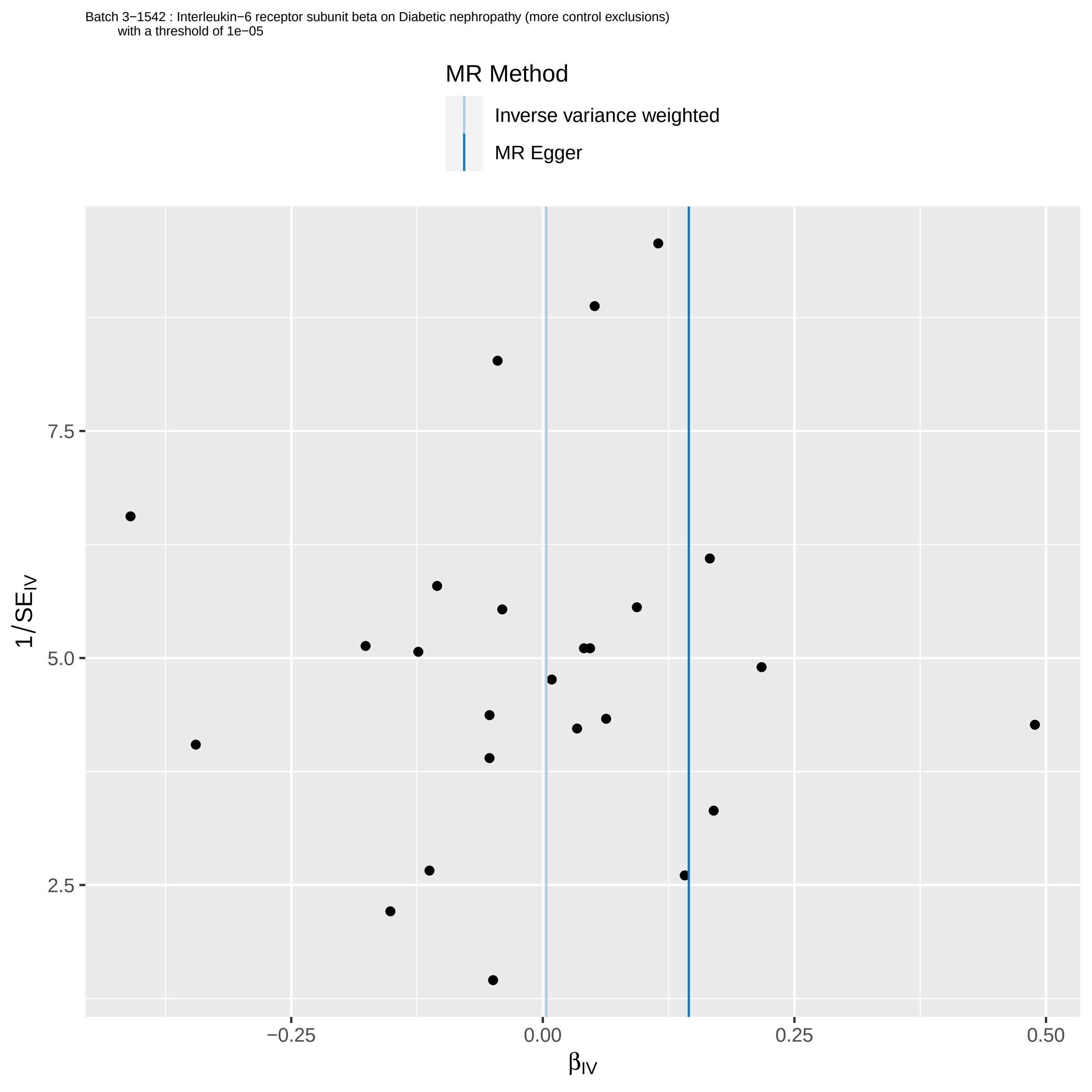

Batch 3−1542 : Interleukin−6 receptor subunit beta on Diabetic nephropathy (more control exclusions)
with a threshold of 1e−05
MR Method
Inverse variance weighted
MR Egger
7.5
5.0
2.5
1/SE_IV
−0.25
0.00
0.25
0.50
β_IV

Batch 3−1543 : Interleukin−7 on Diabetic nephropathy (more control exclusions)
with a threshold of 1e−05

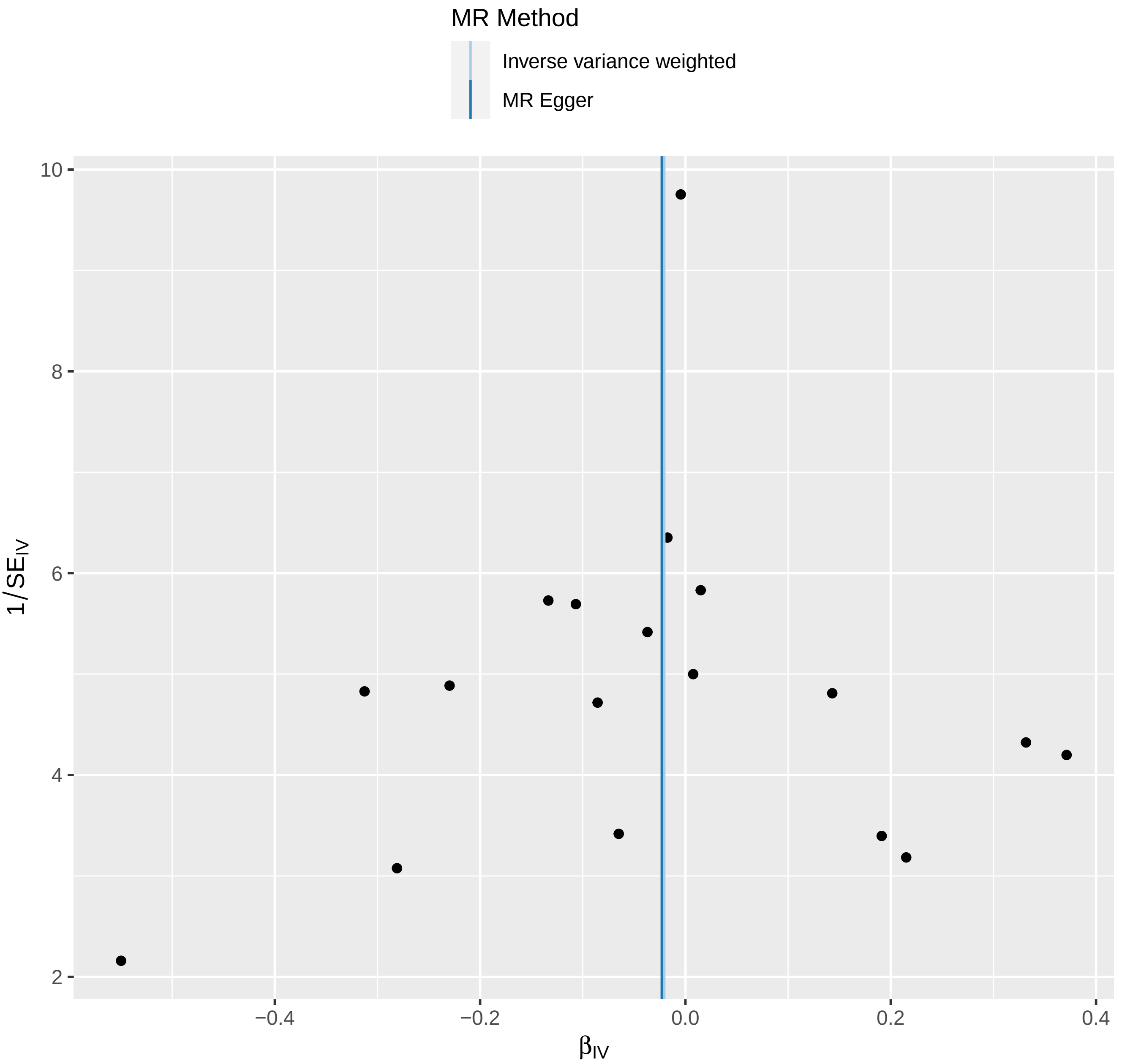

Batch 3−1544 : Interleukin−7 receptor subunit alpha on Diabetic nephropathy (more control exclusions)
with a threshold of 1e−05

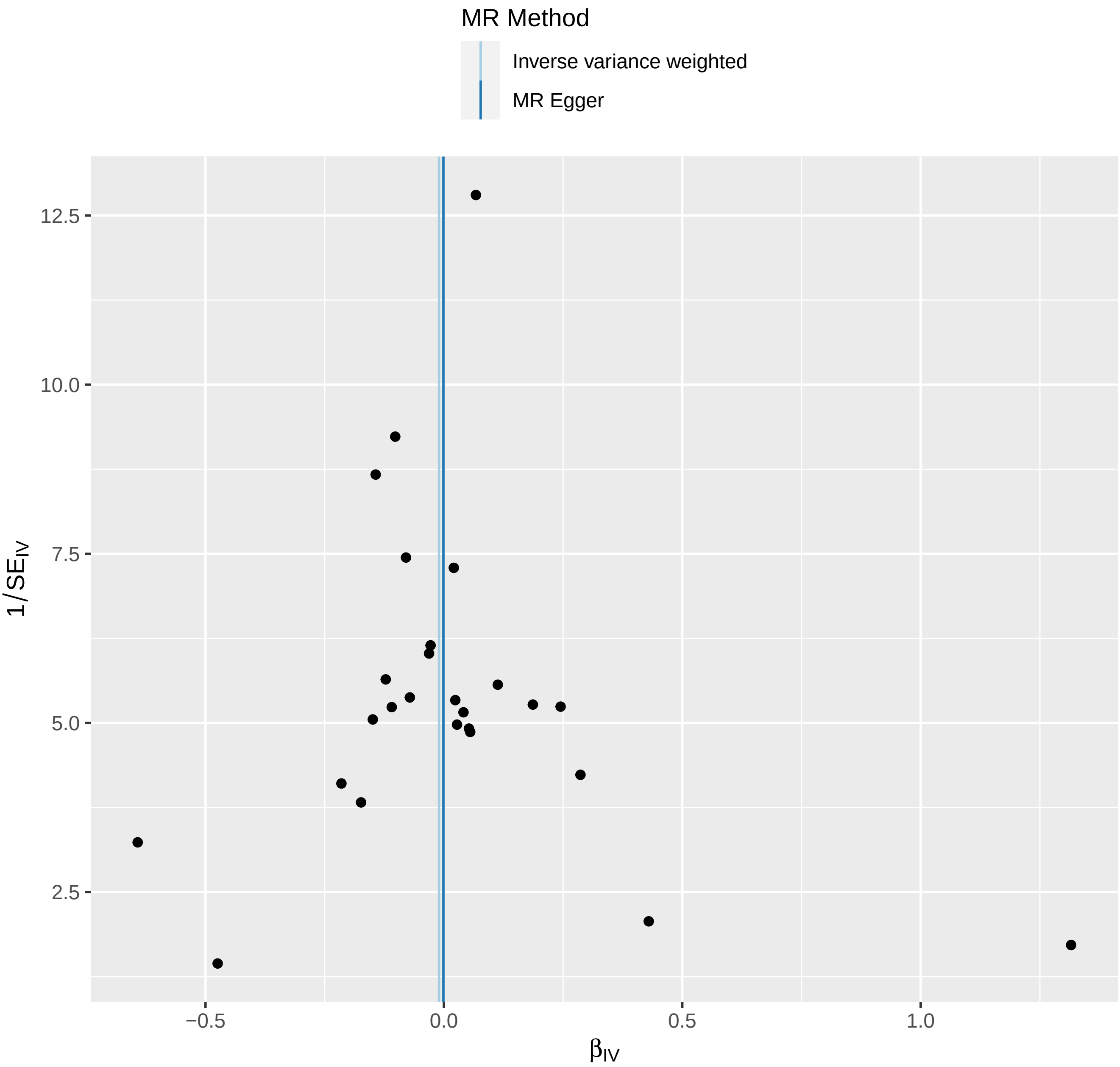

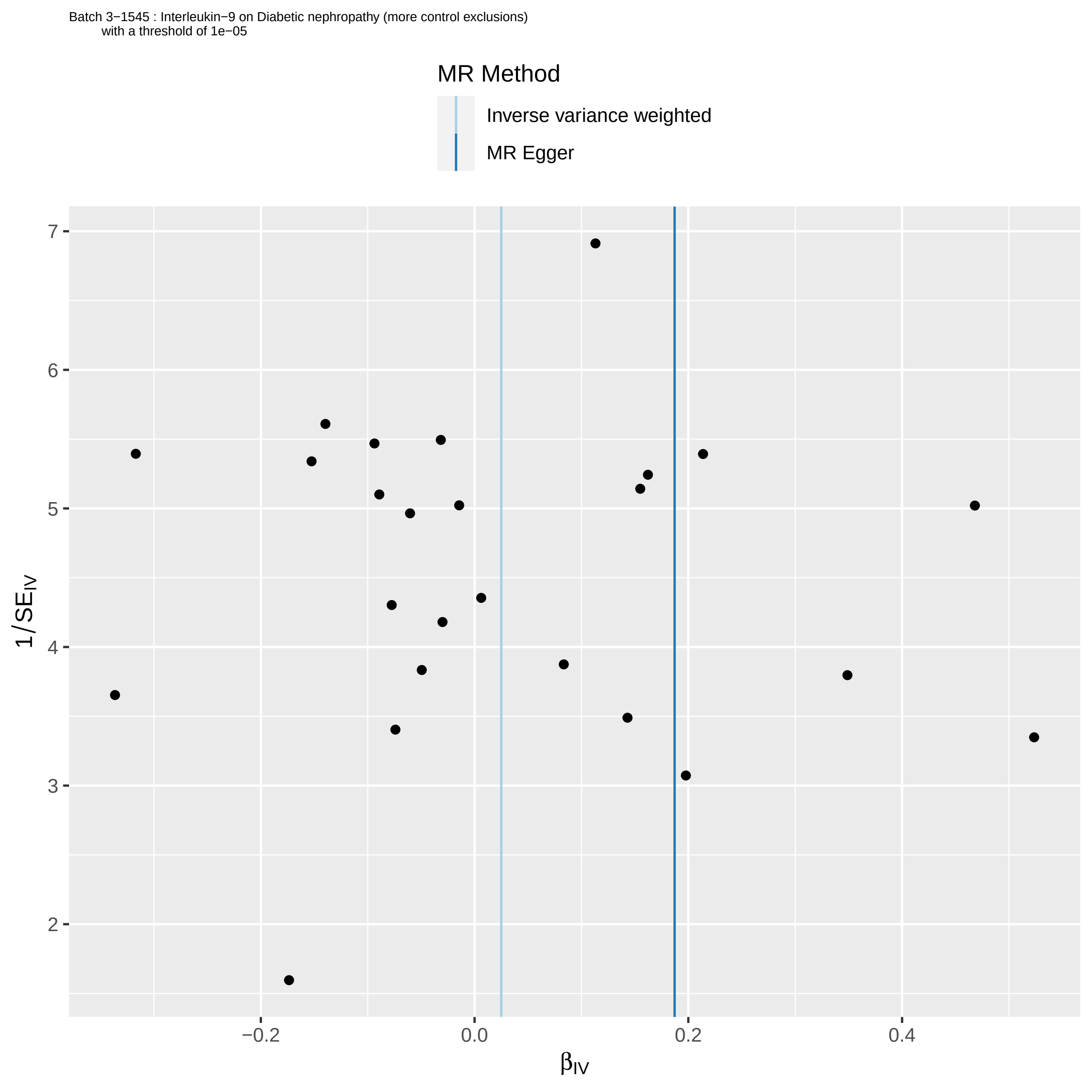

Batch 3−1545 : Interleukin−9 on Diabetic nephropathy (more control exclusions)
with a threshold of 1e−05
MR Method
Inverse variance weighted
MR Egger
7
6
5
4
3
2
1/SE_IV
−0.2
0.0
0.2
0.4
β_IV

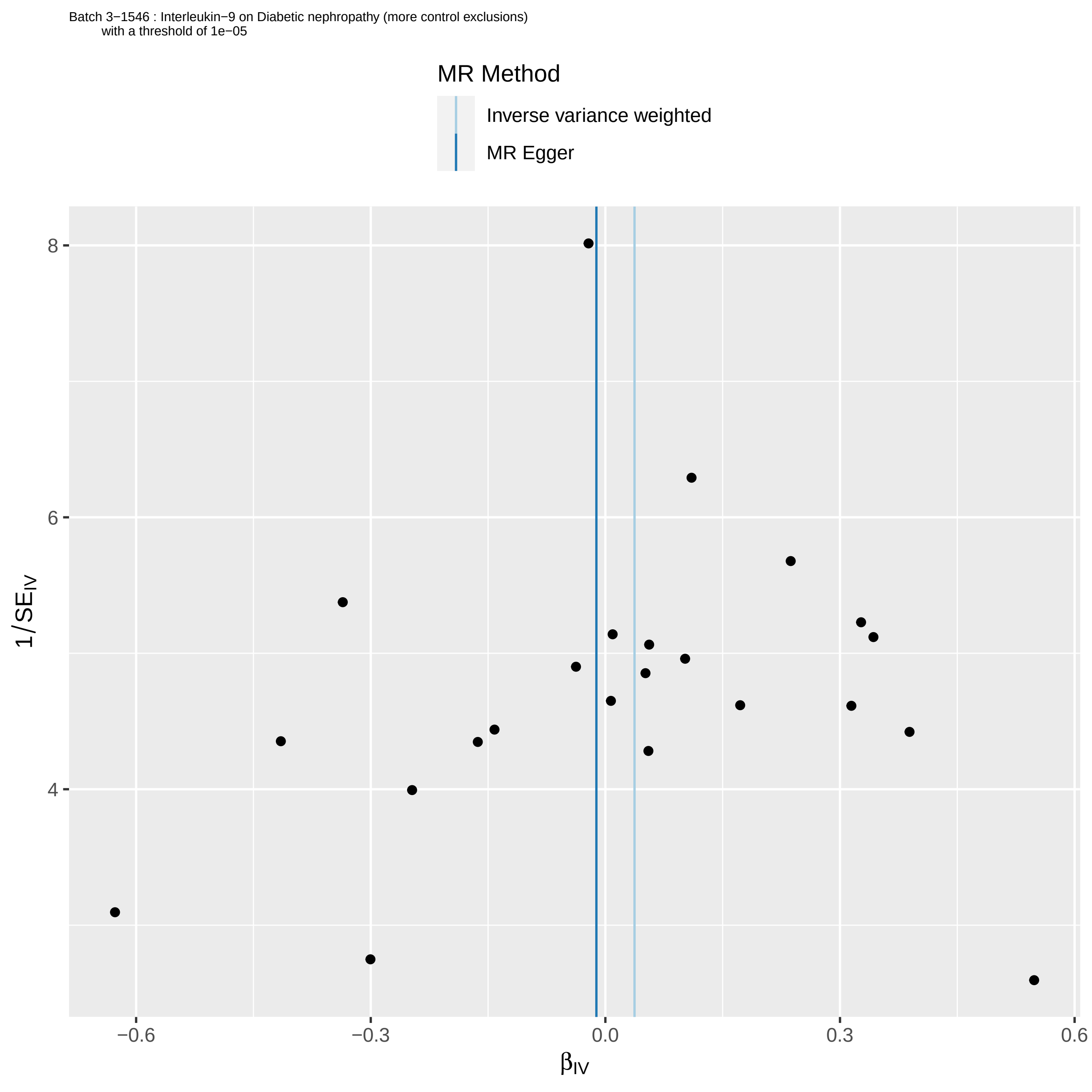

Batch 3−1546 : Interleukin−9 on Diabetic nephropathy (more control exclusions)
with a threshold of 1e−05
MR Method
Inverse variance weighted
MR Egger
8
6
4
1/SE_IV
−0.6
−0.3
0.0
0.3
0.6
β_IV

Batch 3−1547 : Interleukin enhancer−binding factor 3 on Diabetic nephropathy (more control exclusions) with a threshold of 1e−05

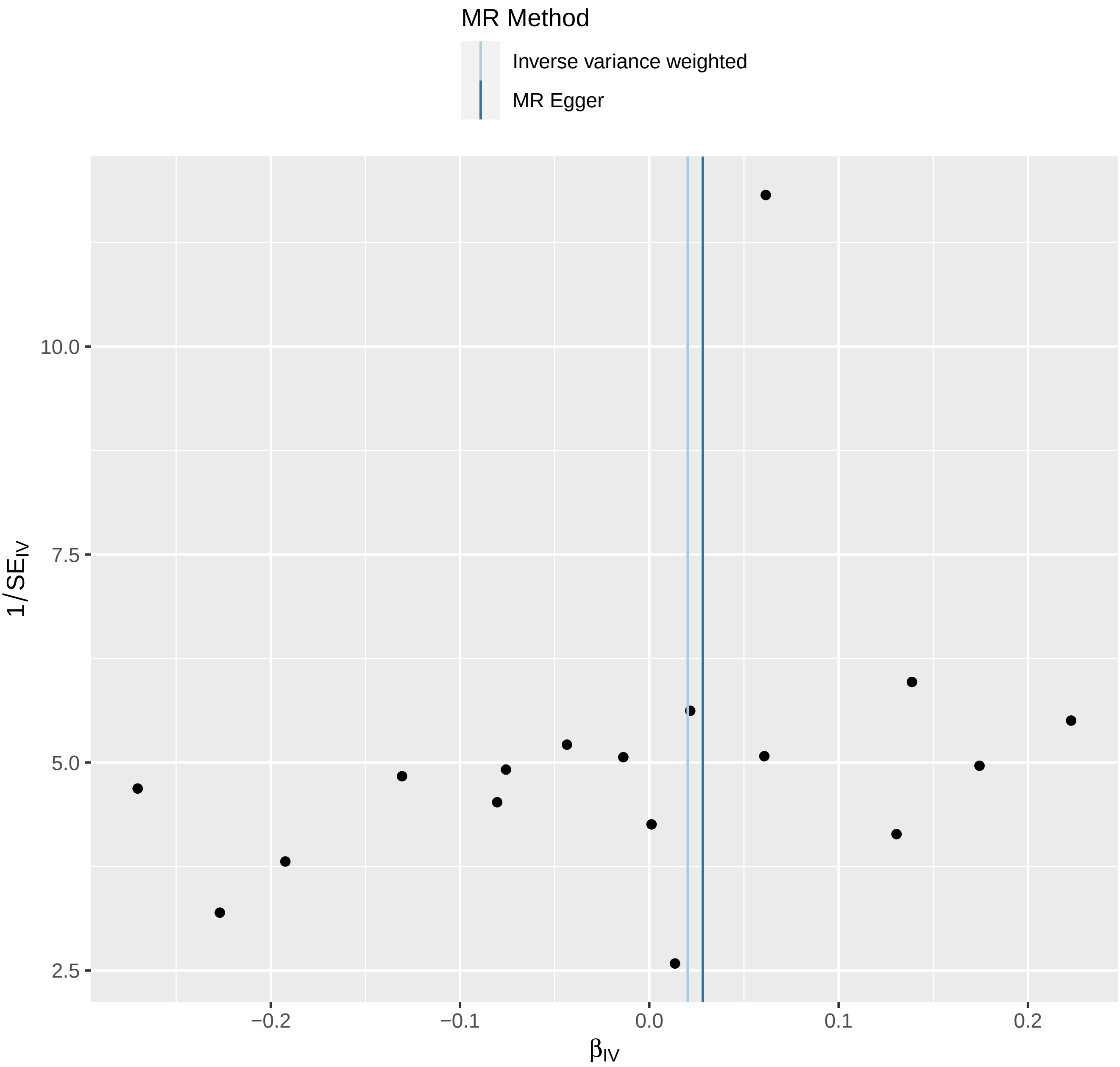

Batch 3−1548 : Inositol monophosphatase 3 on Diabetic nephropathy (more control exclusions)
with a threshold of 1e−05

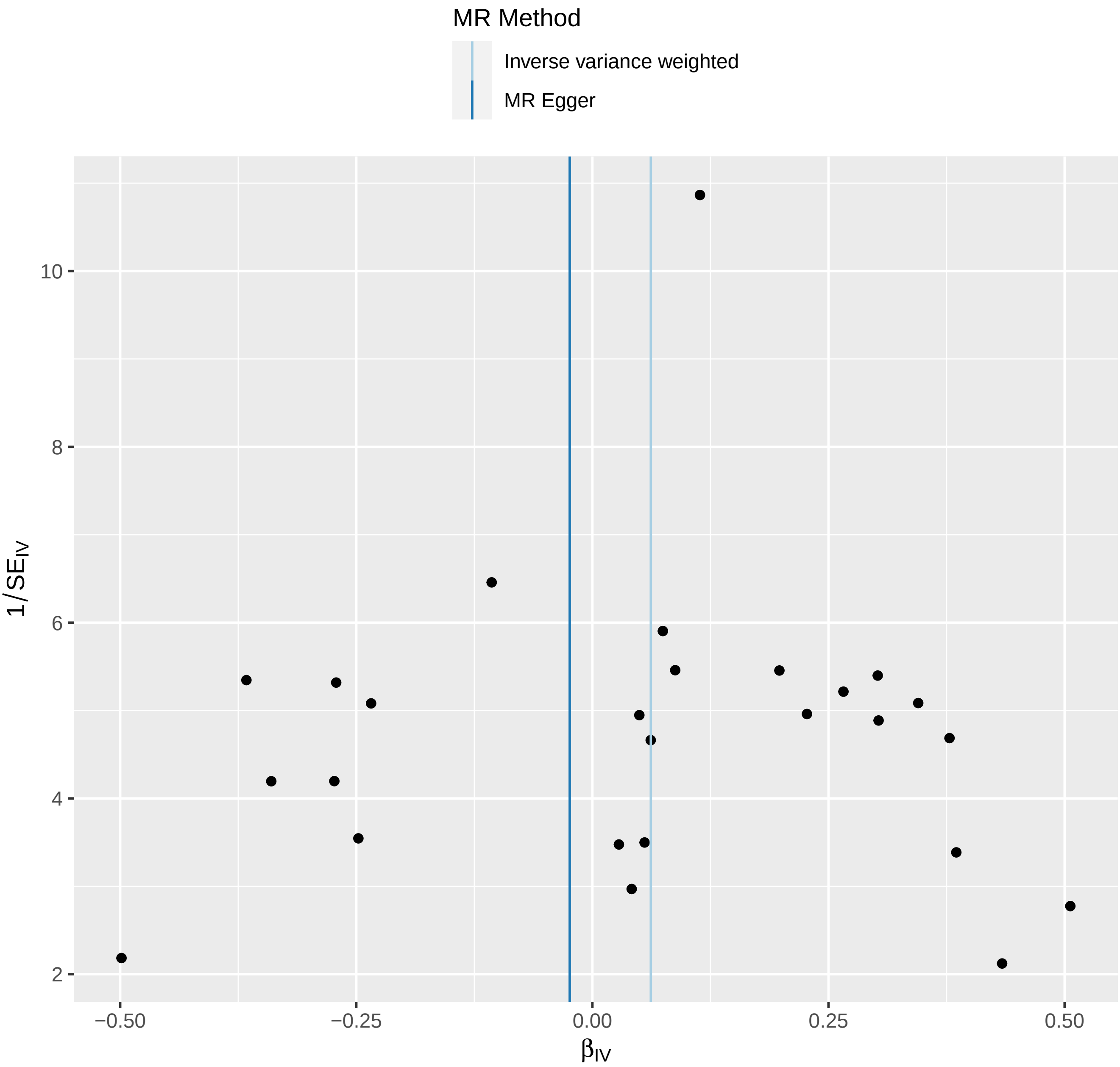

Batch 3−1549 : Inosine−5'−monophosphate dehydrogenase 1 on Diabetic nephropathy (more control exclusions)
with a threshold of 1e−05

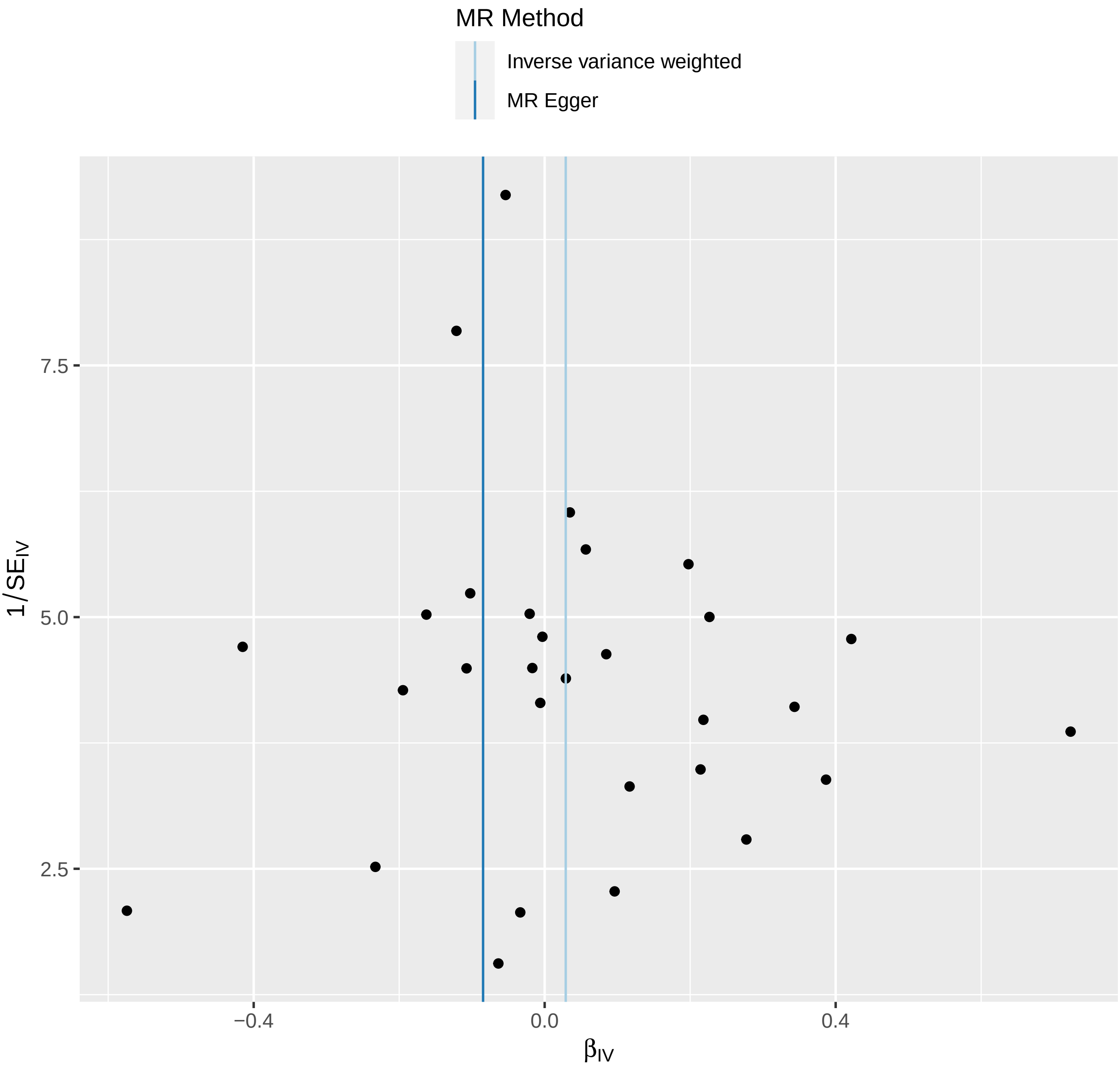

Batch 3−1550 : Inosine−5'−monophosphate dehydrogenase 2 on Diabetic nephropathy (more control exclusions)
with a threshold of 1e−05

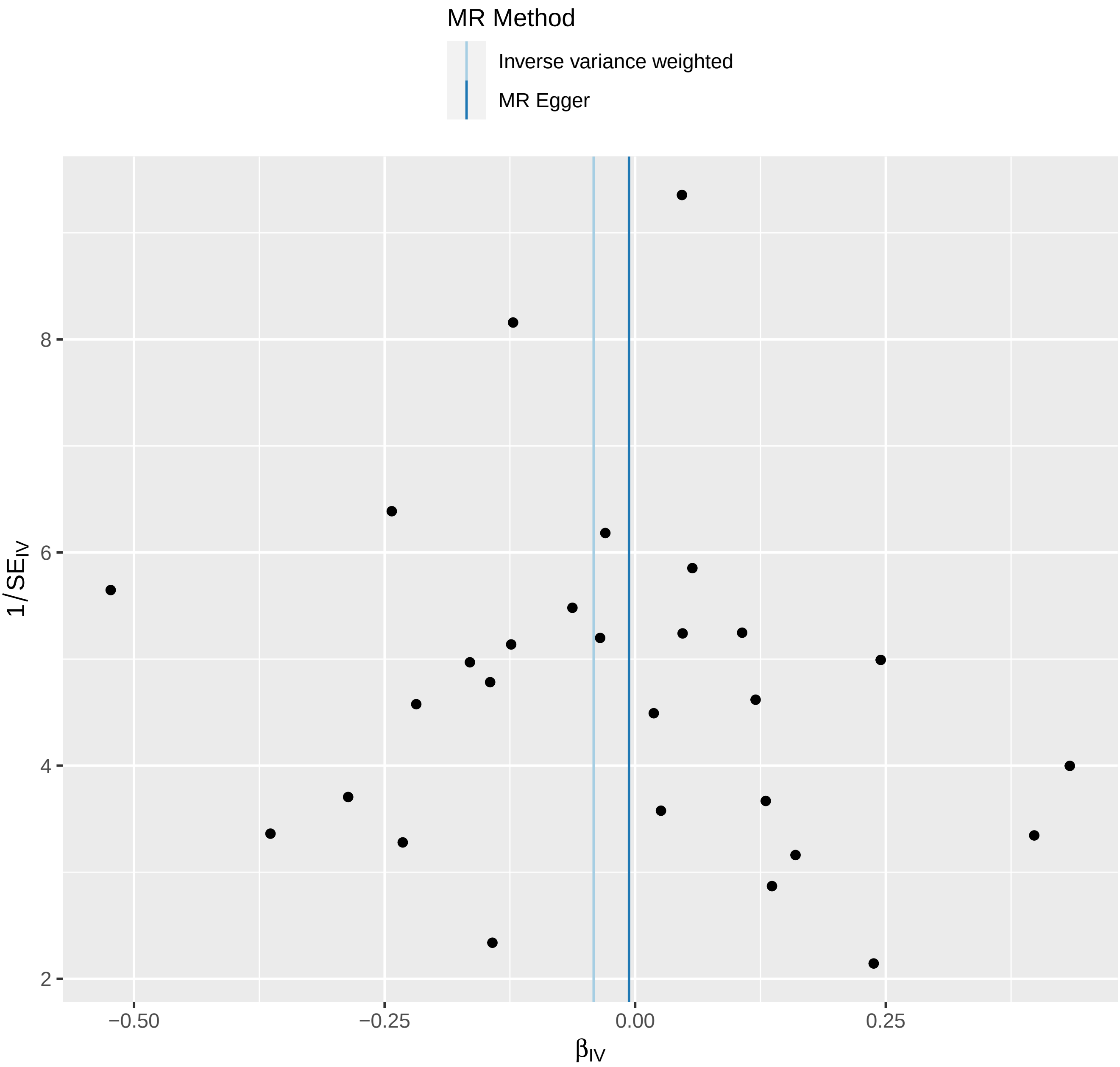

Batch 3−1551 : Alpha−internexin on Diabetic nephropathy (more control exclusions)
with a threshold of 1e−05

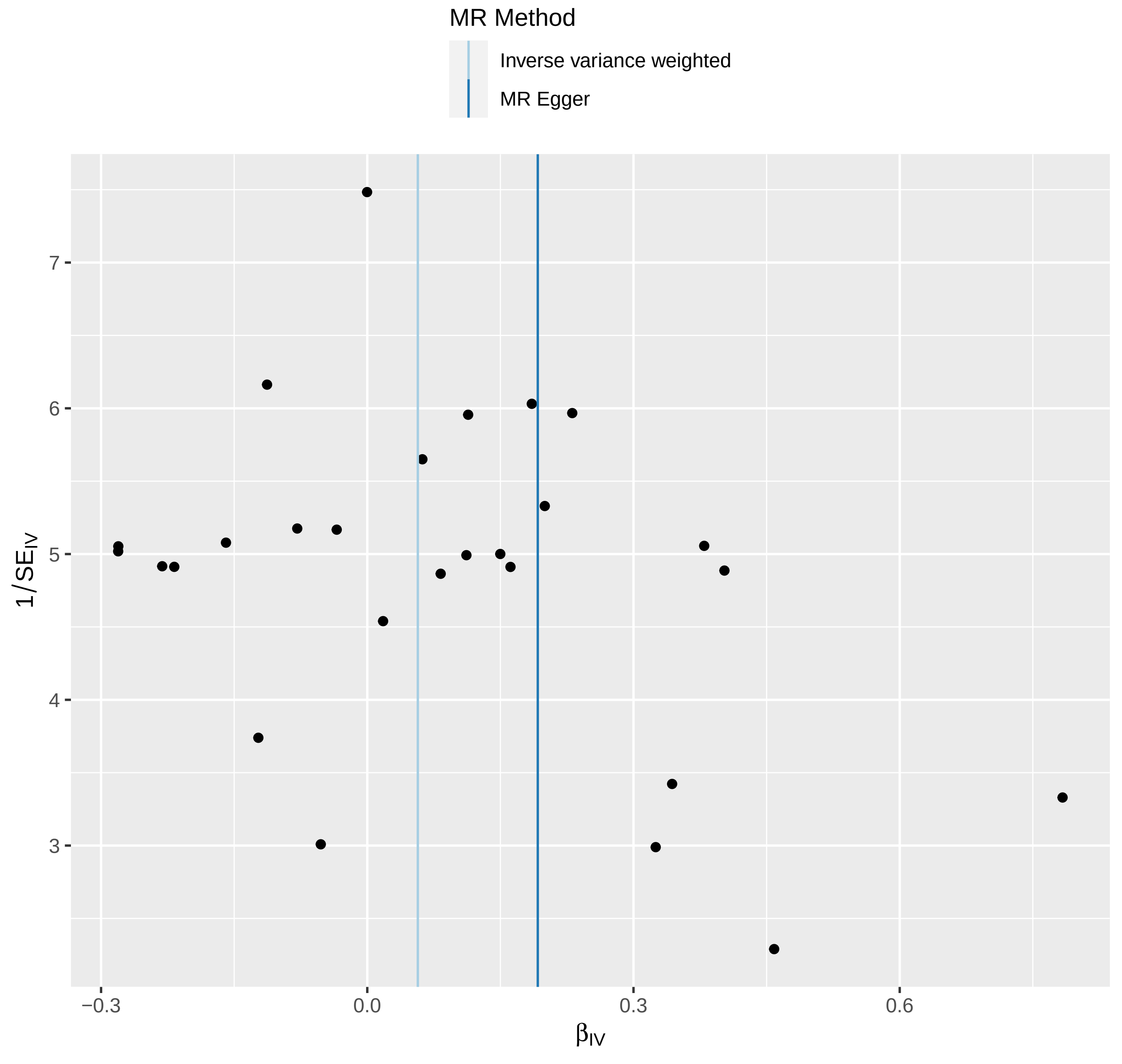

Batch 3−1552 : Inhibitor of growth protein 1 on Diabetic nephropathy (more control exclusions)
with a threshold of 1e−05

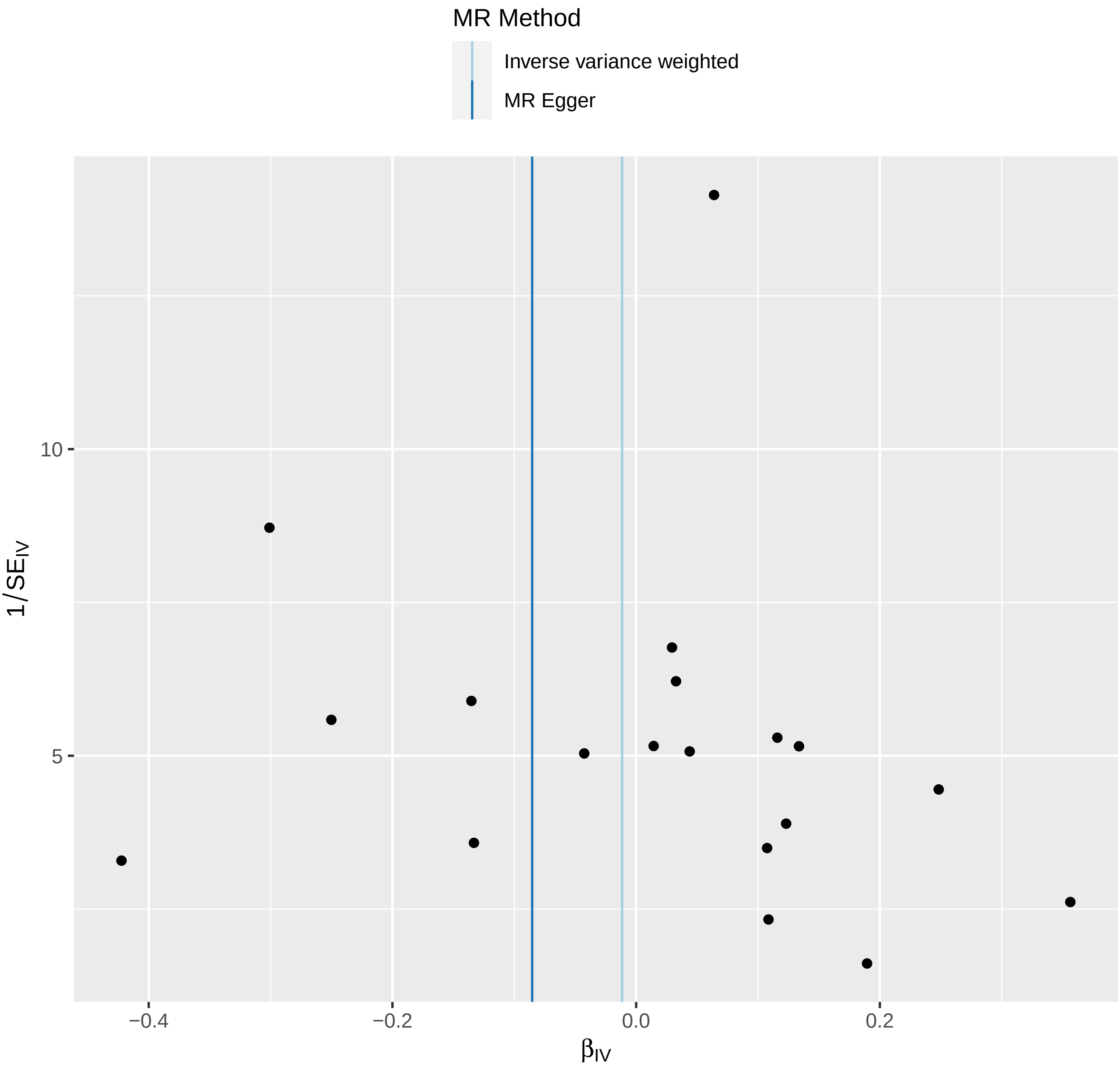

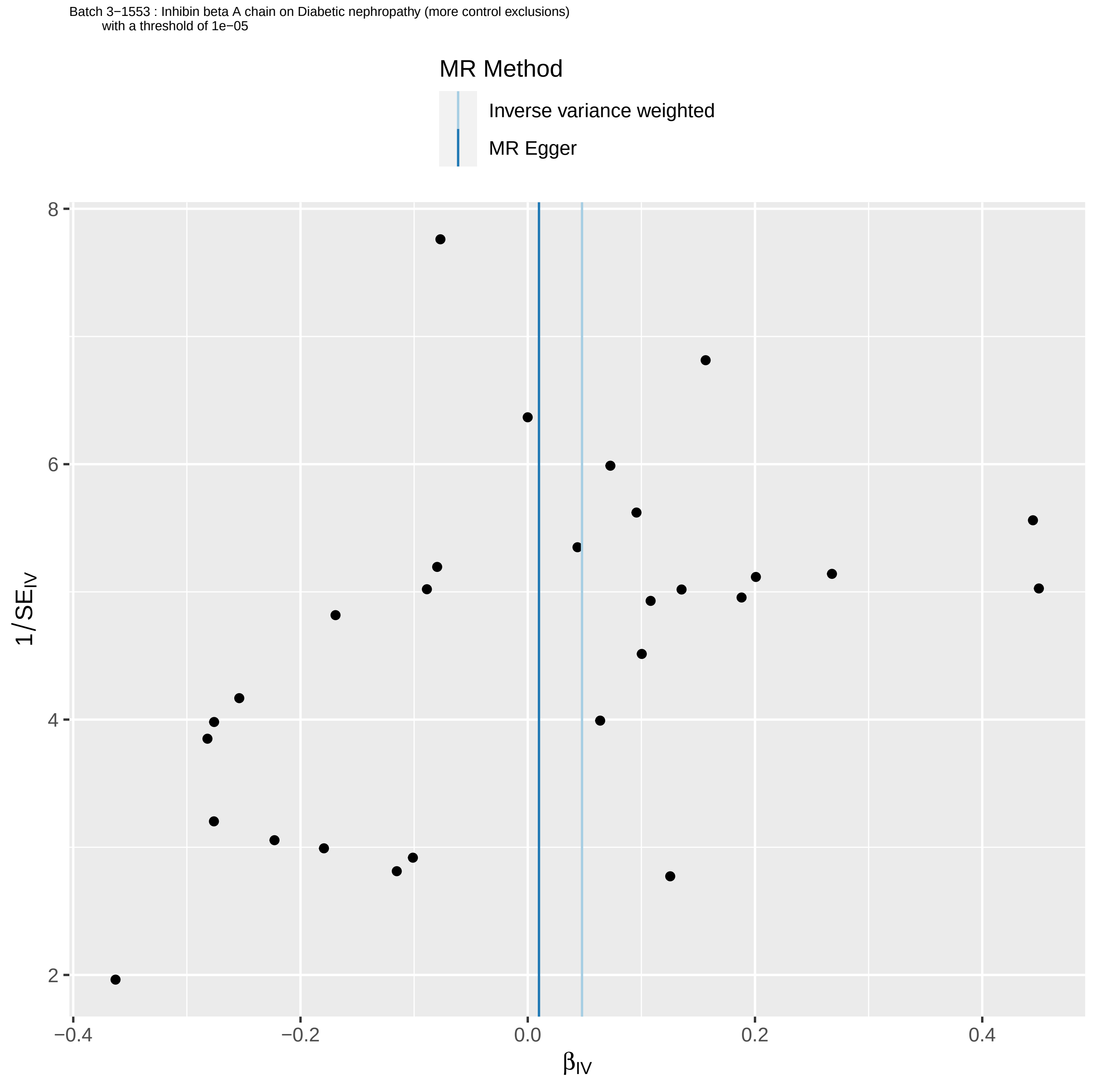
Batch 3−1553 : Inhibin beta A chain on Diabetic nephropathy (more control exclusions)
with a threshold of 1e−05
MR Method
Inverse variance weighted
MR Egger
8
6
4
2
1/SE_IV
−0.4
−0.2
0.0
0.2
0.4
β_IV

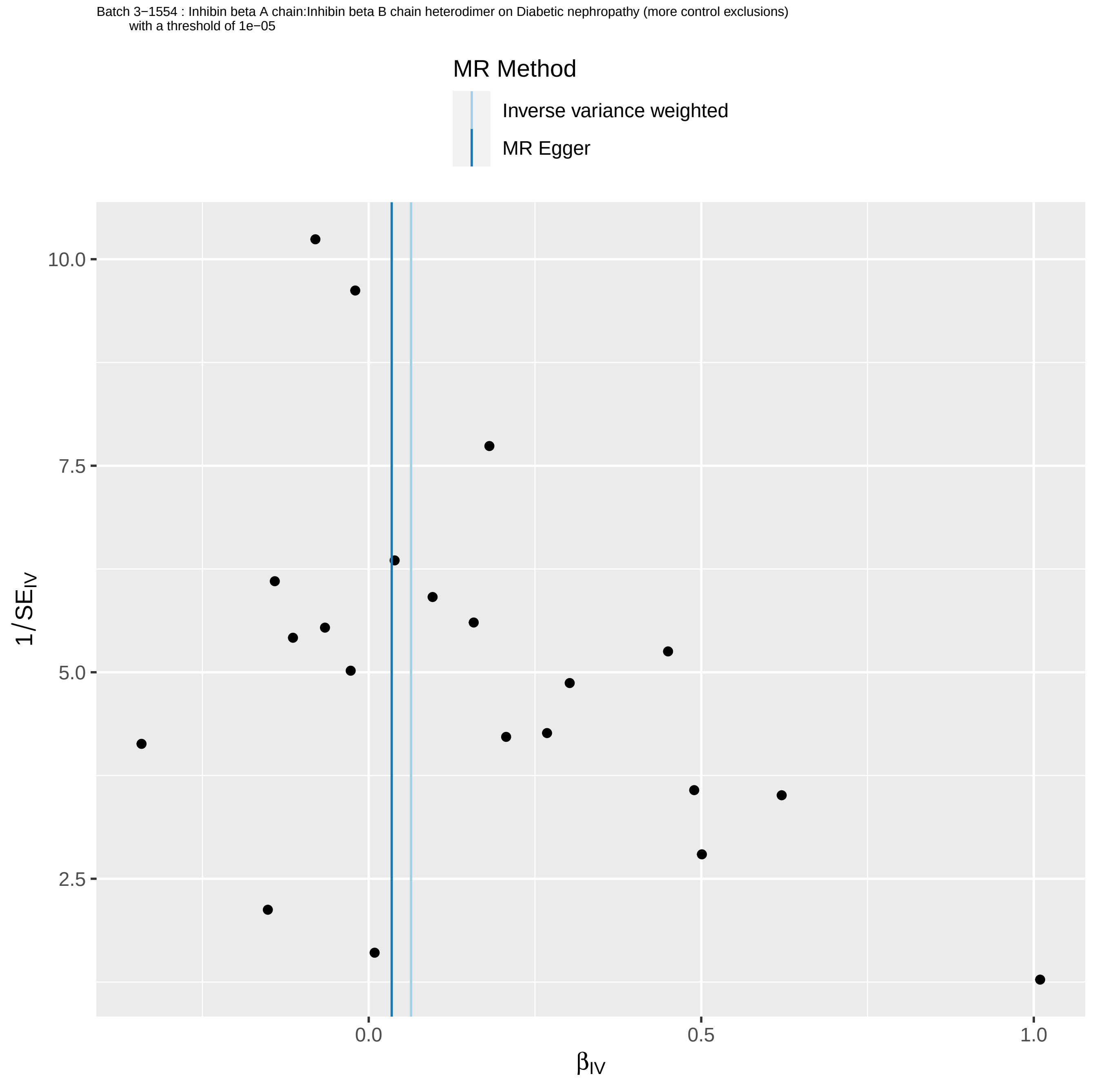

Batch 3−1554 : Inhibin beta A chain:Inhibin beta B chain heterodimer on Diabetic nephropathy (more control exclusions)
with a threshold of 1e−05
MR Method
Inverse variance weighted
MR Egger
10.0
7.5
5.0
2.5
1/SE_IV
0.0
0.5
1.0
β_IV

Batch 3−1555 : Inhibin beta A chain:Inhibin beta B chain heterodimer on Diabetic nephropathy (more control exclusions) with a threshold of 1e−05

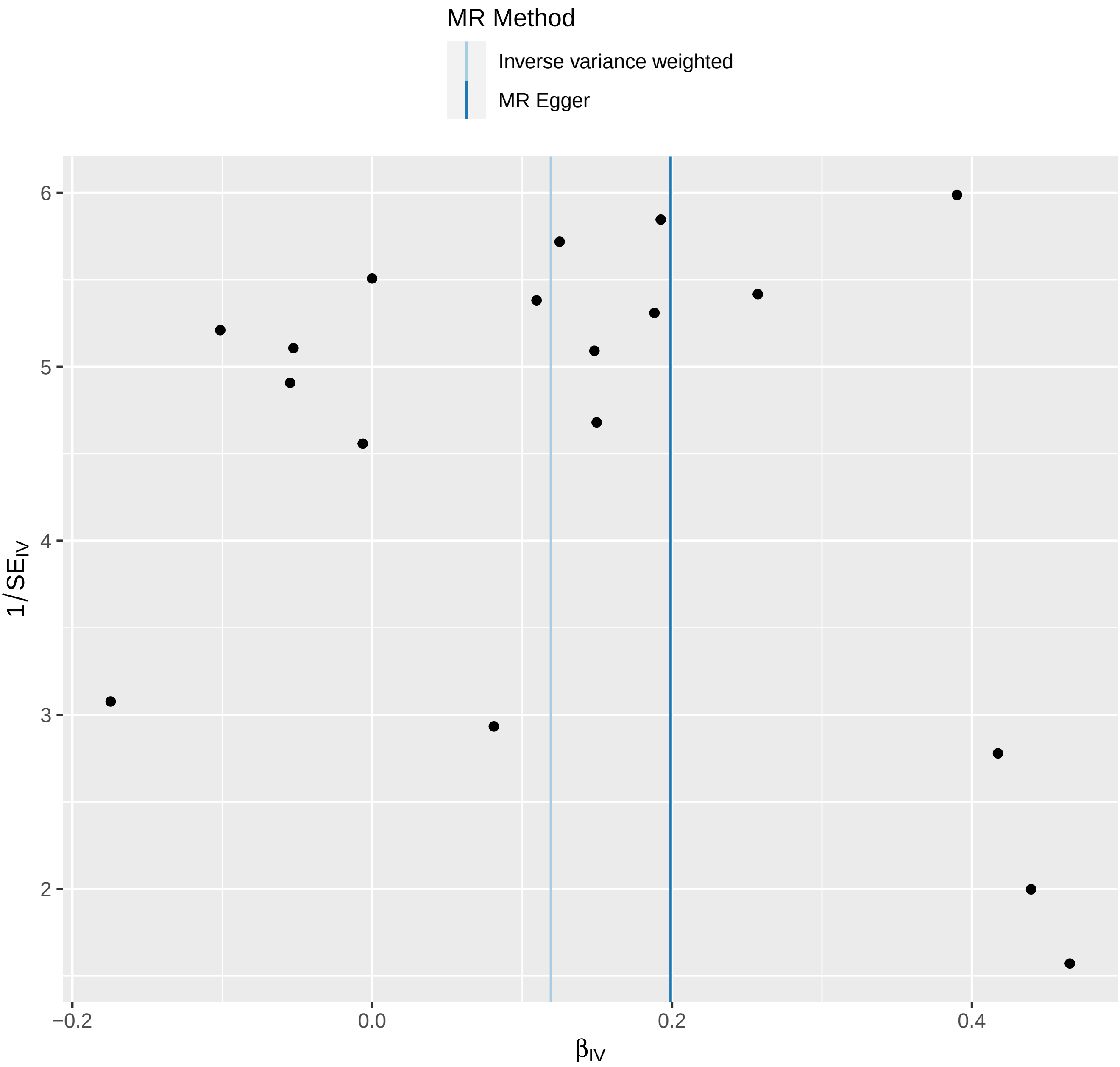

Batch 3−1556 : Inhibin beta C chain on Diabetic nephropathy (more control exclusions)
with a threshold of 1e−05

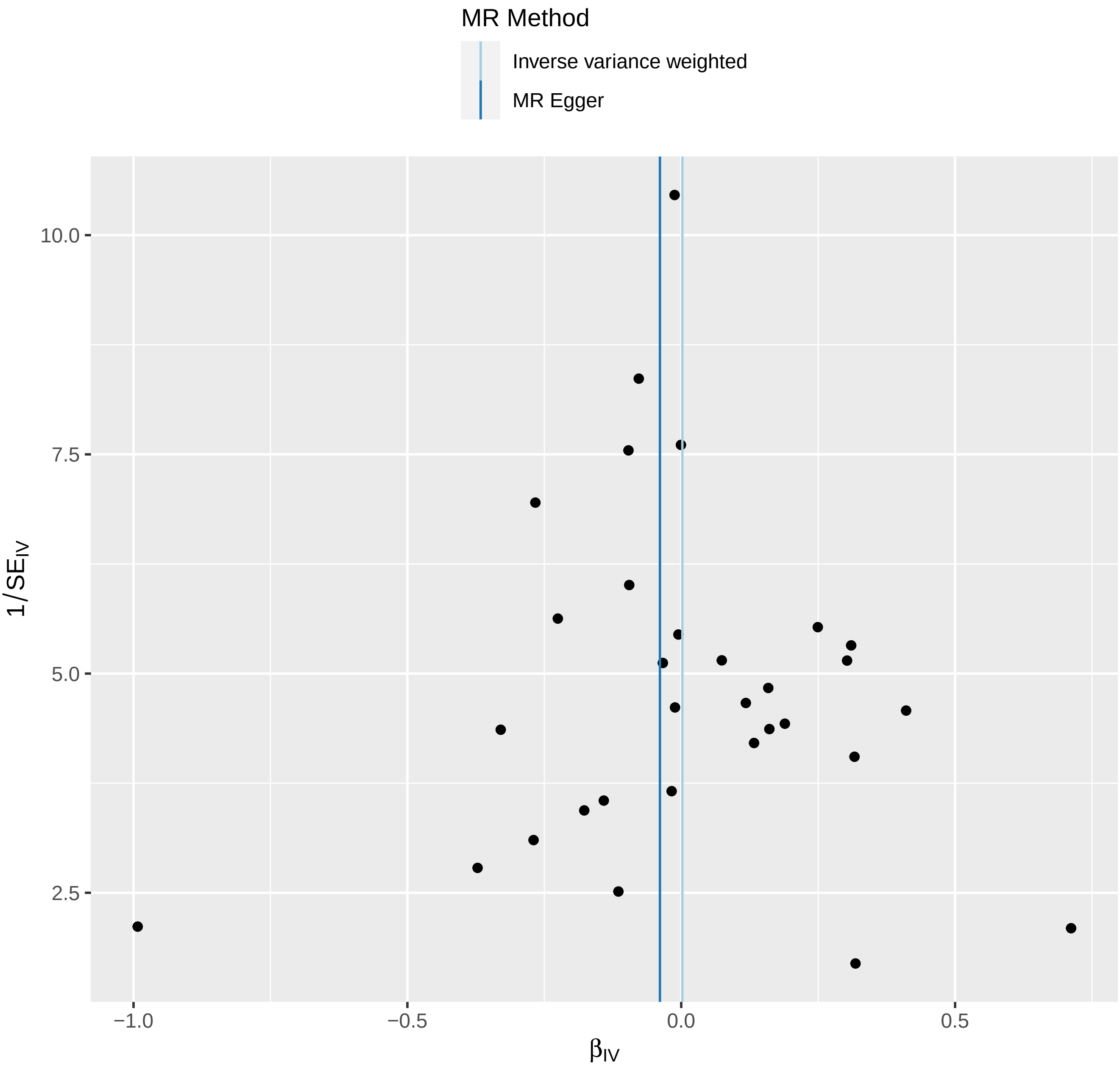

Batch 3−1557 : 72 kDa inositol polyphosphate 5−phosphatase on Diabetic nephropathy (more control exclusions) with a threshold of 1e−05

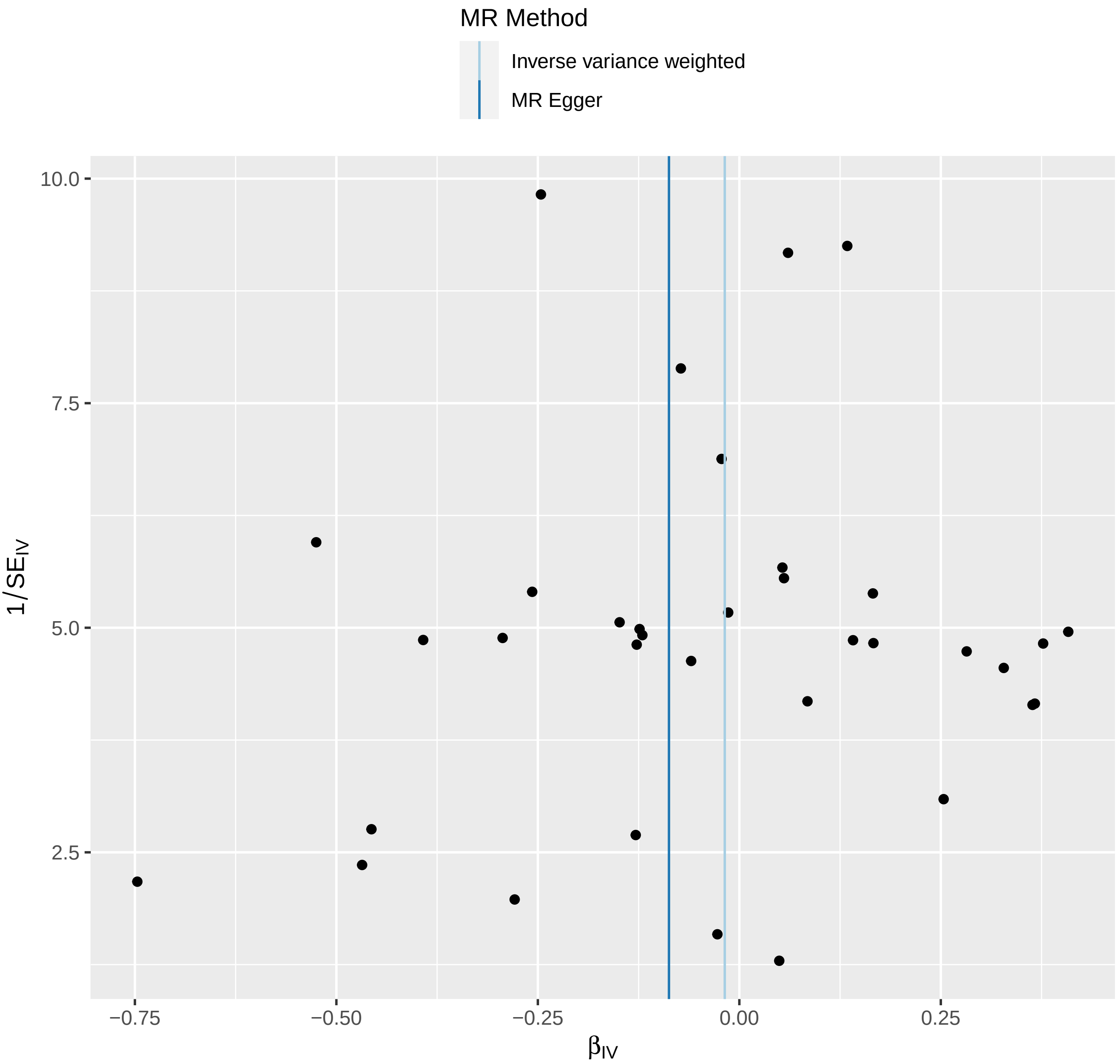

Batch 3−1558 : Insulin on Diabetic nephropathy (more control exclusions)
with a threshold of 1e−05

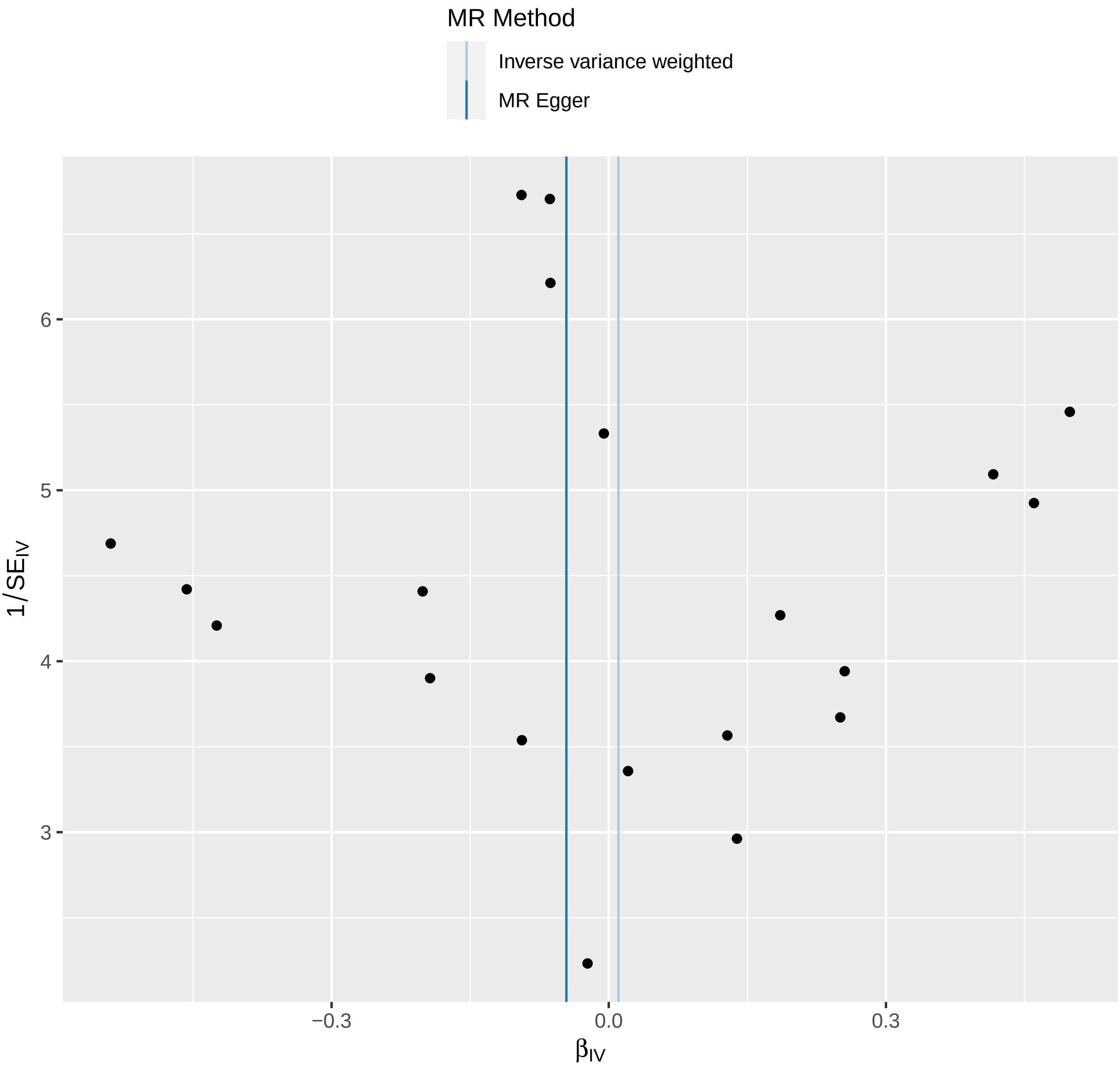

Batch 3−1559 : Insulin−induced gene 1 protein on Diabetic nephropathy (more control exclusions)
with a threshold of 1e−05

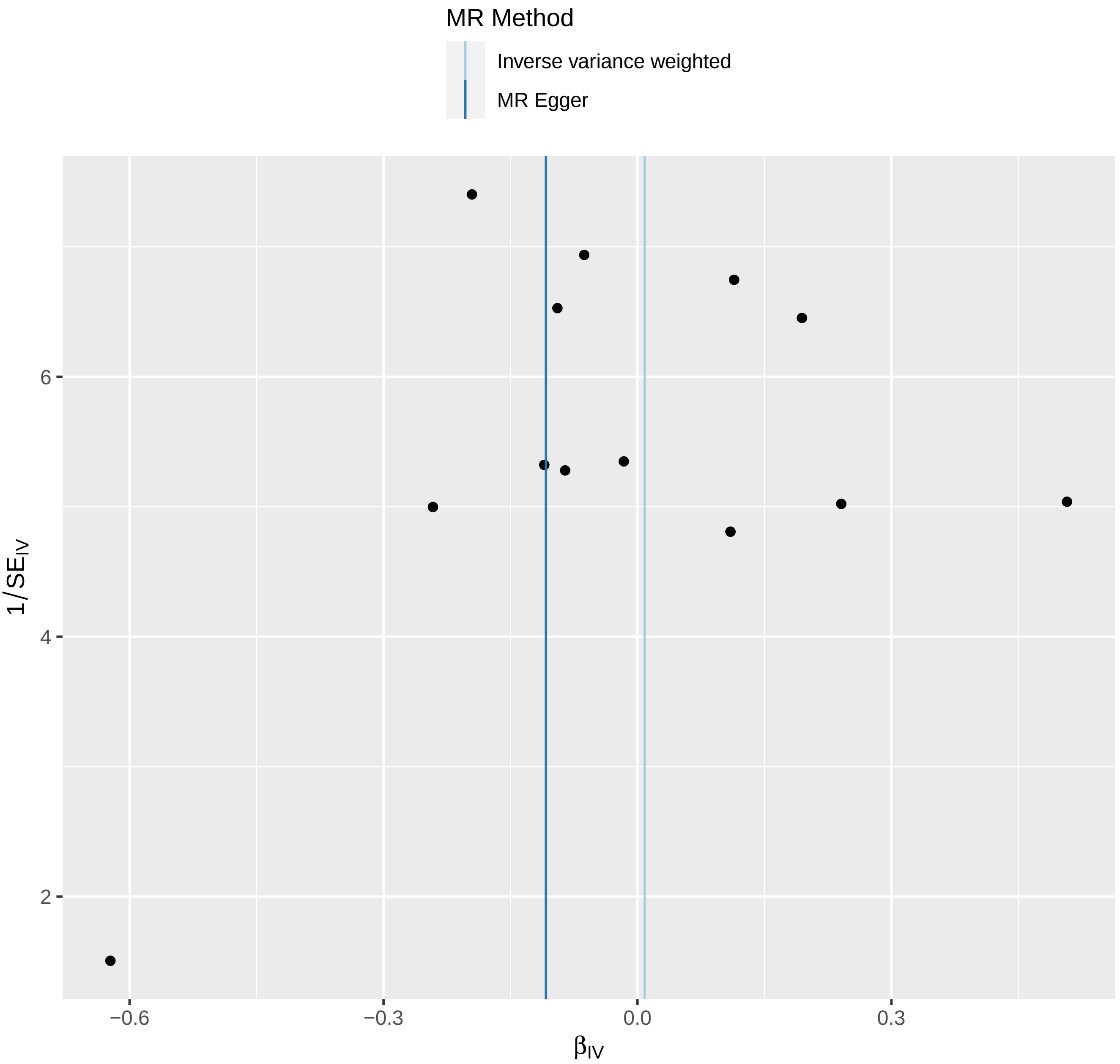

Batch 3−1560 : Insulin−like 3 on Diabetic nephropathy (more control exclusions)
with a threshold of 1e−05

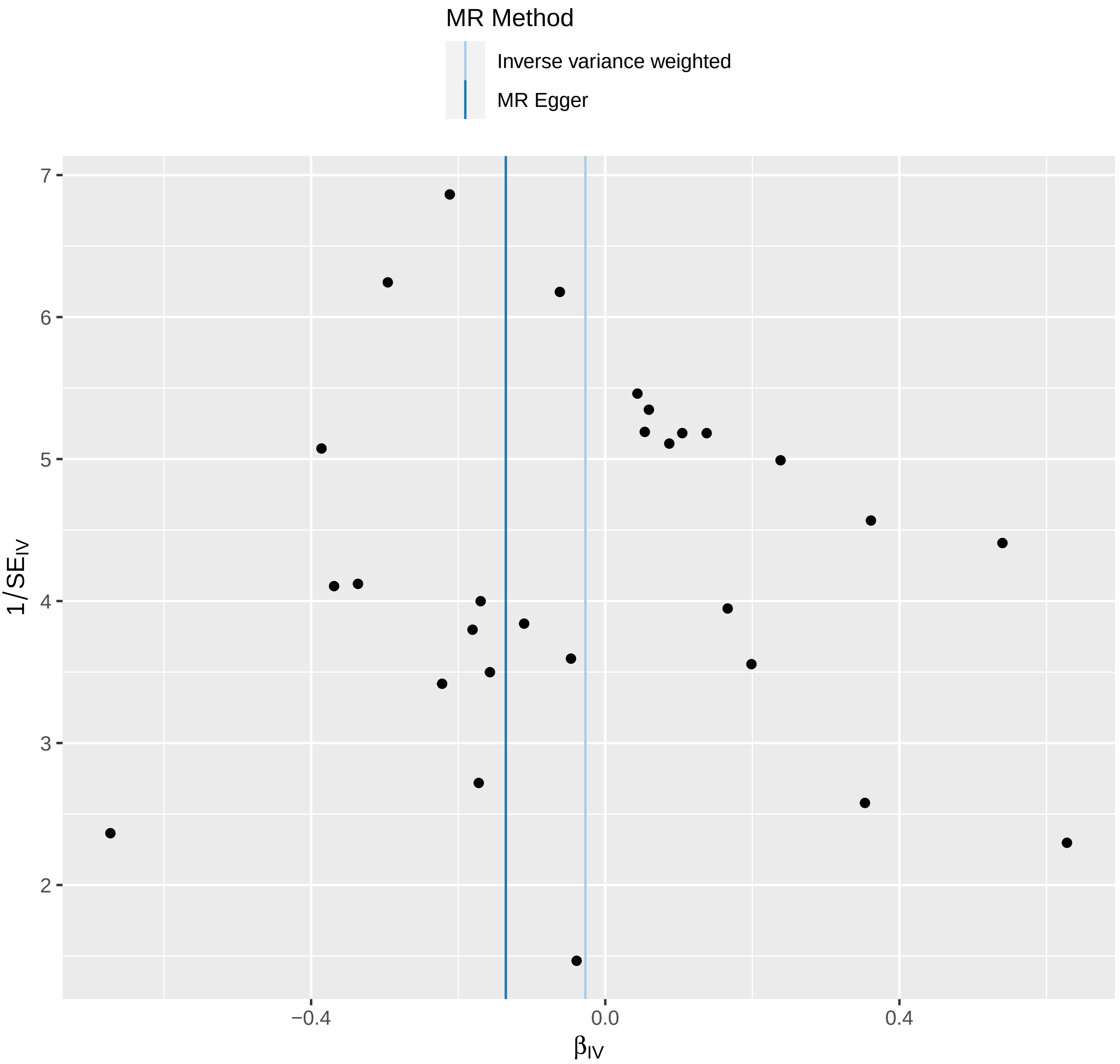

Batch 3−1561 : Early placenta insulin−like peptide on Diabetic nephropathy (more control exclusions)
with a threshold of 1e−05

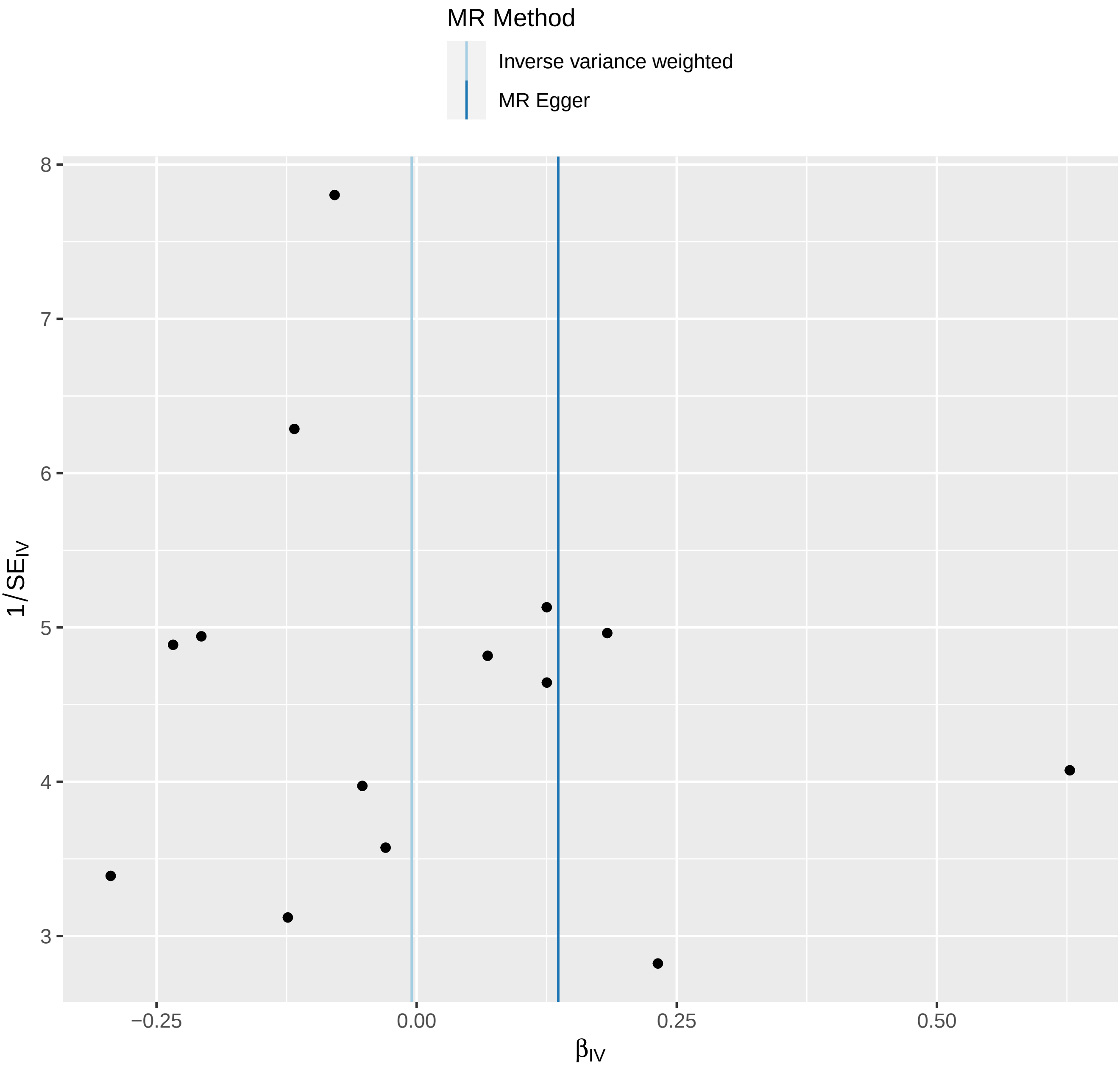

Batch 3−1562 : Insulin−like peptide INSL5 on Diabetic nephropathy (more control exclusions)
with a threshold of 1e−05

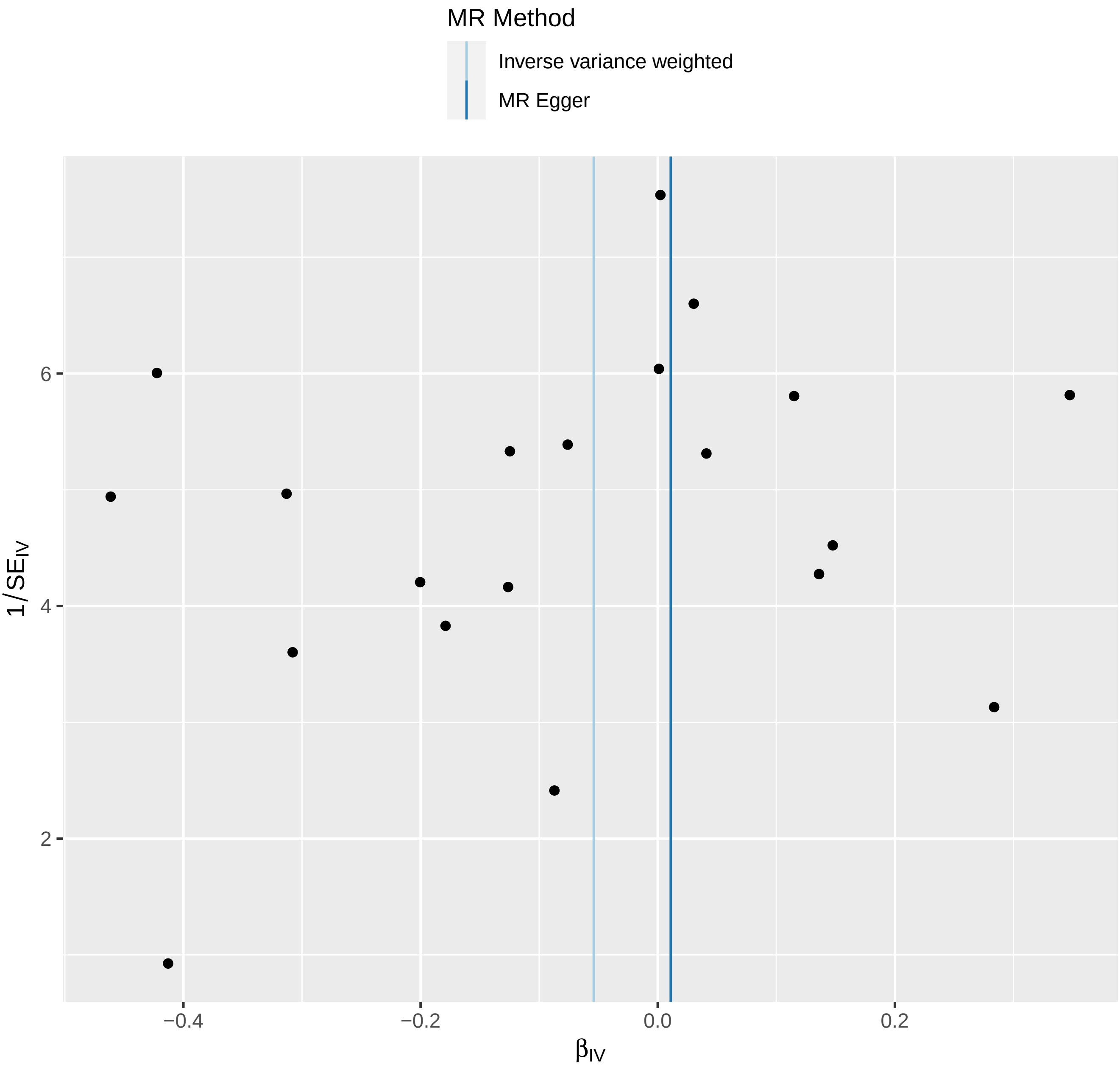

Batch 3−1563 : Insulin−like peptide INSL6 on Diabetic nephropathy (more control exclusions)
with a threshold of 1e−05

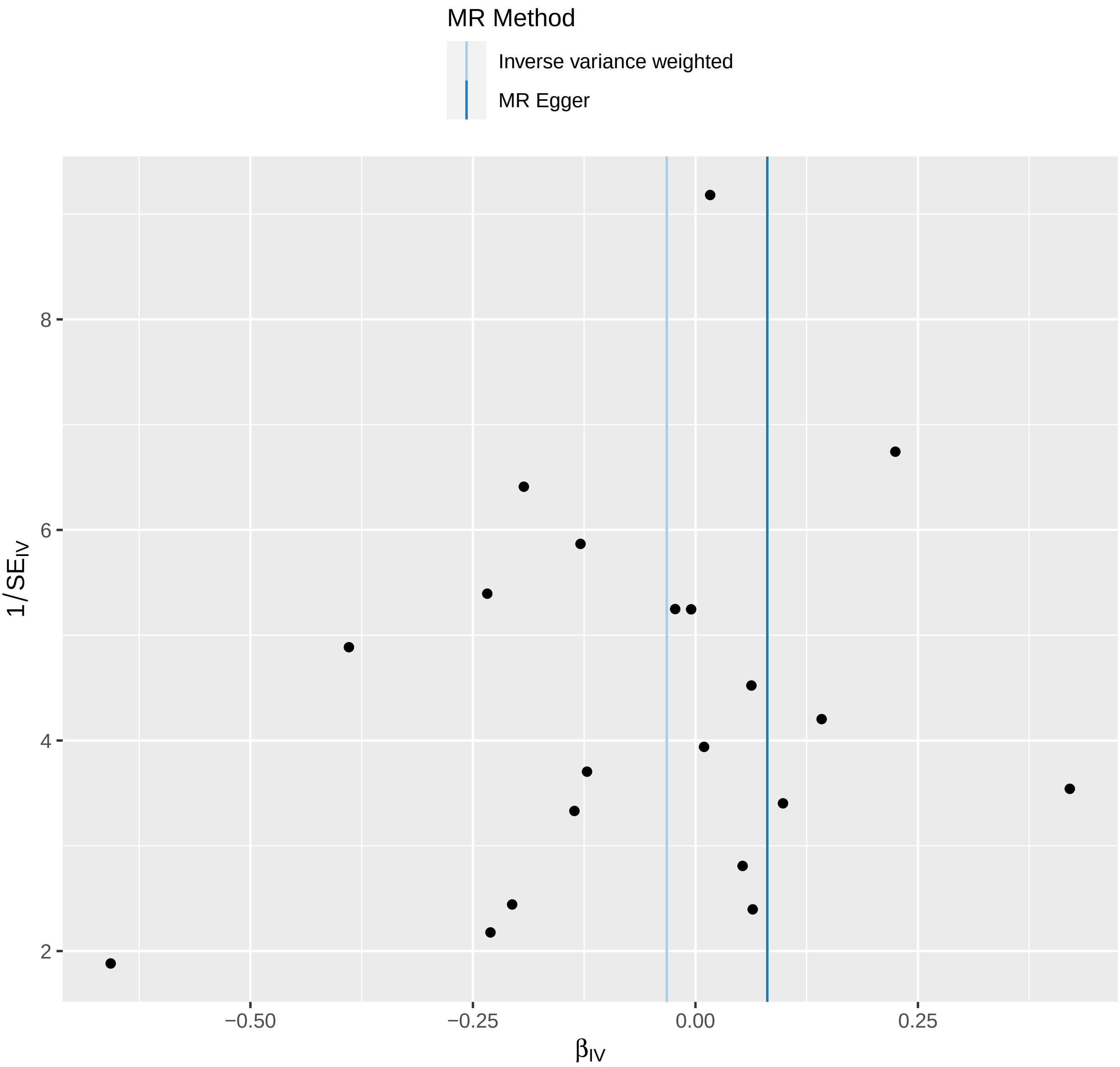

Batch 3−1564 : Insulin receptor on Diabetic nephropathy (more control exclusions)
with a threshold of 1e−05

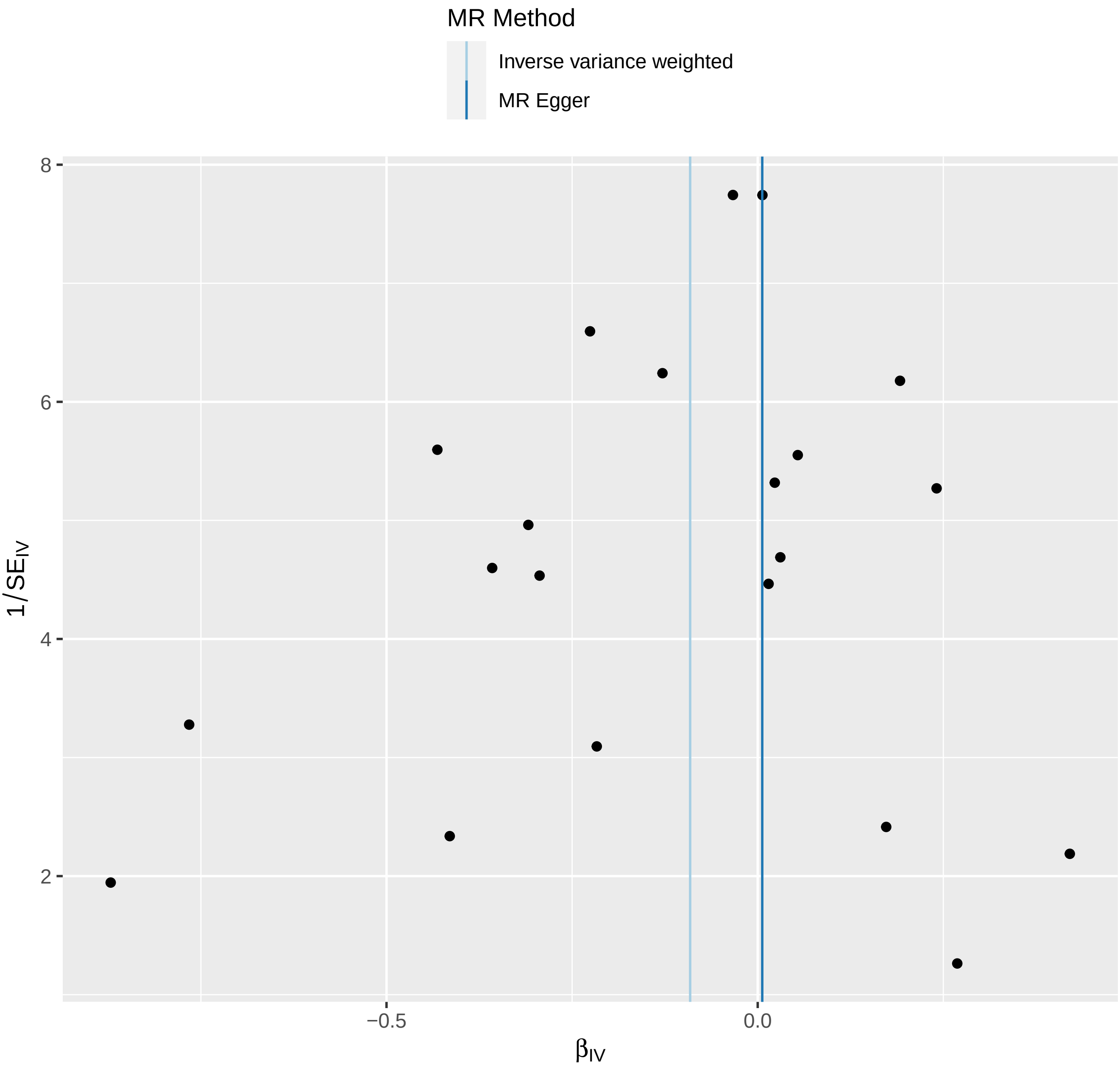

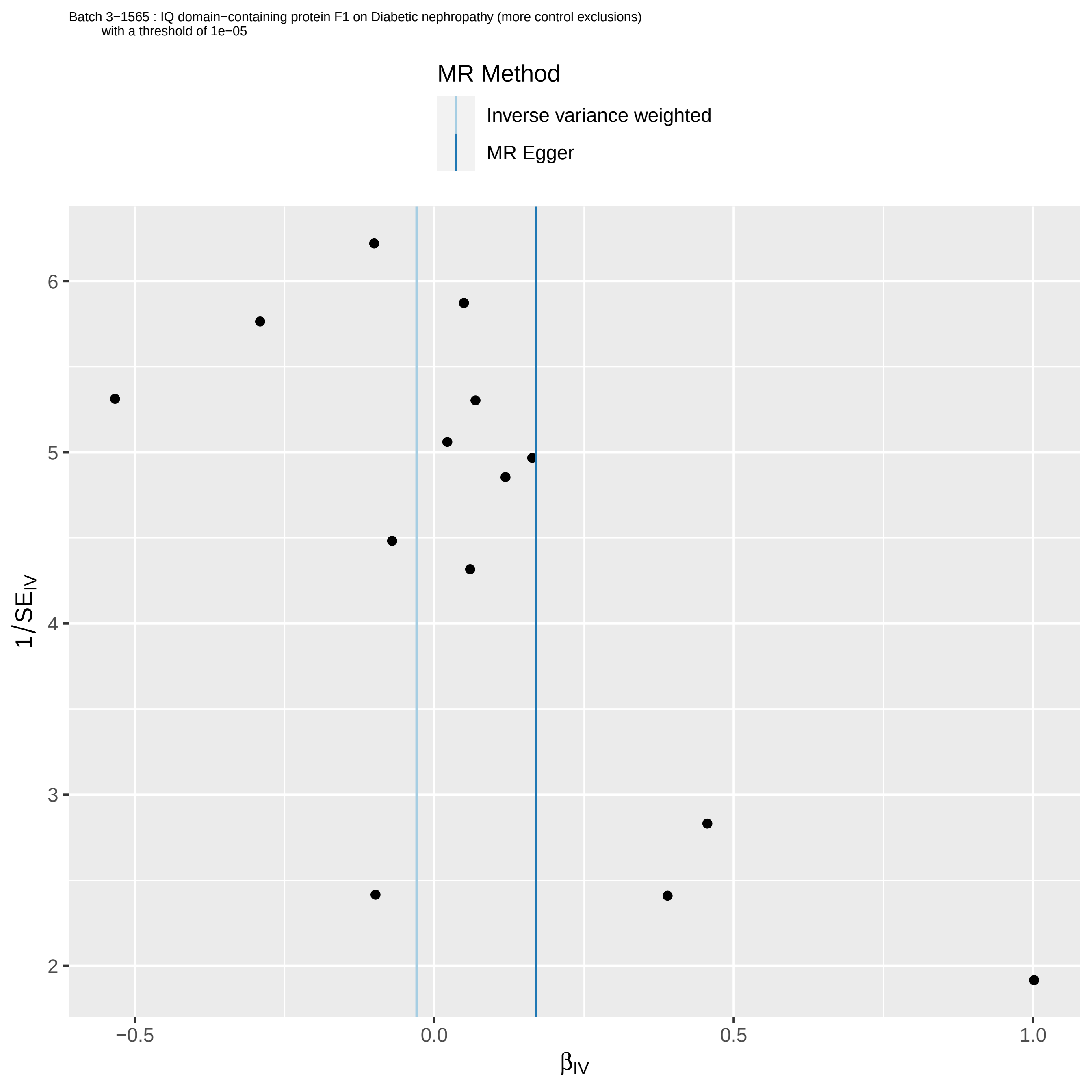
Batch 3−1565 : IQ domain−containing protein F1 on Diabetic nephropathy (more control exclusions)
with a threshold of 1e−05
MR Method
Inverse variance weighted
MR Egger
6
5
4
3
2
1/SE_IV
−0.5
0.0
0.5
1.0
β_IV

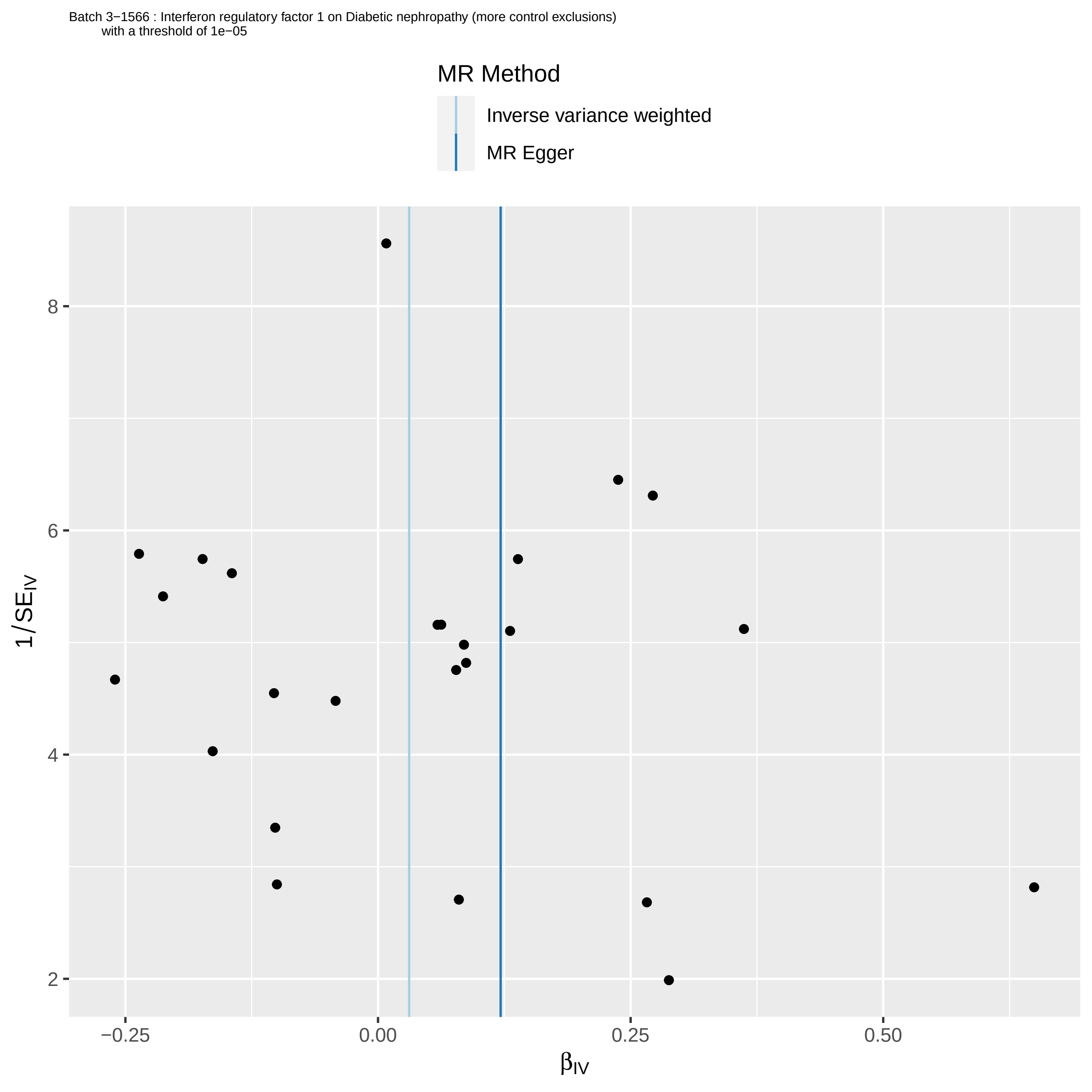

Batch 3−1566 : Interferon regulatory factor 1 on Diabetic nephropathy (more control exclusions)
with a threshold of 1e−05
MR Method
Inverse variance weighted
MR Egger
8
6
4
2
−0.25
0.00
0.25
0.50
1/SE$_{IV}$
β$_{IV}$

Batch 3−1567 : Interferon regulatory factor 1 on Diabetic nephropathy (more control exclusions)
with a threshold of 1e−05

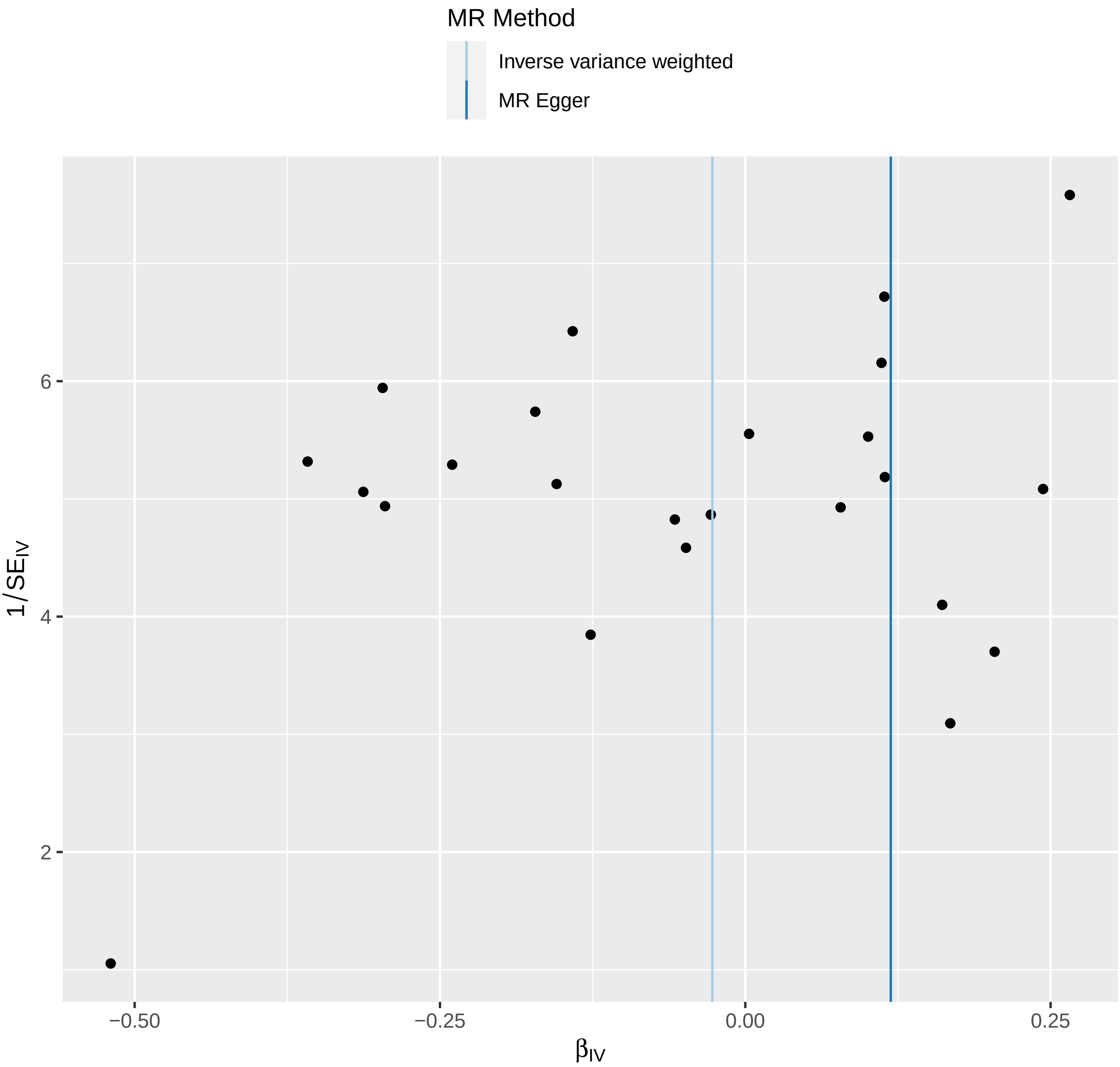

Batch 3−1568 : Interferon regulatory factor 2 on Diabetic nephropathy (more control exclusions)
with a threshold of 1e−05

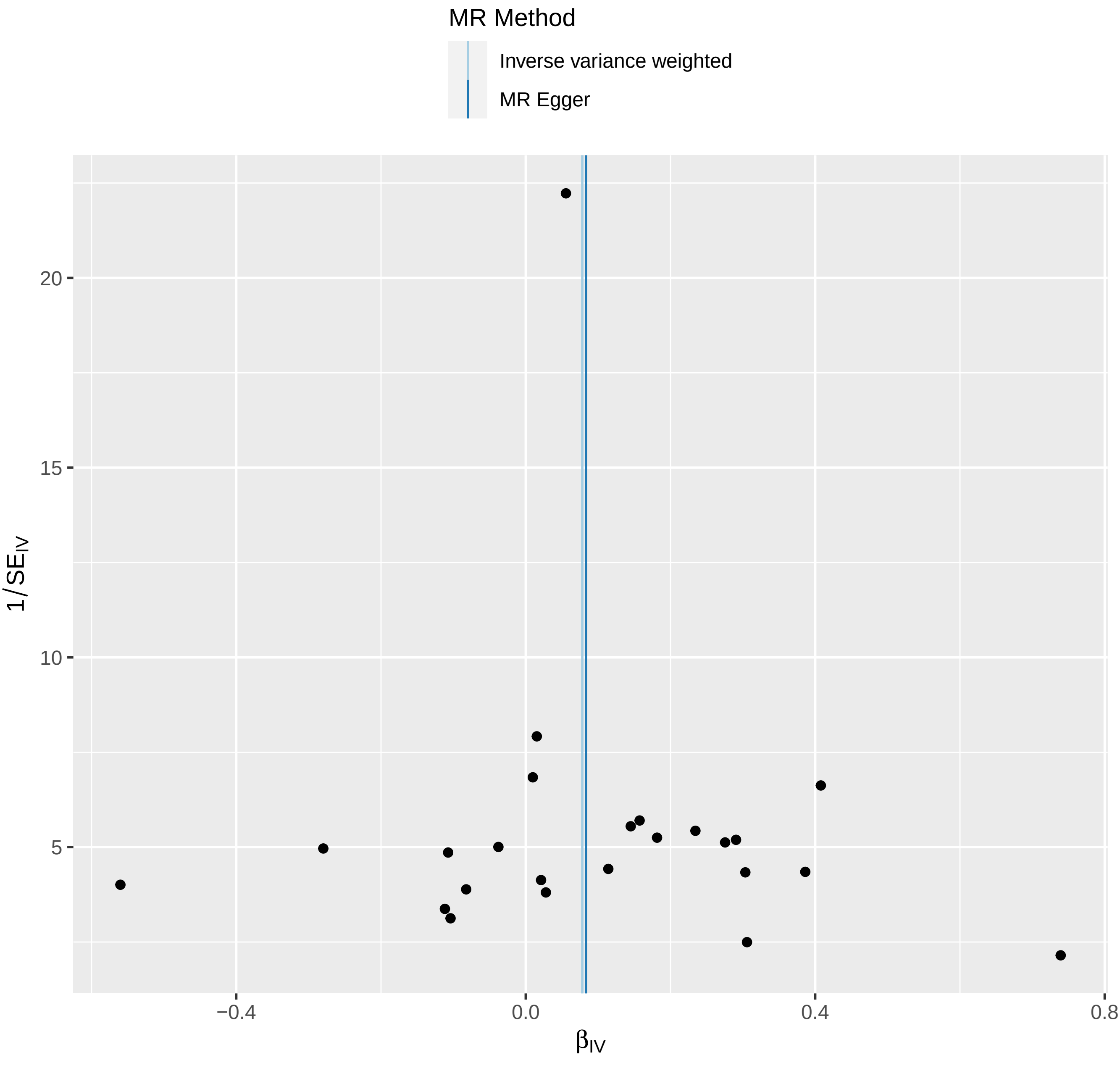

Batch 3−1569 : Interferon regulatory factor 4 on Diabetic nephropathy (more control exclusions)
with a threshold of 1e−05

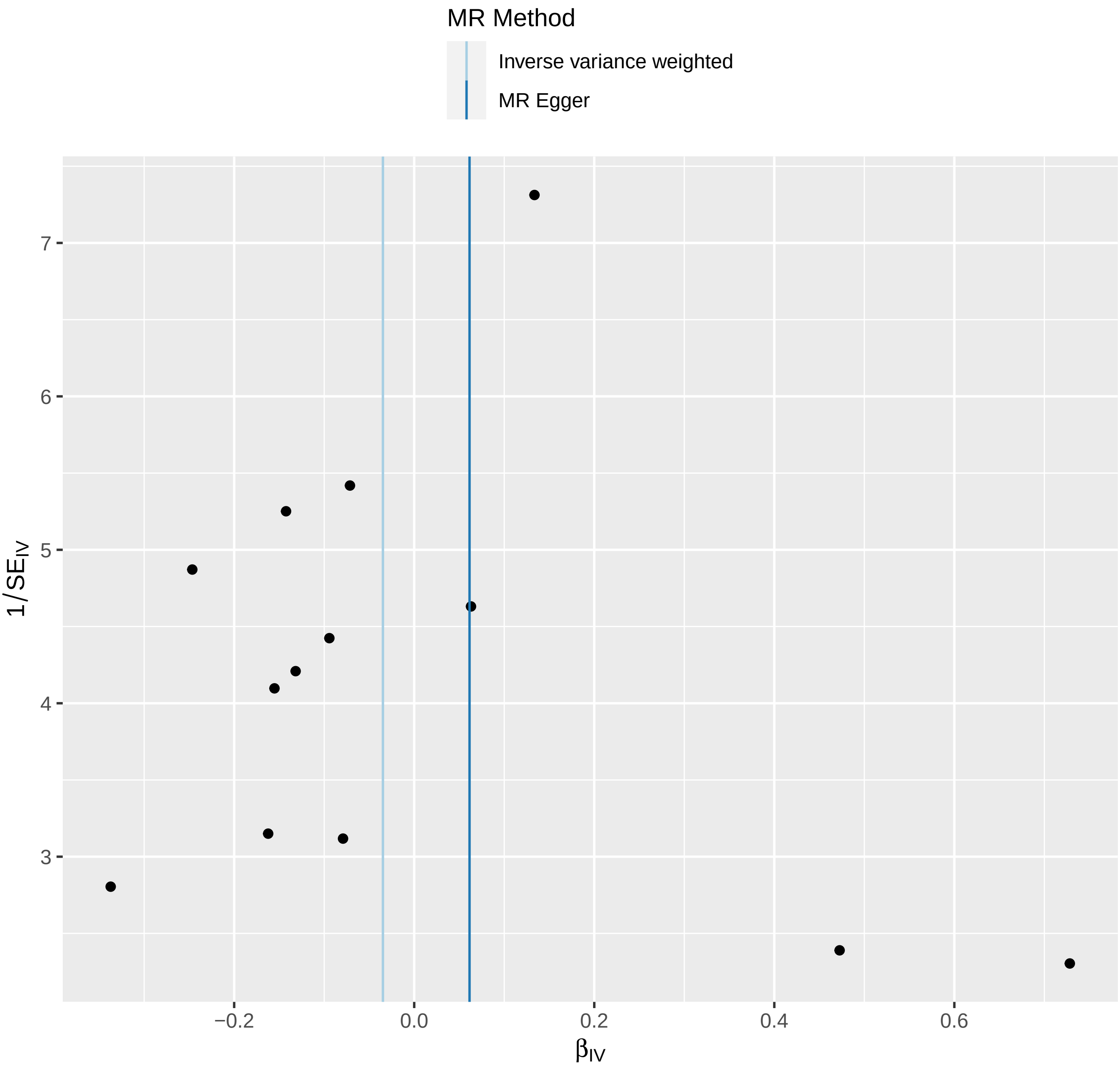

Batch 3−1570 : Interferon regulatory factor 6 on Diabetic nephropathy (more control exclusions)
with a threshold of 1e−05

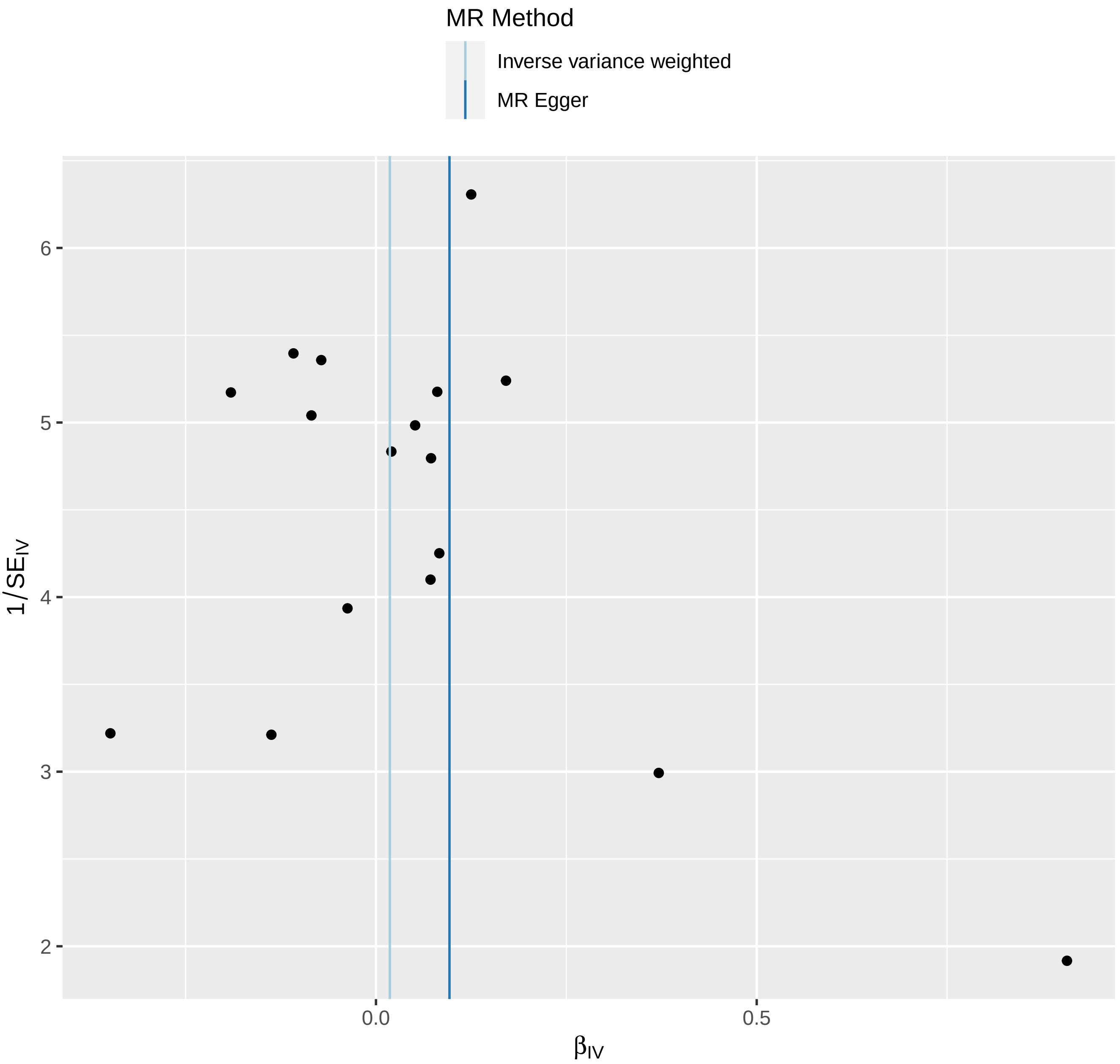

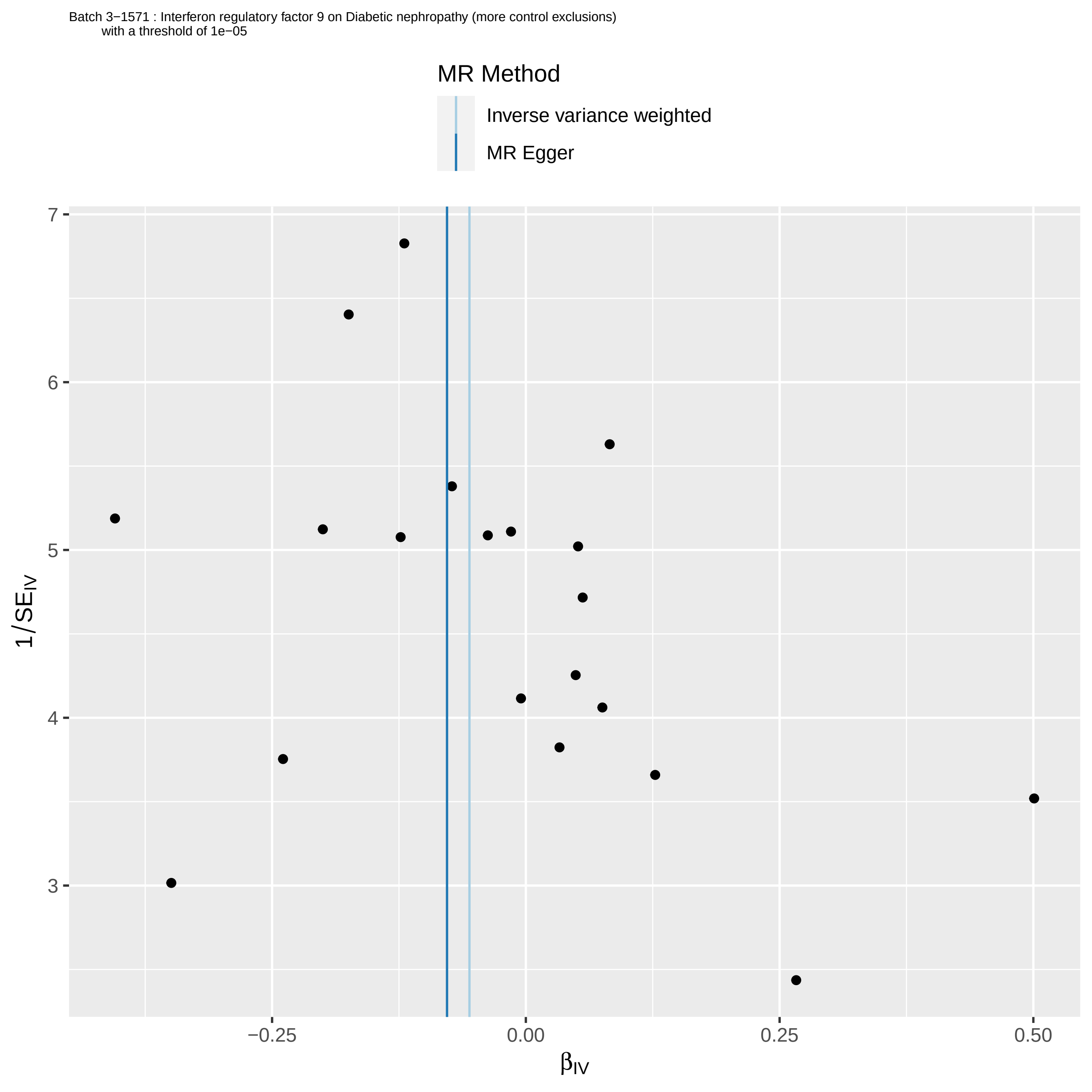

Batch 3−1571 : Interferon regulatory factor 9 on Diabetic nephropathy (more control exclusions)
with a threshold of 1e−05
MR Method
Inverse variance weighted
MR Egger
7
6
5
4
3
1/SE_IV
−0.25
0.00
0.25
0.50
β_IV

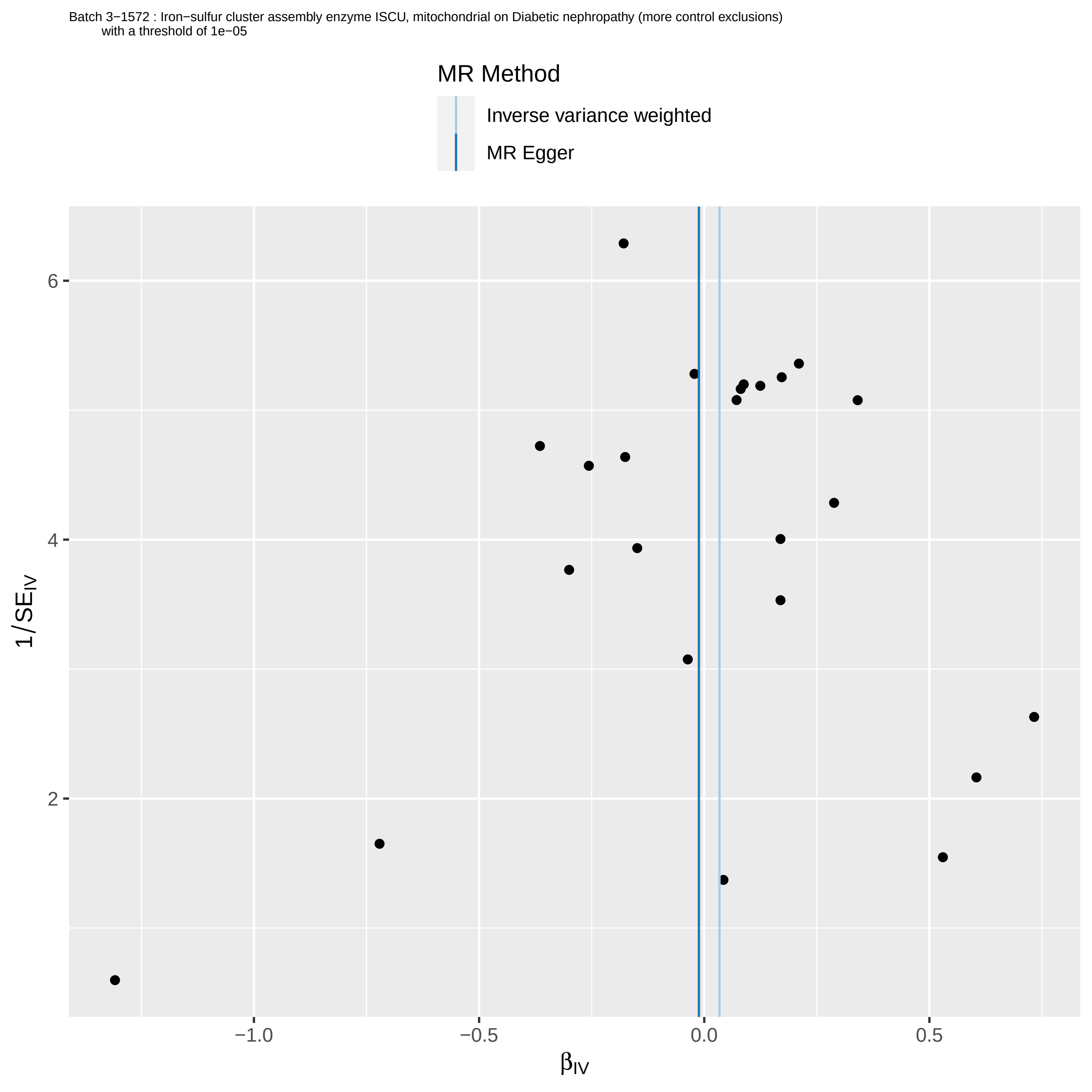

Batch 3−1572 : Iron−sulfur cluster assembly enzyme ISCU, mitochondrial on Diabetic nephropathy (more control exclusions)
with a threshold of 1e−05
MR Method
Inverse variance weighted
MR Egger
6
4
2
1/SE$_{IV}$
−1.0
−0.5
0.0
0.5
β$_{IV}$

Batch 3−1573 : Ubiquitin−like protein ISG15 on Diabetic nephropathy (more control exclusions)
with a threshold of 1e−05

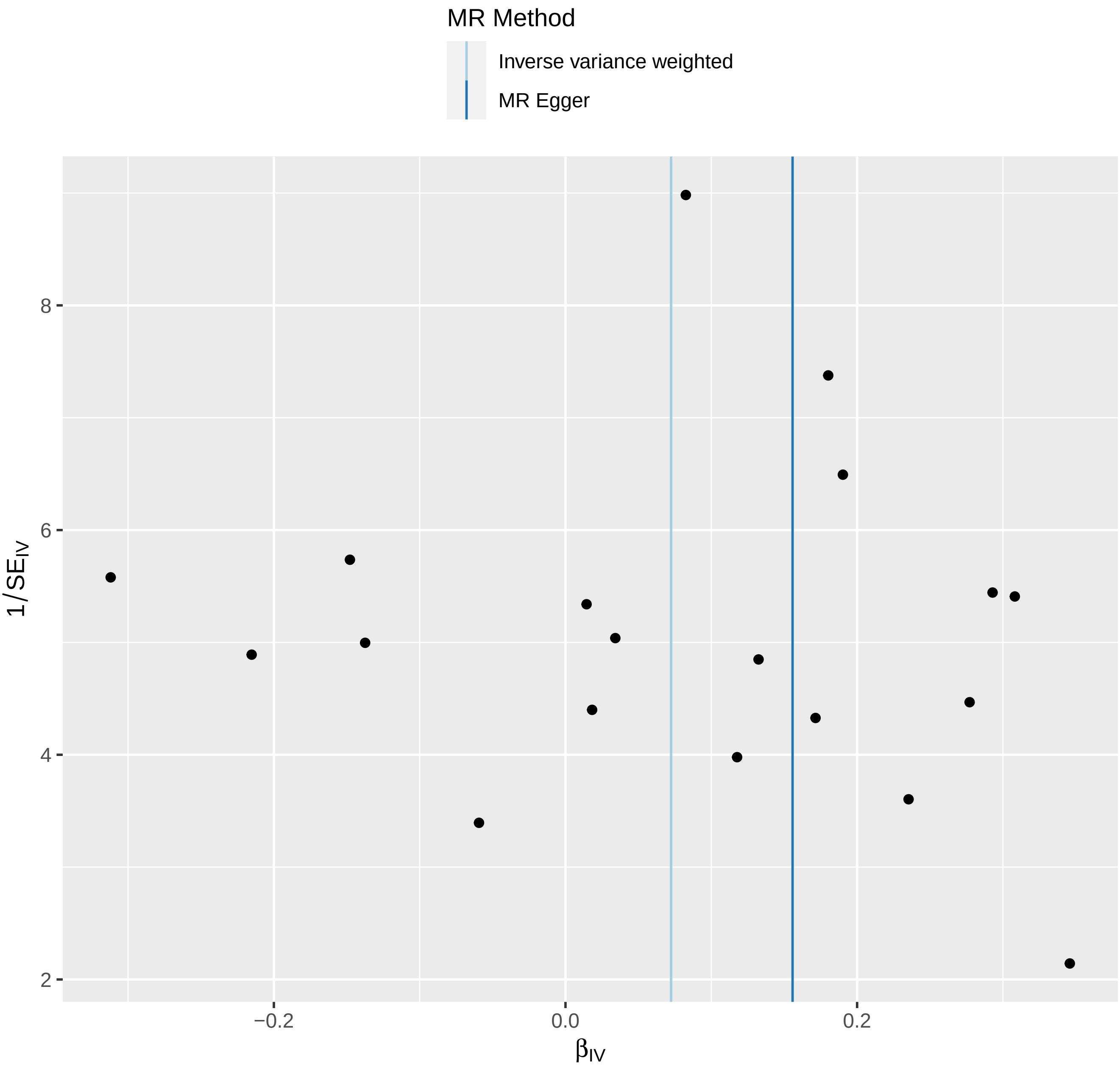

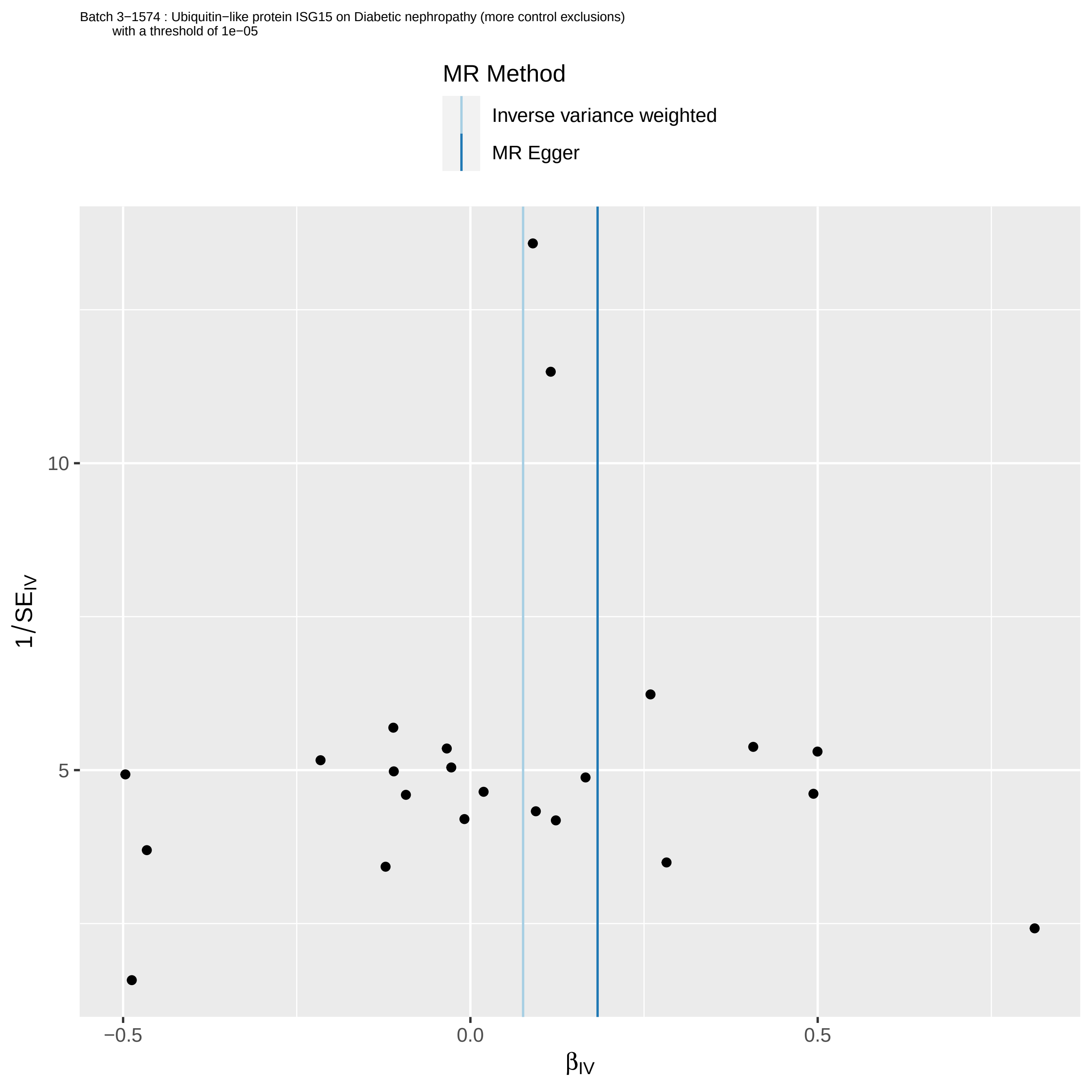

Batch 3−1574 : Ubiquitin−like protein ISG15 on Diabetic nephropathy (more control exclusions)
with a threshold of 1e−05
MR Method
Inverse variance weighted
MR Egger
10
5
1/SE_IV
−0.5
0.0
0.5
β_IV

Batch 3−1575 : Insulin gene enhancer protein ISL−1 on Diabetic nephropathy (more control exclusions)
with a threshold of 1e−05

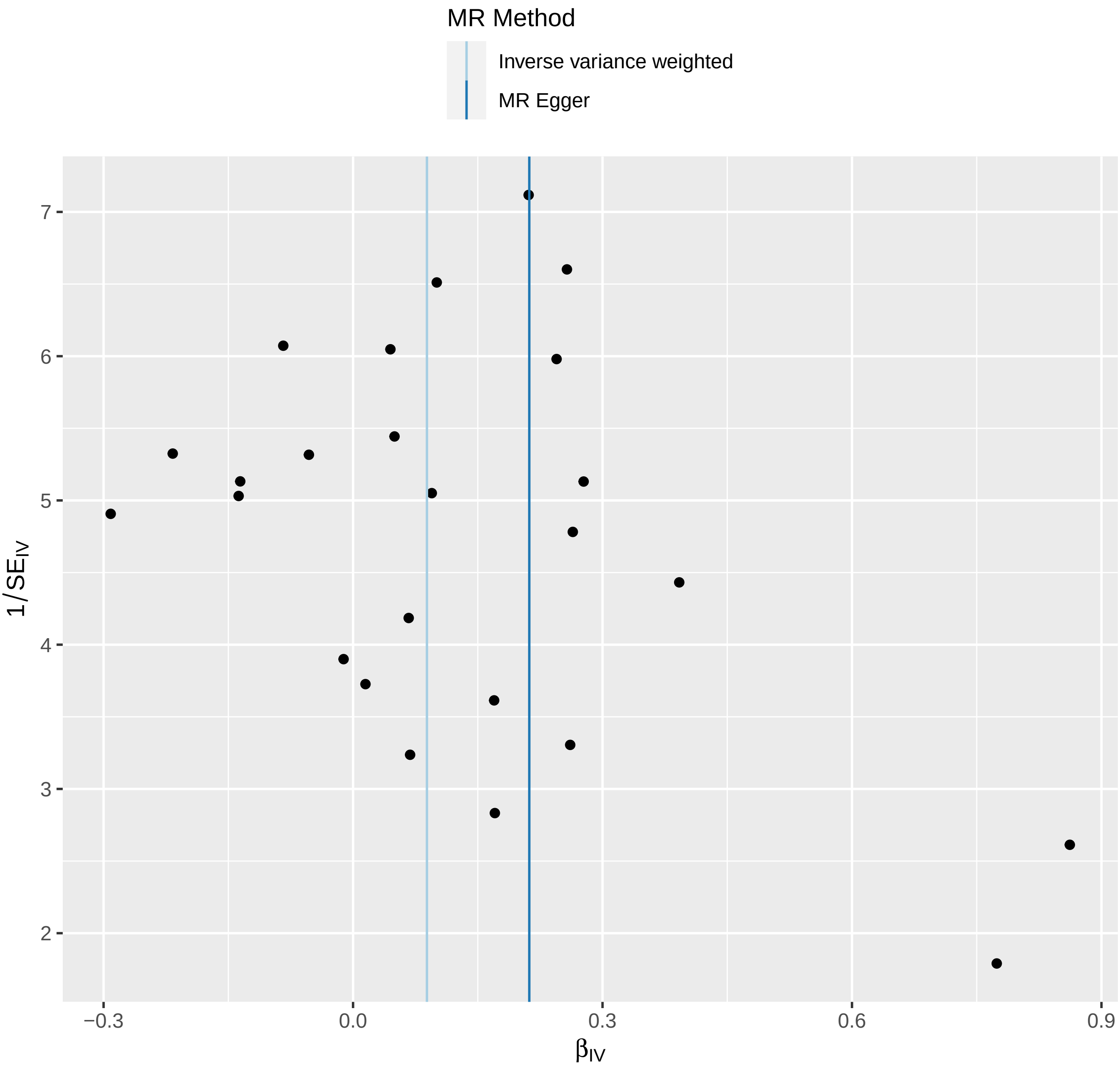

Batch 3−1576 : Immunoglobulin superfamily containing leucine−rich repeat protein 2 on Diabetic nephropathy (more control exclusions) with a threshold of 1e−05

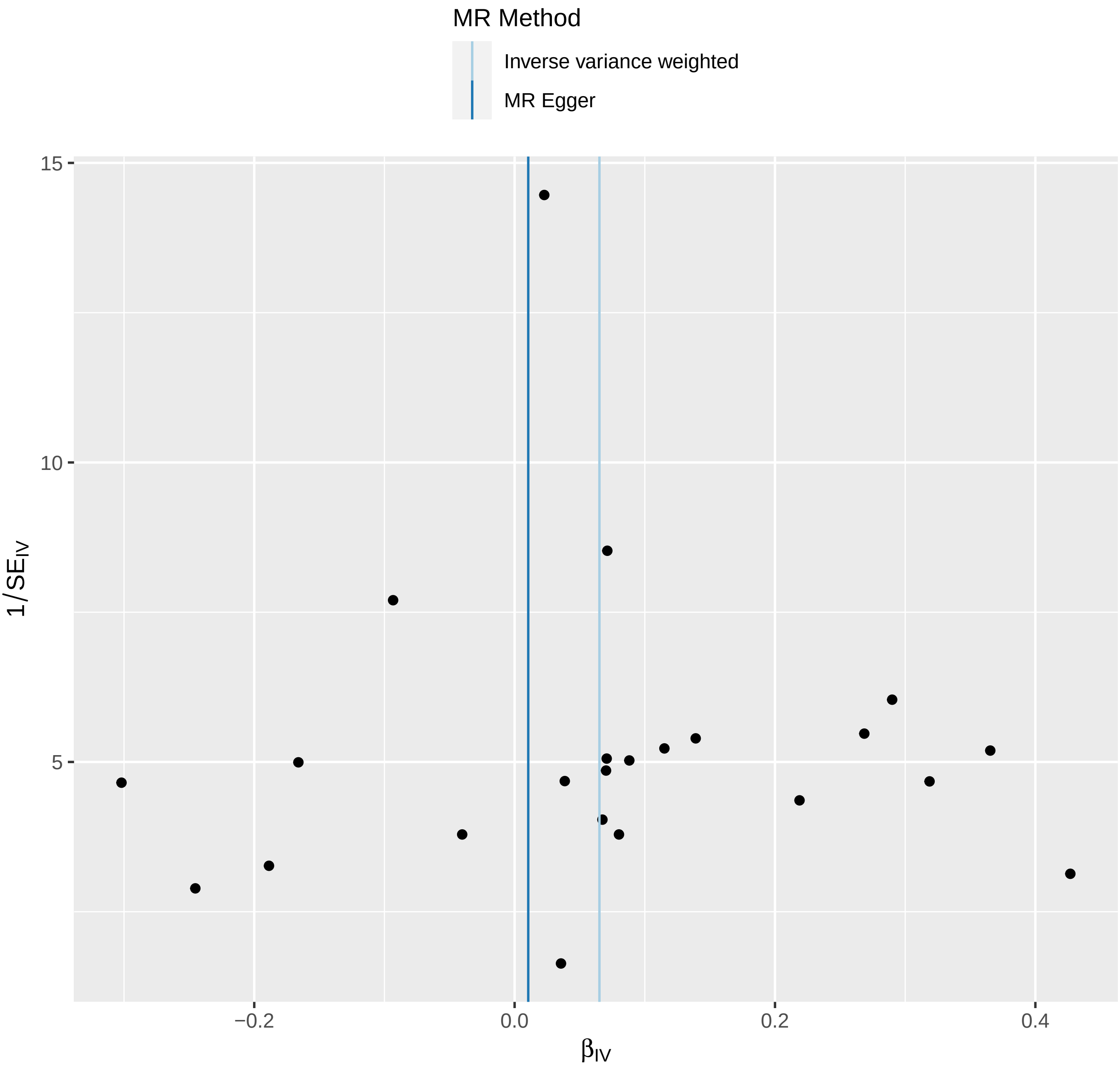

Batch 3−1577 : Isthmin−1 on Diabetic nephropathy (more control exclusions)
with a threshold of 1e−05

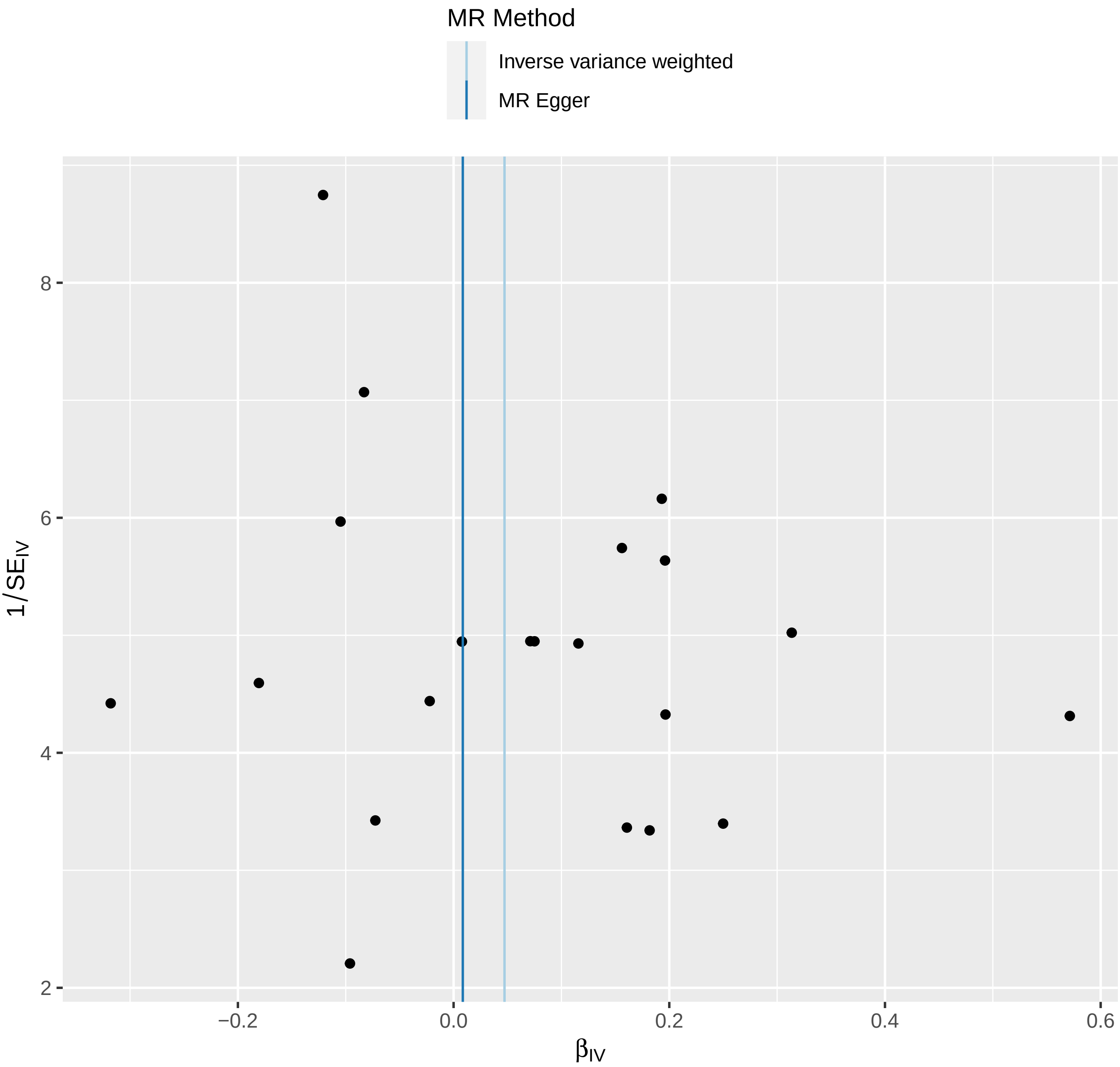

Batch 3−1578 : Isochorismatase domain−containing protein 1 on Diabetic nephropathy (more control exclusions)
with a threshold of 1e−05

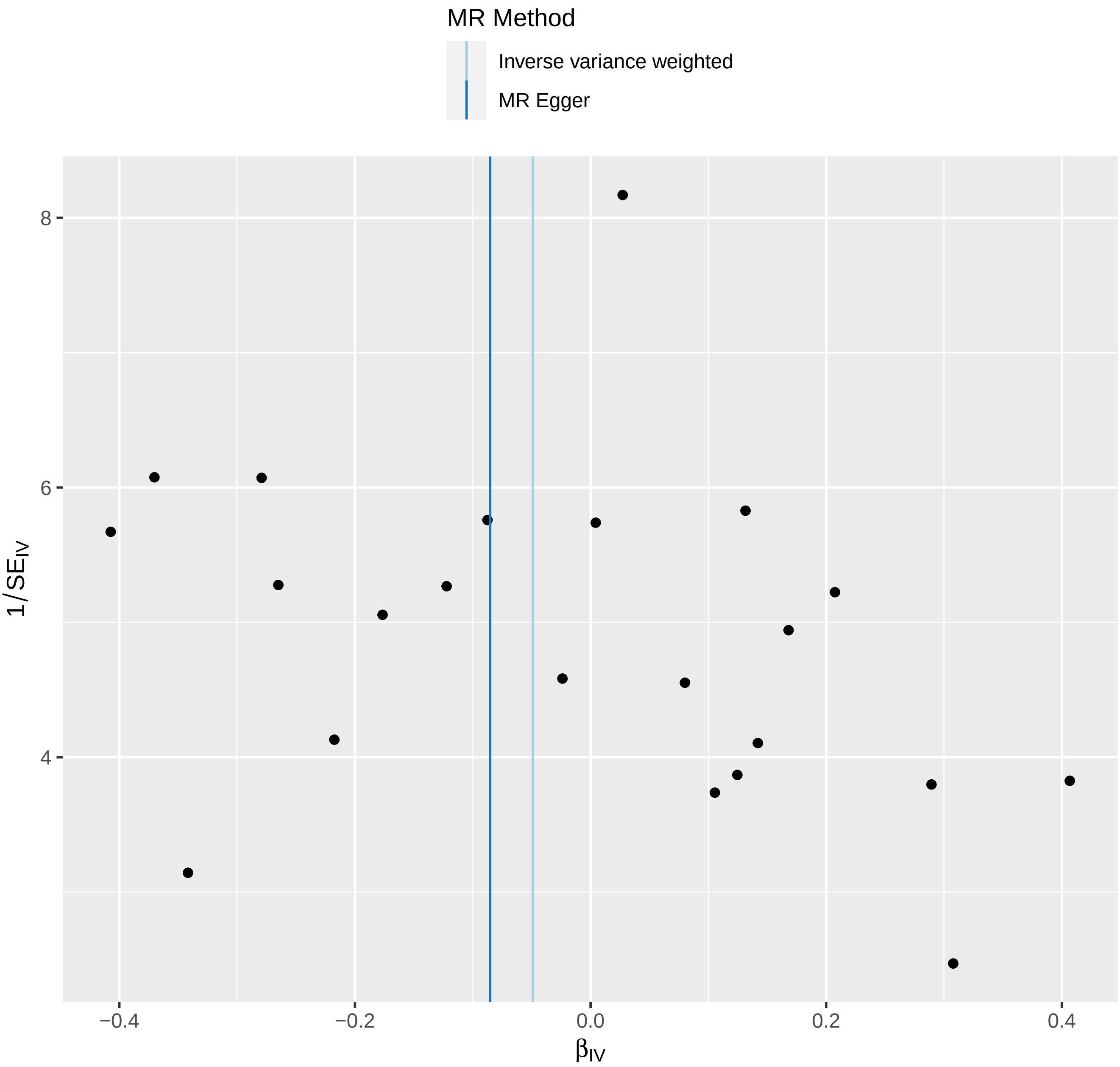

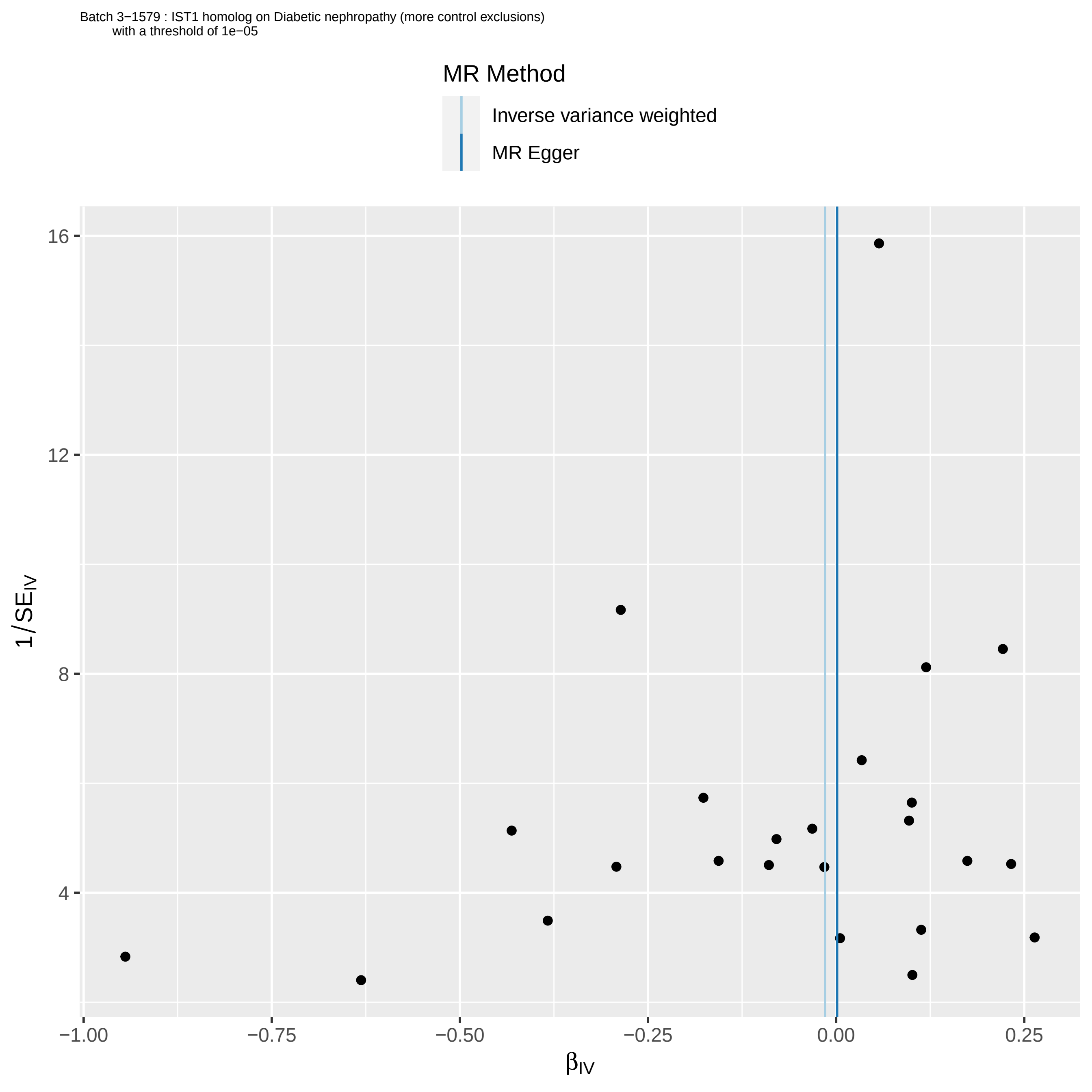

Batch 3−1579 : IST1 homolog on Diabetic nephropathy (more control exclusions)
with a threshold of 1e−05
MR Method
Inverse variance weighted
MR Egger
16
12
8
4
1/SE_IV
−1.00
−0.75
−0.50
−0.25
0.00
0.25
β_IV

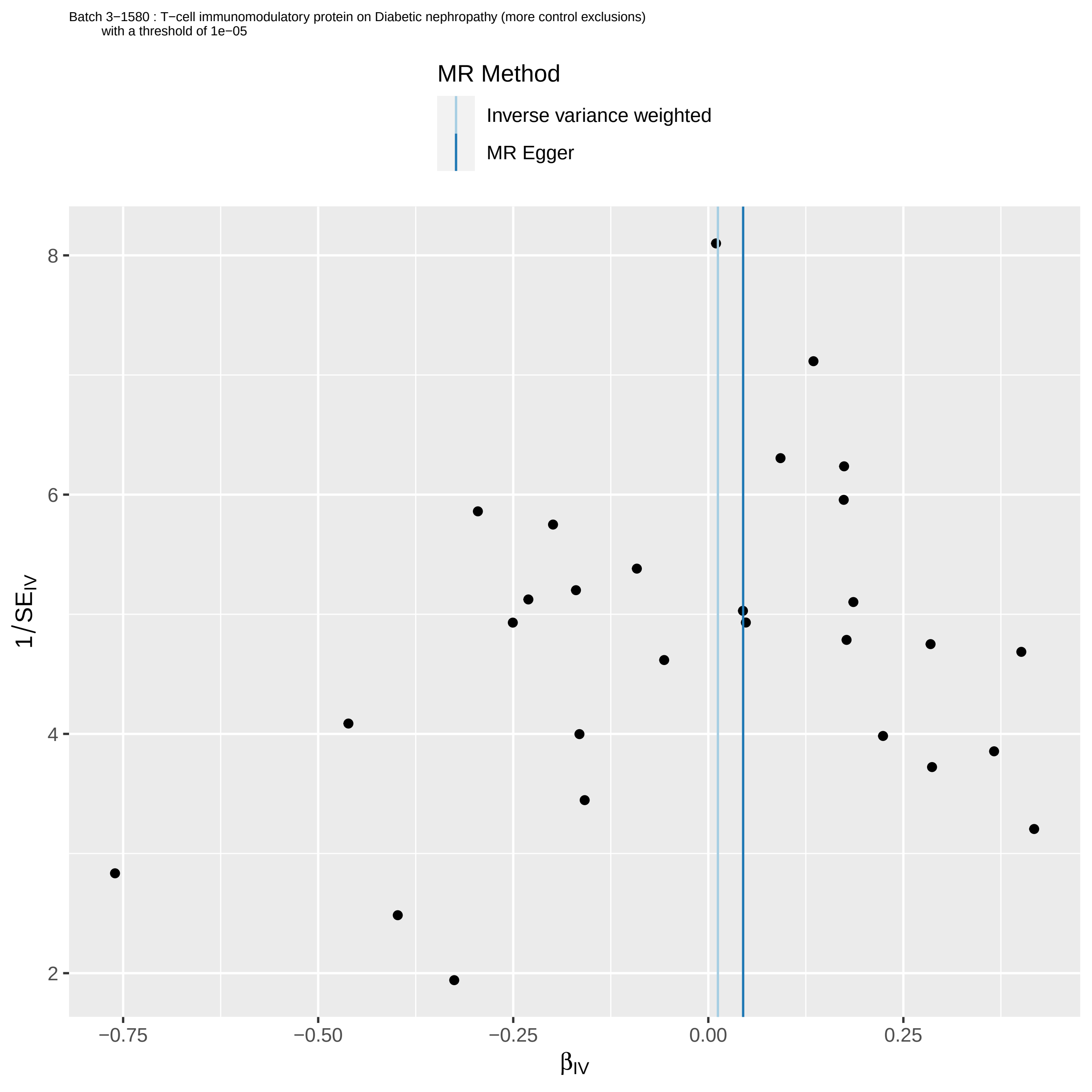
Batch 3−1580 : T−cell immunomodulatory protein on Diabetic nephropathy (more control exclusions)
with a threshold of 1e−05
MR Method
Inverse variance weighted
MR Egger
8
6
4
2
1/SE_IV
−0.75
−0.50
−0.25
0.00
0.25
β_IV

Batch 3−1581 : Integrin alpha−I: beta−1 complex on Diabetic nephropathy (more control exclusions)
with a threshold of 1e−05

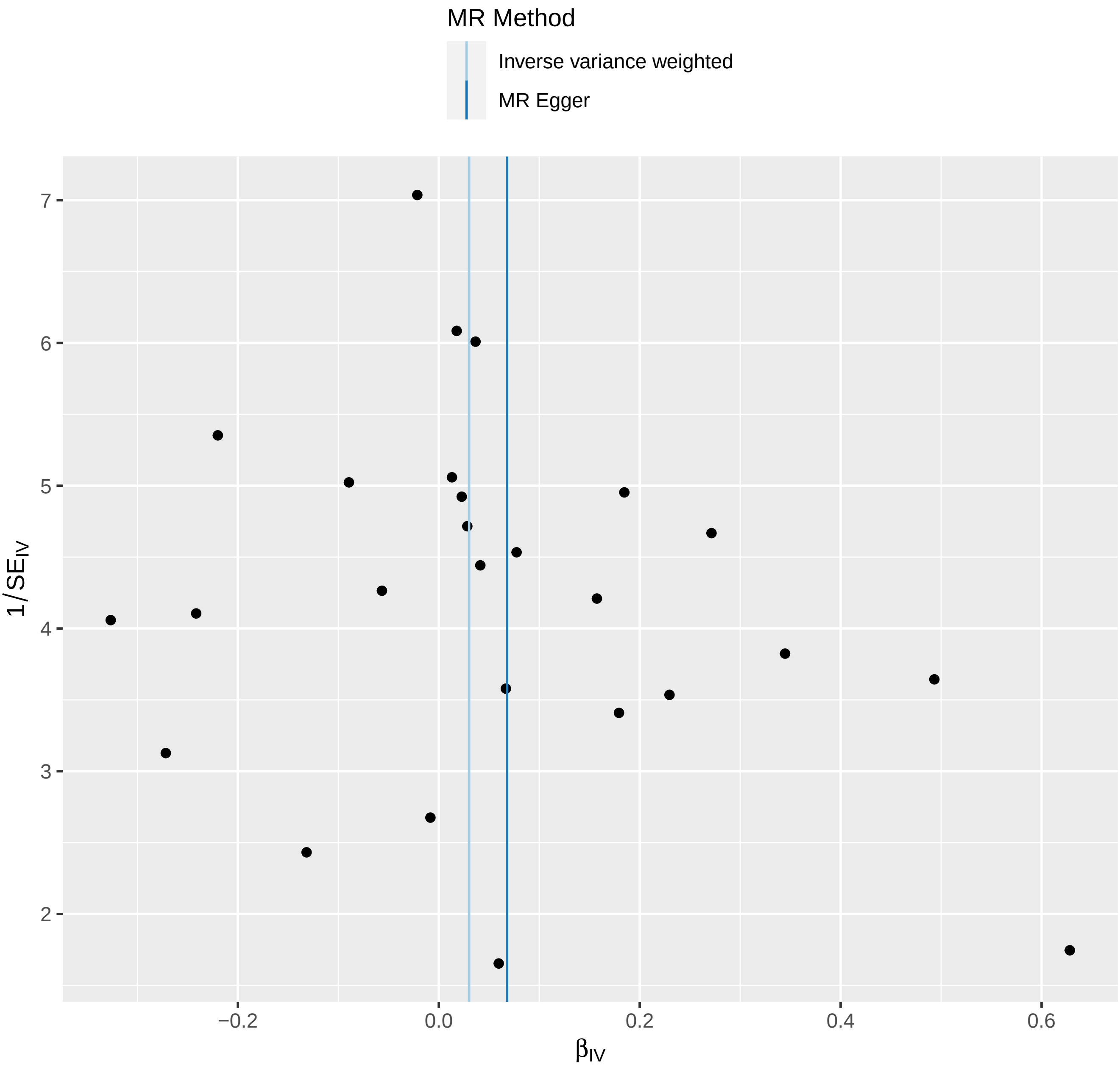

Batch 3−1582 : Integrin alpha−5 on Diabetic nephropathy (more control exclusions)
with a threshold of 1e−05

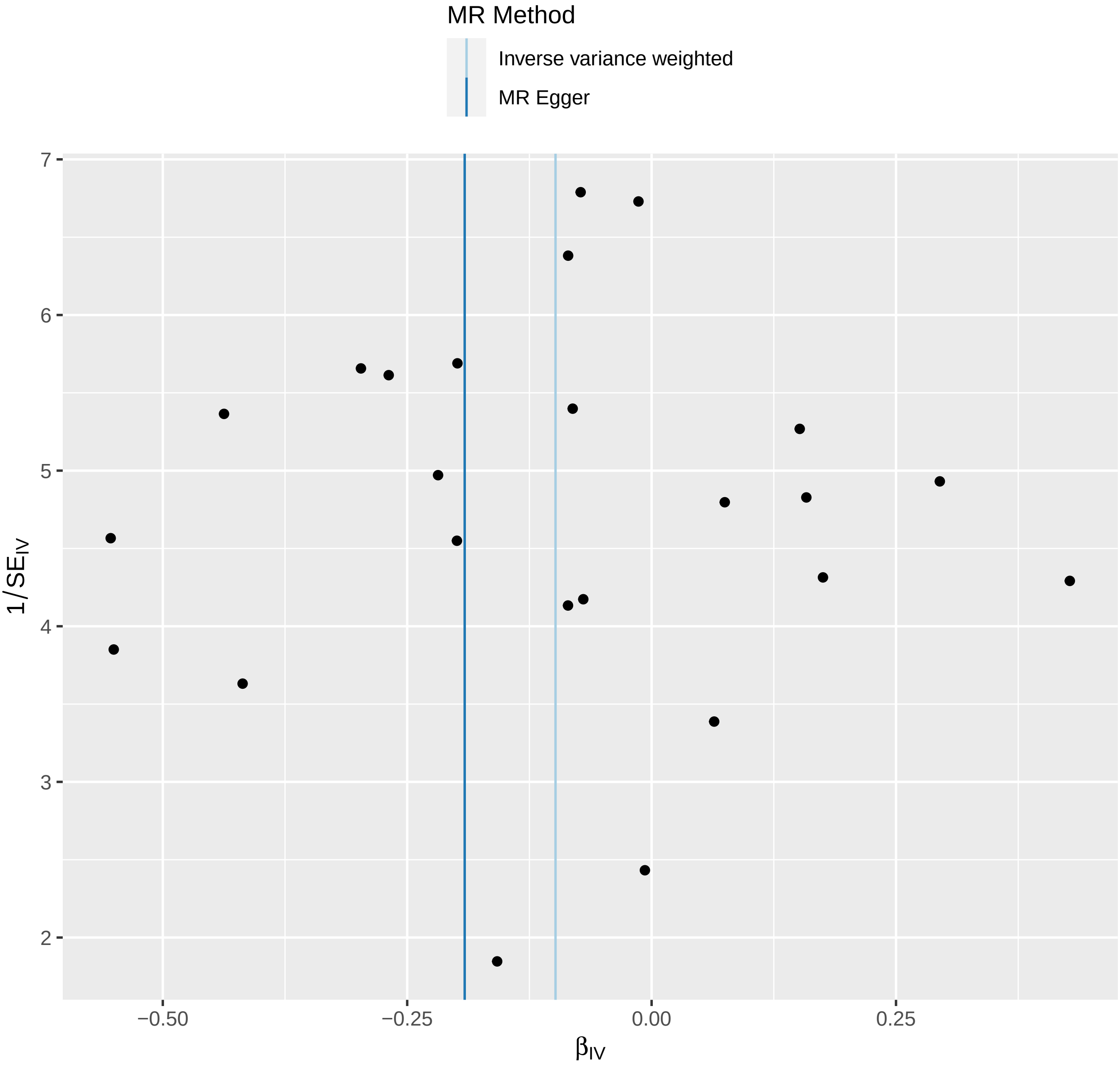

Batch 3−1583 : Integrin alpha−L on Diabetic nephropathy (more control exclusions)
with a threshold of 1e−05

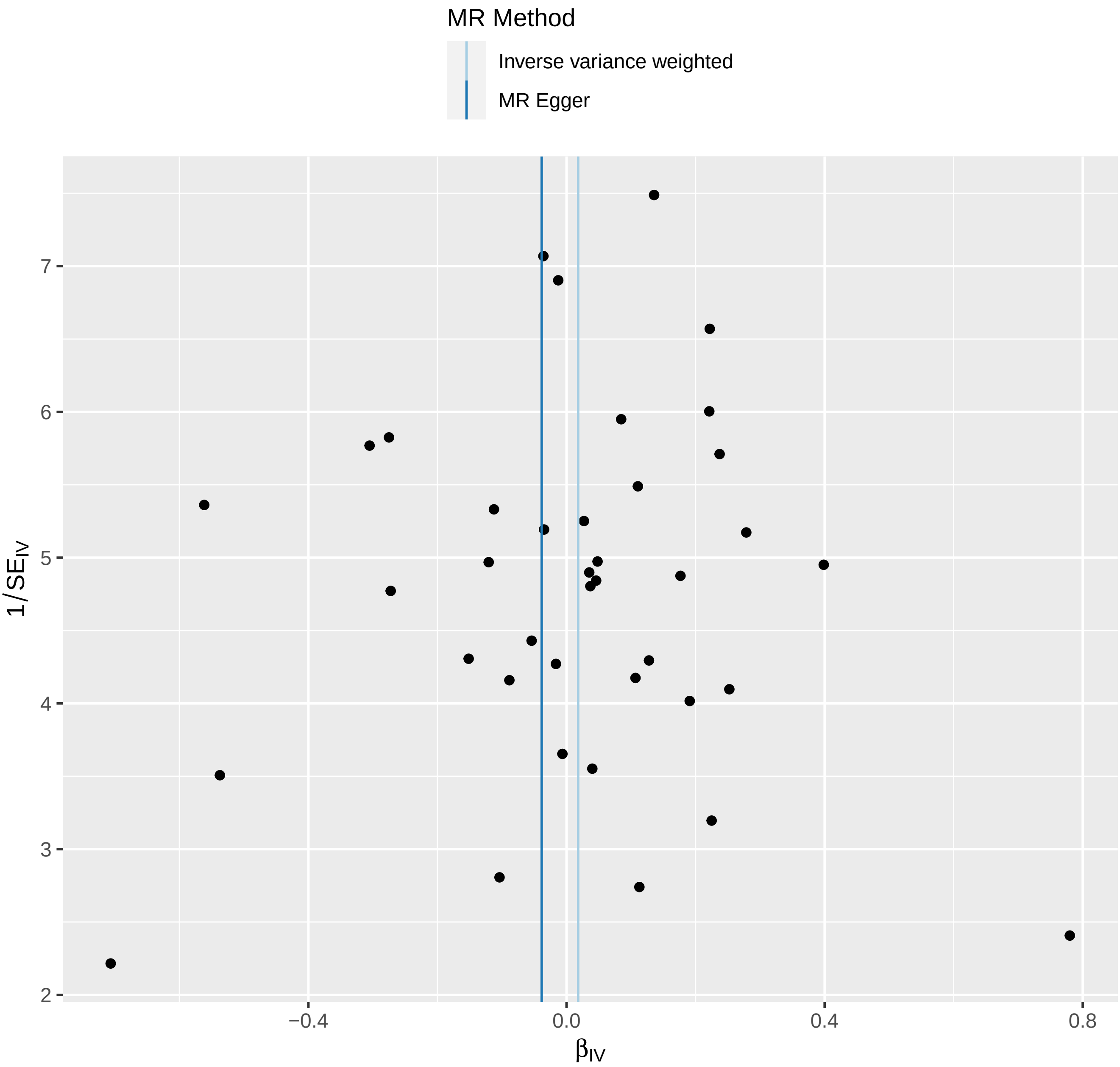

Batch 3−1584 : Integrin alpha−V: beta−5 complex on Diabetic nephropathy (more control exclusions) with a threshold of 1e−05

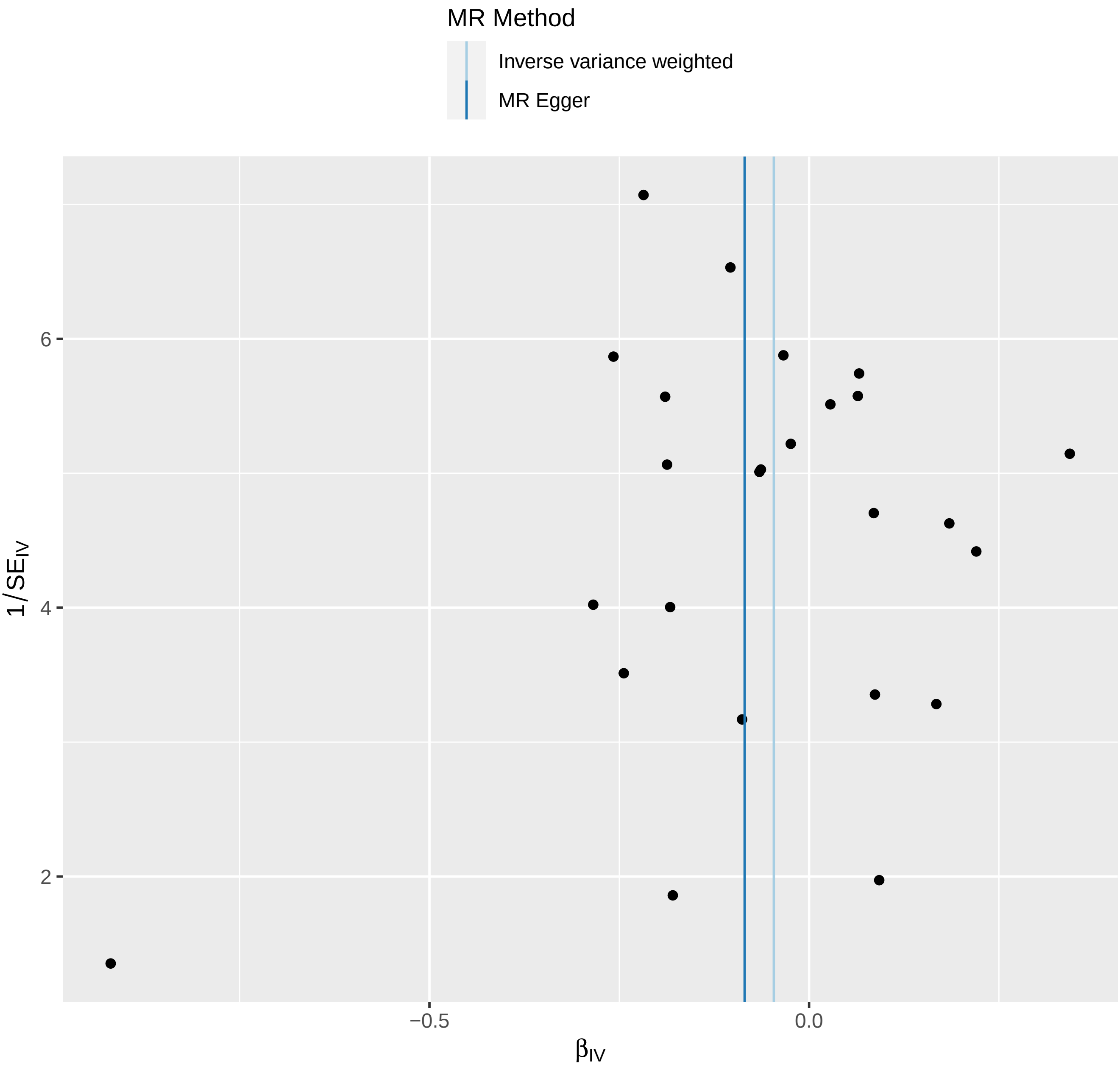

Batch 3−1585 : Integrin beta−7 on Diabetic nephropathy (more control exclusions)
with a threshold of 1e−05

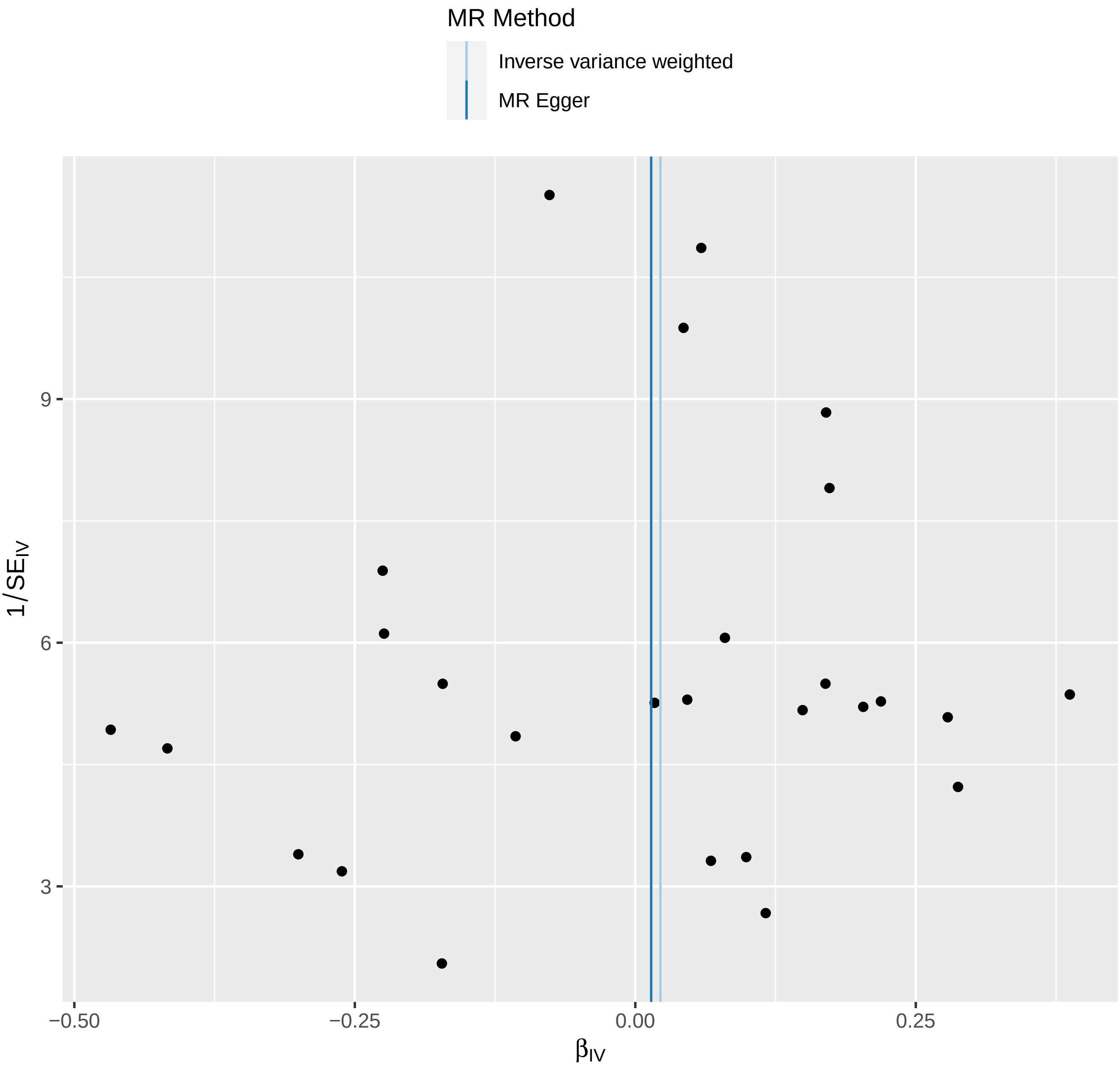

Batch 3−1586 : Inter−alpha−trypsin inhibitor heavy chain H1 on Diabetic nephropathy (more control exclusions)
with a threshold of 1e−05

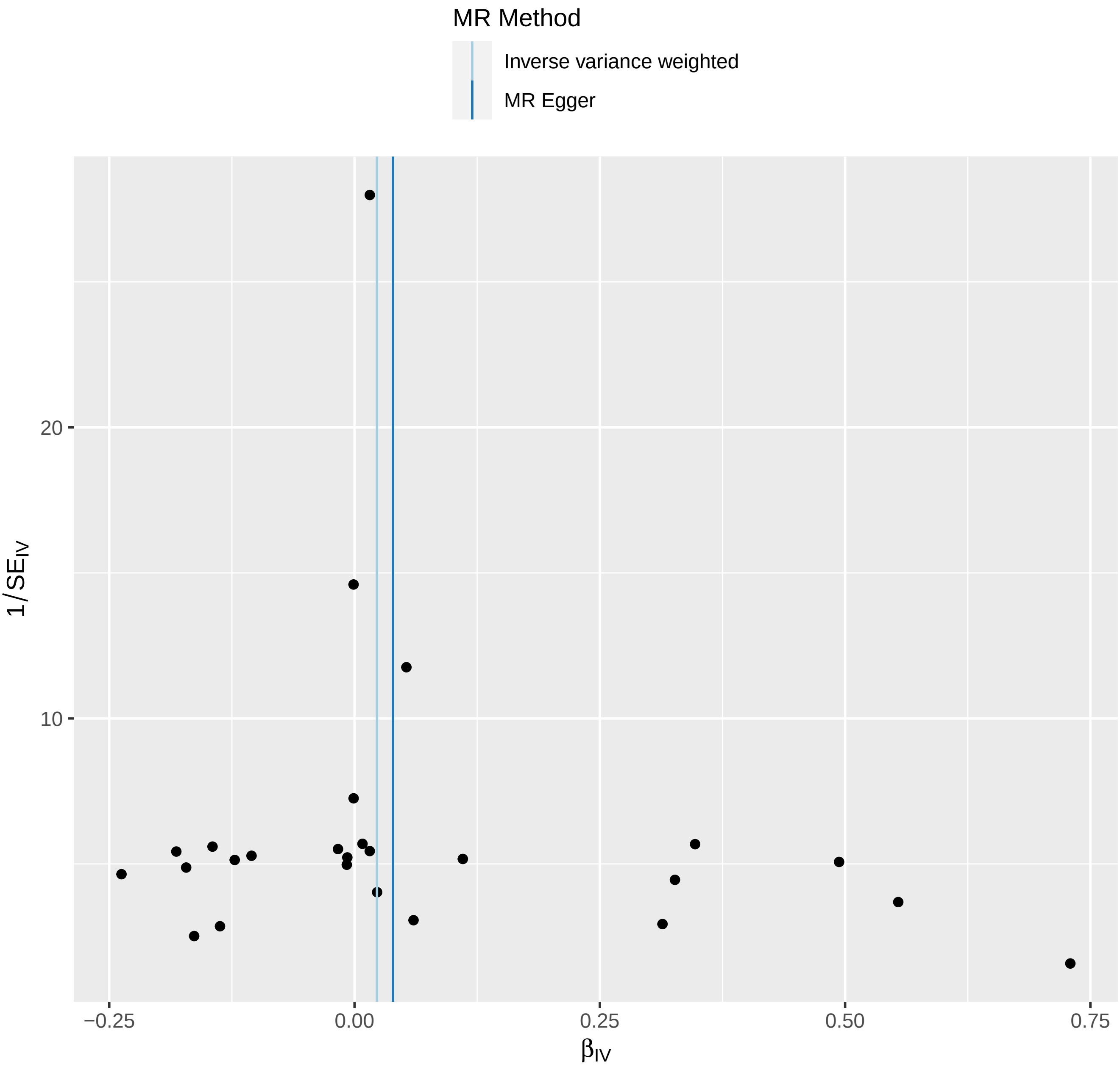

Batch 3−1587 : Inter−alpha−trypsin inhibitor heavy chain H5 on Diabetic nephropathy (more control exclusions) with a threshold of 1e−05

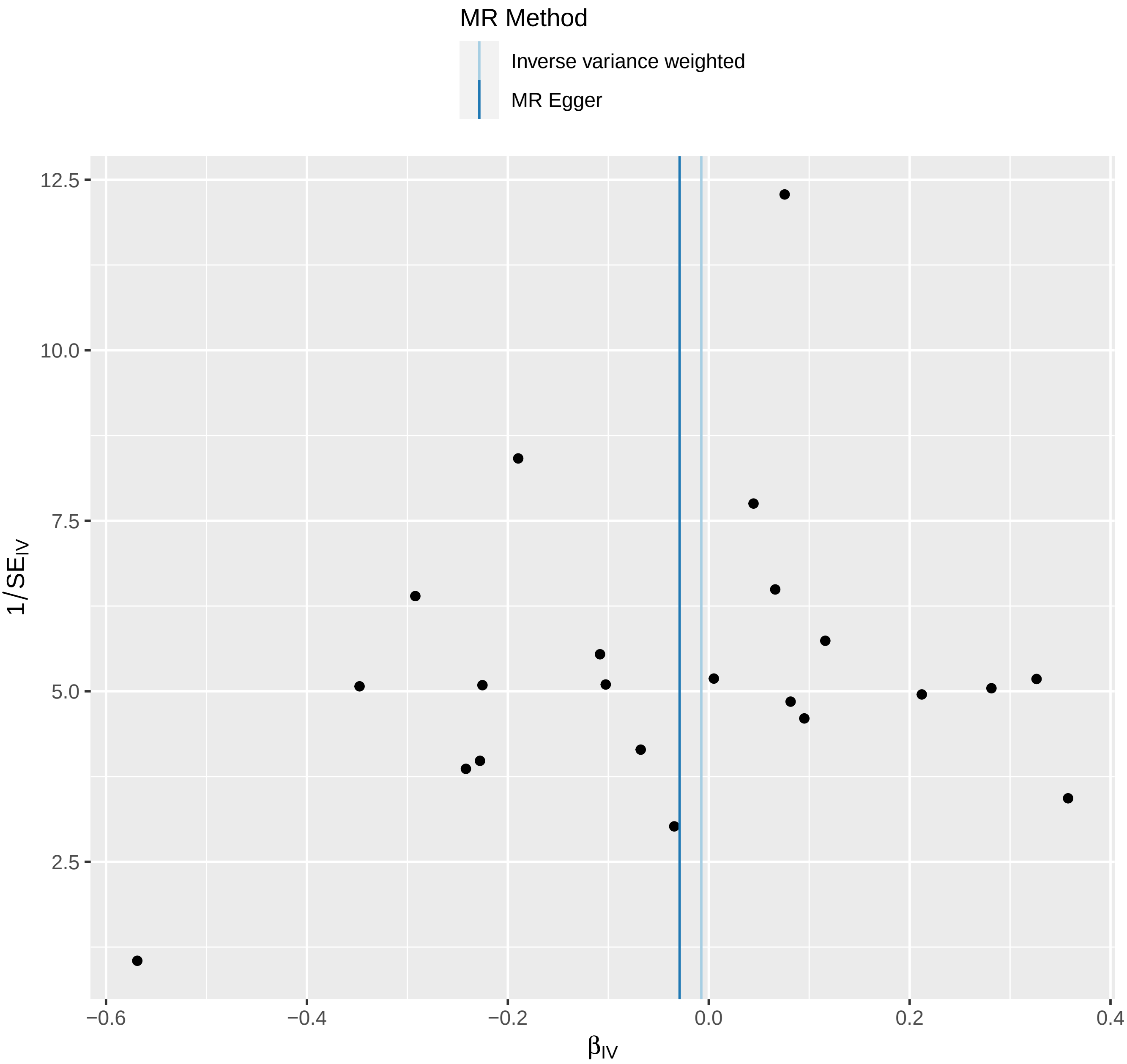

Batch 3−1588 : Integral membrane protein 2A on Diabetic nephropathy (more control exclusions)
with a threshold of 1e−05

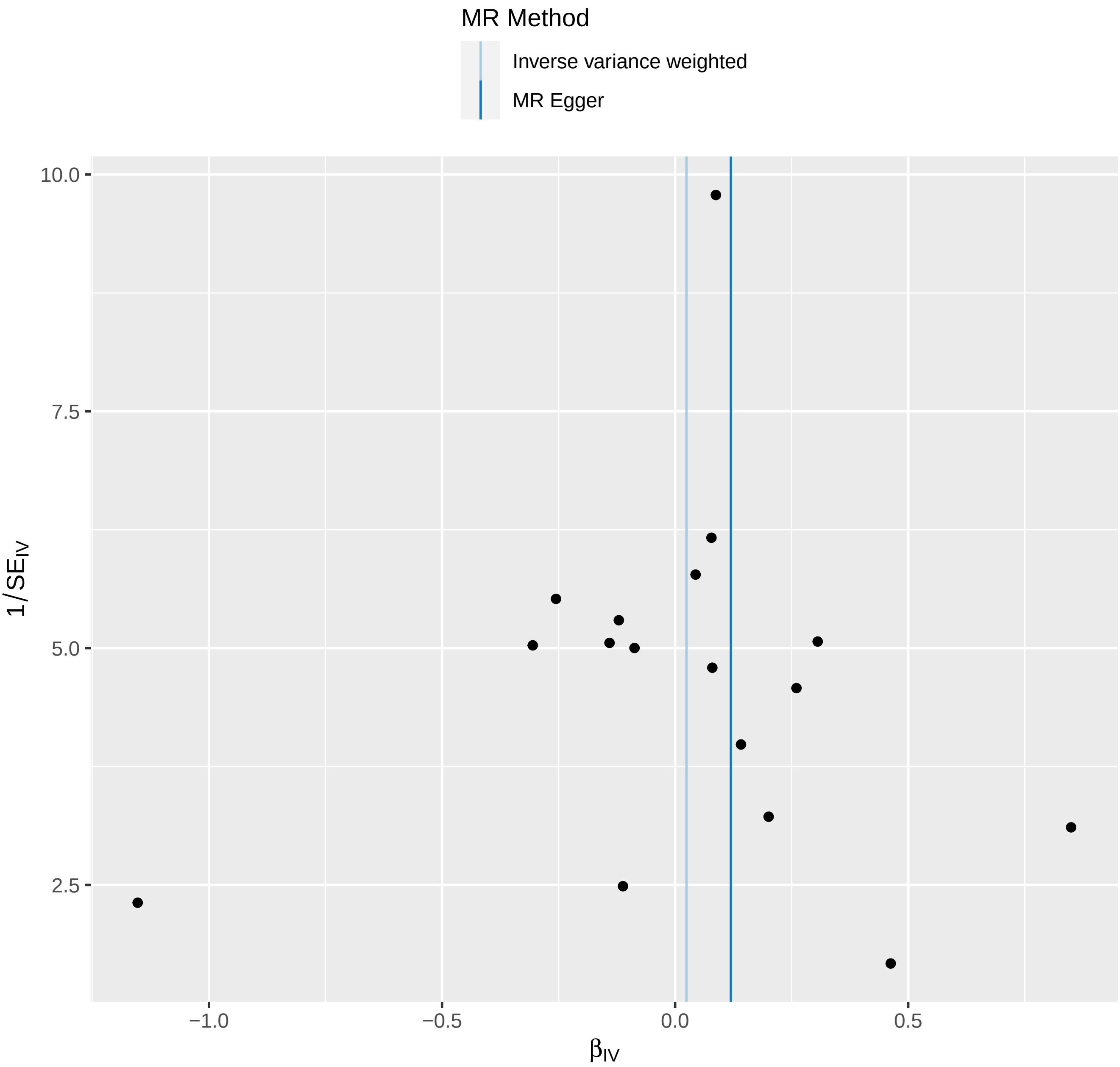

Batch 3−1589 : Integral membrane protein 2A on Diabetic nephropathy (more control exclusions)
with a threshold of 1e−05

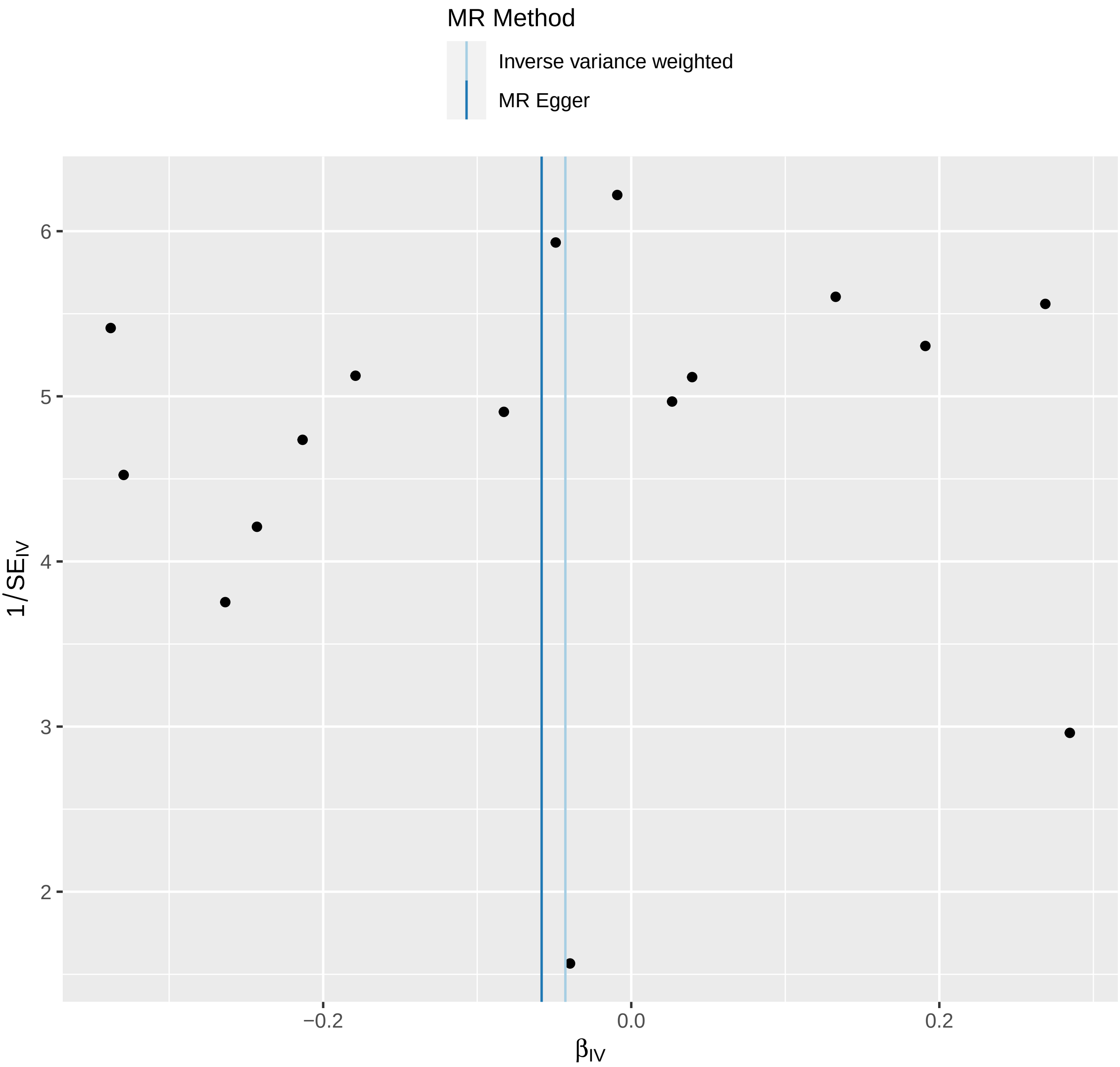

Batch 3−1590 : Integral membrane protein 2B on Diabetic nephropathy (more control exclusions)
with a threshold of 1e−05

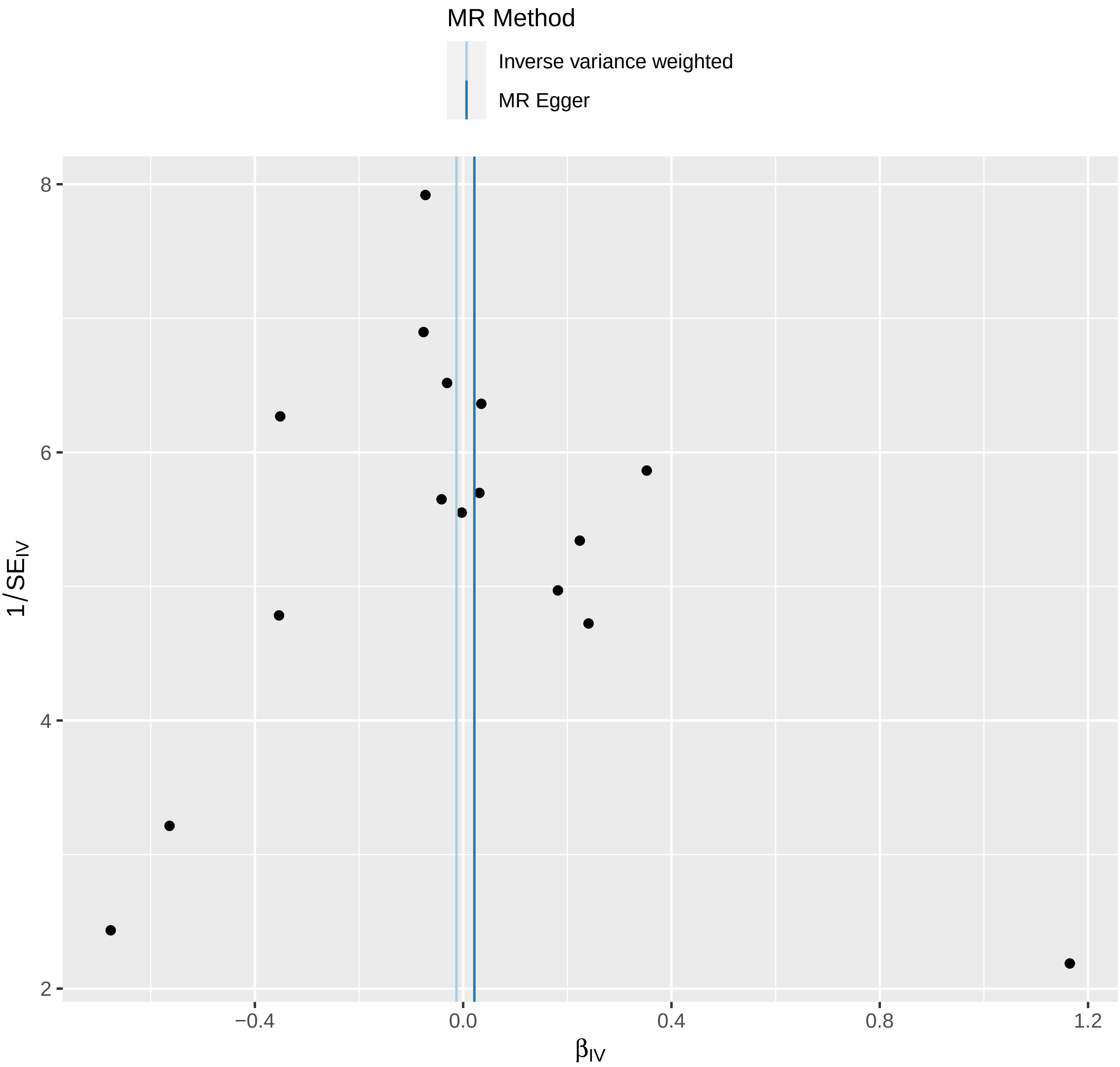

Batch 3−1591 : Integral membrane protein 2C on Diabetic nephropathy (more control exclusions)
with a threshold of 1e−05

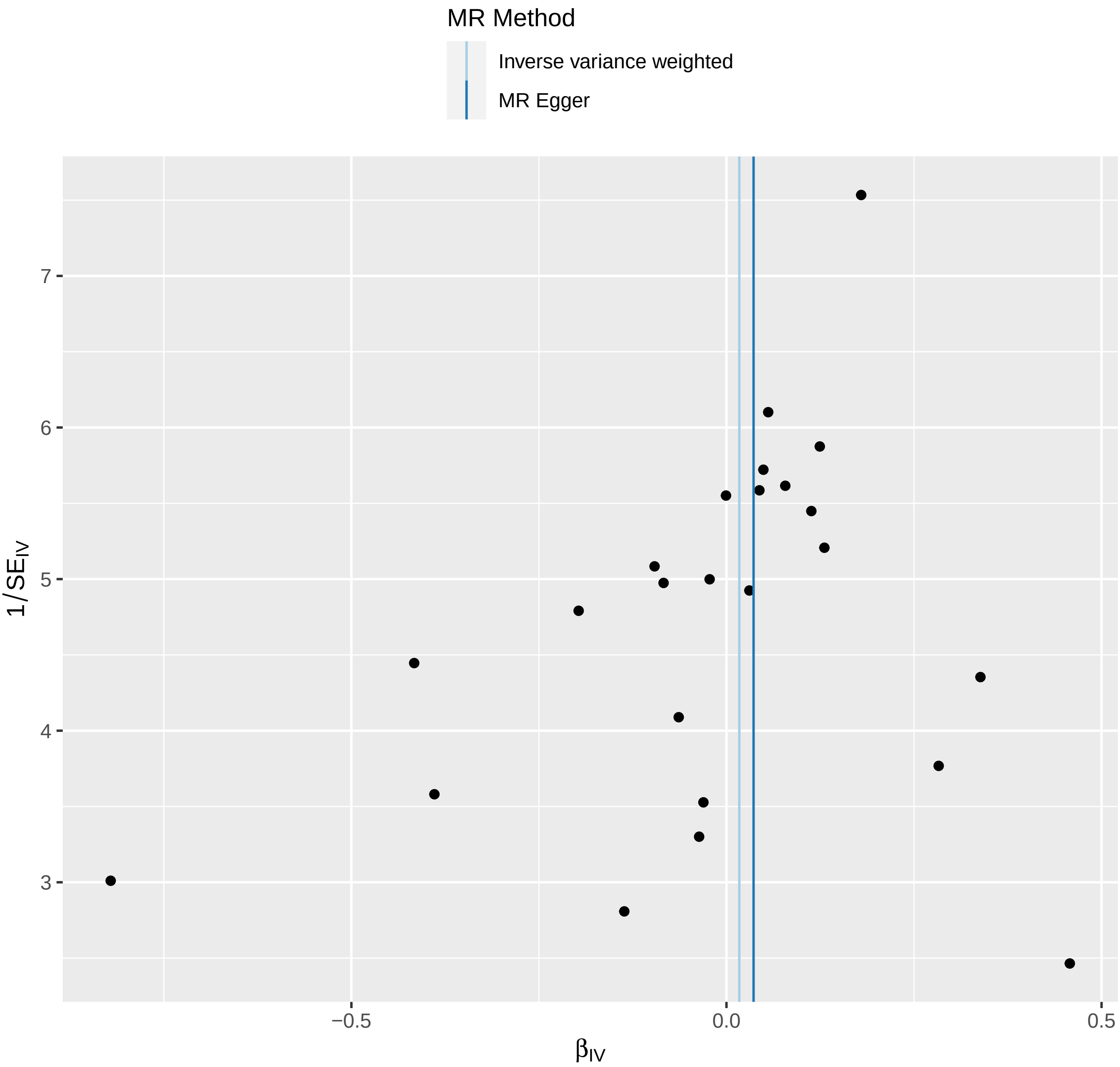

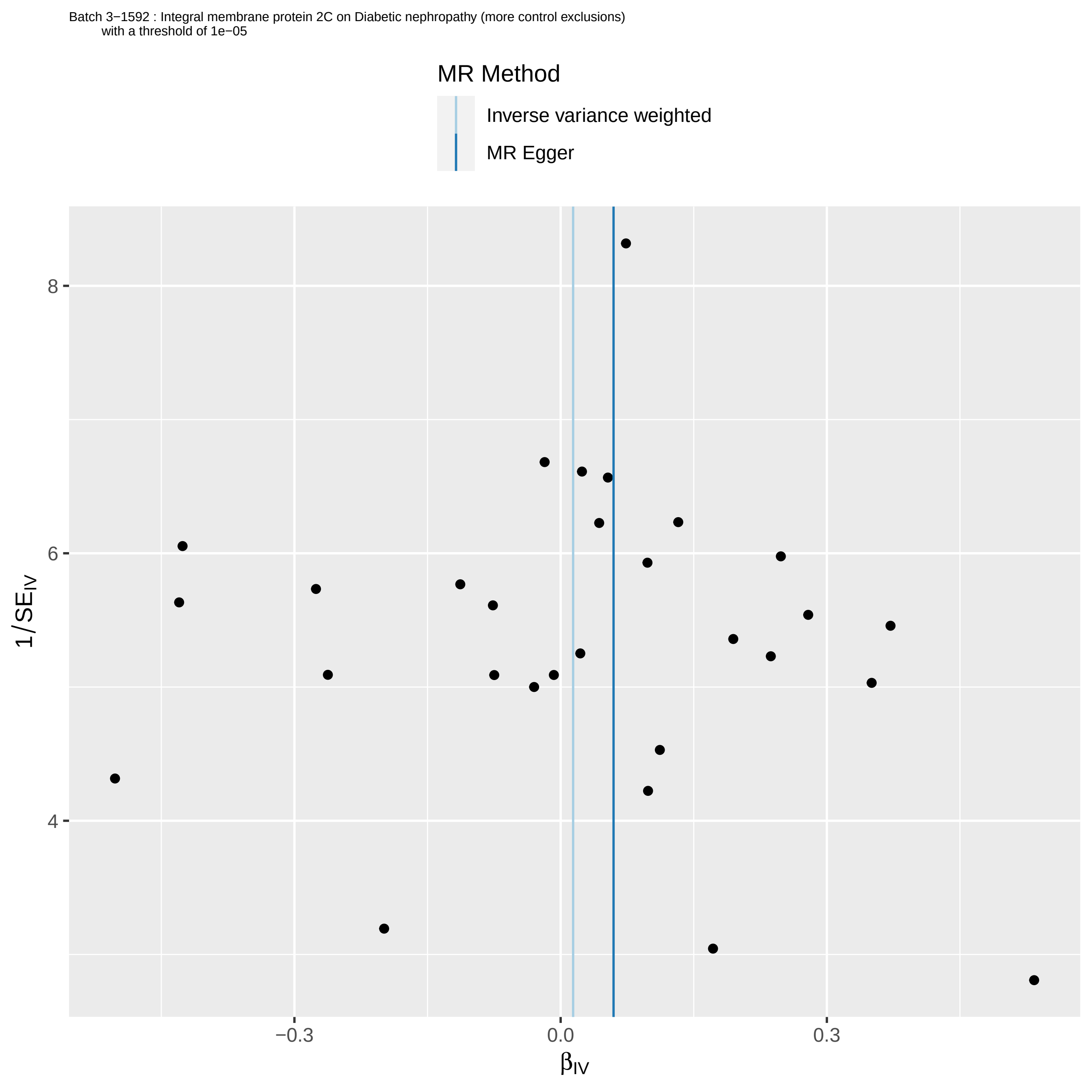
Batch 3−1592 : Integral membrane protein 2C on Diabetic nephropathy (more control exclusions)
with a threshold of 1e−05
MR Method
Inverse variance weighted
MR Egger
8
6
4
1/SE_IV
−0.3
0.0
0.3
β_IV

Batch 3−1593 : Inositol−trisphosphate 3−kinase A on Diabetic nephropathy (more control exclusions)
with a threshold of 1e−05

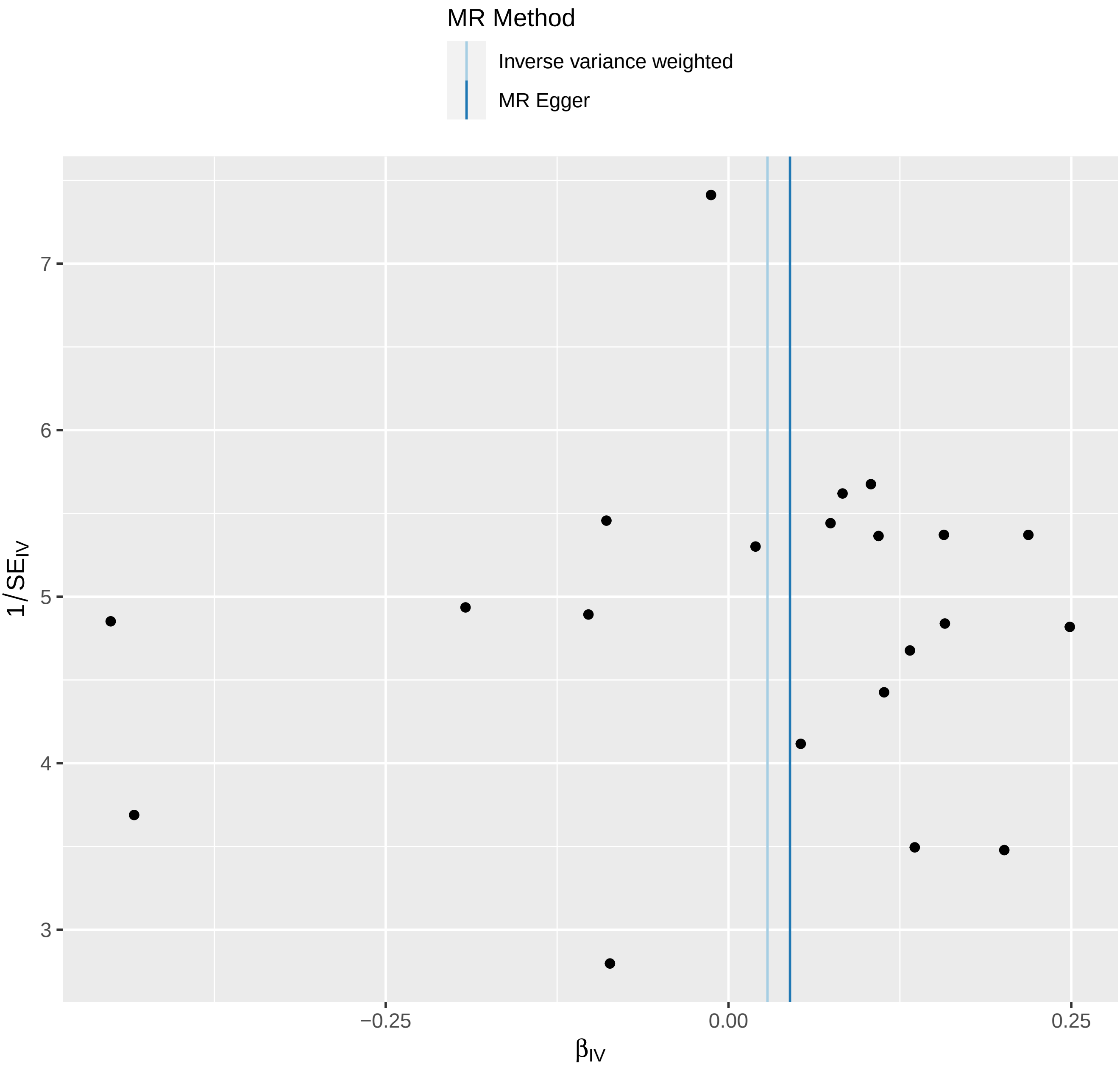

Batch 3−1594 : Inositol 1,4,5−trisphosphate receptor−interacting protein−like 1 on Diabetic nephropathy (more control exclusions) with a threshold of 1e−05

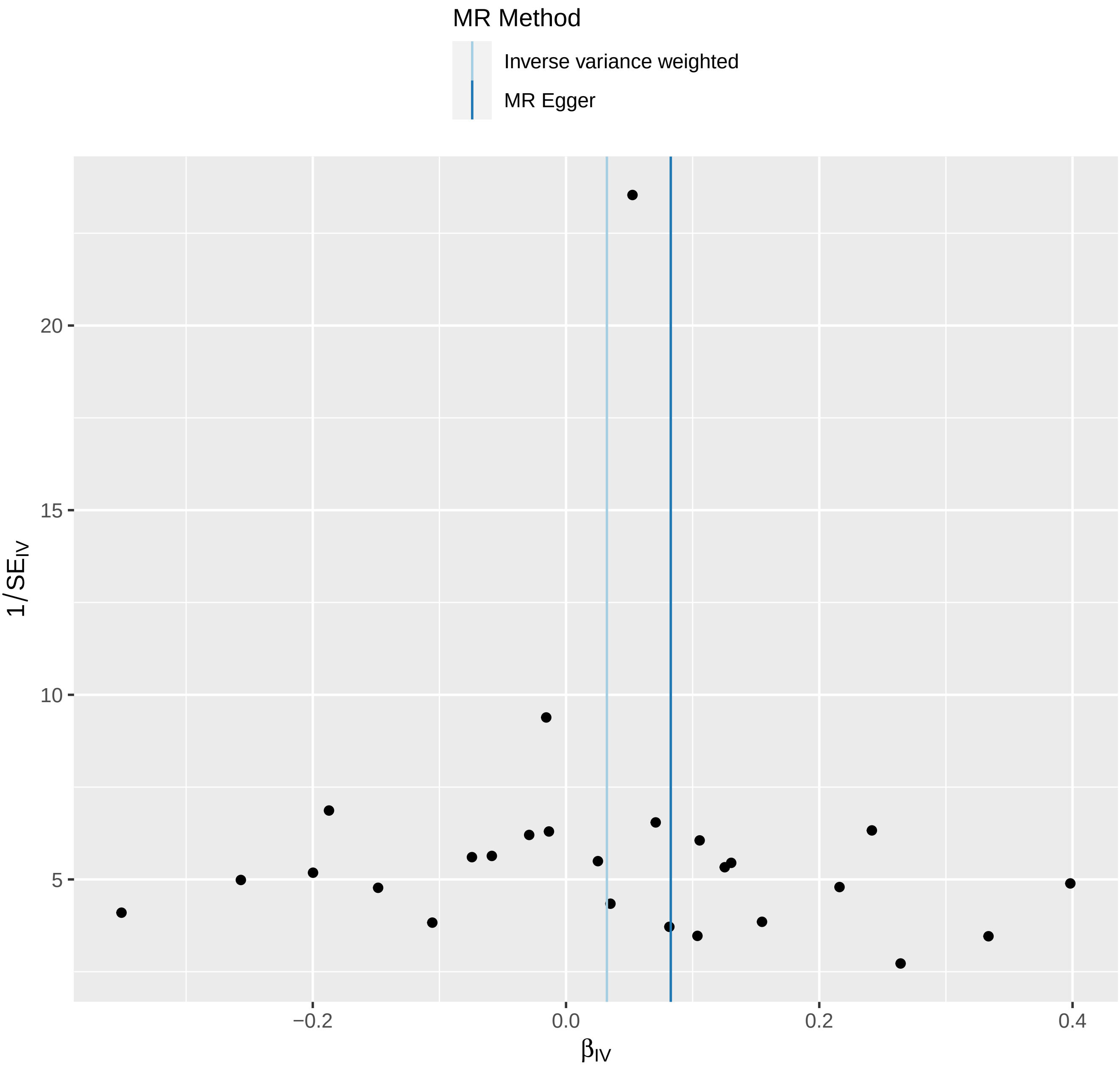

Batch 3−1595 : Intersectin−1 on Diabetic nephropathy (more control exclusions)
with a threshold of 1e−05

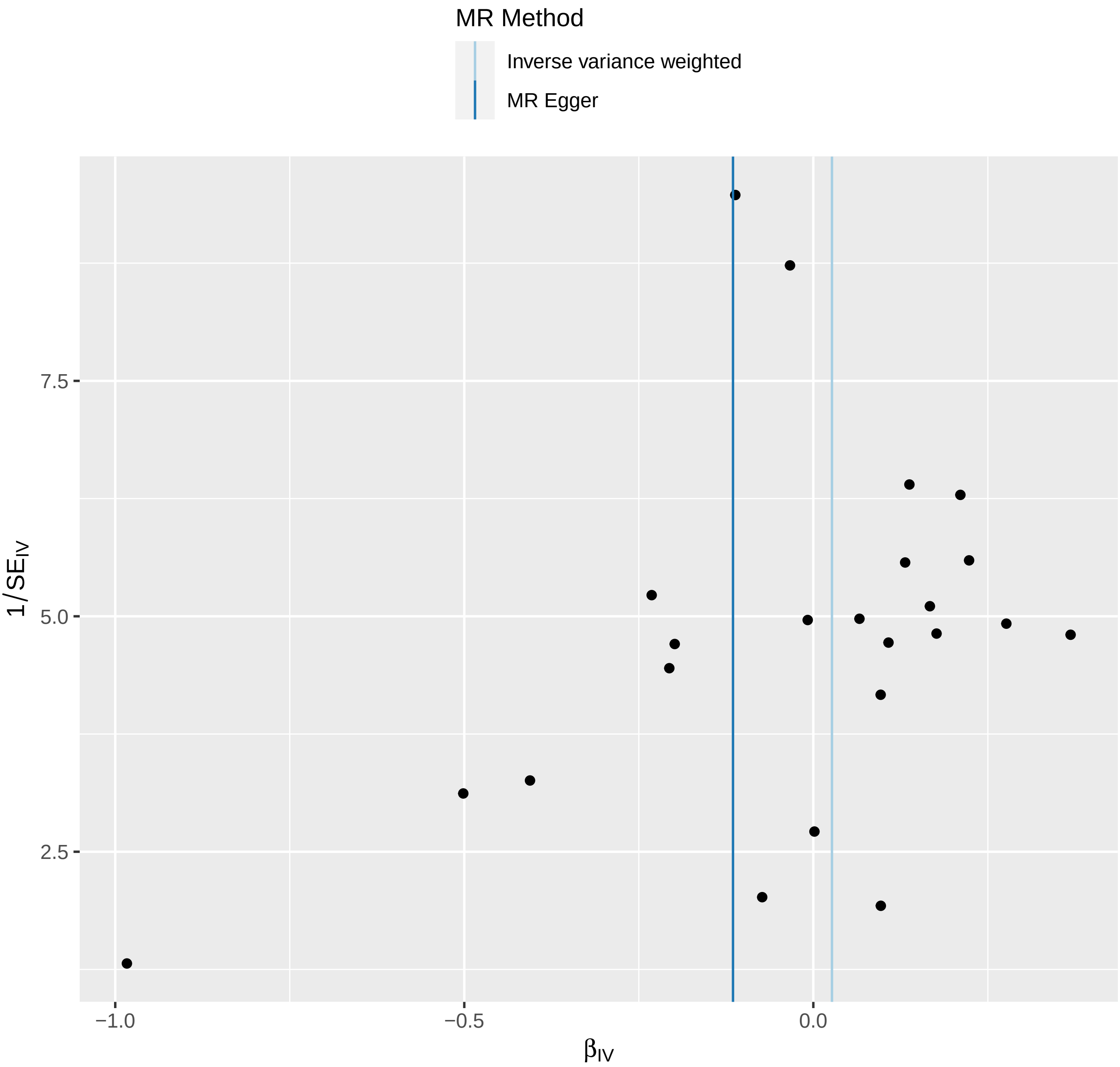

Batch 3−1596 : Protein jagged−1 on Diabetic nephropathy (more control exclusions)
with a threshold of 1e−05

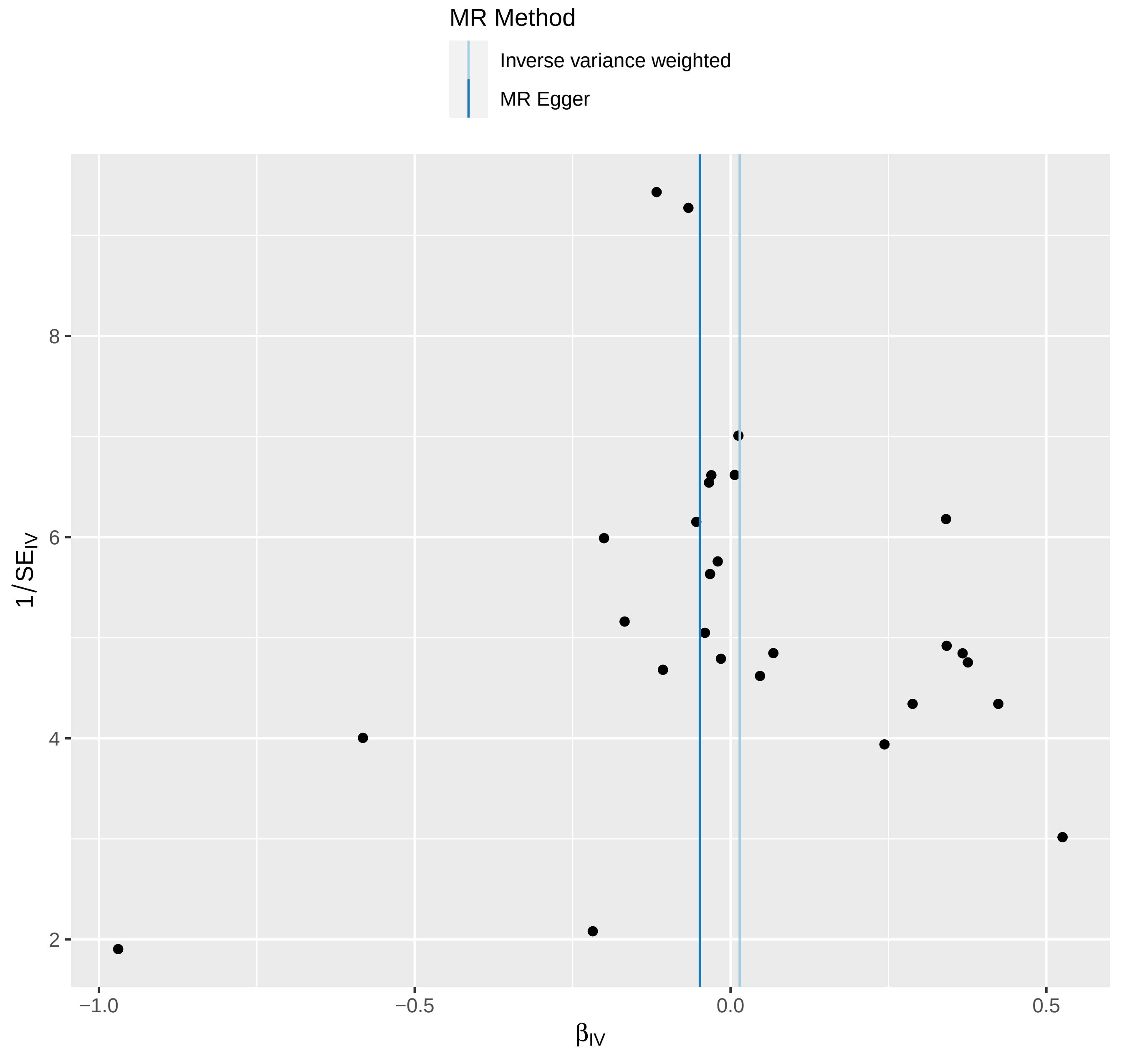

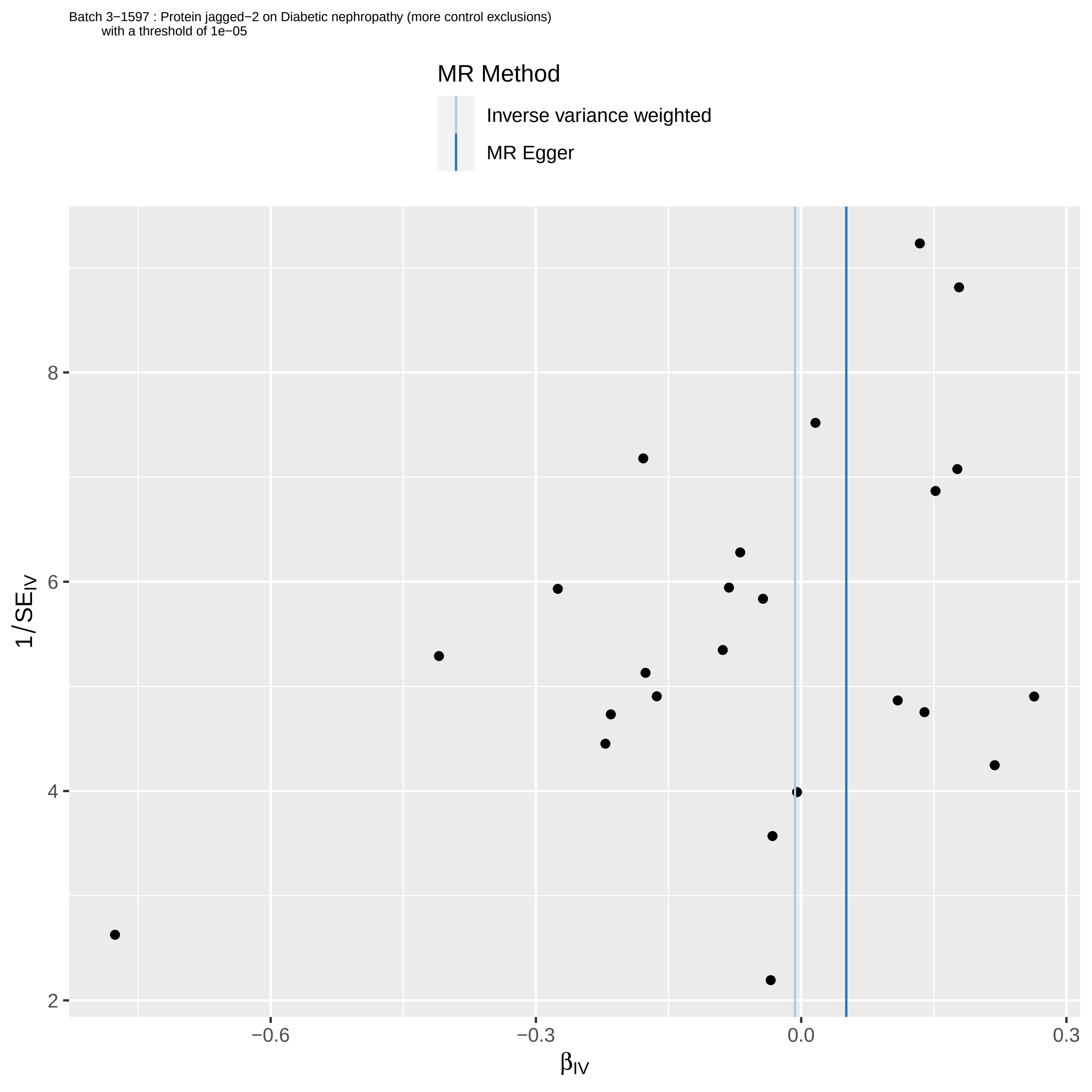

Batch 3−1597 : Protein jagged−2 on Diabetic nephropathy (more control exclusions)
with a threshold of 1e−05
MR Method
Inverse variance weighted
MR Egger
1/SE_IV
8
6
4
2
−0.6
−0.3
0.0
0.3
β_IV

Batch 3−1598 : Tyrosine−protein kinase JAK2 on Diabetic nephropathy (more control exclusions)
with a threshold of 1e−05

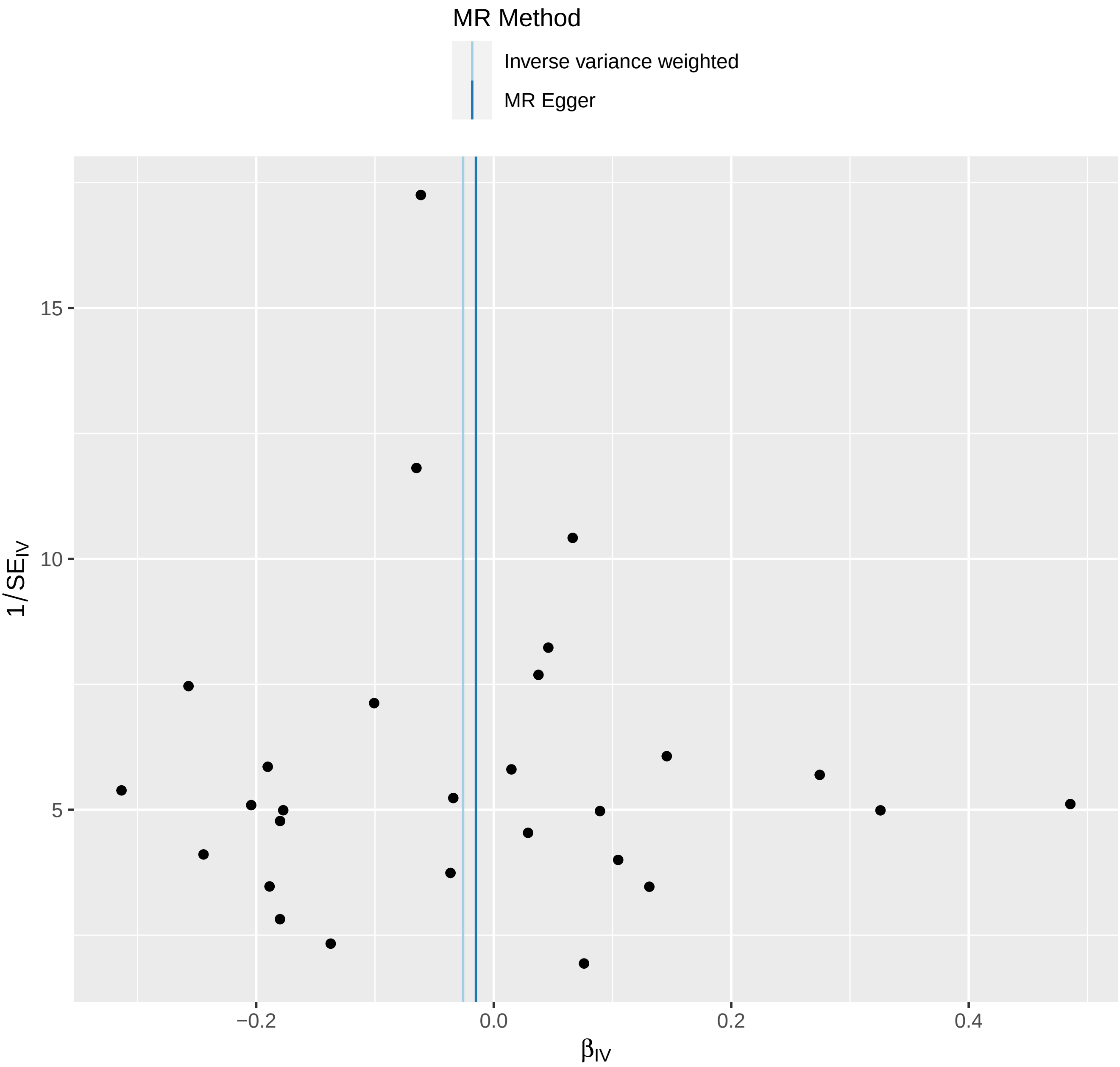

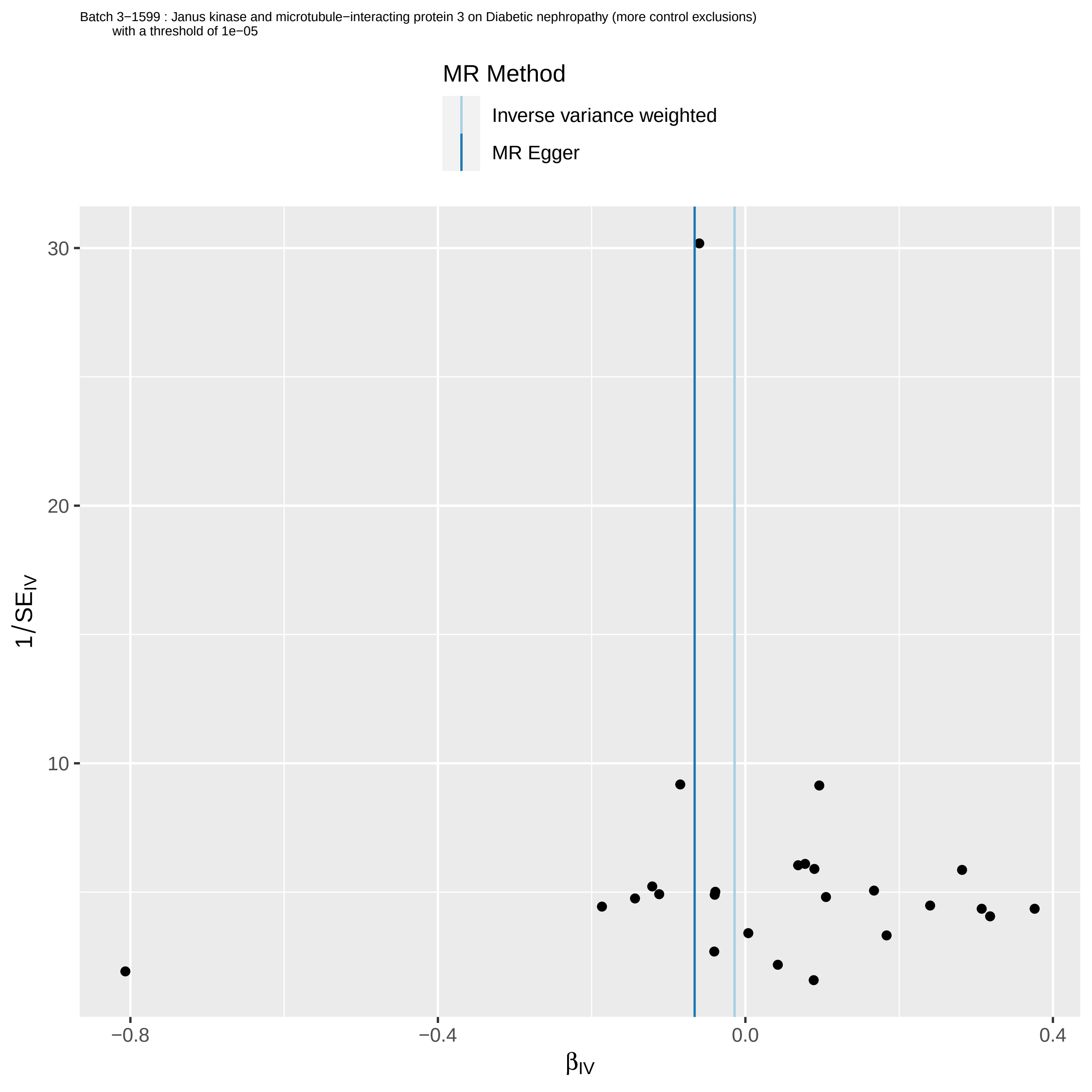

Batch 3−1599 : Janus kinase and microtubule−interacting protein 3 on Diabetic nephropathy (more control exclusions)
with a threshold of 1e−05
MR Method
Inverse variance weighted
MR Egger
30
20
10
1/SE_IV
−0.8
−0.4
0.0
0.4
β_IV

Batch 3−1600 : Junctional adhesion molecule B on Diabetic nephropathy (more control exclusions)
with a threshold of 1e−05

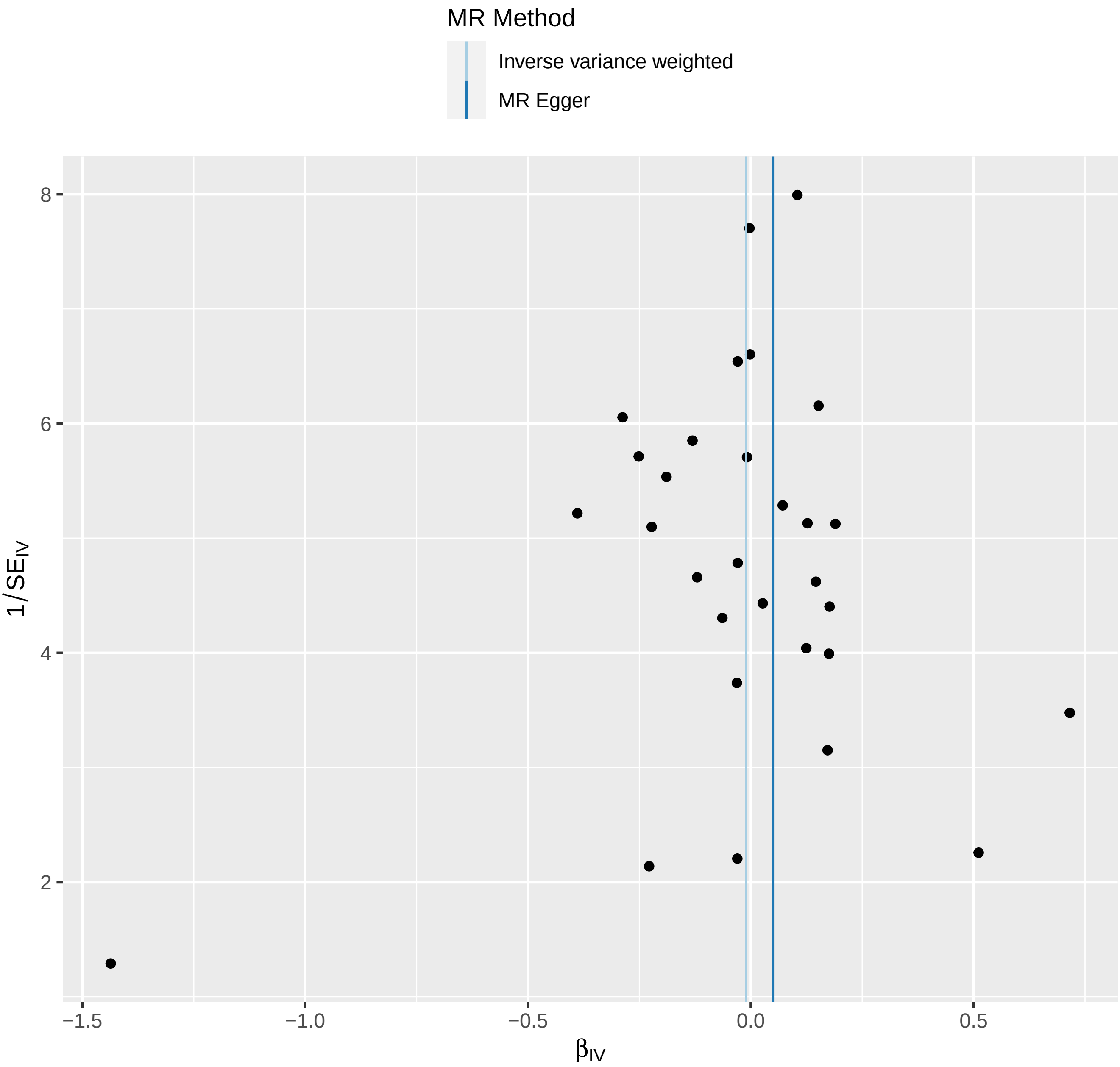

Batch 3−1601 : Junctional adhesion molecule C on Diabetic nephropathy (more control exclusions)
with a threshold of 1e−05

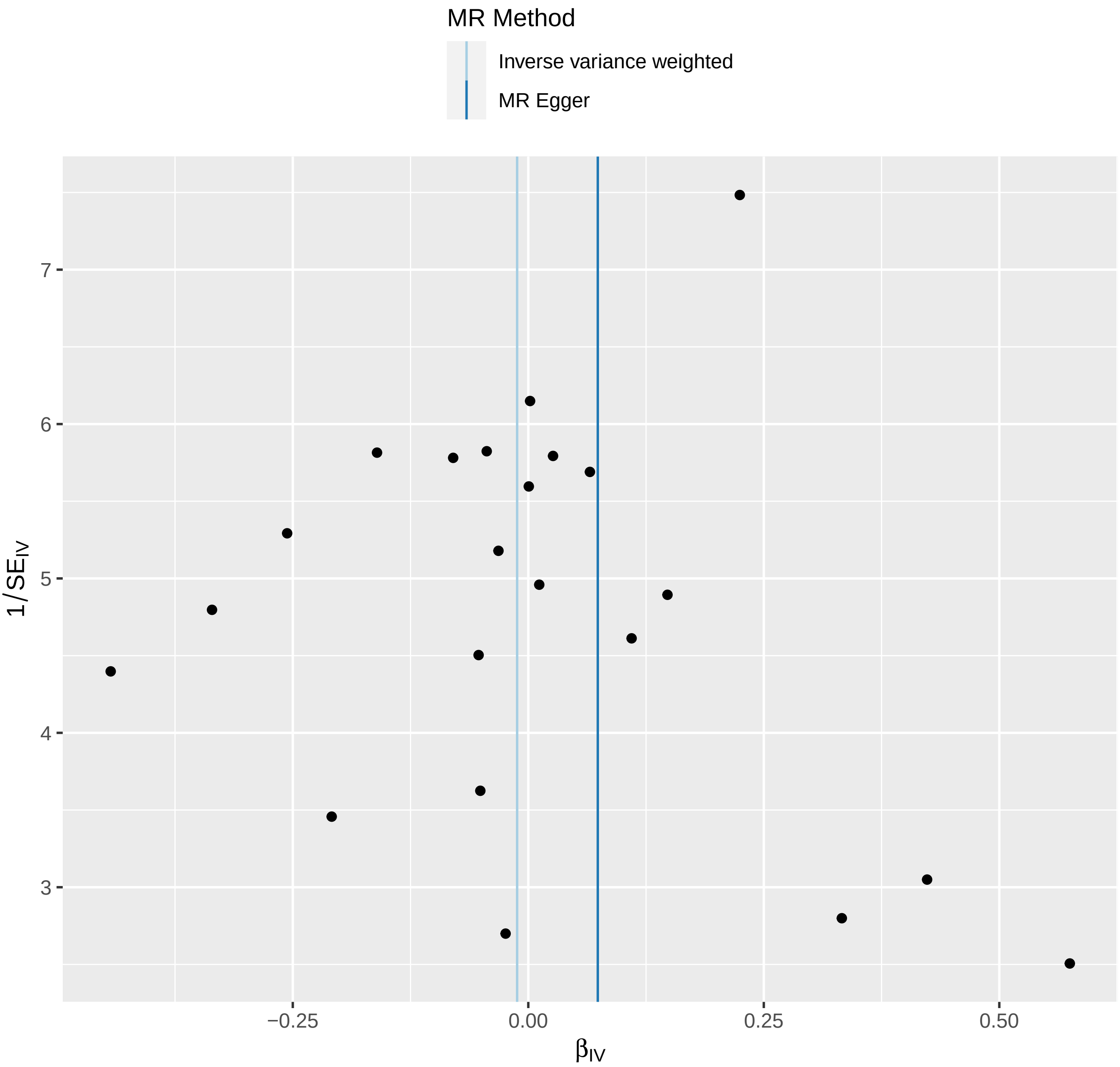

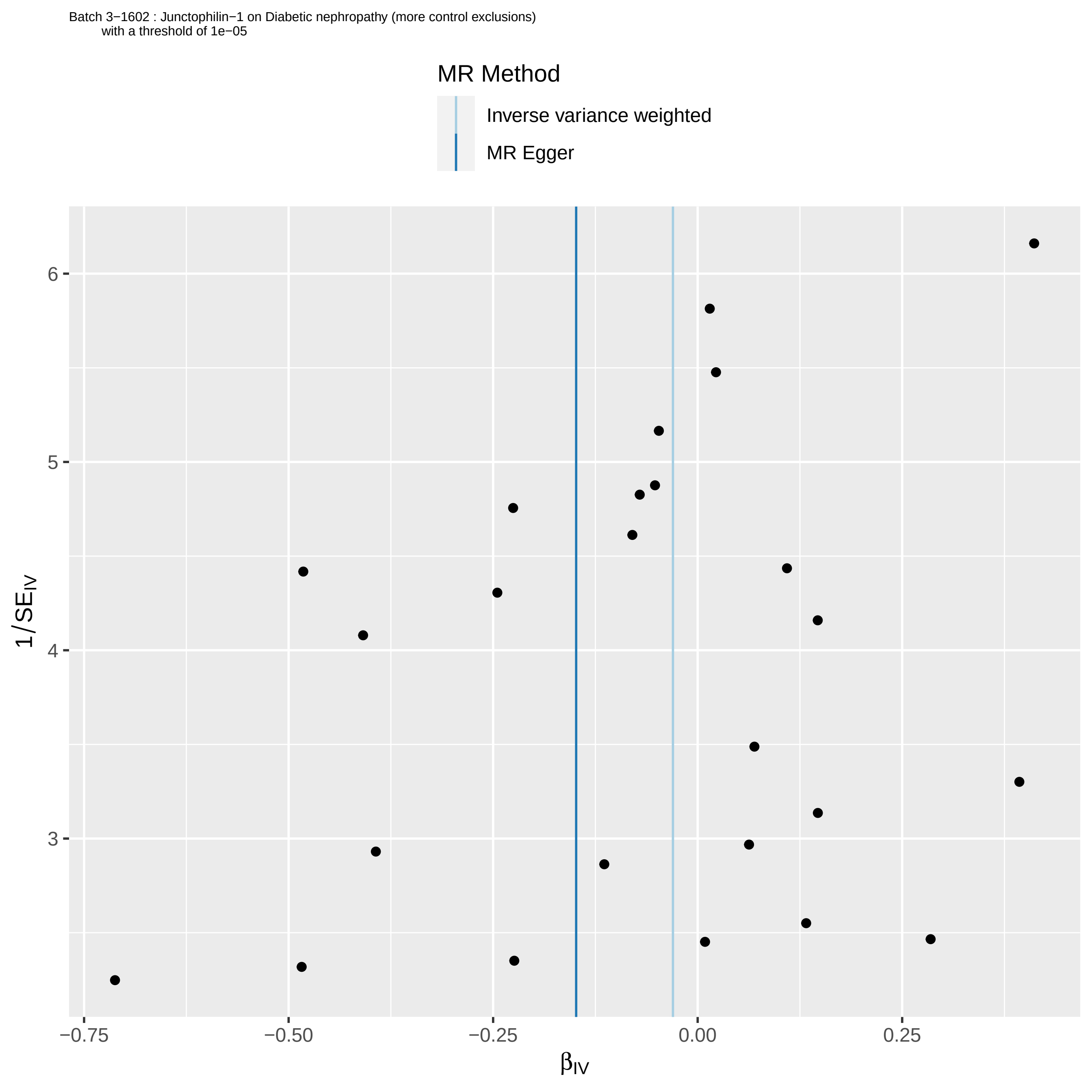

Batch 3−1602 : Junctophilin−1 on Diabetic nephropathy (more control exclusions)
with a threshold of 1e−05
MR Method
Inverse variance weighted
MR Egger
1/SE_IV
β_IV
6
5
4
3
−0.75
−0.50
−0.25
0.00
0.25

Batch 3−1603 : Junctophilin−3 on Diabetic nephropathy (more control exclusions)
with a threshold of 1e−05

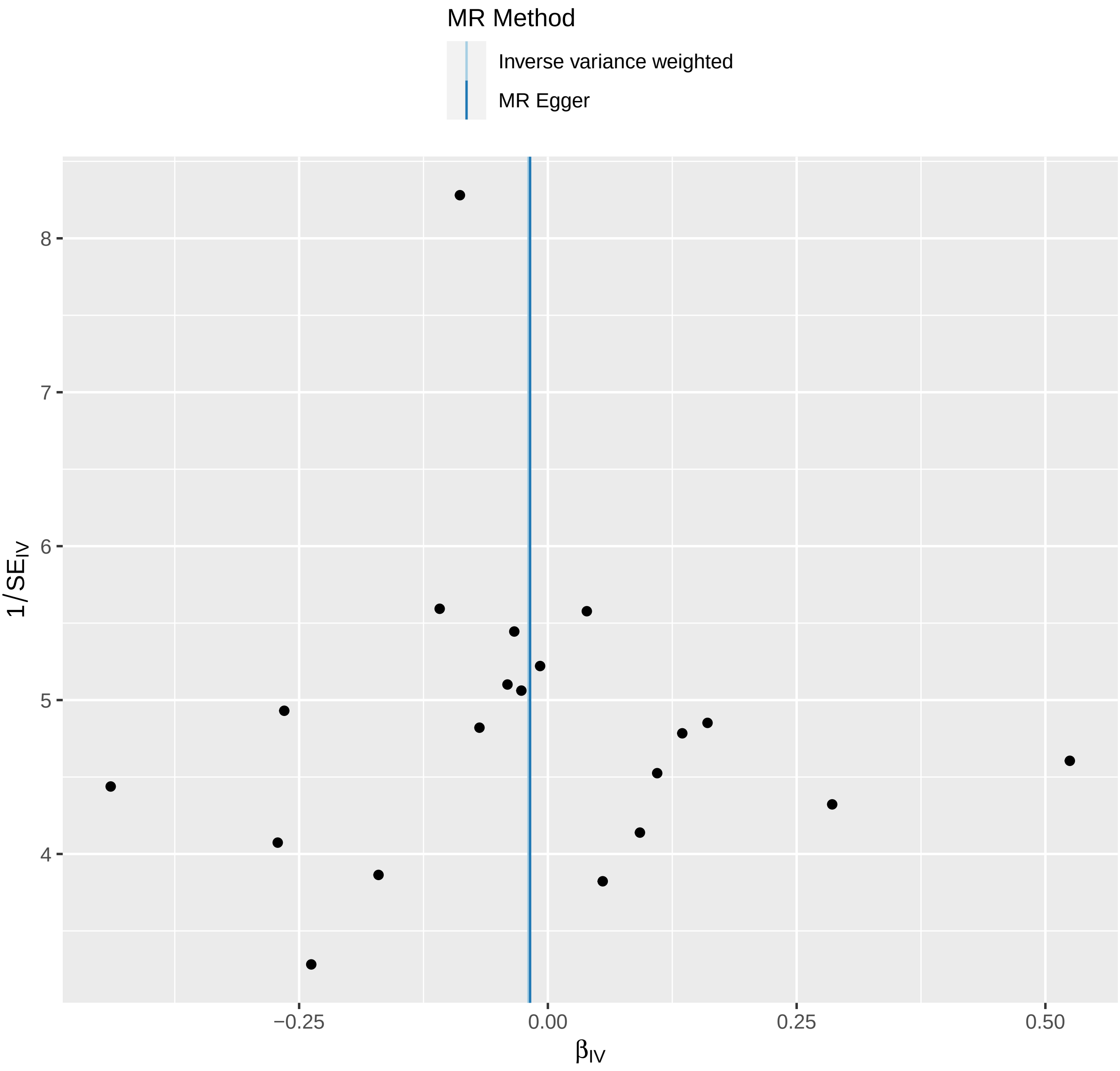

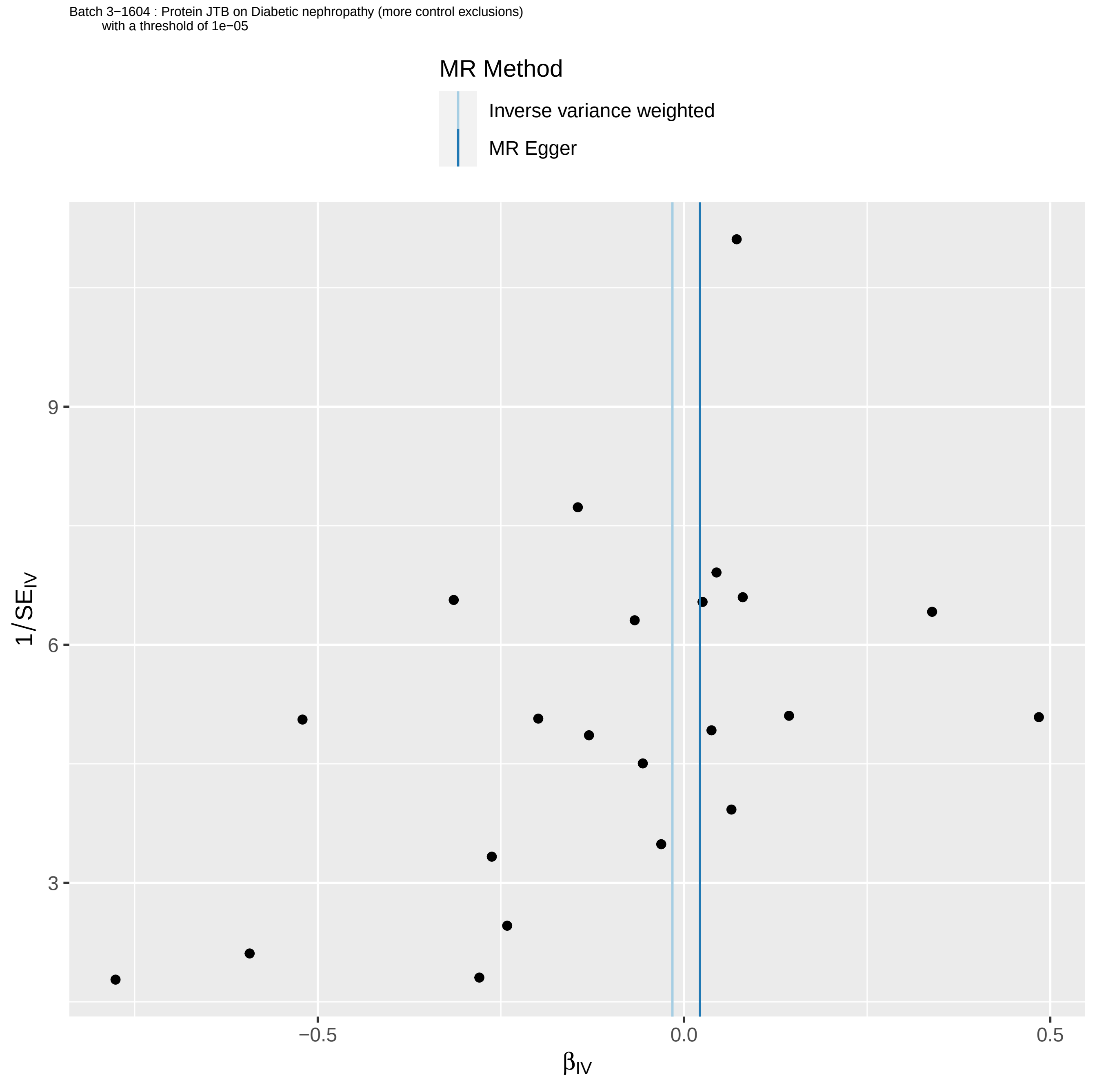

Batch 3−1604 : Protein JTB on Diabetic nephropathy (more control exclusions)
with a threshold of 1e−05
MR Method
Inverse variance weighted
MR Egger
9
6
3
1/SE_IV
−0.5
0.0
0.5
β_IV

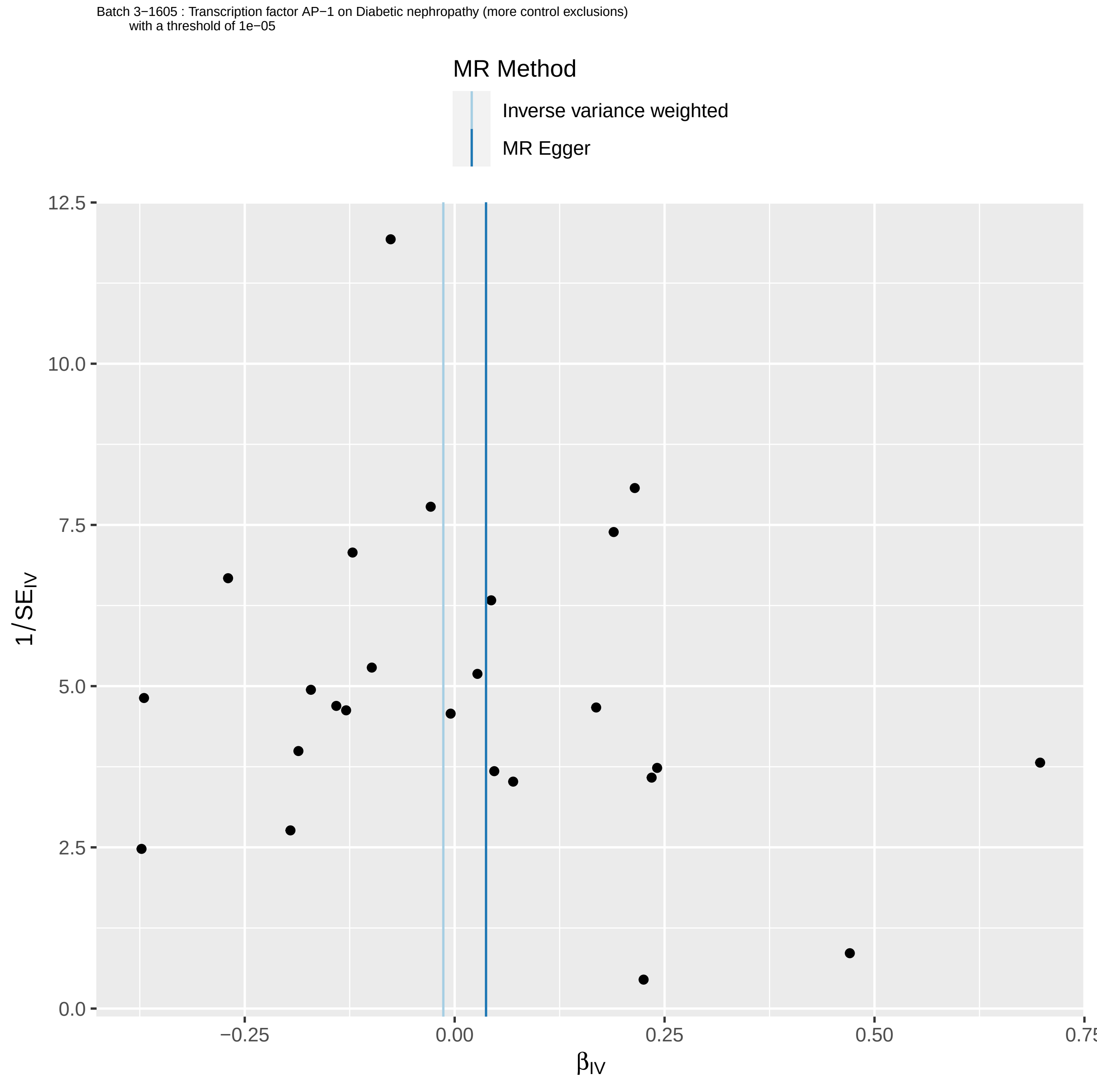

Batch 3−1605 : Transcription factor AP−1 on Diabetic nephropathy (more control exclusions)
with a threshold of 1e−05
MR Method
Inverse variance weighted
MR Egger
12.5
10.0
7.5
5.0
2.5
0.0
1/SE$_{IV}$
−0.25
0.00
0.25
0.50
0.75
β_{IV}

Batch 3−1606 : Anosmin−1 on Diabetic nephropathy (more control exclusions)
with a threshold of 1e−05

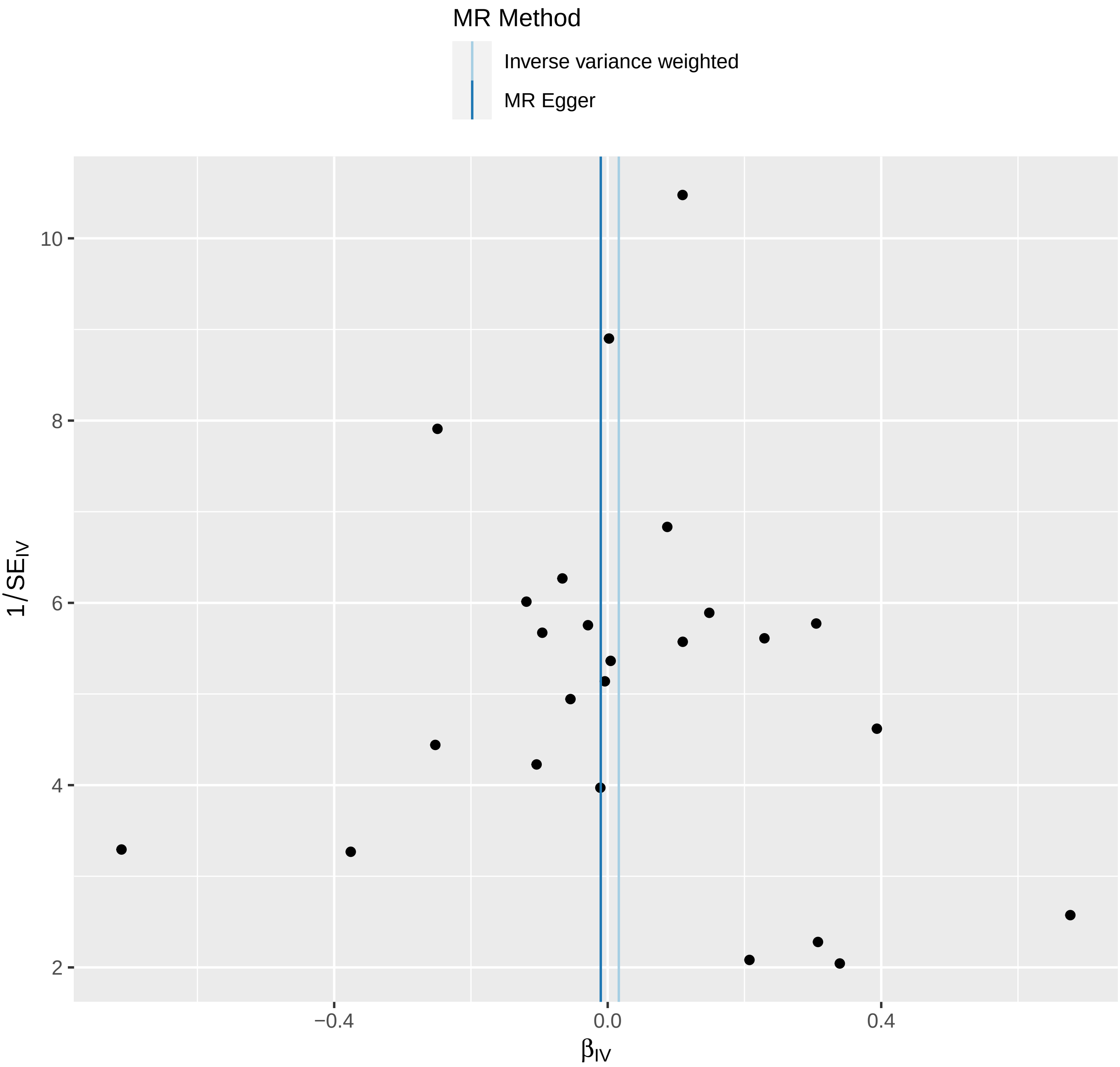

Batch 3−1607 : Histone acetyltransferase KAT6A on Diabetic nephropathy (more control exclusions)
with a threshold of 1e−05

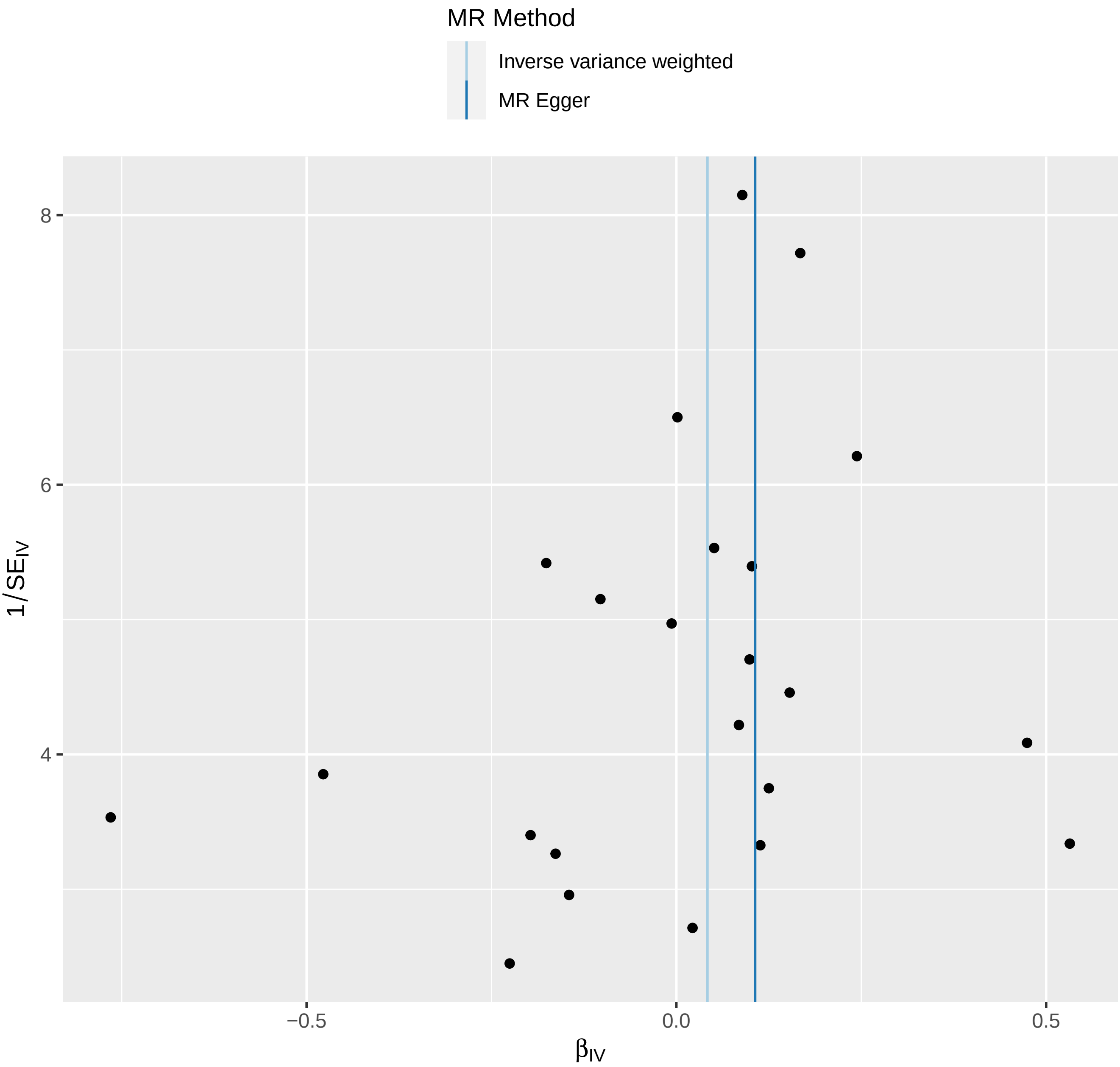

Batch 3−1608 : Potassium voltage−gated channel subfamily A member 10 on Diabetic nephropathy (more control exclusions) with a threshold of 1e−05

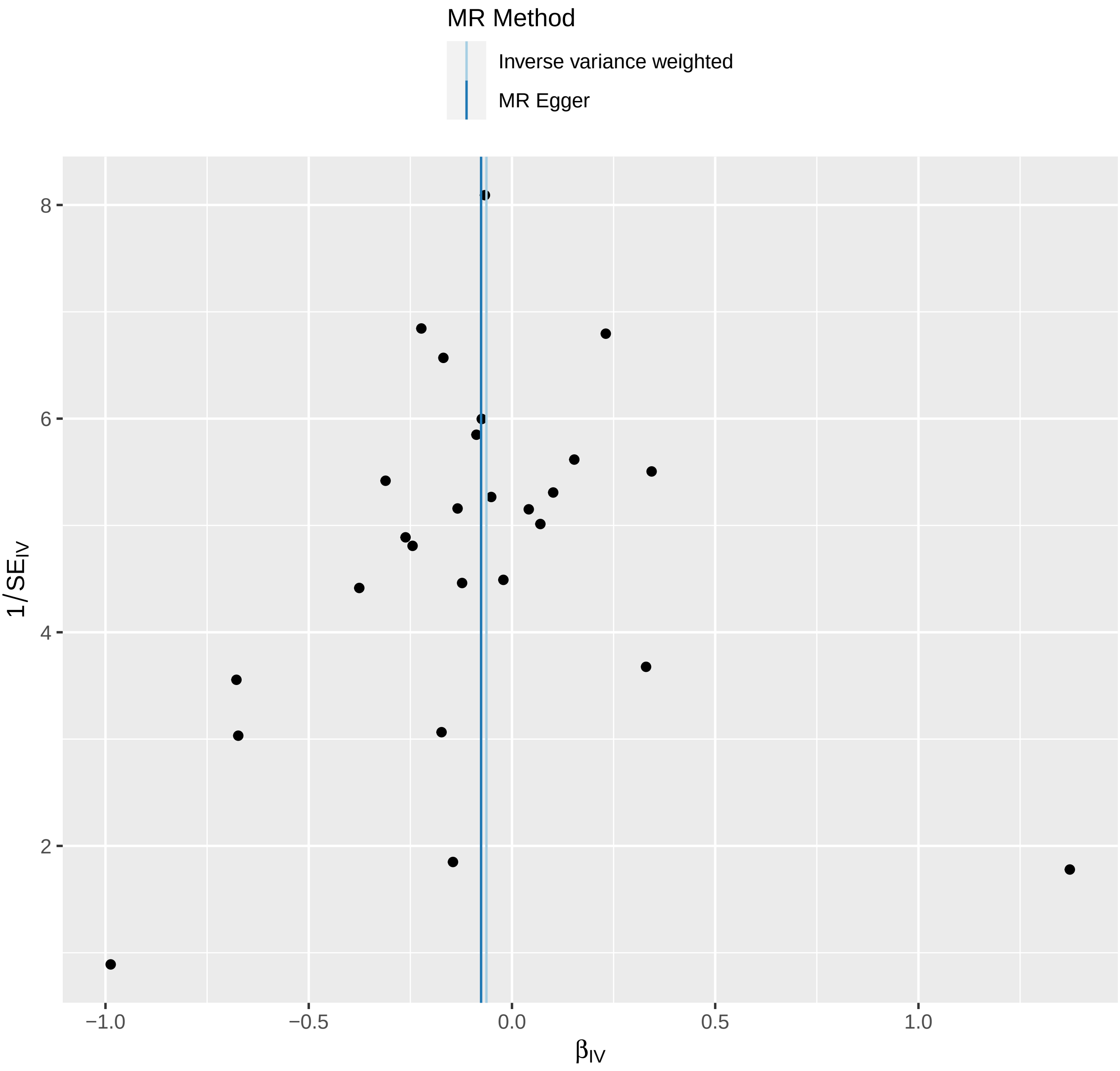

Batch 3−1609 : Voltage−gated potassium channel subunit beta−2 on Diabetic nephropathy (more control exclusions) with a threshold of 1e−05

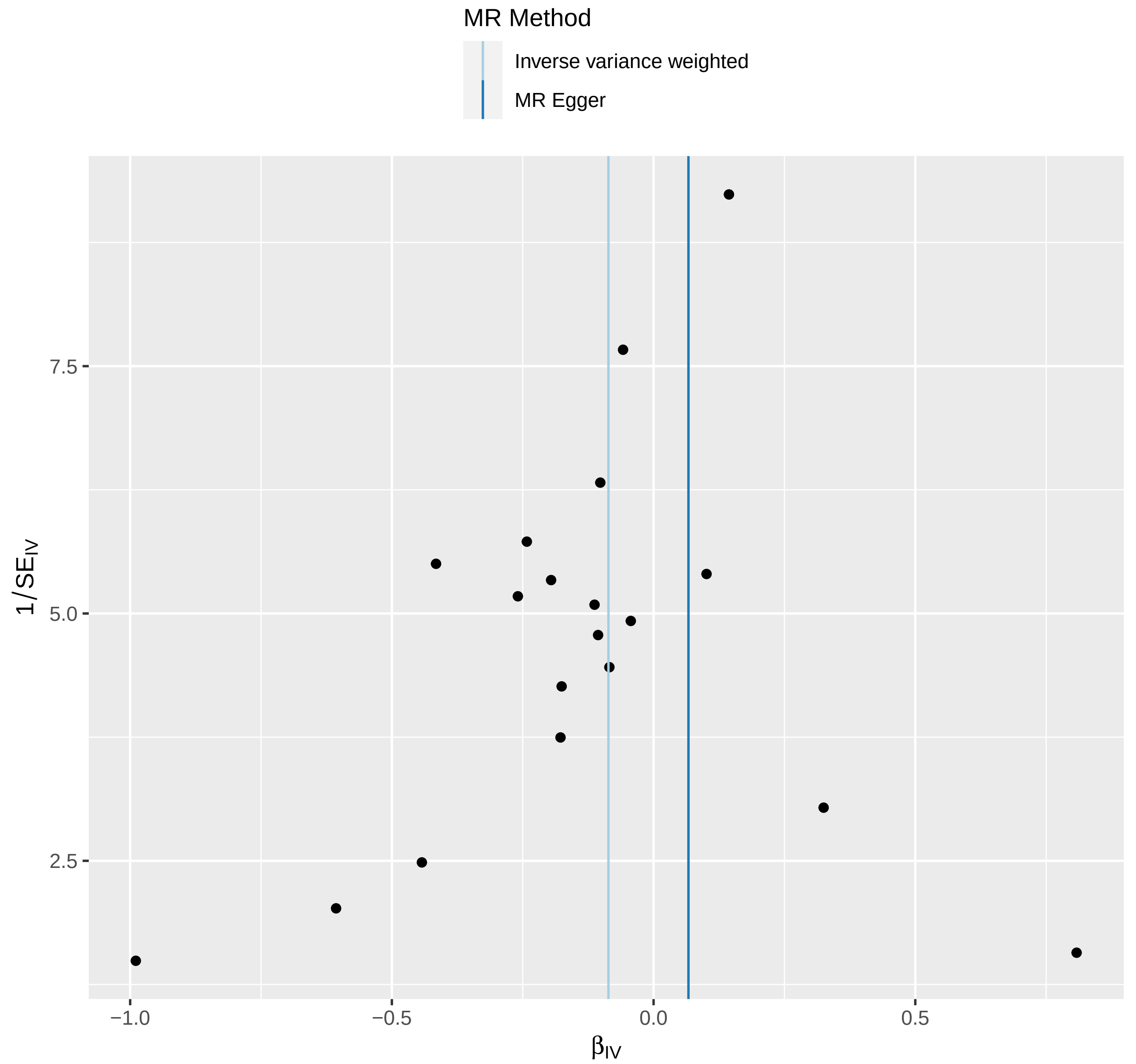

Batch 3−1610 : Potassium voltage−gated channel subfamily E member 2 on Diabetic nephropathy (more control exclusions) with a threshold of 1e−05

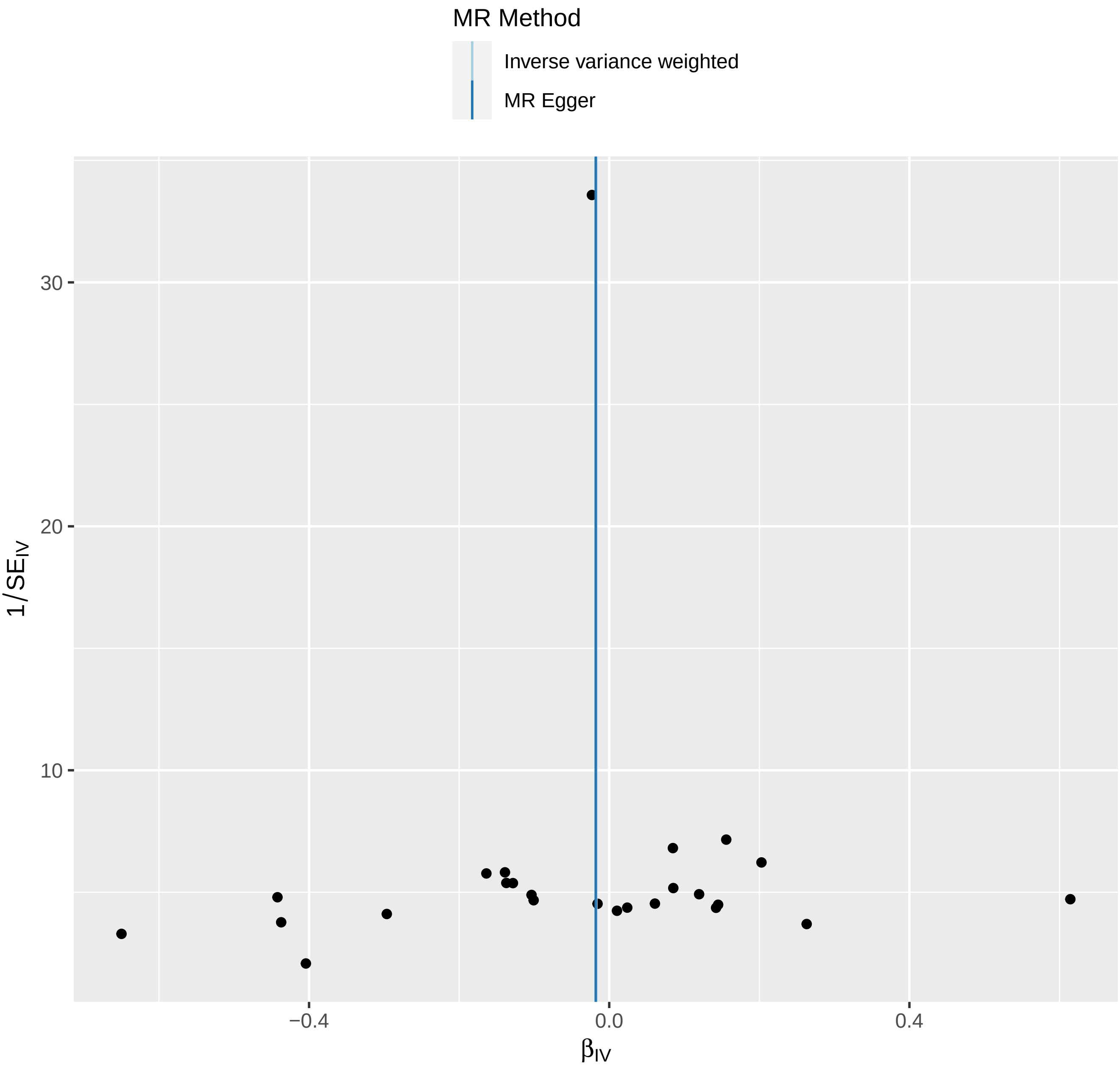

Batch 3−1611 : Potassium voltage−gated channel subfamily E member 3 on Diabetic nephropathy (more control exclusions)
with a threshold of 1e−05

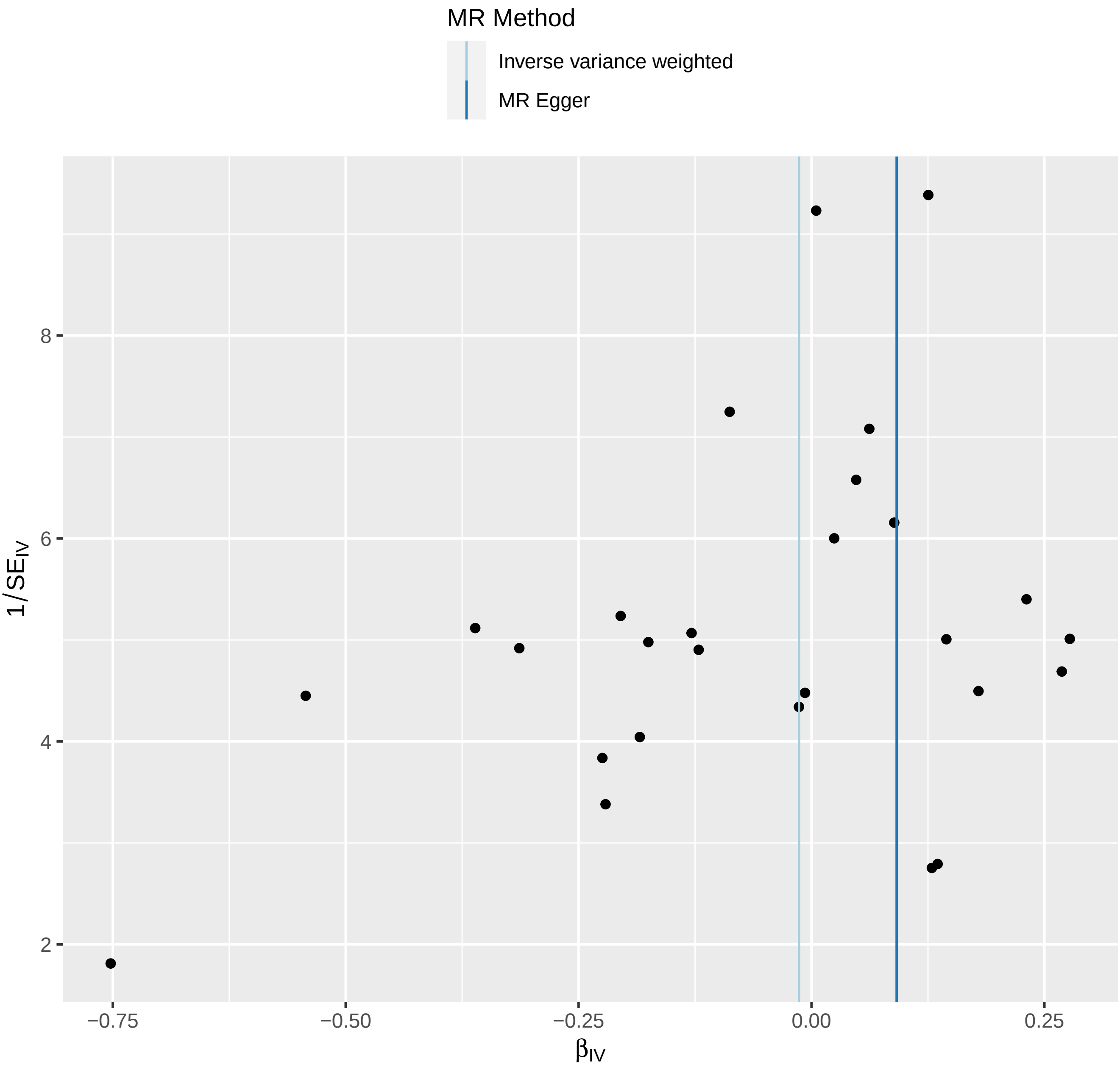

Batch 3−1612 : Potassium voltage−gated channel subfamily E regulatory beta subunit 5 on Diabetic nephropathy (more control exclusions)
with a threshold of 1e−05

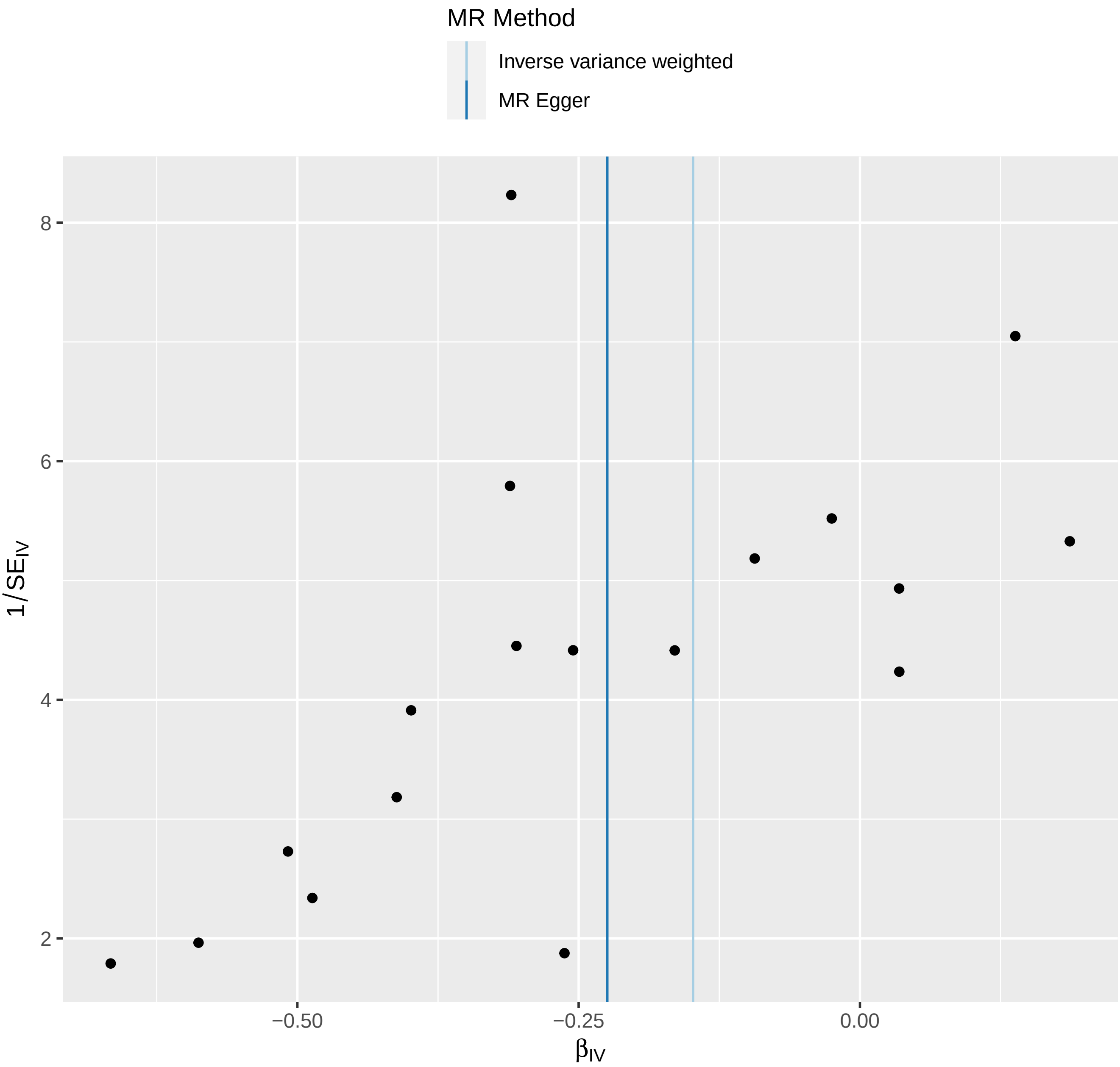

Batch 3−1613 : Potassium voltage−gated channel subfamily E regulatory beta subunit 5 on Diabetic nephropathy (more control exclusions) with a threshold of 1e−05

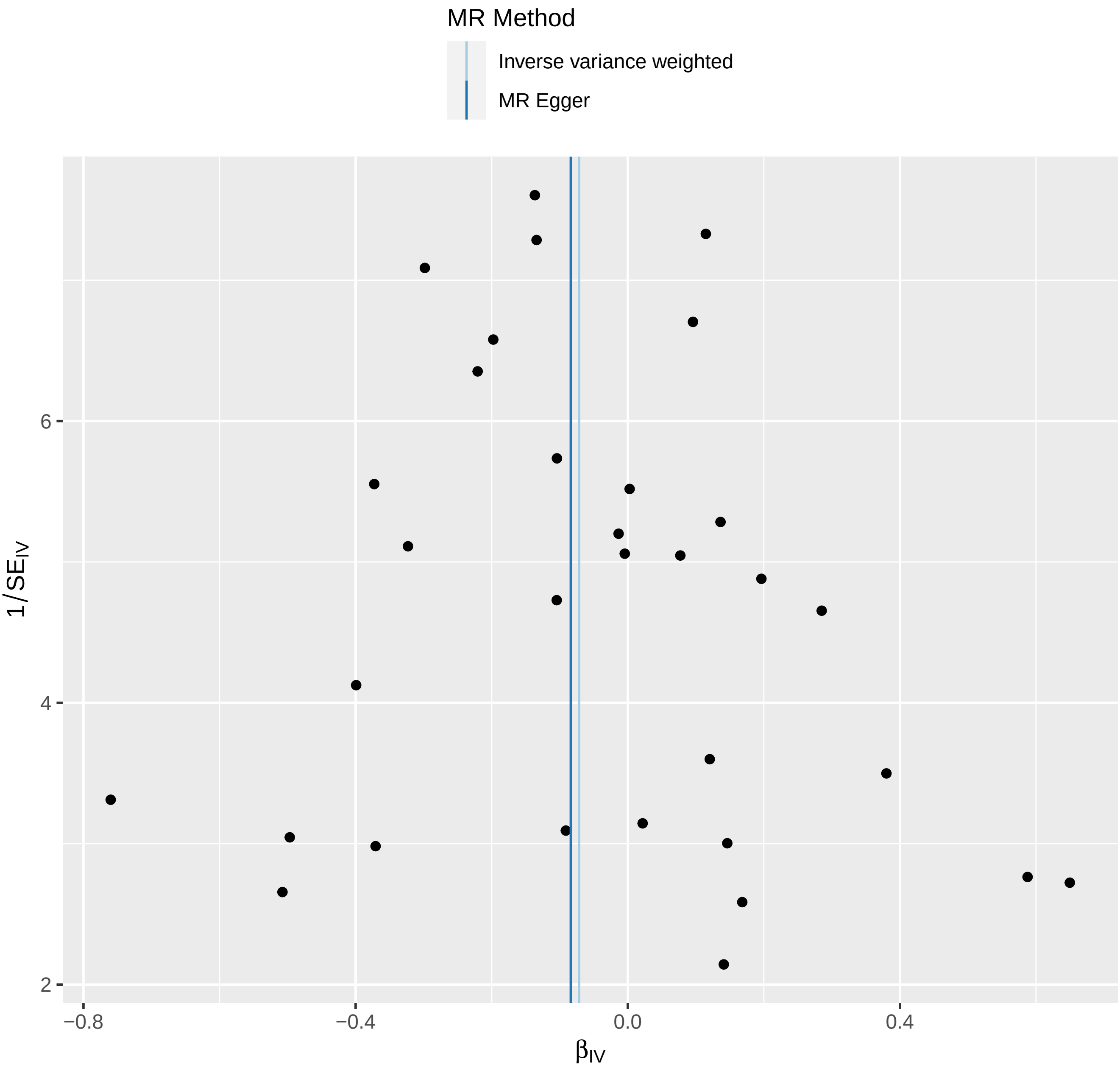

Batch 3−1614 : Potassium voltage−gated channel subfamily F member 1 on Diabetic nephropathy (more control exclusions)
with a threshold of 1e−05

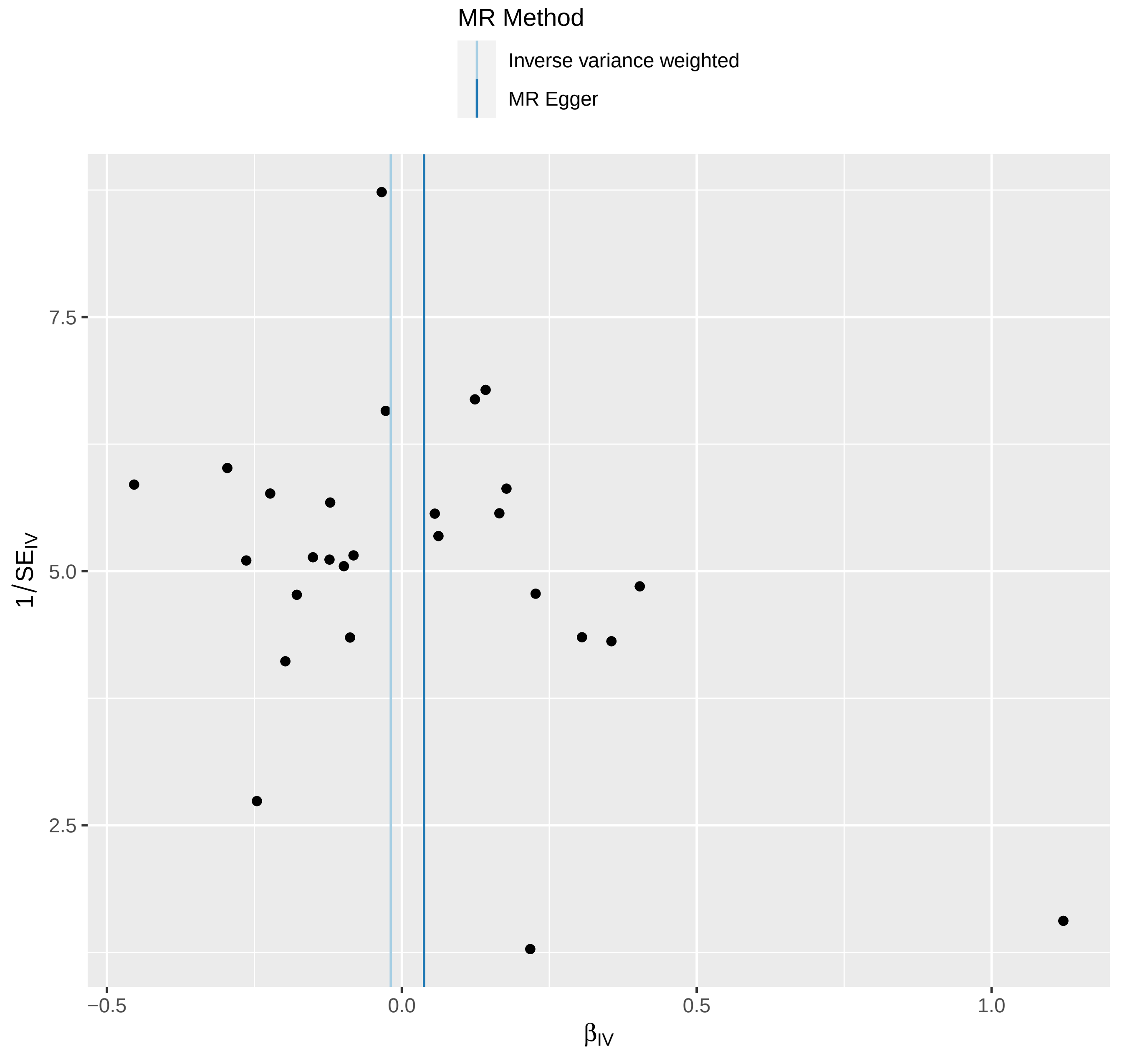

Batch 3−1615 : Potassium voltage−gated channel subfamily G member 4 on Diabetic nephropathy (more control exclusions)
with a threshold of 1e−05

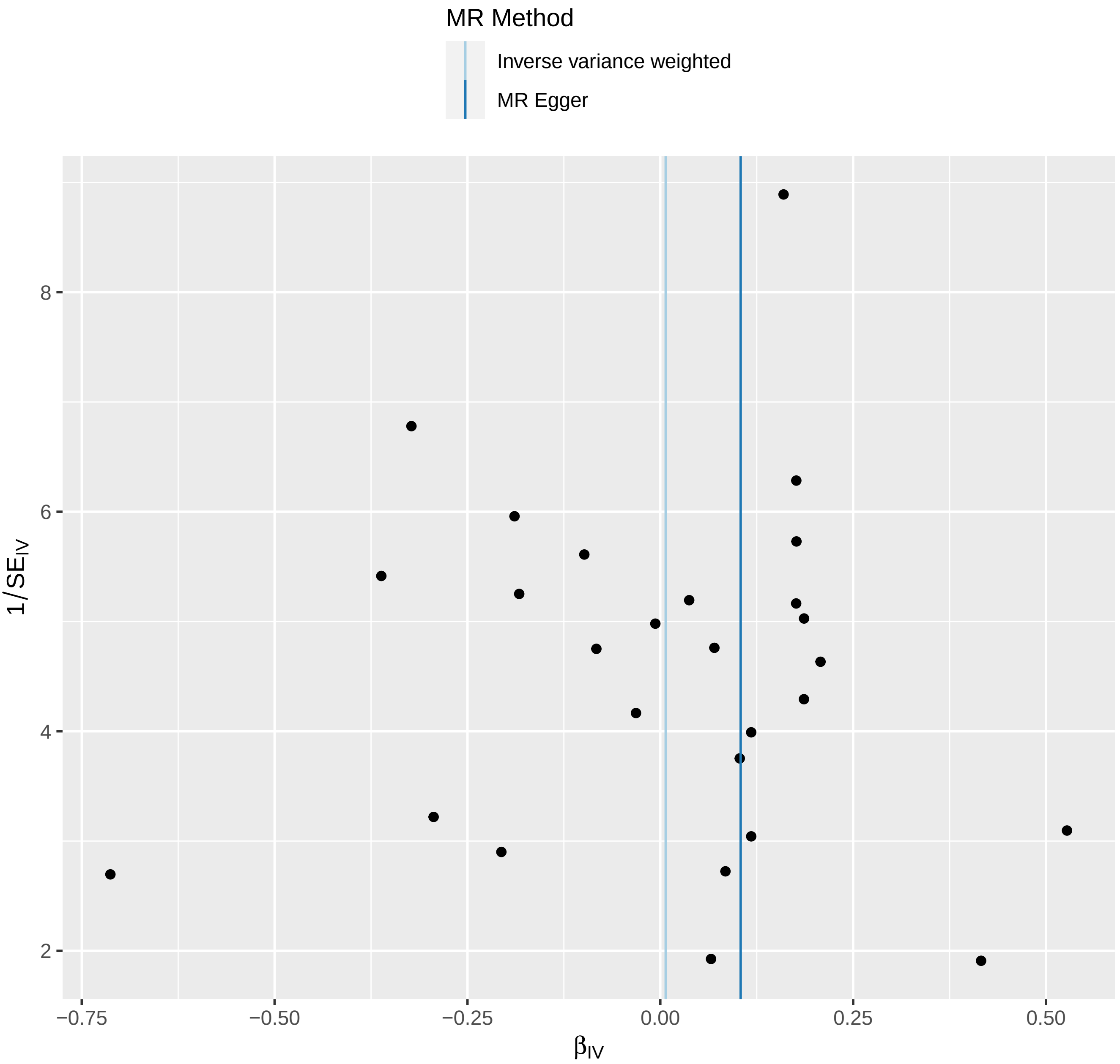

Batch 3−1616 : Kv channel−interacting protein 1 on Diabetic nephropathy (more control exclusions)
with a threshold of 1e−05

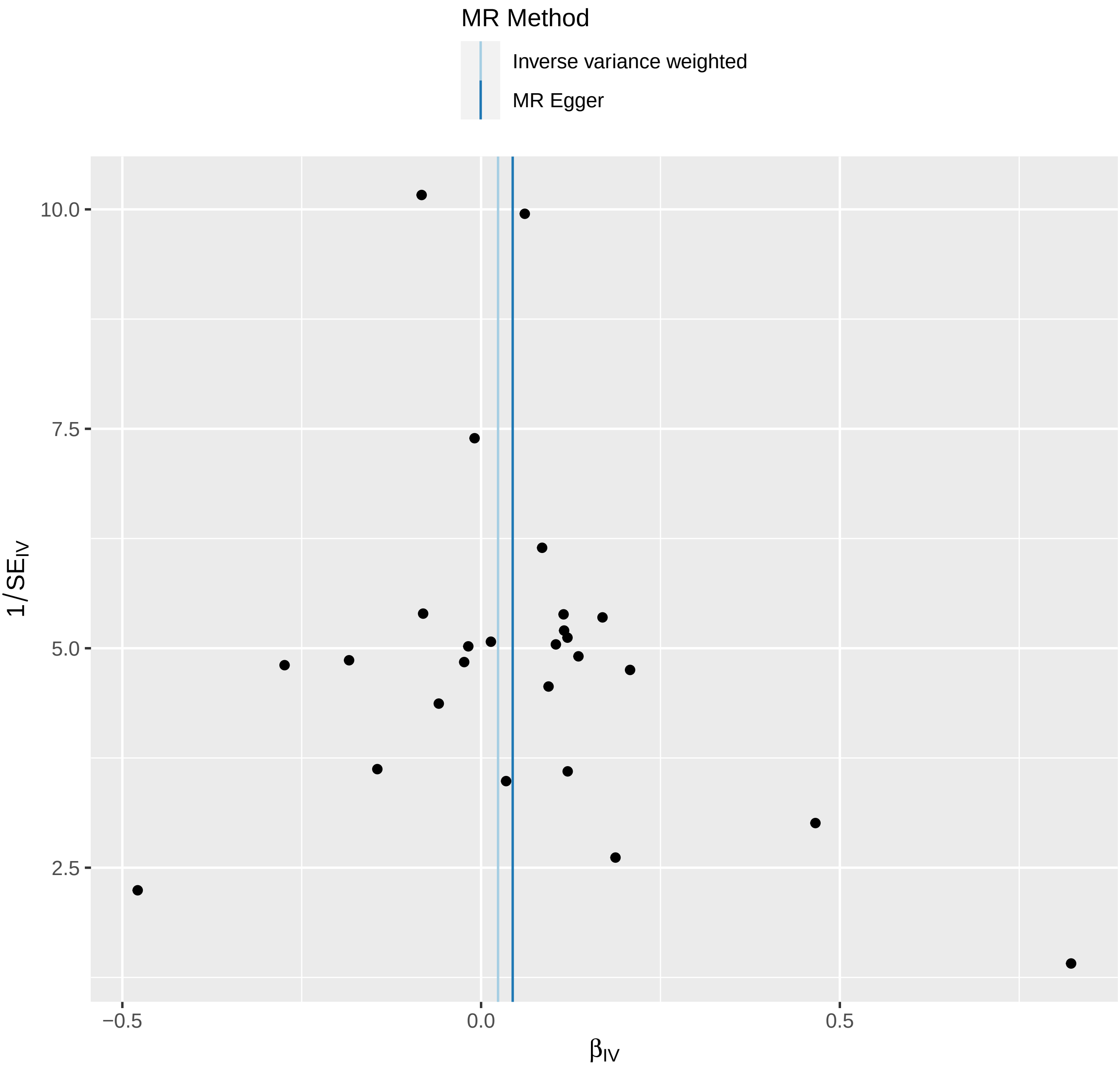

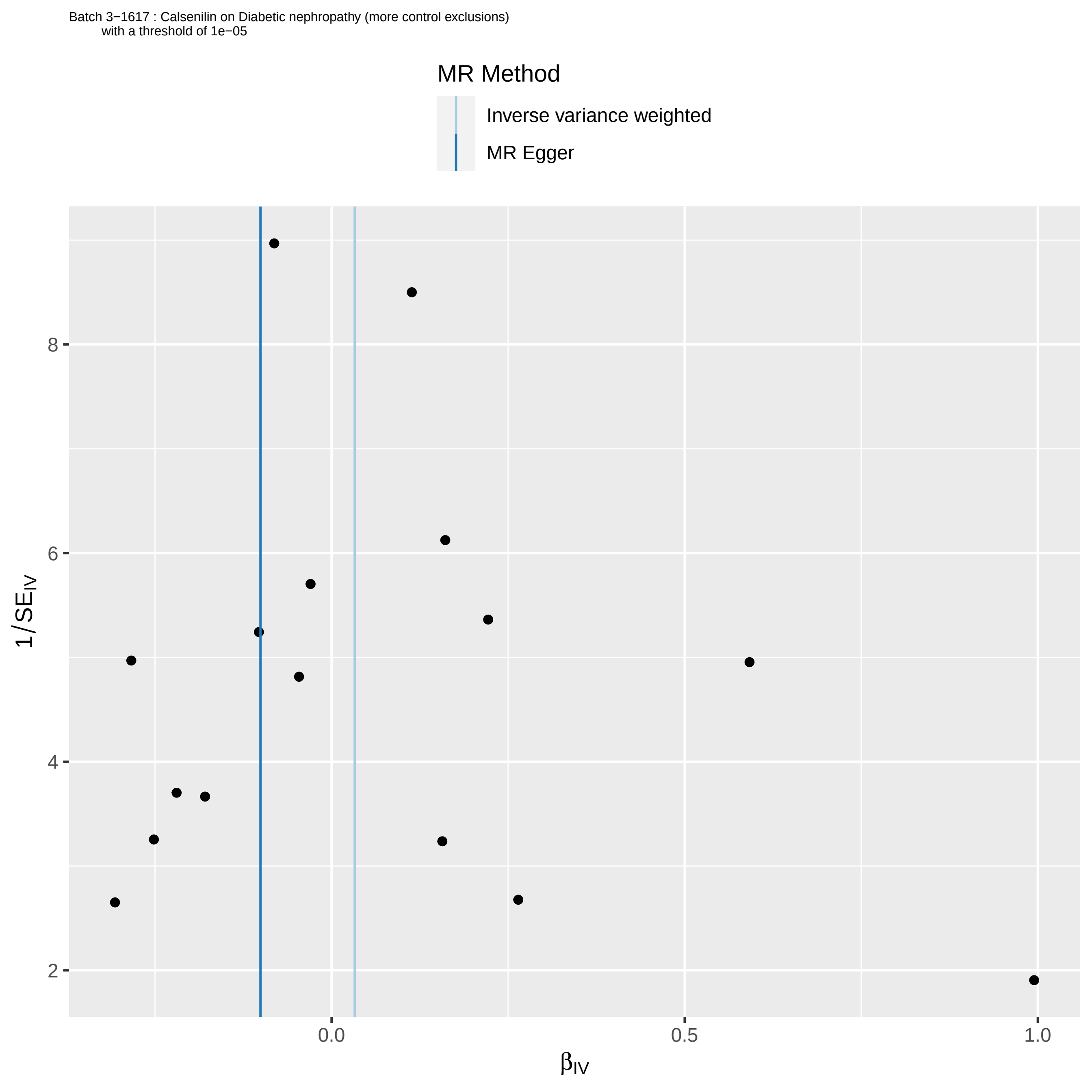
Batch 3−1617 : Calsenilin on Diabetic nephropathy (more control exclusions)
with a threshold of 1e−05
MR Method
Inverse variance weighted
MR Egger
8
6
4
2
$1/SE_{IV}$
0.0
0.5
1.0
β_{IV}

Batch 3−1618 : Small conductance calcium−activated potassium channel protein 1 on Diabetic nephropathy (more control exclusions)
with a threshold of 1e−05

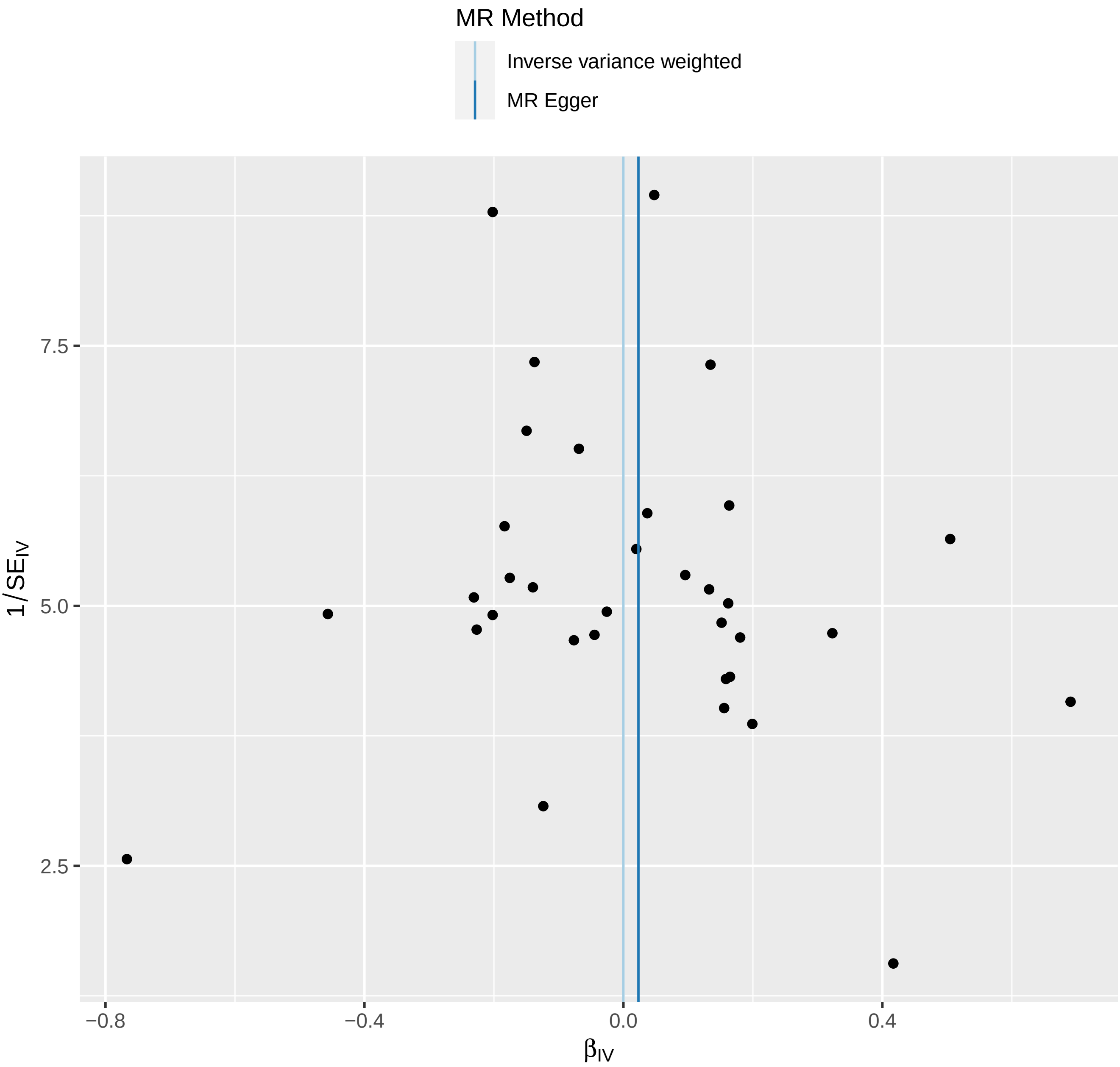

Batch 3−1619 : Keratinocyte−associated transmembrane protein 2 on Diabetic nephropathy (more control exclusions) with a threshold of 1e−05

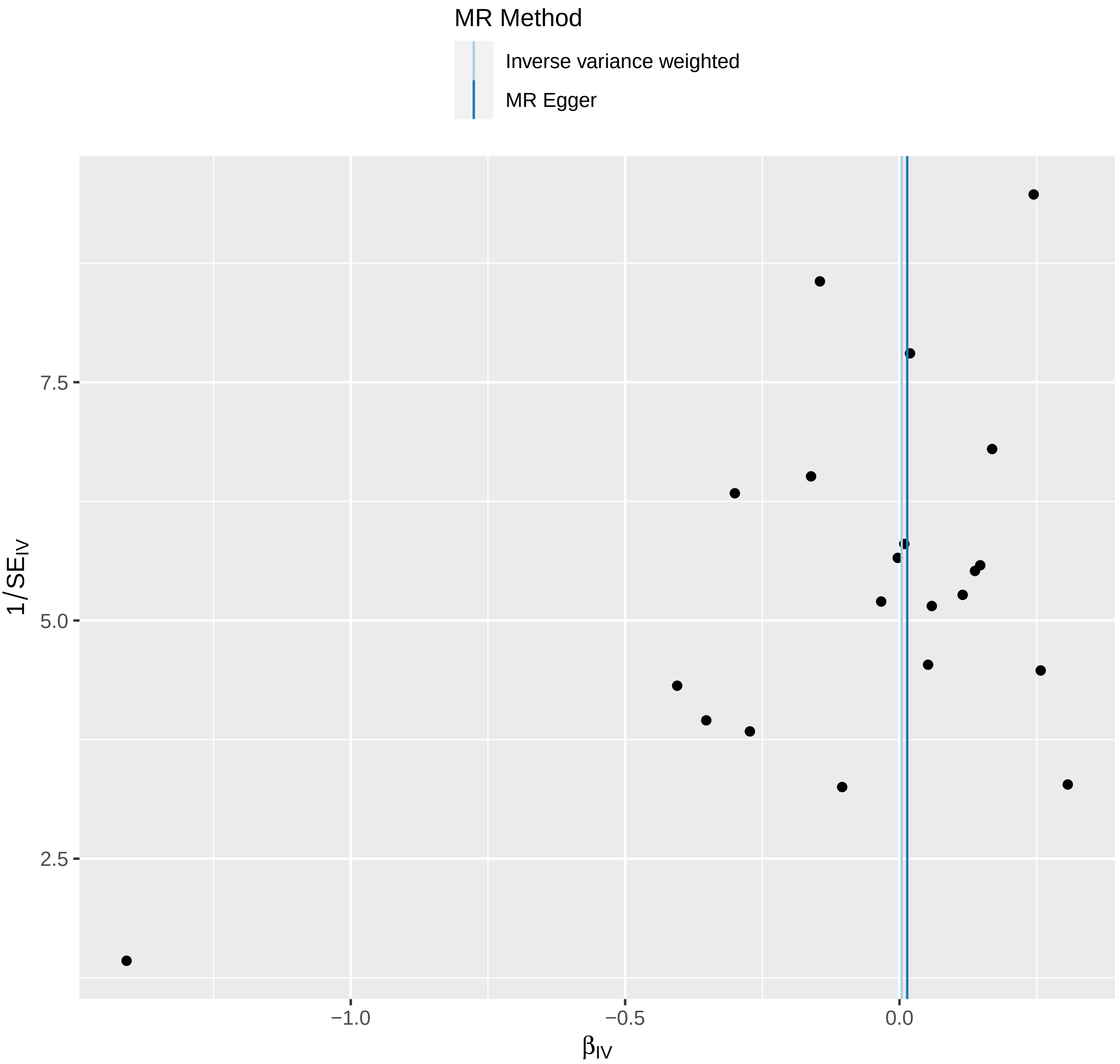

Batch 3−1620 : BTB/POZ domain−containing protein KCTD5 on Diabetic nephropathy (more control exclusions) with a threshold of 1e−05

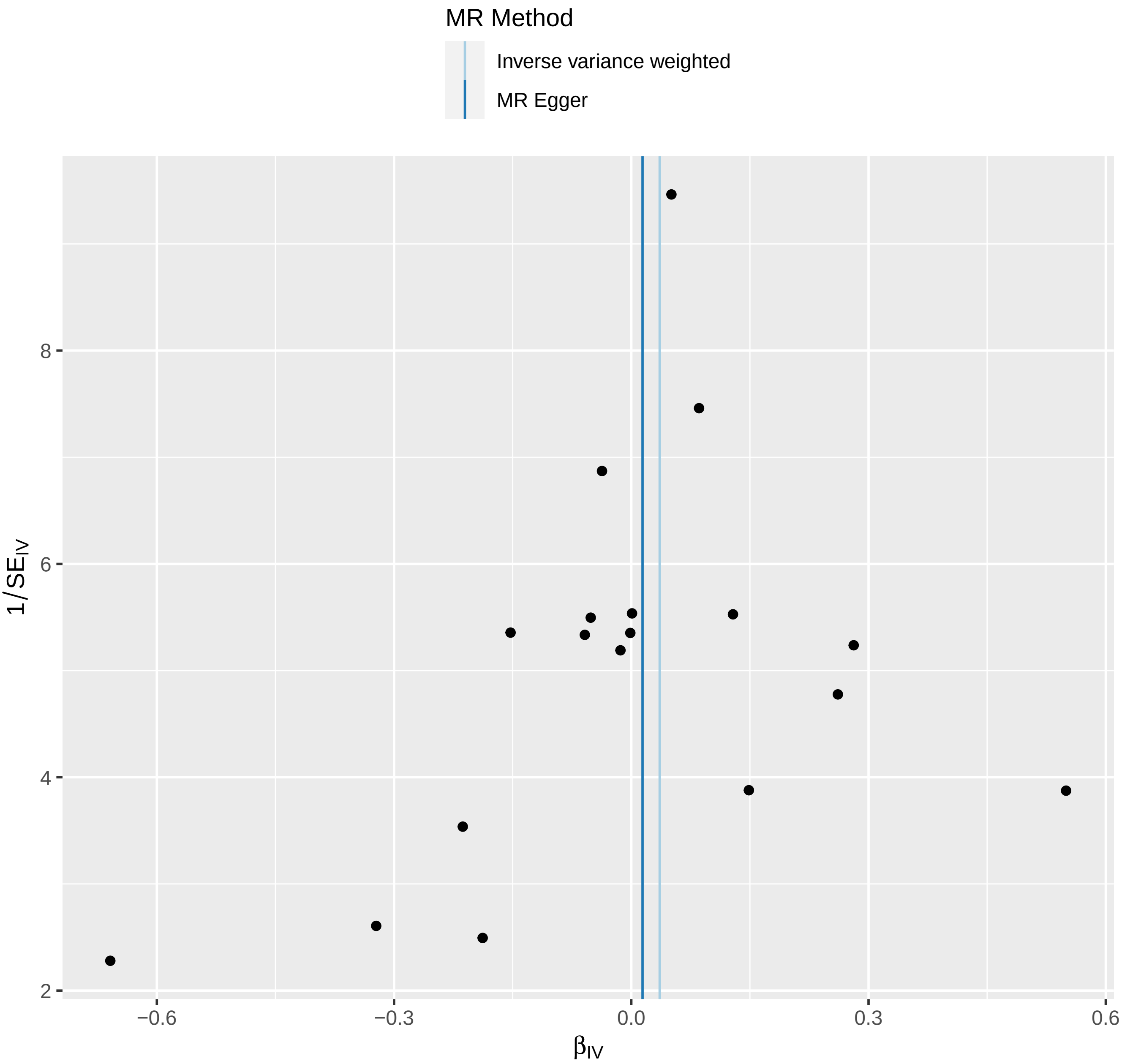

Batch 3−1621 : KDEL motif−containing protein 2 on Diabetic nephropathy (more control exclusions)
with a threshold of 1e−05

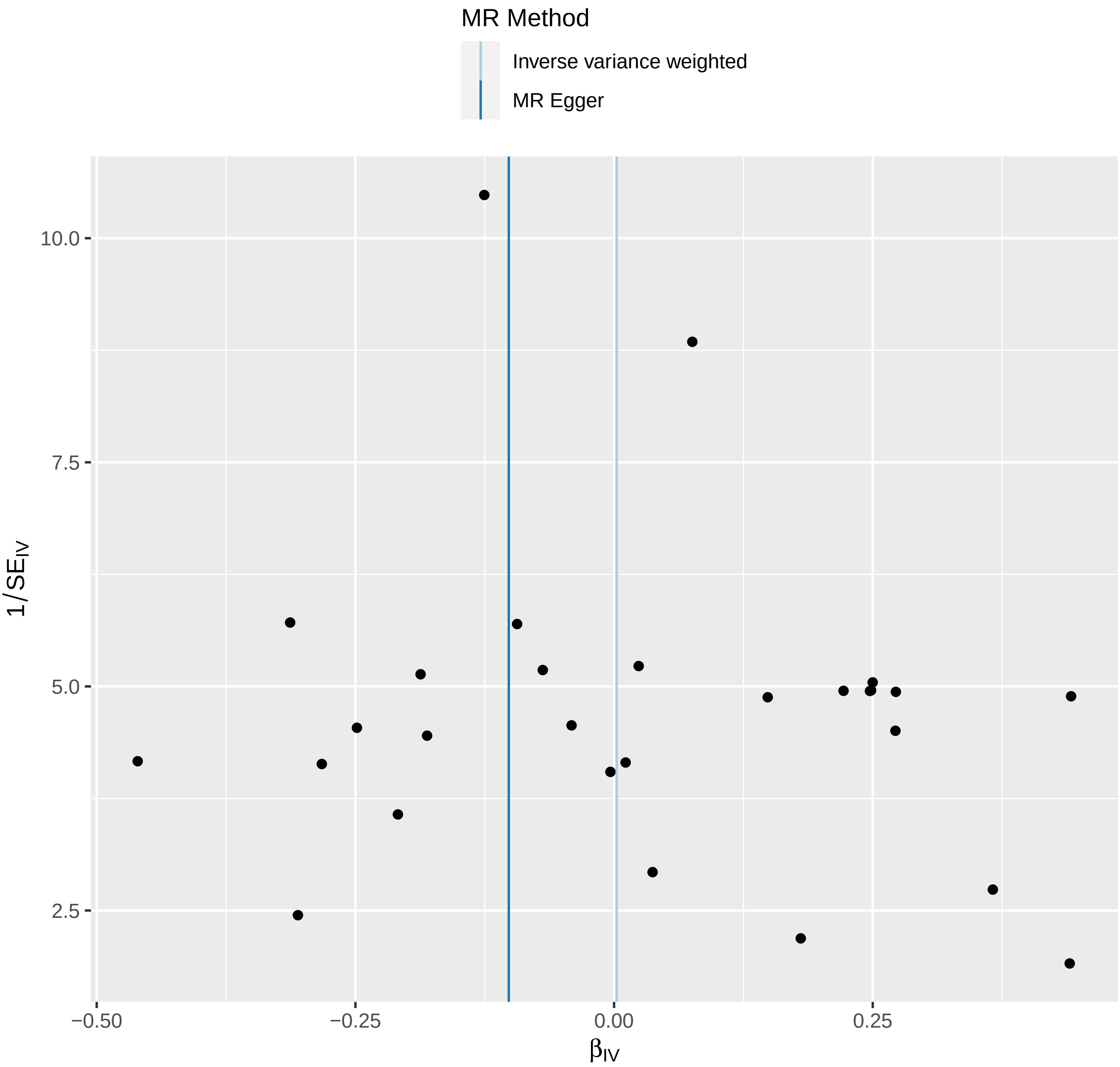

Batch 3−1622 : Vascular endothelial growth factor receptor 2 on Diabetic nephropathy (more control exclusions) with a threshold of 1e−05

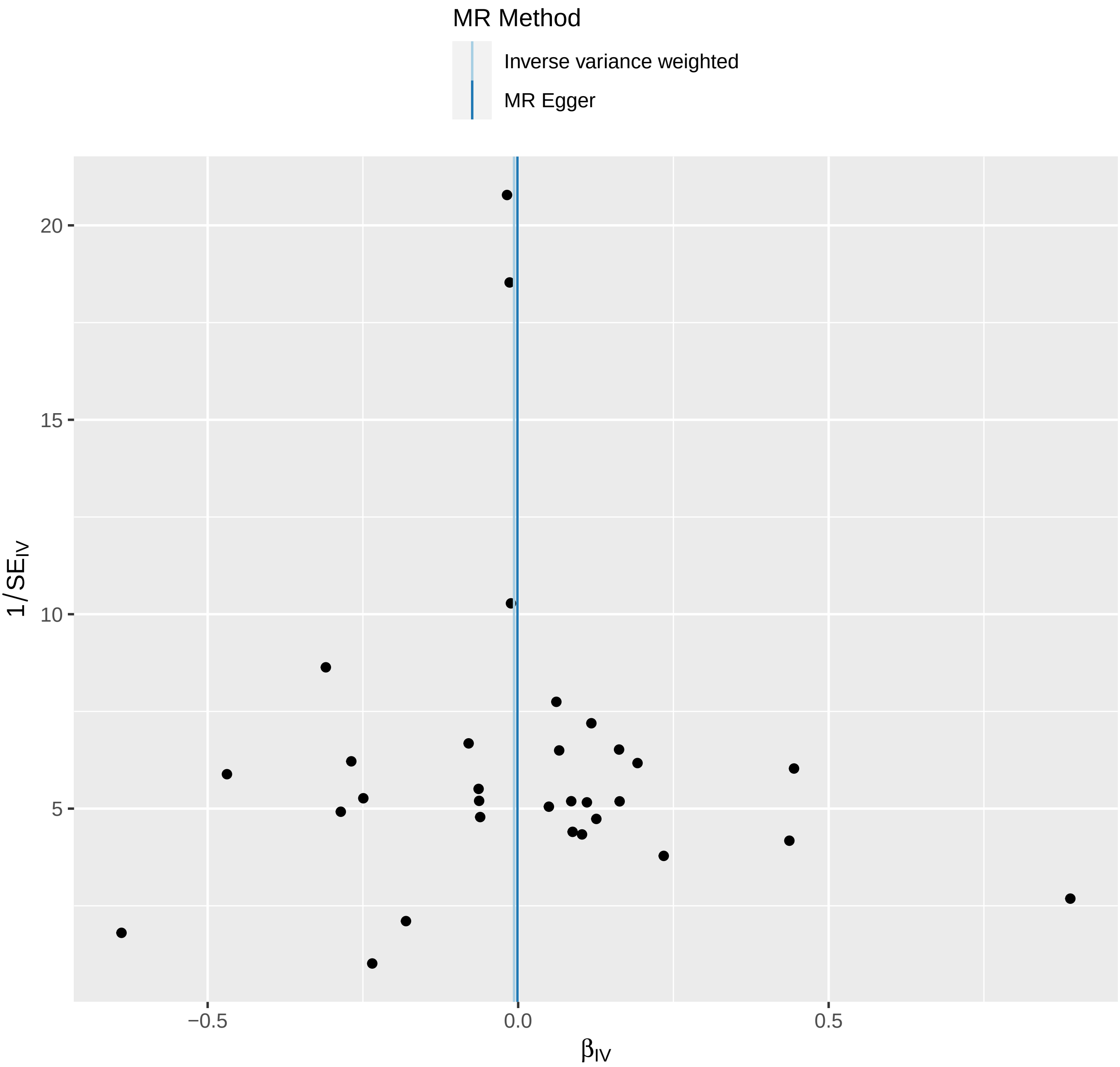

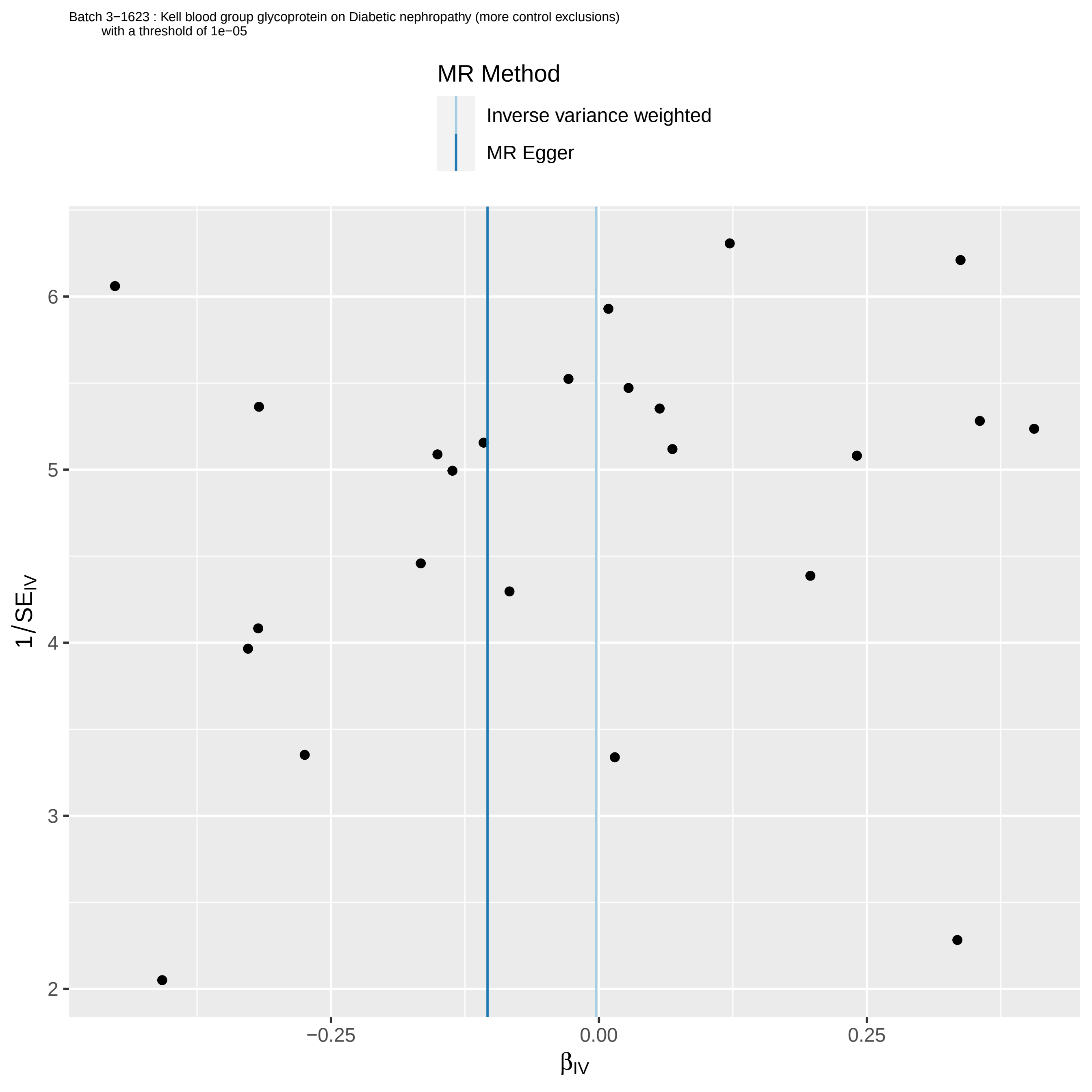

Batch 3−1623 : Kell blood group glycoprotein on Diabetic nephropathy (more control exclusions)
with a threshold of 1e−05
MR Method
Inverse variance weighted
MR Egger
6
5
4
3
2
1/SE_IV
−0.25
0.00
0.25
β_IV

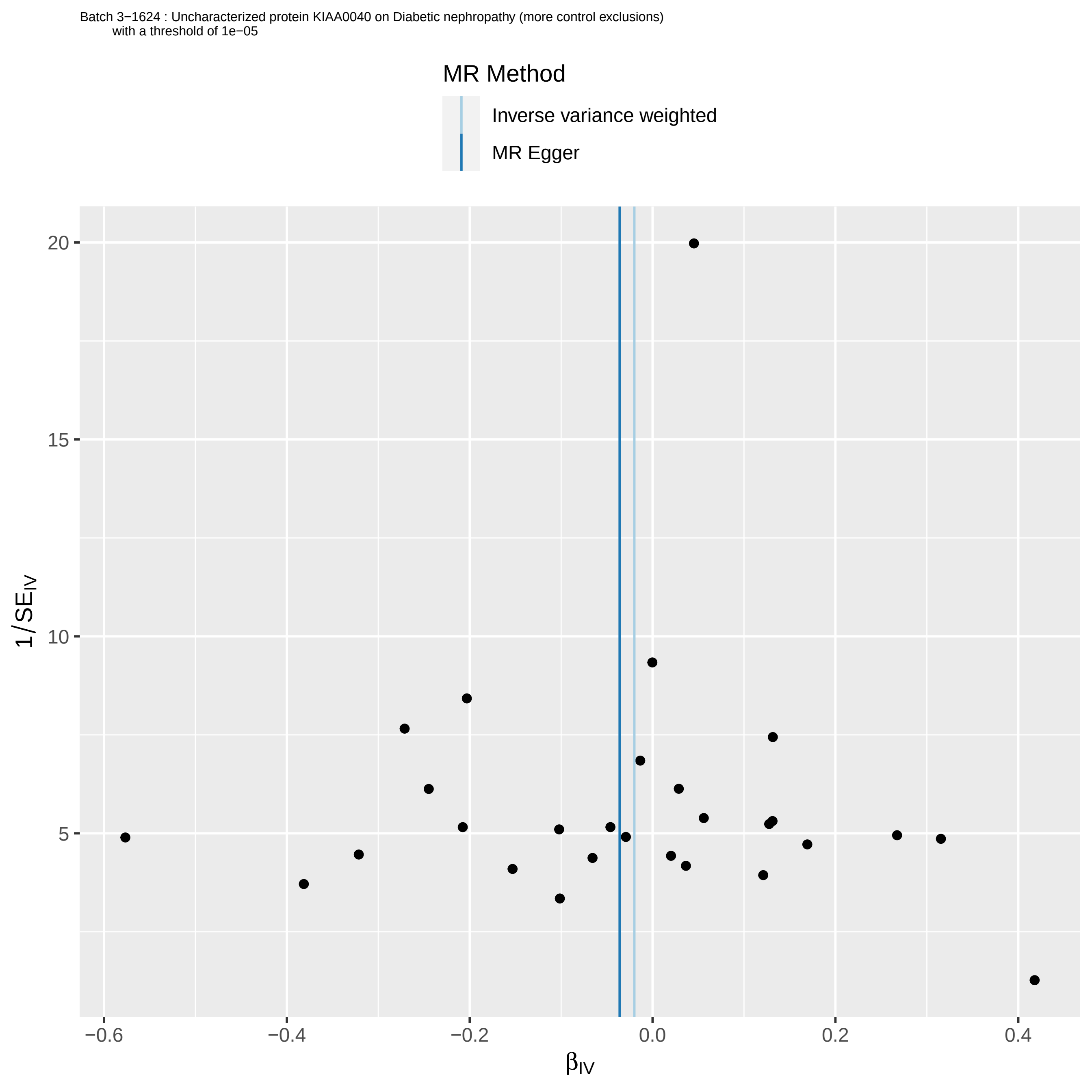

Batch 3−1624 : Uncharacterized protein KIAA0040 on Diabetic nephropathy (more control exclusions)
with a threshold of 1e−05
MR Method
Inverse variance weighted
MR Egger
20
15
10
5
1/SE_IV
−0.6
−0.4
−0.2
0.0
0.2
0.4
β_IV

Batch 3−1625 : Dyslexia−associated protein KIAA0319 on Diabetic nephropathy (more control exclusions)
with a threshold of 1e−05

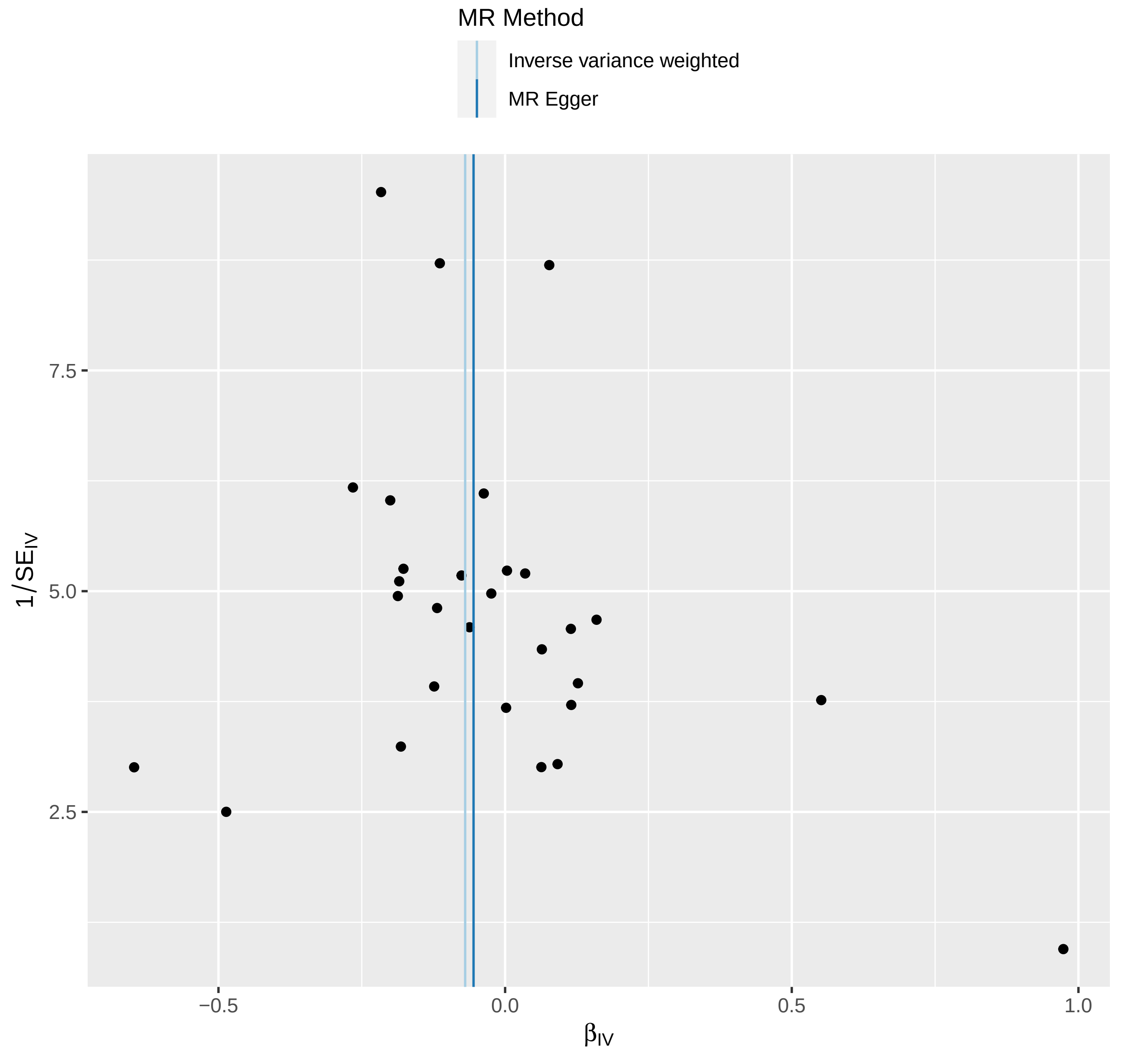

Batch 3−1626 : Dyslexia−associated protein KIAA0319−like protein on Diabetic nephropathy (more control exclusions) with a threshold of 1e−05

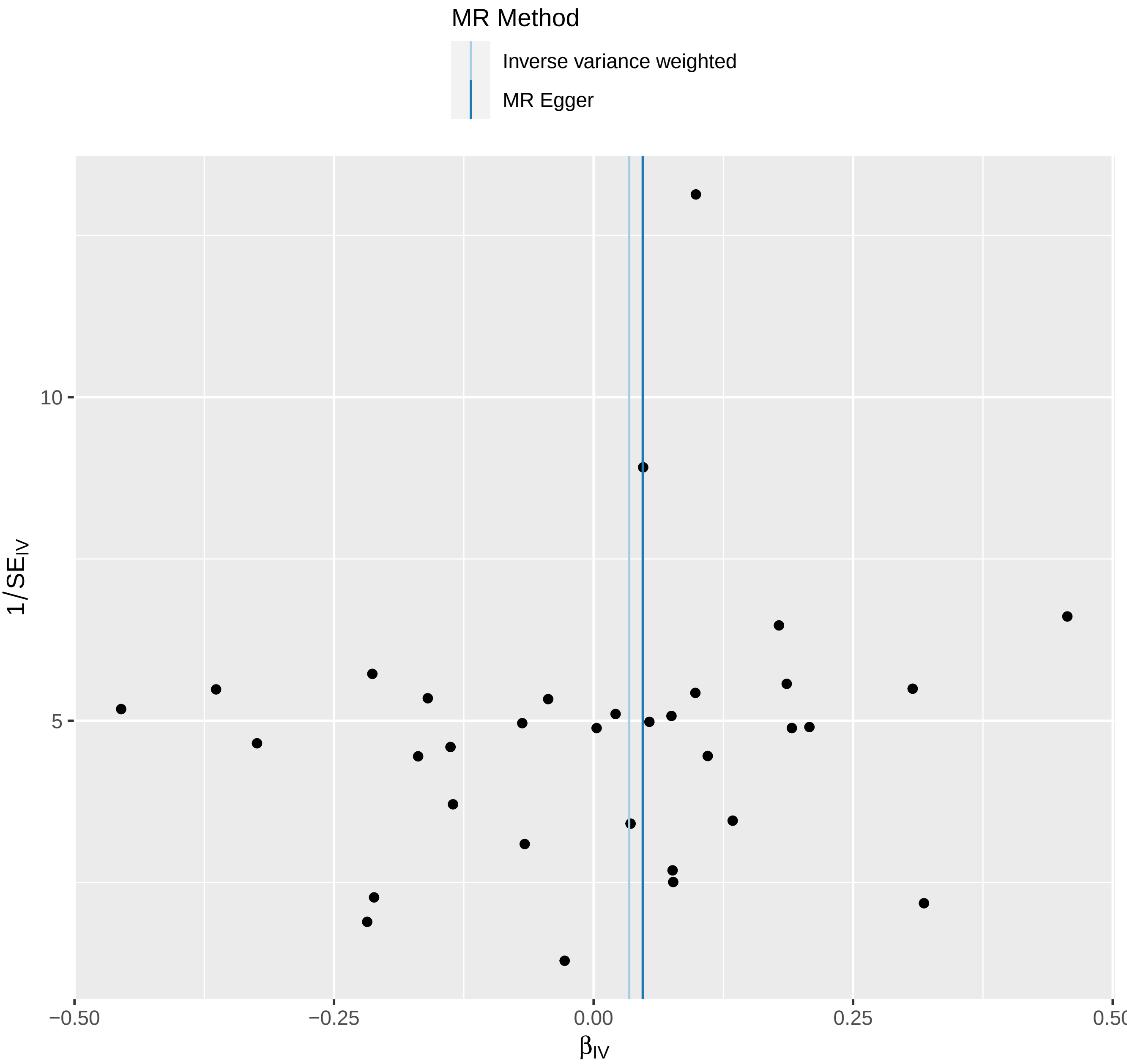

Batch 3−1627 : UPF0258 protein KIAA1024 on Diabetic nephropathy (more control exclusions)
with a threshold of 1e−05

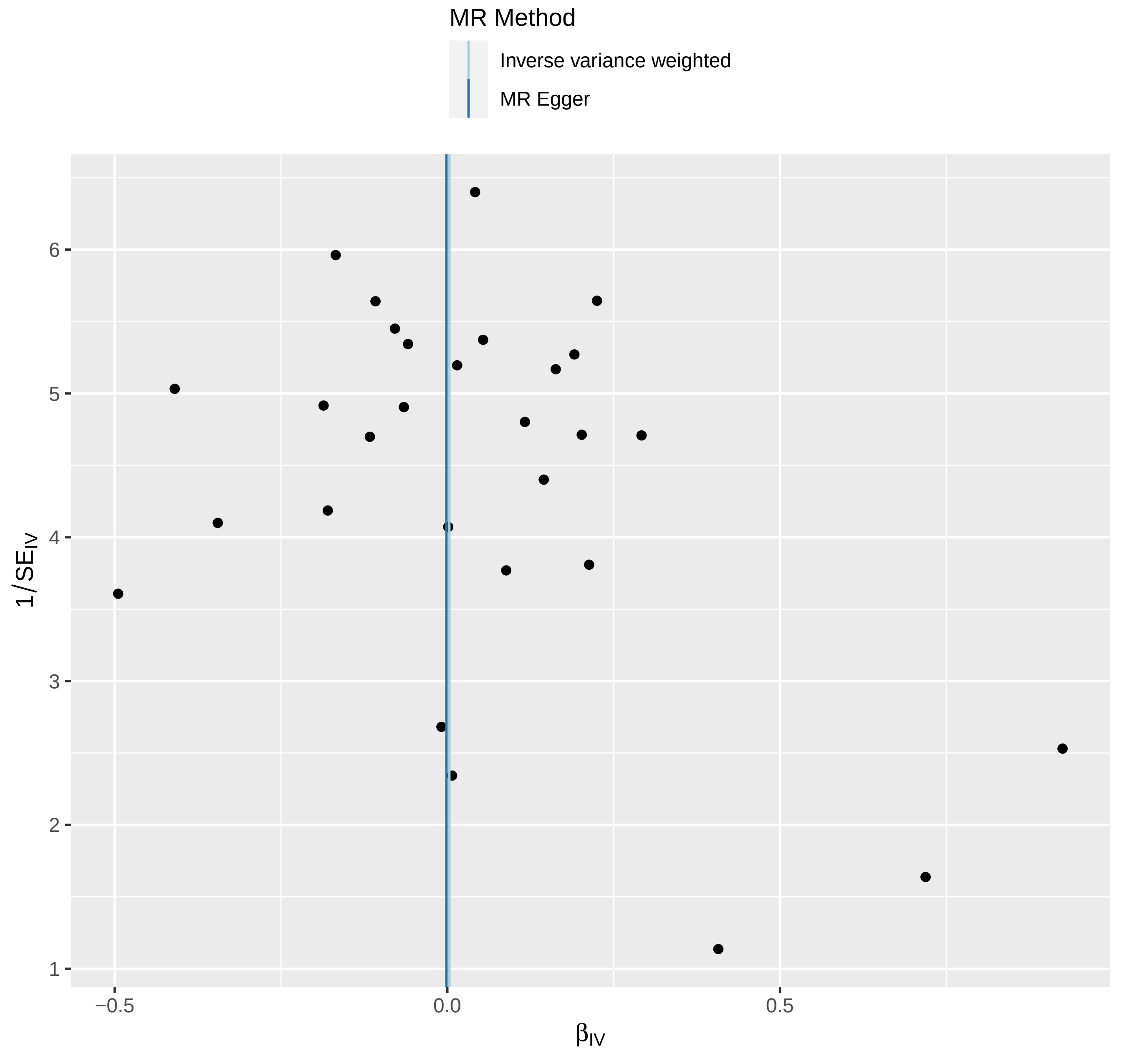

Batch 3−1628 : Uncharacterized family 31 glucosidase KIAA1161 on Diabetic nephropathy (more control exclusions)
with a threshold of 1e−05

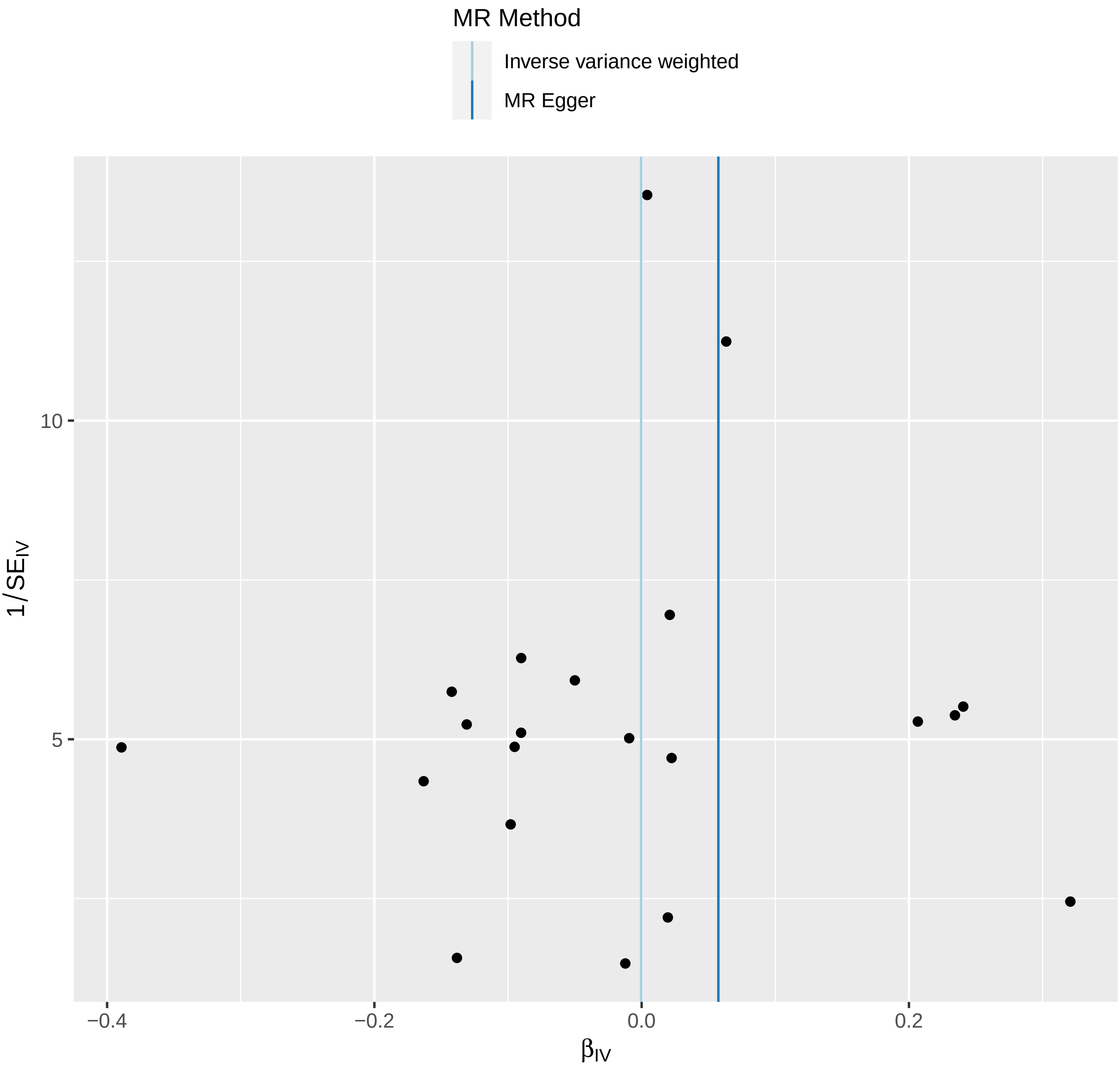

Batch 3−1629 : Uncharacterized family 31 glucosidase KIAA1161 on Diabetic nephropathy (more control exclusions) with a threshold of 1e−05

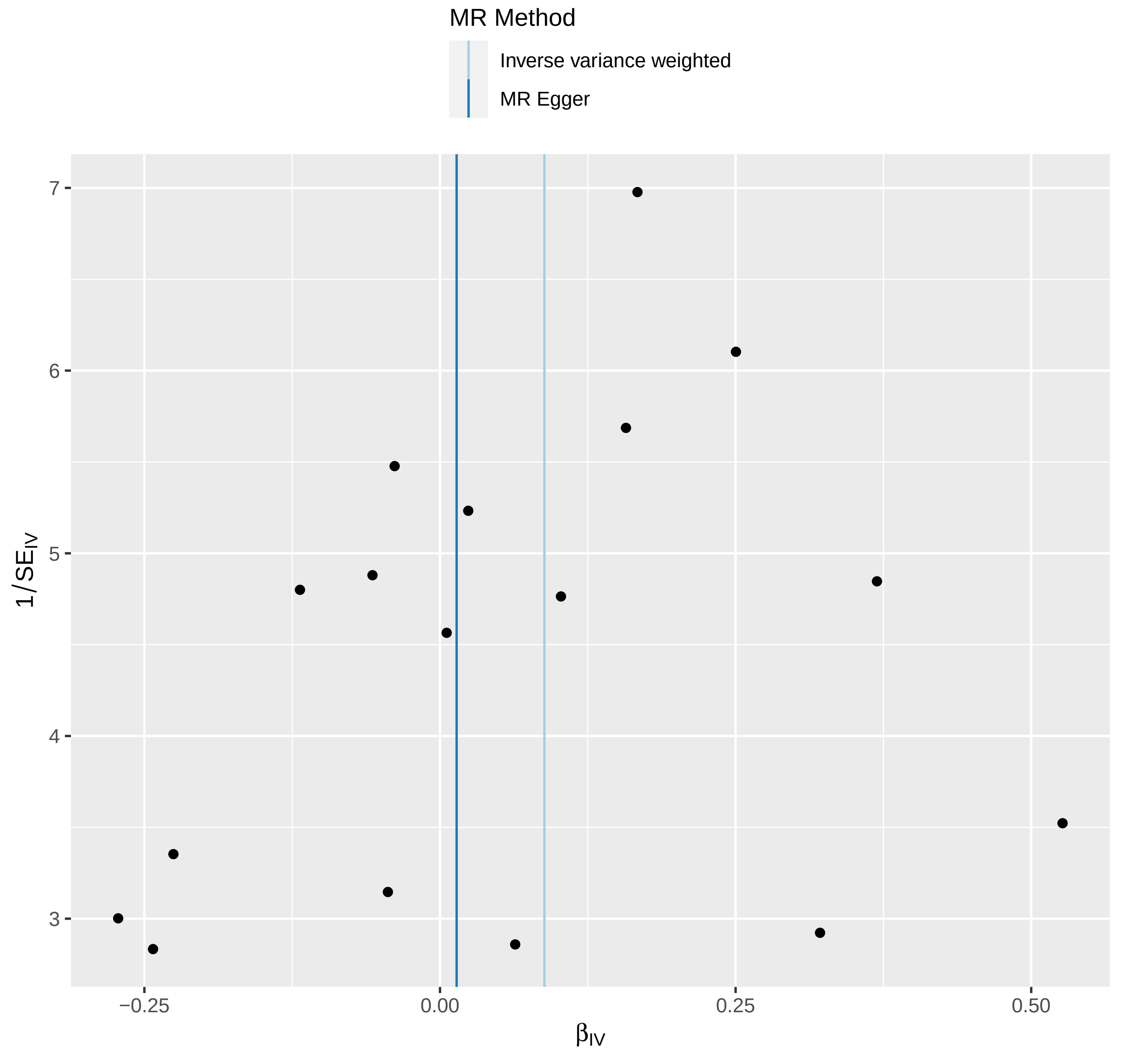

Batch 3−1630 : UPF0577 protein KIAA1324 on Diabetic nephropathy (more control exclusions)
with a threshold of 1e−05

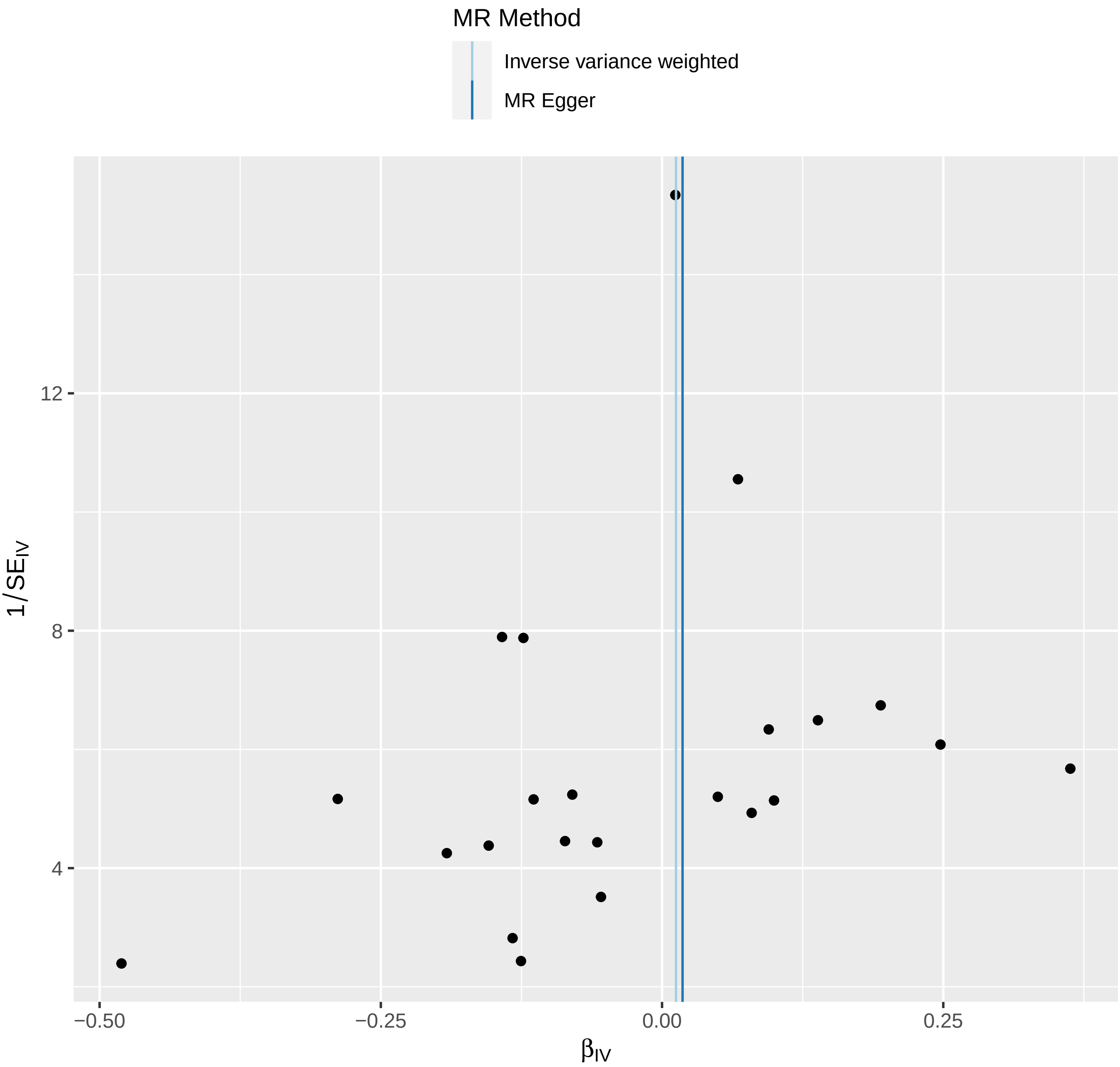

Batch 3−1631 : UPF0577 protein KIAA1324 on Diabetic nephropathy (more control exclusions)
with a threshold of 1e−05

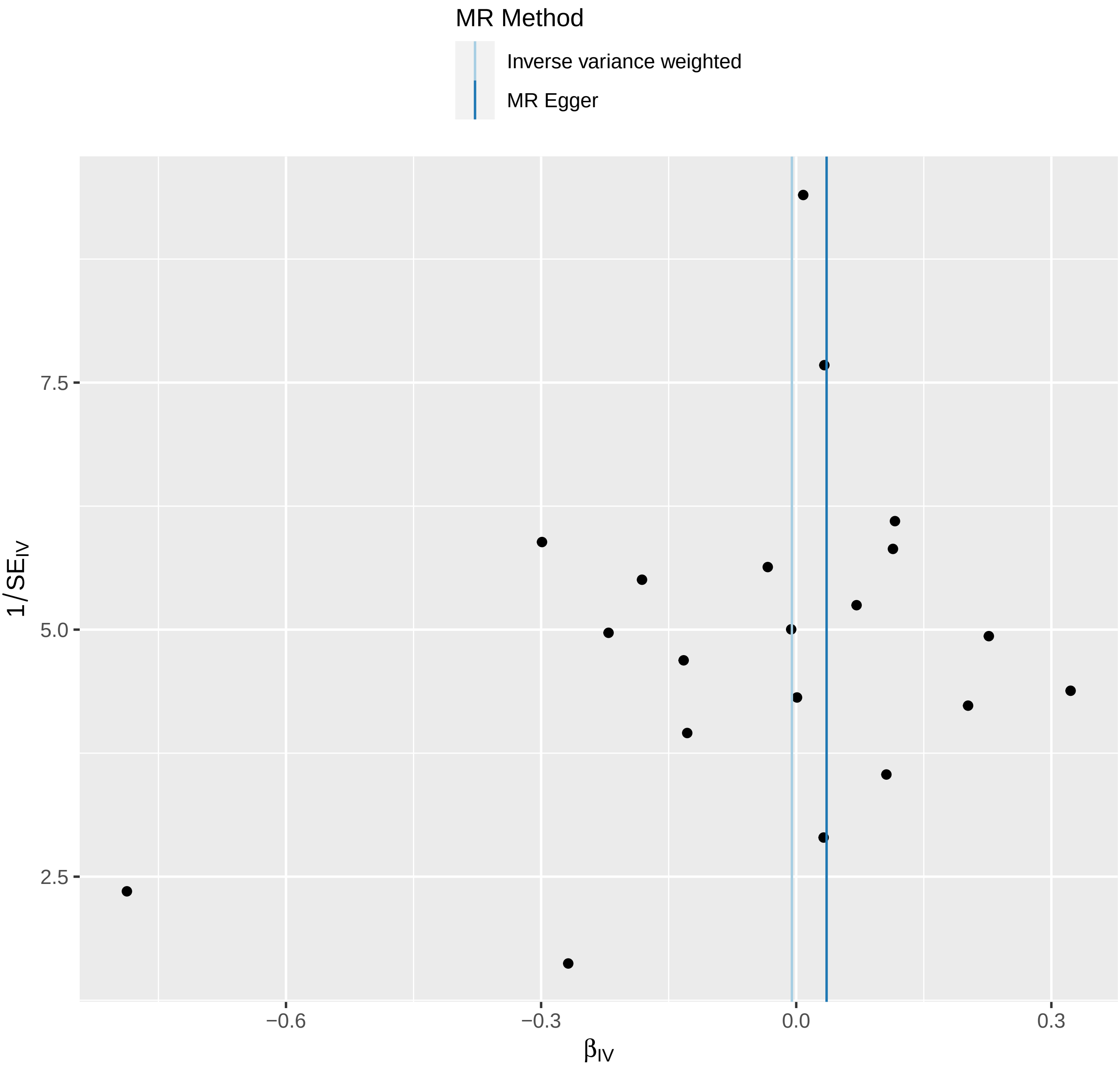

Batch 3−1632 : UPF0577 protein KIAA1324−like on Diabetic nephropathy (more control exclusions)
with a threshold of 1e−05

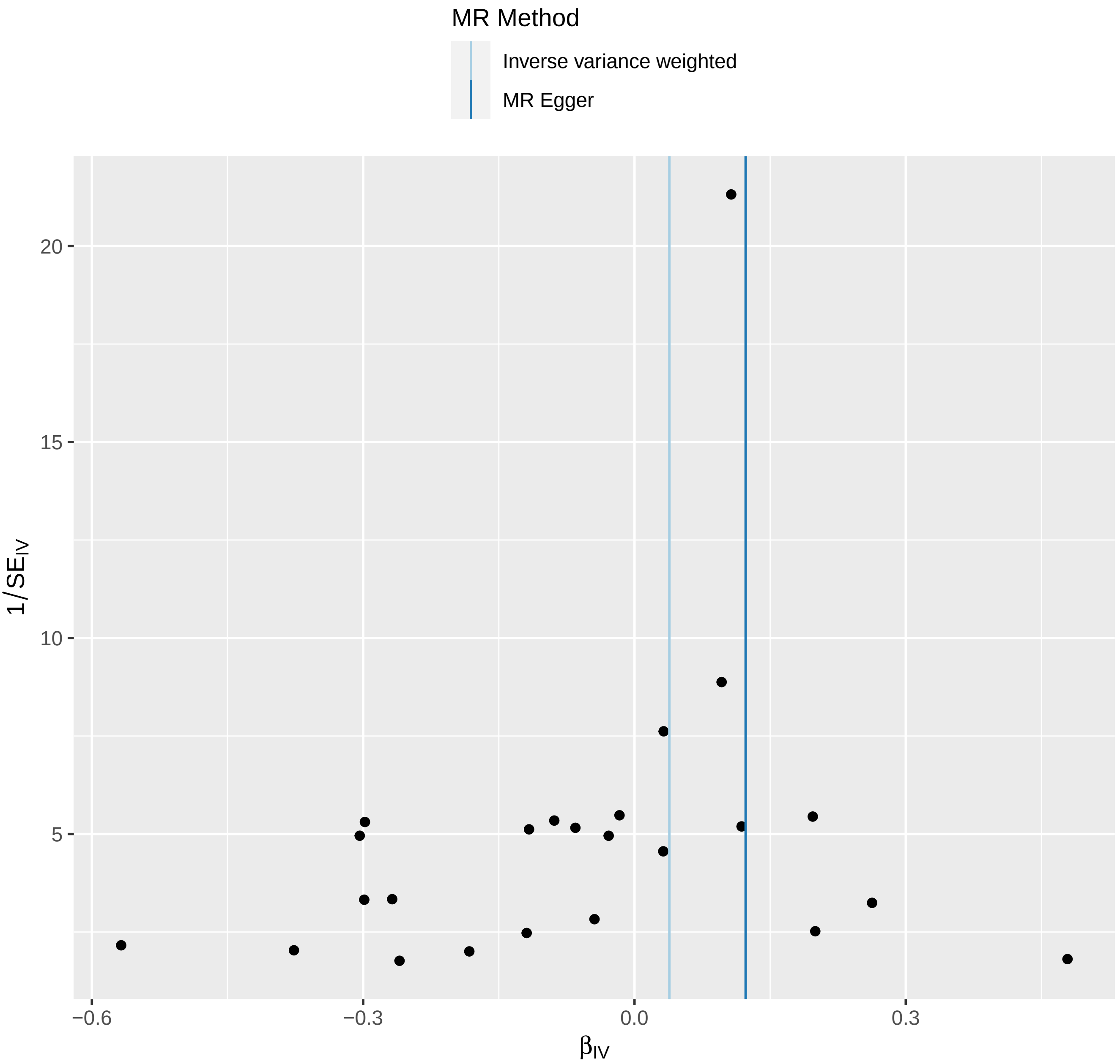

Batch 3−1633 : Uncharacterized protein KIAA1467 on Diabetic nephropathy (more control exclusions)
with a threshold of 1e−05

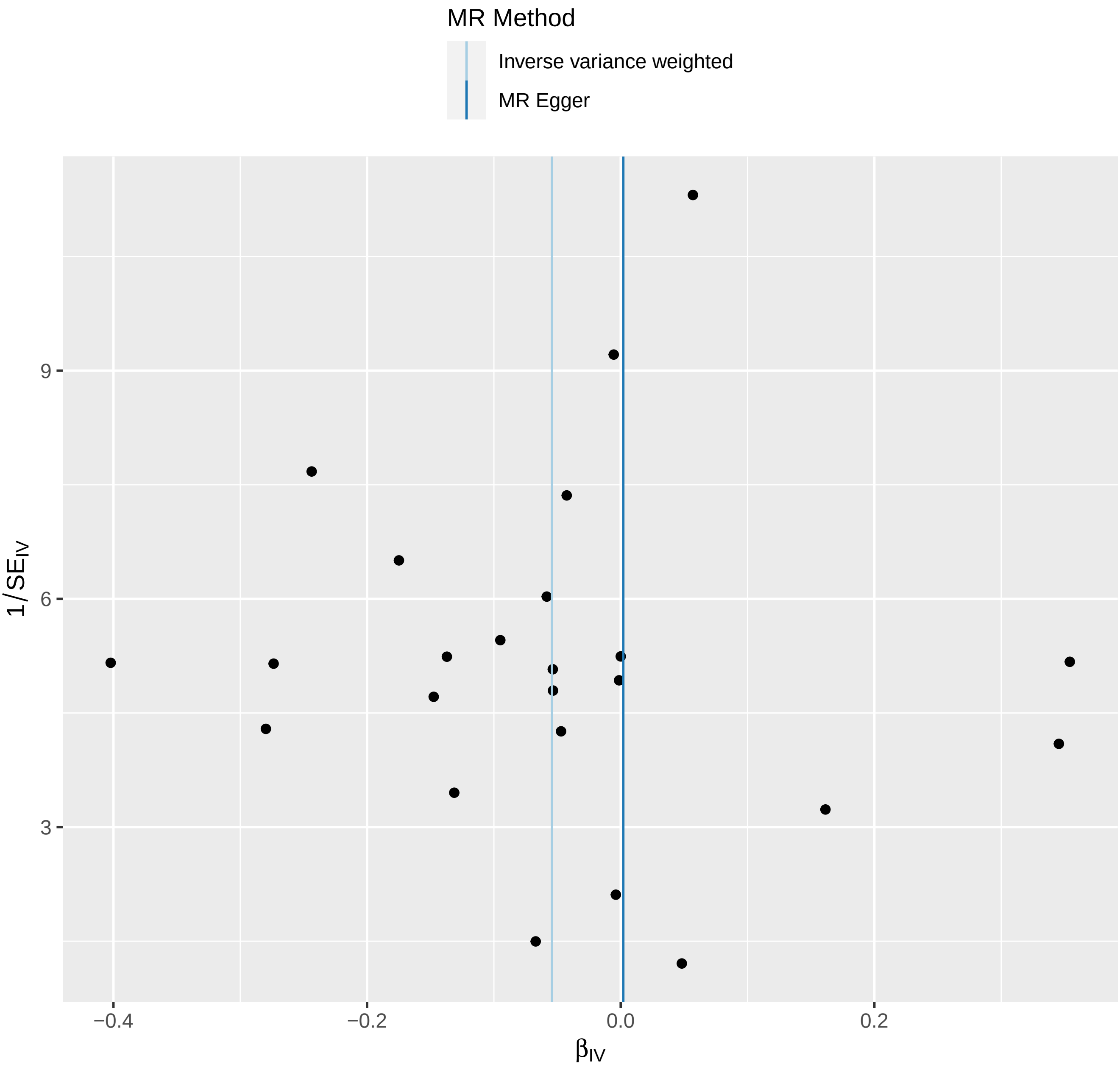

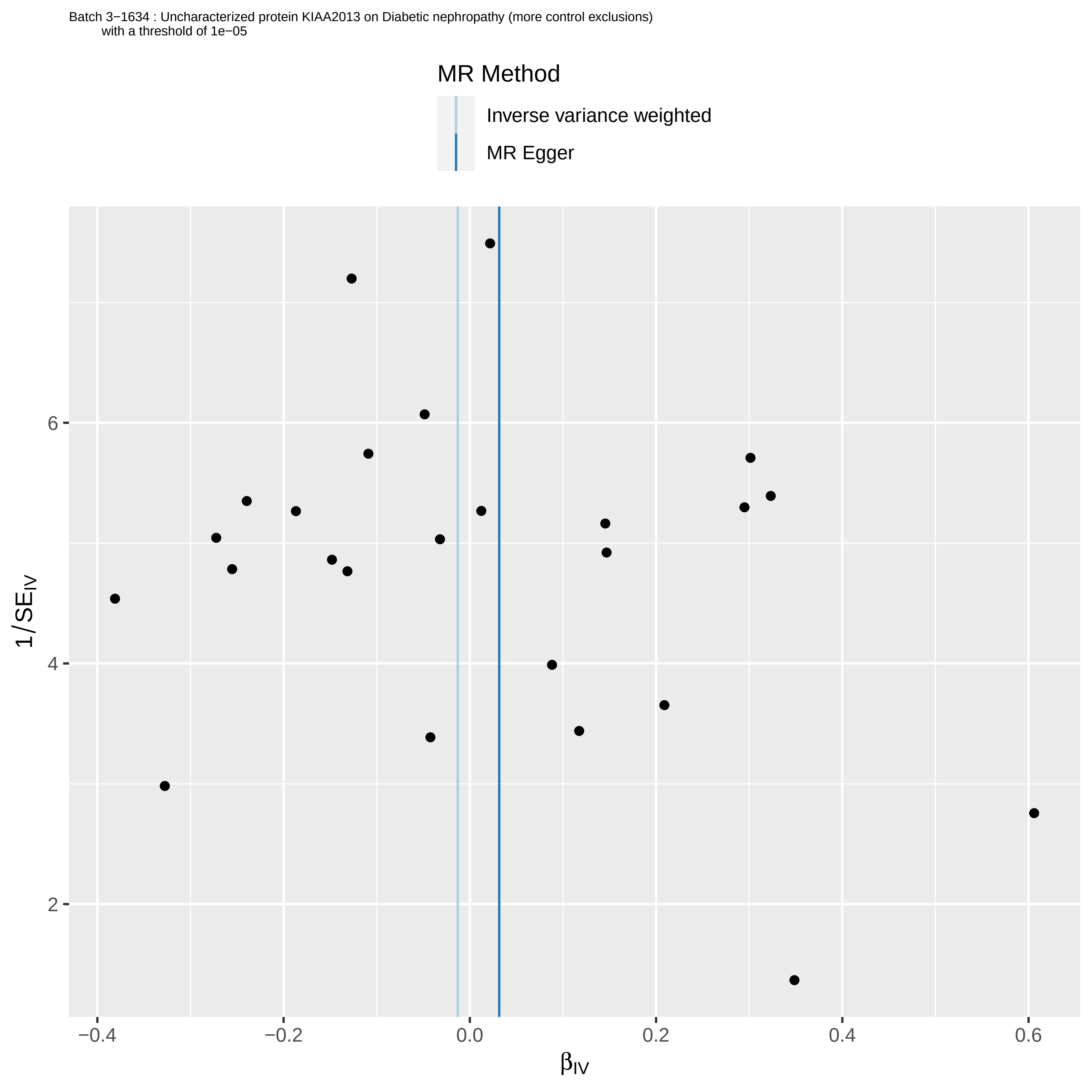
Batch 3−1634 : Uncharacterized protein KIAA2013 on Diabetic nephropathy (more control exclusions)
with a threshold of 1e−05
MR Method
Inverse variance weighted
MR Egger
6
4
2
1/SE_IV
−0.4
−0.2
0.0
0.2
0.4
0.6
β_IV

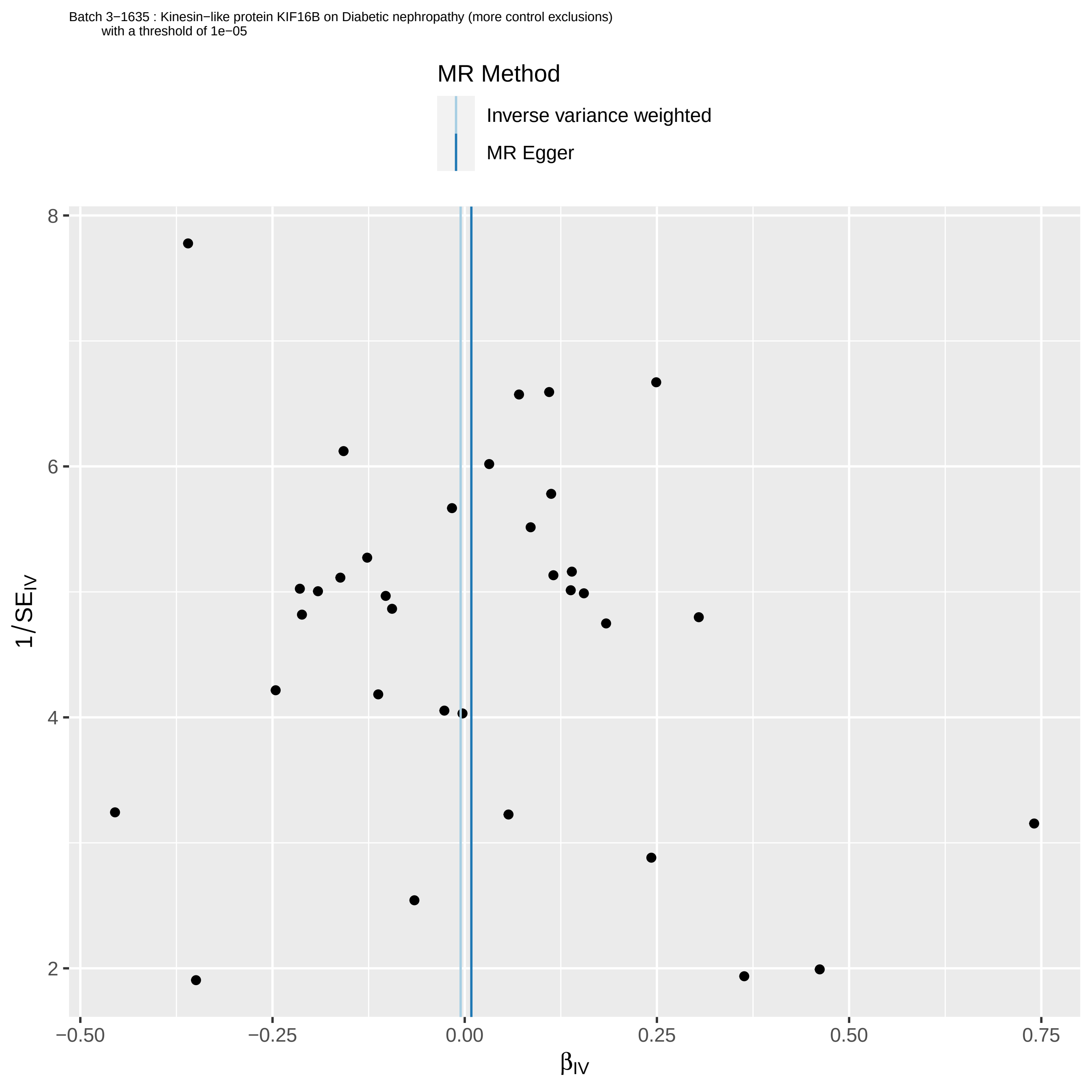

Batch 3−1635 : Kinesin−like protein KIF16B on Diabetic nephropathy (more control exclusions)
with a threshold of 1e−05
MR Method
Inverse variance weighted
MR Egger
8
6
4
2
1/SE_IV
−0.50
−0.25
0.00
0.25
0.50
0.75
β_IV

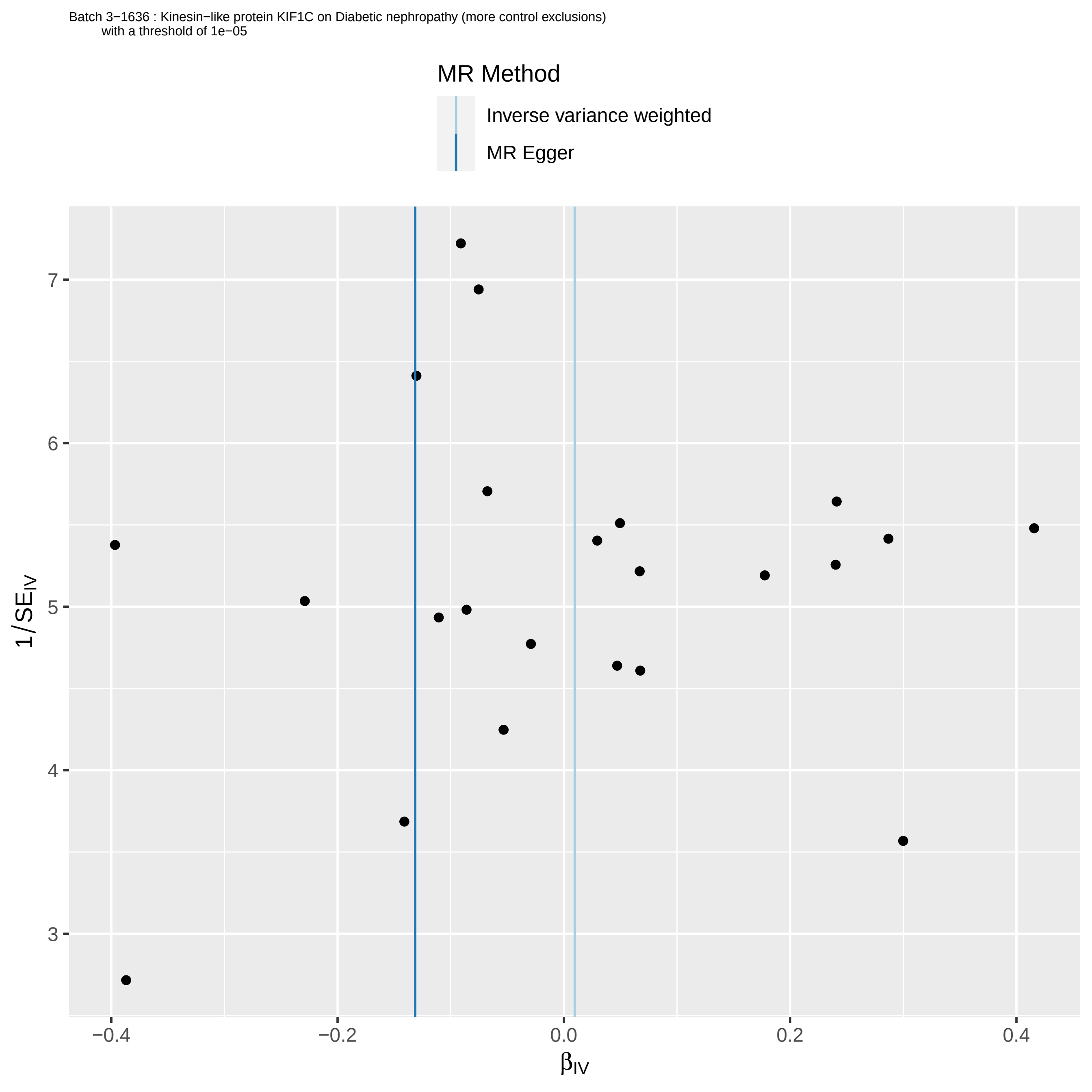
Batch 3−1636 : Kinesin−like protein KIF1C on Diabetic nephropathy (more control exclusions)
with a threshold of 1e−05
MR Method
Inverse variance weighted
MR Egger
1/SE_IV
β_IV
7
6
5
4
3
−0.4
−0.2
0.0
0.2
0.4

Batch 3−1637 : Kinesin−like protein KIF23 on Diabetic nephropathy (more control exclusions)
with a threshold of 1e−05

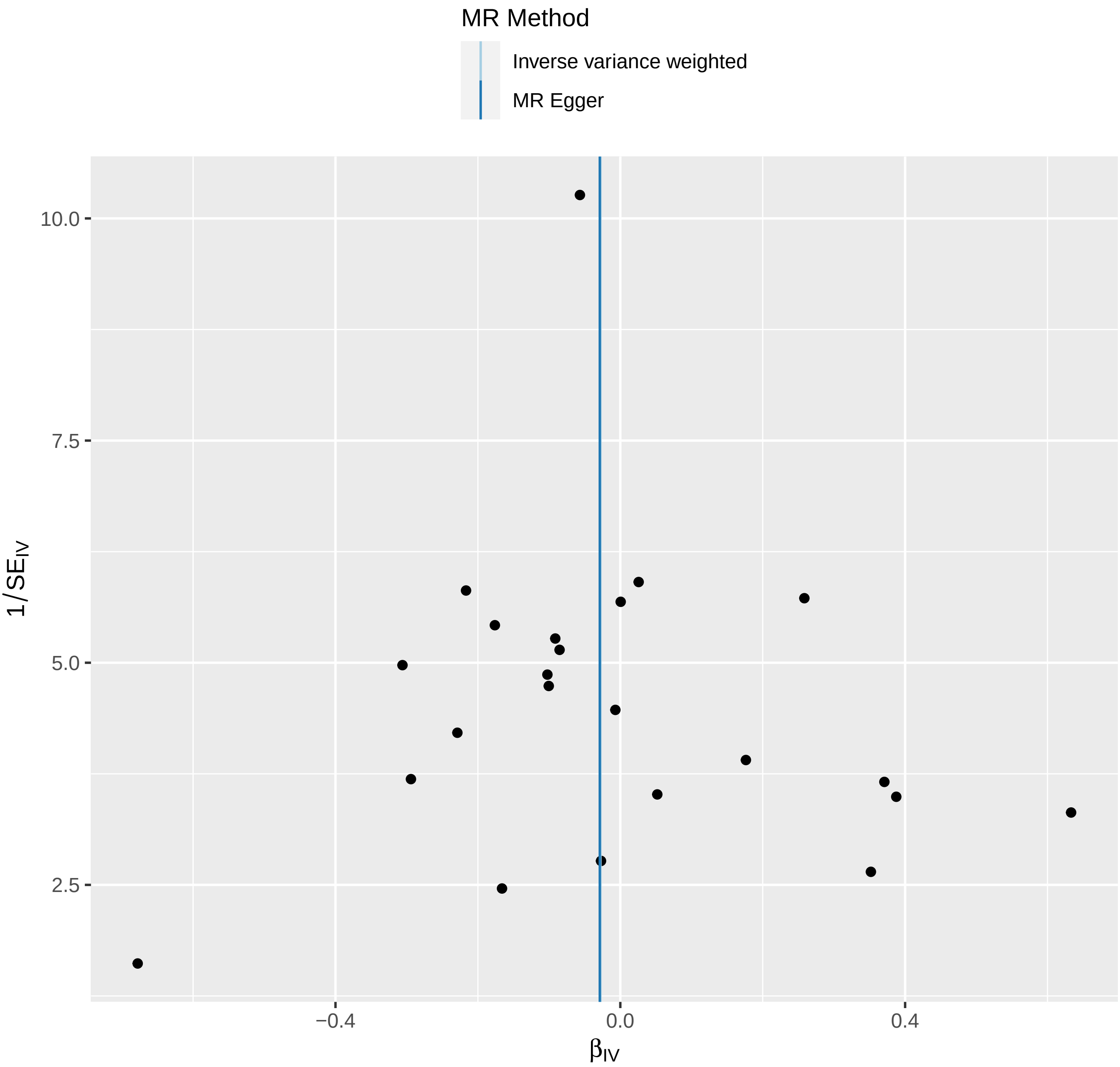

Batch 3−1638 : DNA/RNA−binding protein KIN17 on Diabetic nephropathy (more control exclusions)
with a threshold of 1e−05

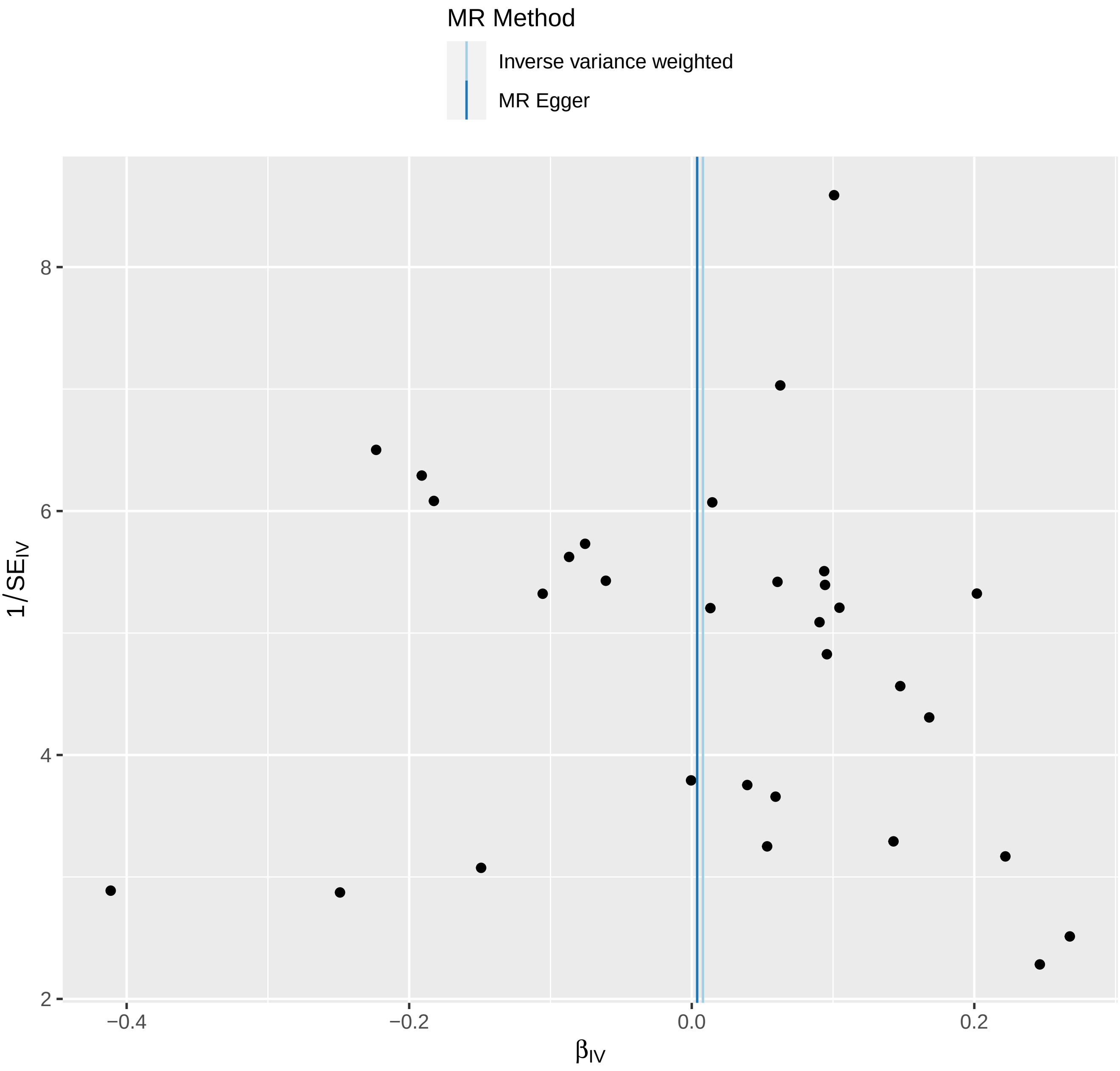

Batch 3−1639 : DNA/RNA−binding protein KIN17 on Diabetic nephropathy (more control exclusions)
with a threshold of 1e−05

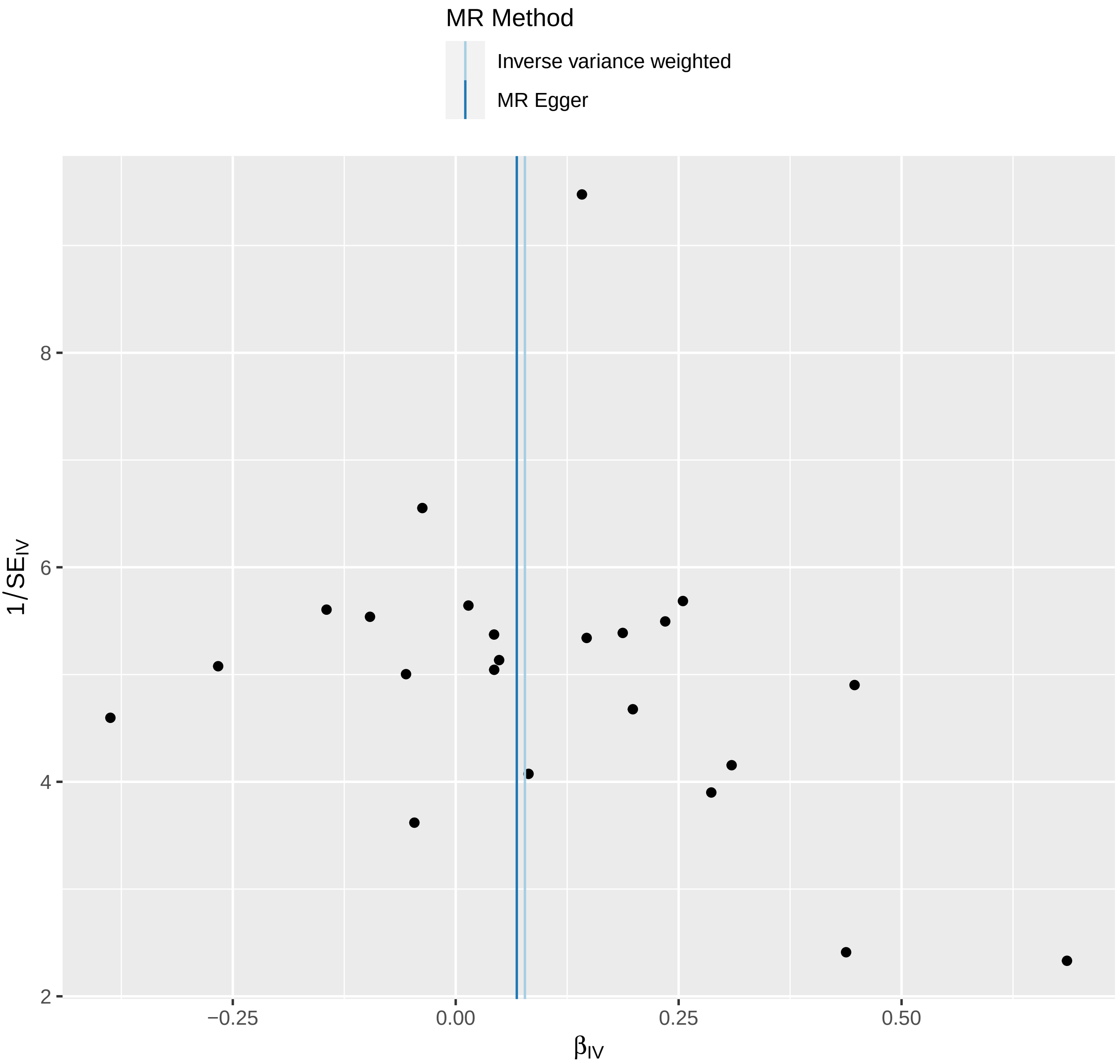

Batch 3−1640 : Killer cell immunoglobulin−like receptor 2DL2 on Diabetic nephropathy (more control exclusions)
with a threshold of 1e−05

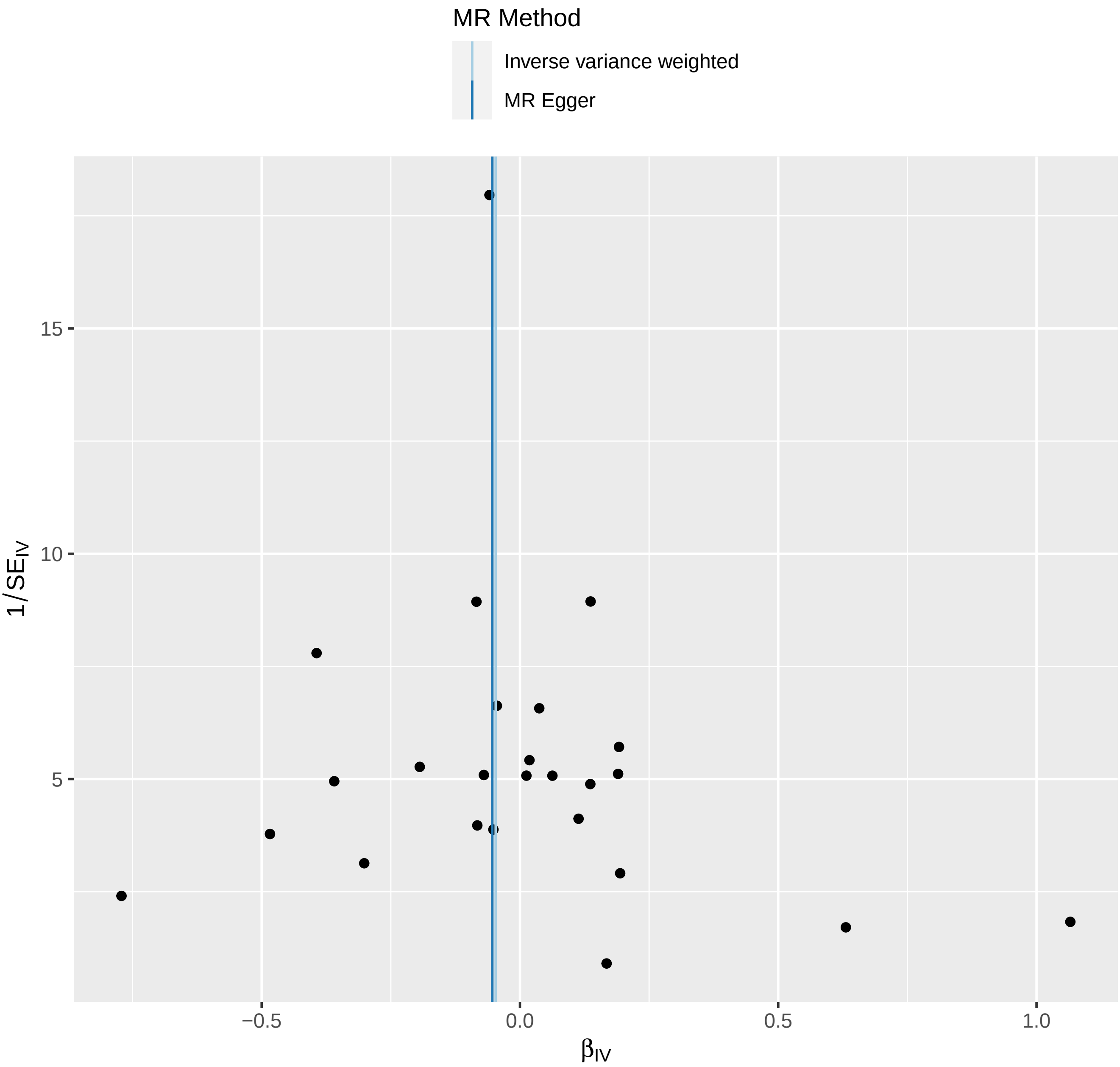

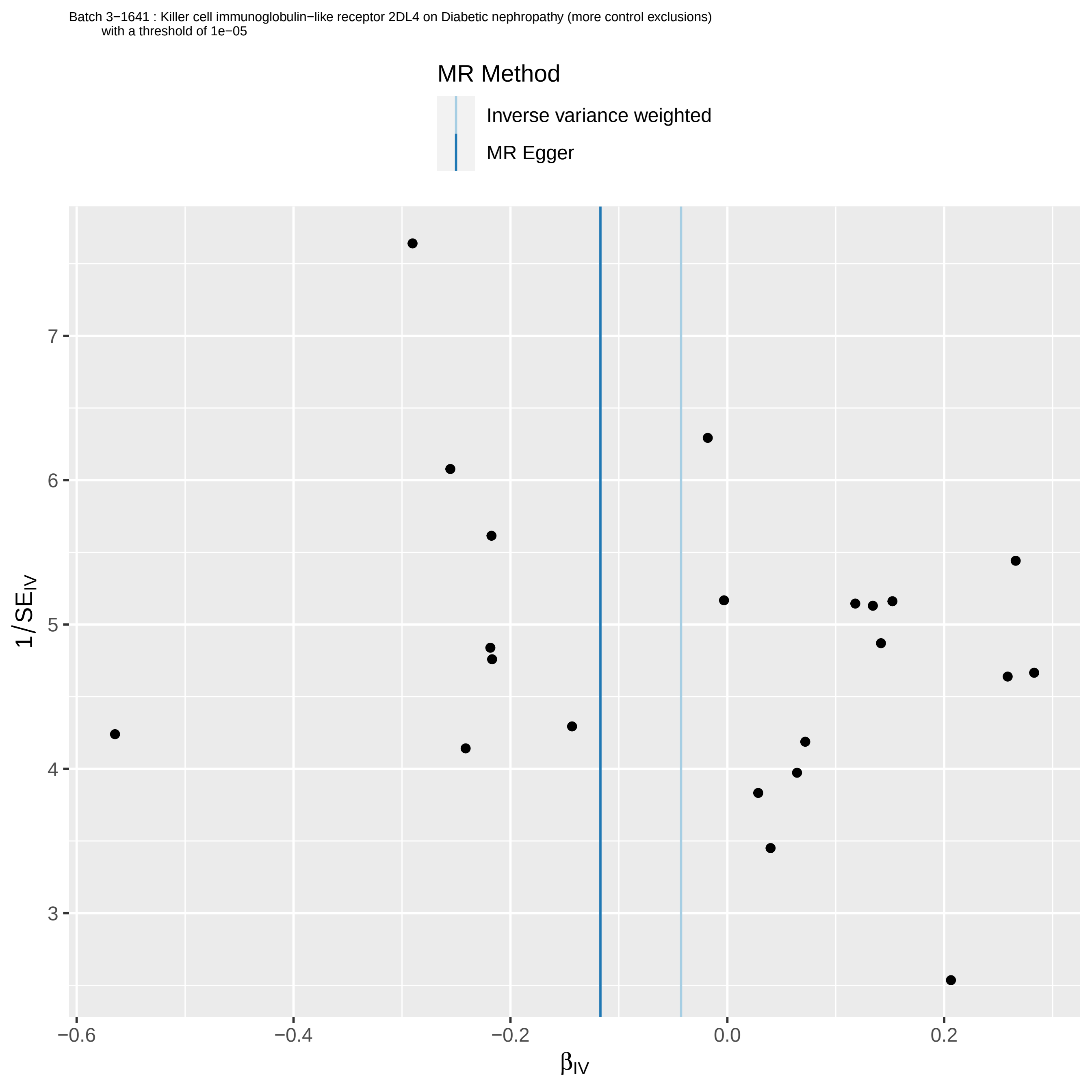
Batch 3−1641 : Killer cell immunoglobulin−like receptor 2DL4 on Diabetic nephropathy (more control exclusions)
with a threshold of 1e−05
MR Method
Inverse variance weighted
MR Egger
1/SE_IV
β_IV
7
6
5
4
3
−0.6
−0.4
−0.2
0.0
0.2

Batch 3−1642 : Killer cell immunoglobulin−like receptor 2DL4 on Diabetic nephropathy (more control exclusions)
with a threshold of 1e−05

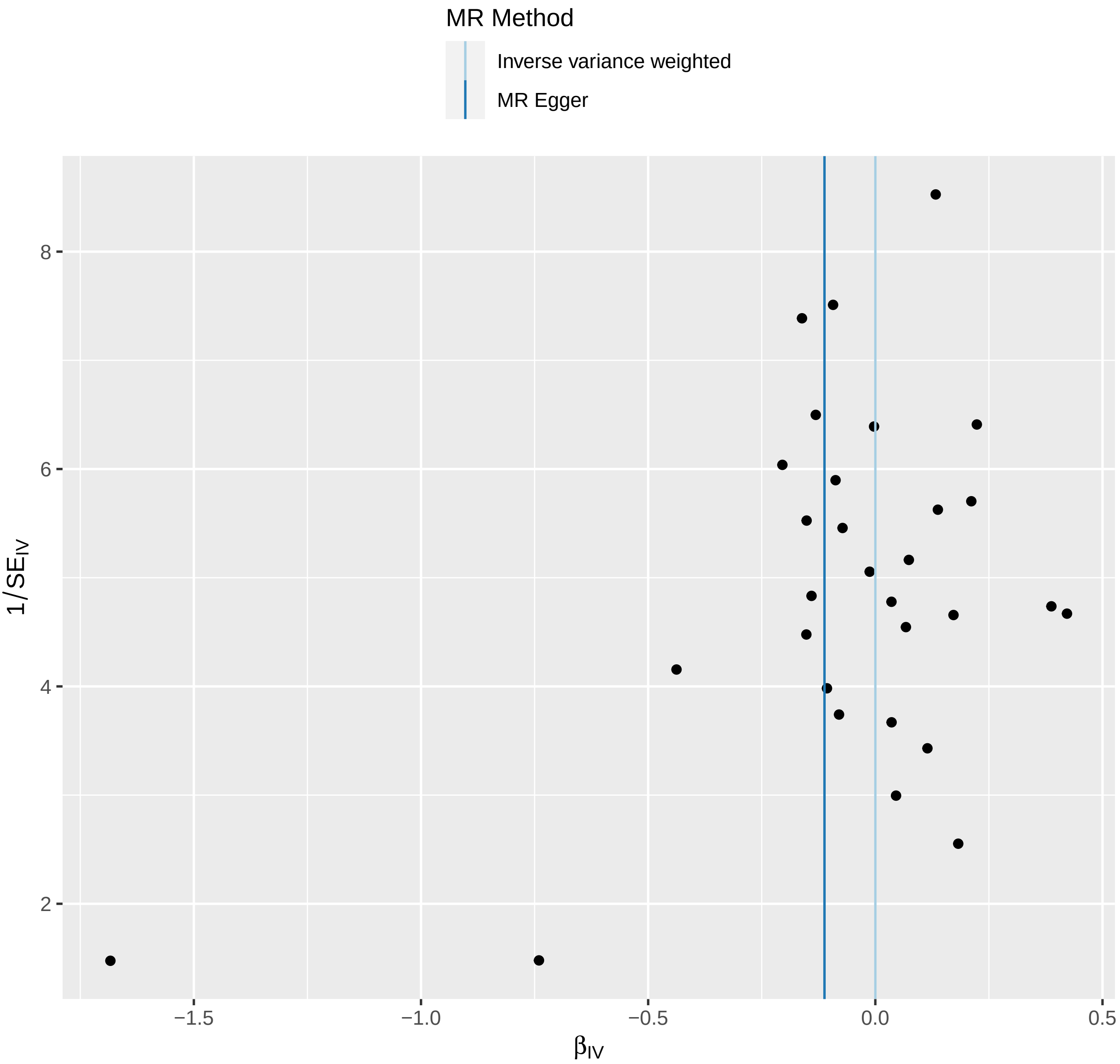

Batch 3−1643 : Killer cell immunoglobulin−like receptor 2DL5A on Diabetic nephropathy (more control exclusions)
with a threshold of 1e−05

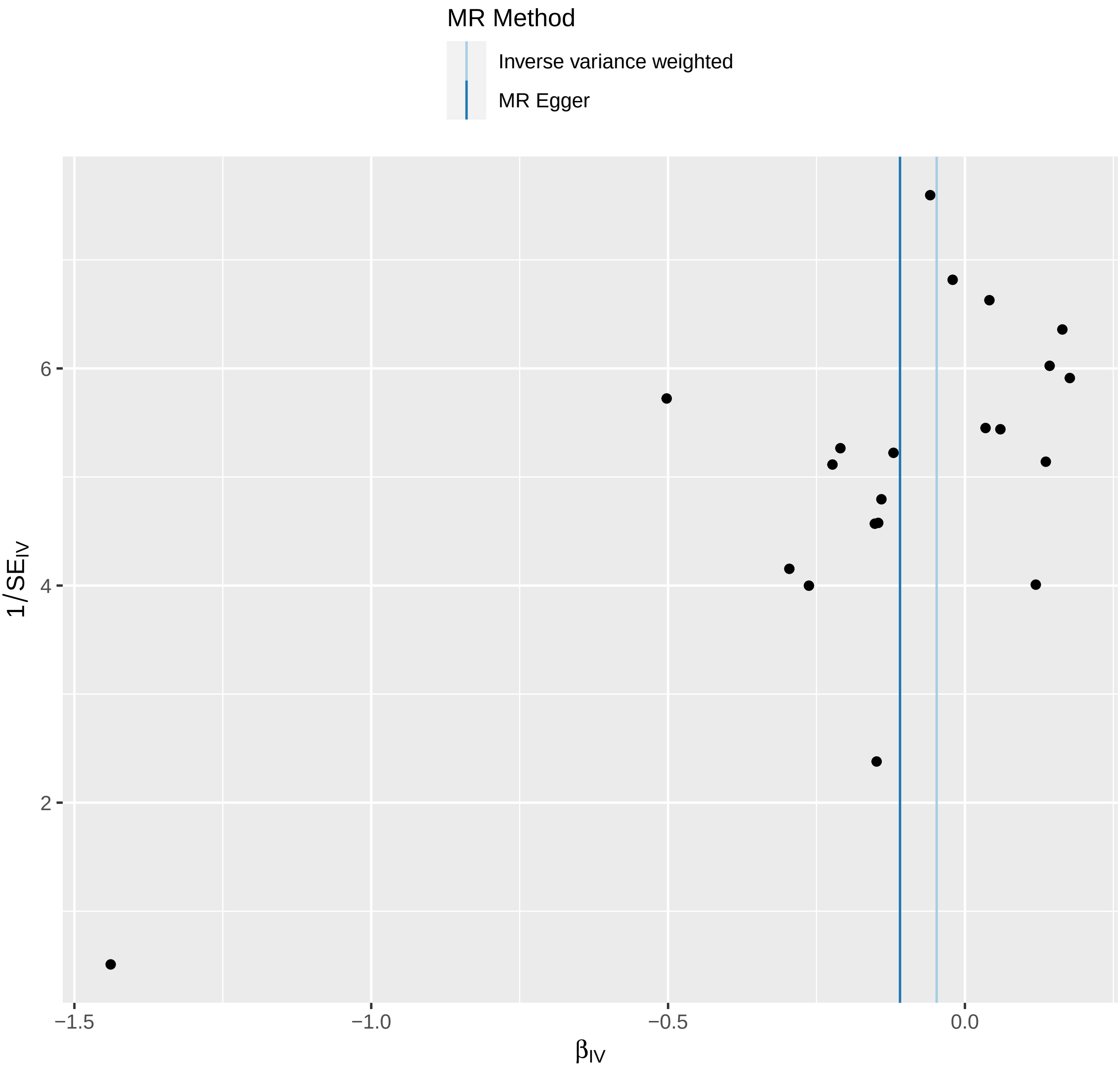

Batch 3−1644 : Killer cell immunoglobulin−like receptor 2DL5A on Diabetic nephropathy (more control exclusions)
with a threshold of 1e−05

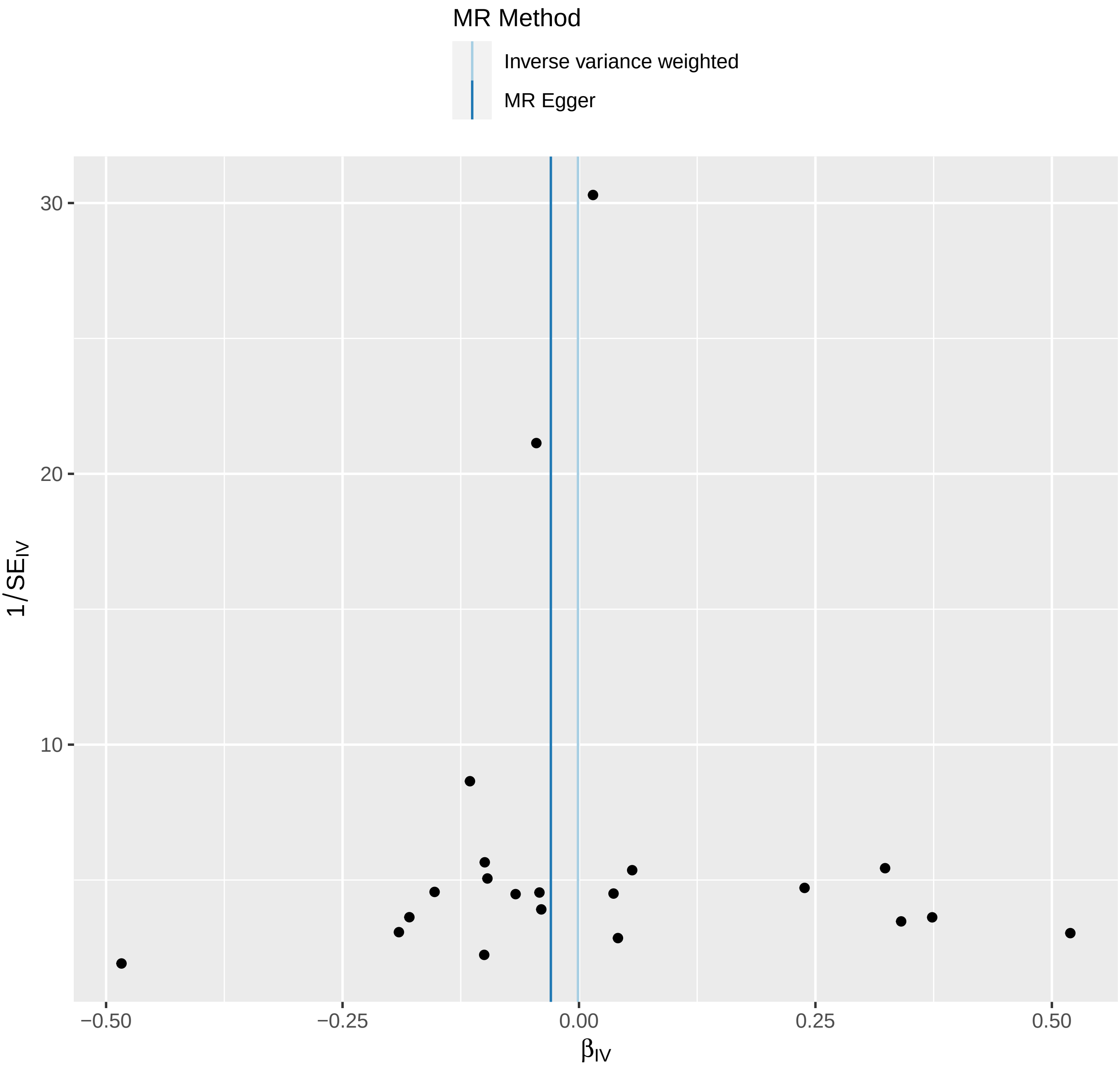

Batch 3−1645 : Killer cell immunoglobulin−like receptor 2DS2 on Diabetic nephropathy (more control exclusions)
with a threshold of 1e−05

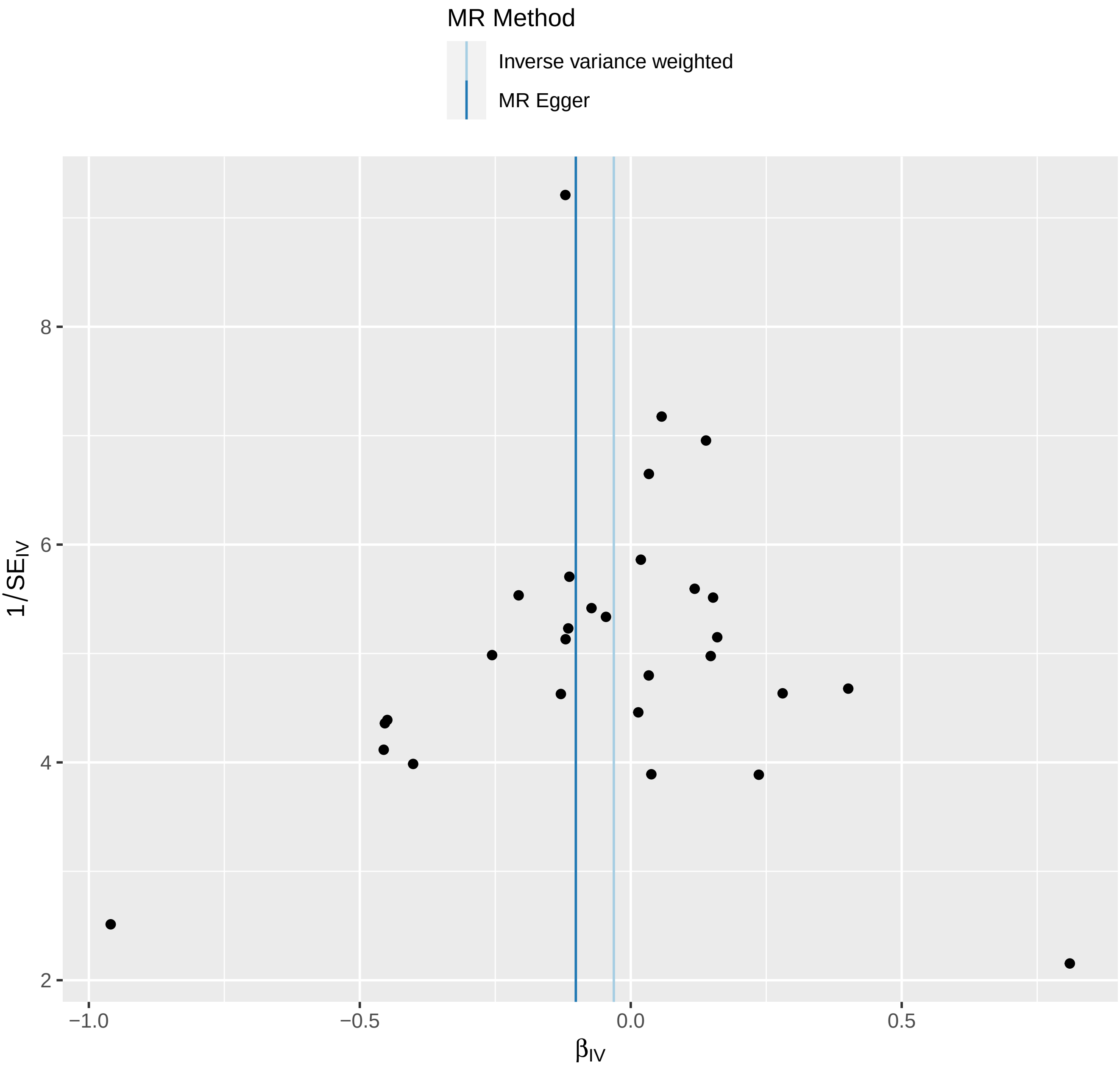

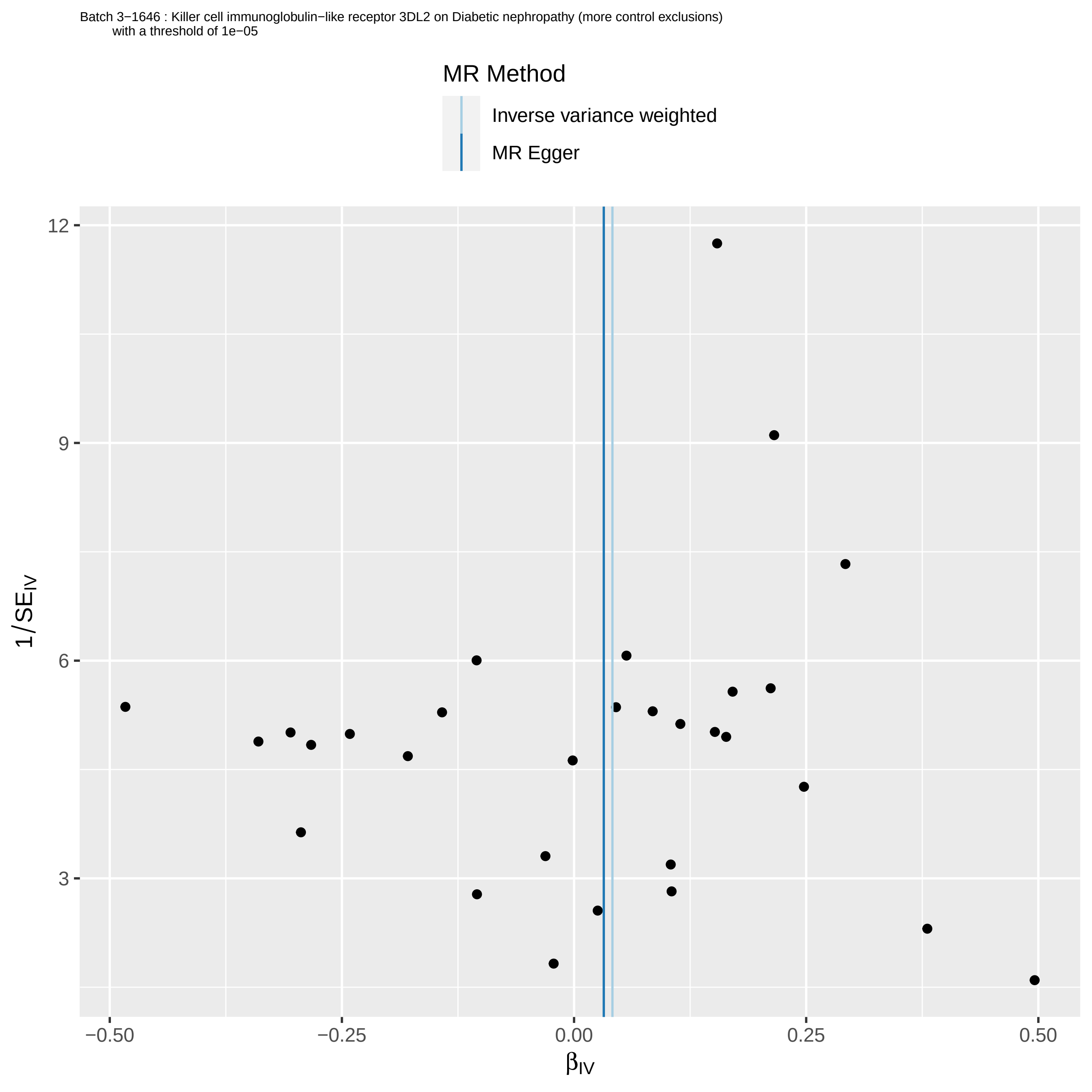

Batch 3−1646 : Killer cell immunoglobulin−like receptor 3DL2 on Diabetic nephropathy (more control exclusions)
with a threshold of 1e−05
MR Method
Inverse variance weighted
MR Egger
12
9
6
3
−0.50
−0.25
0.00
0.25
0.50
1/SE_IV
β_IV

Batch 3−1647 : Killer cell immunoglobulin−like receptor 3DL3 on Diabetic nephropathy (more control exclusions) with a threshold of 1e−05

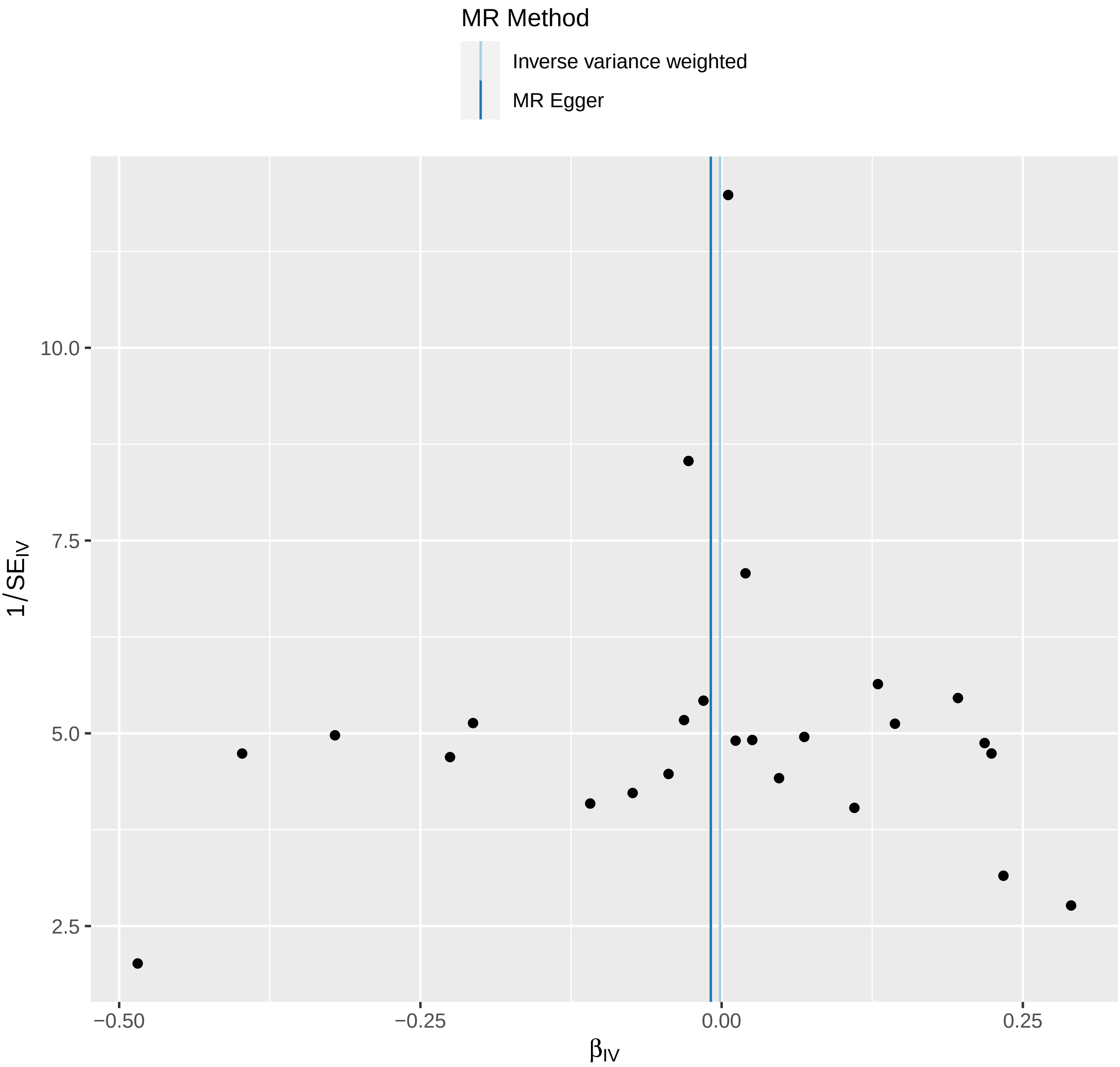

Batch 3−1648 : Killer cell immunoglobulin−like receptor 3DS1 on Diabetic nephropathy (more control exclusions)
with a threshold of 1e−05

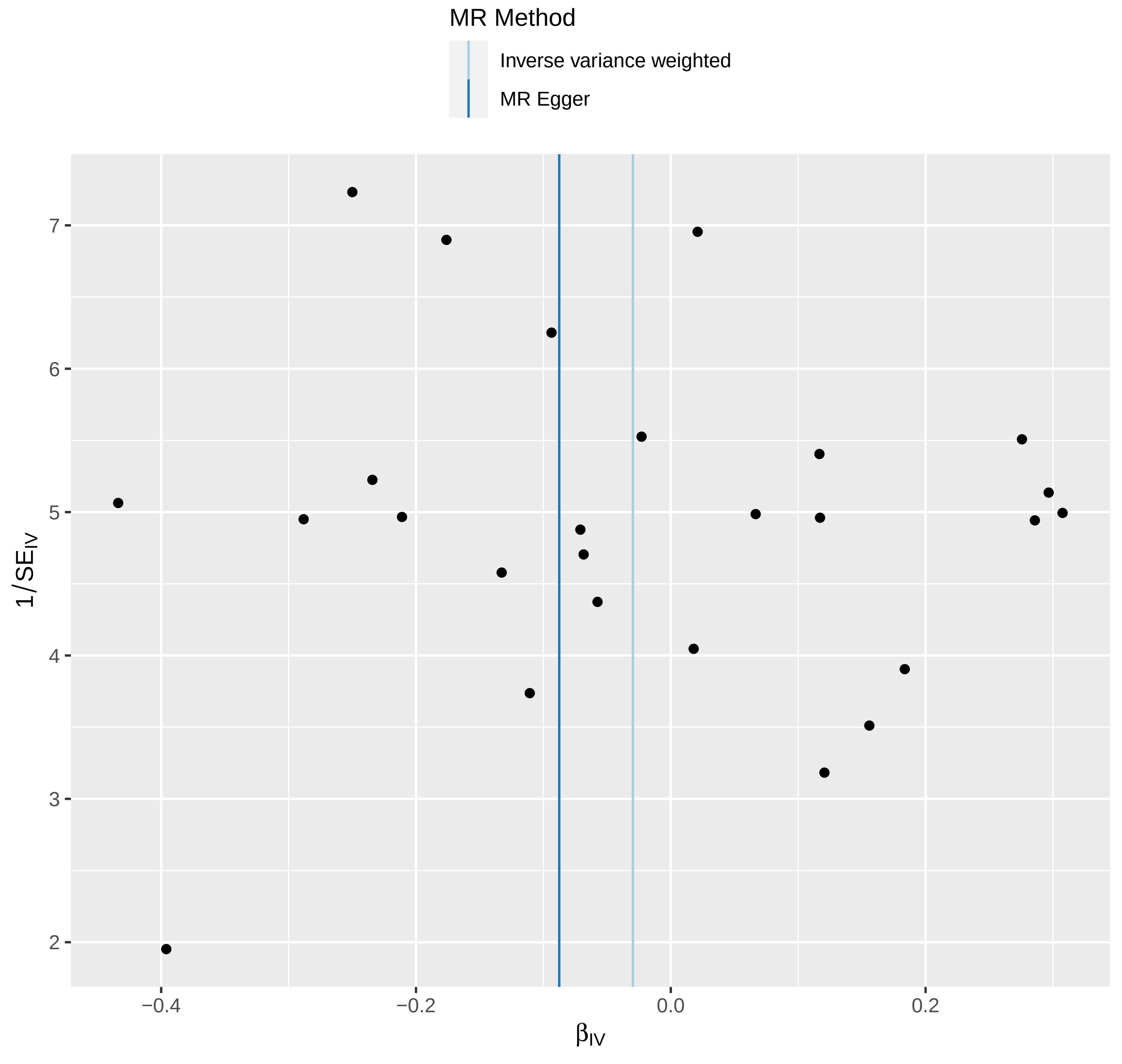

Batch 3−1649 : Kin of IRRE−like protein 2 on Diabetic nephropathy (more control exclusions)
with a threshold of 1e−05

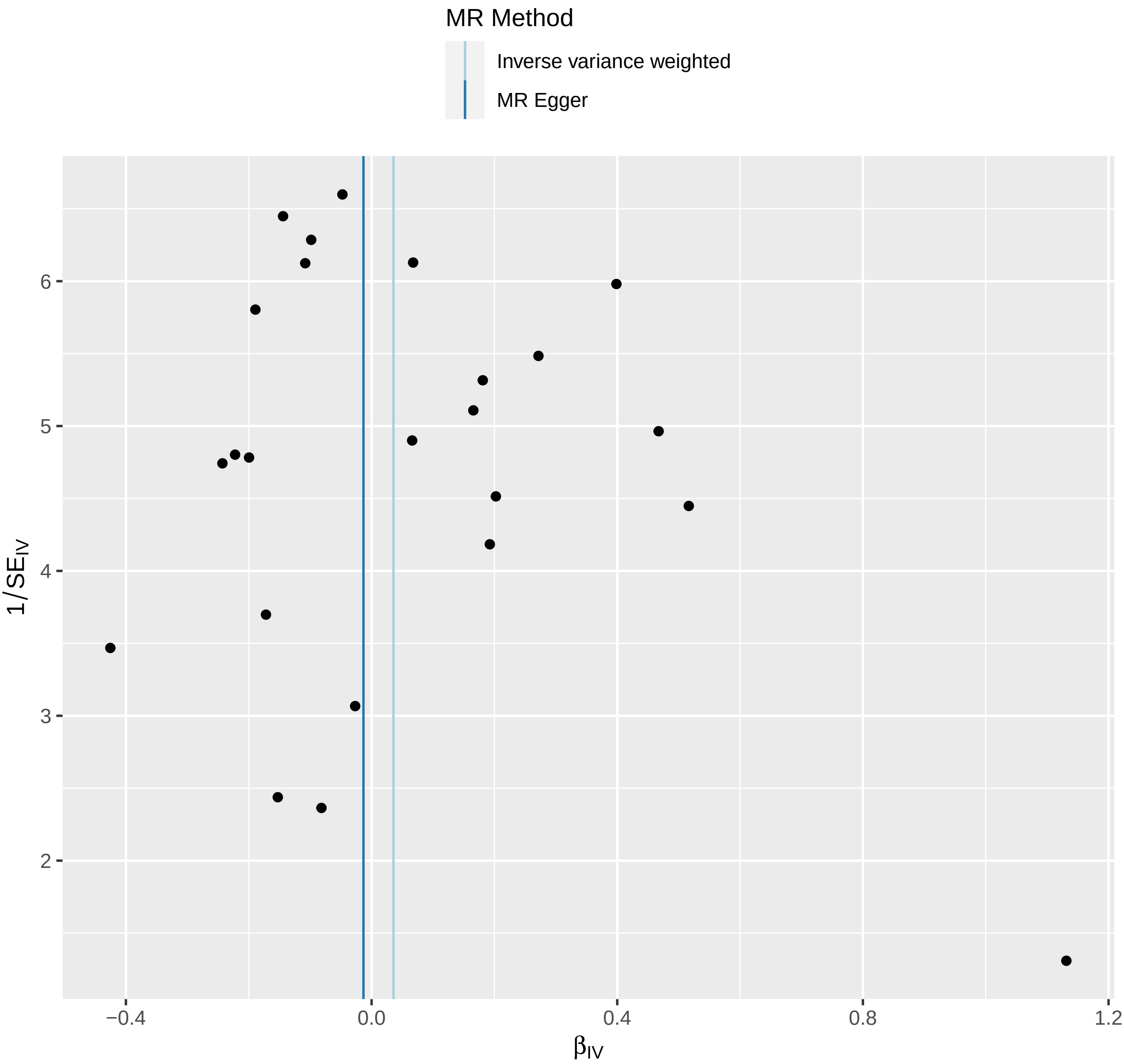

Batch 3−1650 : Kin of IRRE−like protein 2 on Diabetic nephropathy (more control exclusions)
with a threshold of 1e−05

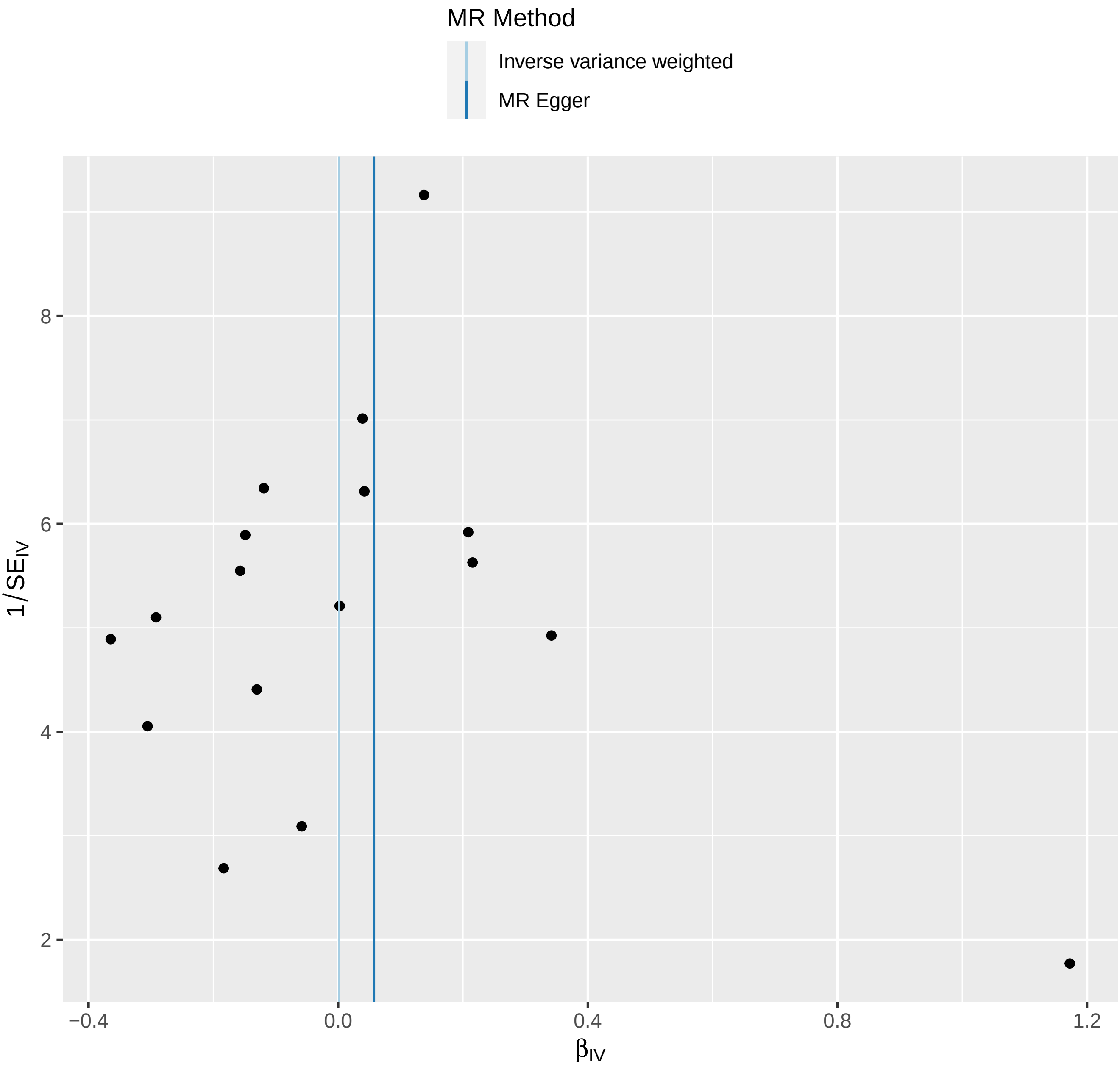

Batch 3−1651 : Kin of IRRE−like protein 3 on Diabetic nephropathy (more control exclusions)
with a threshold of 1e−05

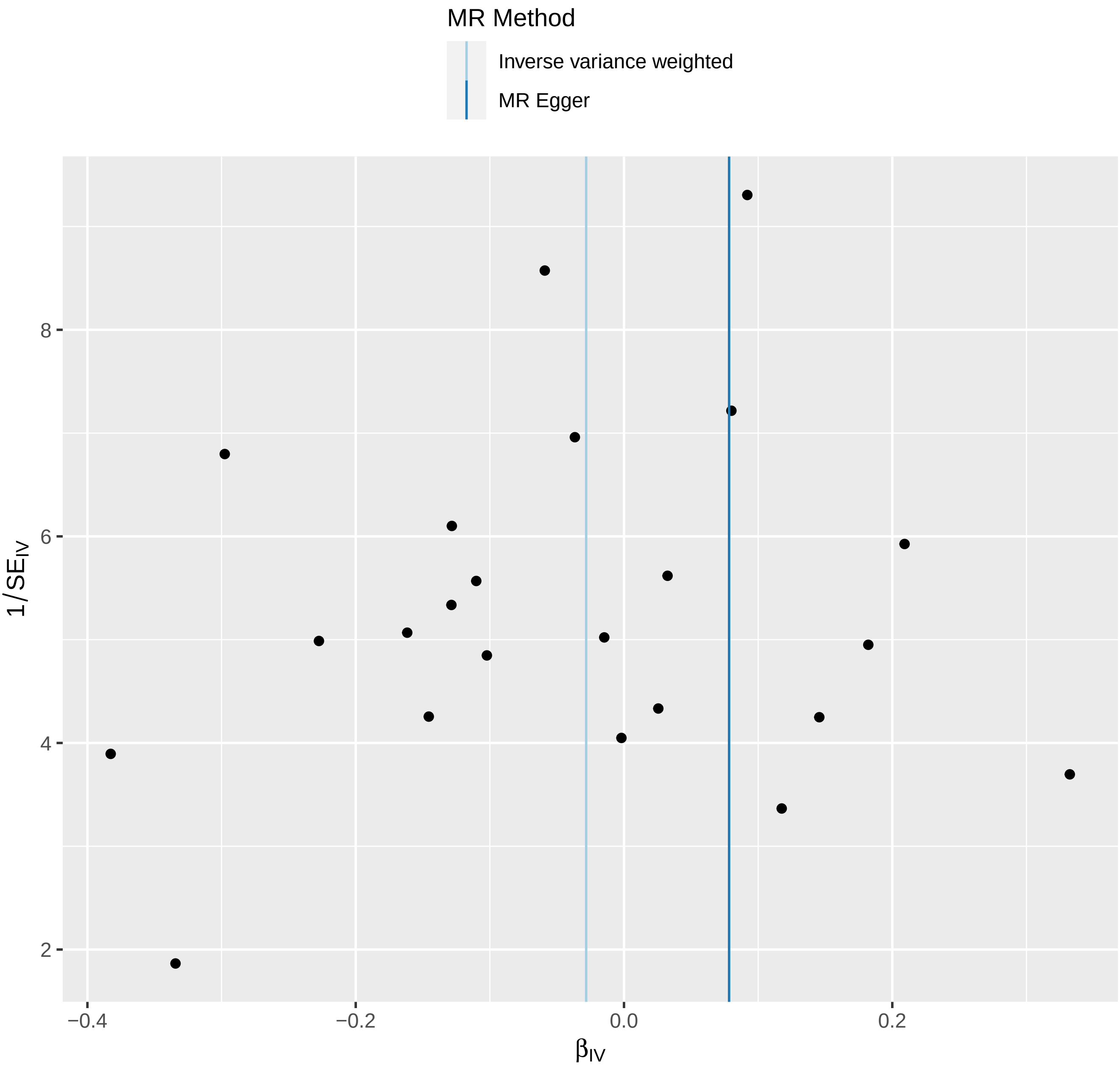

Batch 3−1652 : Mast/stem cell growth factor receptor Kit on Diabetic nephropathy (more control exclusions)
with a threshold of 1e−05

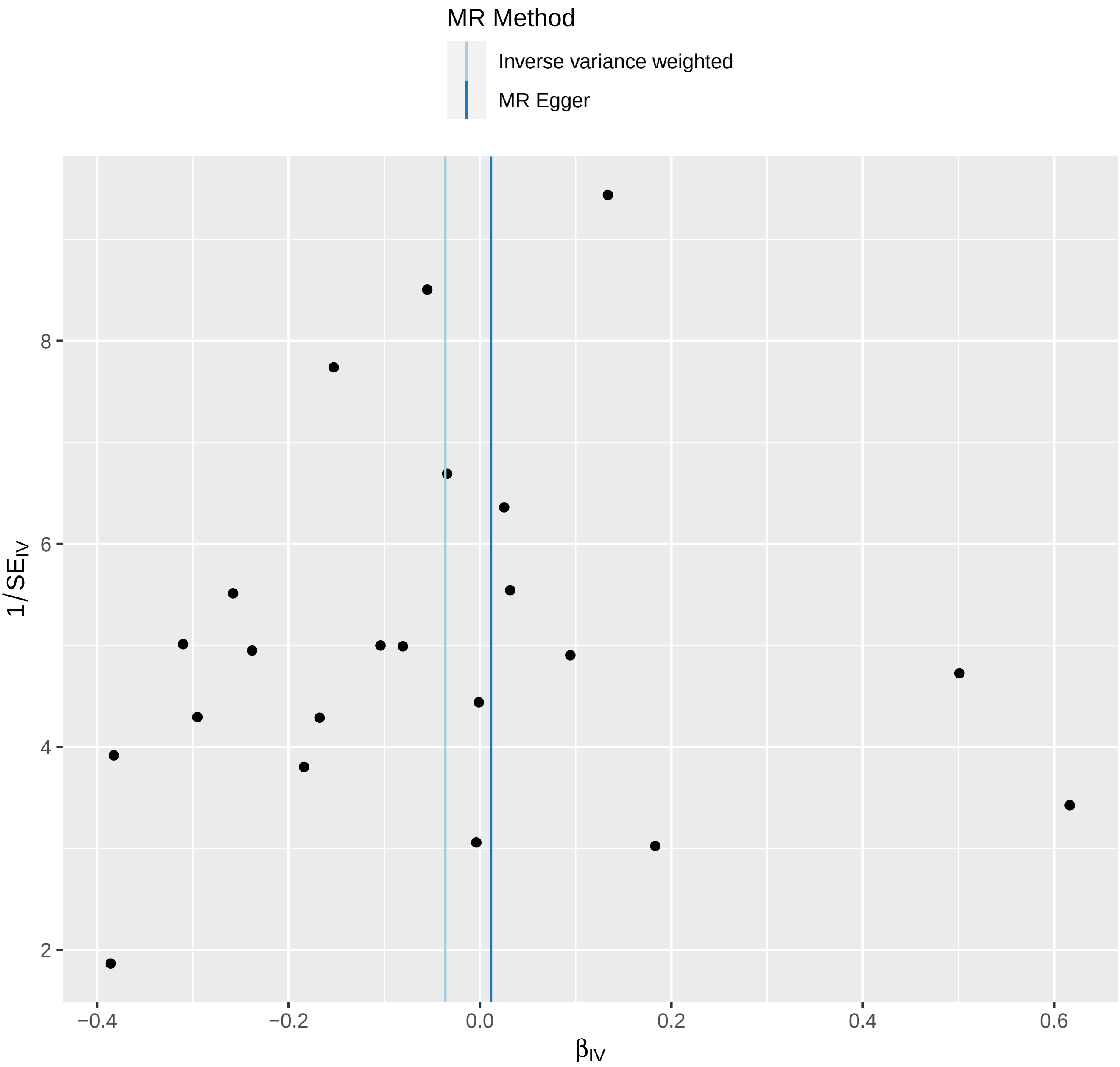

Batch 3−1653 : Kit ligand on Diabetic nephropathy (more control exclusions)
with a threshold of 1e−05

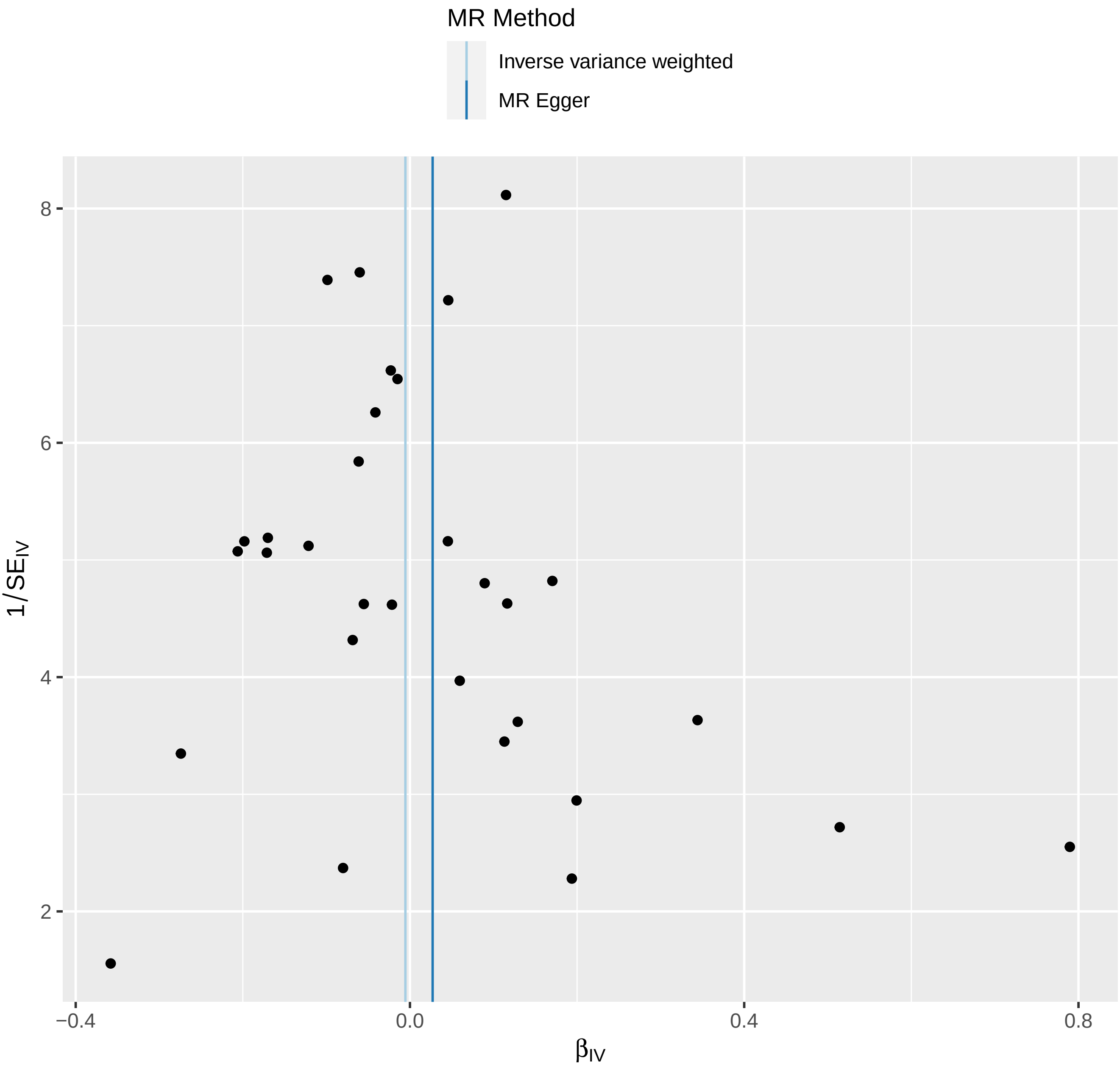

Batch 3−1654 : Kelch−like protein 12 on Diabetic nephropathy (more control exclusions)
with a threshold of 1e−05

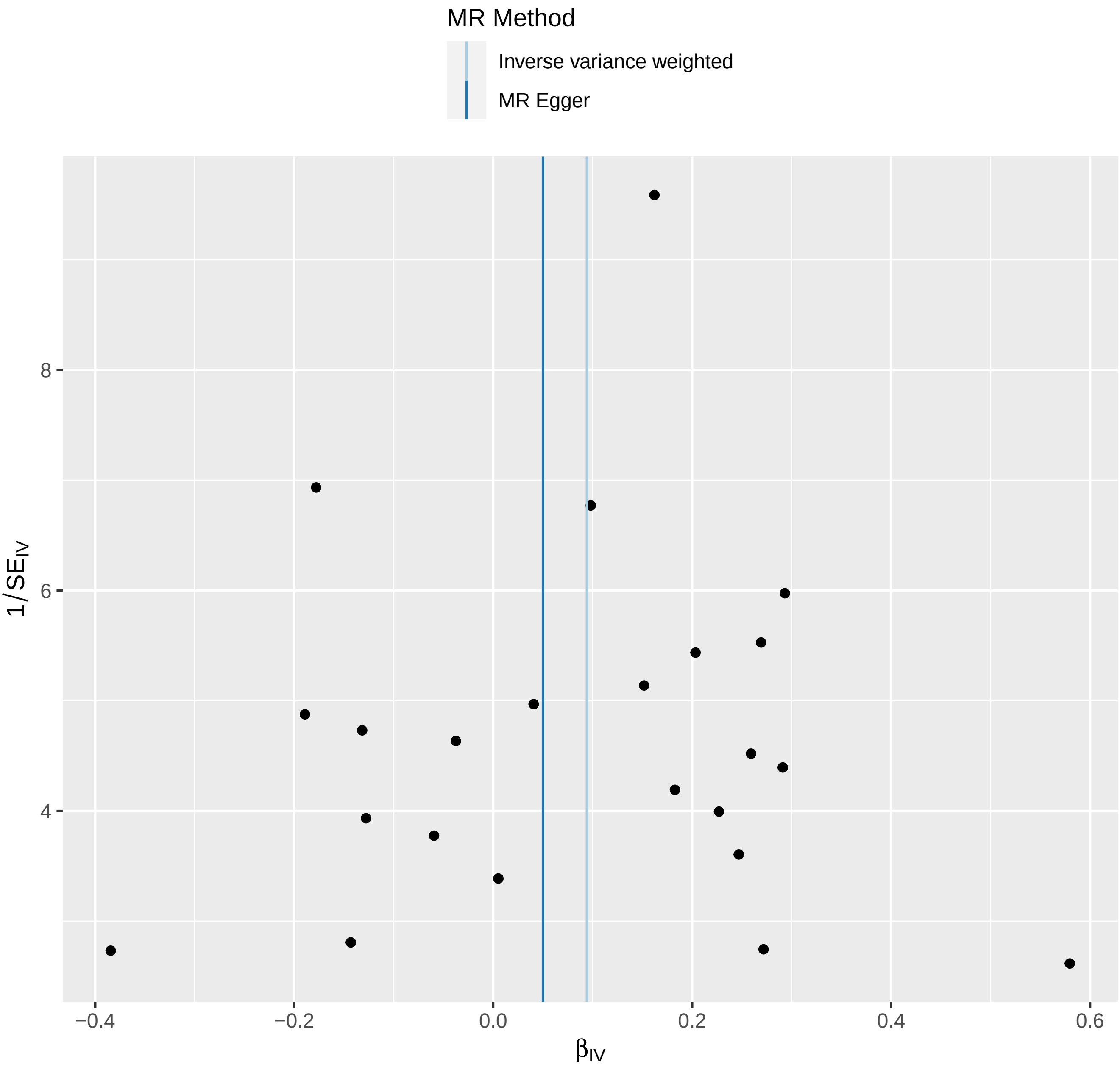

Batch 3−1655 : Kelch−like protein 13 on Diabetic nephropathy (more control exclusions)
with a threshold of 1e−05

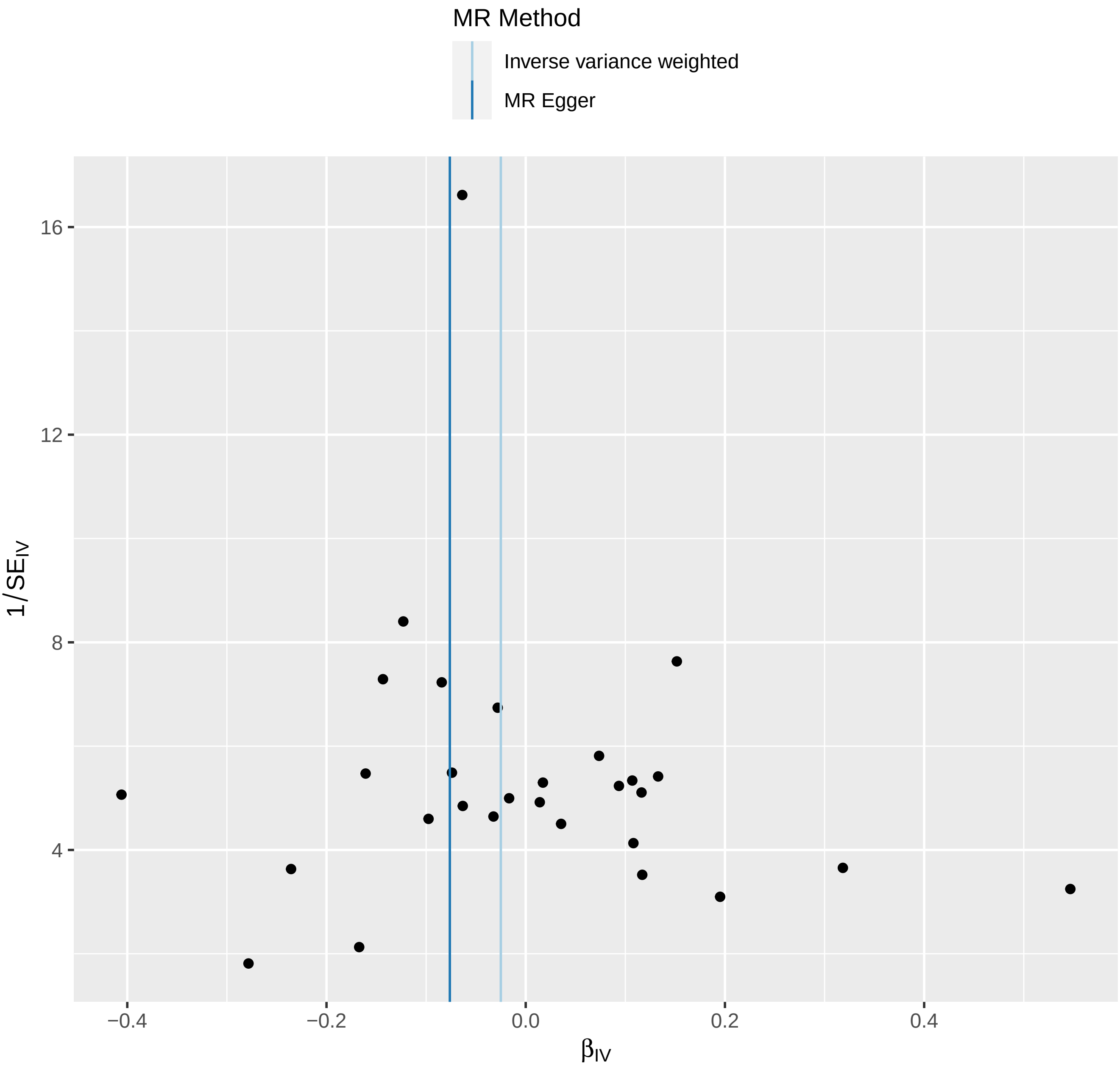

Batch 3−1656 : Kallikrein−11 on Diabetic nephropathy (more control exclusions)
with a threshold of 1e−05

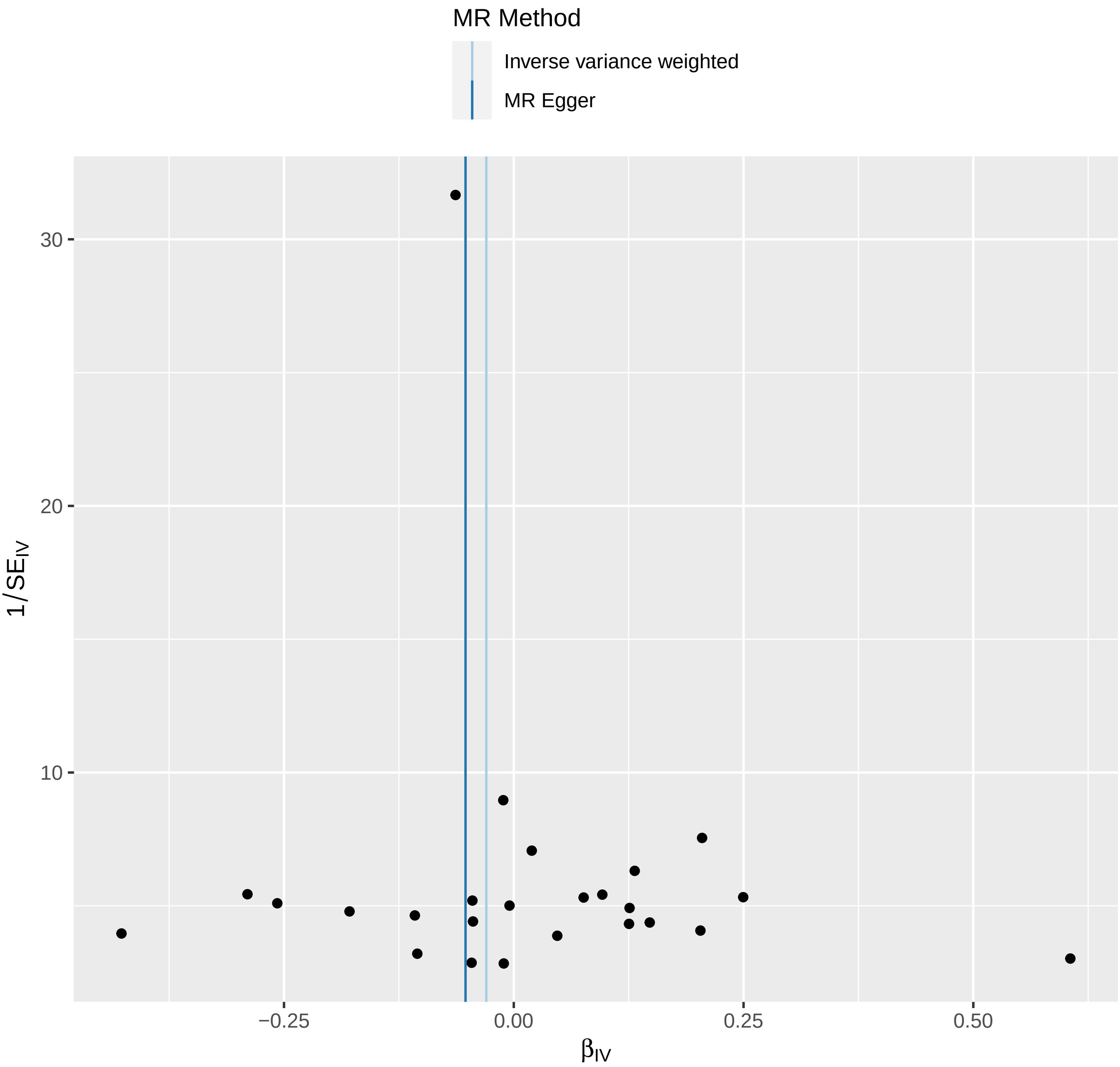

Batch 3−1657 : Kallikrein−12 on Diabetic nephropathy (more control exclusions)
with a threshold of 1e−05

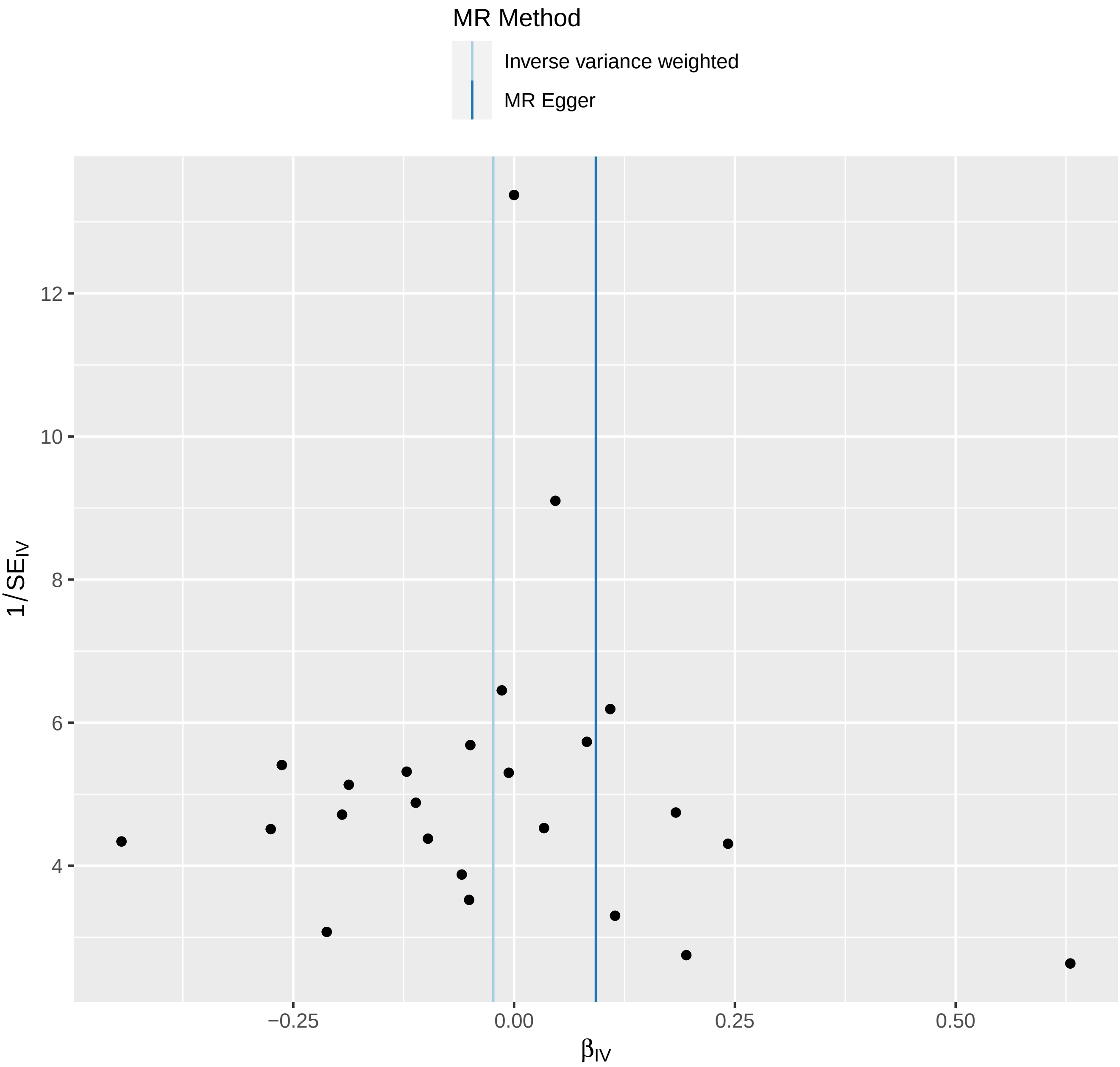

Batch 3−1658 : Kallikrein−14 on Diabetic nephropathy (more control exclusions)
with a threshold of 1e−05

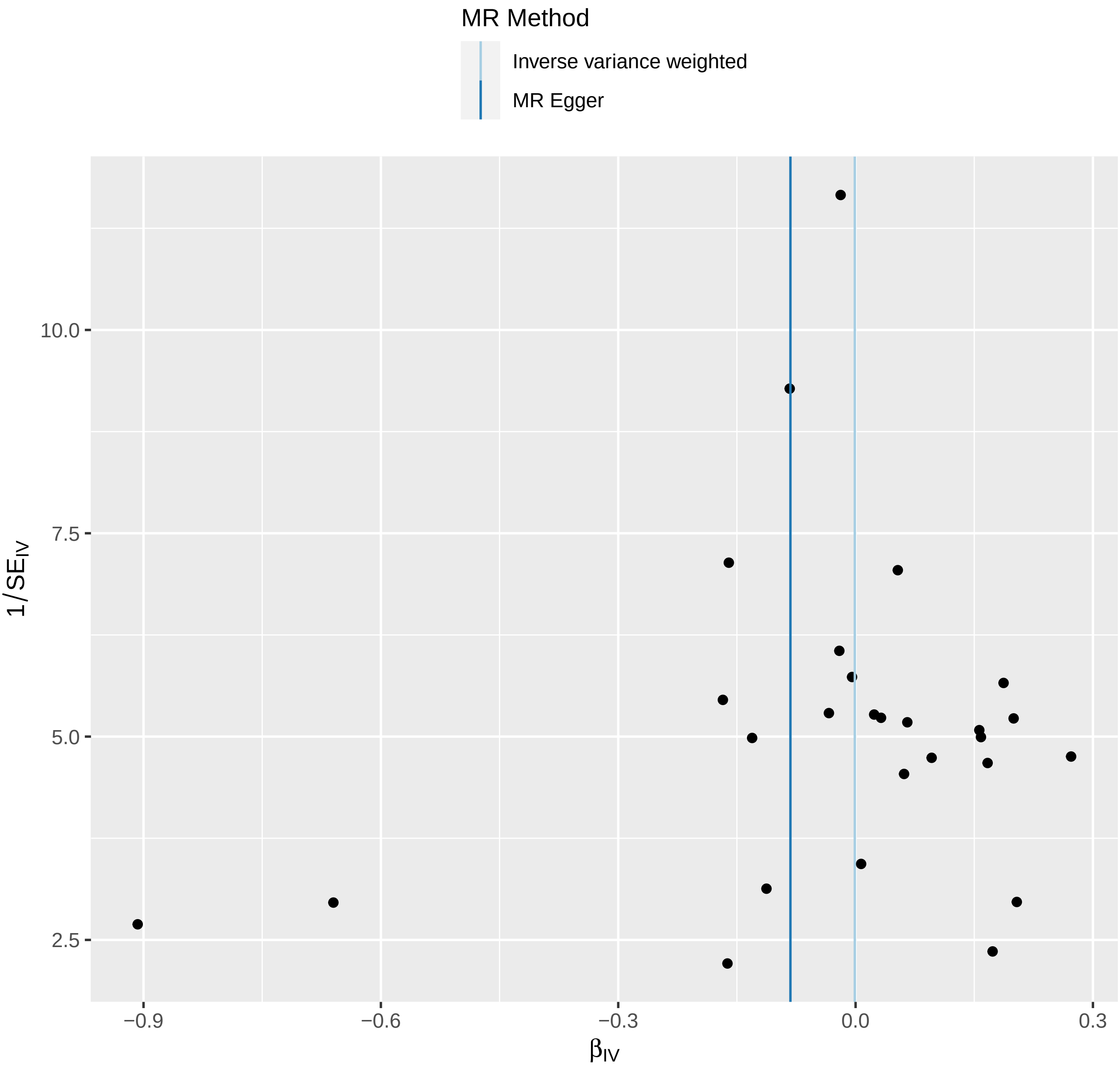

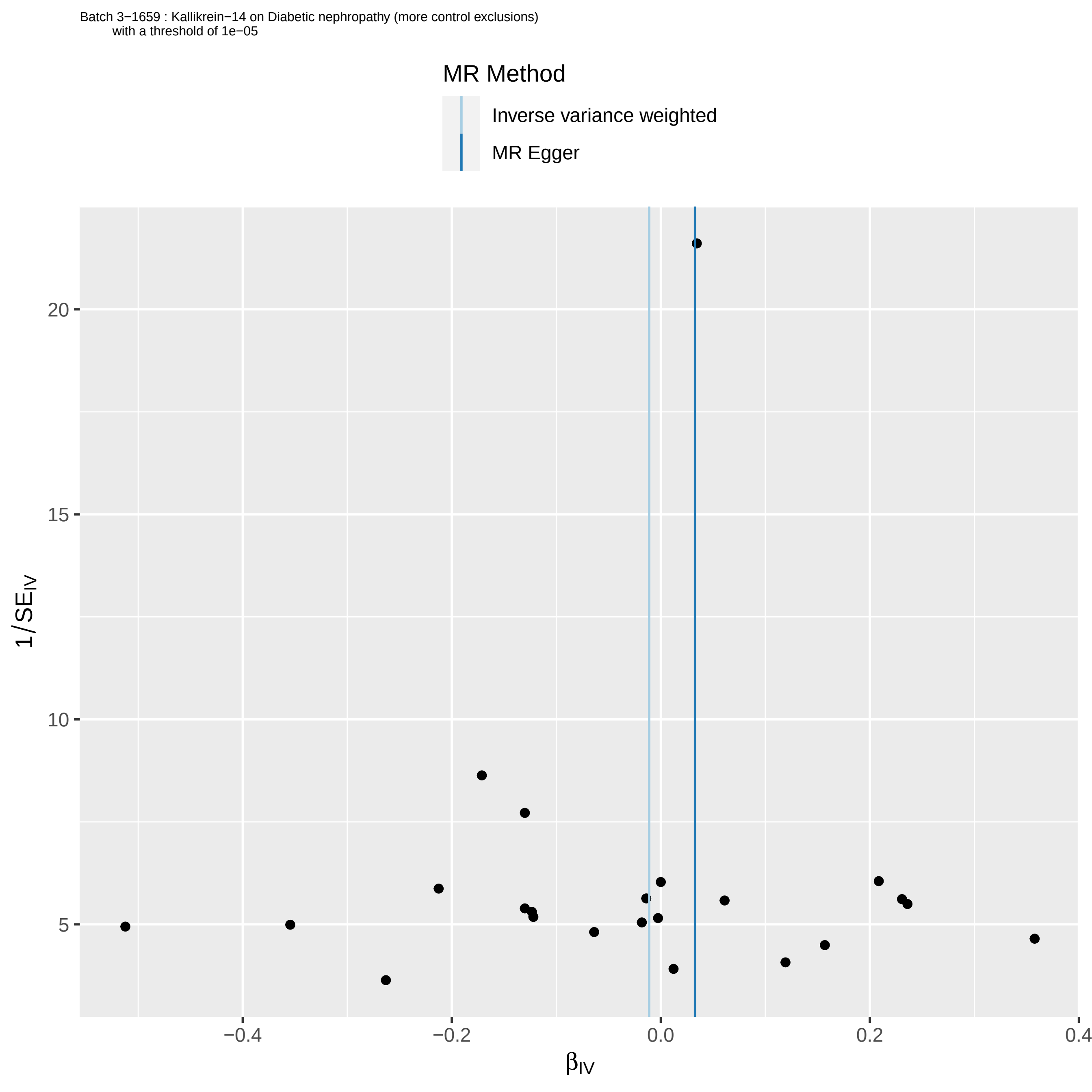

Batch 3−1659 : Kallikrein−14 on Diabetic nephropathy (more control exclusions)
with a threshold of 1e−05
MR Method
Inverse variance weighted
MR Egger
20
15
10
5
1/SE_IV
−0.4
−0.2
0.0
0.2
0.4
β_IV

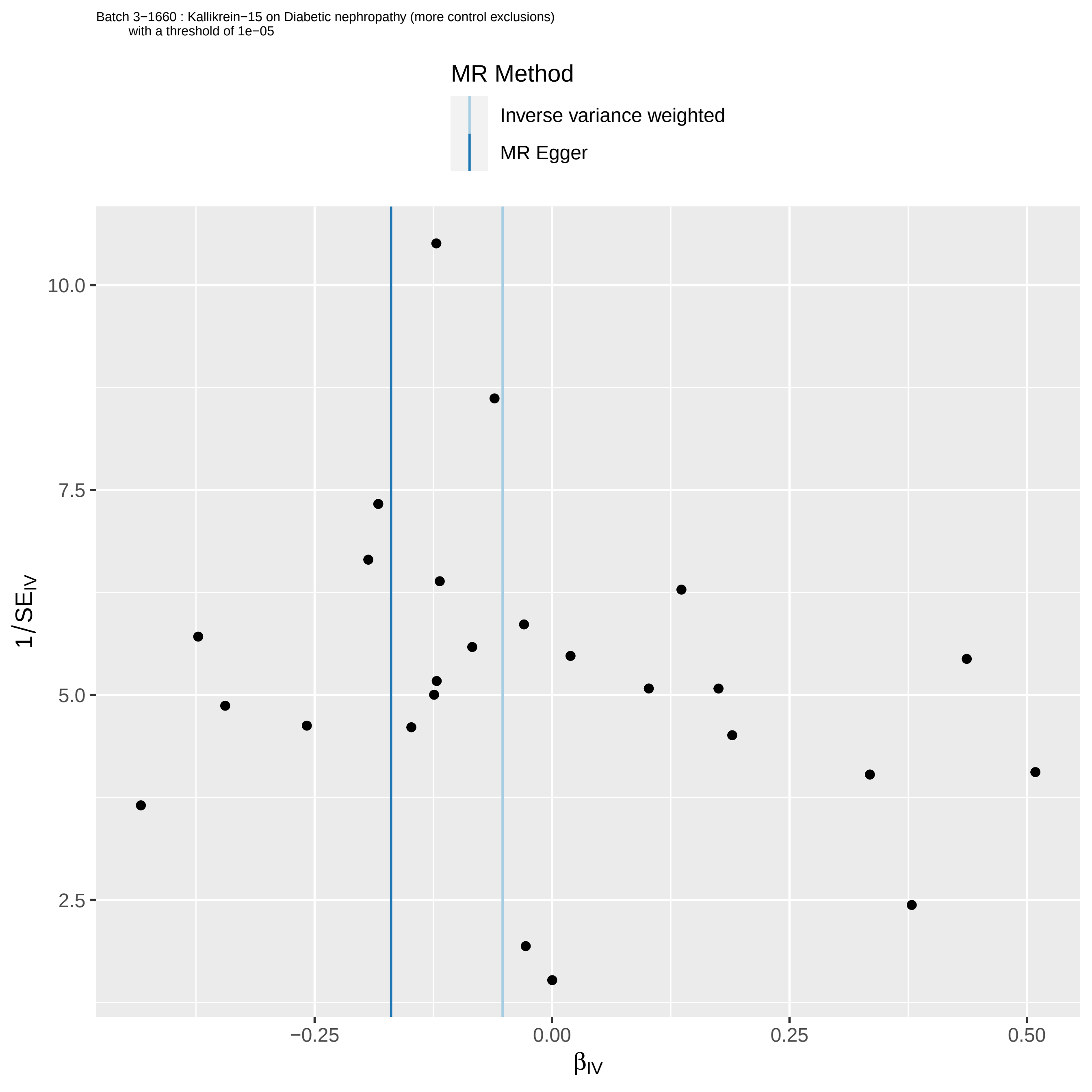

Batch 3−1660 : Kallikrein−15 on Diabetic nephropathy (more control exclusions)
with a threshold of 1e−05
MR Method
Inverse variance weighted
MR Egger
10.0
7.5
5.0
2.5
1/SE$_{IV}$
−0.25
0.00
0.25
0.50
β$_{IV}$

Batch 3−1661 : Prostate−specific antigen on Diabetic nephropathy (more control exclusions)
with a threshold of 1e−05

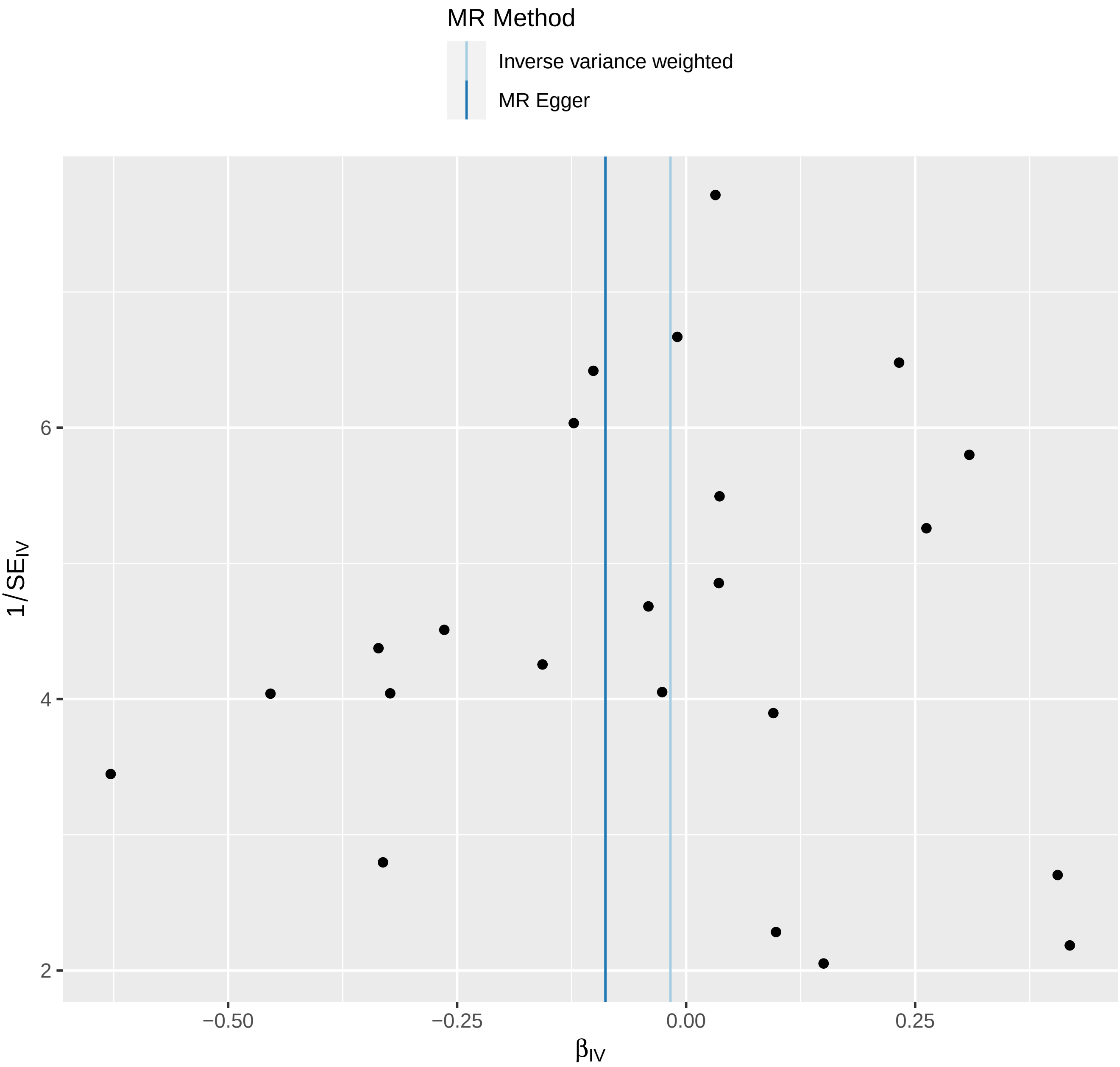

Batch 3−1662 : Kallikrein−4 on Diabetic nephropathy (more control exclusions)
with a threshold of 1e−05

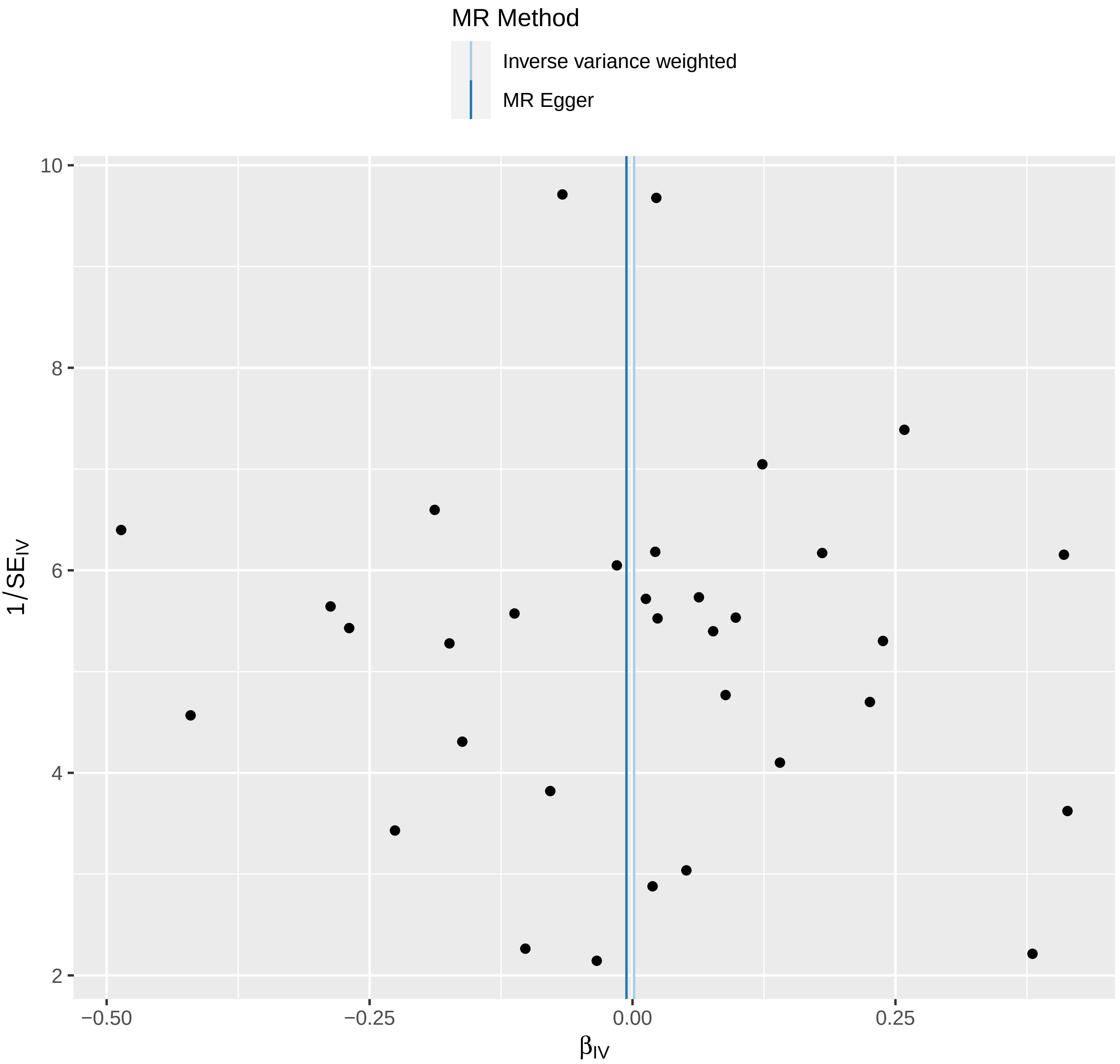

Batch 3−1663 : Kallikrein−5 on Diabetic nephropathy (more control exclusions)
with a threshold of 1e−05

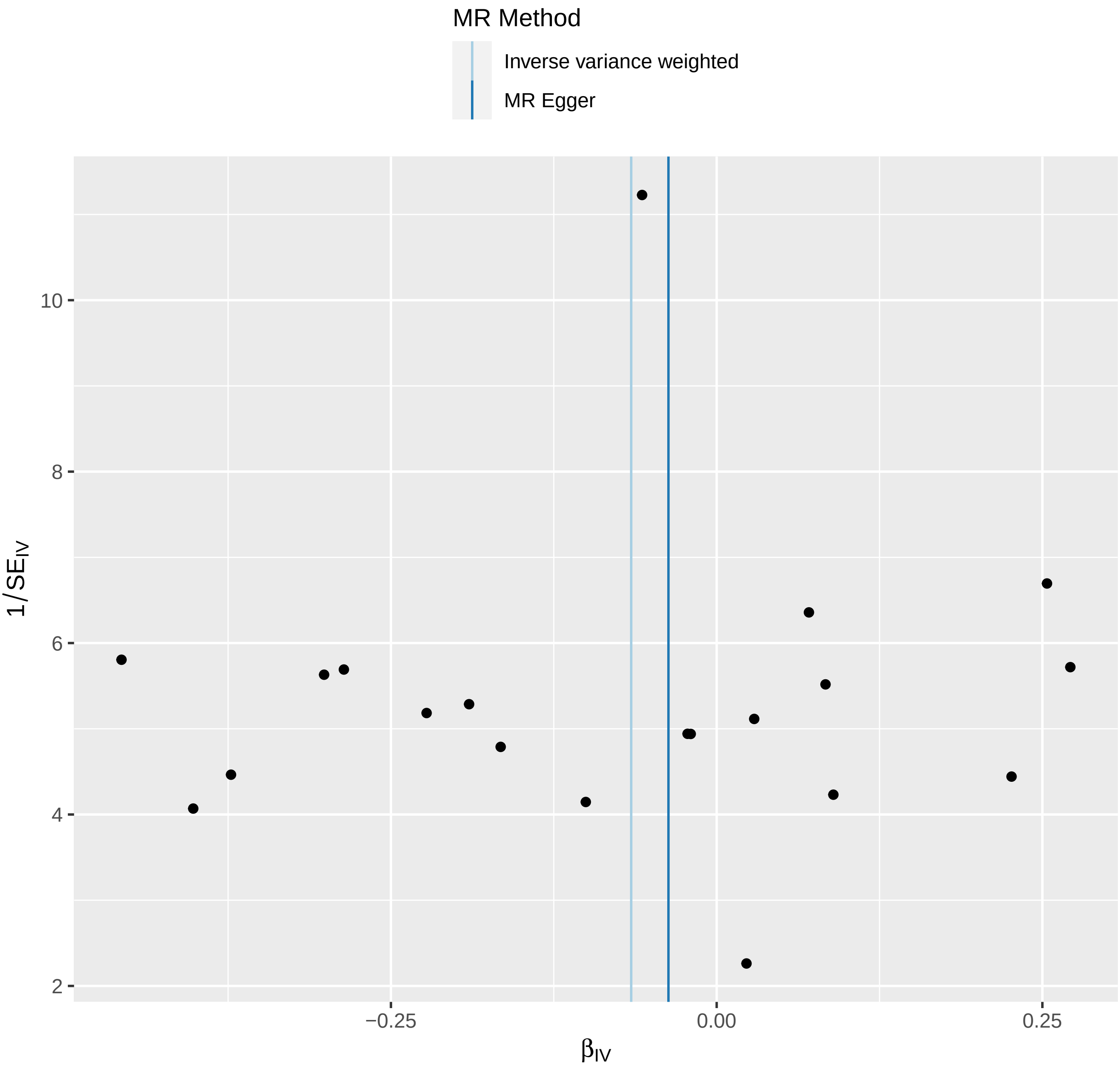

Batch 3−1664 : Kallikrein−5 on Diabetic nephropathy (more control exclusions)
with a threshold of 1e−05

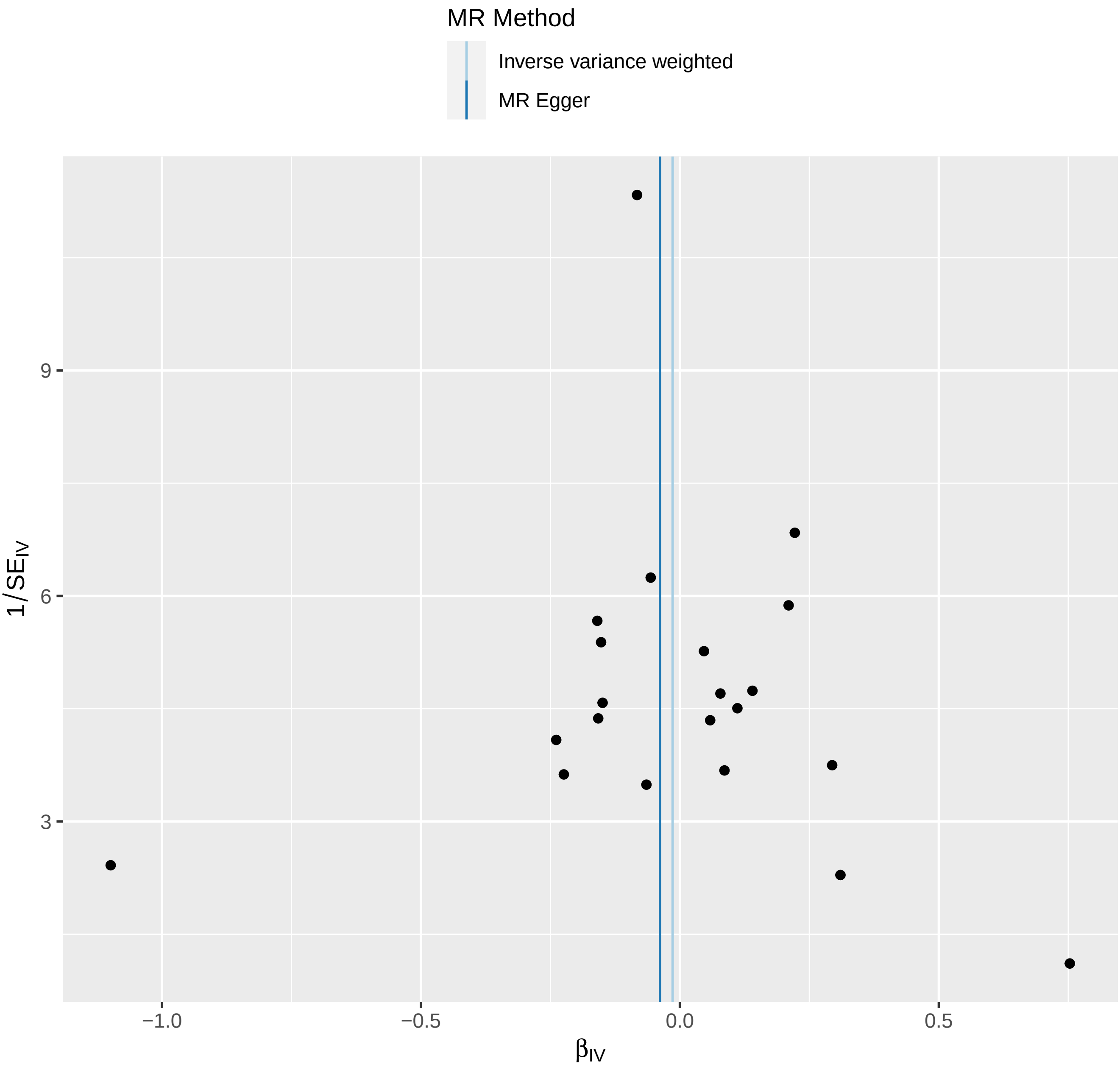

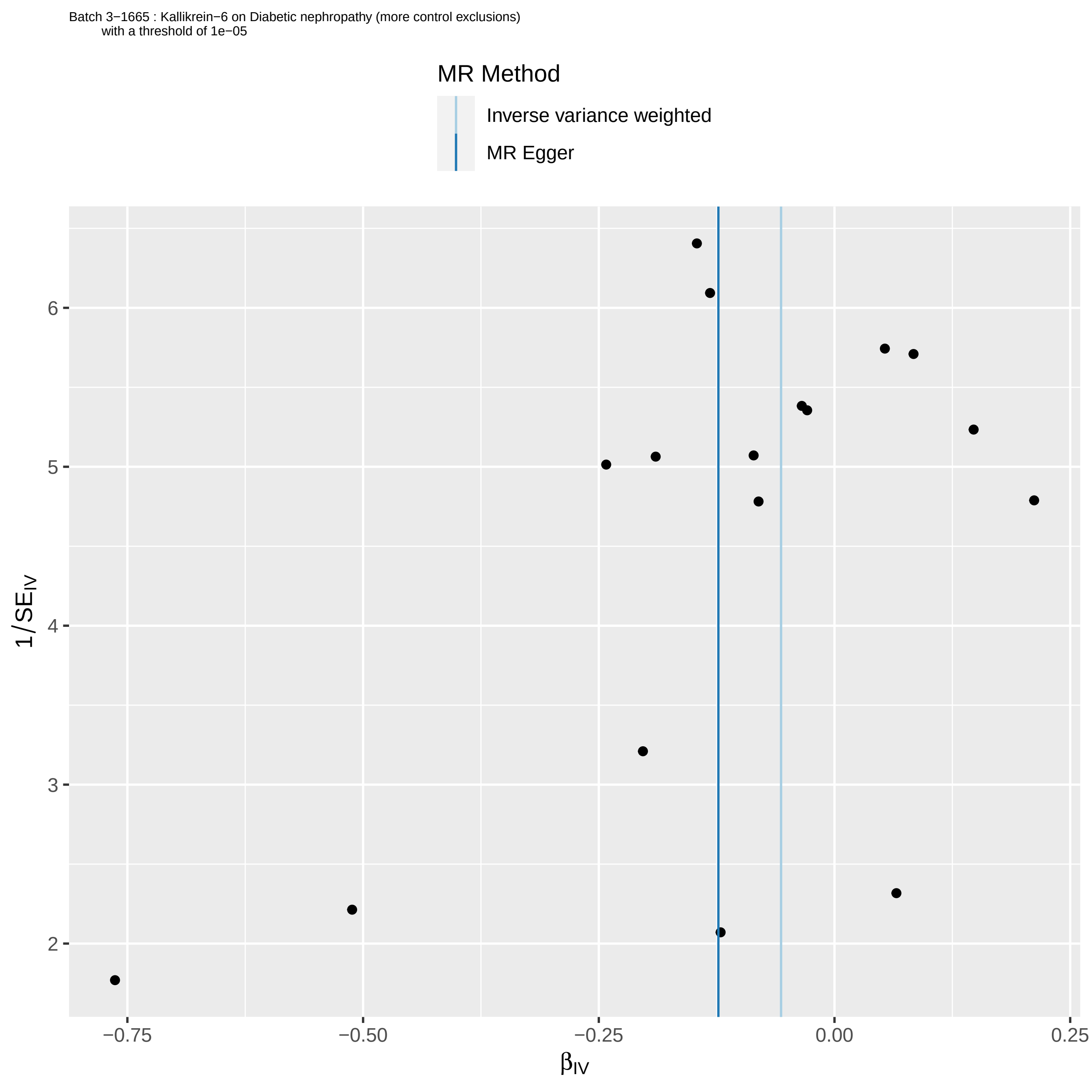
Batch 3−1665 : Kallikrein−6 on Diabetic nephropathy (more control exclusions)
with a threshold of 1e−05
MR Method
Inverse variance weighted
MR Egger
1/SE_IV
β_IV
−0.75
−0.50
−0.25
0.00
0.25
2
3
4
5
6

Batch 3−1666 : Kallikrein−7 on Diabetic nephropathy (more control exclusions)
with a threshold of 1e−05

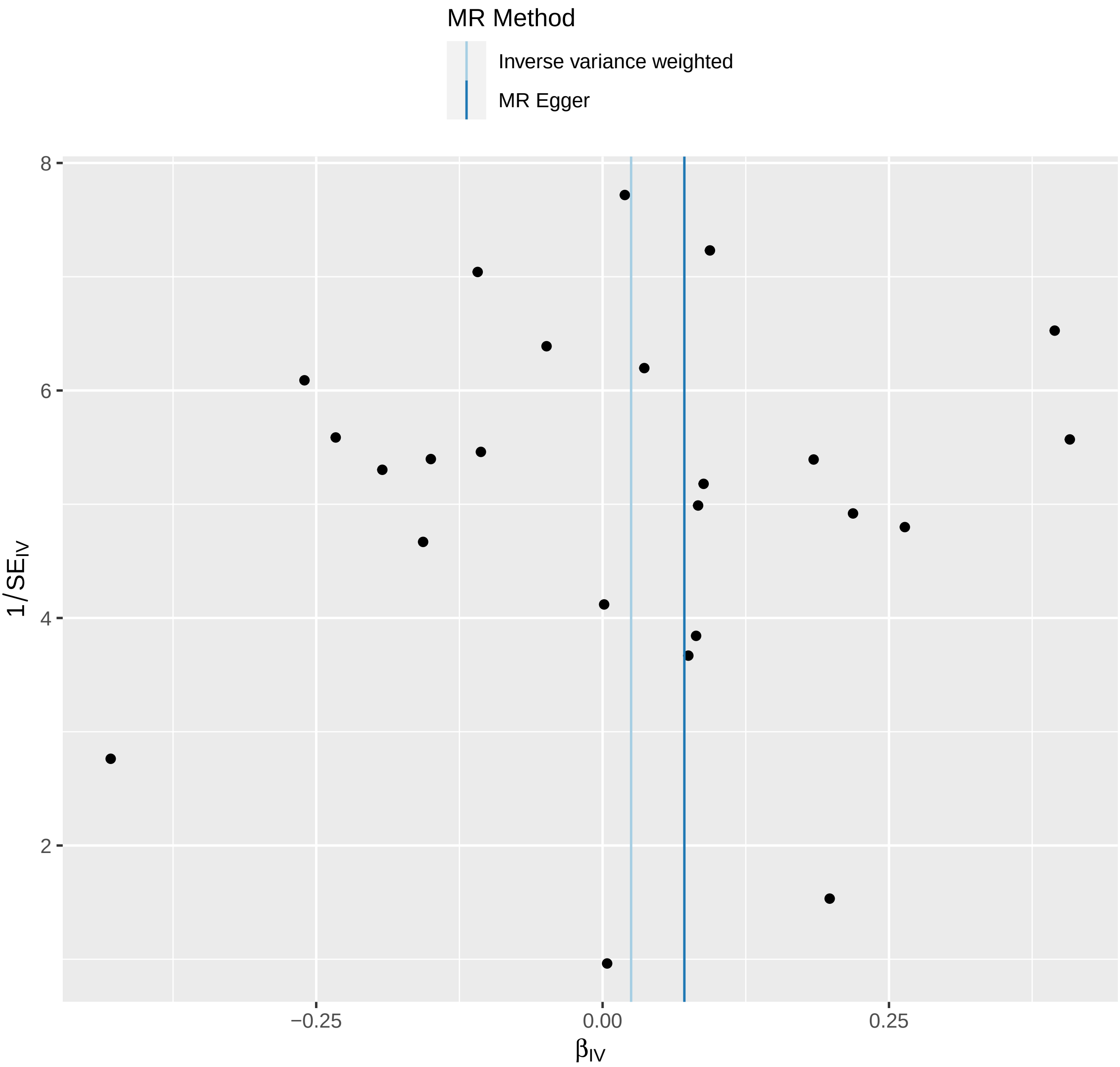

Batch 3−1667 : Kallikrein−8 on Diabetic nephropathy (more control exclusions)
with a threshold of 1e−05

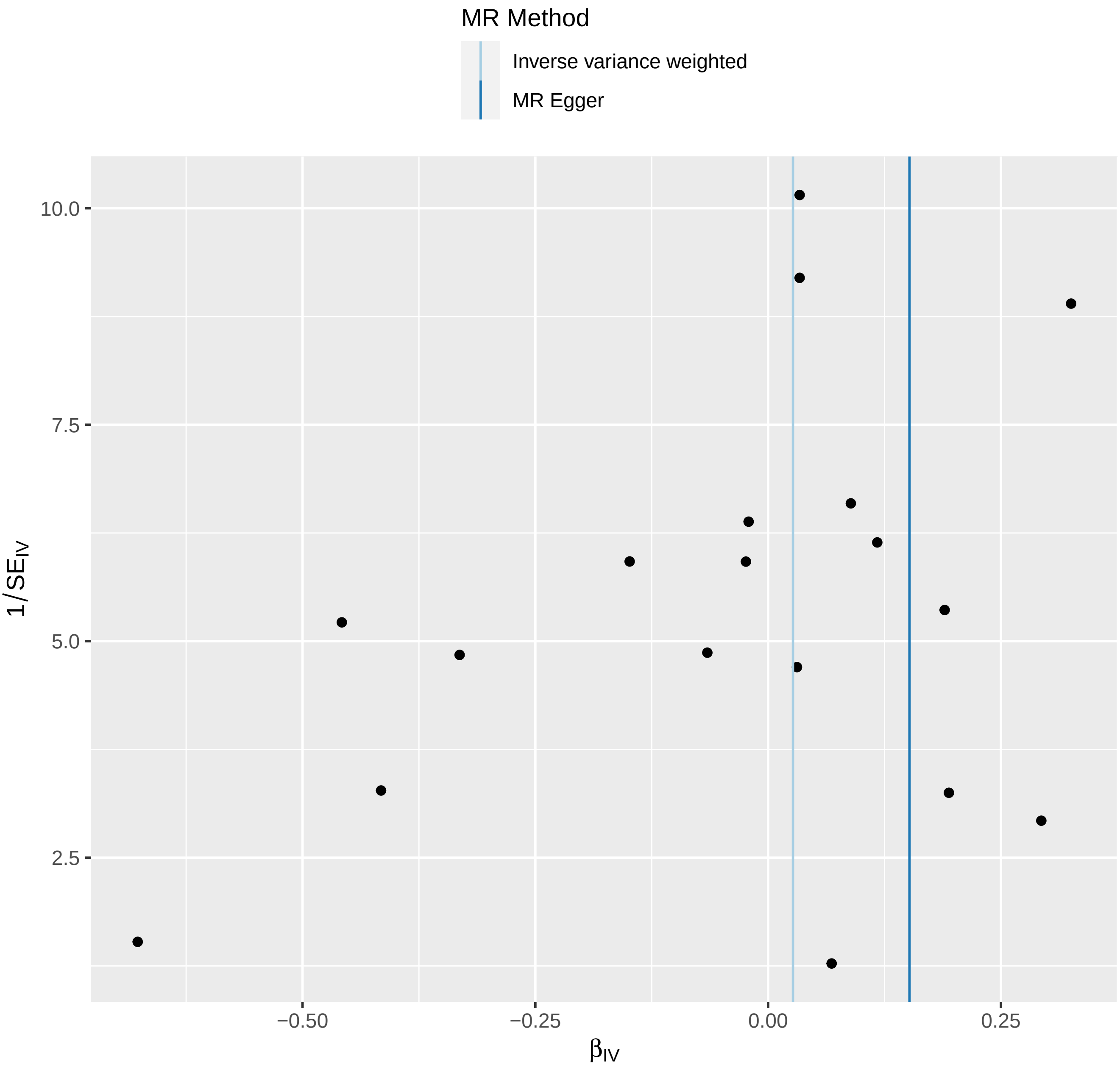

Batch 3−1668 : Kallikrein−8 on Diabetic nephropathy (more control exclusions)
with a threshold of 1e−05

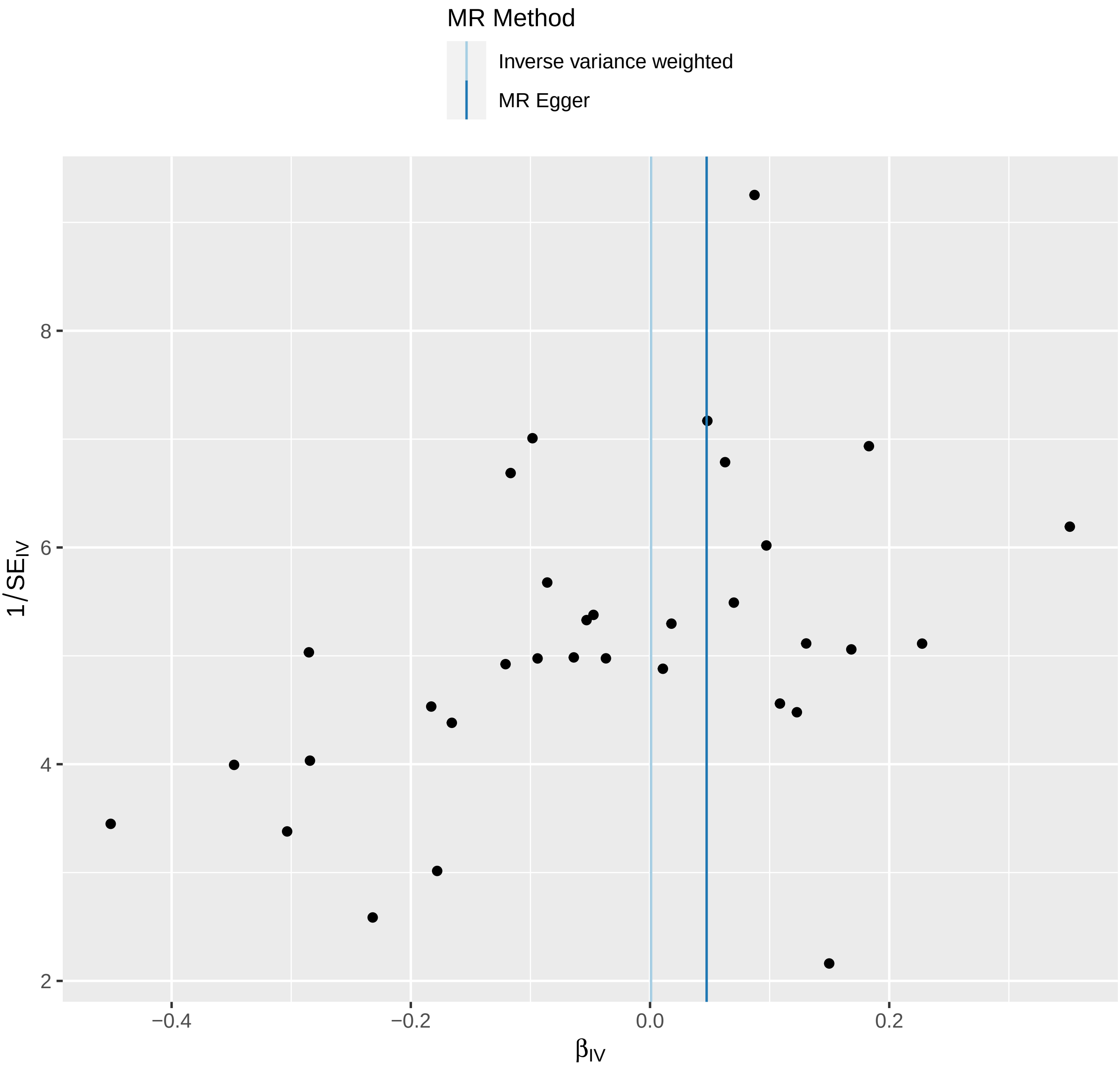

Batch 3−1669 : NKG2−A/NKG2−B type II integral membrane protein on Diabetic nephropathy (more control exclusions) with a threshold of 1e−05

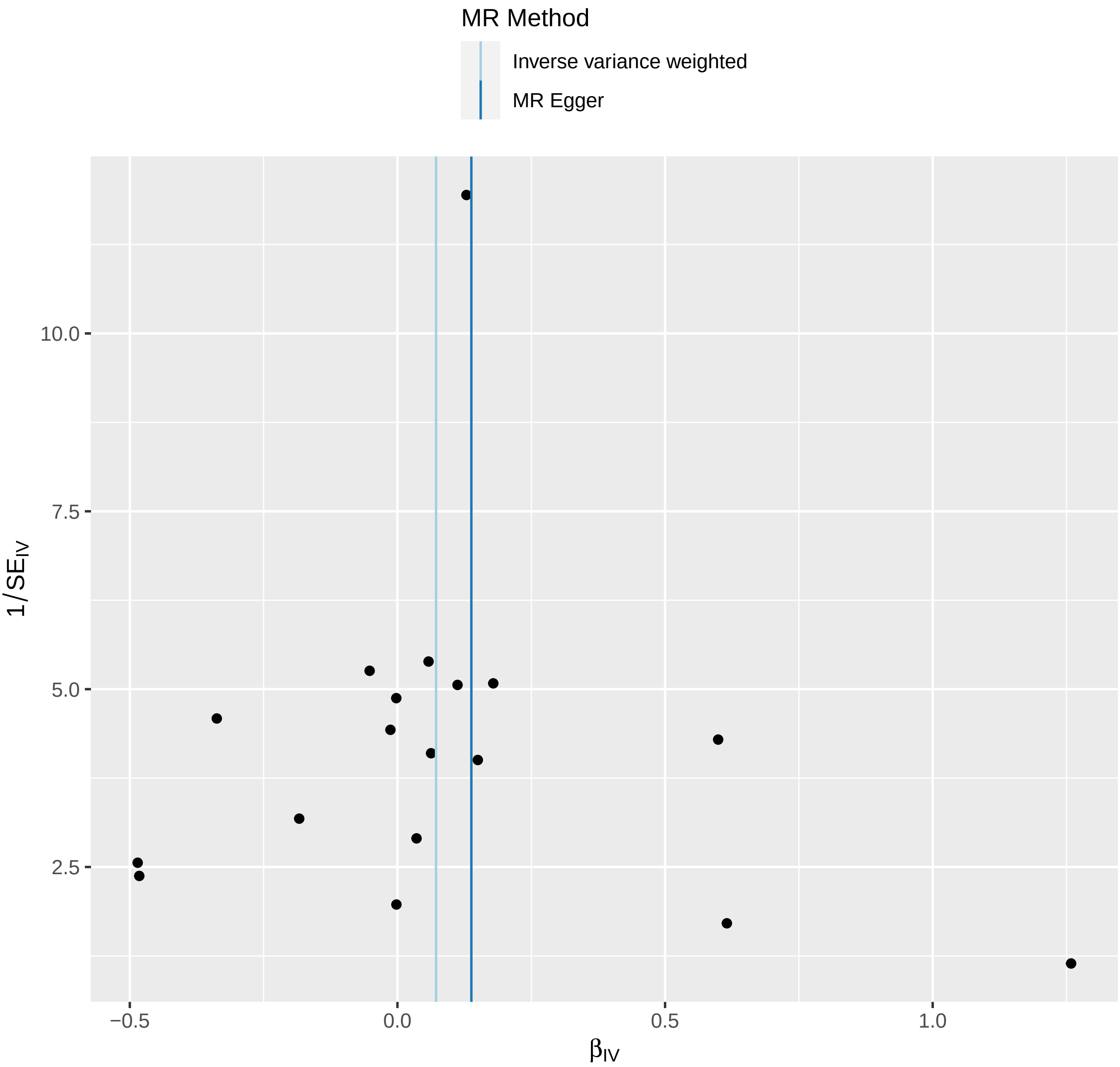

Batch 3−1670 : NKG2−E type II integral membrane protein on Diabetic nephropathy (more control exclusions)
with a threshold of 1e−05

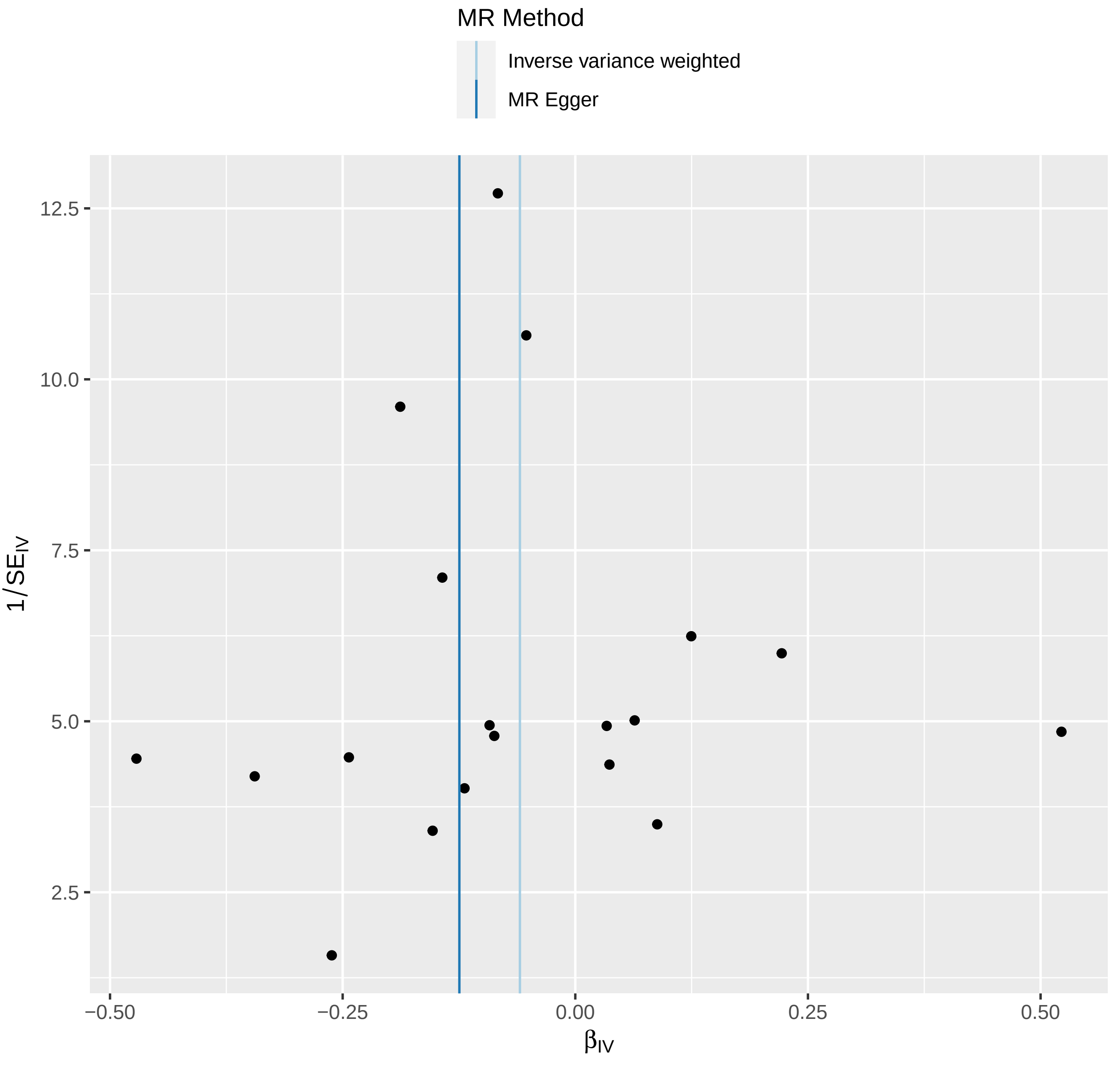

Batch 3−1671 : NKG2−E type II integral membrane protein on Diabetic nephropathy (more control exclusions) with a threshold of 1e−05

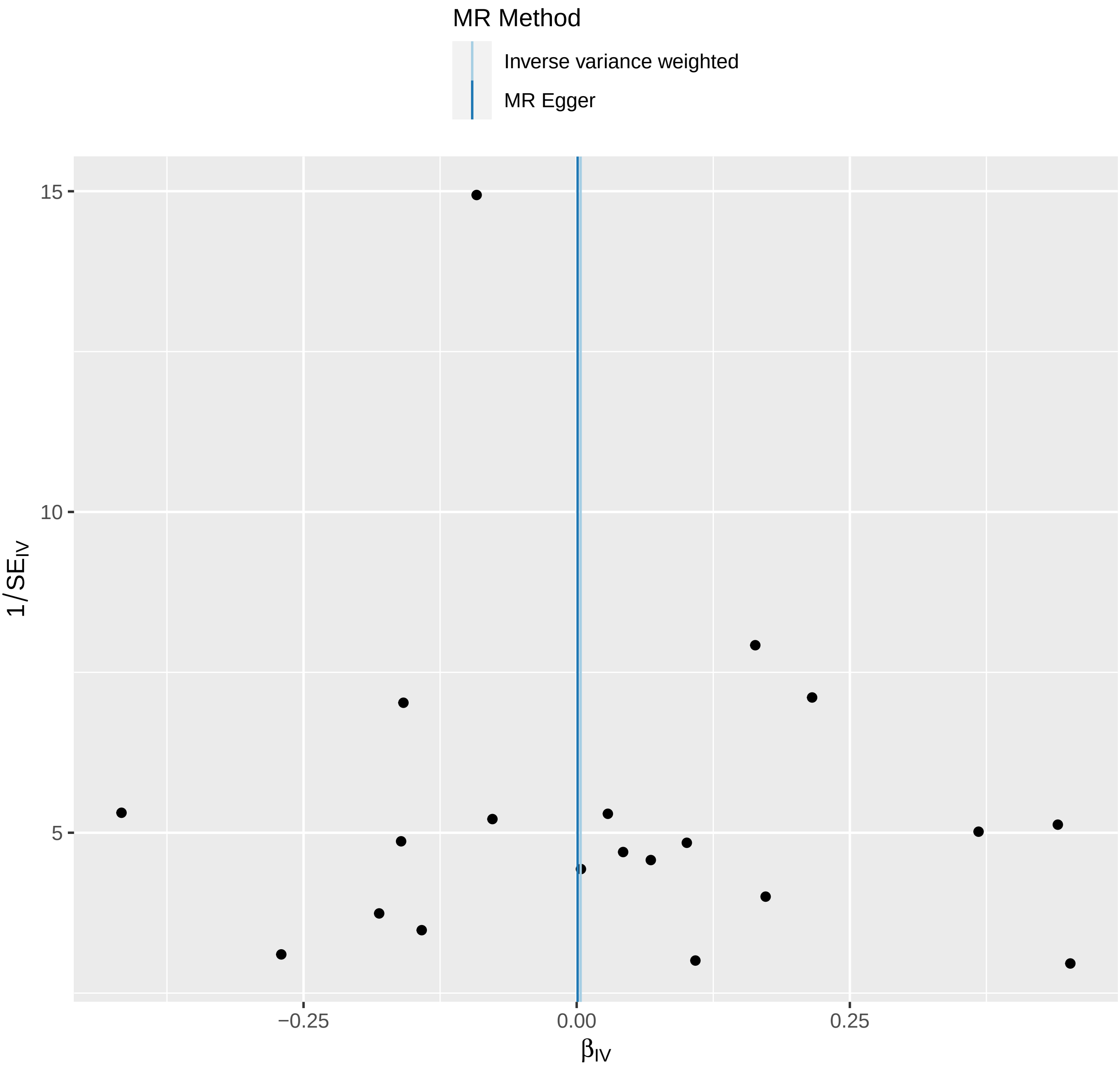

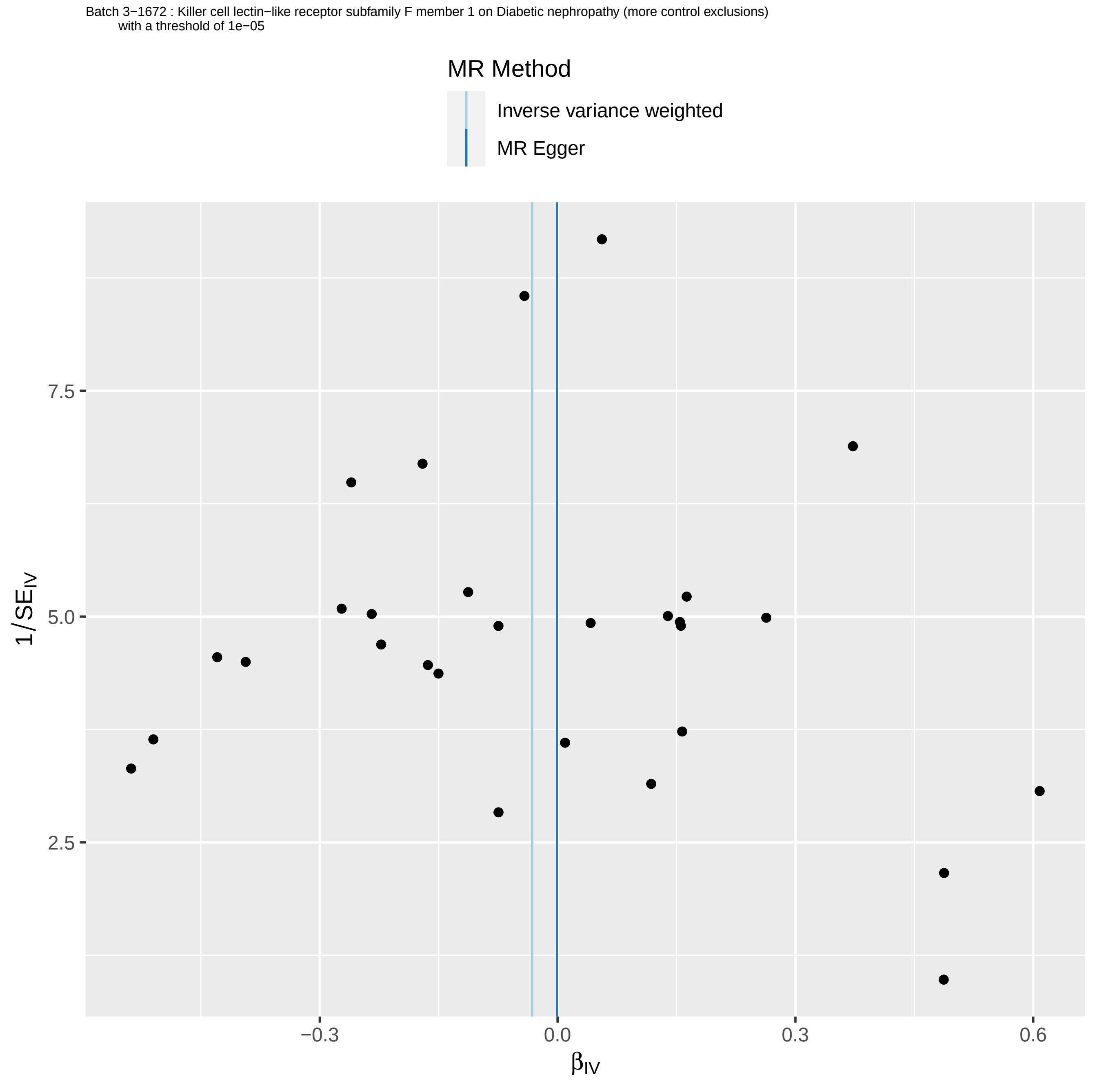

Batch 3−1672 : Killer cell lectin−like receptor subfamily F member 1 on Diabetic nephropathy (more control exclusions)
with a threshold of 1e−05
MR Method
Inverse variance weighted
MR Egger
7.5
5.0
2.5
1/SE_IV
−0.3
0.0
0.3
0.6
β_IV

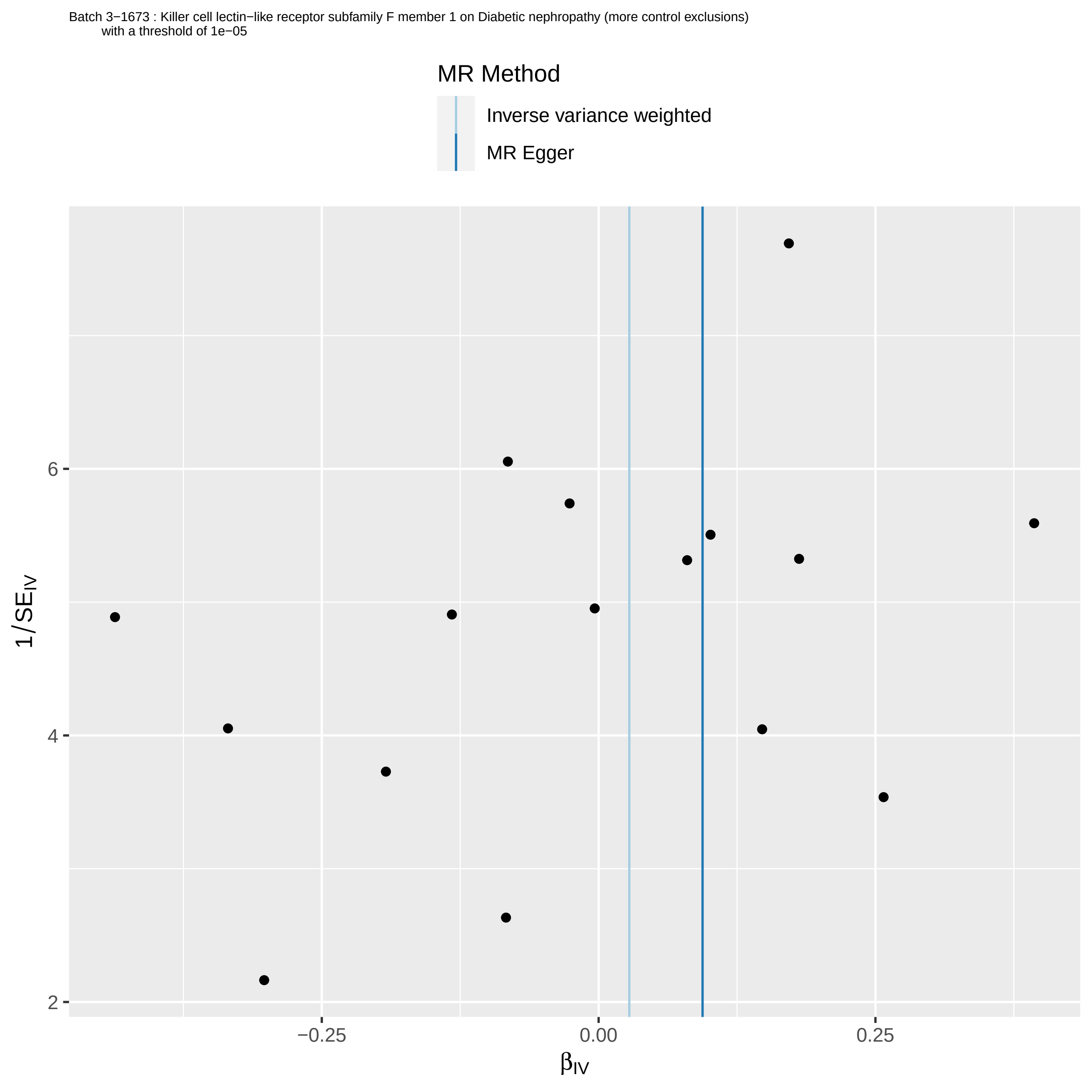
Batch 3−1673 : Killer cell lectin−like receptor subfamily F member 1 on Diabetic nephropathy (more control exclusions)
with a threshold of 1e−05
MR Method
Inverse variance weighted
MR Egger
6
4
2
1/SE_IV
−0.25
0.00
0.25
β_IV

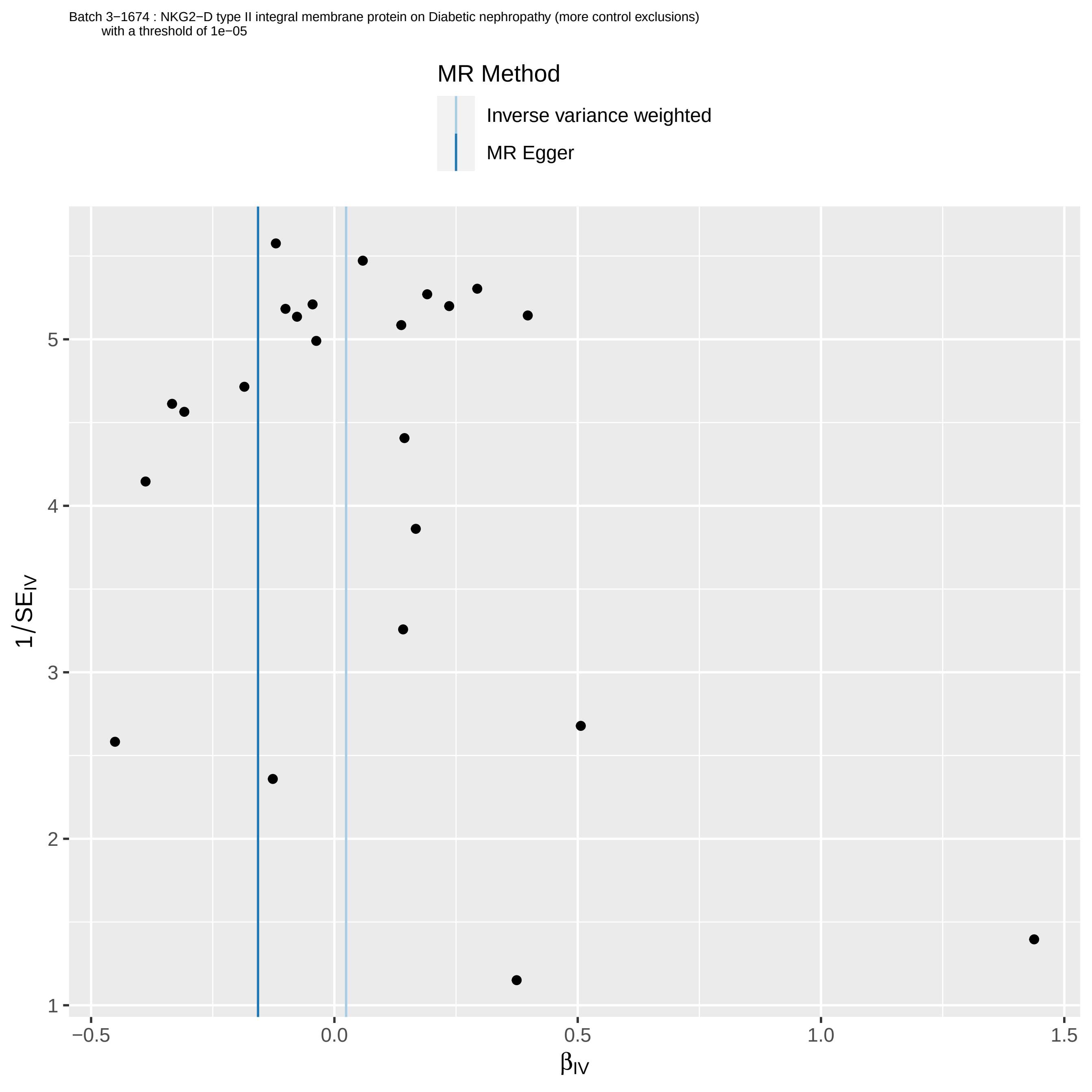
Batch 3−1674 : NKG2−D type II integral membrane protein on Diabetic nephropathy (more control exclusions)
with a threshold of 1e−05
MR Method
Inverse variance weighted
MR Egger
1/SE$_{IV}$
β_{IV}
5
4
3
2
1
−0.5
0.0
0.5
1.0
1.5

Batch 3−1675 : NKG2−D type II integral membrane protein on Diabetic nephropathy (more control exclusions) with a threshold of 1e−05

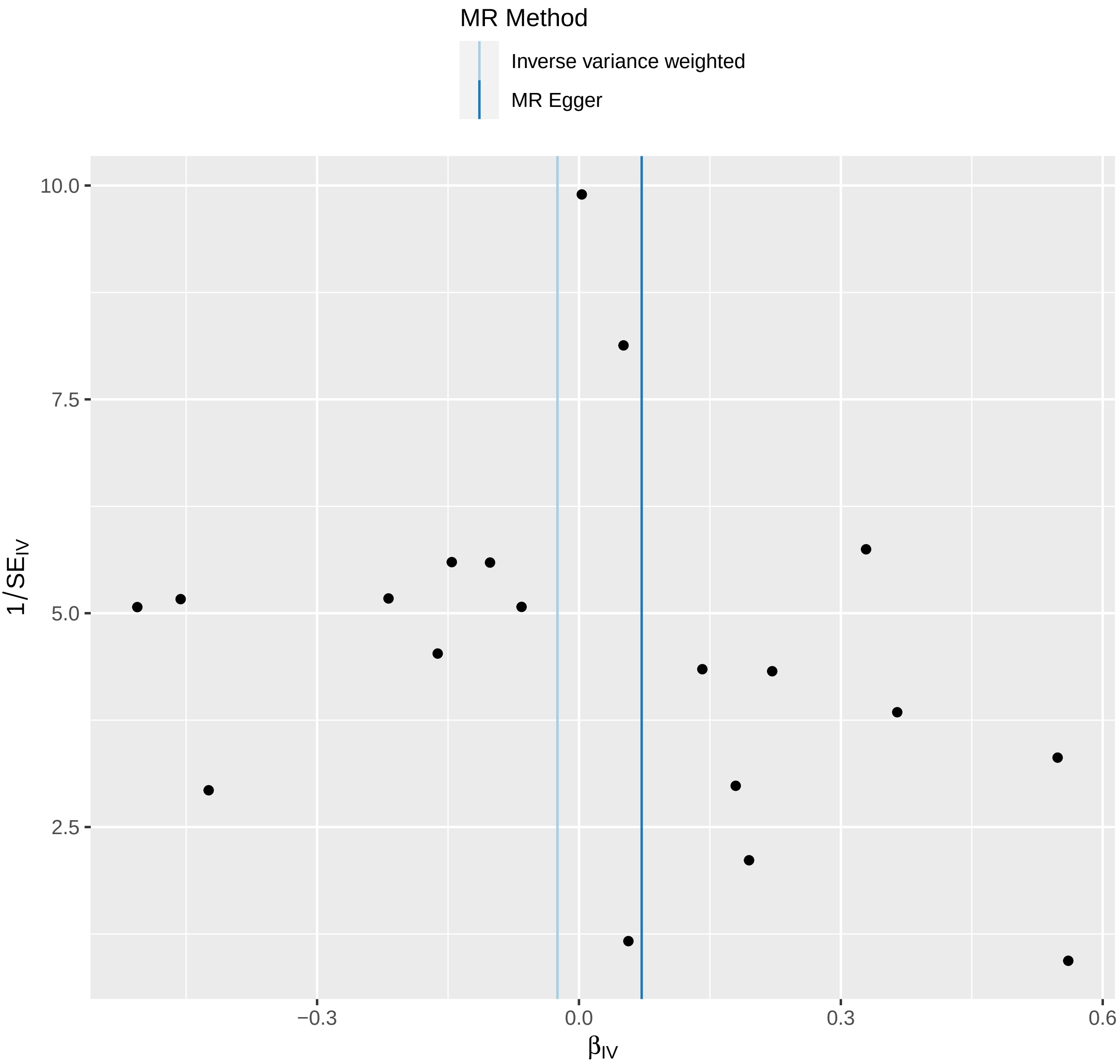

Batch 3−1676 : Histone−lysine N−methyltransferase 2C on Diabetic nephropathy (more control exclusions)
with a threshold of 1e−05

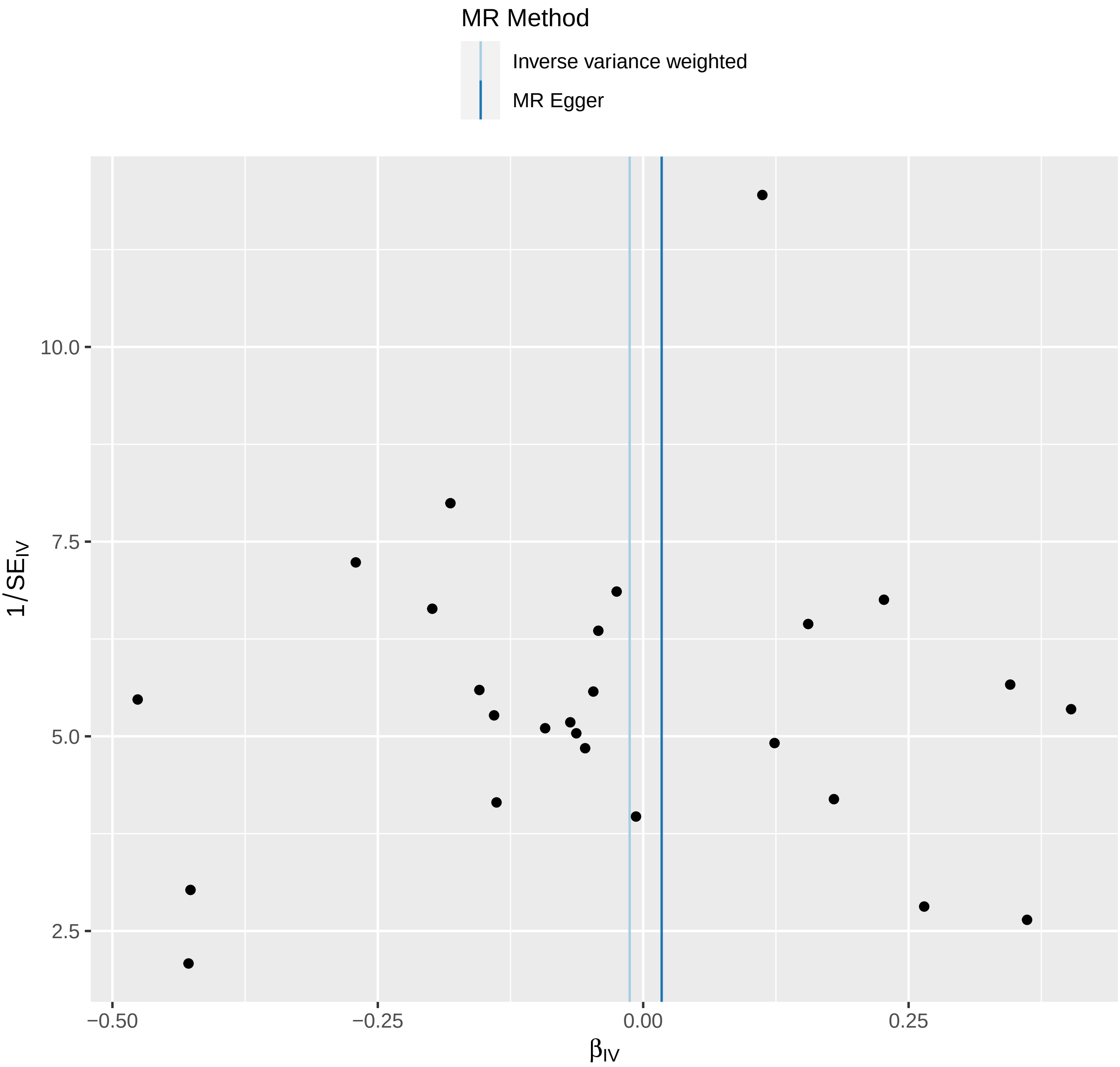

Batch 3−1677 : Importin subunit alpha−1 on Diabetic nephropathy (more control exclusions)
with a threshold of 1e−05

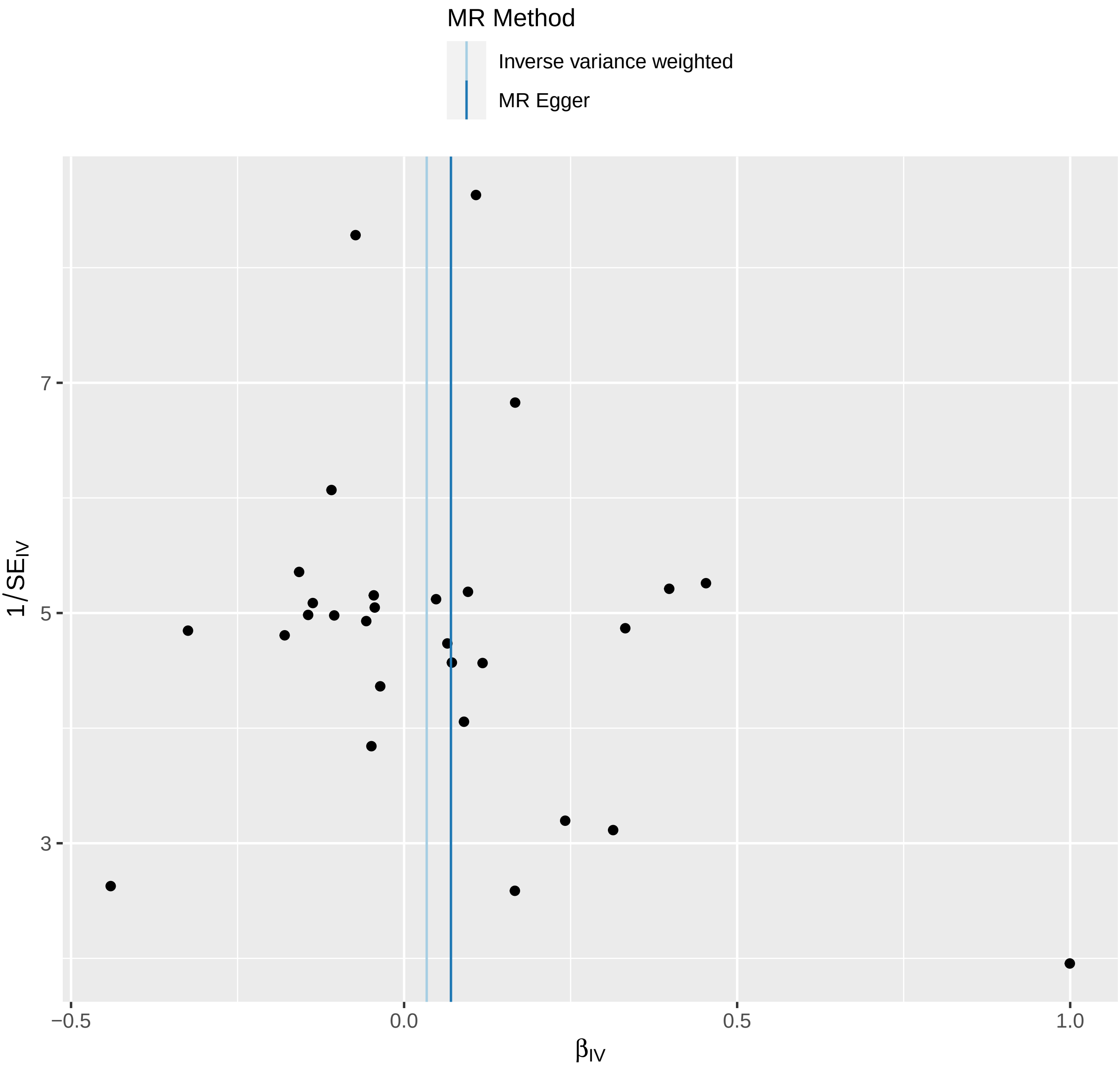

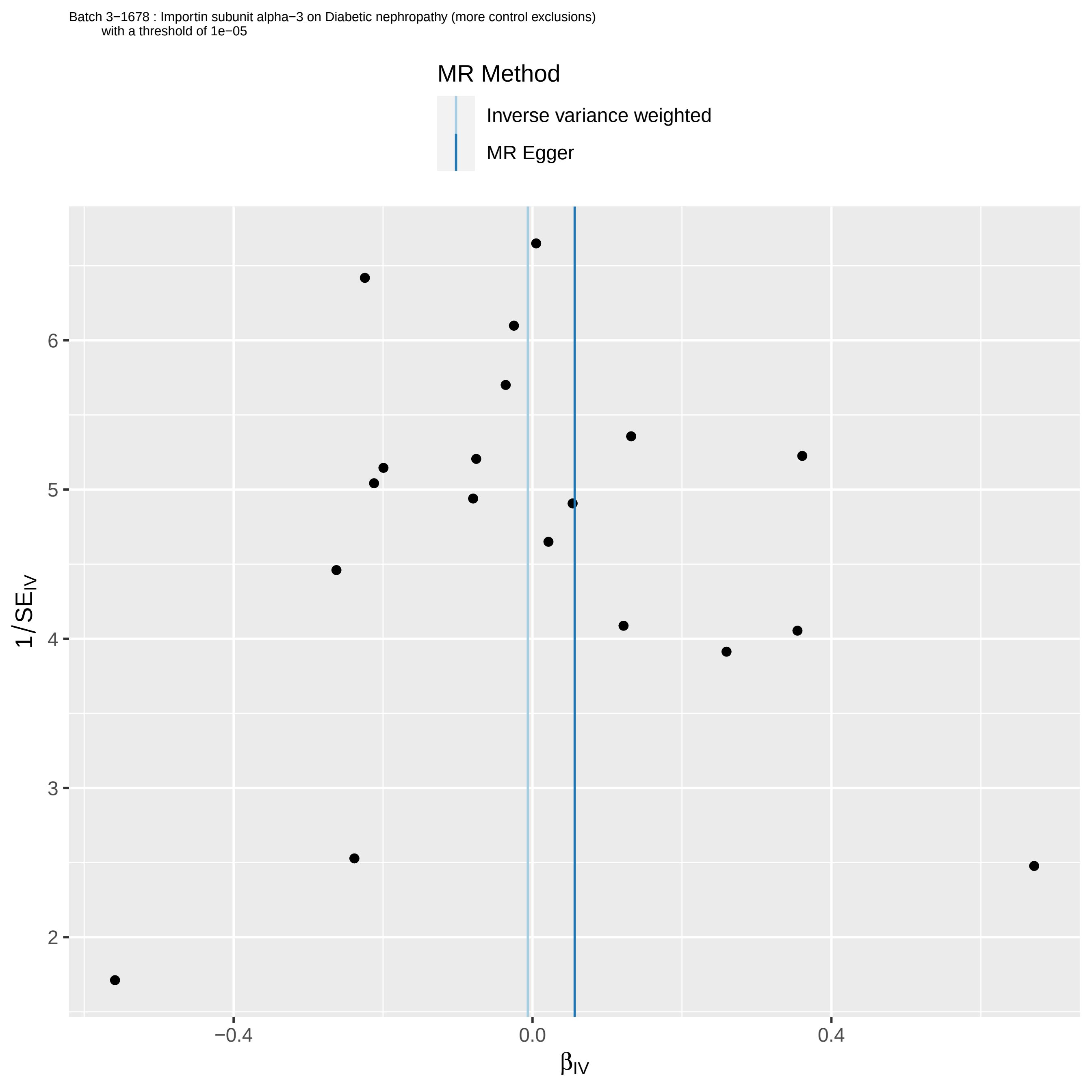

Batch 3−1678 : Importin subunit alpha−3 on Diabetic nephropathy (more control exclusions)
with a threshold of 1e−05
MR Method
Inverse variance weighted
MR Egger
6
5
4
3
2
1/SE_IV
−0.4
0.0
0.4
β_IV

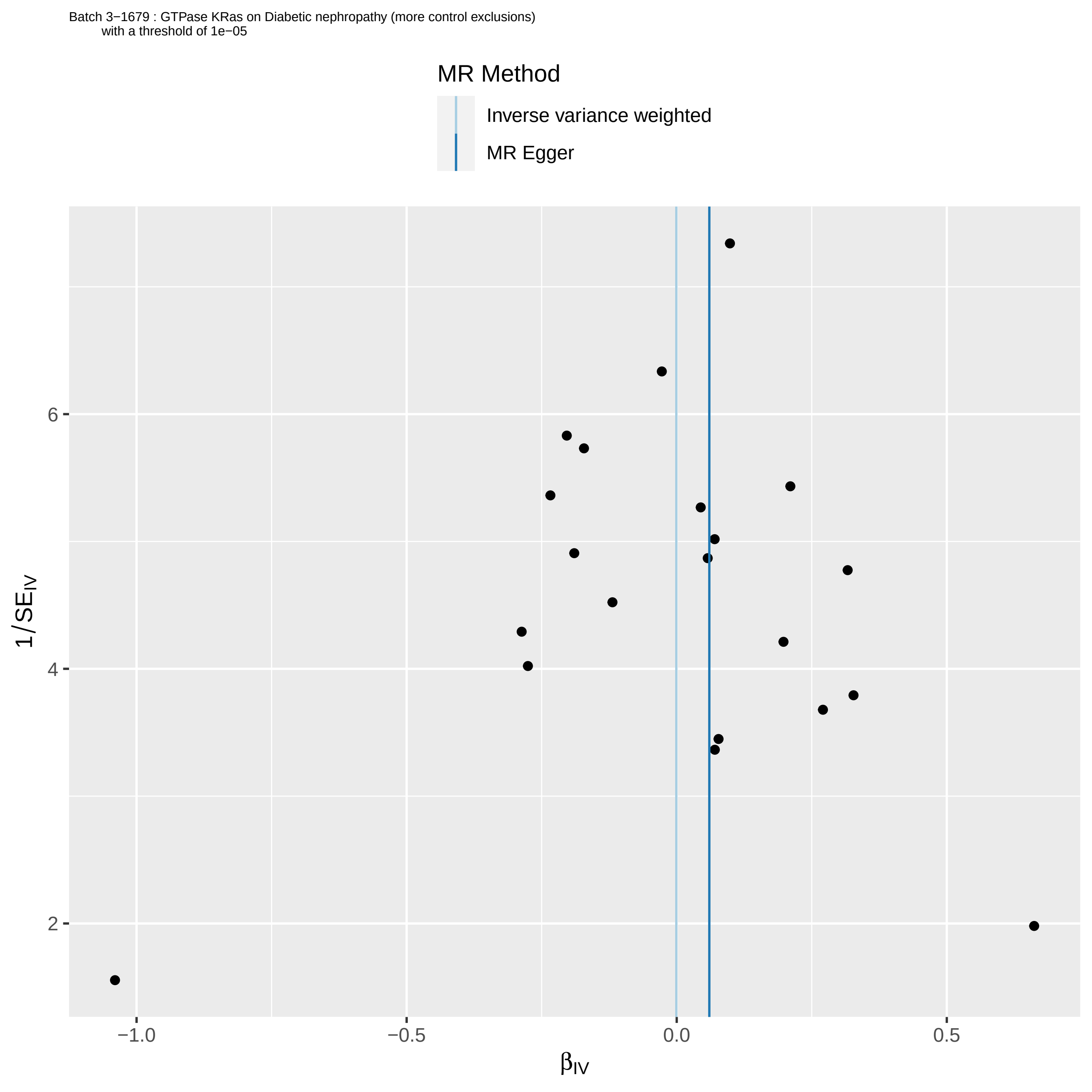

Batch 3−1679 : GTPase KRas on Diabetic nephropathy (more control exclusions)
with a threshold of 1e−05
MR Method
Inverse variance weighted
MR Egger
6
4
2
1/SE_IV
−1.0
−0.5
0.0
0.5
β_IV

Batch 3−1680 : Kremen protein 1 on Diabetic nephropathy (more control exclusions)
with a threshold of 1e−05

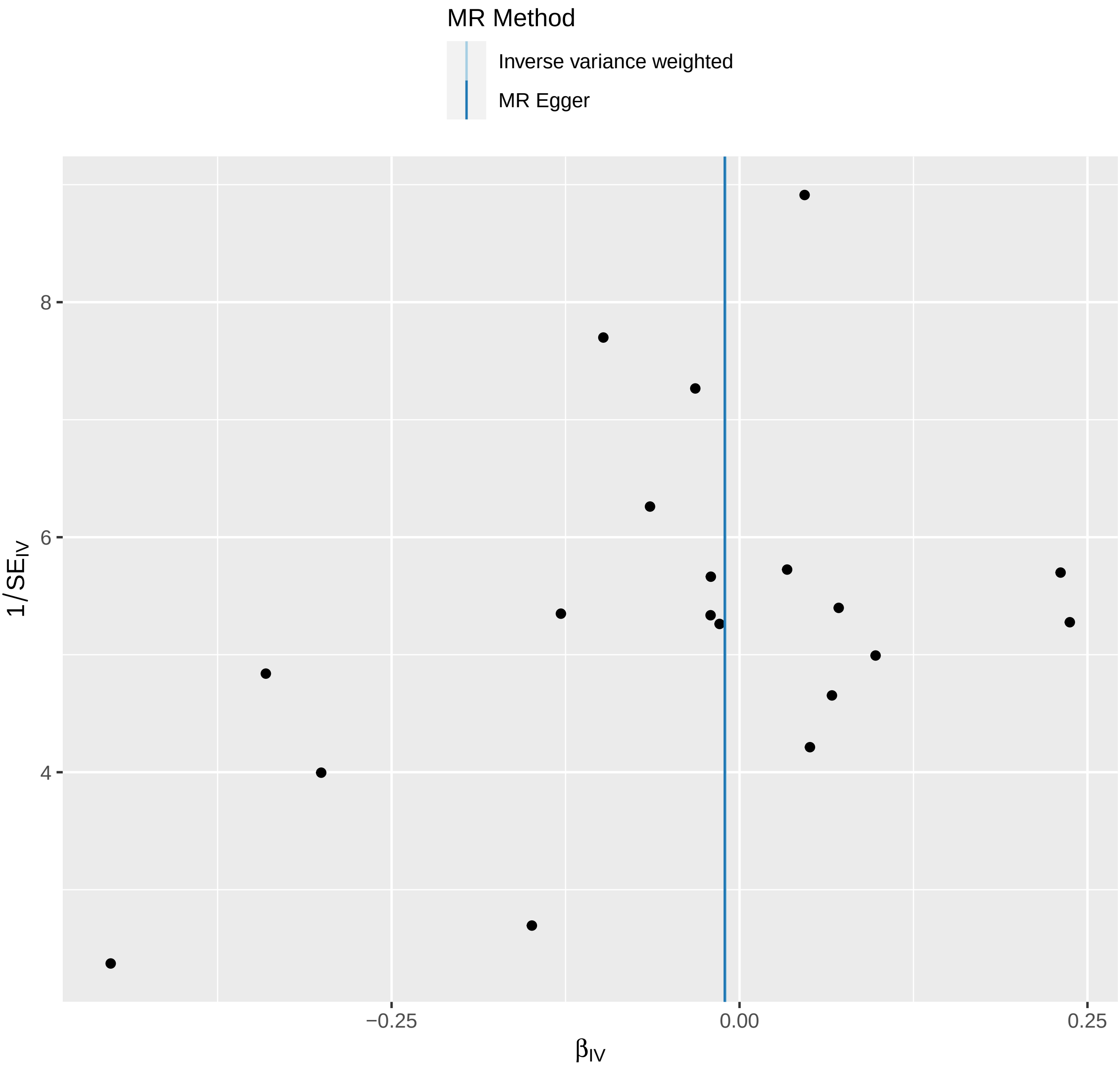

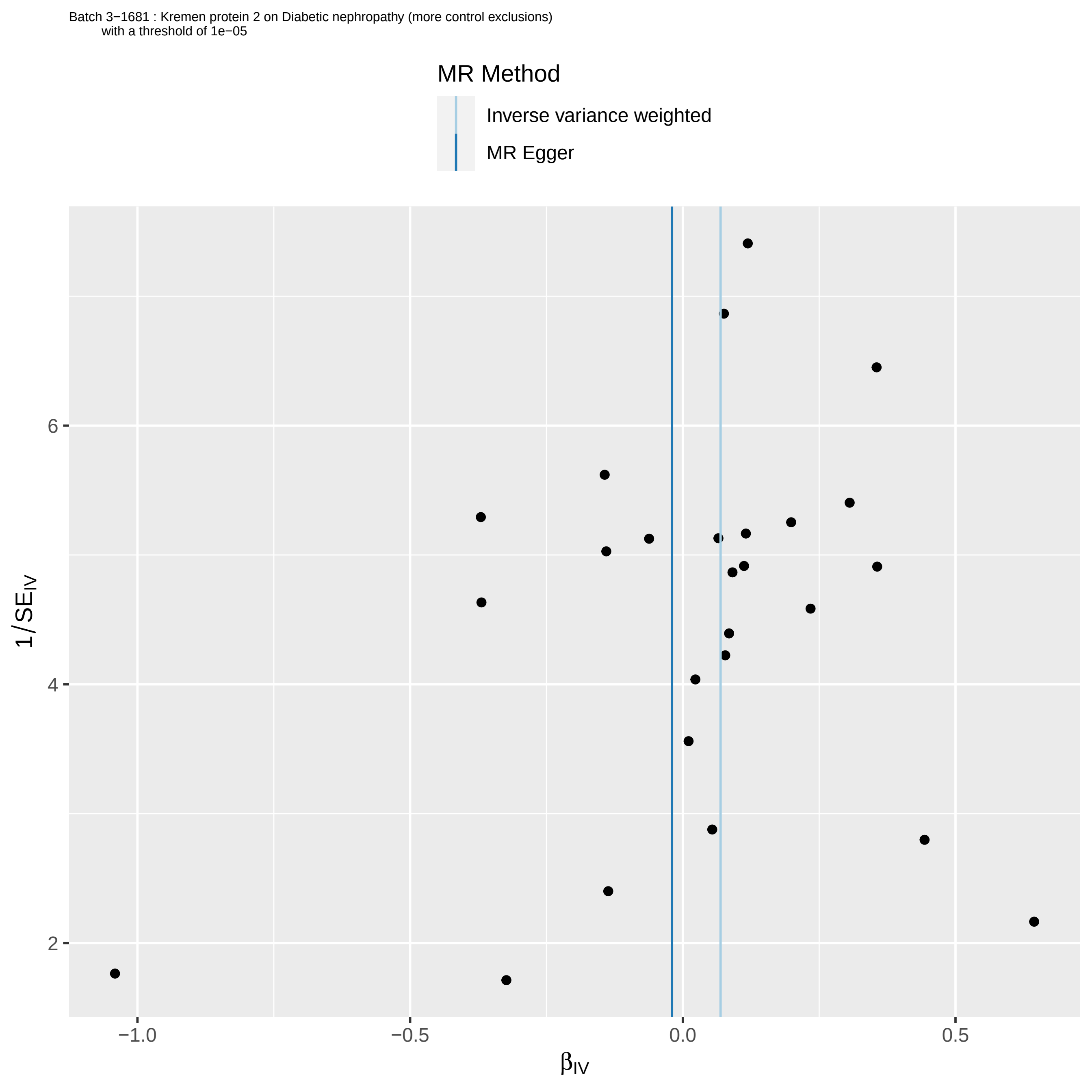
Batch 3−1681 : Kremen protein 2 on Diabetic nephropathy (more control exclusions)
with a threshold of 1e−05
MR Method
Inverse variance weighted
MR Egger
6
4
2
1/SE_IV
−1.0
−0.5
0.0
0.5
β_IV

Batch 3−1682 : Keratin, type I cytoskeletal 17 on Diabetic nephropathy (more control exclusions)
with a threshold of 1e−05

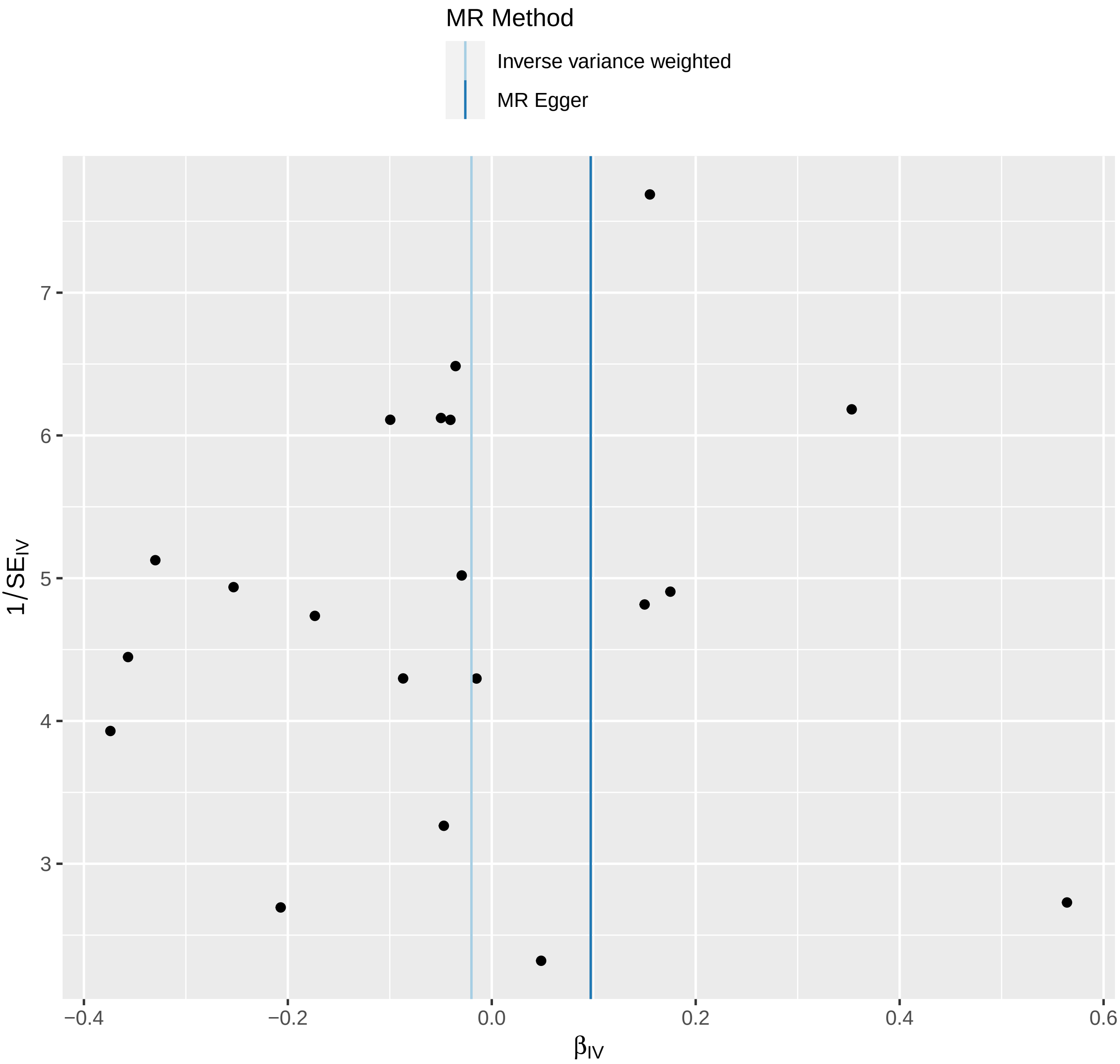

Batch 3−1683 : Keratin, type I cytoskeletal 18 on Diabetic nephropathy (more control exclusions)
with a threshold of 1e−05

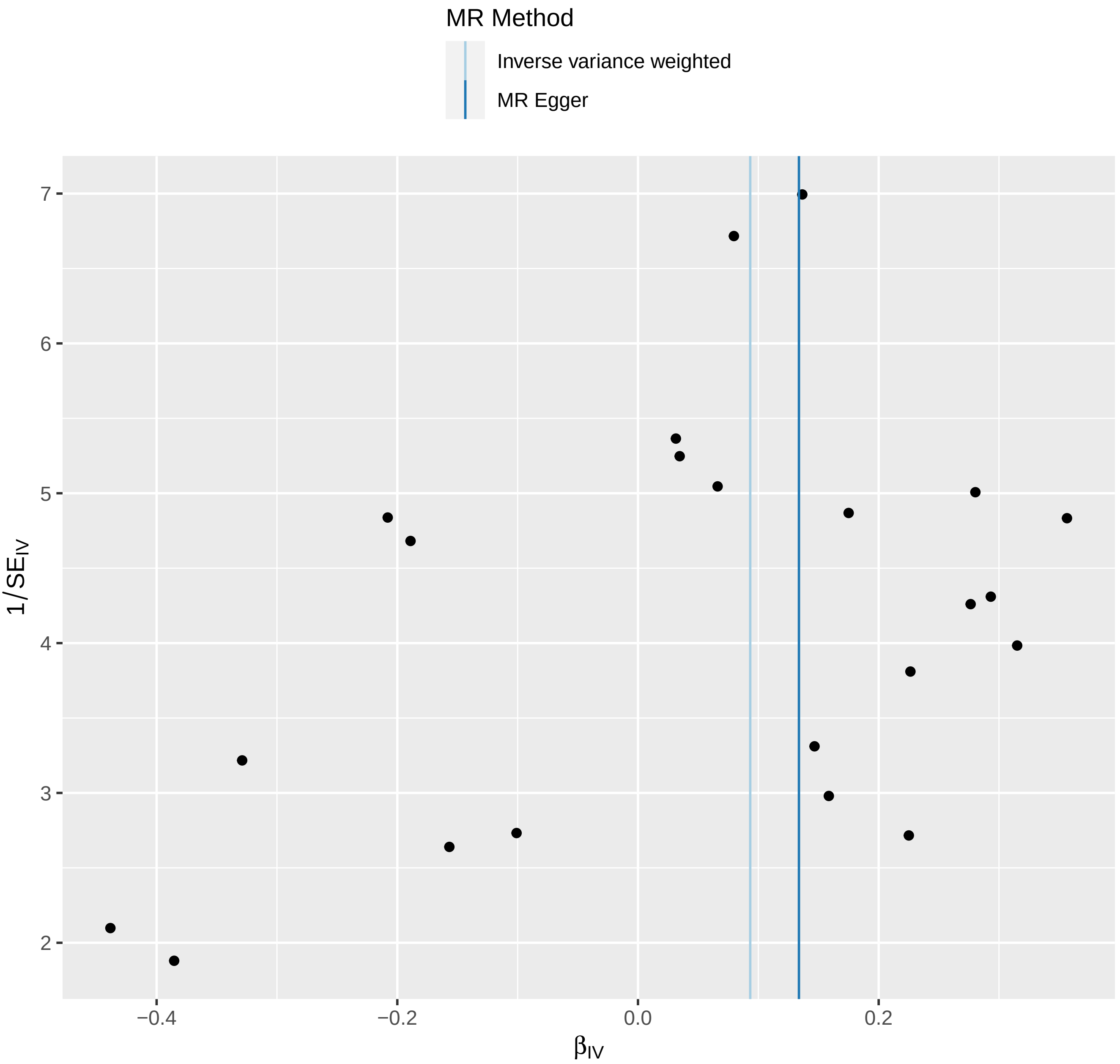

Batch 3−1684 : Keratin, type I cytoskeletal 20 on Diabetic nephropathy (more control exclusions) with a threshold of 1e−05

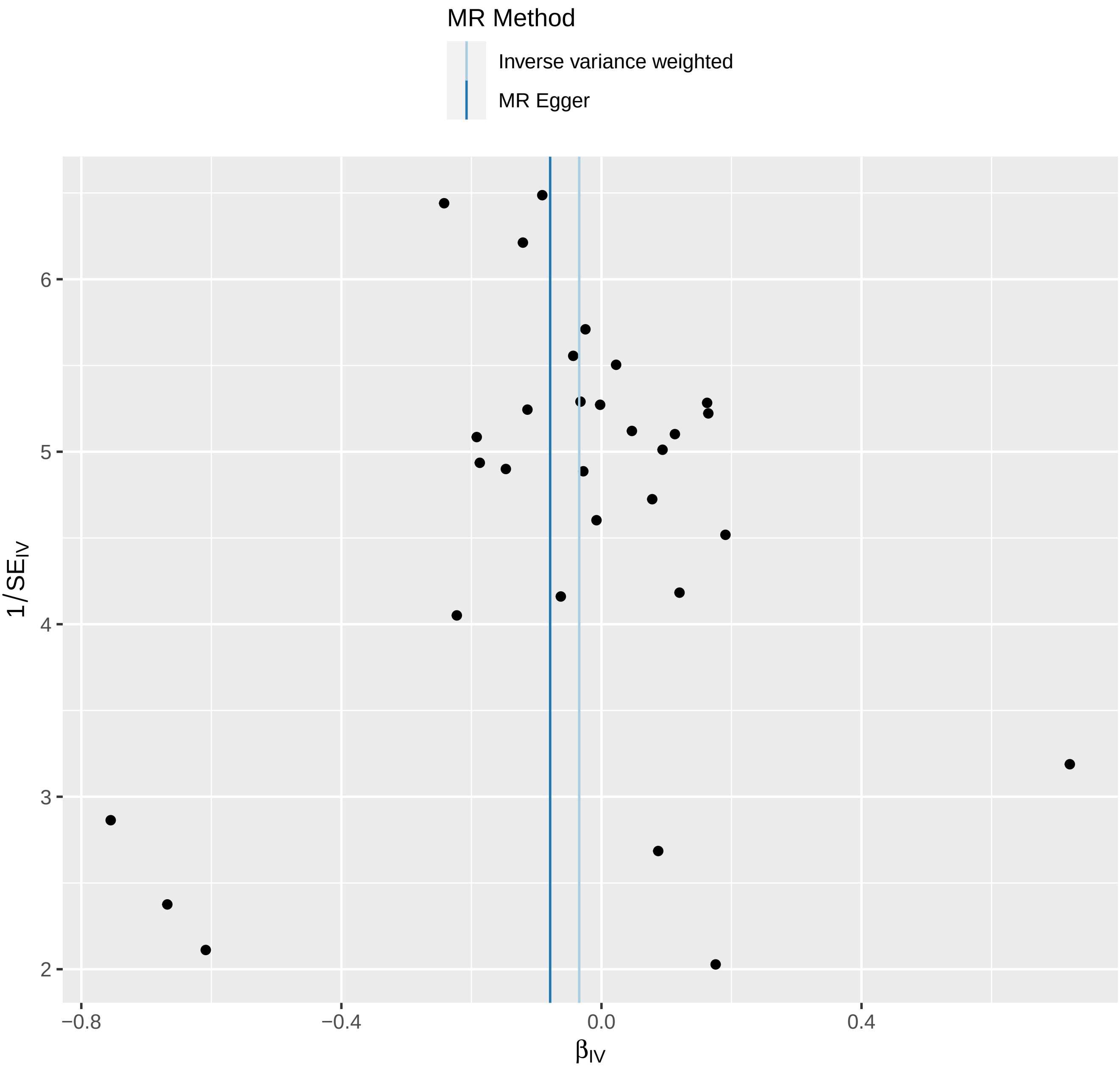

Batch 3−1685 : Keratin, type II cytoskeletal 5 on Diabetic nephropathy (more control exclusions)
with a threshold of 1e−05

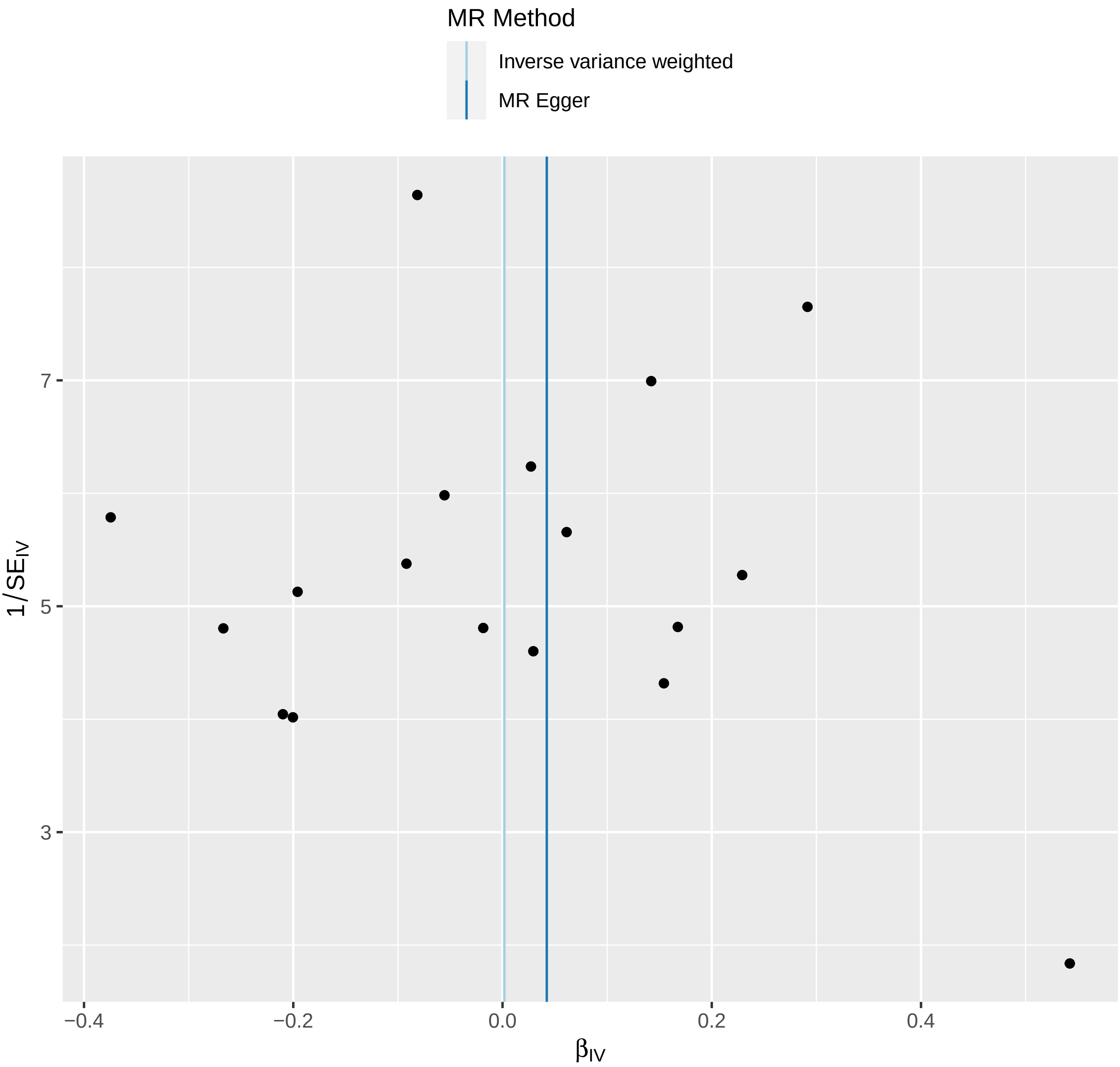

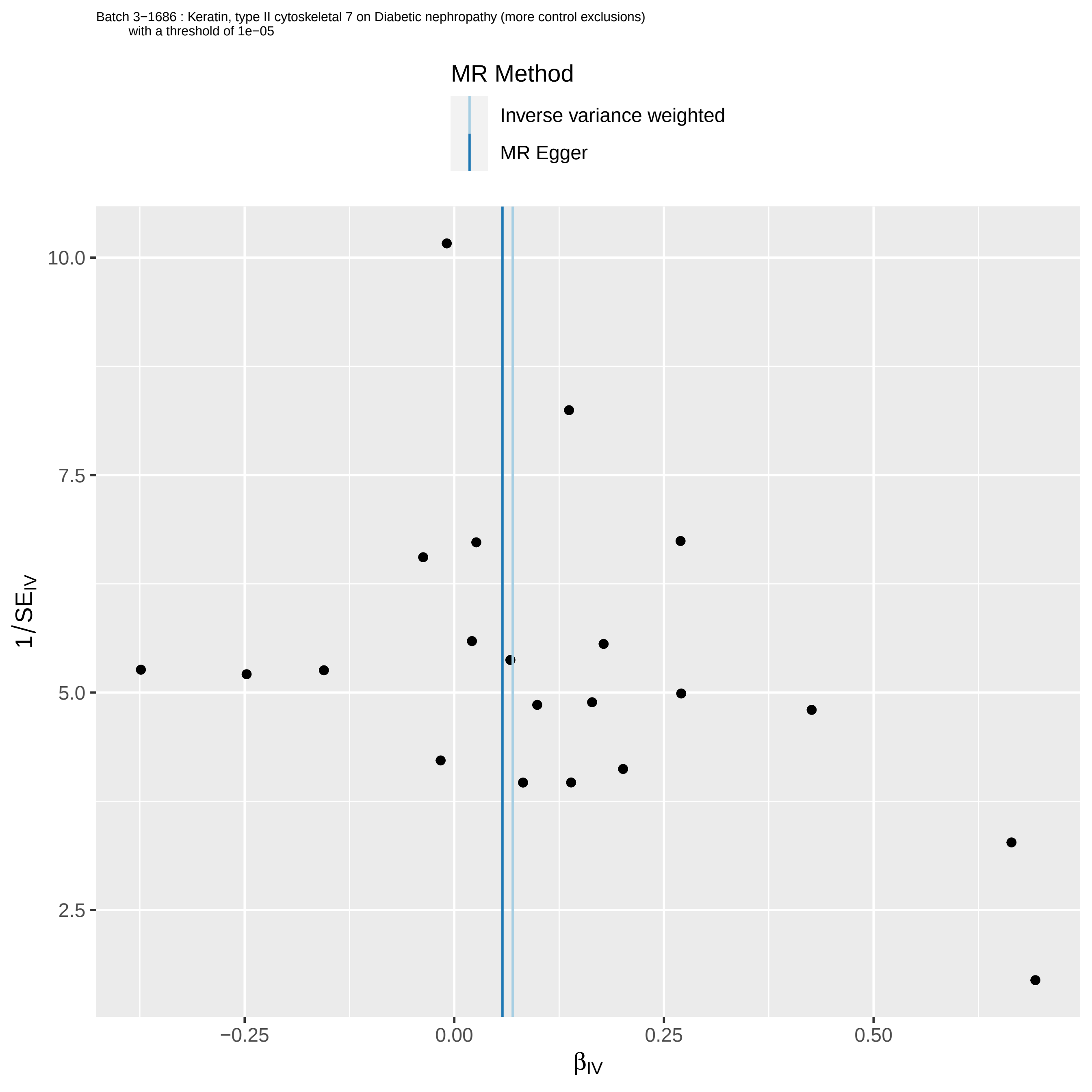
Batch 3−1686 : Keratin, type II cytoskeletal 7 on Diabetic nephropathy (more control exclusions)
with a threshold of 1e−05
MR Method
Inverse variance weighted
MR Egger
10.0
7.5
5.0
2.5
1/SE_IV
−0.25
0.00
0.25
0.50
β_IV

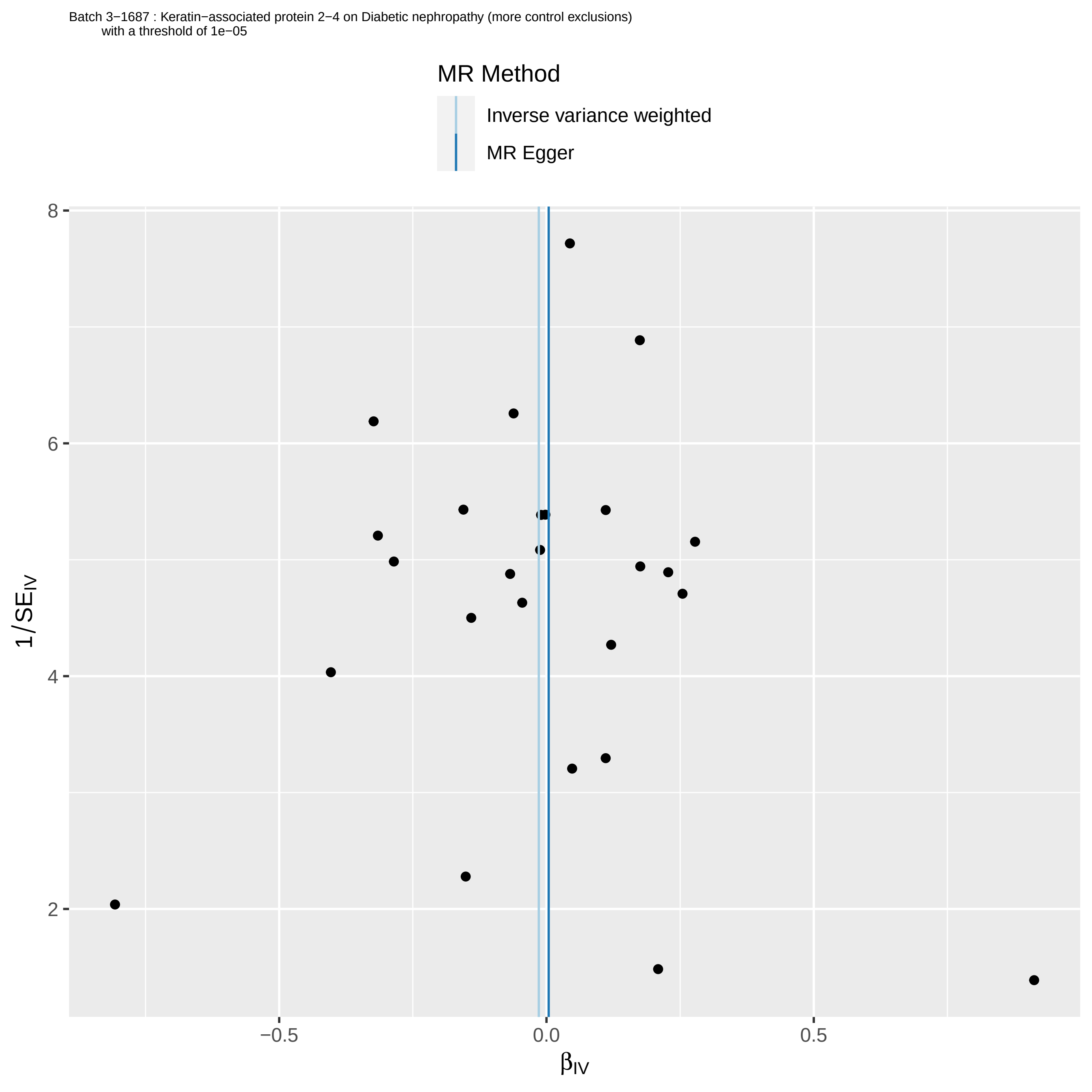
Batch 3−1687 : Keratin−associated protein 2−4 on Diabetic nephropathy (more control exclusions)
with a threshold of 1e−05
MR Method
Inverse variance weighted
MR Egger
8
6
4
2
1/SE_IV
−0.5
0.0
0.5
β_IV

Batch 3−1688 : Keratinocyte differentiation−associated protein on Diabetic nephropathy (more control exclusions) with a threshold of 1e−05

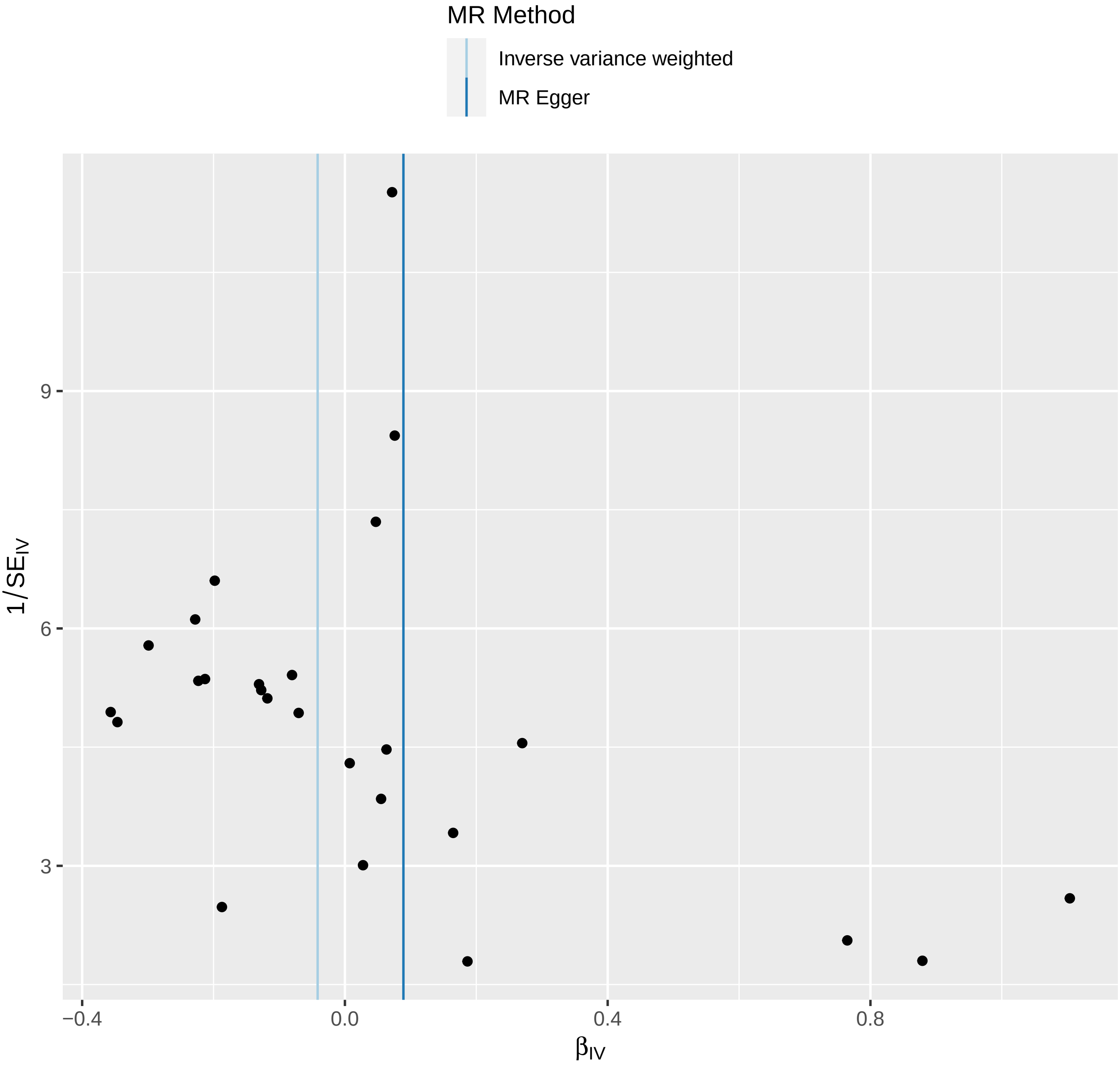

Batch 3−1689 : Kynureninase on Diabetic nephropathy (more control exclusions)
with a threshold of 1e−05

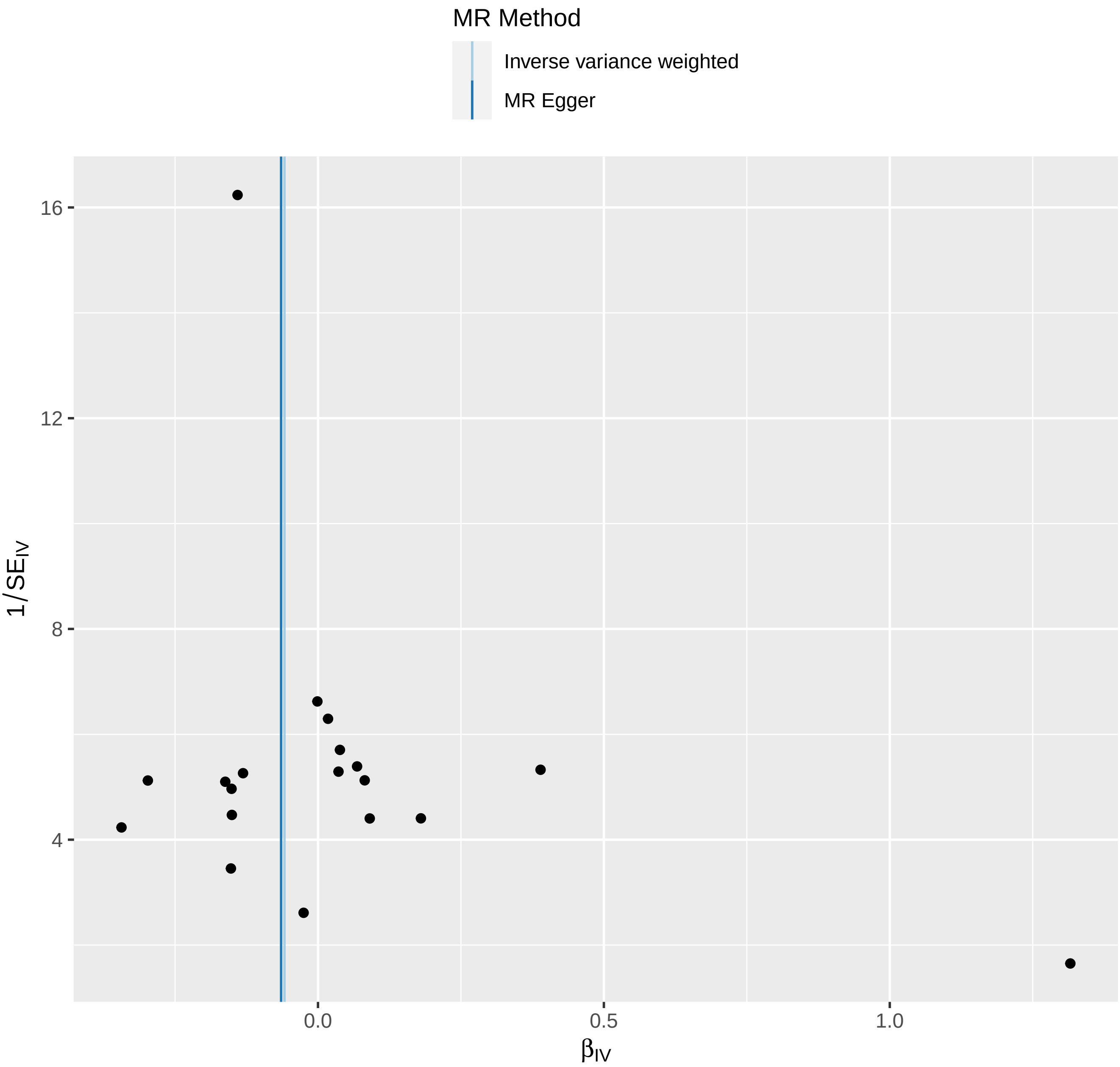

Batch 3−1690 : Neural cell adhesion molecule L1 on Diabetic nephropathy (more control exclusions)
with a threshold of 1e−05

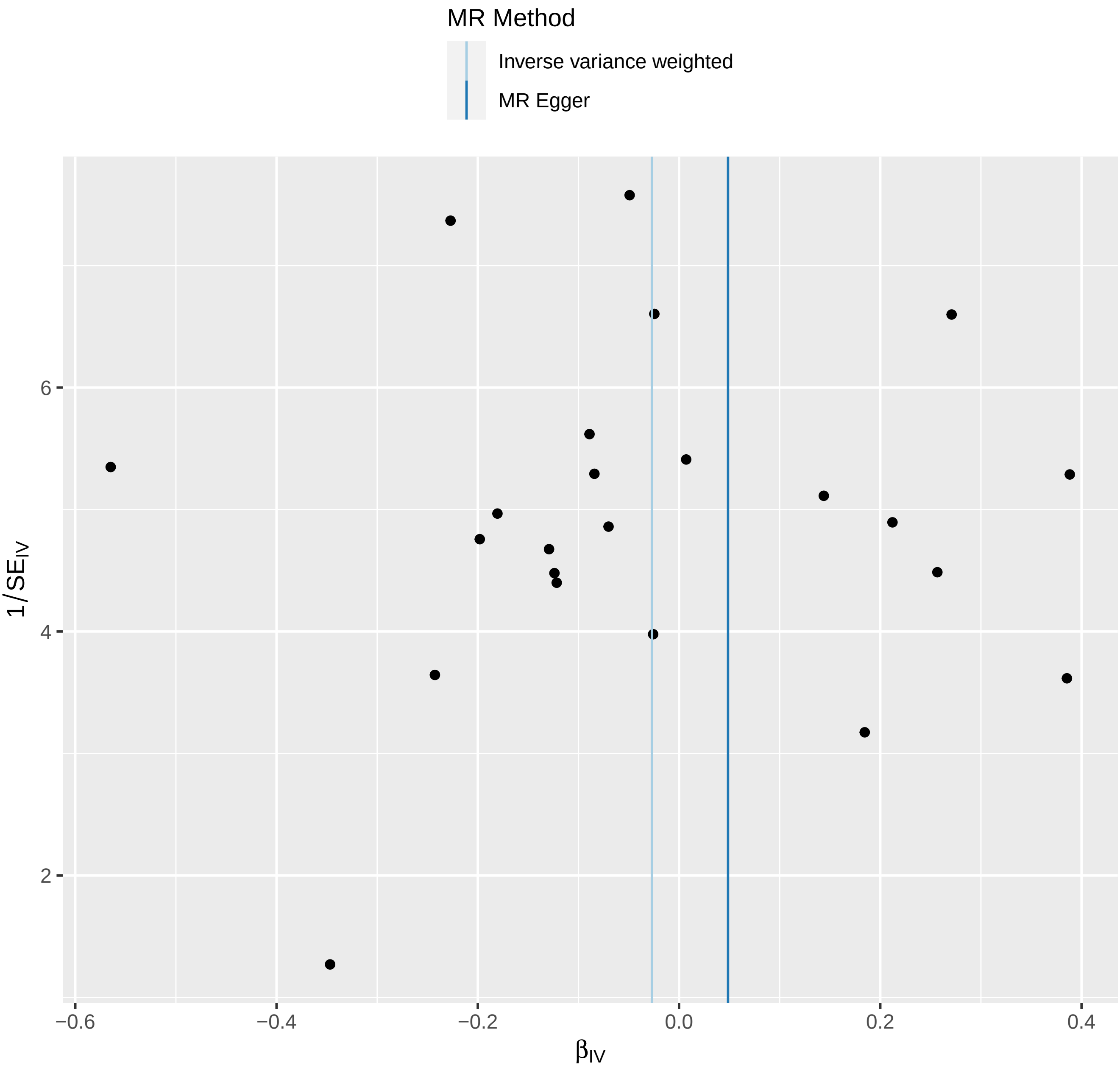

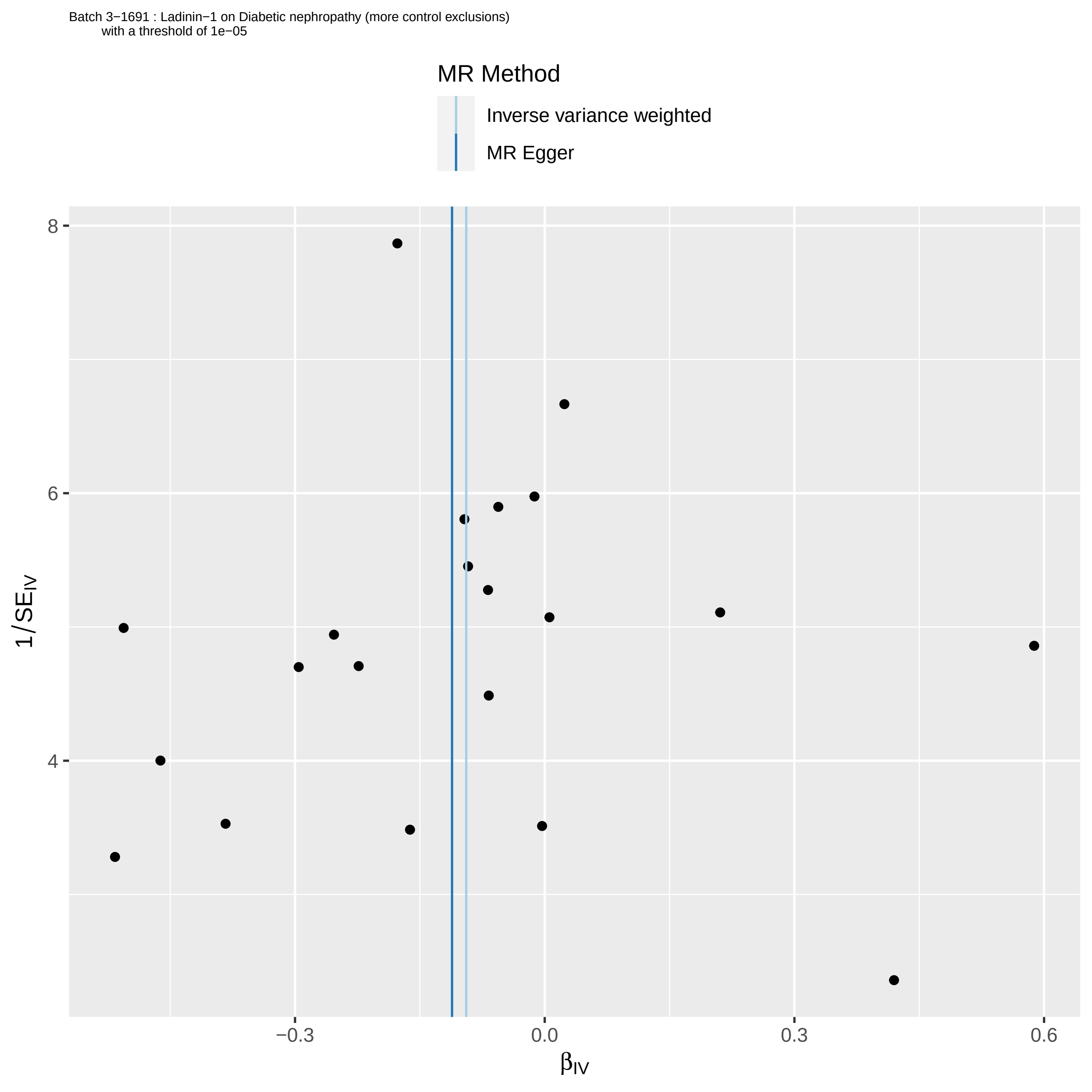
Batch 3−1691 : Ladinin−1 on Diabetic nephropathy (more control exclusions)
with a threshold of 1e−05
MR Method
Inverse variance weighted
MR Egger
8
6
4
1/SE_IV
−0.3
0.0
0.3
0.6
β_IV

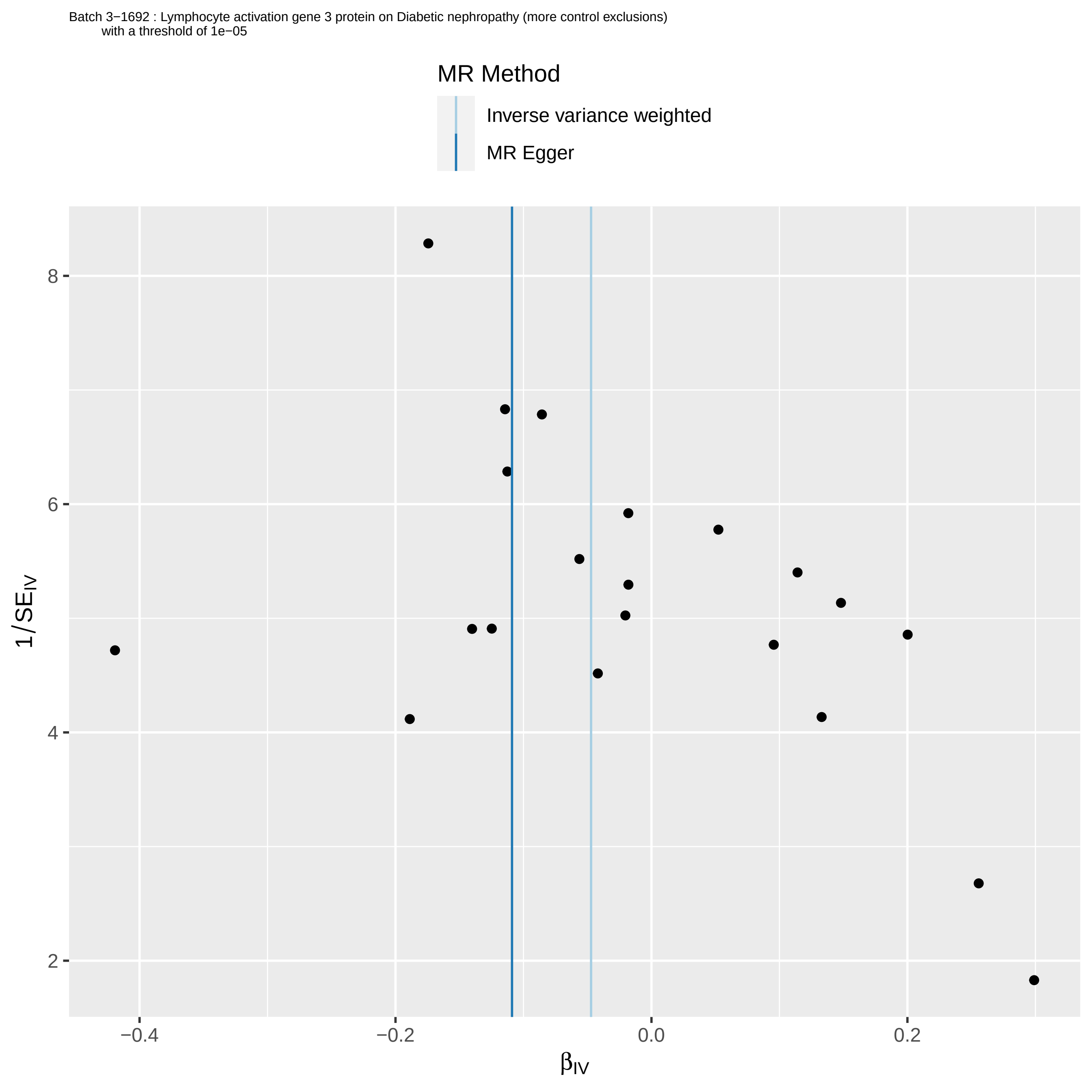

Batch 3−1692 : Lymphocyte activation gene 3 protein on Diabetic nephropathy (more control exclusions)
with a threshold of 1e−05
MR Method
Inverse variance weighted
MR Egger
8
6
4
2
−0.4
−0.2
0.0
0.2
$1/SE_{IV}$
β_{IV}

Batch 3−1693 : Lymphocyte activation gene 3 protein on Diabetic nephropathy (more control exclusions) with a threshold of 1e−05

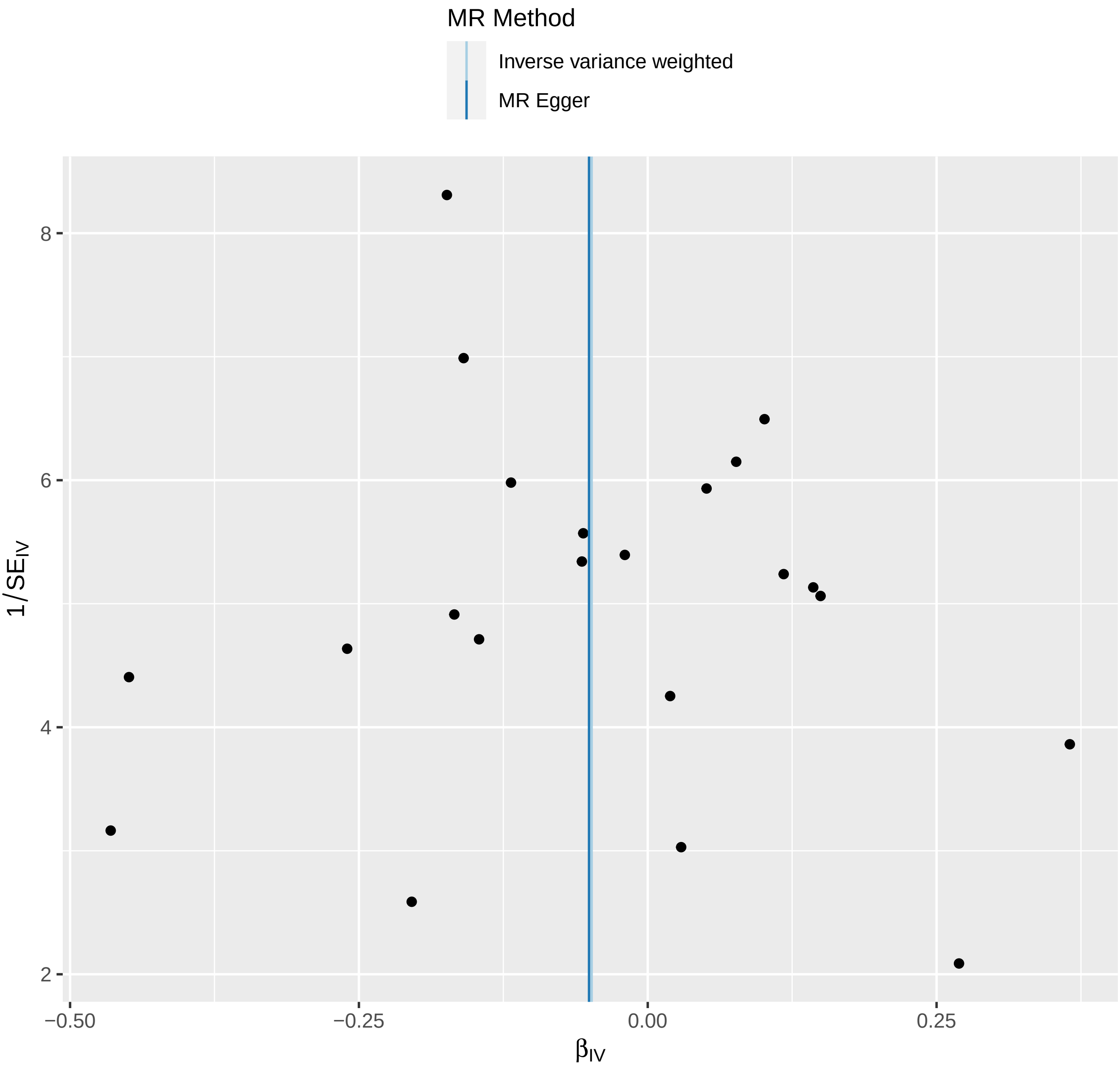

Batch 3−1694 : Leukocyte−associated immunoglobulin−like receptor 1 on Diabetic nephropathy (more control exclusions) with a threshold of 1e−05

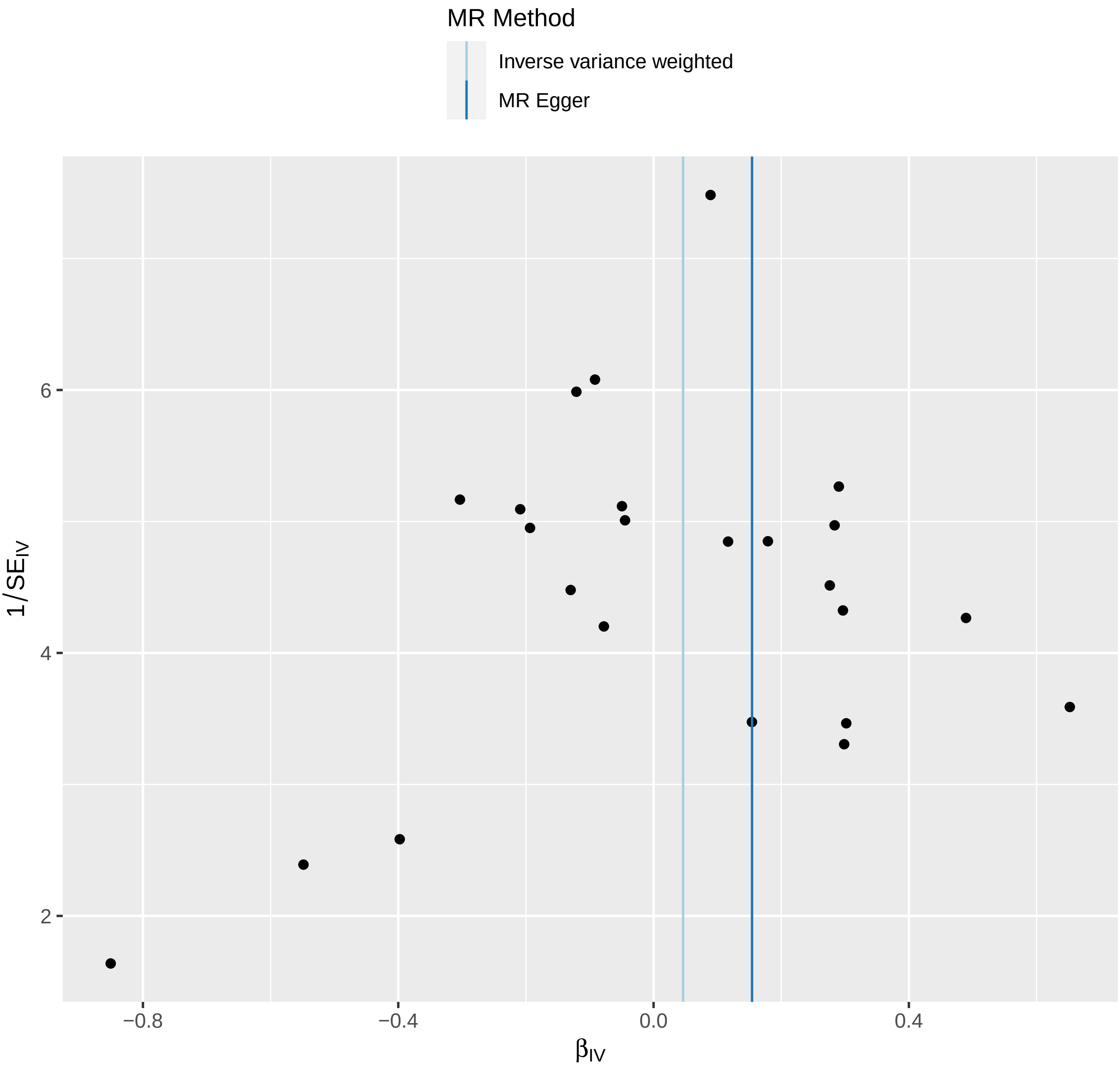

Batch 3−1695 : Laminin on Diabetic nephropathy (more control exclusions)
with a threshold of 1e−05

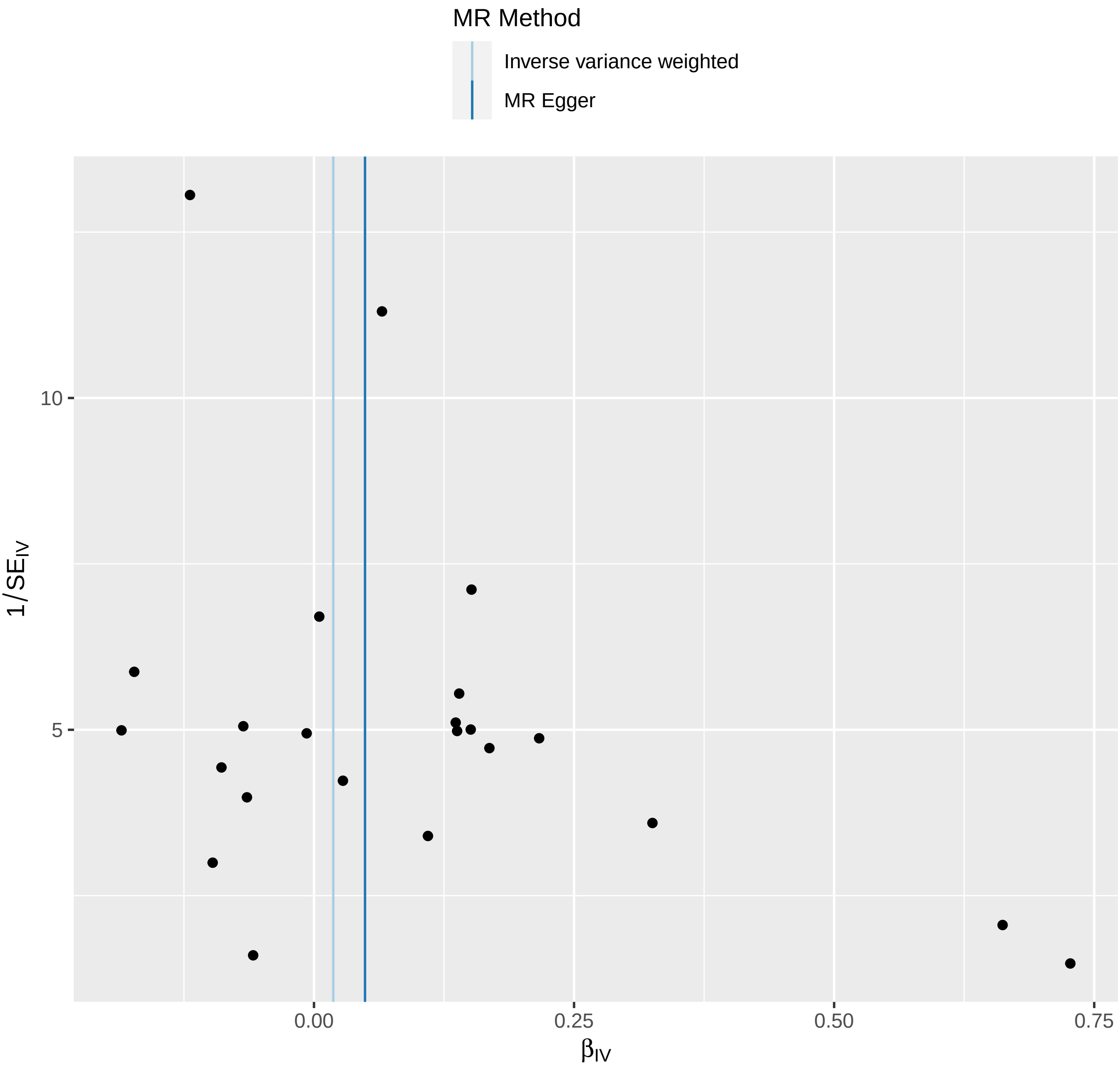

Batch 3−1696 : Laminin subunit alpha−4 on Diabetic nephropathy (more control exclusions)
with a threshold of 1e−05

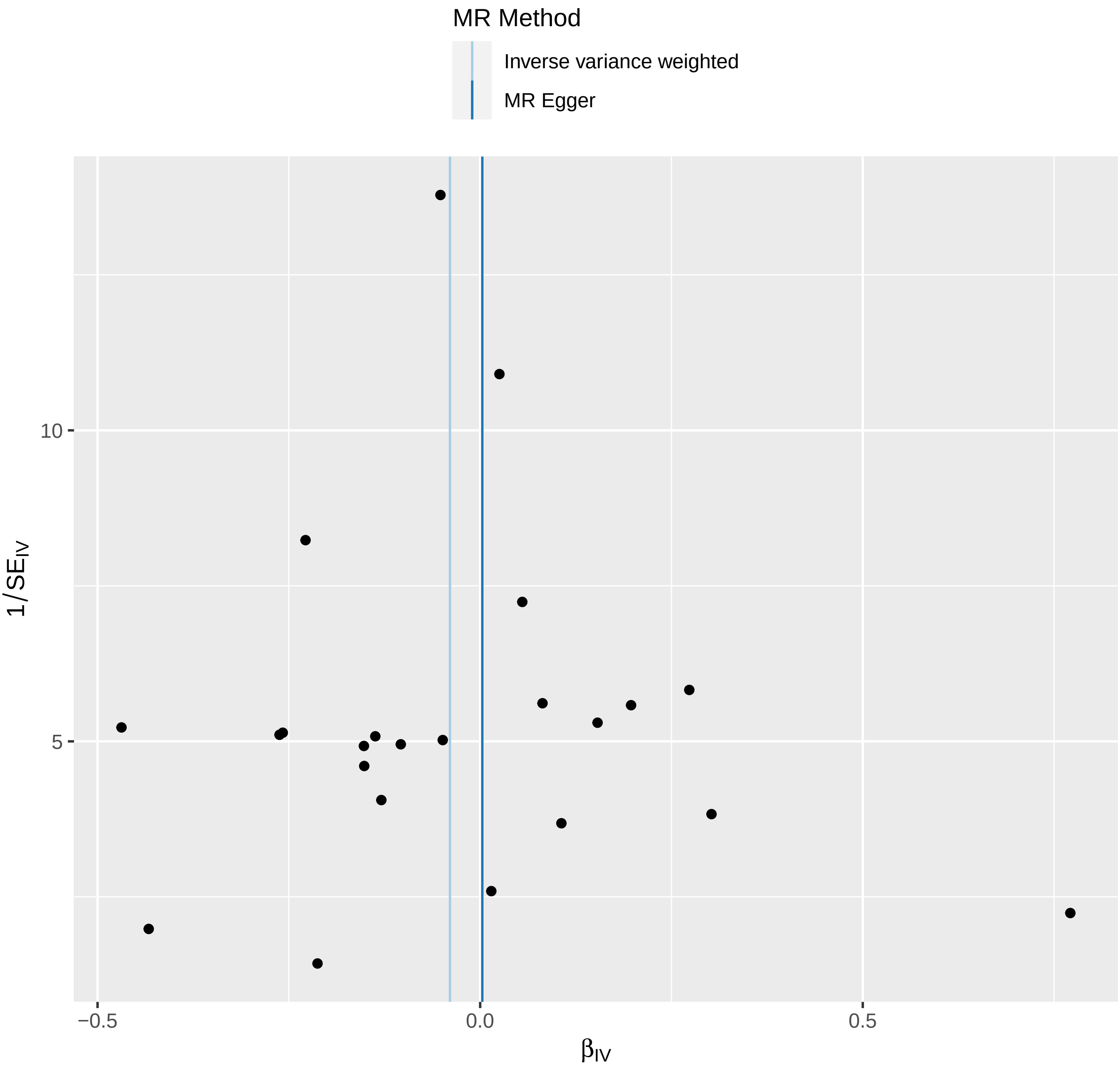

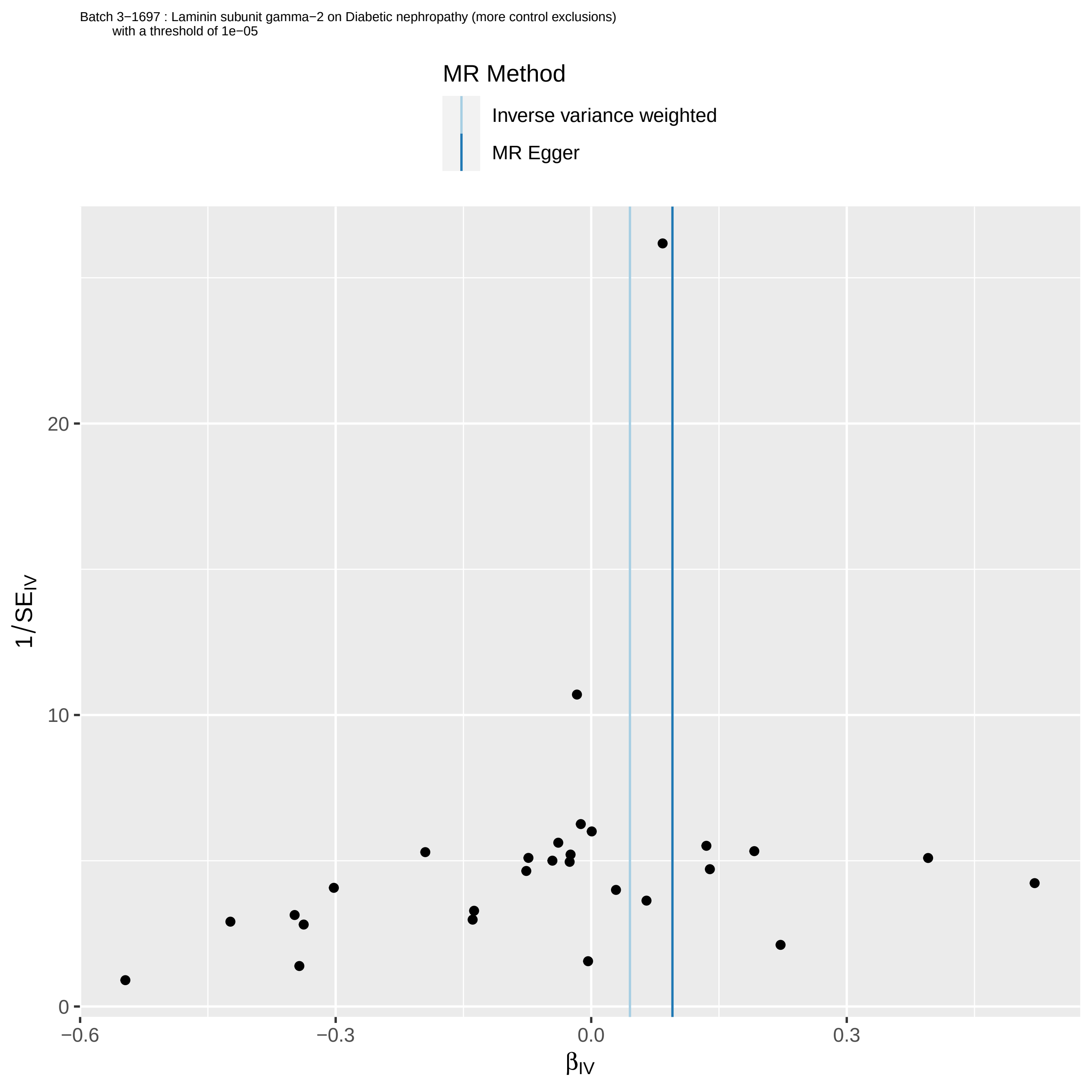
Batch 3−1697 : Laminin subunit gamma−2 on Diabetic nephropathy (more control exclusions)
with a threshold of 1e−05
MR Method
Inverse variance weighted
MR Egger
20
10
0
1/SE_IV
−0.6
−0.3
0.0
0.3
β_IV

Batch 3−1698 : Lysosome−associated membrane glycoprotein 3 on Diabetic nephropathy (more control exclusions)
with a threshold of 1e−05

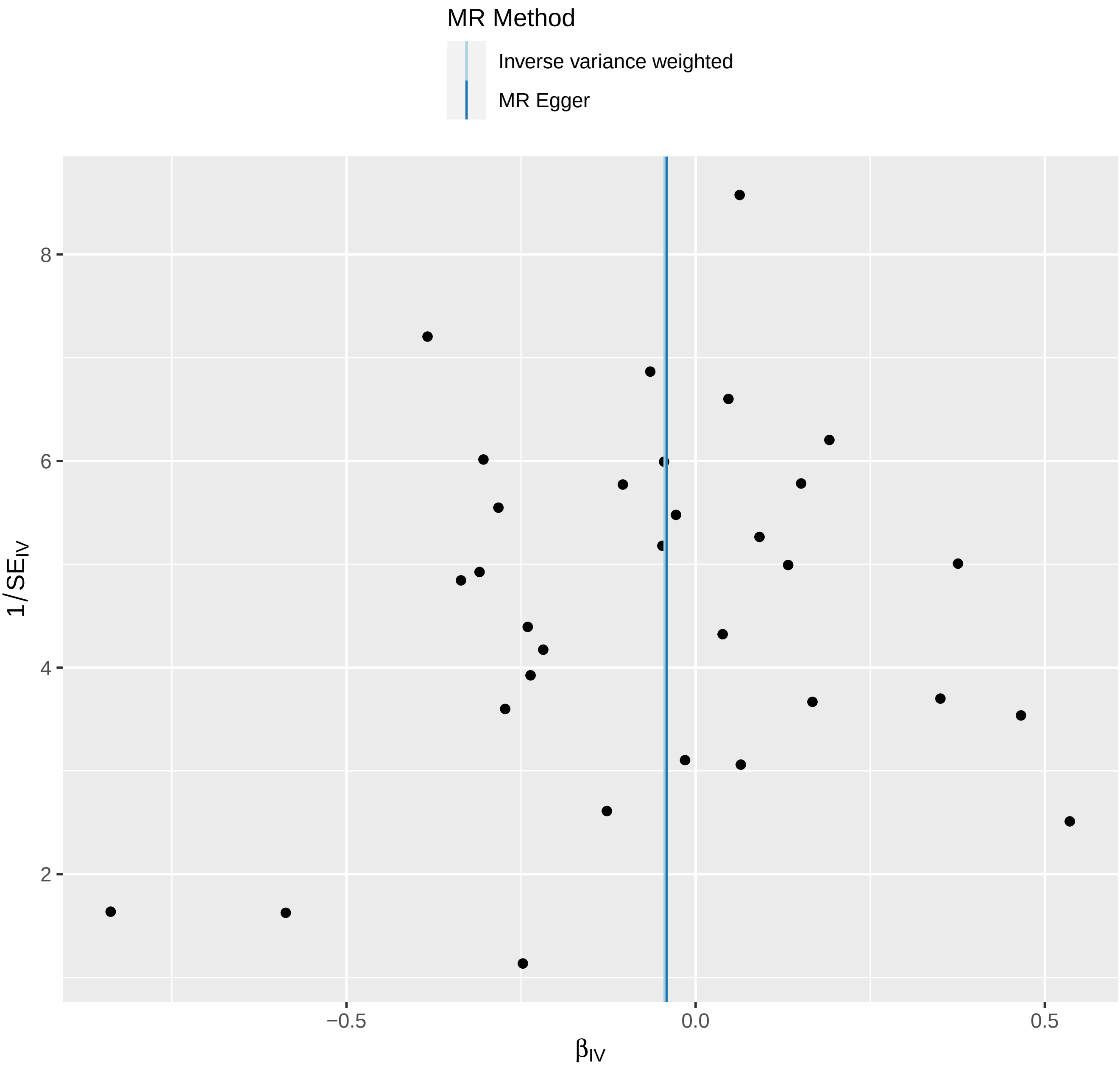

Batch 3−1699 : Ragulator complex protein LAMTOR3 on Diabetic nephropathy (more control exclusions)
with a threshold of 1e−05

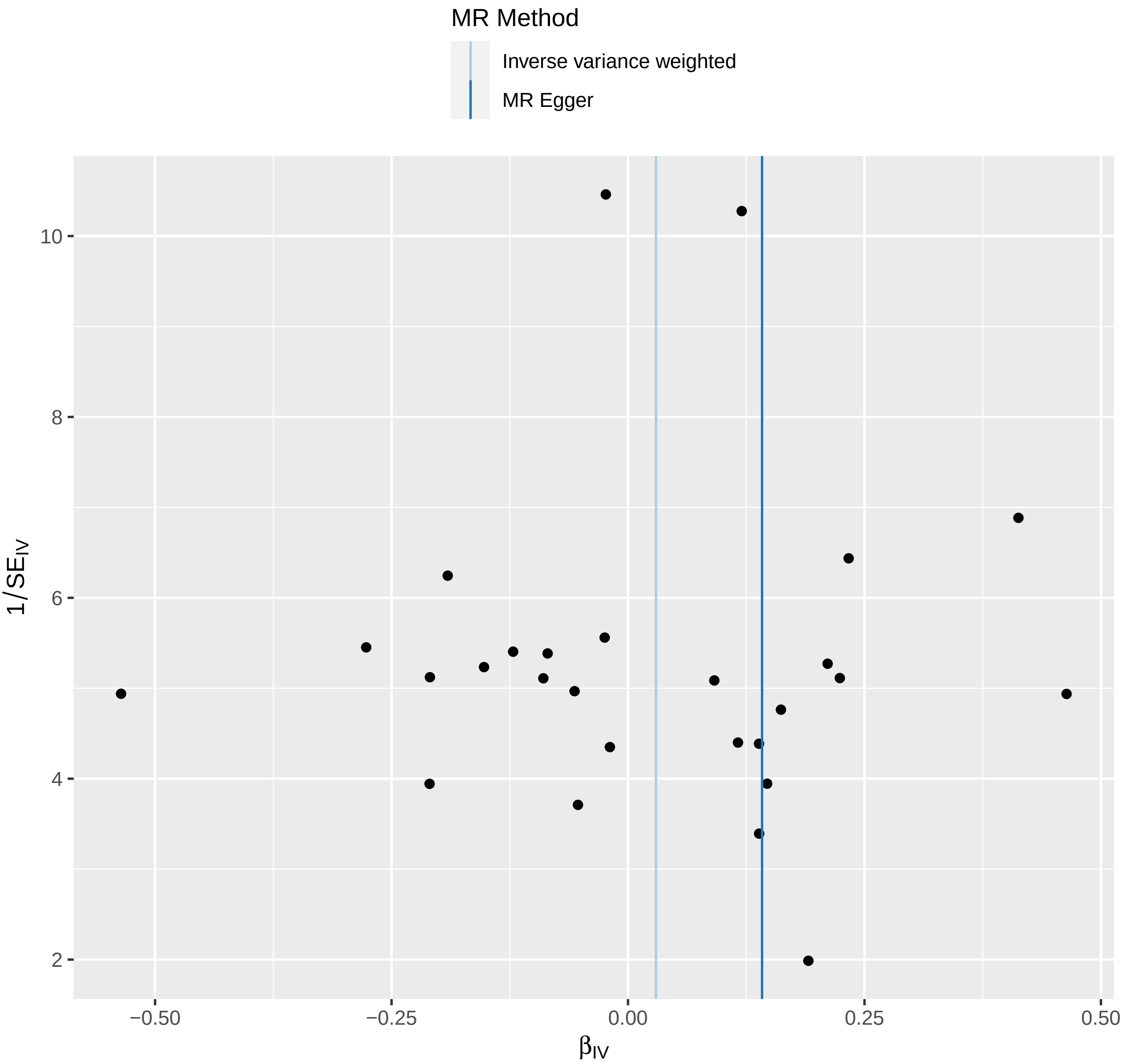

Batch 3−1700 : Glycosyltransferase−like protein LARGE1 on Diabetic nephropathy (more control exclusions) with a threshold of 1e−05

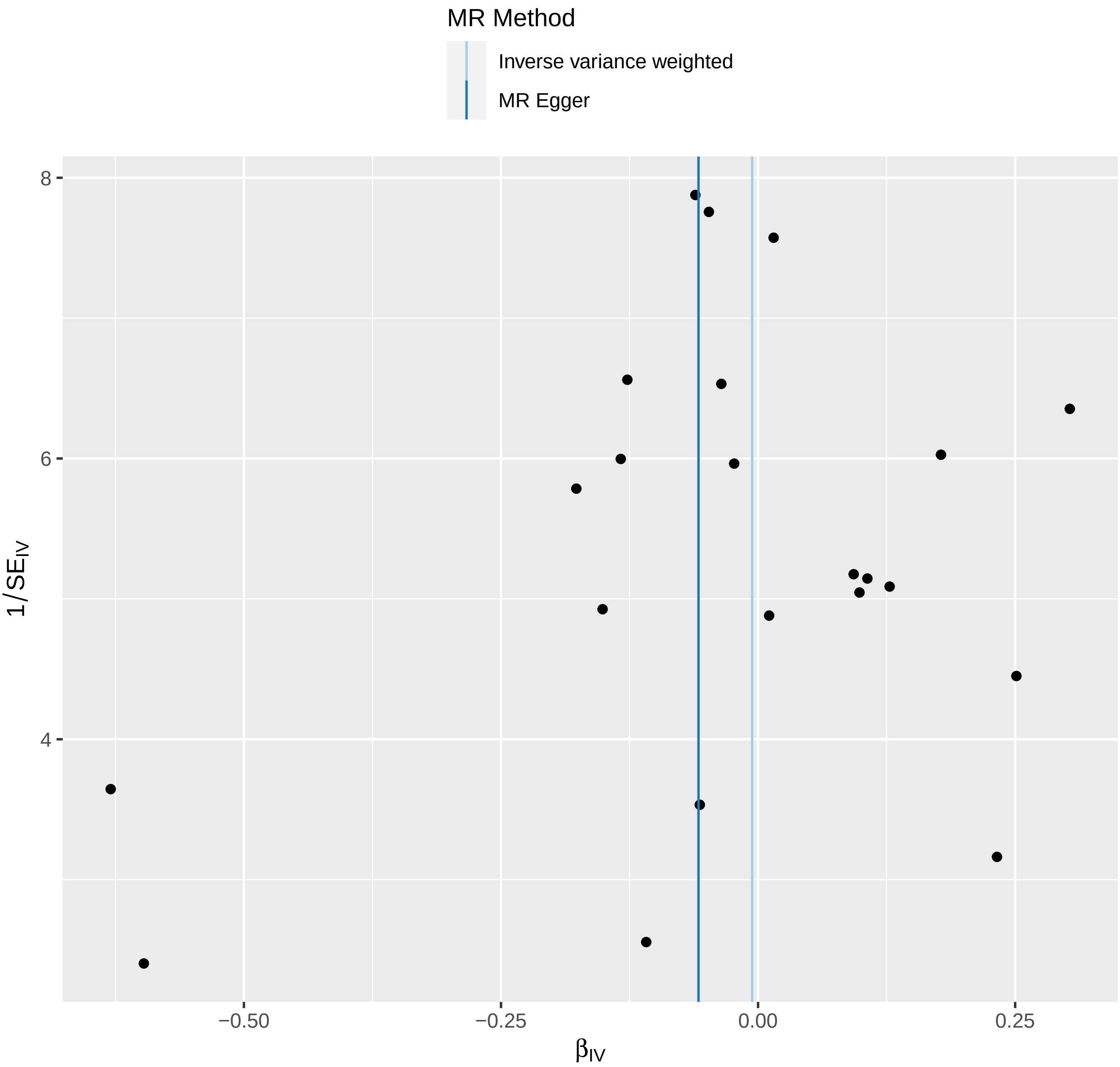

Batch 3−1701 : Linker for activation of T−cells family member 1 on Diabetic nephropathy (more control exclusions)
with a threshold of 1e−05

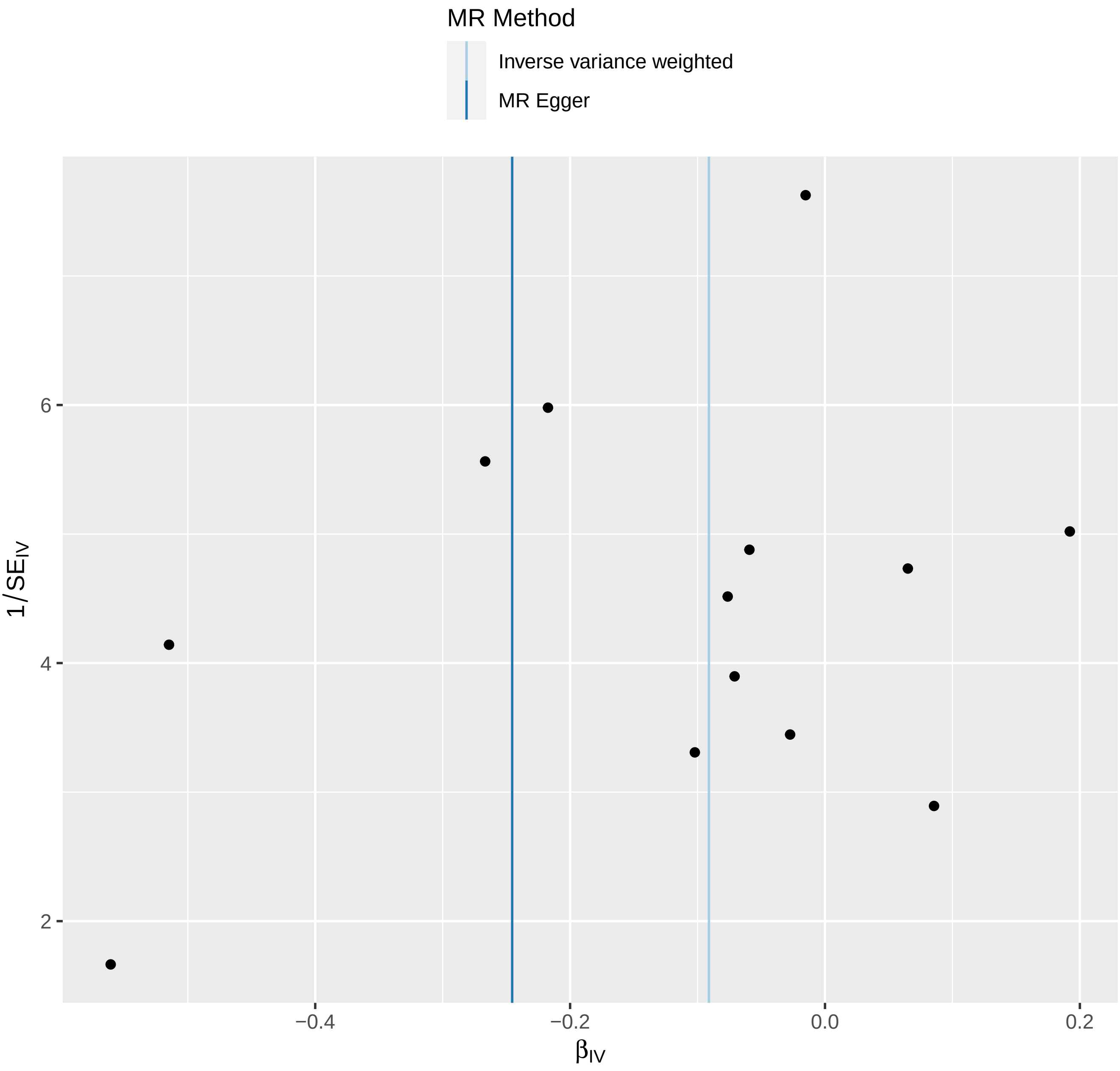

Batch 3−1702 : Linker for activation of T−cells family member 2 on Diabetic nephropathy (more control exclusions) with a threshold of 1e−05

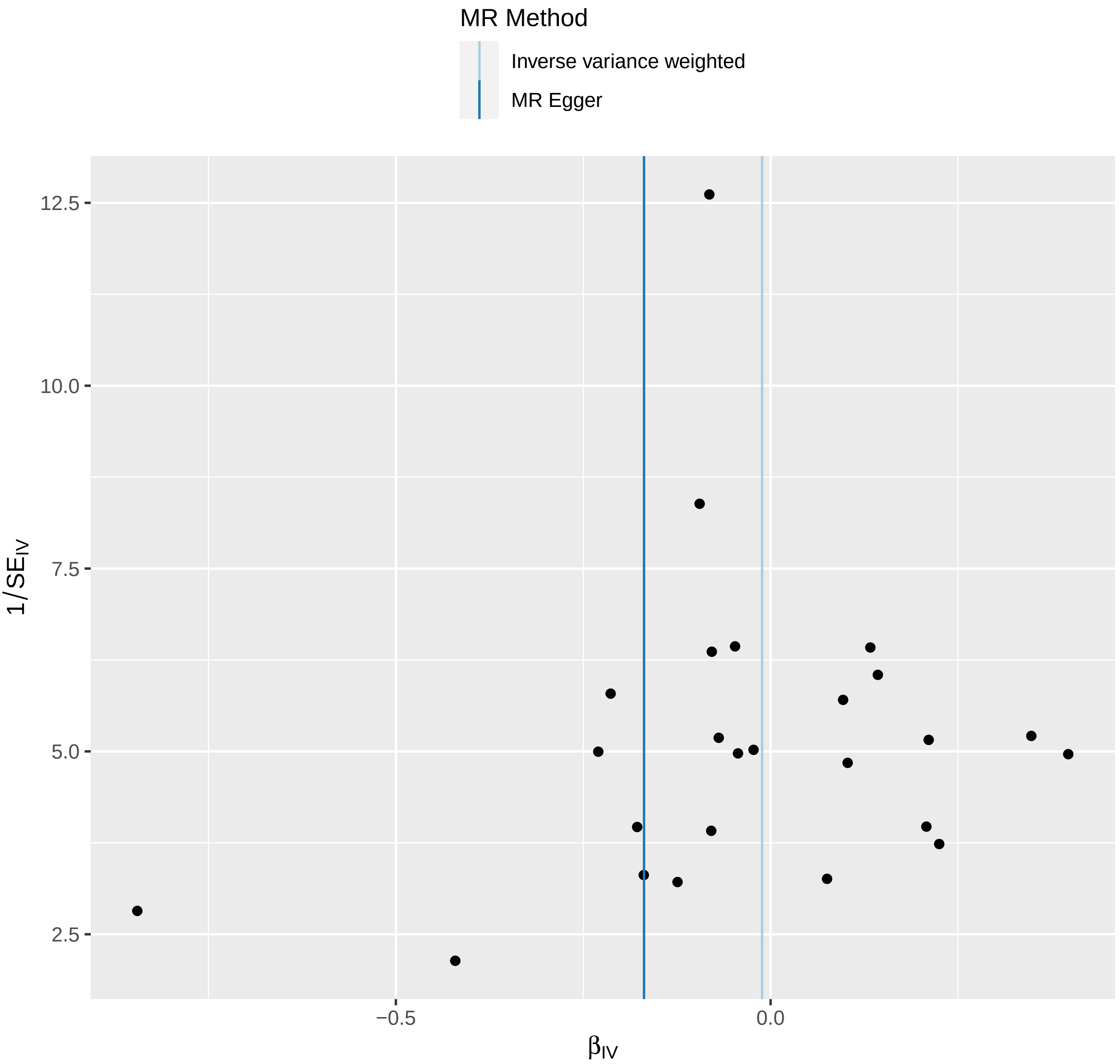

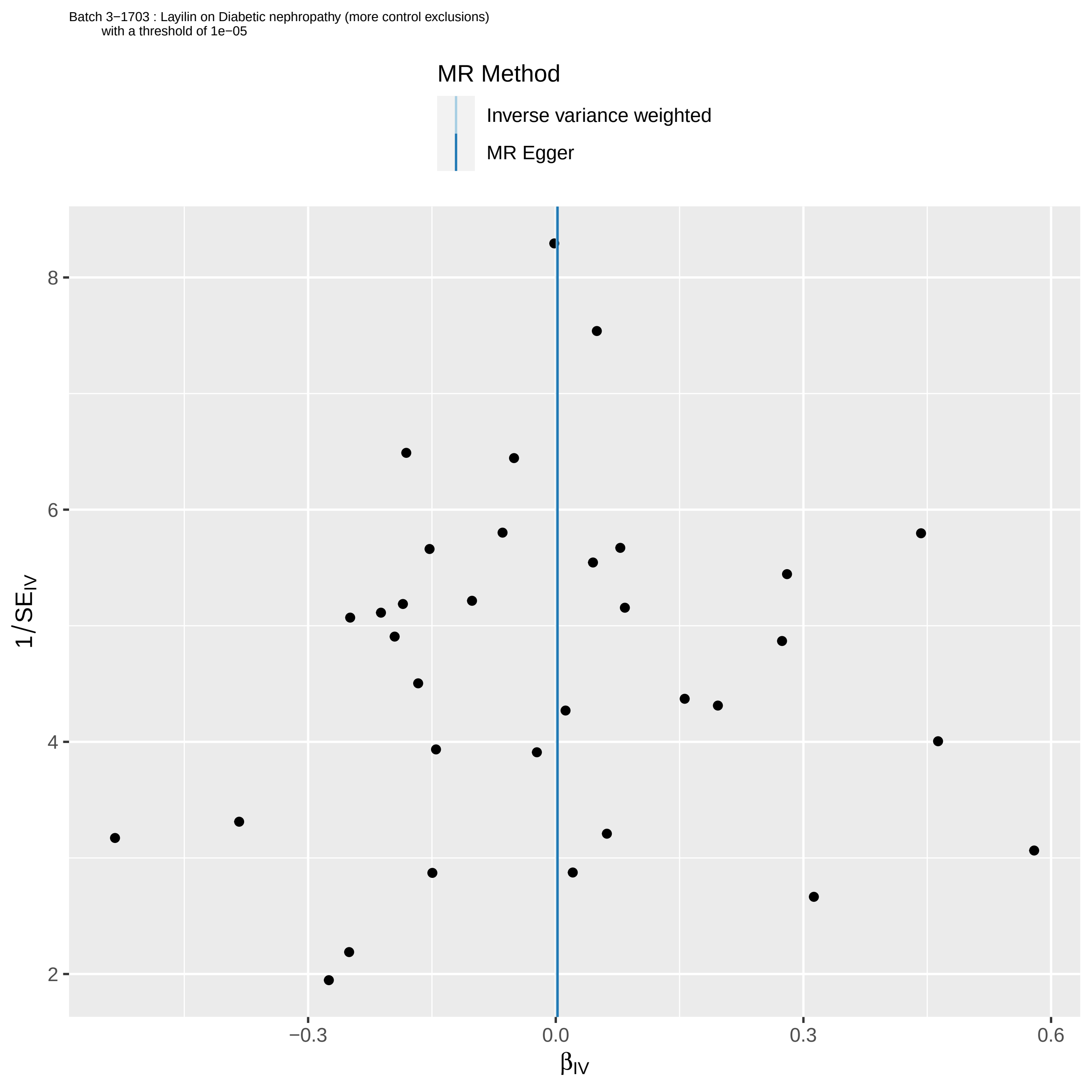
Batch 3−1703 : Layilin on Diabetic nephropathy (more control exclusions)
with a threshold of 1e−05
MR Method
Inverse variance weighted
MR Egger
8
6
4
2
1/SE_IV
−0.3
0.0
0.3
0.6
β_IV

Batch 3−1704 : Tyrosine−protein kinase Lck on Diabetic nephropathy (more control exclusions)
with a threshold of 1e−05

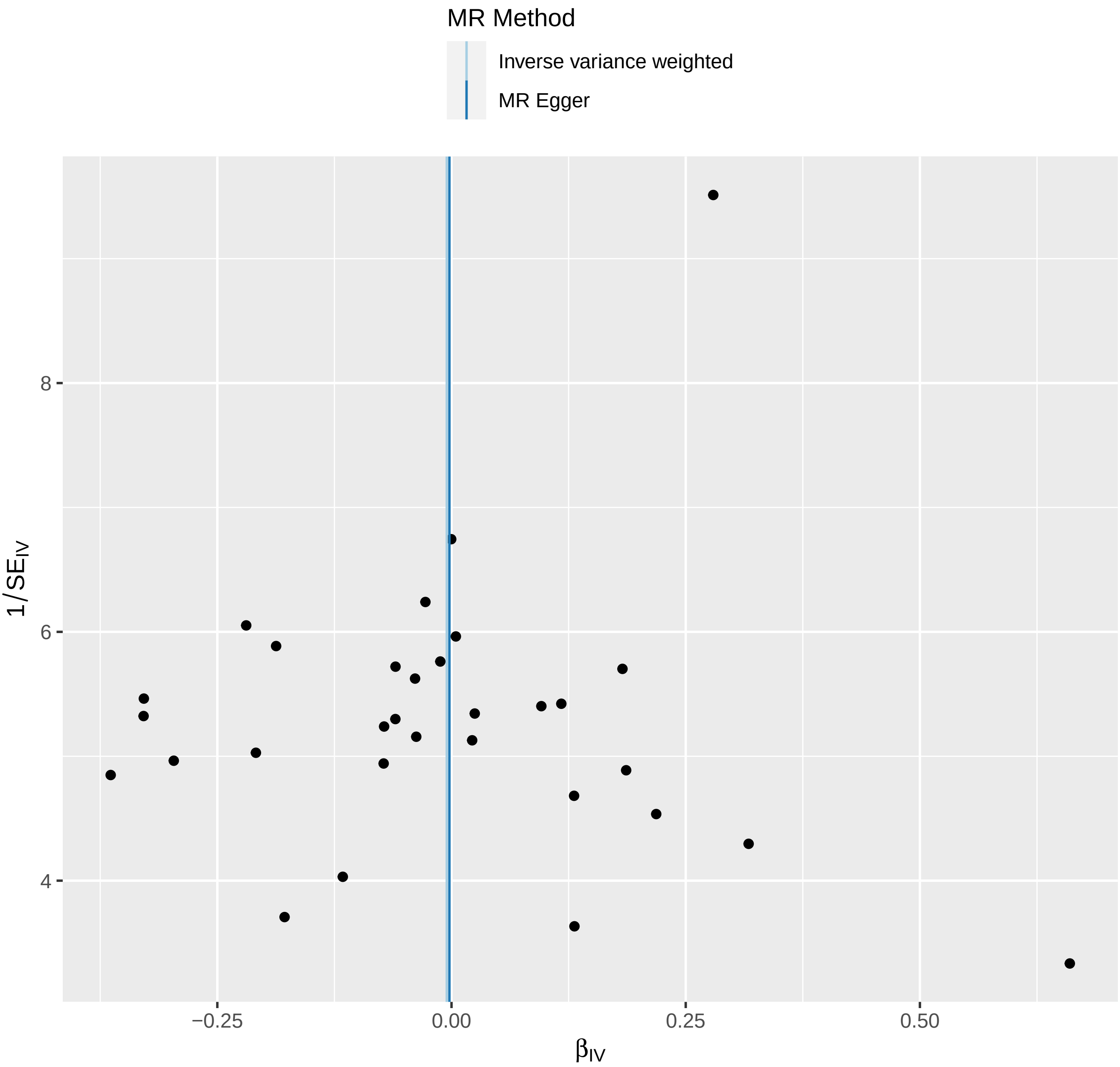

Batch 3−1705 : Tyrosine−protein kinase Lck on Diabetic nephropathy (more control exclusions)
with a threshold of 1e−05

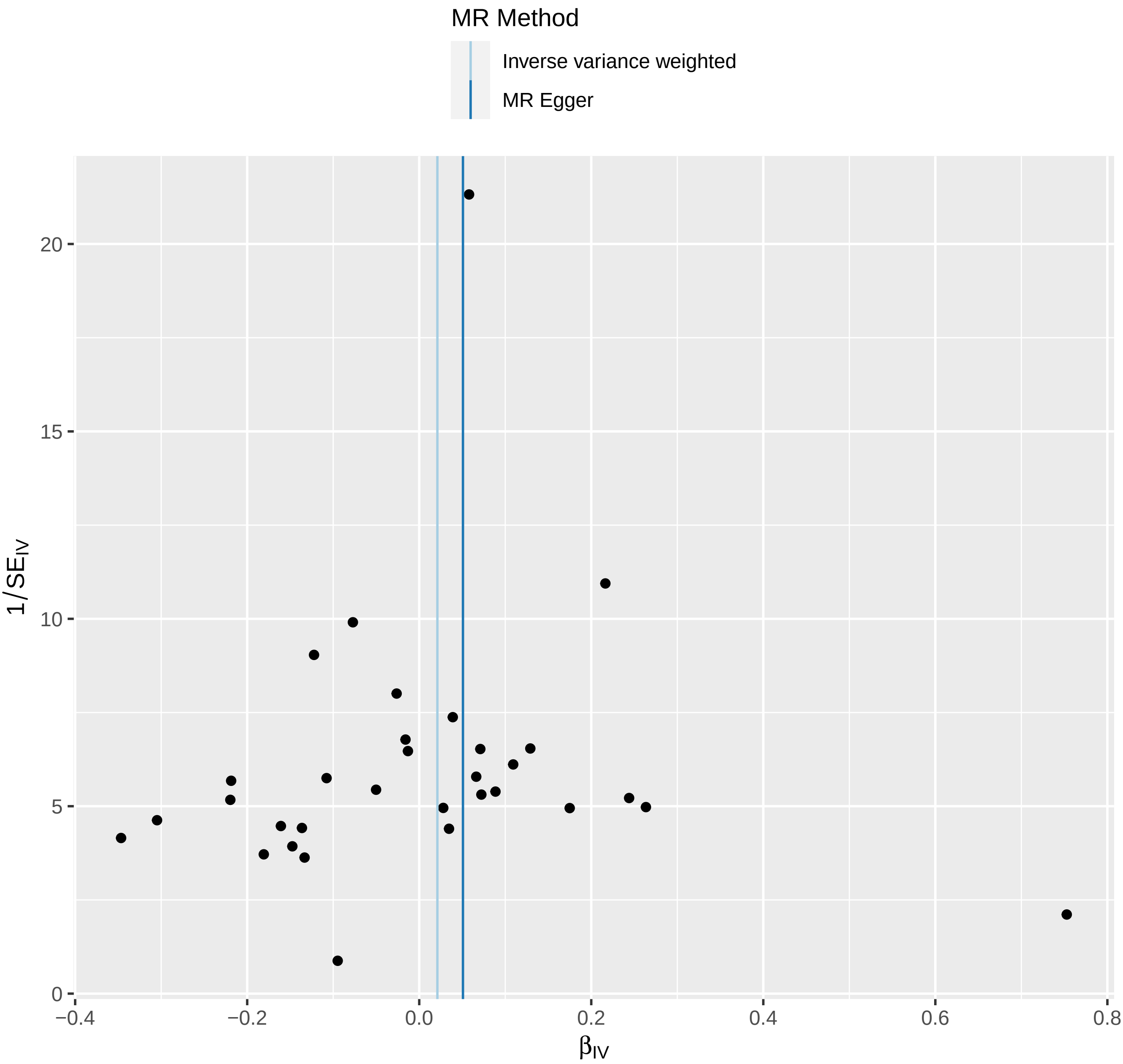

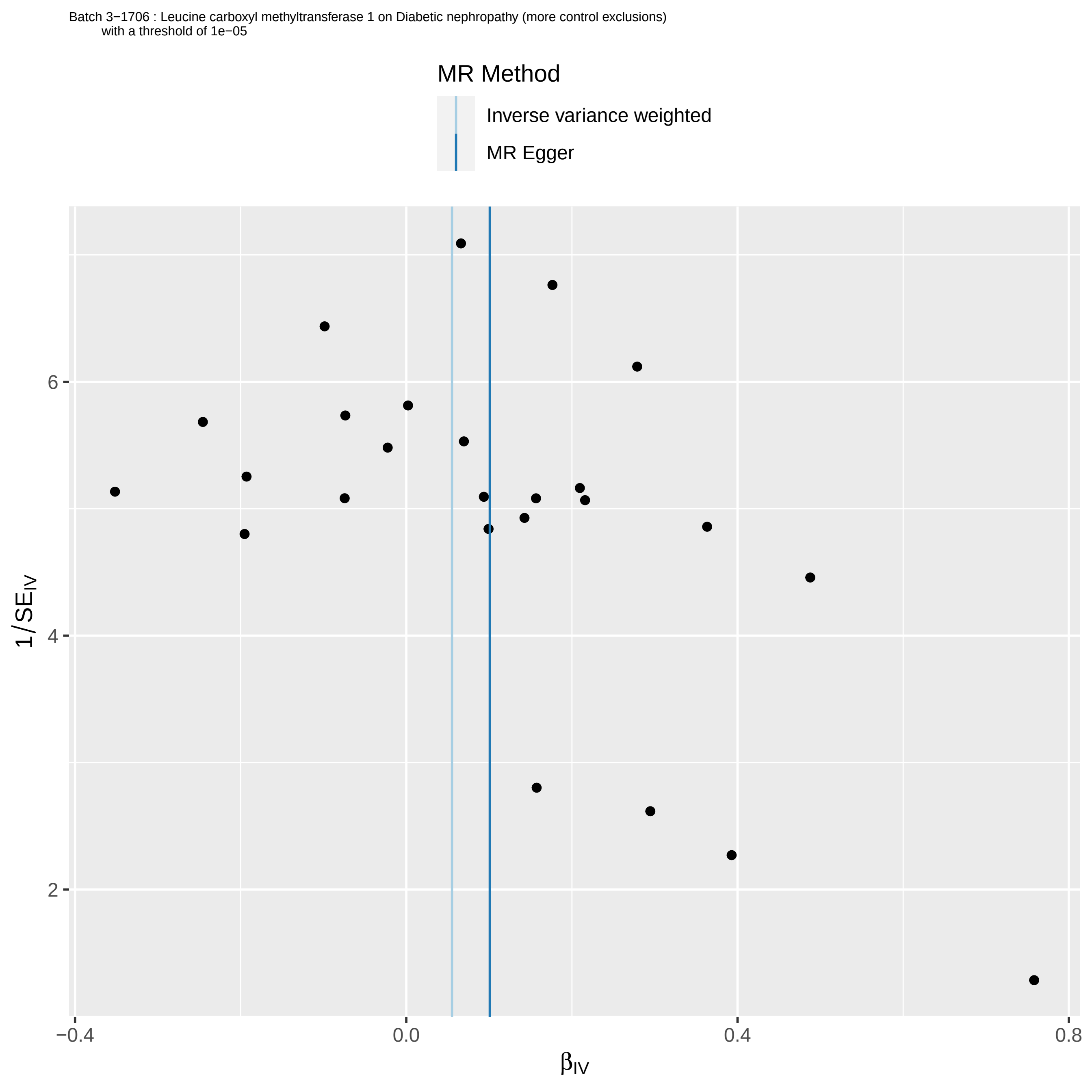
Batch 3−1706 : Leucine carboxyl methyltransferase 1 on Diabetic nephropathy (more control exclusions)
with a threshold of 1e−05
MR Method
Inverse variance weighted
MR Egger
6
4
2
1/SE_IV
−0.4
0.0
0.4
0.8
β_IV

Batch 3−1707 : Epididymal−specific lipocalin−10 on Diabetic nephropathy (more control exclusions)
with a threshold of 1e−05

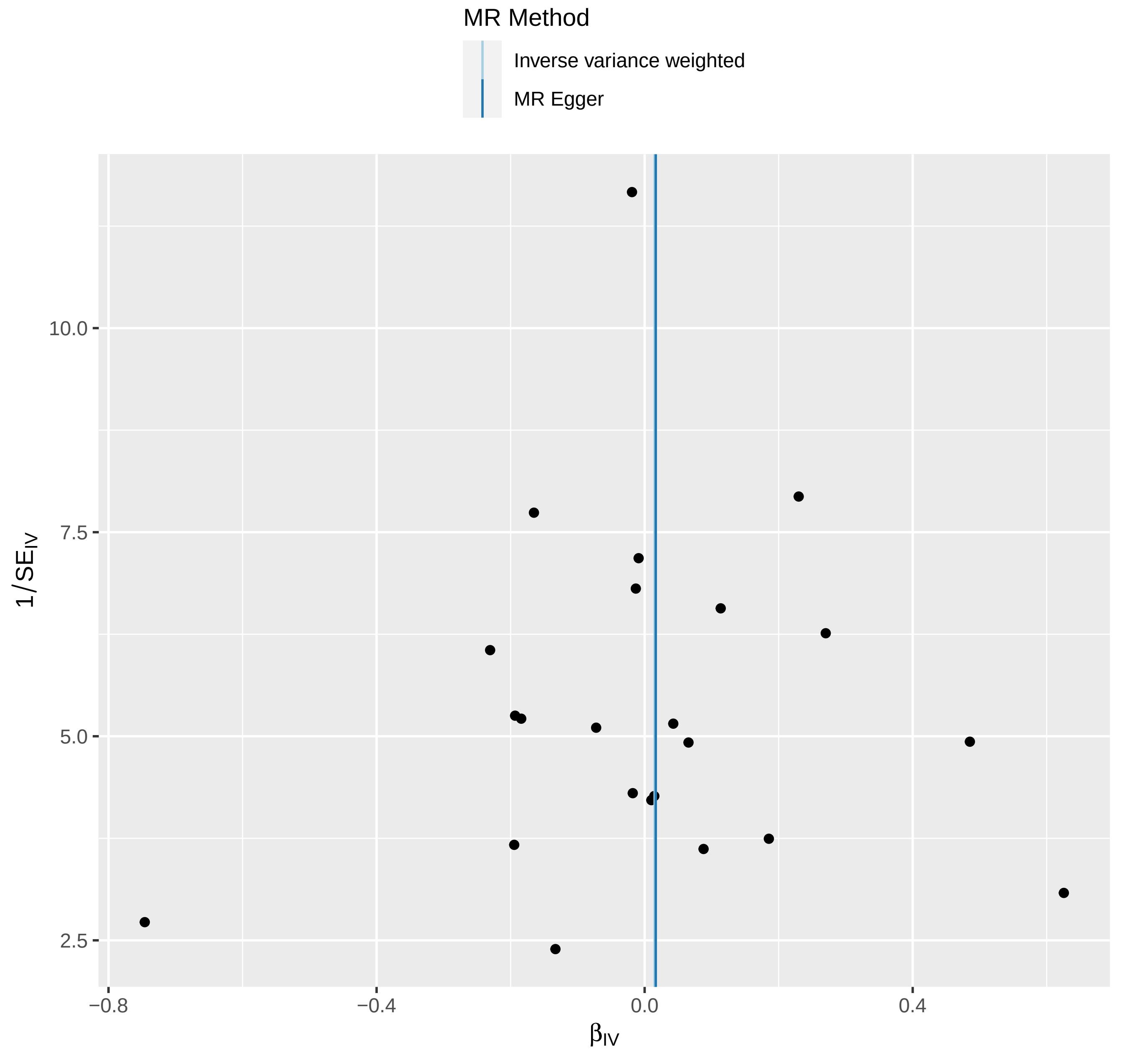

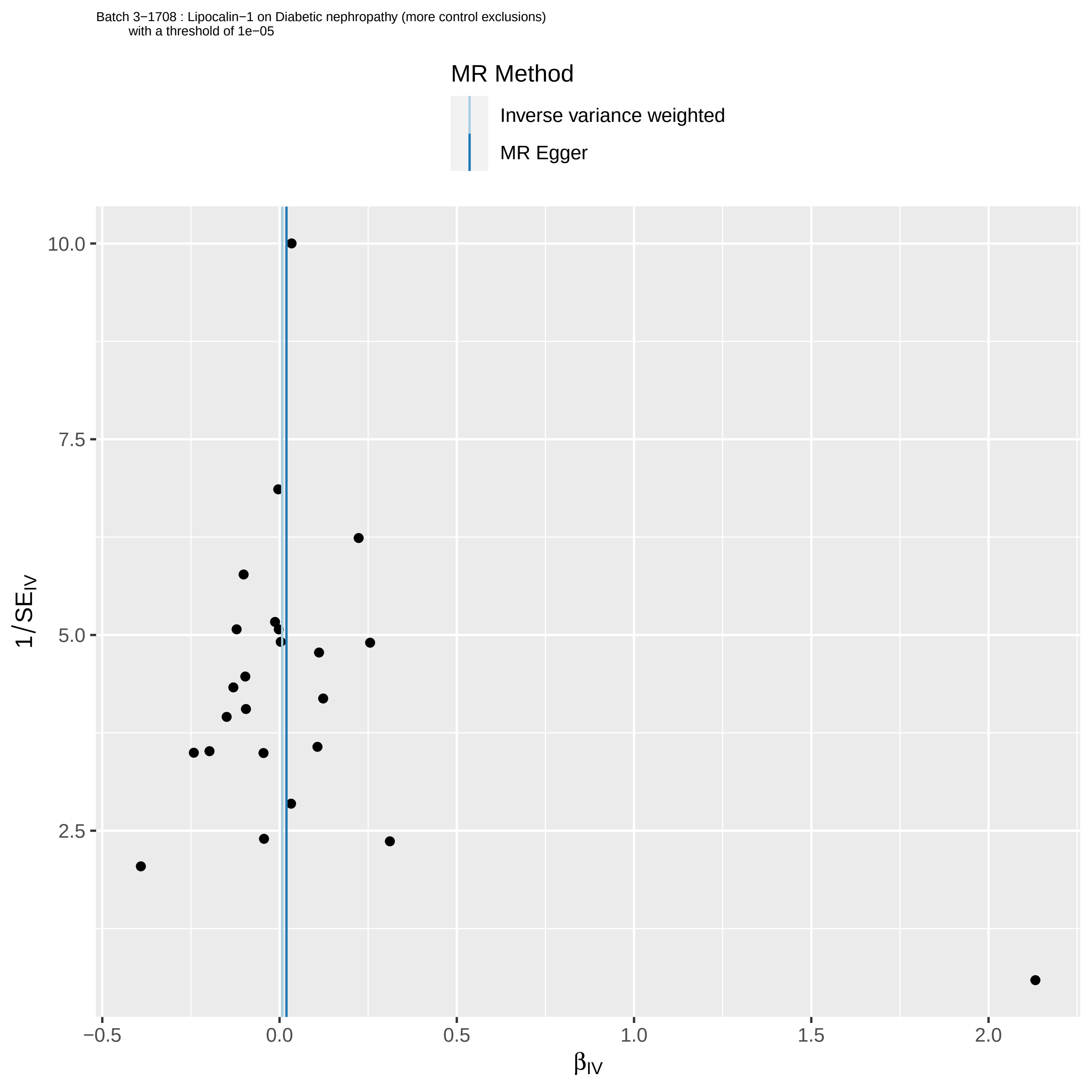
Batch 3−1708 : Lipocalin−1 on Diabetic nephropathy (more control exclusions)
with a threshold of 1e−05
MR Method
Inverse variance weighted
MR Egger
10.0
7.5
5.0
2.5
1/SE$_{IV}$
−0.5
0.0
0.5
1.0
1.5
2.0
β$_{IV}$

Batch 3−1709 : Neutrophil gelatinase−associated lipocalin on Diabetic nephropathy (more control exclusions)
with a threshold of 1e−05

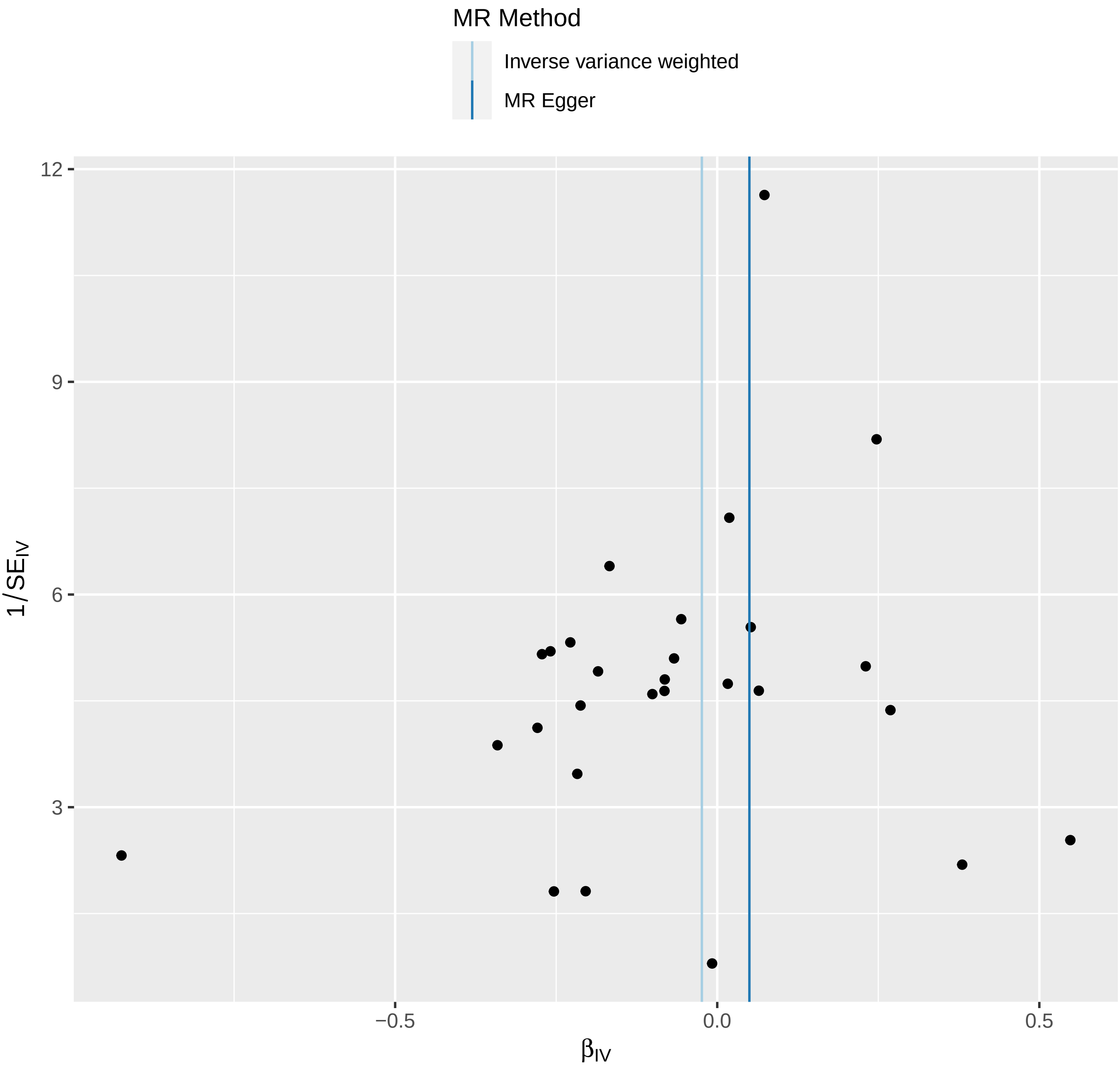

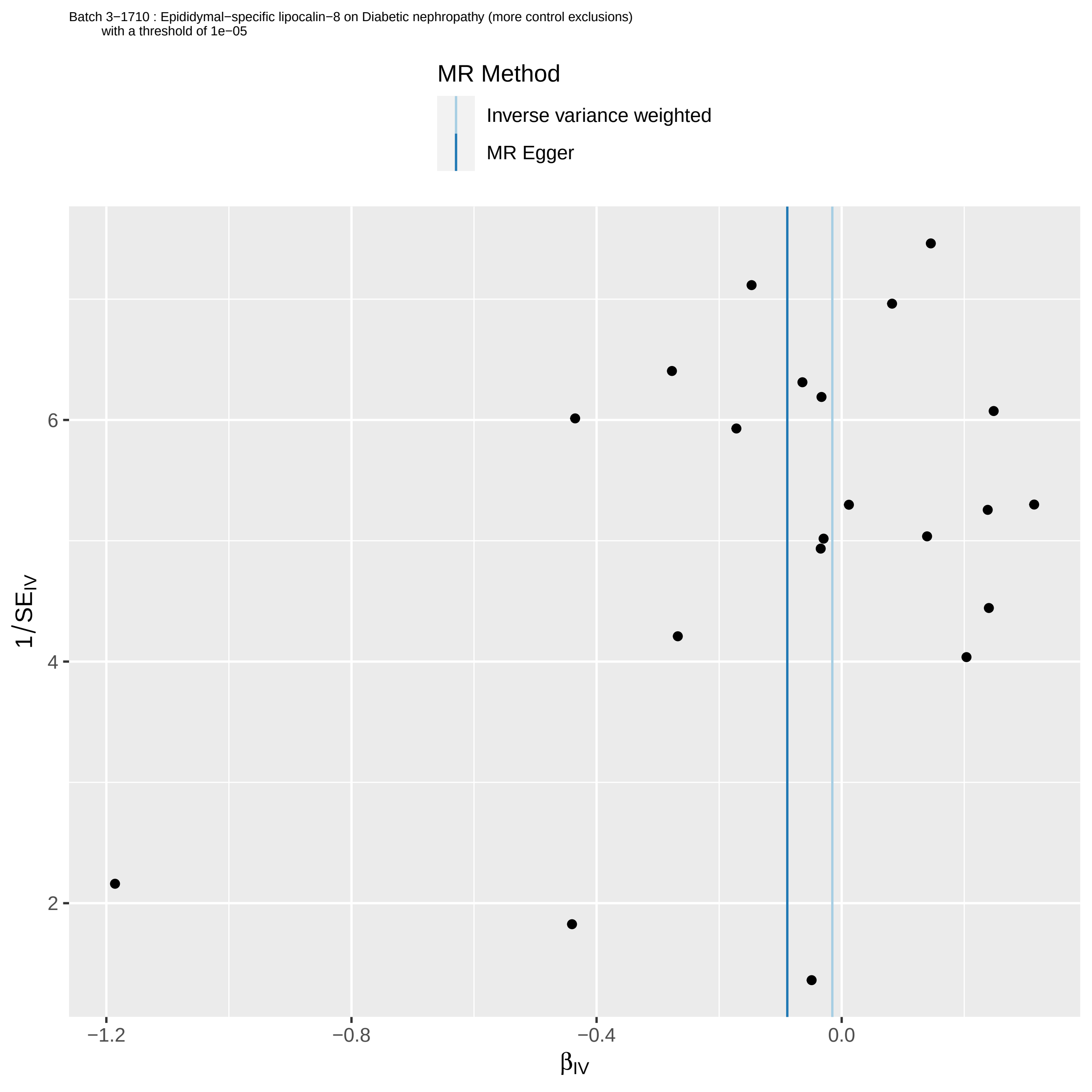

Batch 3−1710 : Epididymal−specific lipocalin−8 on Diabetic nephropathy (more control exclusions)
with a threshold of 1e−05
MR Method
Inverse variance weighted
MR Egger
6
4
2
1/SE$_{IV}$
−1.2
−0.8
−0.4
0.0
β_{IV}

Batch 3−1711 : Ligand−dependent nuclear receptor corepressor−like protein on Diabetic nephropathy (more control exclusions)
with a threshold of 1e−05

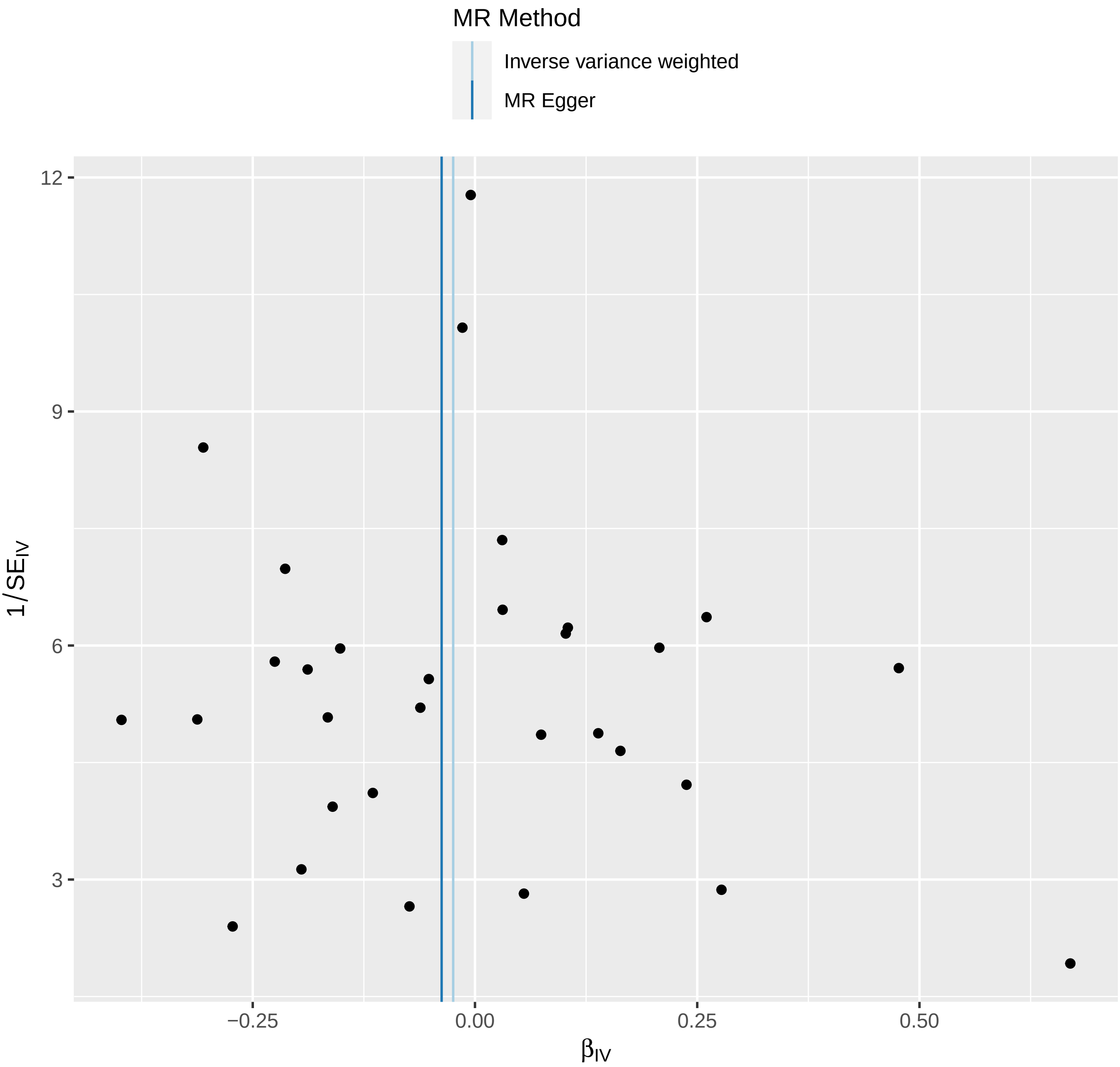

Batch 3−1712 : Plastin−2 on Diabetic nephropathy (more control exclusions)
with a threshold of 1e−05

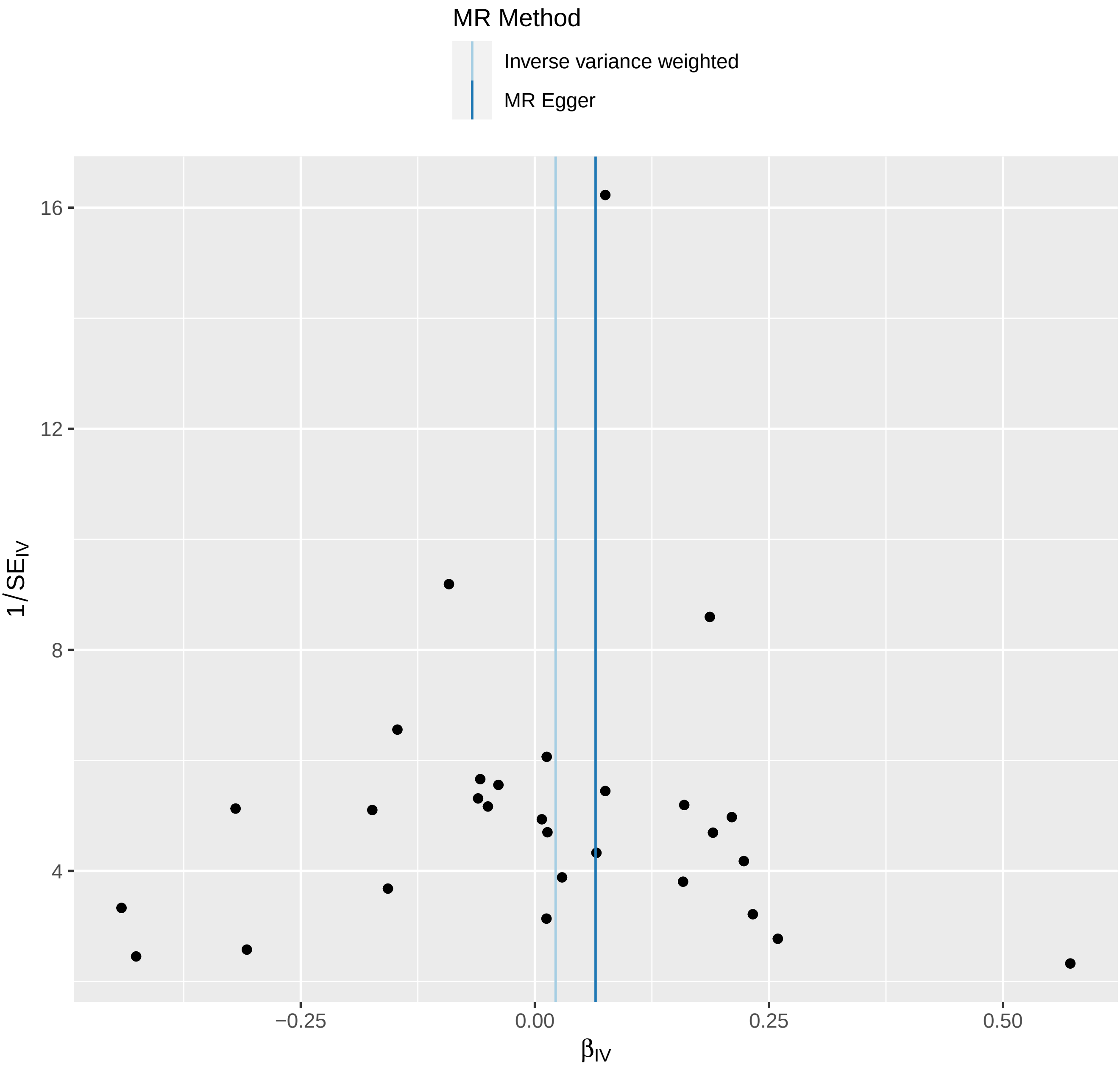

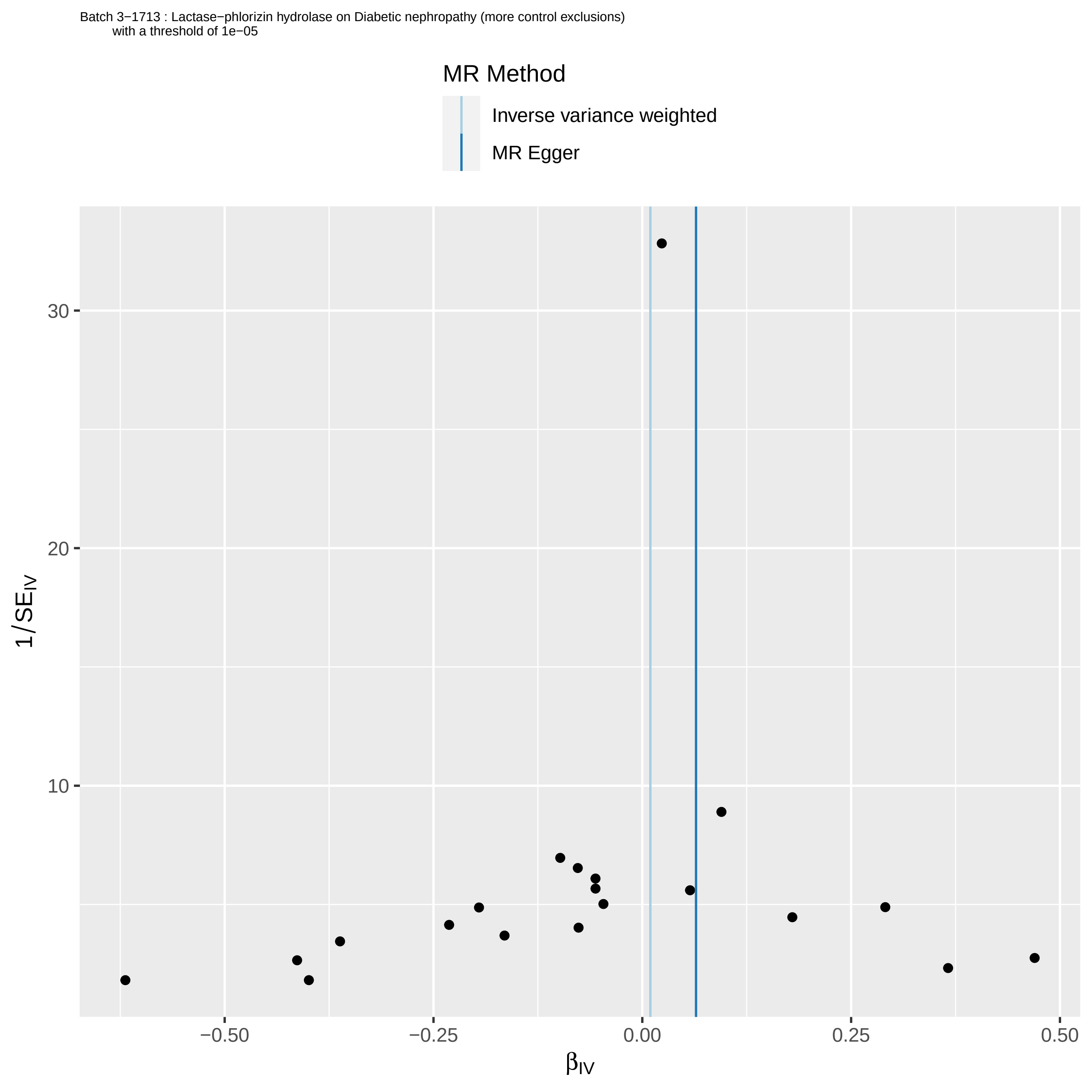
Batch 3−1713 : Lactase−phlorizin hydrolase on Diabetic nephropathy (more control exclusions)
with a threshold of 1e−05
MR Method
Inverse variance weighted
MR Egger
30
20
10
1/SE_IV
−0.50
−0.25
0.00
0.25
0.50
β_IV

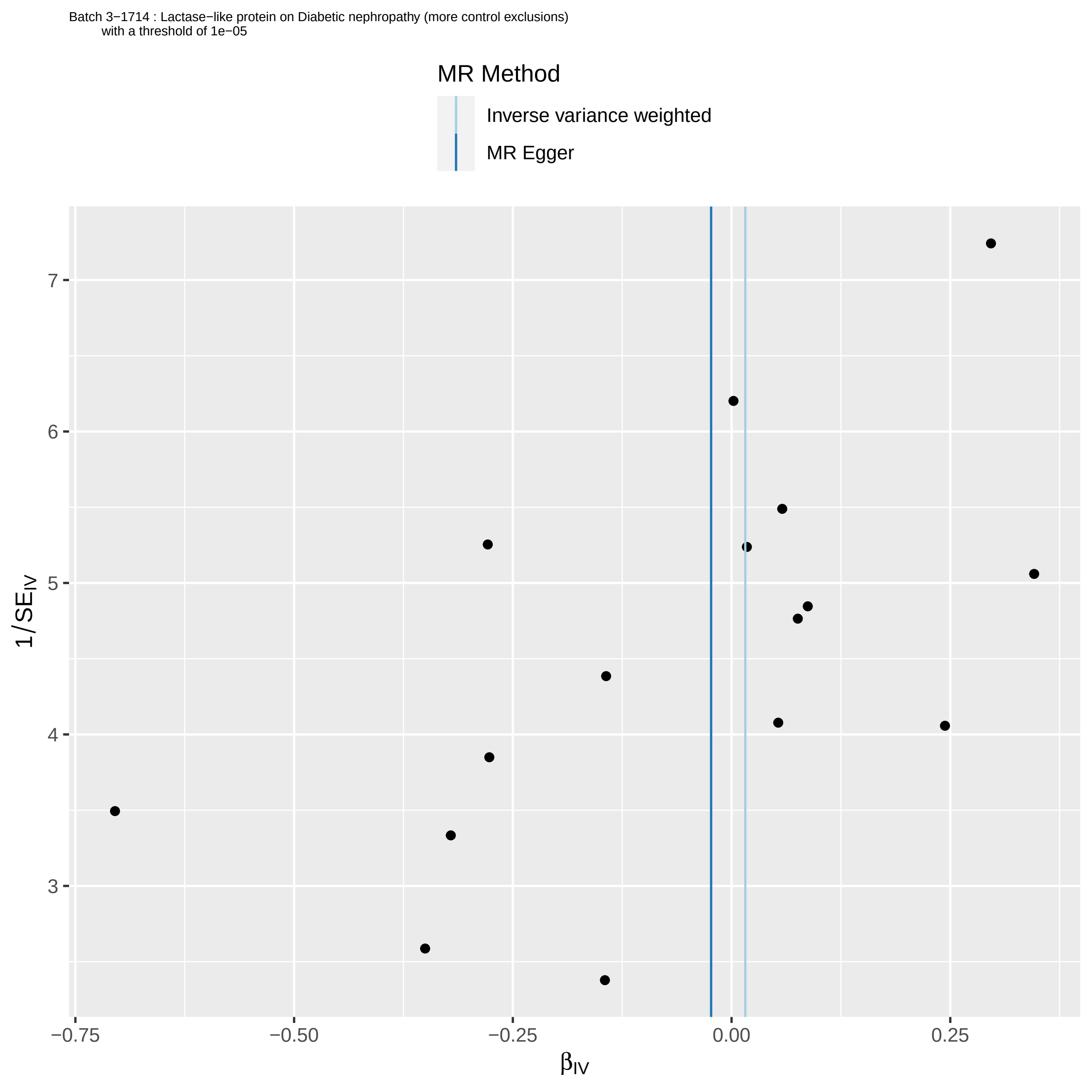
Batch 3−1714 : Lactase−like protein on Diabetic nephropathy (more control exclusions)
with a threshold of 1e−05
MR Method
Inverse variance weighted
MR Egger
7
6
5
4
3
1/SE_IV
−0.75
−0.50
−0.25
0.00
0.25
β_IV

Batch 3−1715 : L−lactate dehydrogenase B chain on Diabetic nephropathy (more control exclusions)
with a threshold of 1e−05

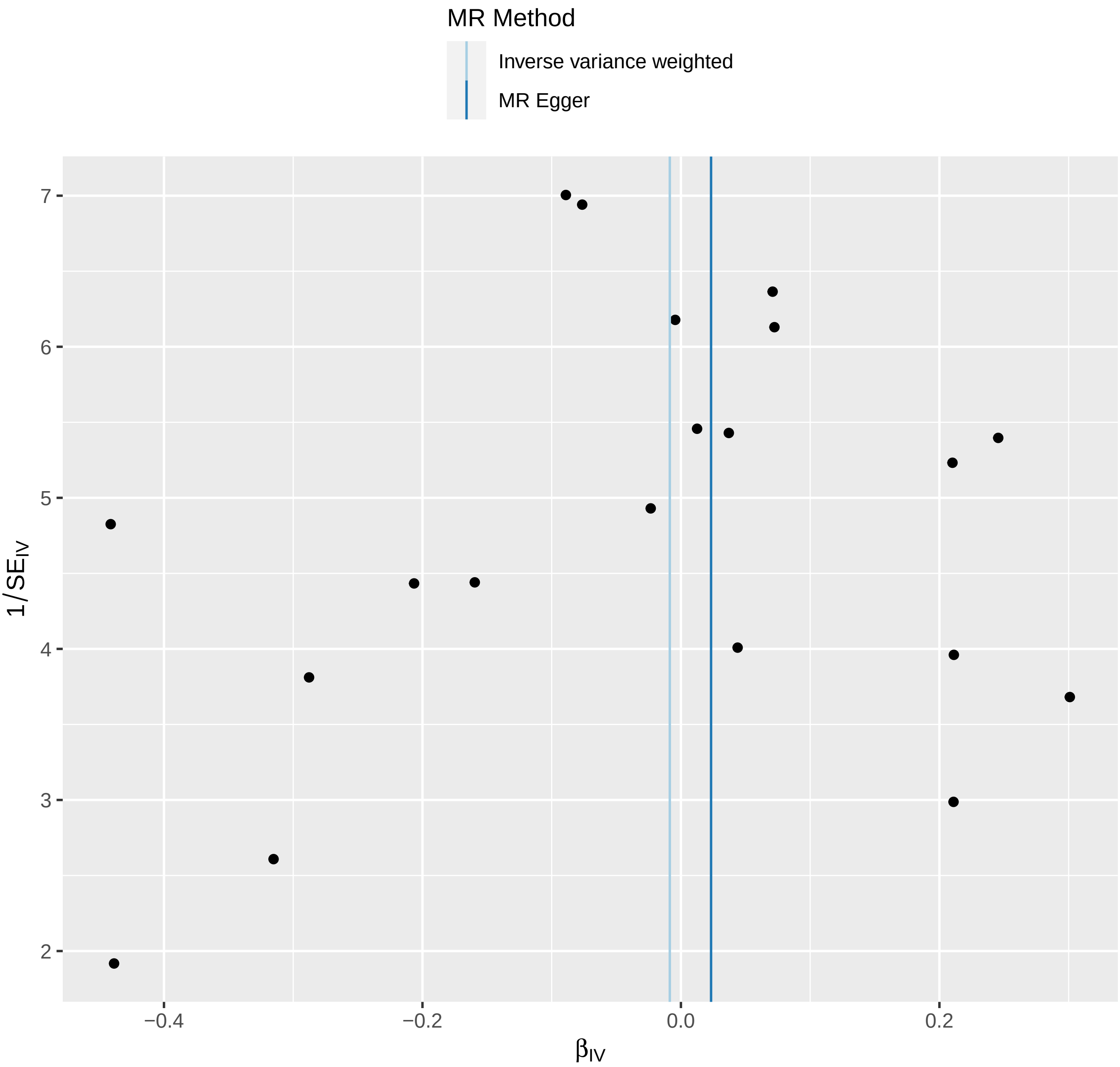

Batch 3−1716 : L−lactate dehydrogenase C chain on Diabetic nephropathy (more control exclusions)
with a threshold of 1e−05

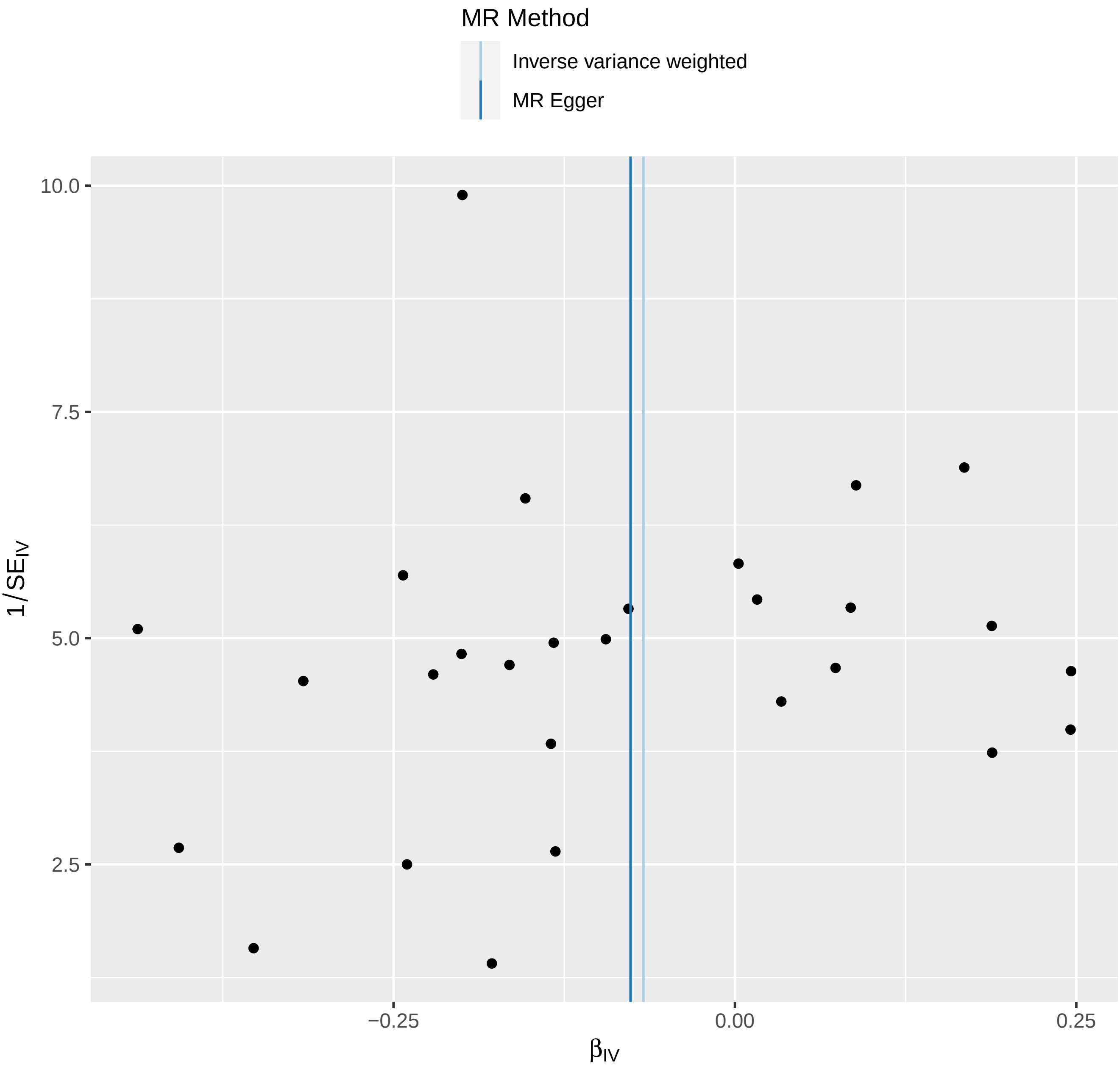

Batch 3−1717 : Low−density lipoprotein receptor on Diabetic nephropathy (more control exclusions)
with a threshold of 1e−05

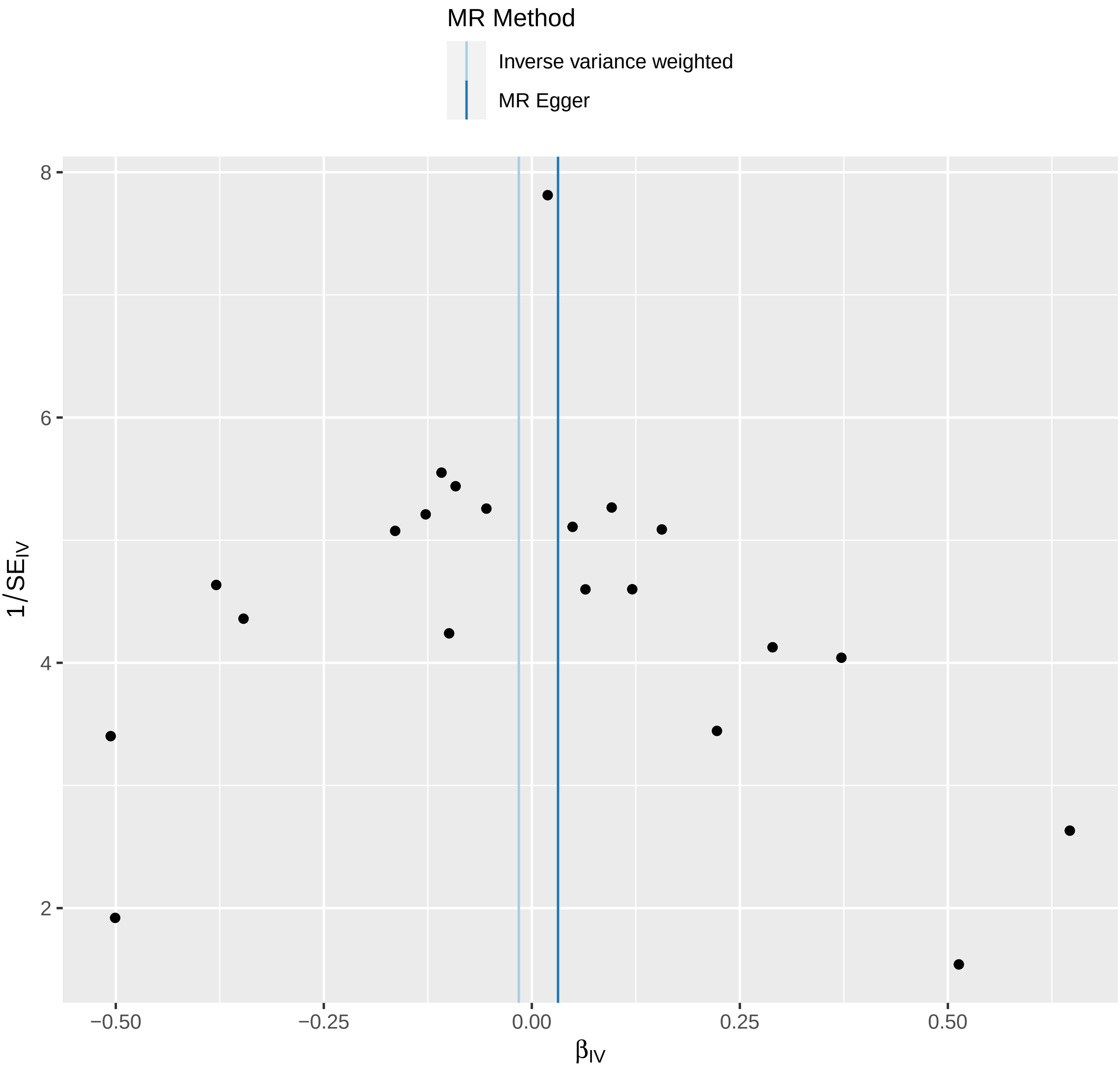

Batch 3−1718 : Protein LDOC1 on Diabetic nephropathy (more control exclusions)
with a threshold of 1e−05

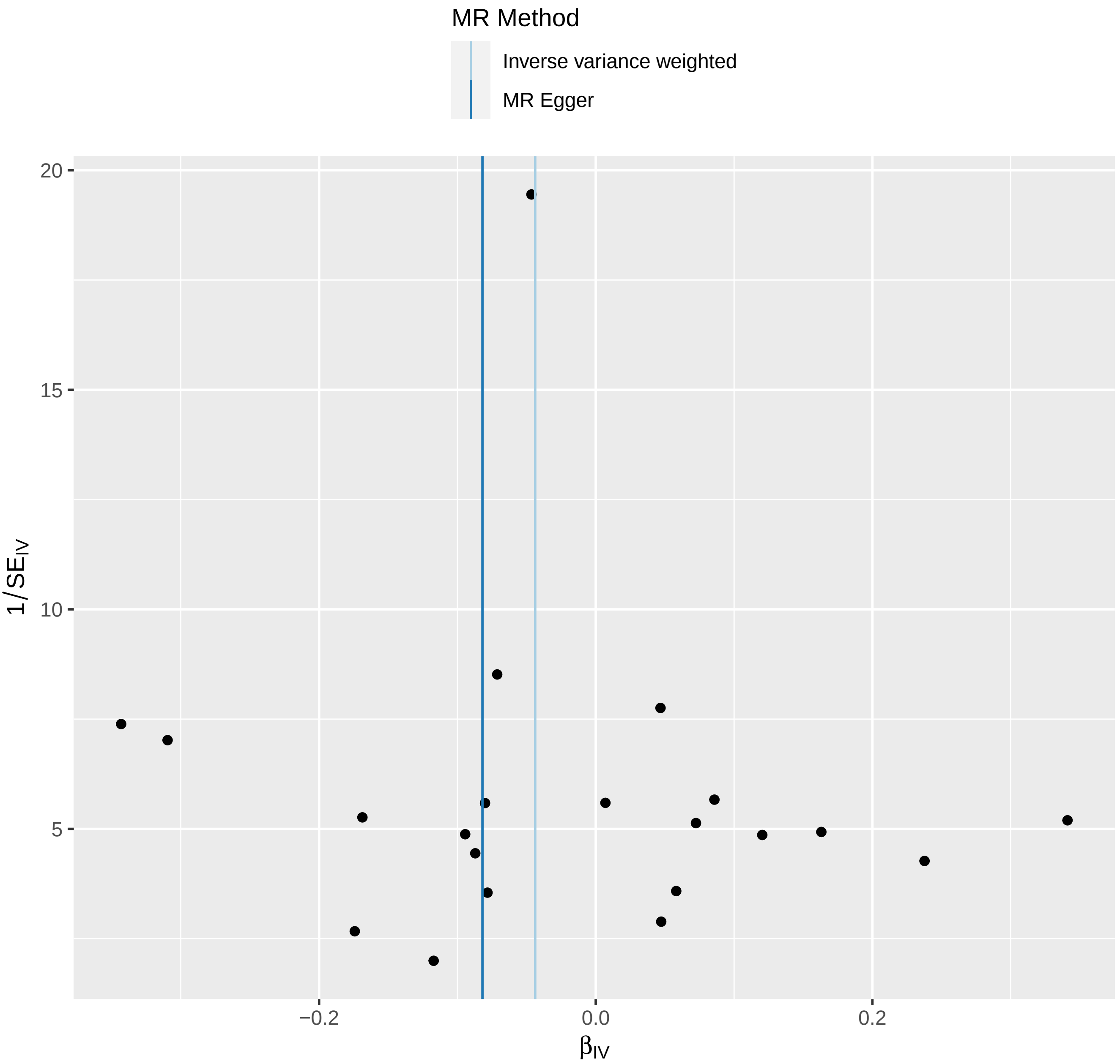

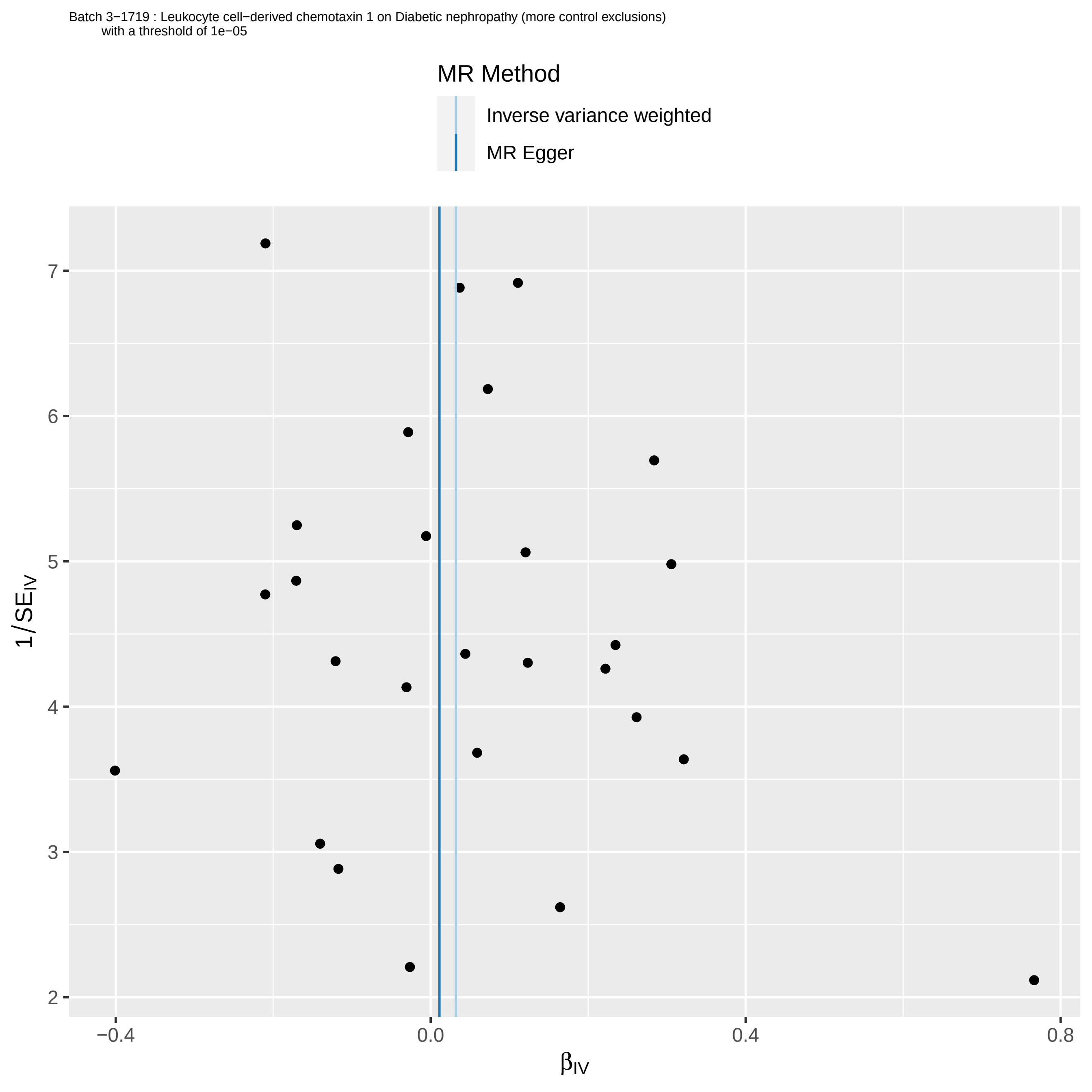
Batch 3−1719 : Leukocyte cell−derived chemotaxin 1 on Diabetic nephropathy (more control exclusions)
with a threshold of 1e−05
MR Method
Inverse variance weighted
MR Egger
7
6
5
4
3
2
$1/SE_{IV}$
−0.4
0.0
0.4
0.8
β_{IV}

Batch 3−1720 : LEM domain−containing protein 1 on Diabetic nephropathy (more control exclusions) with a threshold of 1e−05

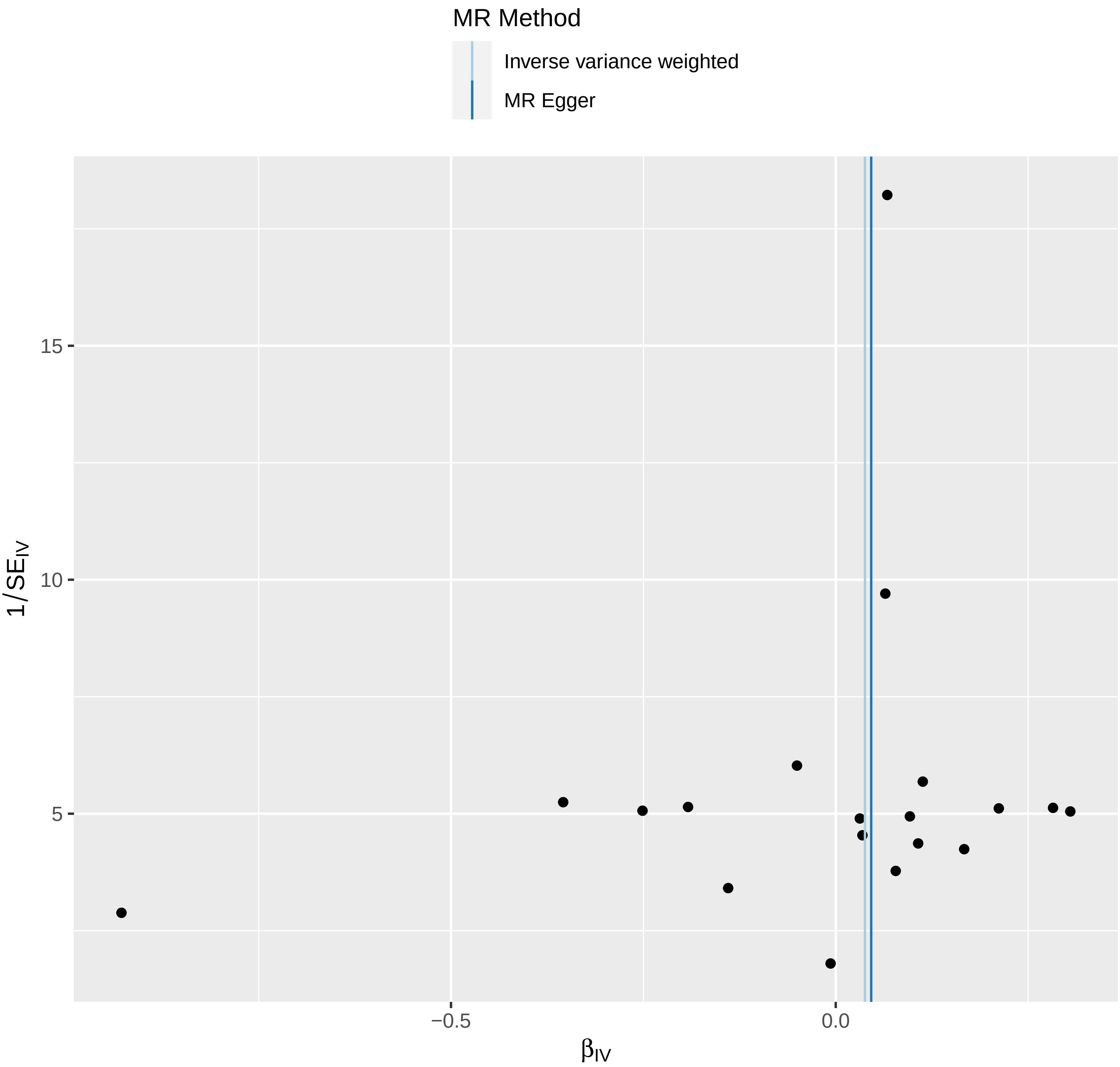

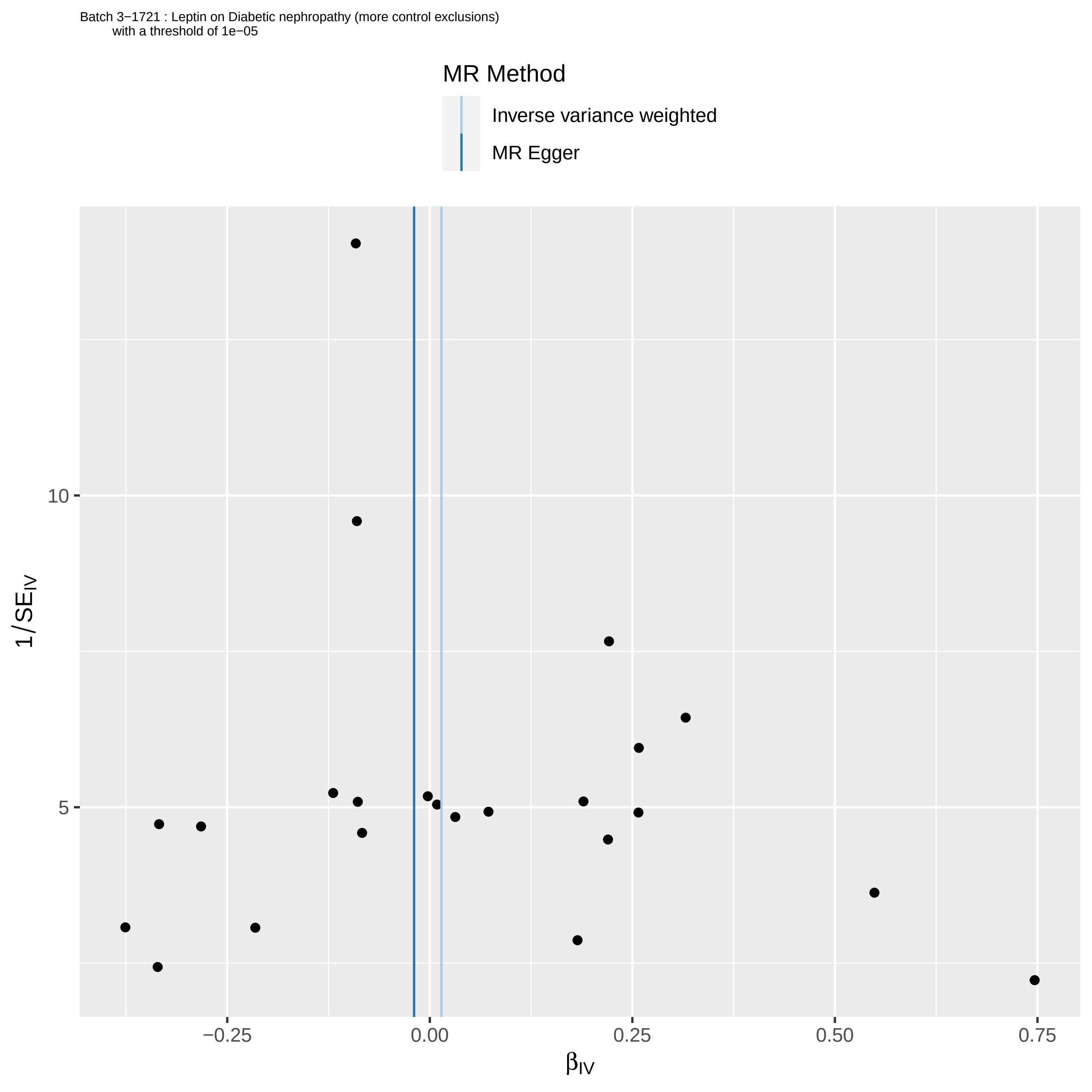

Batch 3−1721 : Leptin on Diabetic nephropathy (more control exclusions)
with a threshold of 1e−05
MR Method
Inverse variance weighted
MR Egger
10
5
1/SE_IV
−0.25
0.00
0.25
0.50
0.75
β_IV

Batch 3−1722 : Leptin on Diabetic nephropathy (more control exclusions)
with a threshold of 1e−05

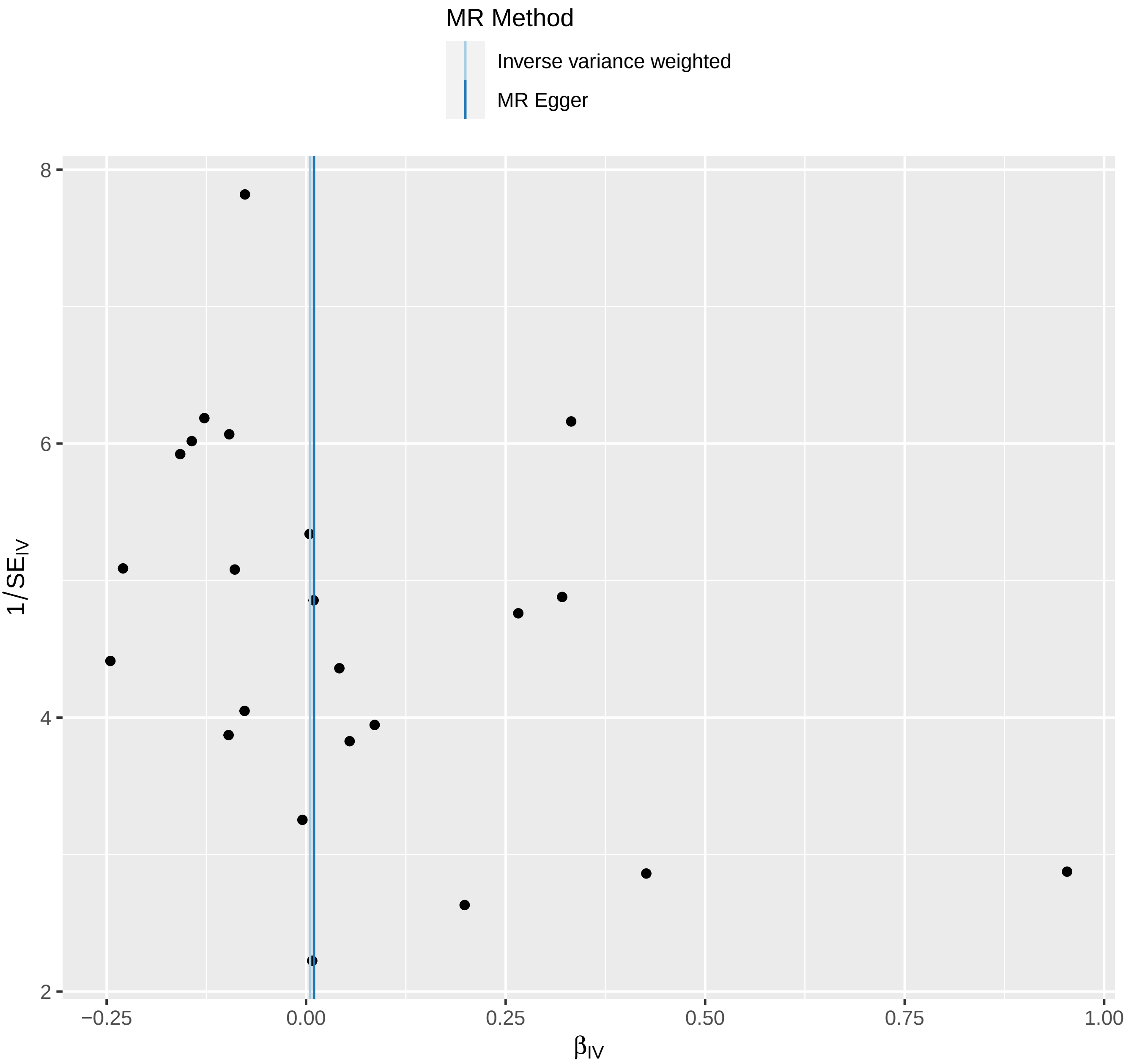

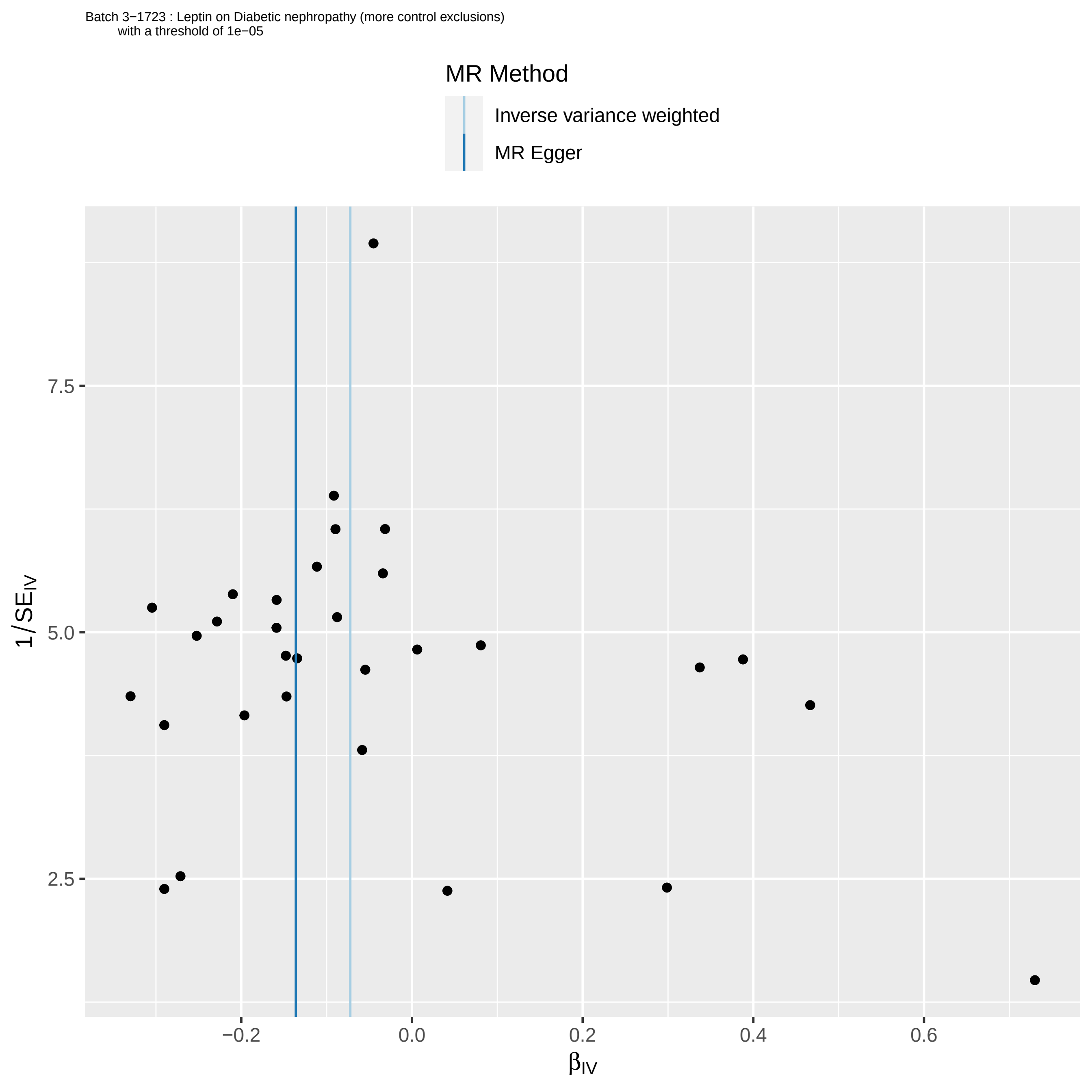

Batch 3−1723 : Leptin on Diabetic nephropathy (more control exclusions)
with a threshold of 1e−05
MR Method
Inverse variance weighted
MR Egger
7.5
5.0
2.5
1/SE$_{IV}$
−0.2
0.0
0.2
0.4
0.6
β$_{IV}$

Batch 3−1724 : Leptin receptor on Diabetic nephropathy (more control exclusions)
with a threshold of 1e−05

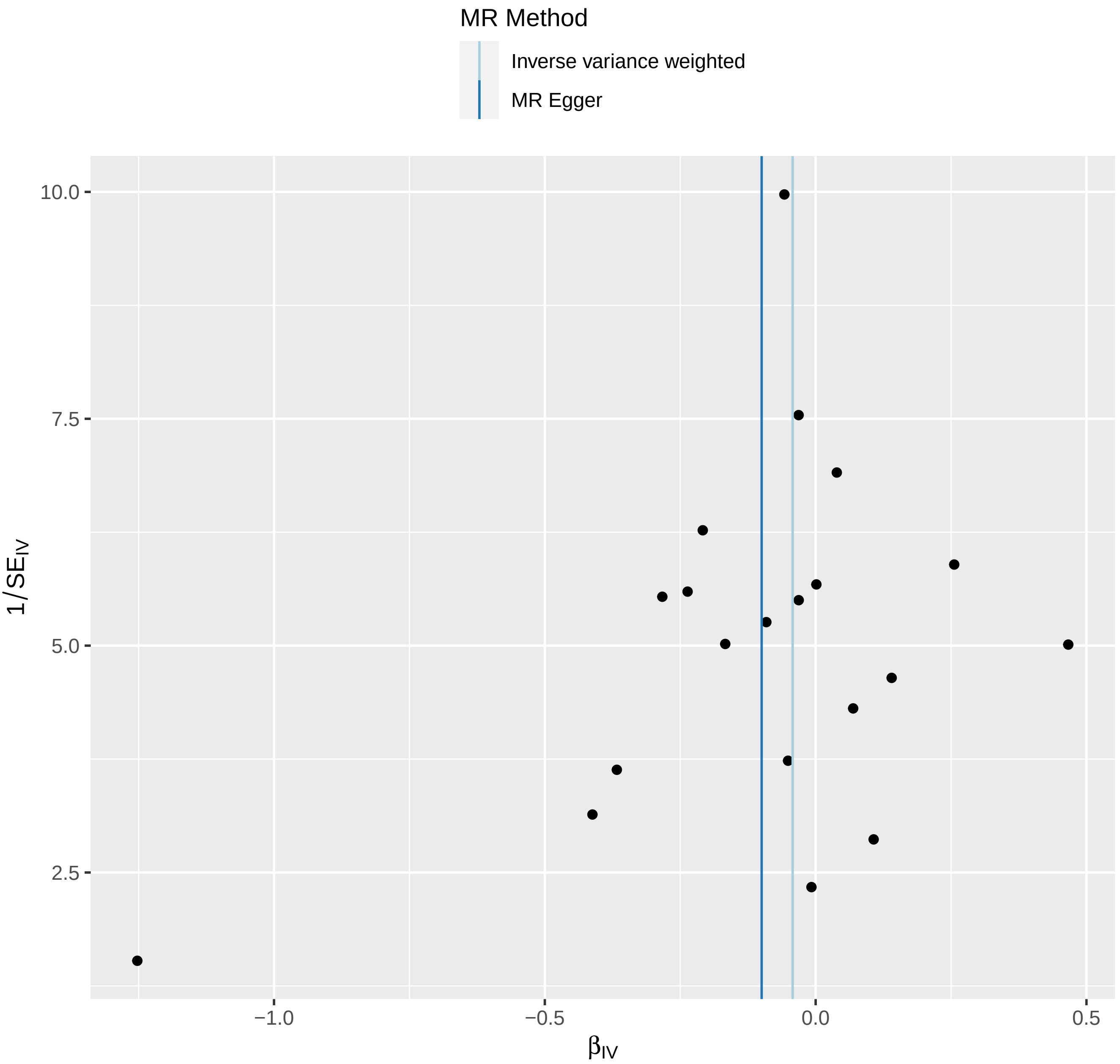

Batch 3−1725 : Leptin receptor on Diabetic nephropathy (more control exclusions)
with a threshold of 1e−05

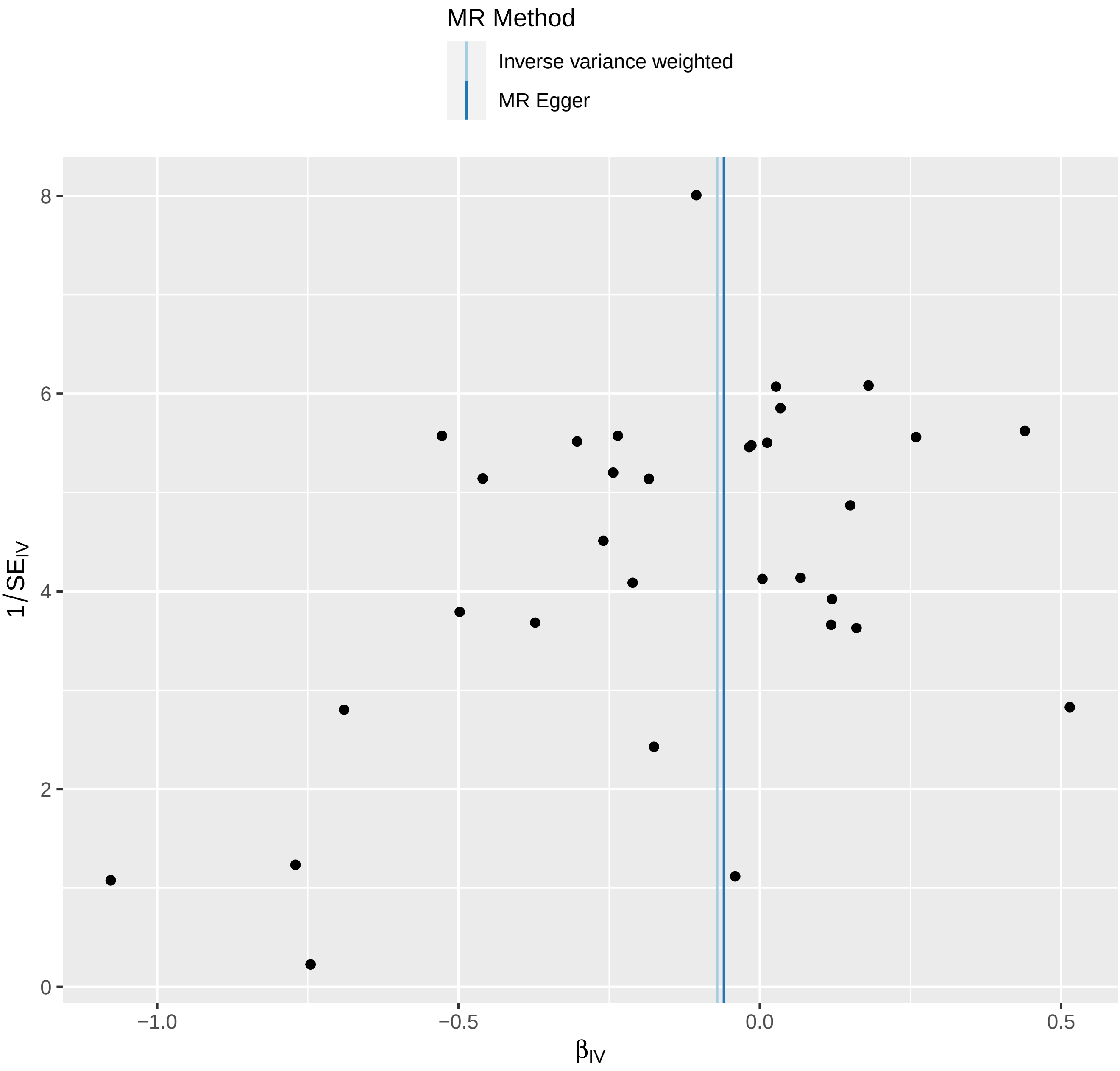

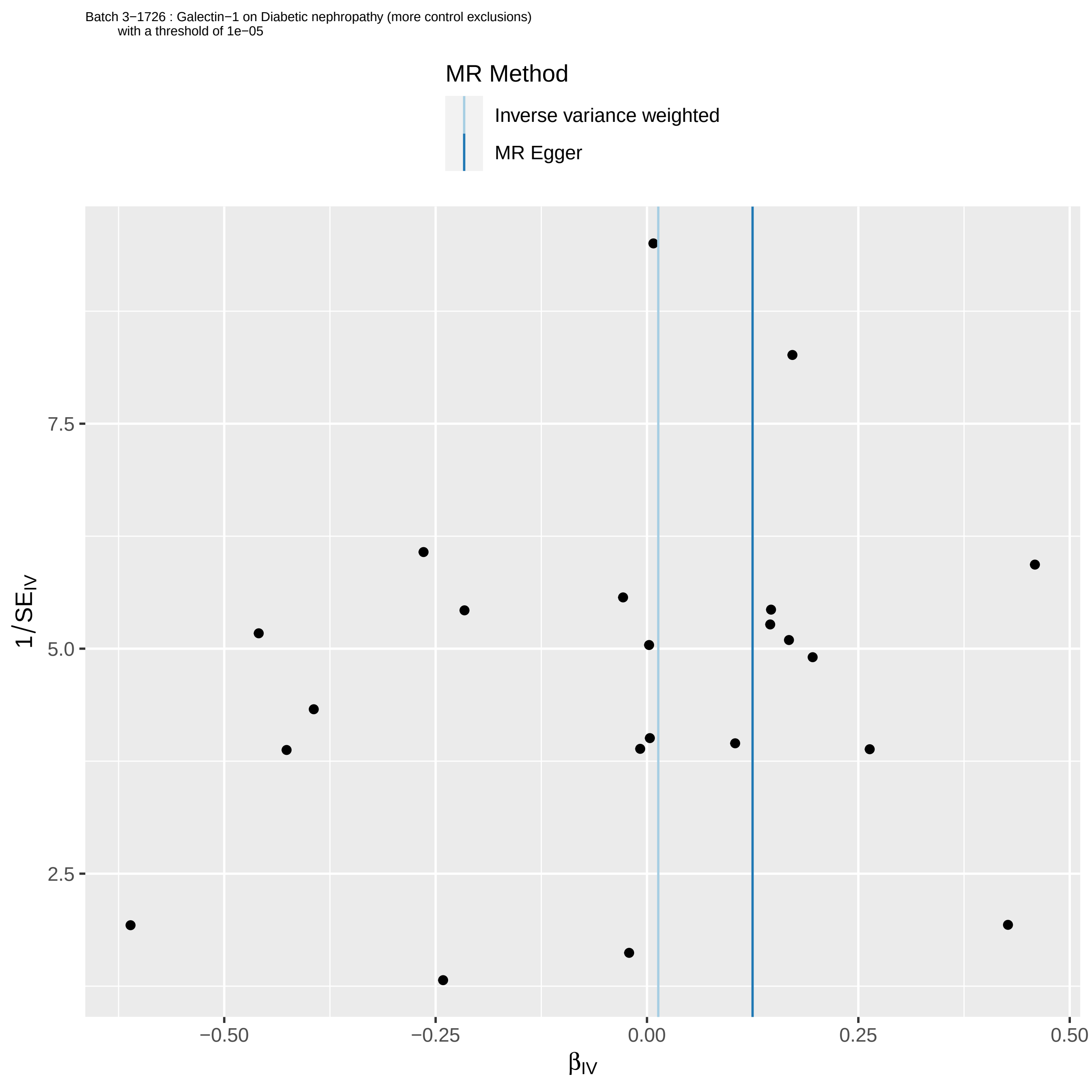

Batch 3−1726 : Galectin−1 on Diabetic nephropathy (more control exclusions)
with a threshold of 1e−05
MR Method
Inverse variance weighted
MR Egger
7.5
5.0
2.5
1/SE_IV
−0.50
−0.25
0.00
0.25
0.50
β_IV

Batch 3−1727 : Galectin−2 on Diabetic nephropathy (more control exclusions)
with a threshold of 1e−05

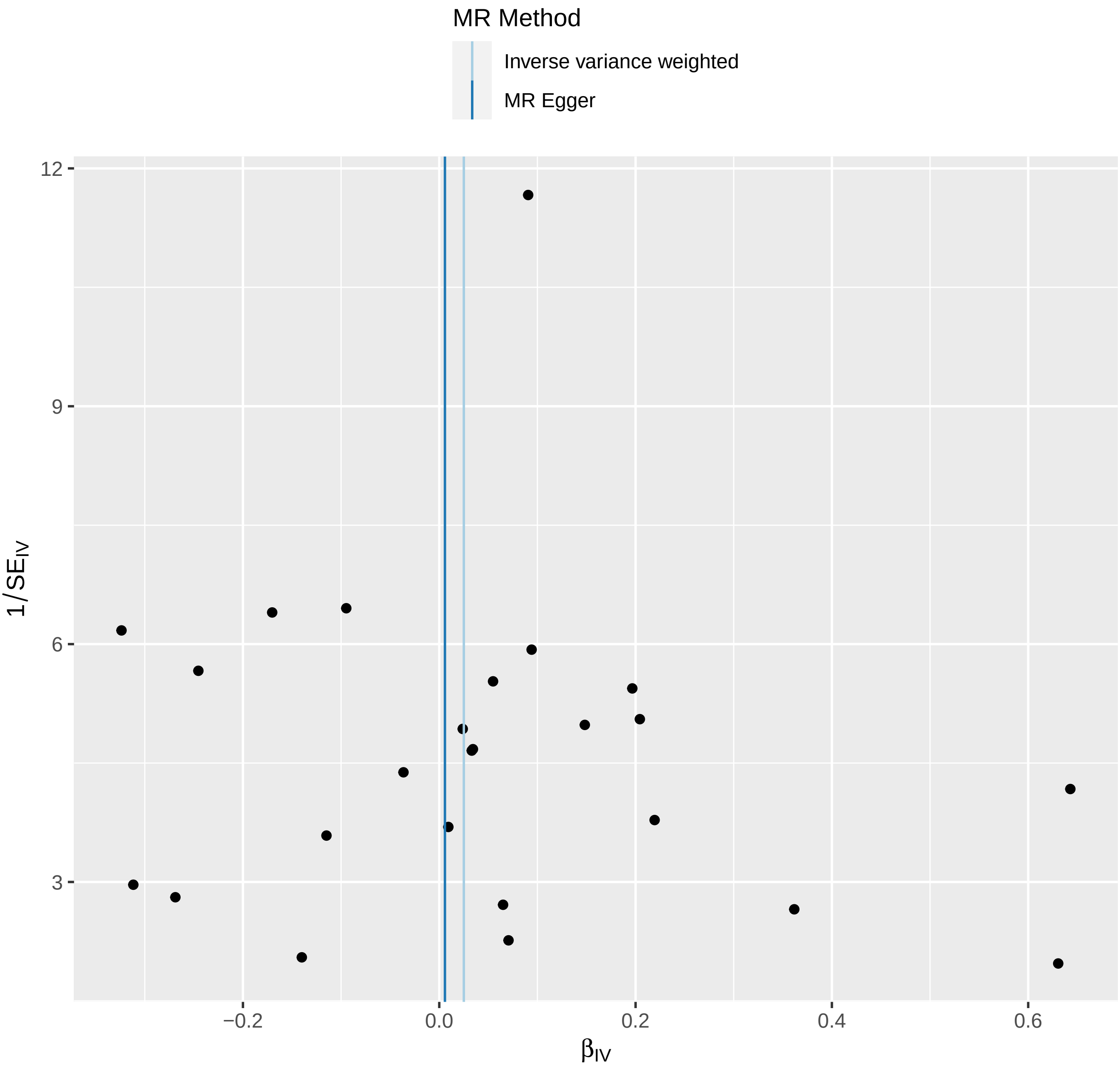

Batch 3−1728 : Galectin−4 on Diabetic nephropathy (more control exclusions)
with a threshold of 1e−05

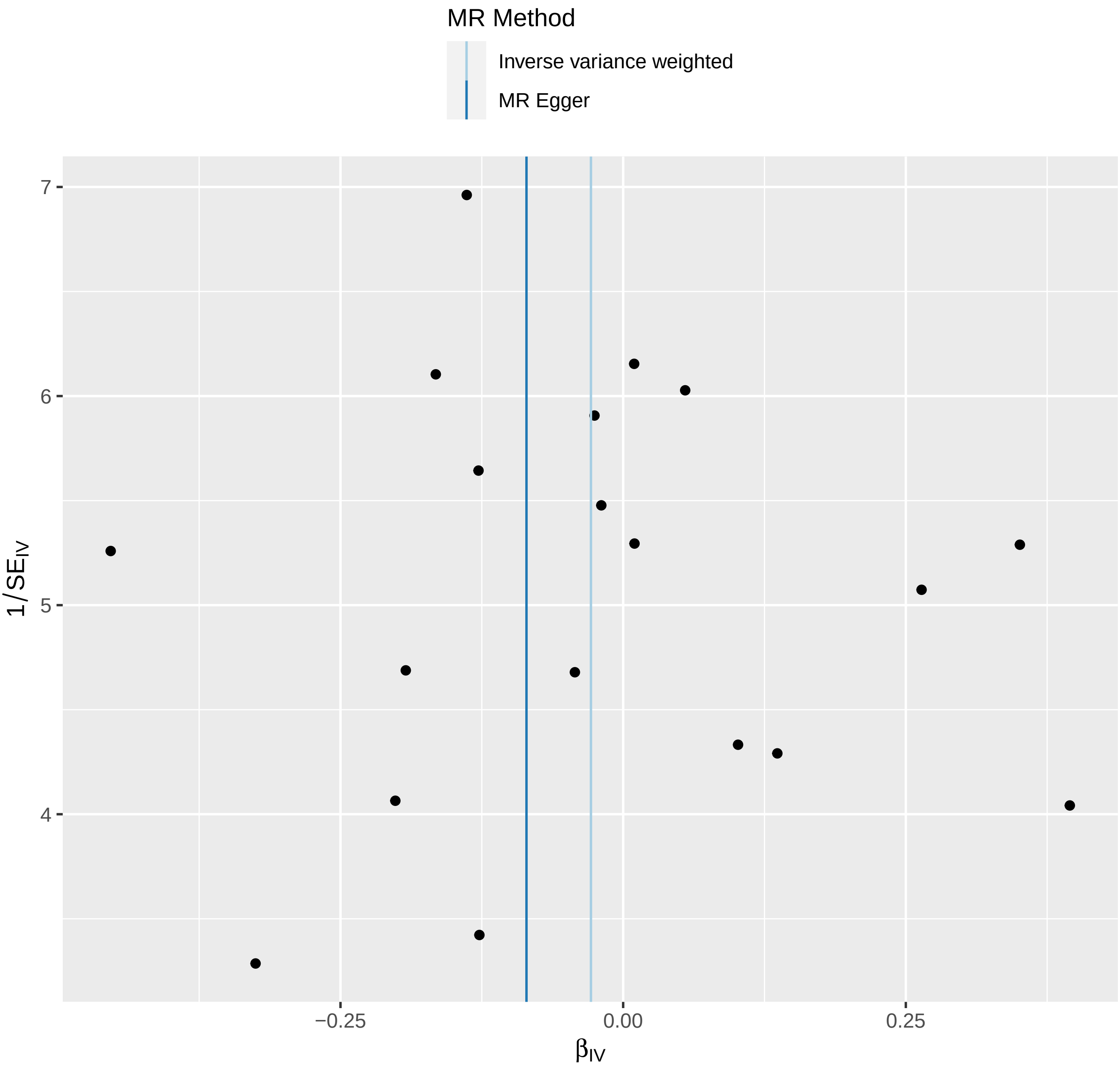

Batch 3−1729 : Galectin−7 on Diabetic nephropathy (more control exclusions)
with a threshold of 1e−05

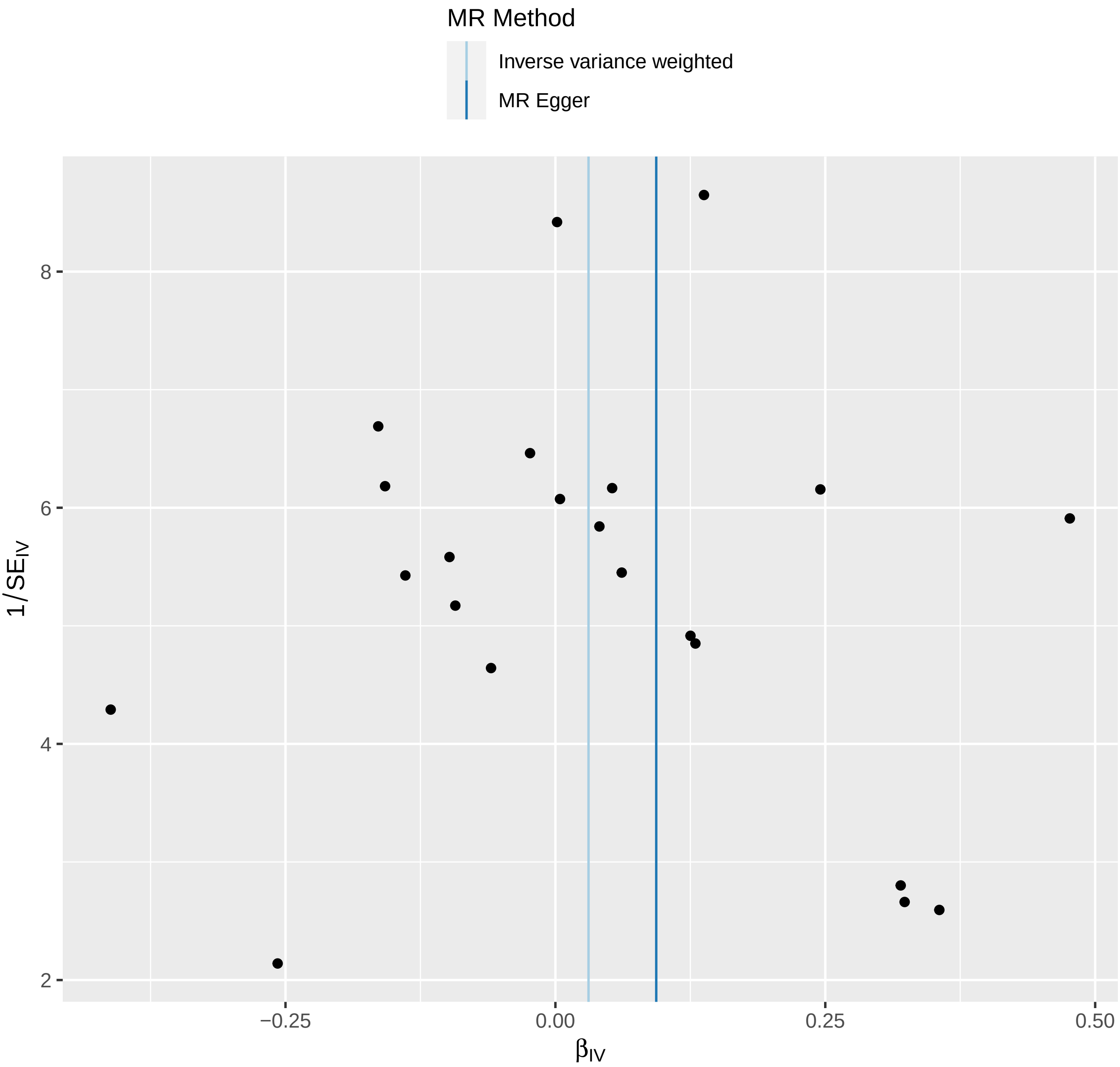

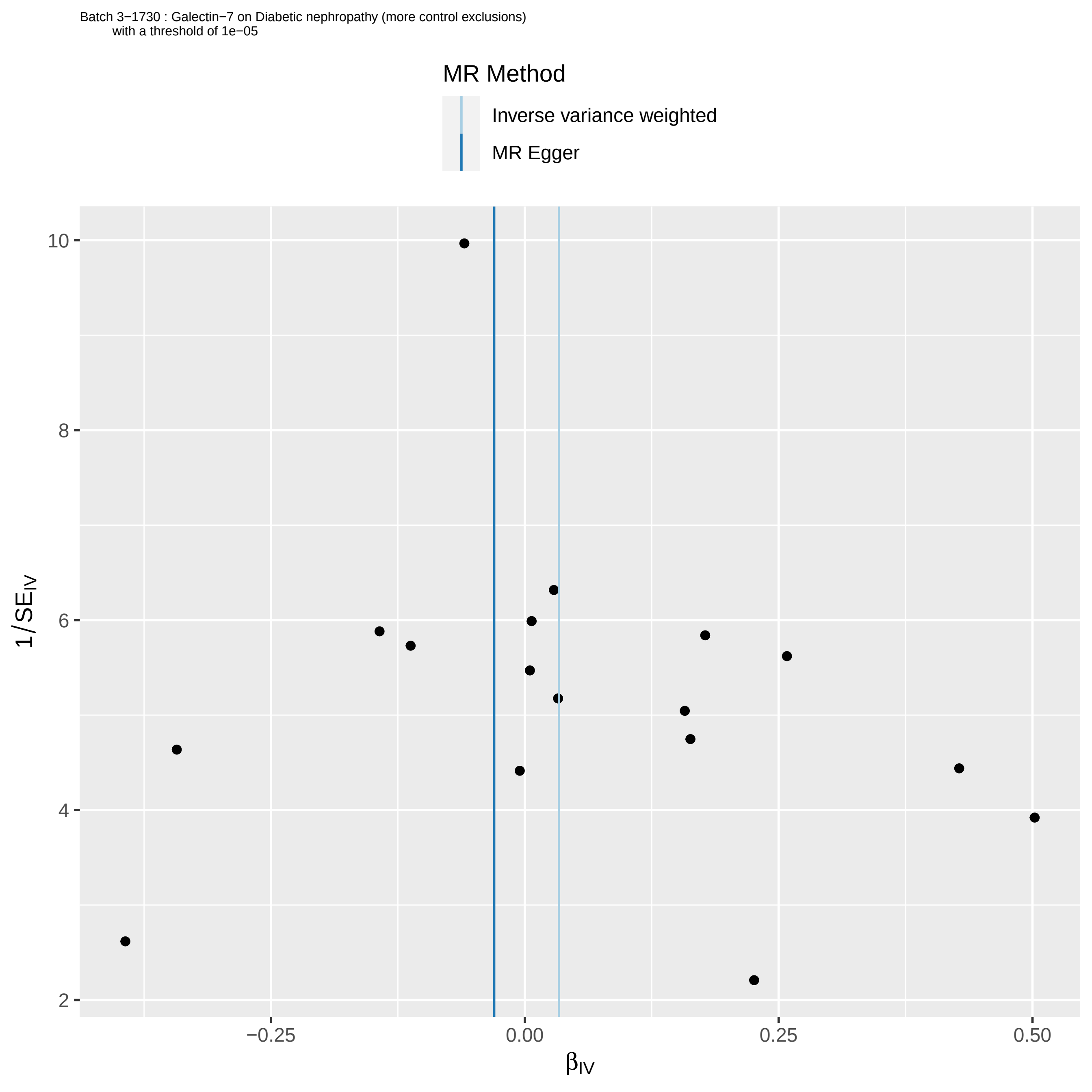

Batch 3−1730 : Galectin−7 on Diabetic nephropathy (more control exclusions)
with a threshold of 1e−05
MR Method
Inverse variance weighted
MR Egger
10
8
6
4
2
1/SE_IV
−0.25
0.00
0.25
0.50
β_IV

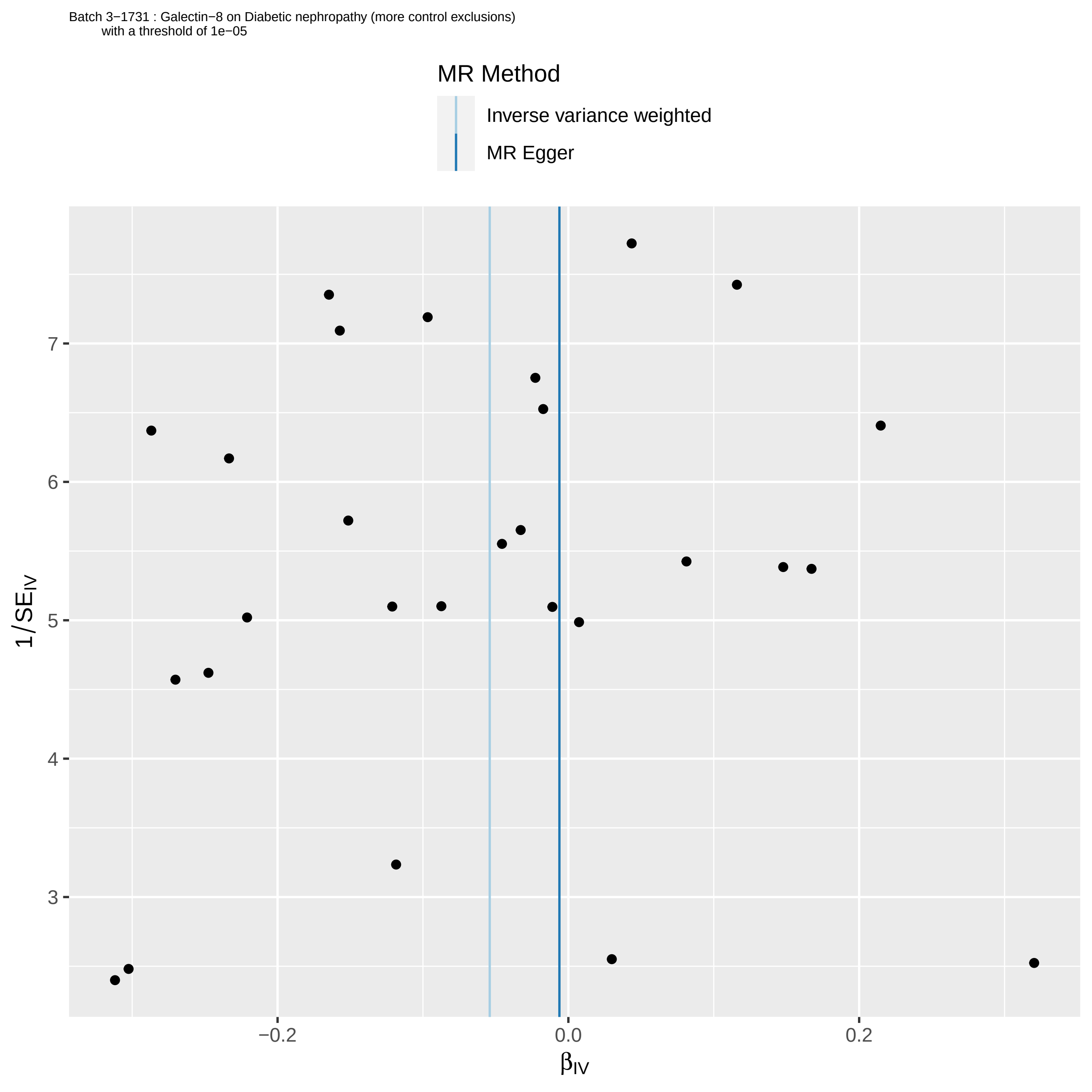
Batch 3−1731 : Galectin−8 on Diabetic nephropathy (more control exclusions)
with a threshold of 1e−05
MR Method
Inverse variance weighted
MR Egger
1/SE_IV
β_IV
−0.2
0.0
0.2
3
4
5
6
7

Batch 3−1732 : Galectin−9 on Diabetic nephropathy (more control exclusions)
with a threshold of 1e−05

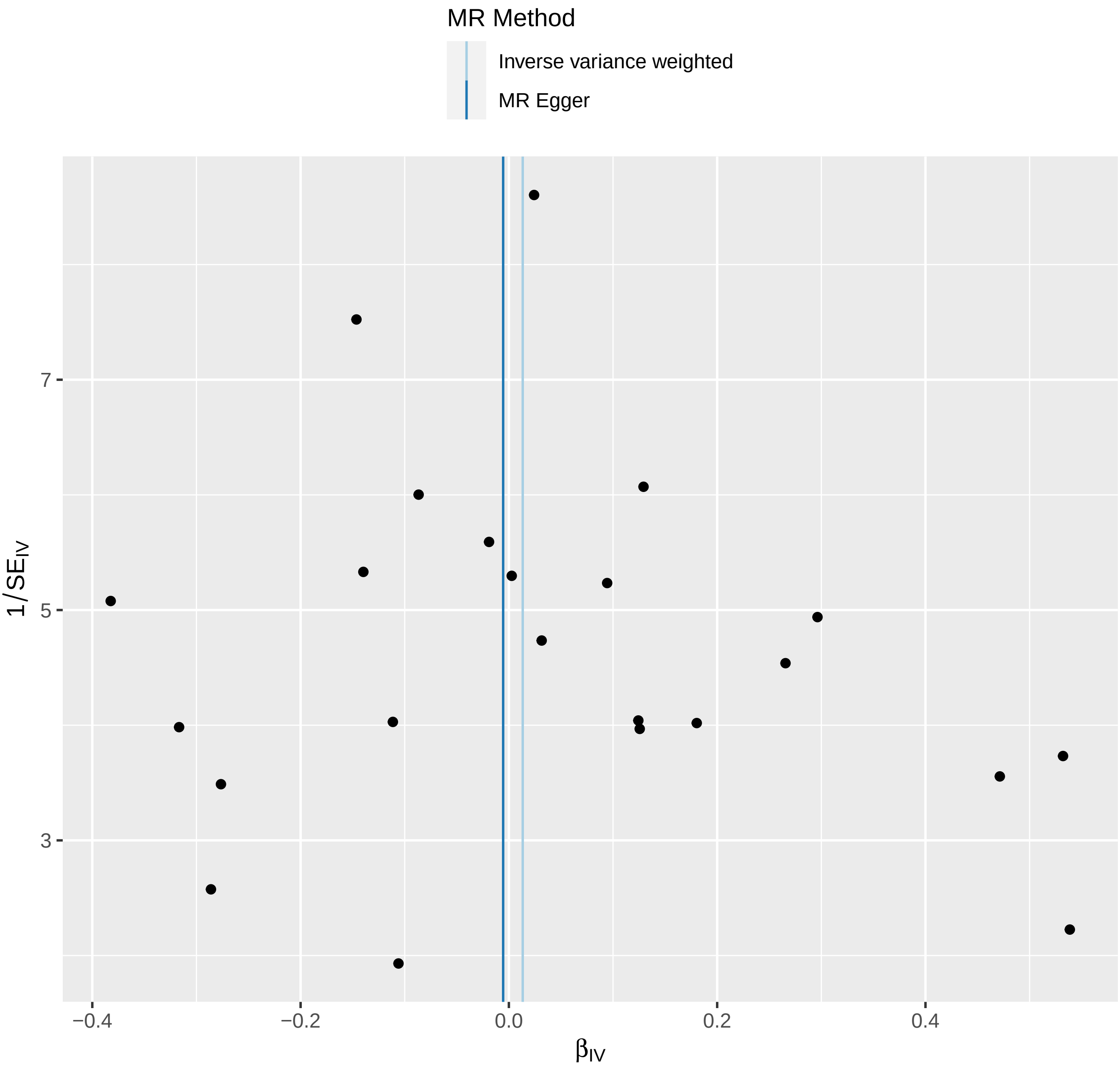

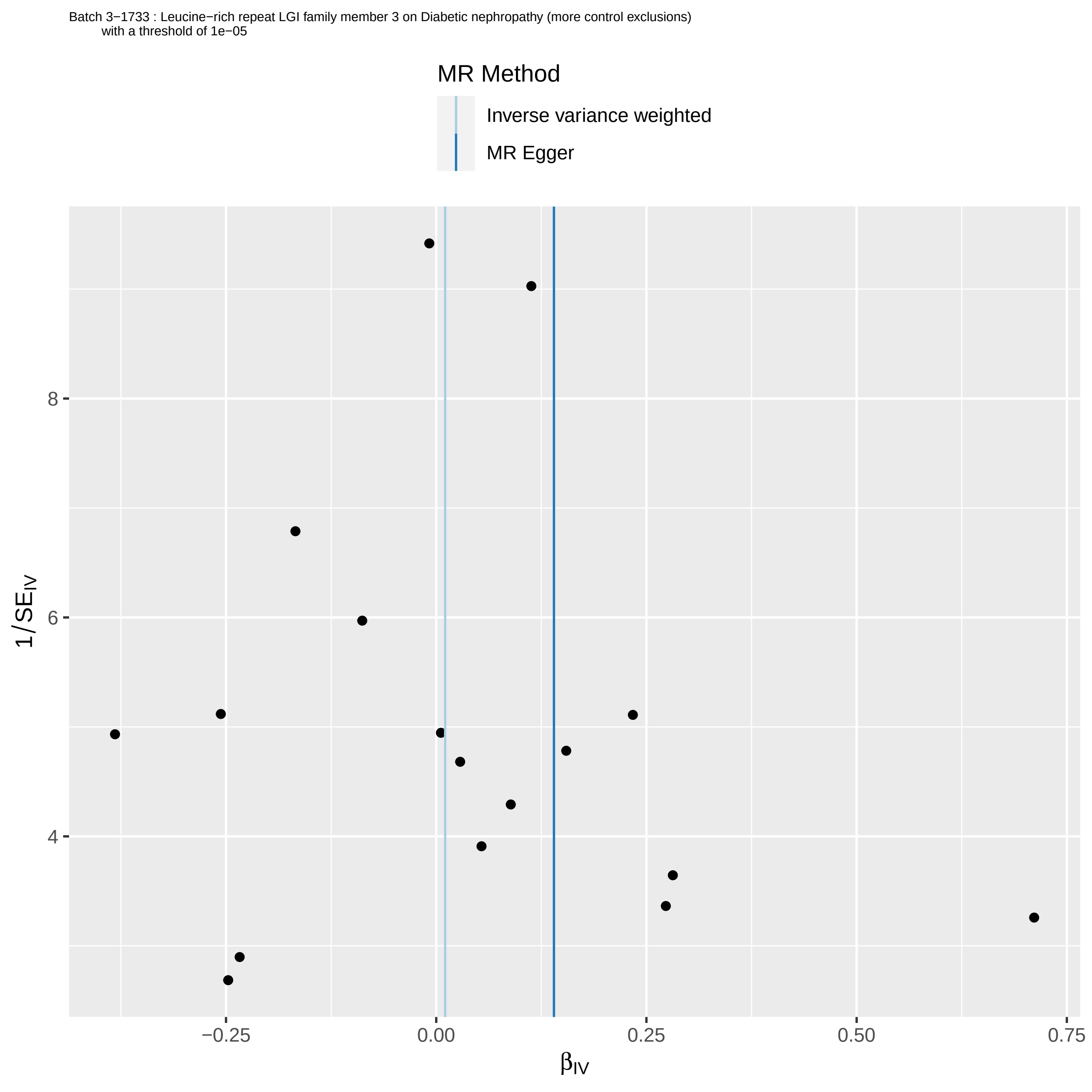

Batch 3−1733 : Leucine−rich repeat LGI family member 3 on Diabetic nephropathy (more control exclusions)
with a threshold of 1e−05
MR Method
Inverse variance weighted
MR Egger
1/SE_IV
8
6
4
−0.25
0.00
0.25
0.50
0.75
β_IV

Batch 3−1734 : Legumain on Diabetic nephropathy (more control exclusions)
with a threshold of 1e−05

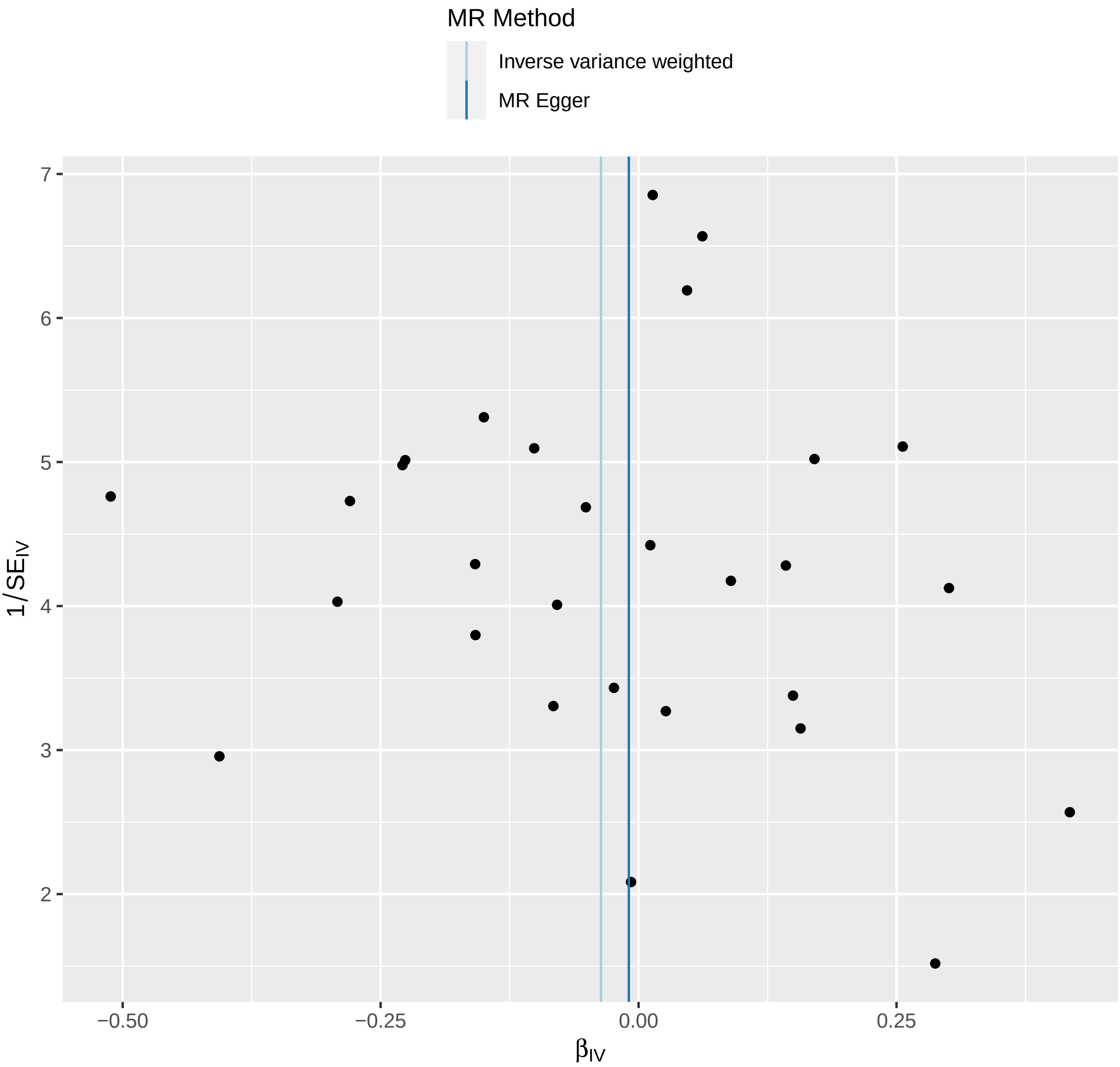

Batch 3−1735 : Lutropin subunit beta on Diabetic nephropathy (more control exclusions)
with a threshold of 1e−05

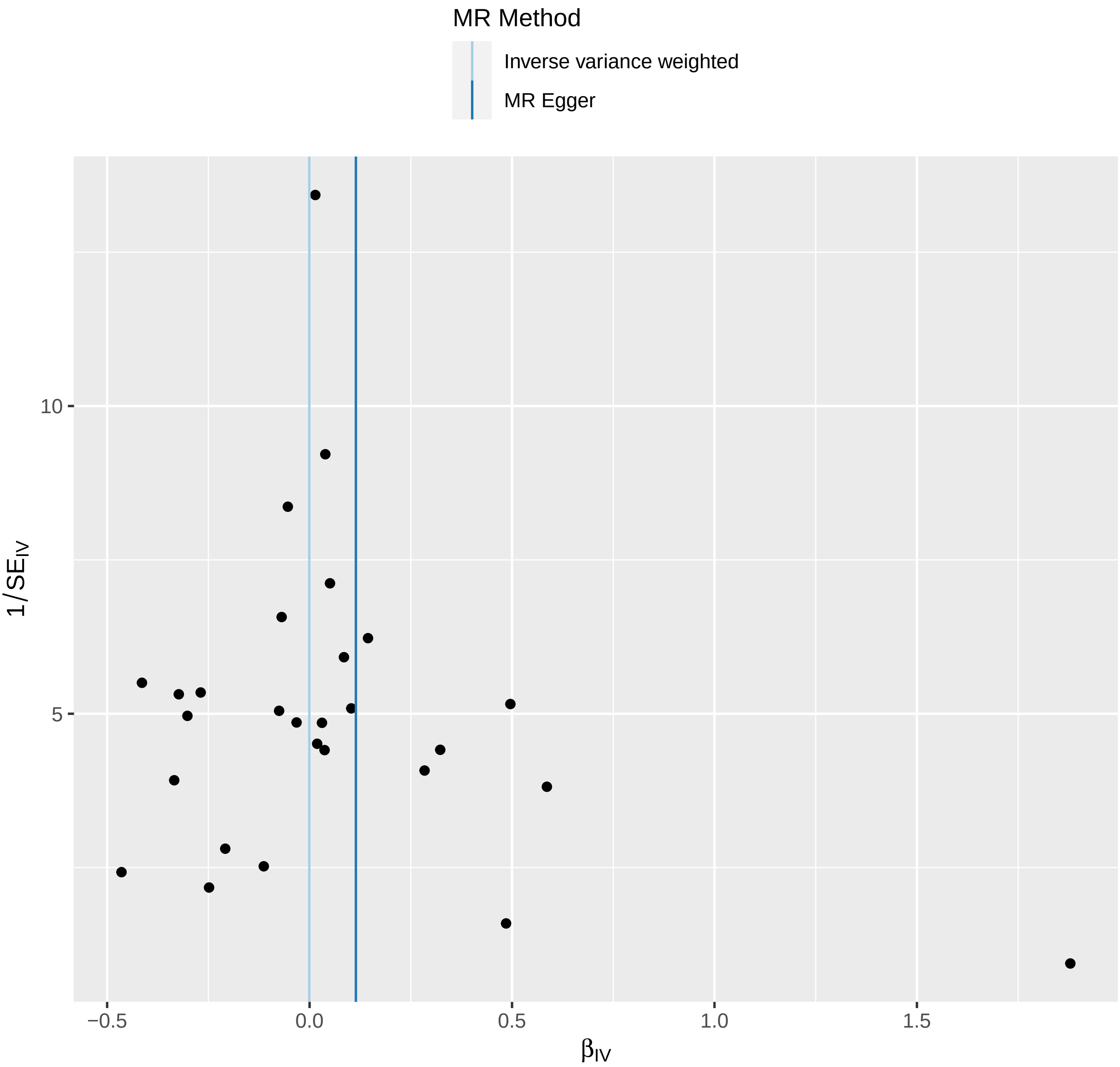

Batch 3−1736 : Leukemia inhibitory factor on Diabetic nephropathy (more control exclusions)
with a threshold of 1e−05

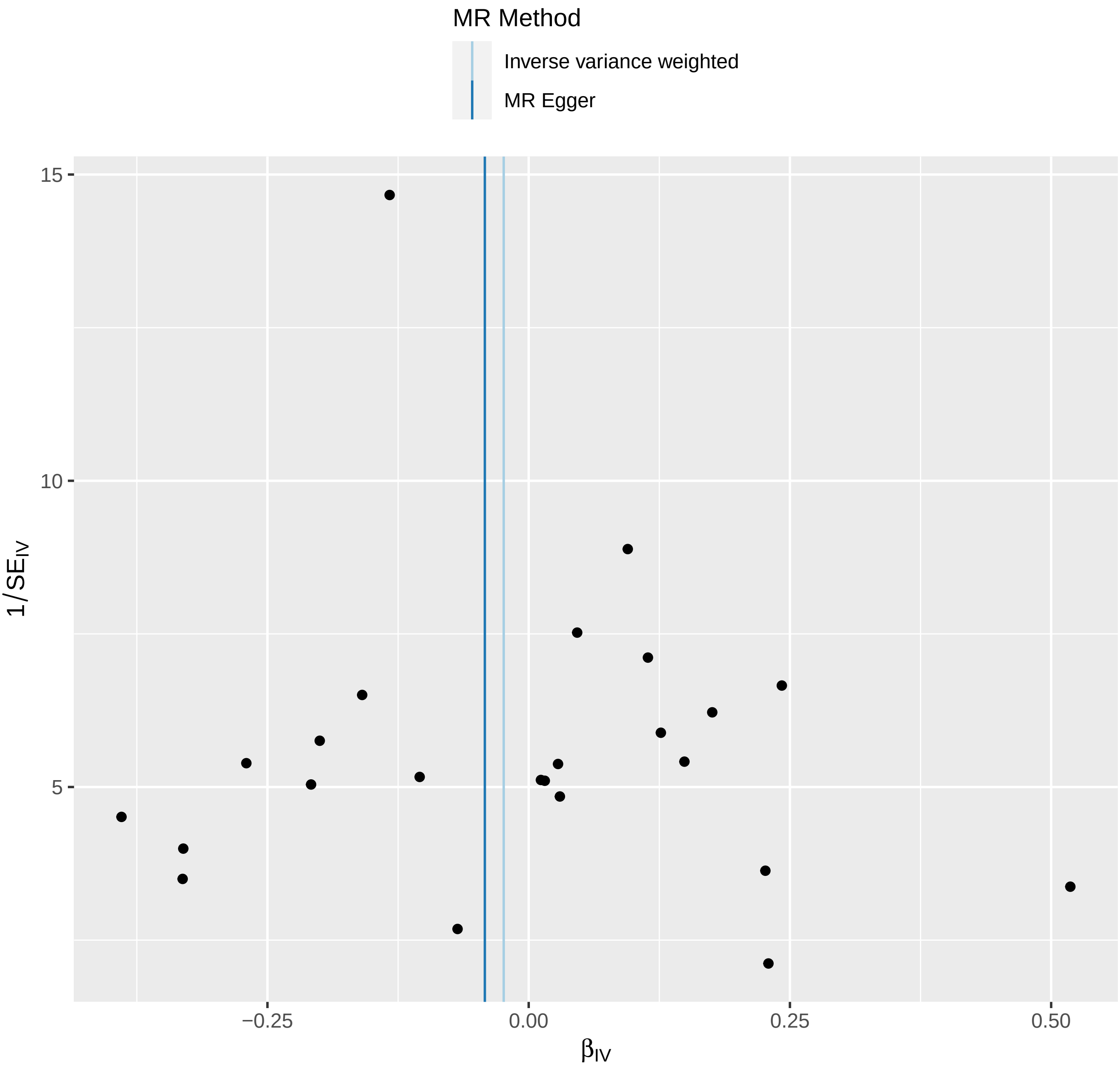

Batch 3−1737 : Leukemia inhibitory factor receptor on Diabetic nephropathy (more control exclusions)
with a threshold of 1e−05

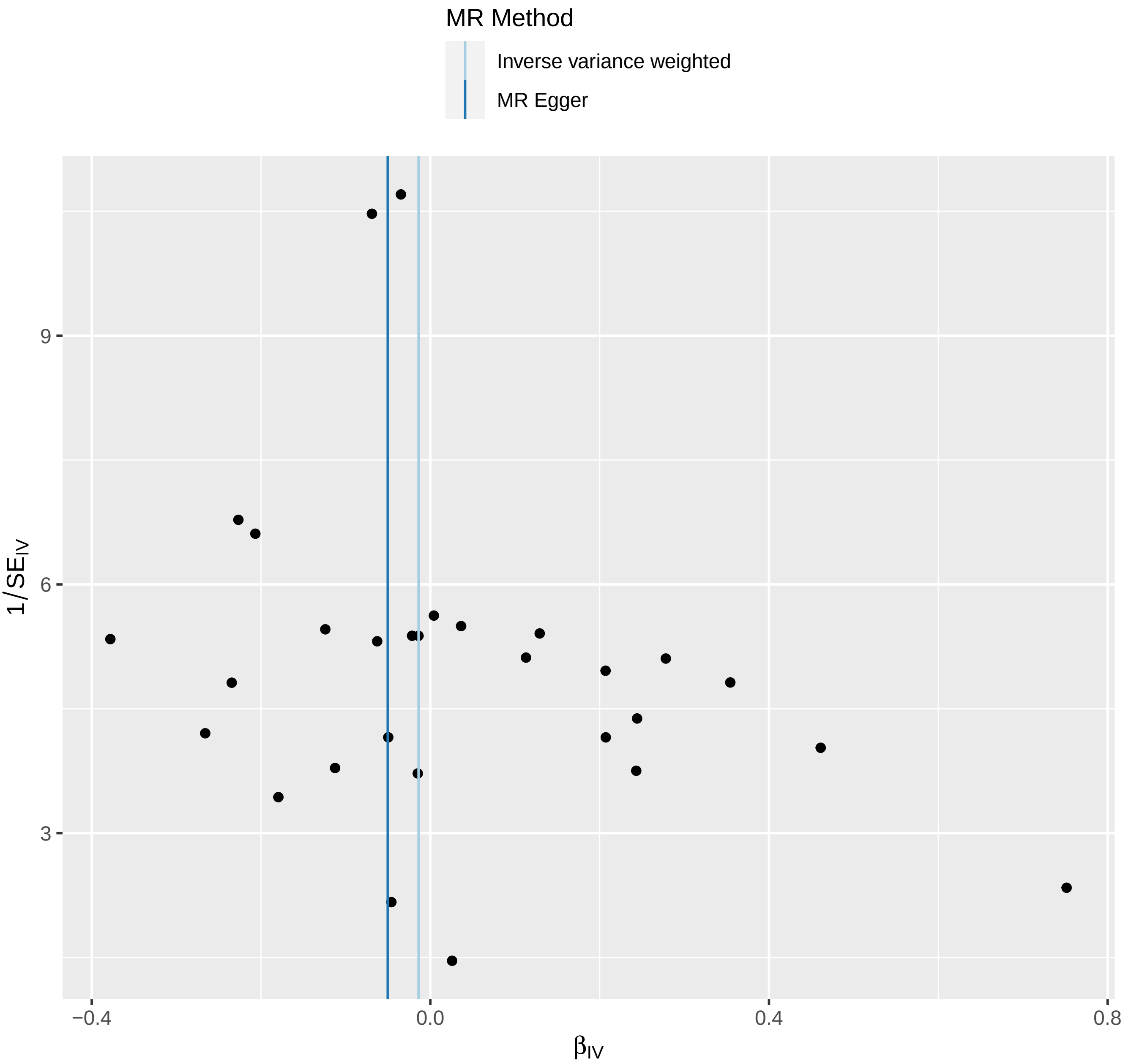

Batch 3−1738 : Leukocyte immunoglobulin−like receptor subfamily A member 4 on Diabetic nephropathy (more control exclusions)
with a threshold of 1e−05

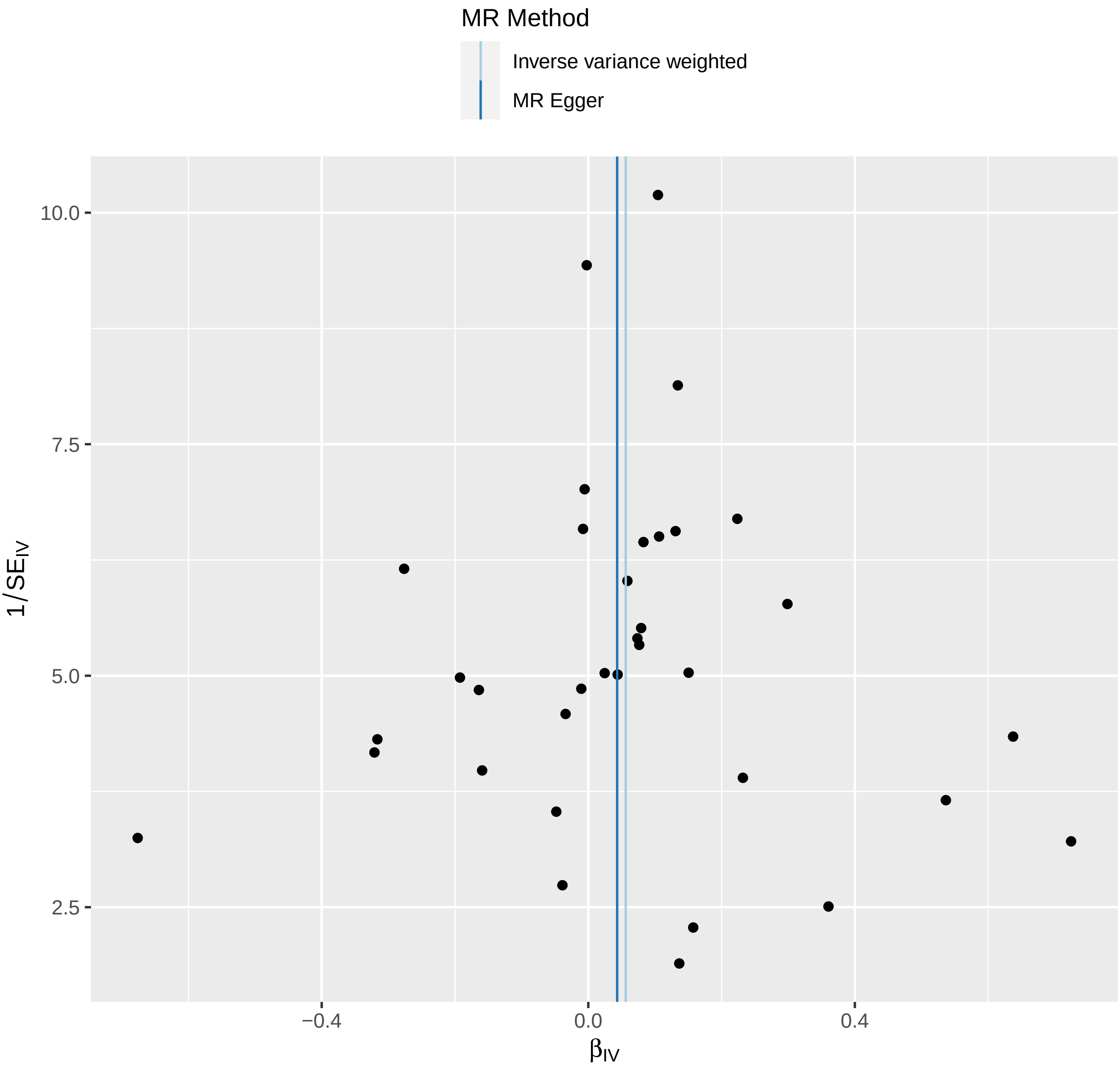

Batch 3−1739 : Leukocyte immunoglobulin−like receptor subfamily A member 5 on Diabetic nephropathy (more control exclusions) with a threshold of 1e−05

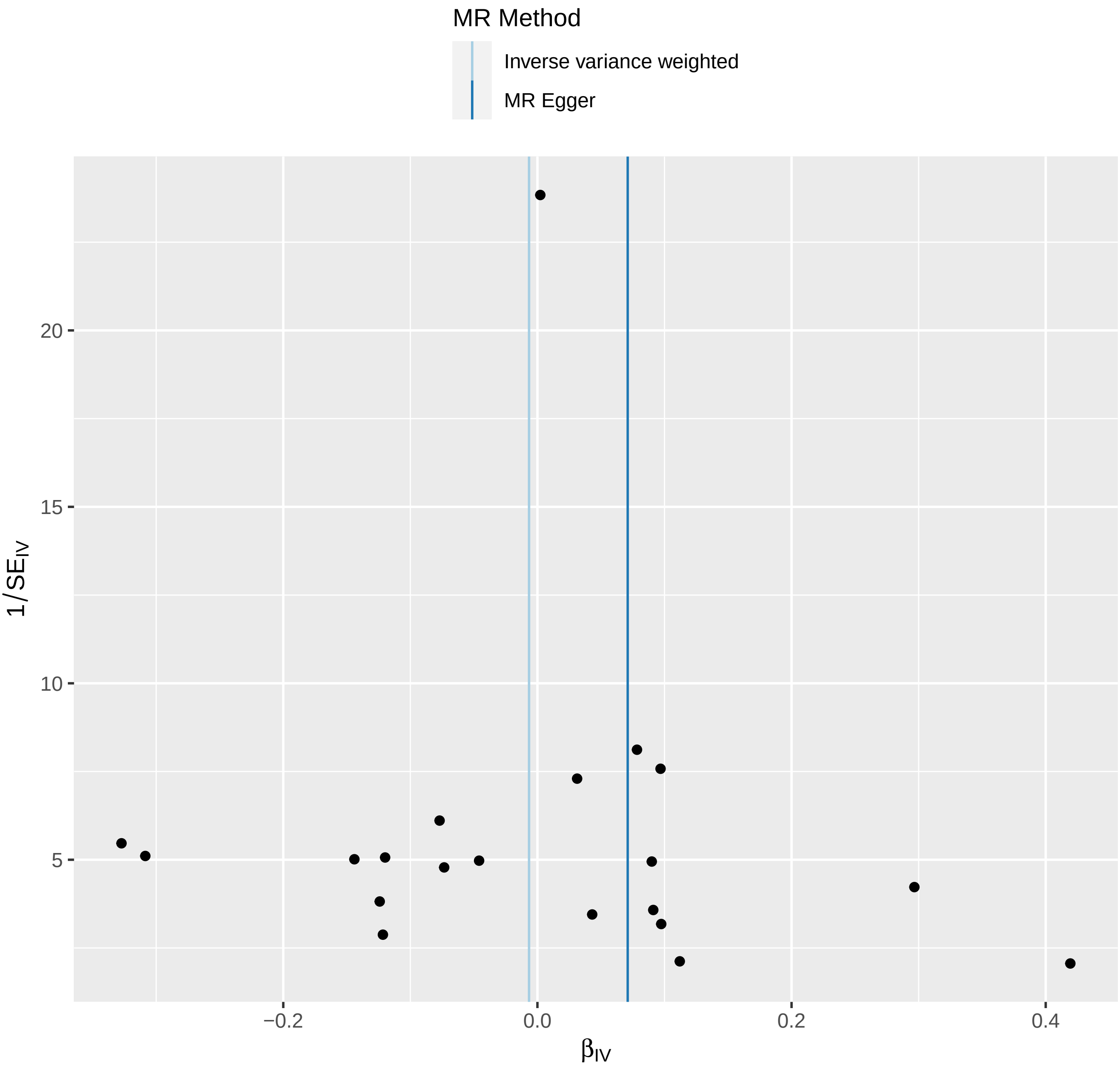

Batch 3−1740 : Leukocyte immunoglobulin−like receptor subfamily A member 5 on Diabetic nephropathy (more control exclusions) with a threshold of 1e−05

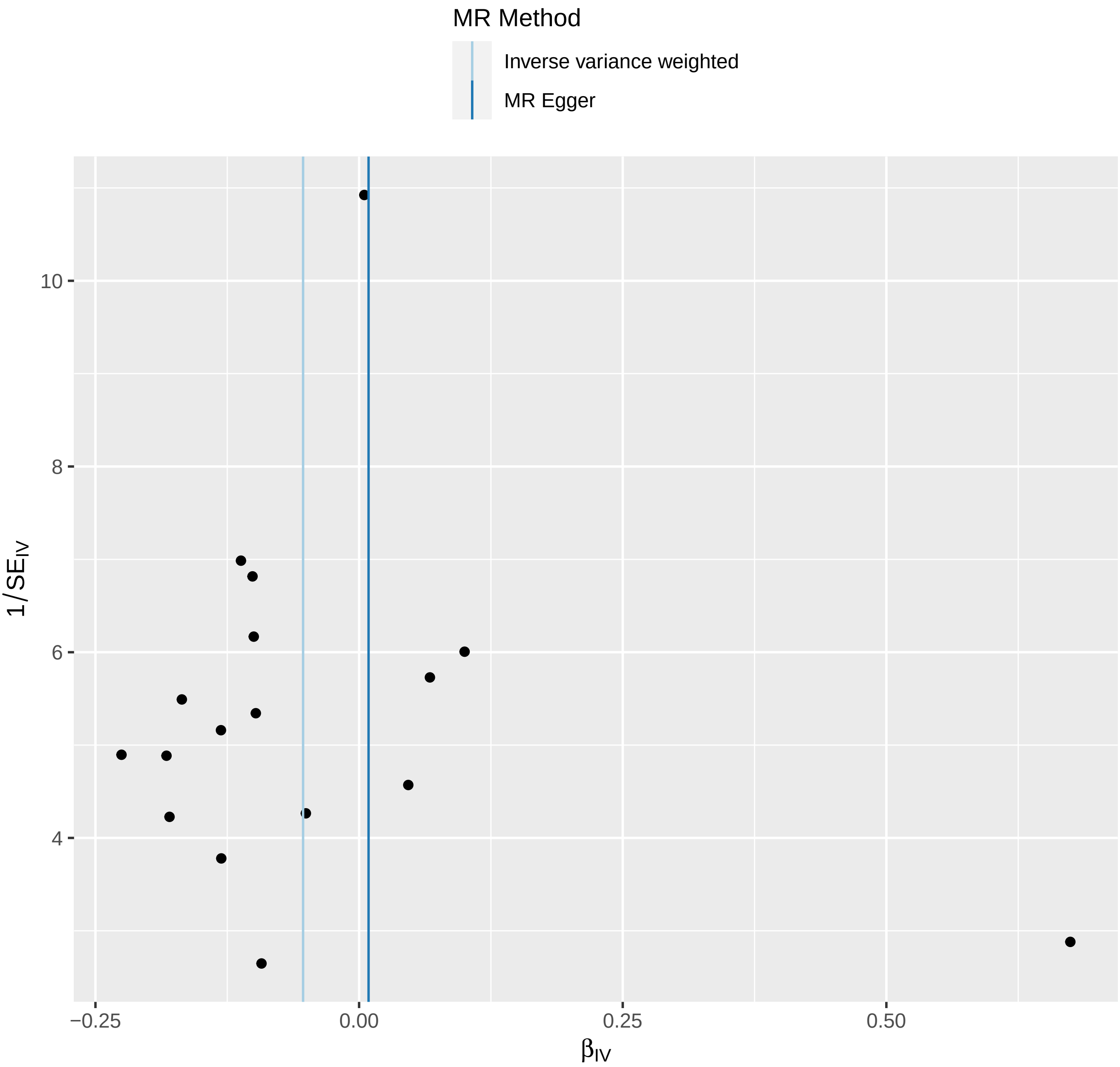

Batch 3−1741 : Leukocyte immunoglobulin−like receptor subfamily A member 6 on Diabetic nephropathy (more control exclusions) with a threshold of 1e−05

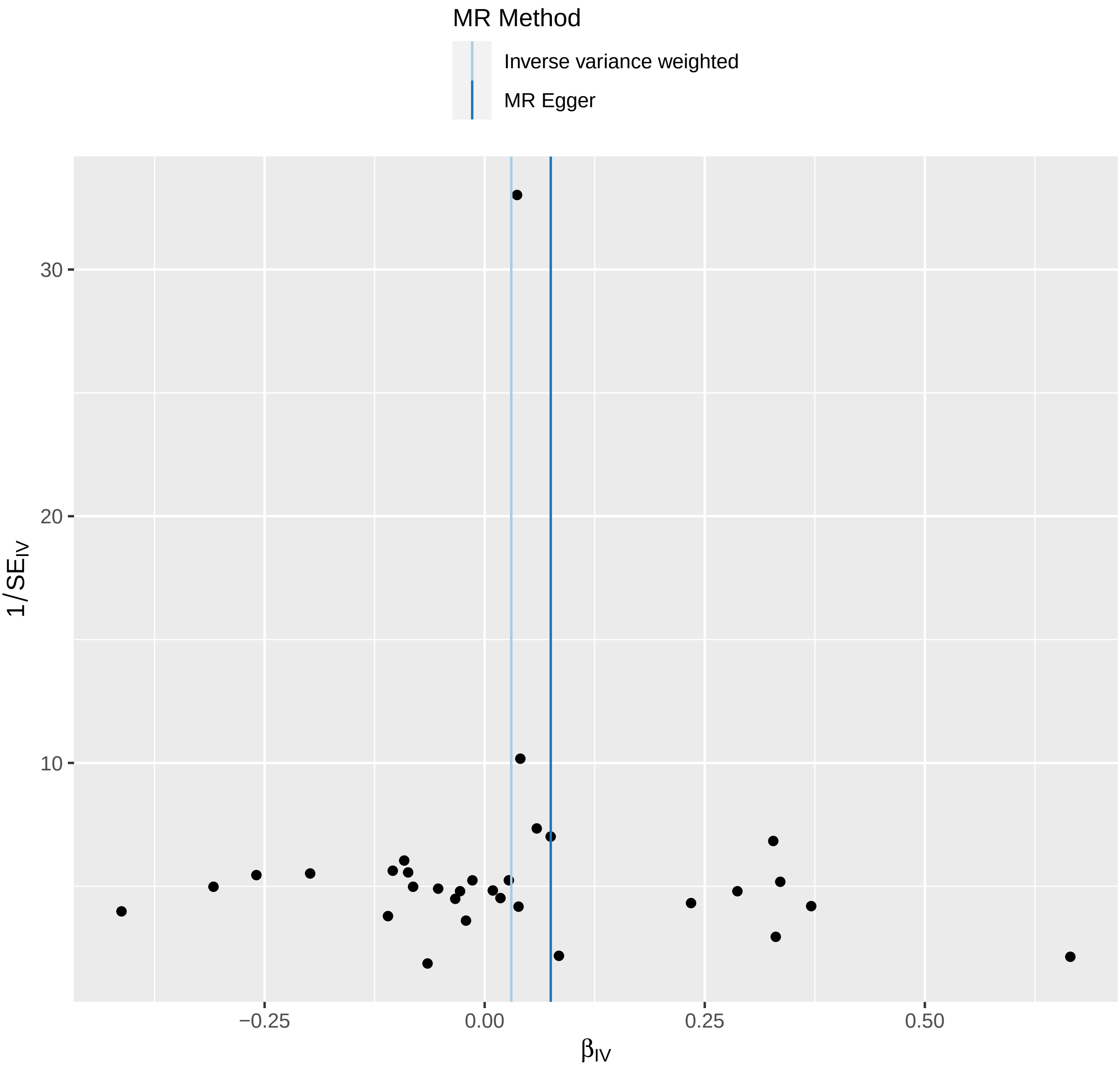

Batch 3−1742 : Leukocyte immunoglobulin−like receptor subfamily B member 1 on Diabetic nephropathy (more control exclusions)
with a threshold of 1e−05

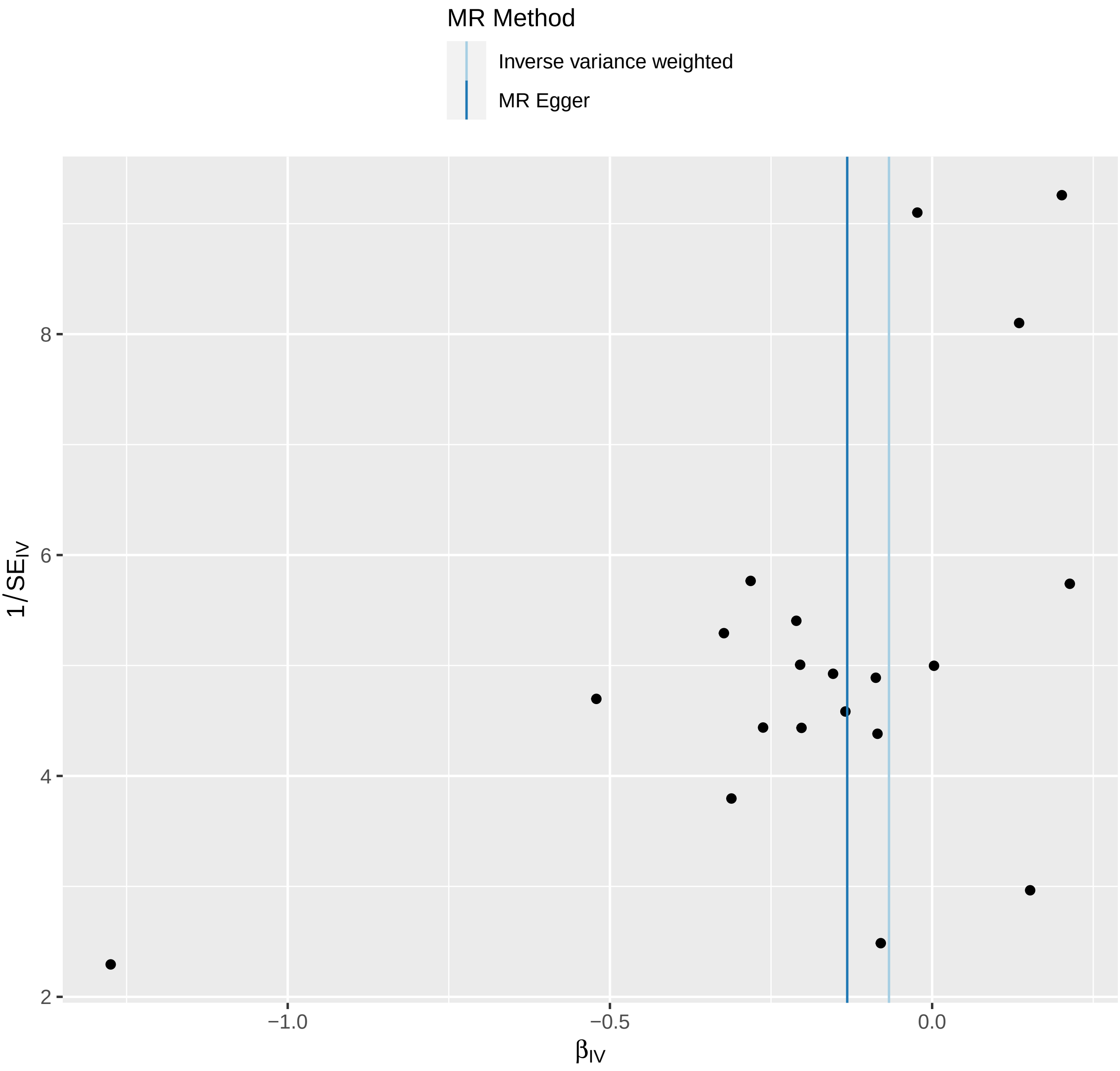

Batch 3−1743 : Leukocyte immunoglobulin−like receptor subfamily B member 2 on Diabetic nephropathy (more control exclusions) with a threshold of 1e−05

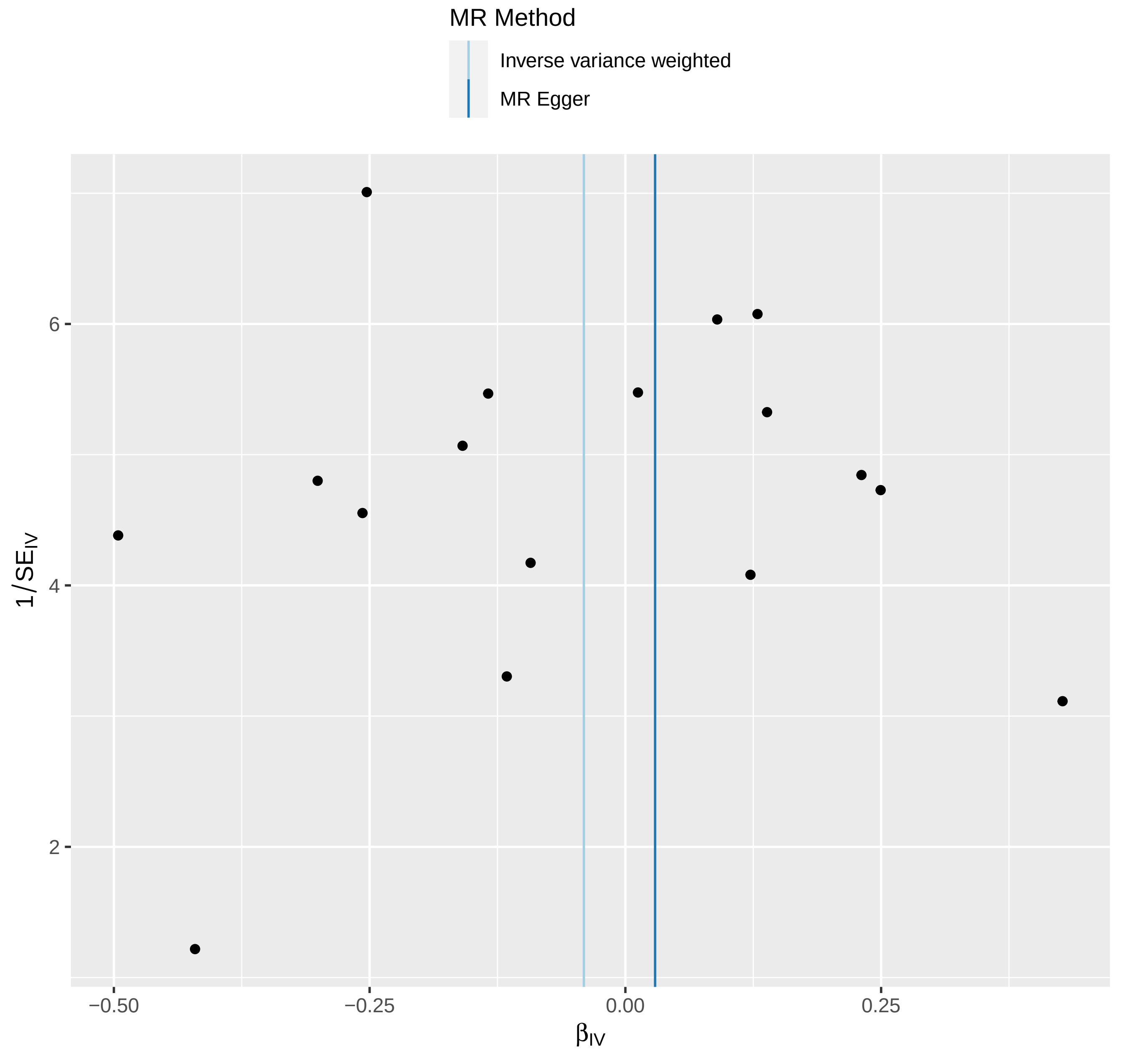

Batch 3−1744 : Leukocyte immunoglobulin−like receptor subfamily B member 2 on Diabetic nephropathy (more control exclusions) with a threshold of 1e−05

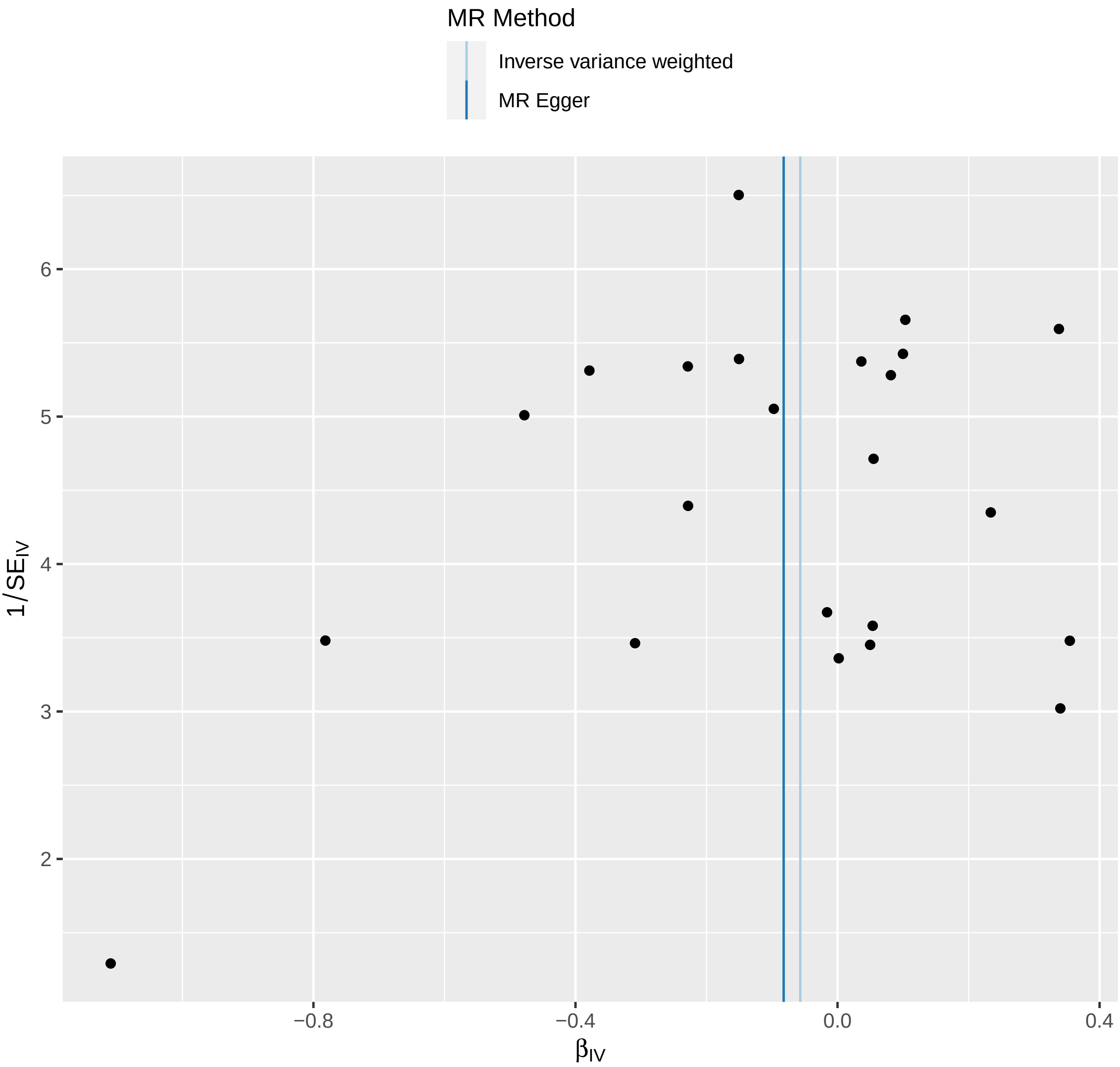

Batch 3−1745 : Leukocyte immunoglobulin−like receptor subfamily B member 3 on Diabetic nephropathy (more control exclusions)
with a threshold of 1e−05

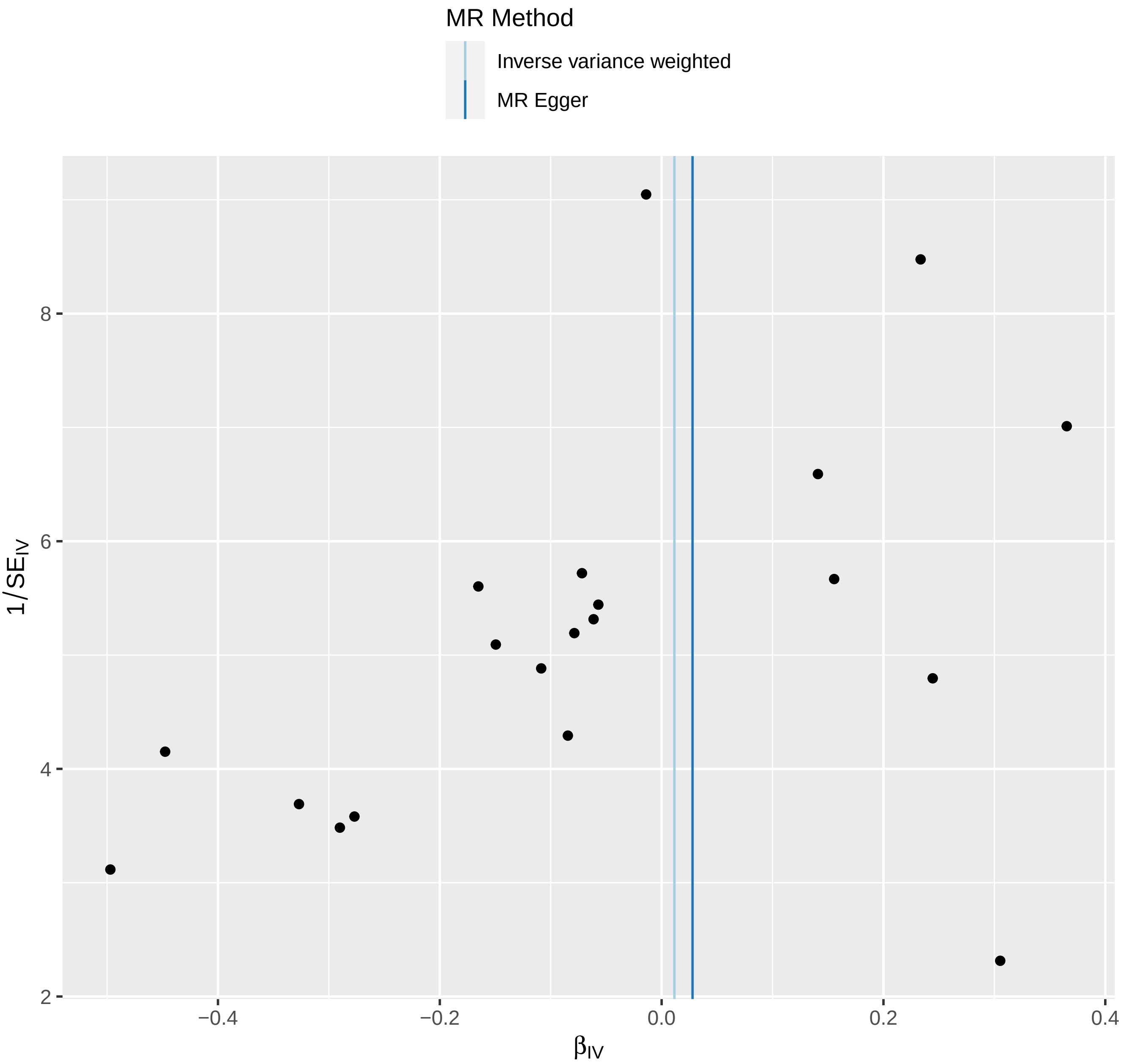

Batch 3−1746 : Leukocyte immunoglobulin−like receptor subfamily B member 4 on Diabetic nephropathy (more control exclusions) with a threshold of 1e−05

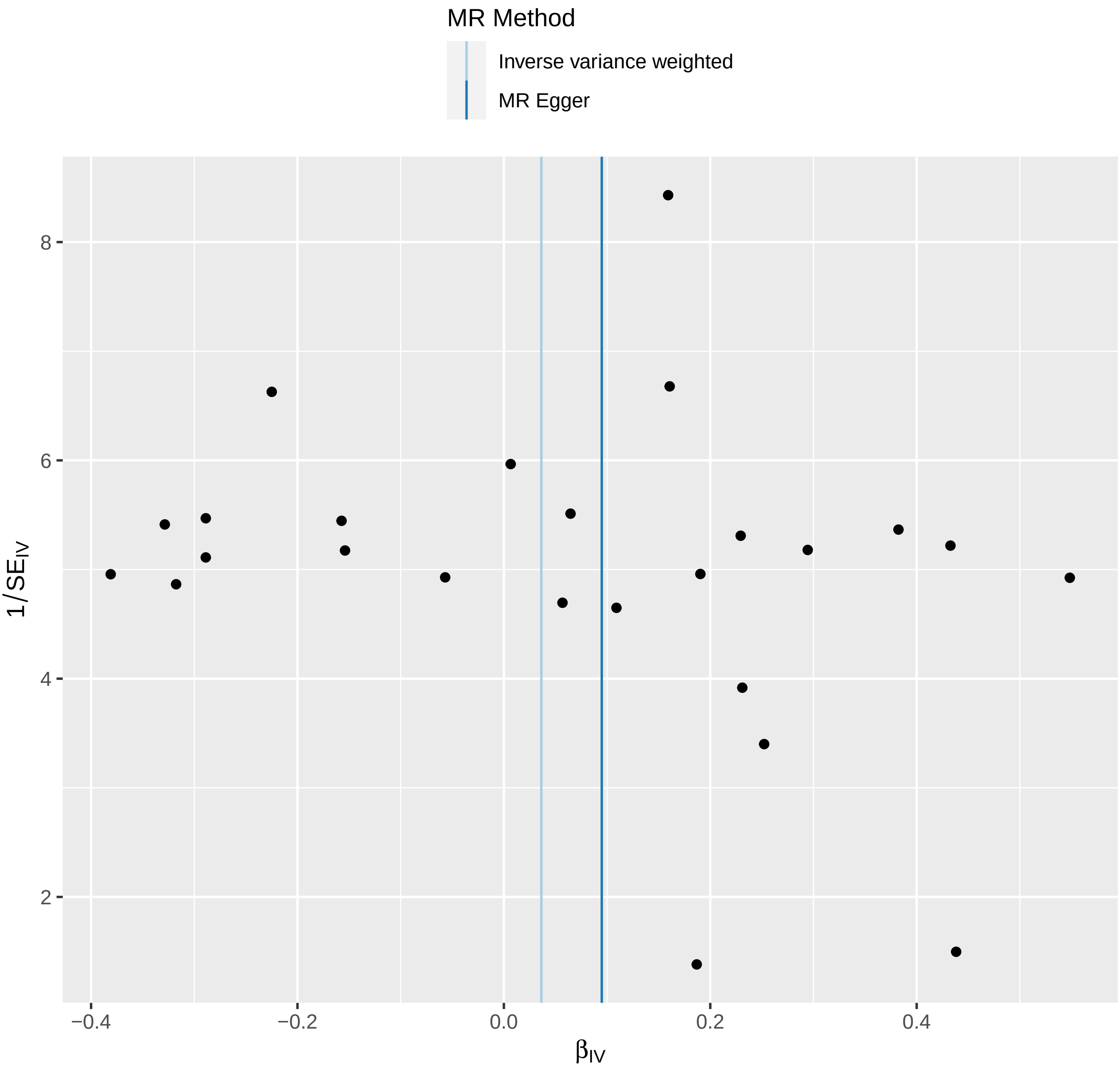

Batch 3−1747 : Leukocyte immunoglobulin−like receptor subfamily B member 5 on Diabetic nephropathy (more control exclusions) with a threshold of 1e−05

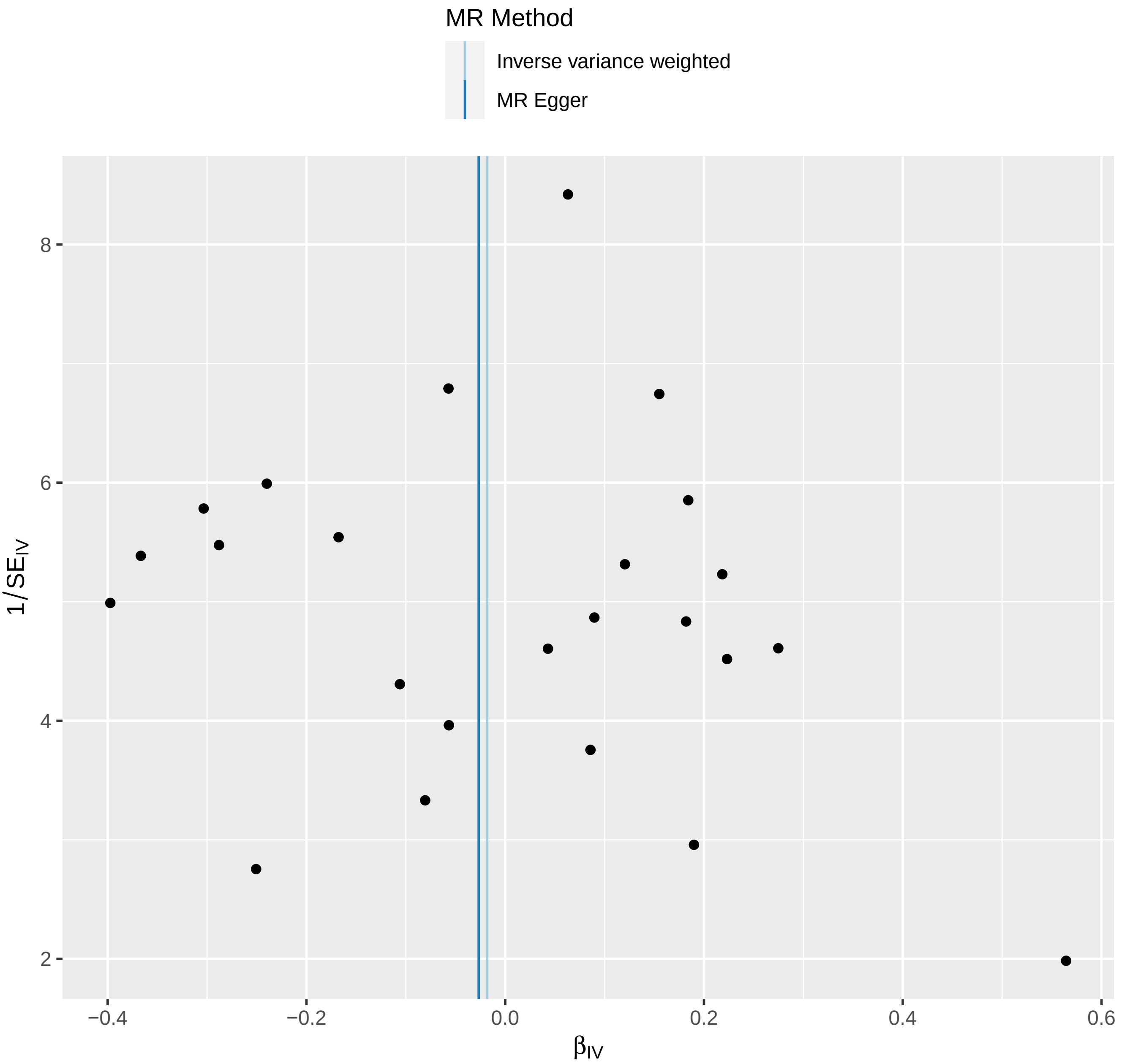

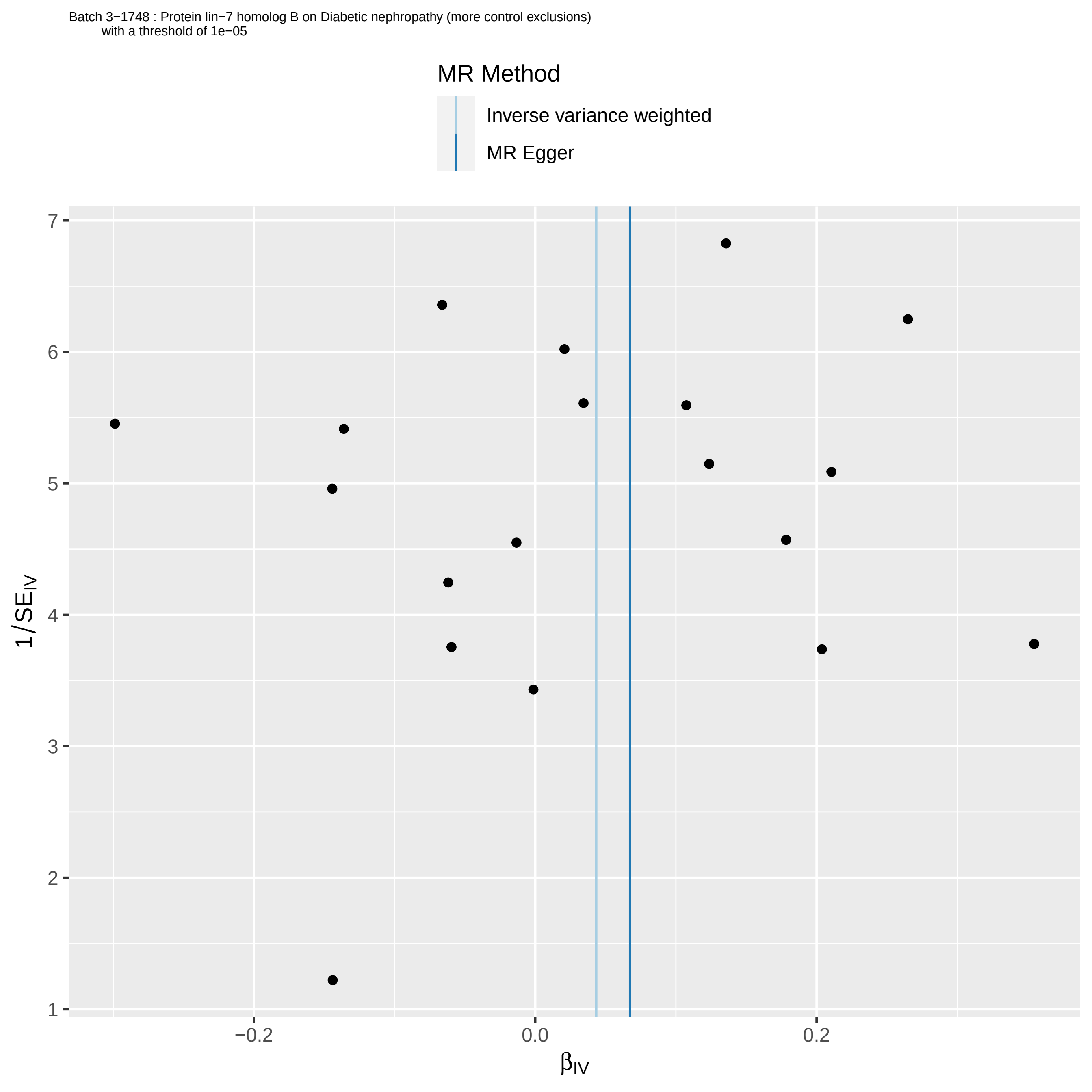

Batch 3−1748 : Protein lin−7 homolog B on Diabetic nephropathy (more control exclusions)
with a threshold of 1e−05
MR Method
Inverse variance weighted
MR Egger
7
6
5
4
3
2
1
1/SE$_{IV}$
−0.2
0.0
0.2
β$_{IV}$

Batch 3−1749 : Leucine−rich repeat and immunoglobulin−like domain−containing nogo receptor−interacting protein 1 on Diabetic nephropathy (more control exclusions) with a threshold of 1e−05

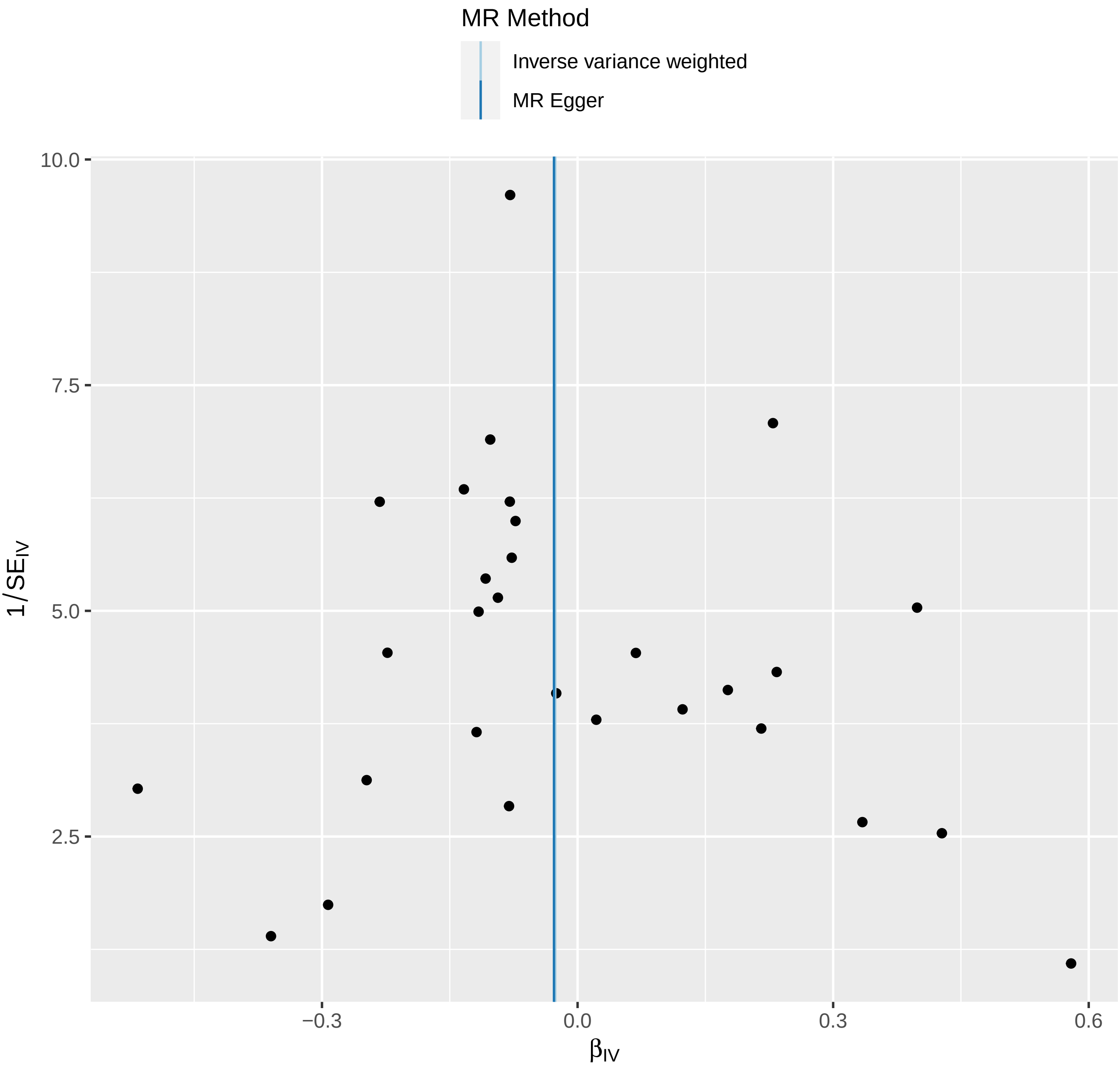

Batch 3−1750 : Leucine−rich repeat and immunoglobulin−like domain−containing nogo receptor−interacting protein 3 on Diabetic nephropathy (more control exclusions) with a threshold of 1e−05

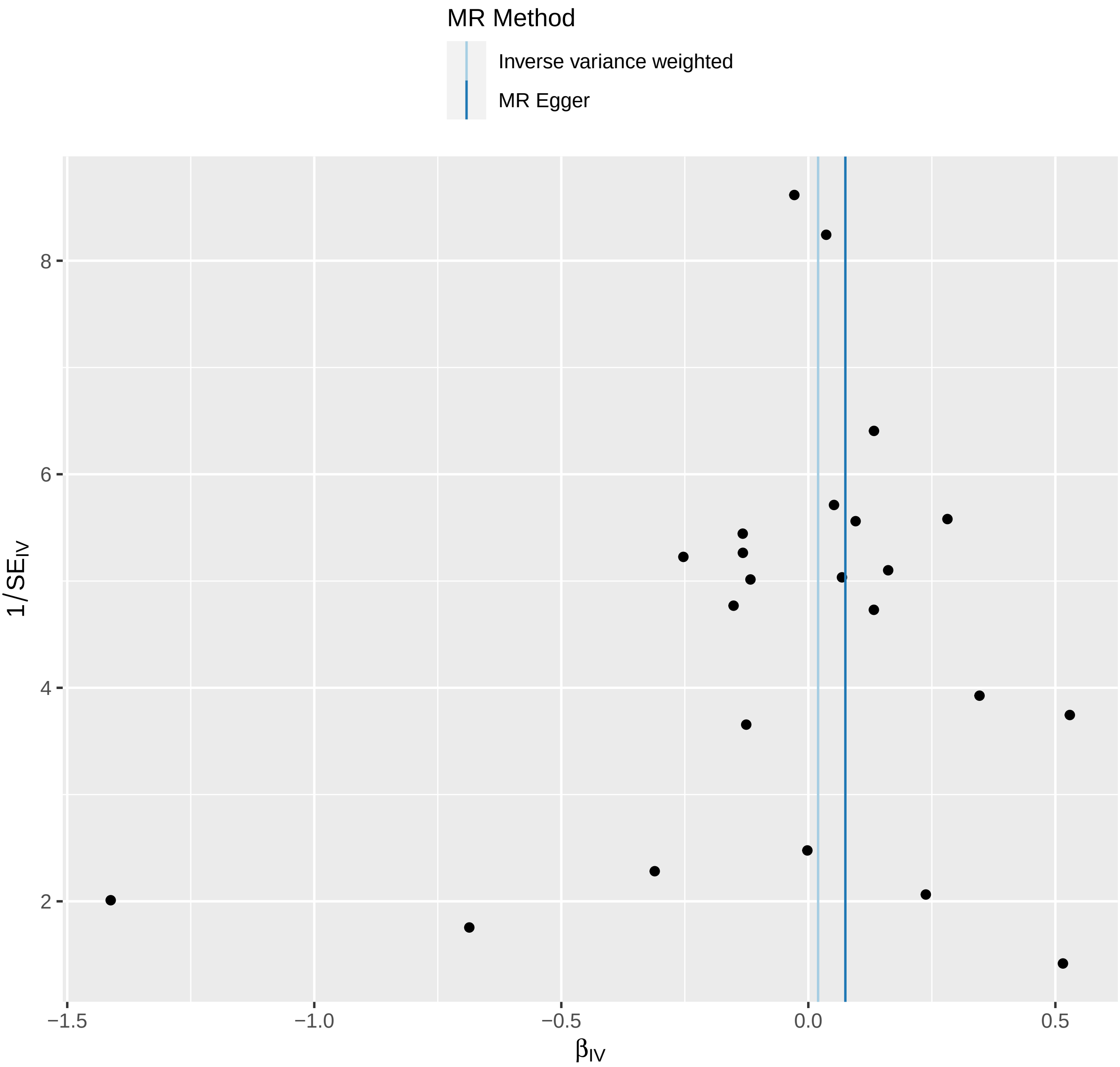

Batch 3−1751 : Gastric triacylglycerol lipase on Diabetic nephropathy (more control exclusions)
with a threshold of 1e−05

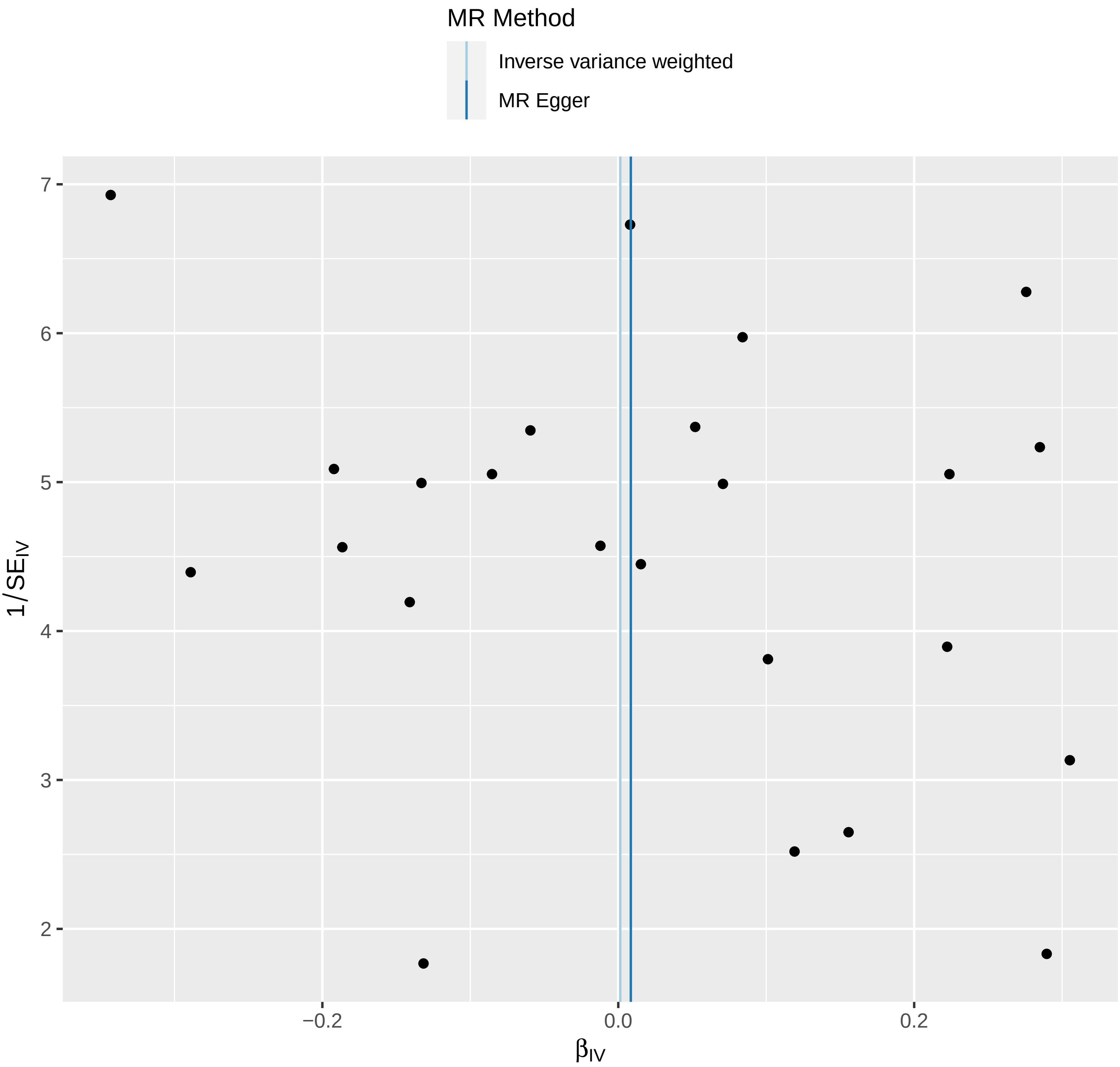

Batch 3−1752 : Lipase member K on Diabetic nephropathy (more control exclusions)
with a threshold of 1e−05

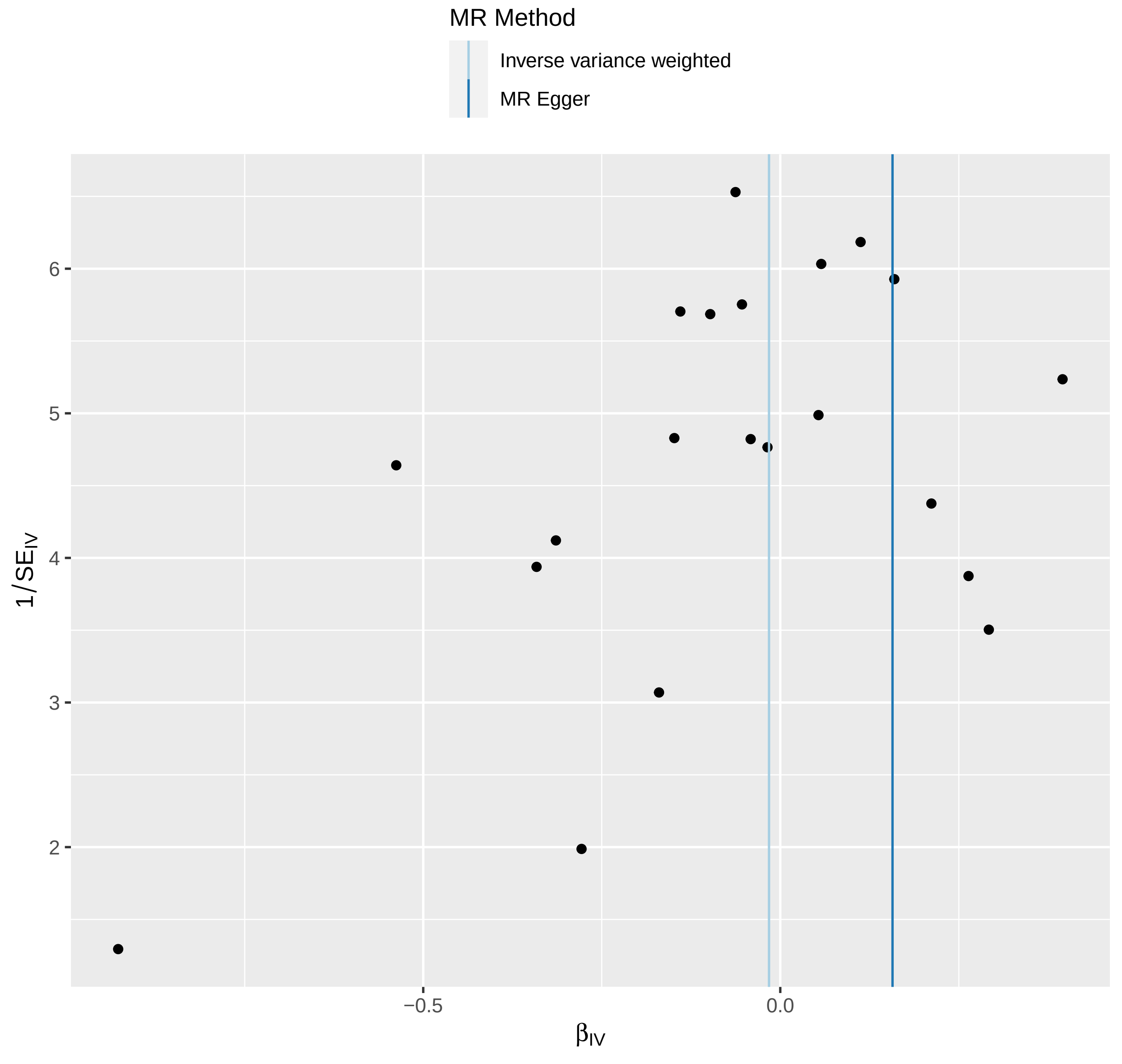

Batch 3−1753 : Lipase member N on Diabetic nephropathy (more control exclusions)
with a threshold of 1e−05

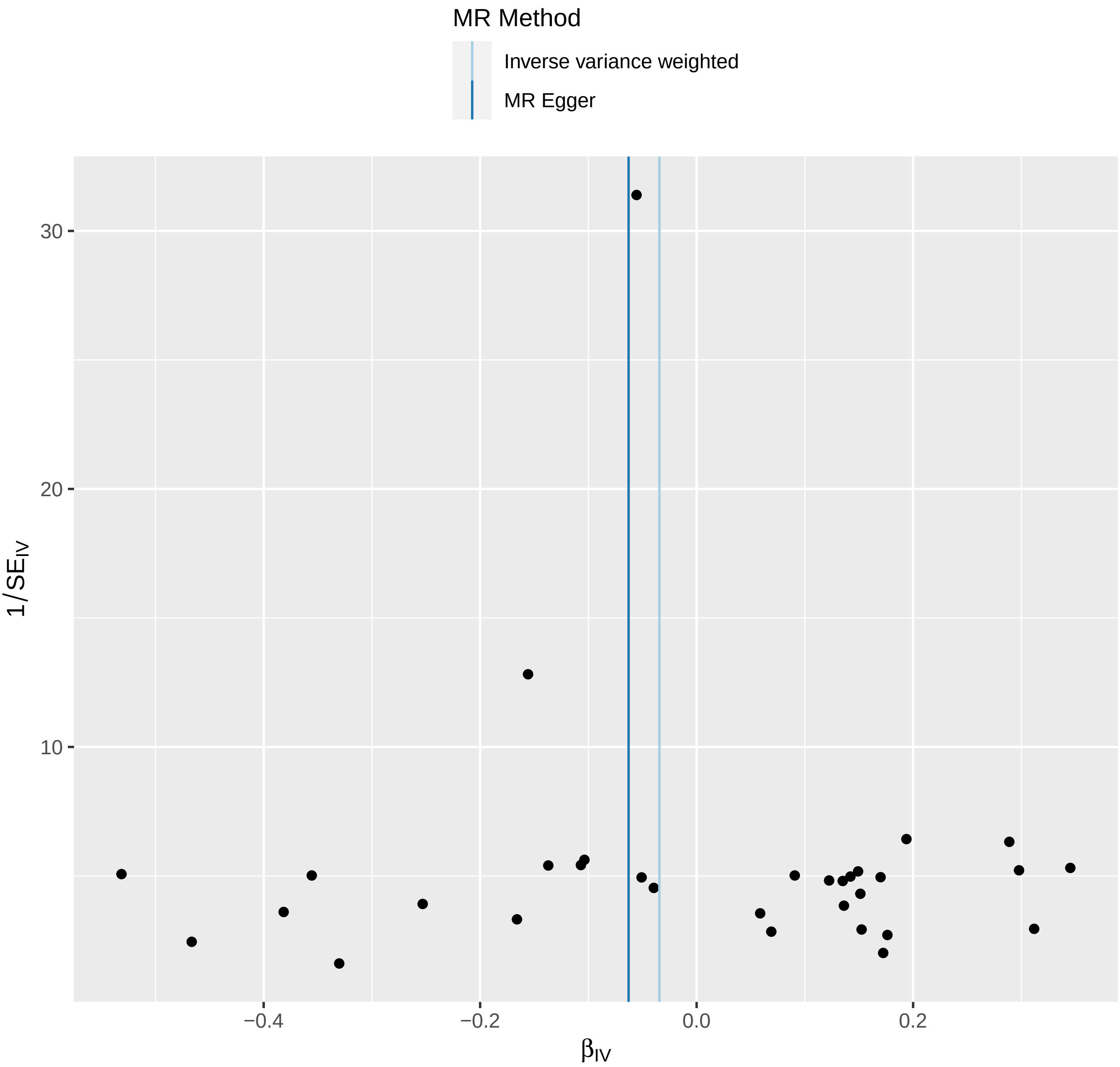

Batch 3−1754 : Vesicular integral−membrane protein VIP36 on Diabetic nephropathy (more control exclusions)
with a threshold of 1e−05

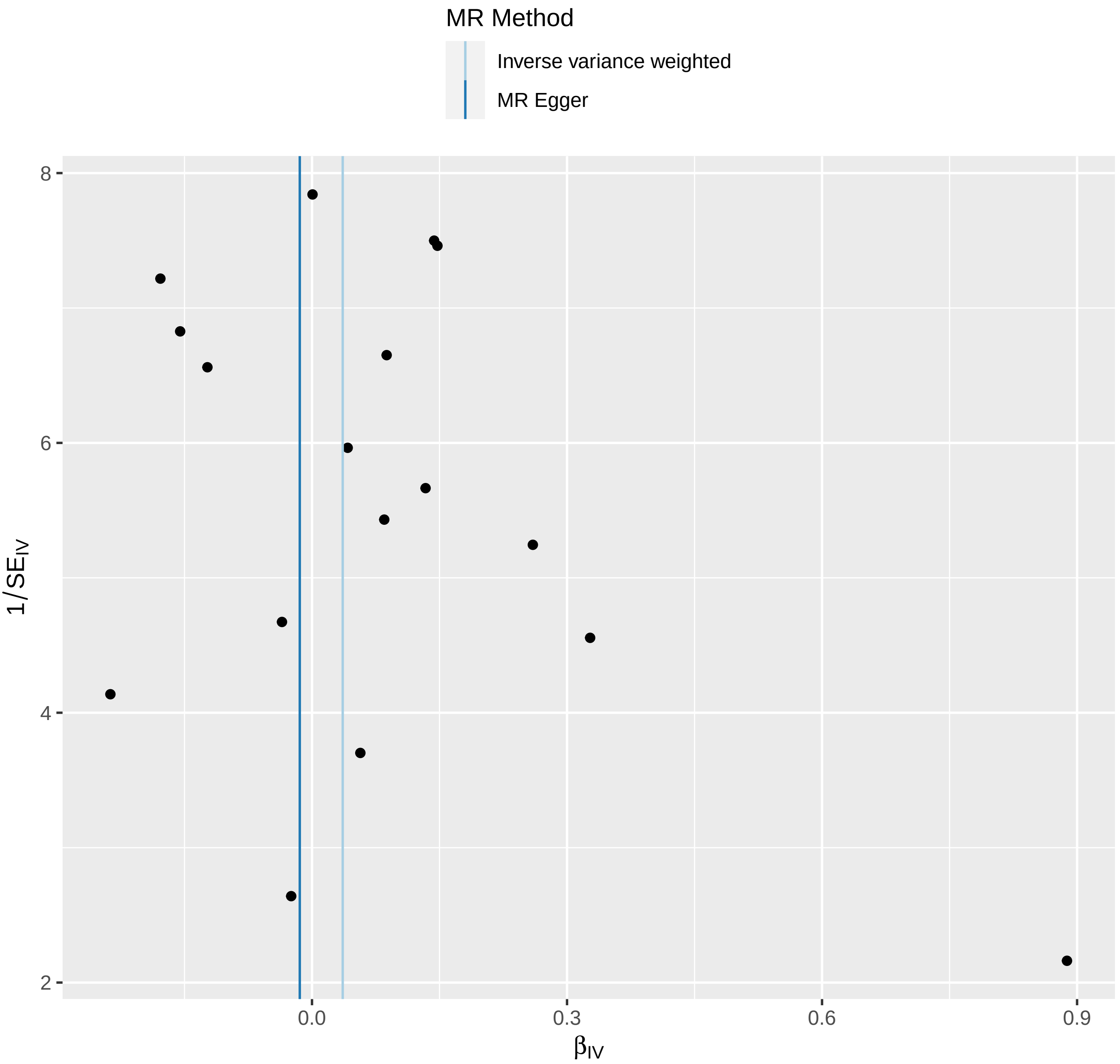

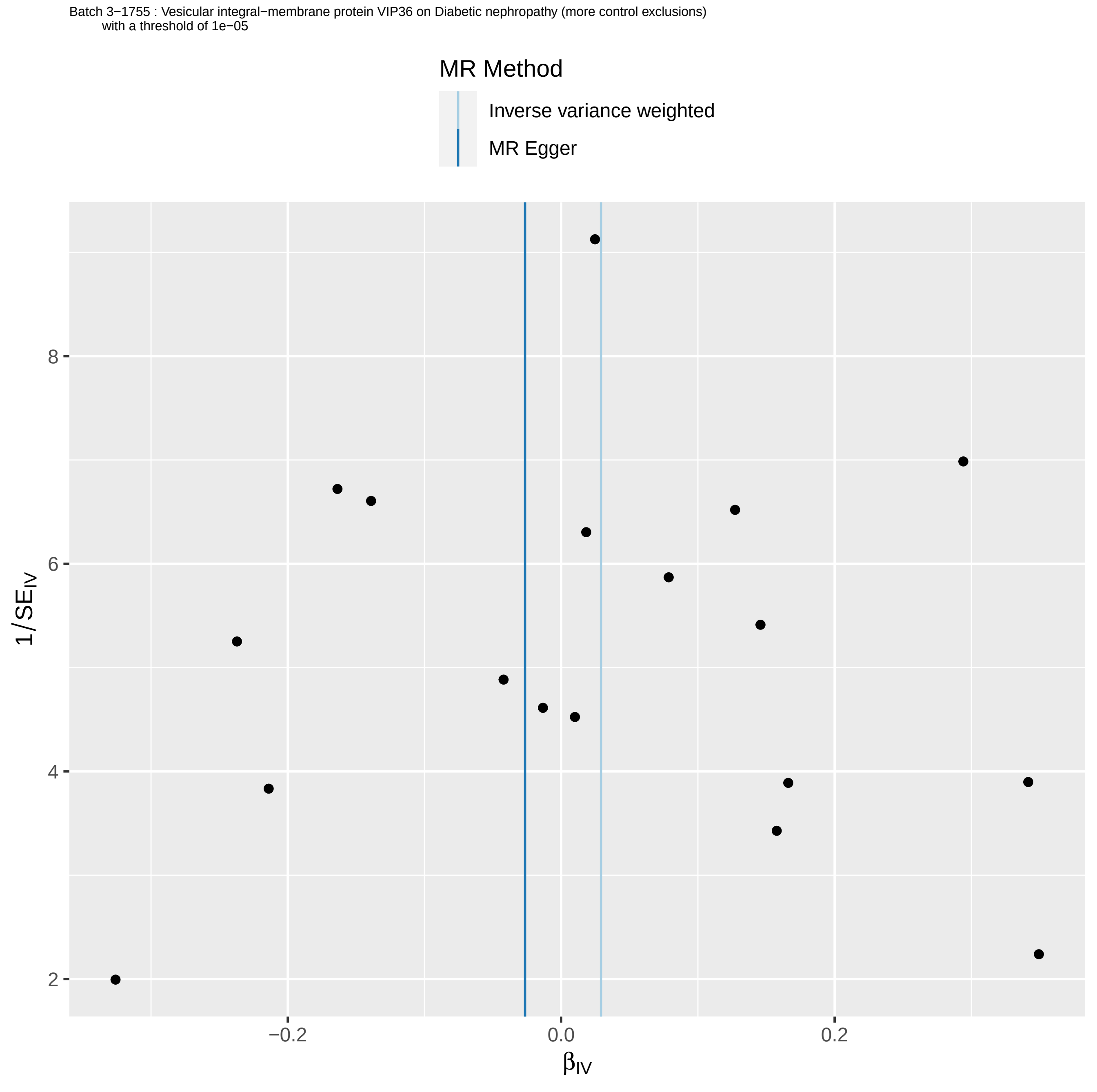

Batch 3−1755 : Vesicular integral−membrane protein VIP36 on Diabetic nephropathy (more control exclusions)
with a threshold of 1e−05
MR Method
Inverse variance weighted
MR Egger
8
6
4
2
1/SE_IV
−0.2
0.0
0.2
β_IV

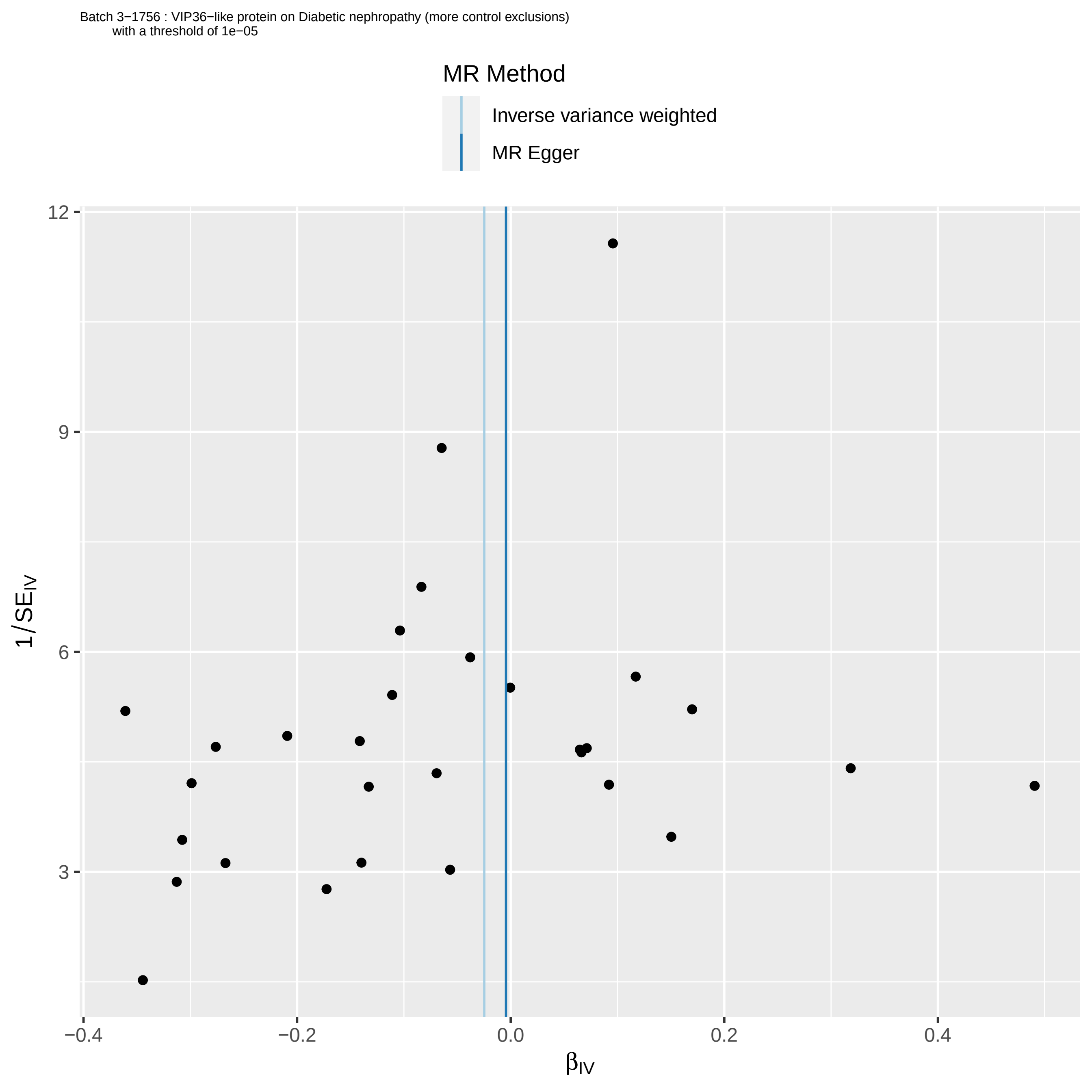
Batch 3−1756 : VIP36−like protein on Diabetic nephropathy (more control exclusions)
with a threshold of 1e−05
MR Method
Inverse variance weighted
MR Egger
12
9
6
3
1/SE_IV
−0.4
−0.2
0.0
0.2
0.4
β_IV

Batch 3−1757 : LIM and cysteine−rich domains protein 1 on Diabetic nephropathy (more control exclusions)
with a threshold of 1e−05

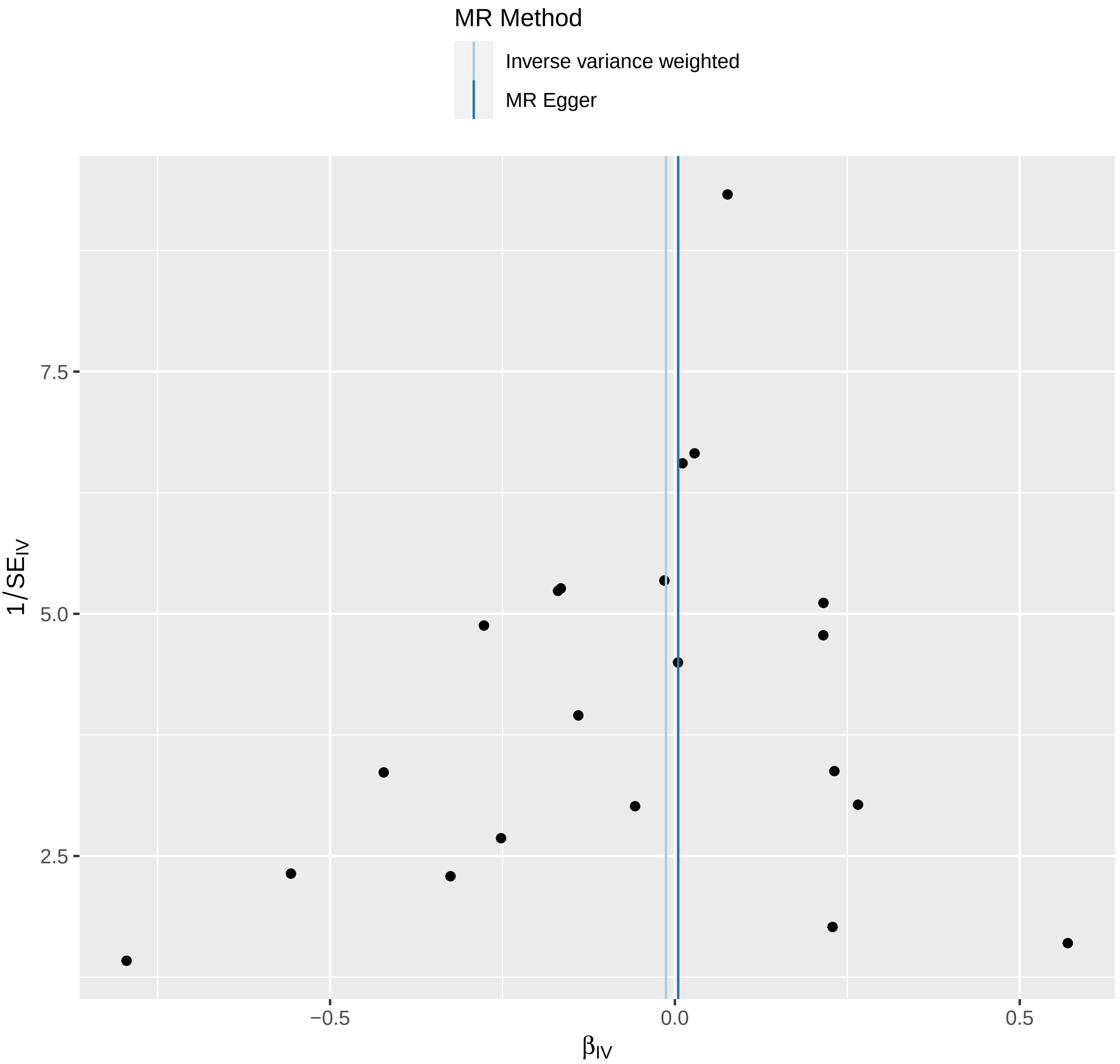

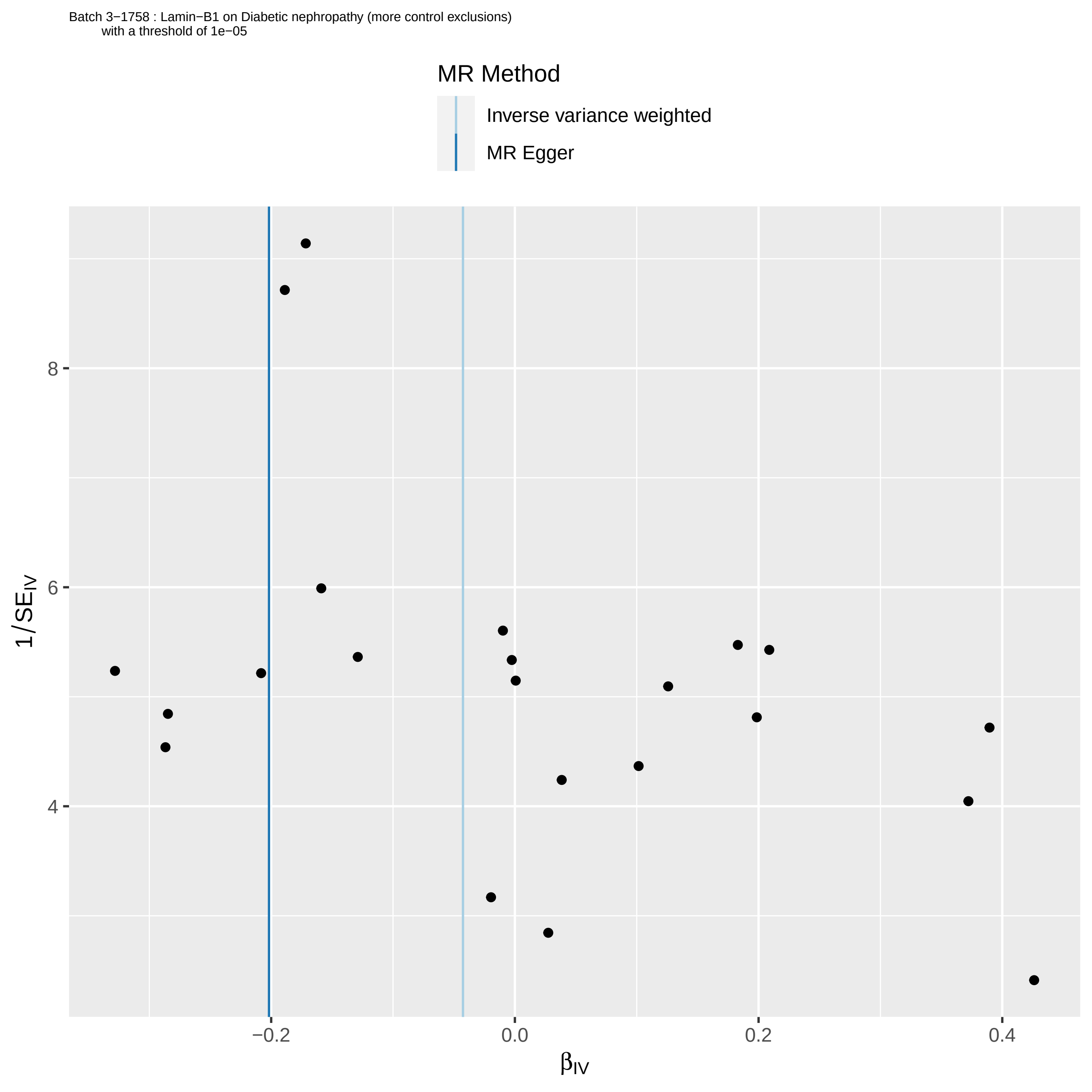

Batch 3−1758 : Lamin−B1 on Diabetic nephropathy (more control exclusions)
with a threshold of 1e−05
MR Method
Inverse variance weighted
MR Egger
8
6
4
$1/SE_{IV}$
−0.2
0.0
0.2
0.4
β_{IV}

Batch 3−1759 : Leiomodin−1 on Diabetic nephropathy (more control exclusions)
with a threshold of 1e−05

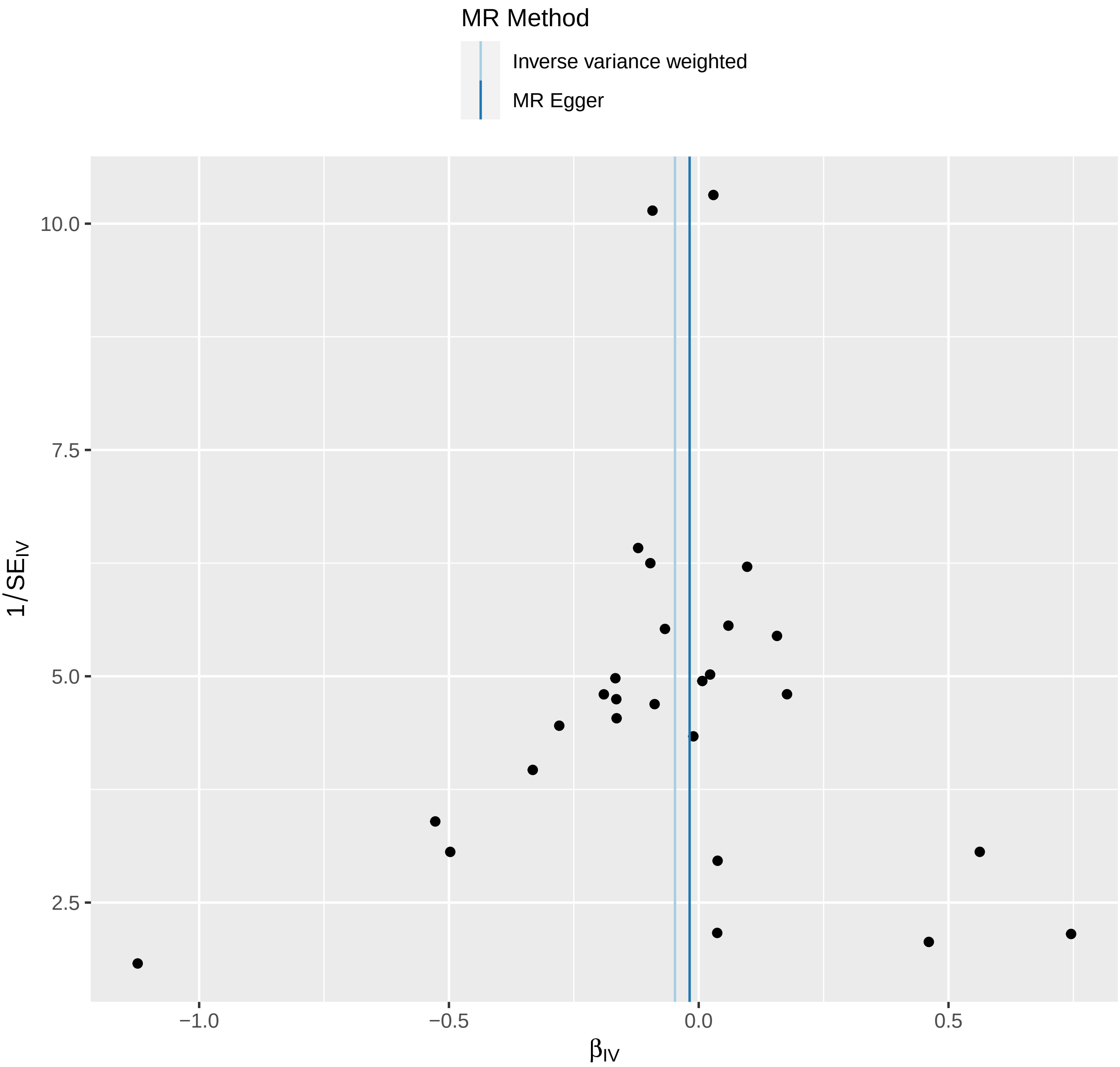

Batch 3−1760 : Ig Kappa chain V−I region HK102− like on Diabetic nephropathy (more control exclusions)
with a threshold of 1e−05

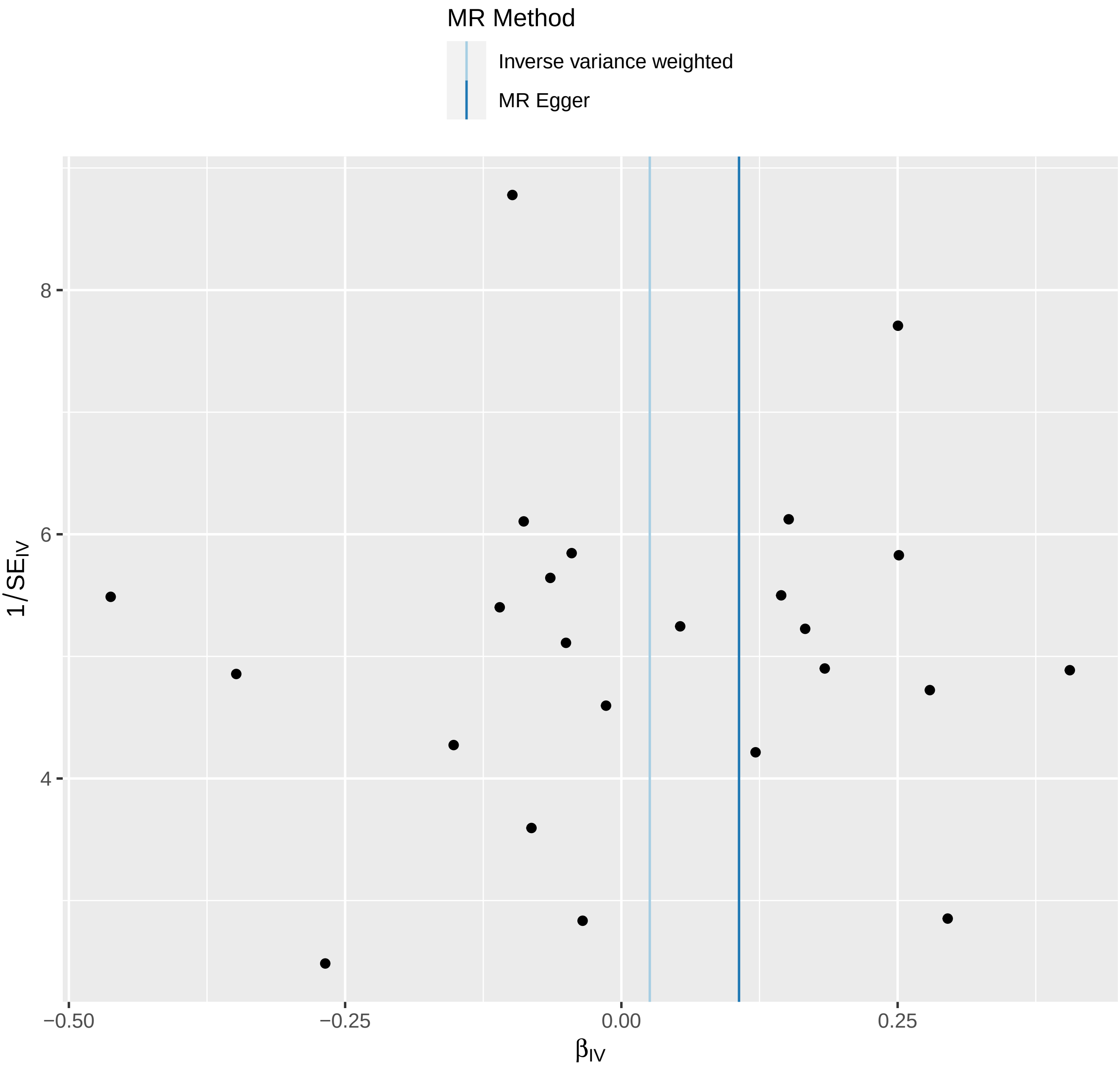

Batch 3−1761 : Lon protease homolog, mitochondrial on Diabetic nephropathy (more control exclusions)
with a threshold of 1e−05

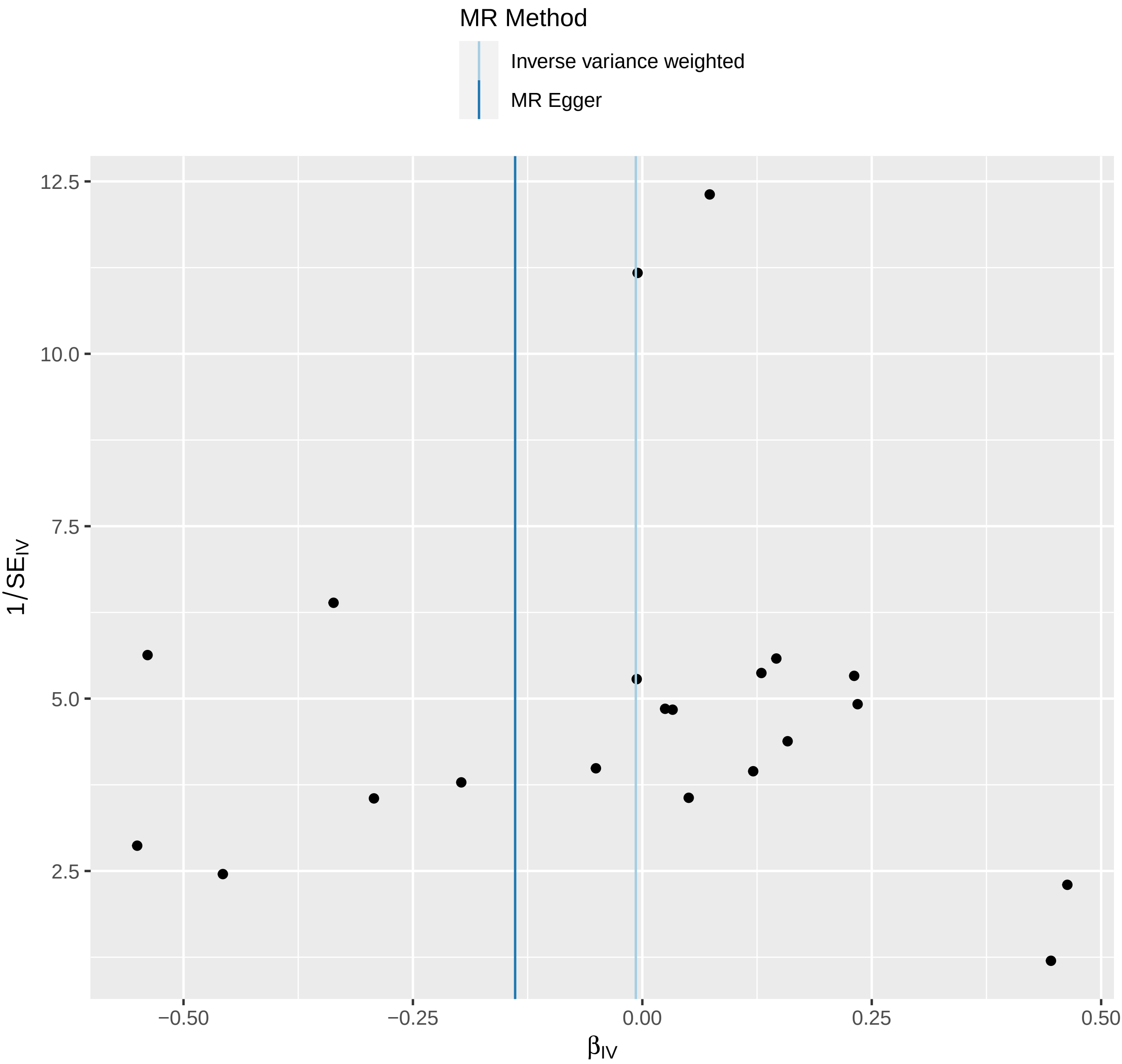

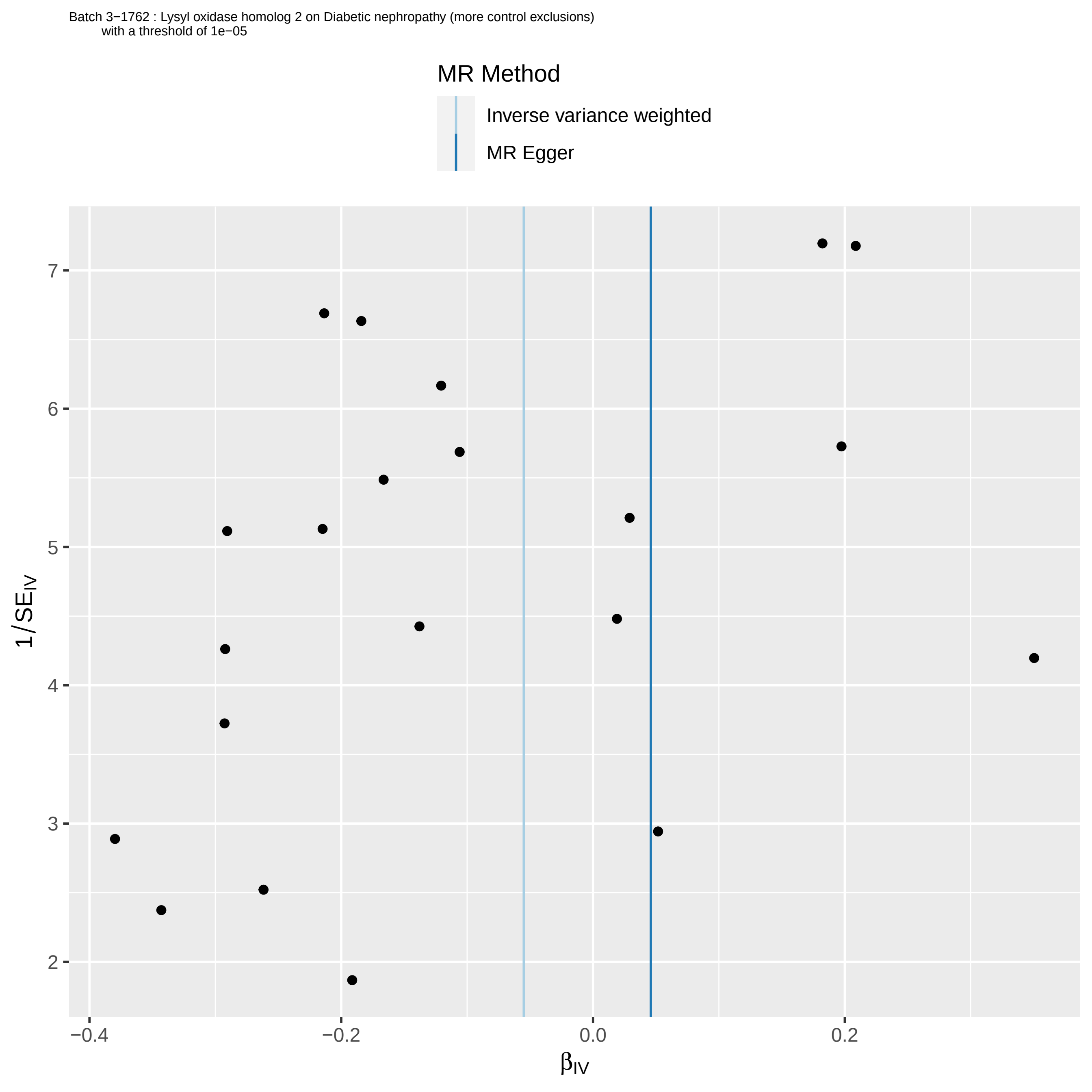

Batch 3−1762 : Lysyl oxidase homolog 2 on Diabetic nephropathy (more control exclusions)
with a threshold of 1e−05
MR Method
Inverse variance weighted
MR Egger
7
6
5
4
3
2
1/SE_IV
−0.4
−0.2
0.0
0.2
β_IV

Batch 3−1763 : Putative apolipoprotein(a)−like protein 2 on Diabetic nephropathy (more control exclusions)
with a threshold of 1e−05

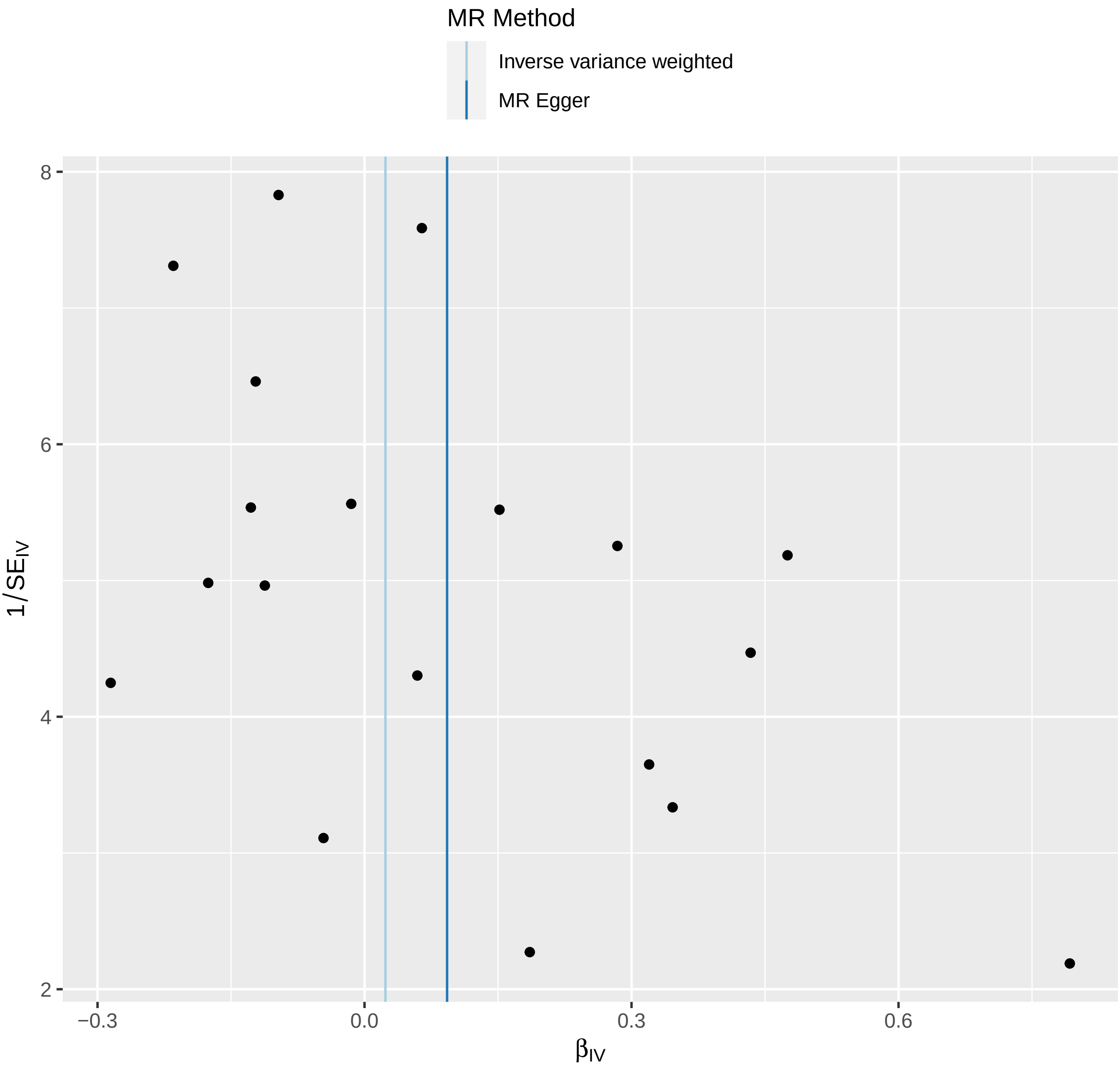

Batch 3−1764 : Lysophosphatidylcholine acyltransferase 2 on Diabetic nephropathy (more control exclusions) with a threshold of 1e−05

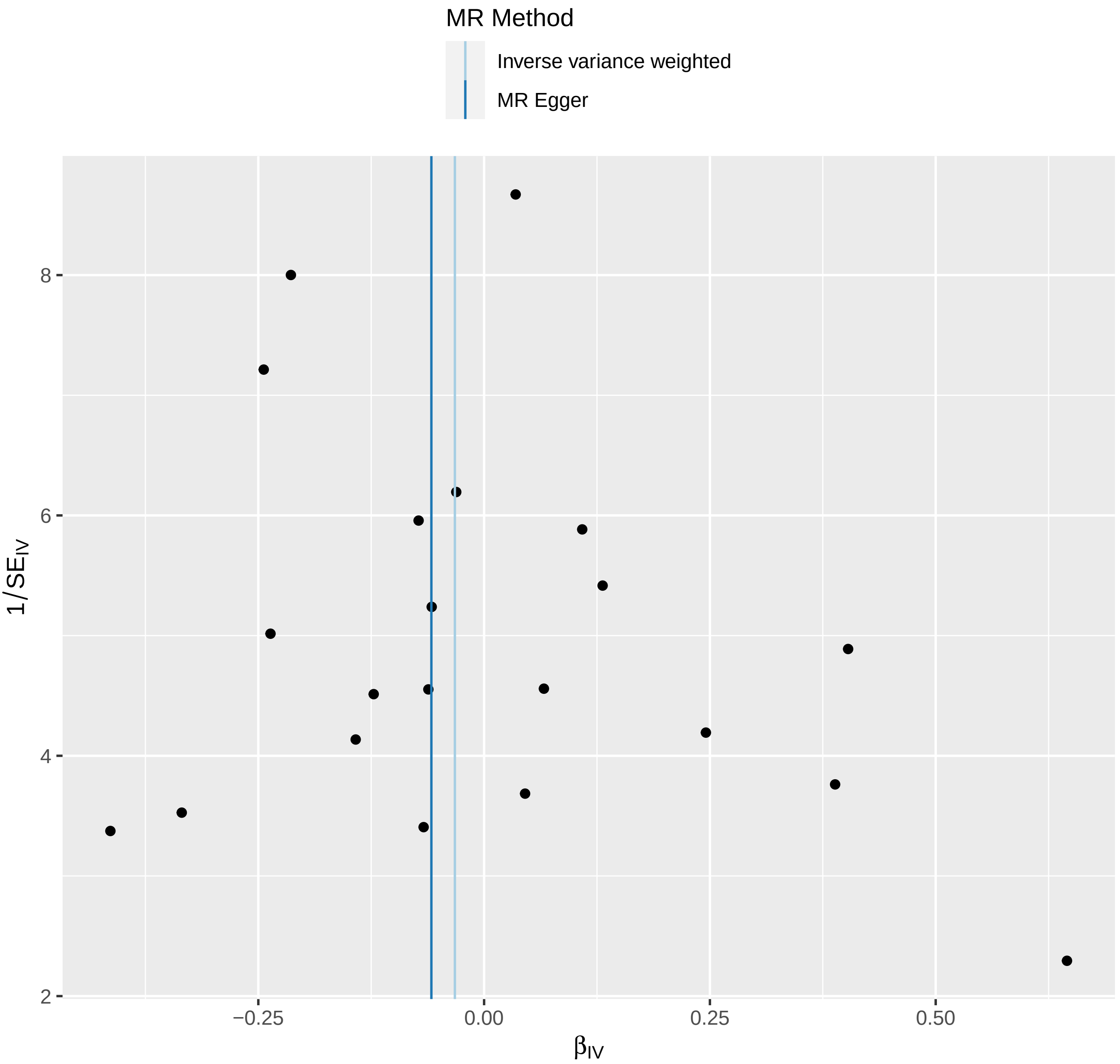

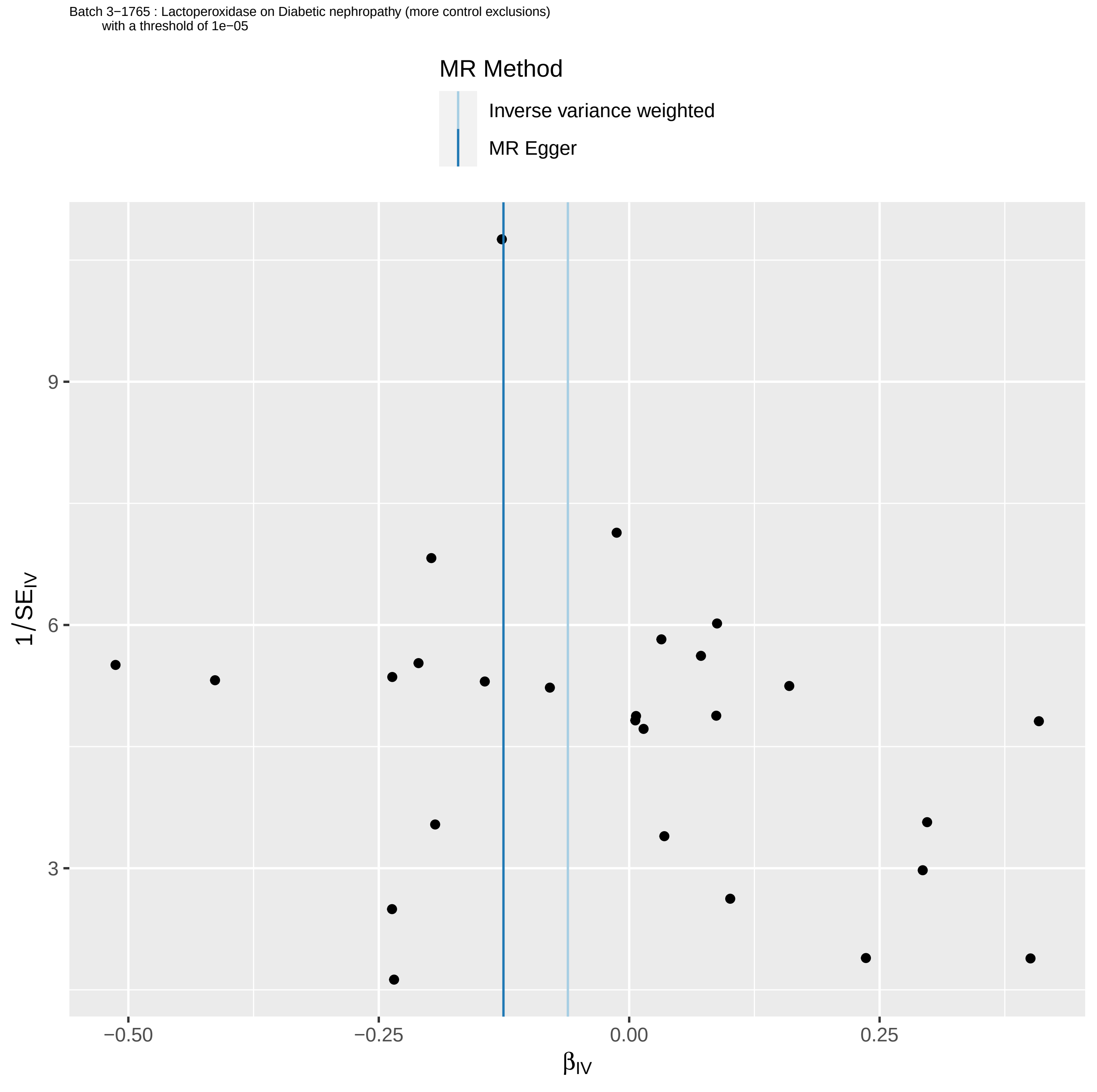
Batch 3−1765 : Lactoperoxidase on Diabetic nephropathy (more control exclusions)
with a threshold of 1e−05
MR Method
Inverse variance weighted
MR Egger
9
6
3
1/SE_IV
−0.50
−0.25
0.00
0.25
β_IV

Batch 3−1766 : Leucine−rich repeat and calponin homology domain−containing protein 4 on Diabetic nephropathy (more control exclusions) with a threshold of 1e−05

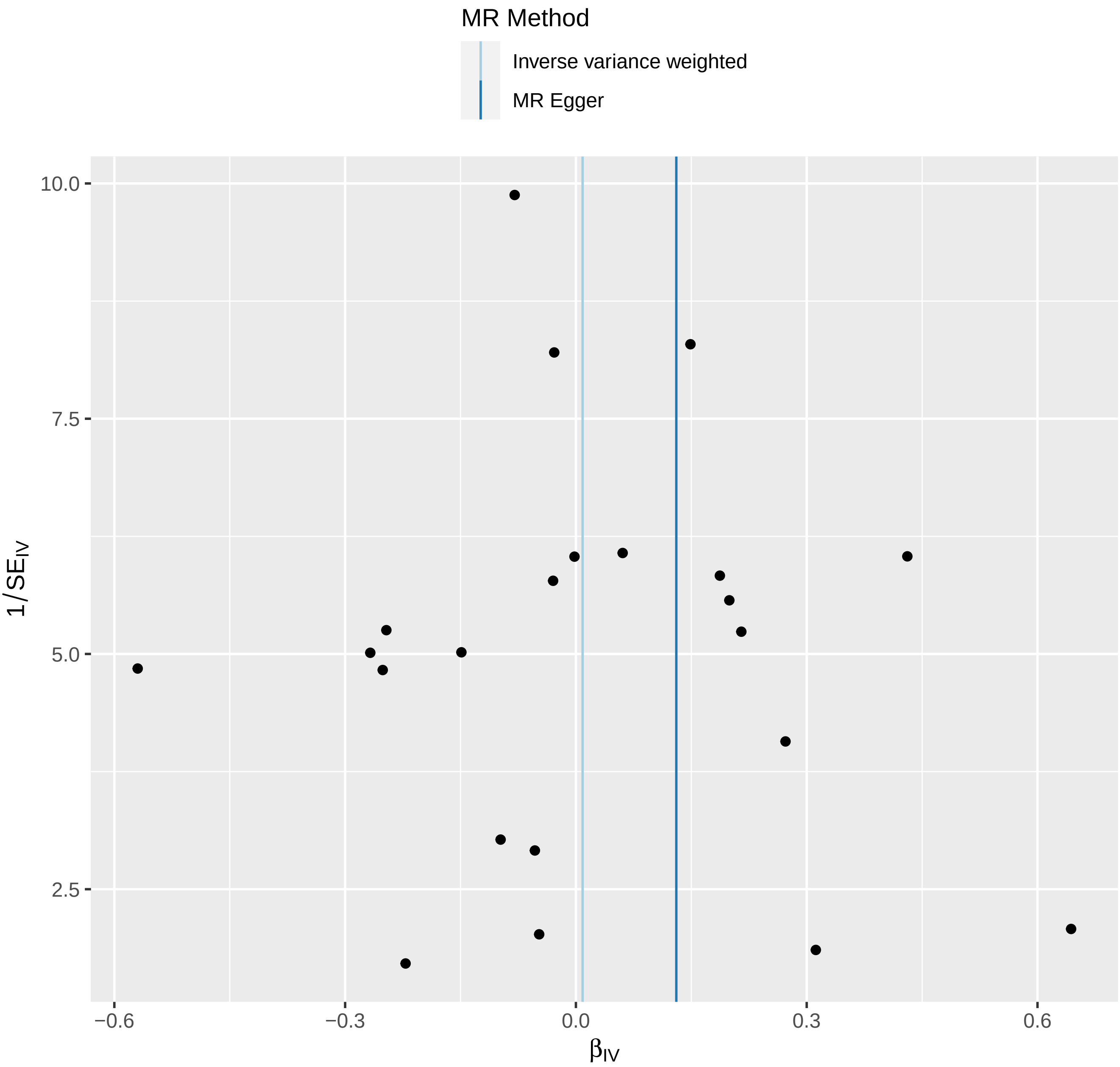

Batch 3−1767 : Leucine−rich repeat and calponin homology domain−containing protein 4 on Diabetic nephropathy (more control exclusions)
with a threshold of 1e−05

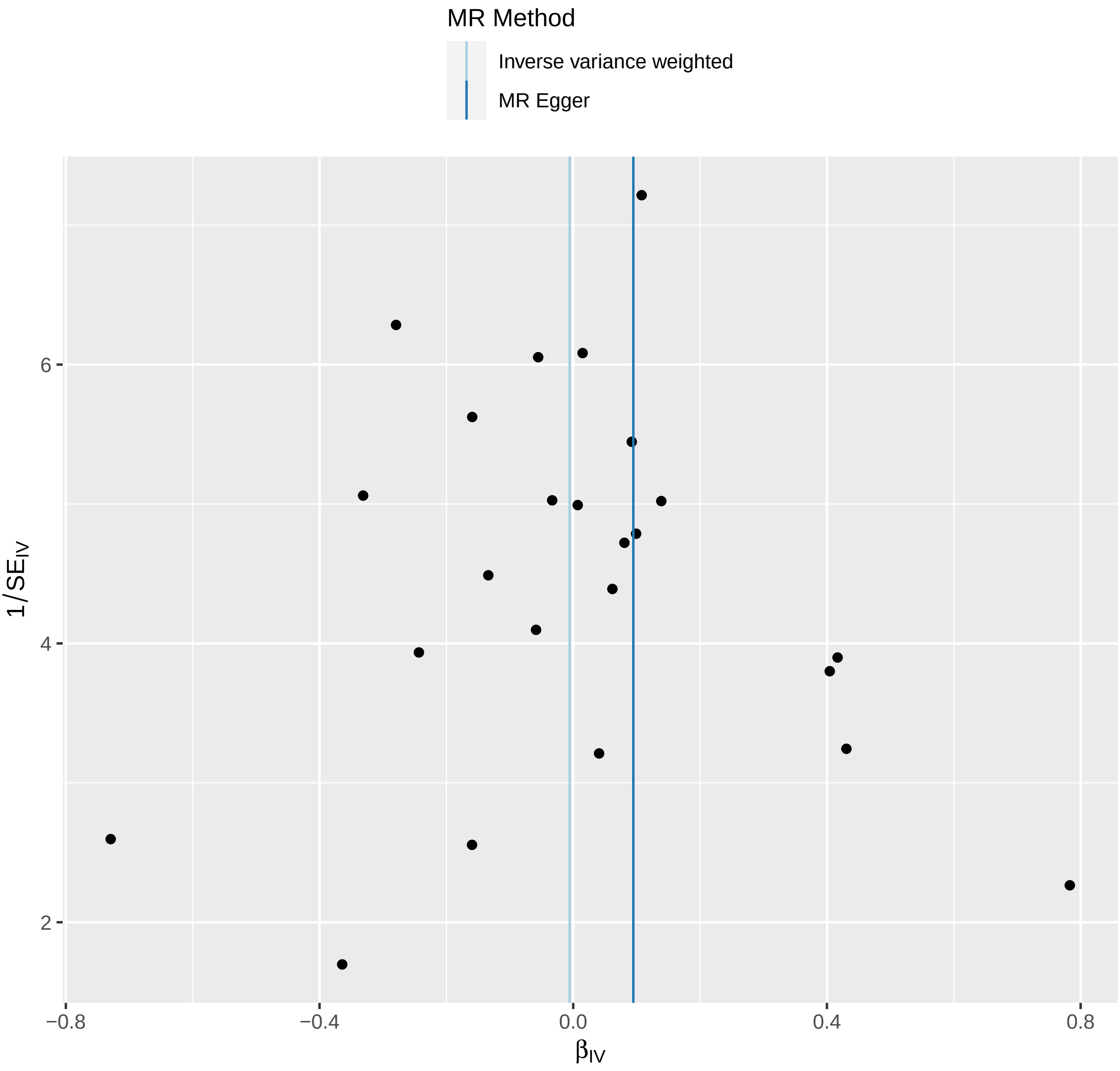

Batch 3−1768 : Leucine−rich repeat and fibronectin type III domain−containing protein 1 on Diabetic nephropathy (more control exclusions) with a threshold of 1e−05

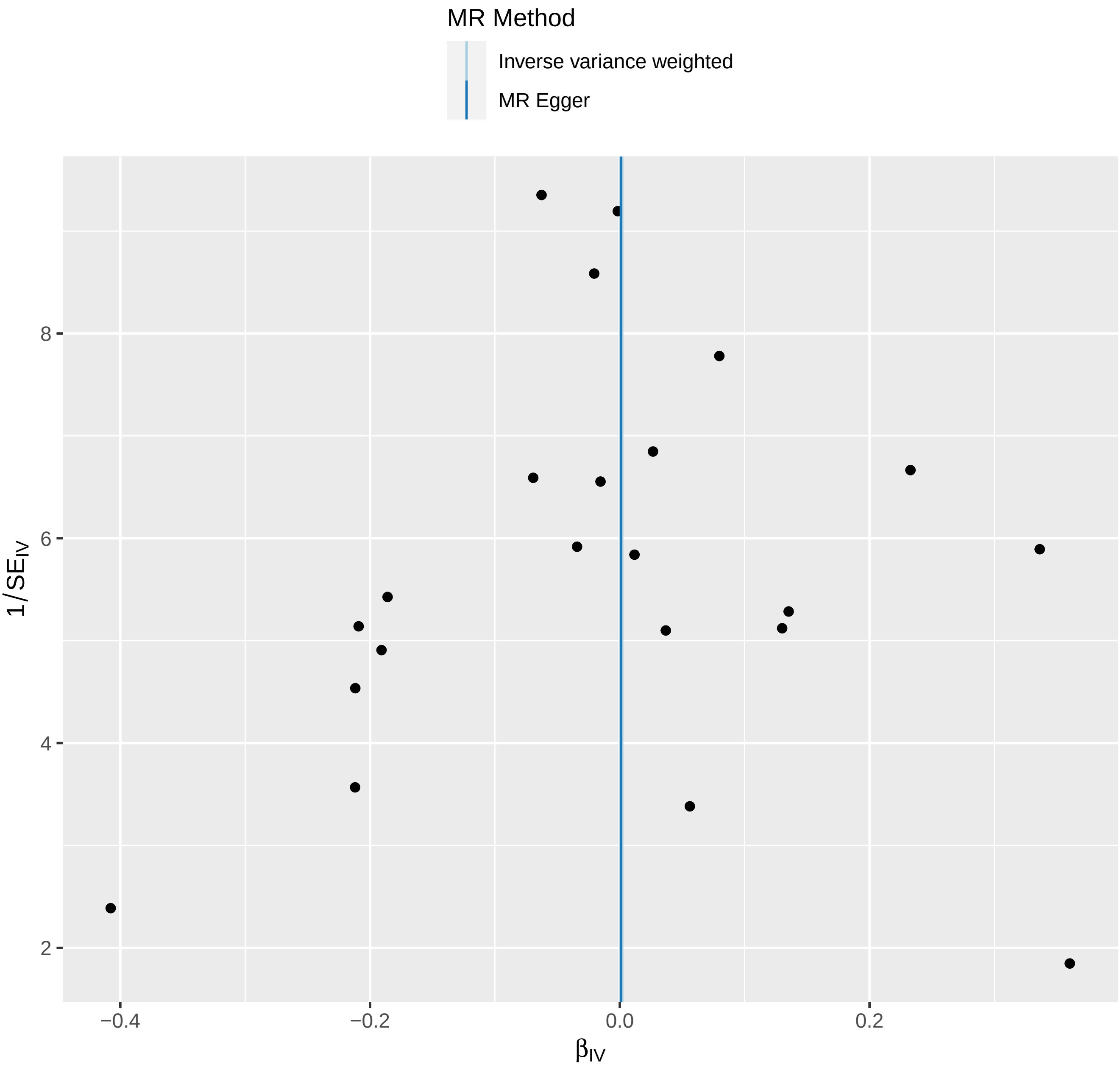

Batch 3−1769 : Leucine−rich repeat and fibronectin type−III domain−containing protein 2 on Diabetic nephropathy (more control exclusions) with a threshold of 1e−05

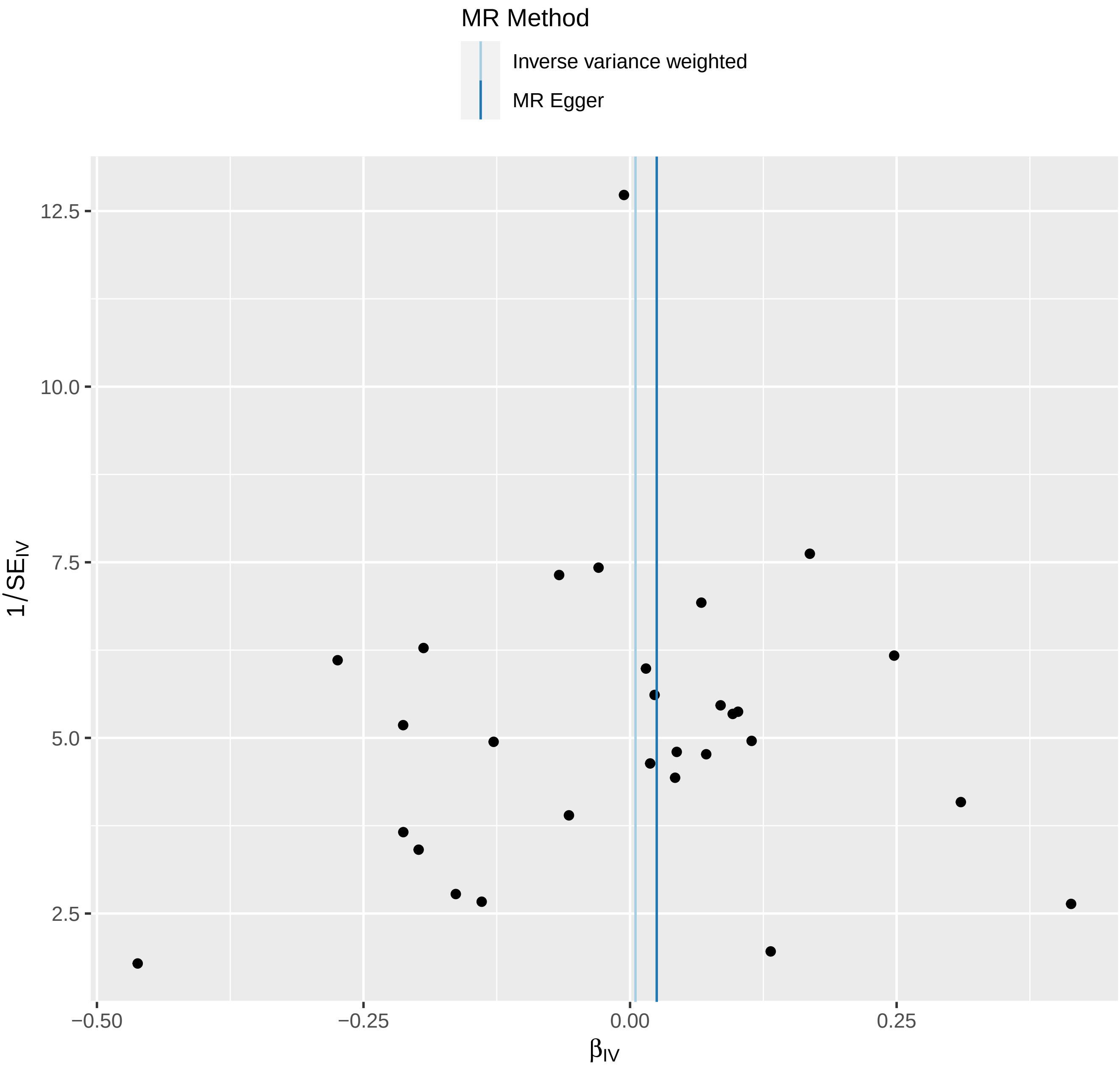

Batch 3−1770 : Leucine−rich repeats and immunoglobulin−like domains protein 3 on Diabetic nephropathy (more control exclusions)
with a threshold of 1e−05

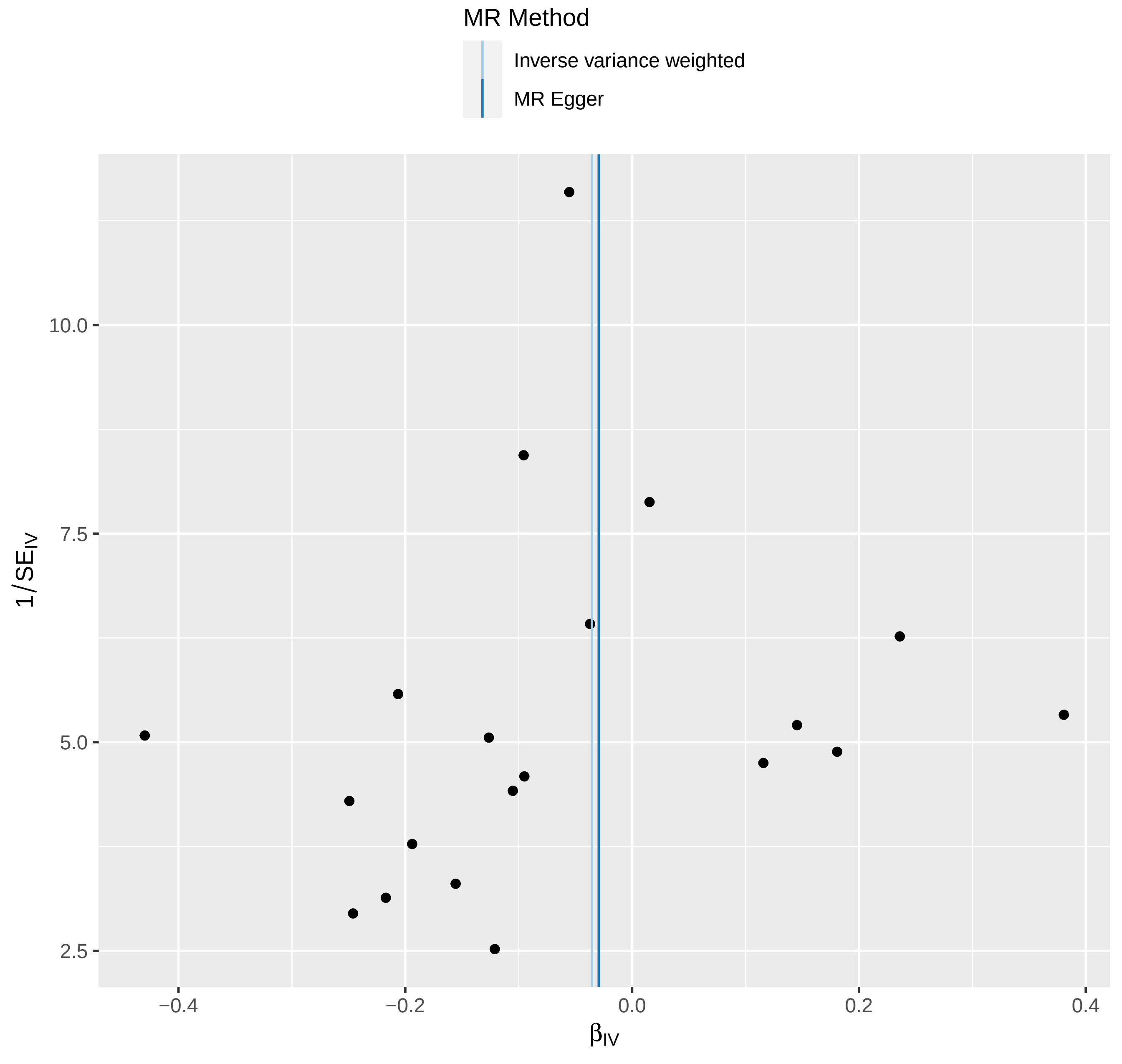

Batch 3−1771 : Leucine−rich repeat, immunoglobulin−like domain and transmembrane domain−containing protein 2 on Diabetic nephropathy (more control exclusions)
with a threshold of 1e−05

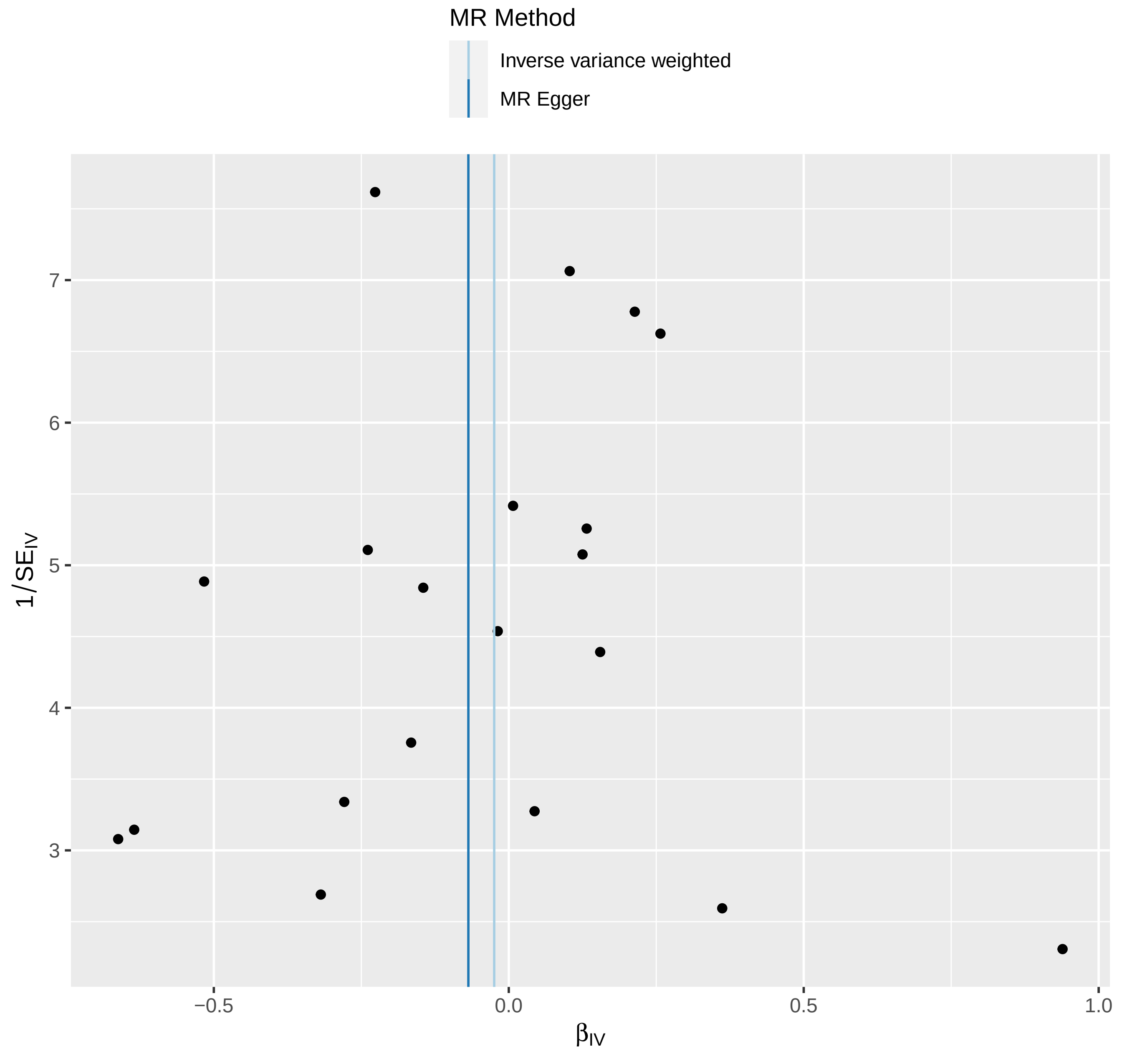

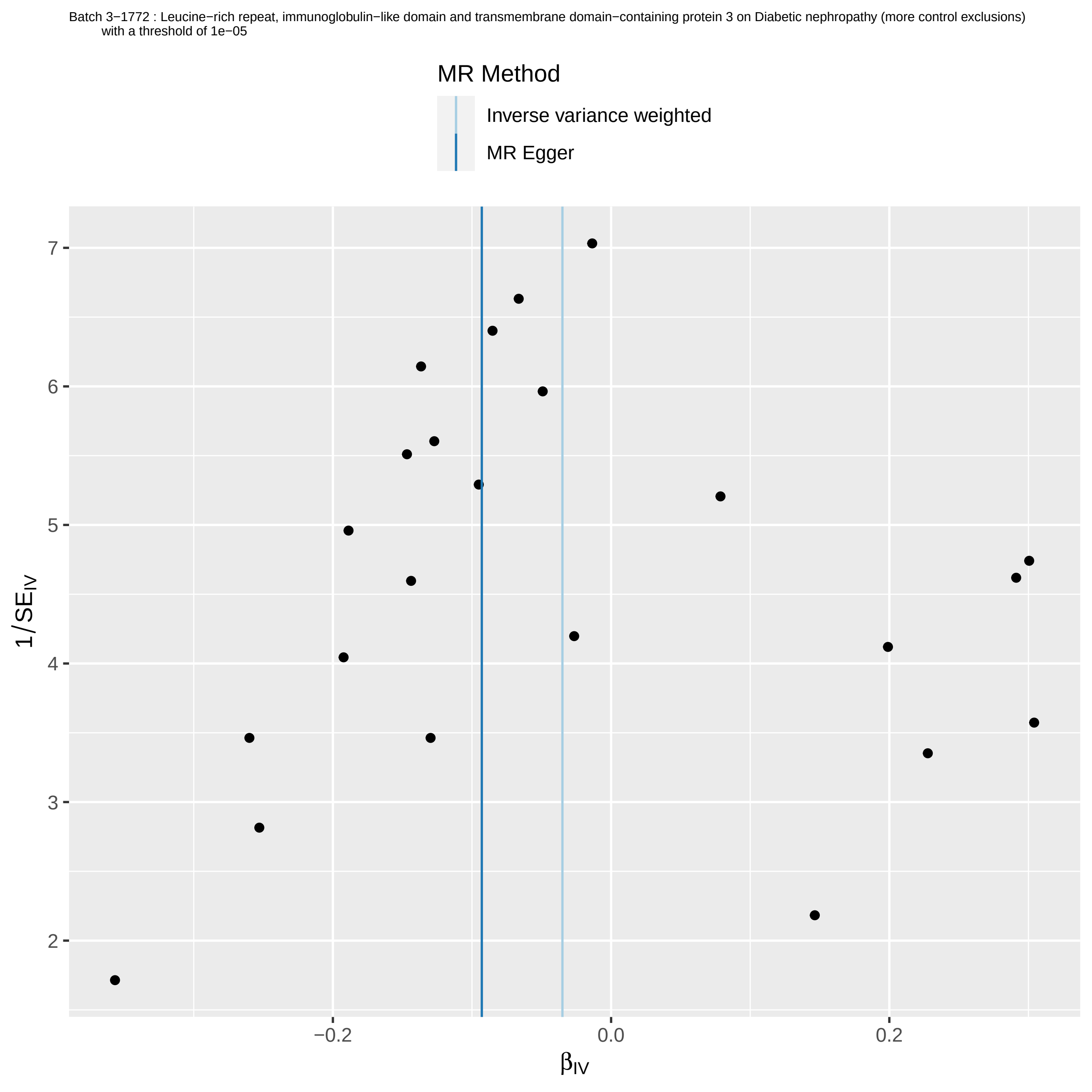

Batch 3−1772 : Leucine−rich repeat, immunoglobulin−like domain and transmembrane domain−containing protein 3 on Diabetic nephropathy (more control exclusions) with a threshold of 1e−05
MR Method
Inverse variance weighted
MR Egger
1/SE_IV
β_IV
7
6
5
4
3
2
−0.2
0.0
0.2

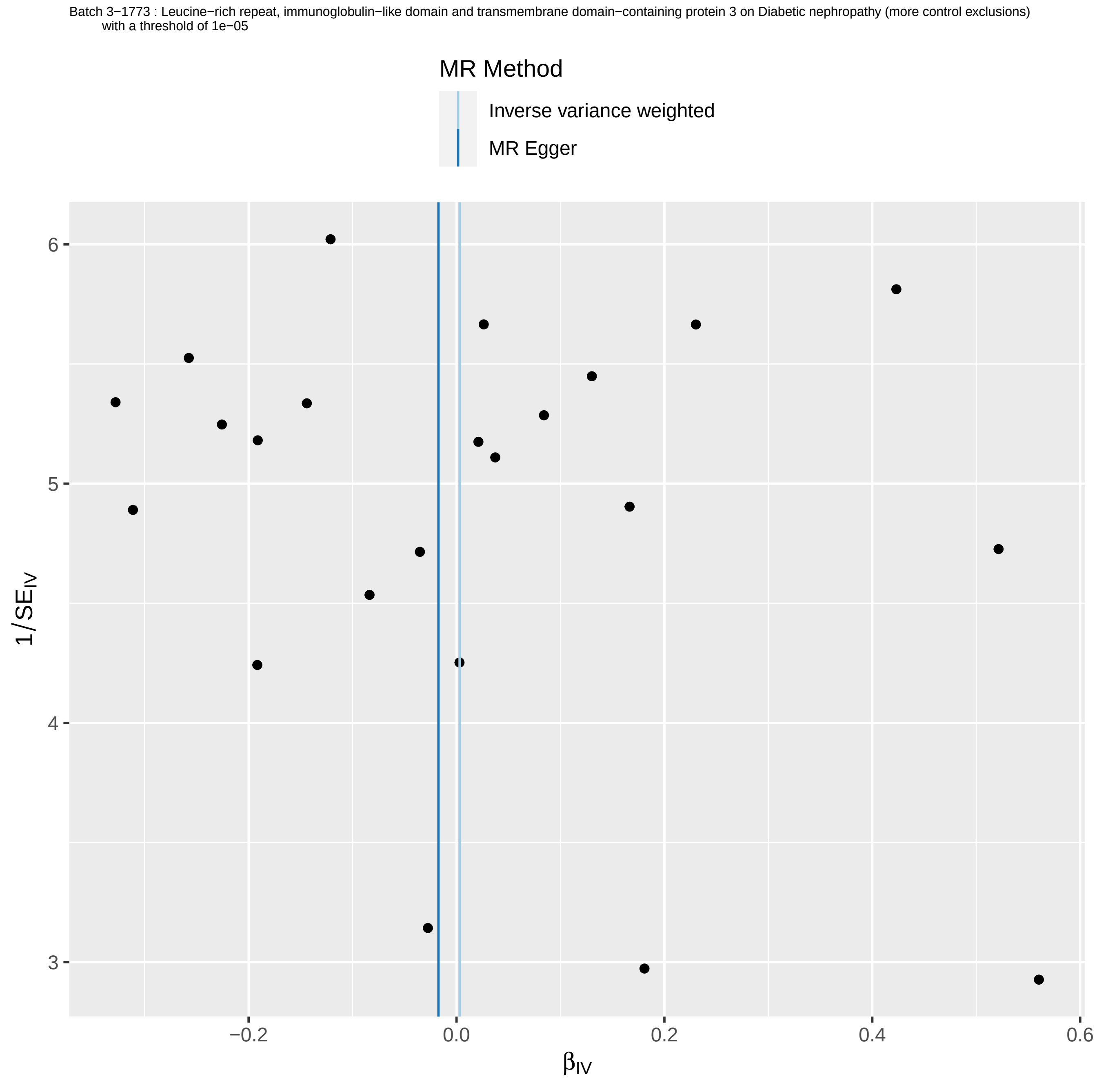
Batch 3−1773 : Leucine−rich repeat, immunoglobulin−like domain and transmembrane domain−containing protein 3 on Diabetic nephropathy (more control exclusions) with a threshold of 1e−05
MR Method
Inverse variance weighted
MR Egger
1/SE_IV
β_IV
6
5
4
3
−0.2
0.0
0.2
0.4
0.6

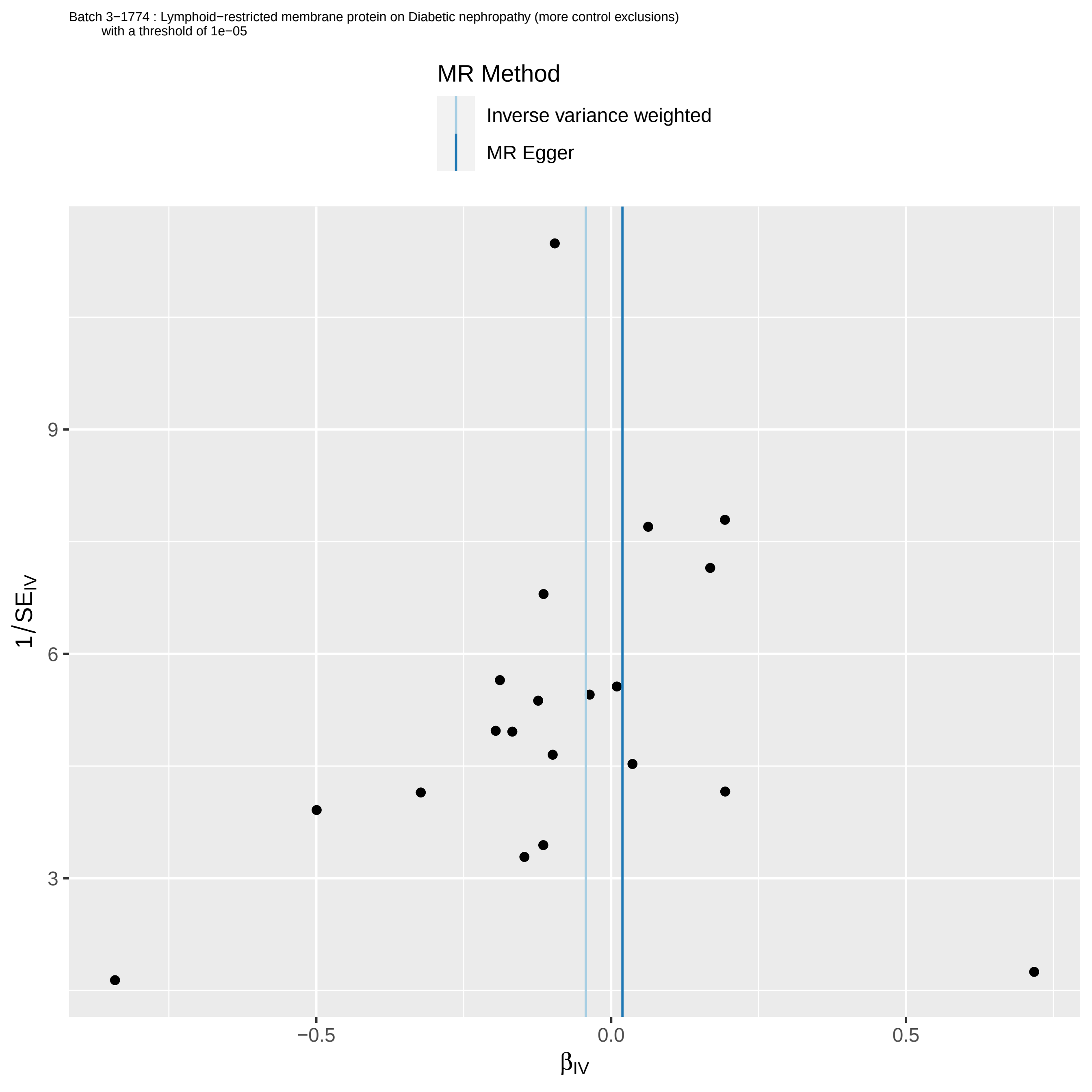

Batch 3−1774 : Lymphoid−restricted membrane protein on Diabetic nephropathy (more control exclusions)
with a threshold of 1e−05
MR Method
Inverse variance weighted
MR Egger
9
6
3
1/SE_IV
−0.5
0.0
0.5
β_IV

Batch 3−1775 : Prolow−density lipoprotein receptor−related protein 1 on Diabetic nephropathy (more control exclusions) with a threshold of 1e−05

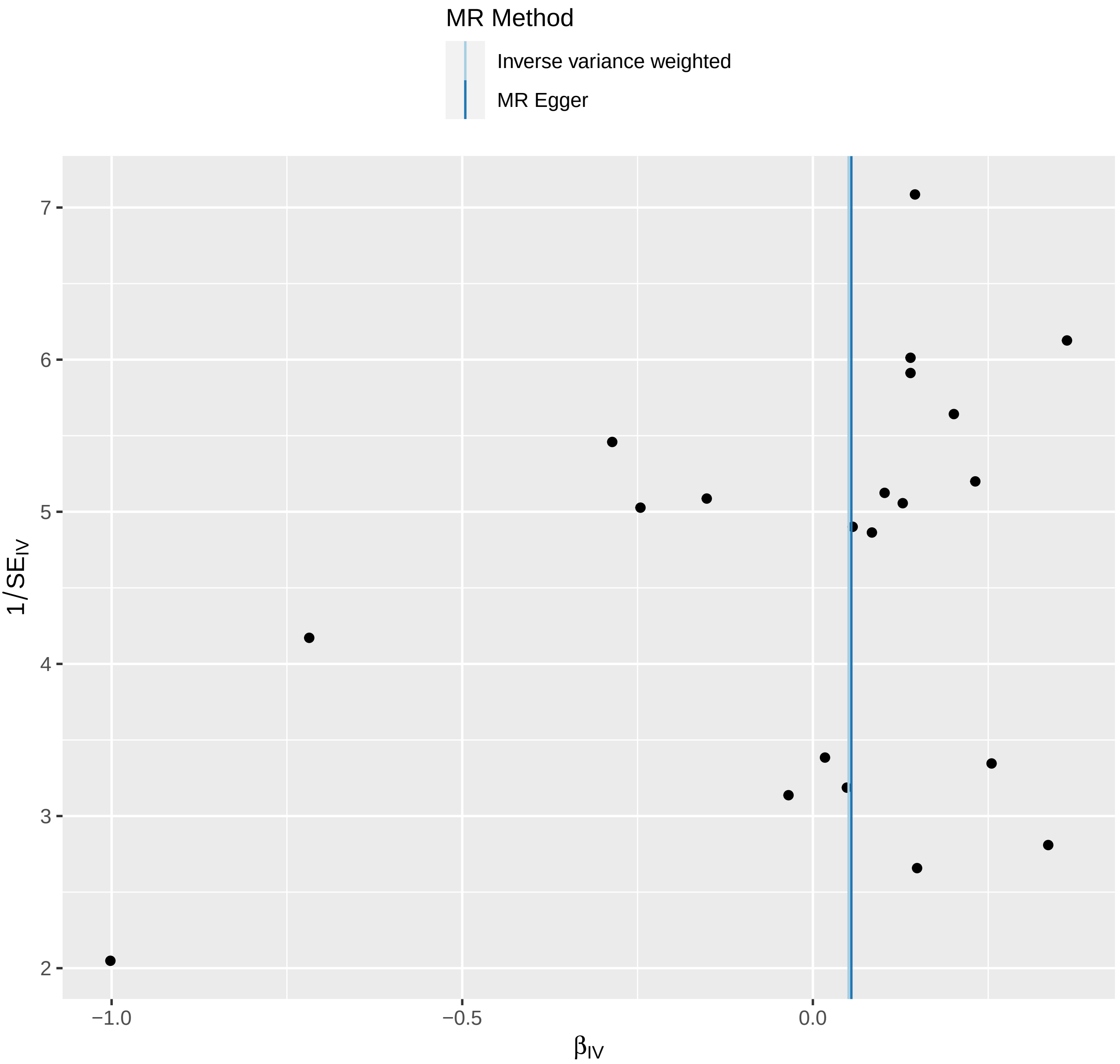

Batch 3−1776 : Low−density lipoprotein receptor−related protein 1, soluble on Diabetic nephropathy (more control exclusions) with a threshold of 1e−05

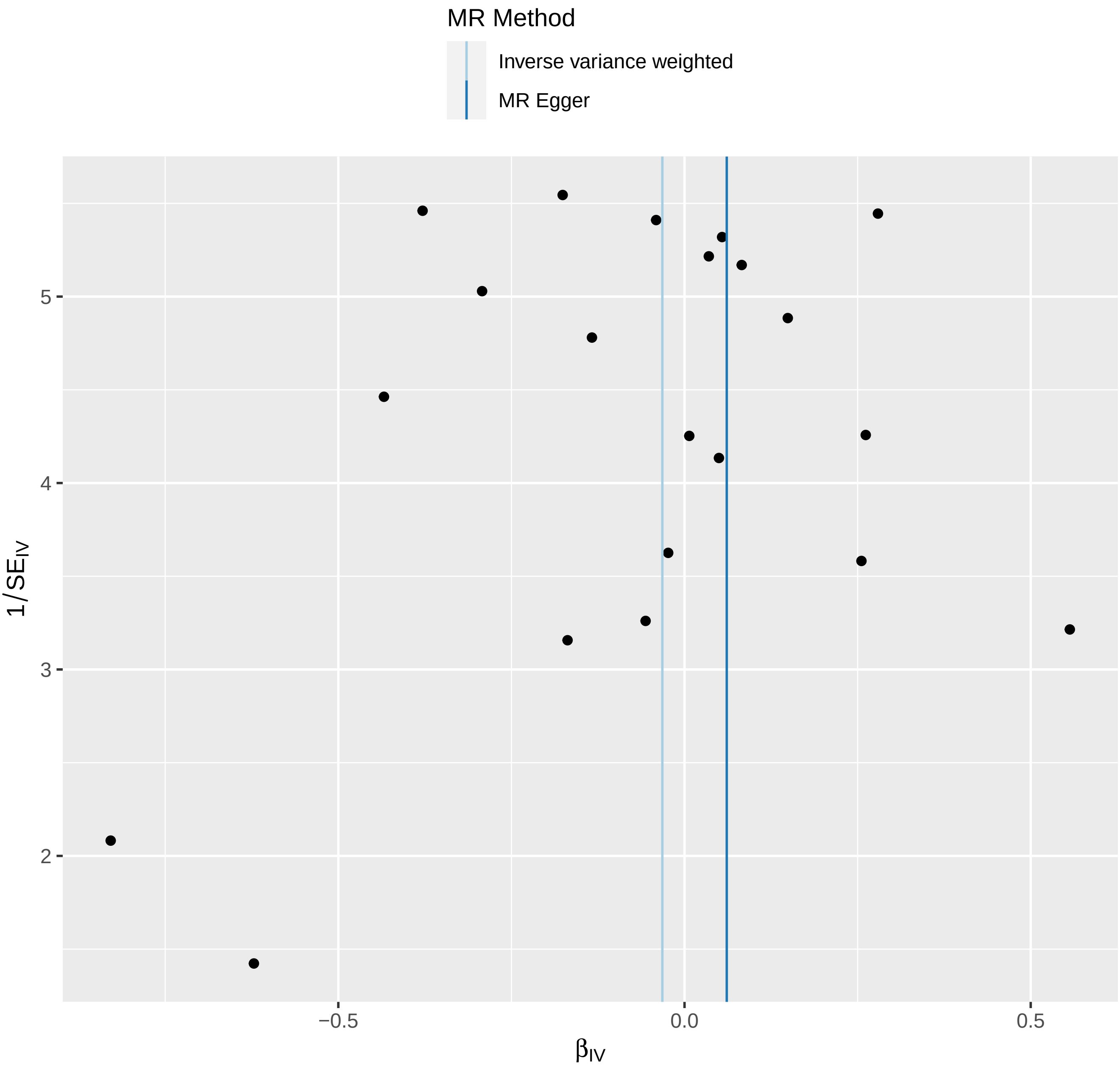

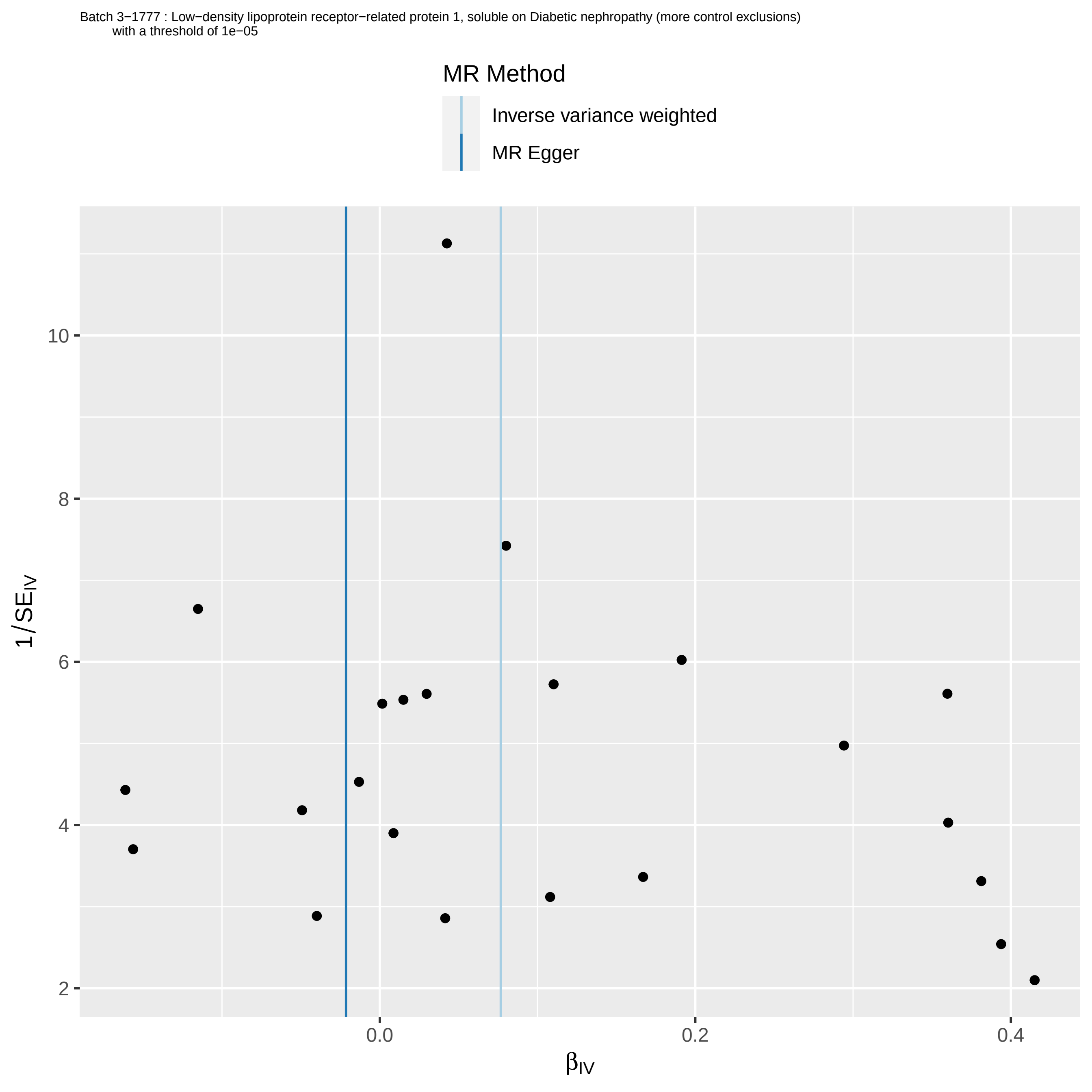
Batch 3−1777 : Low−density lipoprotein receptor−related protein 1, soluble on Diabetic nephropathy (more control exclusions)
with a threshold of 1e−05
MR Method
Inverse variance weighted
MR Egger
10
8
6
4
2
1/SE$_{IV}$
0.0
0.2
0.4
β_{IV}

Batch 3−1778 : Low−density lipoprotein receptor−related protein 1B on Diabetic nephropathy (more control exclusions)
with a threshold of 1e−05

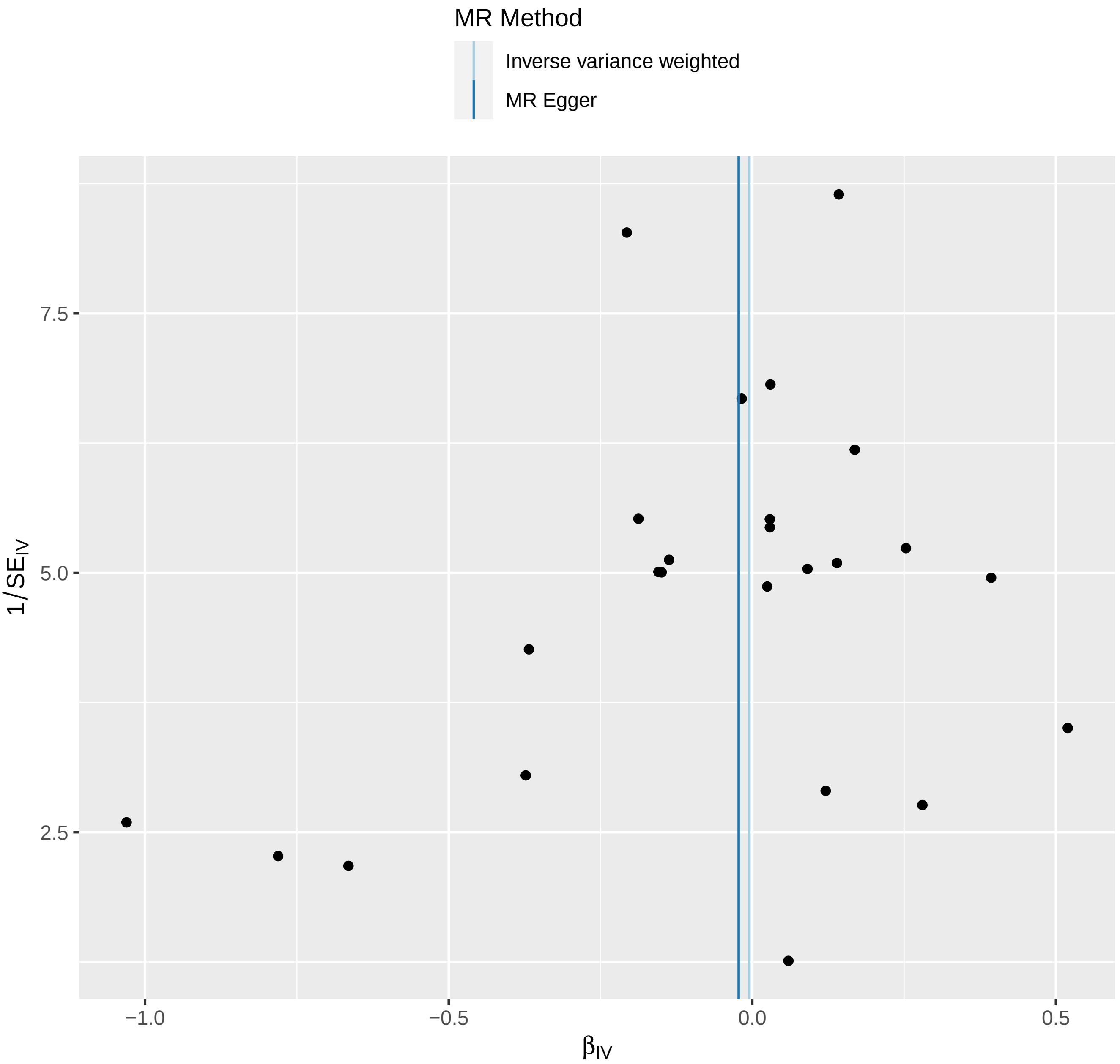

Batch 3−1779 : Low−density lipoprotein receptor−related protein 1B on Diabetic nephropathy (more control exclusions)
with a threshold of 1e−05

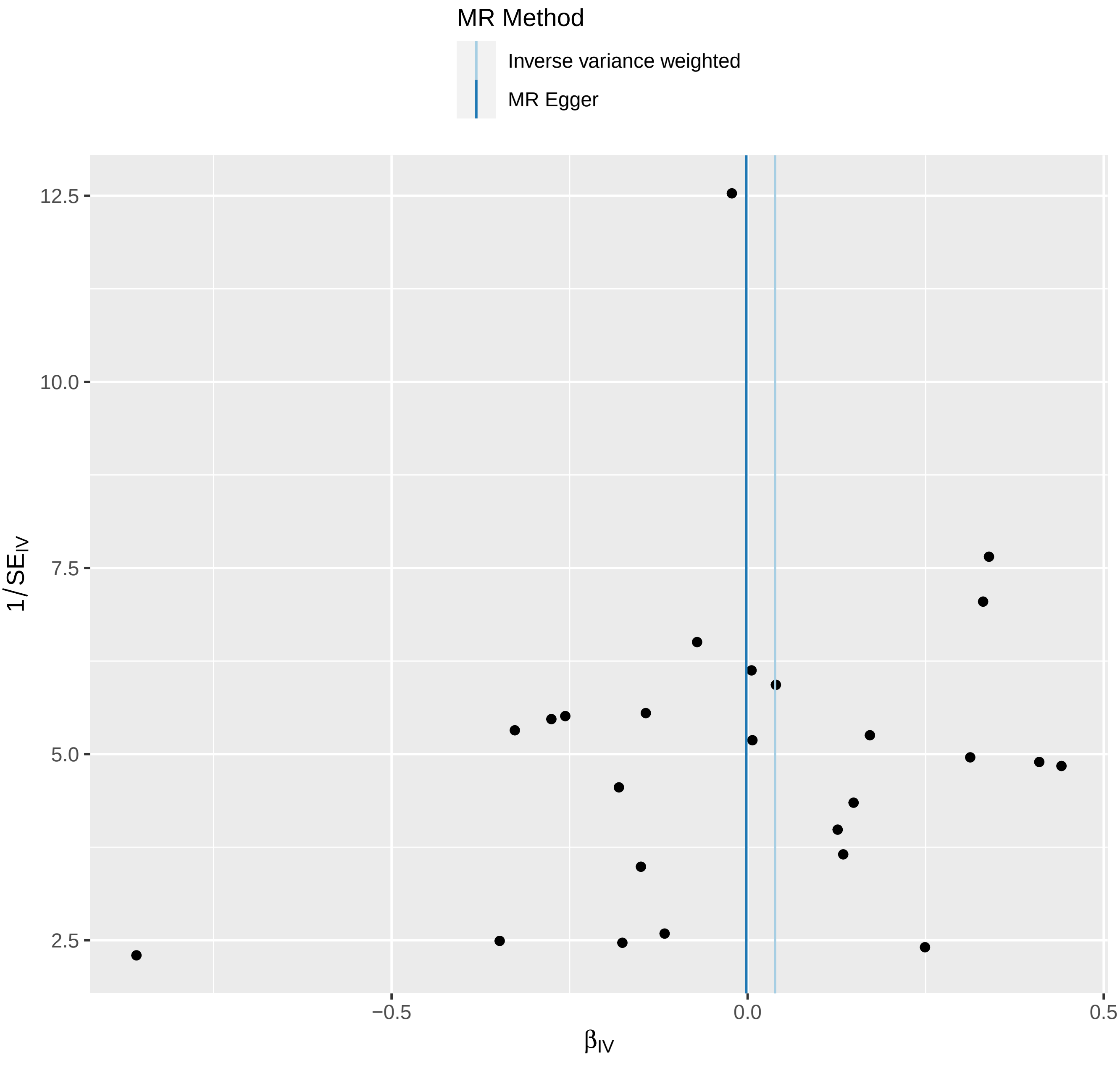

Batch 3−1780 : Low−density lipoprotein receptor−related protein 8 on Diabetic nephropathy (more control exclusions)
with a threshold of 1e−05

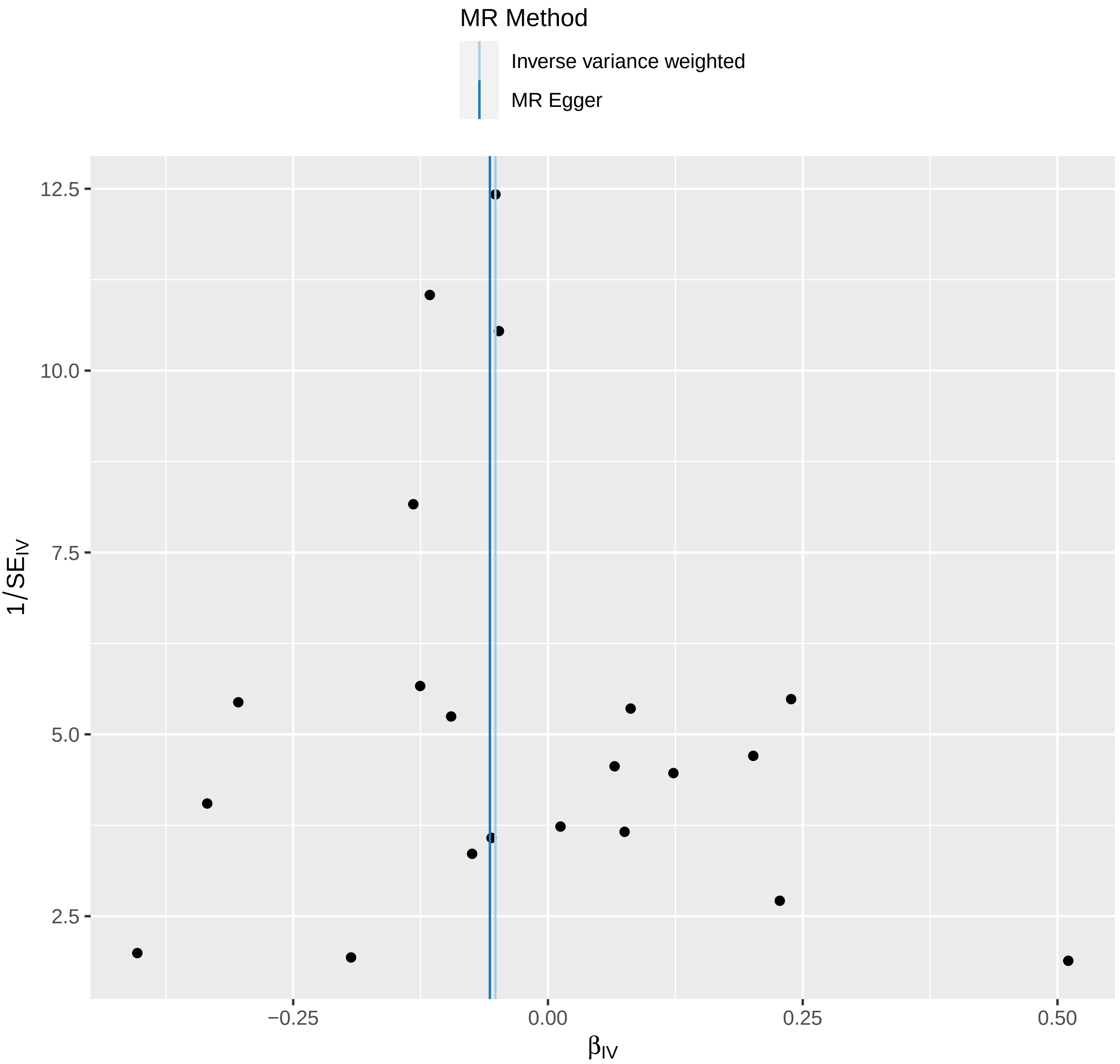

Batch 3−1781 : alpha−2−macroglobulin receptor−associated protein on Diabetic nephropathy (more control exclusions)
with a threshold of 1e−05

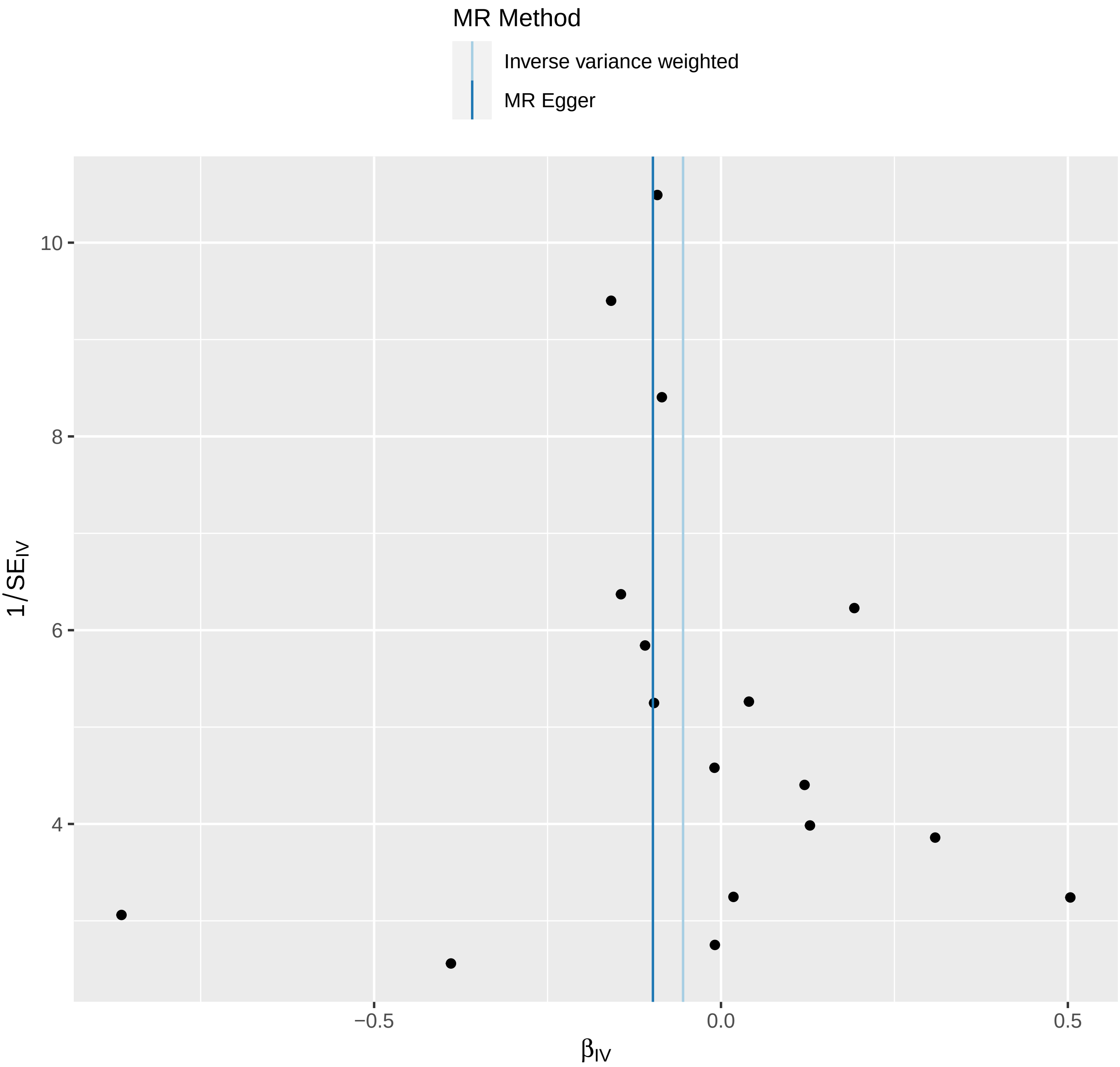

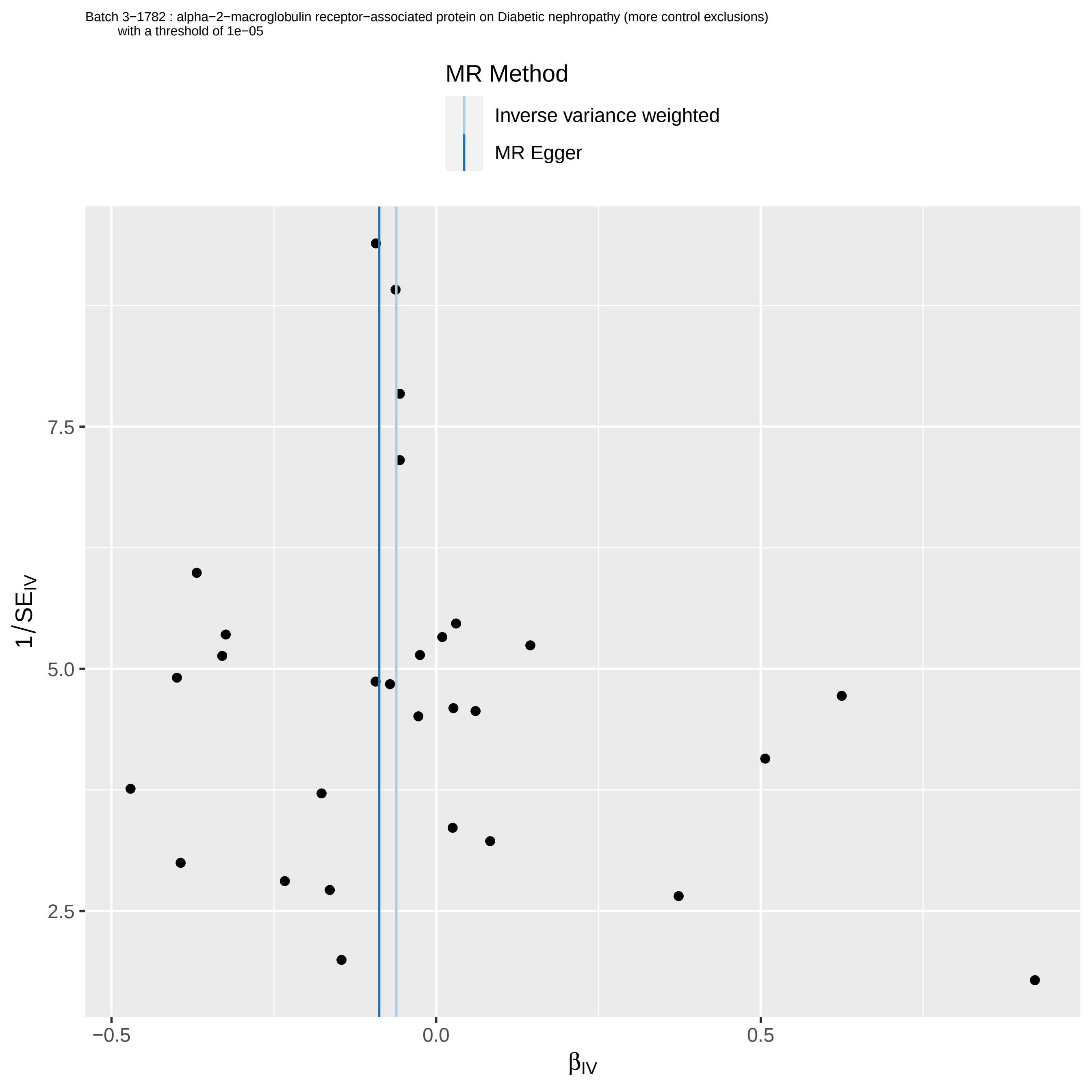

Batch 3−1782 : alpha−2−macroglobulin receptor−associated protein on Diabetic nephropathy (more control exclusions)
with a threshold of 1e−05
MR Method
Inverse variance weighted
MR Egger
7.5
5.0
2.5
1/SE_IV
−0.5
0.0
0.5
β_IV

Batch 3−1783 : Leucine−rich PPR motif−containing protein, mitochondrial on Diabetic nephropathy (more control exclusions)
with a threshold of 1e−05

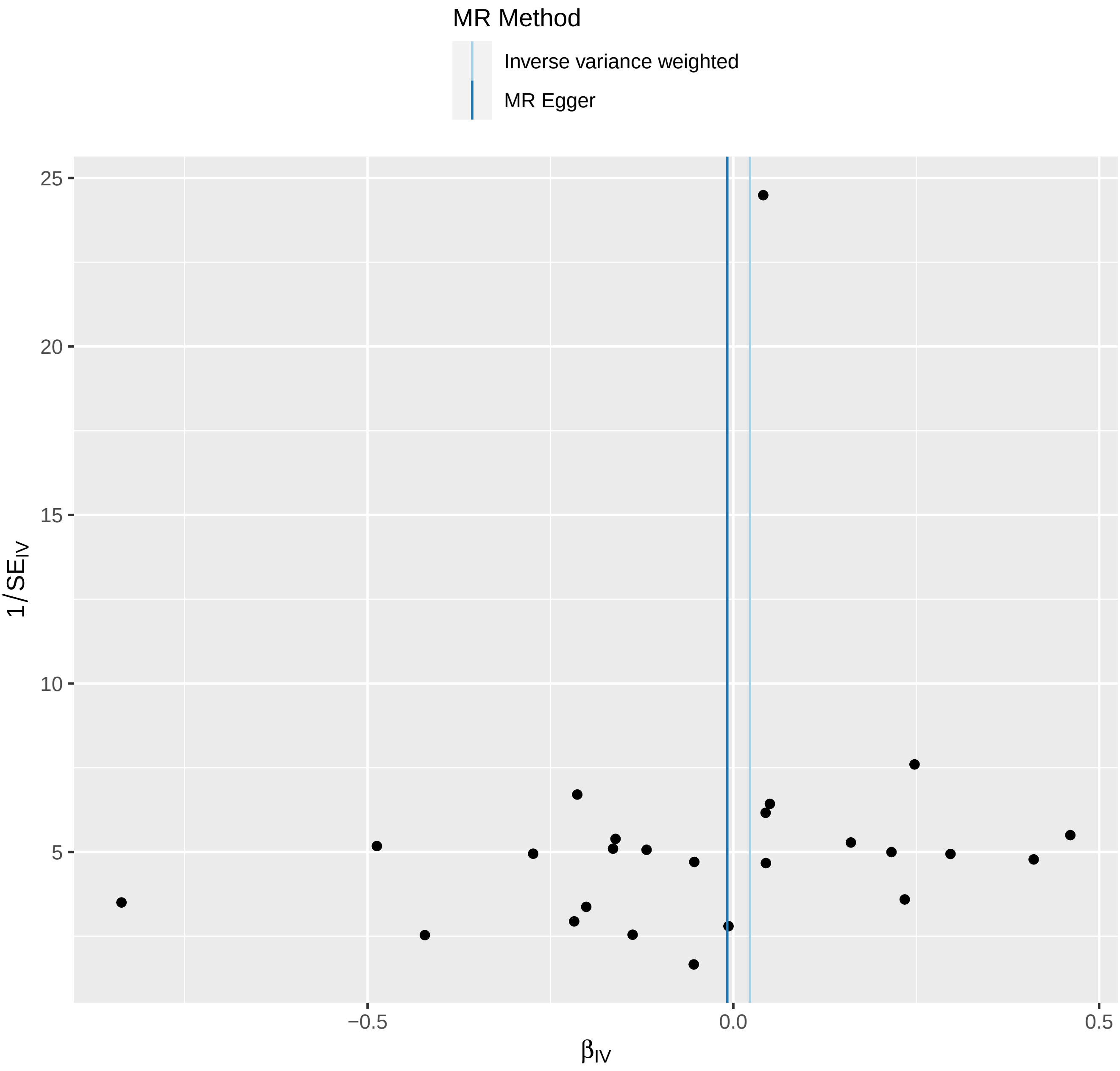

Batch 3−1784 : Leucine−rich repeat−containing protein 15 on Diabetic nephropathy (more control exclusions)
with a threshold of 1e−05

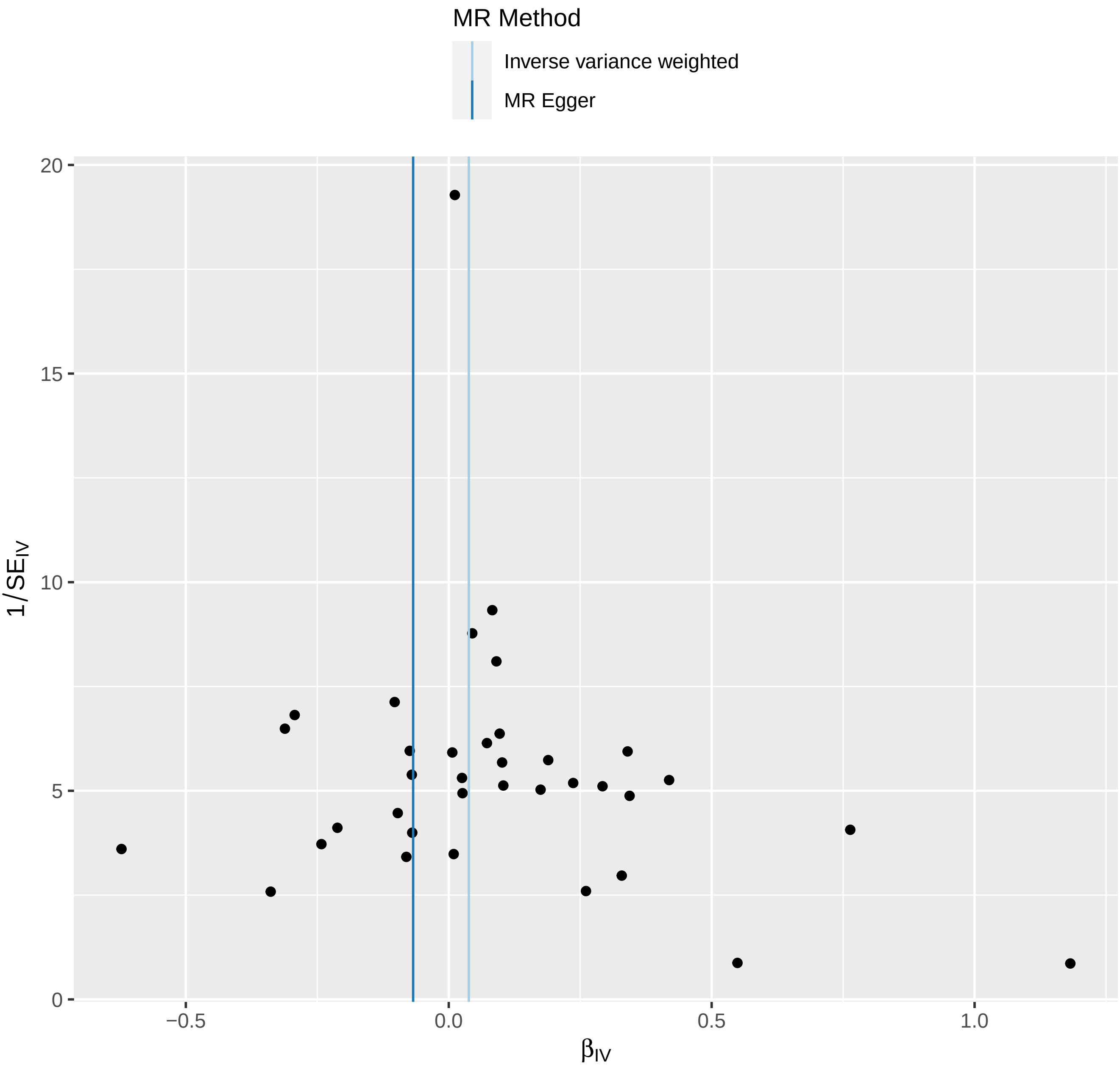

Batch 3−1785 : Leucine−rich repeat−containing protein 19 on Diabetic nephropathy (more control exclusions)
with a threshold of 1e−05

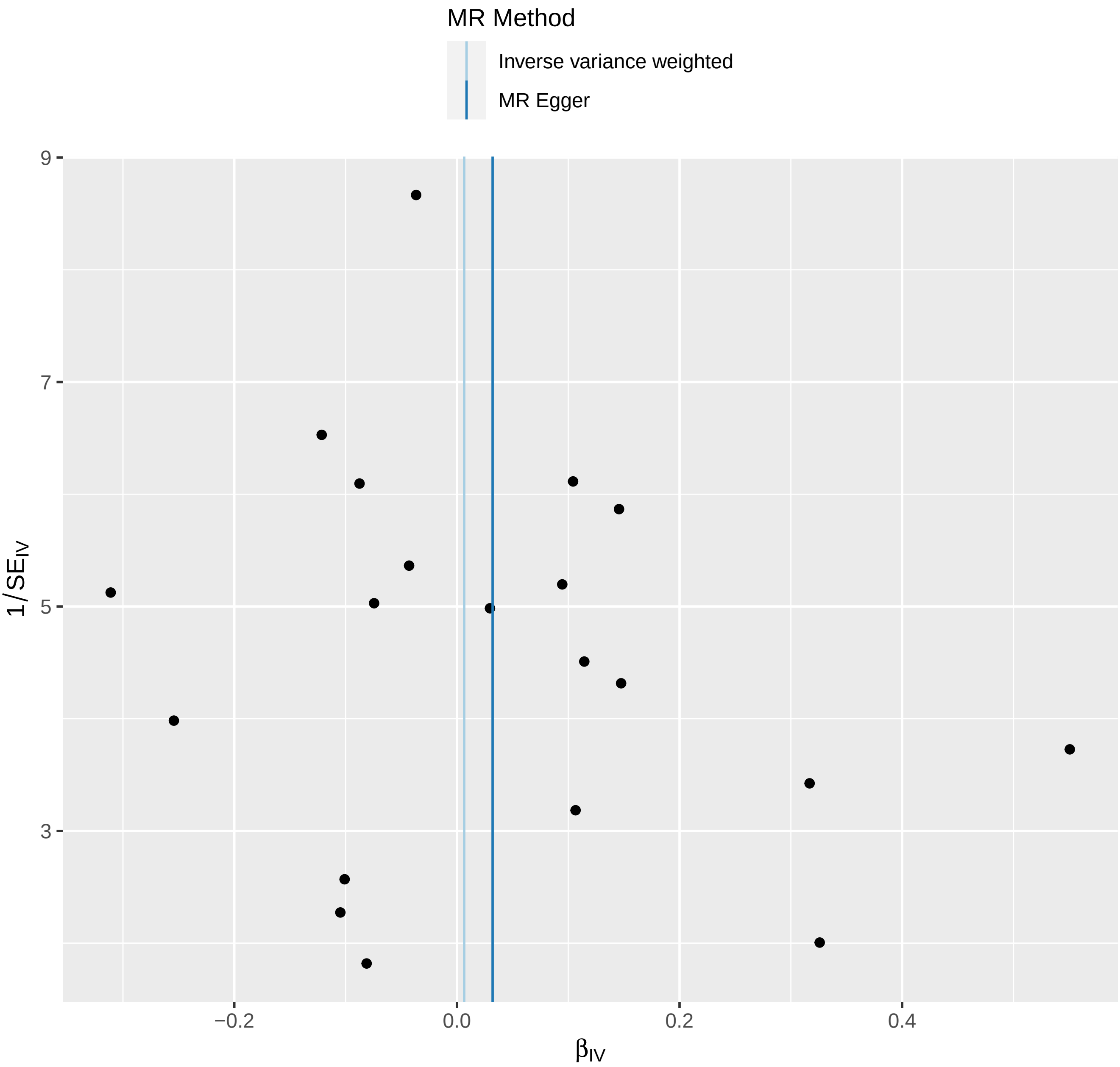

Batch 3−1786 : Leucine−rich repeat−containing protein 3 on Diabetic nephropathy (more control exclusions) with a threshold of 1e−05

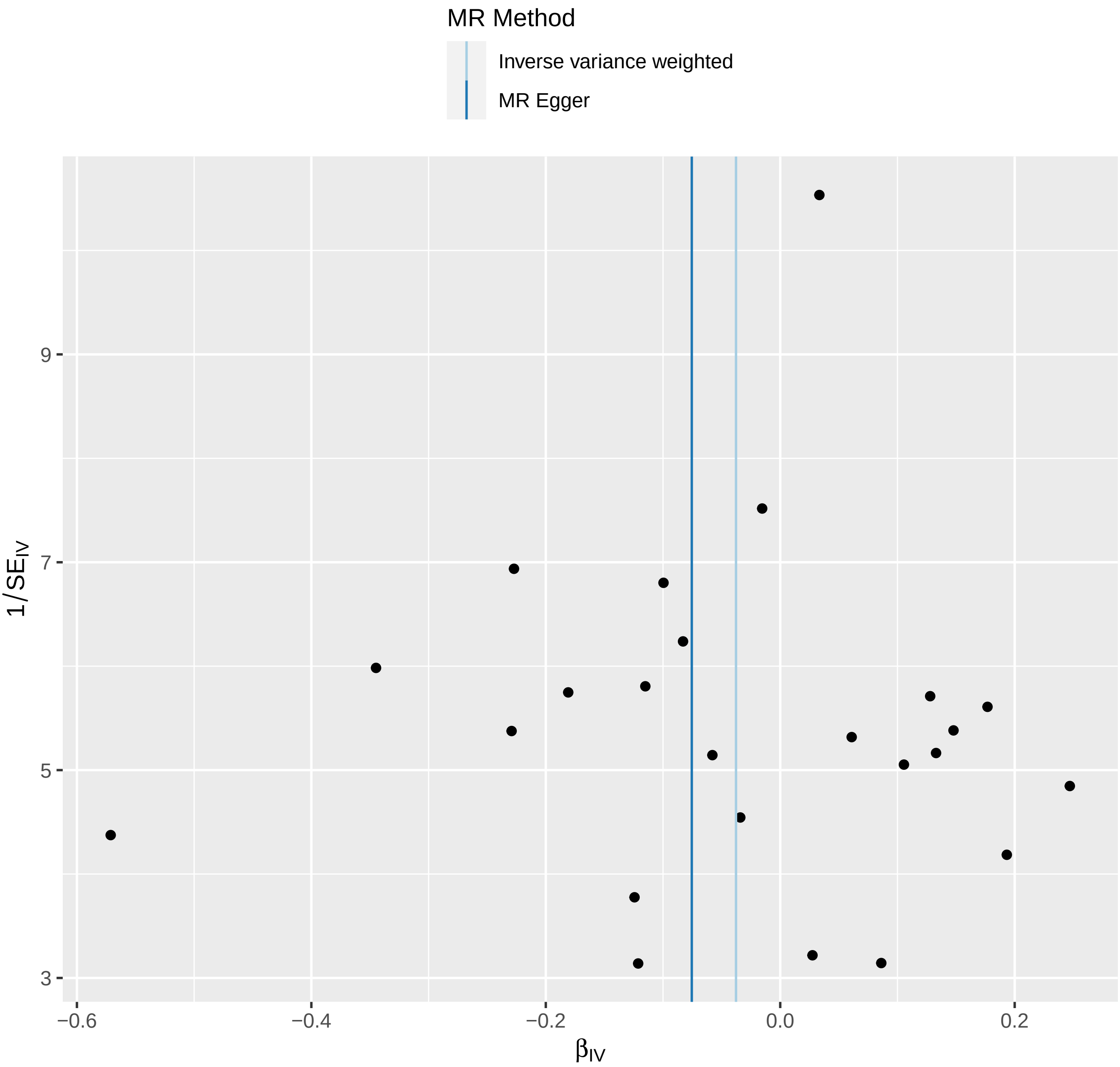

Batch 3−1787 : Leucine−rich repeat−containing protein 37A2 on Diabetic nephropathy (more control exclusions)
with a threshold of 1e−05

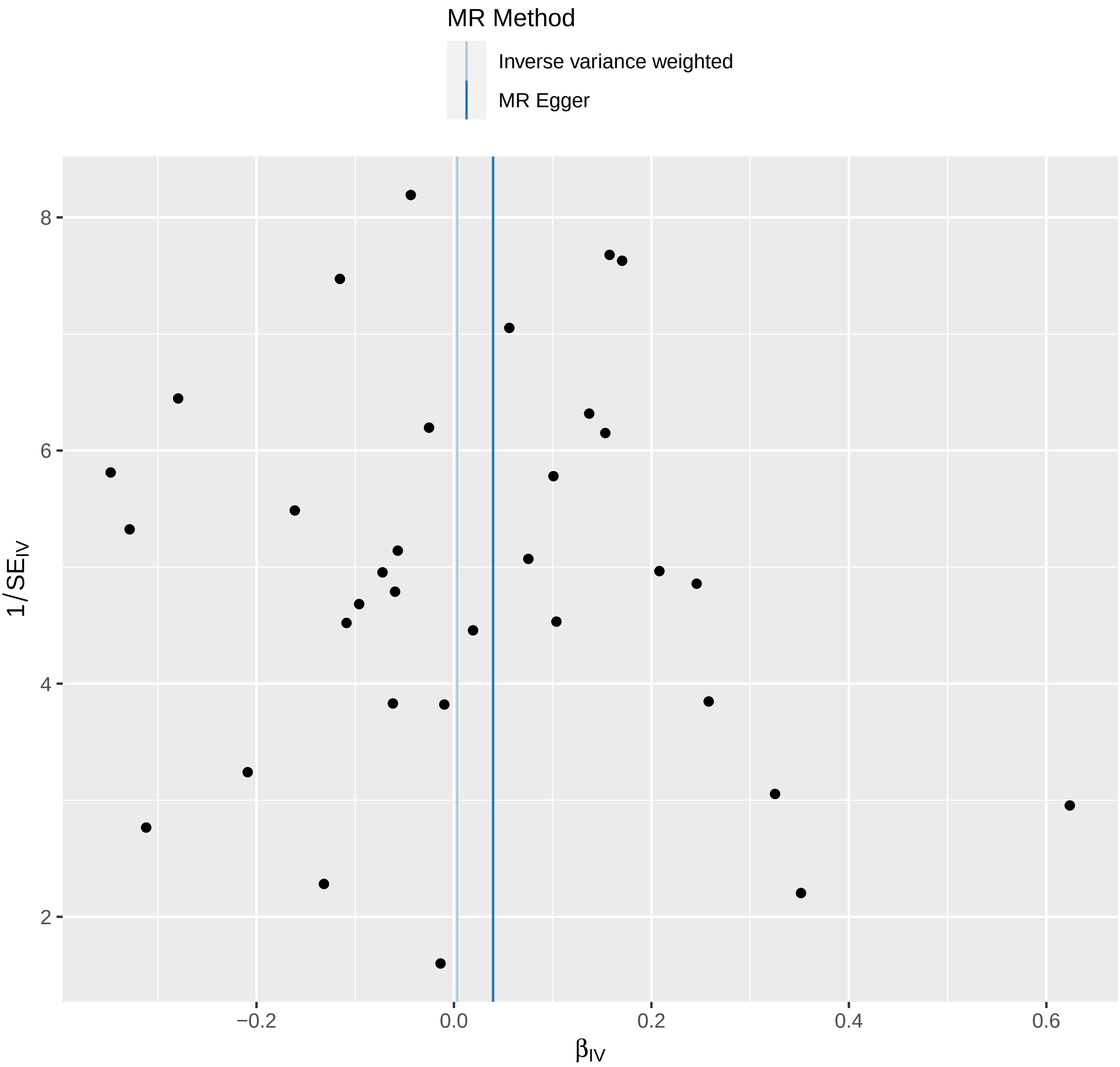

Batch 3−1788 : Leucine−rich repeat−containing protein 4B on Diabetic nephropathy (more control exclusions) with a threshold of 1e−05

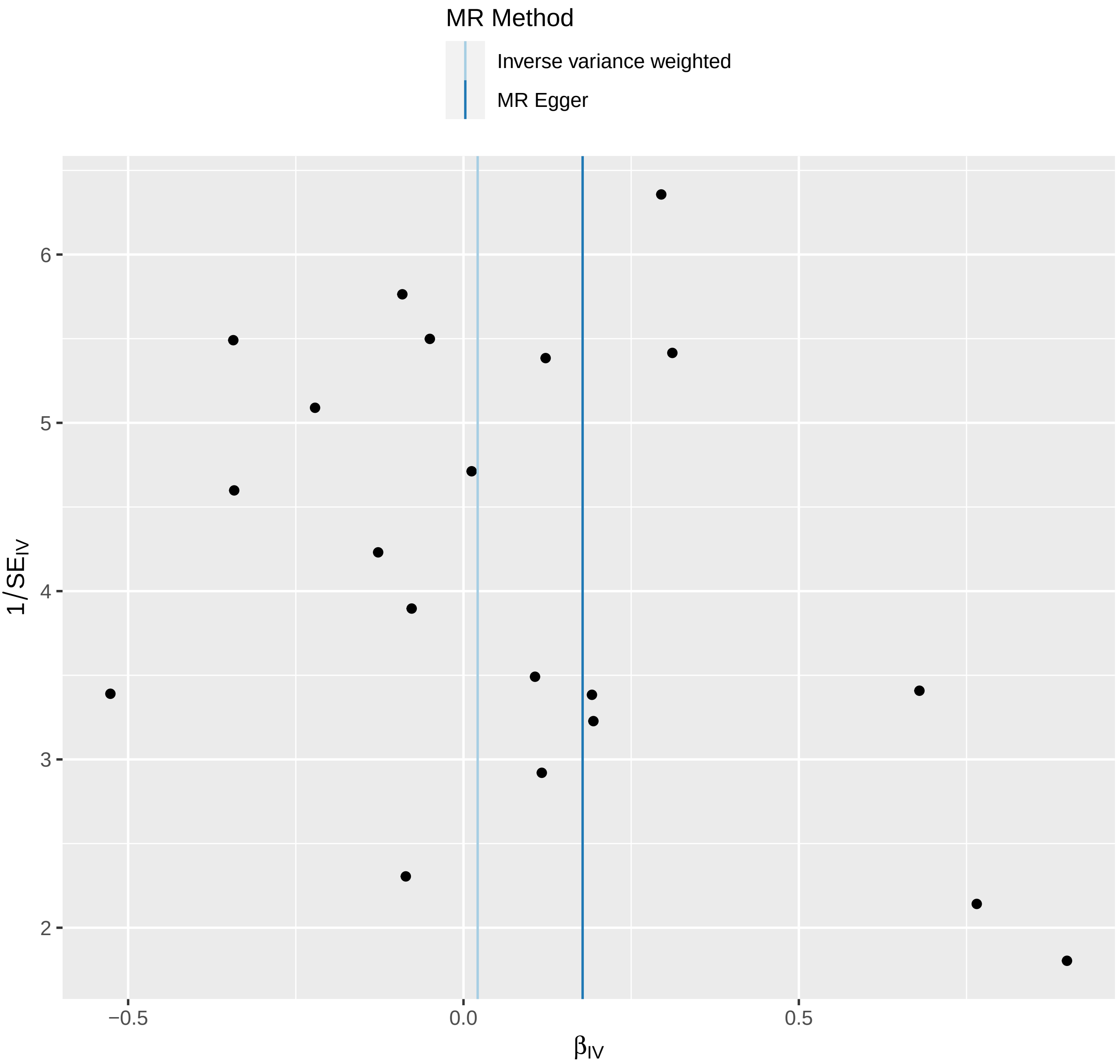

Batch 3−1789 : Leucine−rich repeat−containing protein 4C on Diabetic nephropathy (more control exclusions)
with a threshold of 1e−05

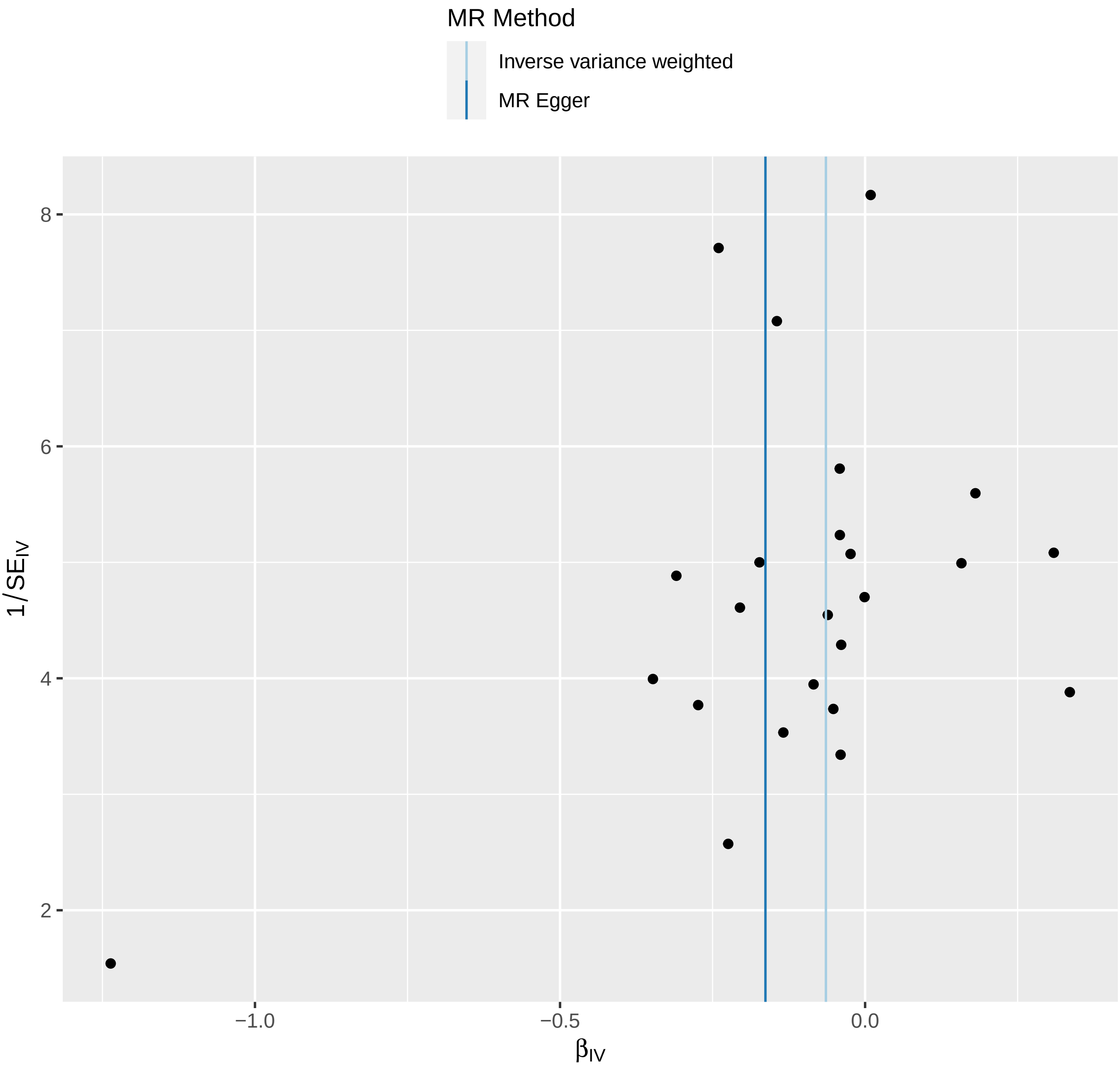

Batch 3−1790 : Leucine−rich repeat−containing protein 74A on Diabetic nephropathy (more control exclusions)
with a threshold of 1e−05

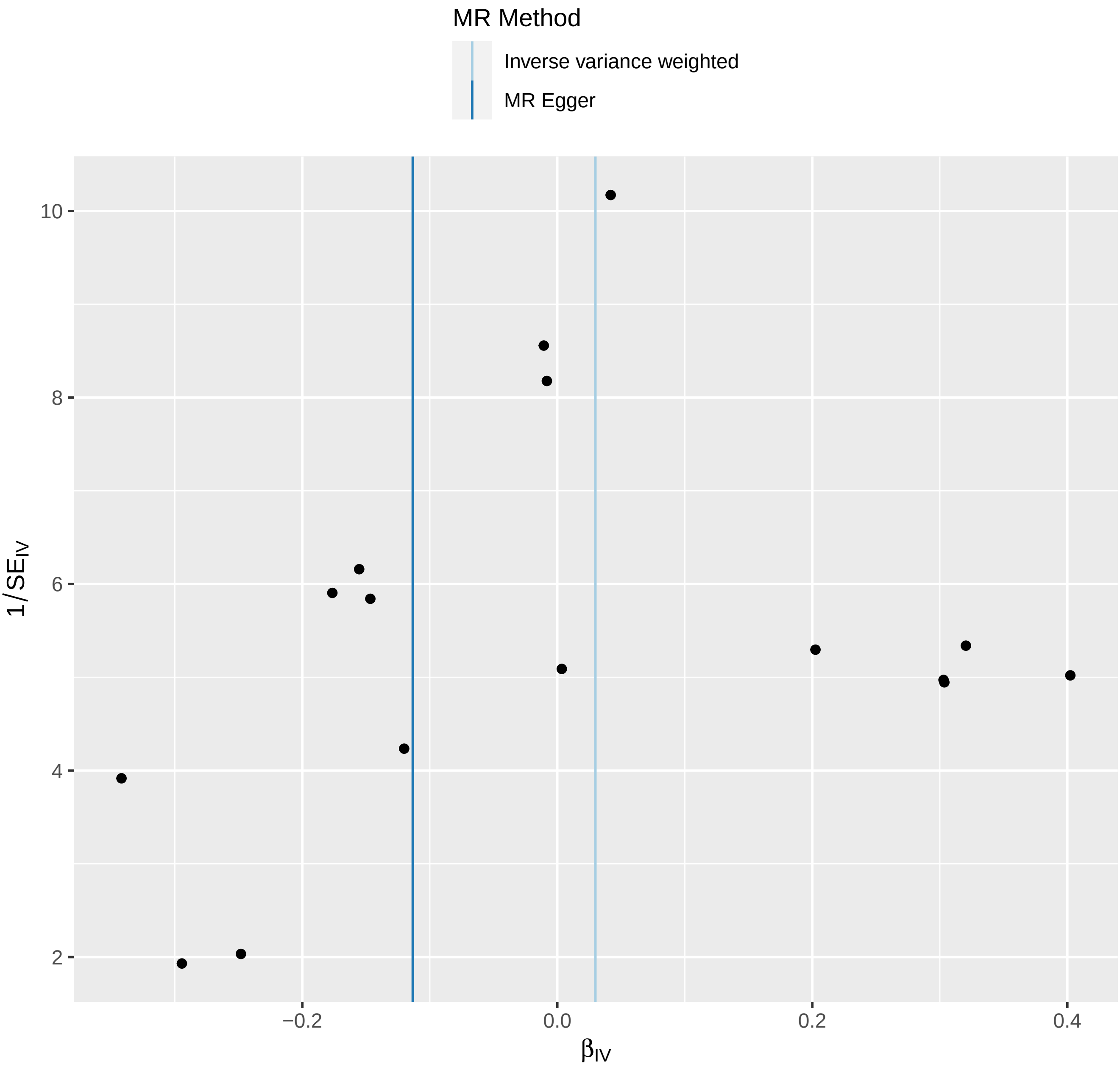

Batch 3−1791 : Leucine−rich repeat serine/threonine−protein kinase 2 on Diabetic nephropathy (more control exclusions)
with a threshold of 1e−05

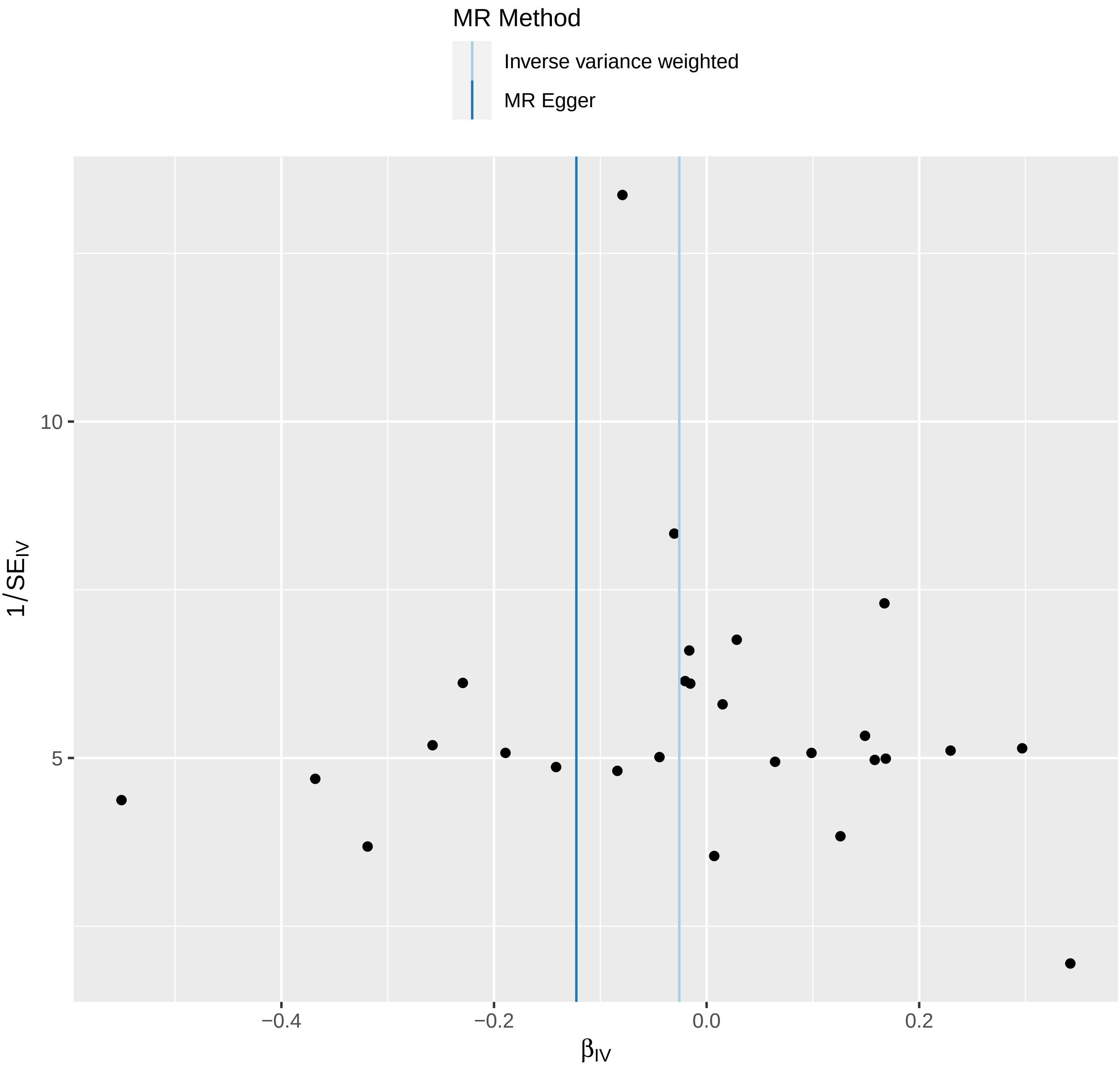

Batch 3−1792 : Leucine−rich repeat neuronal protein 1 on Diabetic nephropathy (more control exclusions)
with a threshold of 1e−05

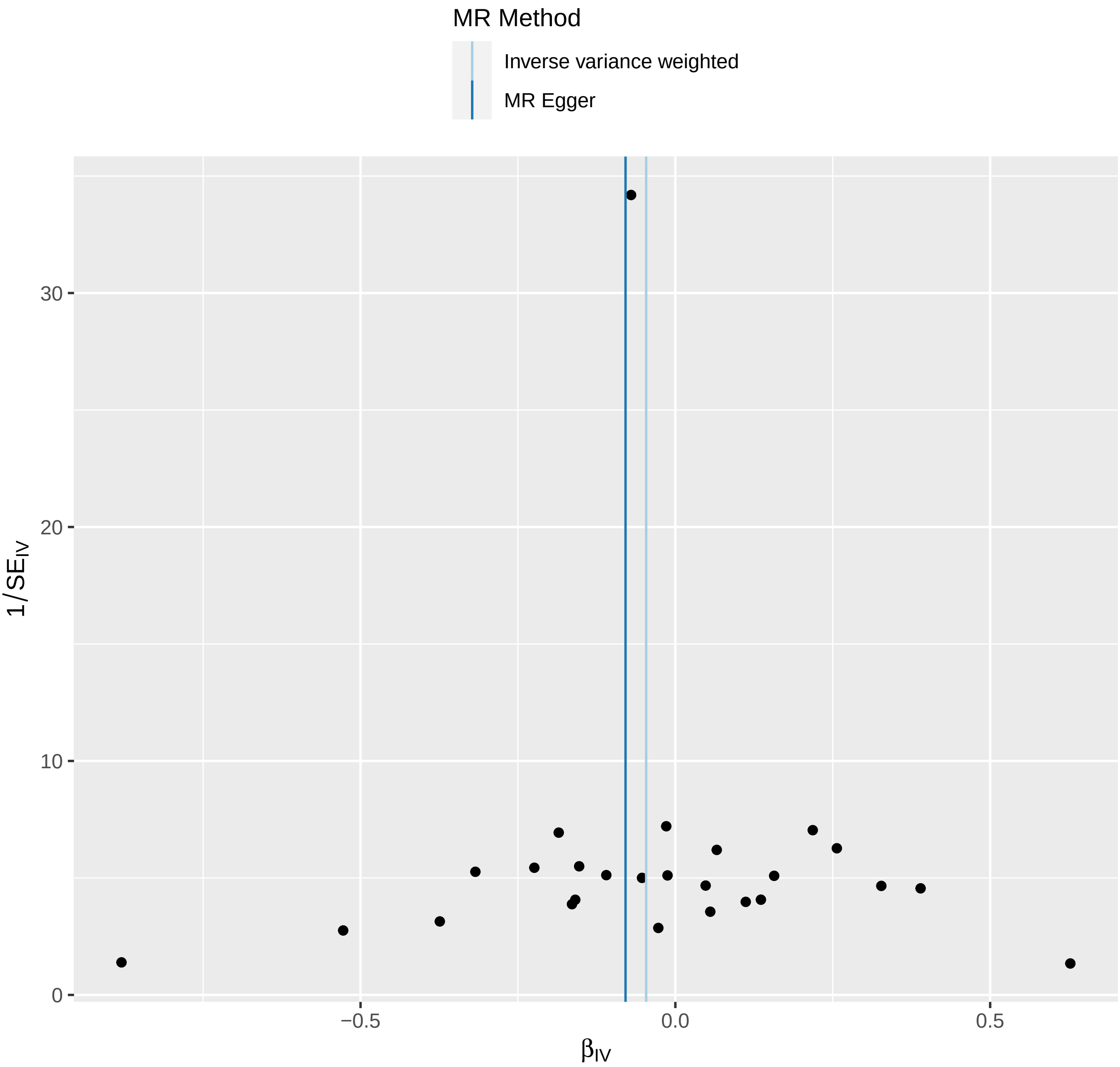

Batch 3−1793 : Leucine−rich repeat neuronal protein 1 on Diabetic nephropathy (more control exclusions)
with a threshold of 1e−05

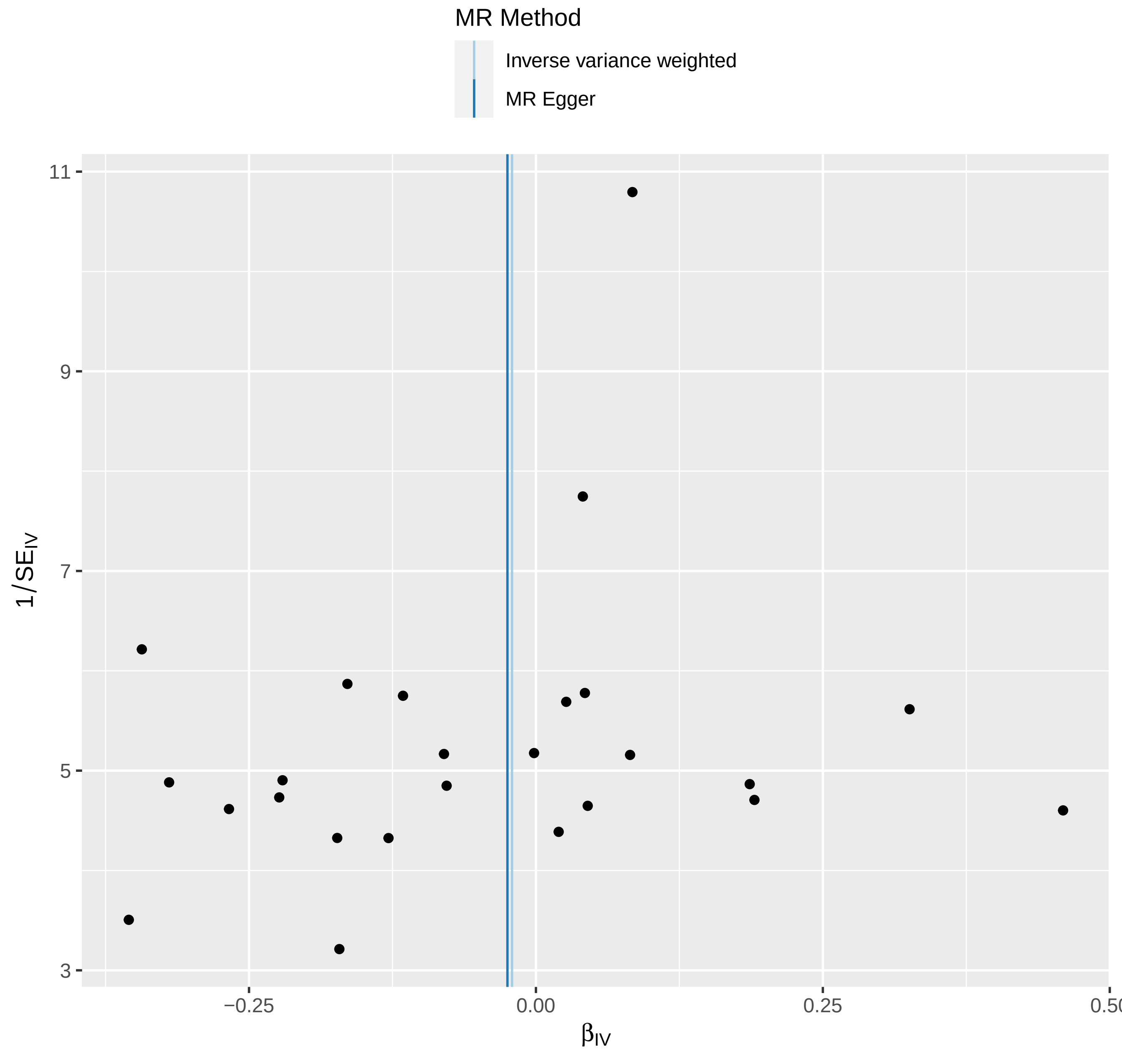

Batch 3−1794 : Leucine−rich repeat neuronal protein 3 on Diabetic nephropathy (more control exclusions)
with a threshold of 1e−05

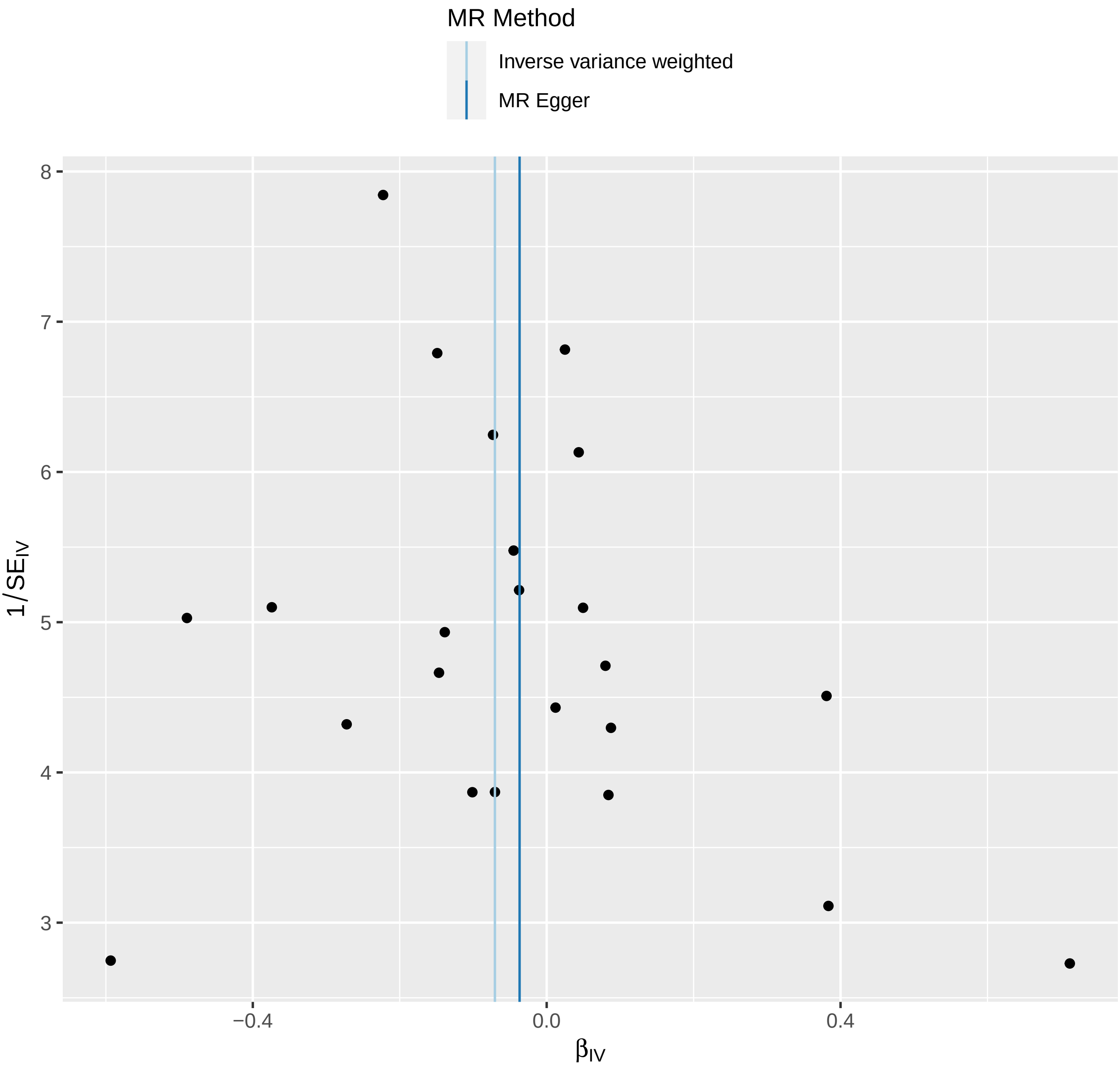

Batch 3−1795 : Leucine−rich repeat transmembrane neuronal protein 1 on Diabetic nephropathy (more control exclusions) with a threshold of 1e−05

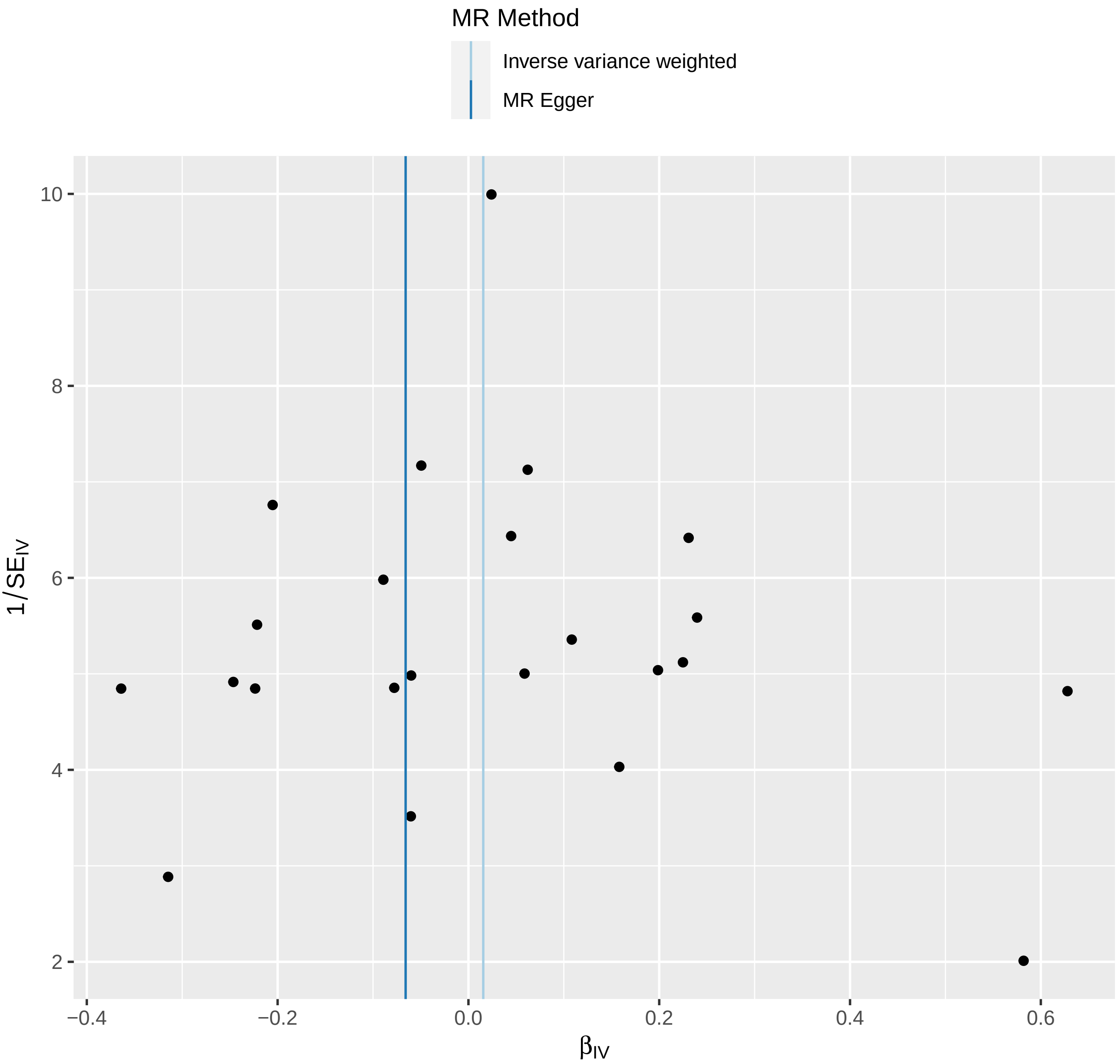

Batch 3−1796 : Leucine−rich repeat transmembrane neuronal protein 2 on Diabetic nephropathy (more control exclusions) with a threshold of 1e−05

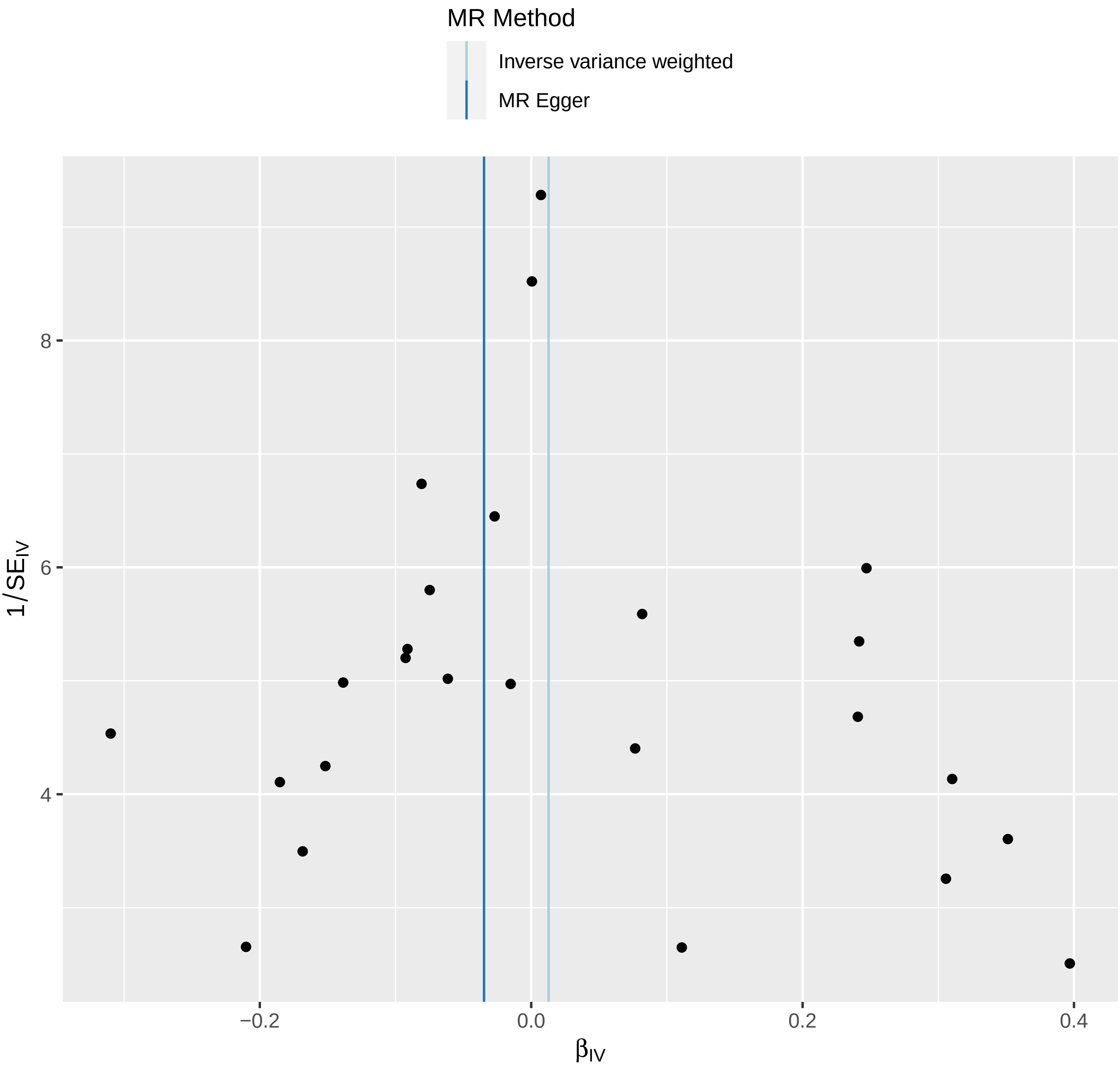

Batch 3−1797 : Leucine−rich repeat transmembrane neuronal protein 4 on Diabetic nephropathy (more control exclusions) with a threshold of 1e−05

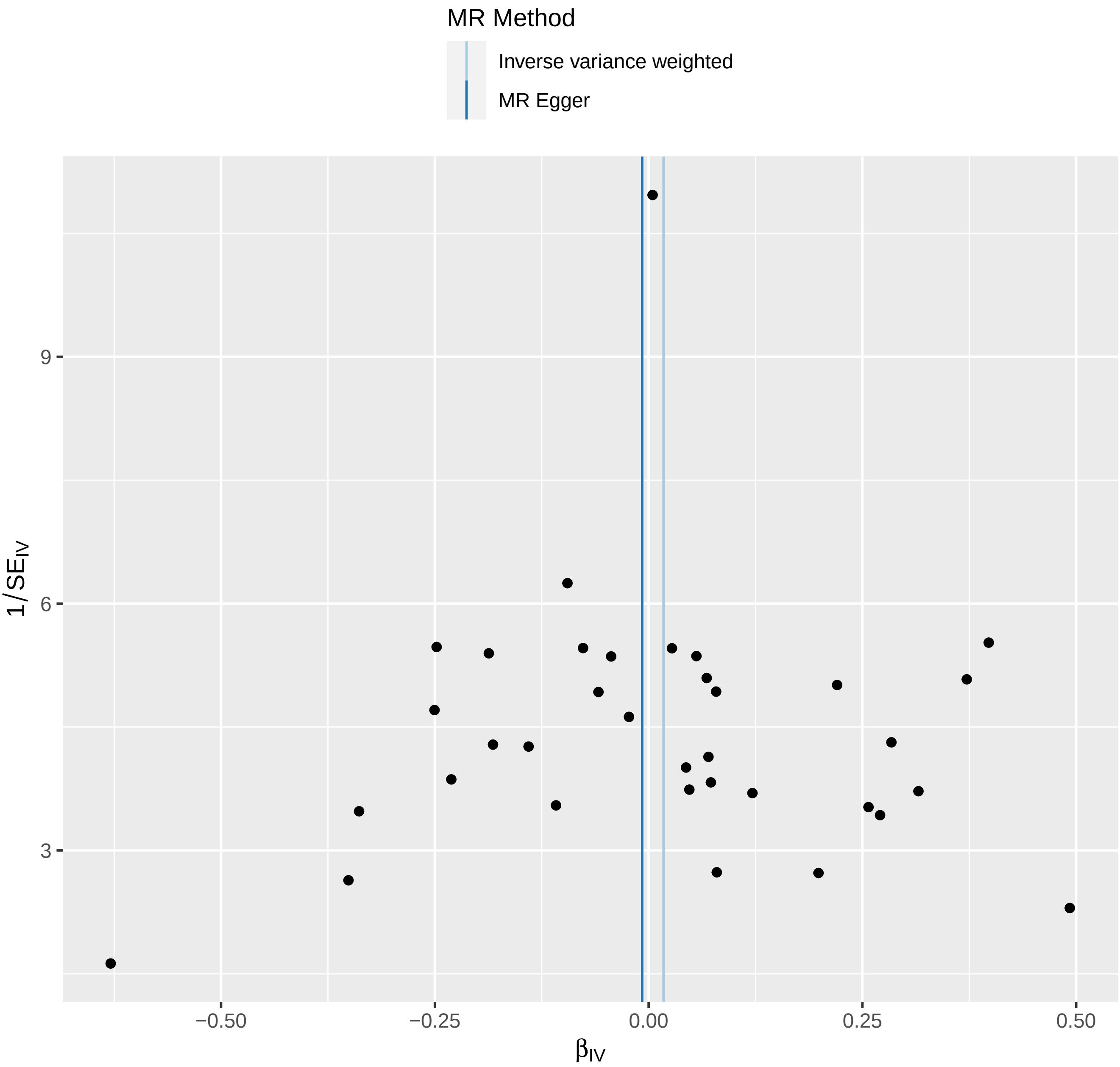

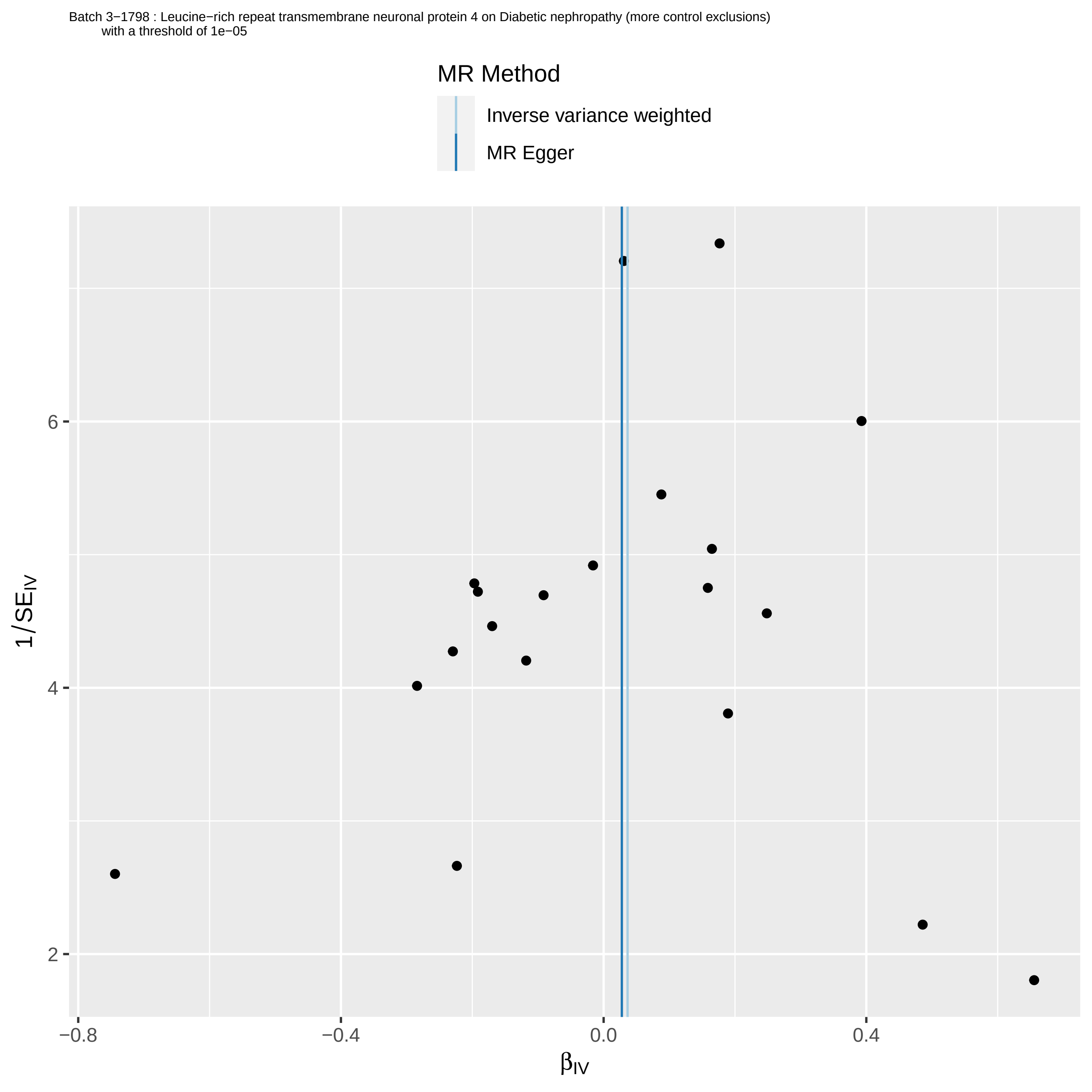

Batch 3−1798 : Leucine−rich repeat transmembrane neuronal protein 4 on Diabetic nephropathy (more control exclusions)
with a threshold of 1e−05
MR Method
Inverse variance weighted
MR Egger
1/SE_IV
β_IV
6
4
2
−0.8
−0.4
0.0
0.4

Batch 3−1799 : Leucine−rich repeat and transmembrane domain−containing protein 1 on Diabetic nephropathy (more control exclusions) with a threshold of 1e−05

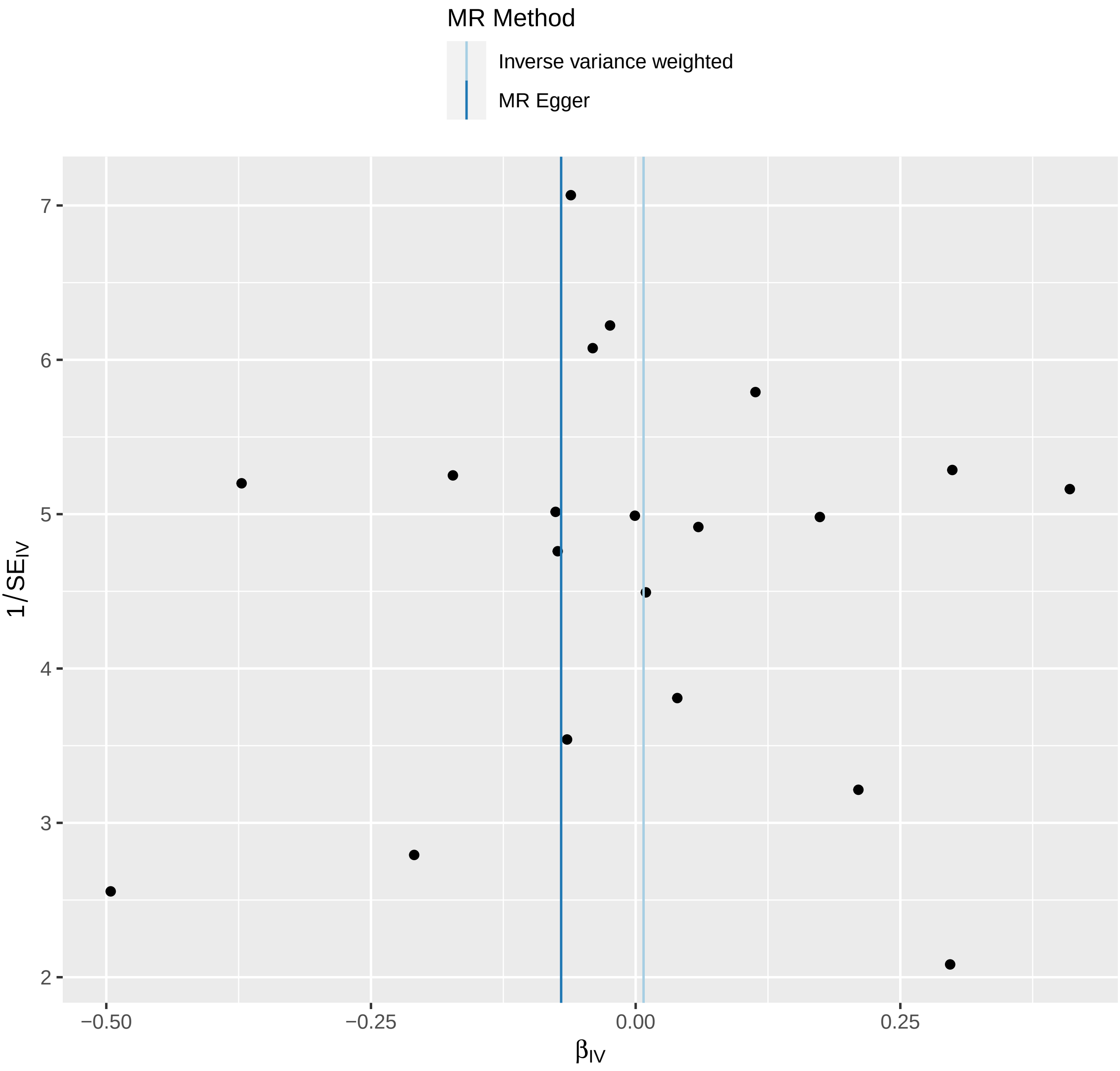

Batch 3−1800 : Leucine−rich repeat and transmembrane domain−containing protein 2 on Diabetic nephropathy (more control exclusions) with a threshold of 1e−05

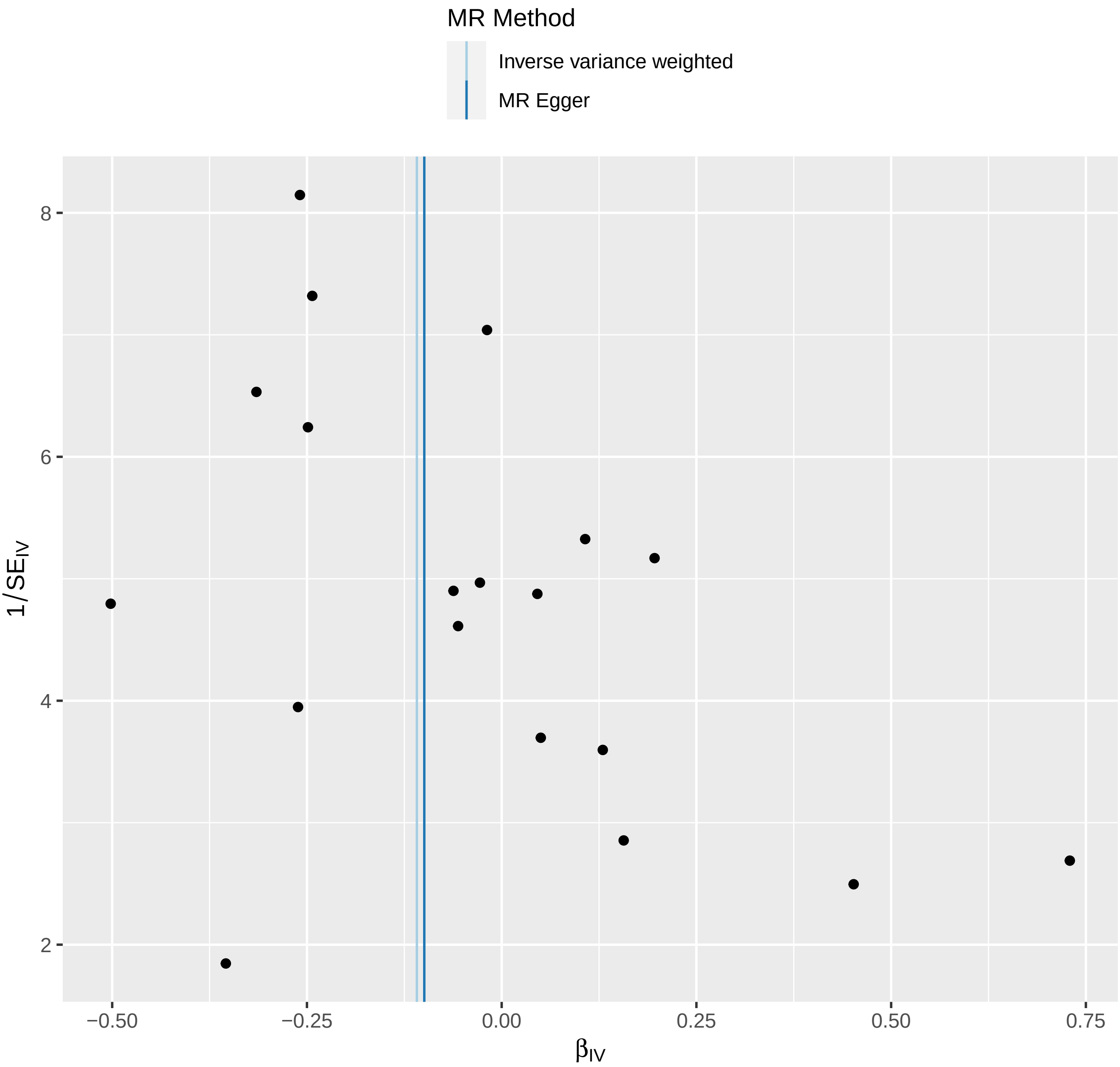

Batch 3−1801 : Limbic system−associated membrane protein on Diabetic nephropathy (more control exclusions)
with a threshold of 1e−05

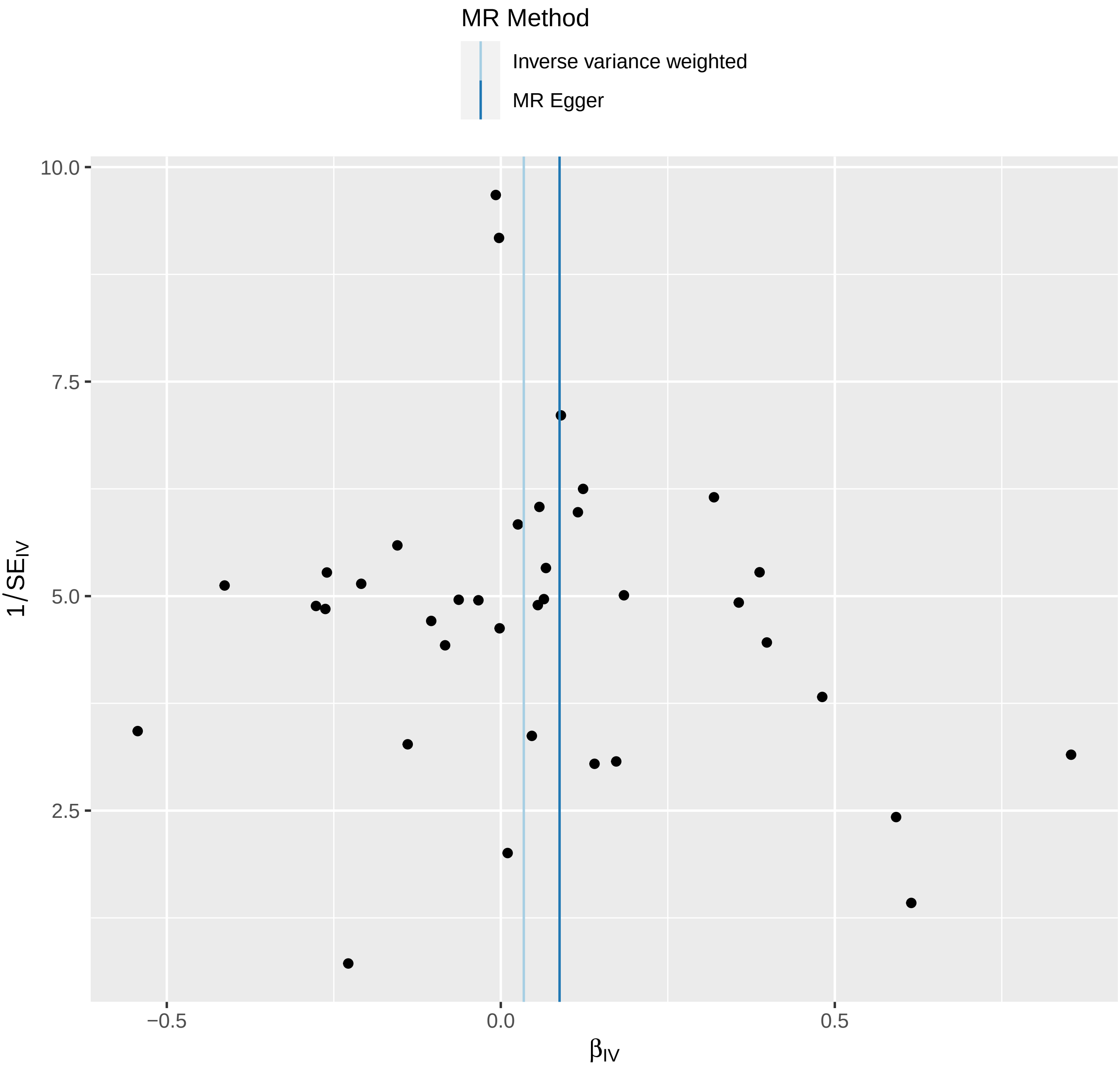

Batch 3−1802 : Leukocyte−specific transcript 1 protein on Diabetic nephropathy (more control exclusions)
with a threshold of 1e−05

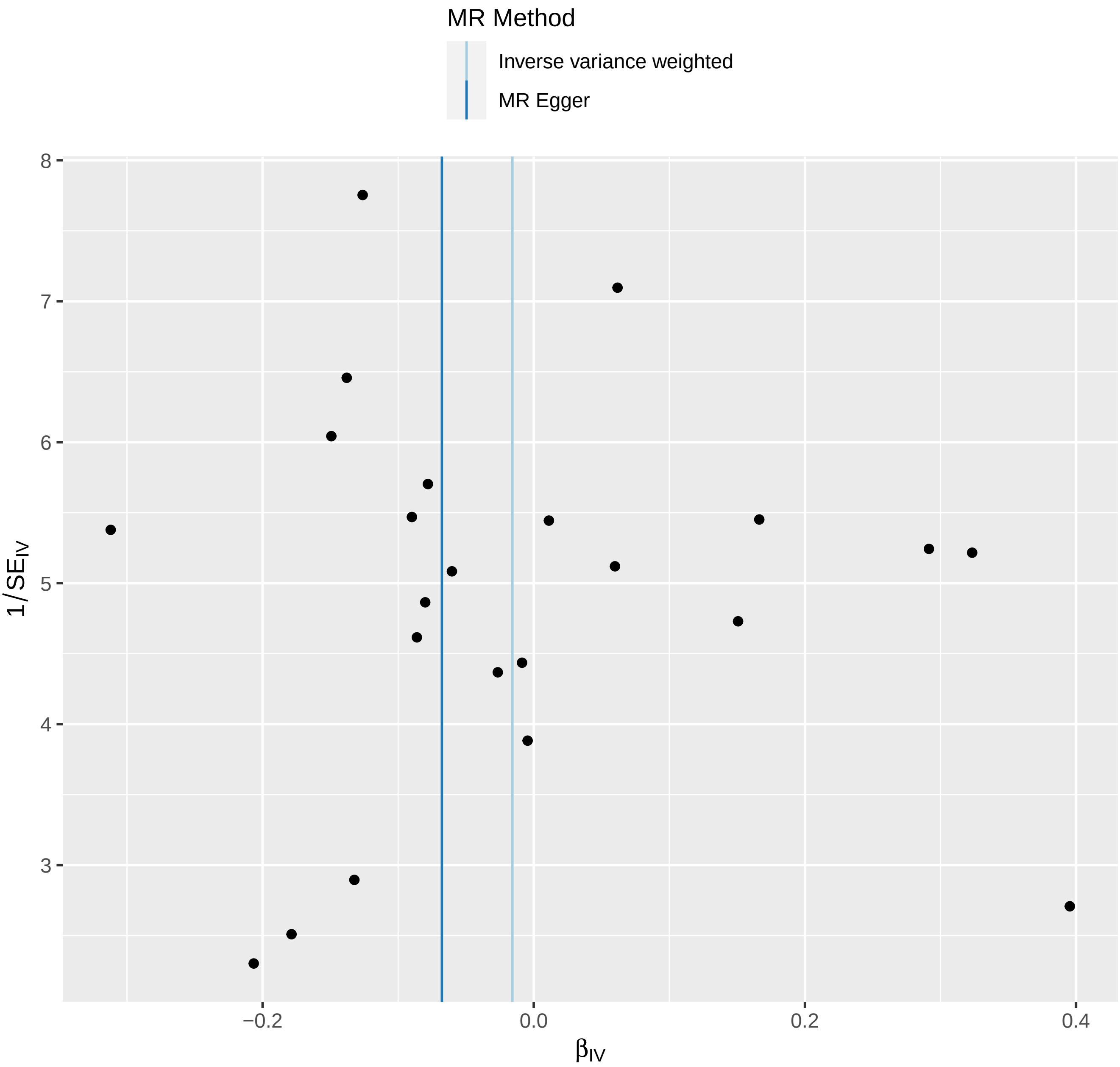

Batch 3−1803 : Lymphotoxin−alpha on Diabetic nephropathy (more control exclusions)
with a threshold of 1e−05

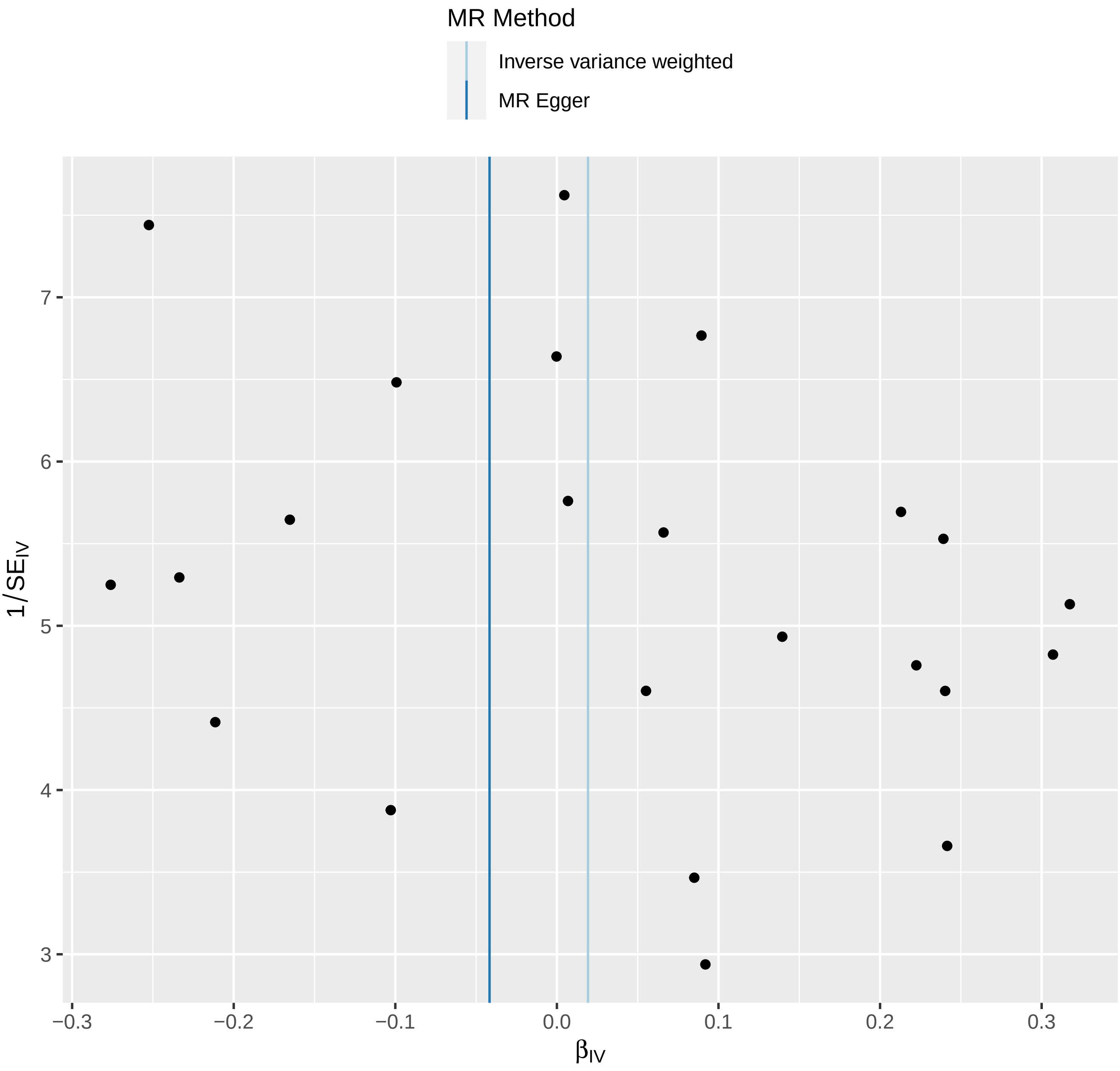

Batch 3−1804 : Leukotriene A−4 hydrolase on Diabetic nephropathy (more control exclusions)
with a threshold of 1e−05

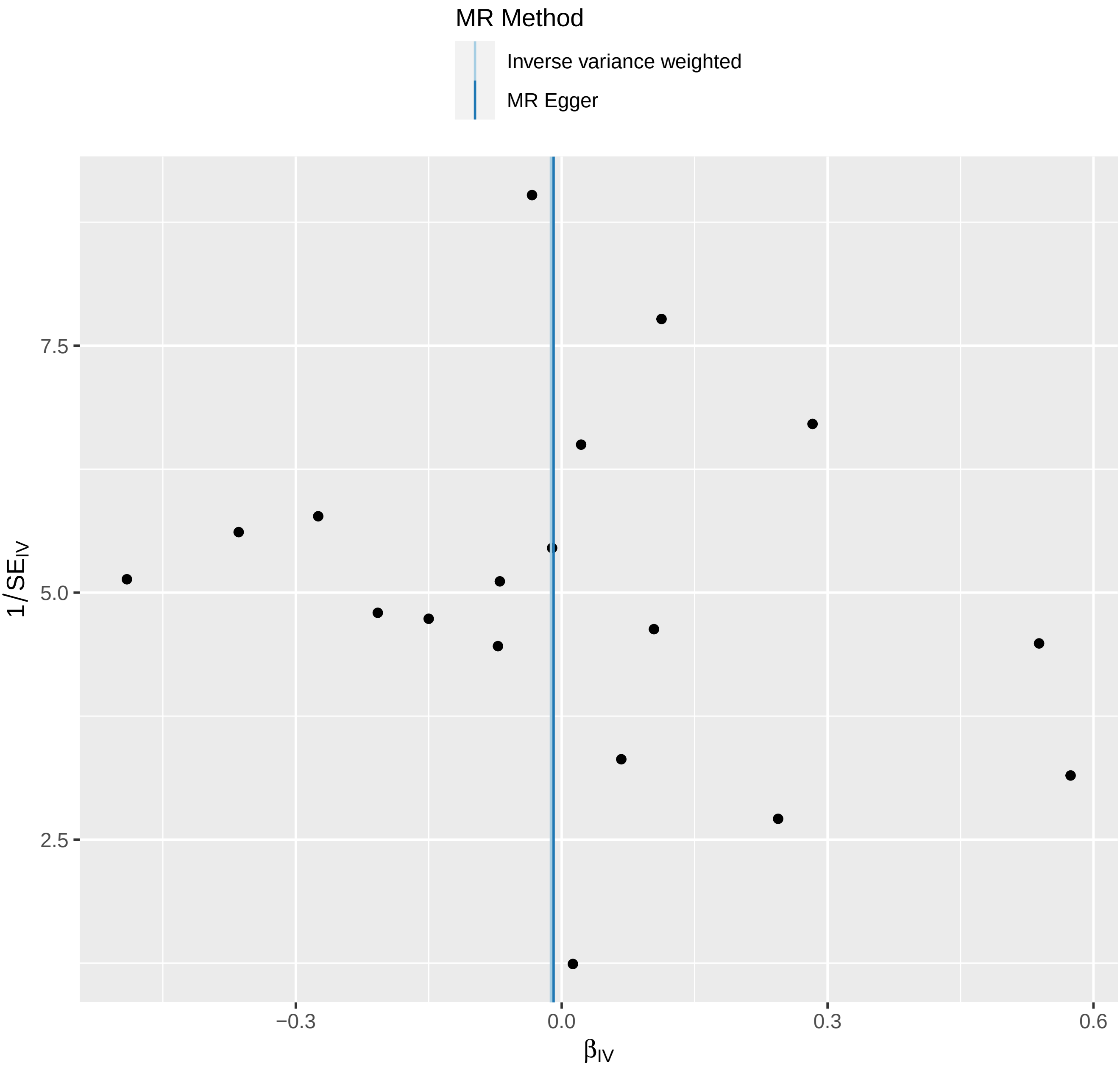

Batch 3−1805 : Lymphotoxin alpha2:beta1 on Diabetic nephropathy (more control exclusions)
with a threshold of 1e−05

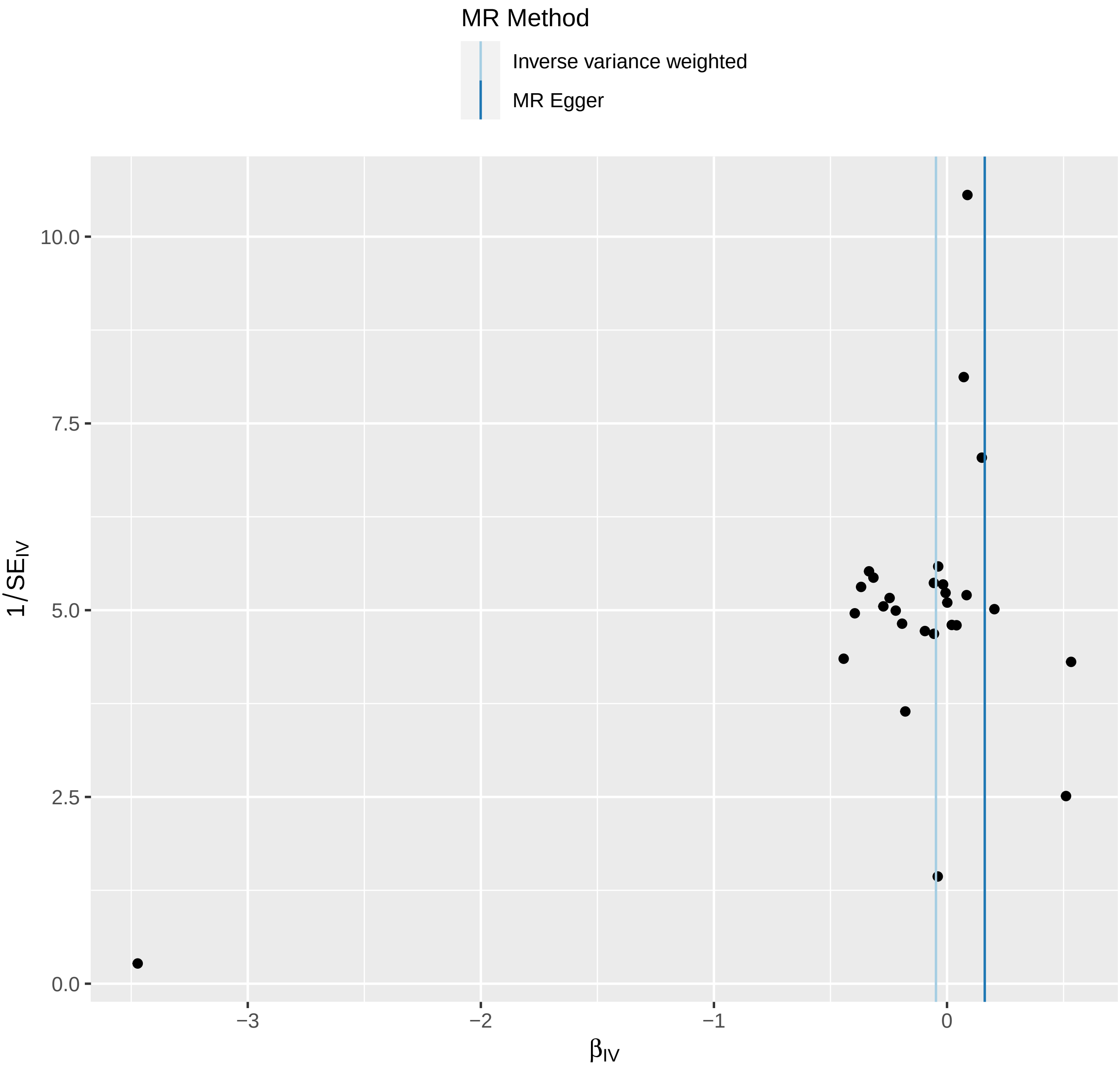

Batch 3−1806 : Latent−transforming growth factor beta−binding protein 4 on Diabetic nephropathy (more control exclusions)
with a threshold of 1e−05

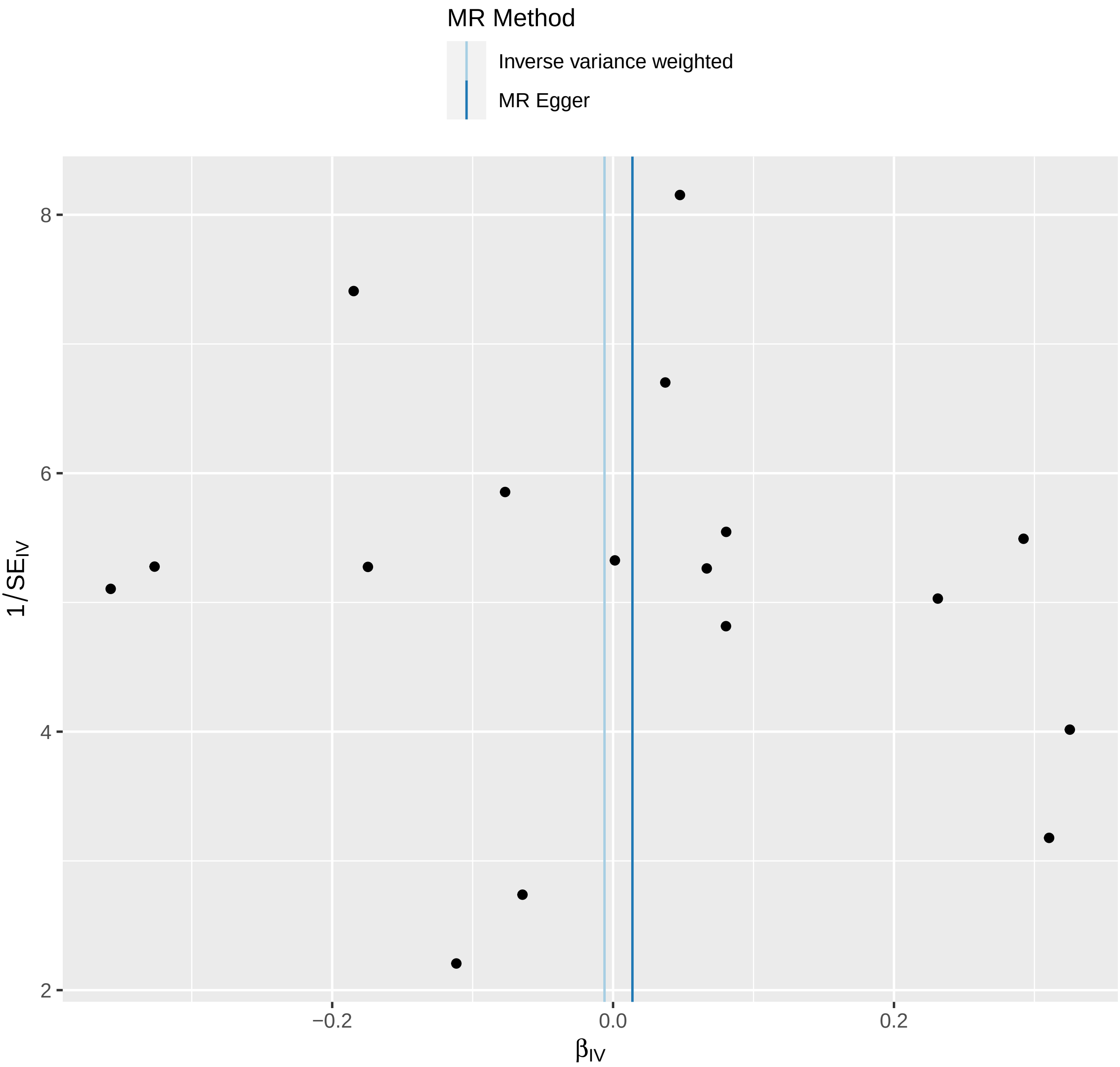

Batch 3−1807 : Tumor necrosis factor receptor superfamily member 3 on Diabetic nephropathy (more control exclusions)
with a threshold of 1e−05

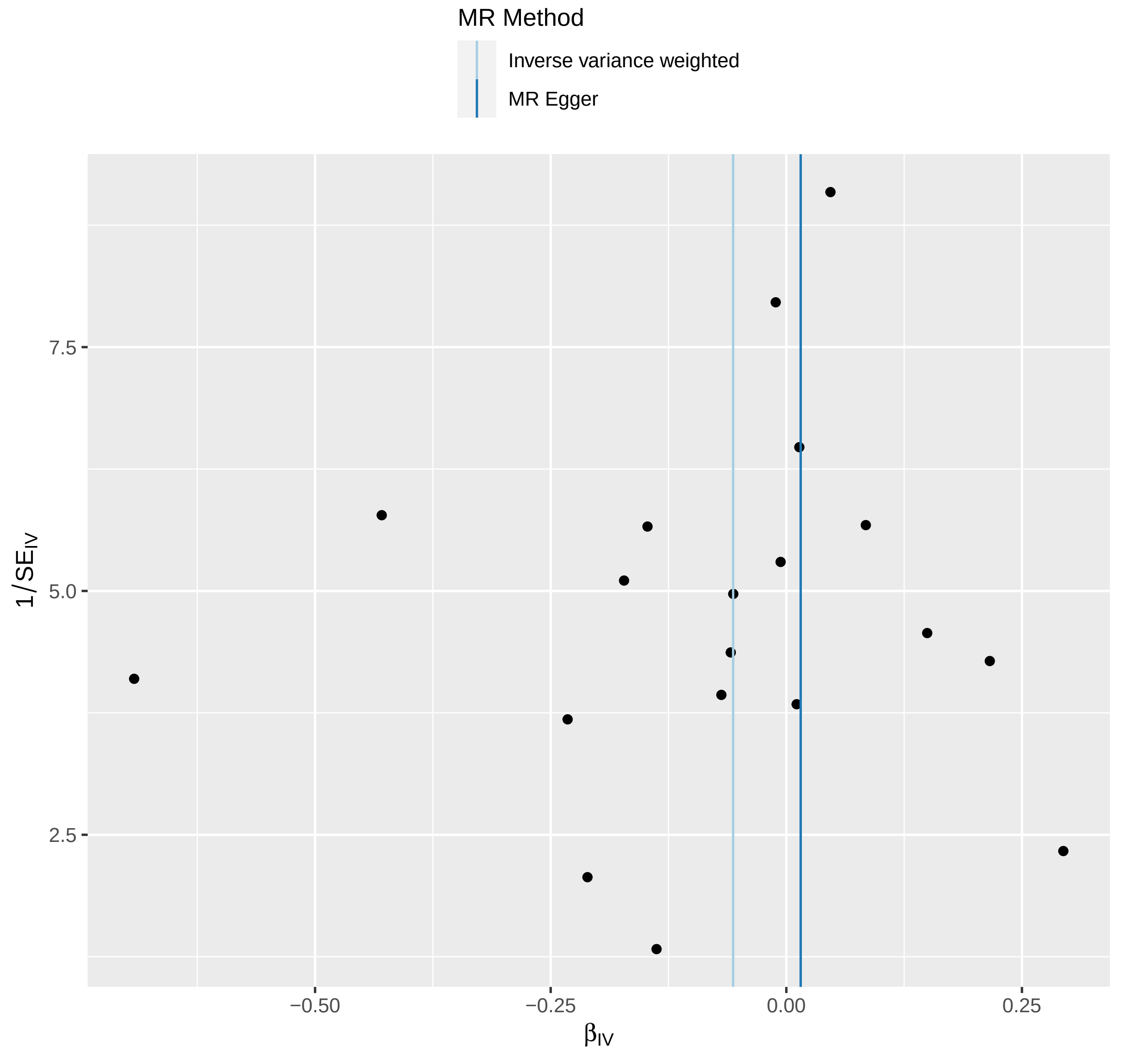

Batch 3−1808 : Lactotransferrin on Diabetic nephropathy (more control exclusions)
with a threshold of 1e−05

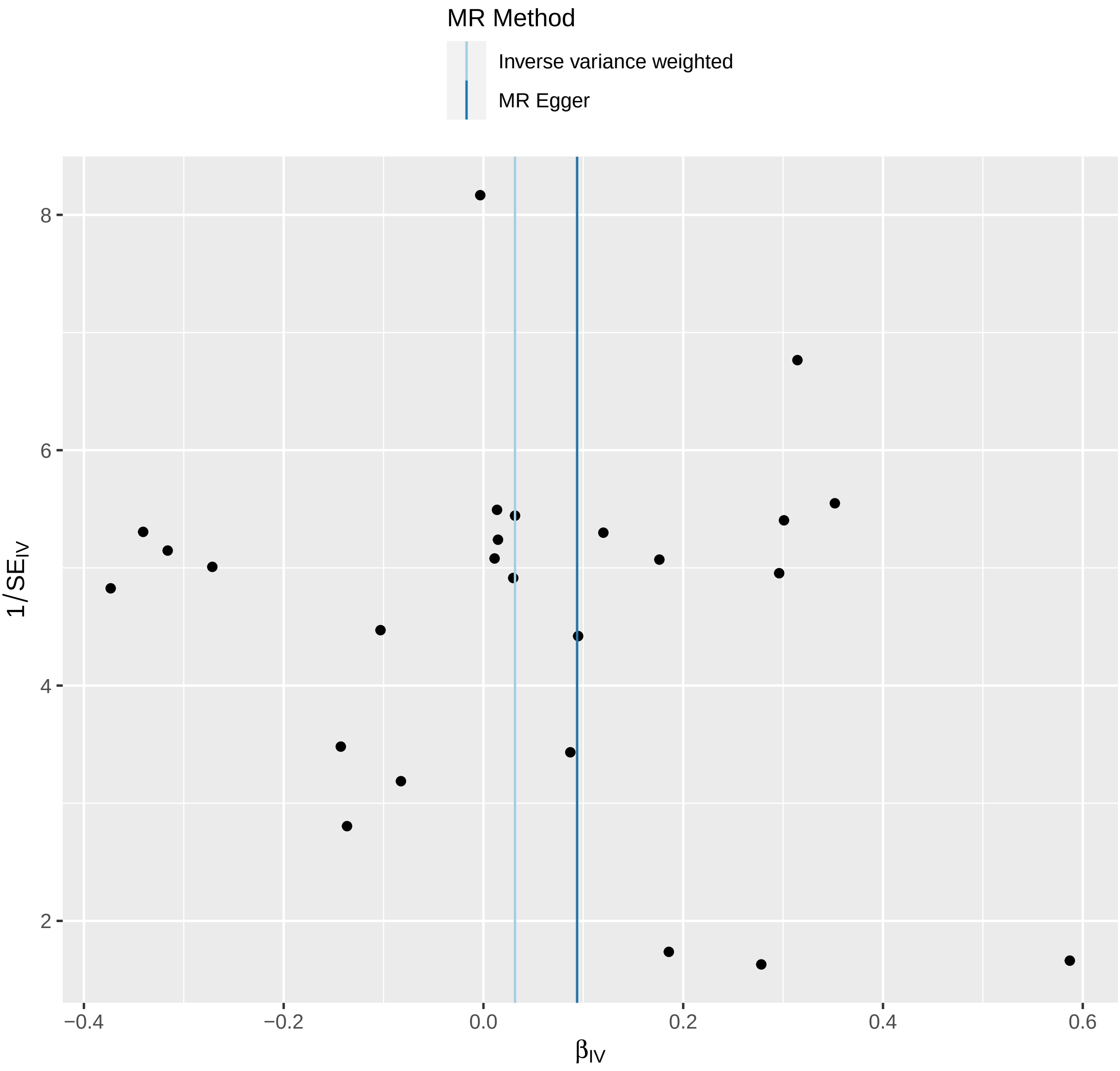

Batch 3−1809 : Lactotransferrin on Diabetic nephropathy (more control exclusions)
with a threshold of 1e−05

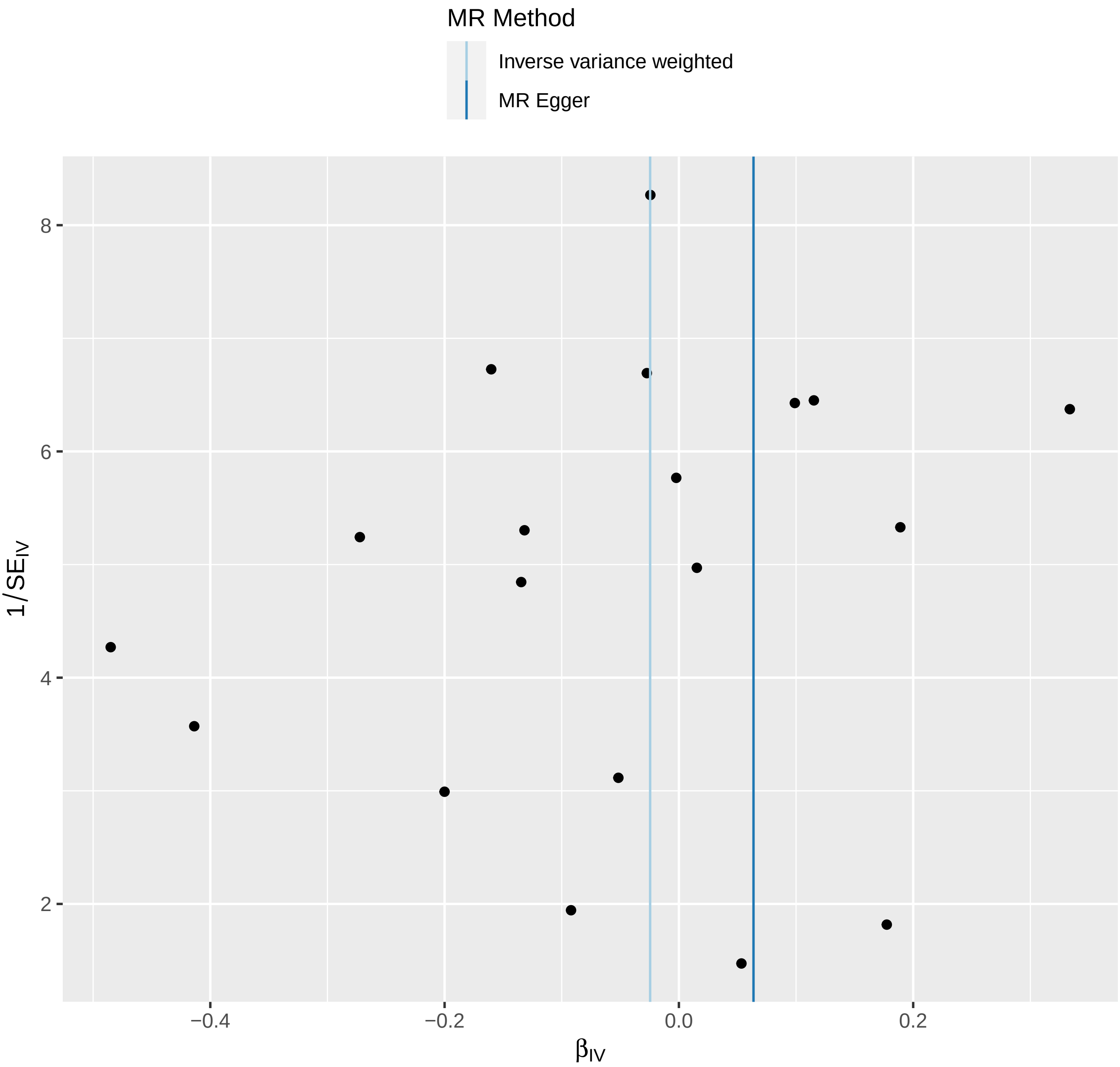

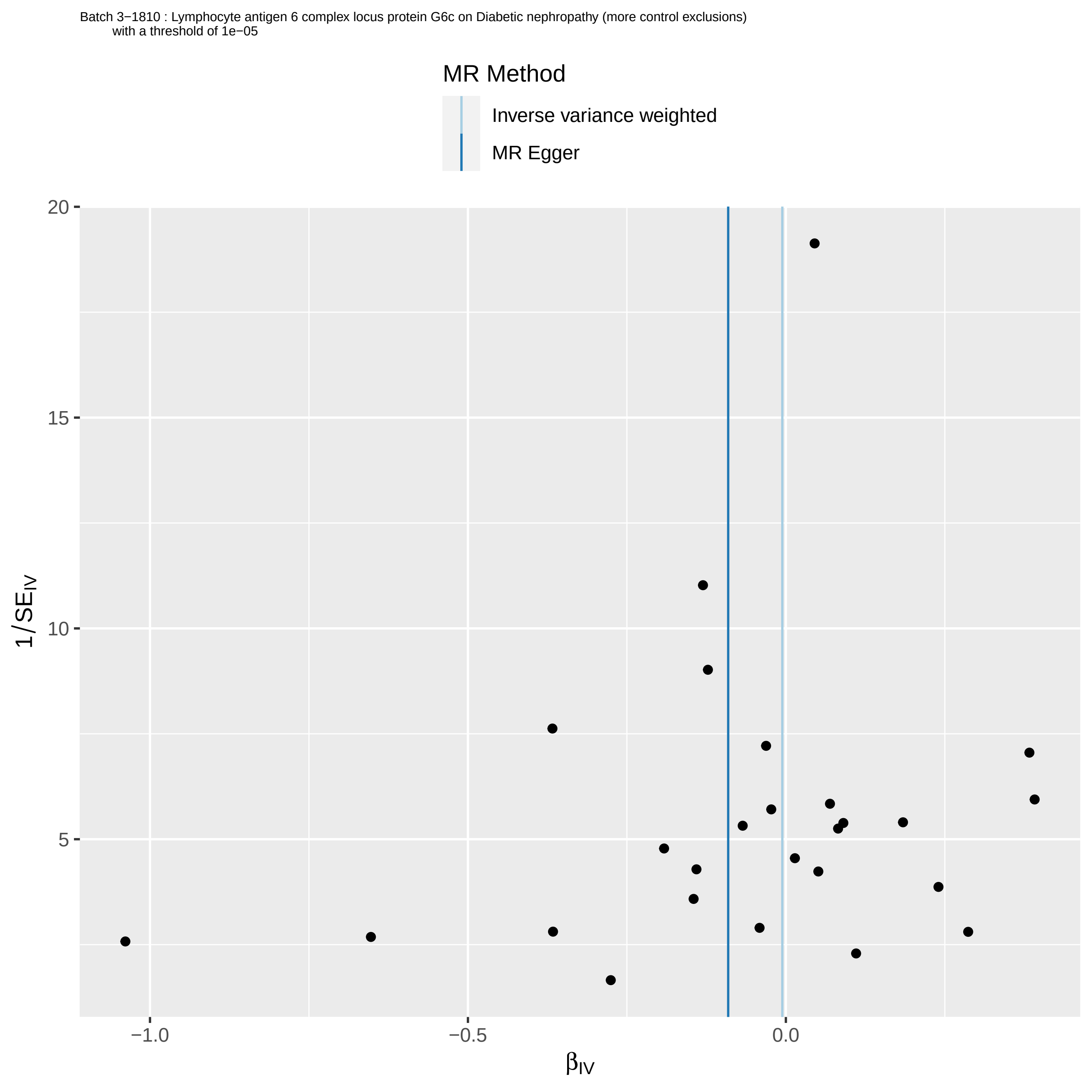

Batch 3−1810 : Lymphocyte antigen 6 complex locus protein G6c on Diabetic nephropathy (more control exclusions)
with a threshold of 1e−05
MR Method
Inverse variance weighted
MR Egger
20
15
10
5
1/SE_IV
−1.0
−0.5
0.0
β_IV

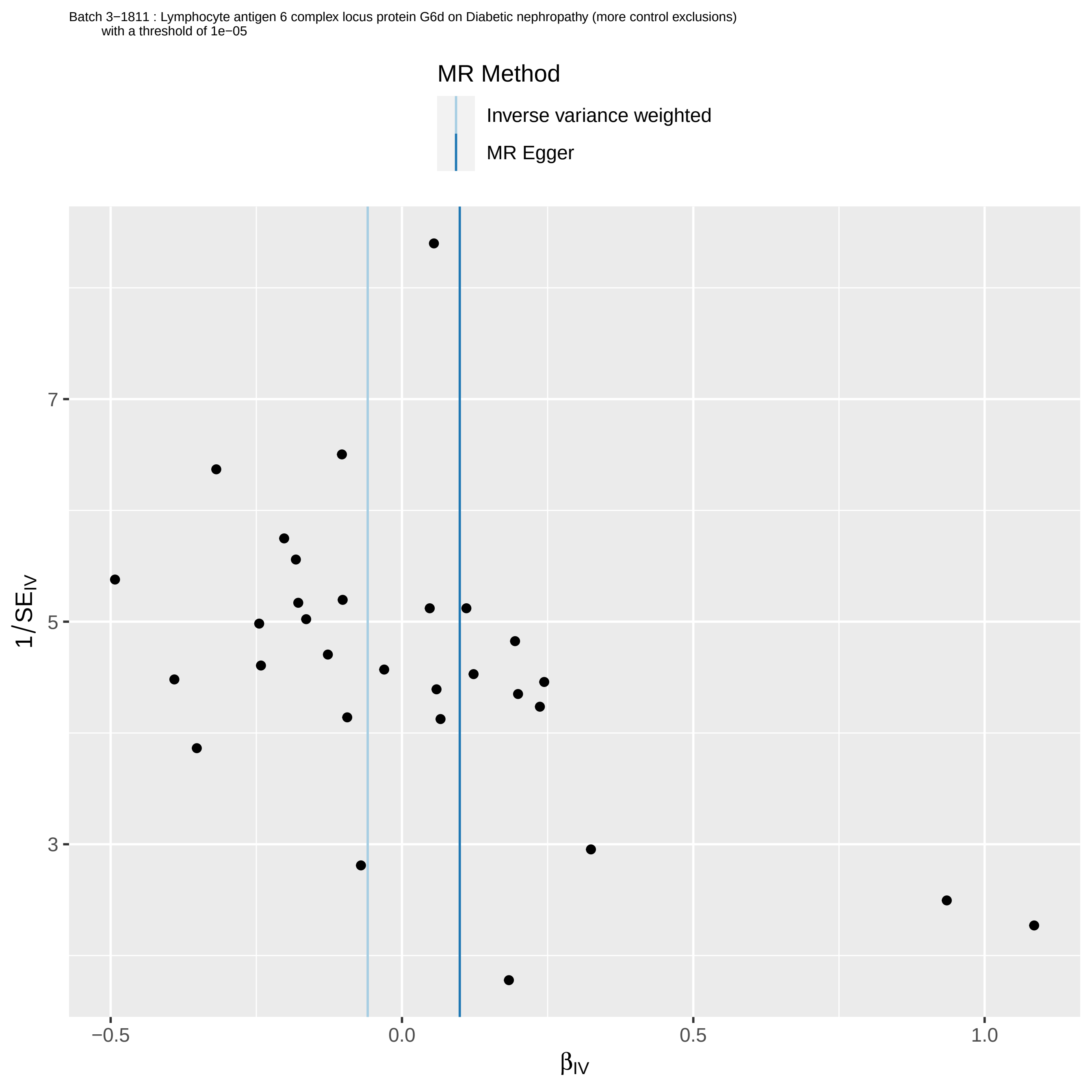

Batch 3−1811 : Lymphocyte antigen 6 complex locus protein G6d on Diabetic nephropathy (more control exclusions)
with a threshold of 1e−05
MR Method
Inverse variance weighted
MR Egger
7
5
3
1/SE_IV
−0.5
0.0
0.5
1.0
β_IV

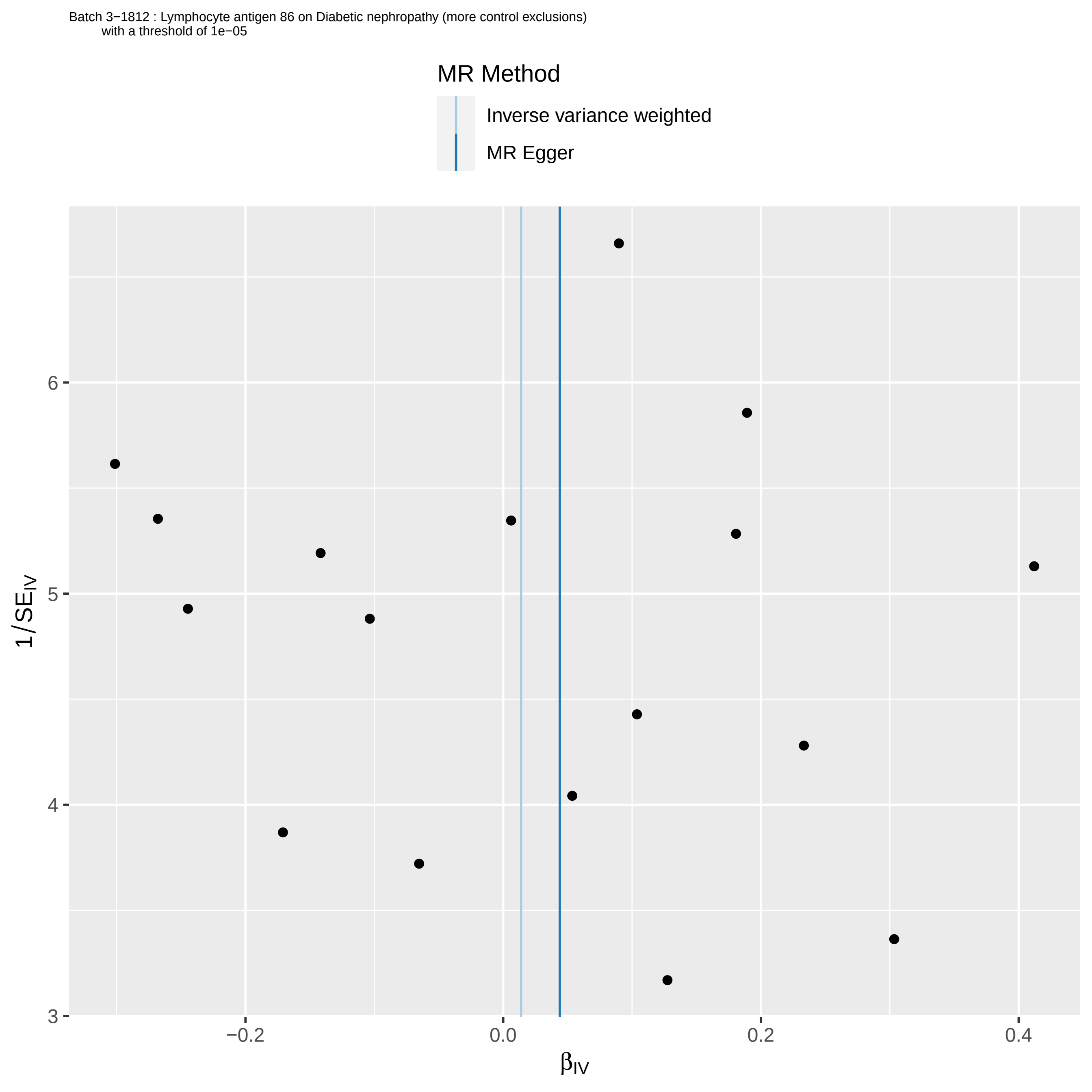

Batch 3−1812 : Lymphocyte antigen 86 on Diabetic nephropathy (more control exclusions)
with a threshold of 1e−05
MR Method
Inverse variance weighted
MR Egger
1/SE$_{IV}$
3
4
5
6
−0.2
0.0
0.2
0.4
β$_{IV}$

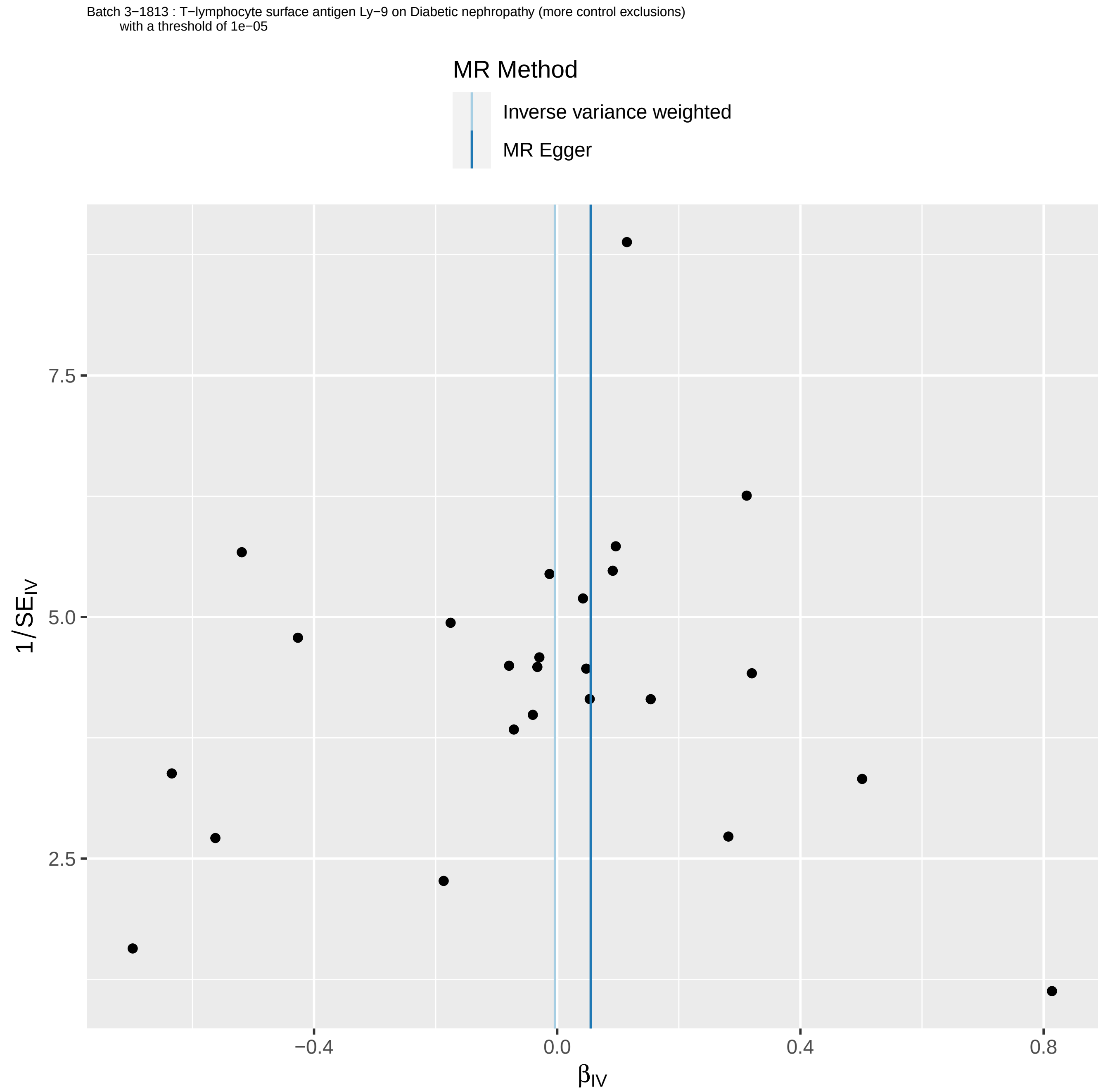
Batch 3−1813 : T−lymphocyte surface antigen Ly−9 on Diabetic nephropathy (more control exclusions)
with a threshold of 1e−05
MR Method
Inverse variance weighted
MR Egger
7.5
5.0
2.5
$1/SE_{IV}$
−0.4
0.0
0.4
0.8
β_{IV}

Batch 3−1814 : Lysozyme g−like protein 1 on Diabetic nephropathy (more control exclusions)
with a threshold of 1e−05

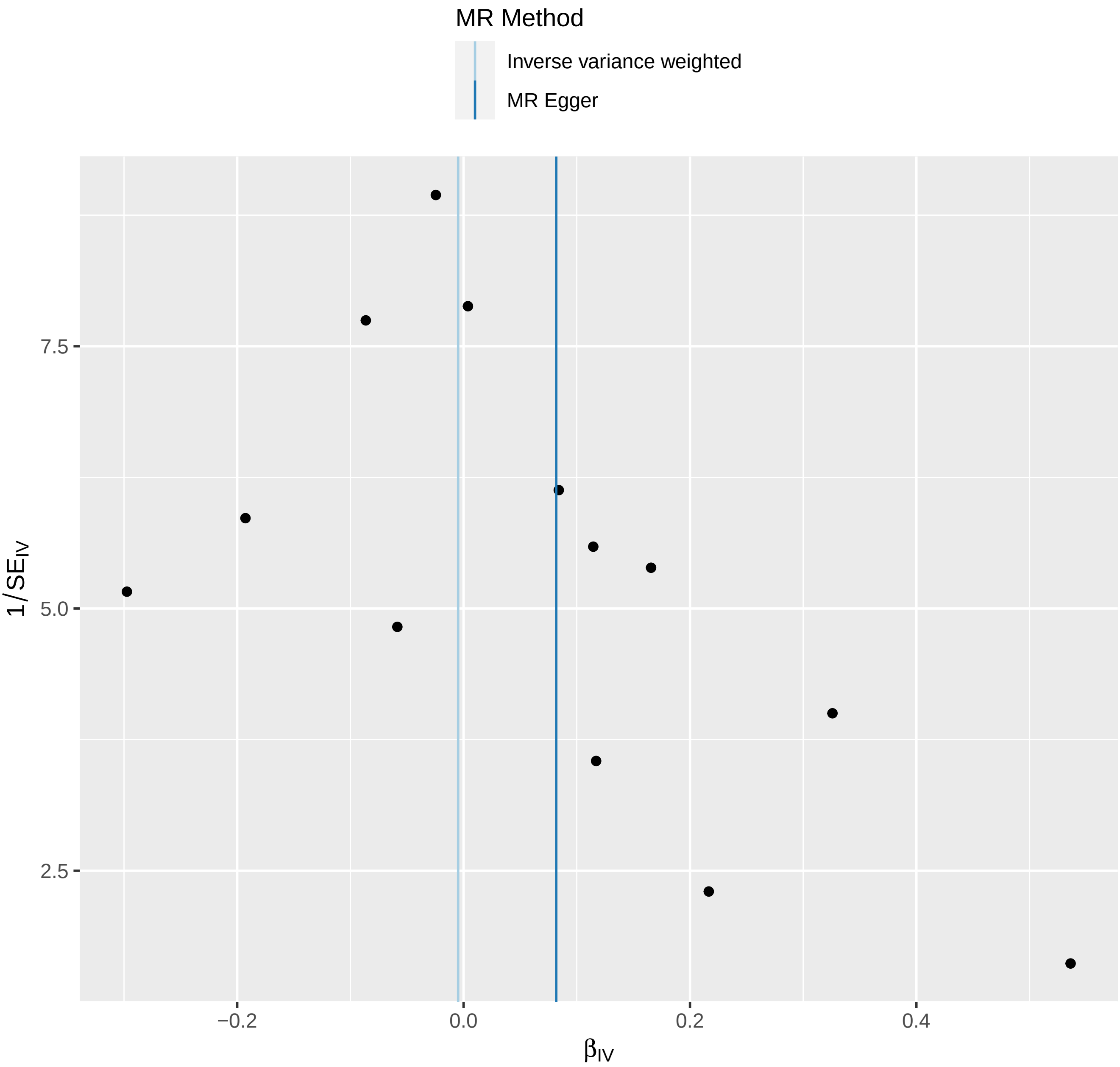

Batch 3−1815 : Tyrosine−protein kinase Lyn, isoform B on Diabetic nephropathy (more control exclusions)
with a threshold of 1e−05

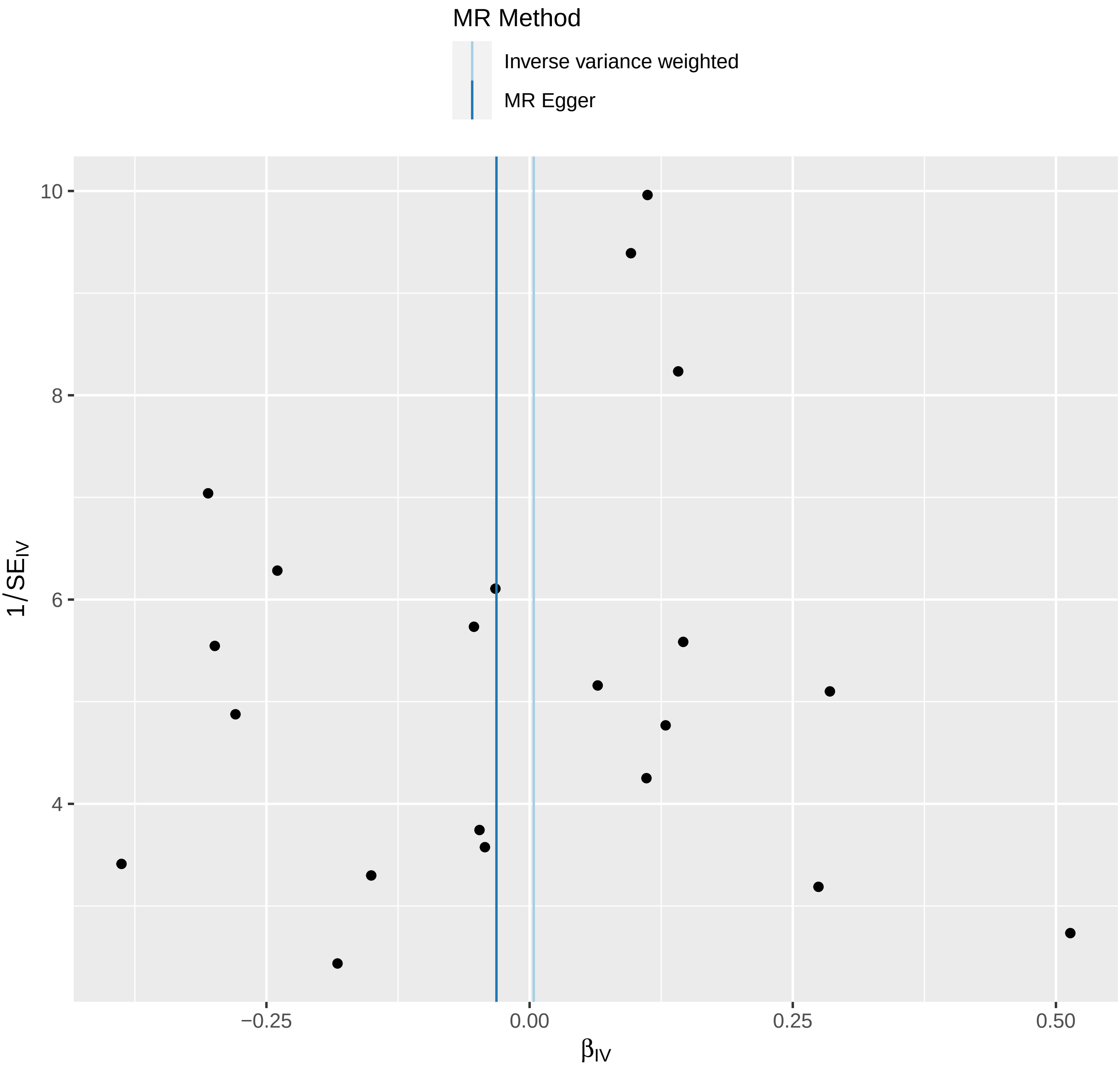

Batch 3−1816 : Tyrosine−protein kinase Lyn on Diabetic nephropathy (more control exclusions)
with a threshold of 1e−05

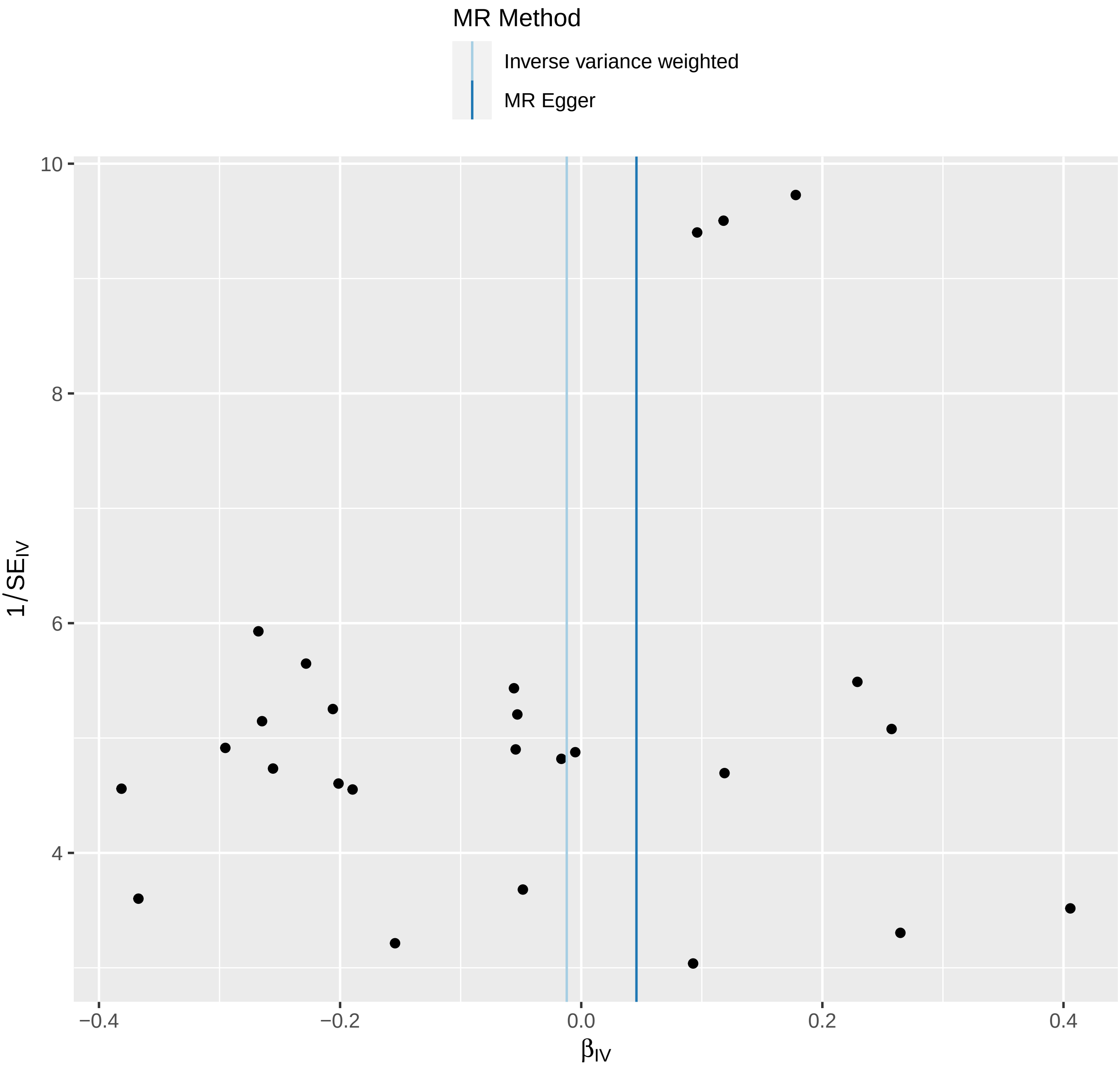

Batch 3−1817 : Ly6/PLAUR domain−containing protein 3 on Diabetic nephropathy (more control exclusions)
with a threshold of 1e−05

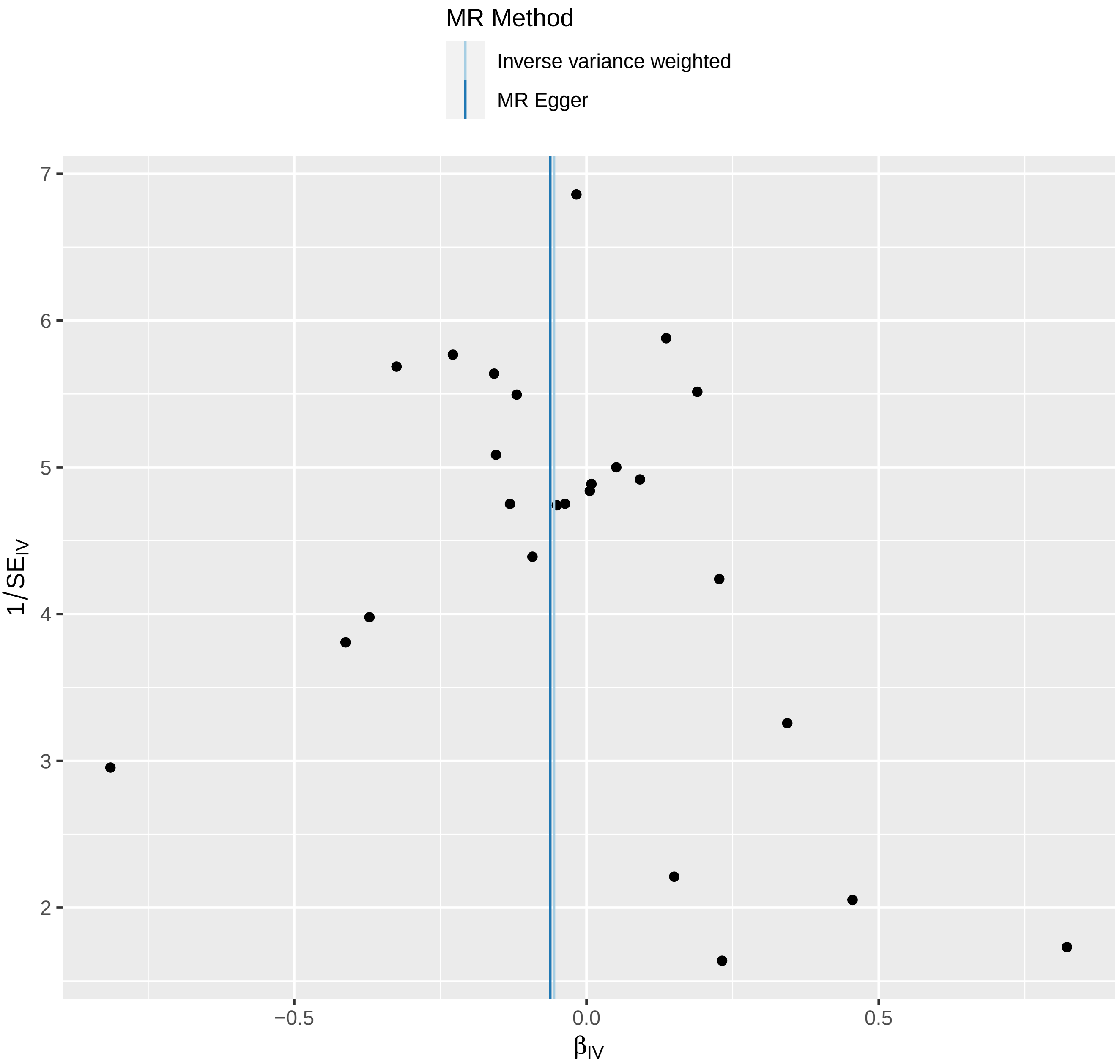

Batch 3−1818 : Lysophospholipase−like protein 1 on Diabetic nephropathy (more control exclusions)
with a threshold of 1e−05

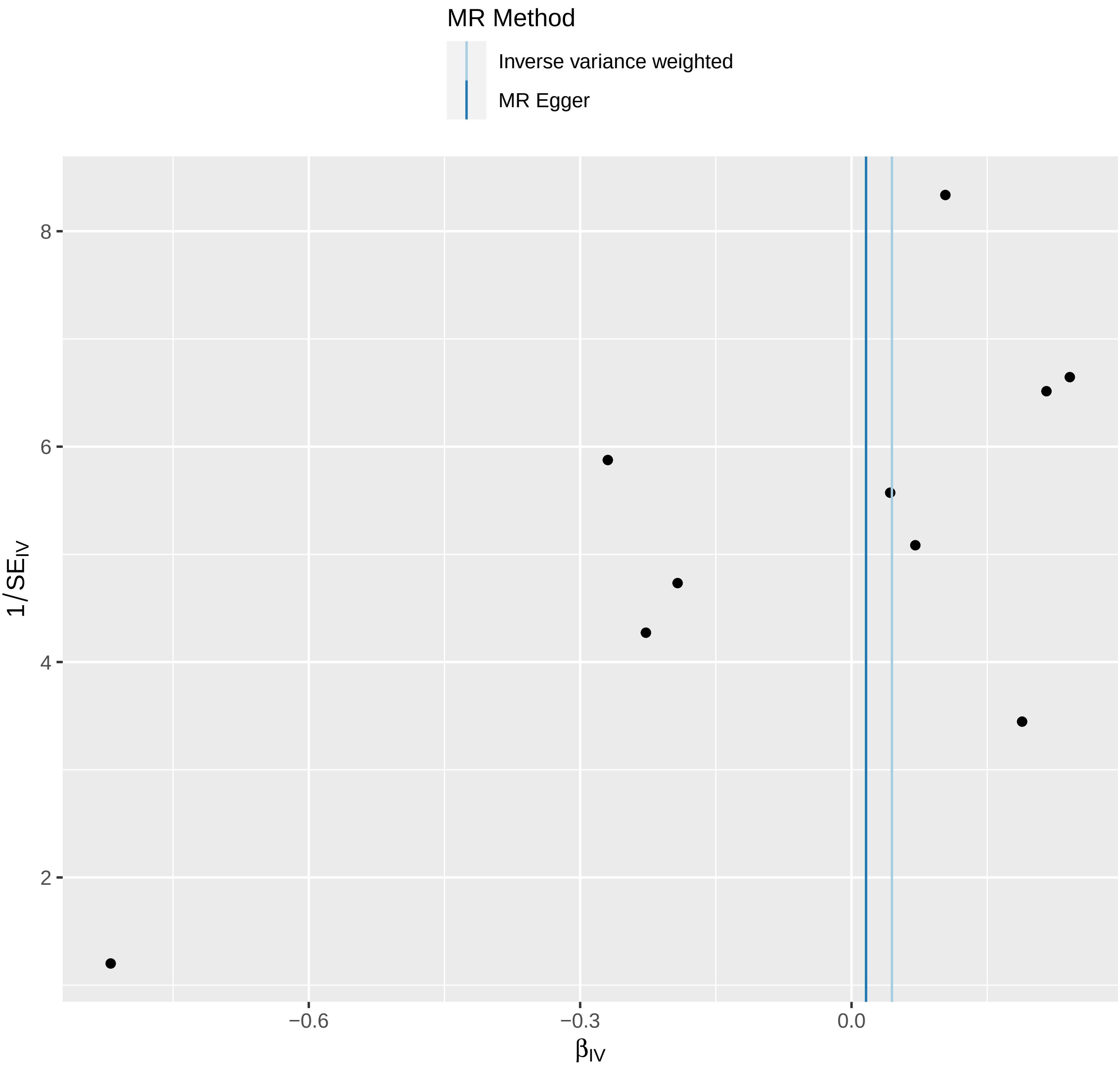

Batch 3−1819 : LysM and putative peptidoglycan−binding domain−containing protein 3 on Diabetic nephropathy (more control exclusions) with a threshold of 1e−05

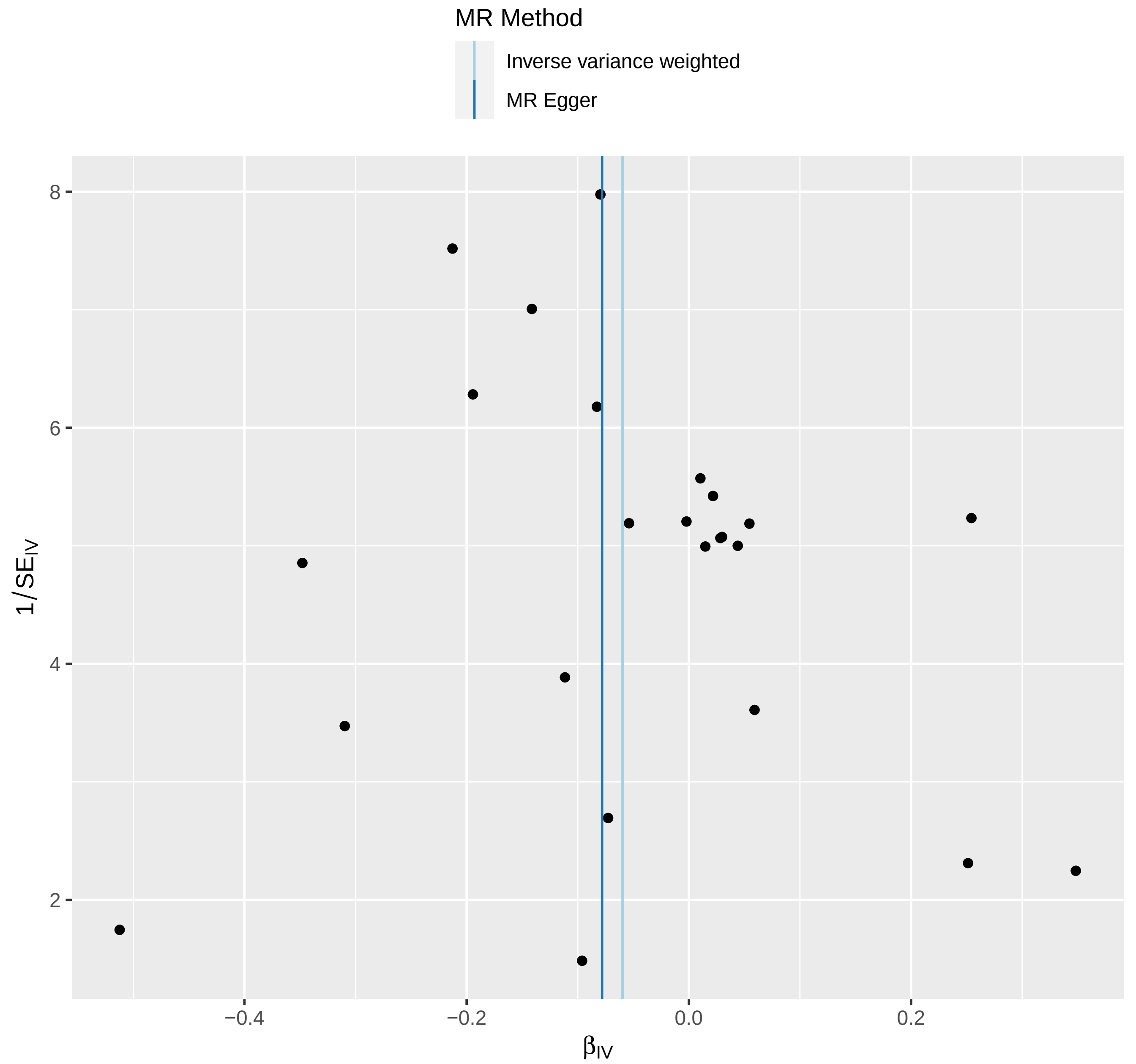

Batch 3−1820 : LysM and putative peptidoglycan−binding domain−containing protein 4 on Diabetic nephropathy (more control exclusions) with a threshold of 1e−05

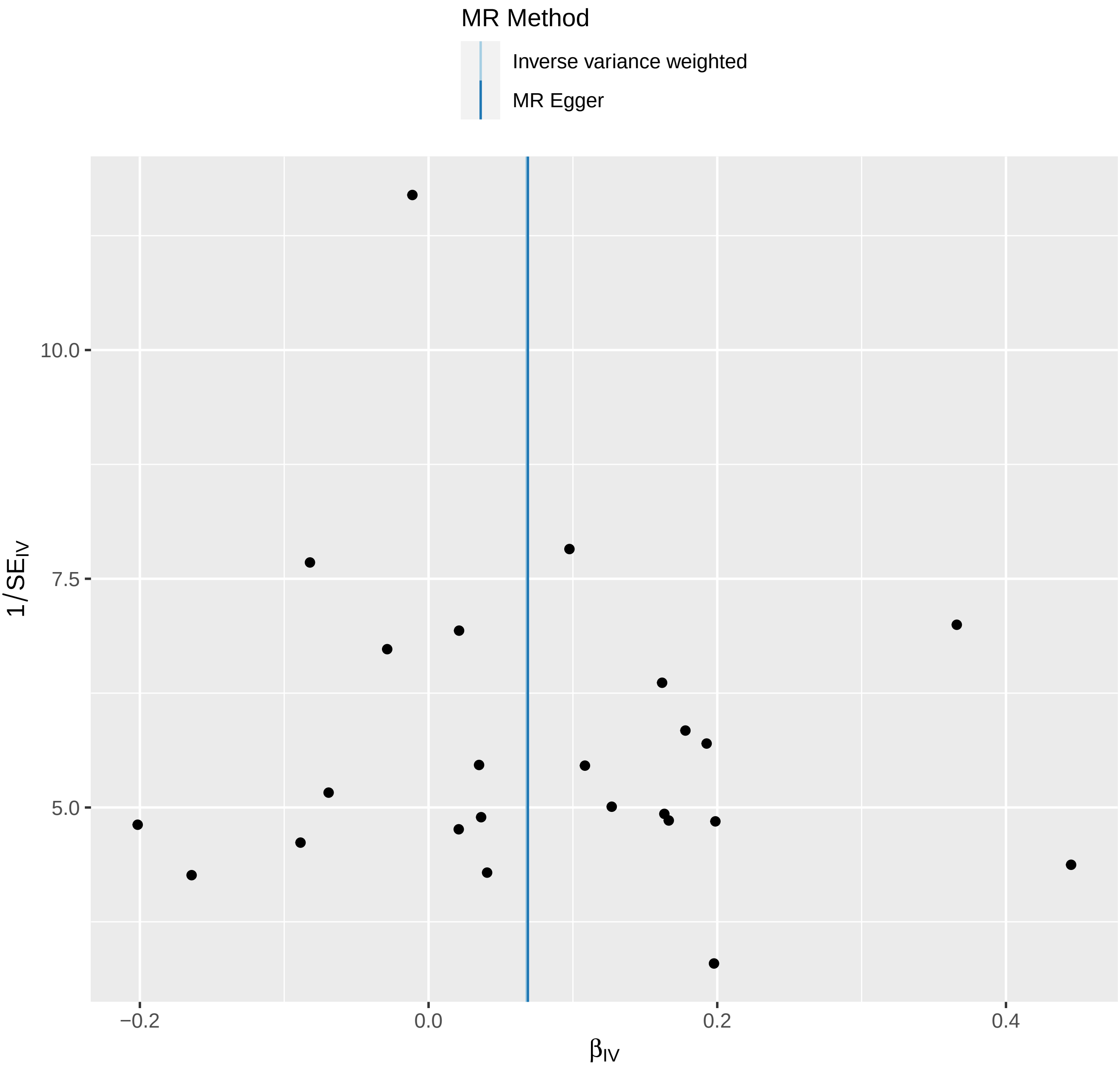

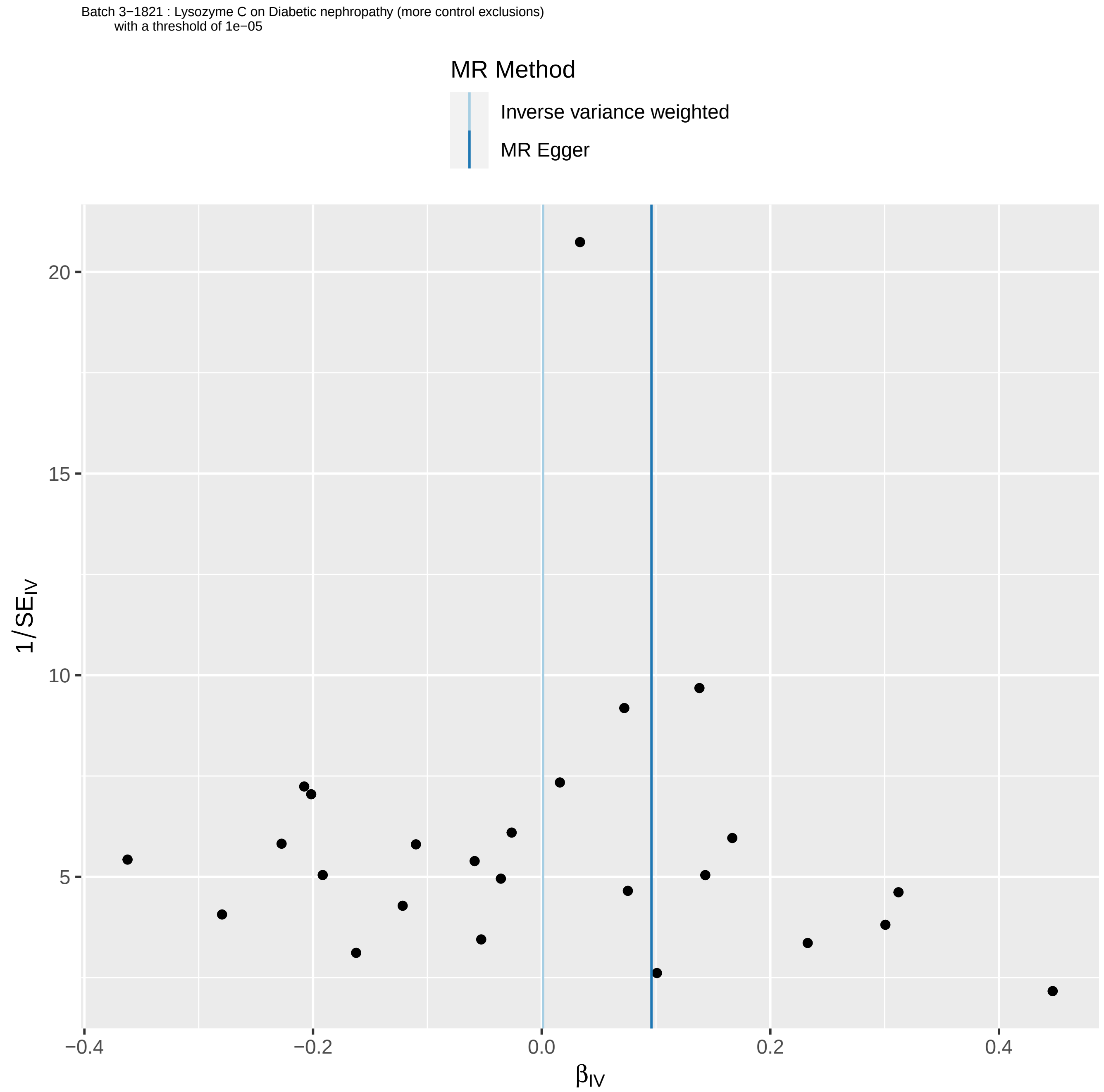

Batch 3−1821 : Lysozyme C on Diabetic nephropathy (more control exclusions)
with a threshold of 1e−05
MR Method
Inverse variance weighted
MR Egger
20
15
10
5
1/SE_IV
−0.4
−0.2
0.0
0.2
0.4
β_IV

Batch 3−1822 : Lysozyme−like protein 2 on Diabetic nephropathy (more control exclusions)
with a threshold of 1e−05

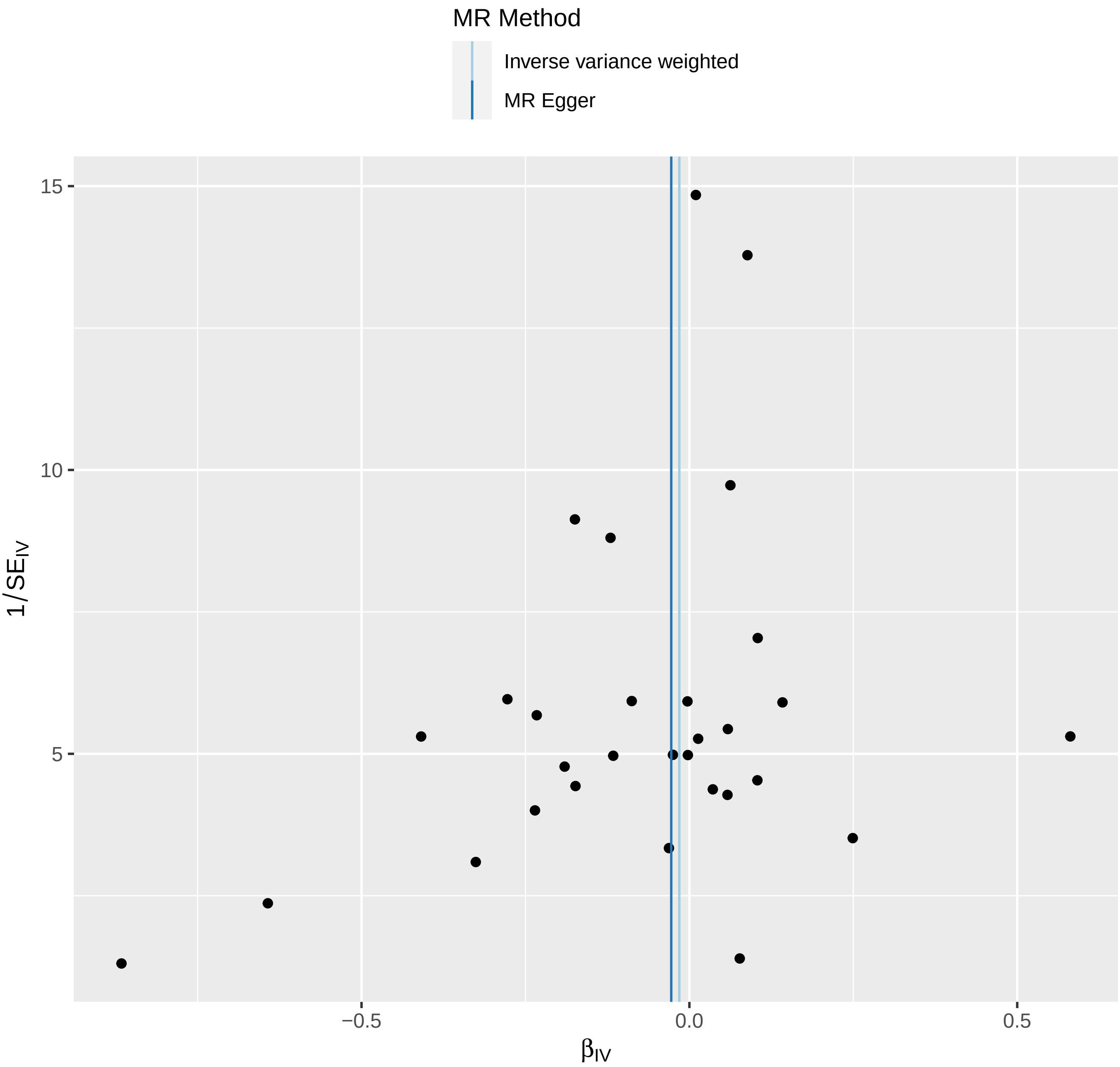

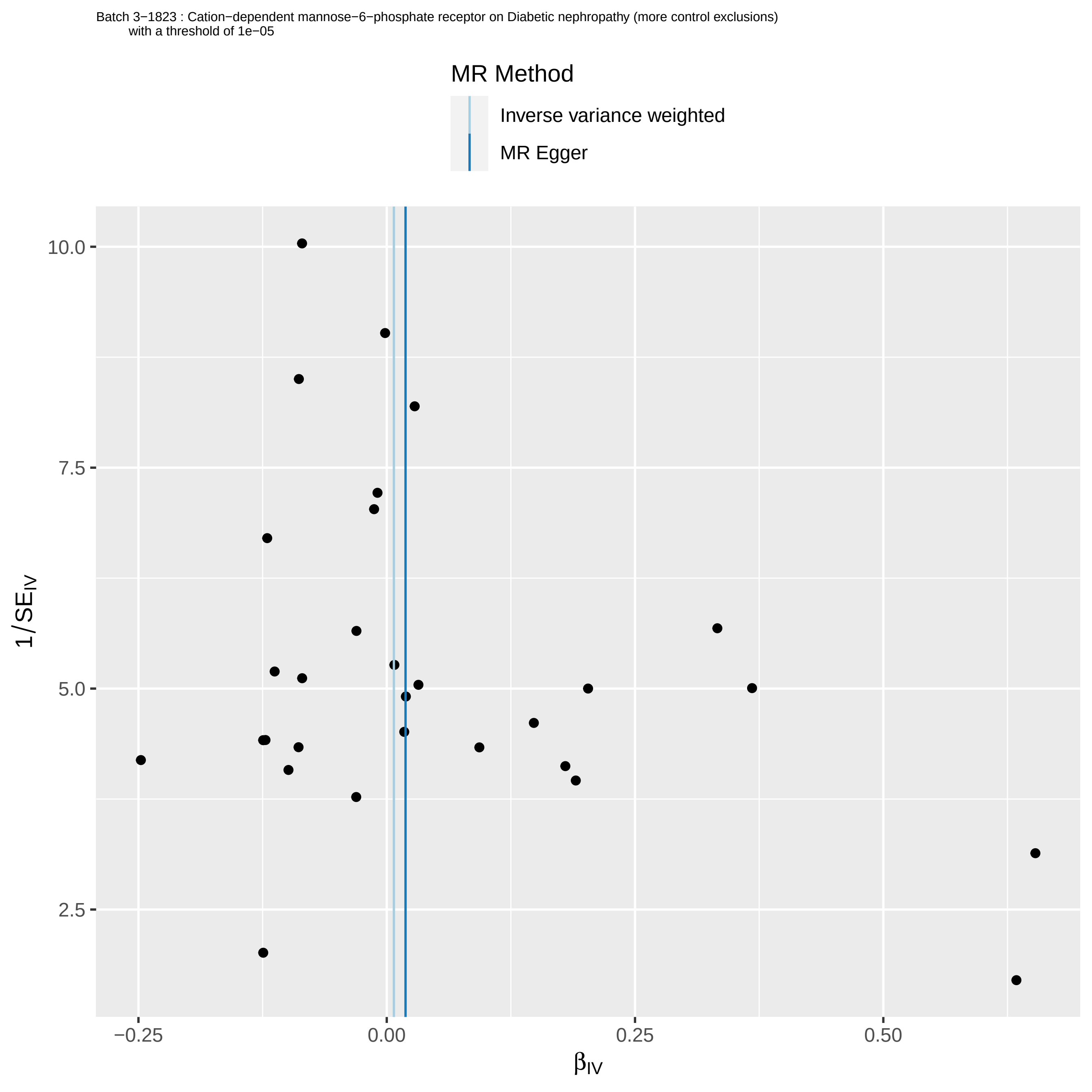

Batch 3−1823 : Cation−dependent mannose−6−phosphate receptor on Diabetic nephropathy (more control exclusions)
with a threshold of 1e−05
MR Method
Inverse variance weighted
MR Egger
10.0
7.5
5.0
2.5
1/SE_IV
−0.25
0.00
0.25
0.50
β_IV

Batch 3−1824 : O−acetyl−ADP−ribose deacetylase MACROD1 on Diabetic nephropathy (more control exclusions) with a threshold of 1e−05

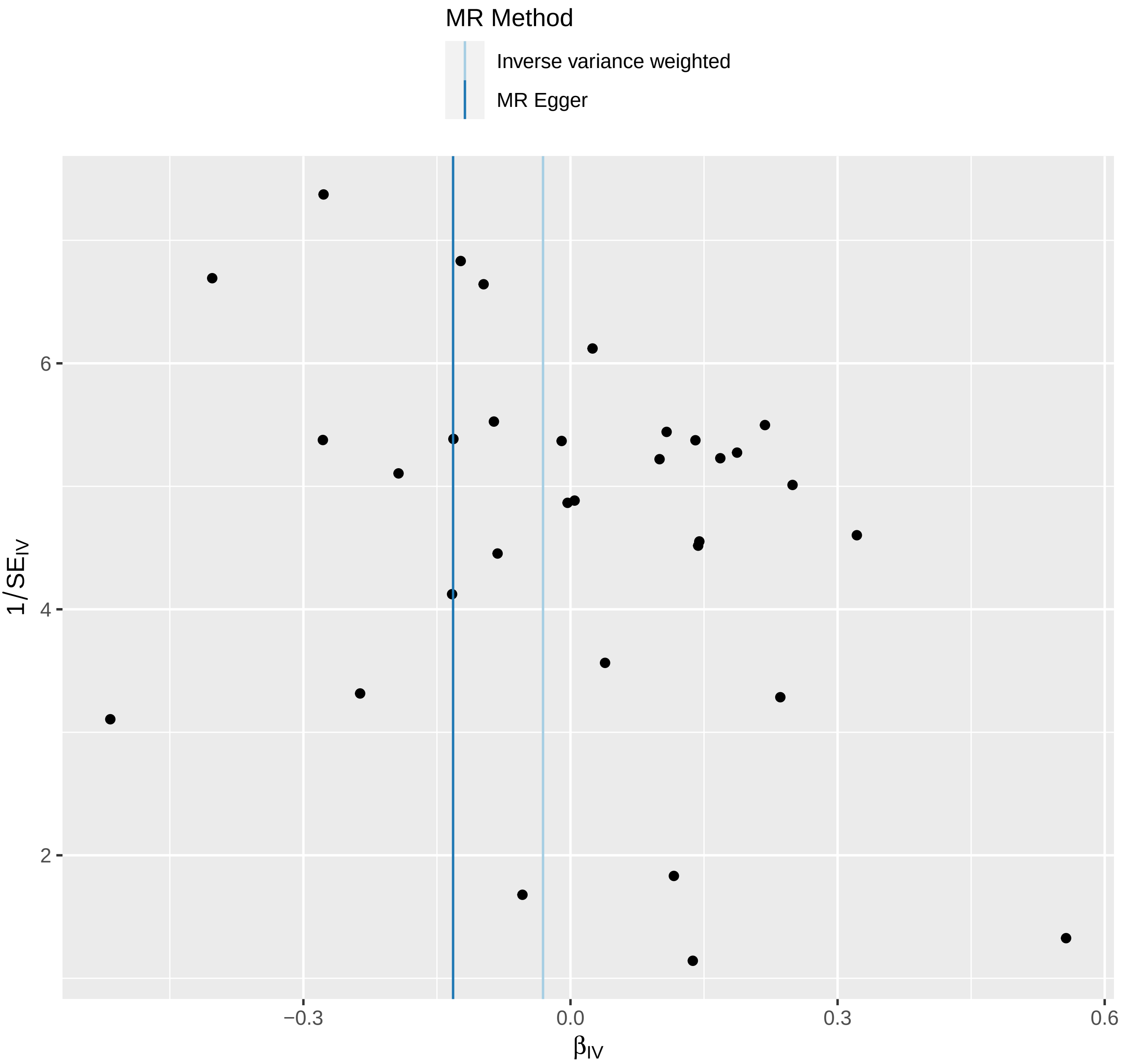

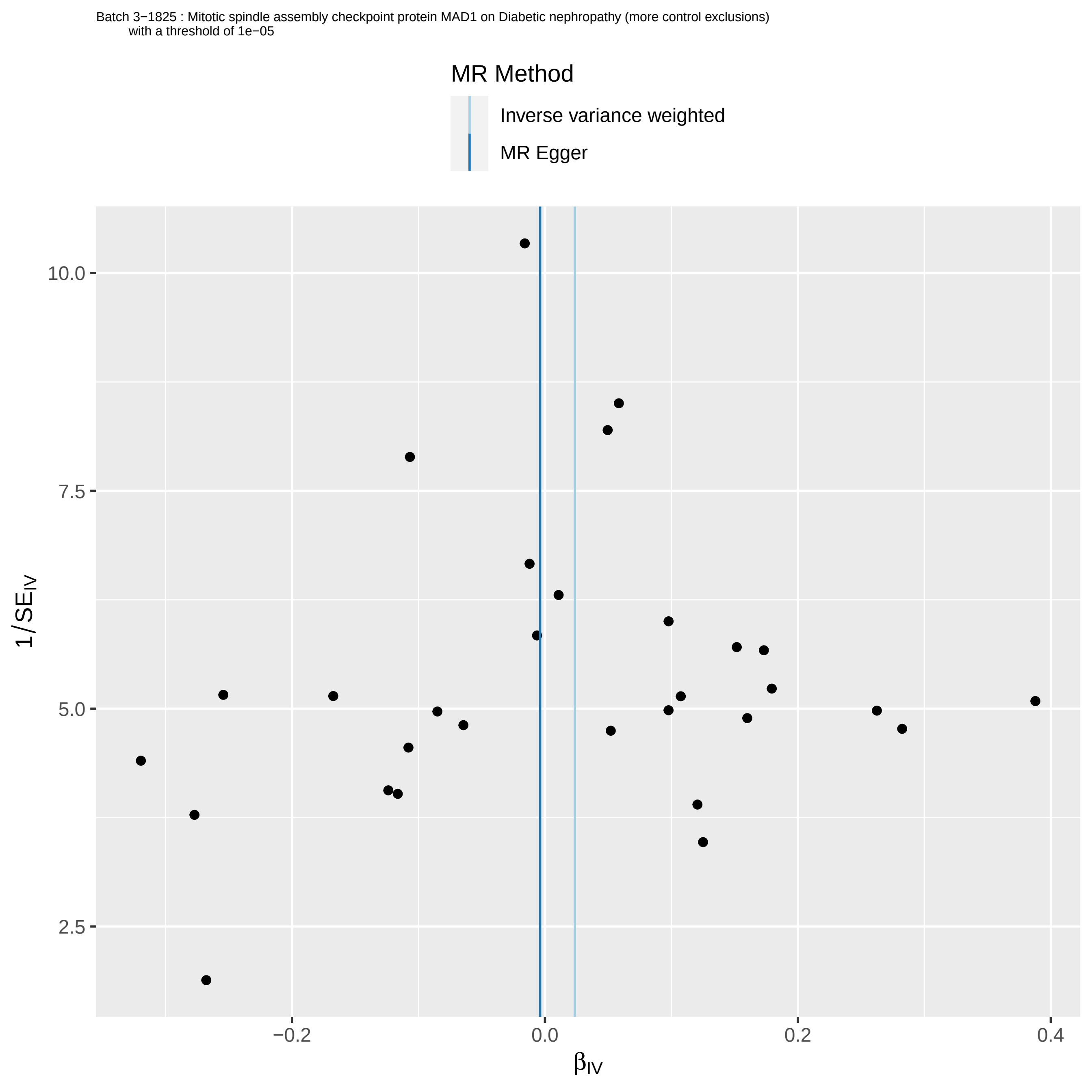

Batch 3−1825 : Mitotic spindle assembly checkpoint protein MAD1 on Diabetic nephropathy (more control exclusions)
with a threshold of 1e−05
MR Method
Inverse variance weighted
MR Egger
10.0
7.5
5.0
2.5
1/SE_IV
−0.2
0.0
0.2
0.4
β_IV

Batch 3−1826 : Mucosal addressin cell adhesion molecule 1 on Diabetic nephropathy (more control exclusions)
with a threshold of 1e−05

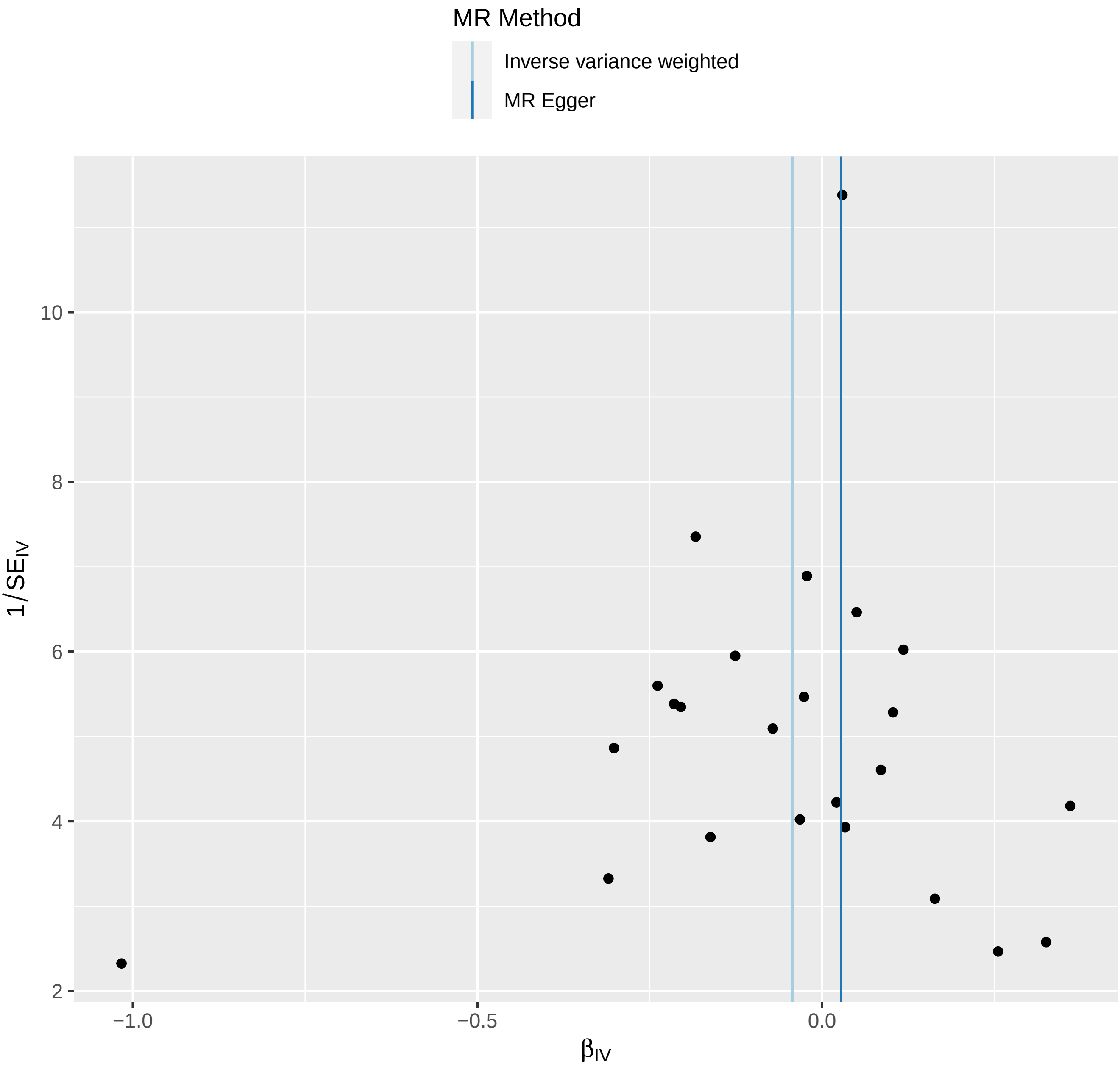

Batch 3−1827 : Melanoma−associated antigen 10 on Diabetic nephropathy (more control exclusions)
with a threshold of 1e−05

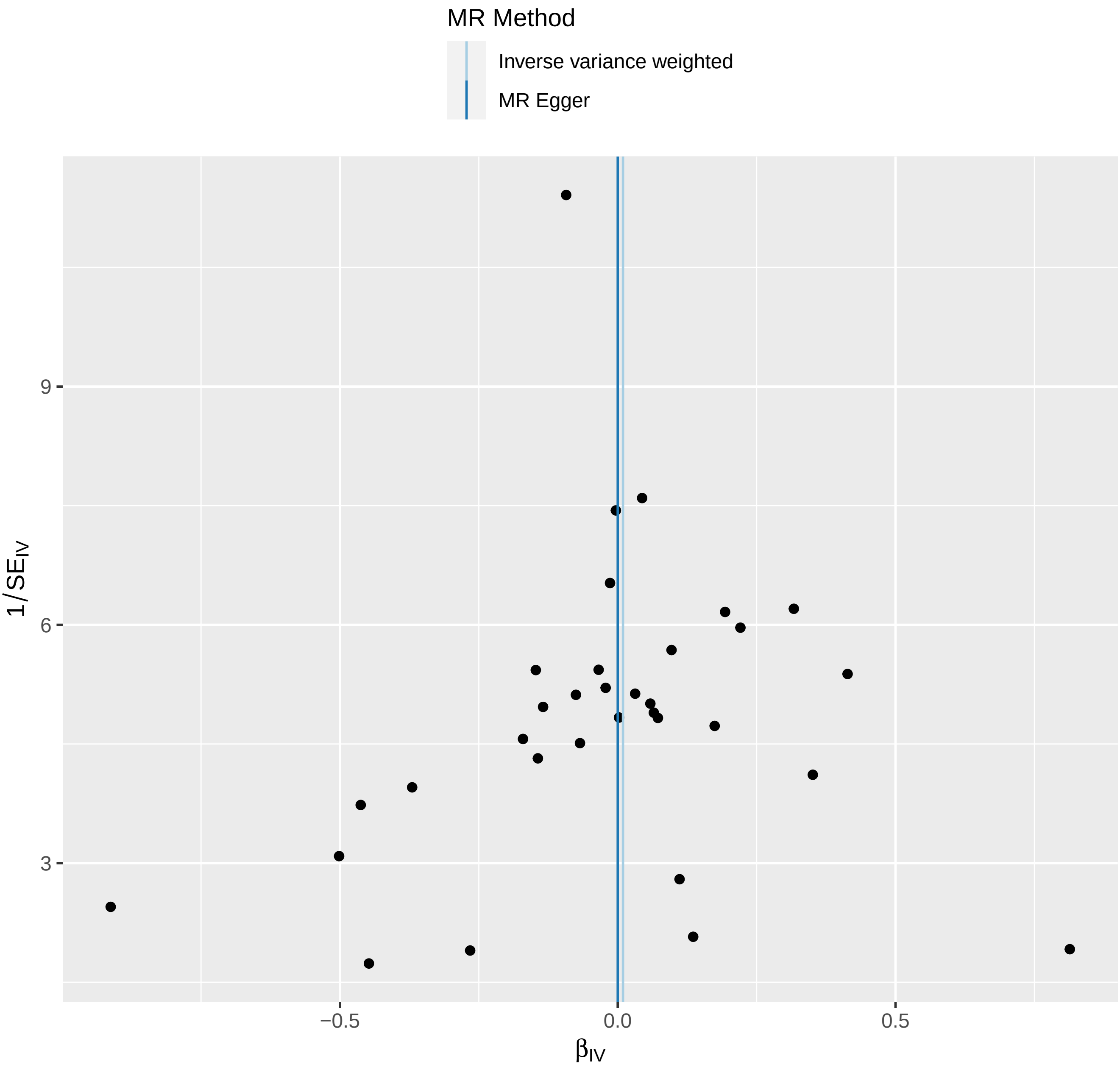

Batch 3−1828 : Melanoma−associated antigen 3 on Diabetic nephropathy (more control exclusions)
with a threshold of 1e−05

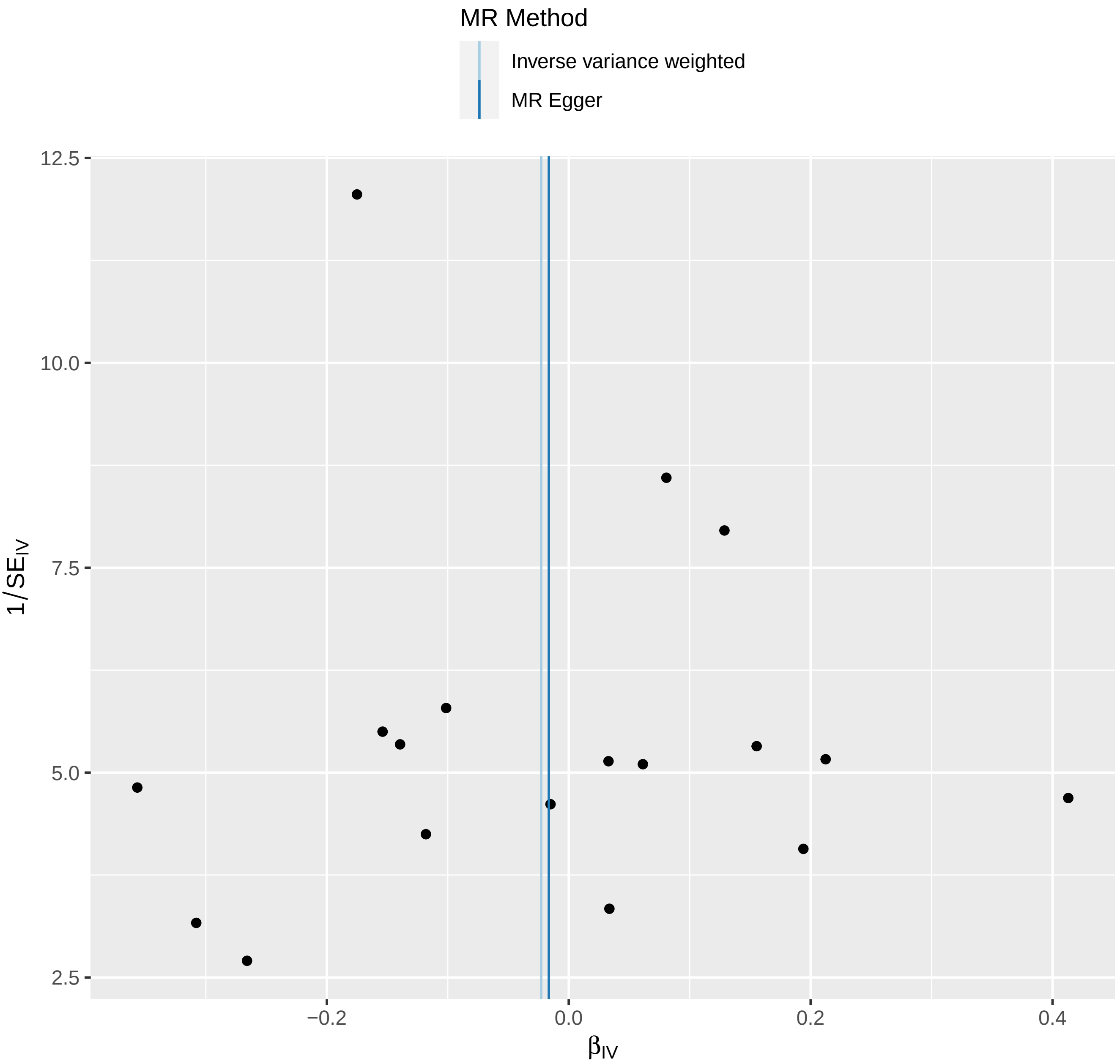

Batch 3−1829 : Melanoma−associated antigen B10 on Diabetic nephropathy (more control exclusions)
with a threshold of 1e−05

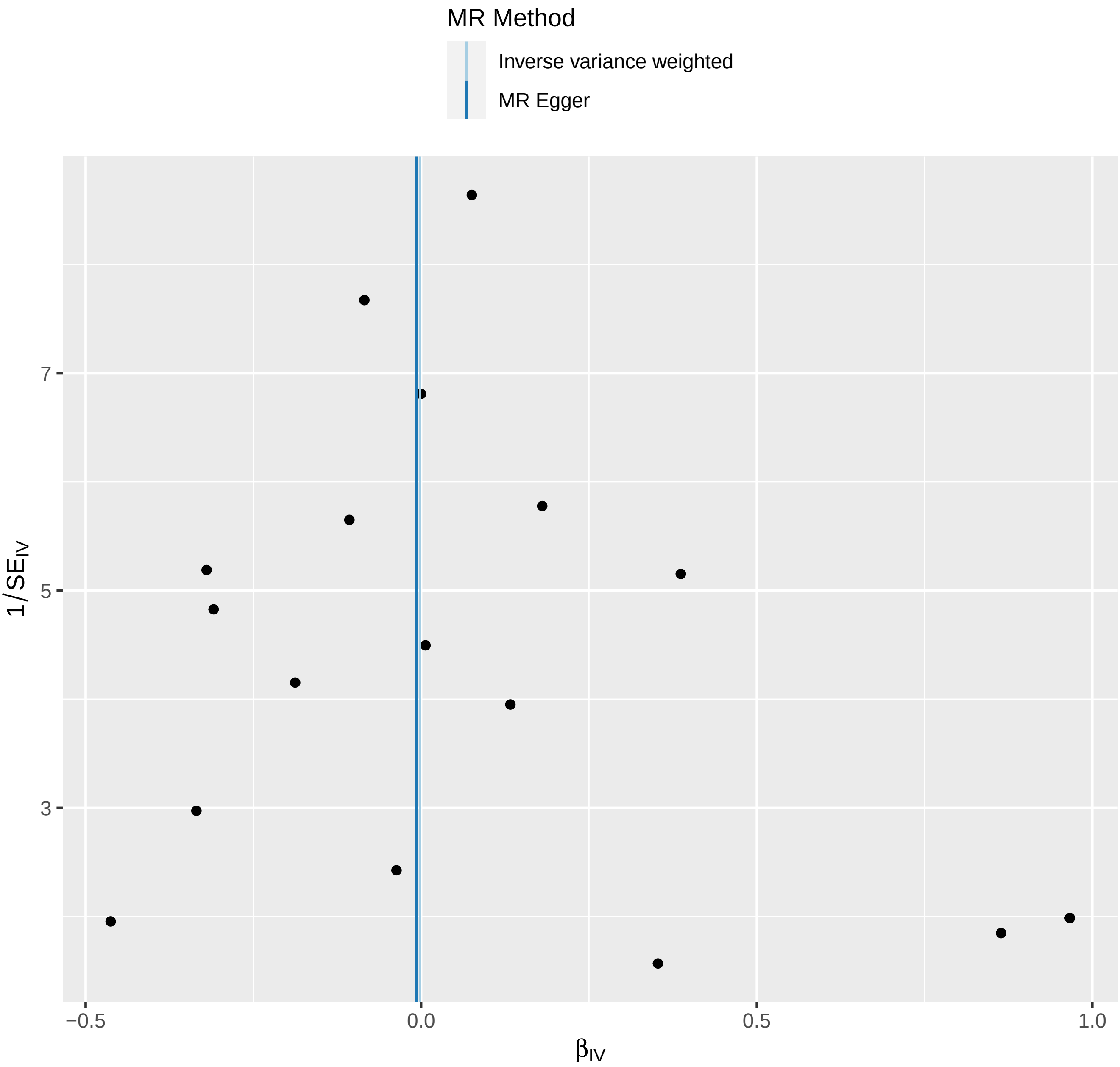

Batch 3−1830 : Membrane−associated guanylate kinase, WW and PDZ domain−containing protein 2 on Diabetic nephropathy (more control exclusions) with a threshold of 1e−05

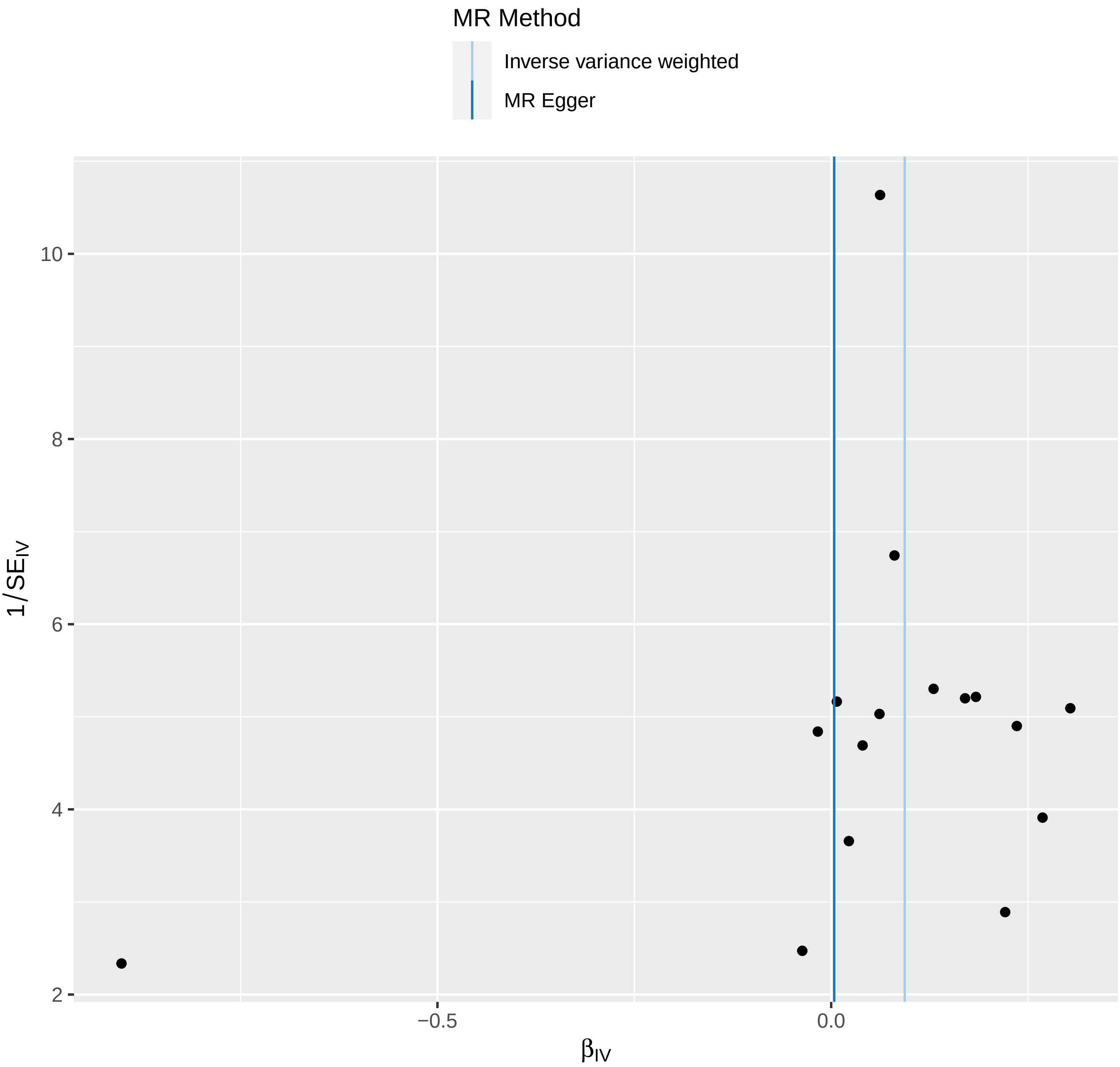

Batch 3−1831 : MAM domain−containing protein 2 on Diabetic nephropathy (more control exclusions)
with a threshold of 1e−05

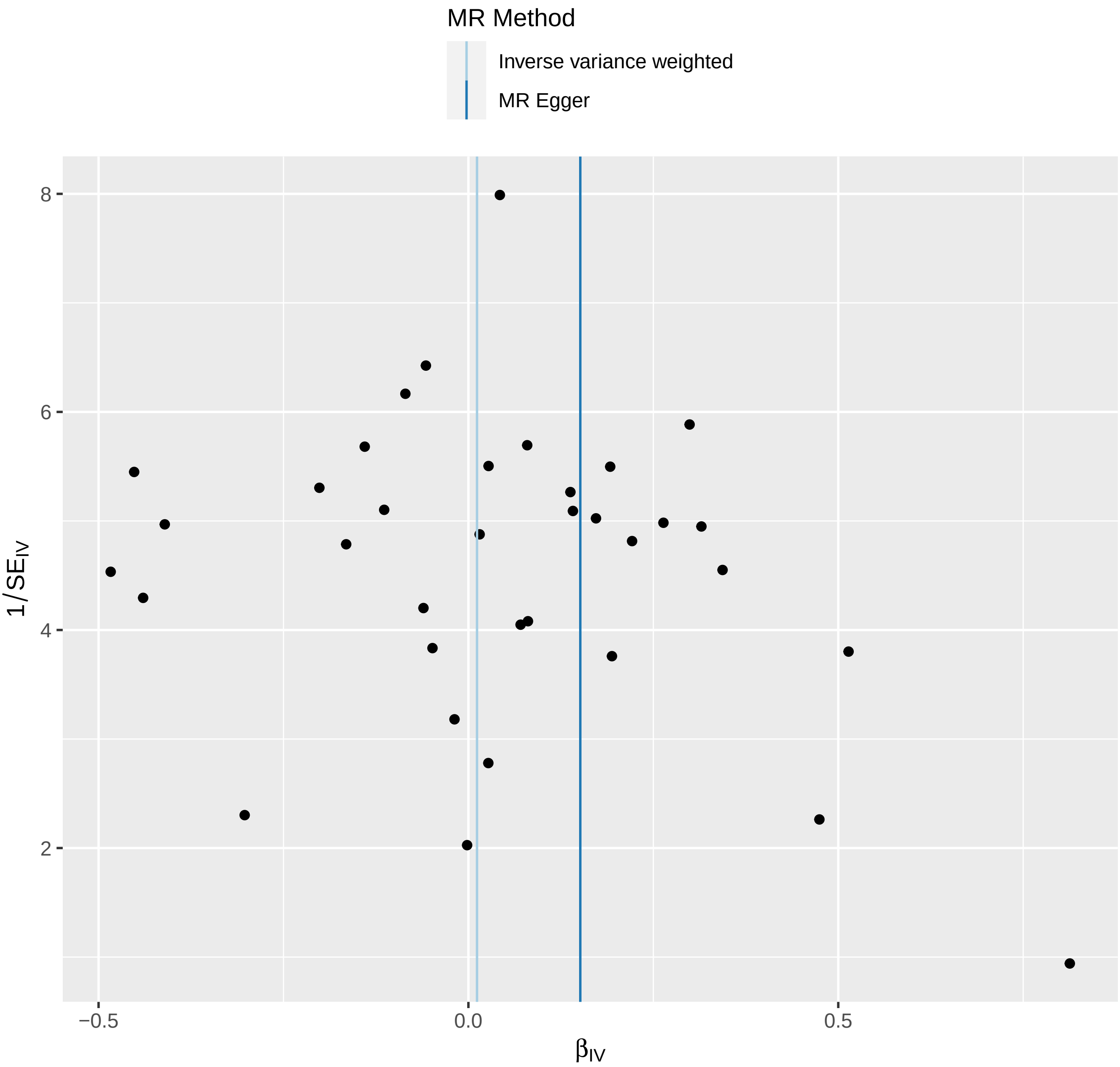

Batch 3−1832 : Mannosyl−oligosaccharide 1,2−alpha−mannosidase IA on Diabetic nephropathy (more control exclusions)
with a threshold of 1e−05

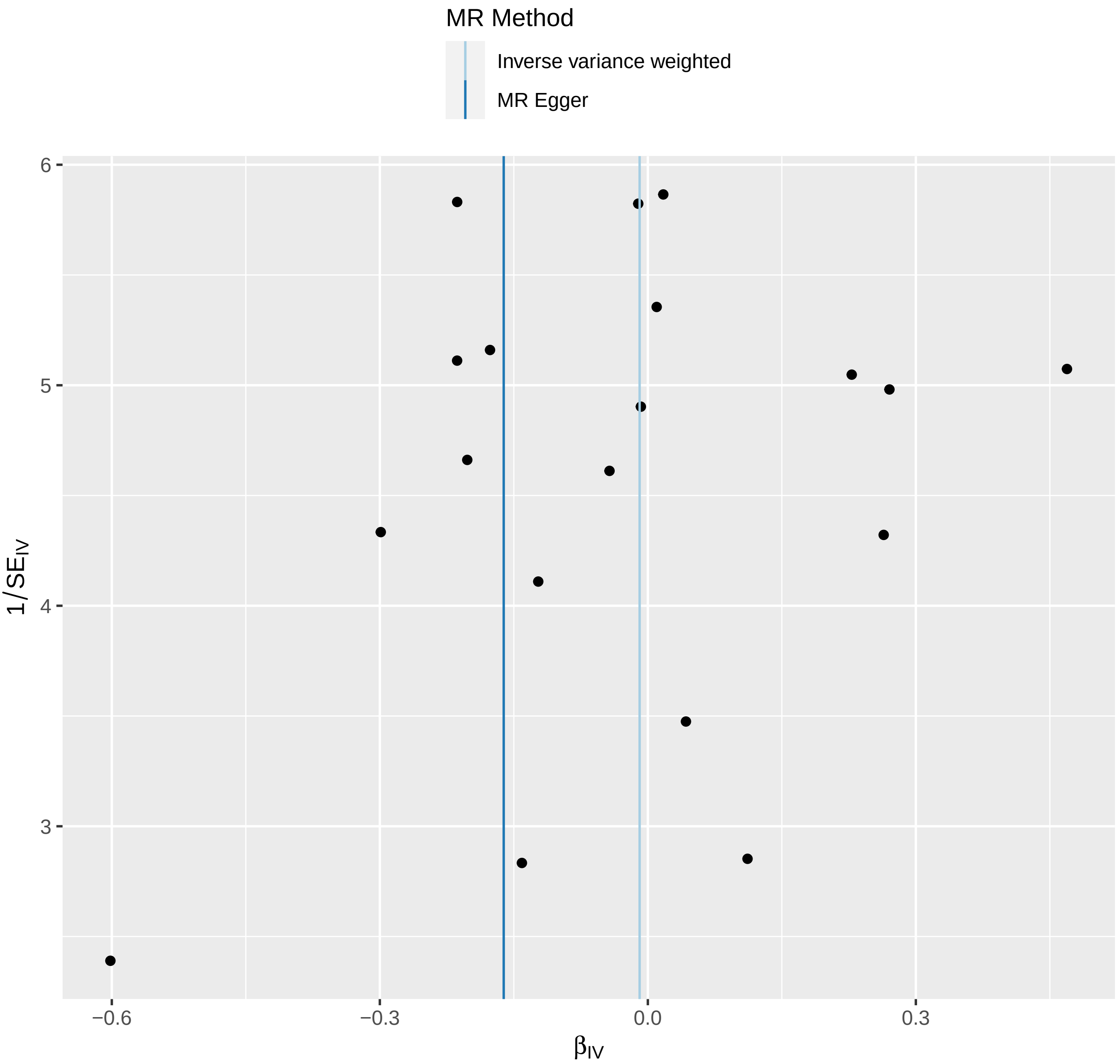

Batch 3−1833 : Mannosyl−oligosaccharide 1,2−alpha−mannosidase IB on Diabetic nephropathy (more control exclusions)
with a threshold of 1e−05

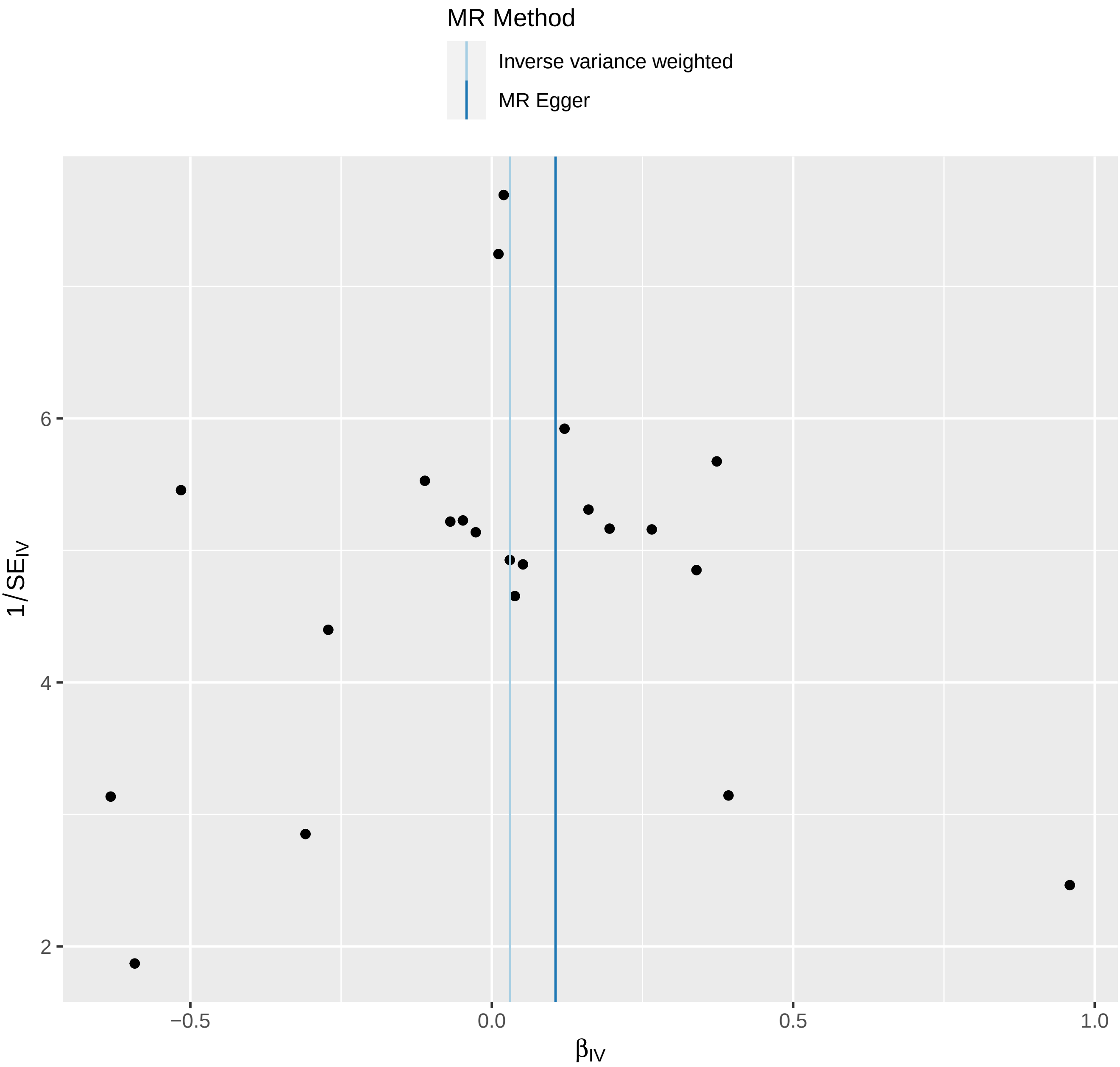

Batch 3−1834 : Endoplasmic reticulum mannosyl−oligosaccharide 1,2−alpha−mannosidase on Diabetic nephropathy (more control exclusions)
with a threshold of 1e−05

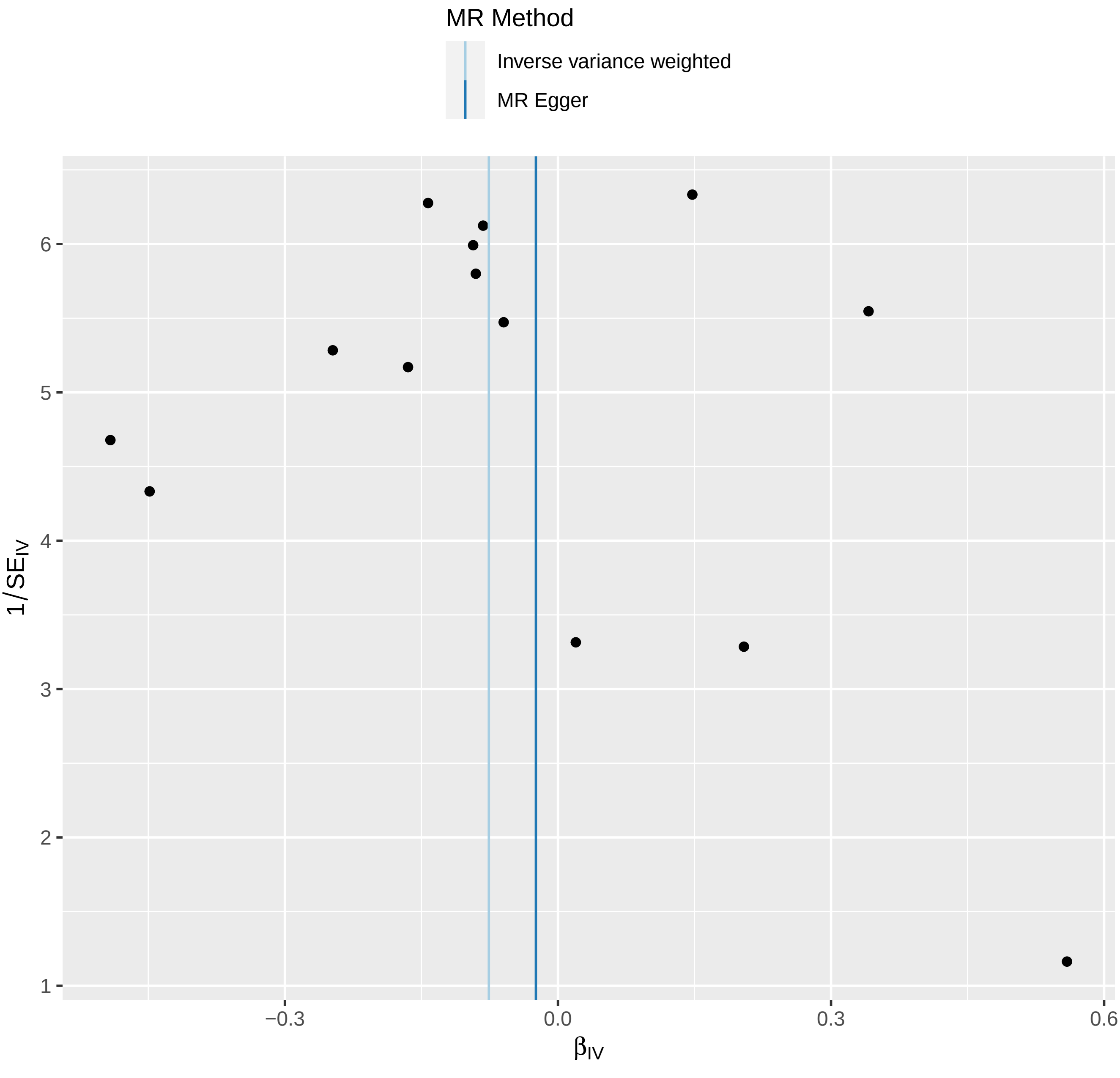

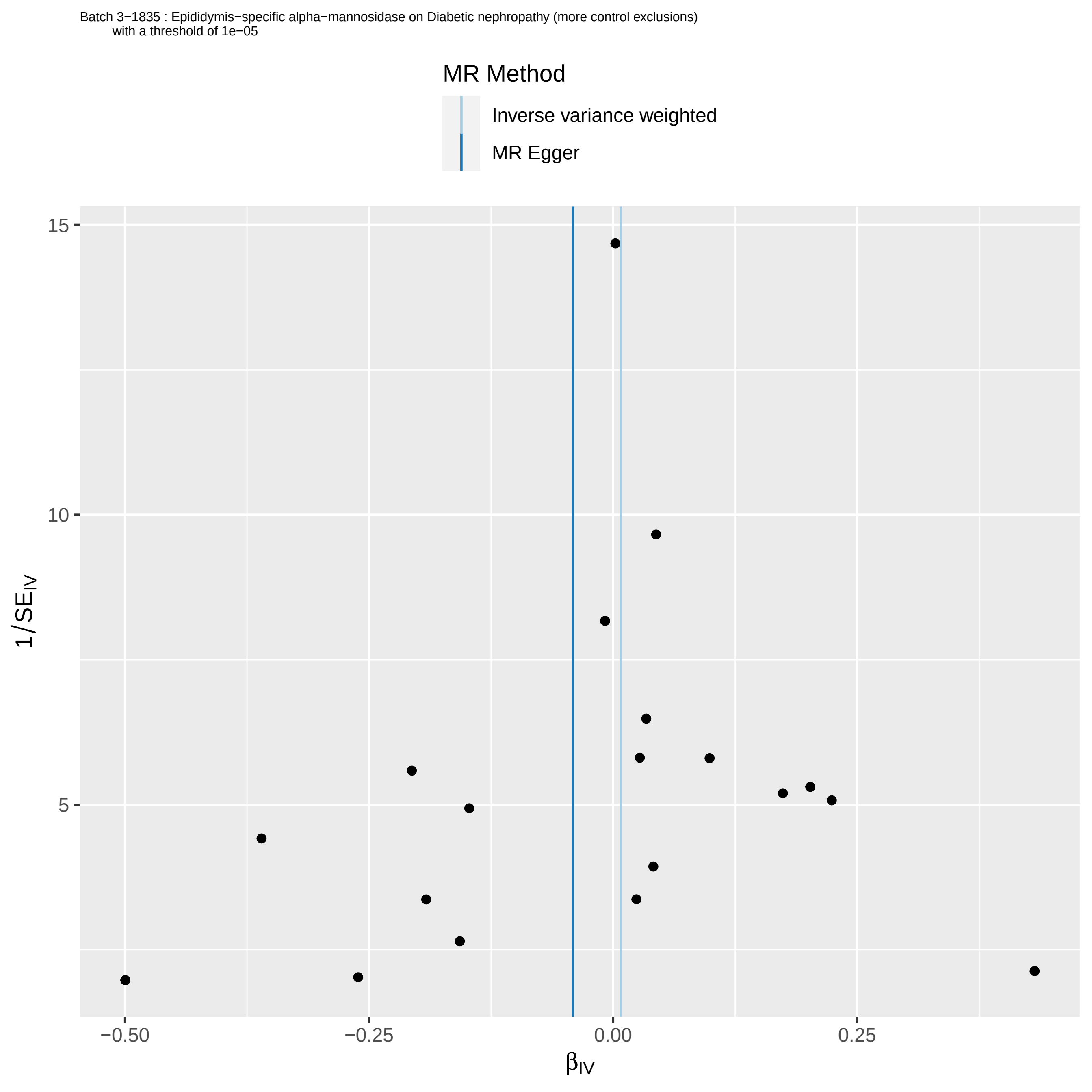
Batch 3−1835 : Epididymis−specific alpha−mannosidase on Diabetic nephropathy (more control exclusions)
with a threshold of 1e−05
MR Method
Inverse variance weighted
MR Egger
15
10
5
1/SE_IV
−0.50
−0.25
0.00
0.25
β_IV

Batch 3−1836 : Beta−mannosidase on Diabetic nephropathy (more control exclusions)
with a threshold of 1e−05

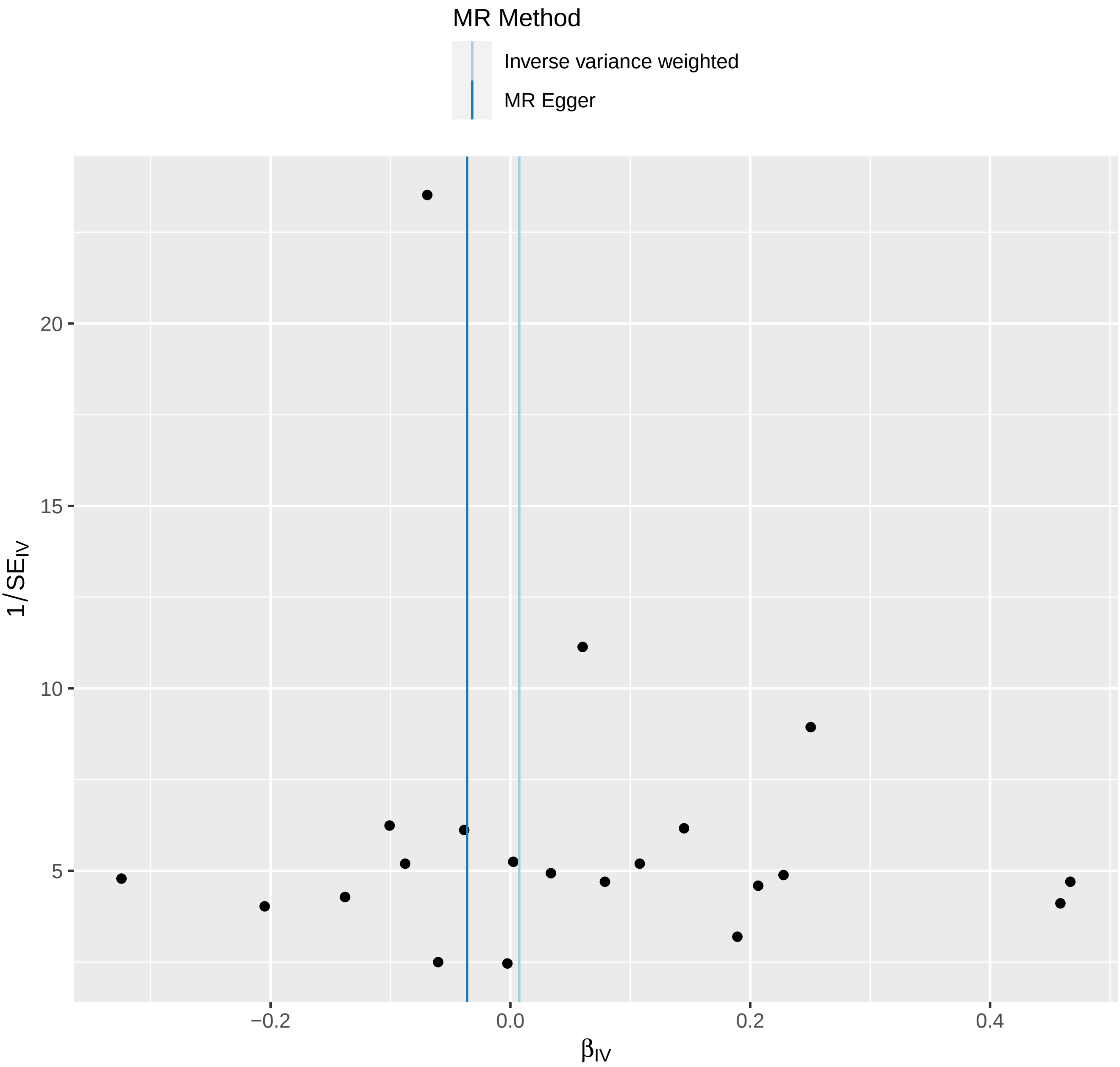

Batch 3−1837 : Glycoprotein endo−alpha−1,2−mannosidase on Diabetic nephropathy (more control exclusions)
with a threshold of 1e−05

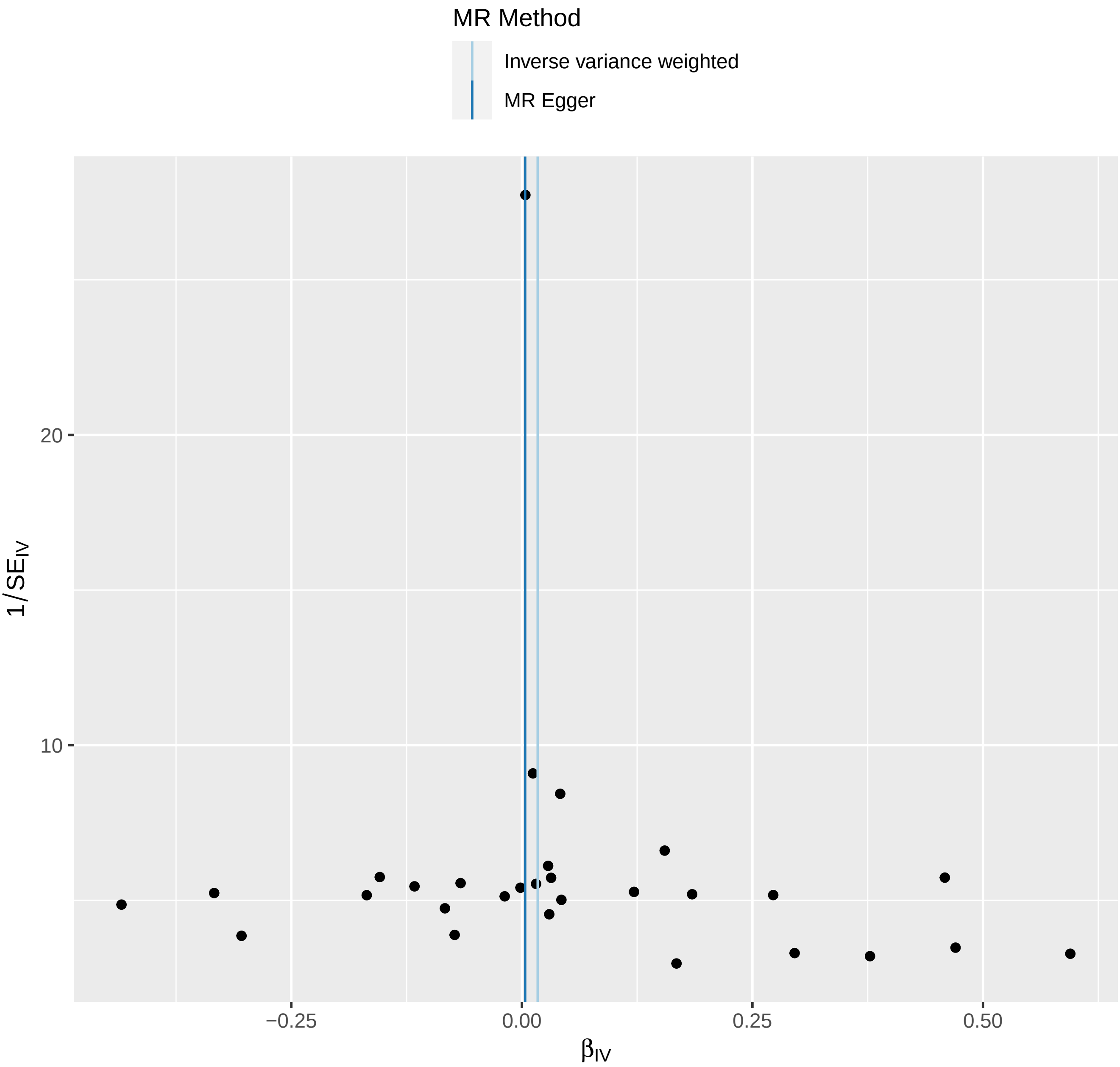

Batch 3−1838 : MANSC domain−containing protein 1 on Diabetic nephropathy (more control exclusions)
with a threshold of 1e−05

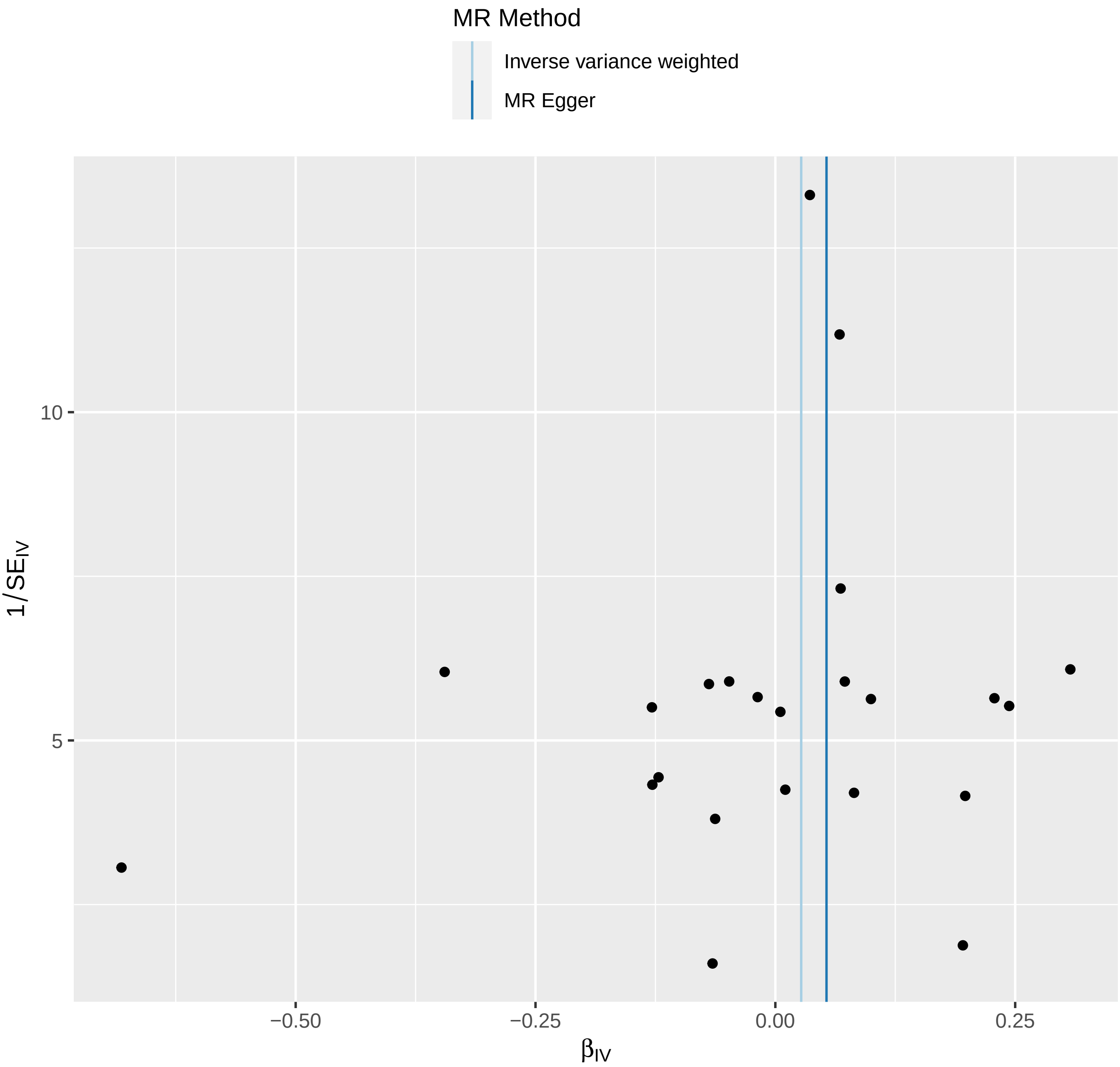

Batch 3−1839 : MANSC domain−containing protein 4 on Diabetic nephropathy (more control exclusions)
with a threshold of 1e−05

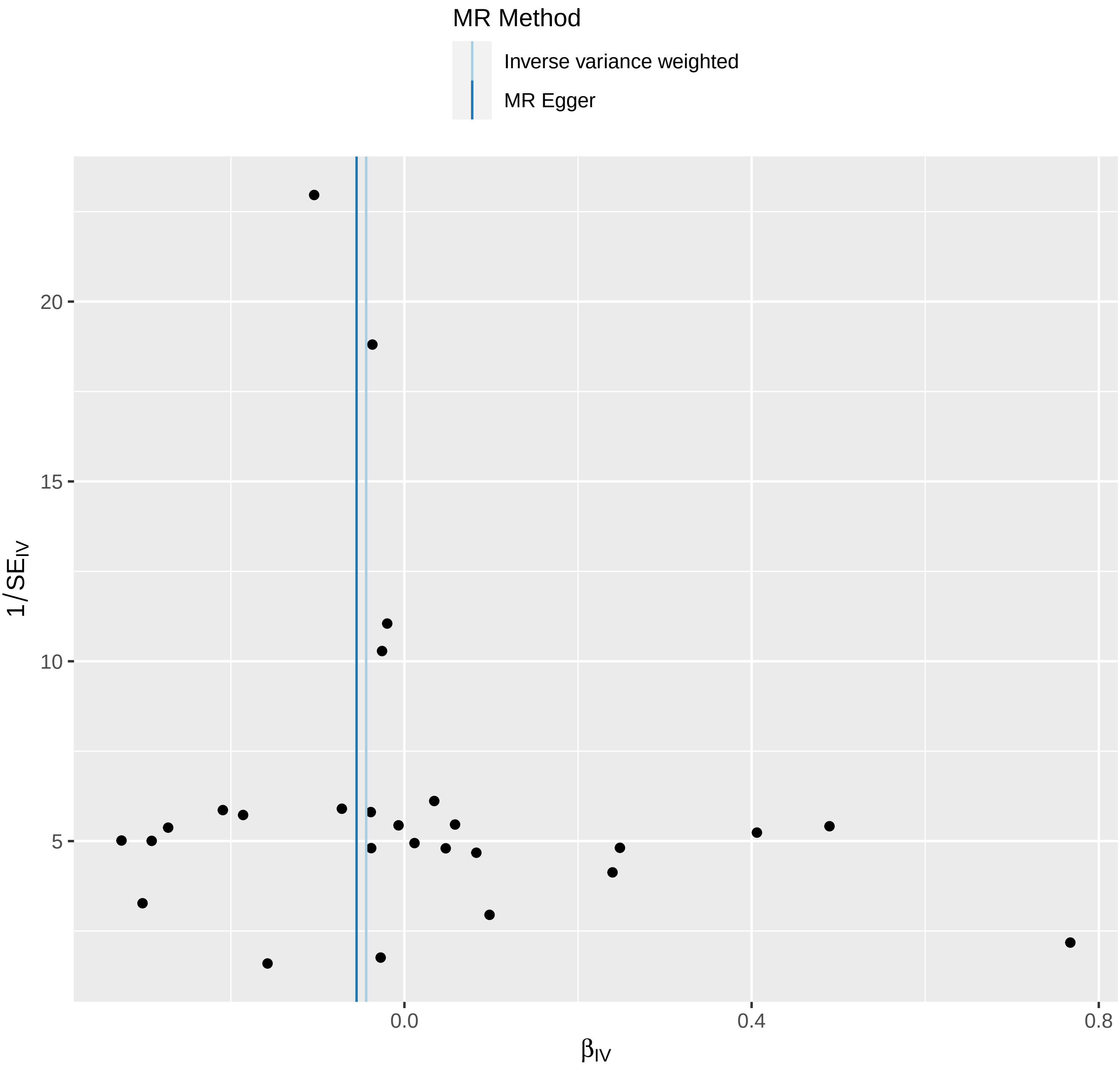

Batch 3−1840 : Dual specificity mitogen−activated protein kinase kinase 1 on Diabetic nephropathy (more control exclusions)
with a threshold of 1e−05

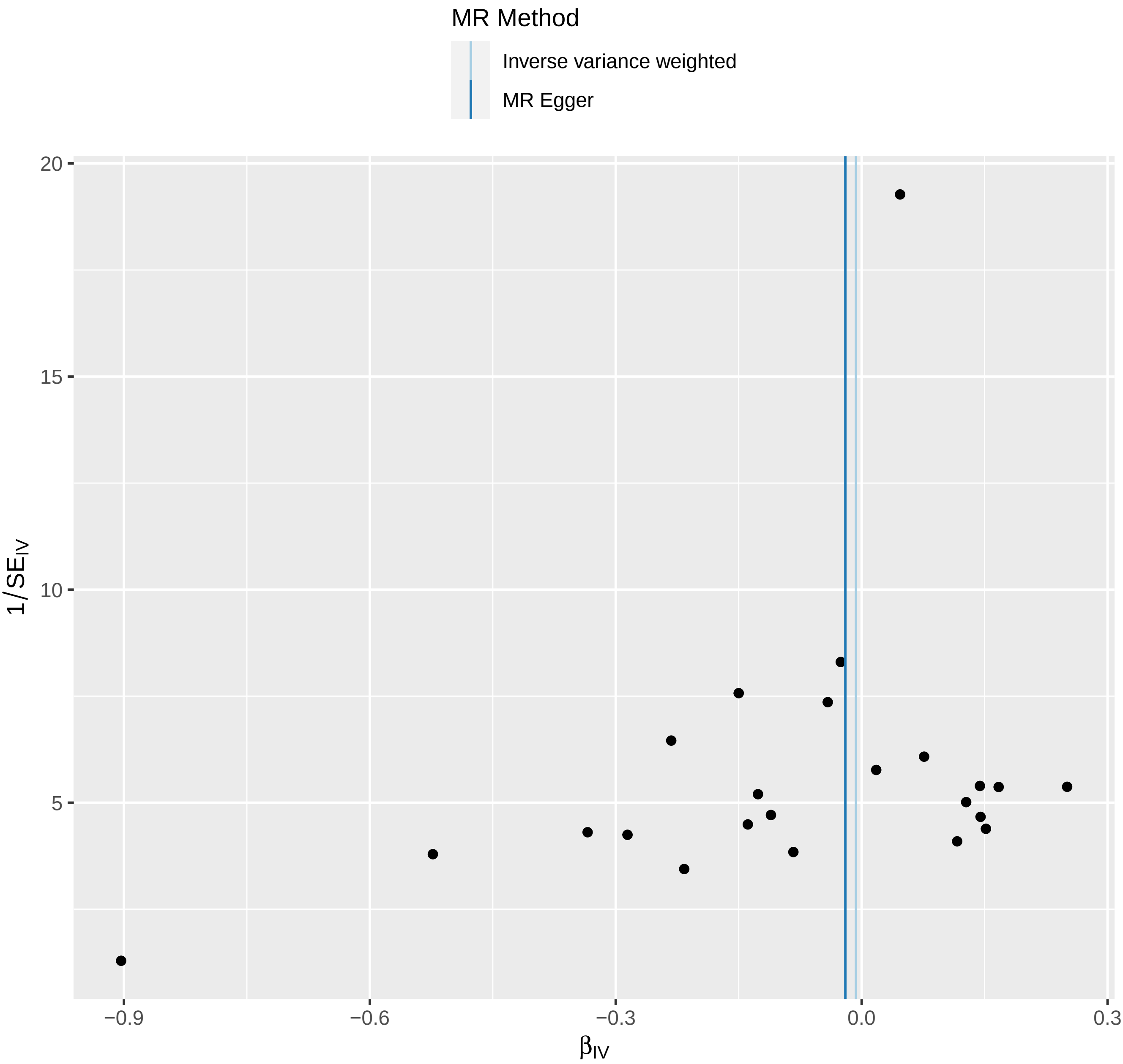

Batch 3−1841 : Dual specificity mitogen−activated protein kinase kinase 3 on Diabetic nephropathy (more control exclusions)
with a threshold of 1e−05

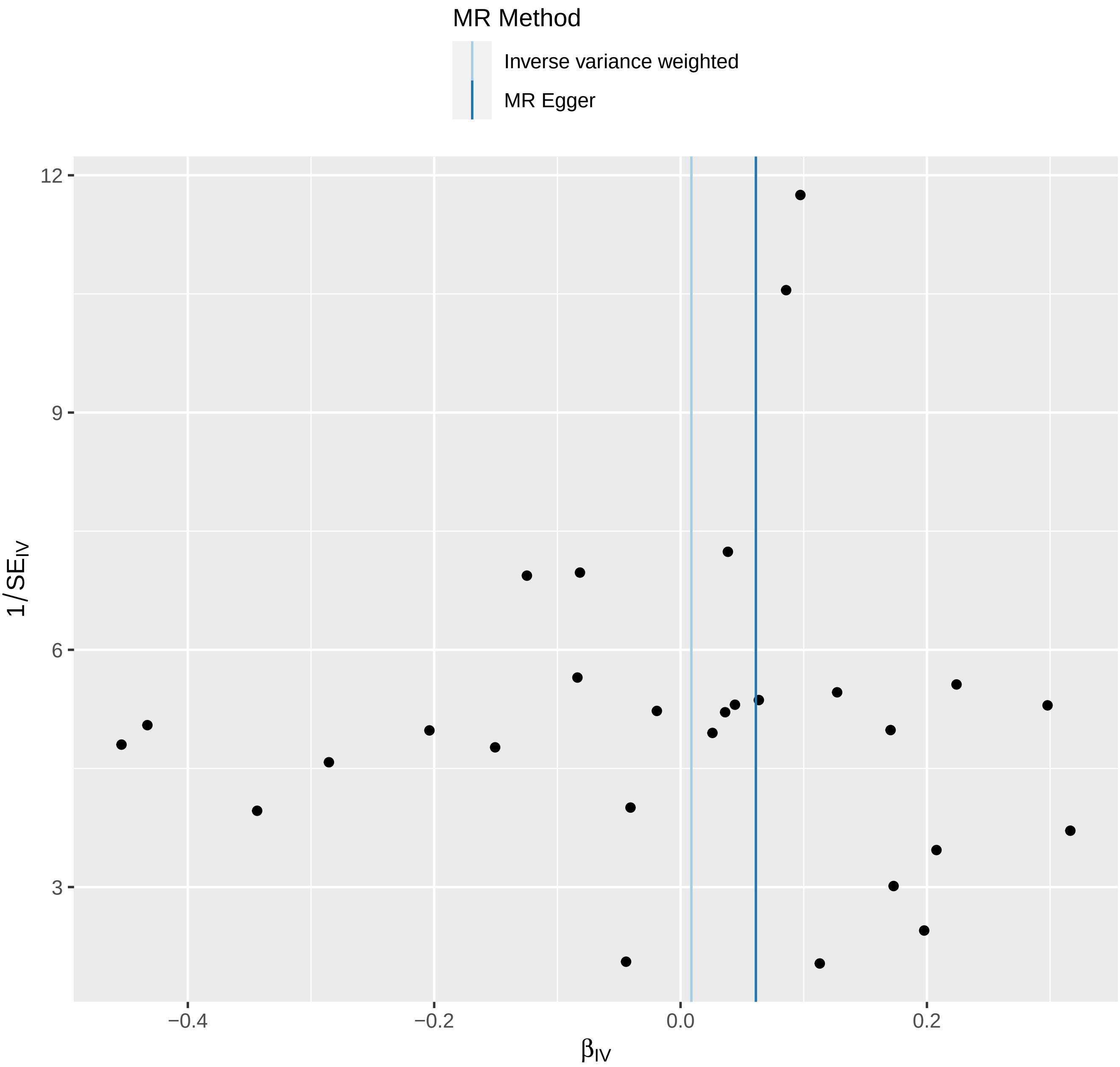

Batch 3−1842 : Dual specificity mitogen−activated protein kinase kinase 4 on Diabetic nephropathy (more control exclusions)
with a threshold of 1e−05

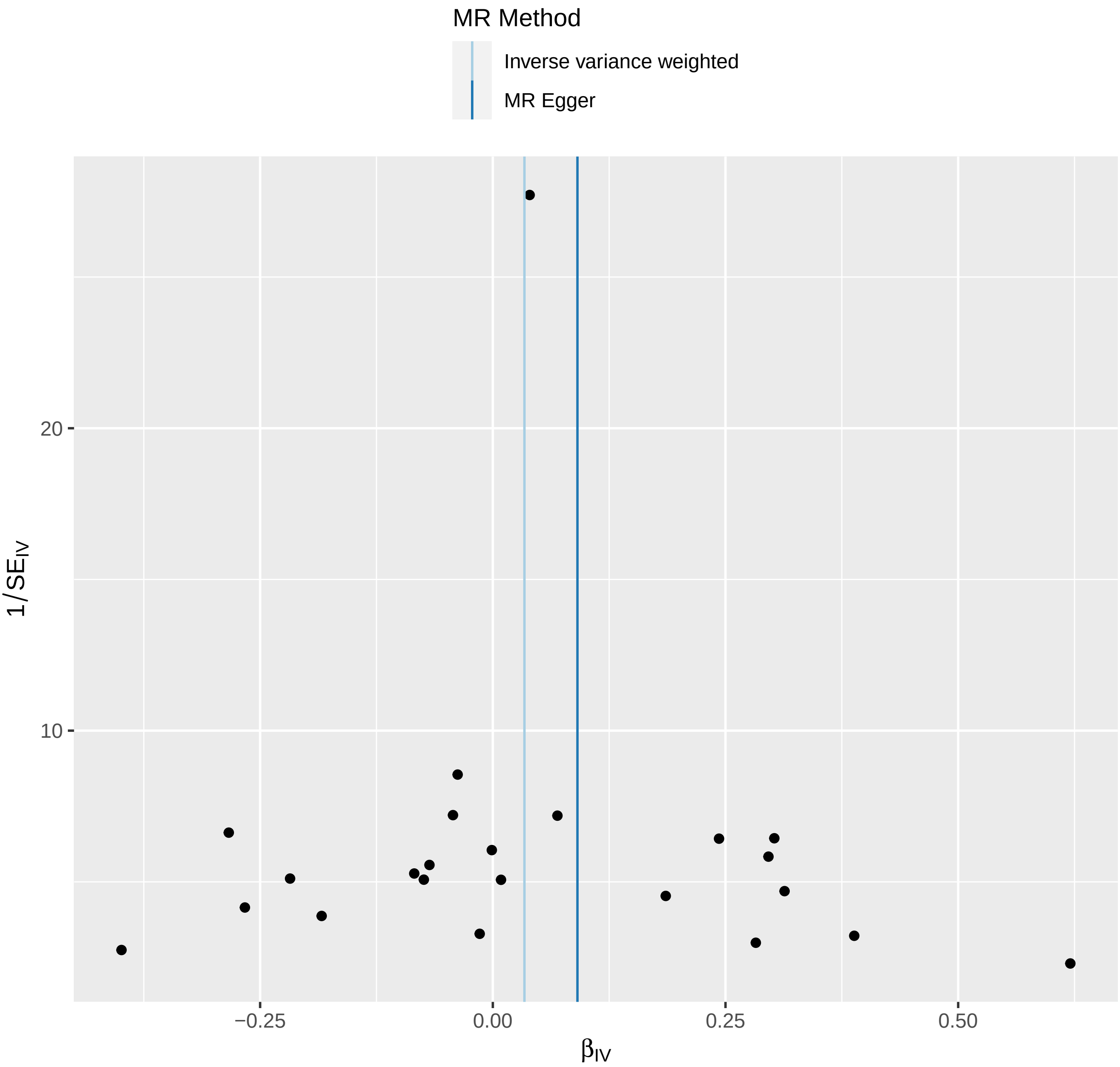

Batch 3−1843 : Mitogen−activated protein kinase kinase kinase 3 on Diabetic nephropathy (more control exclusions)
with a threshold of 1e−05

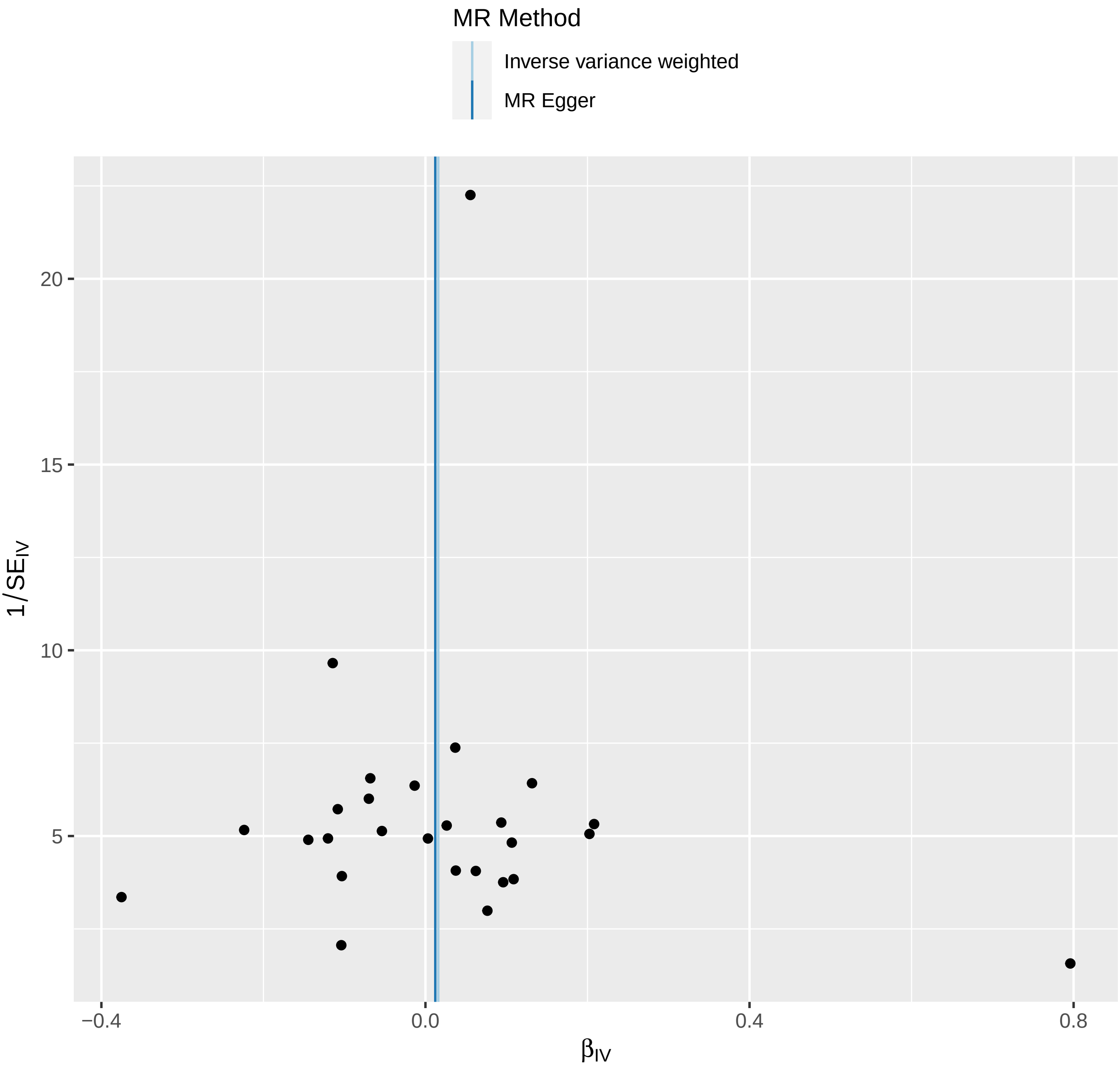

Batch 3−1844 : Mitogen−activated protein kinase kinase kinase 7:TGF−beta−activated kinase 1 and MAP3K7−binding protein 1 fusion on Diabetic nephropathy (more control exclu
with a threshold of 1e−05

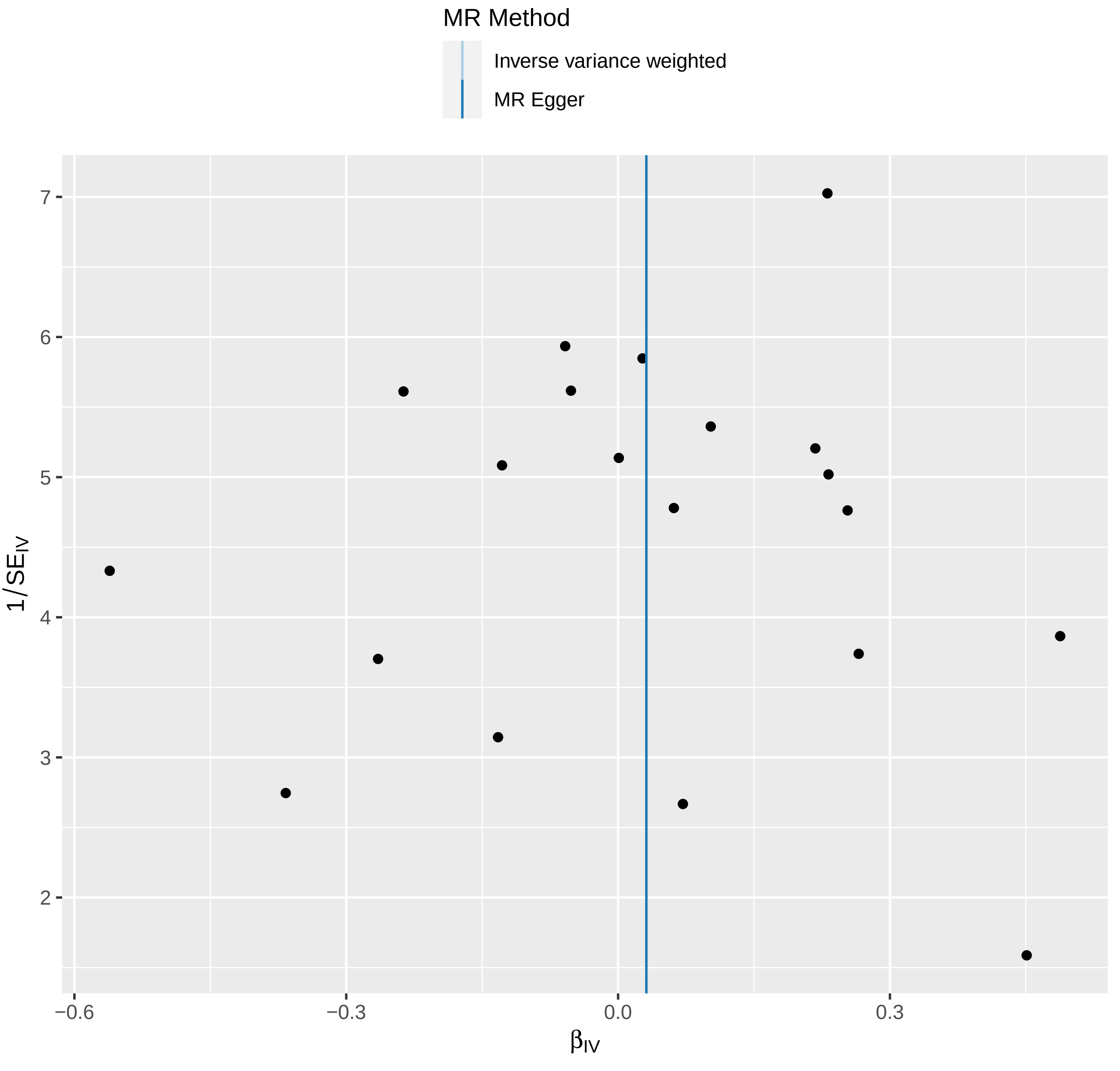

Batch 3−1845 : Mitogen−activated protein kinase 1 on Diabetic nephropathy (more control exclusions)
with a threshold of 1e−05

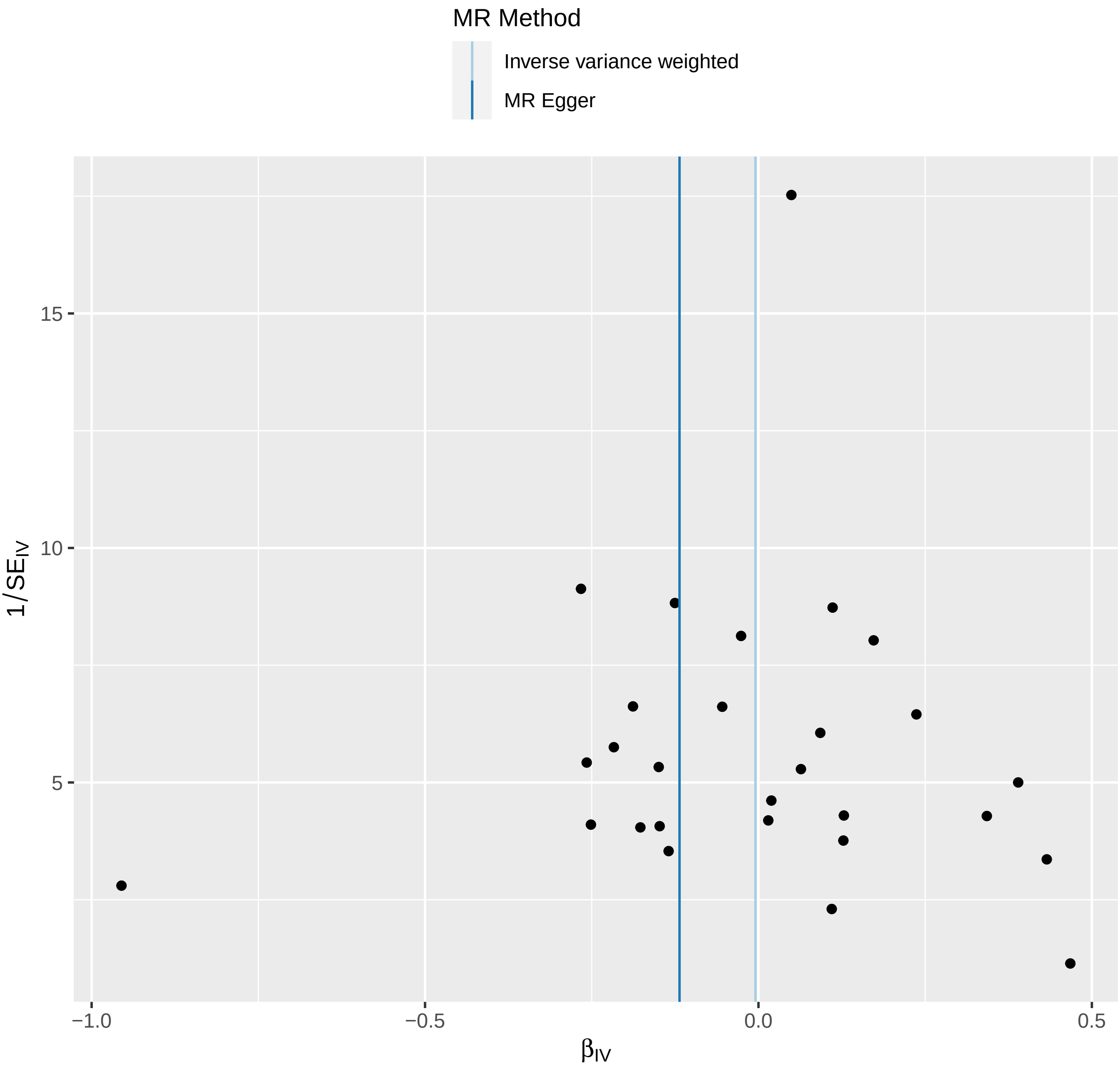

Batch 3−1846 : Mitogen−activated protein kinase 13 on Diabetic nephropathy (more control exclusions)
with a threshold of 1e−05

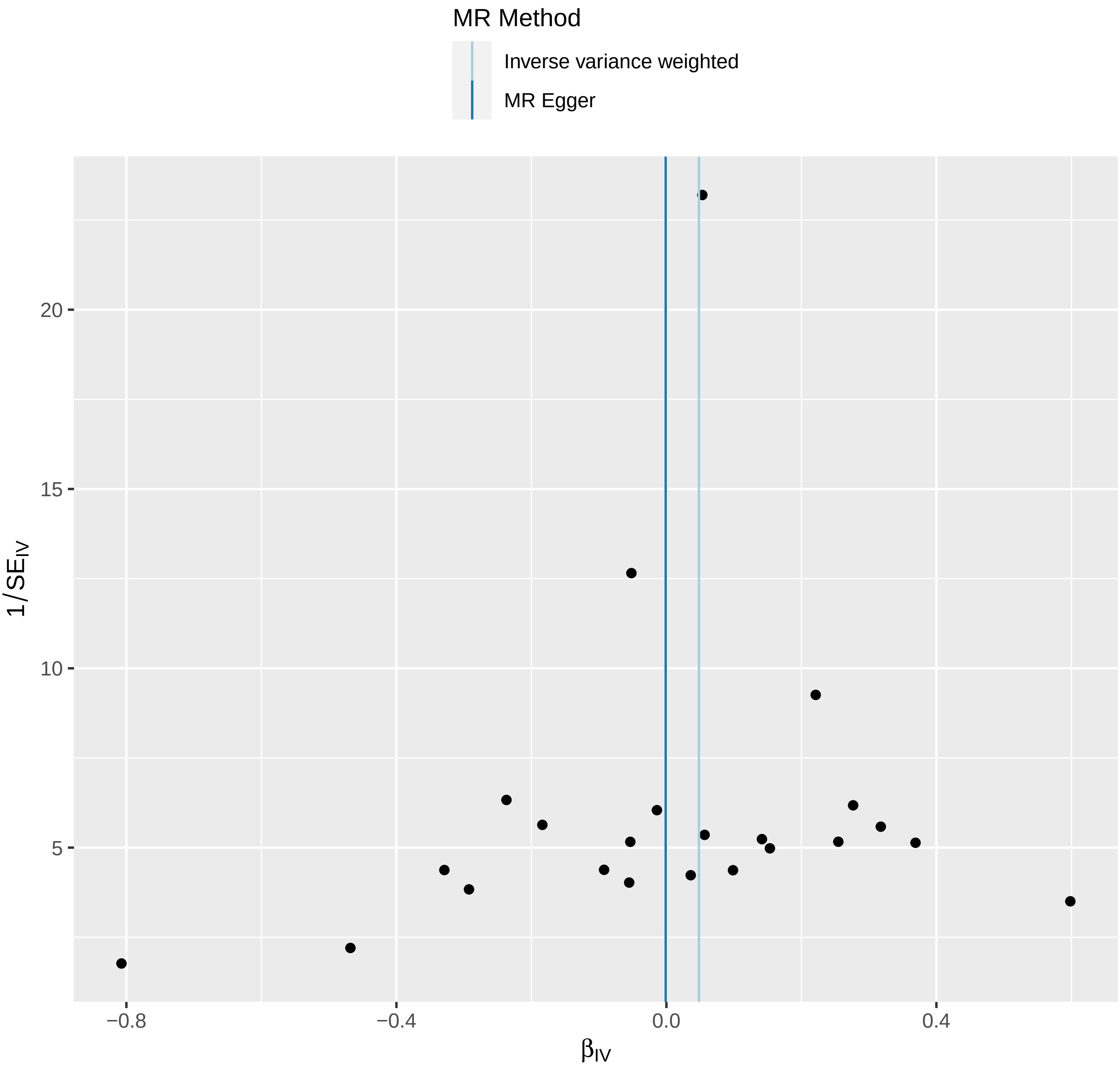

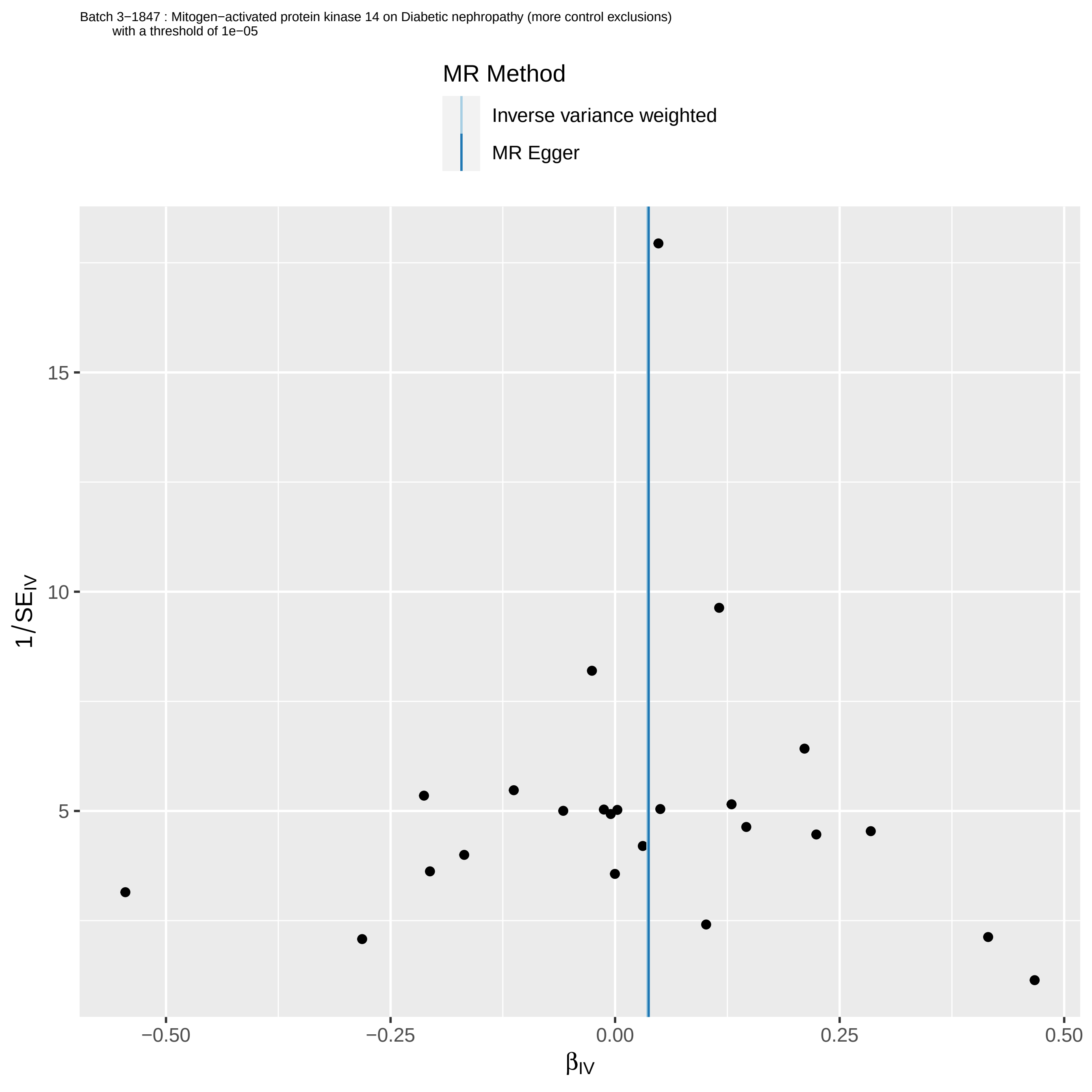

Batch 3−1847 : Mitogen−activated protein kinase 14 on Diabetic nephropathy (more control exclusions)
with a threshold of 1e−05
MR Method
Inverse variance weighted
MR Egger
15
10
5
−0.50
−0.25
0.00
0.25
0.50
1/SE_IV
β_IV

Batch 3−1848 : Mitogen−activated protein kinase 3 on Diabetic nephropathy (more control exclusions) with a threshold of 1e−05

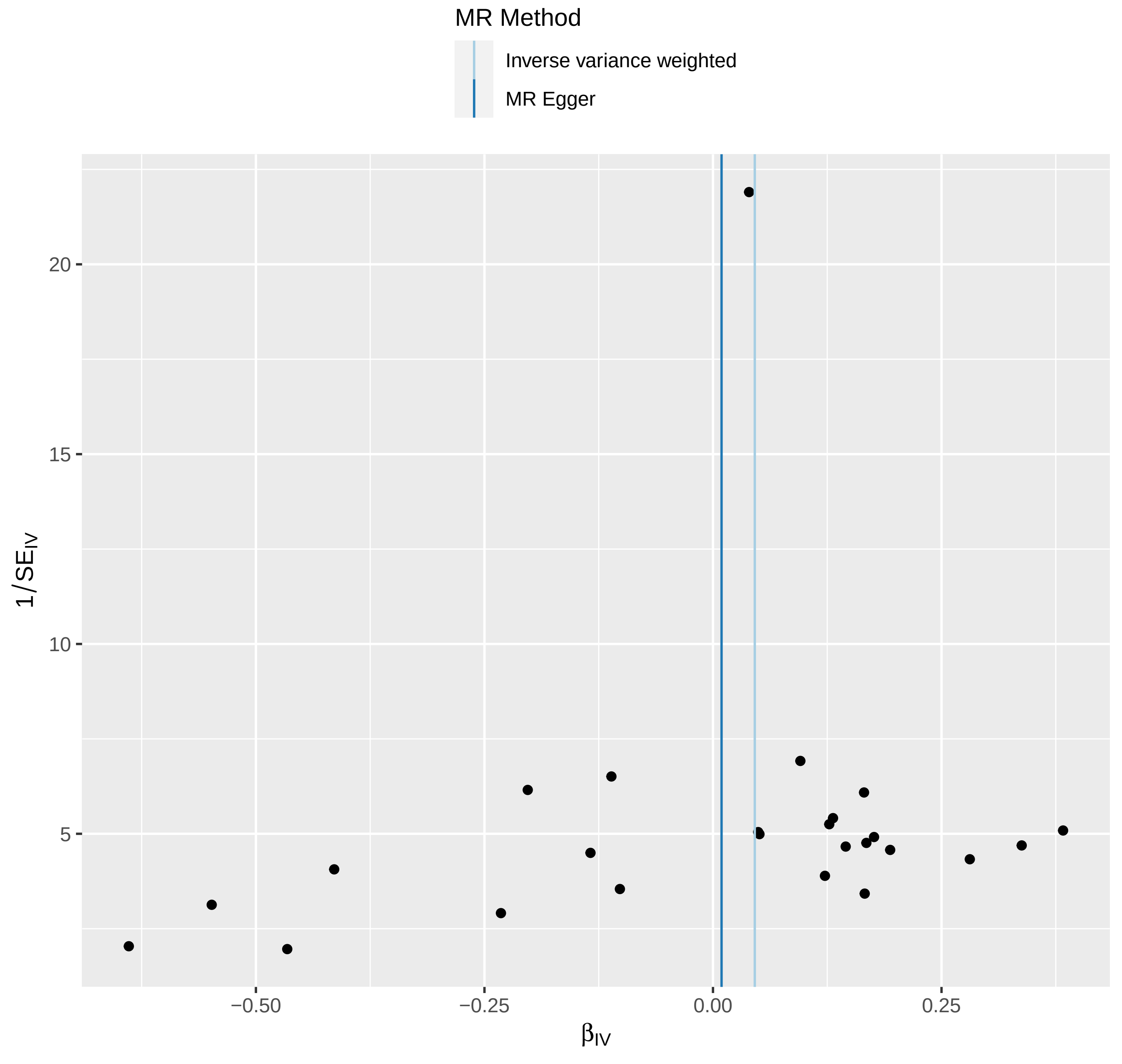

Batch 3−1849 : Mitogen−activated protein kinase 8 on Diabetic nephropathy (more control exclusions)
with a threshold of 1e−05

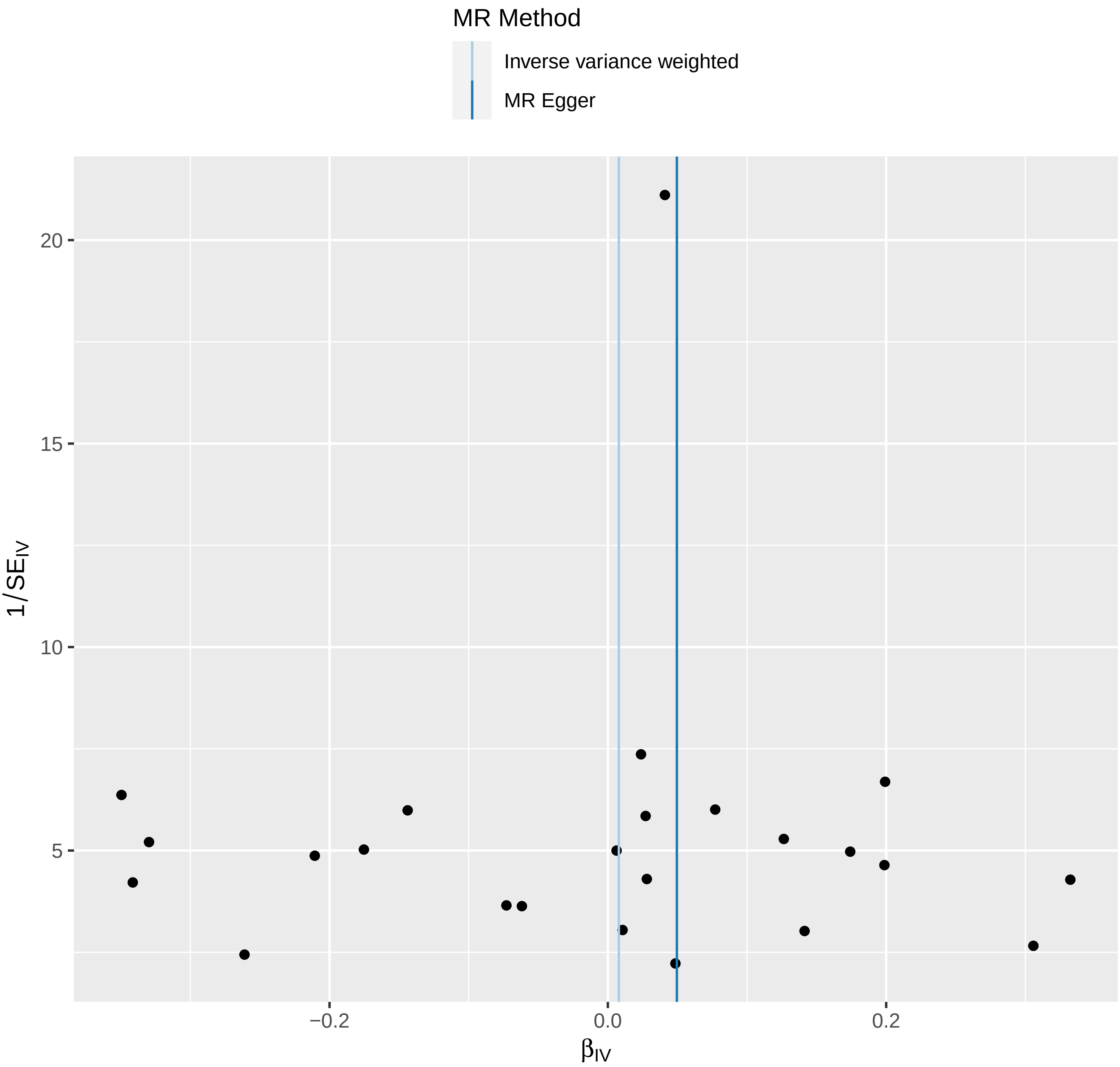

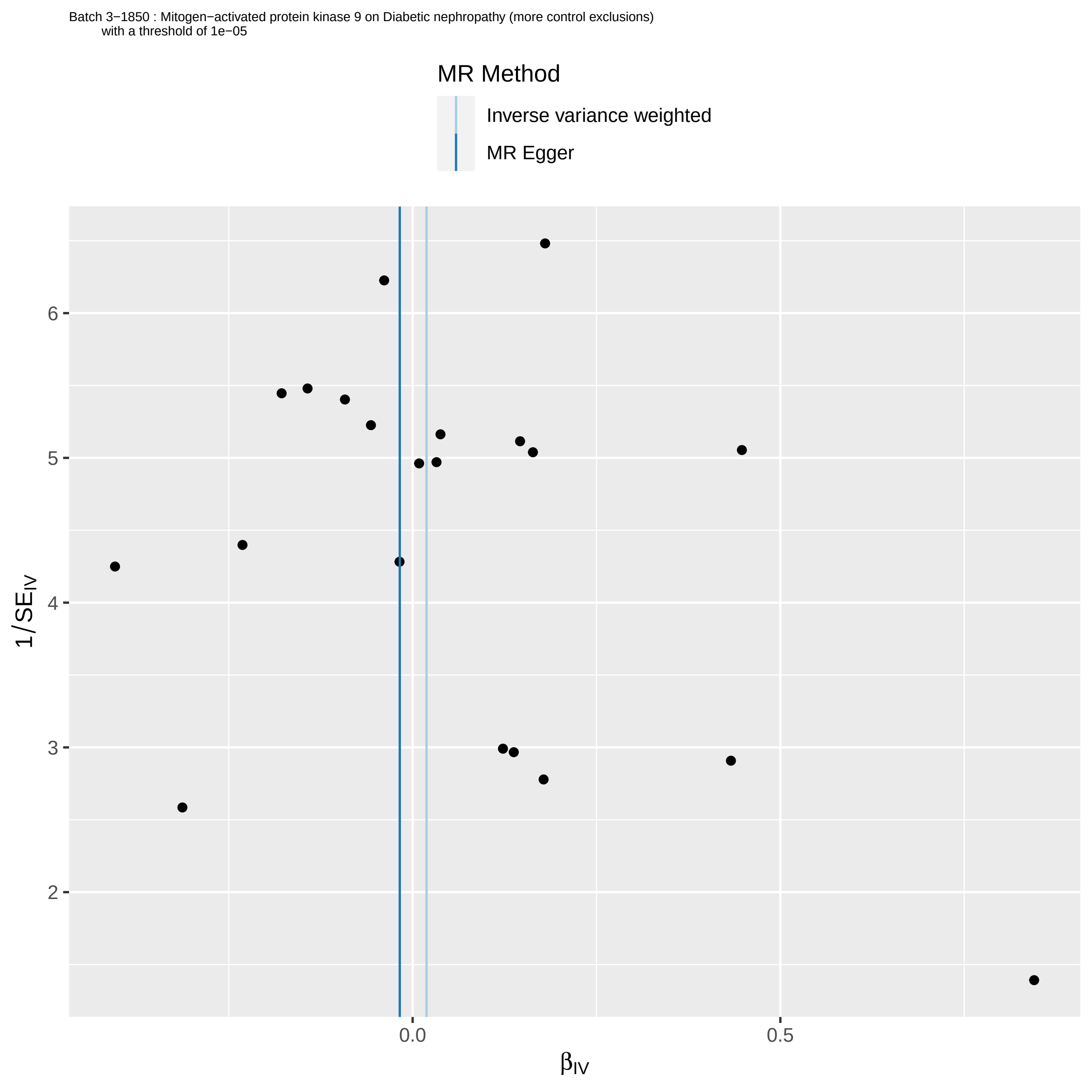
Batch 3−1850 : Mitogen−activated protein kinase 9 on Diabetic nephropathy (more control exclusions)
with a threshold of 1e−05
MR Method
Inverse variance weighted
MR Egger
6
5
4
3
2
1/SE_IV
0.0
0.5
β_IV

Batch 3−1851 : MAP kinase−activated protein kinase 2 on Diabetic nephropathy (more control exclusions)
with a threshold of 1e−05

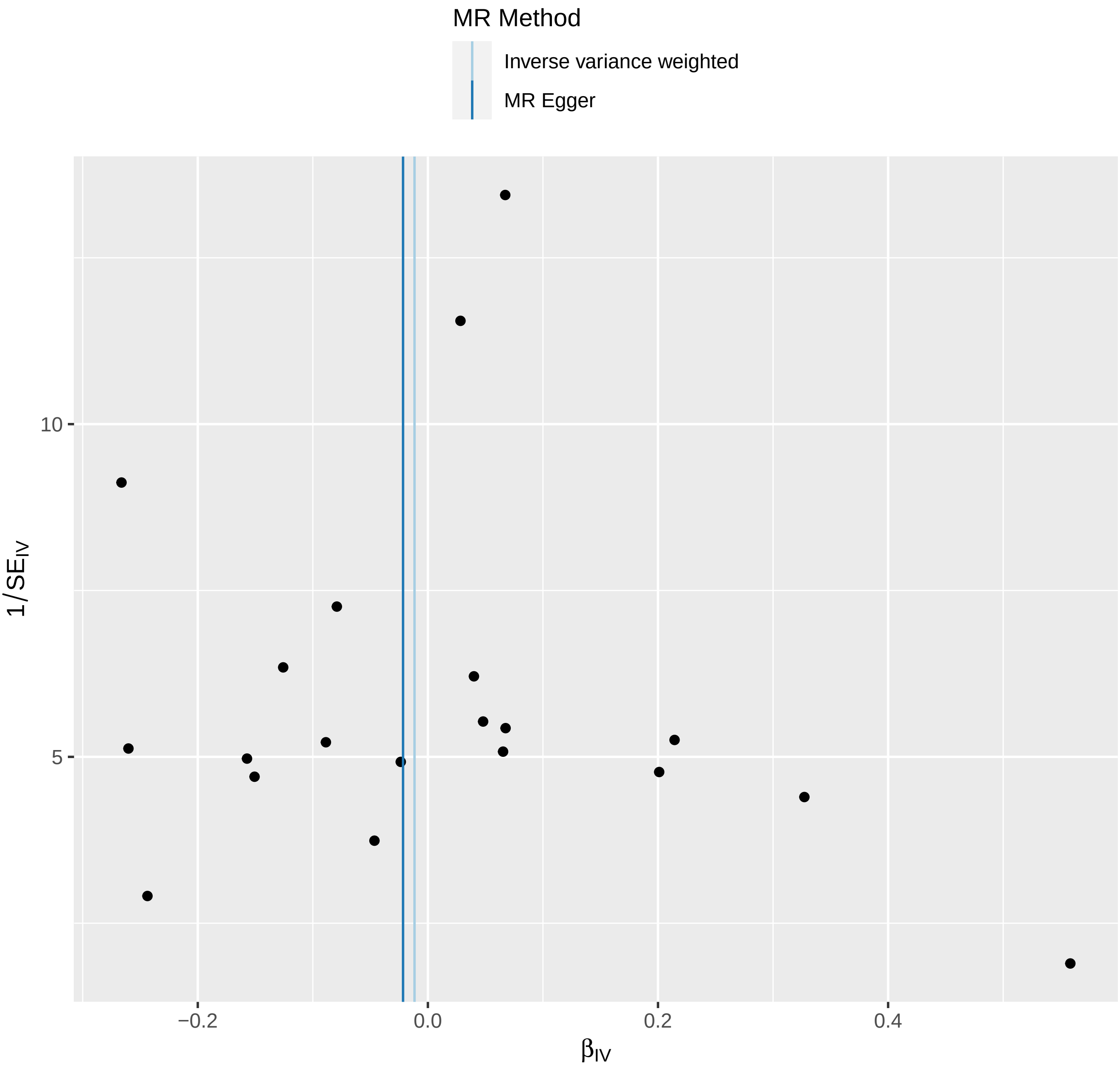

Batch 3−1852 : MAP kinase−activated protein kinase 3 on Diabetic nephropathy (more control exclusions)
with a threshold of 1e−05

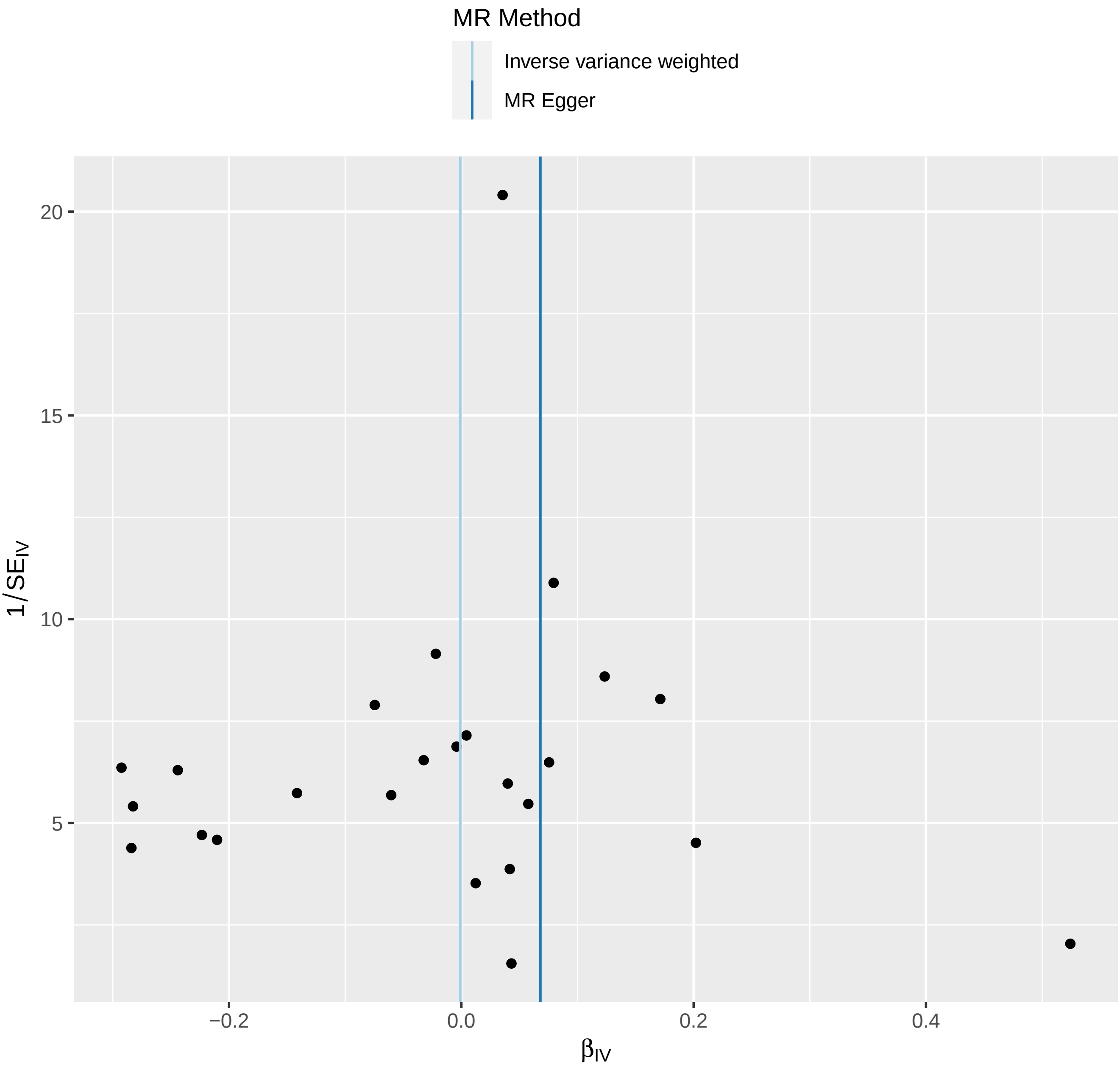

Batch 3−1853 : MAP kinase−activated protein kinase 5 on Diabetic nephropathy (more control exclusions)
with a threshold of 1e−05

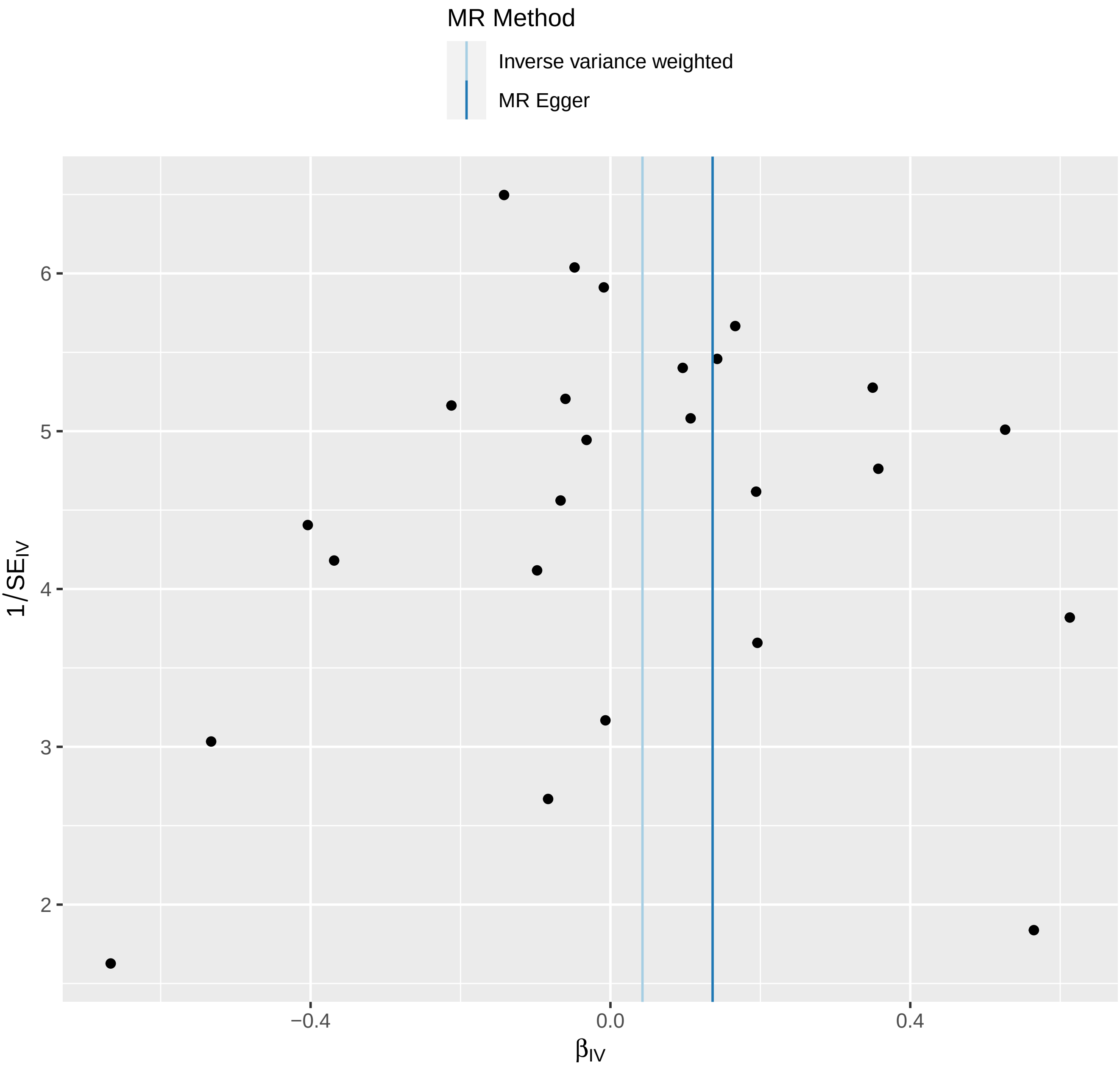

Batch 3−1854 : MAP kinase−activated protein kinase 5 on Diabetic nephropathy (more control exclusions)
with a threshold of 1e−05

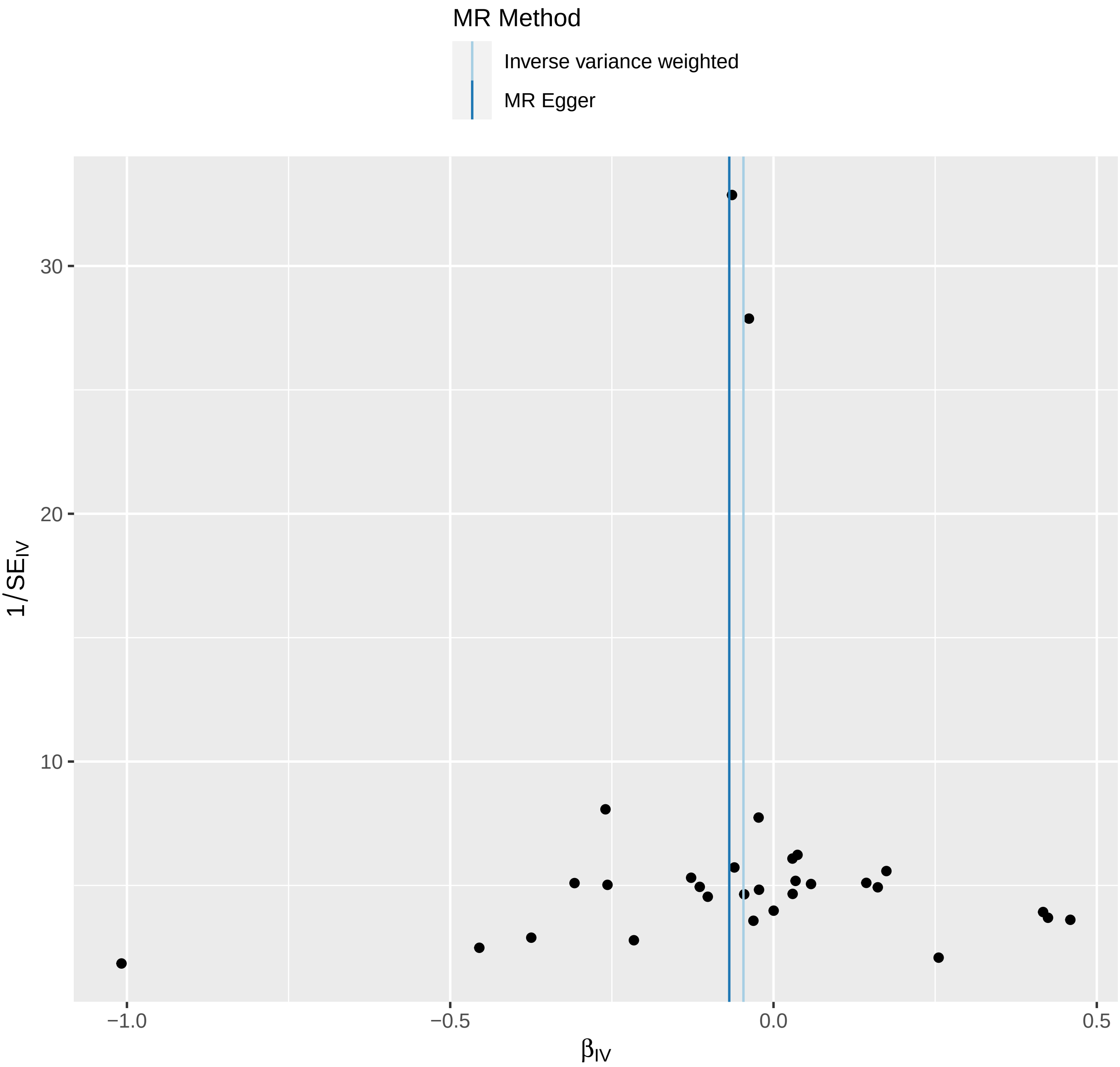

Batch 3−1855 : Mannan−binding lectin serine protease 1 on Diabetic nephropathy (more control exclusions)
with a threshold of 1e−05

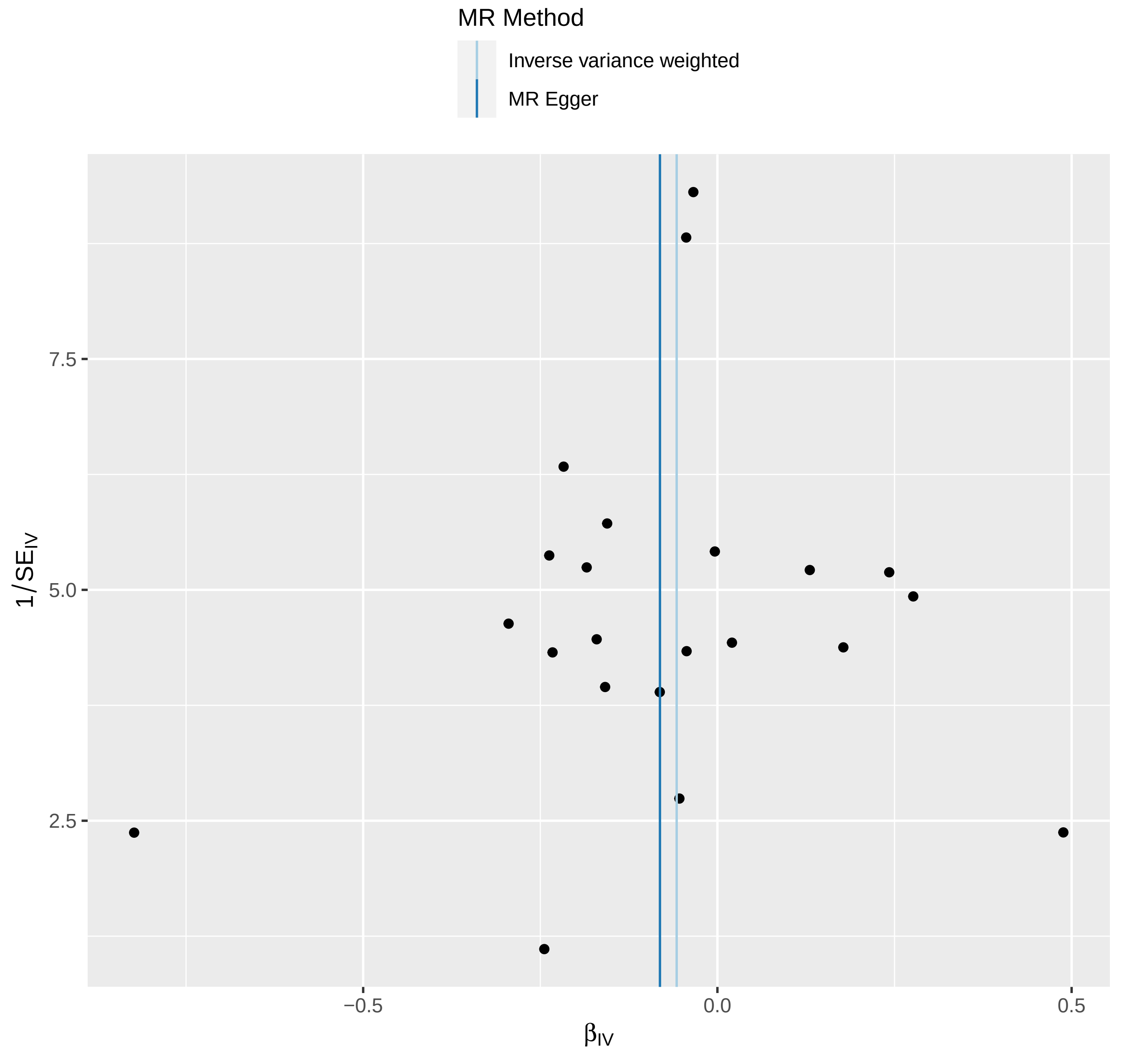

Batch 3−1856 : Megakaryocyte−associated tyrosine−protein kinase on Diabetic nephropathy (more control exclusions) with a threshold of 1e−05

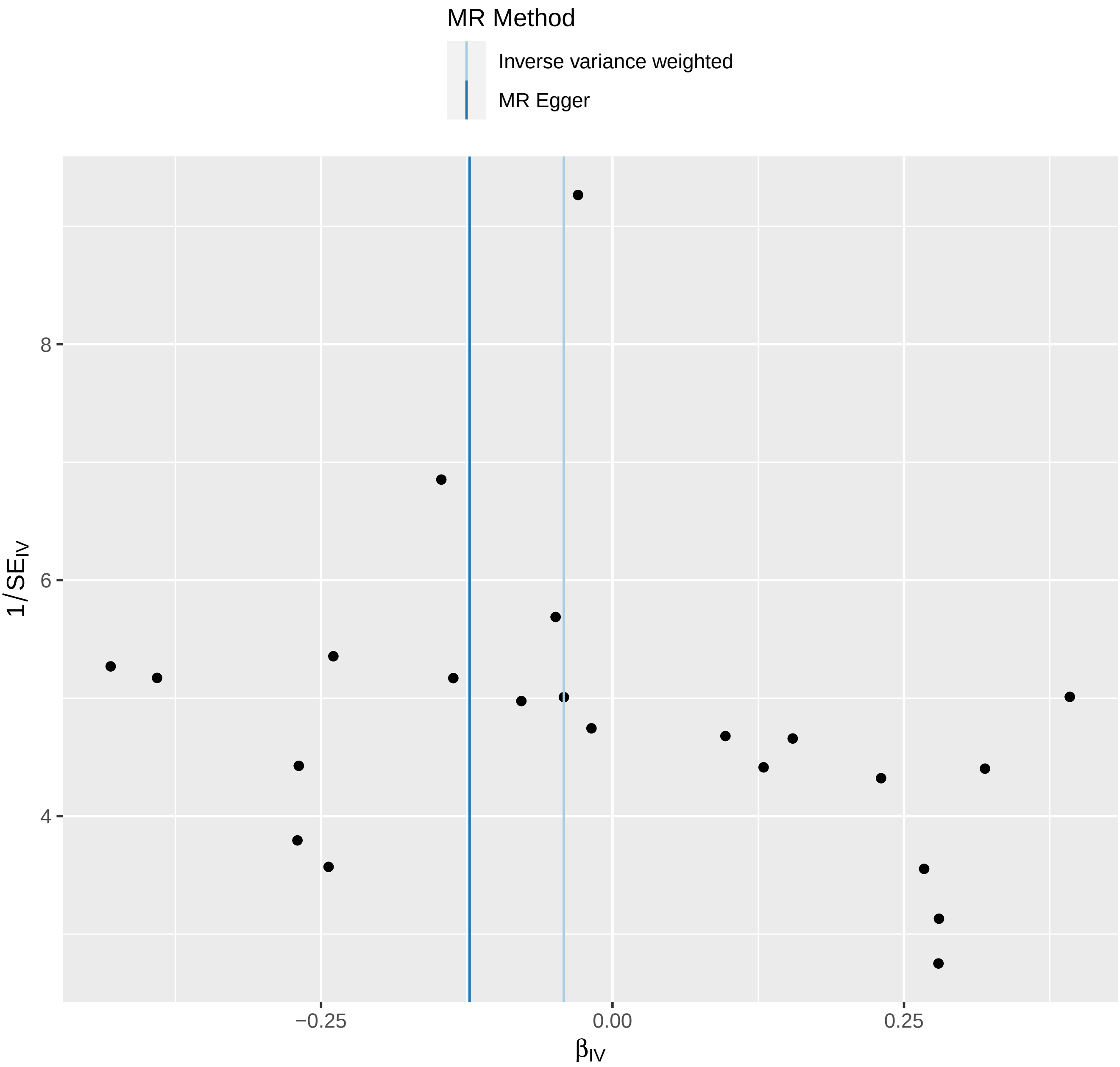

Batch 3−1857 : Matrilin−2 on Diabetic nephropathy (more control exclusions)
with a threshold of 1e−05

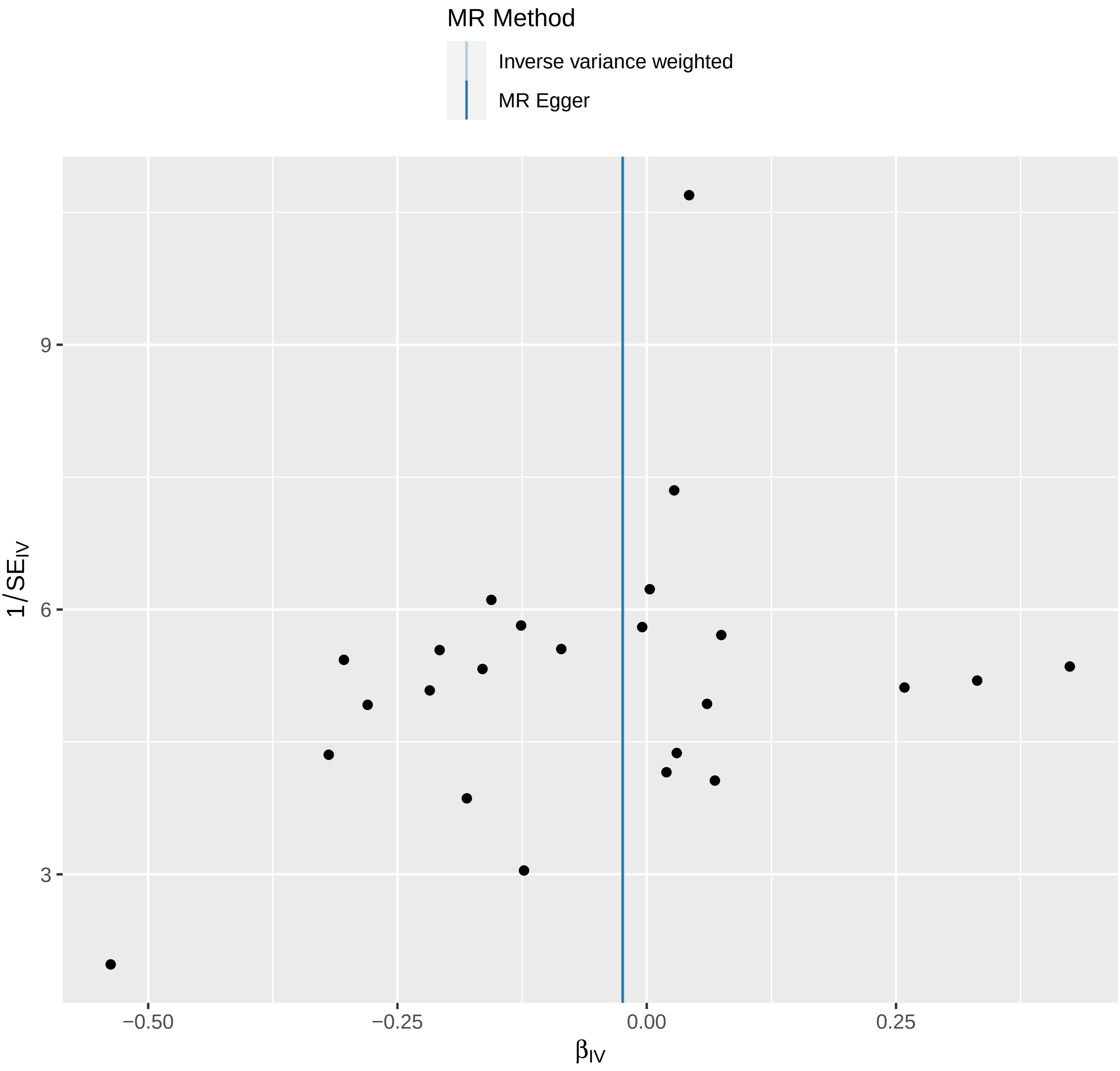

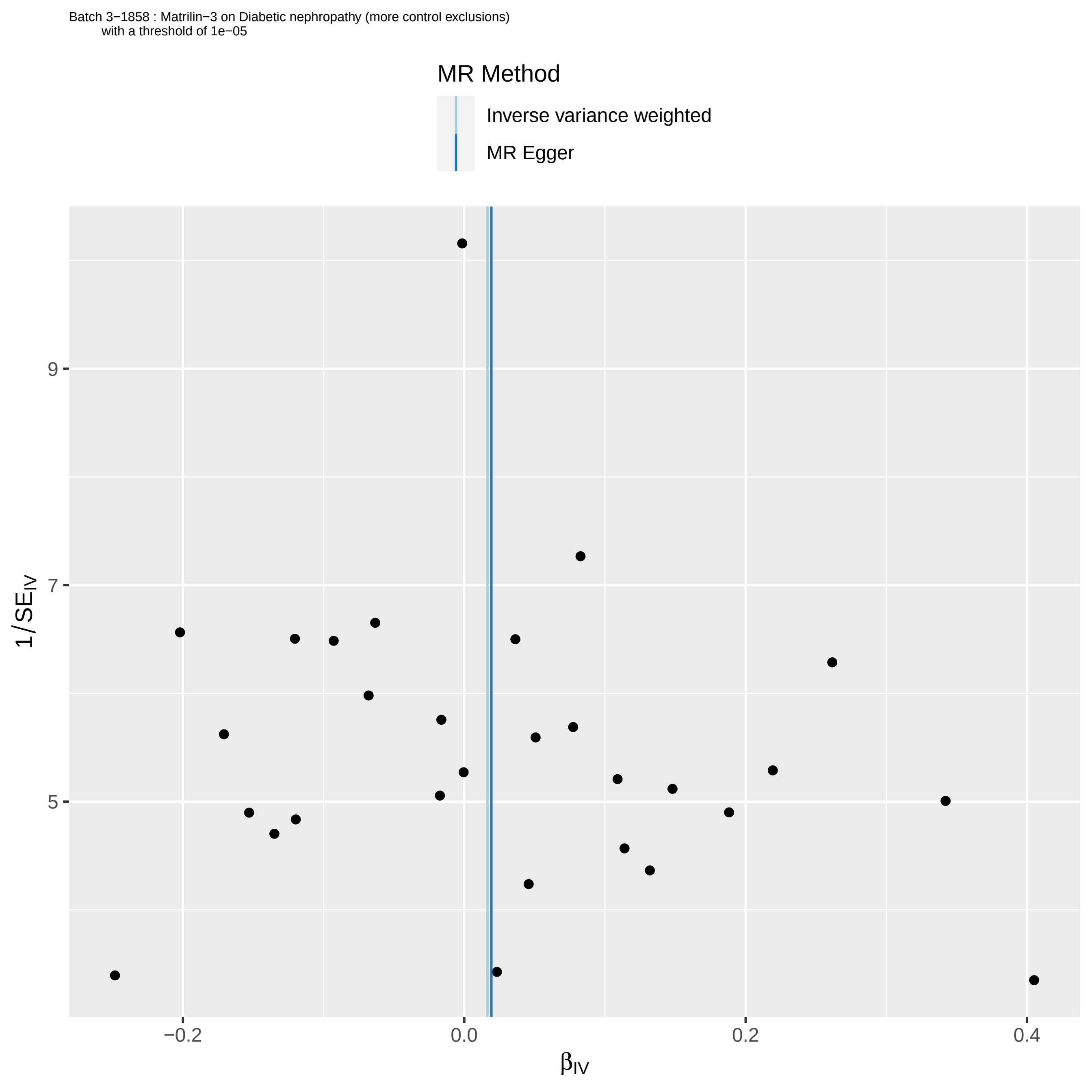
Batch 3−1858 : Matrilin−3 on Diabetic nephropathy (more control exclusions)
with a threshold of 1e−05
MR Method
Inverse variance weighted
MR Egger
9
7
5
1/SE$_{IV}$
−0.2
0.0
0.2
0.4
β_{IV}

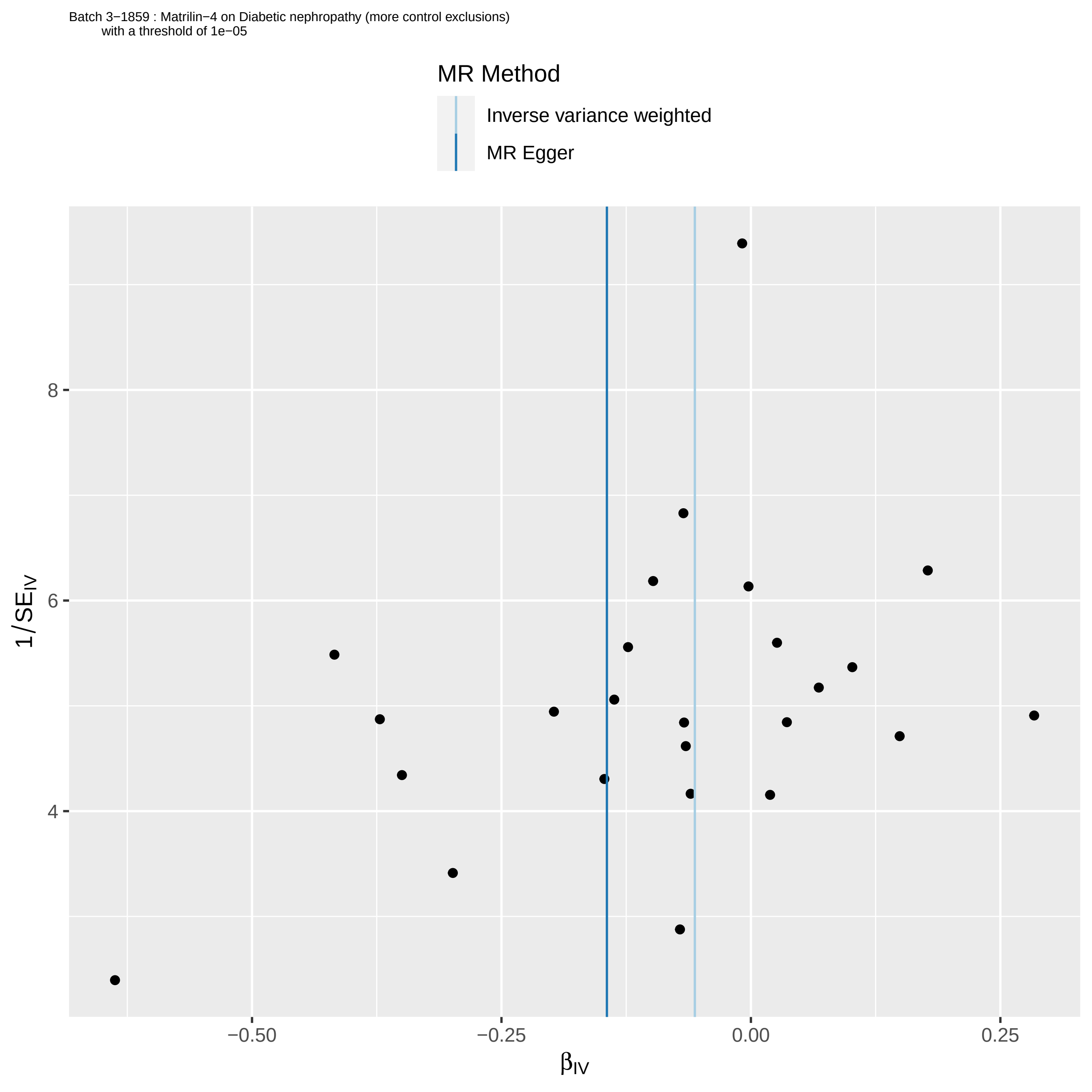
Batch 3−1859 : Matrilin−4 on Diabetic nephropathy (more control exclusions)
with a threshold of 1e−05
MR Method
Inverse variance weighted
MR Egger
8
6
4
$1/SE_{IV}$
−0.50
−0.25
0.00
0.25
β_{IV}

Batch 3−1860 : Myc−associated zinc finger protein on Diabetic nephropathy (more control exclusions) with a threshold of 1e−05

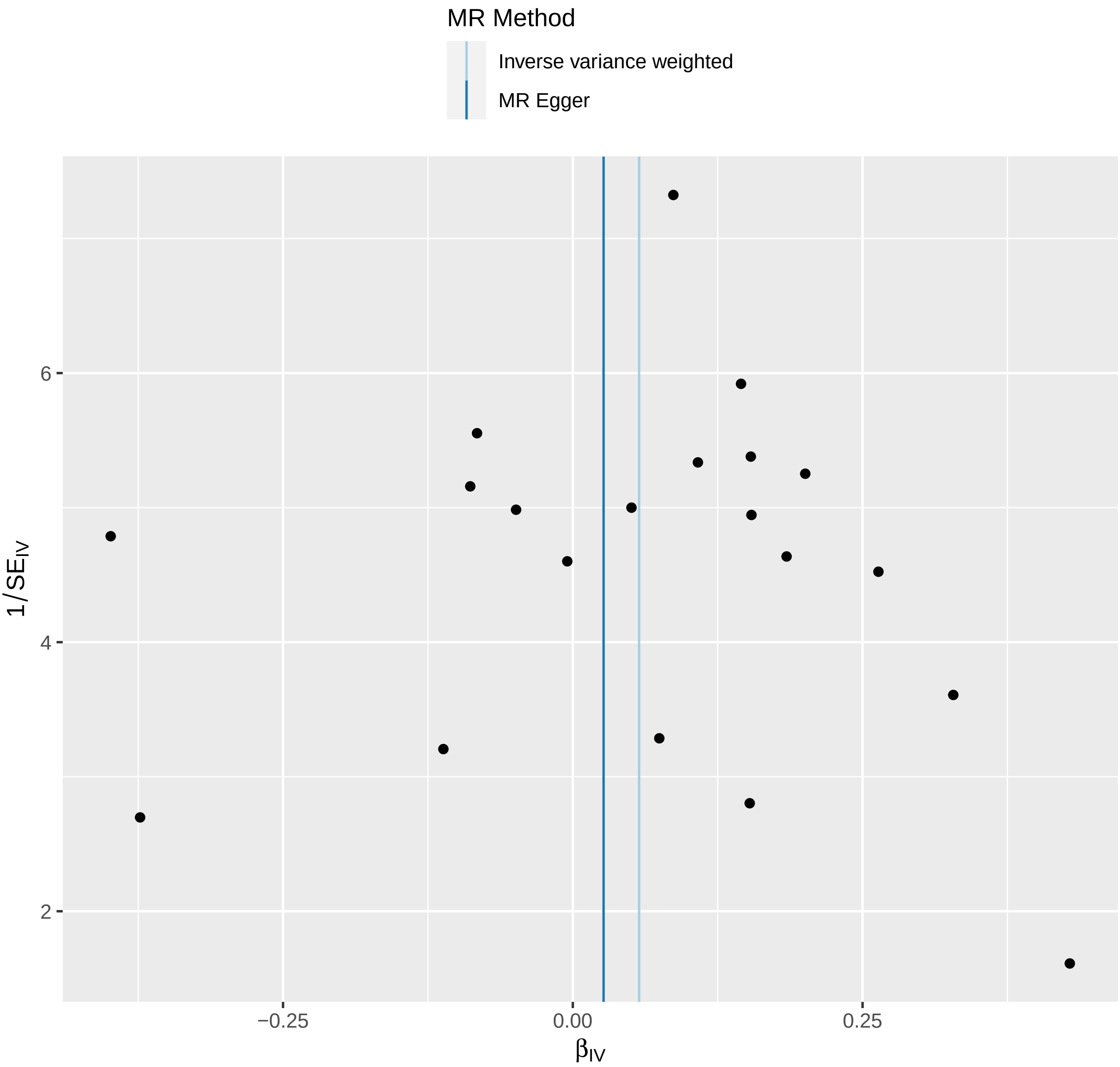

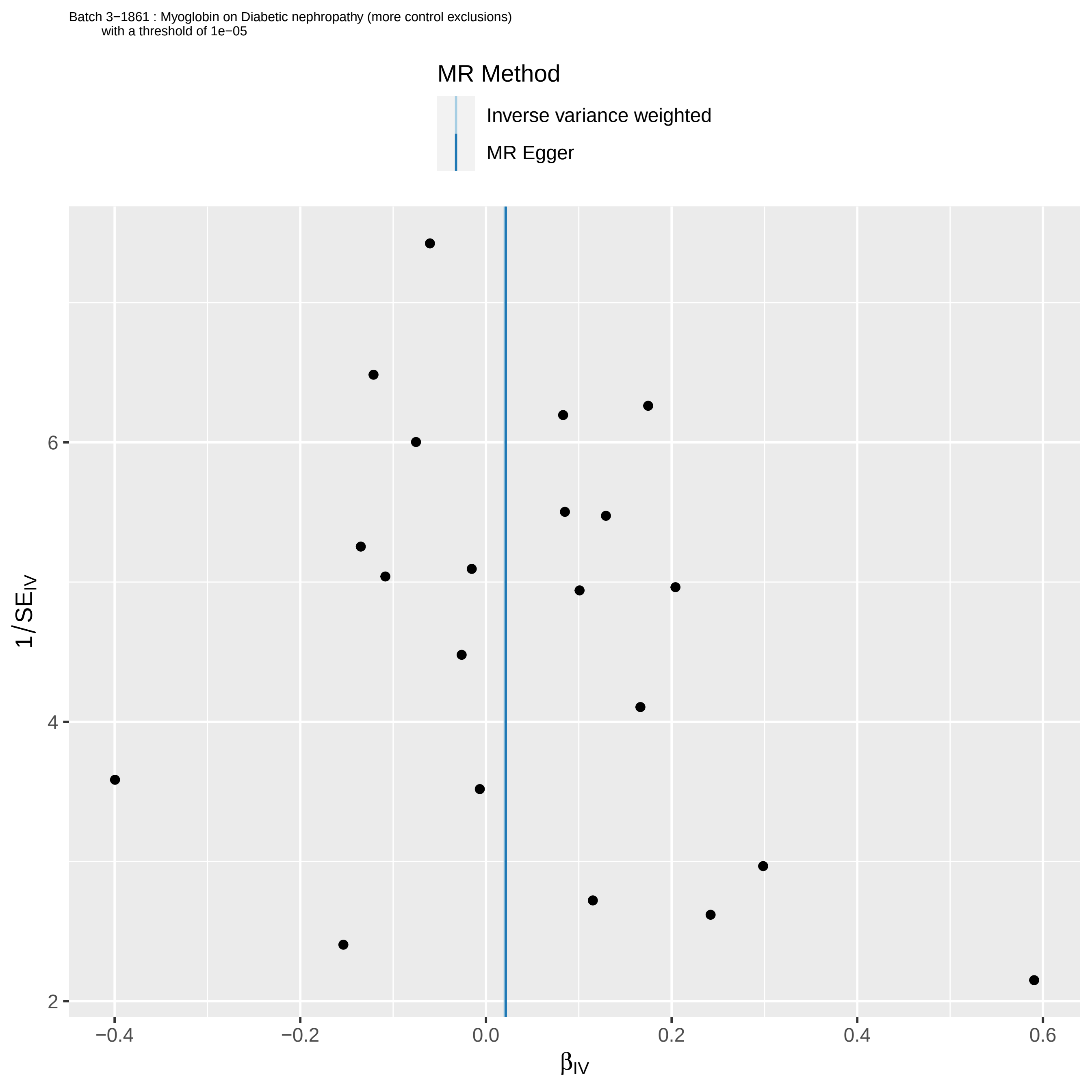
Batch 3−1861 : Myoglobin on Diabetic nephropathy (more control exclusions)
with a threshold of 1e−05
MR Method
Inverse variance weighted
MR Egger
1/SE$_{IV}$
6
4
2
−0.4
−0.2
0.0
0.2
0.4
0.6
β$_{IV}$

Batch 3−1862 : Methyl−CpG−binding domain protein 4 on Diabetic nephropathy (more control exclusions)
with a threshold of 1e−05

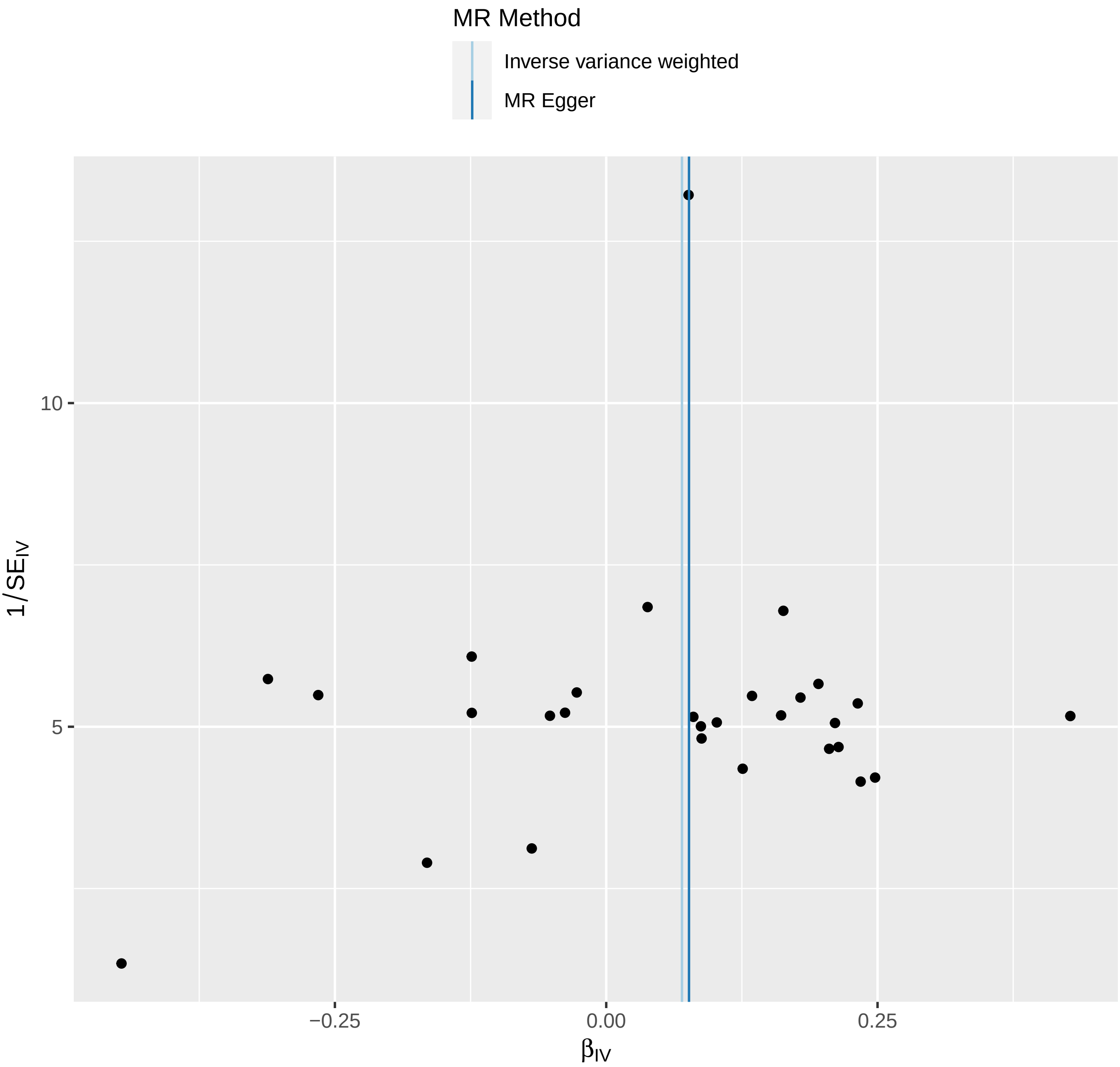

Batch 3−1863 : Mannose−binding protein C on Diabetic nephropathy (more control exclusions)
with a threshold of 1e−05

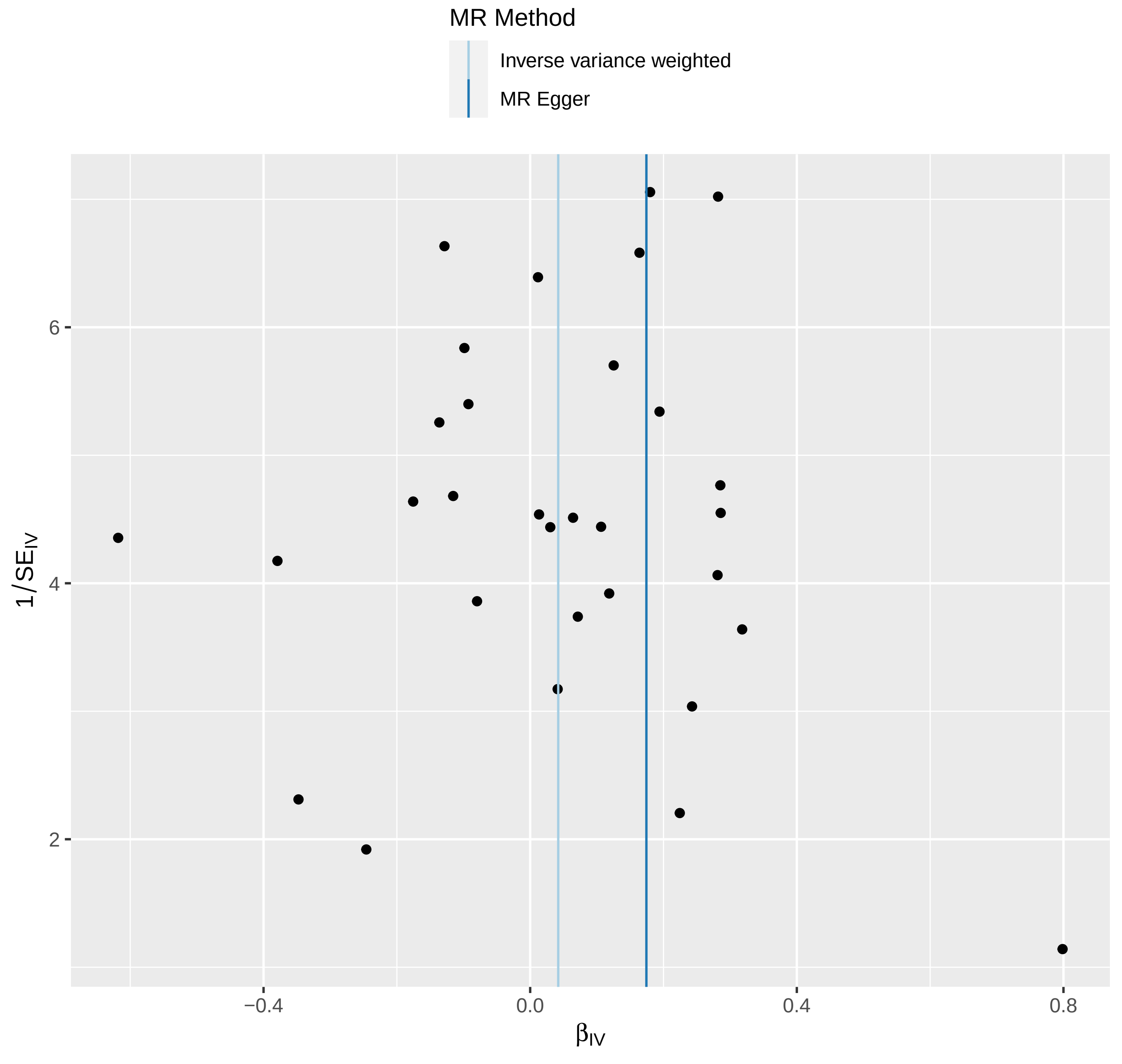

Batch 3−1864 : Methylmalonyl−CoA epimerase, mitochondrial on Diabetic nephropathy (more control exclusions)
with a threshold of 1e−05

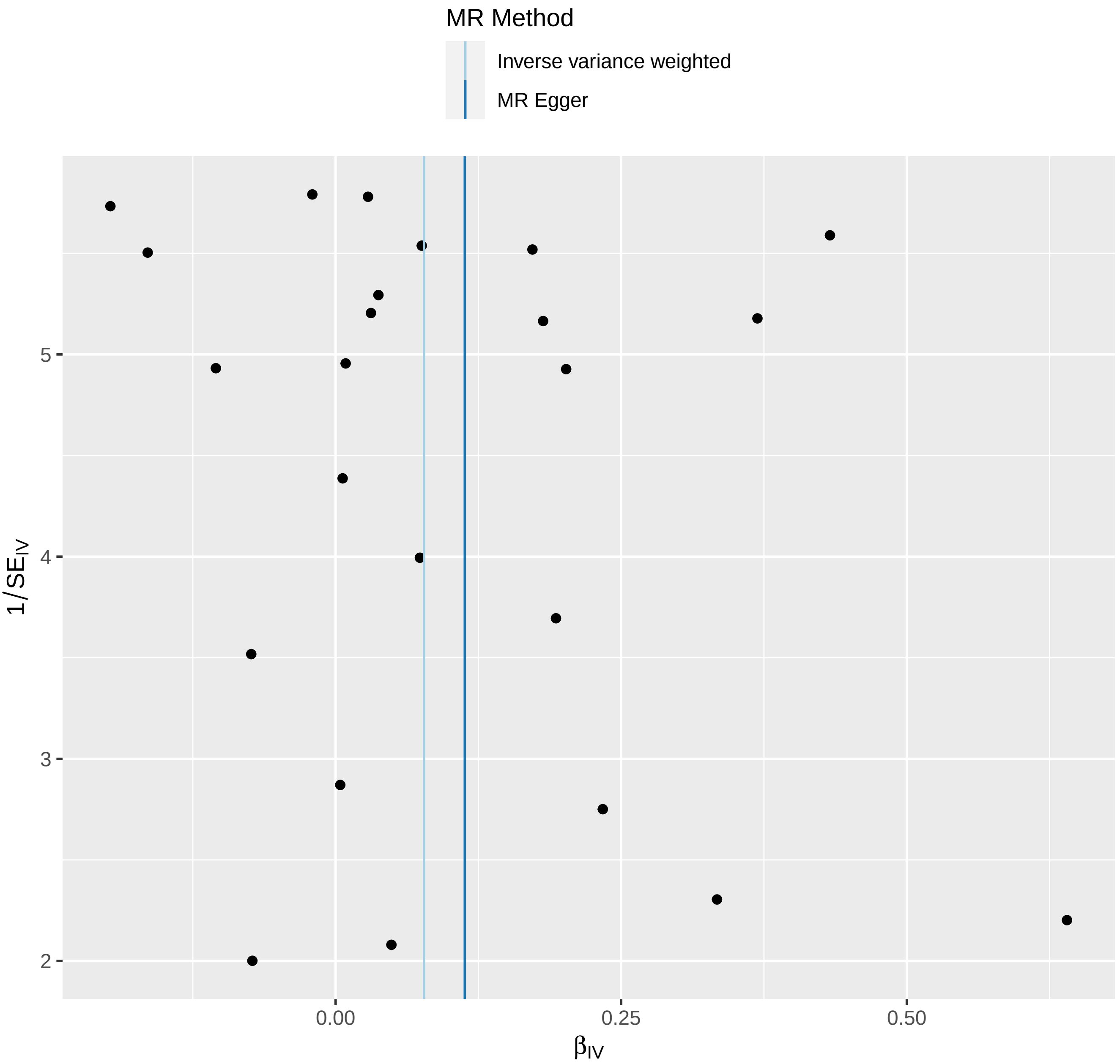

Batch 3−1865 : Mast cell−expressed membrane protein 1 on Diabetic nephropathy (more control exclusions)
with a threshold of 1e−05

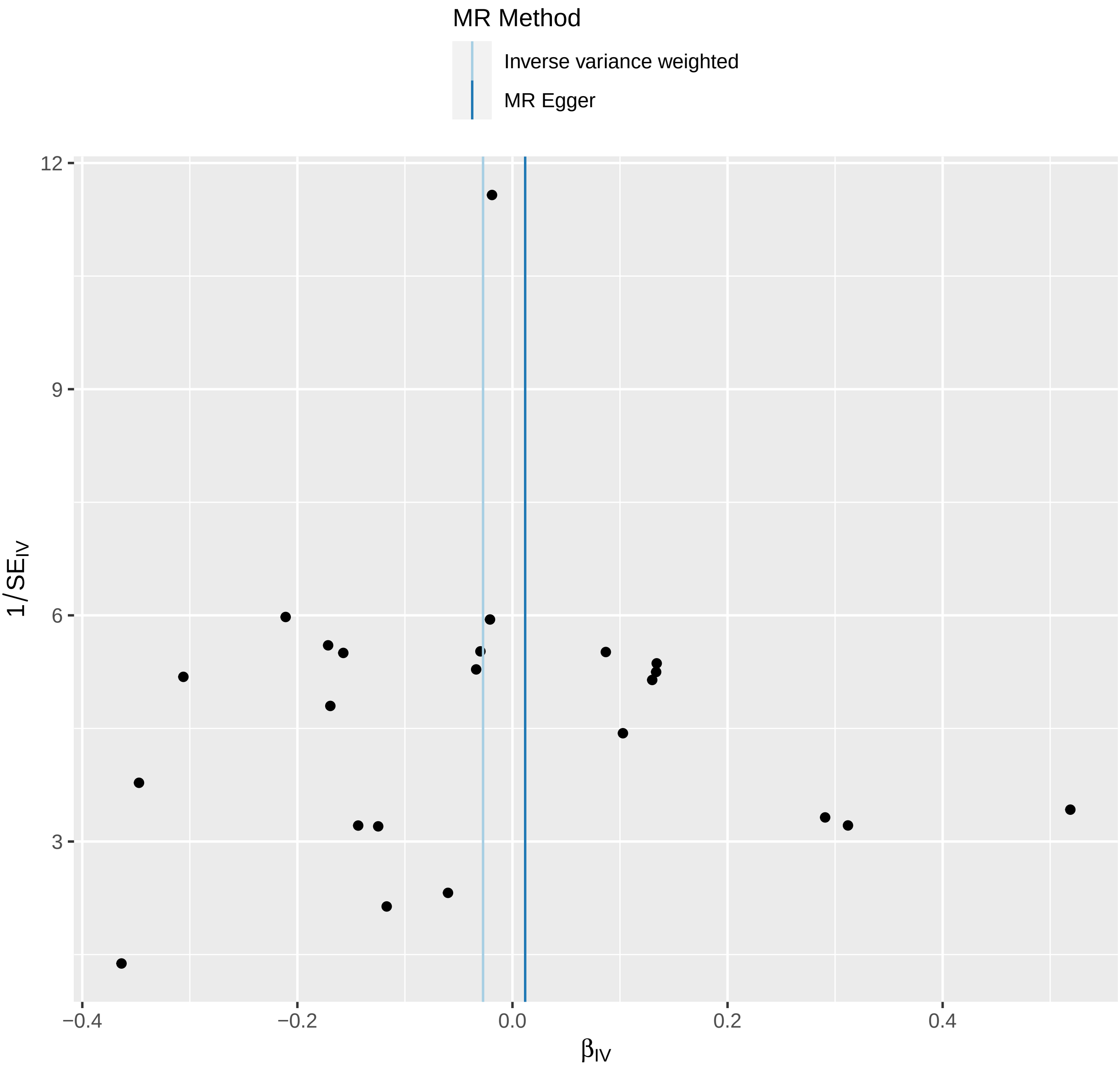

Batch 3−1866 : Guanine nucleotide exchange factor DBS on Diabetic nephropathy (more control exclusions)
with a threshold of 1e−05

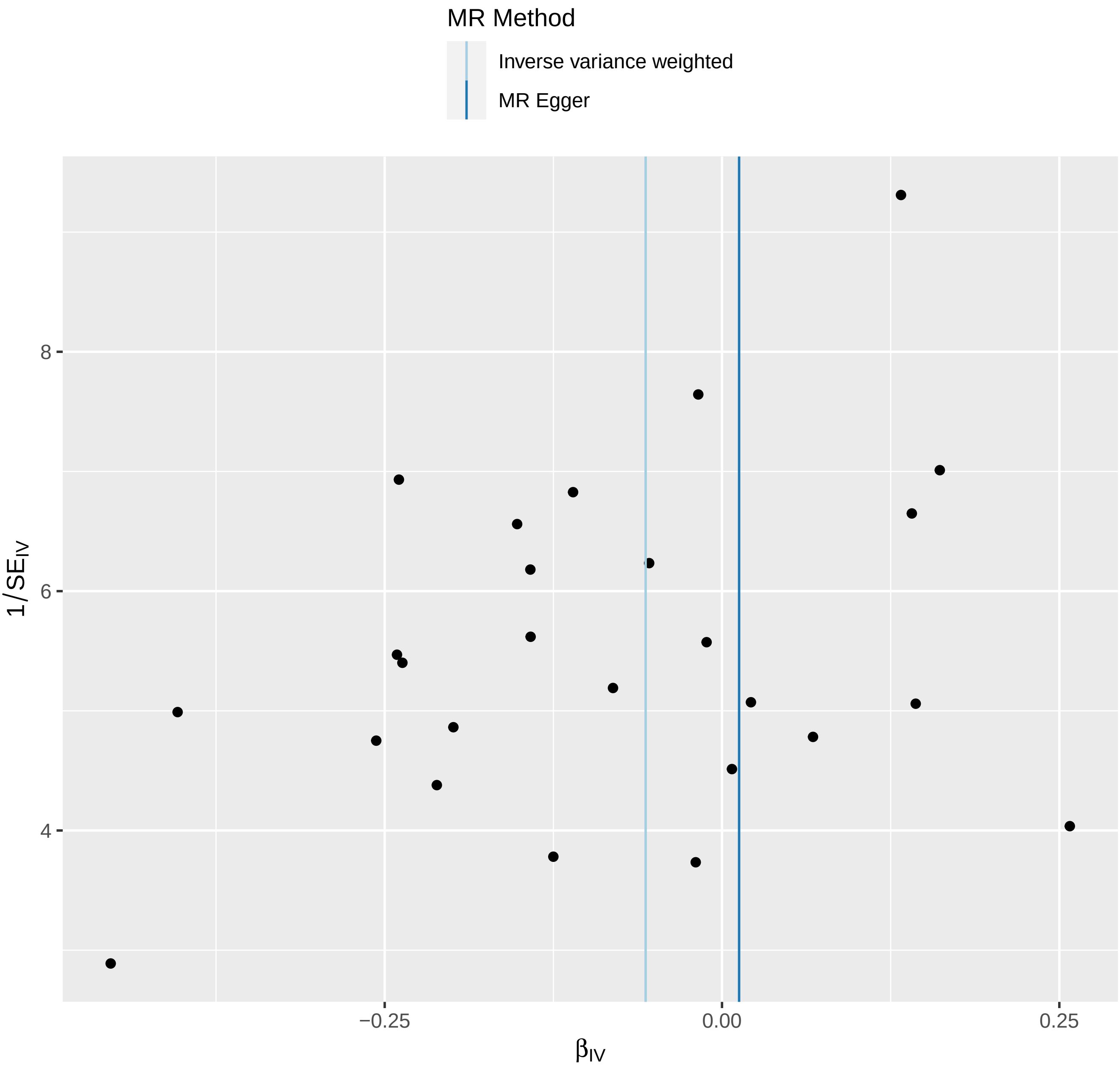

Batch 3−1867 : Multiple coagulation factor deficiency protein 2 on Diabetic nephropathy (more control exclusions)
with a threshold of 1e−05

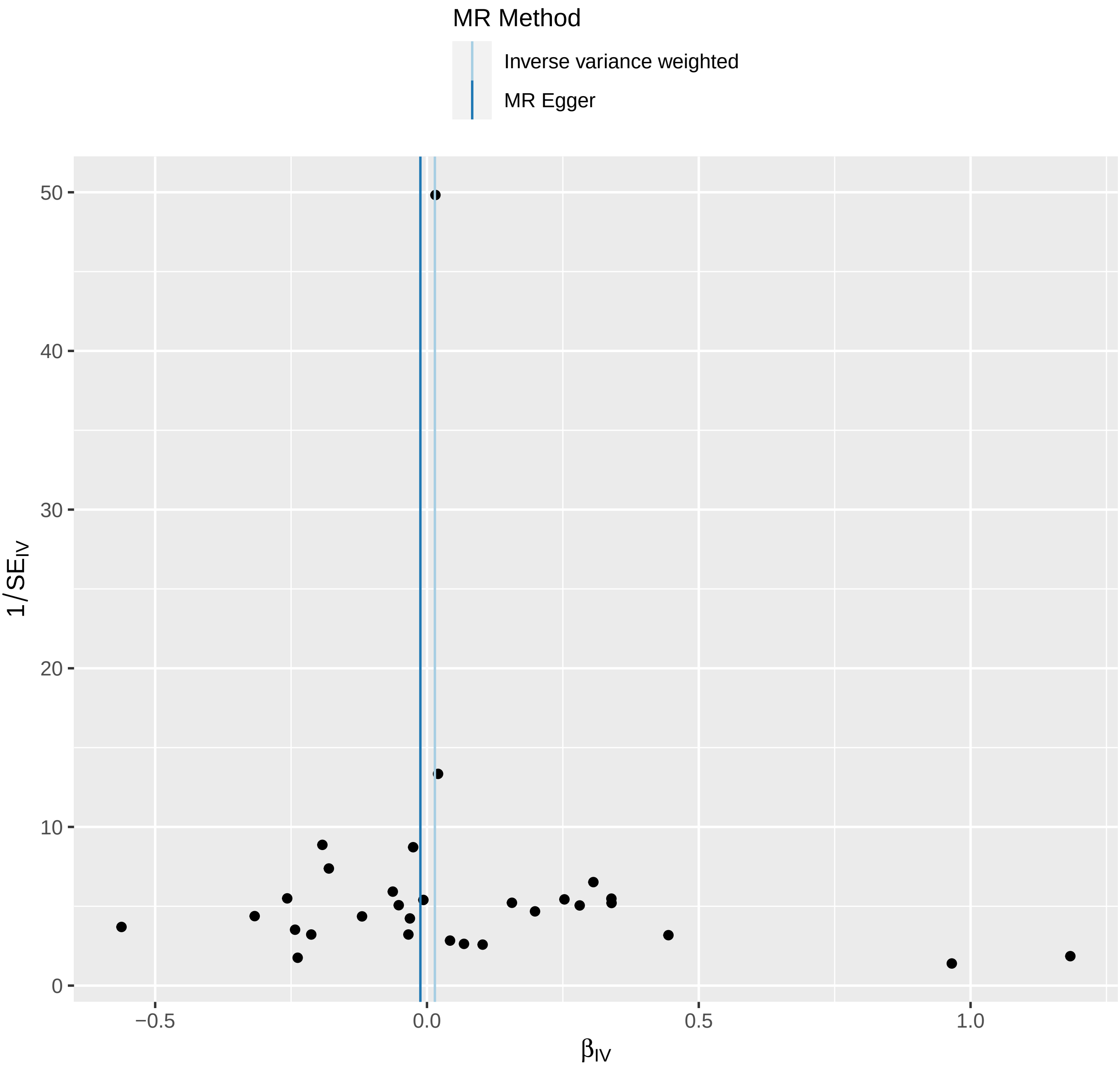

Batch 3−1868 : Induced myeloid leukemia cell differentiation protein Mcl−1 on Diabetic nephropathy (more control exclusions)
with a threshold of 1e−05

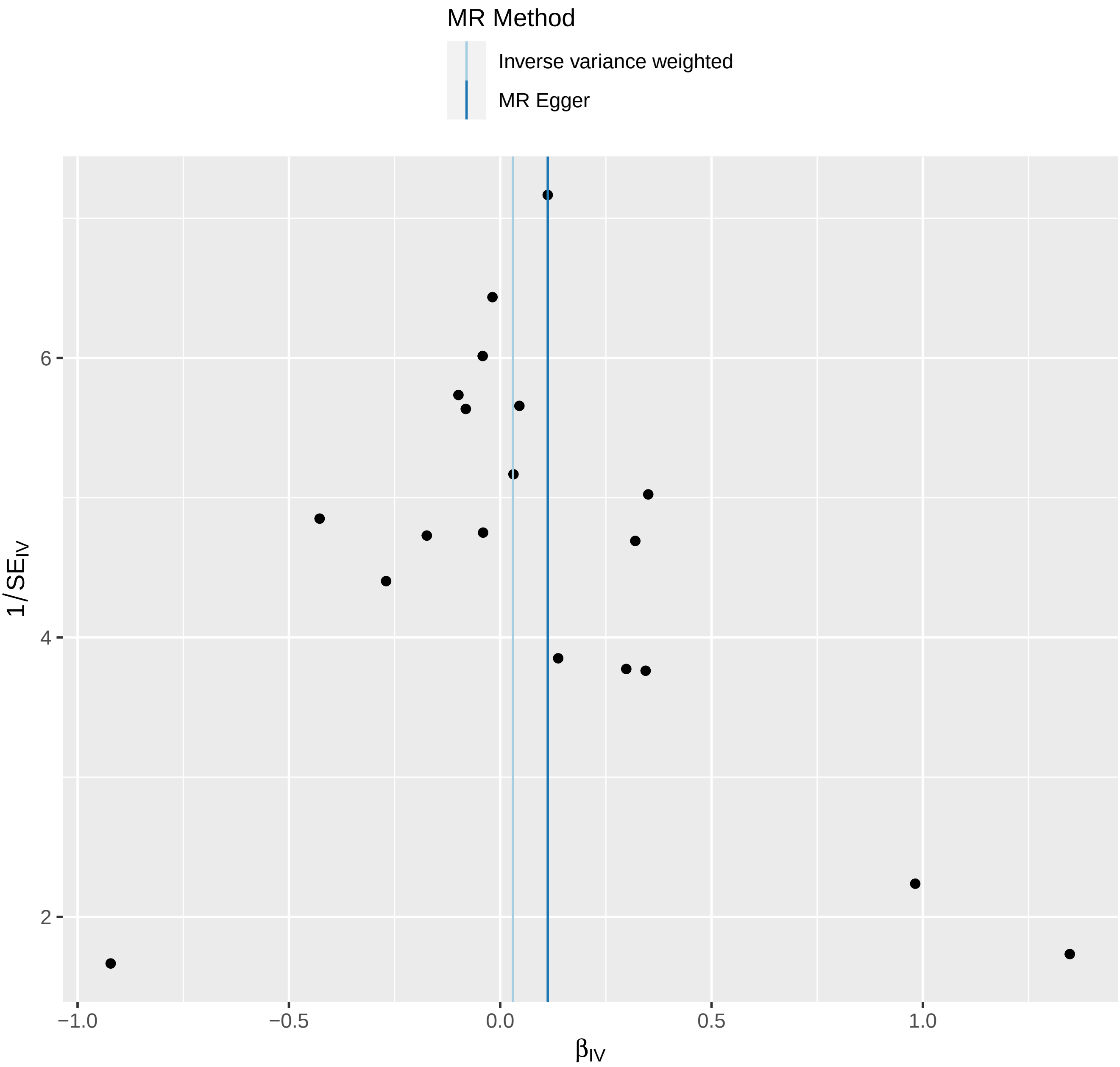

Batch 3−1869 : Malignant T−cell−amplified sequence 1 on Diabetic nephropathy (more control exclusions)
with a threshold of 1e−05

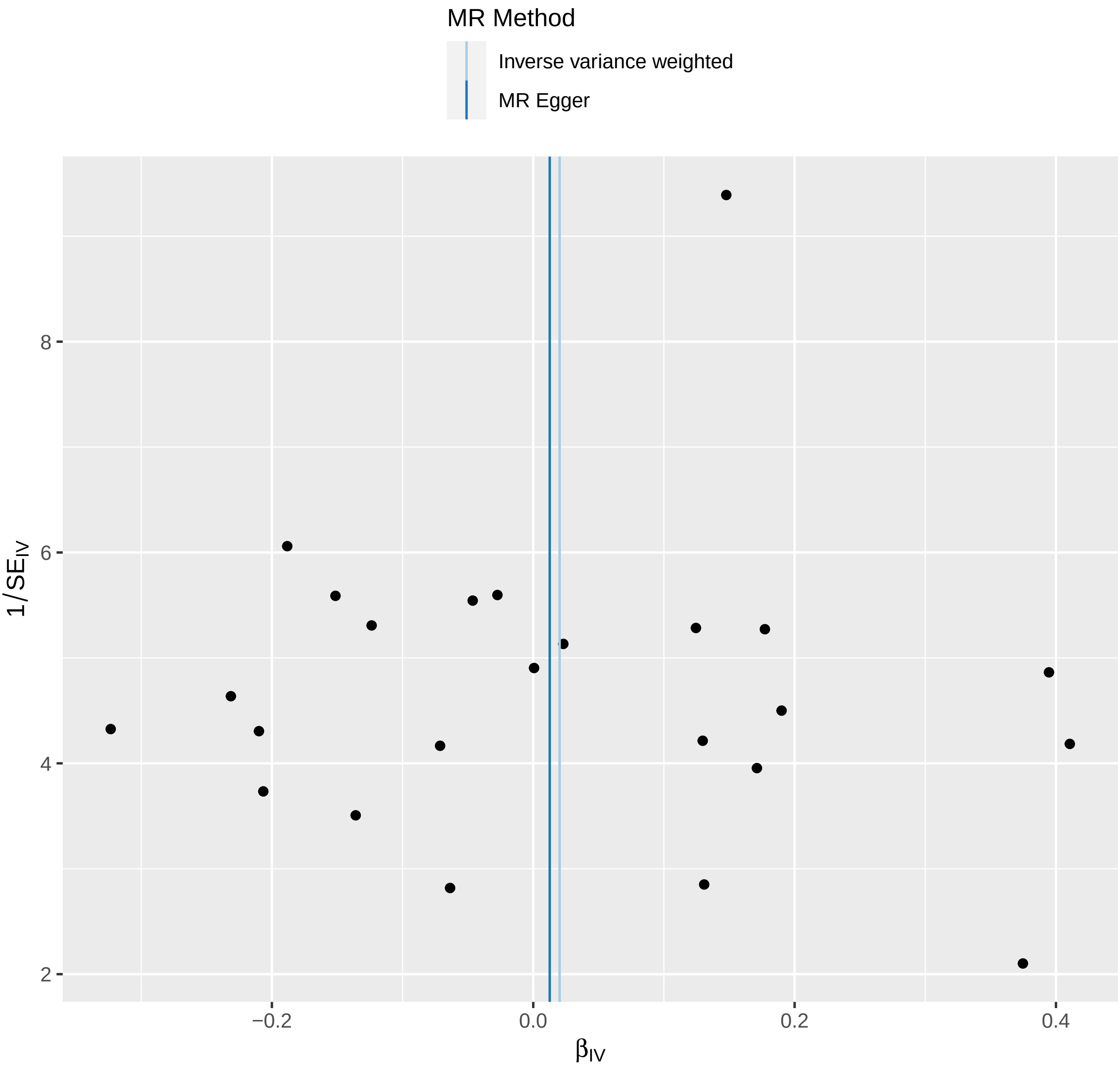

Batch 3−1870 : Malate dehydrogenase, cytoplasmic on Diabetic nephropathy (more control exclusions) with a threshold of 1e−05

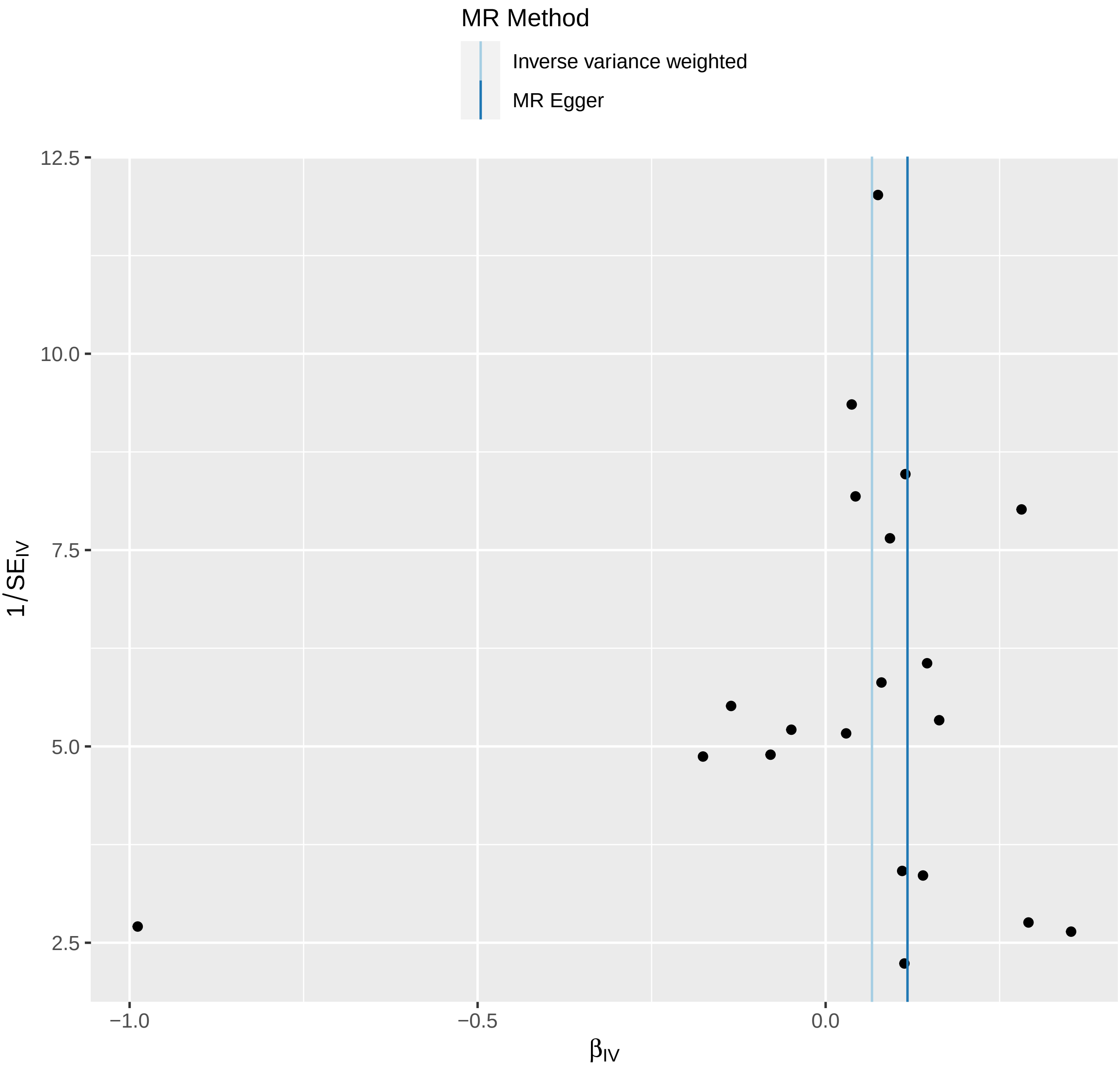

Batch 3−1871 : Midkine on Diabetic nephropathy (more control exclusions)
with a threshold of 1e−05

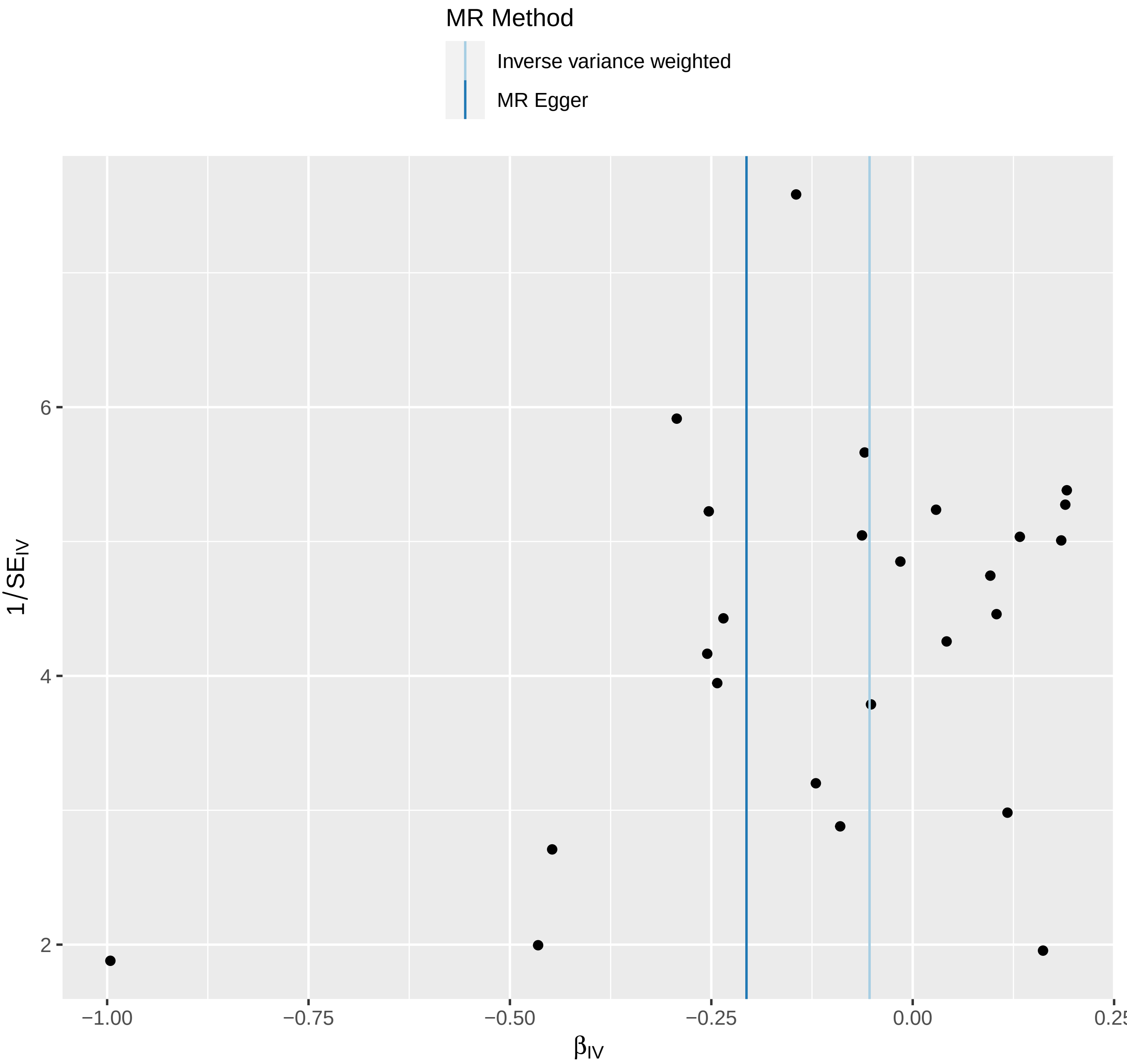

Batch 3−1872 : Nuclear protein MDM1 on Diabetic nephropathy (more control exclusions)
with a threshold of 1e−05

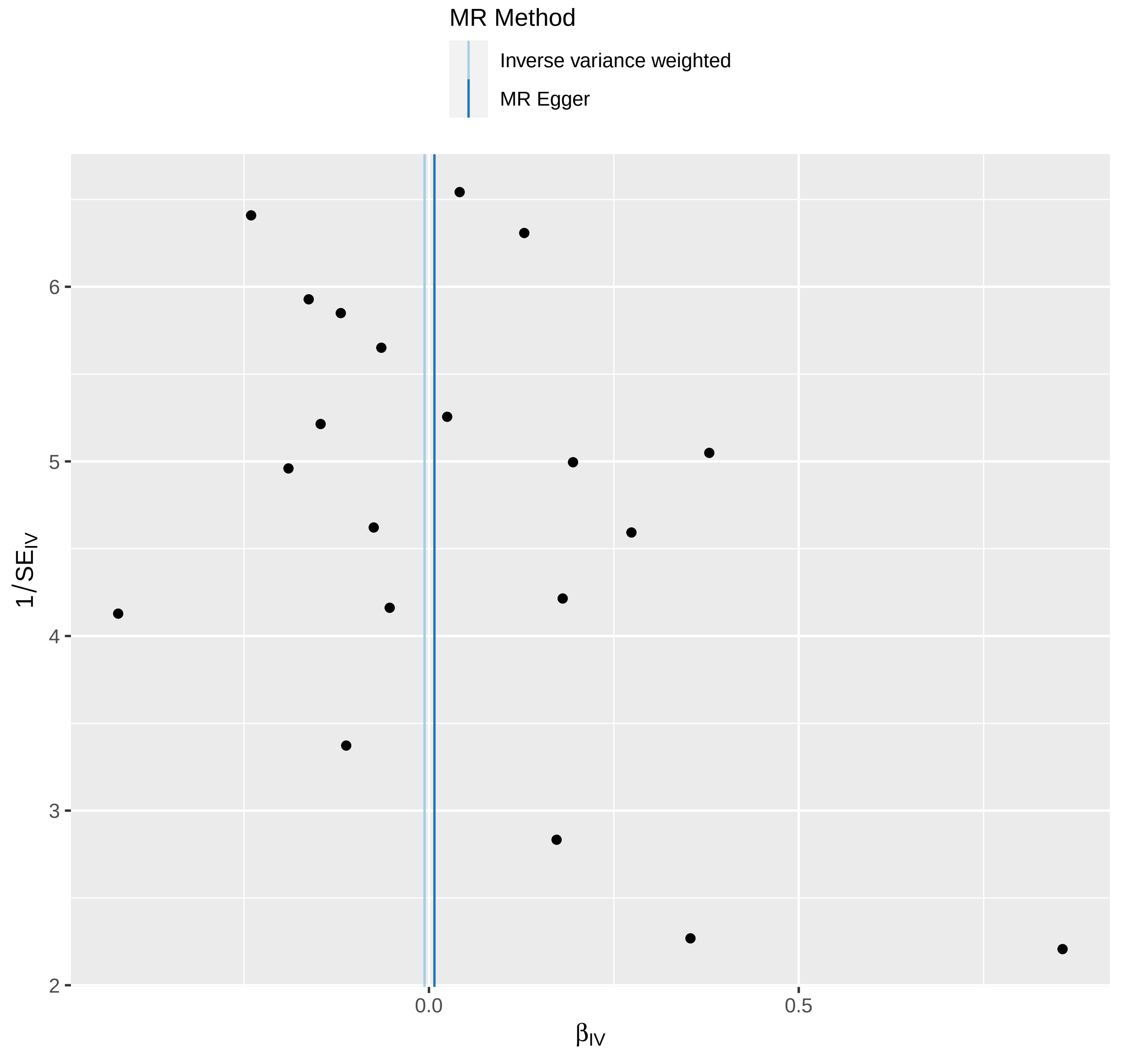

Batch 3−1873 : E3 ubiquitin−protein ligase Mdm2 on Diabetic nephropathy (more control exclusions)
with a threshold of 1e−05

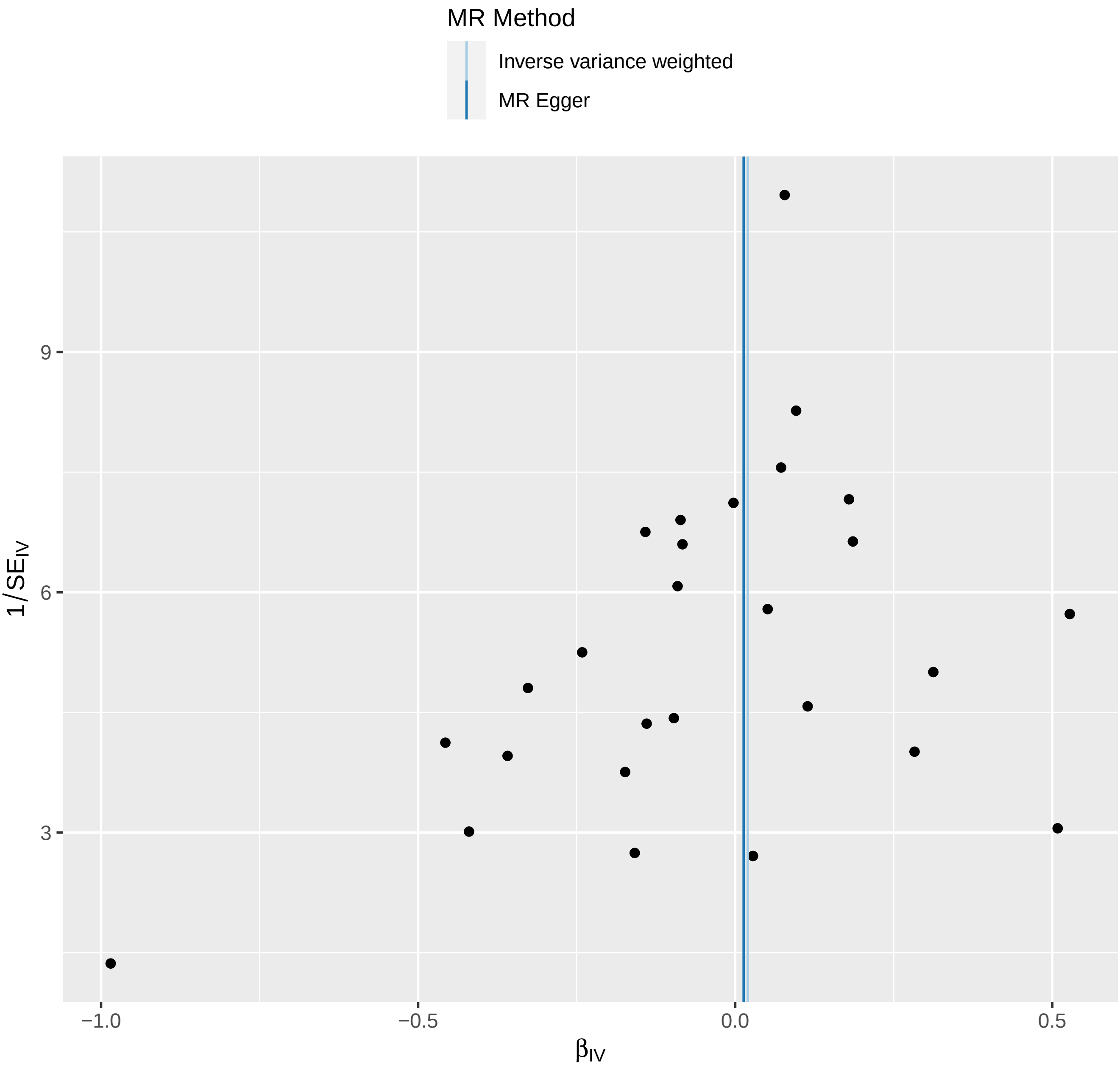

Batch 3−1874 : Mediator of RNA polymerase II transcription subunit 1 on Diabetic nephropathy (more control exclusions)
with a threshold of 1e−05

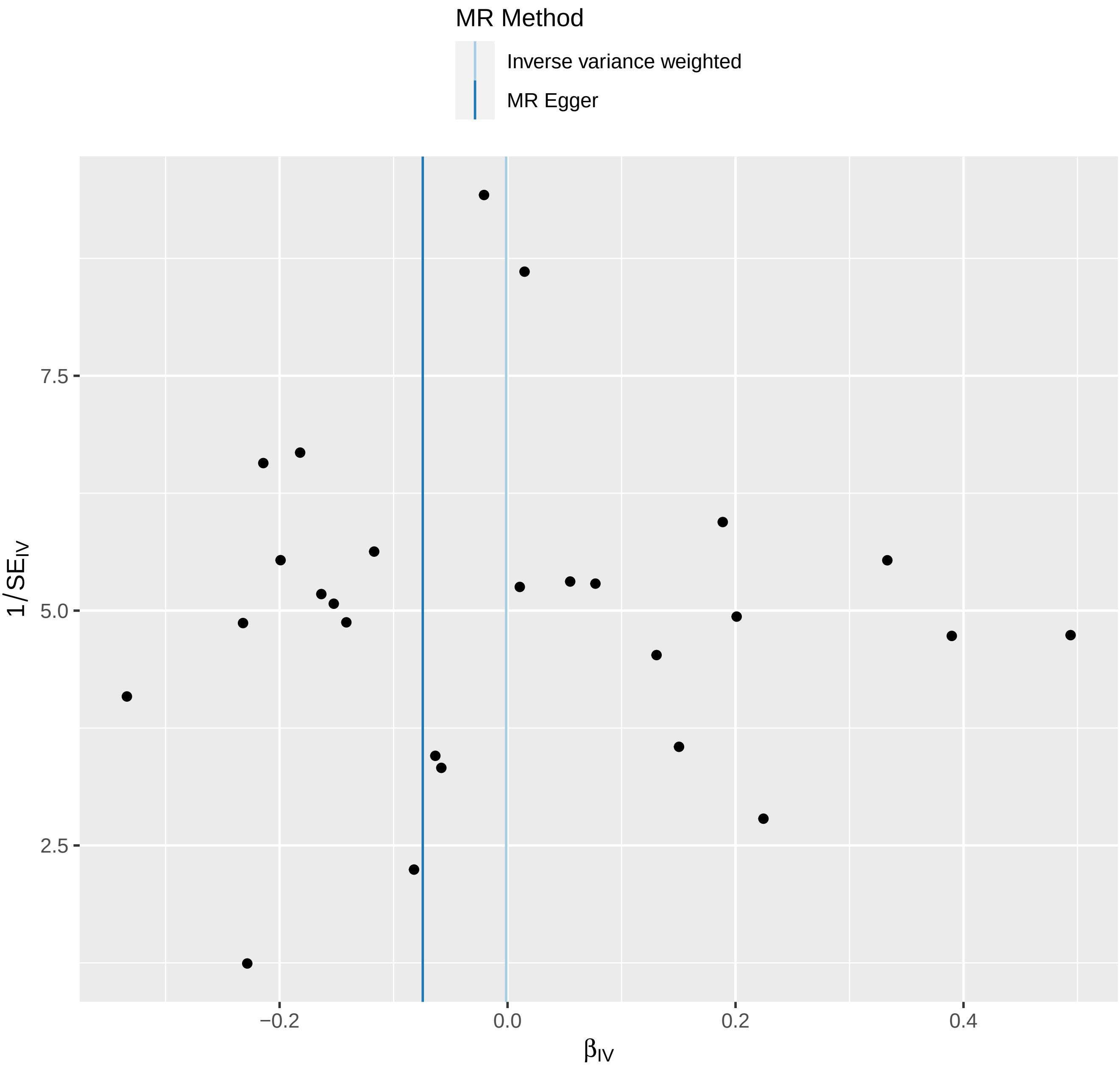

Batch 3−1875 : Mediator of RNA polymerase II transcription subunit 1 on Diabetic nephropathy (more control exclusions)
with a threshold of 1e−05

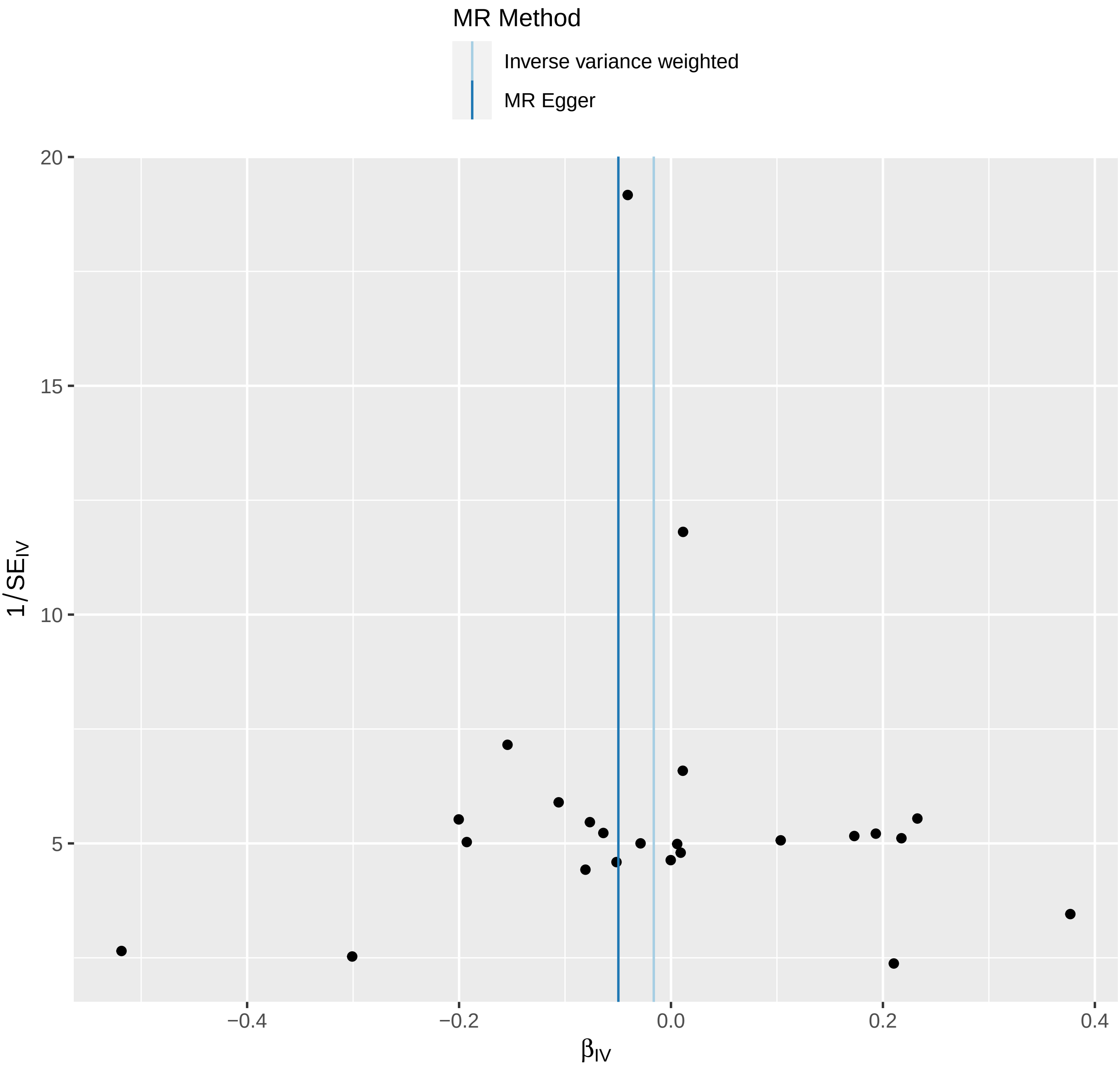

Batch 3−1876 : Mediator of RNA polymerase II transcription subunit 4 on Diabetic nephropathy (more control exclusions)
with a threshold of 1e−05

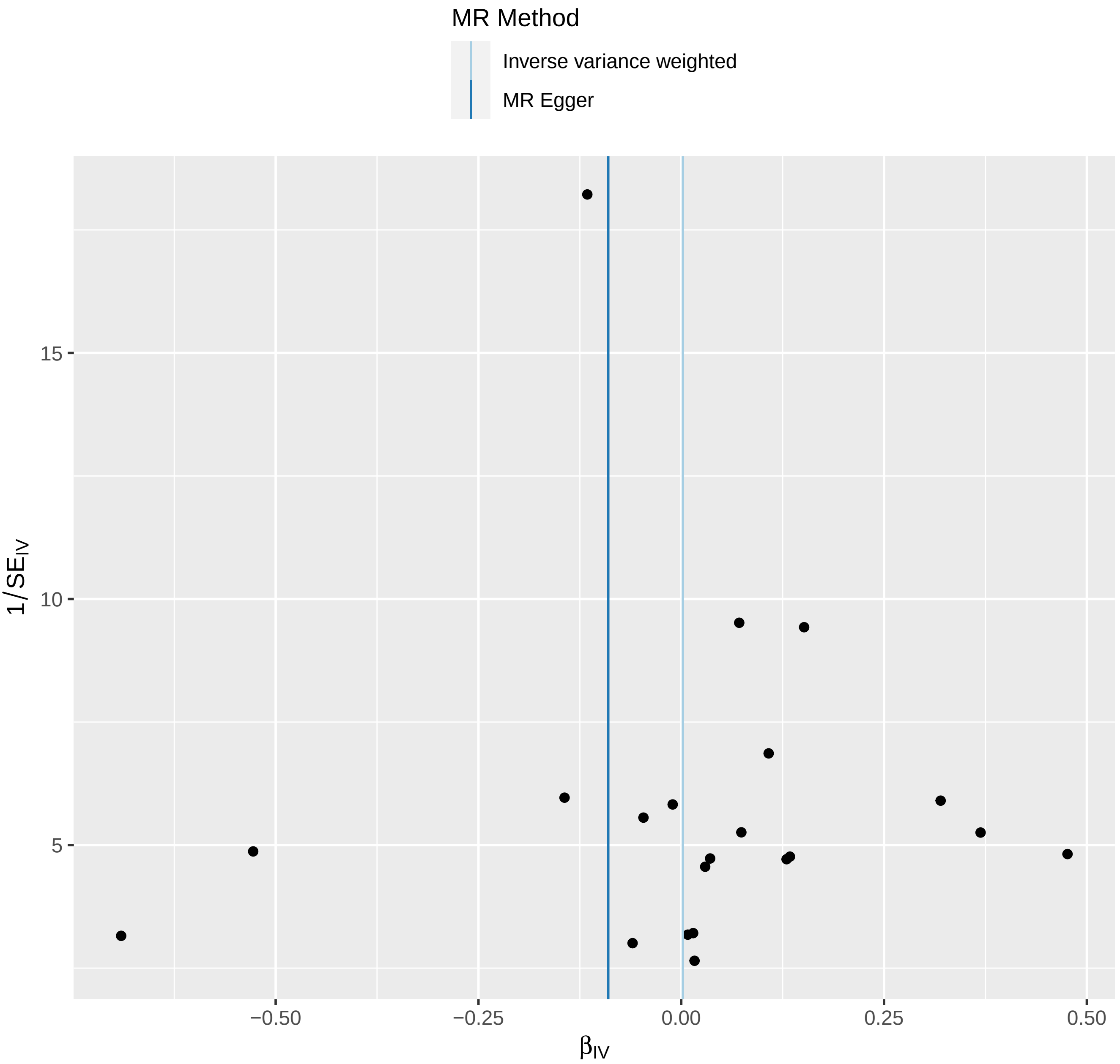

Batch 3−1877 : Myocyte−specific enhancer factor 2C on Diabetic nephropathy (more control exclusions)
with a threshold of 1e−05

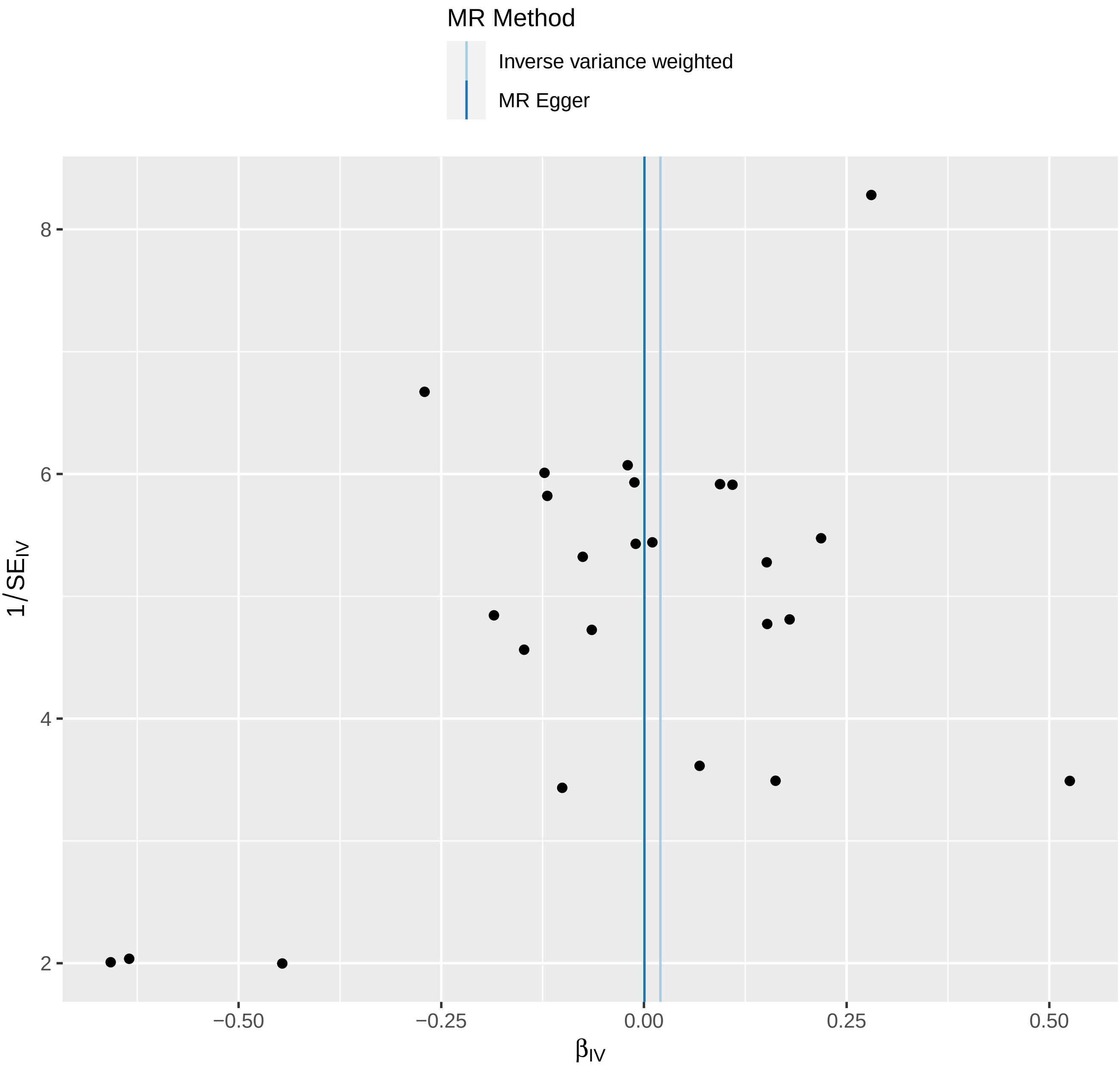

Batch 3−1878 : Multiple epidermal growth factor−like domains protein 10 on Diabetic nephropathy (more control exclusions) with a threshold of 1e−05

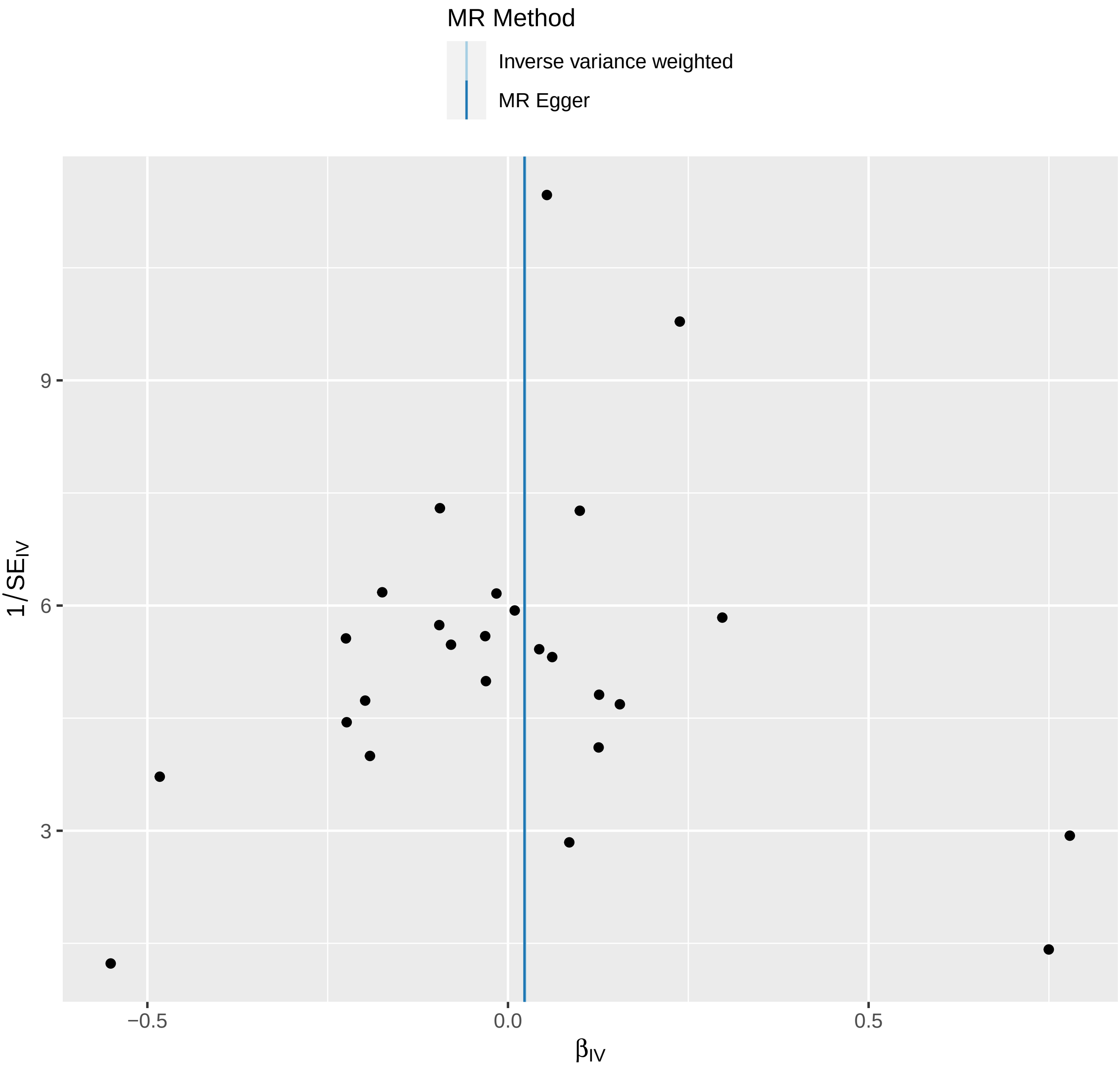

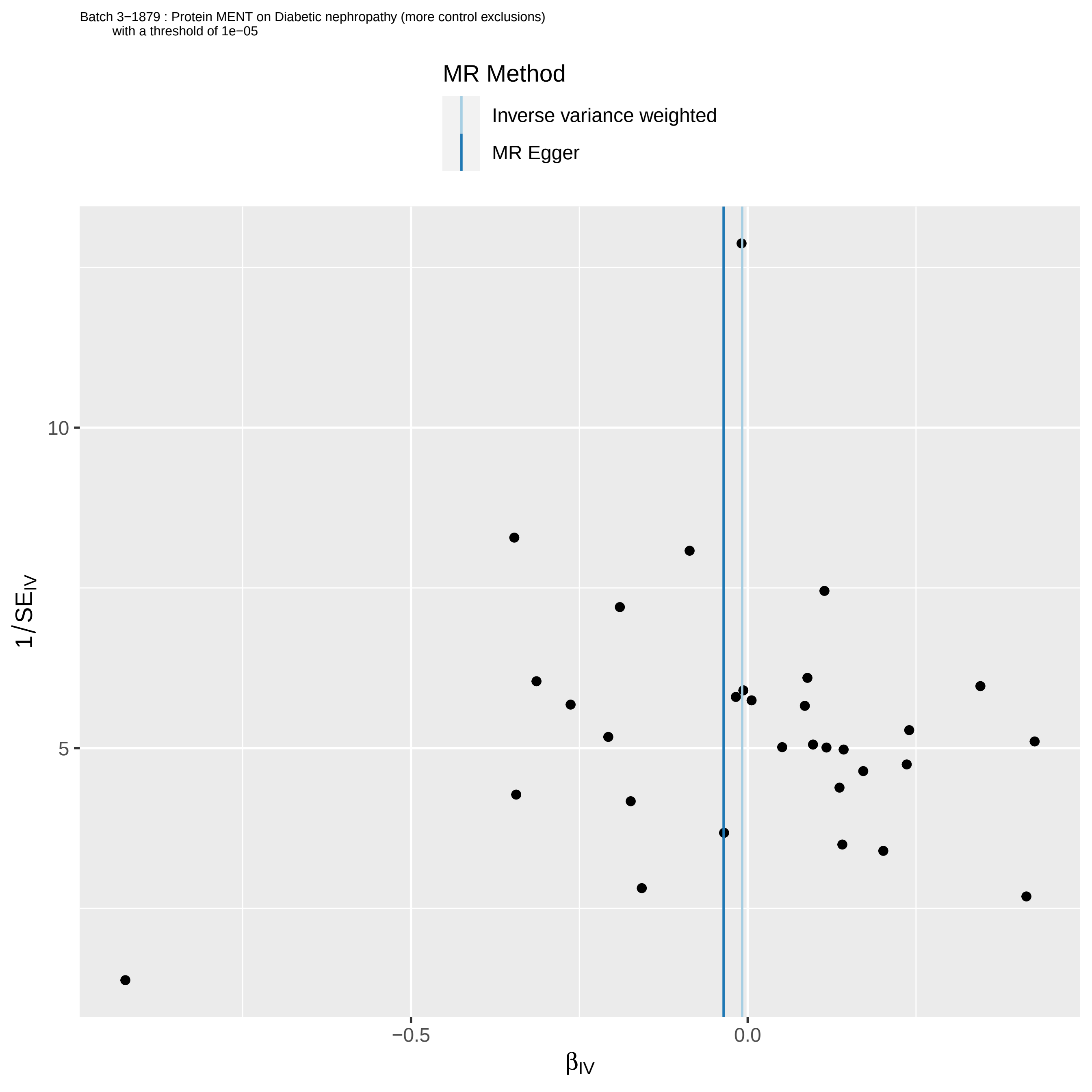

Batch 3−1879 : Protein MENT on Diabetic nephropathy (more control exclusions)
with a threshold of 1e−05
MR Method
Inverse variance weighted
MR Egger
10
5
1/SE_IV
−0.5
0.0
β_IV

Batch 3−1880 : Matrix extracellular phosphoglycoprotein on Diabetic nephropathy (more control exclusions) with a threshold of 1e−05

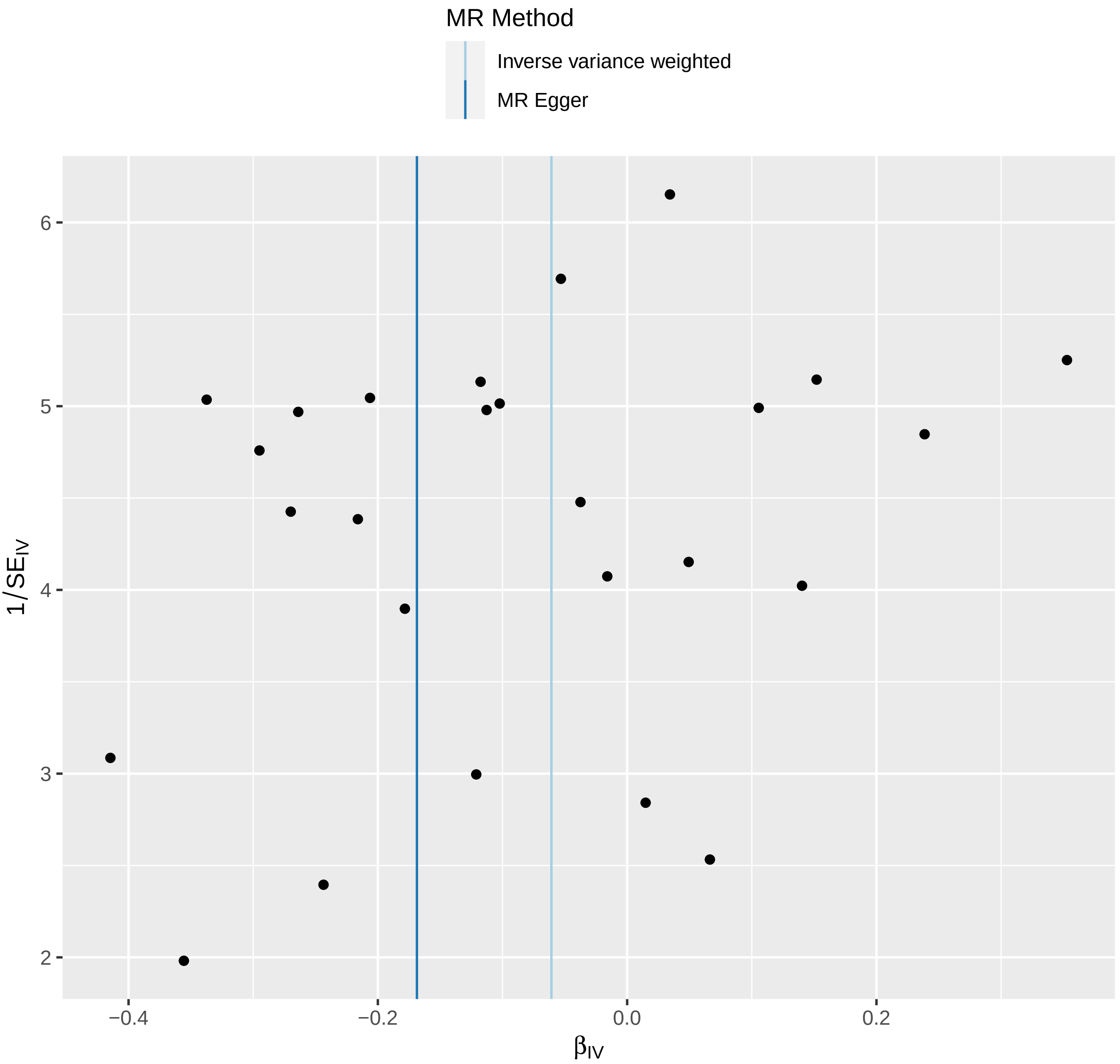

Batch 3−1881 : LDLR chaperone MESD on Diabetic nephropathy (more control exclusions)
with a threshold of 1e−05

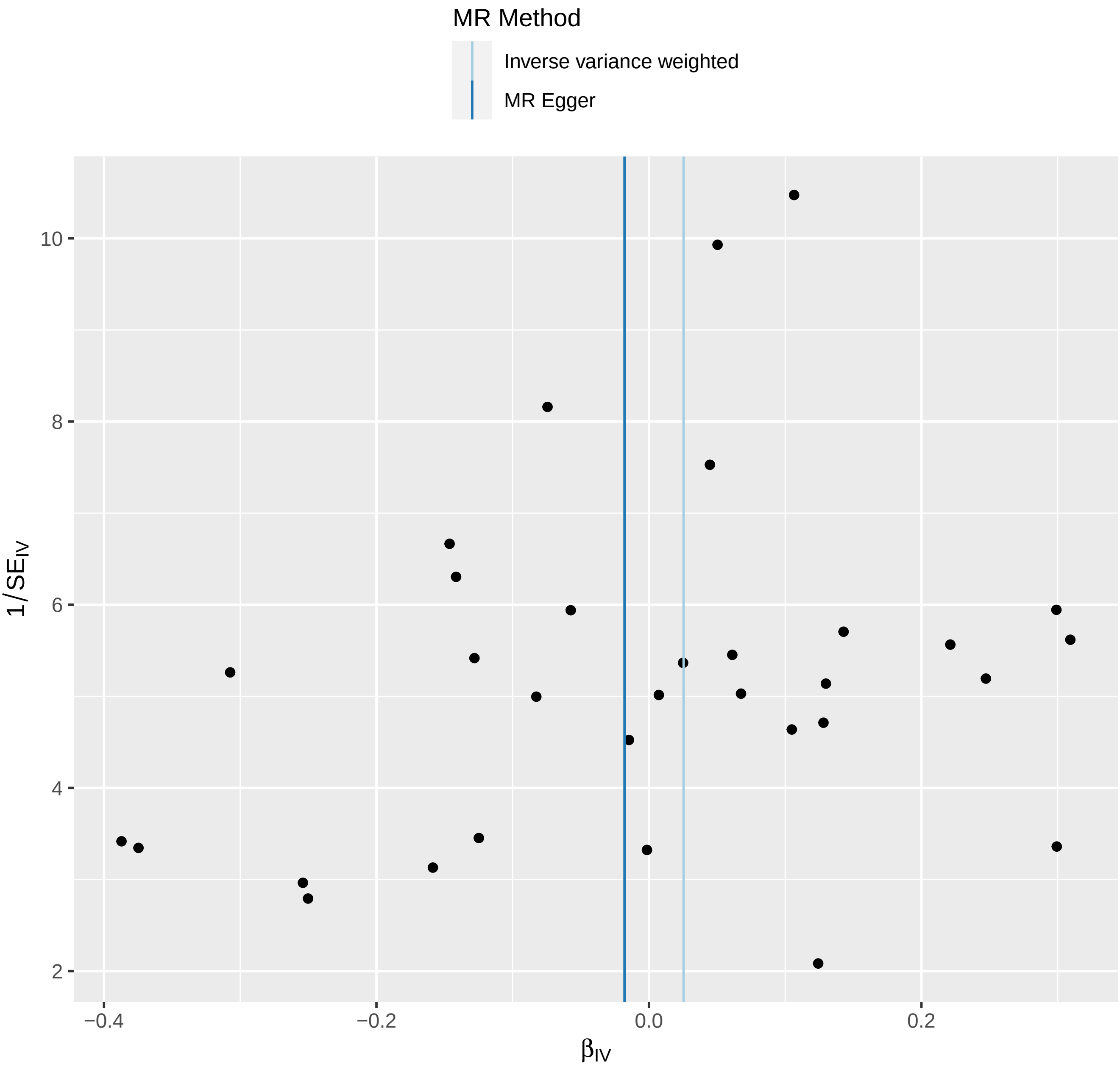

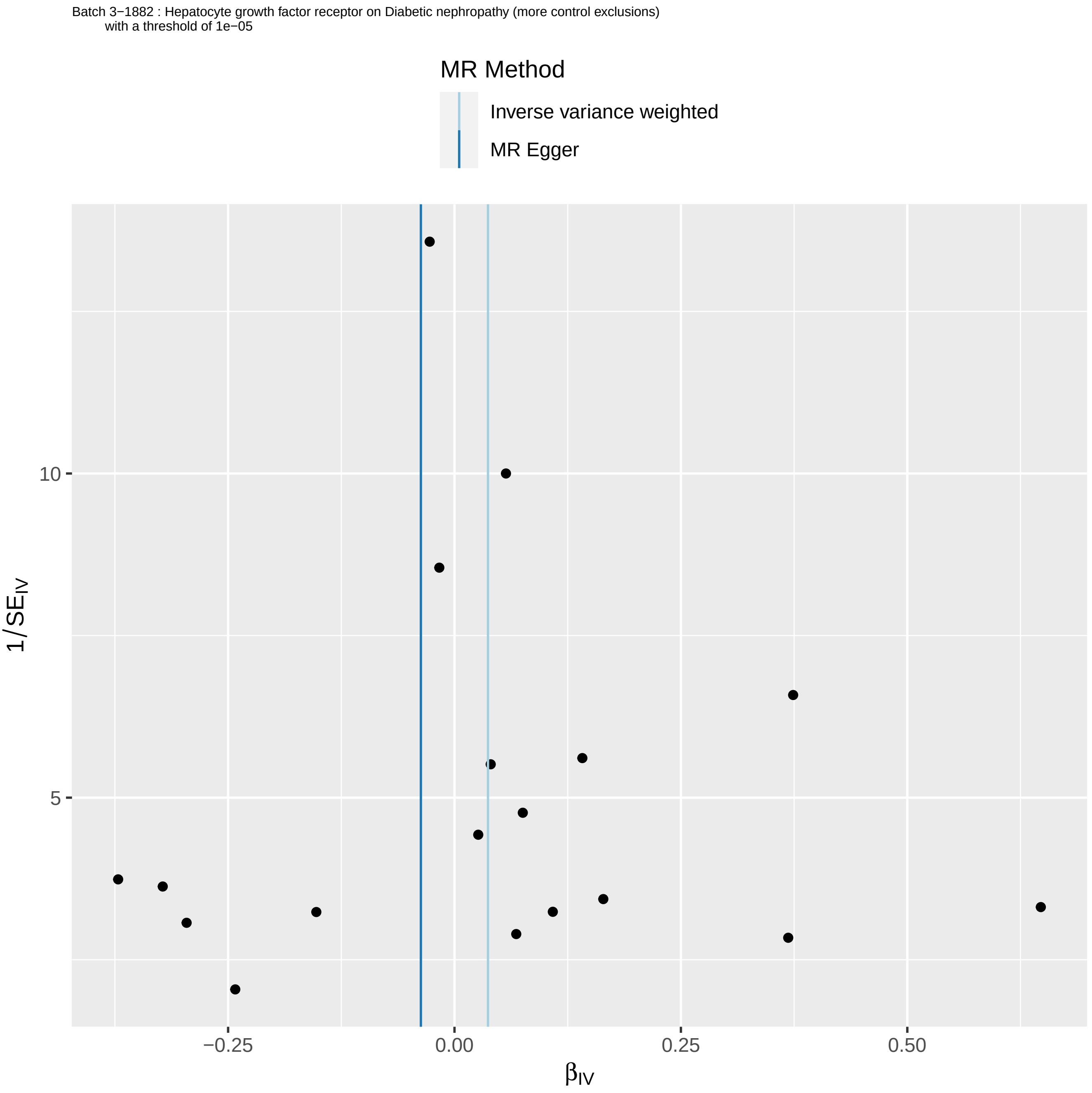

Batch 3−1882 : Hepatocyte growth factor receptor on Diabetic nephropathy (more control exclusions)
with a threshold of 1e−05
MR Method
Inverse variance weighted
MR Egger
10
5
1/SE_IV
−0.25
0.00
0.25
0.50
β_IV

Batch 3−1883 : tRNA (guanine−N(7)−)−methyltransferase on Diabetic nephropathy (more control exclusions)
with a threshold of 1e−05

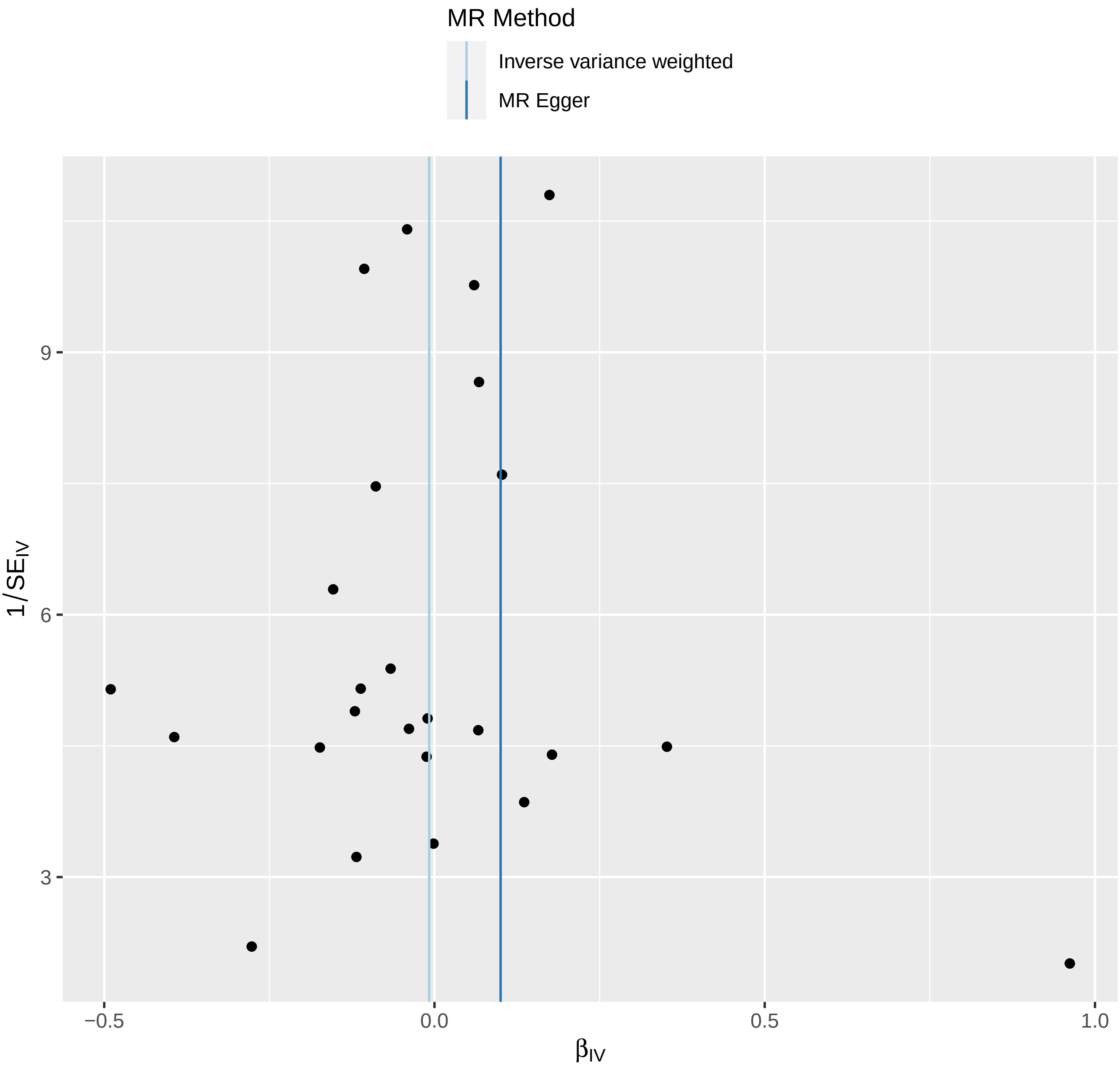

Batch 3−1884 : Microfibrillar−associated protein 1 on Diabetic nephropathy (more control exclusions)
with a threshold of 1e−05

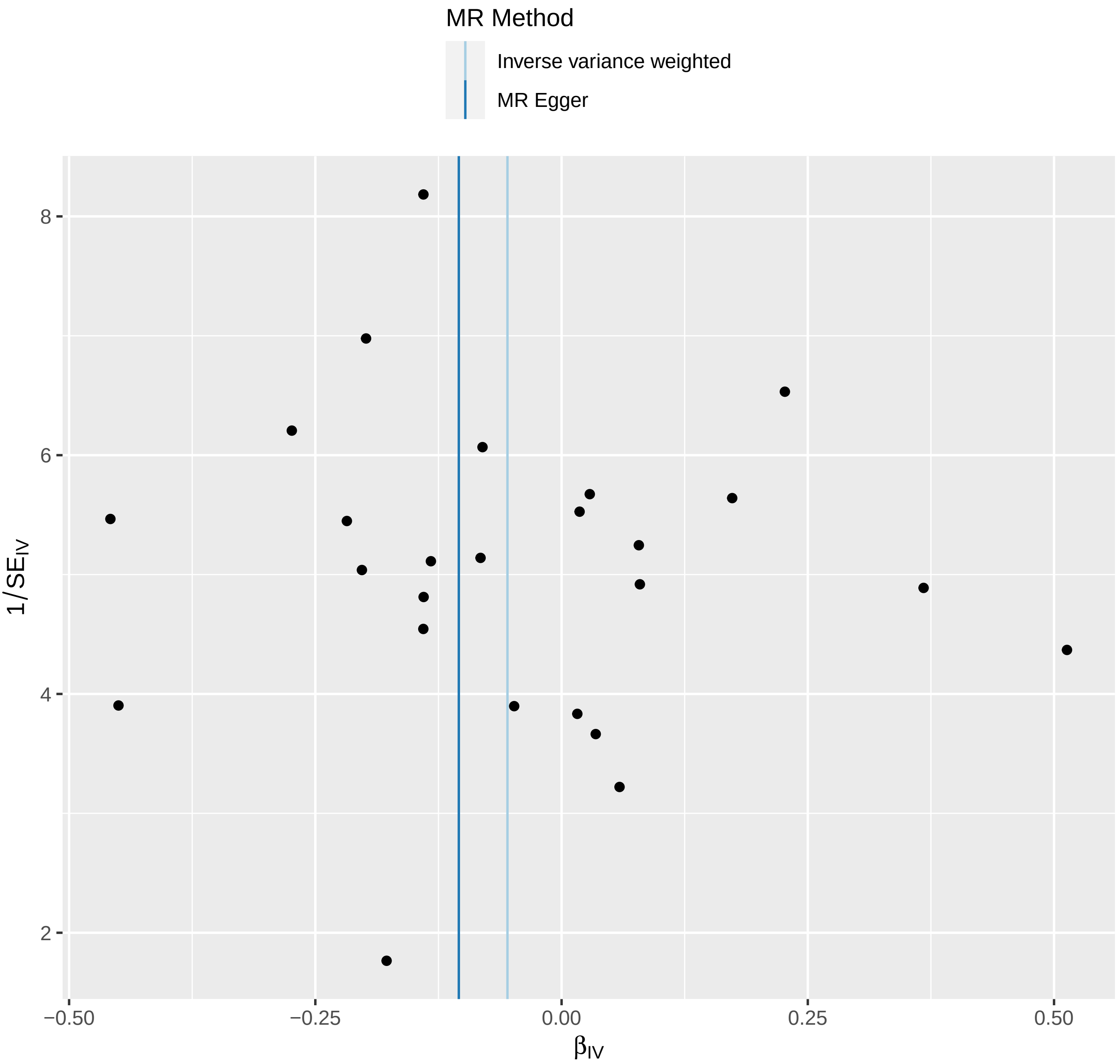

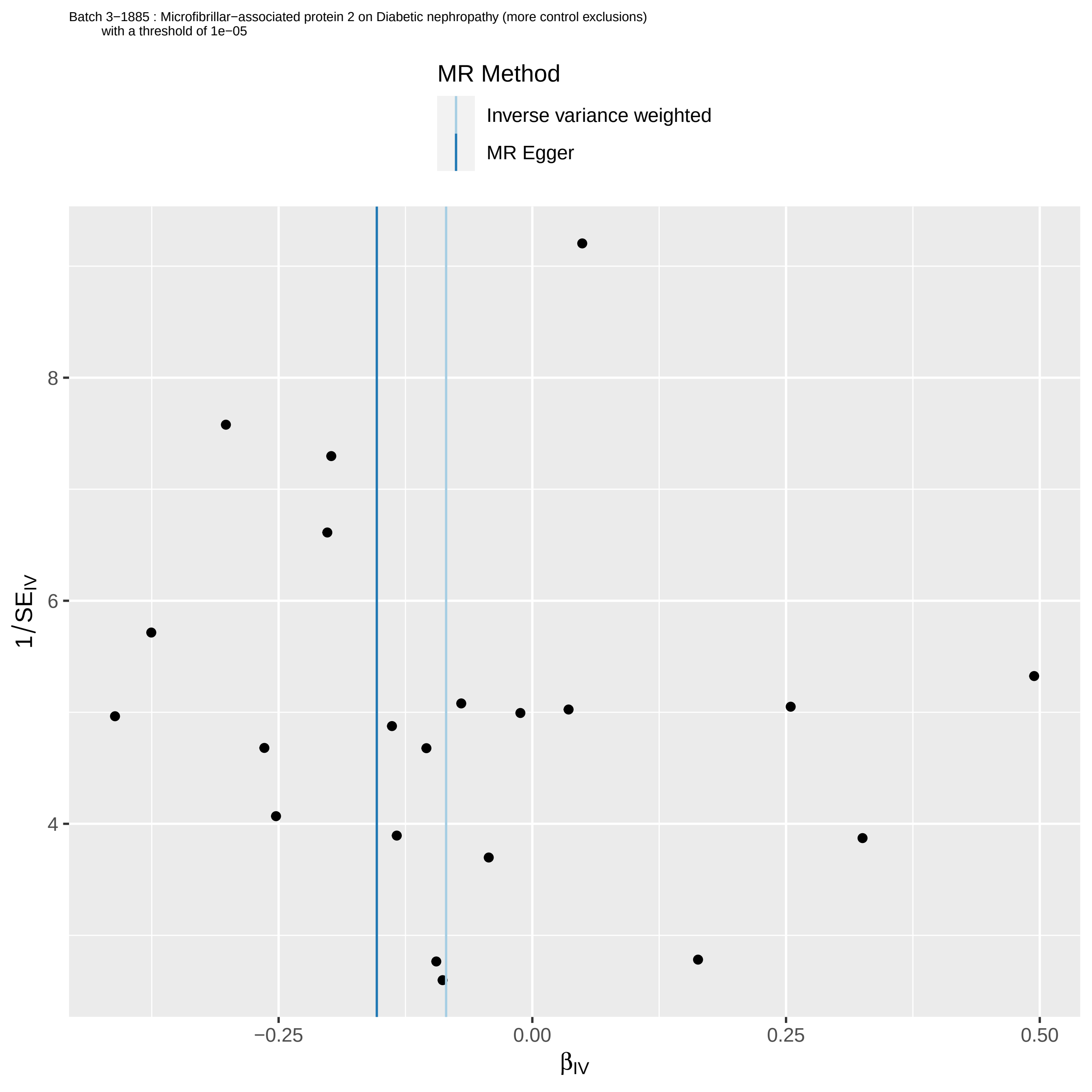
Batch 3−1885 : Microfibrillar−associated protein 2 on Diabetic nephropathy (more control exclusions)
with a threshold of 1e−05
MR Method
Inverse variance weighted
MR Egger
8
6
4
1/SE_IV
−0.25
0.00
0.25
0.50
β_IV

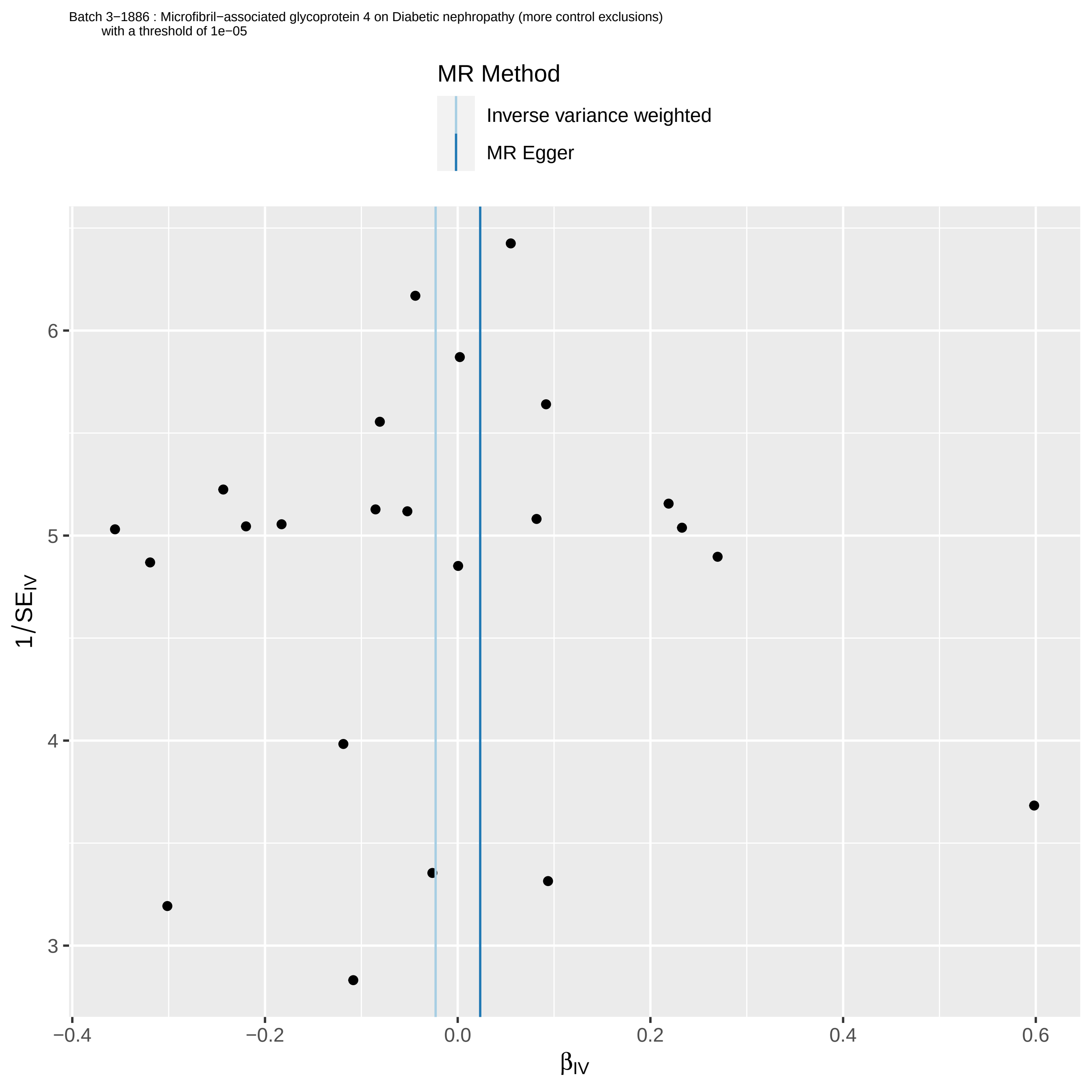
Batch 3−1886 : Microfibril−associated glycoprotein 4 on Diabetic nephropathy (more control exclusions)
with a threshold of 1e−05
MR Method
Inverse variance weighted
MR Egger
6
5
4
3
1/SE_IV
−0.4
−0.2
0.0
0.2
0.4
0.6
β_IV

Batch 3−1887 : Microfibrillar−associated protein 5 on Diabetic nephropathy (more control exclusions)
with a threshold of 1e−05

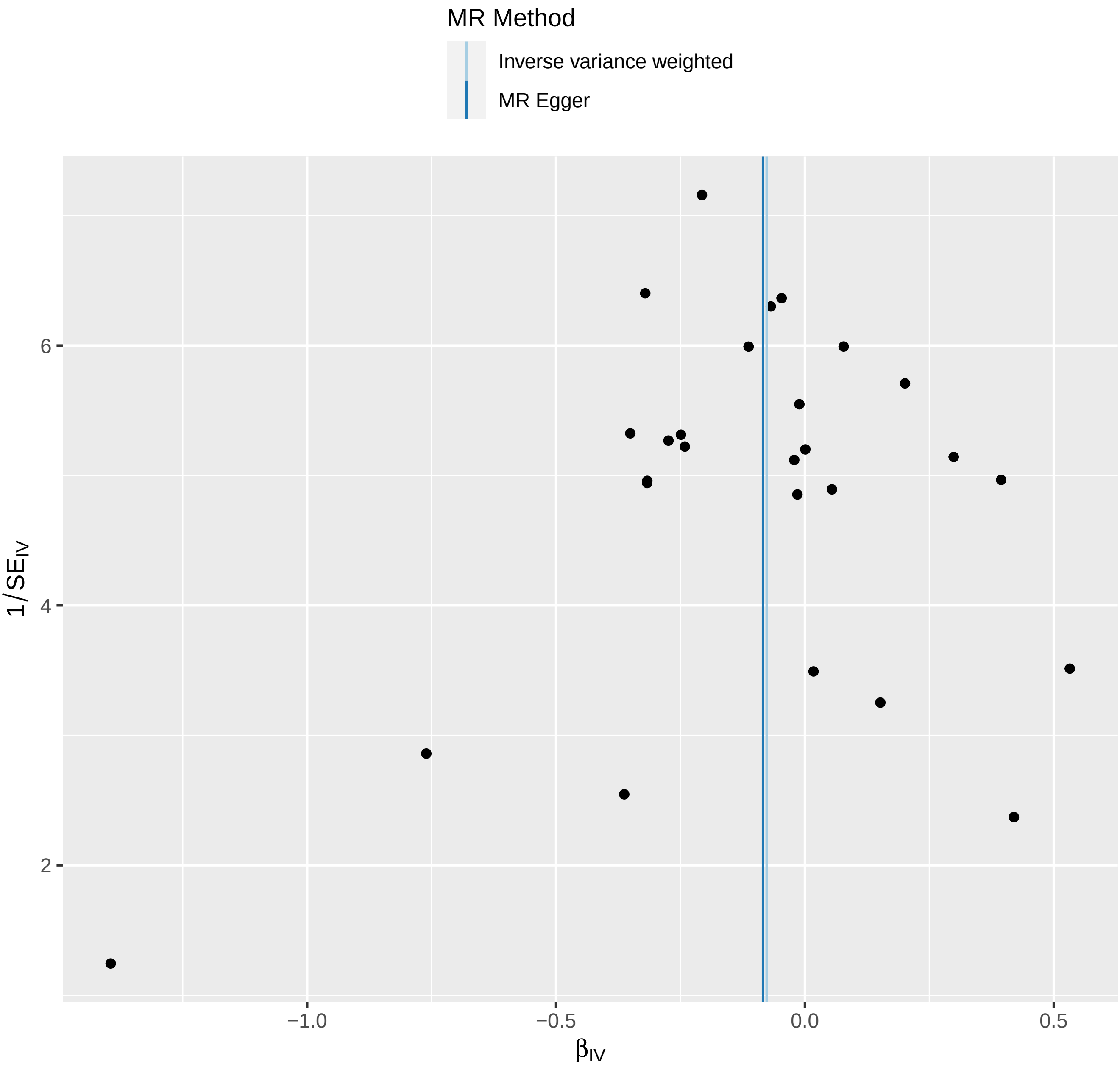

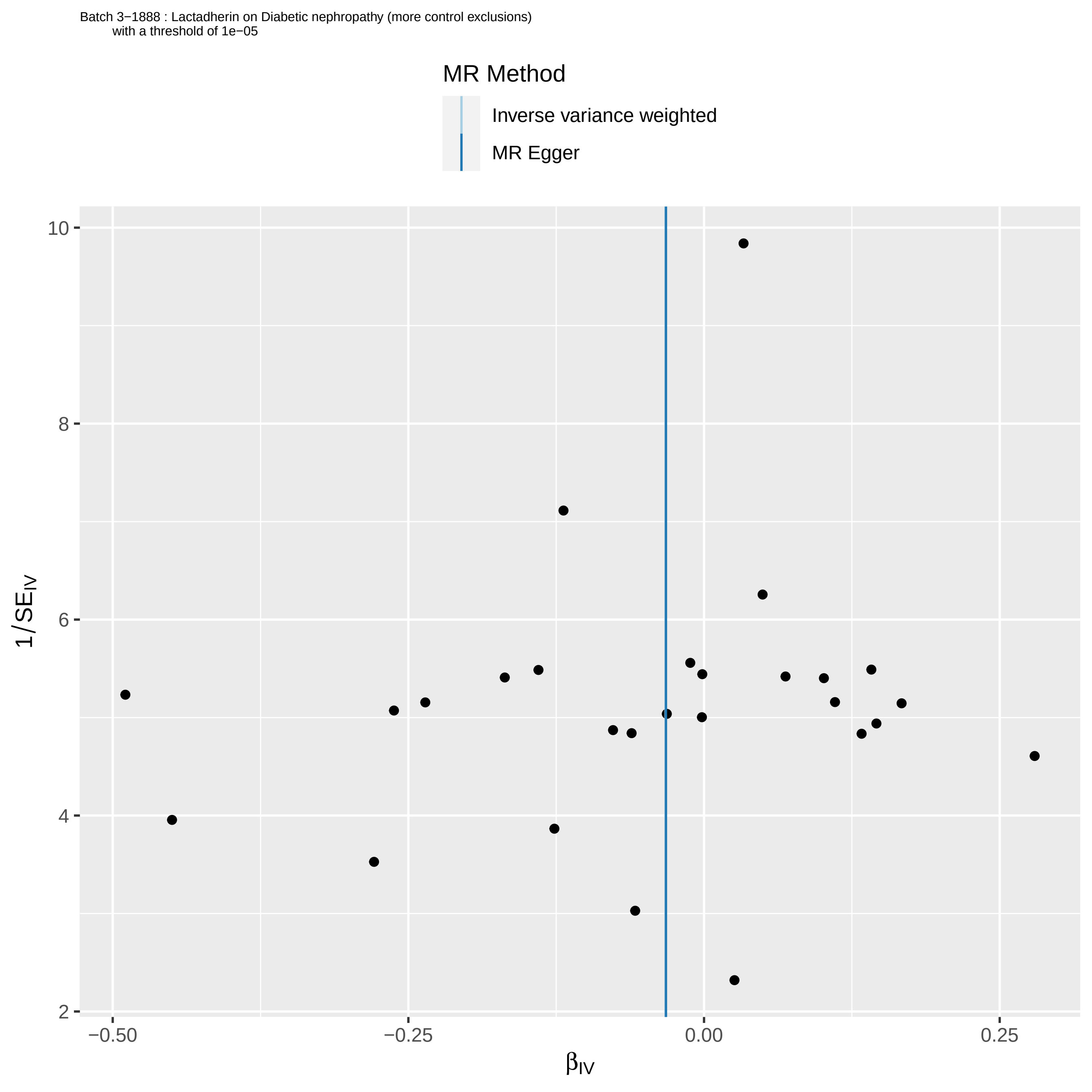

Batch 3−1888 : Lactadherin on Diabetic nephropathy (more control exclusions)
with a threshold of 1e−05
MR Method
Inverse variance weighted
MR Egger
10
8
6
4
2
1/SE_IV
−0.50
−0.25
0.00
0.25
β_IV

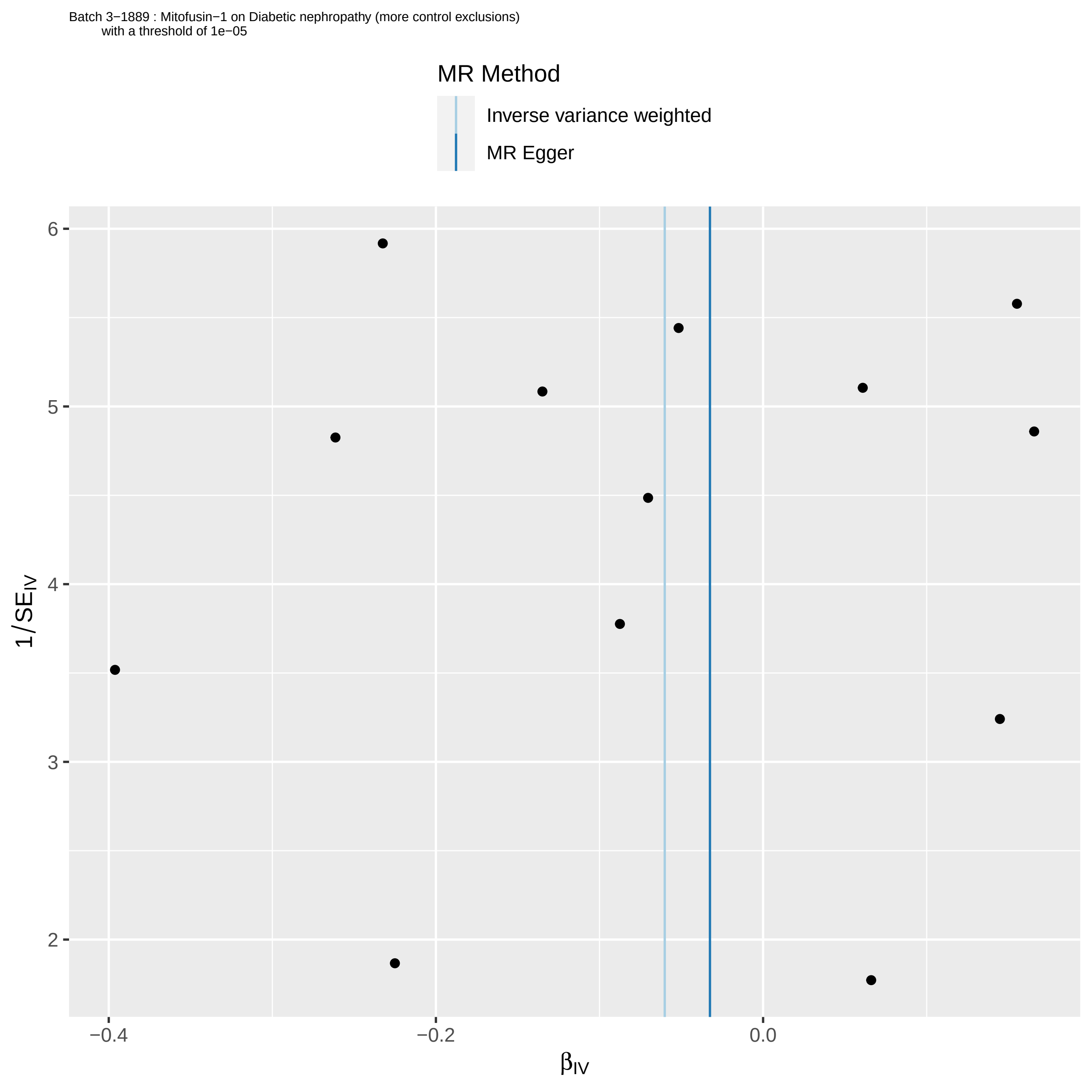

Batch 3−1889 : Mitofusin−1 on Diabetic nephropathy (more control exclusions)
with a threshold of 1e−05
MR Method
Inverse variance weighted
MR Egger
6
5
4
3
2
1/SE_IV
−0.4
−0.2
0.0
β_IV

Batch 3−1890 : Beta−1,3−N−acetylglucosaminyltransferase manic fringe on Diabetic nephropathy (more control exclusions)
with a threshold of 1e−05

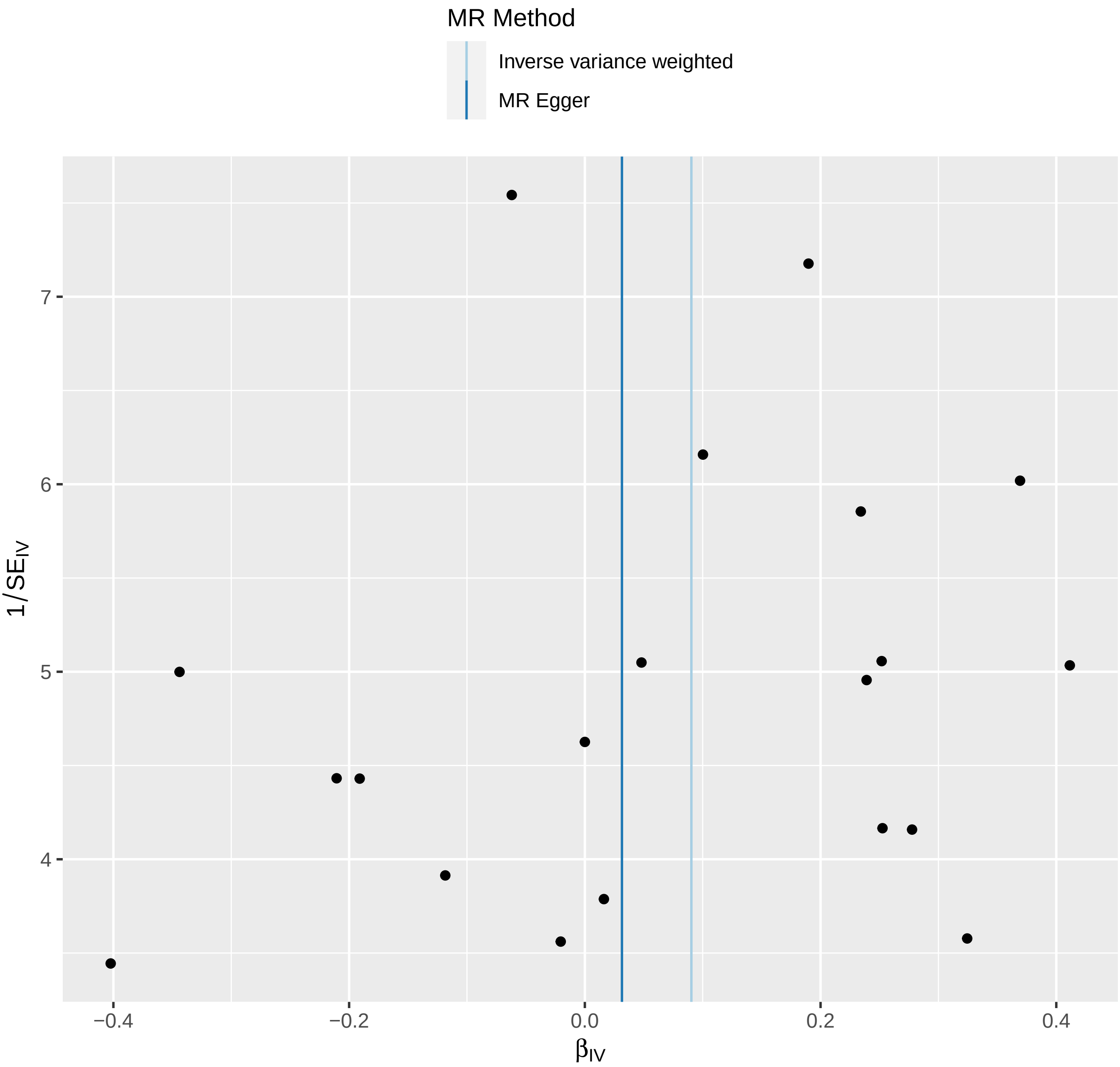

Batch 3−1891 : MAX gene−associated protein on Diabetic nephropathy (more control exclusions)
with a threshold of 1e−05

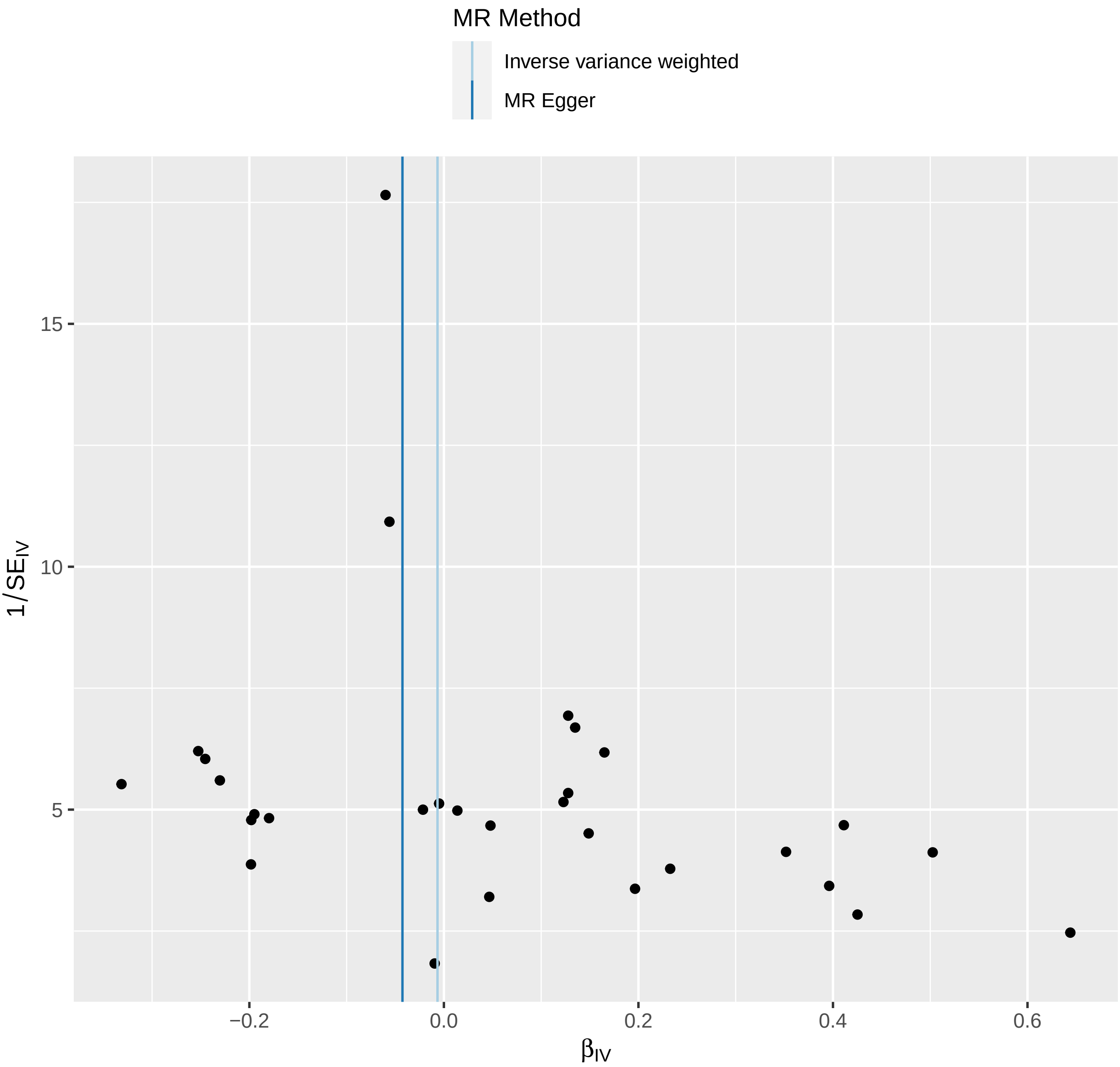

Batch 3−1892 : Alpha−1,3−mannosyl−glycoprotein 2−beta−N−acetylglucosaminyltransferase on Diabetic nephropathy (more control exclusions) with a threshold of 1e−05

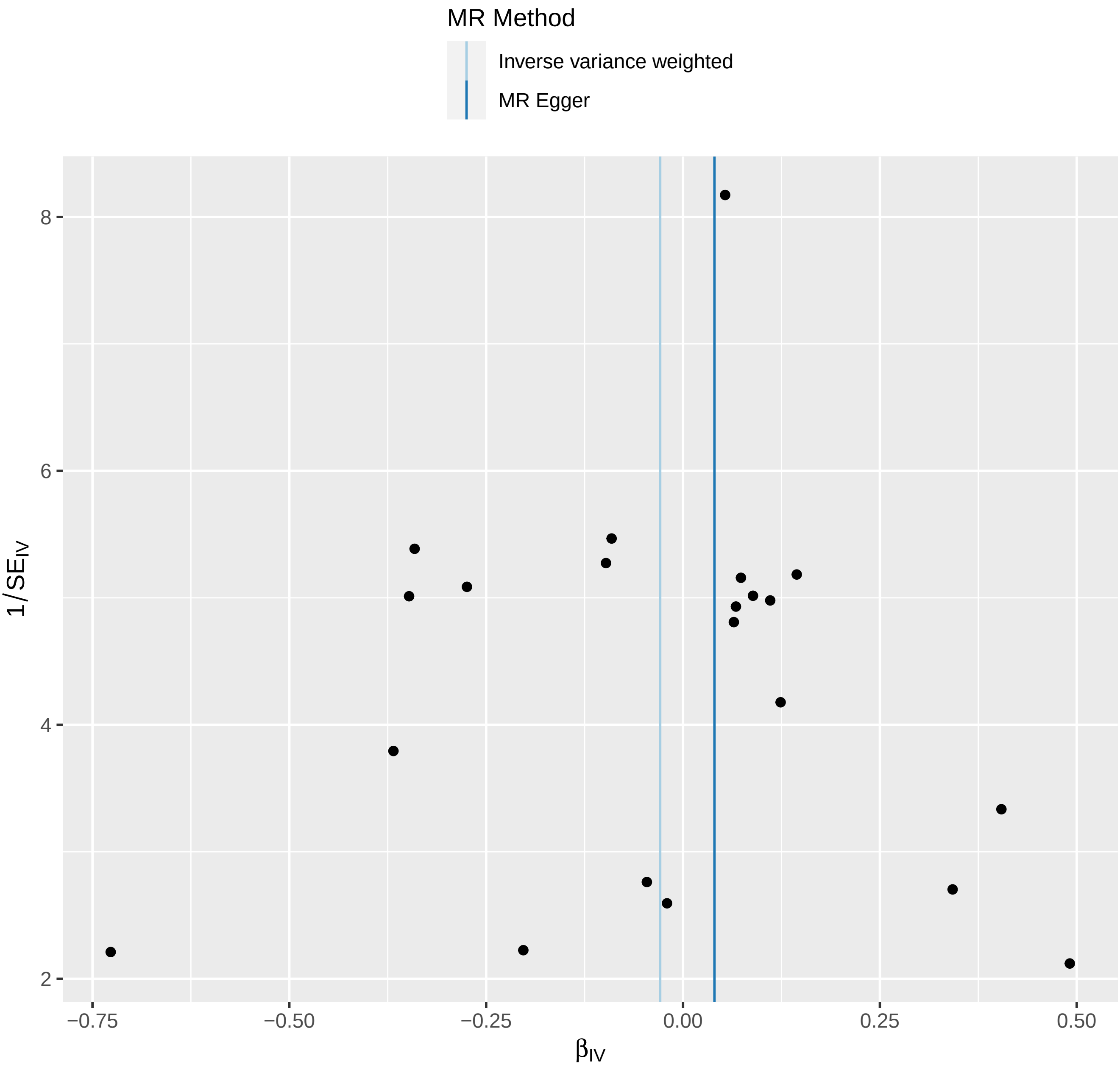

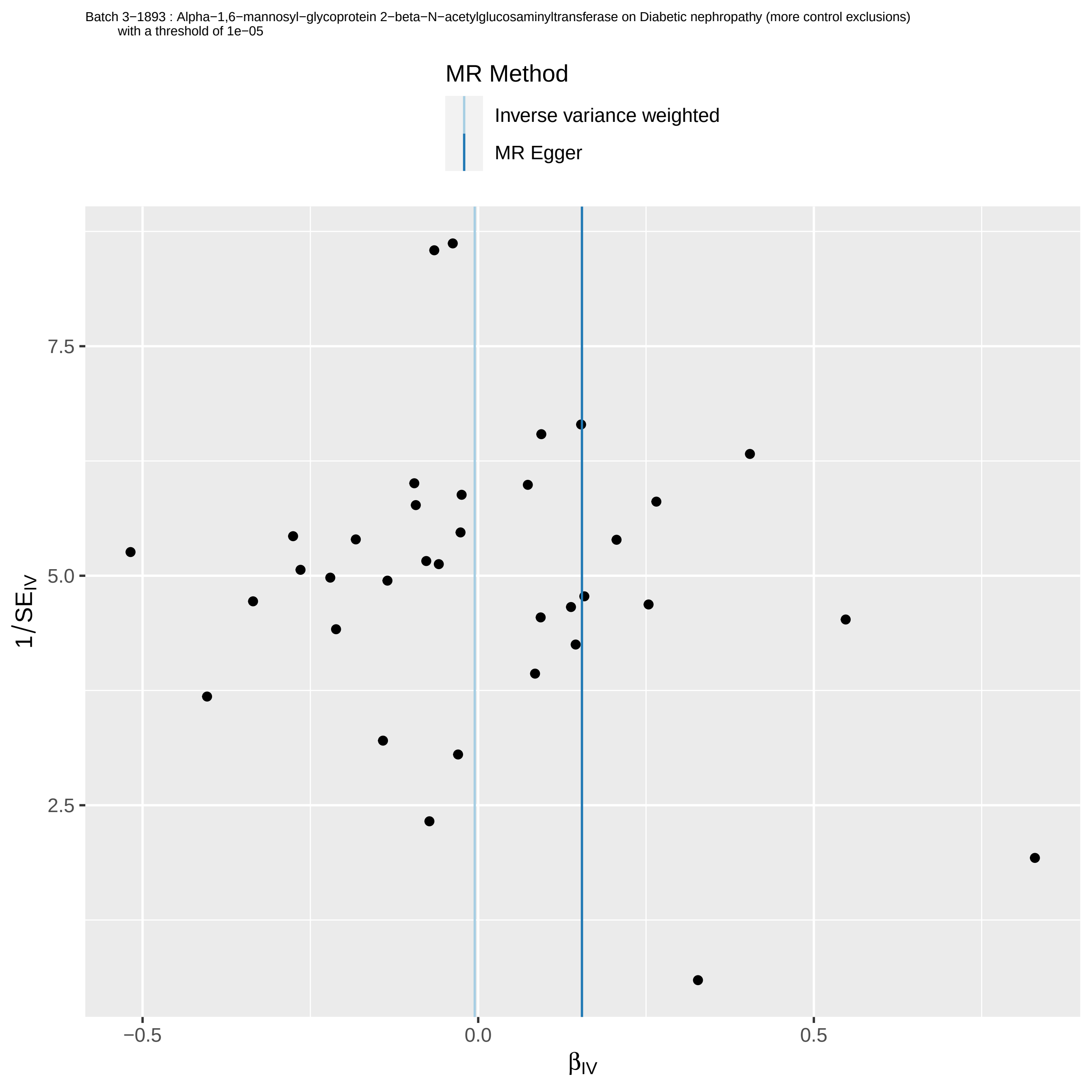

Batch 3−1893 : Alpha−1,6−mannosyl−glycoprotein 2−beta−N−acetylglucosaminyltransferase on Diabetic nephropathy (more control exclusions)
with a threshold of 1e−05
MR Method
Inverse variance weighted
MR Egger
7.5
5.0
2.5
1/SE_IV
−0.5
0.0
0.5
β_IV

Batch 3−1894 : Alpha−1,3−mannosyl−glycoprotein 4−beta−N−acetylglucosaminyltransferase B on Diabetic nephropathy (more control exclusions)
with a threshold of 1e−05

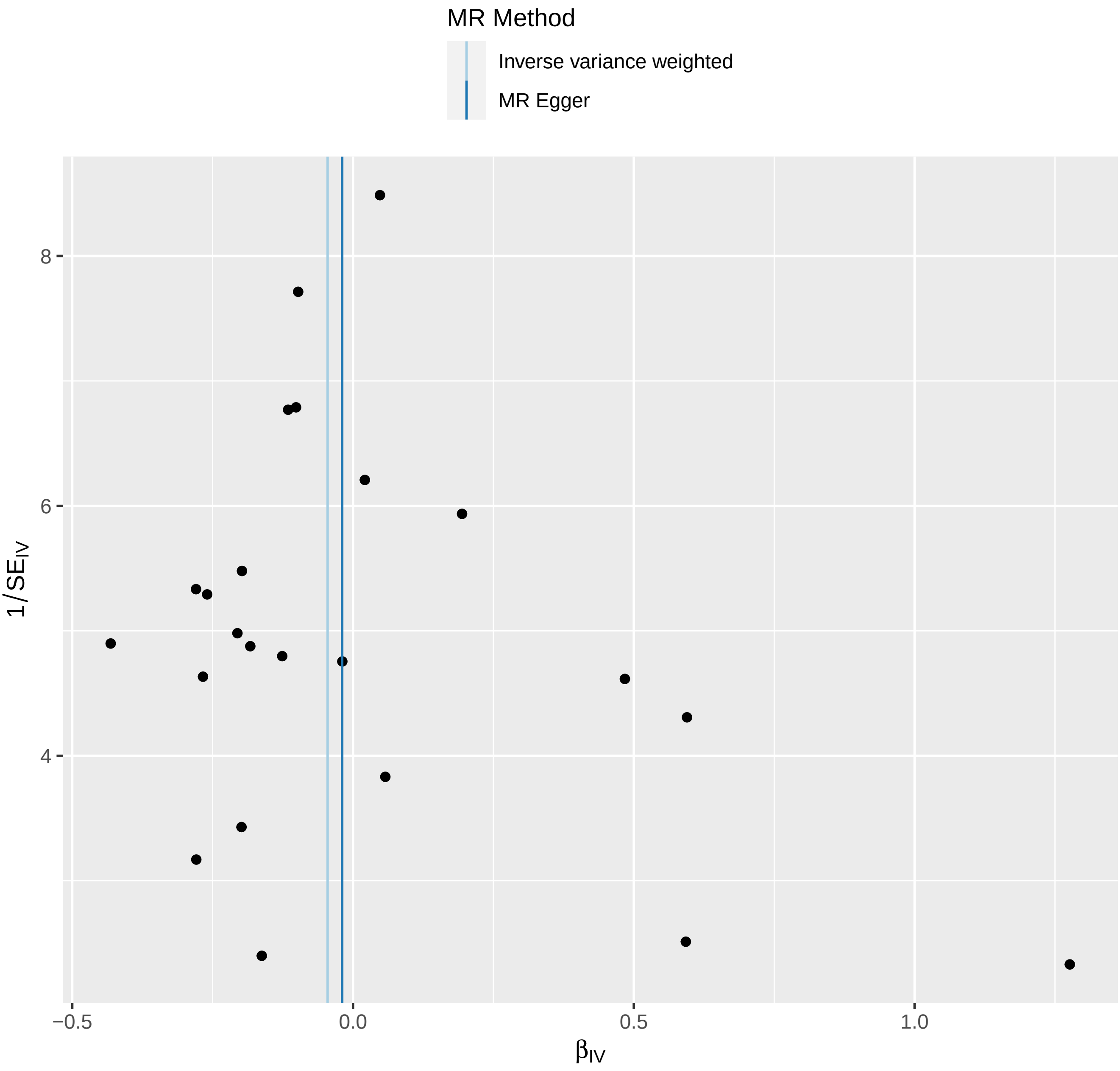

Batch 3−1895 : Alpha−1,3−mannosyl−glycoprotein 4−beta−N−acetylglucosaminyltransferase C on Diabetic nephropathy (more control exclusions) with a threshold of 1e−05

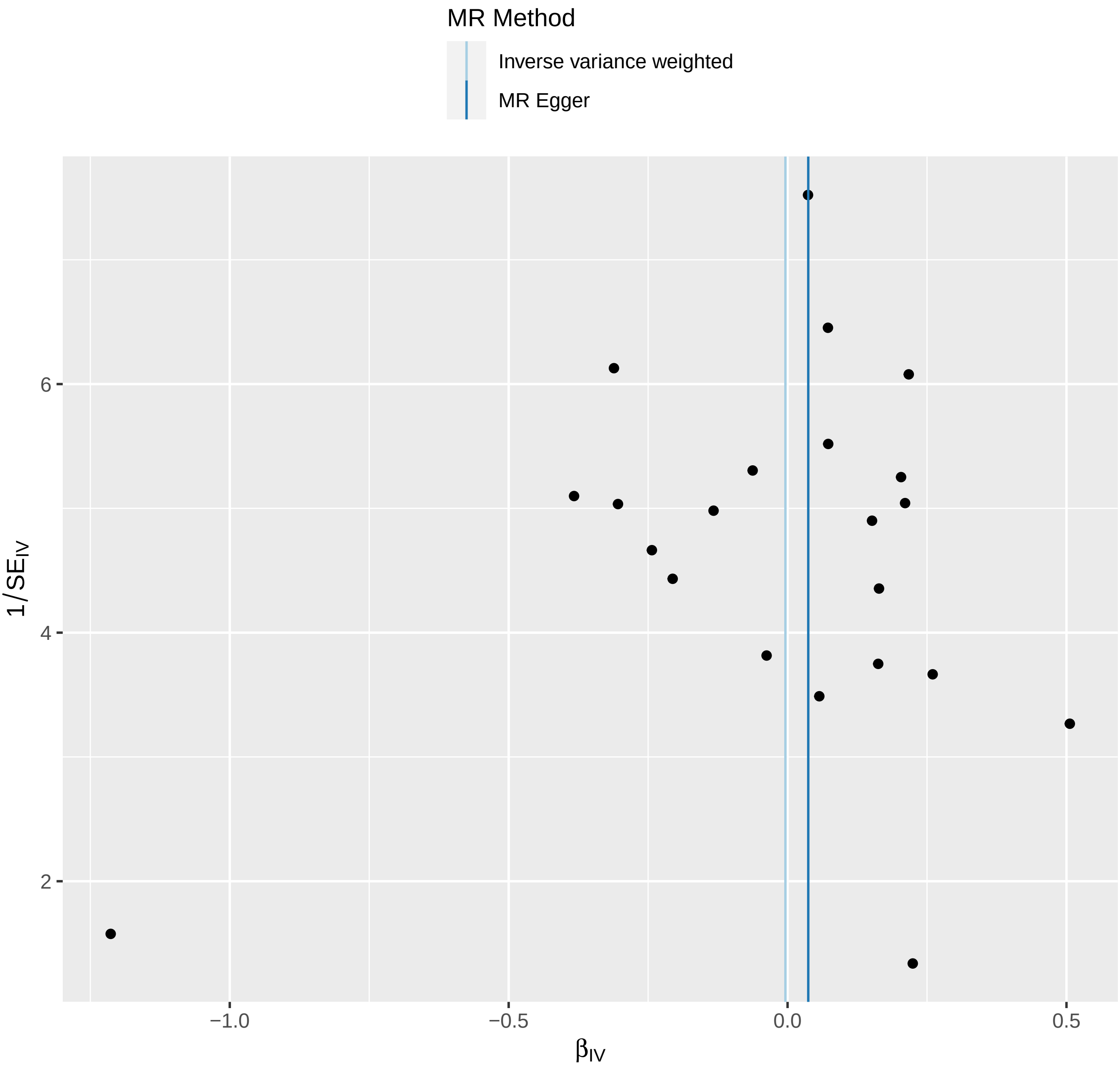

Batch 3−1896 : Melanoma−derived growth regulatory protein on Diabetic nephropathy (more control exclusions)
with a threshold of 1e−05

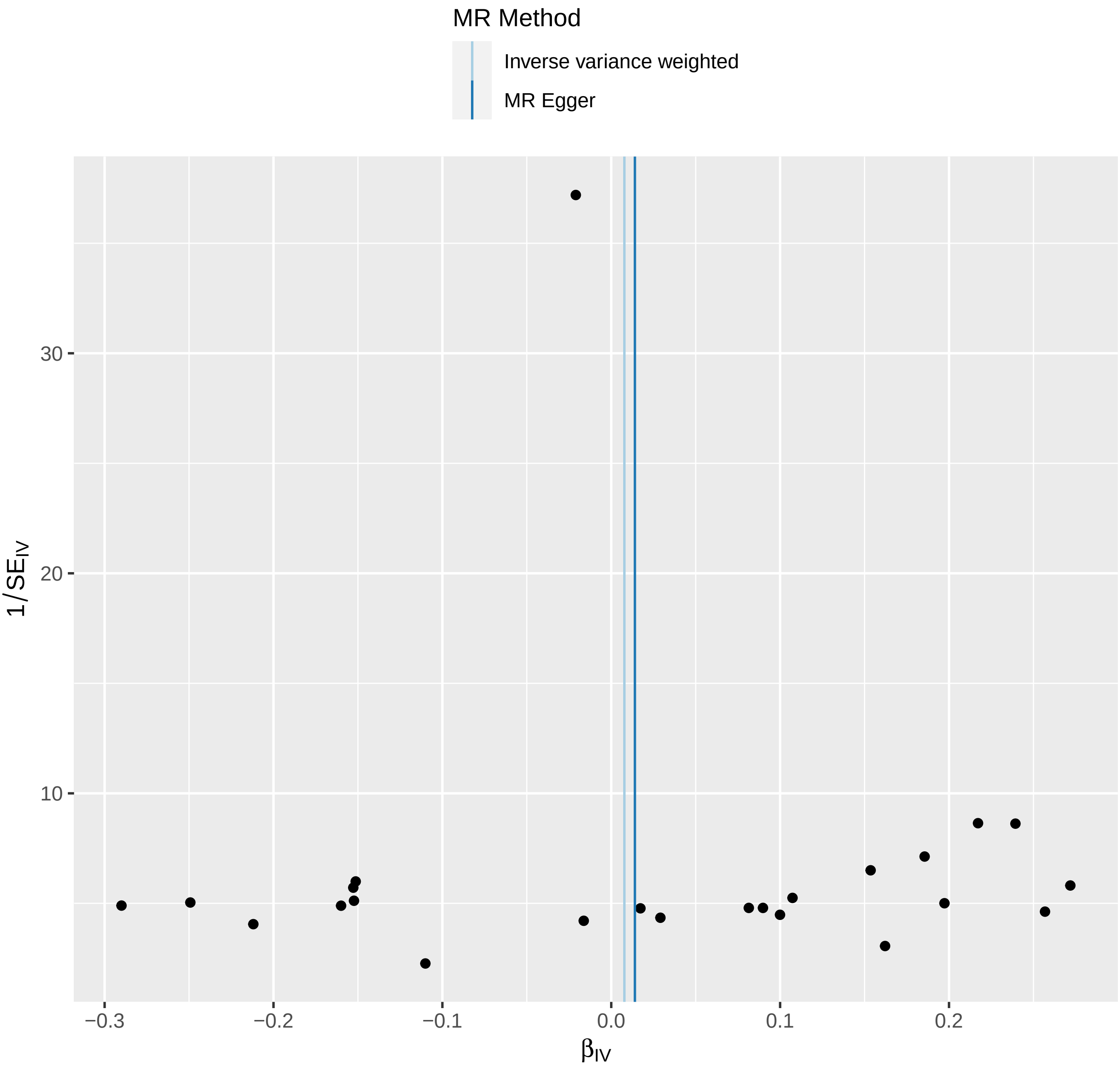

Batch 3−1897 : MICAL−like protein 2 on Diabetic nephropathy (more control exclusions)
with a threshold of 1e−05

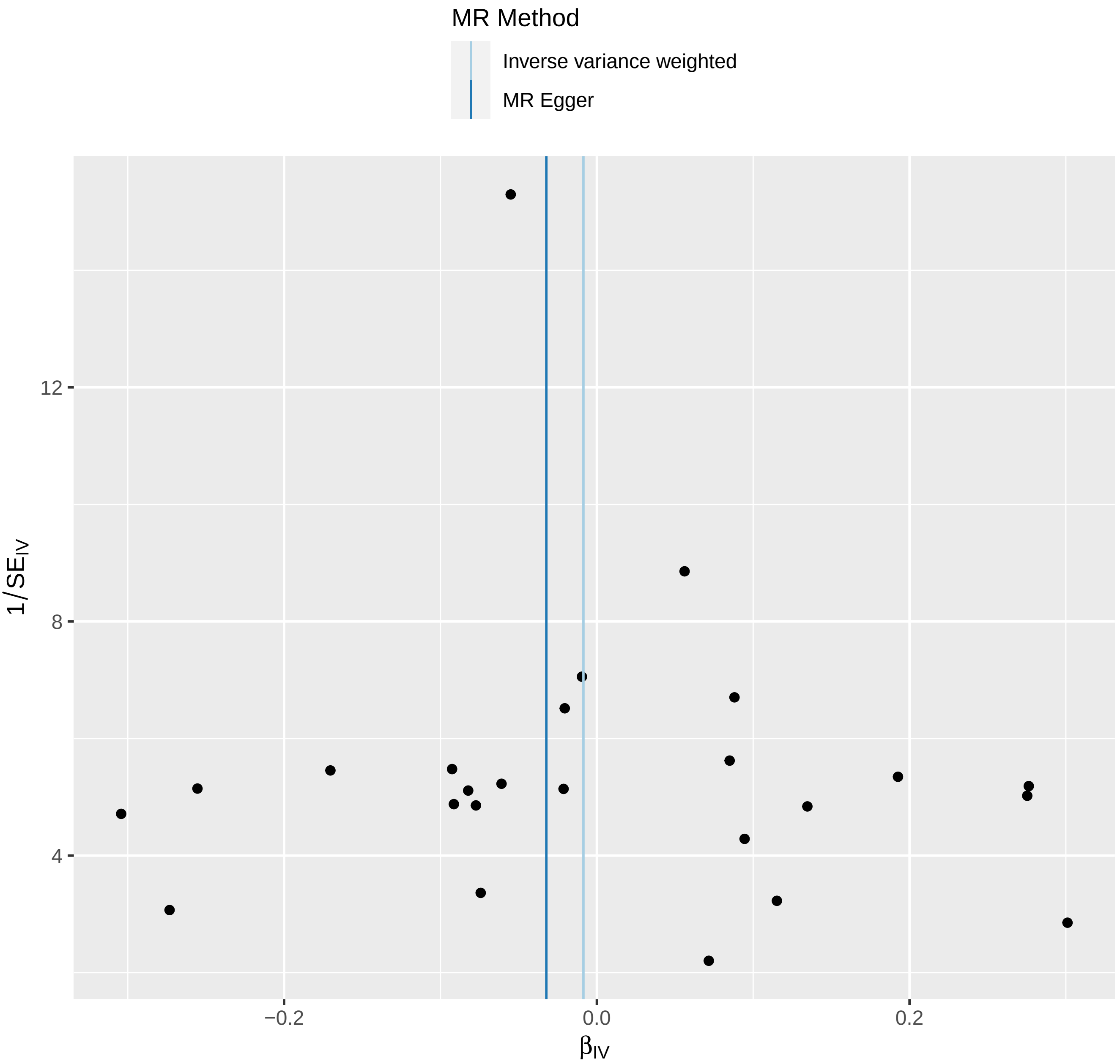

Batch 3−1898 : MHC class I polypeptide−related sequence B on Diabetic nephropathy (more control exclusions) with a threshold of 1e−05

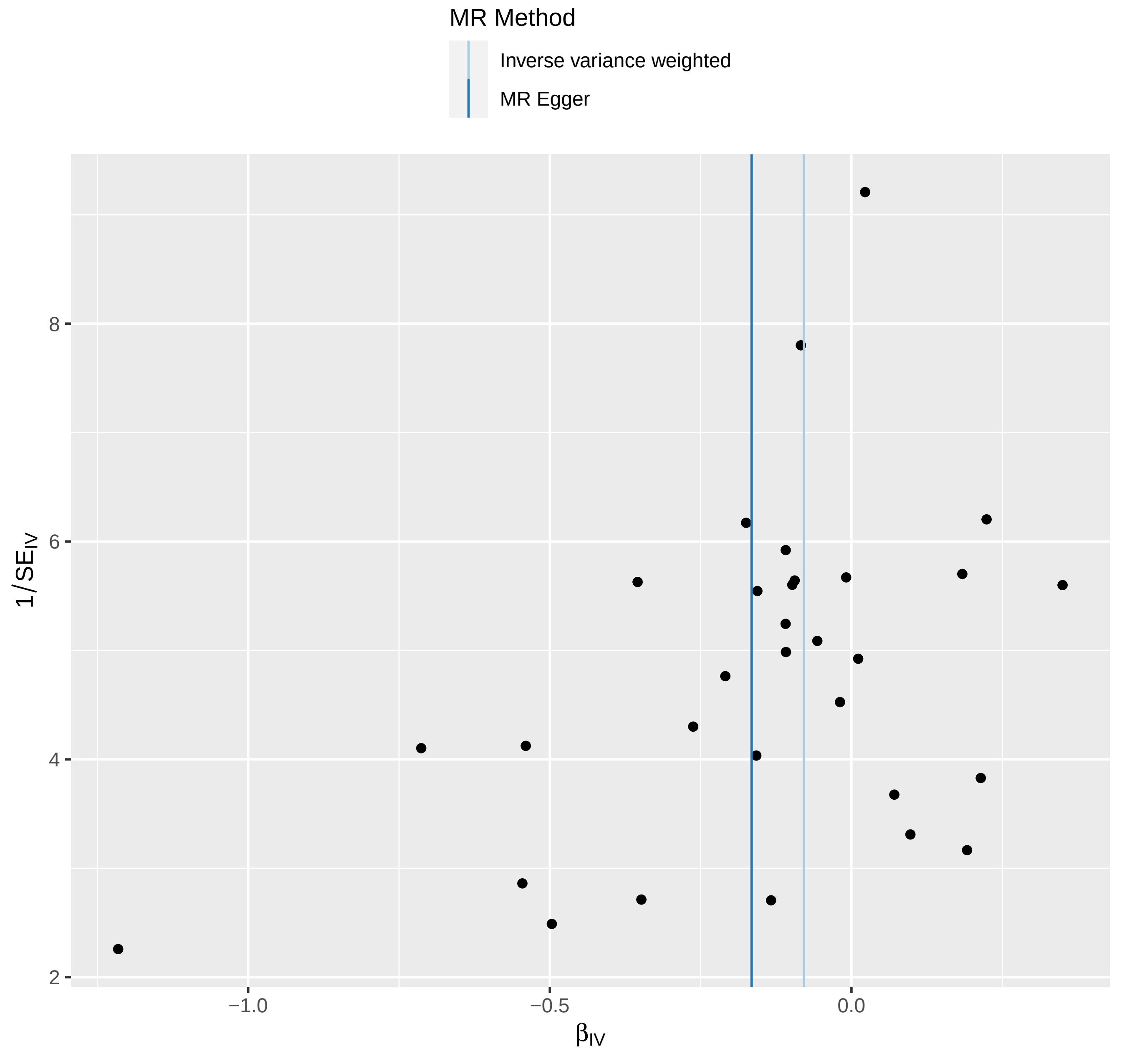

Batch 3−1899 : Probable E3 ubiquitin−protein ligase MID2 on Diabetic nephropathy (more control exclusions)
with a threshold of 1e−05

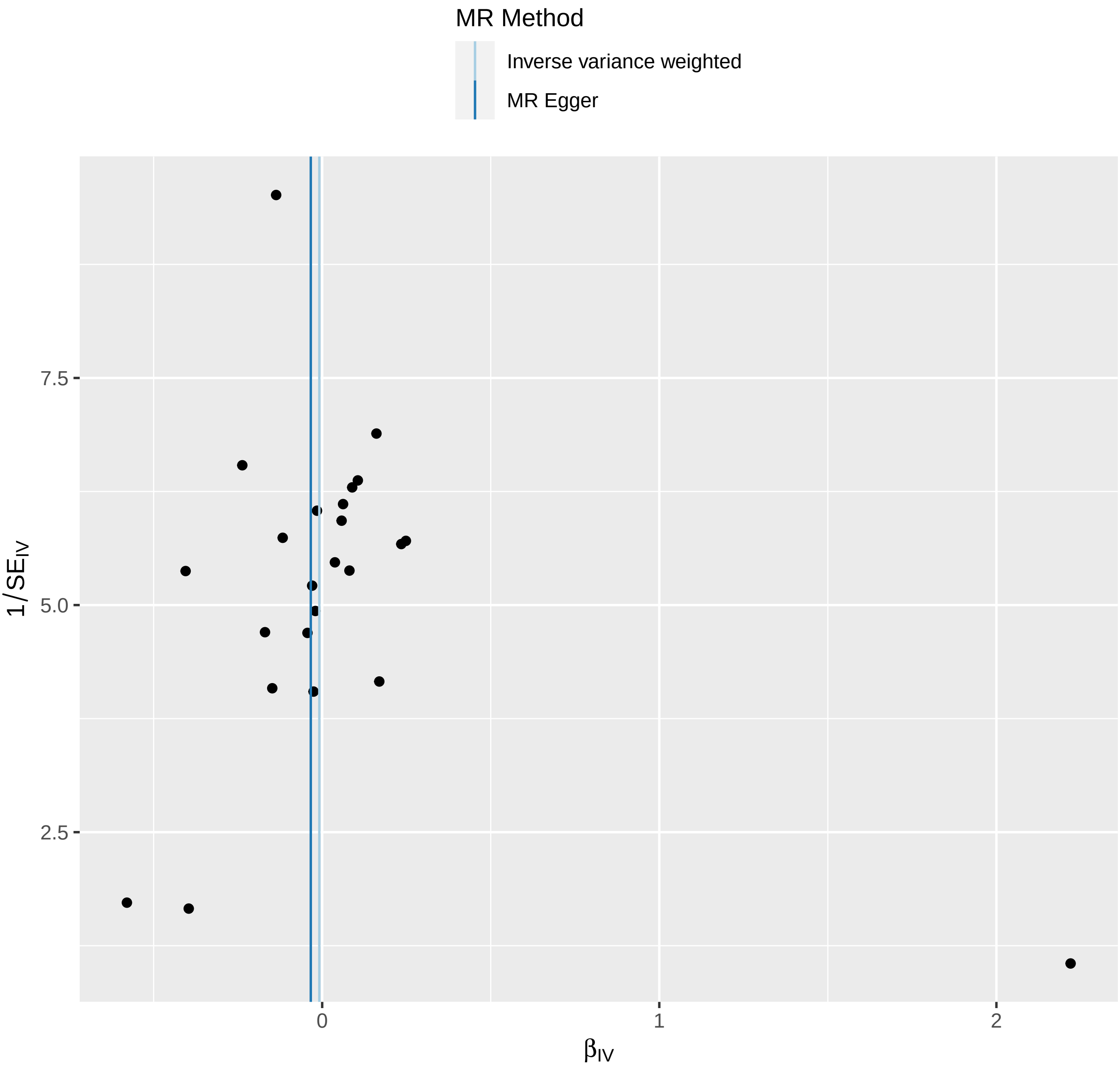

Batch 3−1900 : Macrophage migration inhibitory factor on Diabetic nephropathy (more control exclusions) with a threshold of 1e−05

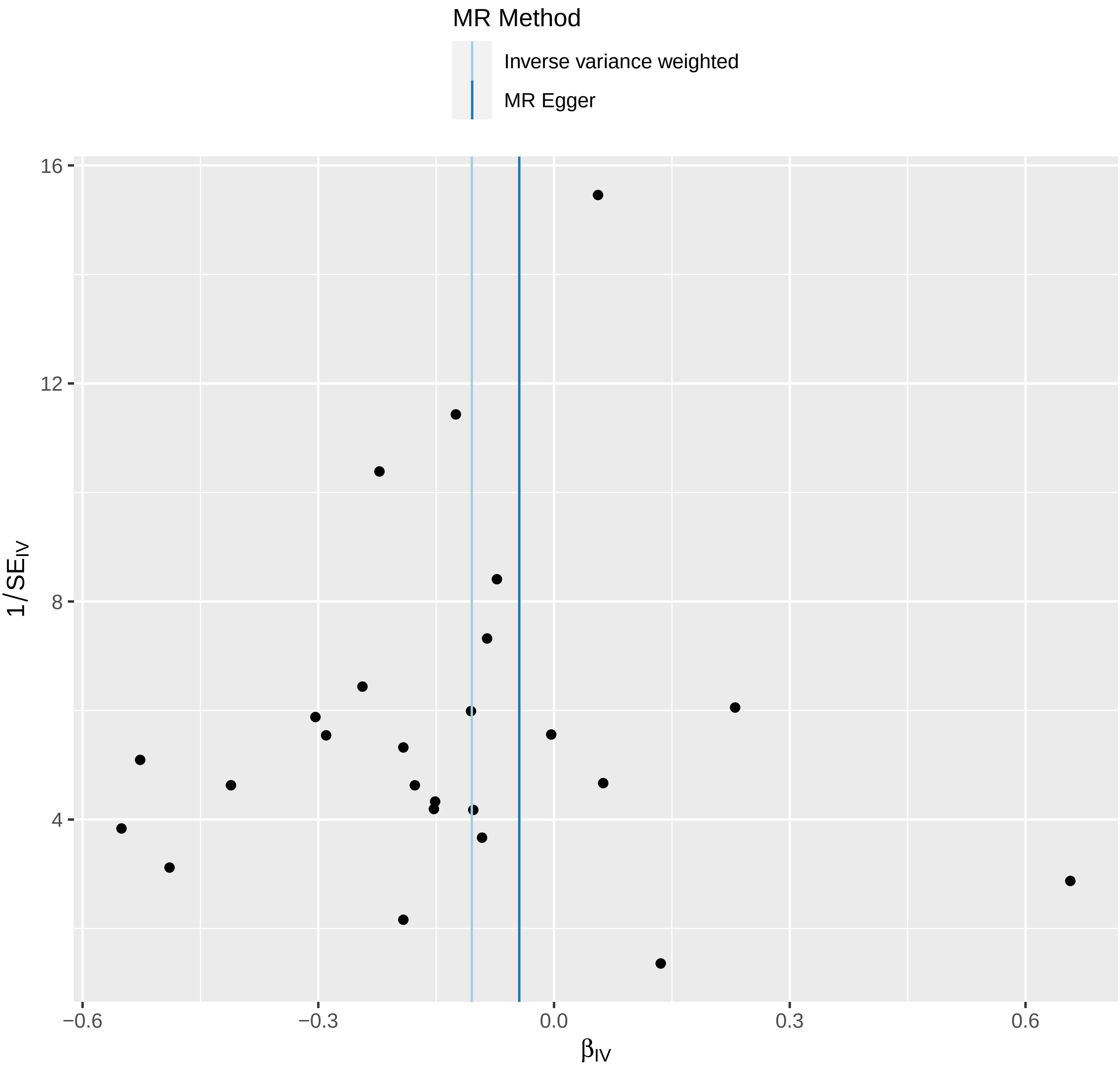

Batch 3−1901 : Allergin−1 on Diabetic nephropathy (more control exclusions)
with a threshold of 1e−05

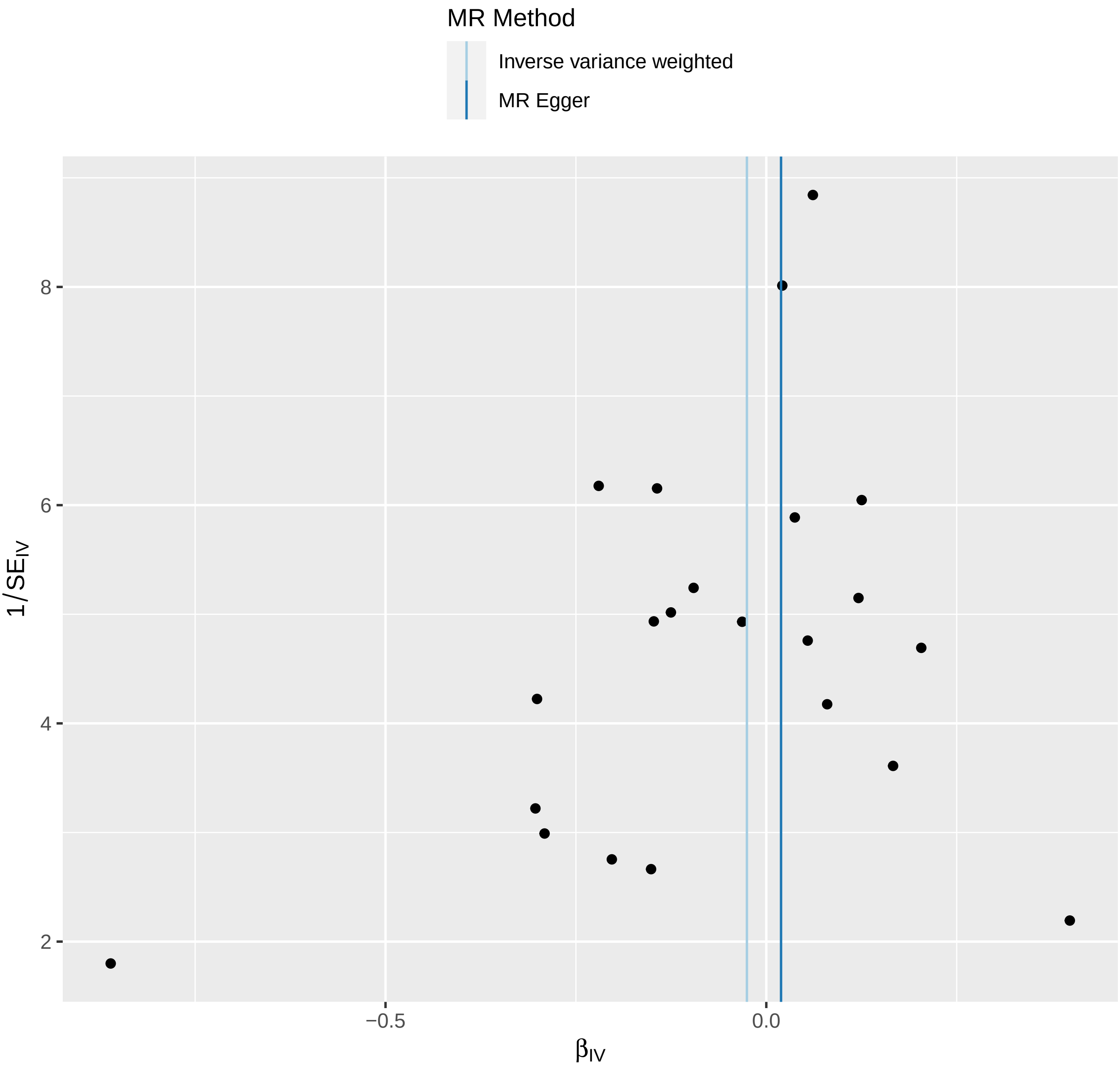

Batch 3−1902 : MICOS complex subunit MIC10 on Diabetic nephropathy (more control exclusions)
with a threshold of 1e−05

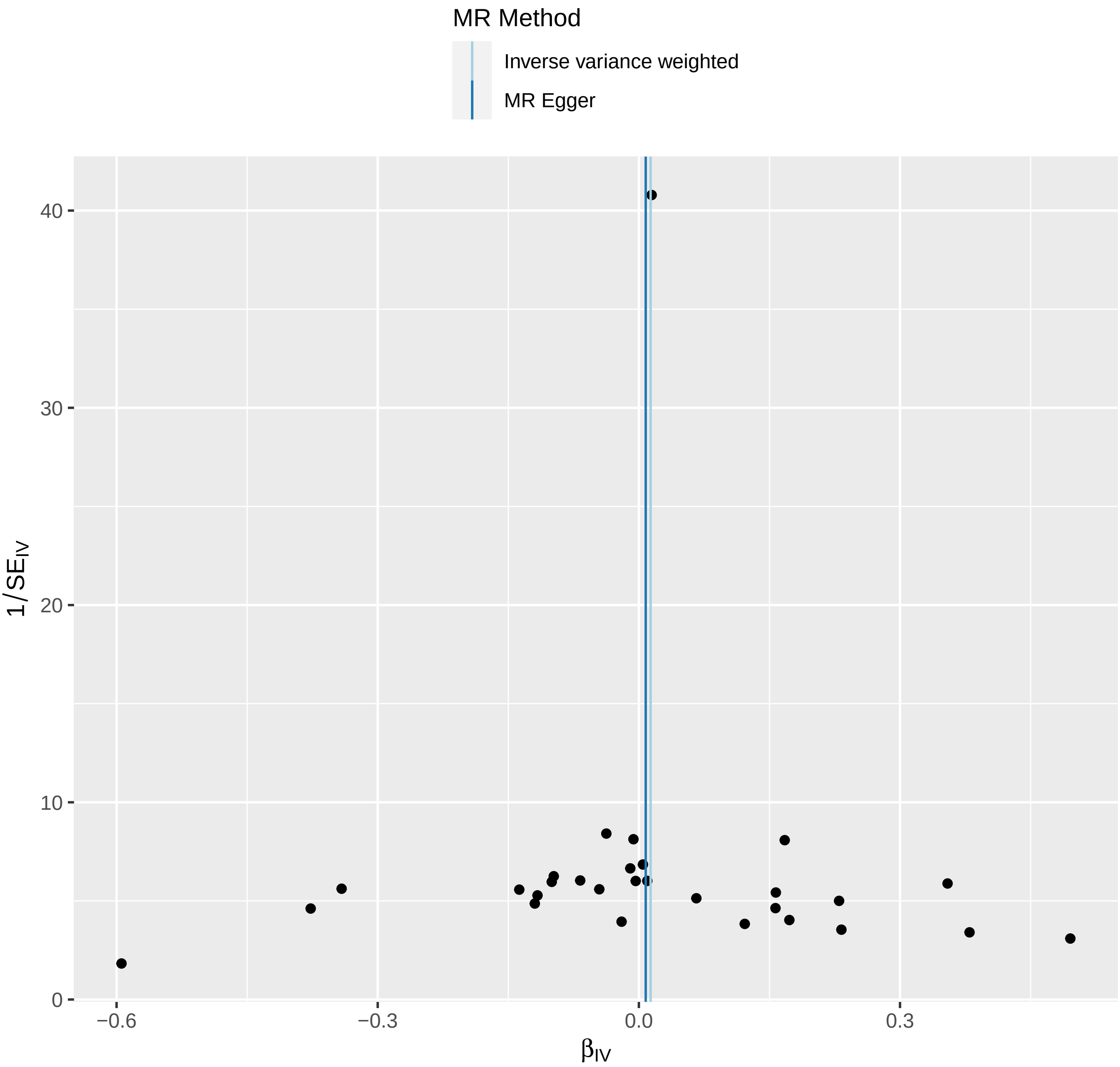

Batch 3−1903 : Multiple inositol polyphosphate phosphatase 1 on Diabetic nephropathy (more control exclusions)
with a threshold of 1e−05

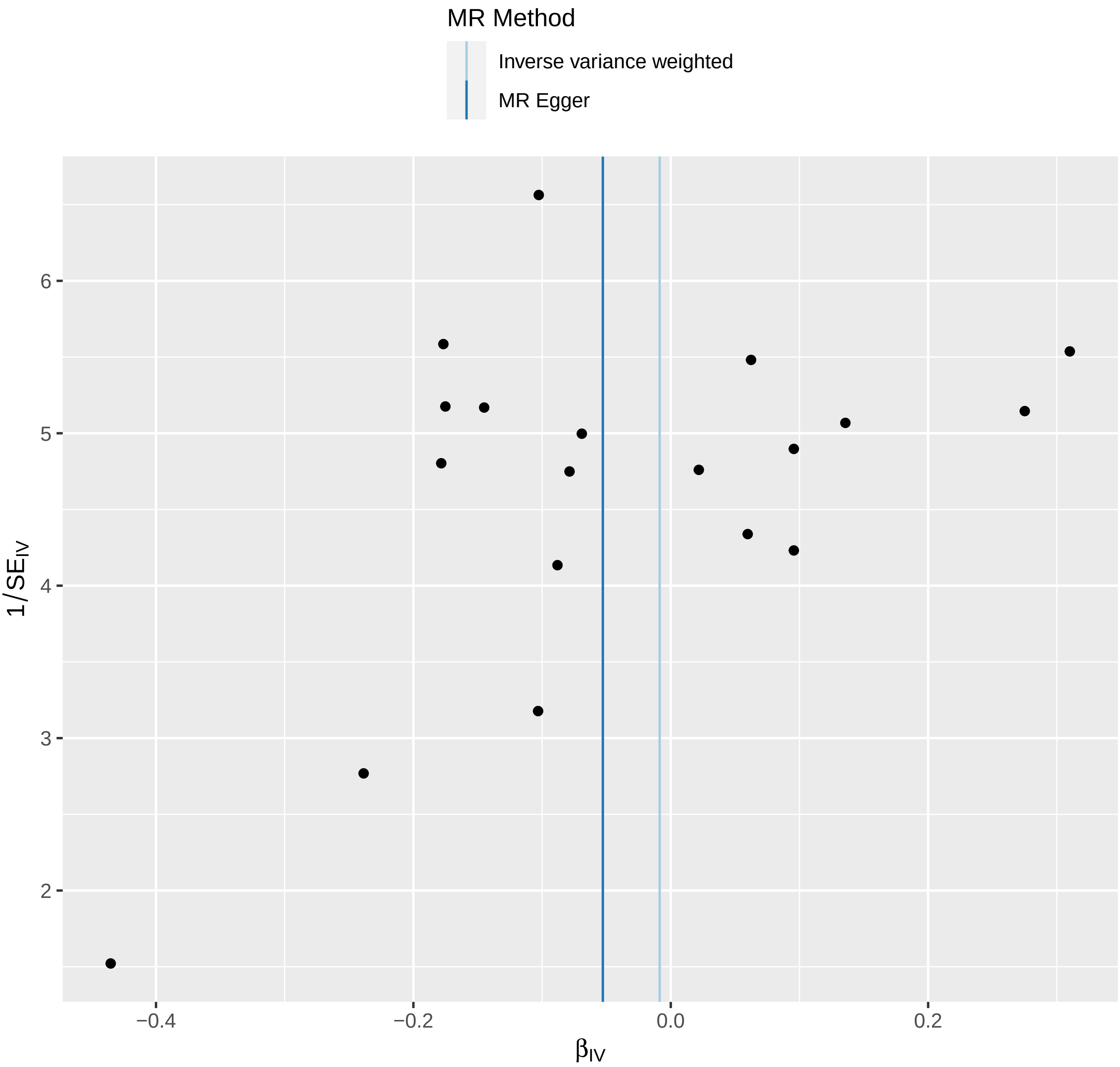

Batch 3−1904 : Malectin on Diabetic nephropathy (more control exclusions)
with a threshold of 1e−05

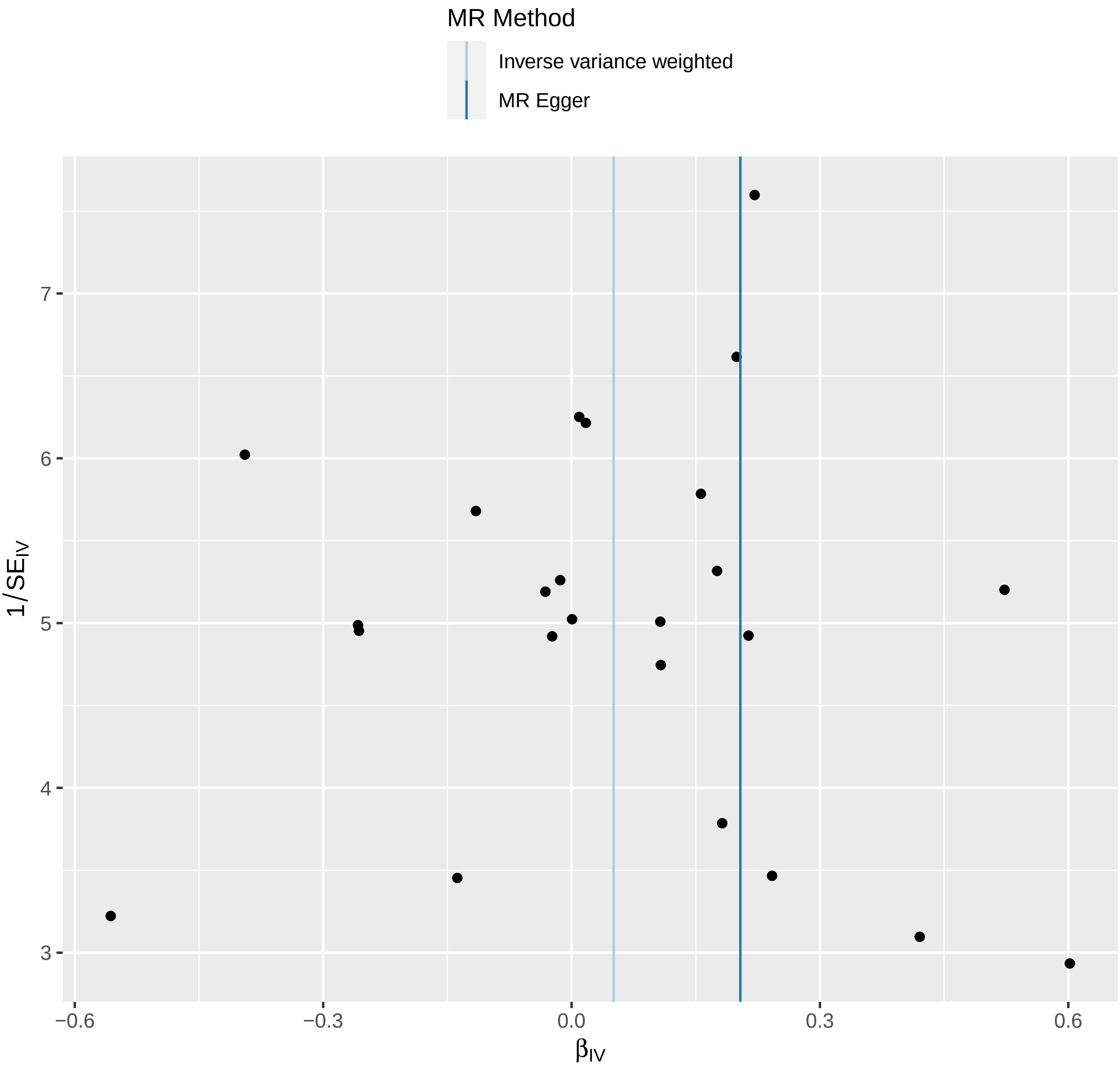

Batch 3−1905 : Histone−lysine N−methyltransferase 2D on Diabetic nephropathy (more control exclusions)
with a threshold of 1e−05

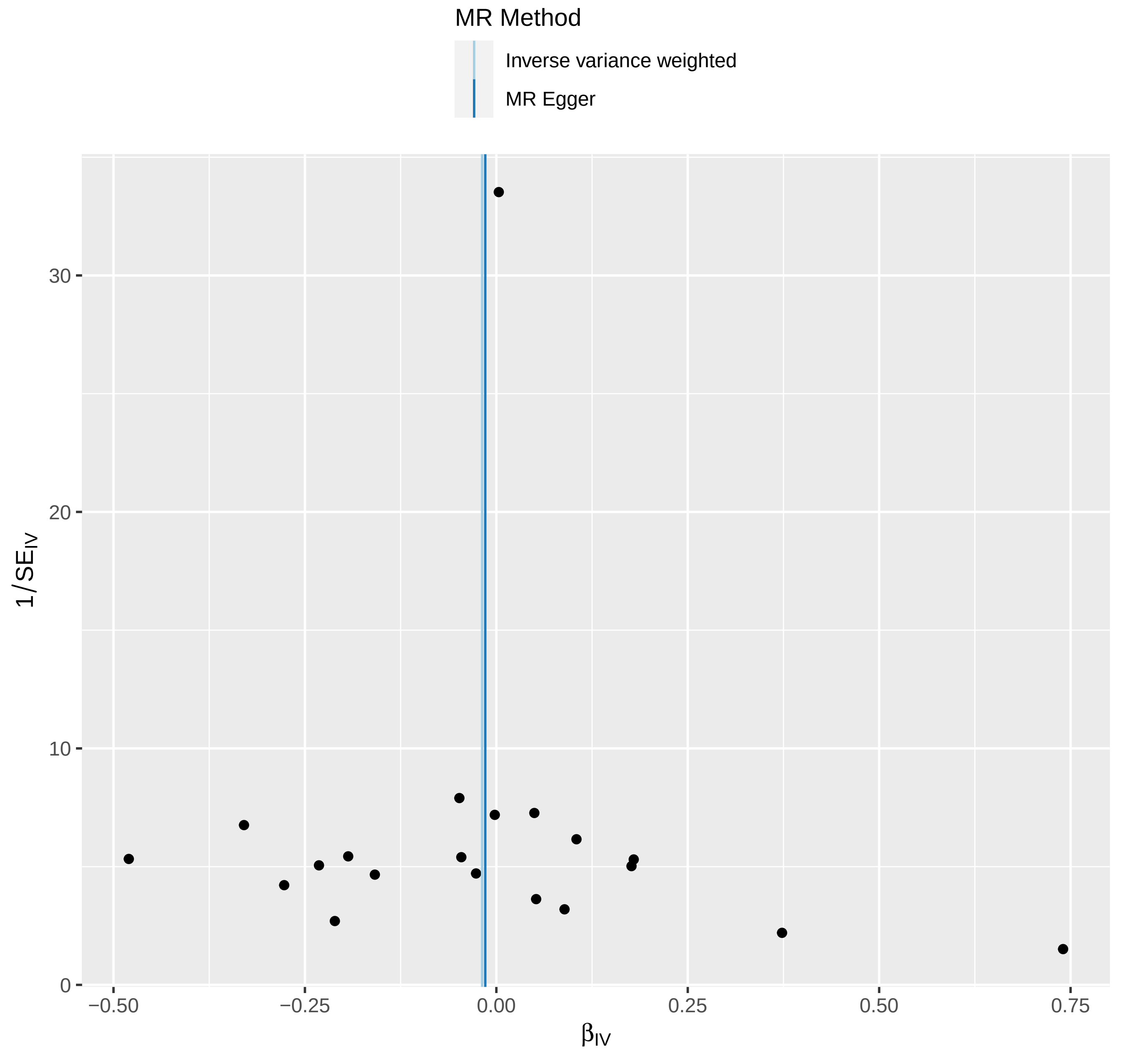

Batch 3−1906 : Promotilin on Diabetic nephropathy (more control exclusions)
with a threshold of 1e−05

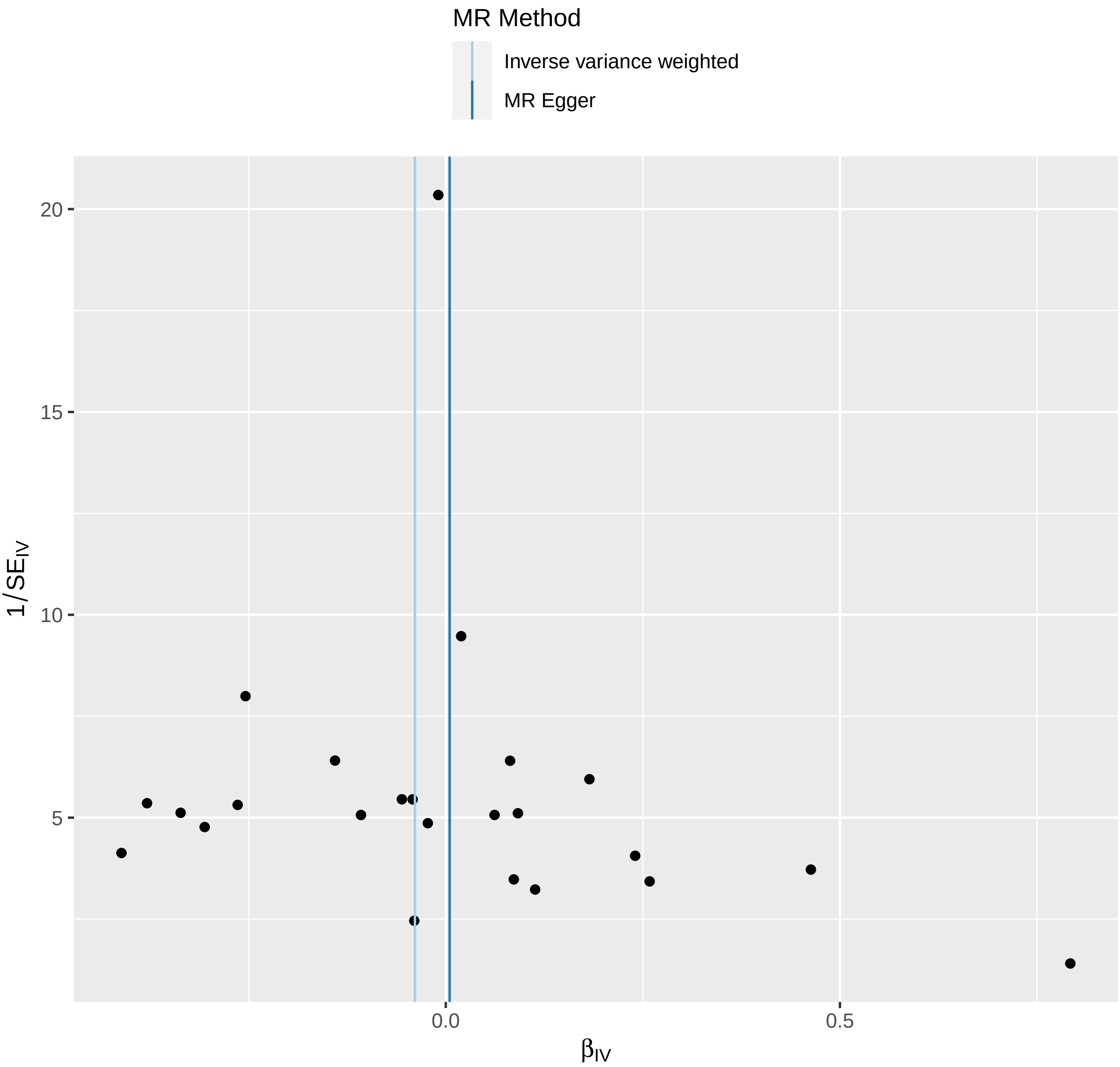

Batch 3−1907 : Malonyl−CoA decarboxylase, mitochondrial on Diabetic nephropathy (more control exclusions)
with a threshold of 1e−05

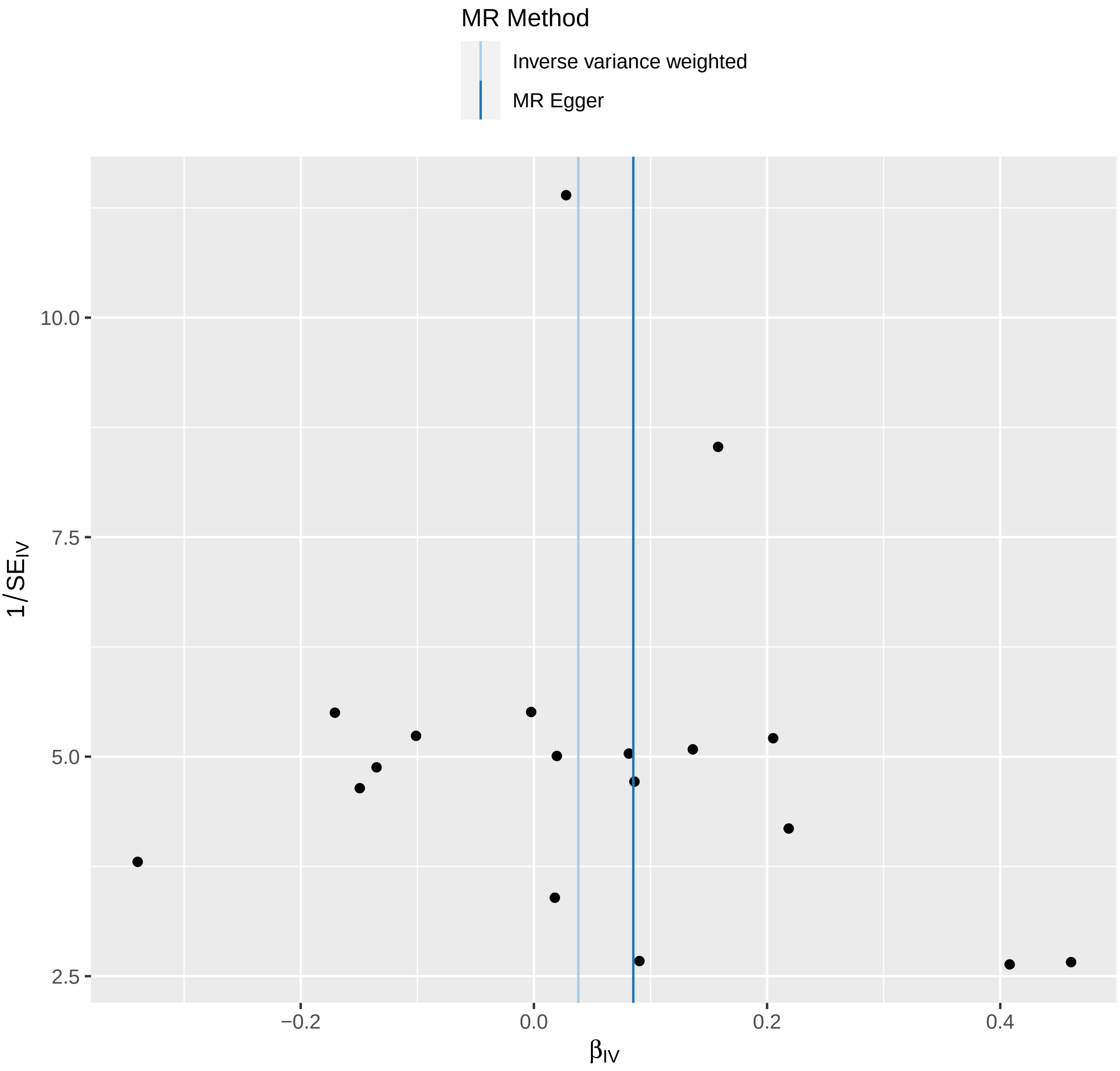

Batch 3−1908 : Membrane metallo−endopeptidase−like 1 on Diabetic nephropathy (more control exclusions)
with a threshold of 1e−05

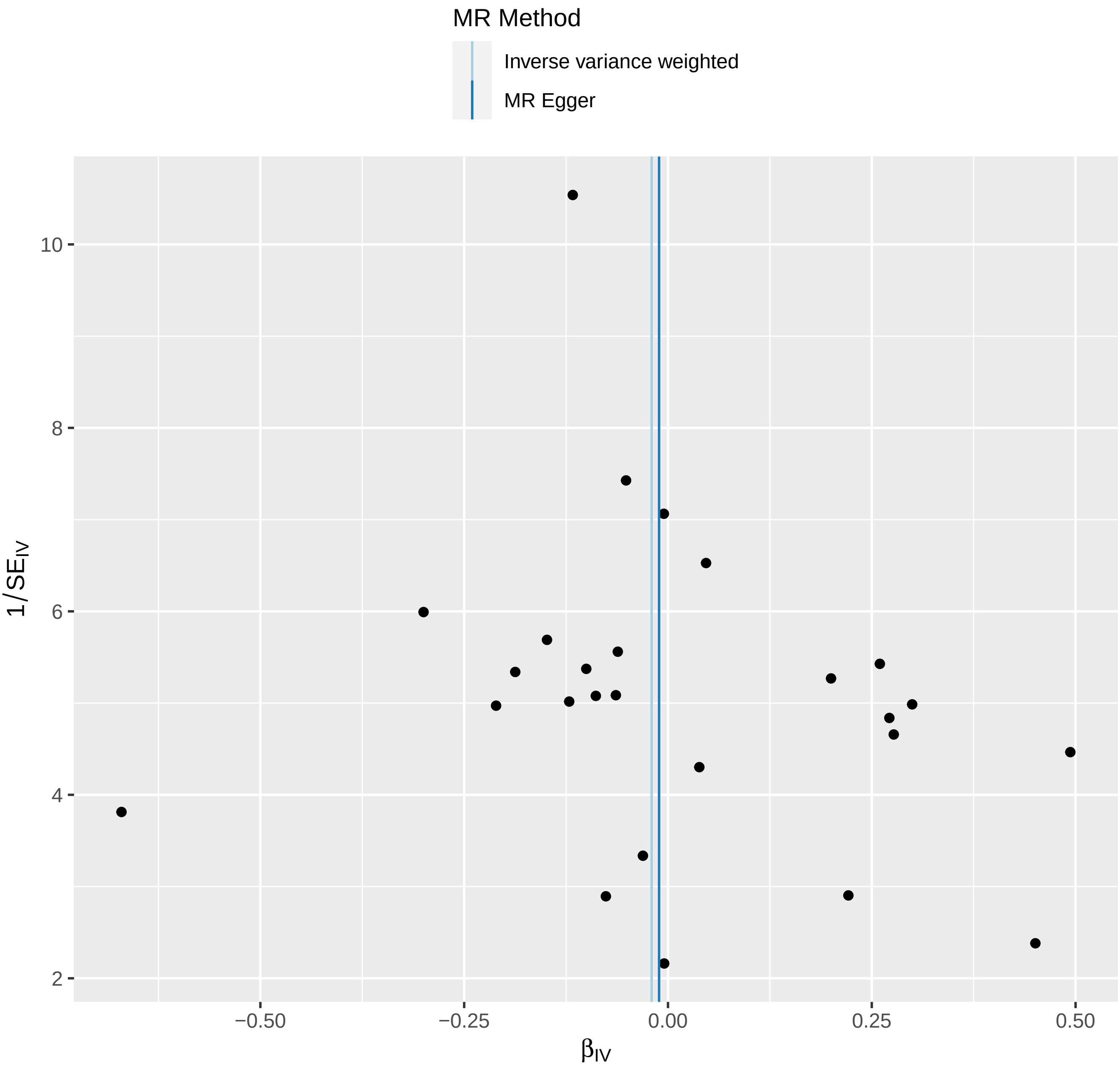

Batch 3−1909 : Membrane magnesium transporter 1 on Diabetic nephropathy (more control exclusions)
with a threshold of 1e−05

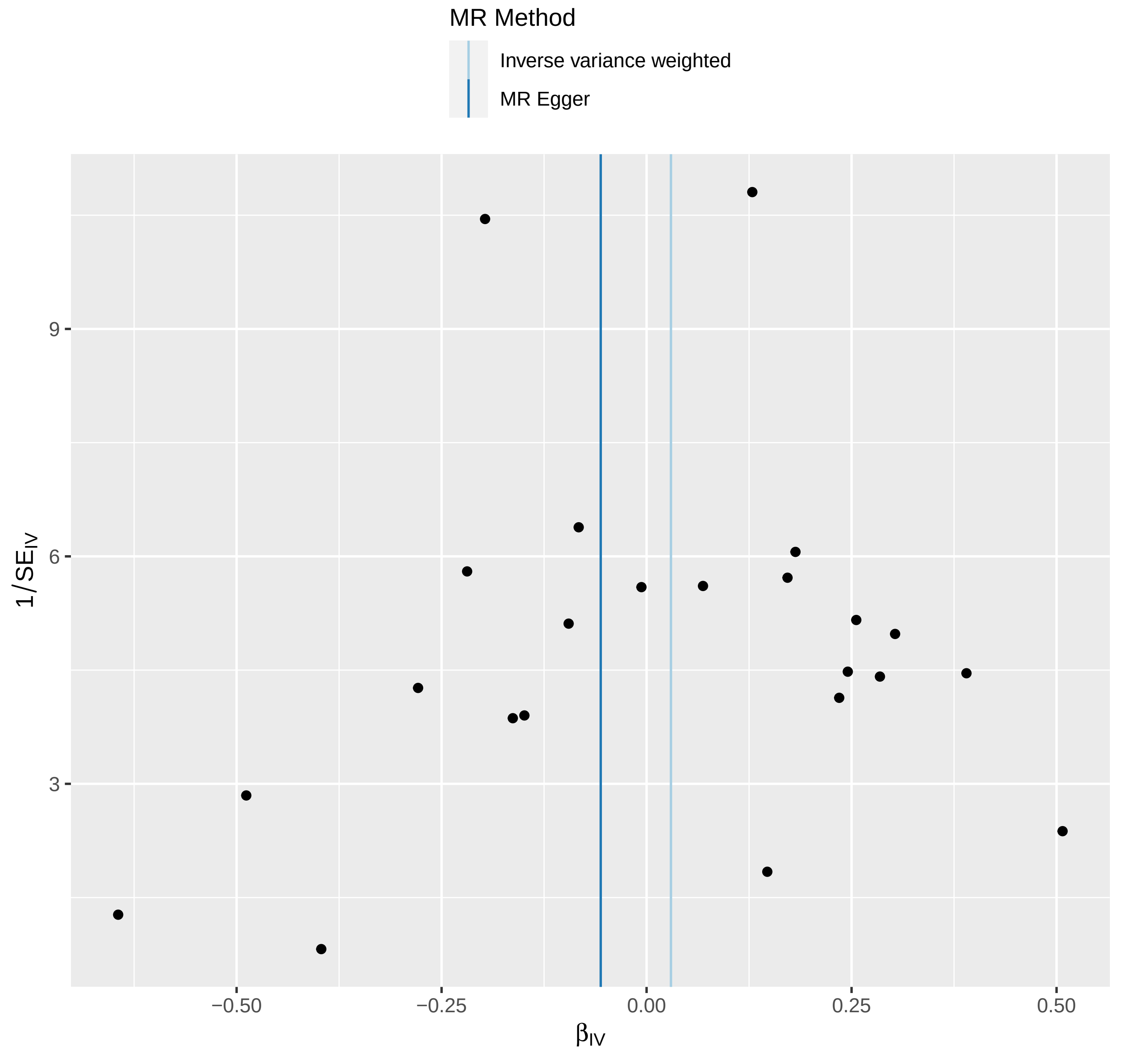

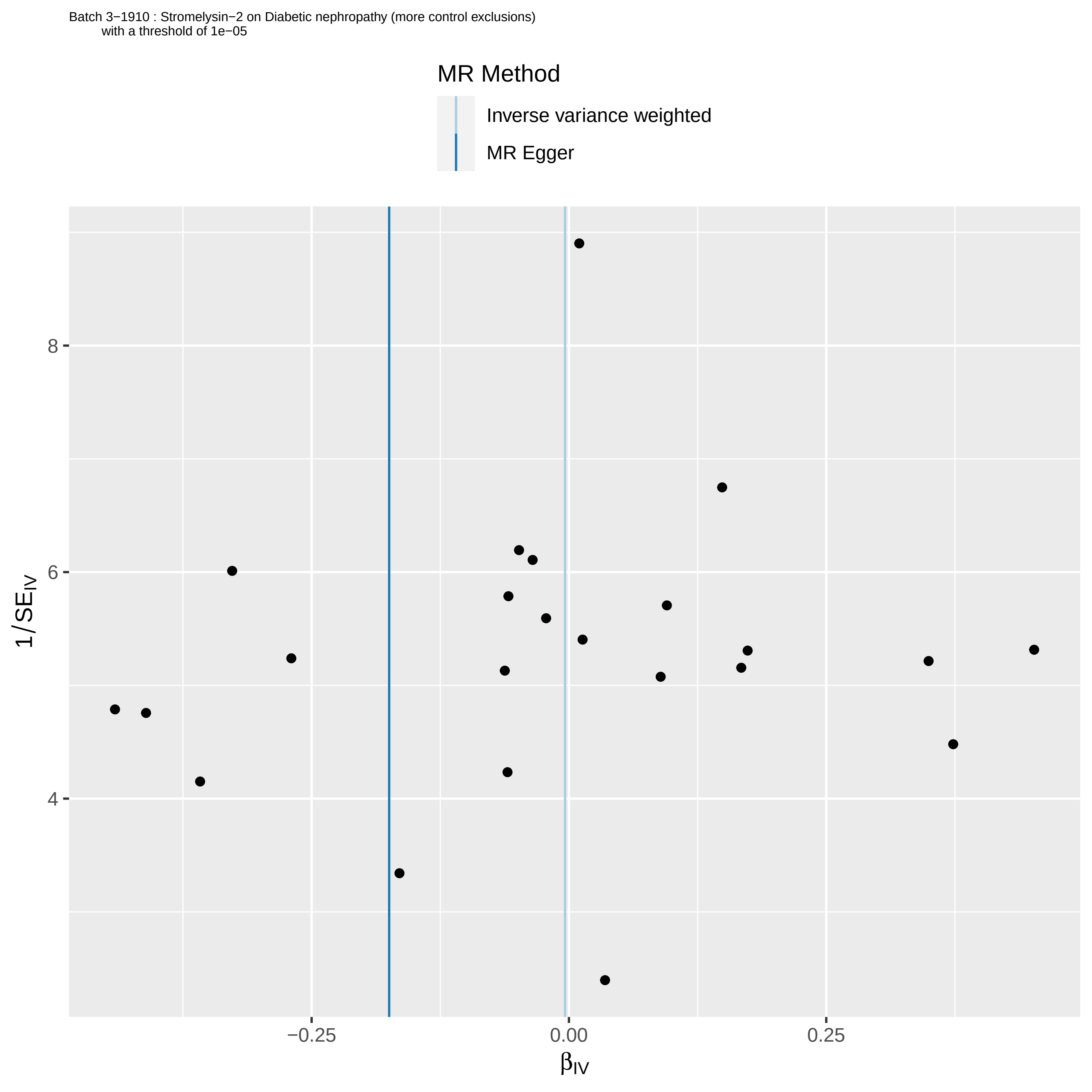
Batch 3−1910 : Stromelysin−2 on Diabetic nephropathy (more control exclusions)
with a threshold of 1e−05
MR Method
Inverse variance weighted
MR Egger
8
6
4
1/SE_IV
−0.25
0.00
0.25
β_IV

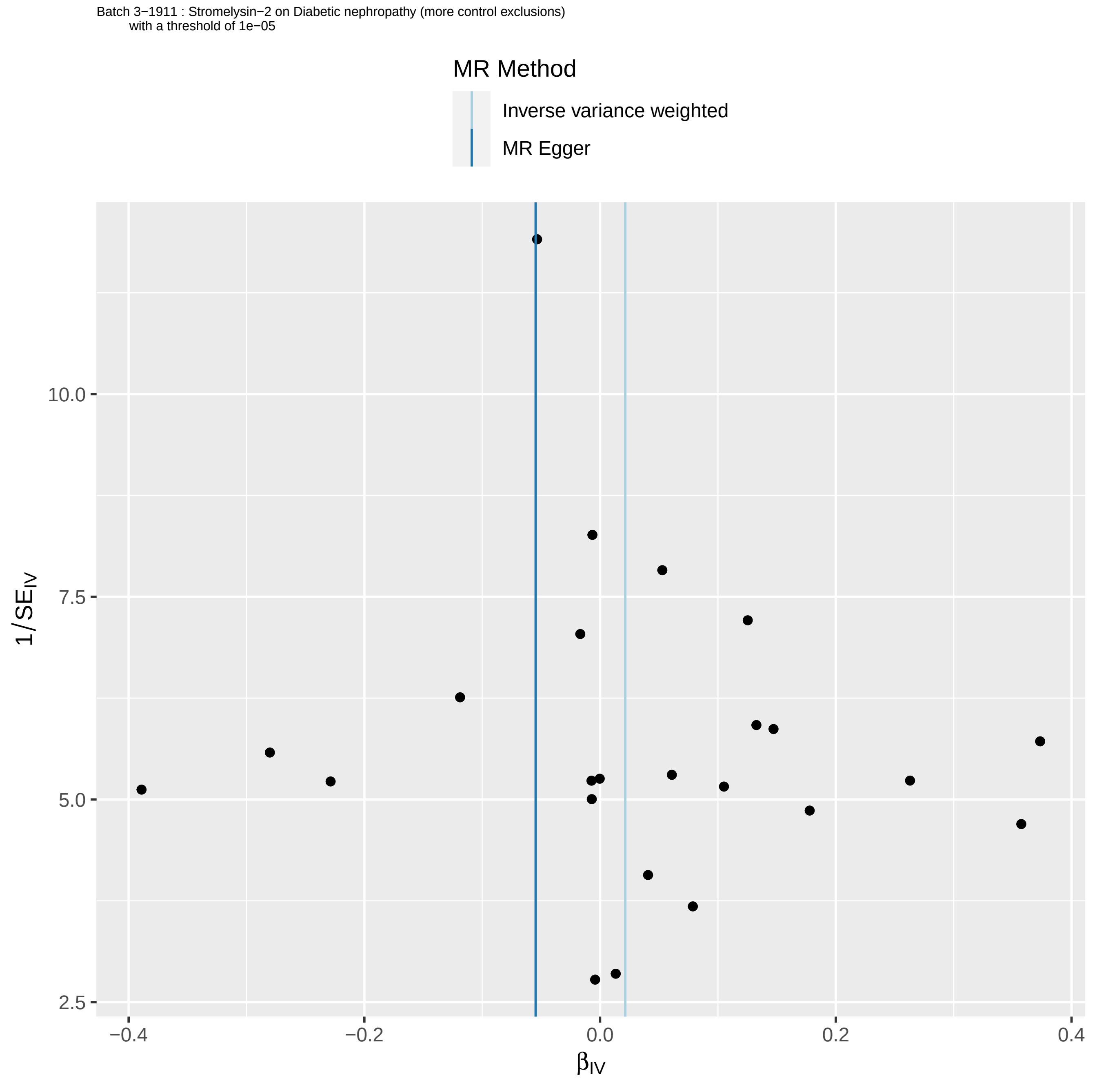

Batch 3−1911 : Stromelysin−2 on Diabetic nephropathy (more control exclusions)
with a threshold of 1e−05
MR Method
Inverse variance weighted
MR Egger
10.0
7.5
5.0
2.5
1/SE_IV
−0.4
−0.2
0.0
0.2
0.4
β_IV

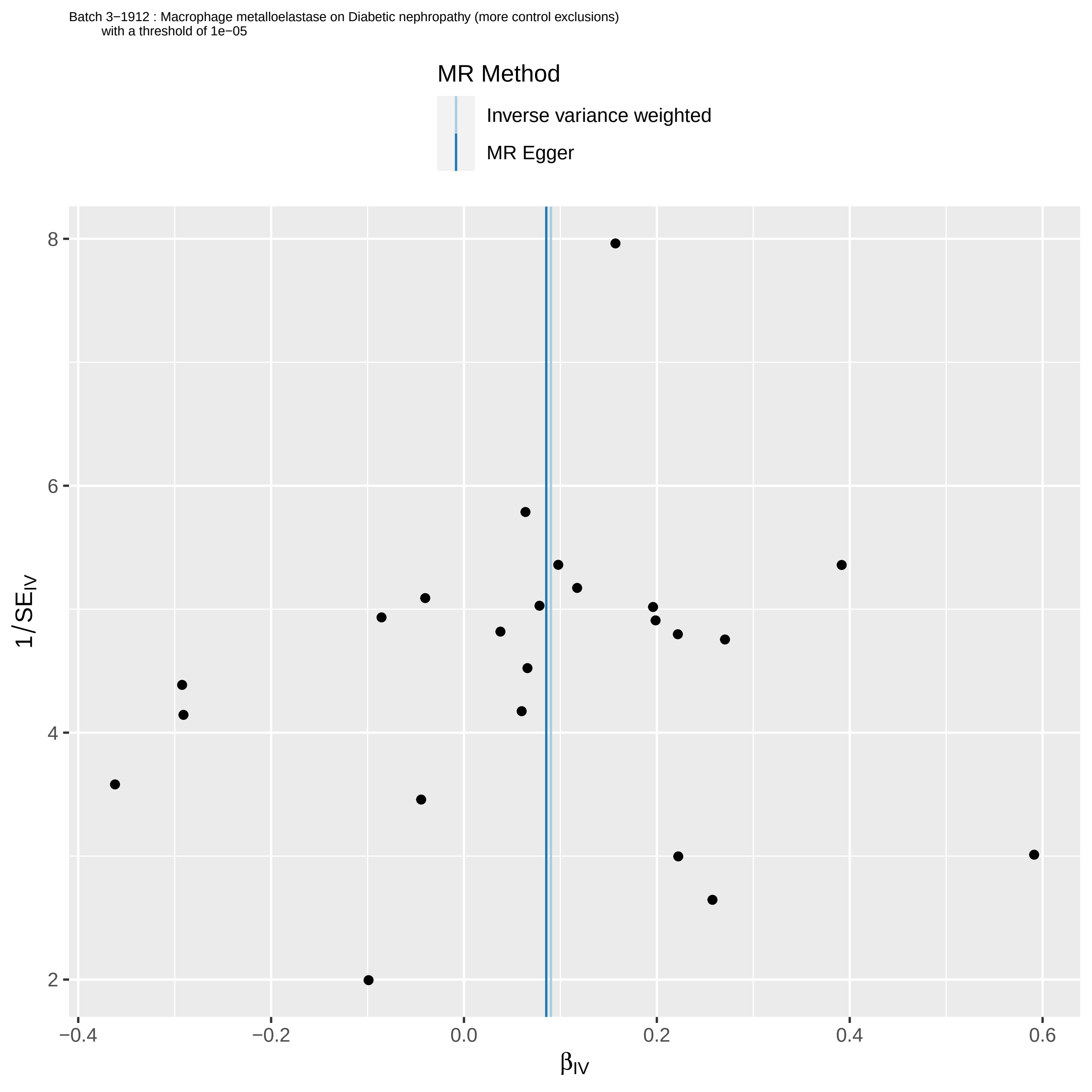

Batch 3−1912 : Macrophage metalloelastase on Diabetic nephropathy (more control exclusions)
with a threshold of 1e−05
MR Method
Inverse variance weighted
MR Egger
8
6
4
2
1/SE_IV
−0.4
−0.2
0.0
0.2
0.4
0.6
β_IV

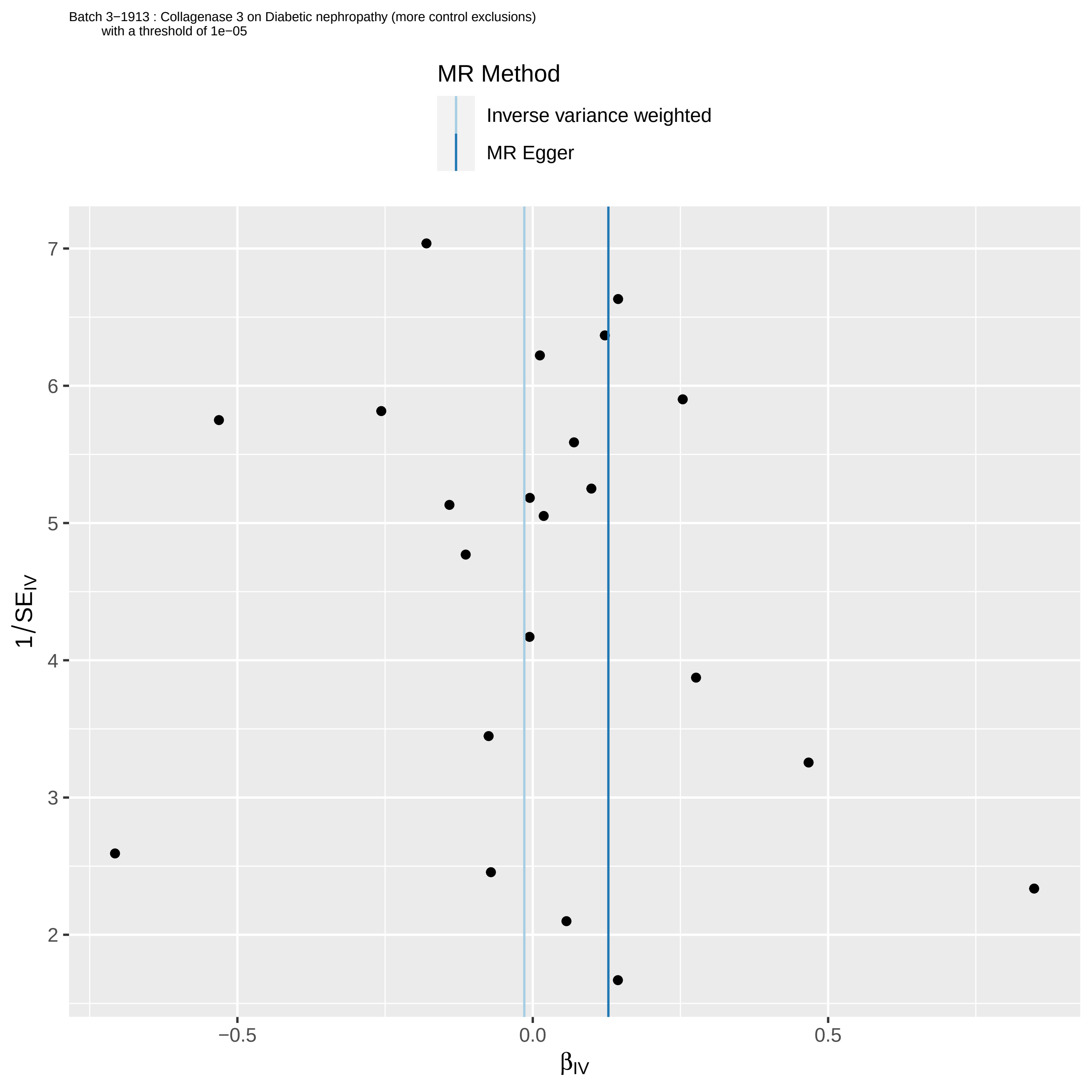
Batch 3−1913 : Collagenase 3 on Diabetic nephropathy (more control exclusions)
with a threshold of 1e−05
MR Method
Inverse variance weighted
MR Egger
7
6
5
4
3
2
1/SE_IV
−0.5
0.0
0.5
β_IV

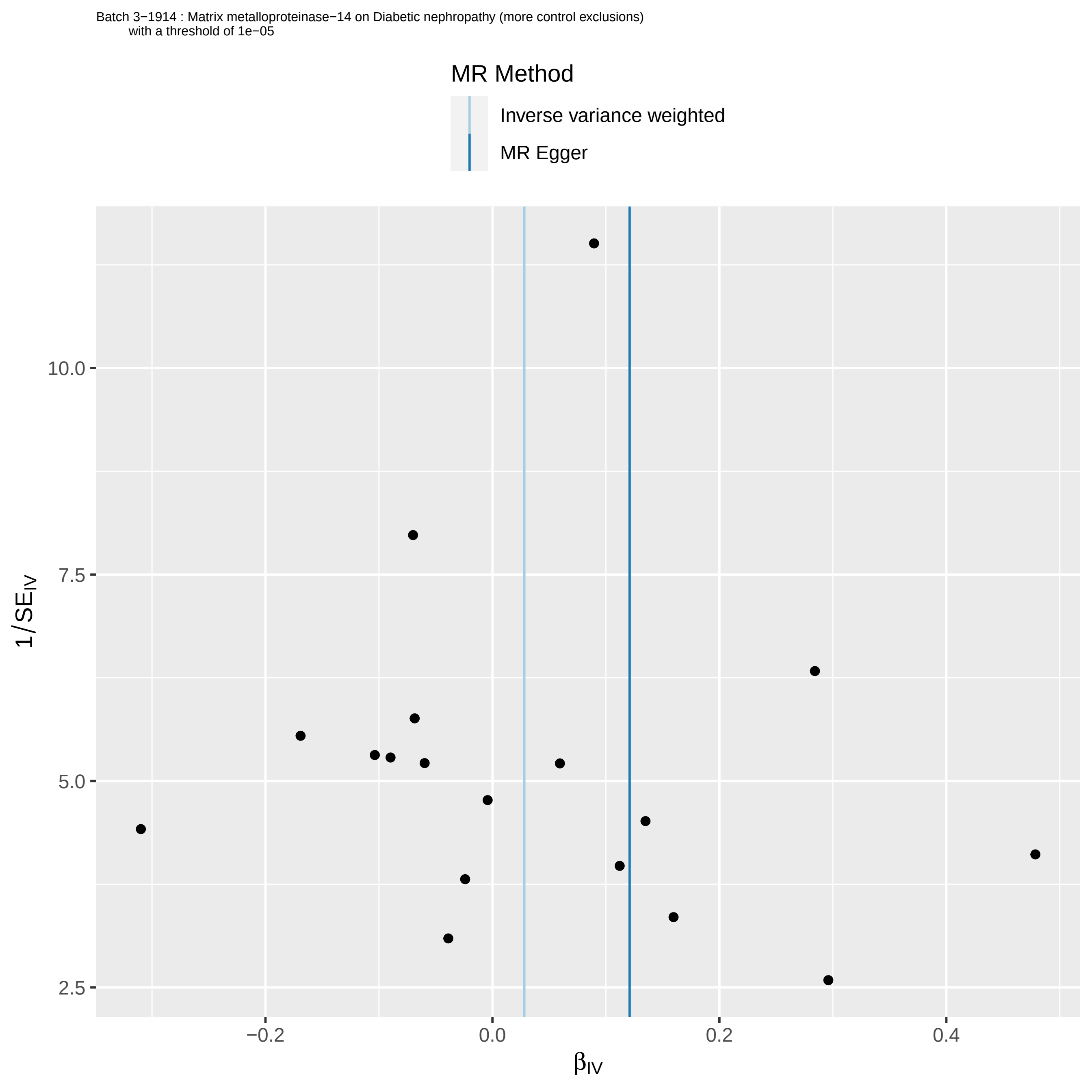
Batch 3−1914 : Matrix metalloproteinase−14 on Diabetic nephropathy (more control exclusions)
with a threshold of 1e−05
MR Method
Inverse variance weighted
MR Egger
10.0
7.5
5.0
2.5
1/SE_IV
−0.2
0.0
0.2
0.4
β_IV

Batch 3−1915 : Matrix metalloproteinase−16 on Diabetic nephropathy (more control exclusions)
with a threshold of 1e−05

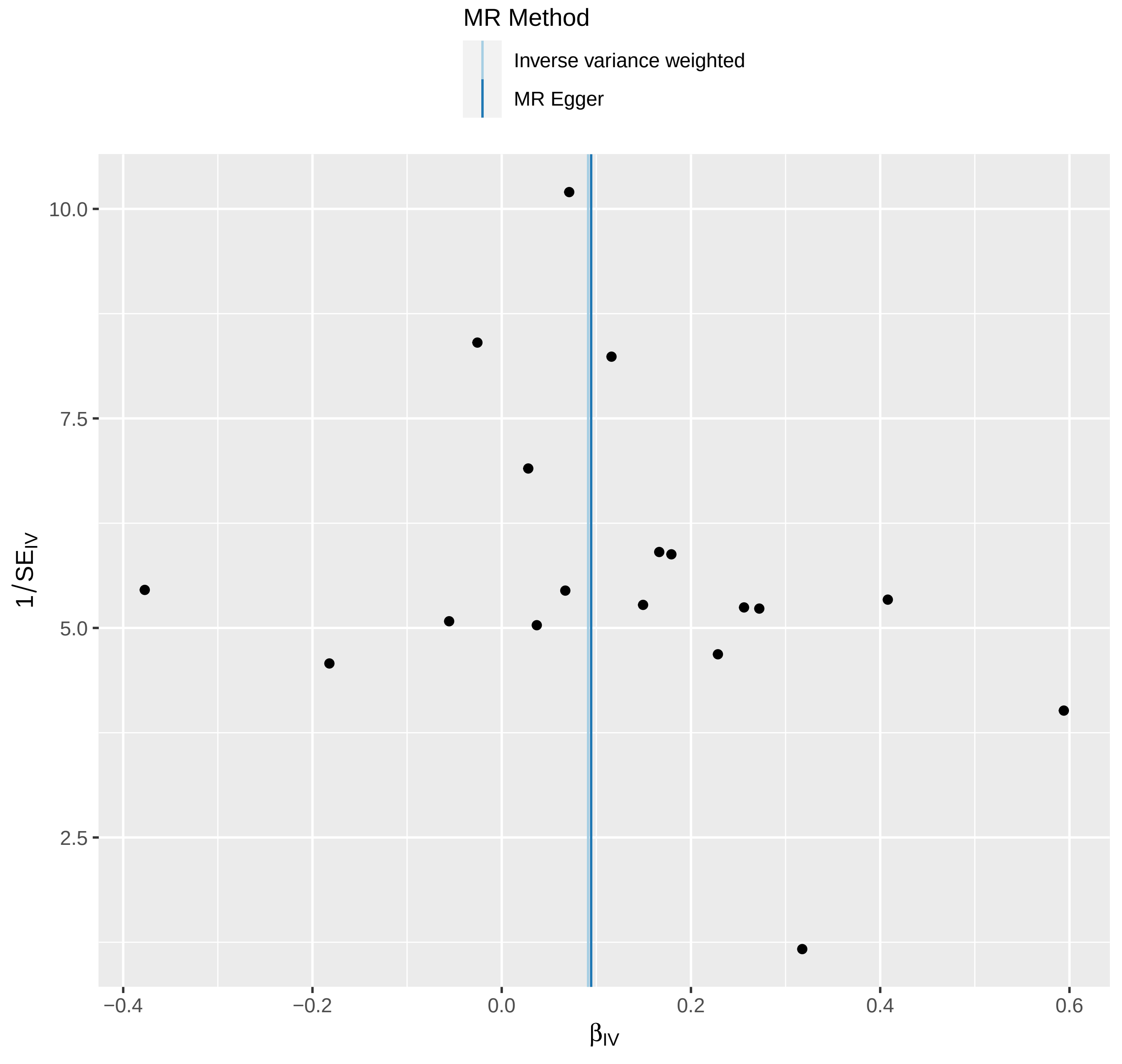

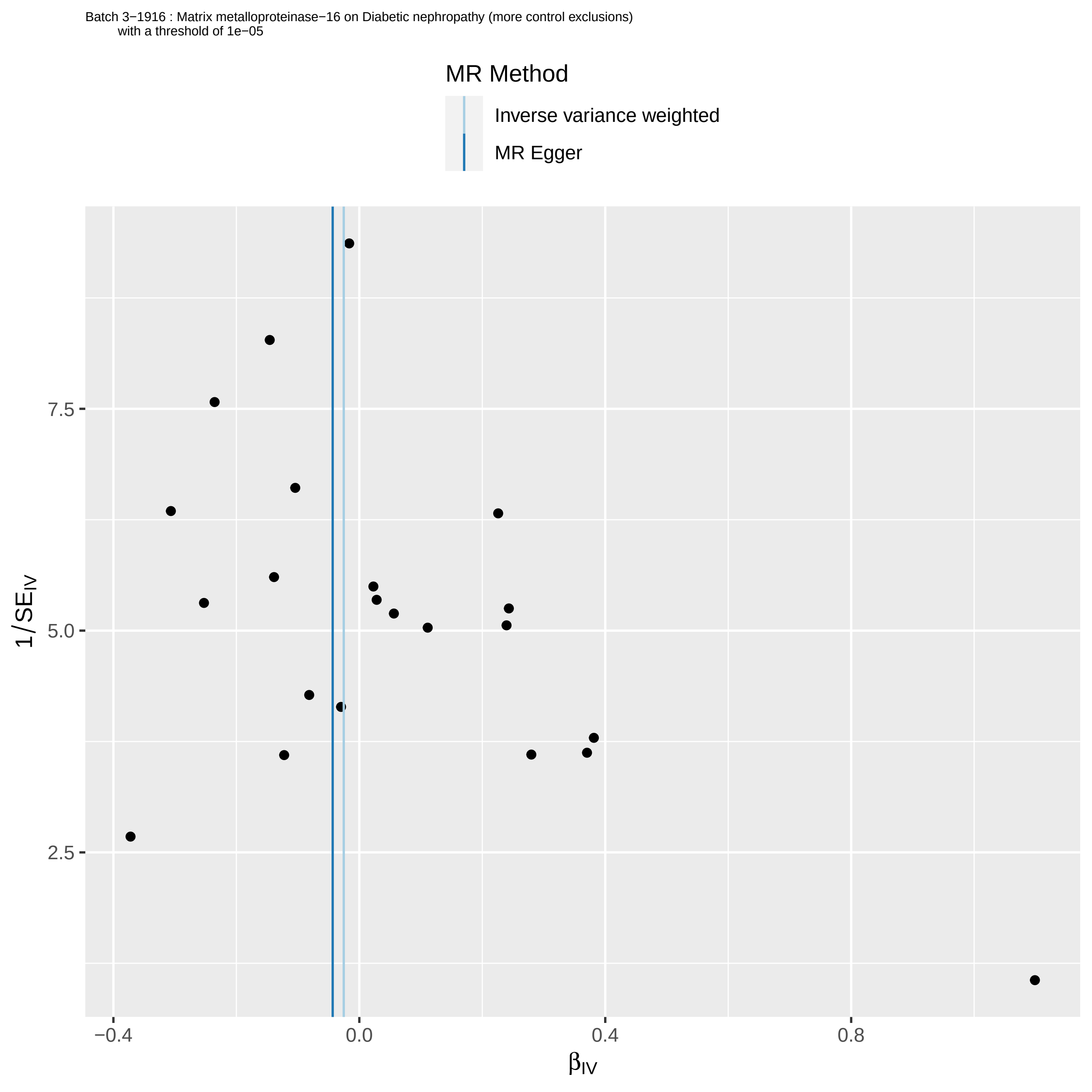

Batch 3−1916 : Matrix metalloproteinase−16 on Diabetic nephropathy (more control exclusions)
with a threshold of 1e−05
MR Method
Inverse variance weighted
MR Egger
7.5
5.0
2.5
1/SE_IV
−0.4
0.0
0.4
0.8
β_IV

Batch 3−1917 : Matrix metalloproteinase−17 on Diabetic nephropathy (more control exclusions)
with a threshold of 1e−05

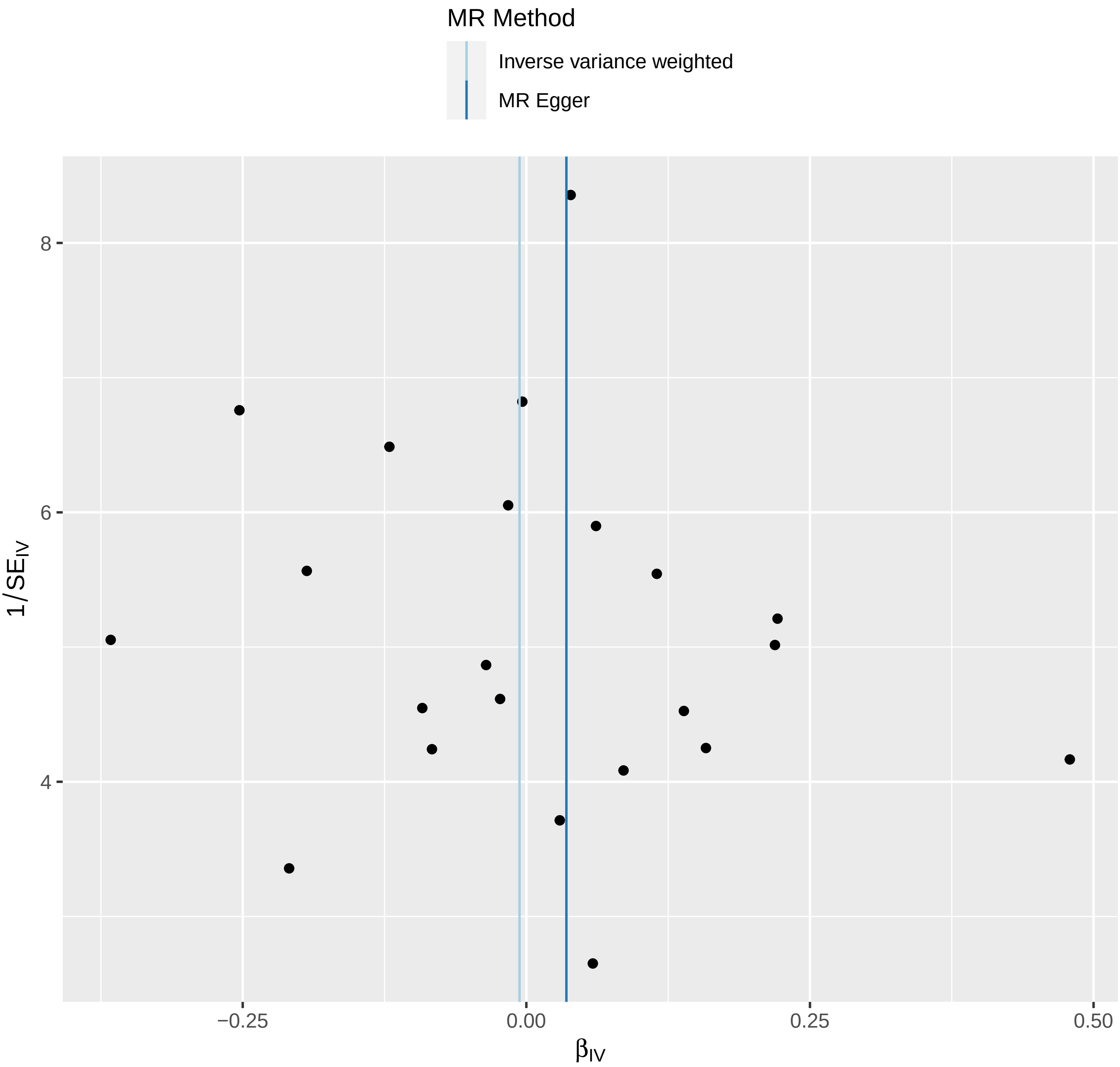

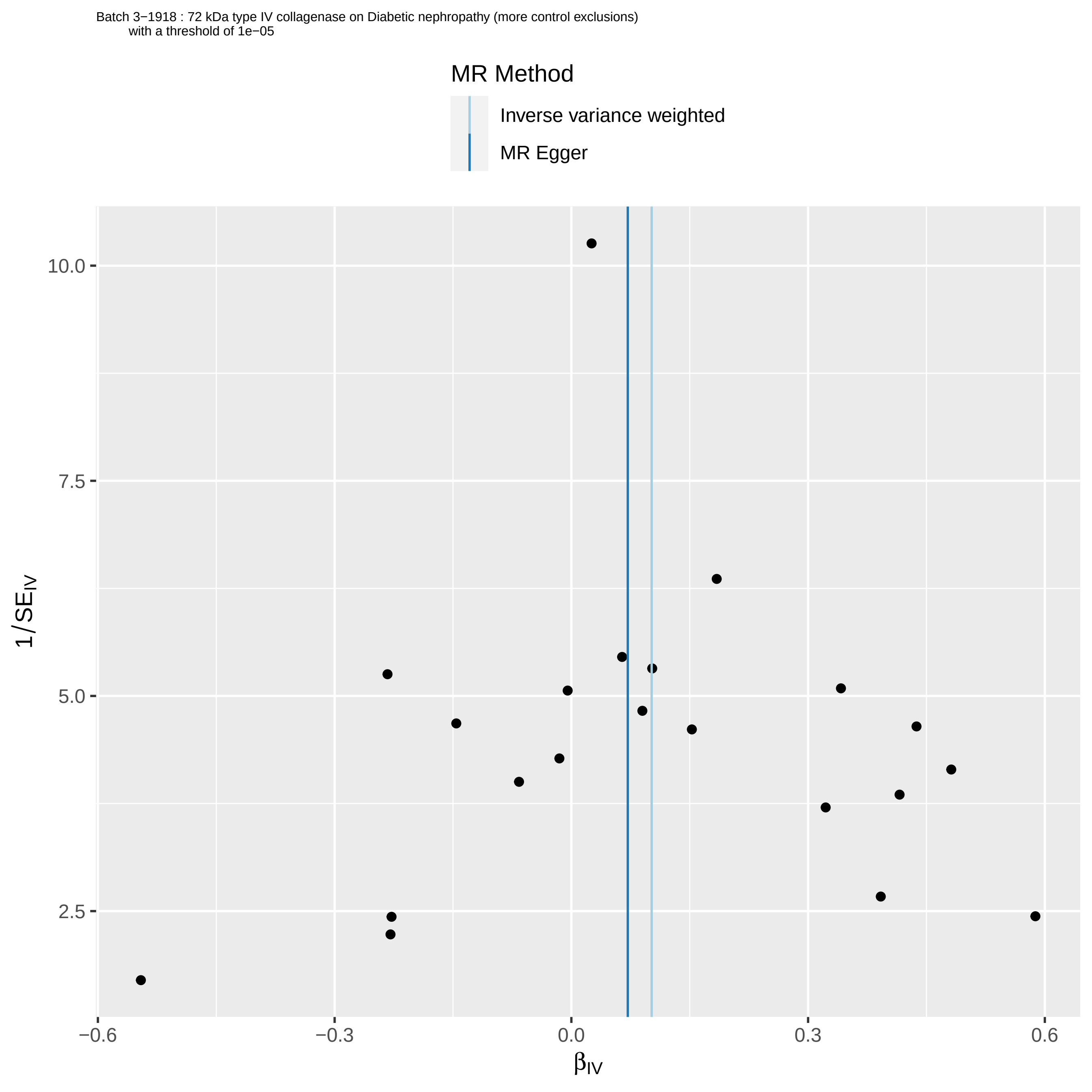

Batch 3−1918 : 72 kDa type IV collagenase on Diabetic nephropathy (more control exclusions)
with a threshold of 1e−05
MR Method
Inverse variance weighted
MR Egger
10.0
7.5
5.0
2.5
1/SE$_{IV}$
−0.6
−0.3
0.0
0.3
0.6
β_{IV}

Batch 3−1919 : Matrilysin on Diabetic nephropathy (more control exclusions)
with a threshold of 1e−05

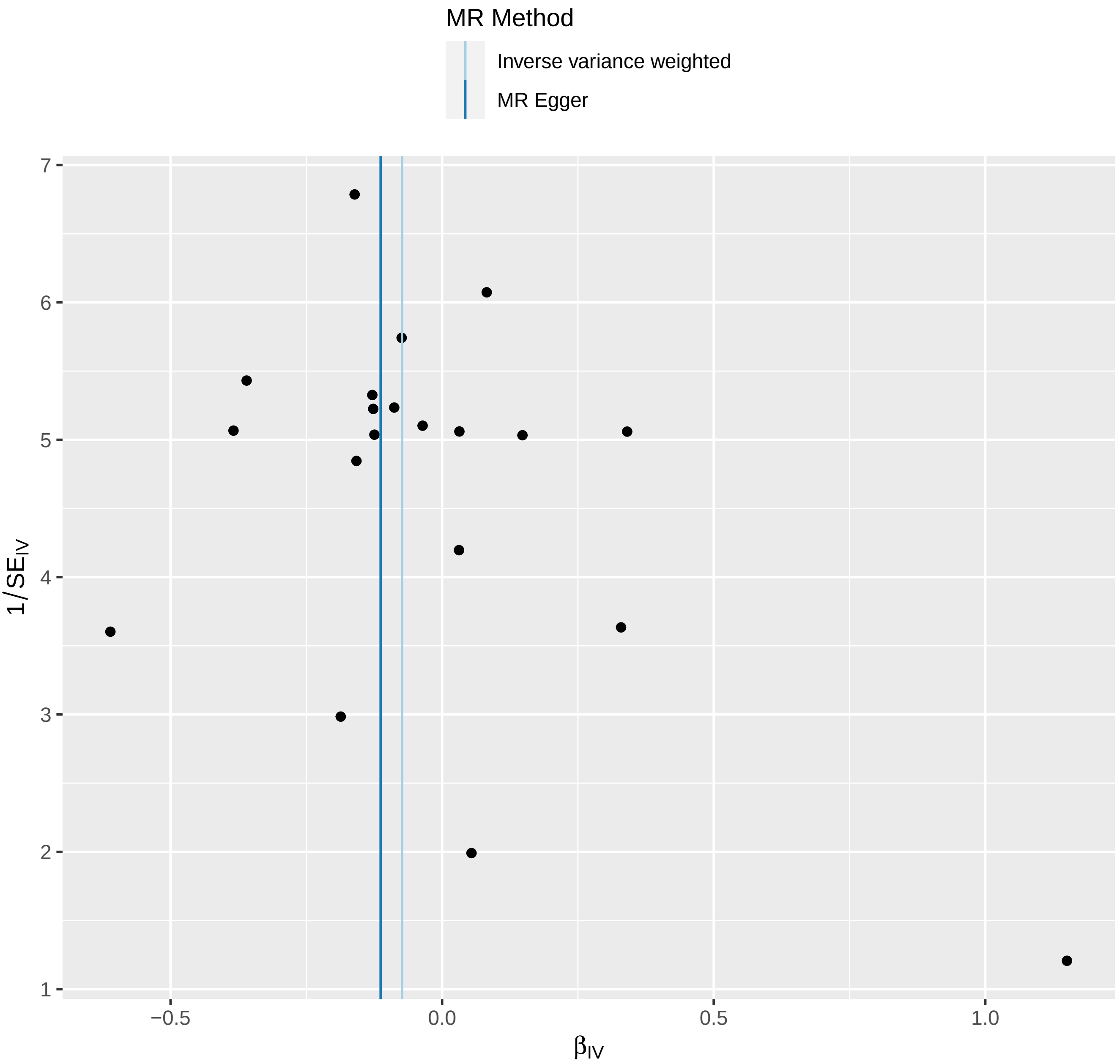

Batch 3−1920 : Neutrophil collagenase on Diabetic nephropathy (more control exclusions)
with a threshold of 1e−05

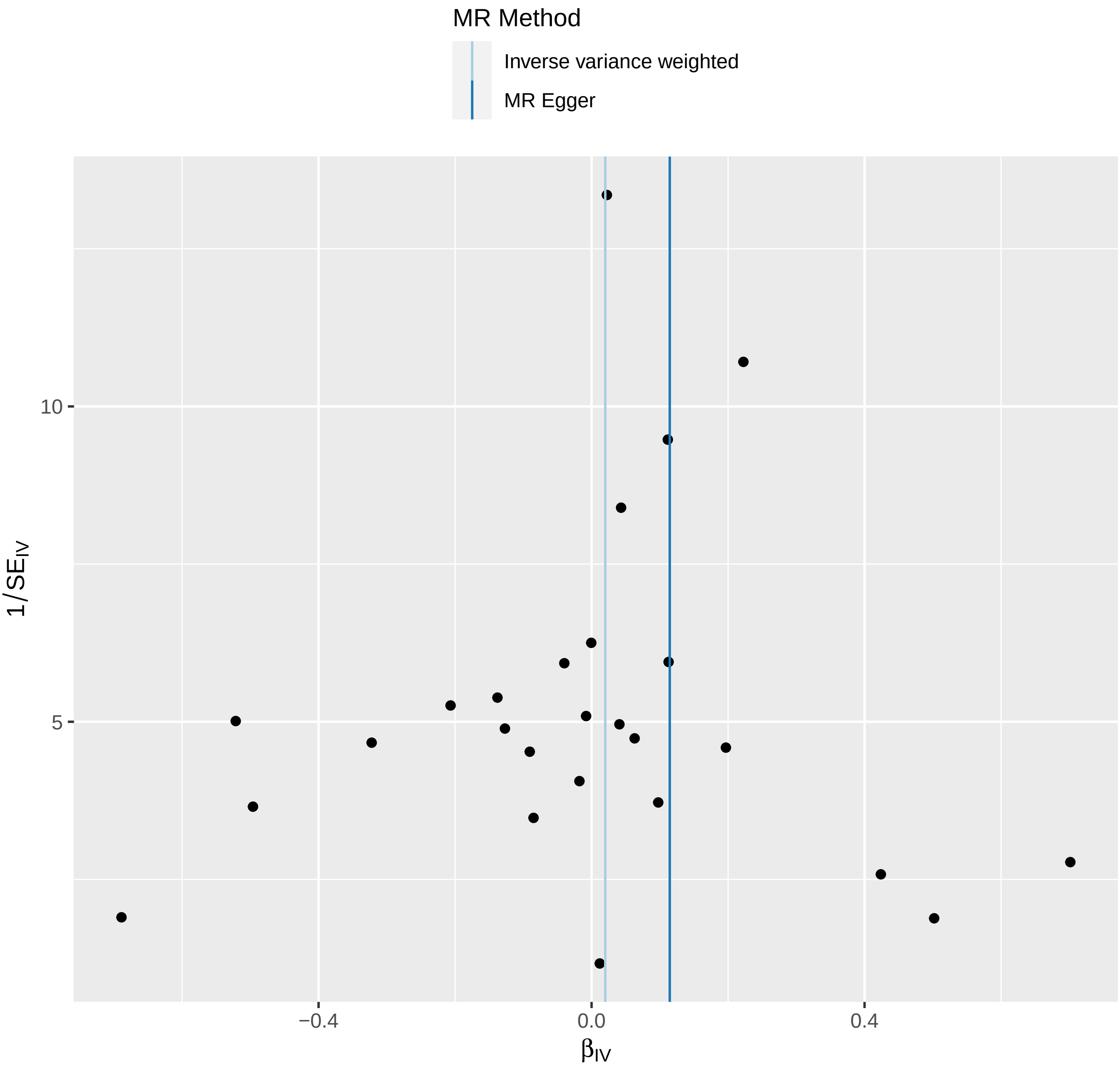

Batch 3−1921 : Matrix metalloproteinase−9 on Diabetic nephropathy (more control exclusions)
with a threshold of 1e−05

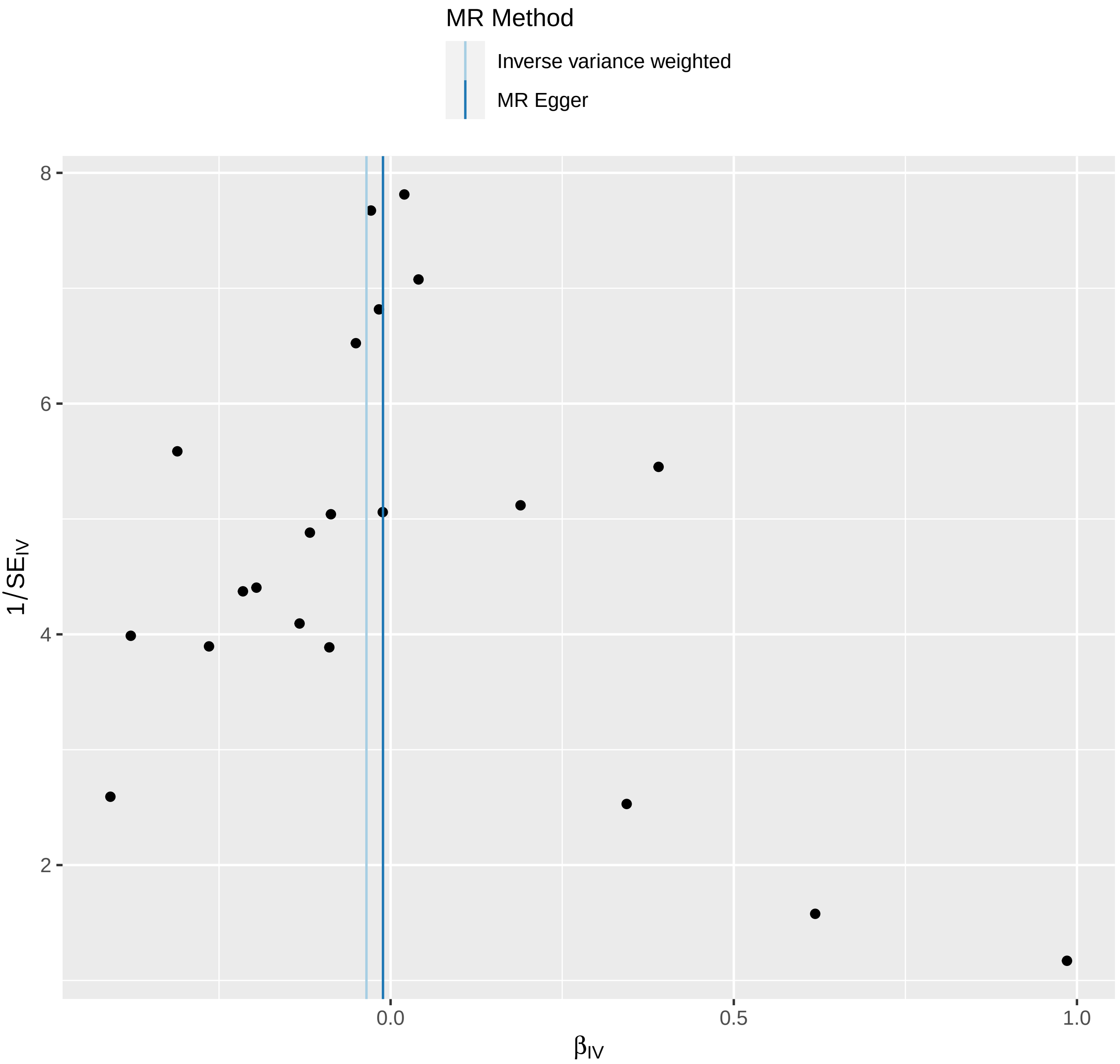

Batch 3−1922 : Multimerin−2 on Diabetic nephropathy (more control exclusions)
with a threshold of 1e−05

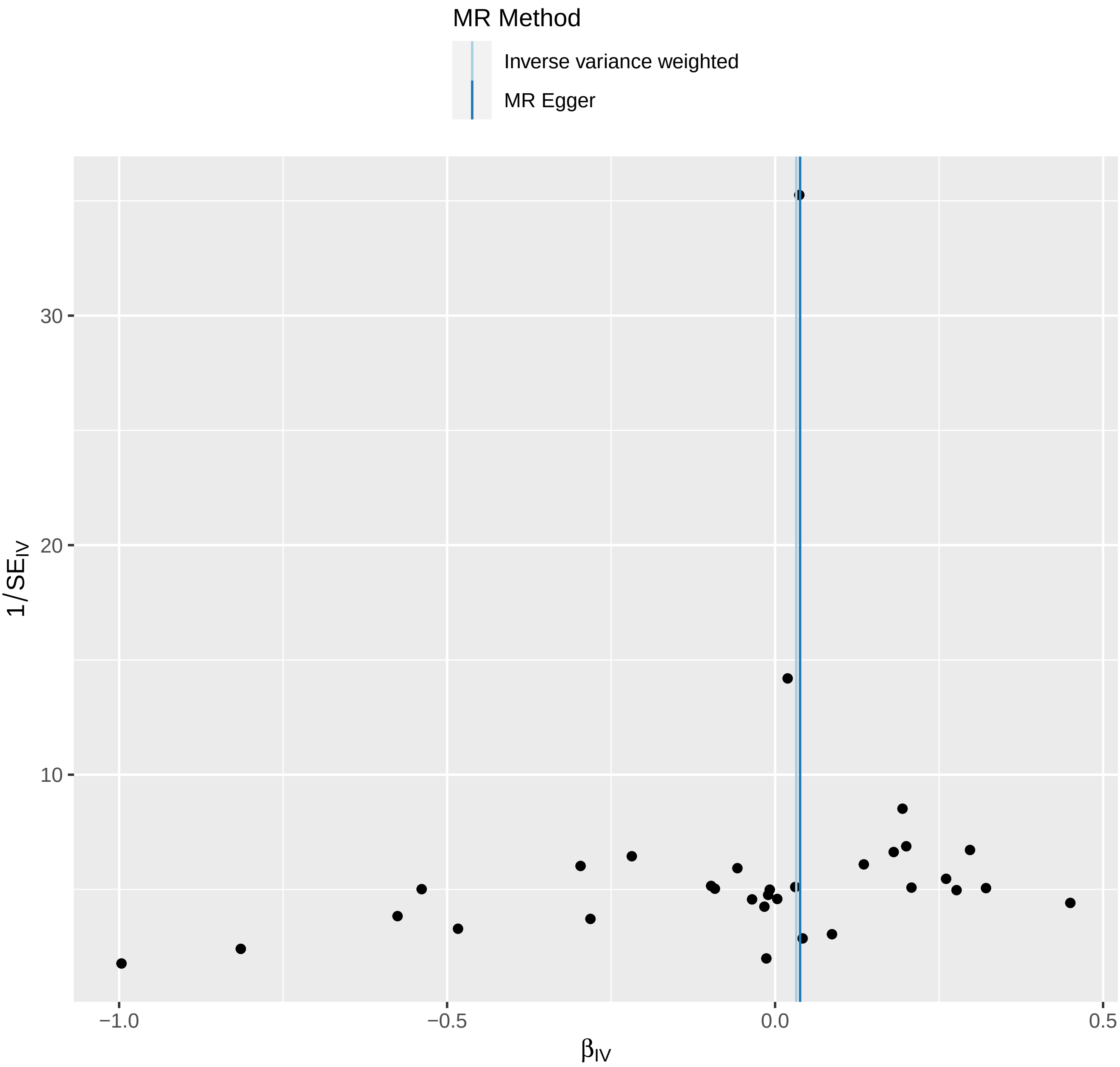

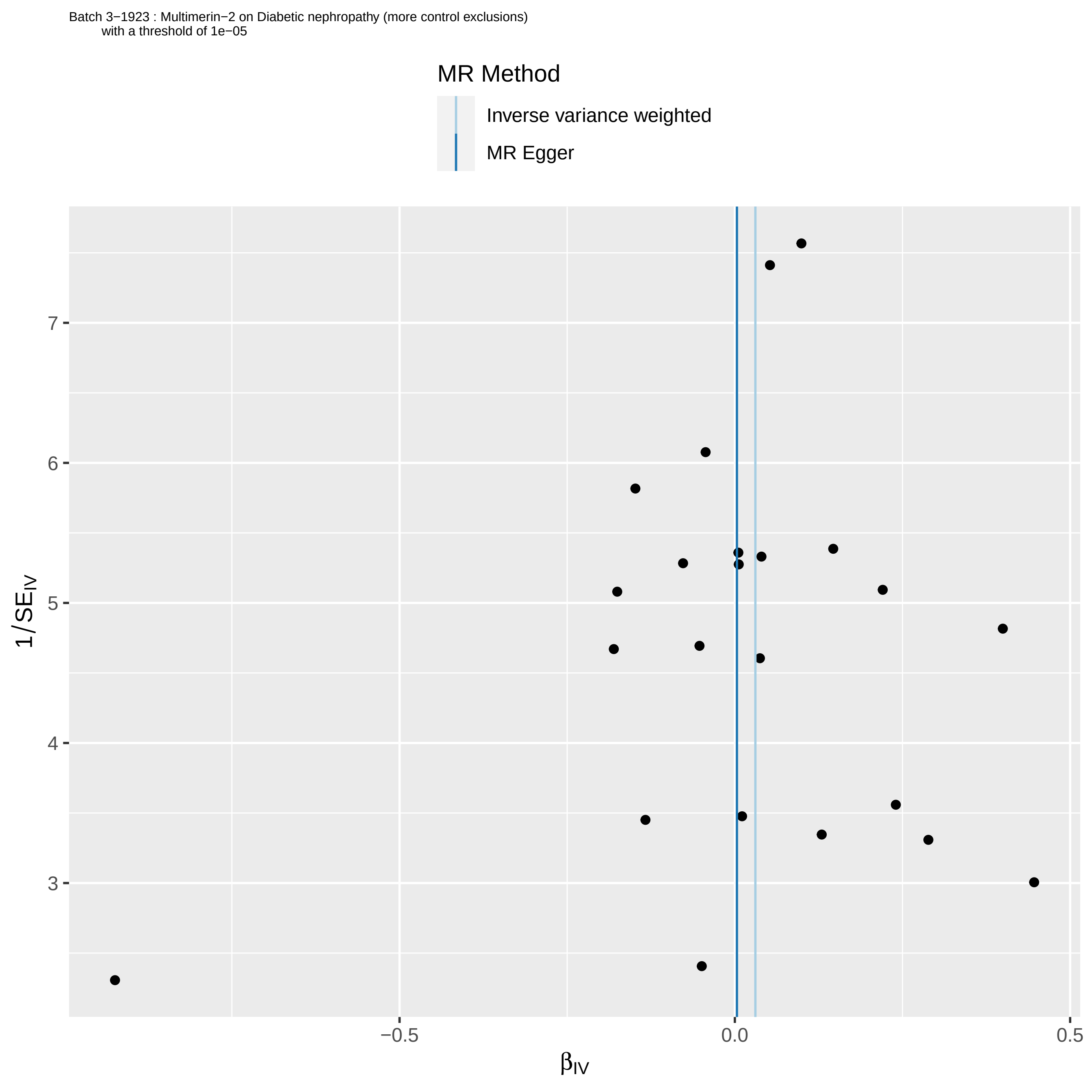
Batch 3−1923 : Multimerin−2 on Diabetic nephropathy (more control exclusions)
with a threshold of 1e−05
MR Method
Inverse variance weighted
MR Egger
7
6
5
4
3
1/SE$_{IV}$
−0.5
0.0
0.5
β$_{IV}$

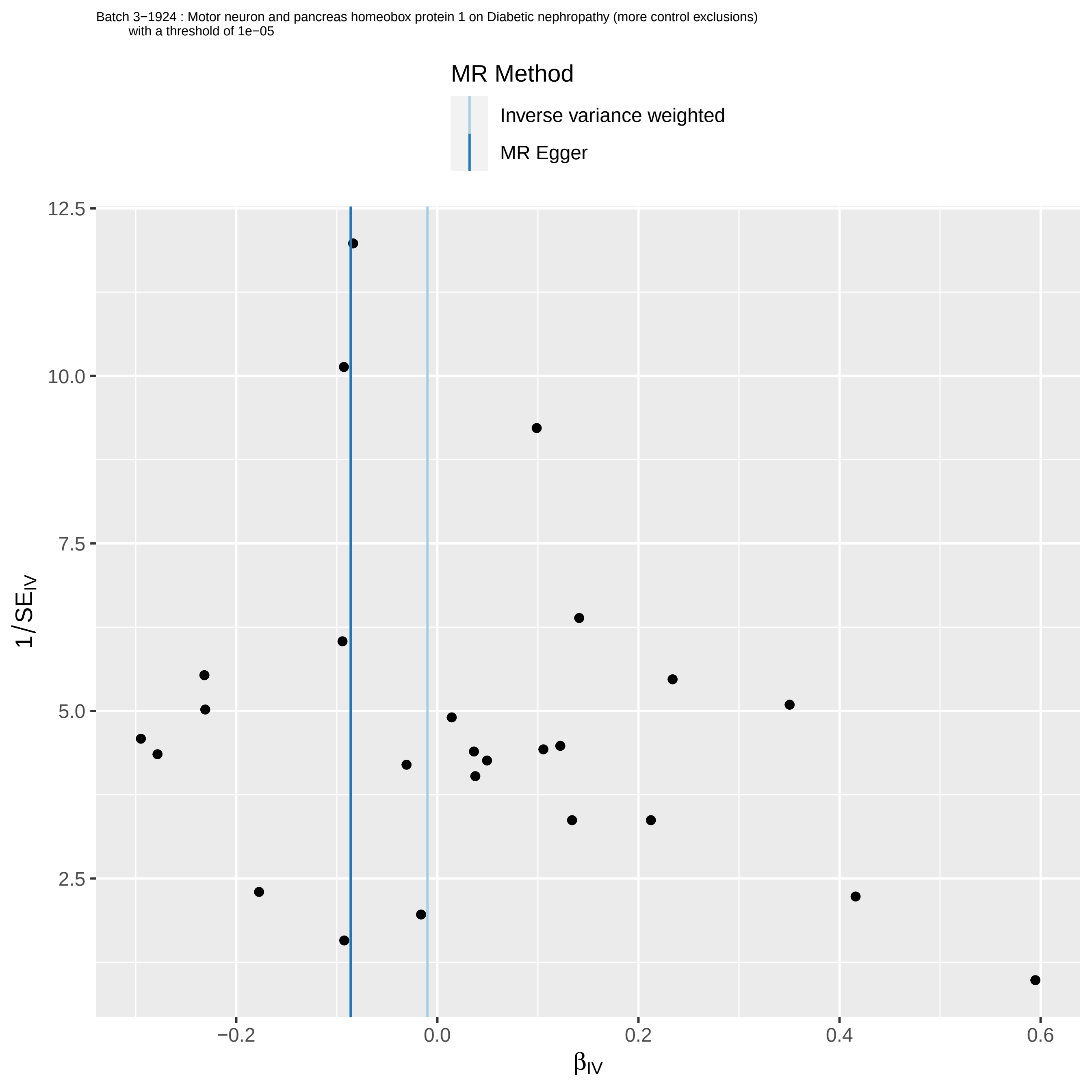
Batch 3−1924 : Motor neuron and pancreas homeobox protein 1 on Diabetic nephropathy (more control exclusions)
with a threshold of 1e−05
MR Method
Inverse variance weighted
MR Egger
12.5
10.0
7.5
5.0
2.5
1/SE_IV
−0.2
0.0
0.2
0.4
0.6
β_IV

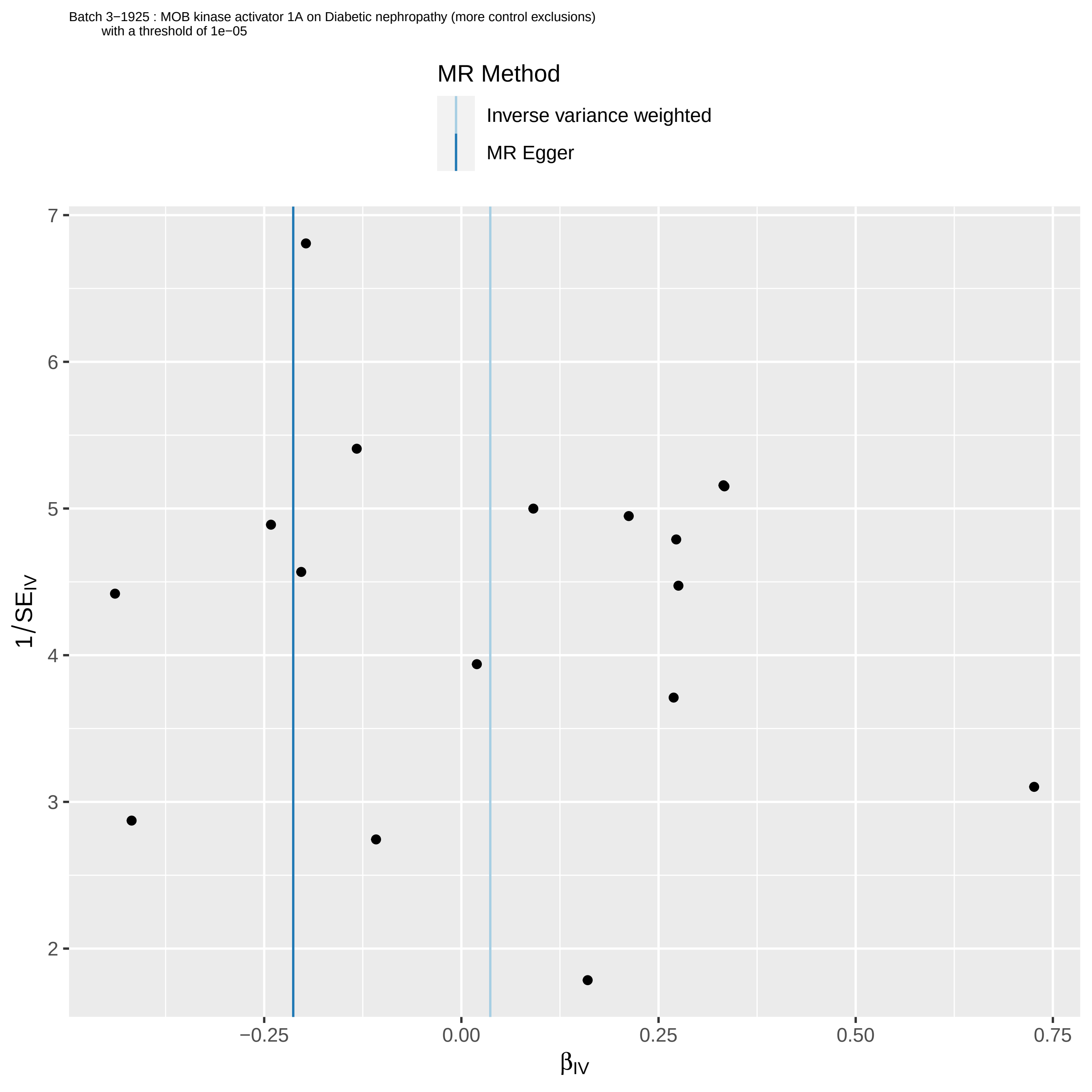
Batch 3−1925 : MOB kinase activator 1A on Diabetic nephropathy (more control exclusions)
with a threshold of 1e−05
MR Method
Inverse variance weighted
MR Egger
7
6
5
4
3
2
1/SE_IV
−0.25
0.00
0.25
0.50
0.75
β_IV

Batch 3−1926 : Adenylyltransferase and sulfurtransferase MOCS3 on Diabetic nephropathy (more control exclusions) with a threshold of 1e−05

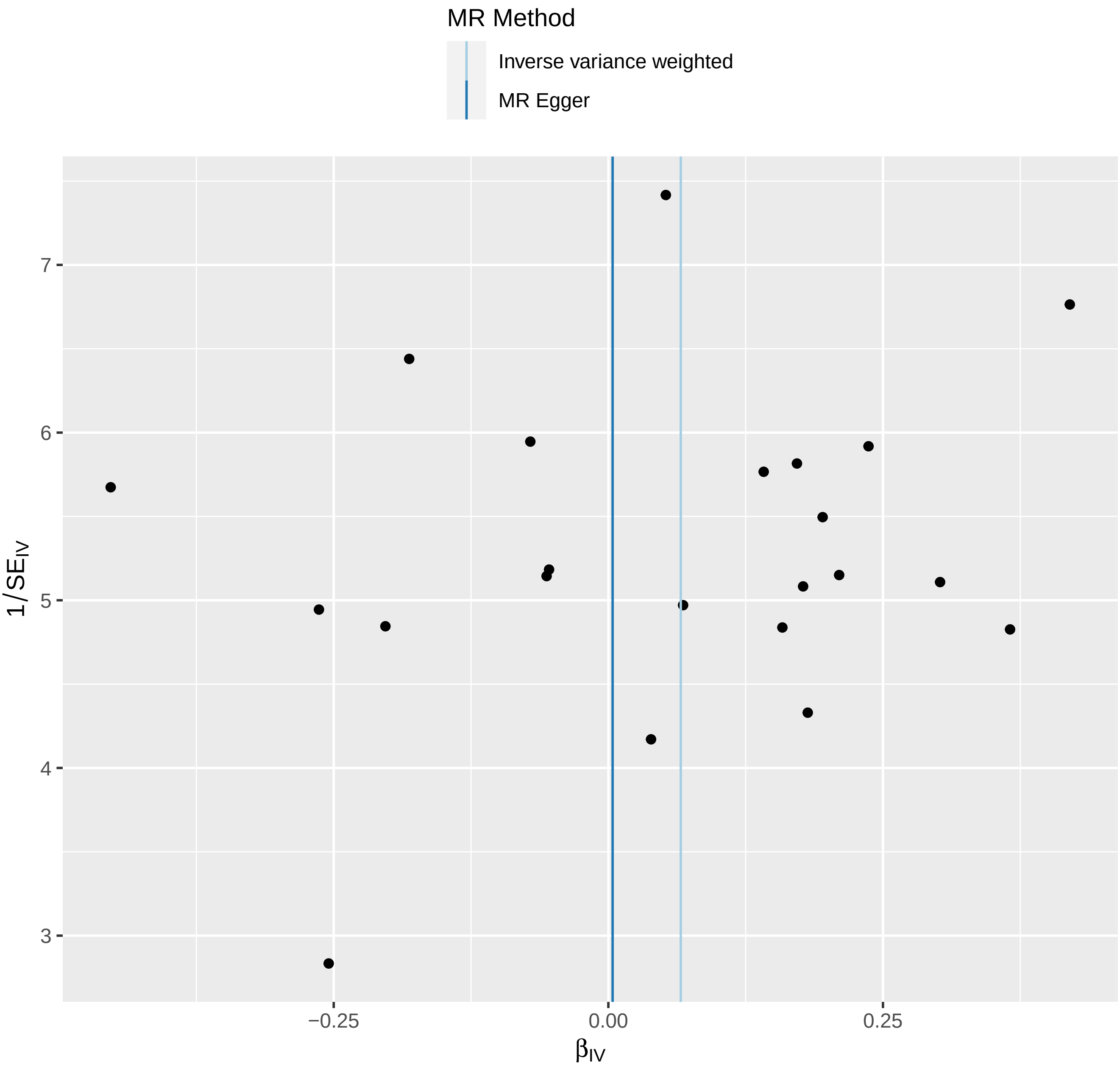

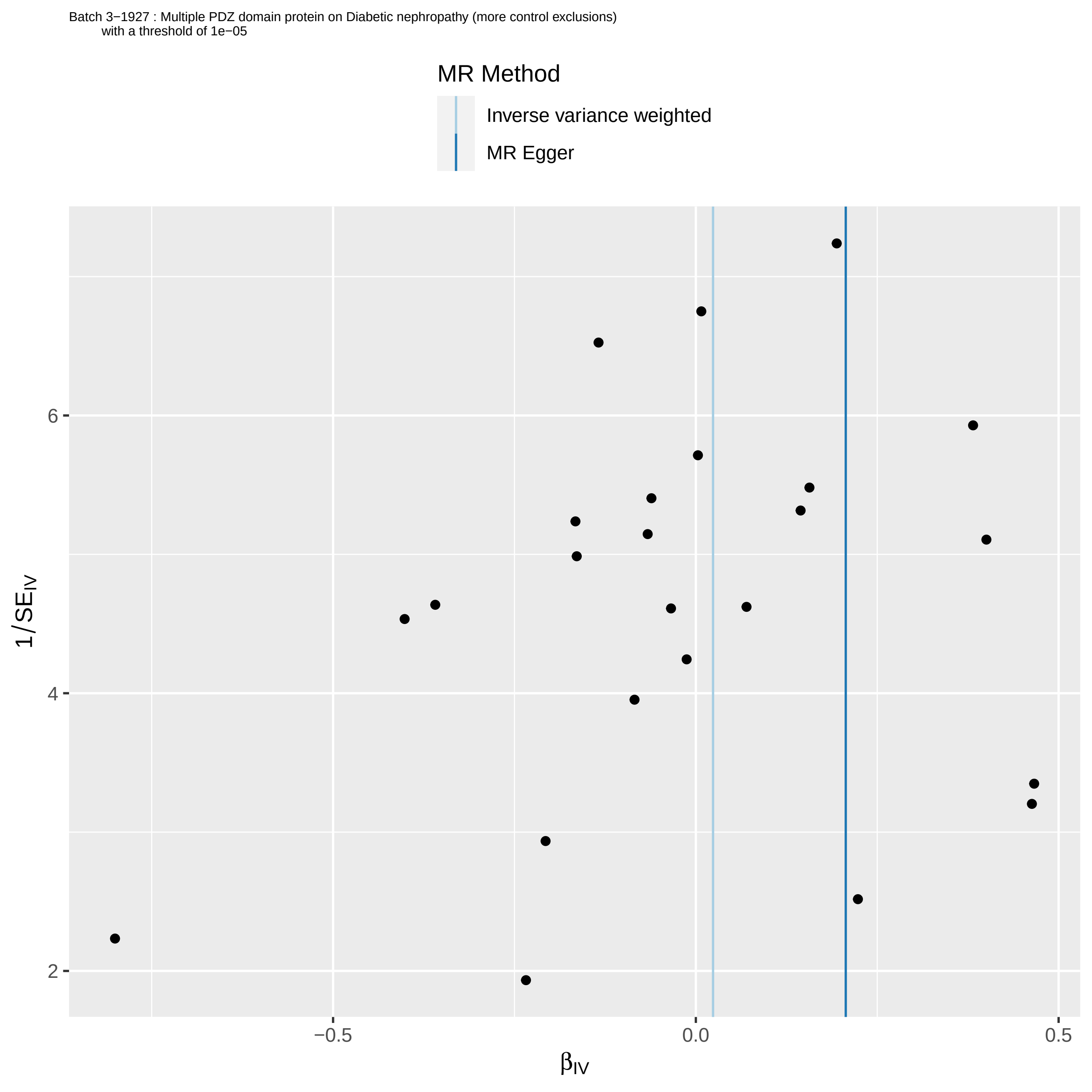
Batch 3−1927 : Multiple PDZ domain protein on Diabetic nephropathy (more control exclusions)
with a threshold of 1e−05
MR Method
Inverse variance weighted
MR Egger
6
4
2
1/SE_IV
−0.5
0.0
0.5
β_IV

Batch 3−1928 : DNA−3−methyladenine glycosylase on Diabetic nephropathy (more control exclusions)
with a threshold of 1e−05

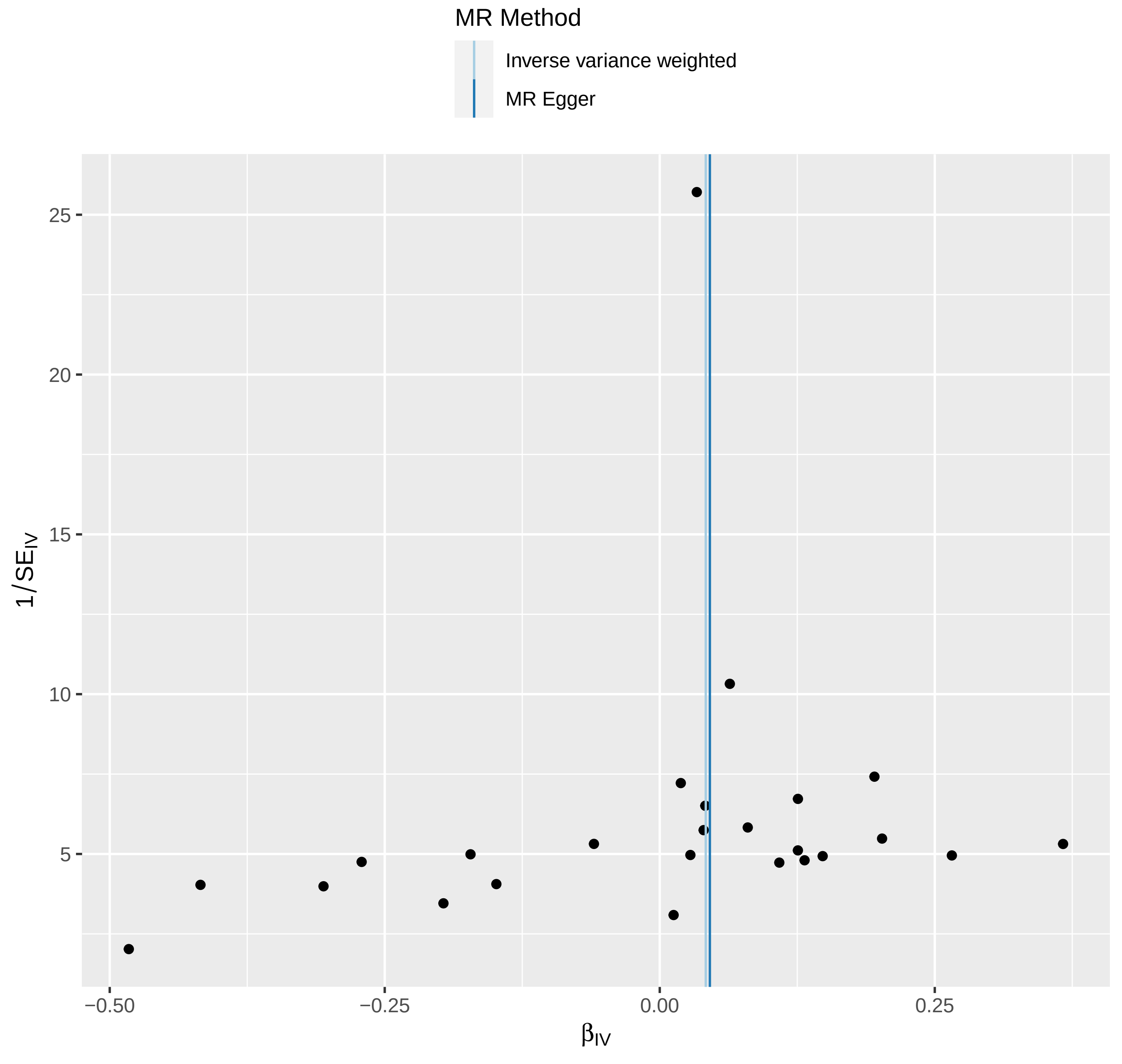

Batch 3−1929 : Thrombopoietin Receptor on Diabetic nephropathy (more control exclusions)
with a threshold of 1e−05

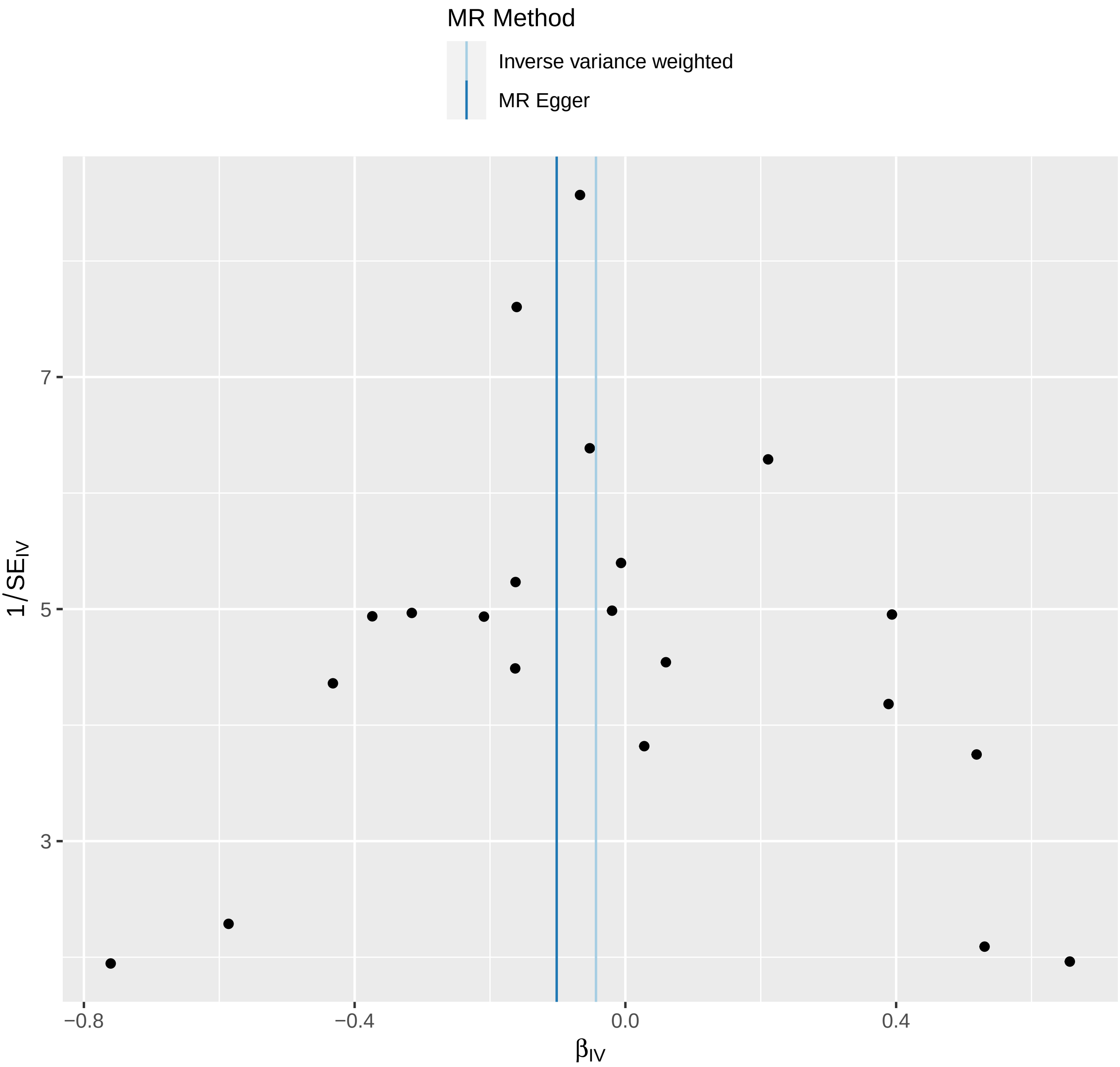

Batch 3−1930 : Myeloperoxidase on Diabetic nephropathy (more control exclusions)
with a threshold of 1e−05

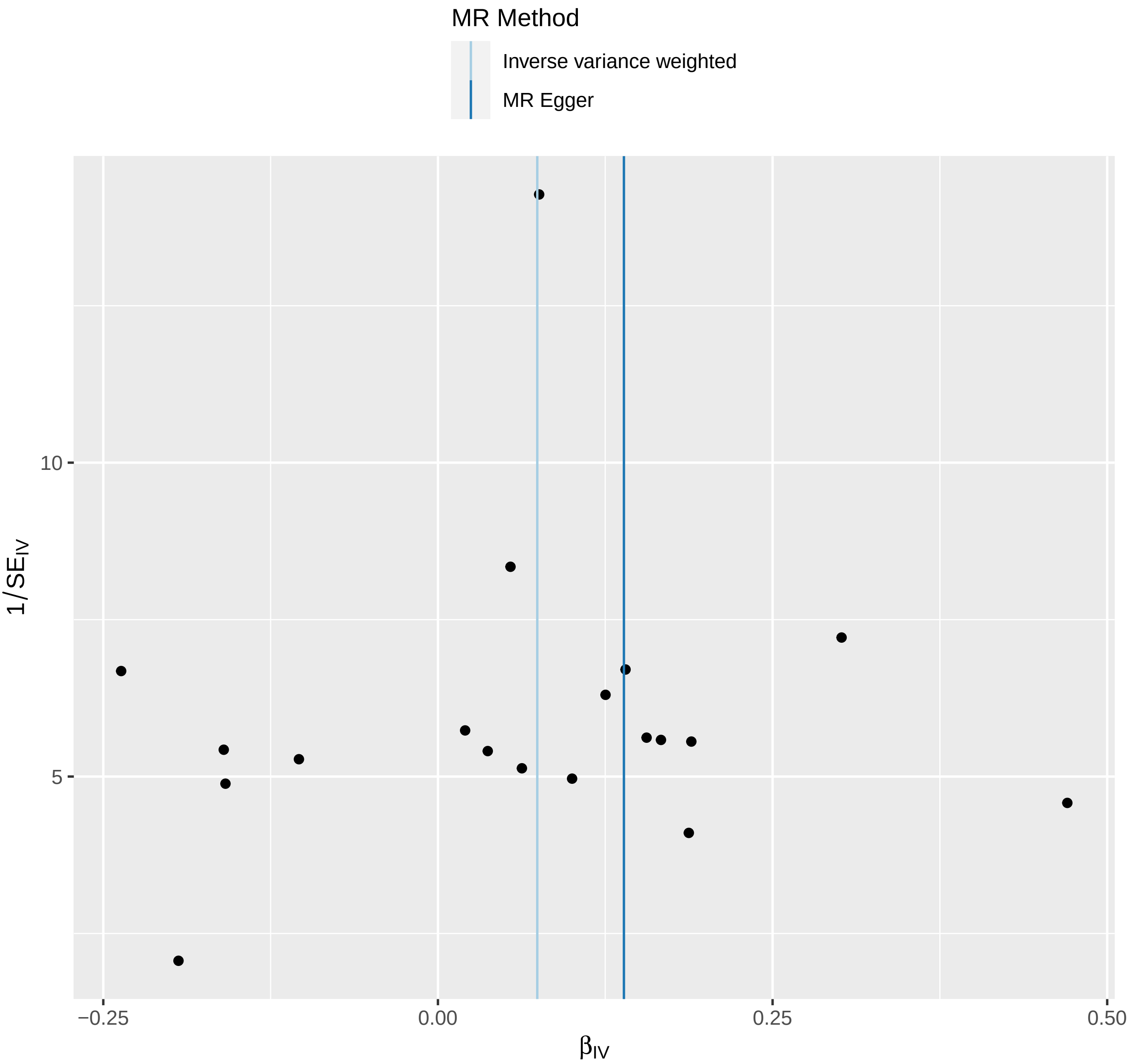

Batch 3−1931 : MAGUK p55 subfamily member 6 on Diabetic nephropathy (more control exclusions)
with a threshold of 1e−05

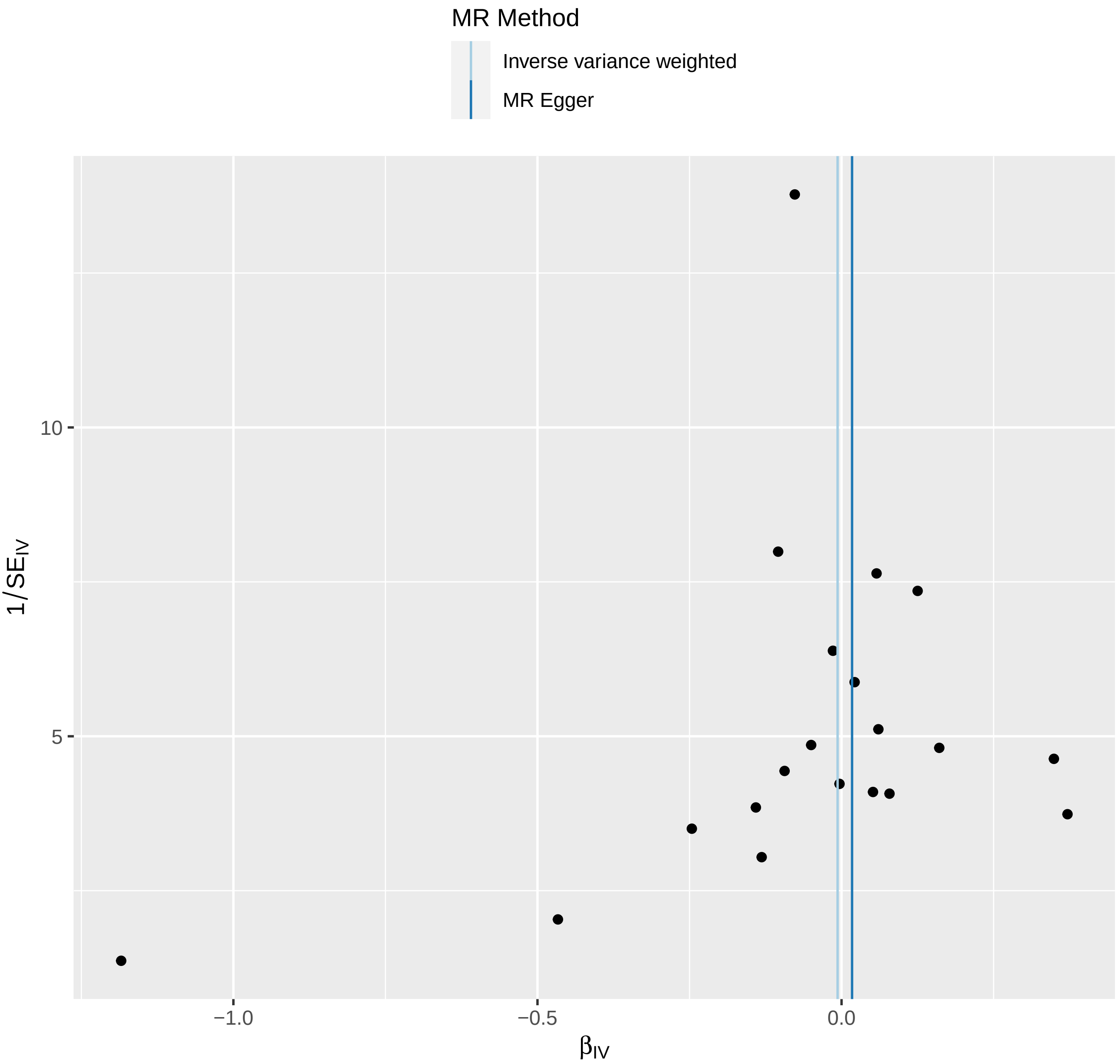

Batch 3−1932 : MAGUK p55 subfamily member 7 on Diabetic nephropathy (more control exclusions)
with a threshold of 1e−05

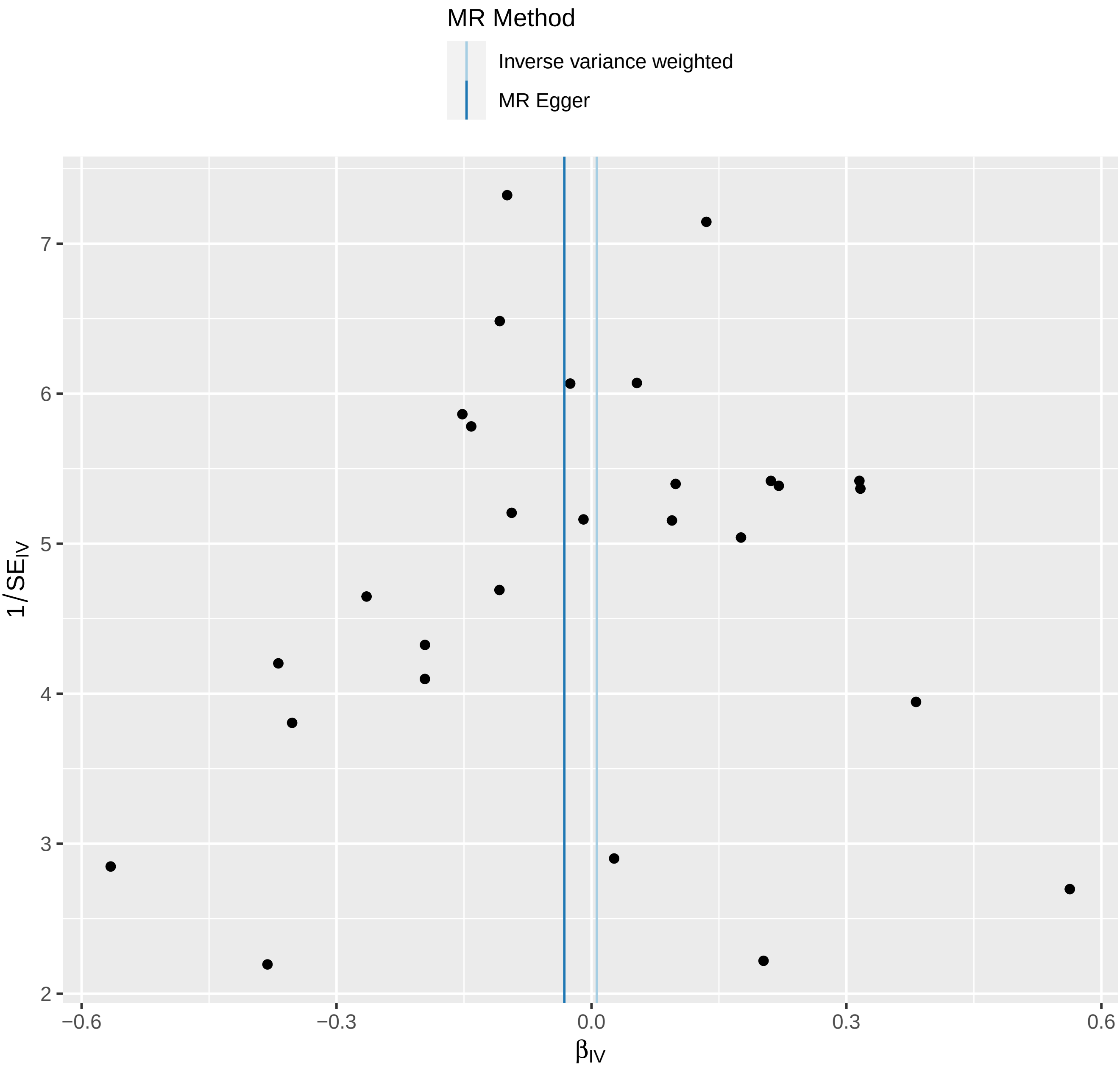

Batch 3−1933 : 3−mercaptopyruvate sulfurtransferase on Diabetic nephropathy (more control exclusions)
with a threshold of 1e−05

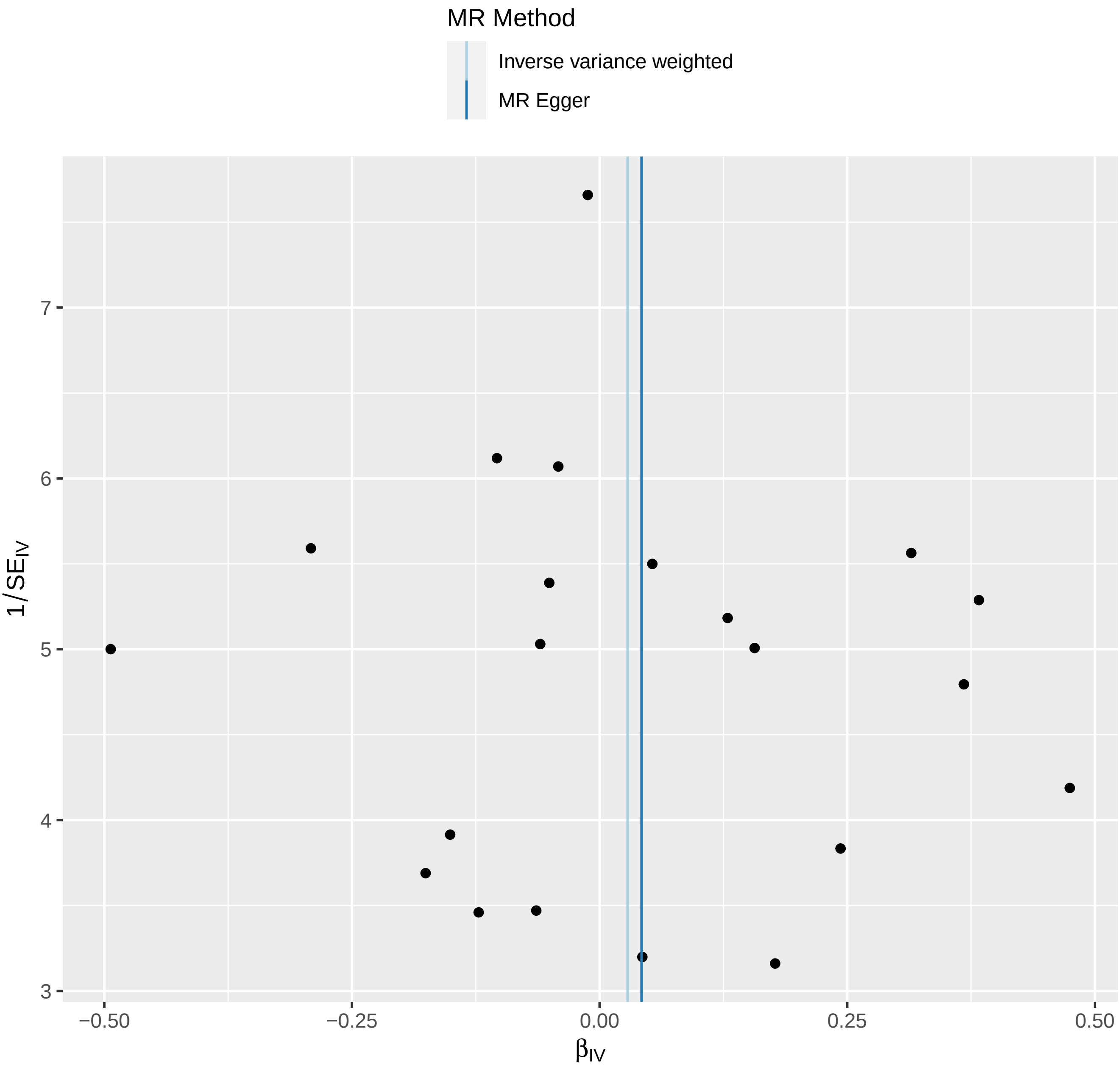

Batch 3−1934 : Myelin protein P0 on Diabetic nephropathy (more control exclusions)
with a threshold of 1e−05

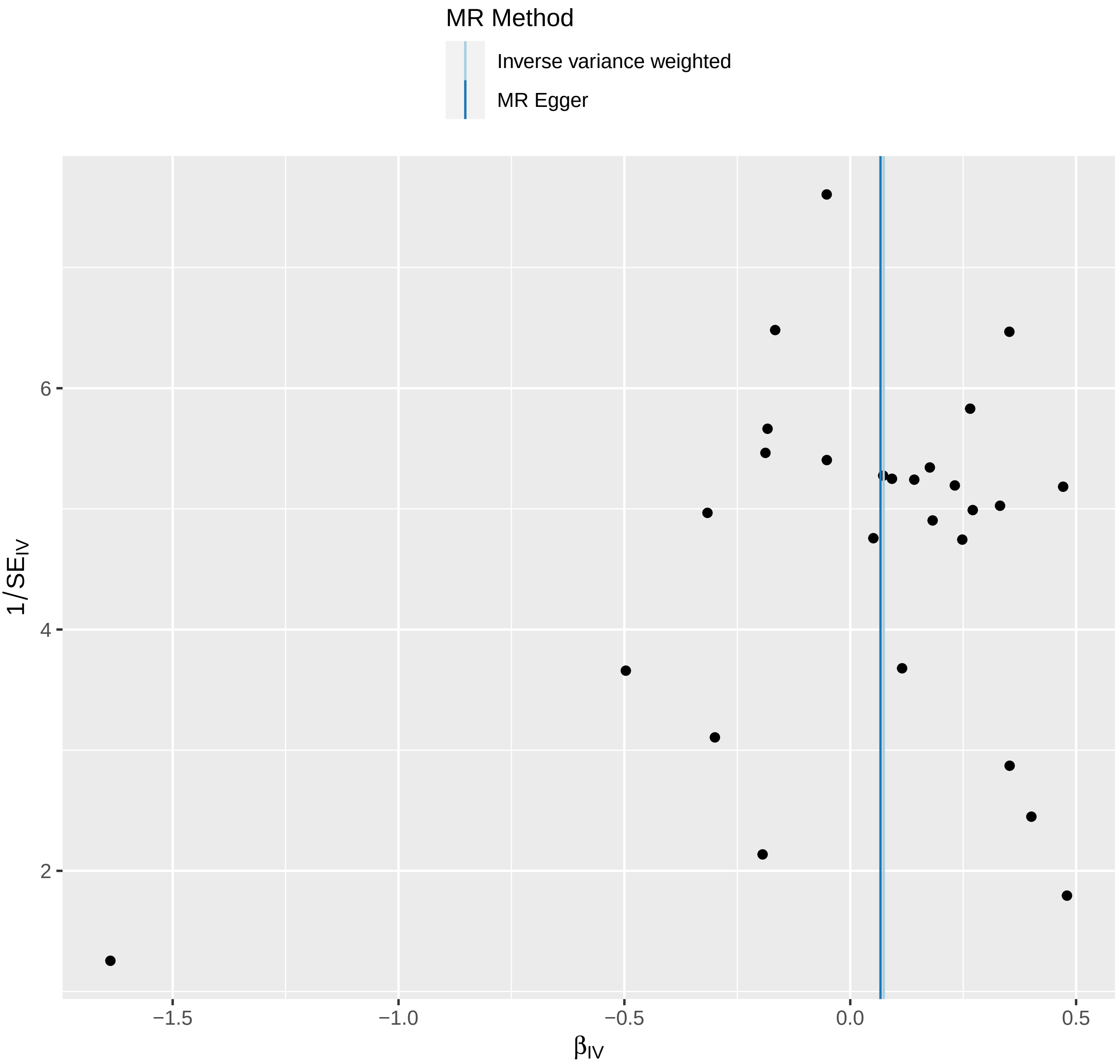

Batch 3−1935 : Melanocortin−2 receptor accessory protein 2 on Diabetic nephropathy (more control exclusions)
with a threshold of 1e−05

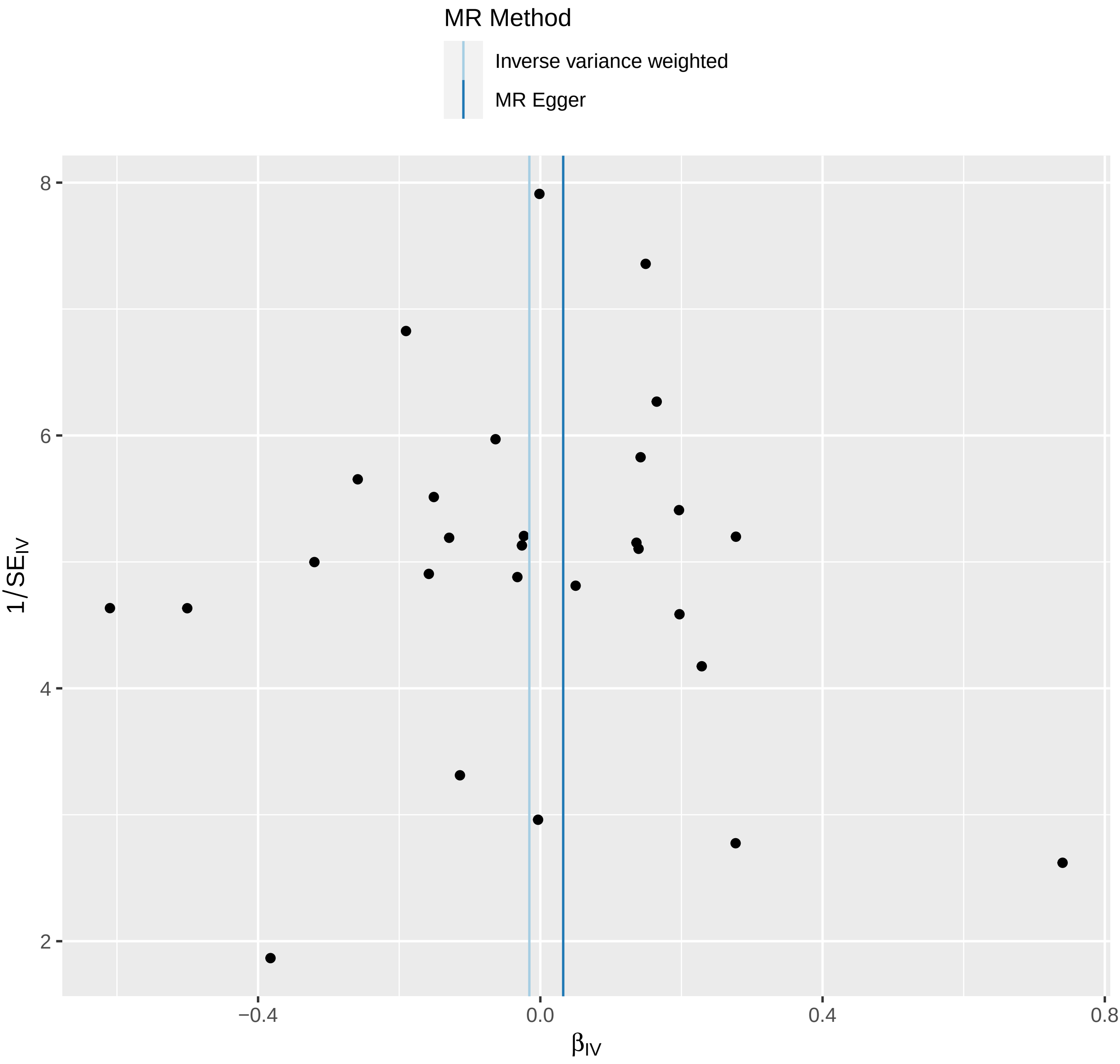

Batch 3−1936 : Melanocortin−2 receptor accessory protein on Diabetic nephropathy (more control exclusions)
with a threshold of 1e−05

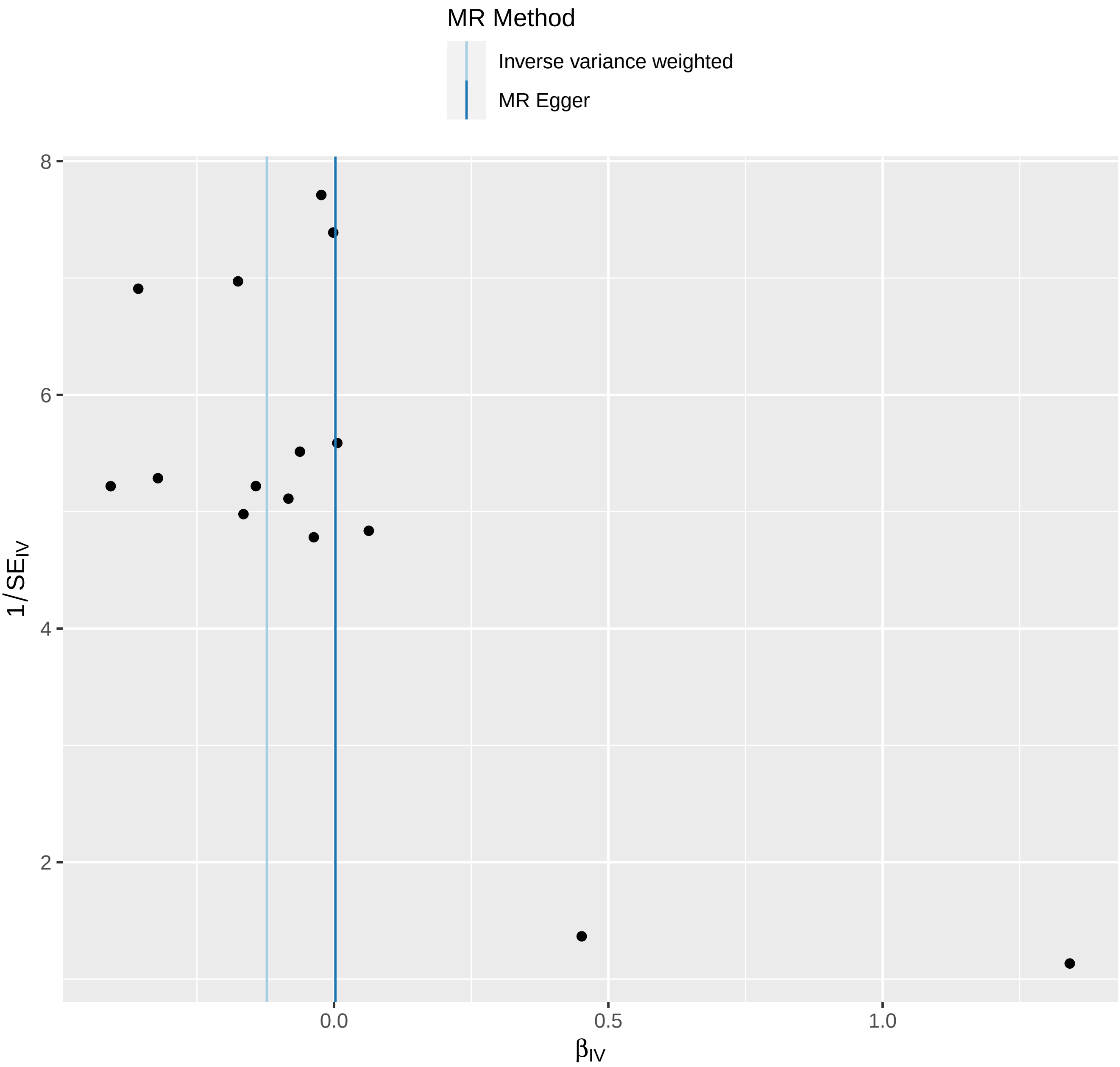

Batch 3−1937 : Macrophage mannose receptor 1 on Diabetic nephropathy (more control exclusions)
with a threshold of 1e−05

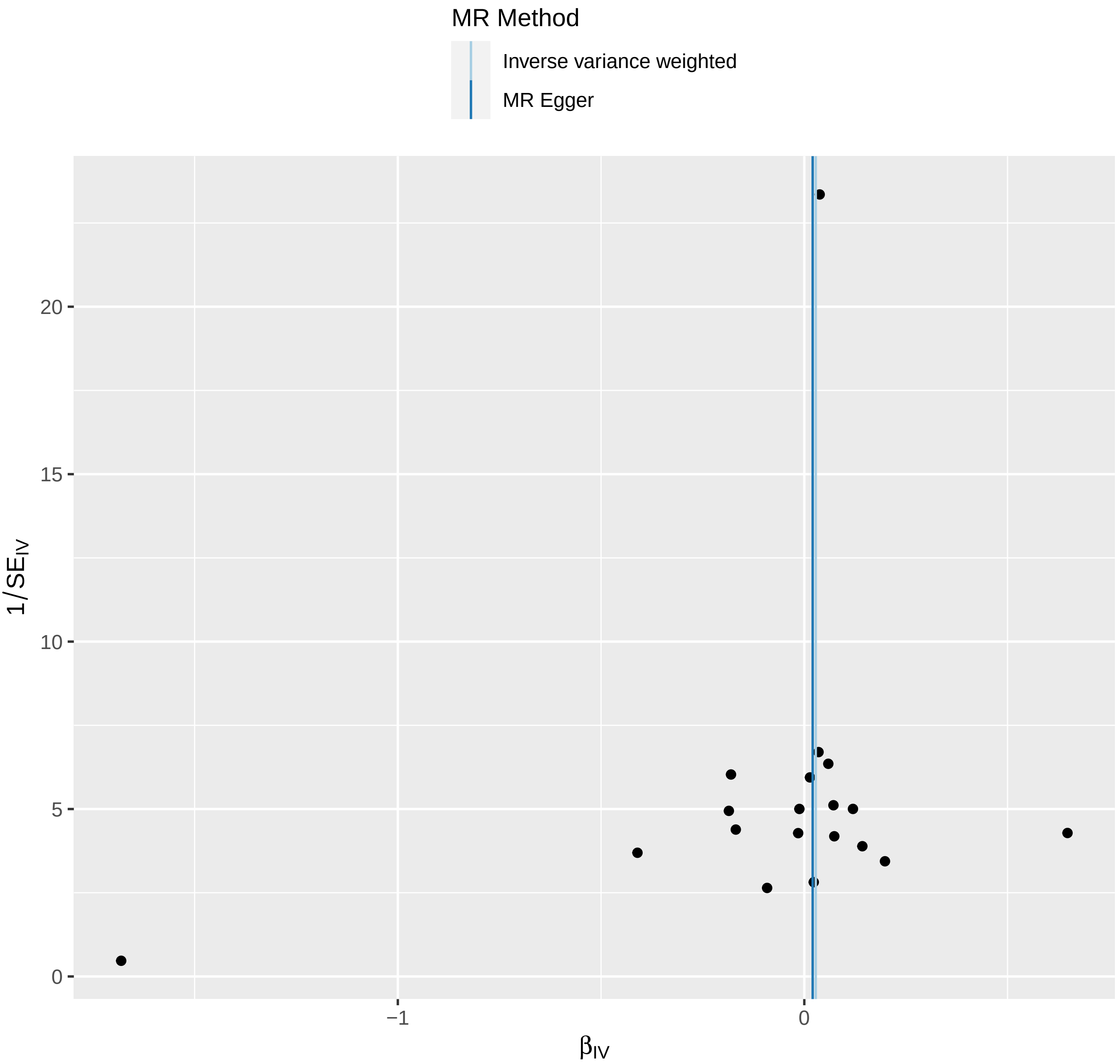

Batch 3−1938 : C−type mannose receptor 2 on Diabetic nephropathy (more control exclusions)
with a threshold of 1e−05

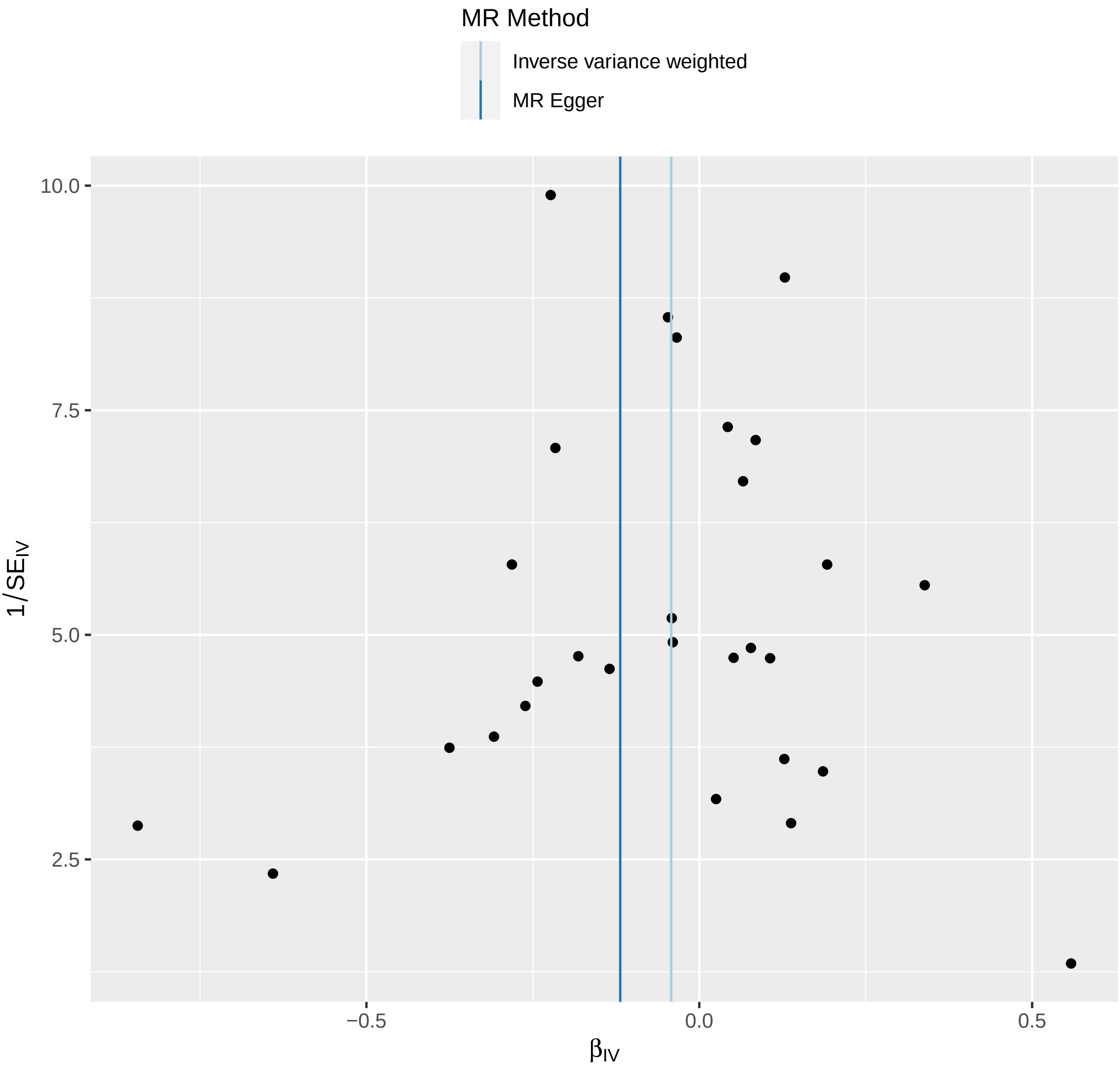

Batch 3−1939 : Double−strand break repair protein MRE11A on Diabetic nephropathy (more control exclusions)
with a threshold of 1e−05

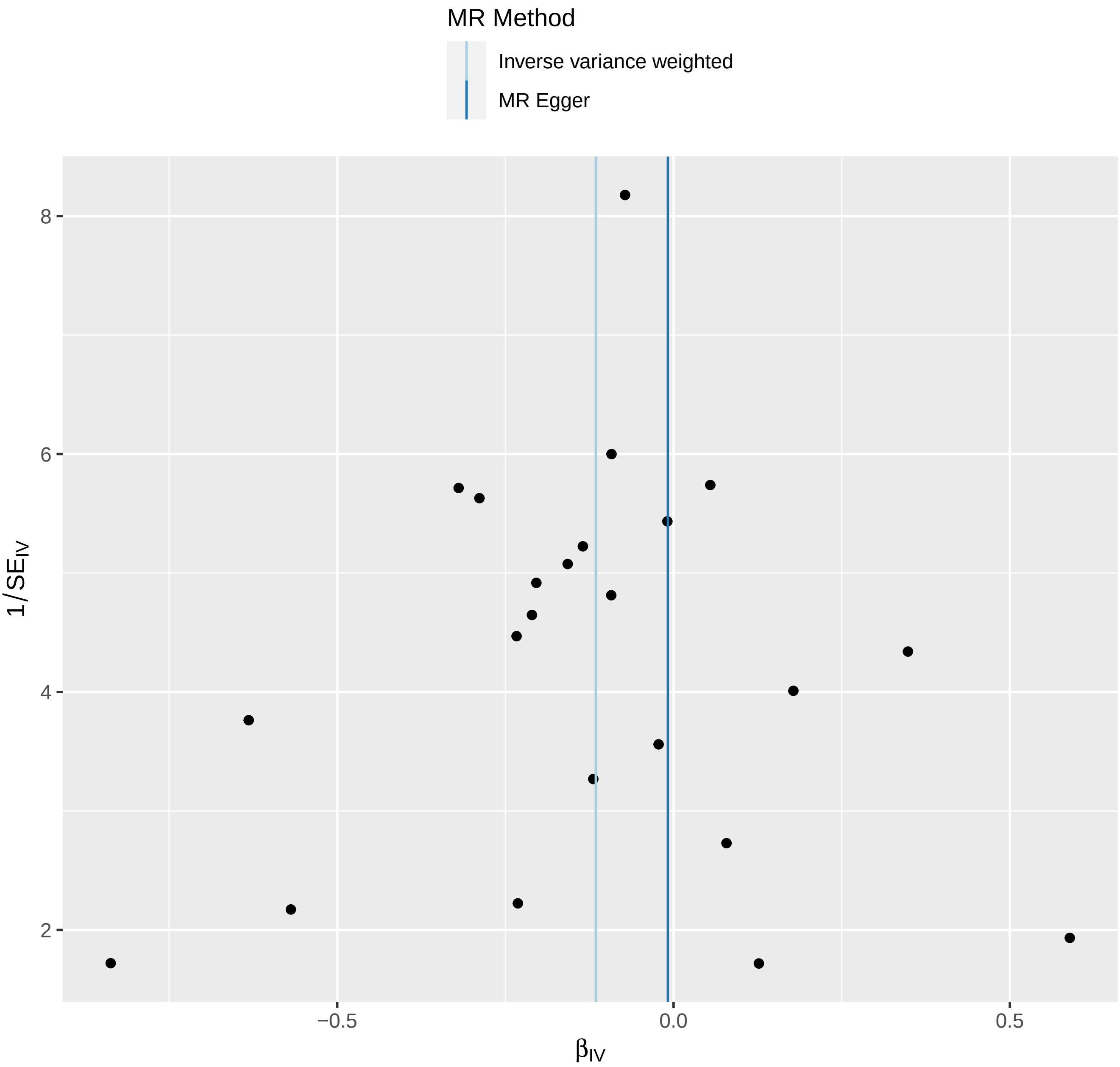

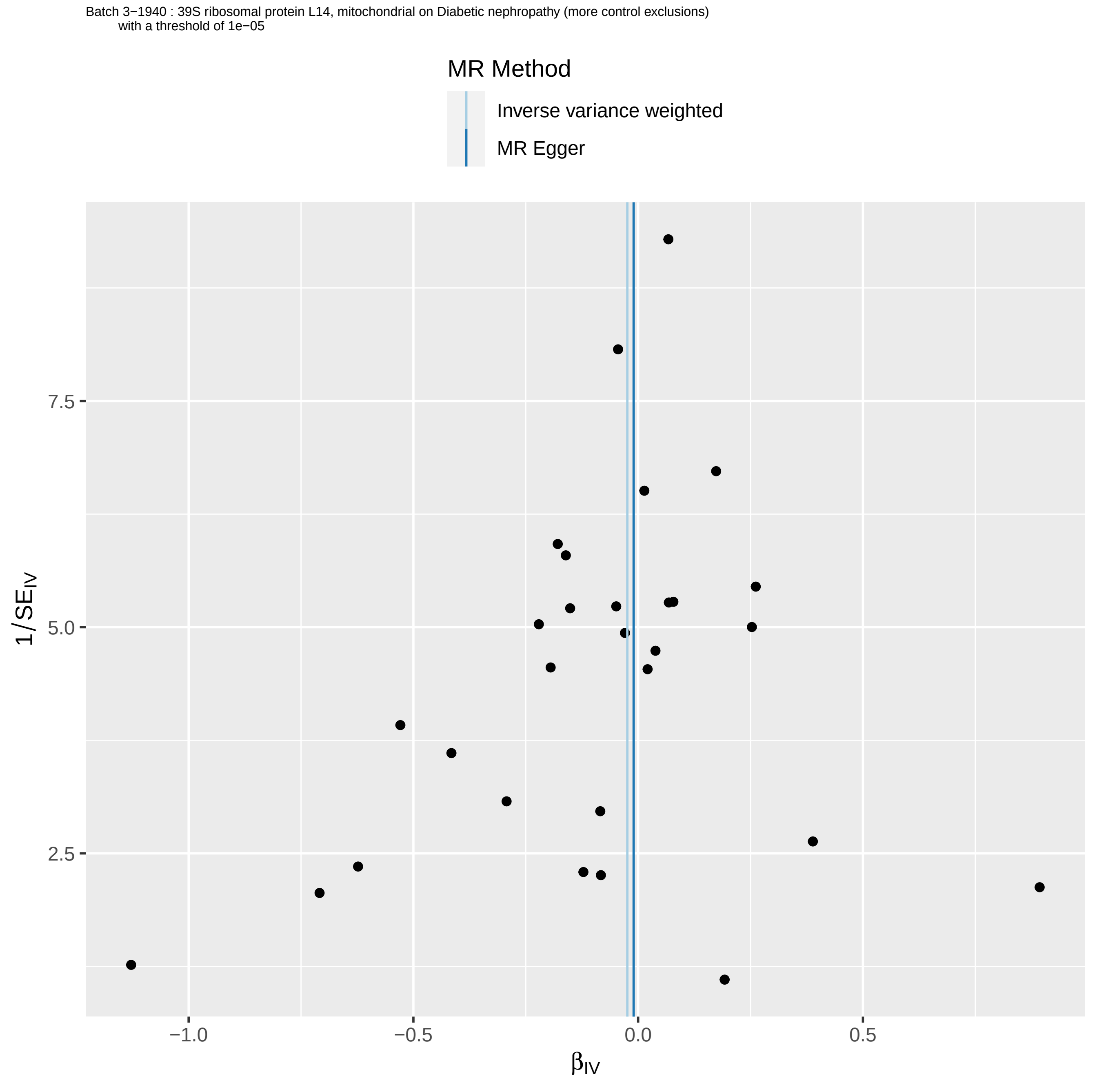
Batch 3−1940 : 39S ribosomal protein L14, mitochondrial on Diabetic nephropathy (more control exclusions)
with a threshold of 1e−05
MR Method
Inverse variance weighted
MR Egger
7.5
5.0
2.5
1/SE_IV
−1.0
−0.5
0.0
0.5
β_IV

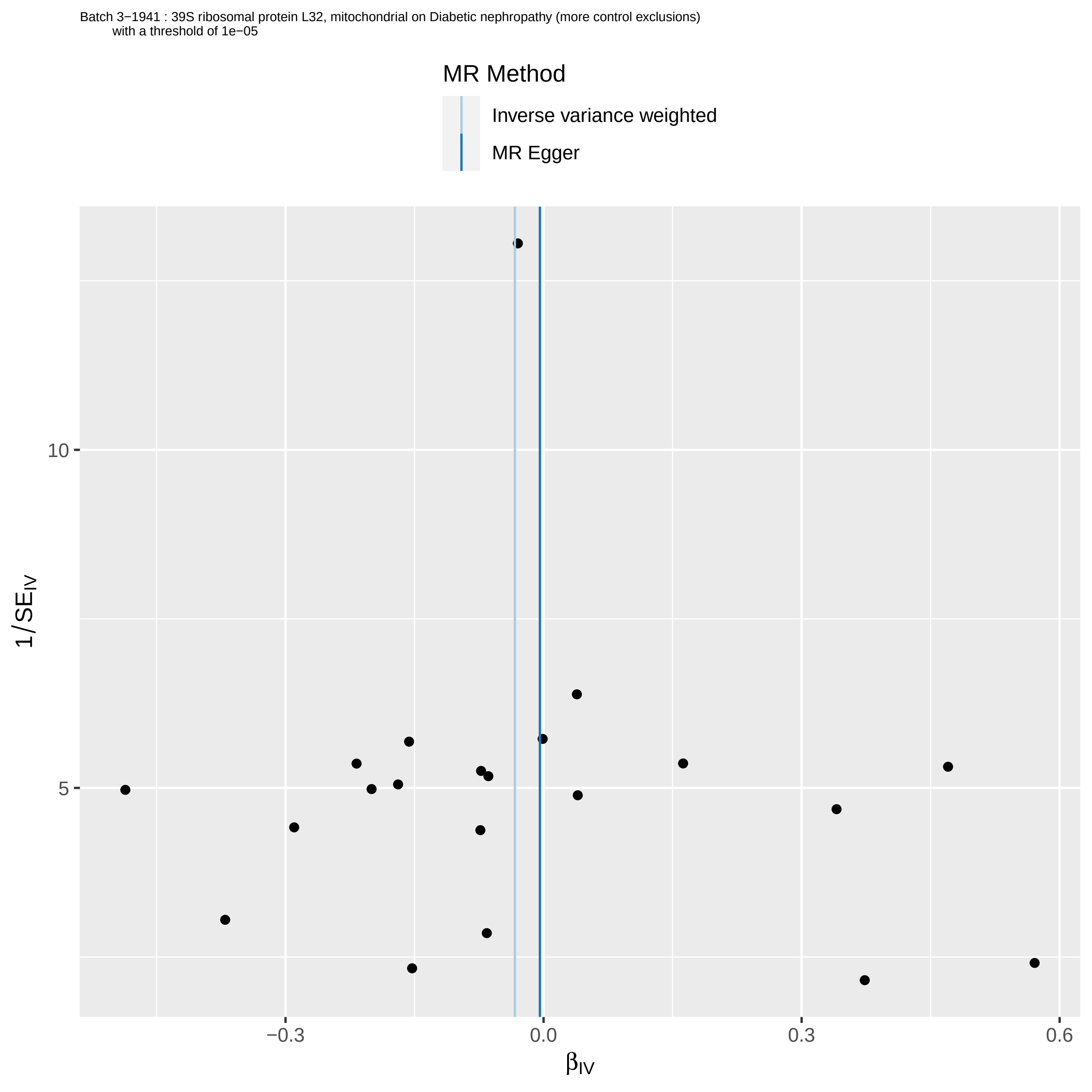

Batch 3−1941 : 39S ribosomal protein L32, mitochondrial on Diabetic nephropathy (more control exclusions)
with a threshold of 1e−05
MR Method
Inverse variance weighted
MR Egger
10
5
1/SE_IV
−0.3
0.0
0.3
0.6
β_IV

Batch 3−1942 : 39S ribosomal protein L33, mitochondrial on Diabetic nephropathy (more control exclusions)
with a threshold of 1e−05

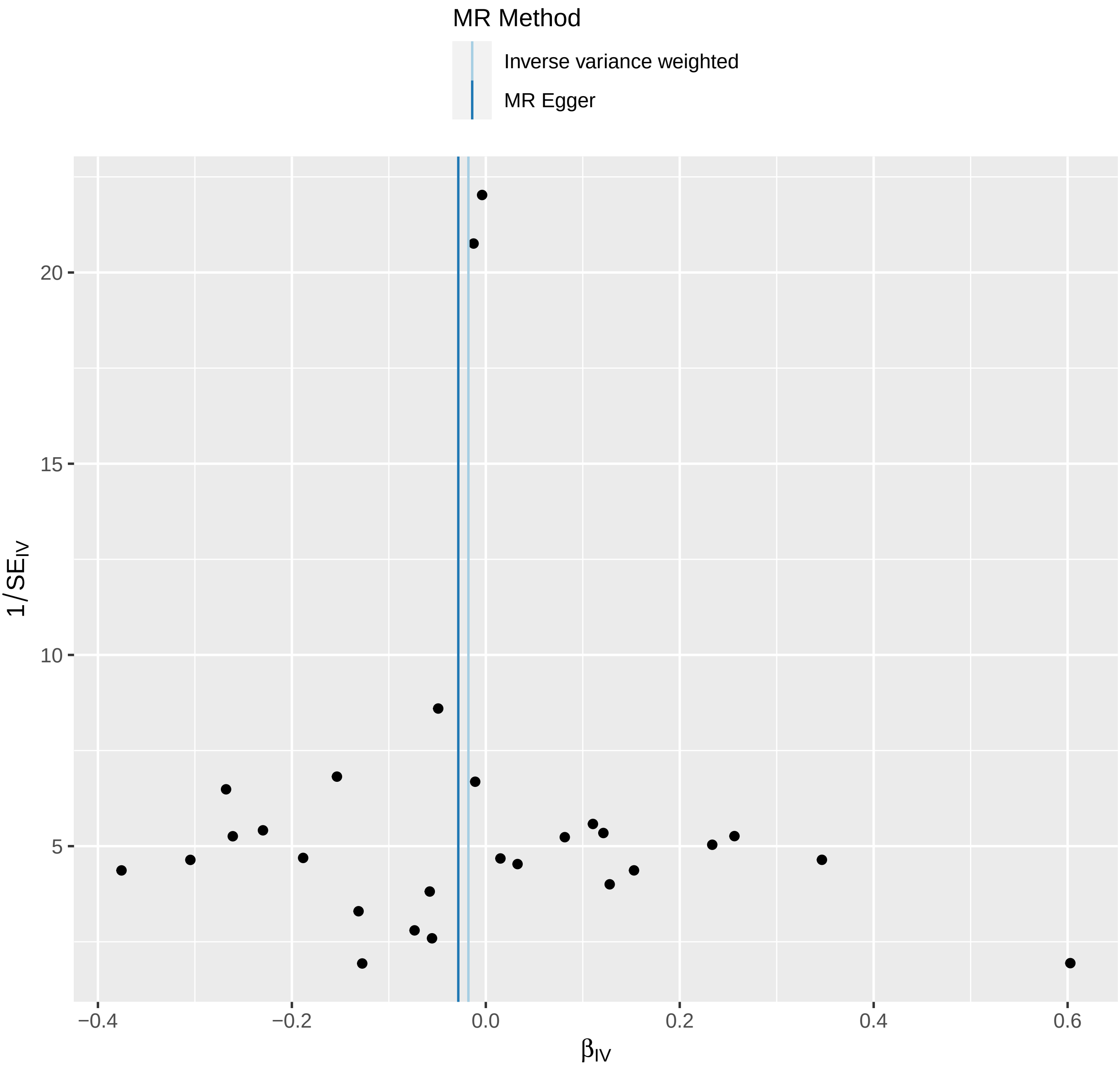

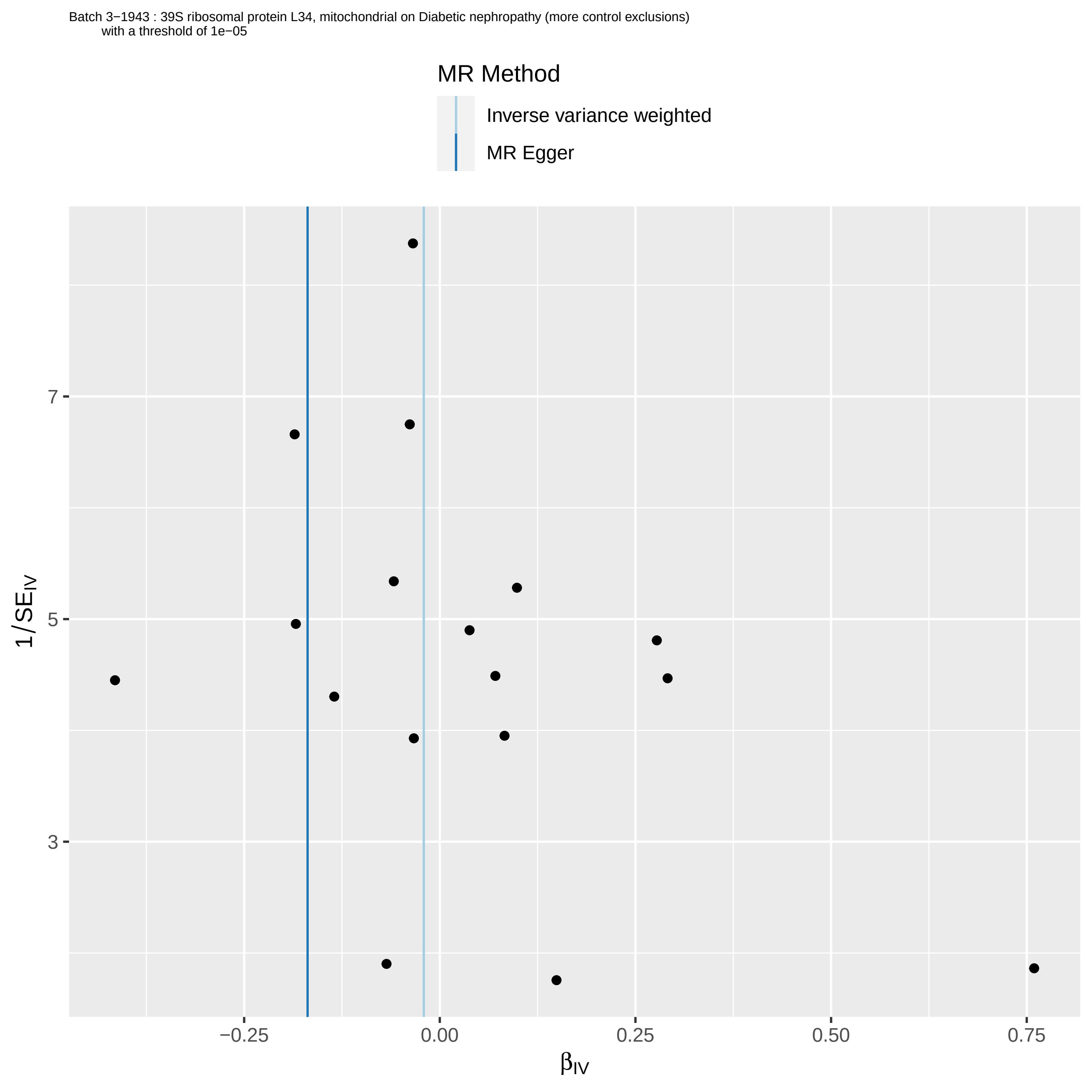
Batch 3−1943 : 39S ribosomal protein L34, mitochondrial on Diabetic nephropathy (more control exclusions)
with a threshold of 1e−05
MR Method
Inverse variance weighted
MR Egger
7
5
3
1/SE_IV
−0.25
0.00
0.25
0.50
0.75
β_IV

Batch 3−1944 : 39S ribosomal protein L52, mitochondrial on Diabetic nephropathy (more control exclusions)
with a threshold of 1e−05

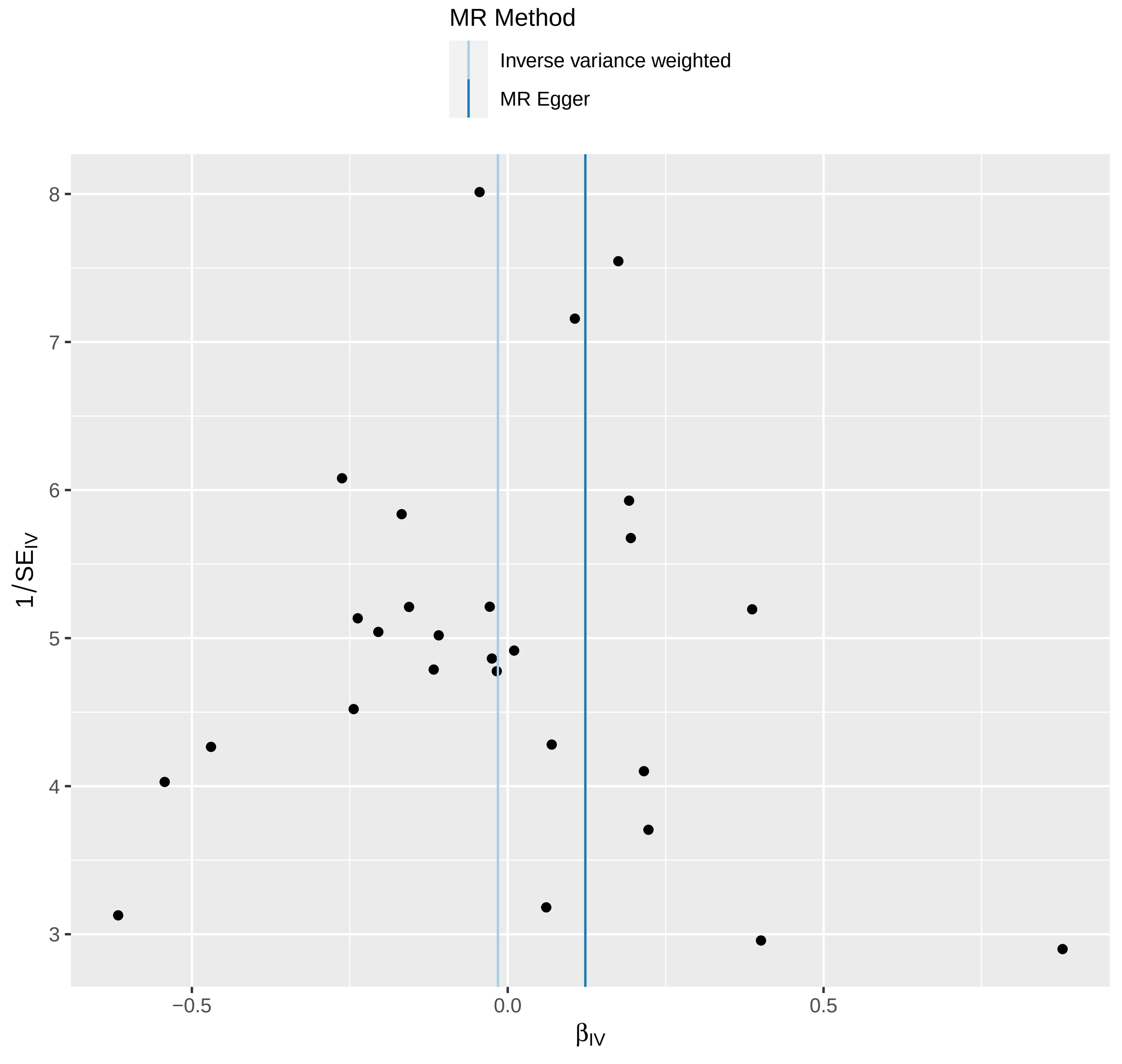

Batch 3−1945 : Ribosome−recycling factor, mitochondrial on Diabetic nephropathy (more control exclusions)
with a threshold of 1e−05

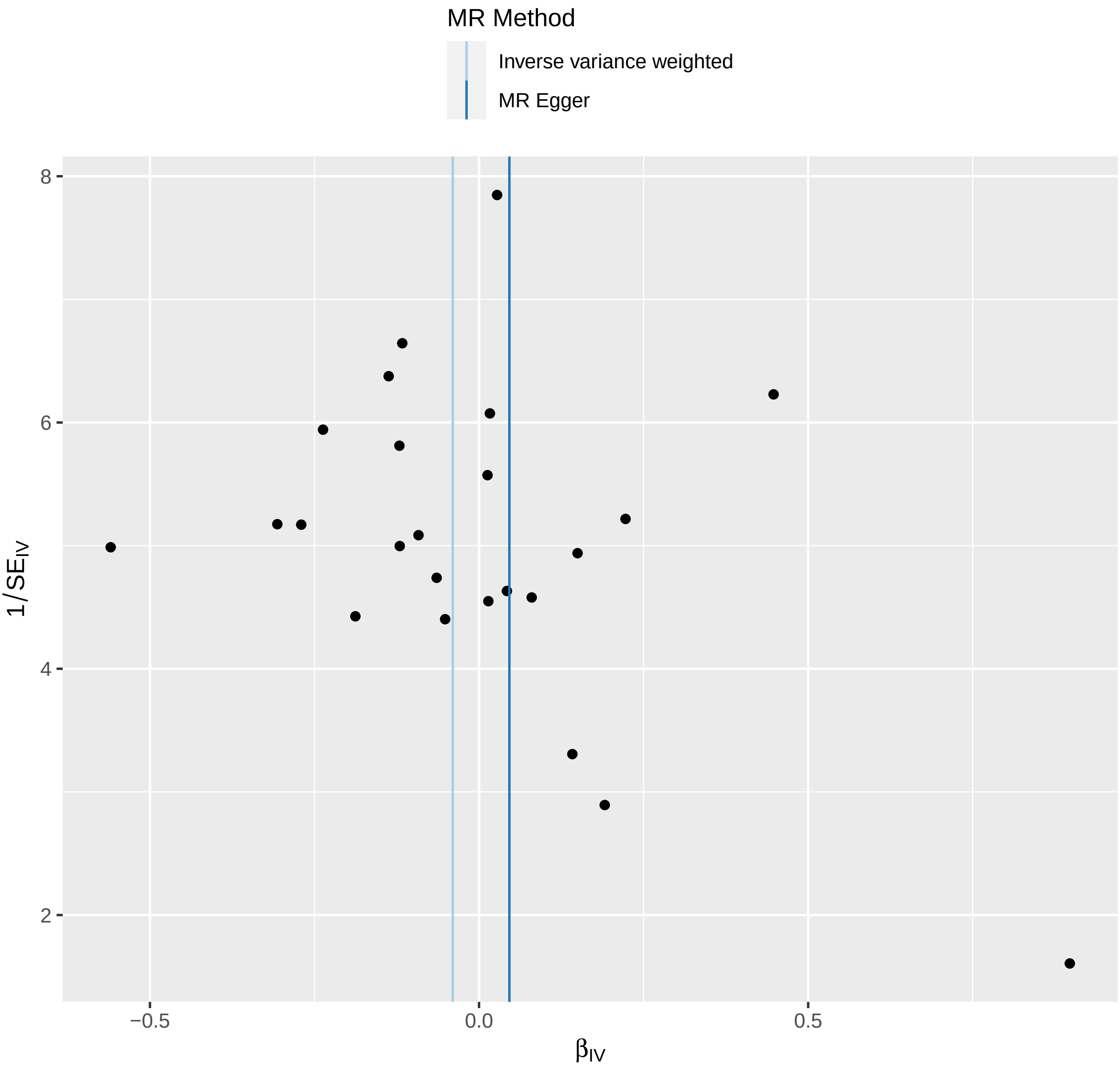

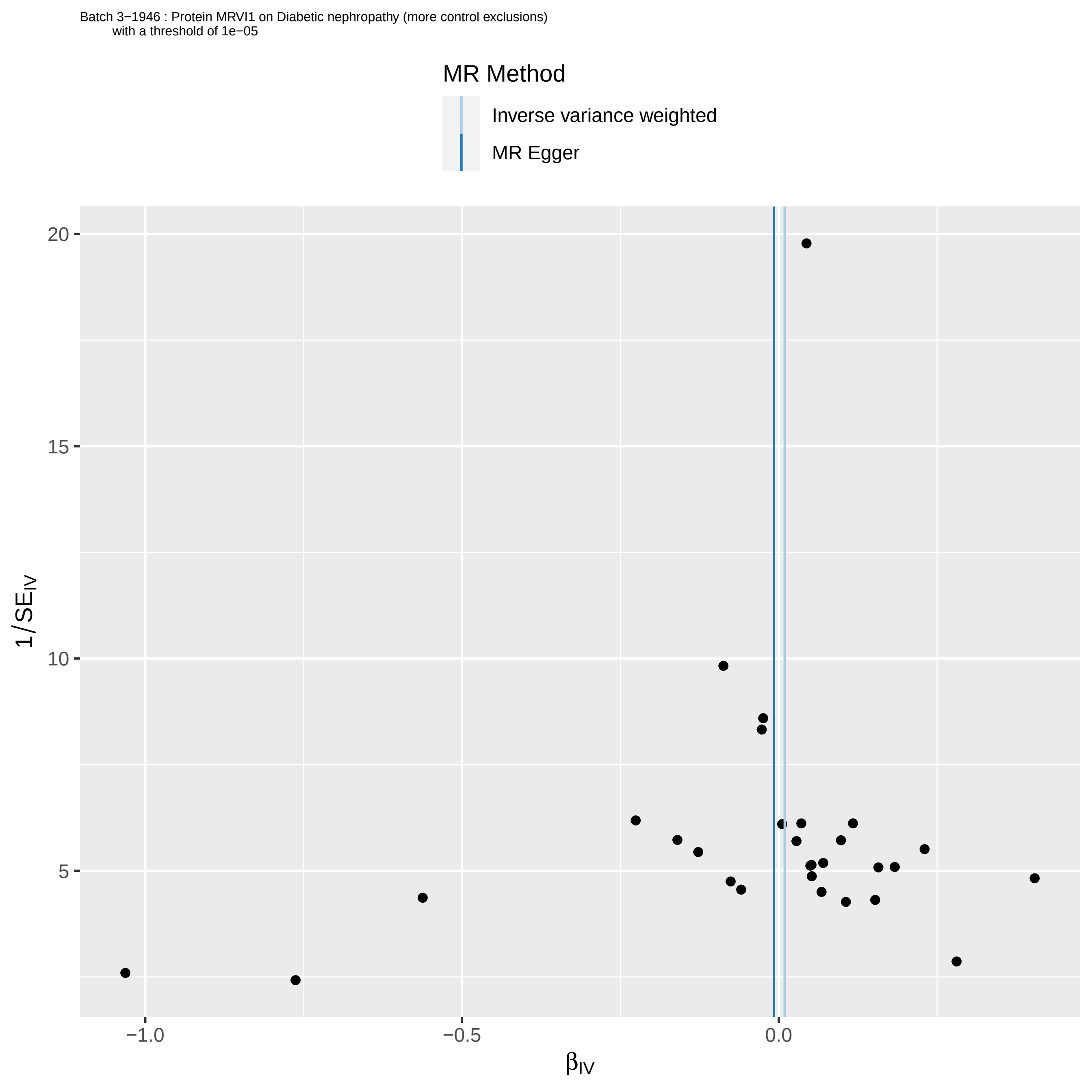

Batch 3−1946 : Protein MRVI1 on Diabetic nephropathy (more control exclusions)
with a threshold of 1e−05
MR Method
Inverse variance weighted
MR Egger
20
15
10
5
$1/SE_{IV}$
−1.0
−0.5
0.0
β_{IV}

Batch 3−1947 : Mesothelin on Diabetic nephropathy (more control exclusions)
with a threshold of 1e−05

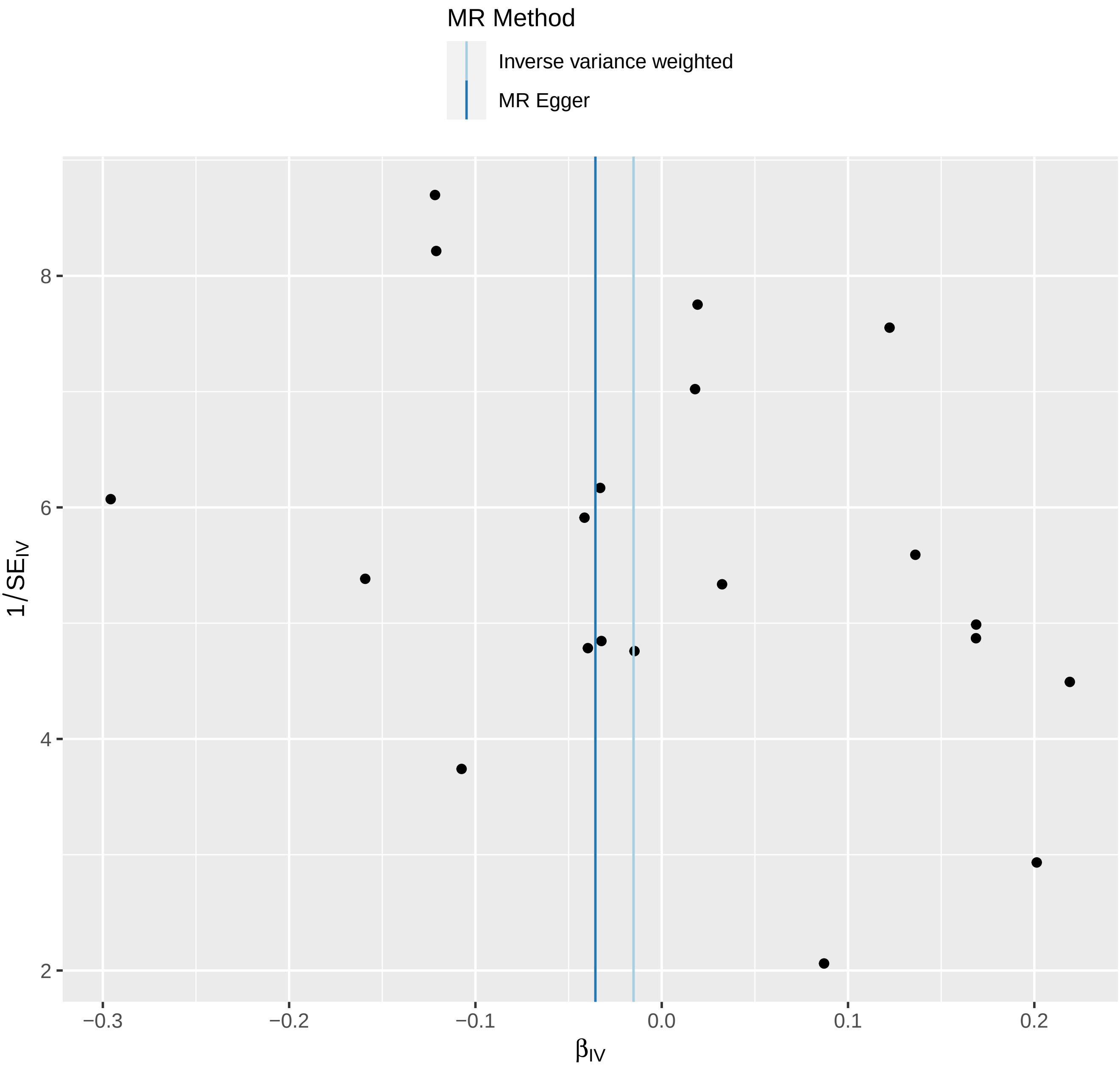

Batch 3−1948 : Mesothelin on Diabetic nephropathy (more control exclusions)
with a threshold of 1e−05

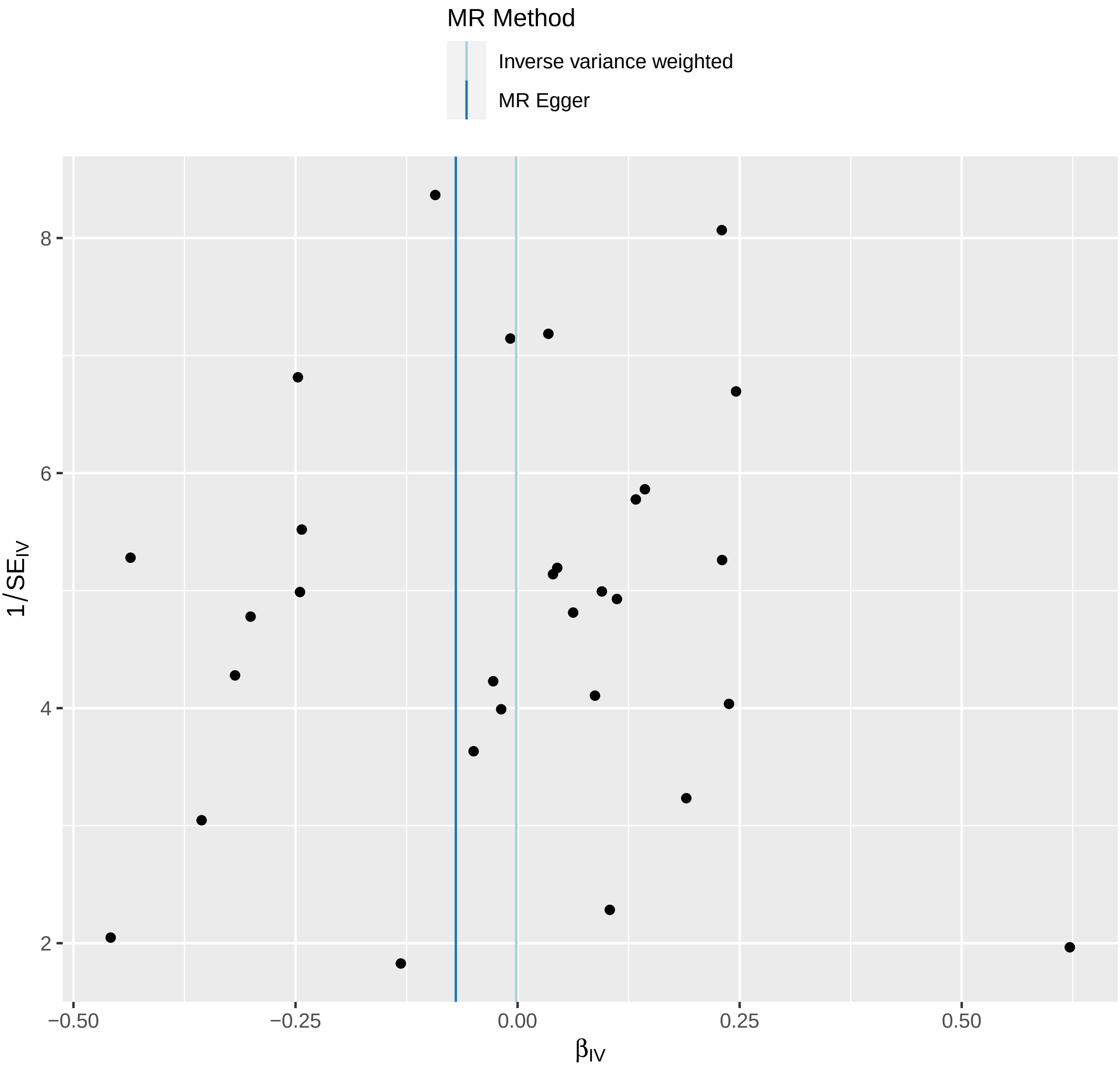

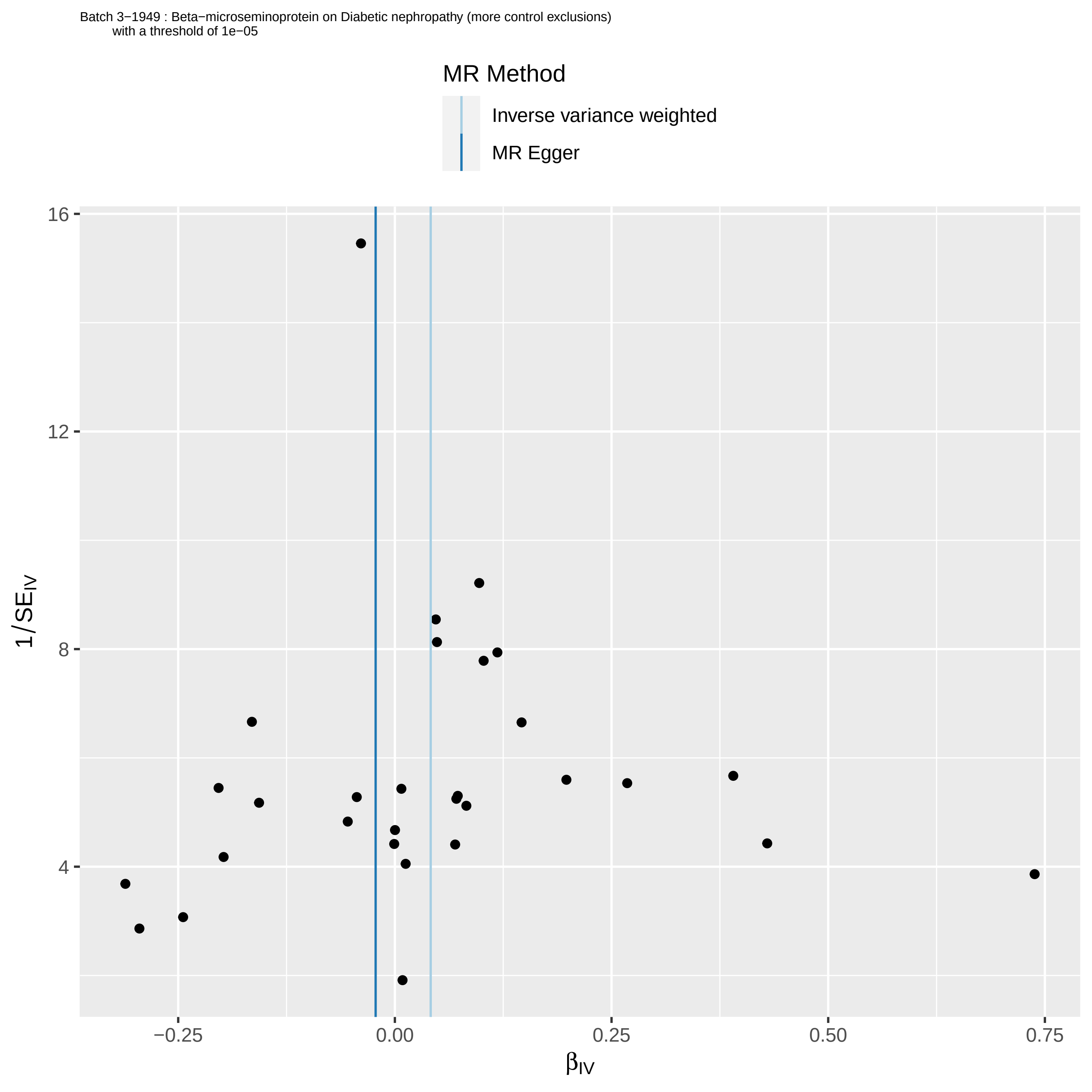

Batch 3−1949 : Beta−microseminoprotein on Diabetic nephropathy (more control exclusions)
with a threshold of 1e−05
MR Method
Inverse variance weighted
MR Egger
16
12
8
4
$1/SE_{IV}$
−0.25
0.00
0.25
0.50
0.75
β_{IV}

Batch 3−1950 : Prostate−associated microseminoprotein on Diabetic nephropathy (more control exclusions)
with a threshold of 1e−05

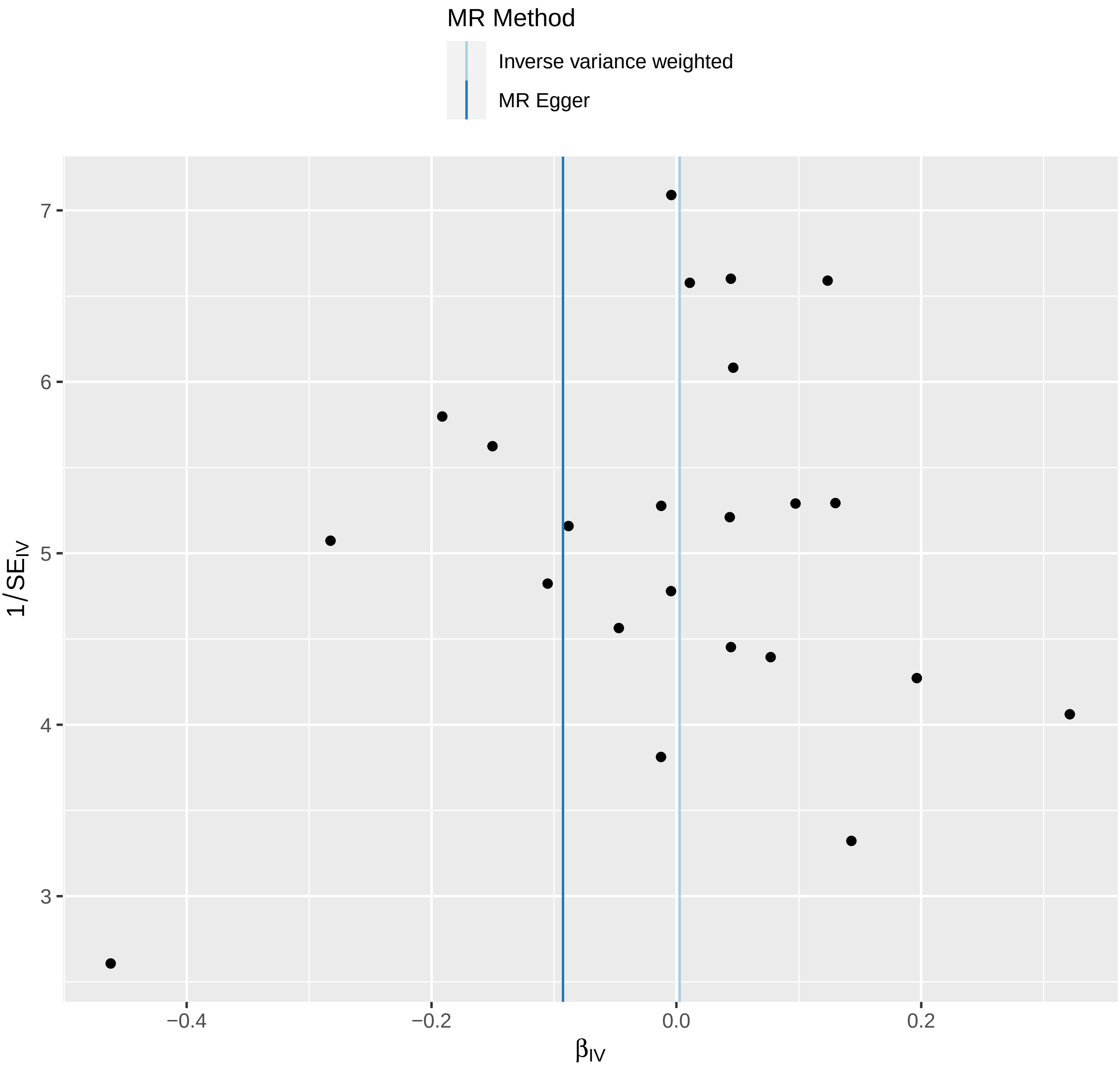

Batch 3−1951 : Macrophage scavenger receptor types I and II on Diabetic nephropathy (more control exclusions)
with a threshold of 1e−05

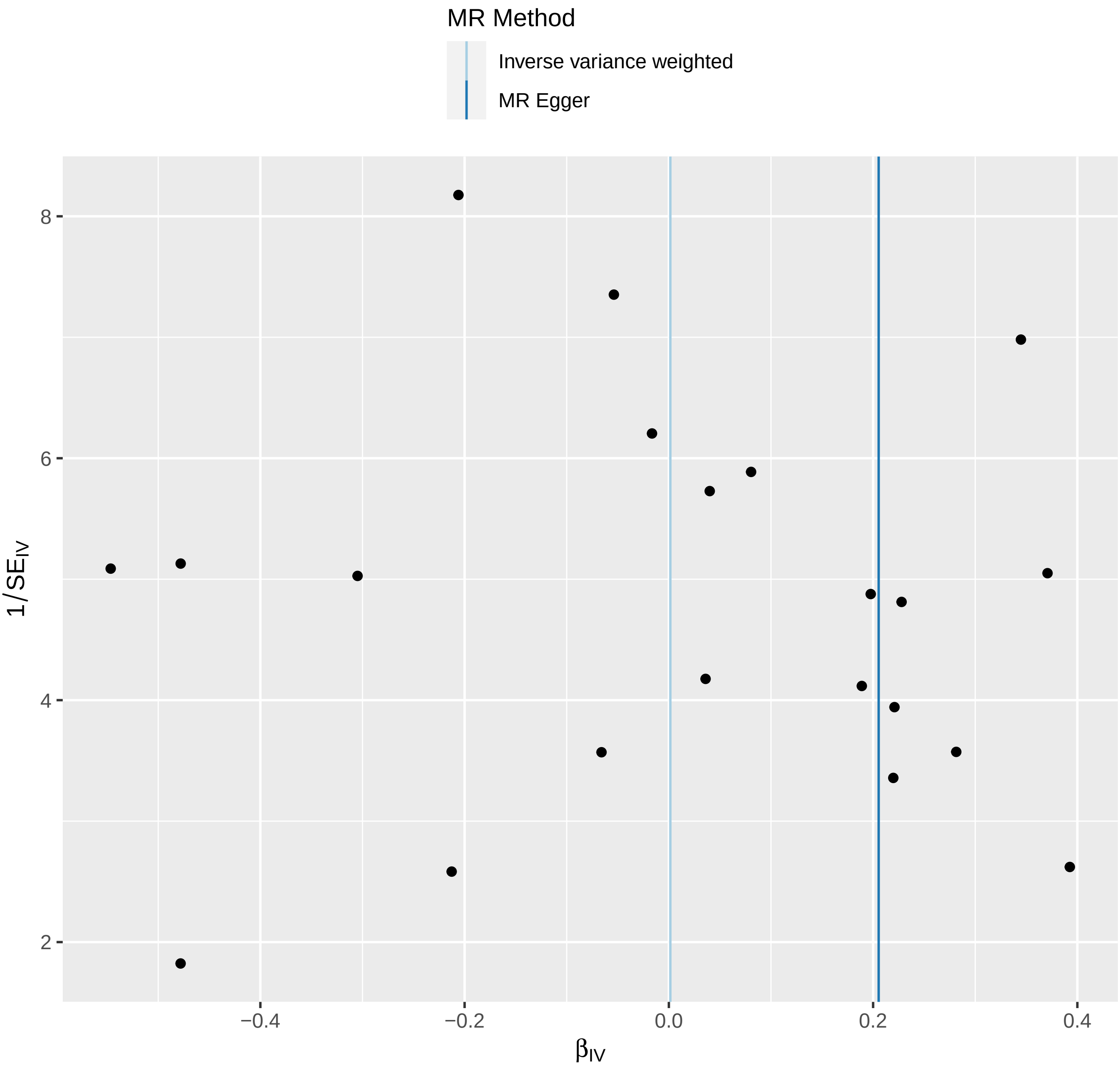

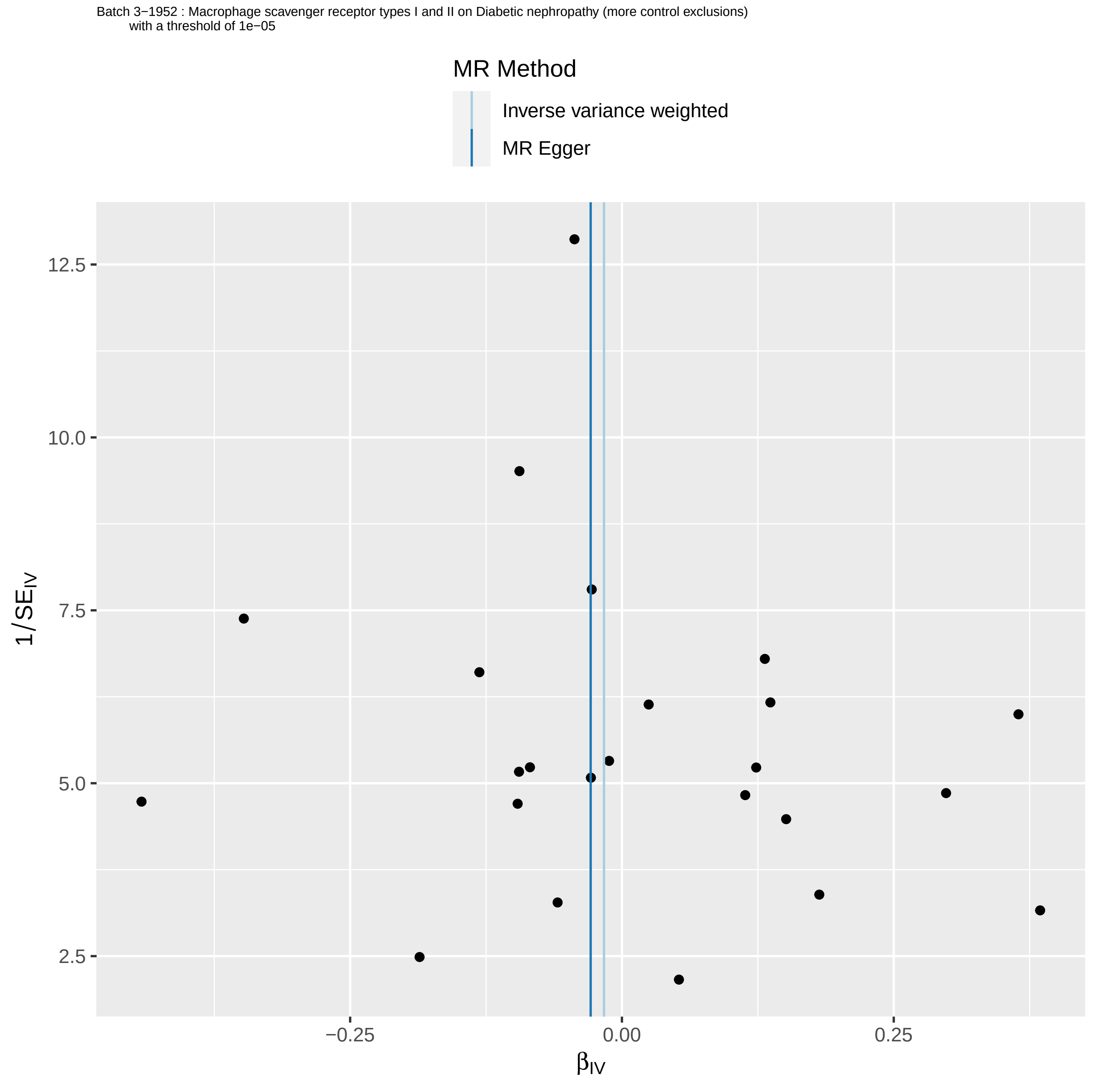
Batch 3−1952 : Macrophage scavenger receptor types I and II on Diabetic nephropathy (more control exclusions)
with a threshold of 1e−05
MR Method
Inverse variance weighted
MR Egger
12.5
10.0
7.5
5.0
2.5
1/SE$_{IV}$
−0.25
0.00
0.25
β$_{IV}$

Batch 3−1953 : Mitochondrial peptide methionine sulfoxide reductase on Diabetic nephropathy (more control exclusions) with a threshold of 1e−05

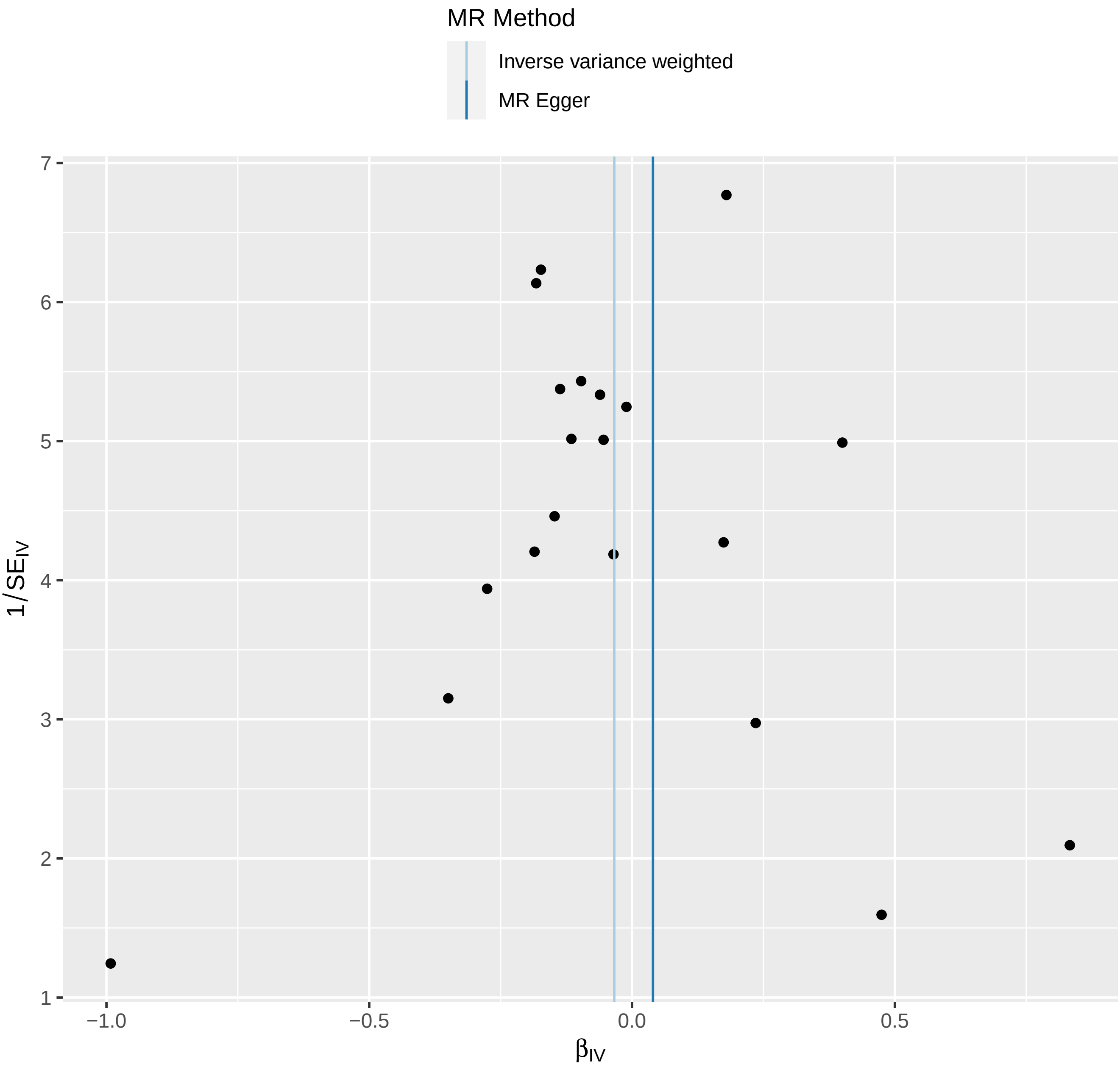

Batch 3−1954 : Methionine−R−sulfoxide reductase B3 on Diabetic nephropathy (more control exclusions)
with a threshold of 1e−05

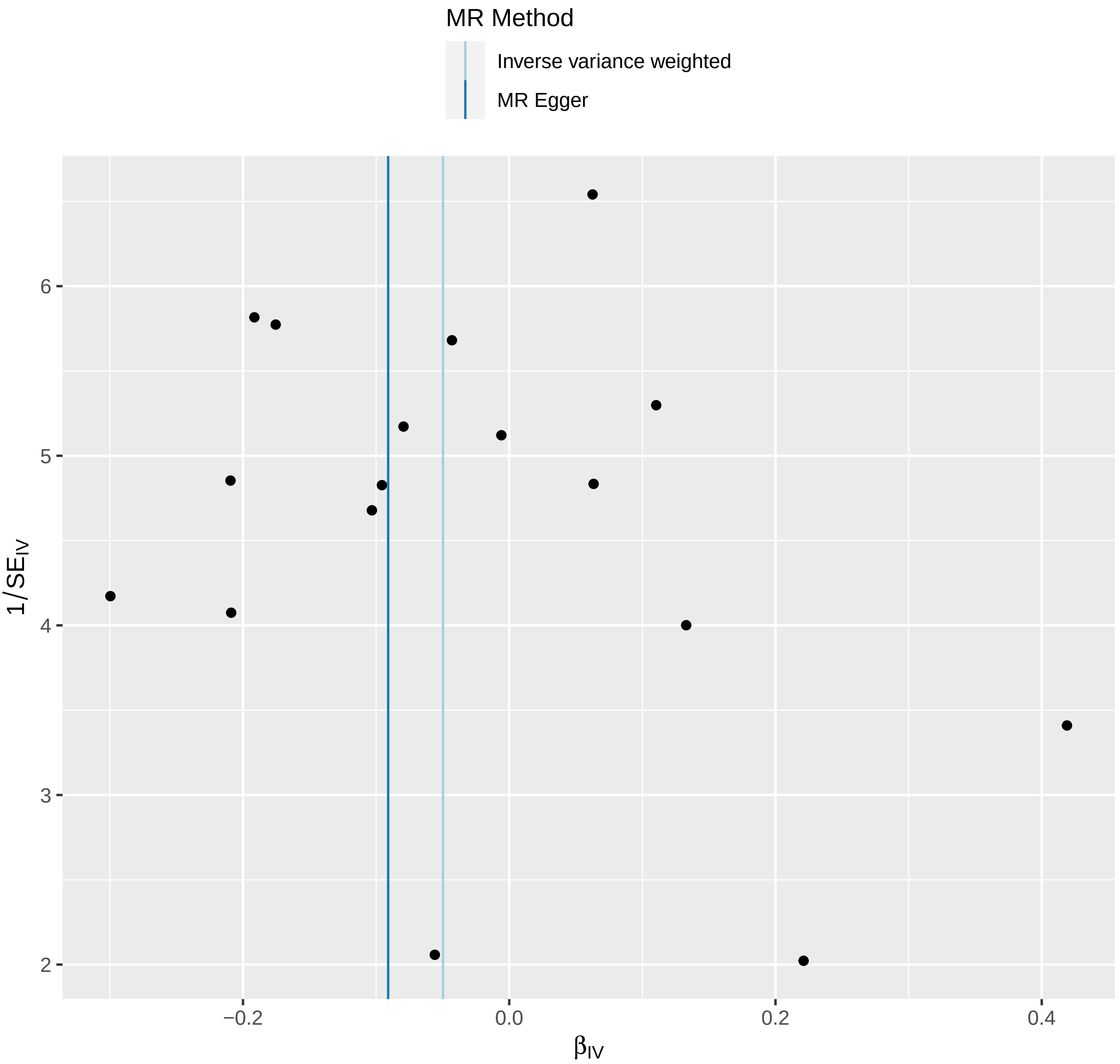

Batch 3−1955 : Macrophage−stimulating protein receptor on Diabetic nephropathy (more control exclusions)
with a threshold of 1e−05

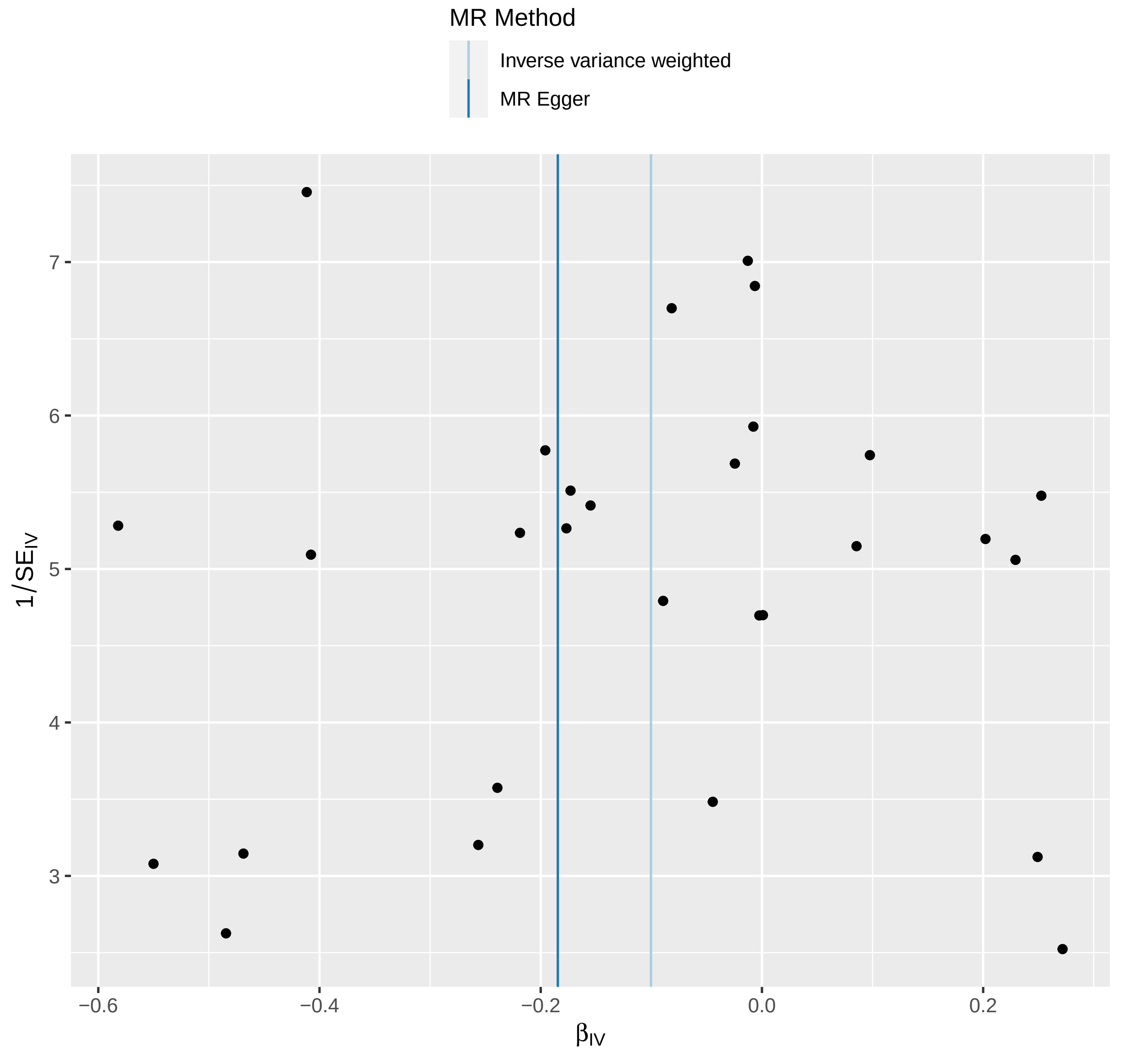

Batch 3−1956 : Growth/differentiation factor 8 on Diabetic nephropathy (more control exclusions)
with a threshold of 1e−05

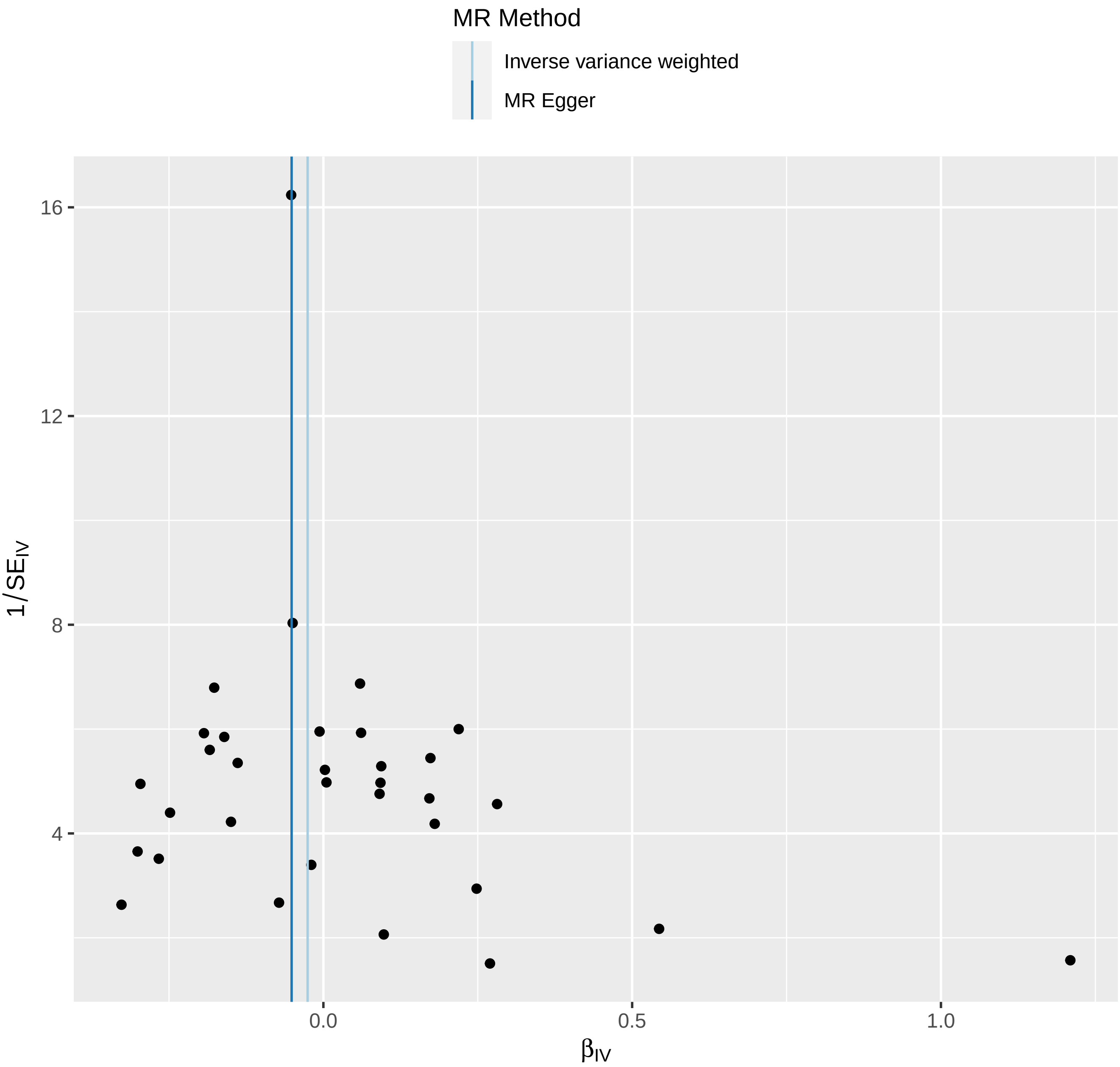

Batch 3−1957 : Growth/differentiation factor 8 on Diabetic nephropathy (more control exclusions)
with a threshold of 1e−05

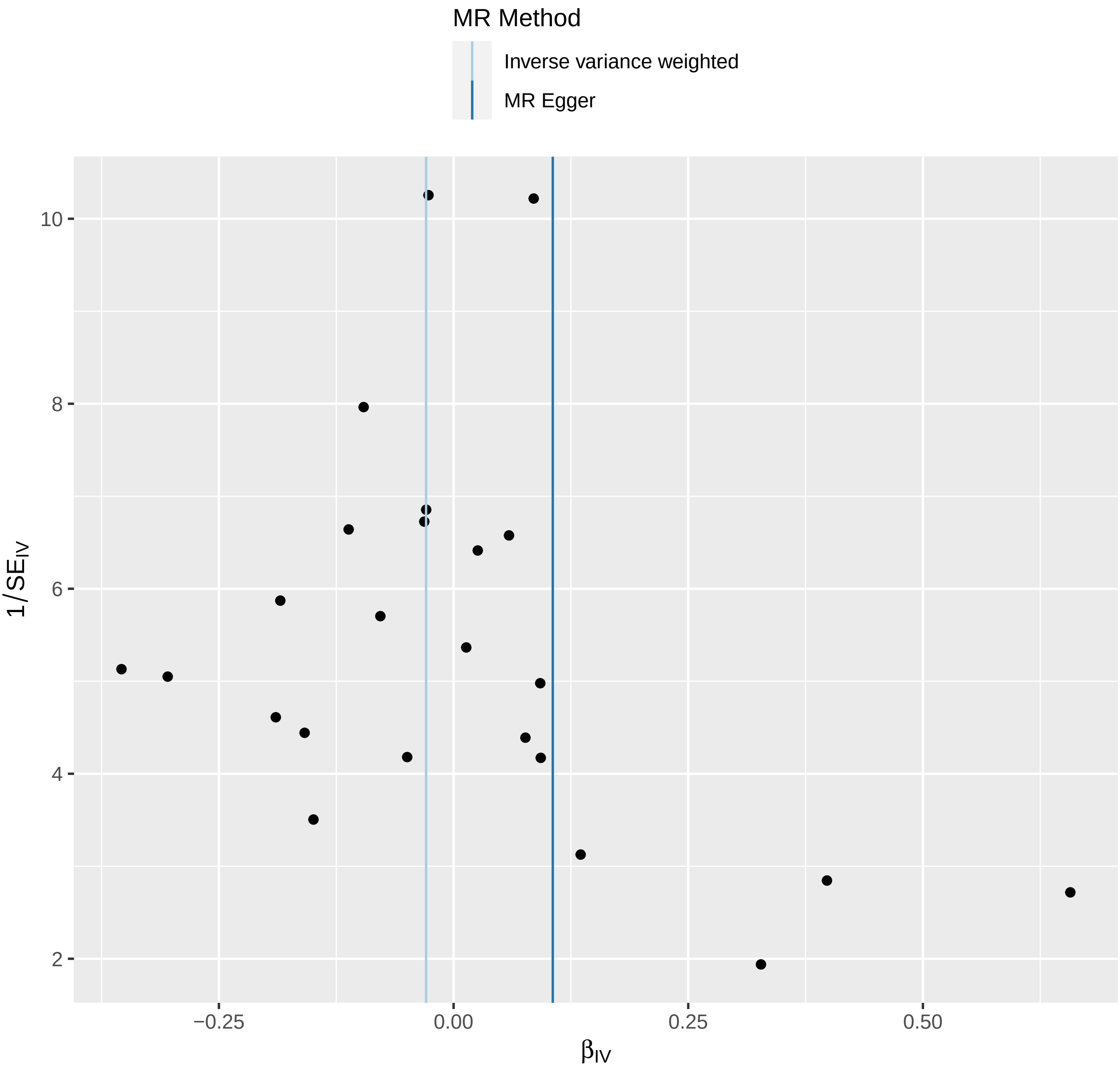

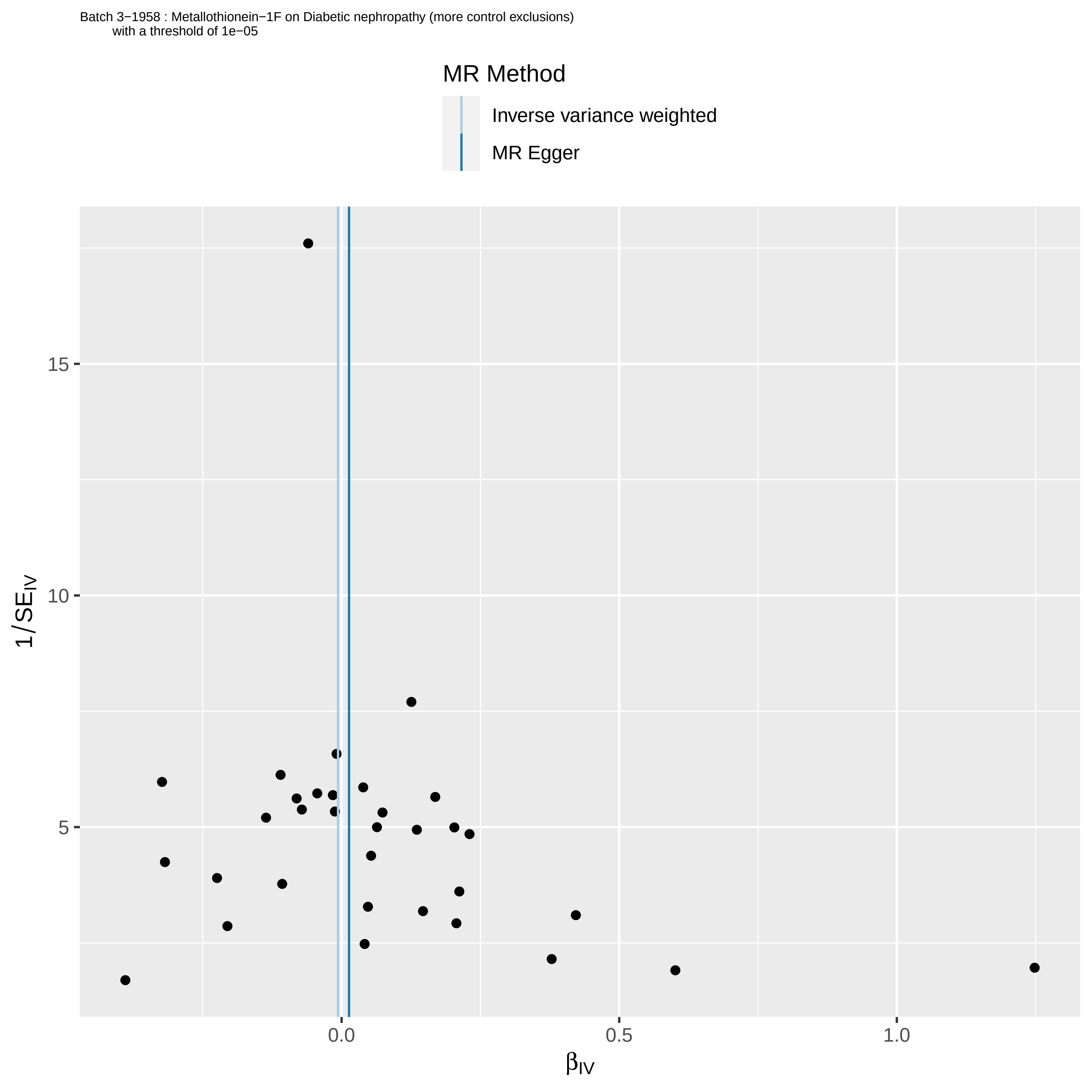

Batch 3−1958 : Metallothionein−1F on Diabetic nephropathy (more control exclusions)
with a threshold of 1e−05
MR Method
Inverse variance weighted
MR Egger
15
10
5
$1/SE_{IV}$
0.0
0.5
1.0
β_{IV}

Batch 3−1959 : S−methyl−5'−thioadenosine phosphorylase on Diabetic nephropathy (more control exclusions)
with a threshold of 1e−05

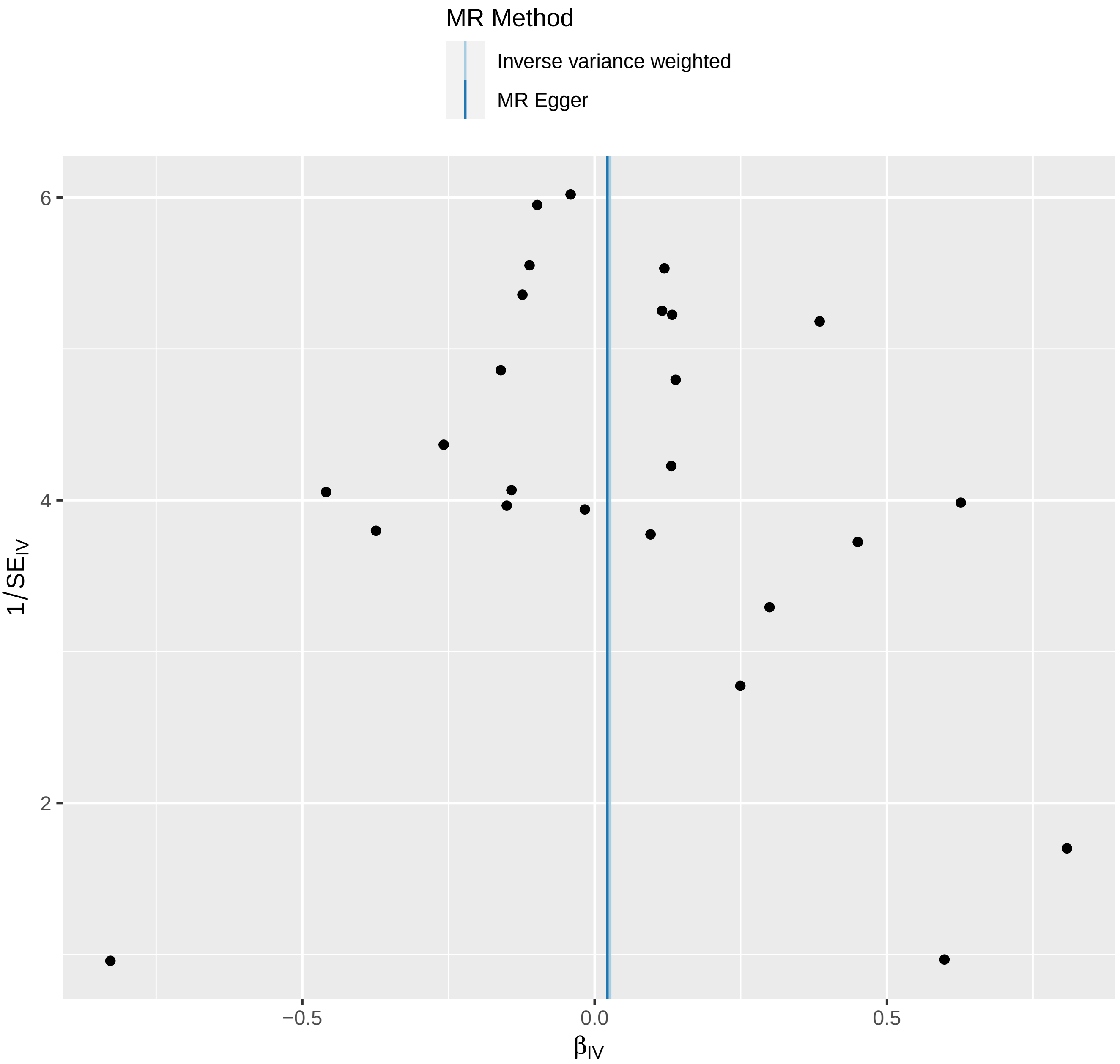

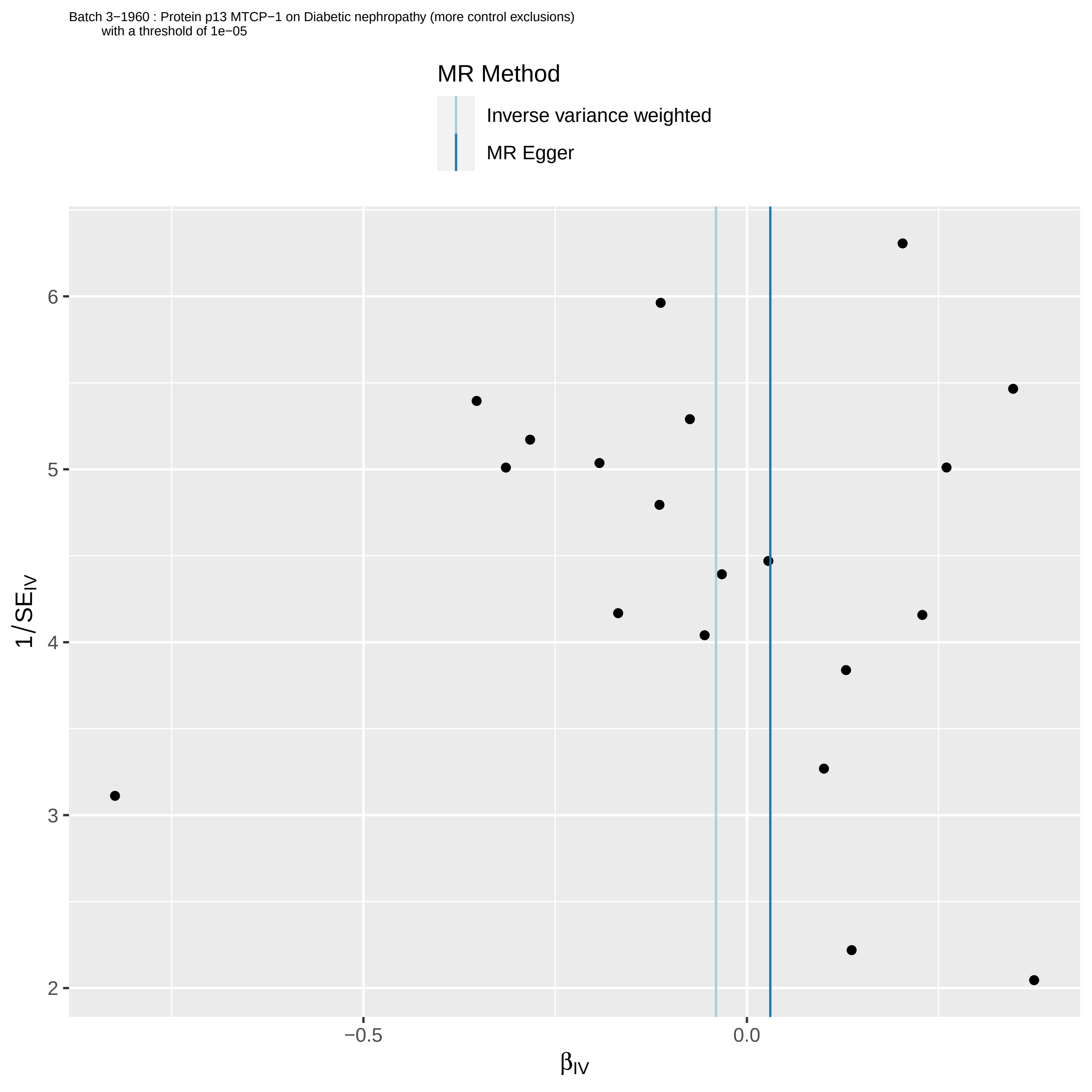

Batch 3−1960 : Protein p13 MTCP−1 on Diabetic nephropathy (more control exclusions)
with a threshold of 1e−05
MR Method
Inverse variance weighted
MR Egger
6
5
4
3
2
1/SE$_{IV}$
−0.5
0.0
β$_{IV}$

Batch 3−1961 : Mitochondrial fission regulator 1 on Diabetic nephropathy (more control exclusions)
with a threshold of 1e−05

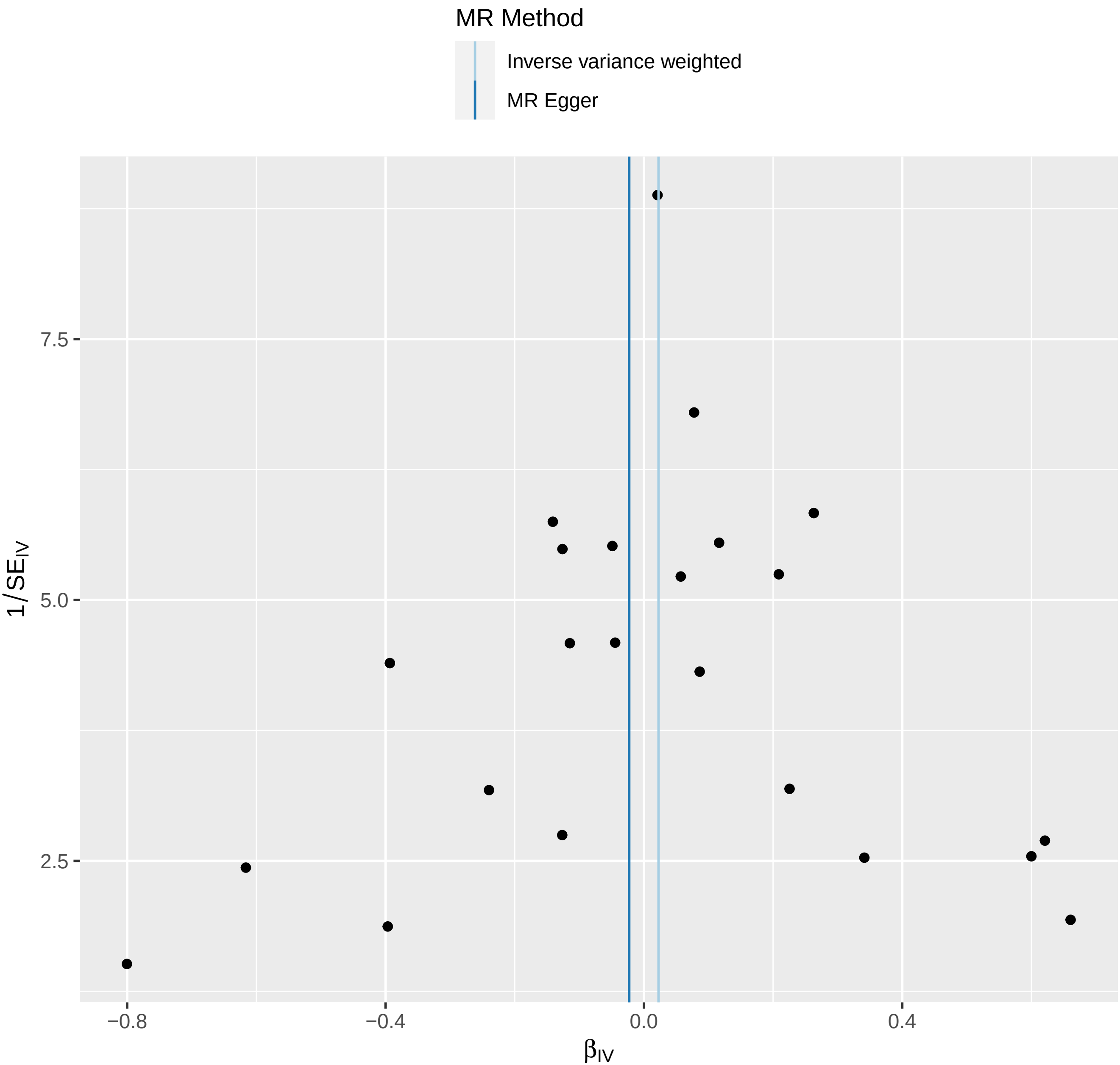

Batch 3−1962 : 5−formyltetrahydrofolate cyclo−ligase on Diabetic nephropathy (more control exclusions) with a threshold of 1e−05

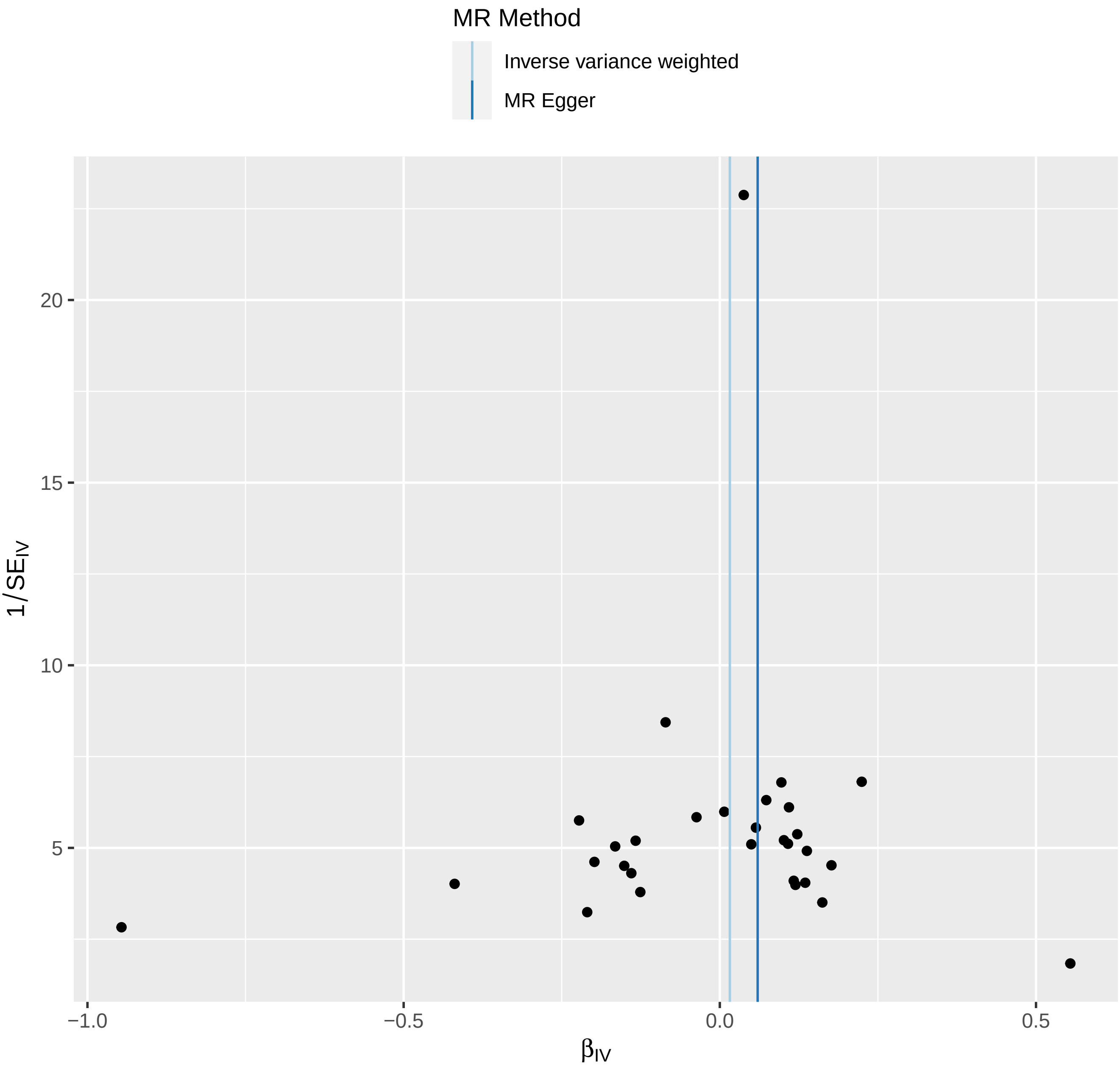

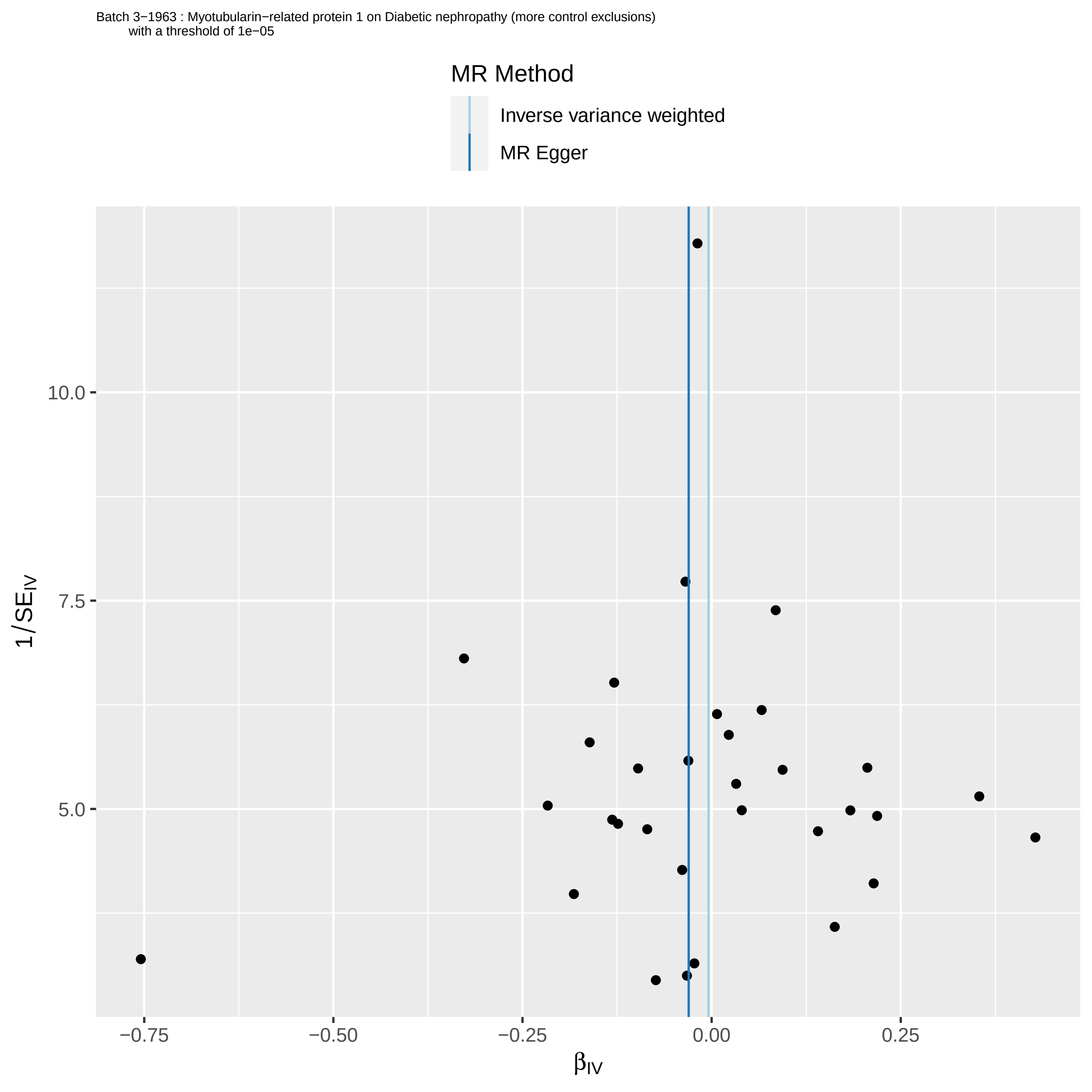
Batch 3−1963 : Myotubularin−related protein 1 on Diabetic nephropathy (more control exclusions)
with a threshold of 1e−05
MR Method
Inverse variance weighted
MR Egger
10.0
7.5
5.0
1/SE$_{IV}$
−0.75
−0.50
−0.25
0.00
0.25
β_{IV}

Batch 3−1964 : Poly(A) RNA polymerase, mitochondrial on Diabetic nephropathy (more control exclusions)
with a threshold of 1e−05

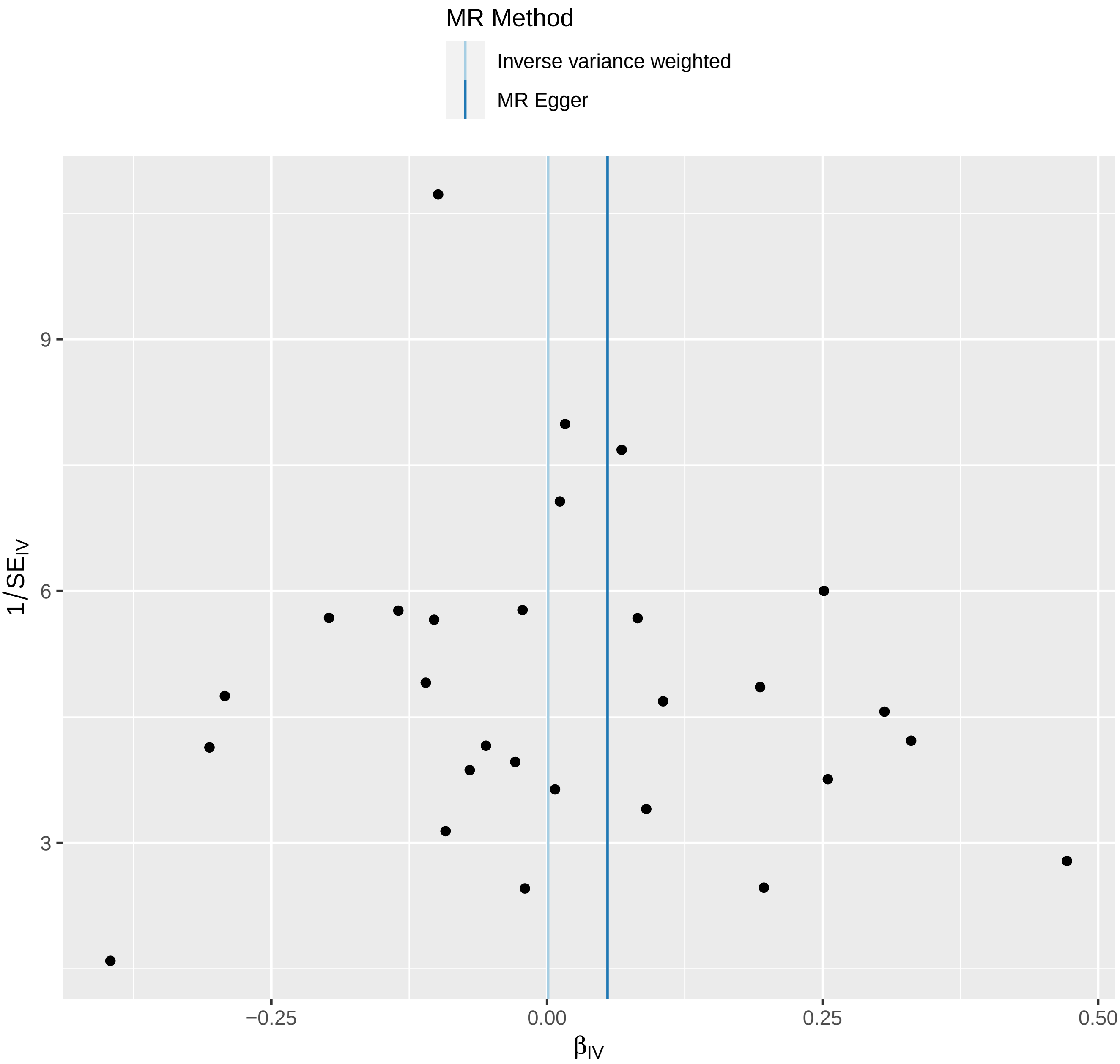

Batch 3−1965 : Peptide chain release factor 1−like, mitochondrial on Diabetic nephropathy (more control exclusions)
with a threshold of 1e−05

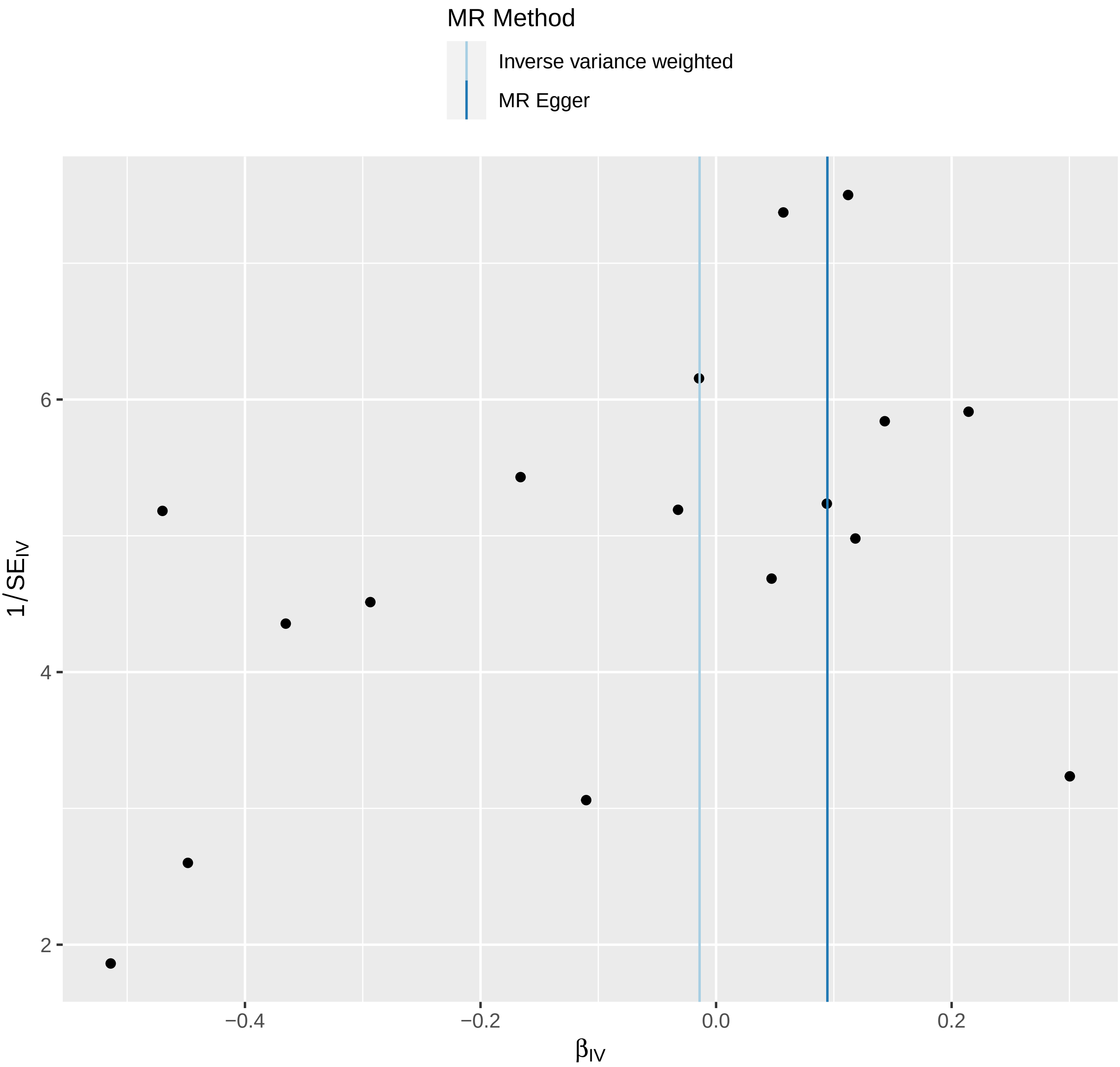

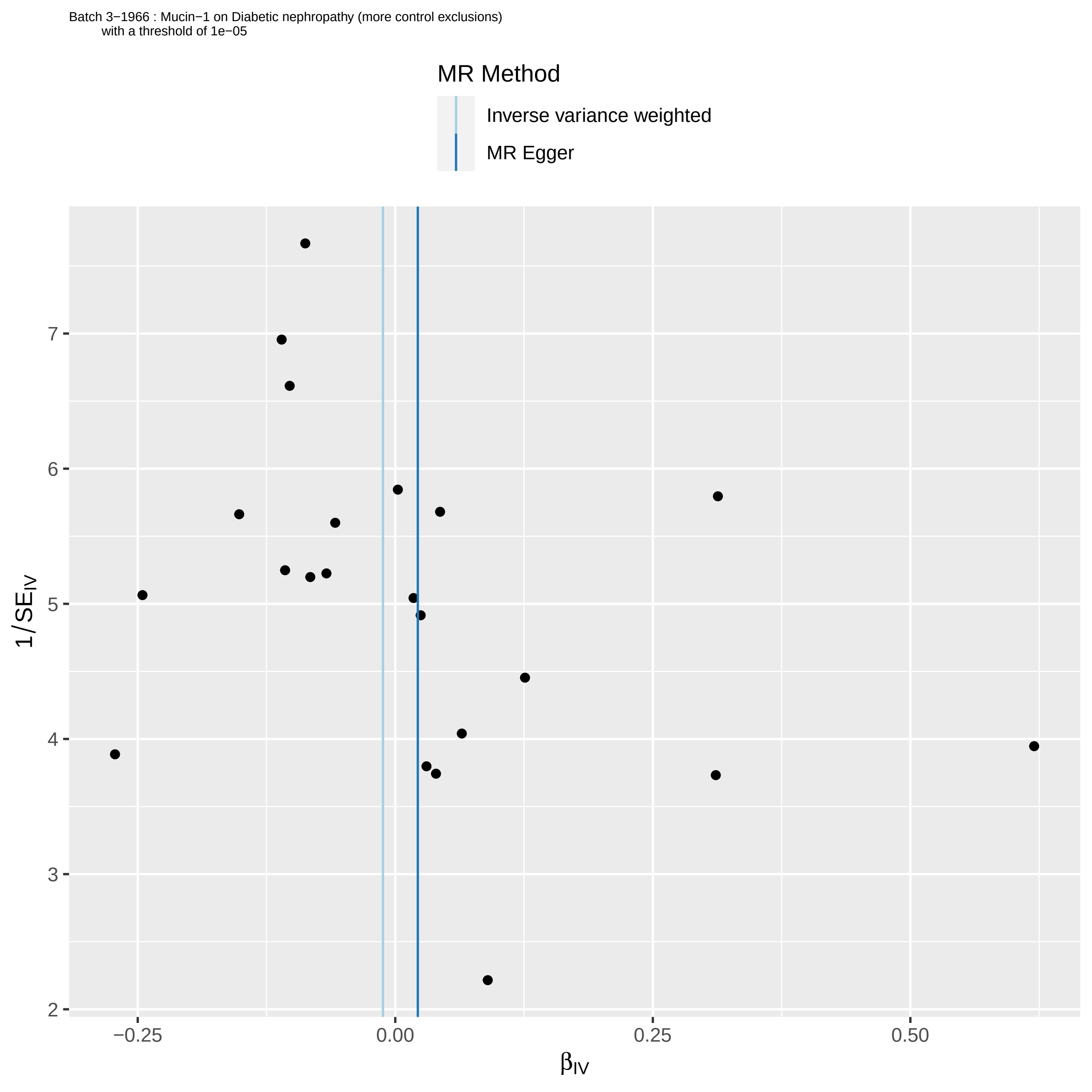

Batch 3−1966 : Mucin−1 on Diabetic nephropathy (more control exclusions)
with a threshold of 1e−05
MR Method
Inverse variance weighted
MR Egger
7
6
5
4
3
2
1/SE$_{IV}$
−0.25
0.00
0.25
0.50
β_{IV}

Batch 3−1967 : Mucin−1 on Diabetic nephropathy (more control exclusions)
with a threshold of 1e−05

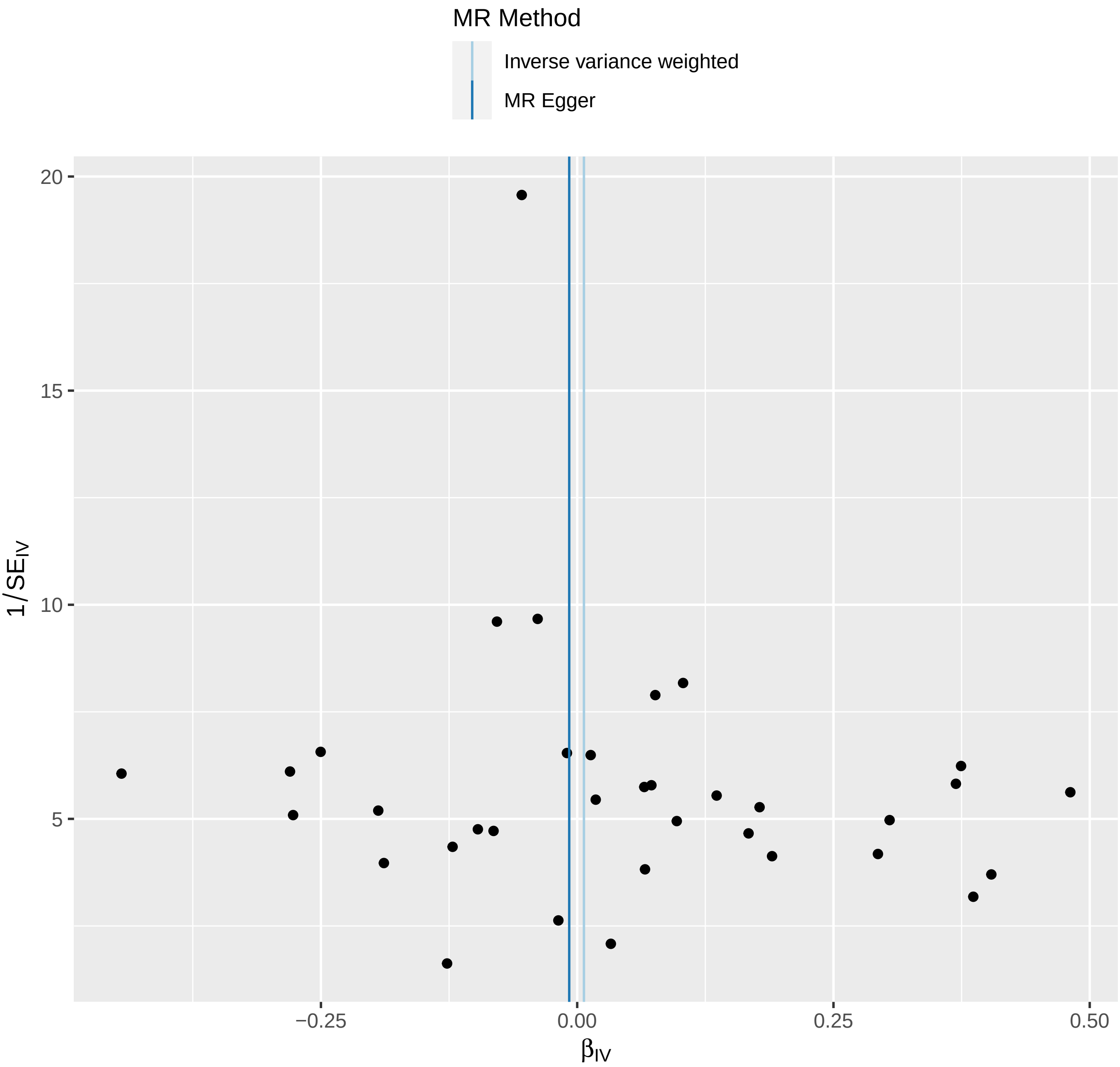

Batch 3−1968 : Mucin−4 on Diabetic nephropathy (more control exclusions)
with a threshold of 1e−05

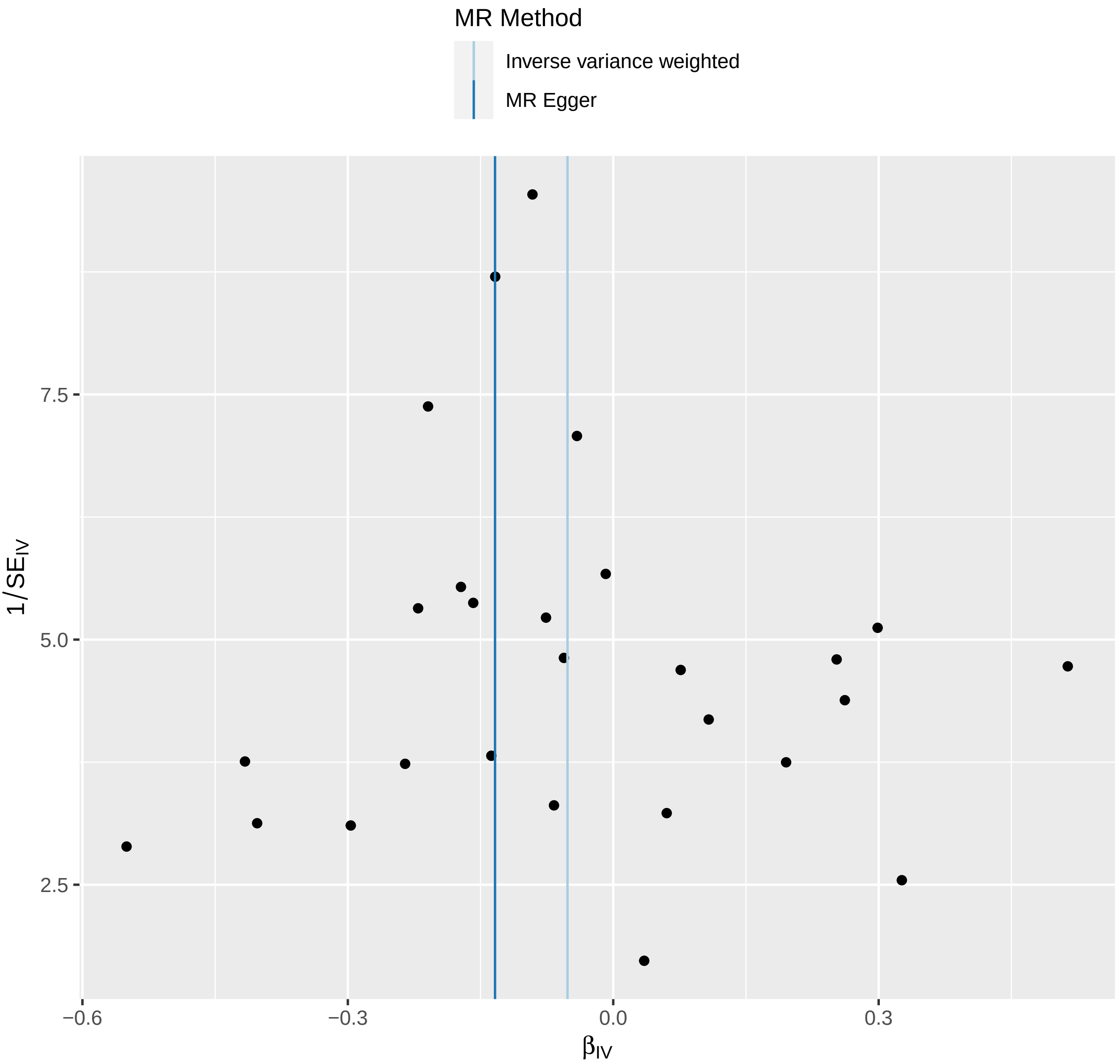

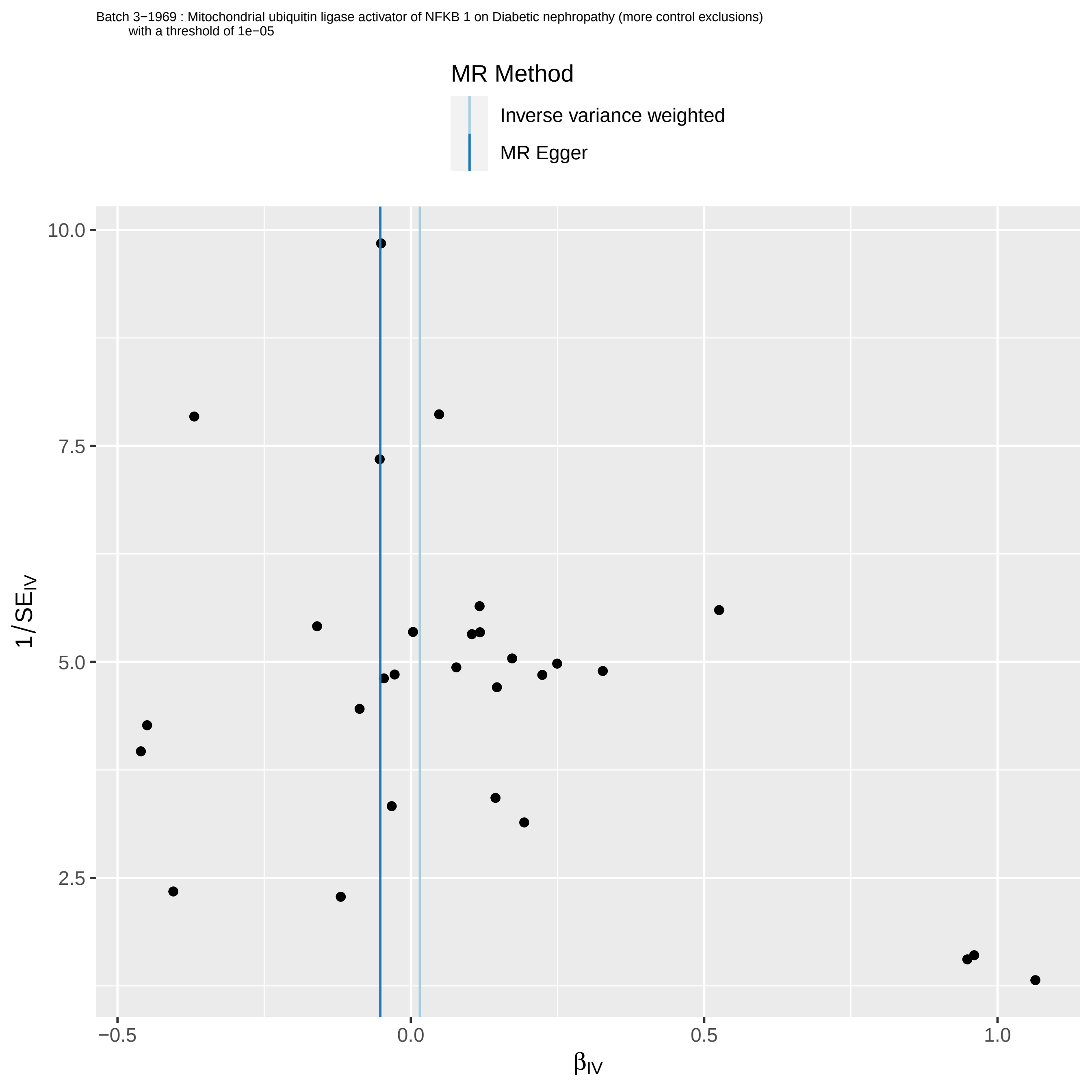
Batch 3−1969 : Mitochondrial ubiquitin ligase activator of NFKB 1 on Diabetic nephropathy (more control exclusions)
with a threshold of 1e−05
MR Method
Inverse variance weighted
MR Egger
10.0
7.5
5.0
2.5
1/SE_IV
−0.5
0.0
0.5
1.0
β_IV

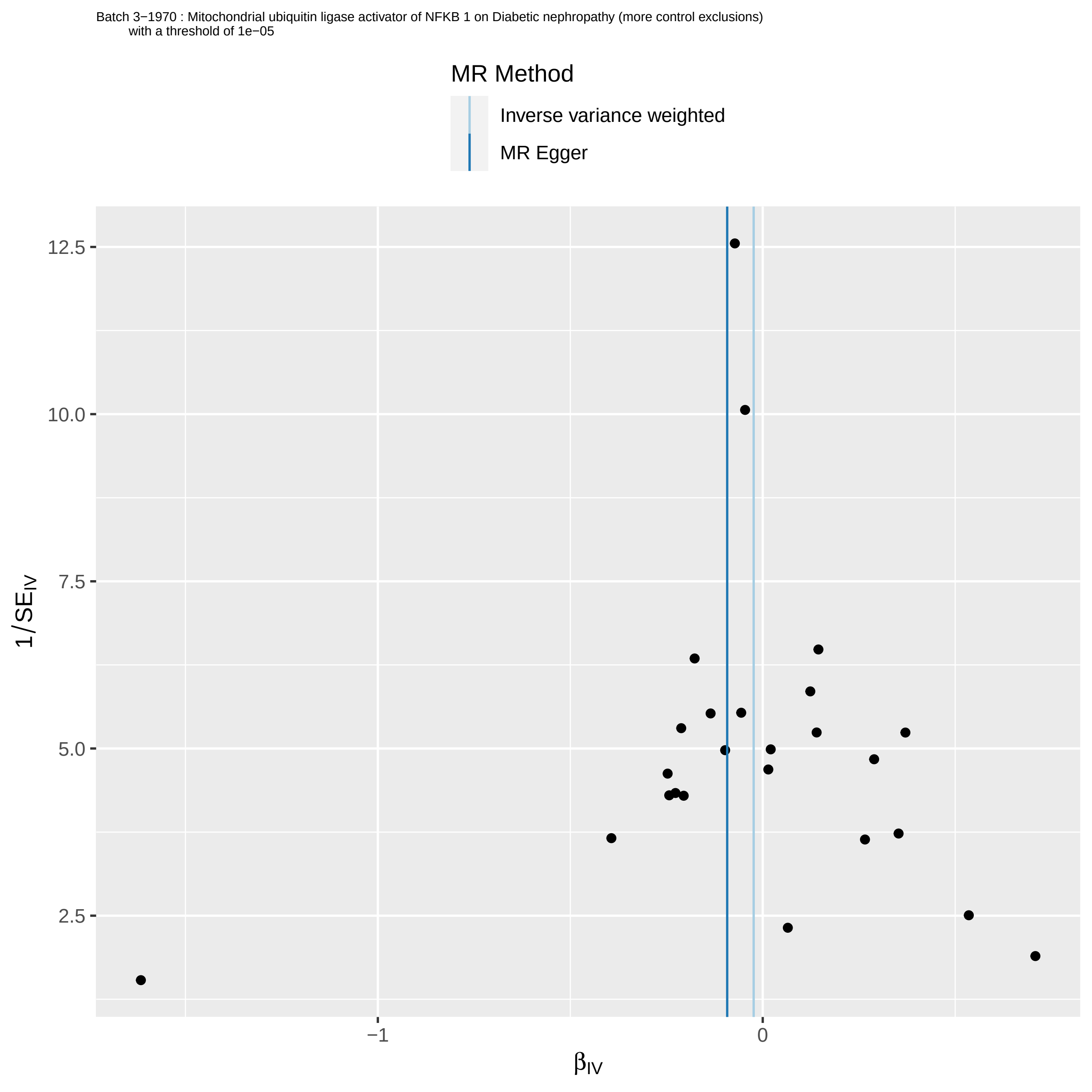

Batch 3−1970 : Mitochondrial ubiquitin ligase activator of NFKB 1 on Diabetic nephropathy (more control exclusions)
with a threshold of 1e−05
MR Method
Inverse variance weighted
MR Egger
12.5
10.0
7.5
5.0
2.5
1/SE_IV
−1
0
β_IV

Batch 3−1971 : Muscle, skeletal receptor tyrosine−protein kinase on Diabetic nephropathy (more control exclusions)
with a threshold of 1e−05

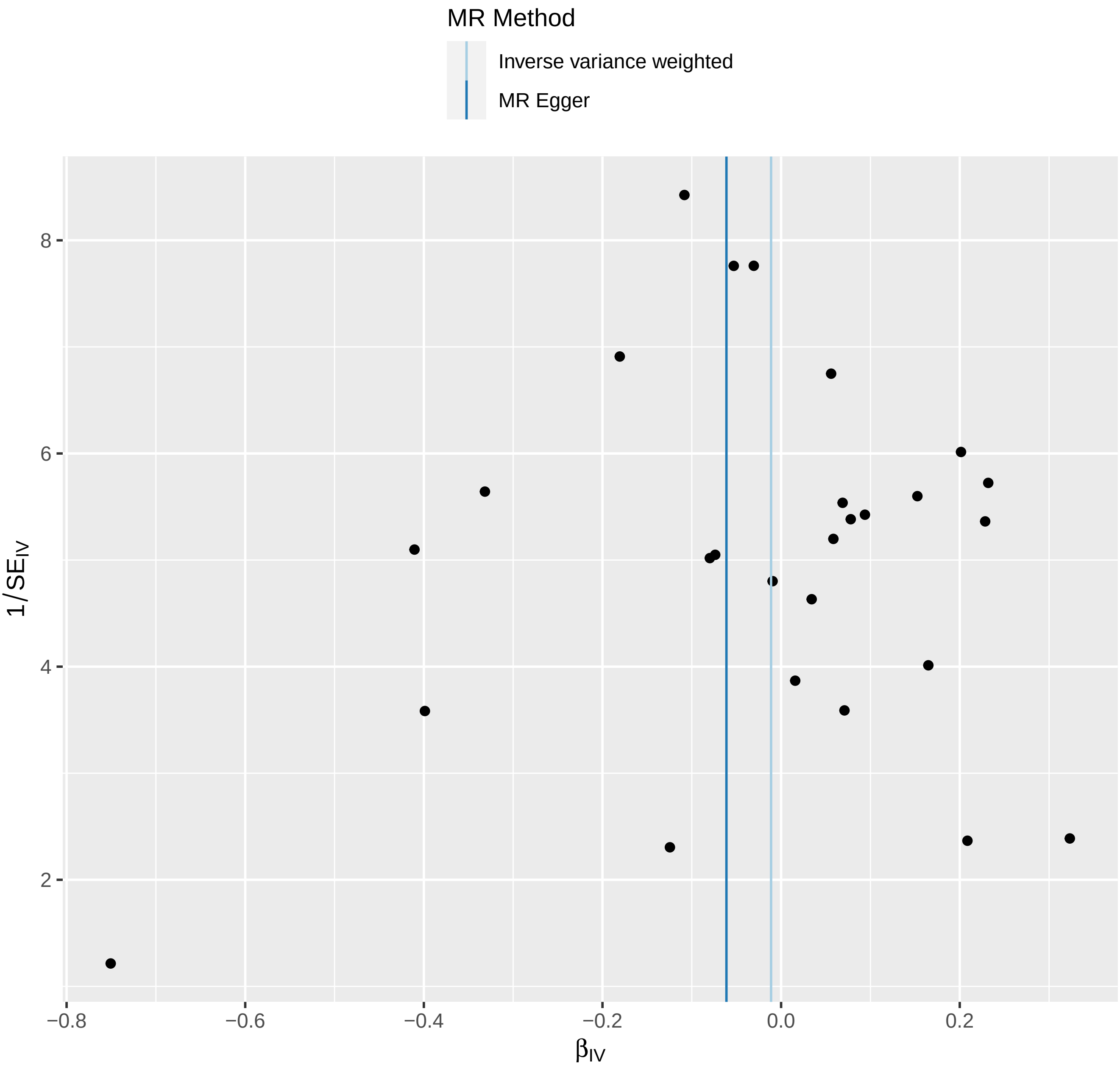

Batch 3−1972 : A/G−specific adenine DNA glycosylase on Diabetic nephropathy (more control exclusions)
with a threshold of 1e−05

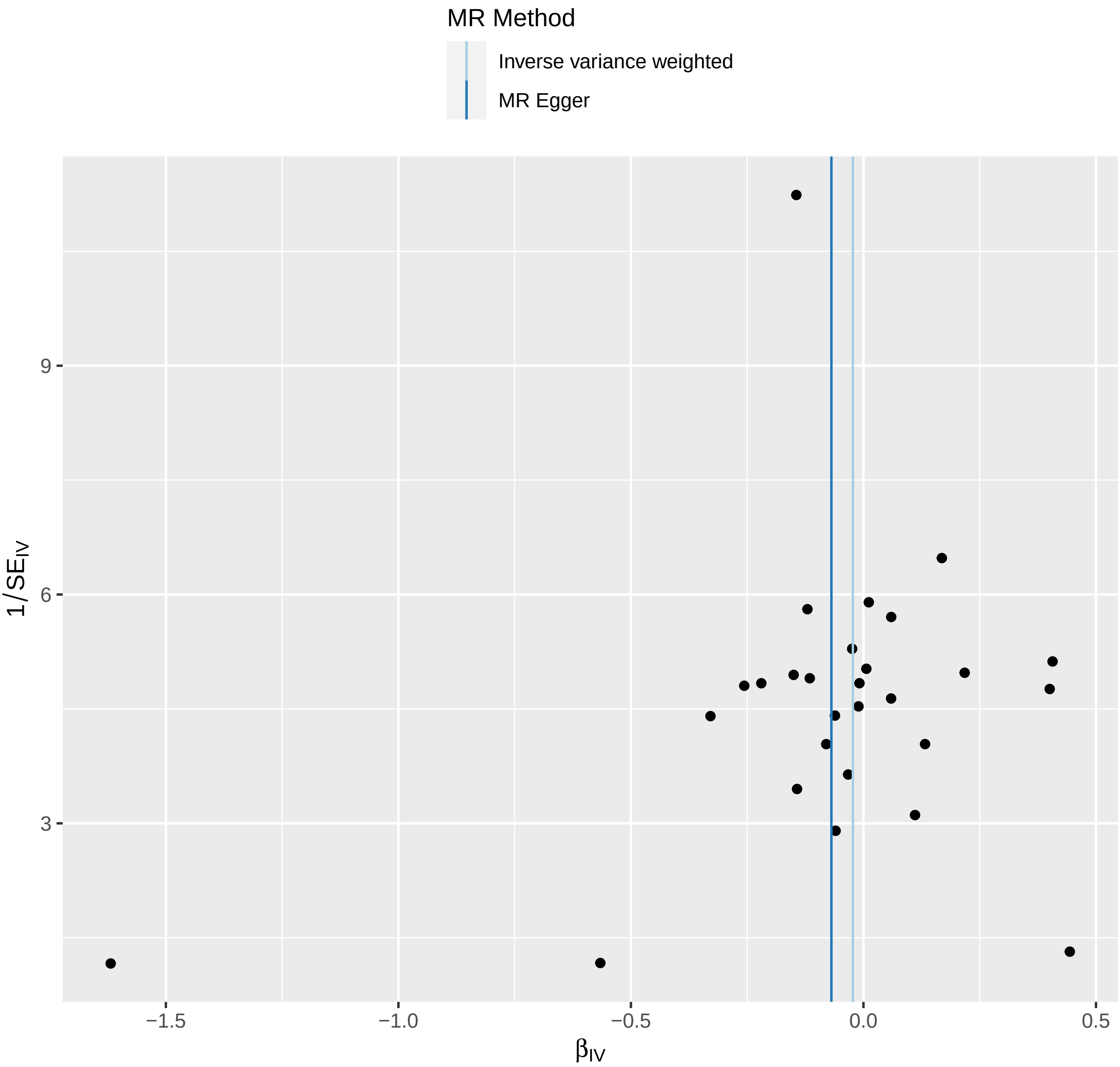

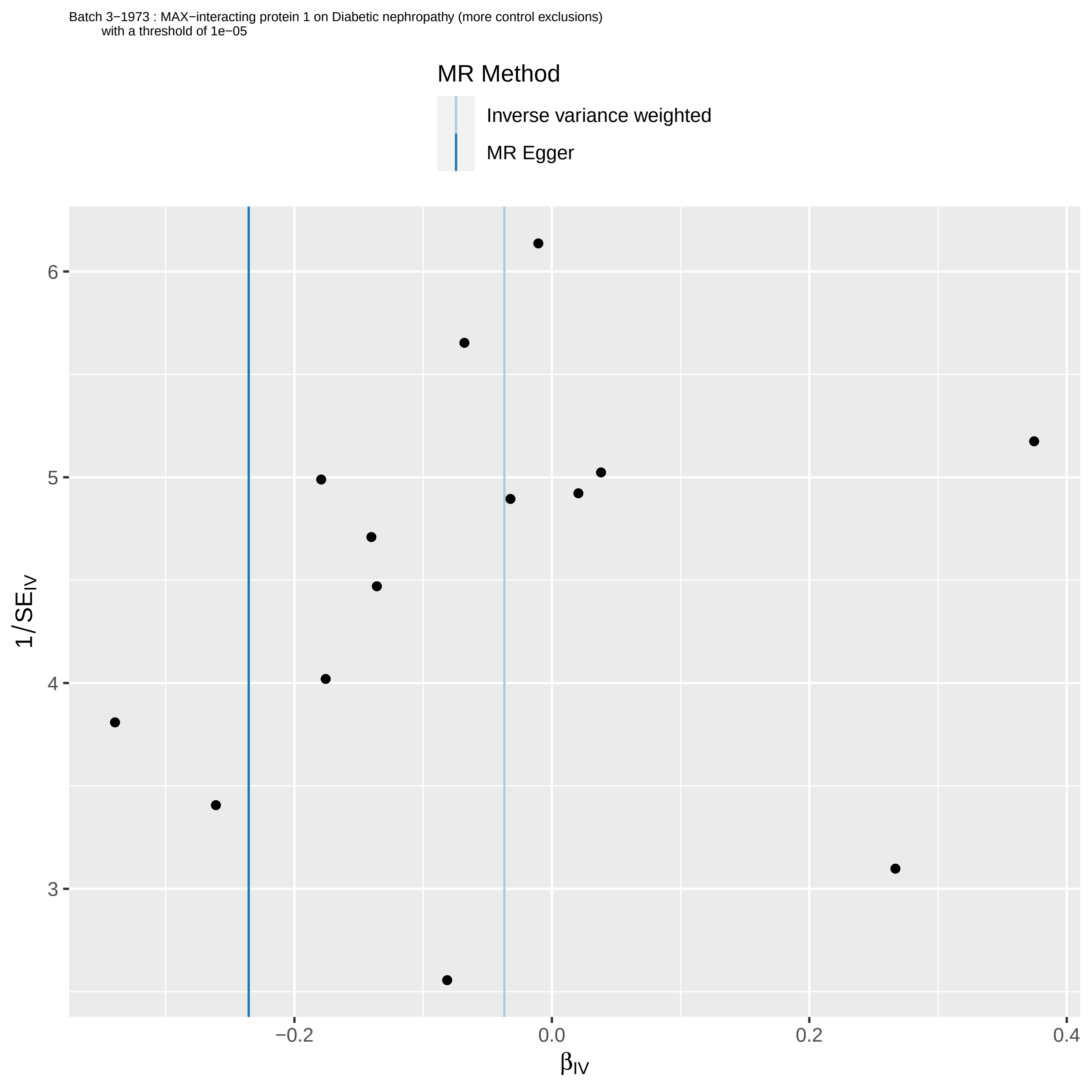

Batch 3−1973 : MAX−interacting protein 1 on Diabetic nephropathy (more control exclusions)
with a threshold of 1e−05
MR Method
Inverse variance weighted
MR Egger
6
5
4
3
$1/SE_{IV}$
−0.2
0.0
0.2
0.4
β_{IV}

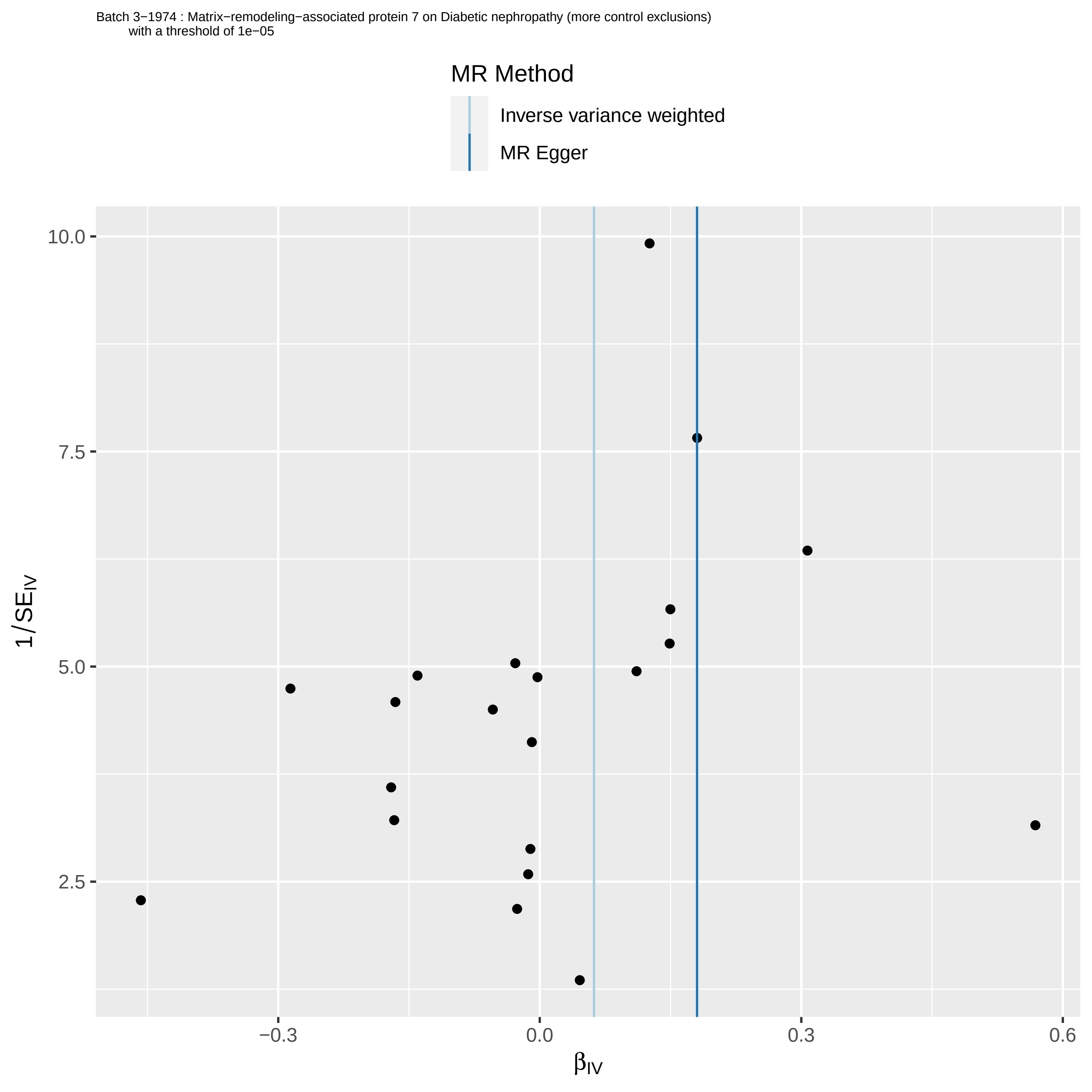
Batch 3−1974 : Matrix−remodeling−associated protein 7 on Diabetic nephropathy (more control exclusions)
with a threshold of 1e−05
MR Method
Inverse variance weighted
MR Egger
10.0
7.5
5.0
2.5
$1/SE_{IV}$
−0.3
0.0
0.3
0.6
β_{IV}

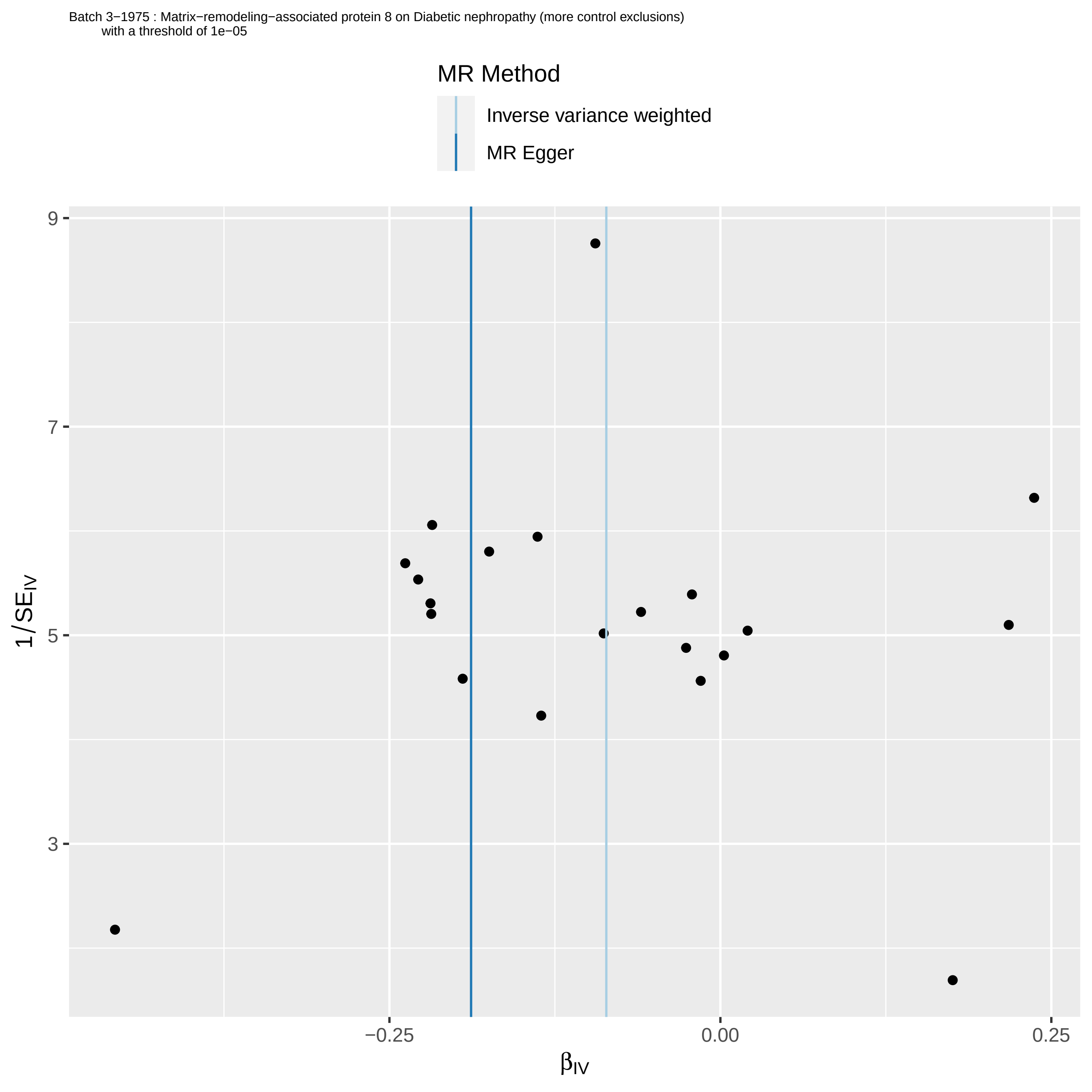
Batch 3−1975 : Matrix−remodeling−associated protein 8 on Diabetic nephropathy (more control exclusions)
with a threshold of 1e−05
MR Method
Inverse variance weighted
MR Egger
9
7
5
3
1/SE_IV
−0.25
0.00
0.25
β_IV

Batch 3−1976 : Matrix−remodeling−associated protein 8 on Diabetic nephropathy (more control exclusions)
with a threshold of 1e−05

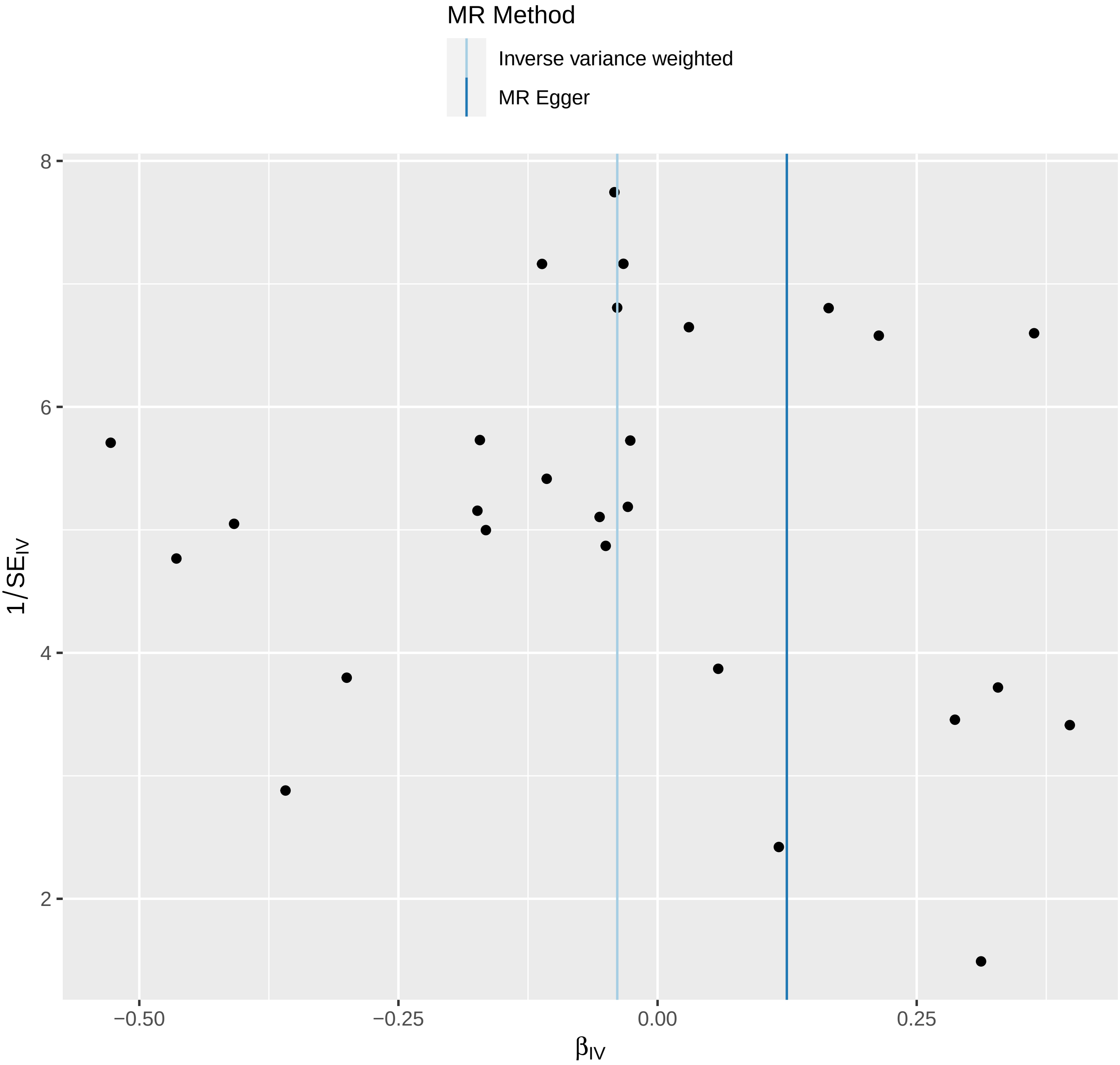

Batch 3−1977 : Myosin−binding protein C, slow−type on Diabetic nephropathy (more control exclusions)
with a threshold of 1e−05

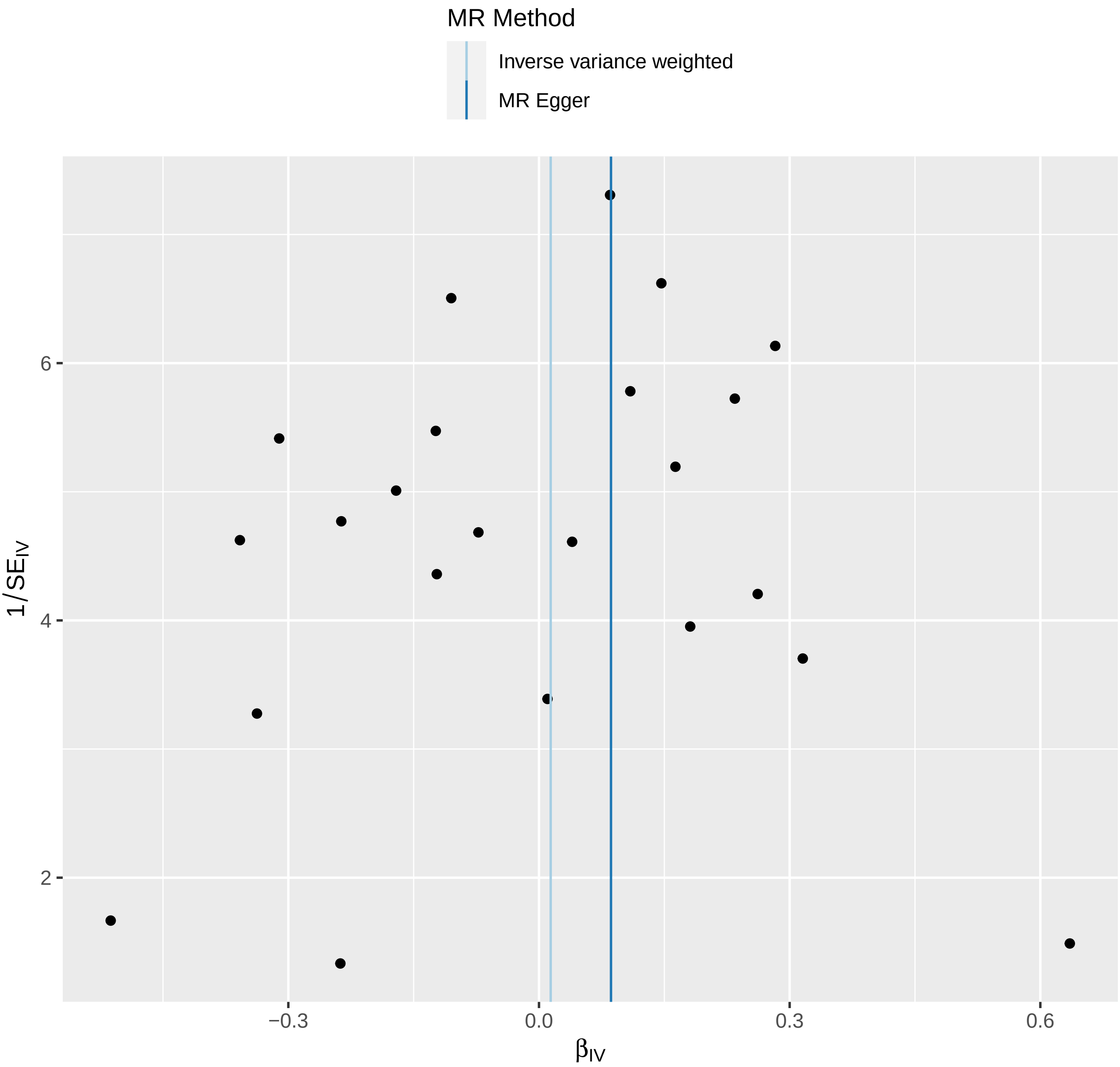

Batch 3−1978 : Myc proto−oncogene protein on Diabetic nephropathy (more control exclusions)
with a threshold of 1e−05

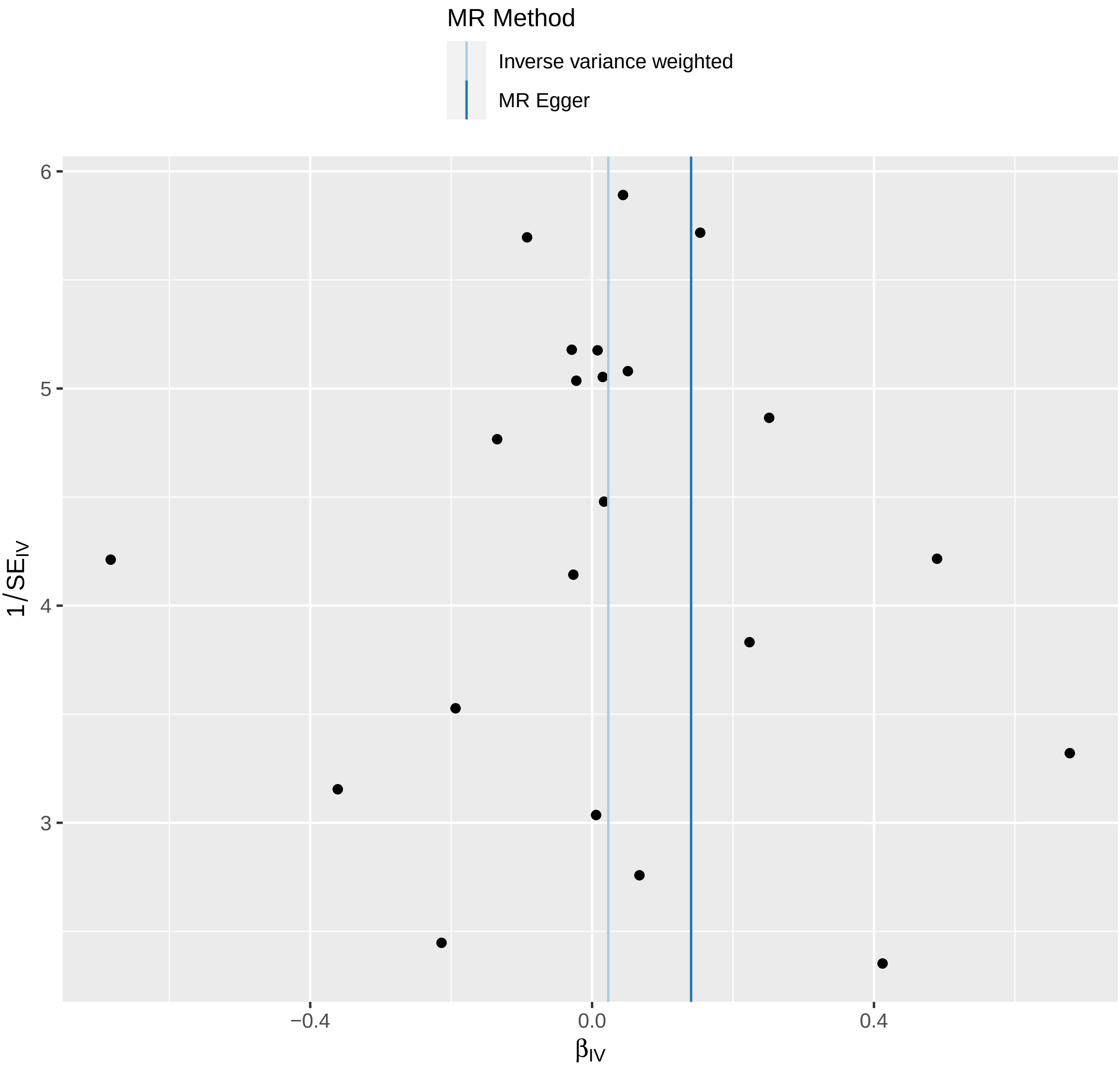

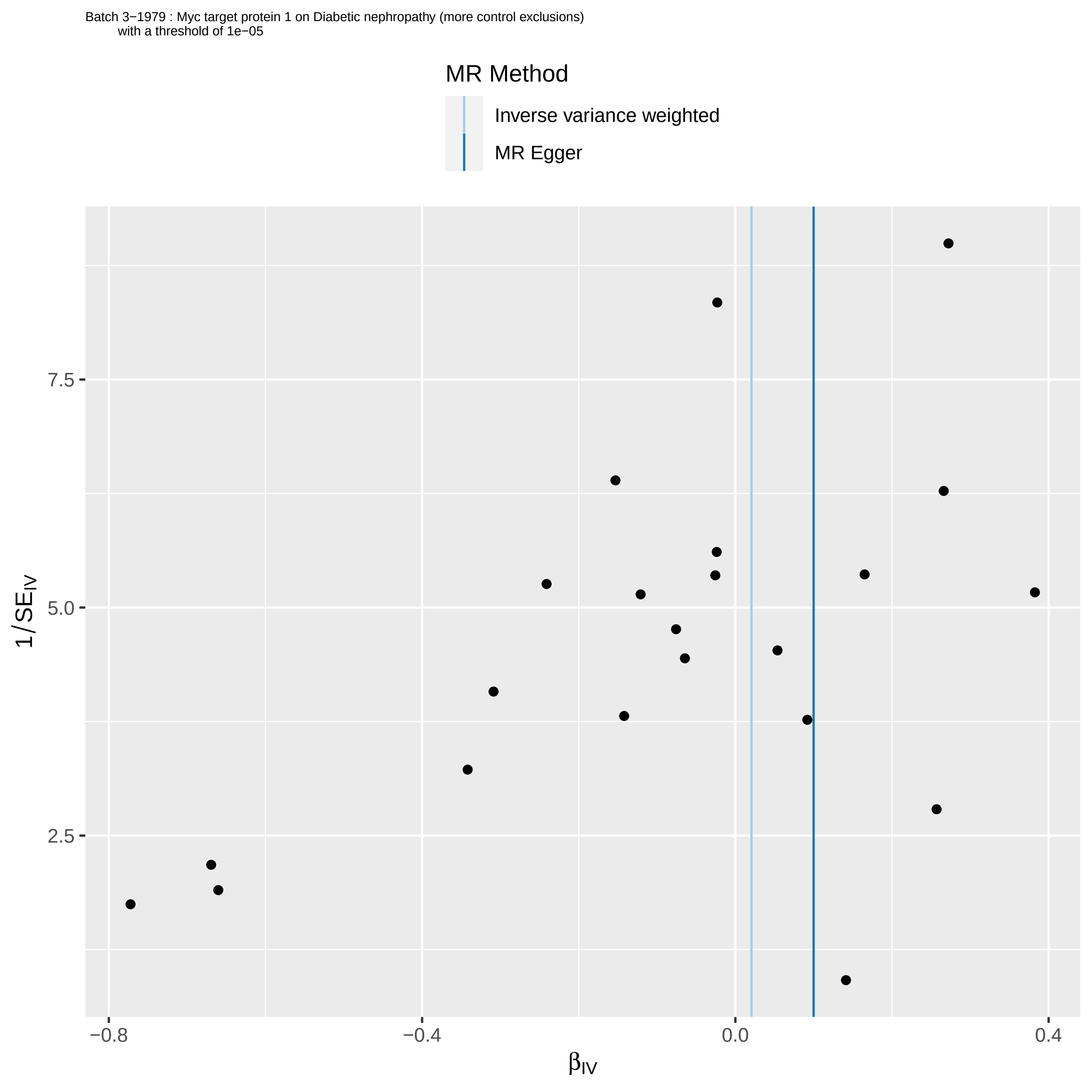

Batch 3−1979 : Myc target protein 1 on Diabetic nephropathy (more control exclusions)
with a threshold of 1e−05
MR Method
Inverse variance weighted
MR Egger
7.5
5.0
2.5
1/SE_IV
−0.8
−0.4
0.0
0.4
β_IV

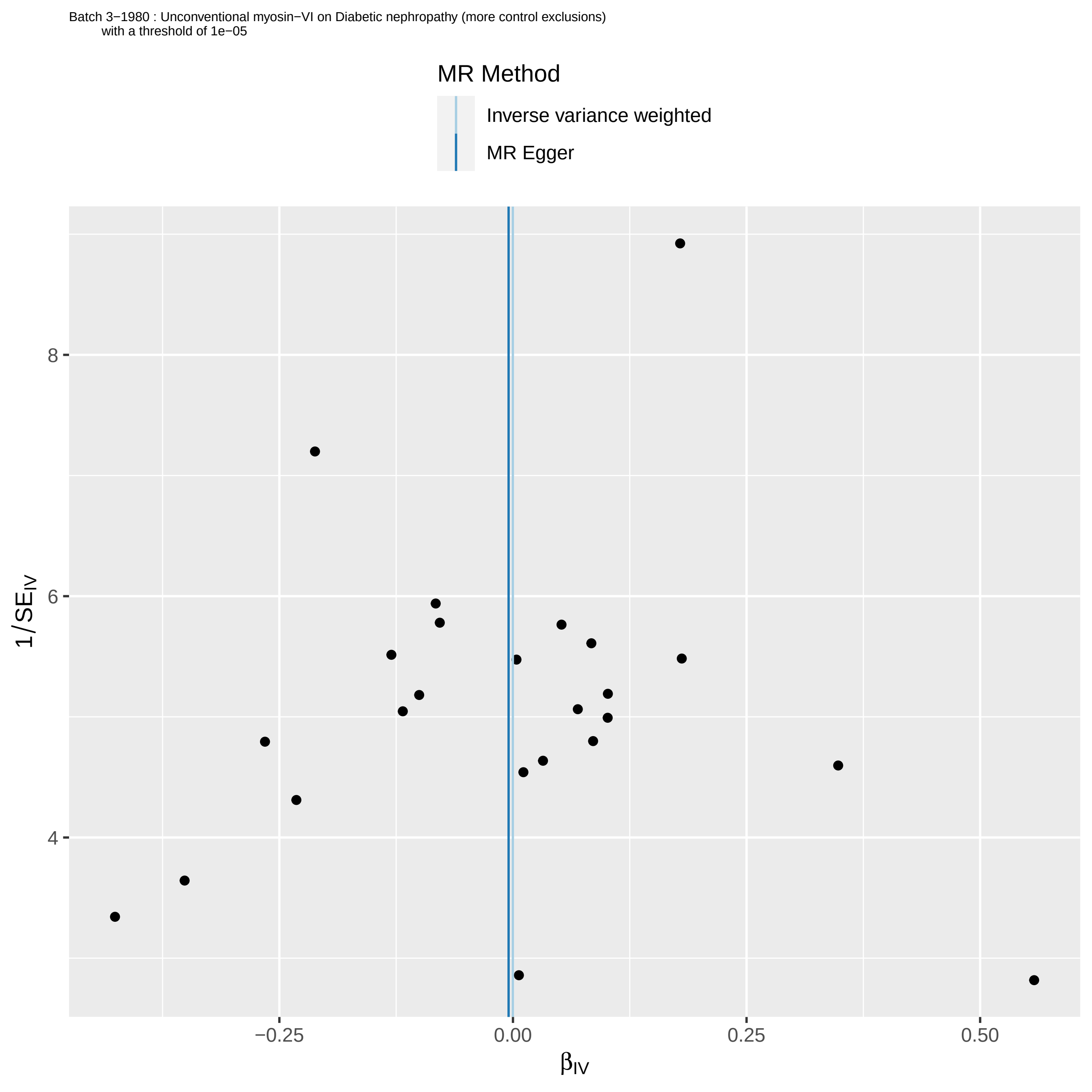

Batch 3−1980 : Unconventional myosin−VI on Diabetic nephropathy (more control exclusions)
with a threshold of 1e−05
MR Method
Inverse variance weighted
MR Egger
8
6
4
1/SE$_{IV}$
−0.25
0.00
0.25
0.50
β$_{IV}$

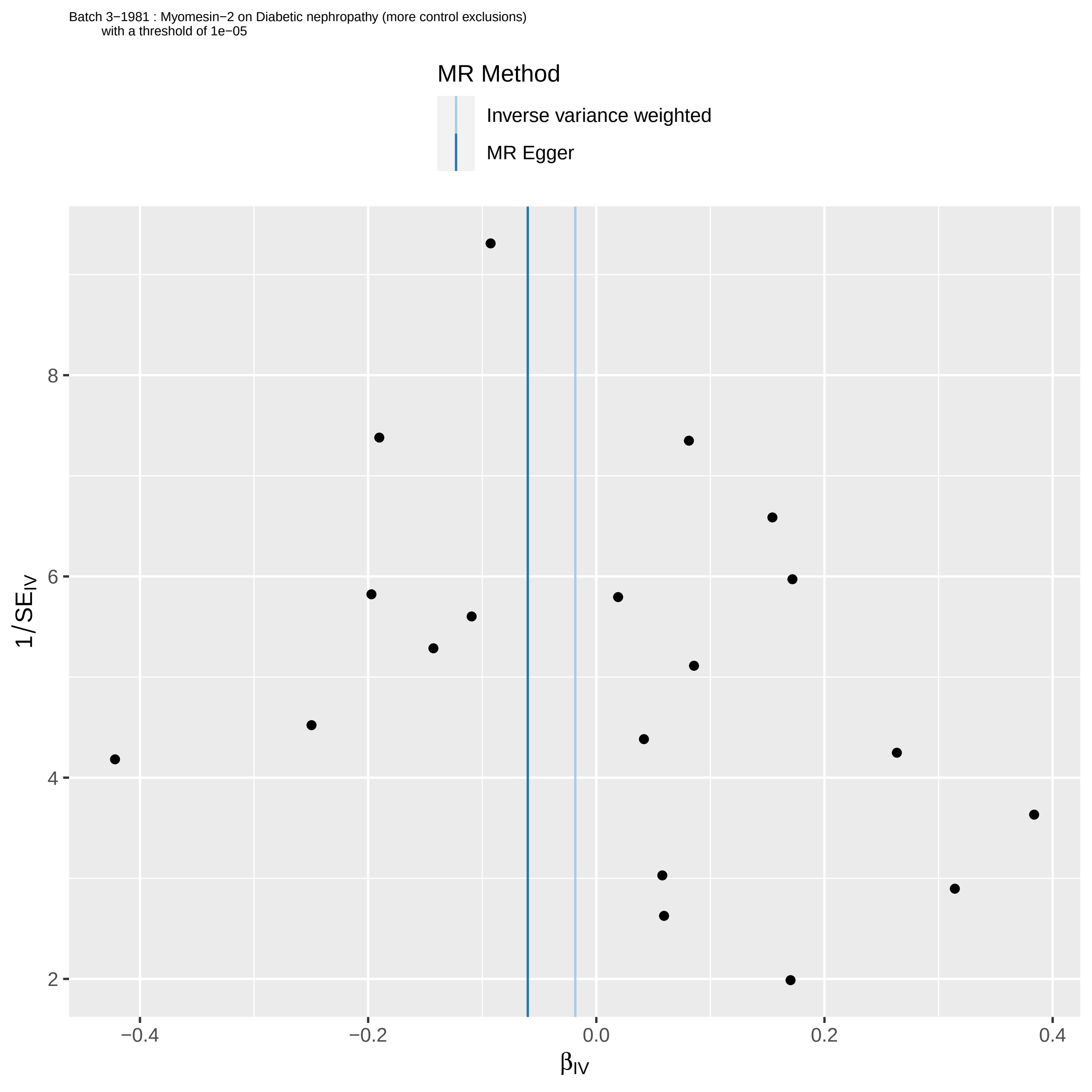

Batch 3−1981 : Myomesin−2 on Diabetic nephropathy (more control exclusions)
with a threshold of 1e−05
MR Method
Inverse variance weighted
MR Egger
8
6
4
2
1/SE_IV
−0.4
−0.2
0.0
0.2
0.4
β_IV

Batch 3−1982 : Myelin regulatory factor on Diabetic nephropathy (more control exclusions)
with a threshold of 1e−05

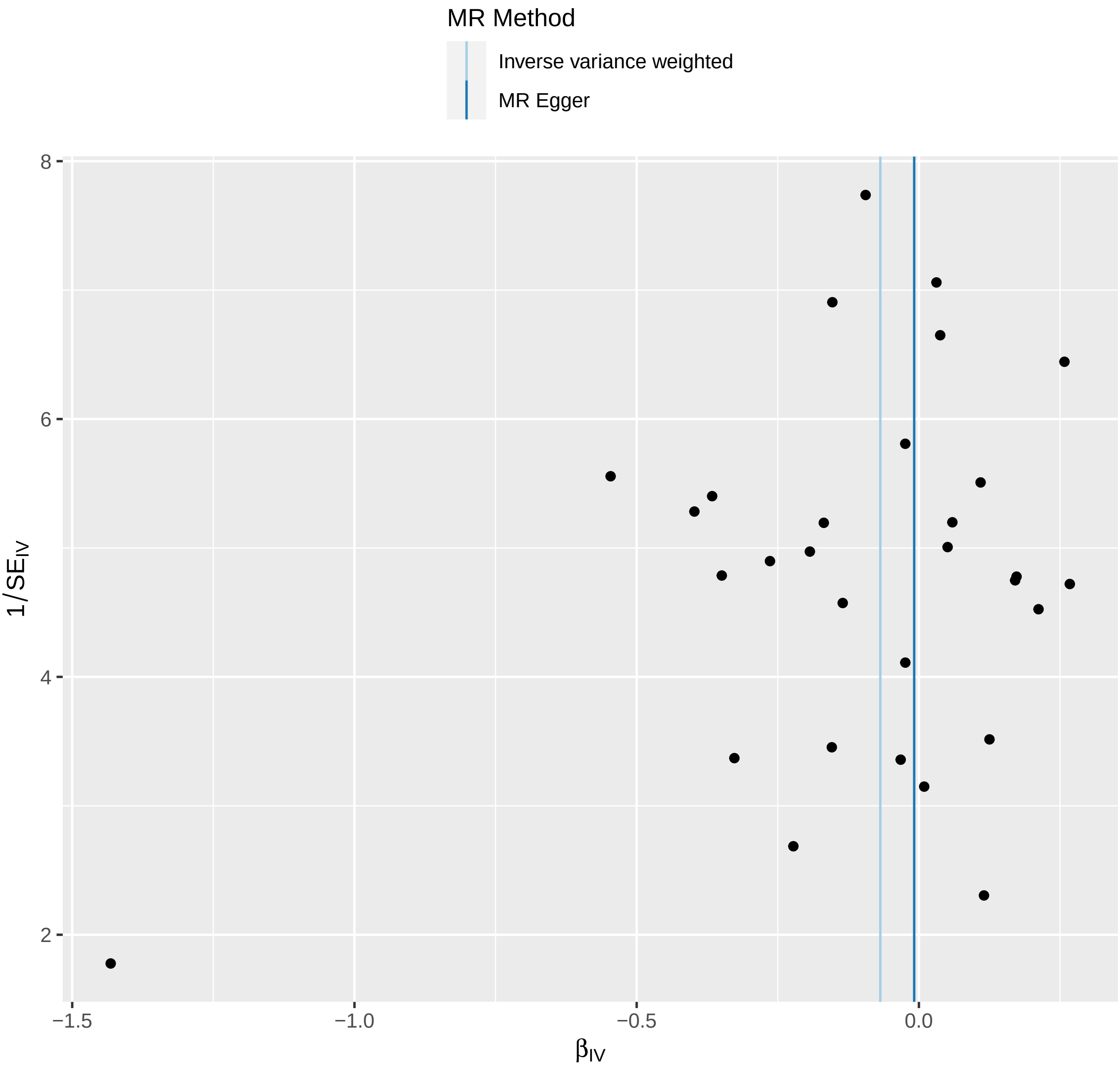

Batch 3−1983 : Histone H2A deubiquitinase MYSM1 on Diabetic nephropathy (more control exclusions)
with a threshold of 1e−05

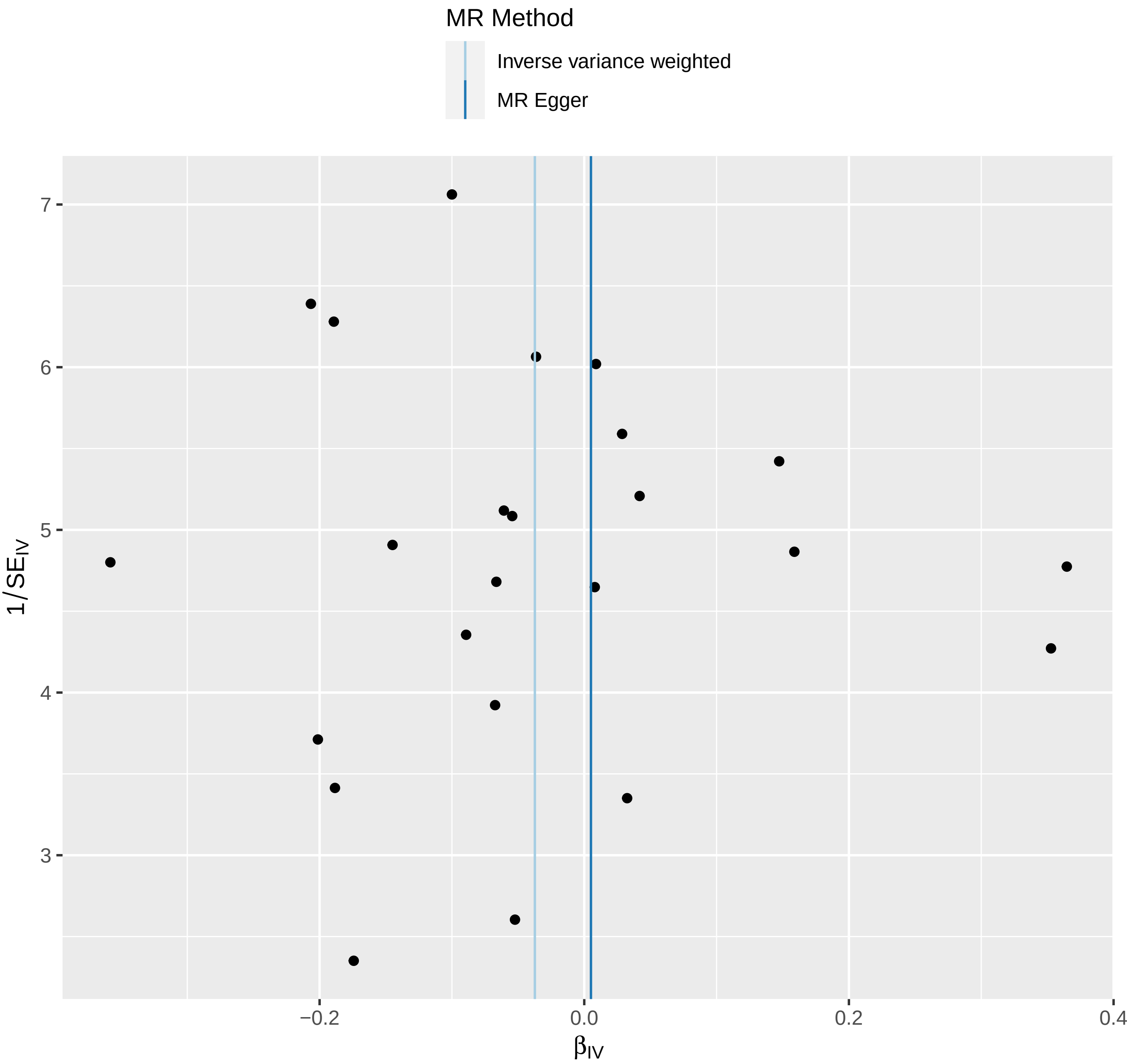

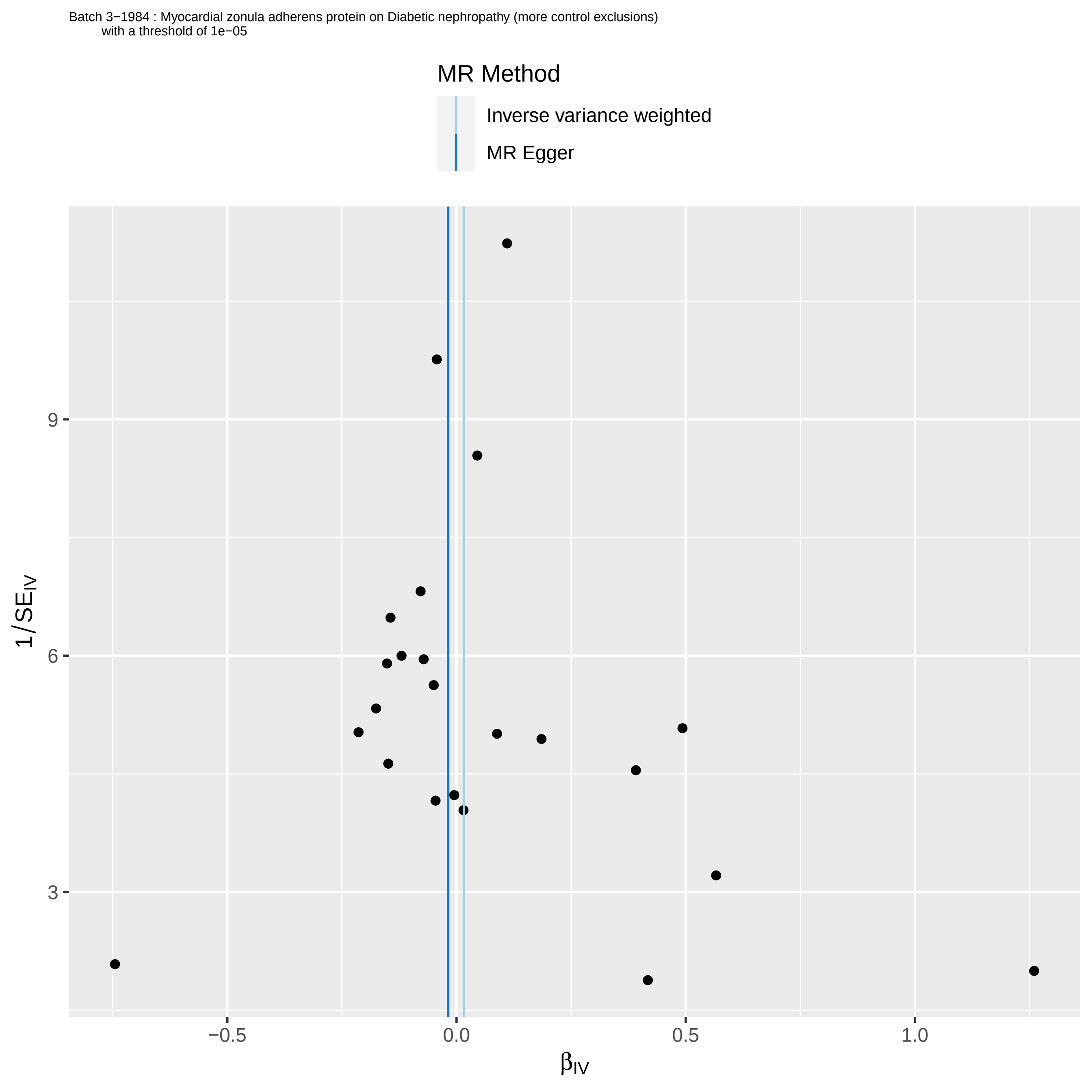

Batch 3−1984 : Myocardial zonula adherens protein on Diabetic nephropathy (more control exclusions)
with a threshold of 1e−05
MR Method
Inverse variance weighted
MR Egger
9
6
3
1/SE_IV
−0.5
0.0
0.5
1.0
β_IV

Batch 3−1985 : Myocardial zonula adherens protein on Diabetic nephropathy (more control exclusions) with a threshold of 1e−05

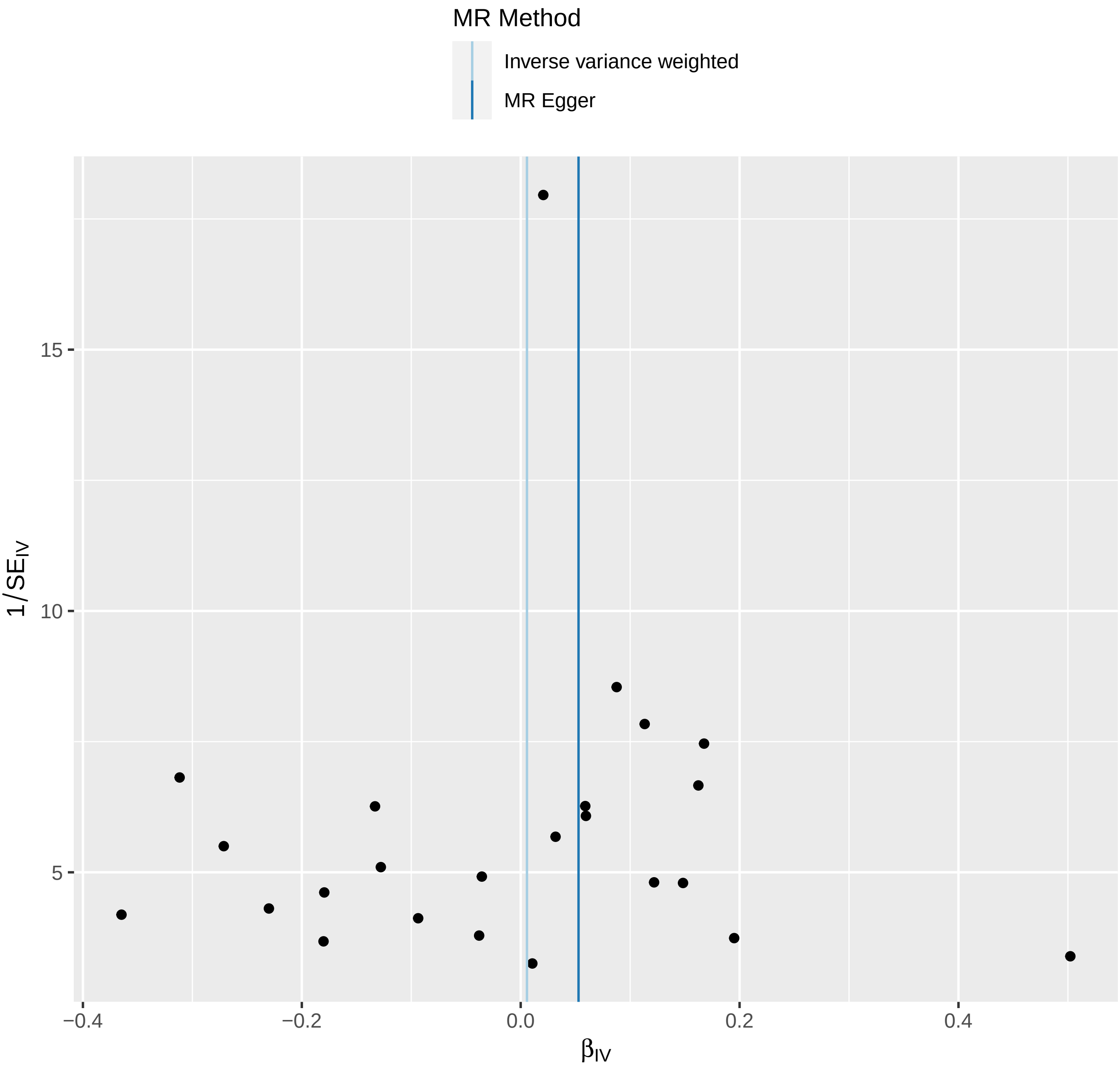

Batch 3−1986 : Myocardial zonula adherens protein on Diabetic nephropathy (more control exclusions)
with a threshold of 1e−05

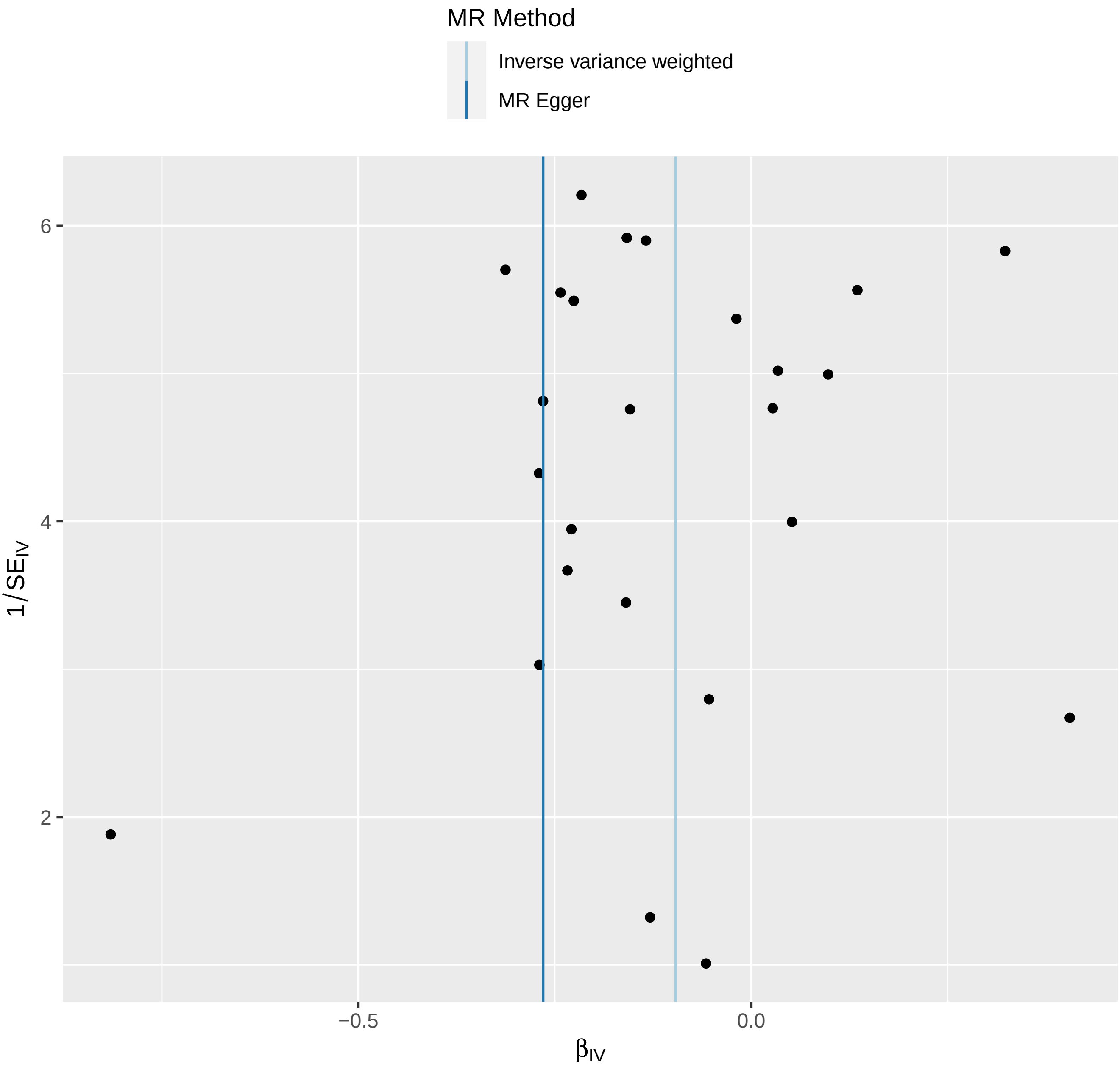

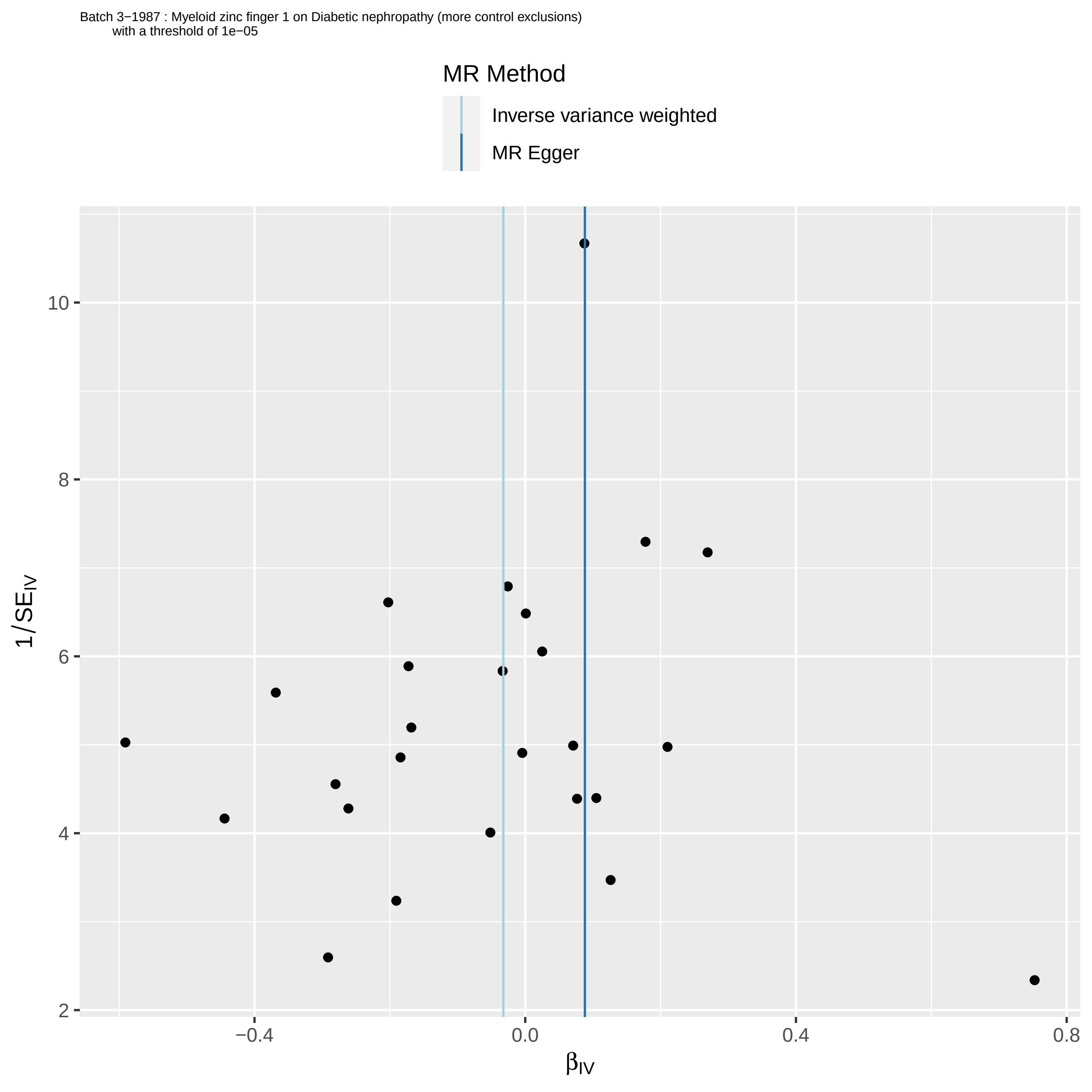
Batch 3−1987 : Myeloid zinc finger 1 on Diabetic nephropathy (more control exclusions)
with a threshold of 1e−05
MR Method
Inverse variance weighted
MR Egger
10
8
6
4
2
−0.4
0.0
0.4
0.8
1/SE_IV
β_IV

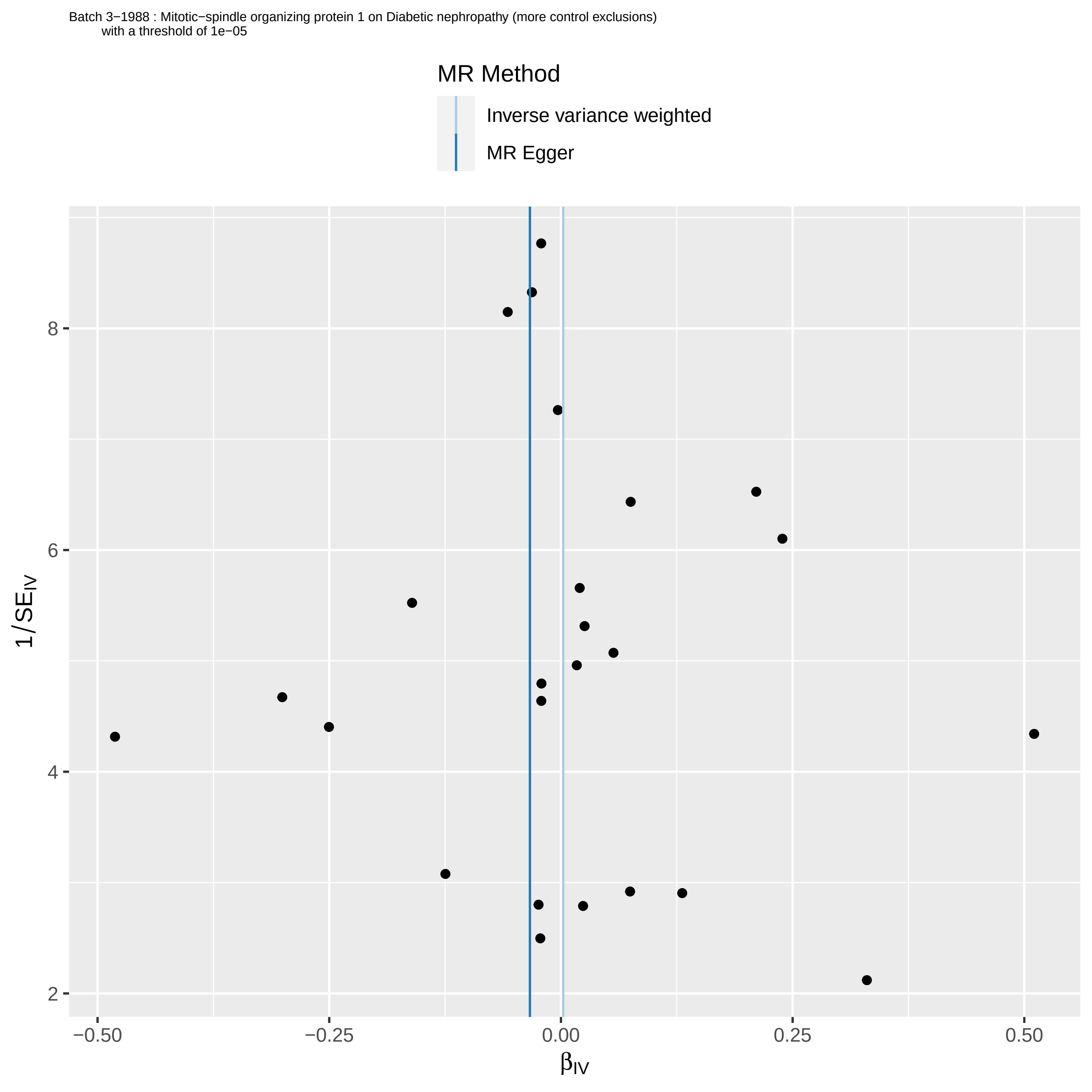

Batch 3−1988 : Mitotic−spindle organizing protein 1 on Diabetic nephropathy (more control exclusions)
with a threshold of 1e−05
MR Method
Inverse variance weighted
MR Egger
1/SE$_{IV}$
8
6
4
2
−0.50
−0.25
0.00
0.25
0.50
β$_{IV}$

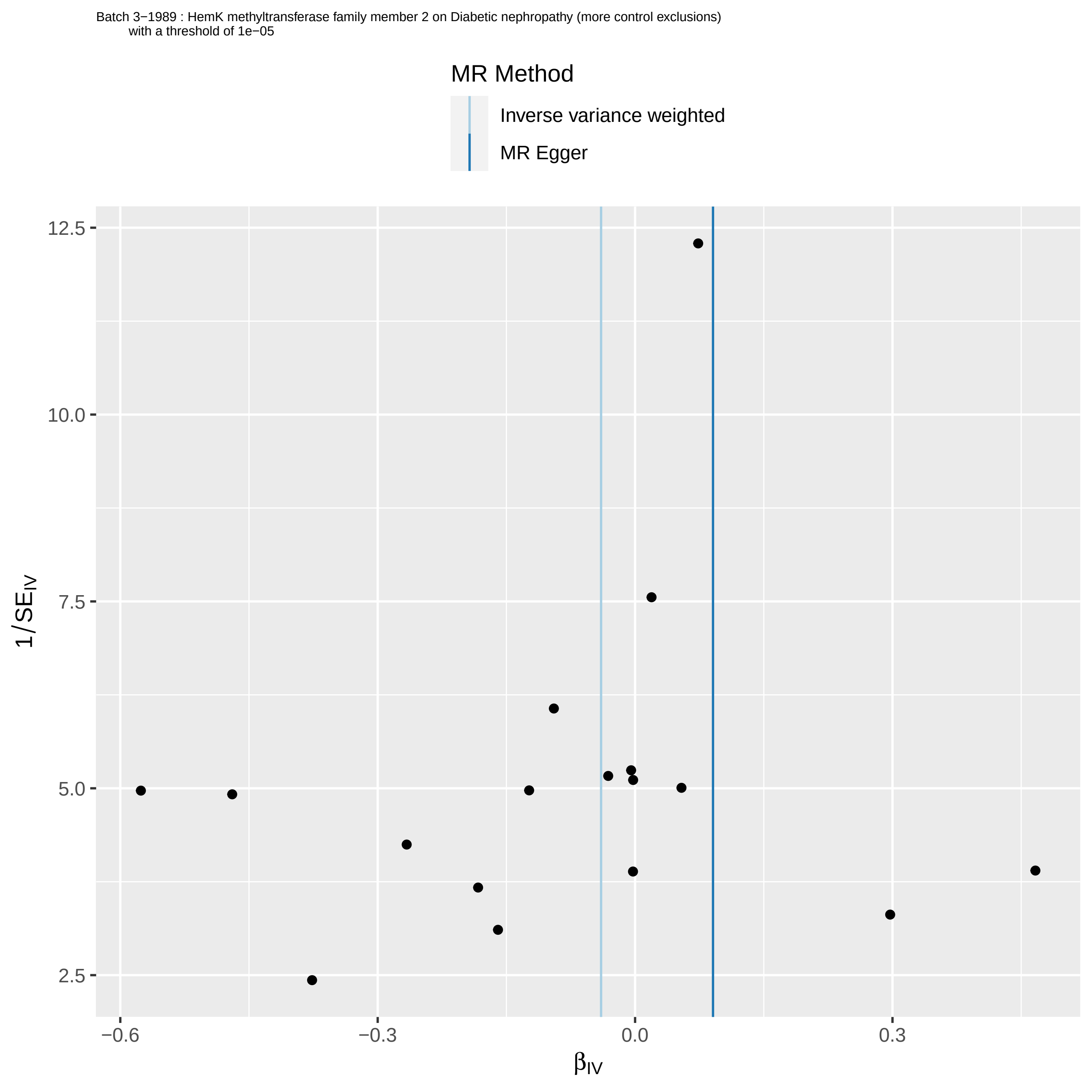

Batch 3−1989 : HemK methyltransferase family member 2 on Diabetic nephropathy (more control exclusions)
with a threshold of 1e−05
MR Method
Inverse variance weighted
MR Egger
12.5
10.0
7.5
5.0
2.5
1/SE_IV
−0.6
−0.3
0.0
0.3
β_IV

Batch 3−1990 : N−acylethanolamine−hydrolyzing acid amidase on Diabetic nephropathy (more control exclusions)
with a threshold of 1e−05

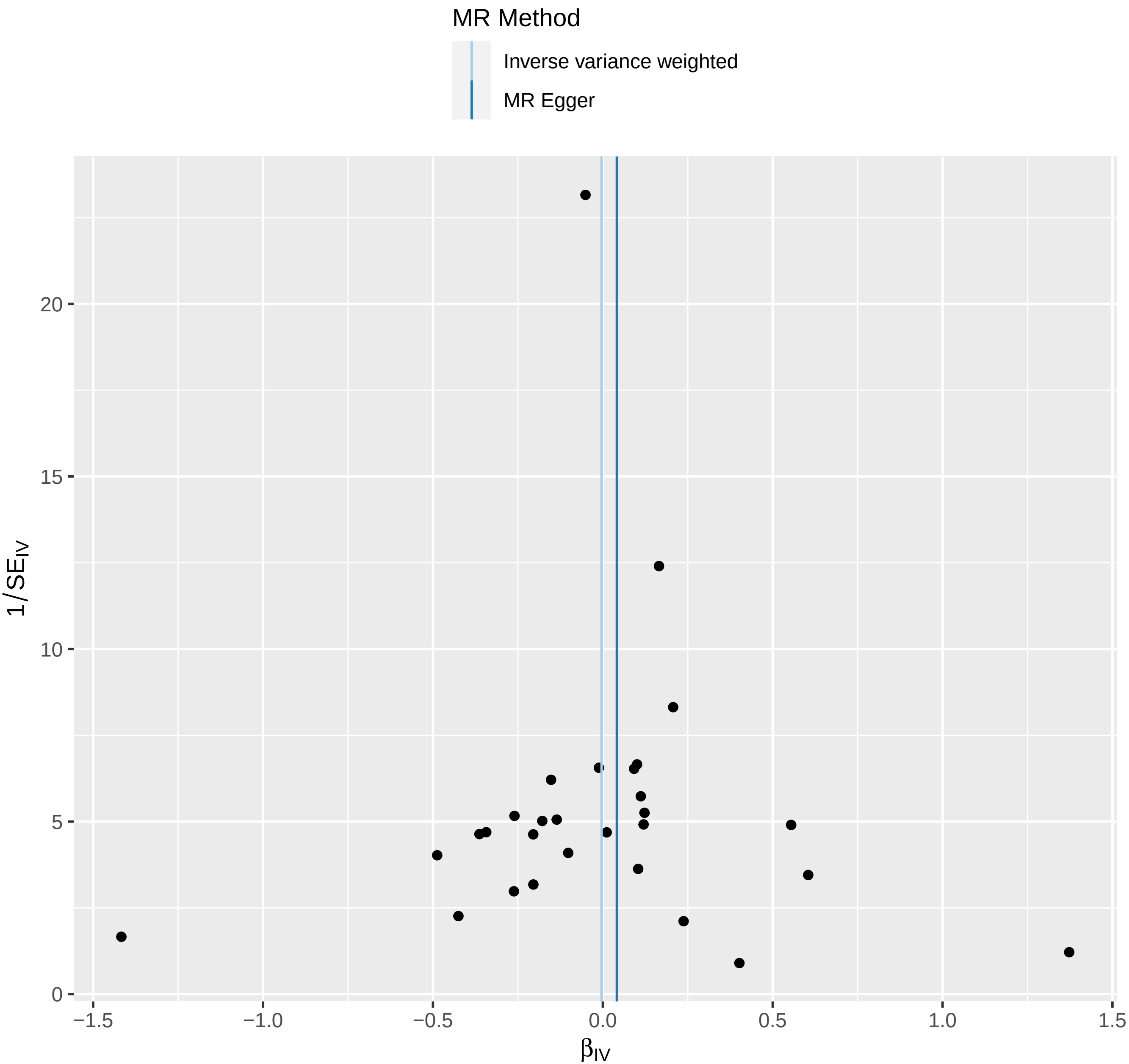

Batch 3−1991 : N−acetylated−alpha−linked acidic dipeptidase 2 on Diabetic nephropathy (more control exclusions) with a threshold of 1e−05

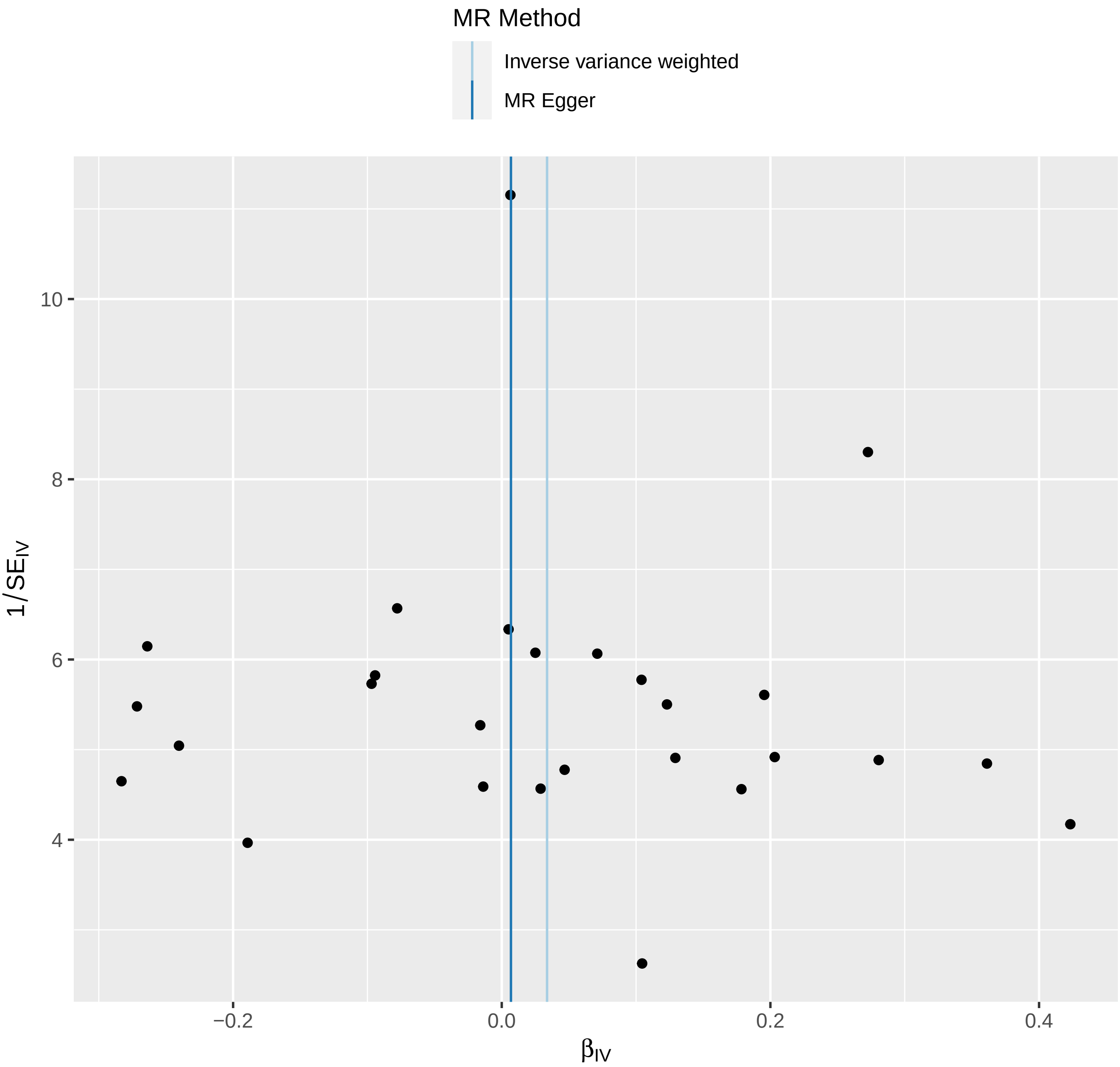

Batch 3−1992 : NGFI−A−binding protein 1 on Diabetic nephropathy (more control exclusions)
with a threshold of 1e−05

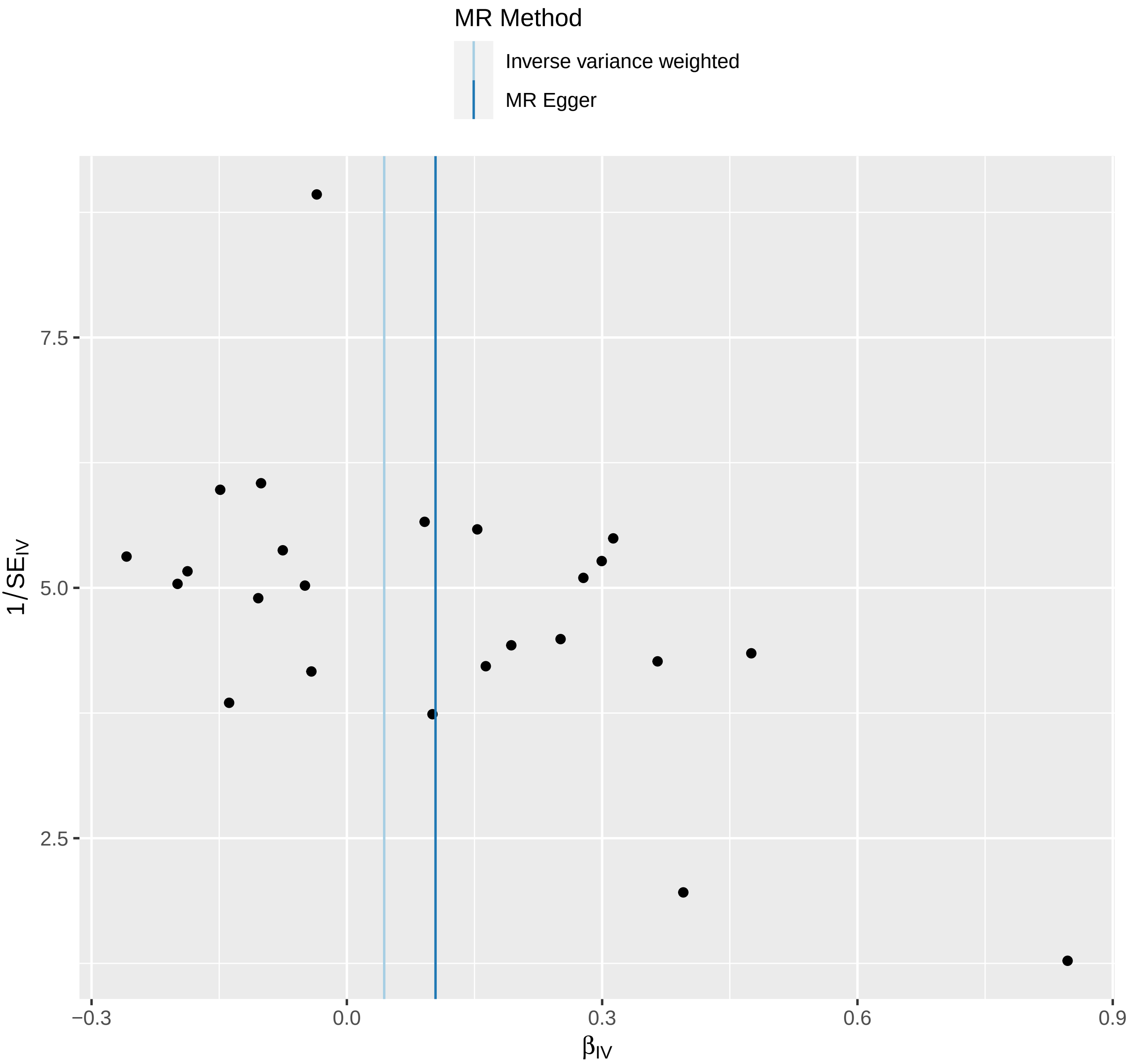

Batch 3−1993 : NGFI−A−binding protein 2 on Diabetic nephropathy (more control exclusions)
with a threshold of 1e−05

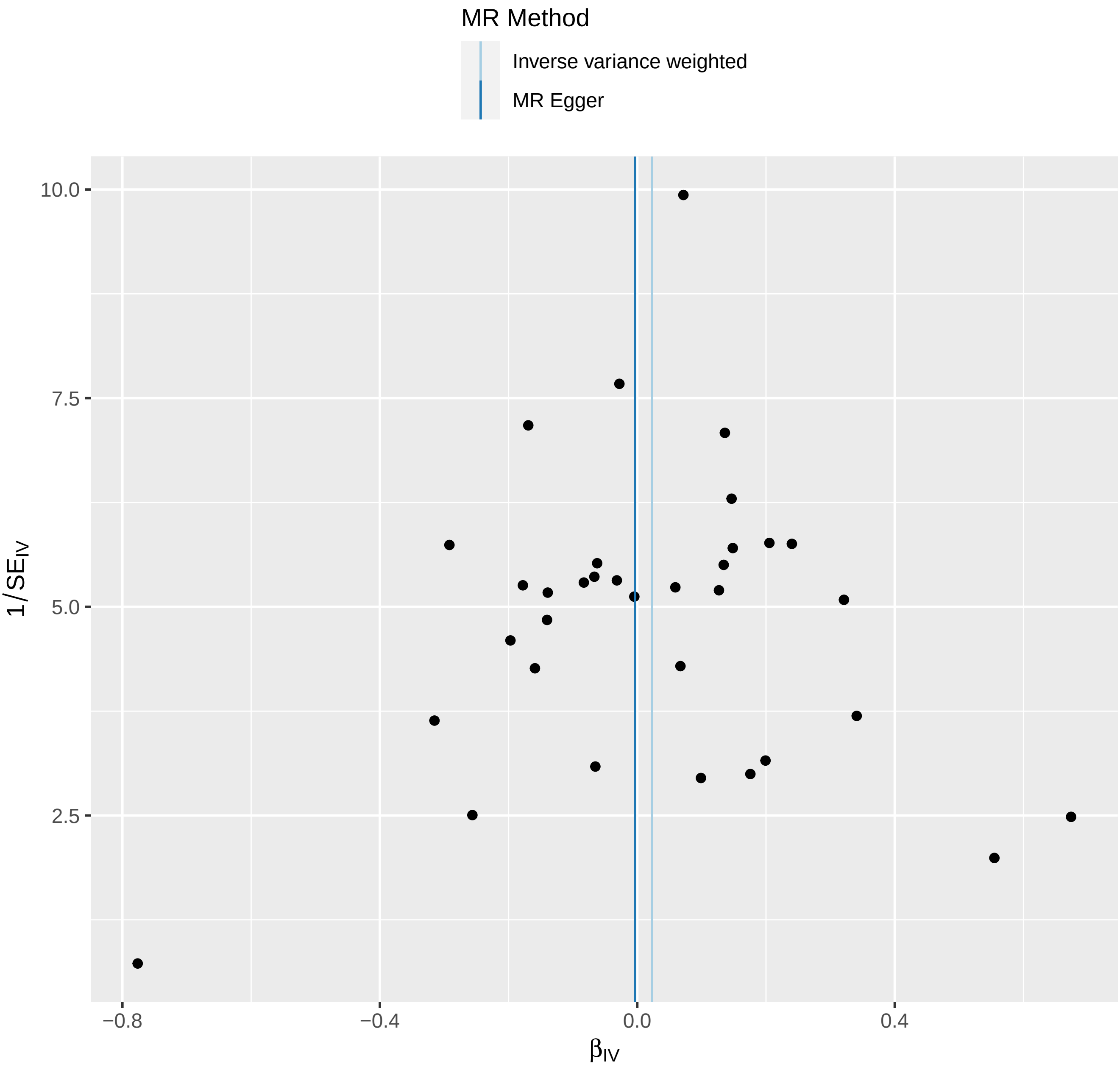

Batch 3−1994 : NAD kinase on Diabetic nephropathy (more control exclusions)
with a threshold of 1e−05

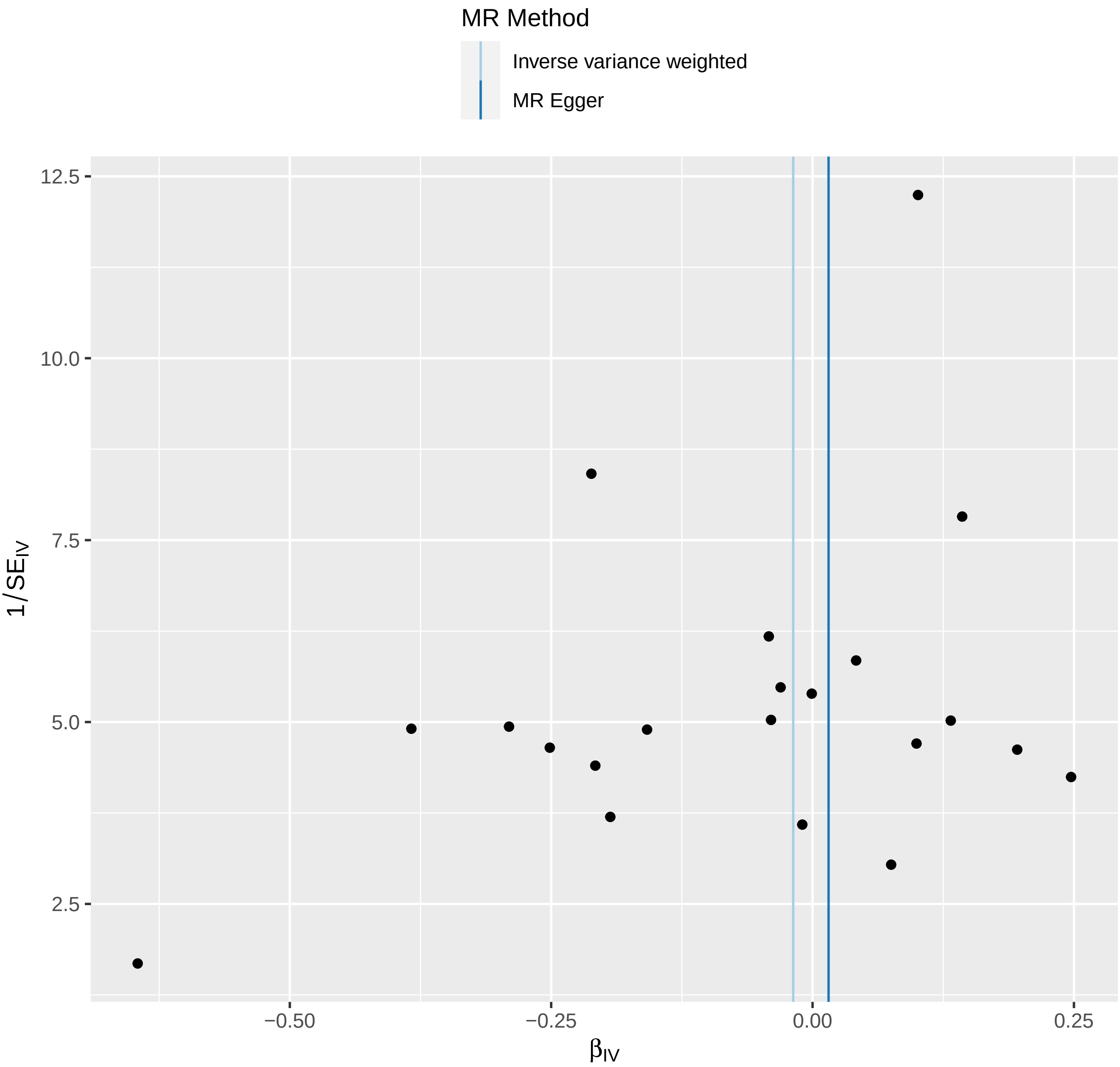

Batch 3−1995 : N−acetyl−D−glucosamine kinase on Diabetic nephropathy (more control exclusions)
with a threshold of 1e−05

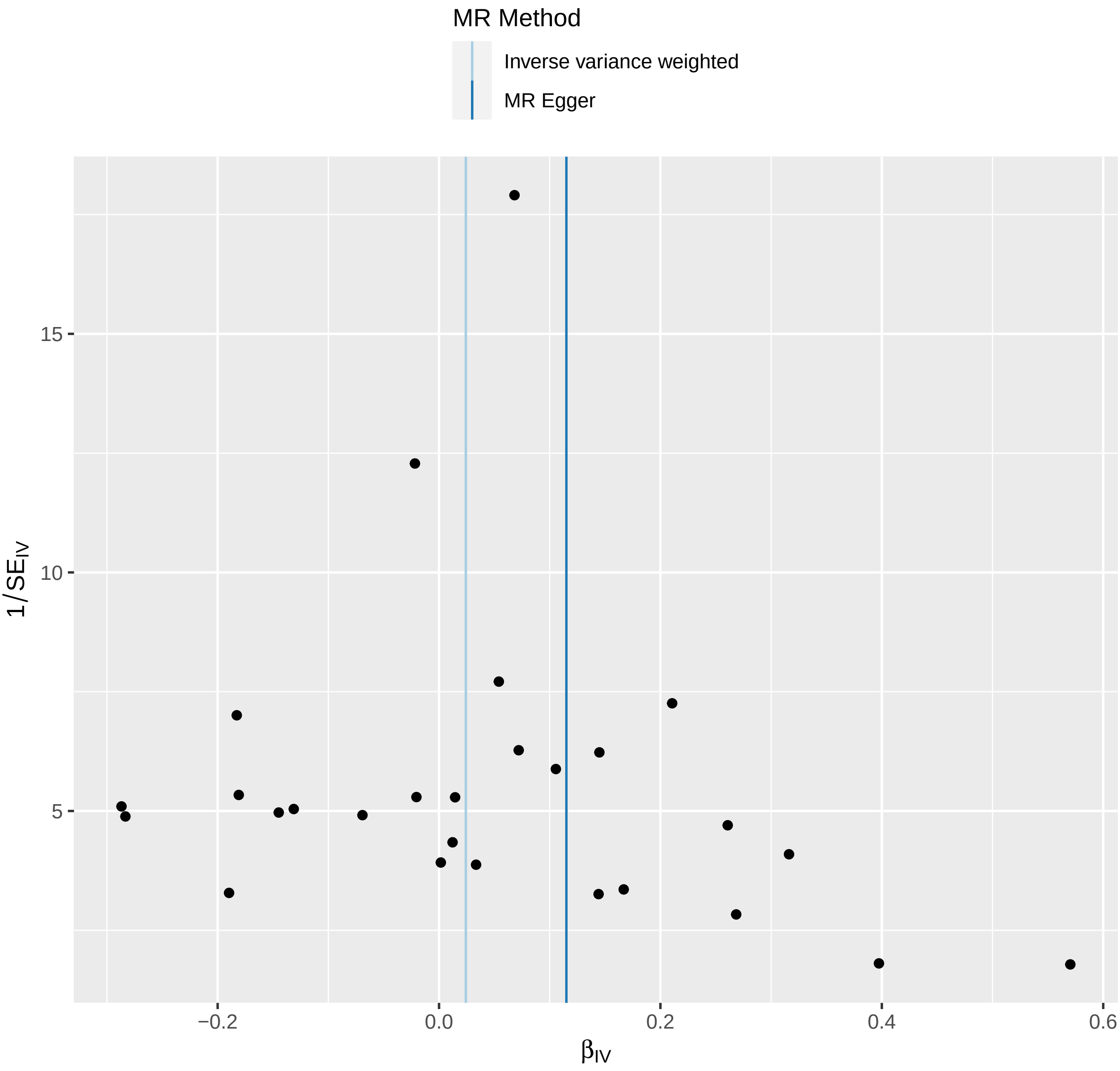

Batch 3−1996 : N−acetylglucosamine−1−phosphodiester alpha−N−acetylglucosaminidase on Diabetic nephropathy (more control exclusions)
with a threshold of 1e−05

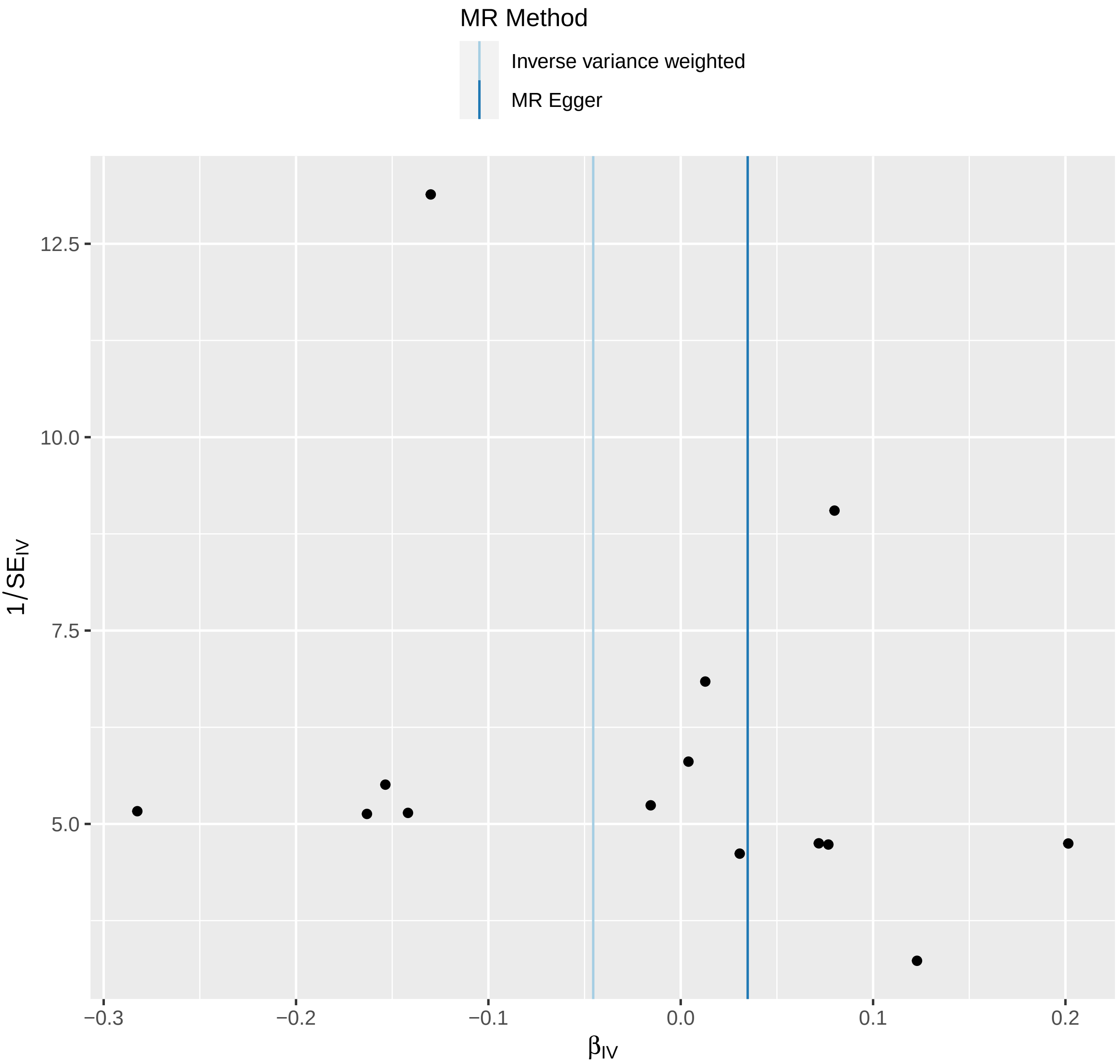

Batch 3−1997 : N−acetylglutamate synthase, mitochondrial on Diabetic nephropathy (more control exclusions)
with a threshold of 1e−05

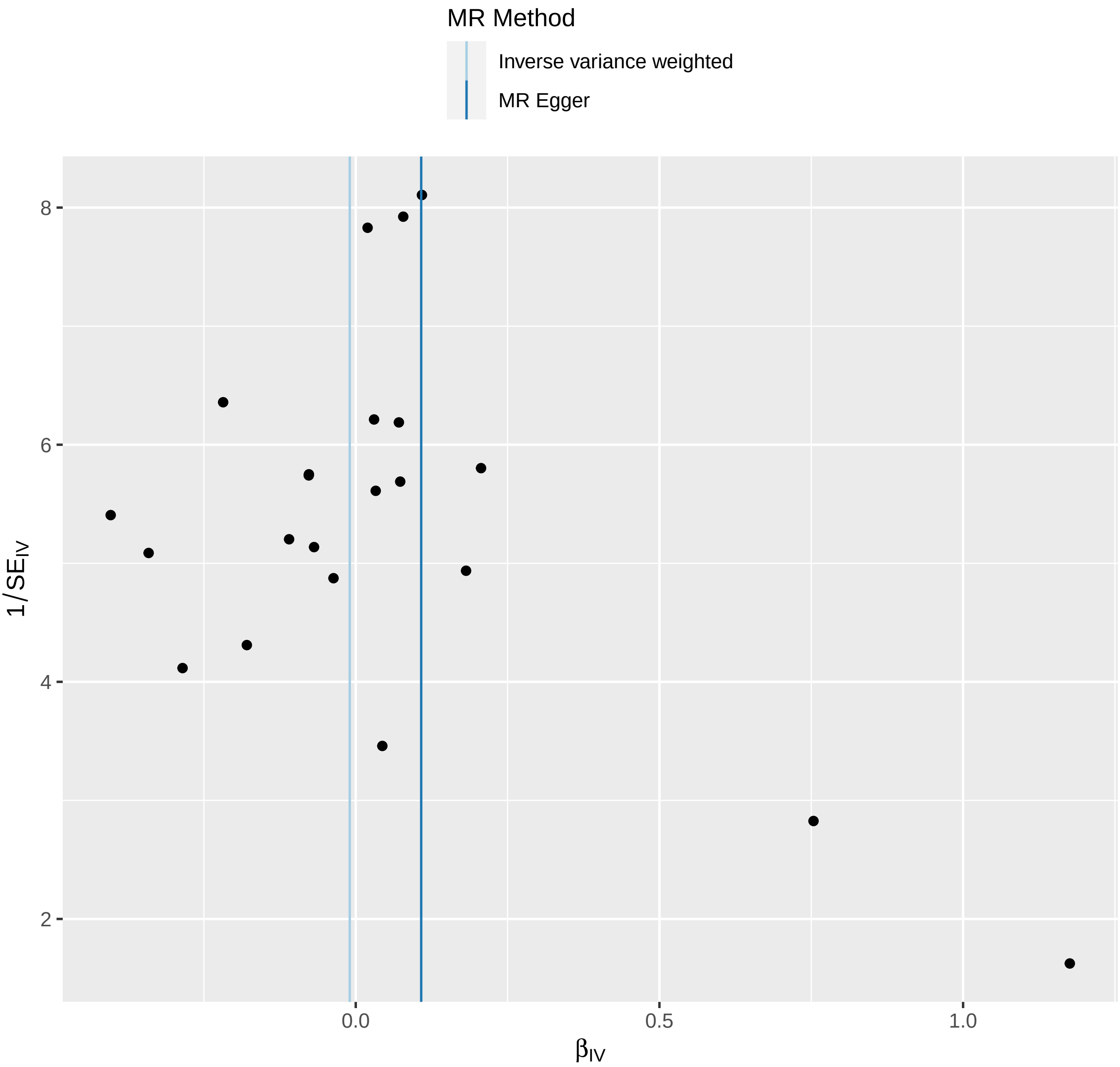

Batch 3−1998 : Nicotinamide phosphoribosyltransferase on Diabetic nephropathy (more control exclusions)
with a threshold of 1e−05

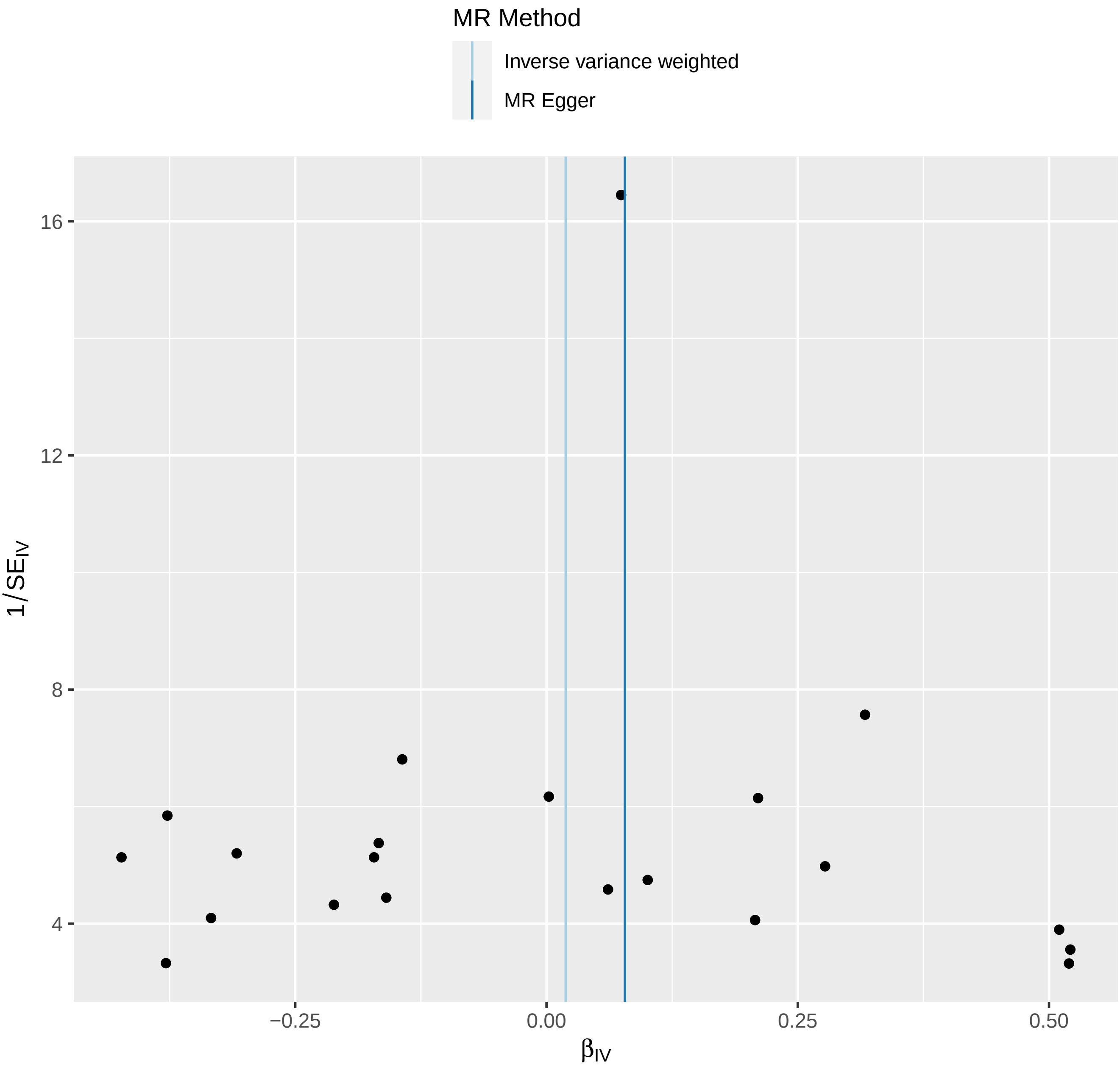

Batch 3−1999 : Nucleosome assembly protein 1−like 2 on Diabetic nephropathy (more control exclusions)
with a threshold of 1e−05

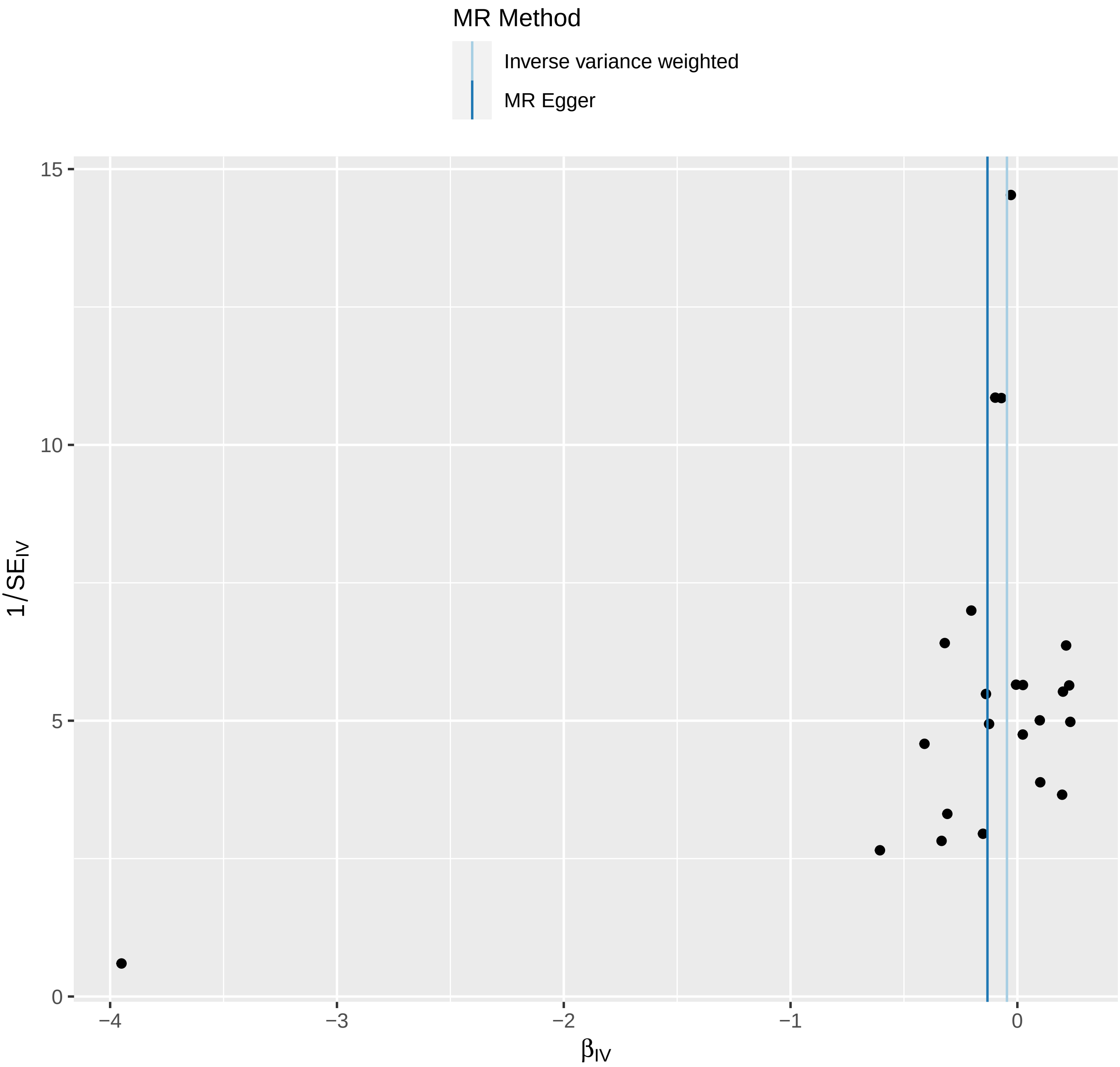

Batch 3−2000 : Beta−soluble NSF attachment protein on Diabetic nephropathy (more control exclusions)
with a threshold of 1e−05

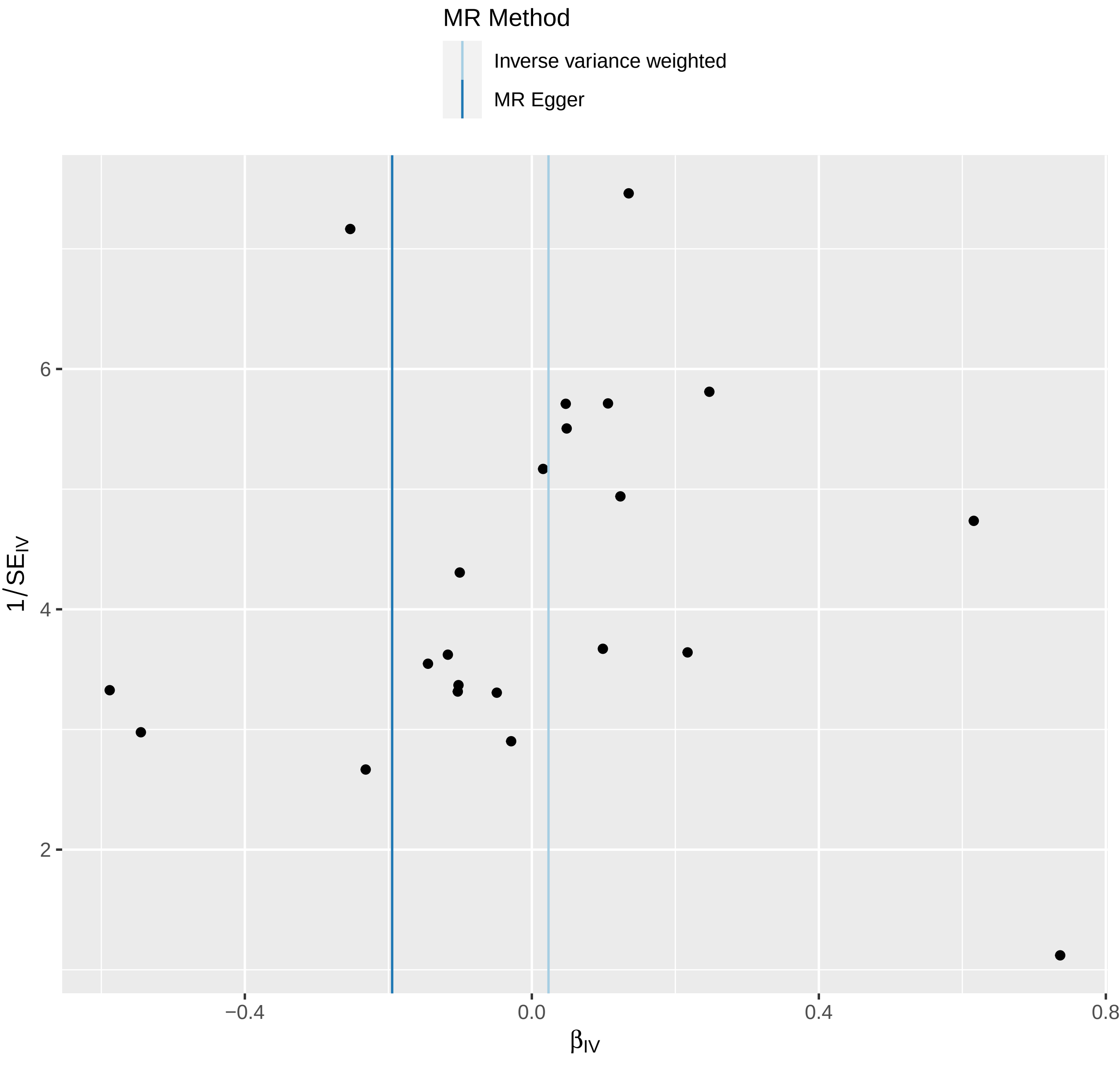

Batch 3−2001 : Arylamine N−acetyltransferase 1 on Diabetic nephropathy (more control exclusions)
with a threshold of 1e−05

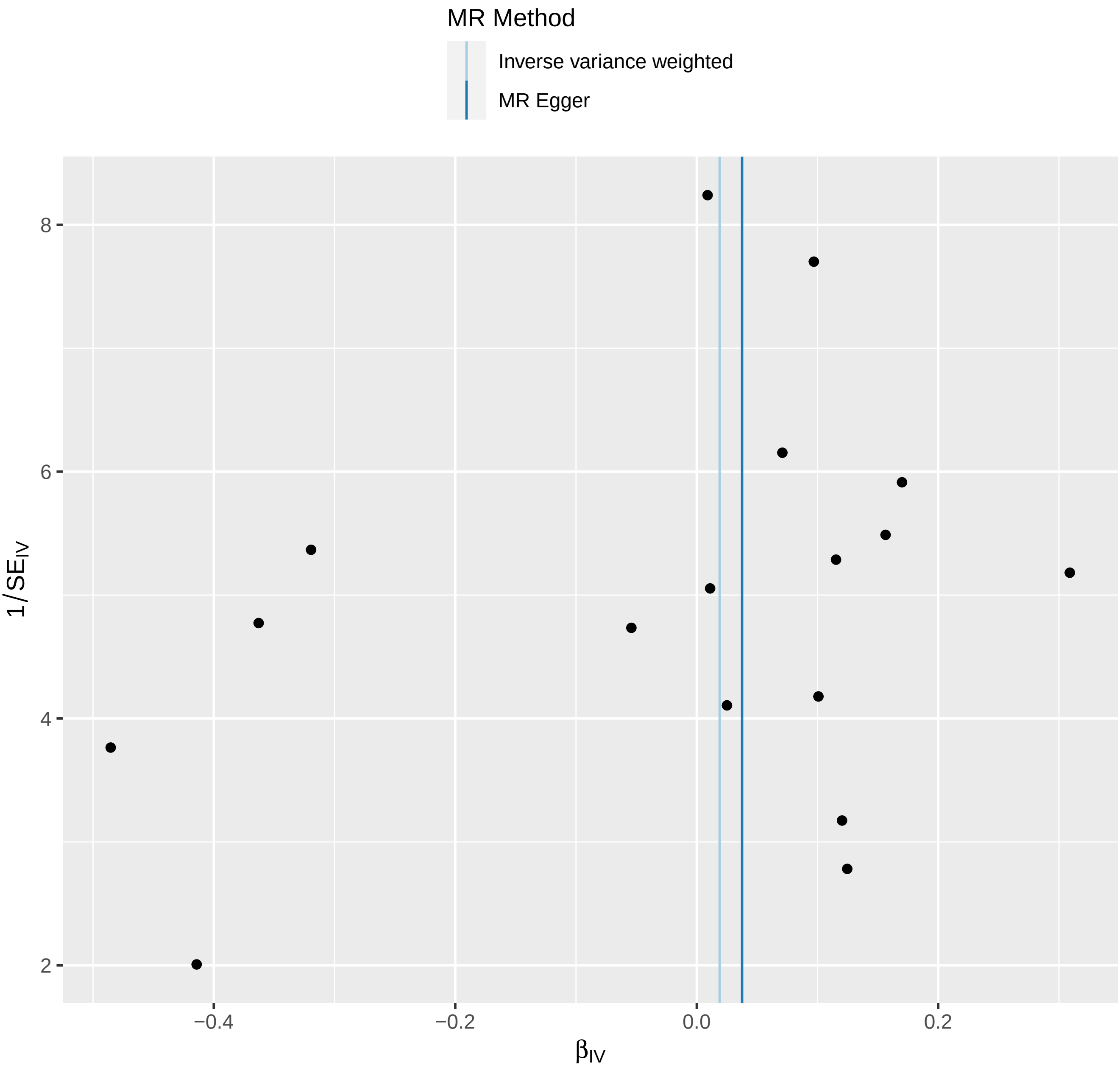

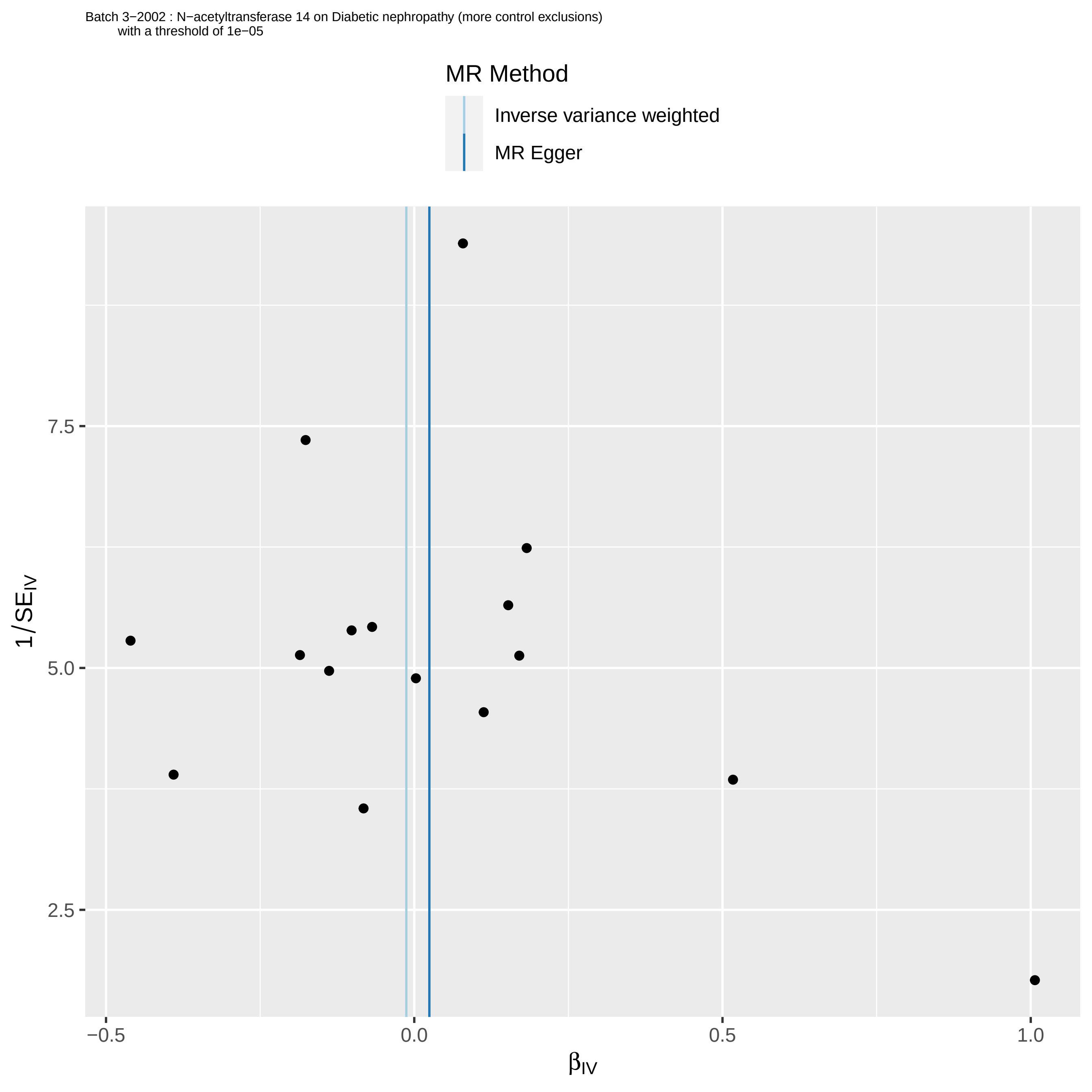

Batch 3−2002 : N−acetyltransferase 14 on Diabetic nephropathy (more control exclusions)
with a threshold of 1e−05
MR Method
Inverse variance weighted
MR Egger
7.5
5.0
2.5
1/SE_IV
−0.5
0.0
0.5
1.0
β_IV

Batch 3−2003 : Neuroblastoma suppressor of tumorigenicity 1 on Diabetic nephropathy (more control exclusions) with a threshold of 1e−05

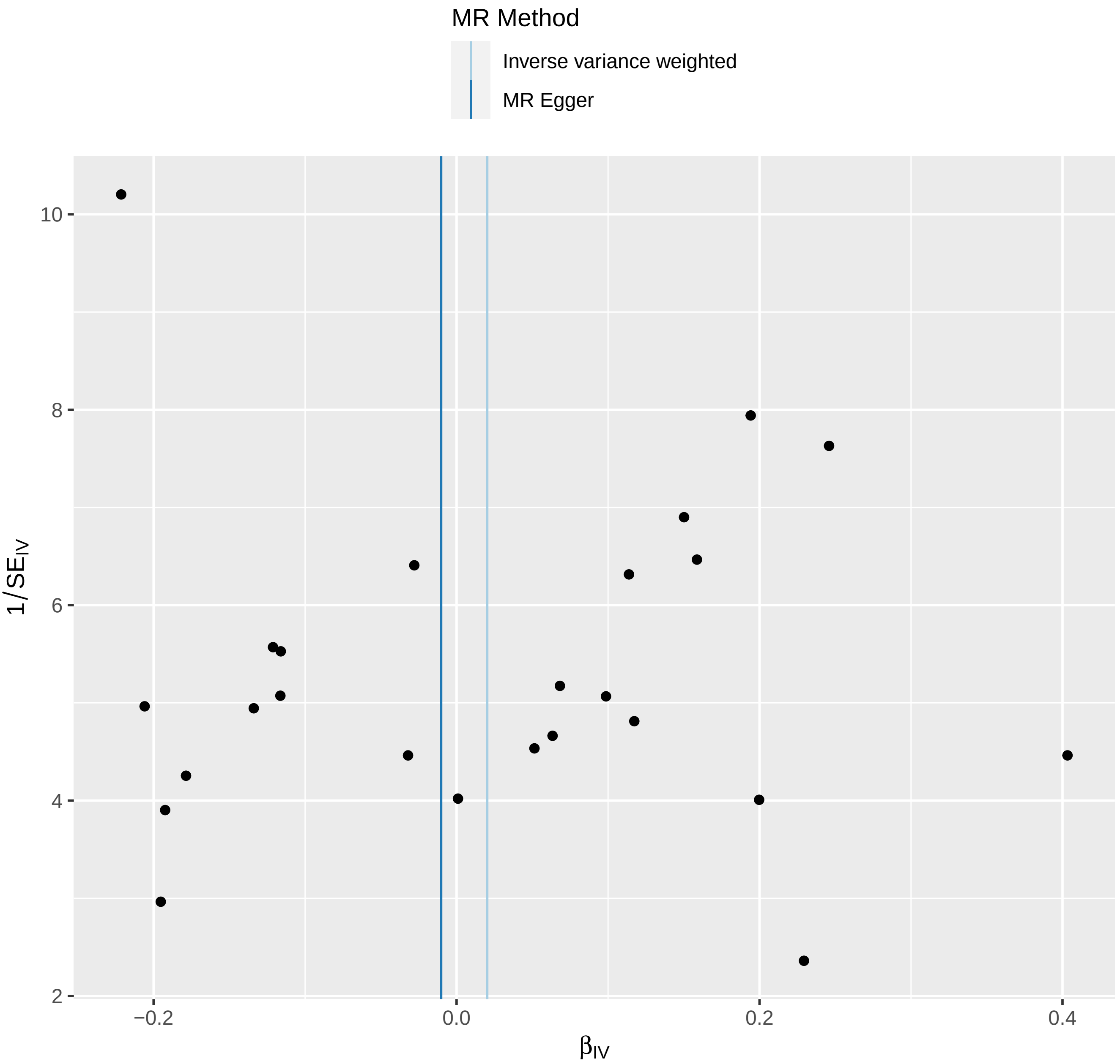

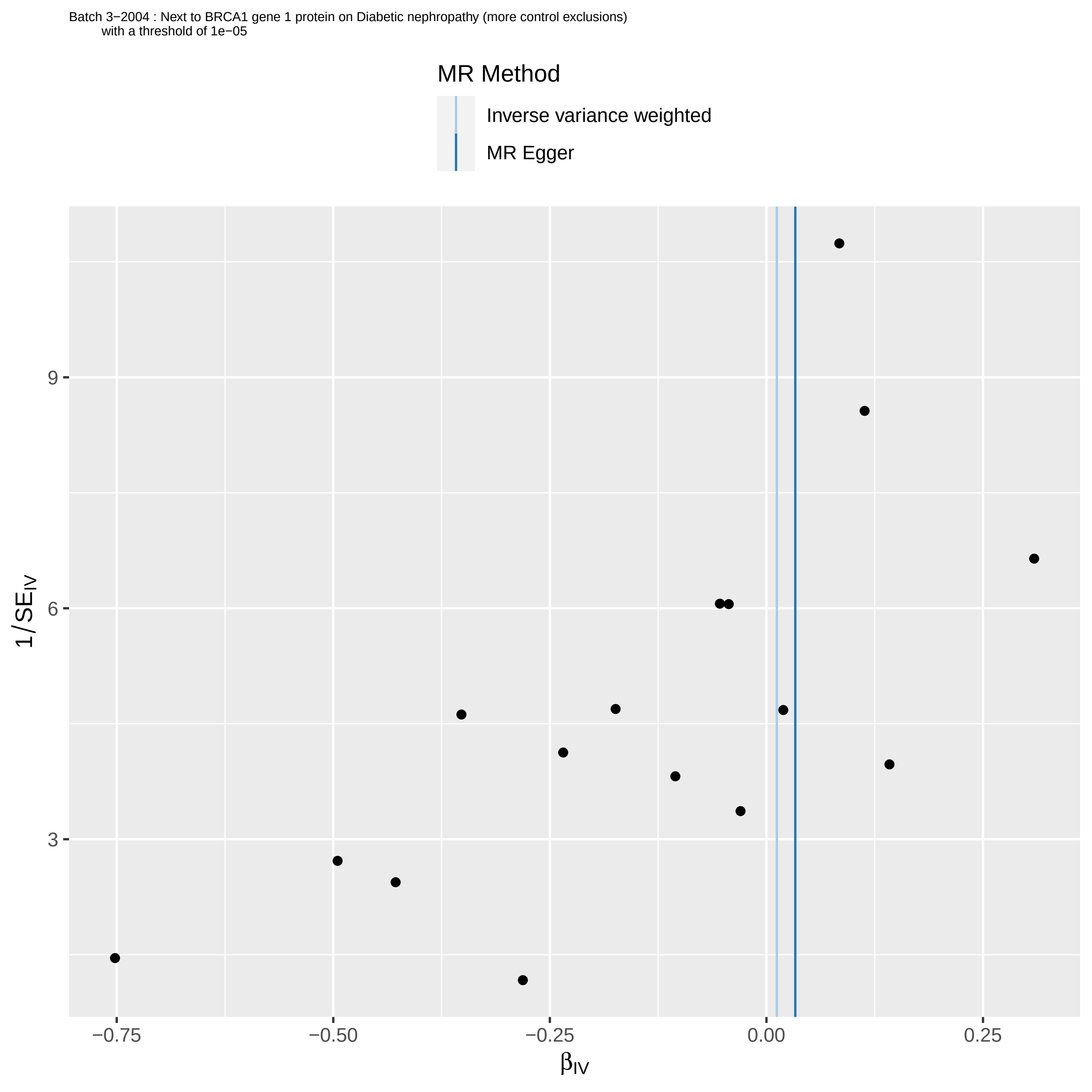
Batch 3−2004 : Next to BRCA1 gene 1 protein on Diabetic nephropathy (more control exclusions)
with a threshold of 1e−05
MR Method
Inverse variance weighted
MR Egger
9
6
3
1/SE_IV
−0.75
−0.50
−0.25
0.00
0.25
β_IV

Batch 3−2005 : Next to BRCA1 gene 1 protein on Diabetic nephropathy (more control exclusions)
with a threshold of 1e−05

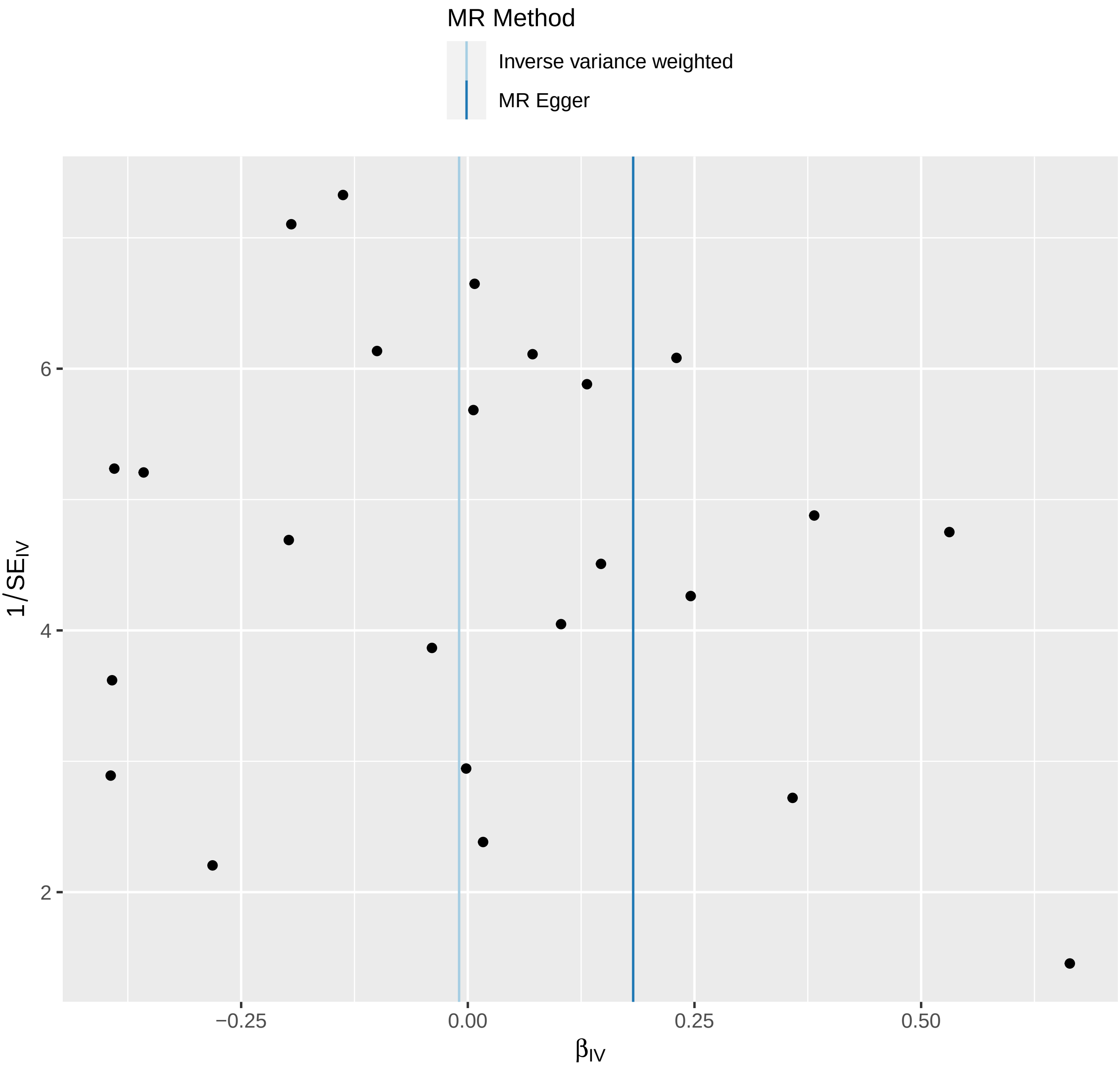

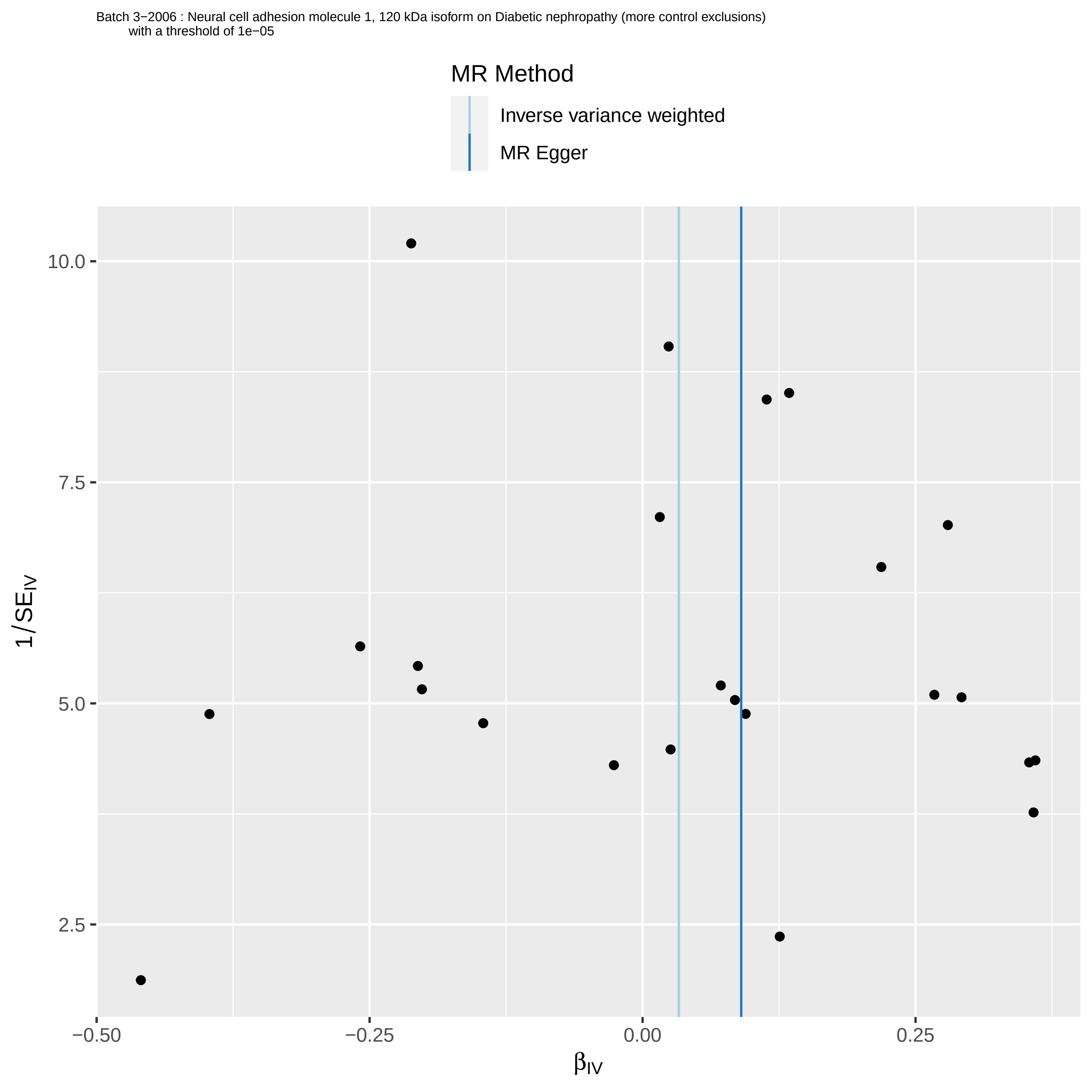
Batch 3−2006 : Neural cell adhesion molecule 1, 120 kDa isoform on Diabetic nephropathy (more control exclusions)
with a threshold of 1e−05
MR Method
Inverse variance weighted
MR Egger
10.0
7.5
5.0
2.5
1/SE_IV
−0.50
−0.25
0.00
0.25
β_IV

Batch 3−2007 : Neural cell adhesion molecule 1 on Diabetic nephropathy (more control exclusions)
with a threshold of 1e−05

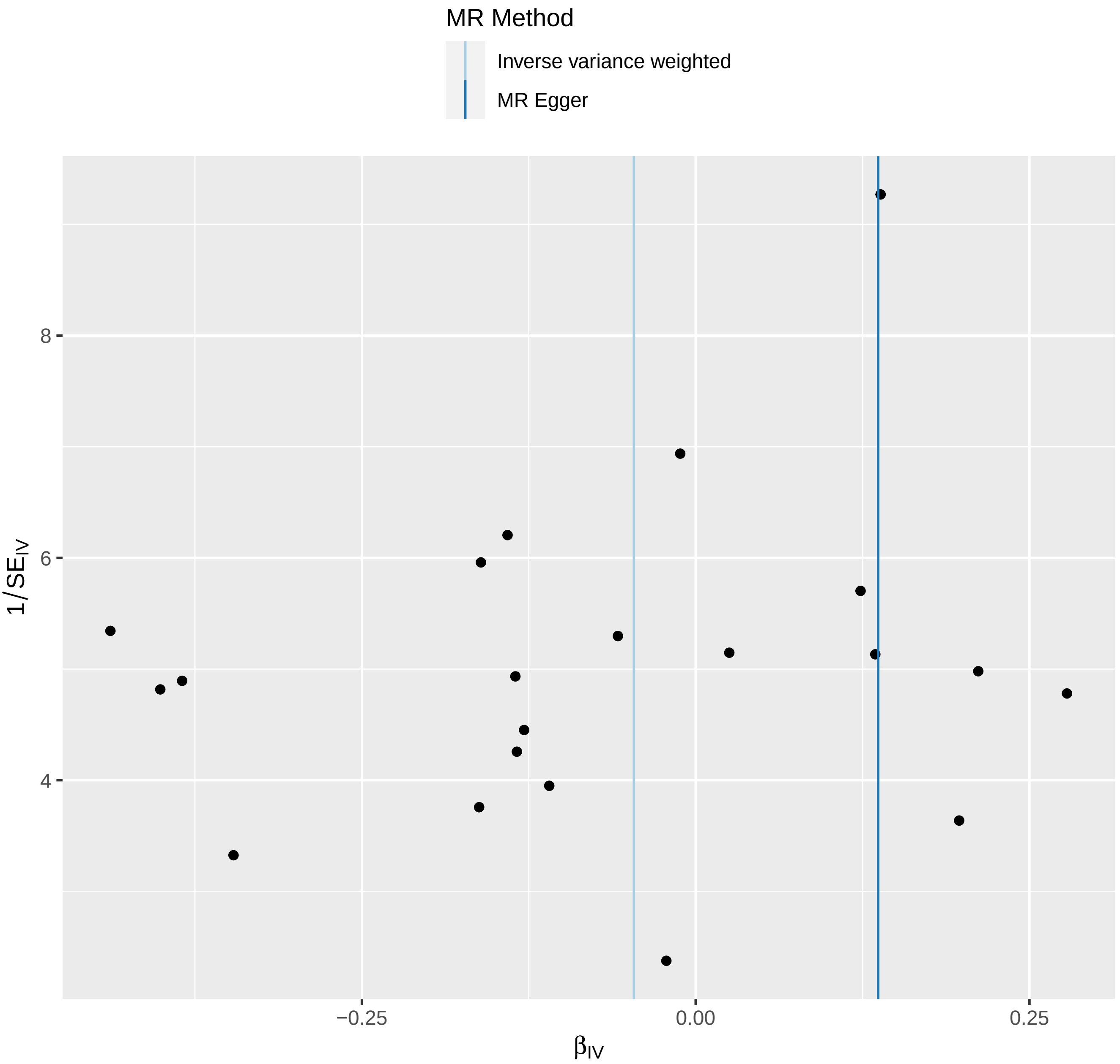

Batch 3−2008 : Neural cell adhesion molecule 2 on Diabetic nephropathy (more control exclusions)
with a threshold of 1e−05

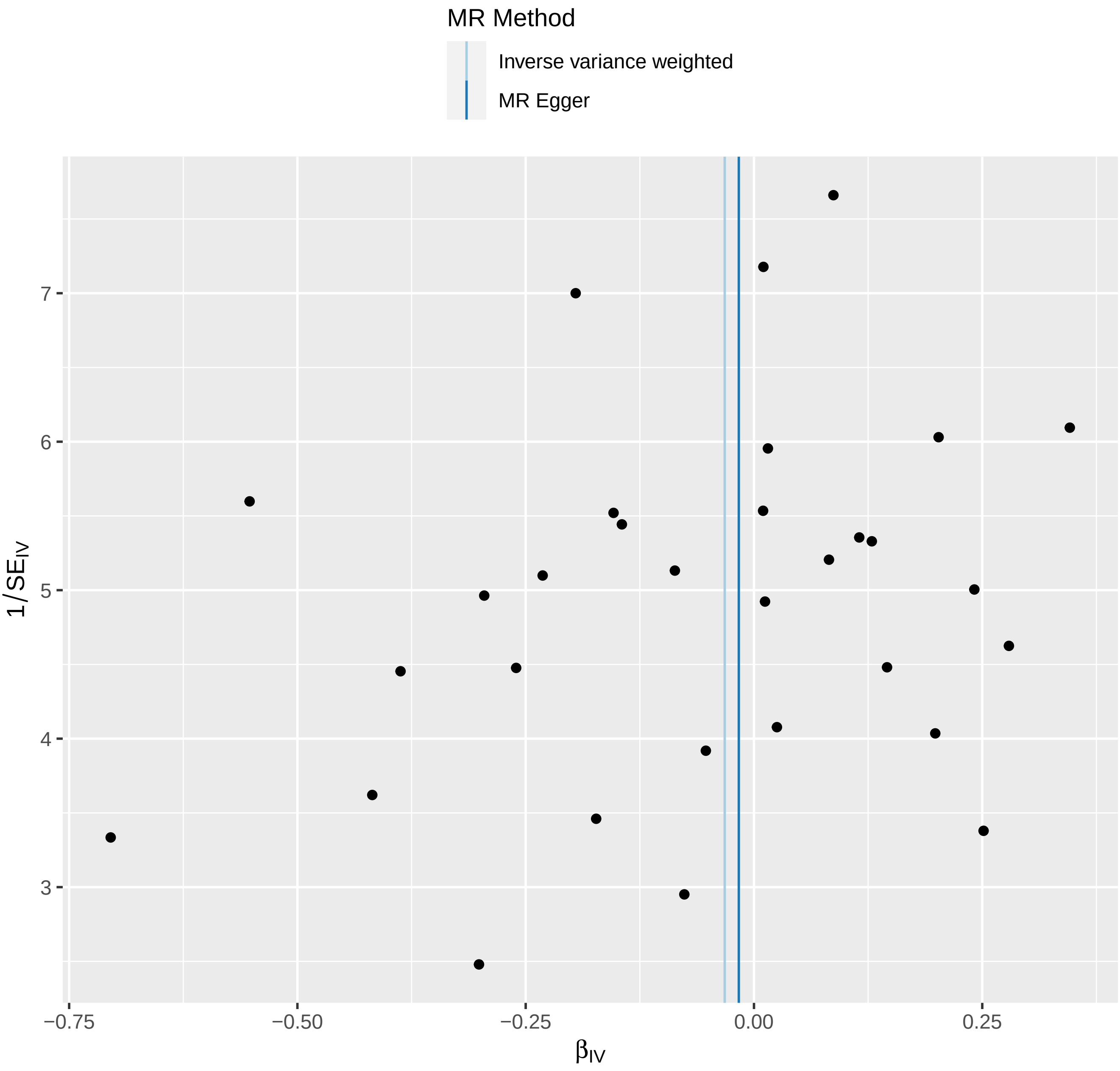

Batch 3−2009 : Neutrophil cytosol factor 2 on Diabetic nephropathy (more control exclusions)
with a threshold of 1e−05

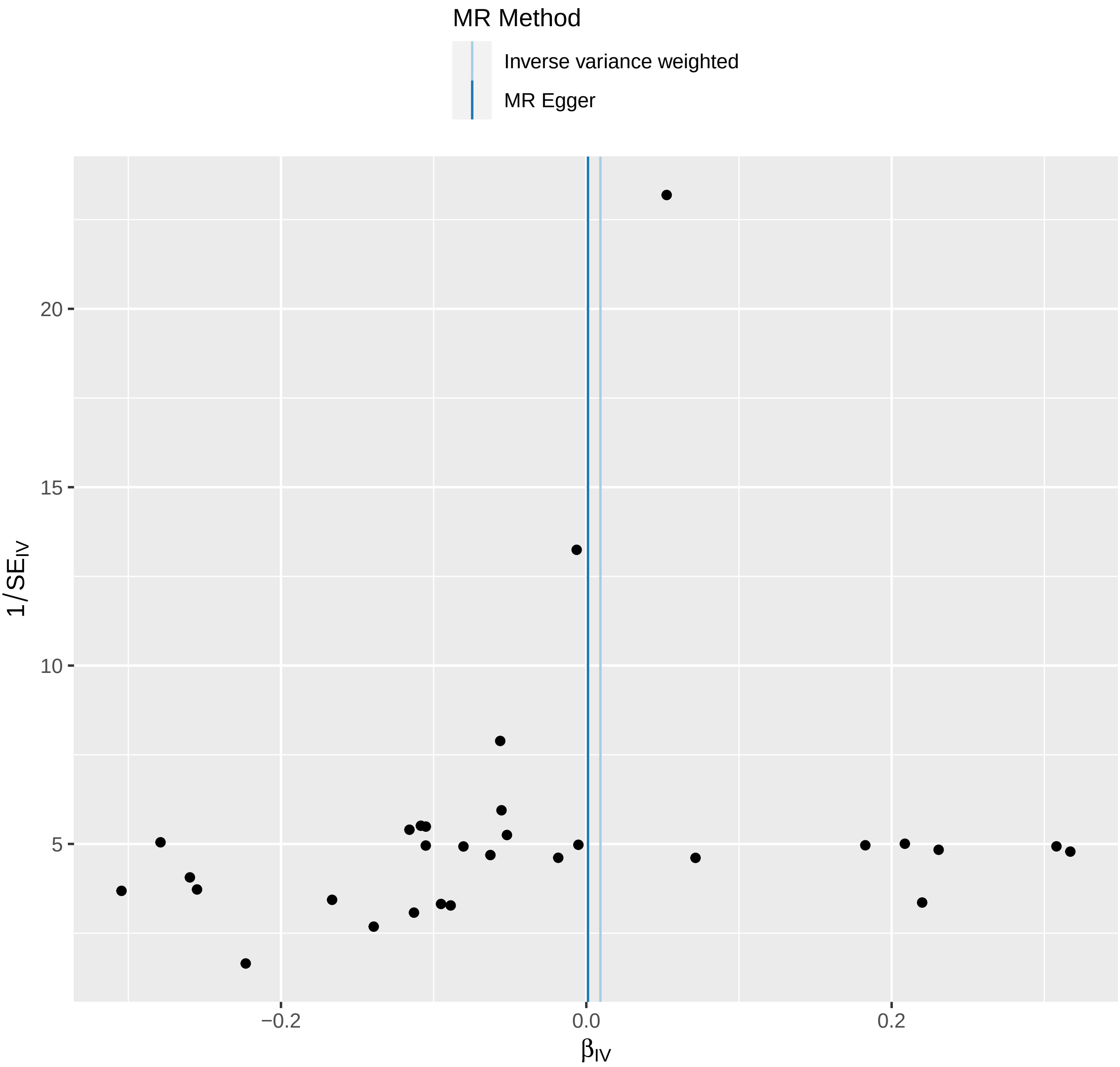

Batch 3−2010 : Nucleolin on Diabetic nephropathy (more control exclusions)
with a threshold of 1e−05

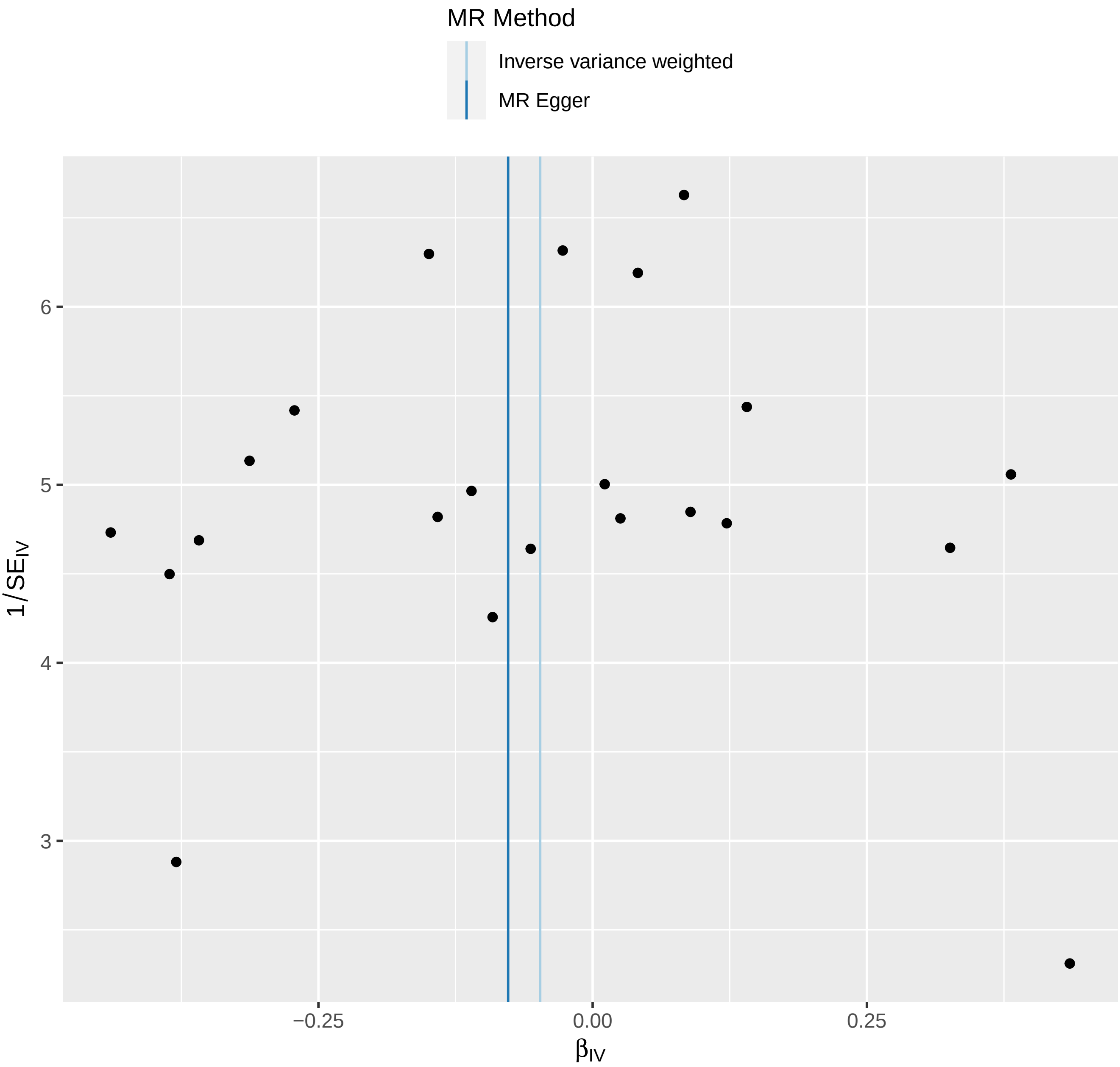

Batch 3−2011 : Noncompact myelin−associated protein on Diabetic nephropathy (more control exclusions)
with a threshold of 1e−05

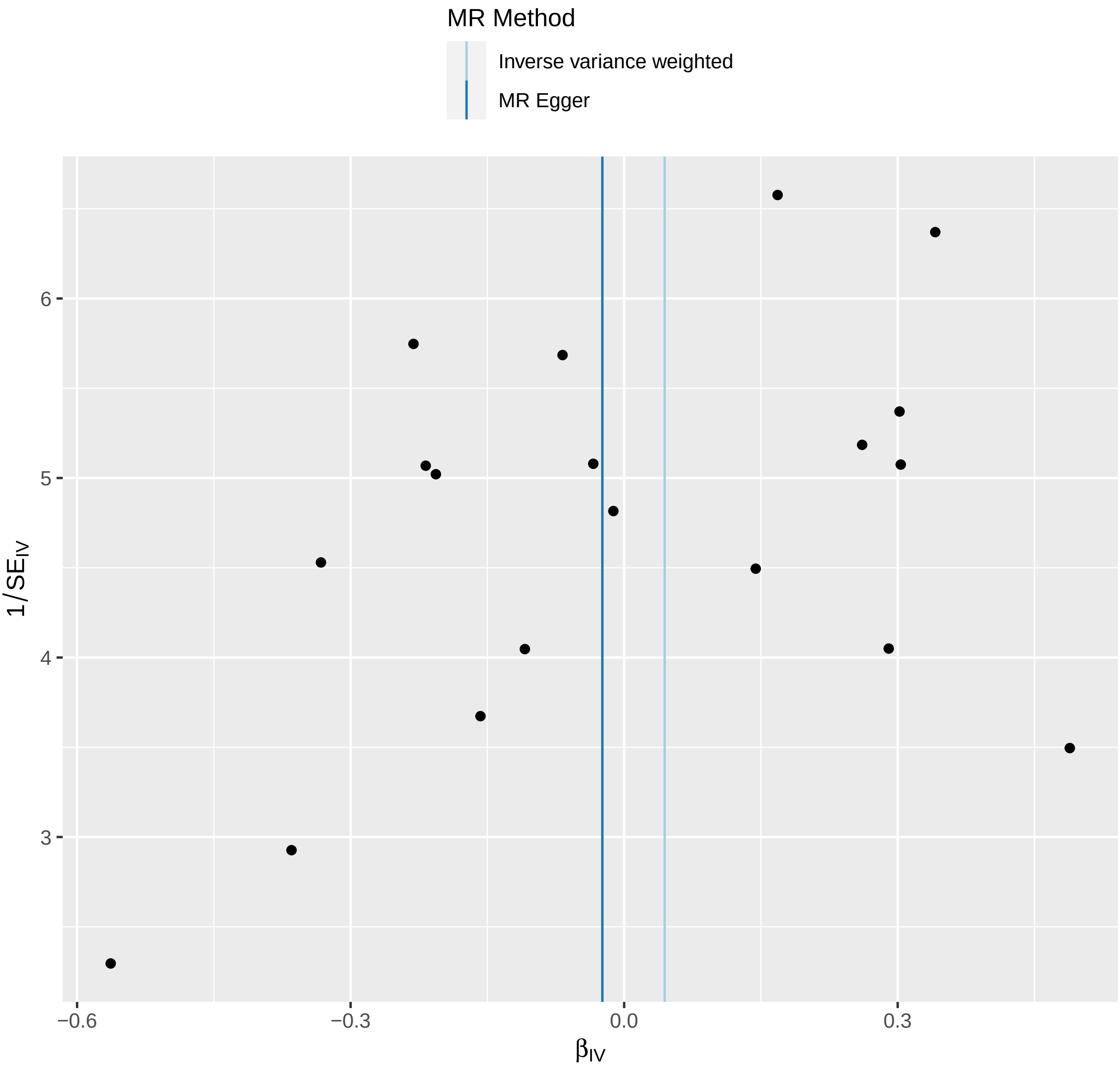

Batch 3−2012 : Nuclear receptor coactivator 2 on Diabetic nephropathy (more control exclusions)
with a threshold of 1e−05

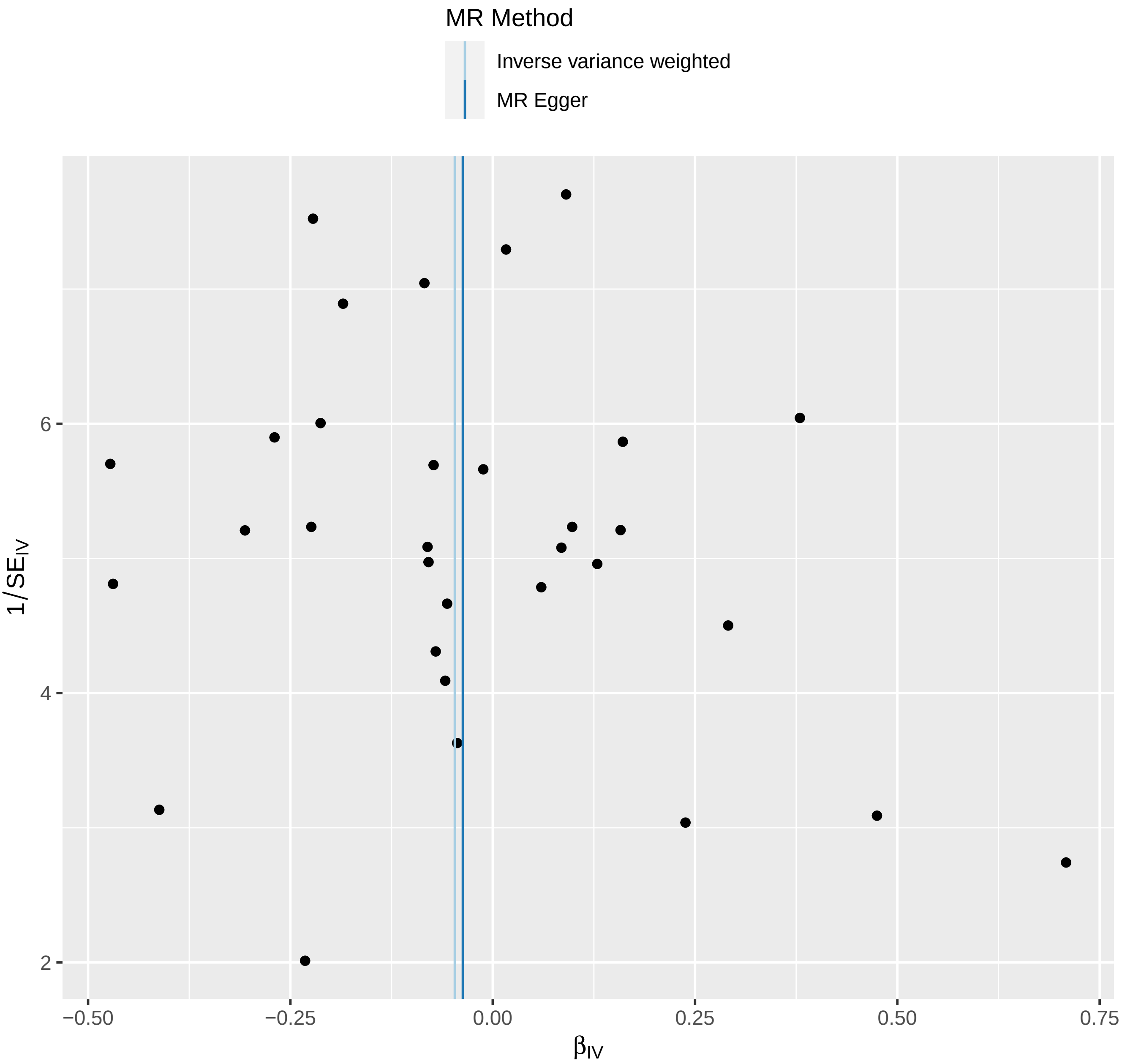

Batch 3−2013 : Natural cytotoxicity triggering receptor 1 on Diabetic nephropathy (more control exclusions)
with a threshold of 1e−05

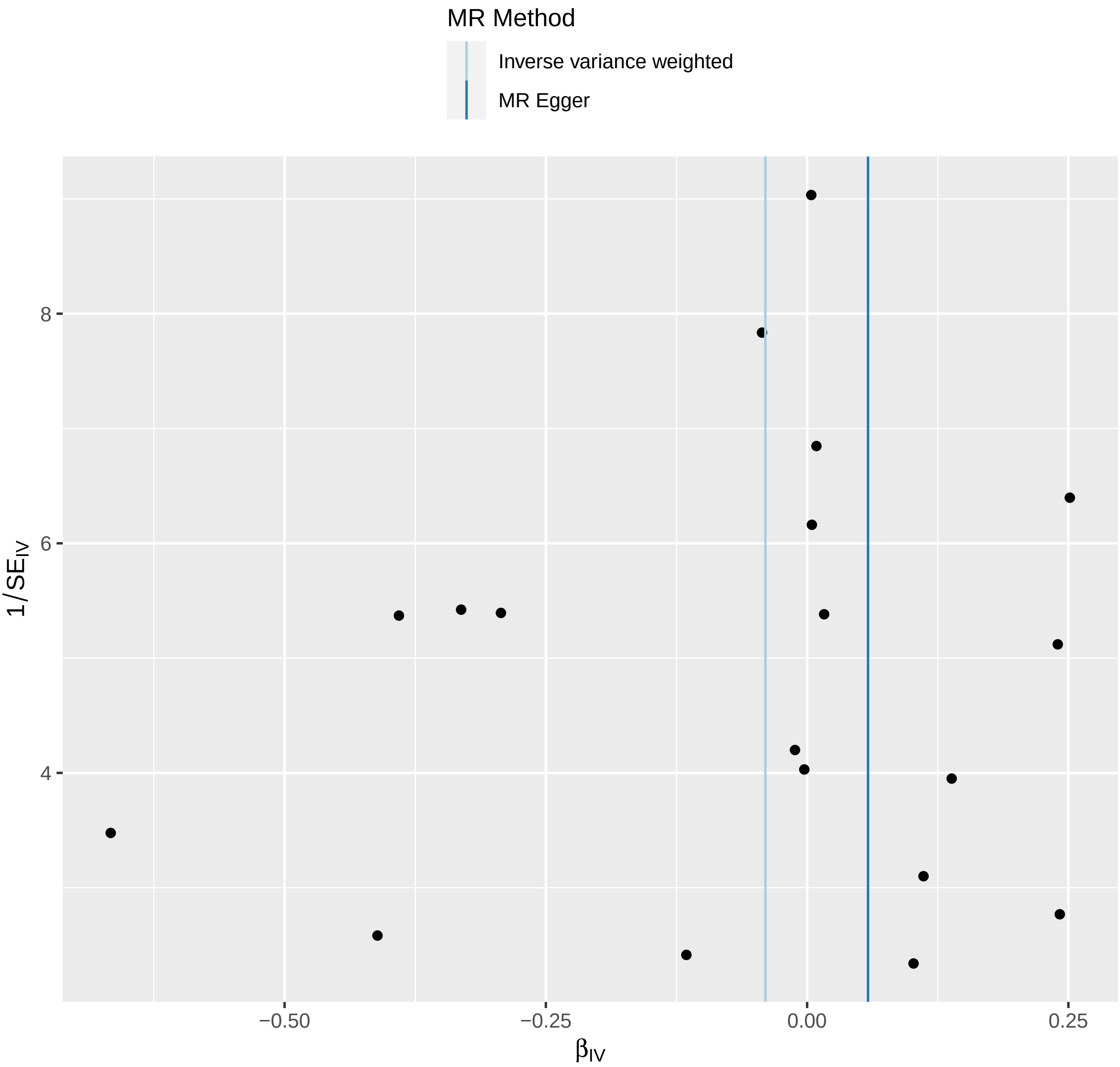

Batch 3−2014 : Natural cytotoxicity triggering receptor 1 on Diabetic nephropathy (more control exclusions)
with a threshold of 1e−05

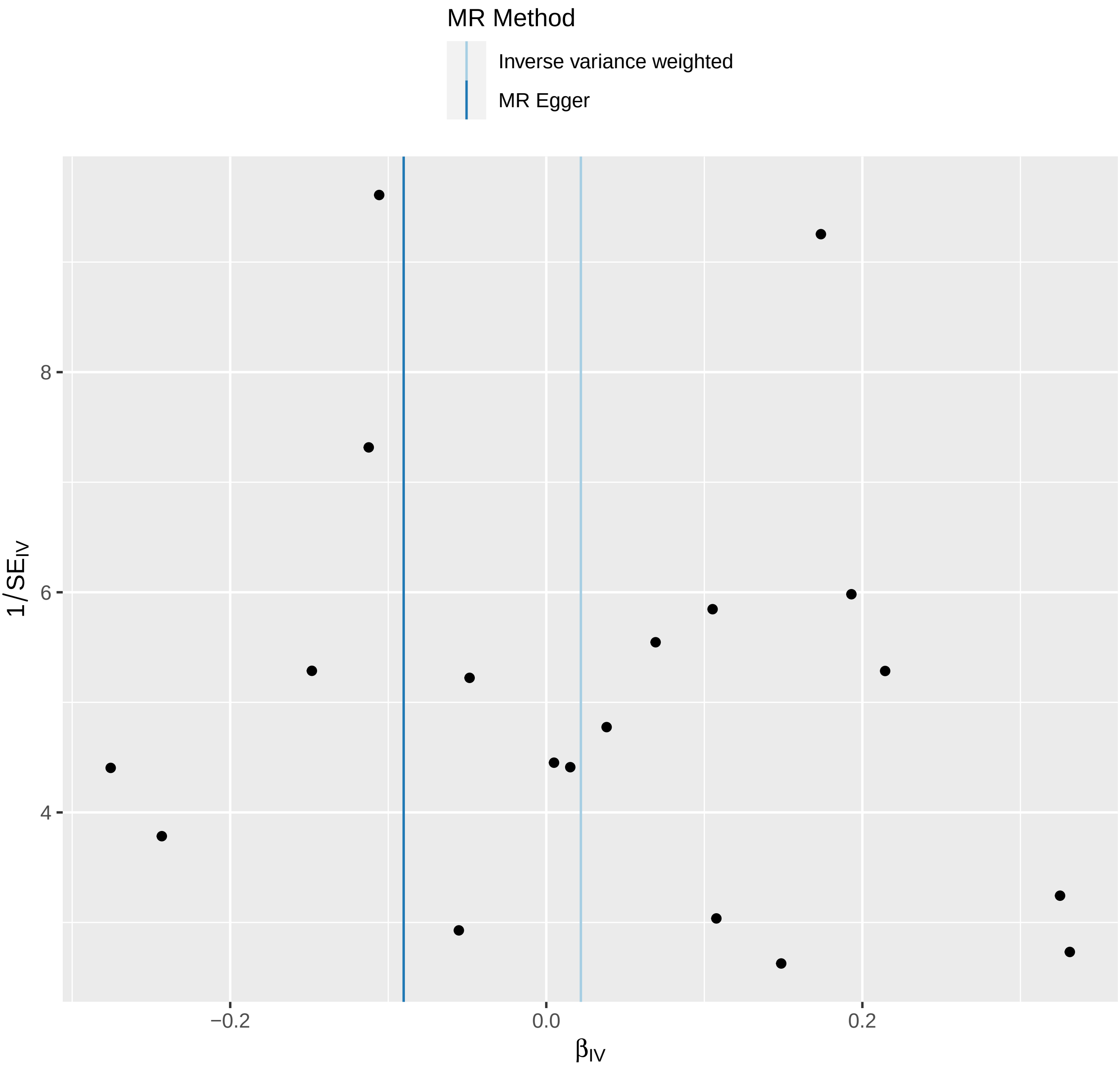

Batch 3−2015 : Natural cytotoxicity triggering receptor 2 on Diabetic nephropathy (more control exclusions)
with a threshold of 1e−05

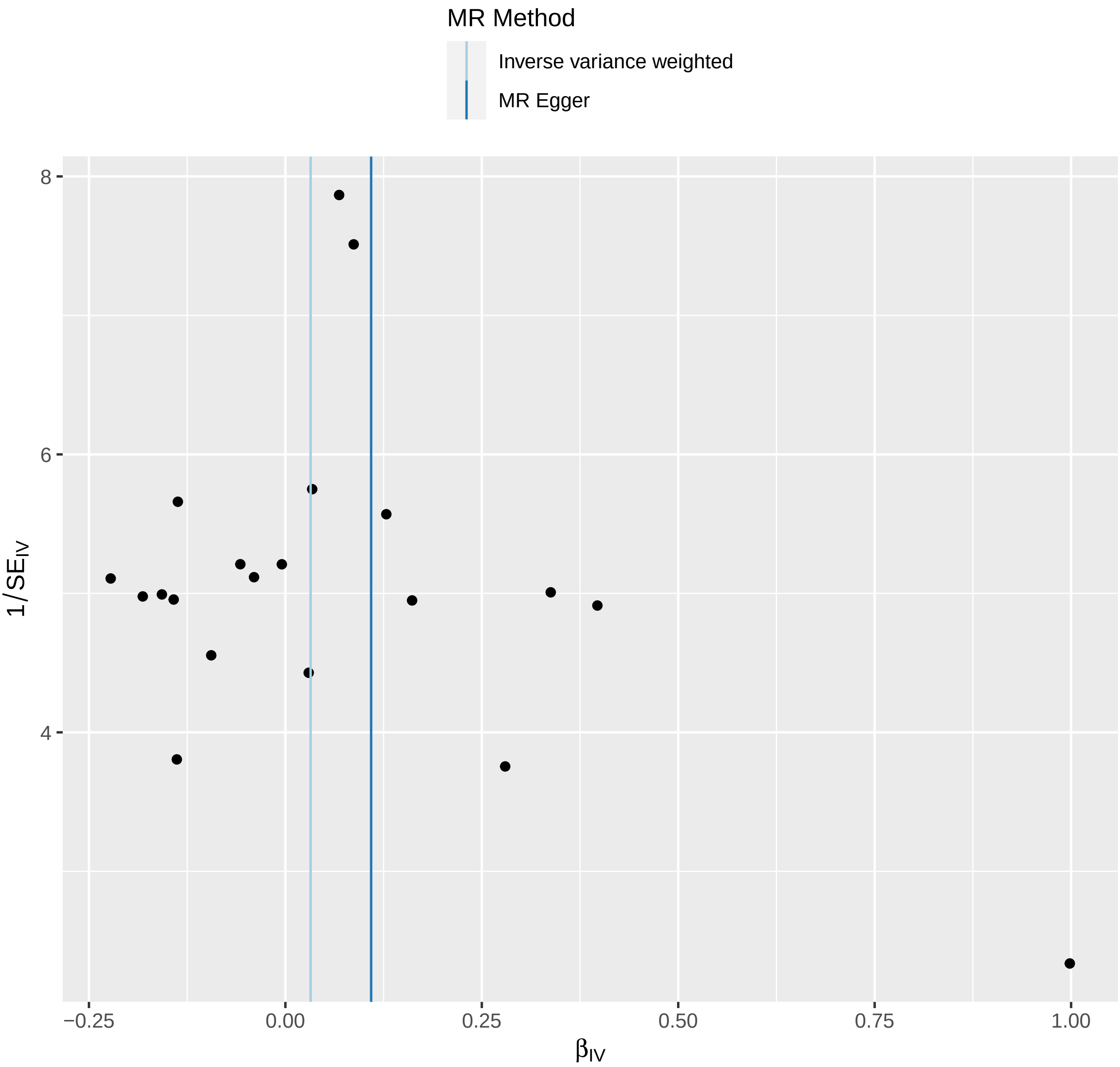

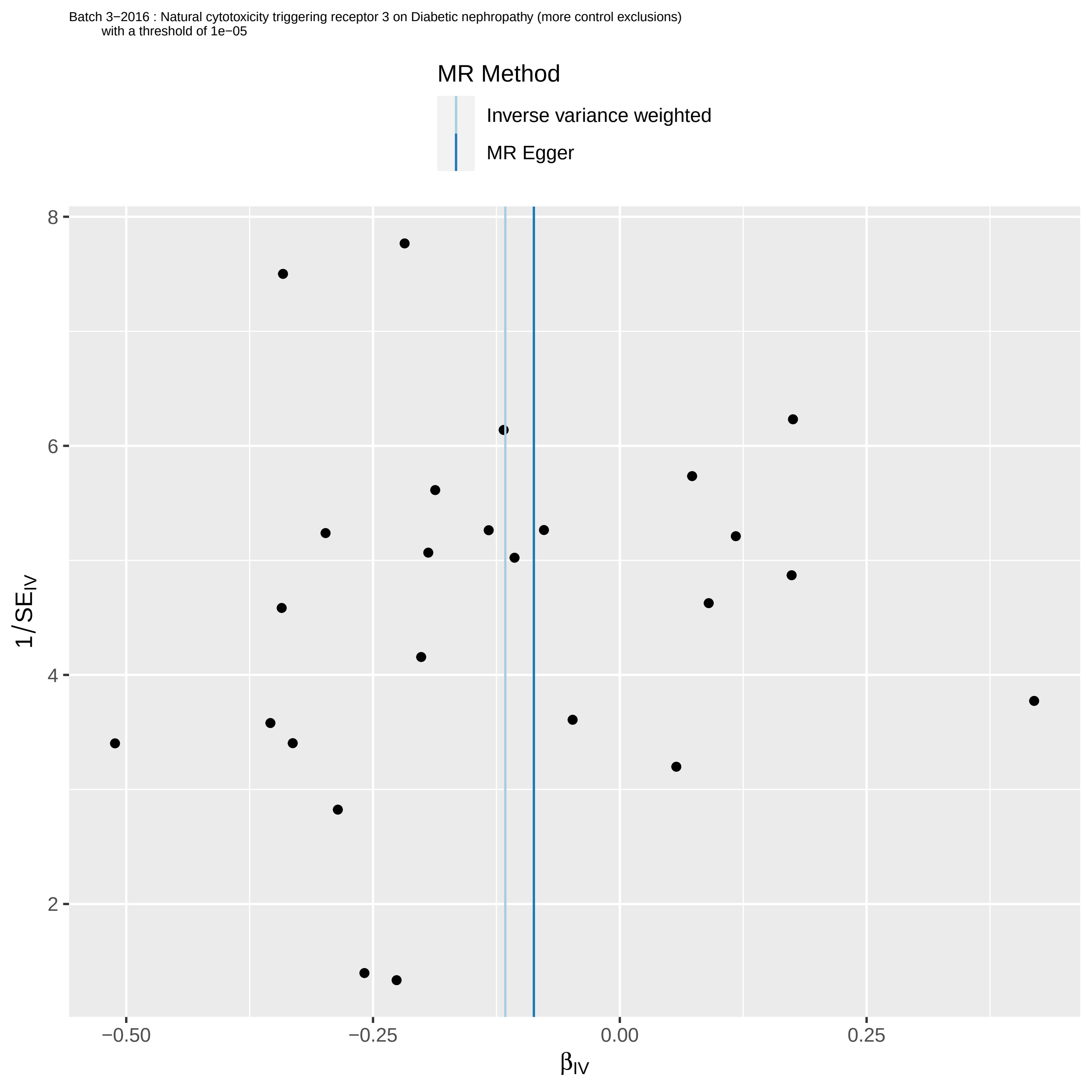
Batch 3−2016 : Natural cytotoxicity triggering receptor 3 on Diabetic nephropathy (more control exclusions)
with a threshold of 1e−05
MR Method
Inverse variance weighted
MR Egger
8
6
4
2
1/SE_IV
−0.50
−0.25
0.00
0.25
β_IV

Batch 3−2017 : Natural cytotoxicity triggering receptor 3 ligand 1 on Diabetic nephropathy (more control exclusions)
with a threshold of 1e−05

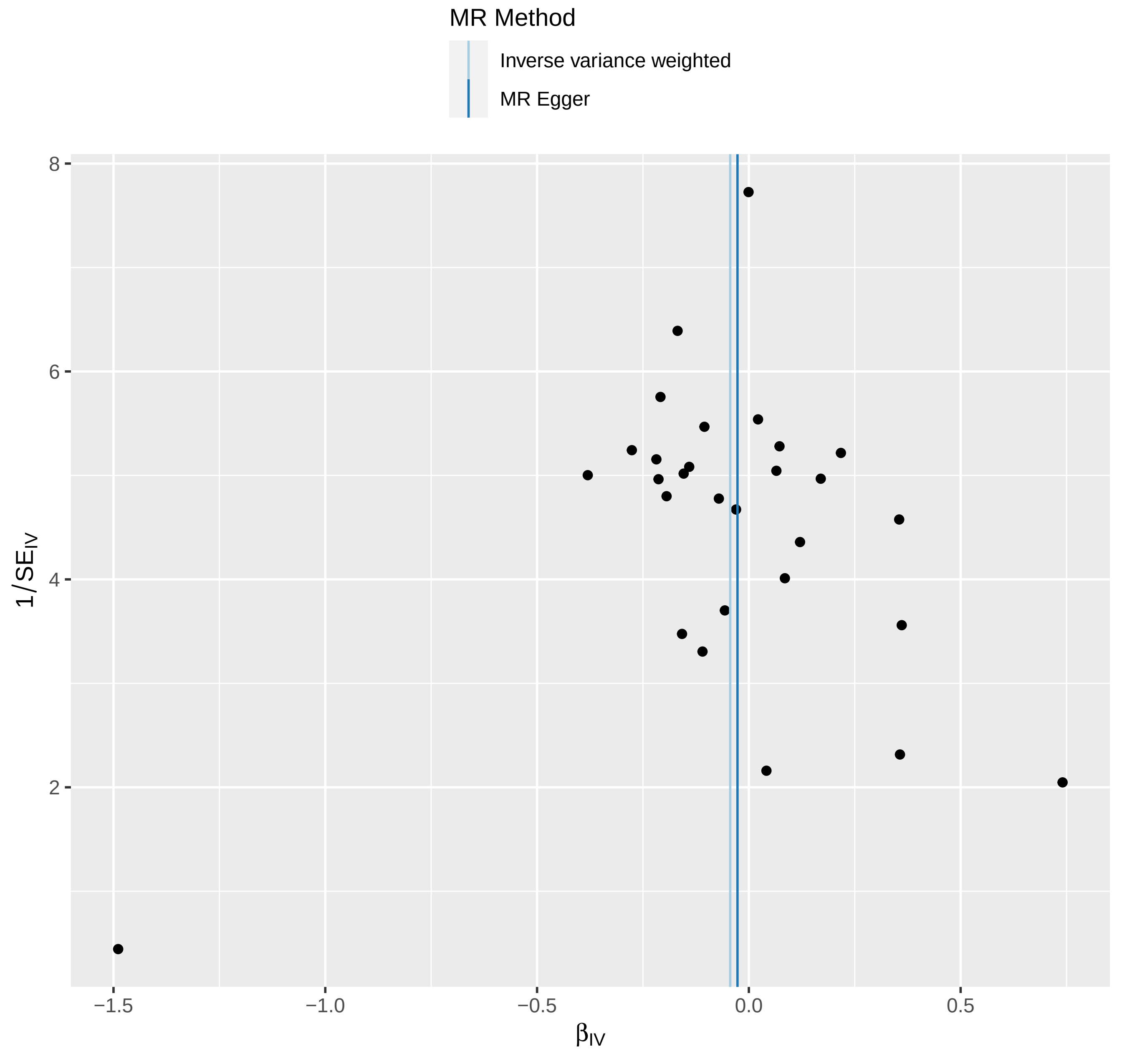

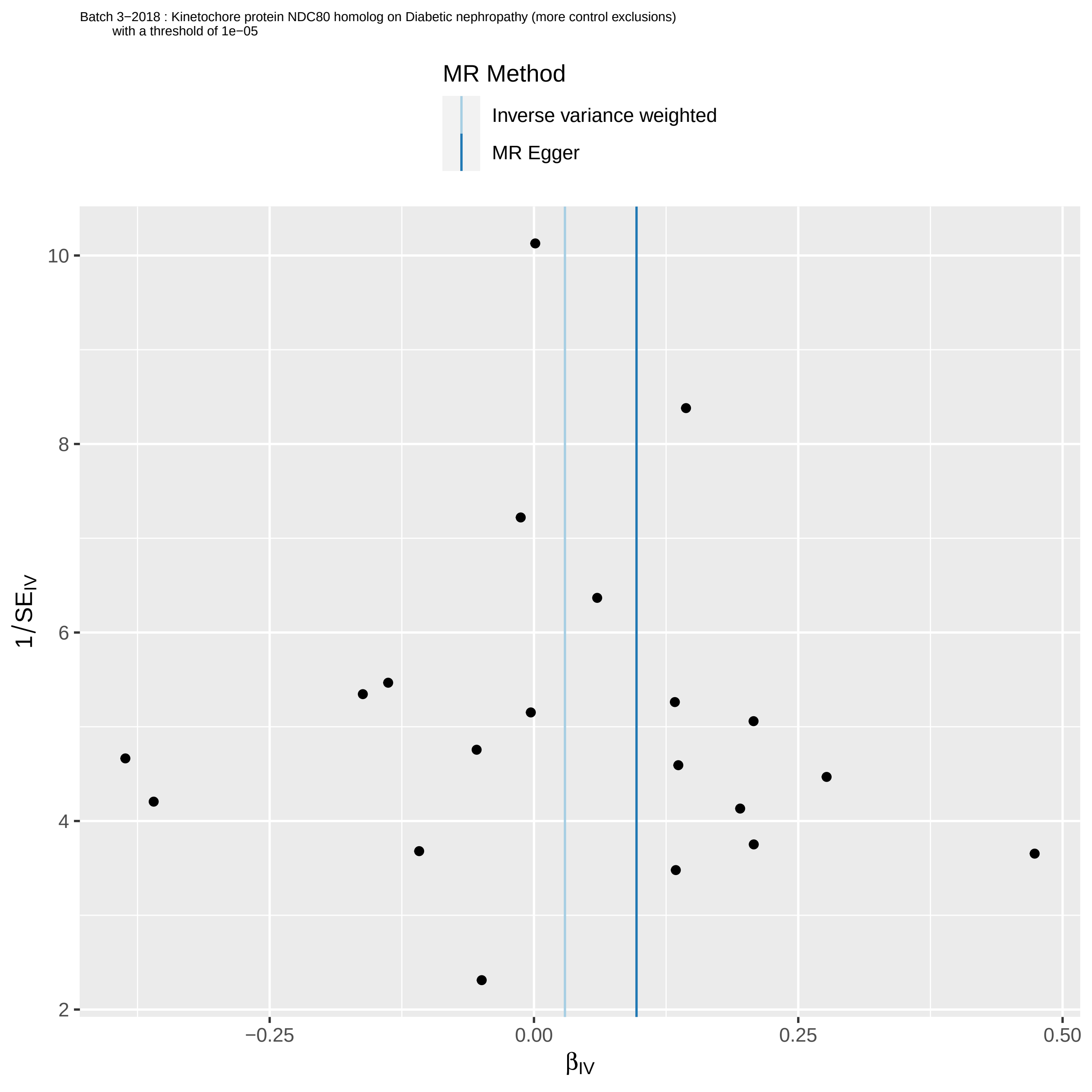

Batch 3−2018 : Kinetochore protein NDC80 homolog on Diabetic nephropathy (more control exclusions)
with a threshold of 1e−05
MR Method
Inverse variance weighted
MR Egger
10
8
6
4
2
1/SE_IV
−0.25
0.00
0.25
0.50
β_IV

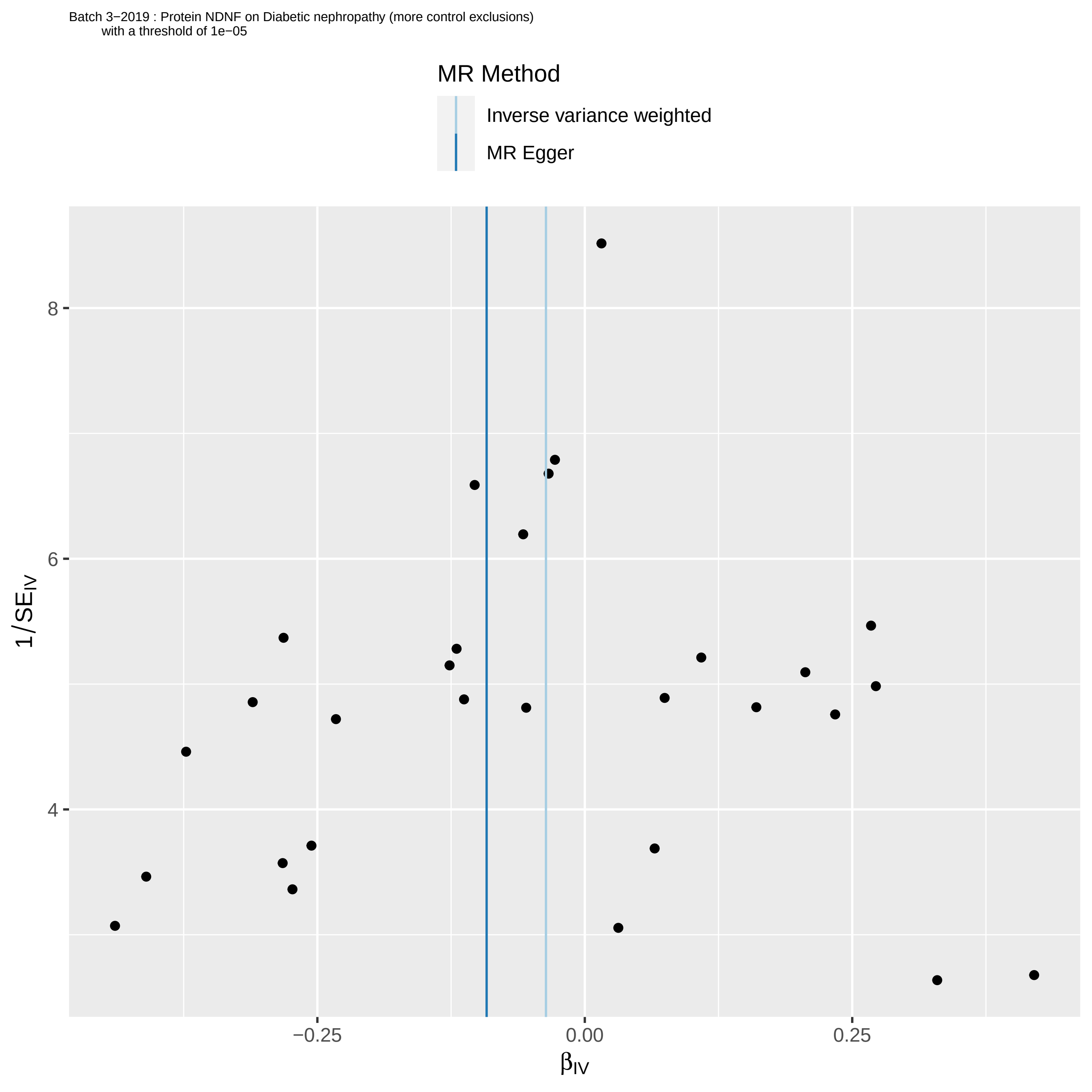
Batch 3−2019 : Protein NDNF on Diabetic nephropathy (more control exclusions)
with a threshold of 1e−05
MR Method
Inverse variance weighted
MR Egger
8
6
4
1/SE_IV
−0.25
0.00
0.25
β_IV

Batch 3−2020 : Protein NDRG4 on Diabetic nephropathy (more control exclusions)
with a threshold of 1e−05

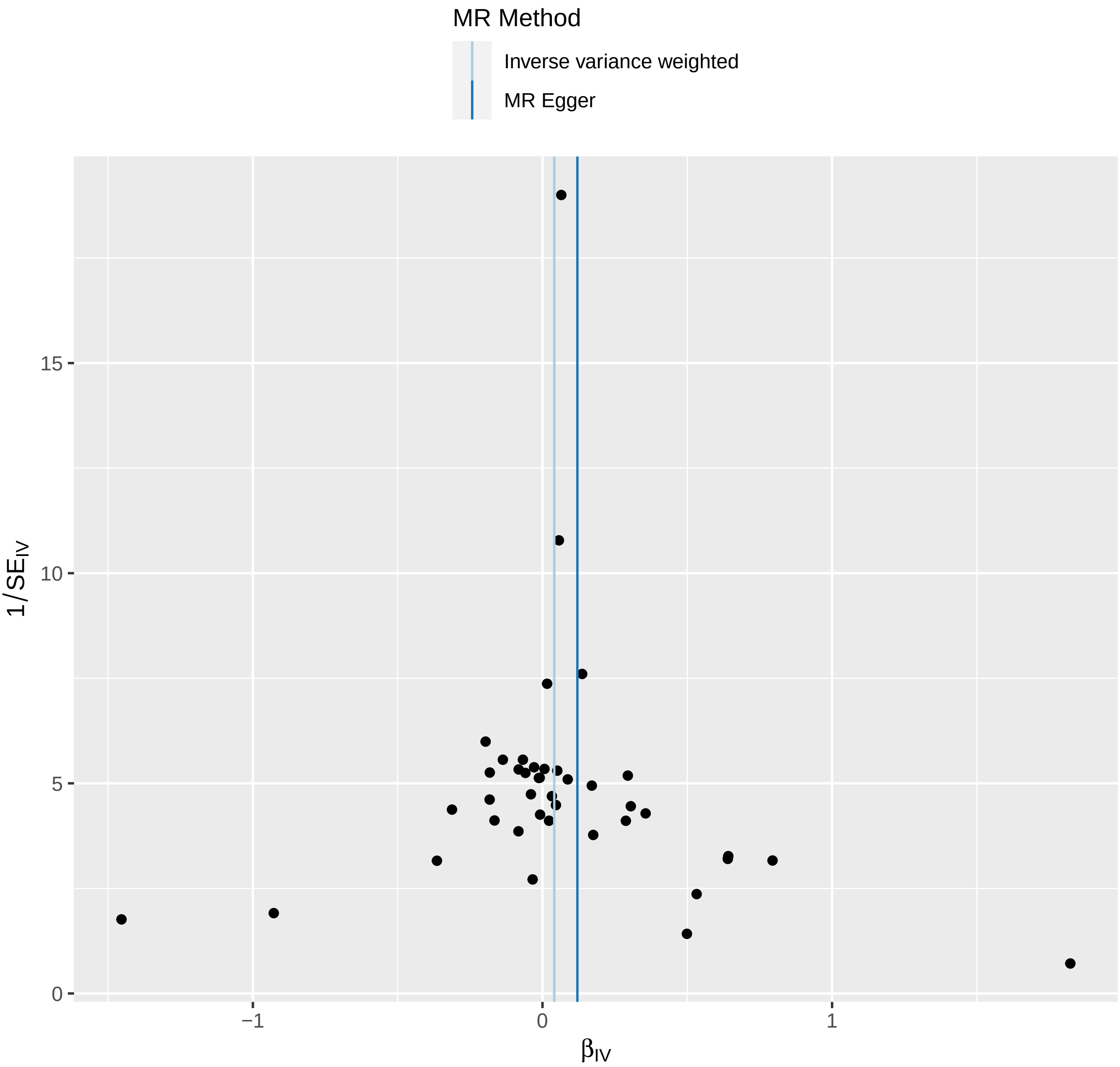

Batch 3−2021 : Bifunctional heparan sulfate N−deacetylase/N−sulfotransferase 1 on Diabetic nephropathy (more control exclusions) with a threshold of 1e−05

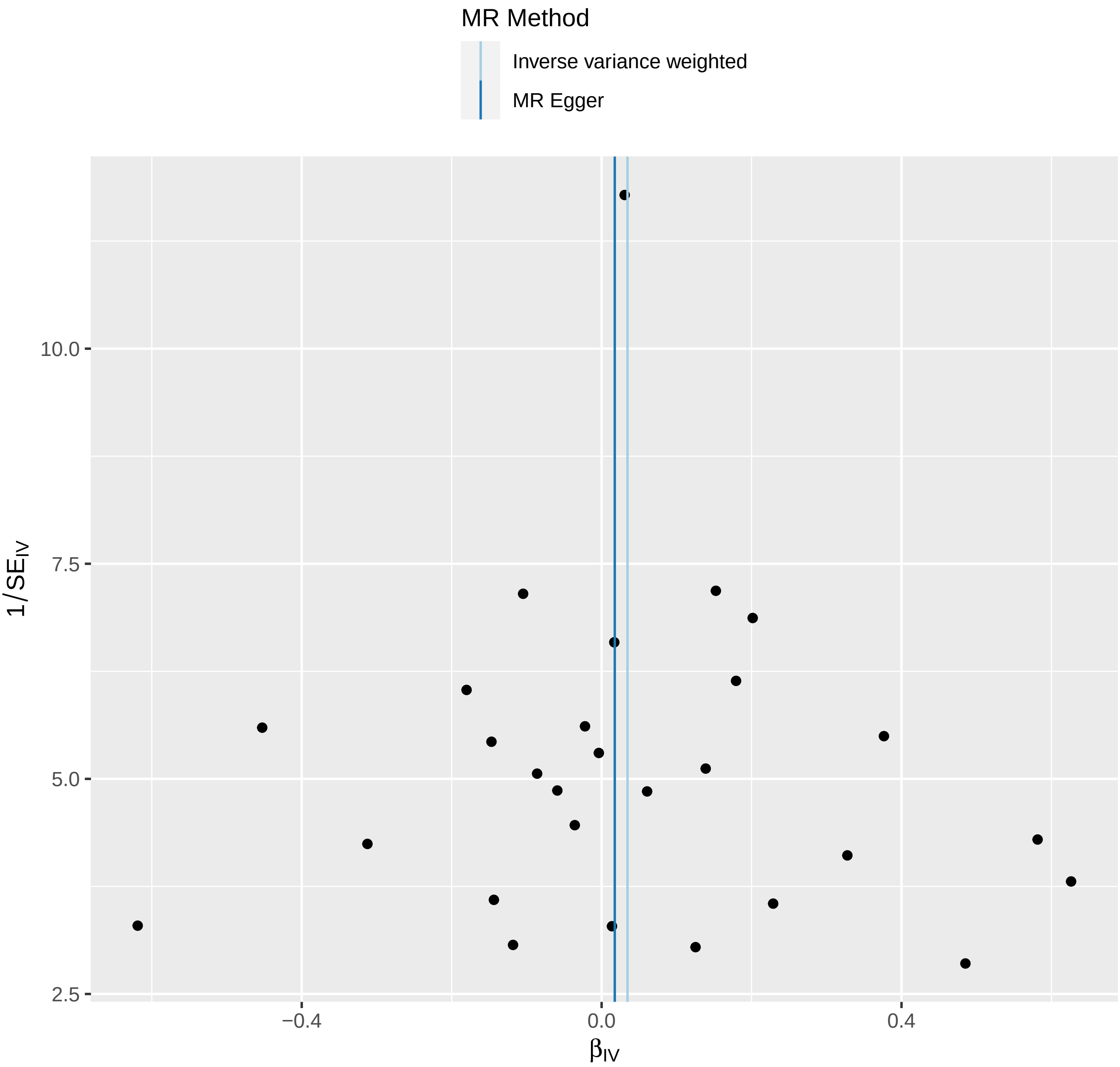

Batch 3−2022 : NADH dehydrogenase [ubiquinone] 1 beta subcomplex subunit 11, mitochondrial on Diabetic nephropathy (more control exclusions) with a threshold of 1e−05

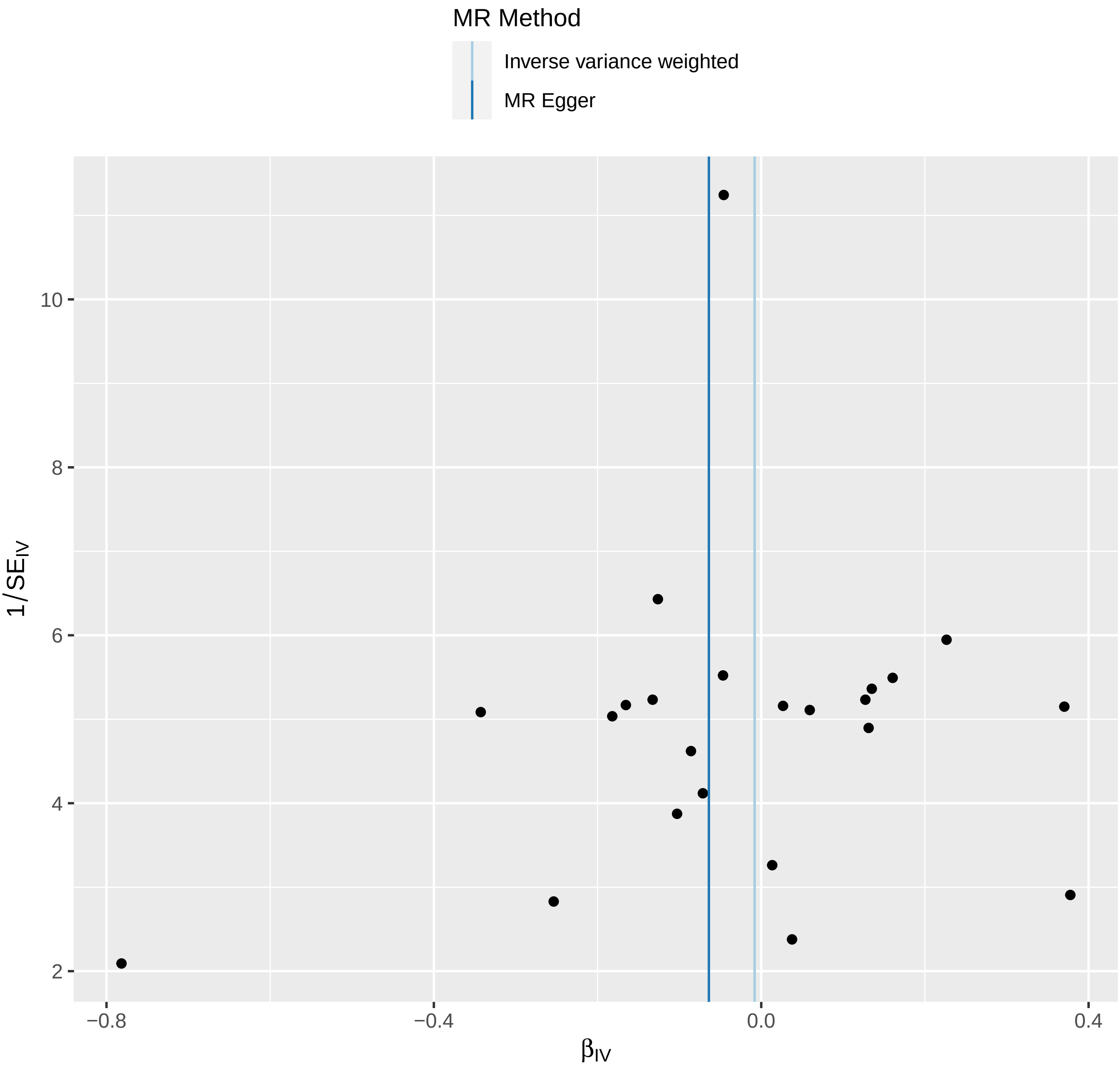

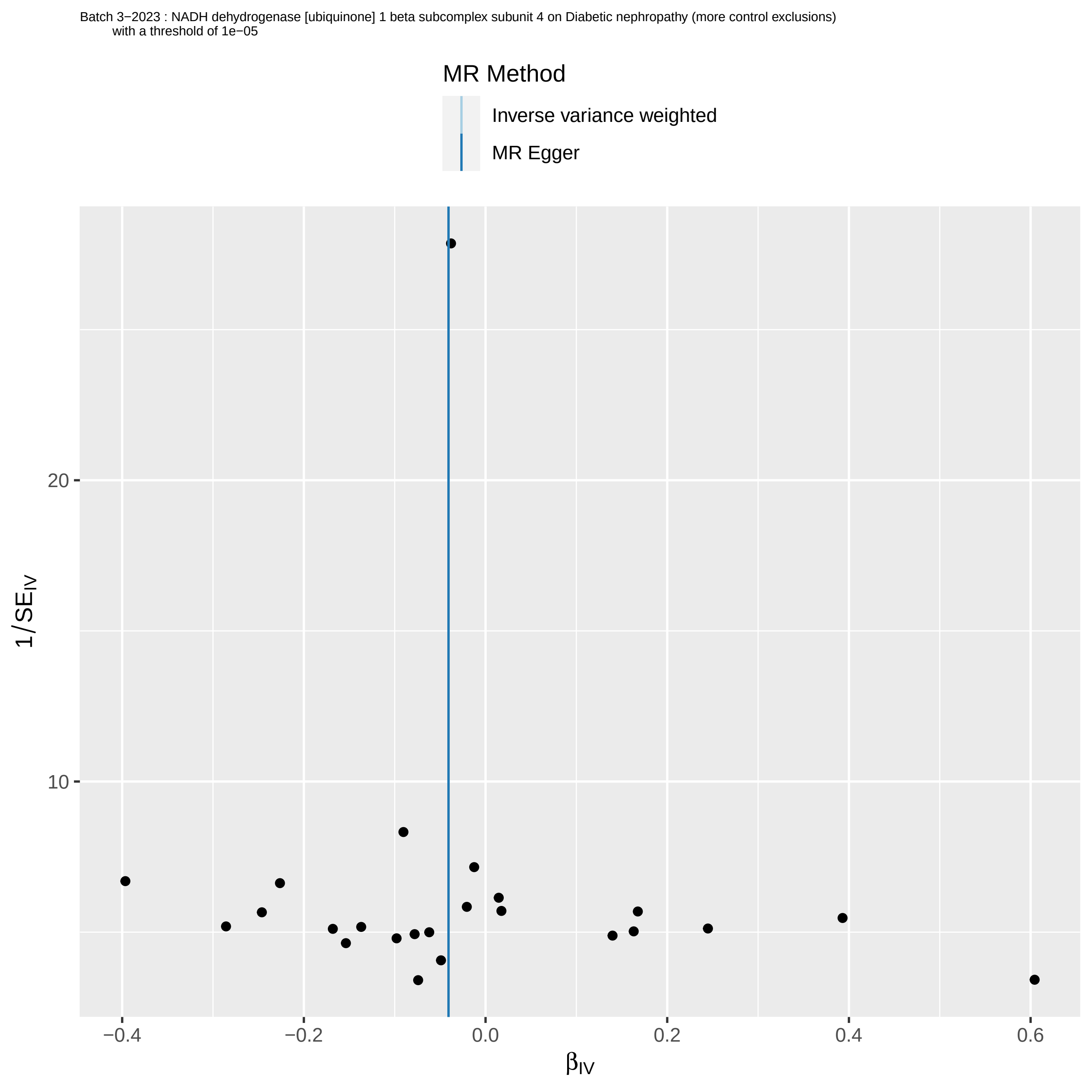
Batch 3−2023 : NADH dehydrogenase [ubiquinone] 1 beta subcomplex subunit 4 on Diabetic nephropathy (more control exclusions)
with a threshold of 1e−05
MR Method
Inverse variance weighted
MR Egger
20
10
1/SE_IV
−0.4
−0.2
0.0
0.2
0.4
0.6
β_IV

Batch 3−2024 : NADH dehydrogenase [ubiquinone] 1 beta subcomplex subunit 8, mitochondrial on Diabetic nephropathy (more control exclusions) with a threshold of 1e−05

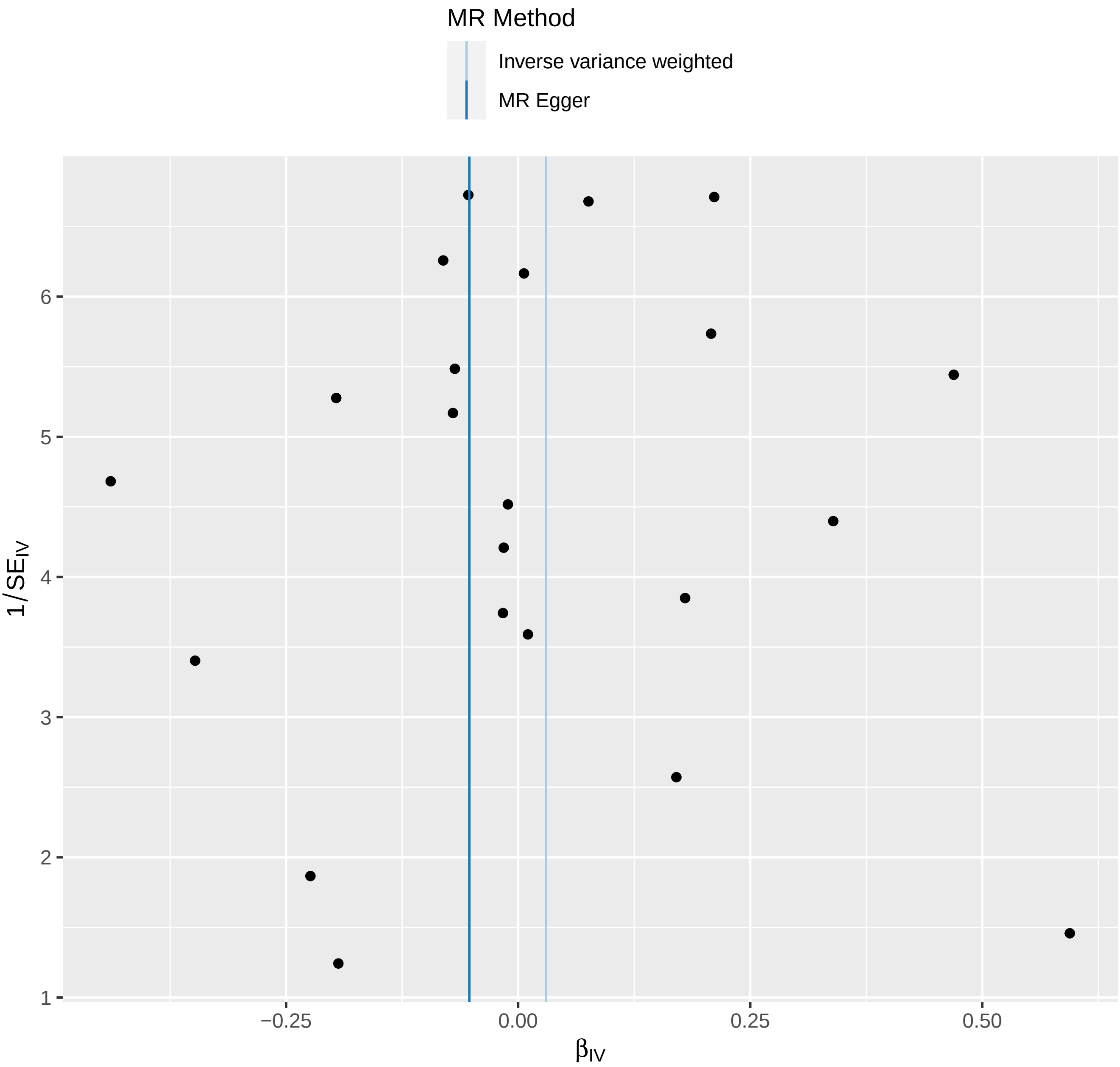

Batch 3−2025 : NADH dehydrogenase [ubiquinone] iron−sulfur protein 4, mitochondrial on Diabetic nephropathy (more control exclusions) with a threshold of 1e−05

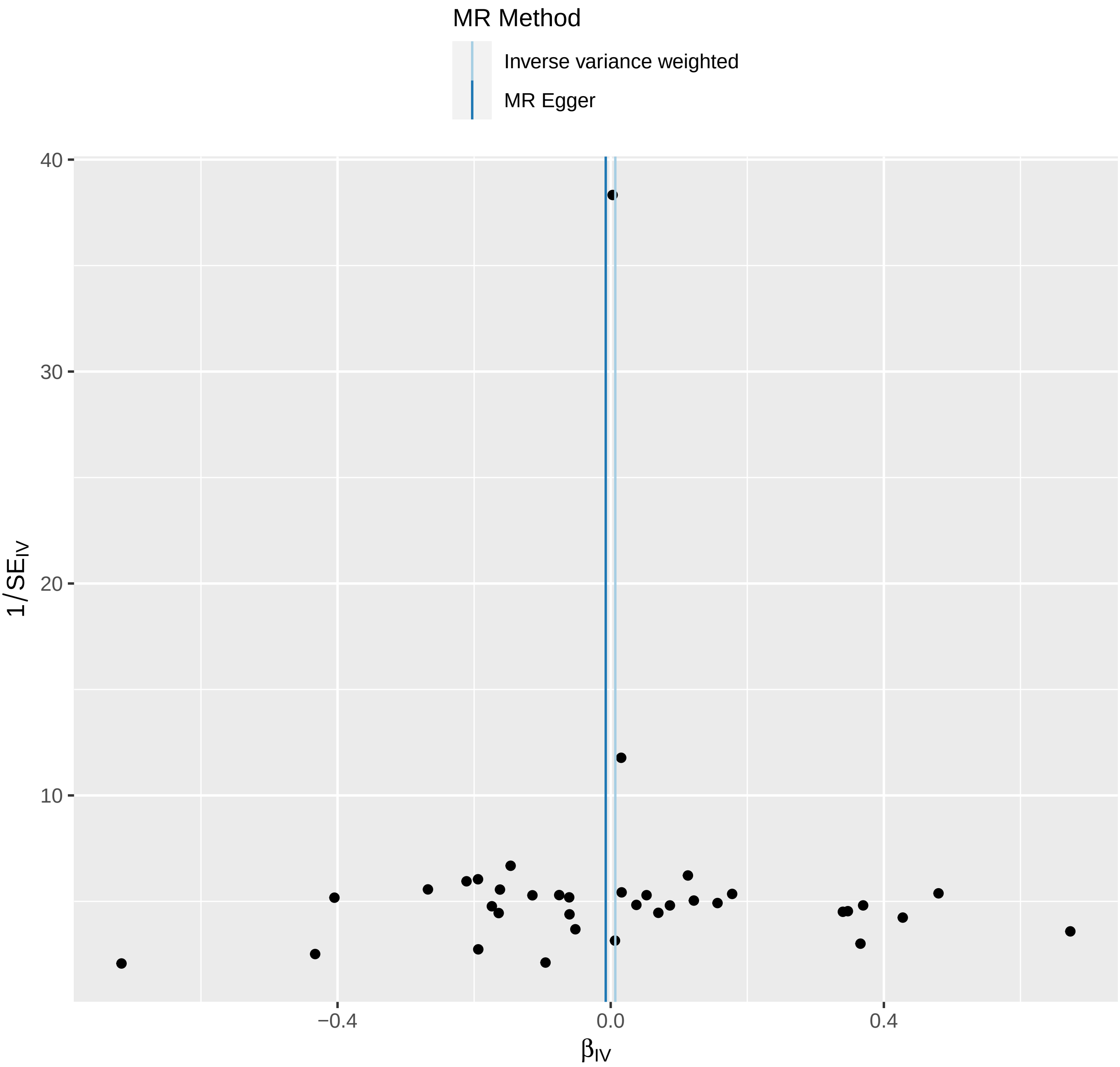

Batch 3−2026 : NADH dehydrogenase [ubiquinone] flavoprotein 2, mitochondrial on Diabetic nephropathy (more control exclusions)
with a threshold of 1e−05

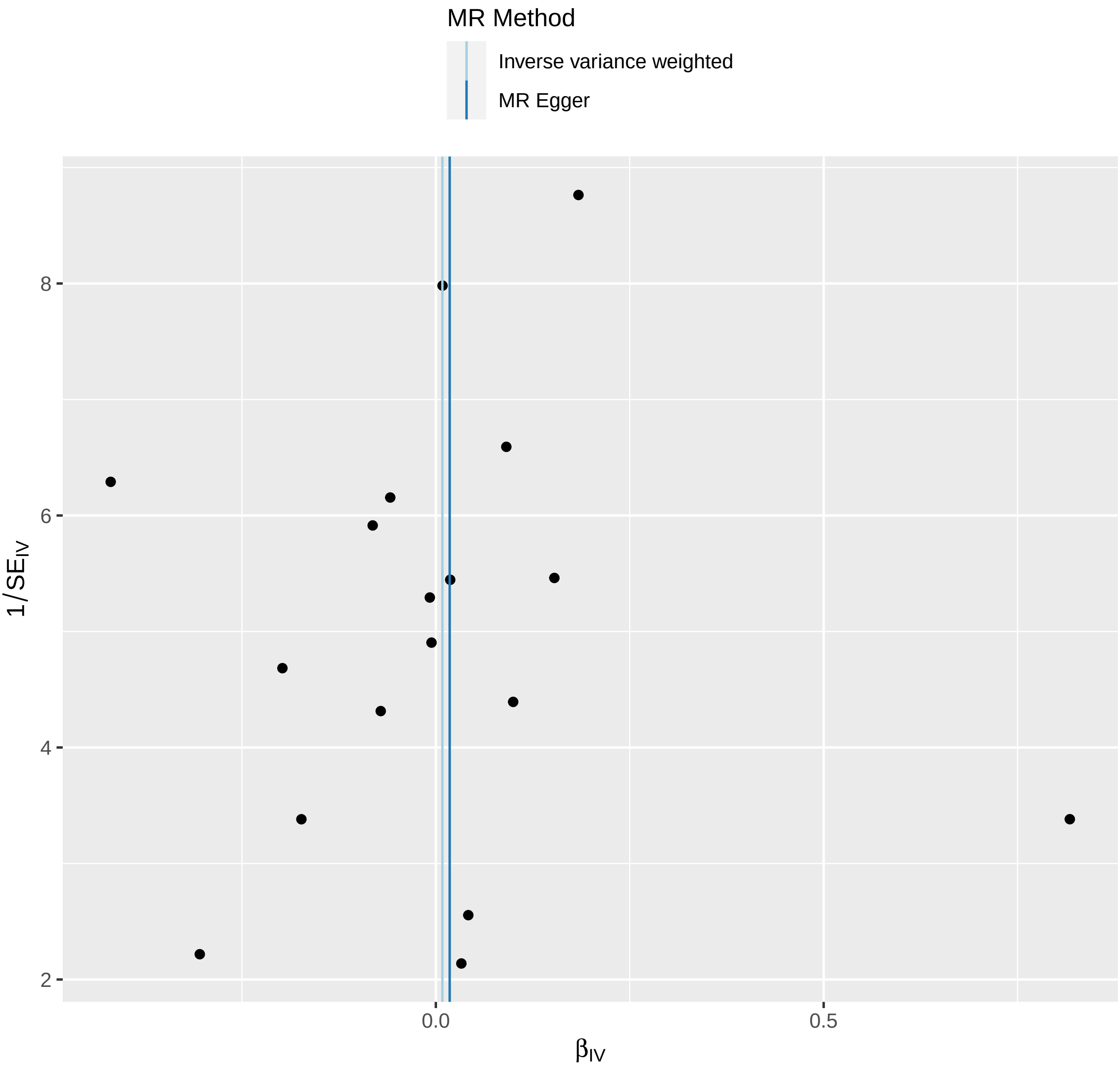

Batch 3−2027 : Neurofilament heavy polypeptide on Diabetic nephropathy (more control exclusions)
with a threshold of 1e−05

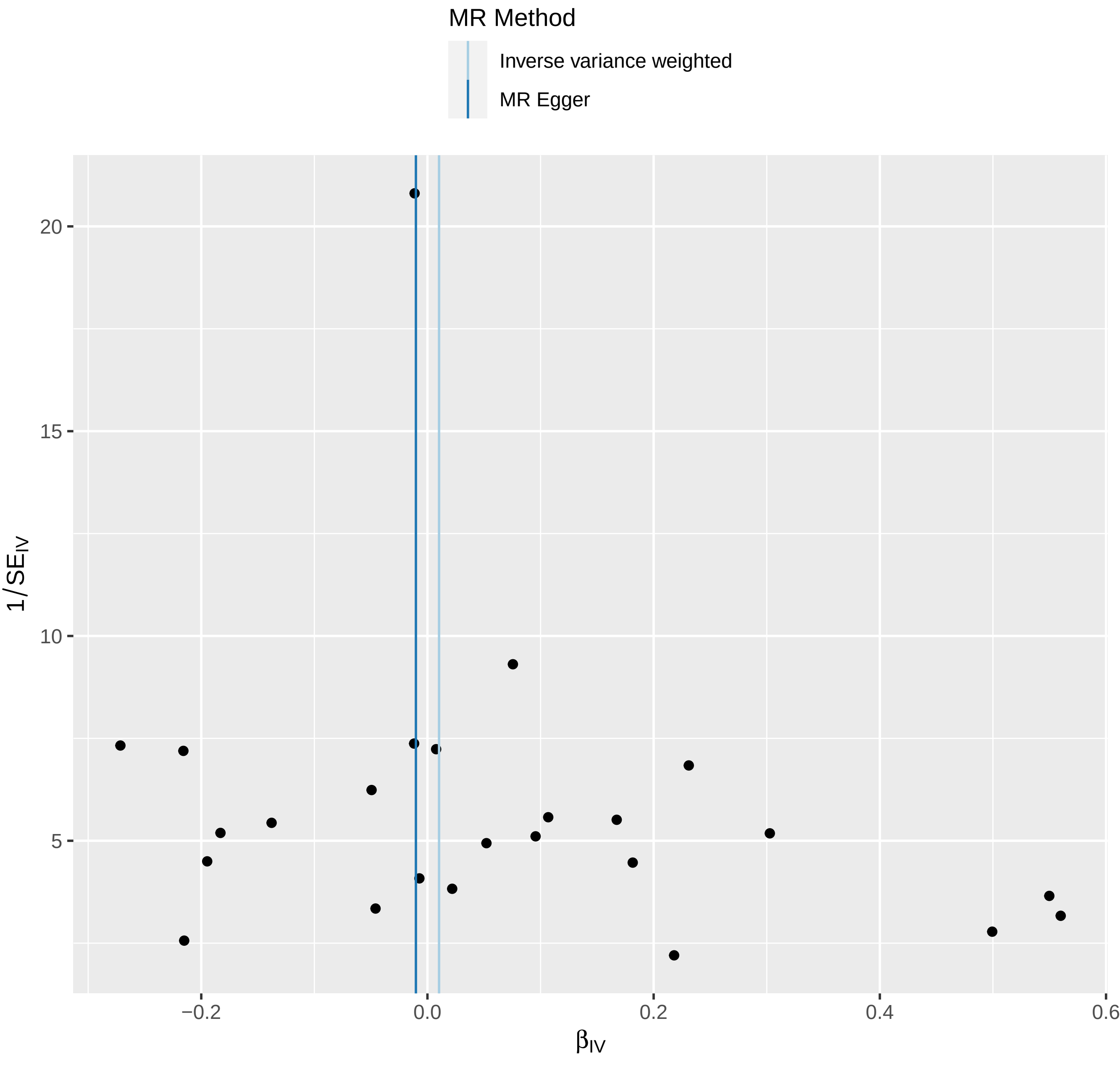

Batch 3−2028 : Neuronal growth regulator 1 on Diabetic nephropathy (more control exclusions)
with a threshold of 1e−05

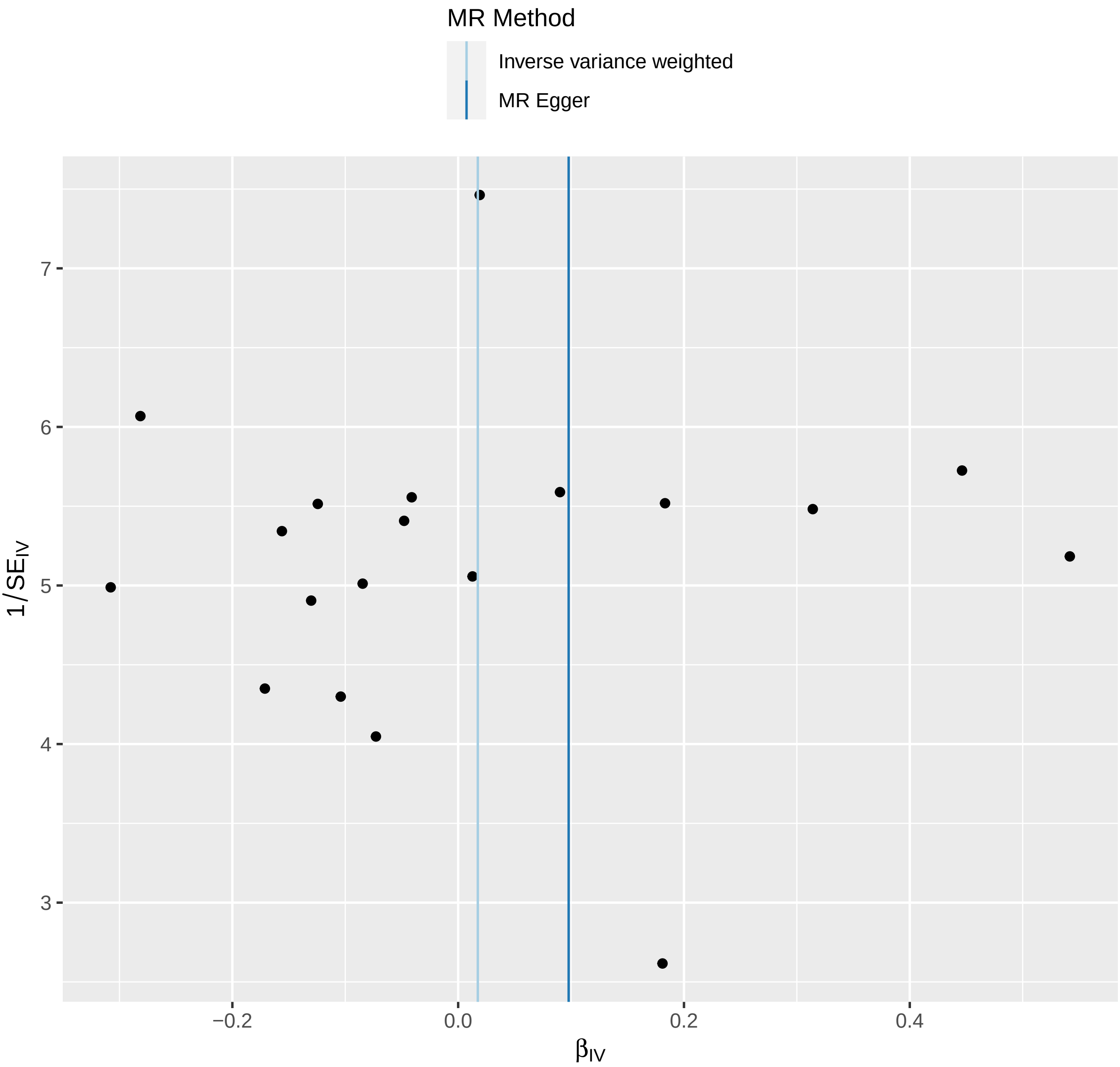

Batch 3−2029 : Neuronal growth regulator 1 on Diabetic nephropathy (more control exclusions)
with a threshold of 1e−05

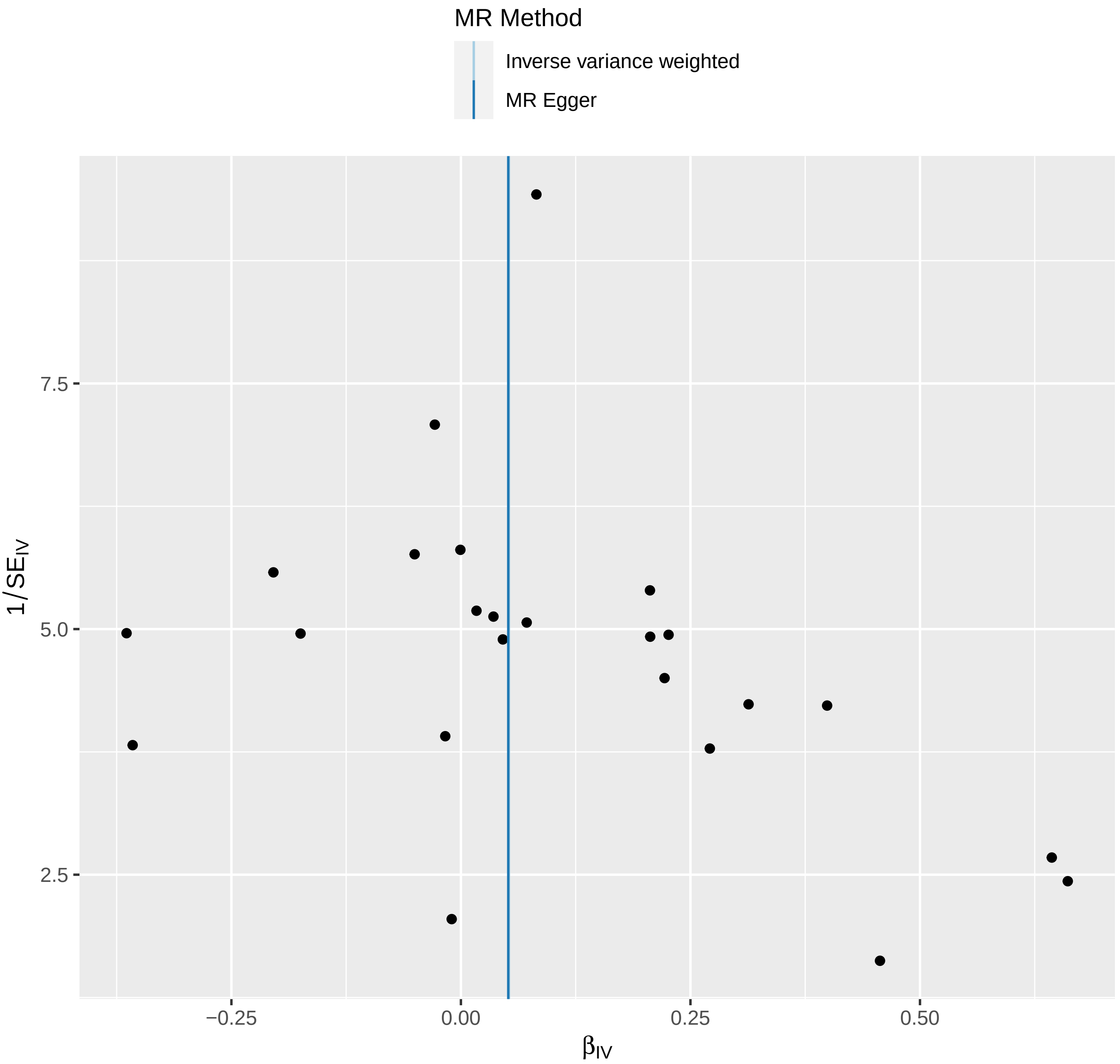

Batch 3−2030 : Protein kinase C−binding protein NELL1 on Diabetic nephropathy (more control exclusions)
with a threshold of 1e−05

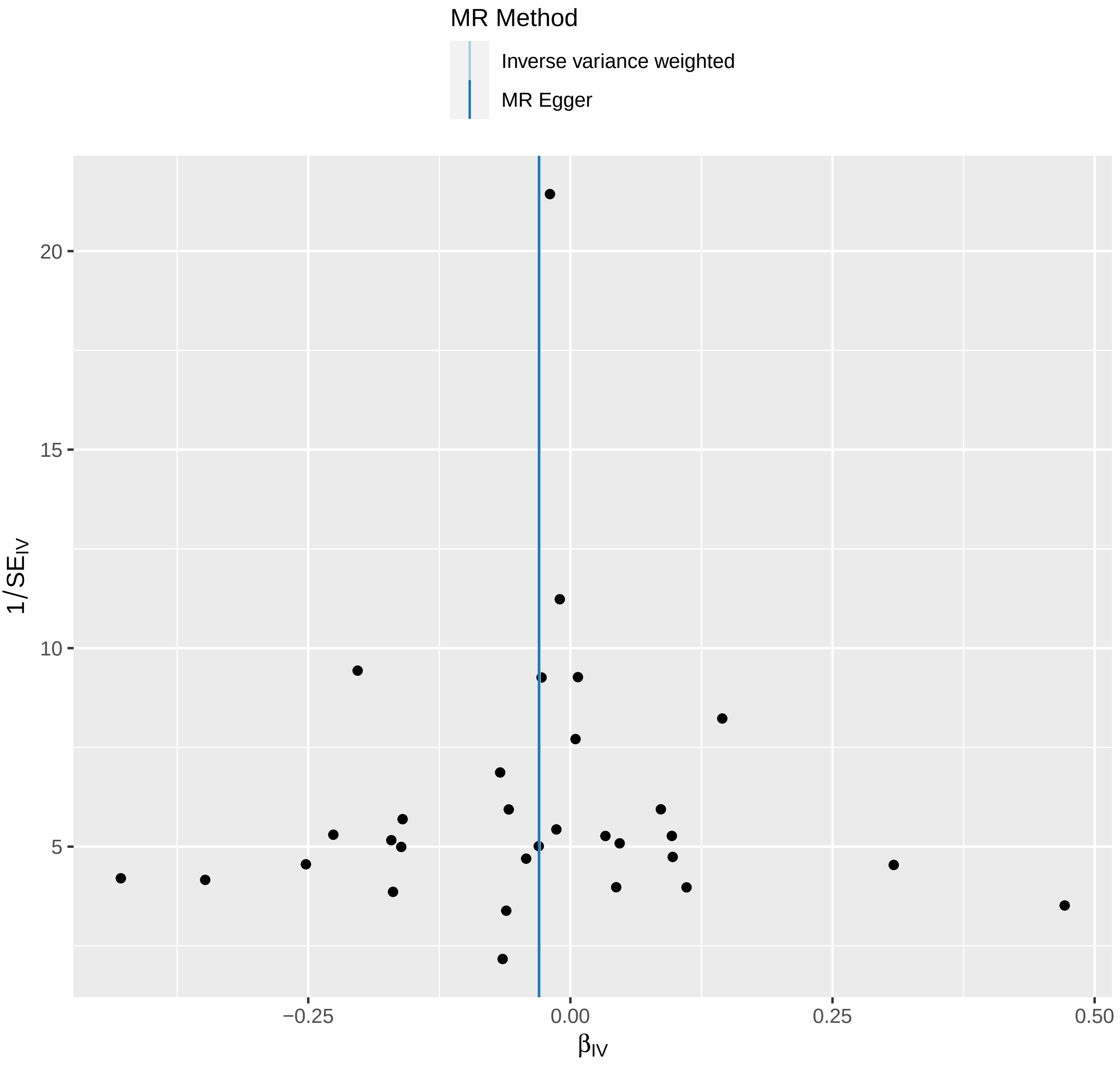

Batch 3−2031 : Protein kinase C−binding protein NELL2 on Diabetic nephropathy (more control exclusions)
with a threshold of 1e−05

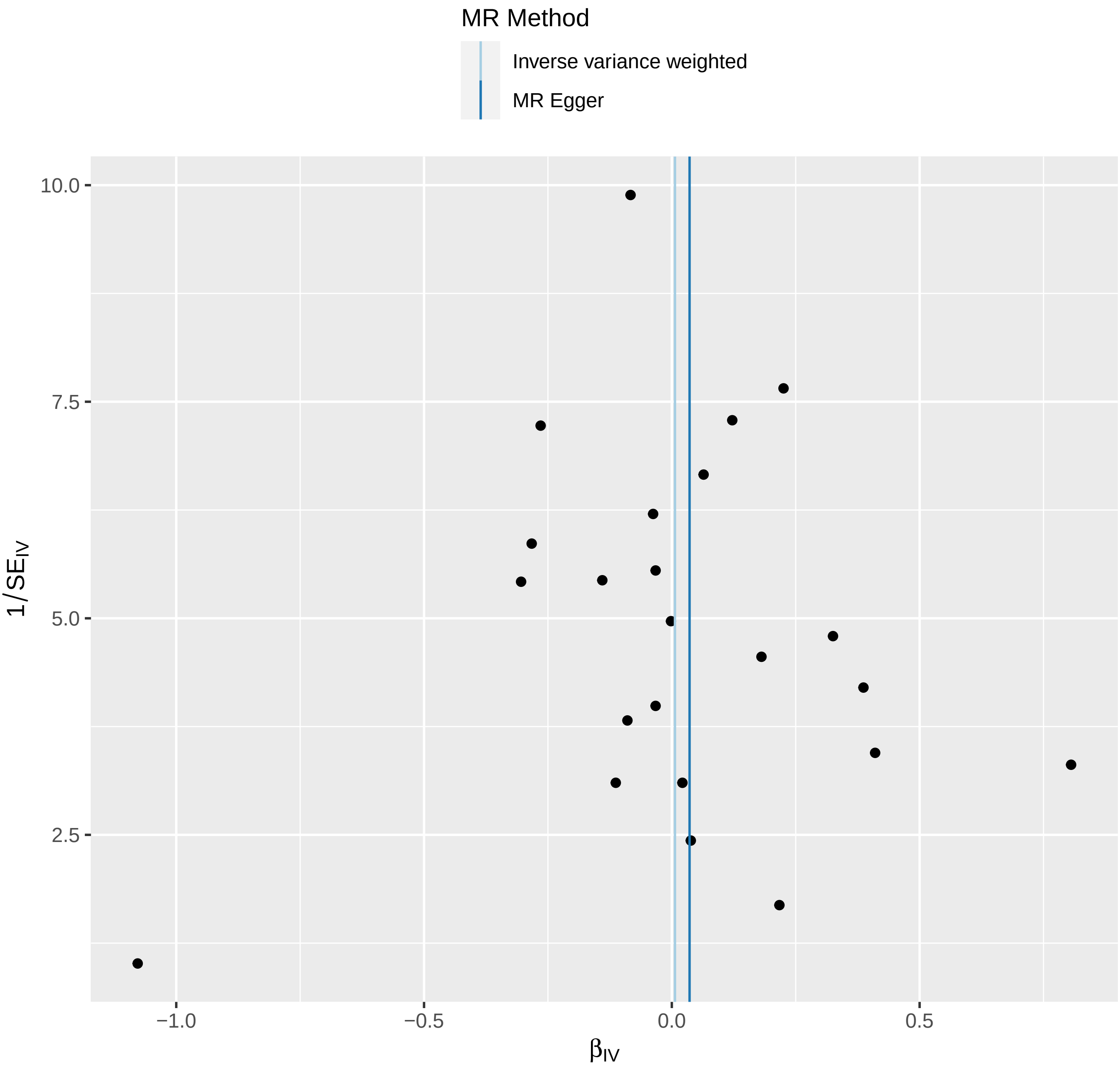

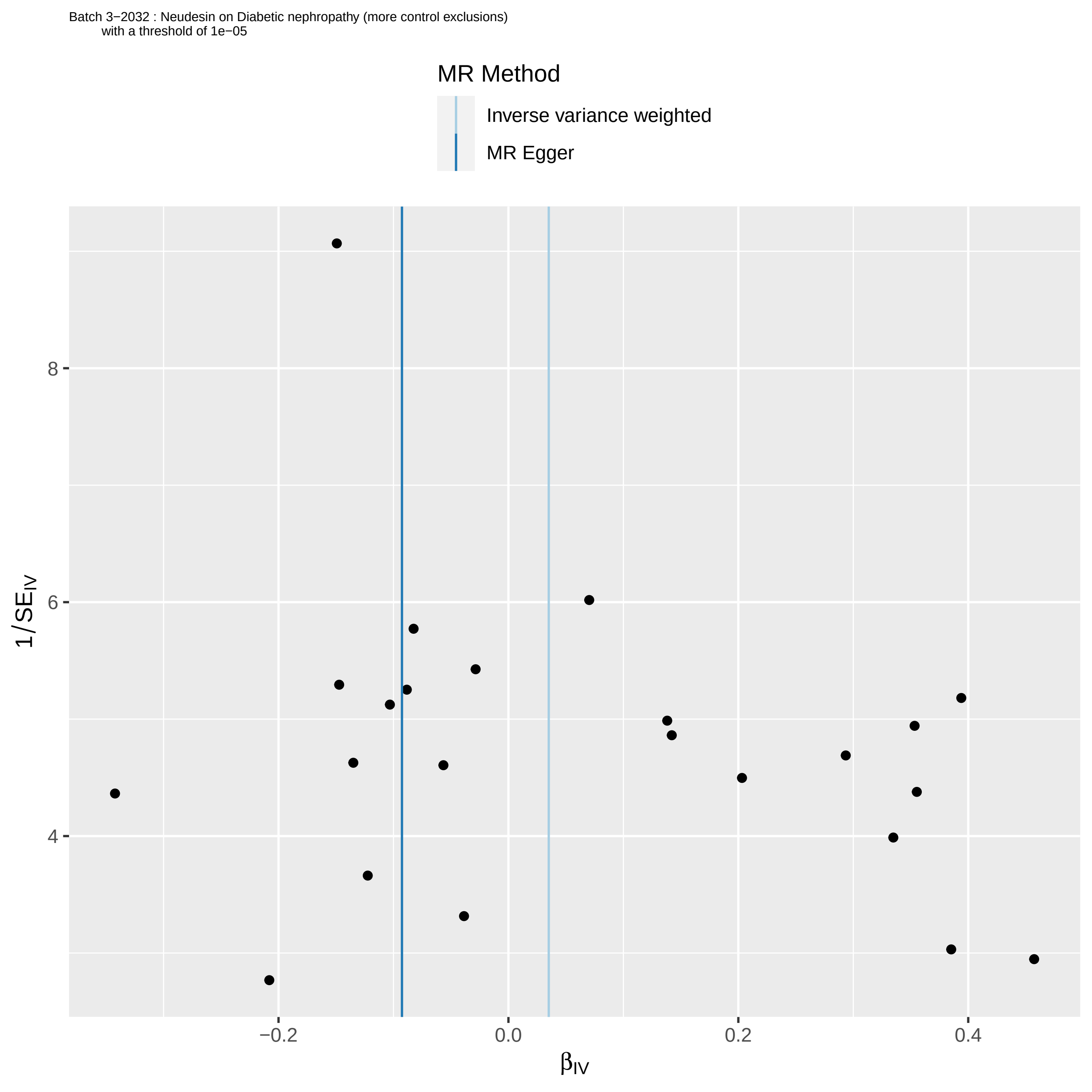
Batch 3−2032 : Neudesin on Diabetic nephropathy (more control exclusions)
with a threshold of 1e−05
MR Method
Inverse variance weighted
MR Egger
8
6
4
1/SE_IV
−0.2
0.0
0.2
0.4
β_IV

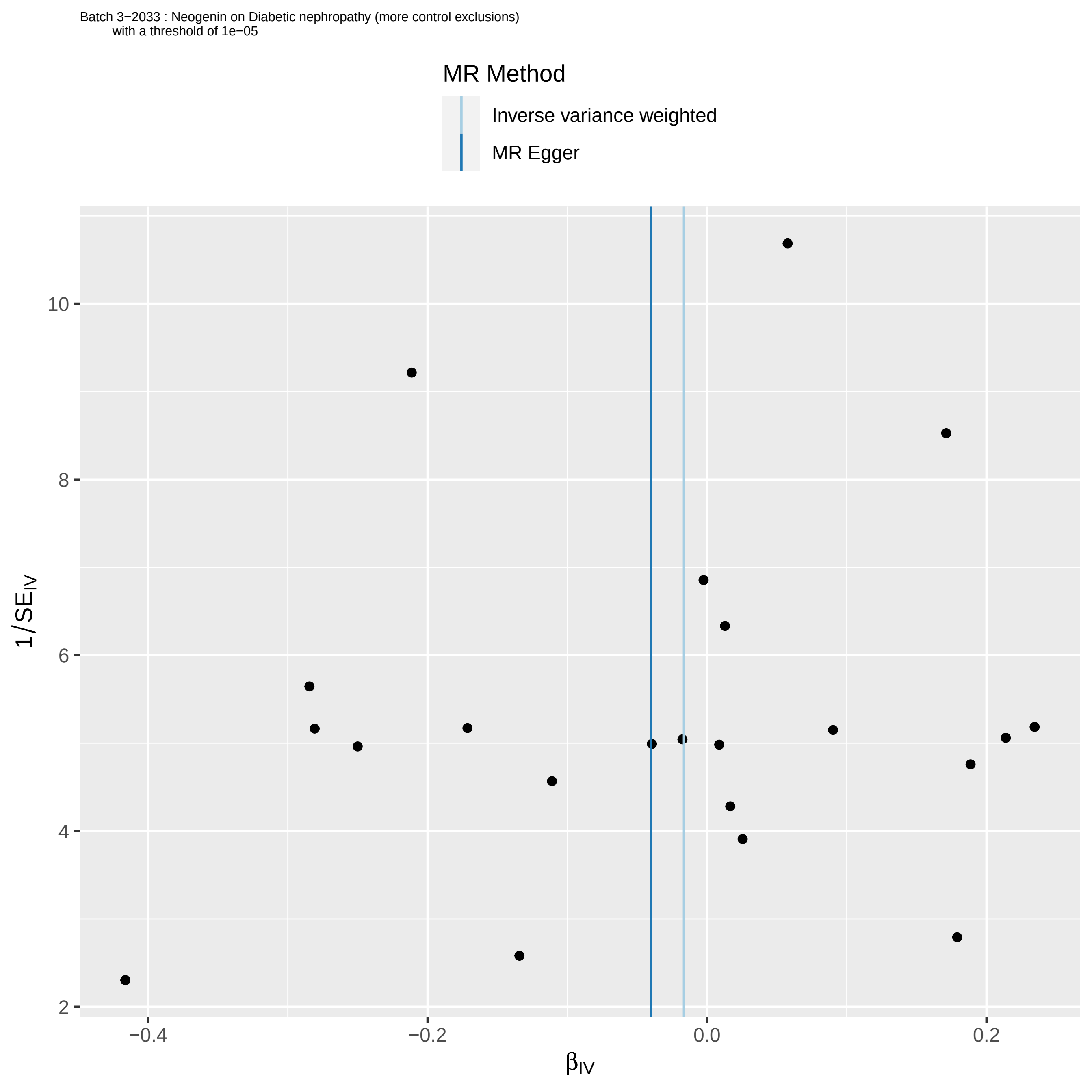
Batch 3−2033 : Neogenin on Diabetic nephropathy (more control exclusions)
with a threshold of 1e−05
MR Method
Inverse variance weighted
MR Egger
1/SE_IV
10
8
6
4
2
−0.4
−0.2
0.0
0.2
β_IV

Batch 3−2034 : Neuroepithelial cell−transforming gene 1 protein on Diabetic nephropathy (more control exclusions)
with a threshold of 1e−05

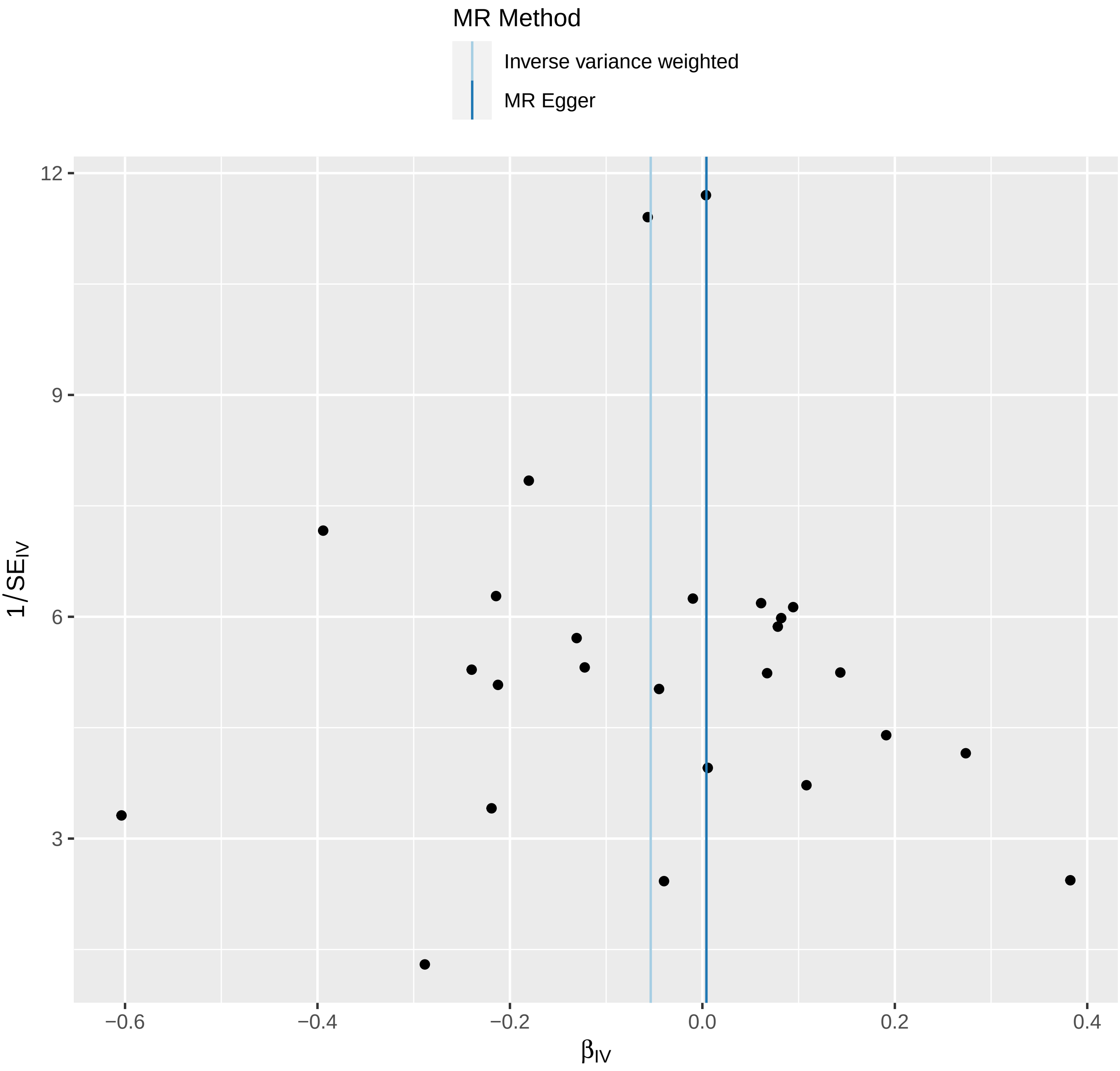

Batch 3−2035 : Neuropilin and tolloid−like protein 1 on Diabetic nephropathy (more control exclusions)
with a threshold of 1e−05

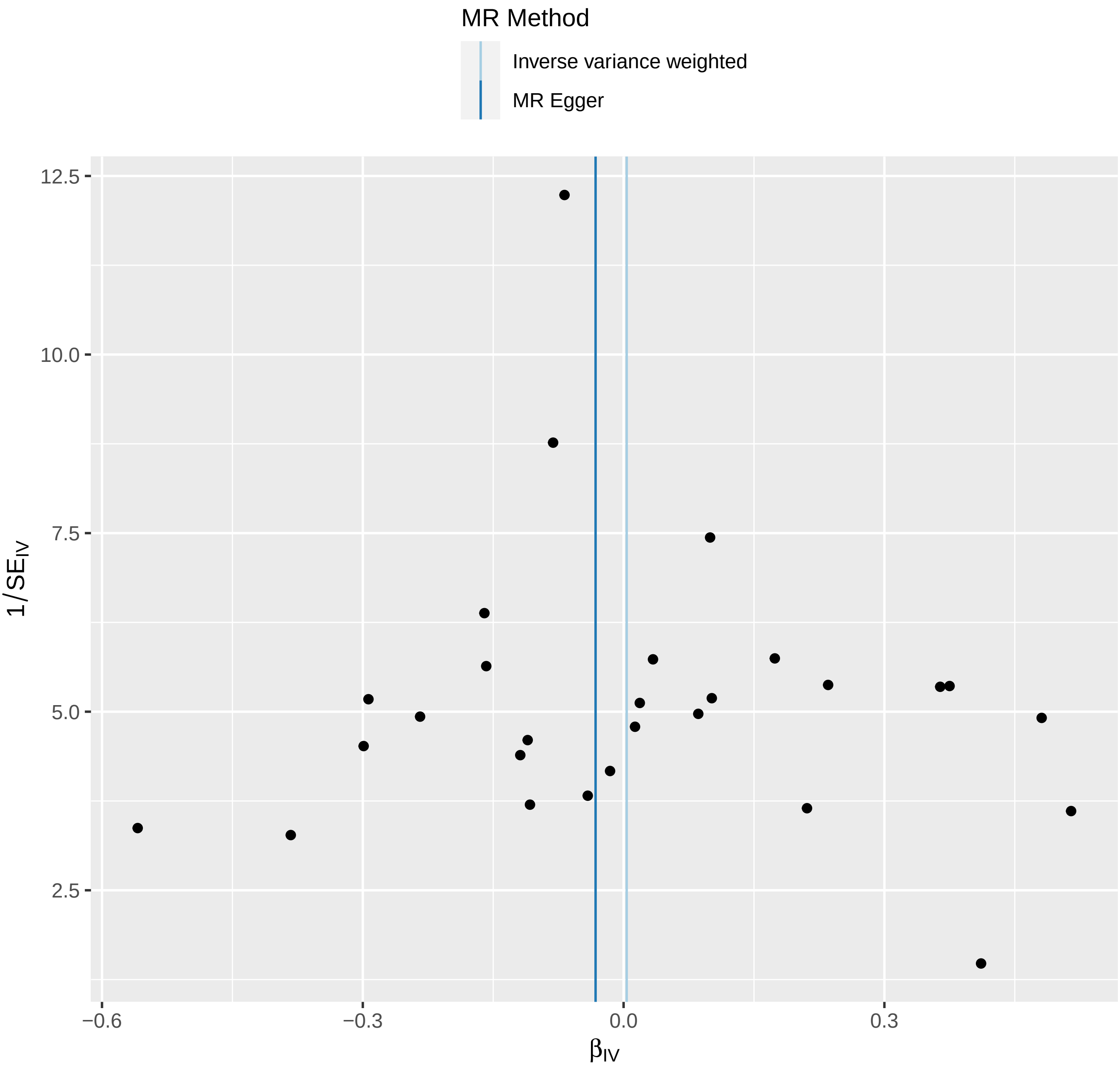

Batch 3−2036 : Neuropilin and tolloid−like protein 2 on Diabetic nephropathy (more control exclusions)
with a threshold of 1e−05

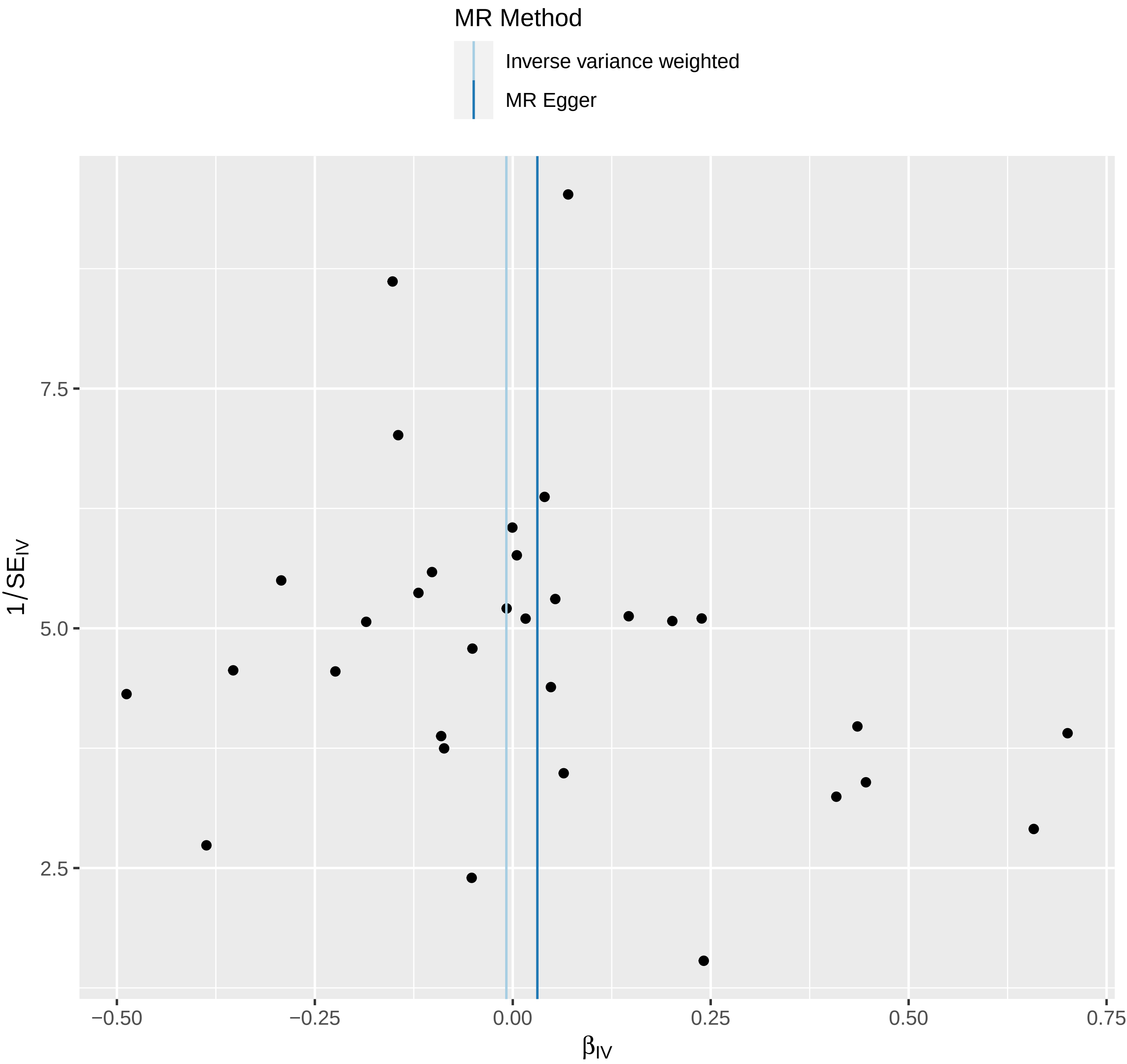

Batch 3−2037 : E3 ubiquitin−protein ligase NEURL1 on Diabetic nephropathy (more control exclusions)
with a threshold of 1e−05

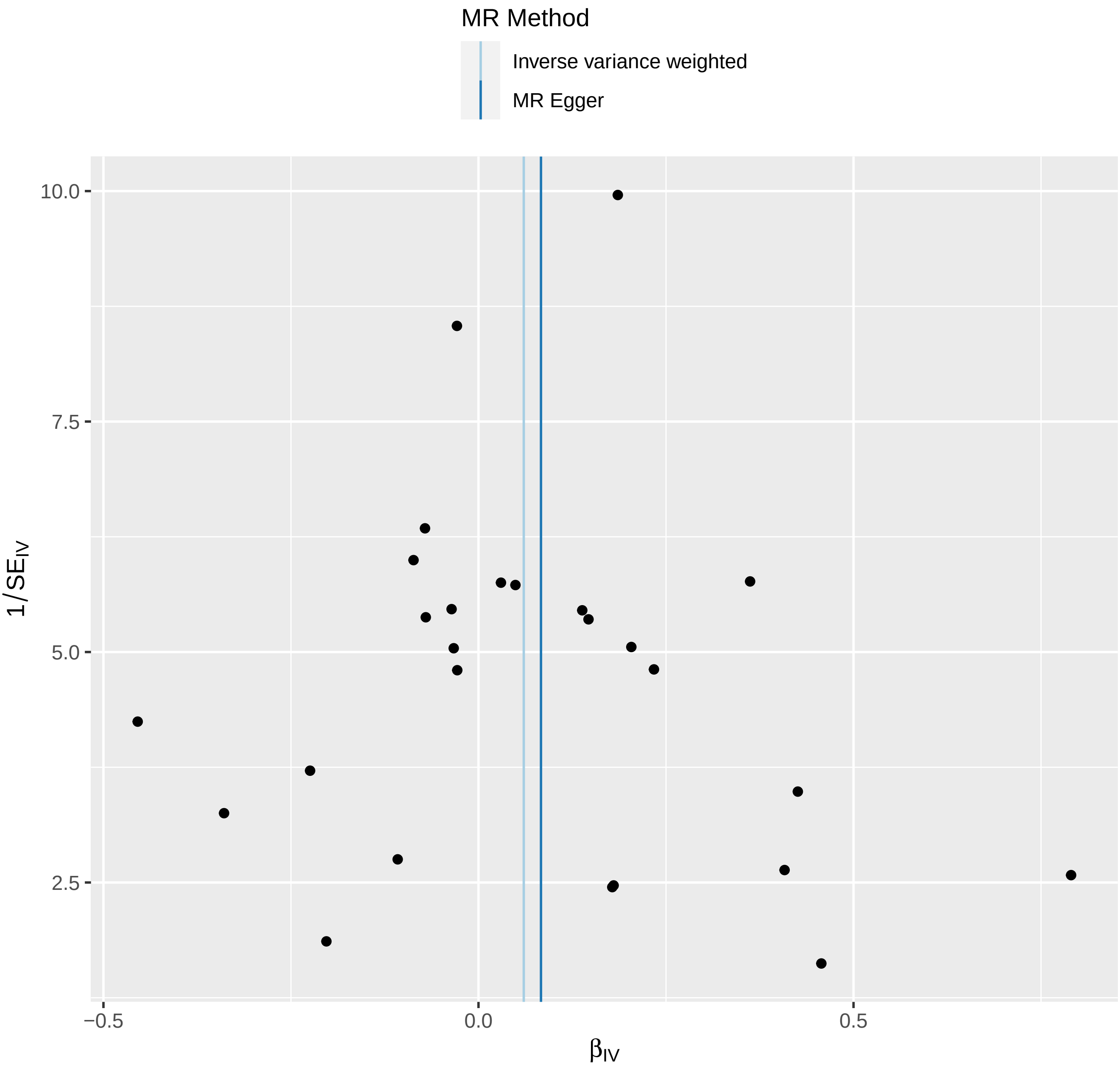

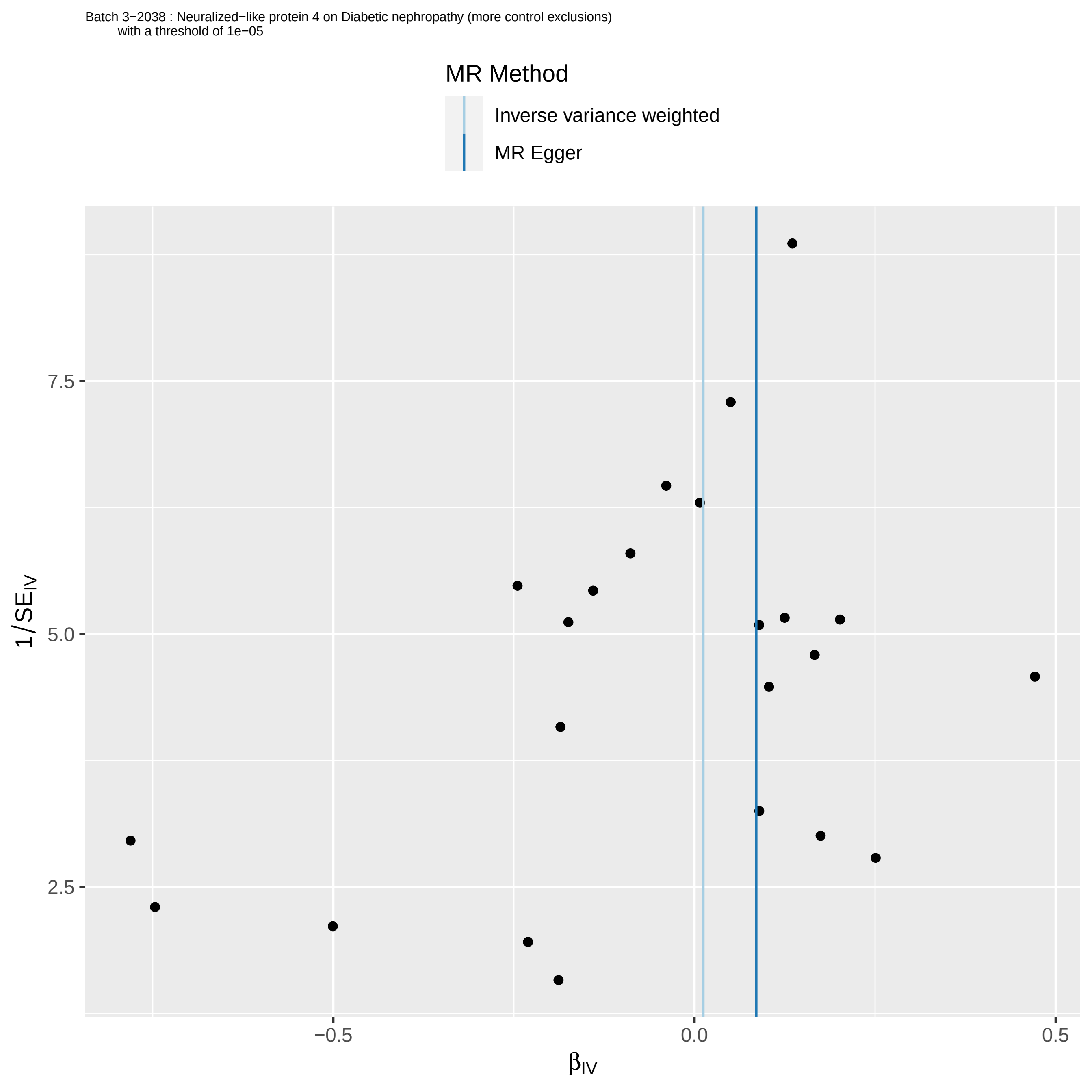

Batch 3−2038 : Neuralized−like protein 4 on Diabetic nephropathy (more control exclusions)
with a threshold of 1e−05
MR Method
Inverse variance weighted
MR Egger
7.5
5.0
2.5
1/SE_IV
−0.5
0.0
0.5
β_IV

Batch 3−2039 : Neurofascin on Diabetic nephropathy (more control exclusions)
with a threshold of 1e−05

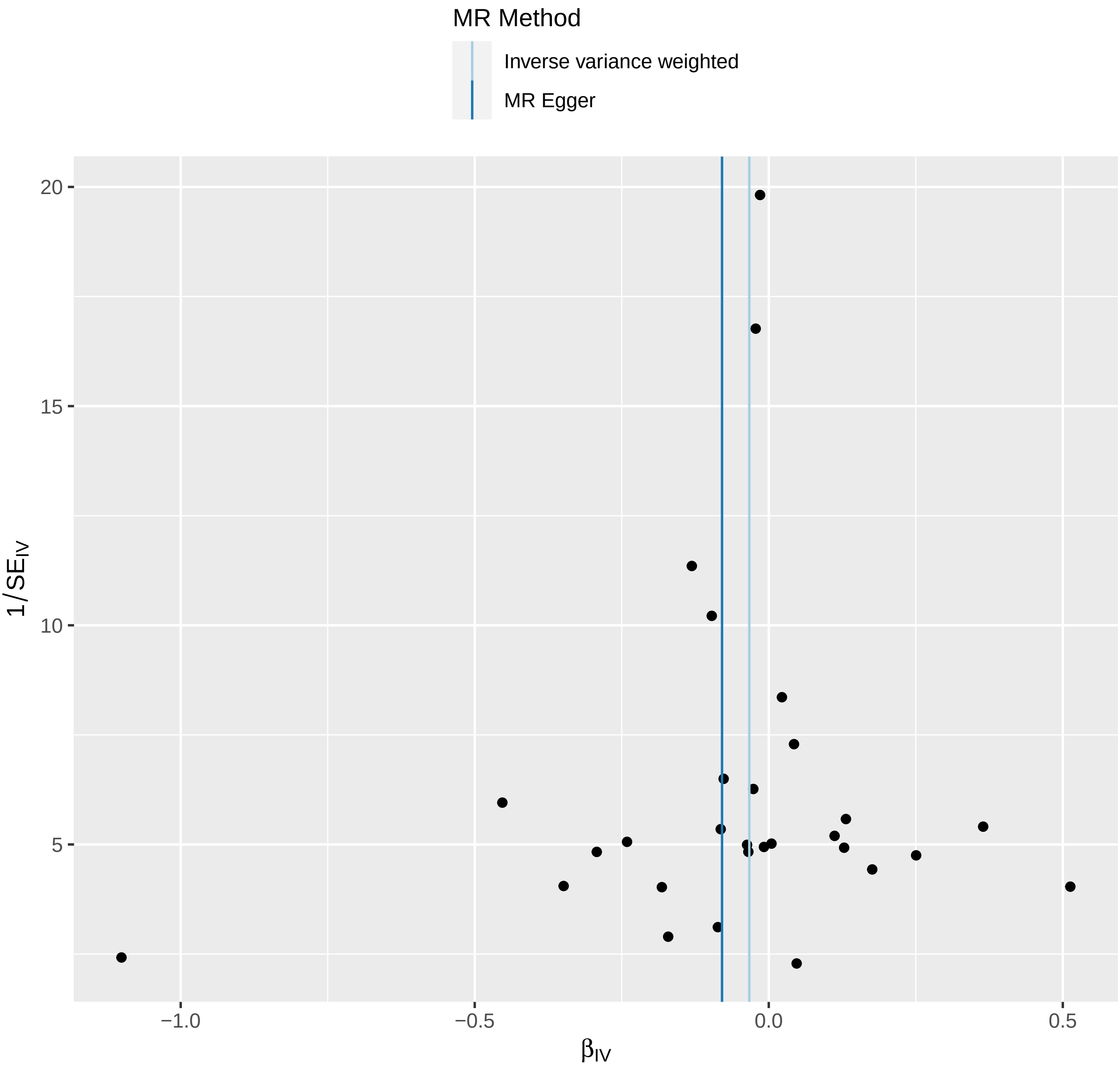

Batch 3−2040 : Nuclear factor erythroid 2−related factor 1 on Diabetic nephropathy (more control exclusions)
with a threshold of 1e−05

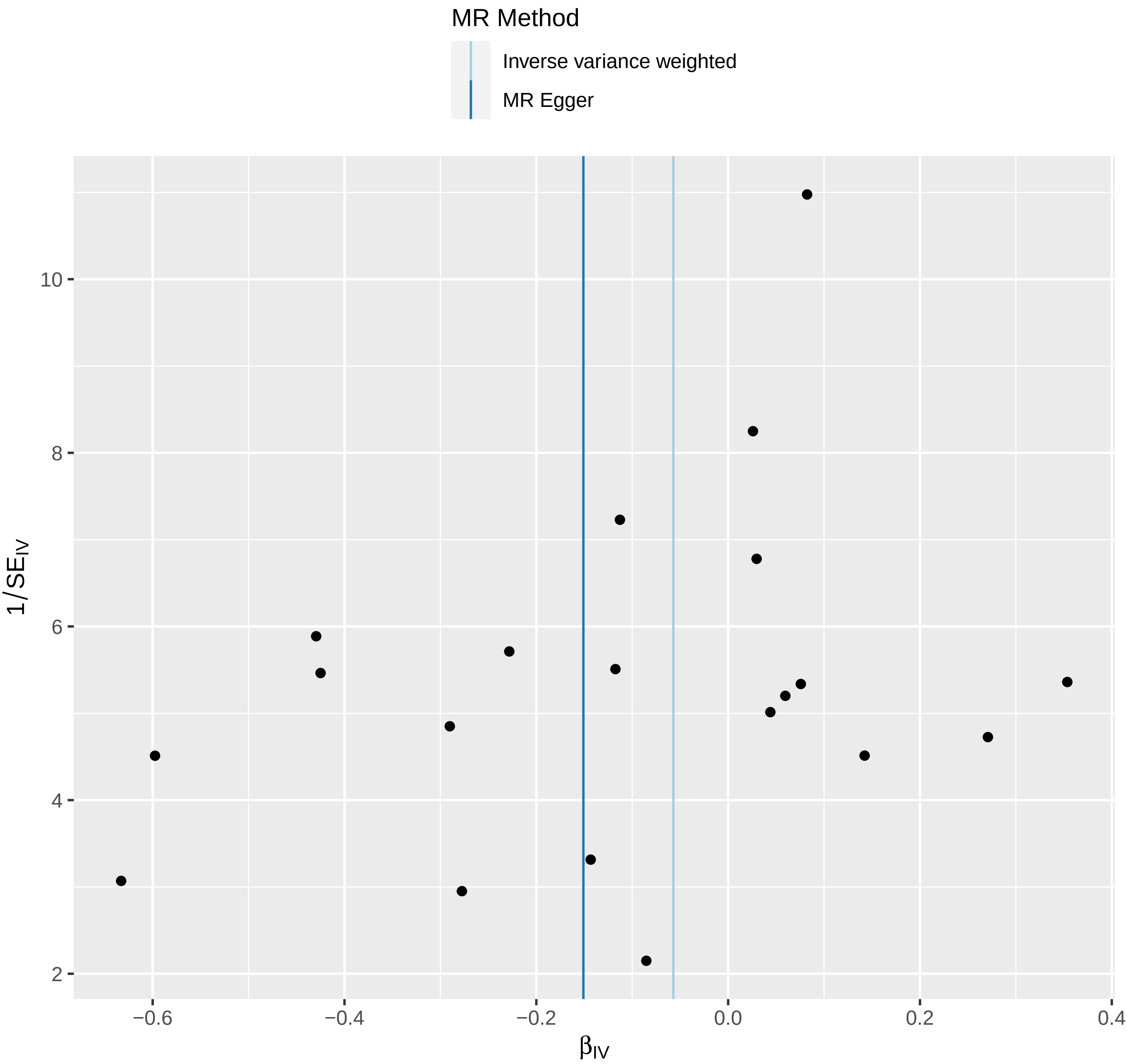

Batch 3−2041 : NFU1 iron−sulfur cluster scaffold homolog, mitochondrial on Diabetic nephropathy (more control exclusions) with a threshold of 1e−05

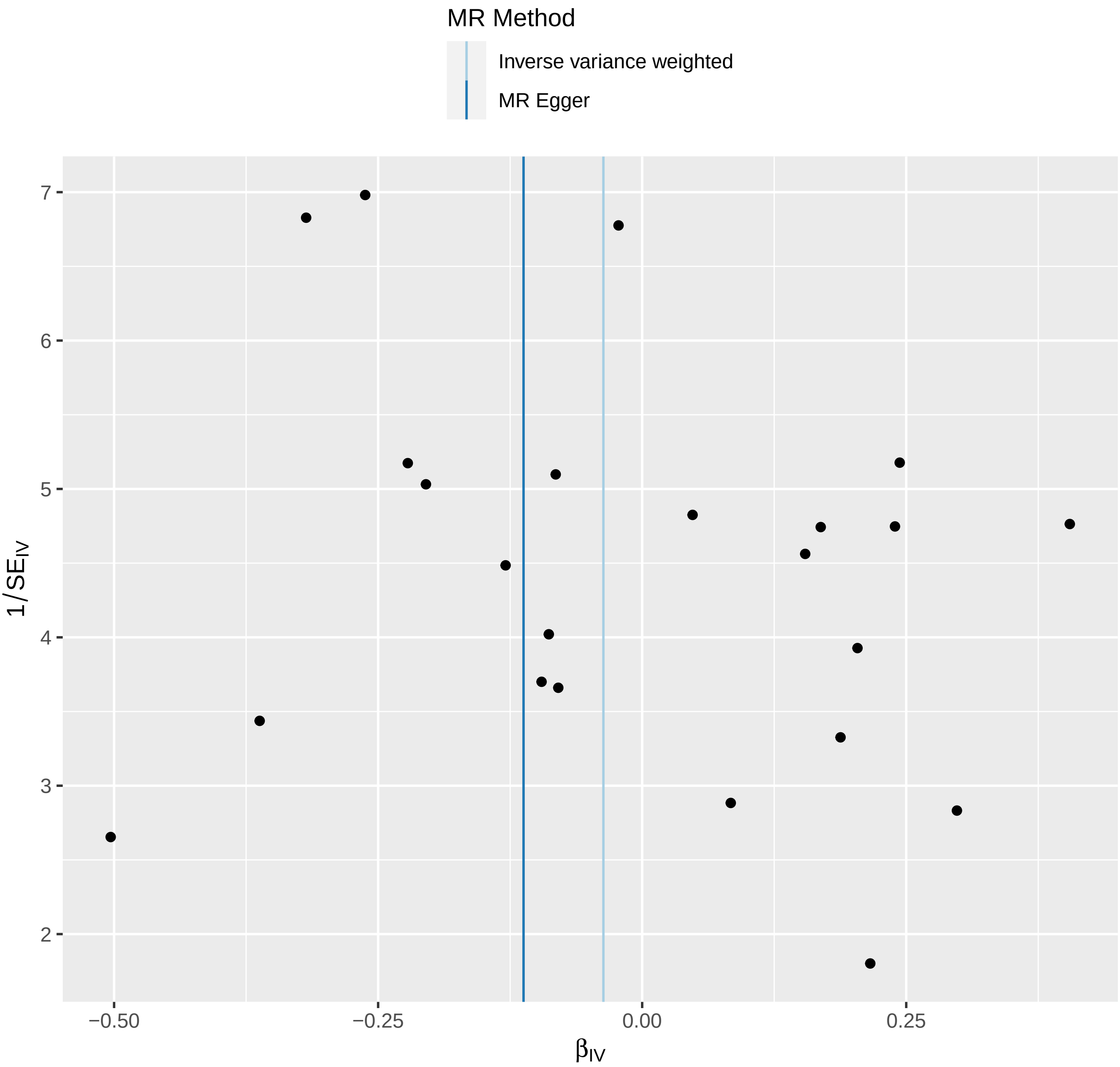

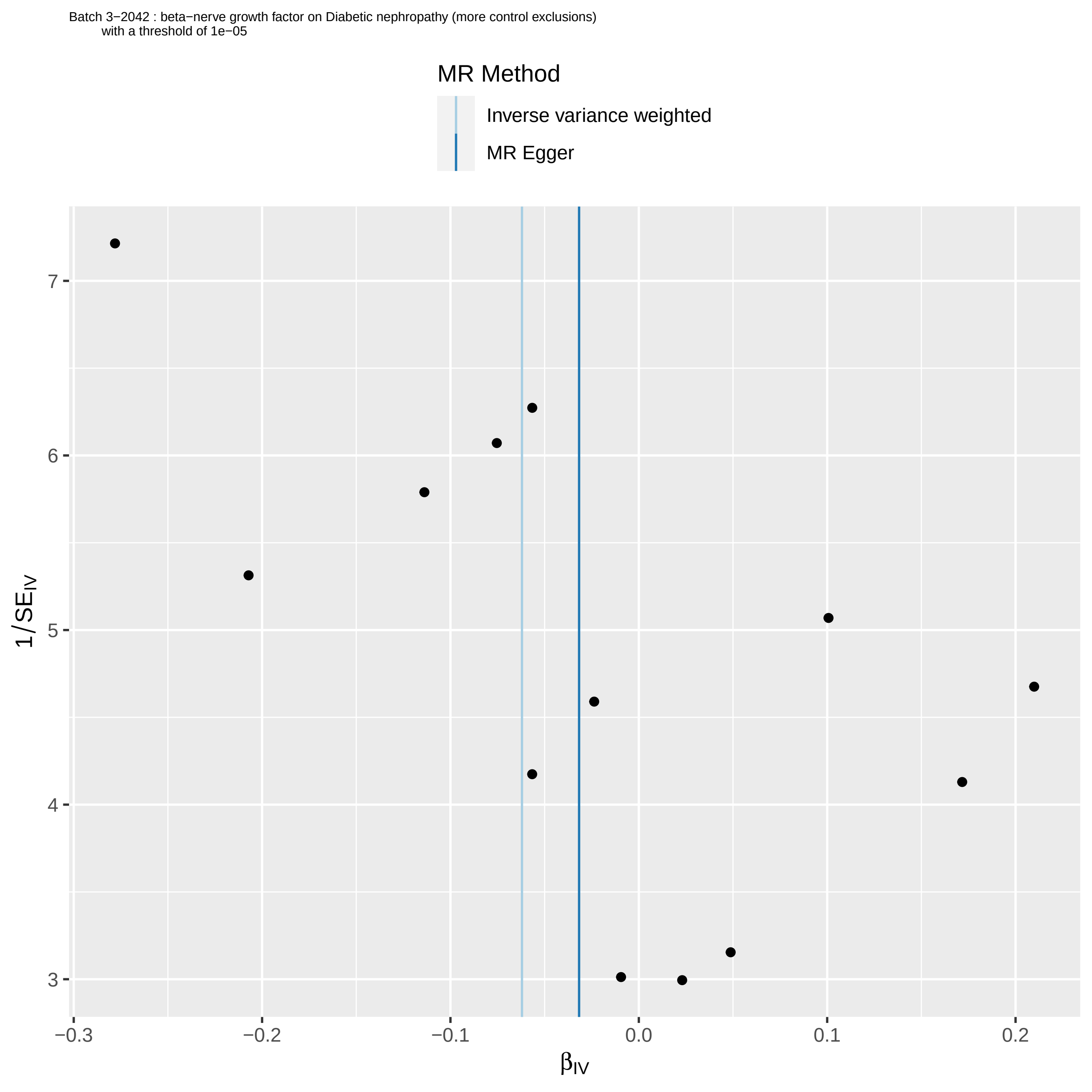
Batch 3−2042 : beta−nerve growth factor on Diabetic nephropathy (more control exclusions)
with a threshold of 1e−05
MR Method
Inverse variance weighted
MR Egger
1/SE_IV
β_IV
−0.3
−0.2
−0.1
0.0
0.1
0.2
3
4
5
6
7

Batch 3−2043 : Tumor necrosis factor receptor superfamily member 16 on Diabetic nephropathy (more control exclusions)
with a threshold of 1e−05

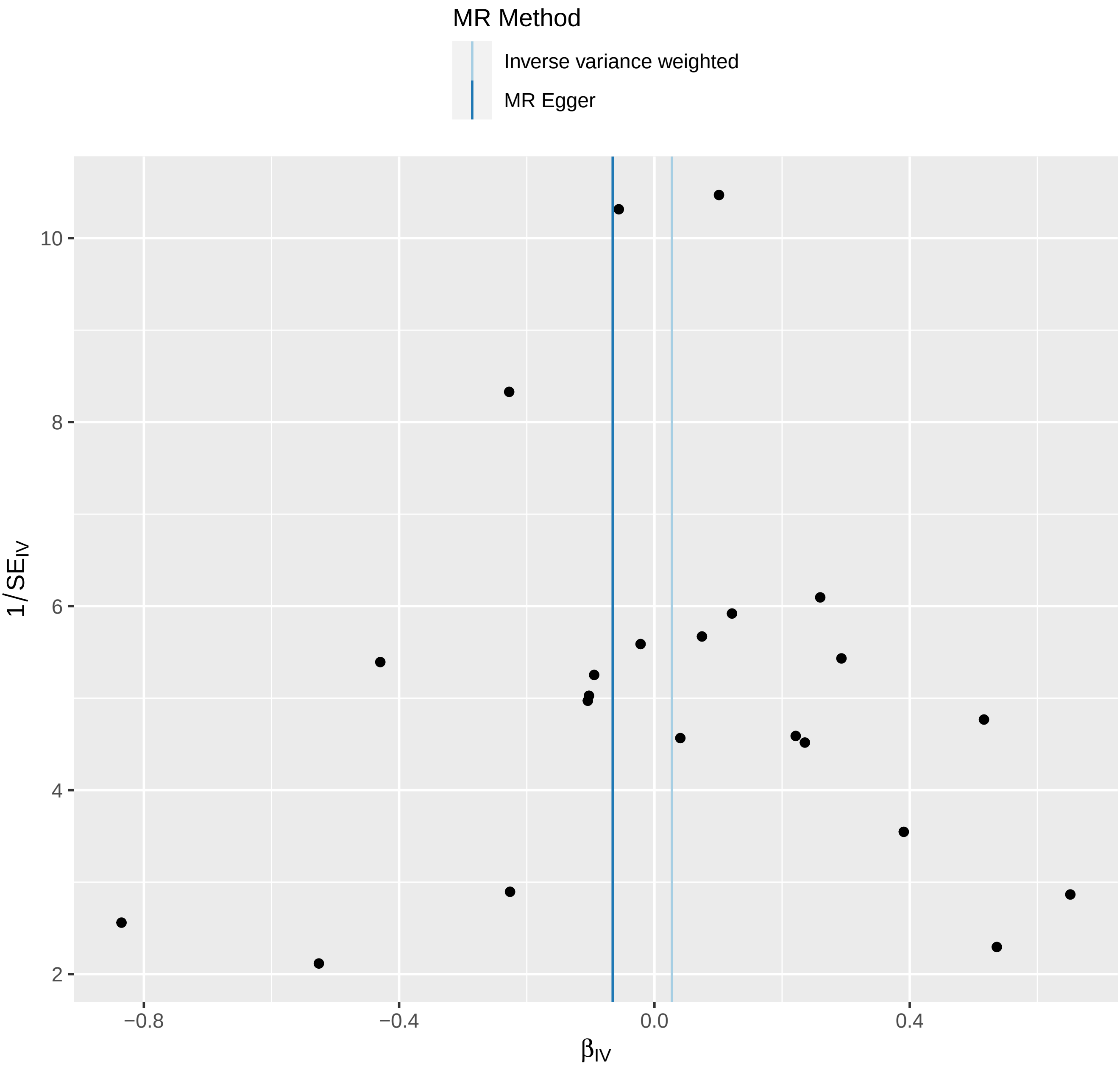

Batch 3−2044 : Tumor necrosis factor receptor superfamily member 16 on Diabetic nephropathy (more control exclusions)
with a threshold of 1e−05

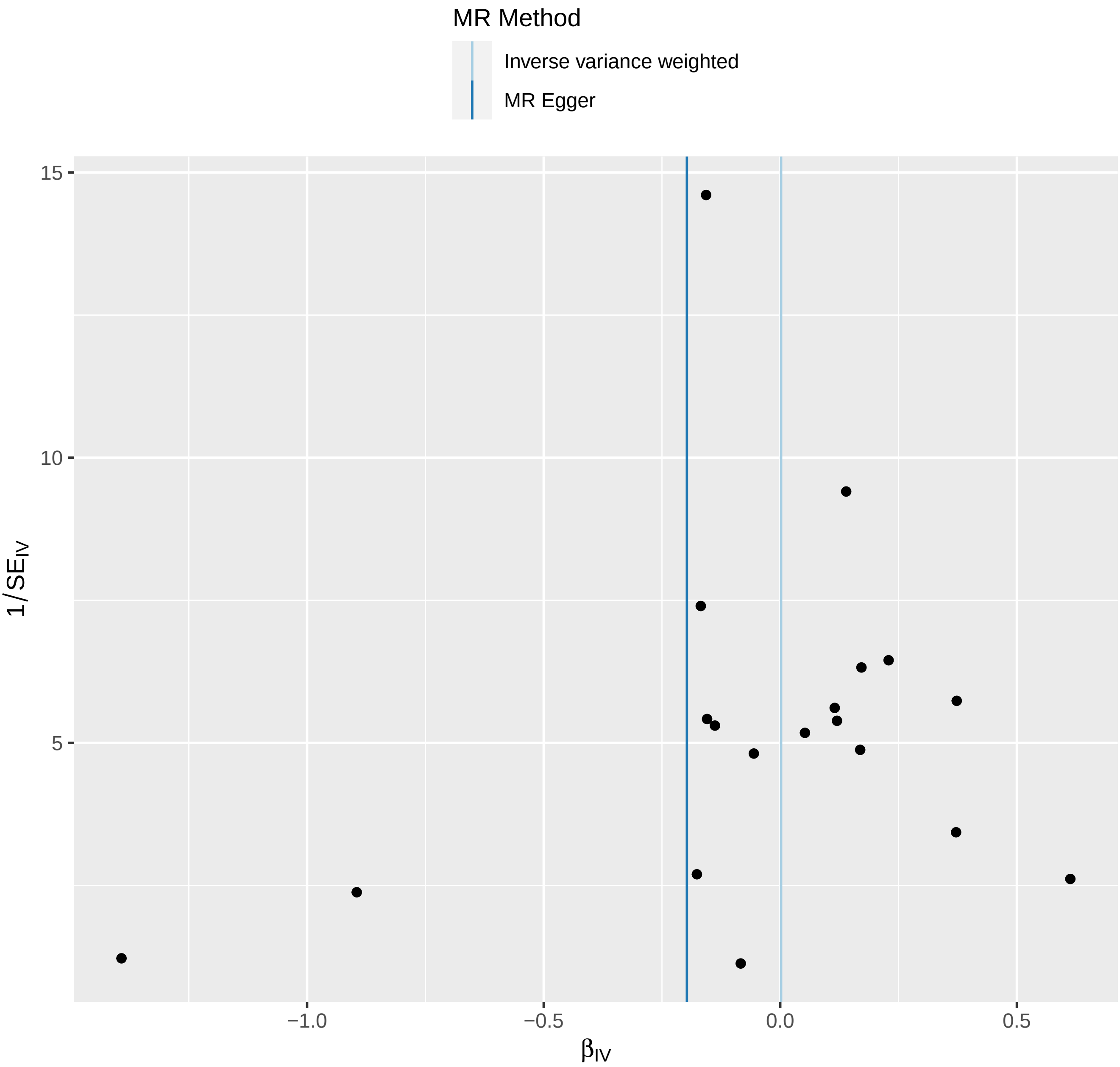

Batch 3−2045 : Neugrin on Diabetic nephropathy (more control exclusions)
with a threshold of 1e−05

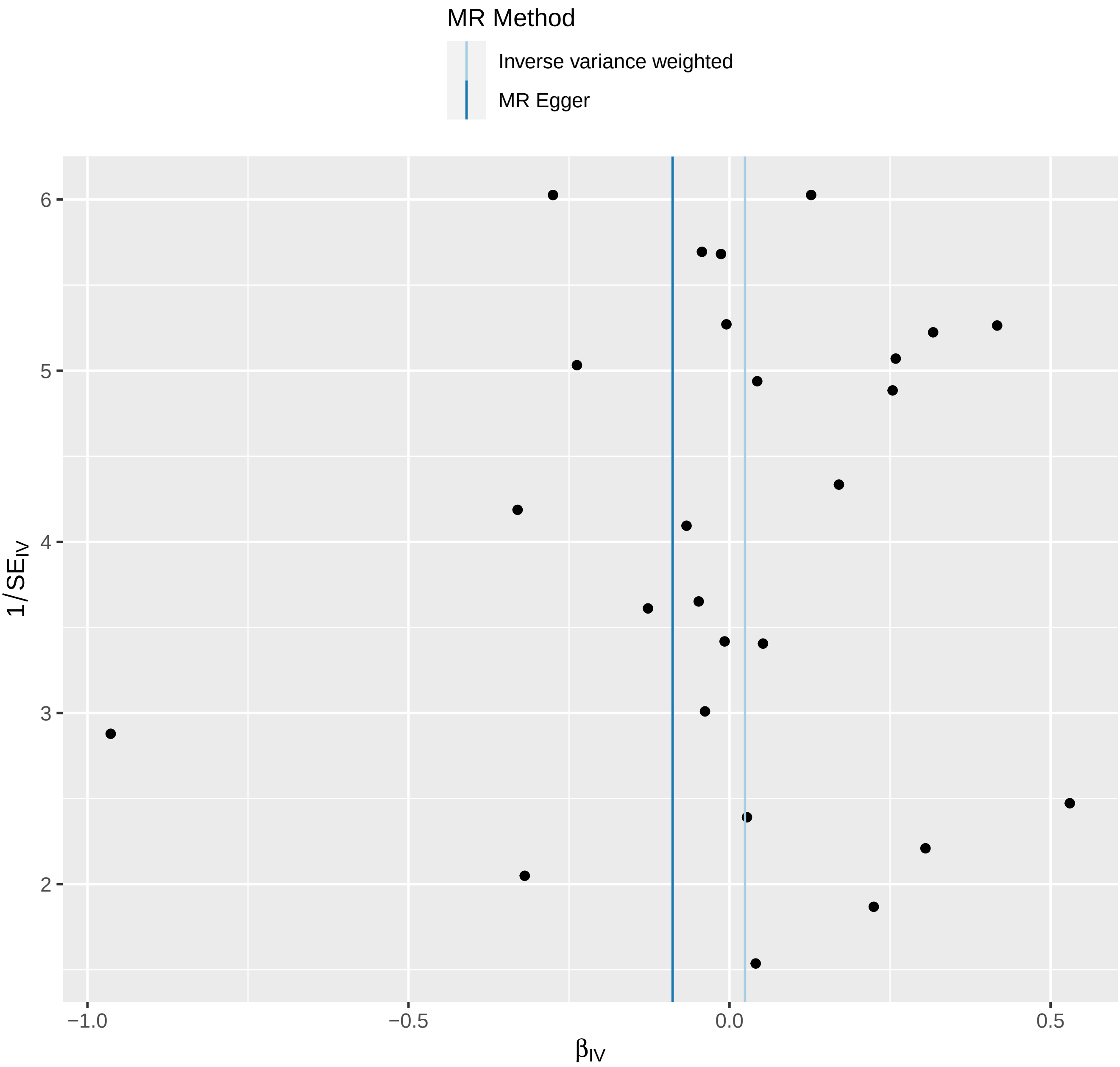

Batch 3−2046 : Non−homologous end−joining factor 1 on Diabetic nephropathy (more control exclusions) with a threshold of 1e−05

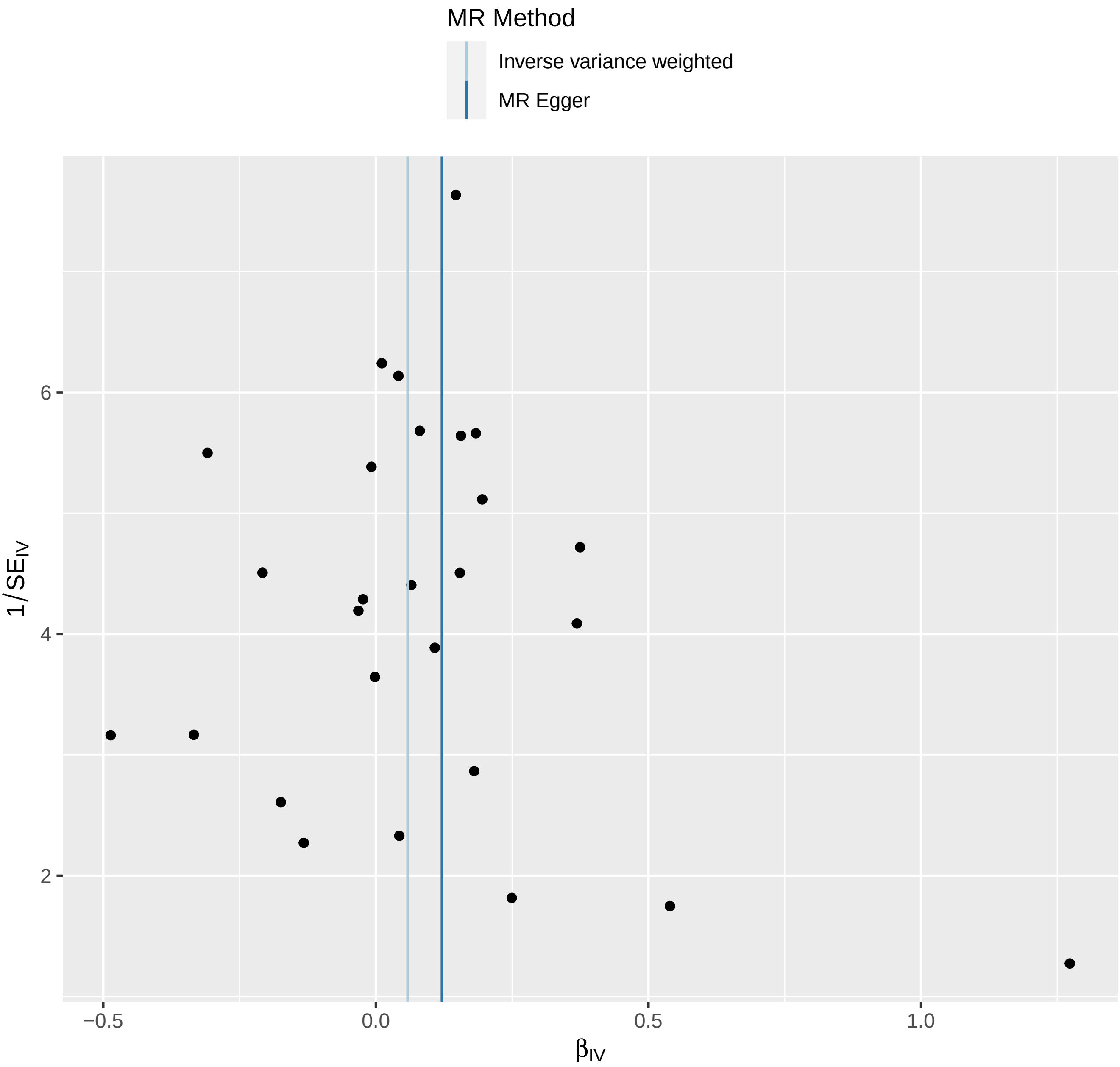

Batch 3−2047 : NHL repeat−containing protein 3 on Diabetic nephropathy (more control exclusions)
with a threshold of 1e−05

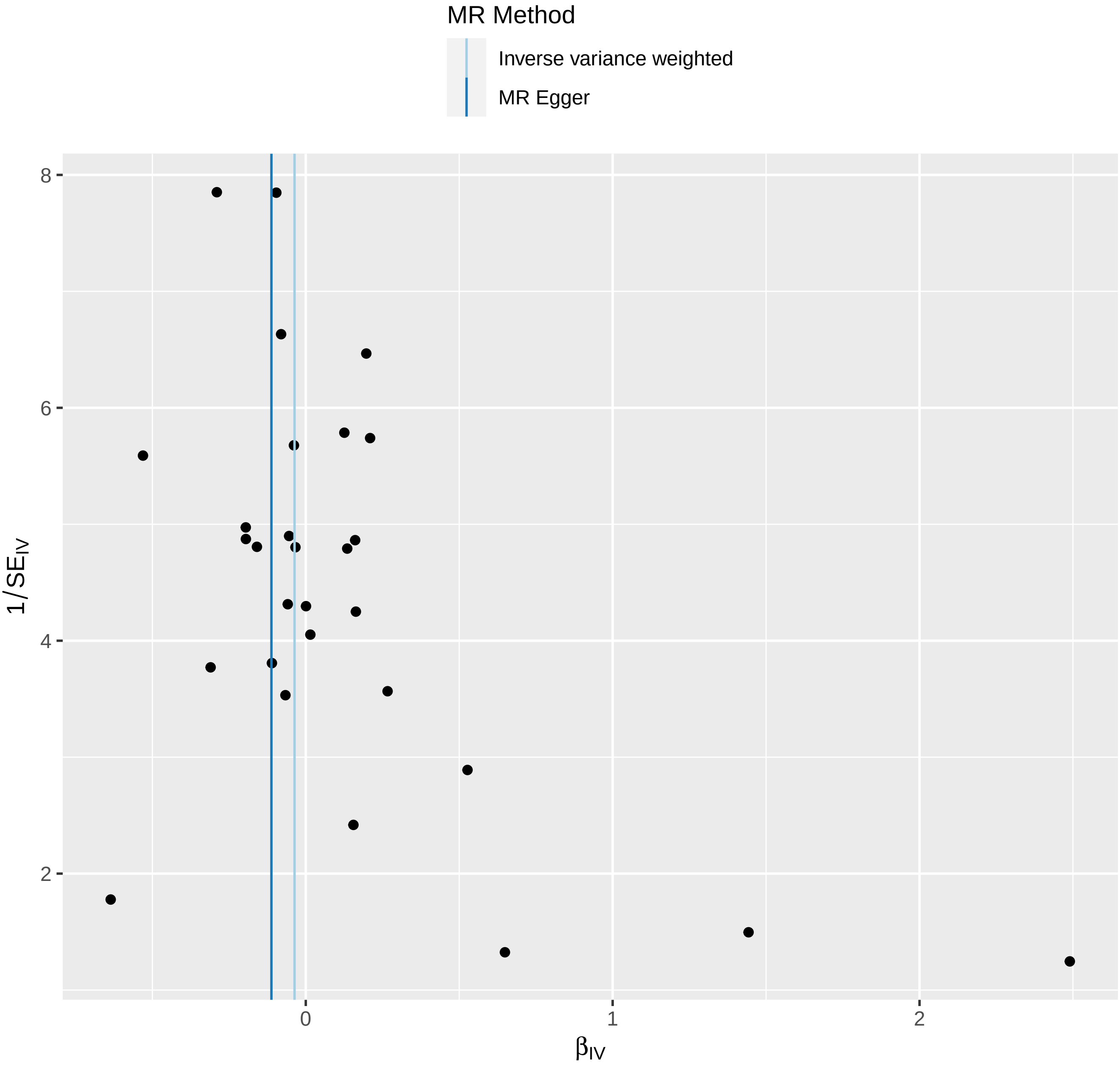

Batch 3−2048 : NHP2−like protein 1 on Diabetic nephropathy (more control exclusions)
with a threshold of 1e−05

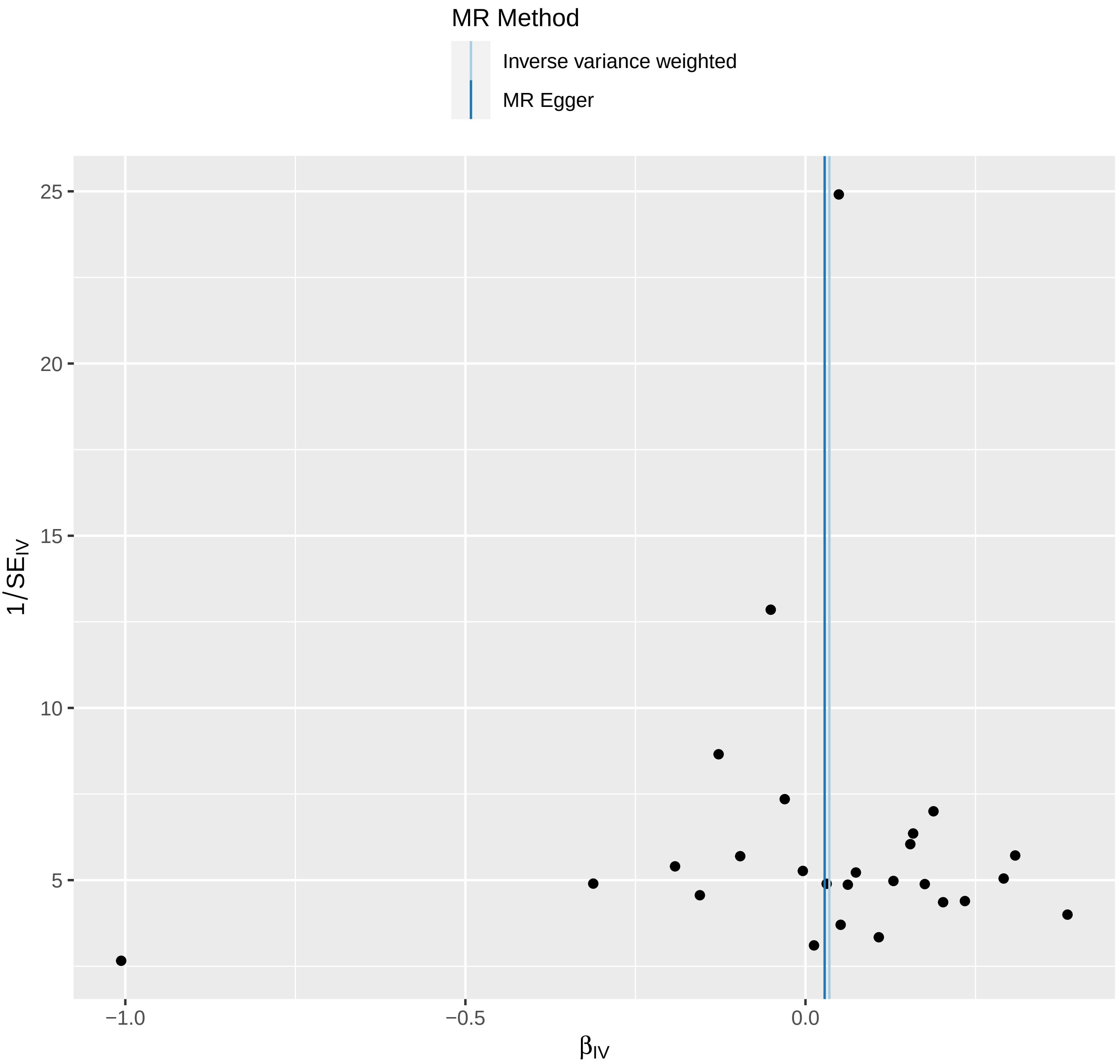

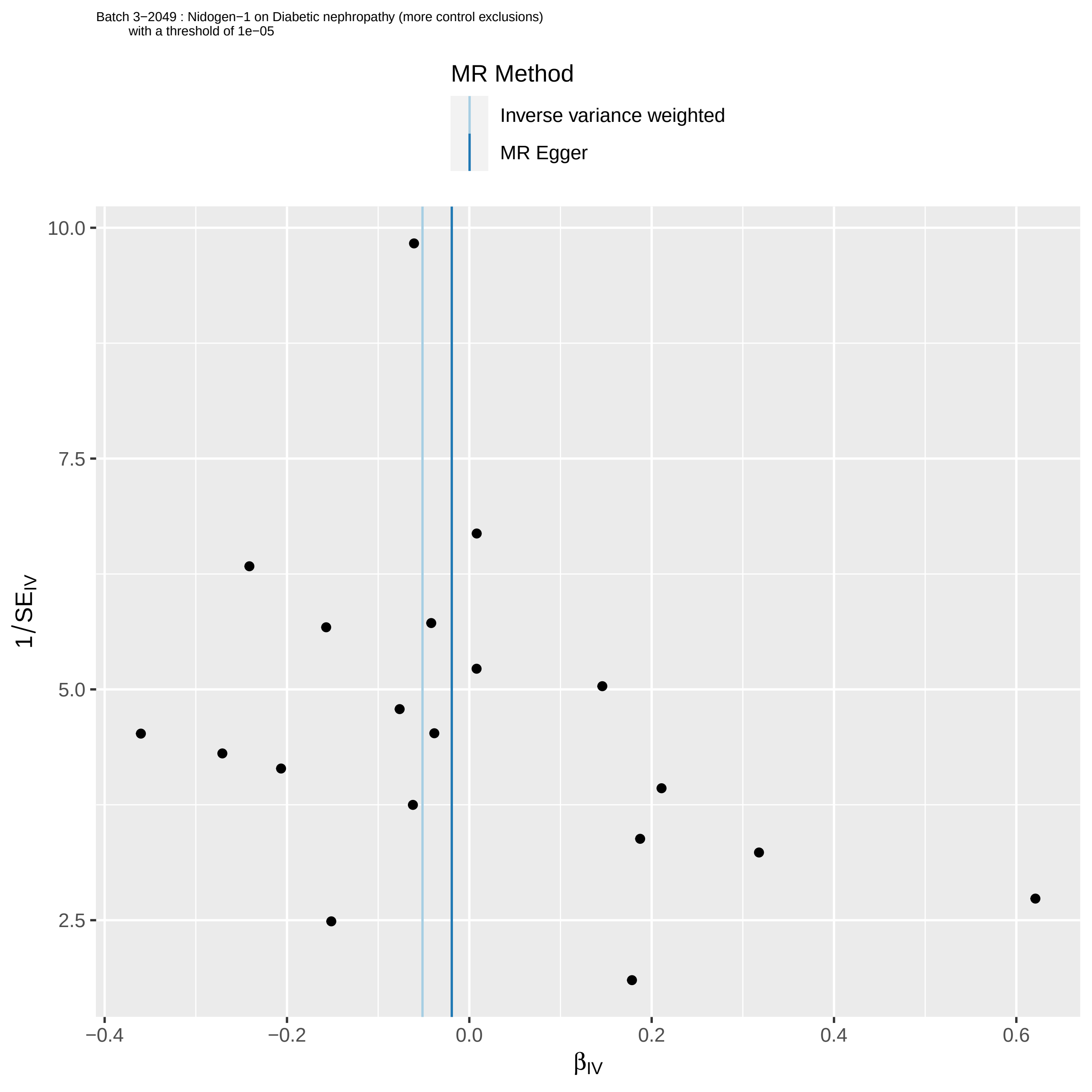

Batch 3−2049 : Nidogen−1 on Diabetic nephropathy (more control exclusions)
with a threshold of 1e−05
MR Method
Inverse variance weighted
MR Egger
10.0
7.5
5.0
2.5
1/SE_IV
−0.4
−0.2
0.0
0.2
0.4
0.6
β_IV

Batch 3−2050 : Nidogen−2 on Diabetic nephropathy (more control exclusions)
with a threshold of 1e−05

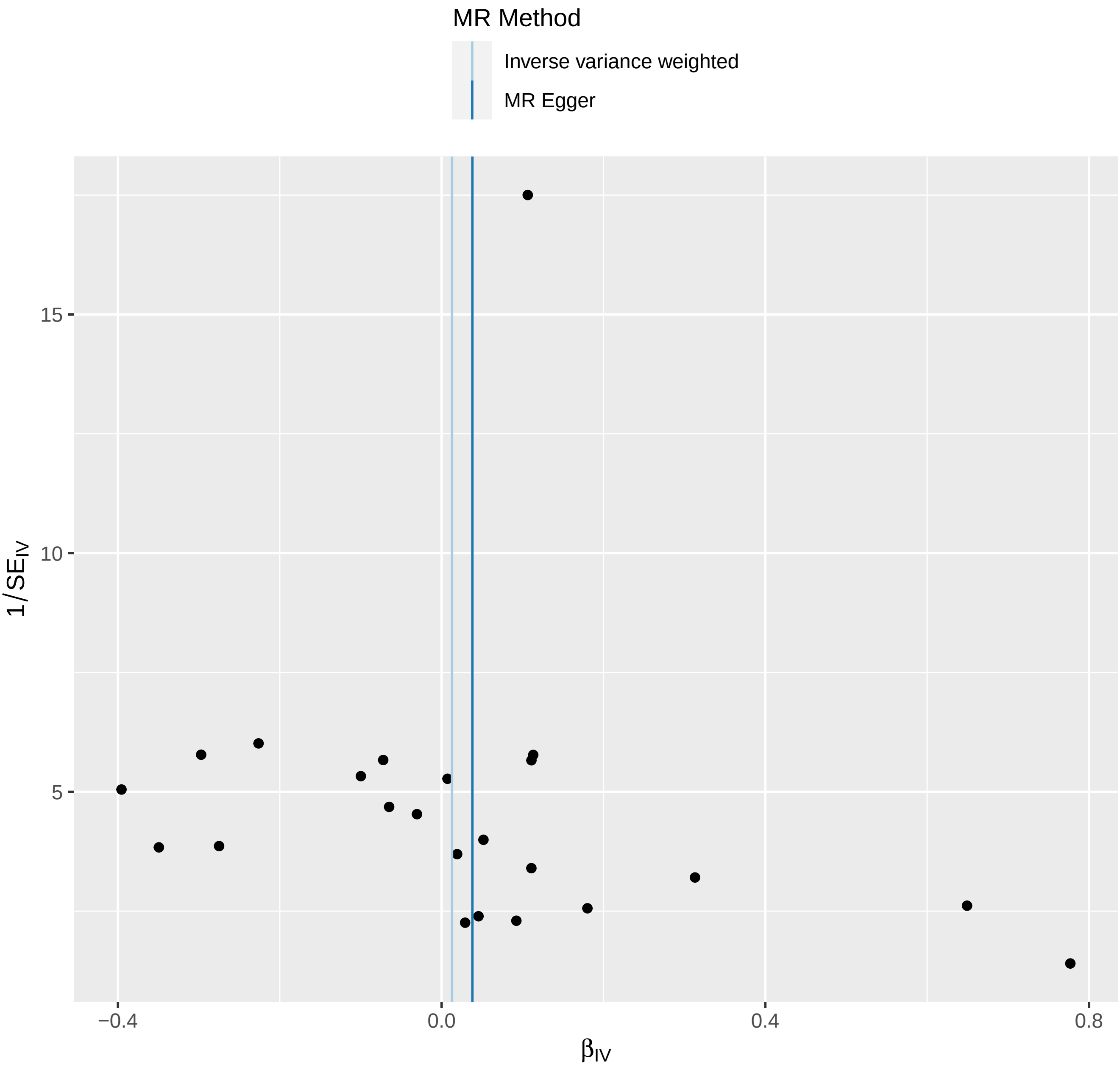

Batch 3−2051 : Magnesium transporter NIPA4 on Diabetic nephropathy (more control exclusions)
with a threshold of 1e−05

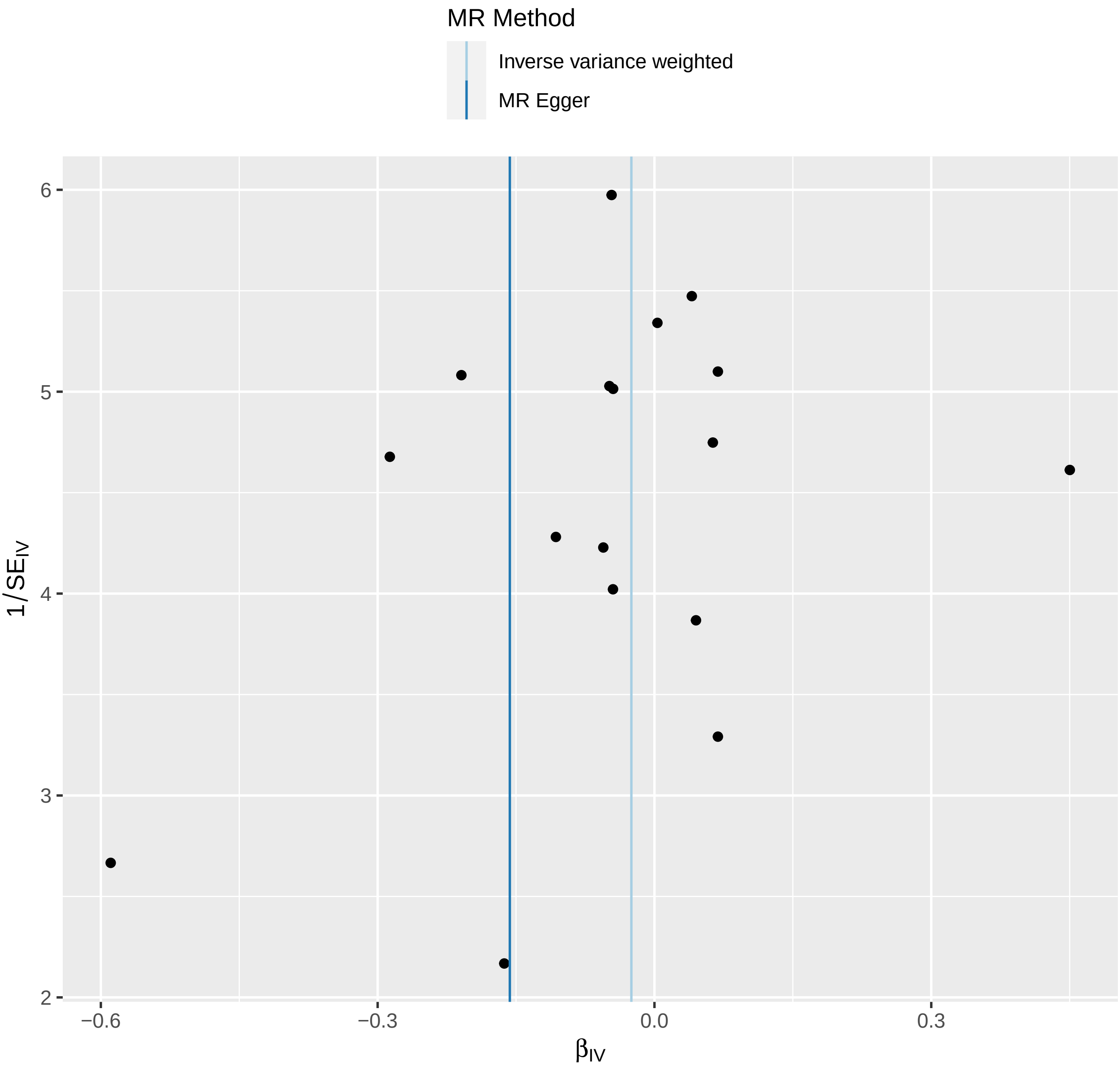

Batch 3−2052 : Nischarin on Diabetic nephropathy (more control exclusions)
with a threshold of 1e−05

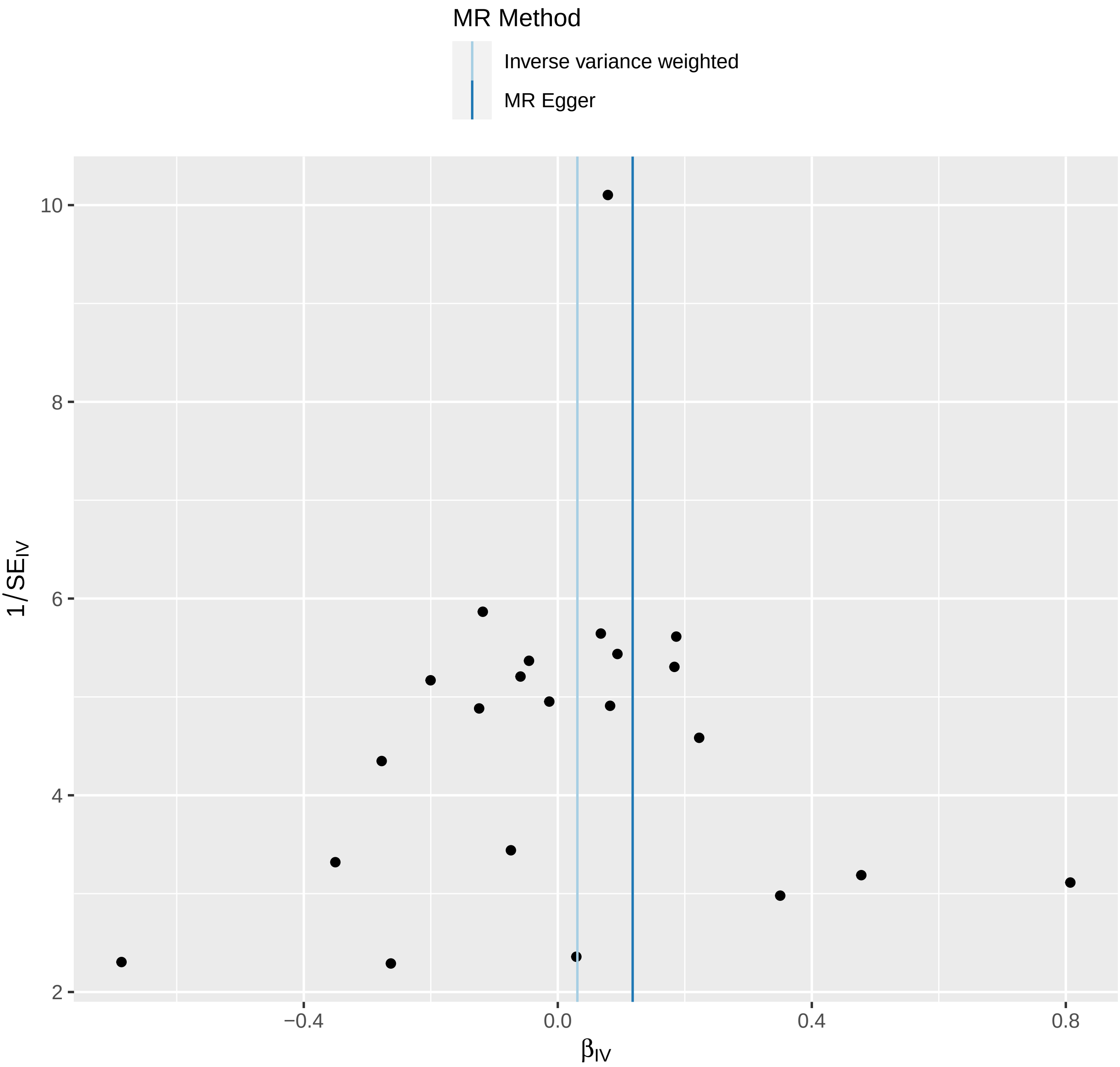

Batch 3−2053 : Neuroligin−1 on Diabetic nephropathy (more control exclusions)
with a threshold of 1e−05

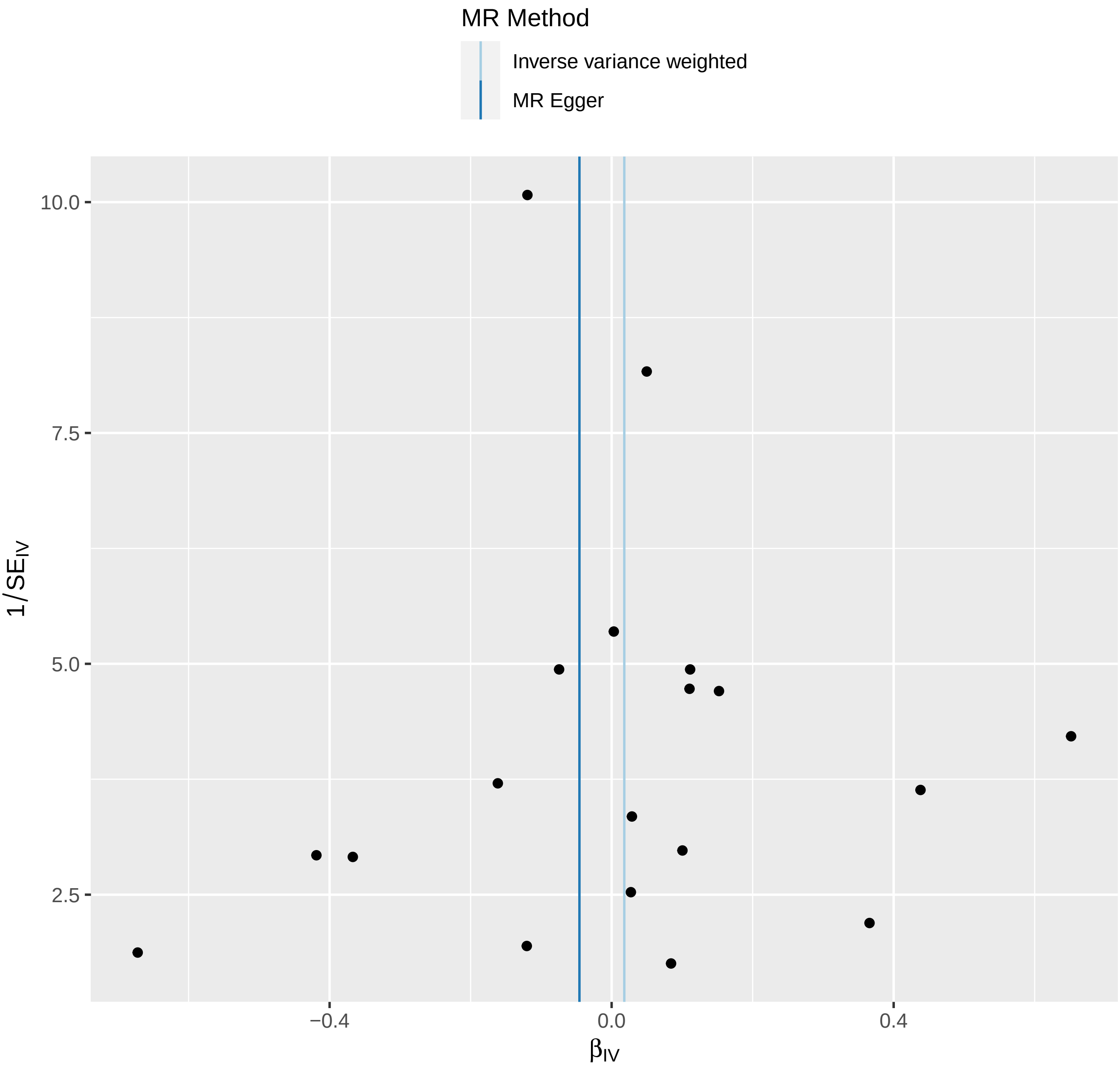

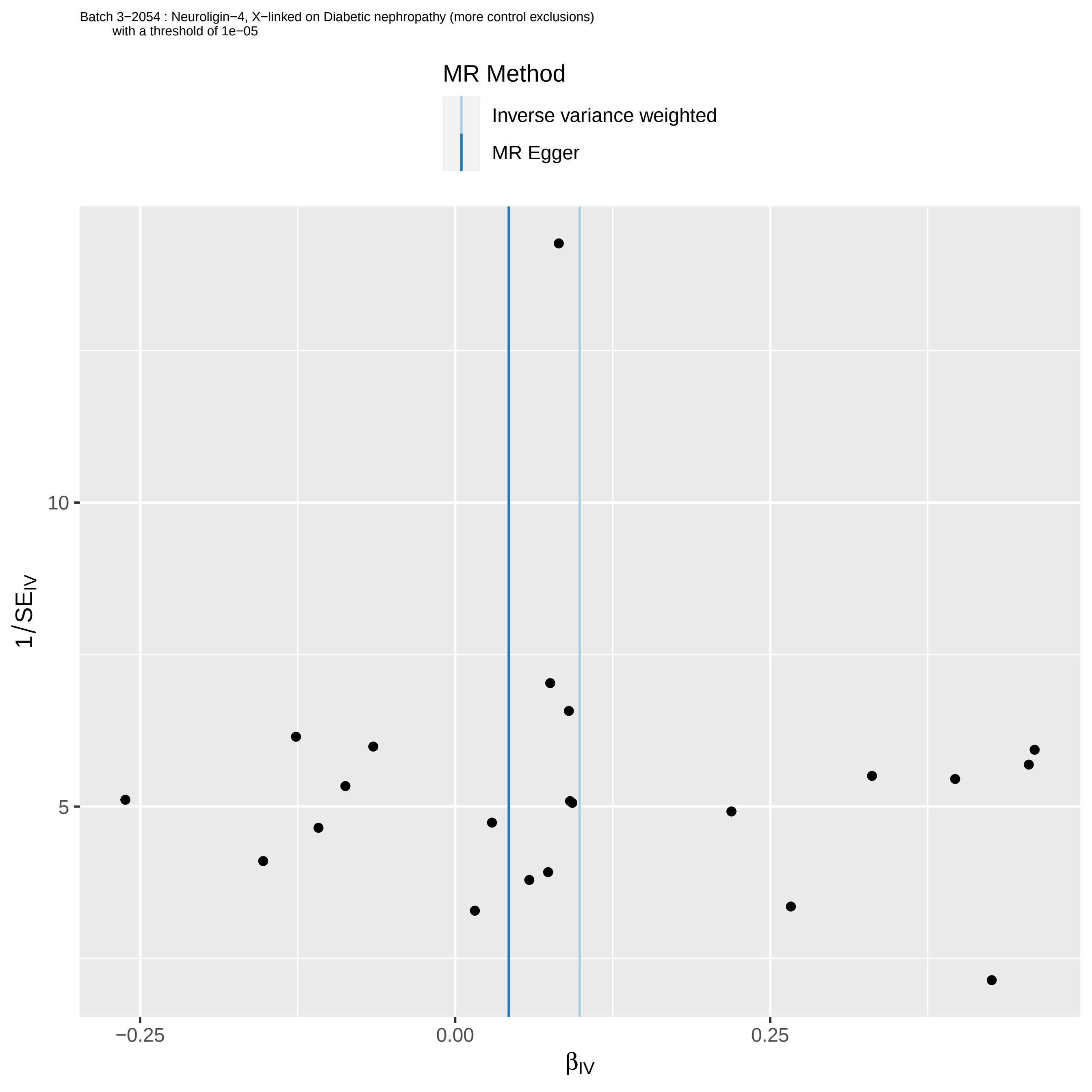

Batch 3−2054 : Neuroligin−4, X−linked on Diabetic nephropathy (more control exclusions)
with a threshold of 1e−05
MR Method
Inverse variance weighted
MR Egger
10
5
1/SE$_{IV}$
−0.25
0.00
0.25
β$_{IV}$

Batch 3−2055 : NACHT, LRR and PYD domains−containing protein 10 on Diabetic nephropathy (more control exclusions) with a threshold of 1e−05

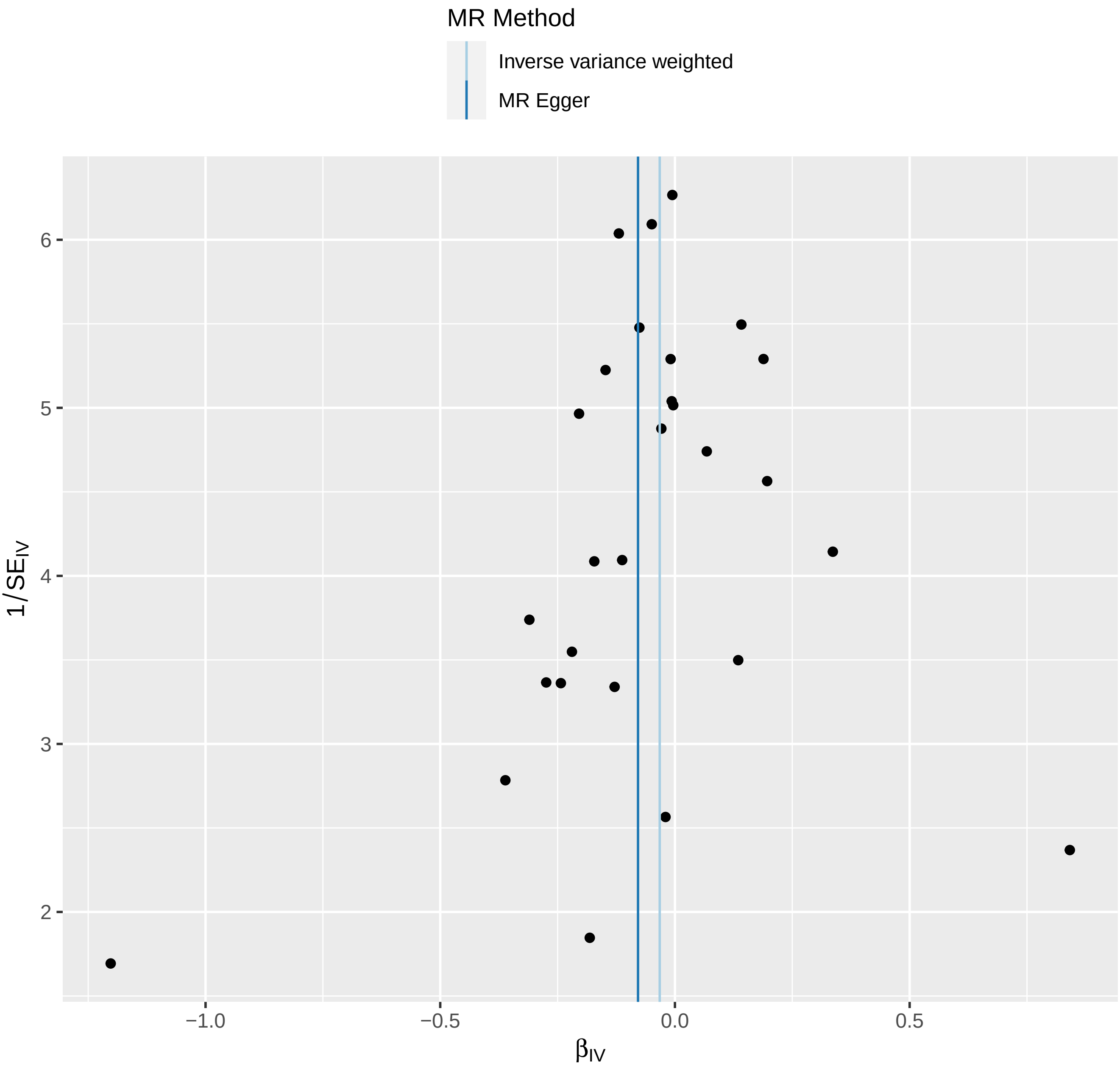

Batch 3−2056 : NACHT, LRR and PYD domains−containing protein 1 on Diabetic nephropathy (more control exclusions) with a threshold of 1e−05

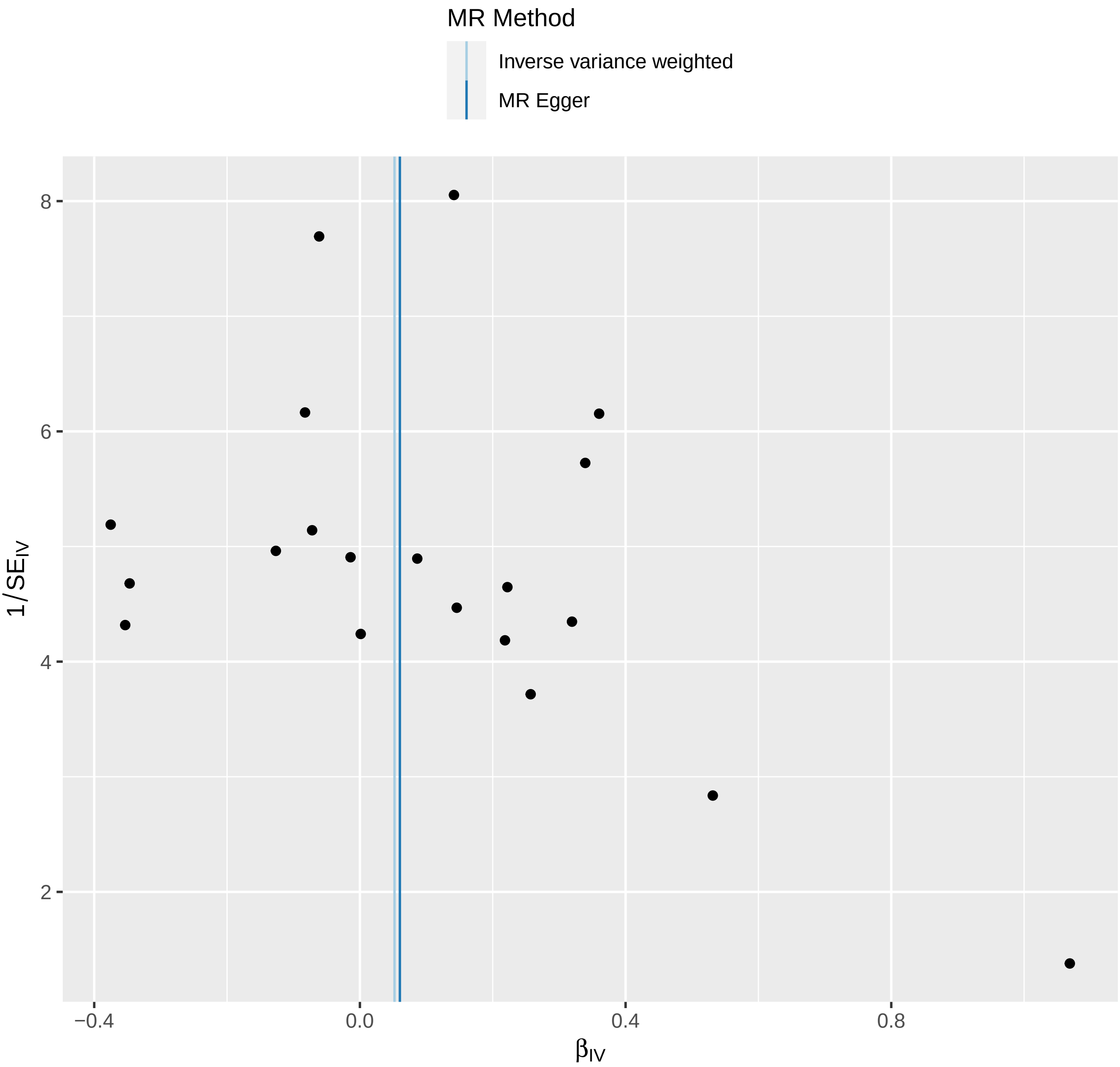

Batch 3−2057 : NACHT, LRR and PYD domains−containing protein 4 on Diabetic nephropathy (more control exclusions)
with a threshold of 1e−05

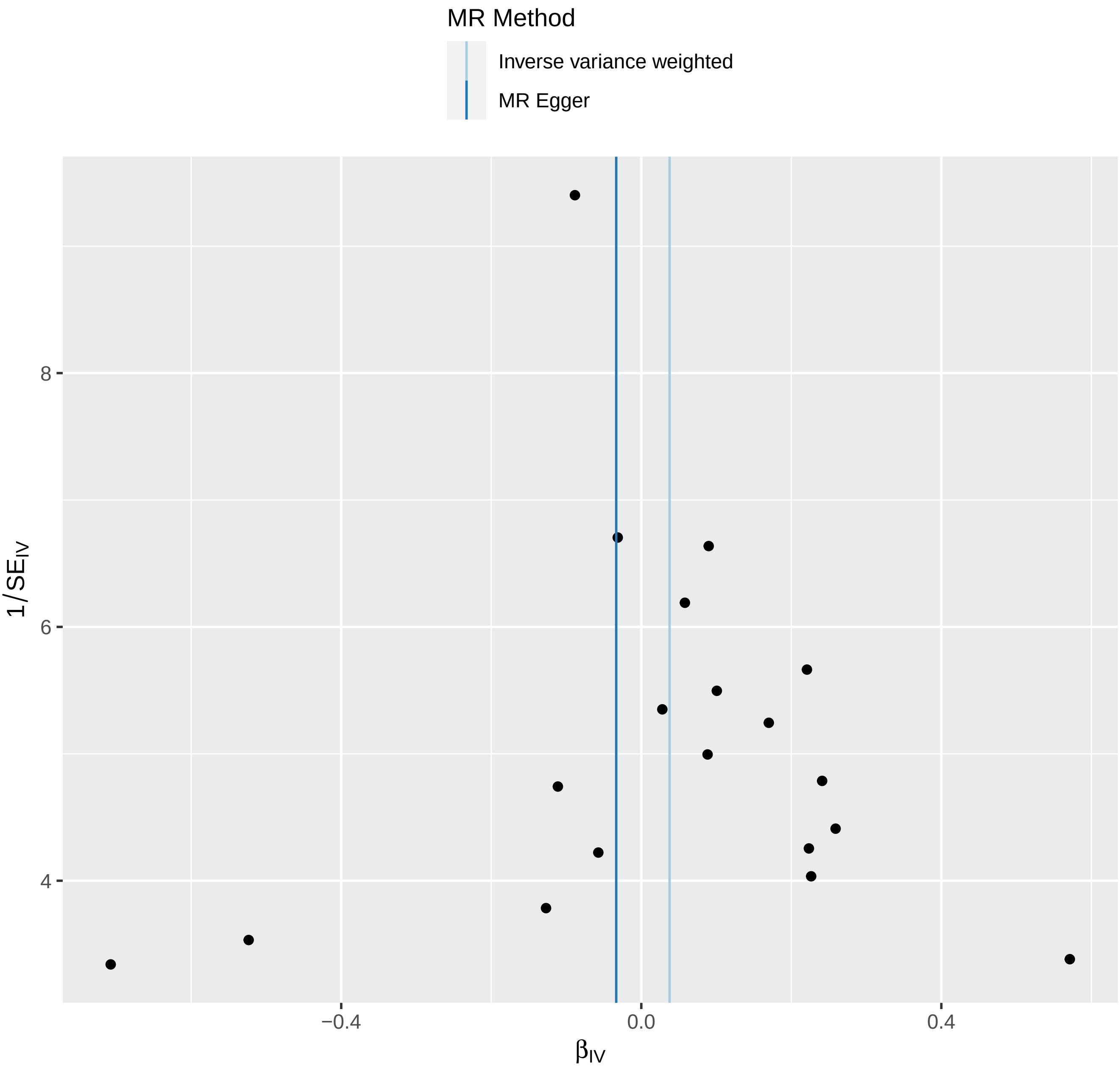

Batch 3−2058 : Neuromedin−B on Diabetic nephropathy (more control exclusions)
with a threshold of 1e−05

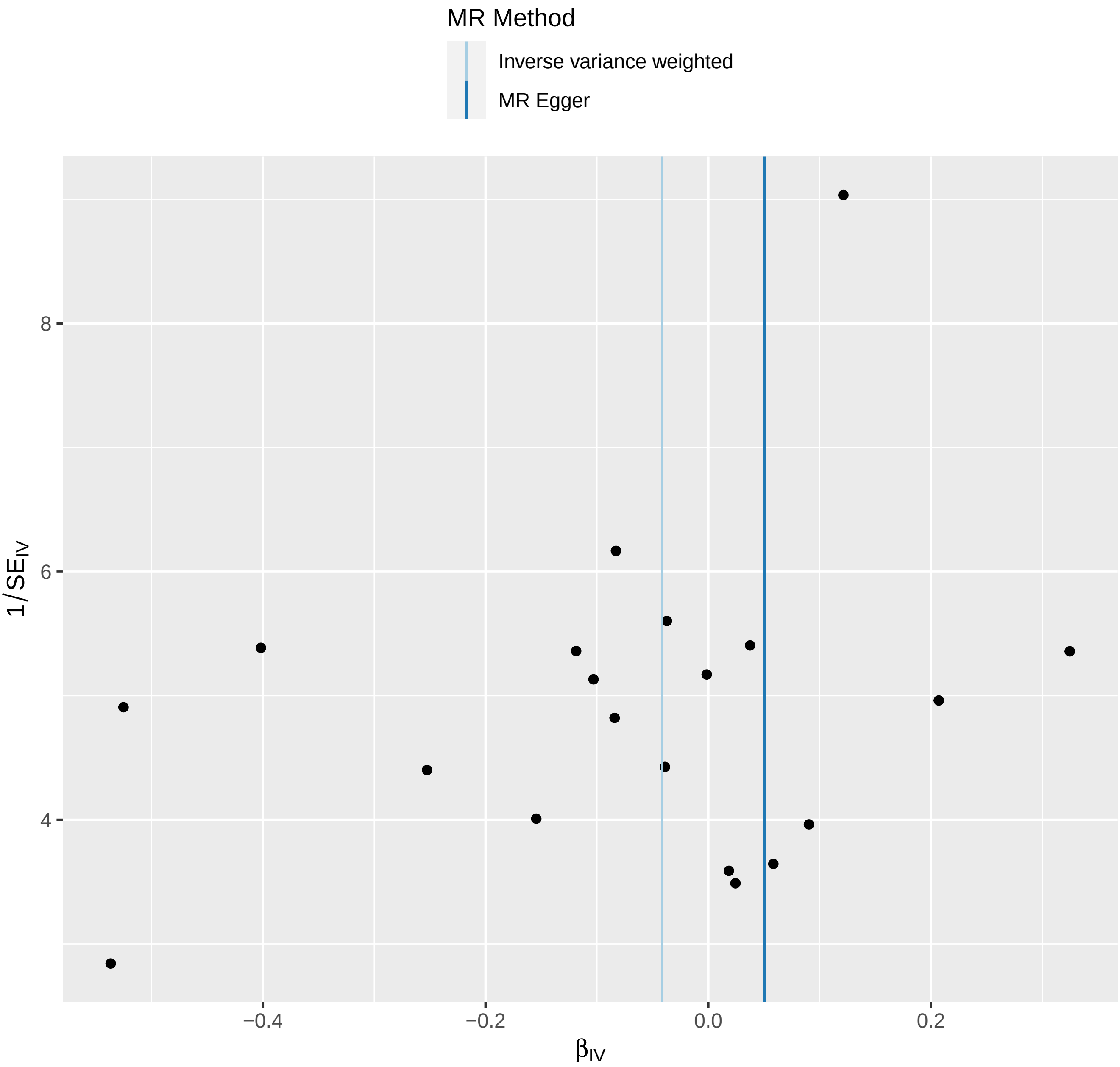

Batch 3−2059 : Nucleoside diphosphate kinase A on Diabetic nephropathy (more control exclusions)
with a threshold of 1e−05

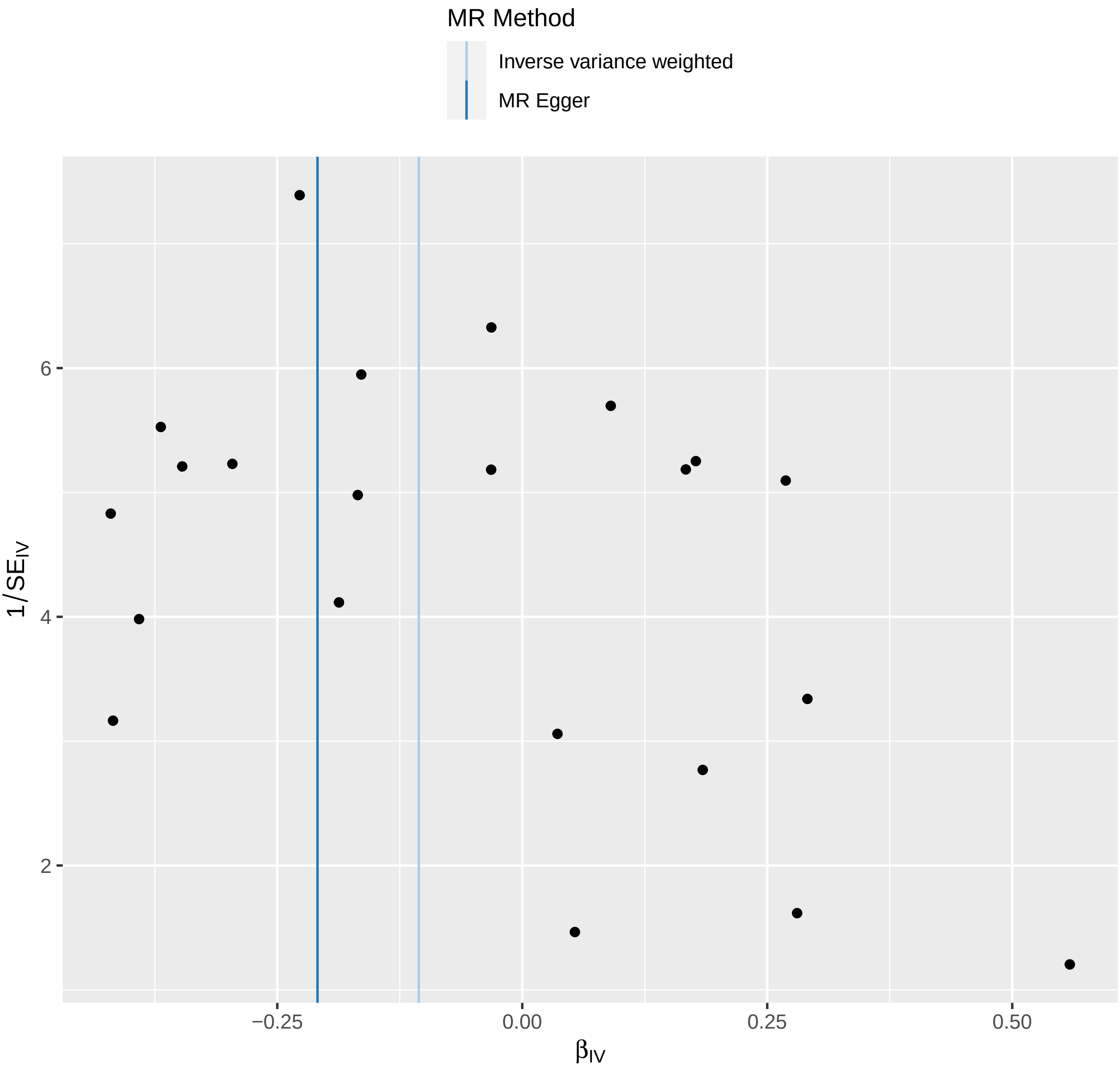

Batch 3−2060 : Nucleoside diphosphate kinase B on Diabetic nephropathy (more control exclusions)
with a threshold of 1e−05

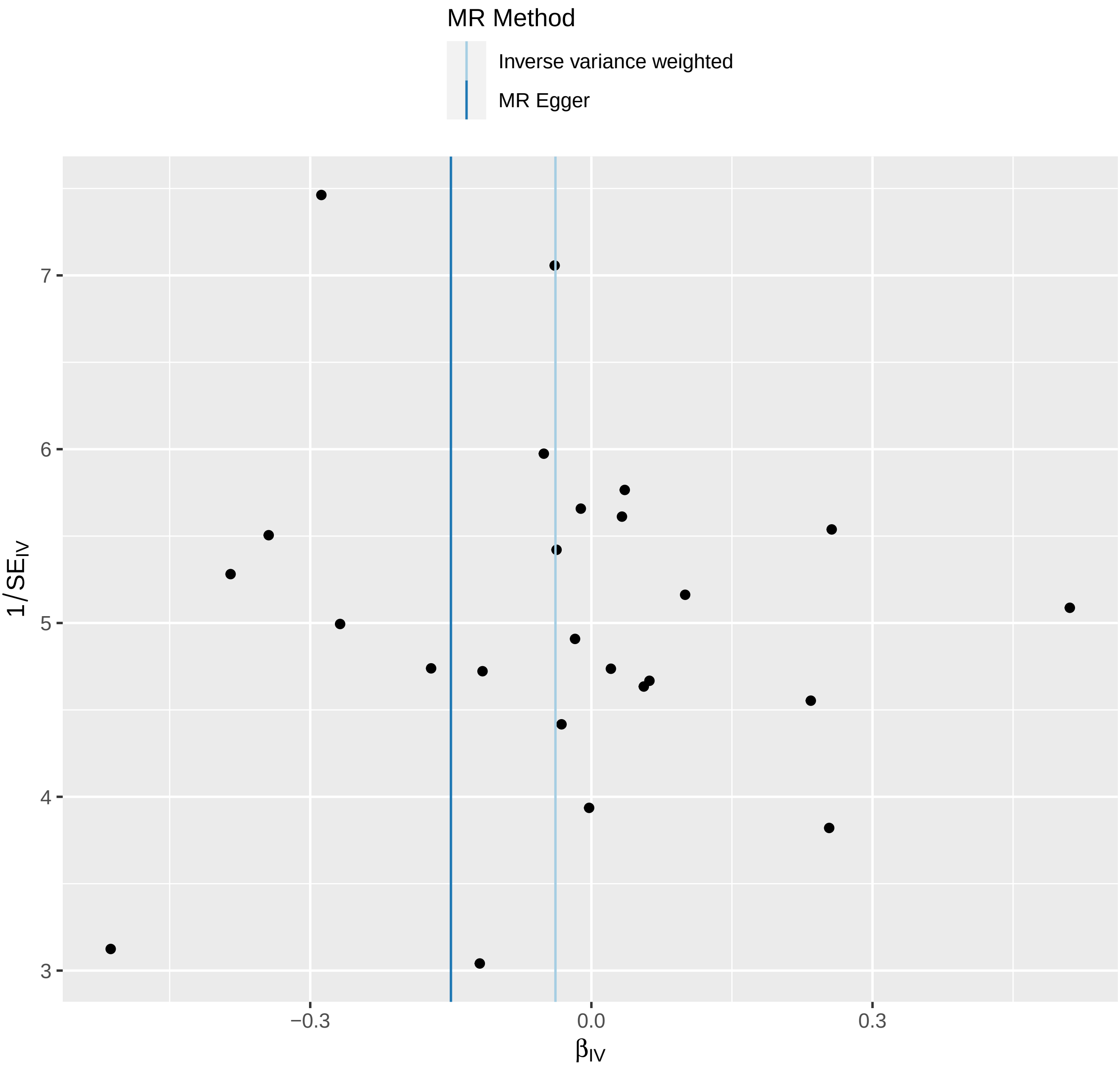

Batch 3−2061 : Normal mucosa of esophagus−specific gene 1 protein on Diabetic nephropathy (more control exclusions)
with a threshold of 1e−05

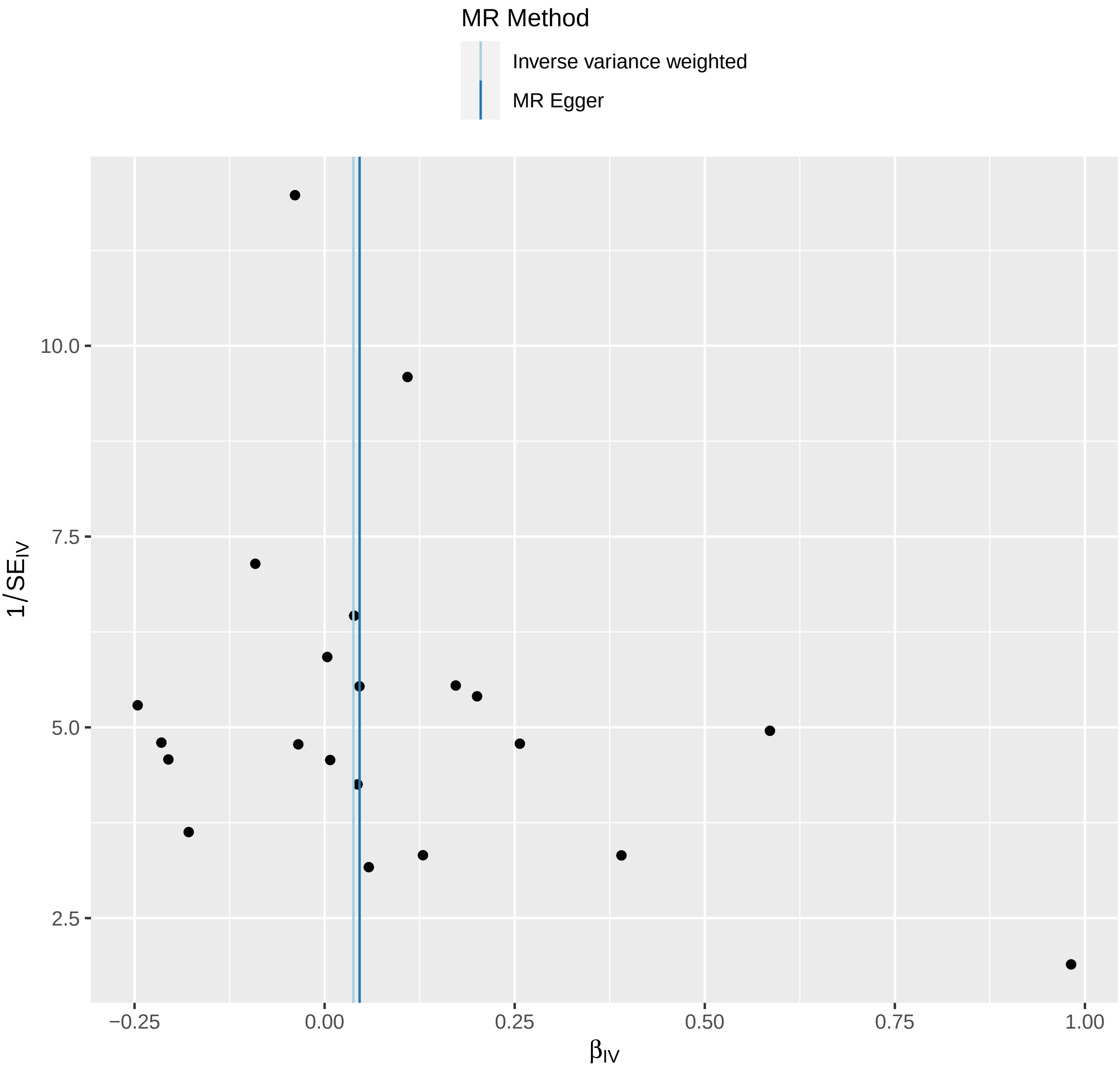

Batch 3−2062 : NmrA−like family domain−containing protein 1 on Diabetic nephropathy (more control exclusions) with a threshold of 1e−05

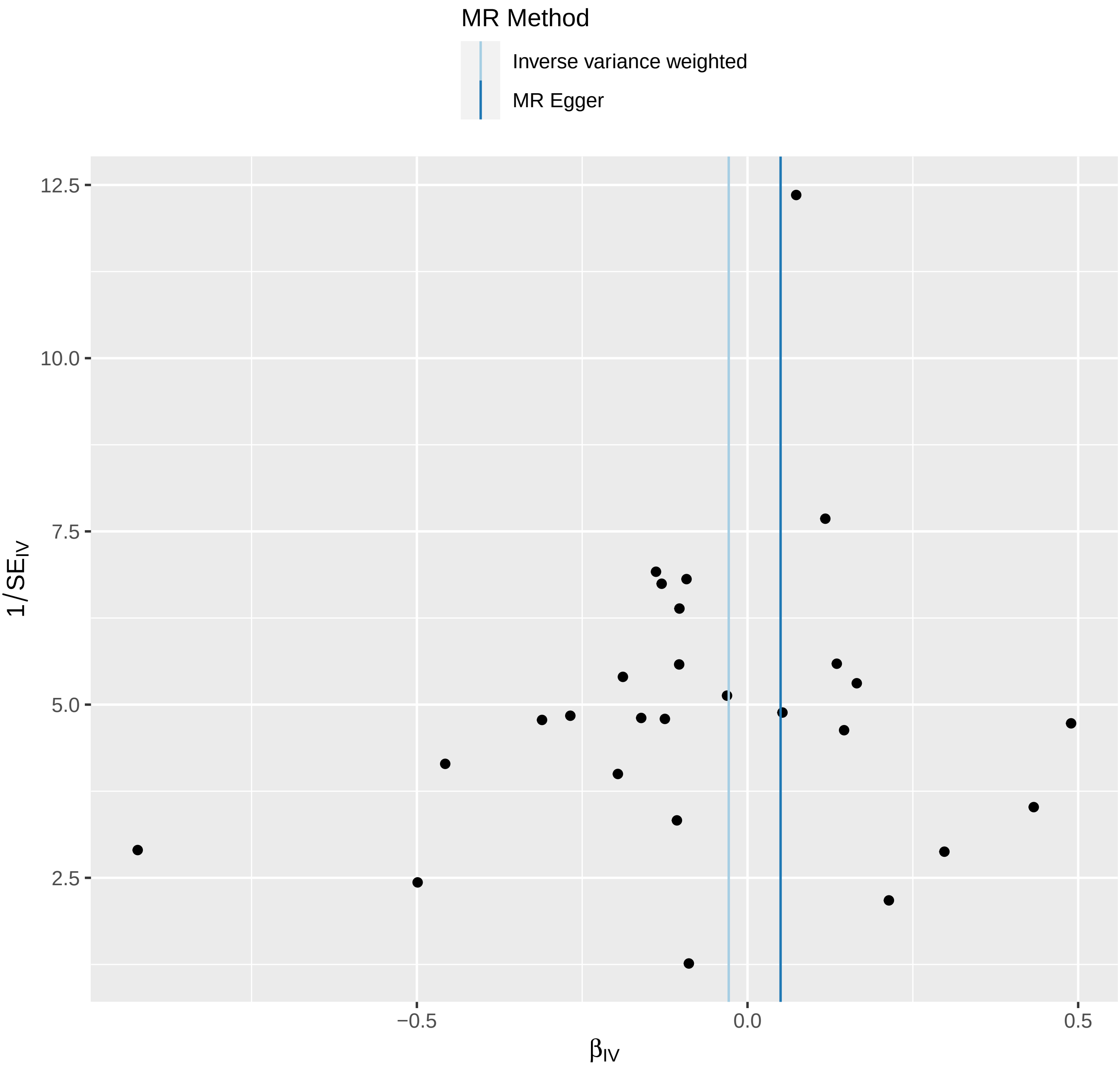

Batch 3−2063 : Glycylpeptide N−tetradecanoyltransferase 1 on Diabetic nephropathy (more control exclusions)
with a threshold of 1e−05

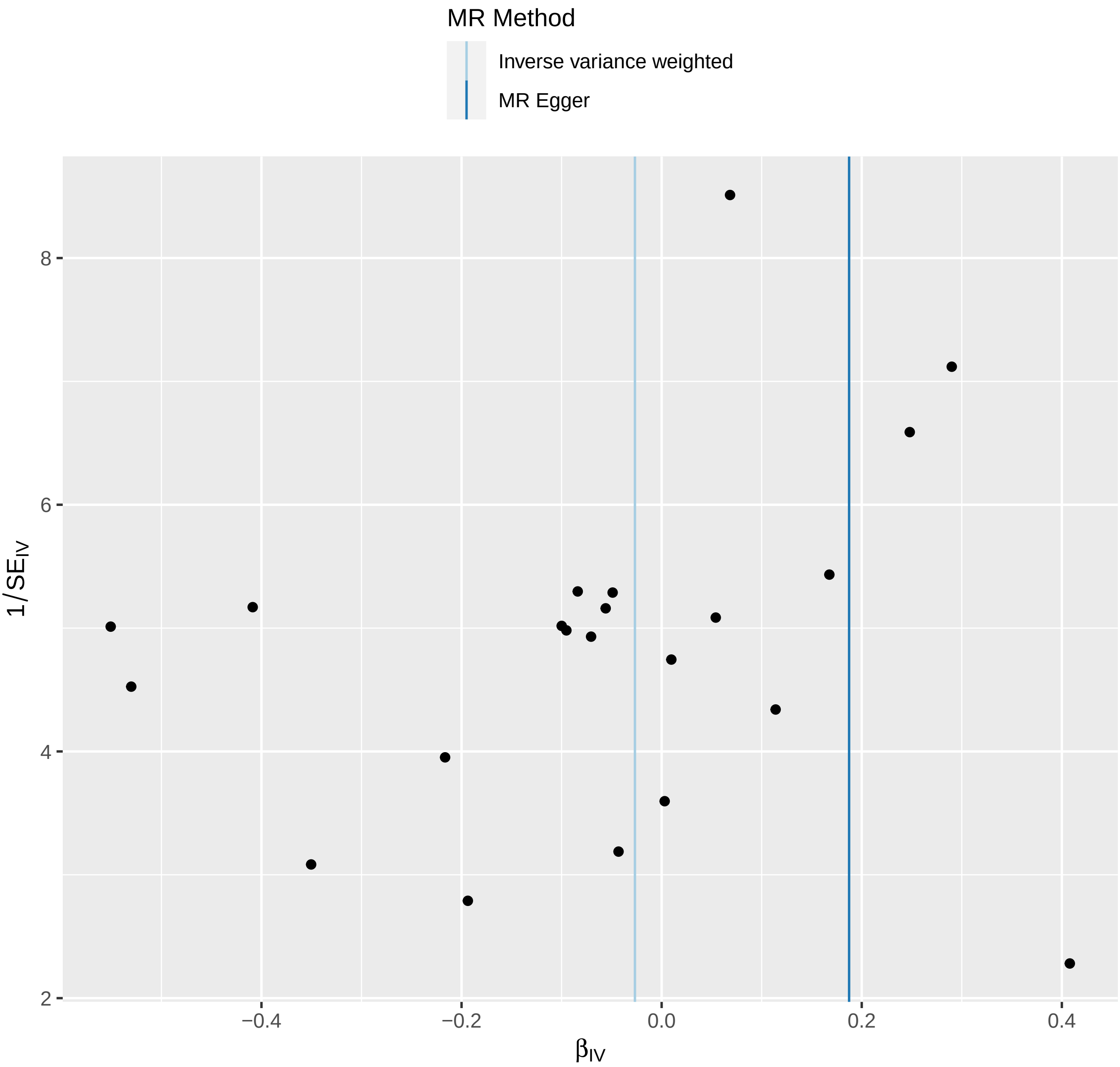

Batch 3−2064 : Noggin on Diabetic nephropathy (more control exclusions)
with a threshold of 1e−05

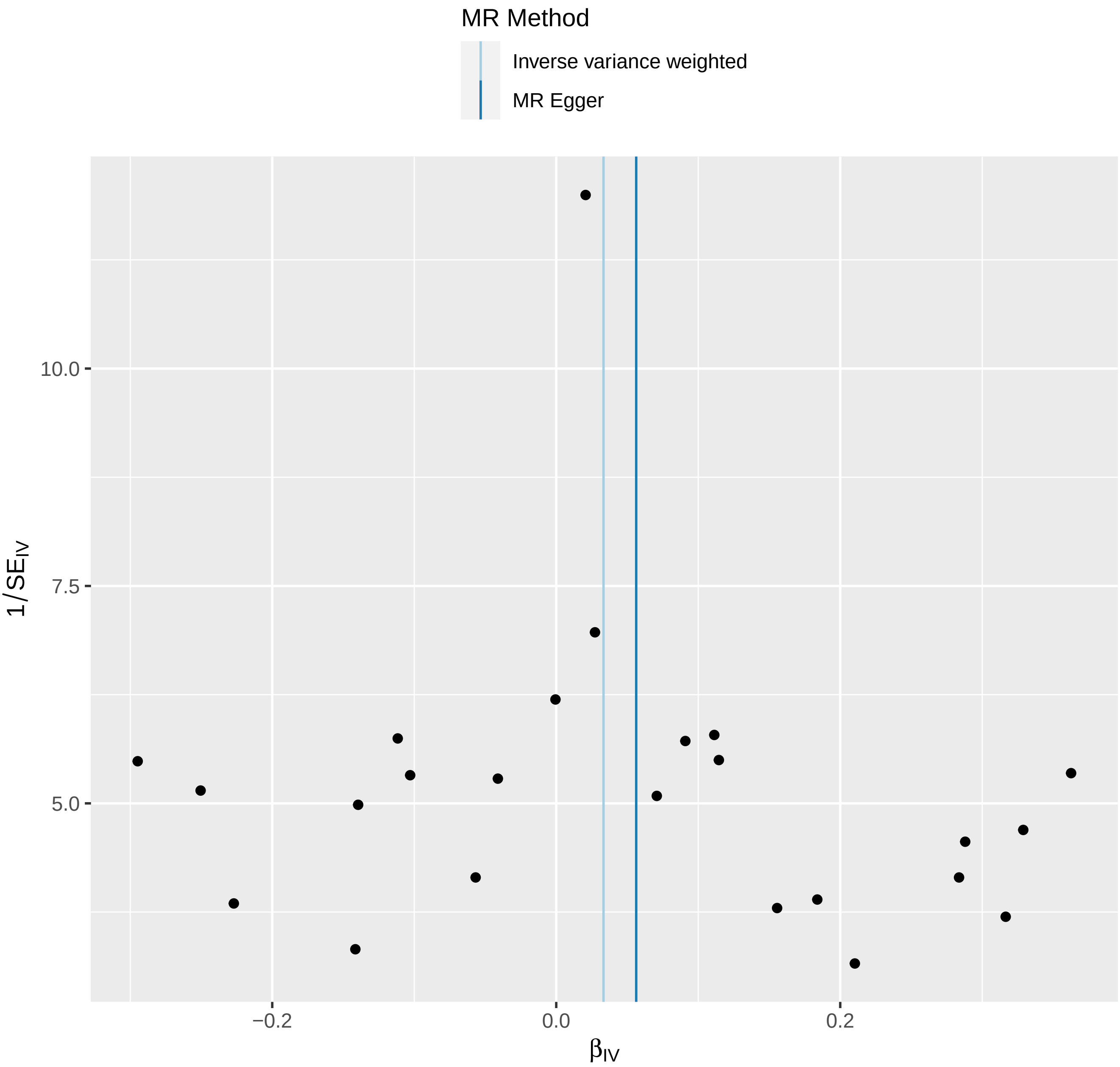

Batch 3−2065 : NoneX on Diabetic nephropathy (more control exclusions)
with a threshold of 1e−05

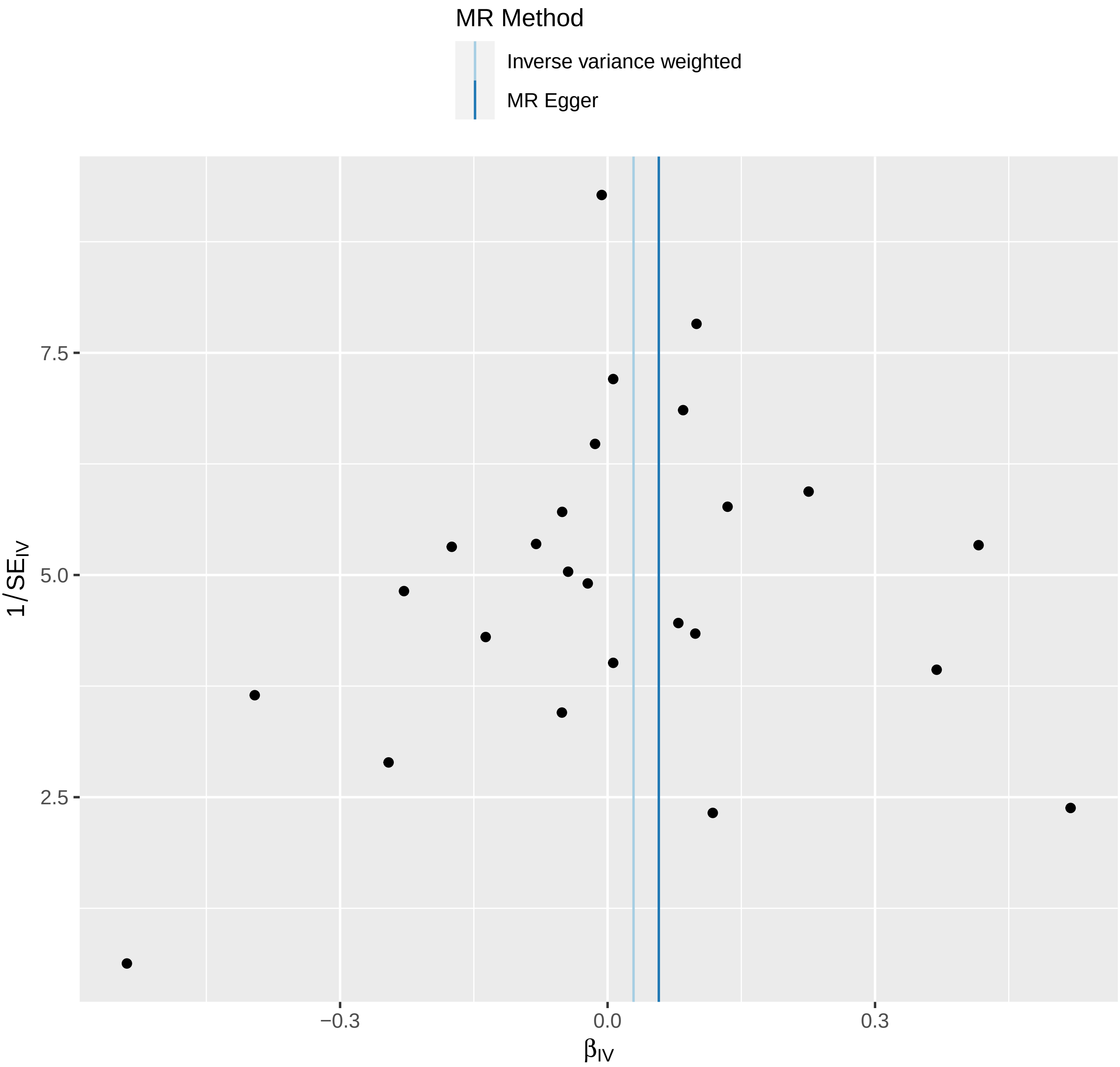

Batch 3−2066 : NoneX on Diabetic nephropathy (more control exclusions)
with a threshold of 1e−05

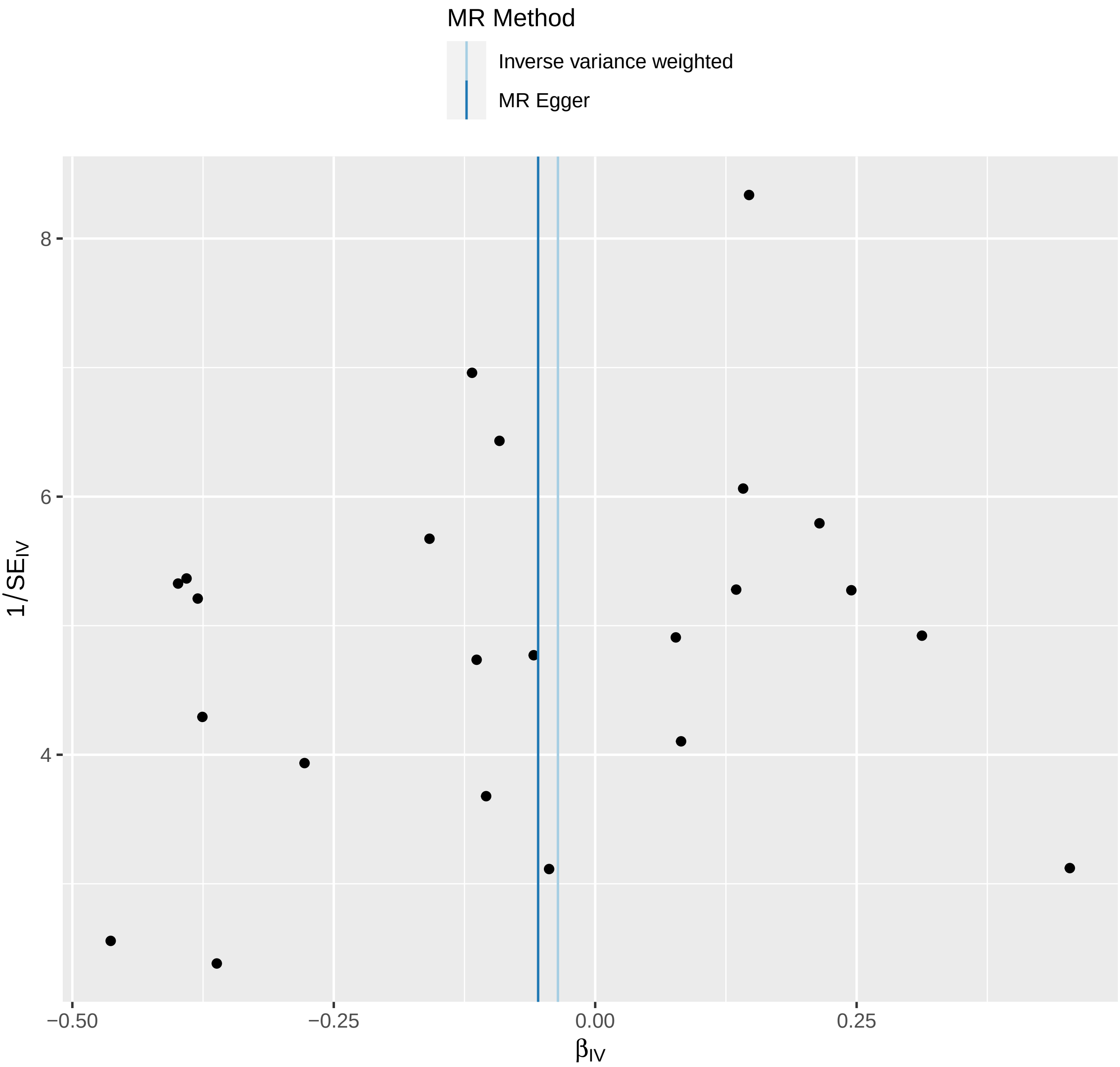

Batch 3−2067 : Neurogenic locus notch homolog protein 1 on Diabetic nephropathy (more control exclusions) with a threshold of 1e−05

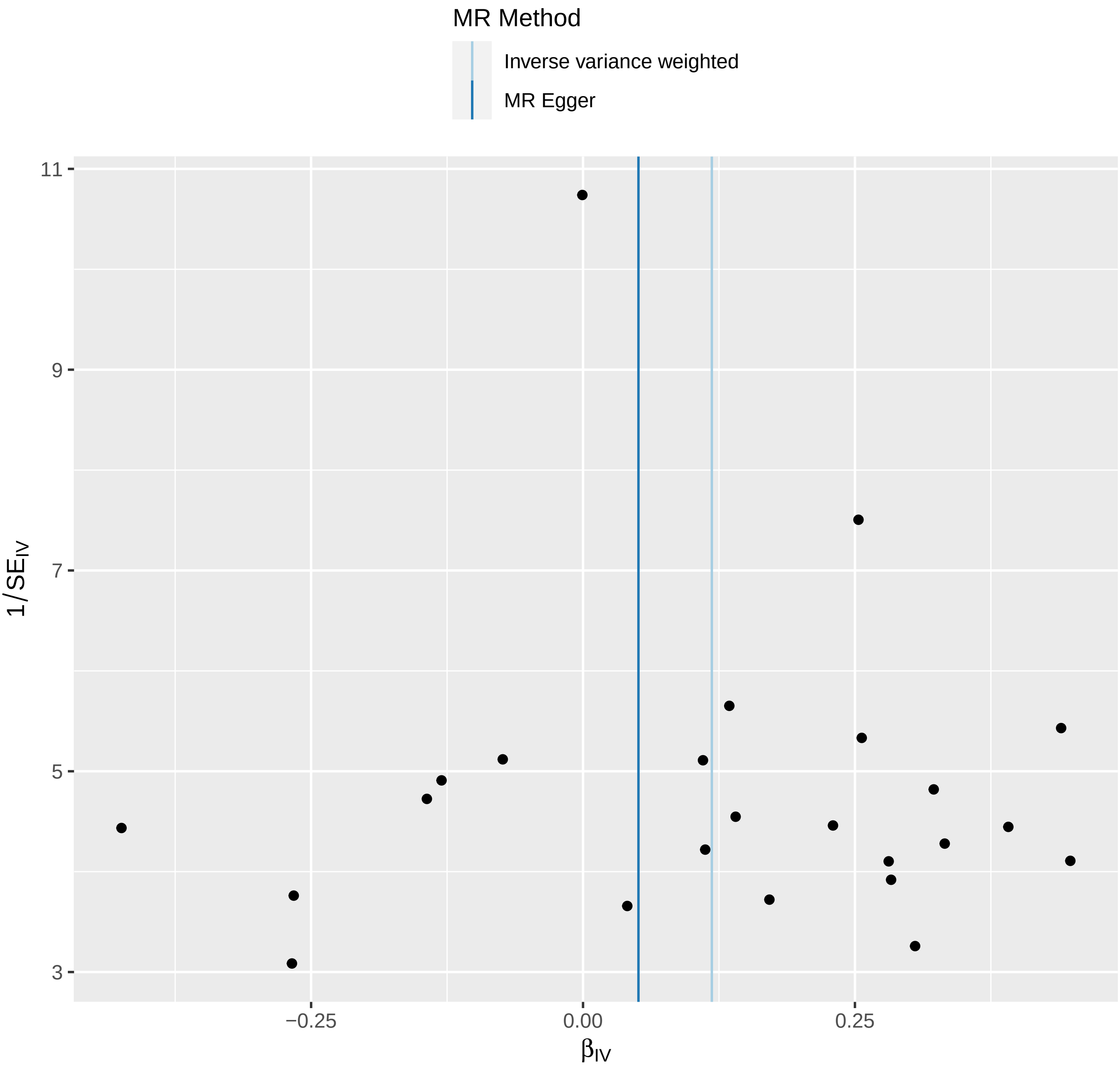

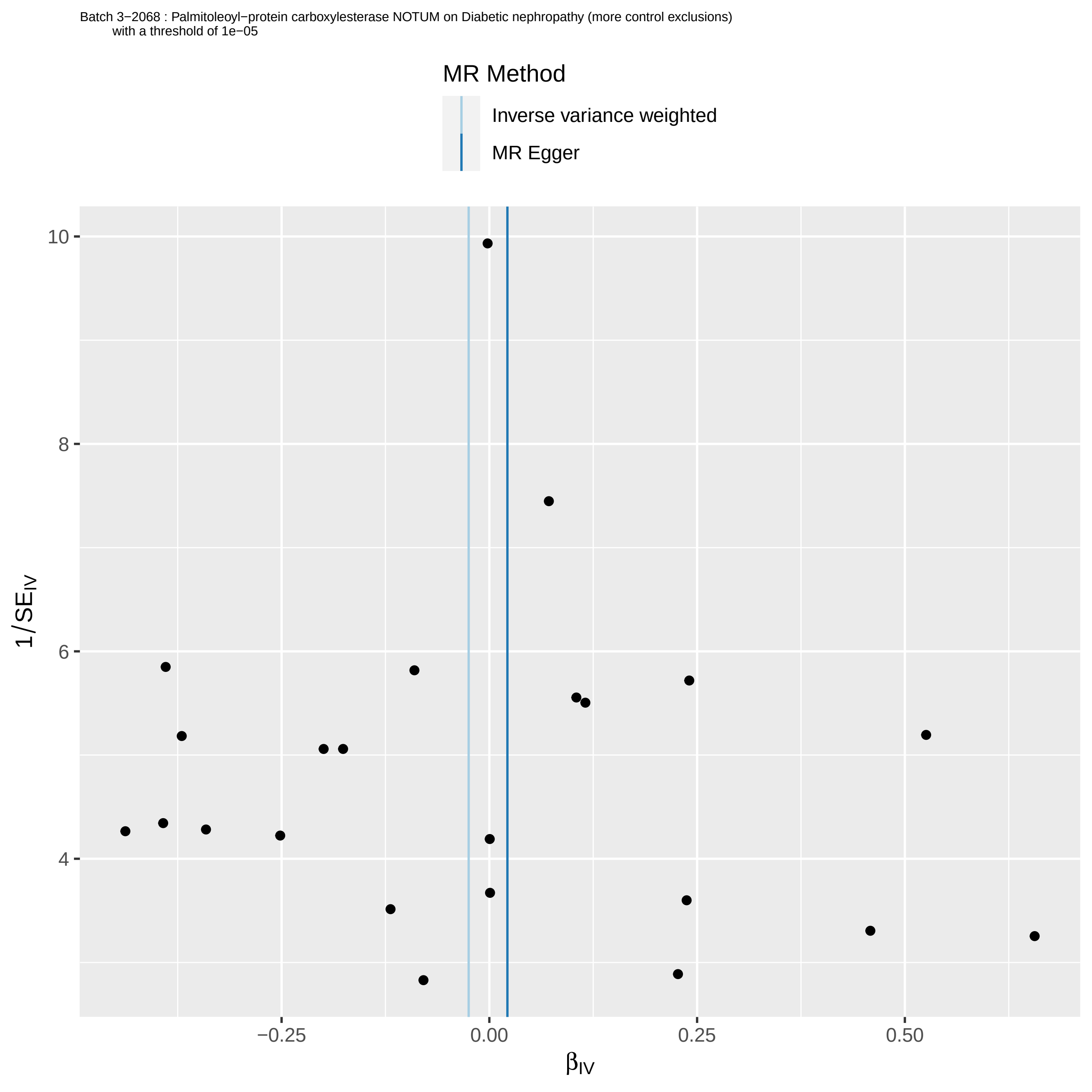

Batch 3−2068 : Palmitoleoyl−protein carboxylesterase NOTUM on Diabetic nephropathy (more control exclusions)
with a threshold of 1e−05
MR Method
Inverse variance weighted
MR Egger
10
8
6
4
1/SE_IV
−0.25
0.00
0.25
0.50
β_IV

Batch 3−2069 : Protein NOV homolog on Diabetic nephropathy (more control exclusions)
with a threshold of 1e−05

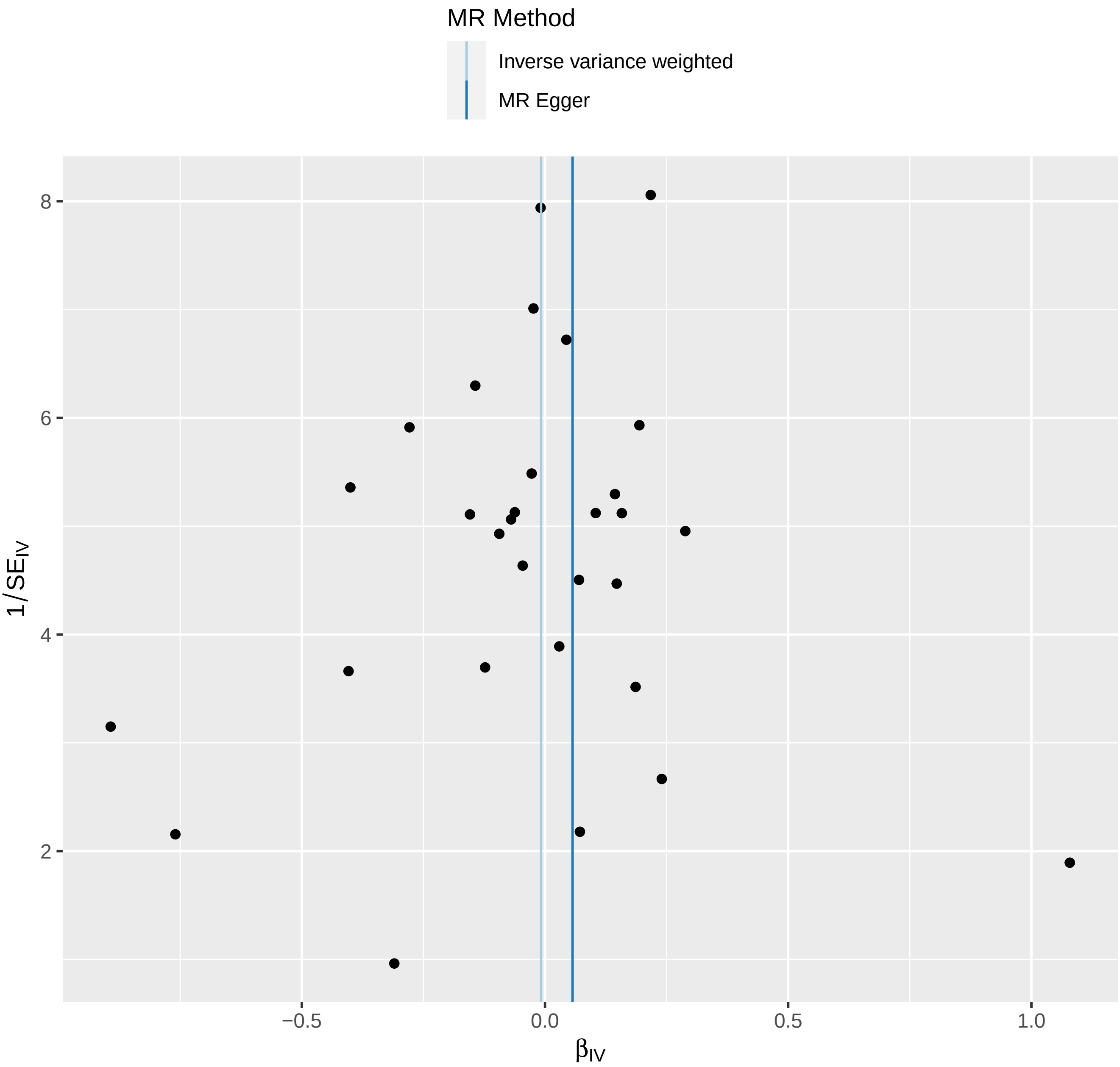

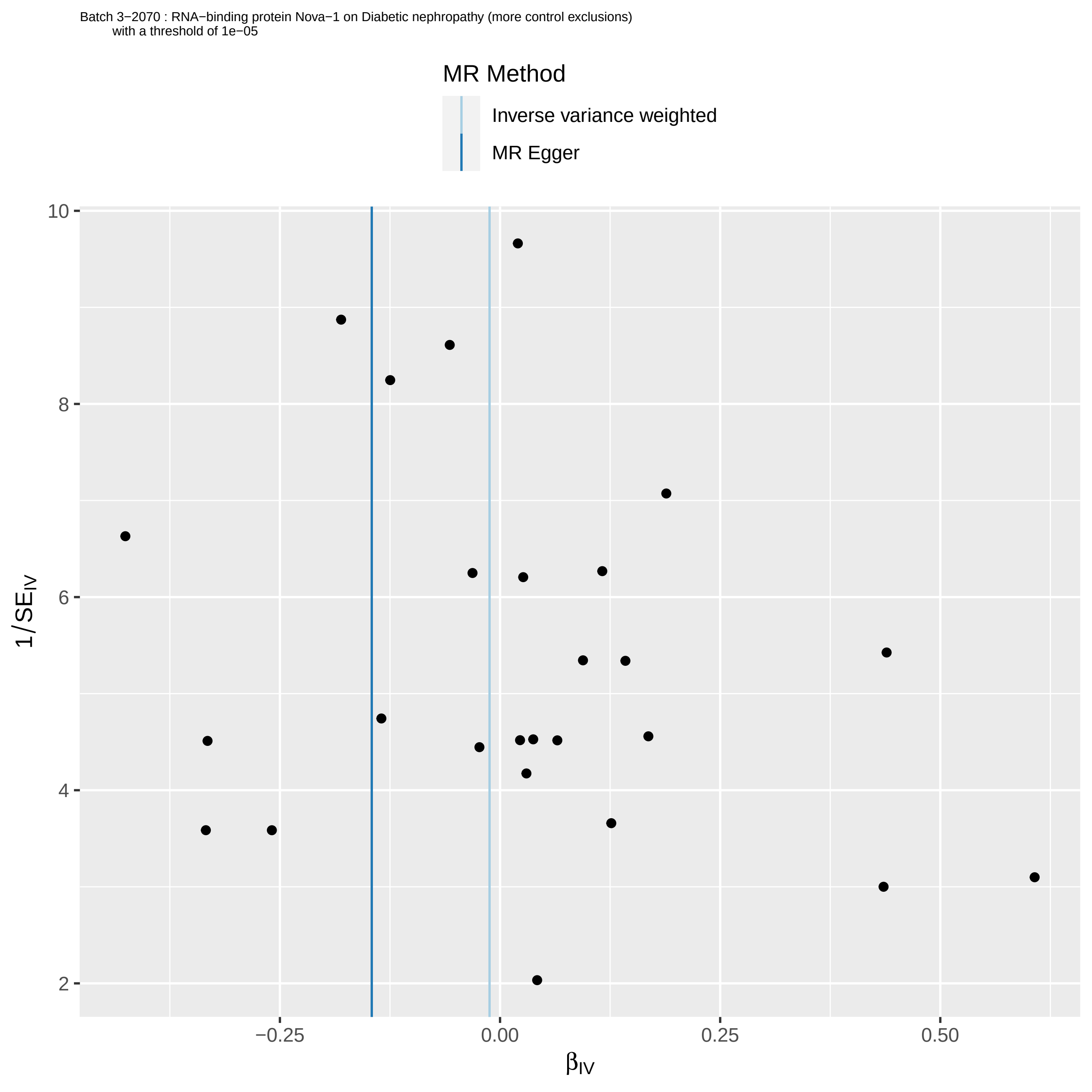
Batch 3−2070 : RNA−binding protein Nova−1 on Diabetic nephropathy (more control exclusions)
with a threshold of 1e−05
MR Method
Inverse variance weighted
MR Egger
10
8
6
4
2
1/SE_IV
−0.25
0.00
0.25
0.50
β_IV

Batch 3−2071 : Epididymal secretory protein E1 on Diabetic nephropathy (more control exclusions)
with a threshold of 1e−05

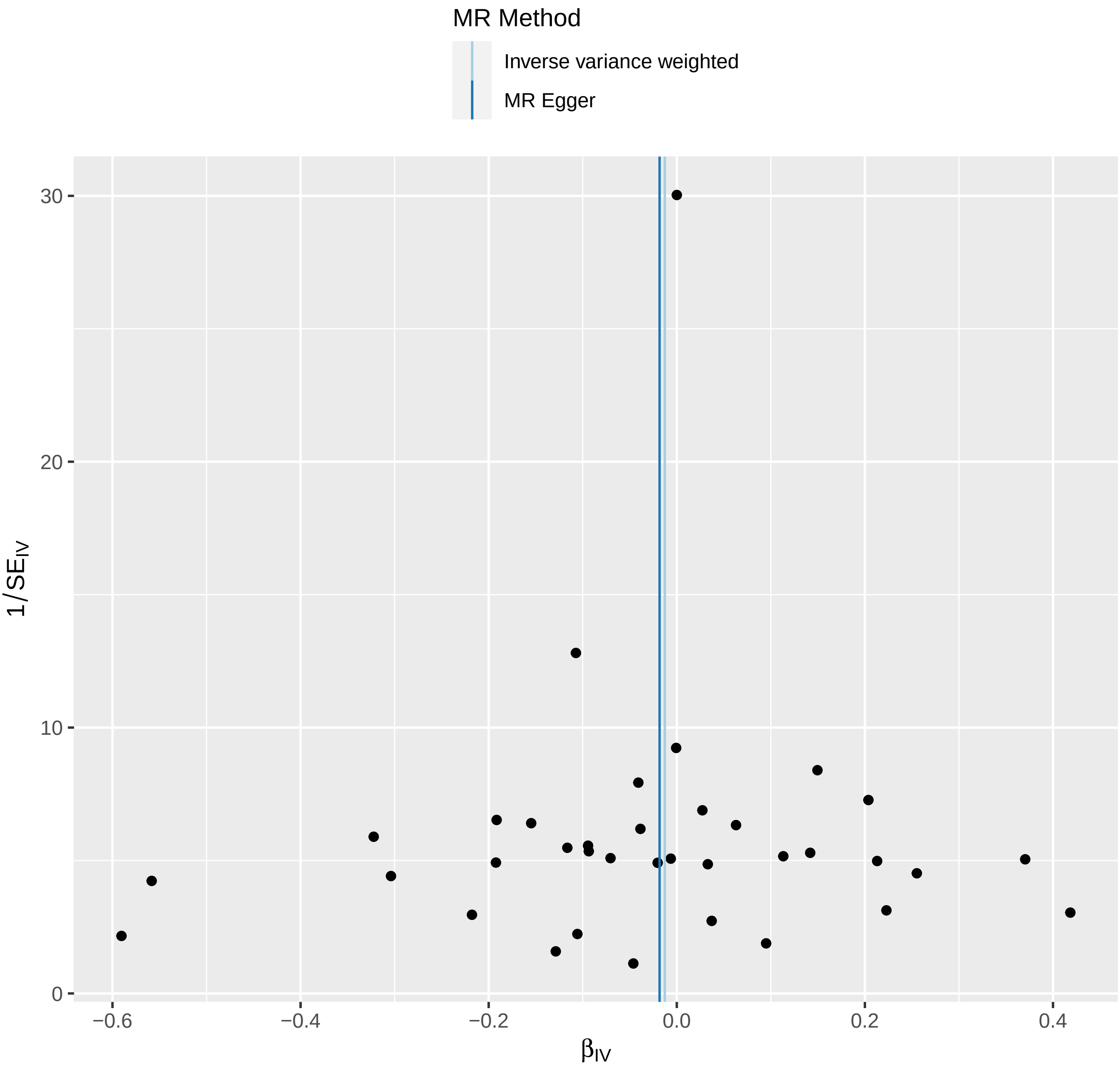

Batch 3−2072 : Neural proliferation differentiation and control protein 1 on Diabetic nephropathy (more control exclusions)
with a threshold of 1e−05

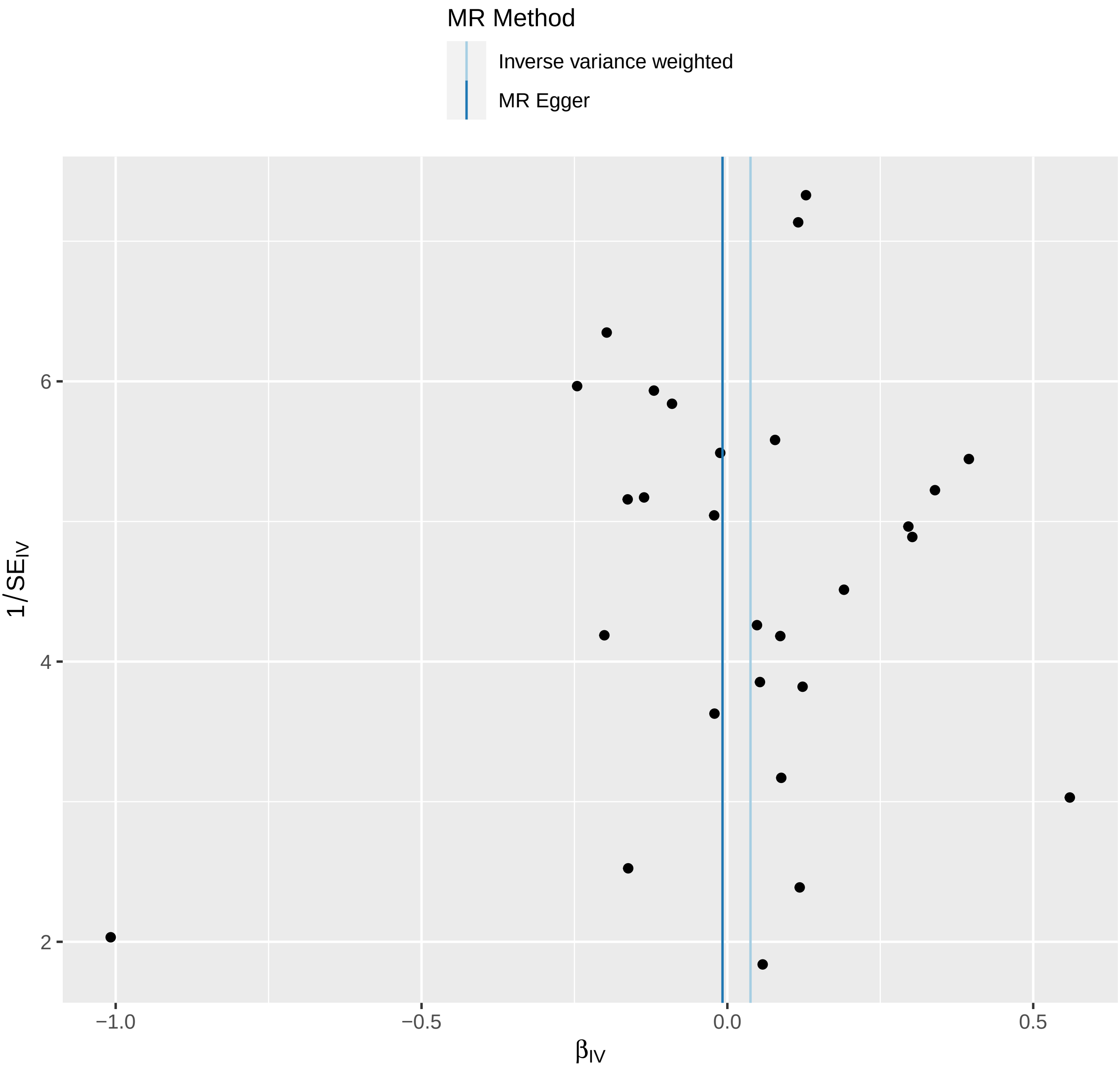

Batch 3−2073 : Pro−FMRFamide−related neuropeptide FF on Diabetic nephropathy (more control exclusions)
with a threshold of 1e−05

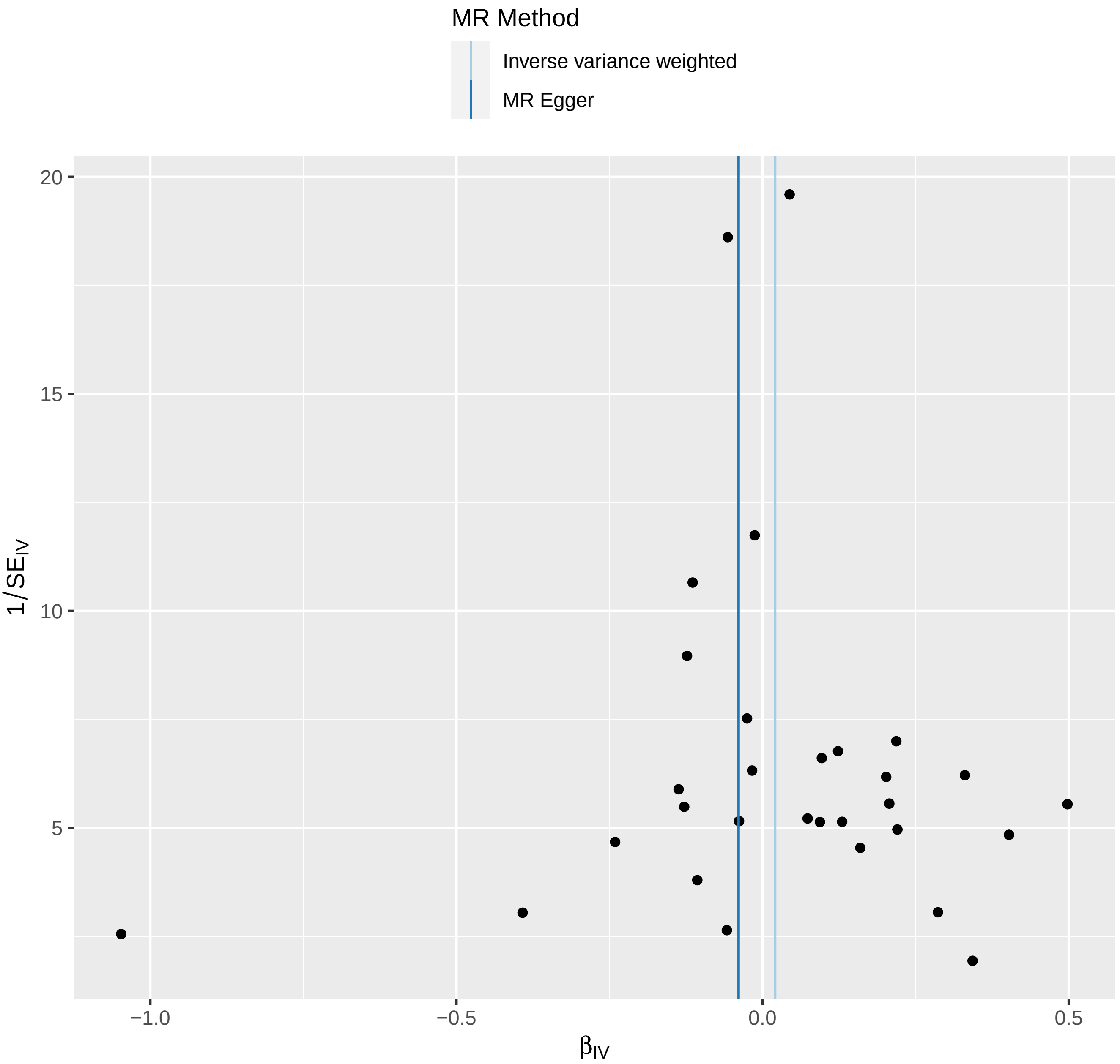

Batch 3−2074 : Nuclear pore complex−interacting protein family member B3 on Diabetic nephropathy (more control exclusions)
with a threshold of 1e−05

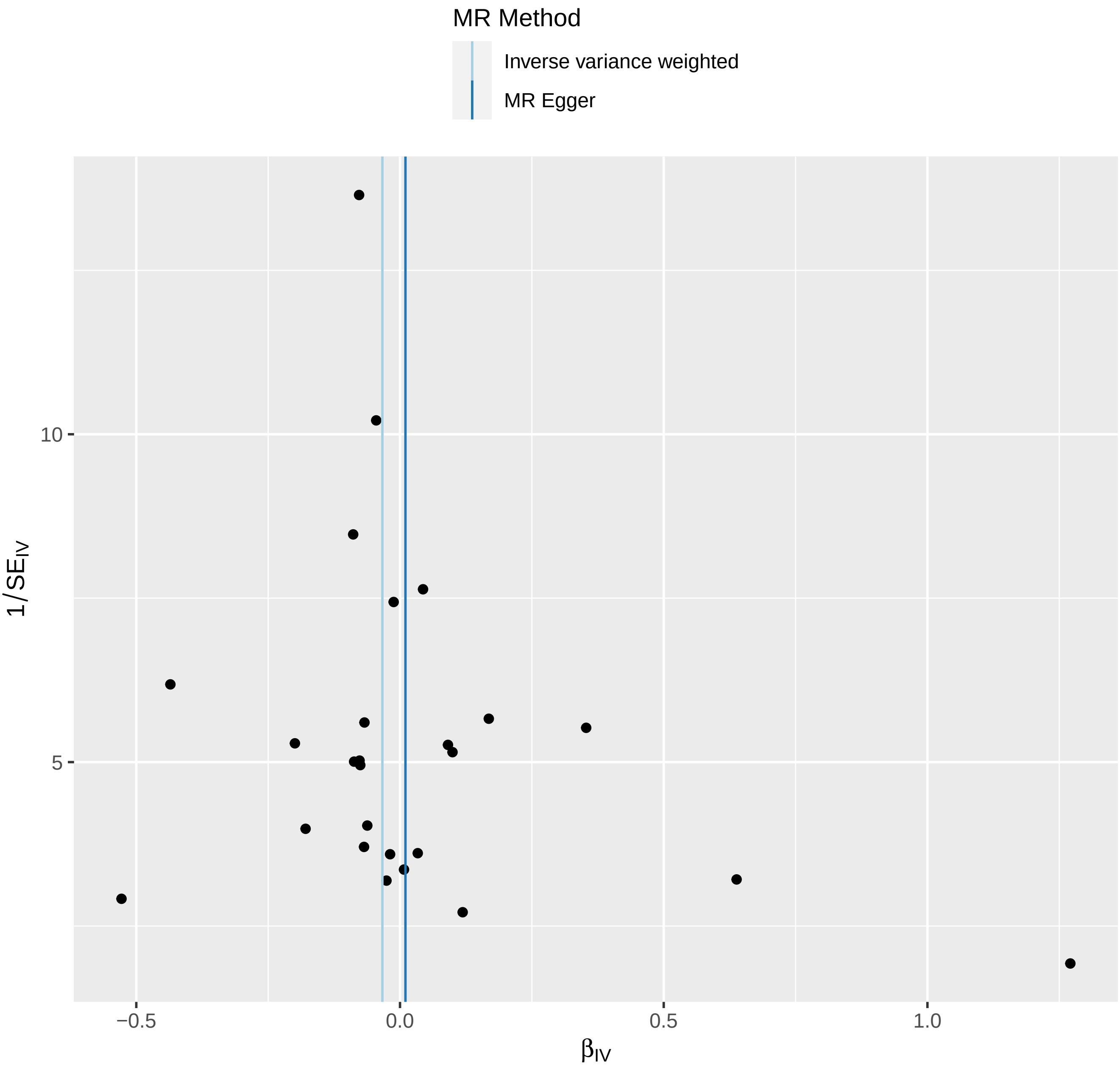

Batch 3−2075 : Nuclear protein localization protein 4 homolog on Diabetic nephropathy (more control exclusions)
with a threshold of 1e−05

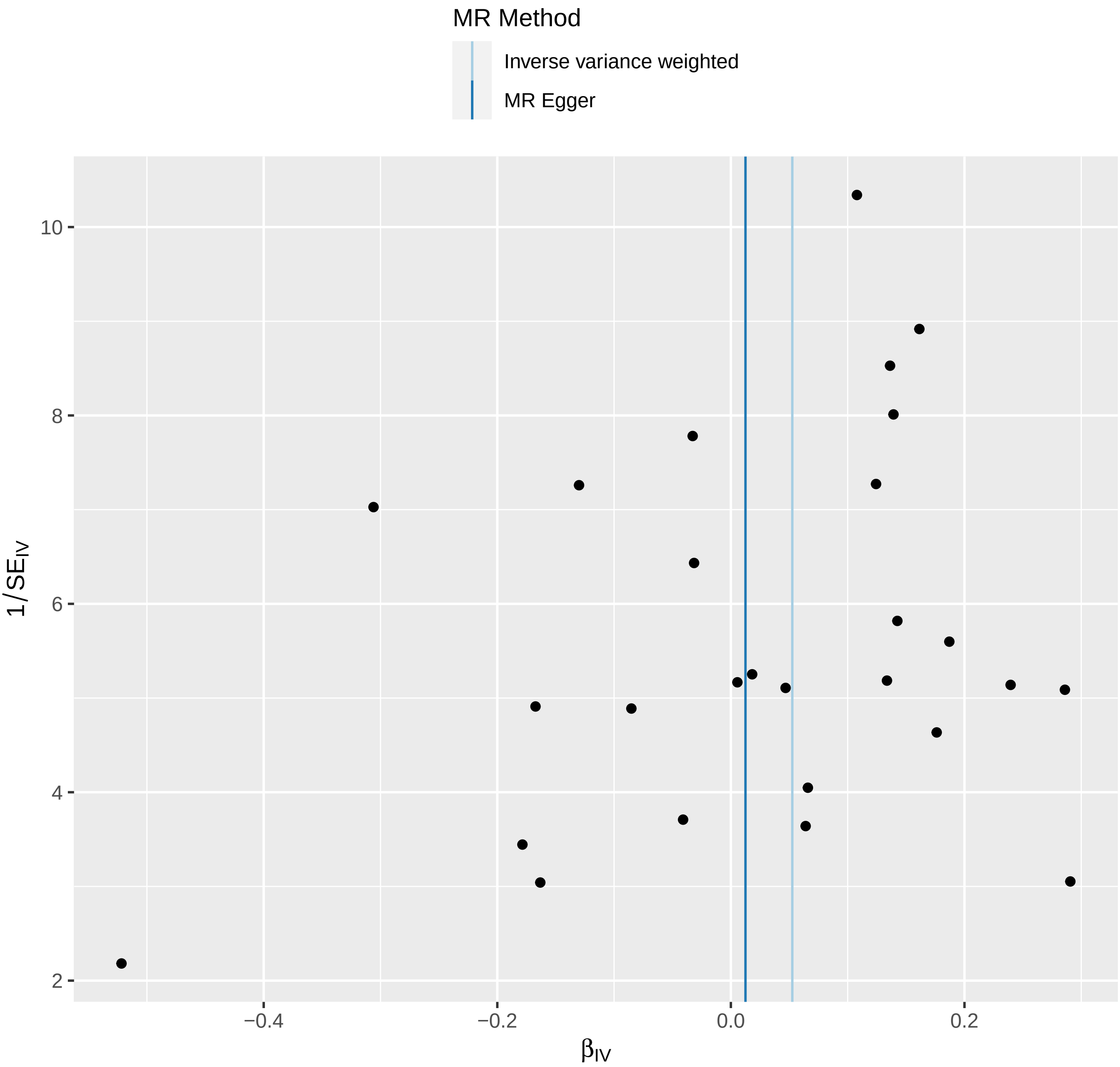

Batch 3−2076 : Atrial natriuretic factor on Diabetic nephropathy (more control exclusions)
with a threshold of 1e−05

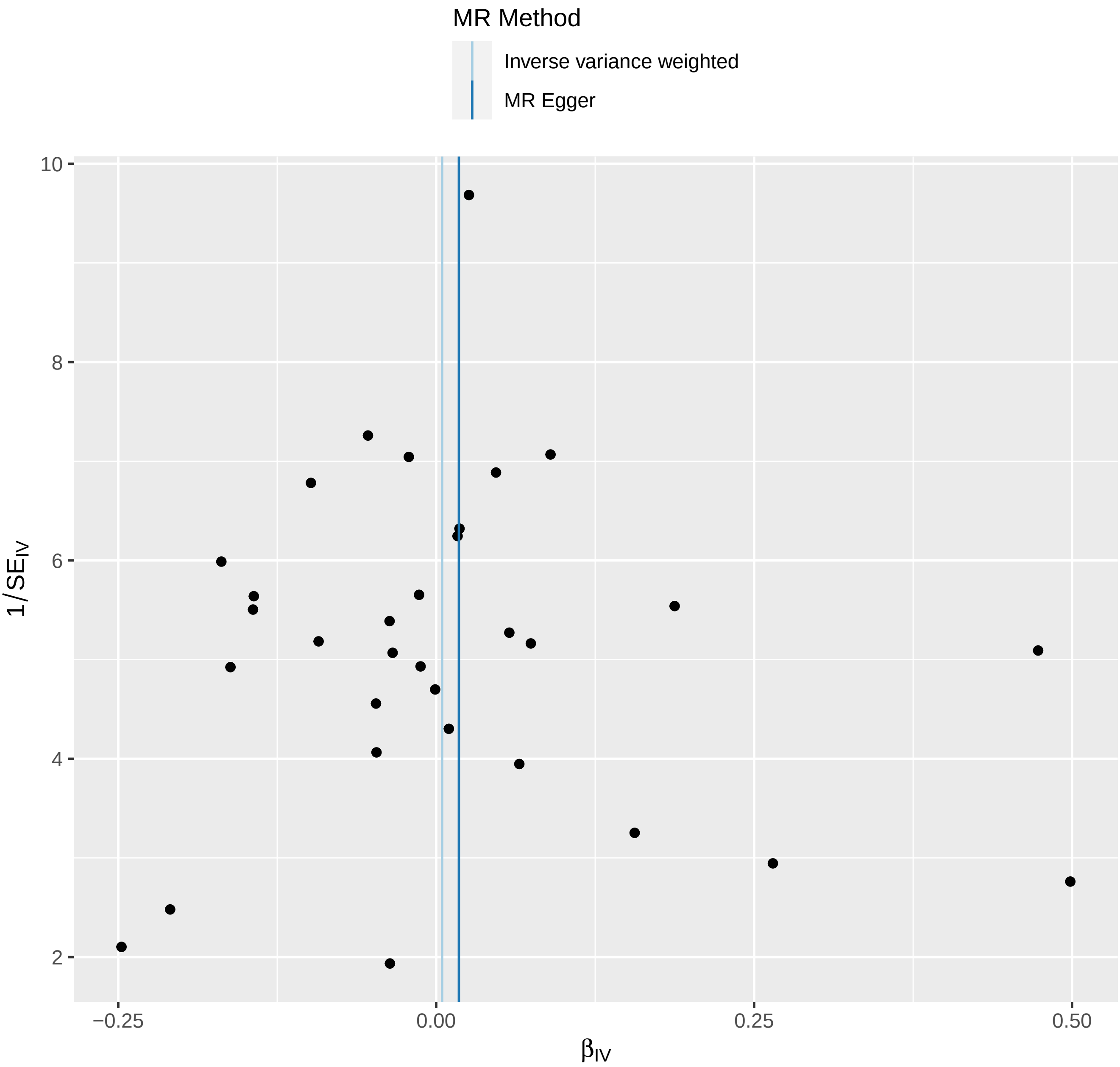

Batch 3−2077 : Brain natriuretic peptide 32 on Diabetic nephropathy (more control exclusions)
with a threshold of 1e−05

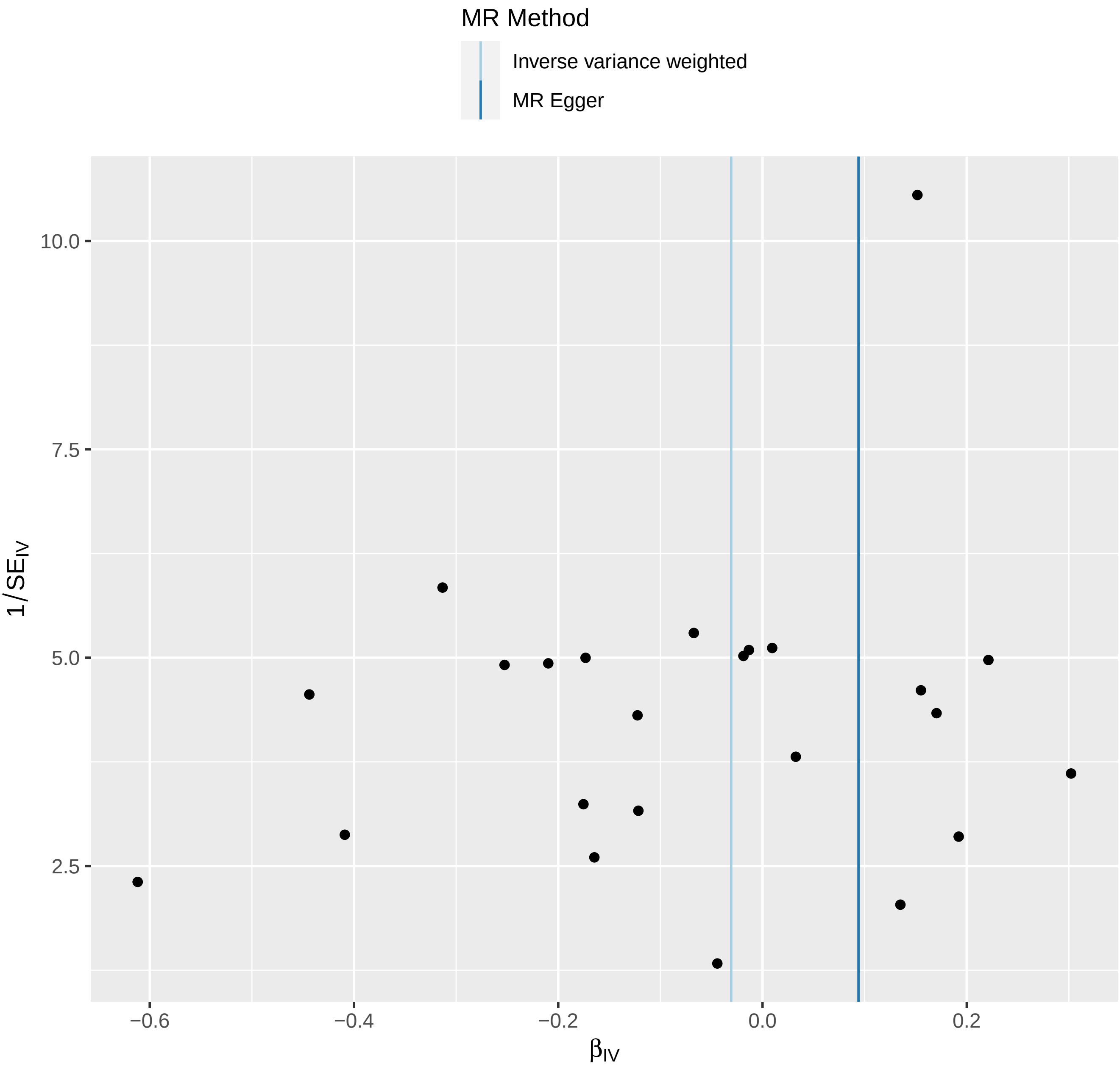

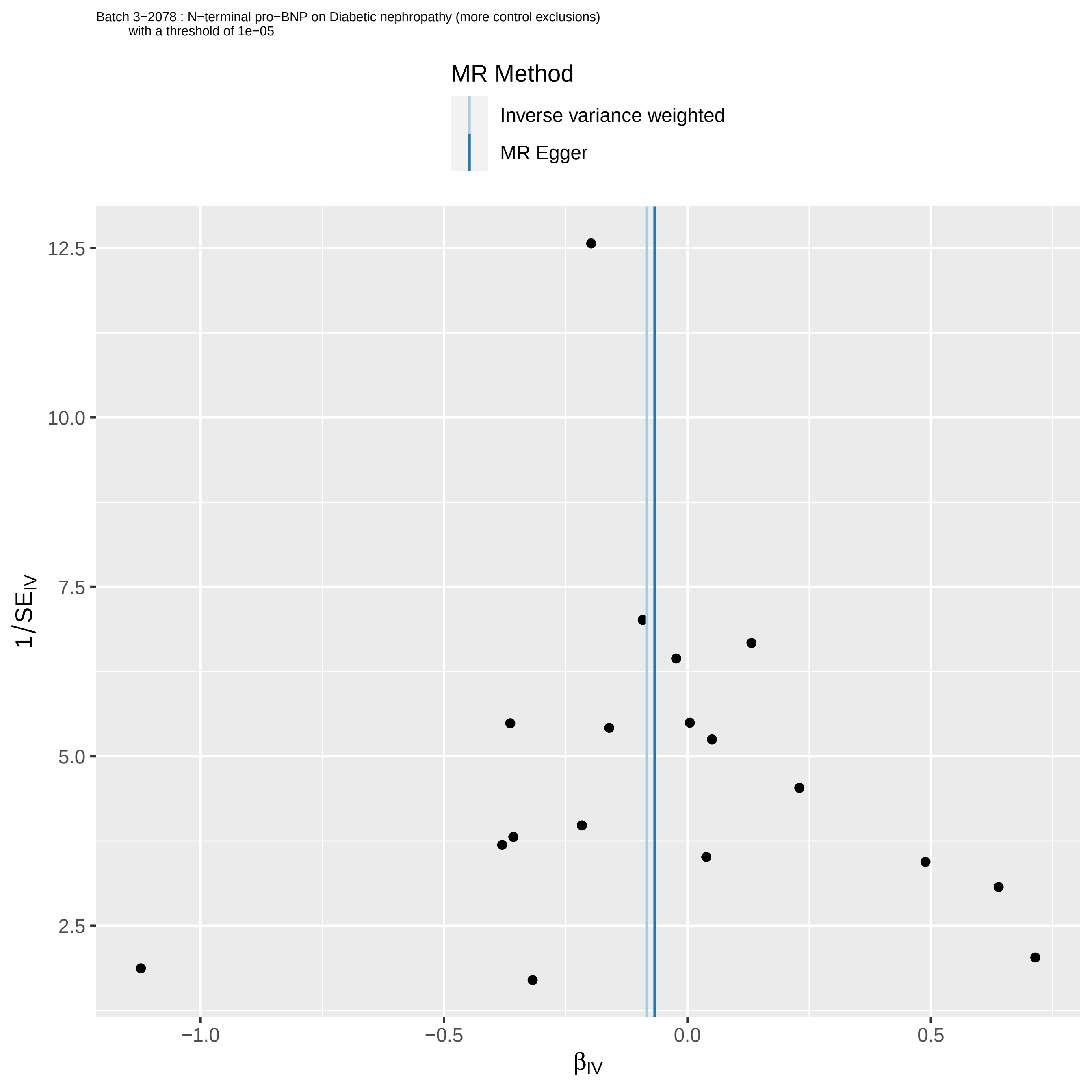
Batch 3−2078 : N−terminal pro−BNP on Diabetic nephropathy (more control exclusions)
with a threshold of 1e−05
MR Method
Inverse variance weighted
MR Egger
12.5
10.0
7.5
5.0
2.5
$1/SE_{IV}$
−1.0
−0.5
0.0
0.5
β_{IV}

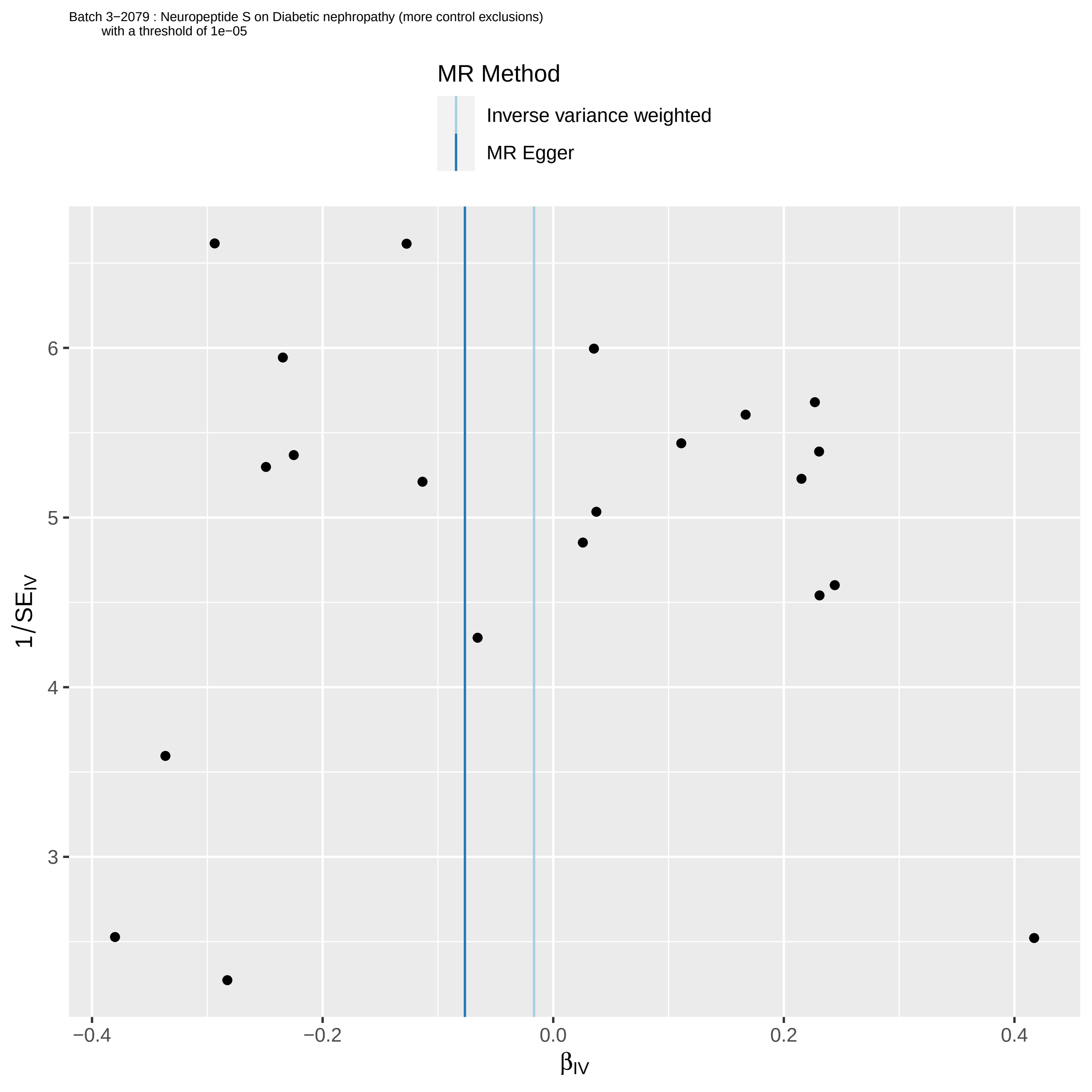
Batch 3−2079 : Neuropeptide S on Diabetic nephropathy (more control exclusions)
with a threshold of 1e−05
MR Method
Inverse variance weighted
MR Egger
6
5
4
3
1/SE_IV
−0.4
−0.2
0.0
0.2
0.4
β_IV

Batch 3−2080 : Neuronal pentraxin−1 on Diabetic nephropathy (more control exclusions)
with a threshold of 1e−05

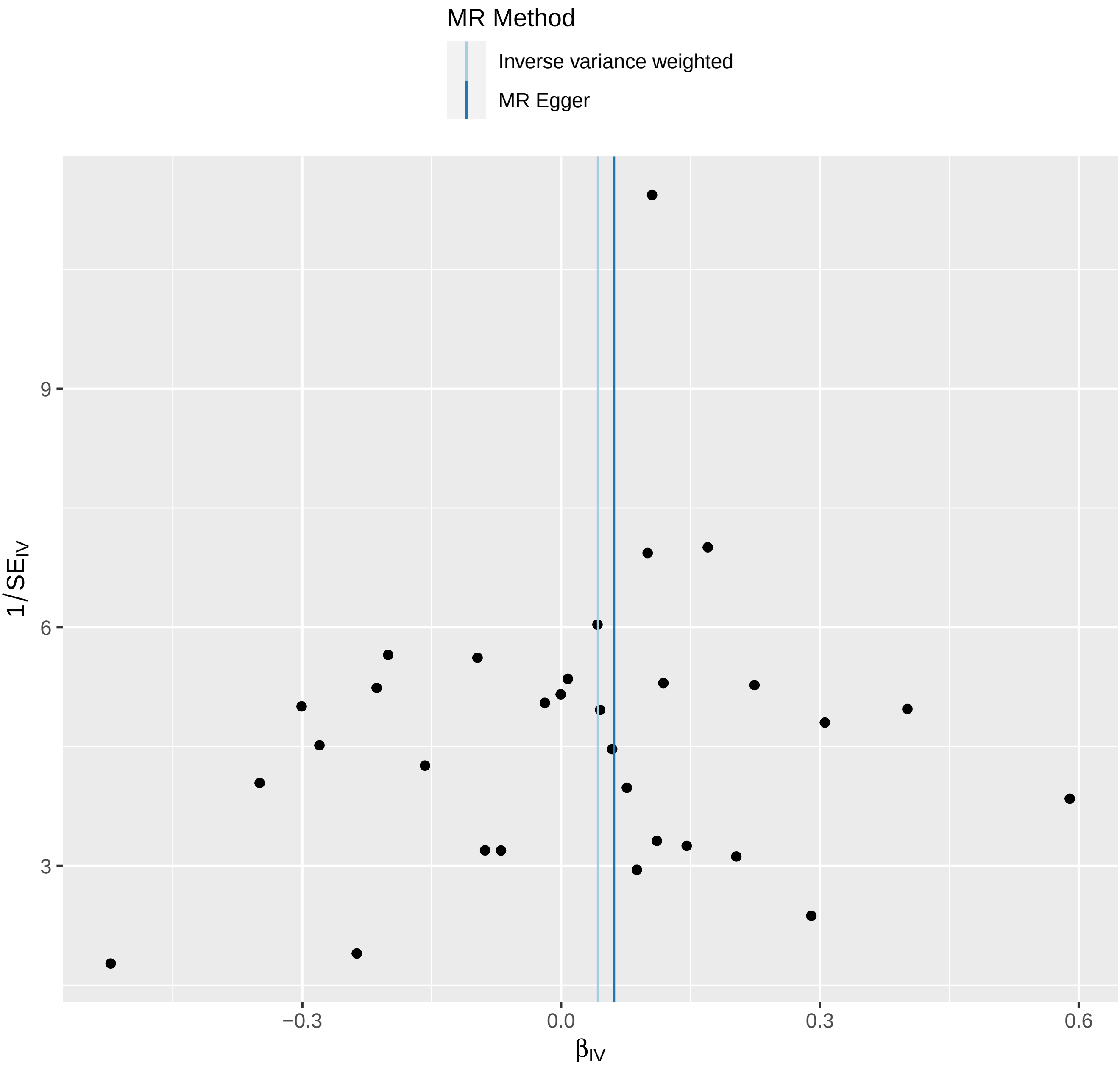

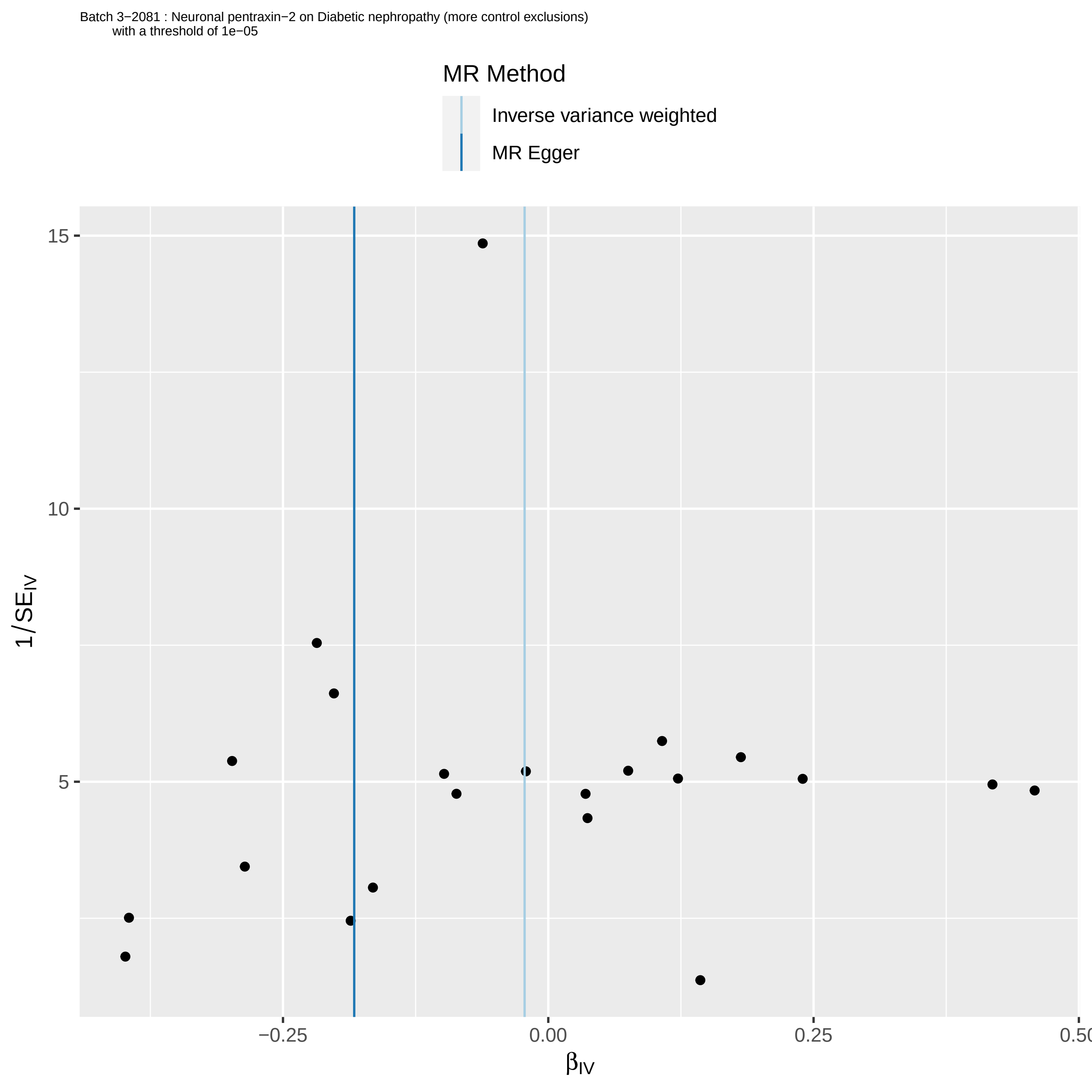

Batch 3−2081 : Neuronal pentraxin−2 on Diabetic nephropathy (more control exclusions)
with a threshold of 1e−05
MR Method
Inverse variance weighted
MR Egger
15
10
5
1/SE_IV
−0.25
0.00
0.25
0.50
β_IV

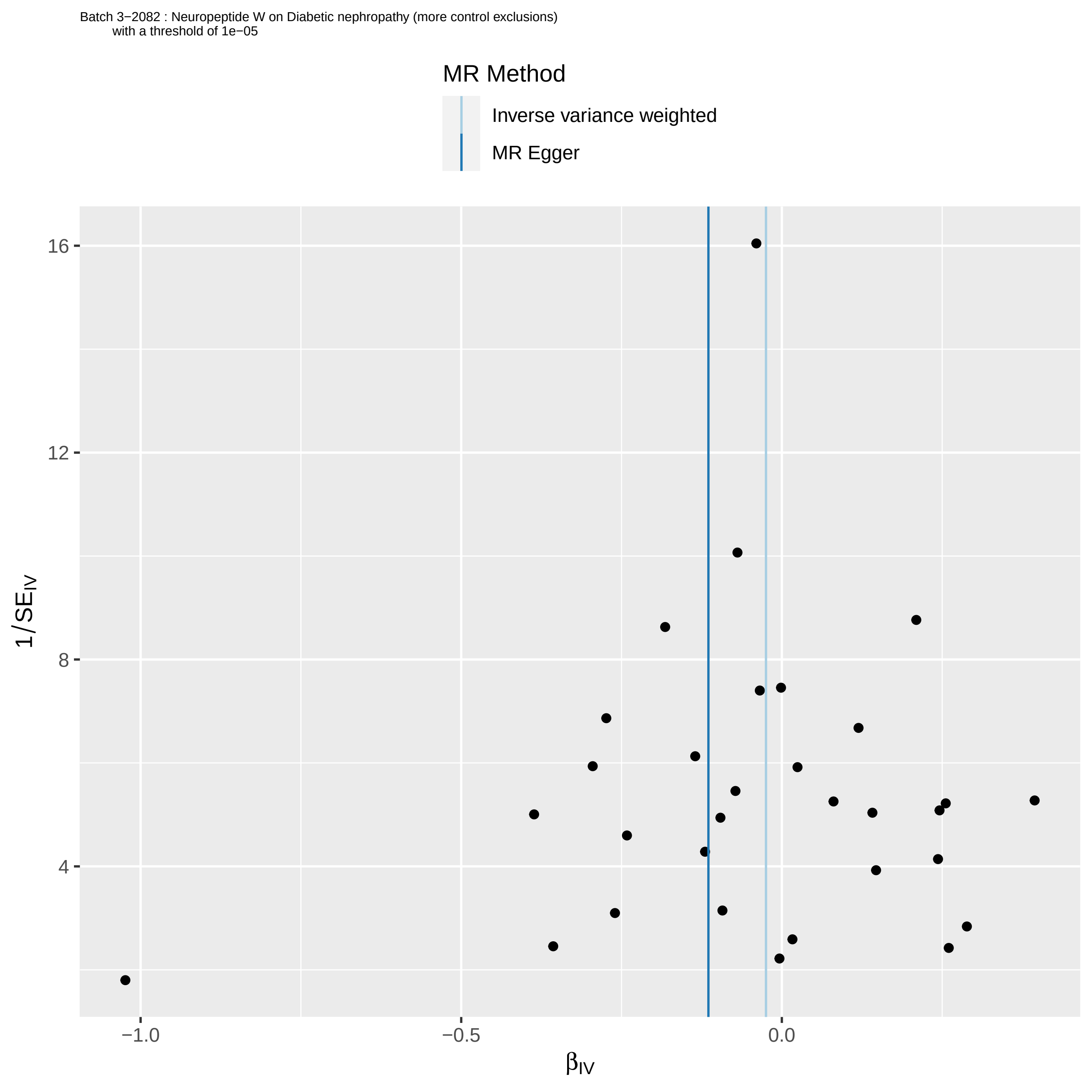

Batch 3−2082 : Neuropeptide W on Diabetic nephropathy (more control exclusions)
with a threshold of 1e−05
MR Method
Inverse variance weighted
MR Egger
16
12
8
4
1/SE_IV
−1.0
−0.5
0.0
β_IV

Batch 3−2083 : NAD(P)H dehydrogenase [quinone] 1 on Diabetic nephropathy (more control exclusions)
with a threshold of 1e−05

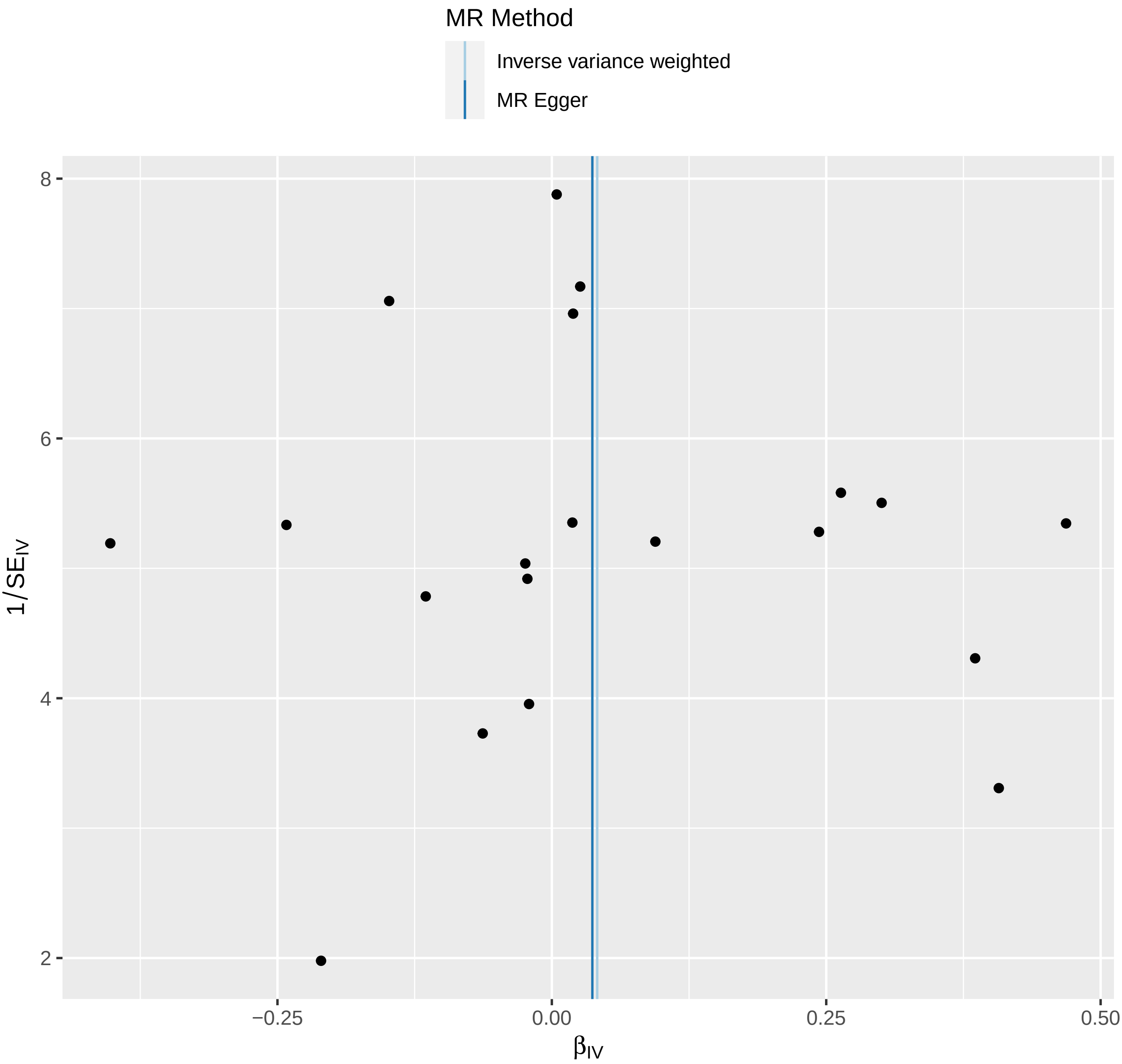

Batch 3−2084 : Ribosyldihydronicotinamide dehydrogenase [quinone] on Diabetic nephropathy (more control exclusions)
with a threshold of 1e−05

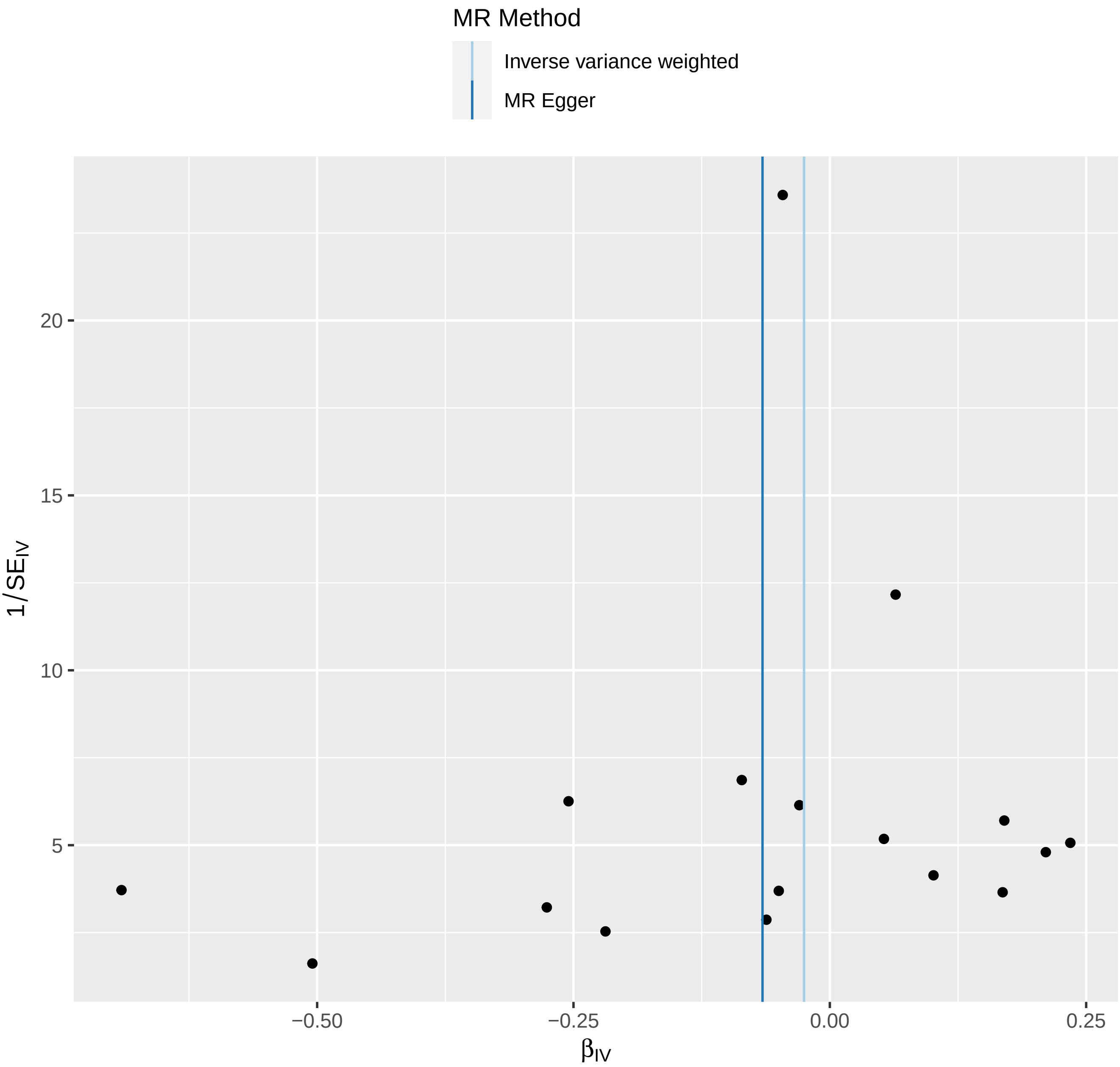

Batch 3−2085 : Nuclear receptor subfamily 1 group D member 1 on Diabetic nephropathy (more control exclusions)
with a threshold of 1e−05

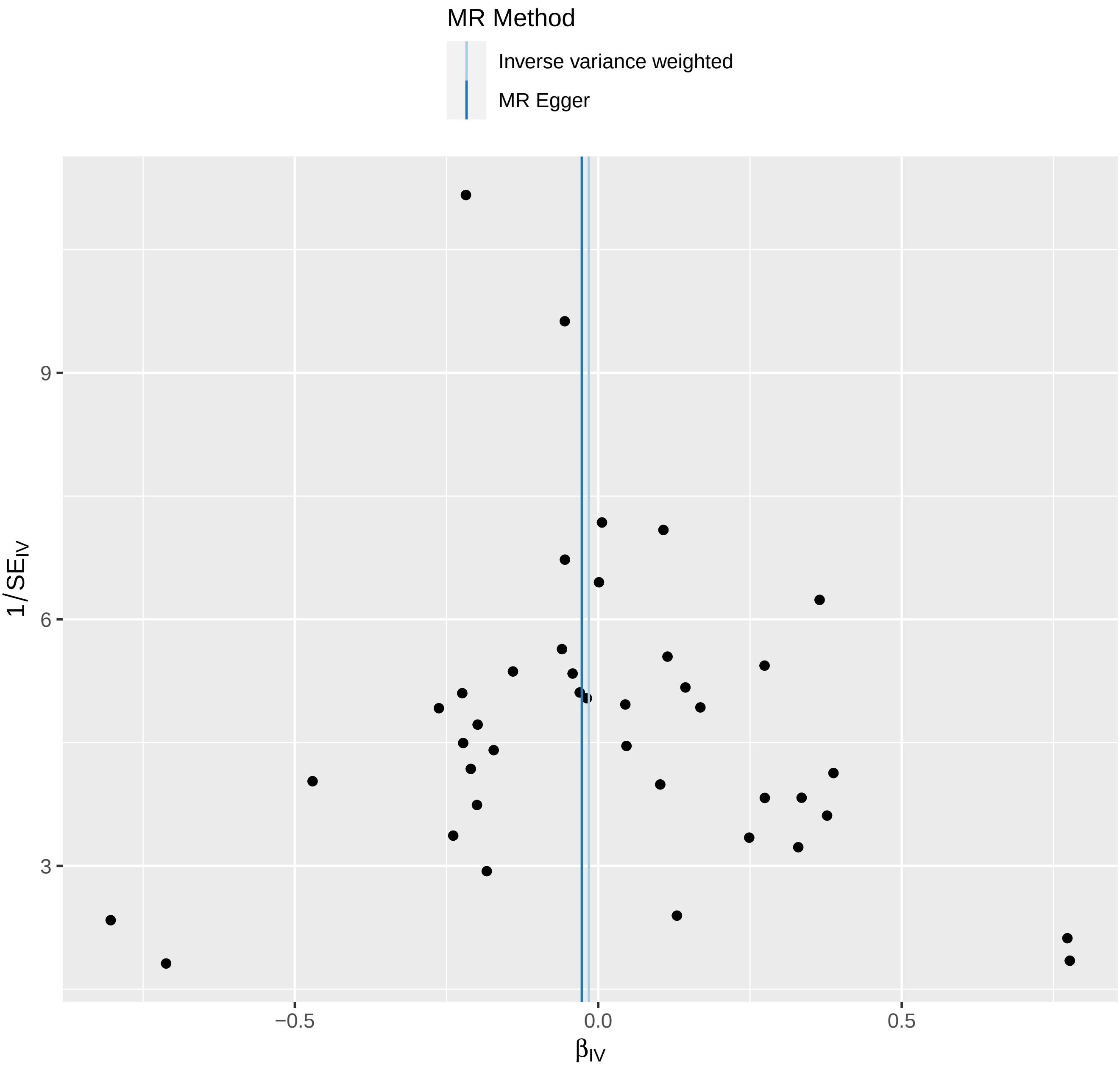

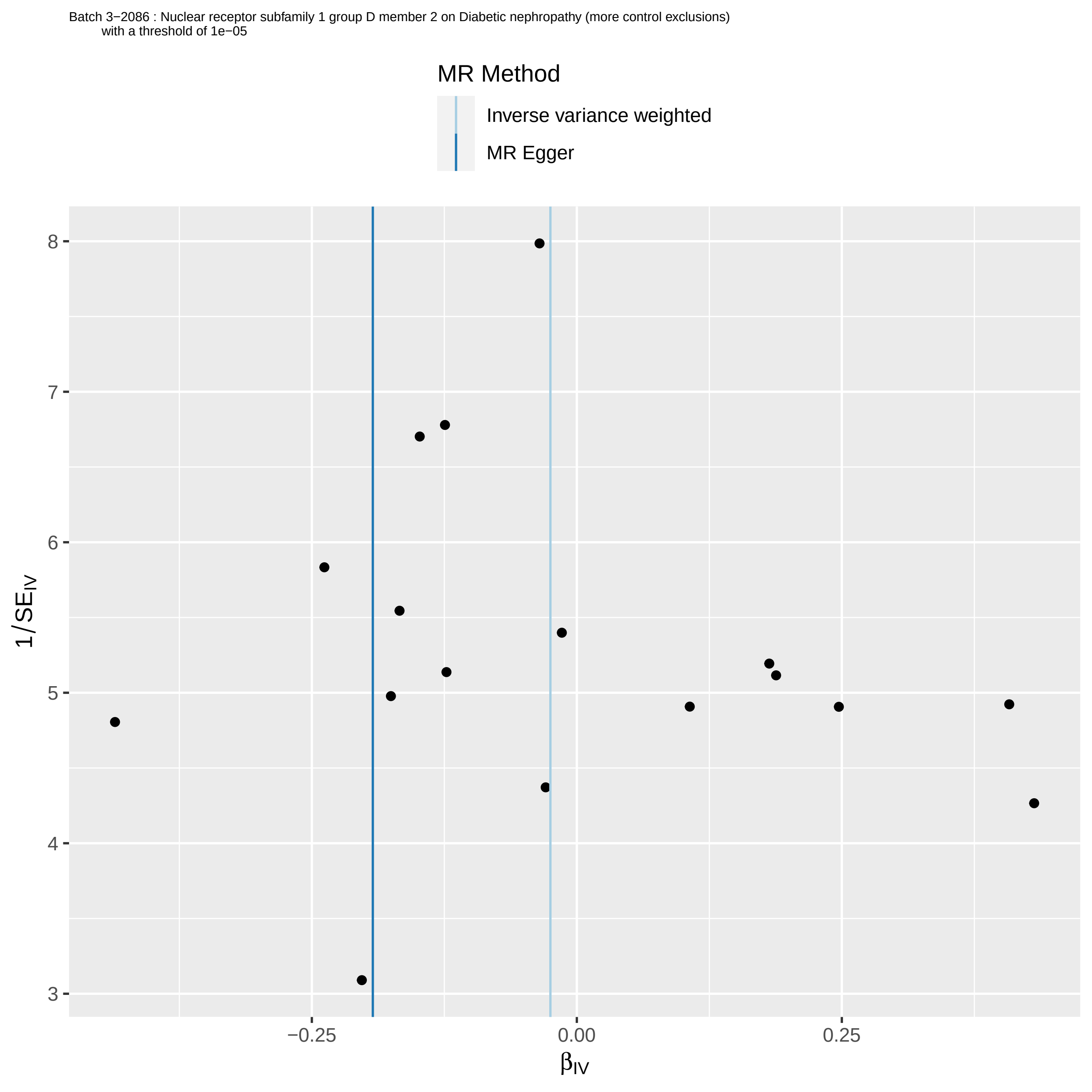
Batch 3−2086 : Nuclear receptor subfamily 1 group D member 2 on Diabetic nephropathy (more control exclusions)
with a threshold of 1e−05
MR Method
Inverse variance weighted
MR Egger
8
7
6
5
4
3
1/SE_IV
−0.25
0.00
0.25
β_IV

Batch 3−2087 : Oxysterols receptor LXR−beta on Diabetic nephropathy (more control exclusions)
with a threshold of 1e−05

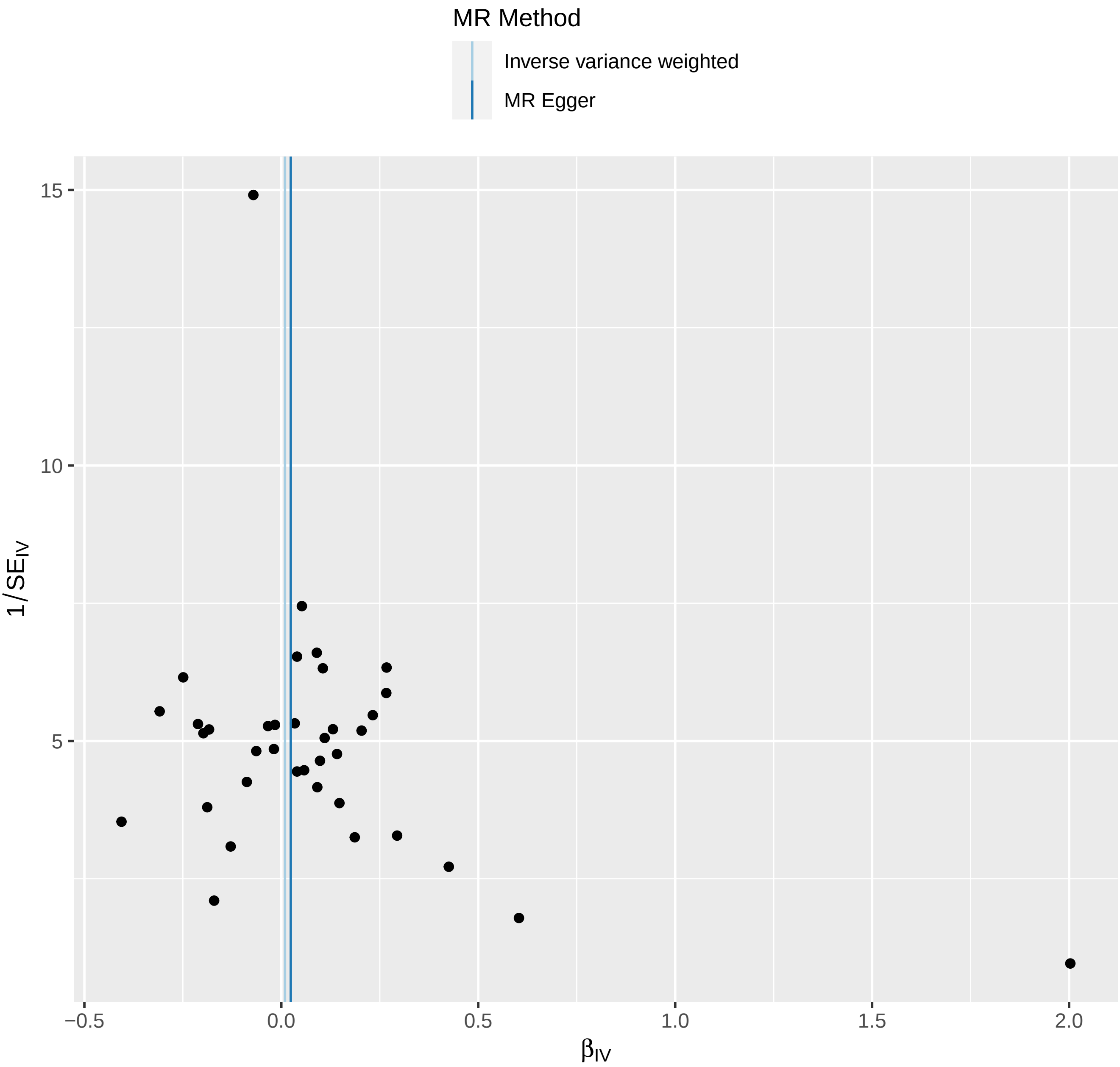

Batch 3−2088 : Glucocorticoid receptor on Diabetic nephropathy (more control exclusions)
with a threshold of 1e−05

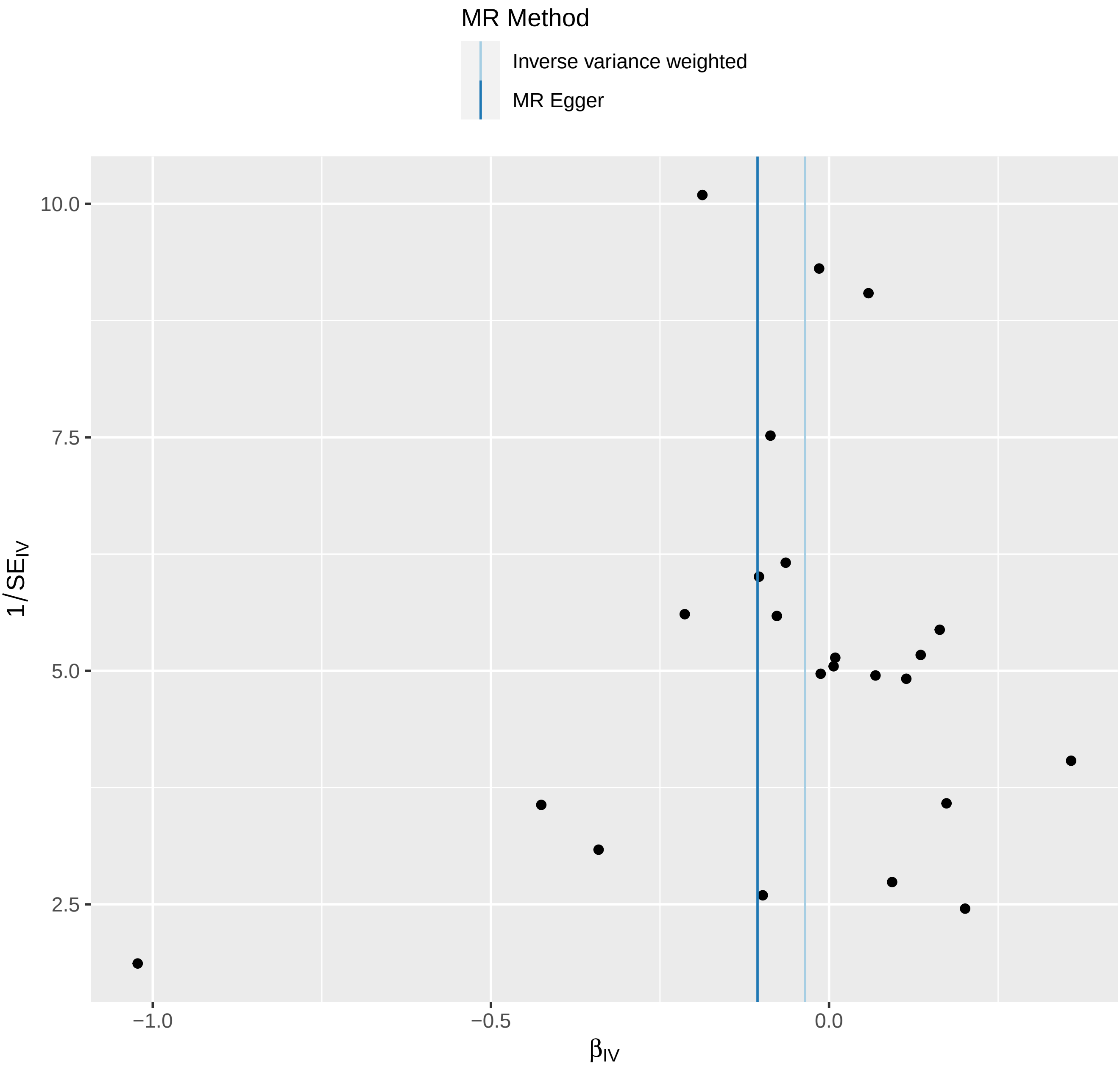

Batch 3−2089 : Mineralocorticoid receptor on Diabetic nephropathy (more control exclusions)
with a threshold of 1e−05

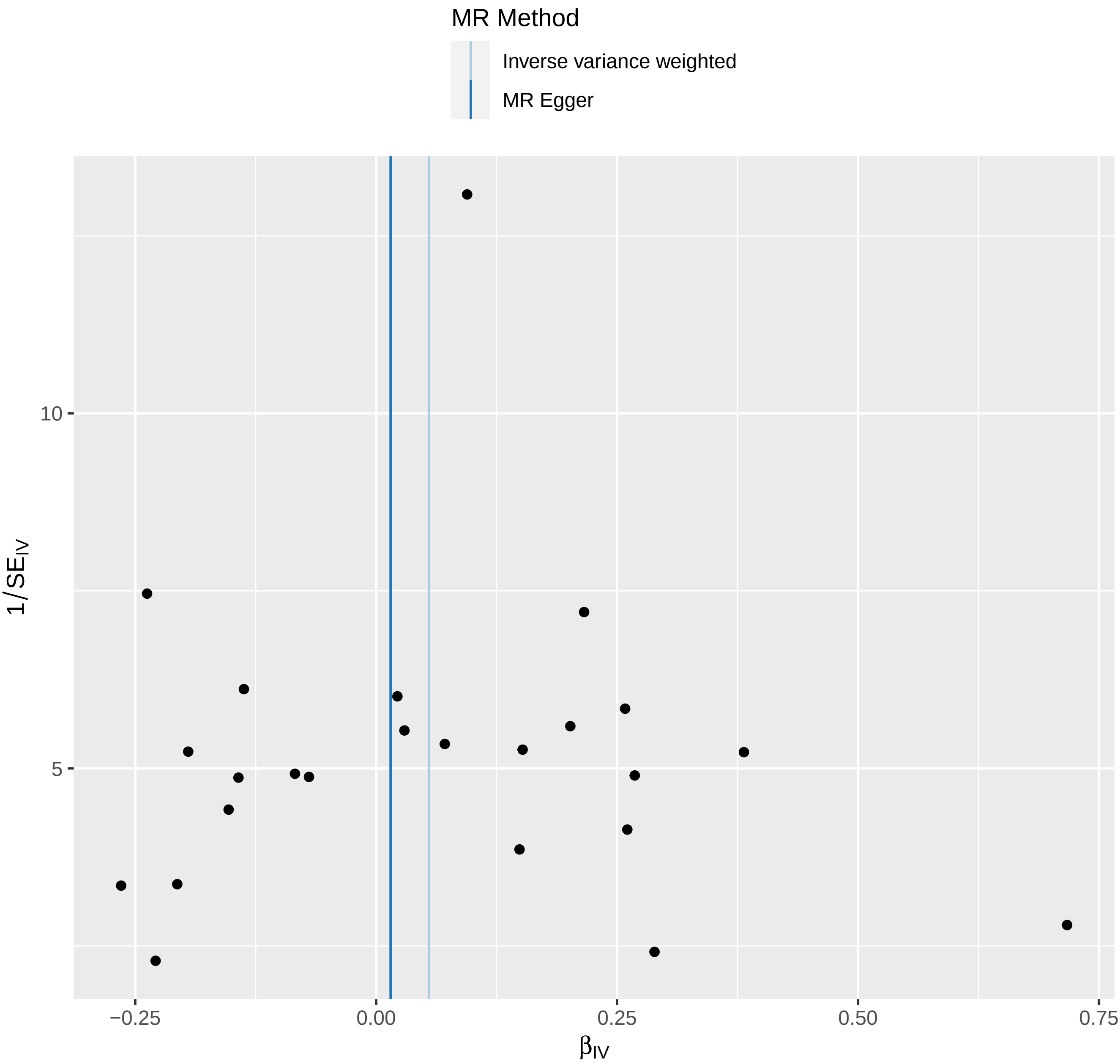

Batch 3−2090 : Nuclear receptor subfamily 4 group A member 1 on Diabetic nephropathy (more control exclusions)
with a threshold of 1e−05

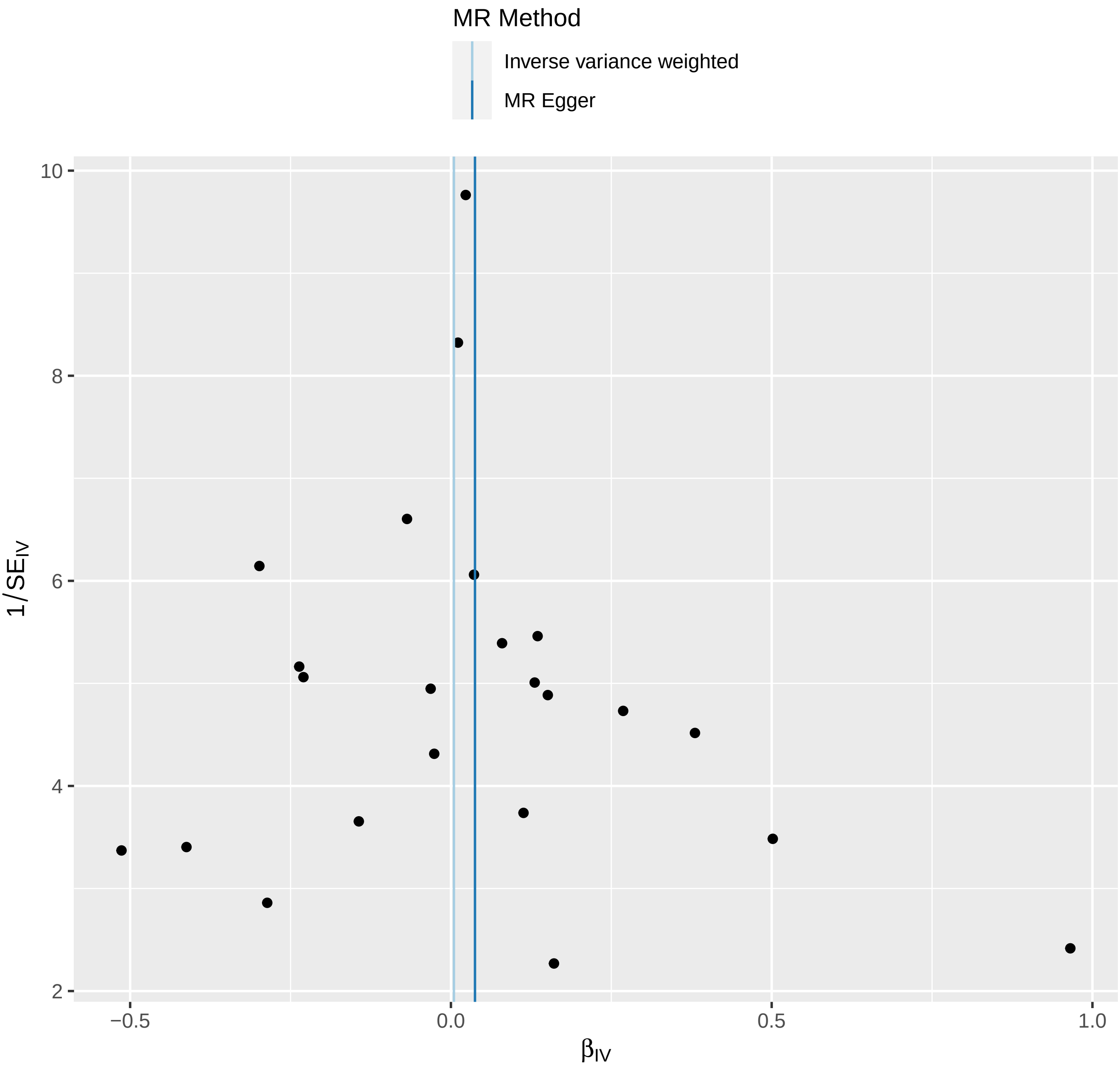

Batch 3−2091 : Nuclear receptor subfamily 5 group A member 2 on Diabetic nephropathy (more control exclusions)
with a threshold of 1e−05

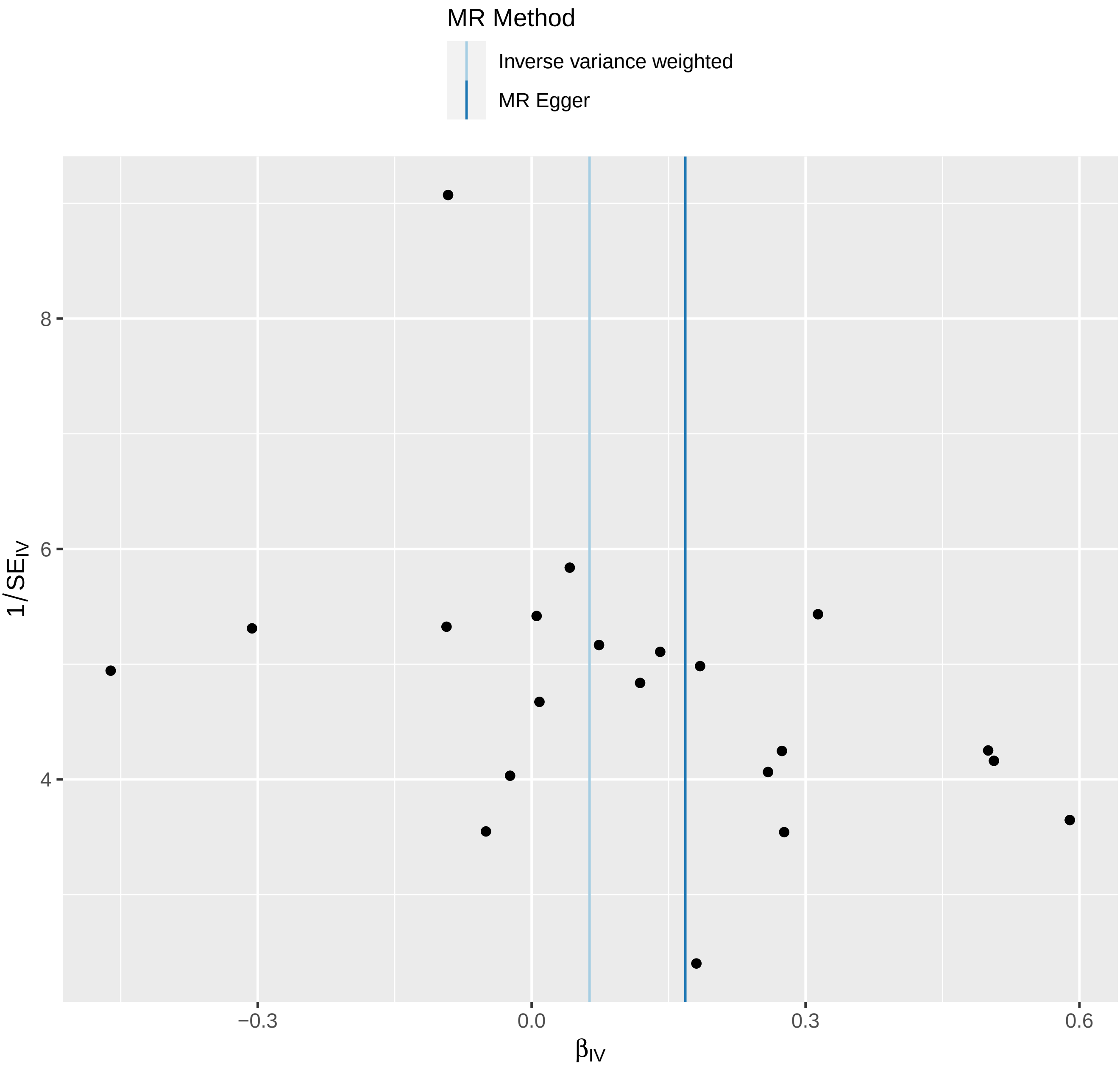

Batch 3−2092 : Nutritionally−regulated adipose and cardiac enriched protein homolog on Diabetic nephropathy (more control exclusions) with a threshold of 1e−05

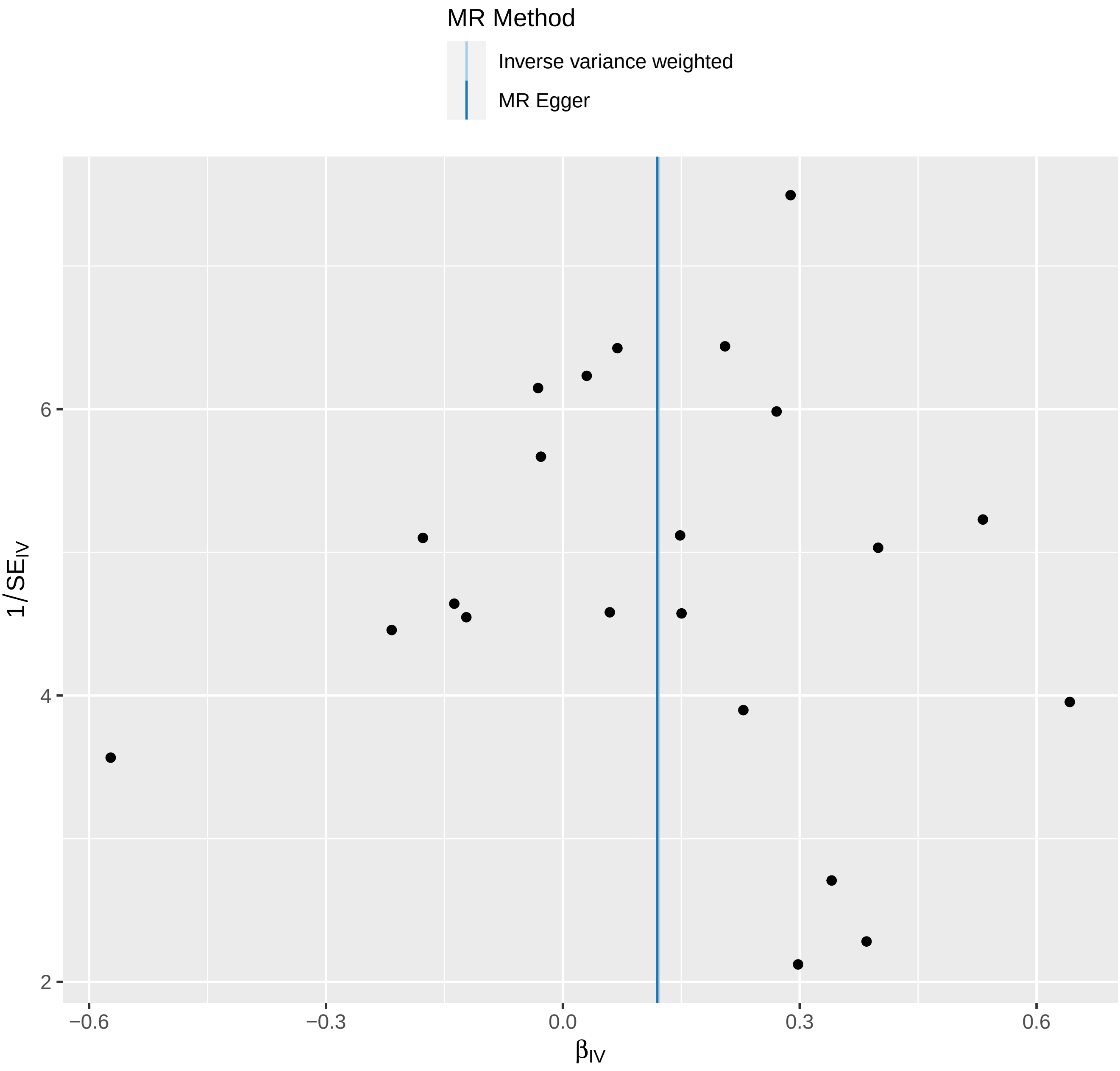

Batch 3−2093 : Nuclear receptor−binding protein on Diabetic nephropathy (more control exclusions)
with a threshold of 1e−05

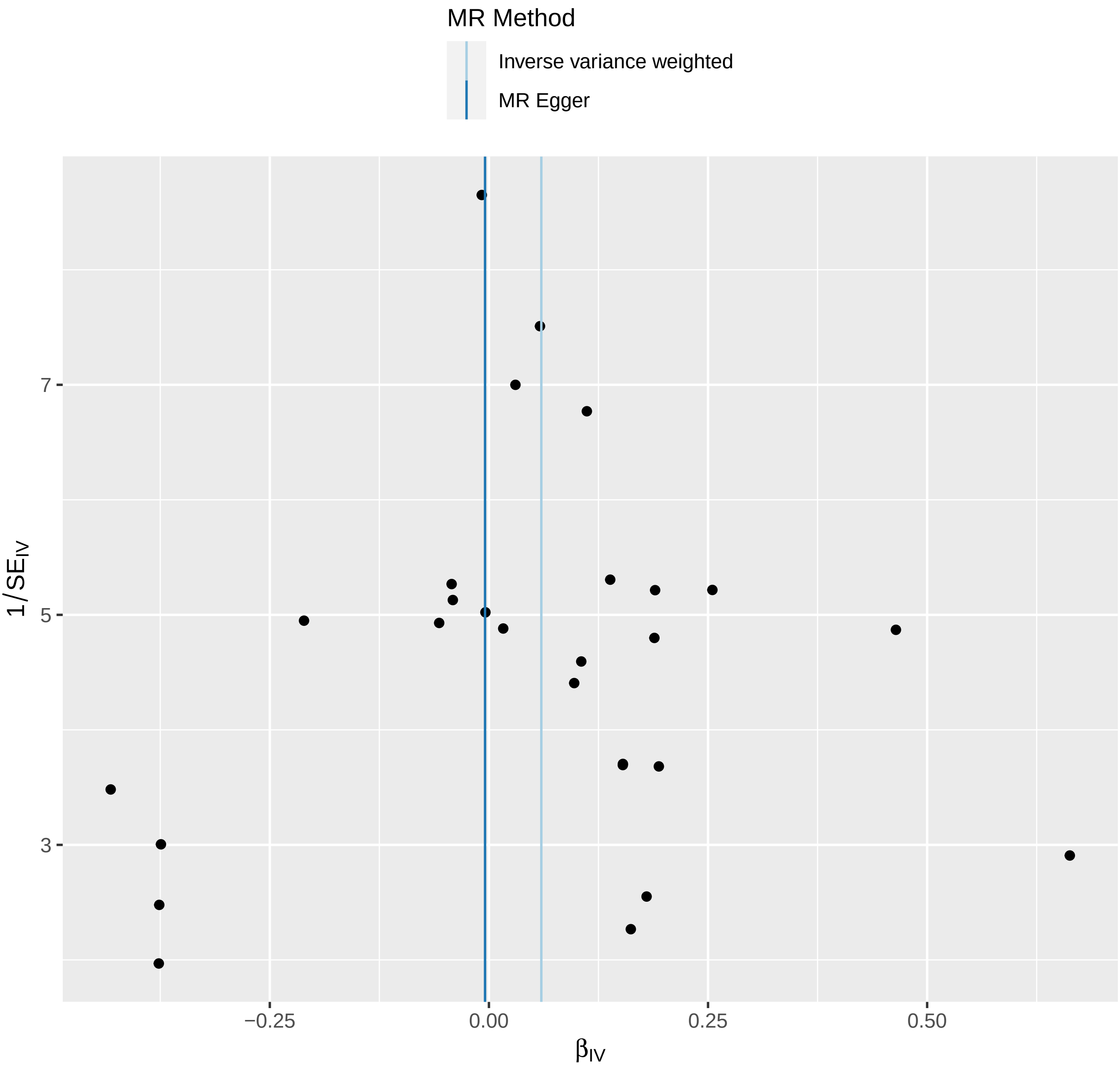

Batch 3−2094 : Neuronal cell adhesion molecule on Diabetic nephropathy (more control exclusions)
with a threshold of 1e−05

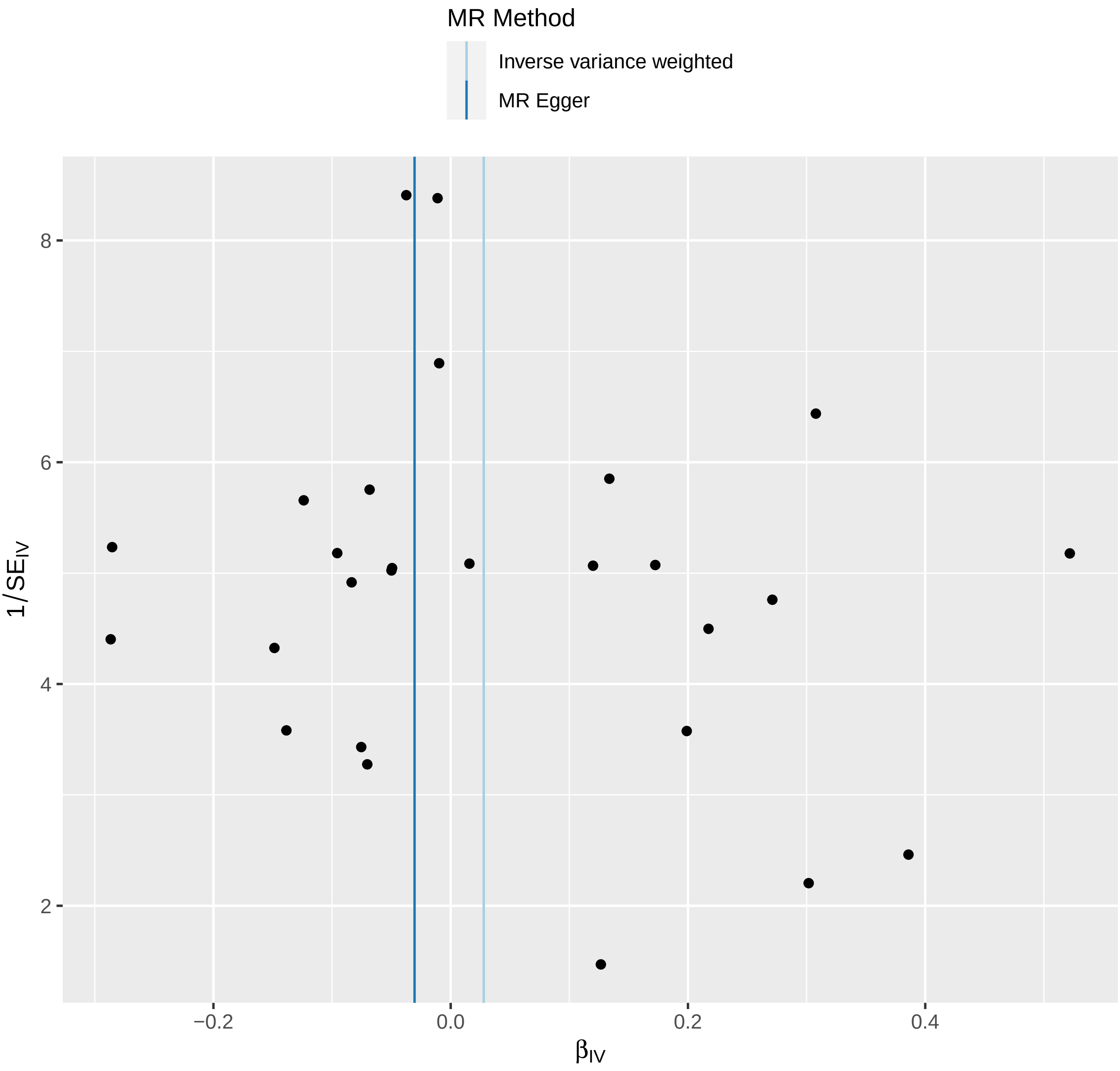

Batch 3−2095 : Neuregulin−1 on Diabetic nephropathy (more control exclusions)
with a threshold of 1e−05

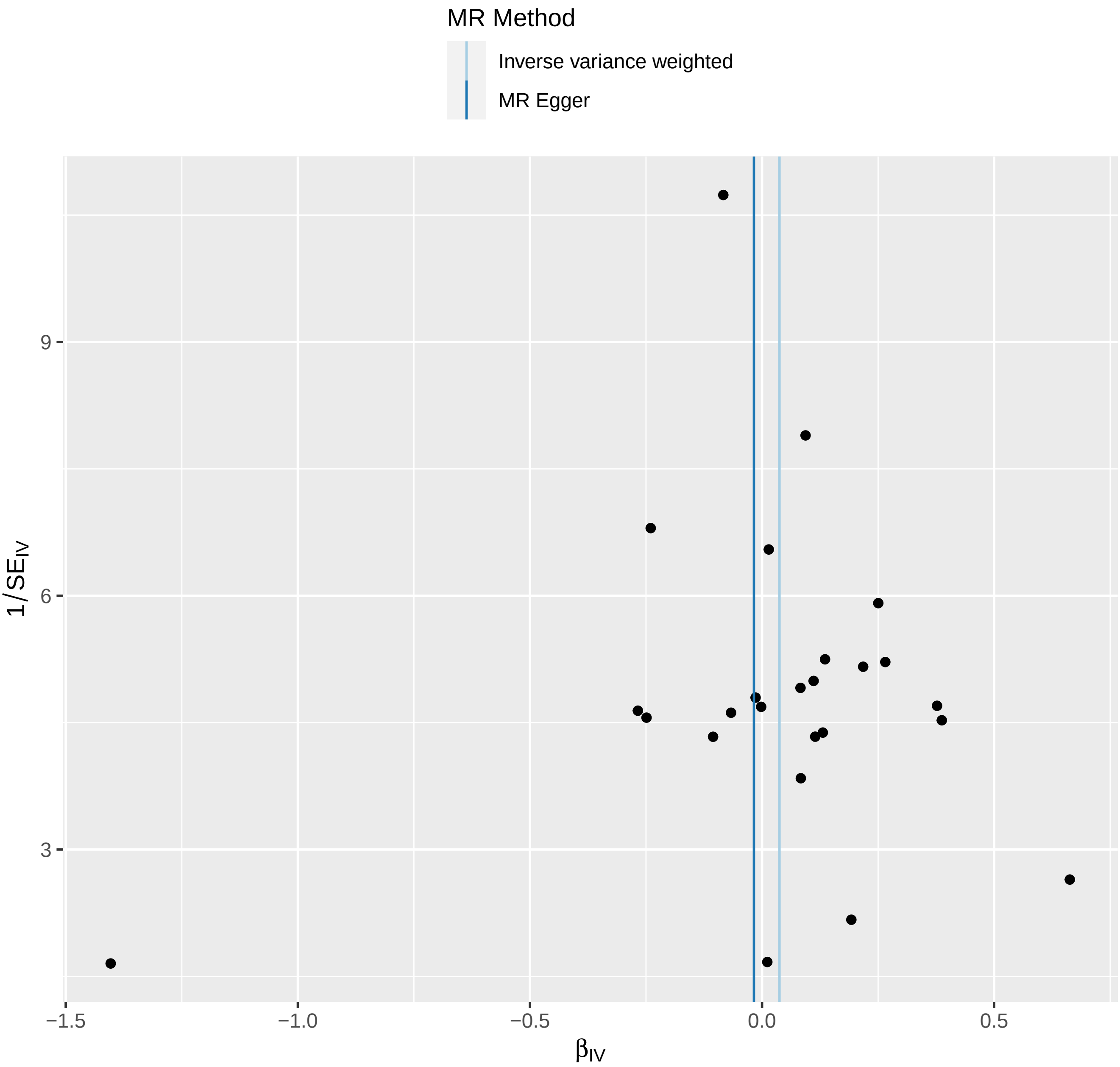

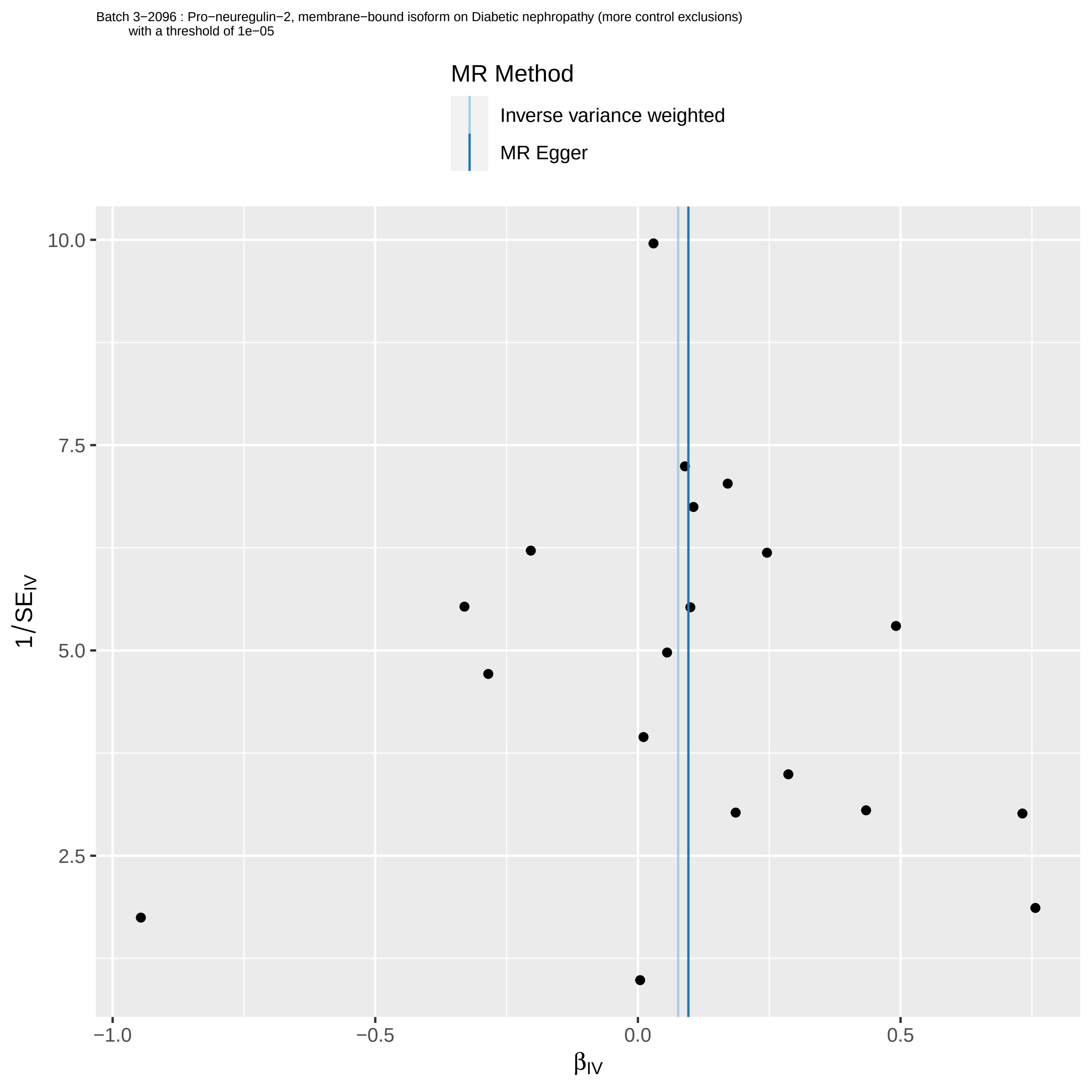

Batch 3−2096 : Pro−neuregulin−2, membrane−bound isoform on Diabetic nephropathy (more control exclusions)
with a threshold of 1e−05
MR Method
Inverse variance weighted
MR Egger
10.0
7.5
5.0
2.5
1/SE_IV
−1.0
−0.5
0.0
0.5
β_IV

Batch 3−2097 : Neuregulin−4 on Diabetic nephropathy (more control exclusions)
with a threshold of 1e−05

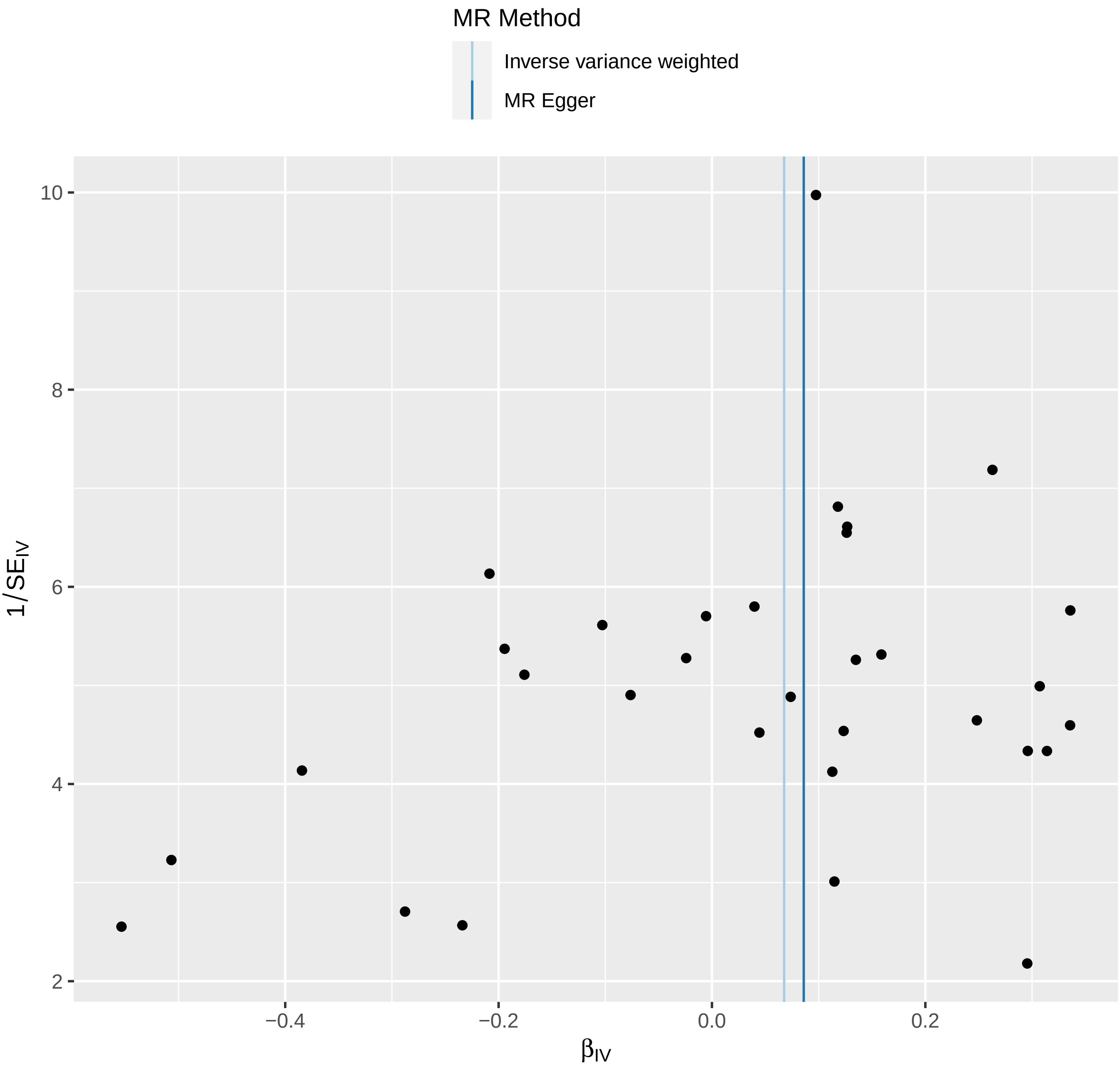

Batch 3−2098 : Neuritin on Diabetic nephropathy (more control exclusions)
with a threshold of 1e−05

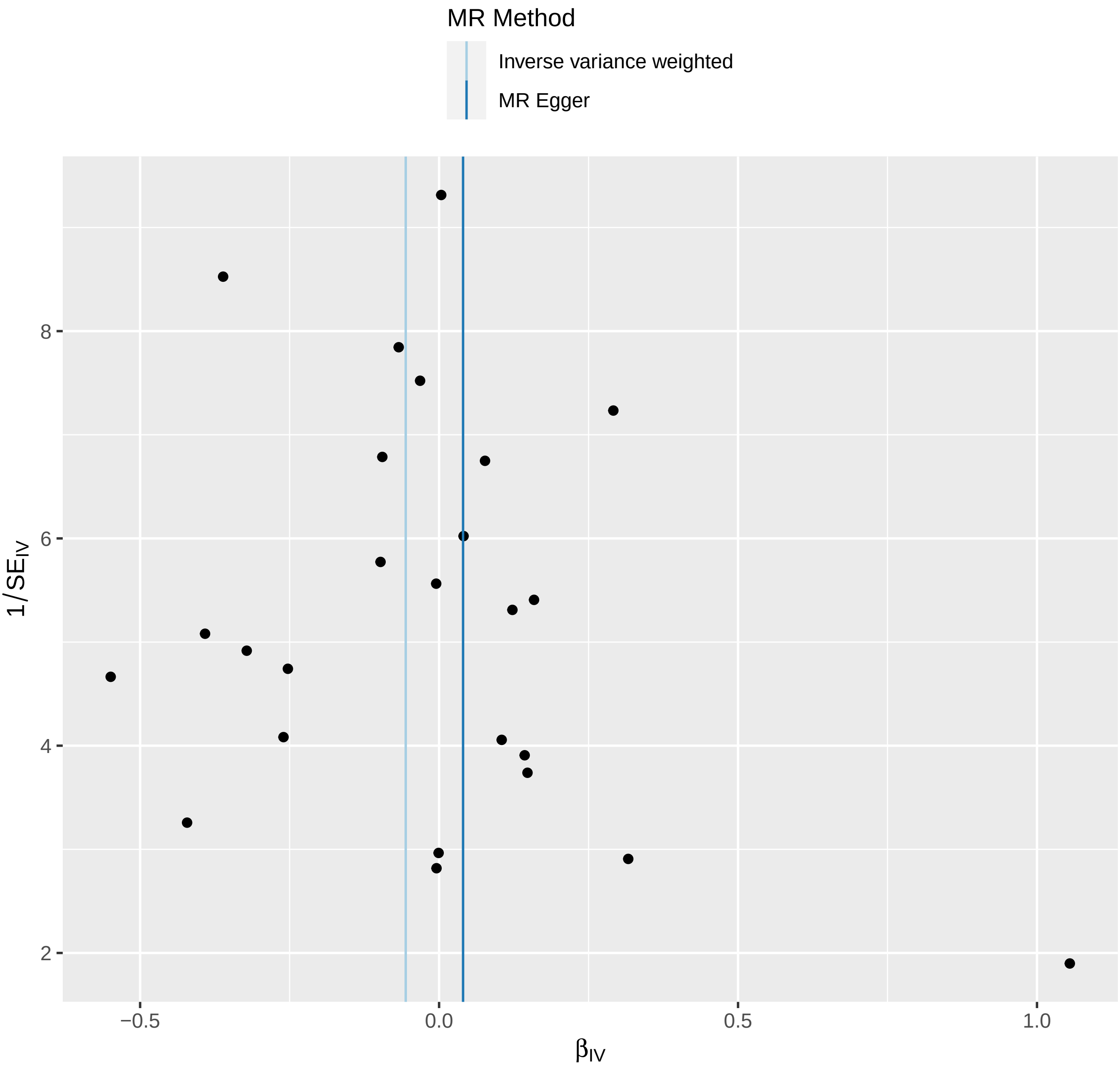

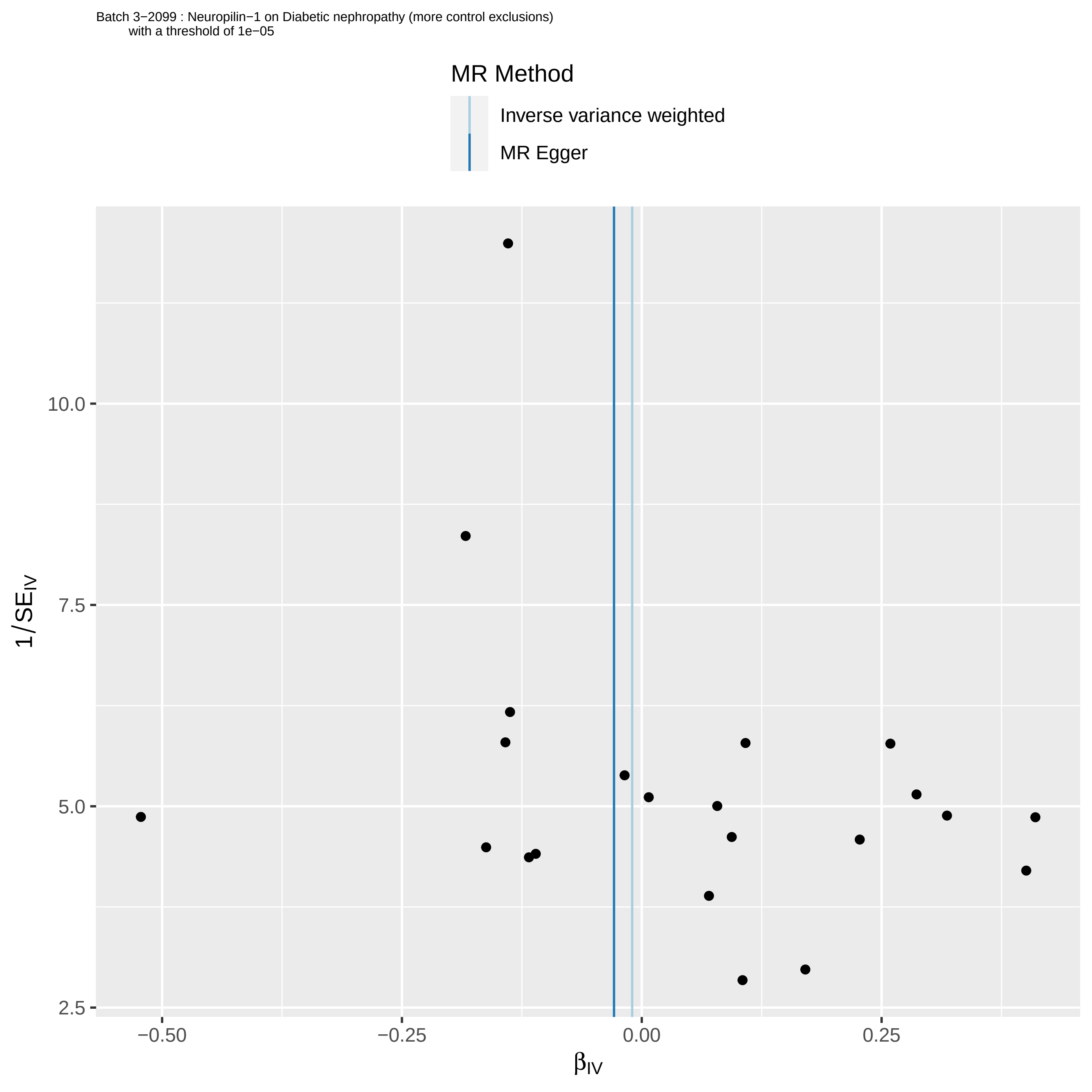

Batch 3−2099 : Neuropilin−1 on Diabetic nephropathy (more control exclusions)
with a threshold of 1e−05
MR Method
Inverse variance weighted
MR Egger
10.0
7.5
5.0
2.5
−0.50
−0.25
0.00
0.25
1/SE_IV
β_IV

Batch 3−2100 : Neuropilin−2 on Diabetic nephropathy (more control exclusions)
with a threshold of 1e−05

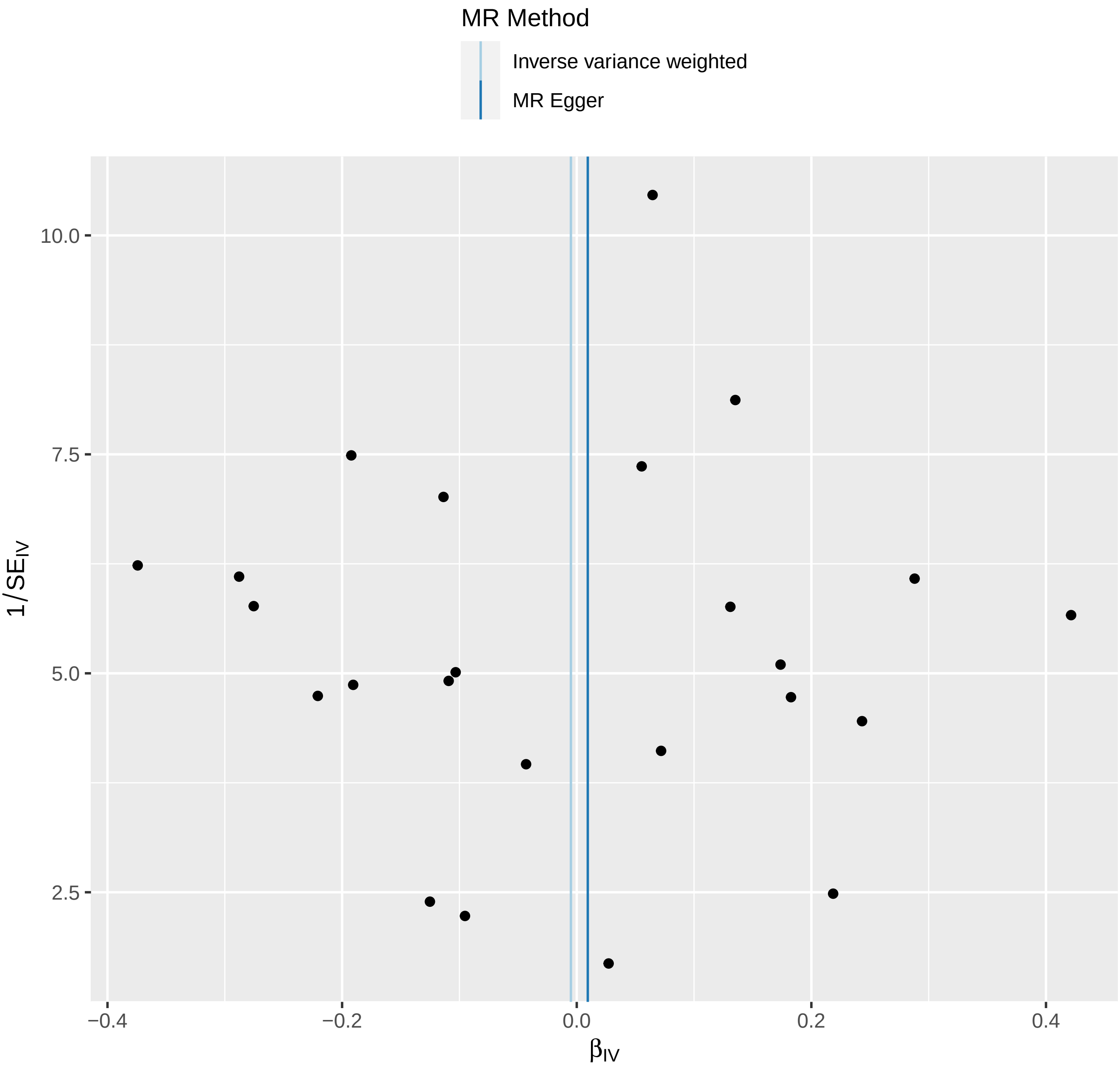

Batch 3−2101 : Neurensin−1 on Diabetic nephropathy (more control exclusions)
with a threshold of 1e−05

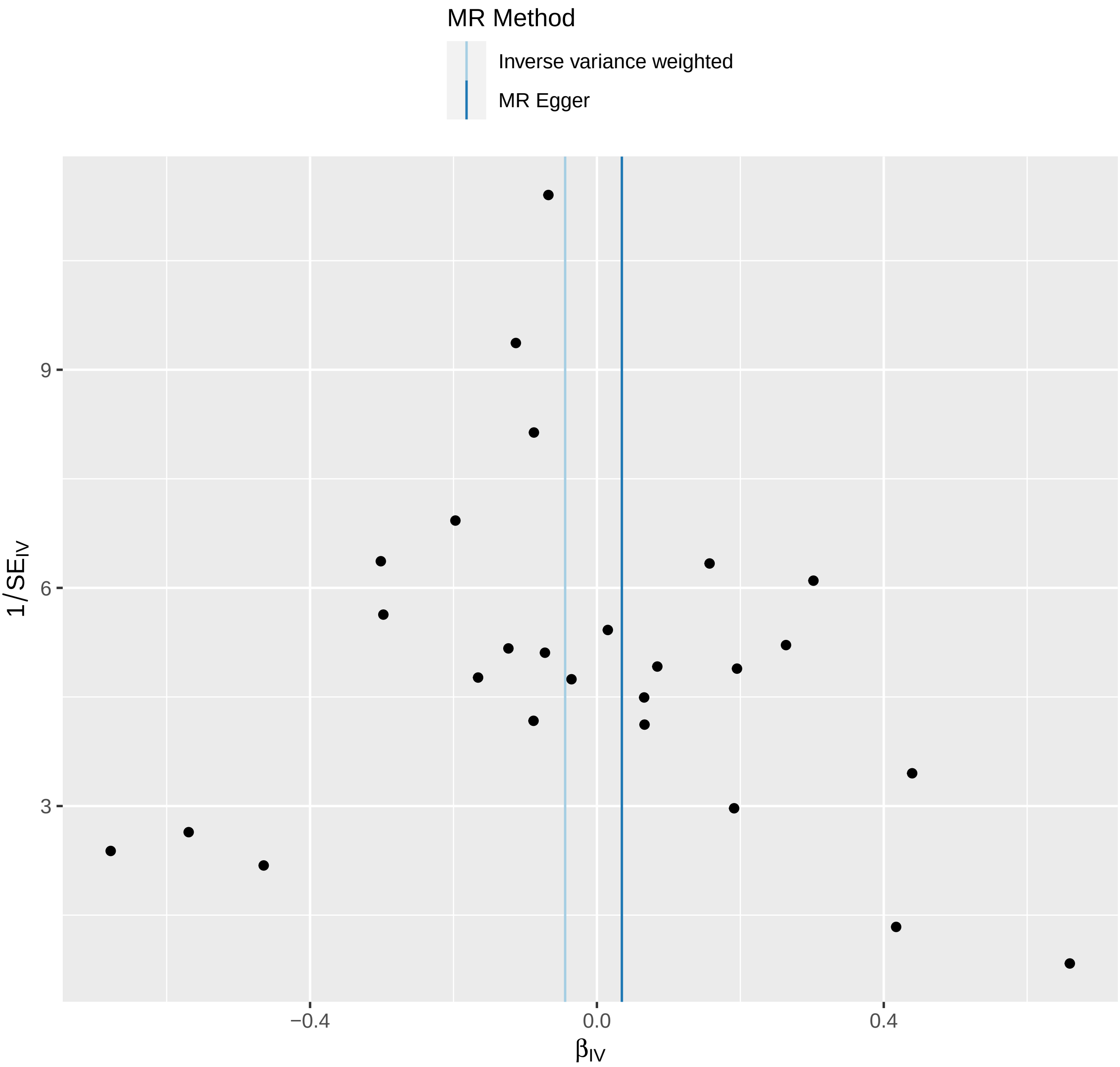

Batch 3−2102 : Neurexin−1−beta on Diabetic nephropathy (more control exclusions)
with a threshold of 1e−05

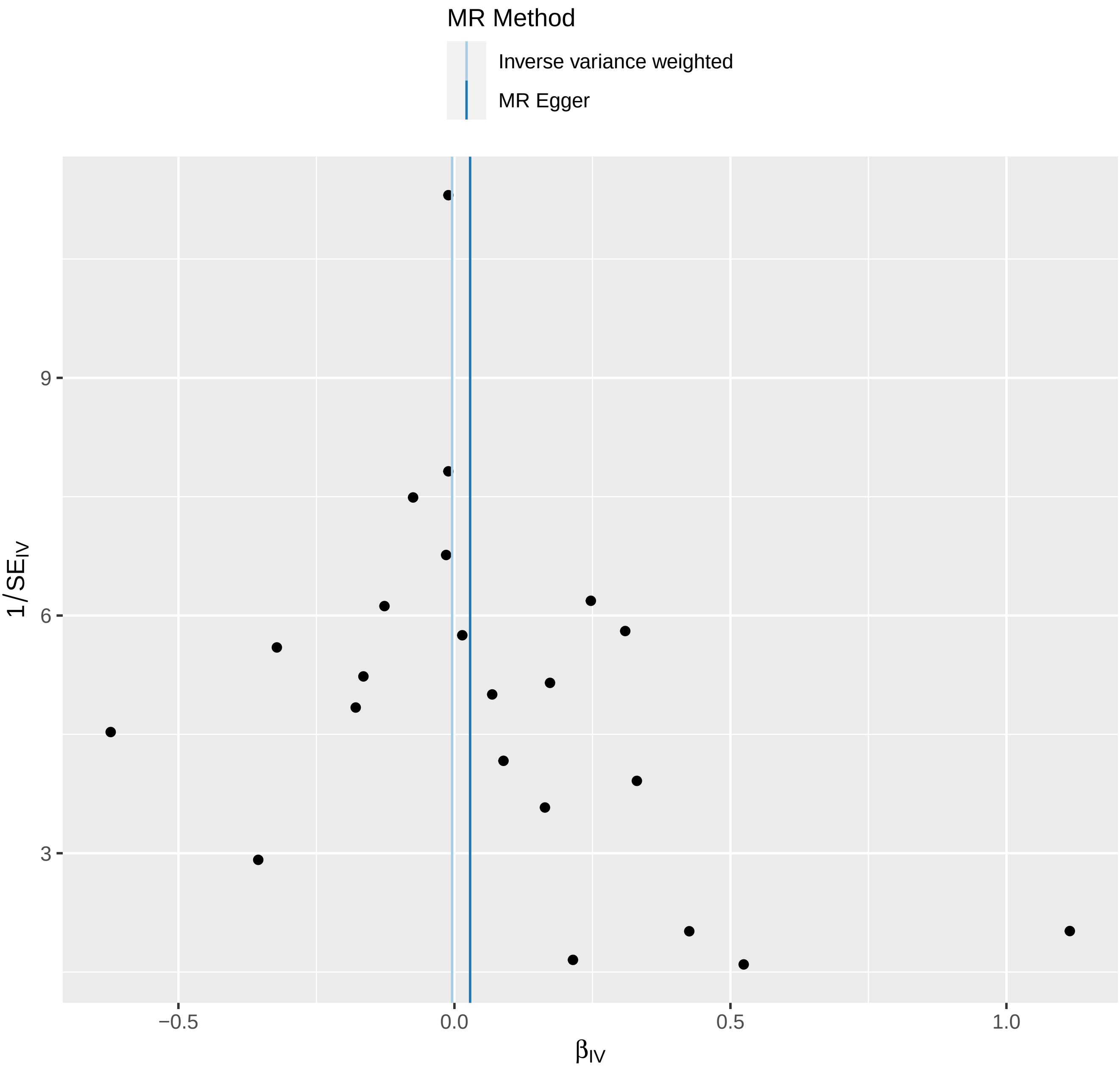

Batch 3−2103 : Neurexin−1 on Diabetic nephropathy (more control exclusions)
with a threshold of 1e−05

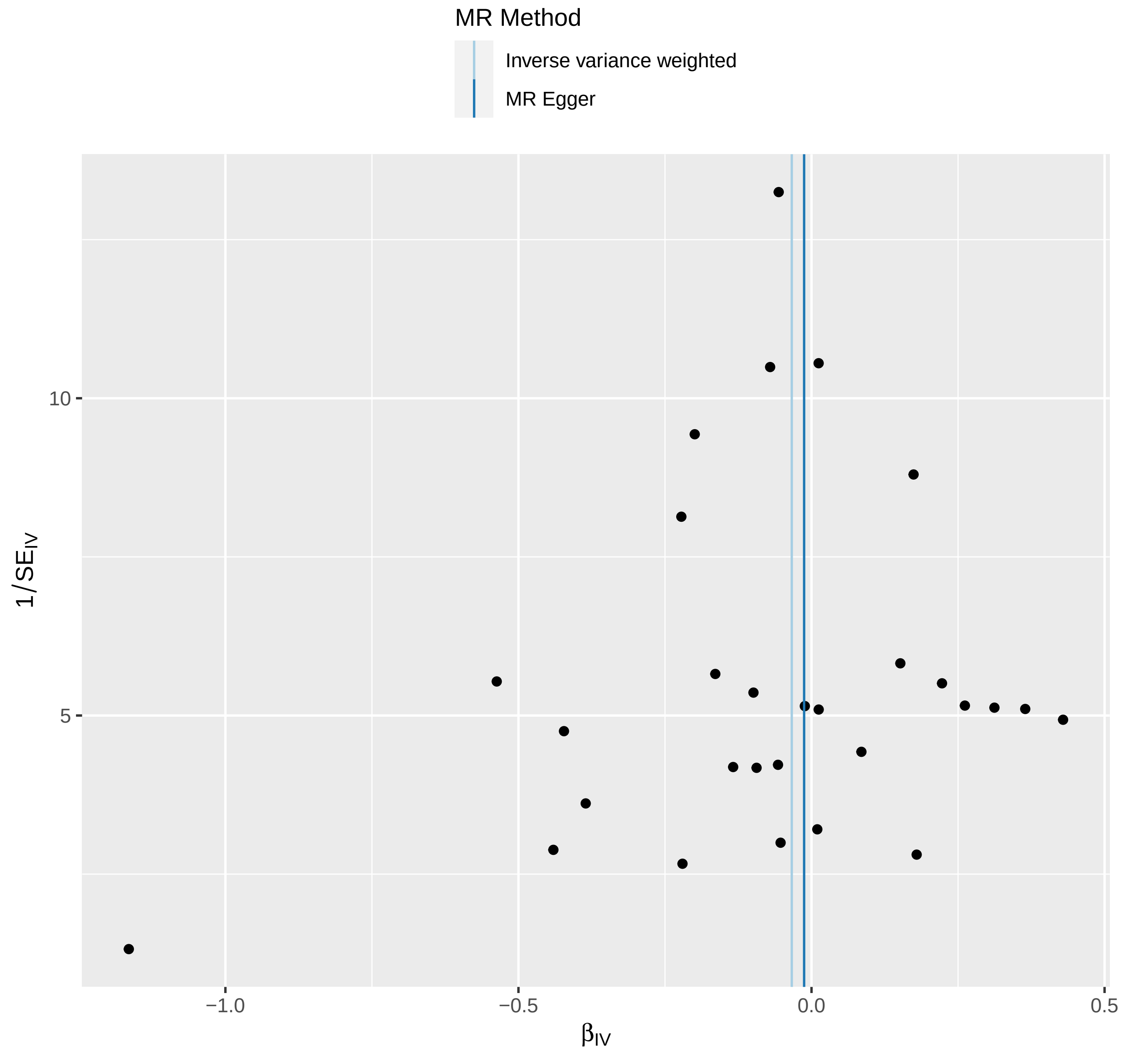

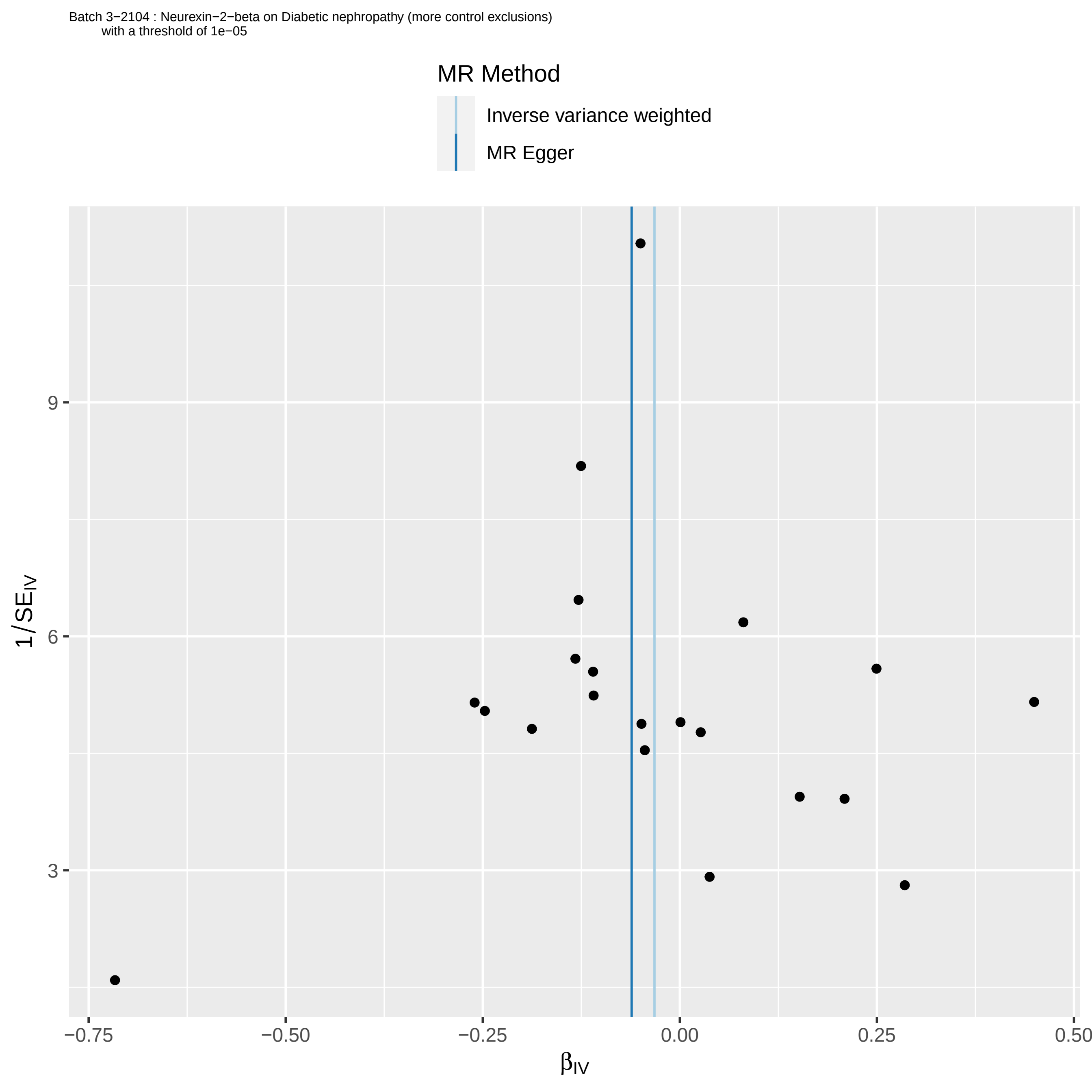

Batch 3−2104 : Neurexin−2−beta on Diabetic nephropathy (more control exclusions)
with a threshold of 1e−05
MR Method
Inverse variance weighted
MR Egger
9
6
3
1/SE_IV
−0.75
−0.50
−0.25
0.00
0.25
0.50
β_IV

Batch 3−2105 : Neurexin−3−beta on Diabetic nephropathy (more control exclusions)
with a threshold of 1e−05

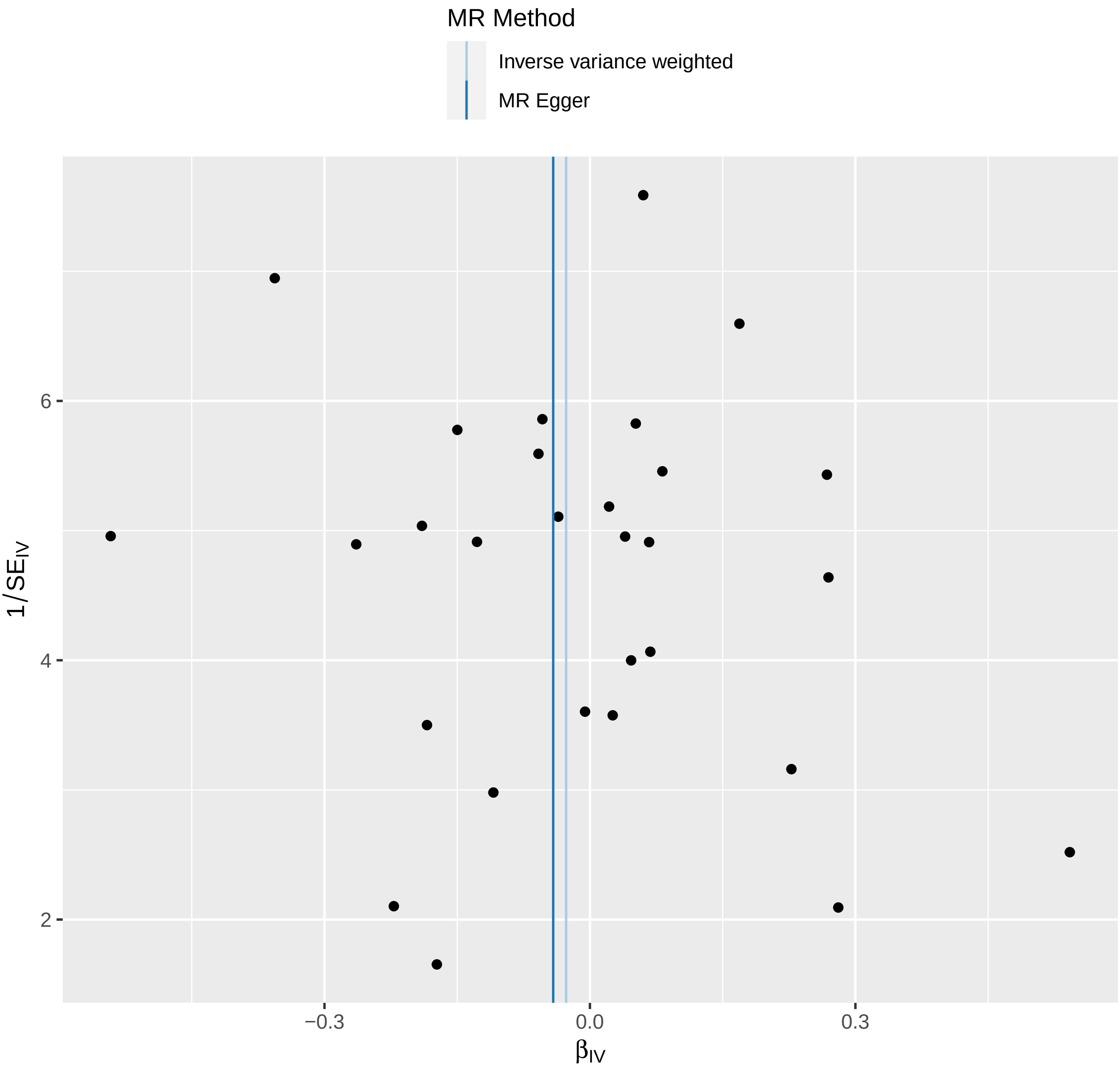

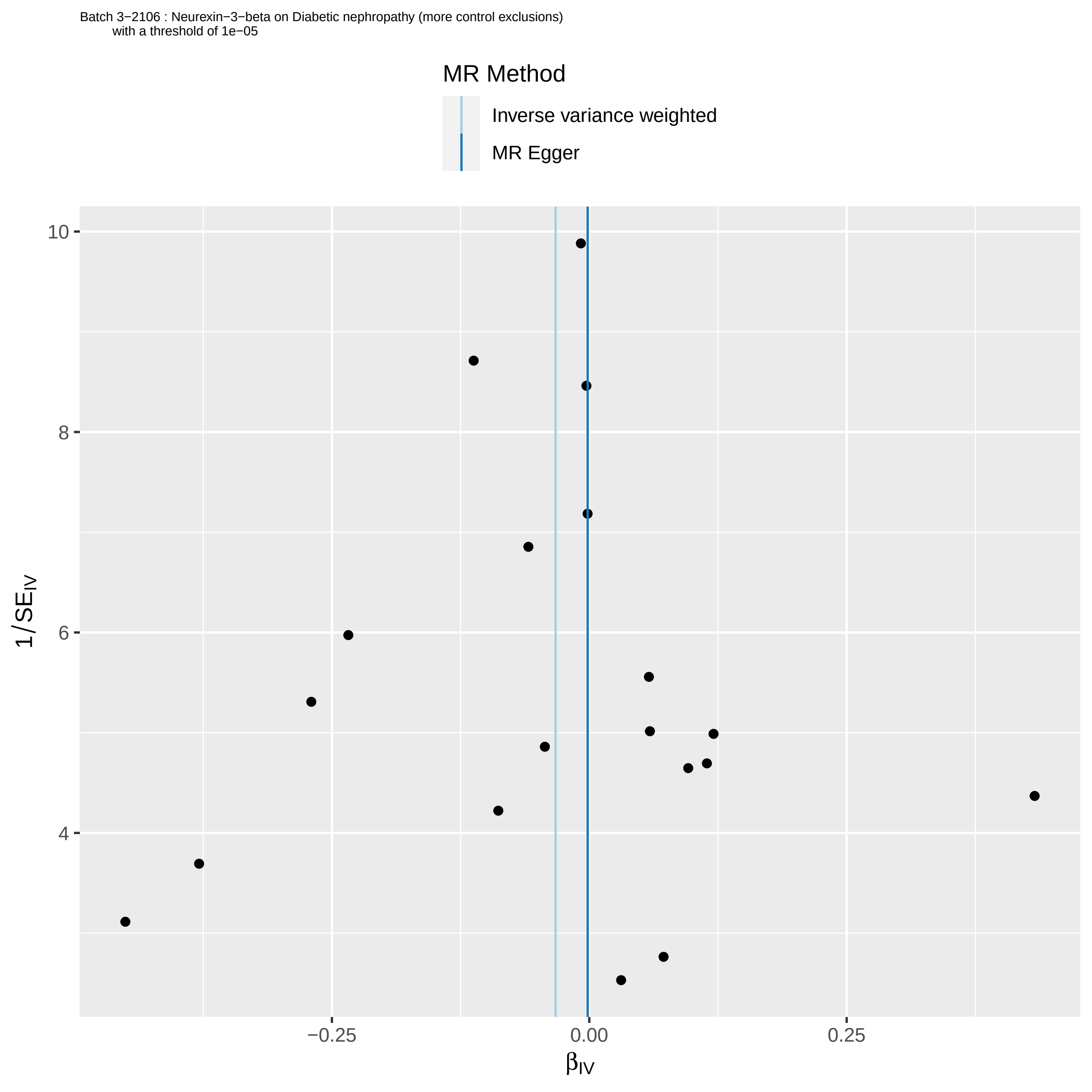

Batch 3−2106 : Neurexin−3−beta on Diabetic nephropathy (more control exclusions)
with a threshold of 1e−05
MR Method
Inverse variance weighted
MR Egger
10
8
6
4
1/SE_IV
−0.25
0.00
0.25
β_IV

Batch 3−2107 : Neurexin−3−beta on Diabetic nephropathy (more control exclusions)
with a threshold of 1e−05

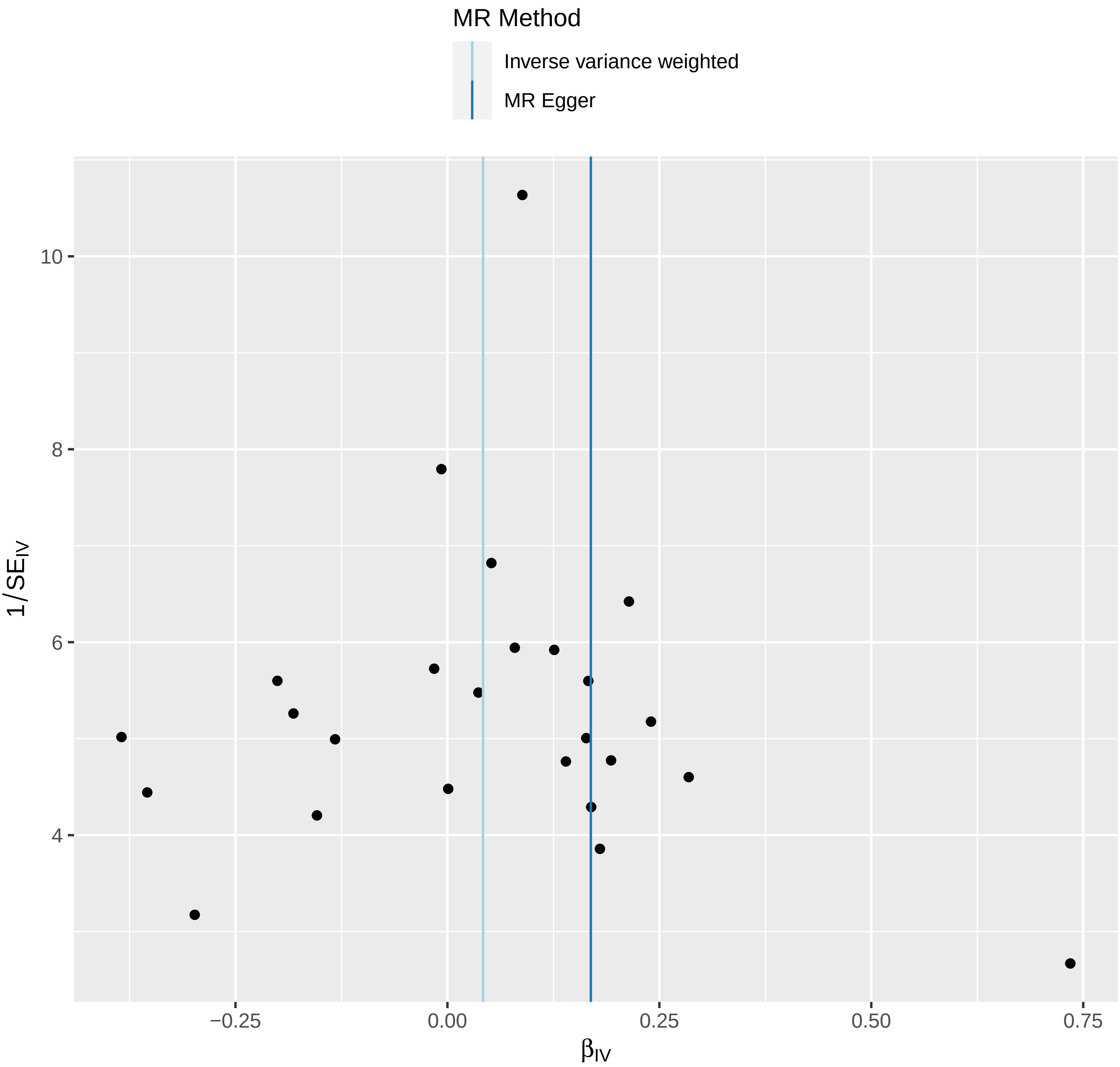

Batch 3−2108 : Sterol−4−alpha−carboxylate 3−dehydrogenase, decarboxylating on Diabetic nephropathy (more control exclusions) with a threshold of 1e−05

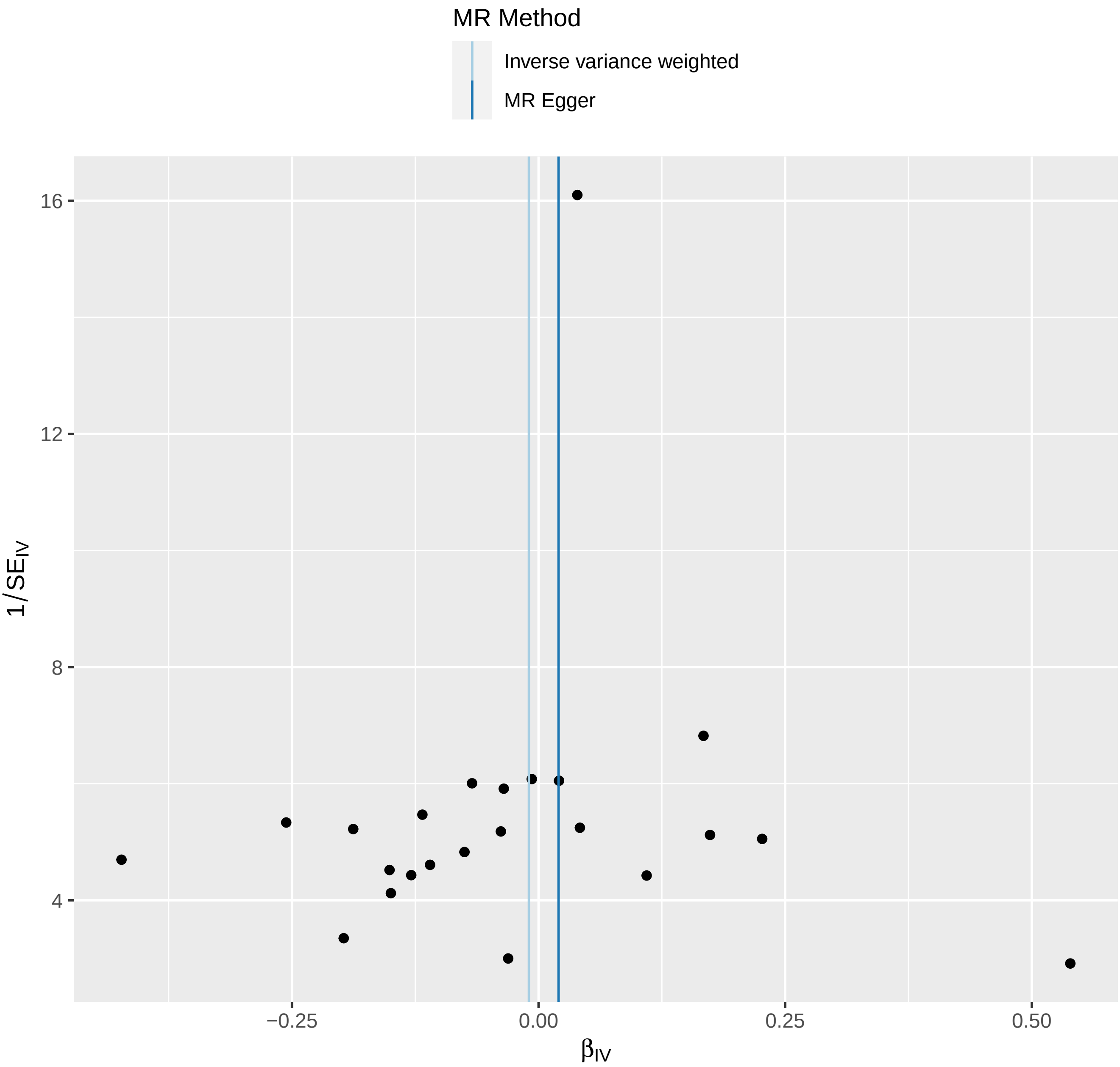

Batch 3−2109 : Vesicle−fusing ATPase on Diabetic nephropathy (more control exclusions)
with a threshold of 1e−05

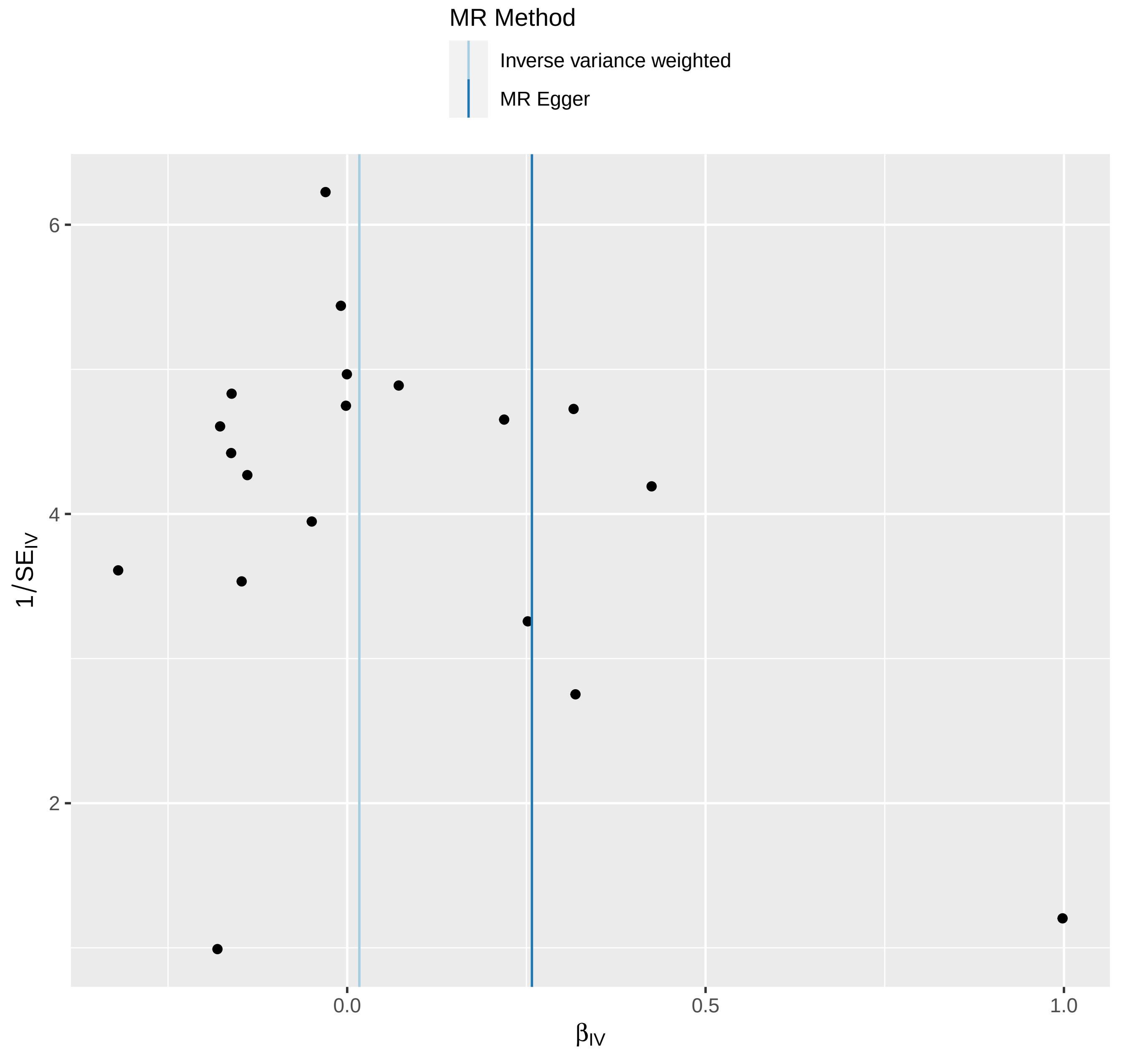

Batch 3−2110 : Neuron−specific protein family member 1 on Diabetic nephropathy (more control exclusions)
with a threshold of 1e−05

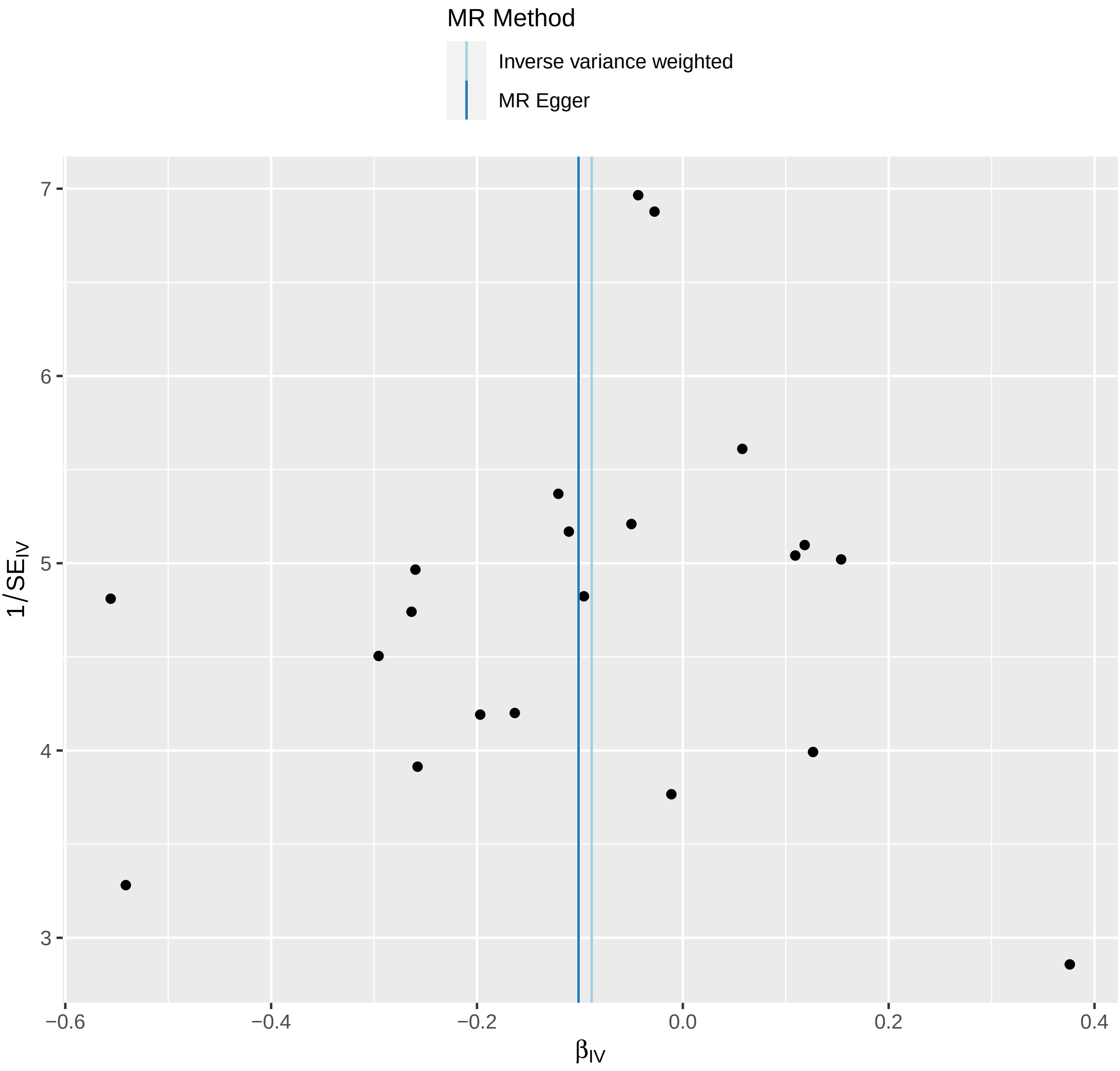

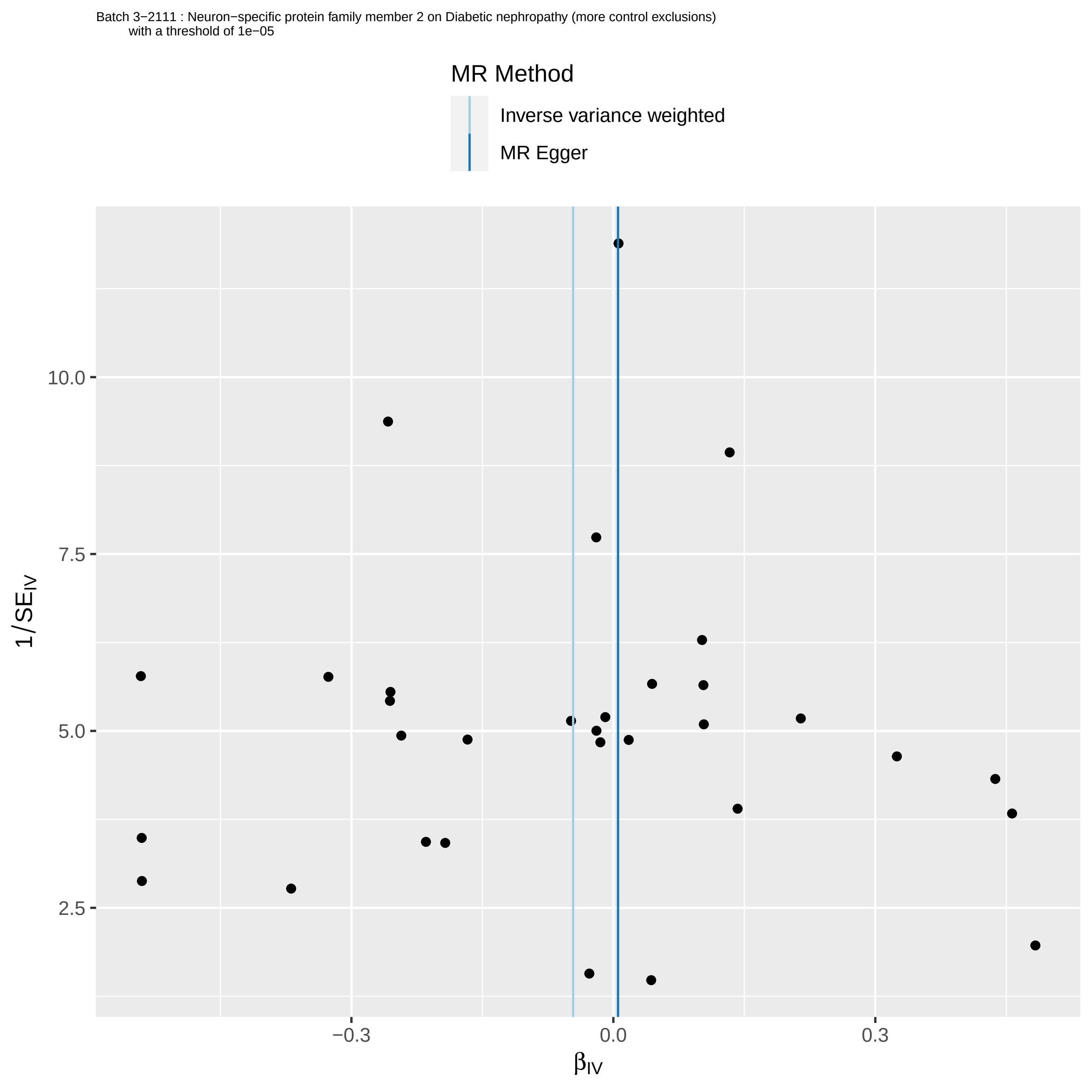
Batch 3−2111 : Neuron−specific protein family member 2 on Diabetic nephropathy (more control exclusions)
with a threshold of 1e−05
MR Method
Inverse variance weighted
MR Egger
10.0
7.5
5.0
2.5
$1/SE_{IV}$
−0.3
0.0
0.3
β_{IV}

Batch 3−2112 : 5'(3')−deoxyribonucleotidase, cytosolic type on Diabetic nephropathy (more control exclusions)
with a threshold of 1e−05

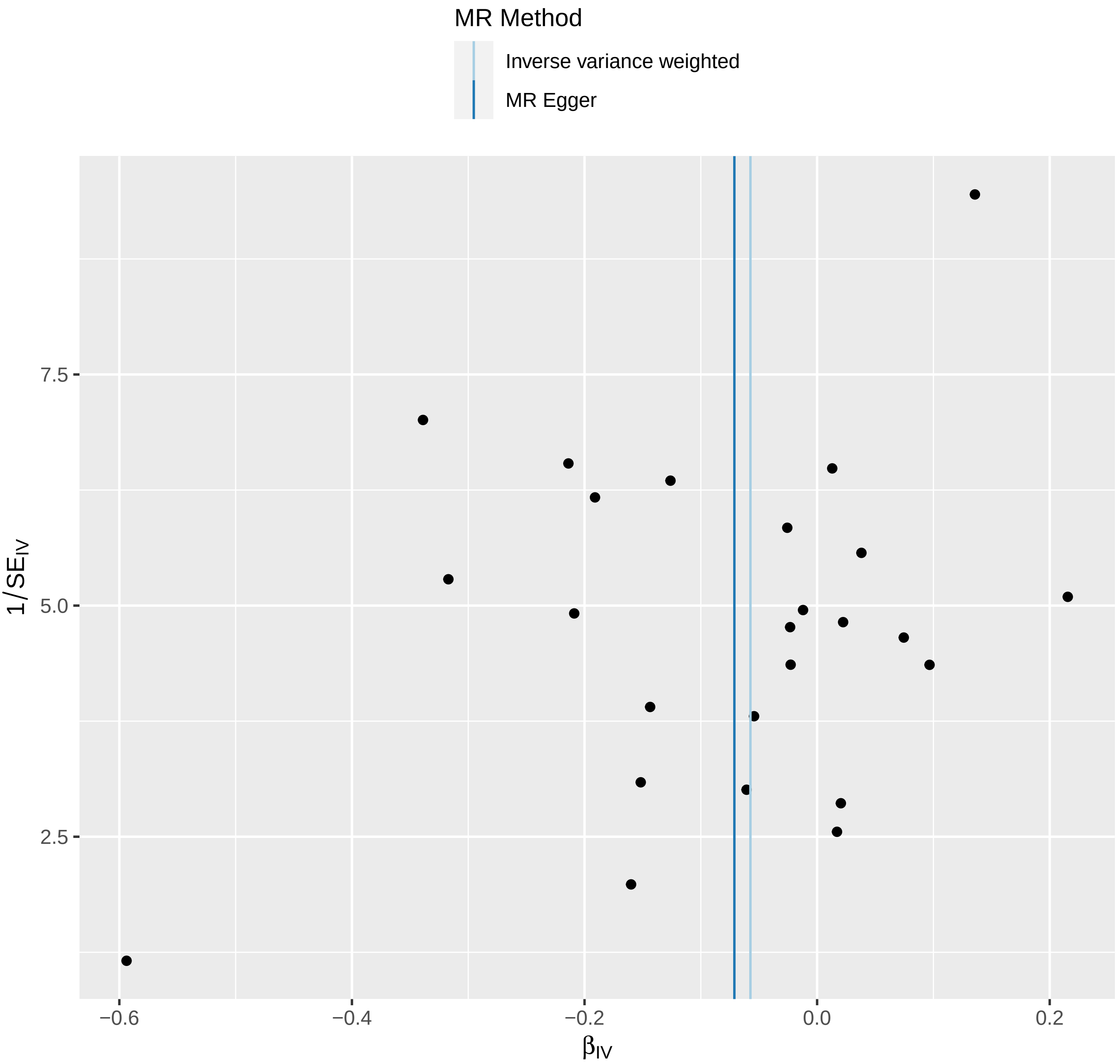

Batch 3−2113 : 5'−Nucleotidase on Diabetic nephropathy (more control exclusions)
with a threshold of 1e−05

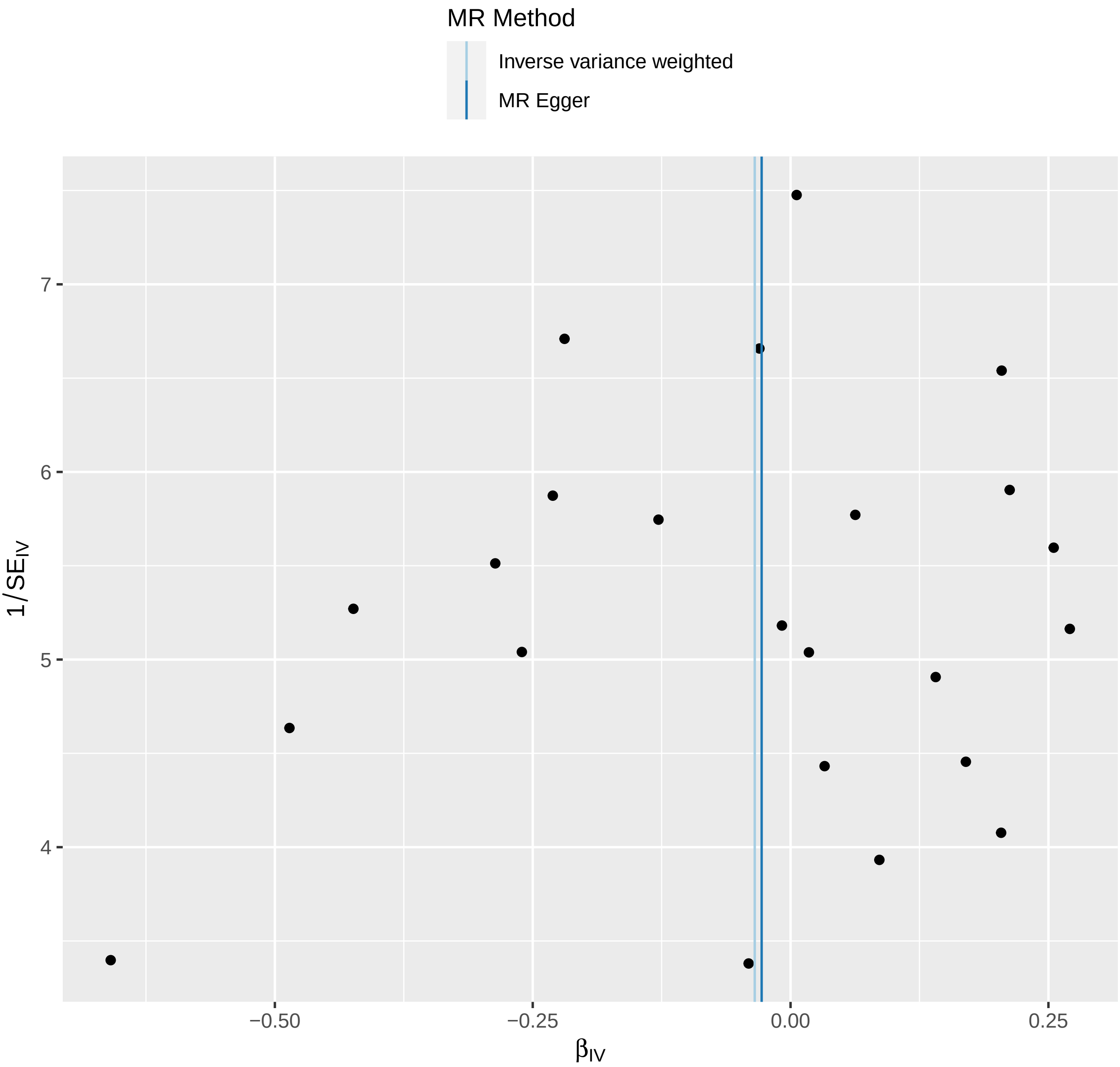

Batch 3−2114 : Neurotrophin−3 on Diabetic nephropathy (more control exclusions)
with a threshold of 1e−05

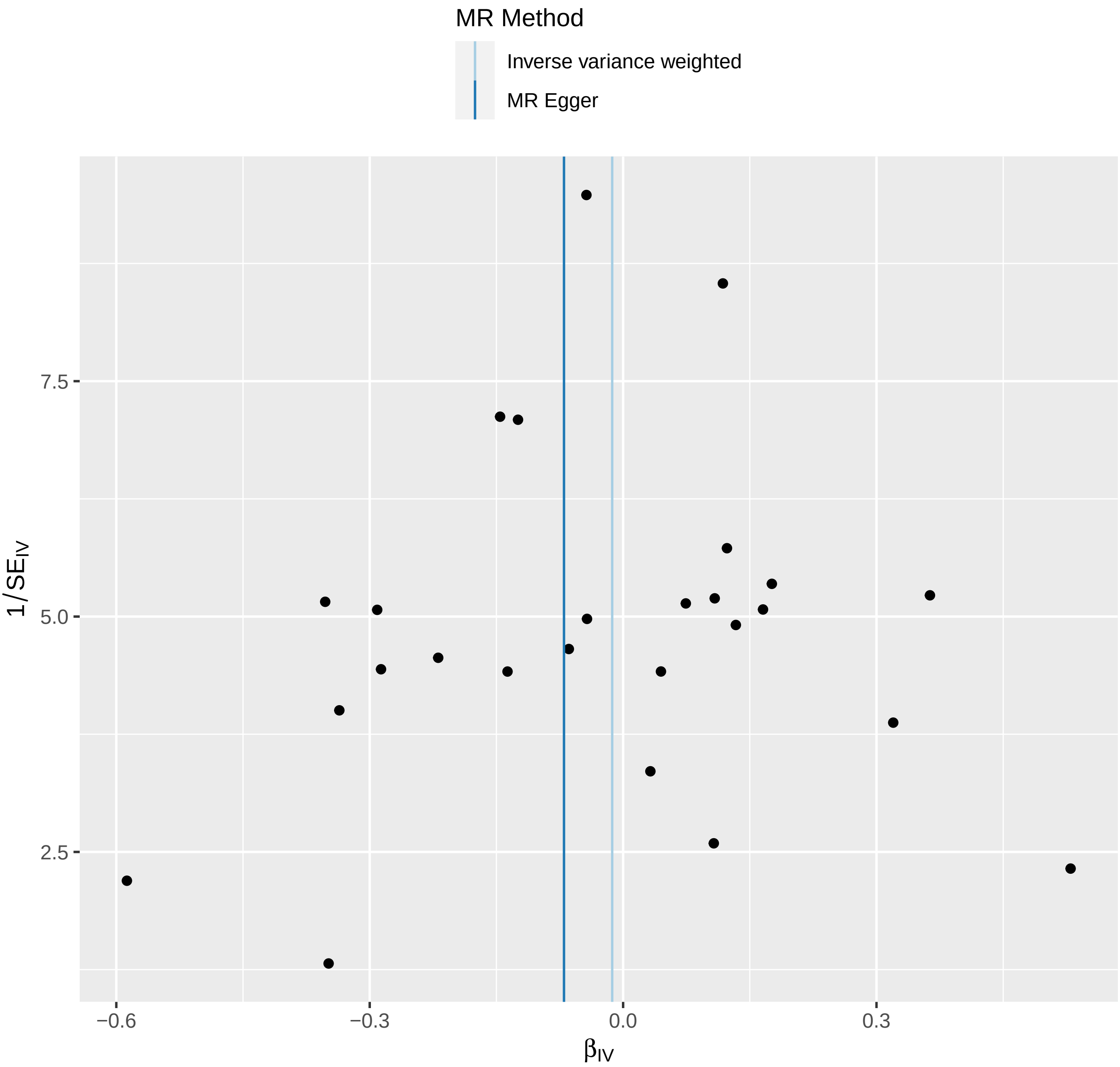

Batch 3−2115 : Neurotrophin−4 on Diabetic nephropathy (more control exclusions)
with a threshold of 1e−05

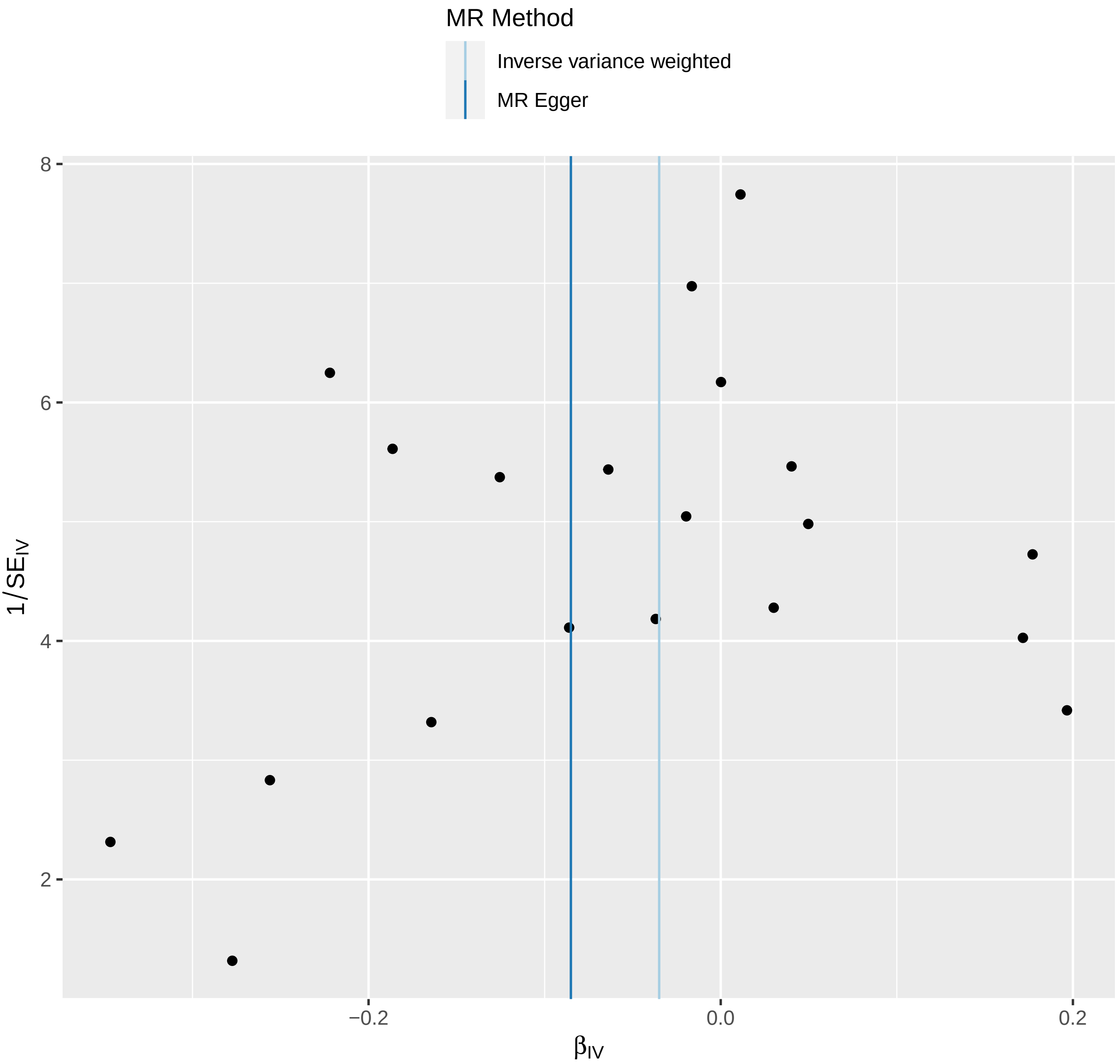

Batch 3−2116 : N−terminal Xaa−Pro−Lys N−methyltransferase 1 on Diabetic nephropathy (more control exclusions)
with a threshold of 1e−05

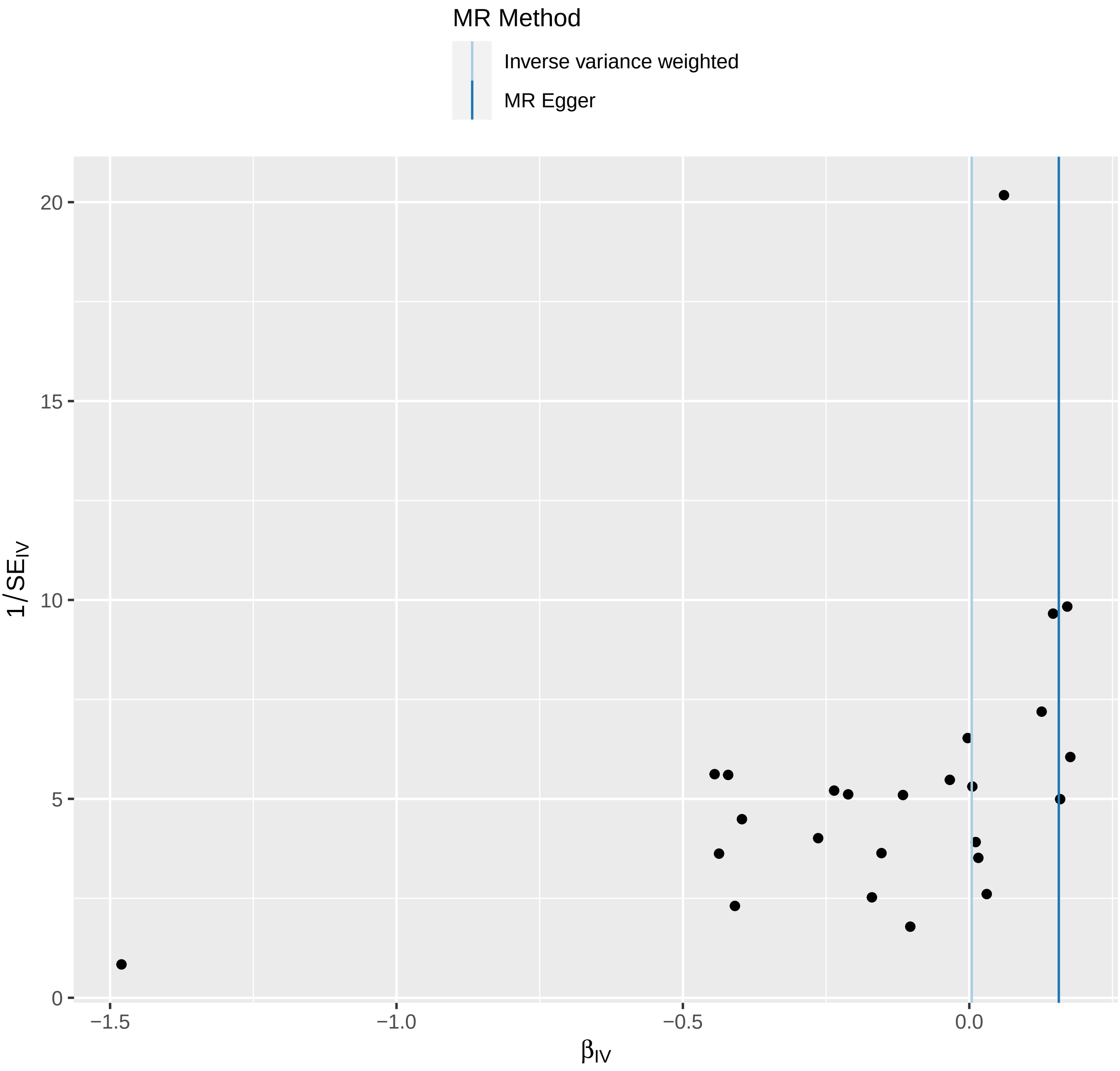

Batch 3−2117 : Netrin−1 on Diabetic nephropathy (more control exclusions)
with a threshold of 1e−05

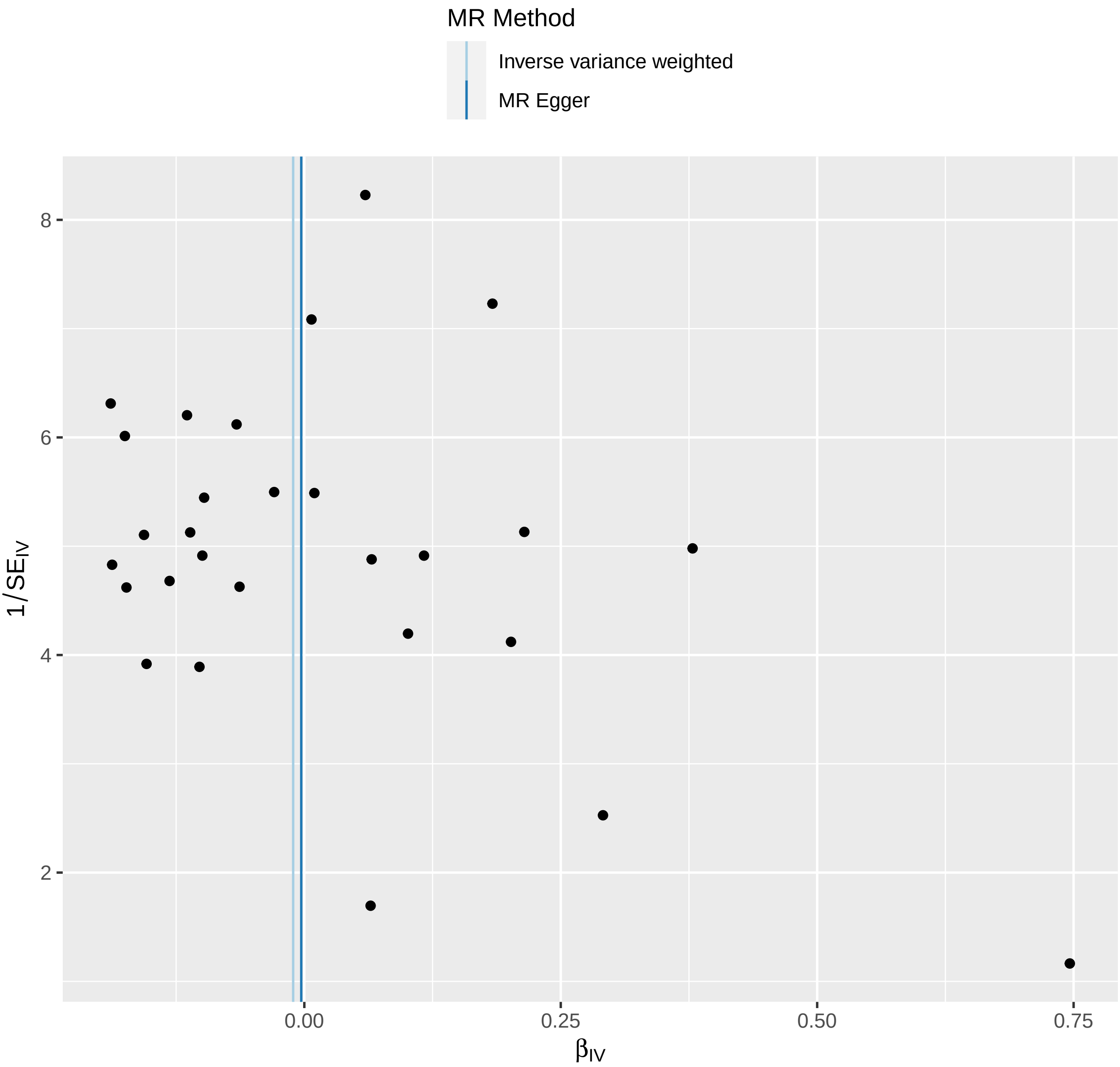

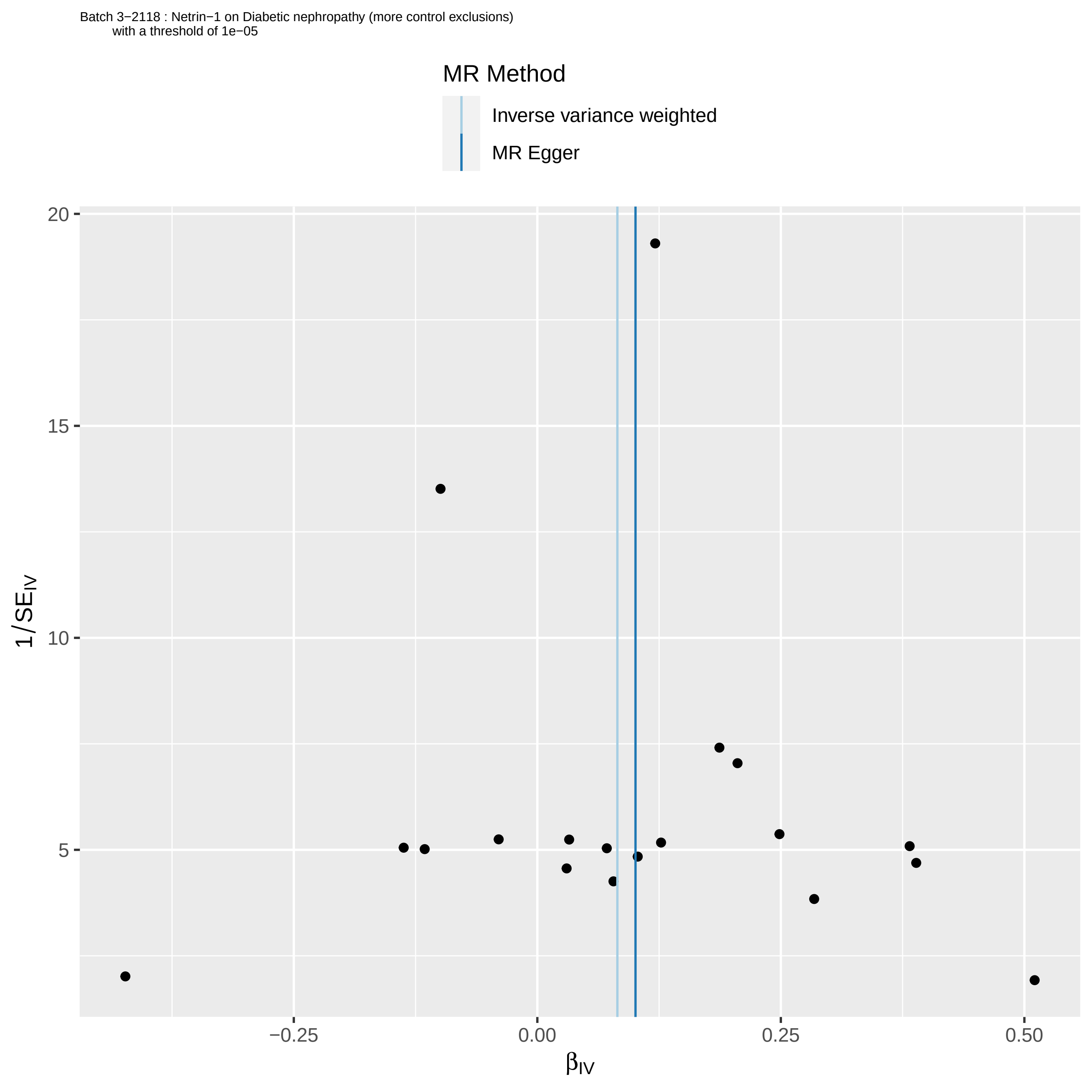
Batch 3−2118 : Netrin−1 on Diabetic nephropathy (more control exclusions)
with a threshold of 1e−05
MR Method
Inverse variance weighted
MR Egger
20
15
10
5
1/SE_IV
−0.25
0.00
0.25
0.50
β_IV

Batch 3−2119 : Netrin−4 on Diabetic nephropathy (more control exclusions)
with a threshold of 1e−05

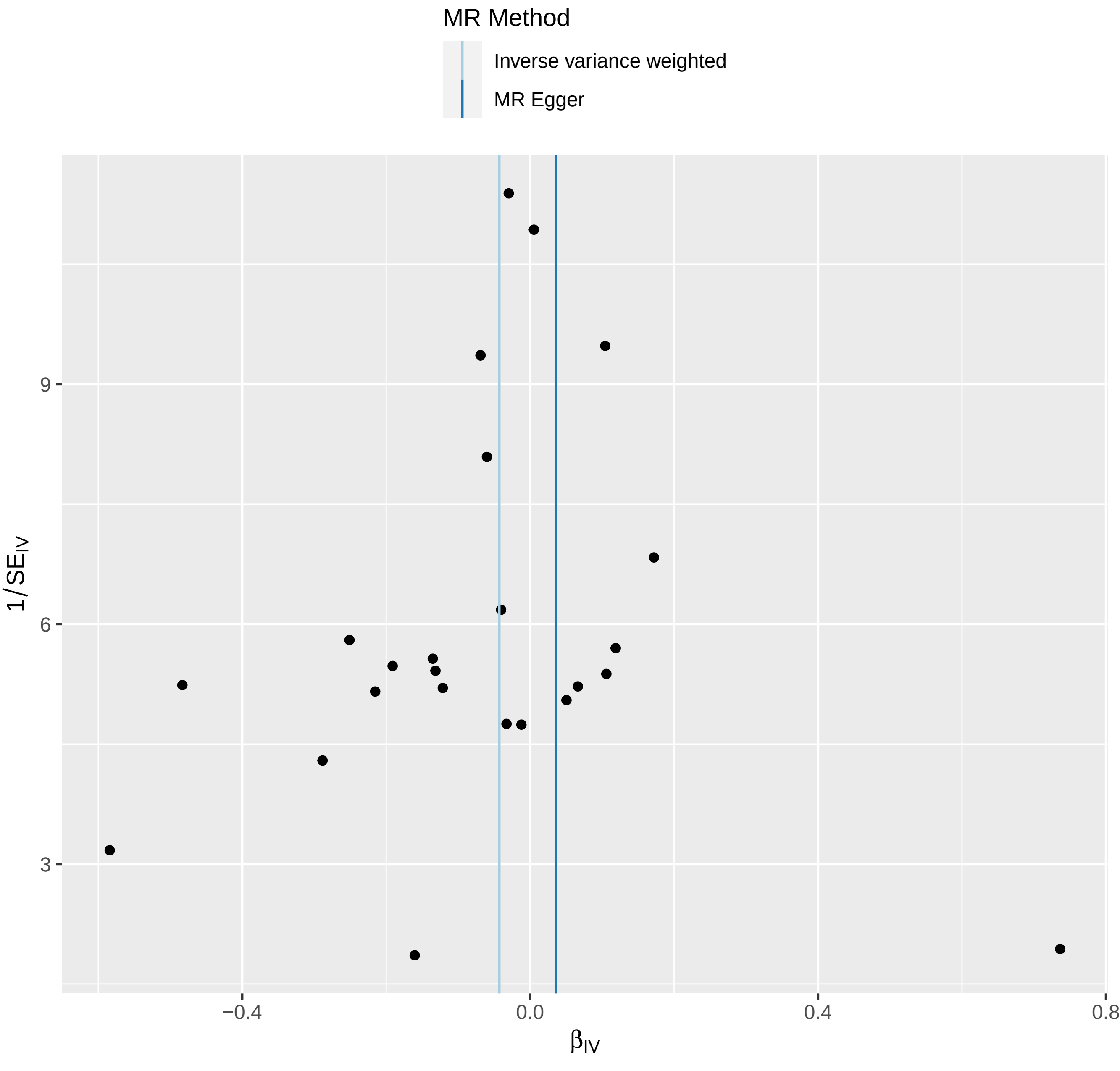

Batch 3−2120 : Netrin−G1 on Diabetic nephropathy (more control exclusions)
with a threshold of 1e−05

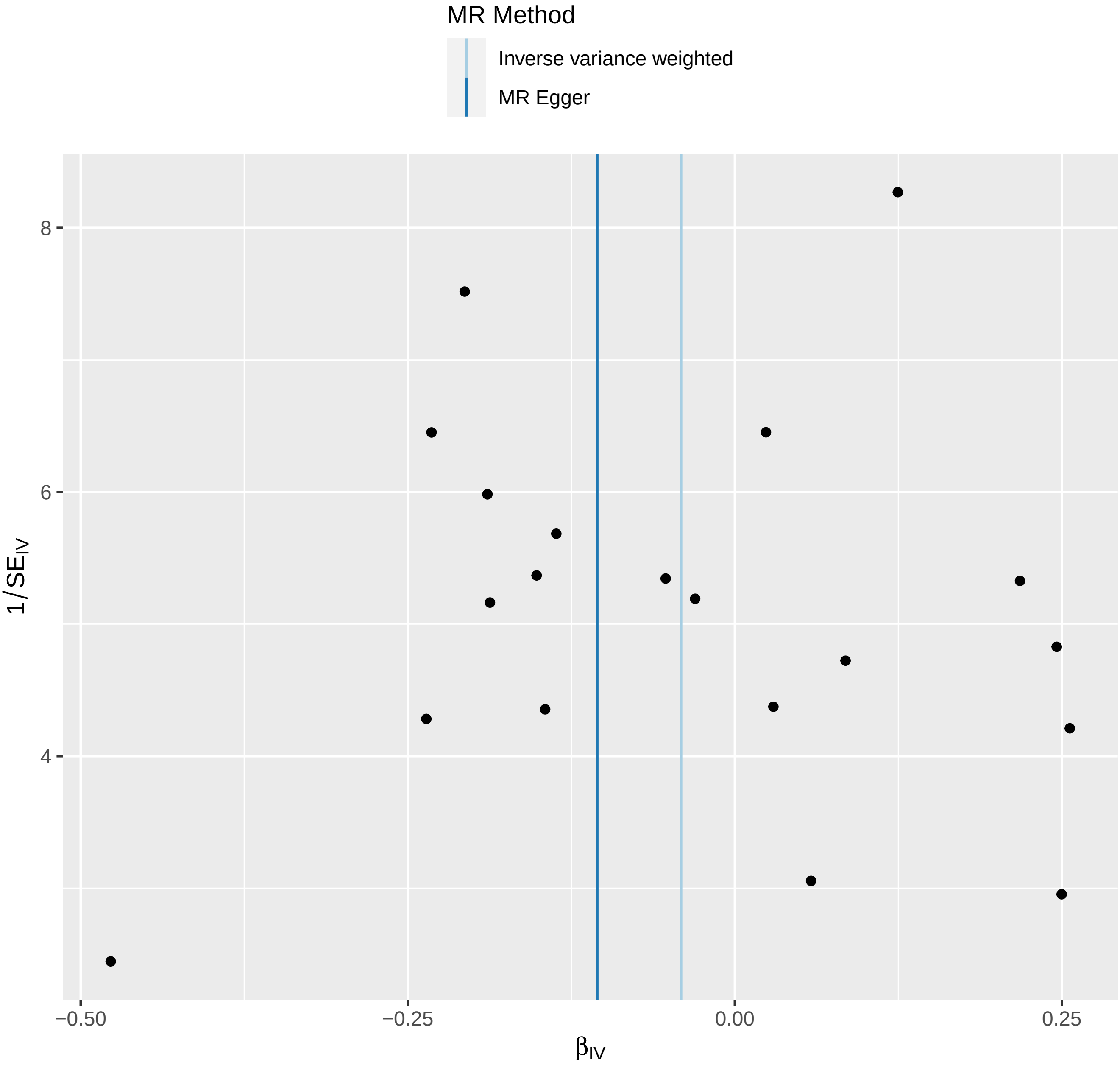

Batch 3−2121 : High affinity nerve growth factor receptor on Diabetic nephropathy (more control exclusions)
with a threshold of 1e−05

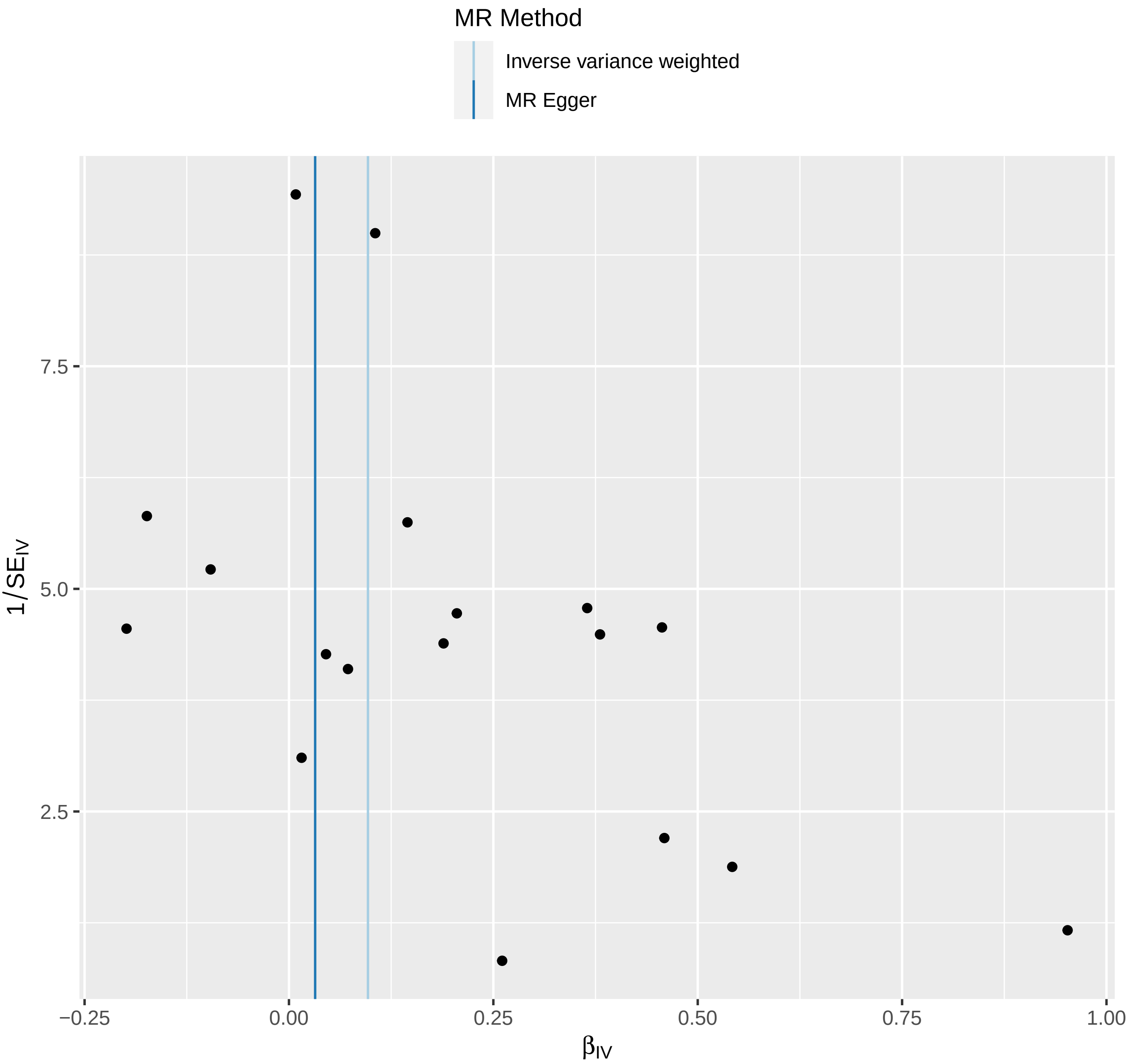

Batch 3−2122 : BDNF/NT−3 growth factors receptor on Diabetic nephropathy (more control exclusions)
with a threshold of 1e−05

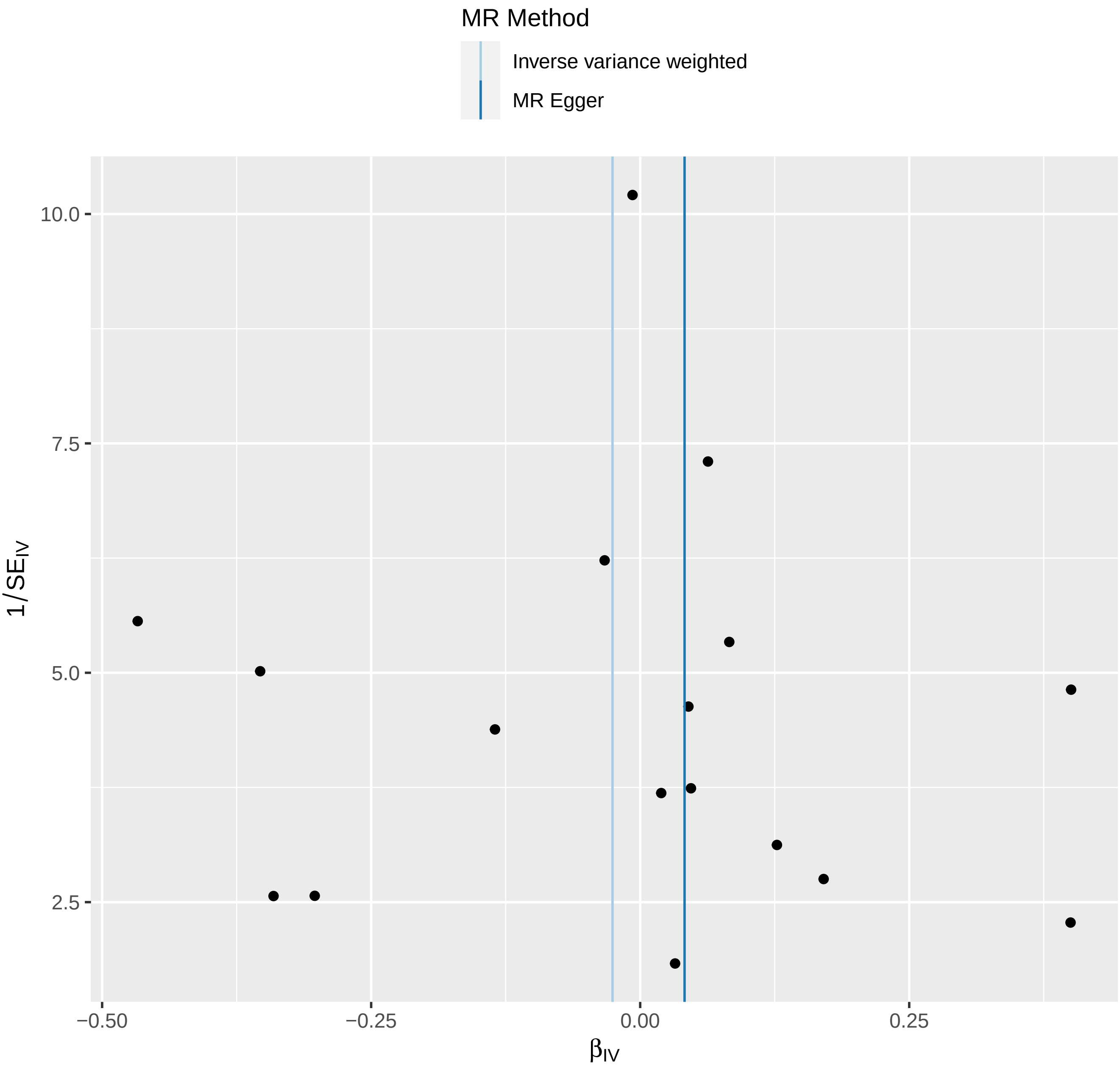

Batch 3−2123 : NT−3 growth factor receptor on Diabetic nephropathy (more control exclusions)
with a threshold of 1e−05

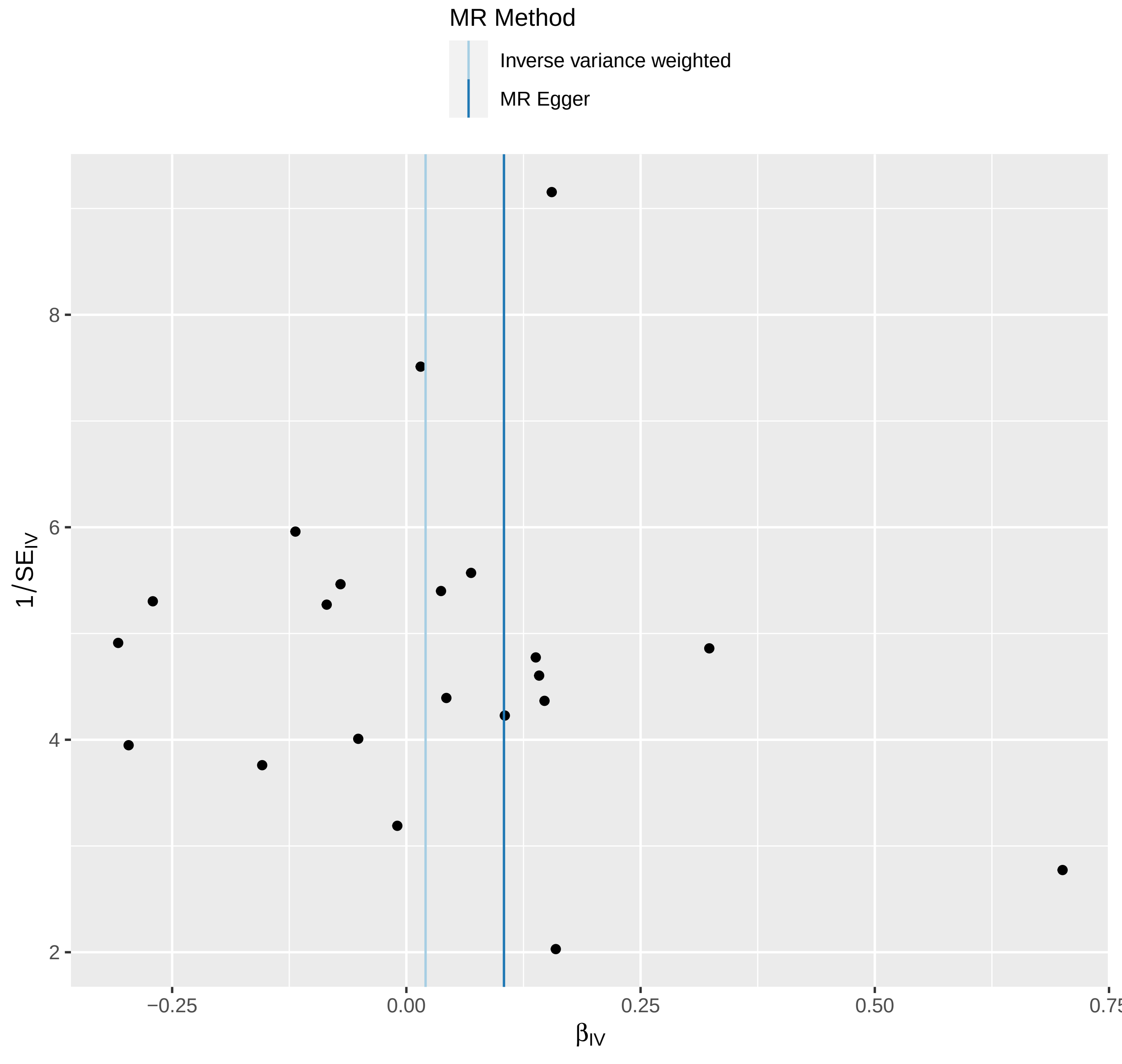

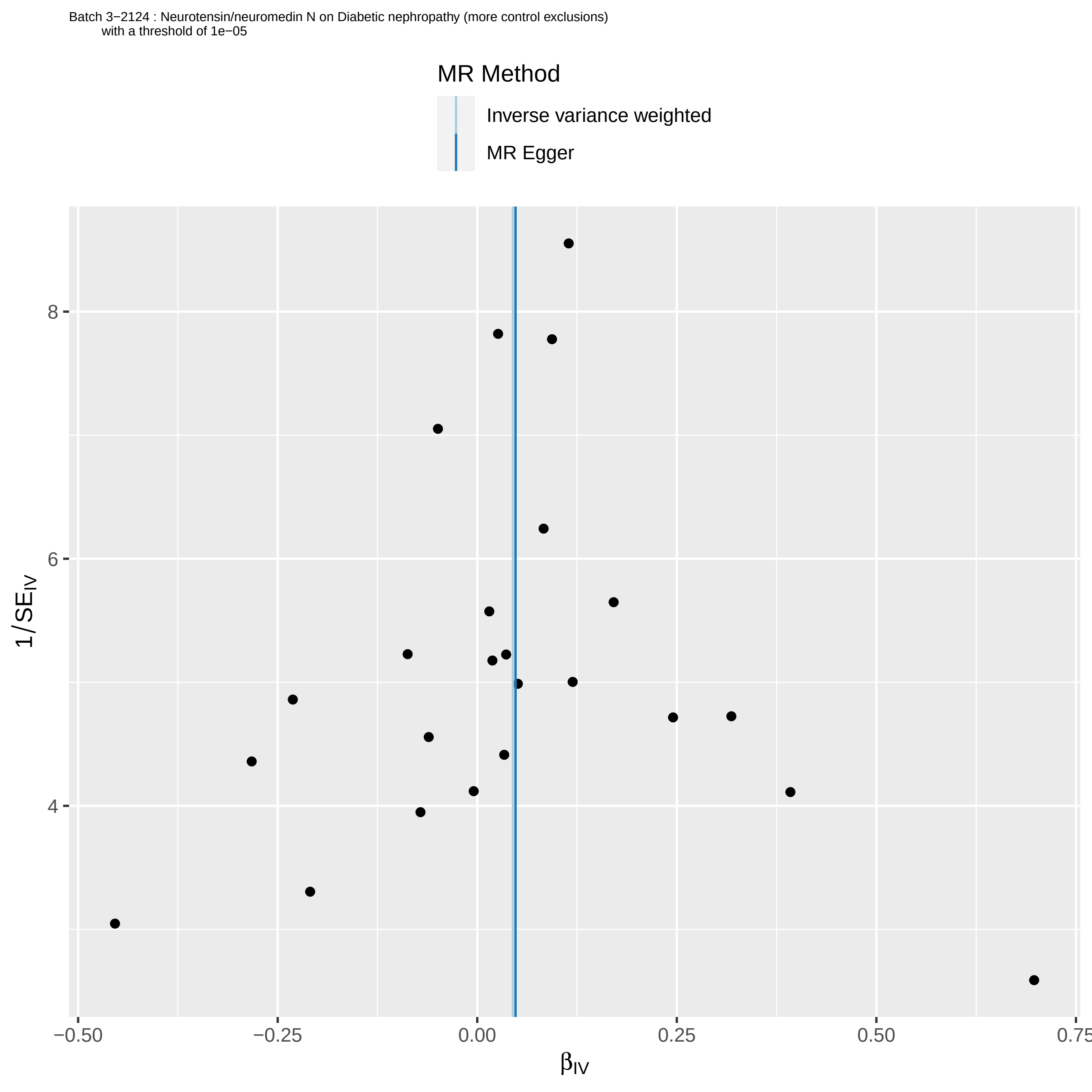

Batch 3−2124 : Neurotensin/neuromedin N on Diabetic nephropathy (more control exclusions)
with a threshold of 1e−05
MR Method
Inverse variance weighted
MR Egger
8
6
4
1/SE_IV
−0.50
−0.25
0.00
0.25
0.50
0.75
β_IV

Batch 3−2125 : Nuclear migration protein nudC on Diabetic nephropathy (more control exclusions)
with a threshold of 1e−05

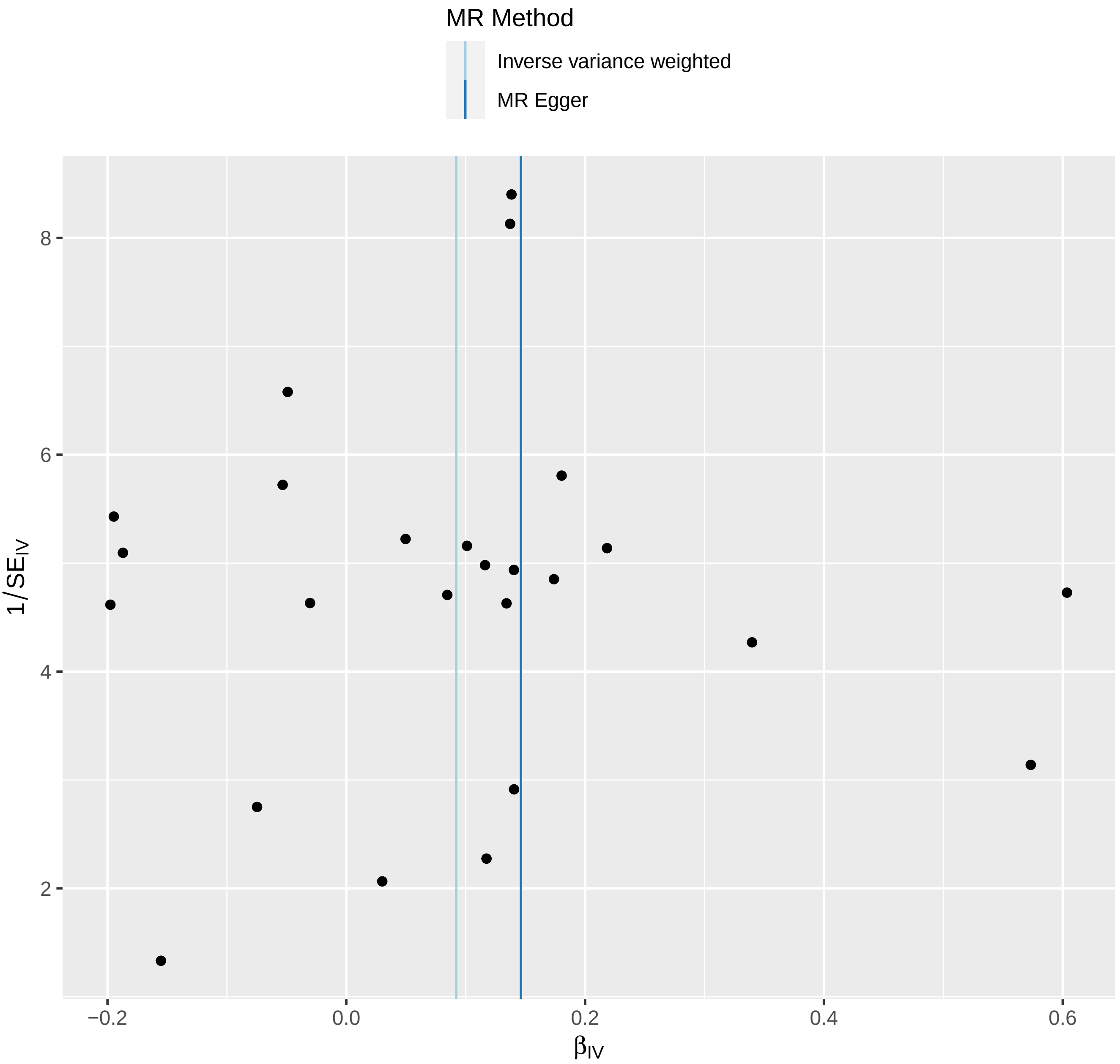

Batch 3−2126 : Peroxisomal NADH pyrophosphatase NUDT12 on Diabetic nephropathy (more control exclusions)
with a threshold of 1e−05

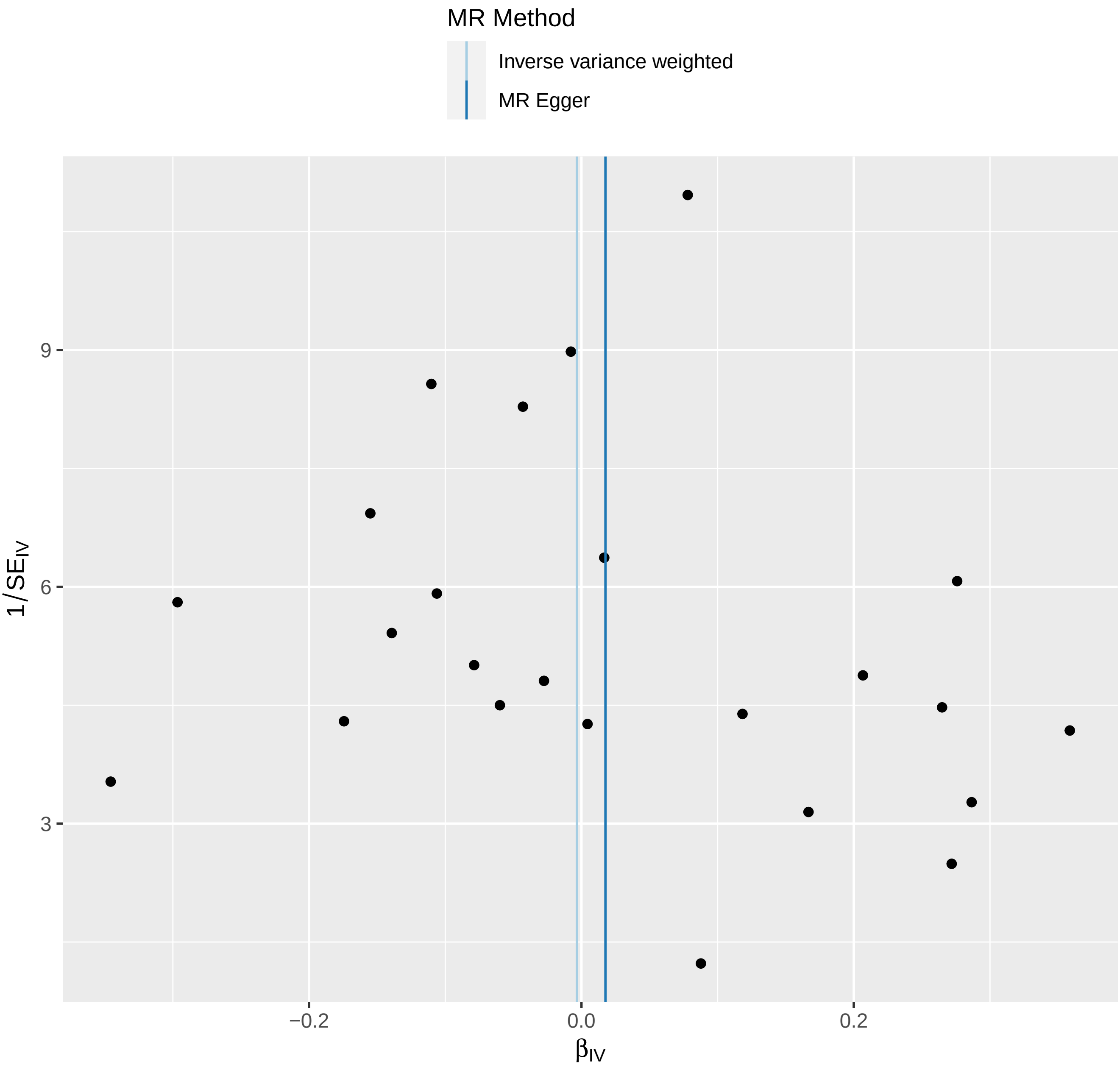

Batch 3−2127 : Protein syndesmos on Diabetic nephropathy (more control exclusions)
with a threshold of 1e−05

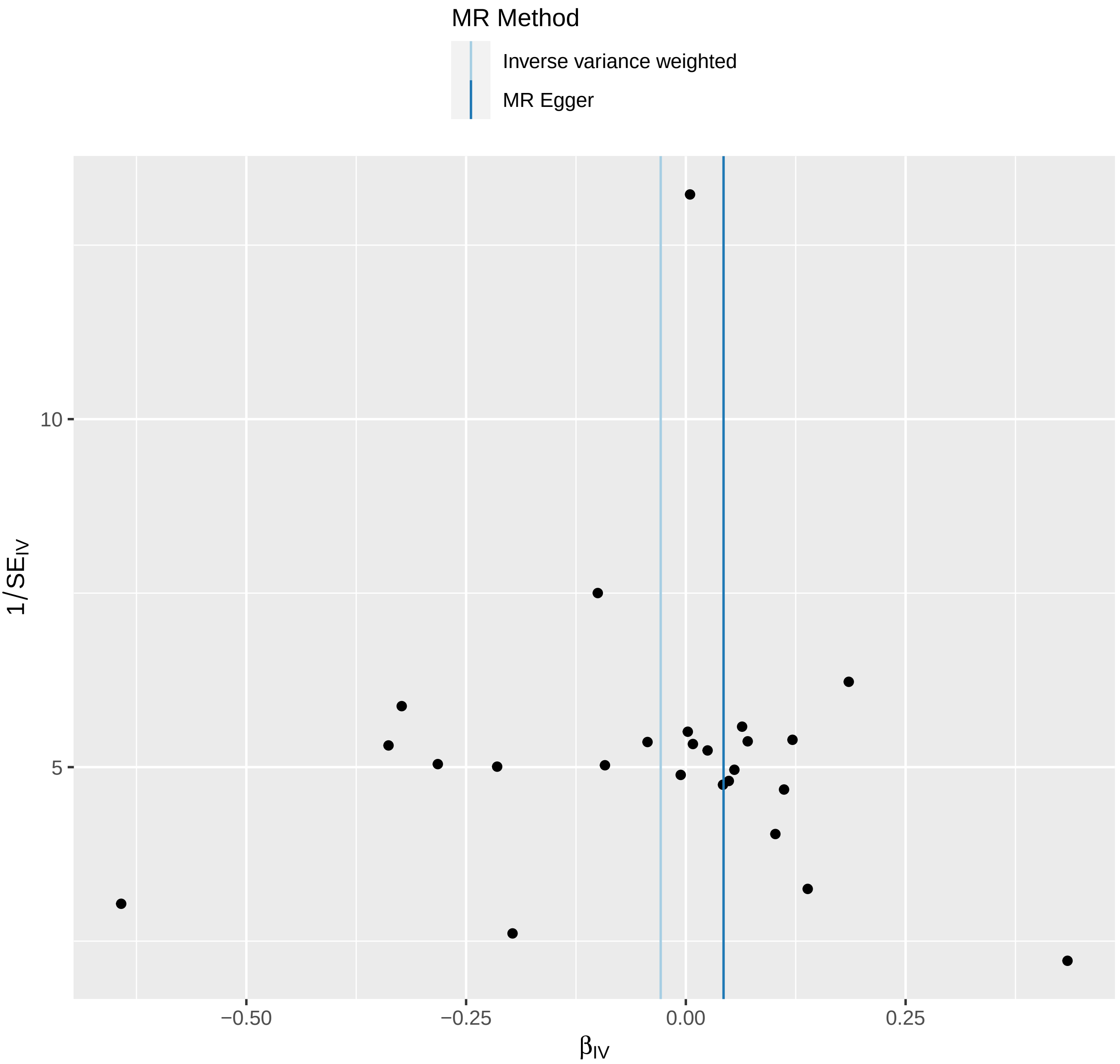

Batch 3−2128 : Nucleoside diphosphate−linked moiety X motif 8, mitochondrial on Diabetic nephropathy (more control exclusions) with a threshold of 1e−05

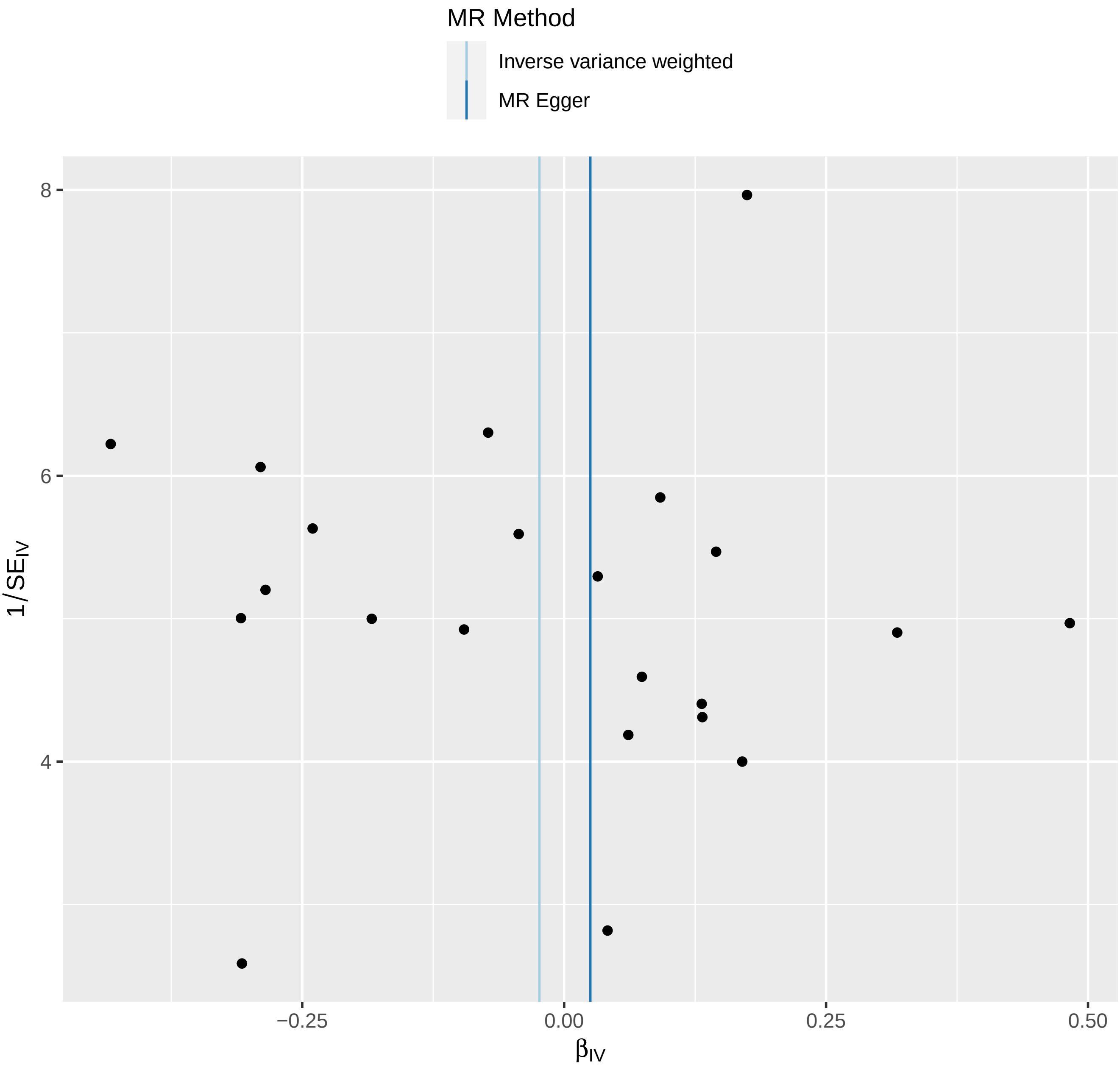

Batch 3−2129 : ADP−ribose pyrophosphatase, mitochondrial on Diabetic nephropathy (more control exclusions)
with a threshold of 1e−05

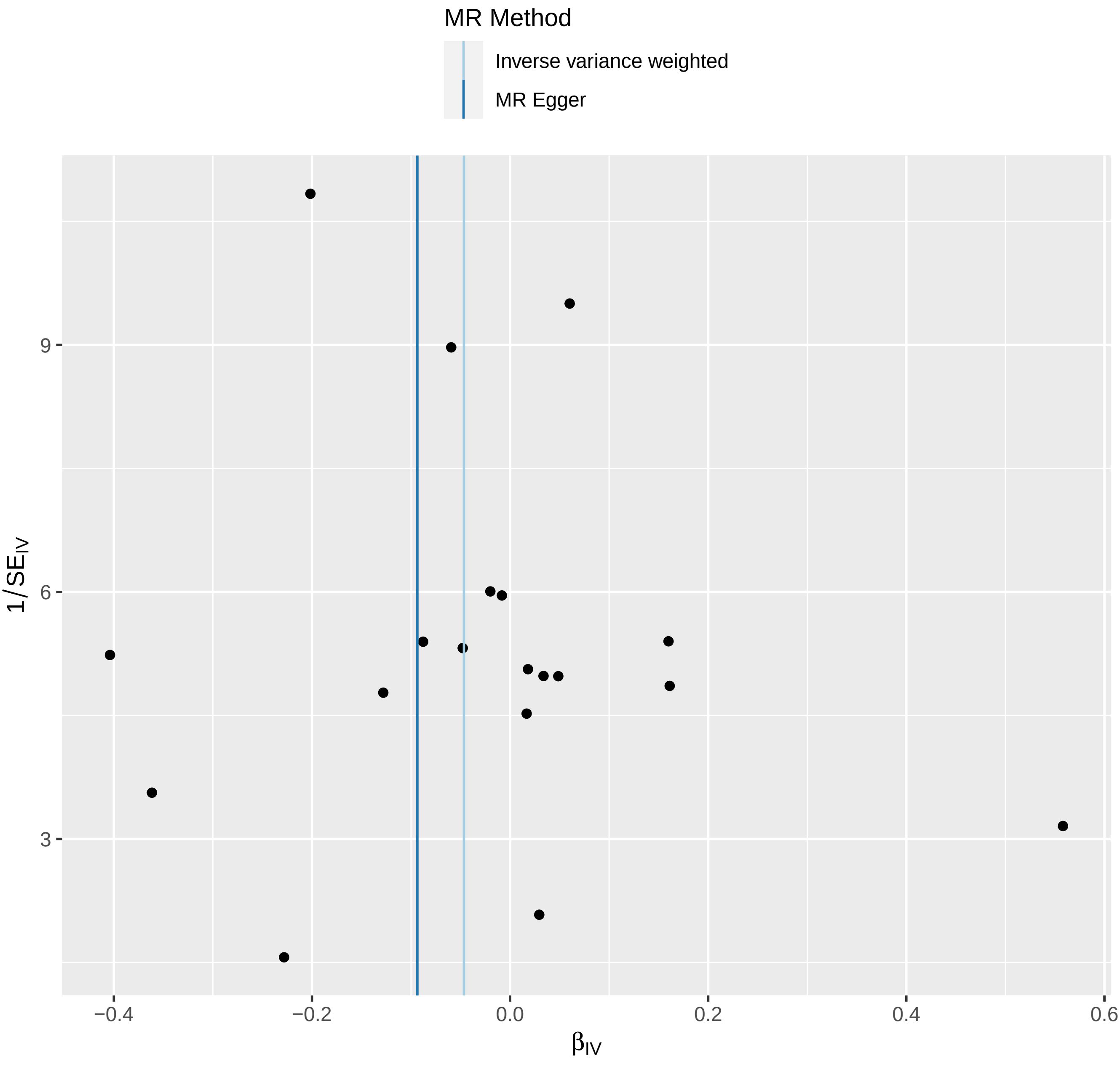

Batch 3−2130 : Nuclear pore membrane glycoprotein 210−like on Diabetic nephropathy (more control exclusions)
with a threshold of 1e−05

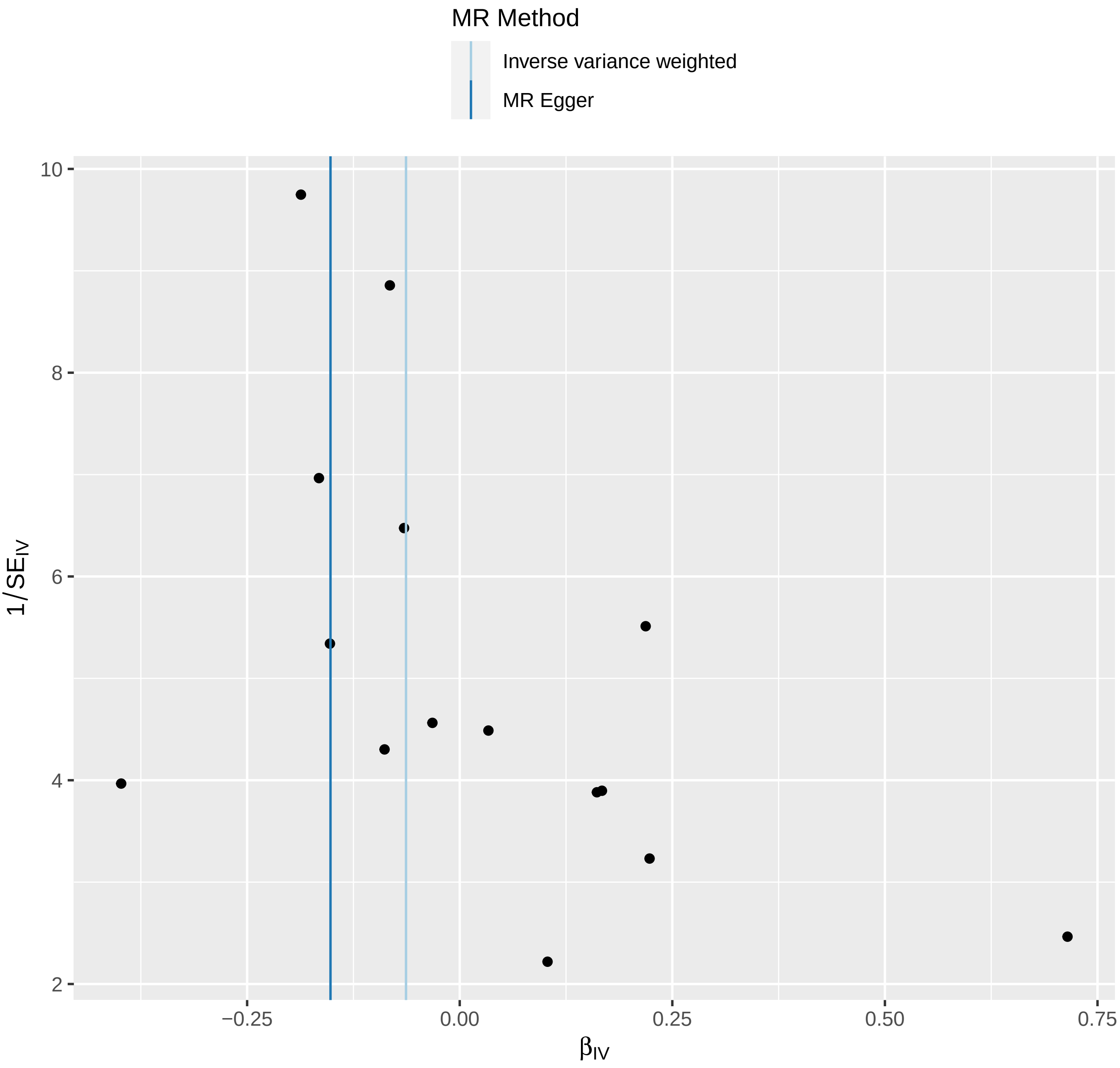

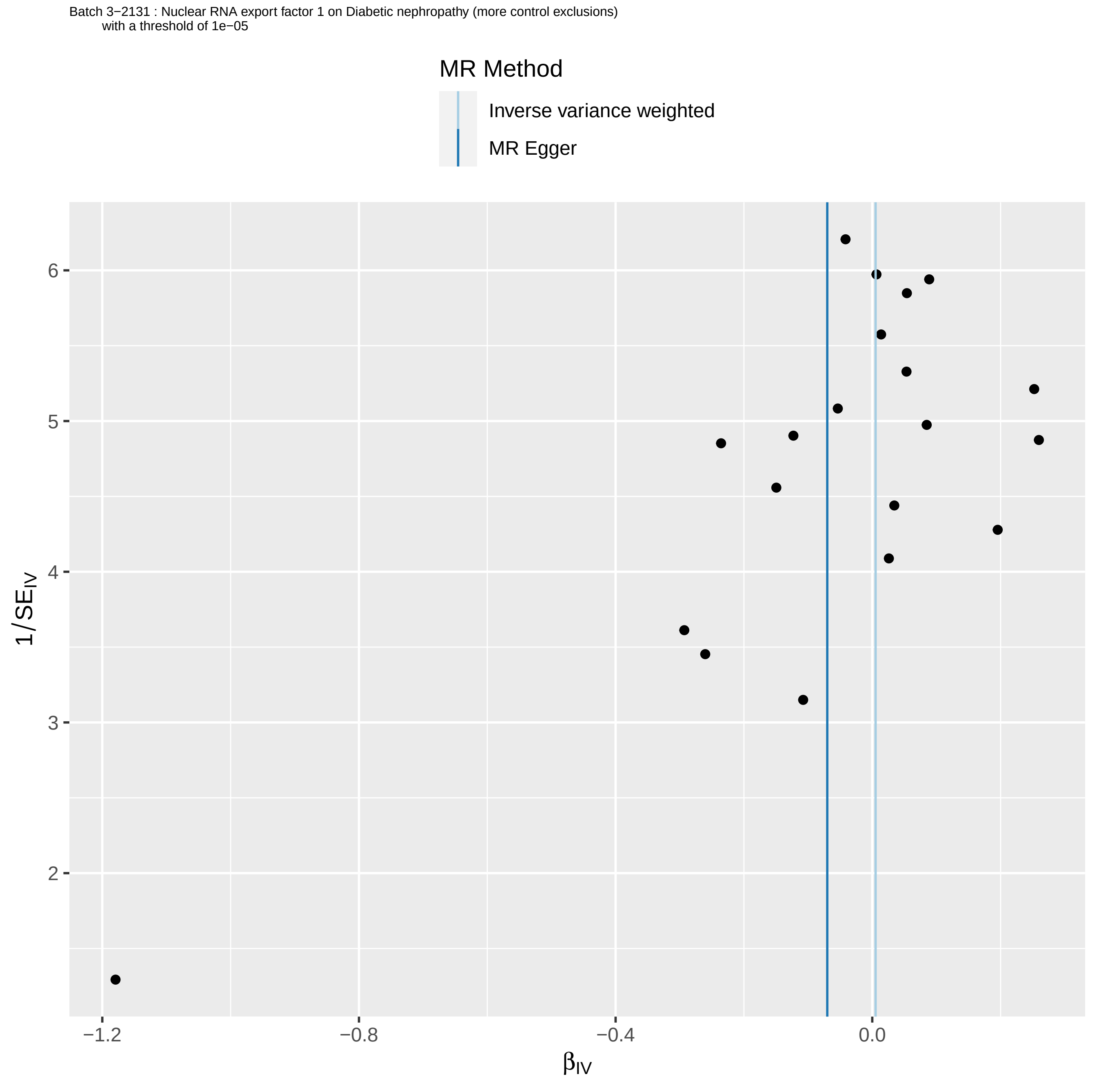
Batch 3−2131 : Nuclear RNA export factor 1 on Diabetic nephropathy (more control exclusions)
with a threshold of 1e−05
MR Method
Inverse variance weighted
MR Egger
6
5
4
3
2
1/SE$_{IV}$
−1.2
−0.8
−0.4
0.0
β$_{IV}$

Batch 3−2132 : Neurexophilin−1 on Diabetic nephropathy (more control exclusions)
with a threshold of 1e−05

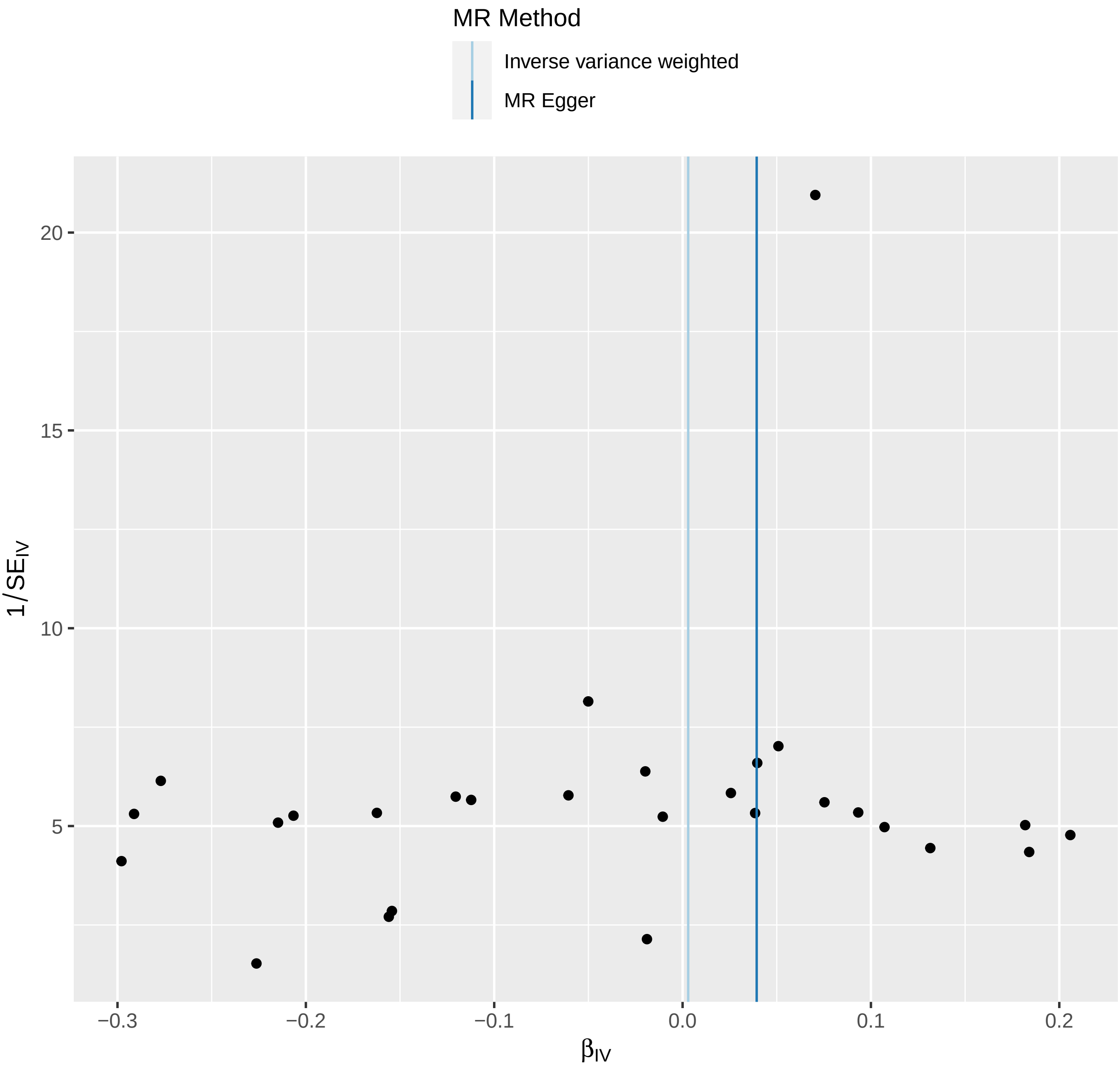

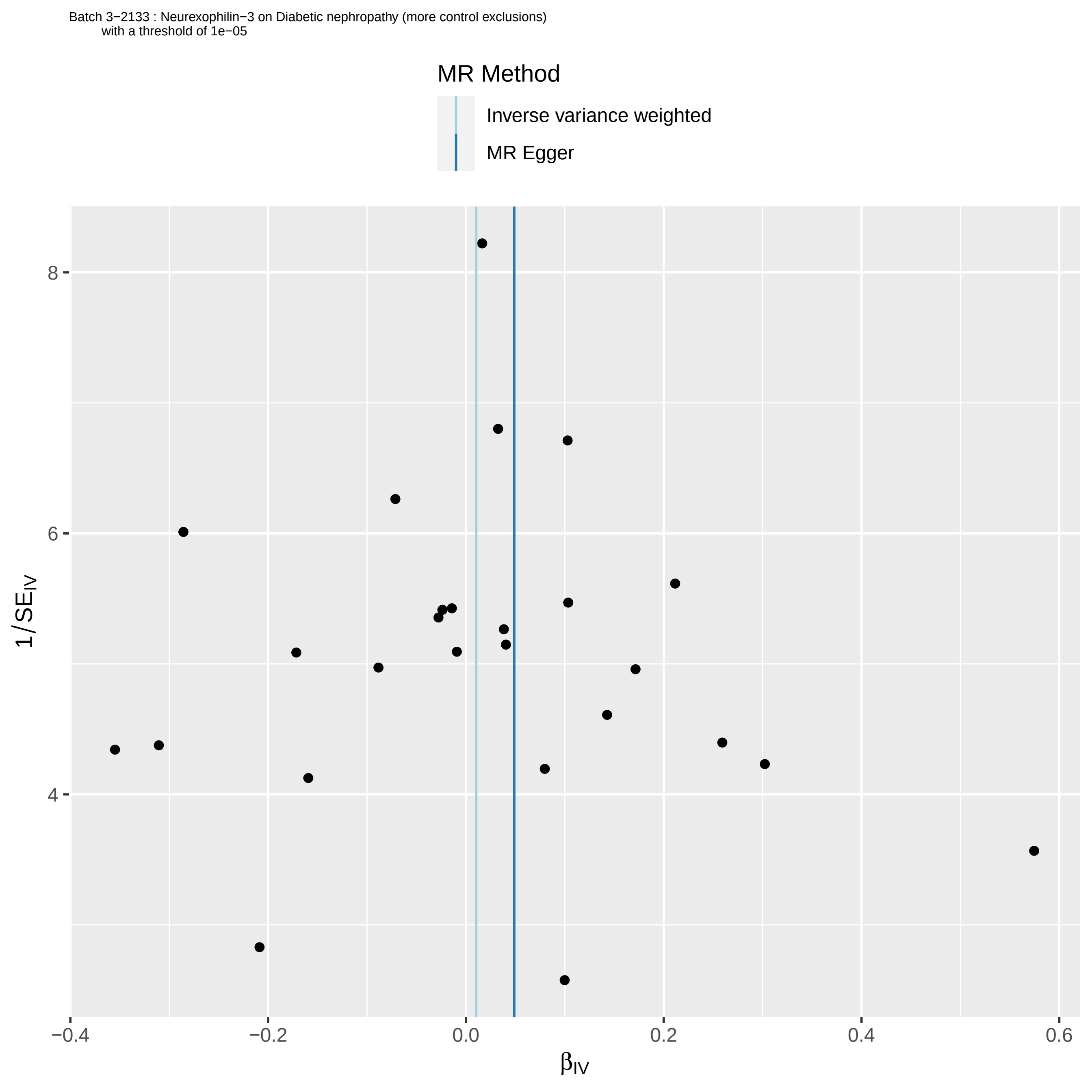
Batch 3−2133 : Neurexophilin−3 on Diabetic nephropathy (more control exclusions)
with a threshold of 1e−05
MR Method
Inverse variance weighted
MR Egger
8
6
4
1/SE_IV
−0.4
−0.2
0.0
0.2
0.4
0.6
β_IV

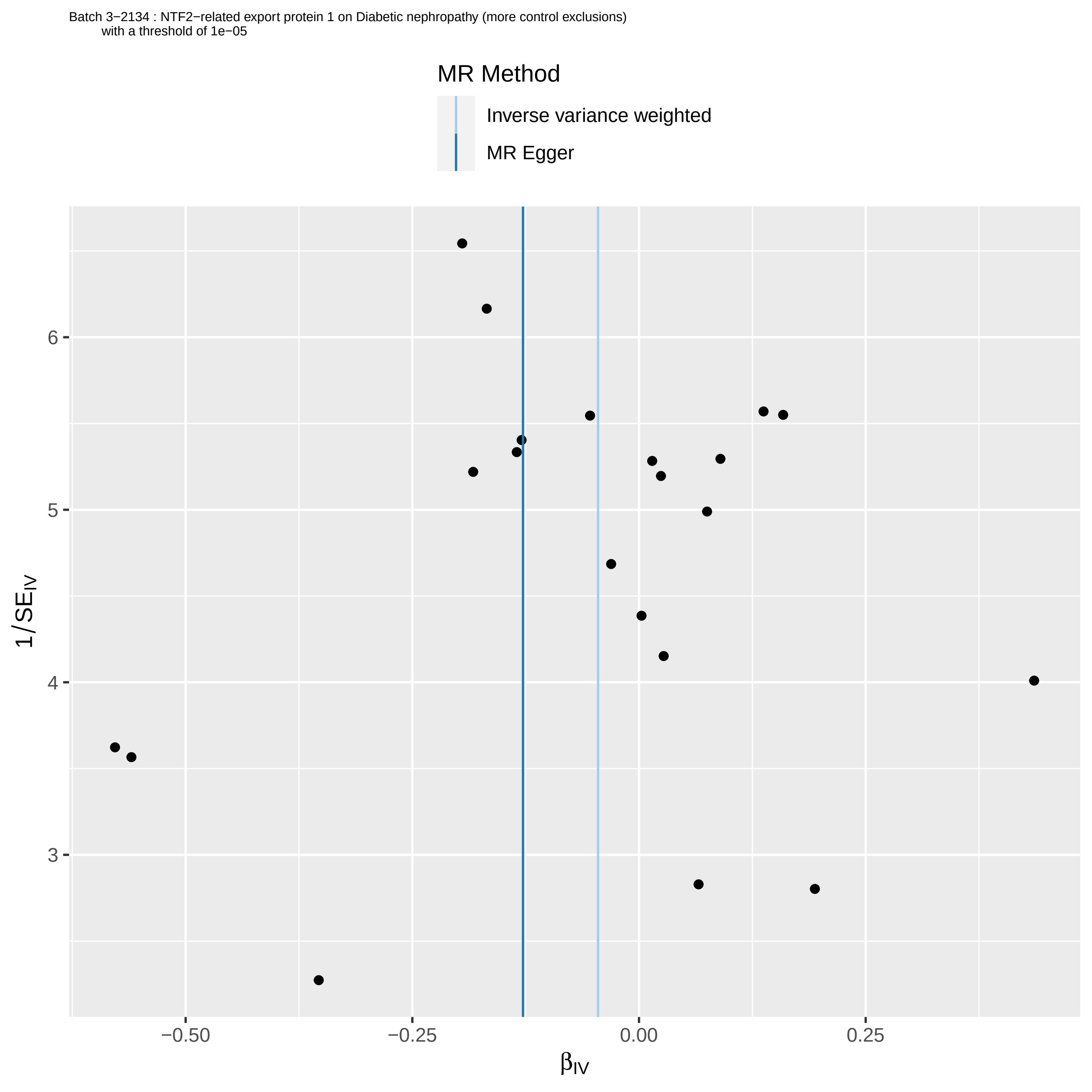
Batch 3−2134 : NTF2−related export protein 1 on Diabetic nephropathy (more control exclusions)
with a threshold of 1e−05
MR Method
Inverse variance weighted
MR Egger
6
5
4
3
1/SE$_{IV}$
−0.50
−0.25
0.00
0.25
β$_{IV}$

Batch 3−2135 : Out at first protein homolog on Diabetic nephropathy (more control exclusions)
with a threshold of 1e−05

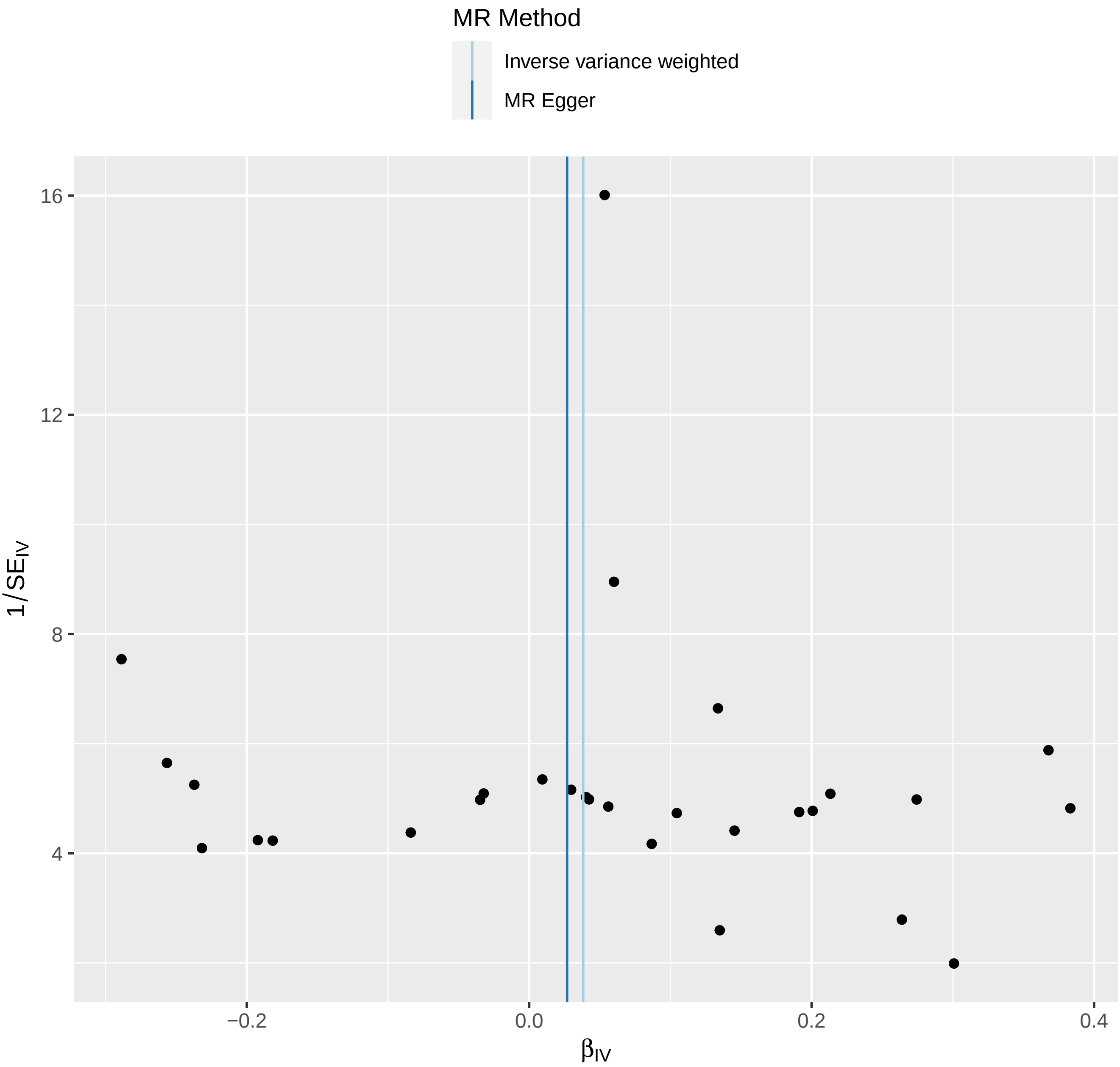

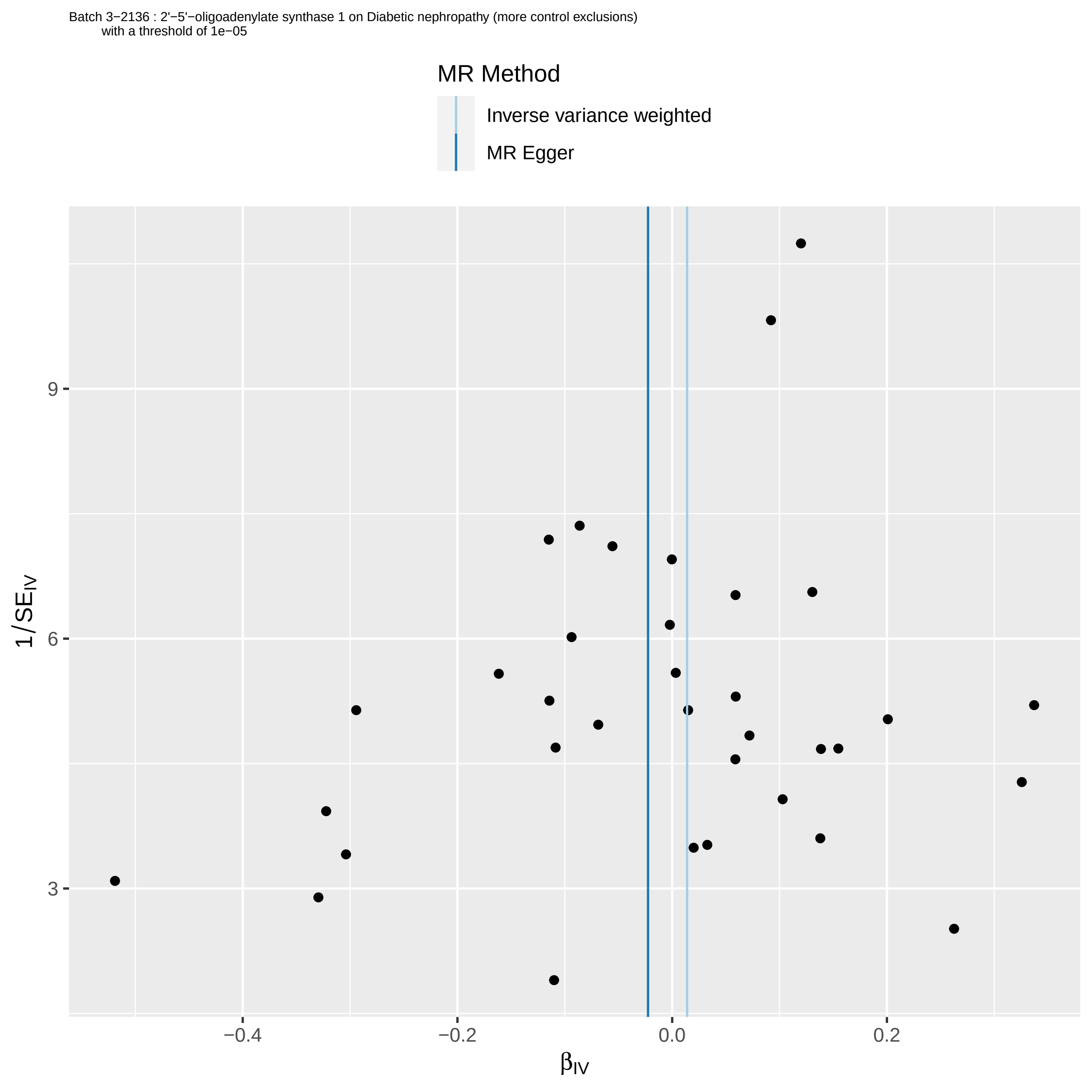
Batch 3−2136 : 2'−5'−oligoadenylate synthase 1 on Diabetic nephropathy (more control exclusions)
with a threshold of 1e−05
MR Method
Inverse variance weighted
MR Egger
9
6
3
1/SE_IV
−0.4
−0.2
0.0
0.2
β_IV

Batch 3−2137 : Ornithine decarboxylase antizyme 1 on Diabetic nephropathy (more control exclusions)
with a threshold of 1e−05

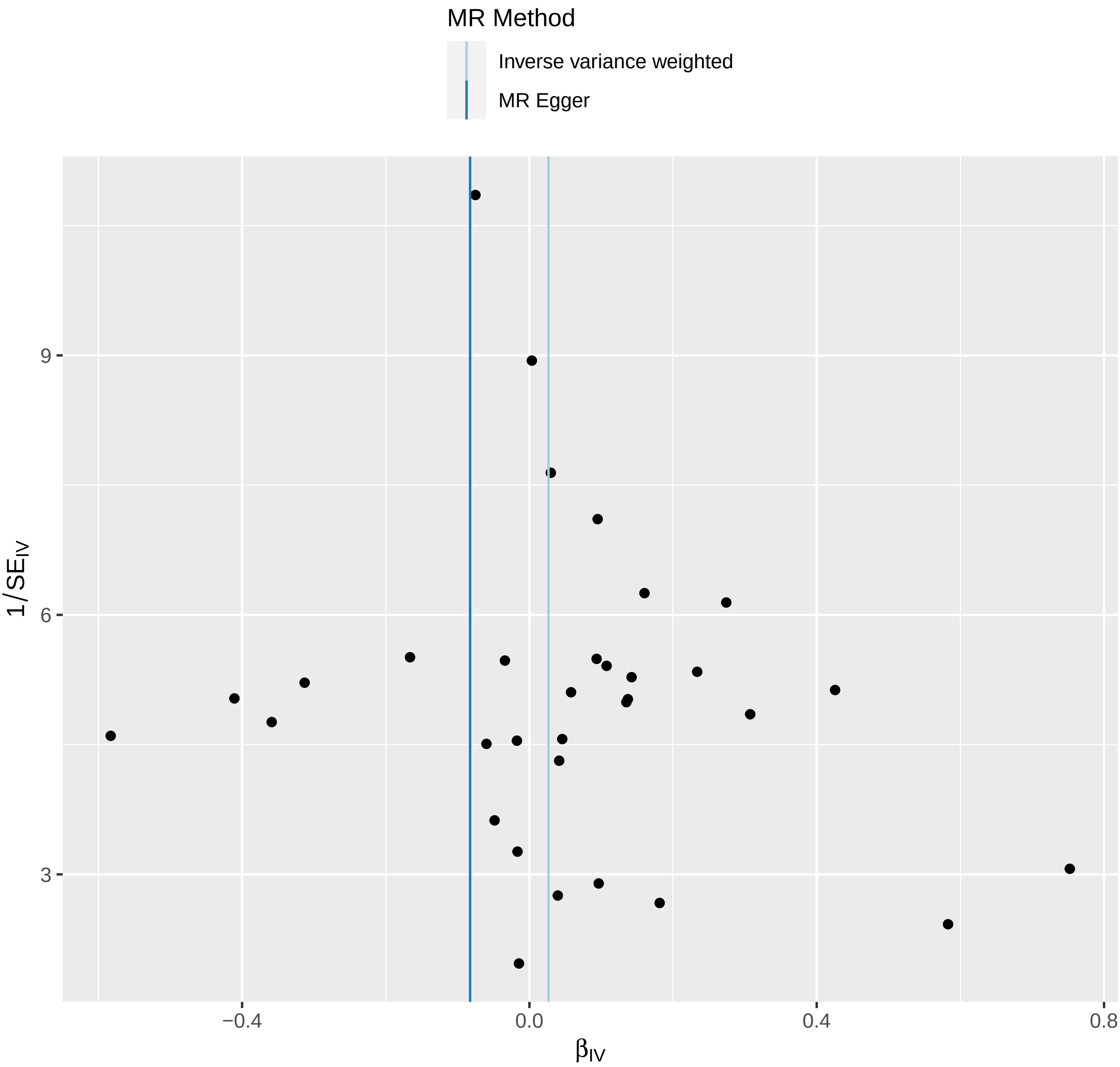

Batch 3−2138 : Odorant−binding protein 2a on Diabetic nephropathy (more control exclusions)
with a threshold of 1e−05

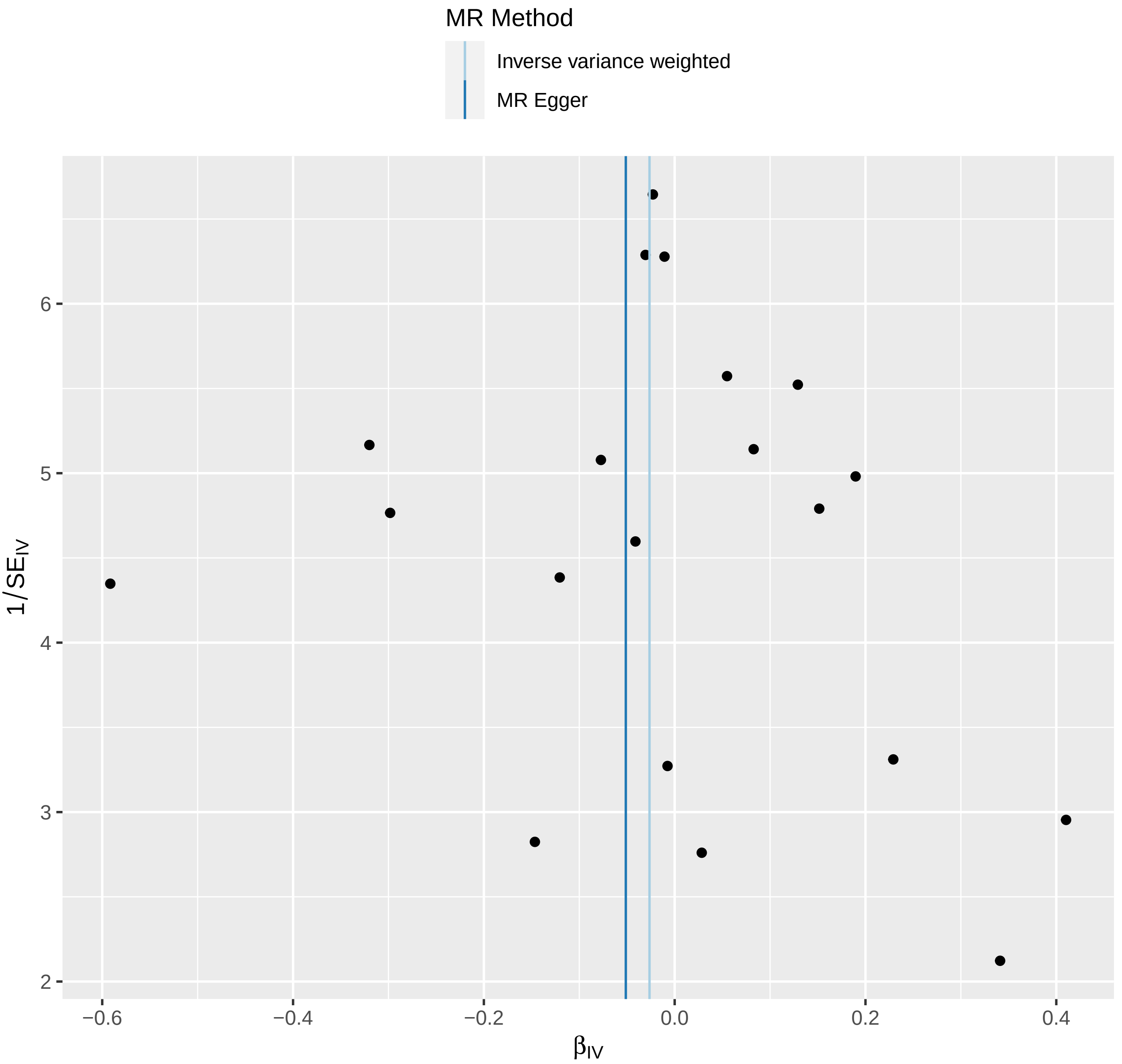

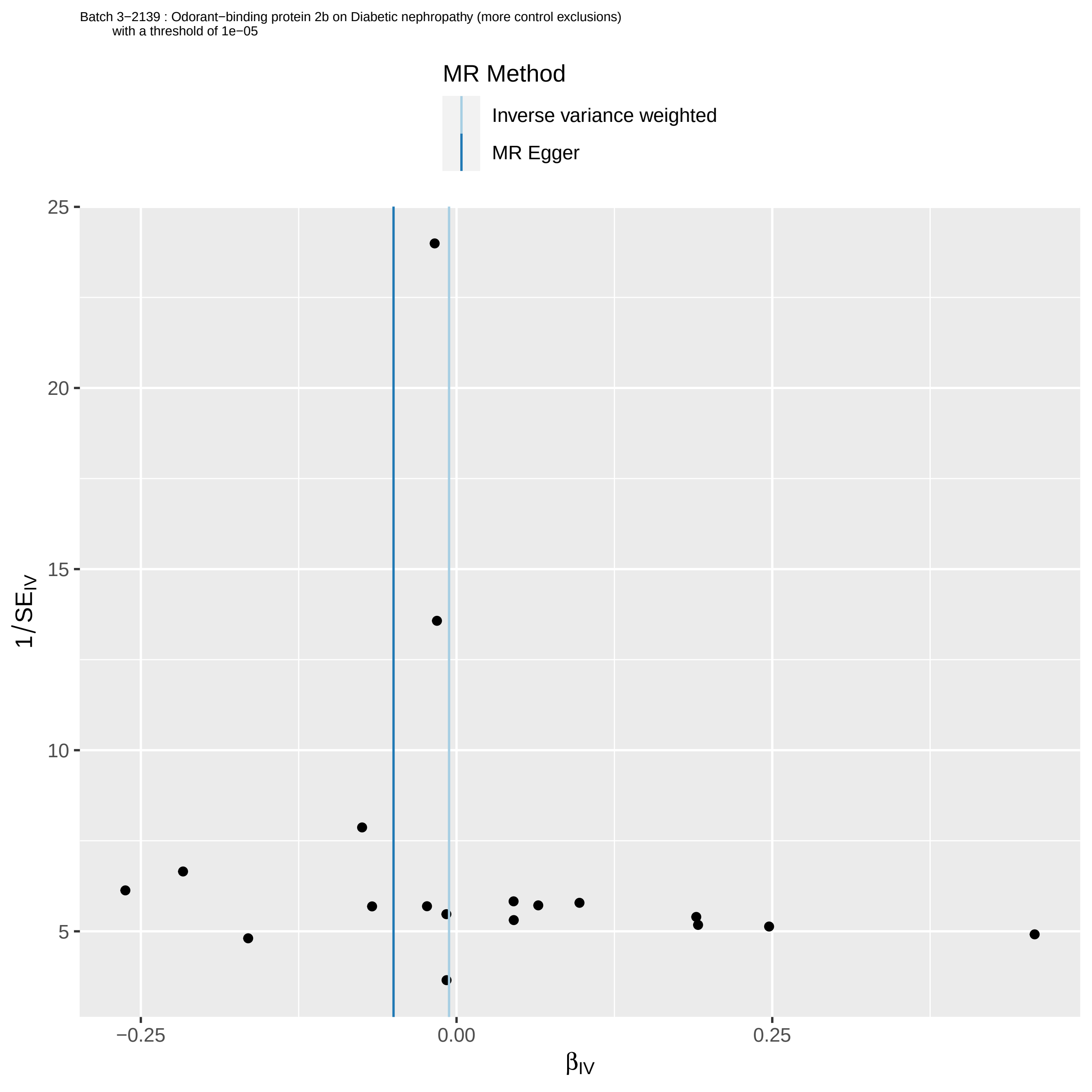
Batch 3−2139 : Odorant−binding protein 2b on Diabetic nephropathy (more control exclusions)
with a threshold of 1e−05
MR Method
Inverse variance weighted
MR Egger
25
20
15
10
5
1/SE_IV
−0.25
0.00
0.25
β_IV

Batch 3−2140 : OCIA domain−containing protein 1 on Diabetic nephropathy (more control exclusions)
with a threshold of 1e−05

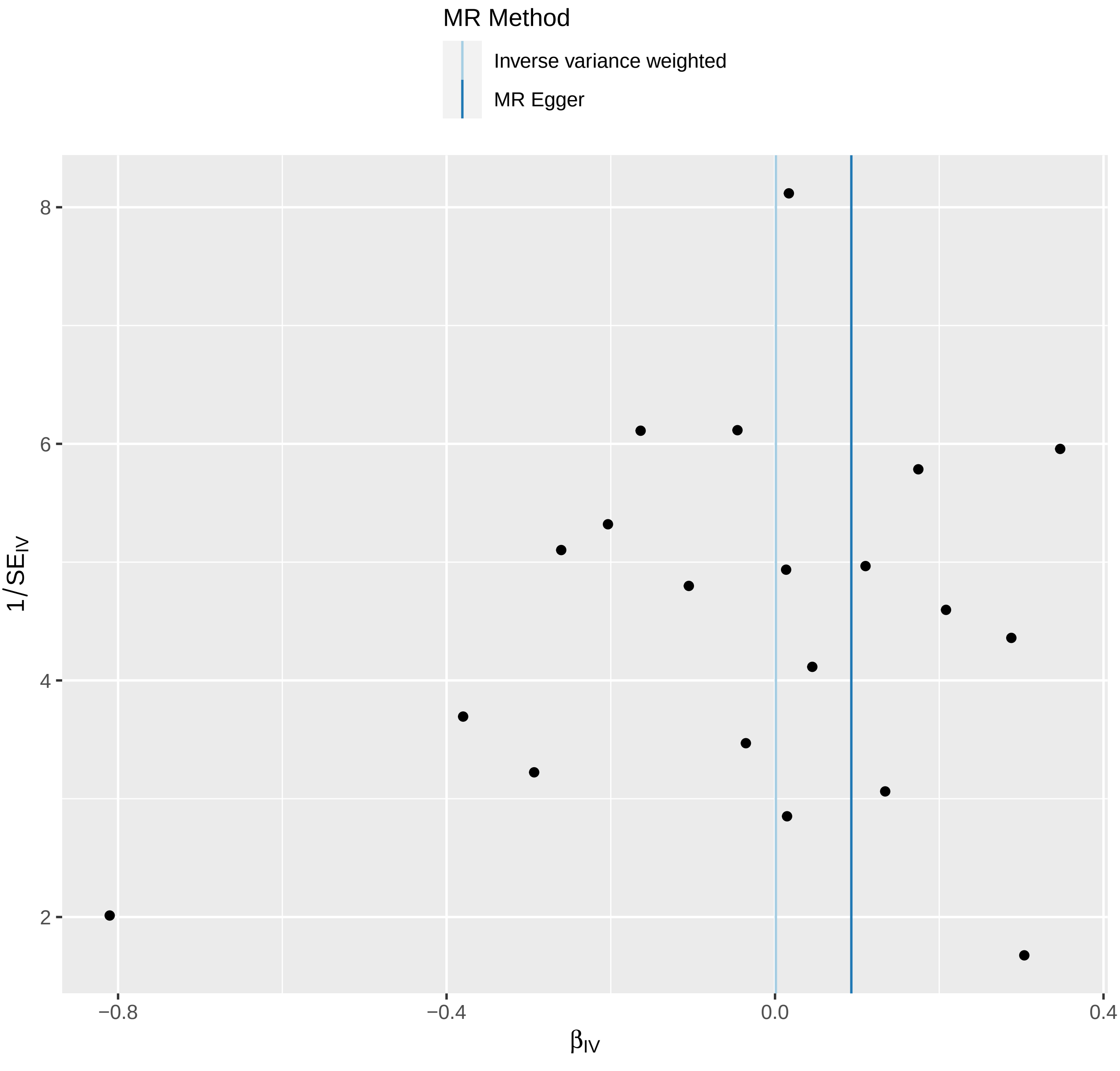

Batch 3−2141 : Inositol polyphosphate 5−phosphatase OCRL−1 on Diabetic nephropathy (more control exclusions) with a threshold of 1e−05

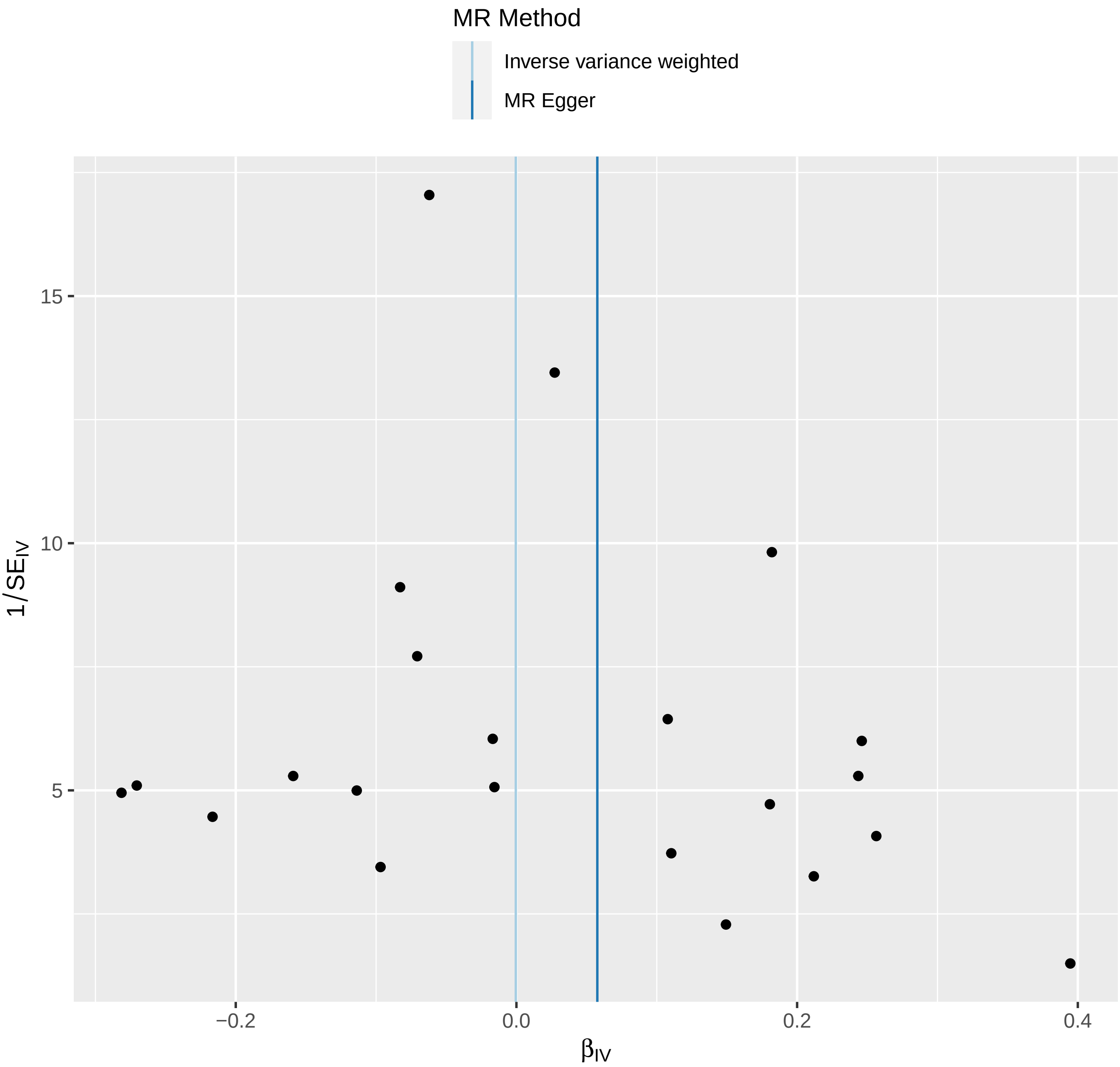

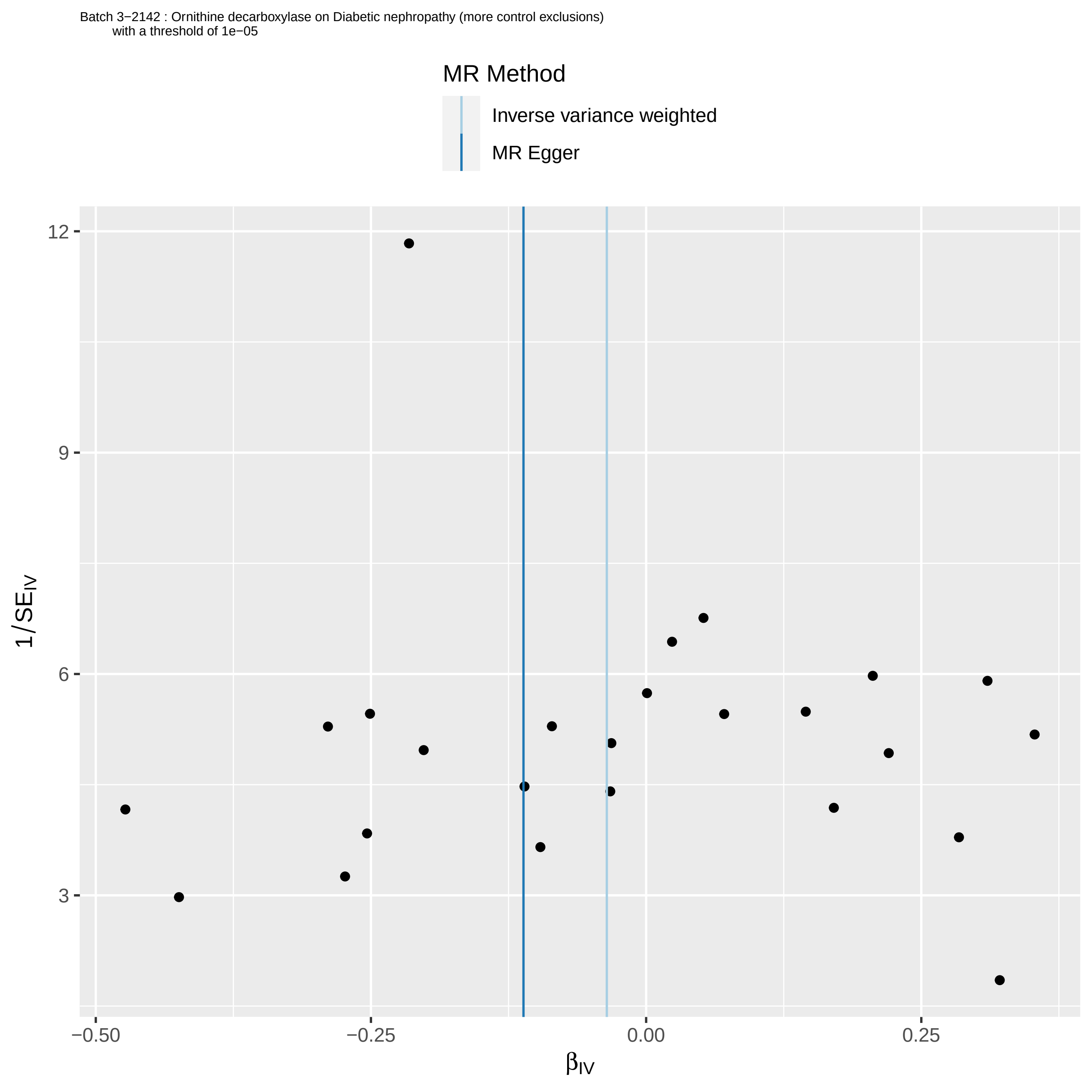

Batch 3−2142 : Ornithine decarboxylase on Diabetic nephropathy (more control exclusions)
with a threshold of 1e−05
MR Method
Inverse variance weighted
MR Egger
12
9
6
3
1/SE_IV
−0.50
−0.25
0.00
0.25
β_IV

Batch 3−2143 : Obg−like ATPase 1 on Diabetic nephropathy (more control exclusions)
with a threshold of 1e−05

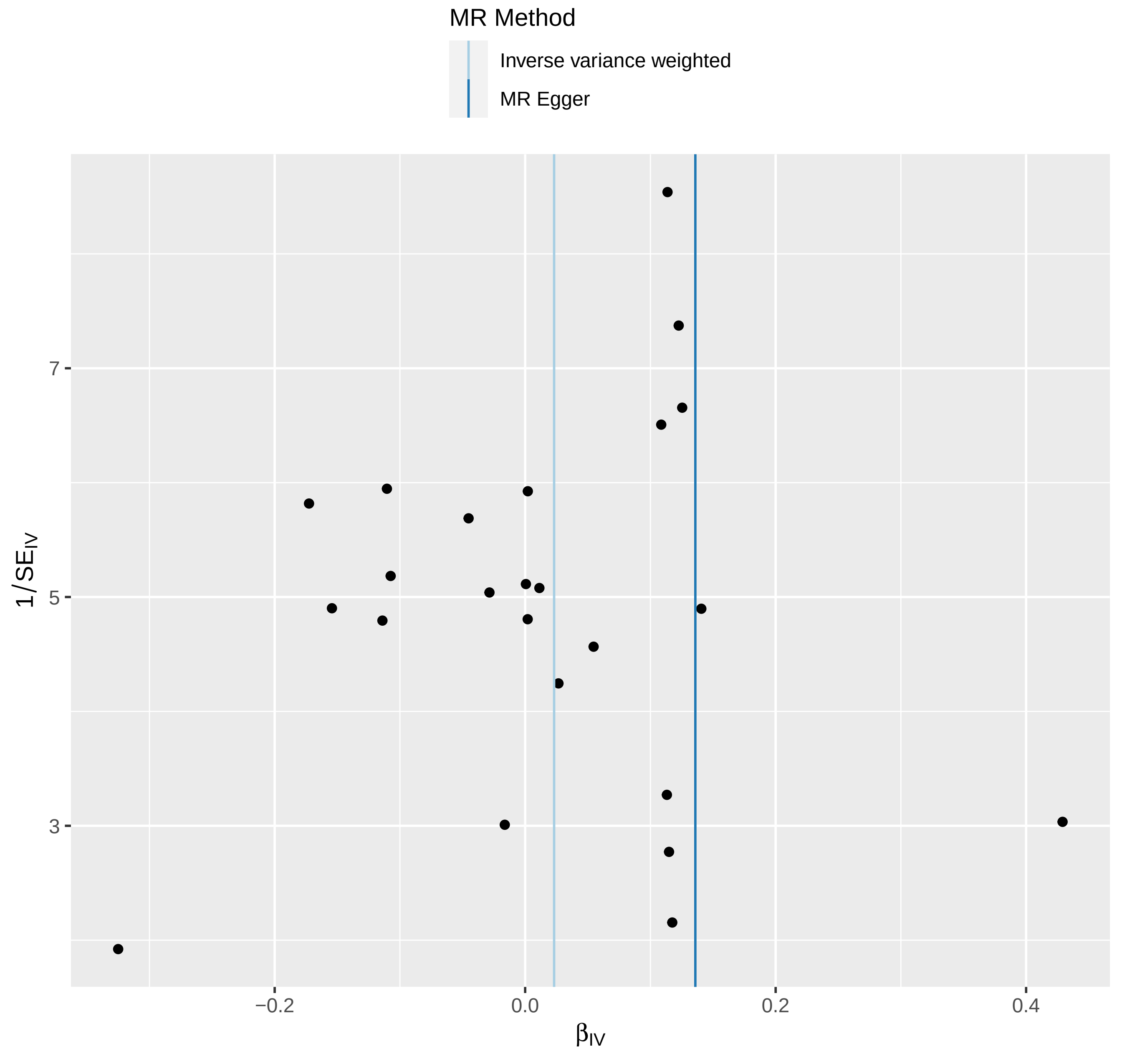

Batch 3−2144 : Olfactomedin−like protein 3 on Diabetic nephropathy (more control exclusions)
with a threshold of 1e−05

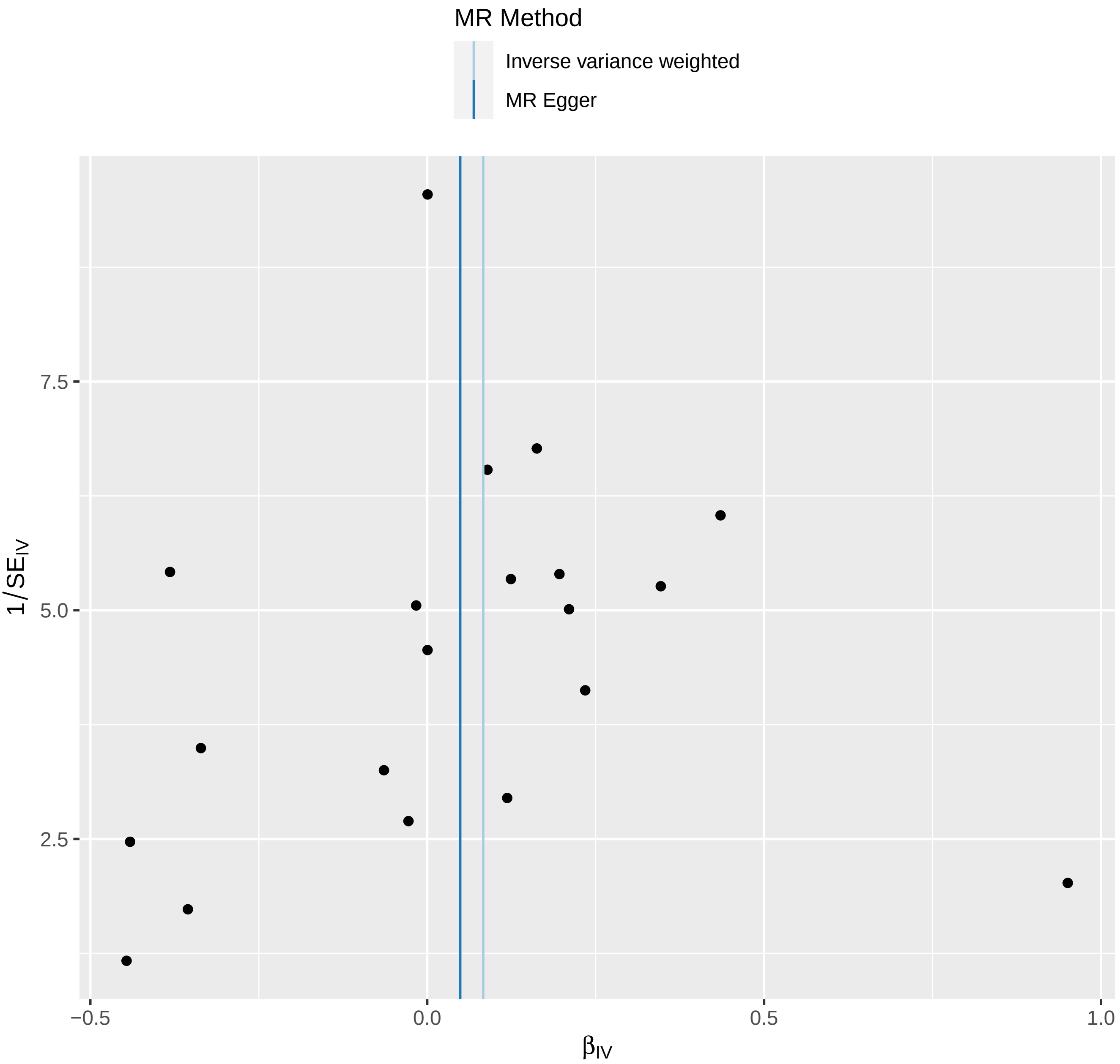

Batch 3−2145 : Olfactomedin−like protein 3 on Diabetic nephropathy (more control exclusions)
with a threshold of 1e−05

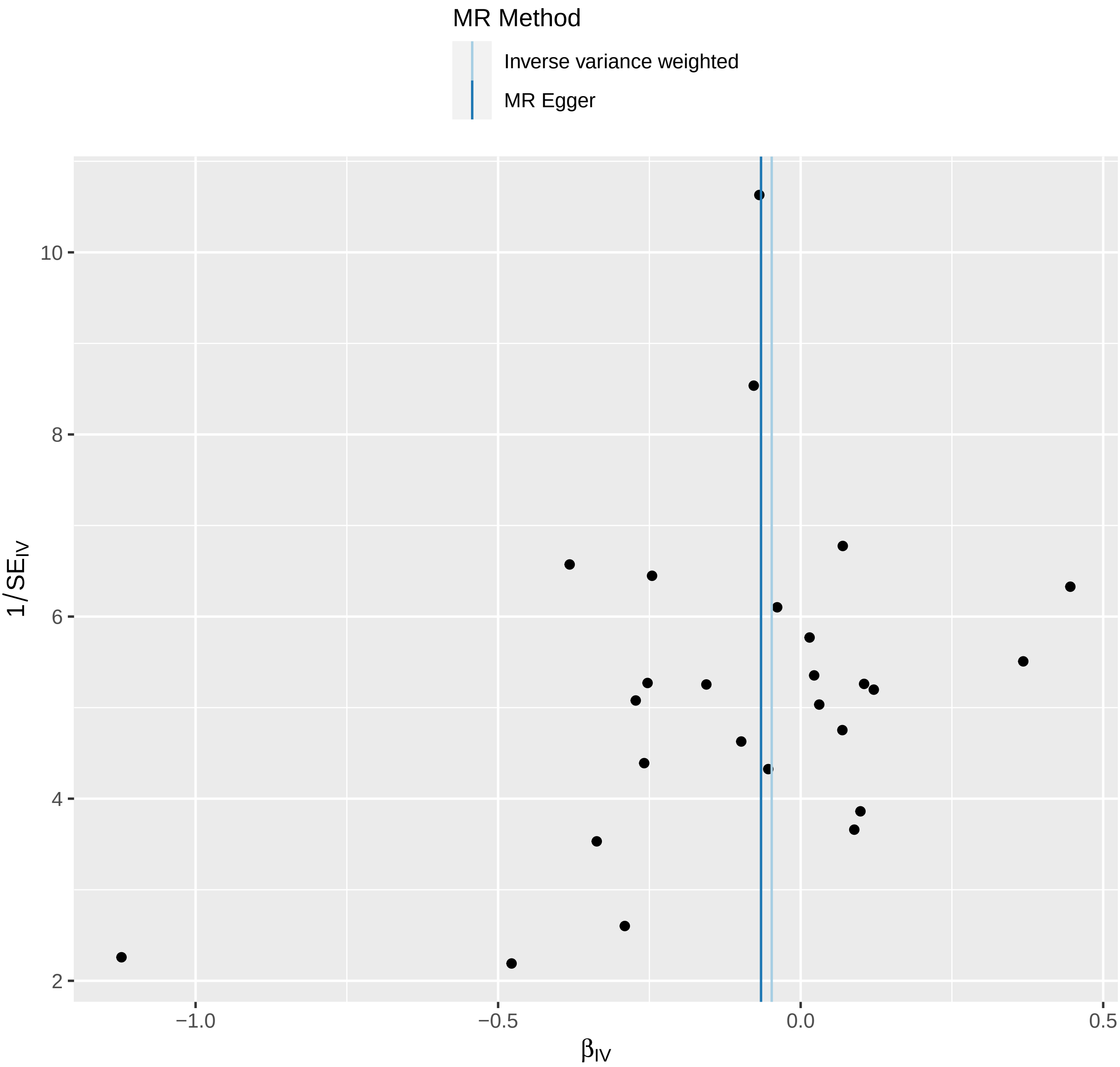

Batch 3−2146 : Oxidized low−density lipoprotein receptor 1 on Diabetic nephropathy (more control exclusions) with a threshold of 1e−05

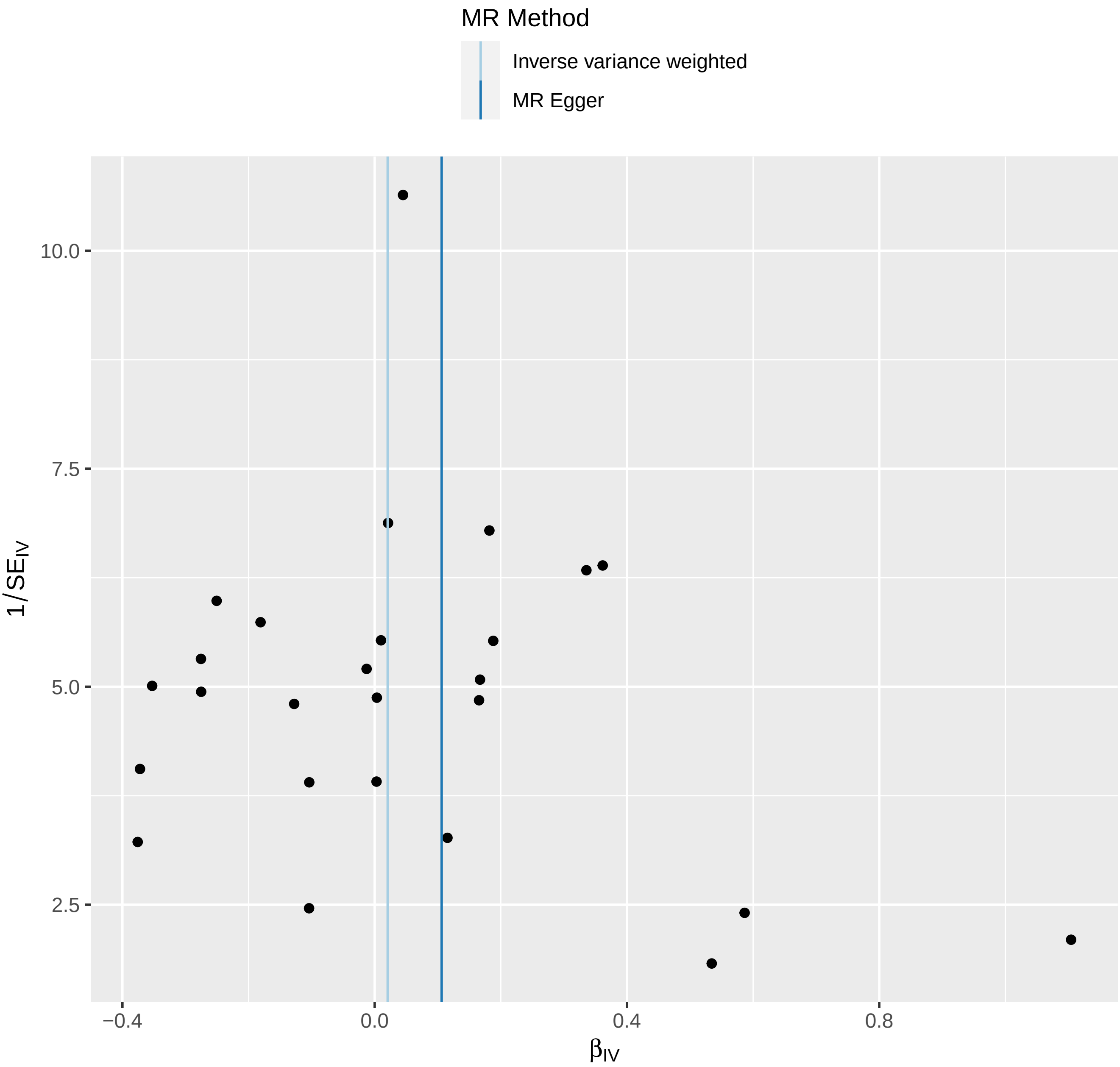

Batch 3−2147 : Osteomodulin on Diabetic nephropathy (more control exclusions)
with a threshold of 1e−05

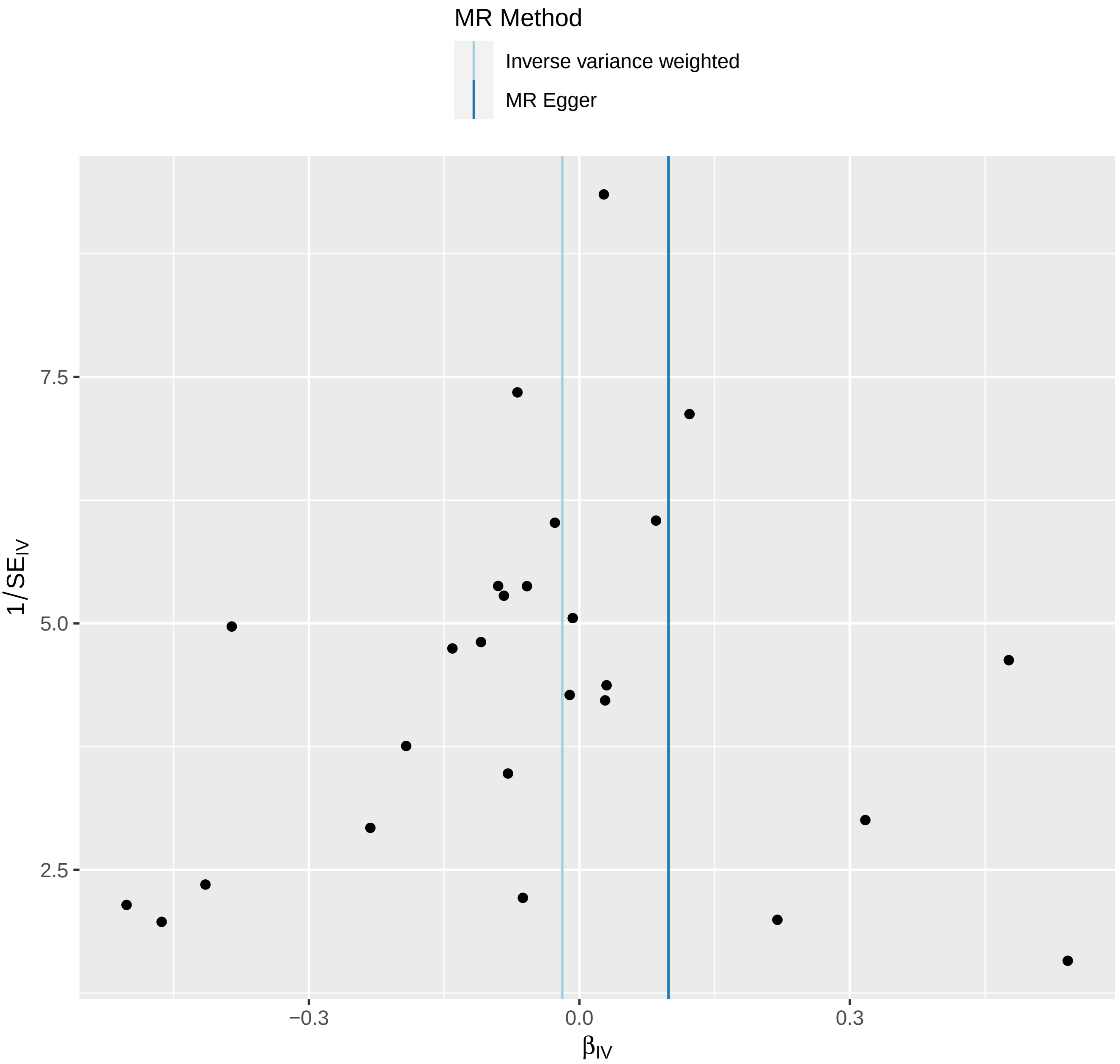

Batch 3−2148 : Opalin on Diabetic nephropathy (more control exclusions)
with a threshold of 1e−05

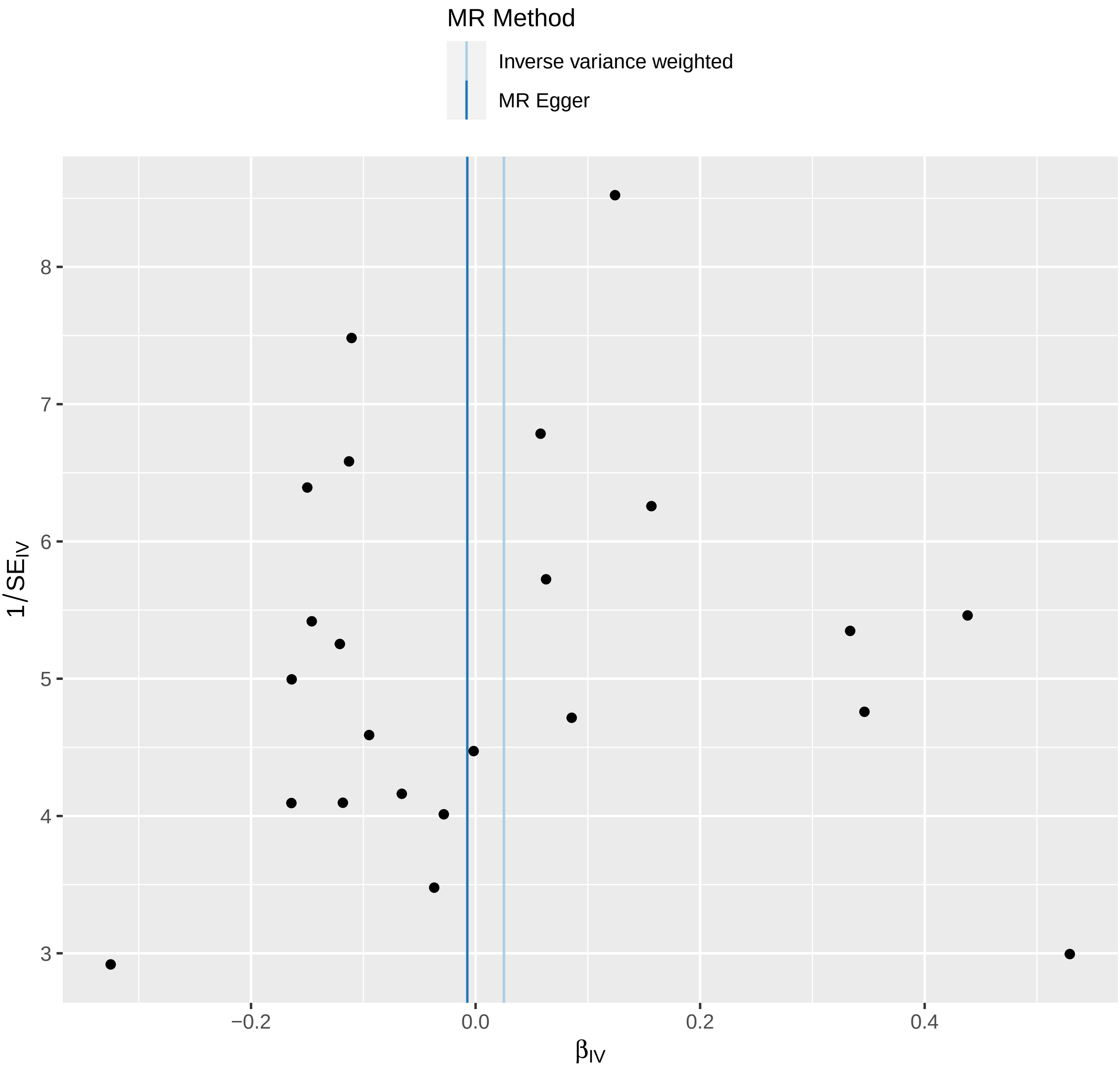

Batch 3−2149 : Opioid−binding protein/cell adhesion molecule on Diabetic nephropathy (more control exclusions)
with a threshold of 1e−05

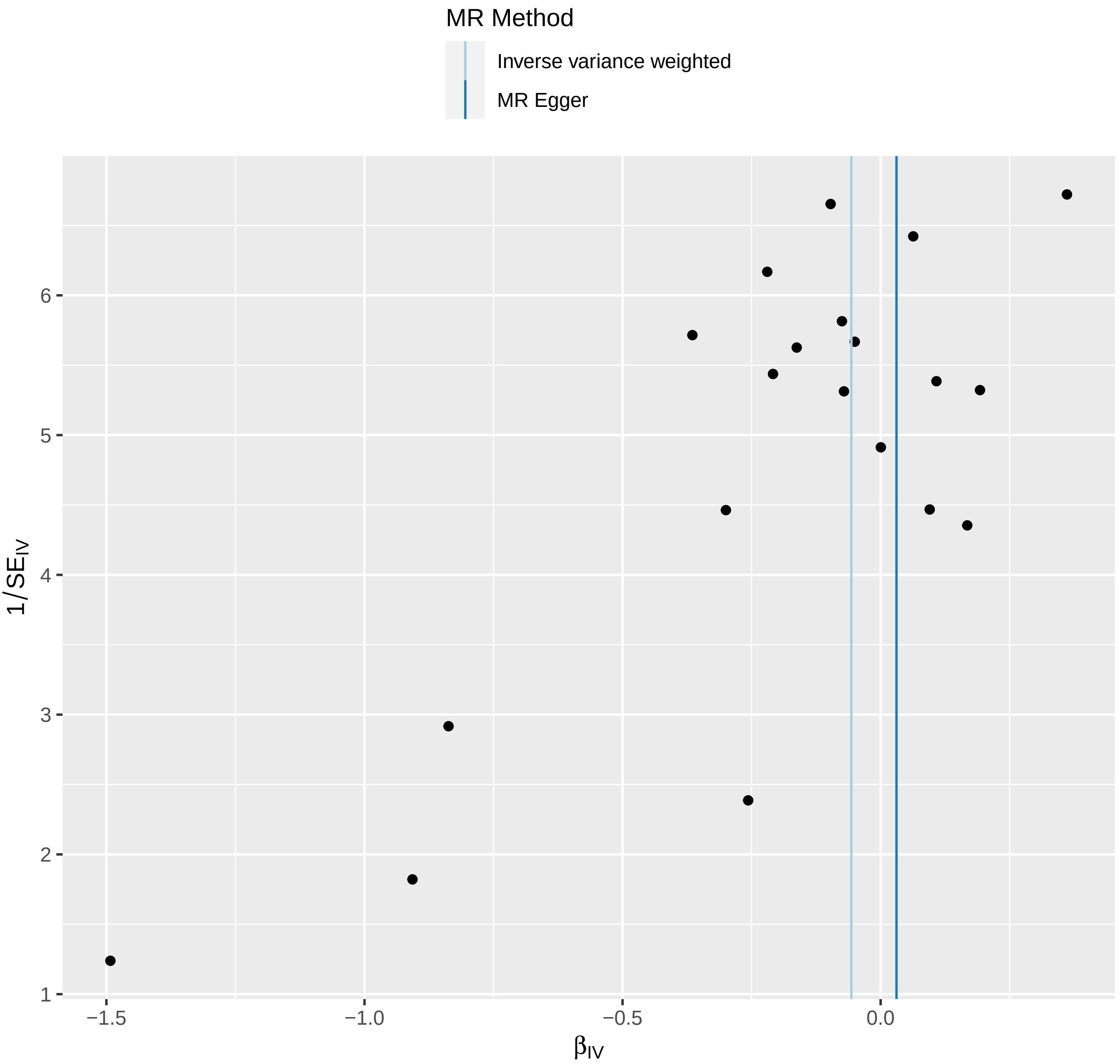

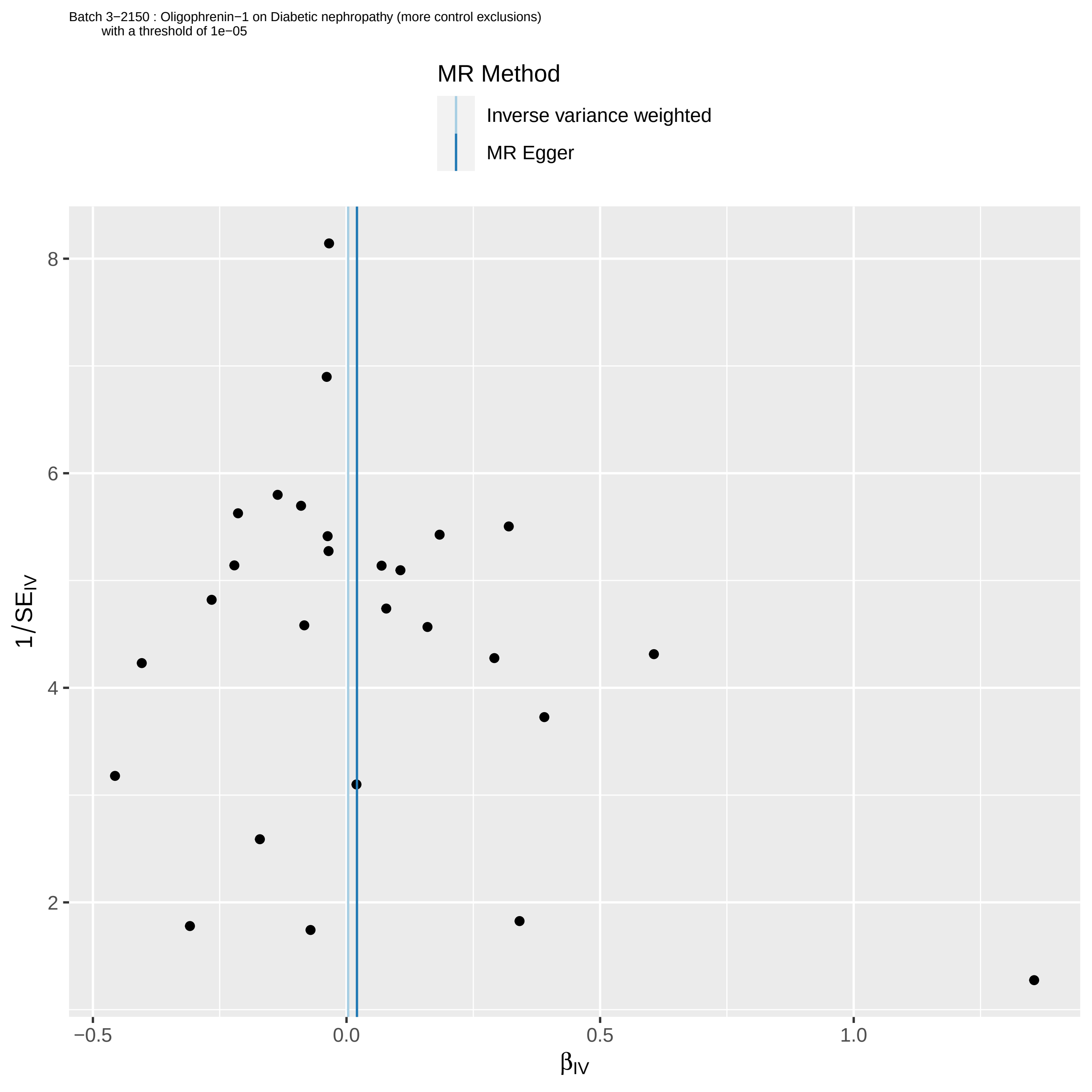

Batch 3−2150 : Oligophrenin−1 on Diabetic nephropathy (more control exclusions)
with a threshold of 1e−05
MR Method
Inverse variance weighted
MR Egger
8
6
4
2
1/SE$_{IV}$
−0.5
0.0
0.5
1.0
β$_{IV}$

Batch 3−2151 : Origin recognition complex subunit 6 on Diabetic nephropathy (more control exclusions)
with a threshold of 1e−05

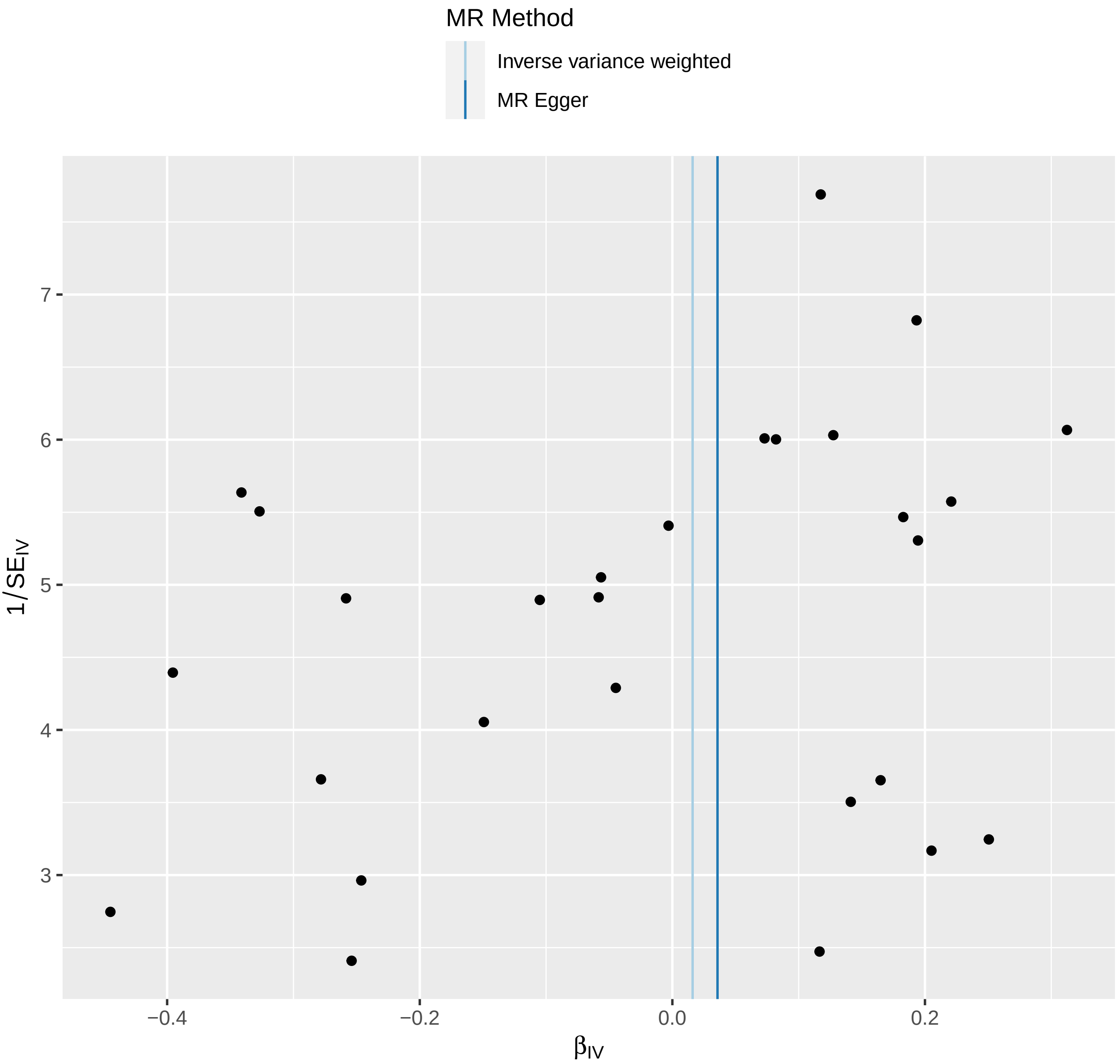

Batch 3−2152 : Oxysterol−binding protein−related protein 11 on Diabetic nephropathy (more control exclusions) with a threshold of 1e−05

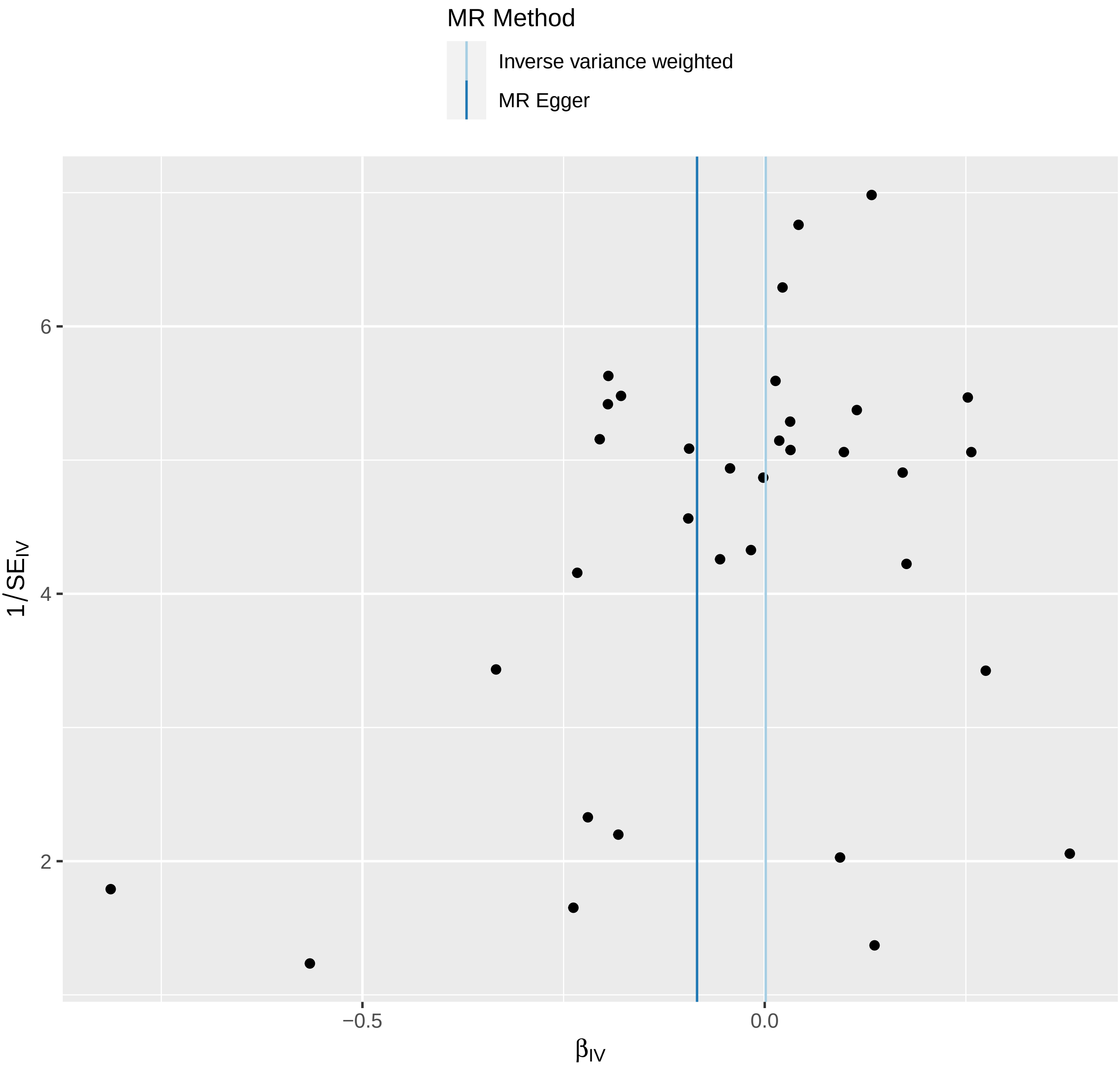

Batch 3−2153 : Osteoclast−associated immunoglobulin−like receptor on Diabetic nephropathy (more control exclusions)
with a threshold of 1e−05

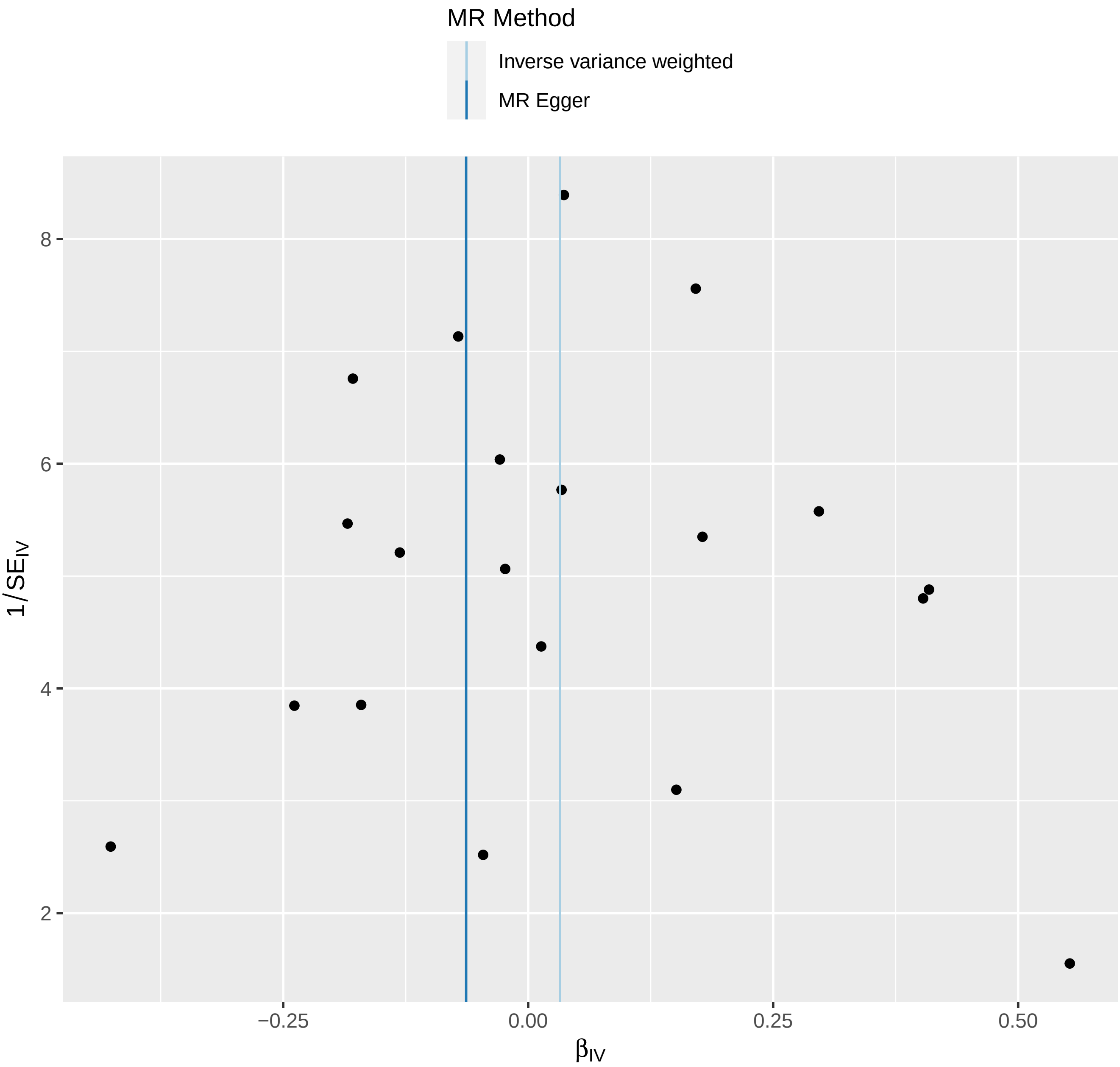

Batch 3−2154 : Oncostatin−M on Diabetic nephropathy (more control exclusions)
with a threshold of 1e−05

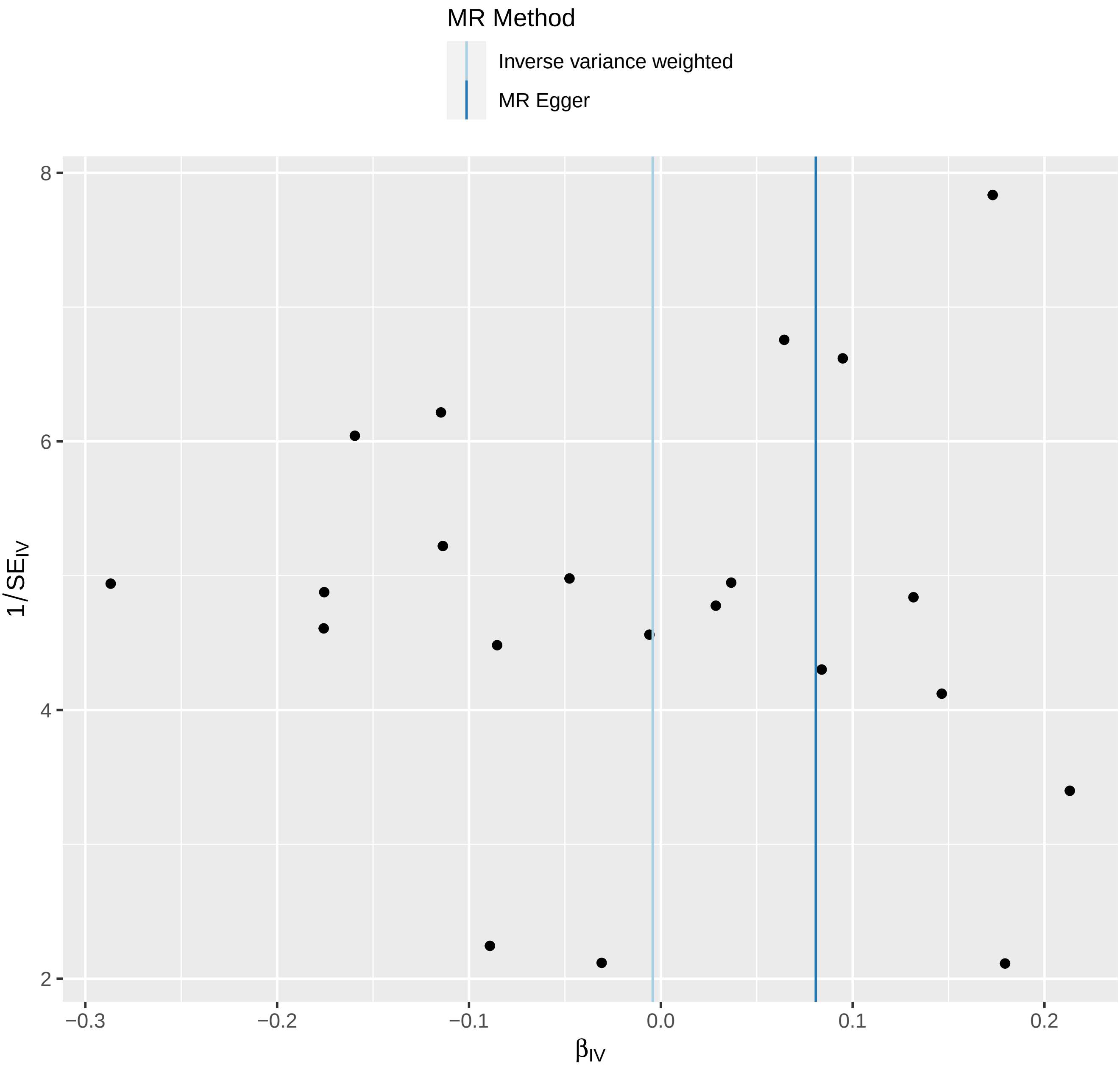

Batch 3−2155 : Oncostatin−M on Diabetic nephropathy (more control exclusions)
with a threshold of 1e−05

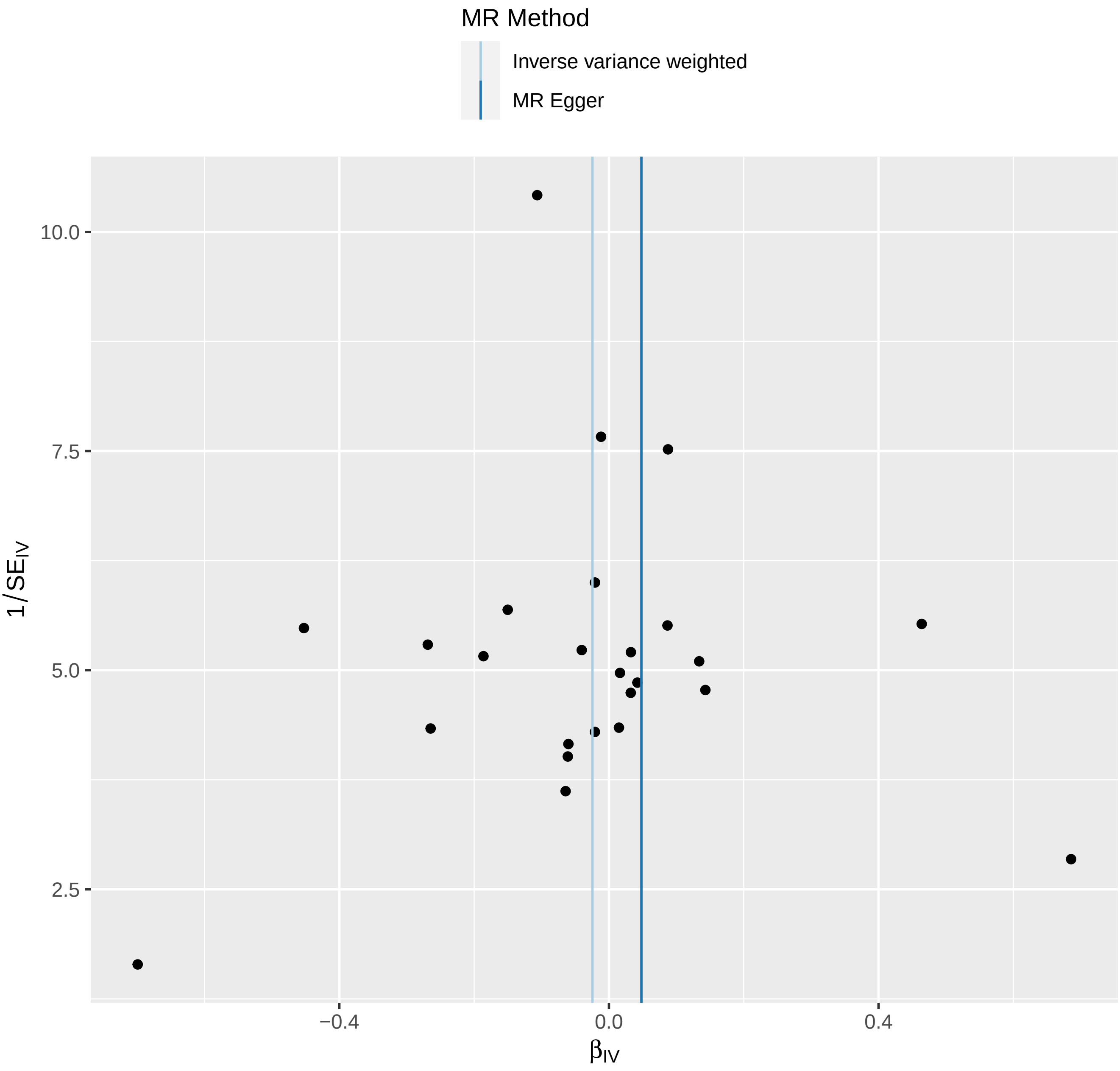

Batch 3−2156 : Oncostatin−M−specific receptor subunit beta on Diabetic nephropathy (more control exclusions) with a threshold of 1e−05

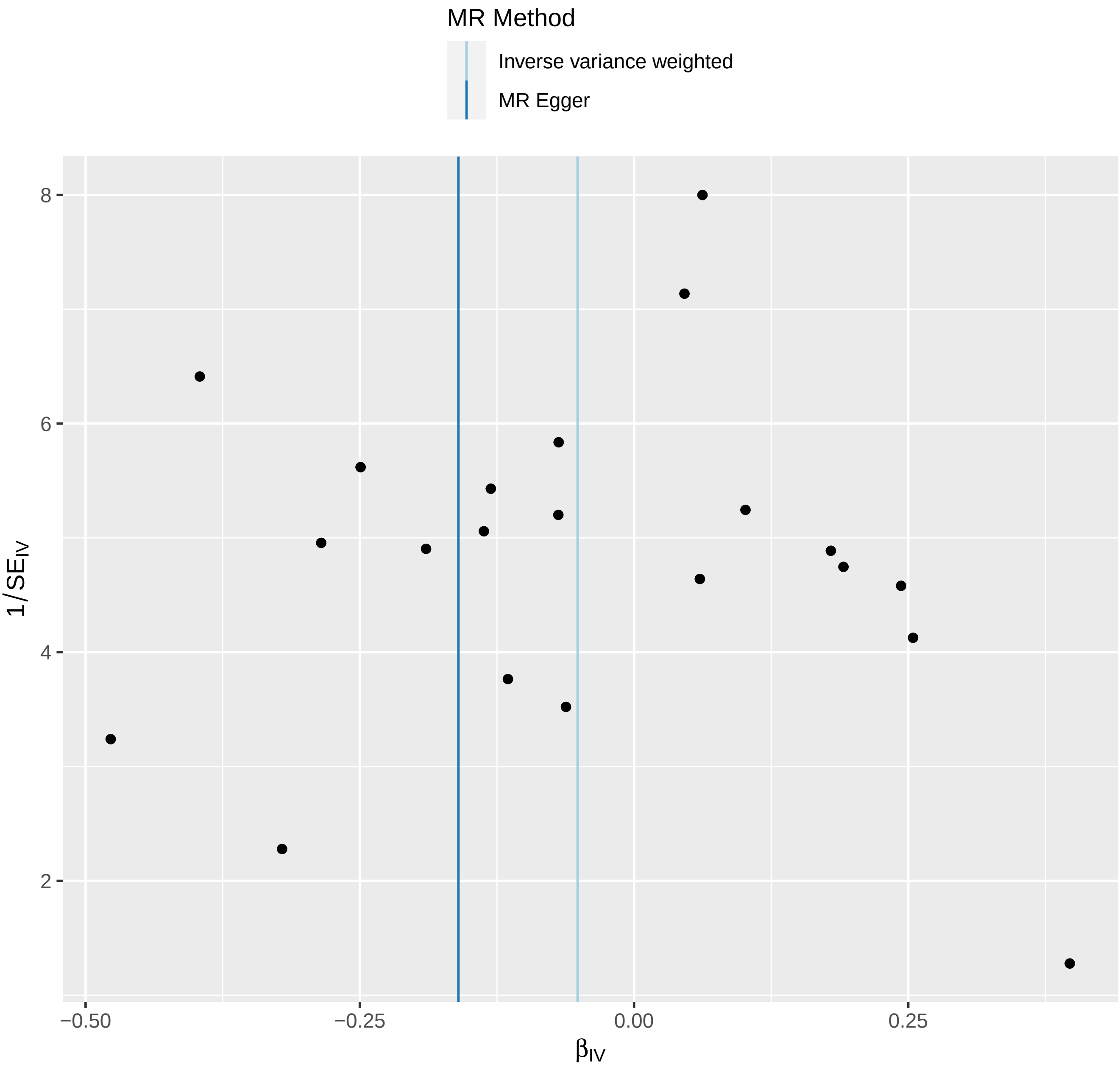

Batch 3−2157 : Osteocrin on Diabetic nephropathy (more control exclusions)
with a threshold of 1e−05

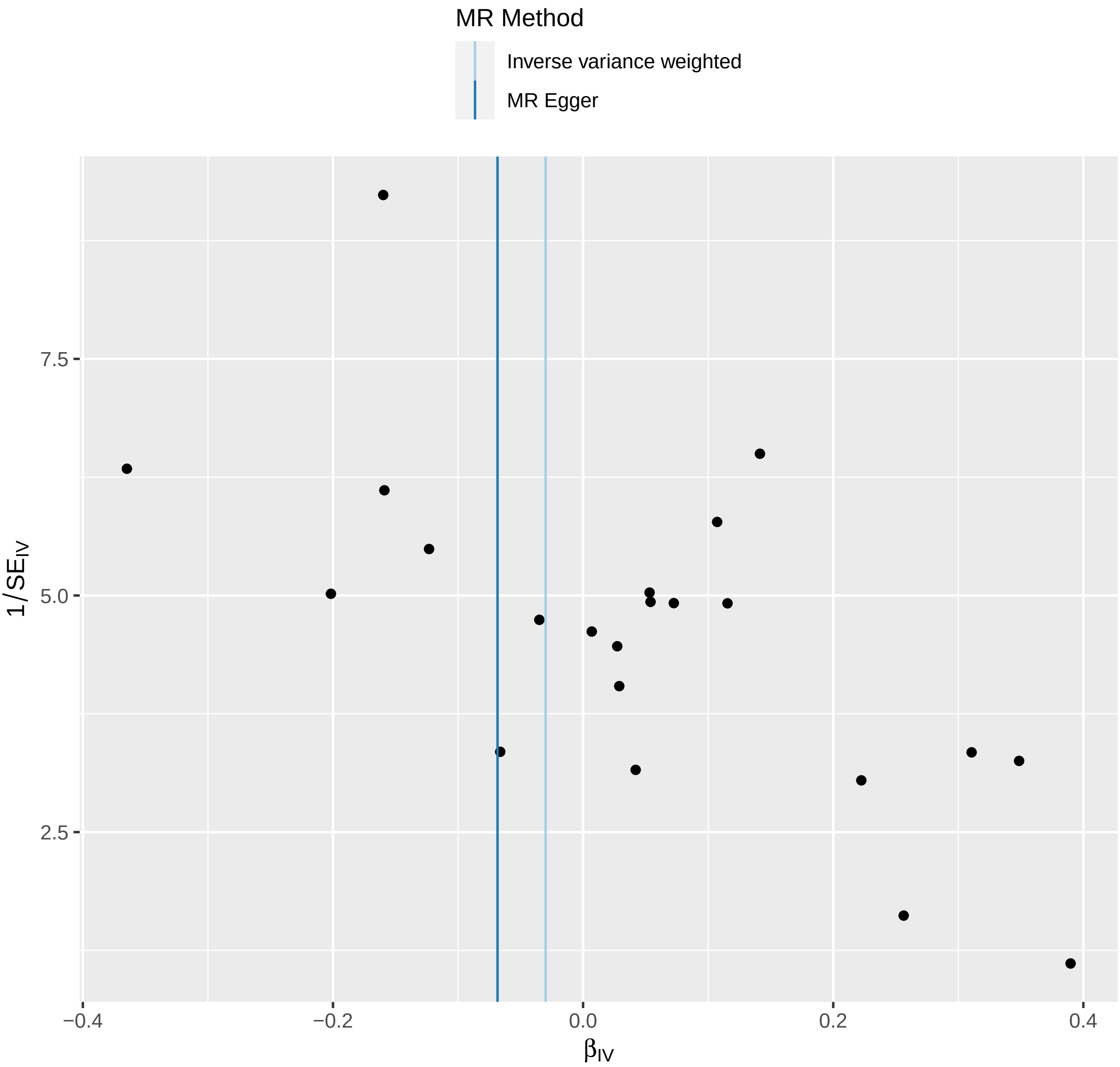

Batch 3−2158 : Ubiquitin thioesterase OTUB2 on Diabetic nephropathy (more control exclusions)
with a threshold of 1e−05

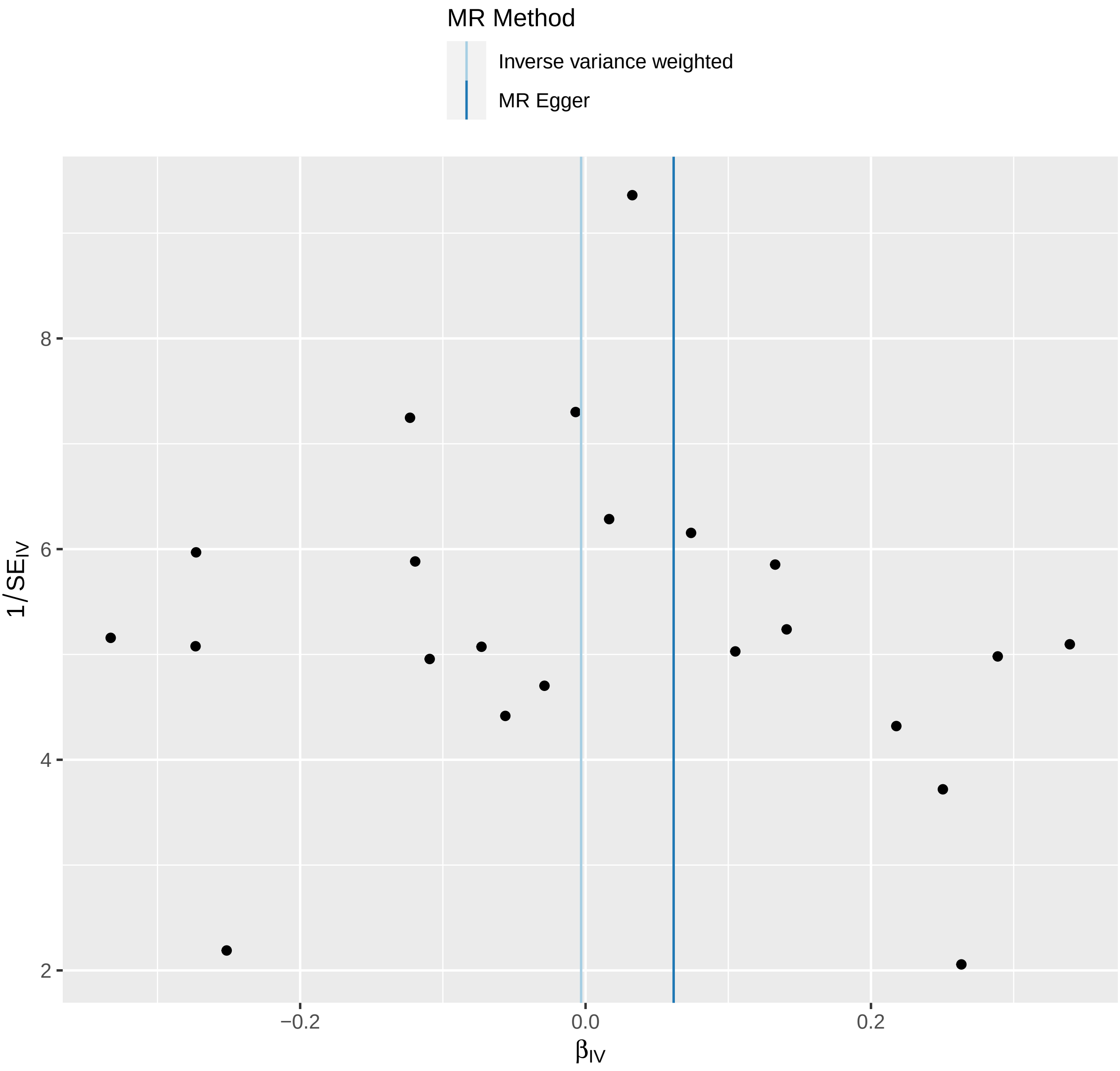

Batch 3−2159 : Oxytocin−neurophysin 1 on Diabetic nephropathy (more control exclusions)
with a threshold of 1e−05

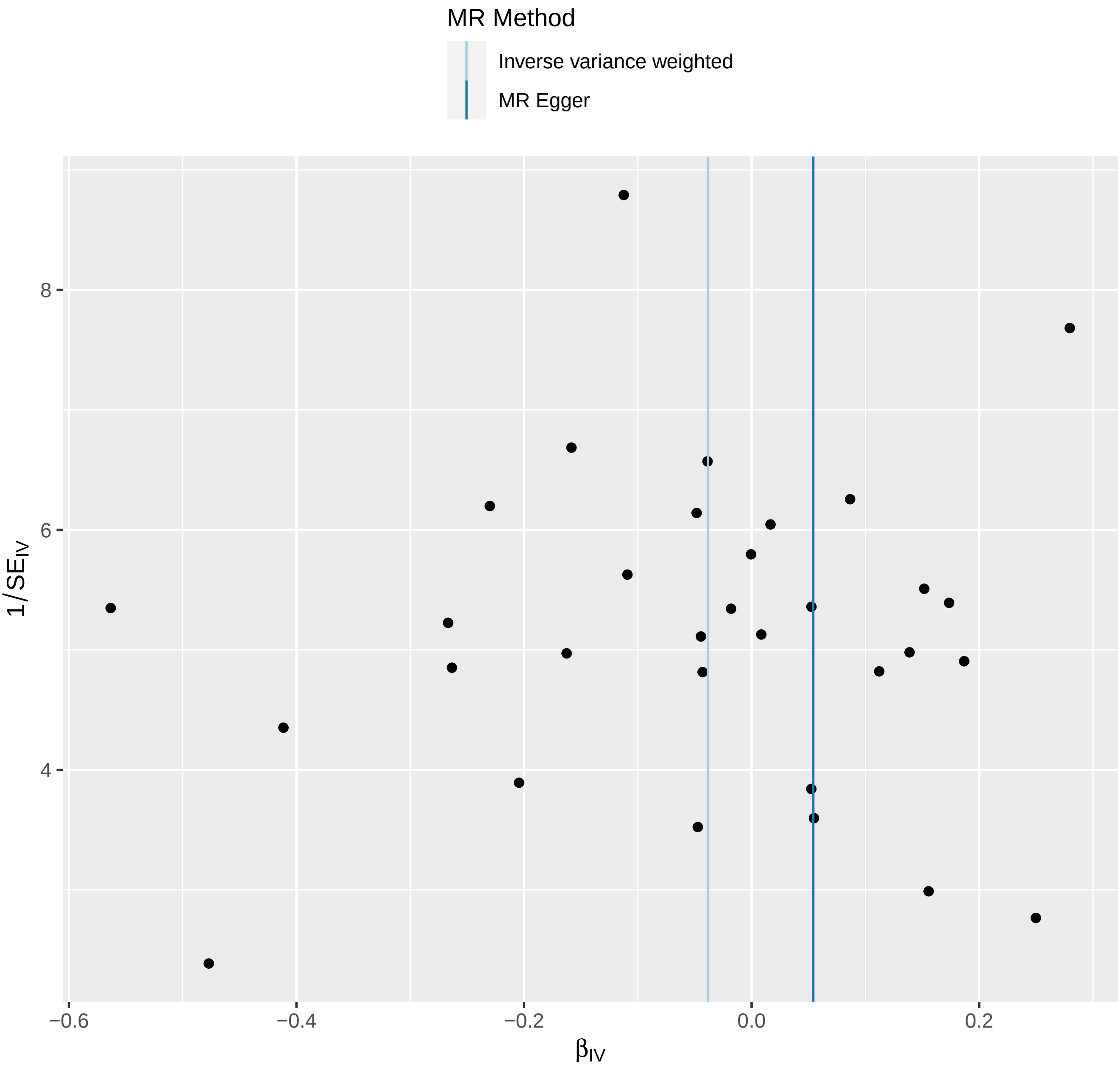

Batch 3−2160 : P2X purinoceptor 6 on Diabetic nephropathy (more control exclusions)
with a threshold of 1e−05

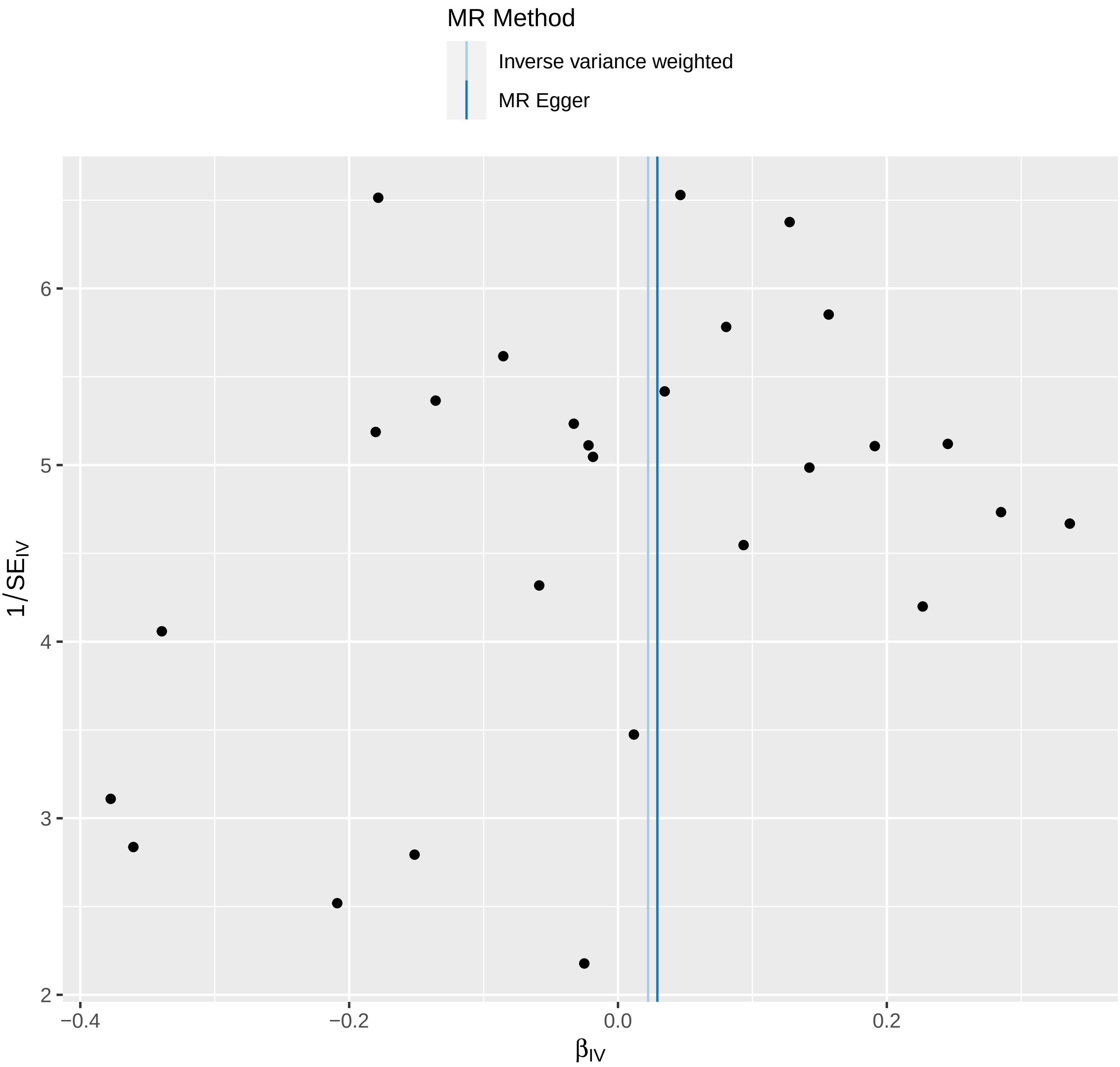

Batch 3−2161 : Prolyl 4−hydroxylase subunit alpha−1 on Diabetic nephropathy (more control exclusions)
with a threshold of 1e−05

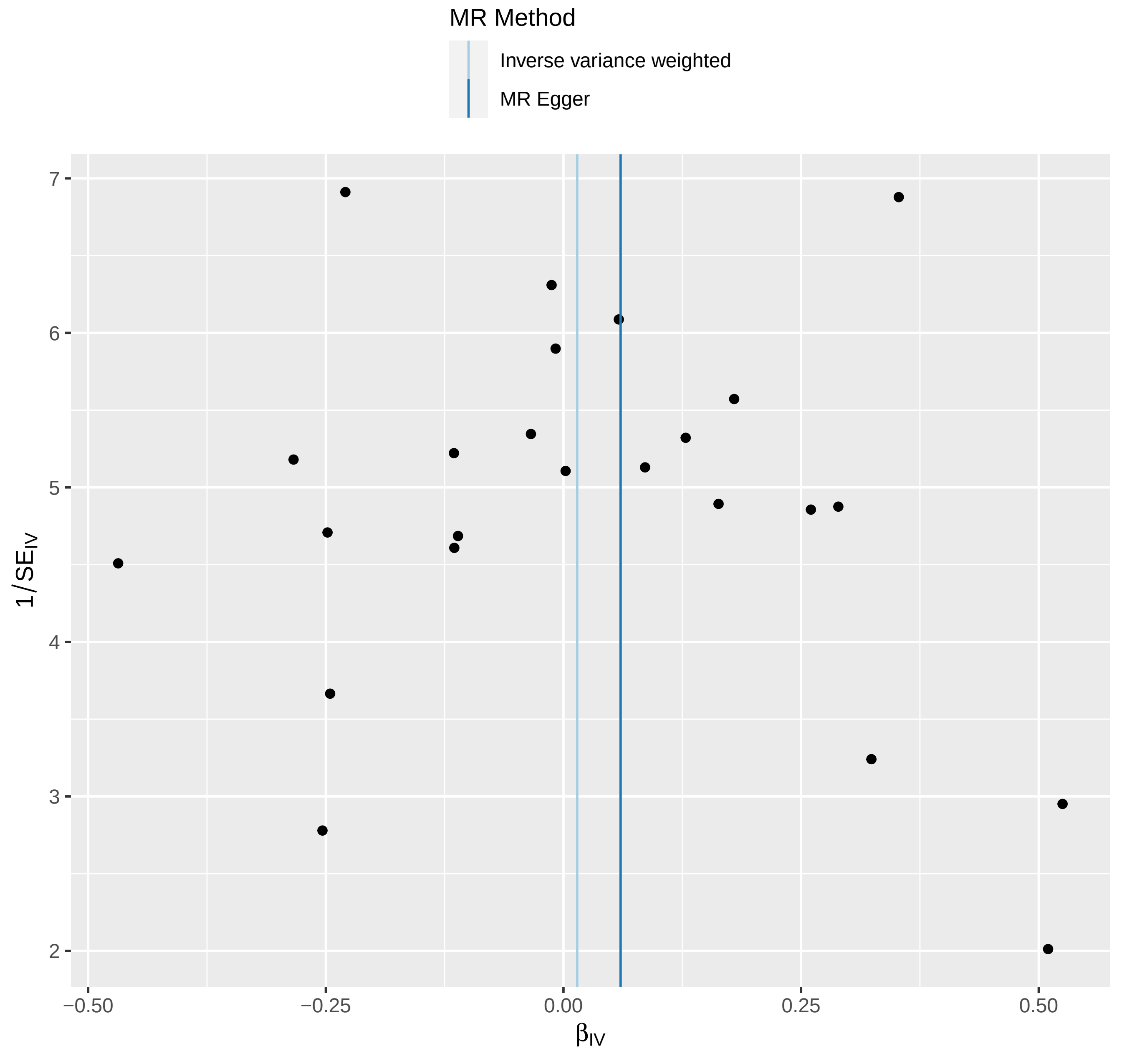

Batch 3−2162 : Prolyl 4−hydroxylase subunit alpha−2 on Diabetic nephropathy (more control exclusions)
with a threshold of 1e−05

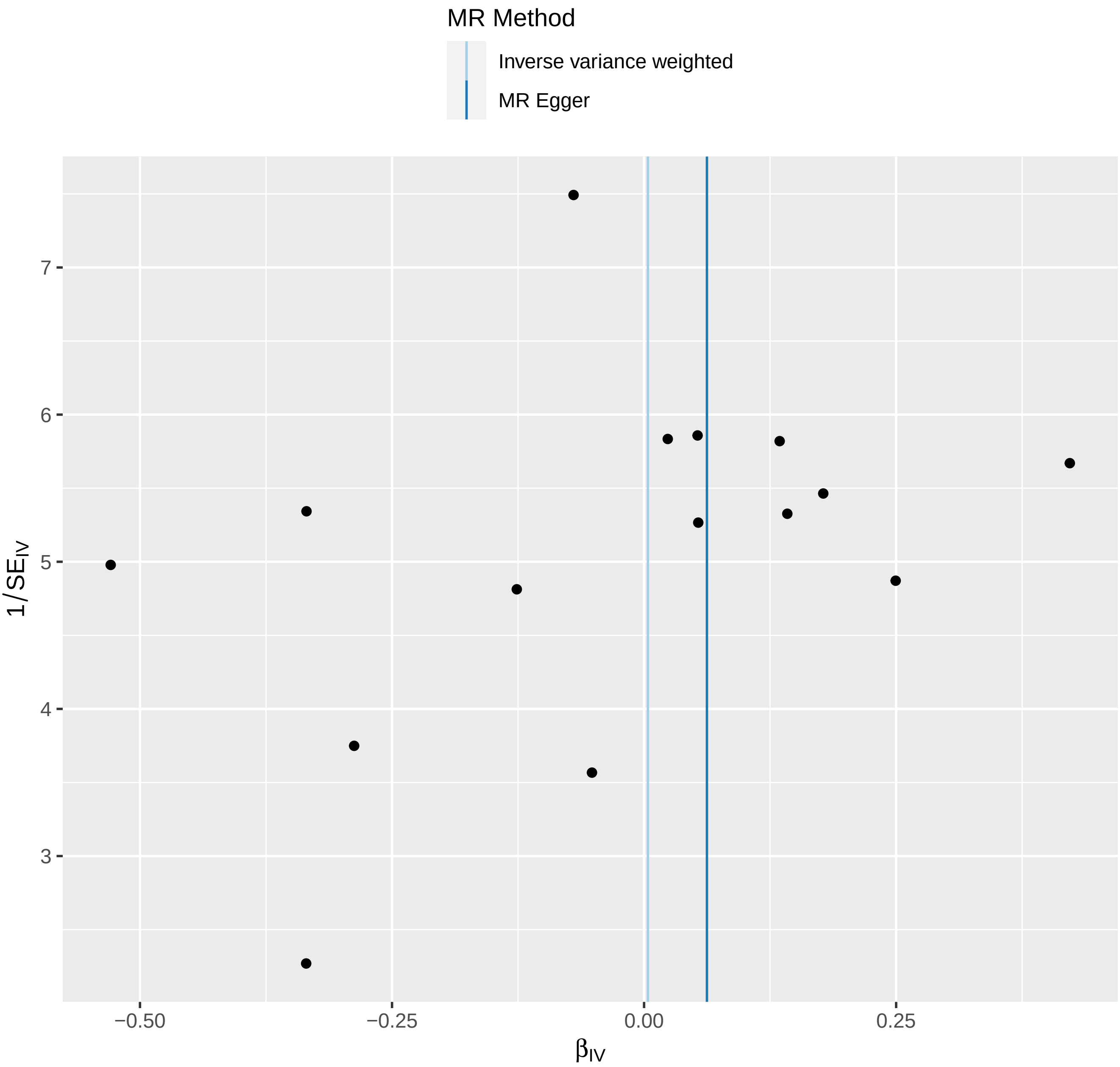

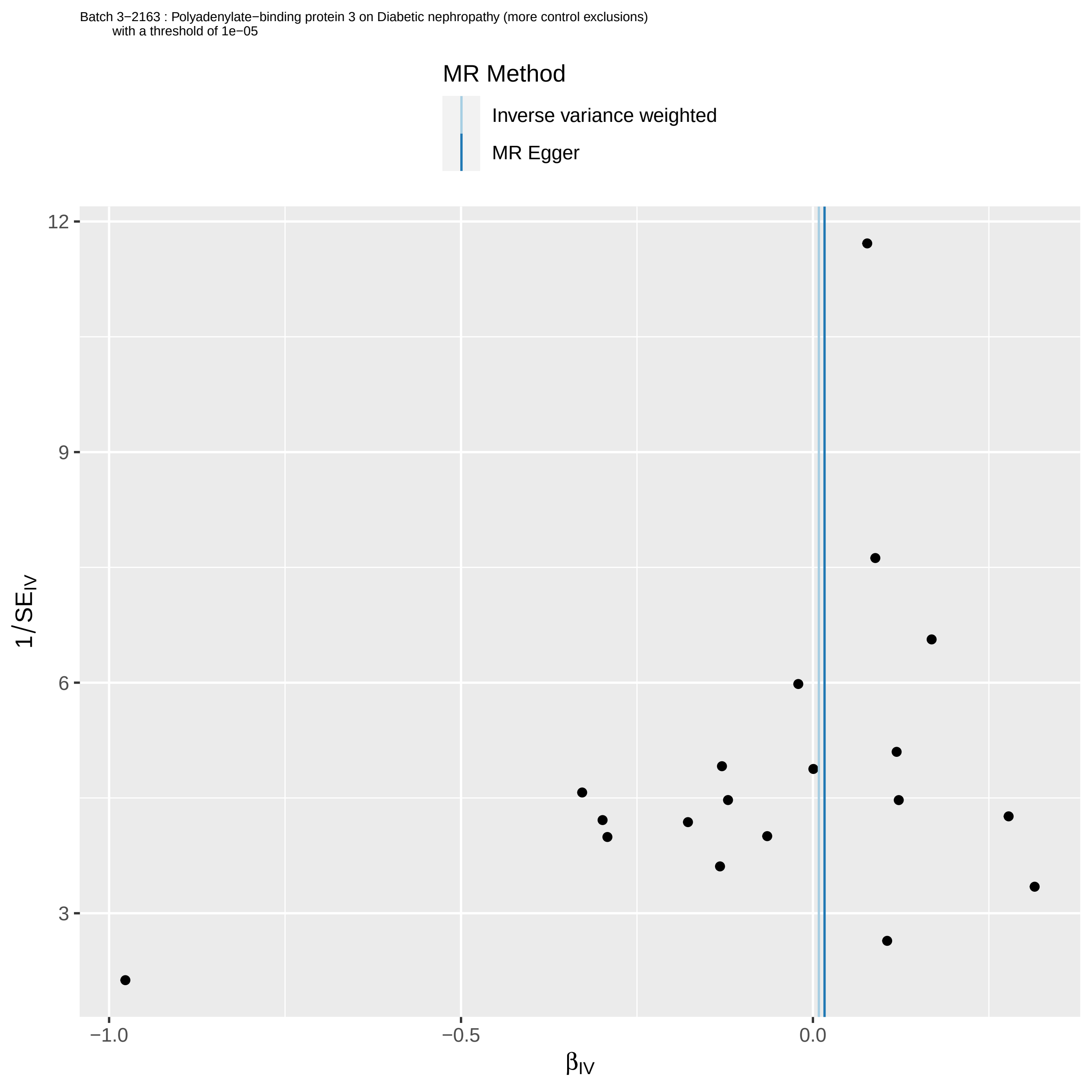
Batch 3−2163 : Polyadenylate−binding protein 3 on Diabetic nephropathy (more control exclusions)
with a threshold of 1e−05
MR Method
Inverse variance weighted
MR Egger
12
9
6
3
1/SE$_{IV}$
−1.0
−0.5
0.0
β$_{IV}$

Batch 3−2164 : Polyadenylate−binding protein 4 on Diabetic nephropathy (more control exclusions)
with a threshold of 1e−05

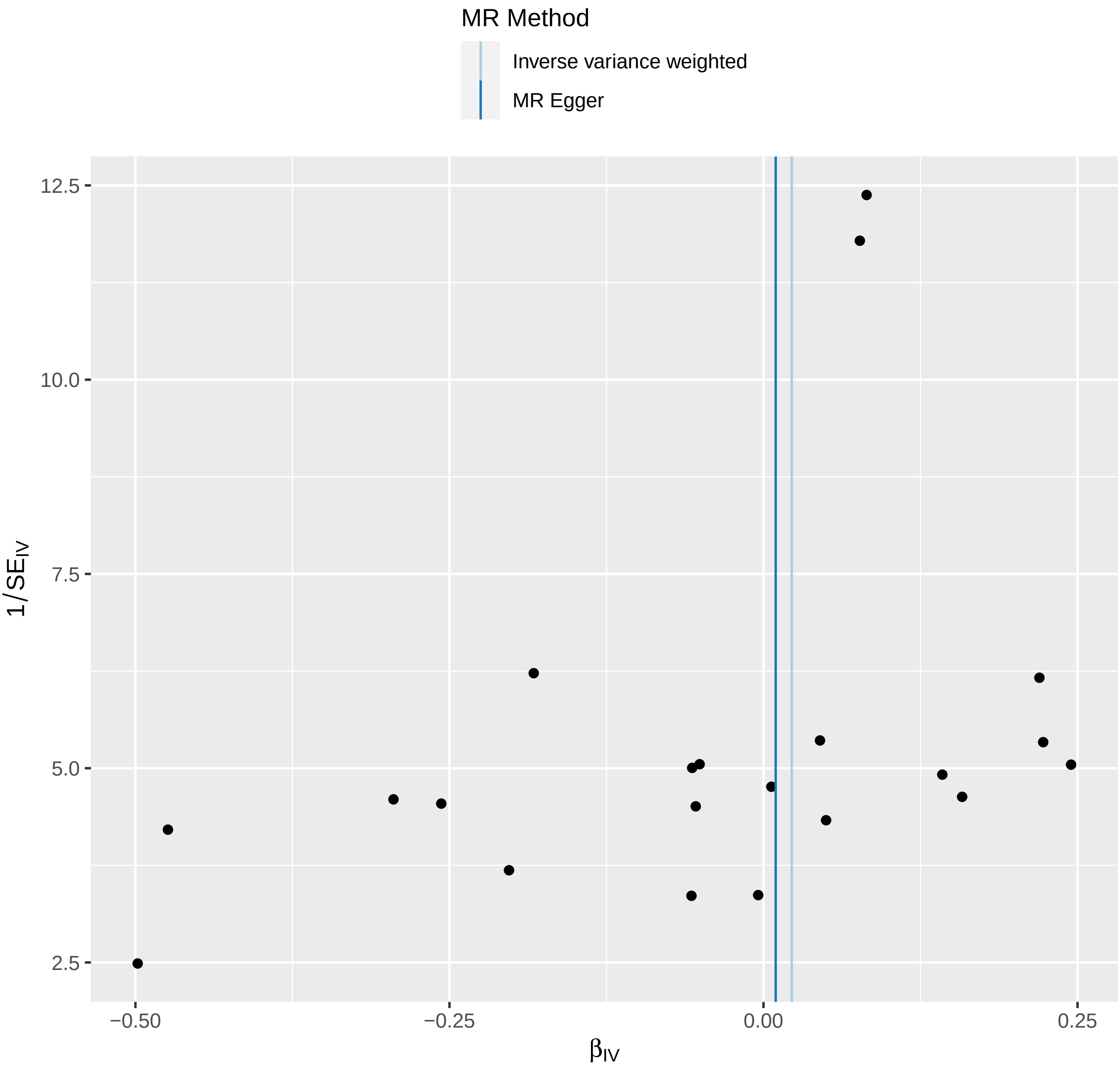

Batch 3−2165 : Marginal zone B− and B1−cell−specific protein on Diabetic nephropathy (more control exclusions) with a threshold of 1e−05

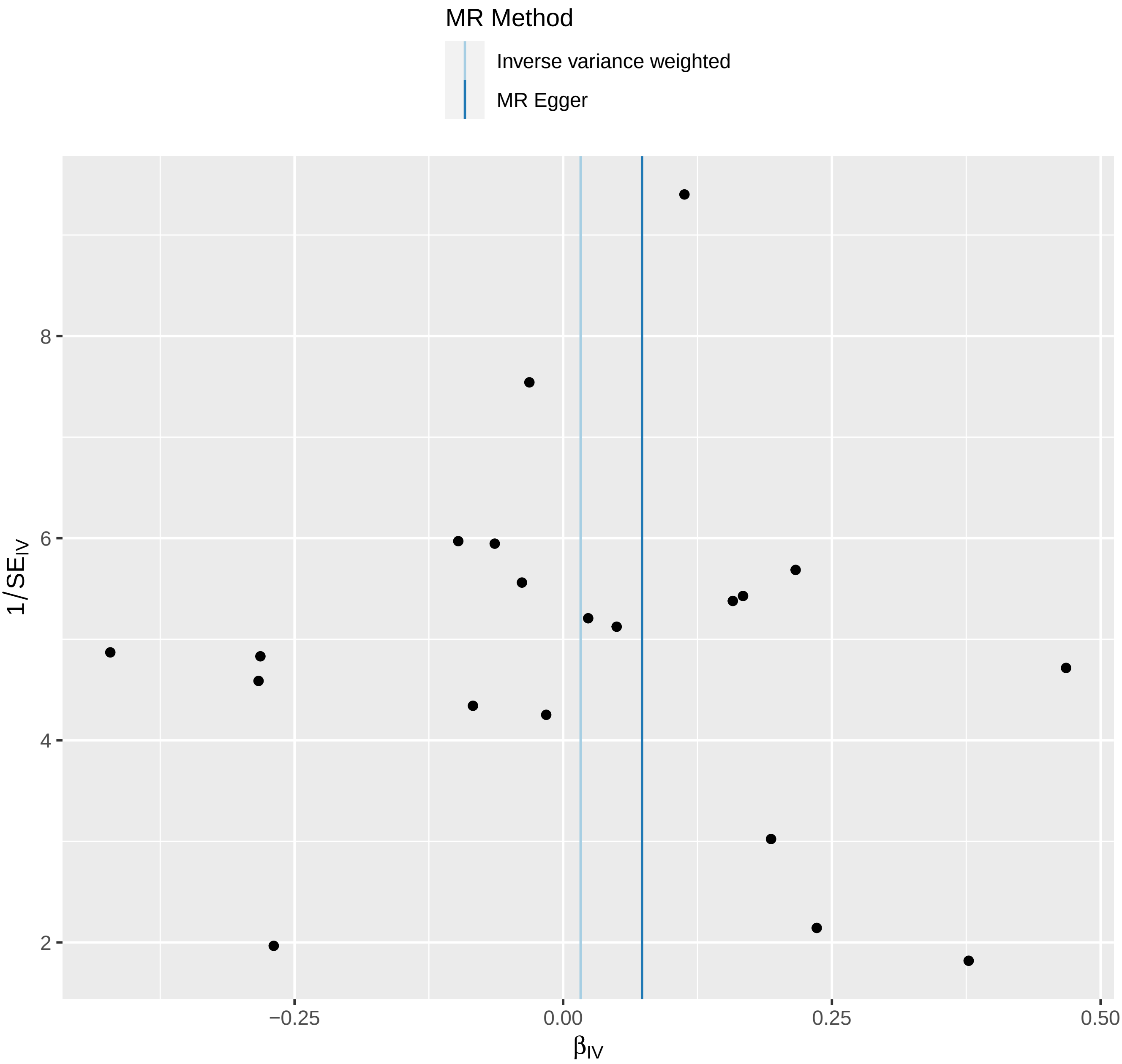

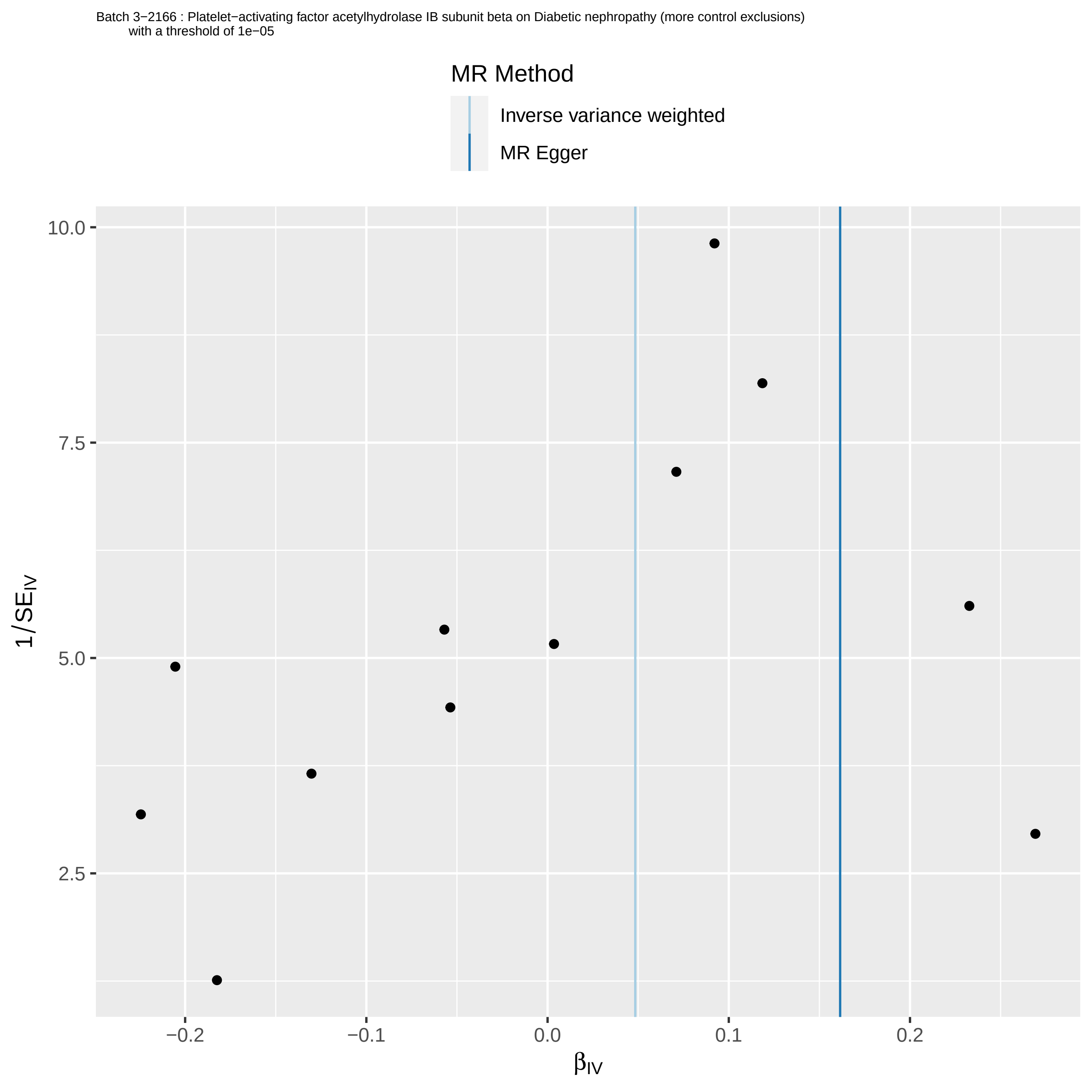
Batch 3−2166 : Platelet−activating factor acetylhydrolase IB subunit beta on Diabetic nephropathy (more control exclusions)
with a threshold of 1e−05
MR Method
Inverse variance weighted
MR Egger
10.0
7.5
5.0
2.5
1/SE_IV
−0.2
−0.1
0.0
0.1
0.2
β_IV

Batch 3−2167 : Multifunctional protein ADE2 on Diabetic nephropathy (more control exclusions)
with a threshold of 1e−05

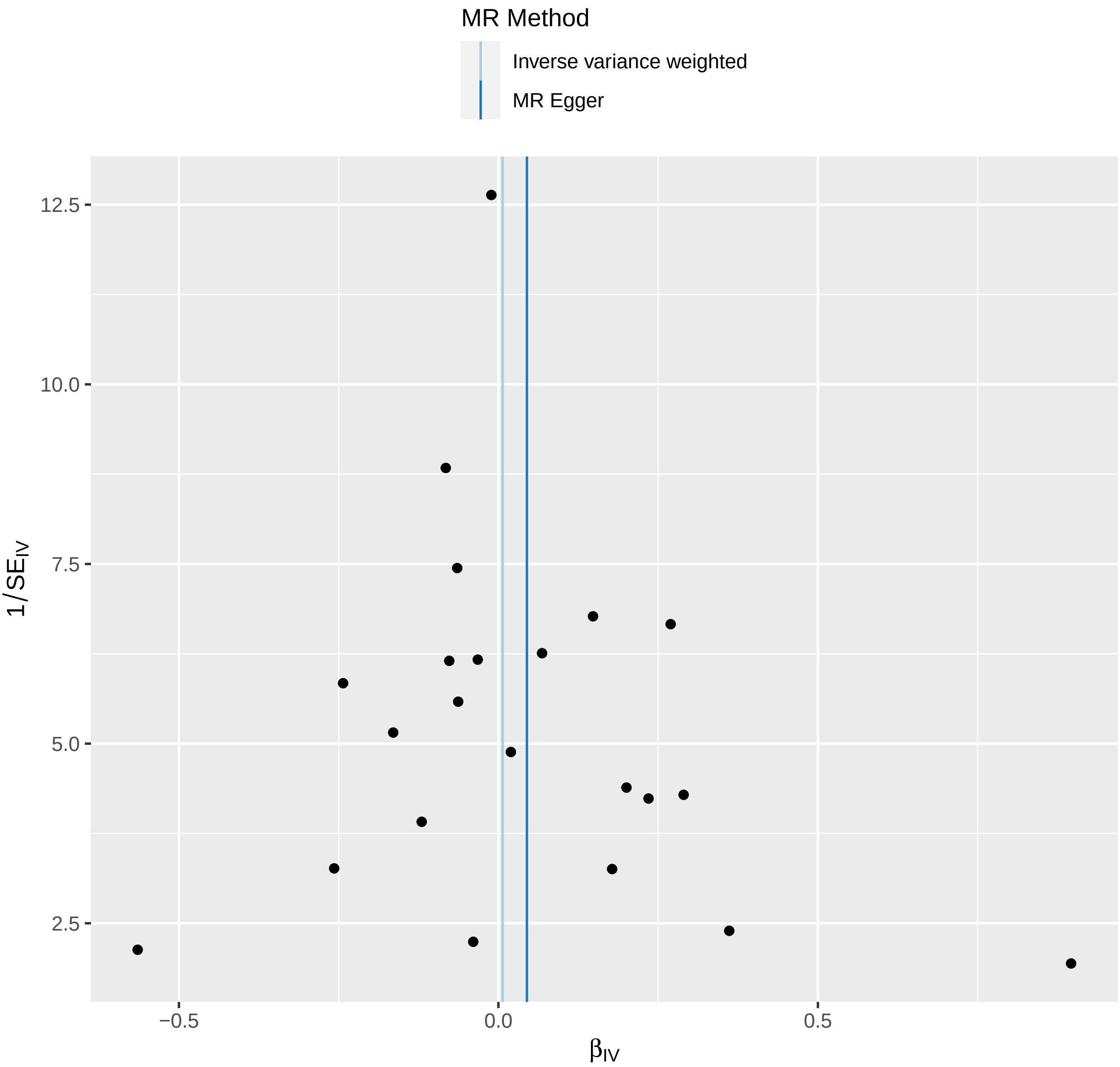

Batch 3−2168 : Polyadenylate−binding protein−interacting protein 1 on Diabetic nephropathy (more control exclusions)
with a threshold of 1e−05

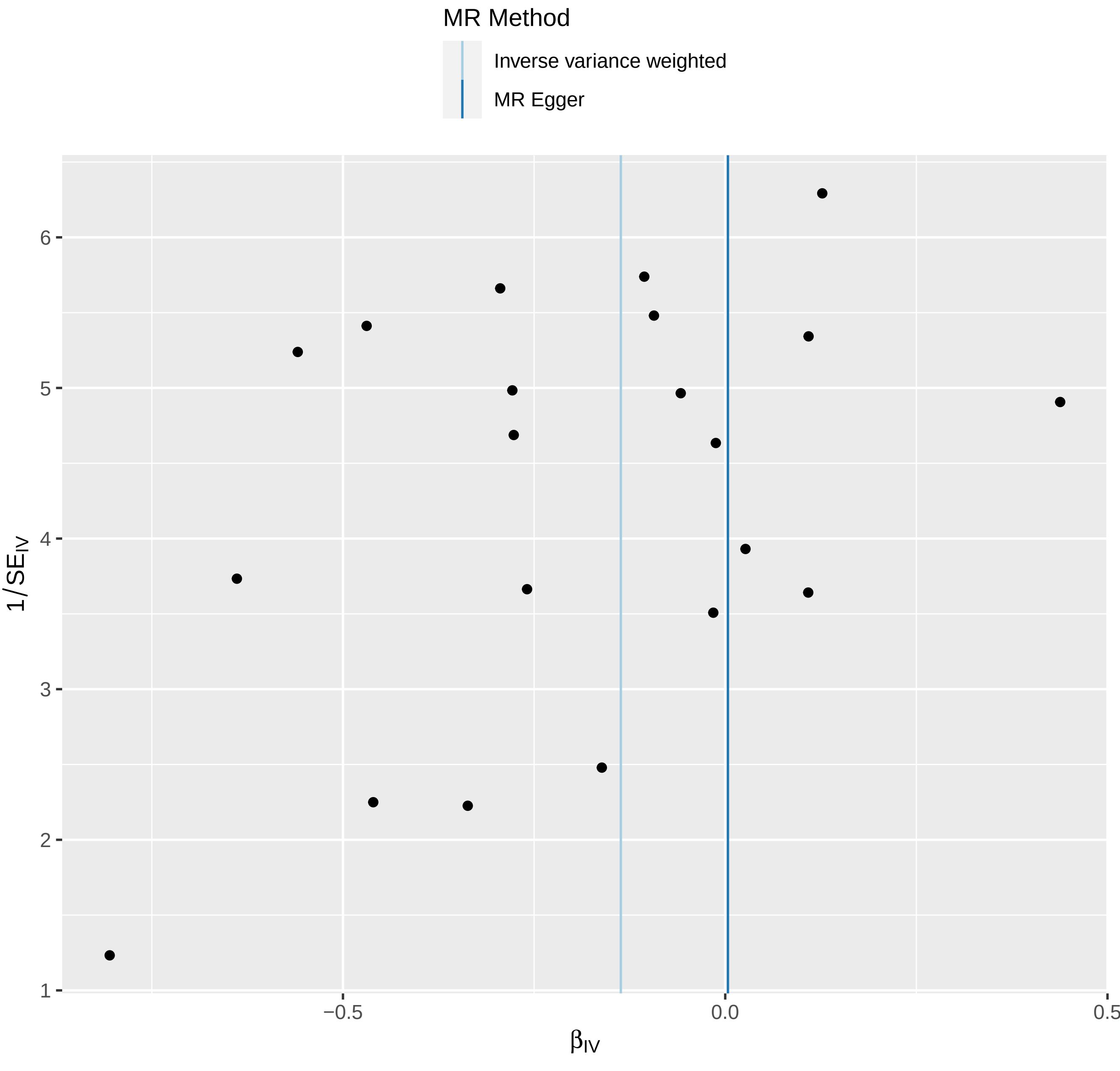

Batch 3−2169 : Serine/threonine−protein kinase PAK 3 on Diabetic nephropathy (more control exclusions)
with a threshold of 1e−05

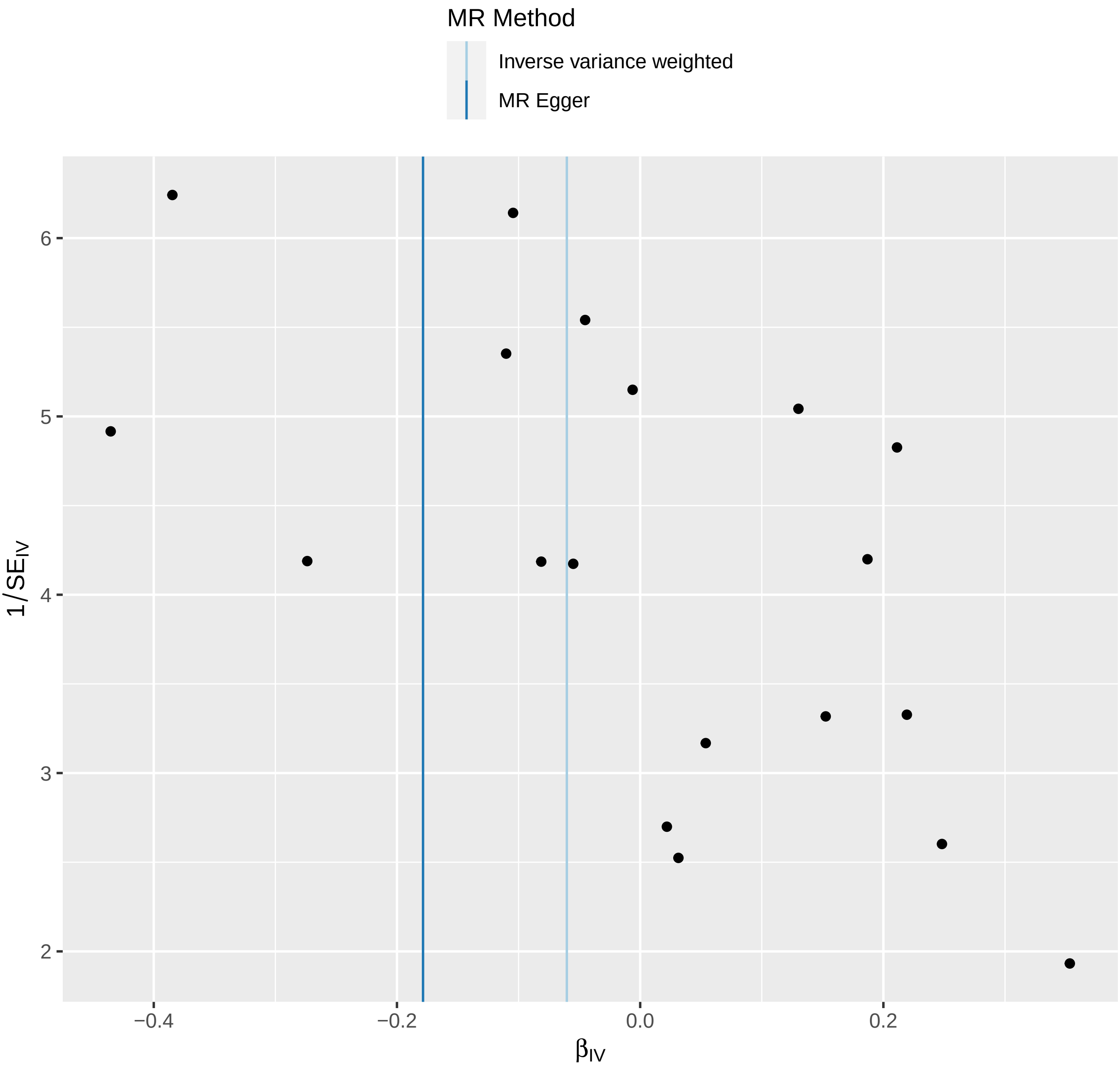

Batch 3−2170 : Serine/threonine−protein kinase PAK 4 on Diabetic nephropathy (more control exclusions)
with a threshold of 1e−05

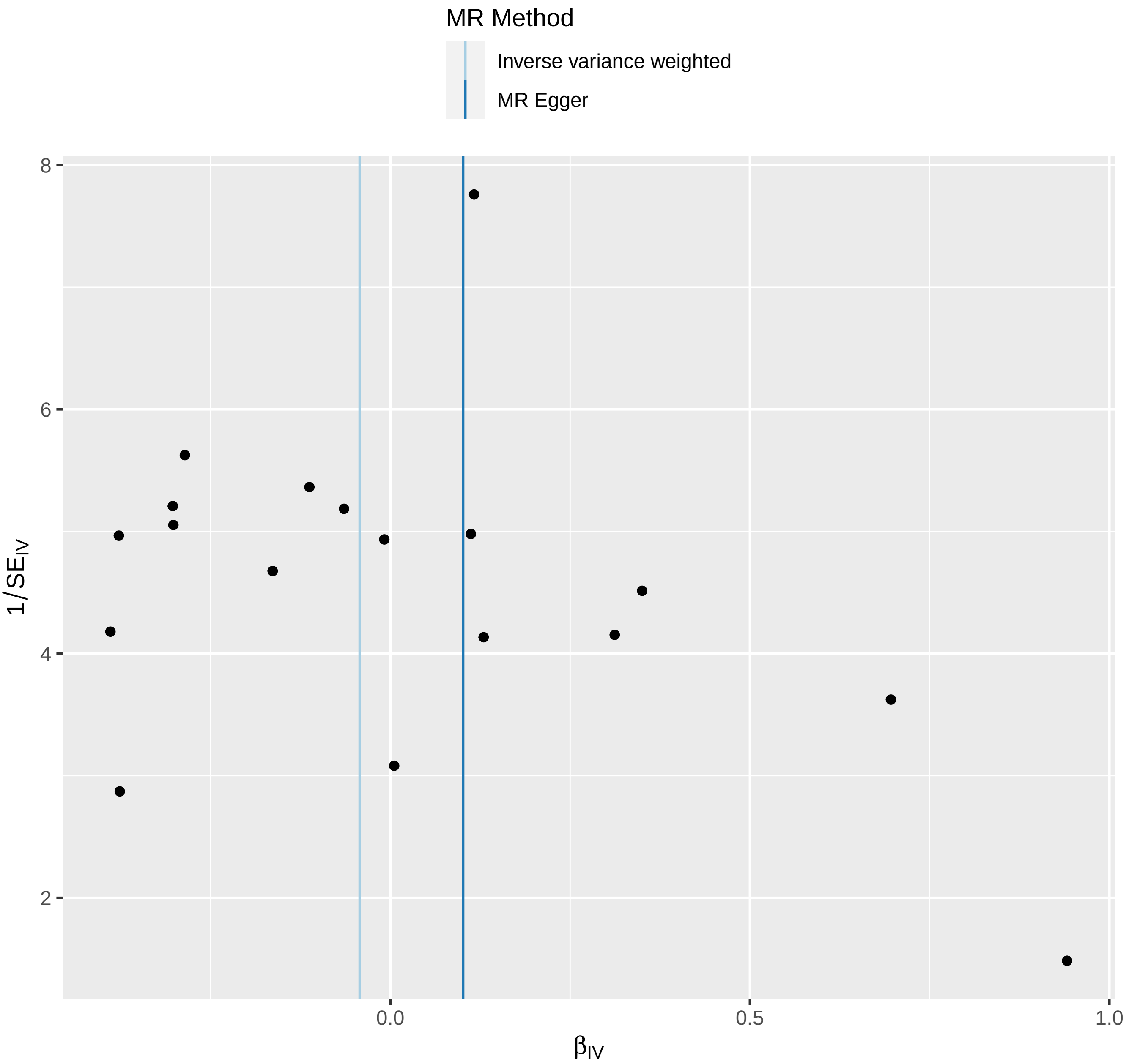

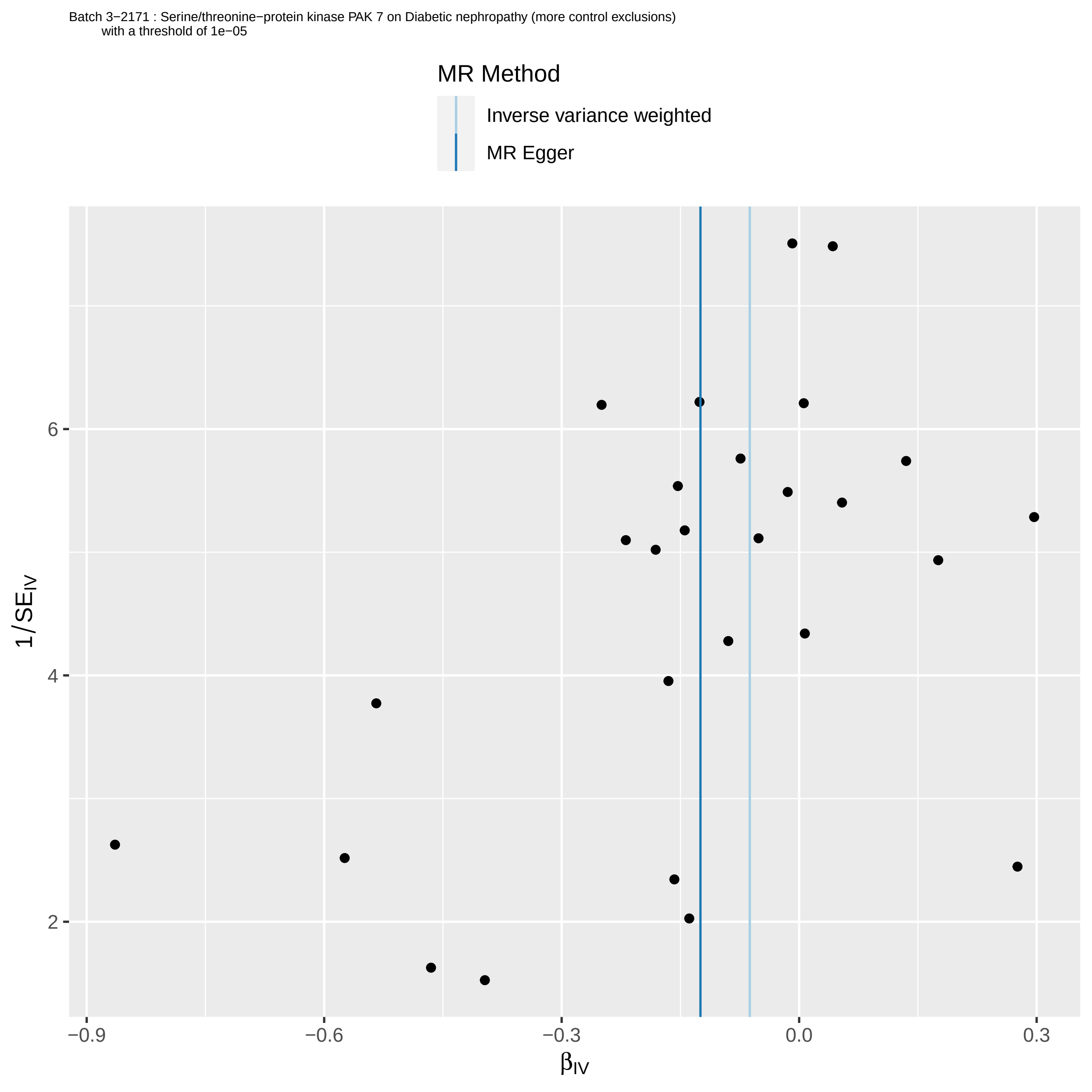
Batch 3−2171 : Serine/threonine−protein kinase PAK 7 on Diabetic nephropathy (more control exclusions)
with a threshold of 1e−05
MR Method
Inverse variance weighted
MR Egger
6
4
2
1/SE$_{IV}$
−0.9
−0.6
−0.3
0.0
0.3
β$_{IV}$

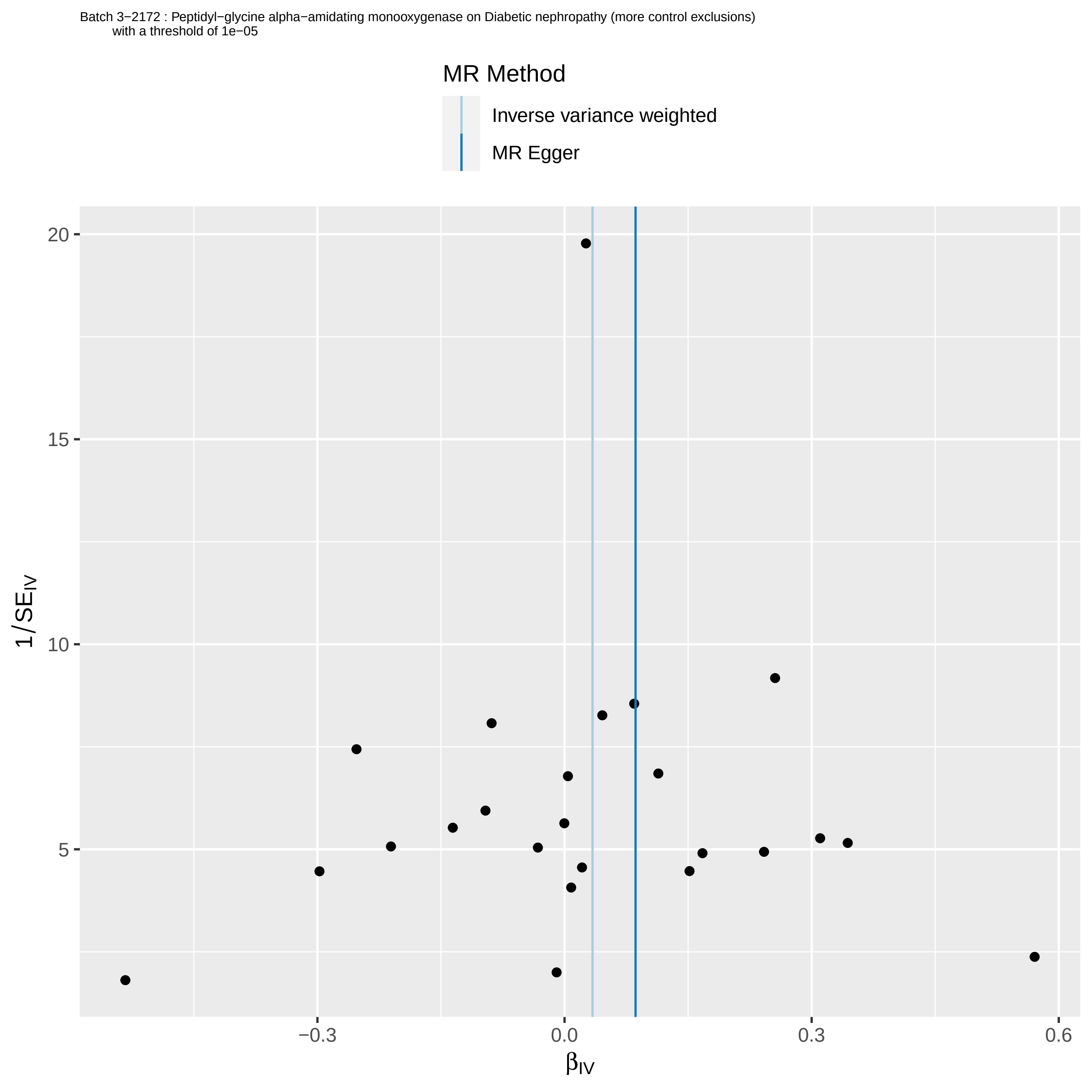
Batch 3−2172 : Peptidyl−glycine alpha−amidating monooxygenase on Diabetic nephropathy (more control exclusions)
with a threshold of 1e−05
MR Method
Inverse variance weighted
MR Egger
20
15
10
5
1/SE_IV
−0.3
0.0
0.3
0.6
β_IV

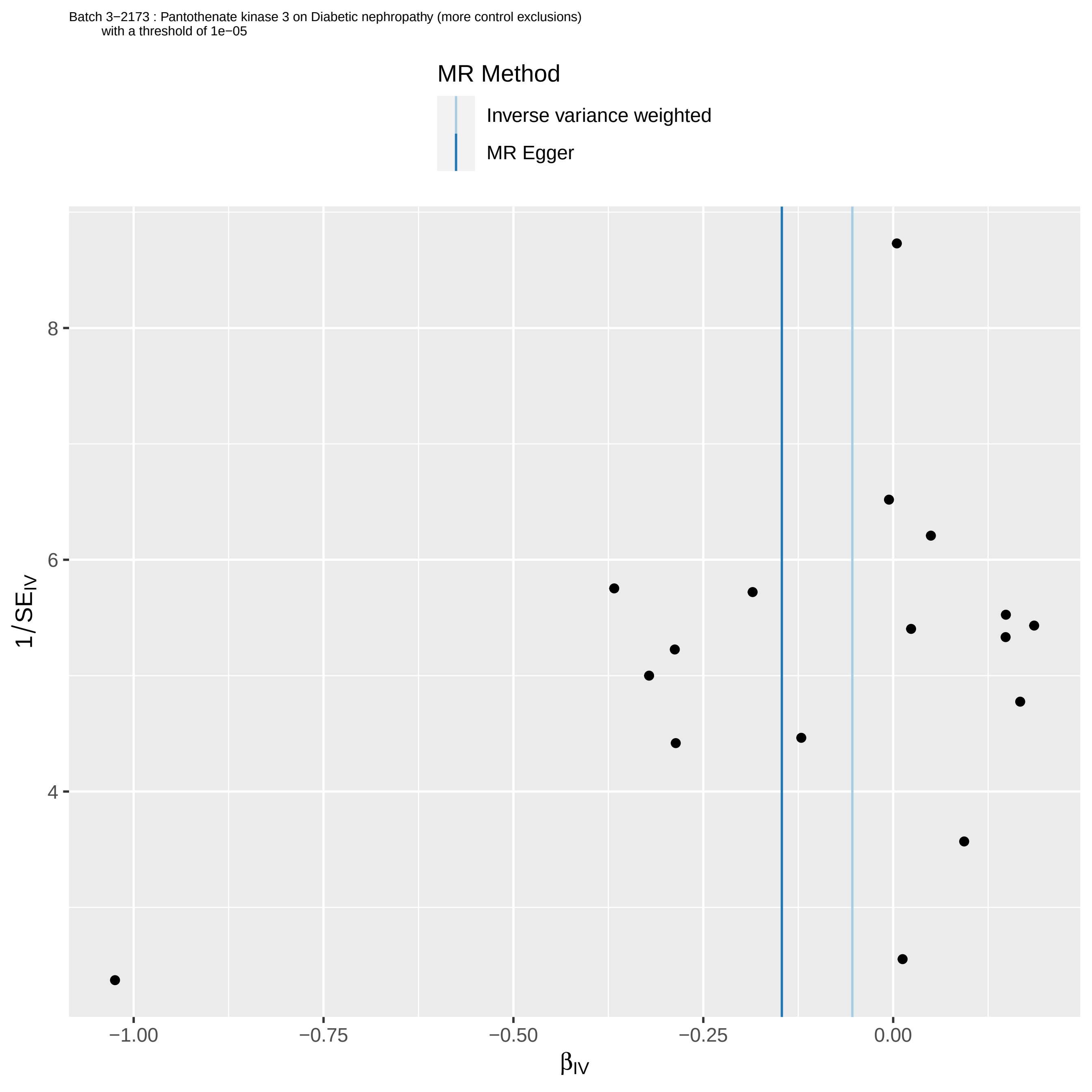
Batch 3−2173 : Pantothenate kinase 3 on Diabetic nephropathy (more control exclusions)
with a threshold of 1e−05
MR Method
Inverse variance weighted
MR Egger
8
6
4
1/SE_IV
−1.00
−0.75
−0.50
−0.25
0.00
β_IV

Batch 3−2174 : Papilin on Diabetic nephropathy (more control exclusions)
with a threshold of 1e−05

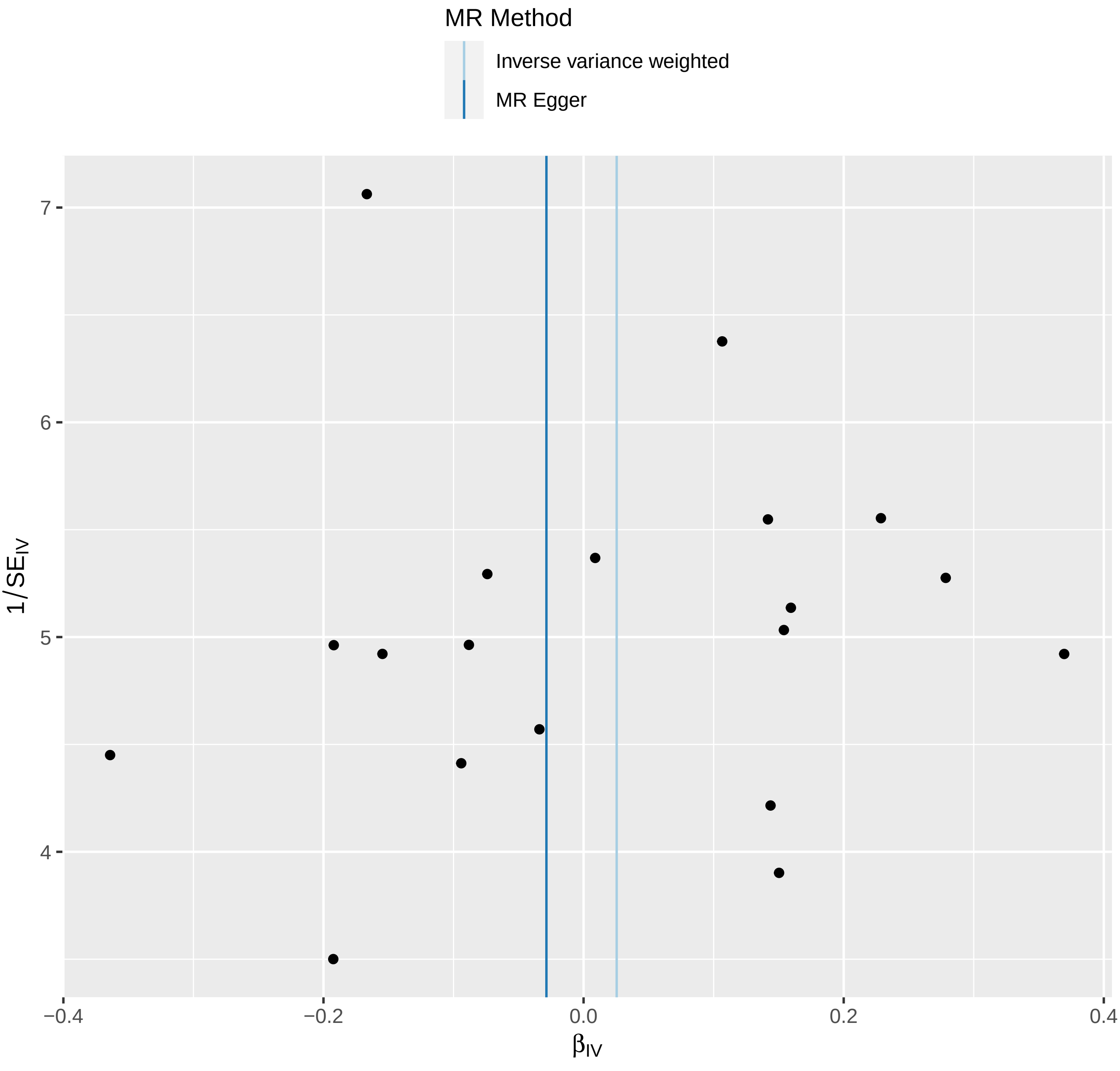

Batch 3−2175 : Poly(A) polymerase gamma on Diabetic nephropathy (more control exclusions)
with a threshold of 1e−05

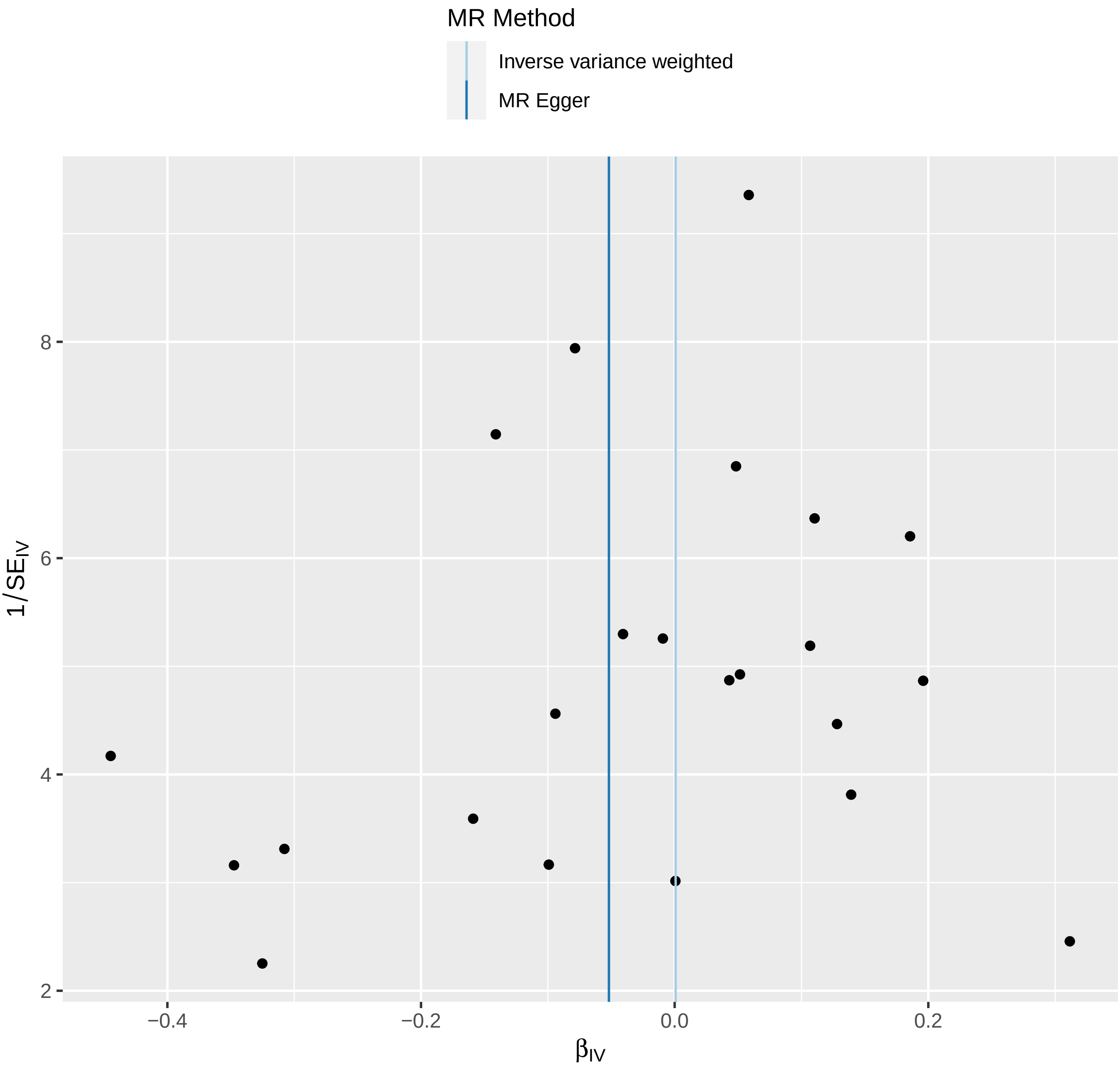

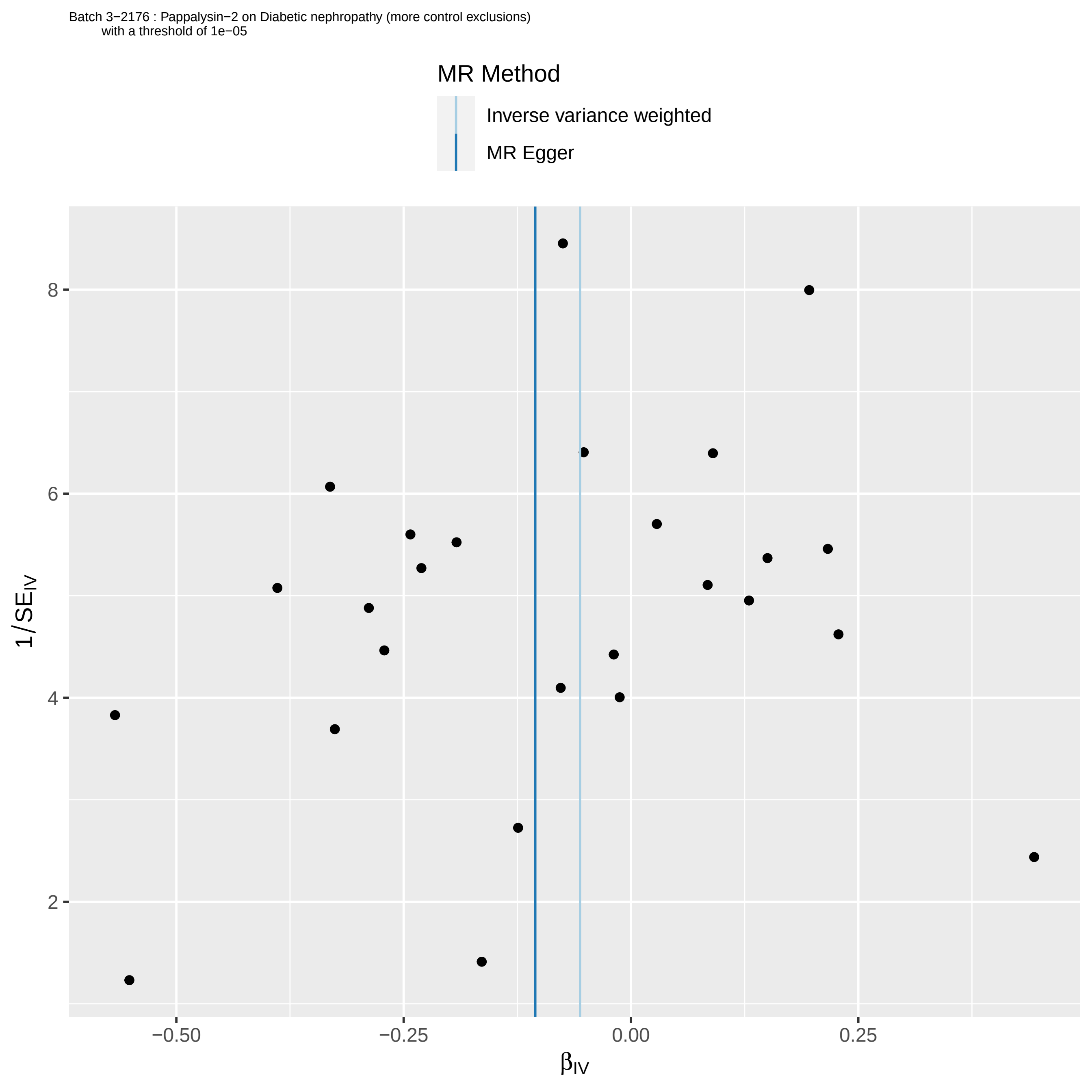
Batch 3−2176 : Pappalysin−2 on Diabetic nephropathy (more control exclusions)
with a threshold of 1e−05
MR Method
Inverse variance weighted
MR Egger
8
6
4
2
1/SE$_{IV}$
−0.50
−0.25
0.00
0.25
β$_{IV}$

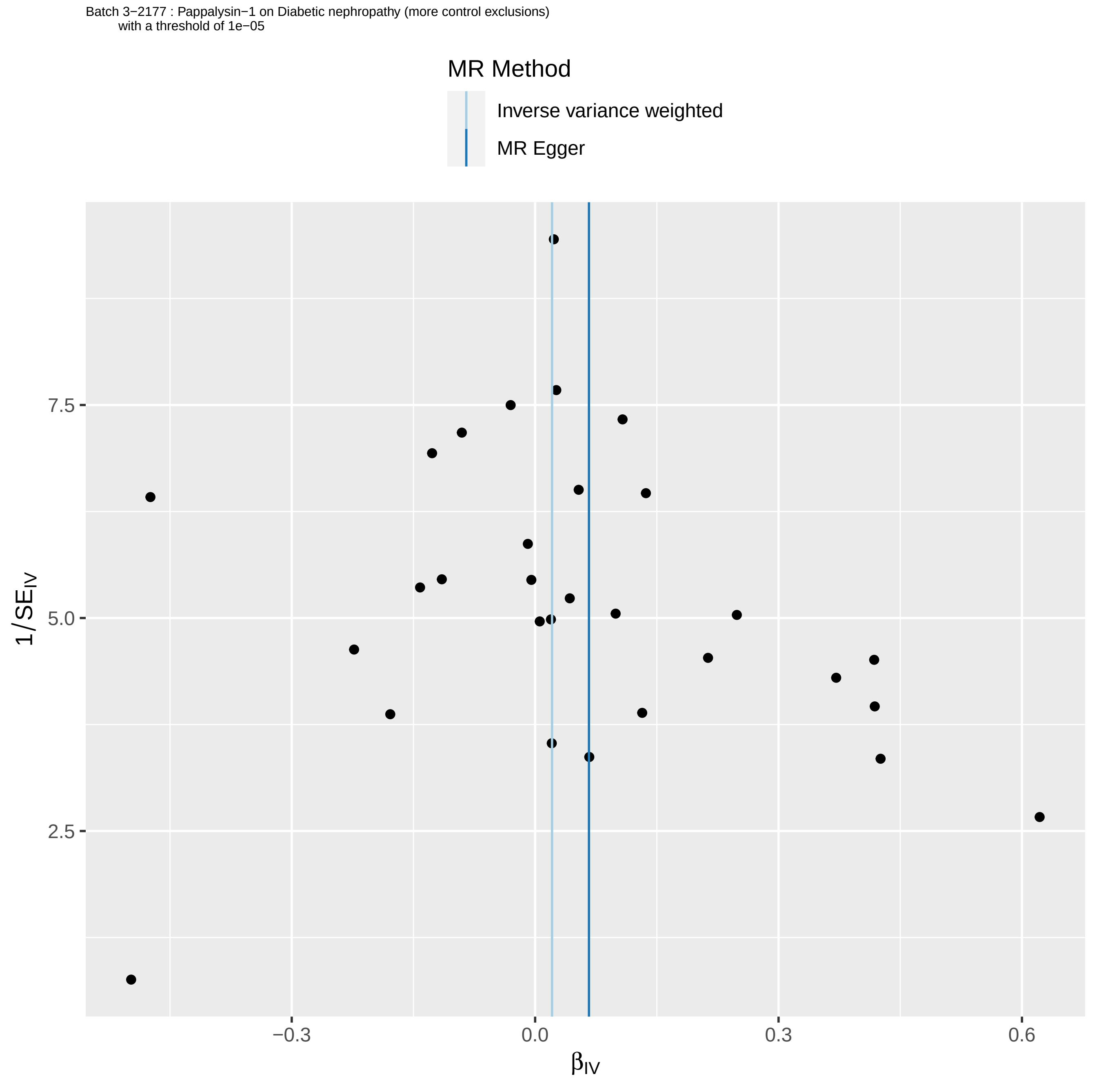
Batch 3−2177 : Pappalysin−1 on Diabetic nephropathy (more control exclusions)
with a threshold of 1e−05
MR Method
Inverse variance weighted
MR Egger
7.5
5.0
2.5
1/SE_IV
−0.3
0.0
0.3
0.6
β_IV

Batch 3−2178 : Bifunctional 3'−phosphoadenosine 5'−phosphosulfate synthase 1 on Diabetic nephropathy (more control exclusions) with a threshold of 1e−05

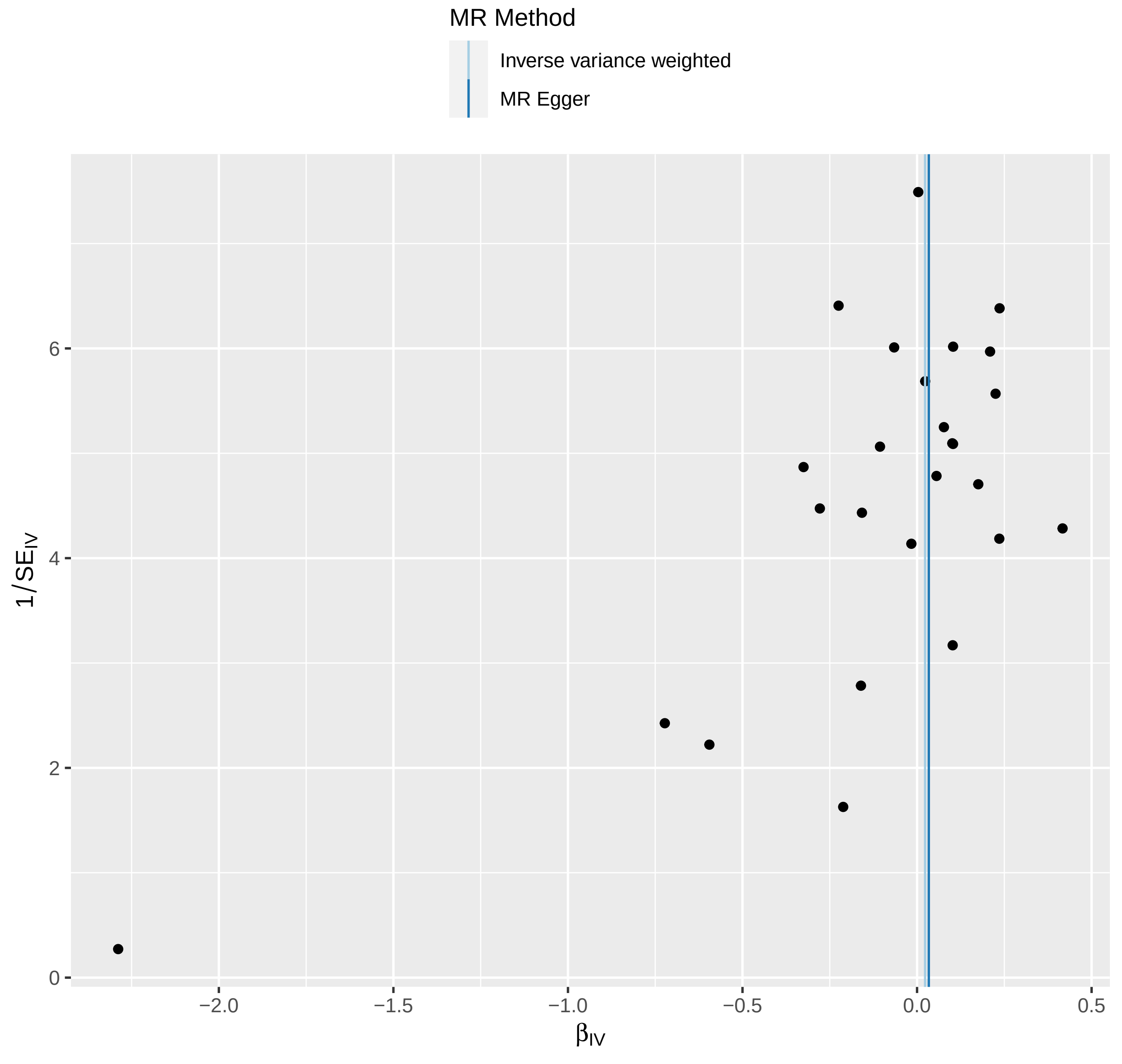

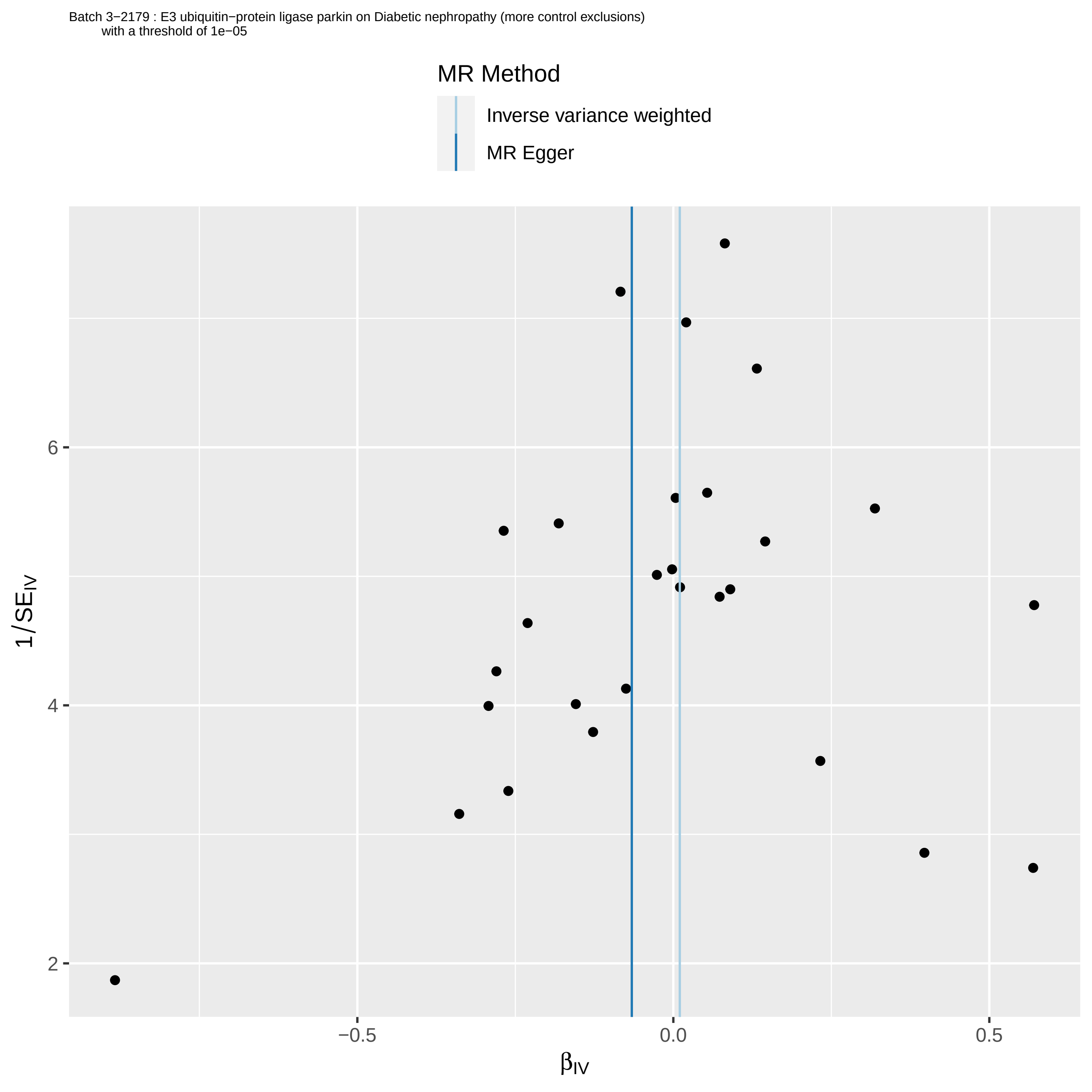
Batch 3−2179 : E3 ubiquitin−protein ligase parkin on Diabetic nephropathy (more control exclusions)
with a threshold of 1e−05
MR Method
Inverse variance weighted
MR Egger
6
4
2
1/SE_IV
−0.5
0.0
0.5
β_IV

Batch 3−2180 : Protein deglycase DJ−1 on Diabetic nephropathy (more control exclusions)
with a threshold of 1e−05

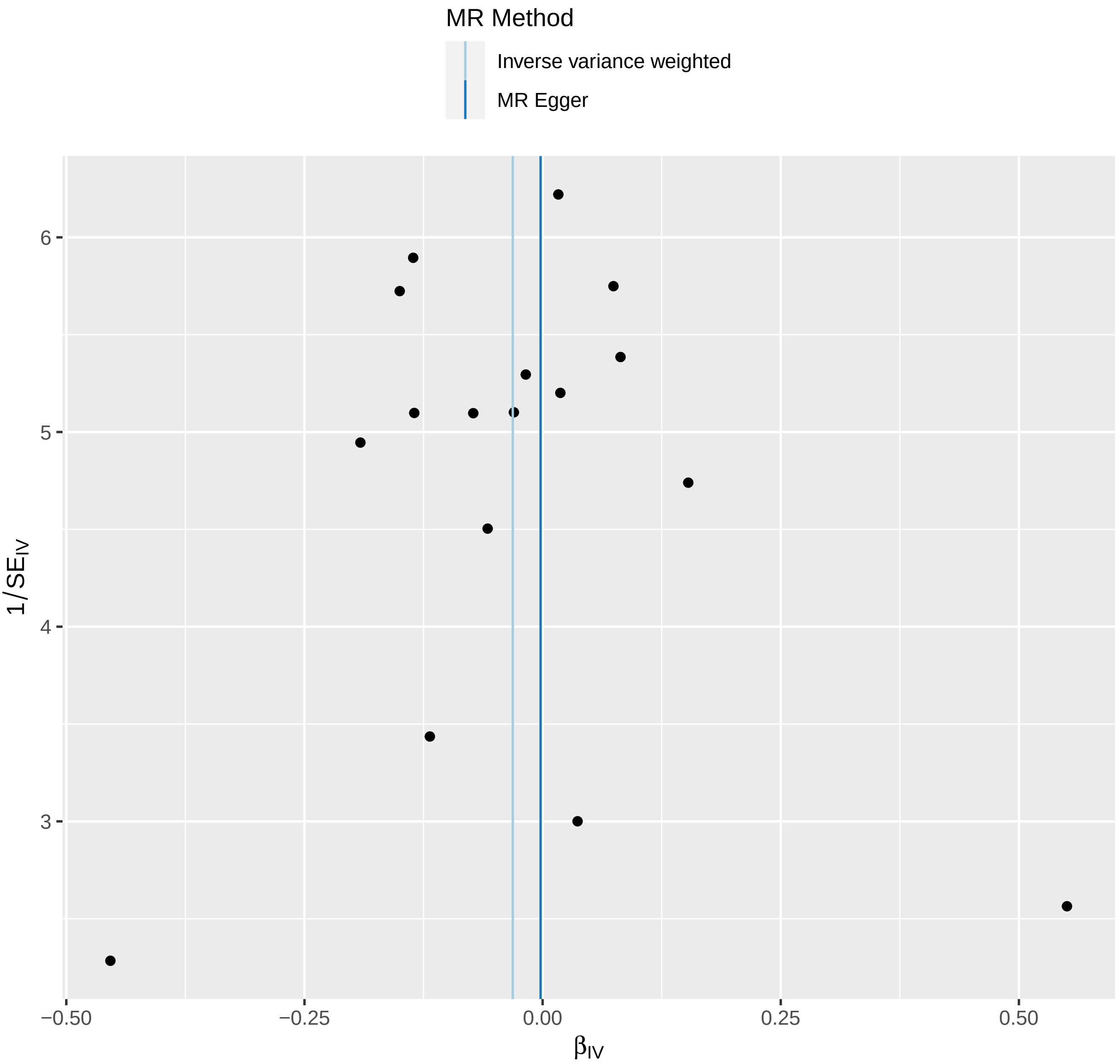

Batch 3−2181 : Protein deglycase DJ−1 on Diabetic nephropathy (more control exclusions)
with a threshold of 1e−05

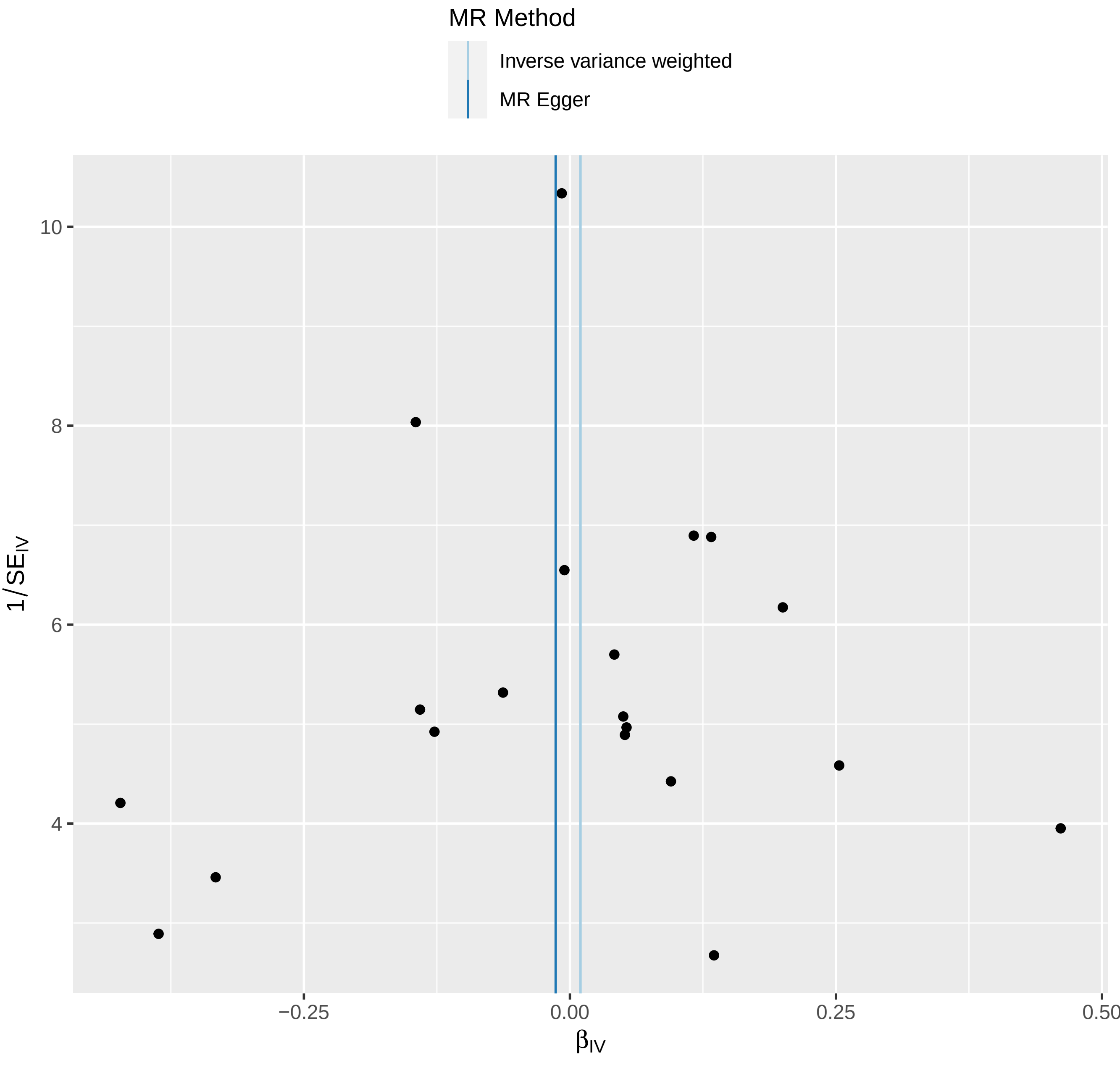

Batch 3−2182 : Poly [ADP−ribose] polymerase 1 on Diabetic nephropathy (more control exclusions)
with a threshold of 1e−05

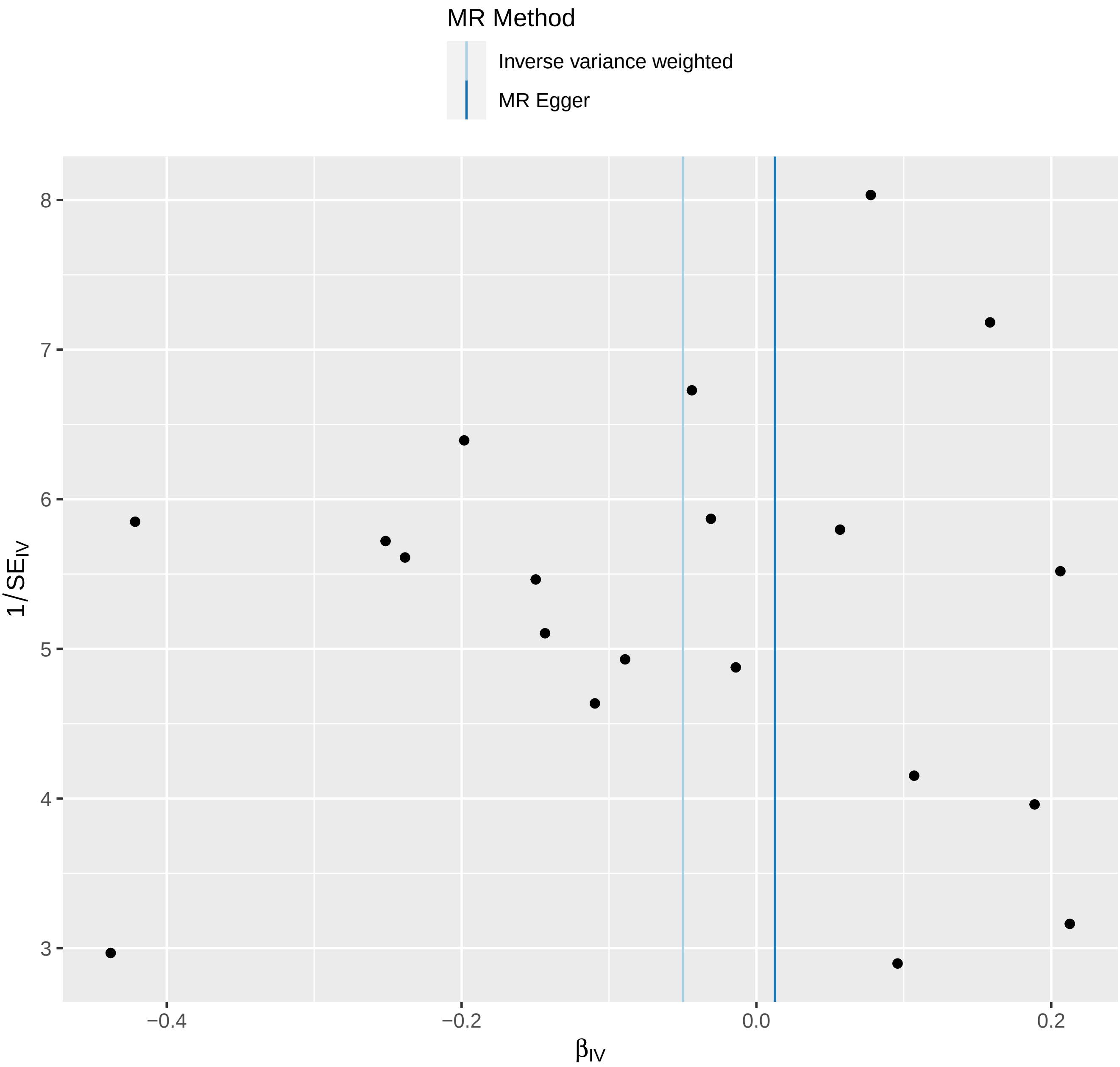

Batch 3−2183 : Poly [ADP−ribose] polymerase 11 on Diabetic nephropathy (more control exclusions)
with a threshold of 1e−05

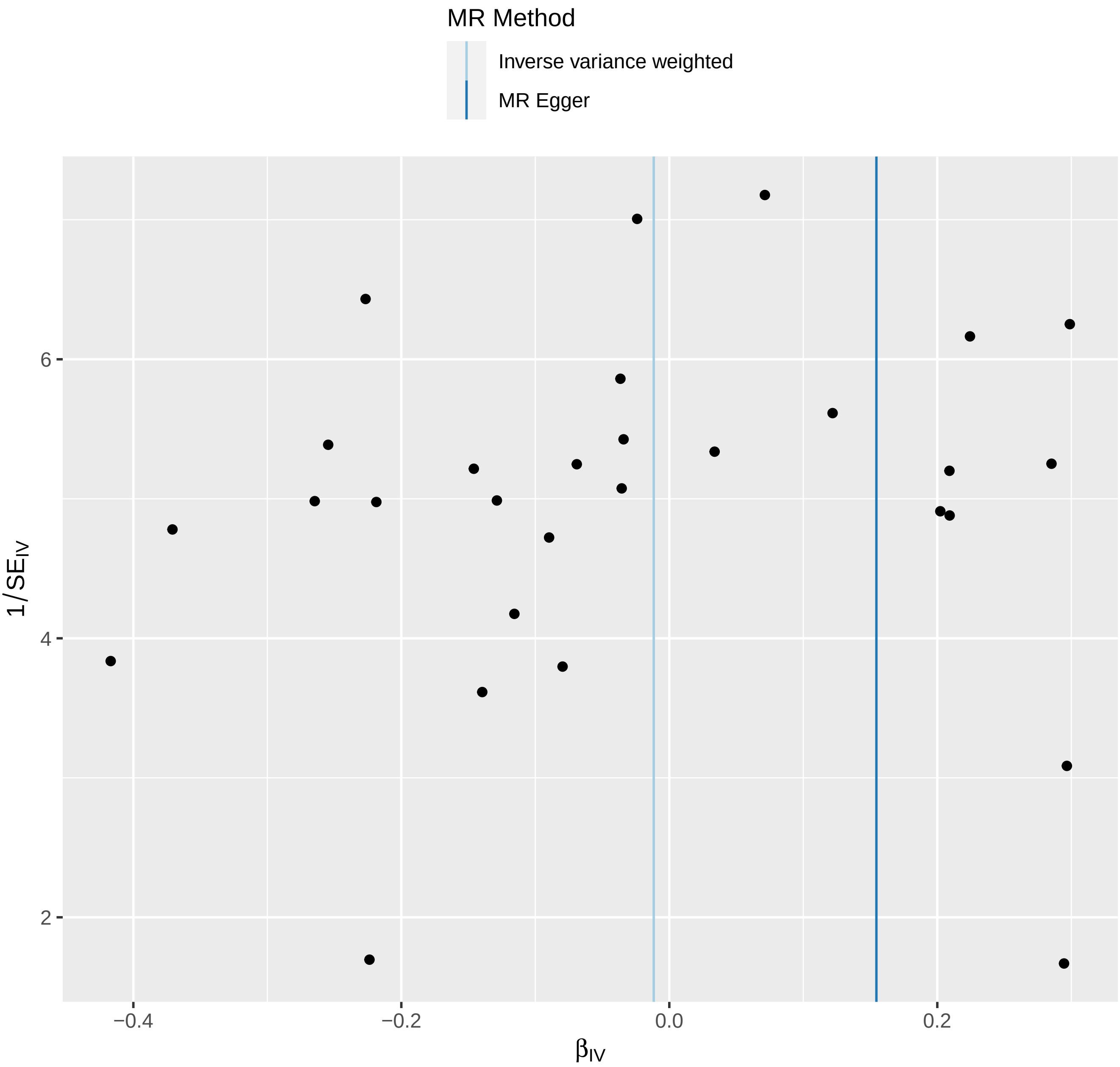

Batch 3−2184 : Mono [ADP−ribose] polymerase PARP16 on Diabetic nephropathy (more control exclusions)
with a threshold of 1e−05

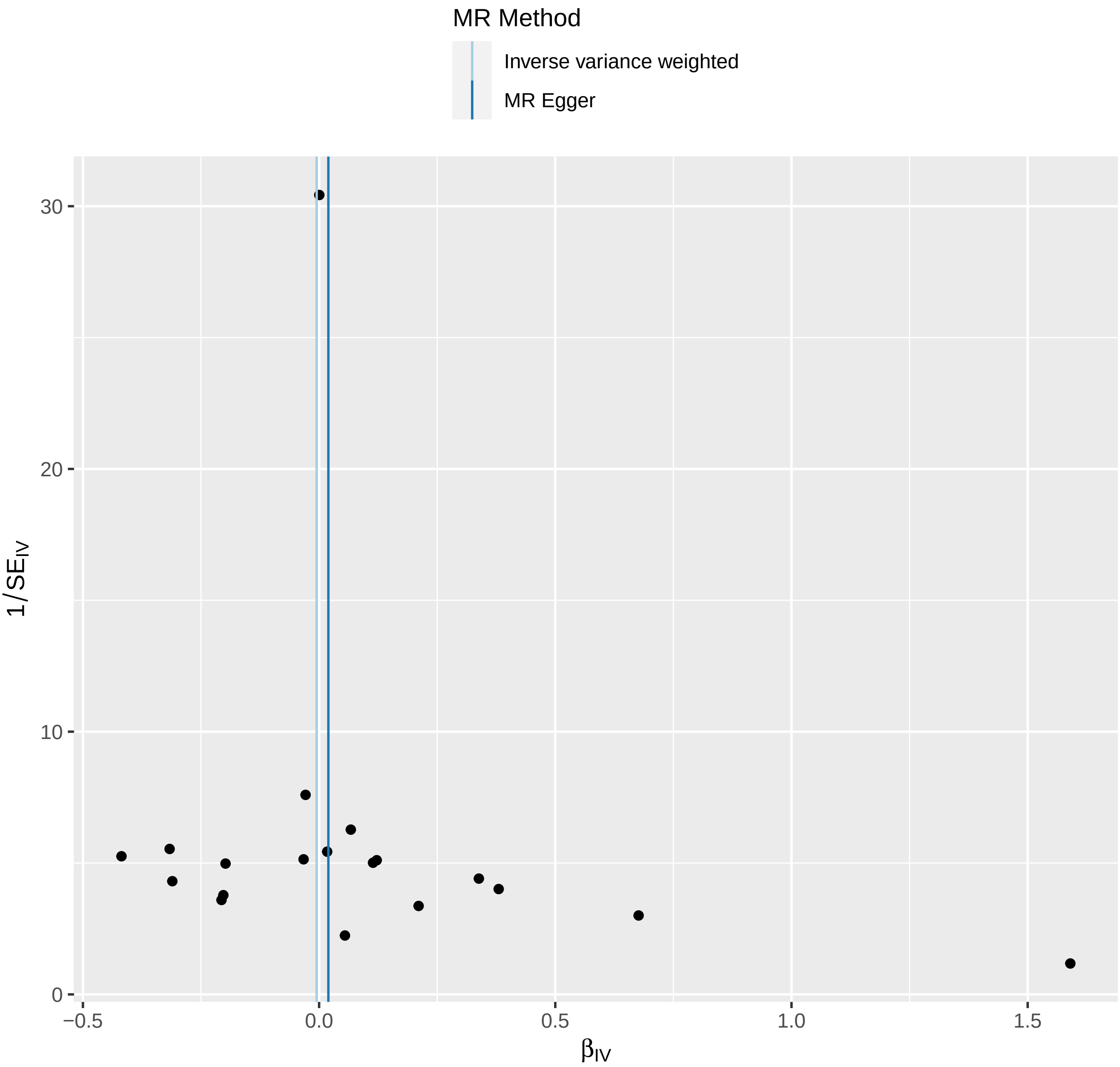

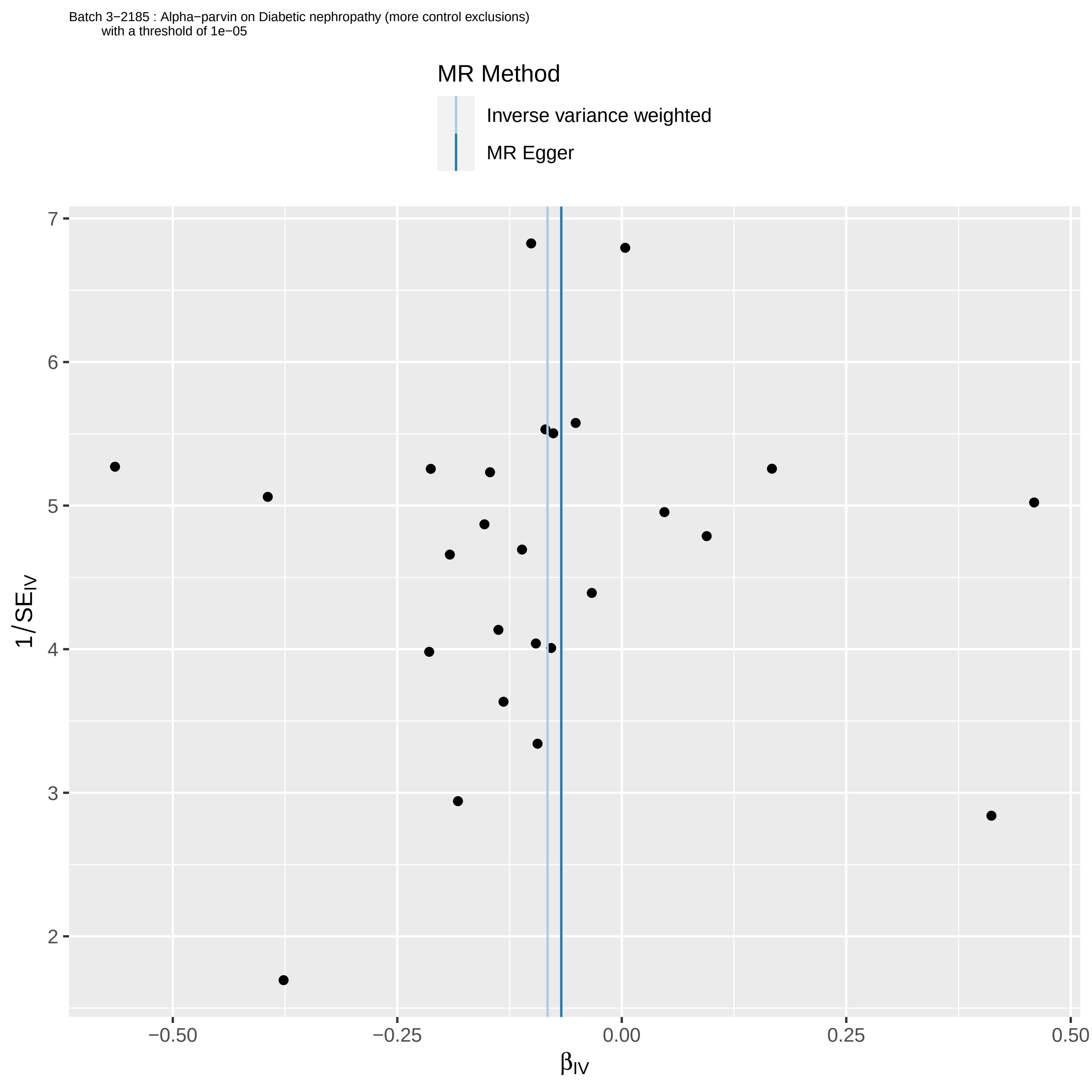

Batch 3−2185 : Alpha−parvin on Diabetic nephropathy (more control exclusions)
with a threshold of 1e−05
MR Method
Inverse variance weighted
MR Egger
1/SE$_{IV}$
7
6
5
4
3
2
−0.50
−0.25
0.00
0.25
0.50
β$_{IV}$

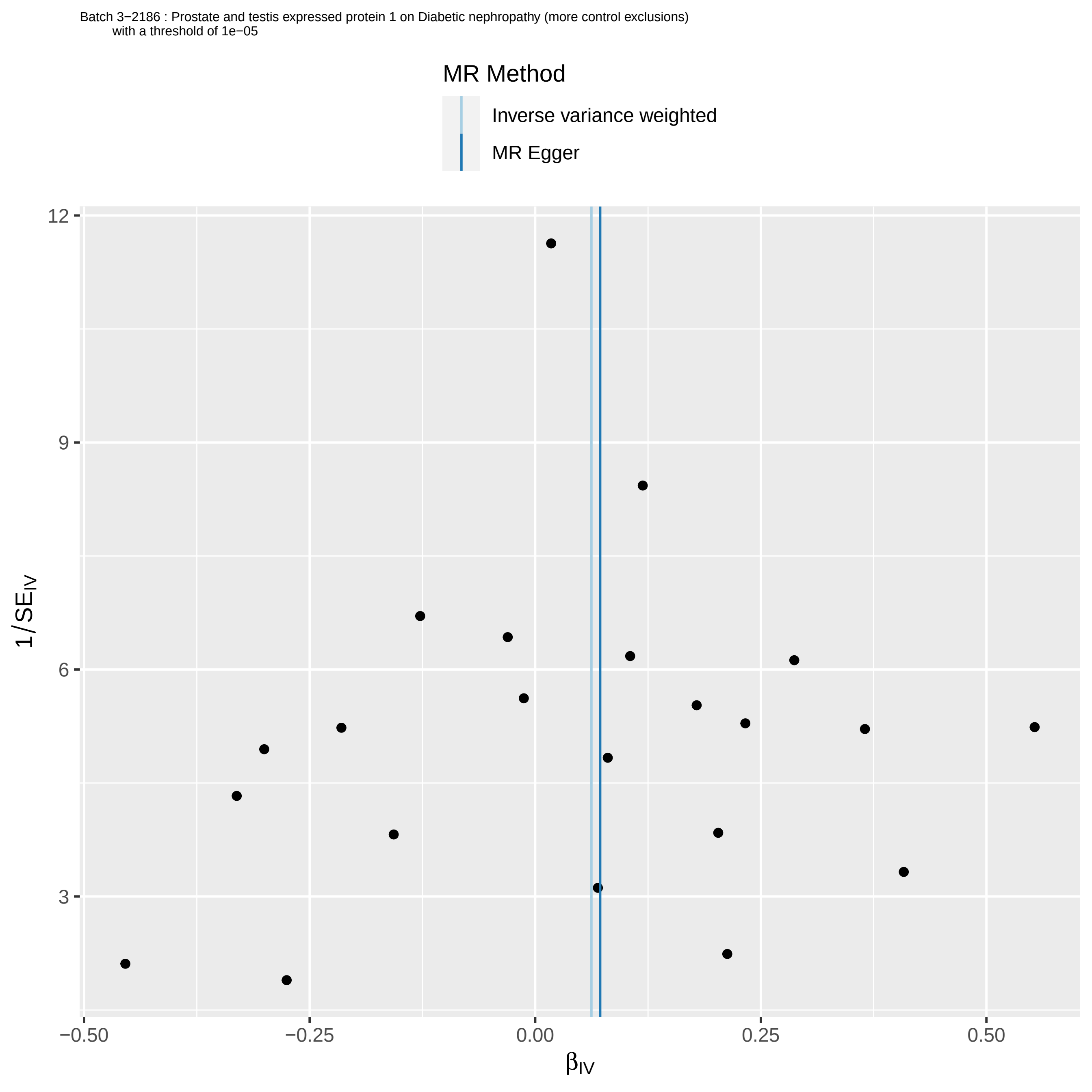
Batch 3−2186 : Prostate and testis expressed protein 1 on Diabetic nephropathy (more control exclusions)
with a threshold of 1e−05
MR Method
Inverse variance weighted
MR Egger
12
9
6
3
1/SE_IV
−0.50
−0.25
0.00
0.25
0.50
β_IV

Batch 3−2187 : Prostate and testis expressed protein 4 on Diabetic nephropathy (more control exclusions)
with a threshold of 1e−05

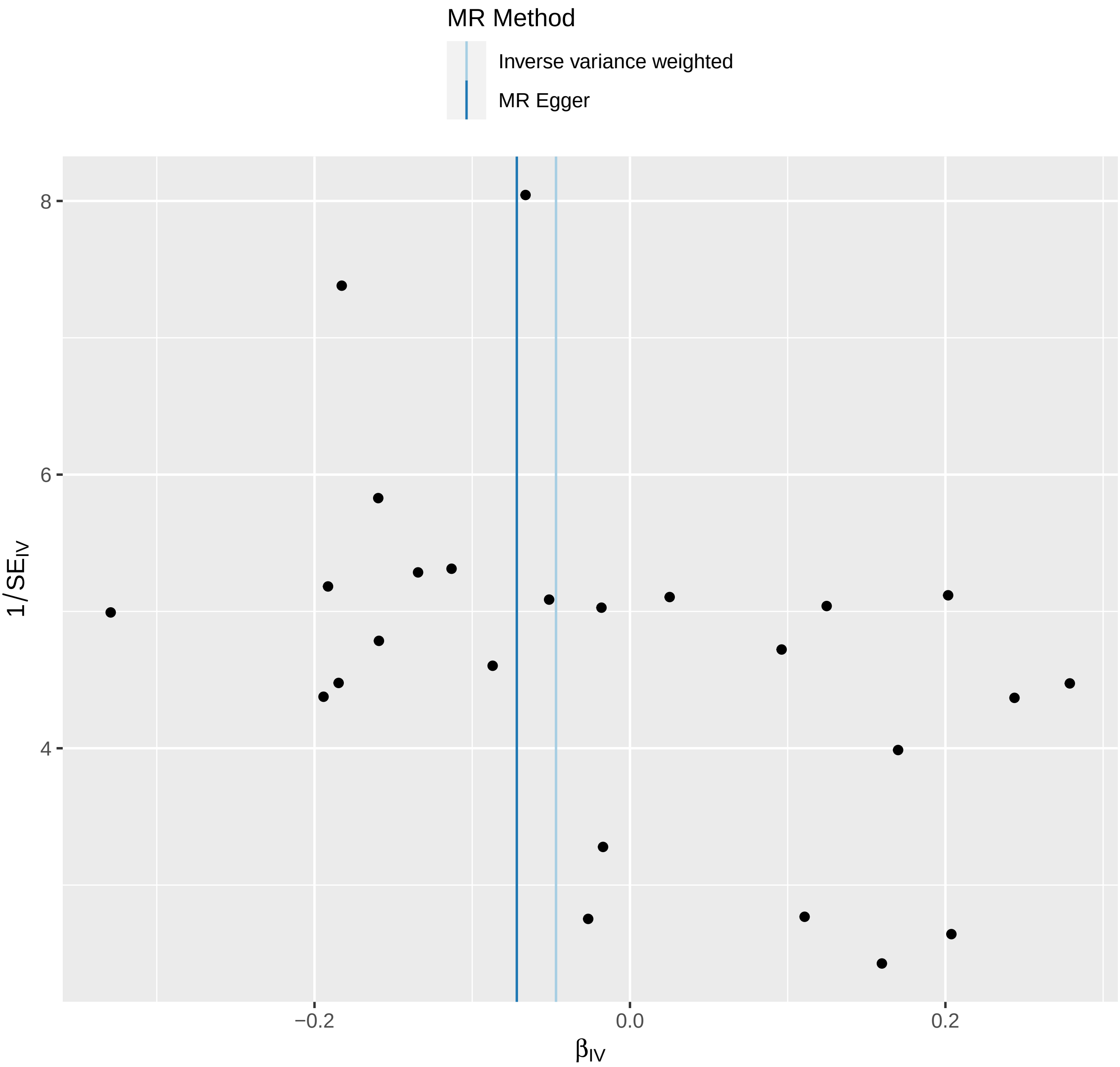

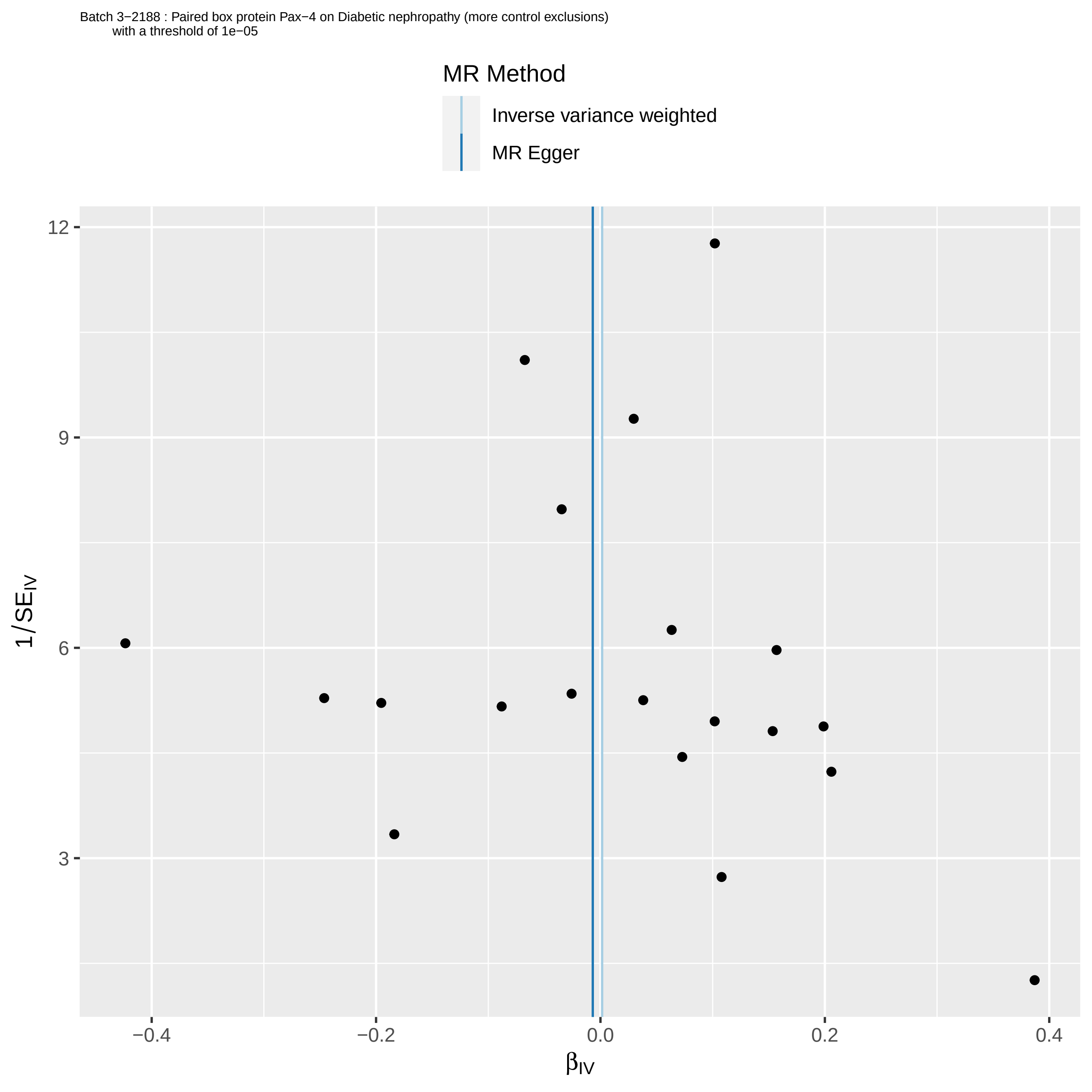

Batch 3−2188 : Paired box protein Pax−4 on Diabetic nephropathy (more control exclusions)
with a threshold of 1e−05
MR Method
Inverse variance weighted
MR Egger
12
9
6
3
1/SE_IV
−0.4
−0.2
0.0
0.2
0.4
β_IV

Batch 3−2189 : PAX−interacting protein 1 on Diabetic nephropathy (more control exclusions)
with a threshold of 1e−05

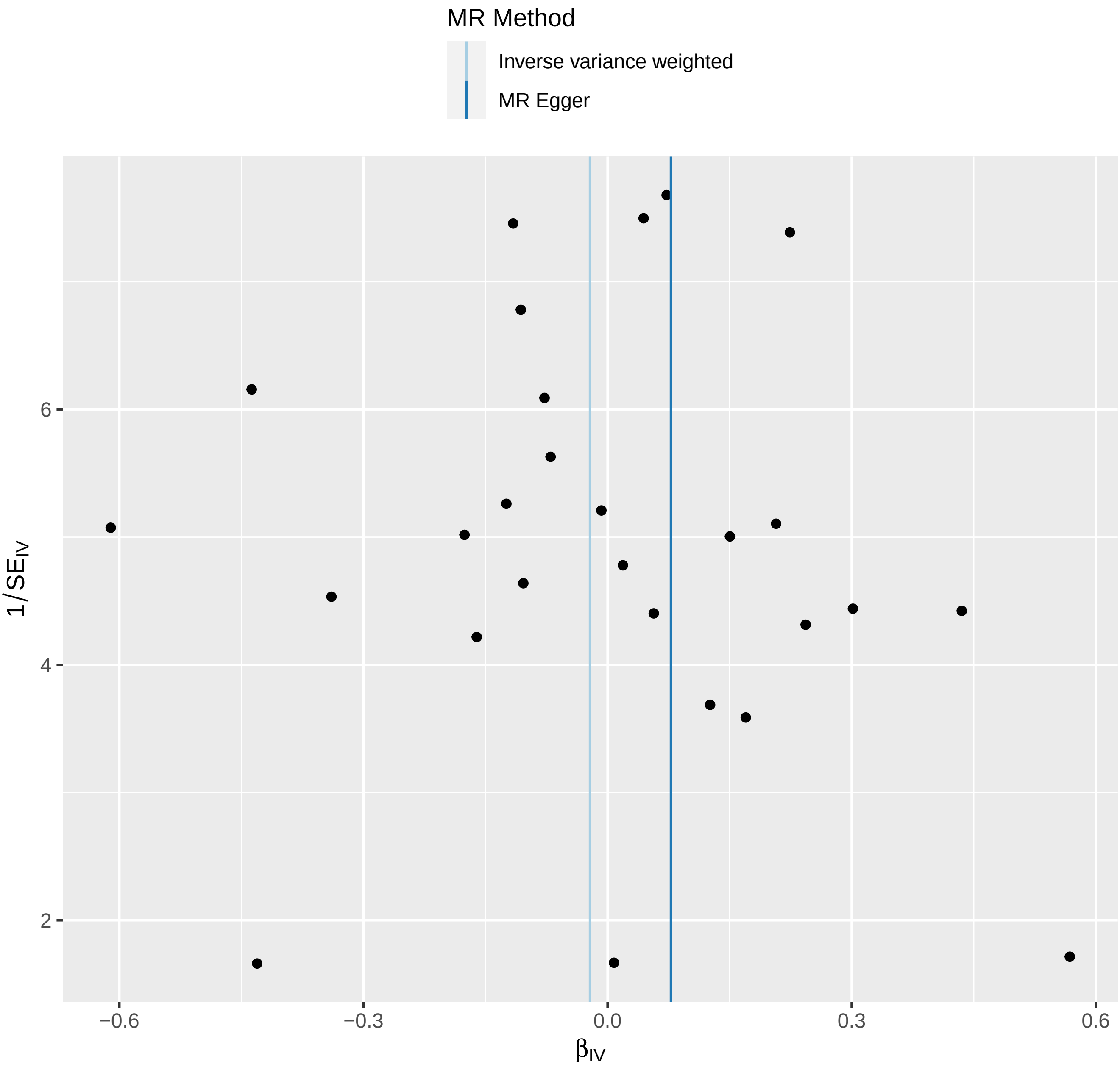

Batch 3−2190 : Pyruvate carboxylase, mitochondrial on Diabetic nephropathy (more control exclusions)
with a threshold of 1e−05

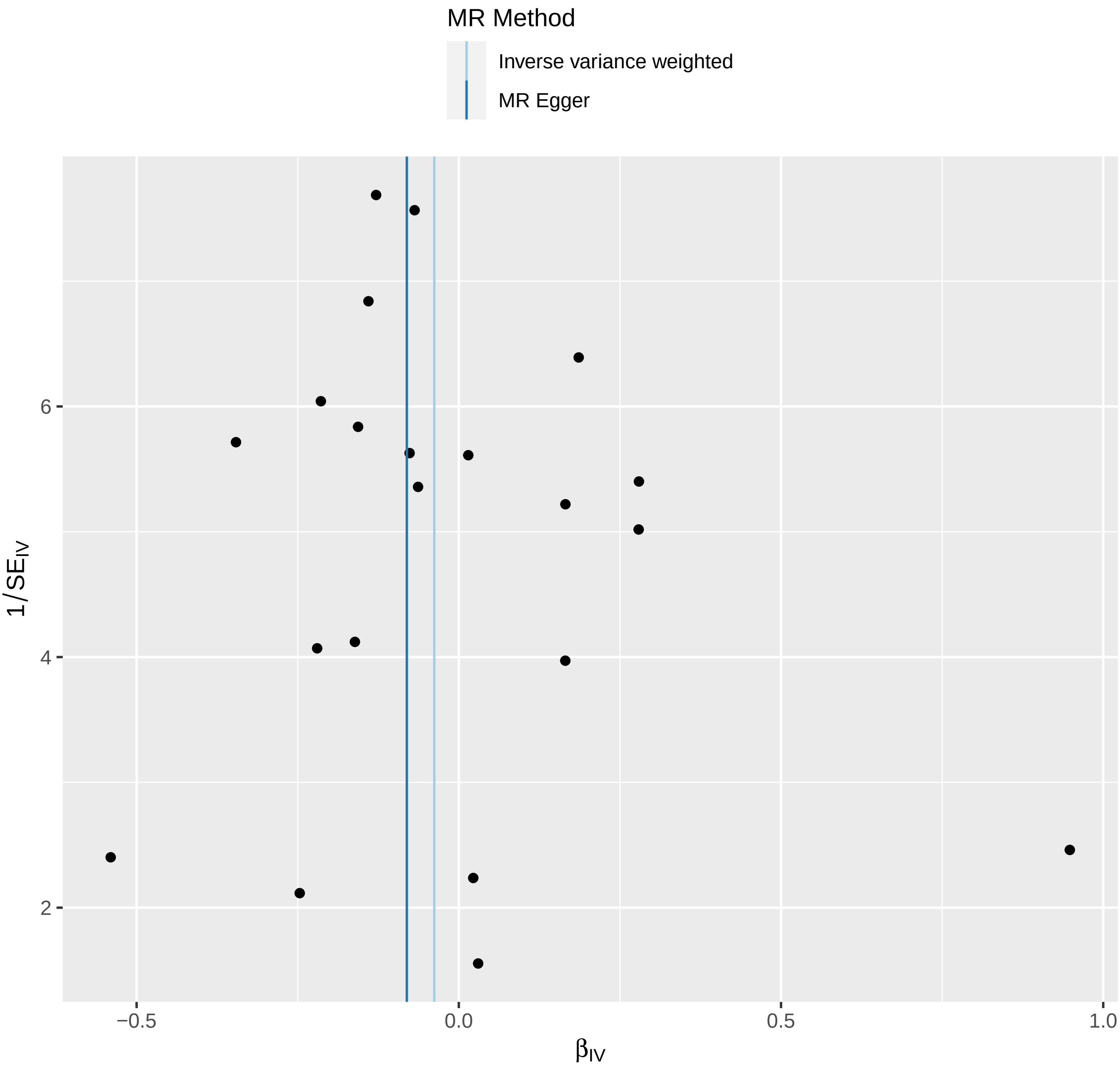

Batch 3−2191 : Pterin−4−alpha−carbinolamine dehydratase on Diabetic nephropathy (more control exclusions)
with a threshold of 1e−05

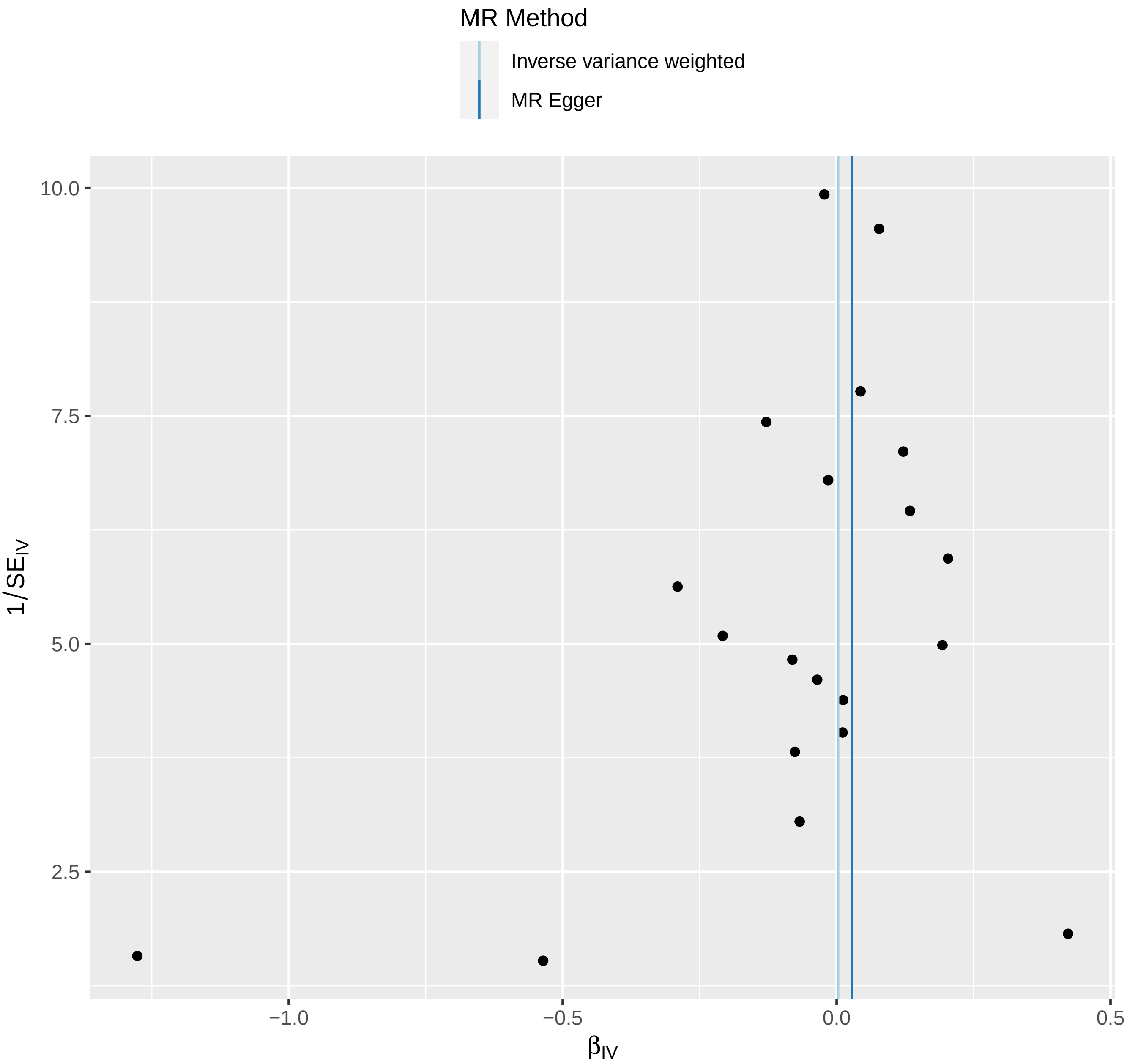

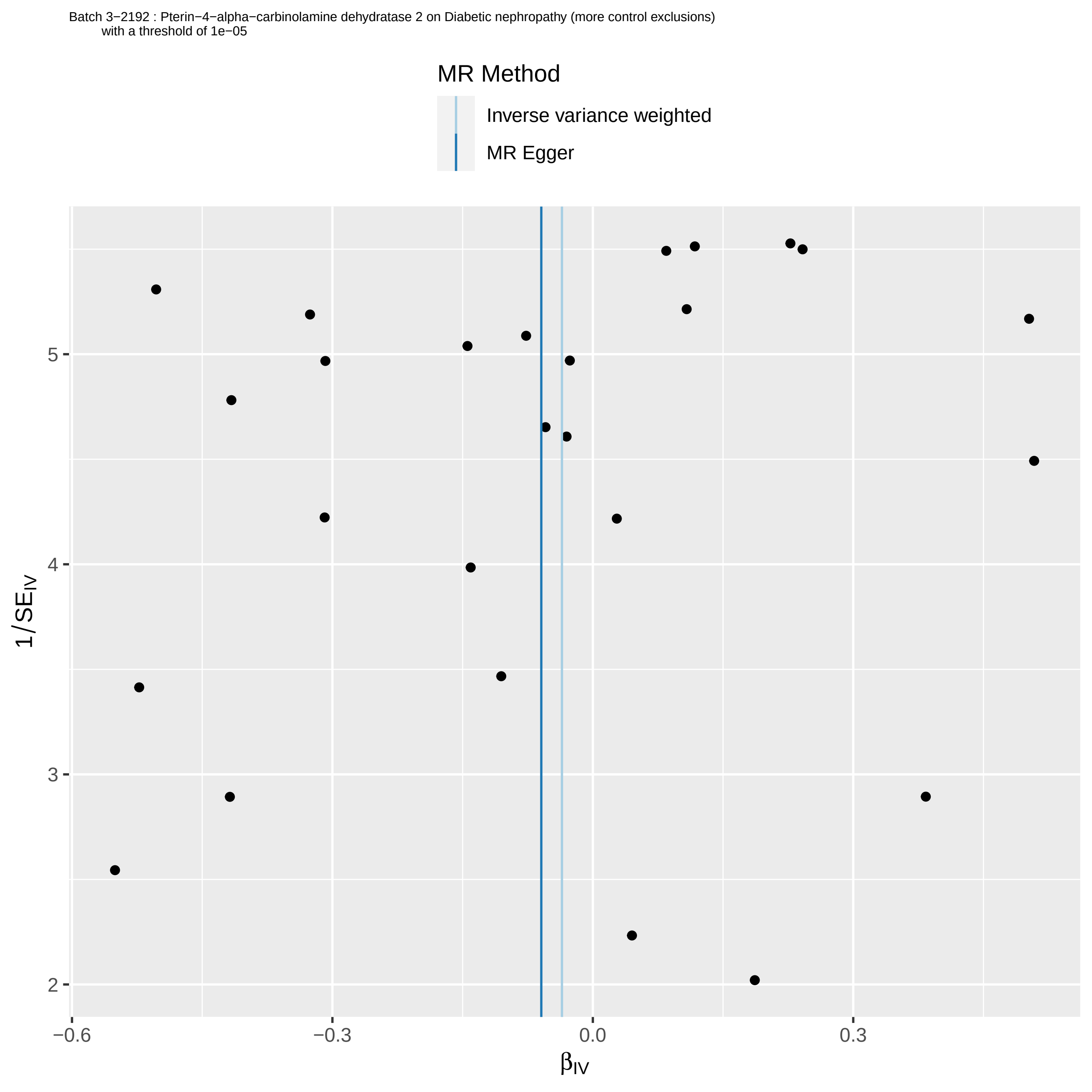
Batch 3−2192 : Pterin−4−alpha−carbinolamine dehydratase 2 on Diabetic nephropathy (more control exclusions)
with a threshold of 1e−05
MR Method
Inverse variance weighted
MR Egger
5
4
3
2
1/SE_IV
−0.6
−0.3
0.0
0.3
β_IV

Batch 3−2193 : Poly(rC)−binding protein 1 on Diabetic nephropathy (more control exclusions)
with a threshold of 1e−05

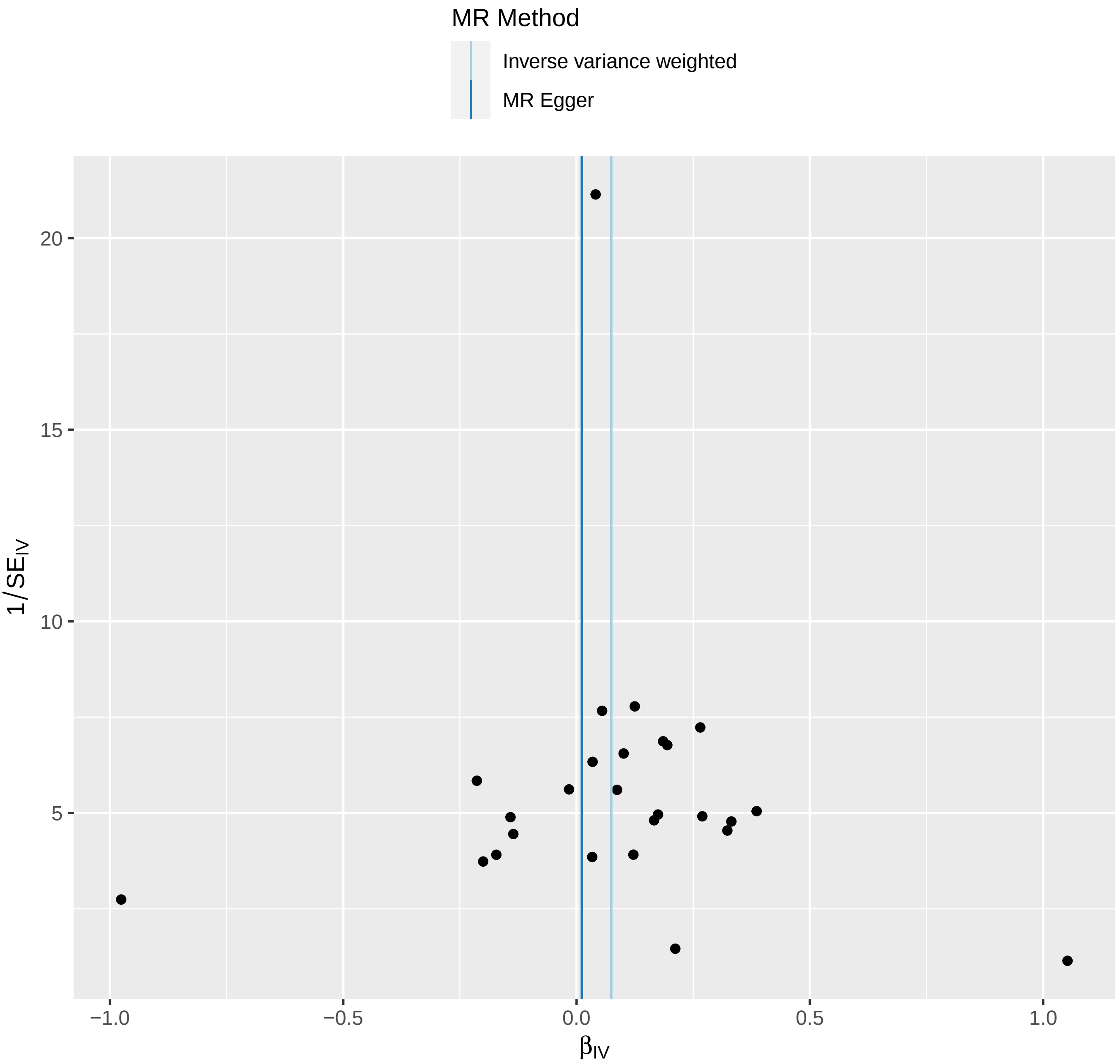

Batch 3−2194 : Protocadherin−10 on Diabetic nephropathy (more control exclusions)
with a threshold of 1e−05

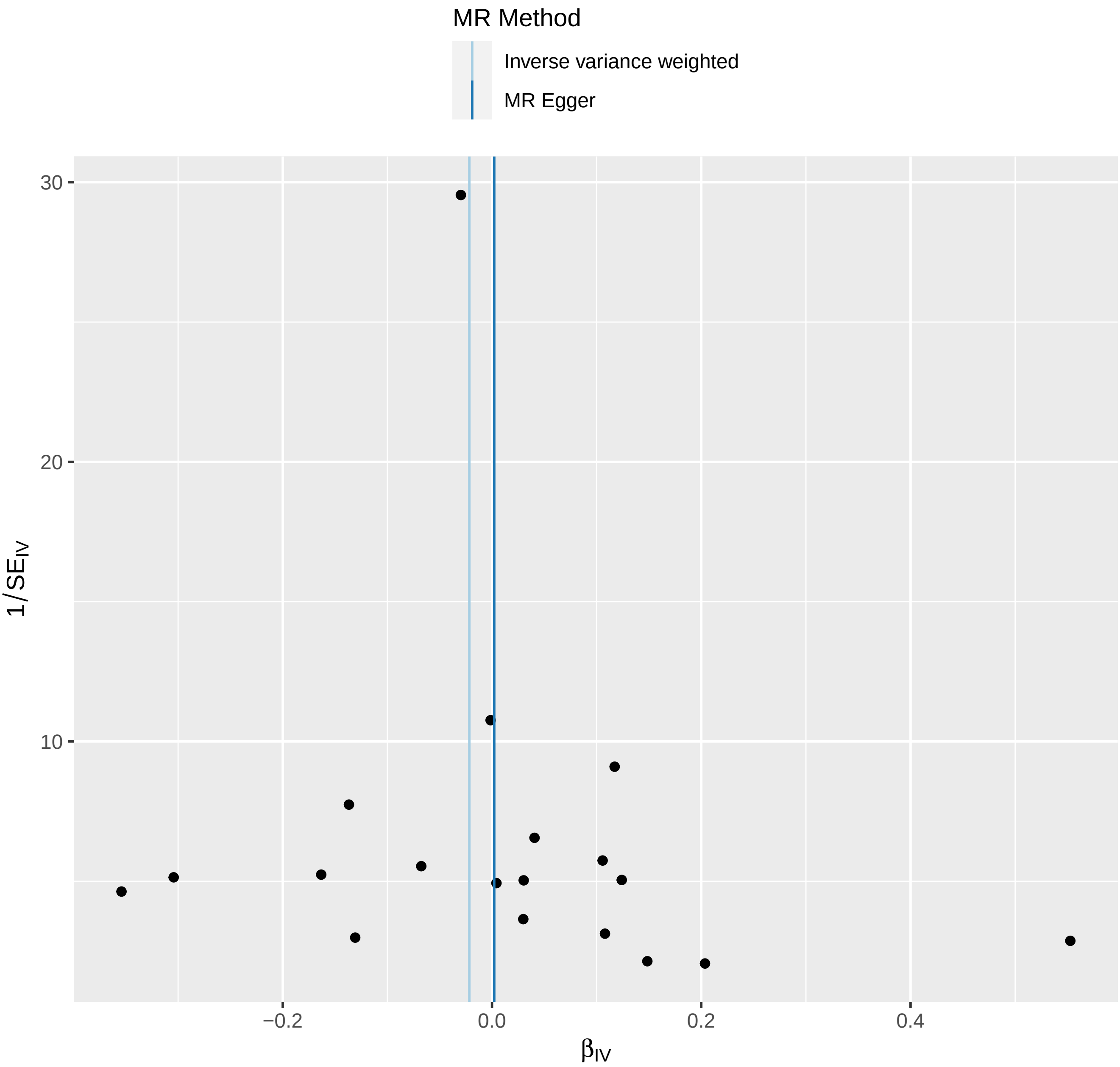

Batch 3−2195 : Protocadherin−10 on Diabetic nephropathy (more control exclusions)
with a threshold of 1e−05

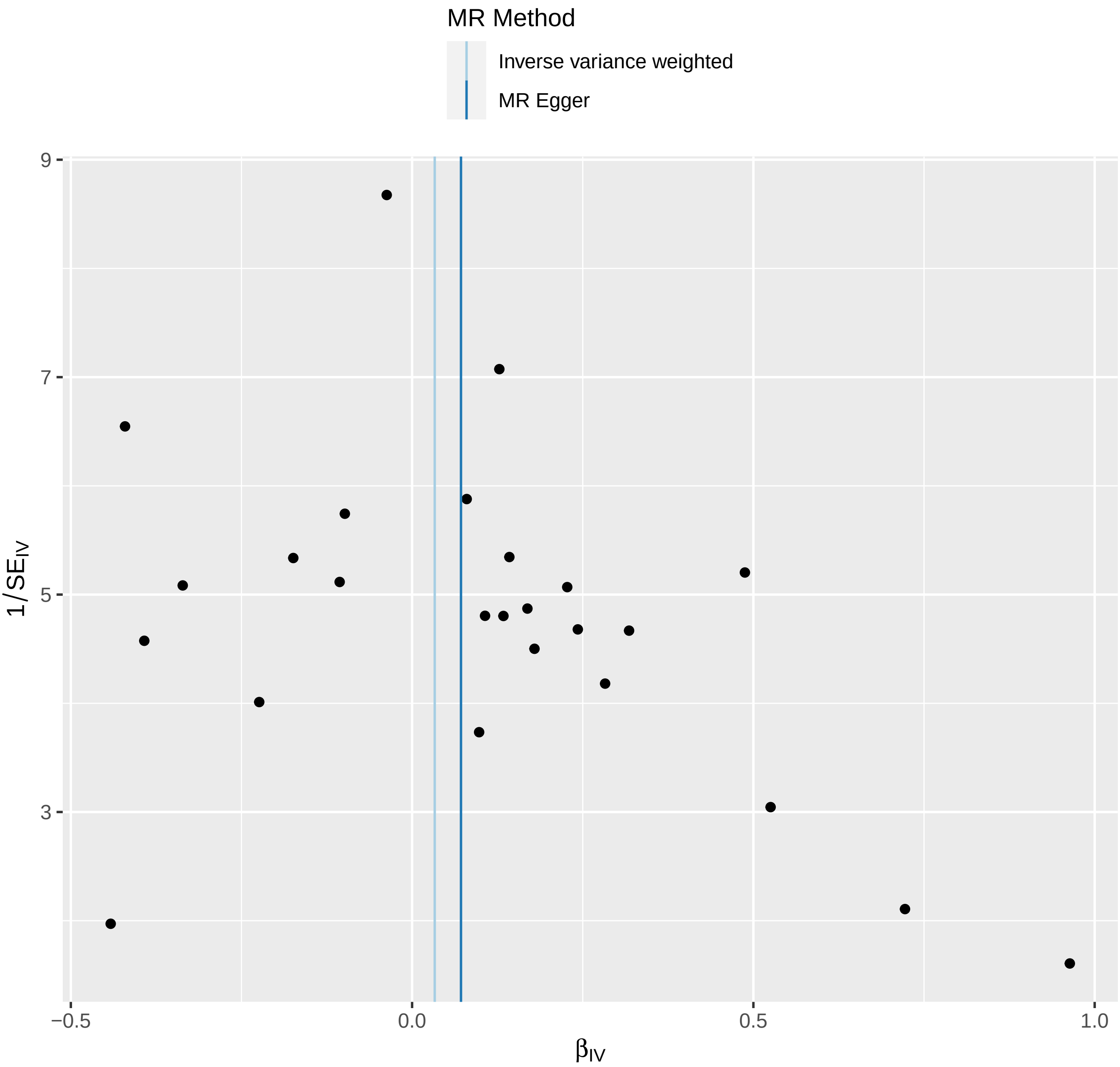

Batch 3−2196 : Protocadherin−15 on Diabetic nephropathy (more control exclusions)
with a threshold of 1e−05

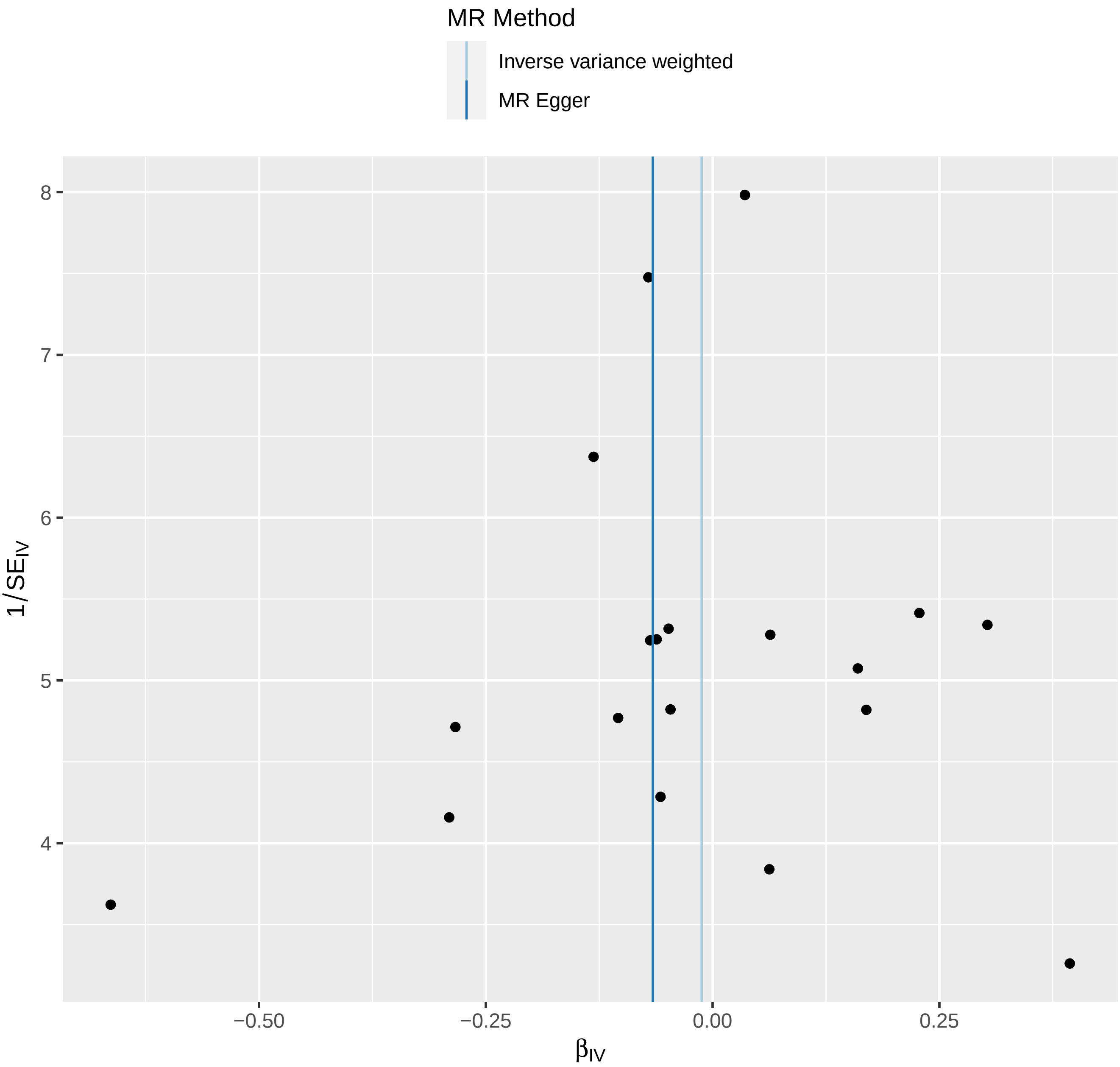

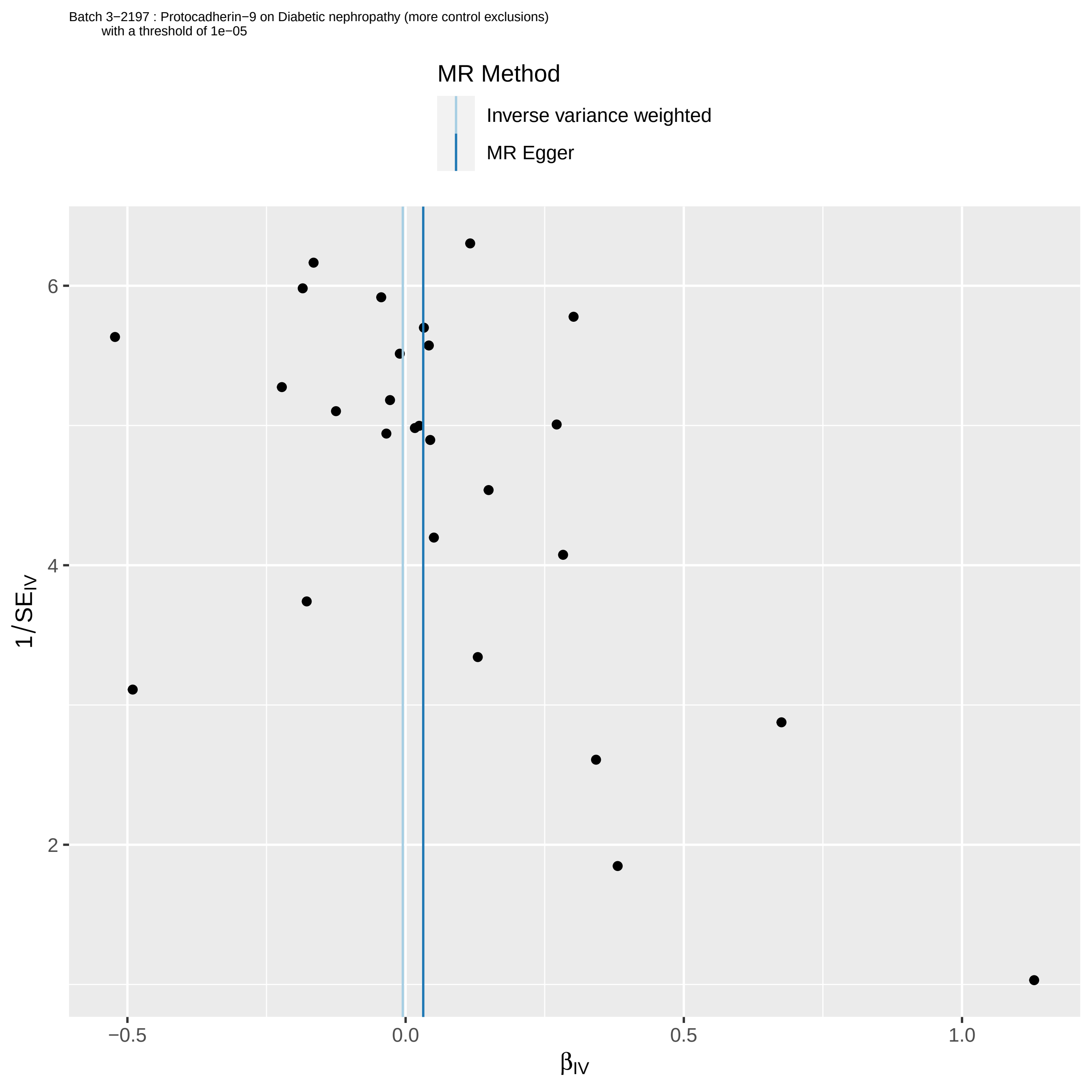
Batch 3−2197 : Protocadherin−9 on Diabetic nephropathy (more control exclusions)
with a threshold of 1e−05
MR Method
Inverse variance weighted
MR Egger
6
4
2
$1/SE_{IV}$
−0.5
0.0
0.5
1.0
β_{IV}

Batch 3−2198 : Protocadherin alpha−4 on Diabetic nephropathy (more control exclusions)
with a threshold of 1e−05

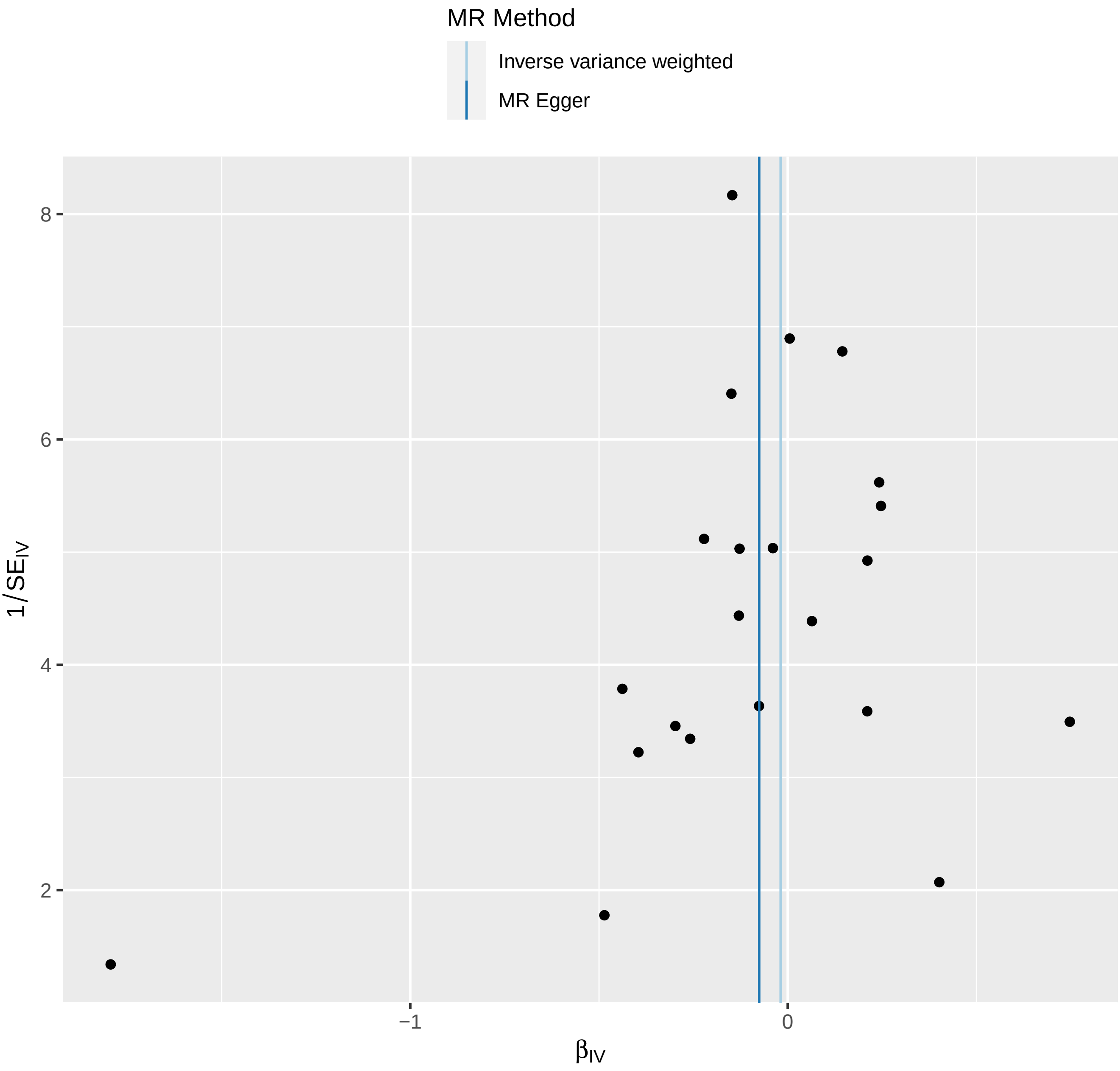

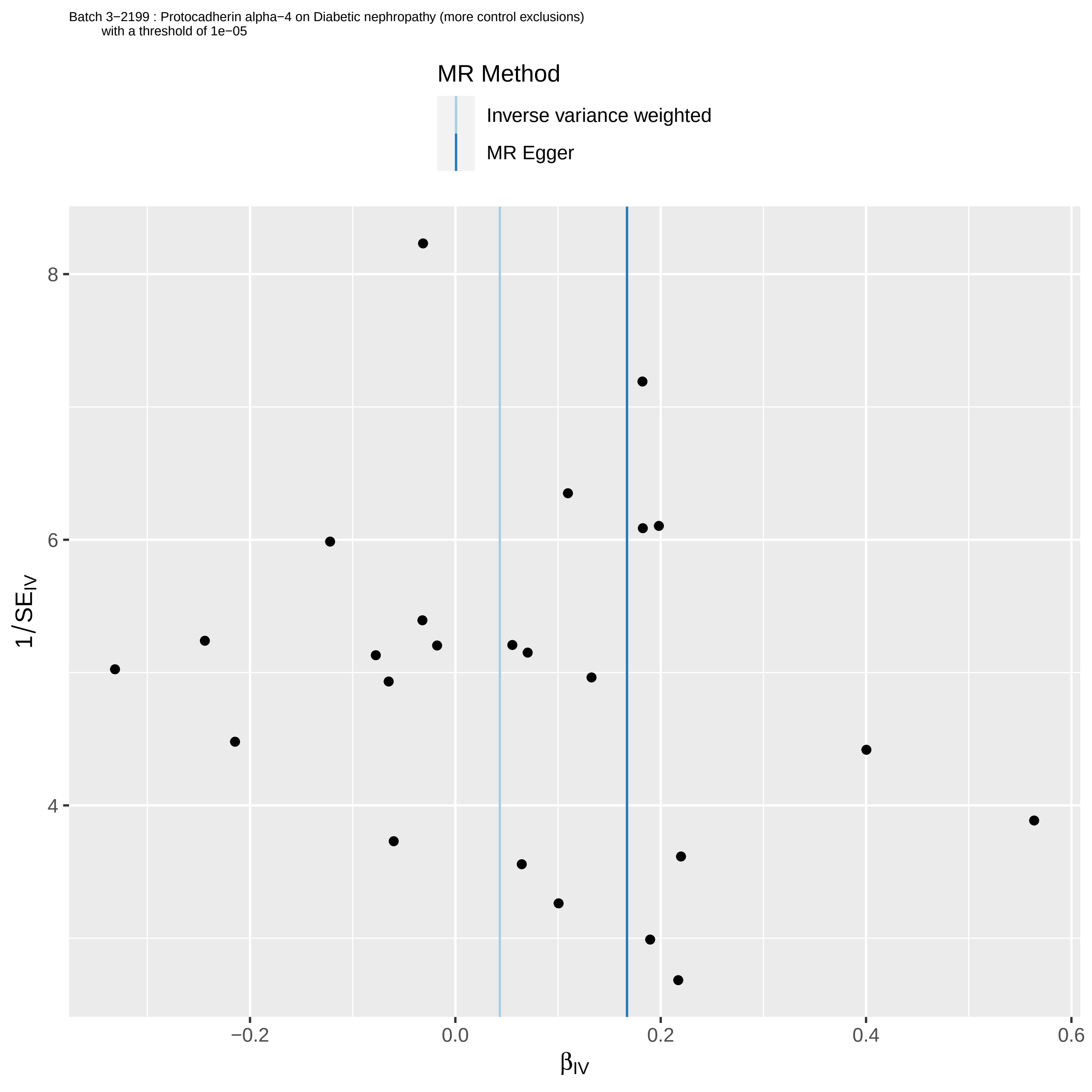
Batch 3−2199 : Protocadherin alpha−4 on Diabetic nephropathy (more control exclusions)
with a threshold of 1e−05
MR Method
Inverse variance weighted
MR Egger
8
6
4
1/SE_IV
−0.2
0.0
0.2
0.4
0.6
β_IV

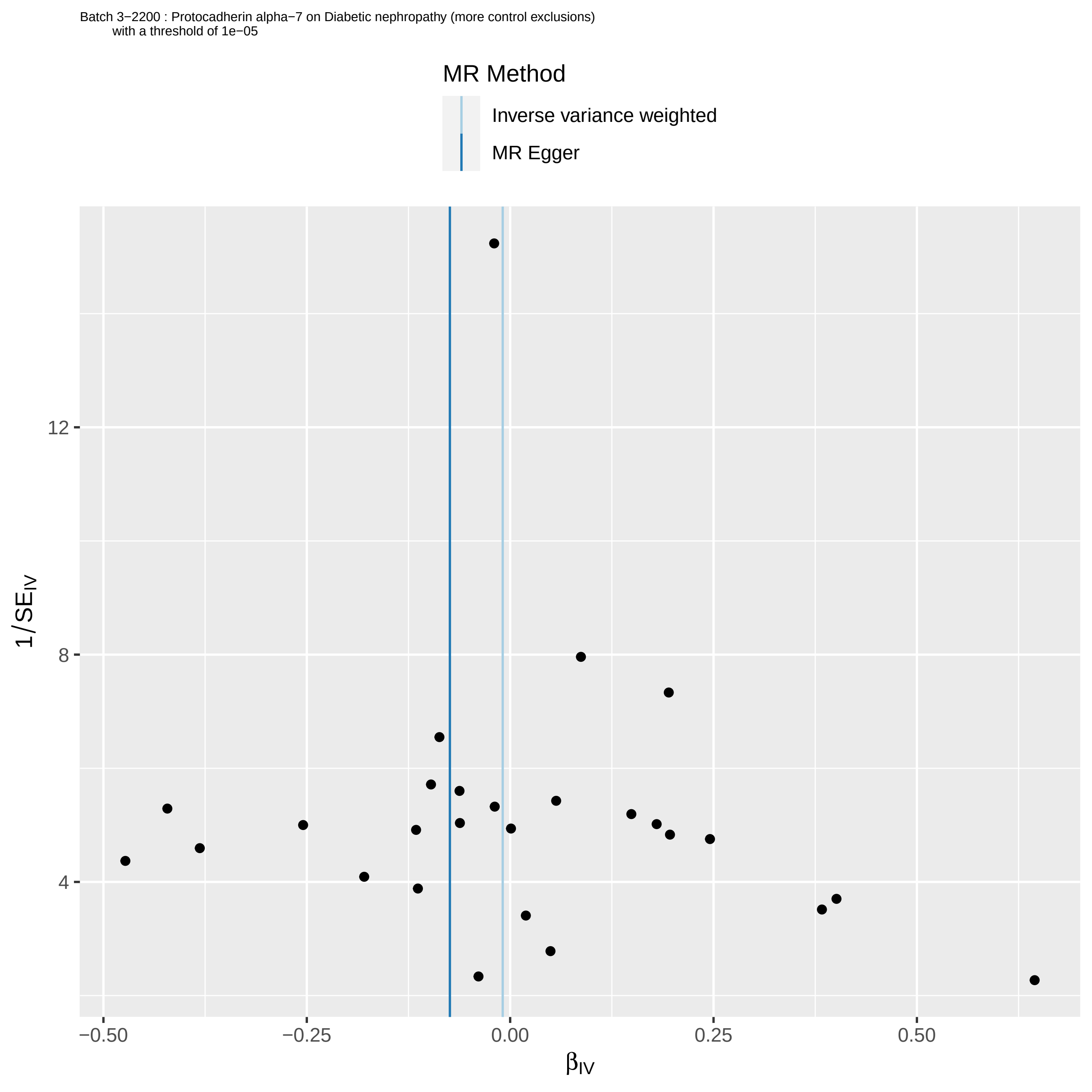
Batch 3−2200 : Protocadherin alpha−7 on Diabetic nephropathy (more control exclusions)
with a threshold of 1e−05
MR Method
Inverse variance weighted
MR Egger
12
8
4
1/SE$_{IV}$
−0.50
−0.25
0.00
0.25
0.50
β$_{IV}$

Batch 3−2201 : Protocadherin alpha−C2 on Diabetic nephropathy (more control exclusions)
with a threshold of 1e−05

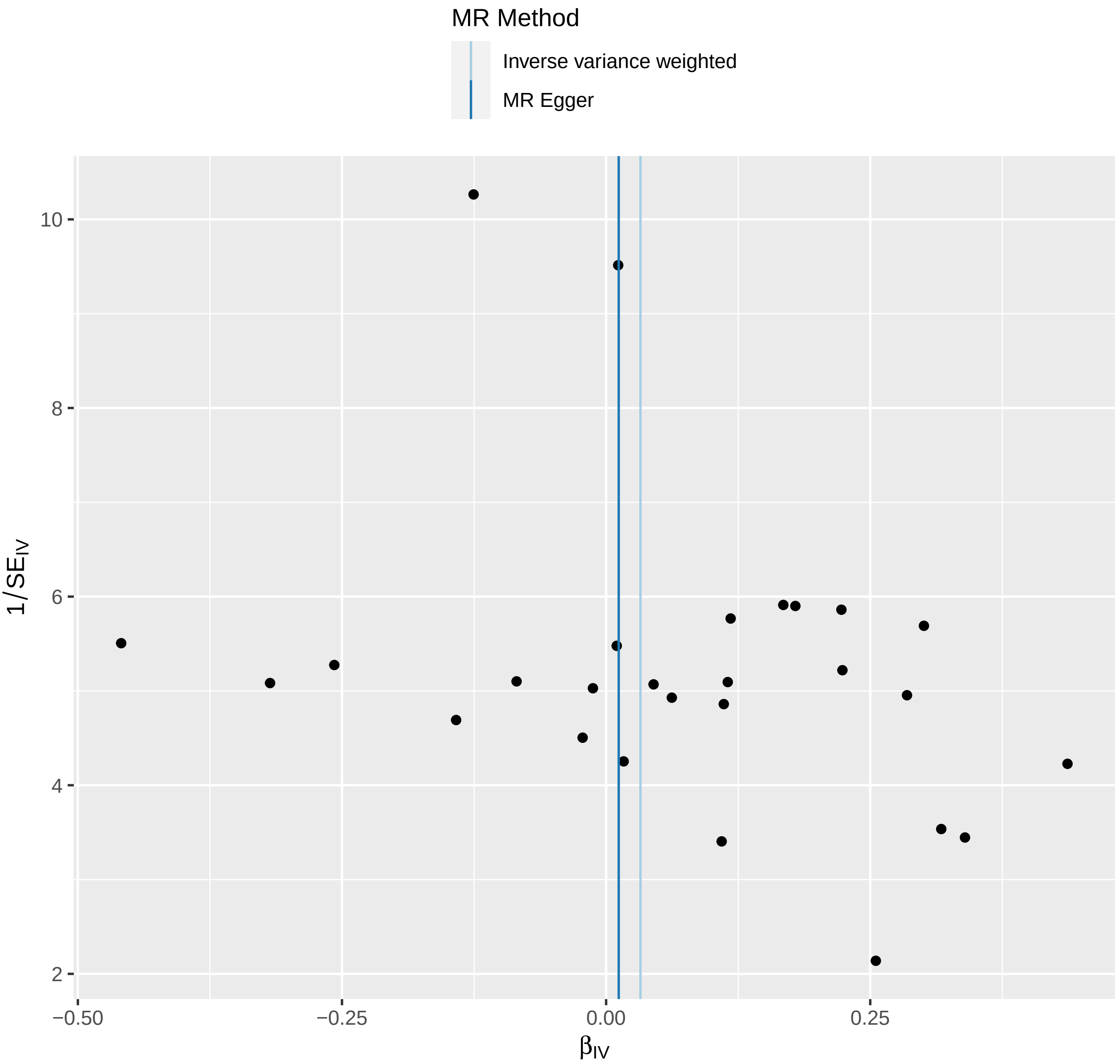

Batch 3−2202 : Protocadherin beta−10 on Diabetic nephropathy (more control exclusions)
with a threshold of 1e−05

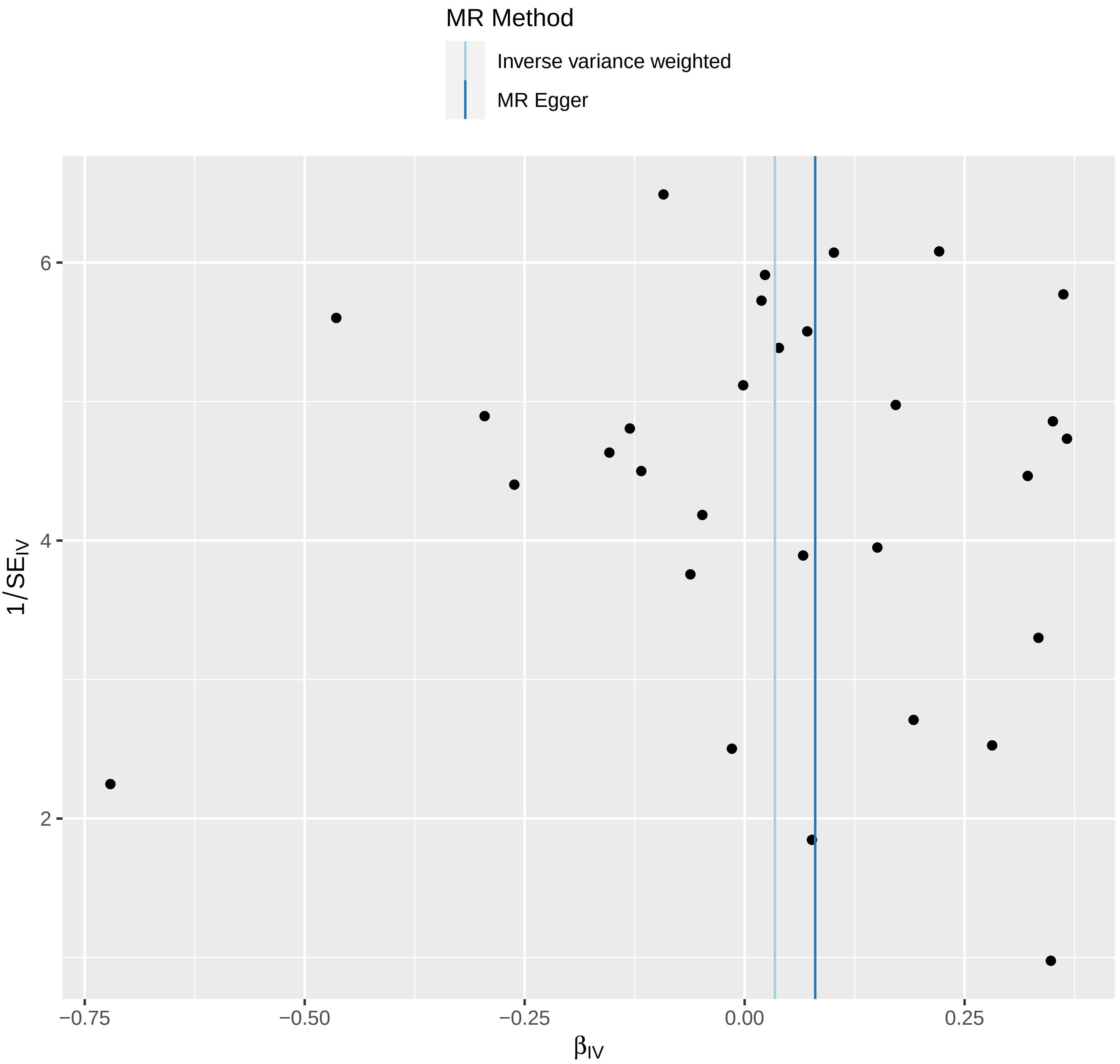

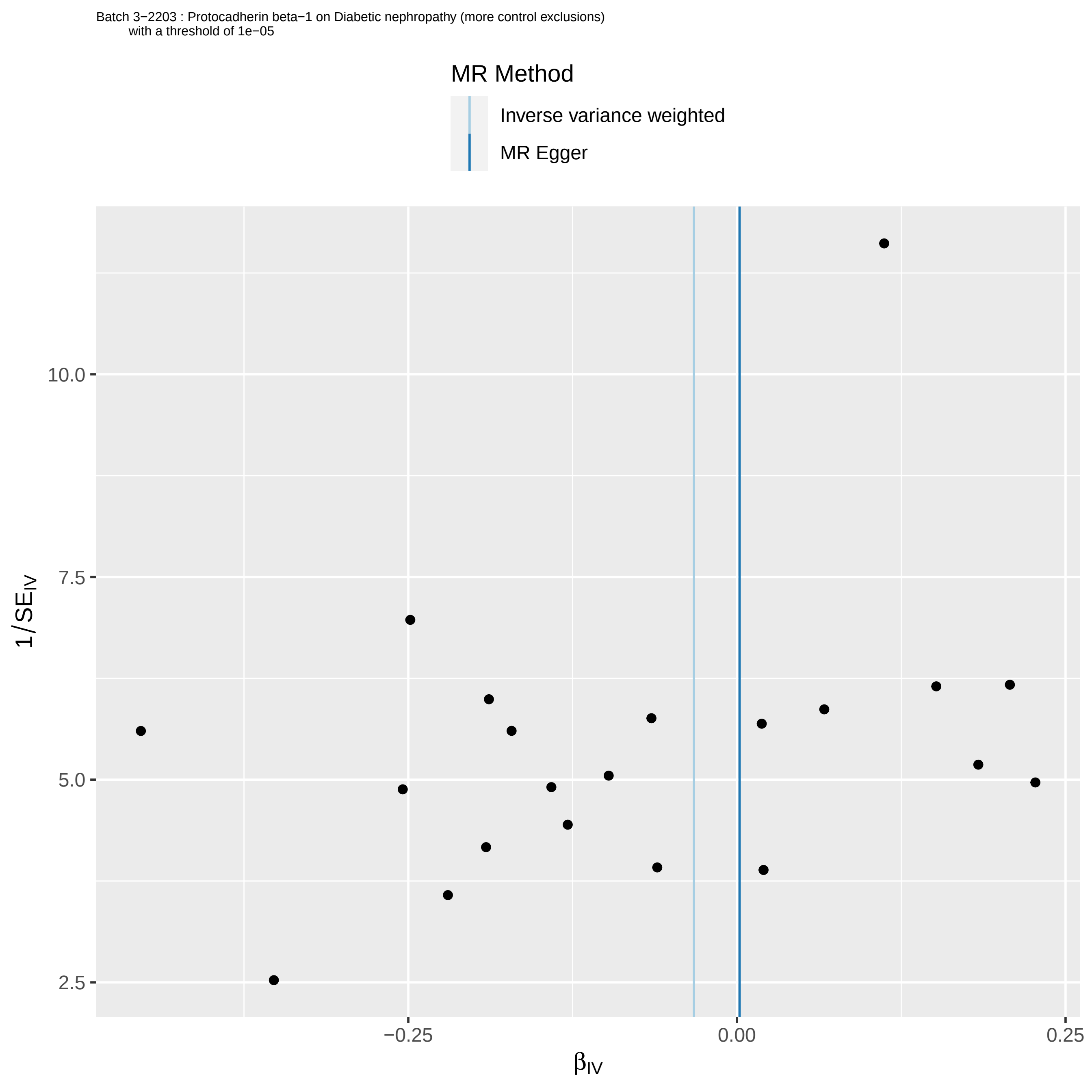
Batch 3−2203 : Protocadherin beta−1 on Diabetic nephropathy (more control exclusions)
with a threshold of 1e−05
MR Method
Inverse variance weighted
MR Egger
10.0
7.5
5.0
2.5
1/SE_IV
−0.25
0.00
0.25
β_IV

Batch 3−2204 : Protocadherin beta−2 on Diabetic nephropathy (more control exclusions)
with a threshold of 1e−05

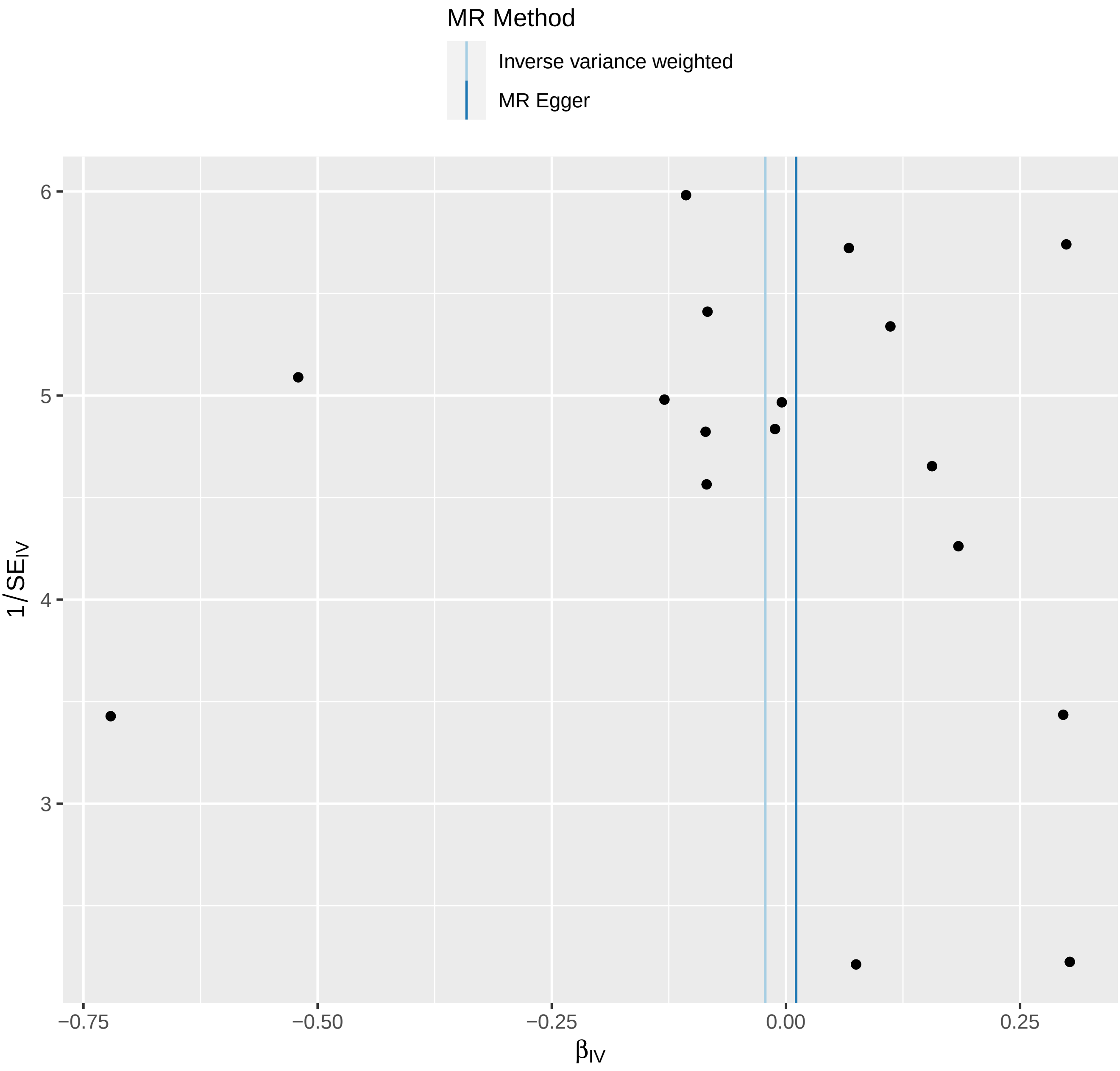

Batch 3−2205 : Protocadherin beta−4 on Diabetic nephropathy (more control exclusions)
with a threshold of 1e−05

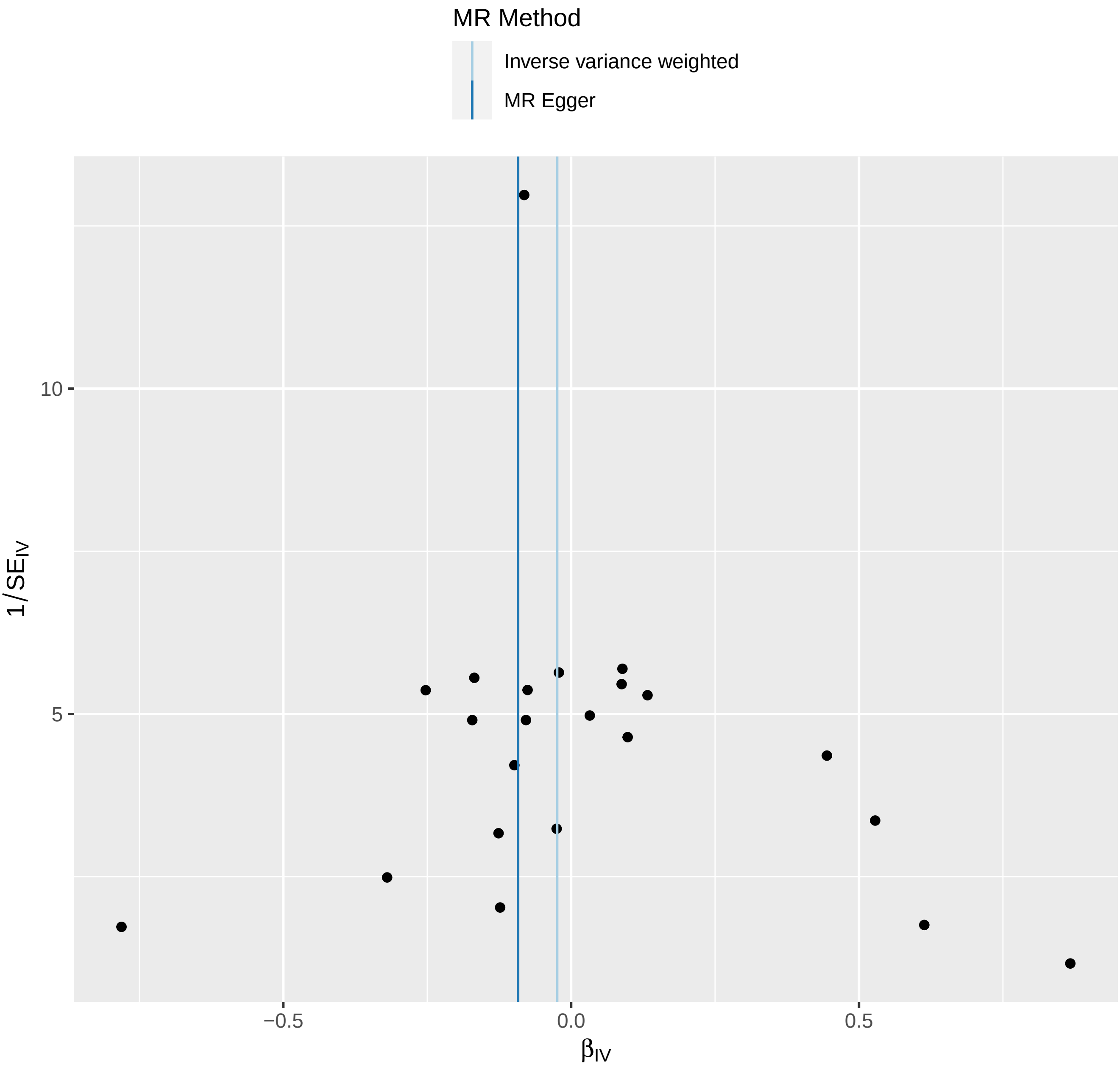

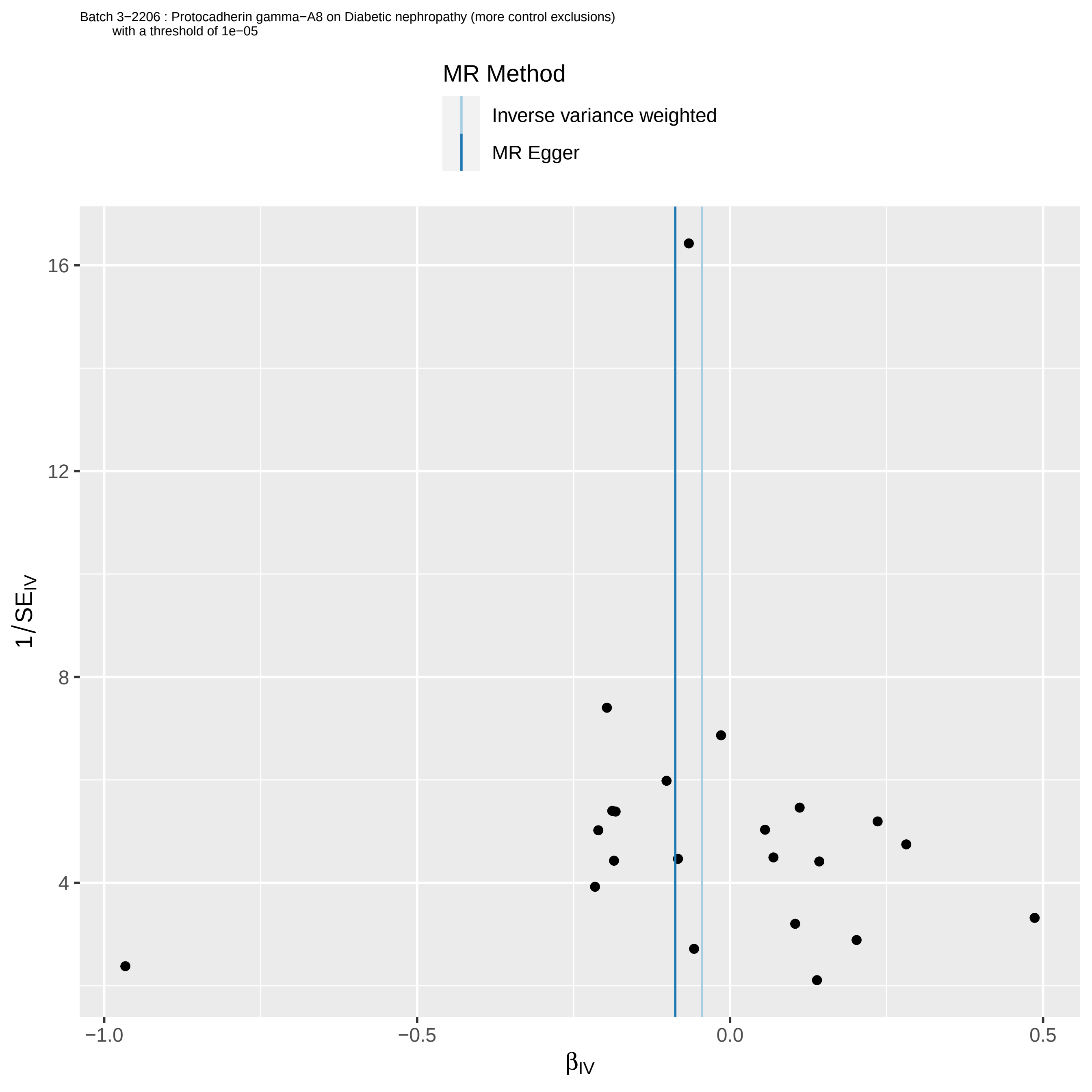
Batch 3−2206 : Protocadherin gamma−A8 on Diabetic nephropathy (more control exclusions)
with a threshold of 1e−05
MR Method
Inverse variance weighted
MR Egger
16
12
8
4
1/SE_IV
−1.0
−0.5
0.0
0.5
β_IV

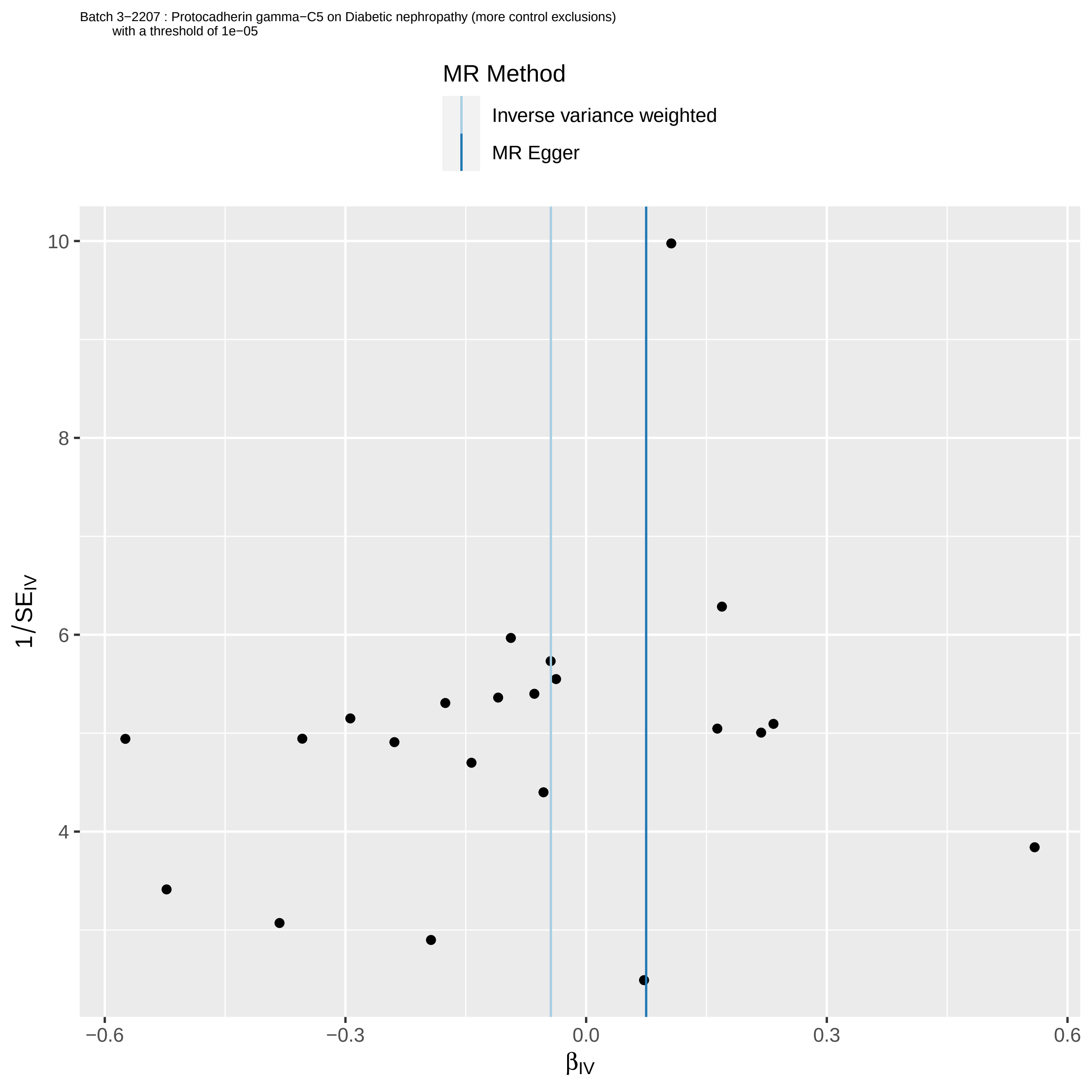

Batch 3−2207 : Protocadherin gamma−C5 on Diabetic nephropathy (more control exclusions)
with a threshold of 1e−05
MR Method
Inverse variance weighted
MR Egger
10
8
6
4
1/SE_IV
−0.6
−0.3
0.0
0.3
0.6
β_IV

Batch 3−2208 : Proliferating cell nuclear antigen on Diabetic nephropathy (more control exclusions)
with a threshold of 1e−05

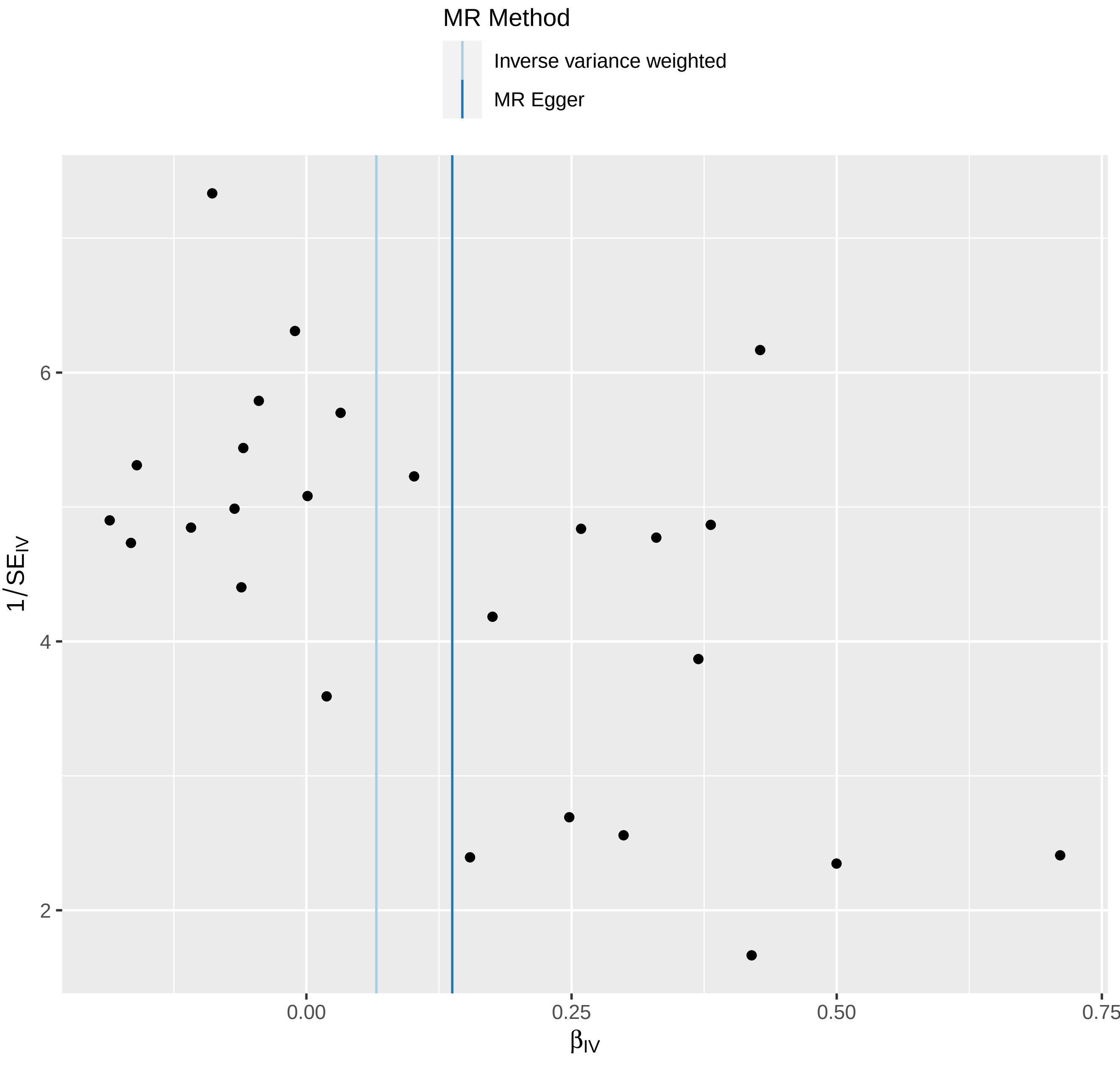

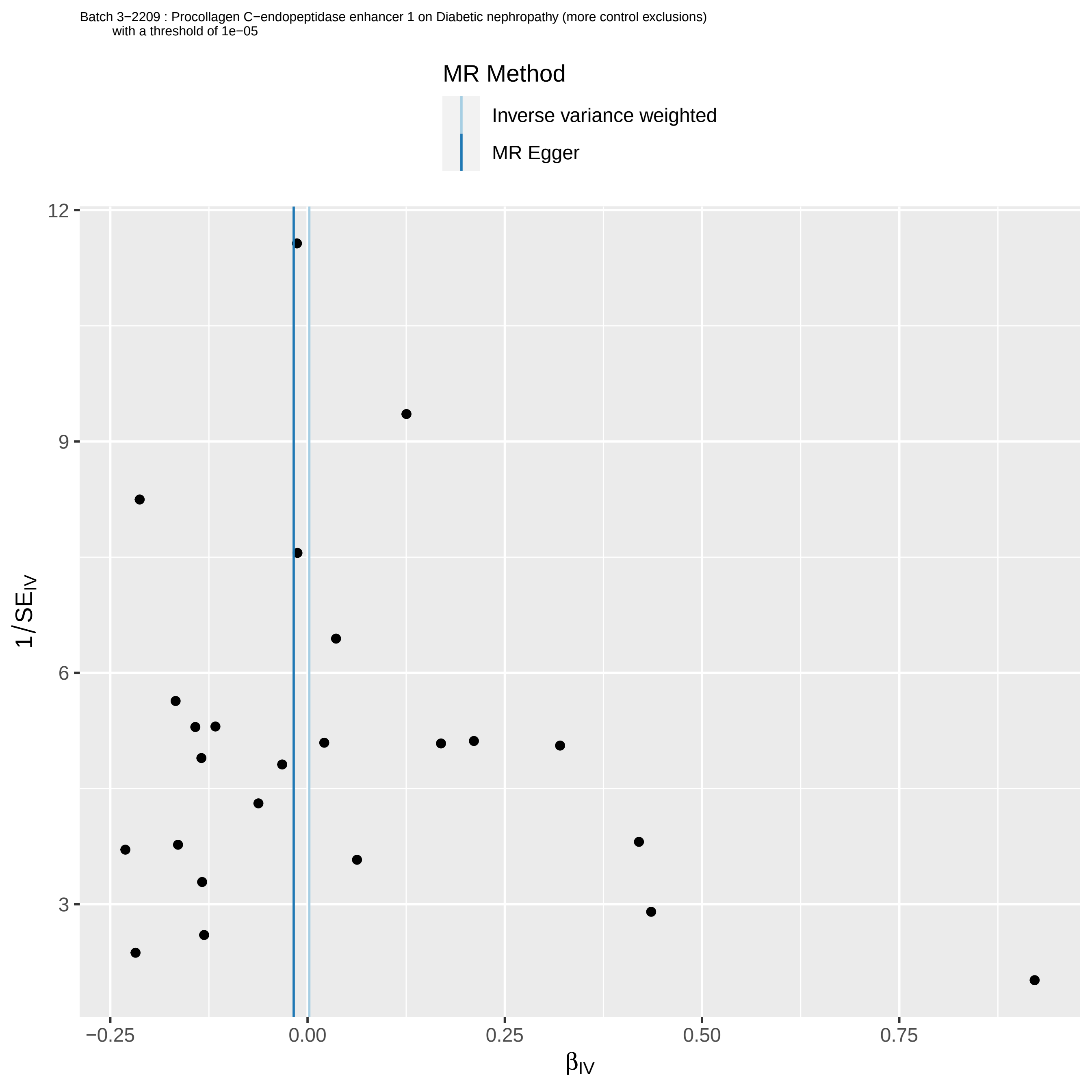
Batch 3−2209 : Procollagen C−endopeptidase enhancer 1 on Diabetic nephropathy (more control exclusions)
with a threshold of 1e−05
MR Method
Inverse variance weighted
MR Egger
12
9
6
3
1/SE_IV
−0.25
0.00
0.25
0.50
0.75
β_IV

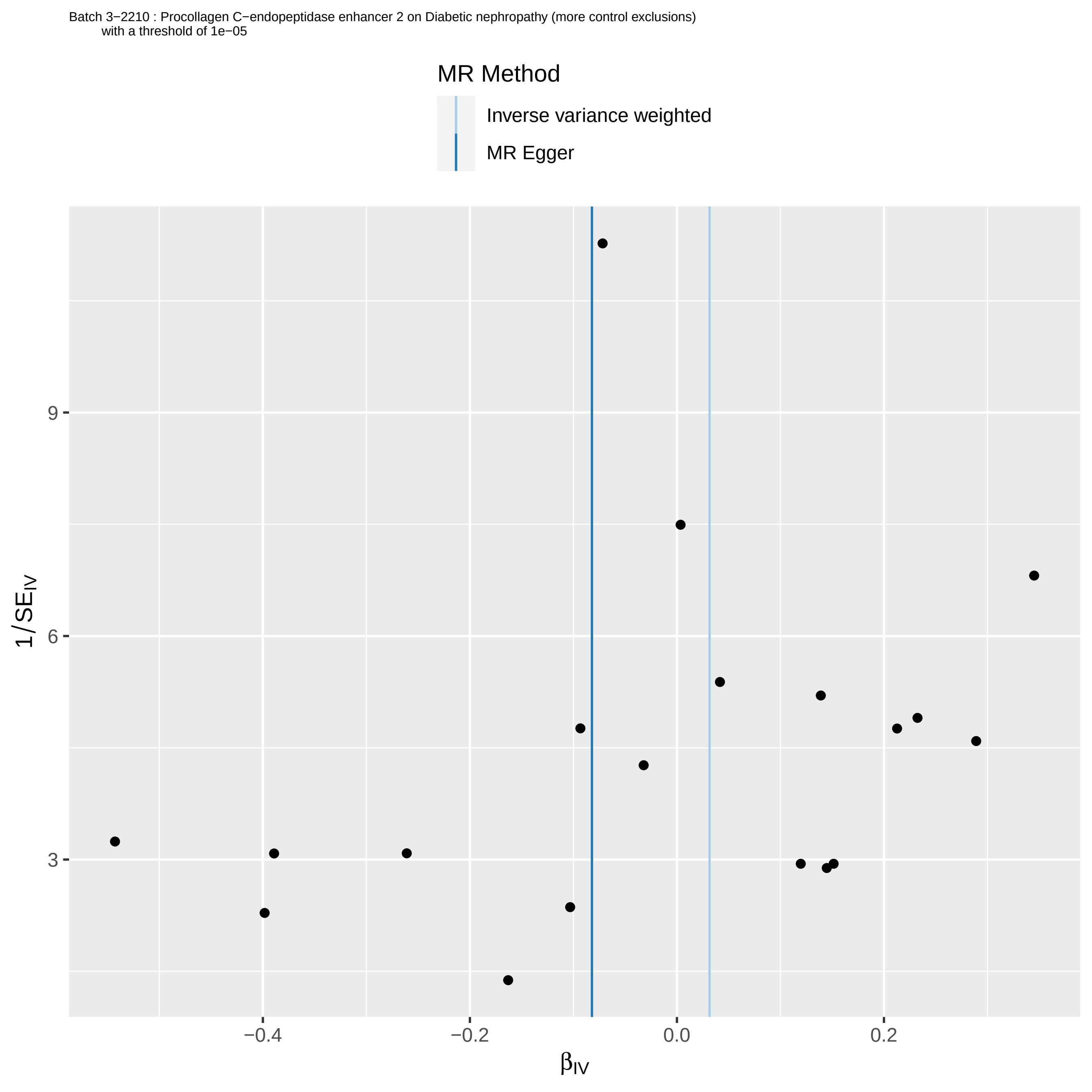

Batch 3−2210 : Procollagen C−endopeptidase enhancer 2 on Diabetic nephropathy (more control exclusions)
with a threshold of 1e−05
MR Method
Inverse variance weighted
MR Egger
9
6
3
1/SE_IV
−0.4
−0.2
0.0
0.2
β_IV

Batch 3−2211 : Neuroendocrine convertase 1 on Diabetic nephropathy (more control exclusions)
with a threshold of 1e−05

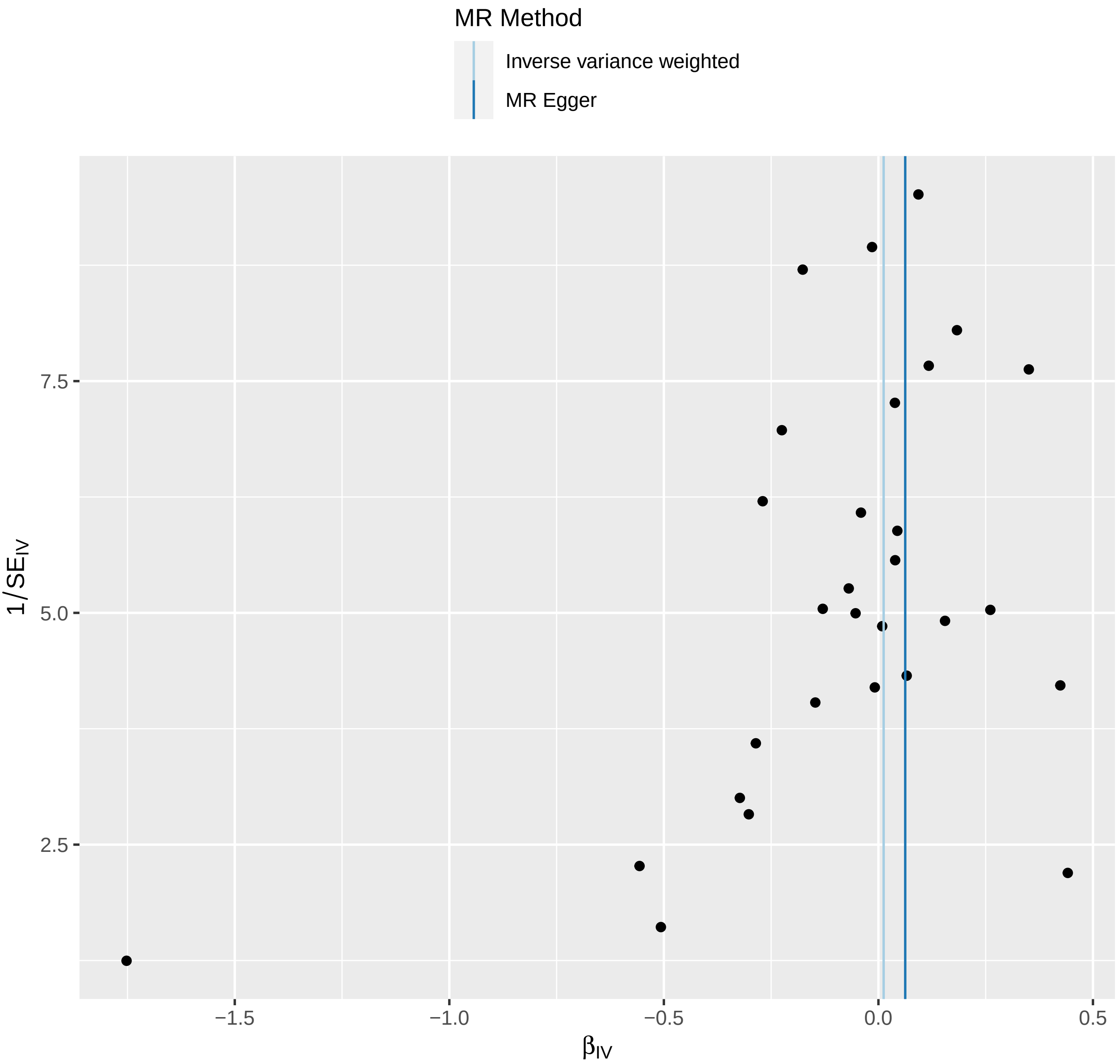

Batch 3−2212 : Proprotein convertase subtilisin/kexin type 7 on Diabetic nephropathy (more control exclusions)
with a threshold of 1e−05

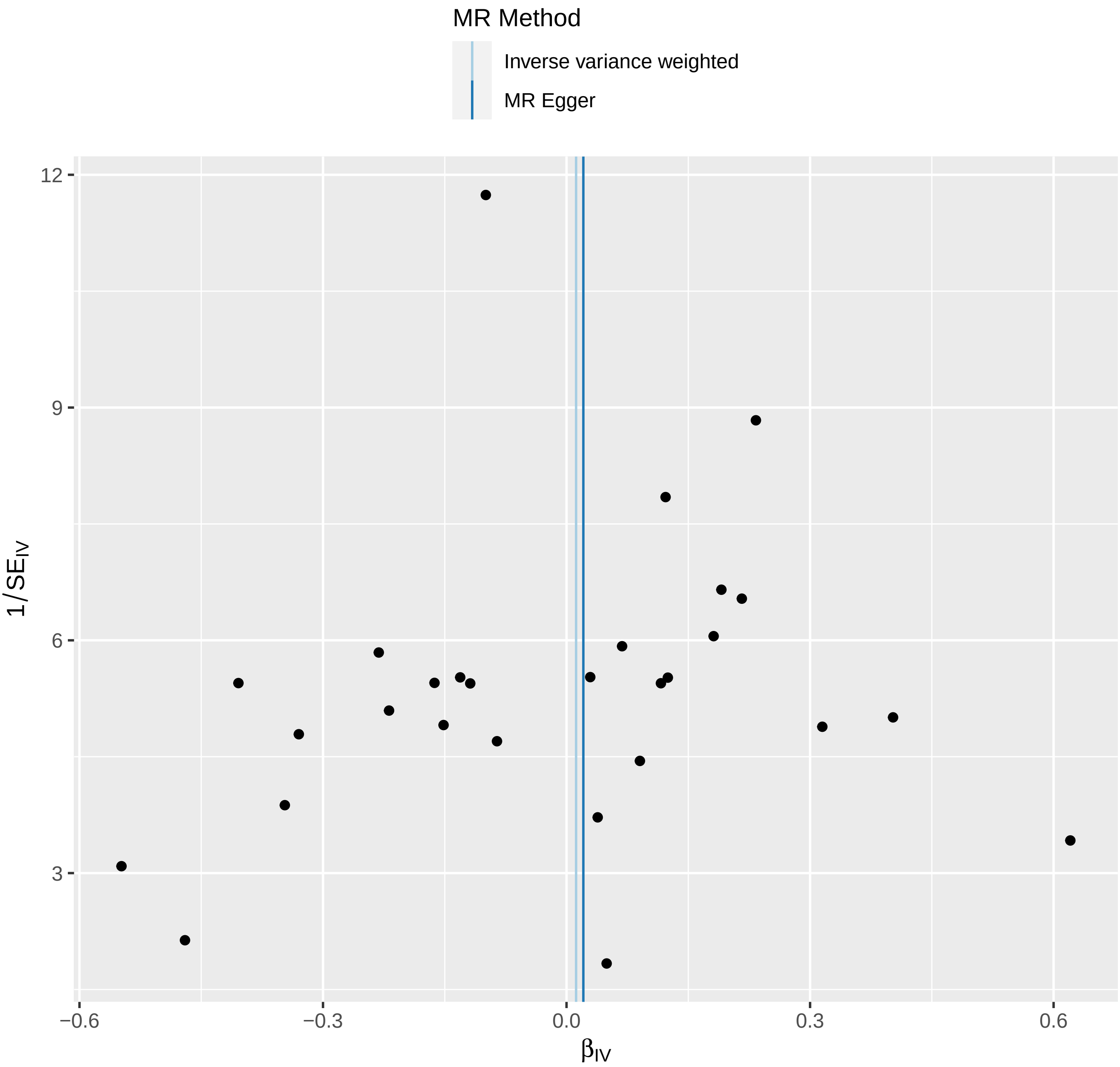

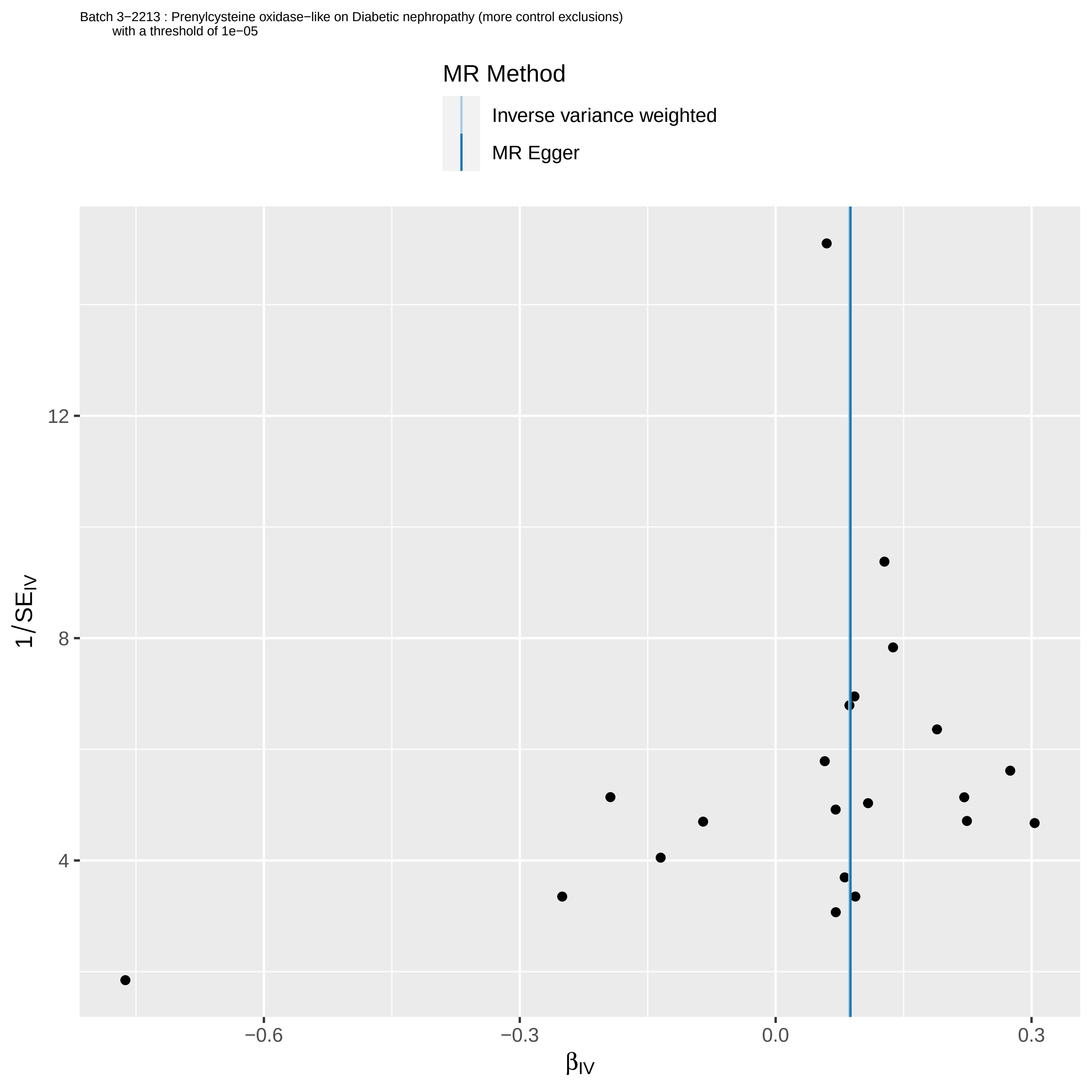

Batch 3−2213 : Prenylcysteine oxidase−like on Diabetic nephropathy (more control exclusions)
with a threshold of 1e−05
MR Method
Inverse variance weighted
MR Egger
12
8
4
1/SE$_{IV}$
−0.6
−0.3
0.0
0.3
β_{IV}

Batch 3−2214 : Programmed cell death protein 1 on Diabetic nephropathy (more control exclusions)
with a threshold of 1e−05

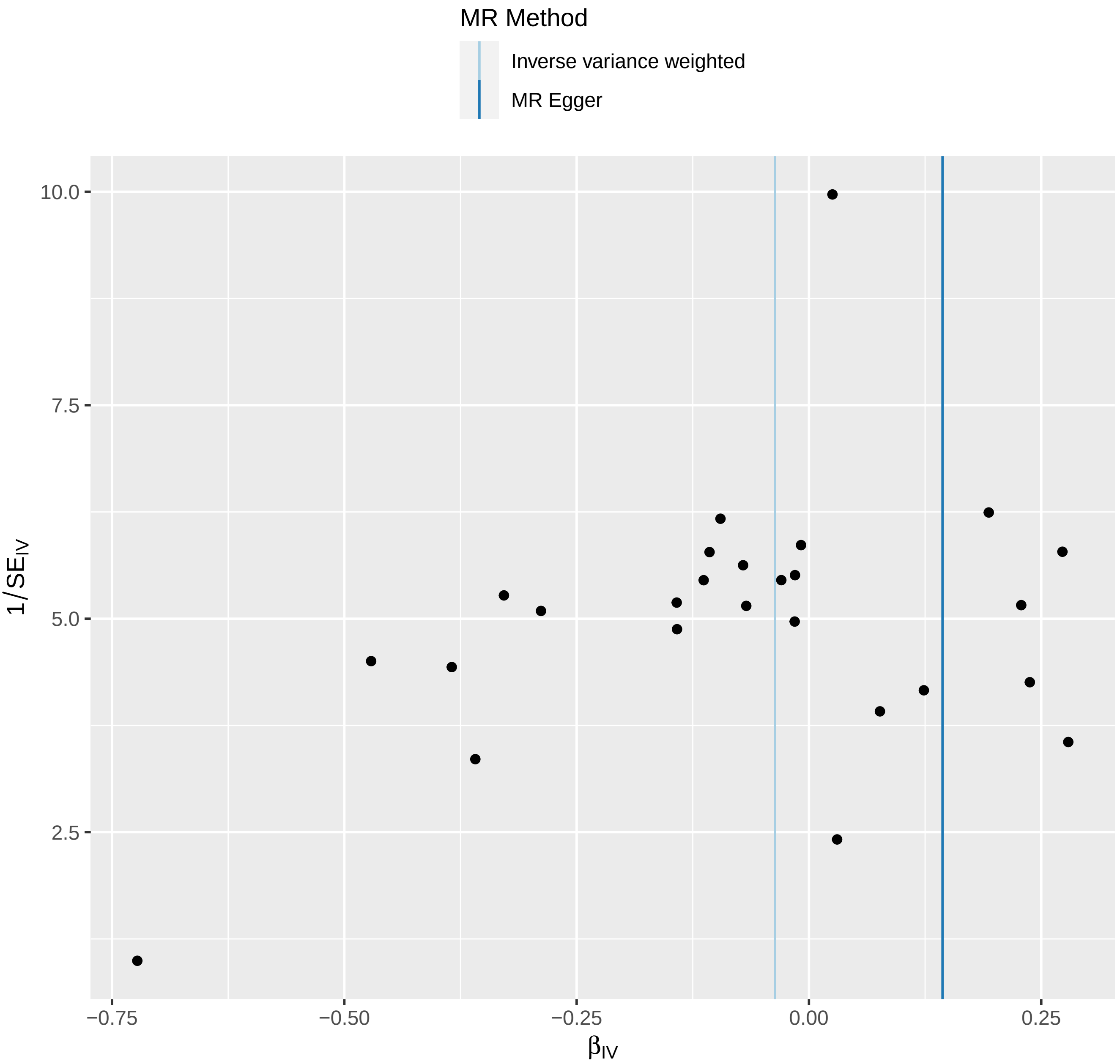

Batch 3−2215 : Programmed cell death 1 ligand 2 on Diabetic nephropathy (more control exclusions)
with a threshold of 1e−05

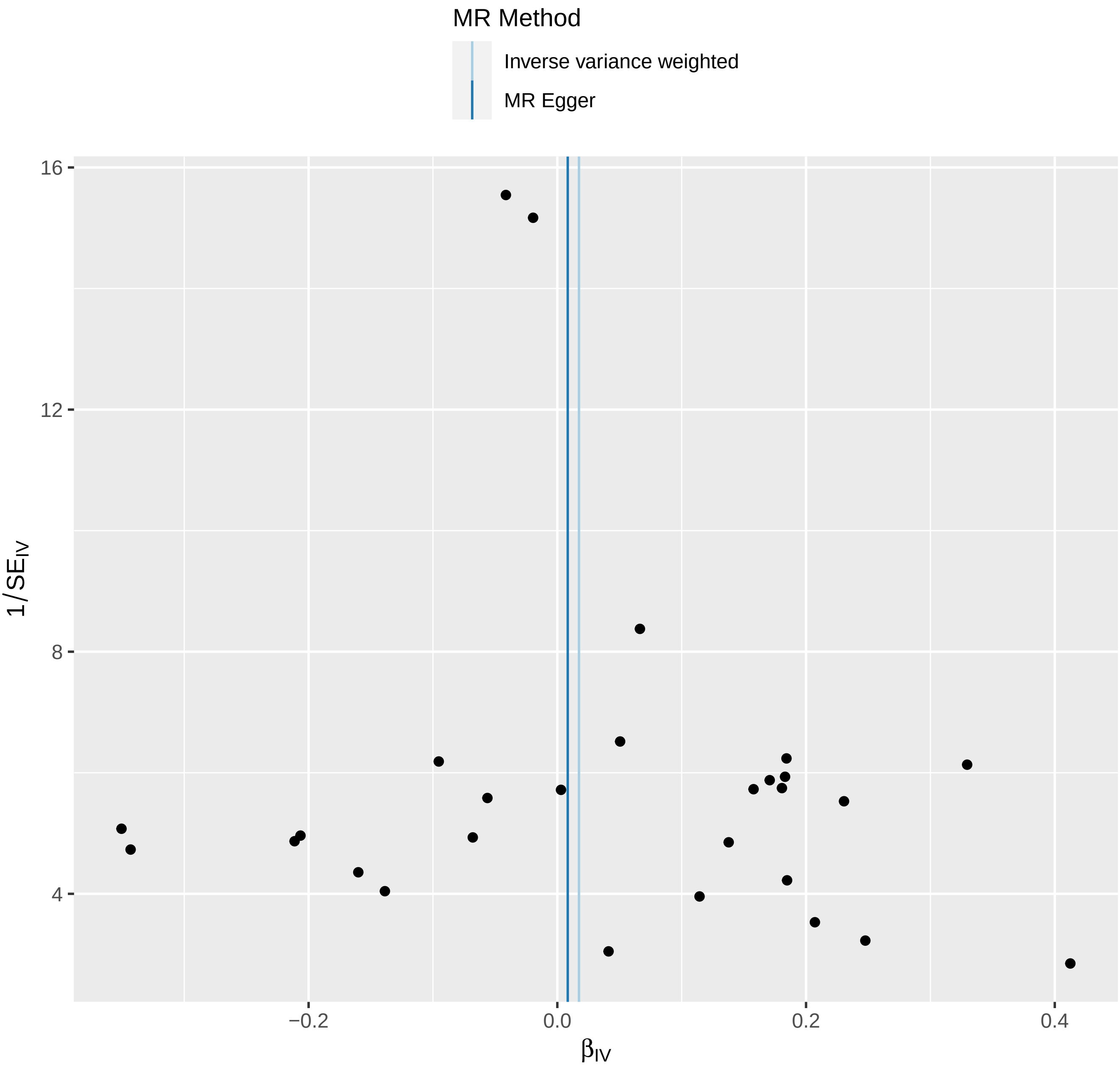

Batch 3−2216 : Programmed cell death protein 5 on Diabetic nephropathy (more control exclusions)
with a threshold of 1e−05

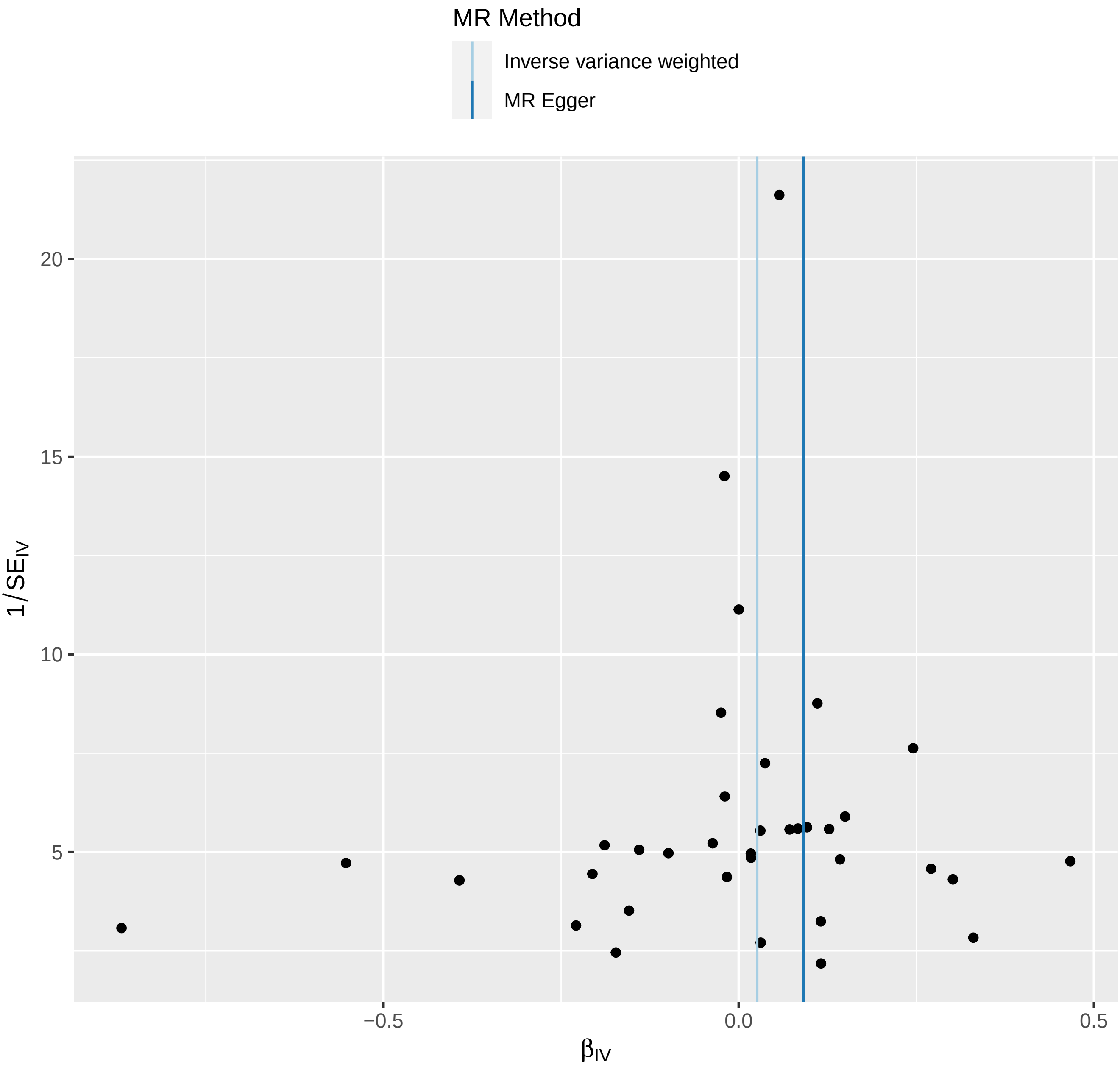

Batch 3−2217 : Phosducin−like protein 2 on Diabetic nephropathy (more control exclusions)
with a threshold of 1e−05

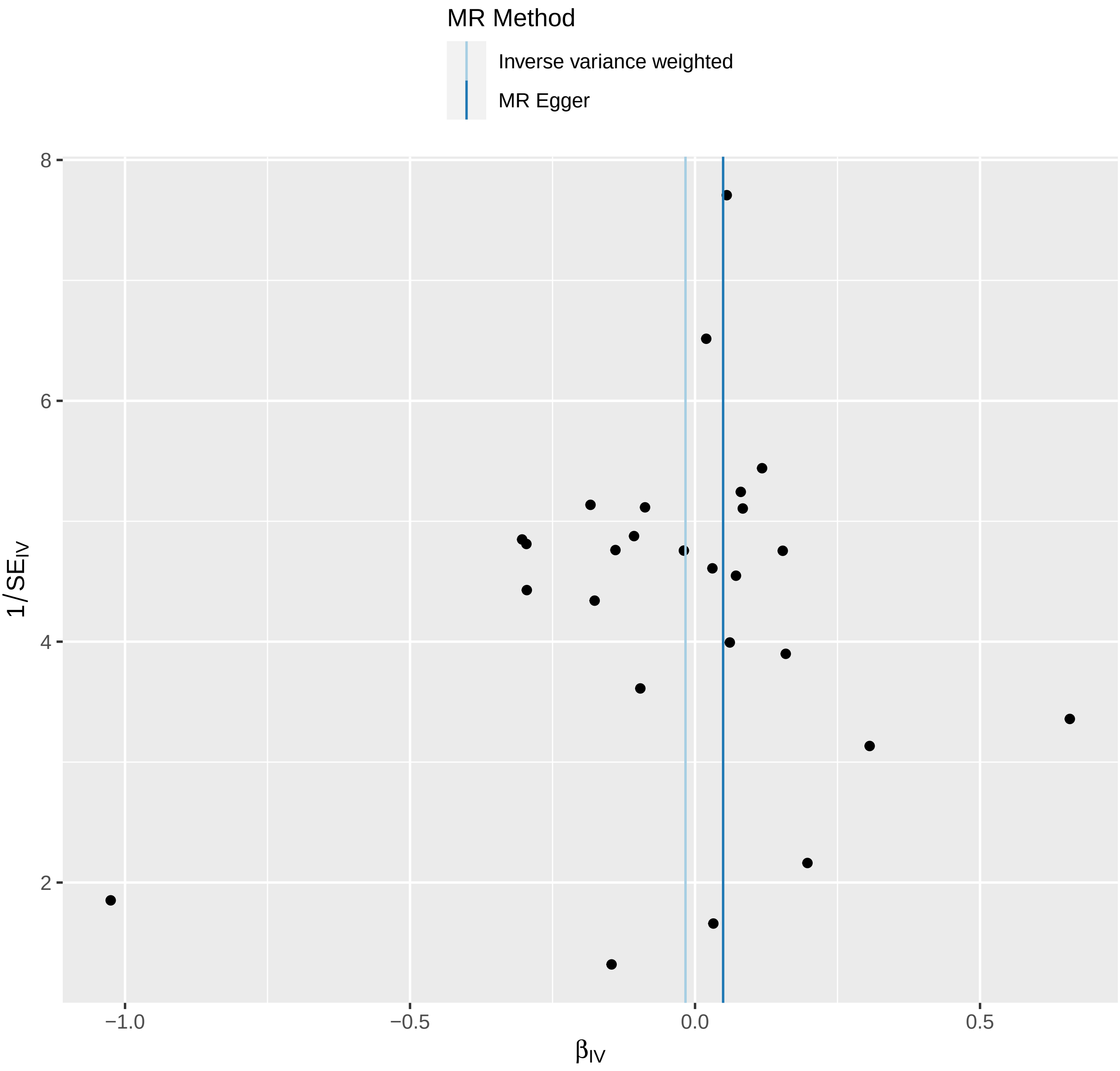

Batch 3−2218 : Calcium/calmodulin−dependent 3',5'−cyclic nucleotide phosphodiesterase 1A on Diabetic nephropathy (more control exclusions) with a threshold of 1e−05

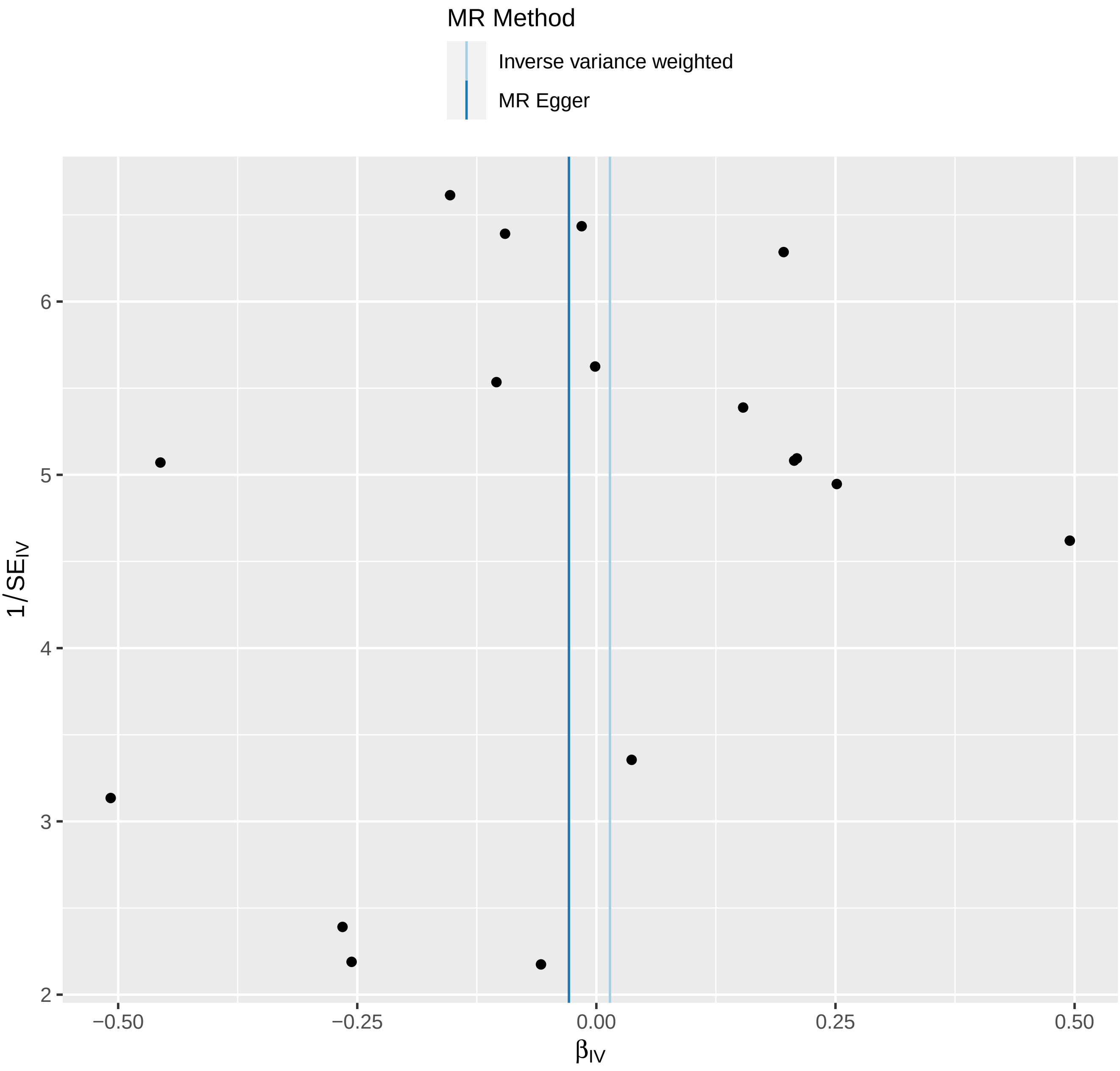

Batch 3−2219 : cGMP−dependent 3',5'−cyclic phosphodiesterase on Diabetic nephropathy (more control exclusions) with a threshold of 1e−05

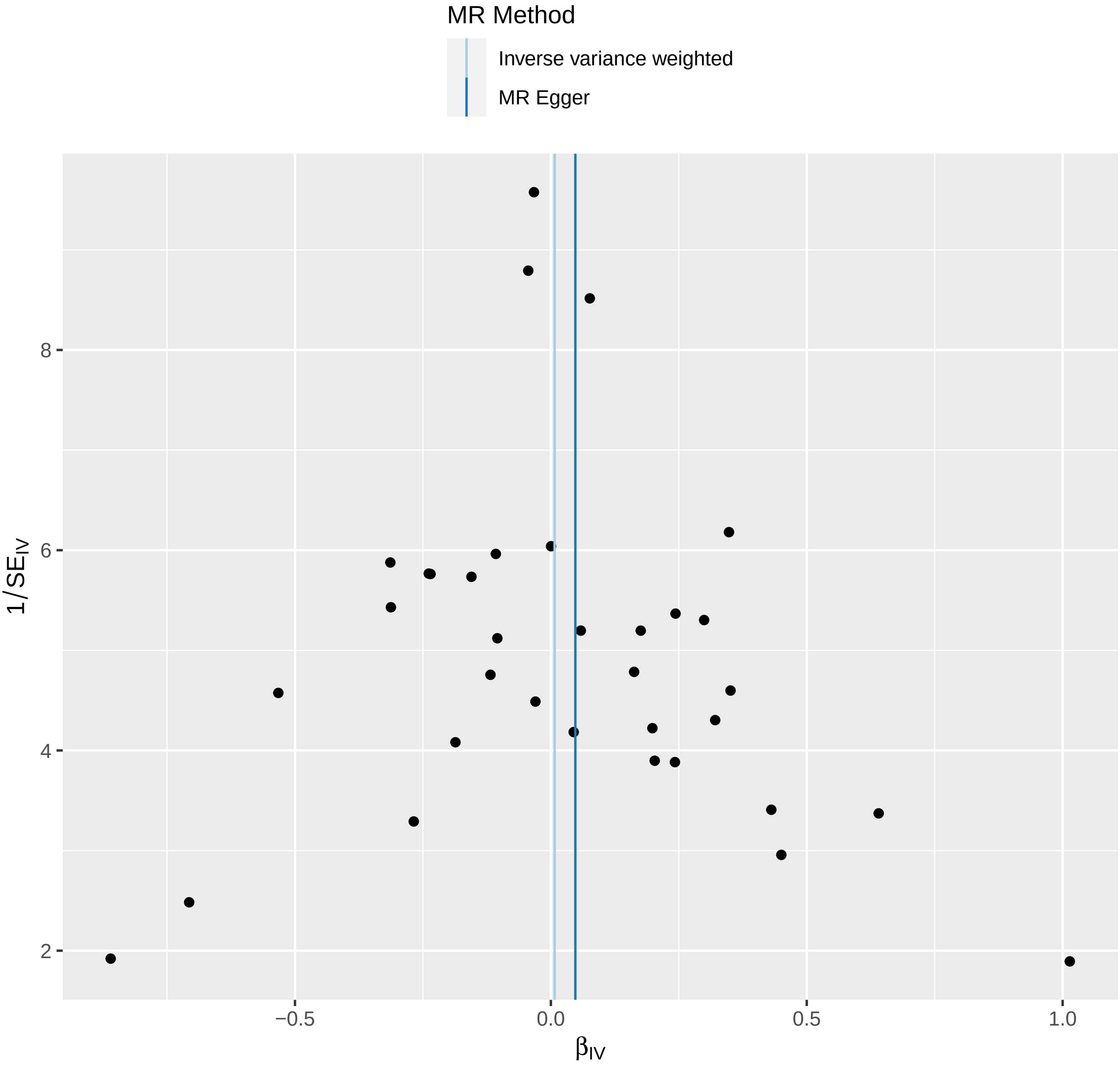

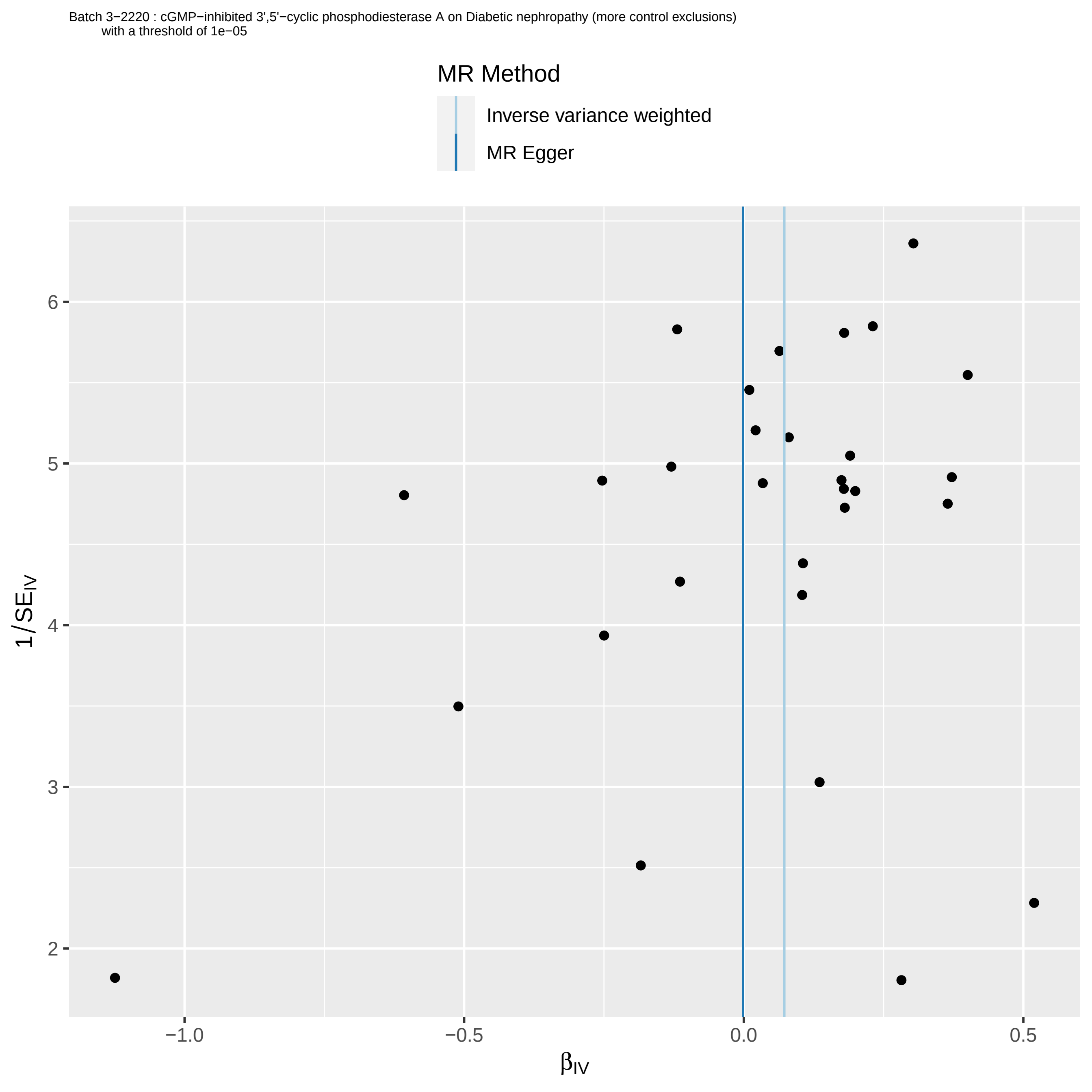
Batch 3−2220 : cGMP−inhibited 3',5'−cyclic phosphodiesterase A on Diabetic nephropathy (more control exclusions)
with a threshold of 1e−05
MR Method
Inverse variance weighted
MR Egger
1/SE_IV
6
5
4
3
2
−1.0
−0.5
0.0
0.5
β_IV

Batch 3−2221 : cAMP−specific 3',5'−cyclic phosphodiesterase 4D on Diabetic nephropathy (more control exclusions)
with a threshold of 1e−05

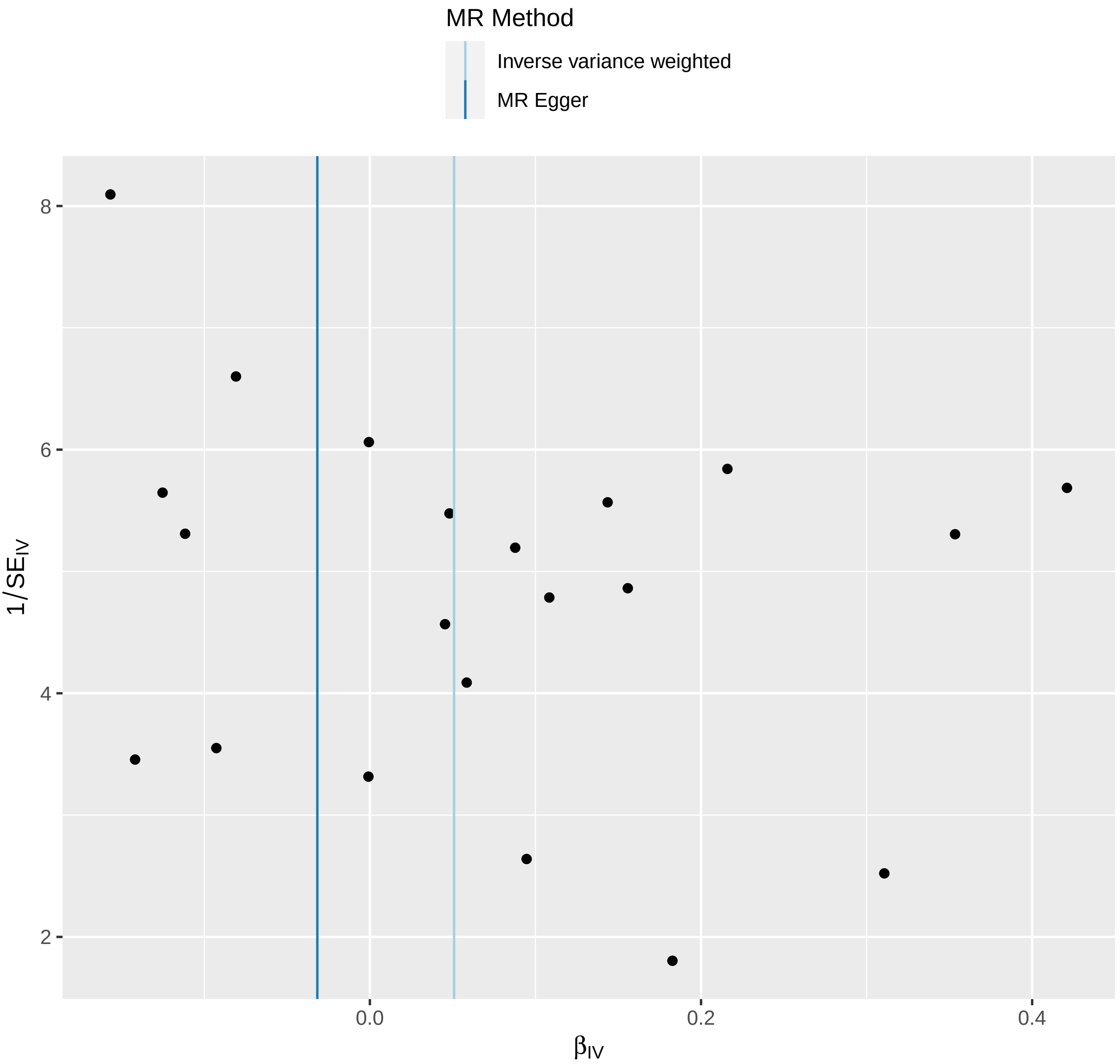

Batch 3−2222 : Retinal rod rhodopsin−sensitive cGMP 3',5'−cyclic phosphodiesterase subunit delta on Diabetic nephropathy (more control exclusions)
with a threshold of 1e−05

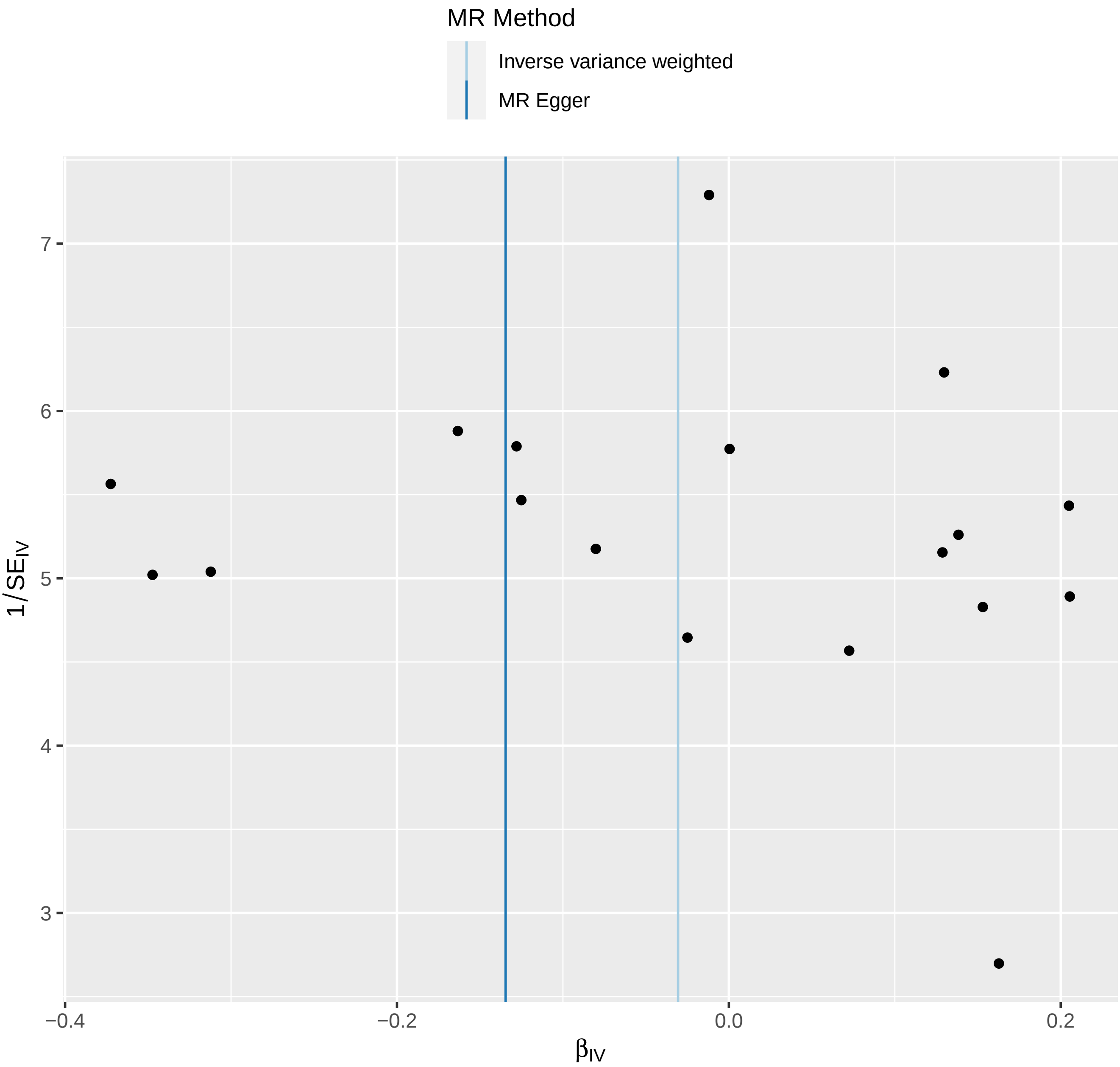

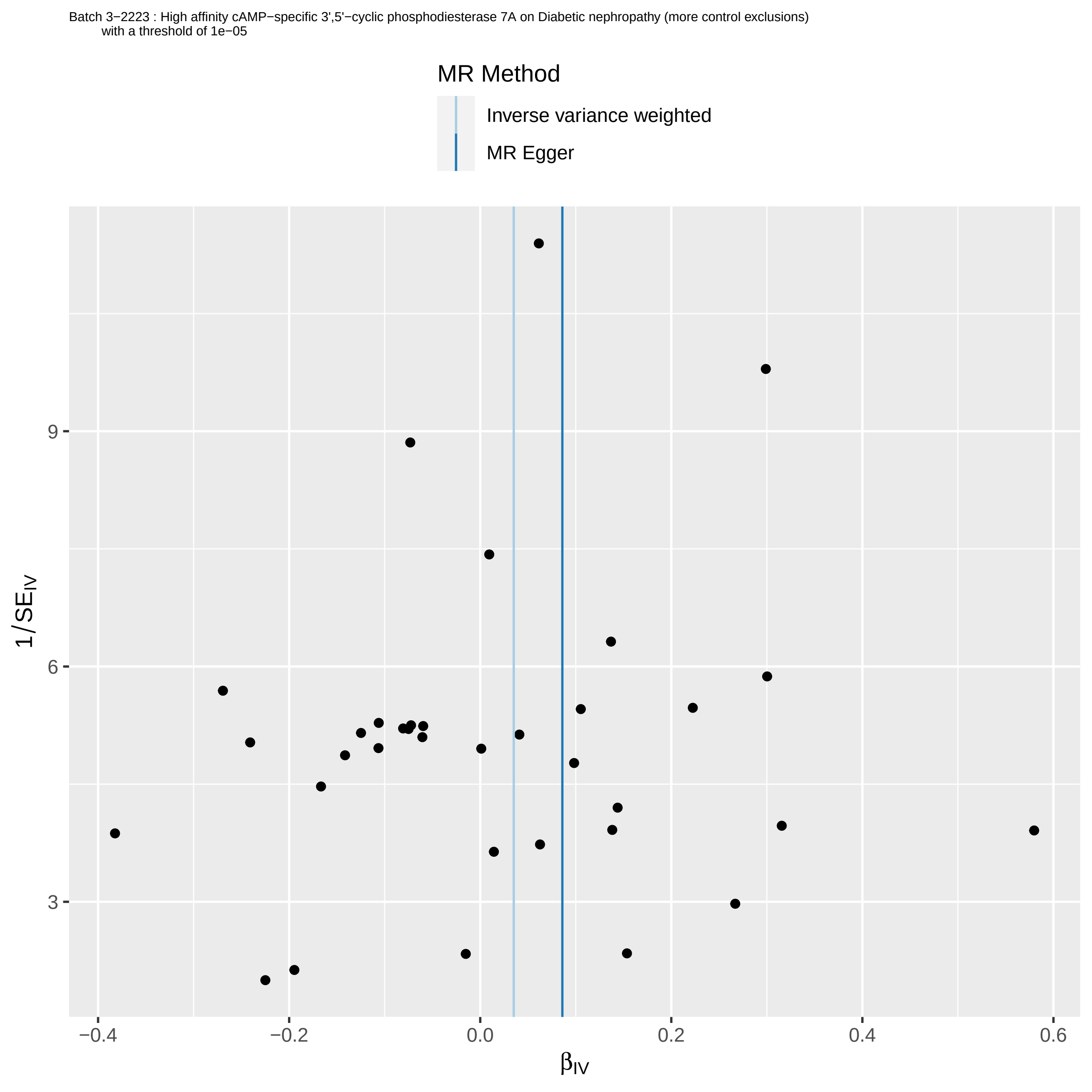
Batch 3−2223 : High affinity cAMP−specific 3',5'−cyclic phosphodiesterase 7A on Diabetic nephropathy (more control exclusions)
with a threshold of 1e−05
MR Method
Inverse variance weighted
MR Egger
9
6
3
$1/SE_{IV}$
−0.4
−0.2
0.0
0.2
0.4
0.6
β_{IV}

Batch 3−2224 : High affinity cGMP−specific 3',5'−cyclic phosphodiesterase 9A on Diabetic nephropathy (more control exclusions)
with a threshold of 1e−05

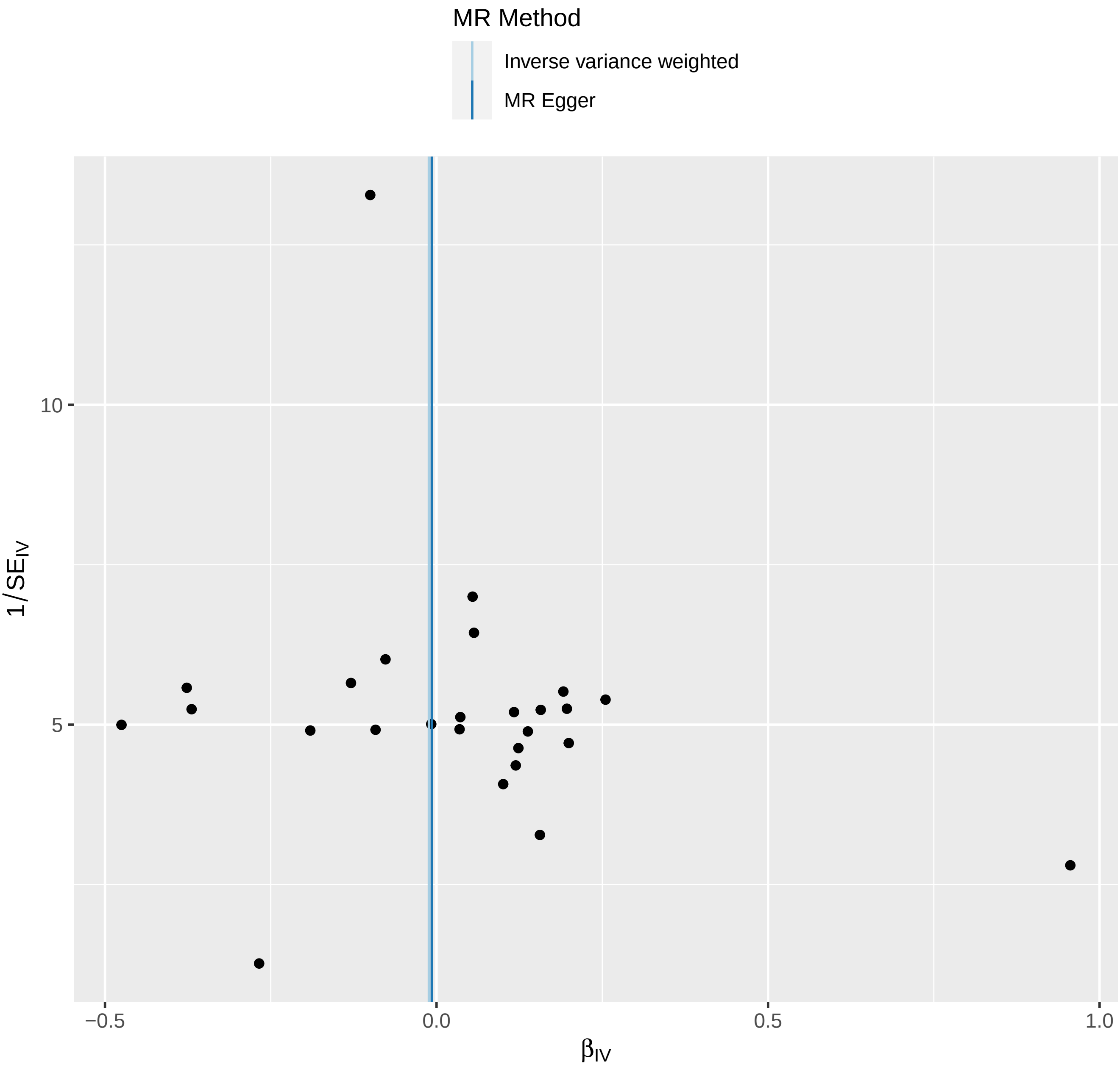

Batch 3−2225 : Platelet−derived growth factor subunit A on Diabetic nephropathy (more control exclusions)
with a threshold of 1e−05

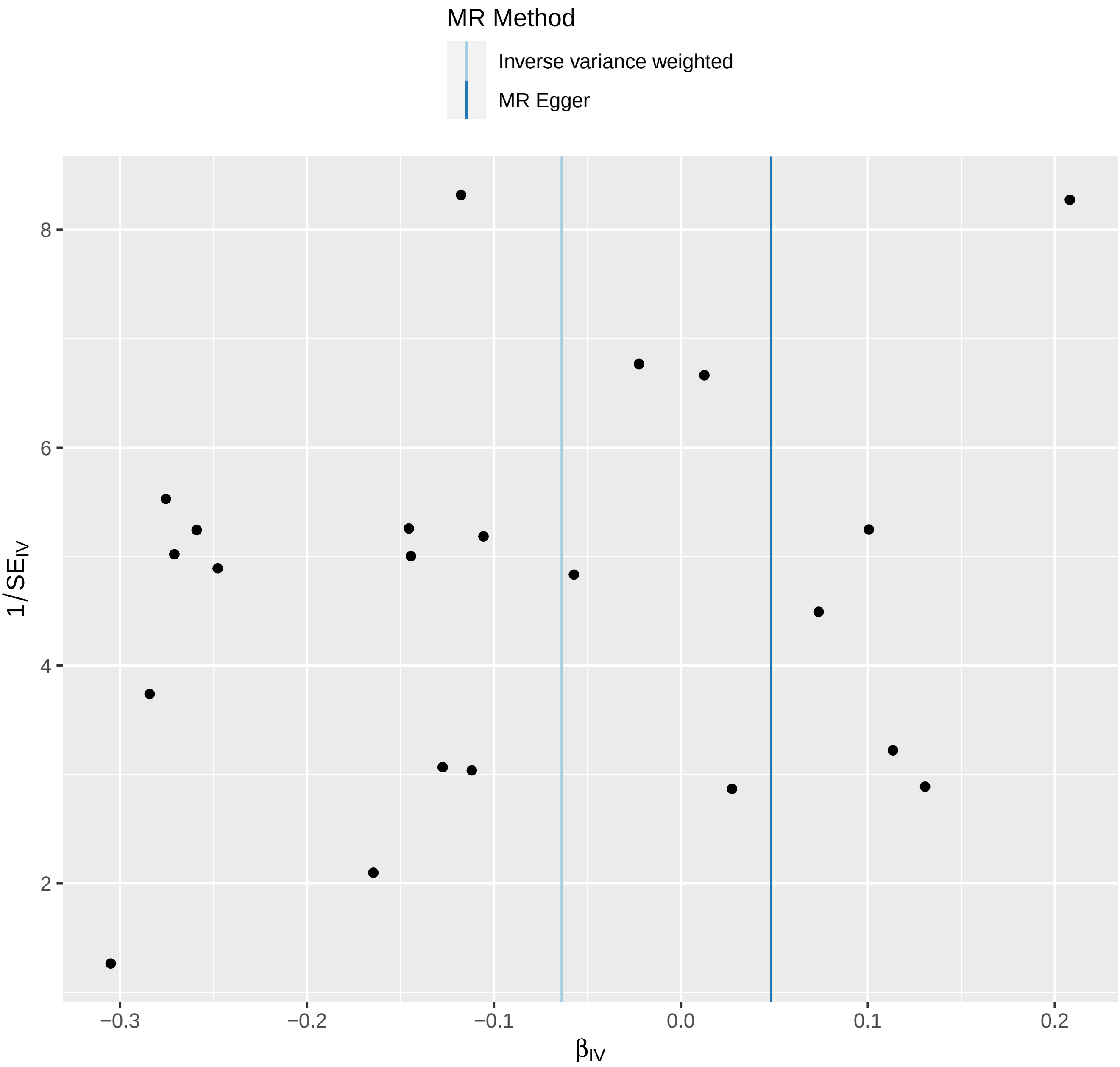

Batch 3−2226 : Platelet−derived growth factor subunit B on Diabetic nephropathy (more control exclusions)
with a threshold of 1e−05

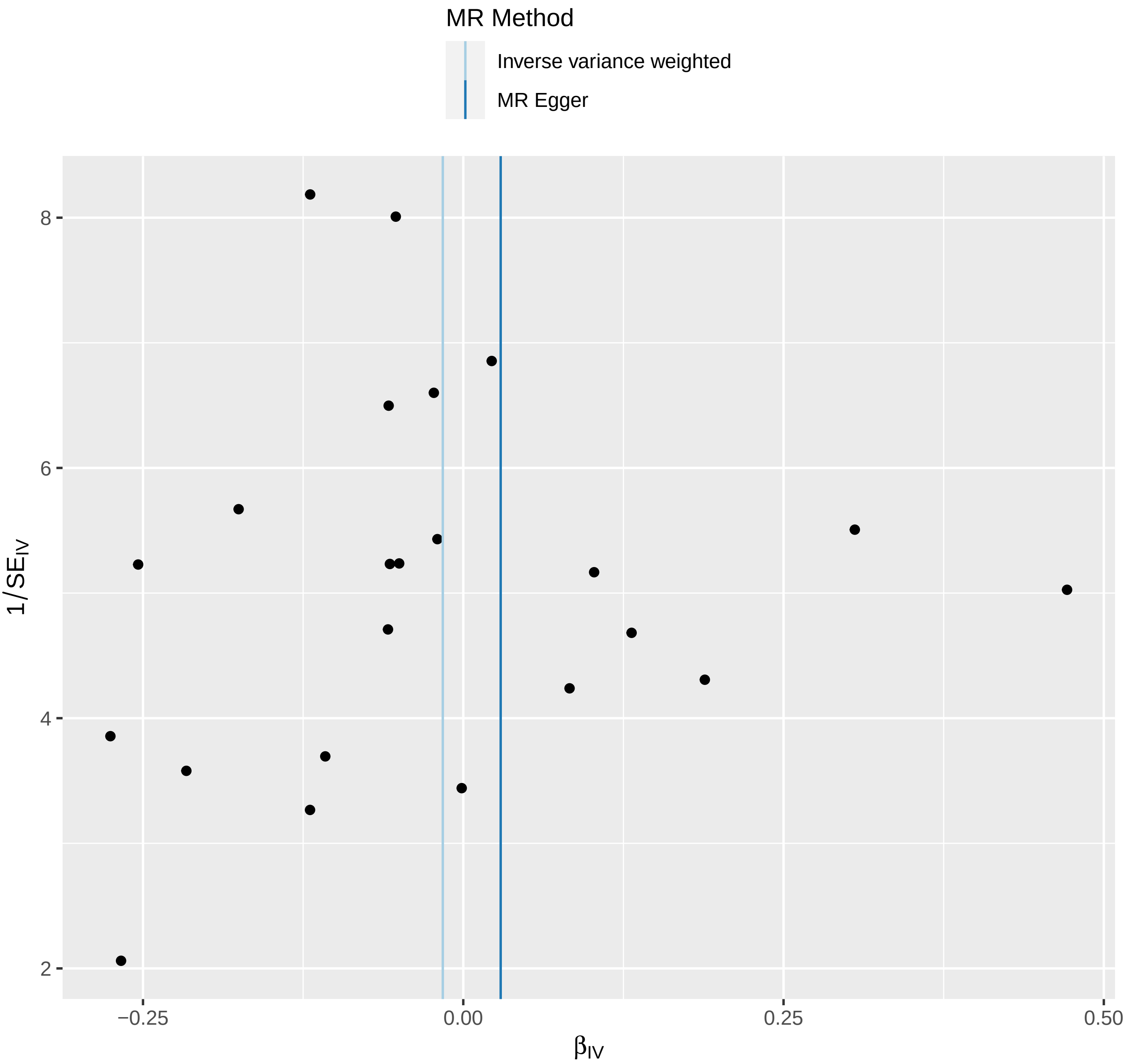

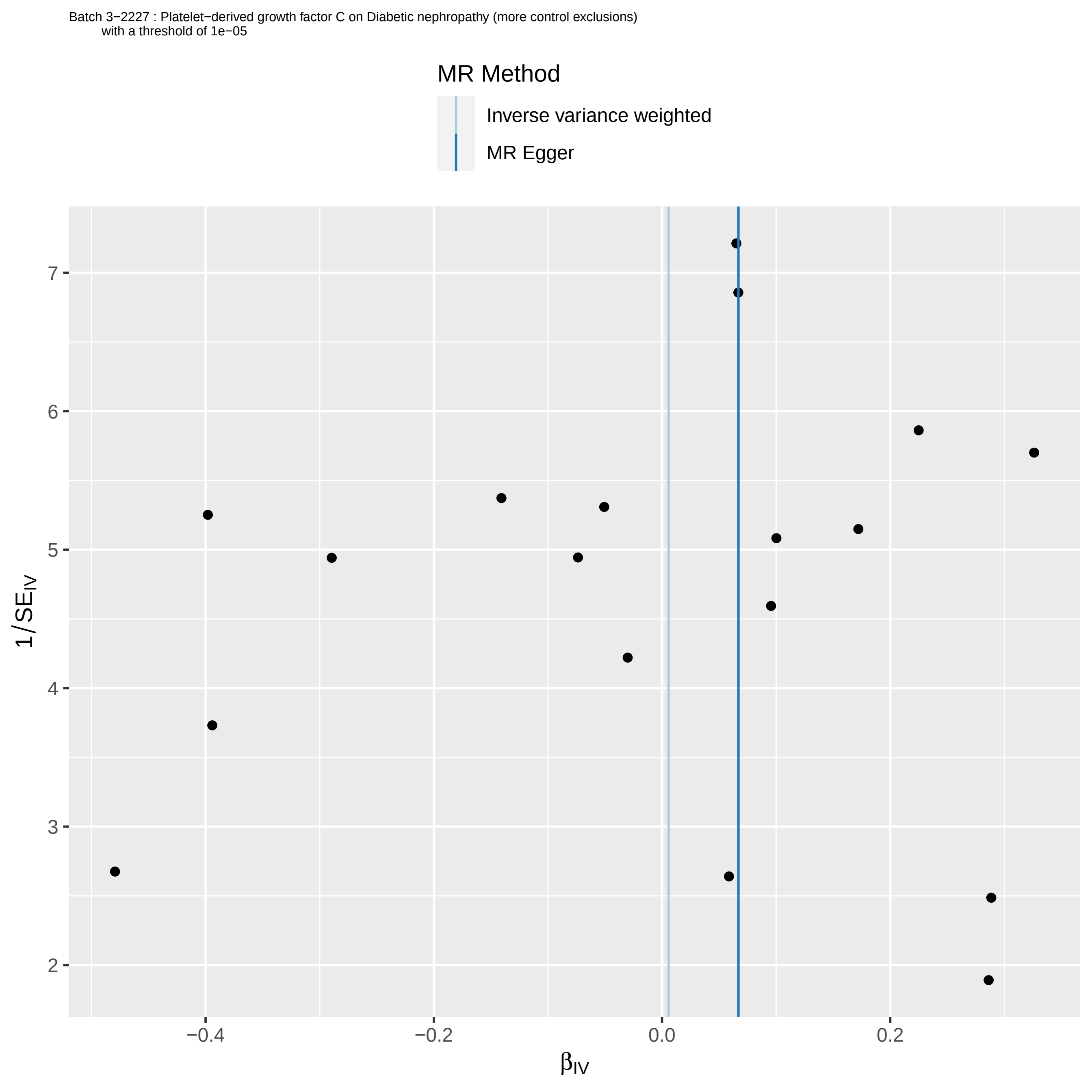

Batch 3−2227 : Platelet−derived growth factor C on Diabetic nephropathy (more control exclusions)
with a threshold of 1e−05
MR Method
Inverse variance weighted
MR Egger
7
6
5
4
3
2
1/SE_IV
−0.4
−0.2
0.0
0.2
β_IV

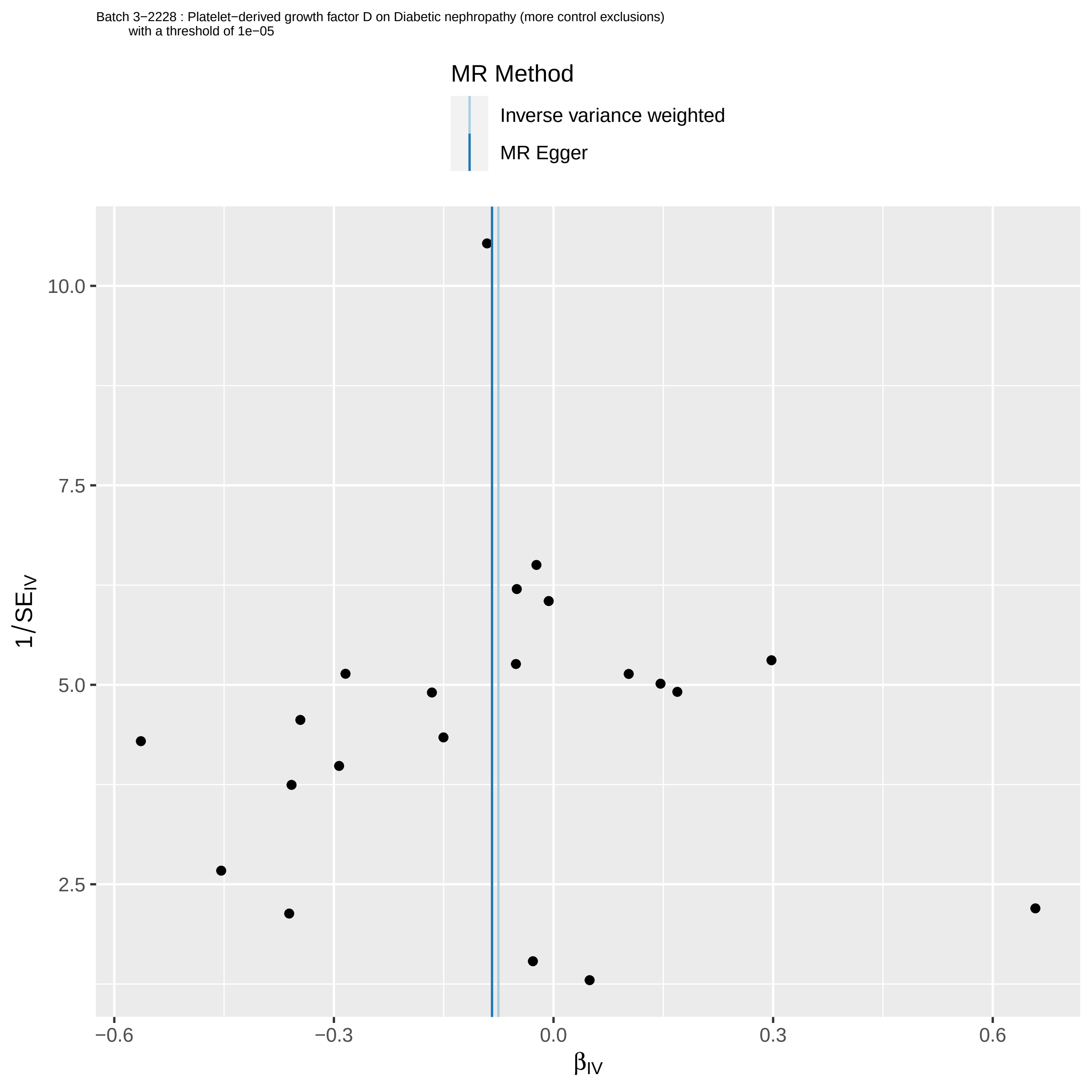

Batch 3−2228 : Platelet−derived growth factor D on Diabetic nephropathy (more control exclusions)
with a threshold of 1e−05
MR Method
Inverse variance weighted
MR Egger
10.0
7.5
5.0
2.5
1/SE_IV
−0.6
−0.3
0.0
0.3
0.6
β_IV

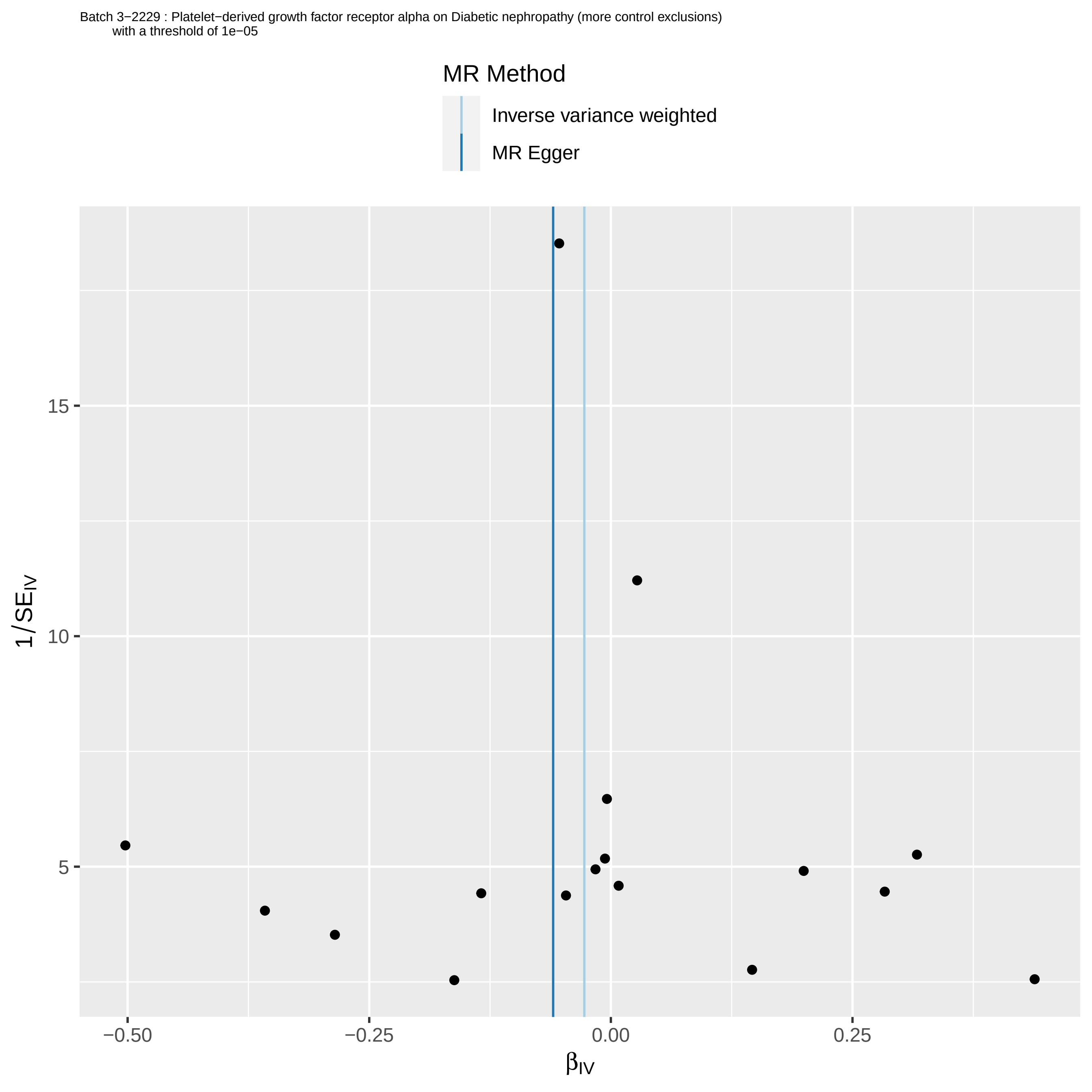
Batch 3−2229 : Platelet−derived growth factor receptor alpha on Diabetic nephropathy (more control exclusions)
with a threshold of 1e−05
MR Method
Inverse variance weighted
MR Egger
15
10
5
1/SE$_{IV}$
−0.50
−0.25
0.00
0.25
β_{IV}

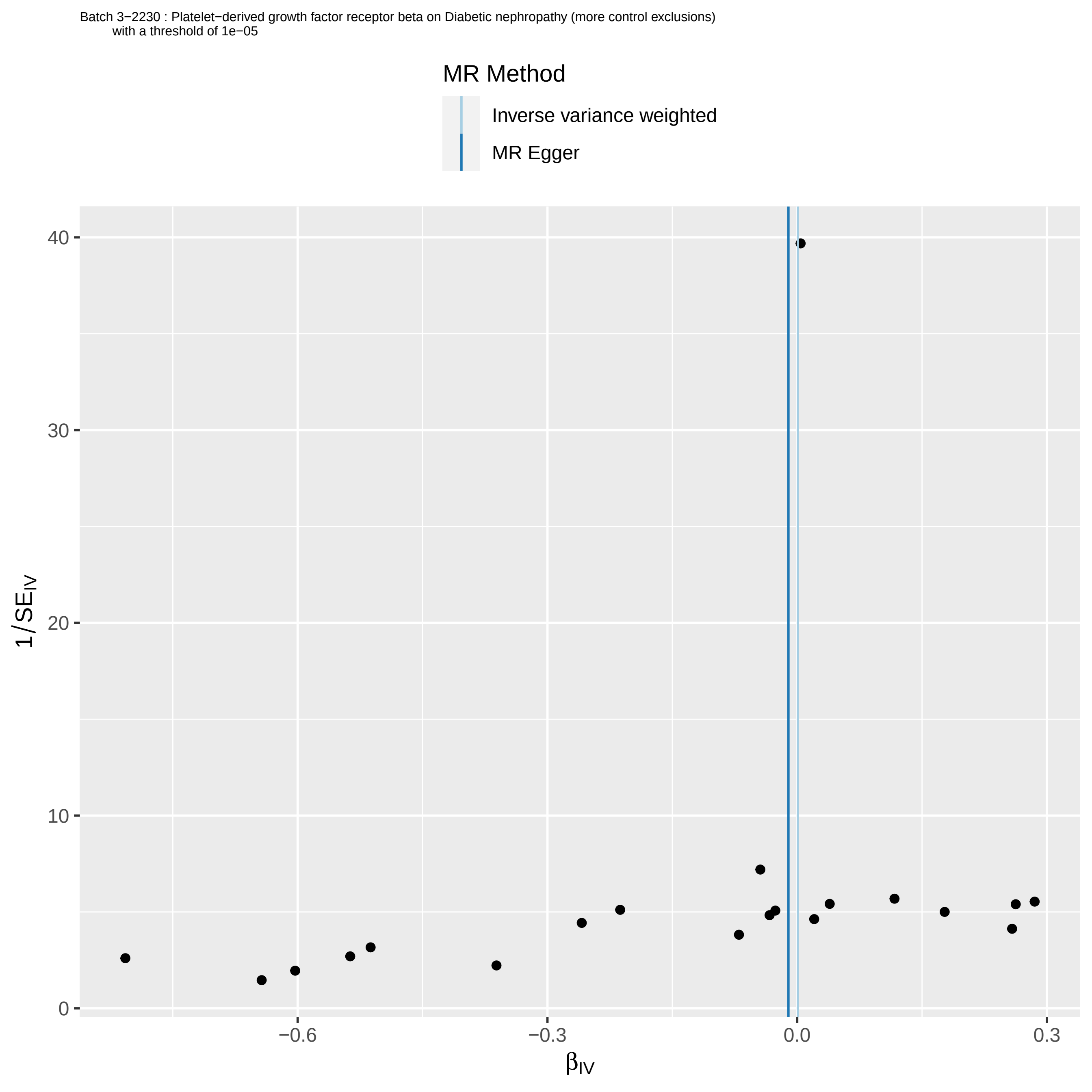
Batch 3−2230 : Platelet−derived growth factor receptor beta on Diabetic nephropathy (more control exclusions)
with a threshold of 1e−05
MR Method
Inverse variance weighted
MR Egger
40
30
20
10
0
1/SE$_{IV}$
−0.6
−0.3
0.0
0.3
β$_{IV}$

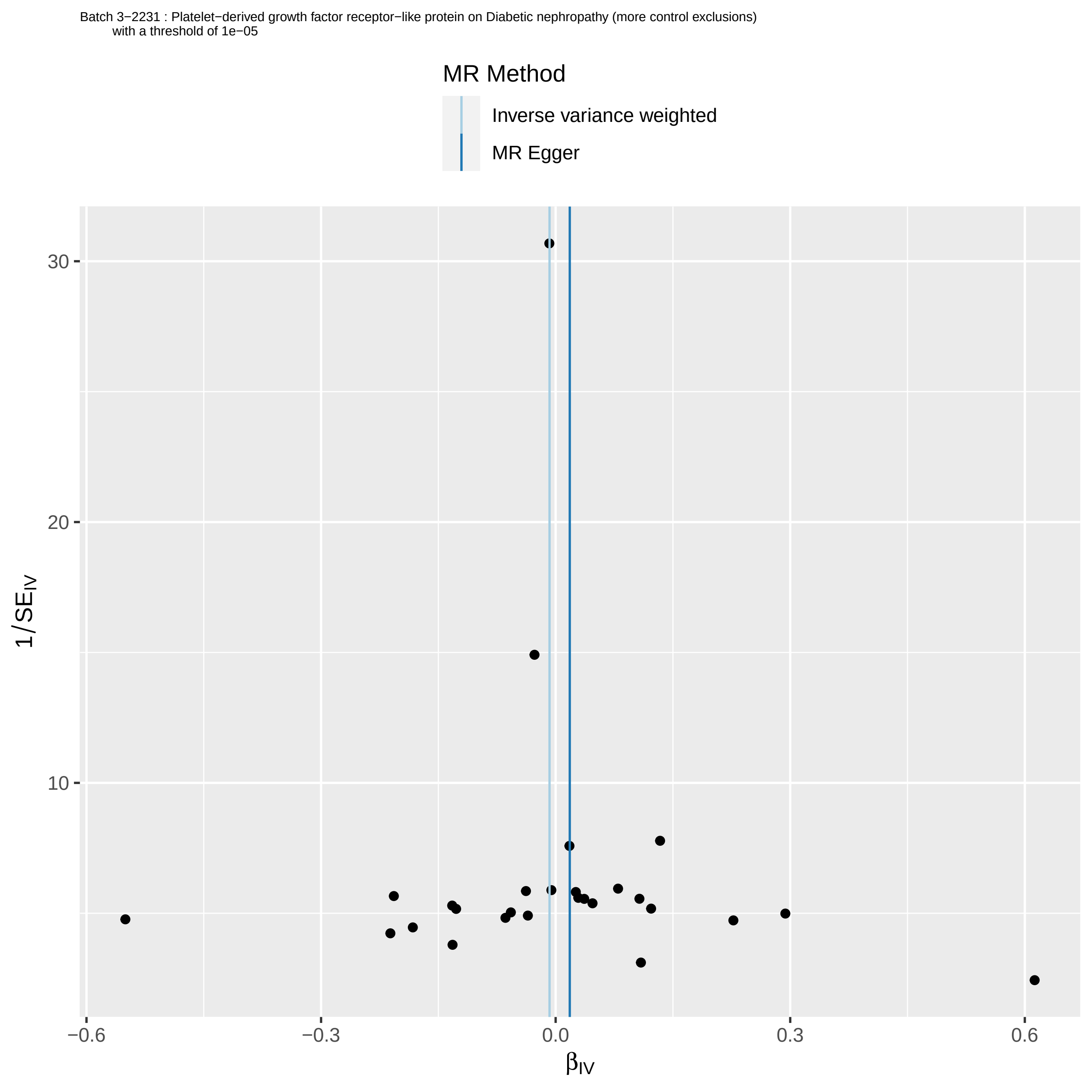
Batch 3−2231 : Platelet−derived growth factor receptor−like protein on Diabetic nephropathy (more control exclusions)
with a threshold of 1e−05
MR Method
Inverse variance weighted
MR Egger
30
20
10
$1/SE_{IV}$
−0.6
−0.3
0.0
0.3
0.6
β_{IV}

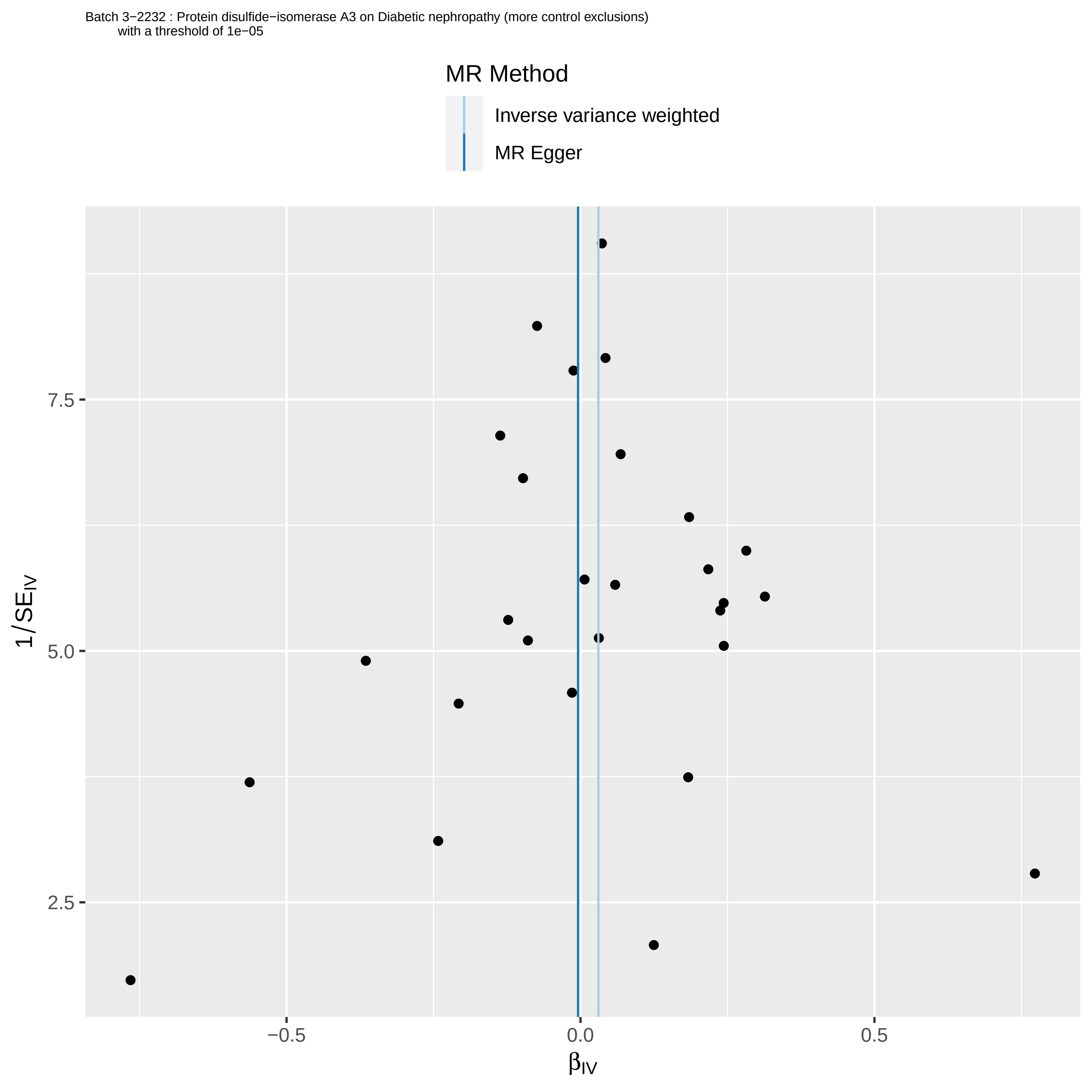

Batch 3−2232 : Protein disulfide−isomerase A3 on Diabetic nephropathy (more control exclusions)
with a threshold of 1e−05
MR Method
Inverse variance weighted
MR Egger
7.5
5.0
2.5
1/SE_IV
−0.5
0.0
0.5
β_IV

Batch 3−2233 : Protein disulfide−isomerase A5 on Diabetic nephropathy (more control exclusions)
with a threshold of 1e−05

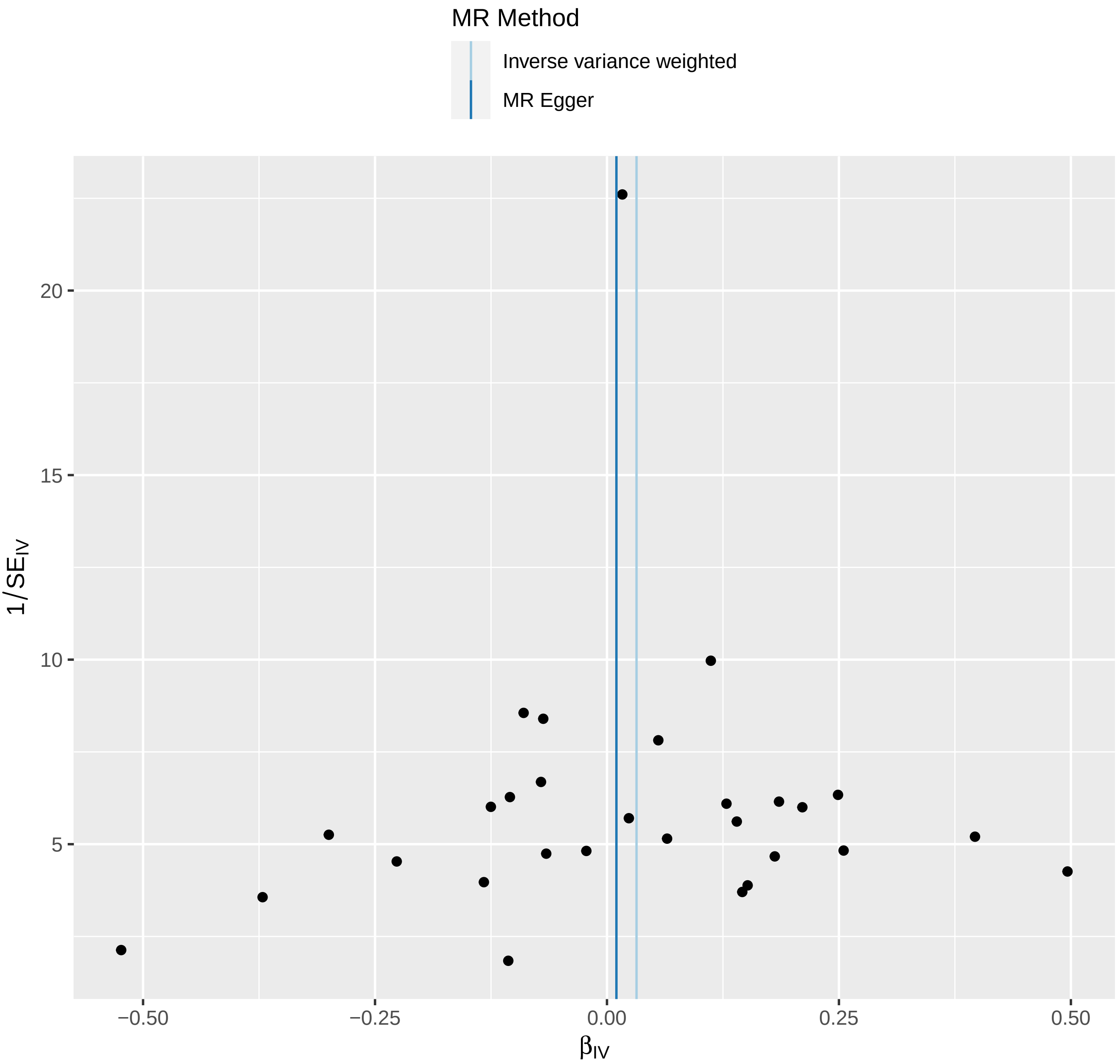

Batch 3−2234 : Protein disulfide−isomerase−like protein of the testis on Diabetic nephropathy (more control exclusions) with a threshold of 1e−05

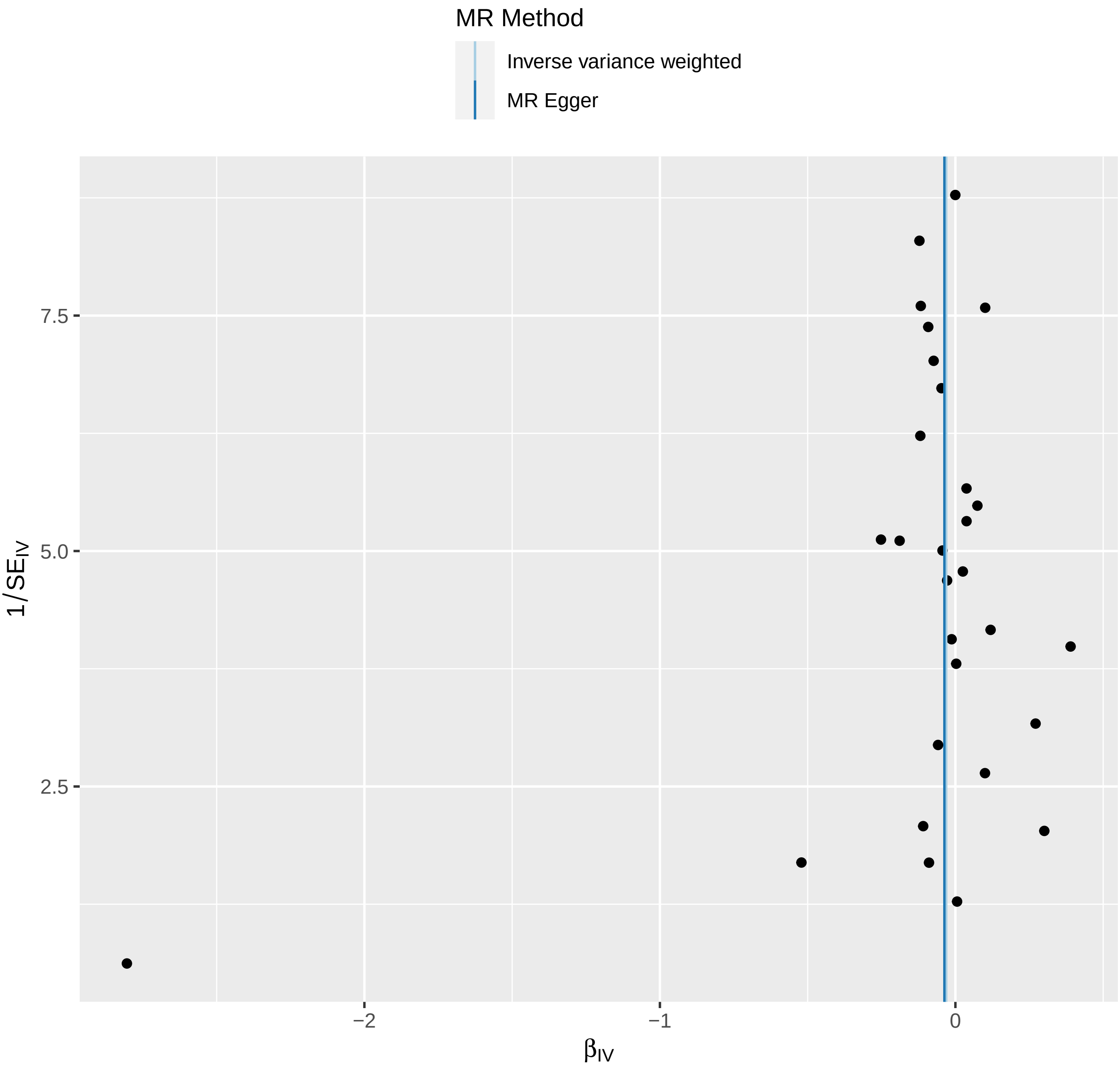

Batch 3−2235 : [Pyruvate dehydrogenase (acetyl−transferring)] kinase isozyme 1, mitochondrial on Diabetic nephropathy (more control exclusions) with a threshold of 1e−05

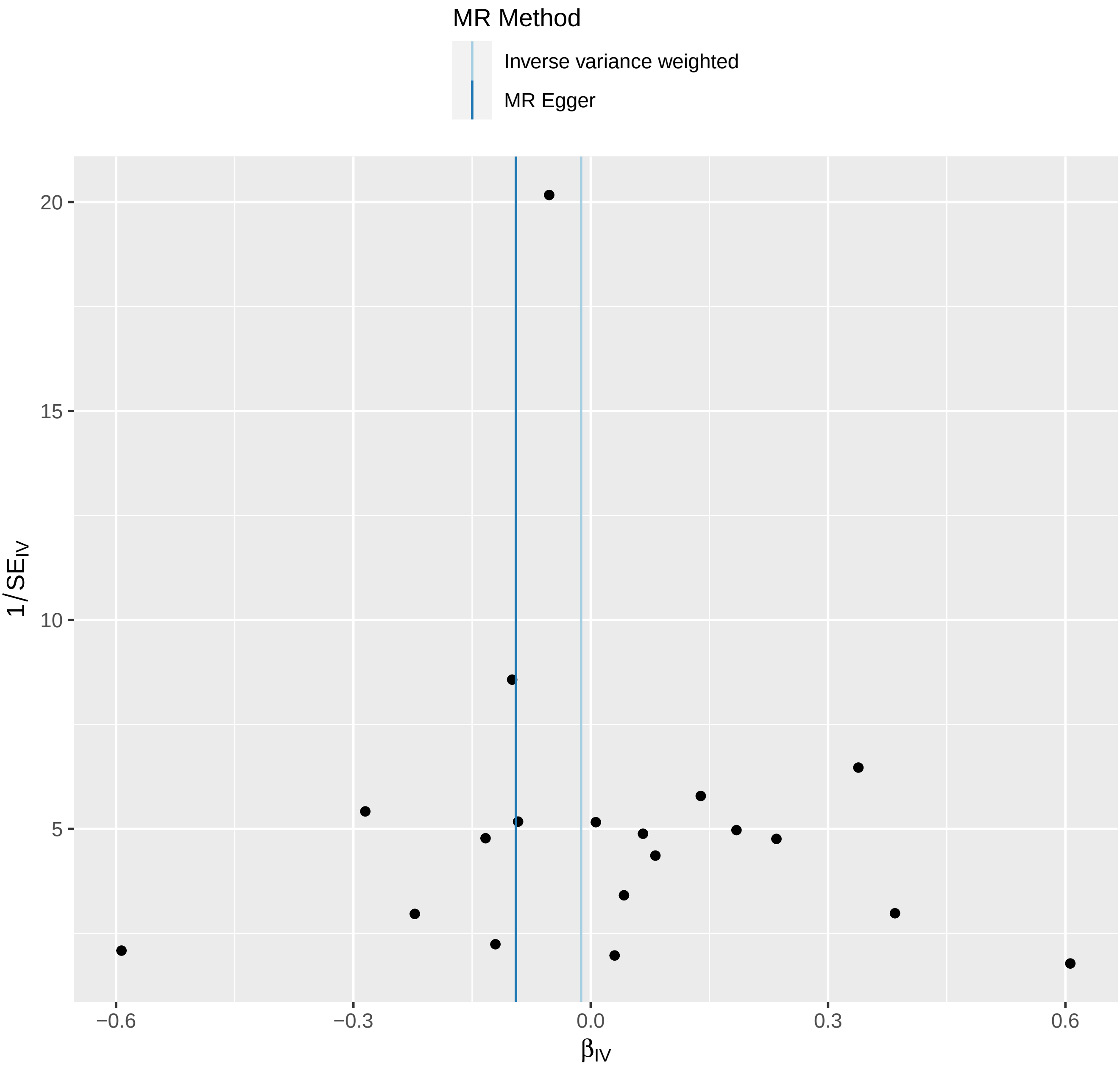

Batch 3−2236 : [Pyruvate dehydrogenase (acetyl−transferring)] kinase isozyme 2, mitochondrial on Diabetic nephropathy (more control exclusions) with a threshold of 1e−05

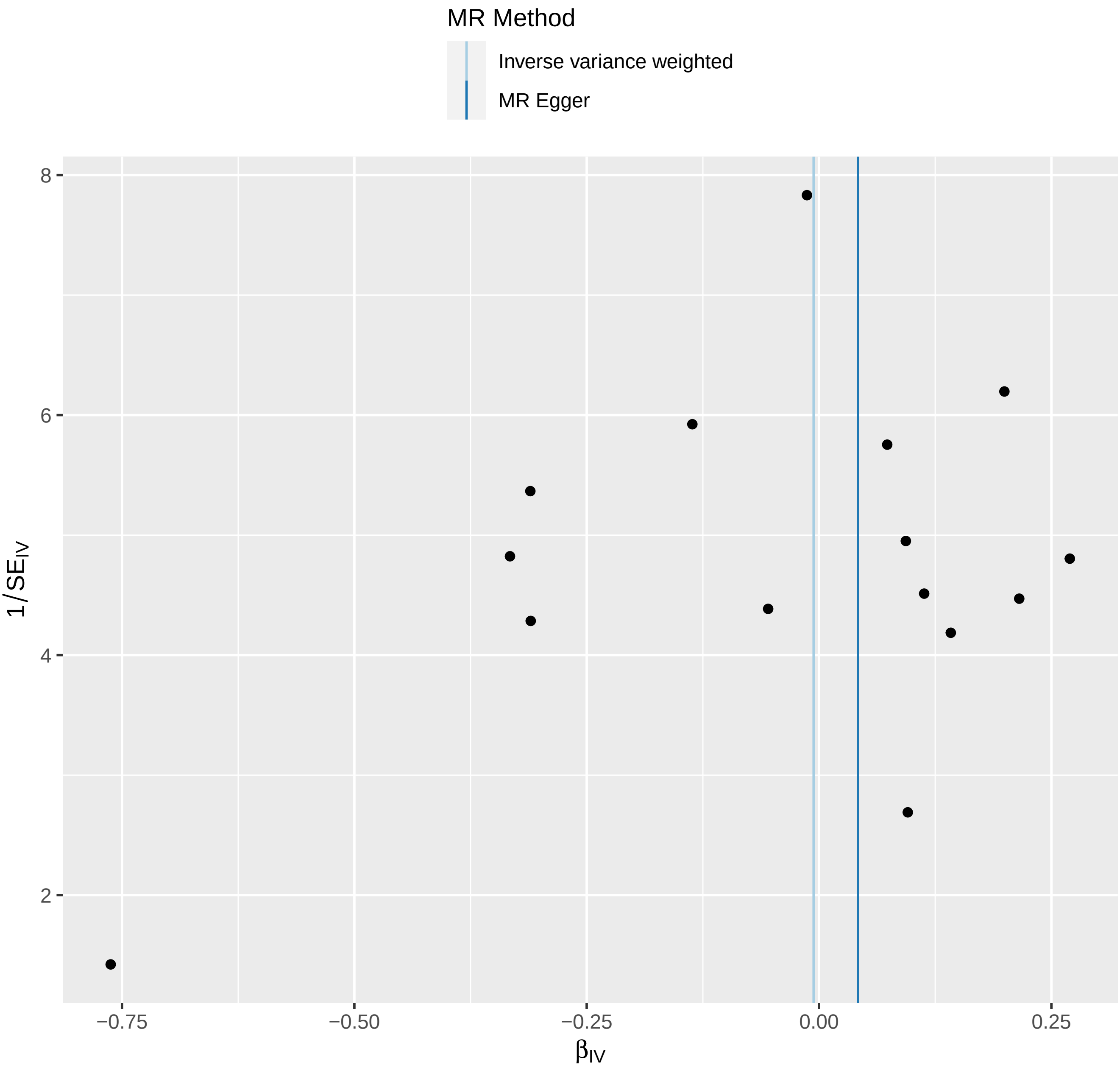

Batch 3−2237 : PDZ and LIM domain protein 4 on Diabetic nephropathy (more control exclusions)
with a threshold of 1e−05

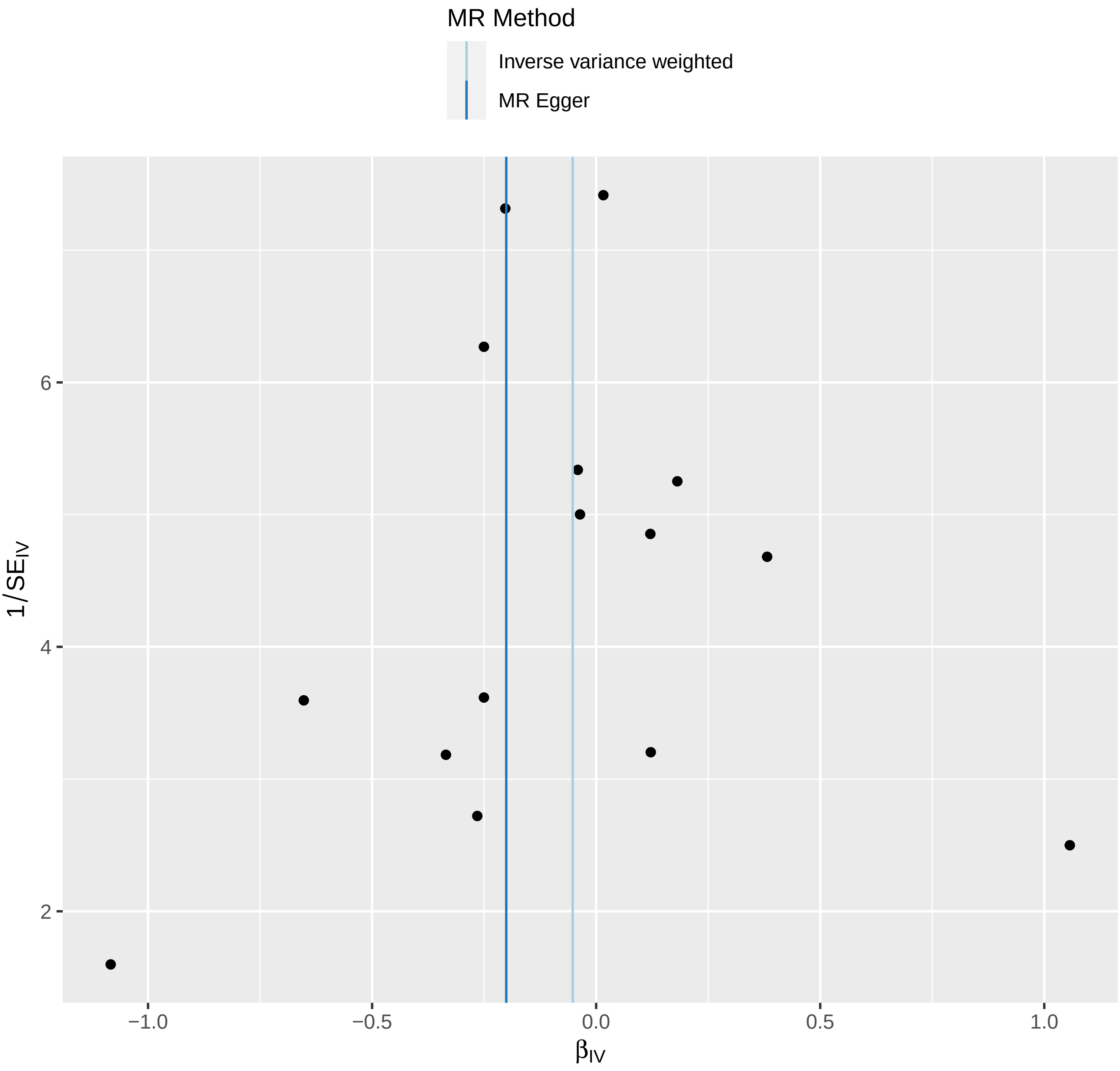

Batch 3−2238 : p53 and DNA damage−regulated protein 1 on Diabetic nephropathy (more control exclusions)
with a threshold of 1e−05

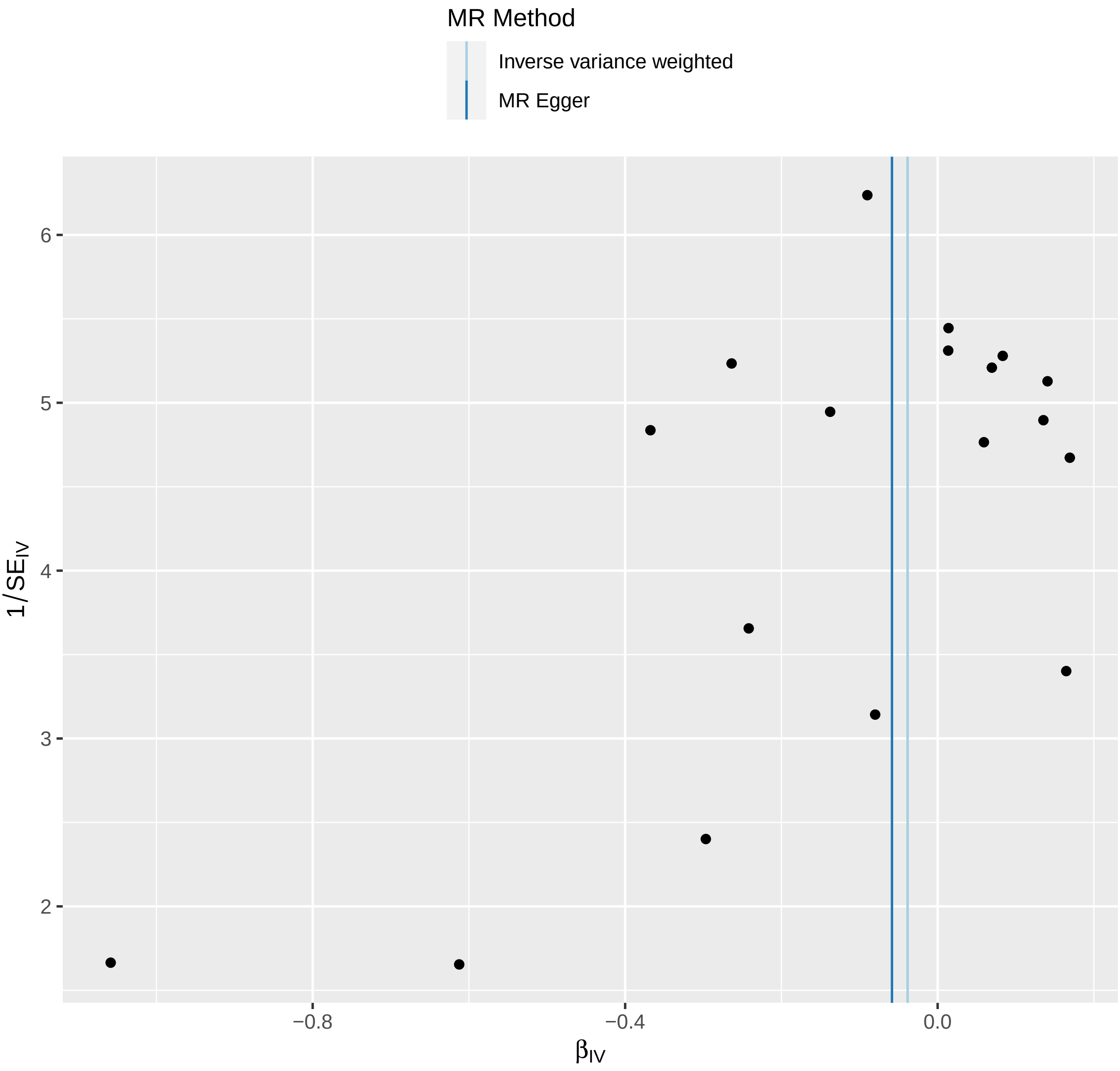

Batch 3−2239 : Pyridoxal kinase on Diabetic nephropathy (more control exclusions)
with a threshold of 1e−05

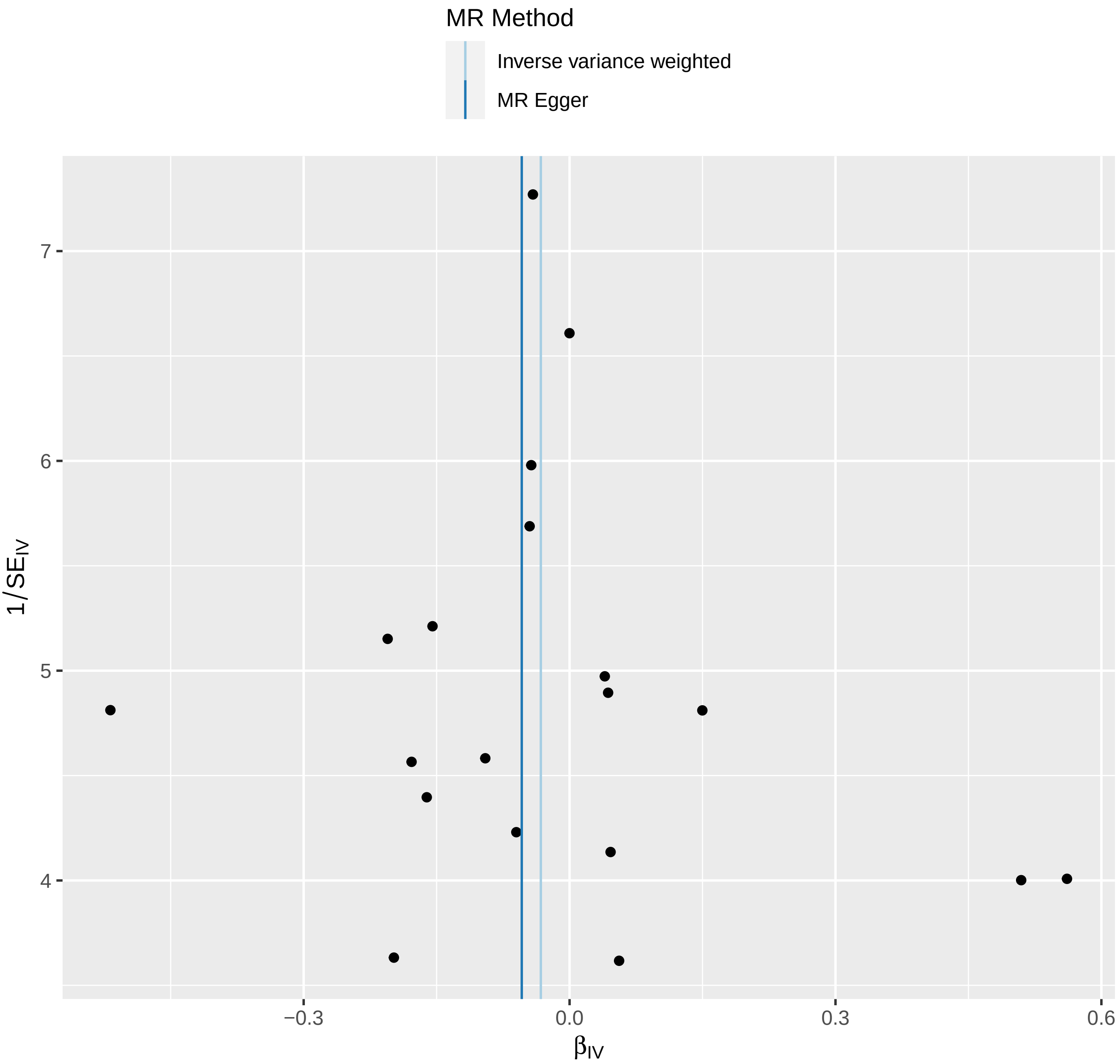

Batch 3−2240 : Pyridoxal phosphate phosphatase on Diabetic nephropathy (more control exclusions)
with a threshold of 1e−05

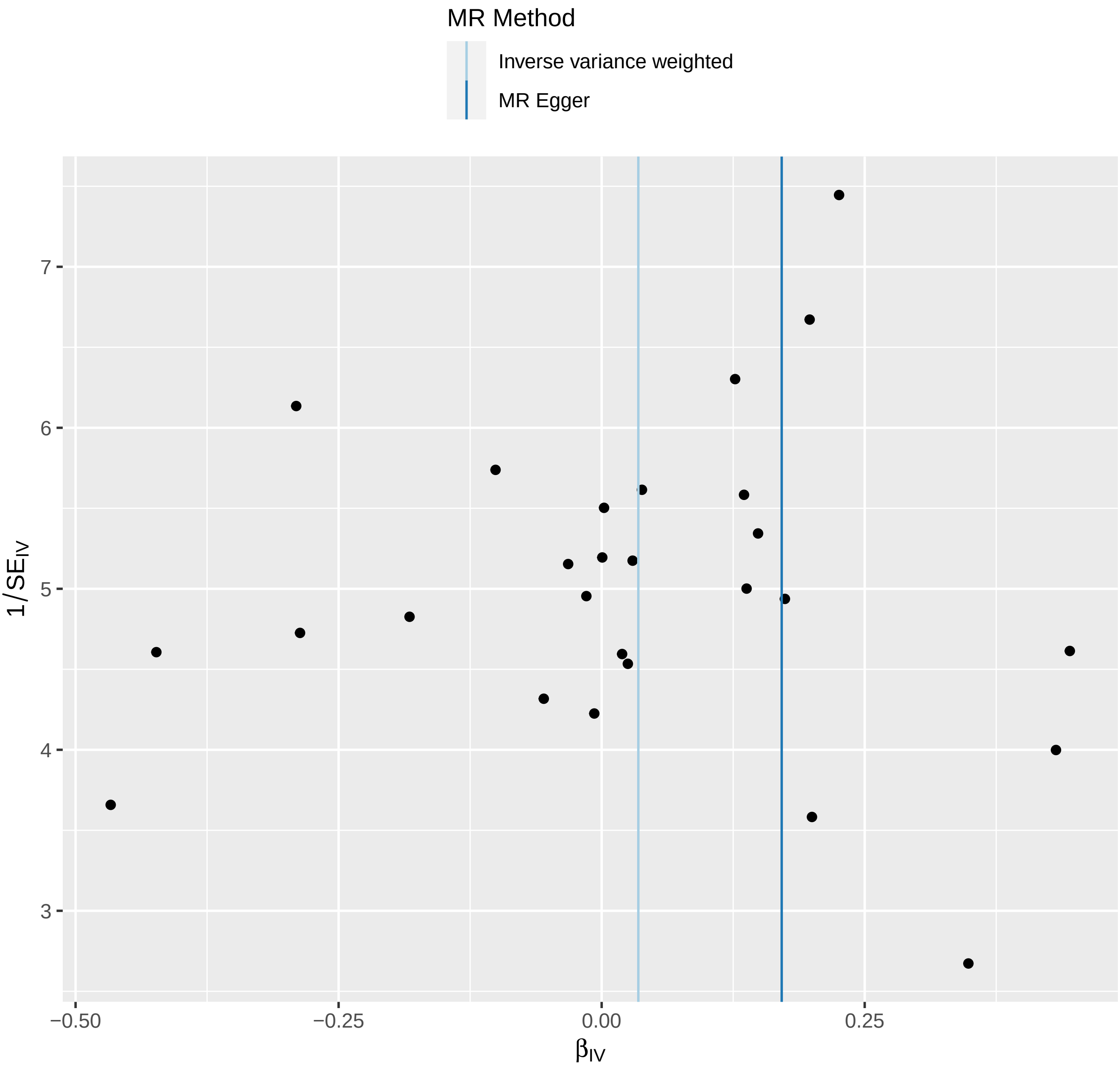

Batch 3−2241 : PDZ domain−containing protein 11 on Diabetic nephropathy (more control exclusions)
with a threshold of 1e−05

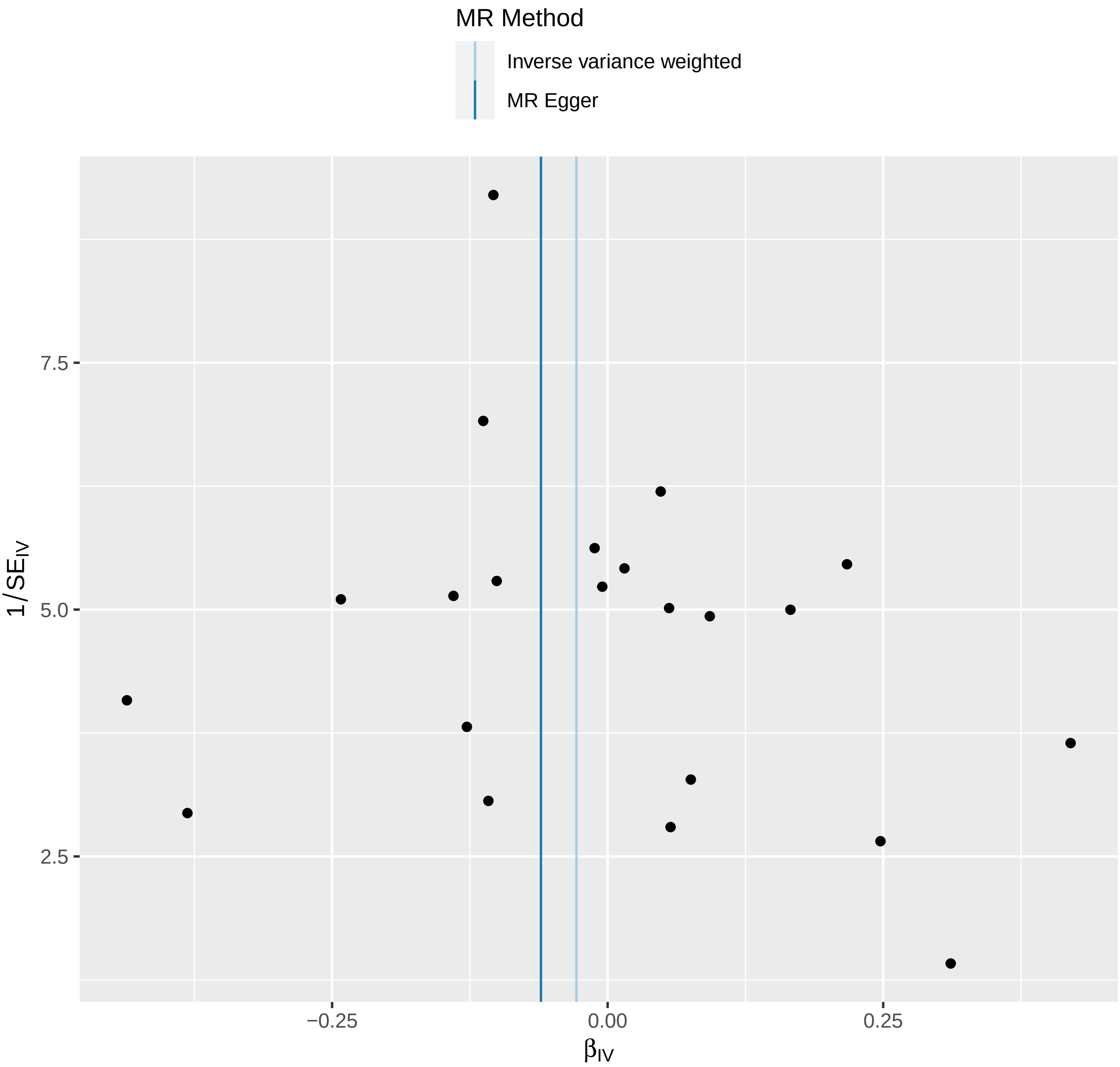

Batch 3−2242 : PDZK1−interacting protein 1 on Diabetic nephropathy (more control exclusions)
with a threshold of 1e−05

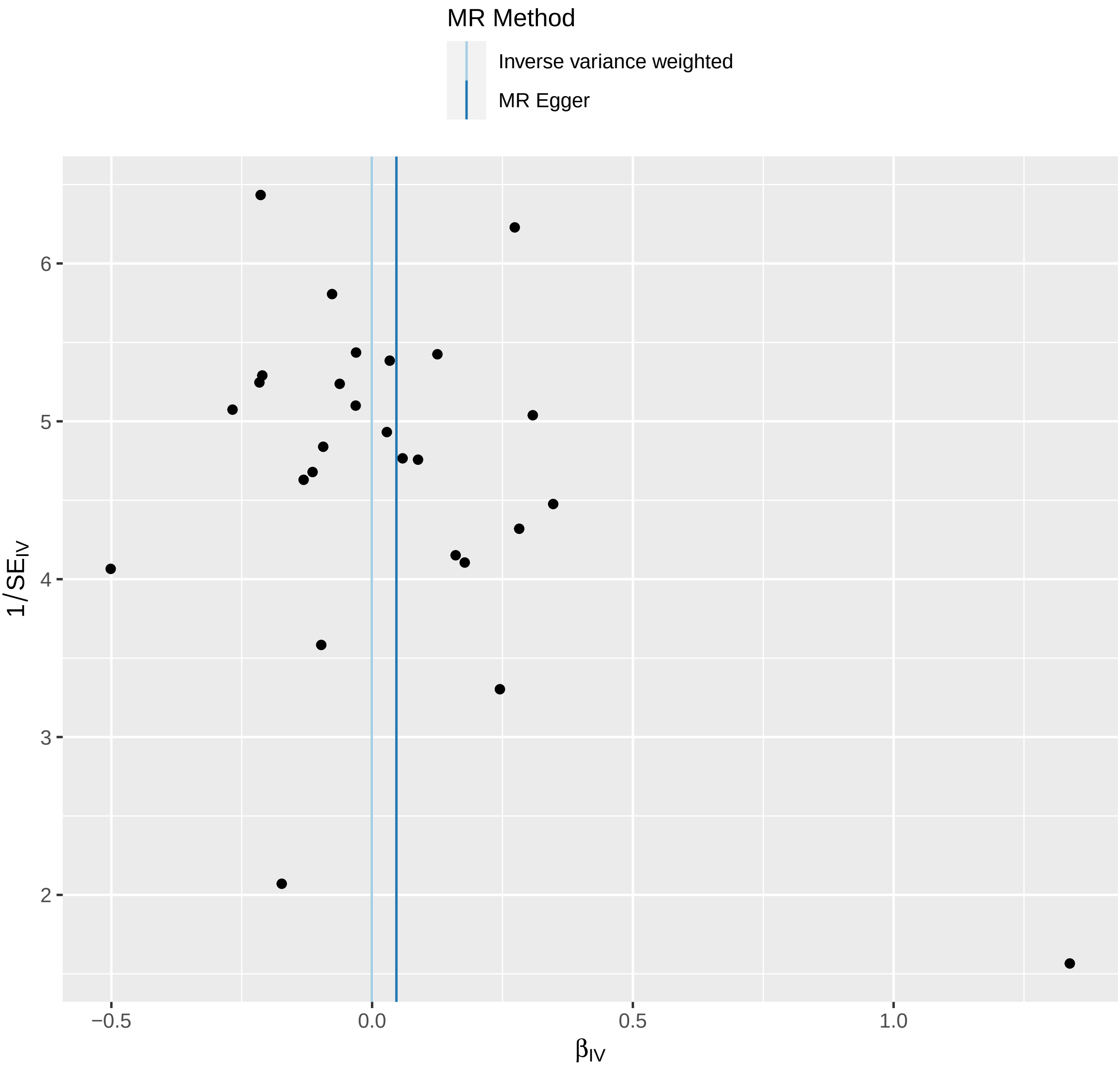

Batch 3−2243 : Platelet endothelial aggregation receptor 1 on Diabetic nephropathy (more control exclusions) with a threshold of 1e−05

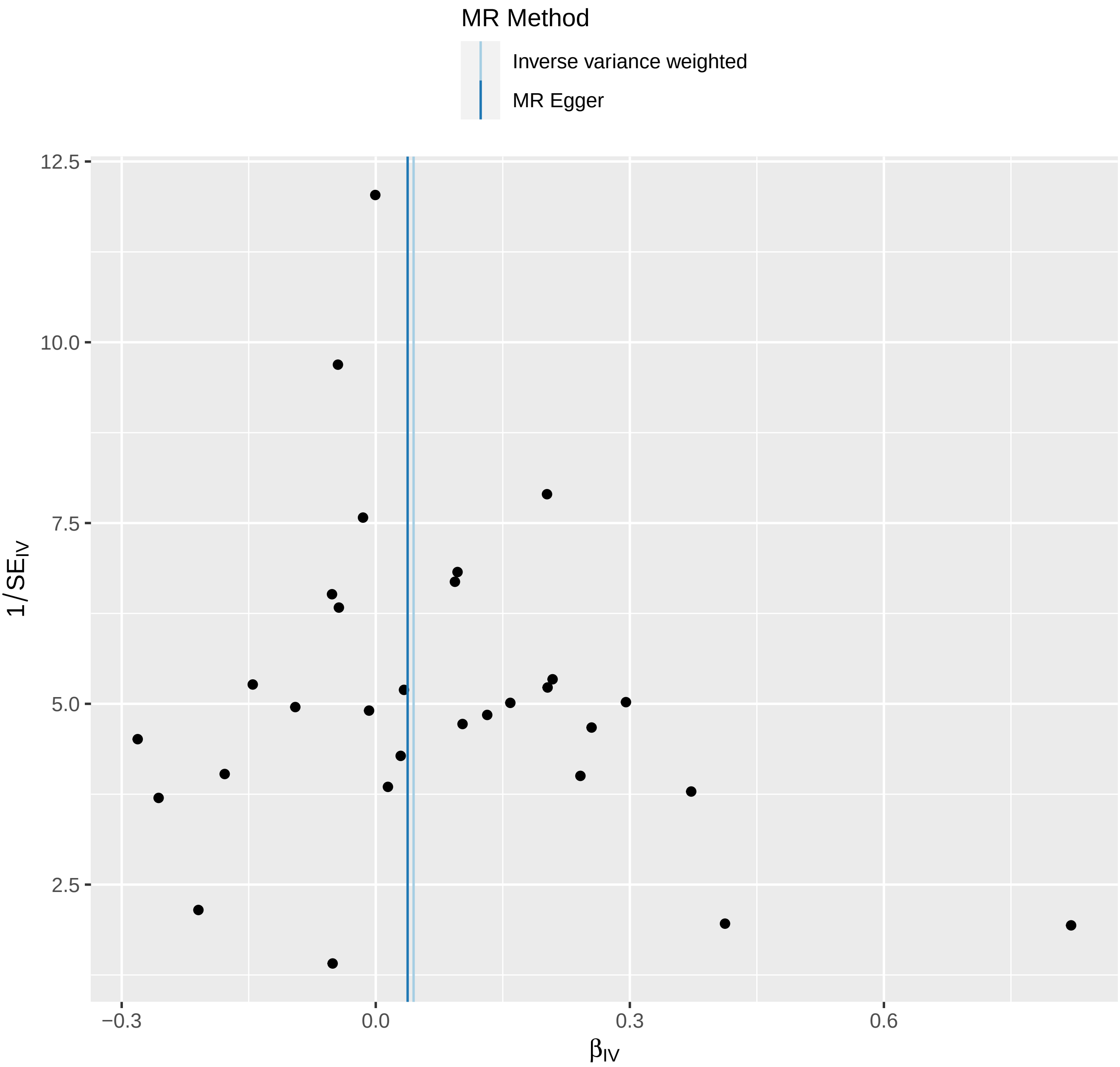

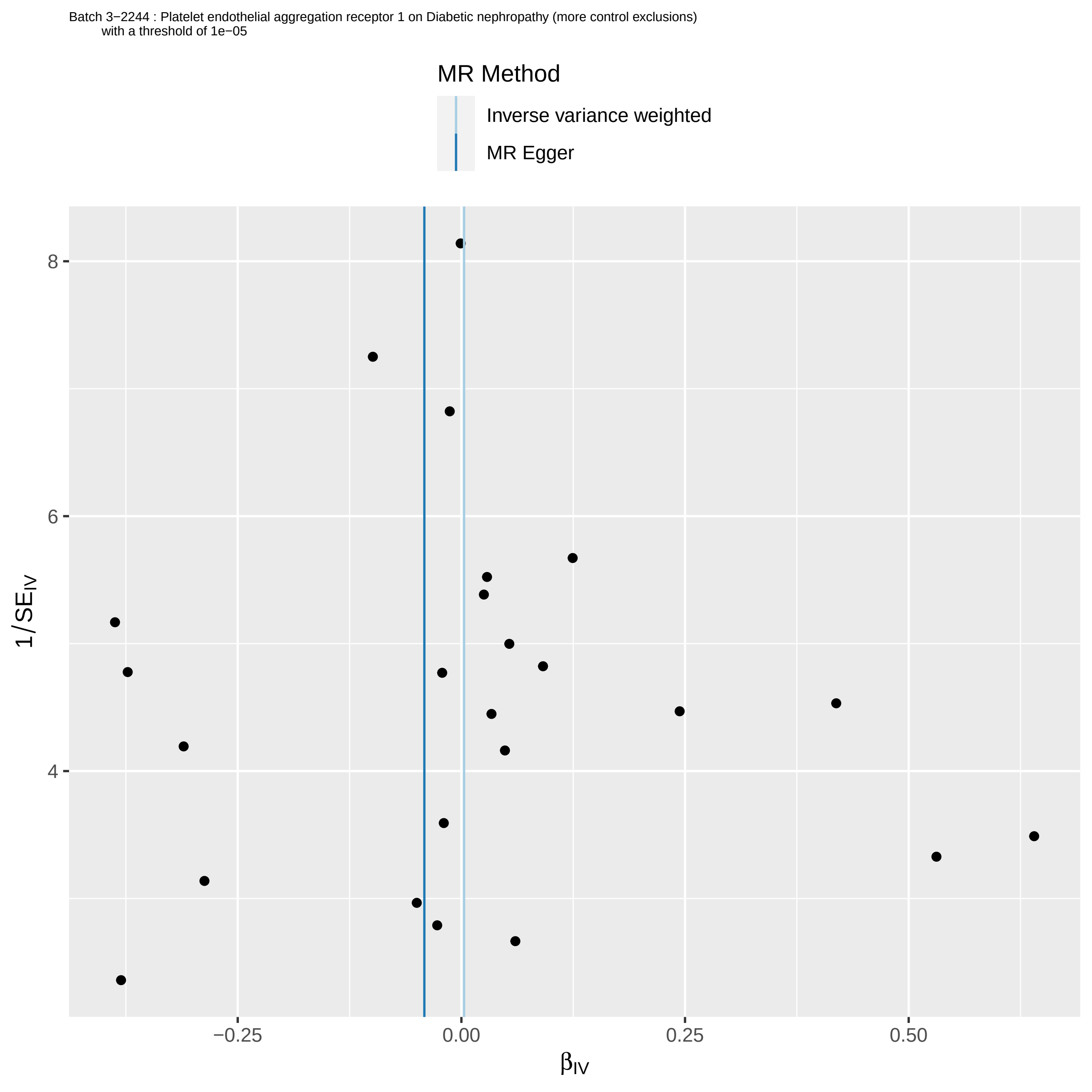
Batch 3−2244 : Platelet endothelial aggregation receptor 1 on Diabetic nephropathy (more control exclusions)
with a threshold of 1e−05
MR Method
Inverse variance weighted
MR Egger
8
6
4
1/SE_IV
−0.25
0.00
0.25
0.50
β_IV

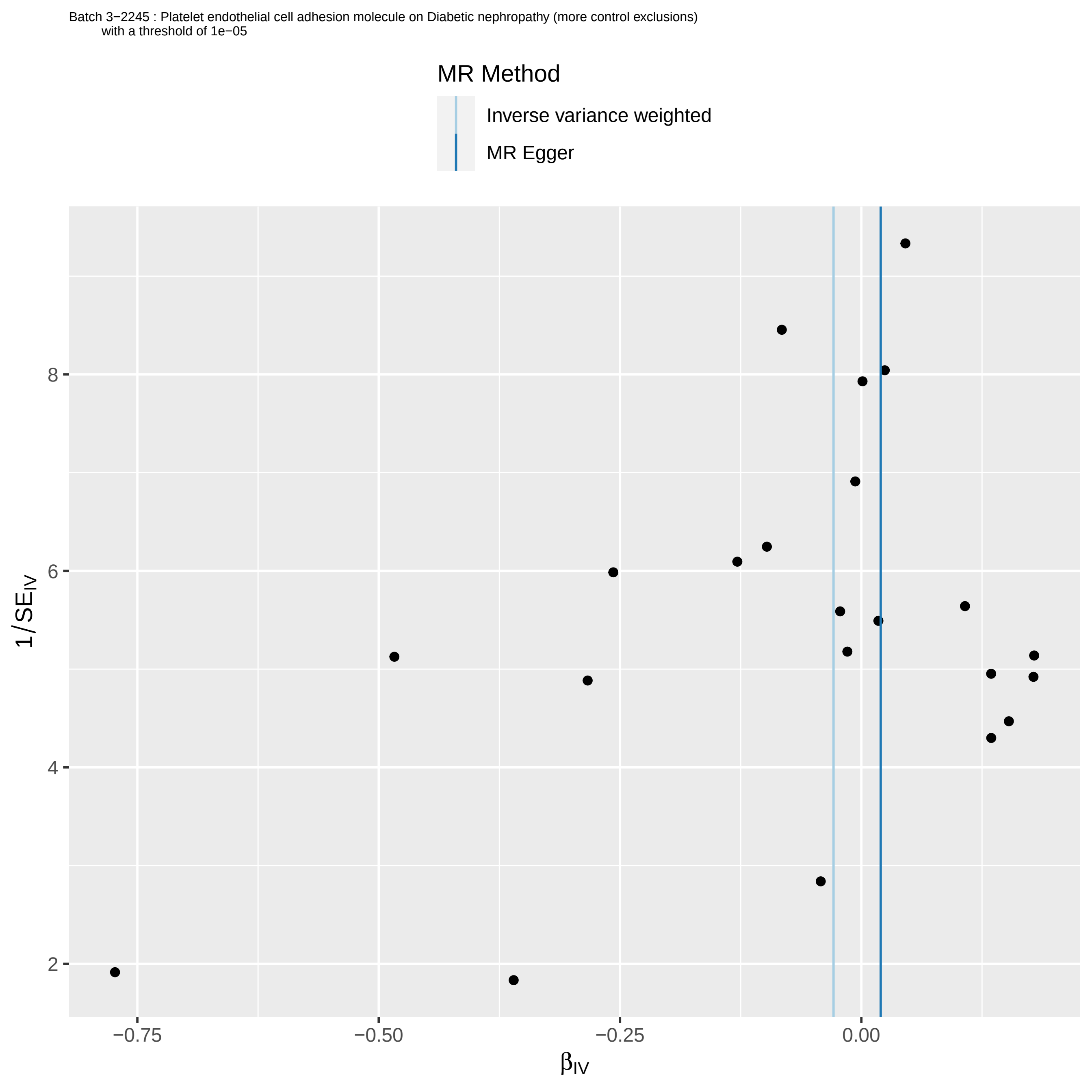
Batch 3−2245 : Platelet endothelial cell adhesion molecule on Diabetic nephropathy (more control exclusions)
with a threshold of 1e−05
MR Method
Inverse variance weighted
MR Egger
1/SEIV
βIV
2
4
6
8
−0.75
−0.50
−0.25
0.00

Batch 3−2246 : E3 ubiquitin−protein ligase pellino homolog 2 on Diabetic nephropathy (more control exclusions)
with a threshold of 1e−05

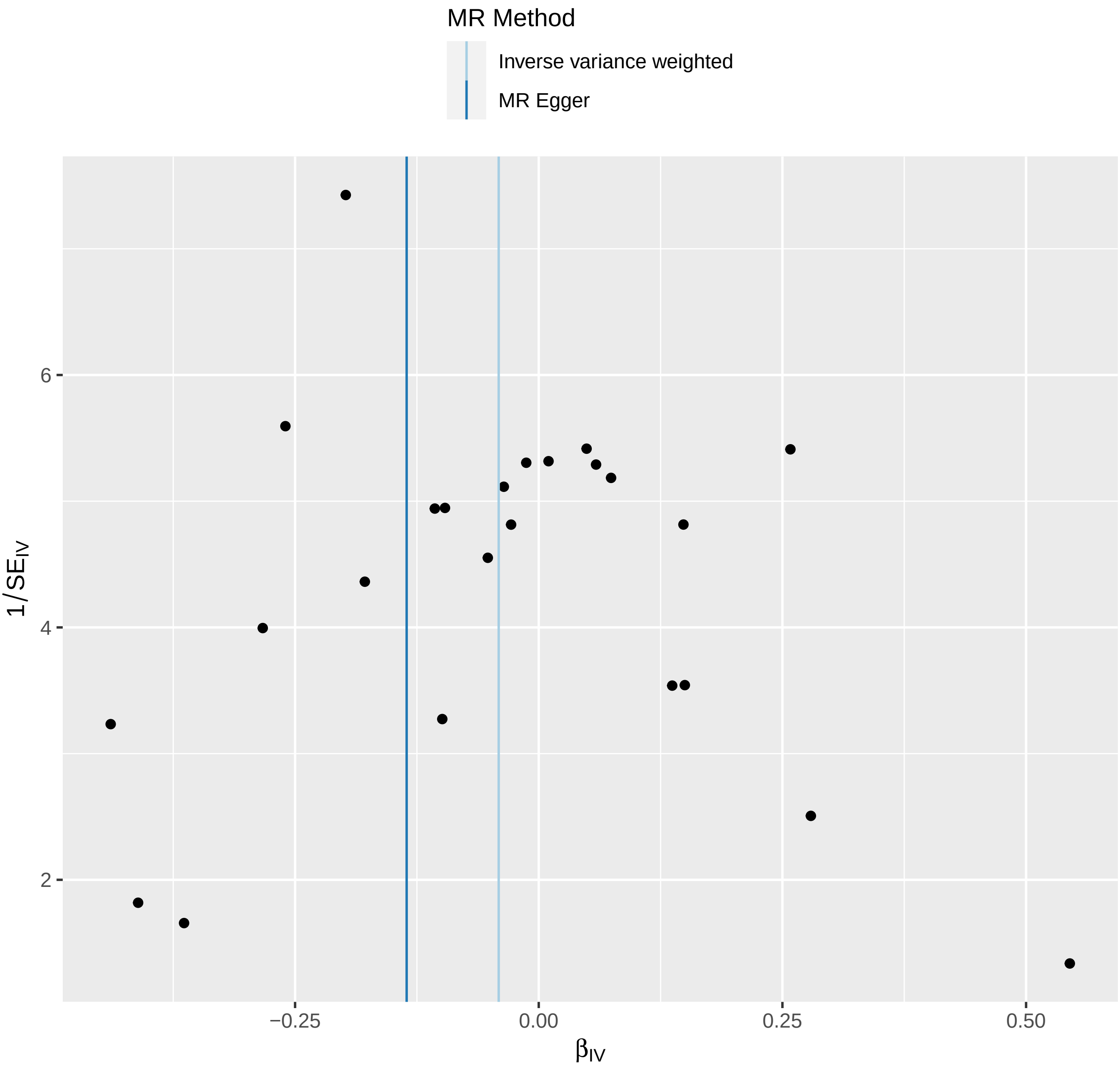

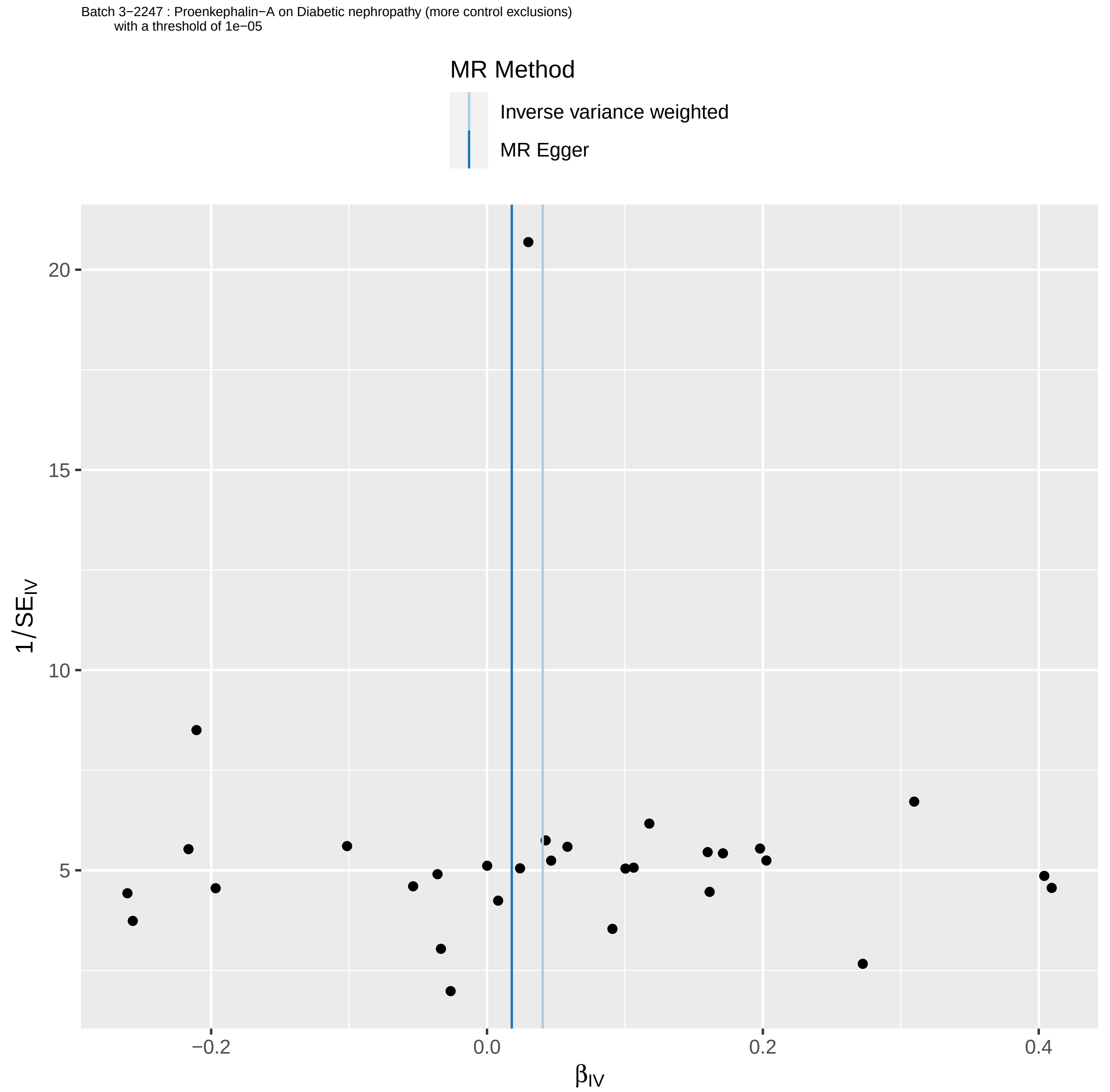

Batch 3−2247 : Proenkephalin−A on Diabetic nephropathy (more control exclusions)
with a threshold of 1e−05
MR Method
Inverse variance weighted
MR Egger
20
15
10
5
1/SE_IV
−0.2
0.0
0.2
0.4
β_IV

Batch 3−2248 : Pescadillo homolog on Diabetic nephropathy (more control exclusions)
with a threshold of 1e−05

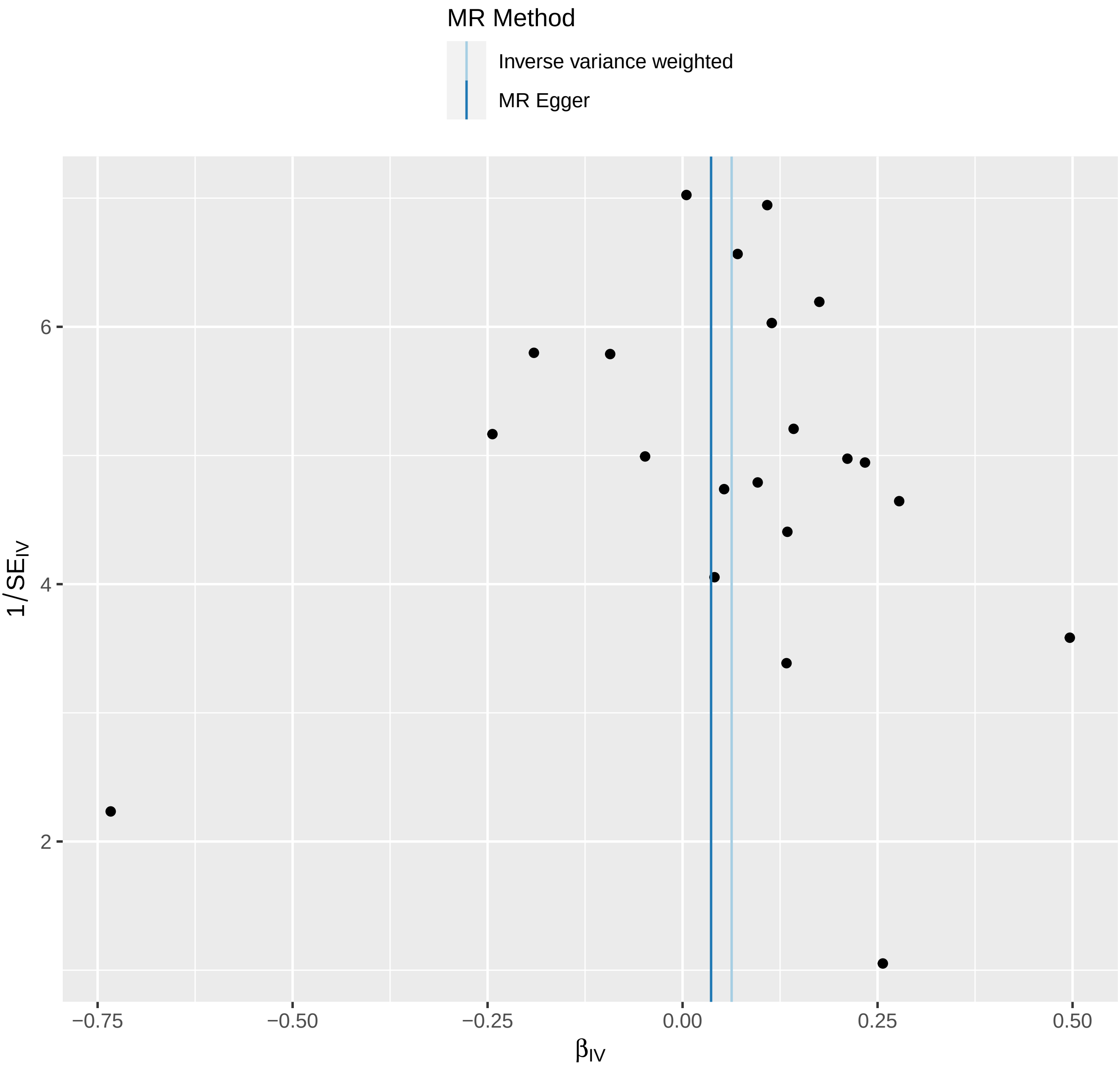

Batch 3−2249 : Peroxisomal membrane protein PEX14 on Diabetic nephropathy (more control exclusions)
with a threshold of 1e−05

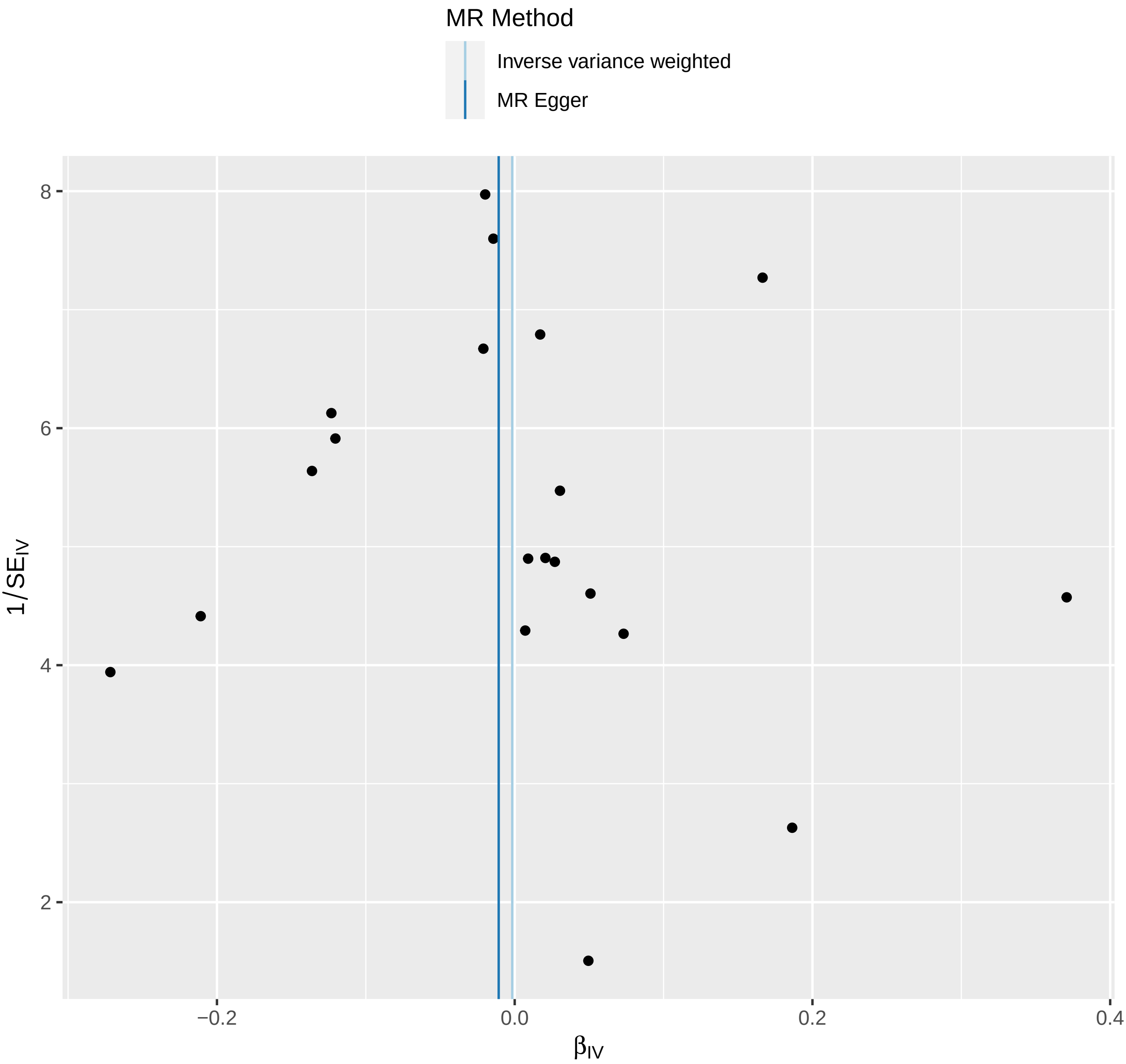

Batch 3−2250 : Peroxisomal targeting signal 1 receptor on Diabetic nephropathy (more control exclusions) with a threshold of 1e−05

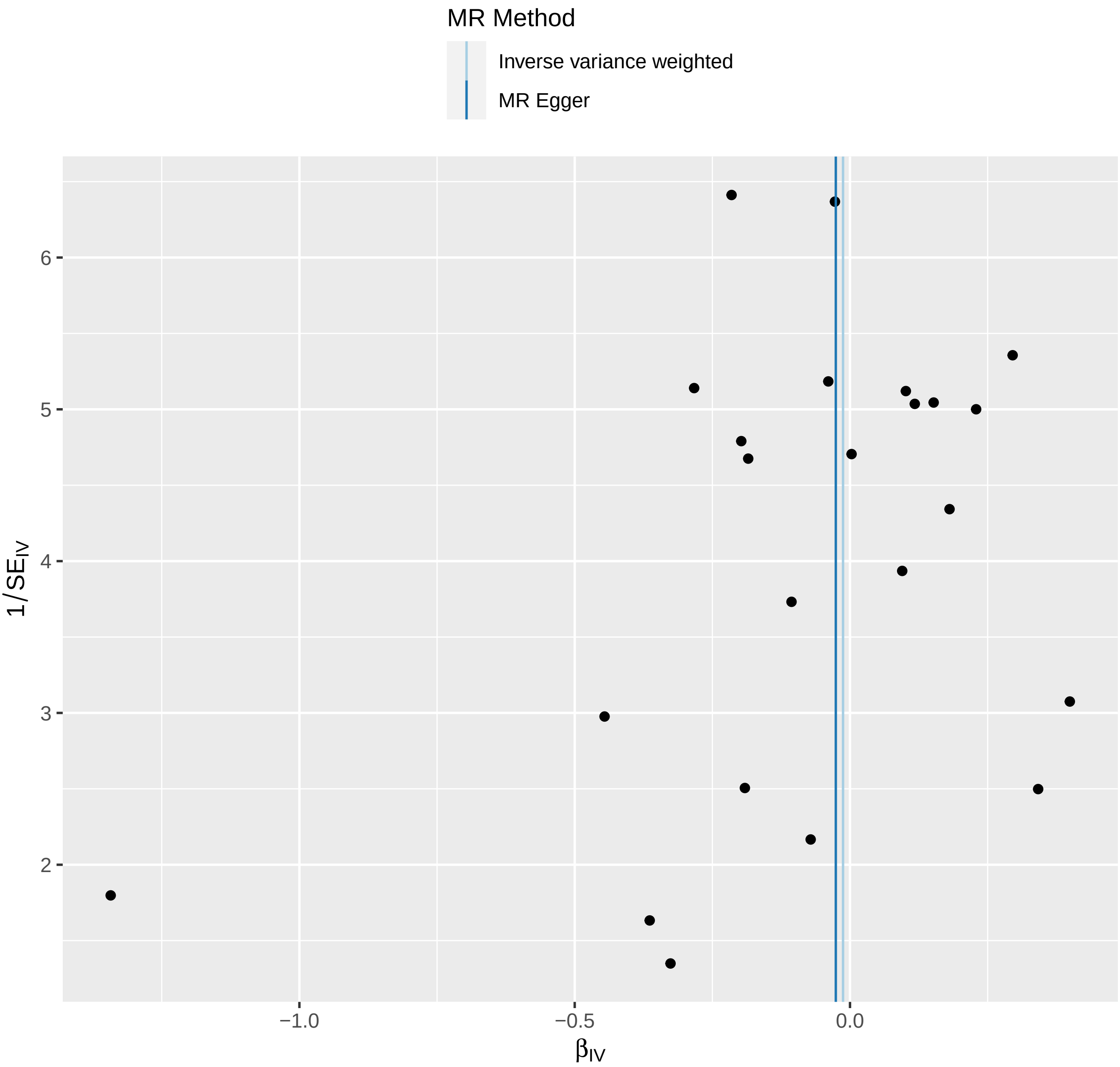

Batch 3−2251 : Platelet factor 4 variant on Diabetic nephropathy (more control exclusions)
with a threshold of 1e−05

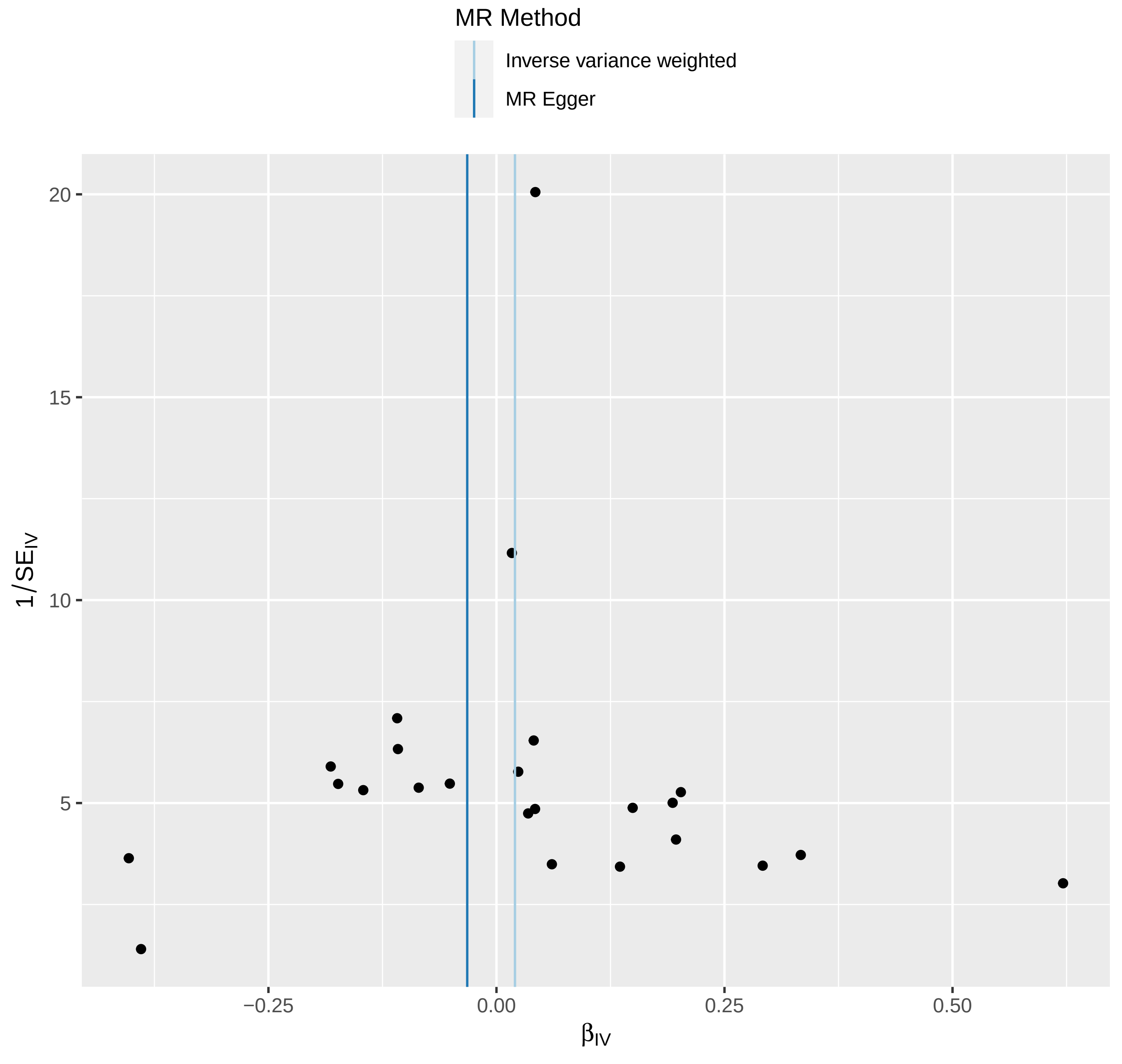

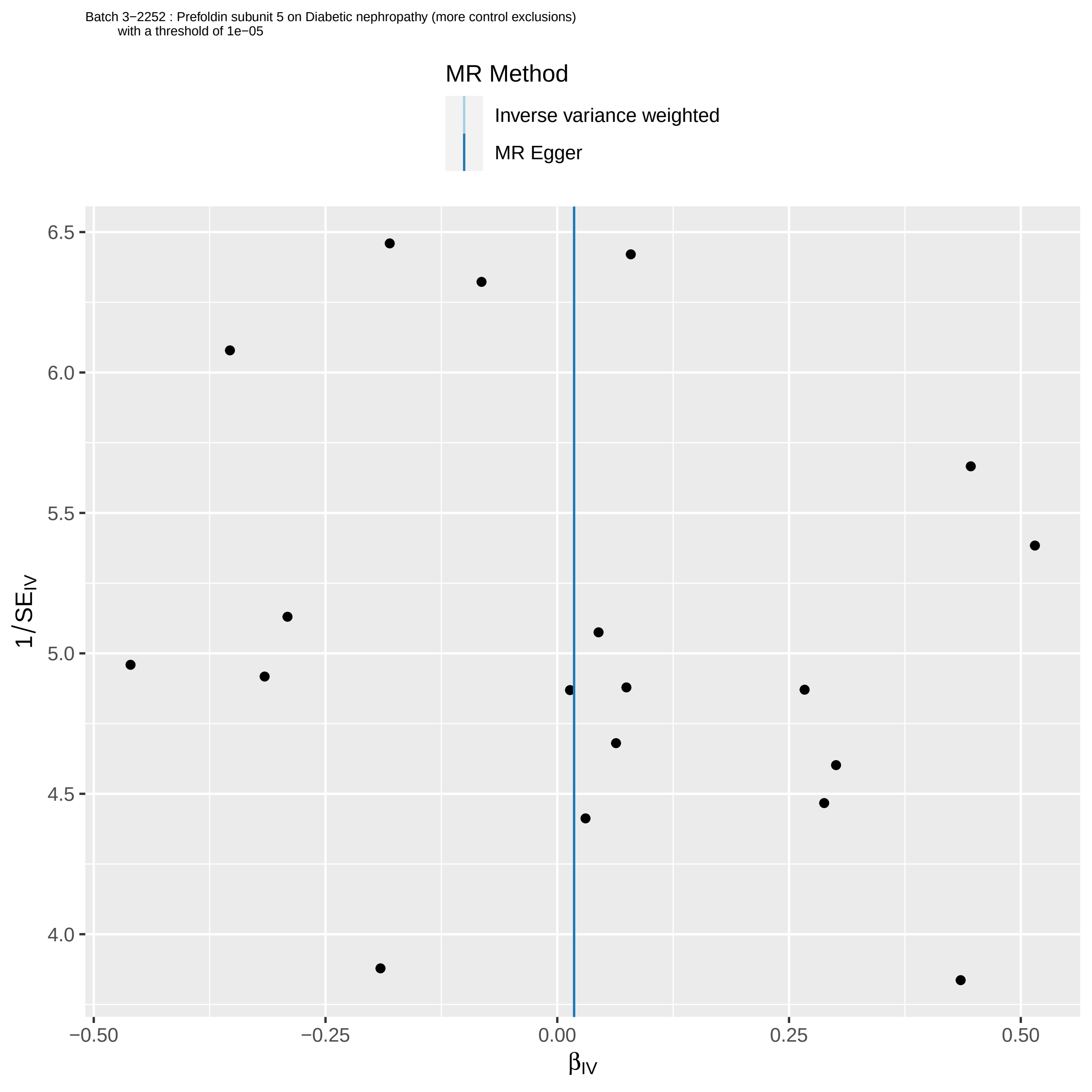
Batch 3−2252 : Prefoldin subunit 5 on Diabetic nephropathy (more control exclusions)
with a threshold of 1e−05
MR Method
Inverse variance weighted
MR Egger
6.5
6.0
5.5
5.0
4.5
4.0
1/SE_IV
−0.50
−0.25
0.00
0.25
0.50
β_IV

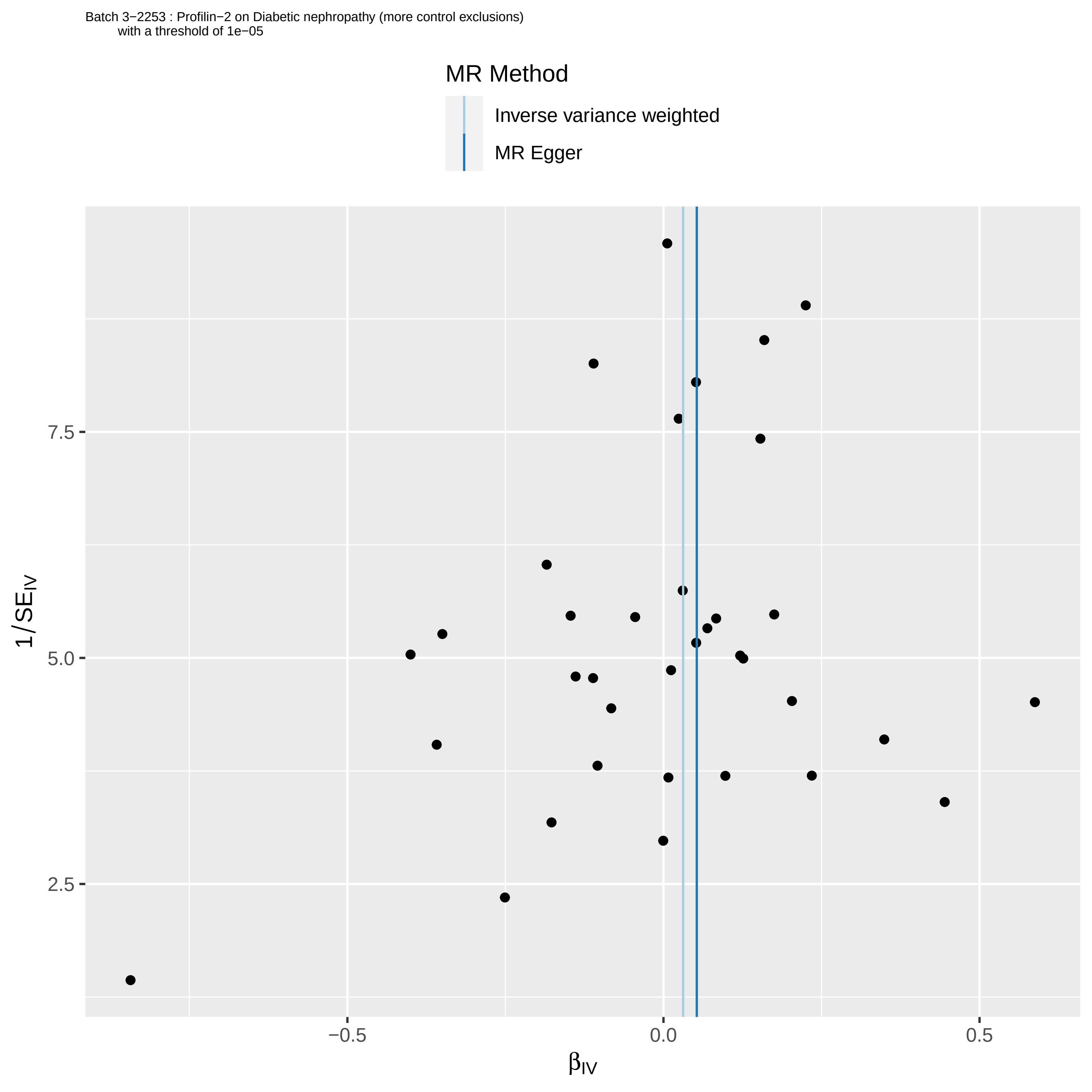

Batch 3−2253 : Profilin−2 on Diabetic nephropathy (more control exclusions)
with a threshold of 1e−05
MR Method
Inverse variance weighted
MR Egger
7.5
5.0
2.5
1/SE_IV
−0.5
0.0
0.5
β_IV

Batch 3−2254 : Placenta growth factor on Diabetic nephropathy (more control exclusions)
with a threshold of 1e−05

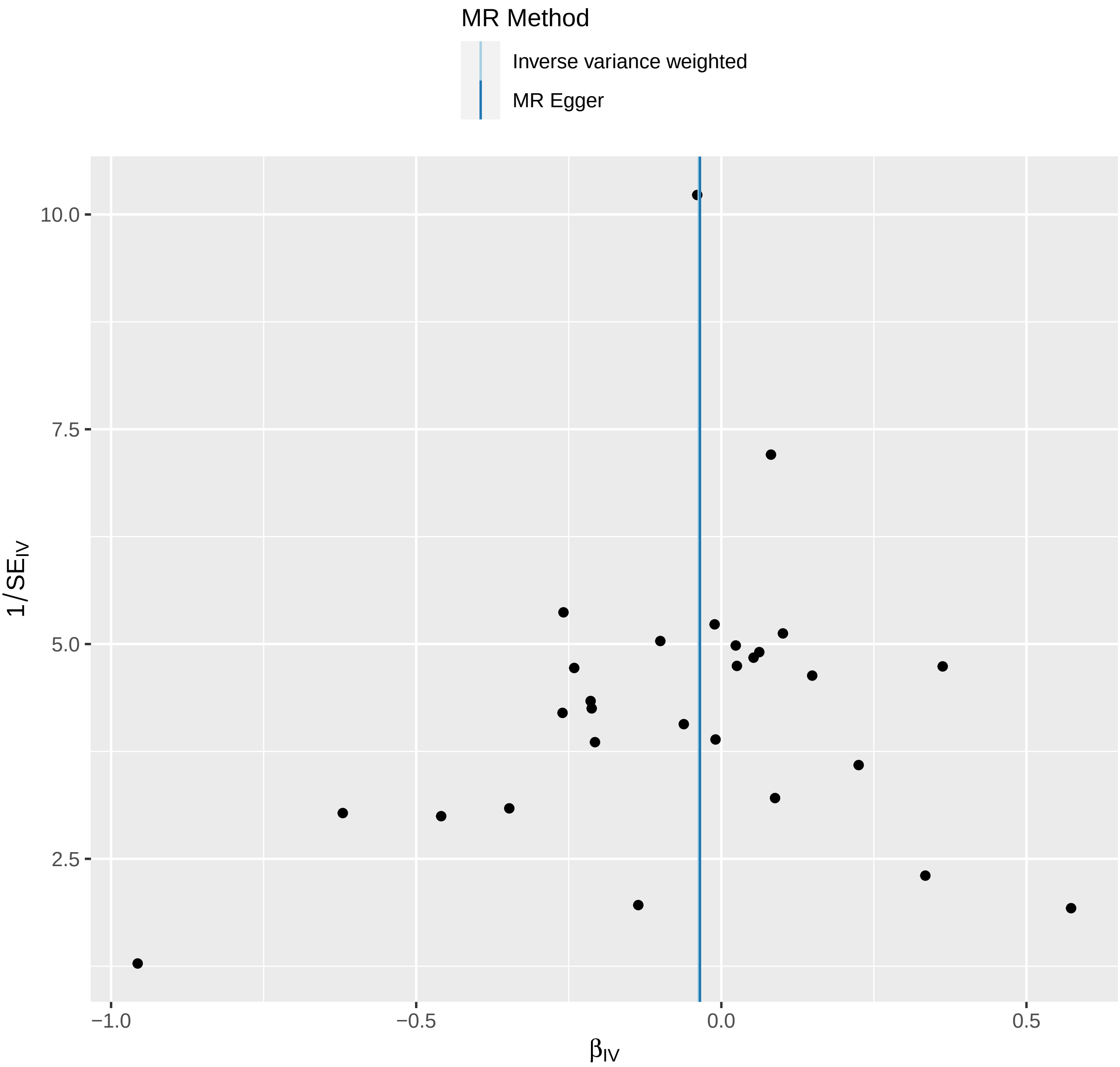

Batch 3−2255 : Phosphoglycerate kinase 1 on Diabetic nephropathy (more control exclusions)
with a threshold of 1e−05

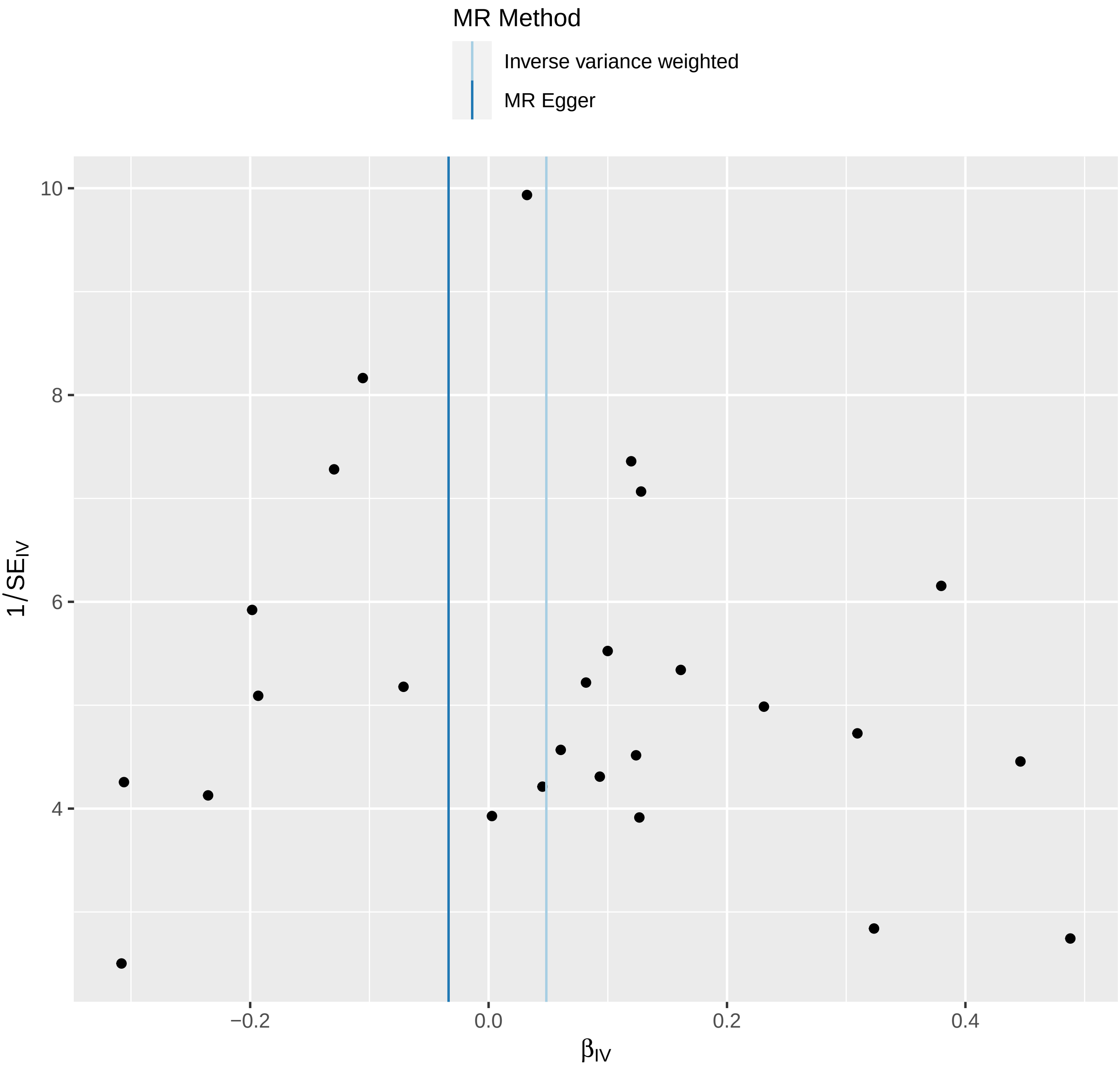

Batch 3−2256 : Phosphoglycerate kinase 2 on Diabetic nephropathy (more control exclusions)
with a threshold of 1e−05

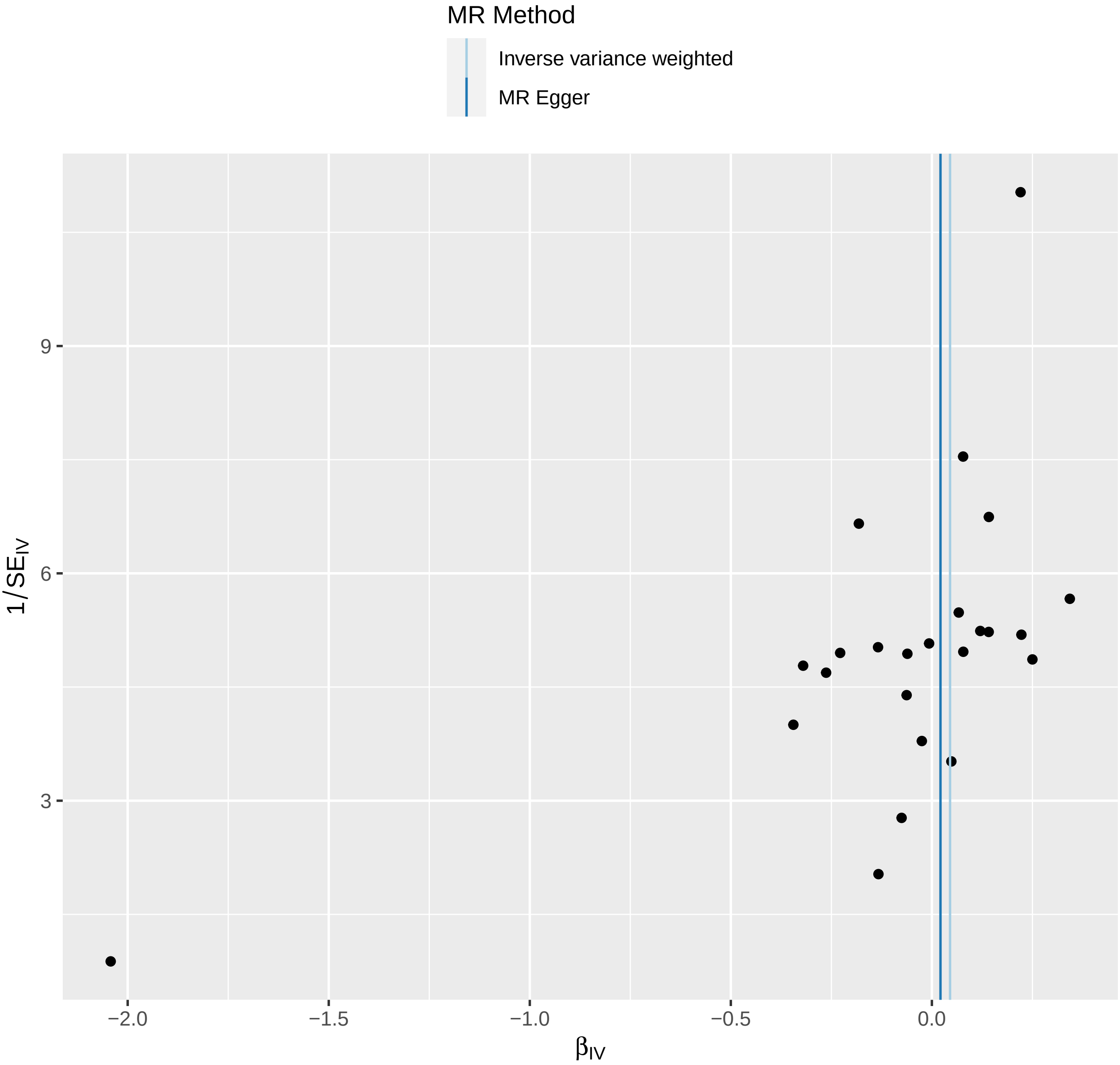

Batch 3−2257 : Peptidoglycan recognition protein 1 on Diabetic nephropathy (more control exclusions)
with a threshold of 1e−05

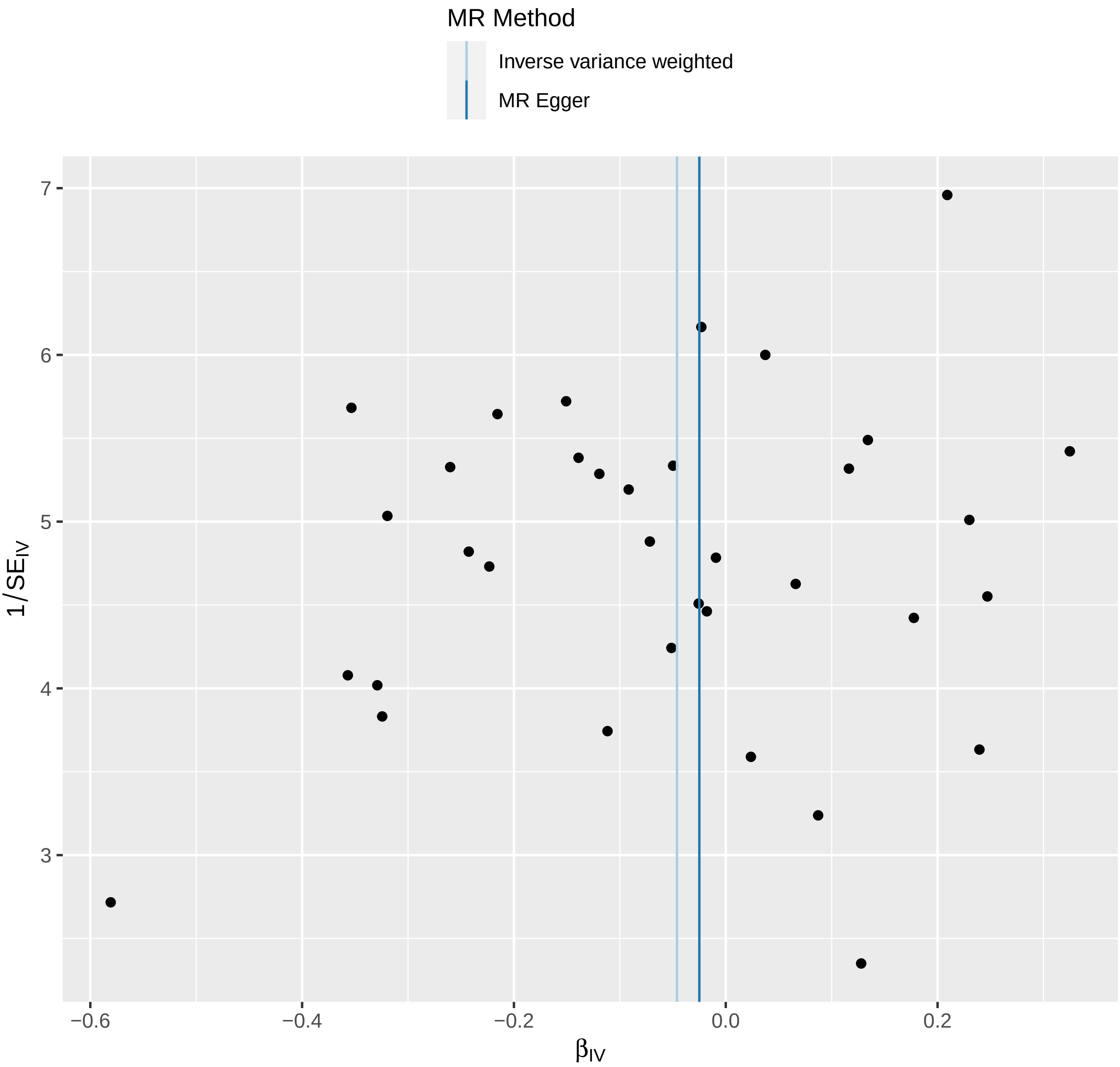

Batch 3−2258 : Phosphoglucomutase−1 on Diabetic nephropathy (more control exclusions)
with a threshold of 1e−05

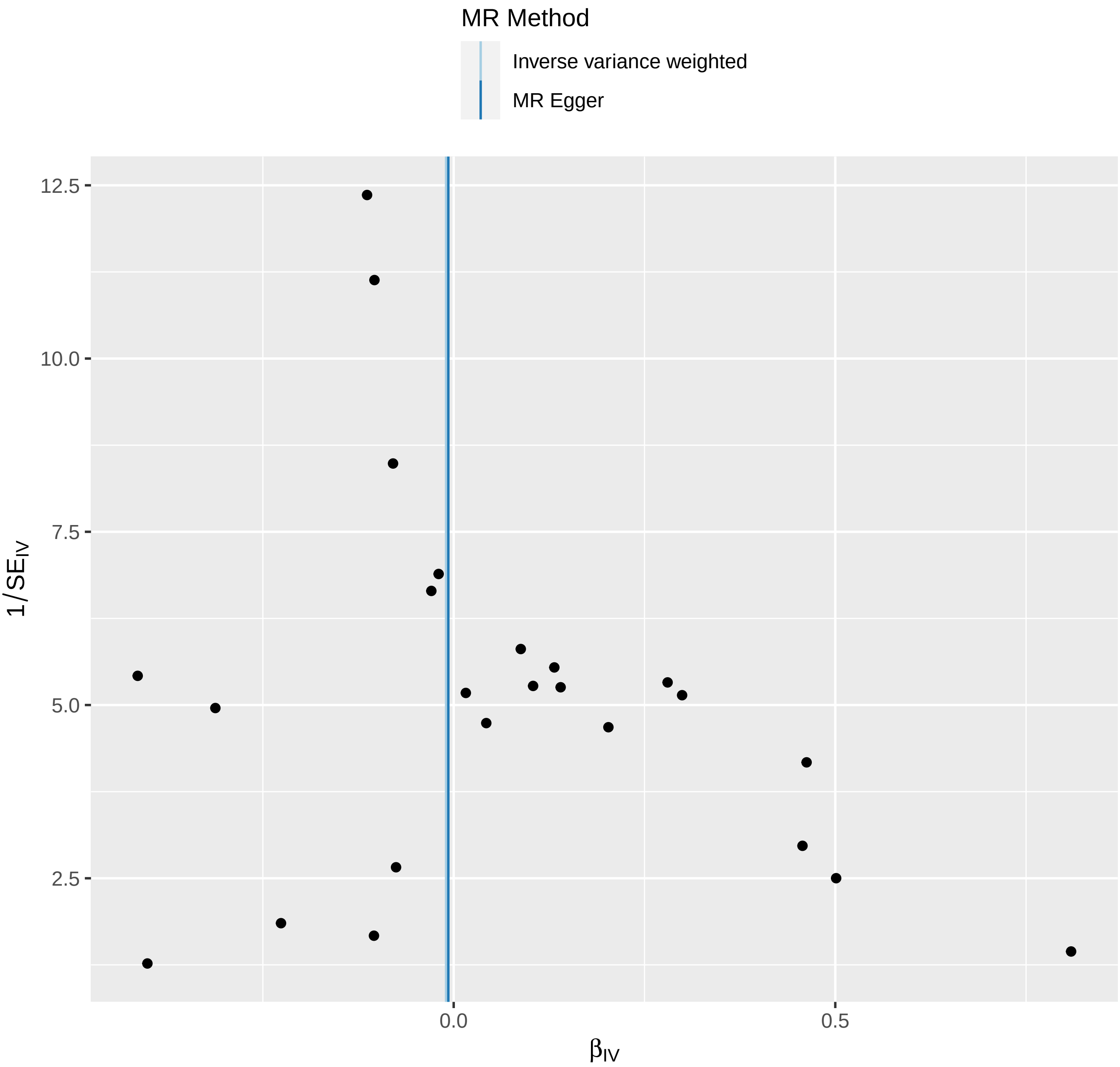

Batch 3−2259 : Membrane−associated progesterone receptor component 1 on Diabetic nephropathy (more control exclusions)
with a threshold of 1e−05

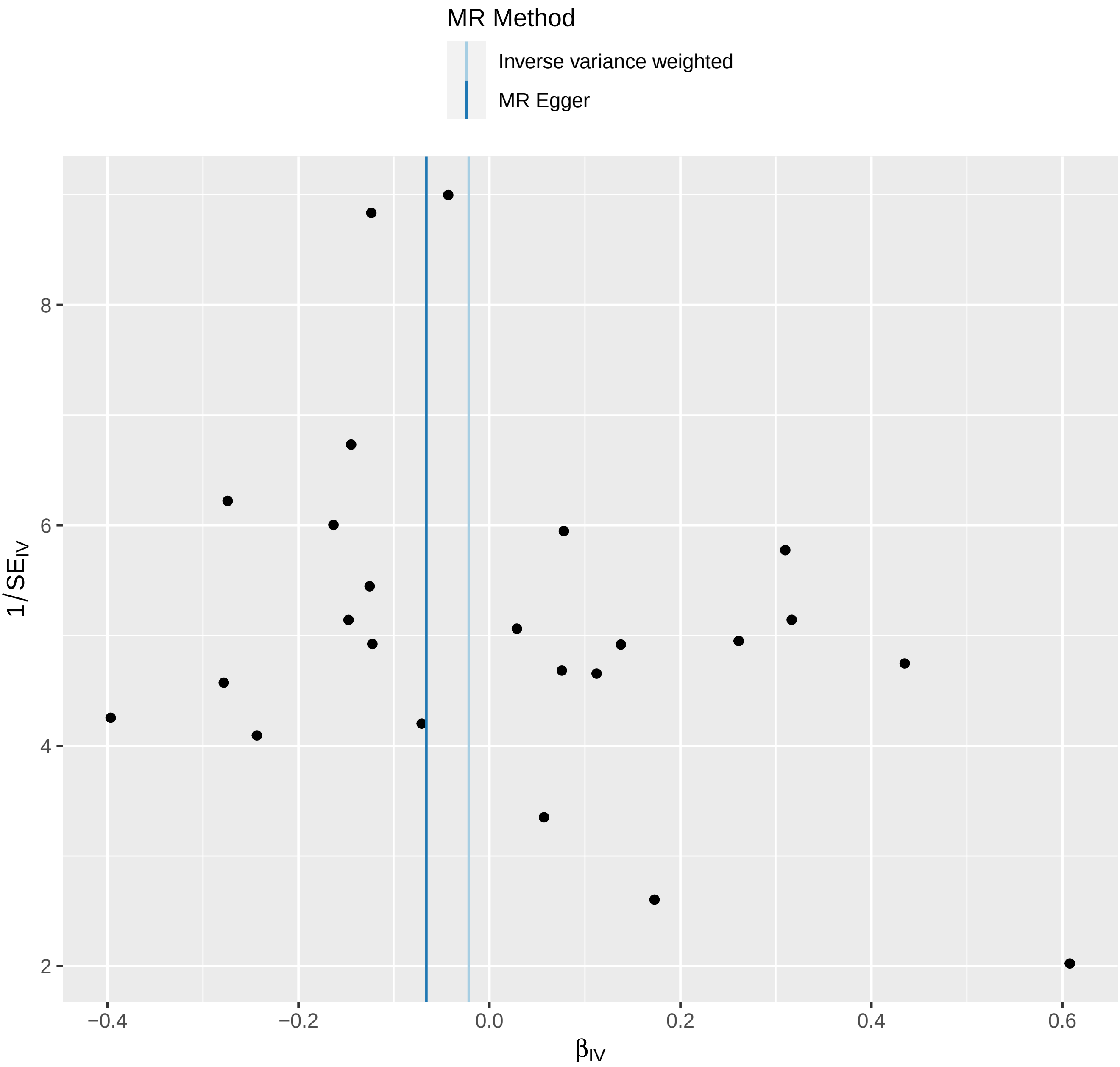

Batch 3−2260 : Membrane−associated progesterone receptor component 2 on Diabetic nephropathy (more control exclusions) with a threshold of 1e−05

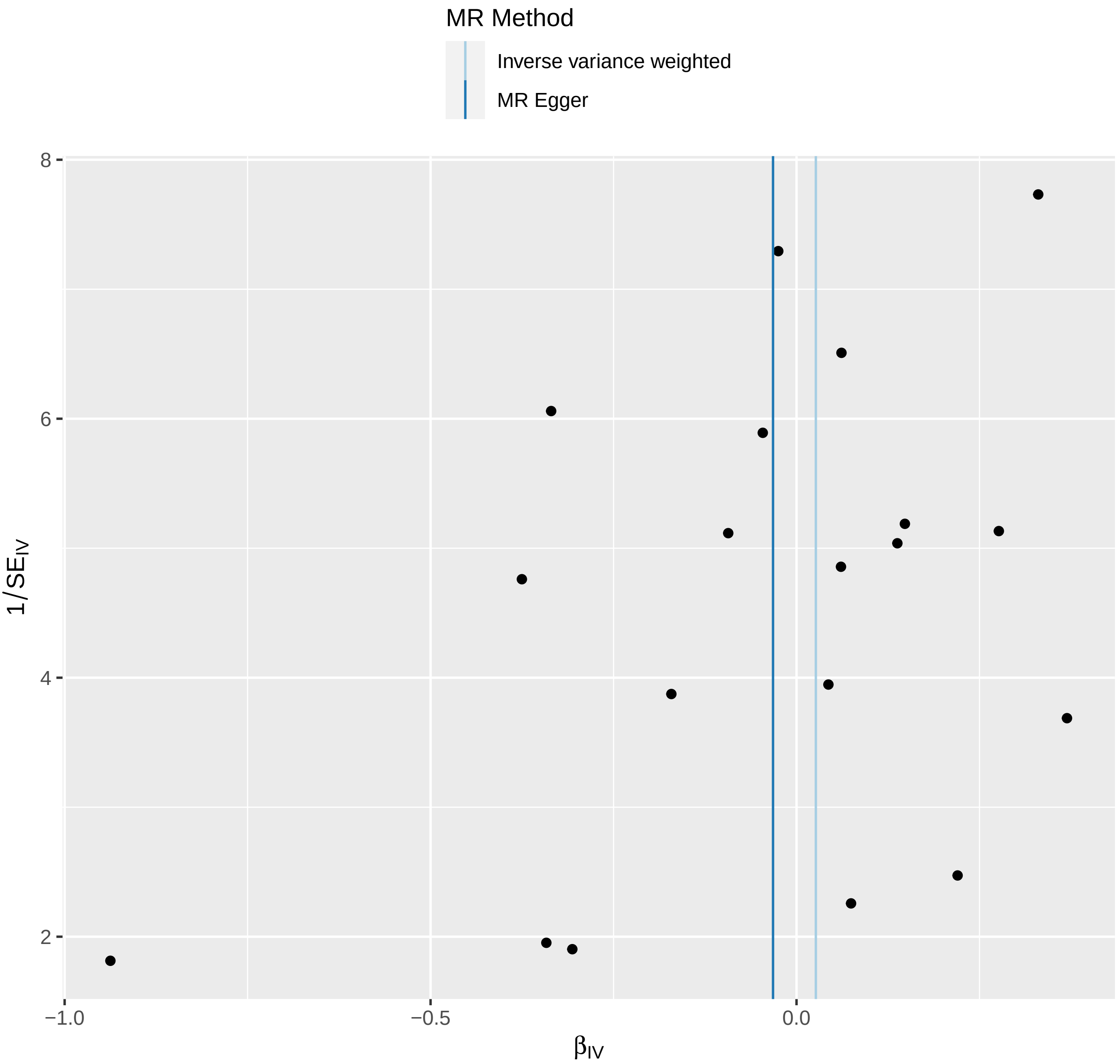

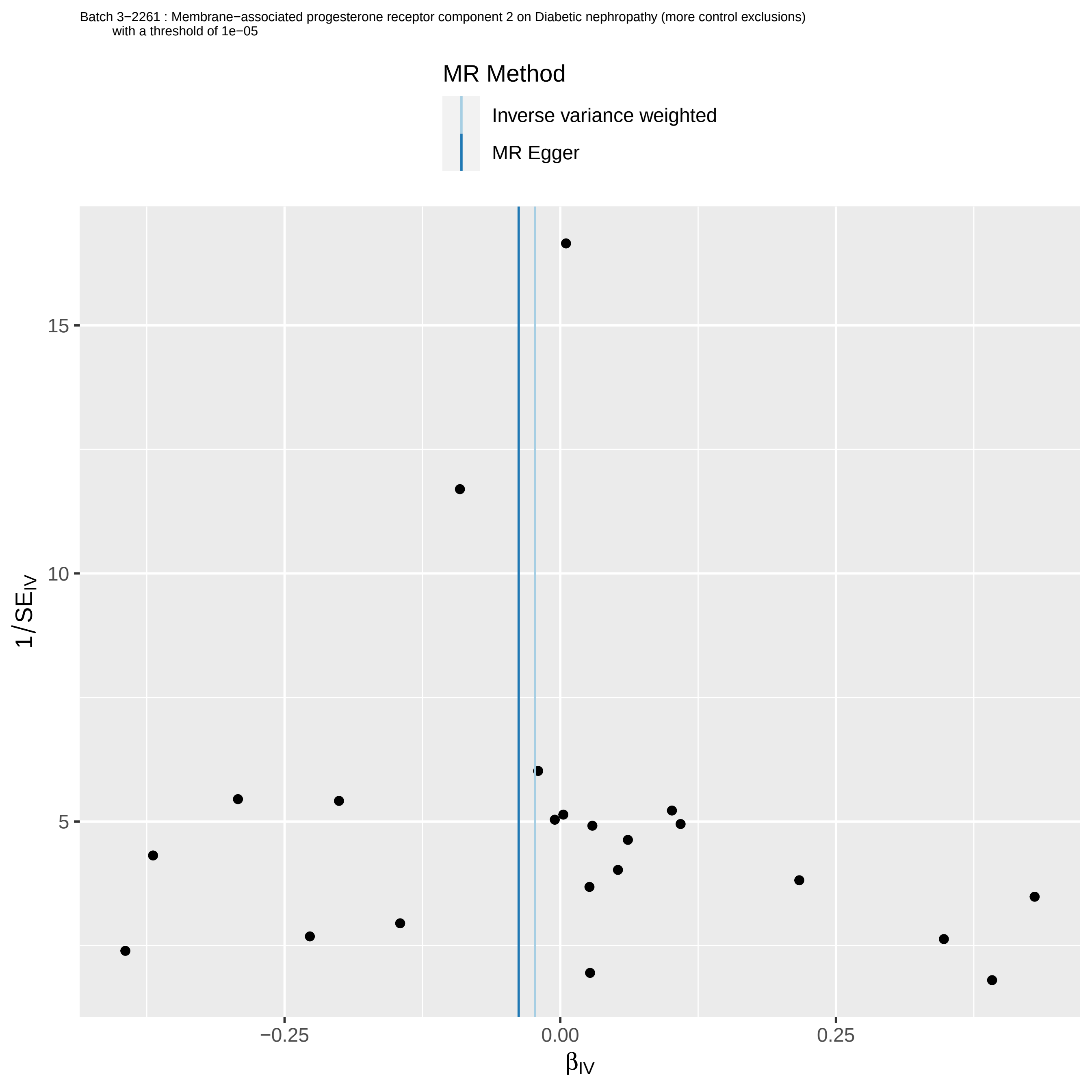
Batch 3−2261 : Membrane−associated progesterone receptor component 2 on Diabetic nephropathy (more control exclusions)
with a threshold of 1e−05
MR Method
Inverse variance weighted
MR Egger
15
10
5
$1/SE_{IV}$
−0.25
0.00
0.25
β_{IV}

Batch 3−2262 : Membrane−associated progesterone receptor component 2 on Diabetic nephropathy (more control exclusions) with a threshold of 1e−05

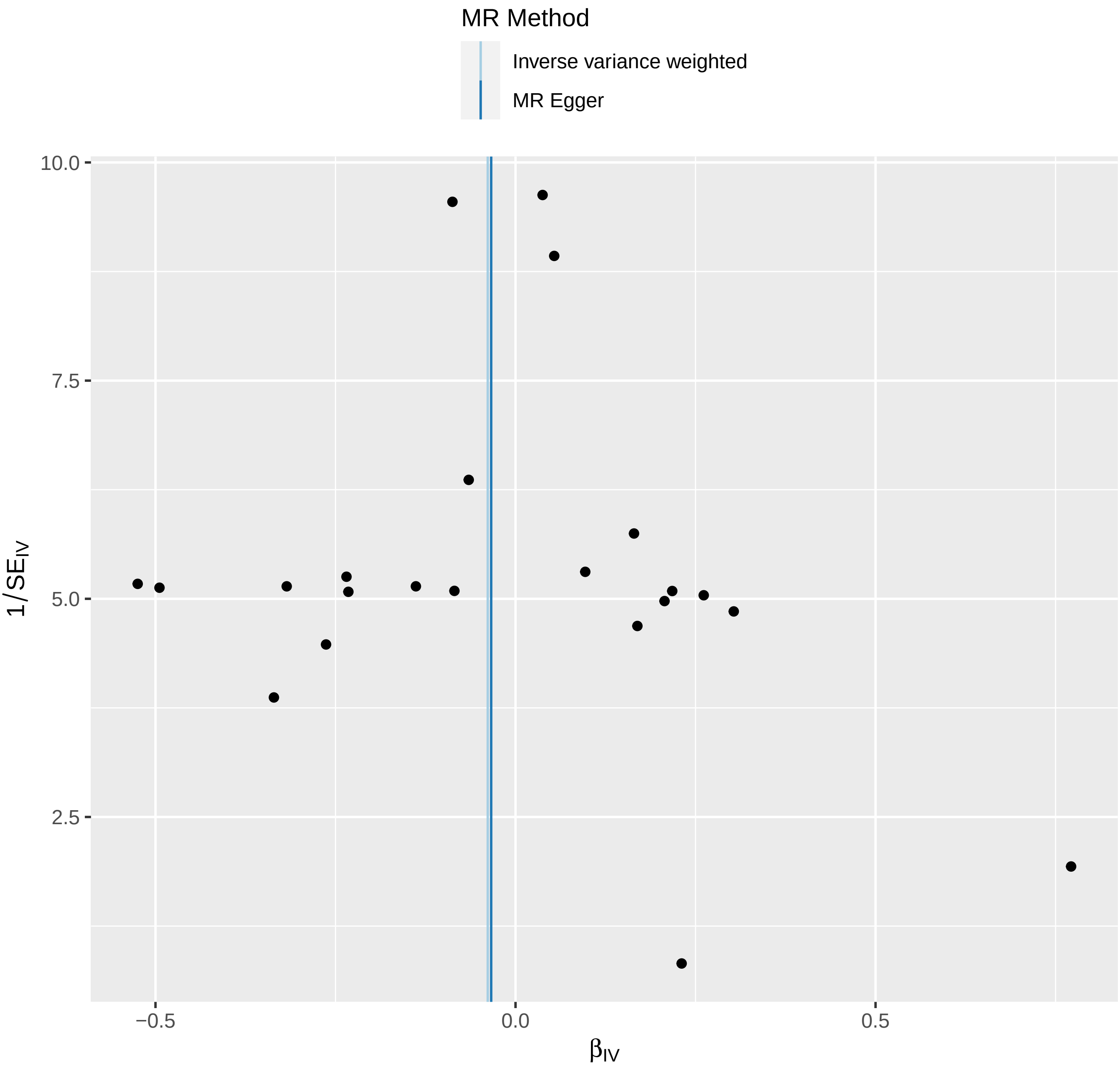

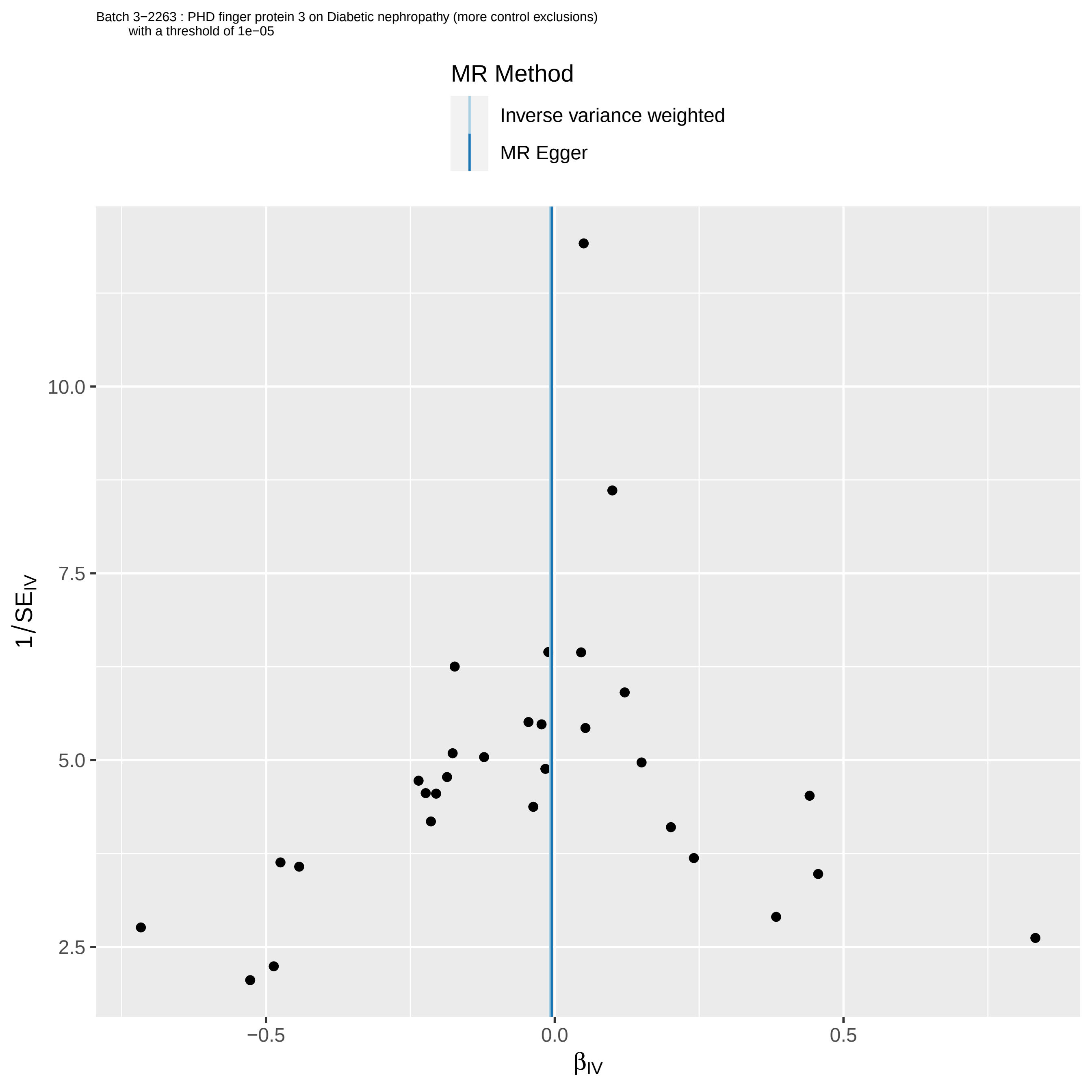

Batch 3−2263 : PHD finger protein 3 on Diabetic nephropathy (more control exclusions)
with a threshold of 1e−05
MR Method
Inverse variance weighted
MR Egger
10.0
7.5
5.0
2.5
1/SE_IV
−0.5
0.0
0.5
β_IV

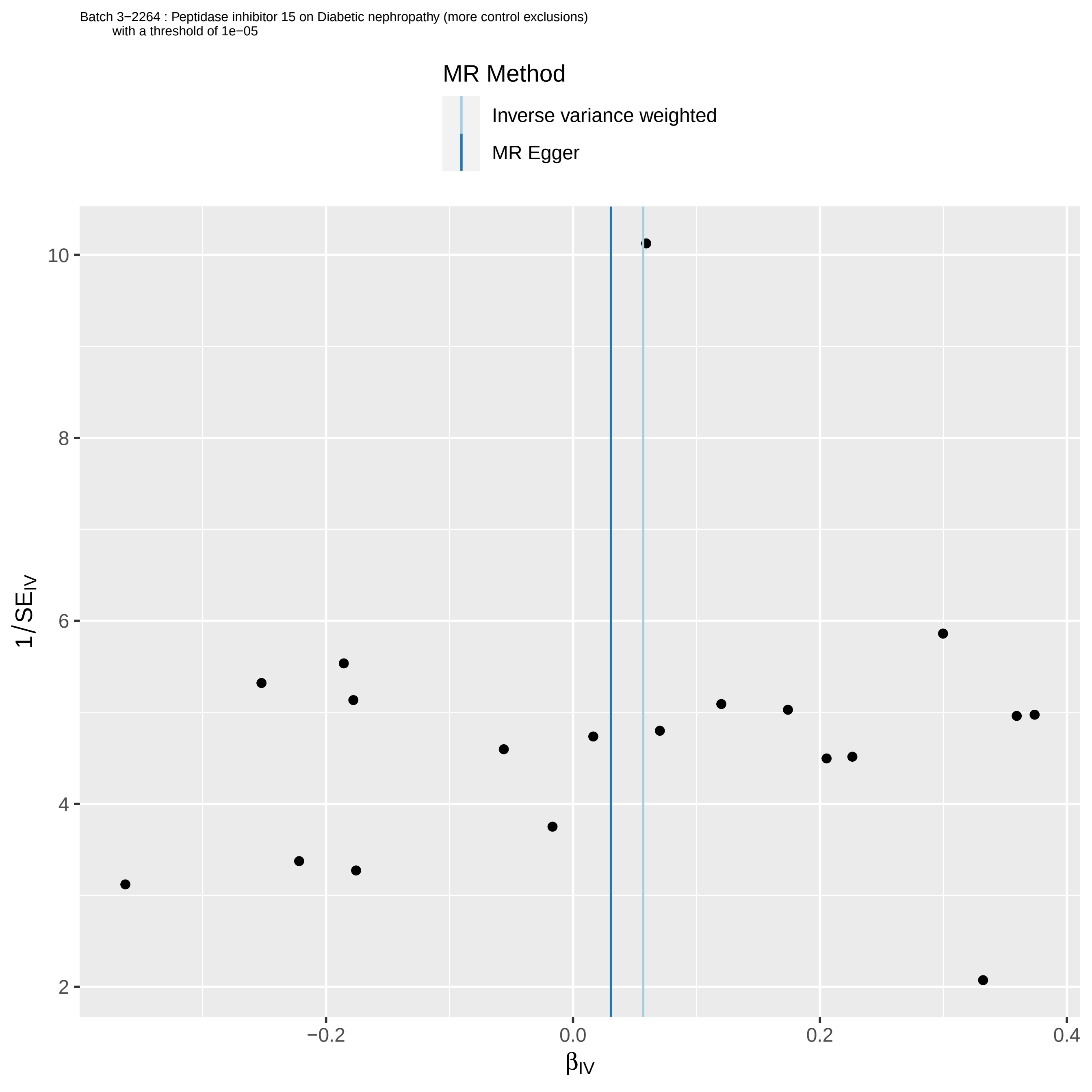
Batch 3−2264 : Peptidase inhibitor 15 on Diabetic nephropathy (more control exclusions)
with a threshold of 1e−05
MR Method
Inverse variance weighted
MR Egger
10
8
6
4
2
1/SE$_{IV}$
−0.2
0.0
0.2
0.4
β_{IV}

Batch 3−2265 : Elafin on Diabetic nephropathy (more control exclusions)
with a threshold of 1e−05

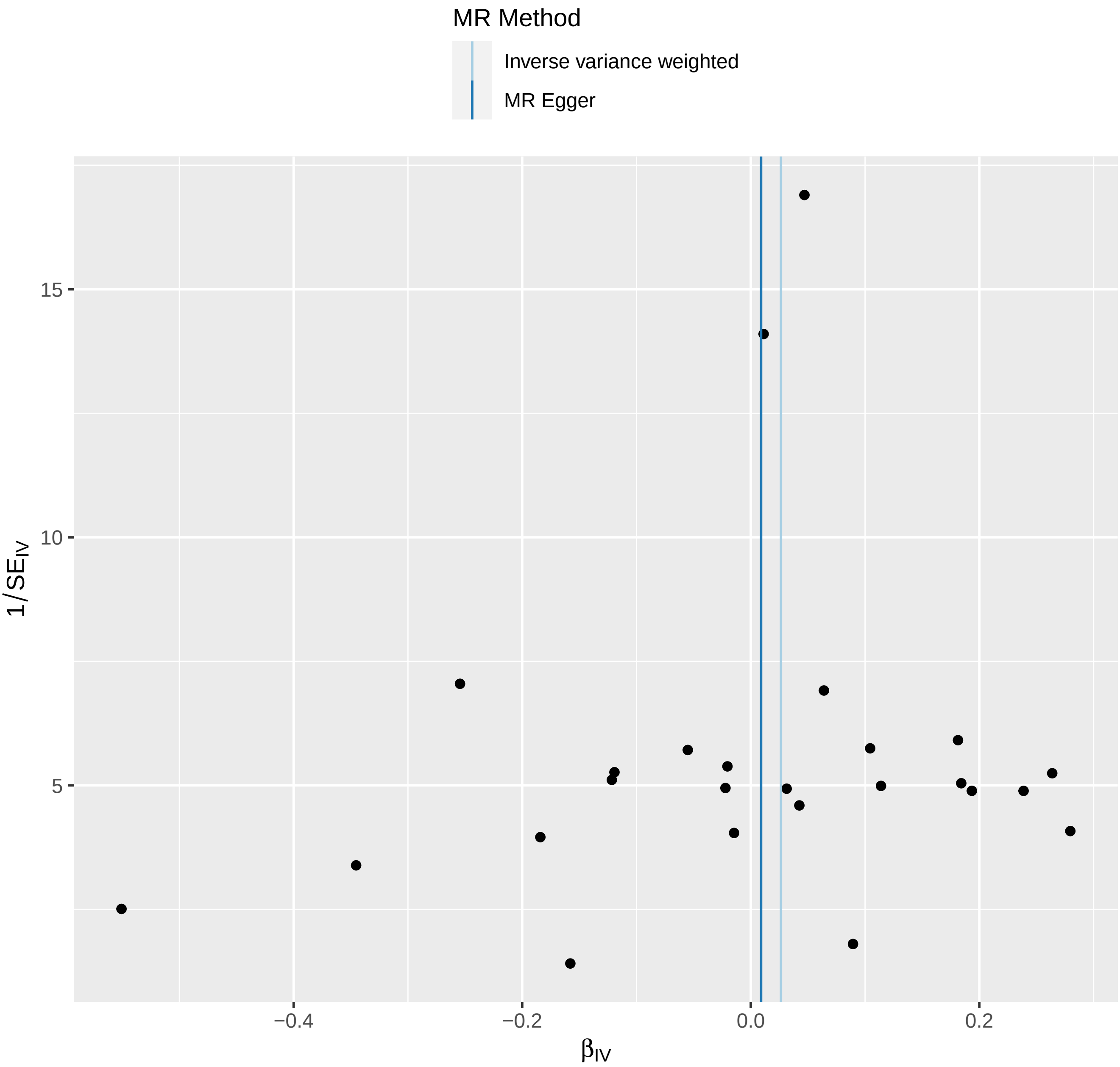

Batch 3−2266 : PILR alpha−associated neural protein on Diabetic nephropathy (more control exclusions)
with a threshold of 1e−05

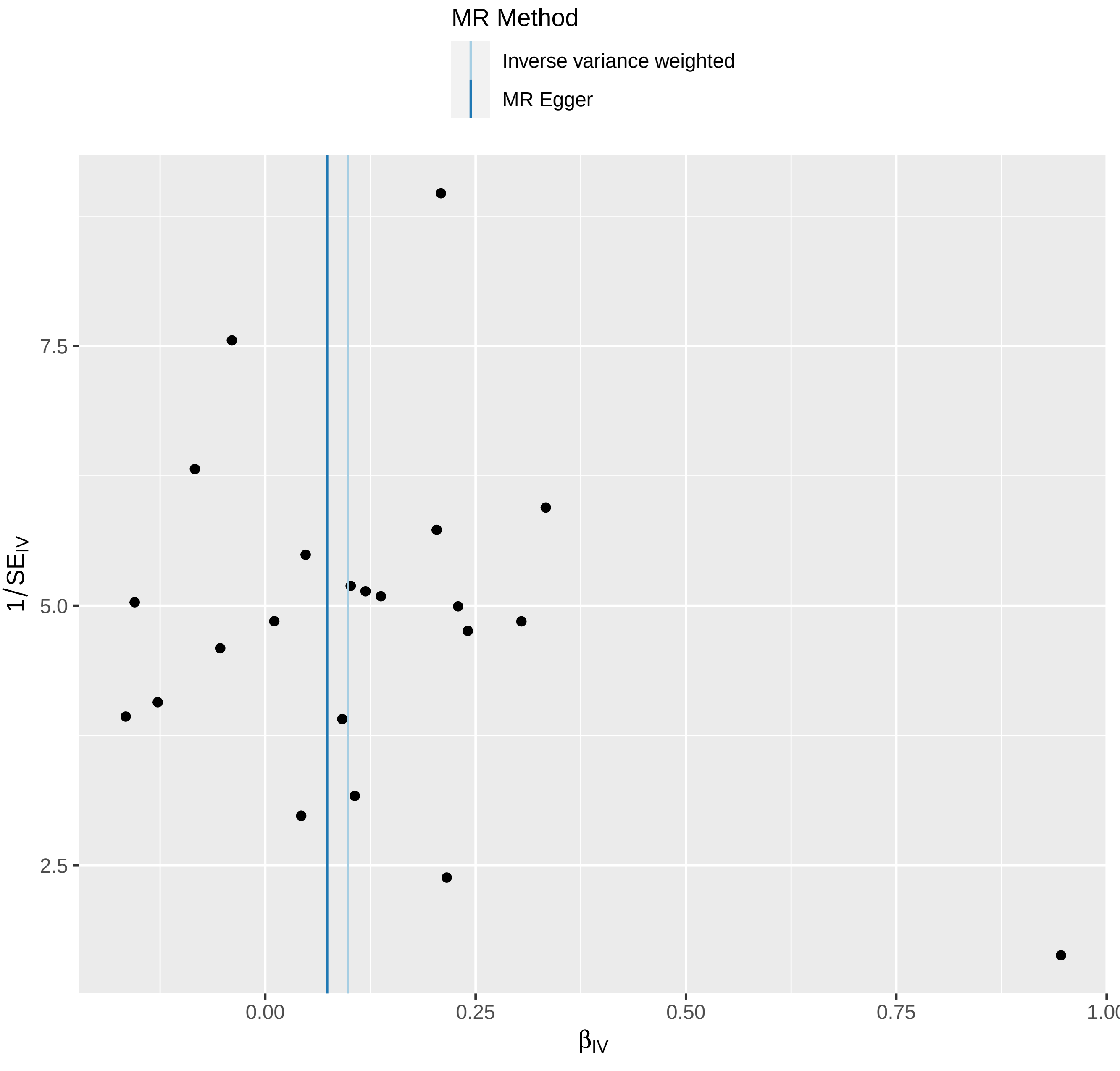

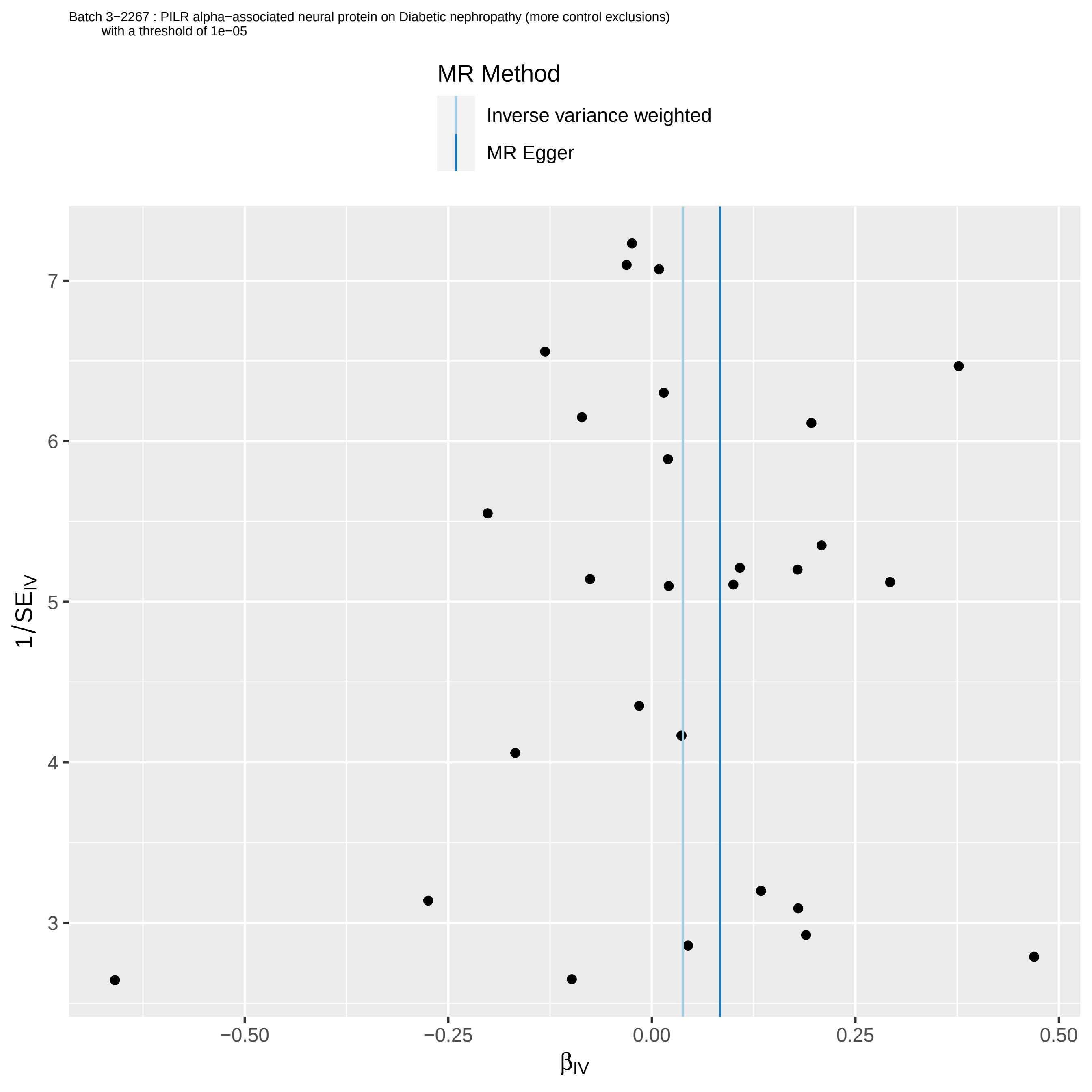
Batch 3−2267 : PILR alpha−associated neural protein on Diabetic nephropathy (more control exclusions)
with a threshold of 1e−05
MR Method
Inverse variance weighted
MR Egger
1/SE_IV
β_IV
7
6
5
4
3
−0.50
−0.25
0.00
0.25
0.50

Batch 3−2268 : Polymeric immunoglobulin receptor on Diabetic nephropathy (more control exclusions)
with a threshold of 1e−05

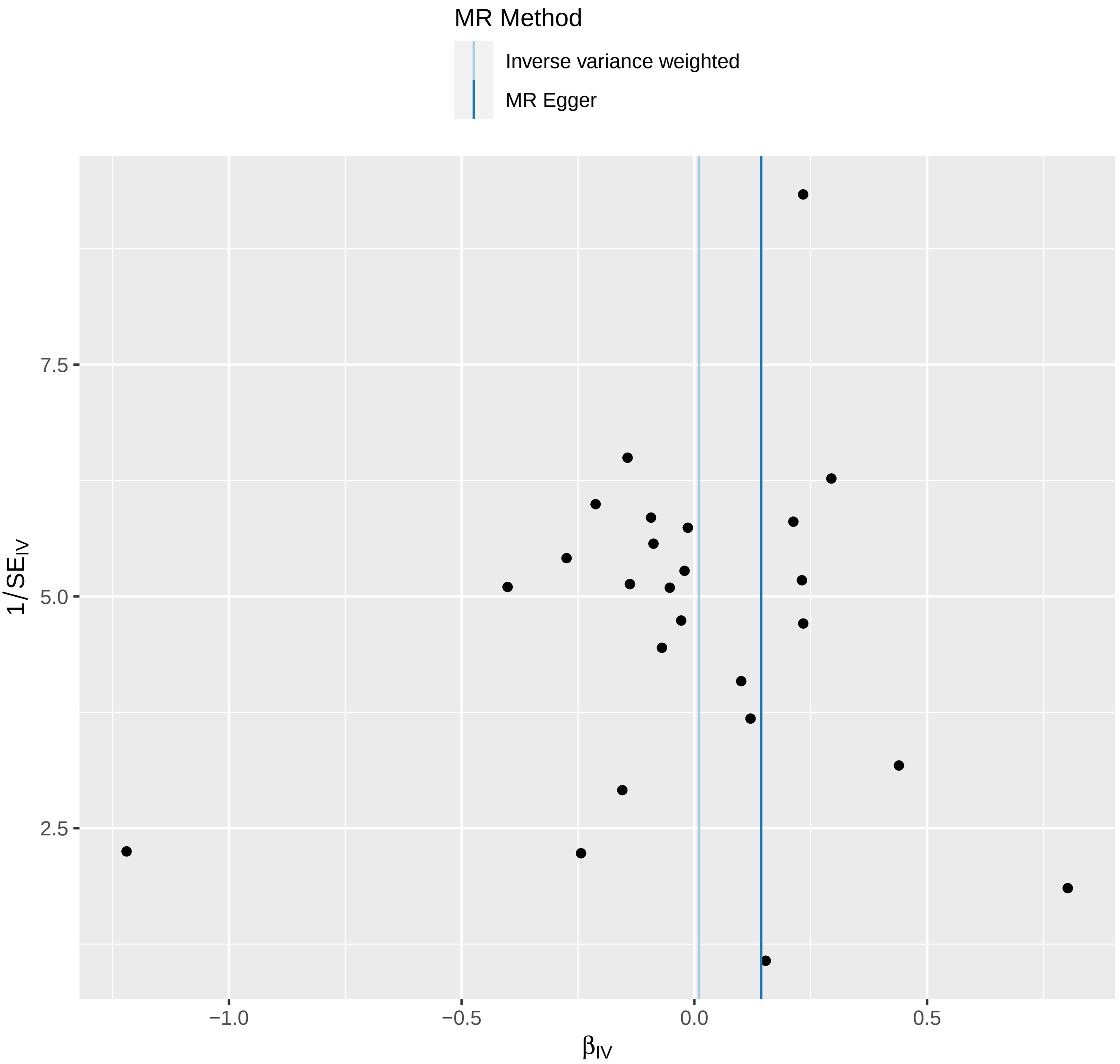

Batch 3−2269 : Phosphatidylinositol 4−phosphate 3−kinase C2 domain−containing subunit alpha on Diabetic nephropathy (more control exclusions) with a threshold of 1e−05

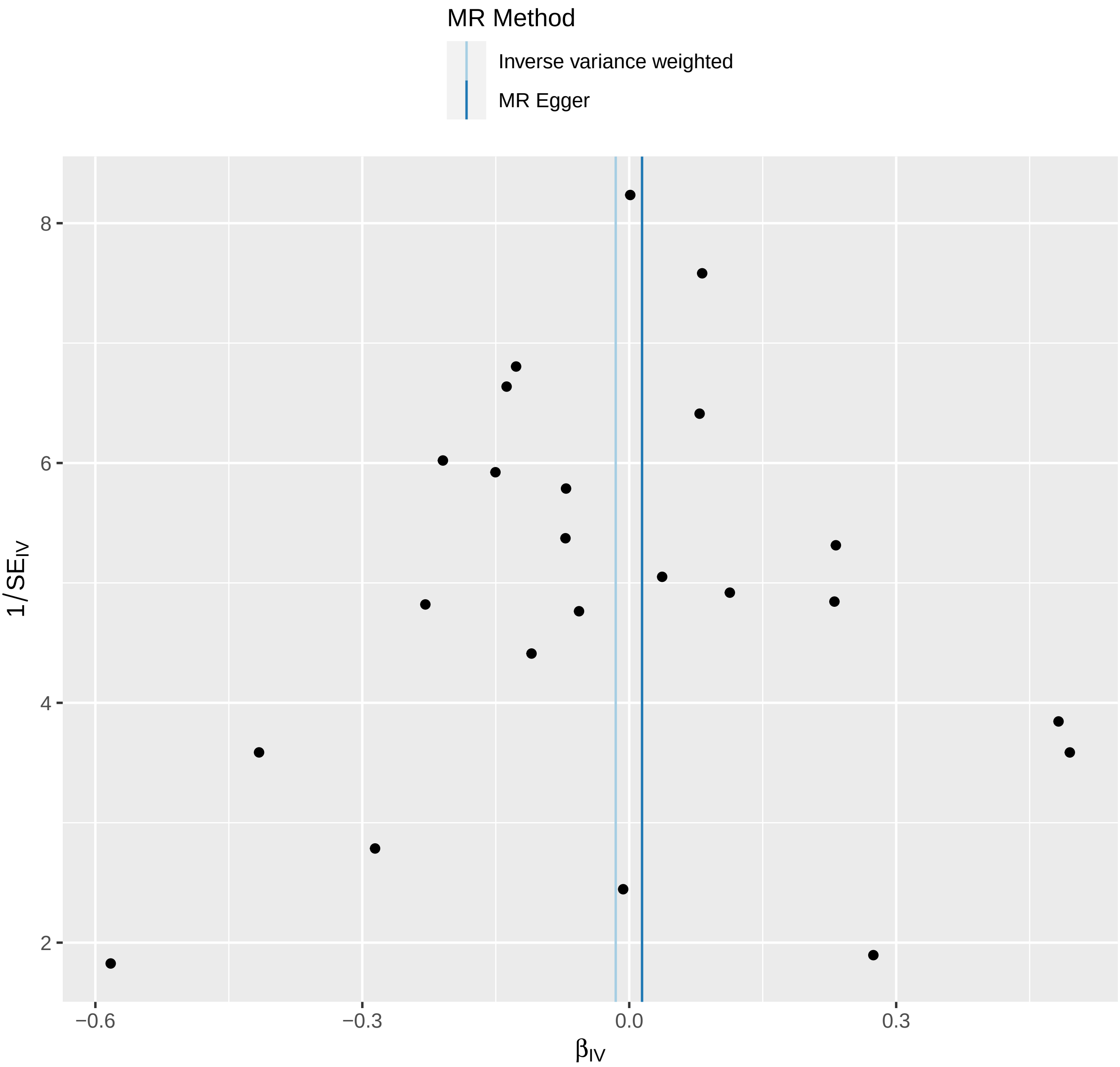

Batch 3−2270 : PIK3CA/PIK3R1 on Diabetic nephropathy (more control exclusions)
with a threshold of 1e−05

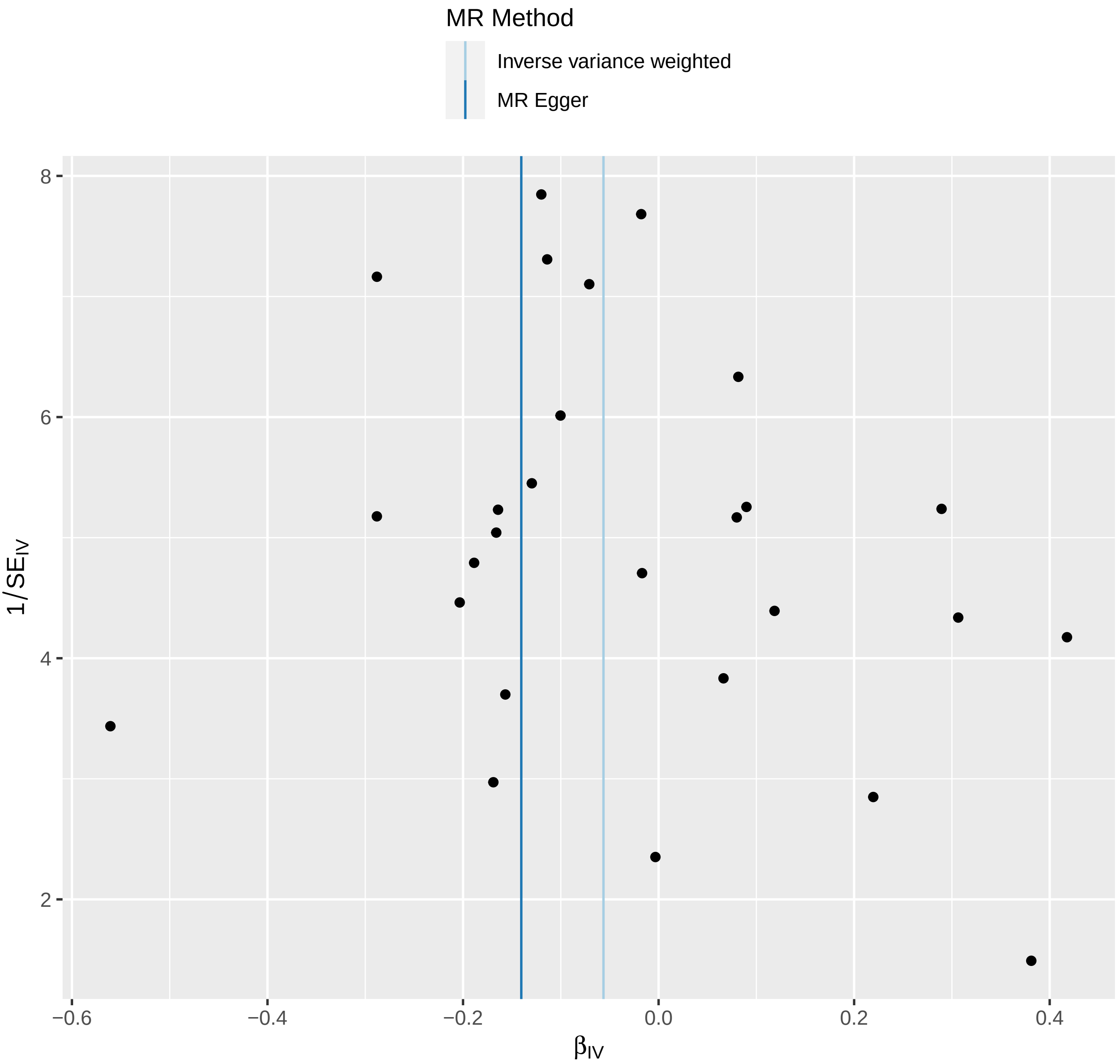

Batch 3−2271 : Phosphatidylinositol 4,5−bisphosphate 3−kinase catalytic subunit gamma isoform on Diabetic nephropathy (more control exclusions) with a threshold of 1e−05

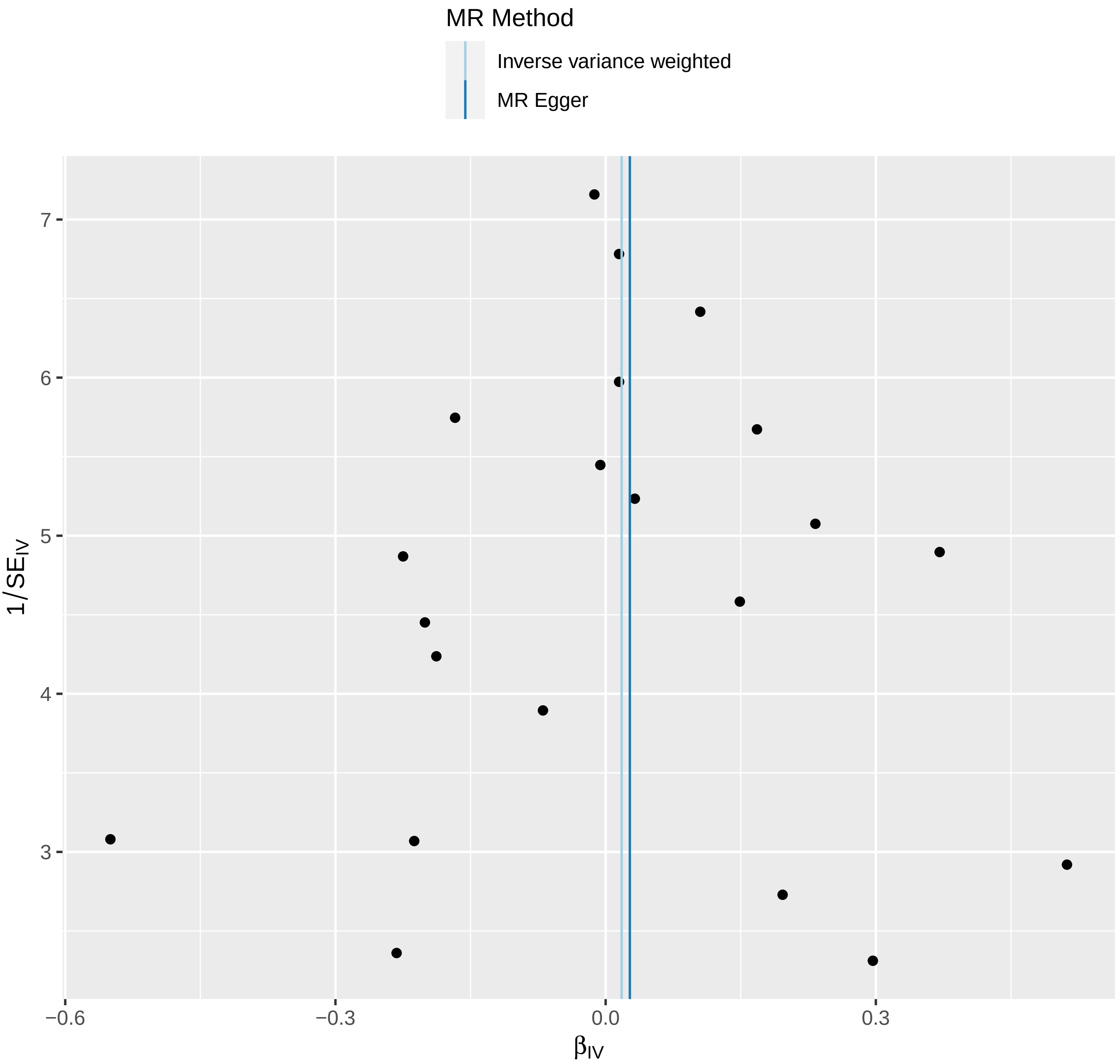

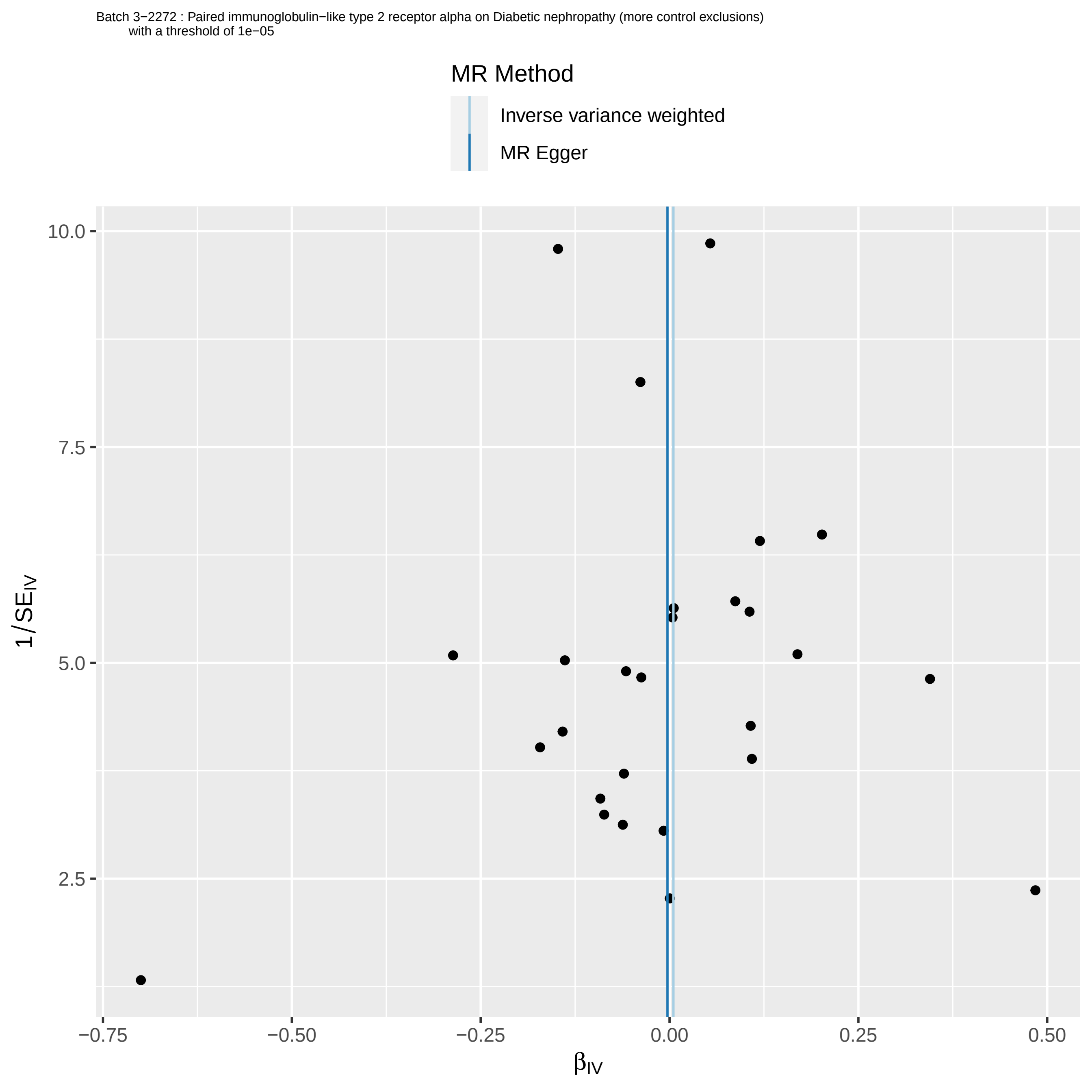

Batch 3−2272 : Paired immunoglobulin−like type 2 receptor alpha on Diabetic nephropathy (more control exclusions)
with a threshold of 1e−05
MR Method
Inverse variance weighted
MR Egger
10.0
7.5
5.0
2.5
1/SE$_{IV}$
−0.75
−0.50
−0.25
0.00
0.25
0.50
β$_{IV}$

Batch 3−2273 : Paired immunoglobulin−like type 2 receptor alpha on Diabetic nephropathy (more control exclusions)
with a threshold of 1e−05

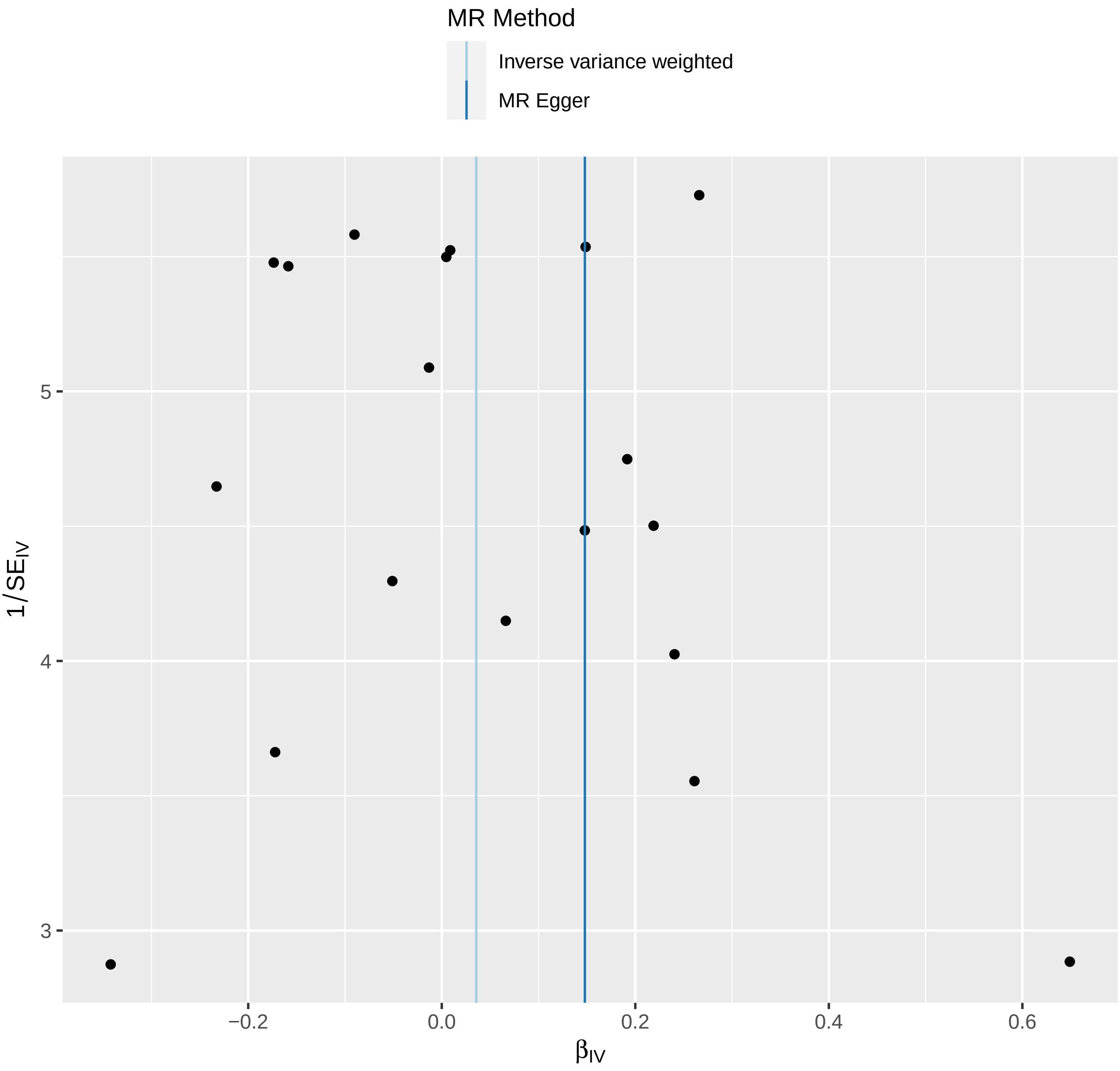

Batch 3−2274 : Serine/threonine−protein kinase pim−1 on Diabetic nephropathy (more control exclusions) with a threshold of 1e−05

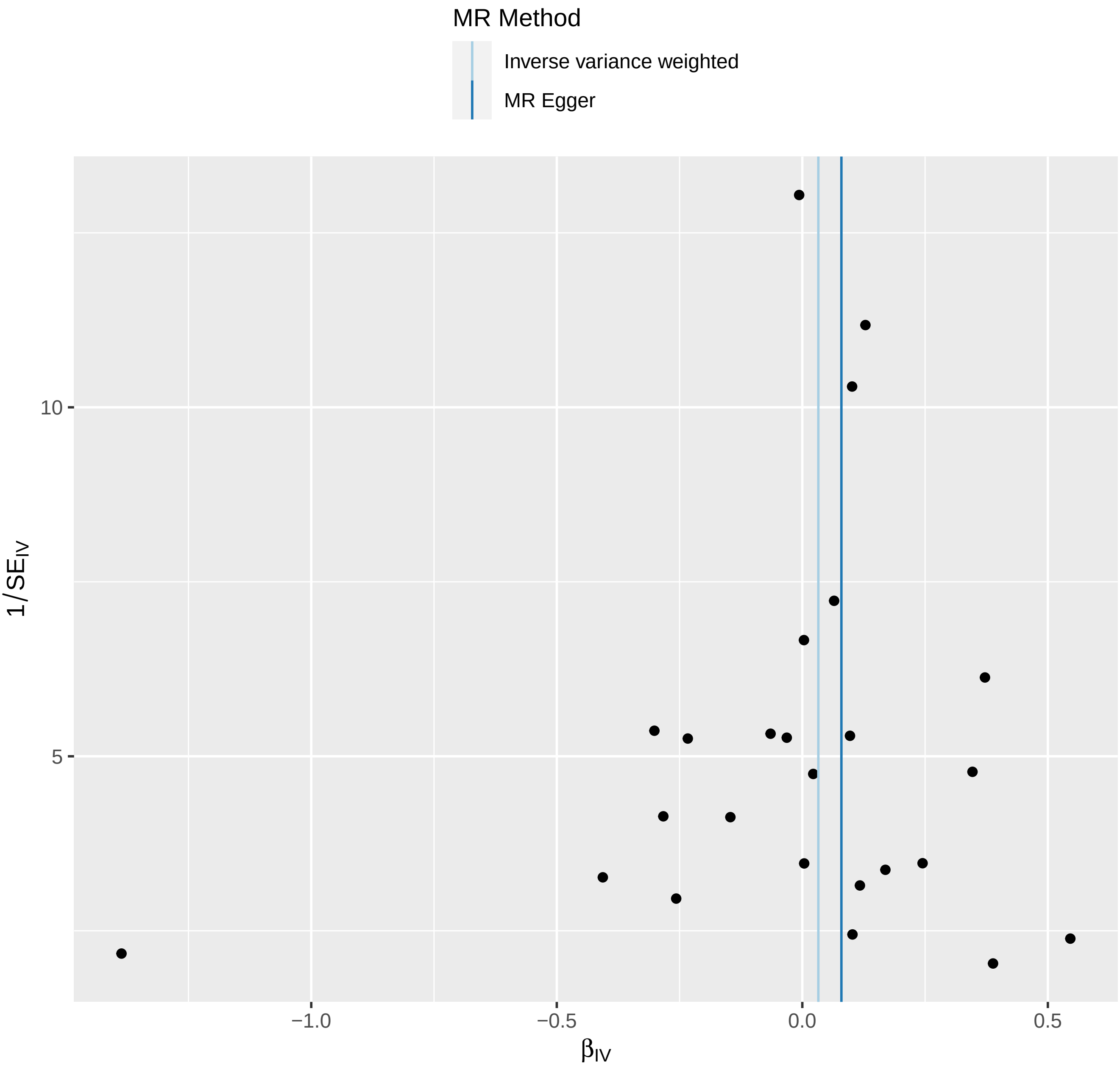

Batch 3−2275 : Peptidyl−prolyl cis−trans isomerase NIMA−interacting 4 on Diabetic nephropathy (more control exclusions) with a threshold of 1e−05

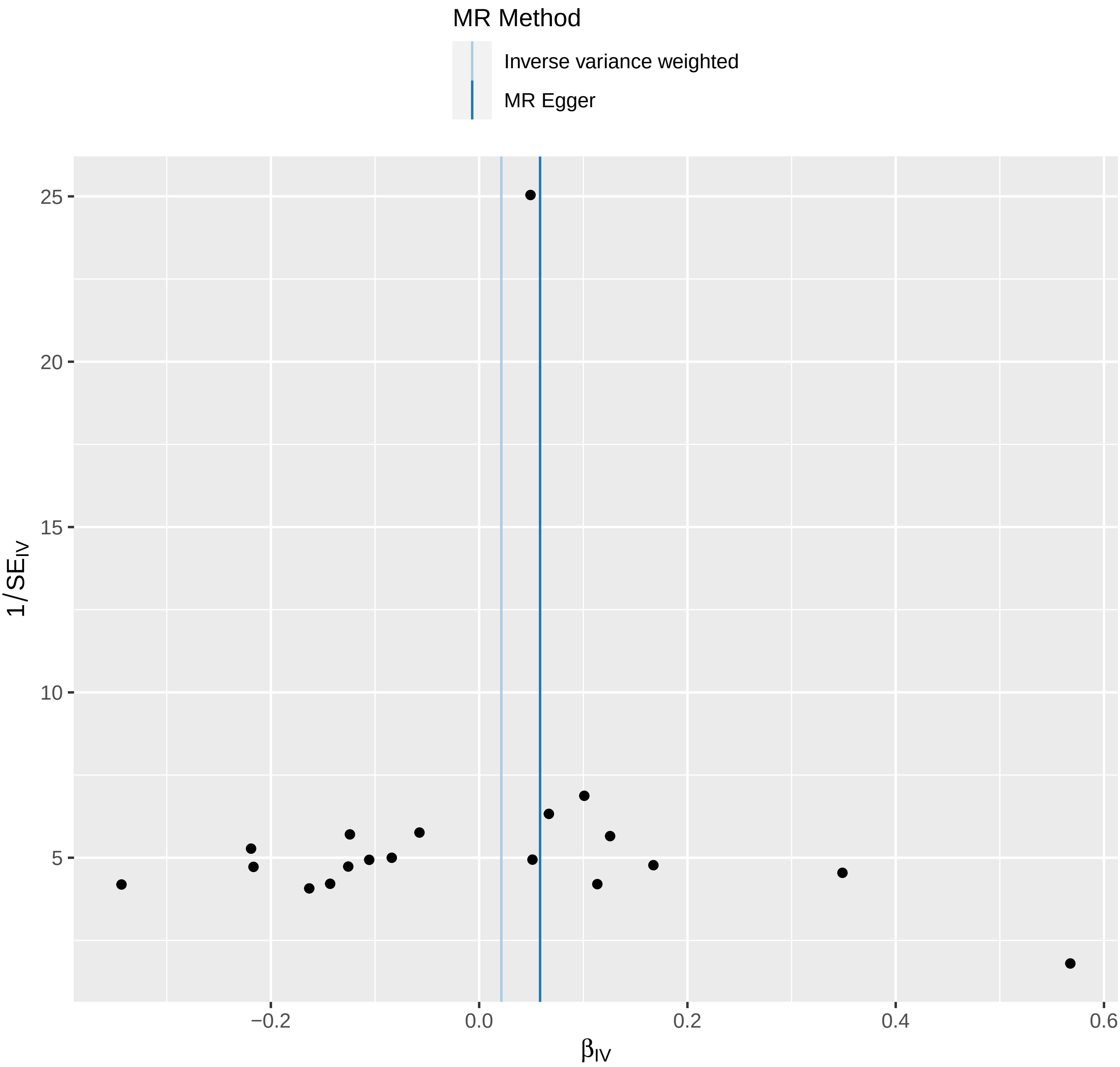

Batch 3−2276 : Phosphatidylinositol 5−phosphate 4−kinase type−2 alpha on Diabetic nephropathy (more control exclusions) with a threshold of 1e−05

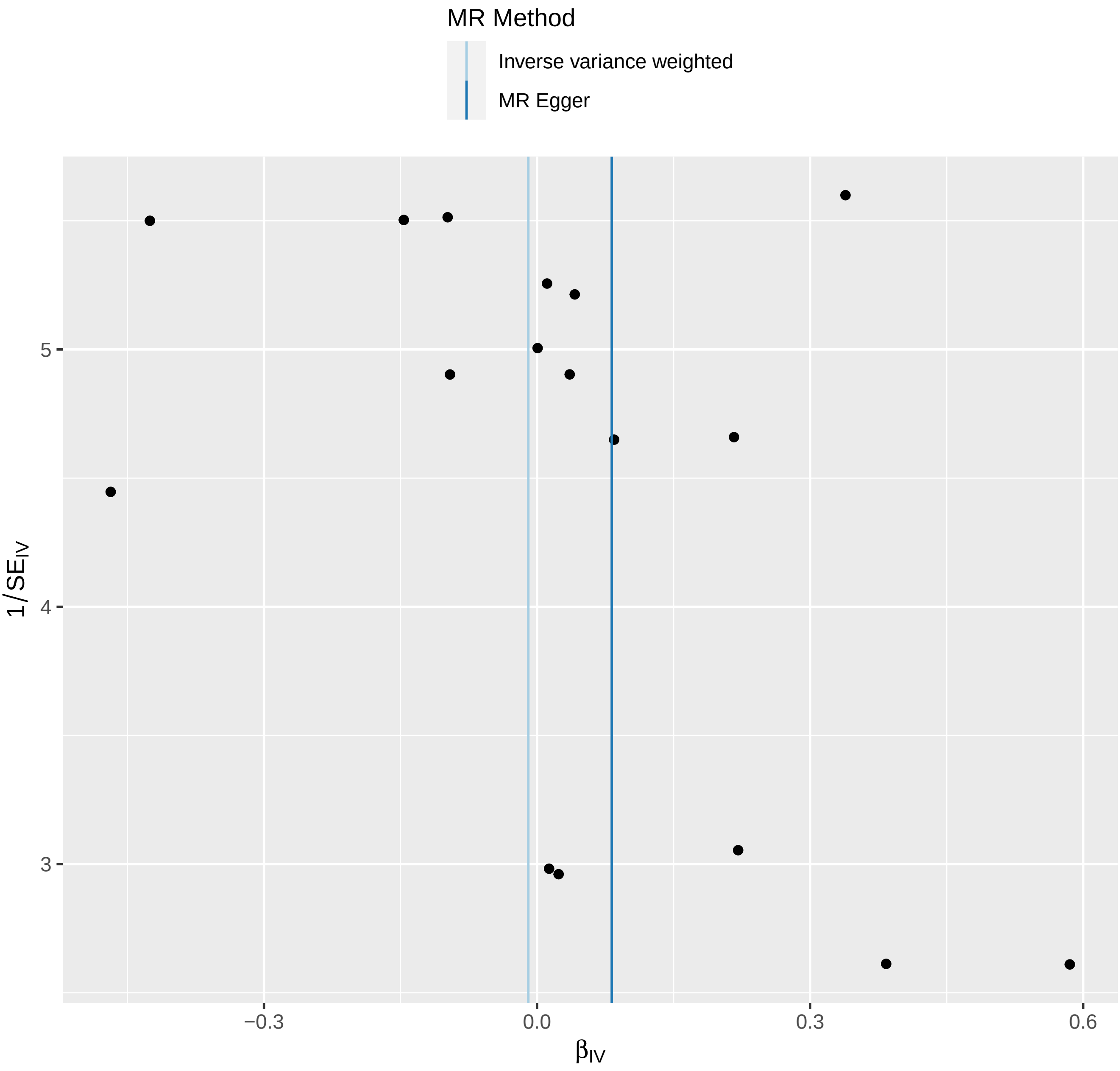

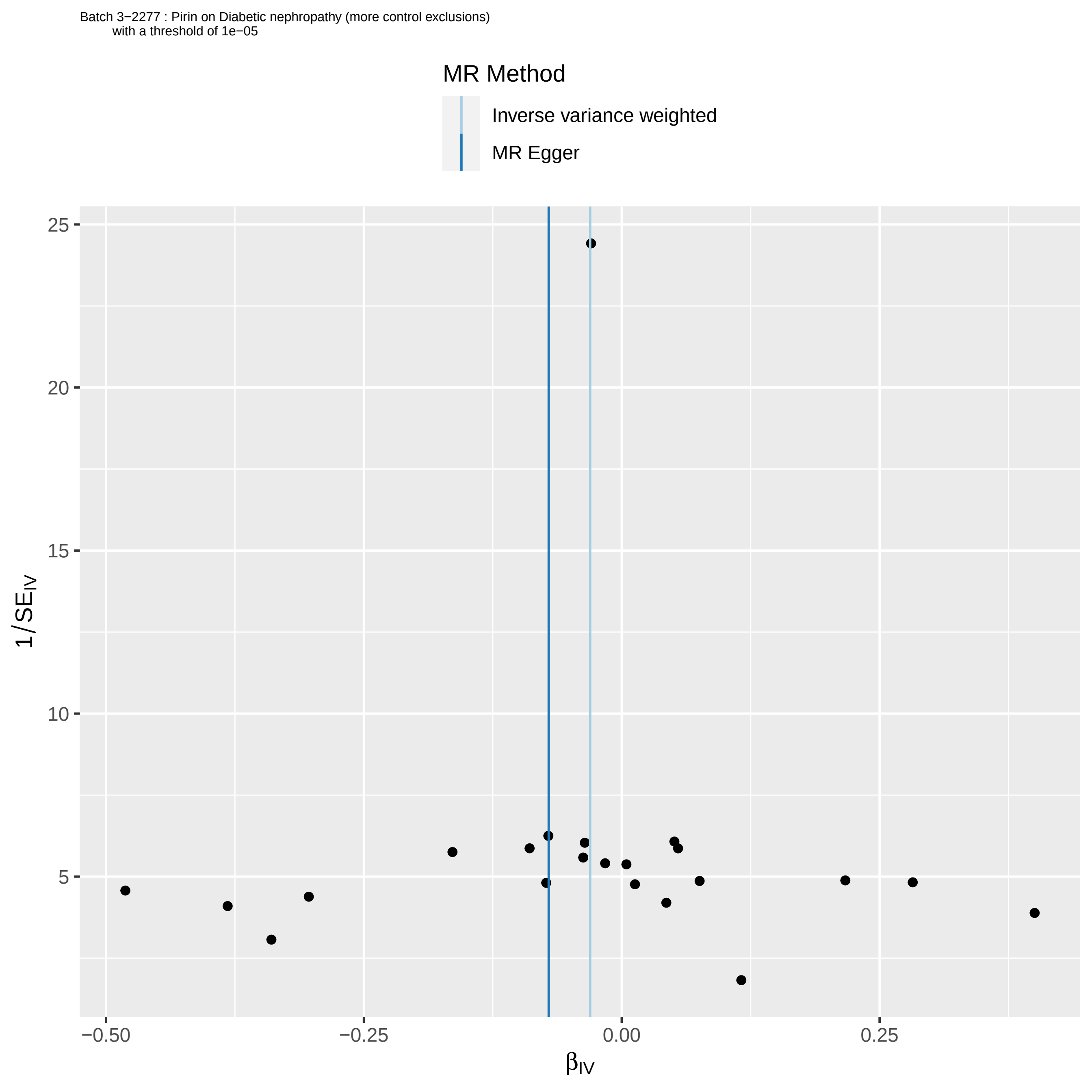
Batch 3−2277 : Pirin on Diabetic nephropathy (more control exclusions)
with a threshold of 1e−05
MR Method
Inverse variance weighted
MR Egger
25
20
15
10
5
1/SE_IV
−0.50
−0.25
0.00
0.25
β_IV

Batch 3−2278 : Phosphatidylinositol transfer protein alpha isoform on Diabetic nephropathy (more control exclusions)
with a threshold of 1e−05

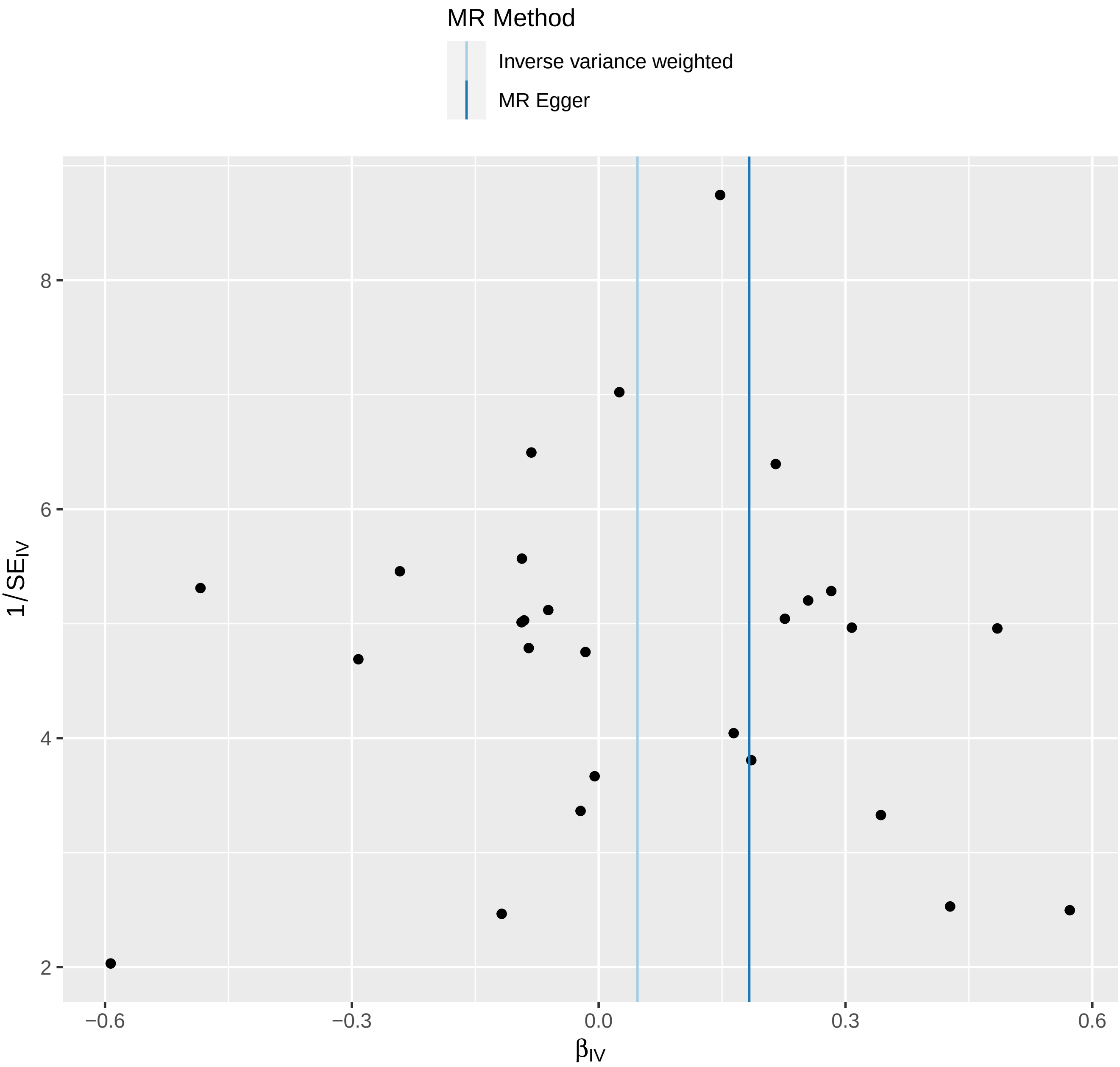

Batch 3−2279 : Phosphatidylinositol transfer protein beta isoform on Diabetic nephropathy (more control exclusions)
with a threshold of 1e−05

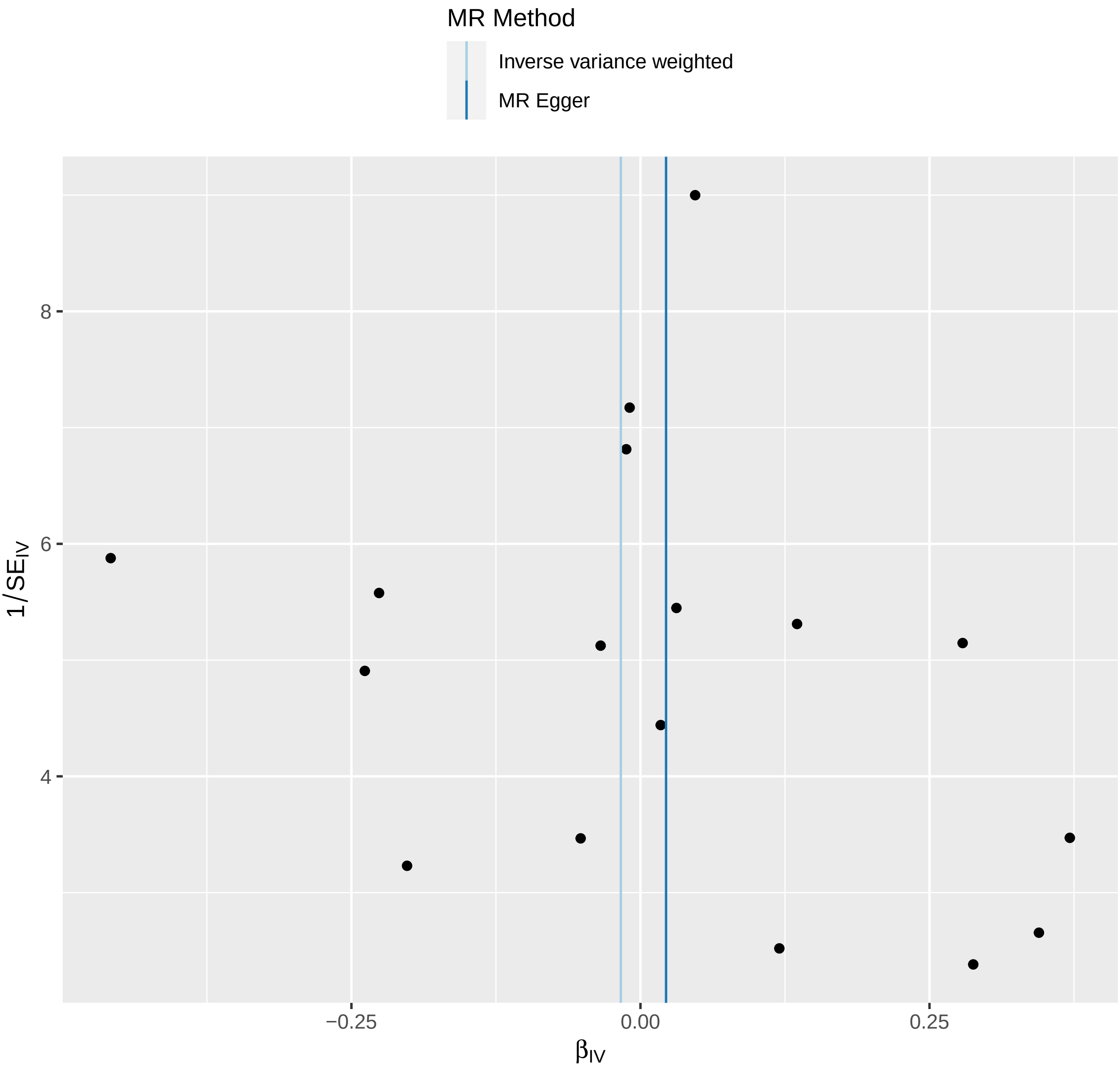

Batch 3−2280 : Piwi−like protein 1 on Diabetic nephropathy (more control exclusions)
with a threshold of 1e−05

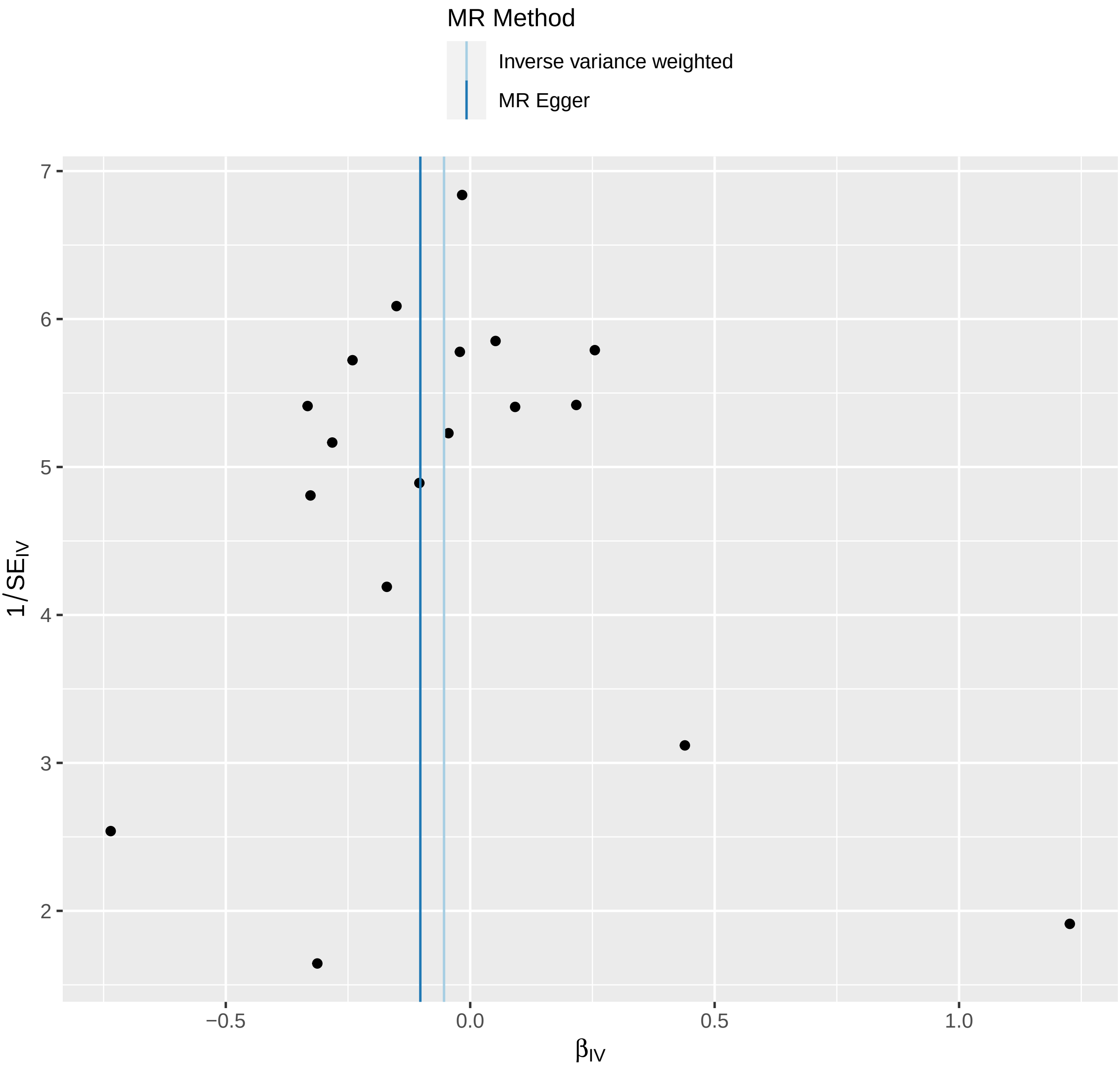

Batch 3−2281 : Polycystin−2 on Diabetic nephropathy (more control exclusions)
with a threshold of 1e−05

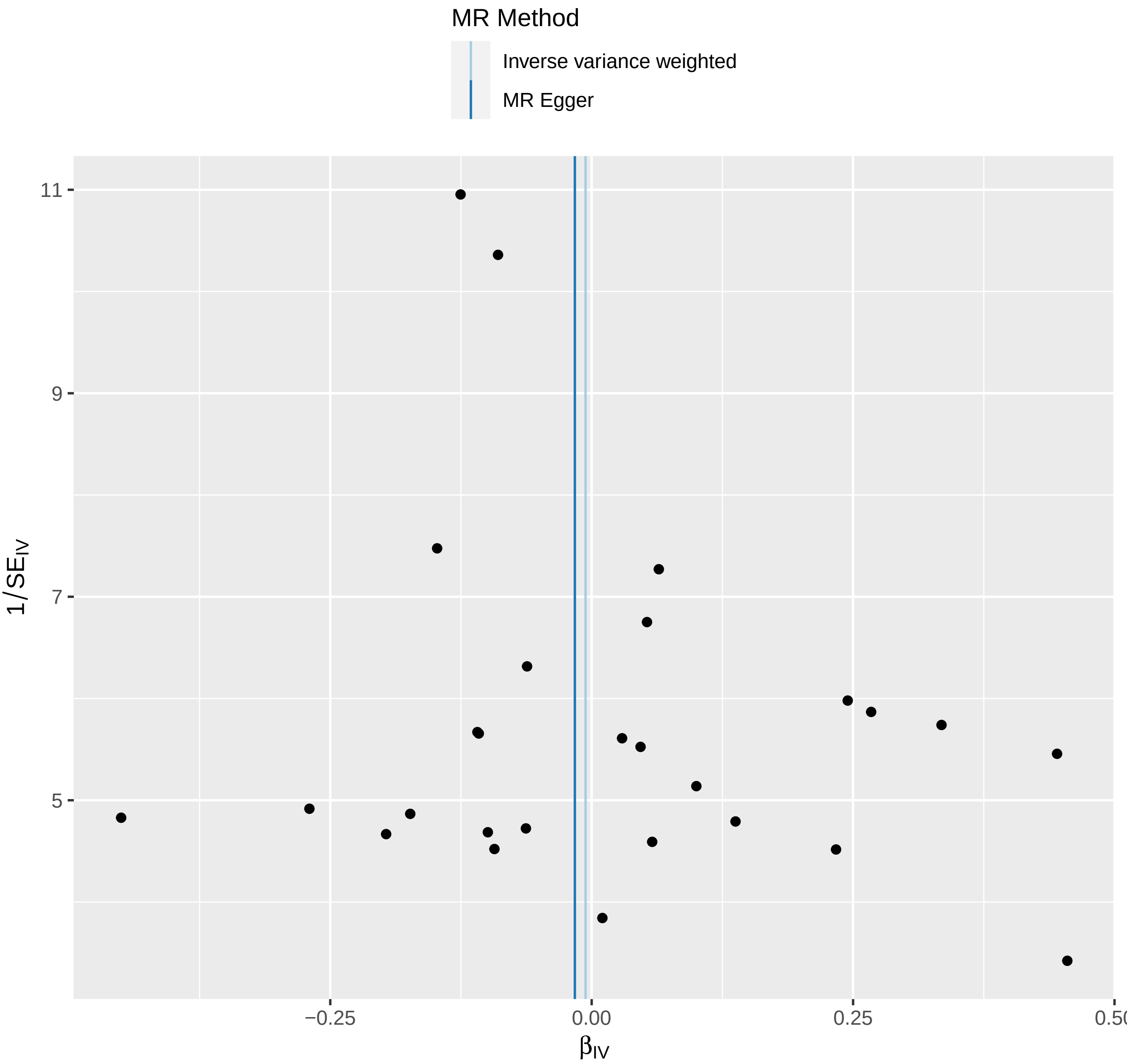

Batch 3−2282 : Pyruvate kinase PKLR on Diabetic nephropathy (more control exclusions)
with a threshold of 1e−05

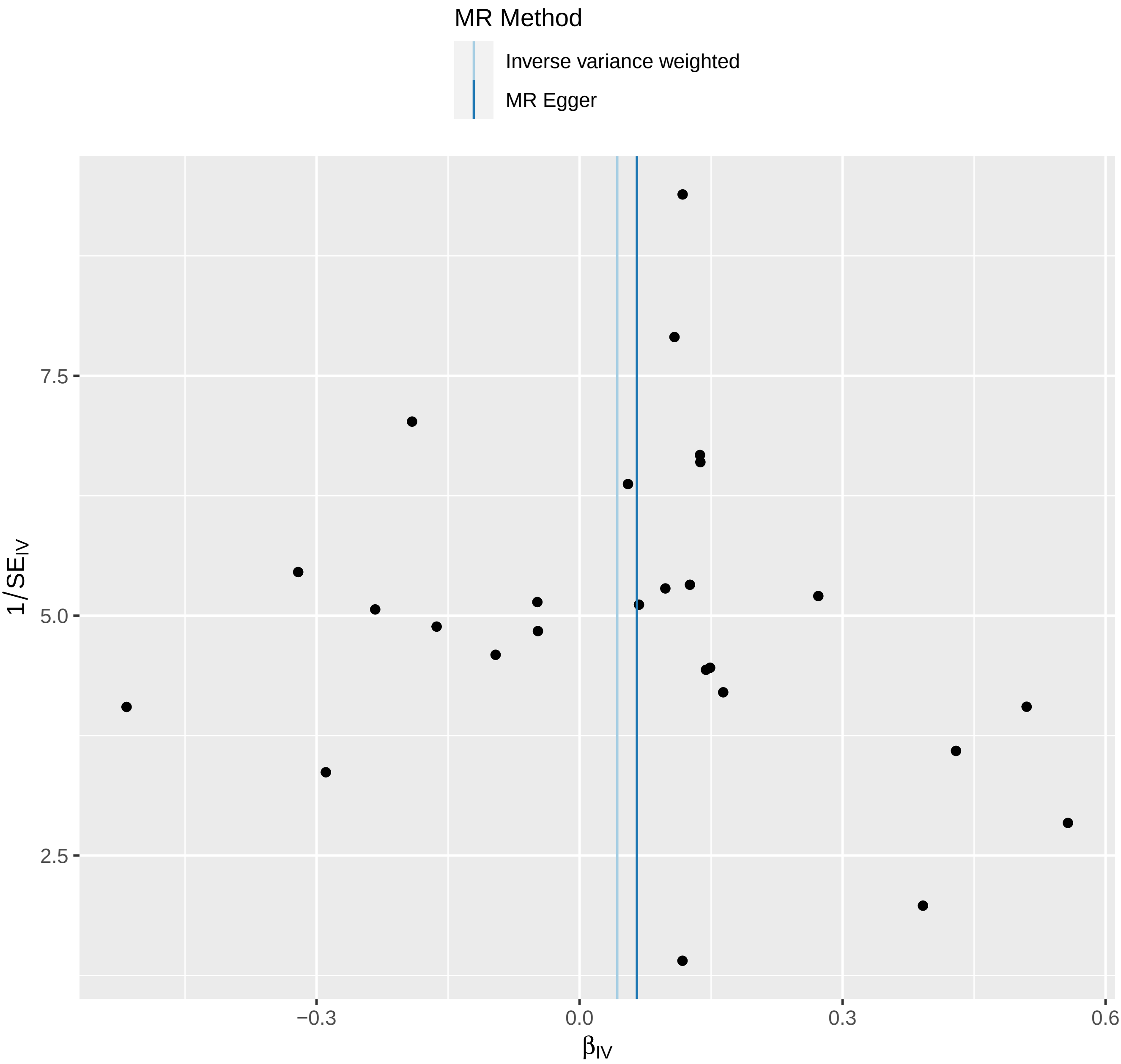

Batch 3−2283 : Group 10 secretory phospholipase A2 on Diabetic nephropathy (more control exclusions)
with a threshold of 1e−05

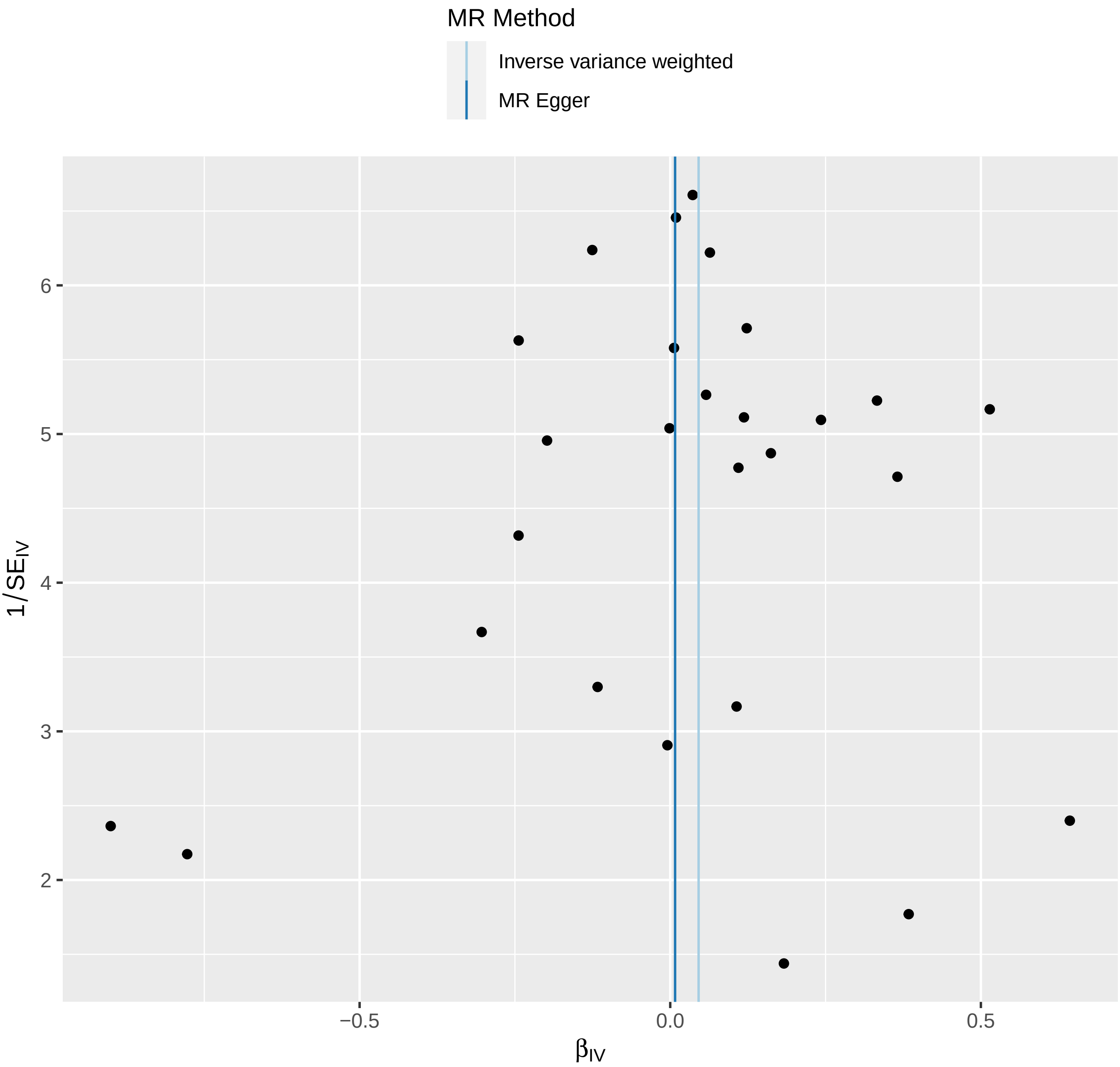

Batch 3−2284 : Group XIIB secretory phospholipase A2−like protein on Diabetic nephropathy (more control exclusions)
with a threshold of 1e−05

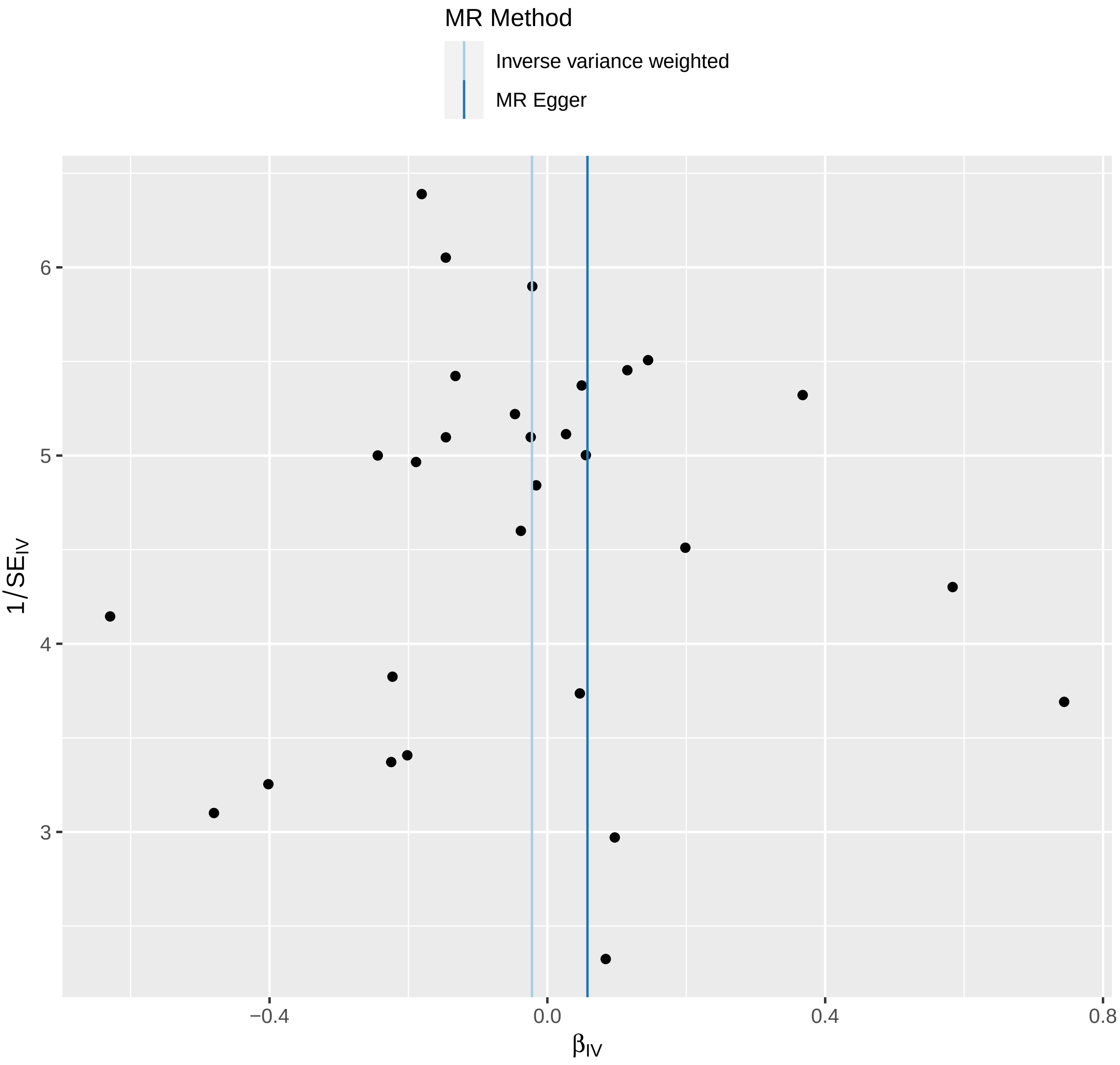

Batch 3−2285 : HRAS−like suppressor 3 on Diabetic nephropathy (more control exclusions)
with a threshold of 1e−05

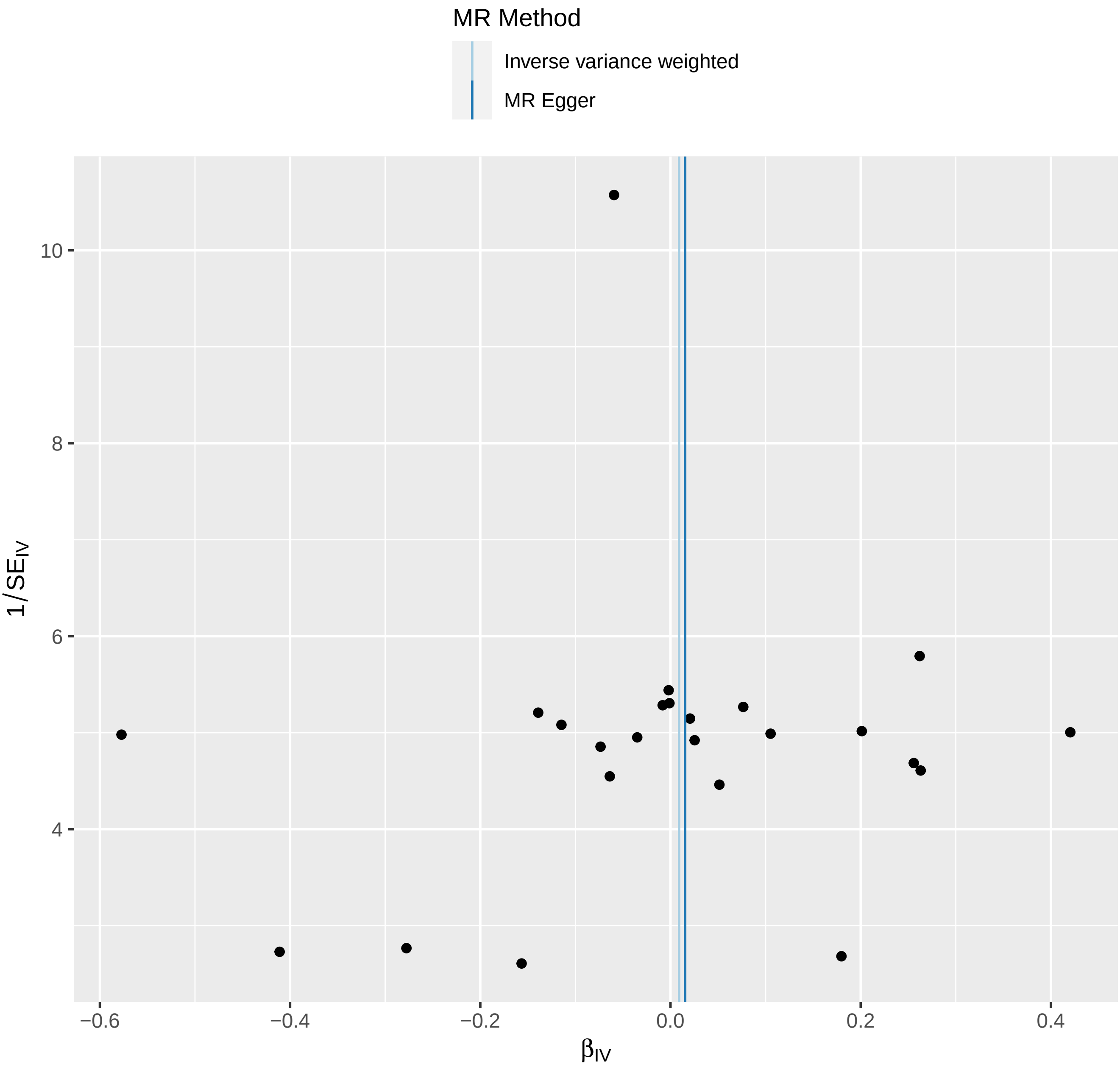

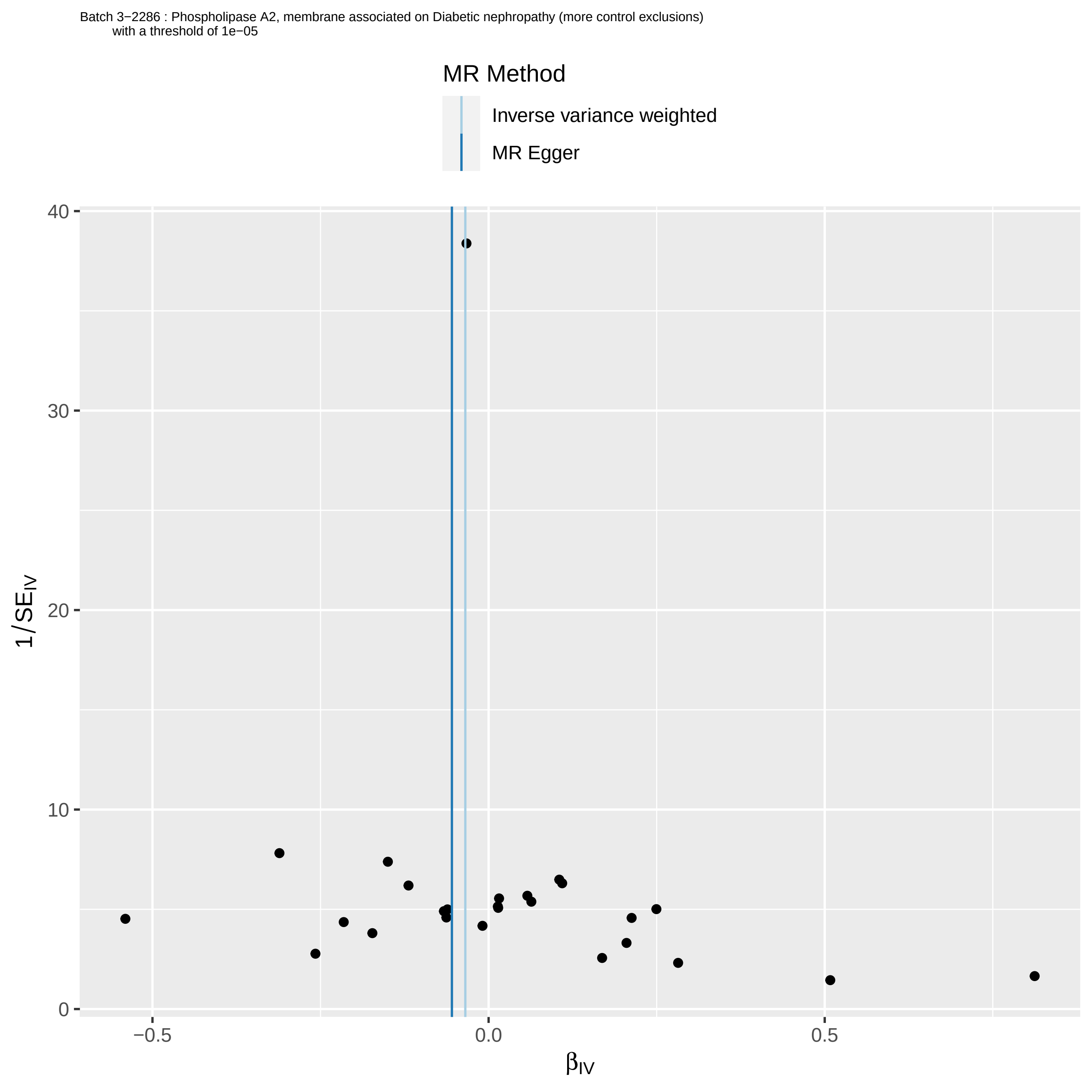

Batch 3−2286 : Phospholipase A2, membrane associated on Diabetic nephropathy (more control exclusions)
with a threshold of 1e−05
MR Method
Inverse variance weighted
MR Egger
40
30
20
10
0
1/SE_IV
−0.5
0.0
0.5
β_IV

Batch 3−2287 : Putative inactive group IIC secretory phospholipase A2 on Diabetic nephropathy (more control exclusions) with a threshold of 1e−05

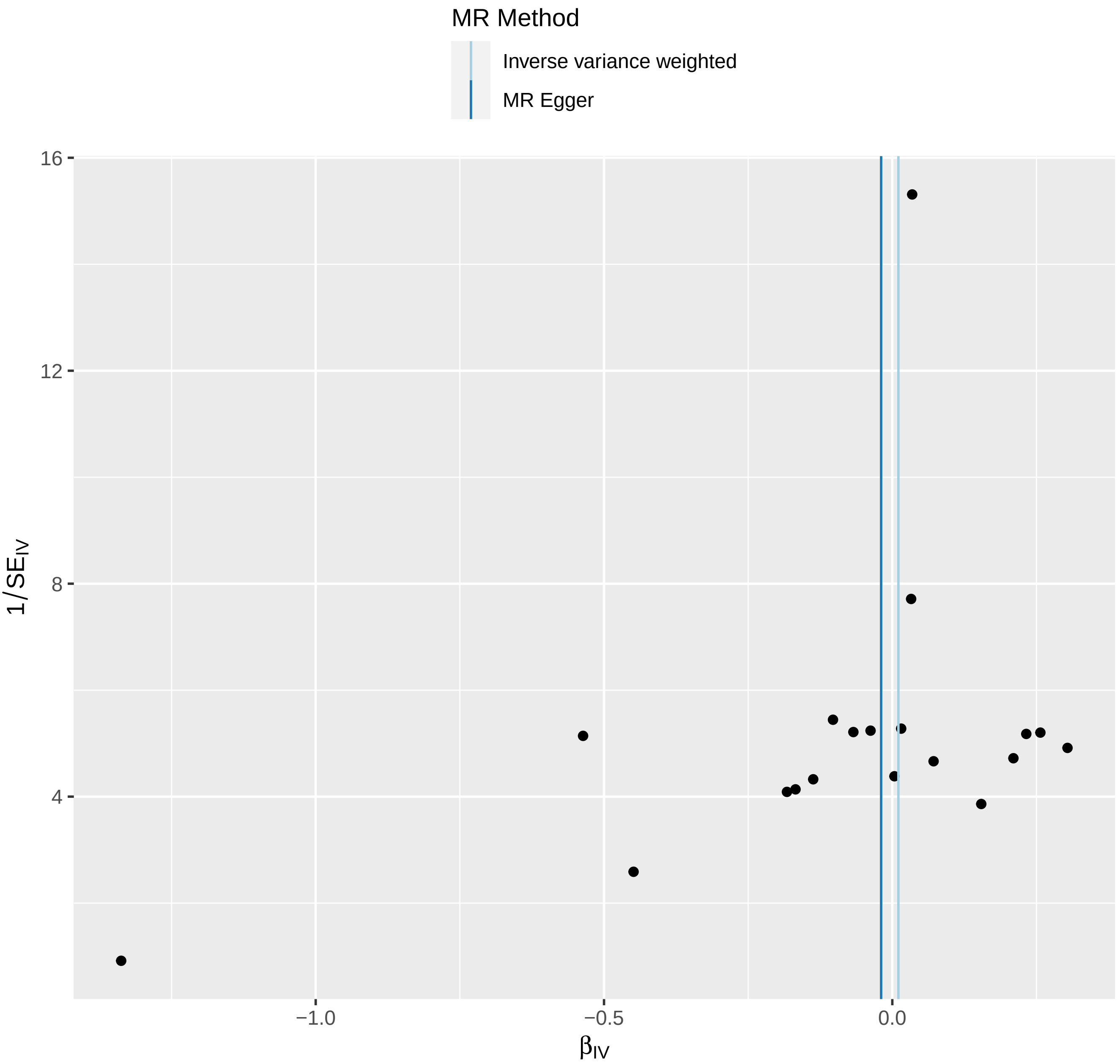

Batch 3−2288 : Group IIE secretory phospholipase A2 on Diabetic nephropathy (more control exclusions)
with a threshold of 1e−05

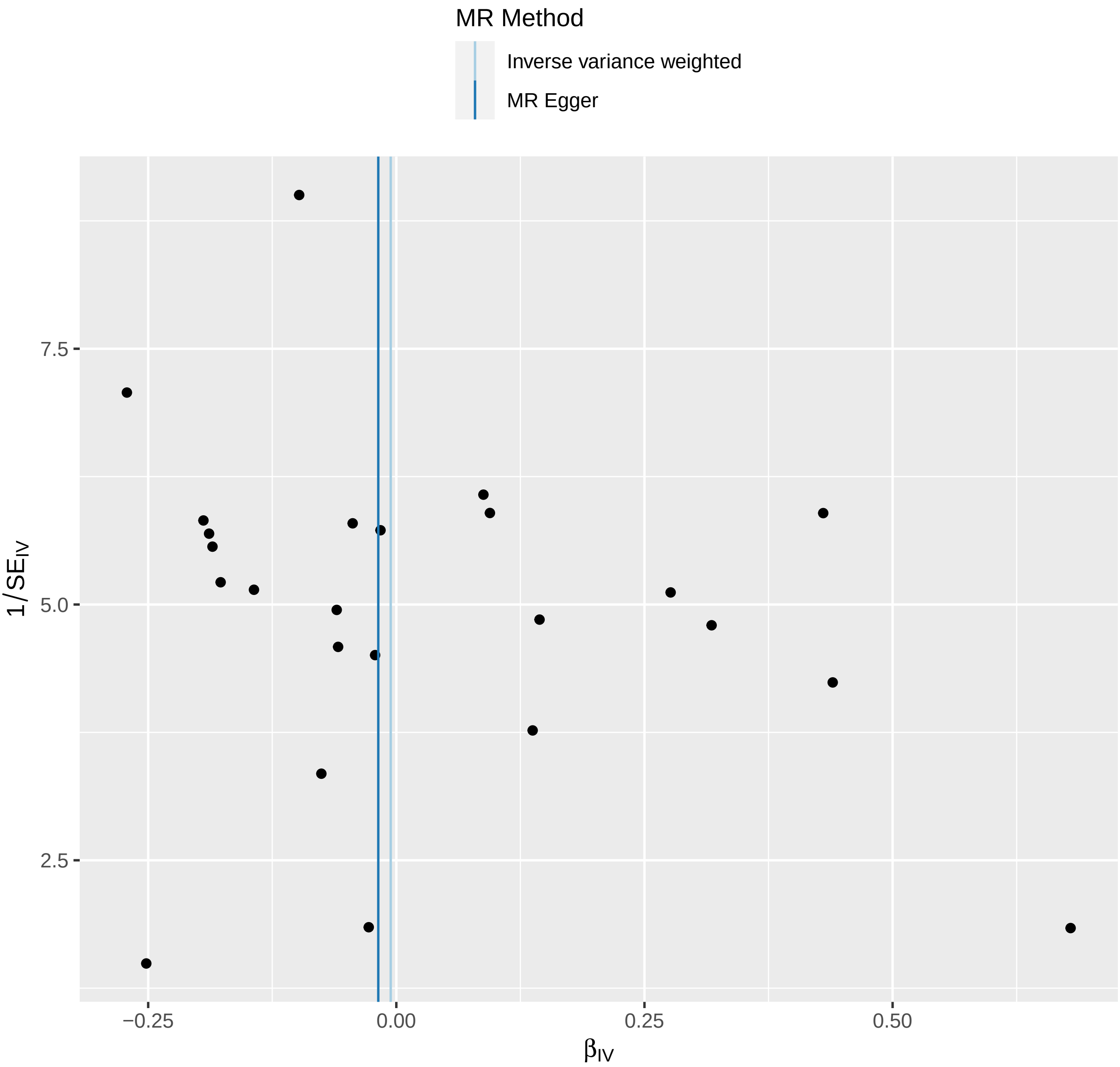

Batch 3−2289 : Group IIE secretory phospholipase A2 on Diabetic nephropathy (more control exclusions)
with a threshold of 1e−05

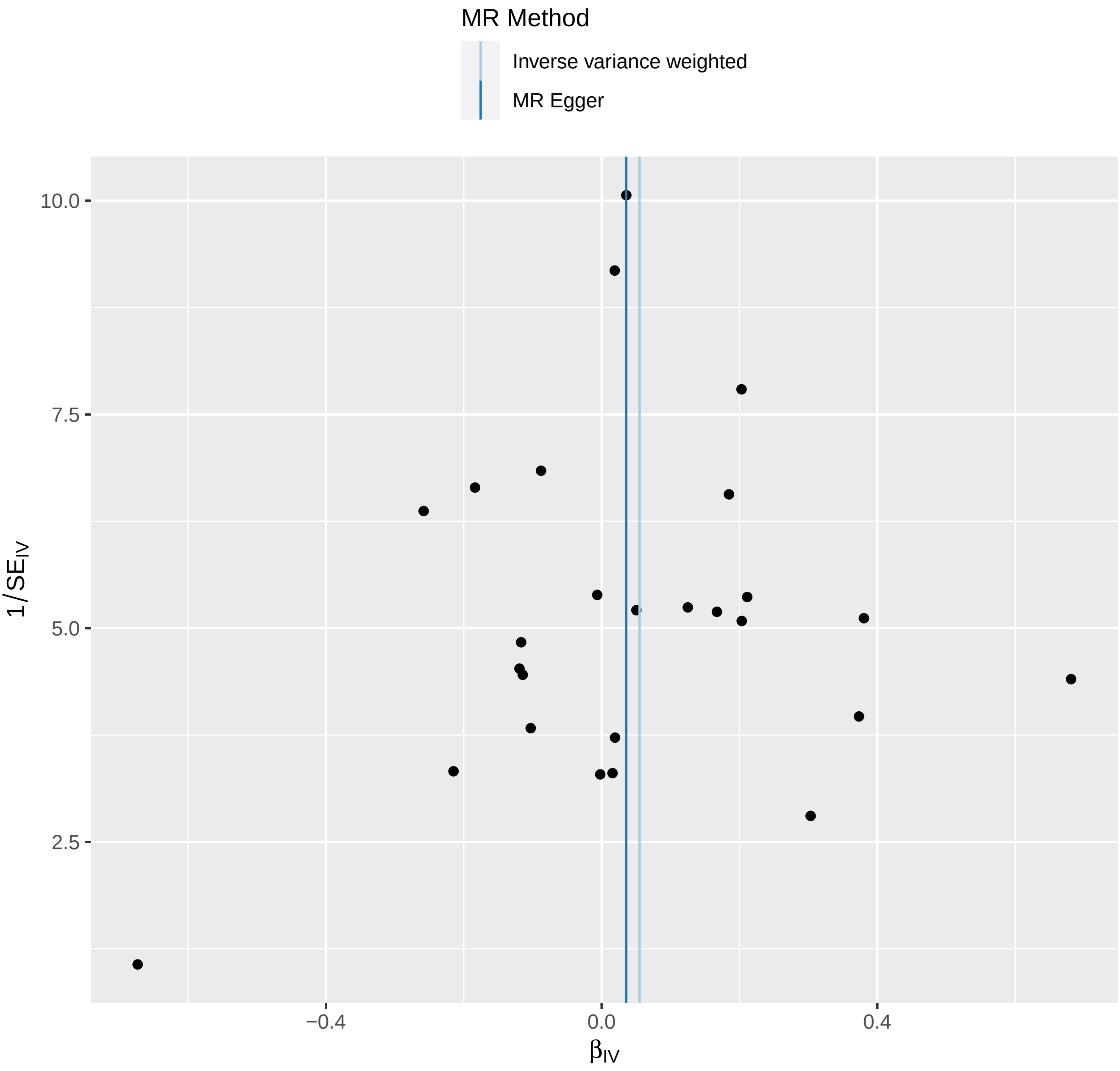

Batch 3−2290 : Calcium−dependent phospholipase A2 on Diabetic nephropathy (more control exclusions)
with a threshold of 1e−05

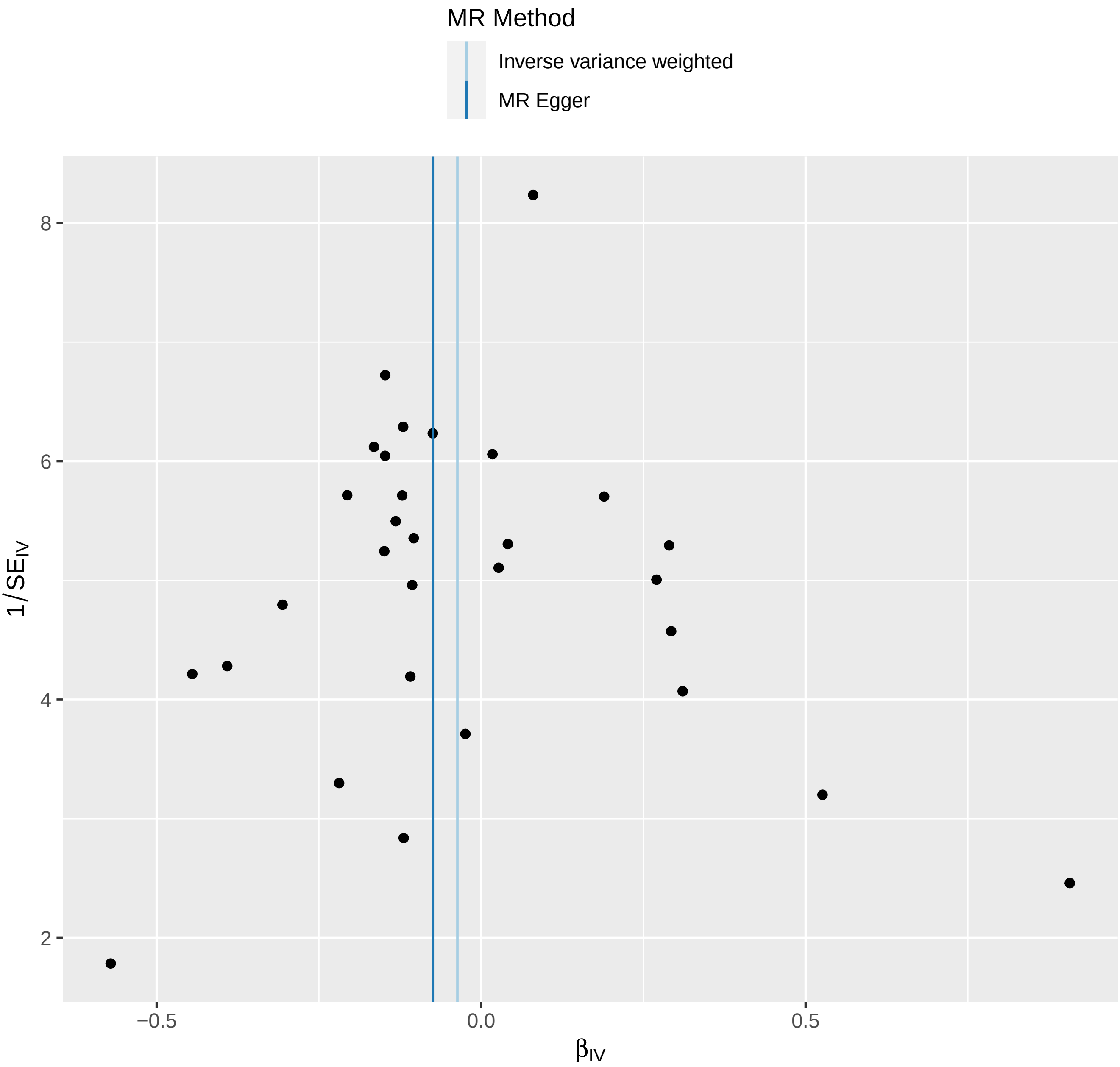

Batch 3−2291 : Secretory phospholipase A2 receptor on Diabetic nephropathy (more control exclusions)
with a threshold of 1e−05

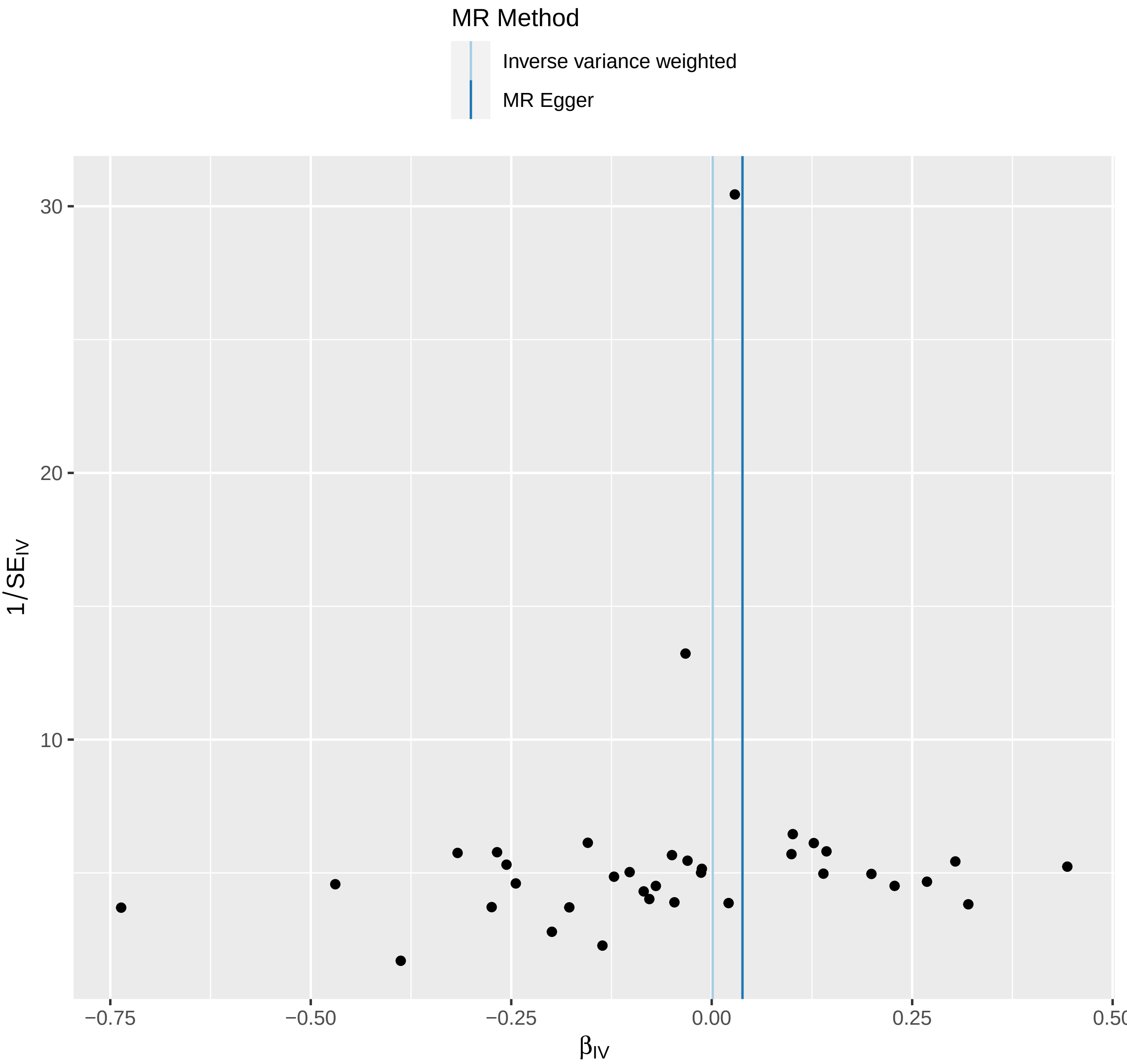

Batch 3−2292 : Urokinase−type plasminogen activator on Diabetic nephropathy (more control exclusions)
with a threshold of 1e−05

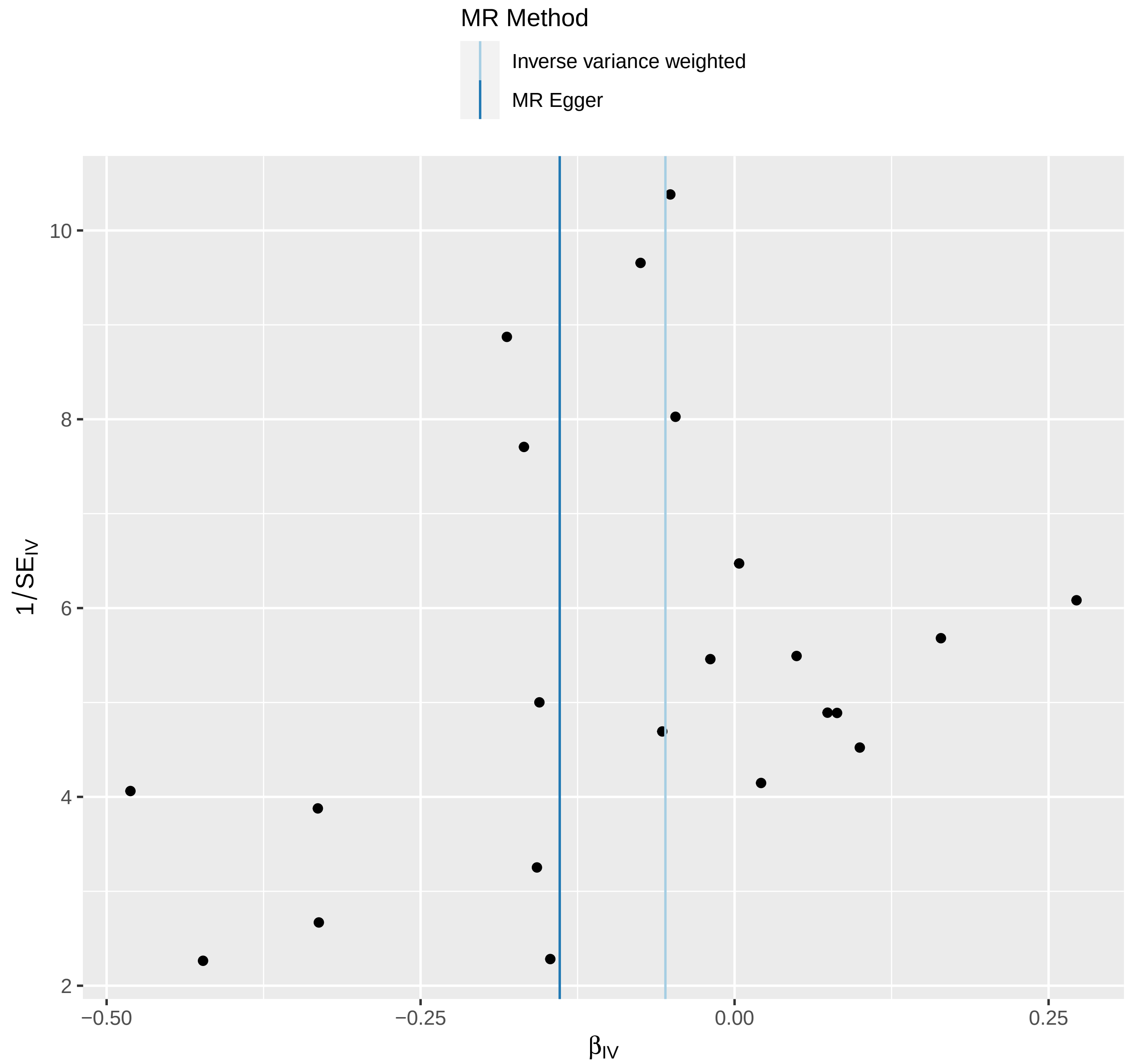

Batch 3−2293 : Urokinase plasminogen activator surface receptor on Diabetic nephropathy (more control exclusions)
with a threshold of 1e−05

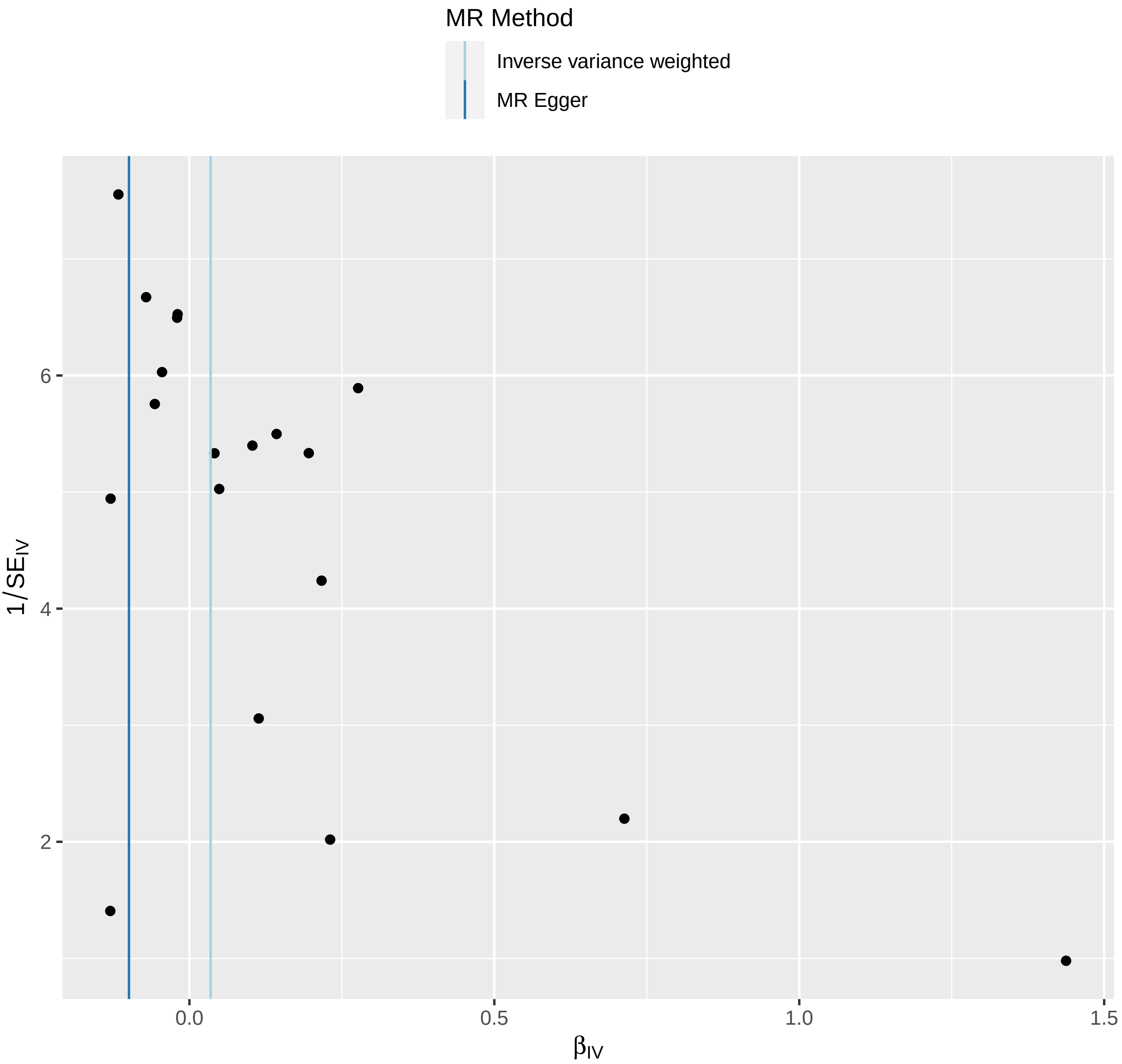

Batch 3−2294 : Phospholipase B−like 1 on Diabetic nephropathy (more control exclusions) with a threshold of 1e−05

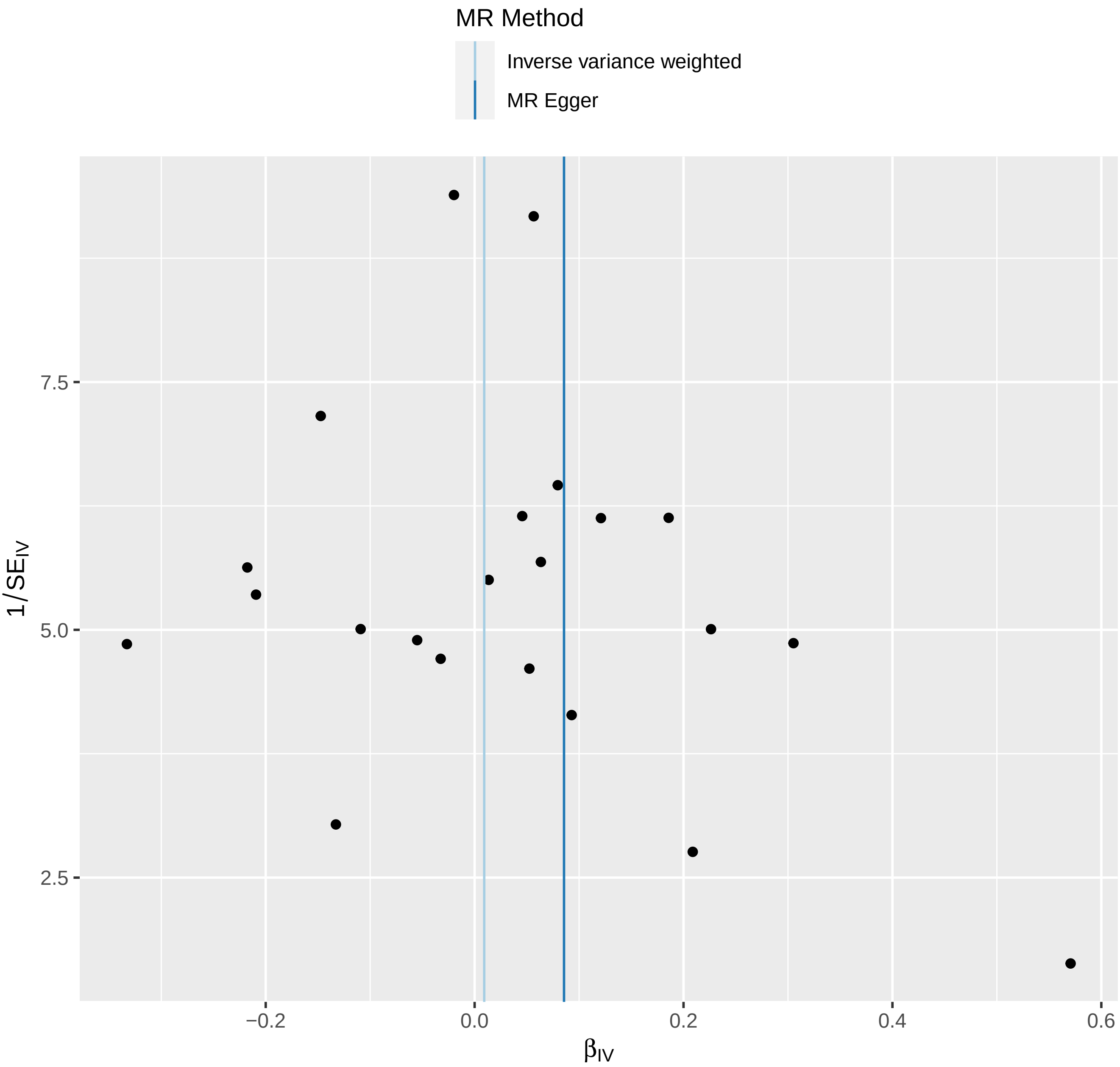

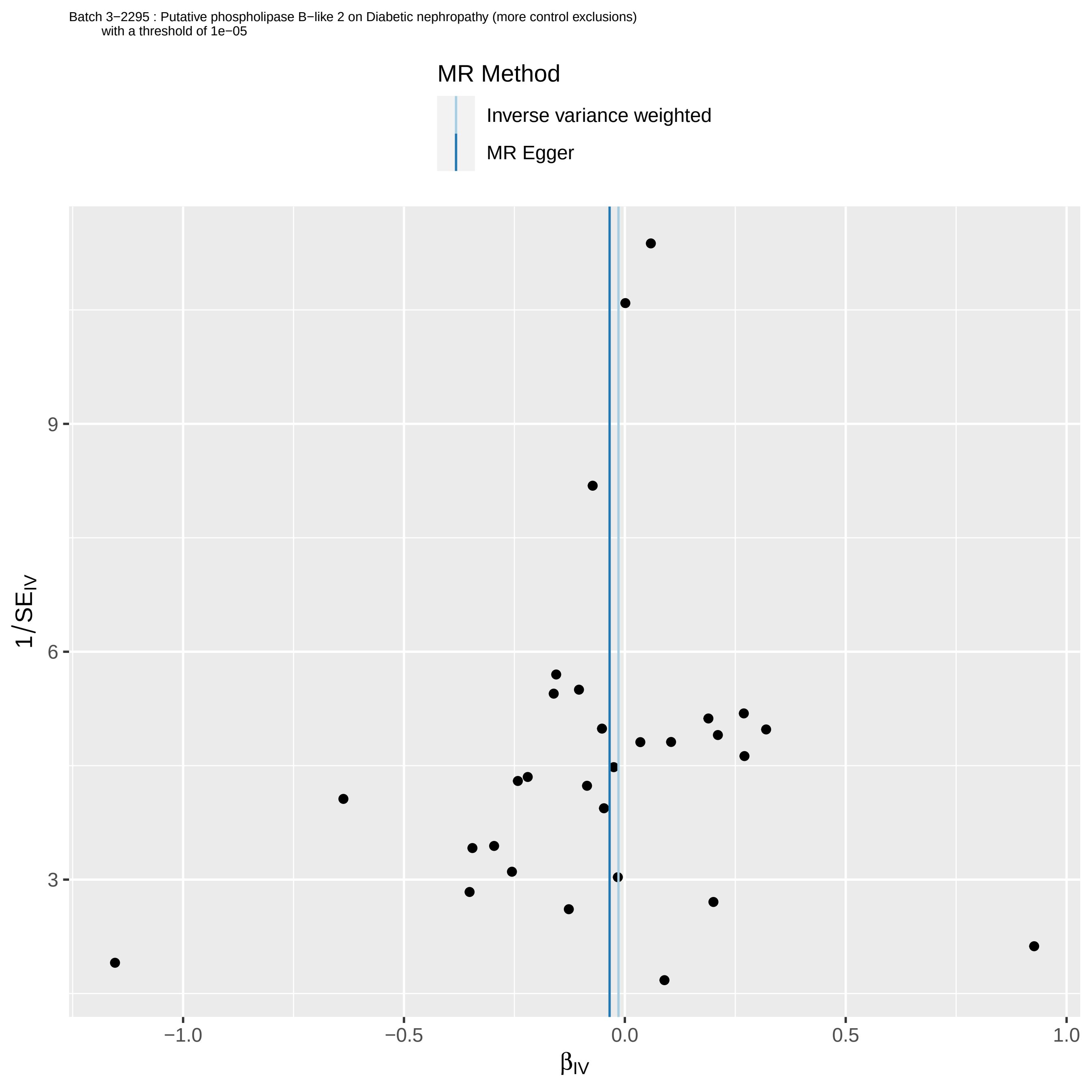

Batch 3−2295 : Putative phospholipase B−like 2 on Diabetic nephropathy (more control exclusions)
with a threshold of 1e−05
MR Method
Inverse variance weighted
MR Egger
9
6
3
$1/SE_{IV}$
−1.0
−0.5
0.0
0.5
1.0
β_{IV}

Batch 3−2296 : 1−phosphatidylinositol 4,5−bisphosphate phosphodiesterase gamma−1 on Diabetic nephropathy (more control exclusions) with a threshold of 1e−05

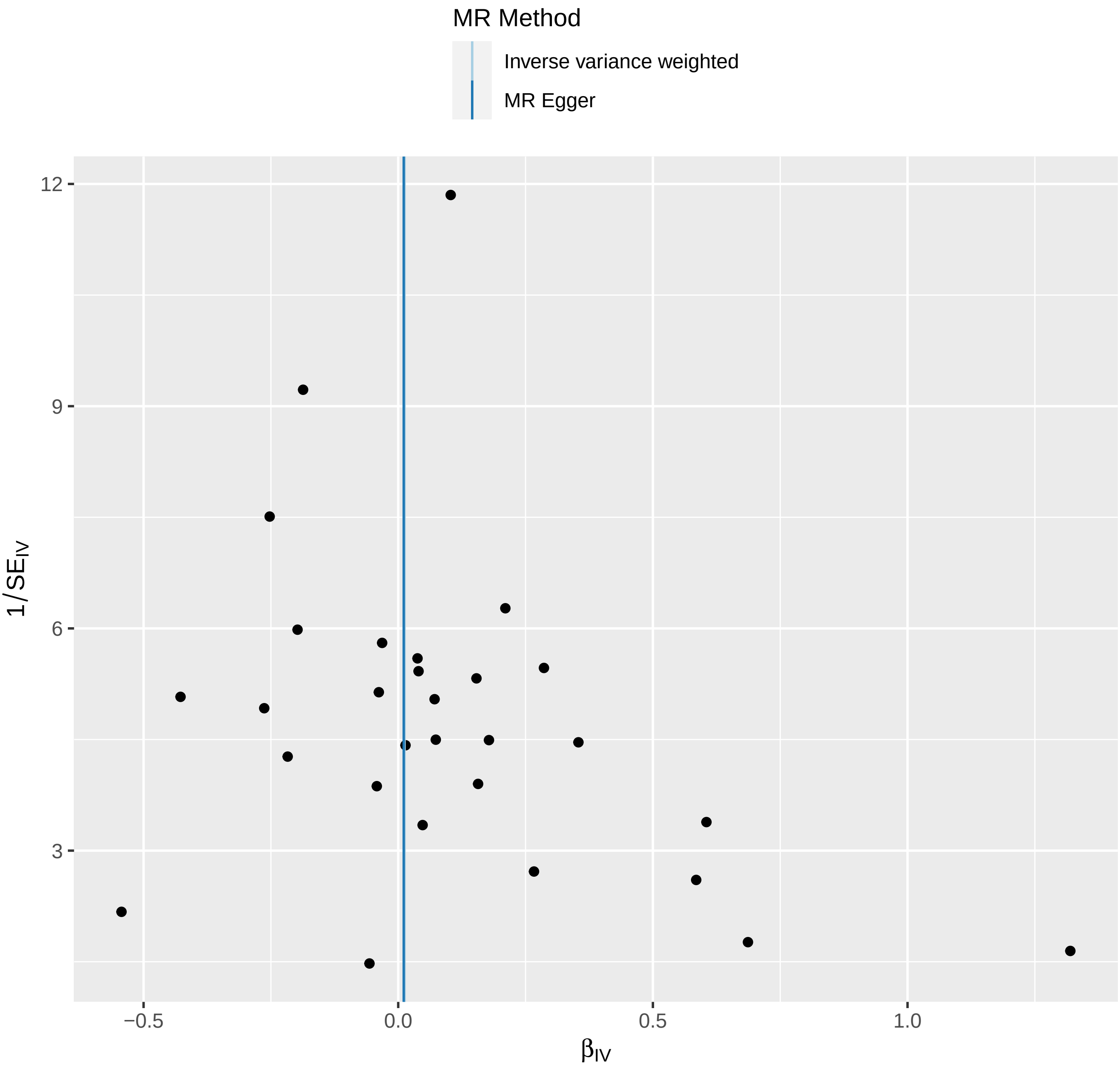

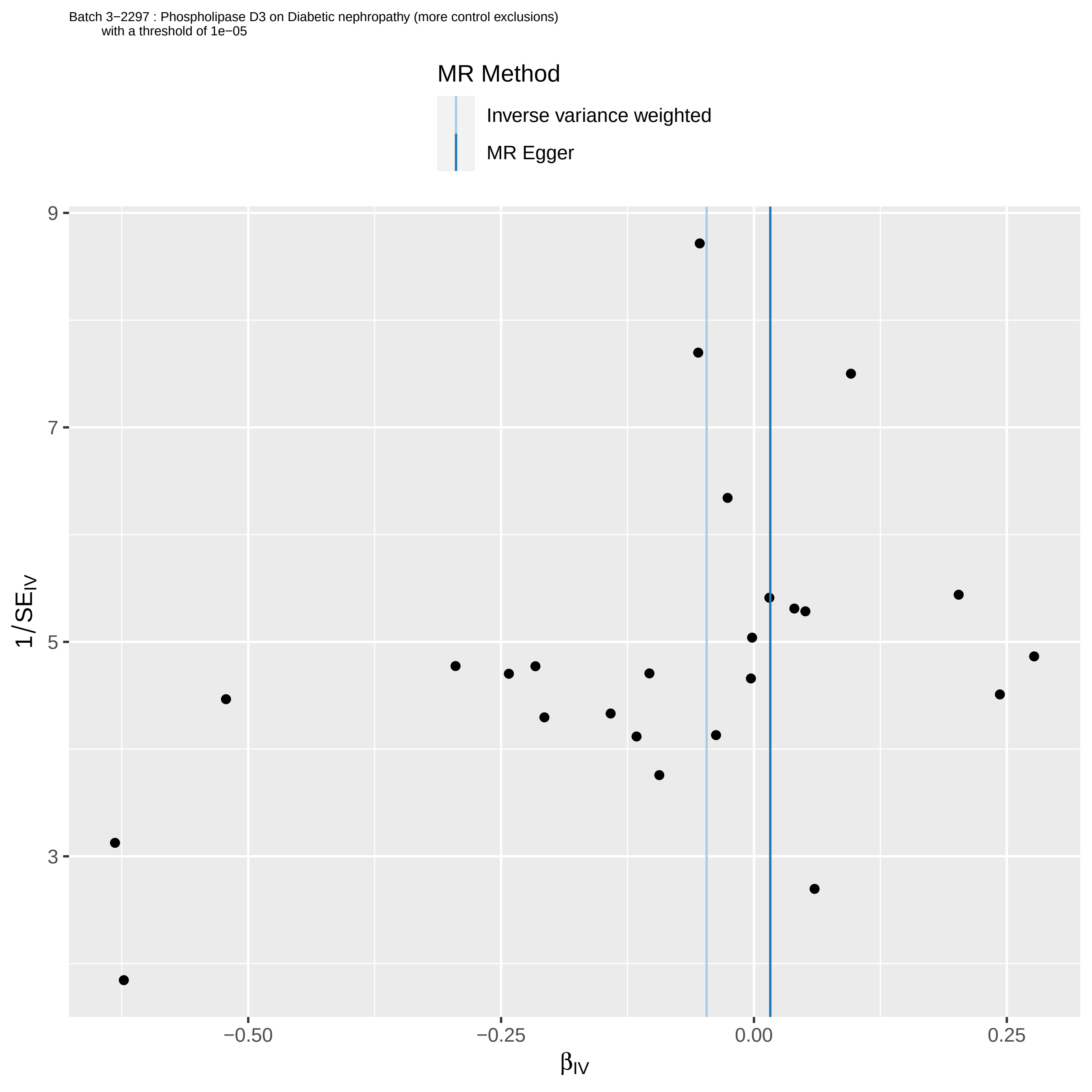
Batch 3−2297 : Phospholipase D3 on Diabetic nephropathy (more control exclusions)
with a threshold of 1e−05
MR Method
Inverse variance weighted
MR Egger
9
7
5
3
1/SE_IV
−0.50
−0.25
0.00
0.25
β_IV

Batch 3−2298 : Inactive phospholipase D5 on Diabetic nephropathy (more control exclusions)
with a threshold of 1e−05

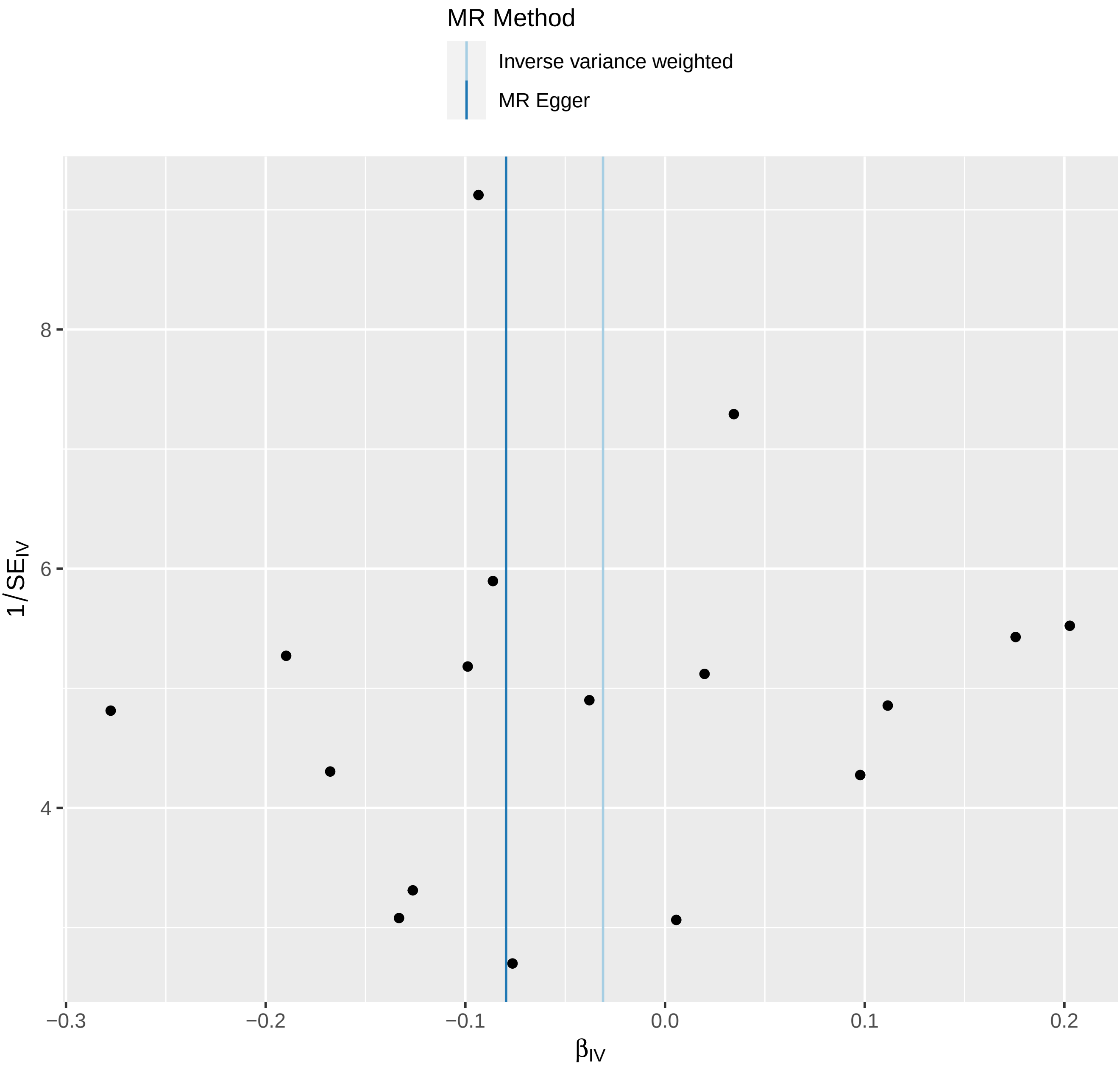

Batch 3−2299 : Pleckstrin homology domain−containing family A member 1 on Diabetic nephropathy (more control exclusions)
with a threshold of 1e−05

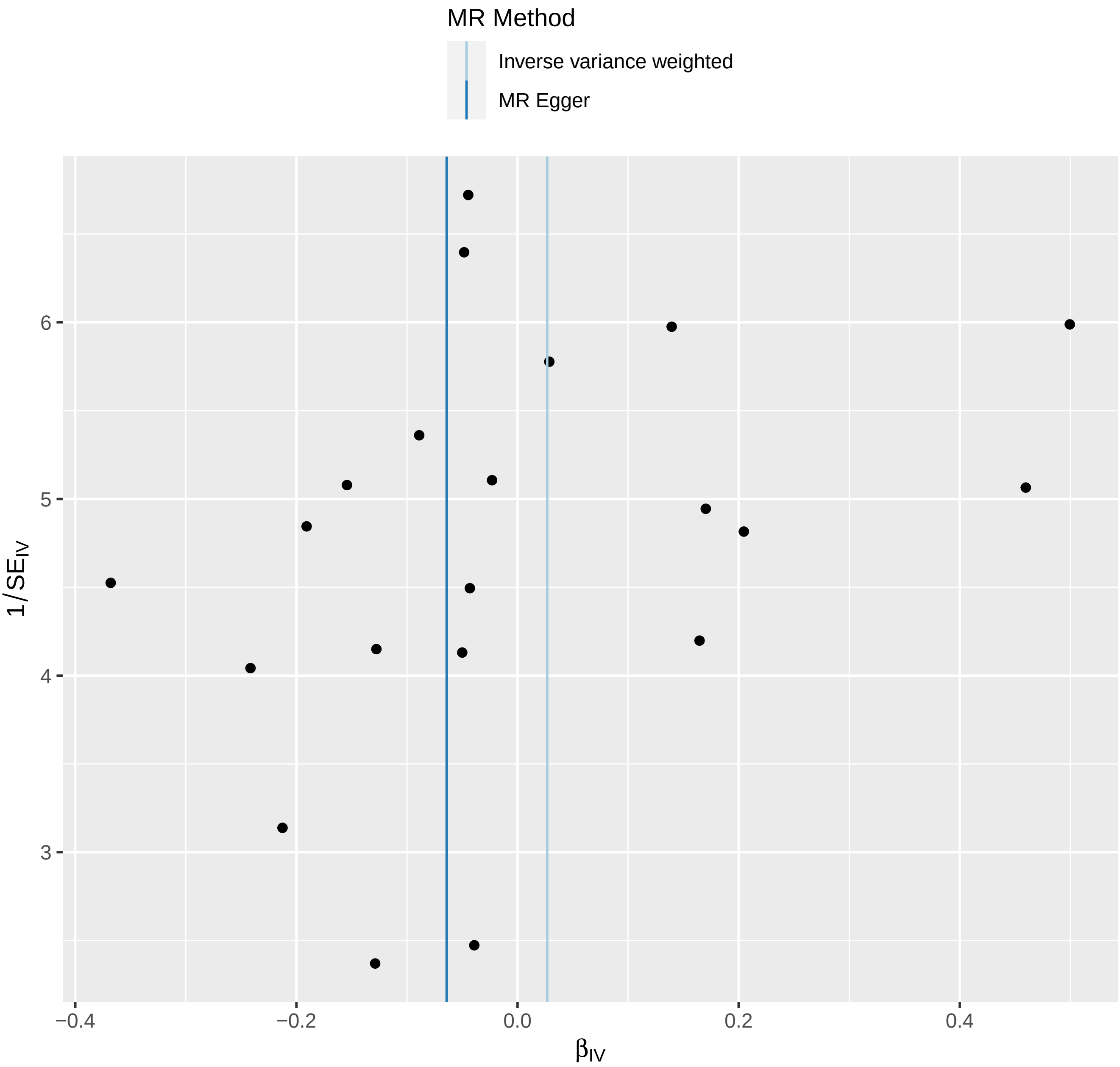

Batch 3−2300 : Pleckstrin homology domain−containing family A member 7 on Diabetic nephropathy (more control exclusions) with a threshold of 1e−05

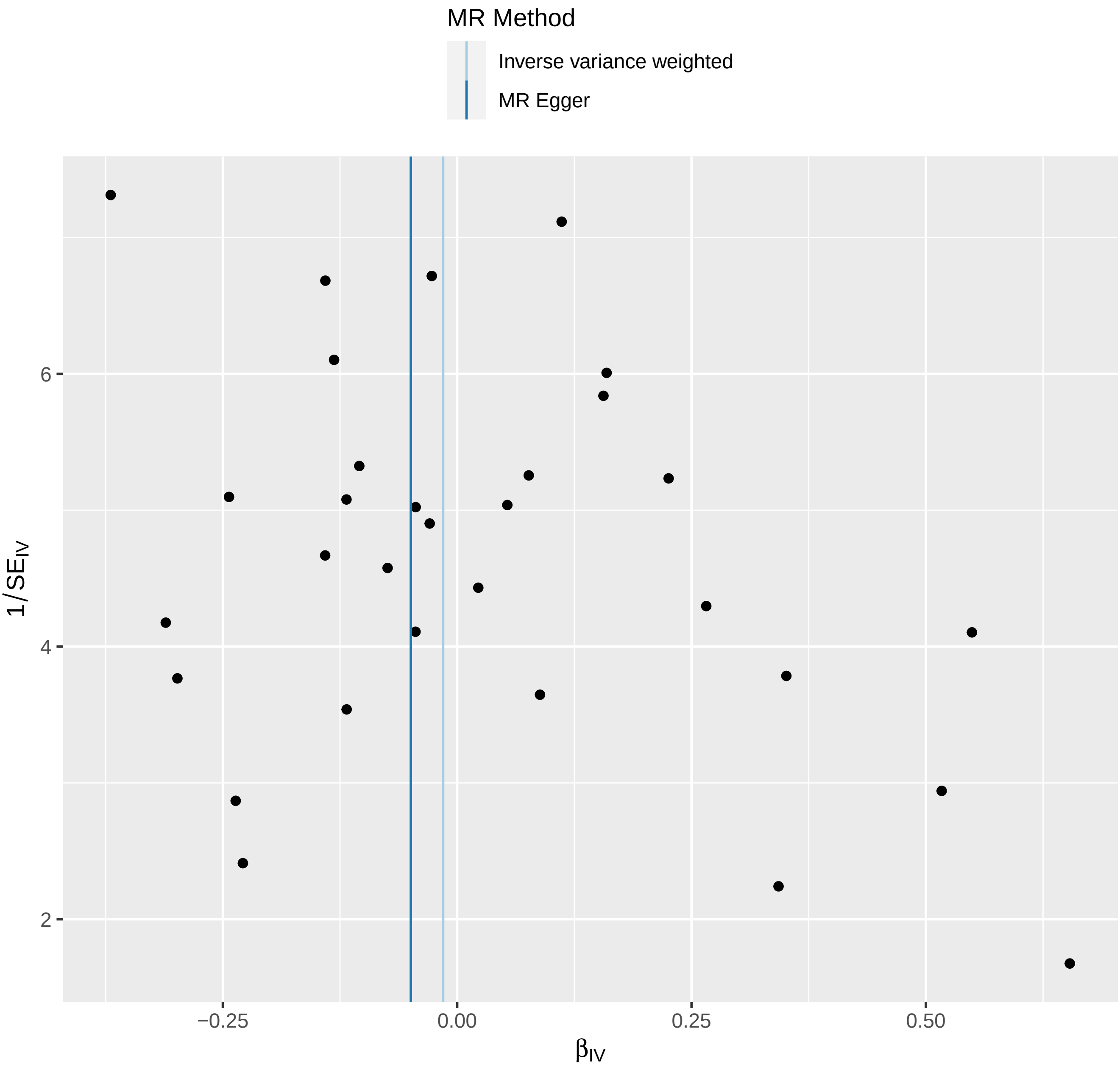

Batch 3−2301 : Angiostatin on Diabetic nephropathy (more control exclusions)
with a threshold of 1e−05

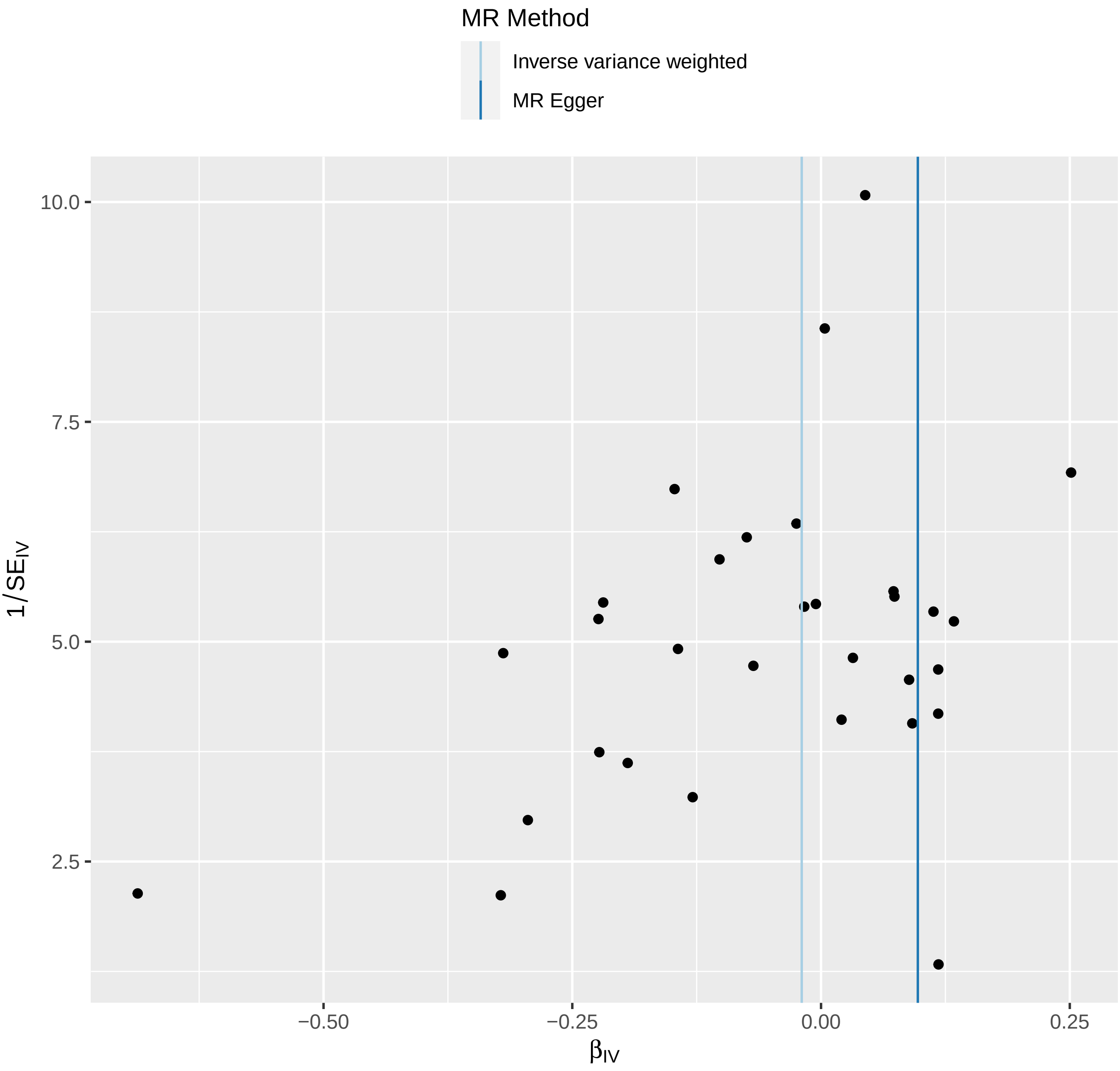

Batch 3−2302 : Plasmin on Diabetic nephropathy (more control exclusions)
with a threshold of 1e−05

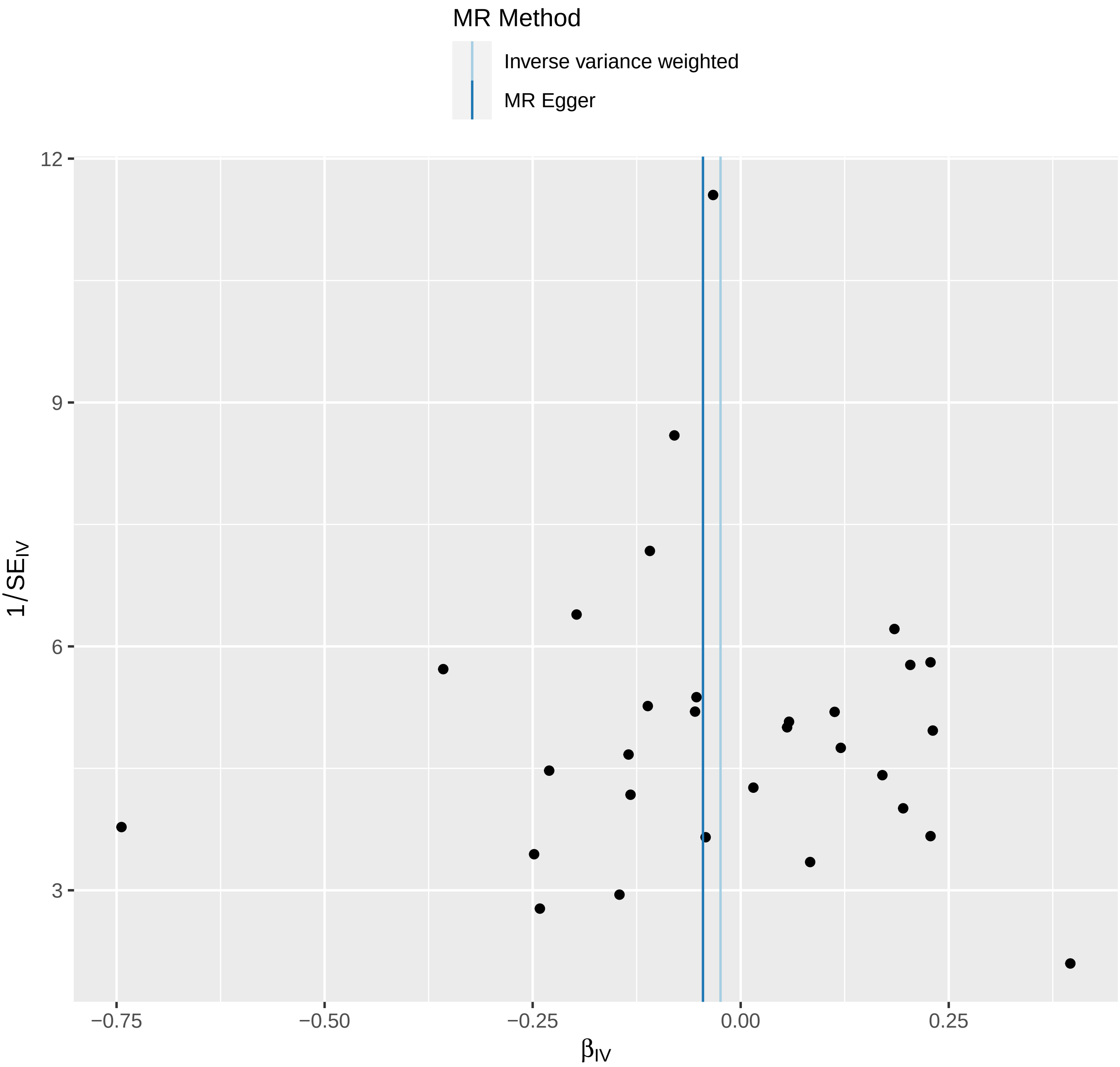

Batch 3−2303 : Serine/threonine−protein kinase PLK1 on Diabetic nephropathy (more control exclusions) with a threshold of 1e−05

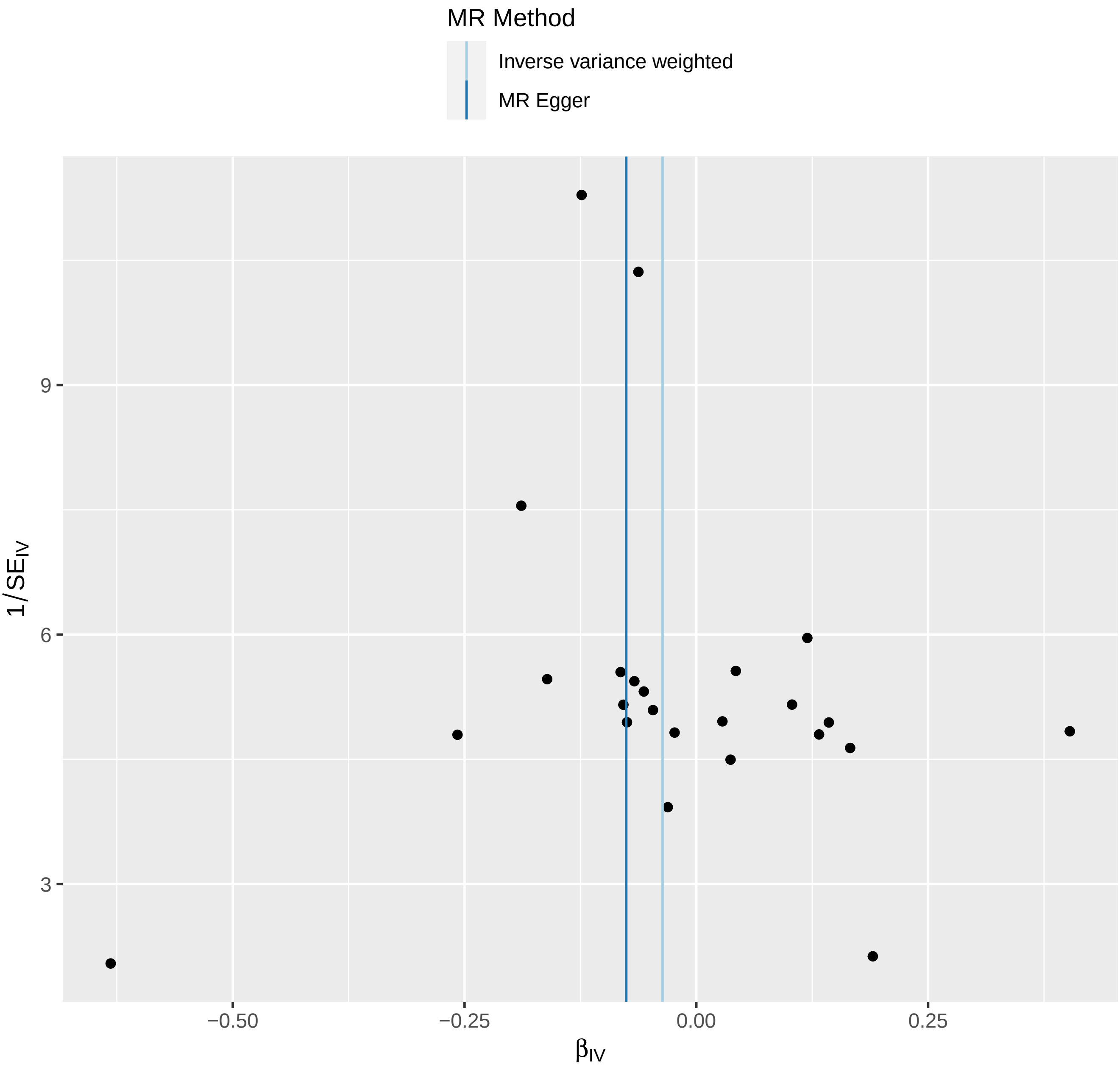

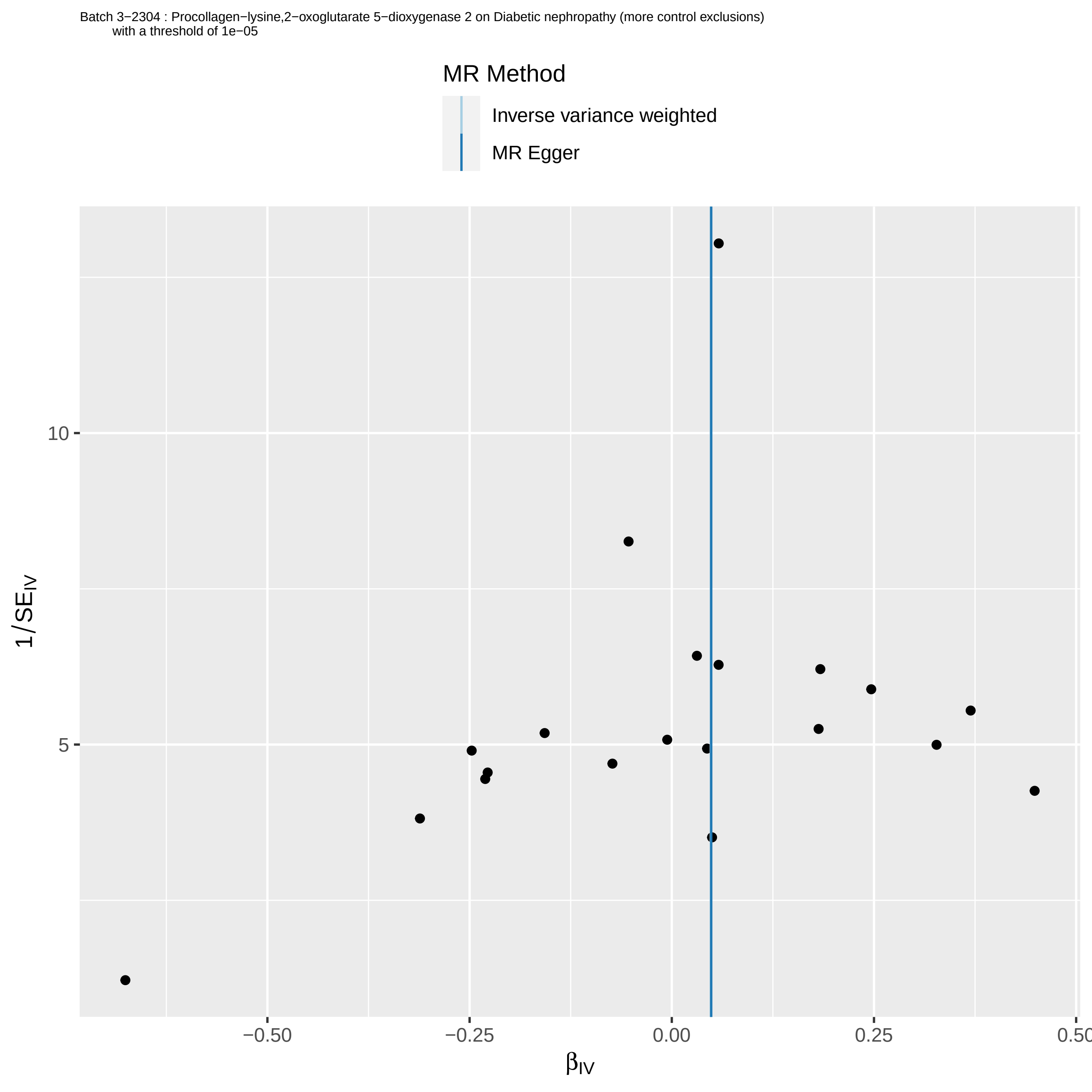

Batch 3−2304 : Procollagen−lysine,2−oxoglutarate 5−dioxygenase 2 on Diabetic nephropathy (more control exclusions)
with a threshold of 1e−05
MR Method
Inverse variance weighted
MR Egger
10
5
1/SE$_{IV}$
−0.50
−0.25
0.00
0.25
0.50
β$_{IV}$

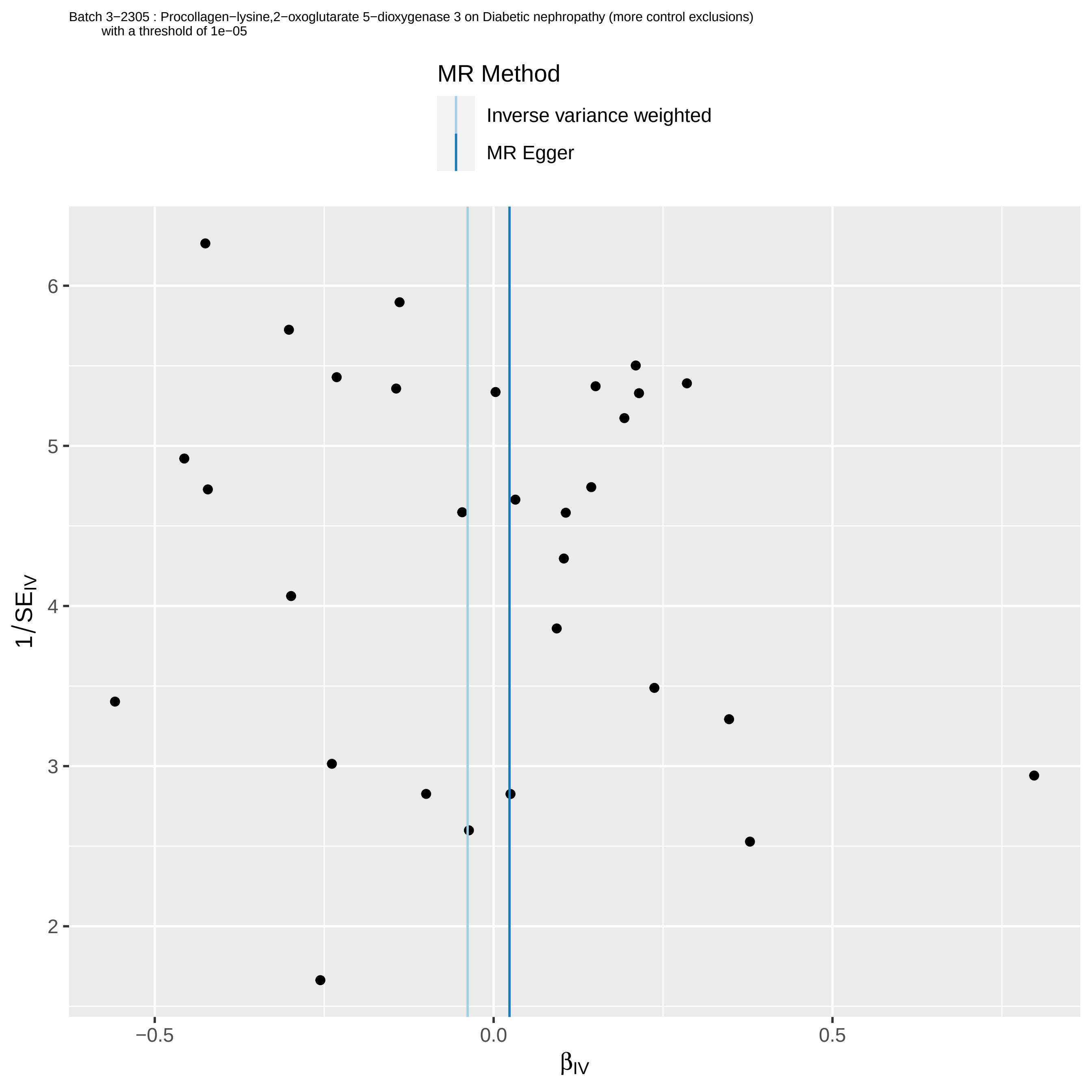
Batch 3−2305 : Procollagen−lysine,2−oxoglutarate 5−dioxygenase 3 on Diabetic nephropathy (more control exclusions)
with a threshold of 1e−05
MR Method
Inverse variance weighted
MR Egger
6
5
4
3
2
1/SE_IV
−0.5
0.0
0.5
β_IV

Batch 3−2306 : Plastin−1 on Diabetic nephropathy (more control exclusions)
with a threshold of 1e−05

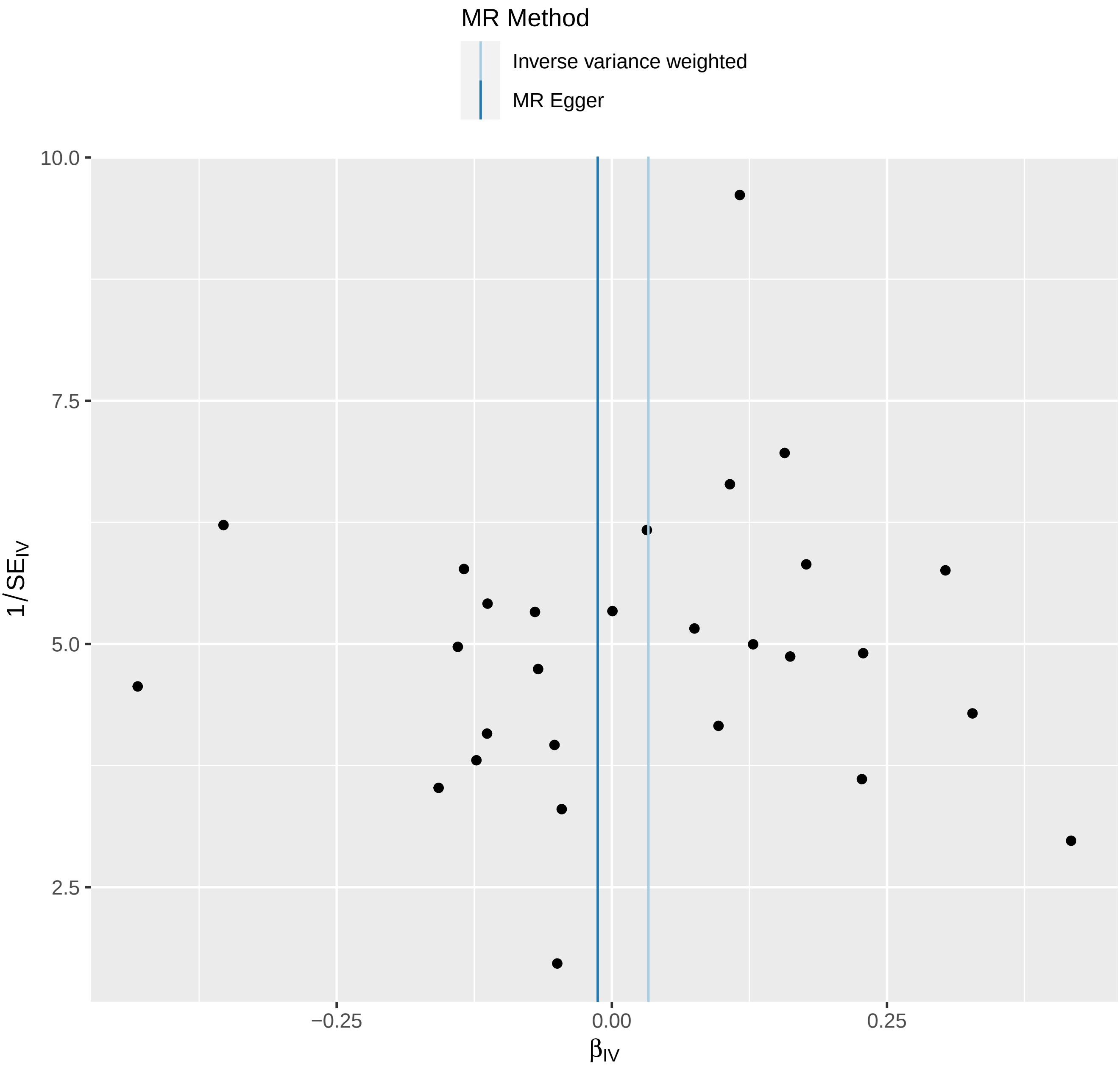

Batch 3−2307 : Plexin−A1 on Diabetic nephropathy (more control exclusions)
with a threshold of 1e−05

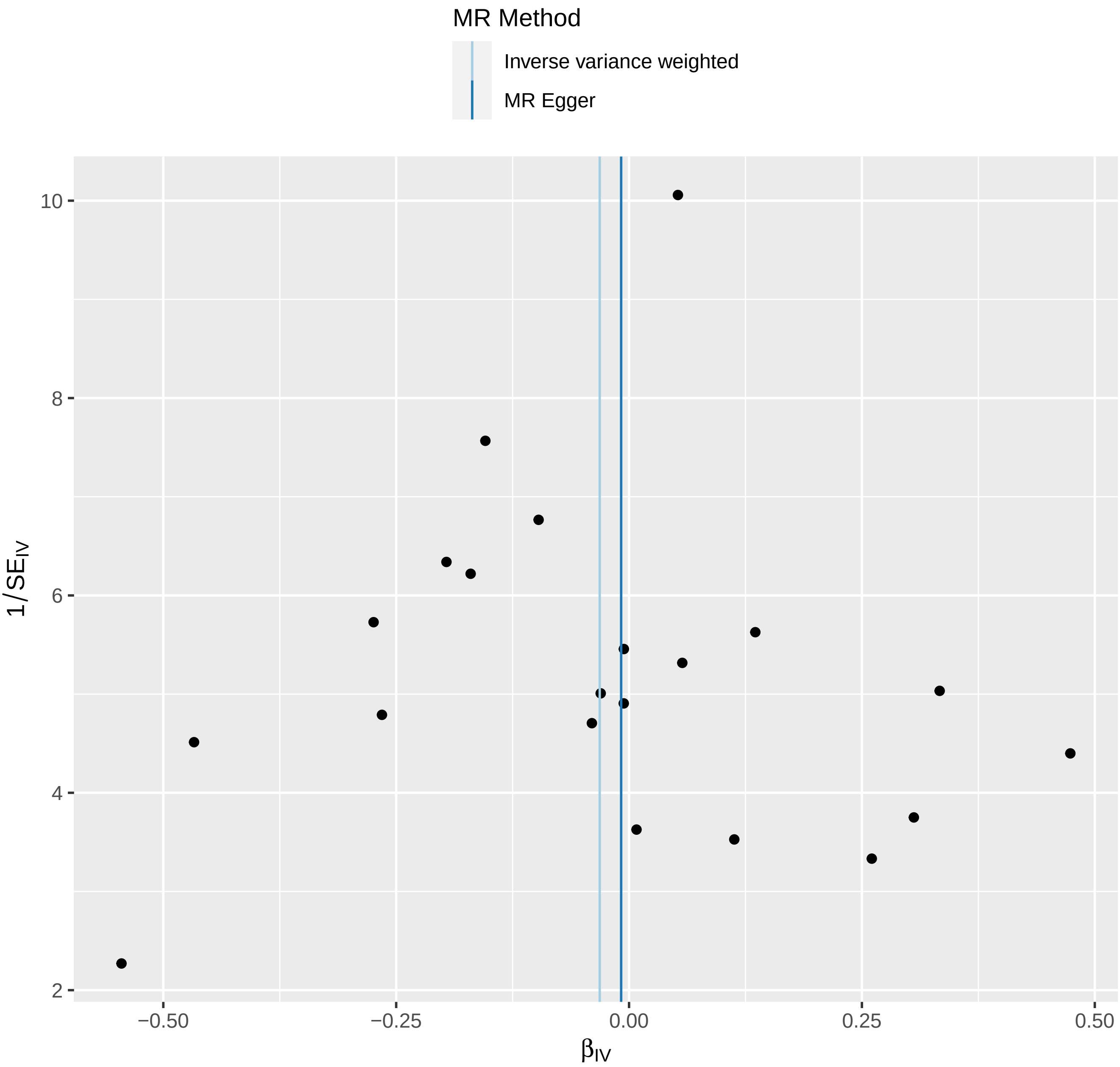

Batch 3−2308 : Plexin−B2 on Diabetic nephropathy (more control exclusions)
with a threshold of 1e−05

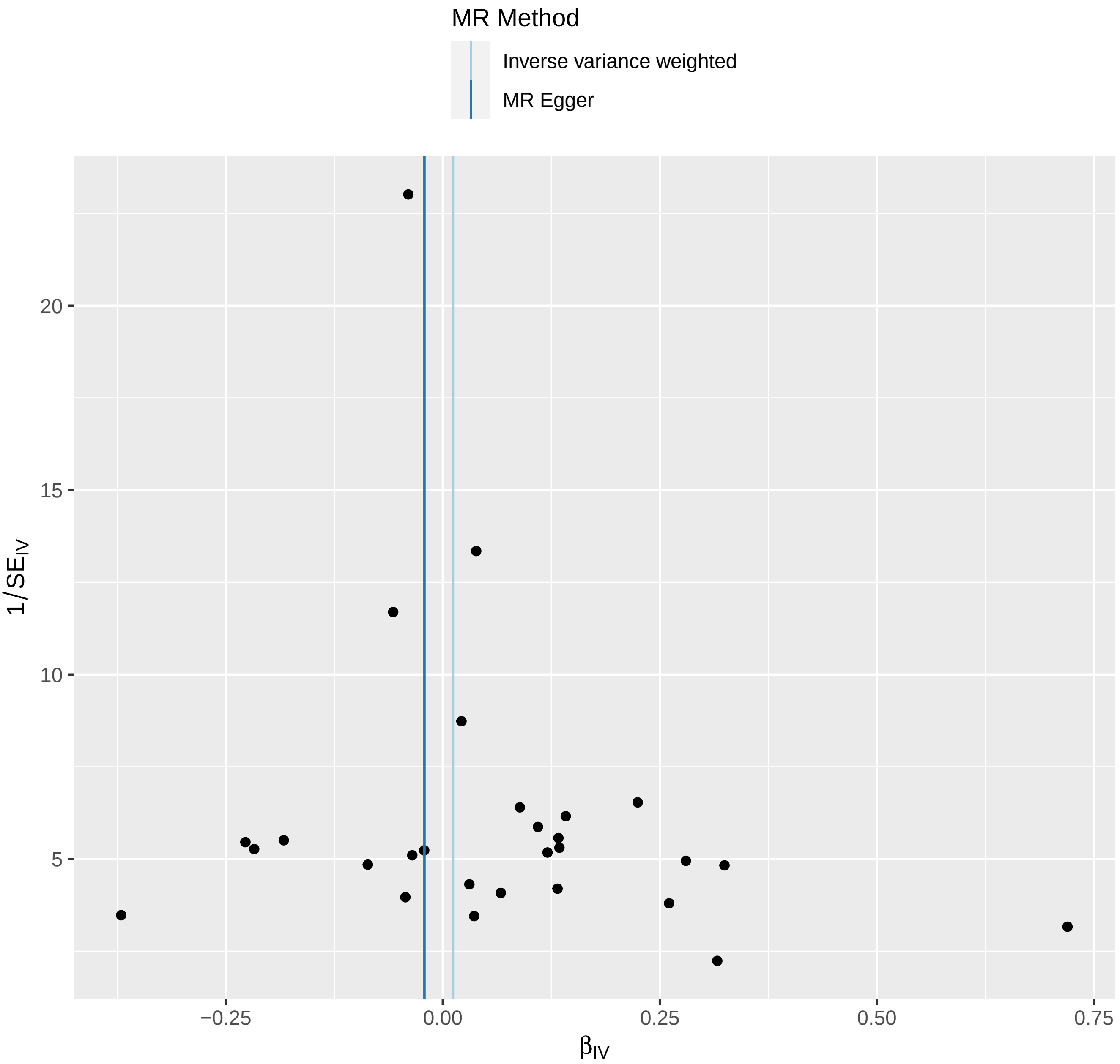

Batch 3−2309 : Plexin−C1 on Diabetic nephropathy (more control exclusions)
with a threshold of 1e−05

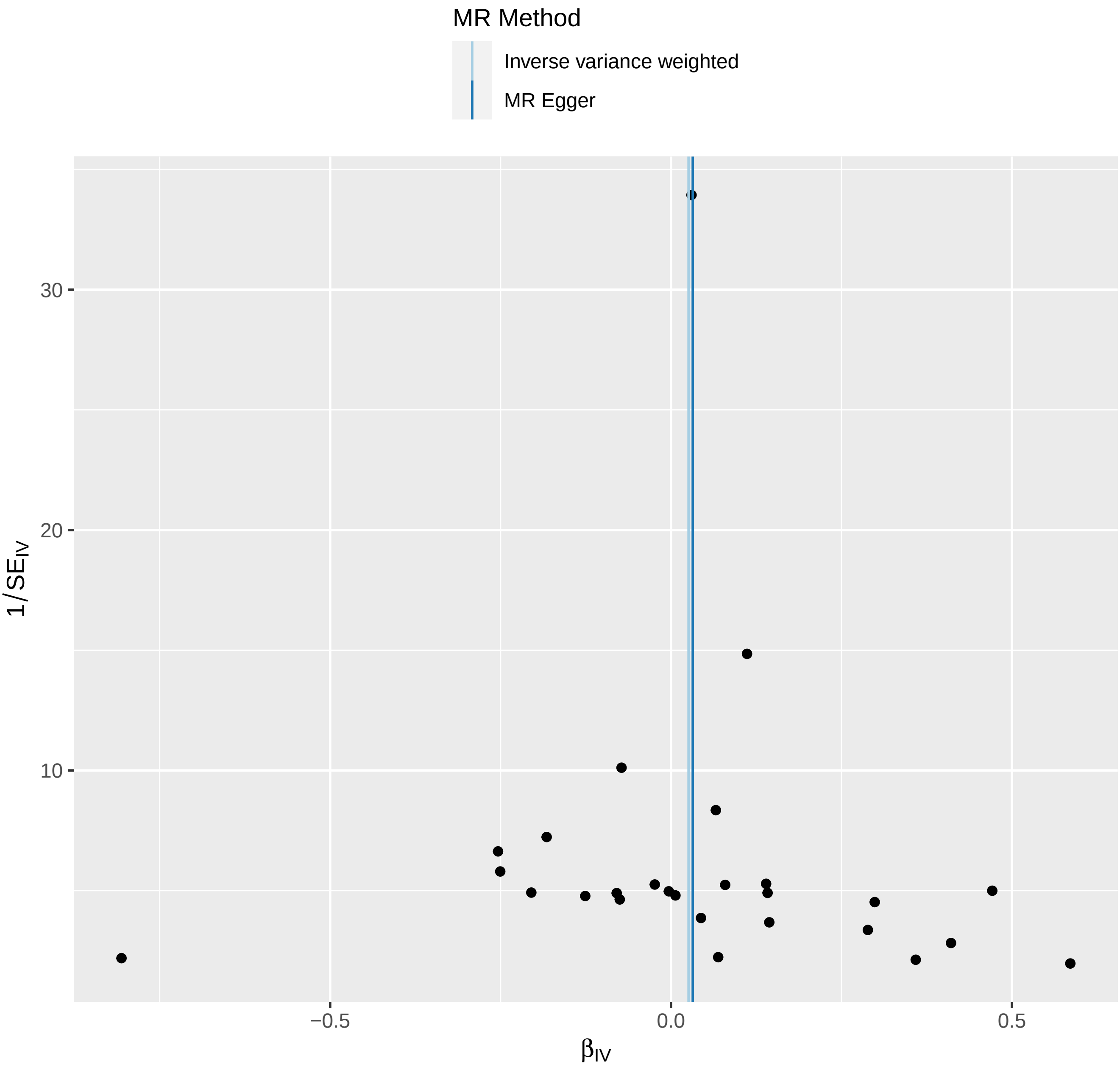

Batch 3−2310 : Melanocyte protein PMEL on Diabetic nephropathy (more control exclusions)
with a threshold of 1e−05

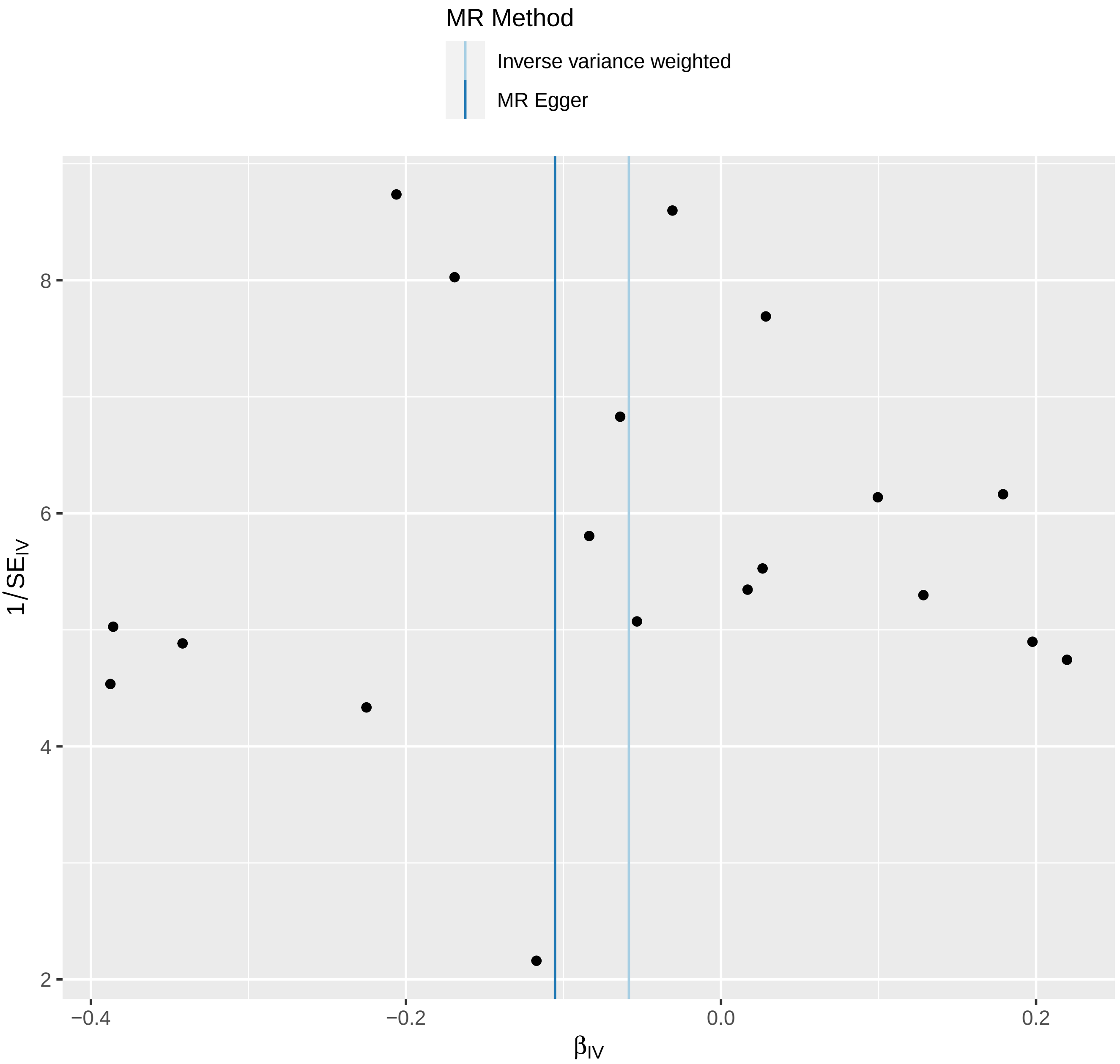

Batch 3−2311 : Protein TMEPAI on Diabetic nephropathy (more control exclusions)
with a threshold of 1e−05

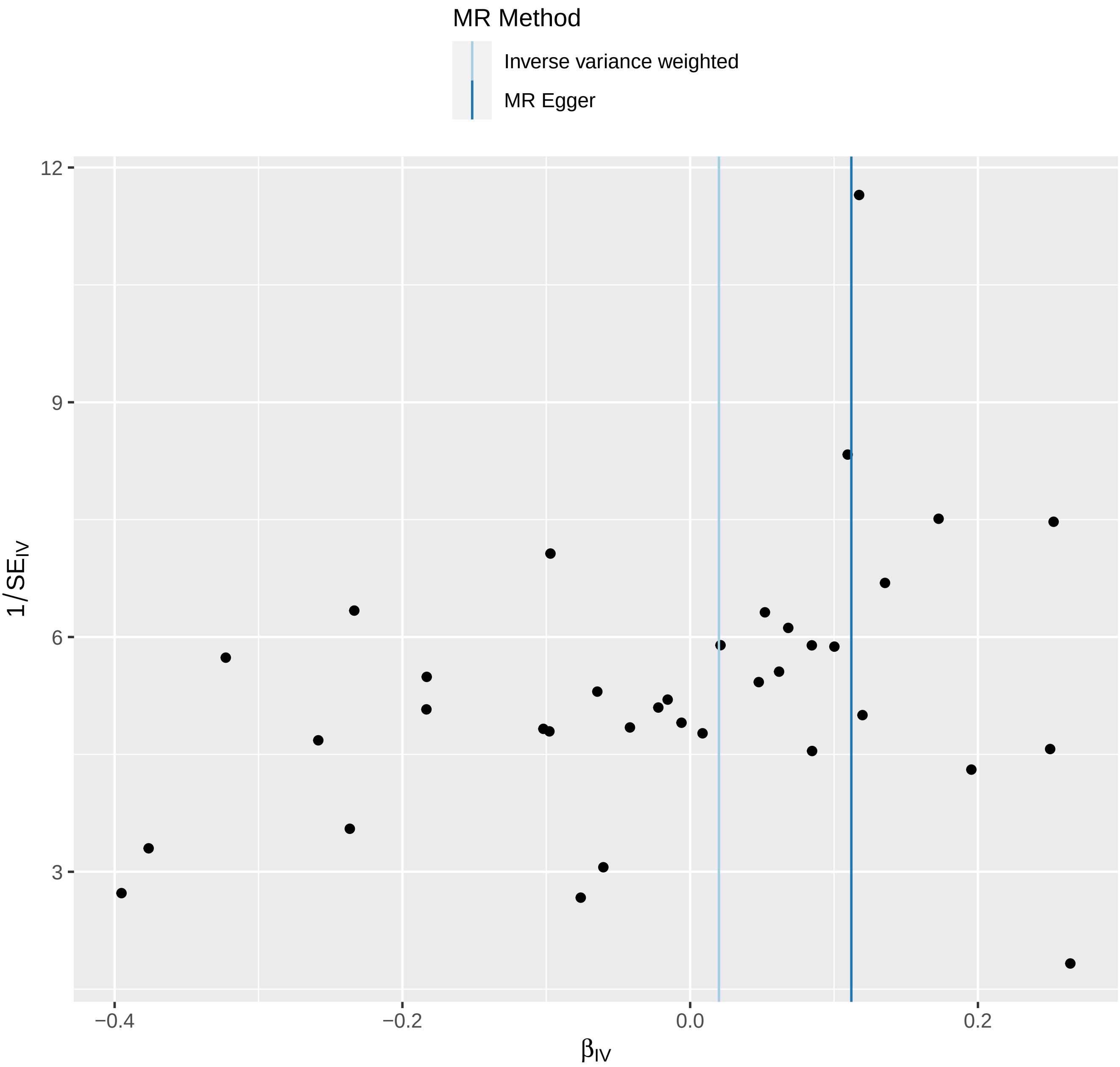

Batch 3−2312 : Mismatch repair endonuclease PMS2 on Diabetic nephropathy (more control exclusions)
with a threshold of 1e−05

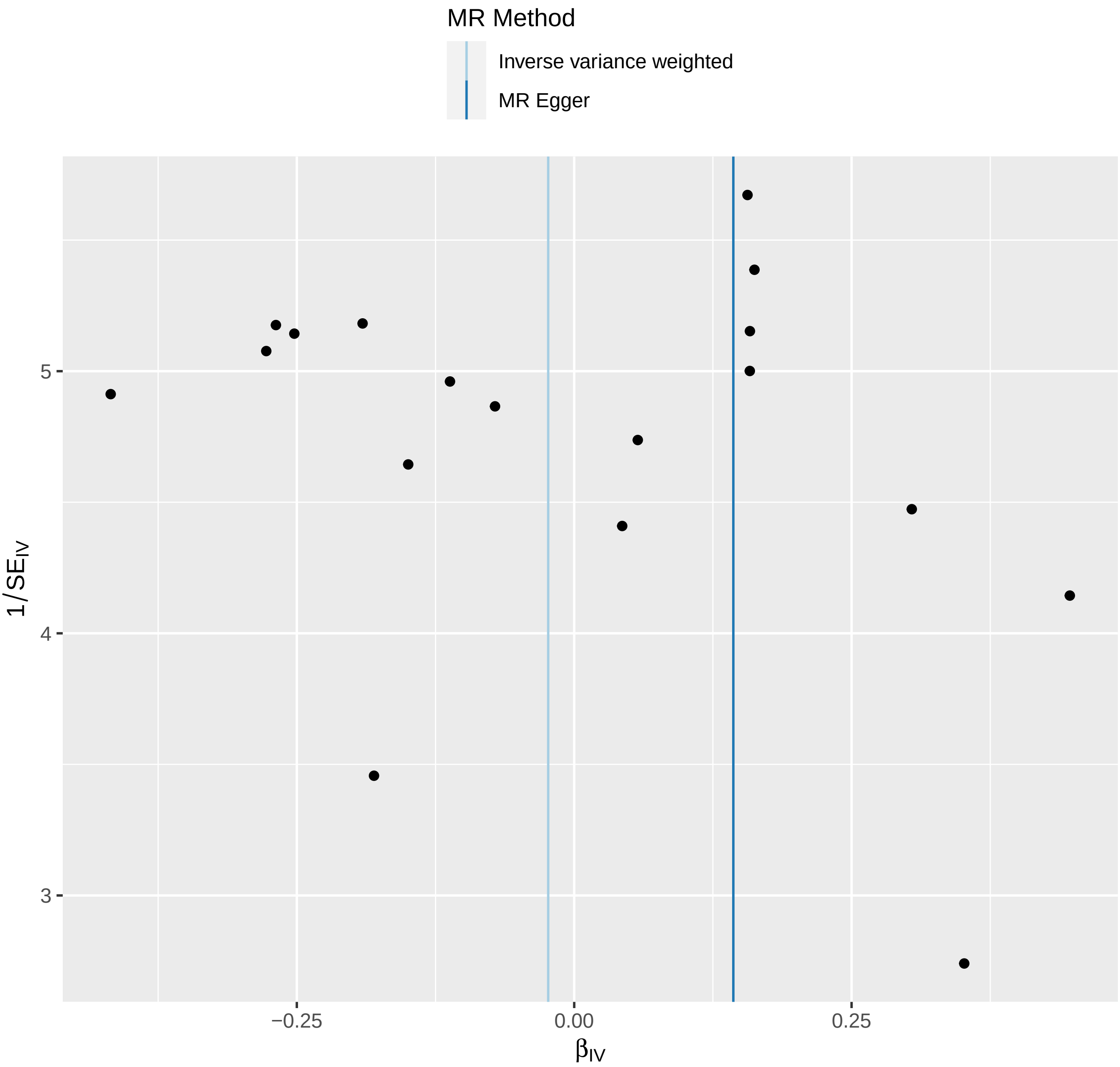

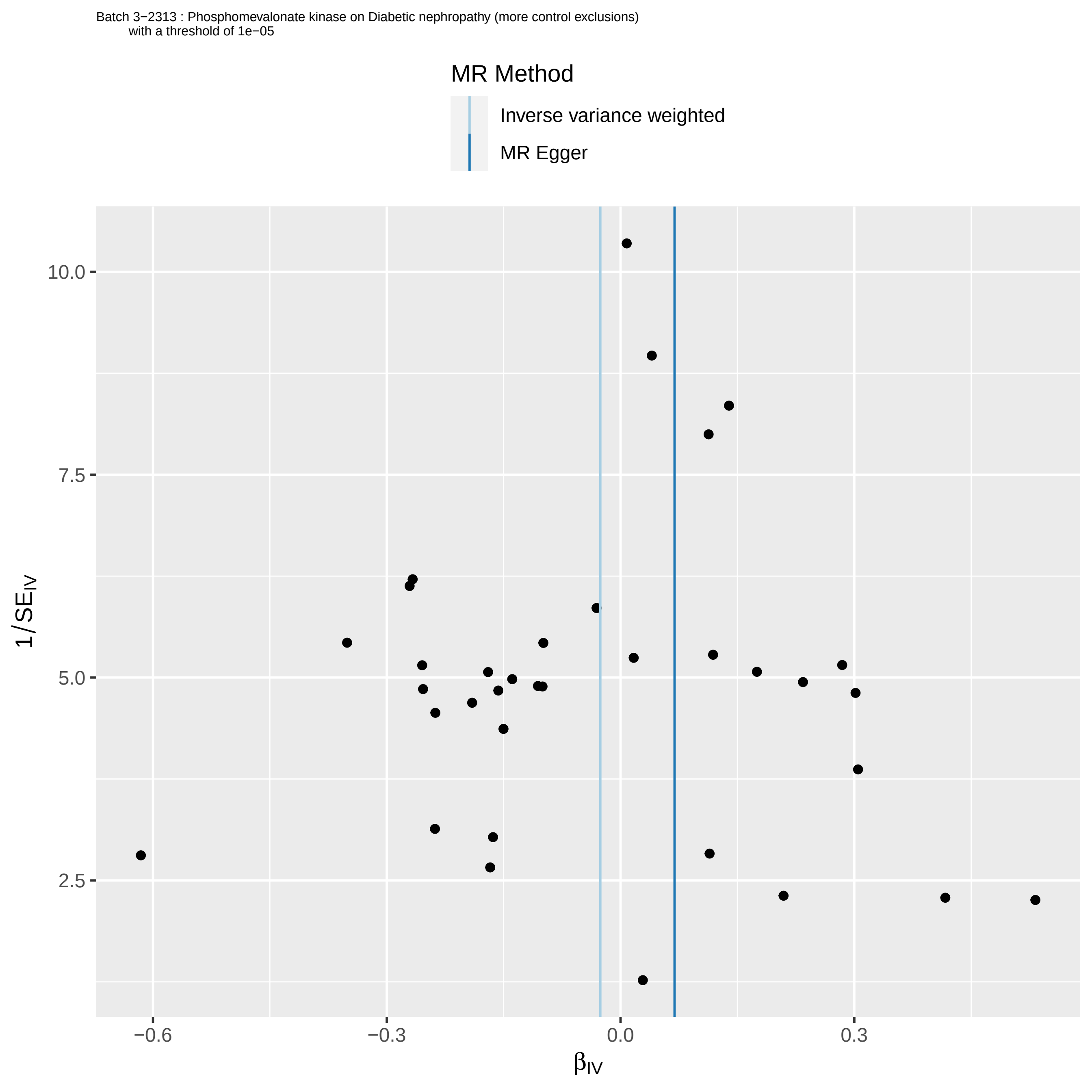

Batch 3−2313 : Phosphomevalonate kinase on Diabetic nephropathy (more control exclusions)
with a threshold of 1e−05
MR Method
Inverse variance weighted
MR Egger
10.0
7.5
5.0
2.5
1/SE_IV
−0.6
−0.3
0.0
0.3
β_IV

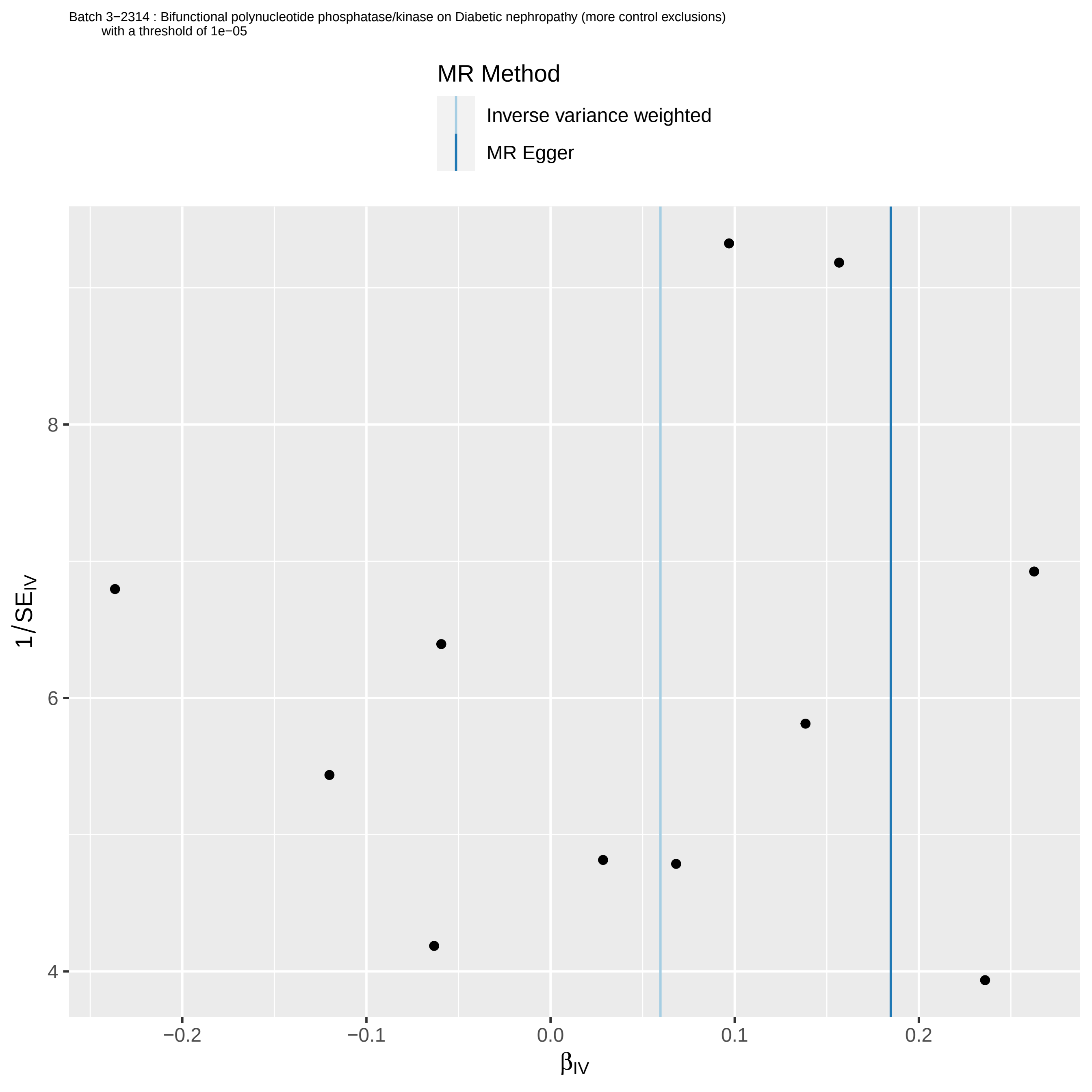
Batch 3−2314 : Bifunctional polynucleotide phosphatase/kinase on Diabetic nephropathy (more control exclusions)
with a threshold of 1e−05
MR Method
Inverse variance weighted
MR Egger
8
6
4
1/SE_IV
−0.2
−0.1
0.0
0.1
0.2
β_IV

Batch 3−2315 : Inactive pancreatic lipase−related protein 1 on Diabetic nephropathy (more control exclusions)
with a threshold of 1e−05

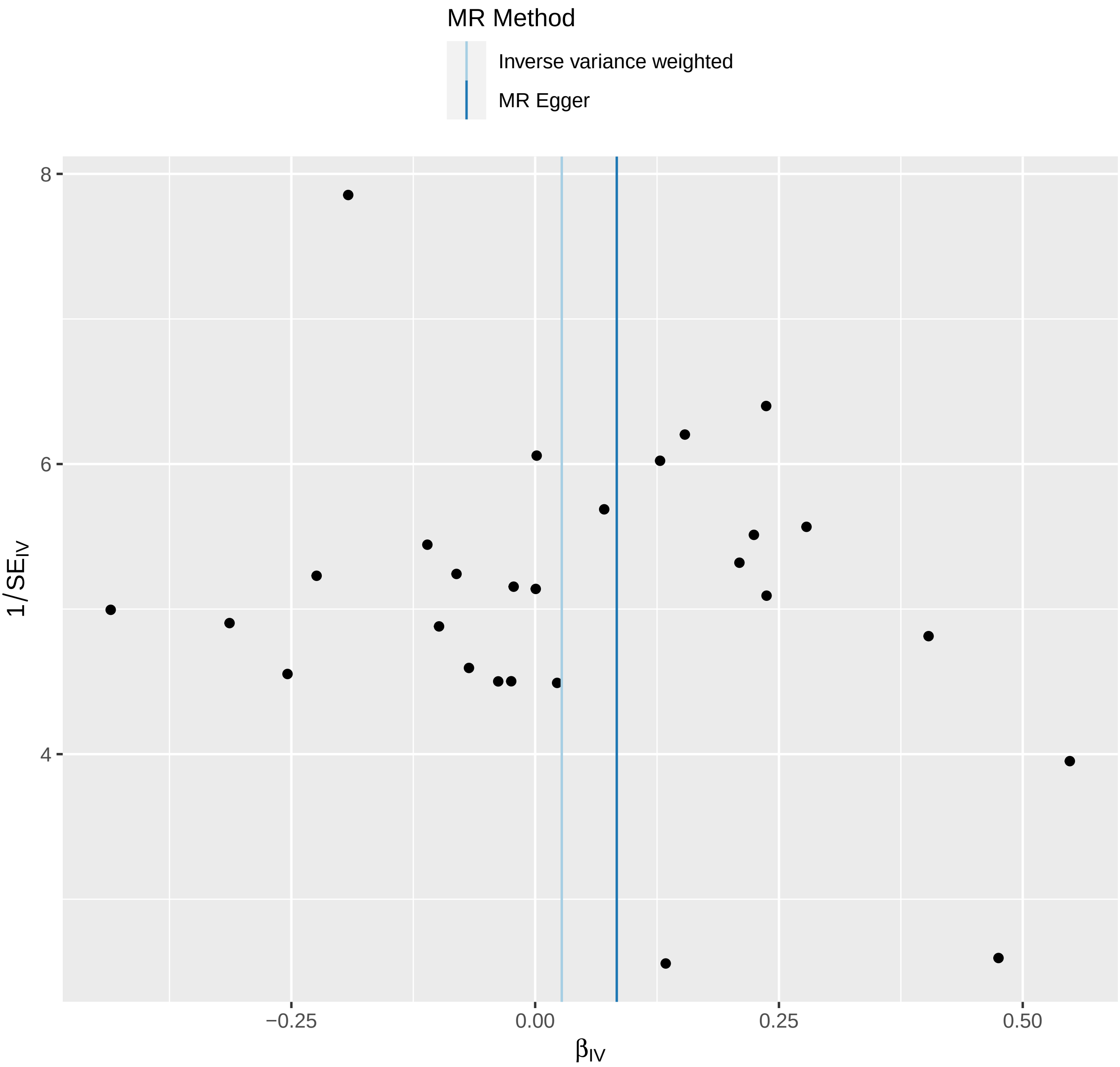

Batch 3−2316 : Purine nucleoside phosphorylase on Diabetic nephropathy (more control exclusions)
with a threshold of 1e−05

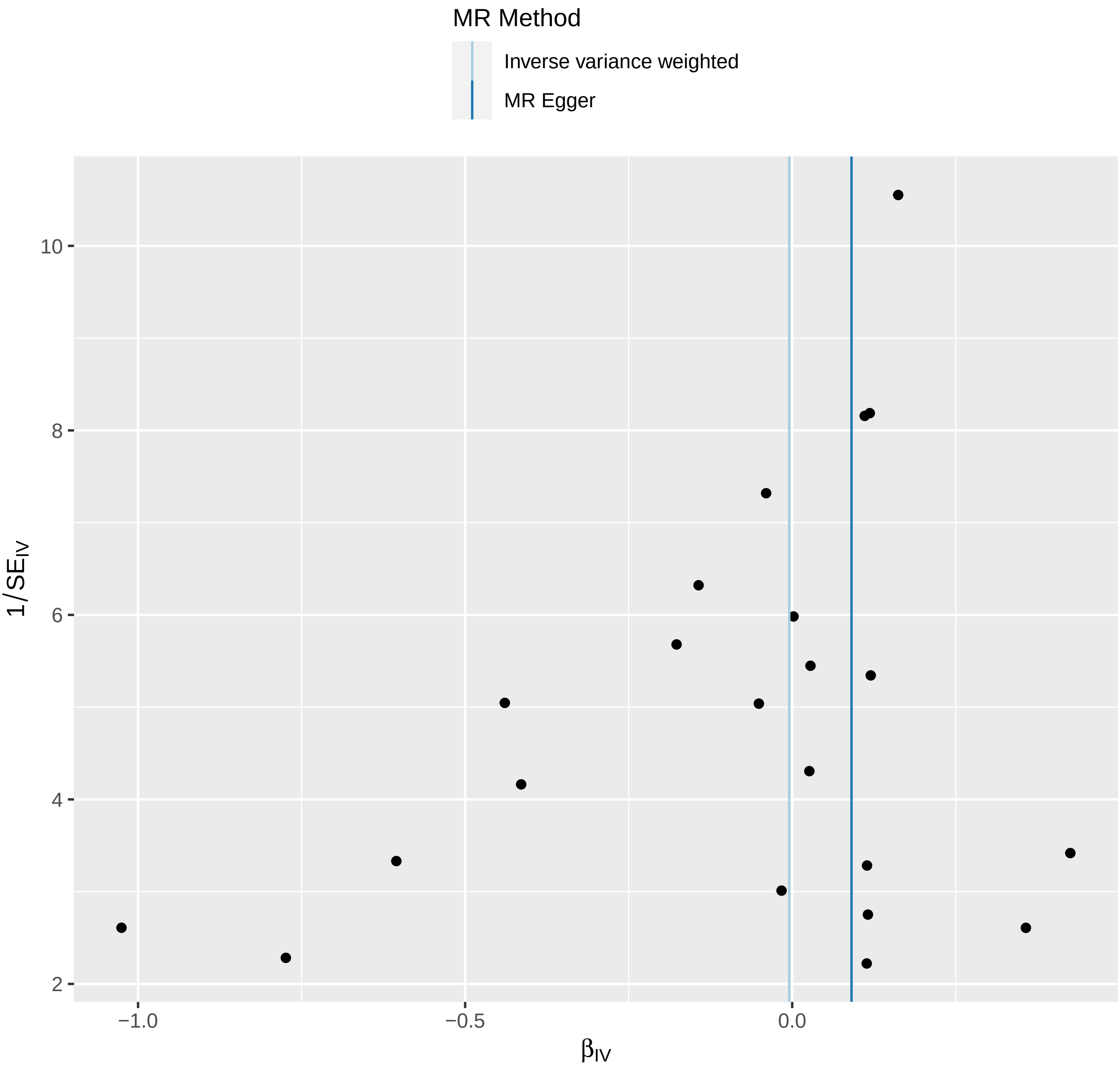

Batch 3−2317 : Podocalyxin on Diabetic nephropathy (more control exclusions)
with a threshold of 1e−05

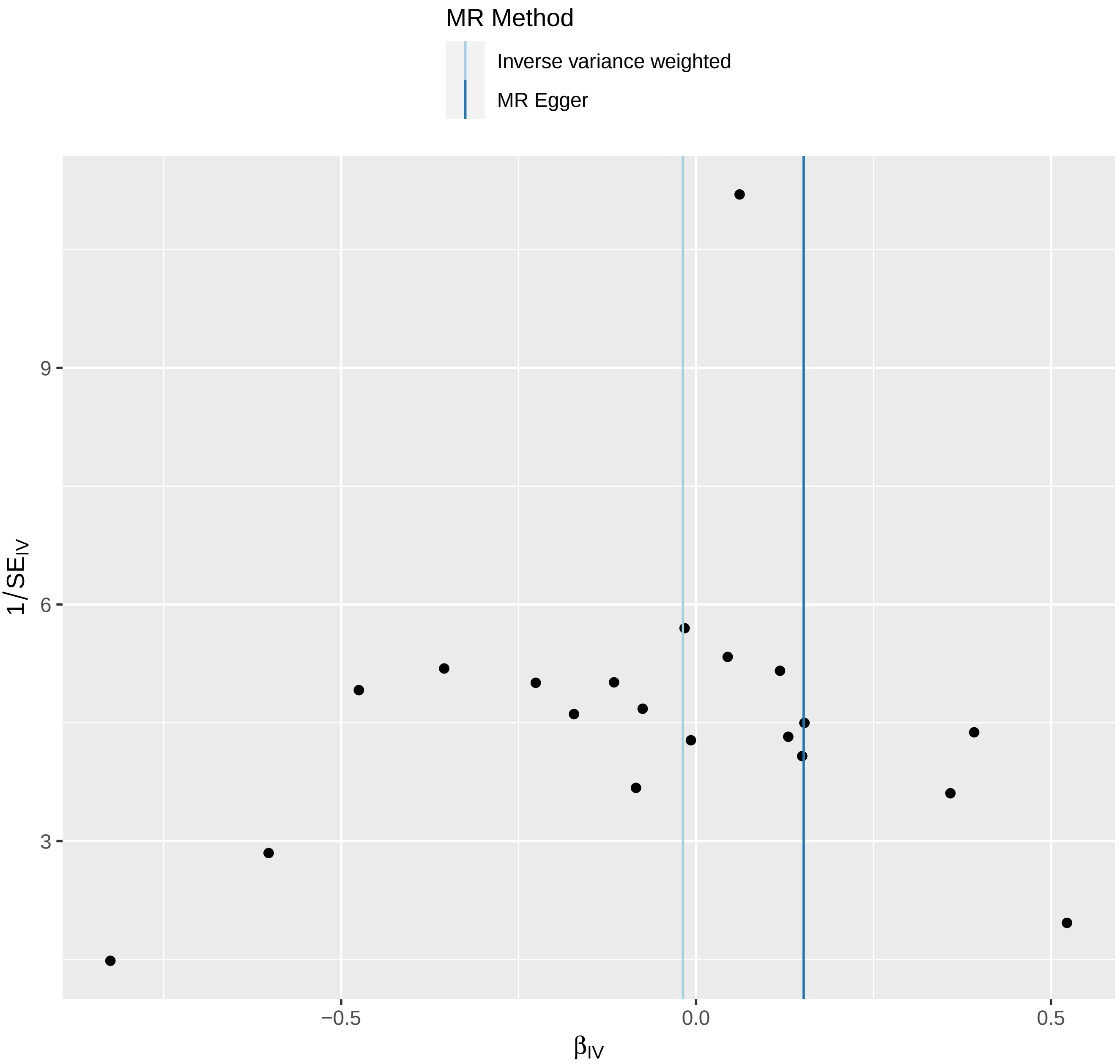

Batch 3−2318 : GDP−fucose protein O−fucosyltransferase 1 on Diabetic nephropathy (more control exclusions)
with a threshold of 1e−05

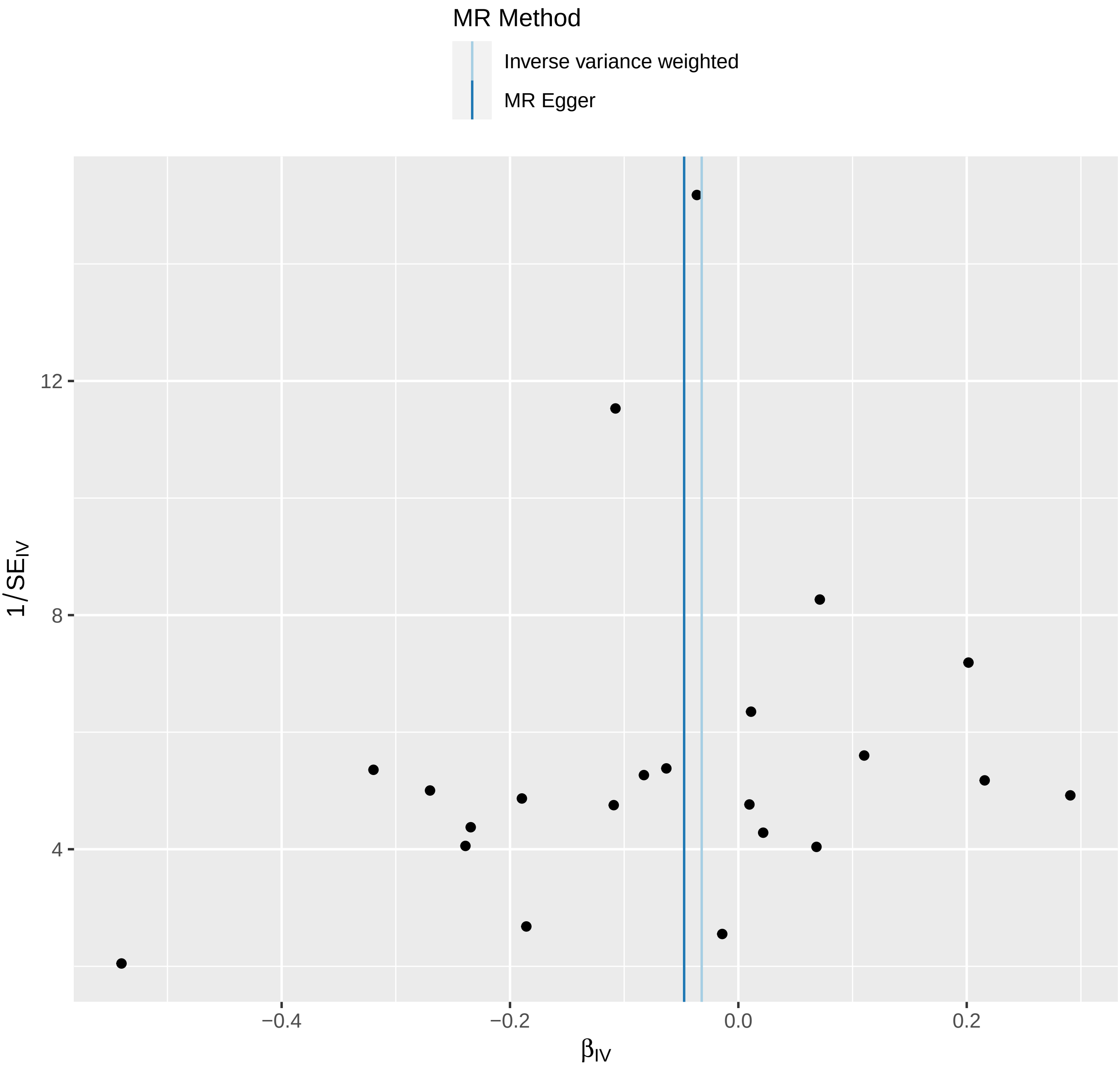

Batch 3−2319 : GDP−fucose protein O−fucosyltransferase 2 on Diabetic nephropathy (more control exclusions) with a threshold of 1e−05

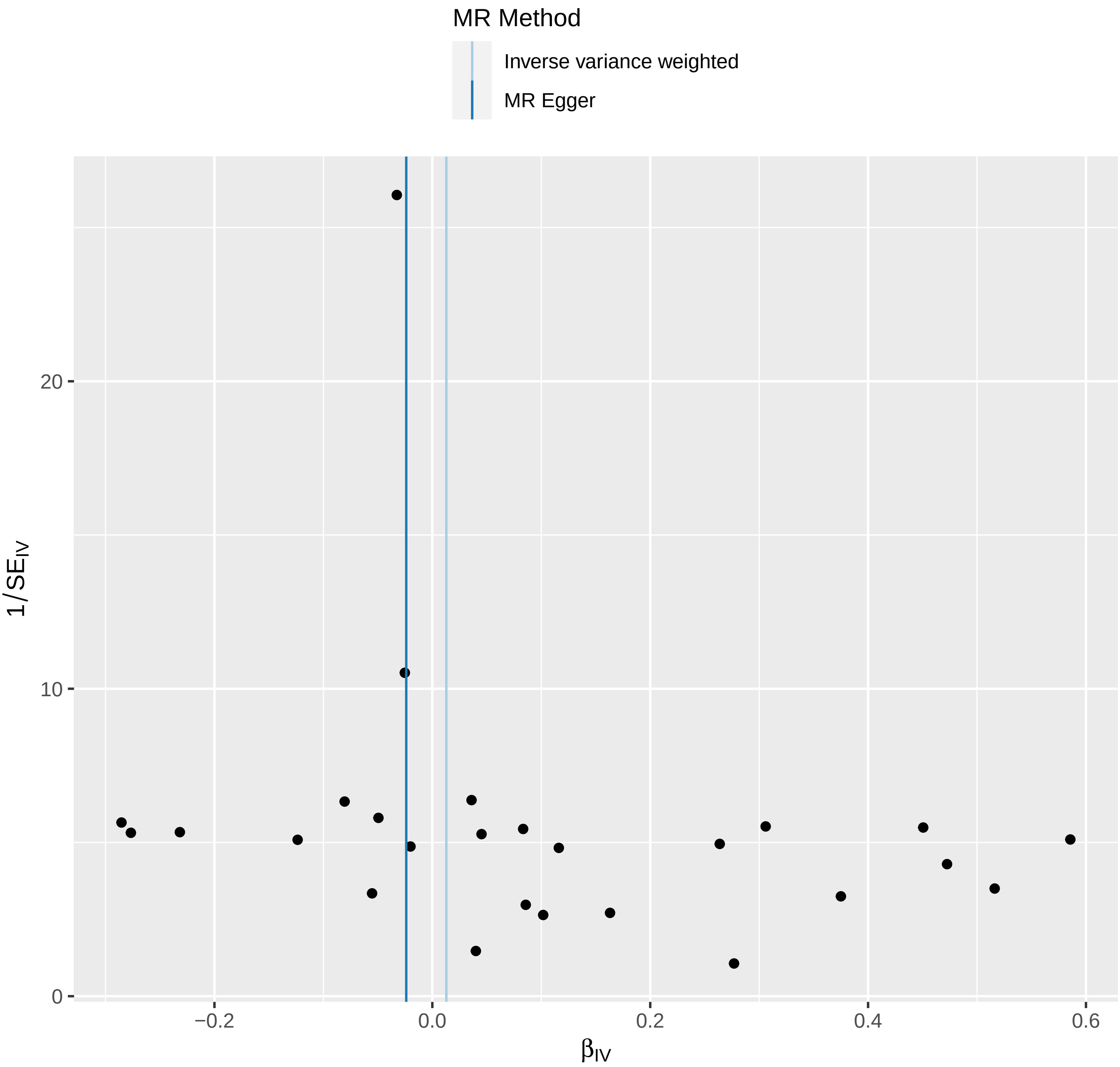

Batch 3−2320 : Protein O−glucosyltransferase 1 on Diabetic nephropathy (more control exclusions)
with a threshold of 1e−05

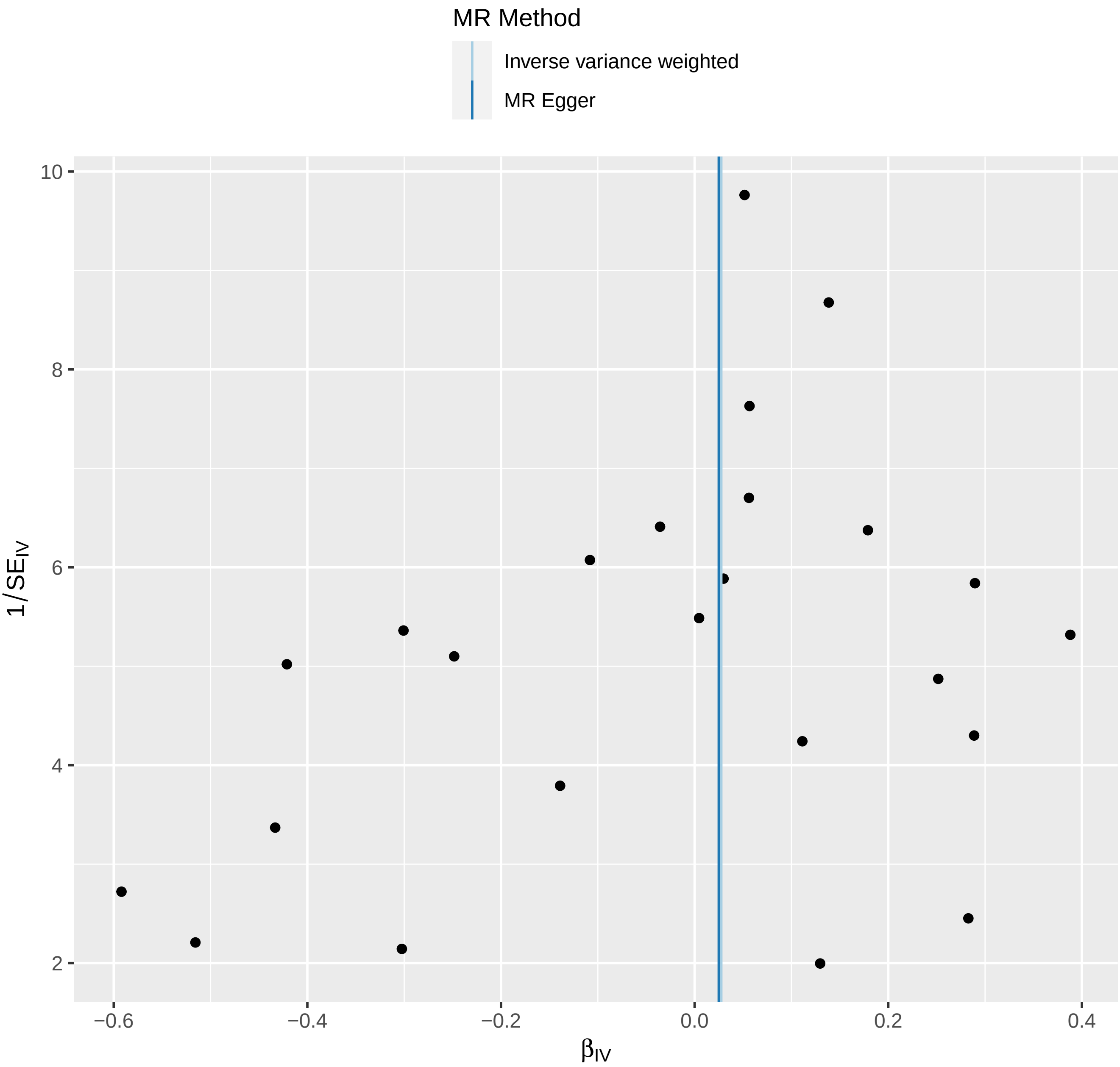

Batch 3−2321 : DNA polymerase eta on Diabetic nephropathy (more control exclusions)
with a threshold of 1e−05

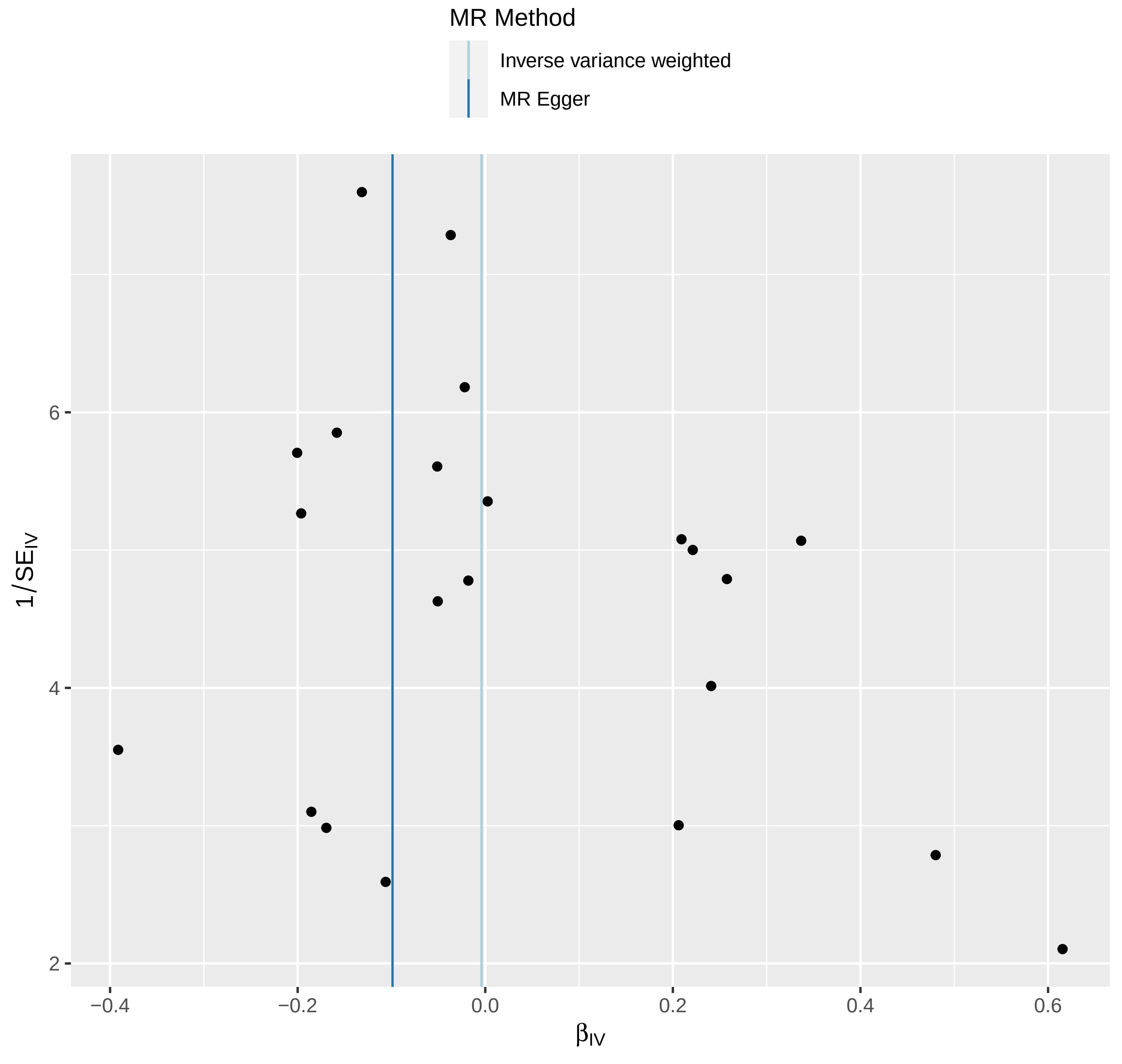

Batch 3−2322 : DNA polymerase iota on Diabetic nephropathy (more control exclusions)
with a threshold of 1e−05

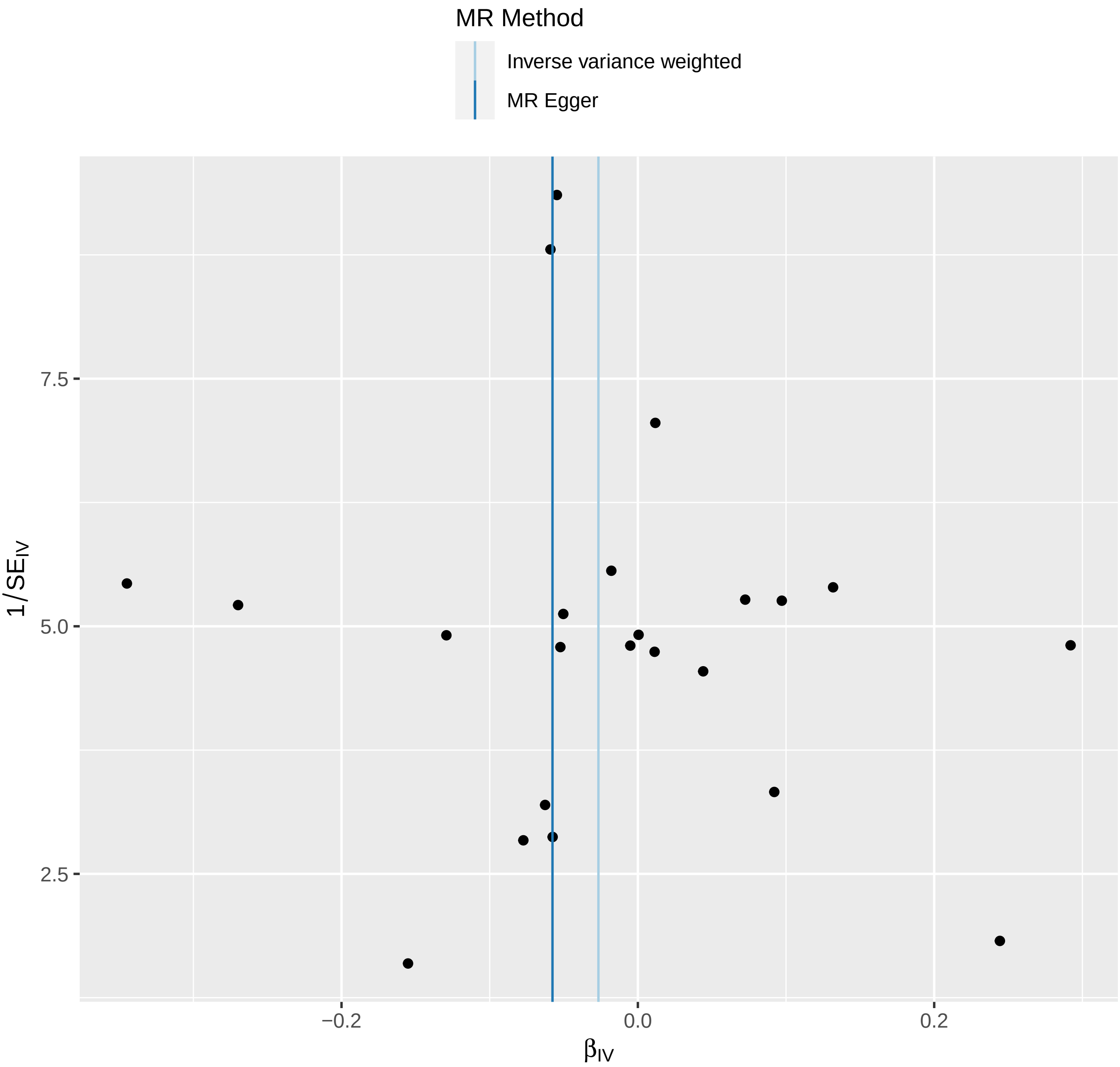

Batch 3−2323 : DNA−directed DNA/RNA polymerase mu on Diabetic nephropathy (more control exclusions)
with a threshold of 1e−05

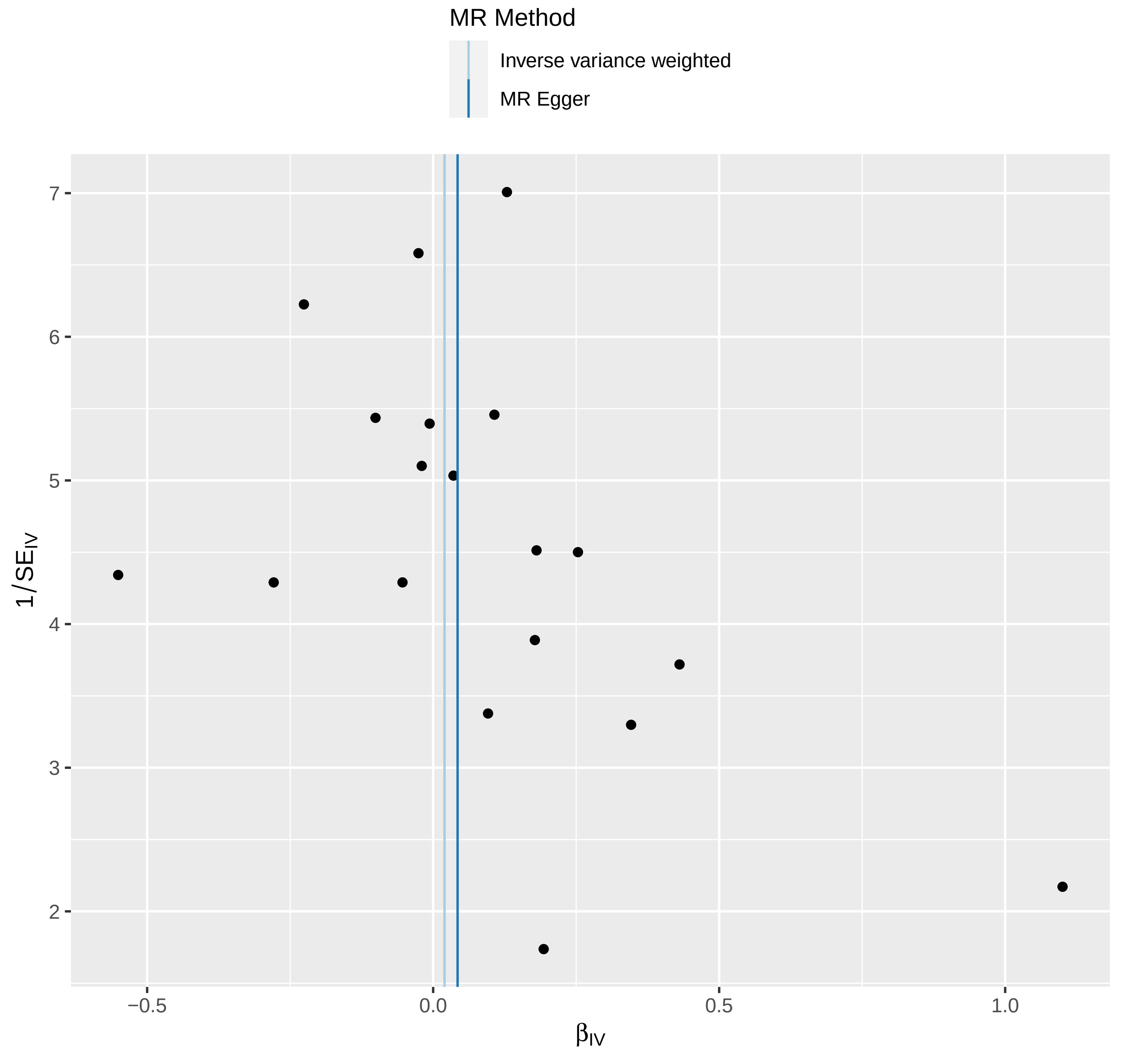

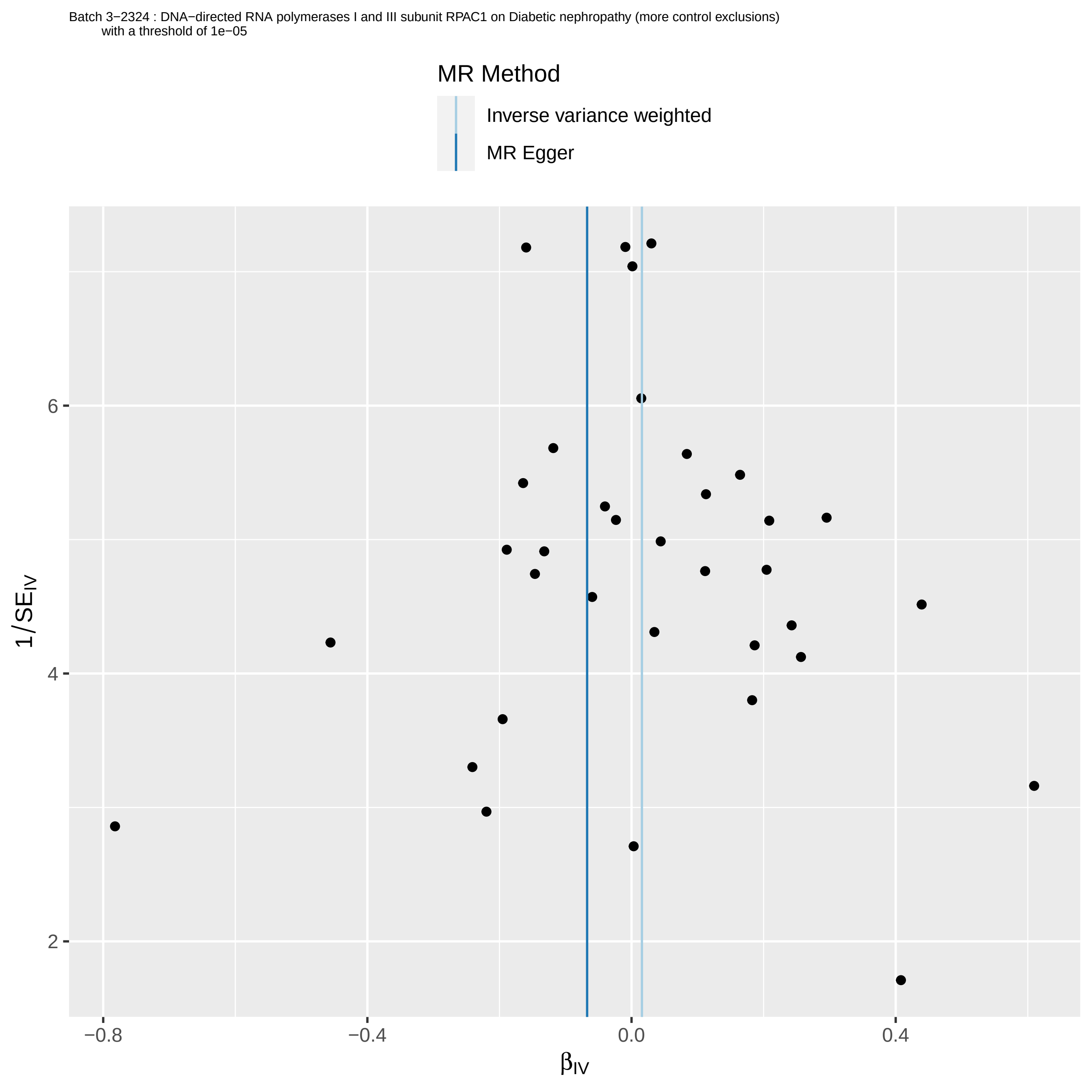

Batch 3−2324 : DNA−directed RNA polymerases I and III subunit RPAC1 on Diabetic nephropathy (more control exclusions)
with a threshold of 1e−05
MR Method
Inverse variance weighted
MR Egger
6
4
2
1/SE_IV
−0.8
−0.4
0.0
0.4
β_IV

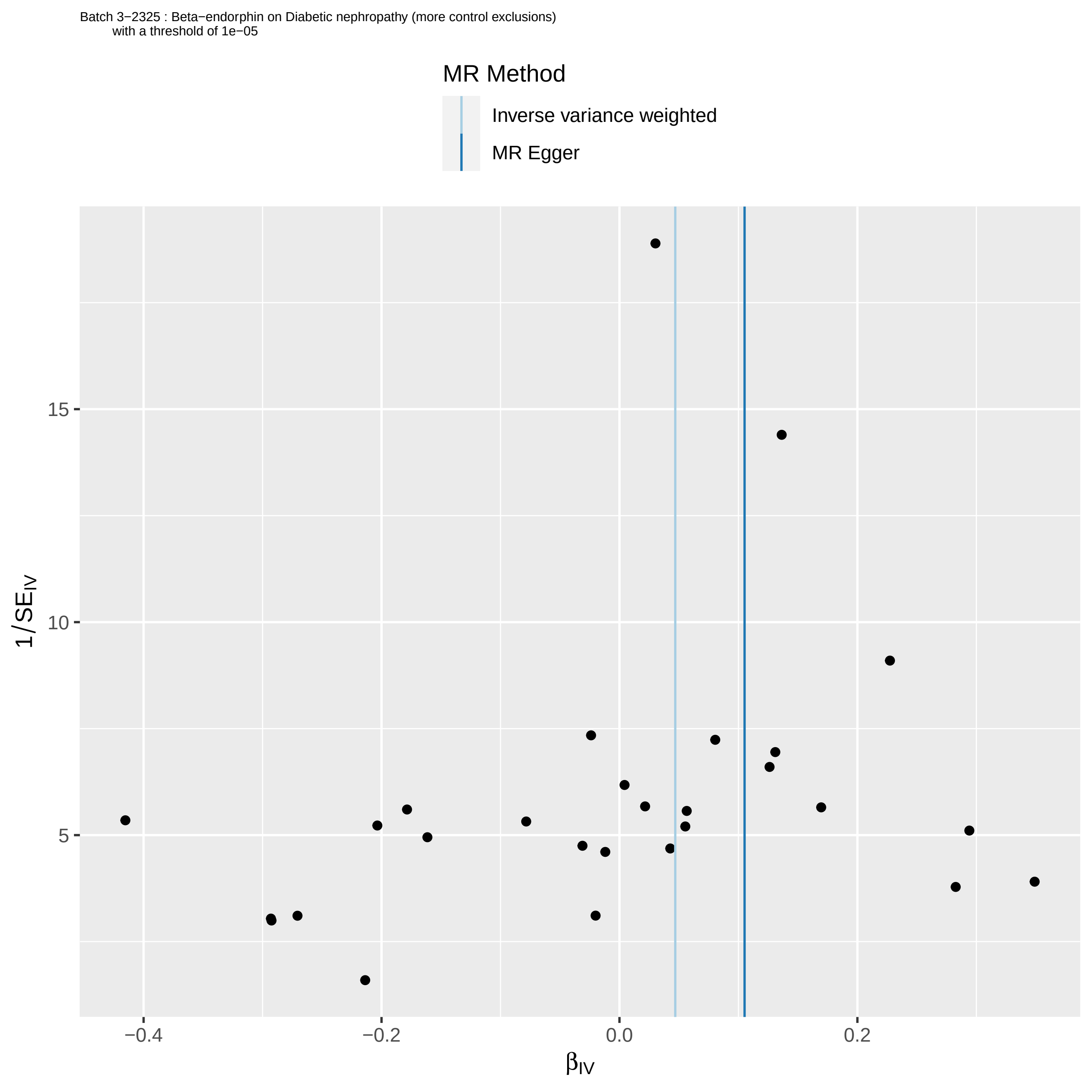

Batch 3−2325 : Beta−endorphin on Diabetic nephropathy (more control exclusions)
with a threshold of 1e−05
MR Method
Inverse variance weighted
MR Egger
15
10
5
1/SE_IV
−0.4
−0.2
0.0
0.2
β_IV

Batch 3−2326 : Corticotropin on Diabetic nephropathy (more control exclusions)
with a threshold of 1e−05

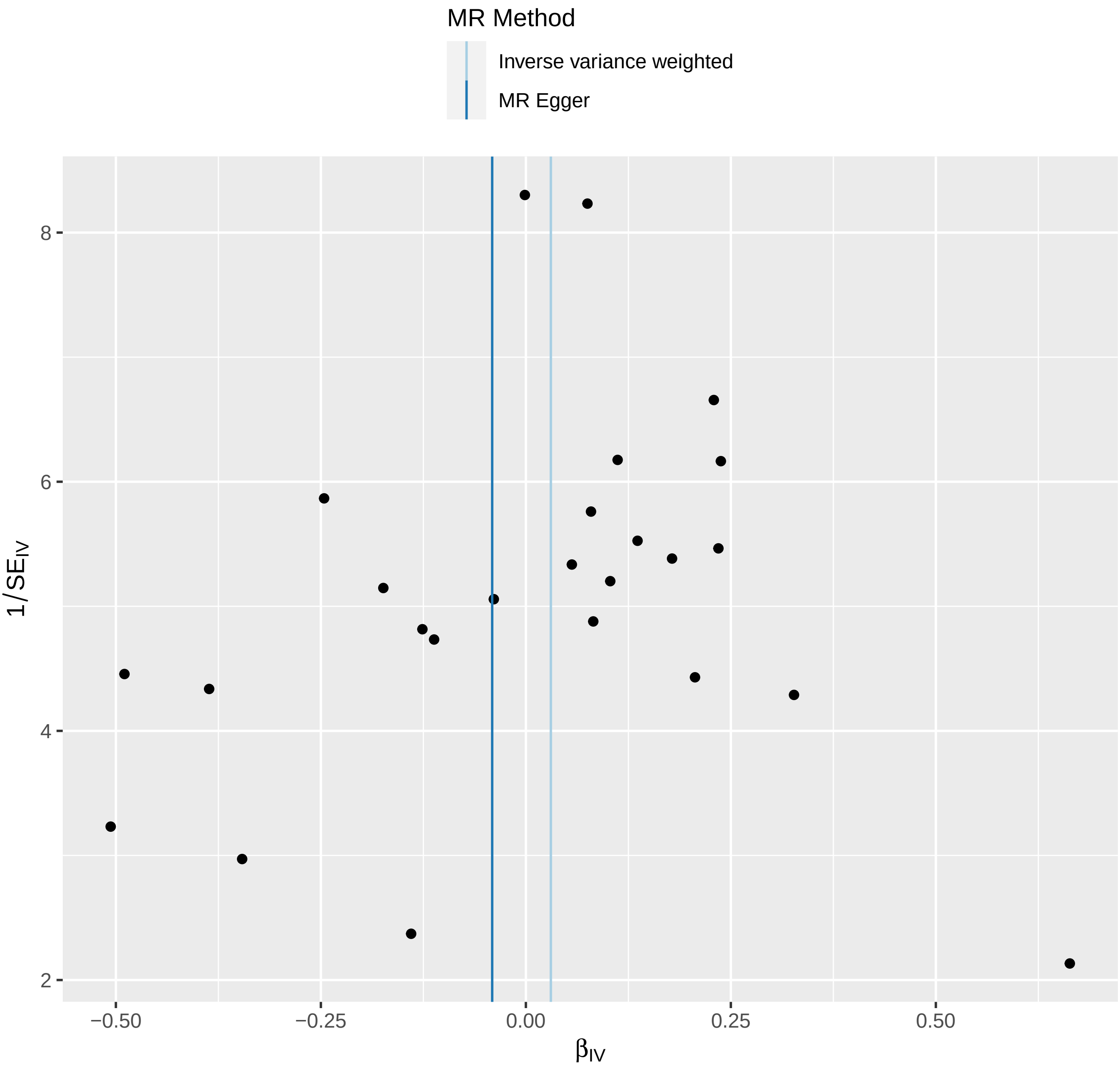

Batch 3−2327 : Pro−opiomelanocortin on Diabetic nephropathy (more control exclusions)
with a threshold of 1e−05

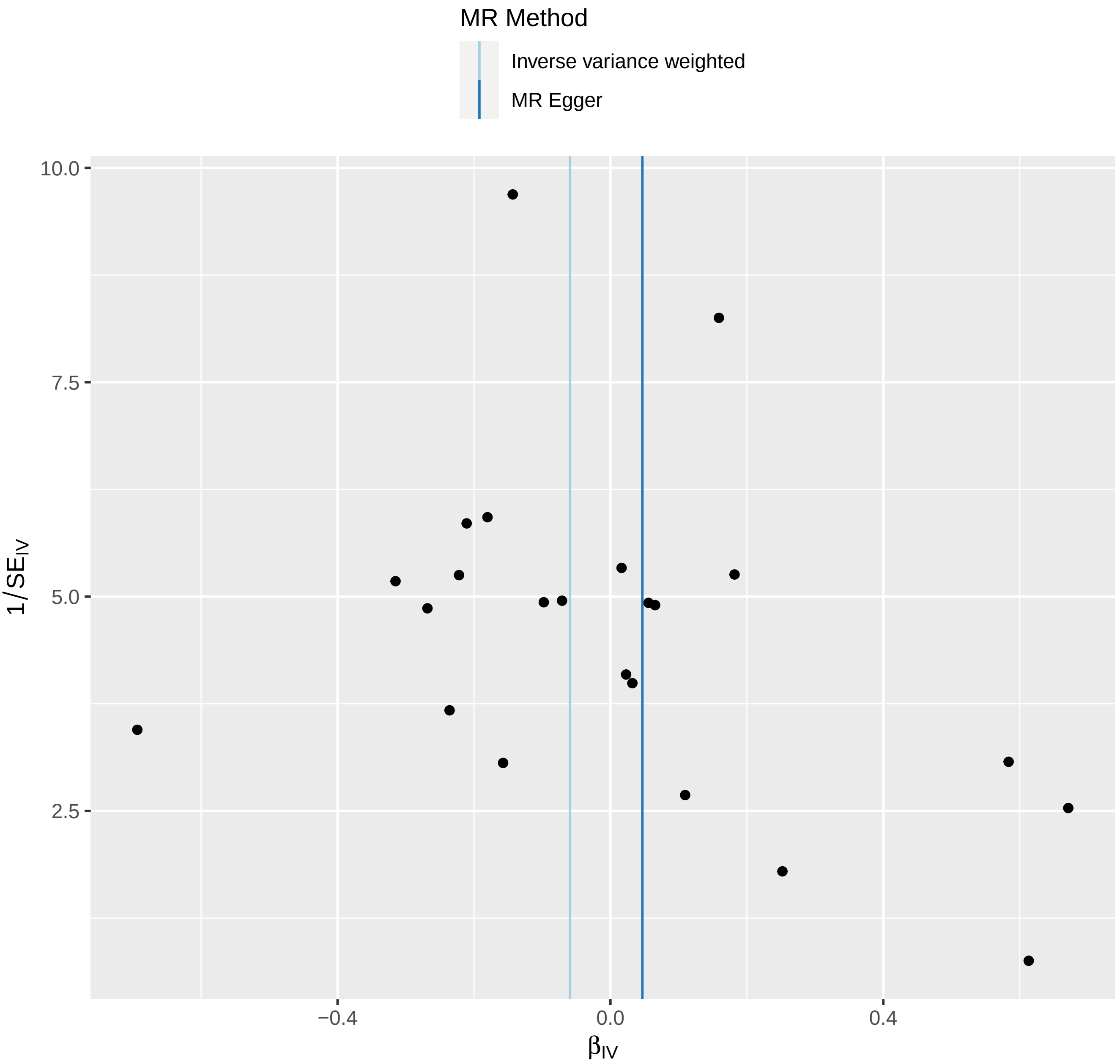

Batch 3−2328 : Protein O−linked−mannose beta−1,2−N−acetylglucosaminyltransferase 1 on Diabetic nephropathy (more control exclusions)
with a threshold of 1e−05

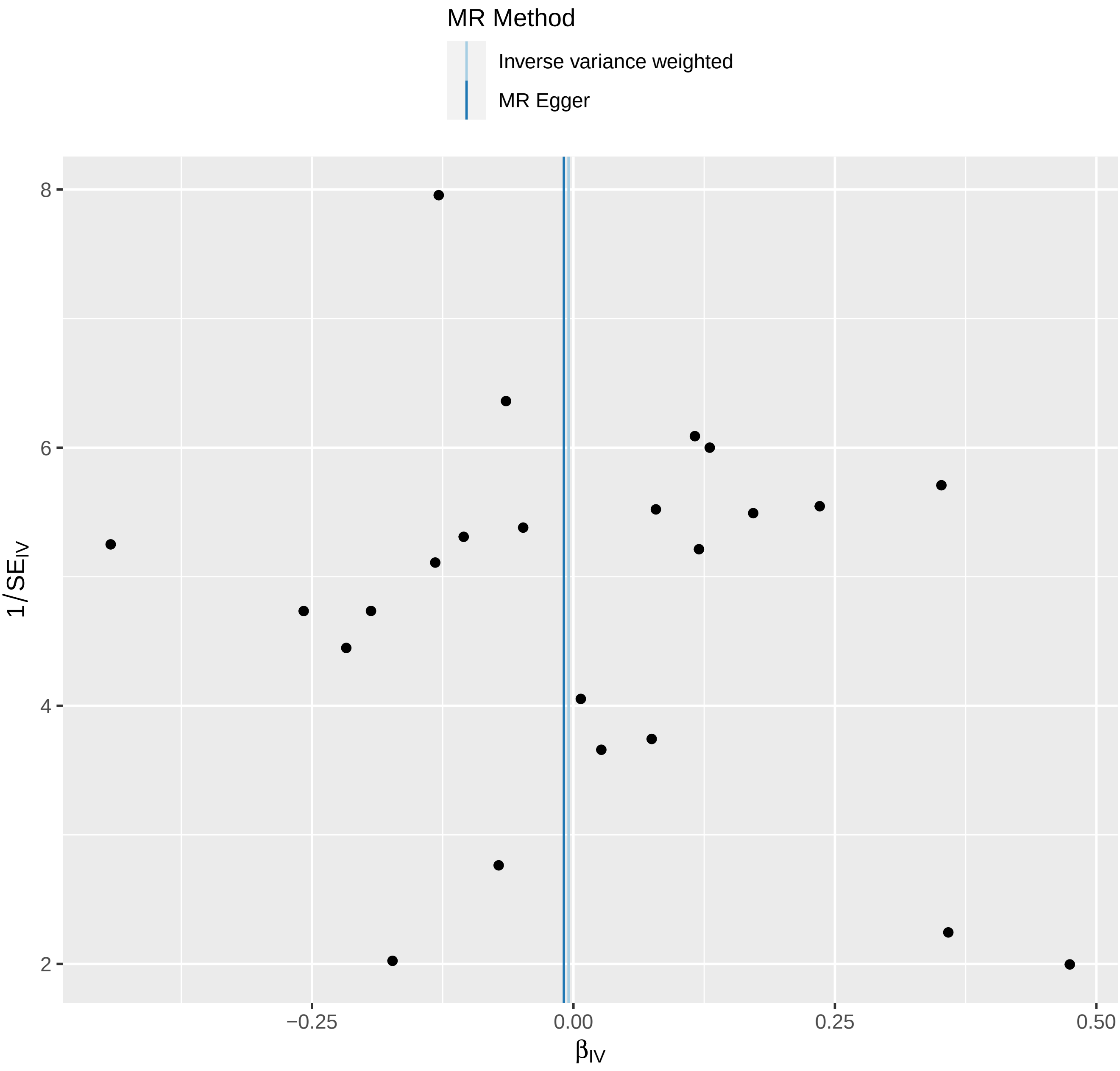

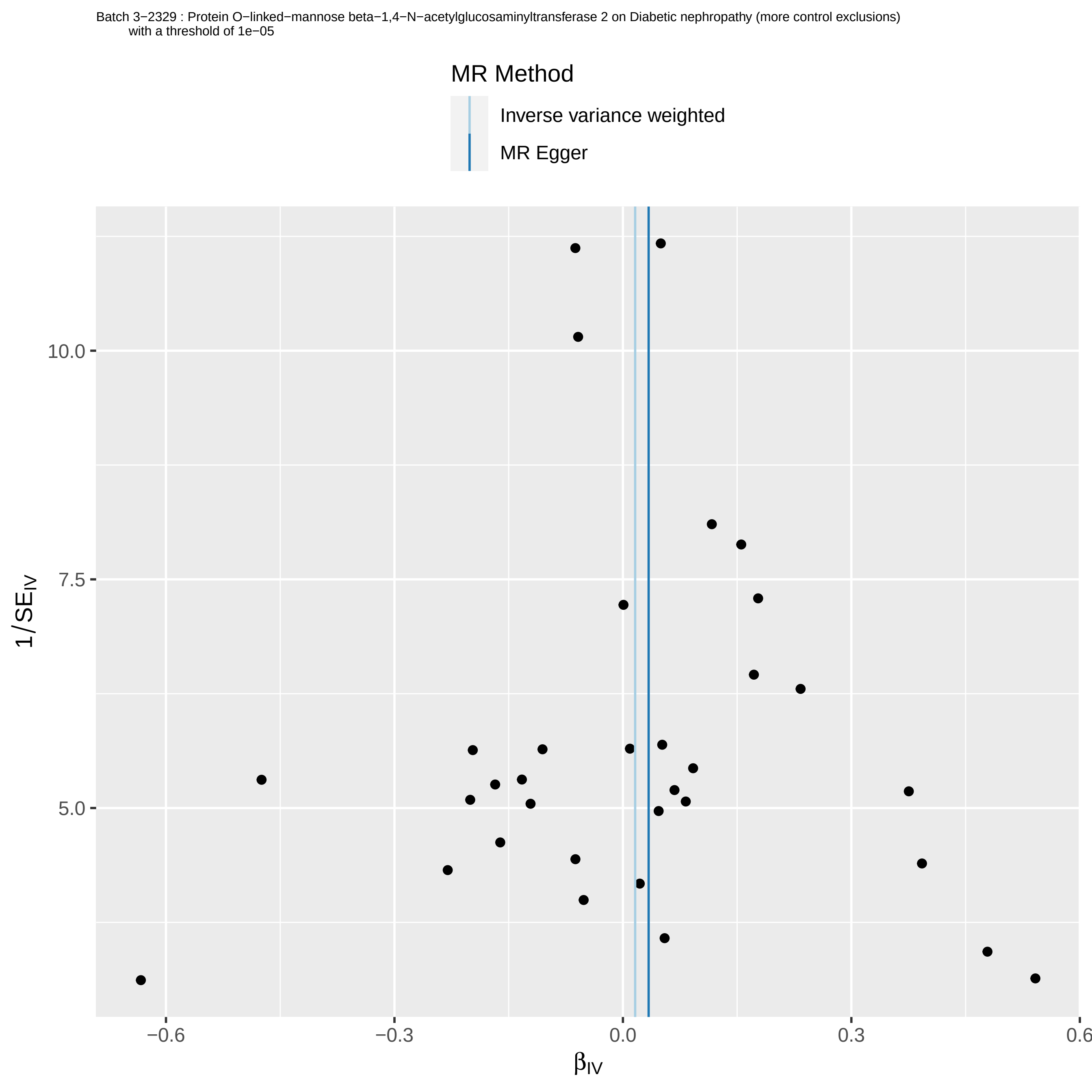

Batch 3−2329 : Protein O−linked−mannose beta−1,4−N−acetylglucosaminyltransferase 2 on Diabetic nephropathy (more control exclusions)
with a threshold of 1e−05
MR Method
Inverse variance weighted
MR Egger
10.0
7.5
5.0
1/SE_IV
−0.6
−0.3
0.0
0.3
0.6
β_IV

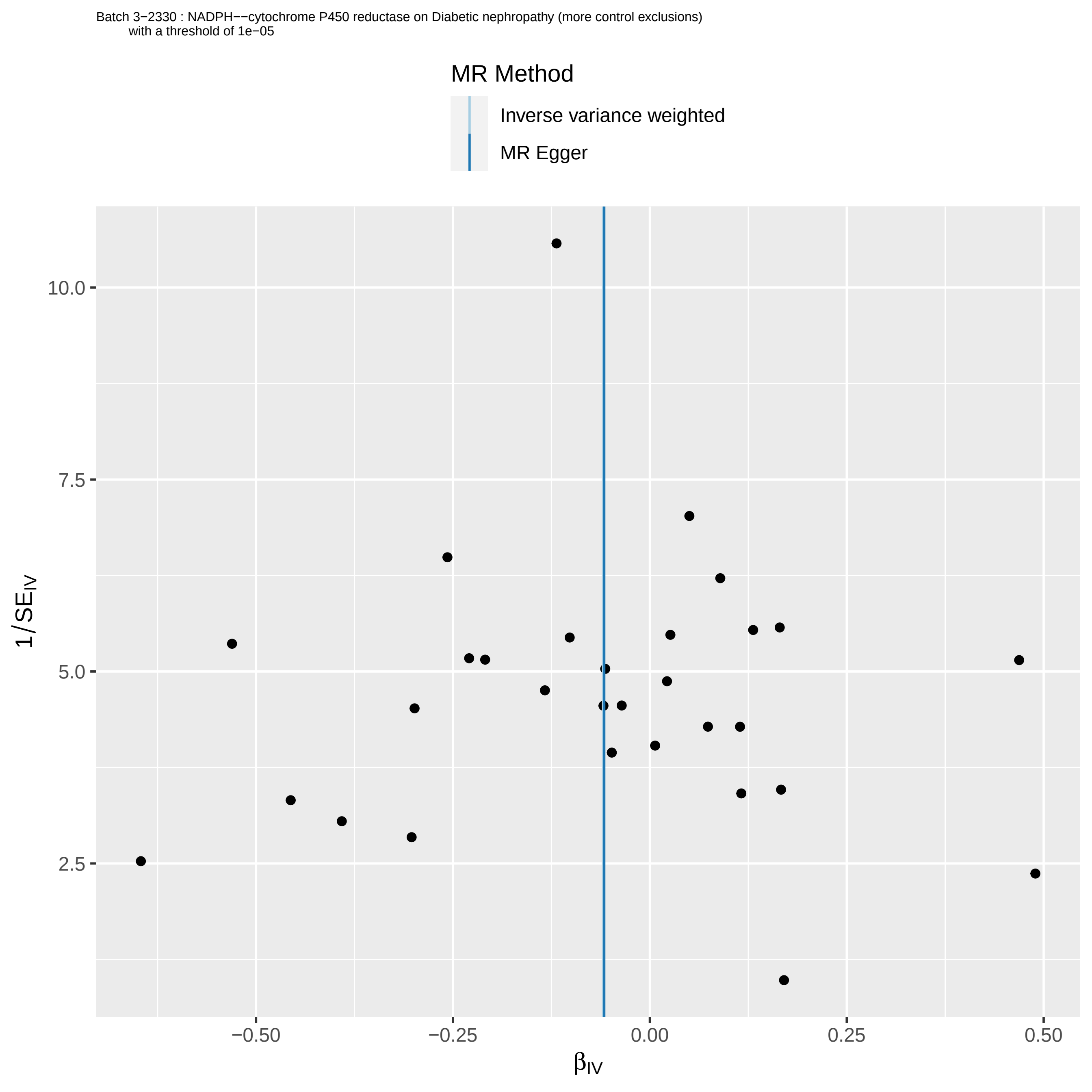
Batch 3−2330 : NADPH−−cytochrome P450 reductase on Diabetic nephropathy (more control exclusions)
with a threshold of 1e−05
MR Method
Inverse variance weighted
MR Egger
10.0
7.5
5.0
2.5
1/SE_IV
−0.50
−0.25
0.00
0.25
0.50
β_IV

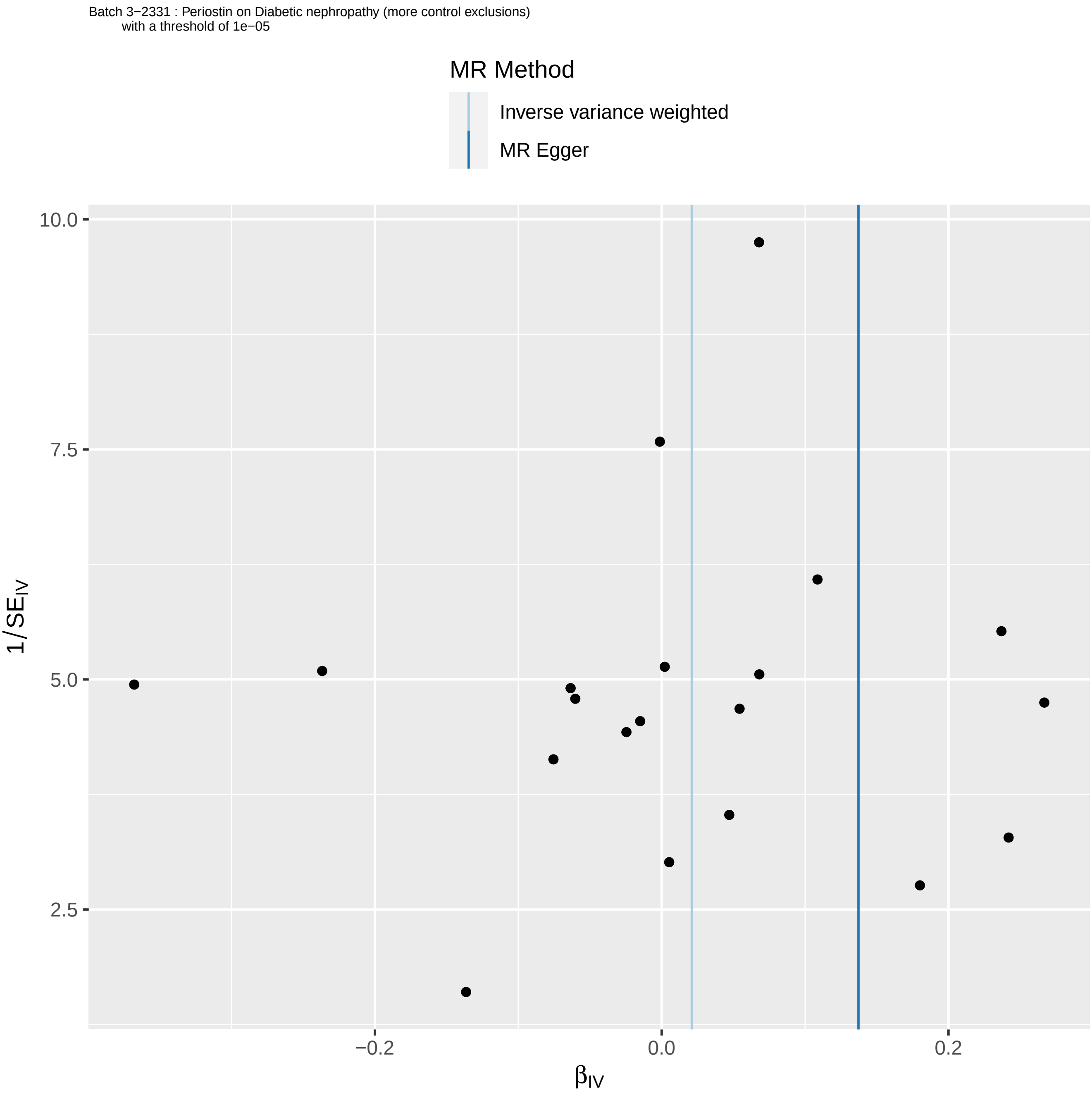
Batch 3−2331 : Periostin on Diabetic nephropathy (more control exclusions)
with a threshold of 1e−05
MR Method
Inverse variance weighted
MR Egger
10.0
7.5
5.0
2.5
$1/SE_{IV}$
−0.2
0.0
0.2
β_{IV}

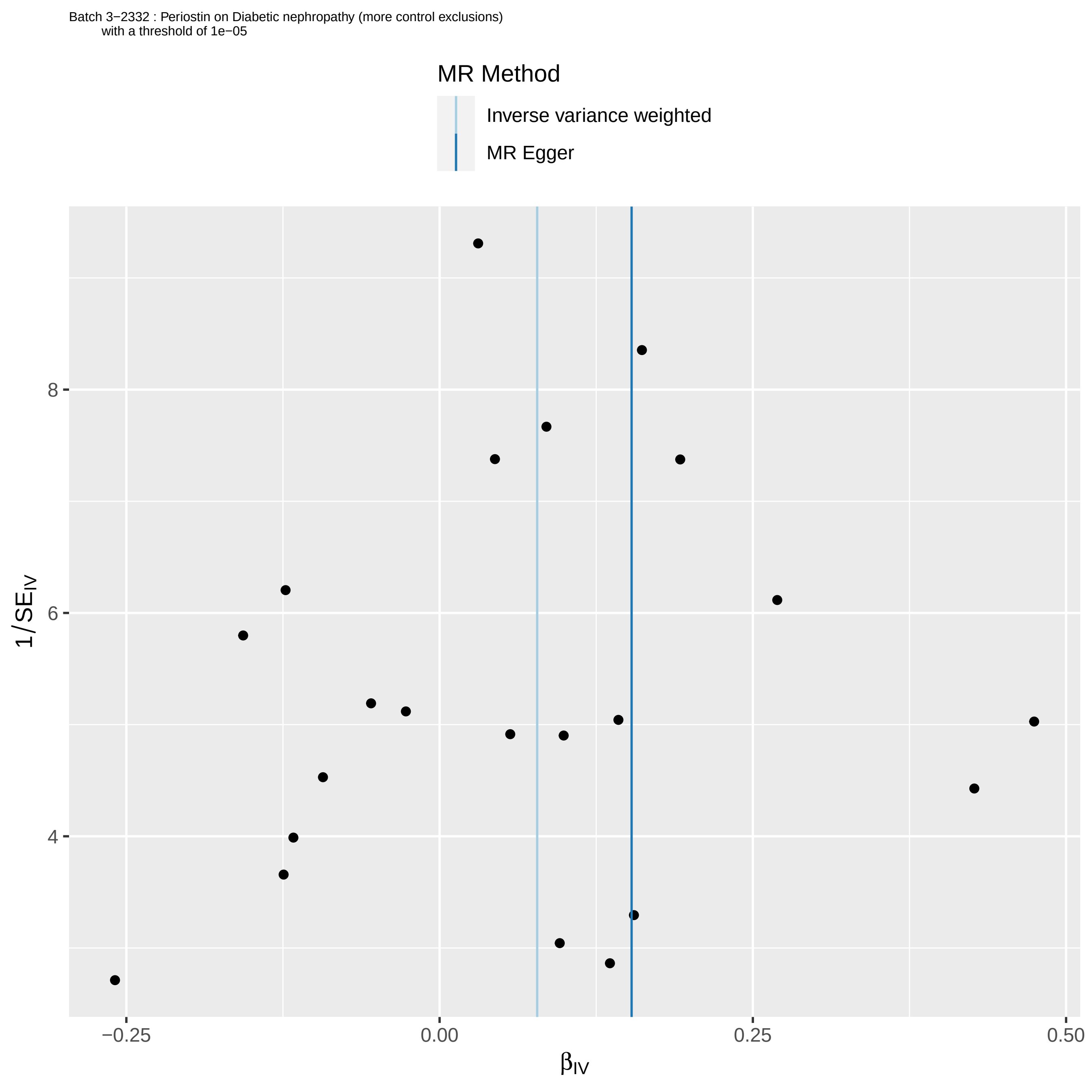

Batch 3−2332 : Periostin on Diabetic nephropathy (more control exclusions)
with a threshold of 1e−05
MR Method
Inverse variance weighted
MR Egger
8
6
4
1/SE_IV
−0.25
0.00
0.25
0.50
β_IV

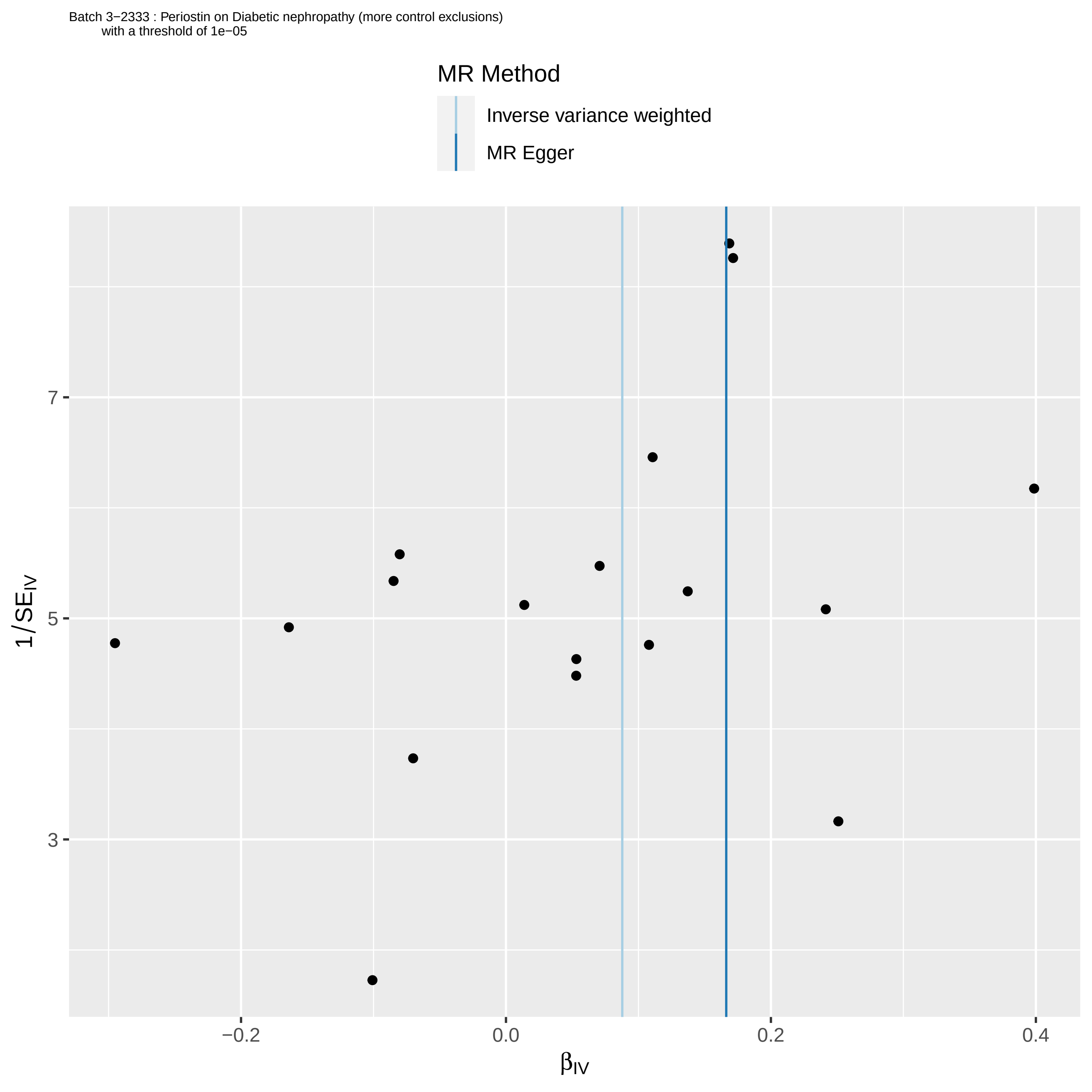
Batch 3−2333 : Periostin on Diabetic nephropathy (more control exclusions)
with a threshold of 1e−05
MR Method
Inverse variance weighted
MR Egger
7
5
3
1/SE_IV
−0.2
0.0
0.2
0.4
β_IV

Batch 3−2334 : POU domain, class 2, transcription factor 1 on Diabetic nephropathy (more control exclusions)
with a threshold of 1e−05

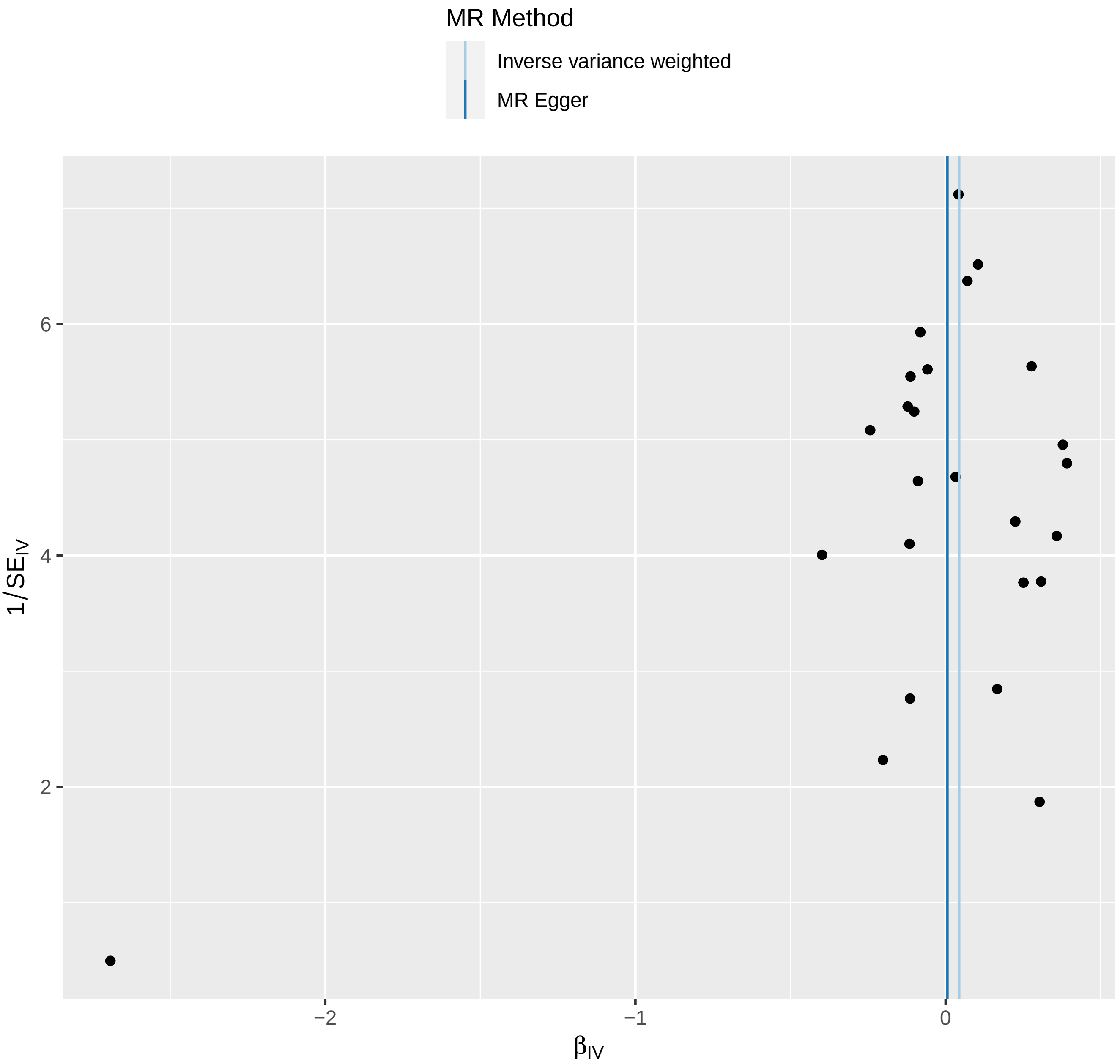

Batch 3−2335 : Phosphatidate phosphatase PPAPDC1A on Diabetic nephropathy (more control exclusions)
with a threshold of 1e−05

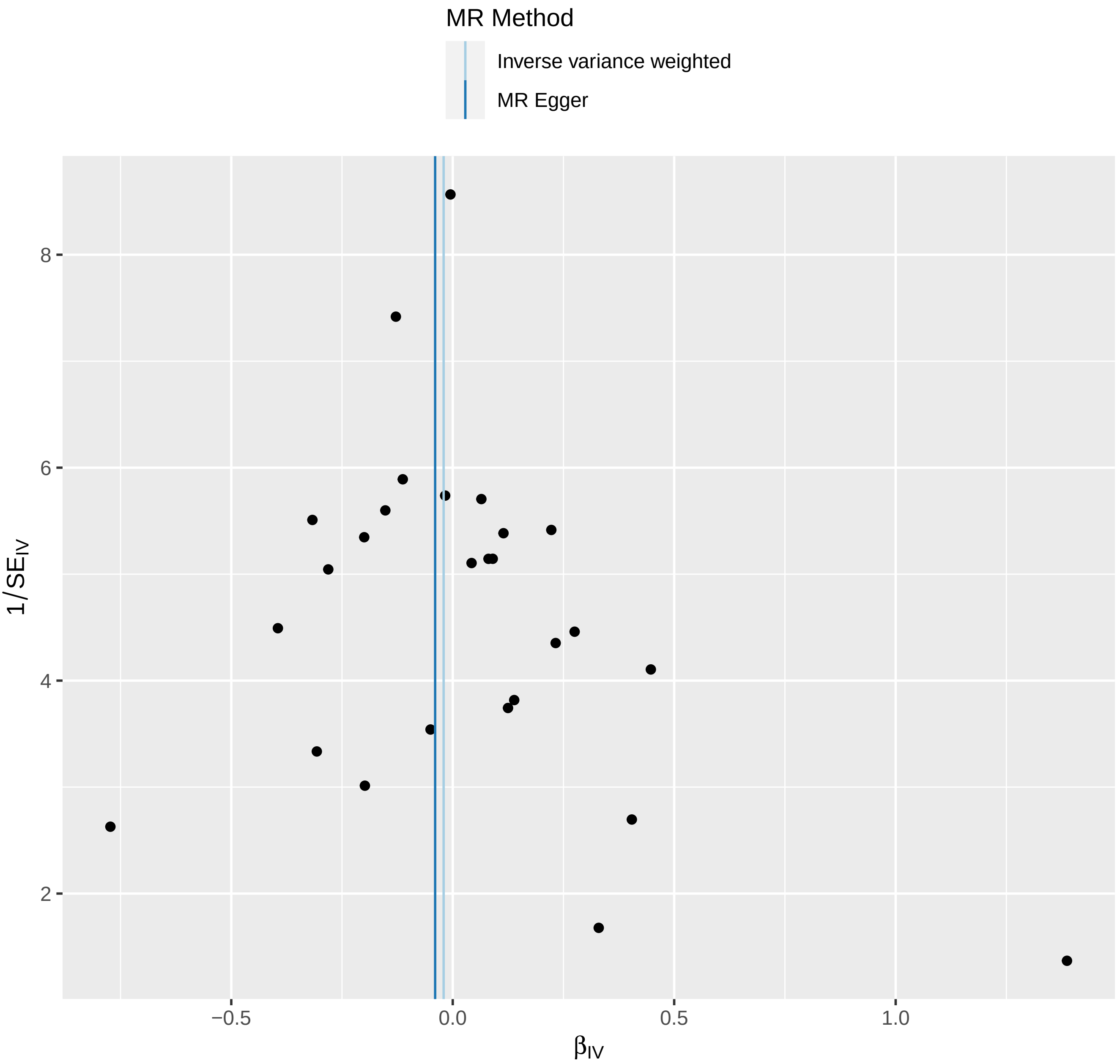

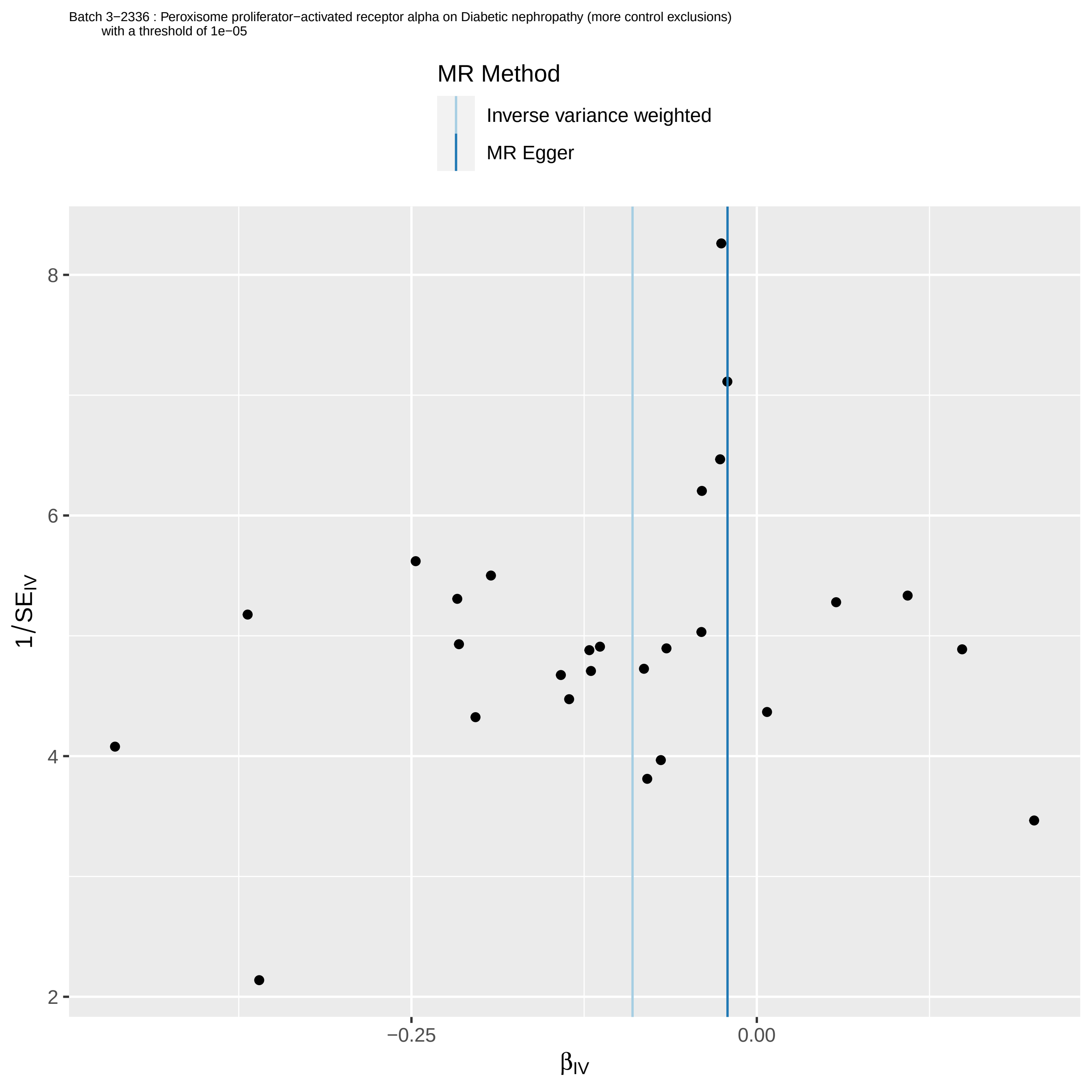

Batch 3−2336 : Peroxisome proliferator−activated receptor alpha on Diabetic nephropathy (more control exclusions)
with a threshold of 1e−05
MR Method
Inverse variance weighted
MR Egger
8
6
4
2
$1/SE_{IV}$
−0.25
0.00
β_{IV}

Batch 3−2337 : Neutrophil−activating peptide 2 on Diabetic nephropathy (more control exclusions)
with a threshold of 1e−05

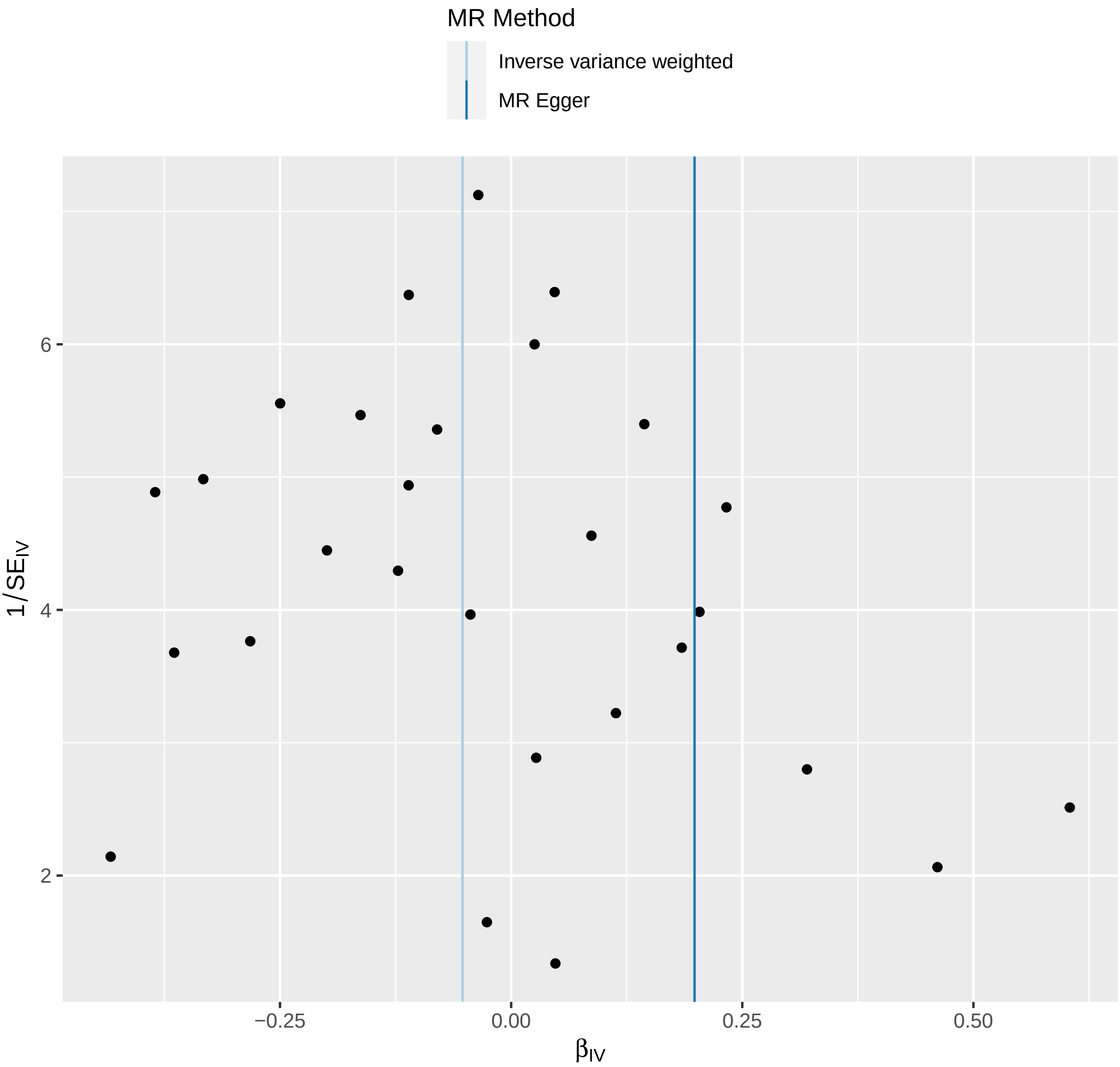

Batch 3−2338 : Phosphopantothenoylcysteine decarboxylase on Diabetic nephropathy (more control exclusions) with a threshold of 1e−05

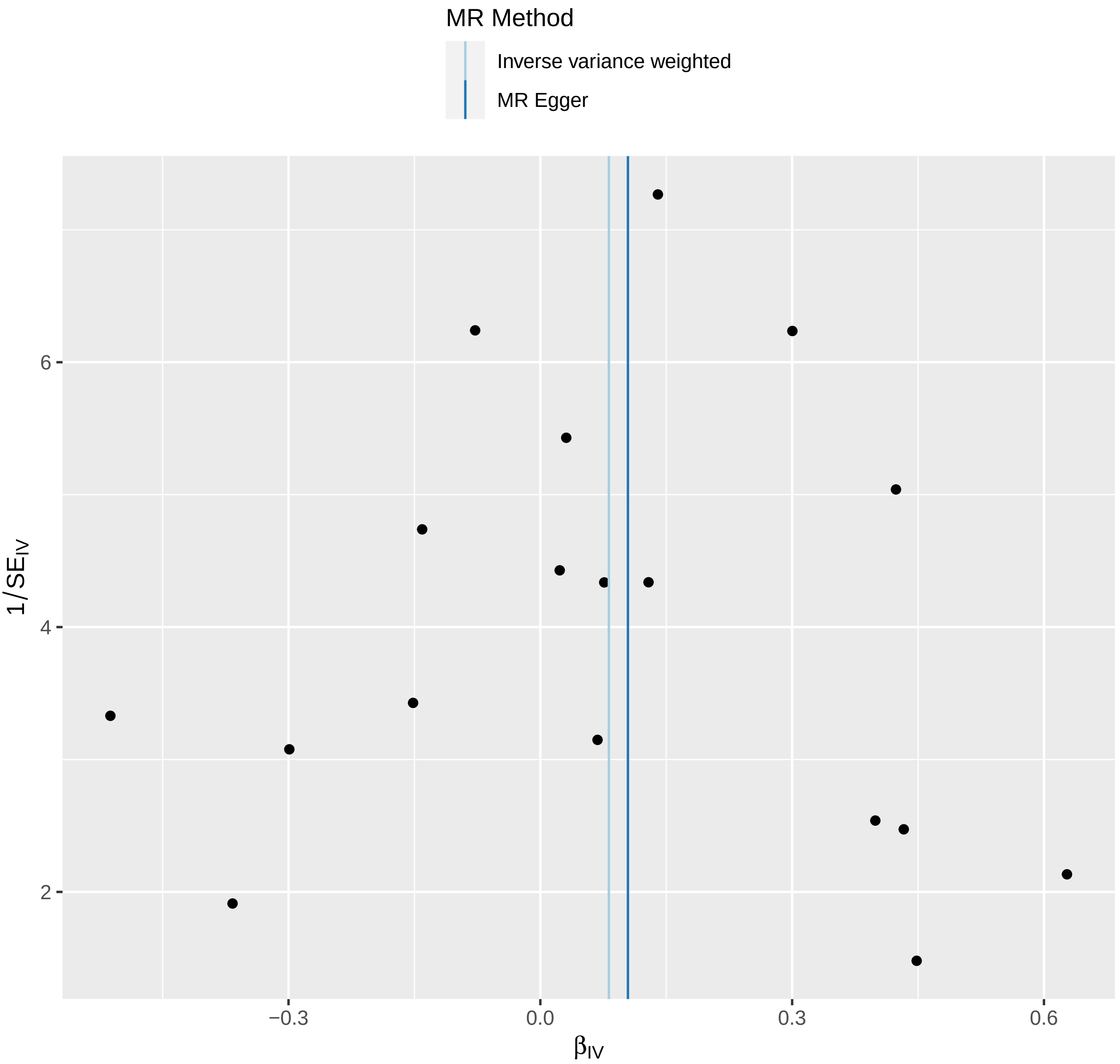

Batch 3−2339 : Peptidyl−prolyl cis−trans isomerase A on Diabetic nephropathy (more control exclusions)
with a threshold of 1e−05

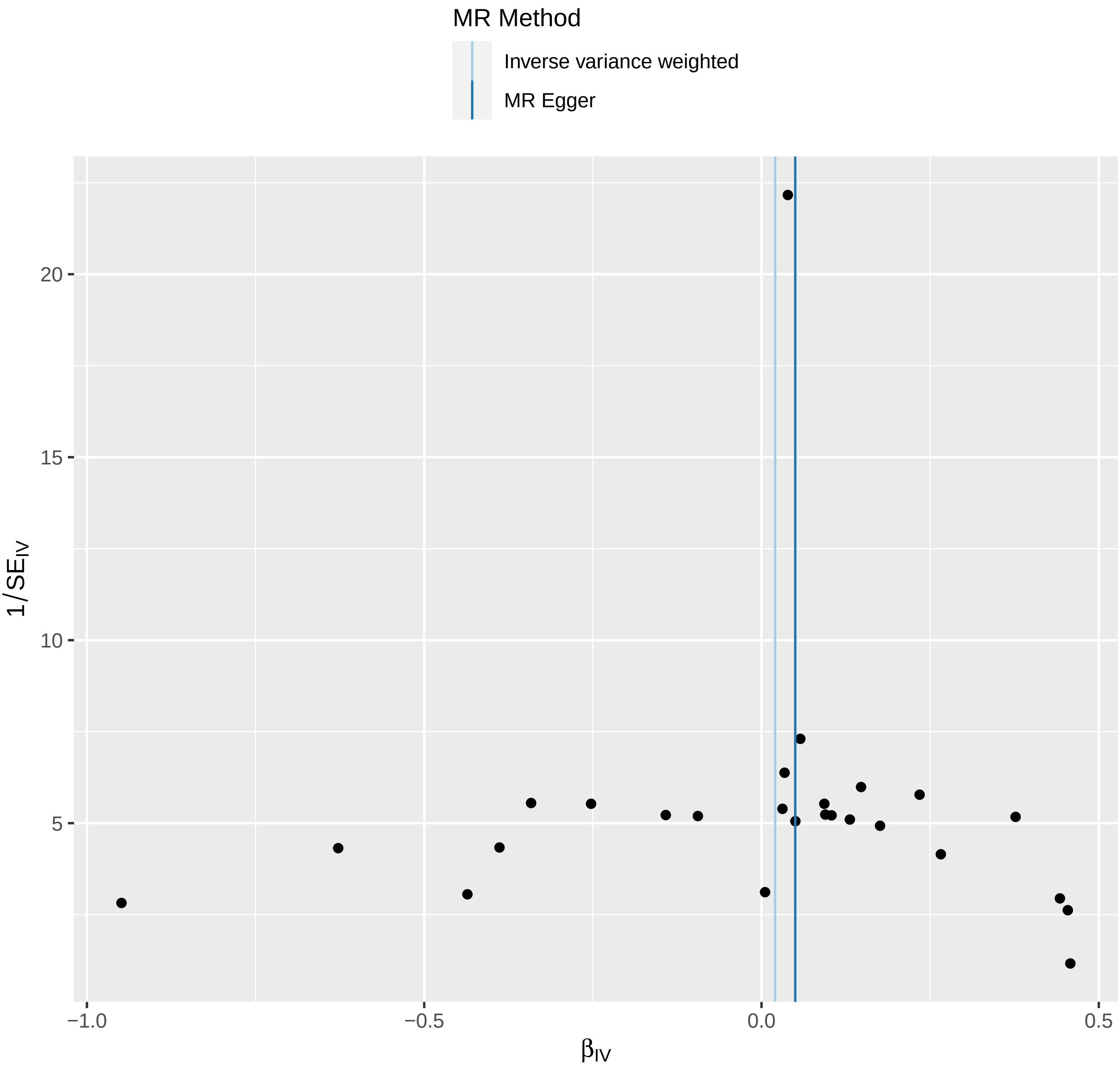

Batch 3−2340 : Peptidyl−prolyl cis−trans isomerase B on Diabetic nephropathy (more control exclusions)
with a threshold of 1e−05

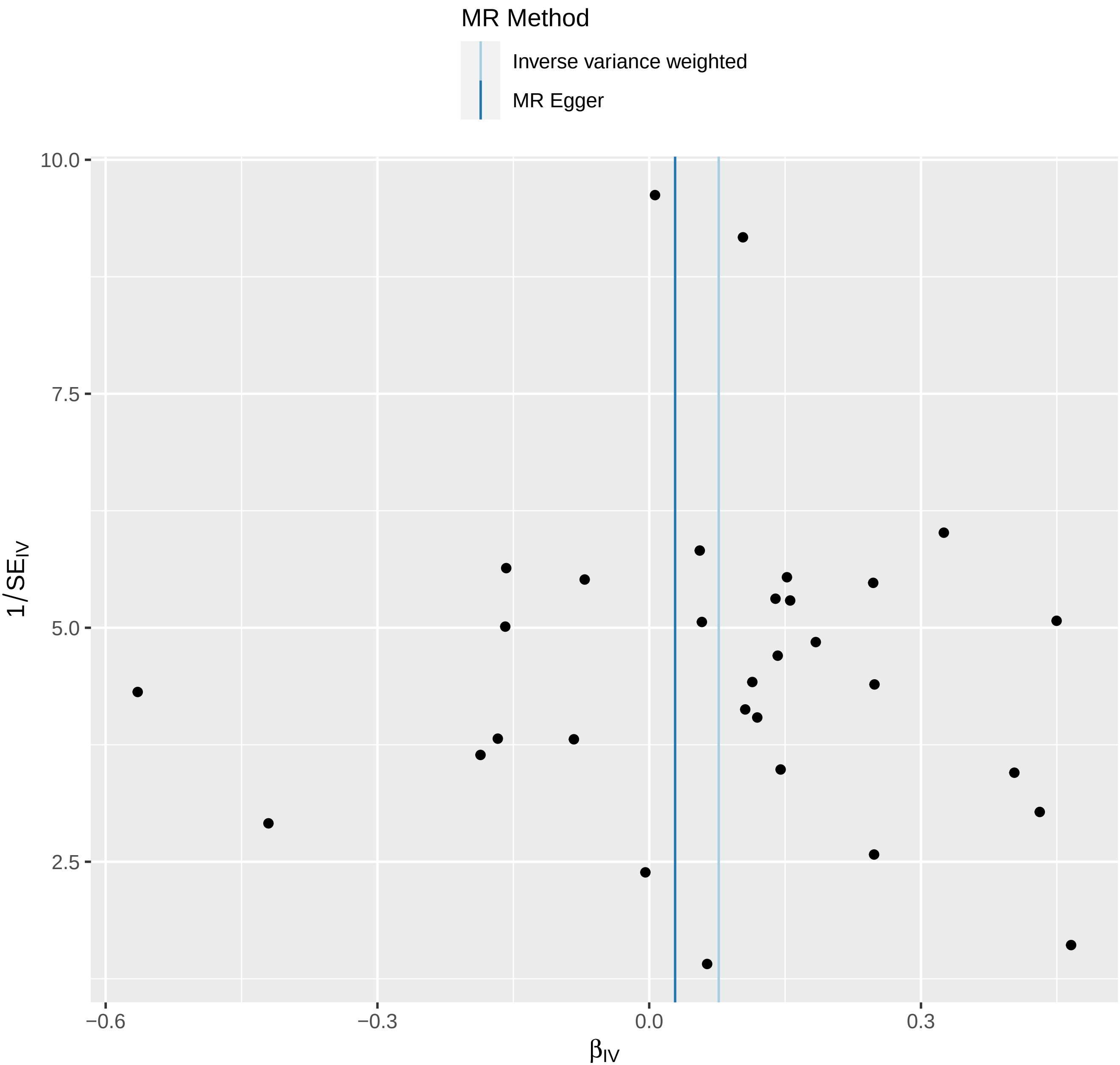

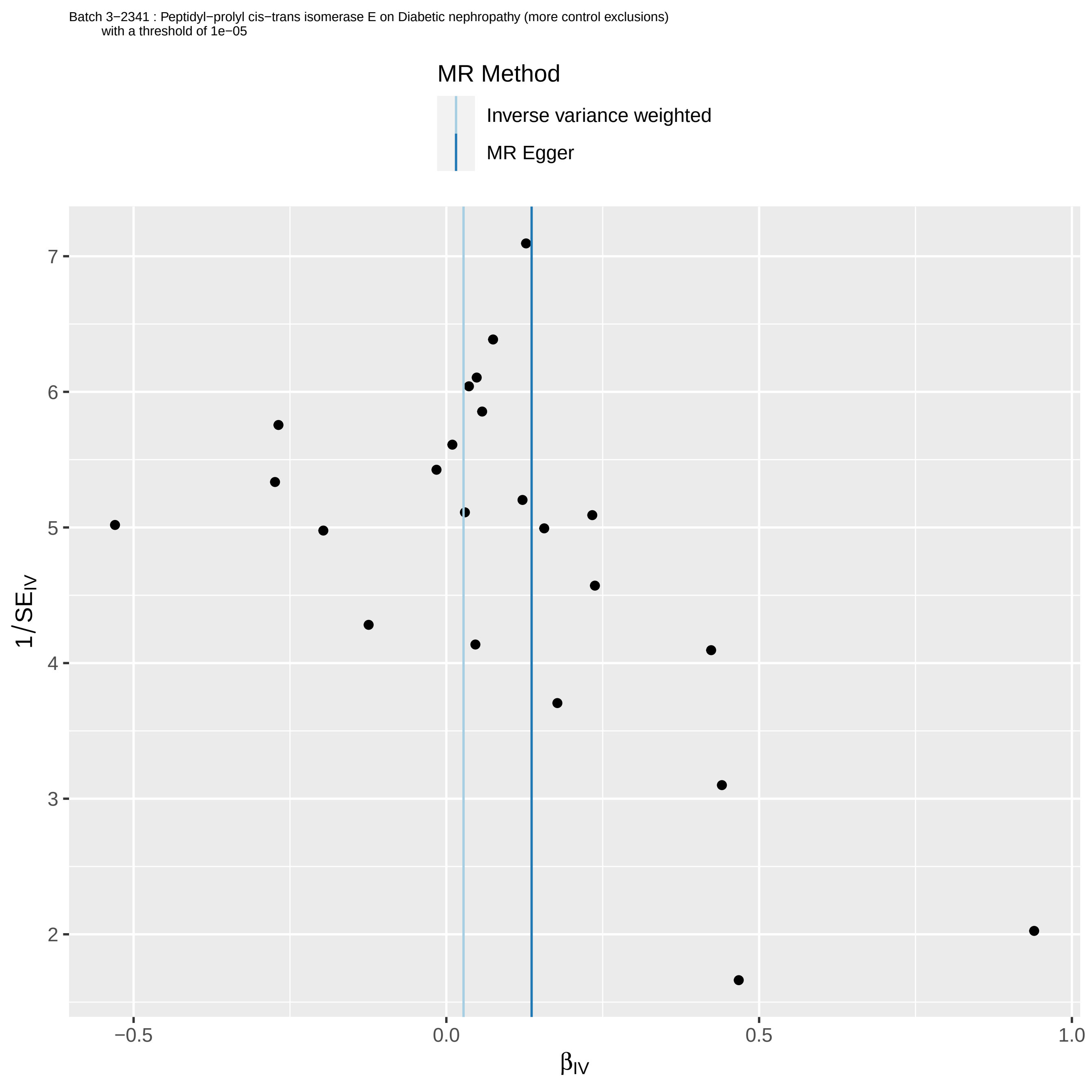

Batch 3−2341 : Peptidyl−prolyl cis−trans isomerase E on Diabetic nephropathy (more control exclusions)
with a threshold of 1e−05
MR Method
Inverse variance weighted
MR Egger
1/SE$_{IV}$
β$_{IV}$
7
6
5
4
3
2
−0.5
0.0
0.5
1.0

Batch 3−2342 : Peptidyl−prolyl cis−trans isomerase H on Diabetic nephropathy (more control exclusions)
with a threshold of 1e−05

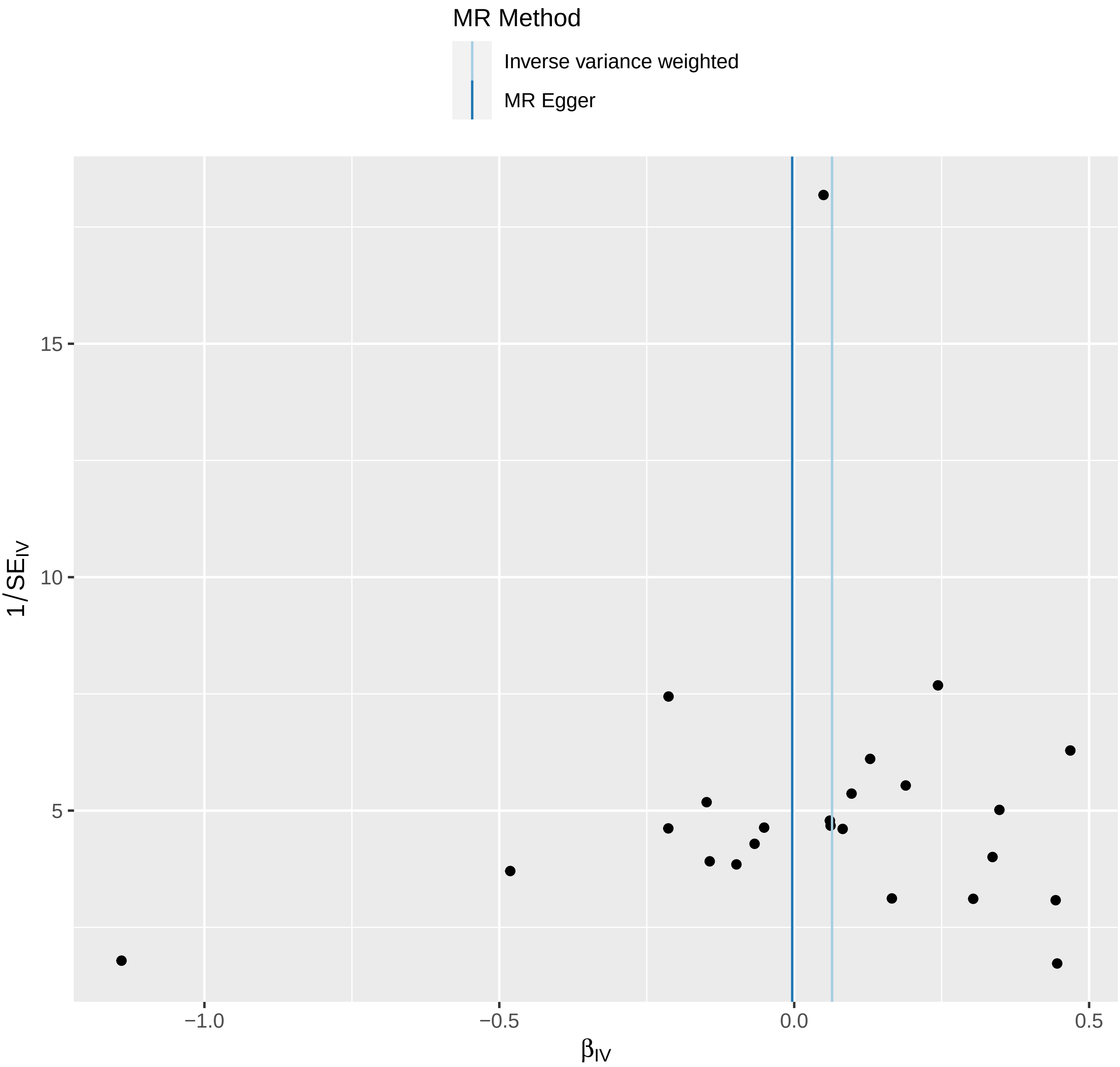

Batch 3−2343 : Peptidyl−prolyl cis−trans isomerase−like 1 on Diabetic nephropathy (more control exclusions) with a threshold of 1e−05

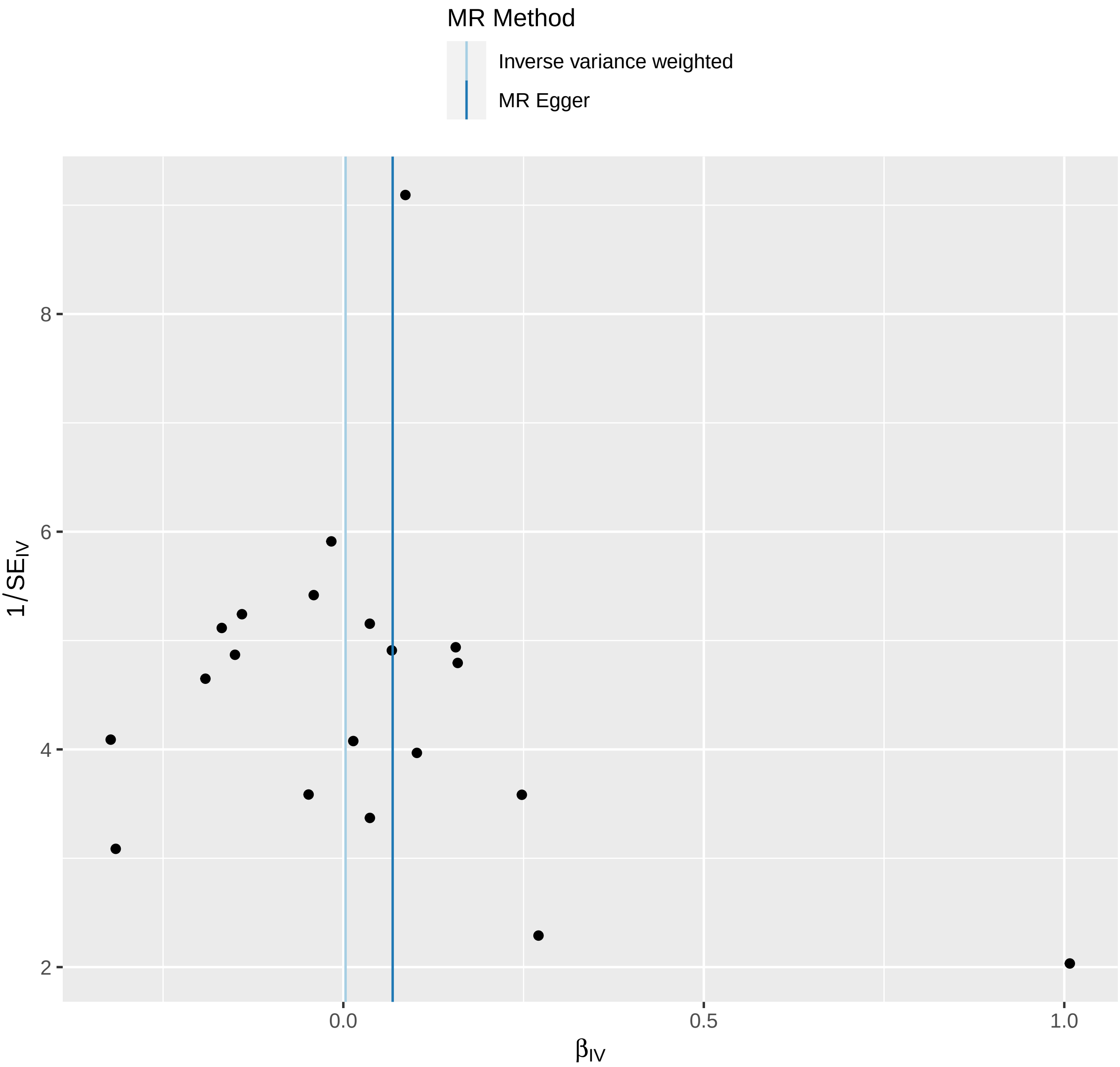

Batch 3−2344 : Peptidyl−prolyl cis−trans isomerase−like 2 on Diabetic nephropathy (more control exclusions)
with a threshold of 1e−05

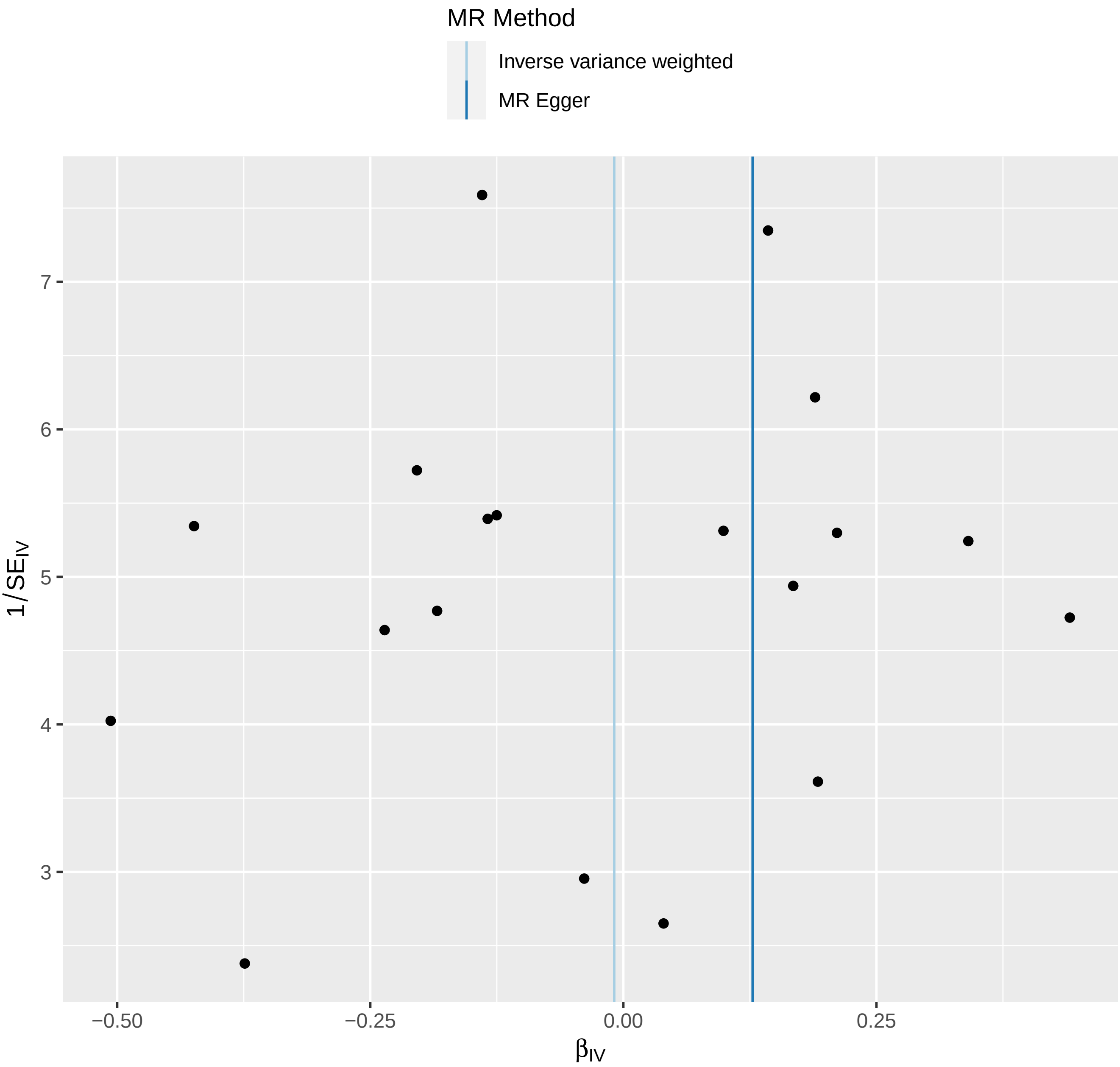

Batch 3−2345 : Protein phosphatase 1A on Diabetic nephropathy (more control exclusions)
with a threshold of 1e−05

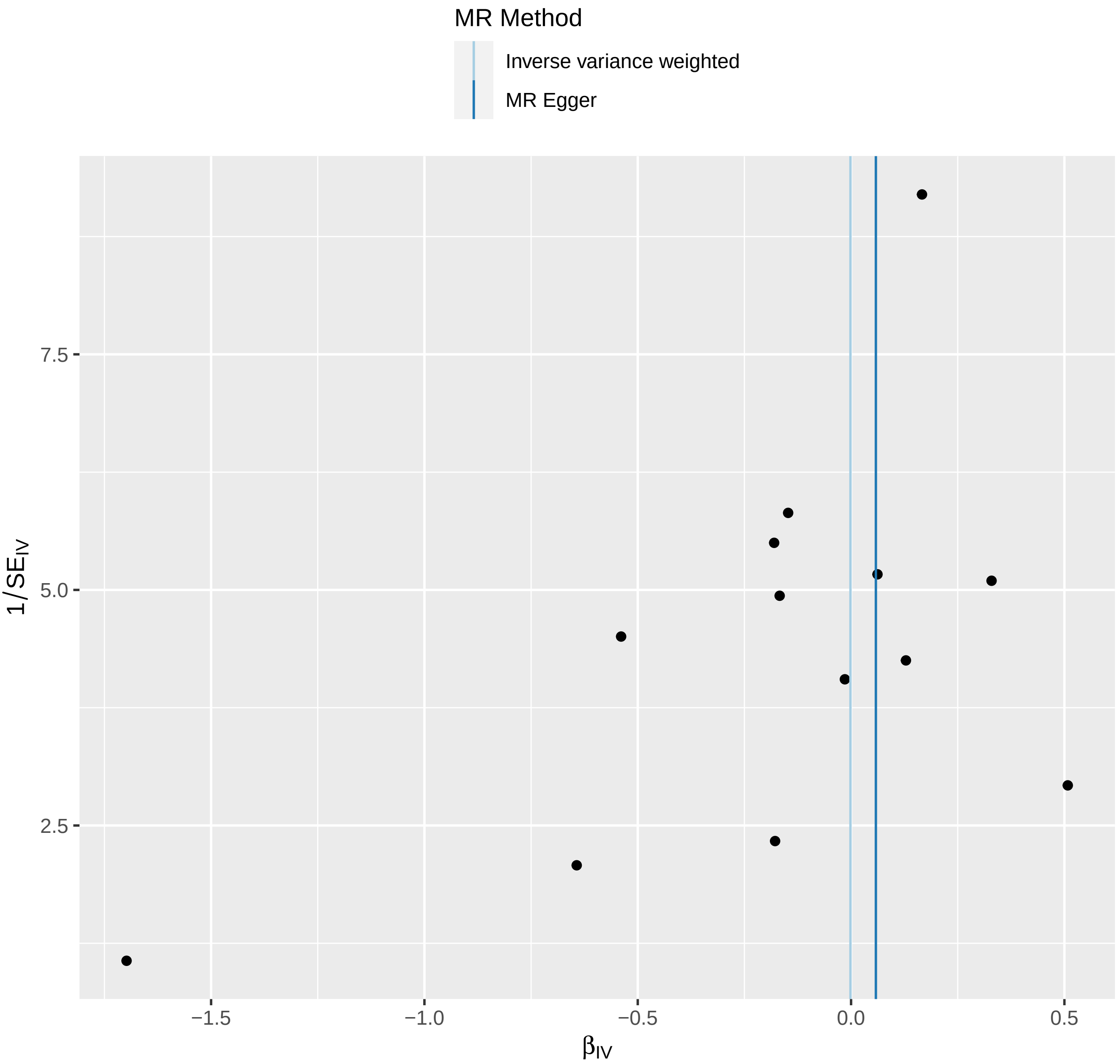

Batch 3−2346 : Protein phosphatase 1L on Diabetic nephropathy (more control exclusions)
with a threshold of 1e−05

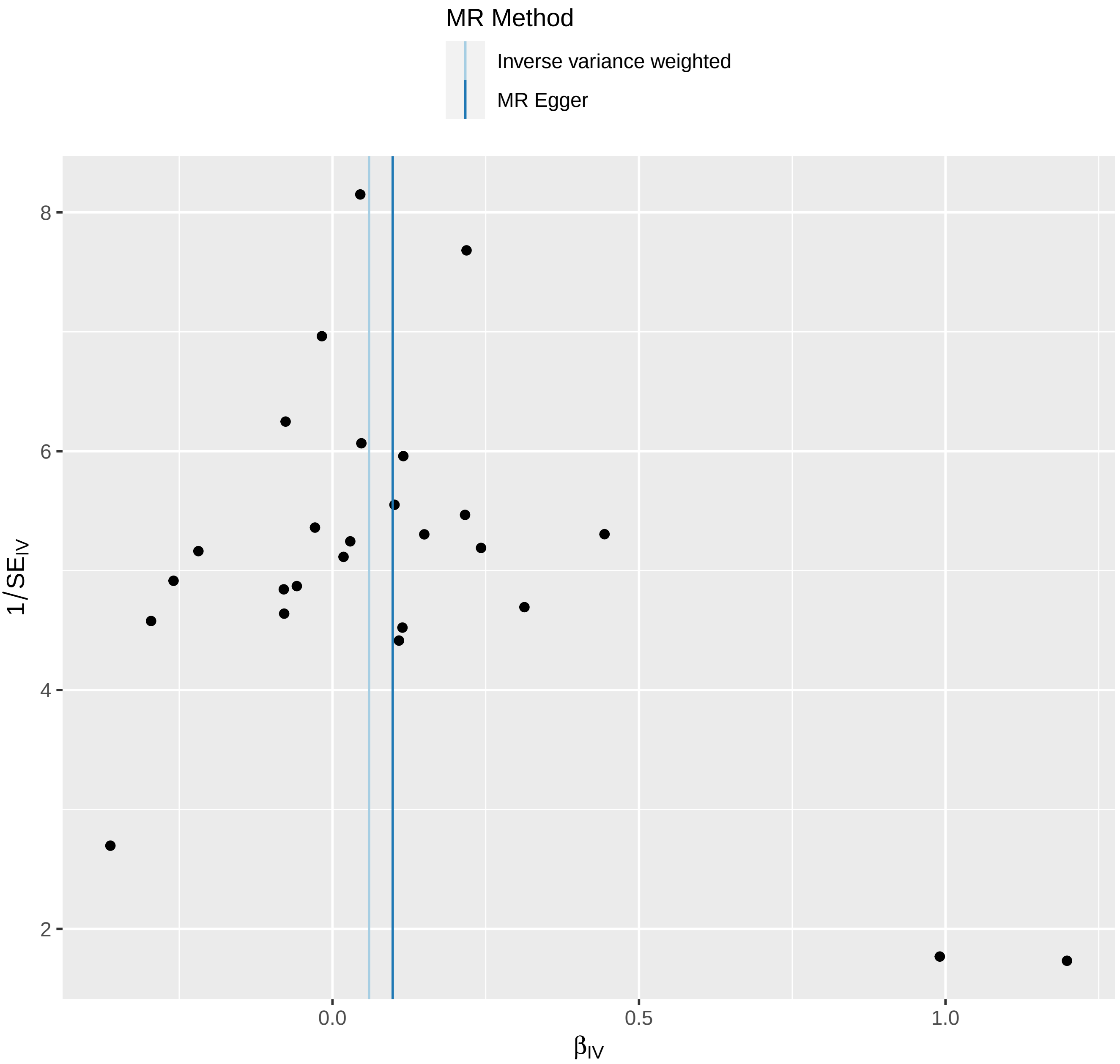

Batch 3−2347 : Protein phosphatase 1 regulatory subunit 3B on Diabetic nephropathy (more control exclusions)
with a threshold of 1e−05

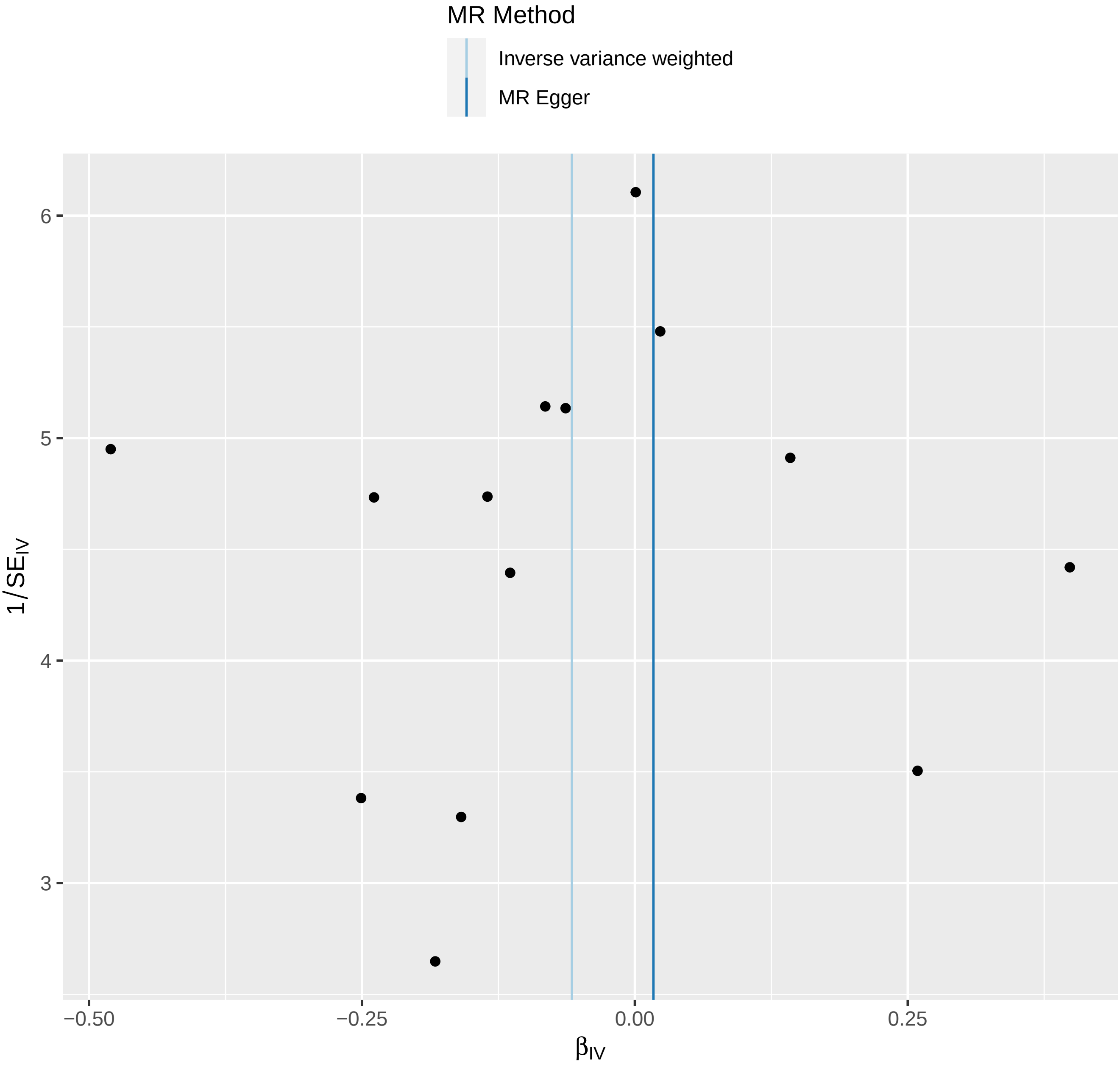

Batch 3−2348 : Serine/threonine−protein phosphatase 2A 65 kDa regulatory subunit A alpha isoform on Diabetic nephropathy (more control exclusions) with a threshold of 1e−05

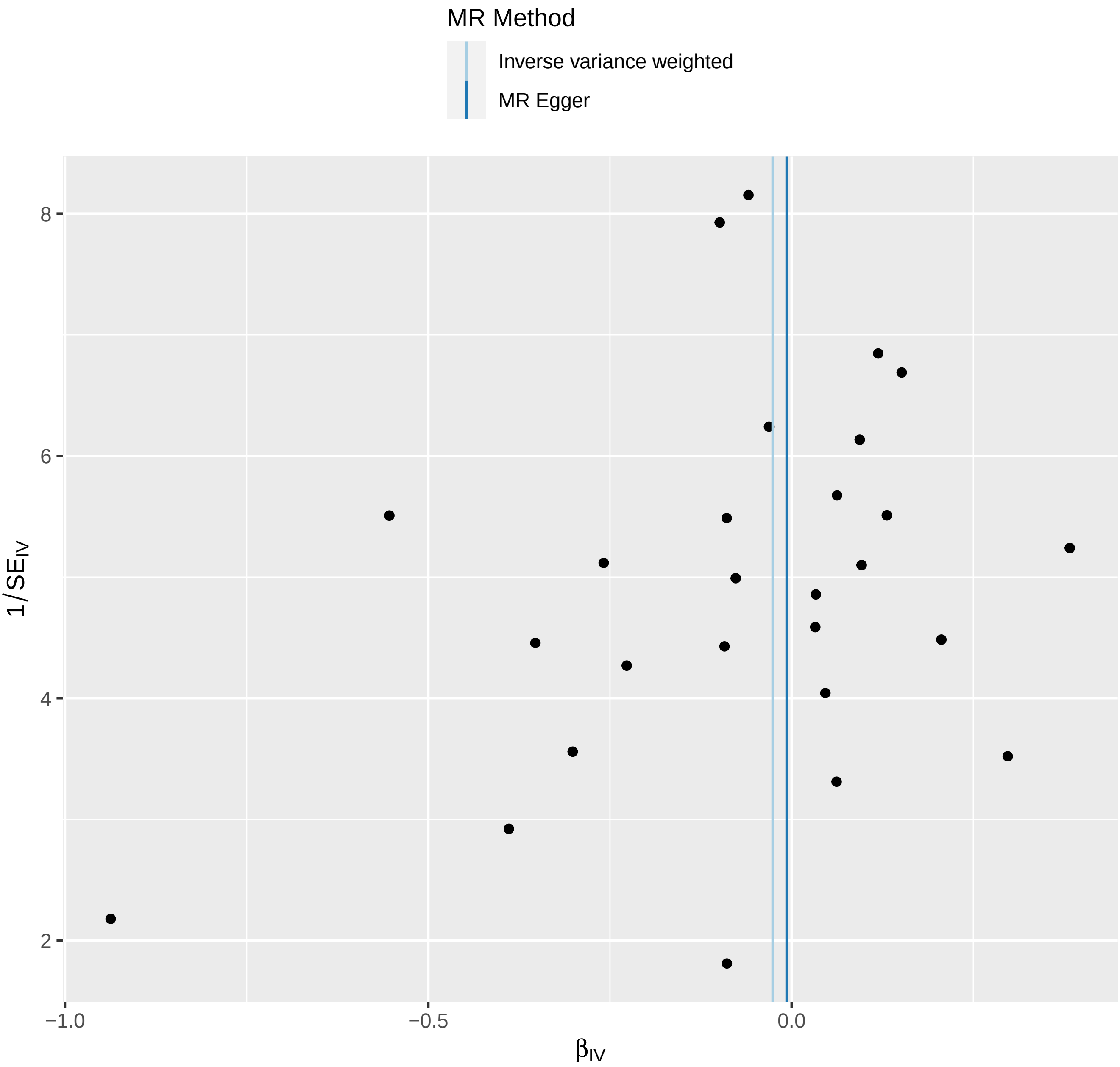

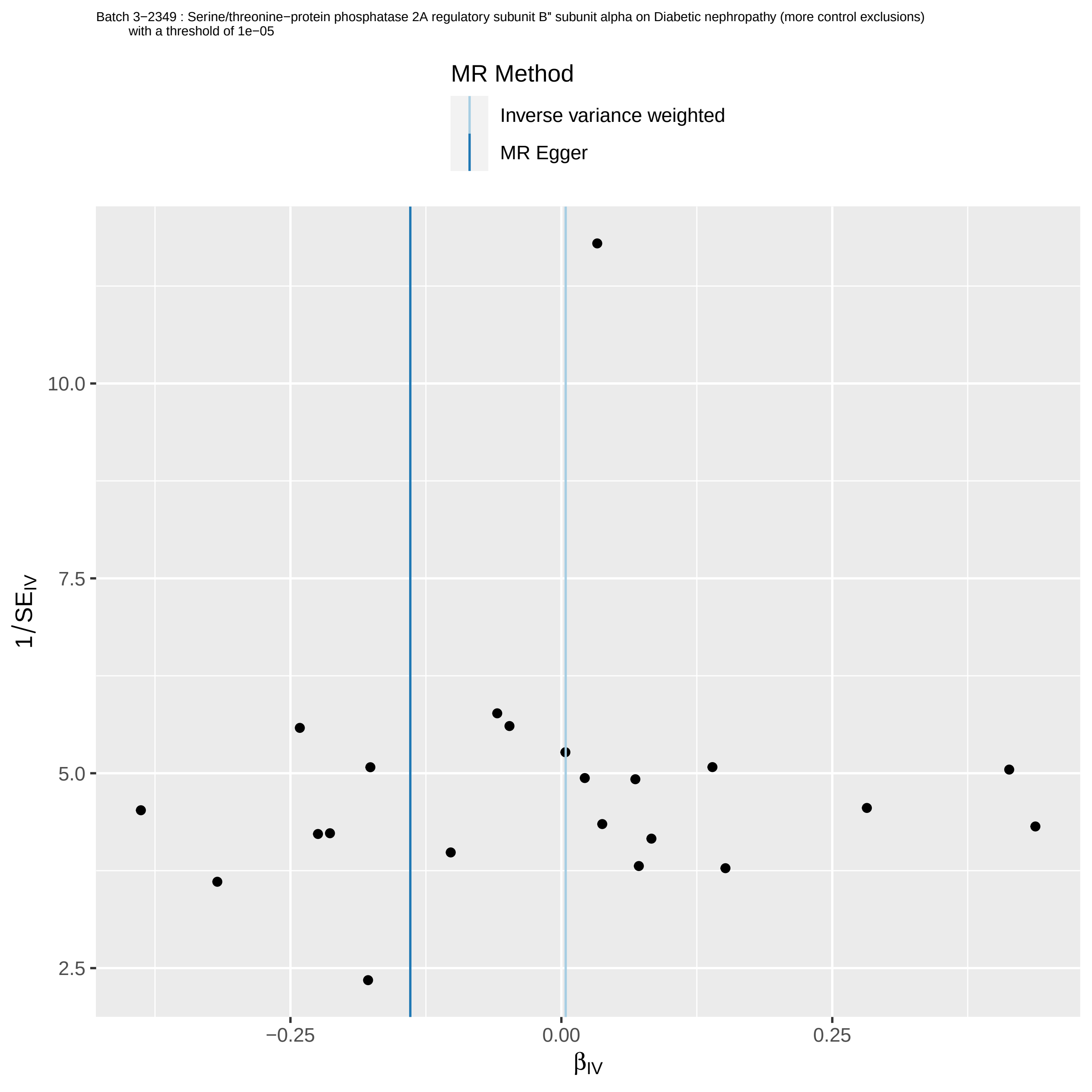
Batch 3−2349 : Serine/threonine−protein phosphatase 2A regulatory subunit B'' subunit alpha on Diabetic nephropathy (more control exclusions) with a threshold of 1e−05
MR Method
Inverse variance weighted
MR Egger
10.0
7.5
5.0
2.5
1/SE$_{IV}$
−0.25
0.00
0.25
β$_{IV}$

Batch 3−2350 : Calcineurin subunit B type 1 on Diabetic nephropathy (more control exclusions)
with a threshold of 1e−05

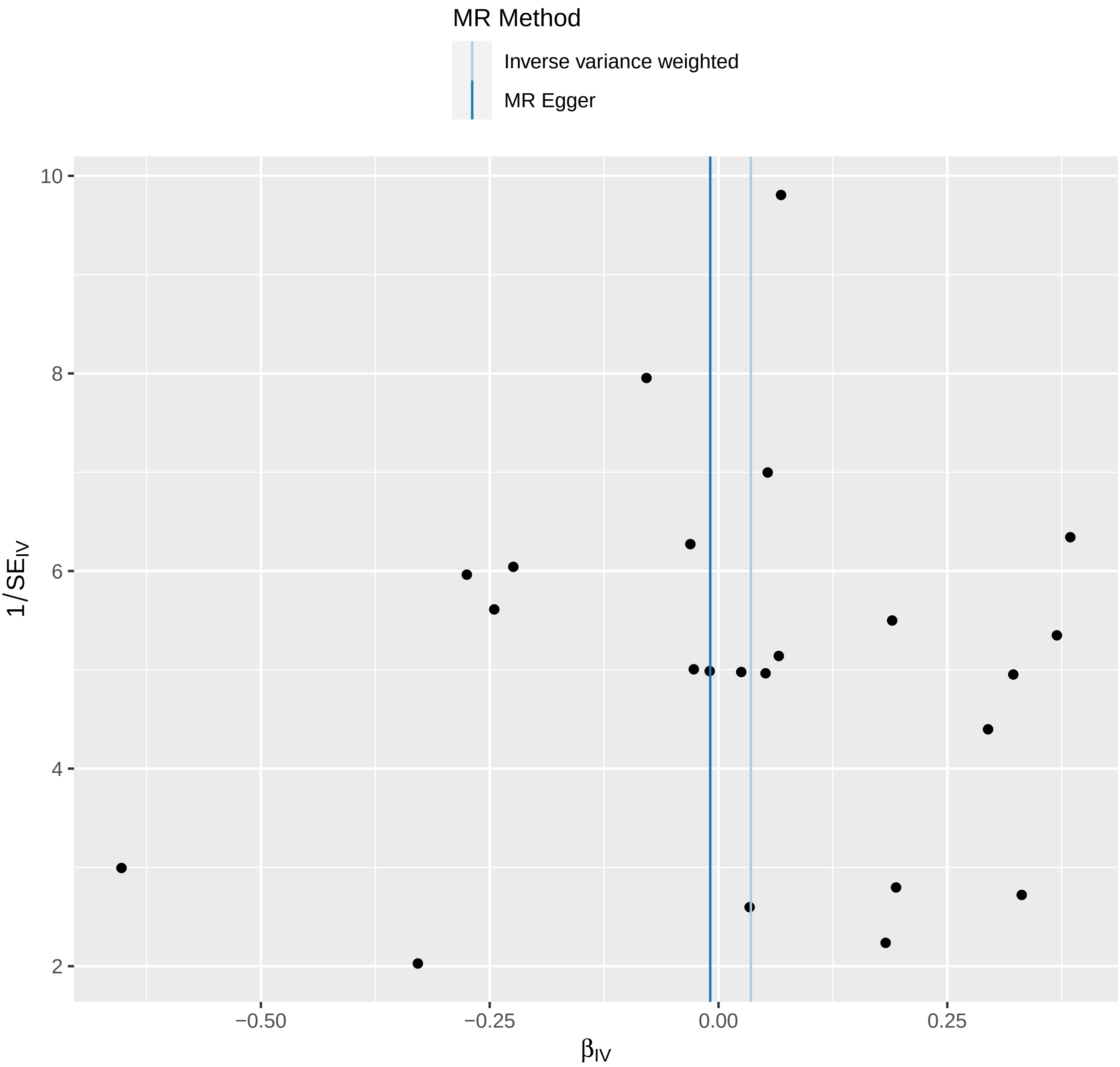

Batch 3−2351 : Palmitoyl−protein thioesterase 1 on Diabetic nephropathy (more control exclusions)
with a threshold of 1e−05

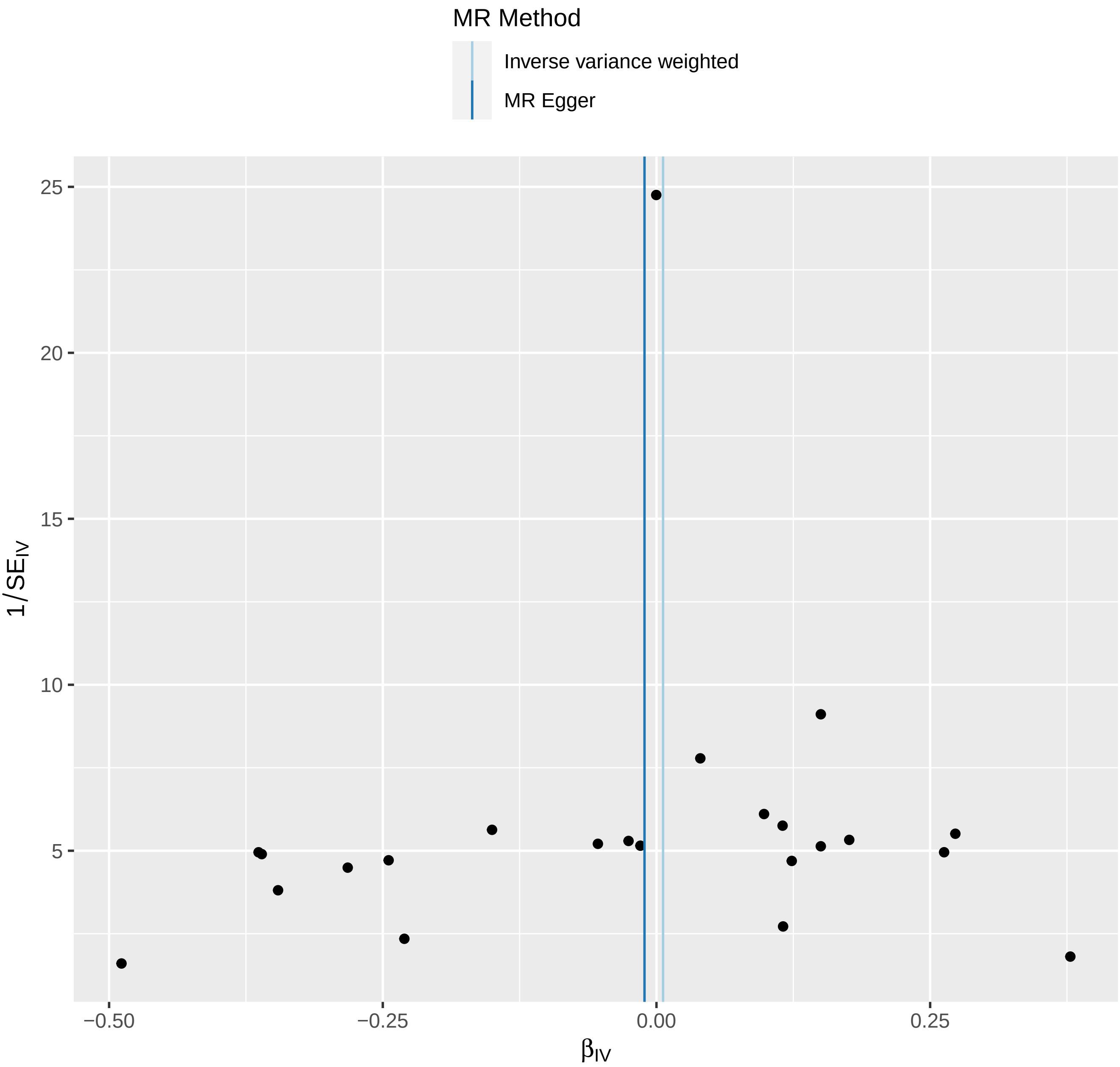

Batch 3−2352 : Pancreatic hormone on Diabetic nephropathy (more control exclusions)
with a threshold of 1e−05

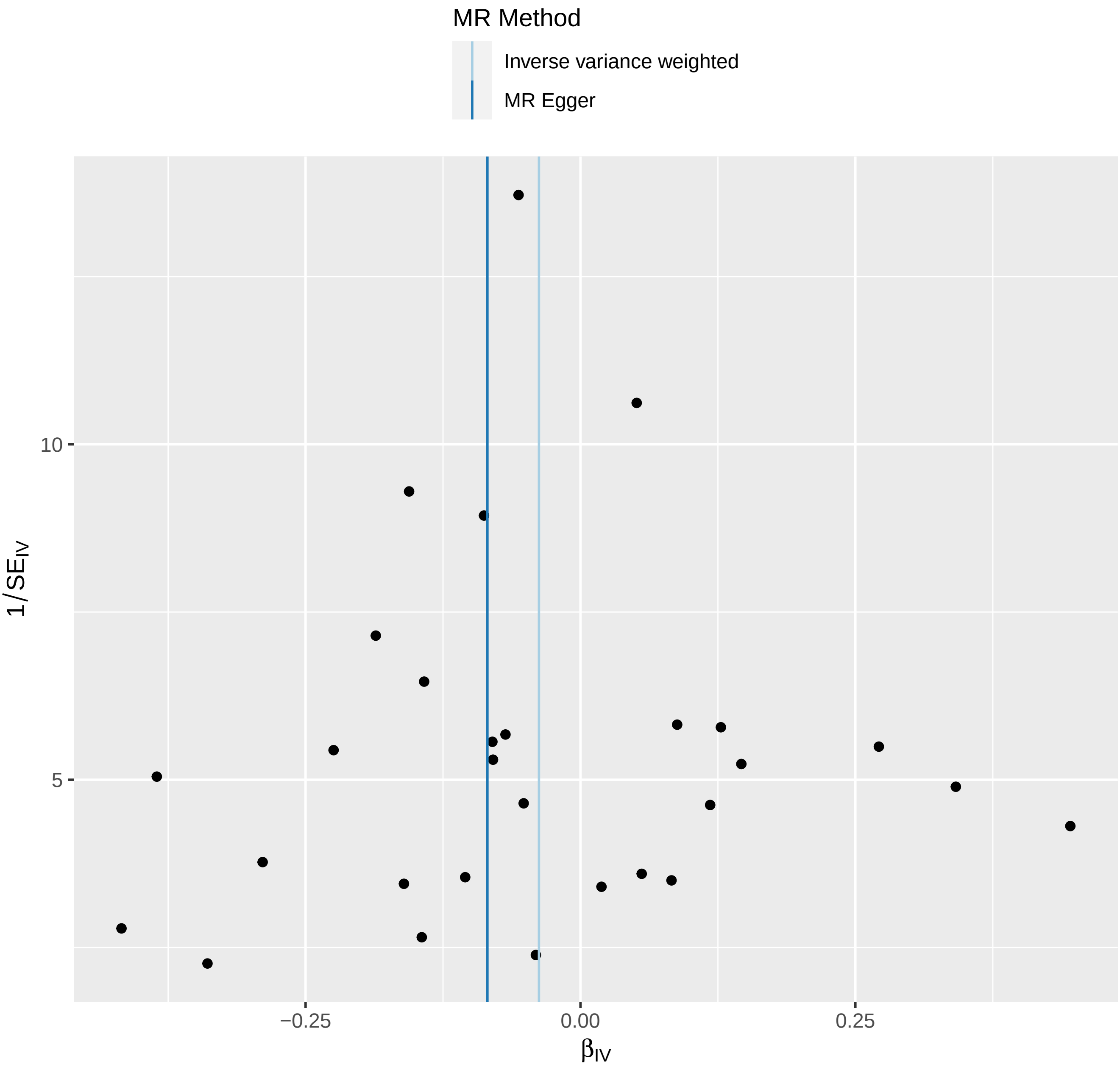

Batch 3−2353 : Putative uncharacterized protein PQLC2L on Diabetic nephropathy (more control exclusions)
with a threshold of 1e−05

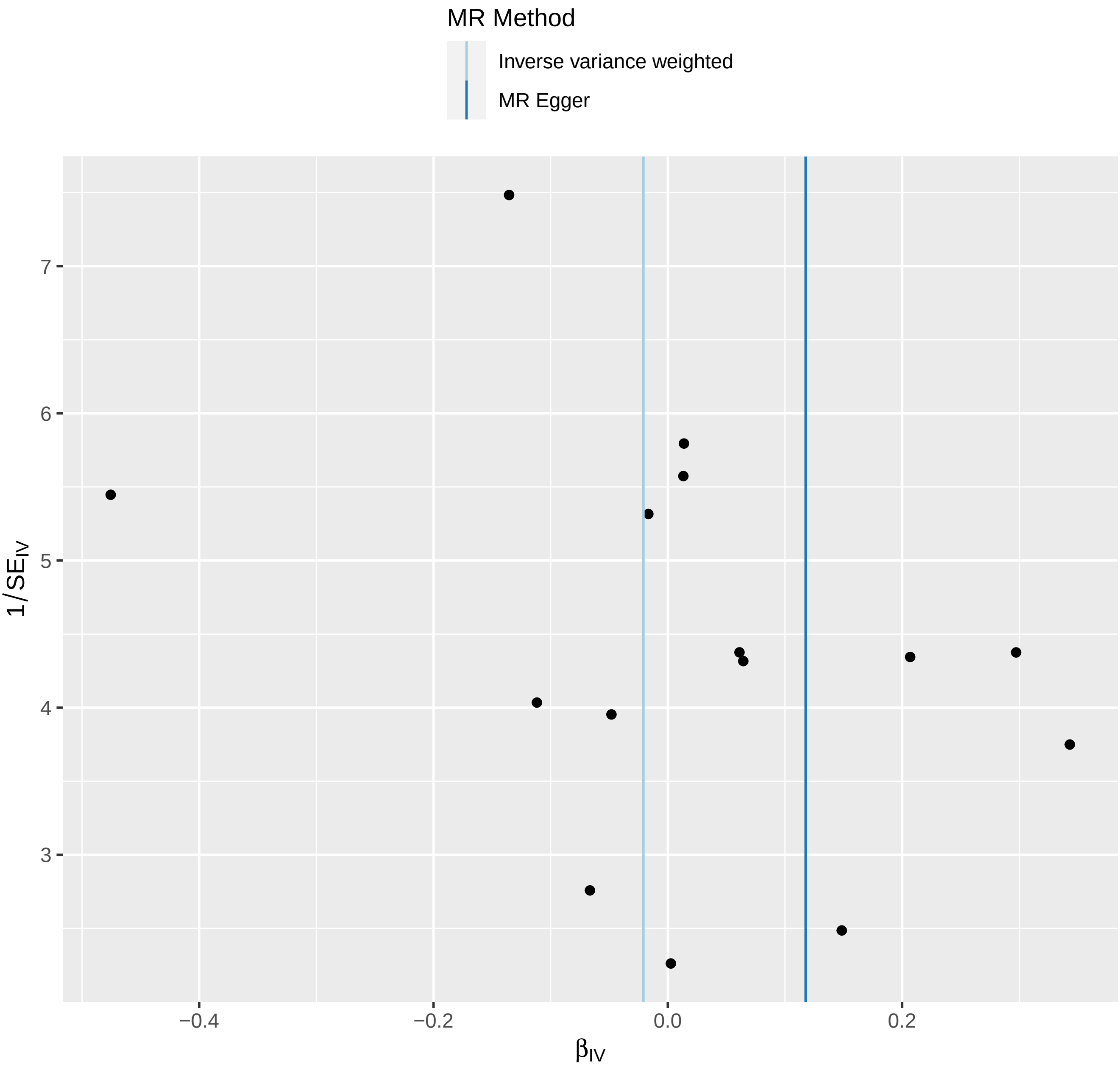

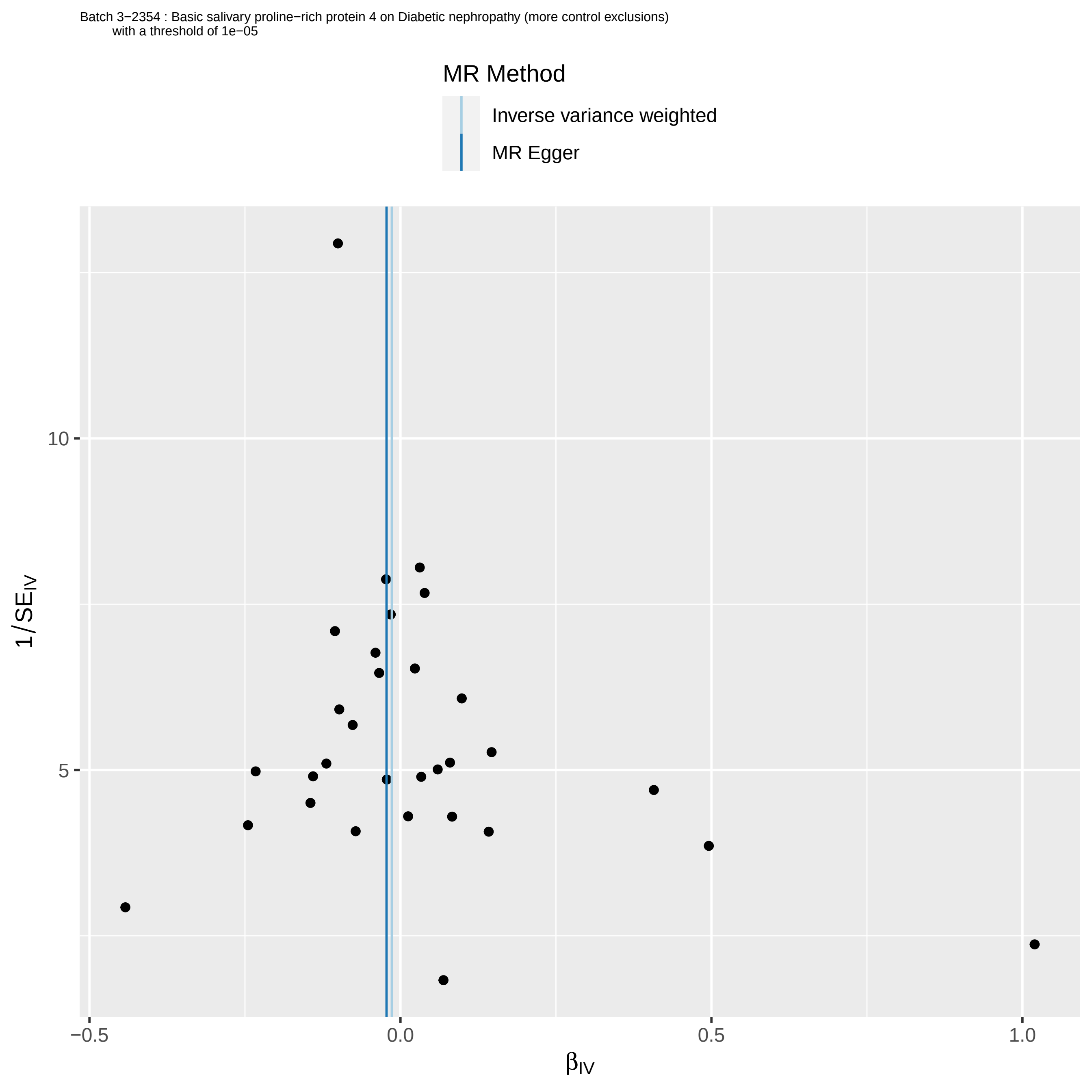

Batch 3−2354 : Basic salivary proline−rich protein 4 on Diabetic nephropathy (more control exclusions)
with a threshold of 1e−05
MR Method
Inverse variance weighted
MR Egger
10
5
1/SE_IV
−0.5
0.0
0.5
1.0
β_IV

Batch 3−2355 : Protein regulator of cytokinesis 1 on Diabetic nephropathy (more control exclusions)
with a threshold of 1e−05

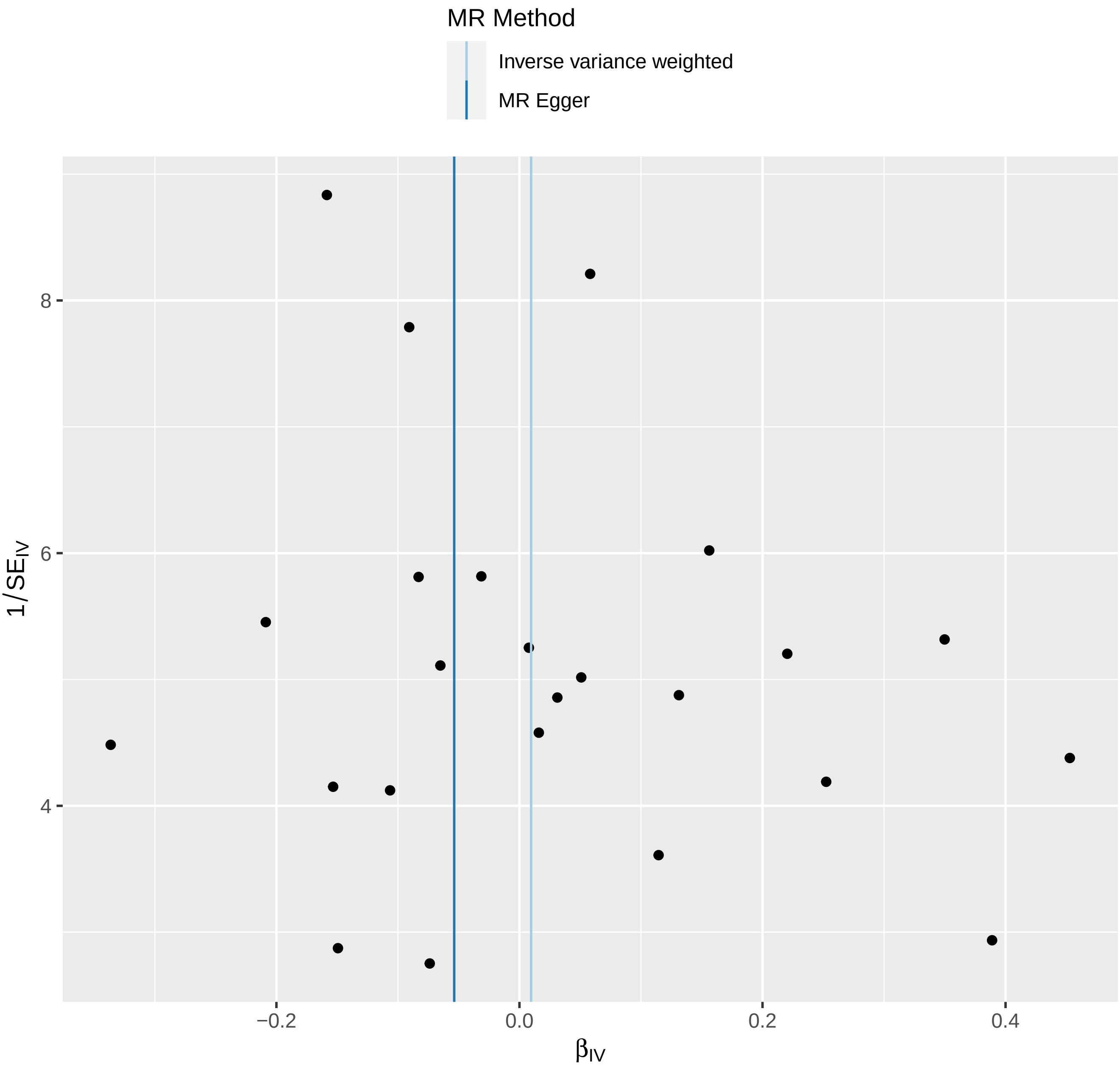

Batch 3−2356 : Lysosomal Pro−X carboxypeptidase on Diabetic nephropathy (more control exclusions)
with a threshold of 1e−05

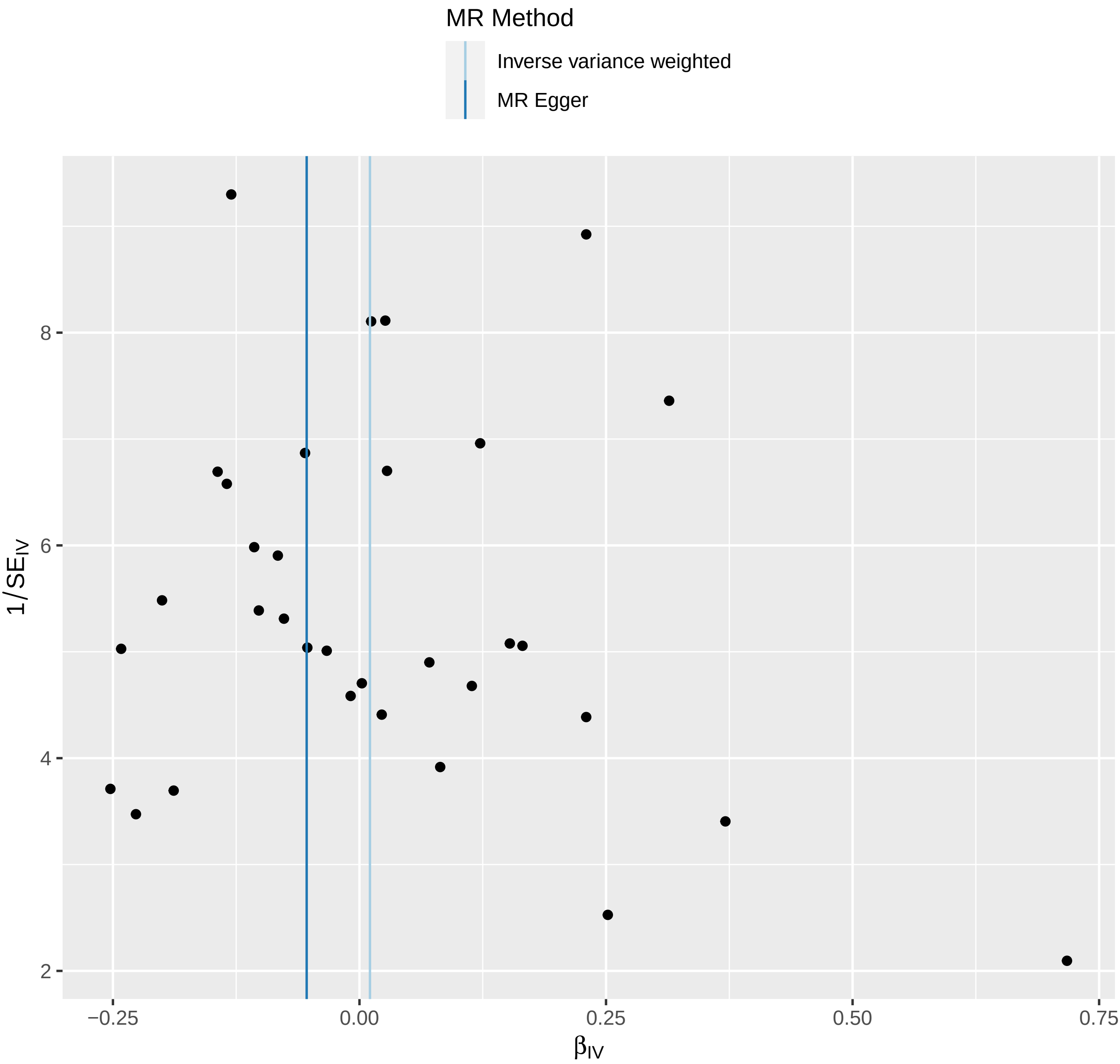

Batch 3−2357 : PR domain zinc finger protein 1 on Diabetic nephropathy (more control exclusions)
with a threshold of 1e−05

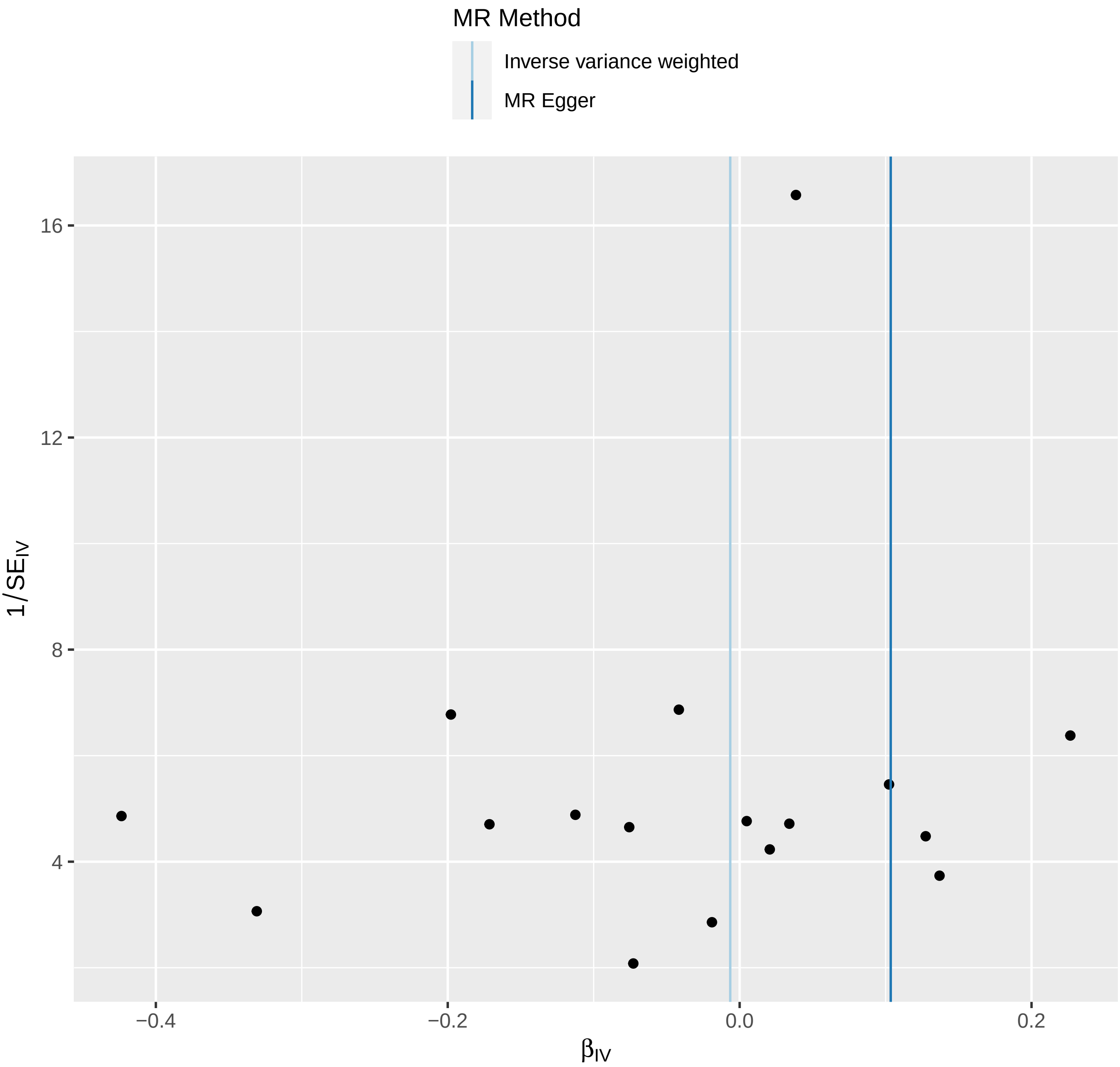

Batch 3−2358 : PR domain zinc finger protein 4 on Diabetic nephropathy (more control exclusions)
with a threshold of 1e−05

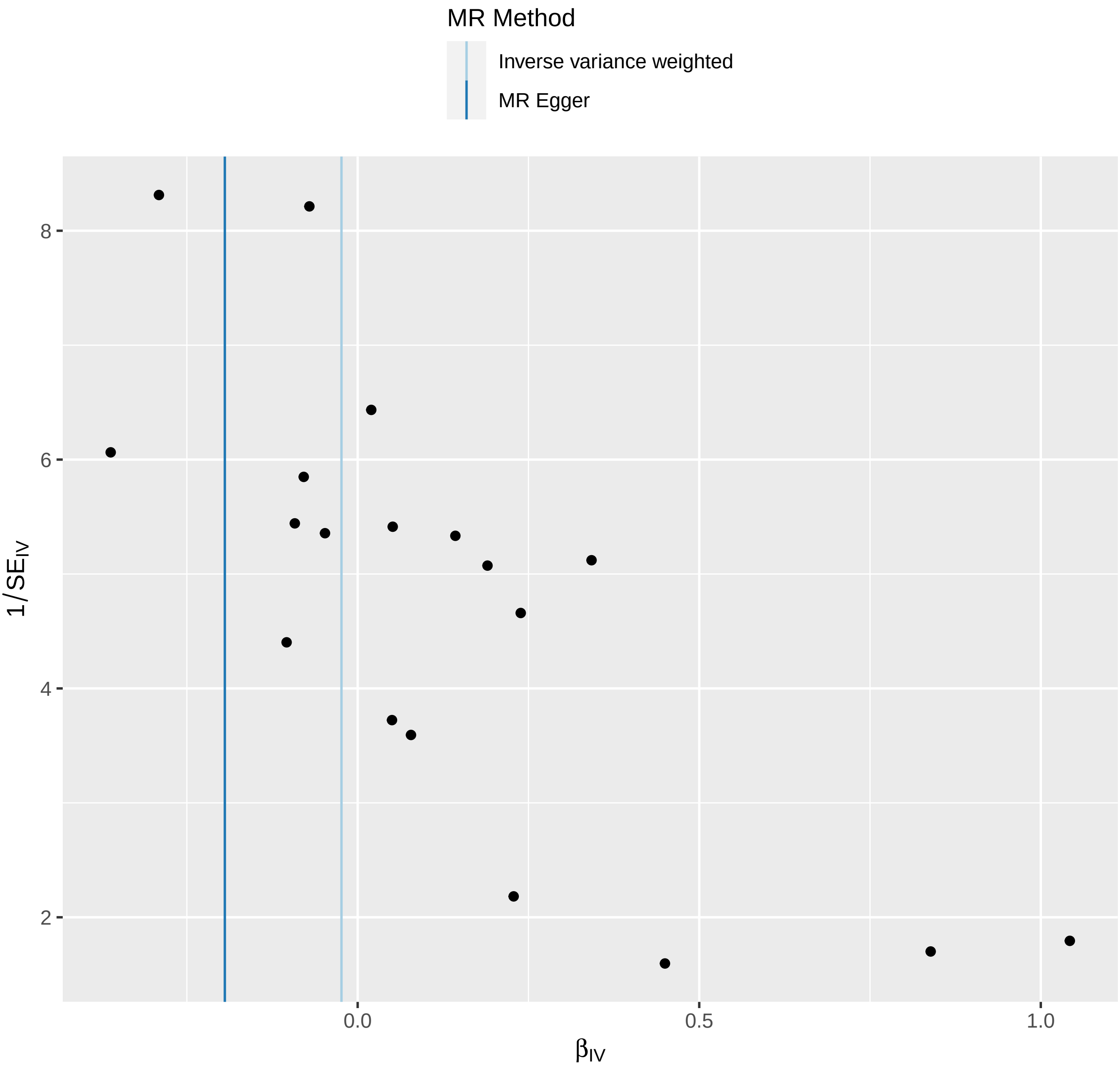

Batch 3−2359 : Peroxiredoxin−1 on Diabetic nephropathy (more control exclusions)
with a threshold of 1e−05

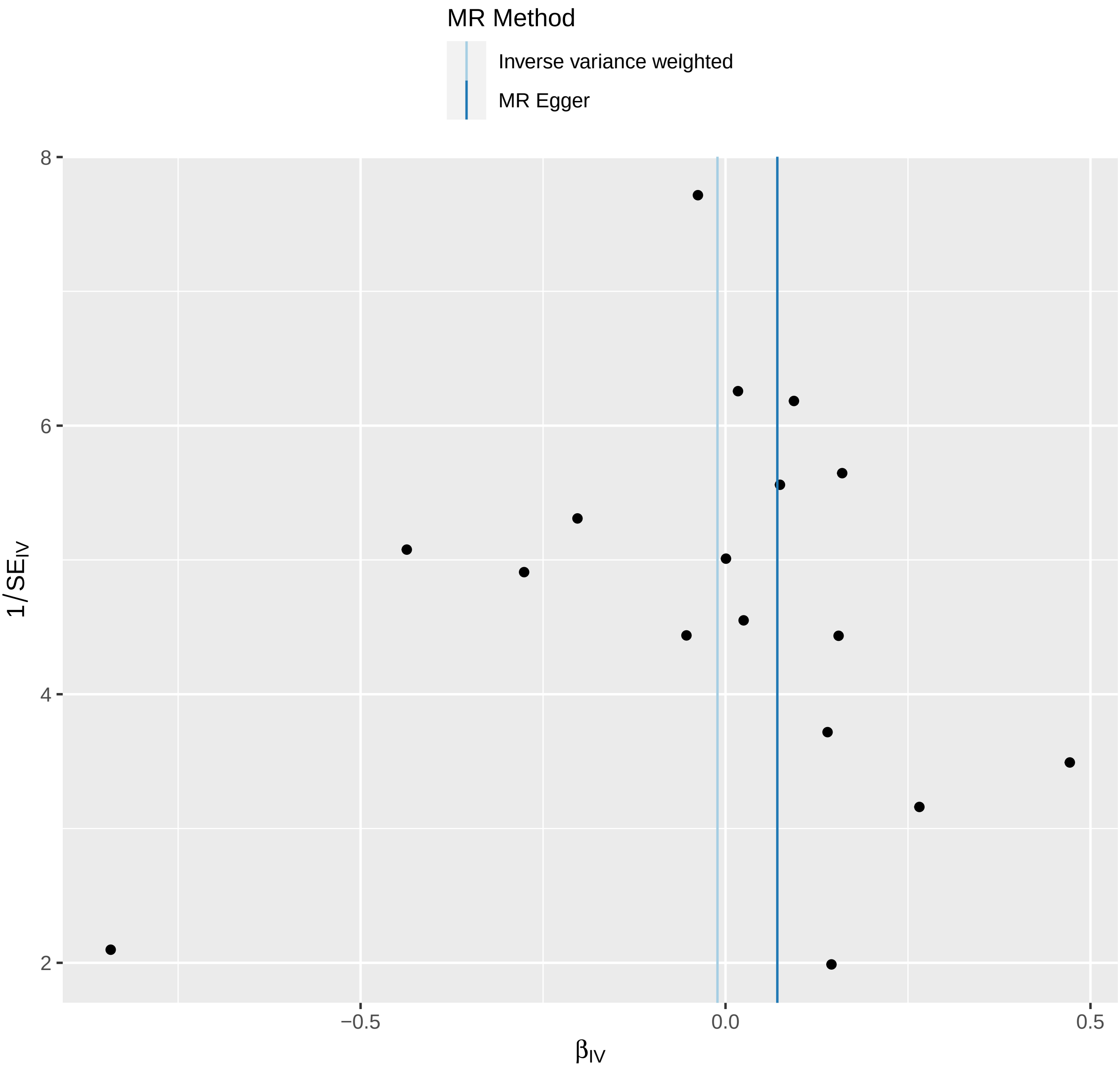

Batch 3−2360 : Peroxiredoxin−4 on Diabetic nephropathy (more control exclusions)
with a threshold of 1e−05

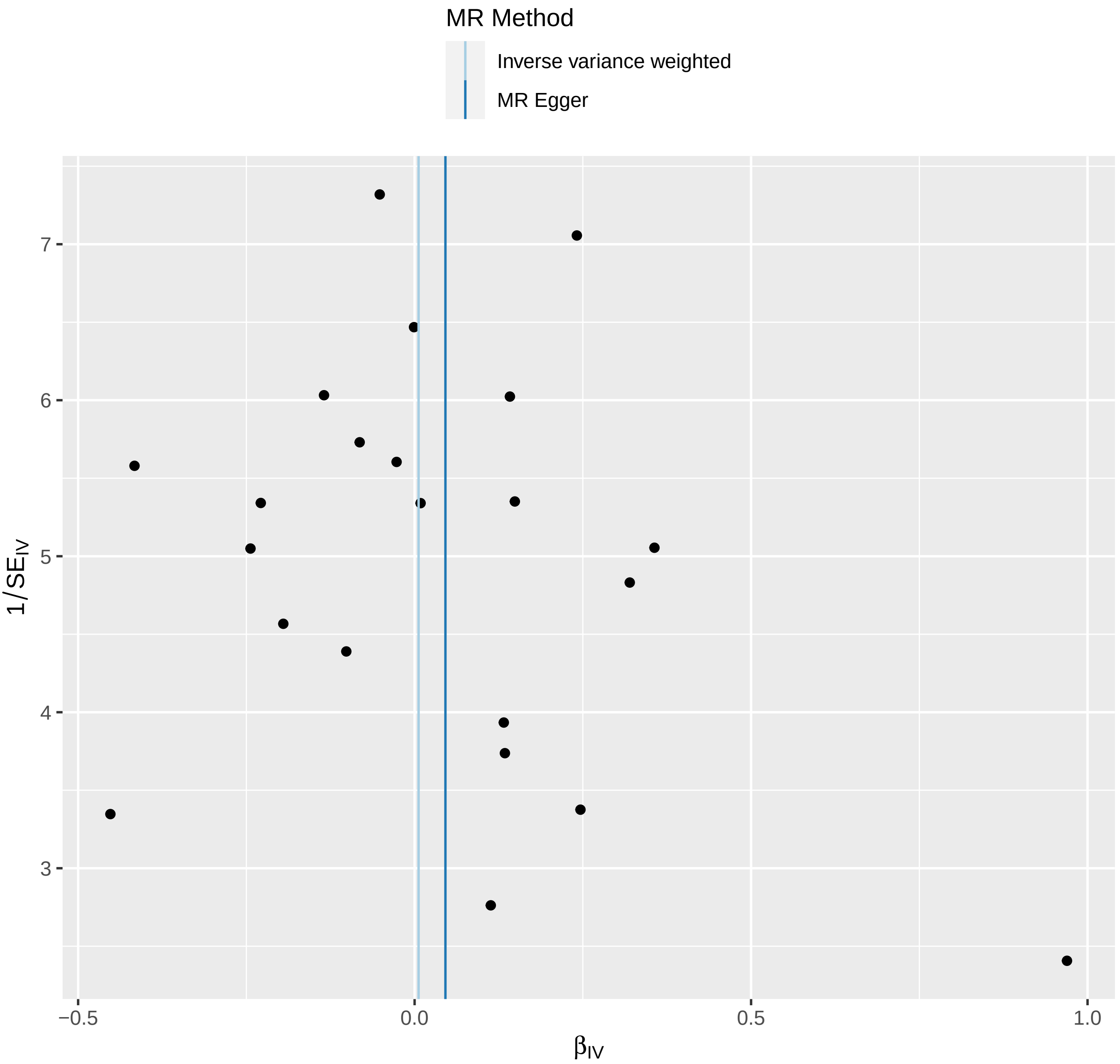

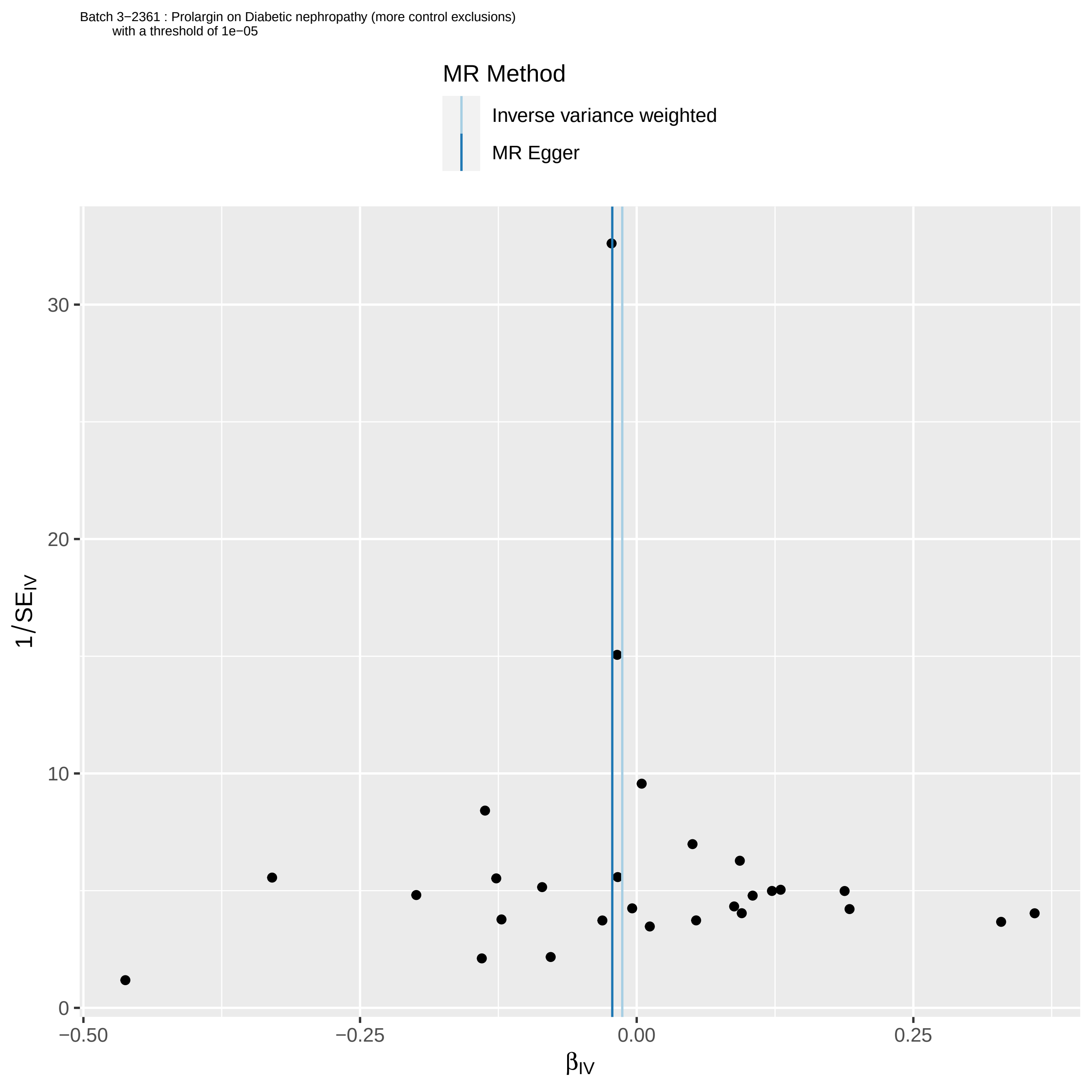
Batch 3−2361 : Prolargin on Diabetic nephropathy (more control exclusions)
with a threshold of 1e−05
MR Method
Inverse variance weighted
MR Egger
30
20
10
0
1/SE_IV
−0.50
−0.25
0.00
0.25
β_IV

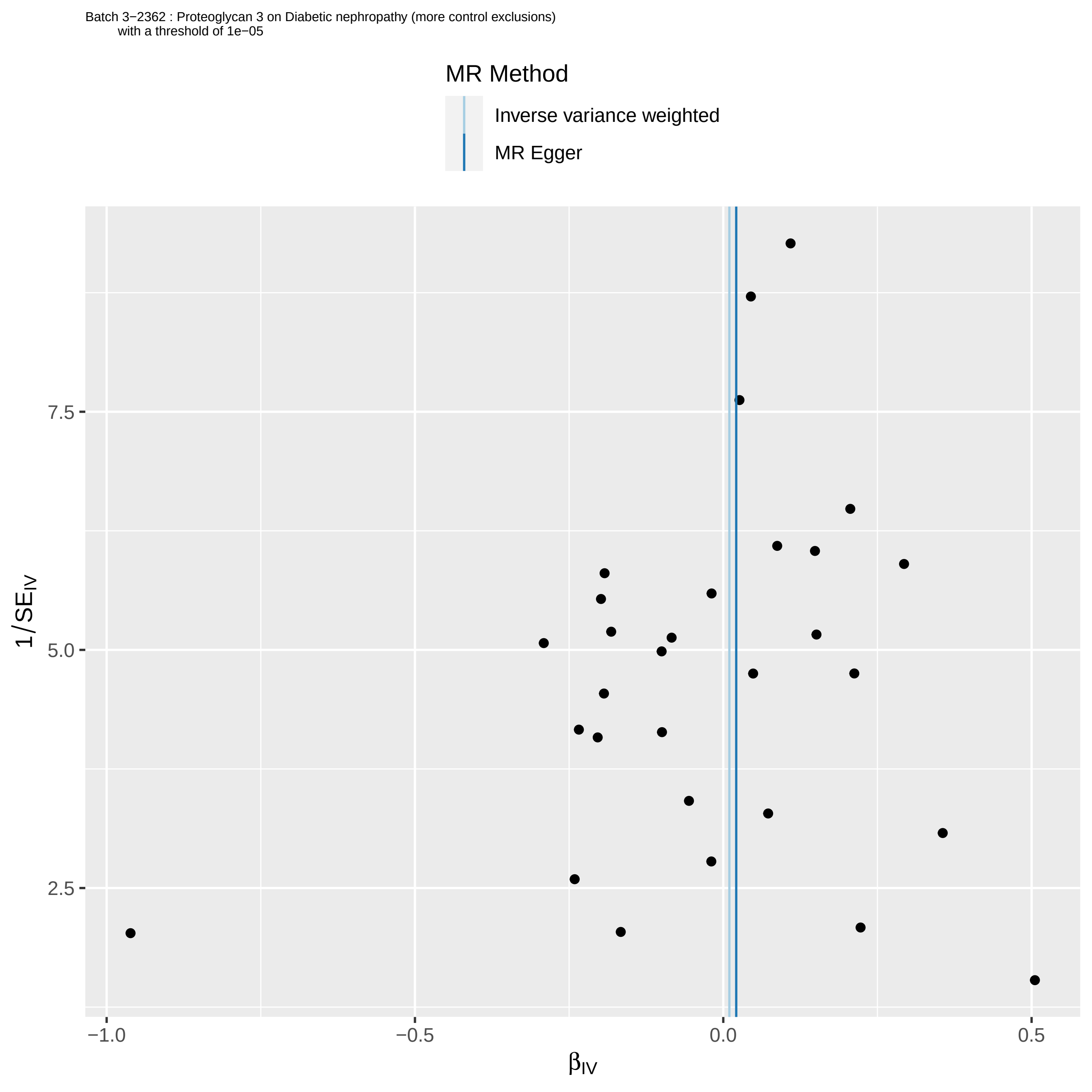

Batch 3−2362 : Proteoglycan 3 on Diabetic nephropathy (more control exclusions)
with a threshold of 1e−05
MR Method
Inverse variance weighted
MR Egger
7.5
5.0
2.5
1/SE_IV
−1.0
−0.5
0.0
0.5
β_IV

Batch 3−2363 : Salivary acidic proline−rich phosphoprotein 1/2 on Diabetic nephropathy (more control exclusions)
with a threshold of 1e−05

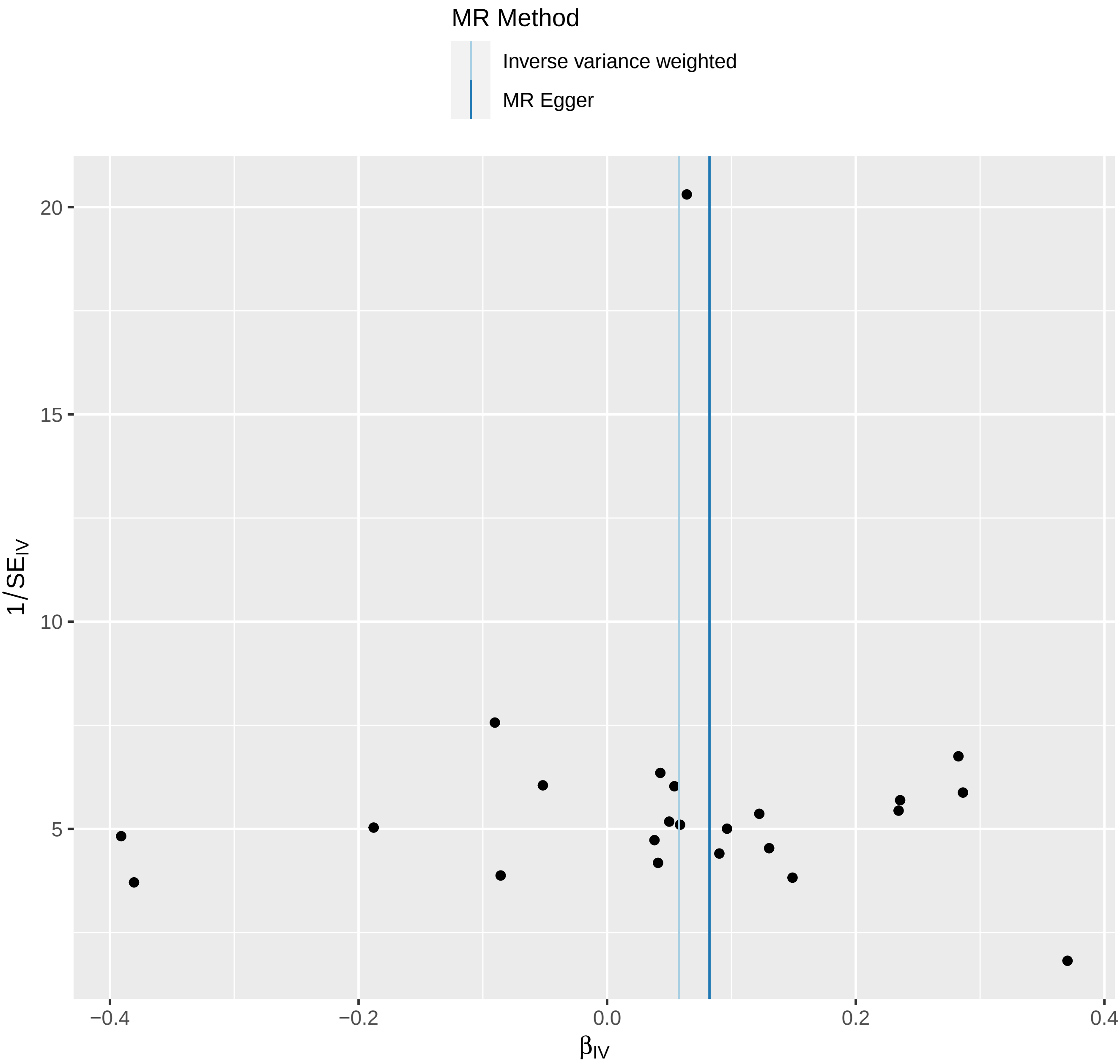

Batch 3−2364 : DNA primase small subunit on Diabetic nephropathy (more control exclusions)
with a threshold of 1e−05

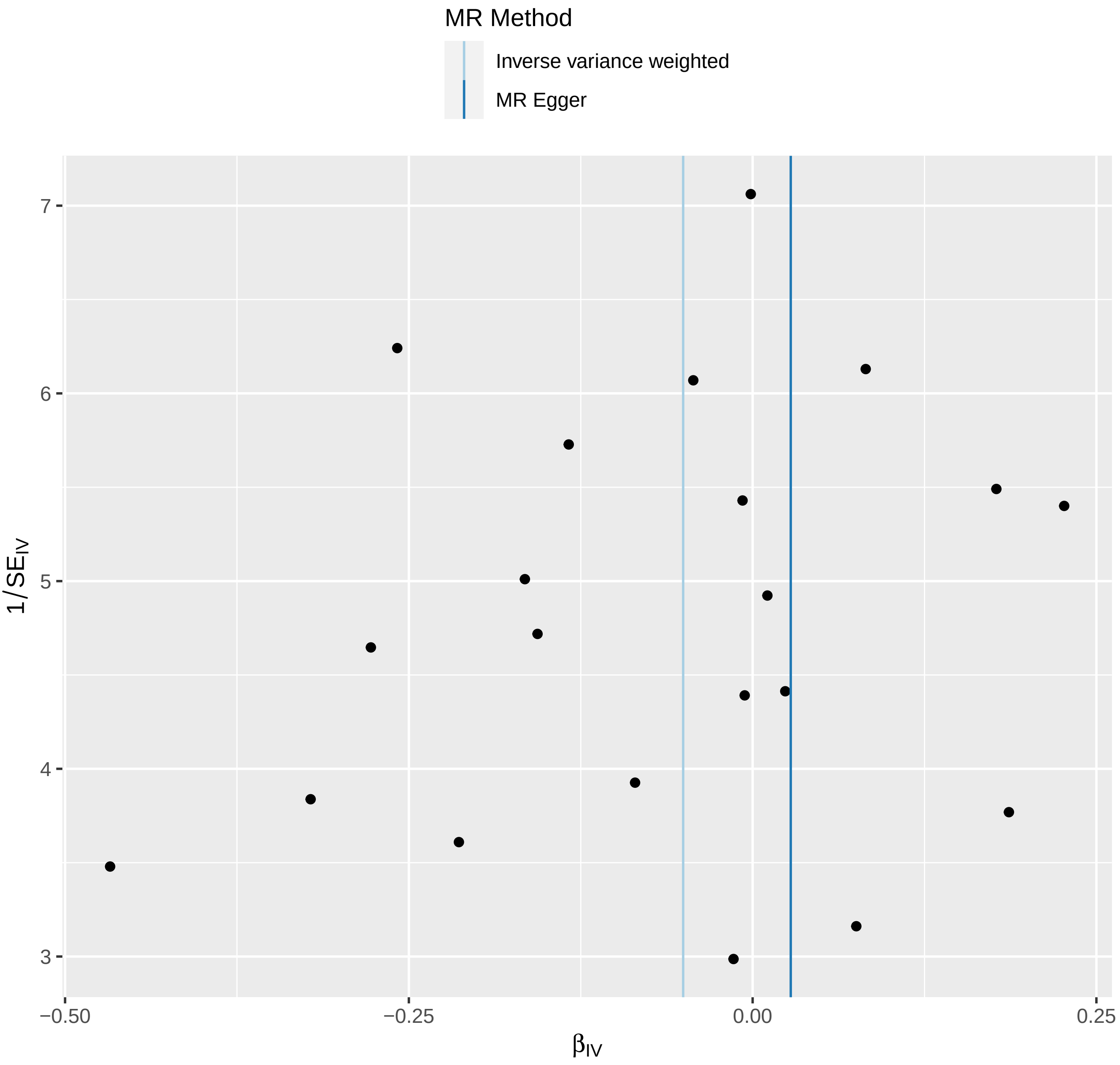

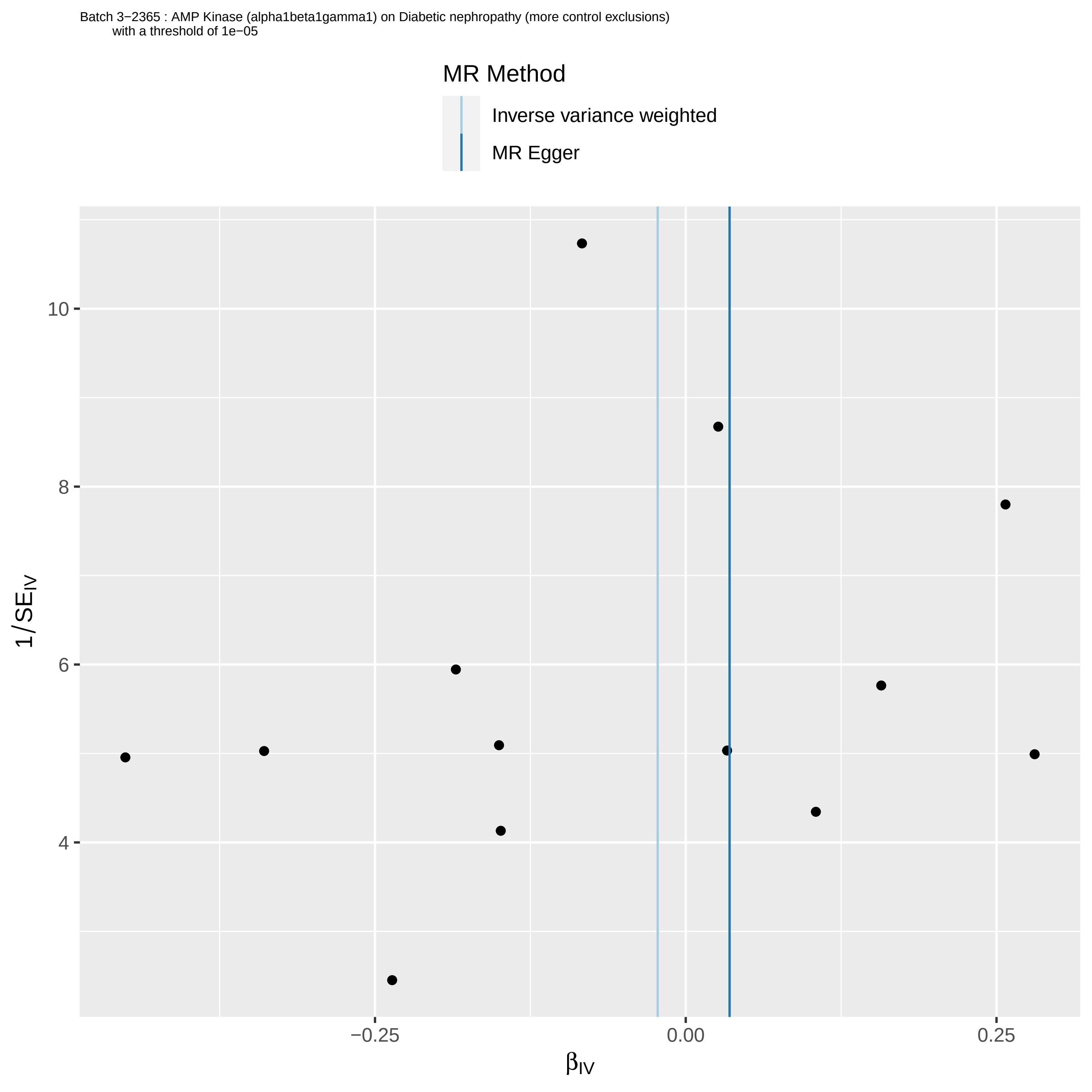

Batch 3−2365 : AMP Kinase (alpha1beta1gamma1) on Diabetic nephropathy (more control exclusions)
with a threshold of 1e−05
MR Method
Inverse variance weighted
MR Egger
10
8
6
4
1/SE_IV
−0.25
0.00
0.25
β_IV

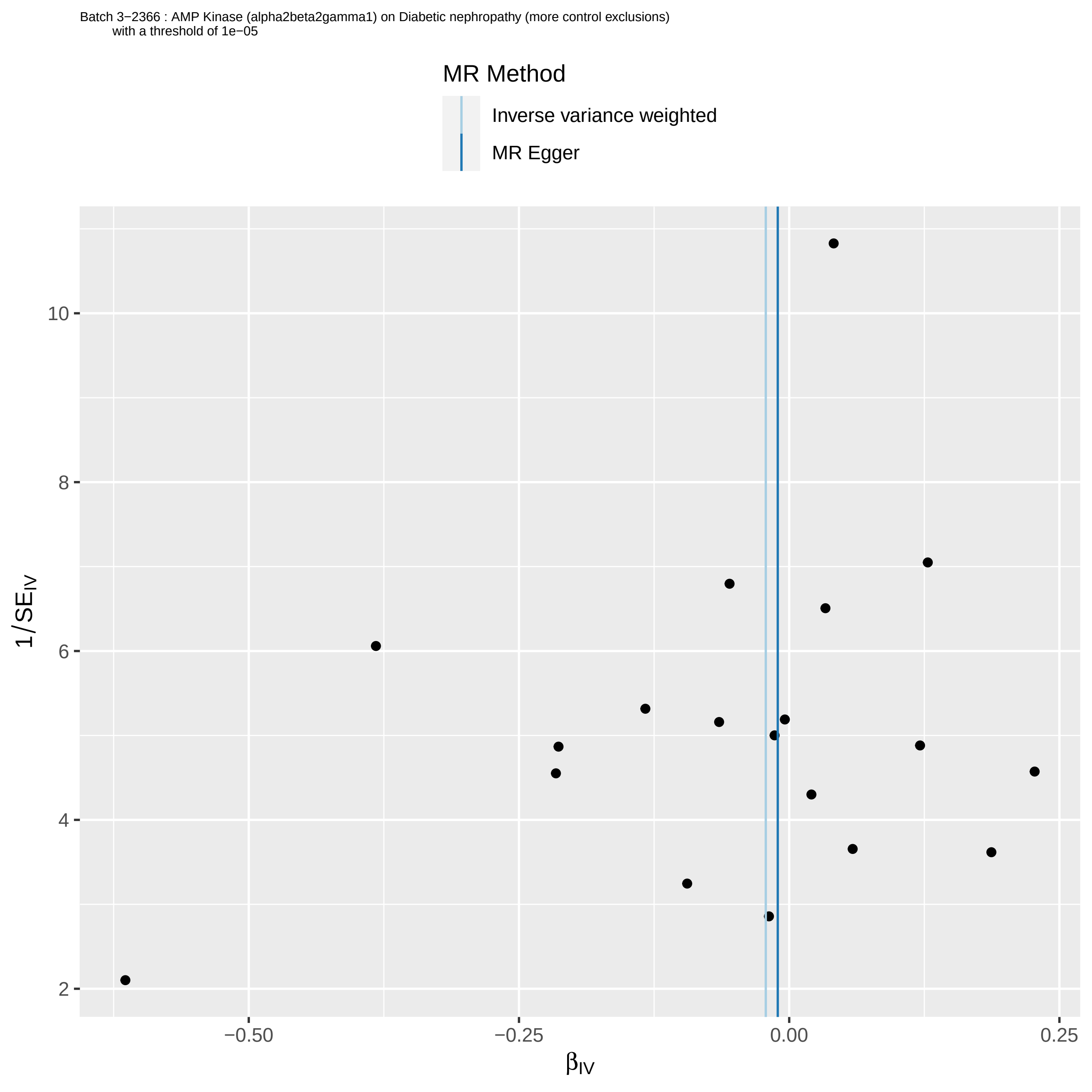
Batch 3−2366 : AMP Kinase (alpha2beta2gamma1) on Diabetic nephropathy (more control exclusions)
with a threshold of 1e−05
MR Method
Inverse variance weighted
MR Egger
1/SE_IV
10
8
6
4
2
−0.50
−0.25
0.00
0.25
β_IV

Batch 3−2367 : cAMP−dependent protein kinase catalytic subunit alpha on Diabetic nephropathy (more control exclusions) with a threshold of 1e−05

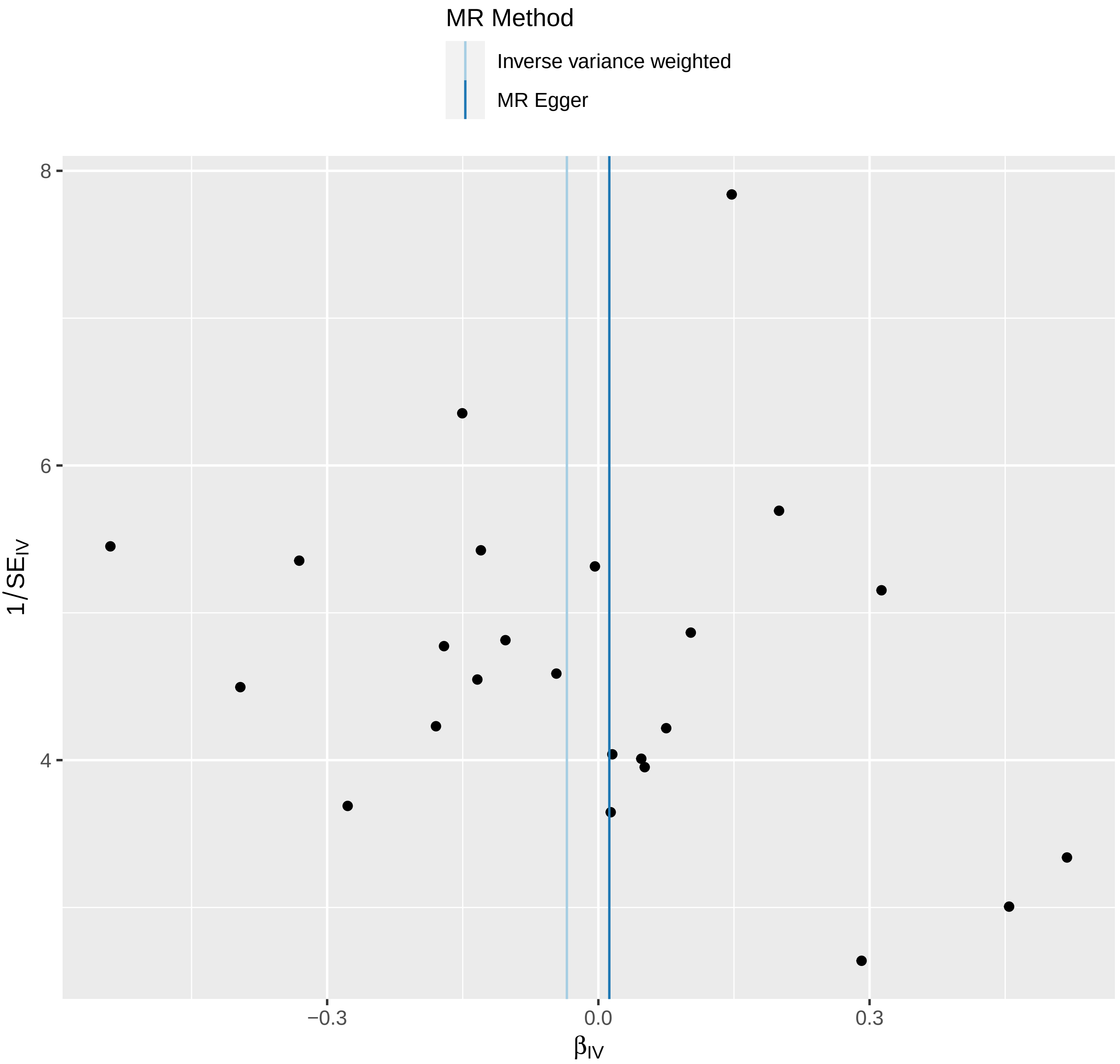

Batch 3−2368 : cAMP−dependent protein kinase type I−beta regulatory subunit on Diabetic nephropathy (more control exclusions)
with a threshold of 1e−05

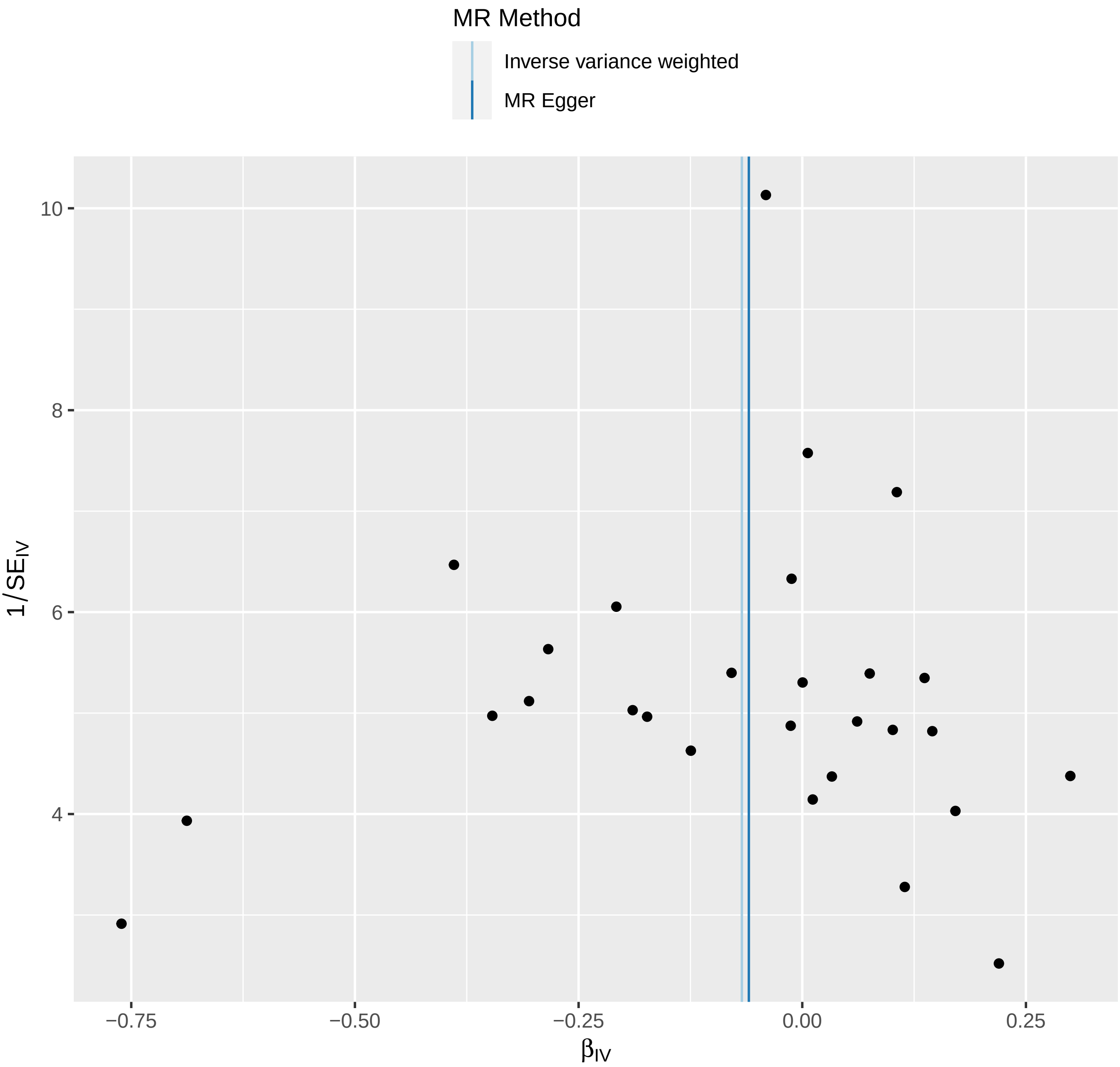

Batch 3−2369 : Protein kinase C alpha type on Diabetic nephropathy (more control exclusions)
with a threshold of 1e−05

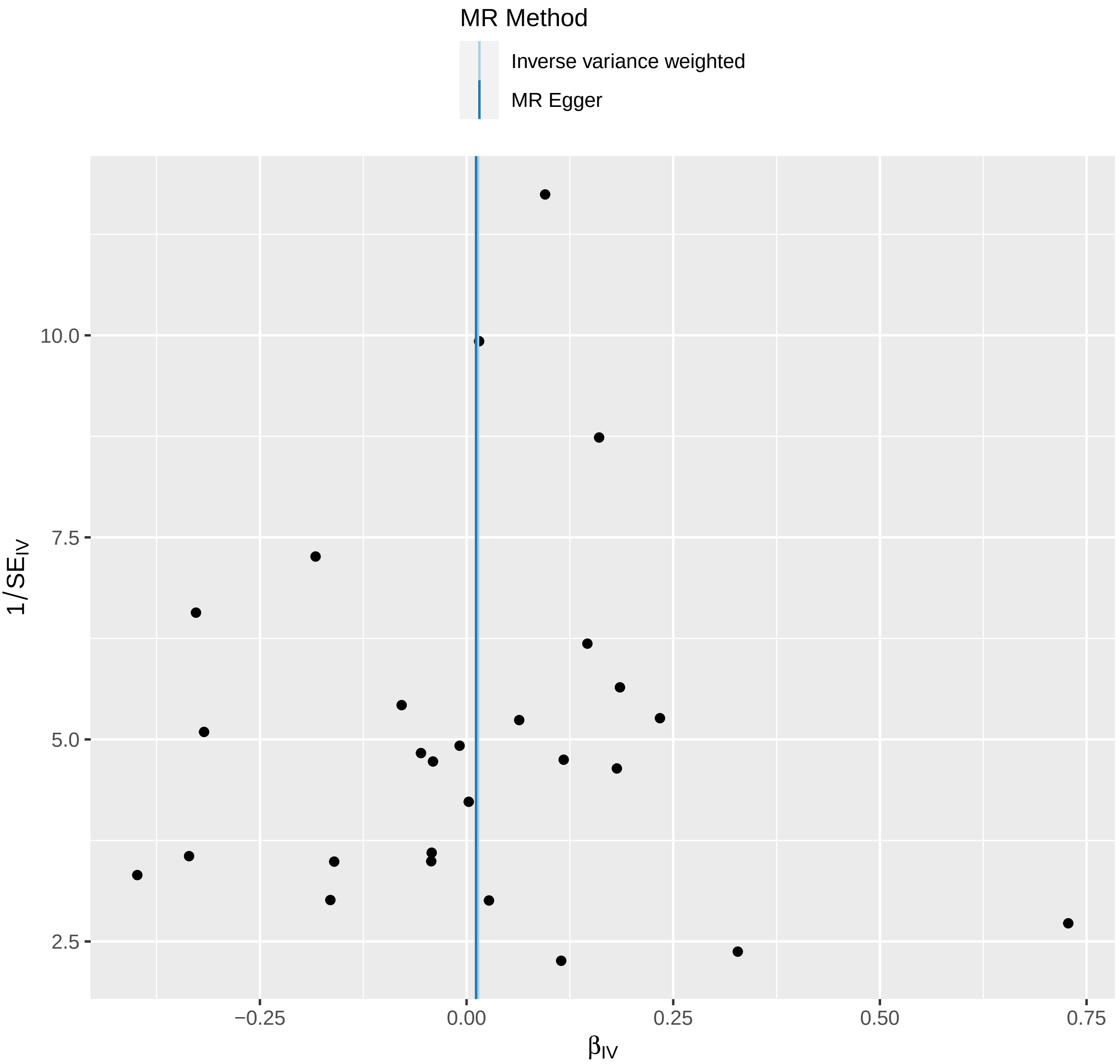

Batch 3−2370 : Protein kinase C beta type (splice variant beta−II) on Diabetic nephropathy (more control exclusions) with a threshold of 1e−05

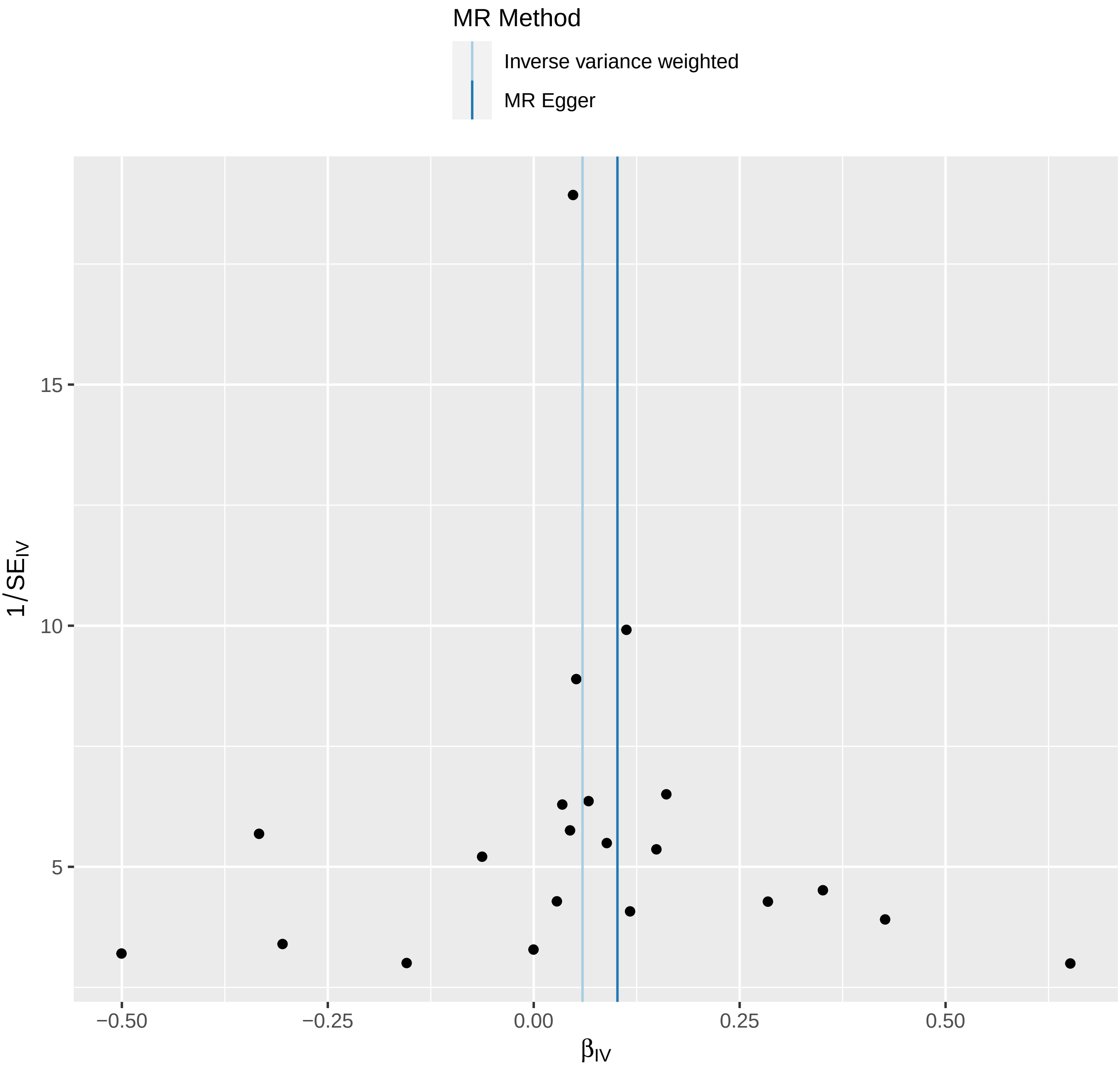

Batch 3−2371 : Protein kinase C iota type on Diabetic nephropathy (more control exclusions)
with a threshold of 1e−05

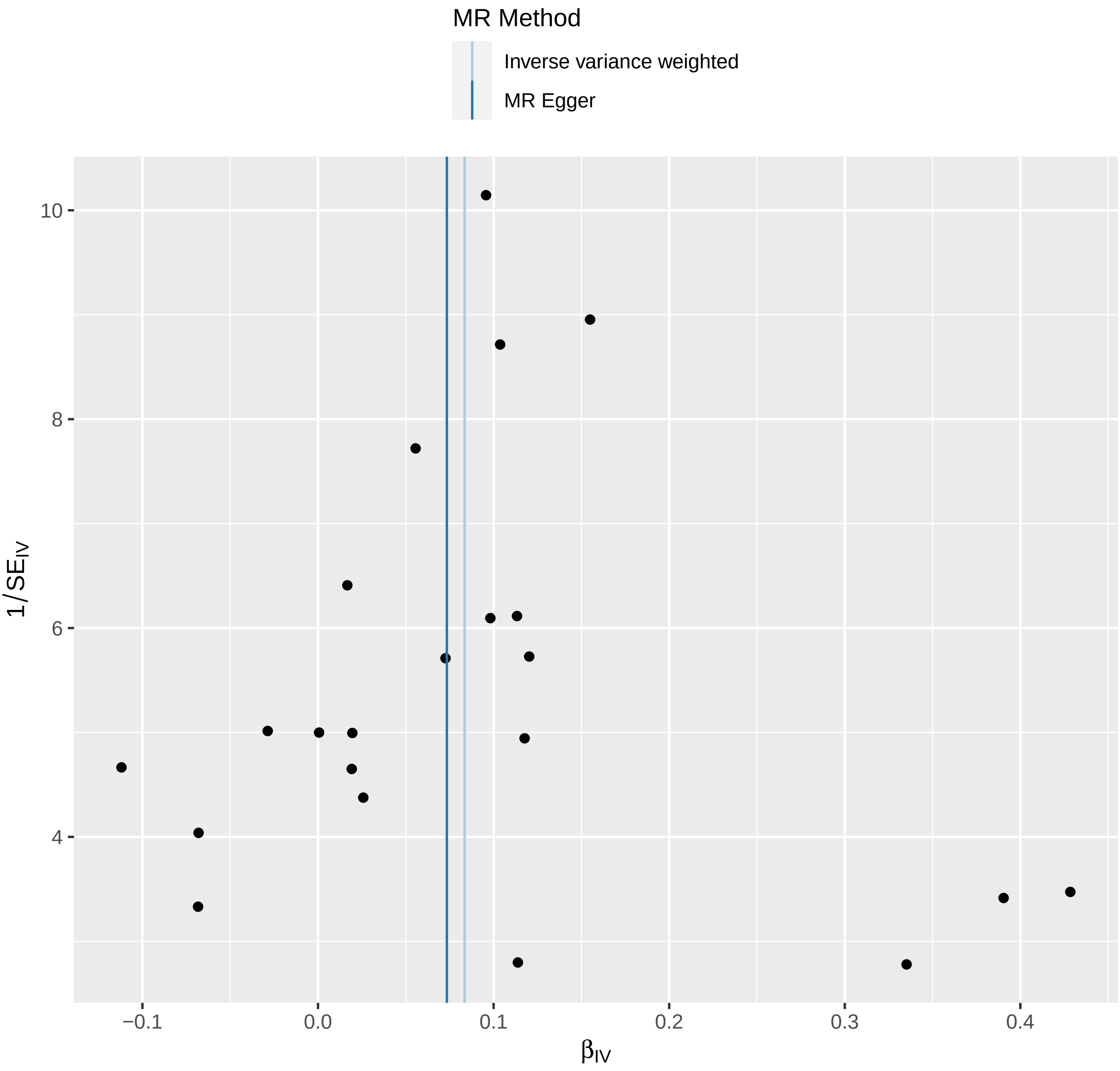

Batch 3−2372 : Glucosidase 2 subunit beta on Diabetic nephropathy (more control exclusions)
with a threshold of 1e−05

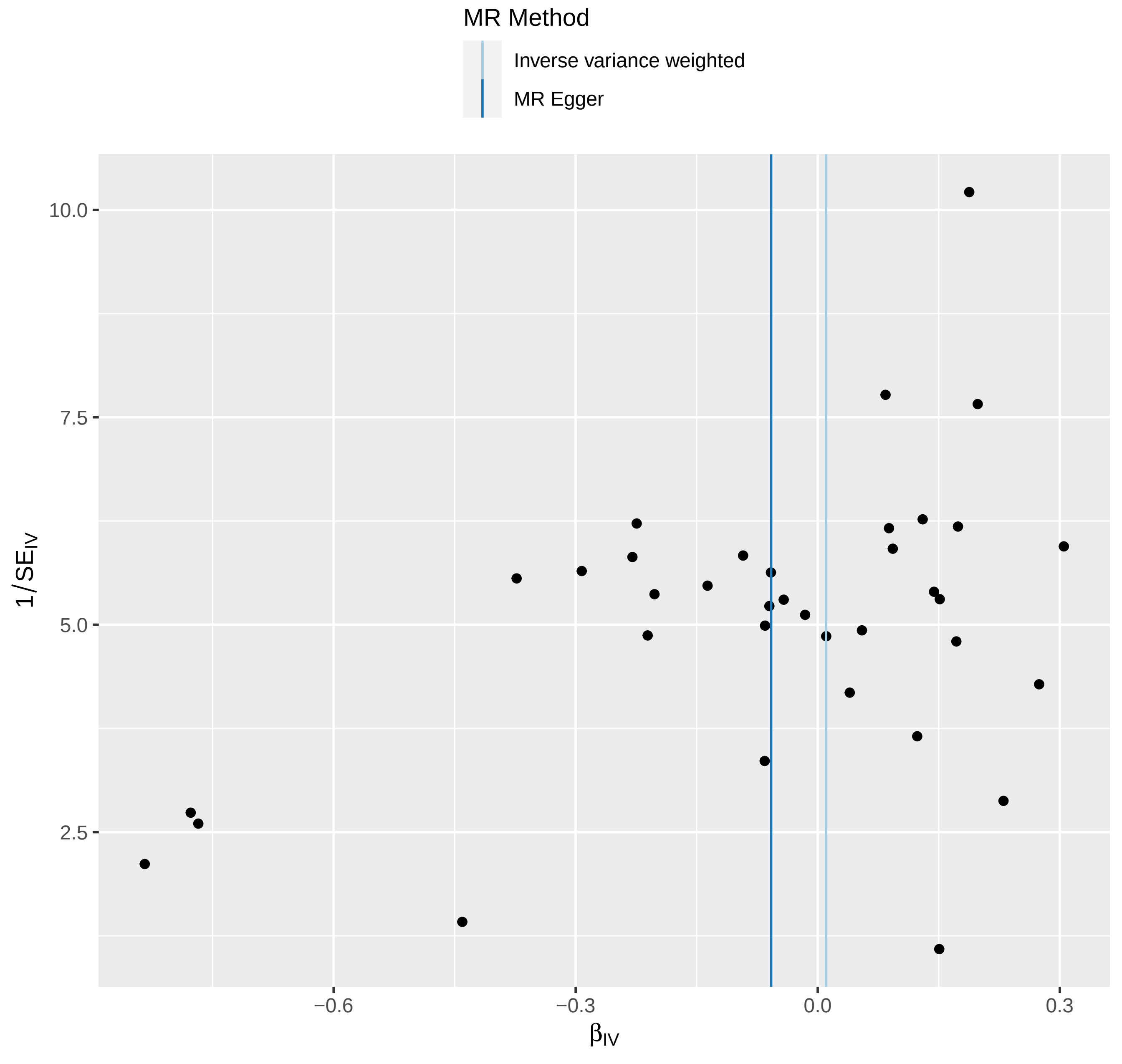

Batch 3−2373 : Protein kinase C zeta type on Diabetic nephropathy (more control exclusions)
with a threshold of 1e−05

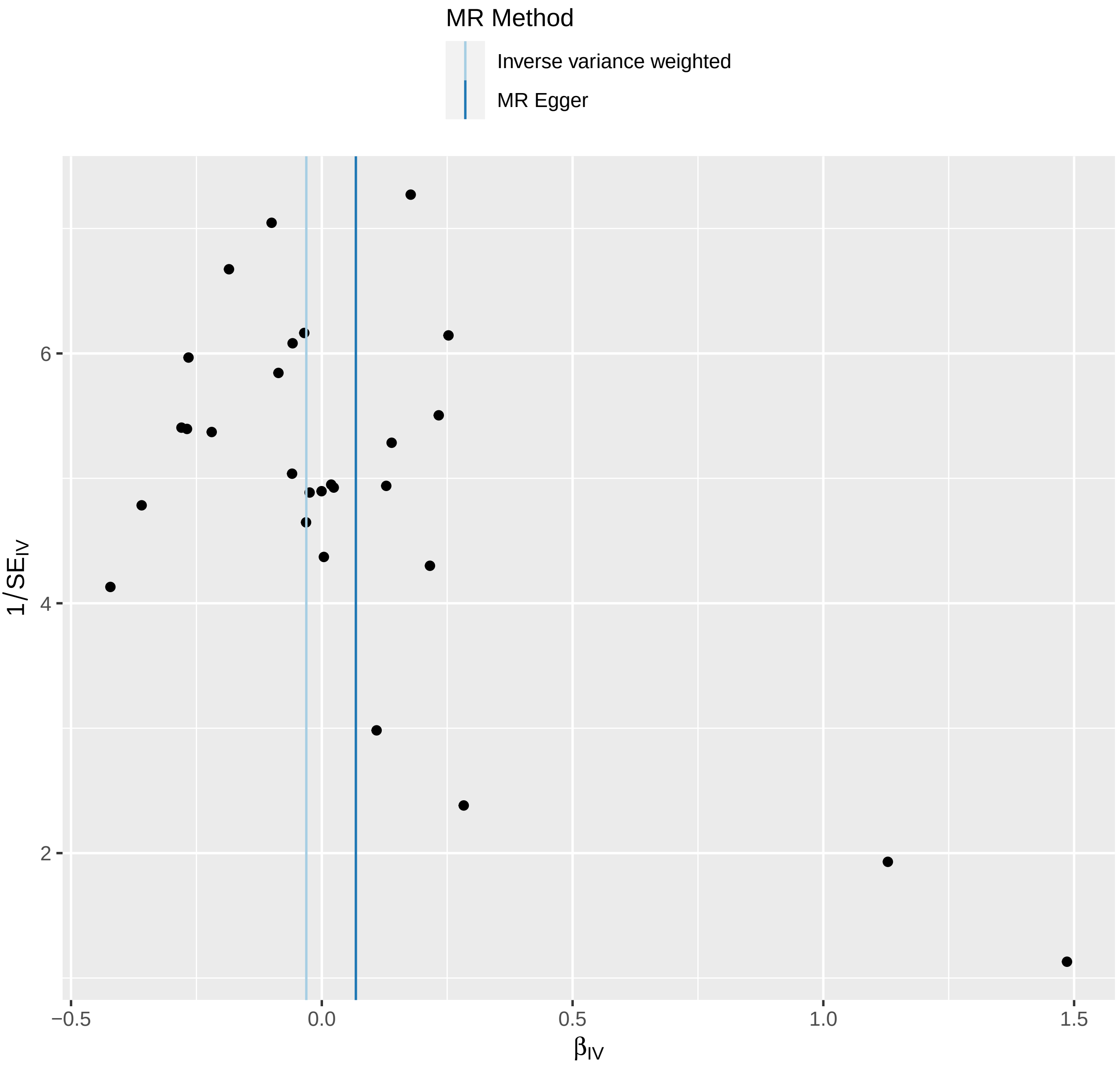

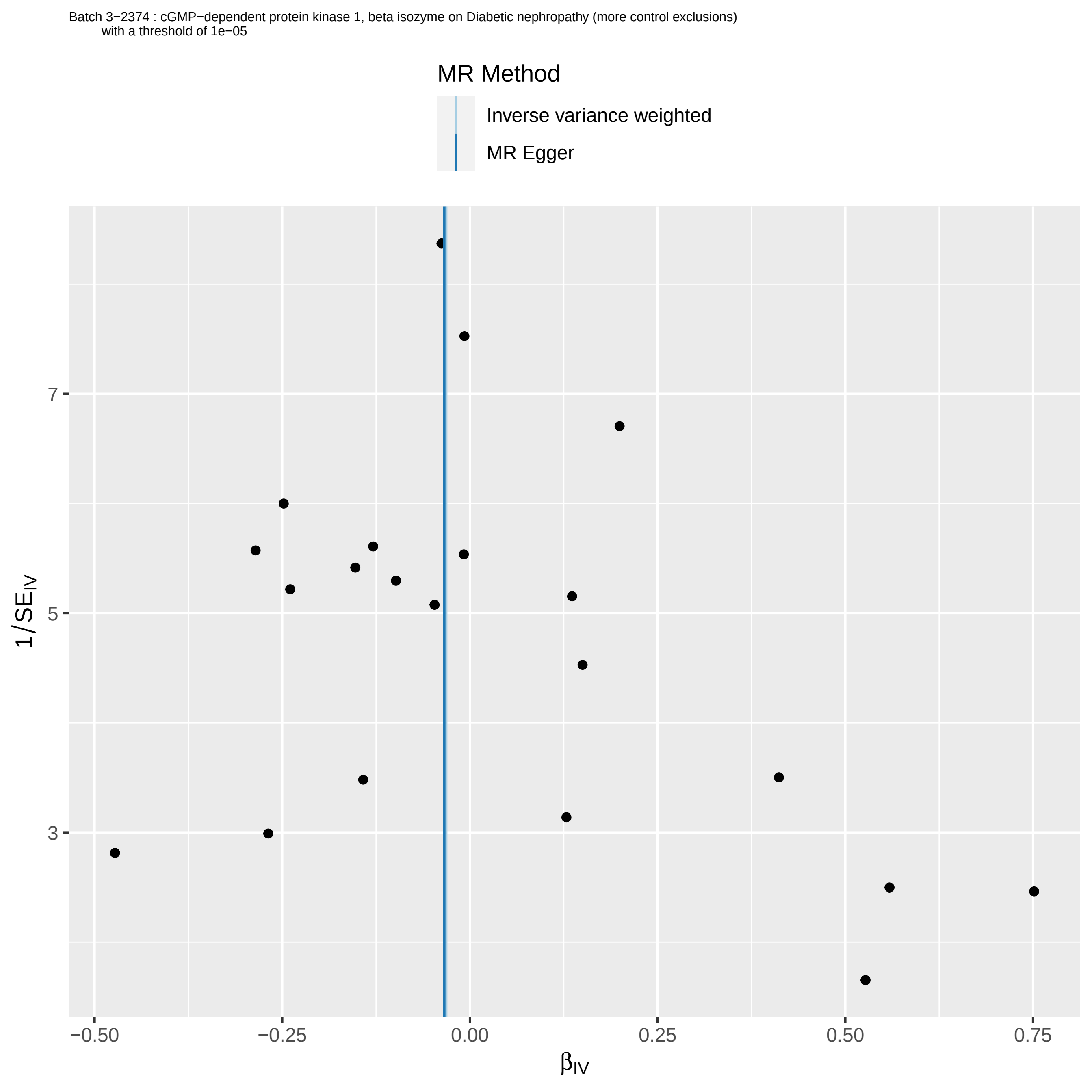
Batch 3−2374 : cGMP−dependent protein kinase 1, beta isozyme on Diabetic nephropathy (more control exclusions)
with a threshold of 1e−05
MR Method
Inverse variance weighted
MR Egger
7
5
3
1/SE_IV
−0.50
−0.25
0.00
0.25
0.50
0.75
β_IV

Batch 3−2375 : Prolactin on Diabetic nephropathy (more control exclusions)
with a threshold of 1e−05

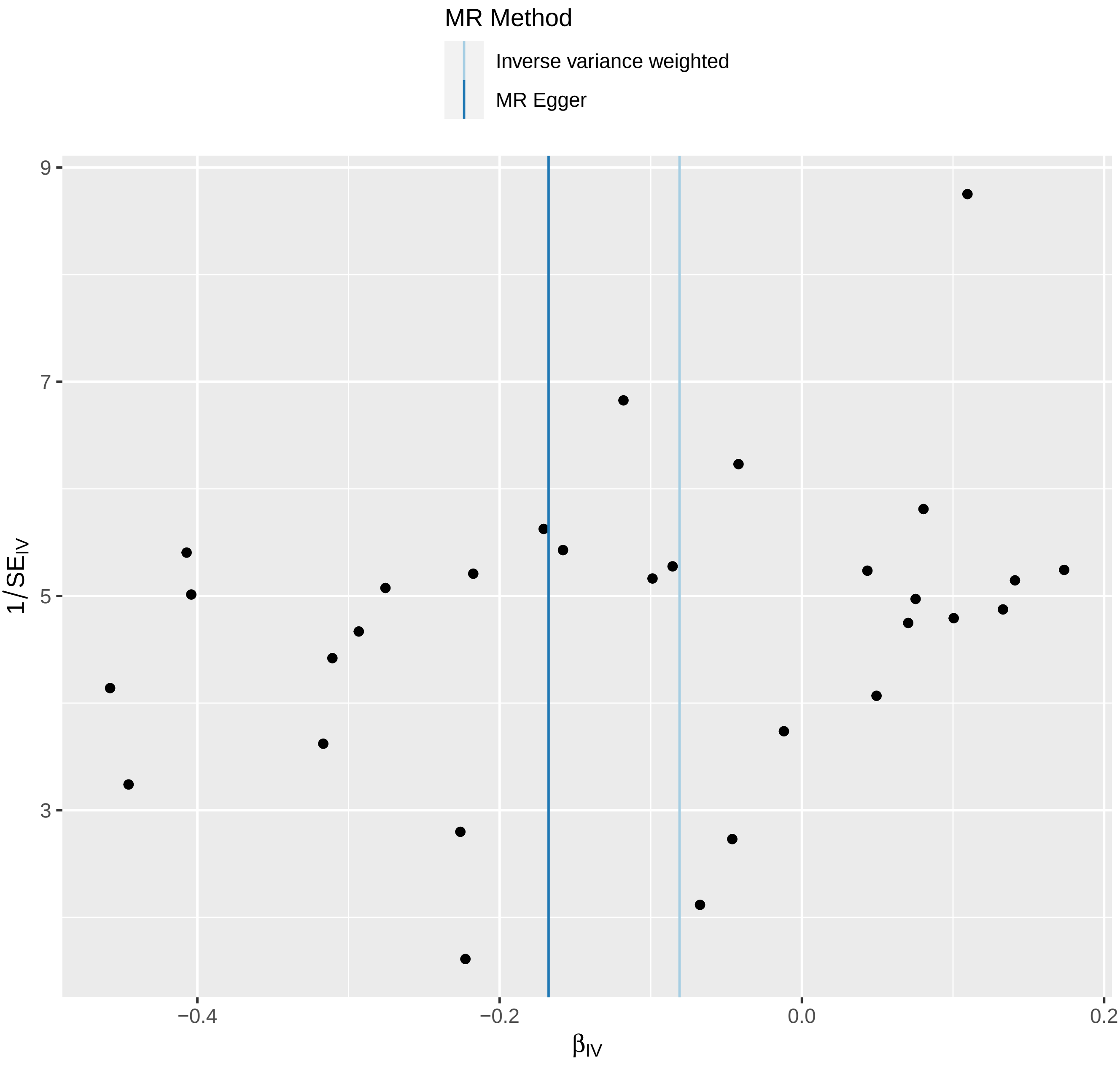

Batch 3−2376 : Prolactin−releasing peptide on Diabetic nephropathy (more control exclusions)
with a threshold of 1e−05

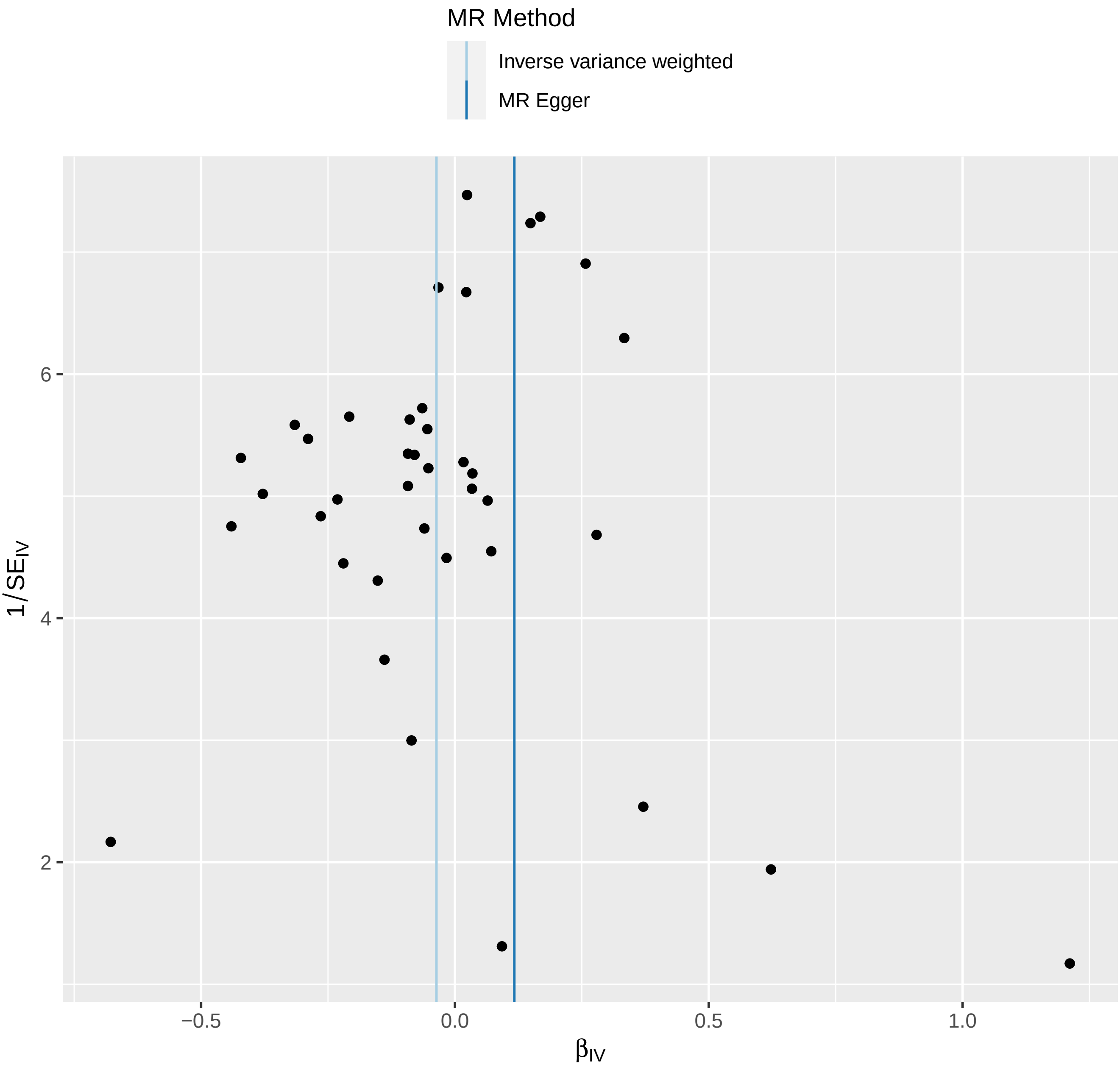

Batch 3−2377 : Prolactin receptor on Diabetic nephropathy (more control exclusions)
with a threshold of 1e−05

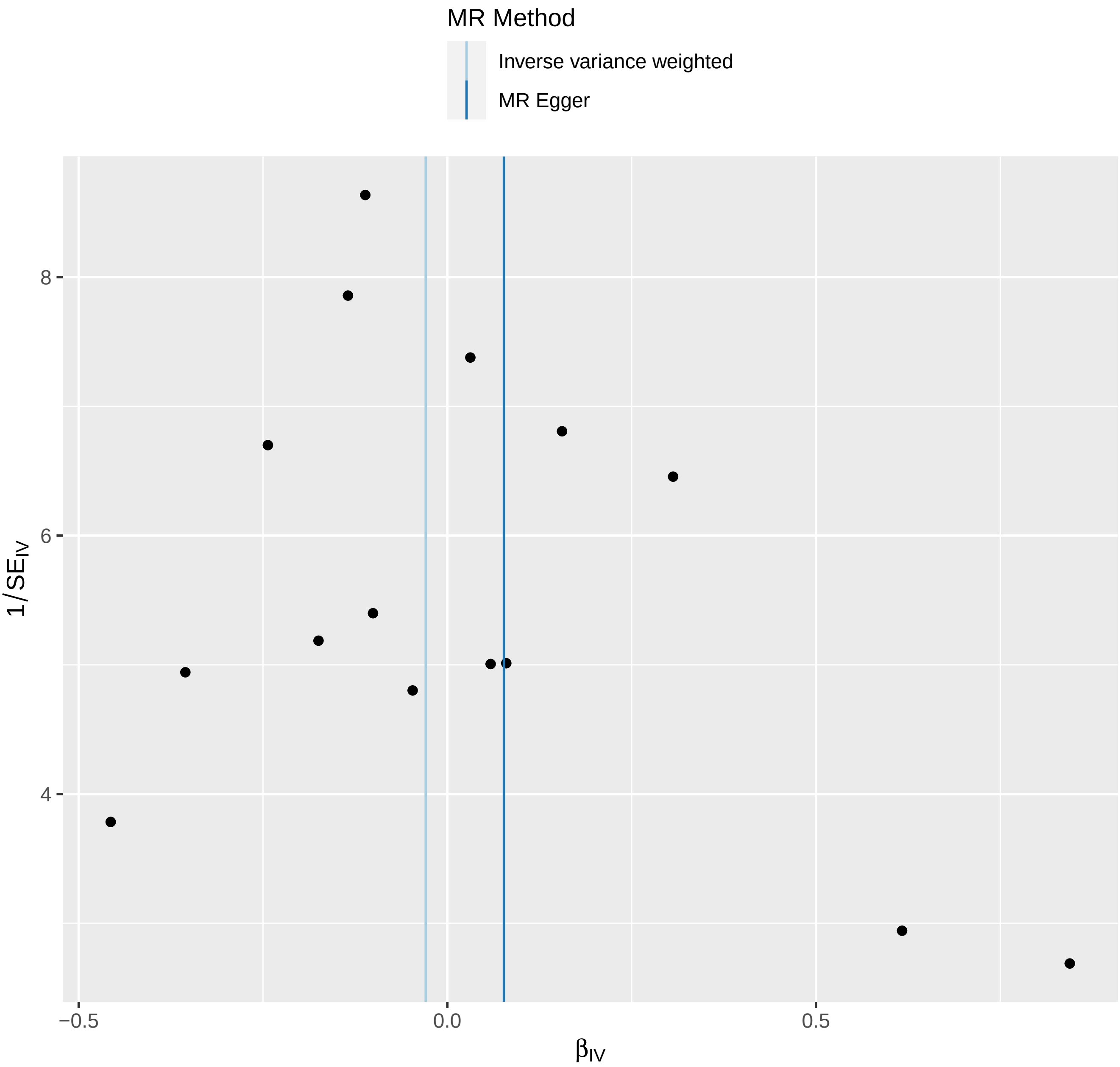

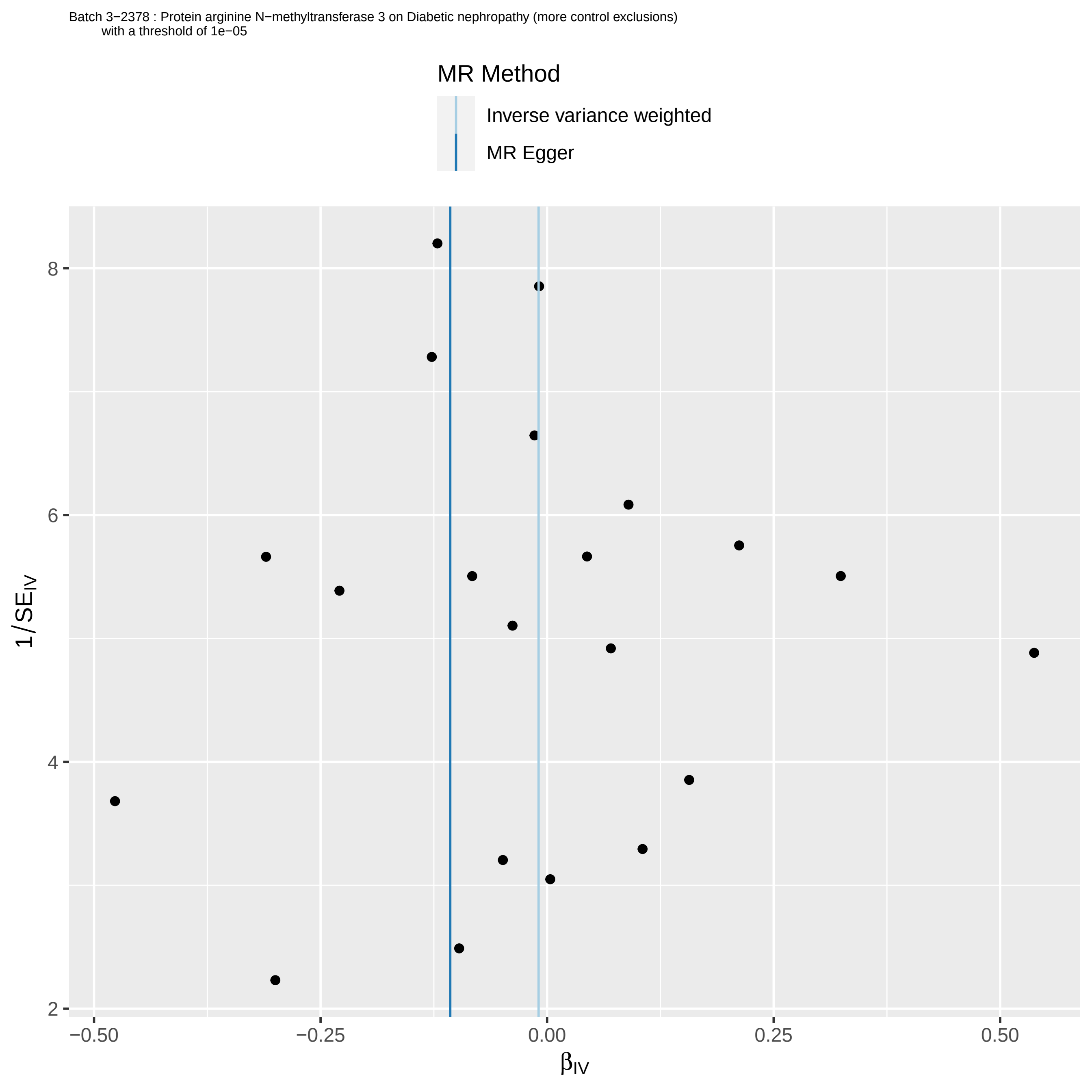

Batch 3−2378 : Protein arginine N−methyltransferase 3 on Diabetic nephropathy (more control exclusions)
with a threshold of 1e−05
MR Method
Inverse variance weighted
MR Egger
8
6
4
2
1/SE_IV
−0.50
−0.25
0.00
0.25
0.50
β_IV

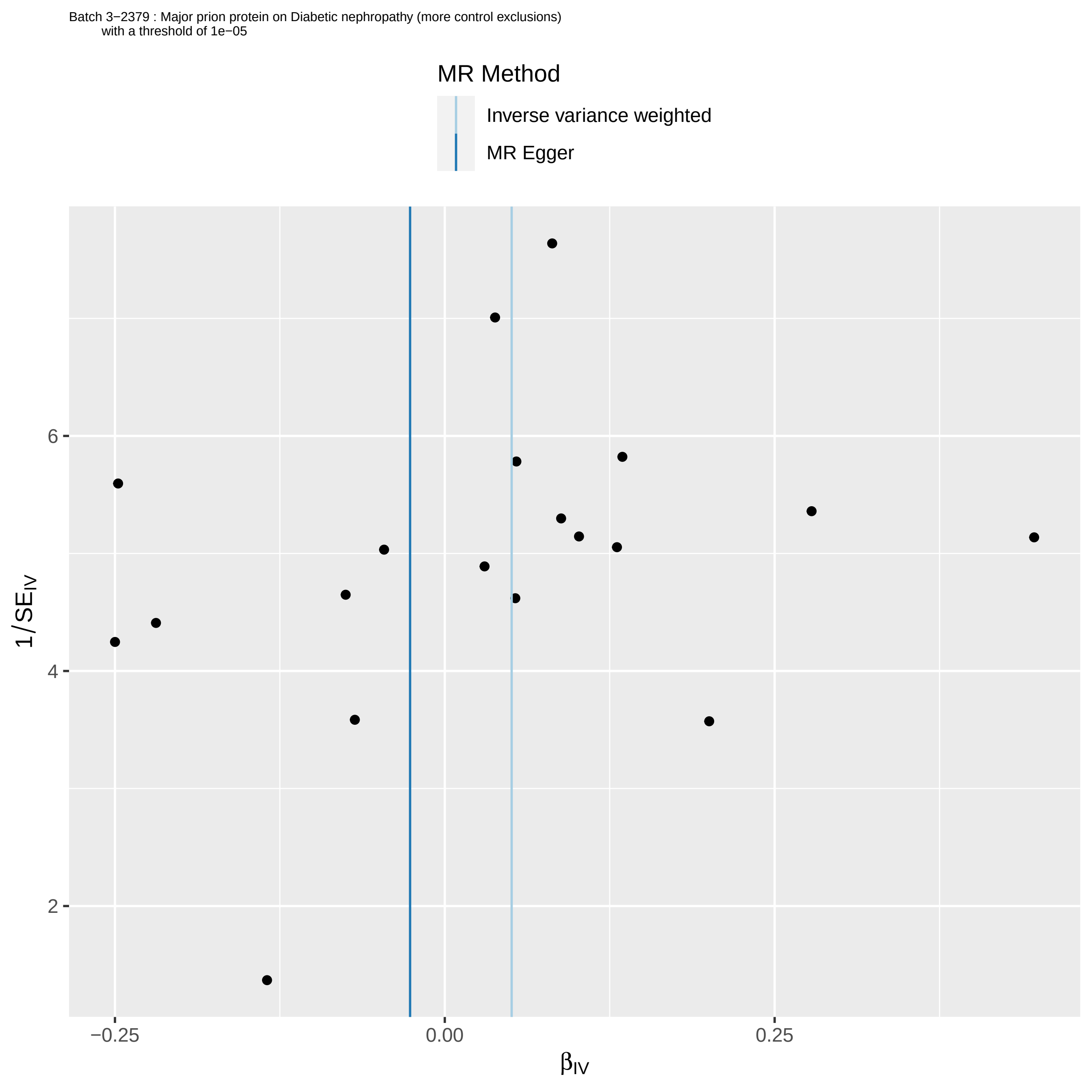

Batch 3−2379 : Major prion protein on Diabetic nephropathy (more control exclusions)
with a threshold of 1e−05
MR Method
Inverse variance weighted
MR Egger
6
4
2
1/SE$_{IV}$
−0.25
0.00
0.25
β$_{IV}$

Batch 3−2380 : Activated Protein C on Diabetic nephropathy (more control exclusions)
with a threshold of 1e−05

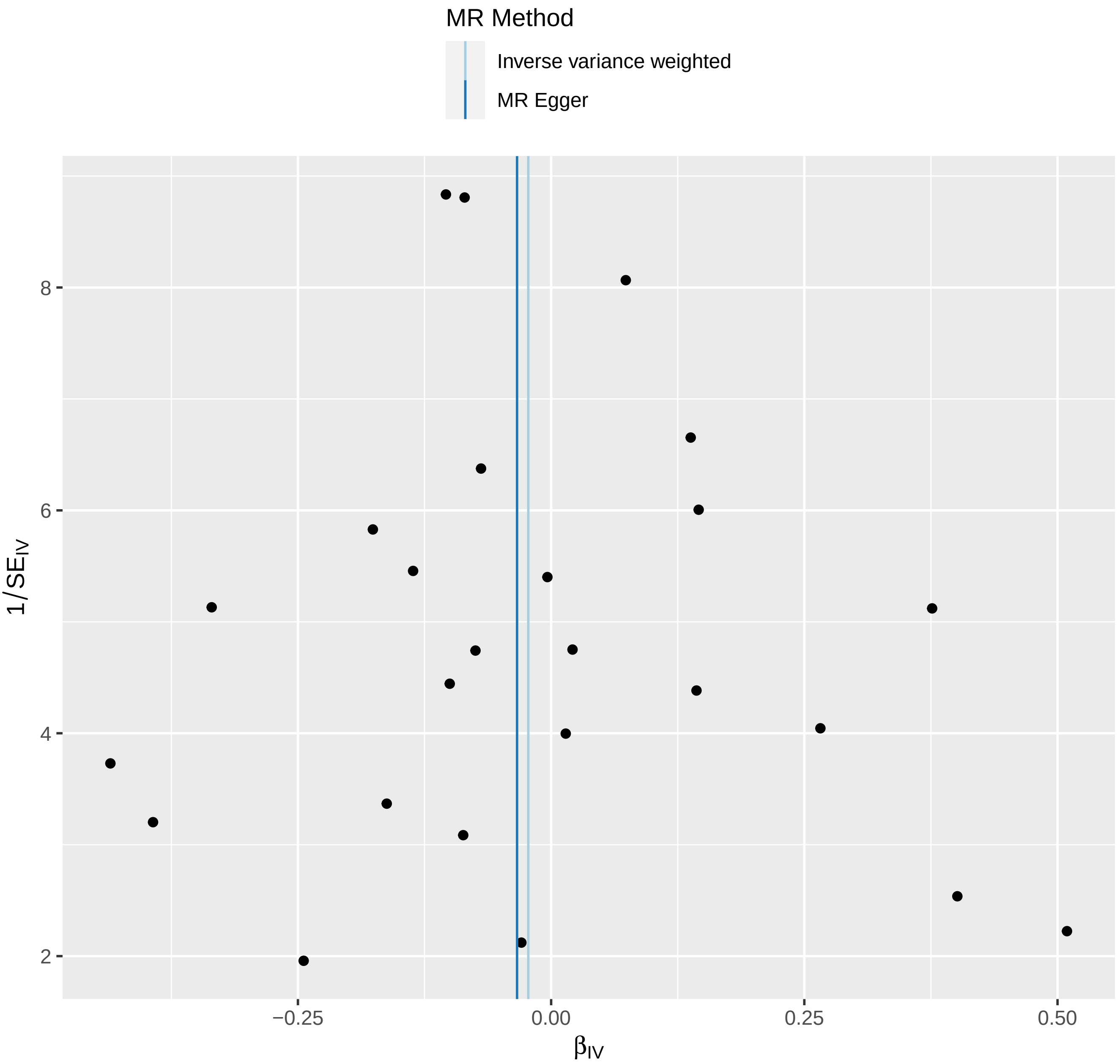

Batch 3−2381 : Activated Protein C on Diabetic nephropathy (more control exclusions)
with a threshold of 1e−05

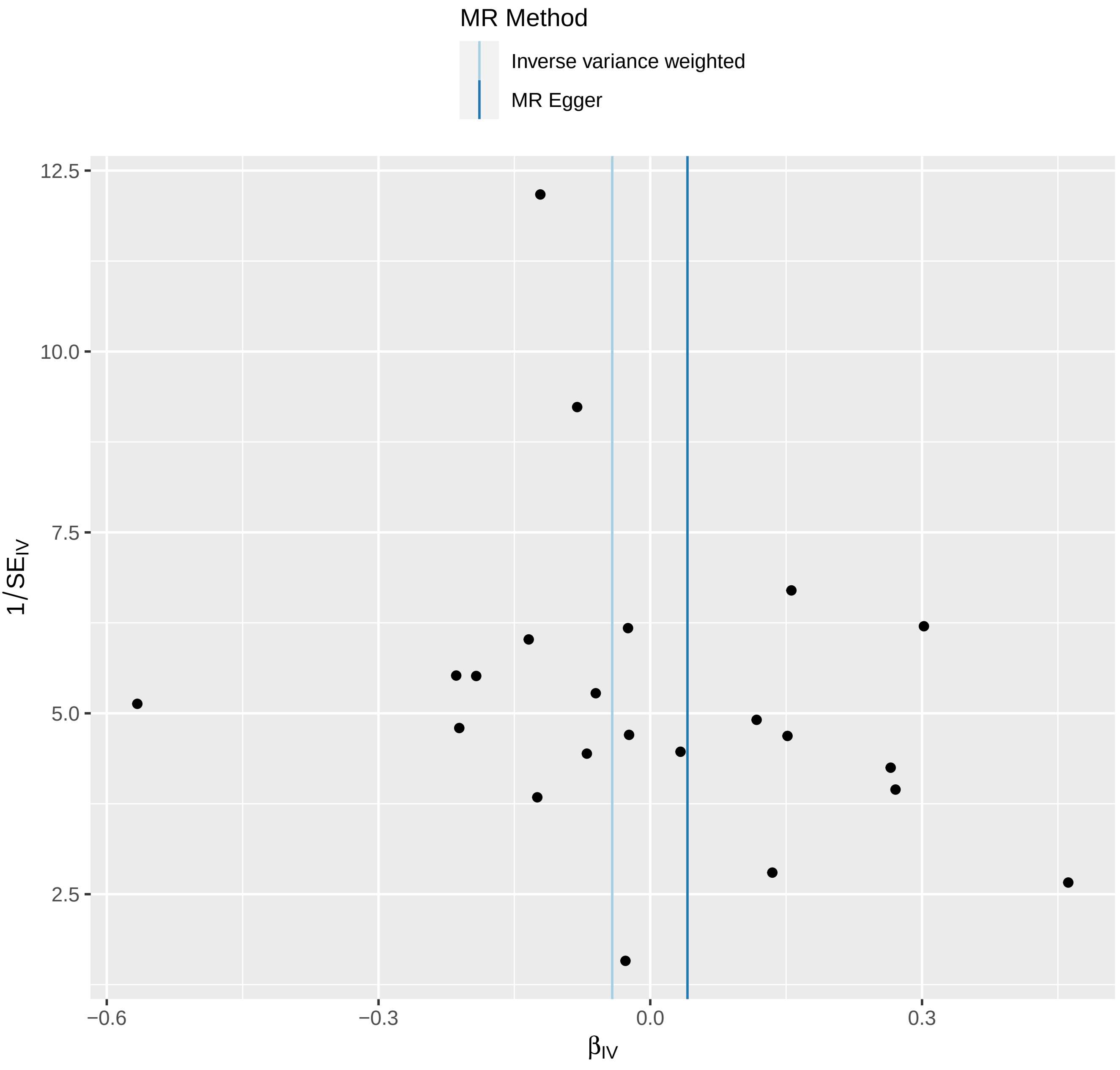

Batch 3−2382 : Prokineticin−1 on Diabetic nephropathy (more control exclusions)
with a threshold of 1e−05

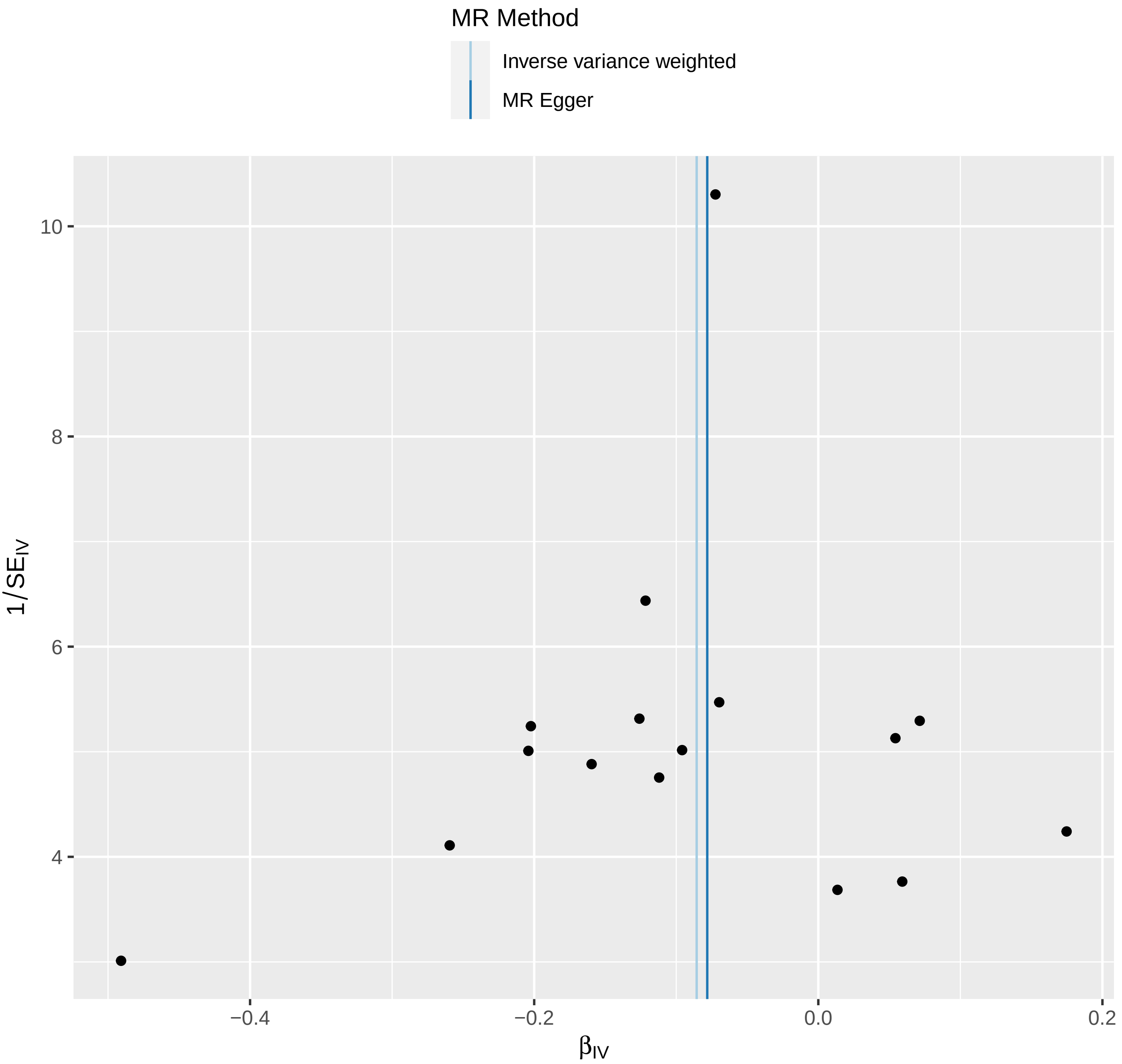

Batch 3−2383 : Proline−rich protein 1 on Diabetic nephropathy (more control exclusions)
with a threshold of 1e−05

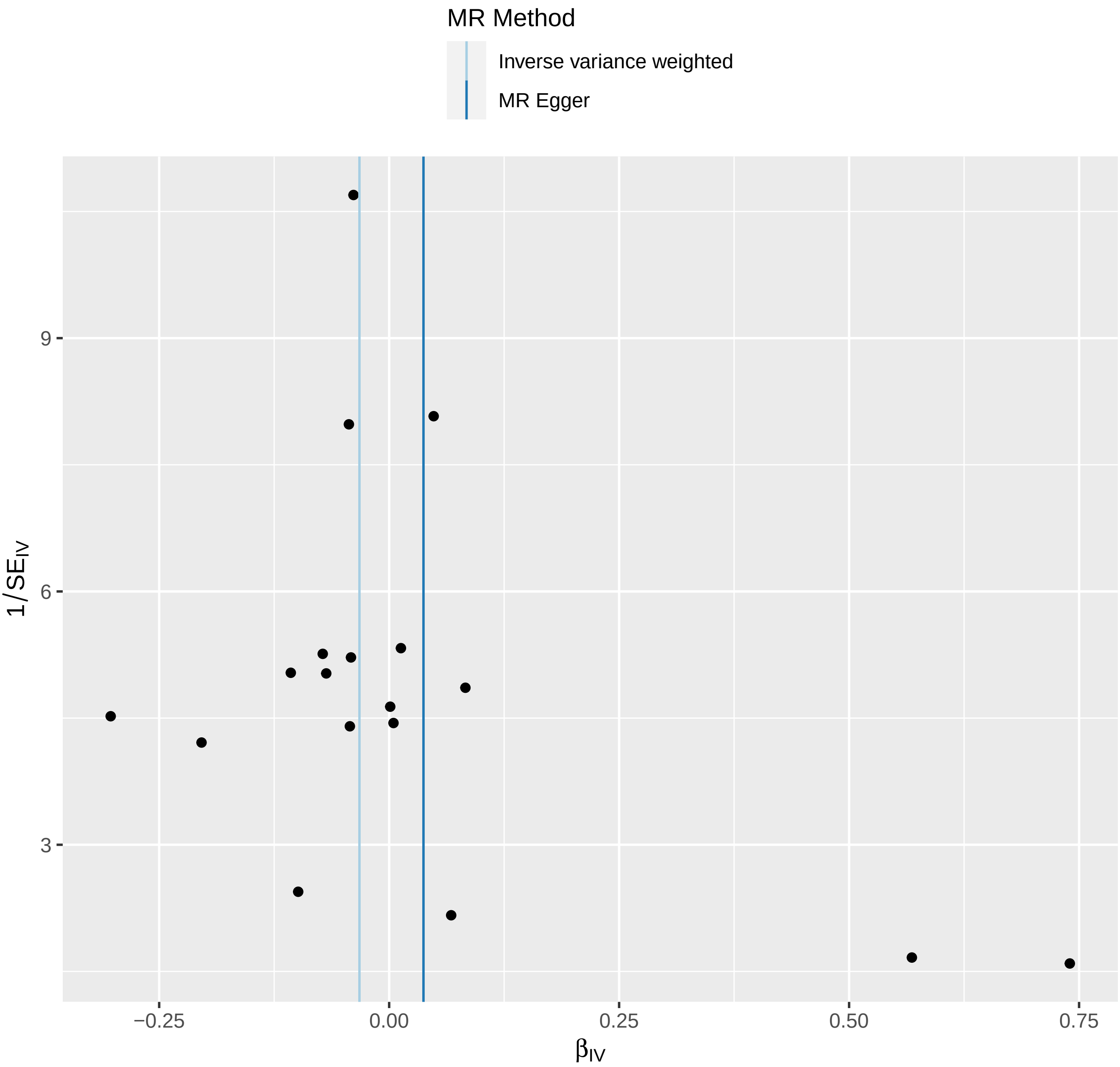

Batch 3−2384 : Pre−mRNA−processing factor 6 on Diabetic nephropathy (more control exclusions)
with a threshold of 1e−05

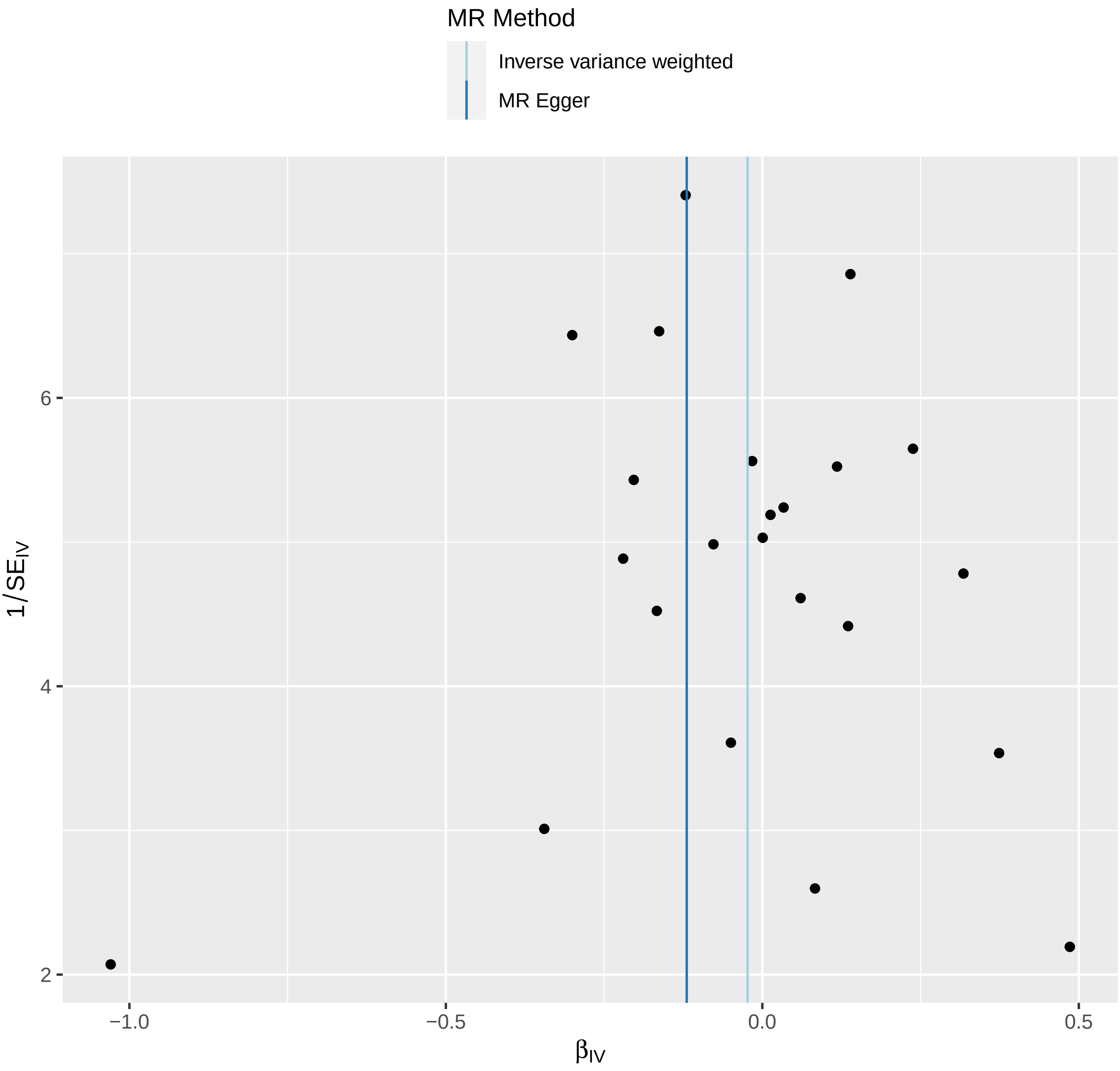

Batch 3−2385 : Phosphoribosyl pyrophosphate synthase−associated protein 1 on Diabetic nephropathy (more control exclusions) with a threshold of 1e−05

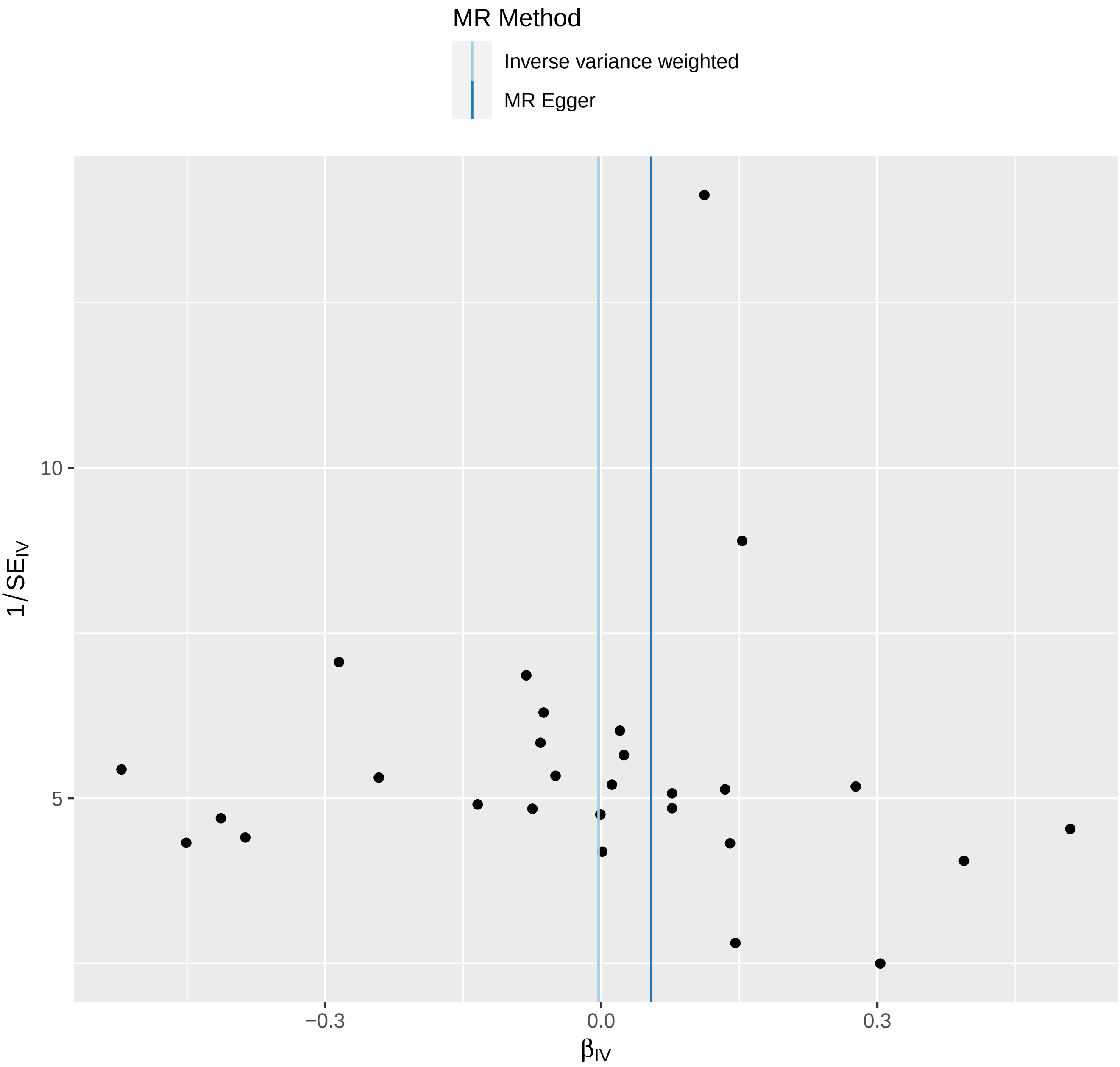

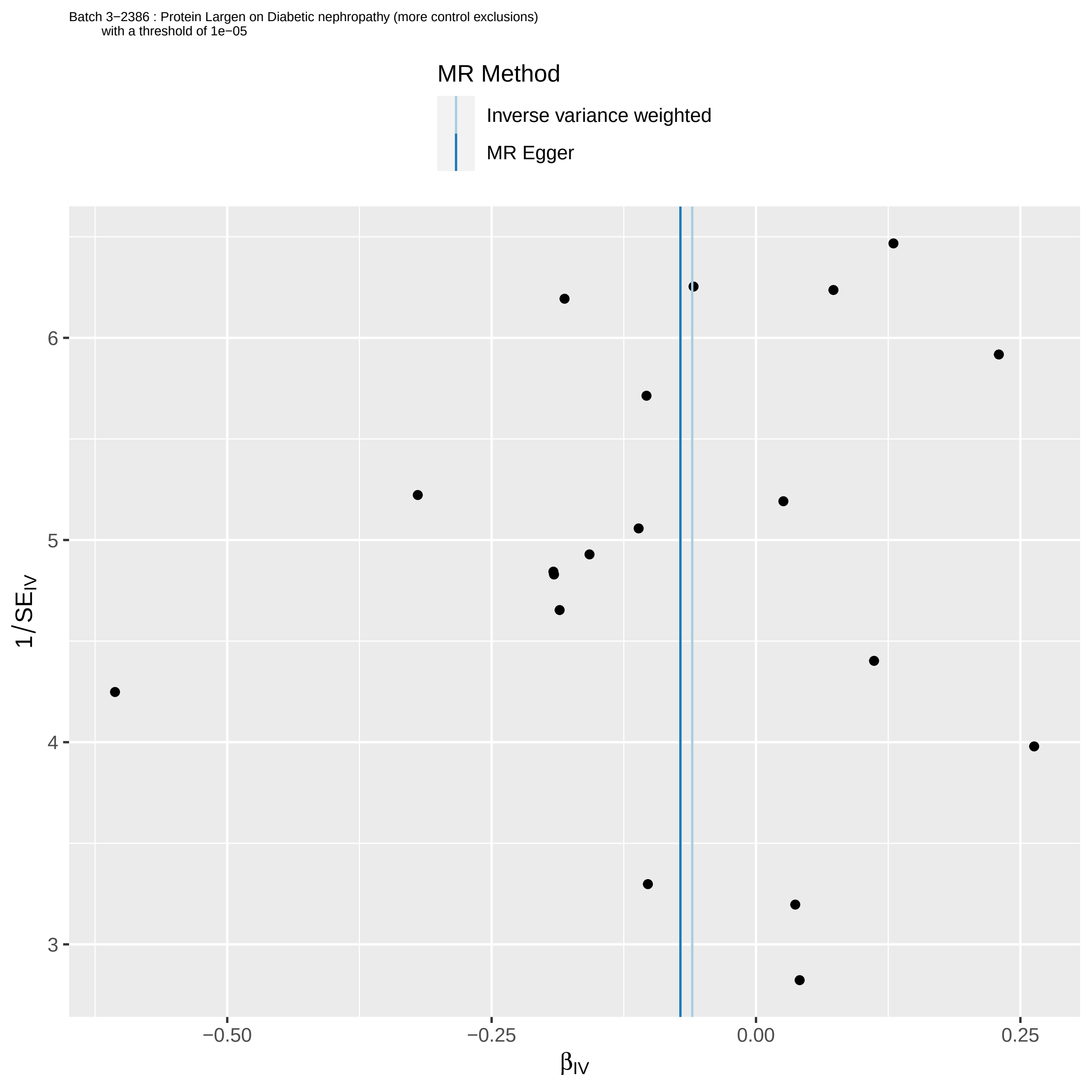

Batch 3−2386 : Protein Largen on Diabetic nephropathy (more control exclusions)
with a threshold of 1e−05
MR Method
Inverse variance weighted
MR Egger
1/SE$_{IV}$
6
5
4
3
−0.50
−0.25
0.00
0.25
β$_{IV}$

Batch 3−2387 : Transmembrane gamma−carboxyglutamic acid protein 1 on Diabetic nephropathy (more control exclusions)
with a threshold of 1e−05

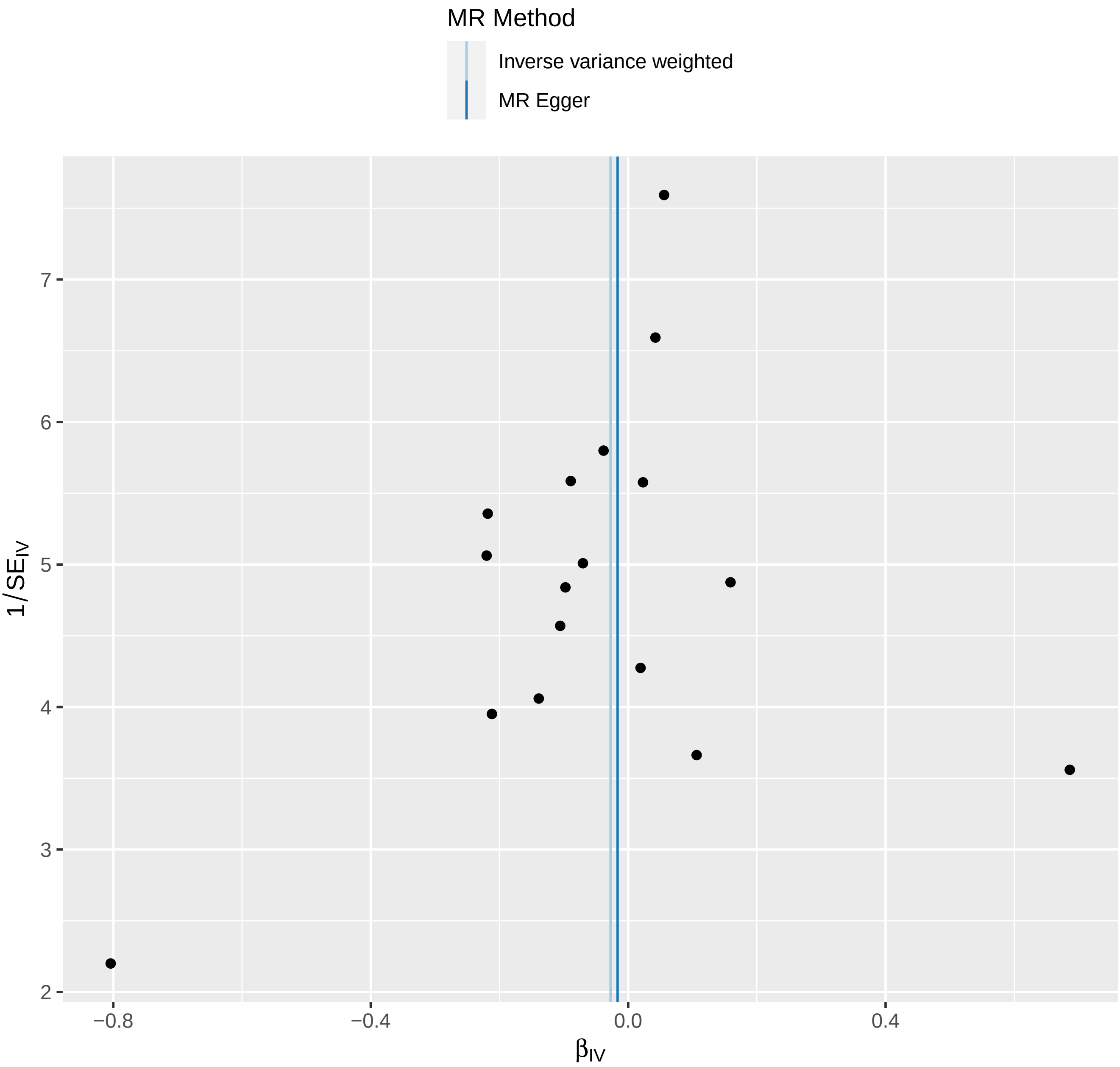

Batch 3−2388 : Transmembrane gamma−carboxyglutamic acid protein 1 on Diabetic nephropathy (more control exclusions)
with a threshold of 1e−05

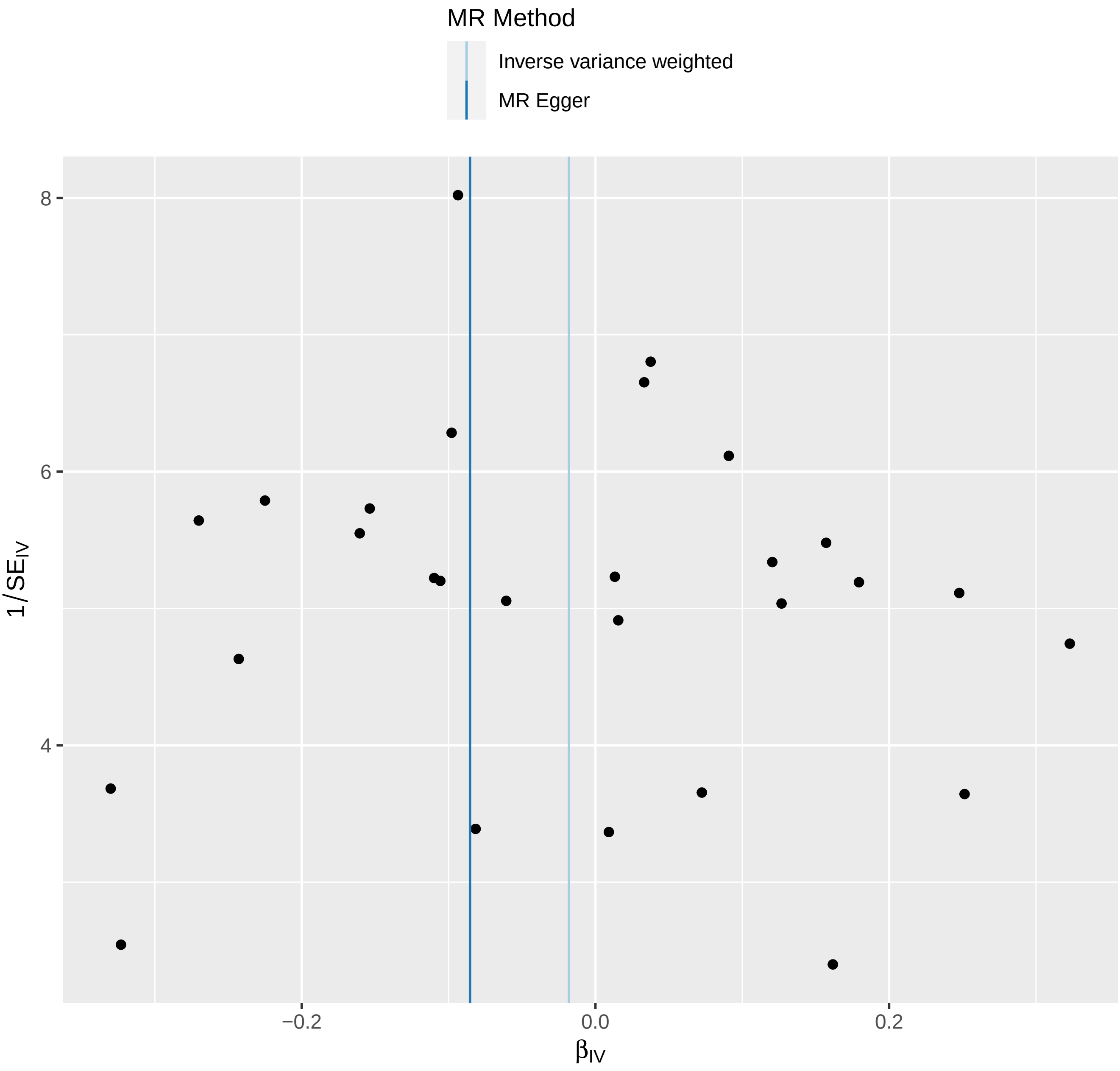

Batch 3−2389 : Transmembrane gamma−carboxyglutamic acid protein 4 on Diabetic nephropathy (more control exclusions) with a threshold of 1e−05

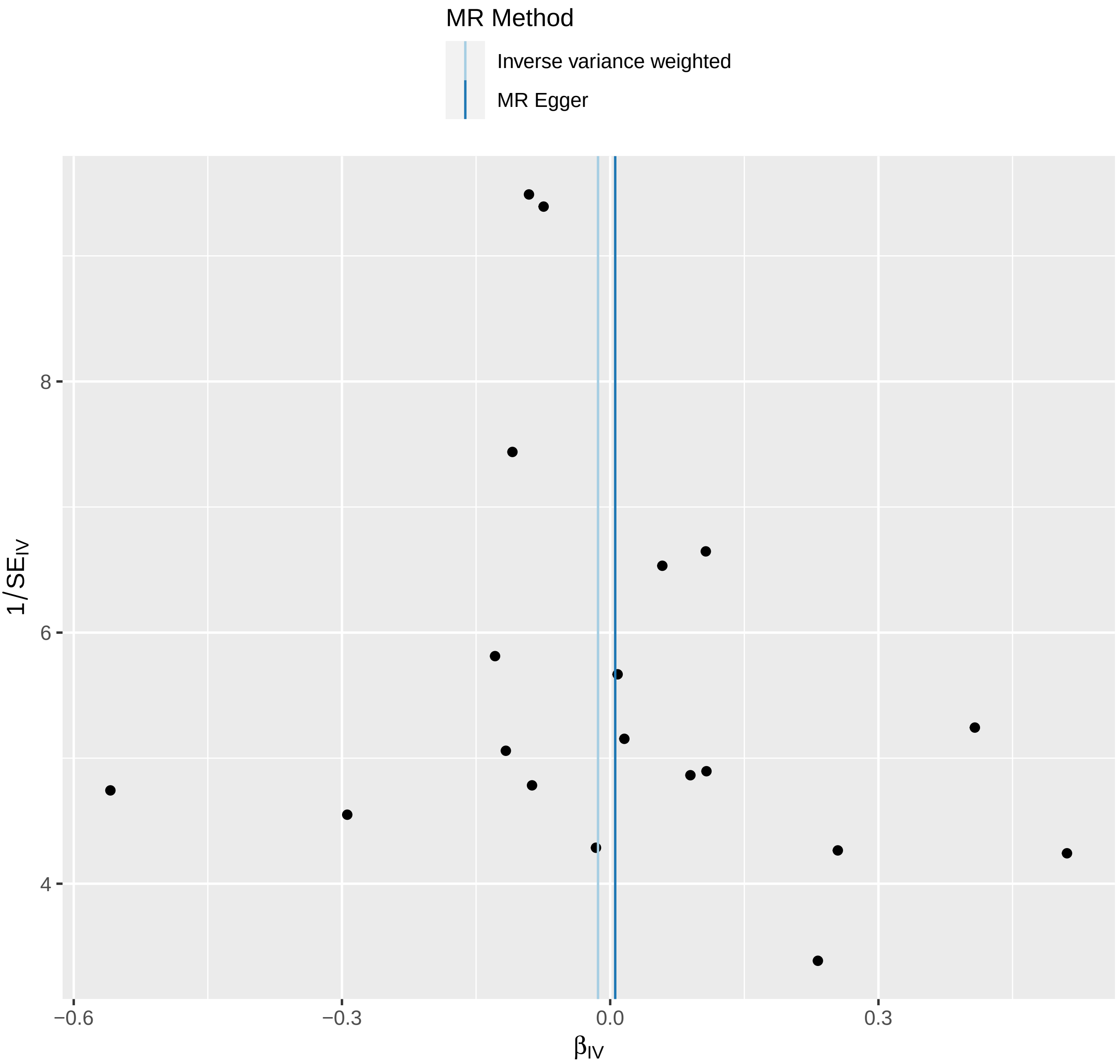

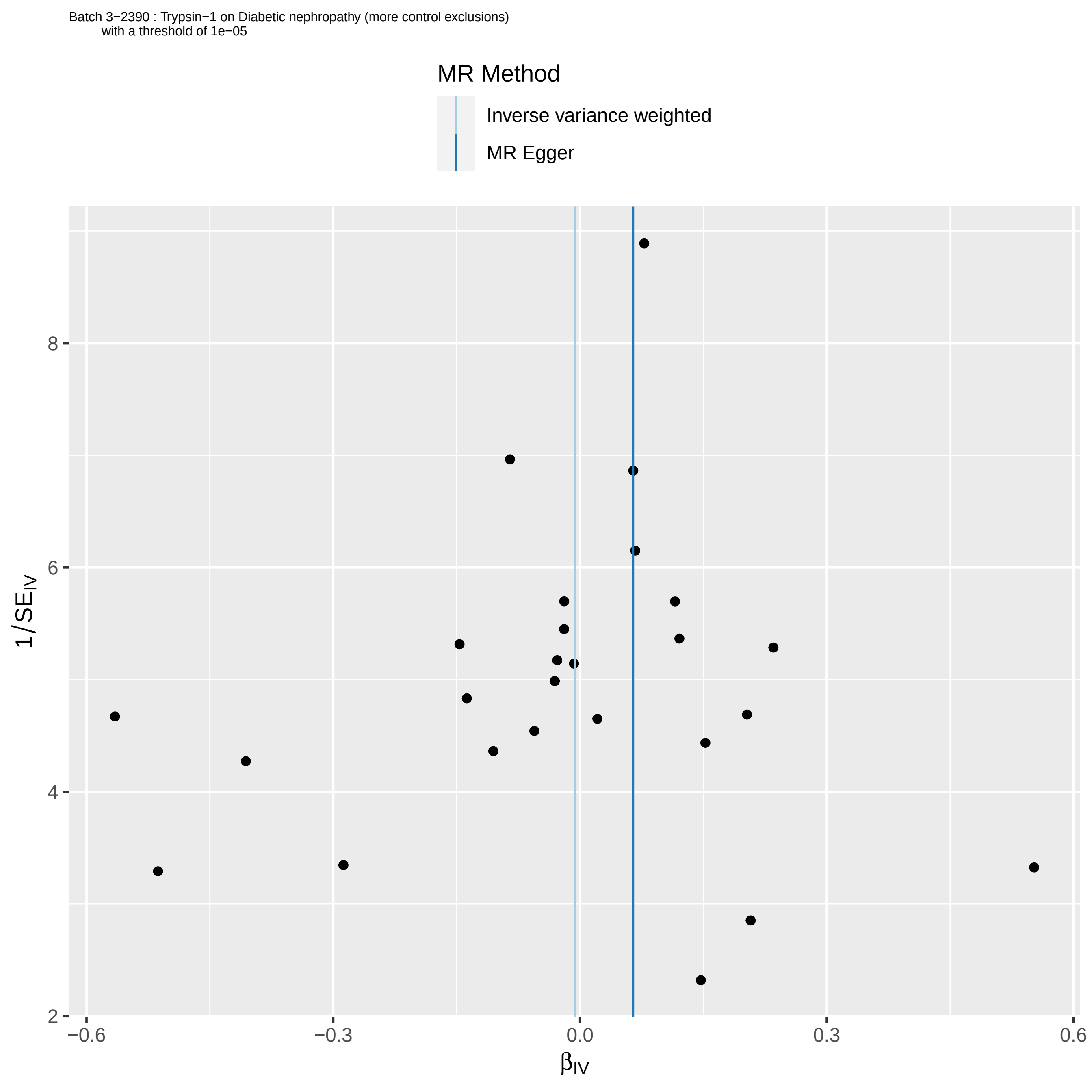

Batch 3−2390 : Trypsin−1 on Diabetic nephropathy (more control exclusions)
with a threshold of 1e−05
MR Method
Inverse variance weighted
MR Egger
1/SE_IV
β_IV
8
6
4
2
−0.6
−0.3
0.0
0.3
0.6

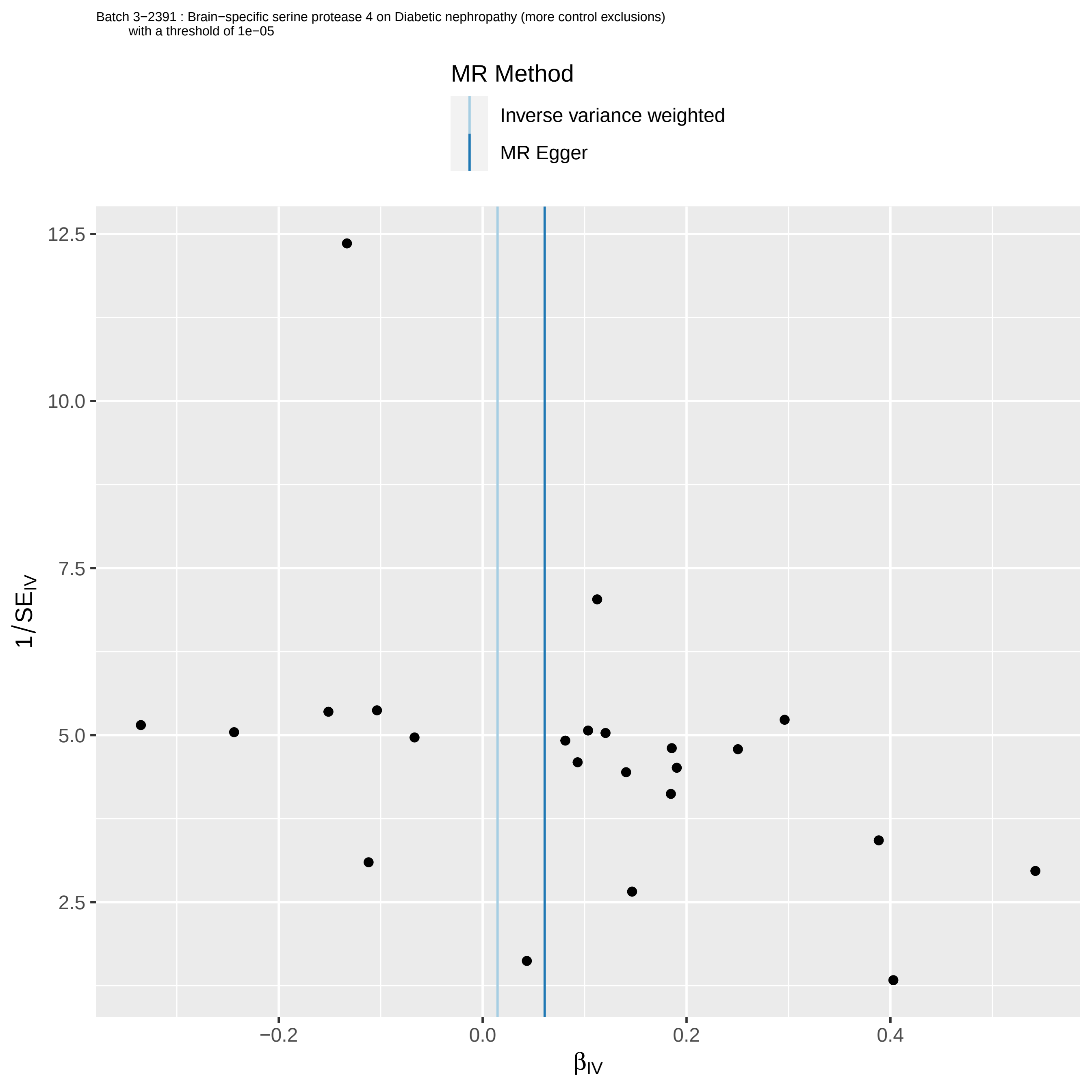
Batch 3−2391 : Brain−specific serine protease 4 on Diabetic nephropathy (more control exclusions)
with a threshold of 1e−05
MR Method
Inverse variance weighted
MR Egger
12.5
10.0
7.5
5.0
2.5
1/SE_IV
−0.2
0.0
0.2
0.4
β_IV

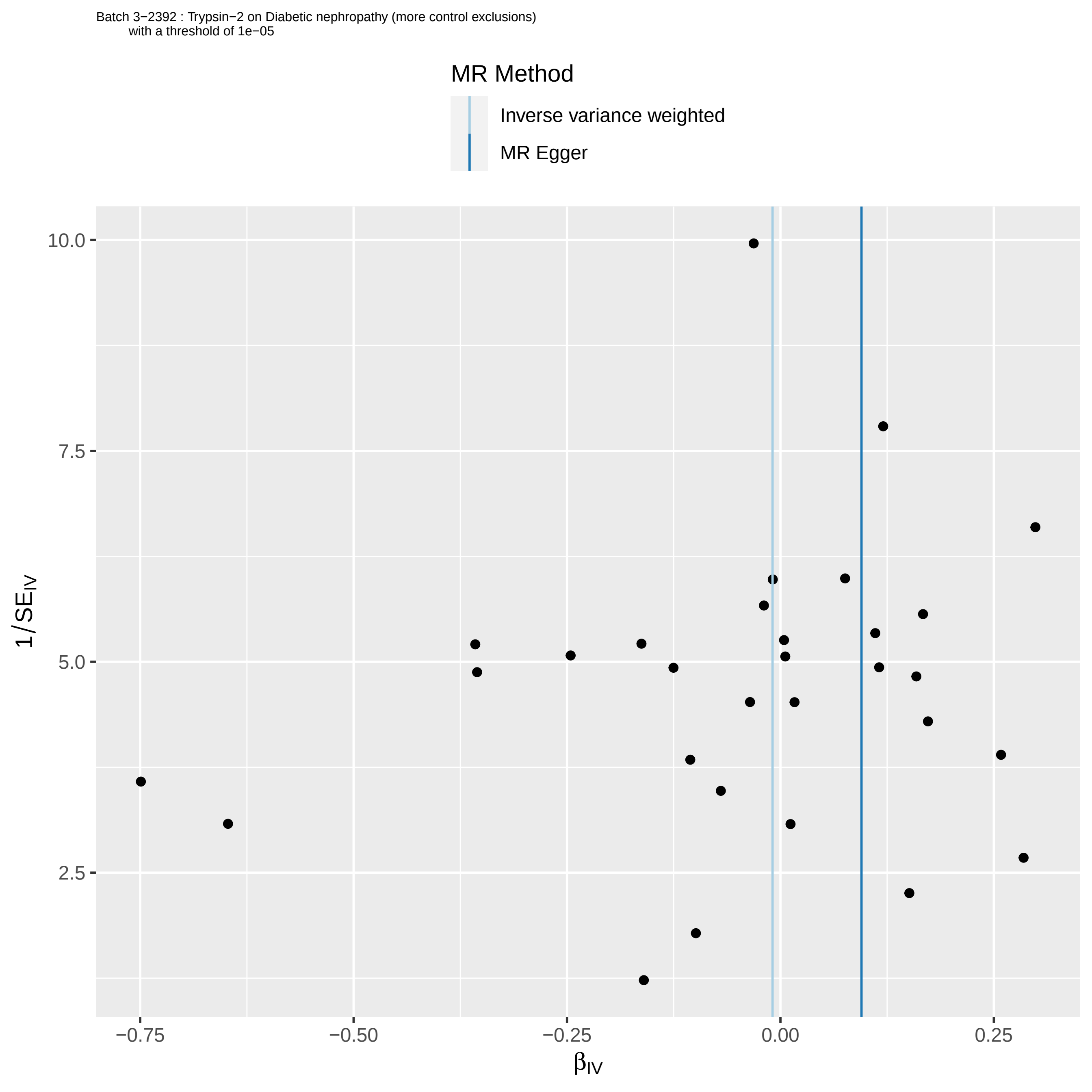

Batch 3−2392 : Trypsin−2 on Diabetic nephropathy (more control exclusions)
with a threshold of 1e−05
MR Method
Inverse variance weighted
MR Egger
10.0
7.5
5.0
2.5
1/SE$_{IV}$
−0.75
−0.50
−0.25
0.00
0.25
β_{IV}

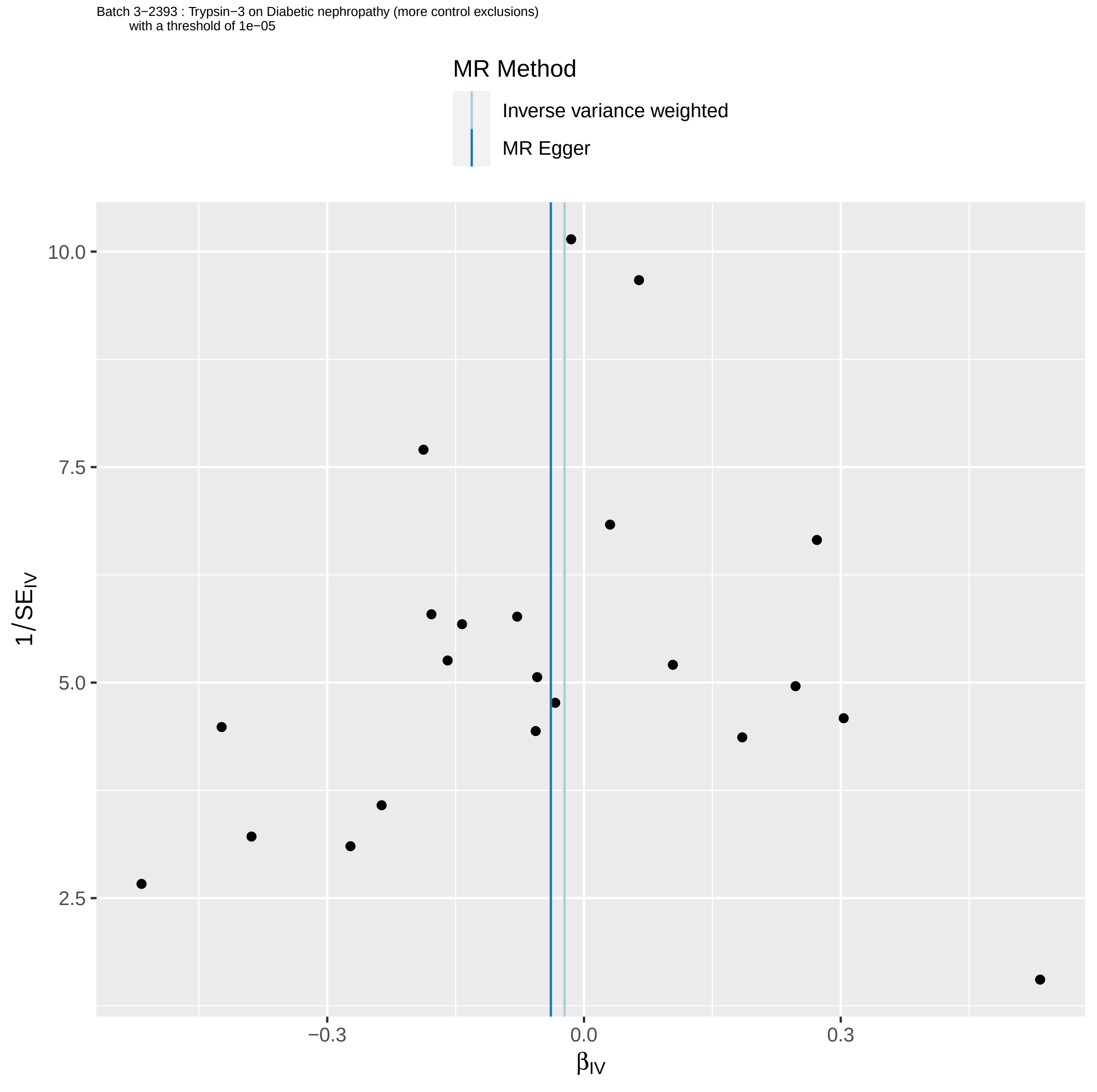
Batch 3−2393 : Trypsin−3 on Diabetic nephropathy (more control exclusions)
with a threshold of 1e−05
MR Method
Inverse variance weighted
MR Egger
10.0
7.5
5.0
2.5
1/SE$_{IV}$
−0.3
0.0
0.3
β$_{IV}$

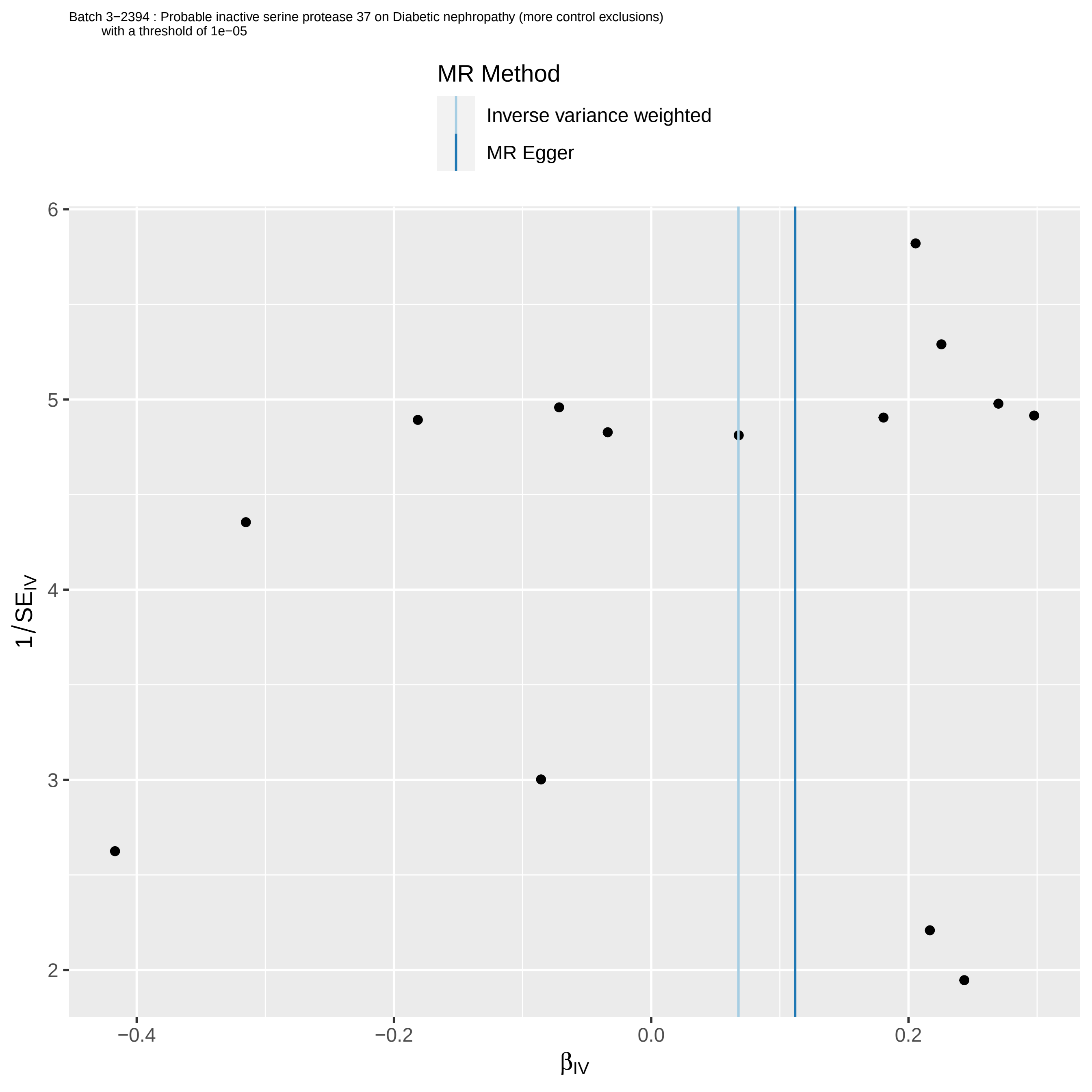

Batch 3−2394 : Probable inactive serine protease 37 on Diabetic nephropathy (more control exclusions)
with a threshold of 1e−05
MR Method
Inverse variance weighted
MR Egger
6
5
4
3
2
1/SE_IV
−0.4
−0.2
0.0
0.2
β_IV

Batch 3−2395 : Myeloblastin on Diabetic nephropathy (more control exclusions)
with a threshold of 1e−05

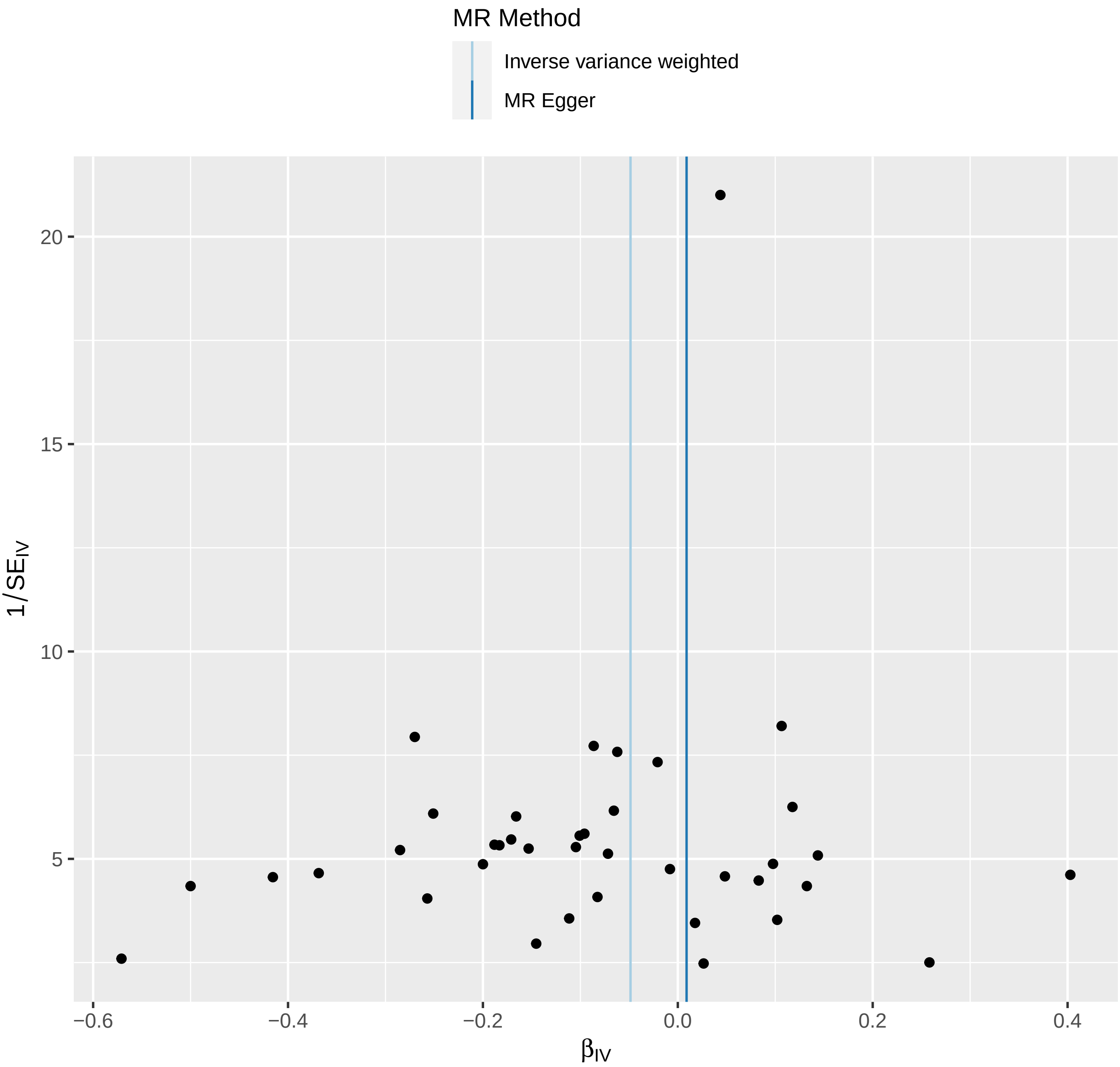

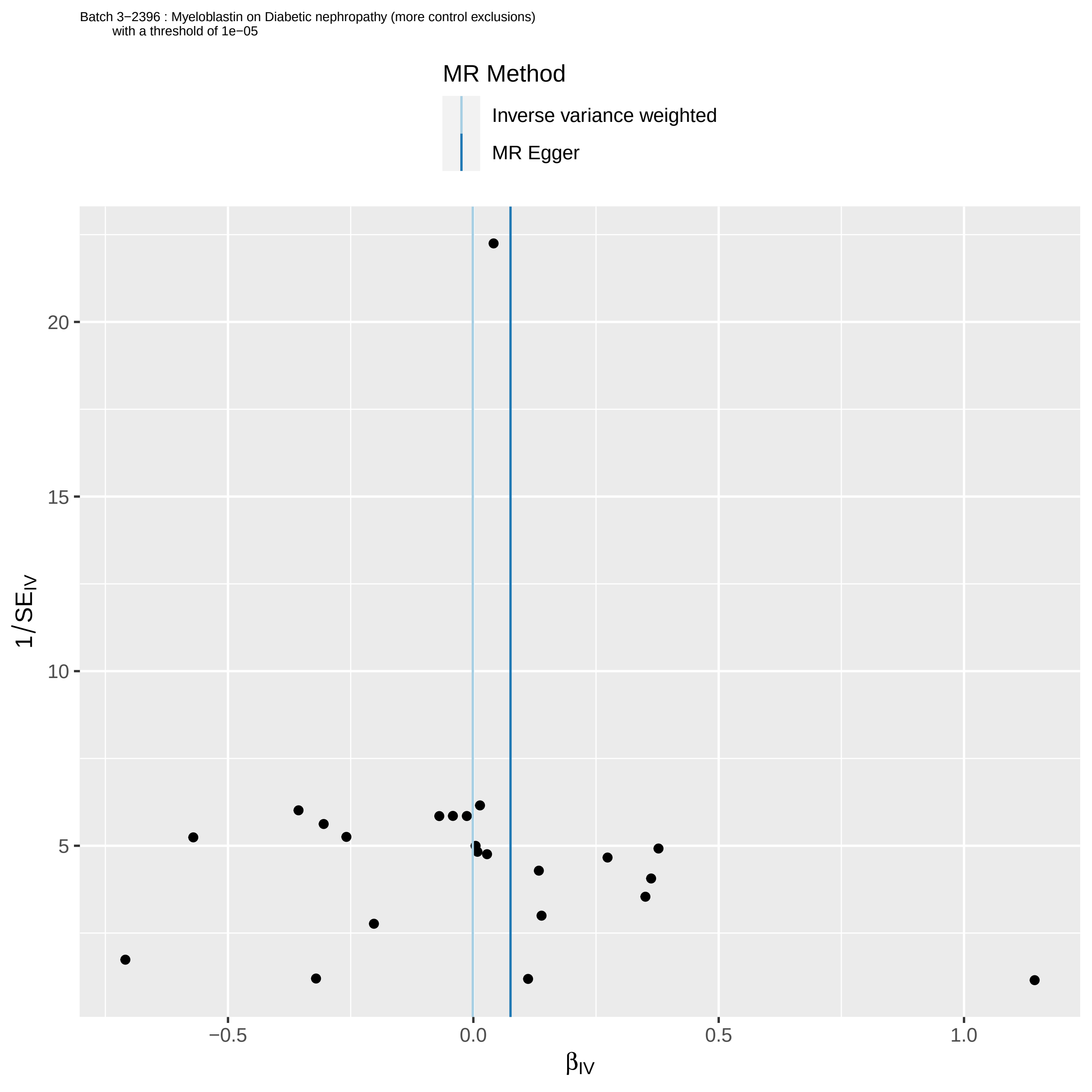
Batch 3−2396 : Myeloblastin on Diabetic nephropathy (more control exclusions)
with a threshold of 1e−05
MR Method
Inverse variance weighted
MR Egger
20
15
10
5
1/SE$_{IV}$
−0.5
0.0
0.5
1.0
β$_{IV}$

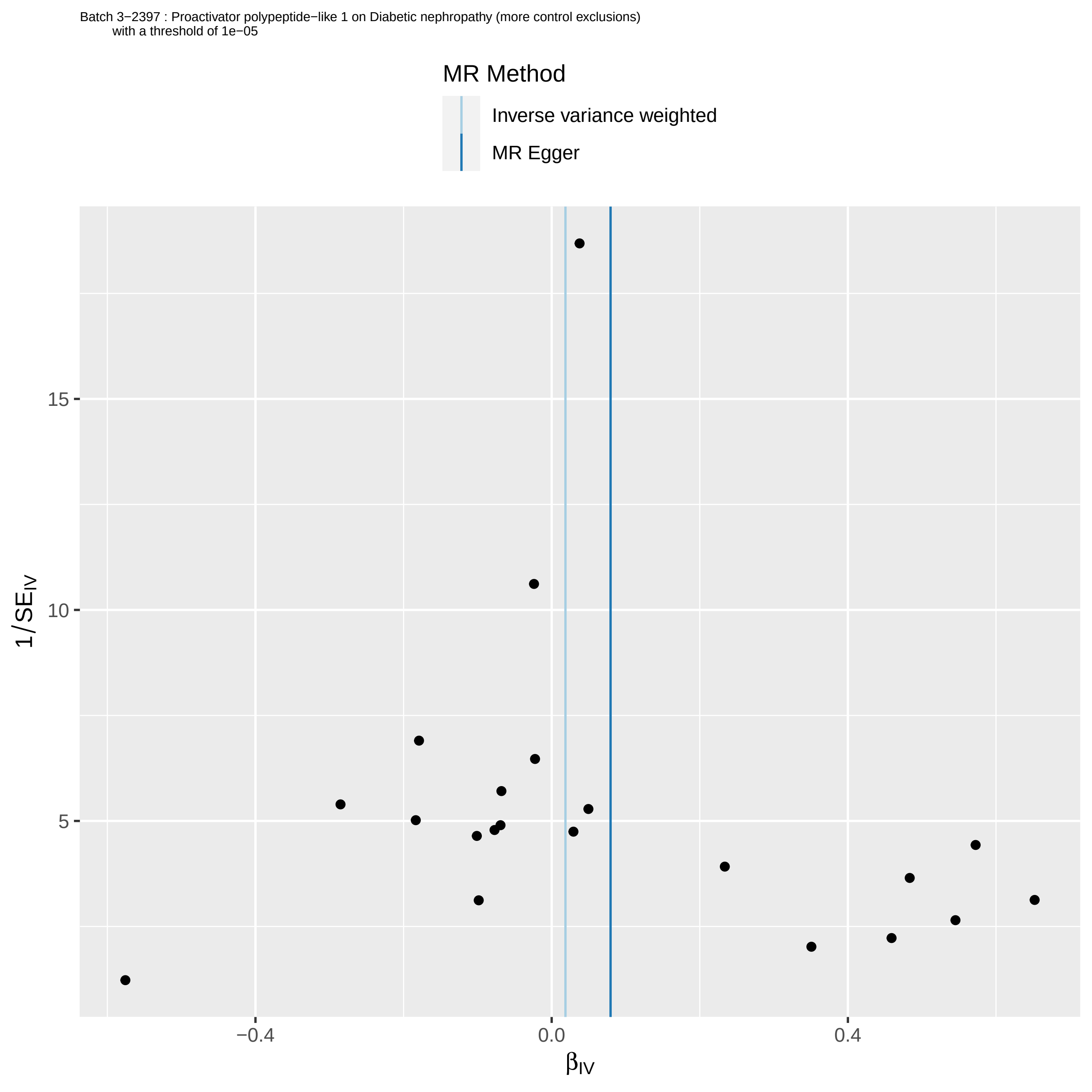
Batch 3−2397 : Proactivator polypeptide−like 1 on Diabetic nephropathy (more control exclusions)
with a threshold of 1e−05
MR Method
Inverse variance weighted
MR Egger
15
10
5
1/SE$_{IV}$
−0.4
0.0
0.4
β$_{IV}$

Batch 3−2398 : PH and SEC7 domain−containing protein 1 on Diabetic nephropathy (more control exclusions)
with a threshold of 1e−05

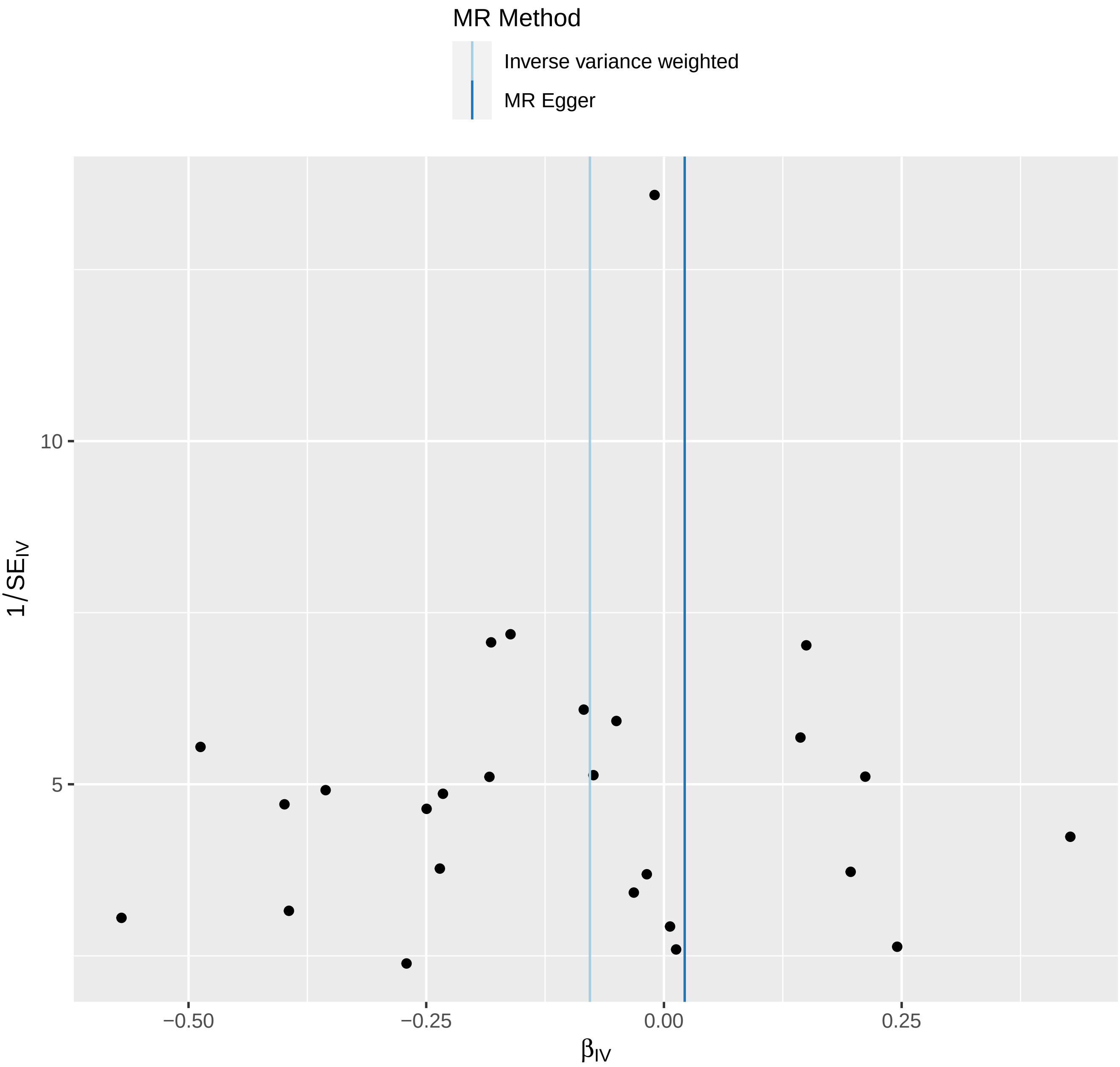

Batch 3−2399 : PH and SEC7 domain−containing protein 2 on Diabetic nephropathy (more control exclusions)
with a threshold of 1e−05

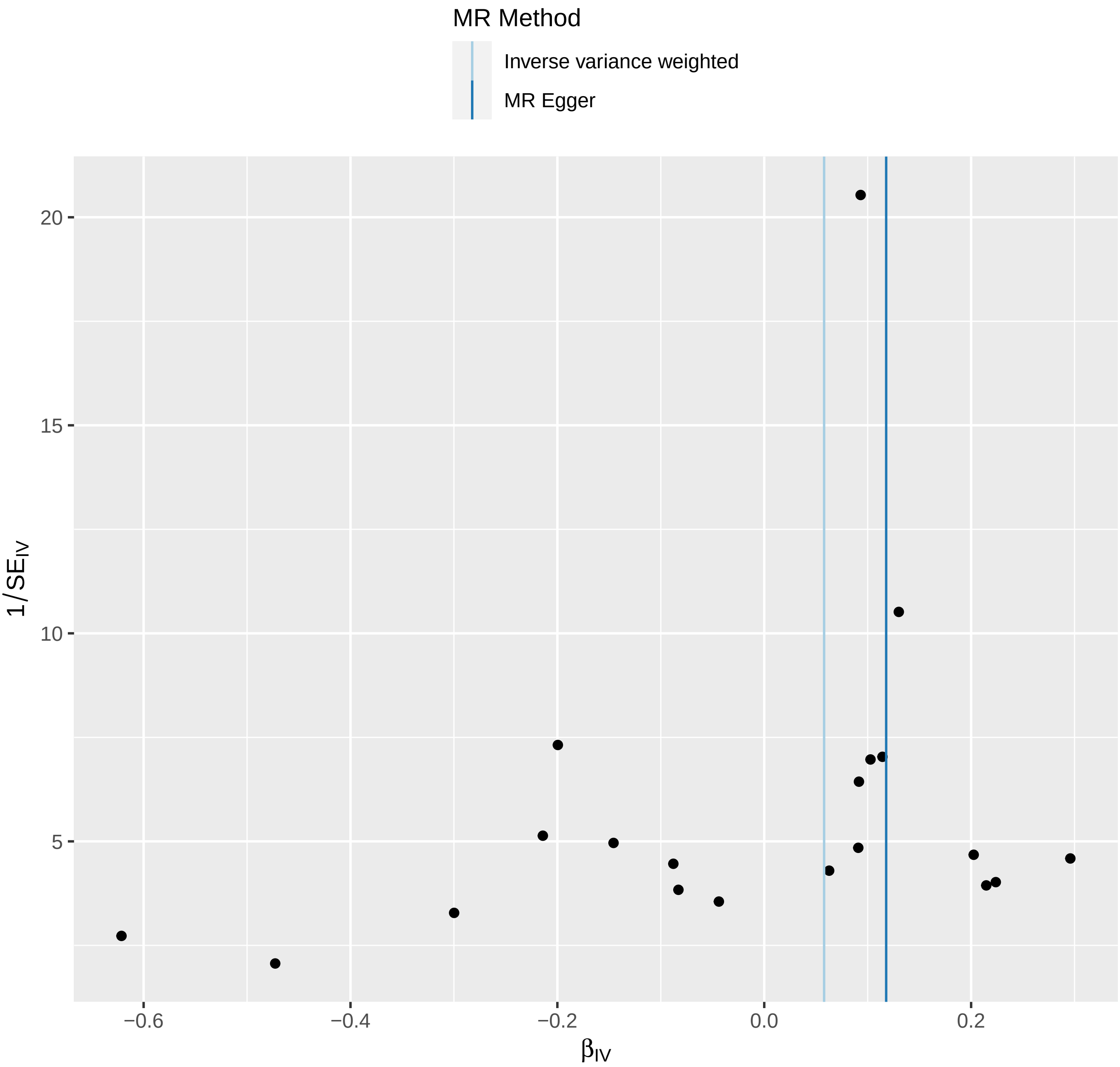

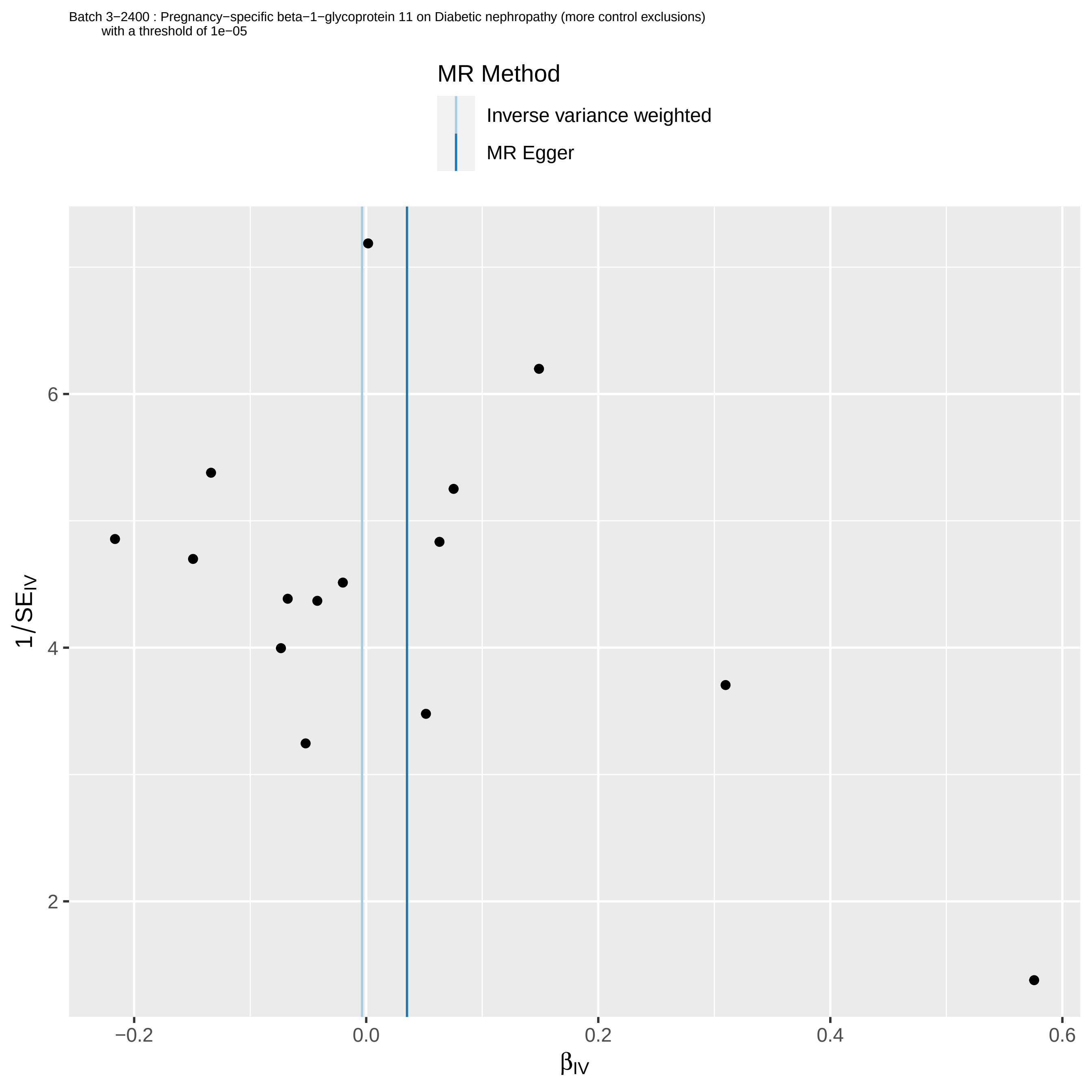
Batch 3−2400 : Pregnancy−specific beta−1−glycoprotein 11 on Diabetic nephropathy (more control exclusions)
with a threshold of 1e−05
MR Method
Inverse variance weighted
MR Egger
6
4
2
1/SE_IV
−0.2
0.0
0.2
0.4
0.6
β_IV

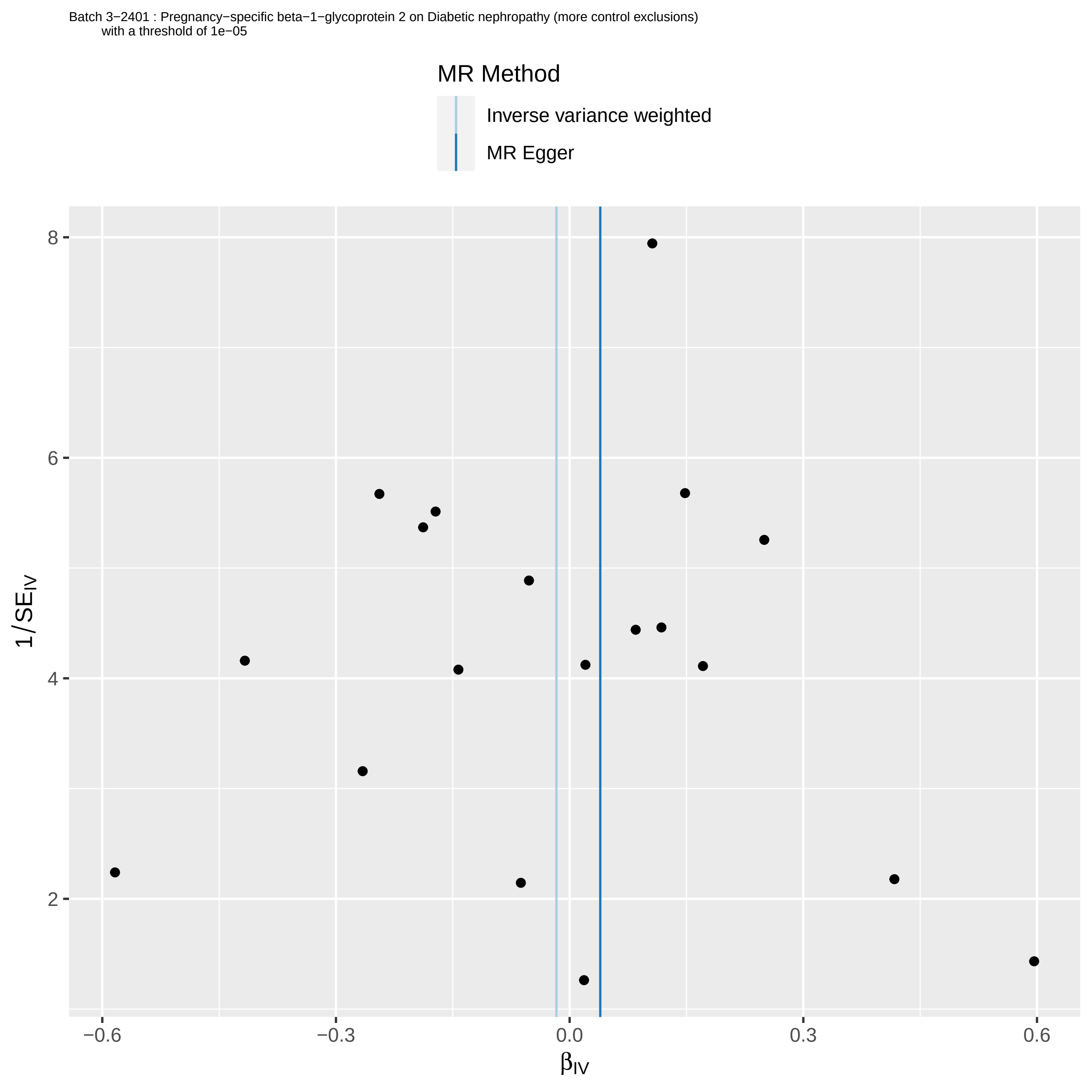
Batch 3−2401 : Pregnancy−specific beta−1−glycoprotein 2 on Diabetic nephropathy (more control exclusions)
with a threshold of 1e−05
MR Method
Inverse variance weighted
MR Egger
8
6
4
2
$1/SE_{IV}$
−0.6
−0.3
0.0
0.3
0.6
β_{IV}

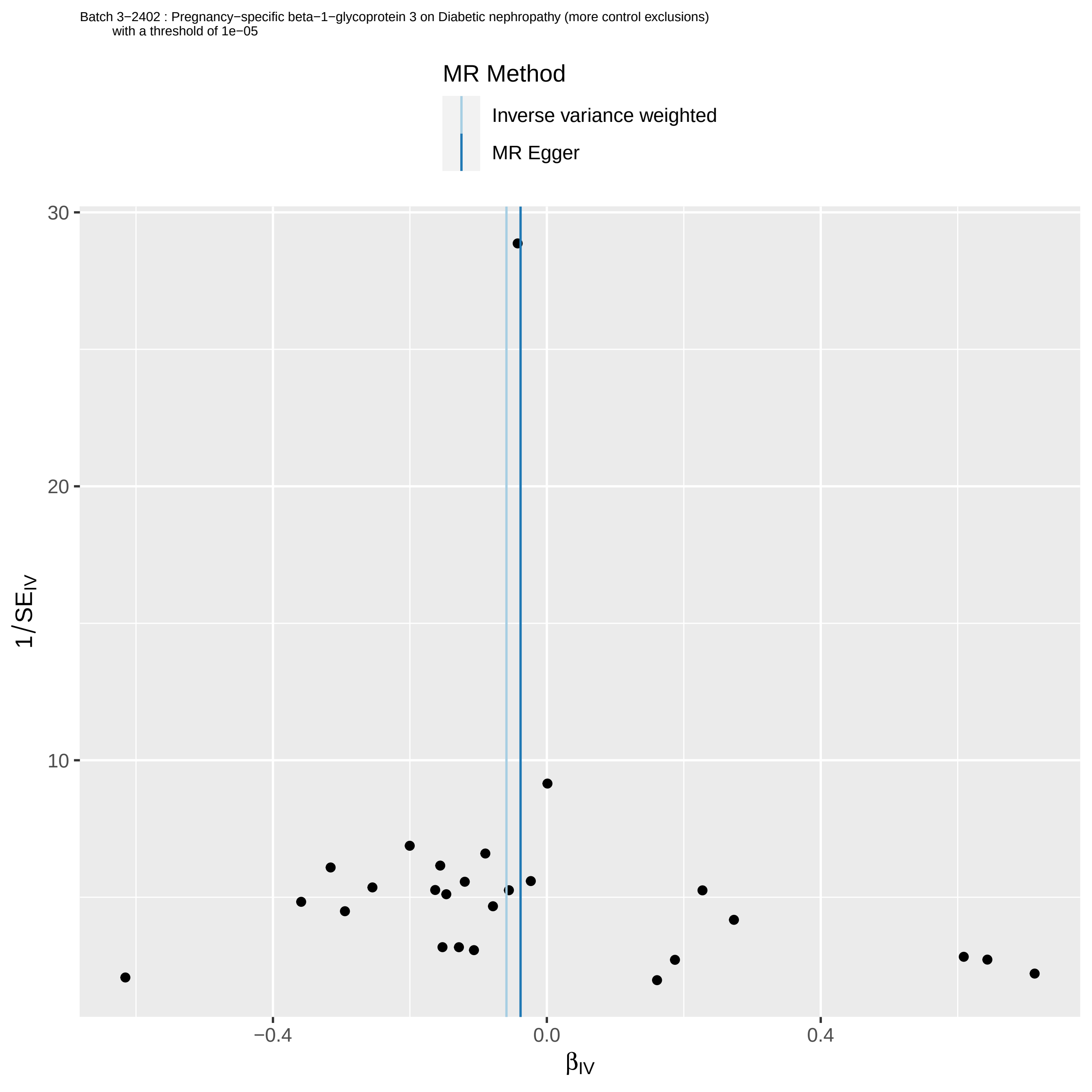
Batch 3−2402 : Pregnancy−specific beta−1−glycoprotein 3 on Diabetic nephropathy (more control exclusions)
with a threshold of 1e−05
MR Method
Inverse variance weighted
MR Egger
30
20
10
1/SE_IV
−0.4
0.0
0.4
β_IV

Batch 3−2403 : Pregnancy−specific beta−1−glycoprotein 4 on Diabetic nephropathy (more control exclusions)
with a threshold of 1e−05

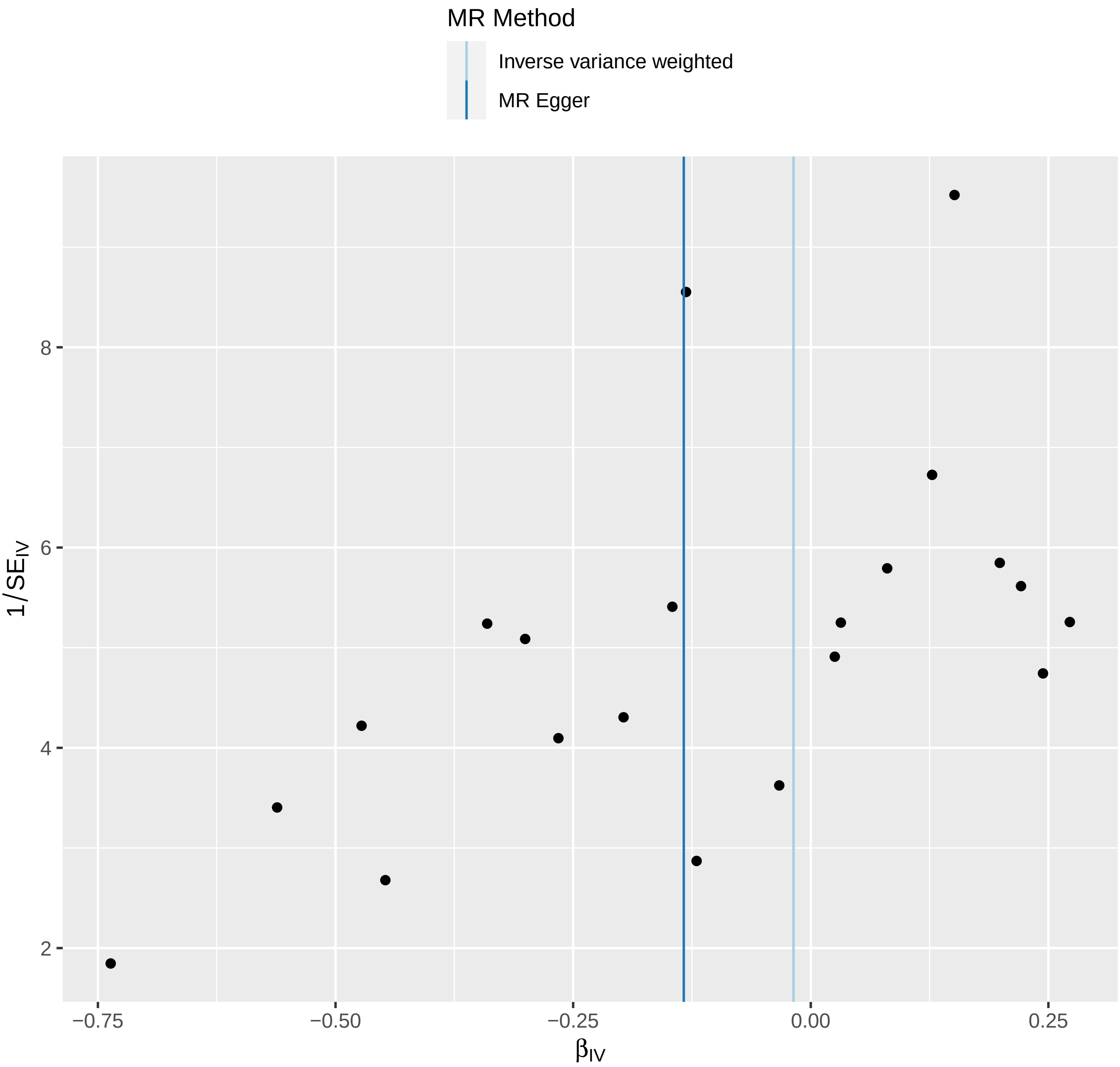

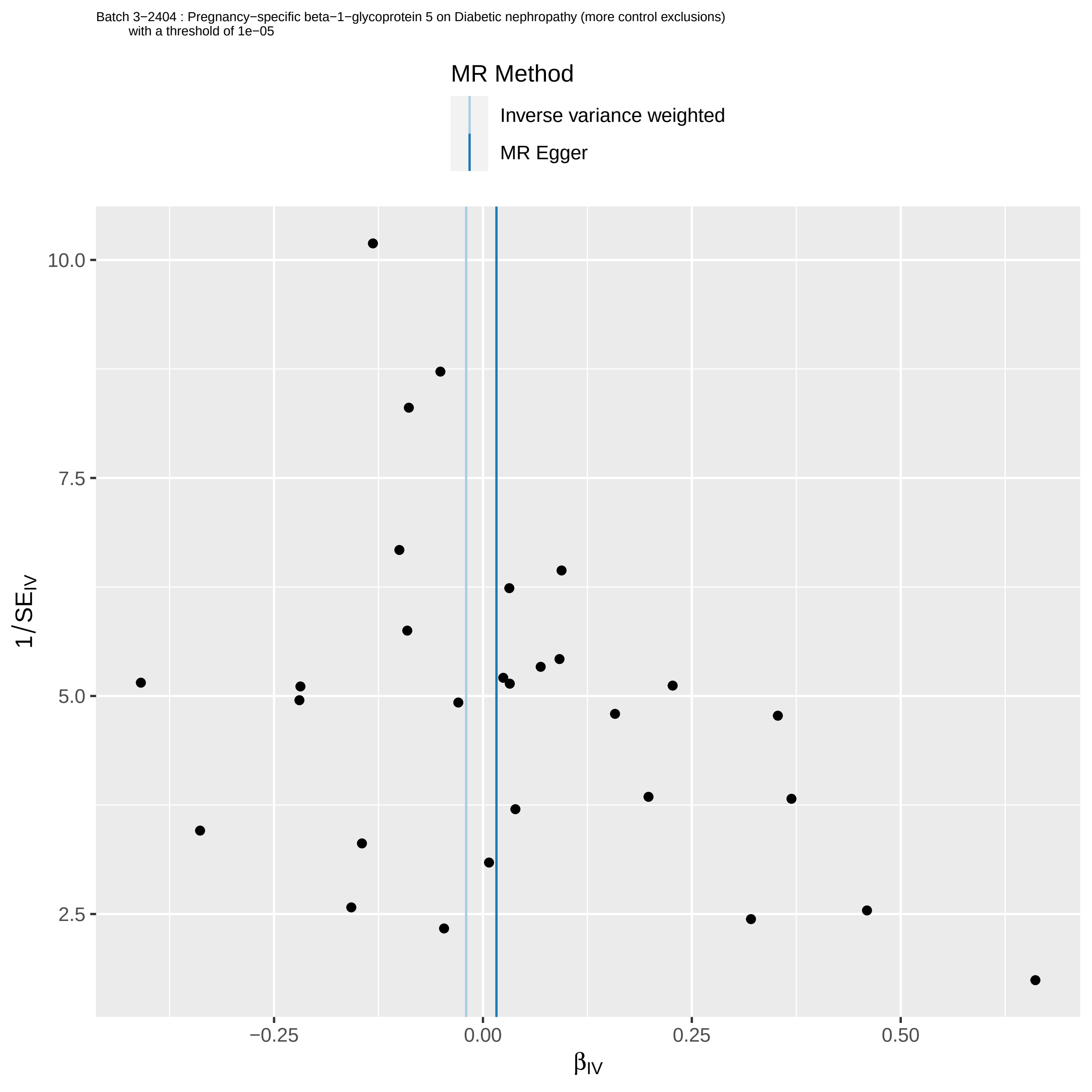

Batch 3−2404 : Pregnancy−specific beta−1−glycoprotein 5 on Diabetic nephropathy (more control exclusions)
with a threshold of 1e−05
MR Method
Inverse variance weighted
MR Egger
10.0
7.5
5.0
2.5
1/SE_IV
−0.25
0.00
0.25
0.50
β_IV

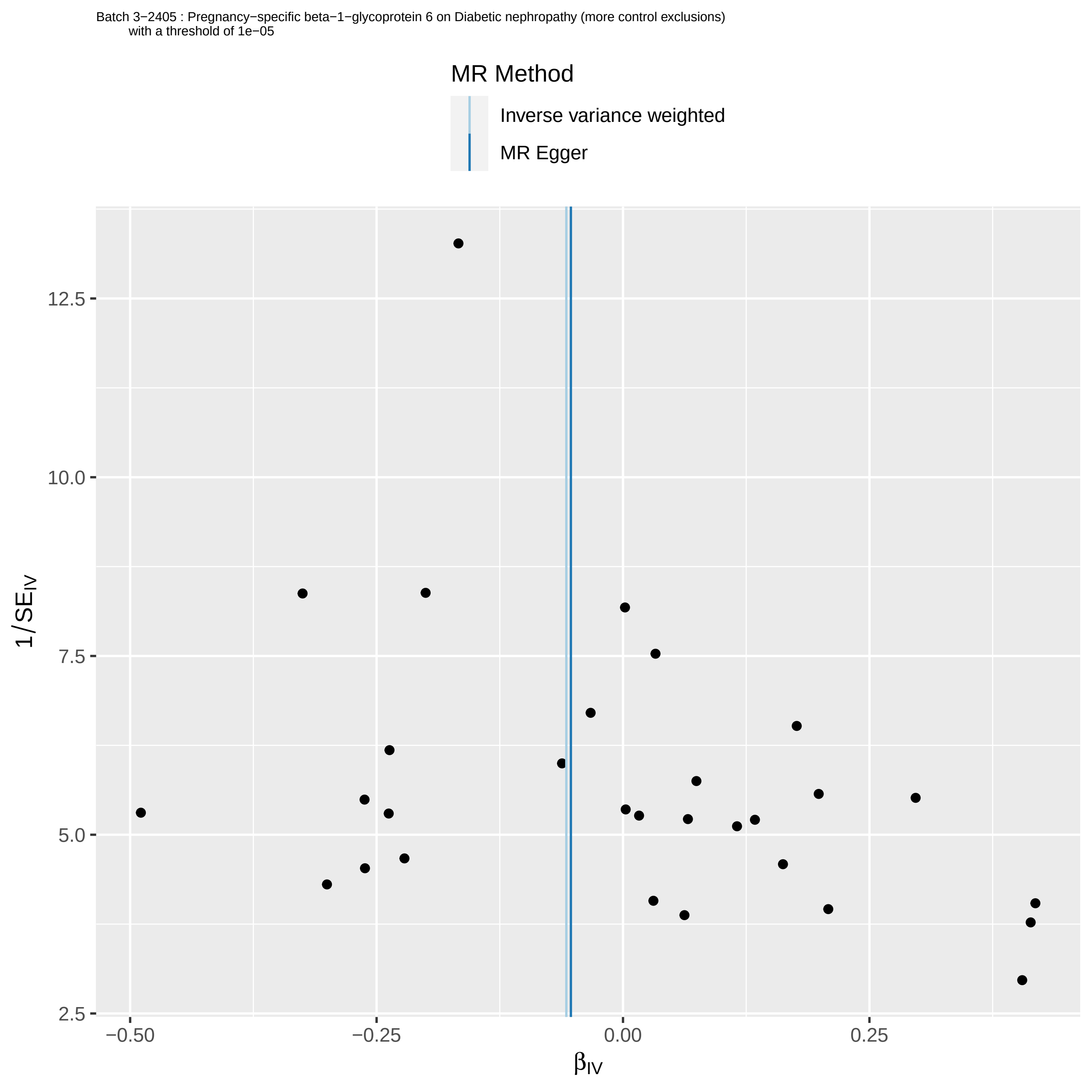

Batch 3−2405 : Pregnancy−specific beta−1−glycoprotein 6 on Diabetic nephropathy (more control exclusions)
with a threshold of 1e−05
MR Method
Inverse variance weighted
MR Egger
1/SE_IV
12.5
10.0
7.5
5.0
2.5
−0.50
−0.25
0.00
0.25
β_IV

Batch 3−2406 : Putative pregnancy−specific beta−1−glycoprotein 7 on Diabetic nephropathy (more control exclusions)
with a threshold of 1e−05

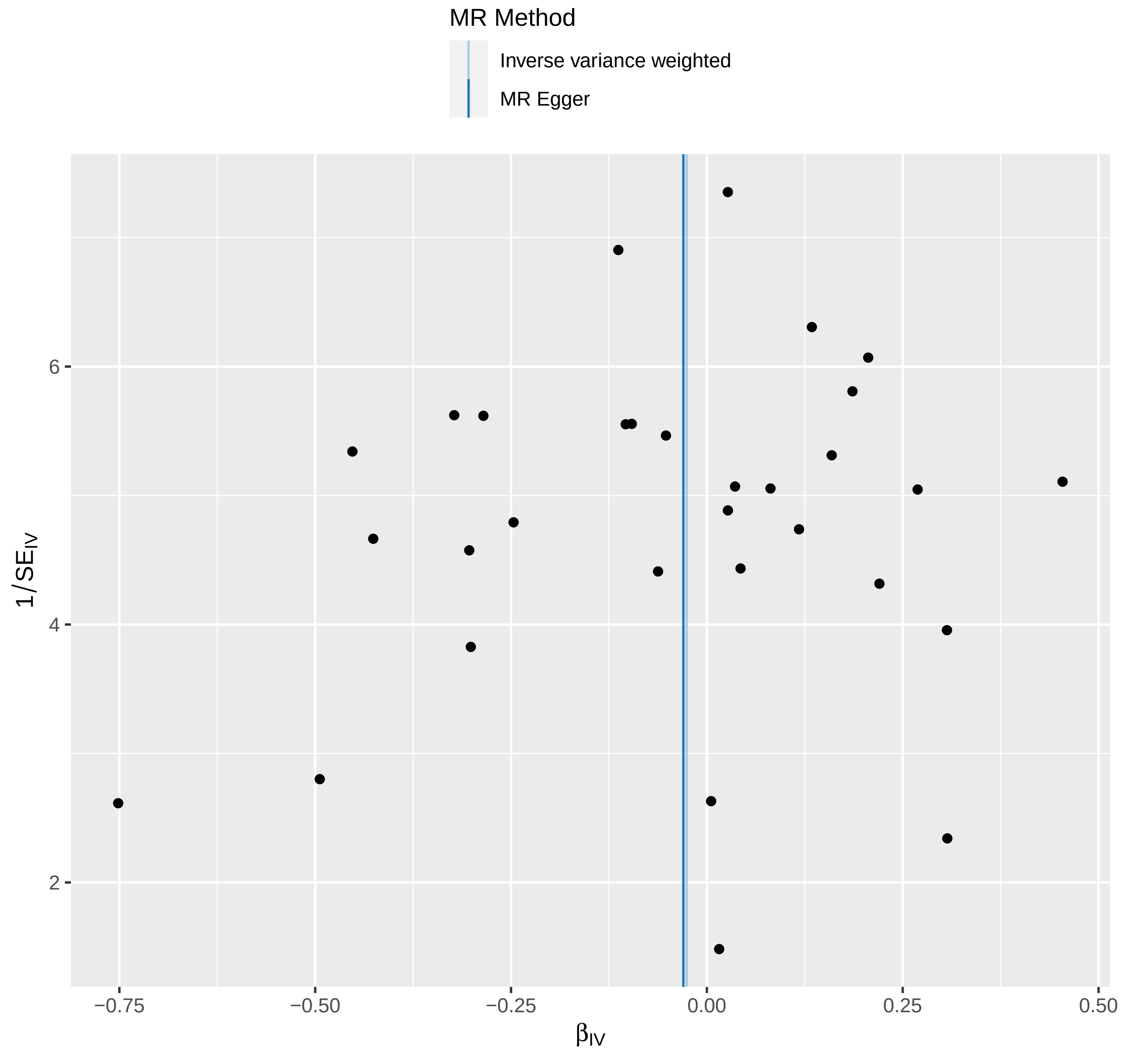

Batch 3−2407 : Pregnancy−specific beta−1−glycoprotein 8 on Diabetic nephropathy (more control exclusions)
with a threshold of 1e−05

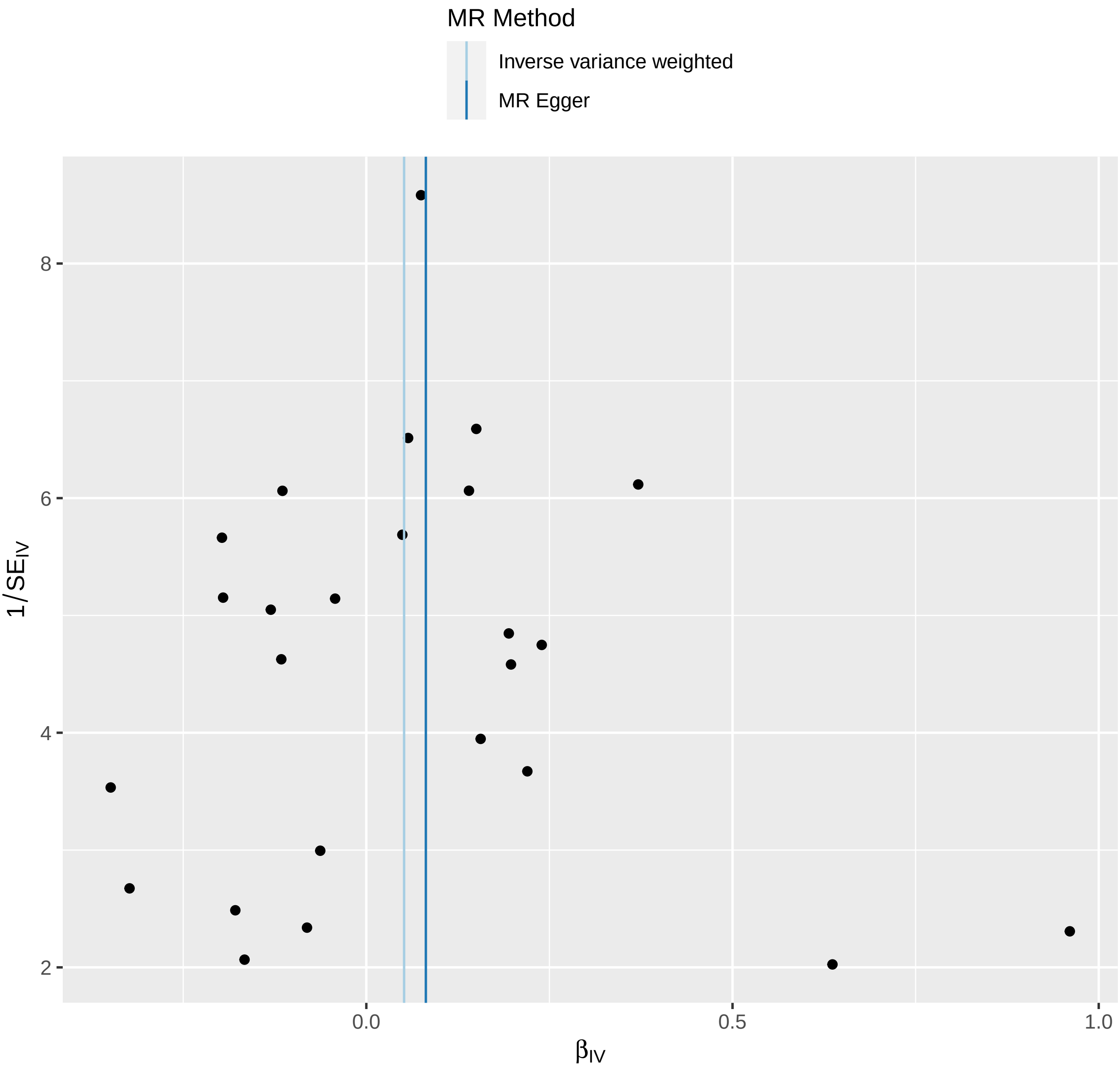

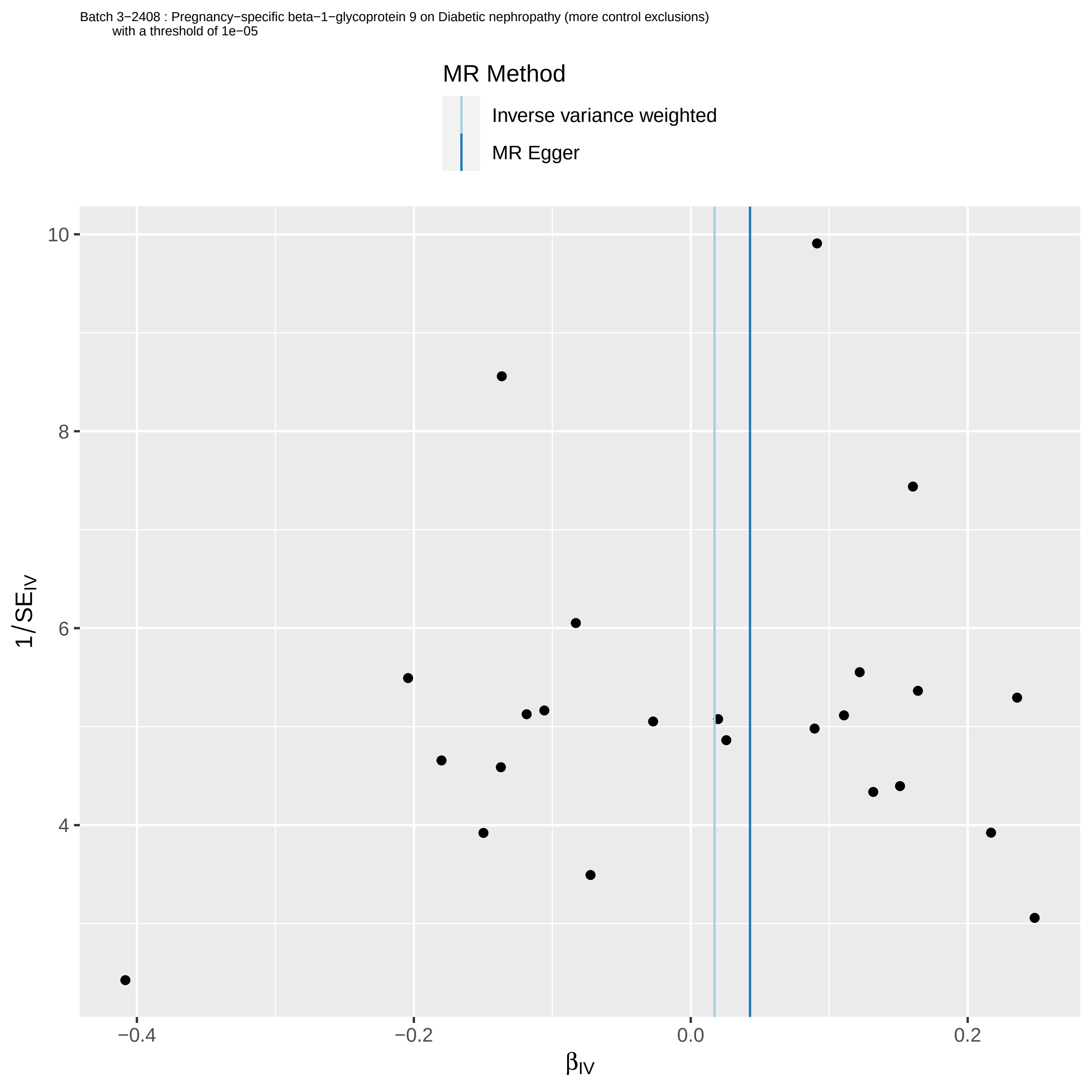
Batch 3−2408 : Pregnancy−specific beta−1−glycoprotein 9 on Diabetic nephropathy (more control exclusions)
with a threshold of 1e−05
MR Method
Inverse variance weighted
MR Egger
10
8
6
4
1/SE_IV
−0.4
−0.2
0.0
0.2
β_IV

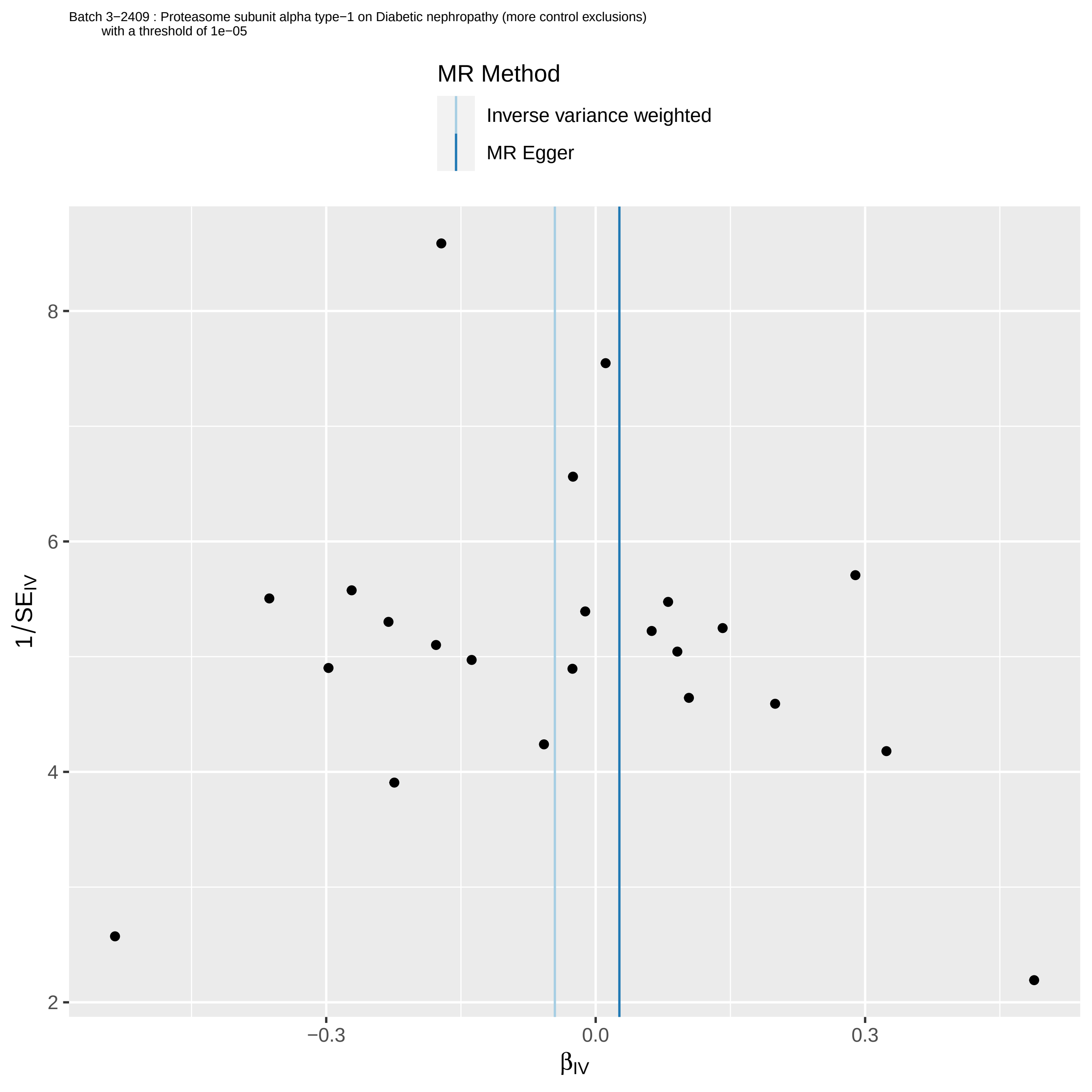

Batch 3−2409 : Proteasome subunit alpha type−1 on Diabetic nephropathy (more control exclusions)
with a threshold of 1e−05
MR Method
Inverse variance weighted
MR Egger
8
6
4
2
1/SE_IV
−0.3
0.0
0.3
β_IV

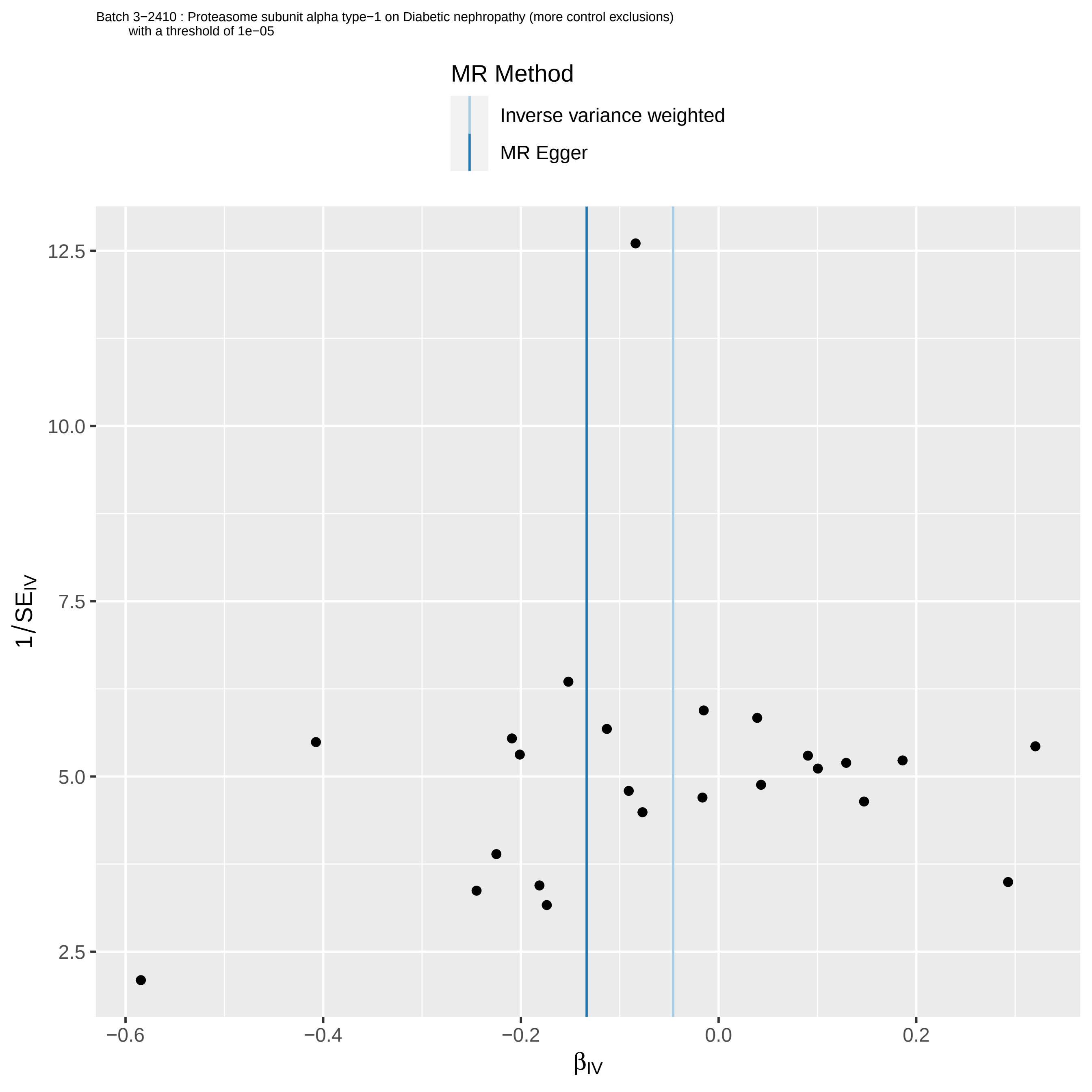
Batch 3−2410 : Proteasome subunit alpha type−1 on Diabetic nephropathy (more control exclusions)
with a threshold of 1e−05
MR Method
Inverse variance weighted
MR Egger
12.5
10.0
7.5
5.0
2.5
1/SE$_{IV}$
−0.6
−0.4
−0.2
0.0
0.2
β$_{IV}$

Batch 3−2411 : Proteasome subunit alpha type−2 on Diabetic nephropathy (more control exclusions)
with a threshold of 1e−05

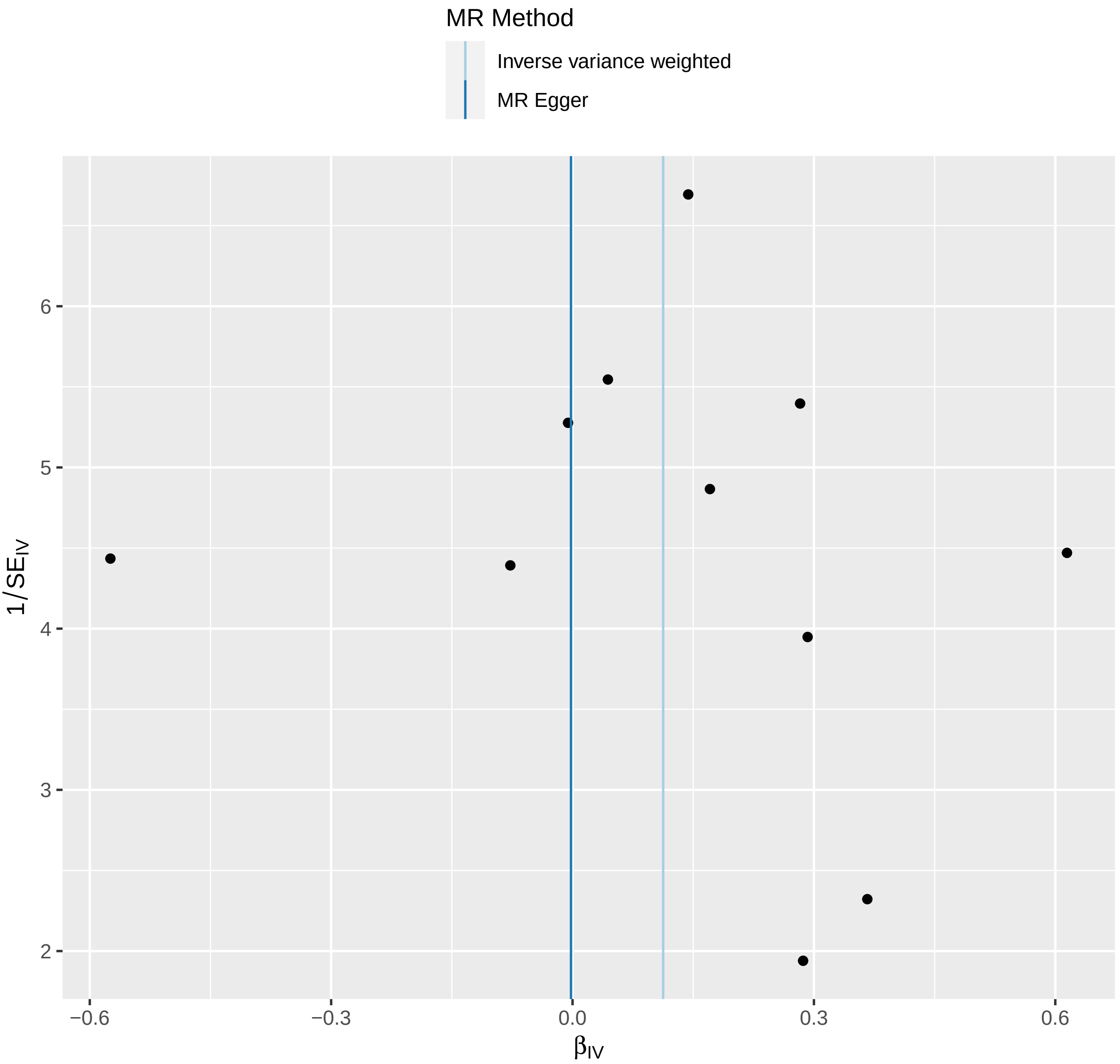

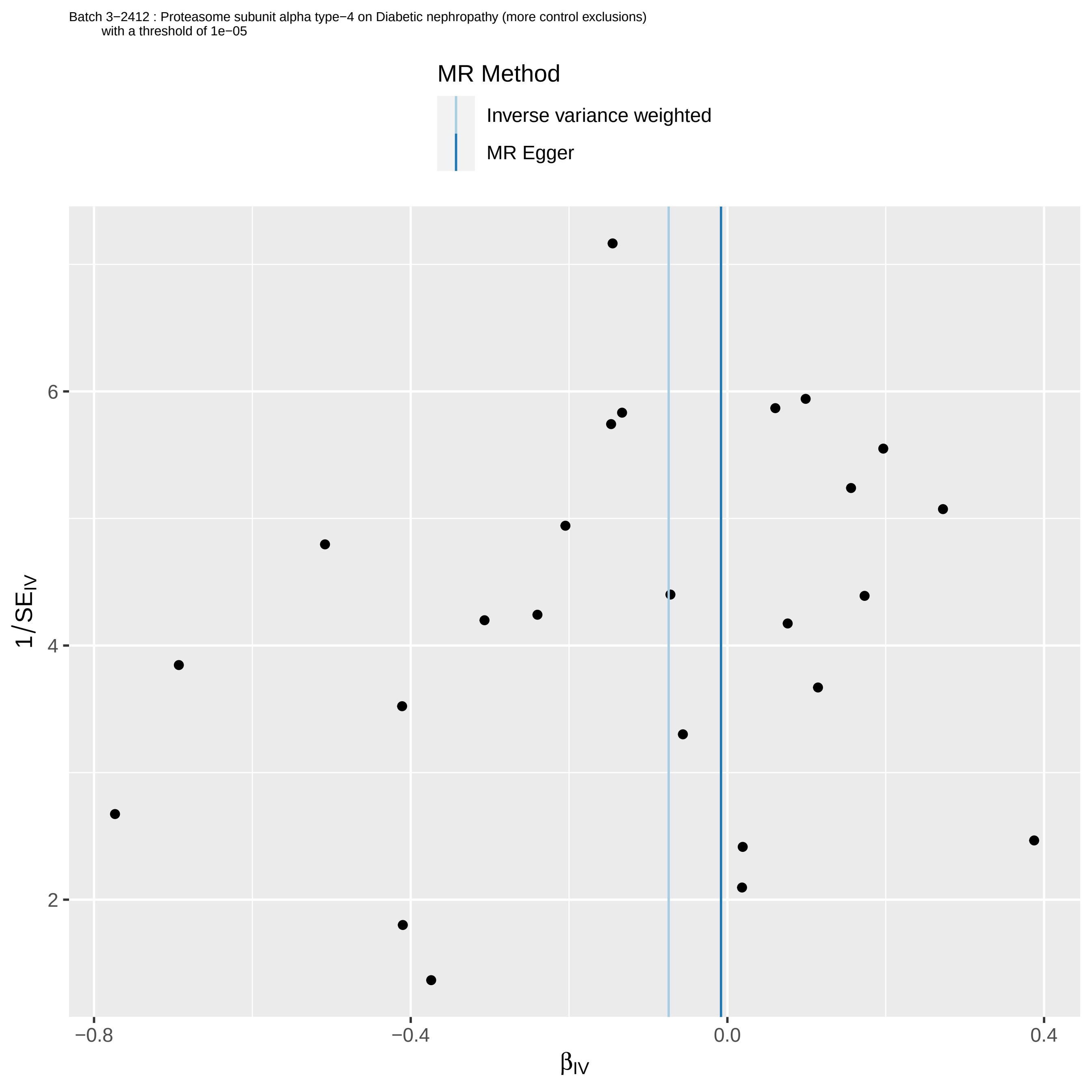

Batch 3−2412 : Proteasome subunit alpha type−4 on Diabetic nephropathy (more control exclusions)
with a threshold of 1e−05
MR Method
Inverse variance weighted
MR Egger
6
4
2
1/SE_IV
−0.8
−0.4
0.0
0.4
β_IV

Batch 3−2413 : Proteasome subunit alpha type−6 on Diabetic nephropathy (more control exclusions)
with a threshold of 1e−05

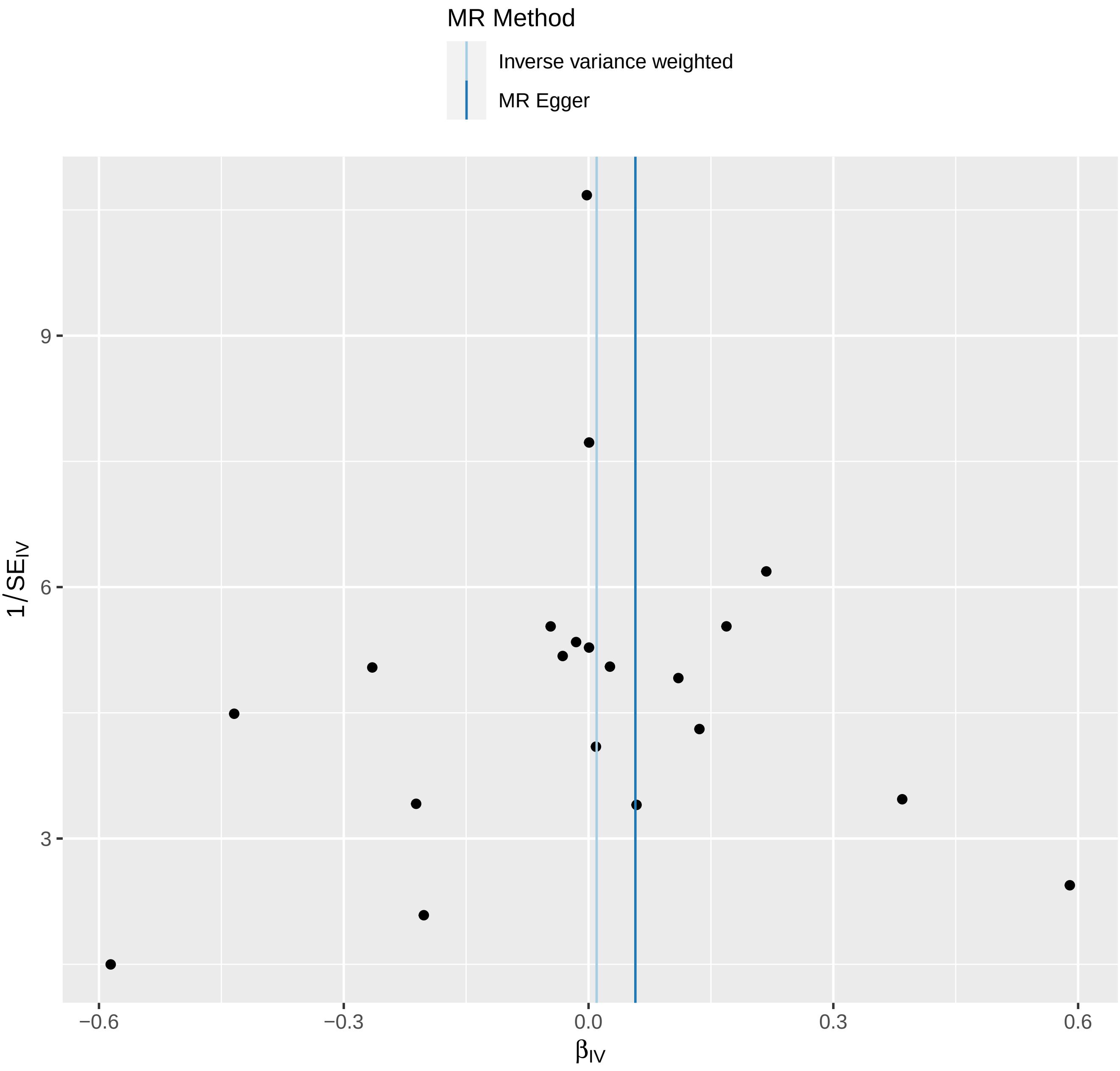

Batch 3−2414 : Proteasome subunit beta type−5 on Diabetic nephropathy (more control exclusions)
with a threshold of 1e−05

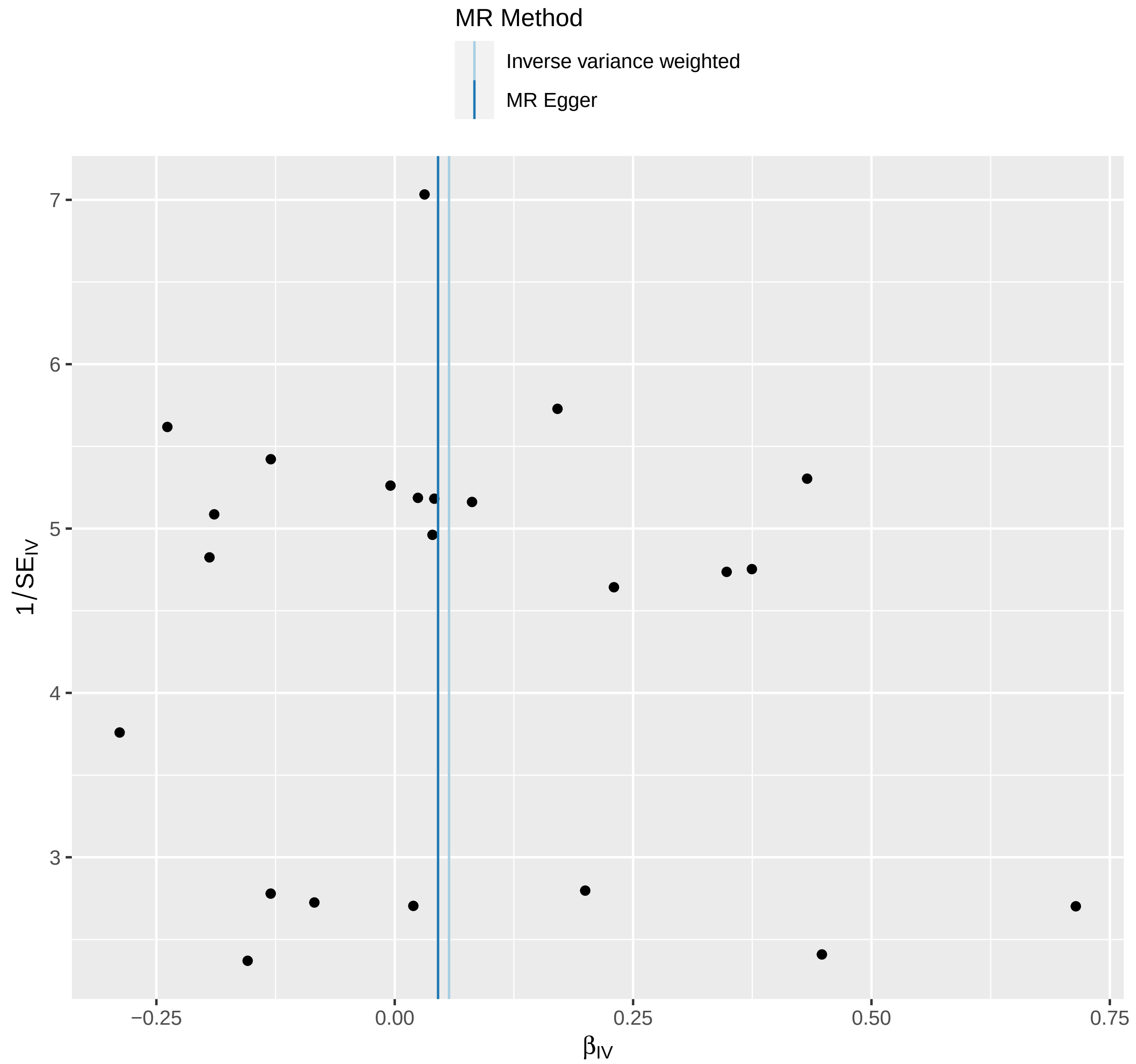

Batch 3−2415 : 26S proteasome non−ATPase regulatory subunit 11 on Diabetic nephropathy (more control exclusions) with a threshold of 1e−05

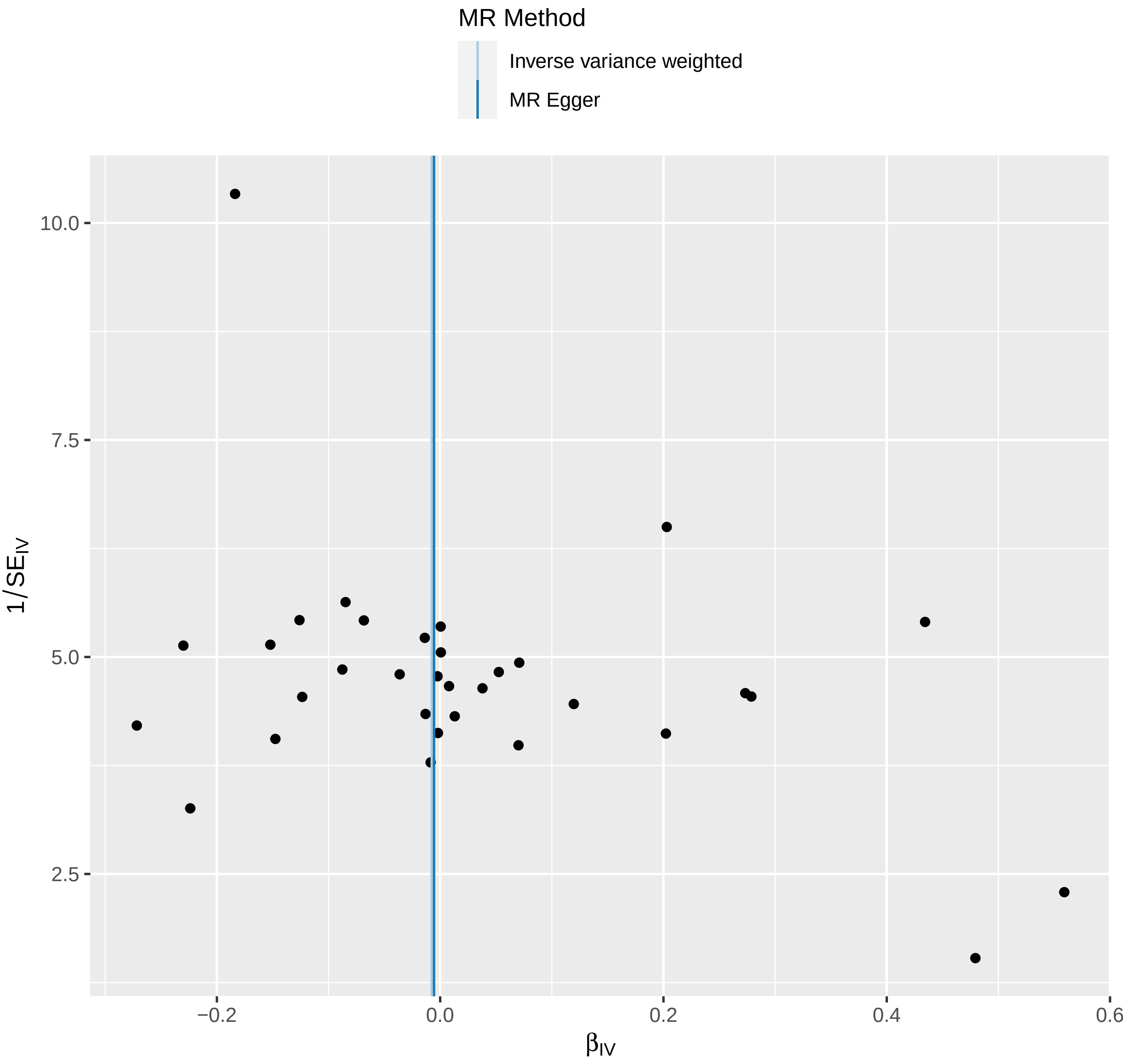

Batch 3−2416 : 26S proteasome non−ATPase regulatory subunit 1 on Diabetic nephropathy (more control exclusions)
with a threshold of 1e−05

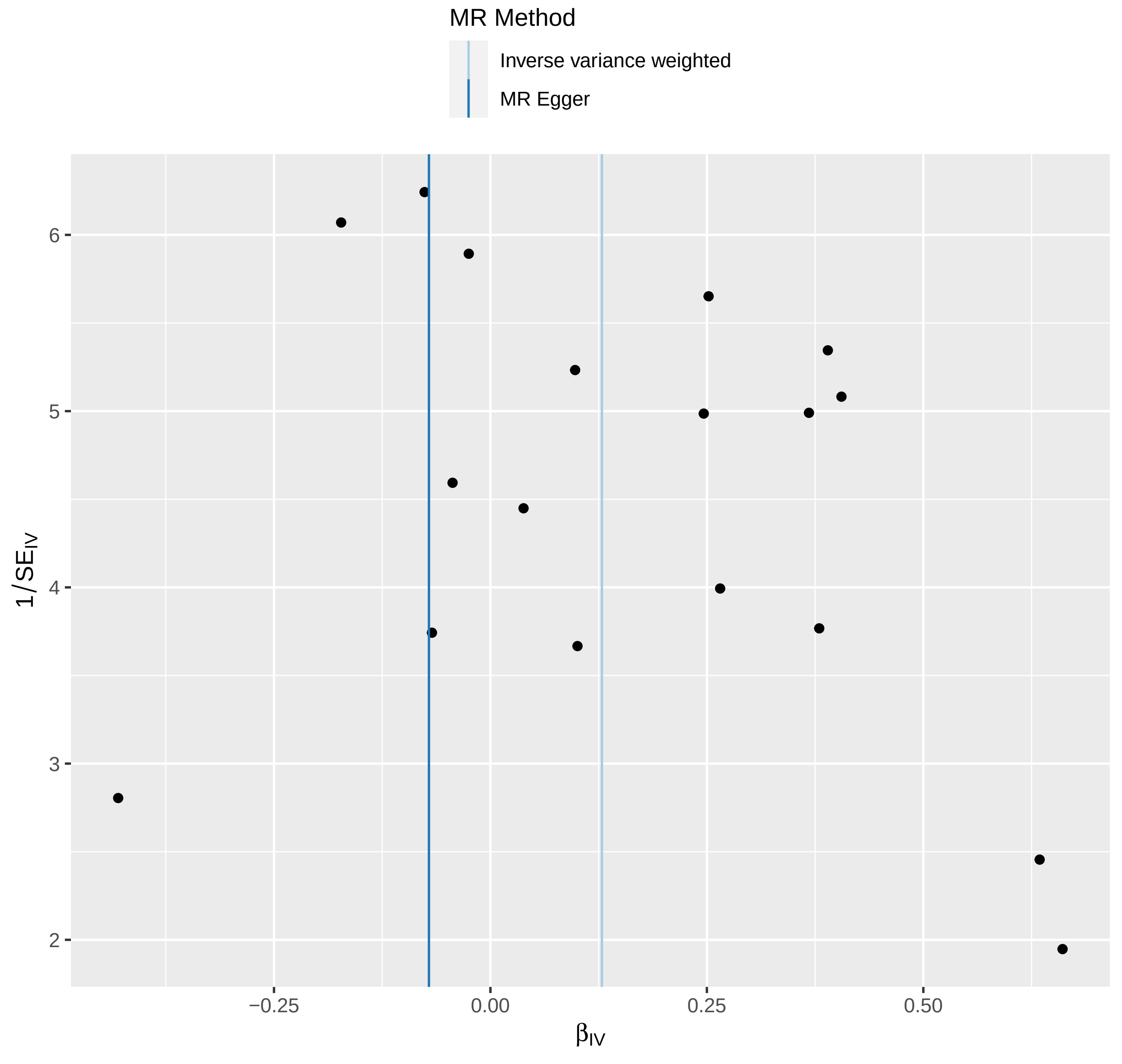

Batch 3−2417 : 26S proteasome non−ATPase regulatory subunit 4 on Diabetic nephropathy (more control exclusions)
with a threshold of 1e−05

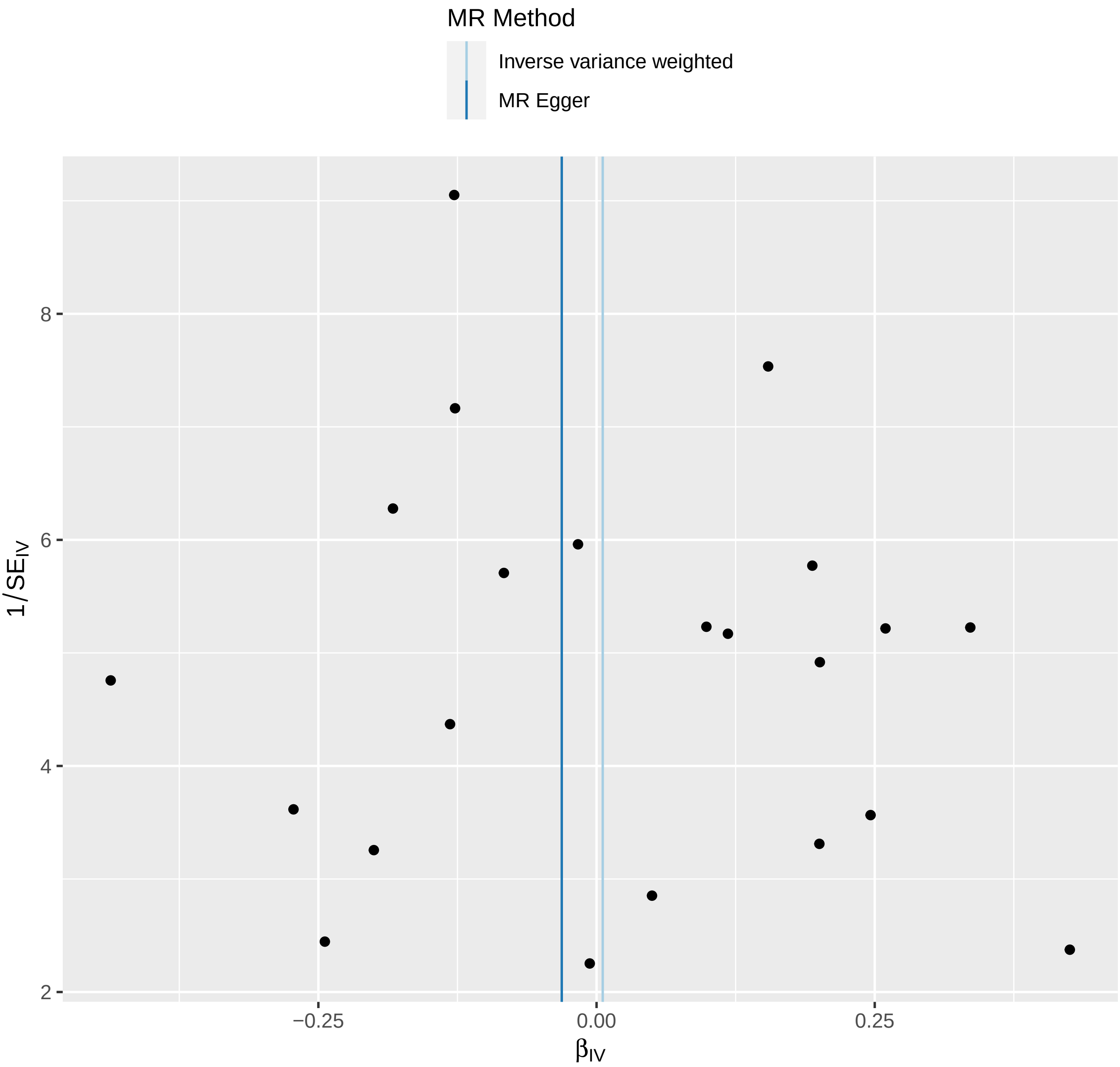

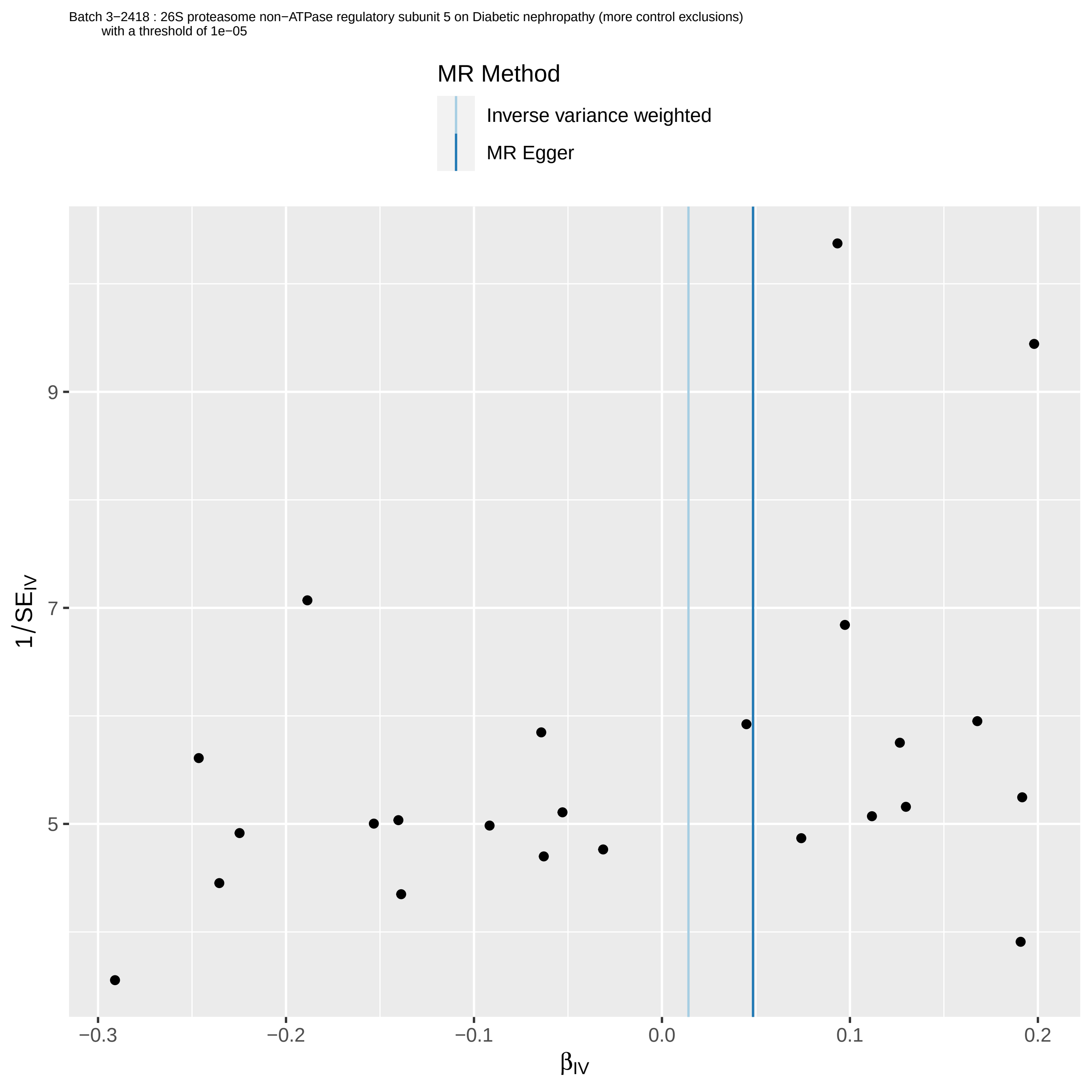

Batch 3−2418 : 26S proteasome non−ATPase regulatory subunit 5 on Diabetic nephropathy (more control exclusions)
with a threshold of 1e−05
MR Method
Inverse variance weighted
MR Egger
9
7
5
1/SE_IV
−0.3
−0.2
−0.1
0.0
0.1
0.2
β_IV

Batch 3−2419 : 26S proteasome non−ATPase regulatory subunit 7 on Diabetic nephropathy (more control exclusions)
with a threshold of 1e−05

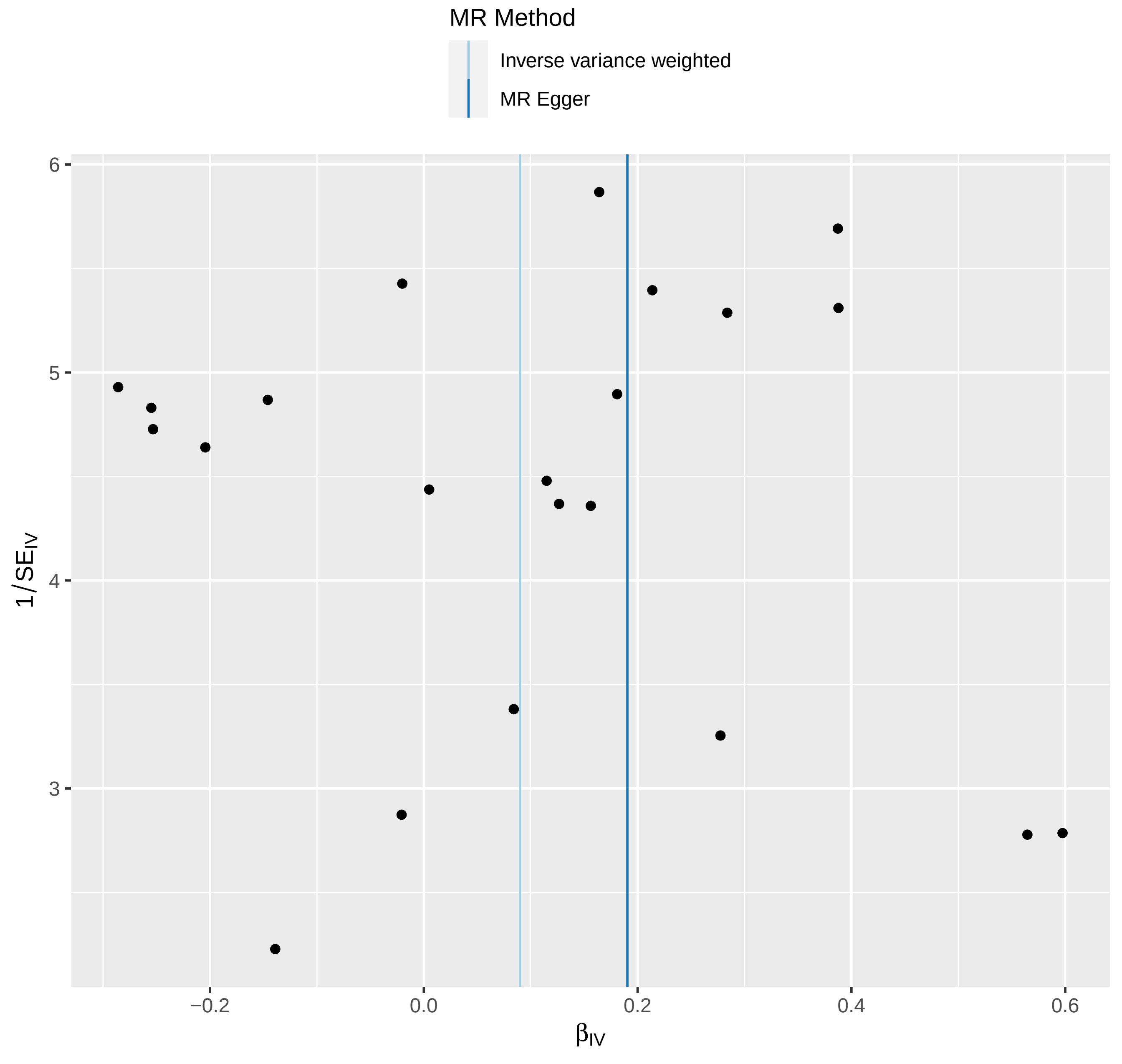

Batch 3−2420 : Proteasome activator complex subunit 1 on Diabetic nephropathy (more control exclusions)
with a threshold of 1e−05

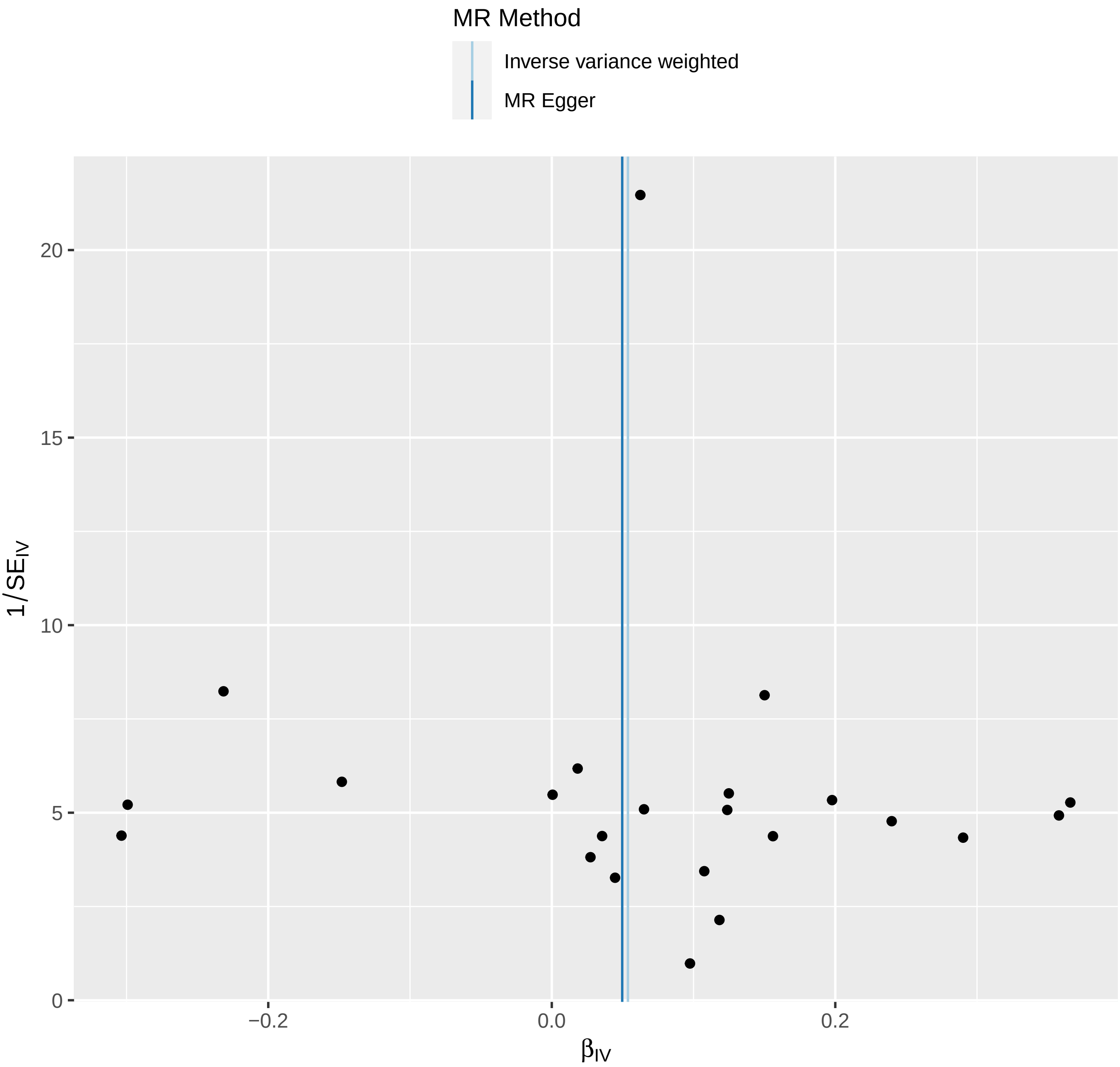

Batch 3−2421 : Proteasome activator complex subunit 3 on Diabetic nephropathy (more control exclusions) with a threshold of 1e−05

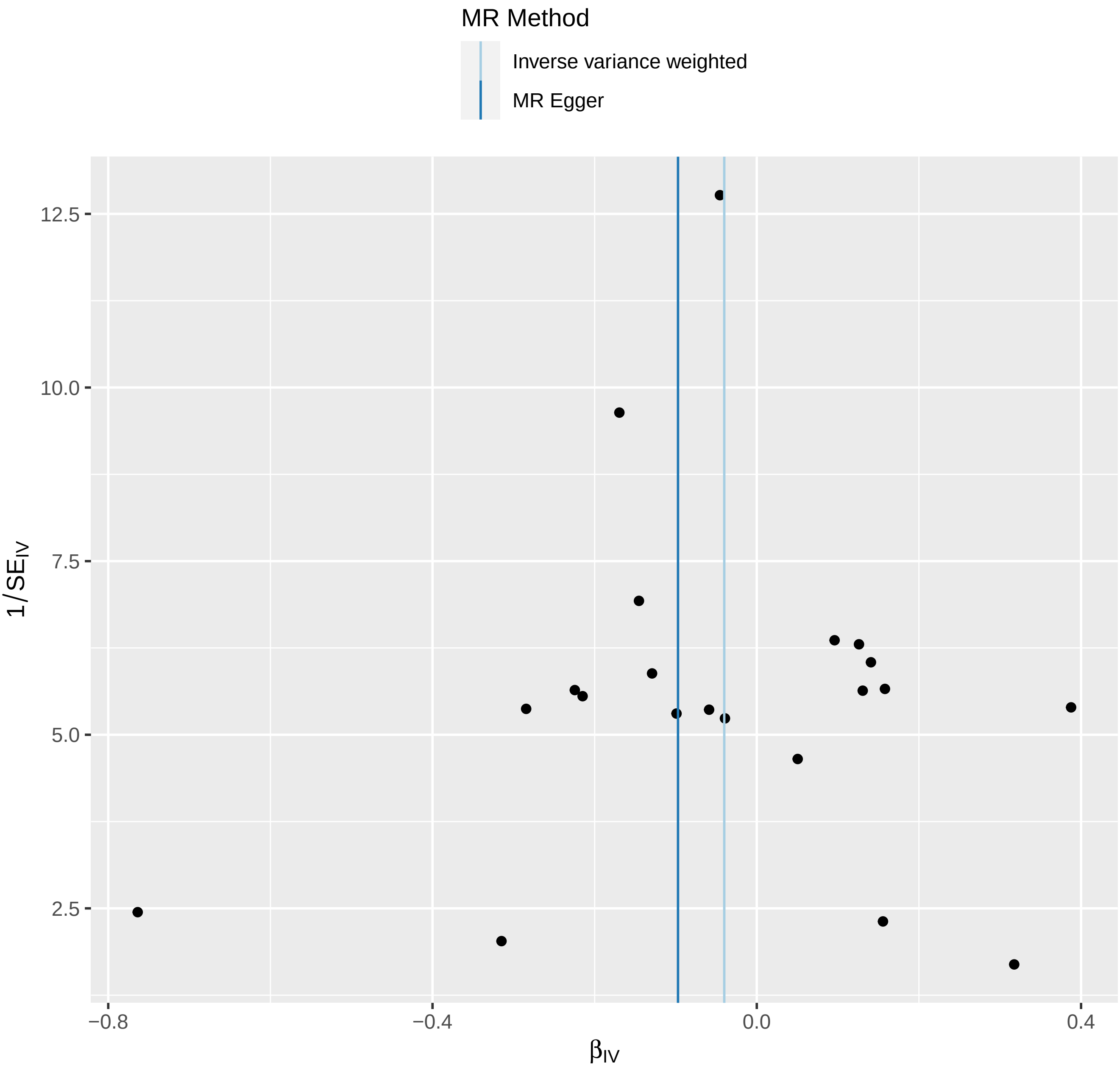

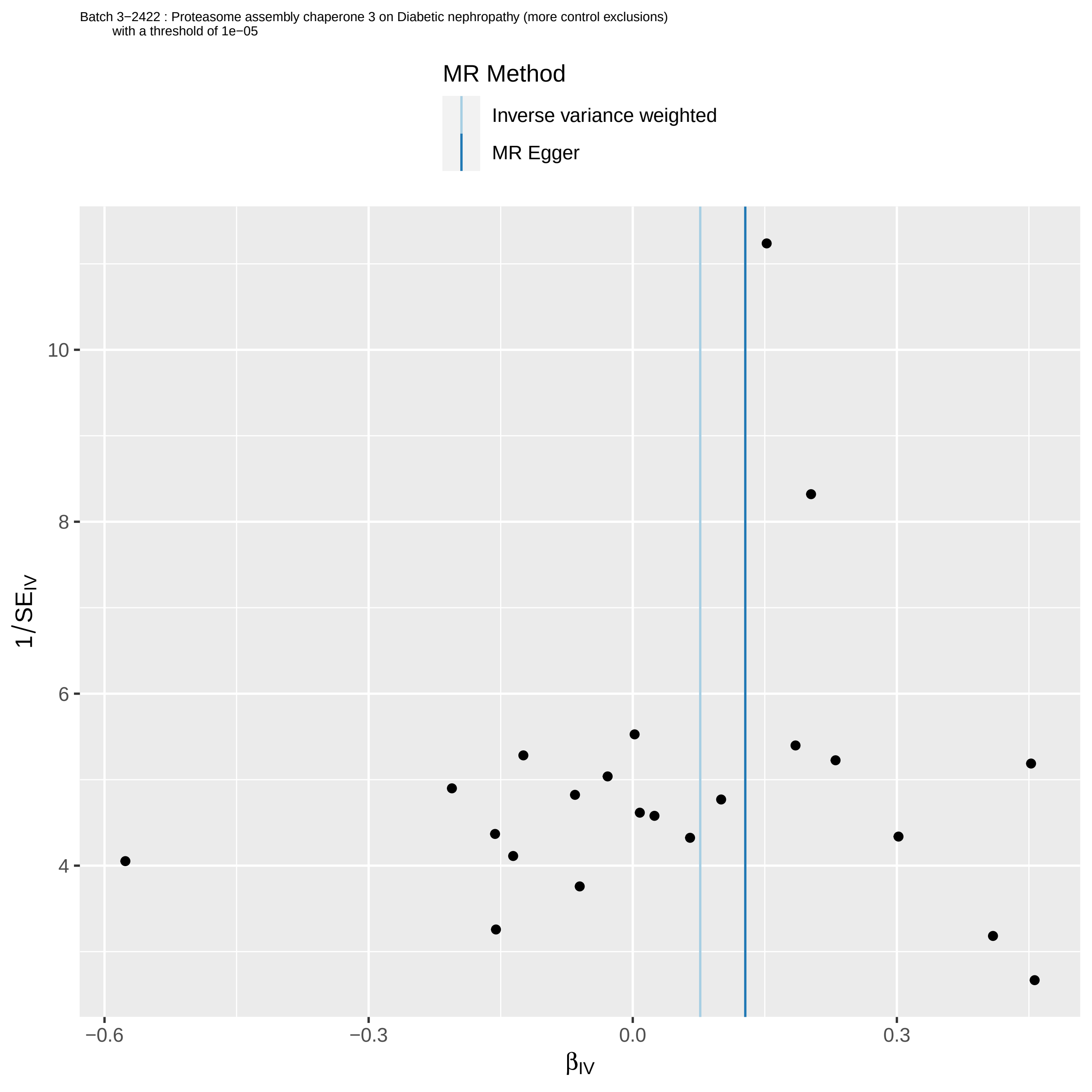

Batch 3−2422 : Proteasome assembly chaperone 3 on Diabetic nephropathy (more control exclusions)
with a threshold of 1e−05
MR Method
Inverse variance weighted
MR Egger
10
8
6
4
1/SE_IV
−0.6
−0.3
0.0
0.3
β_IV

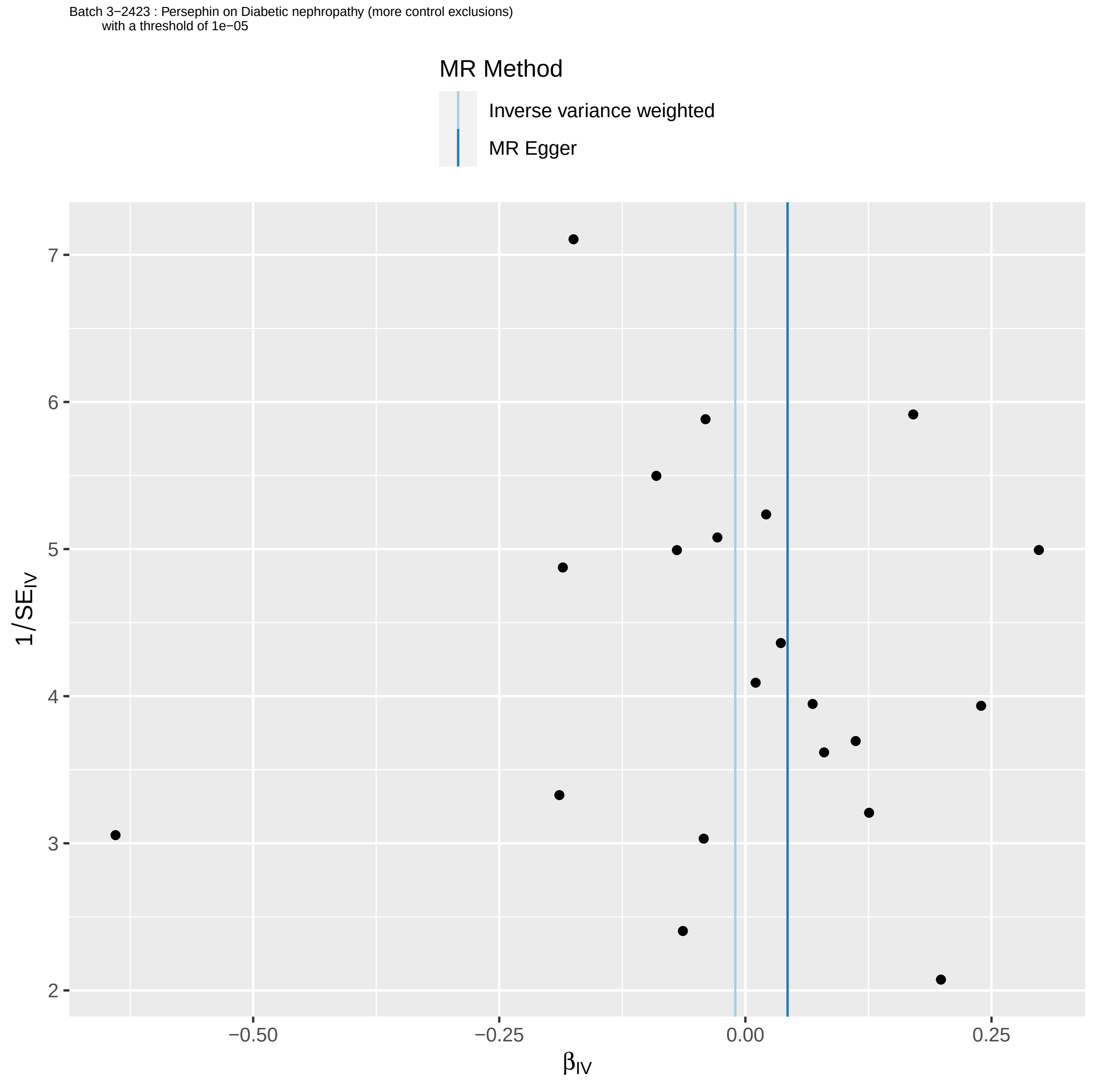
Batch 3−2423 : Persephin on Diabetic nephropathy (more control exclusions)
with a threshold of 1e−05
MR Method
Inverse variance weighted
MR Egger
7
6
5
4
3
2
1/SE_IV
−0.50
−0.25
0.00
0.25
β_IV

Batch 3−2424 : Patched domain−containing protein 3 on Diabetic nephropathy (more control exclusions) with a threshold of 1e−05

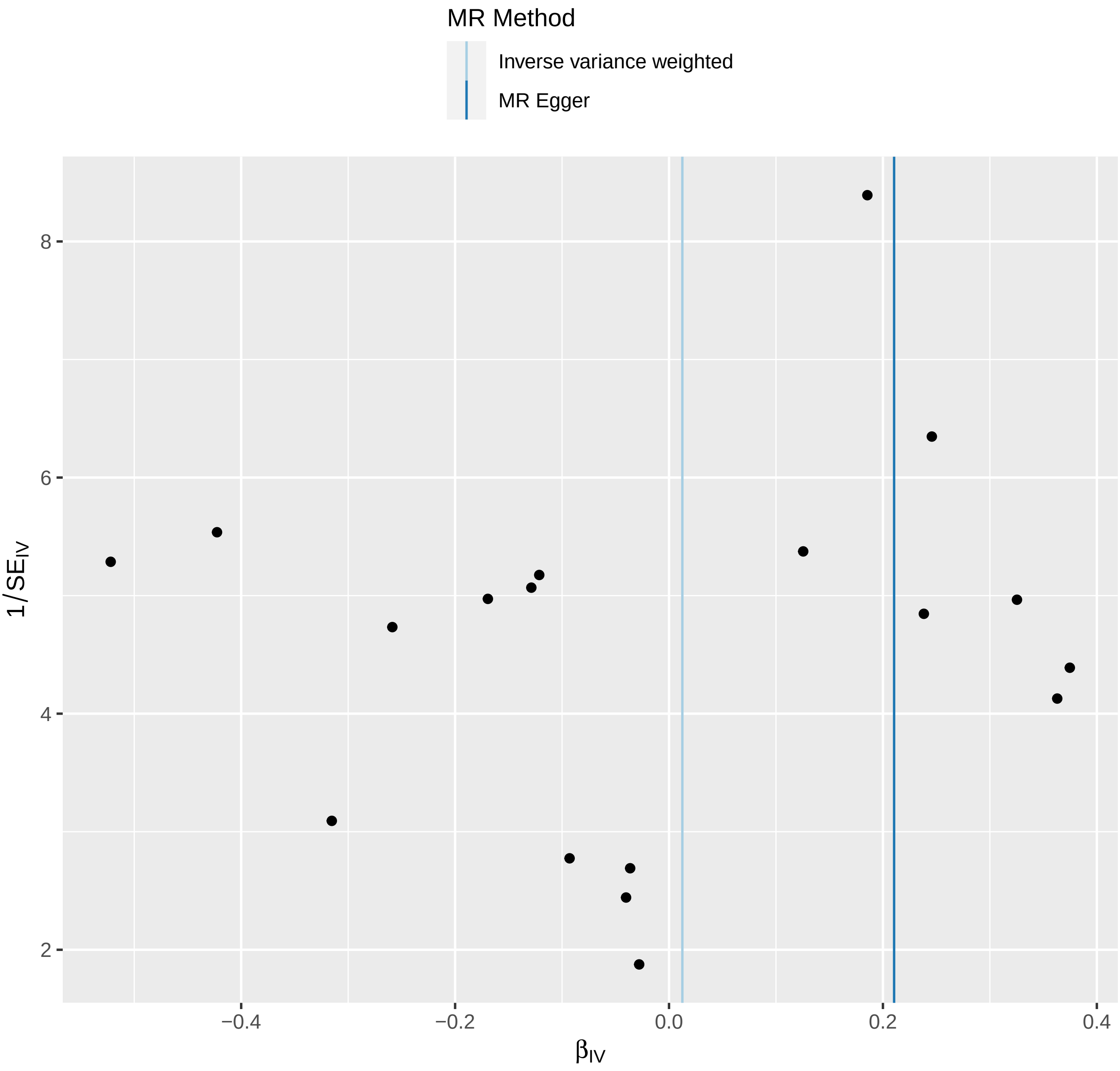

Batch 3−2425 : Phosphatidylinositol 3,4,5−trisphosphate 3−phosphatase and dual−specificity protein phosphatase PTEN on Diabetic nephropathy (more control exclusions) with a threshold of 1e−05

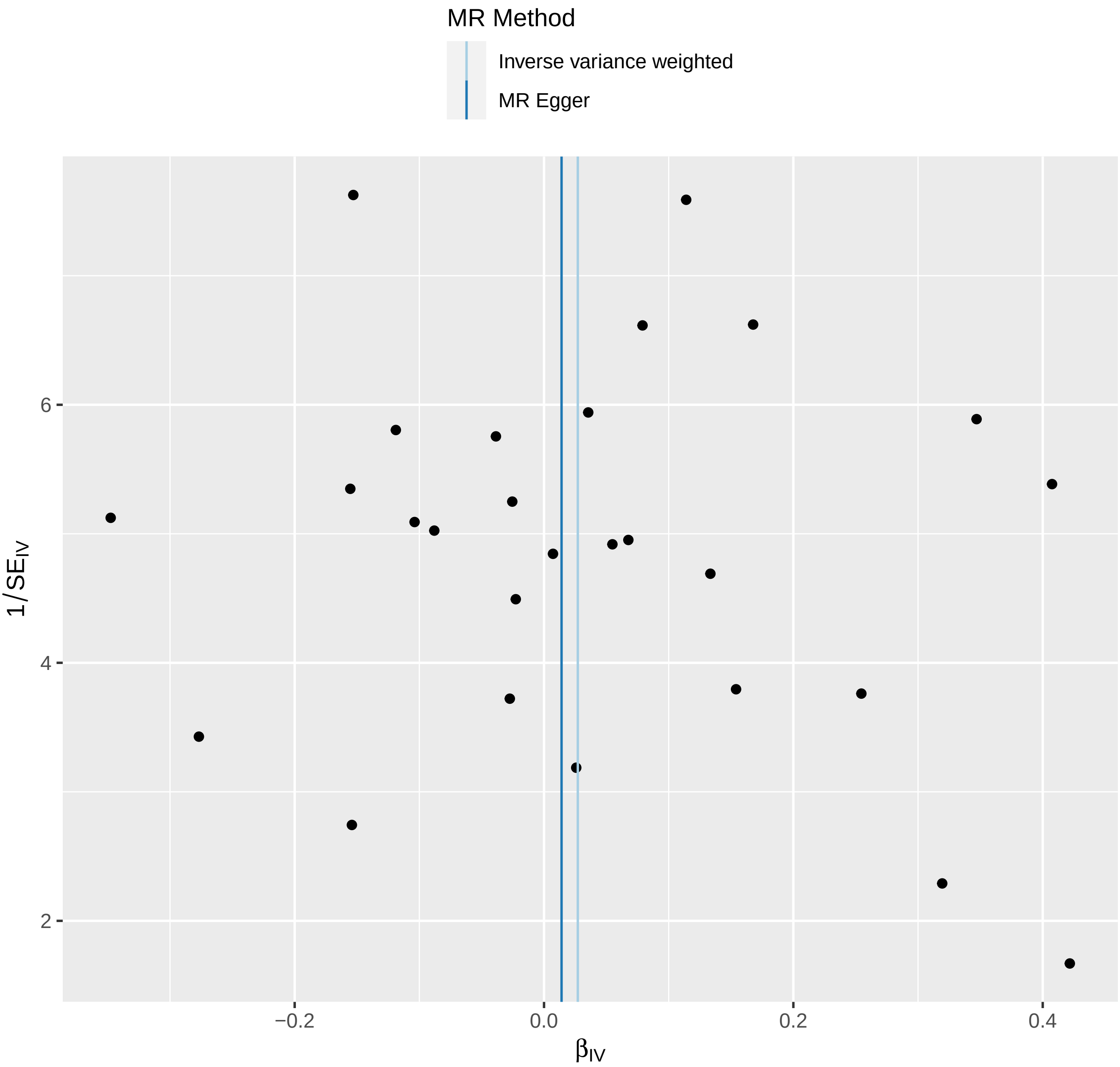

Batch 3−2426 : Prostaglandin−H2 D−isomerase on Diabetic nephropathy (more control exclusions)
with a threshold of 1e−05

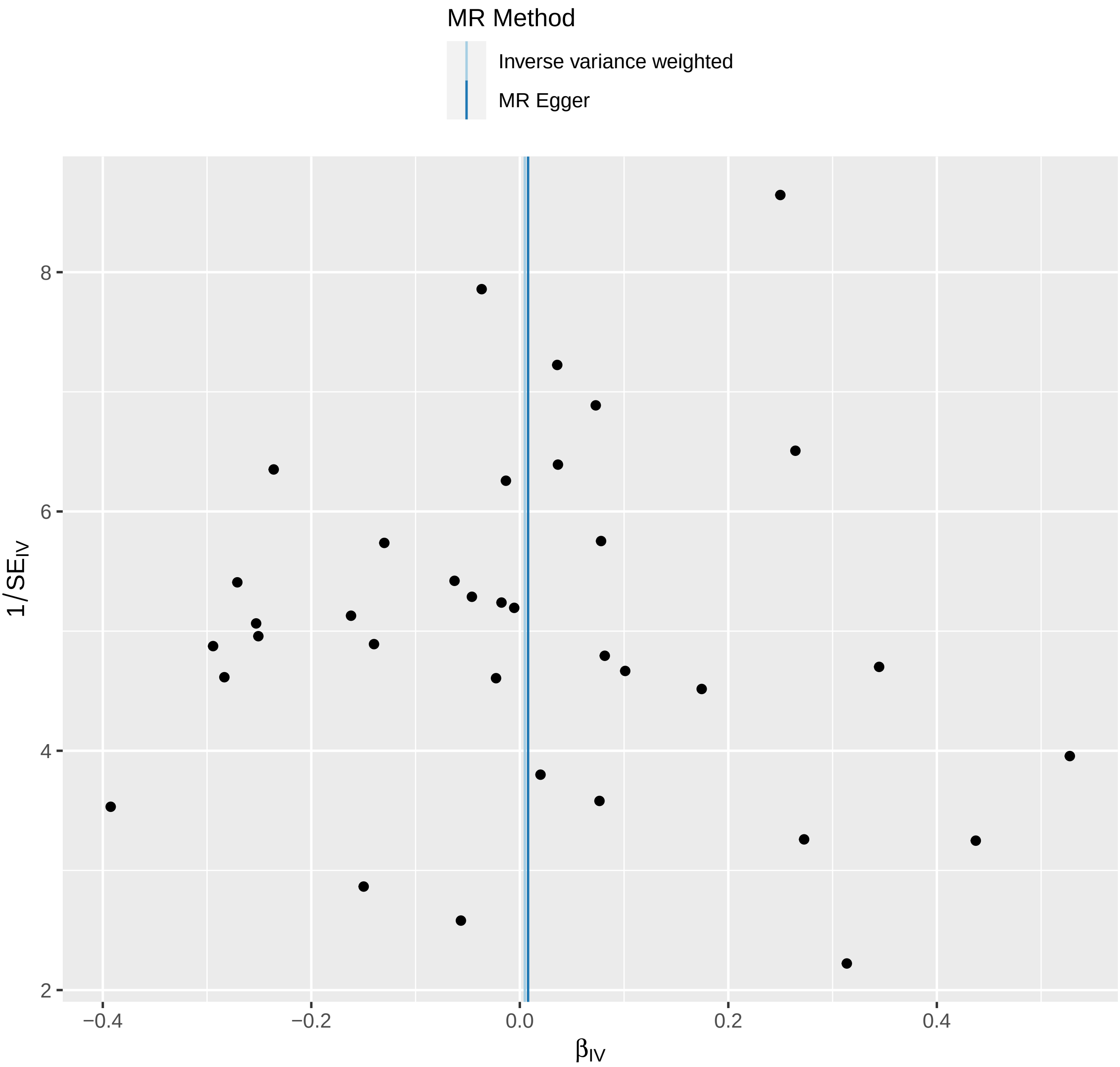

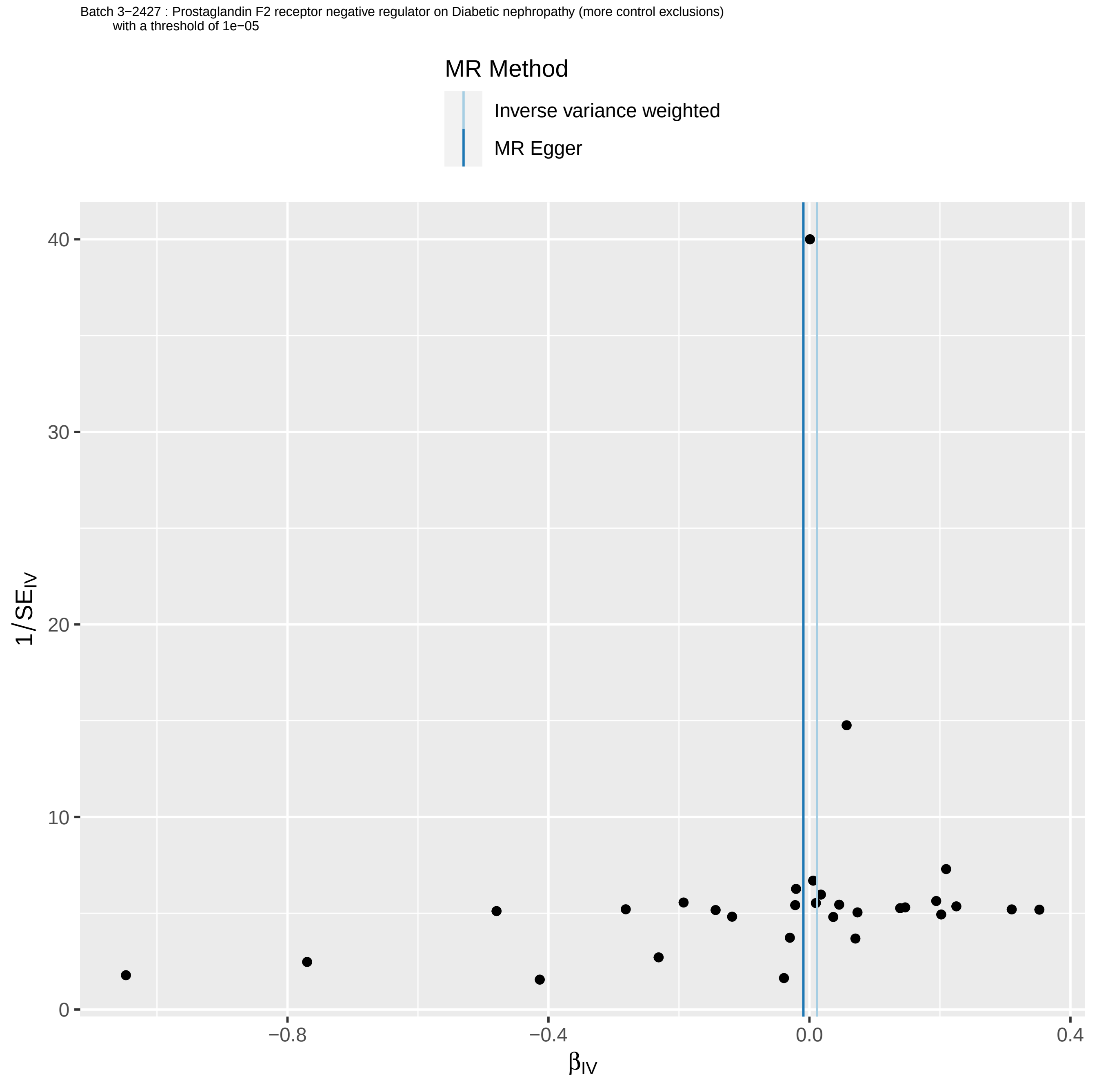
Batch 3−2427 : Prostaglandin F2 receptor negative regulator on Diabetic nephropathy (more control exclusions)
with a threshold of 1e−05
MR Method
Inverse variance weighted
MR Egger
40
30
20
10
0
1/SE$_{IV}$
−0.8
−0.4
0.0
0.4
β$_{IV}$

Batch 3−2428 : Prostaglandin reductase 1 on Diabetic nephropathy (more control exclusions)
with a threshold of 1e−05

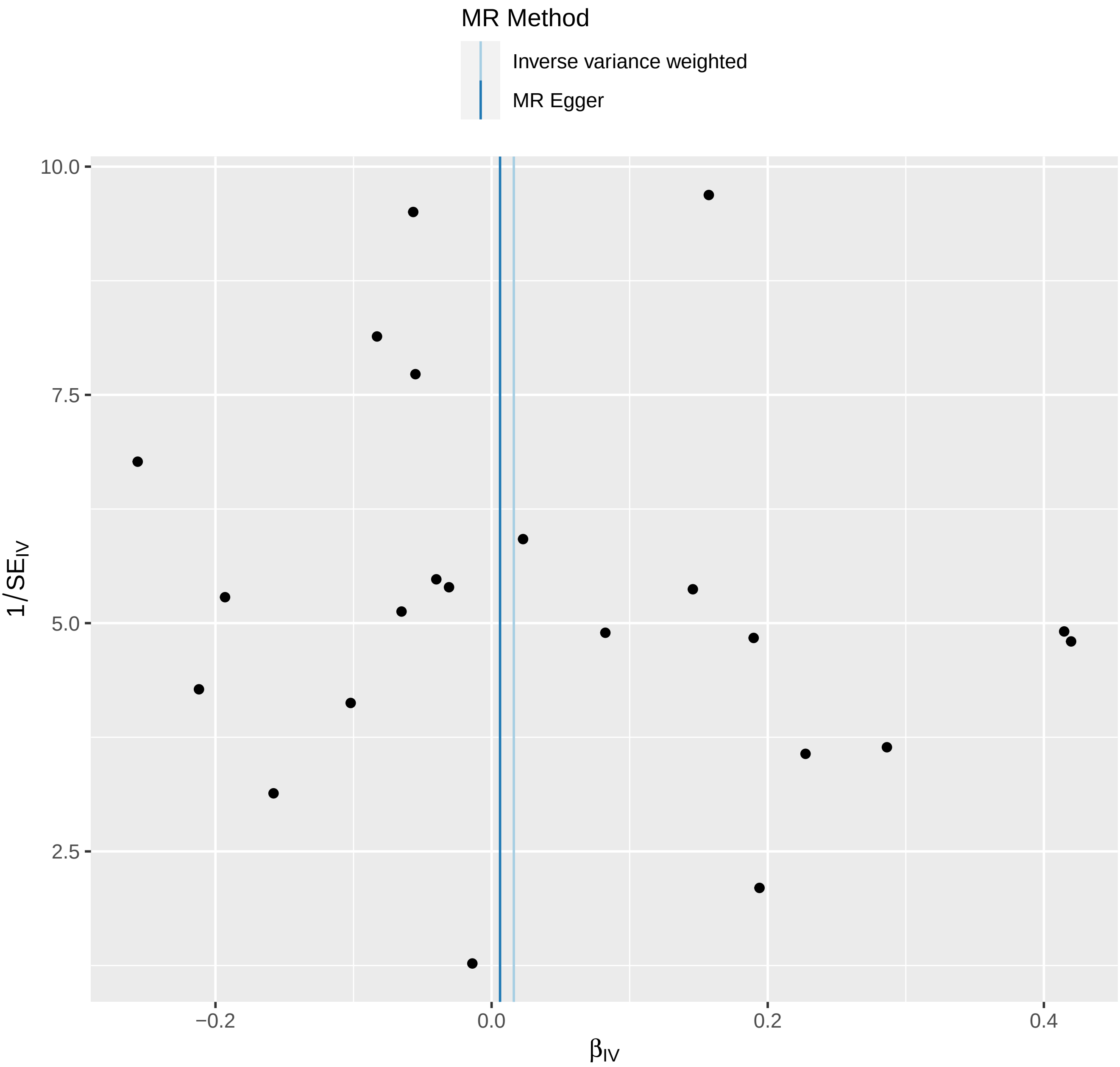

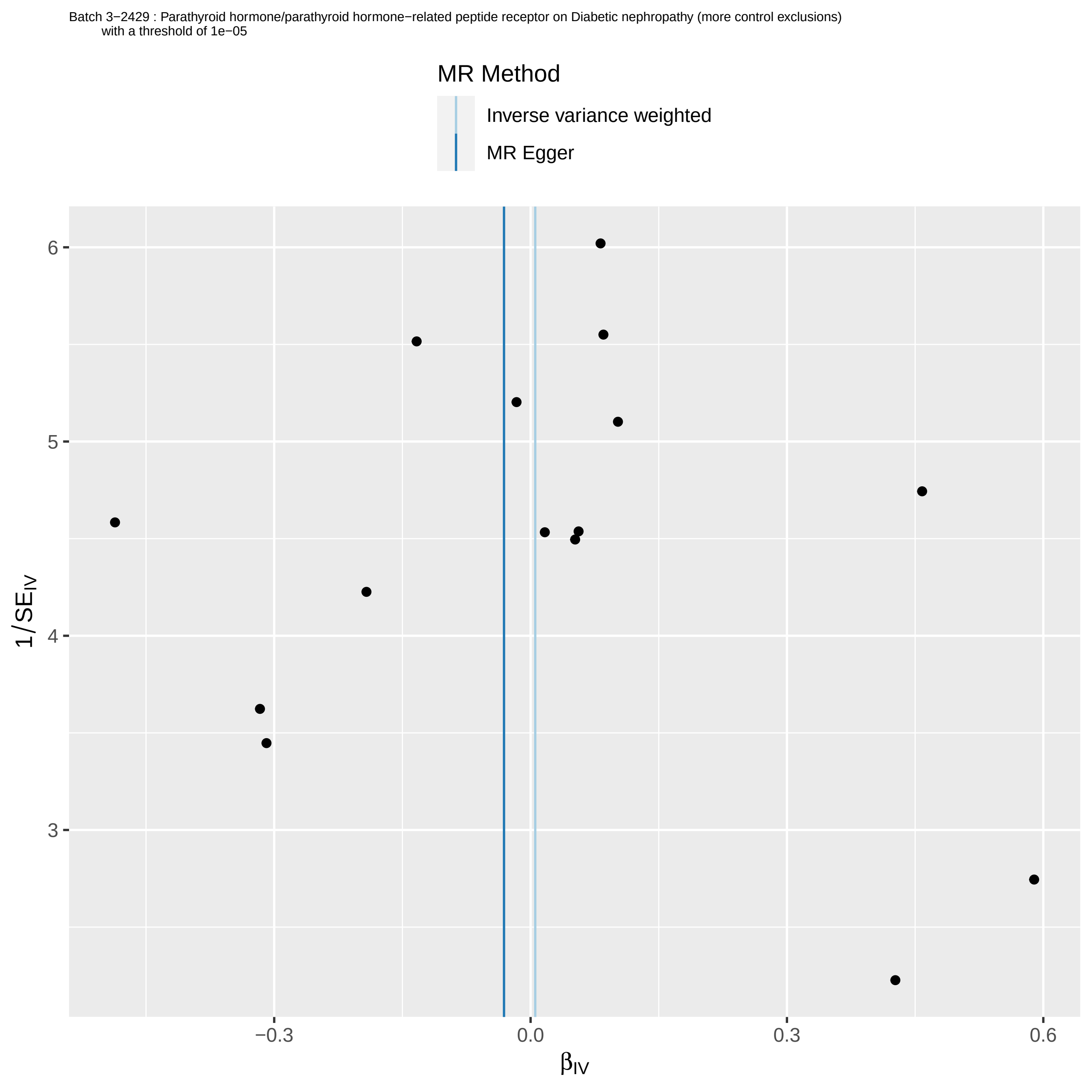
Batch 3−2429 : Parathyroid hormone/parathyroid hormone−related peptide receptor on Diabetic nephropathy (more control exclusions)
with a threshold of 1e−05
MR Method
Inverse variance weighted
MR Egger
6
5
4
3
1/SE_IV
−0.3
0.0
0.3
0.6
β_IV

Batch 3−2430 : Tuberoinfundibular peptide of 39 residues on Diabetic nephropathy (more control exclusions)
with a threshold of 1e−05

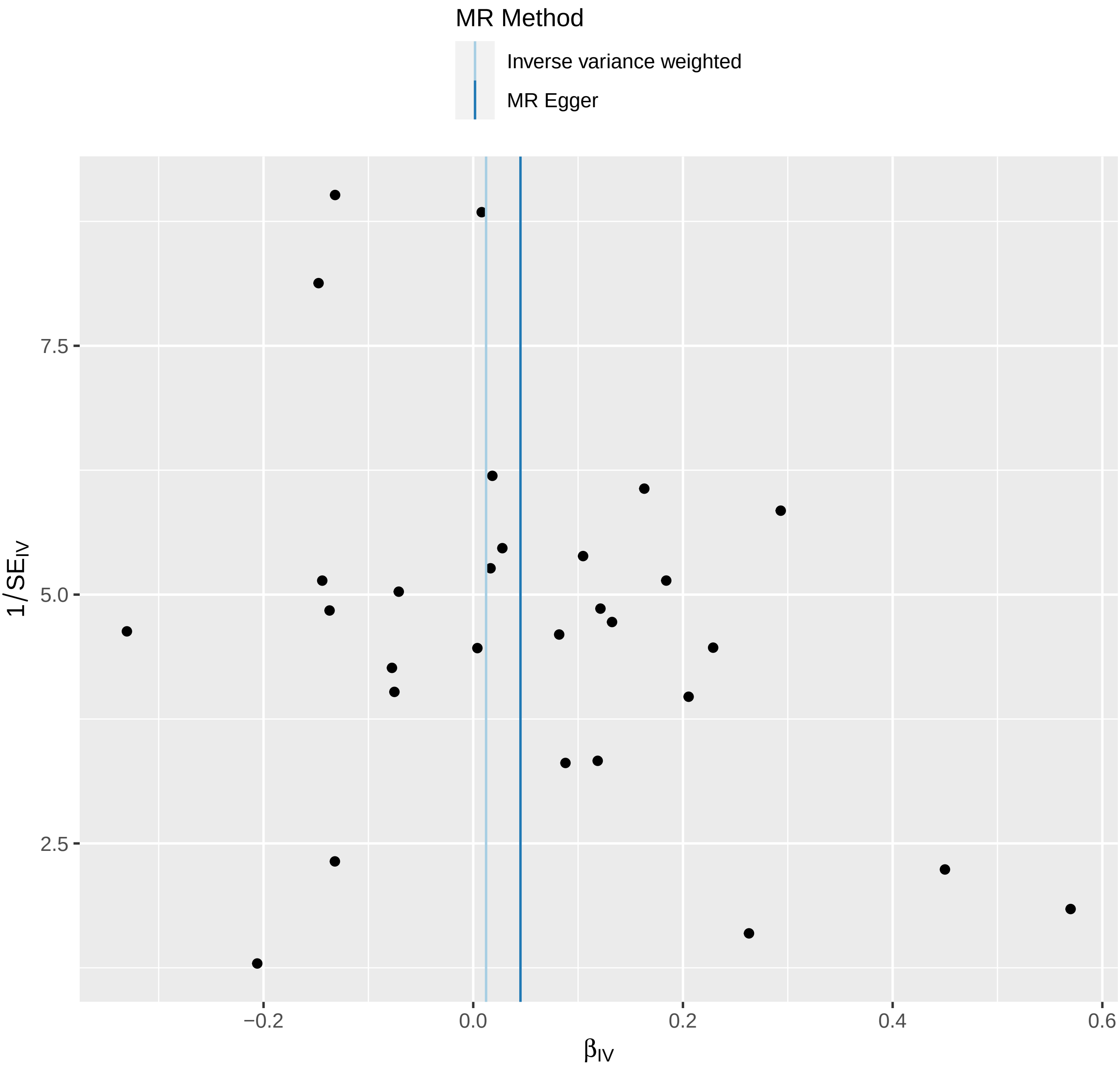

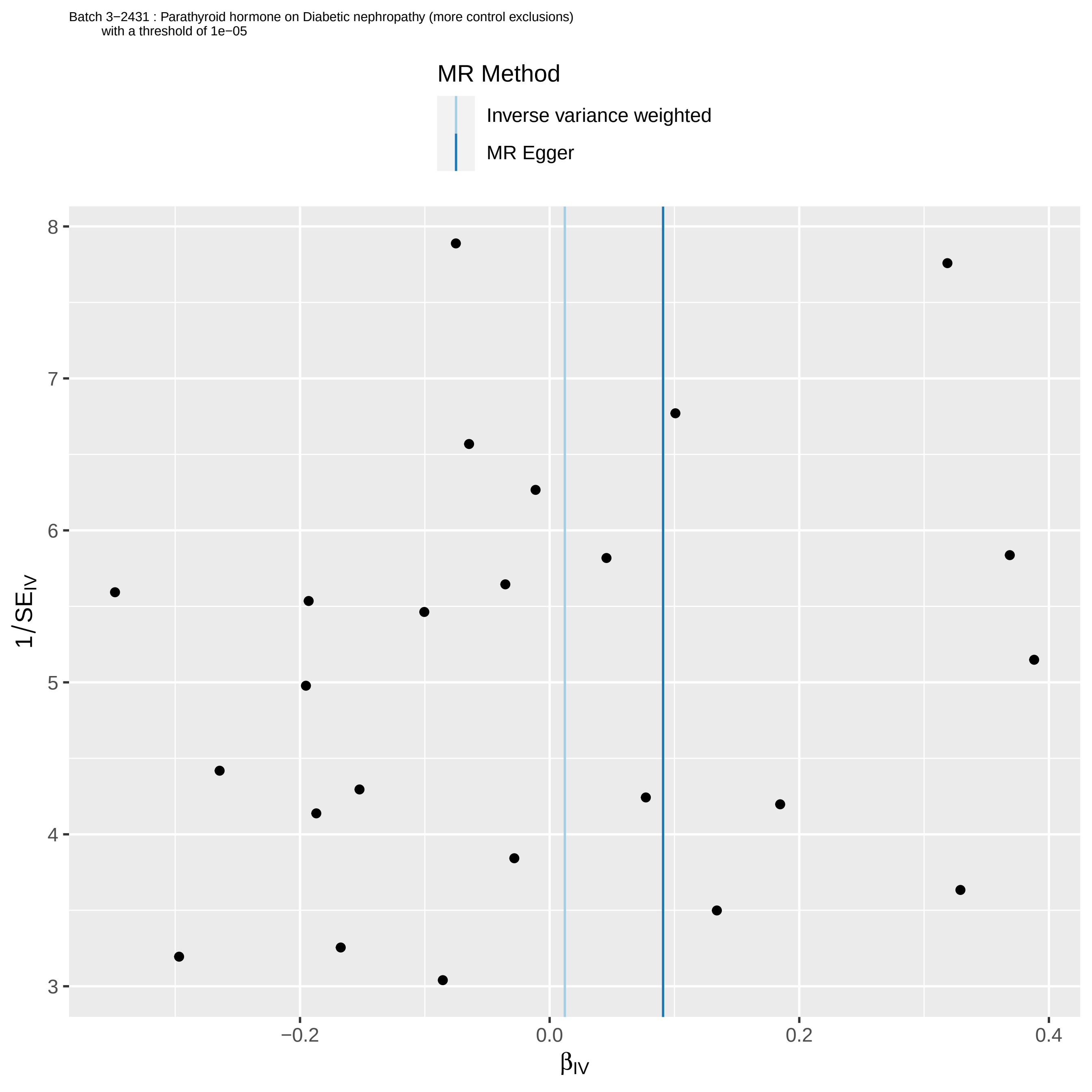

Batch 3−2431 : Parathyroid hormone on Diabetic nephropathy (more control exclusions)
with a threshold of 1e−05
MR Method
Inverse variance weighted
MR Egger
1/SE_IV
β_IV
8
7
6
5
4
3
−0.2
0.0
0.2
0.4

Batch 3−2432 : Parathyroid hormone−related protein on Diabetic nephropathy (more control exclusions)
with a threshold of 1e−05

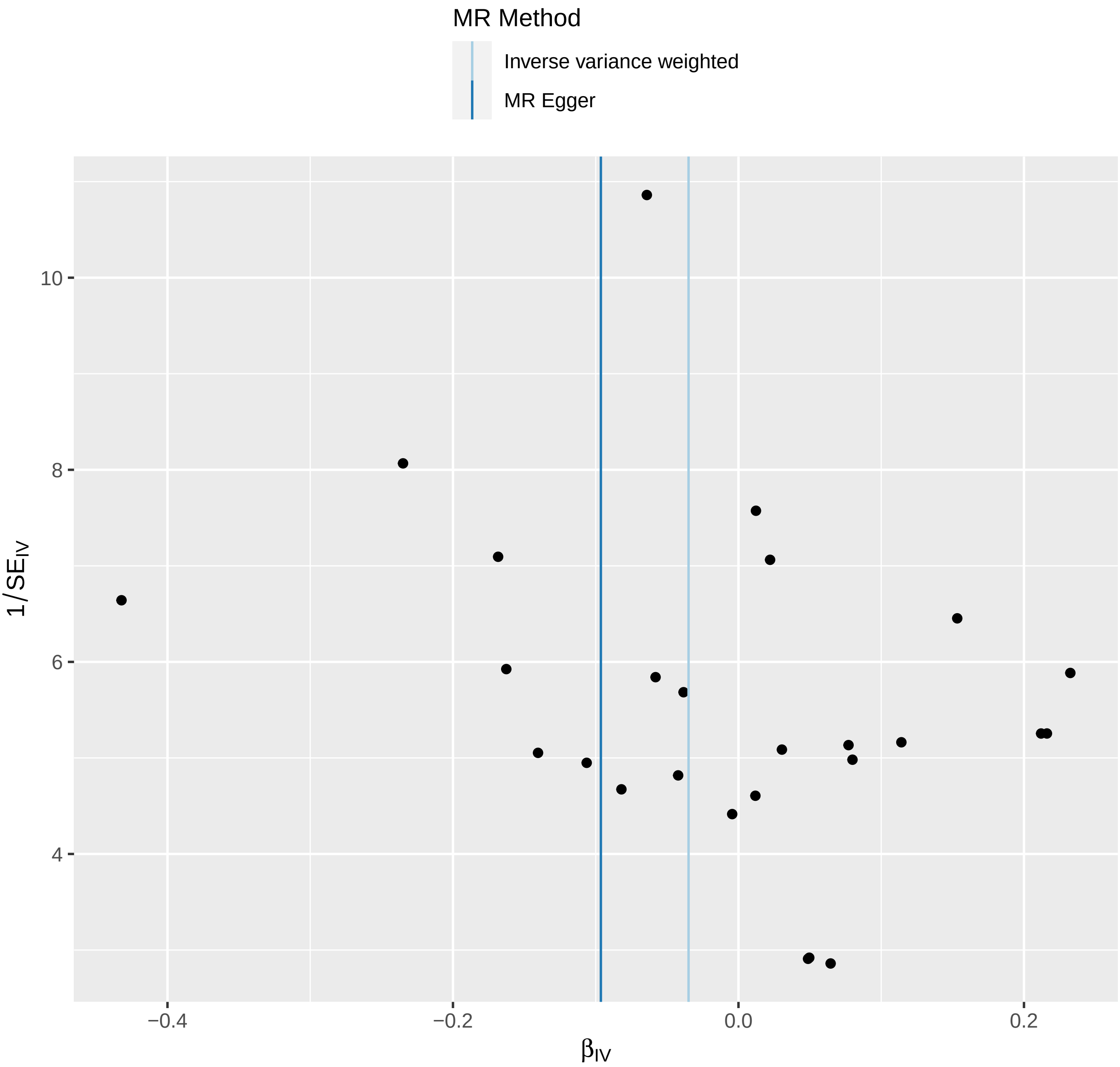

Batch 3−2433 : Protein−tyrosine kinase 2−beta on Diabetic nephropathy (more control exclusions)
with a threshold of 1e−05

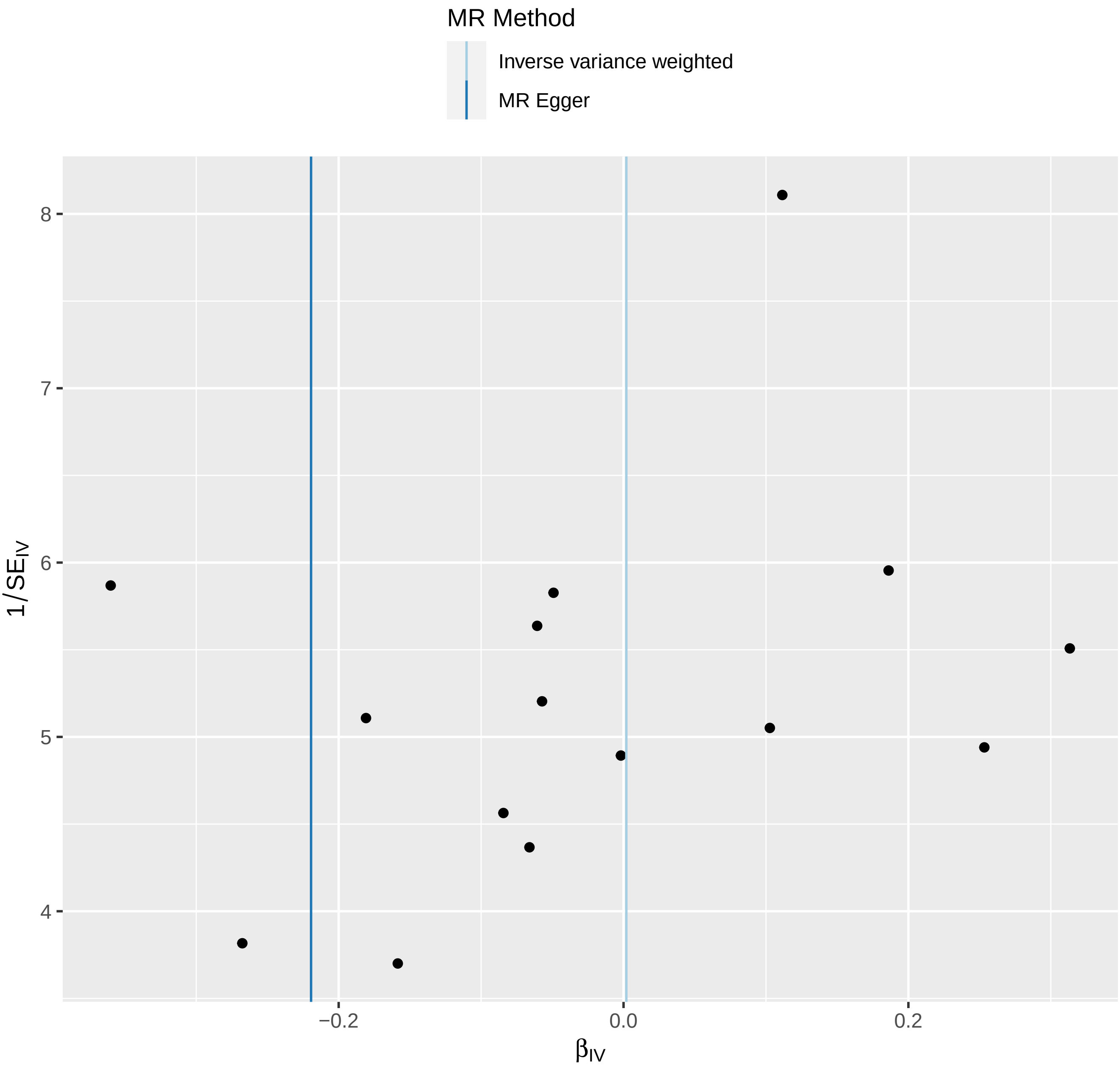

Batch 3−2434 : Protein−tyrosine kinase 6 on Diabetic nephropathy (more control exclusions)
with a threshold of 1e−05

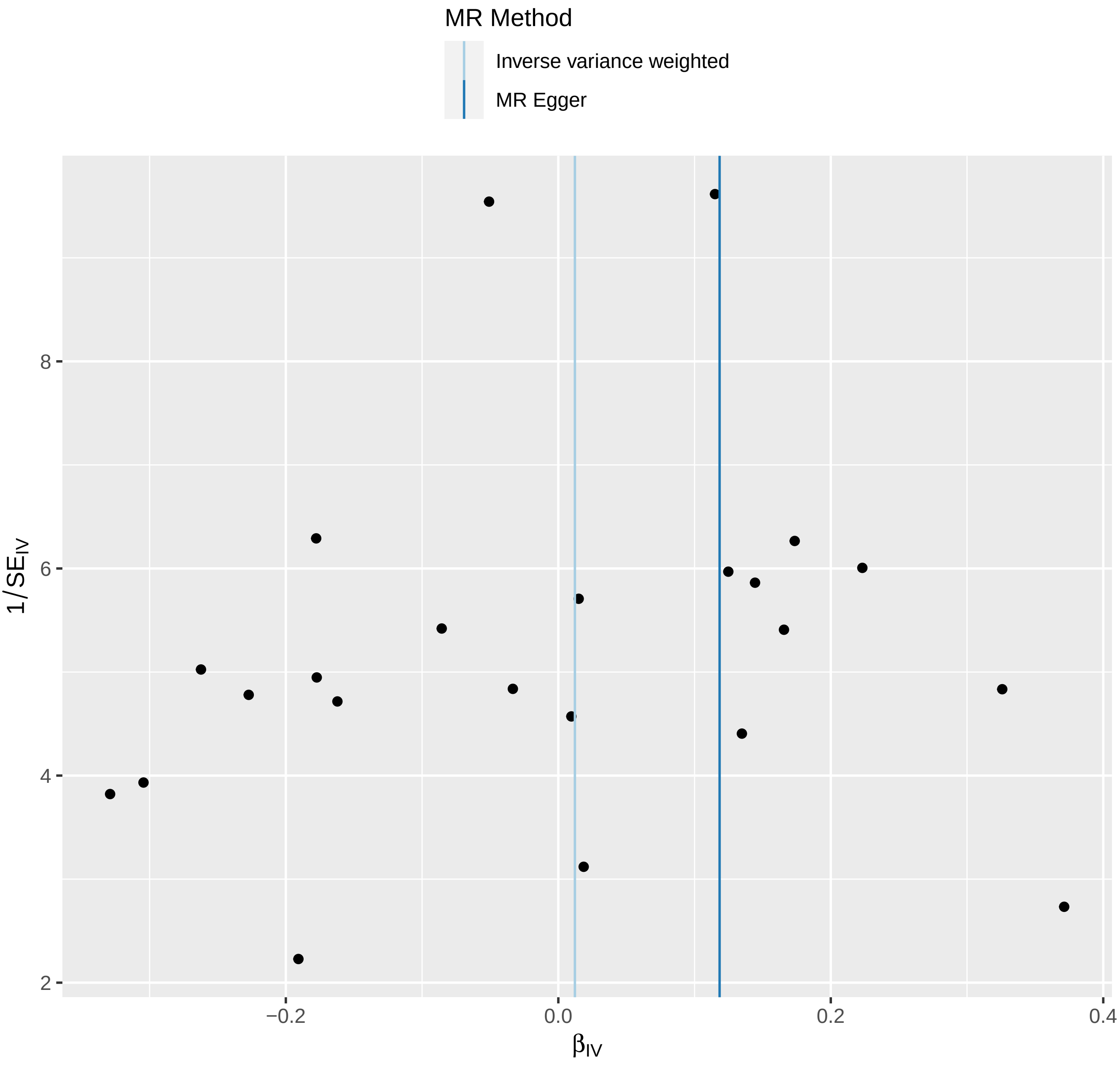

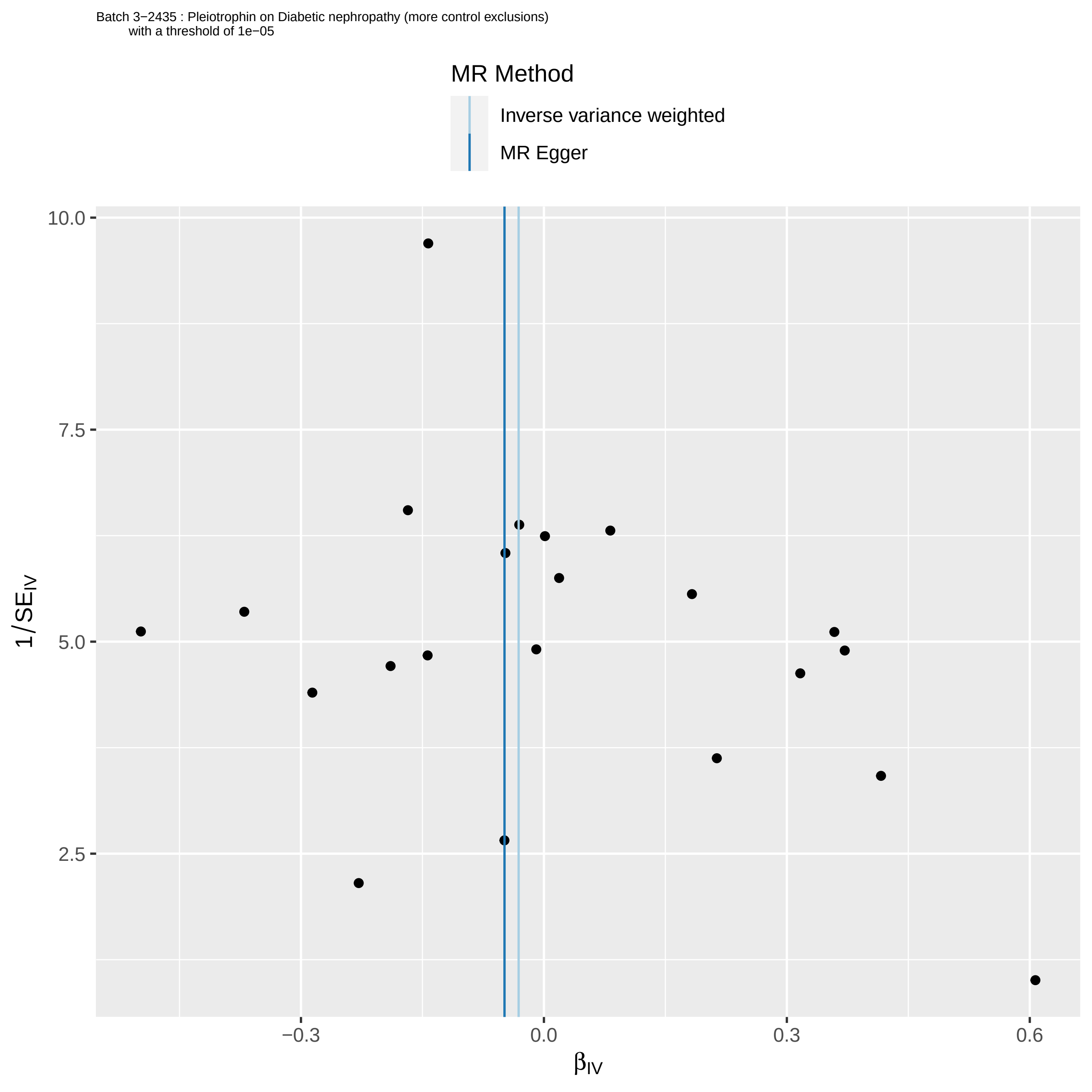
Batch 3−2435 : Pleiotrophin on Diabetic nephropathy (more control exclusions)
with a threshold of 1e−05
MR Method
Inverse variance weighted
MR Egger
10.0
7.5
5.0
2.5
1/SE_IV
−0.3
0.0
0.3
0.6
β_IV

Batch 3−2436 : Protein tyrosine phosphatase type IVA 2 on Diabetic nephropathy (more control exclusions)
with a threshold of 1e−05

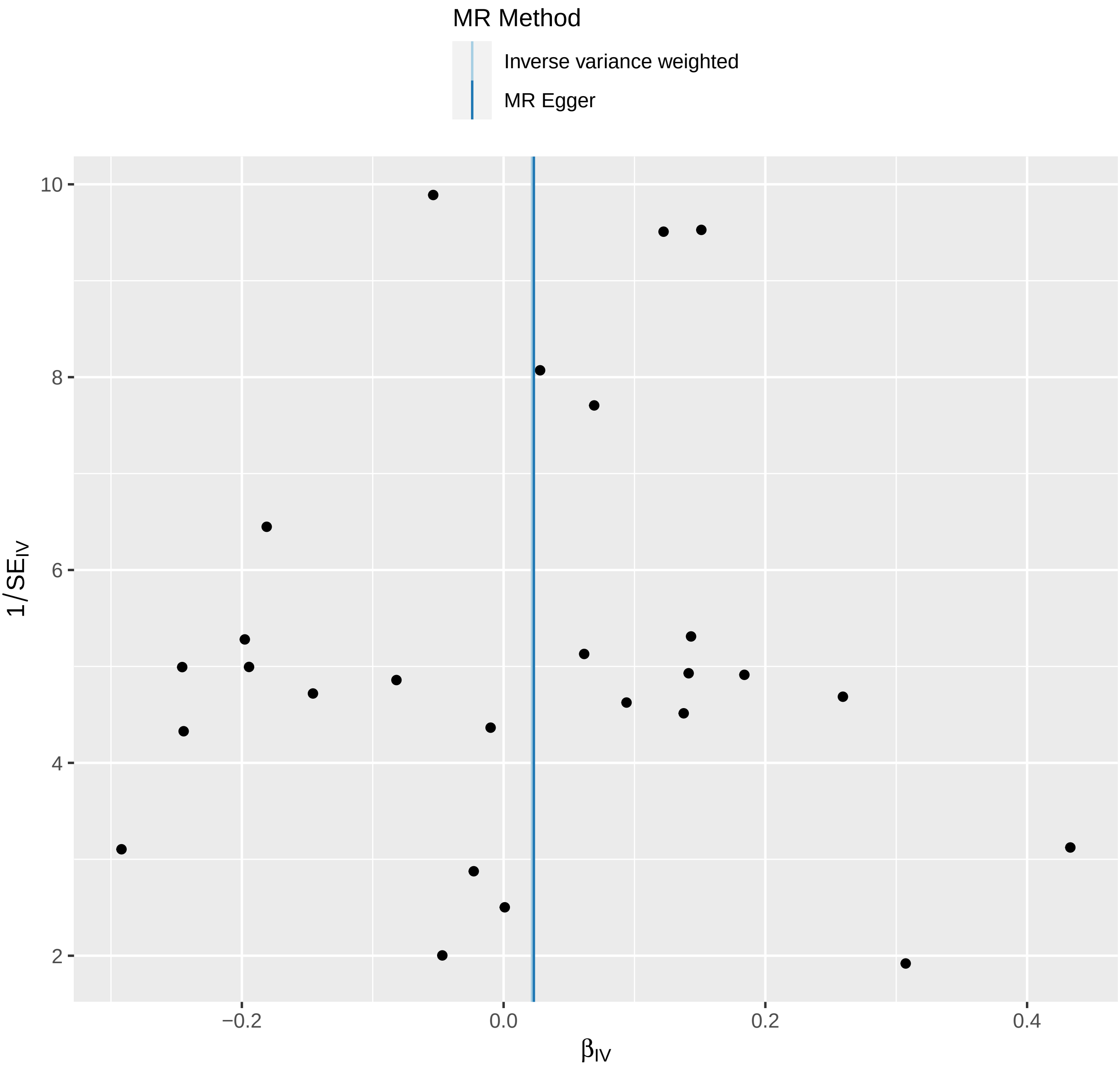

Batch 3−2437 : Tyrosine−protein phosphatase non−receptor type 11 on Diabetic nephropathy (more control exclusions)
with a threshold of 1e−05

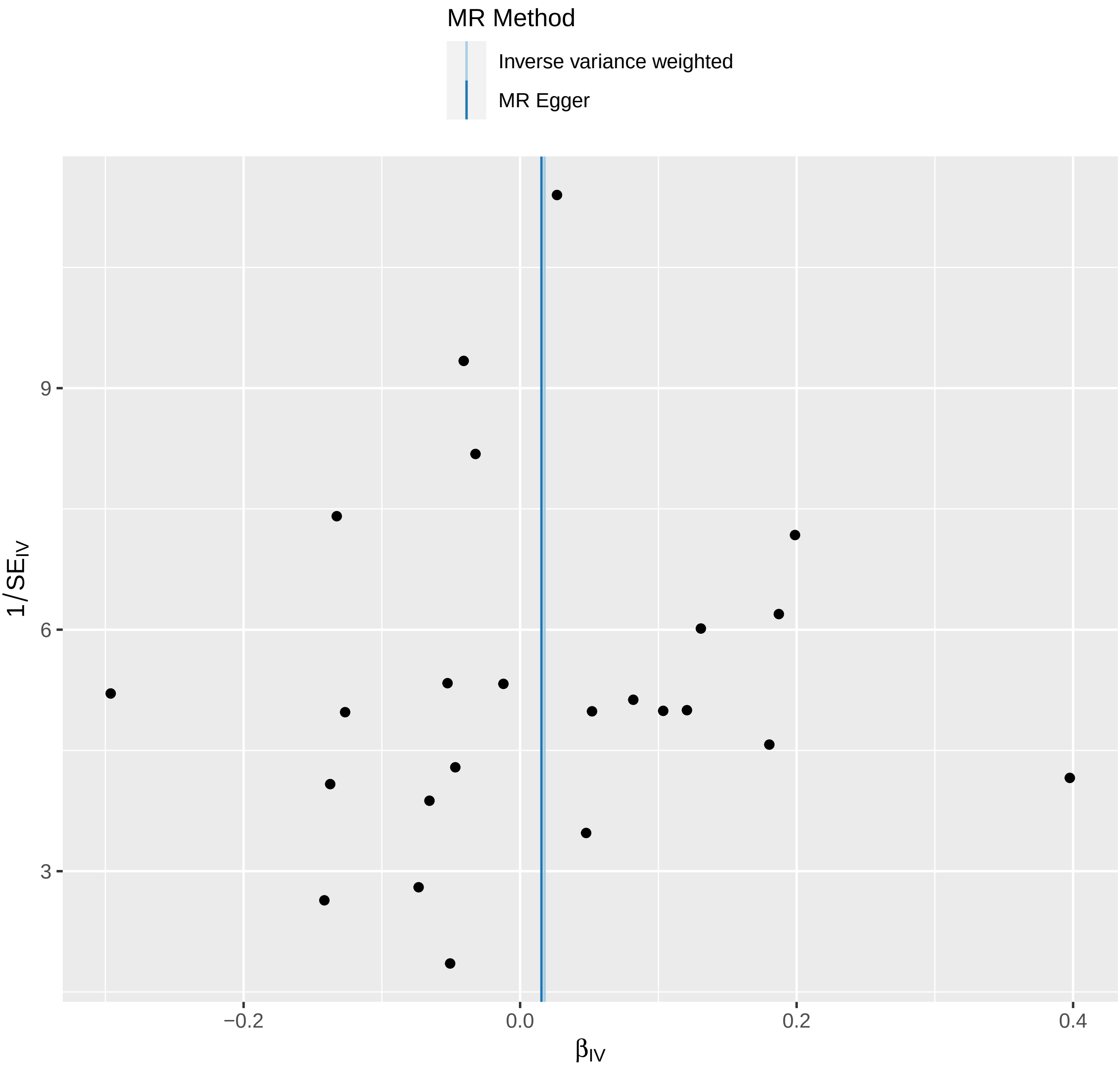

Batch 3−2438 : Tyrosine−protein phosphatase non−receptor type 1 on Diabetic nephropathy (more control exclusions)
with a threshold of 1e−05

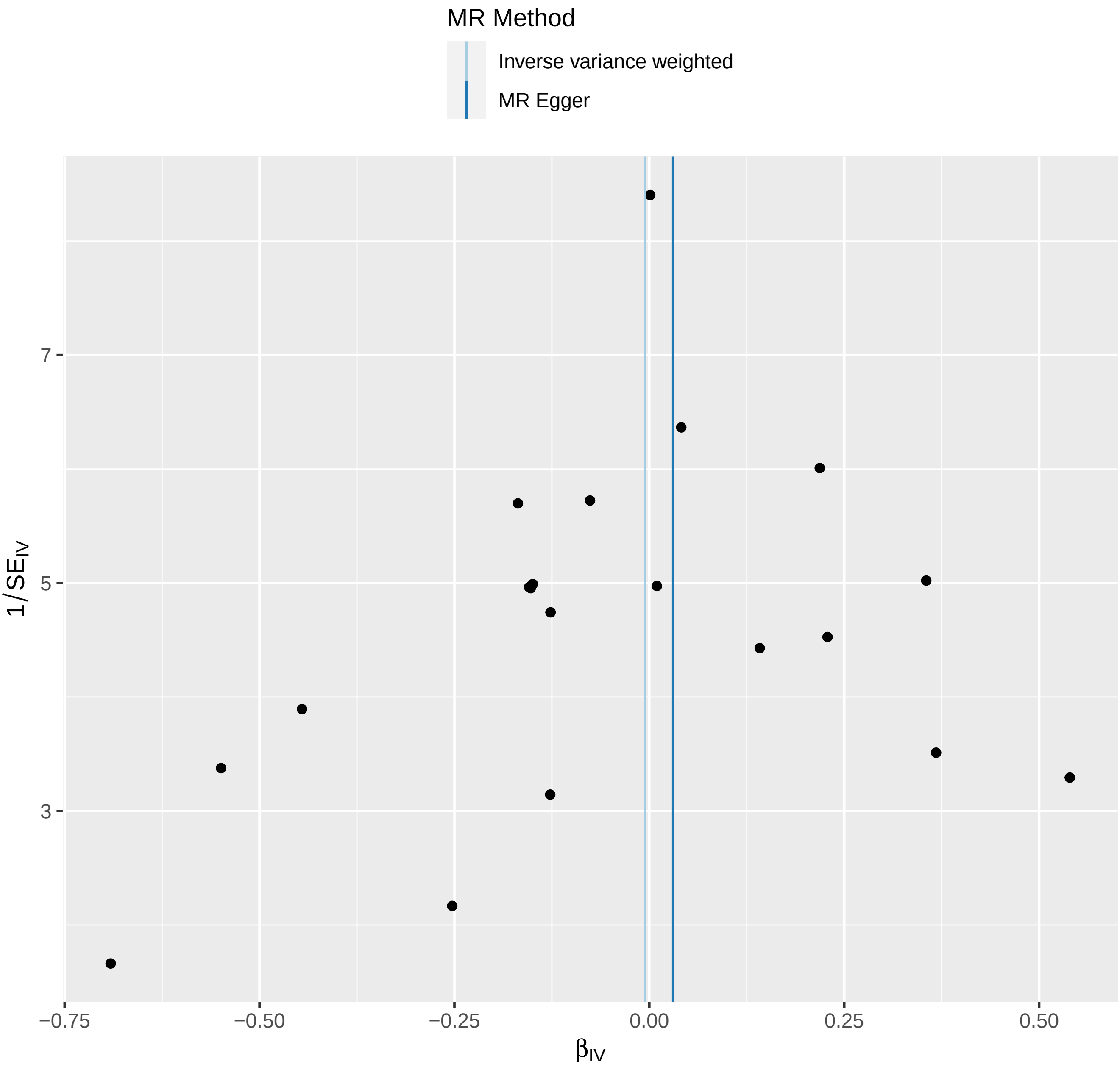

Batch 3−2439 : Tyrosine−protein phosphatase non−receptor type 2 on Diabetic nephropathy (more control exclusions)
with a threshold of 1e−05

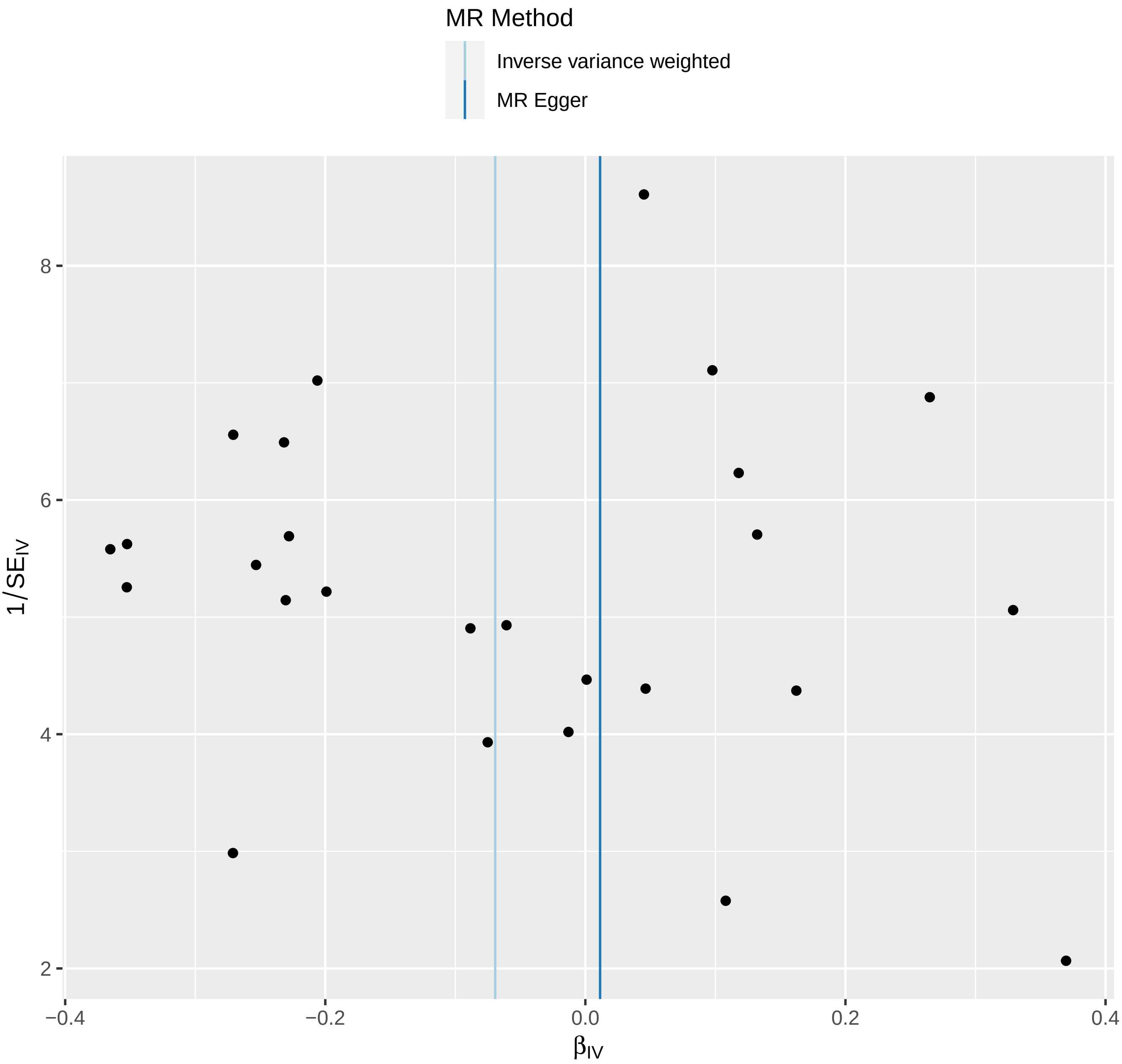

Batch 3−2440 : Tyrosine−protein phosphatase non−receptor type 4 on Diabetic nephropathy (more control exclusions) with a threshold of 1e−05

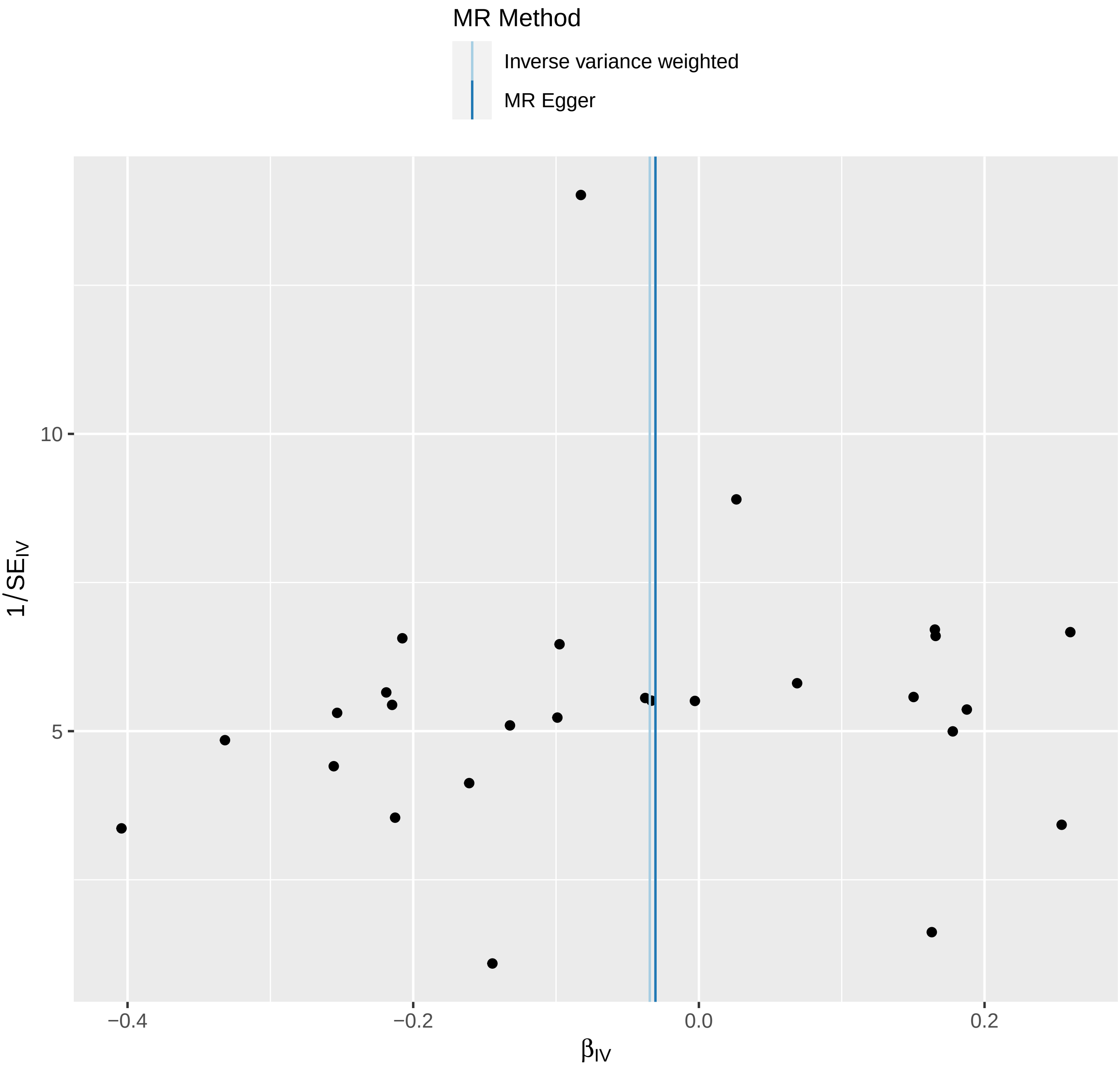

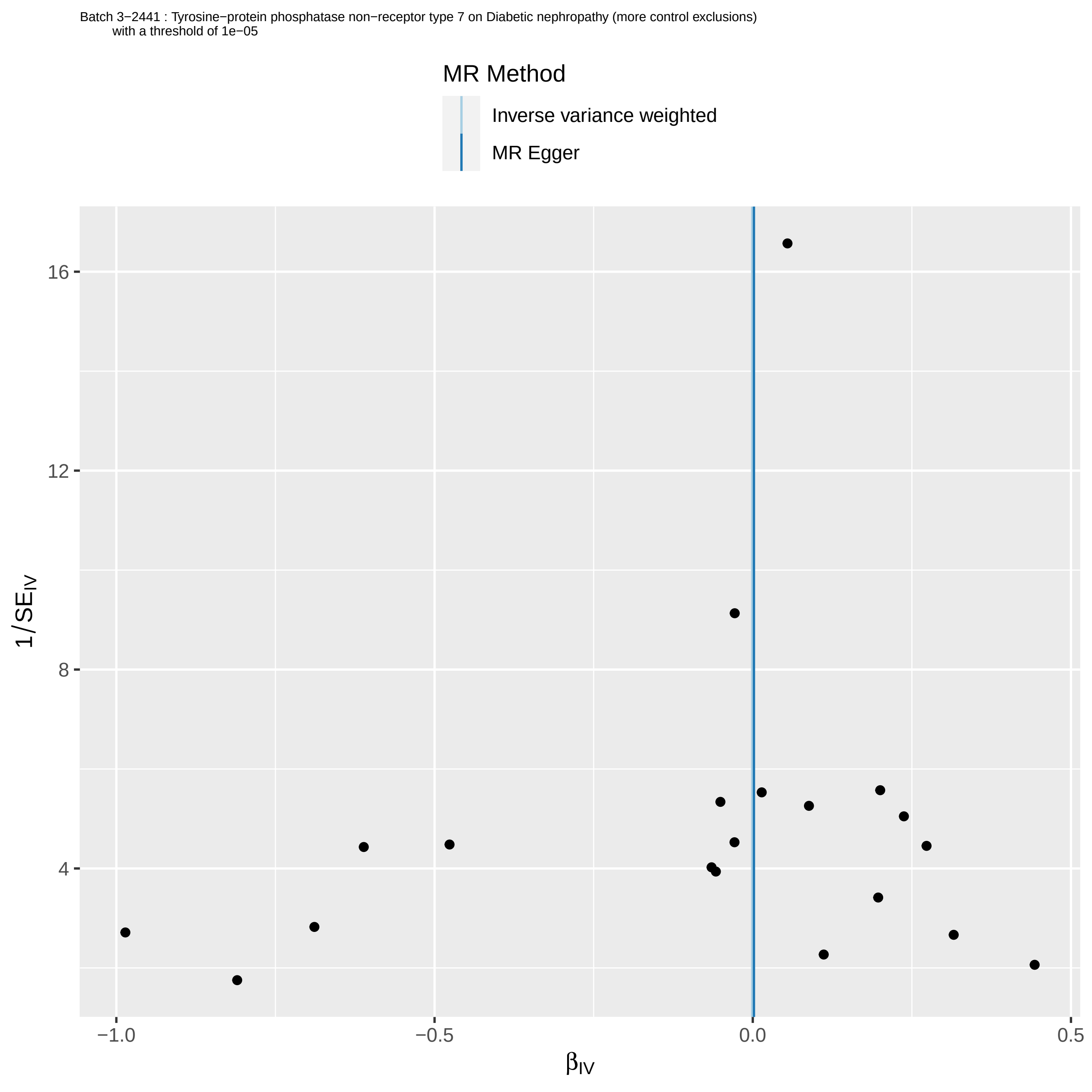

Batch 3−2441 : Tyrosine−protein phosphatase non−receptor type 7 on Diabetic nephropathy (more control exclusions)
with a threshold of 1e−05
MR Method
Inverse variance weighted
MR Egger
16
12
8
4
1/SE_IV
−1.0
−0.5
0.0
0.5
β_IV

Batch 3−2442 : Tyrosine−protein phosphatase non−receptor type 9 on Diabetic nephropathy (more control exclusions) with a threshold of 1e−05

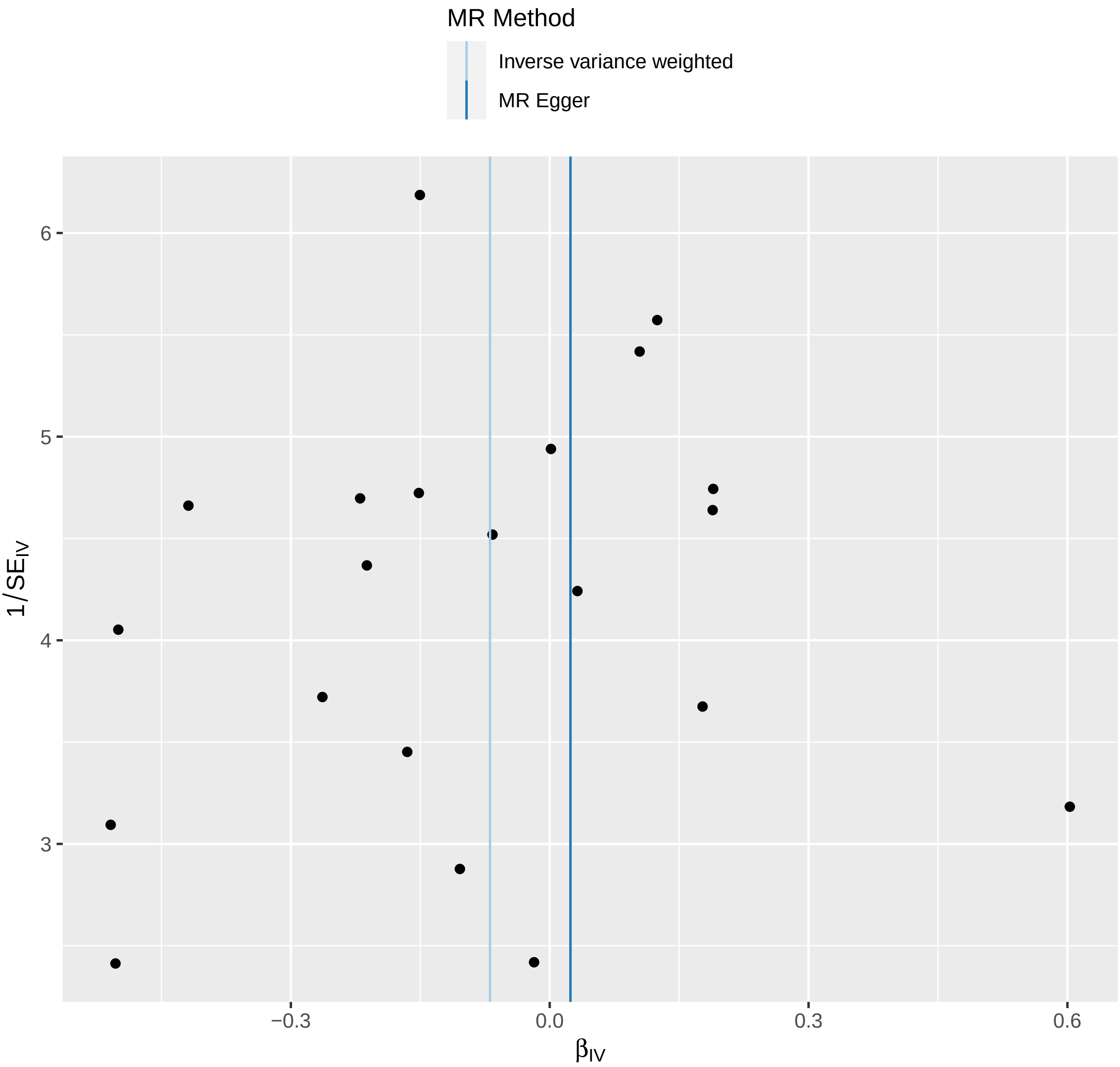

Batch 3−2443 : Receptor−type tyrosine−protein phosphatase delta on Diabetic nephropathy (more control exclusions)
with a threshold of 1e−05

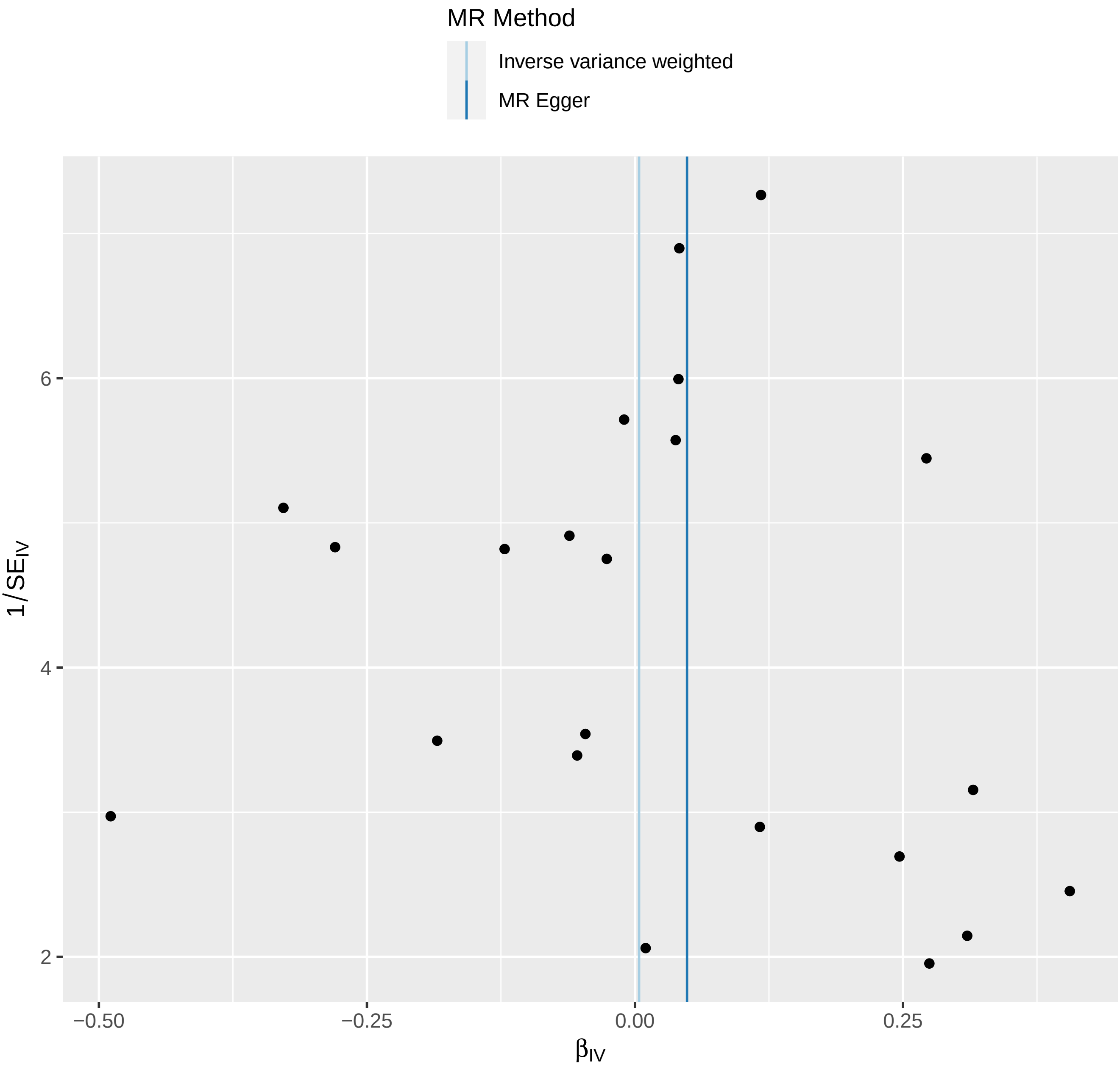

Batch 3−2444 : Receptor−type tyrosine−protein phosphatase H on Diabetic nephropathy (more control exclusions)
with a threshold of 1e−05

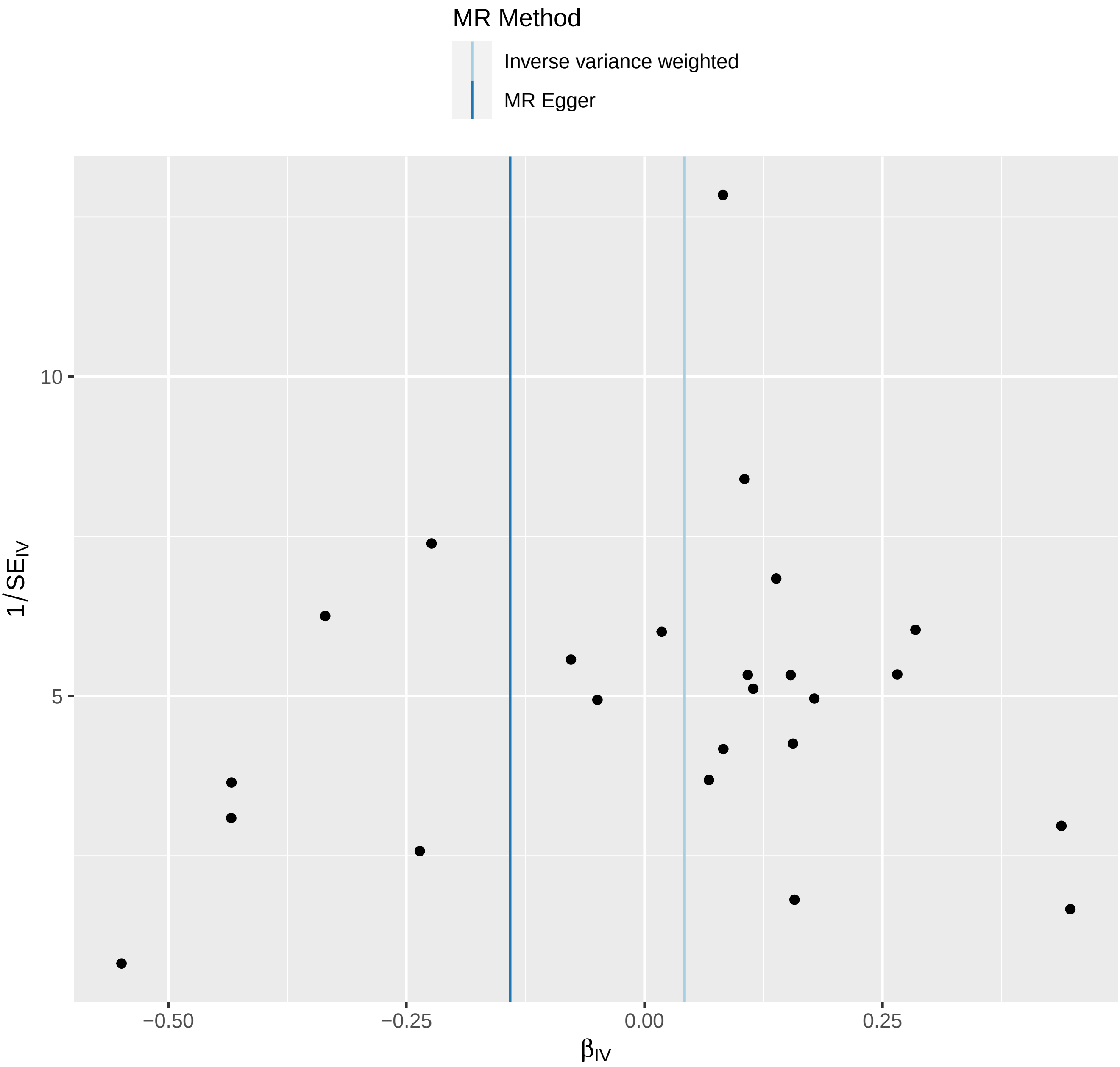

Batch 3−2445 : Receptor−type tyrosine−protein phosphatase H on Diabetic nephropathy (more control exclusions)
with a threshold of 1e−05

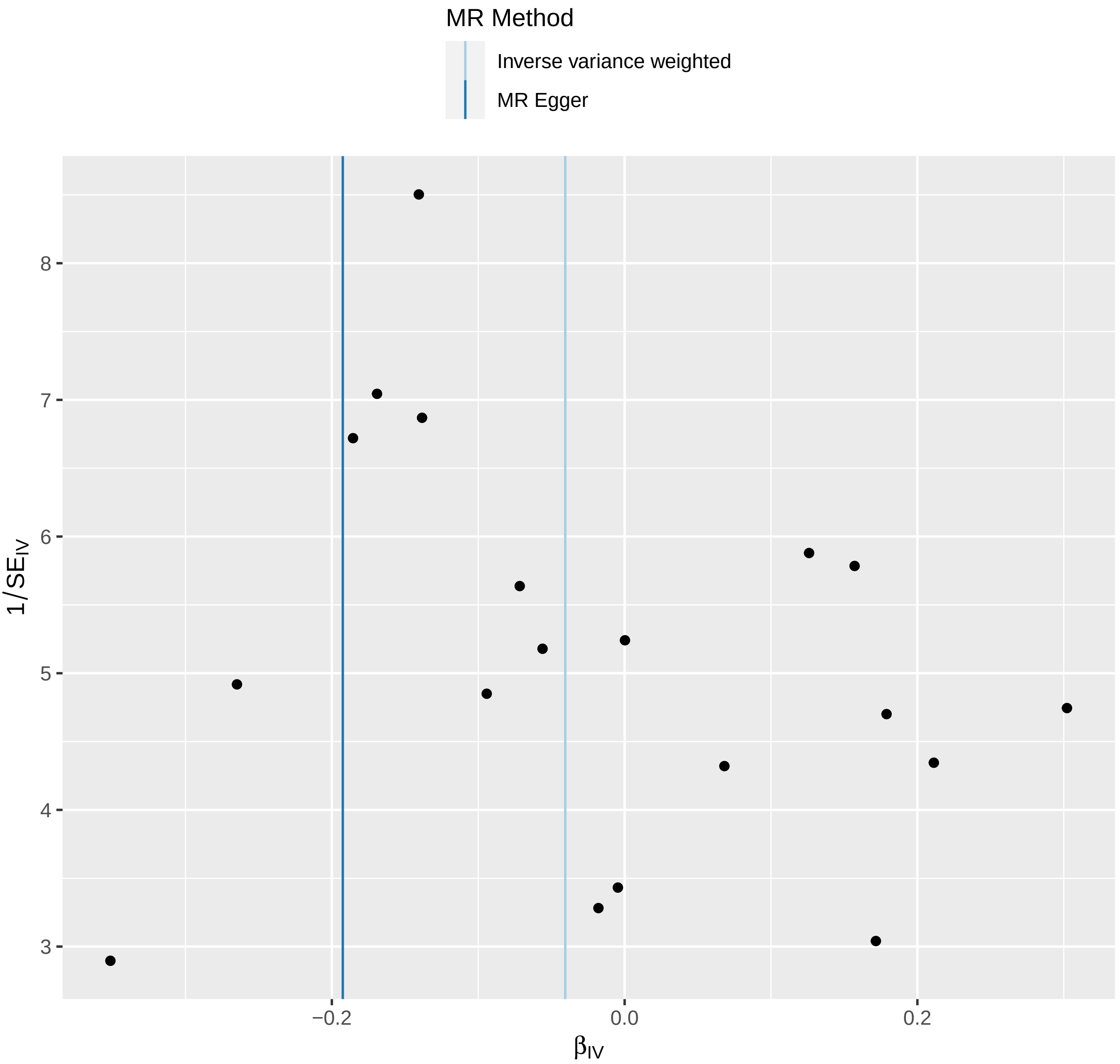

Batch 3−2446 : Receptor−type tyrosine−protein phosphatase eta on Diabetic nephropathy (more control exclusions)
with a threshold of 1e−05

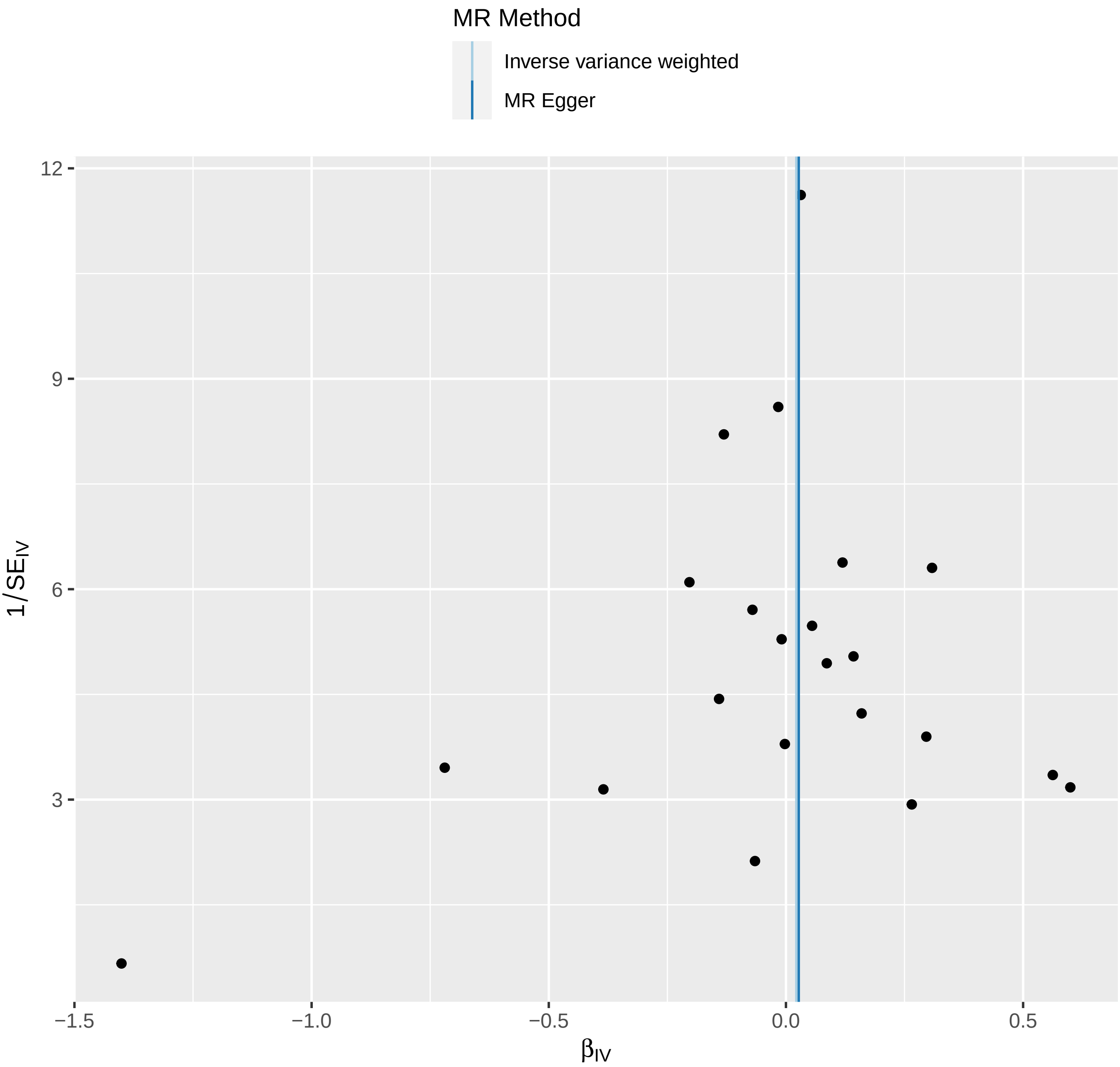

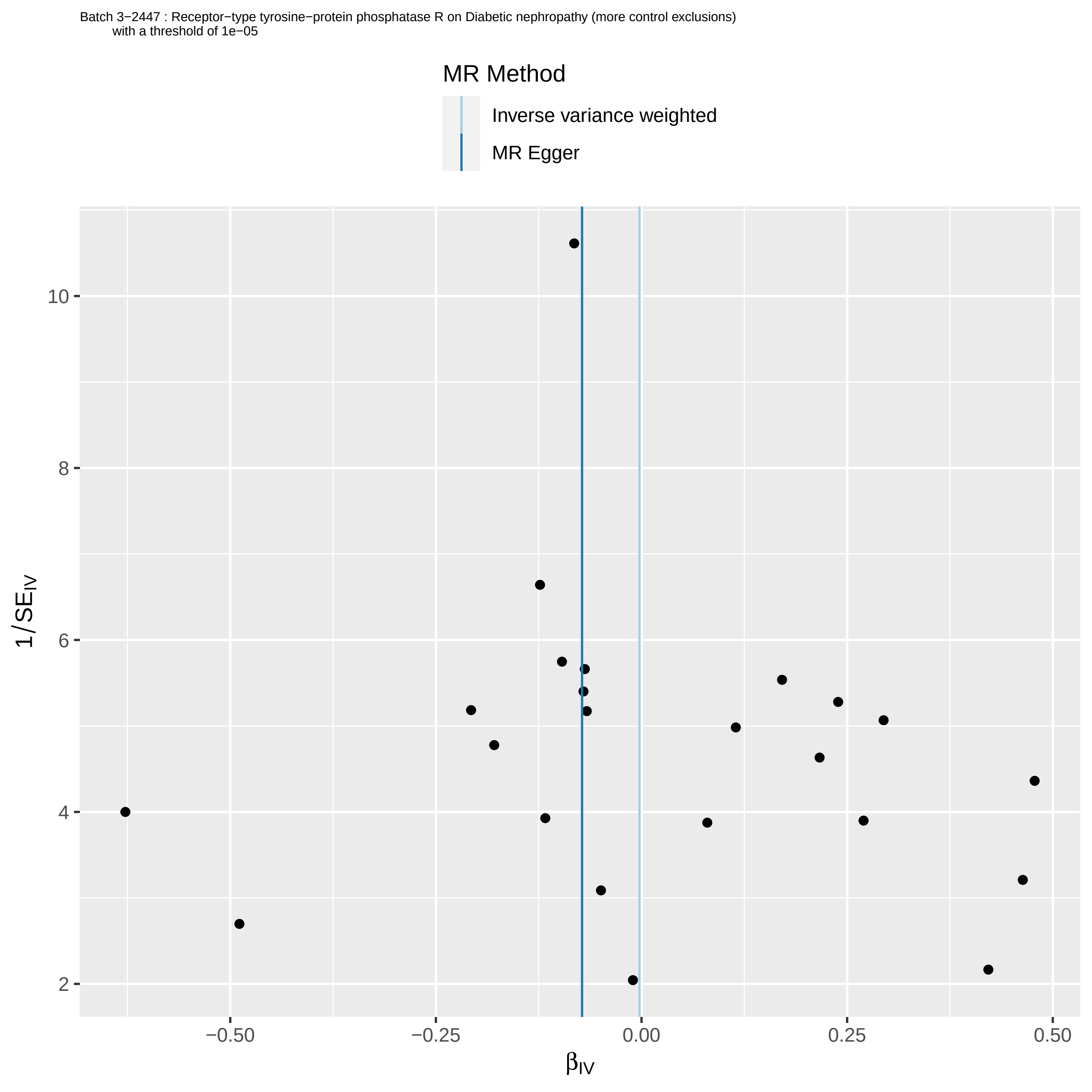
Batch 3−2447 : Receptor−type tyrosine−protein phosphatase R on Diabetic nephropathy (more control exclusions)
with a threshold of 1e−05
MR Method
Inverse variance weighted
MR Egger
10
8
6
4
2
1/SE$_{IV}$
−0.50
−0.25
0.00
0.25
0.50
β$_{IV}$

Batch 3−2448 : Receptor−type tyrosine−protein phosphatase S on Diabetic nephropathy (more control exclusions)
with a threshold of 1e−05

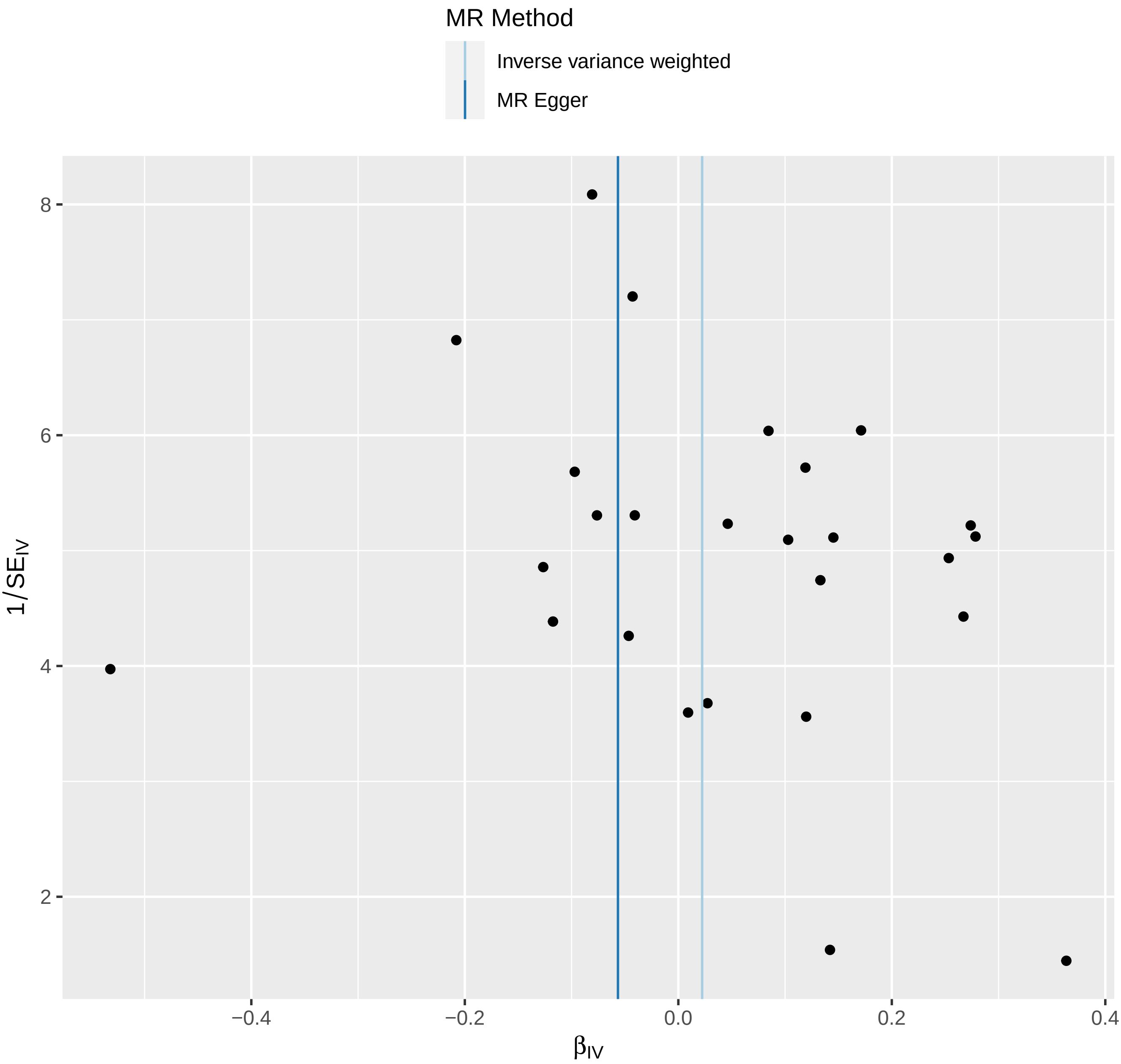

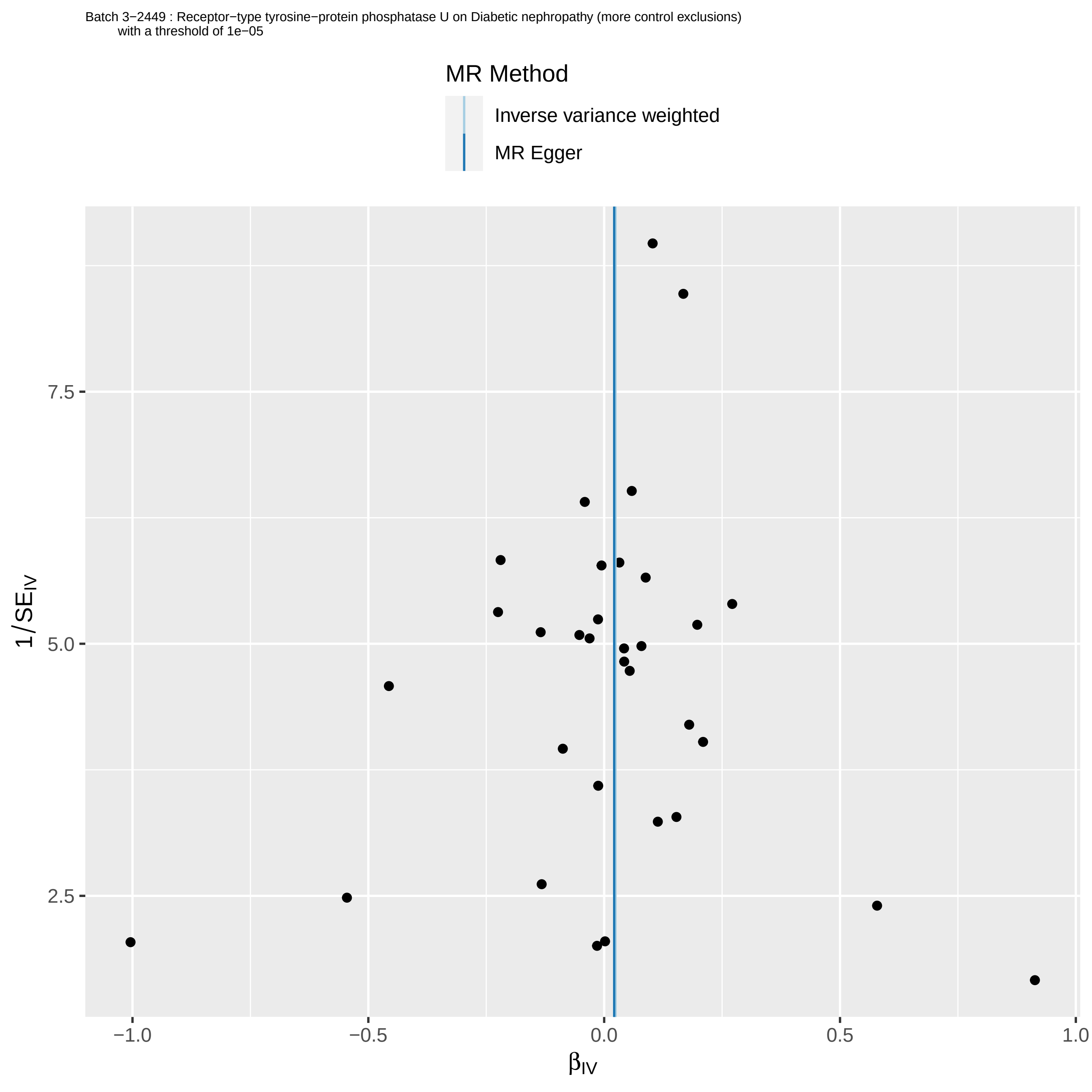

Batch 3−2449 : Receptor−type tyrosine−protein phosphatase U on Diabetic nephropathy (more control exclusions)
with a threshold of 1e−05
MR Method
Inverse variance weighted
MR Egger
7.5
5.0
2.5
1/SE_IV
−1.0
−0.5
0.0
0.5
1.0
β_IV

Batch 3−2450 : 6−pyruvoyl tetrahydrobiopterin synthase on Diabetic nephropathy (more control exclusions)
with a threshold of 1e−05

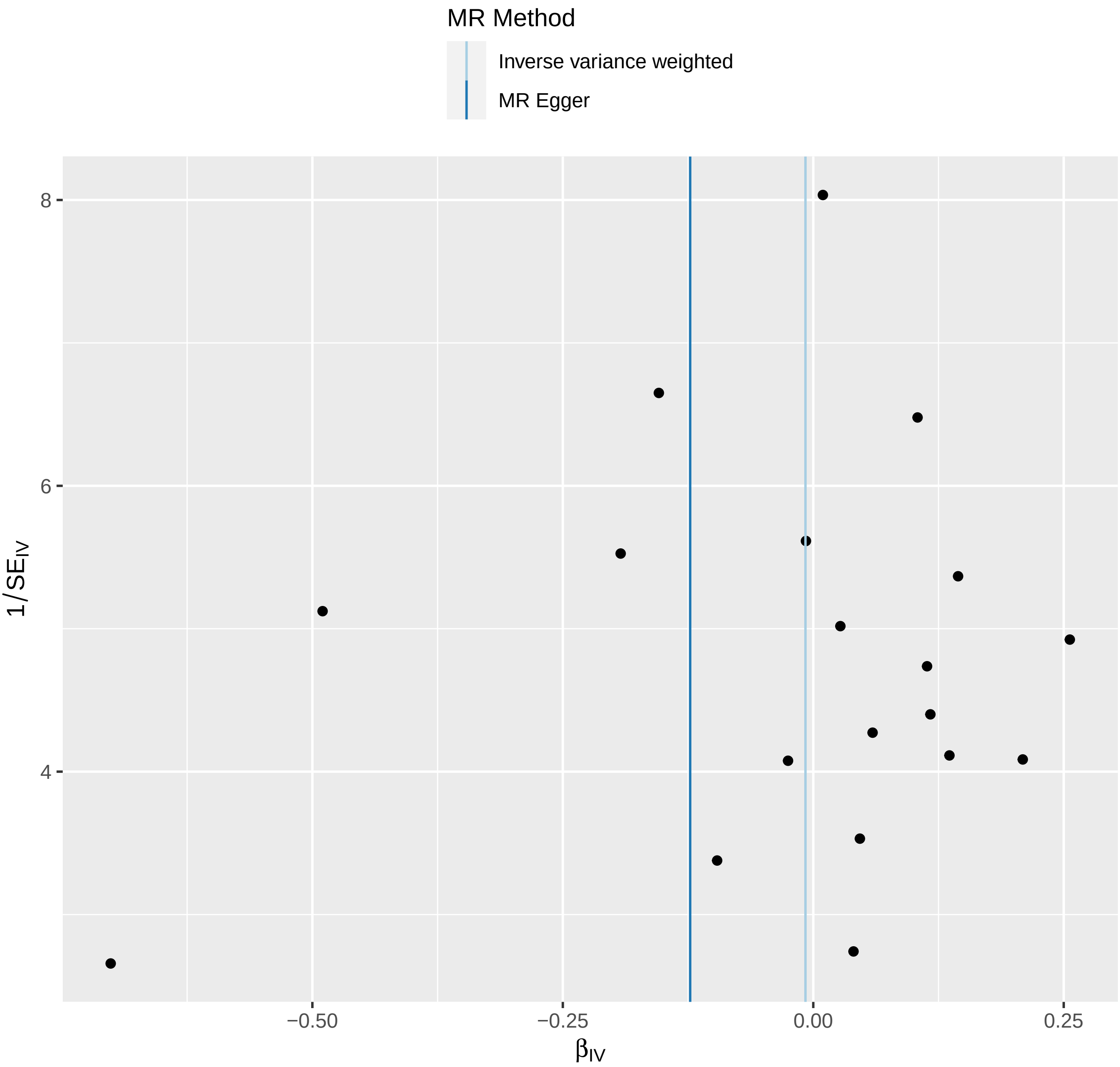

Batch 3−2451 : Pentraxin−related protein PTX3 on Diabetic nephropathy (more control exclusions)
with a threshold of 1e−05

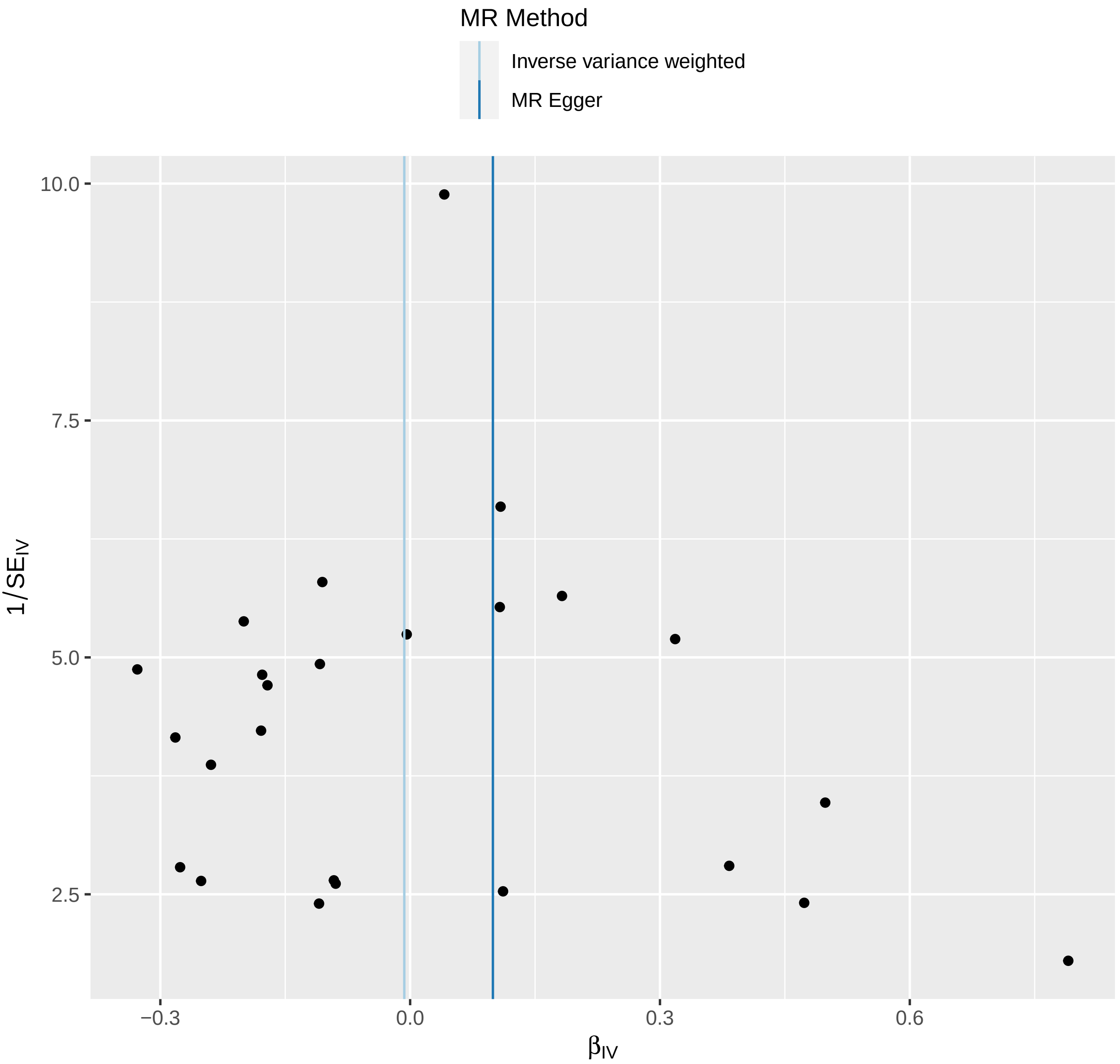

Batch 3−2452 : Poly(U)−binding−splicing factor PUF60 on Diabetic nephropathy (more control exclusions)
with a threshold of 1e−05

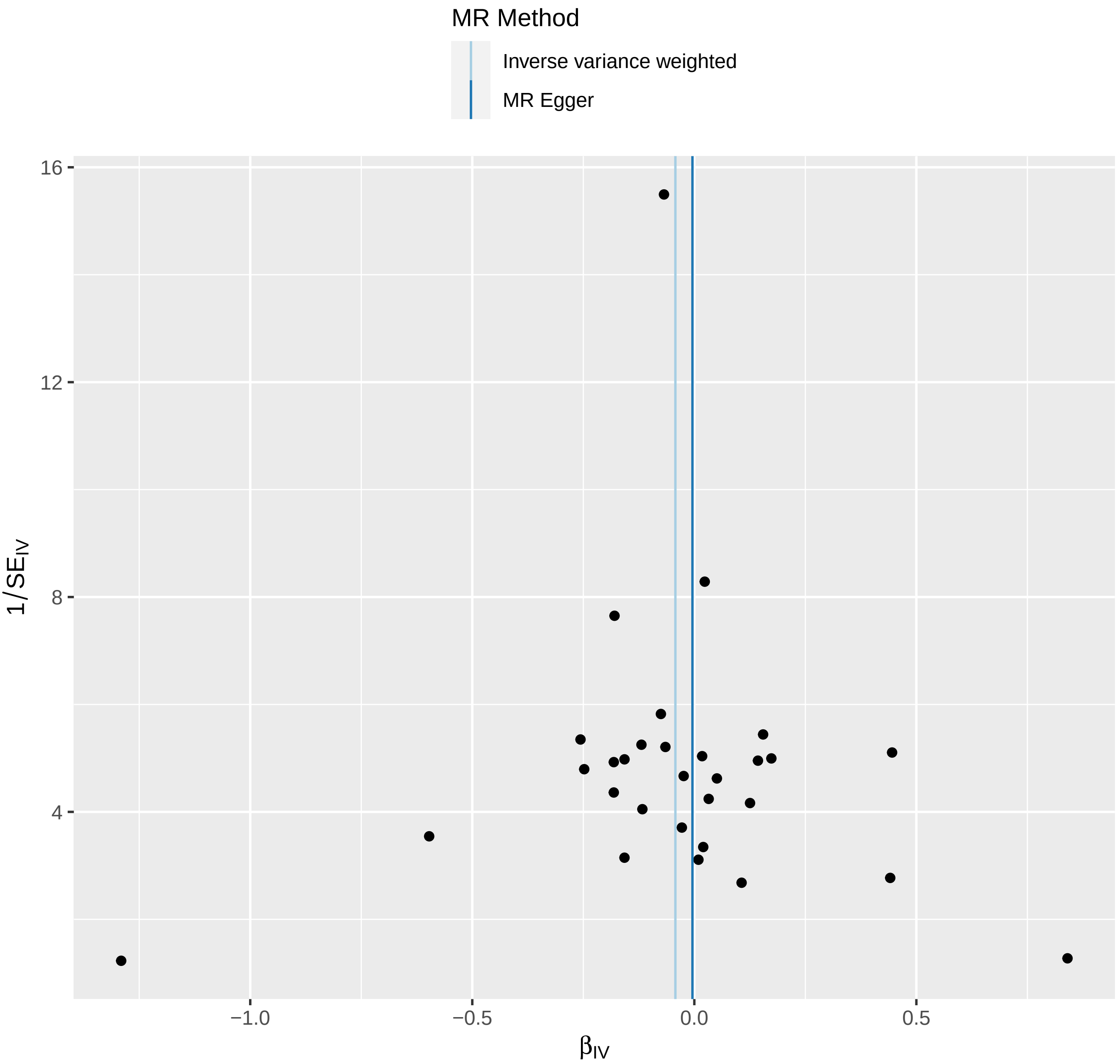

Batch 3−2453 : Transcriptional activator protein Pur−alpha on Diabetic nephropathy (more control exclusions) with a threshold of 1e−05

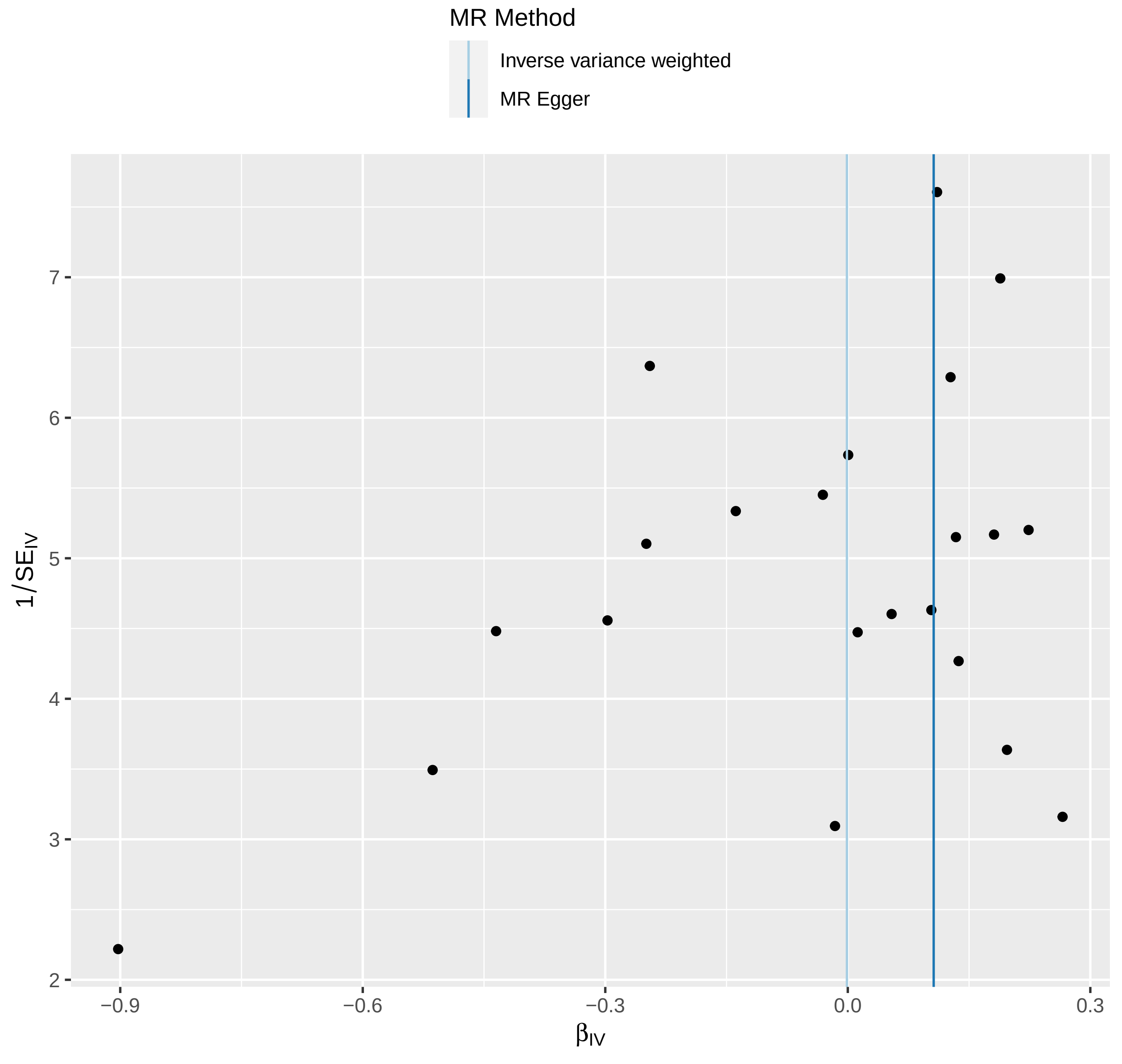

Batch 3−2454 : tRNA pseudouridine synthase A, mitochondrial on Diabetic nephropathy (more control exclusions)
with a threshold of 1e−05

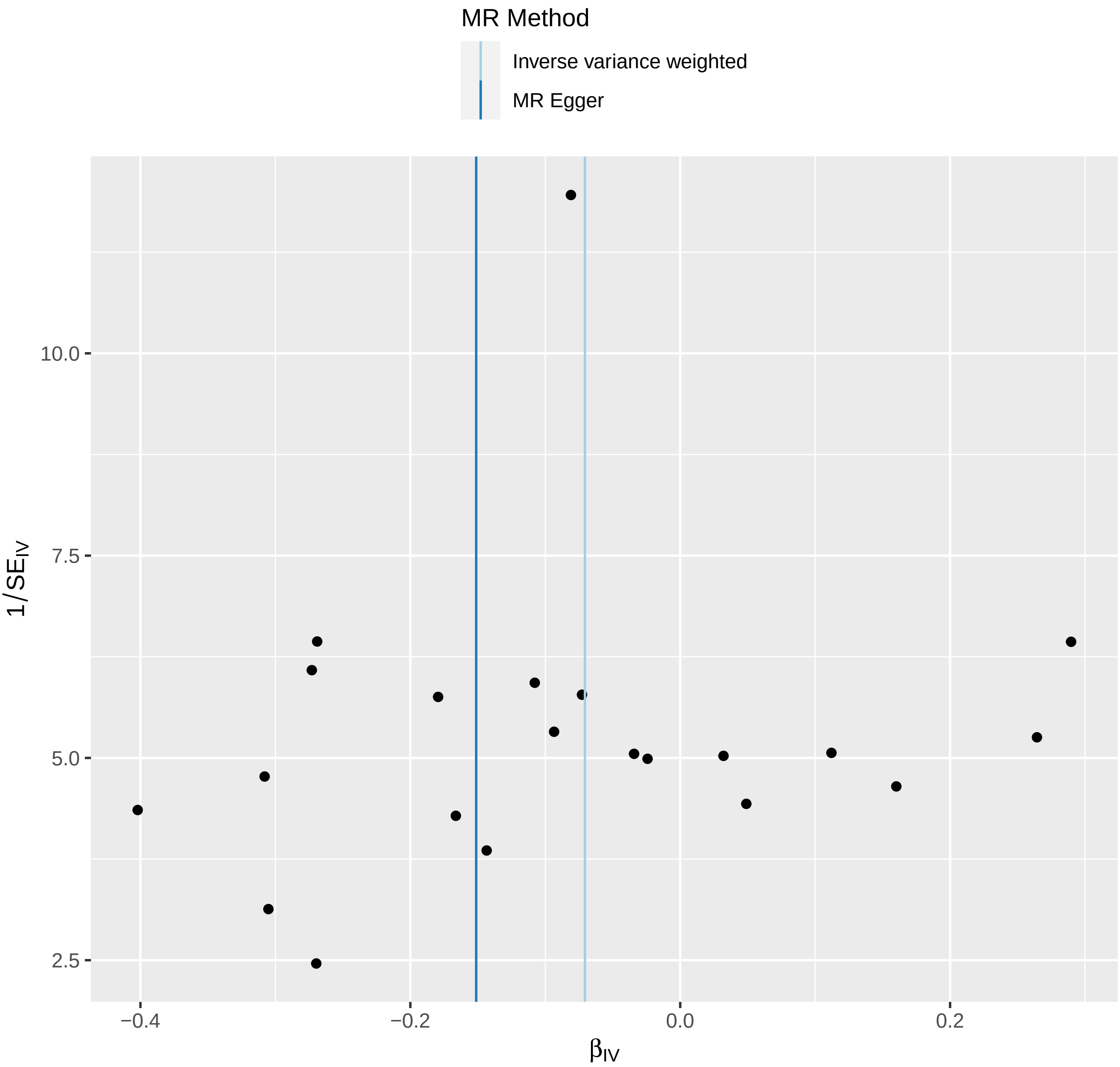

Batch 3−2455 : Poliovirus receptor on Diabetic nephropathy (more control exclusions)
with a threshold of 1e−05

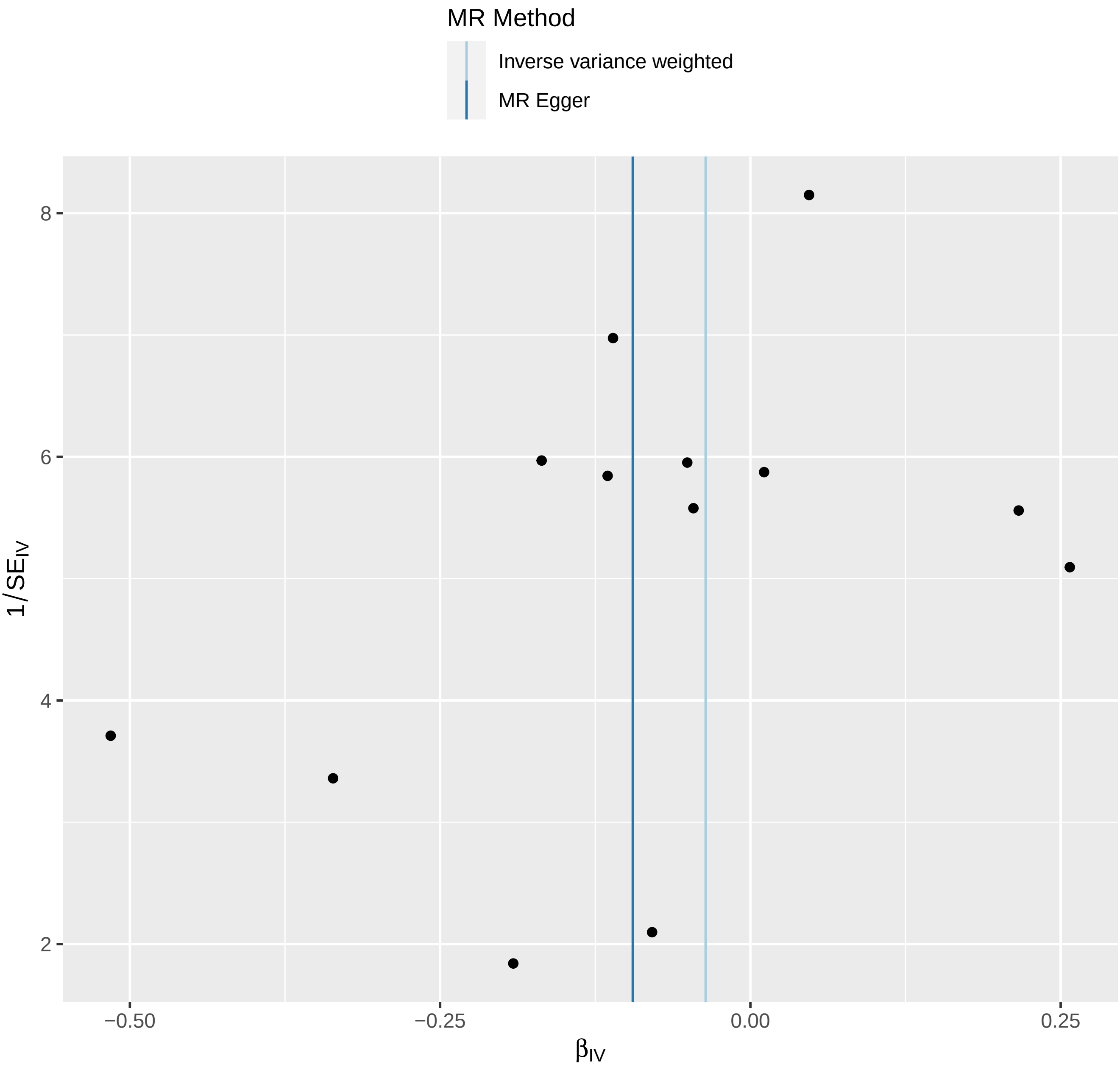

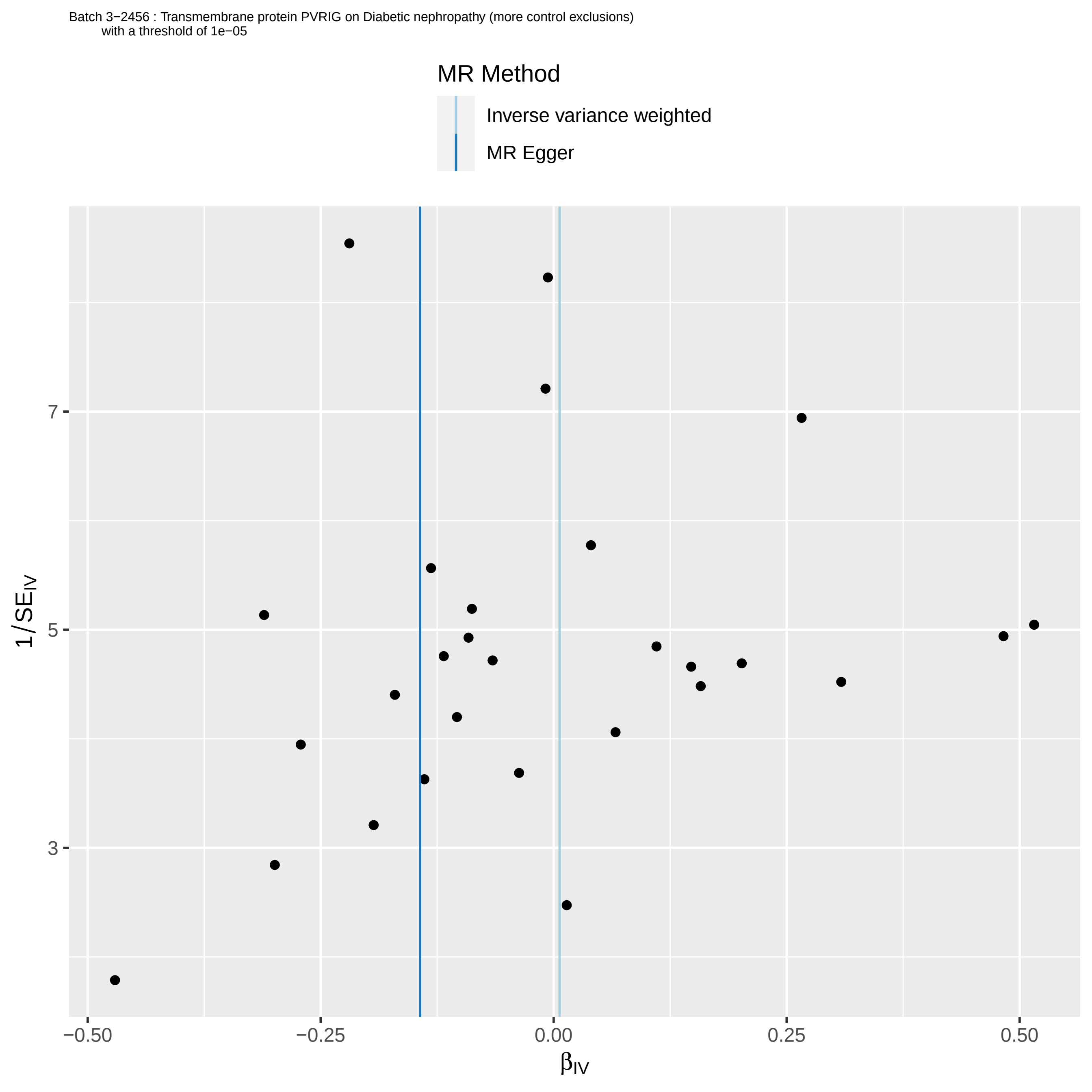
Batch 3−2456 : Transmembrane protein PVRIG on Diabetic nephropathy (more control exclusions)
with a threshold of 1e−05
MR Method
Inverse variance weighted
MR Egger
7
5
3
1/SE$_{IV}$
−0.50
−0.25
0.00
0.25
0.50
β$_{IV}$

Batch 3−2457 : Nectin−2 on Diabetic nephropathy (more control exclusions)
with a threshold of 1e−05

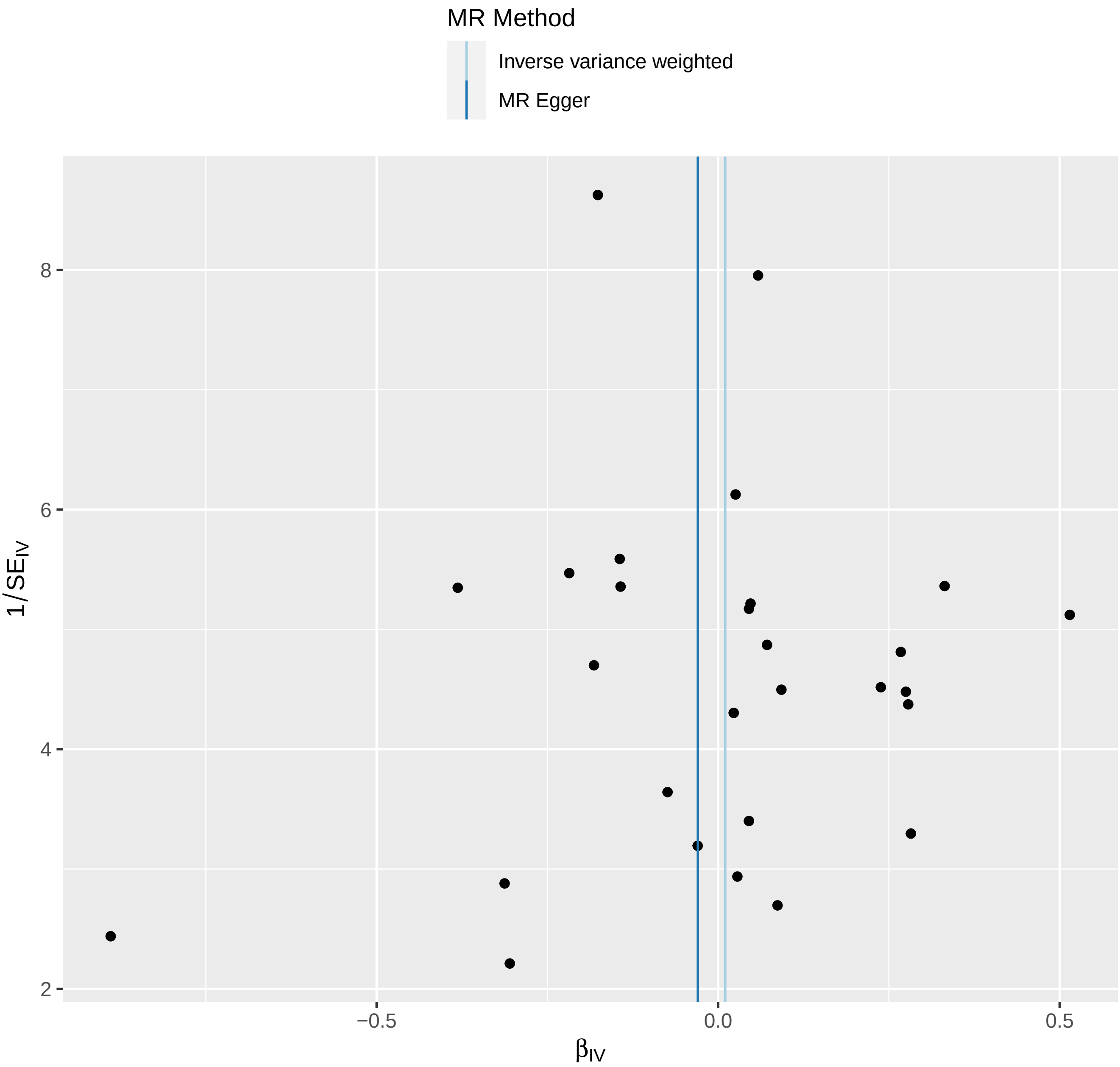

Batch 3−2458 : Nectin−3 on Diabetic nephropathy (more control exclusions)
with a threshold of 1e−05

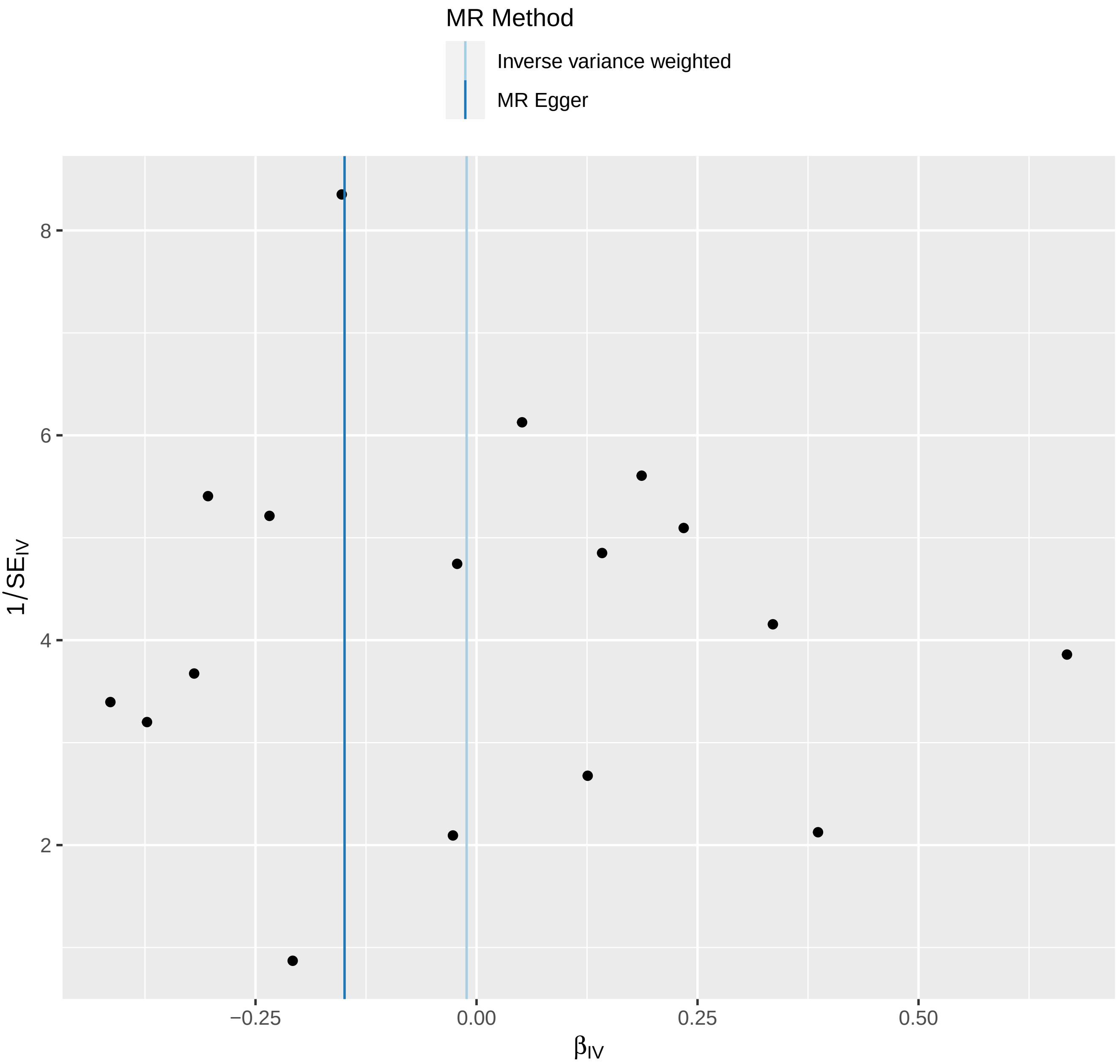

Batch 3−2459 : Nectin−4 on Diabetic nephropathy (more control exclusions)
with a threshold of 1e−05

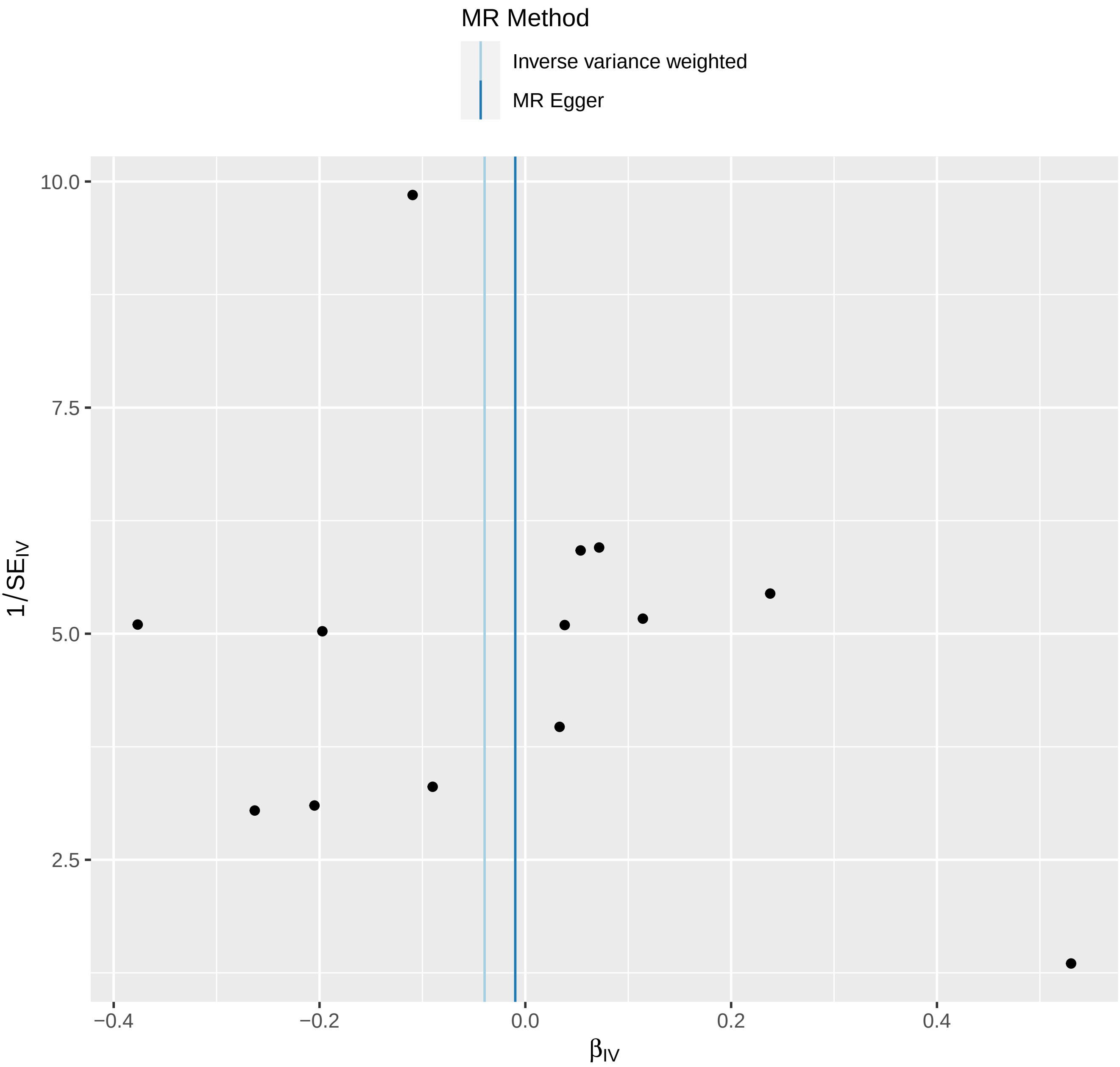

Batch 3−2460 : Peroxidasin−like protein on Diabetic nephropathy (more control exclusions)
with a threshold of 1e−05

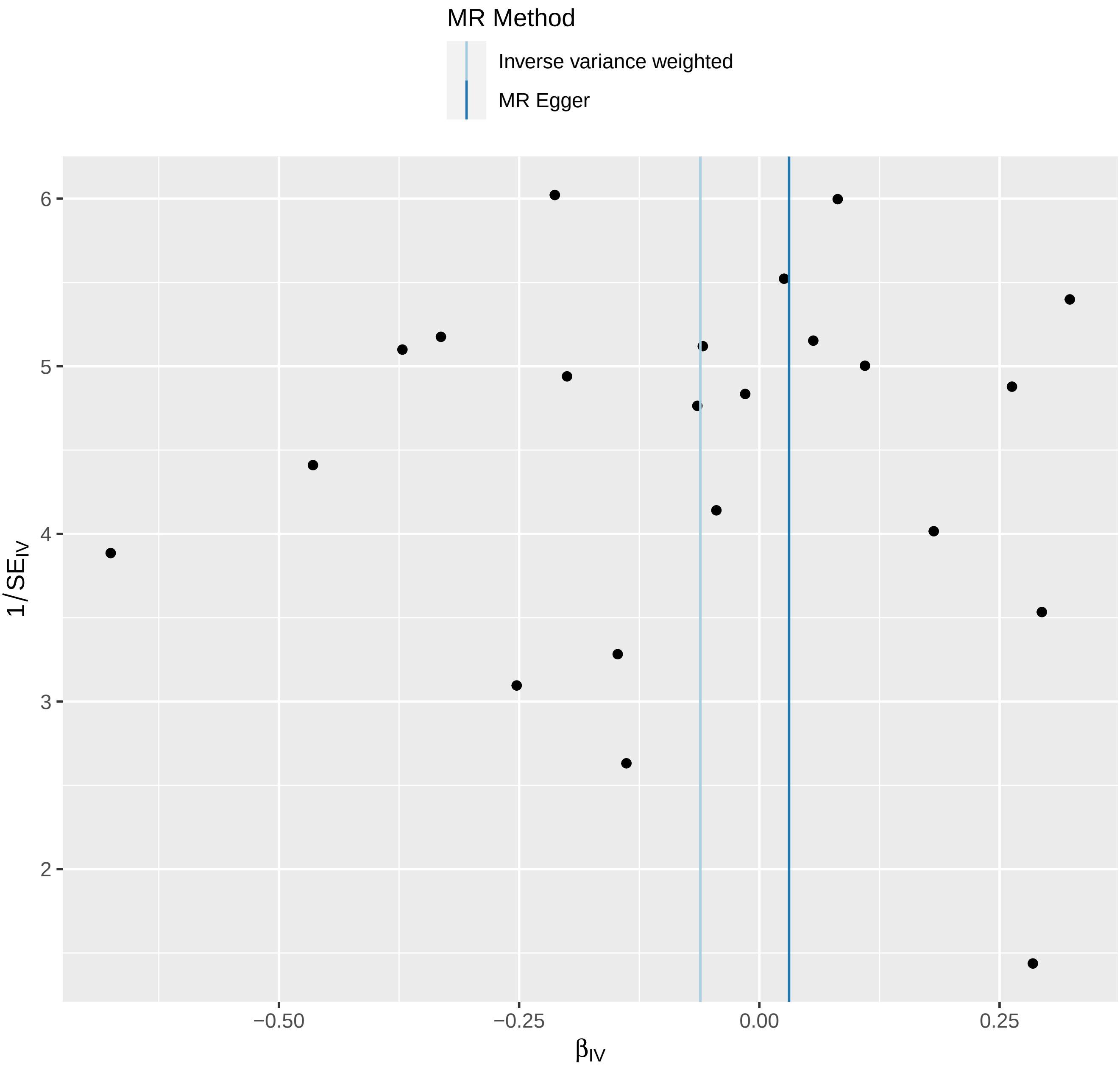

Batch 3−2461 : Pyrin domain−containing protein 1 on Diabetic nephropathy (more control exclusions)
with a threshold of 1e−05

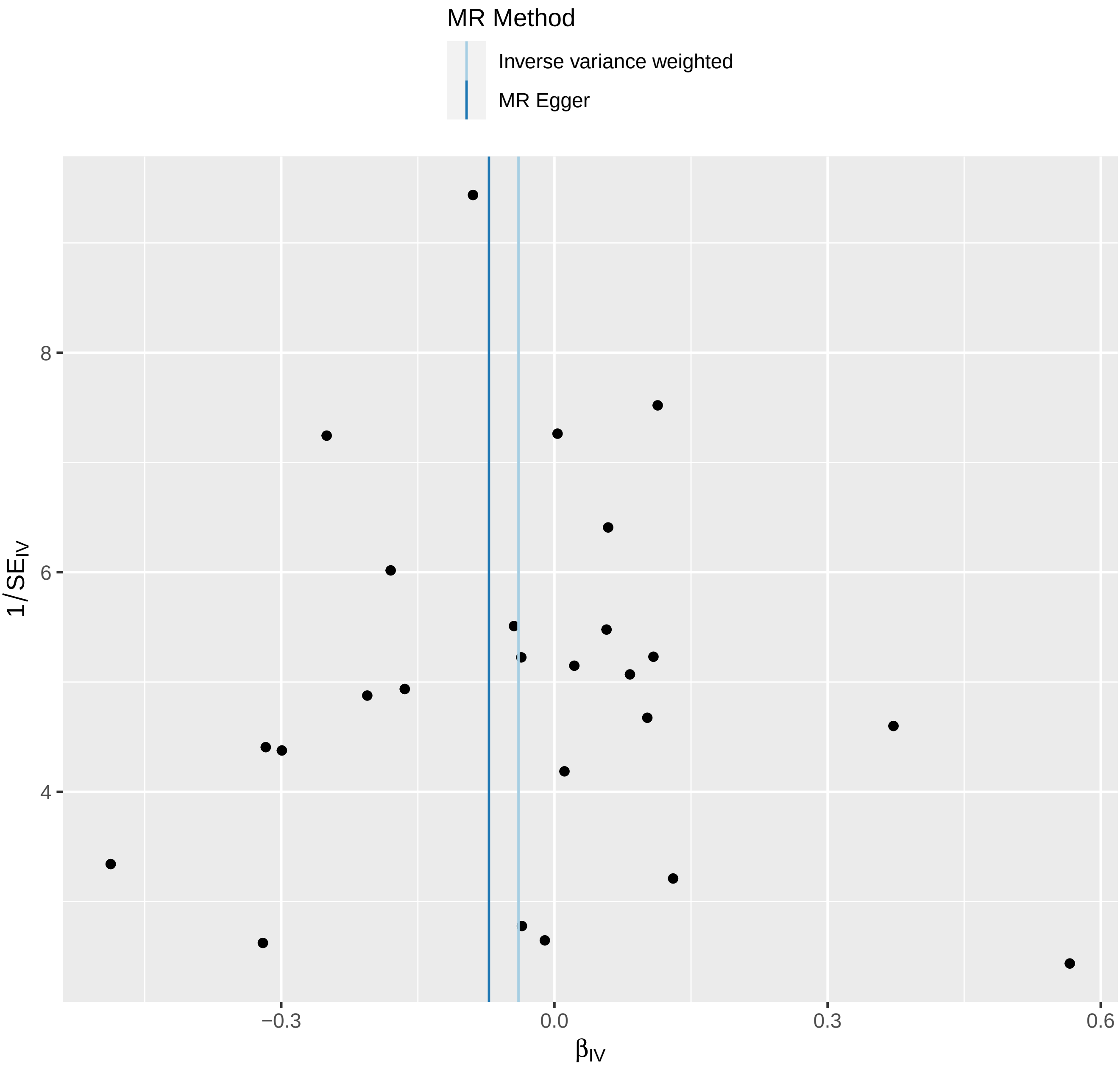

Batch 3−2462 : Glycogen phosphorylase, liver form on Diabetic nephropathy (more control exclusions)
with a threshold of 1e−05

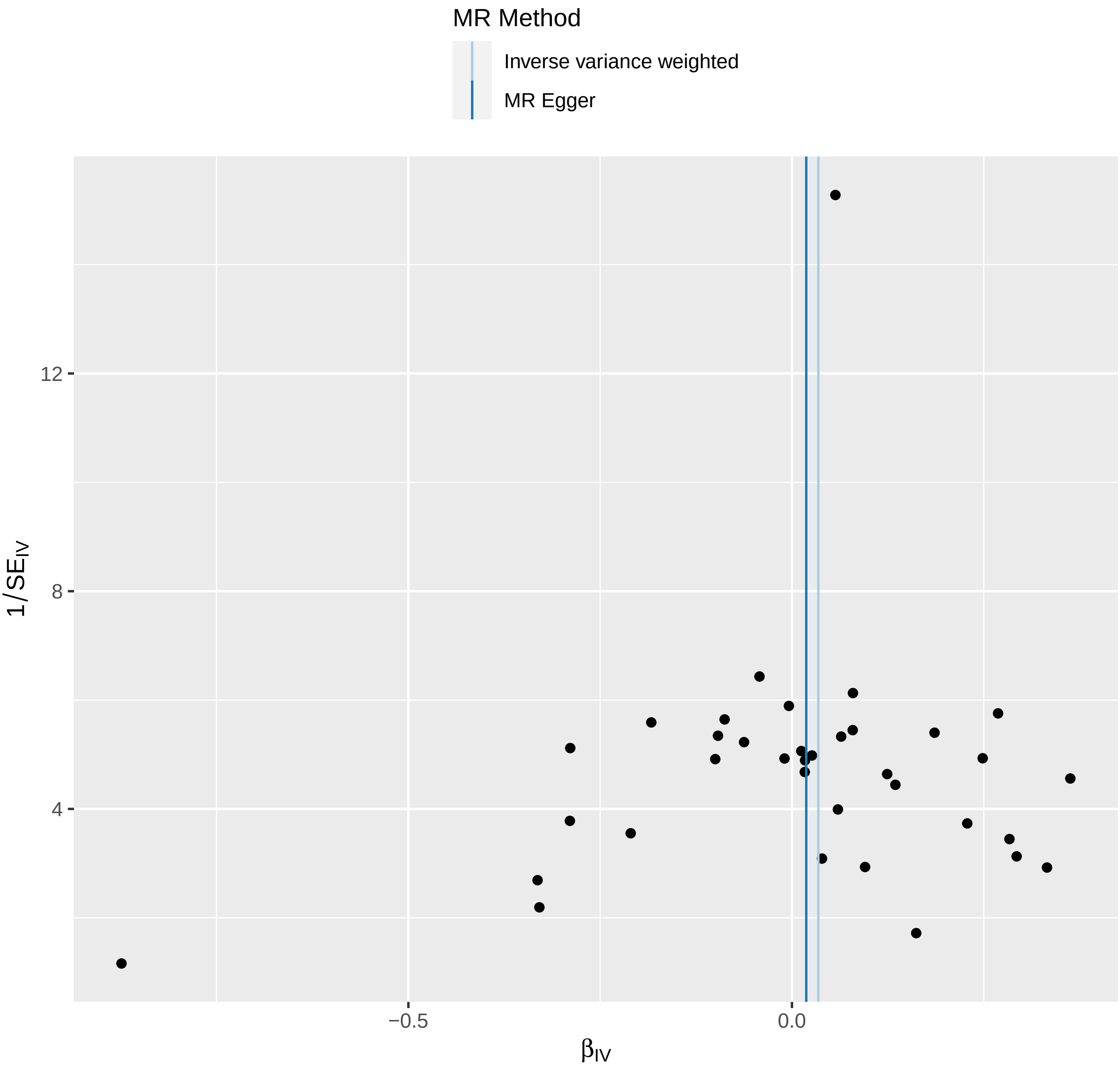

Batch 3−2463 : Peptide YY on Diabetic nephropathy (more control exclusions)
with a threshold of 1e−05

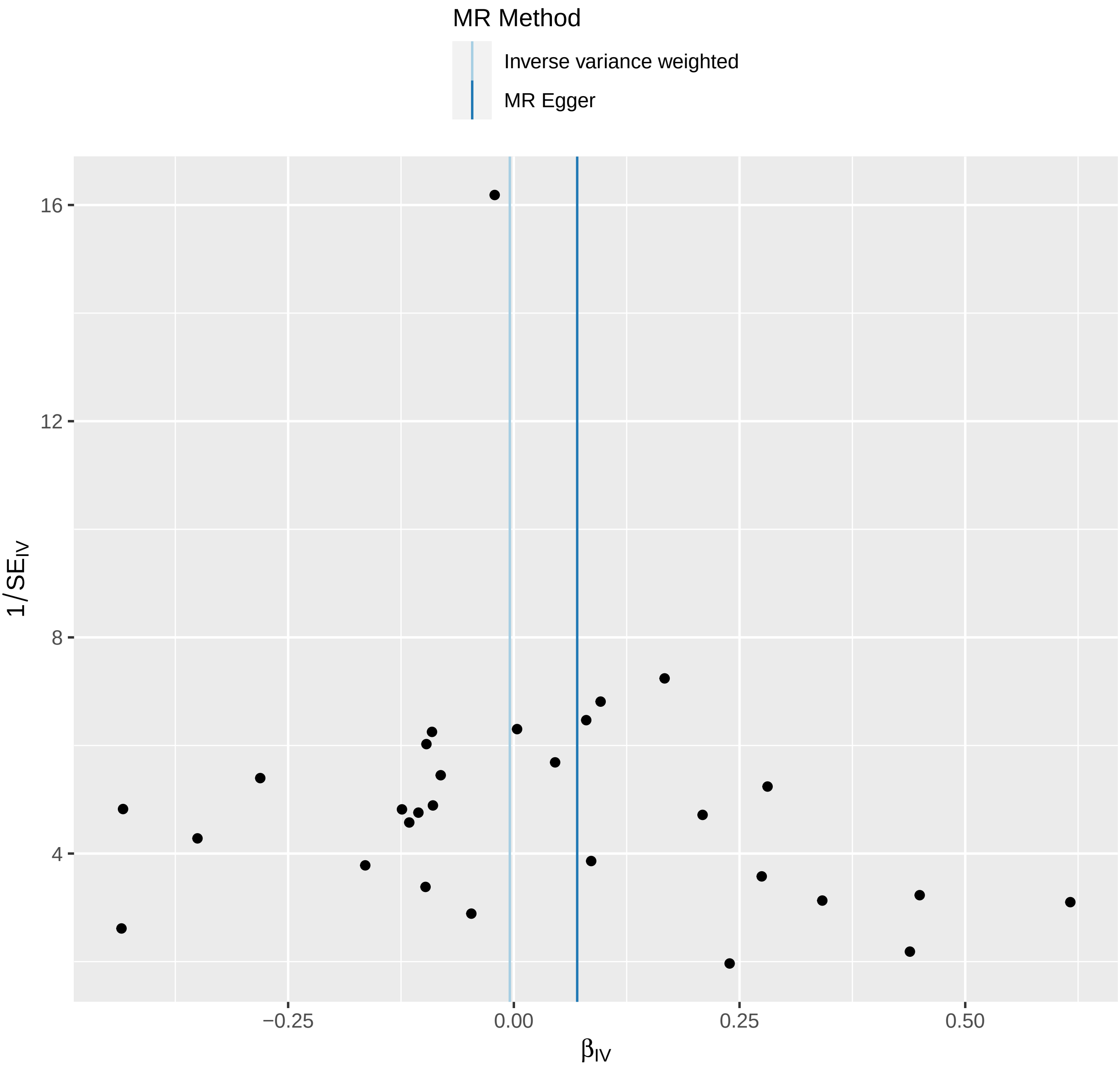

Batch 3−2464 : Dihydropteridine reductase on Diabetic nephropathy (more control exclusions)
with a threshold of 1e−05

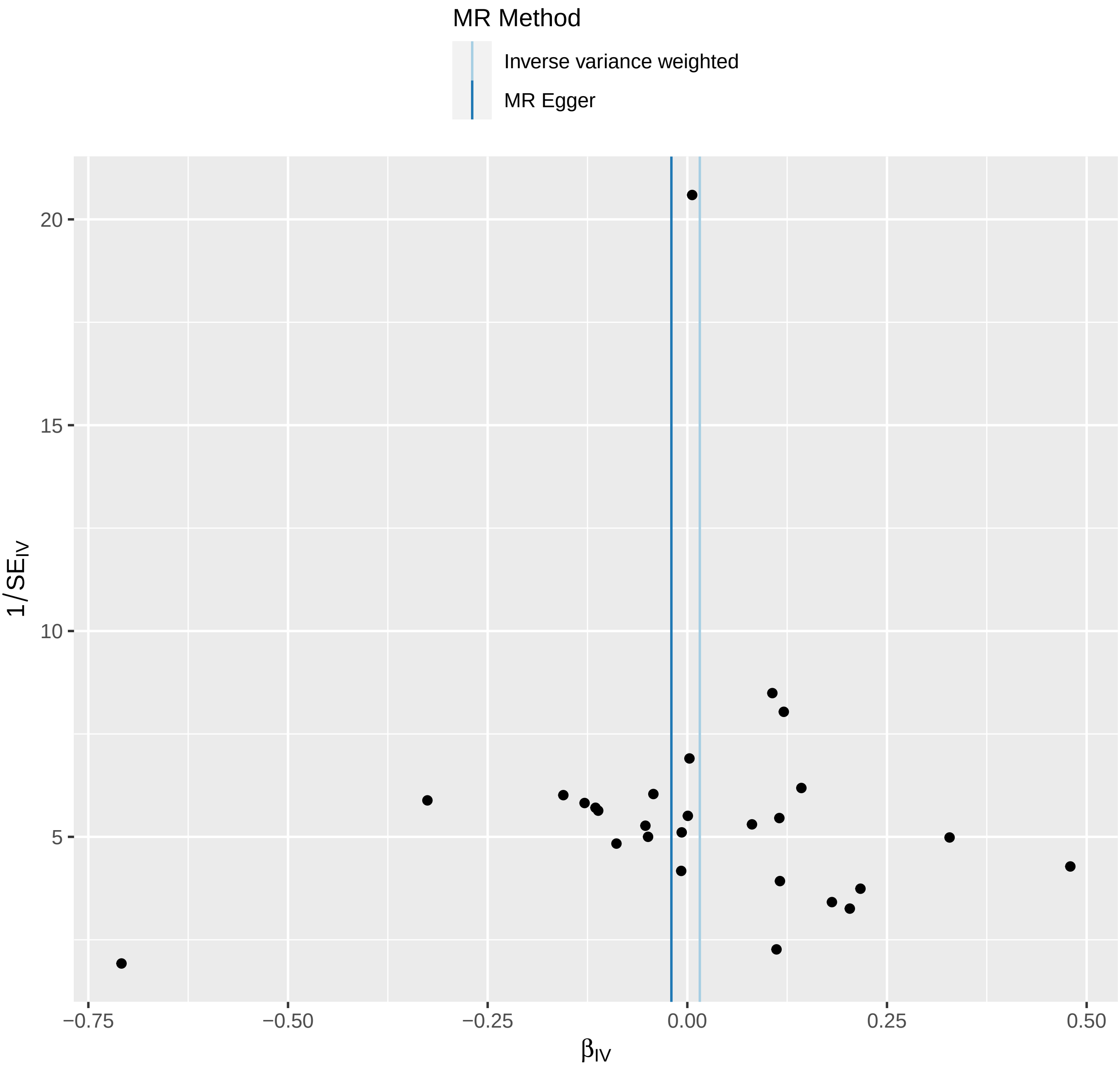

Batch 3−2465 : Glutaminyl−peptide cyclotransferase on Diabetic nephropathy (more control exclusions) with a threshold of 1e−05

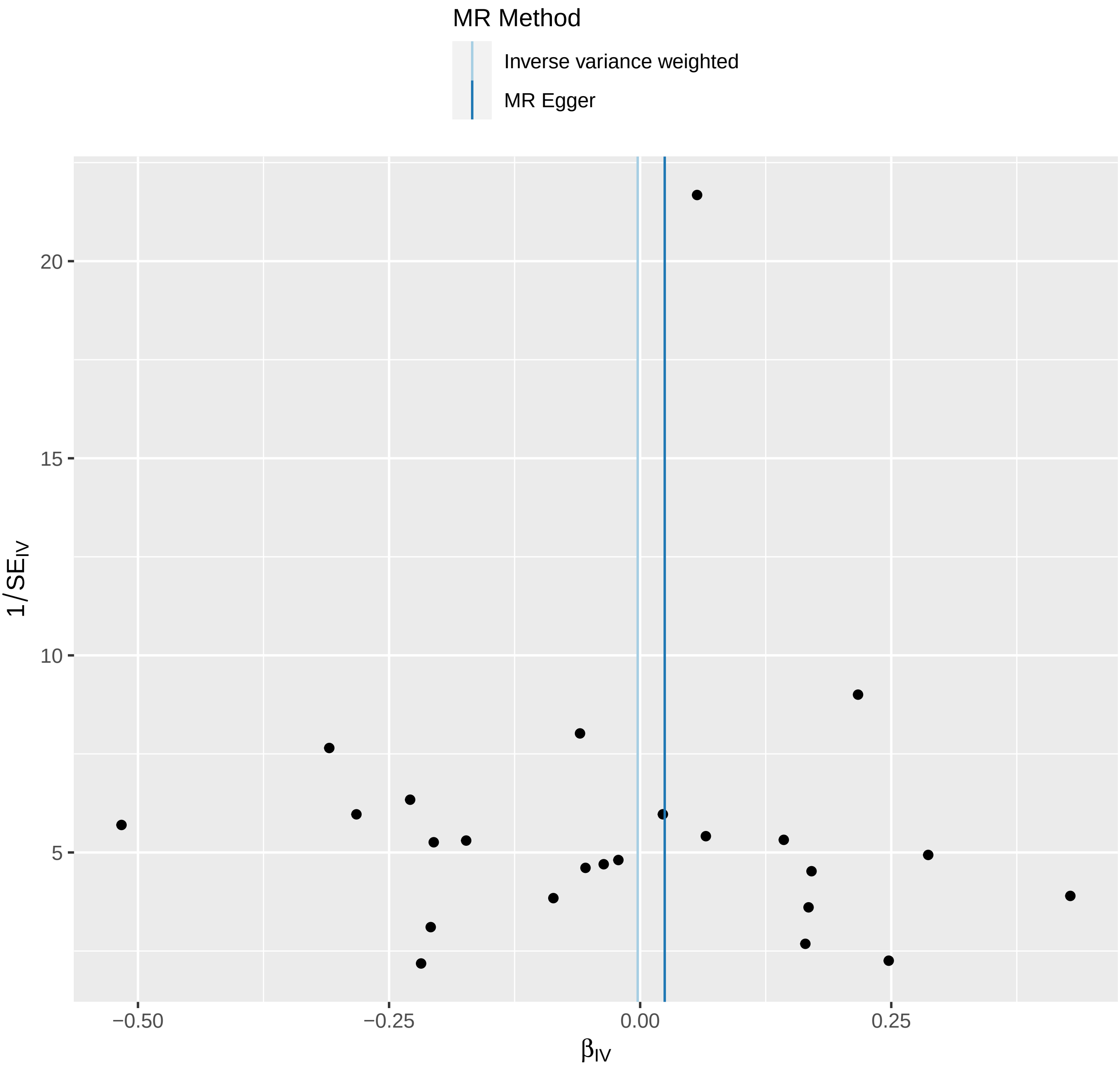

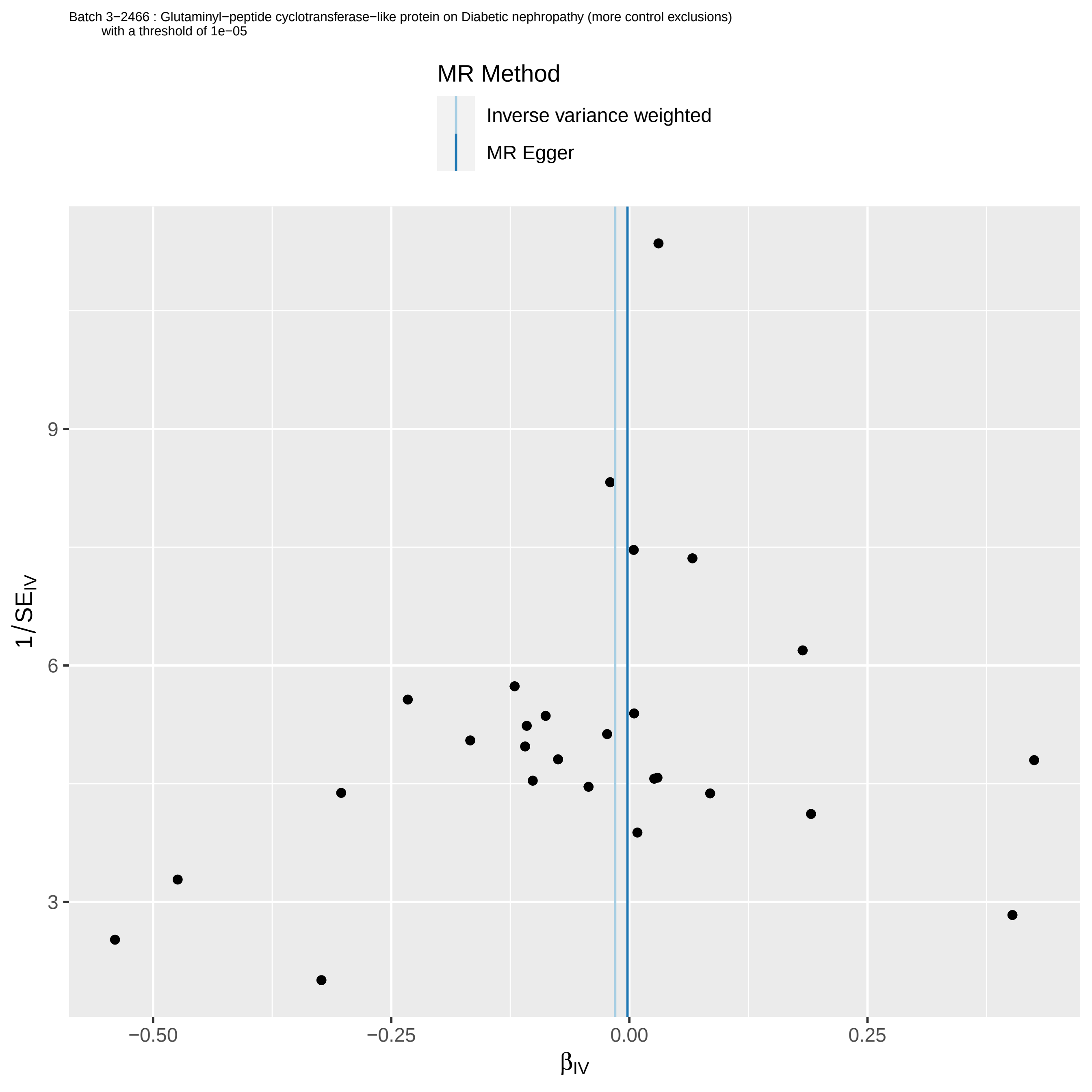

Batch 3−2466 : Glutaminyl−peptide cyclotransferase−like protein on Diabetic nephropathy (more control exclusions)
with a threshold of 1e−05
MR Method
Inverse variance weighted
MR Egger
9
6
3
1/SE_IV
−0.50
−0.25
0.00
0.25
β_IV

Batch 3−2467 : Orexigenic neuropeptide QRFP on Diabetic nephropathy (more control exclusions)
with a threshold of 1e−05

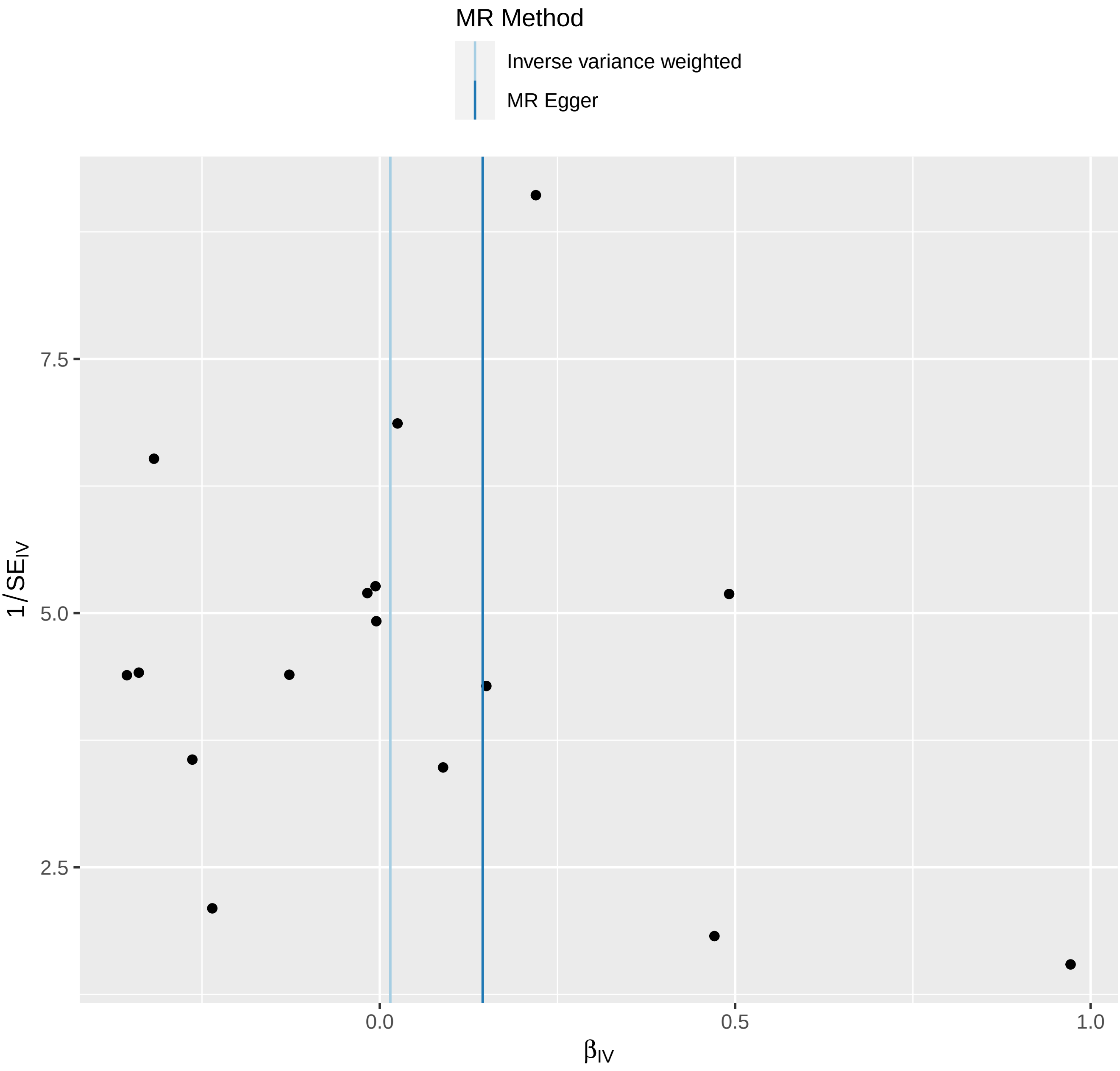

Batch 3−2468 : Sulfhydryl oxidase 1 on Diabetic nephropathy (more control exclusions)
with a threshold of 1e−05

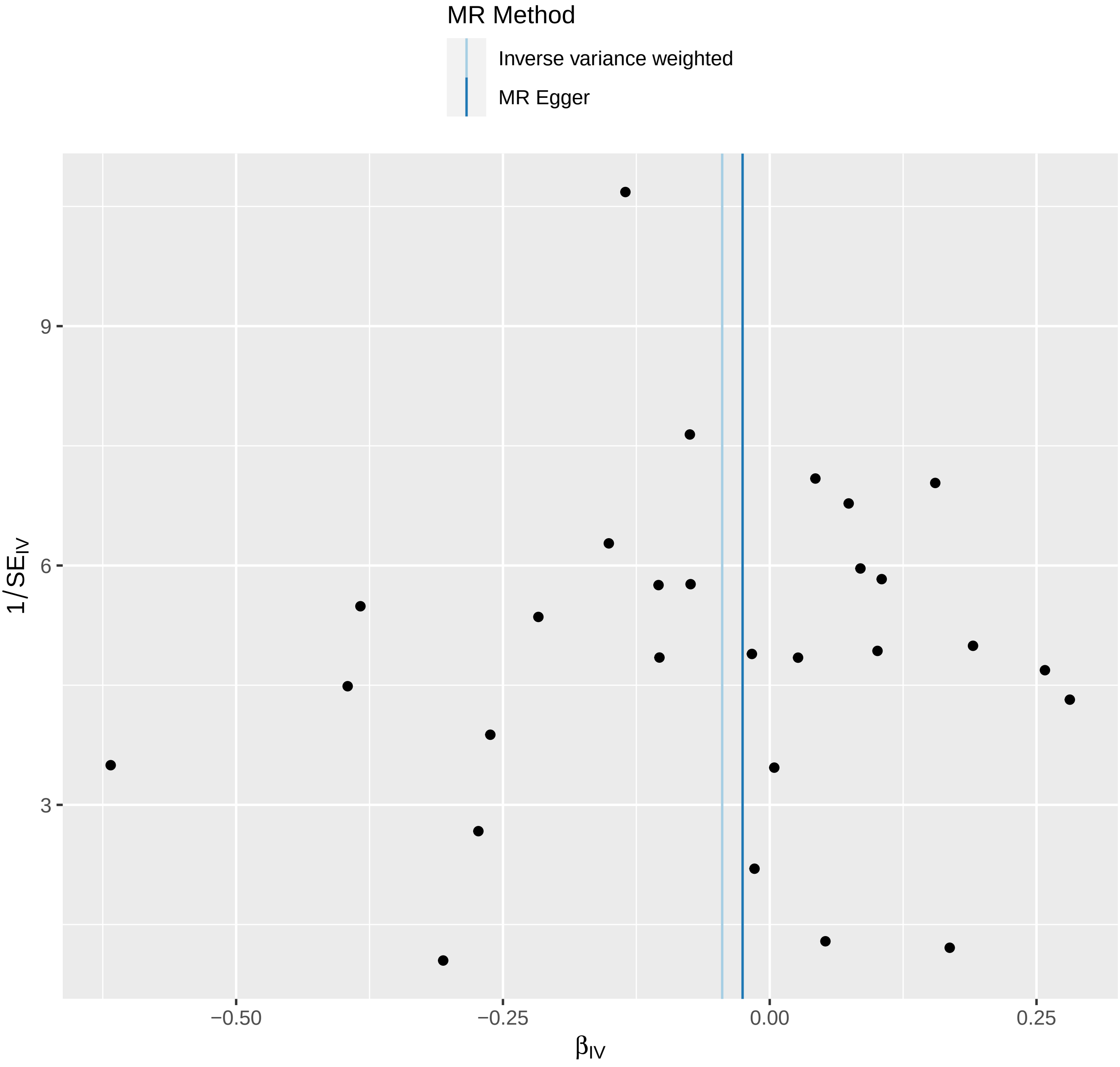

Batch 3−2469 : Sulfhydryl oxidase 1 on Diabetic nephropathy (more control exclusions)
with a threshold of 1e−05

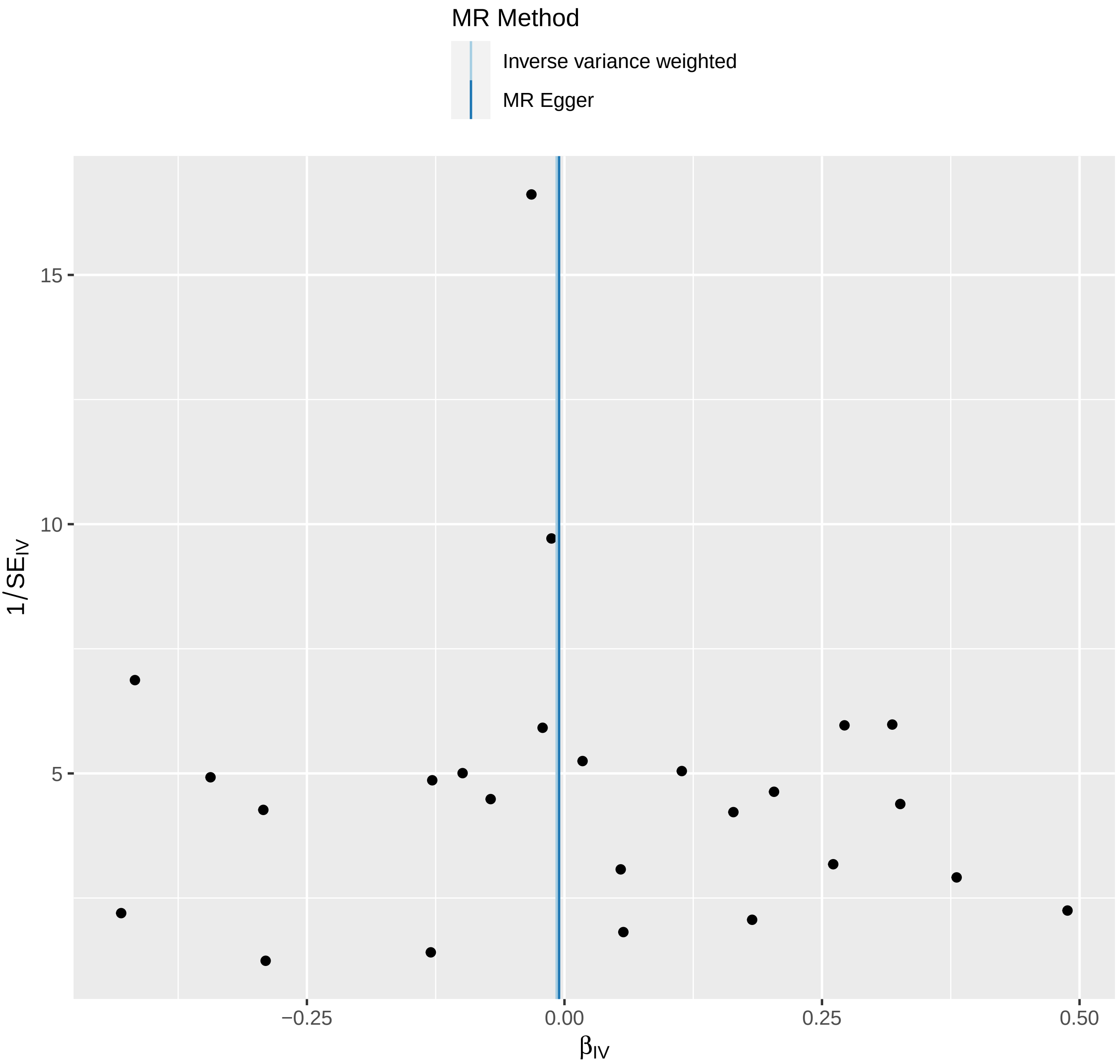

Batch 3−2470 : Sulfhydryl oxidase 2 on Diabetic nephropathy (more control exclusions)
with a threshold of 1e−05

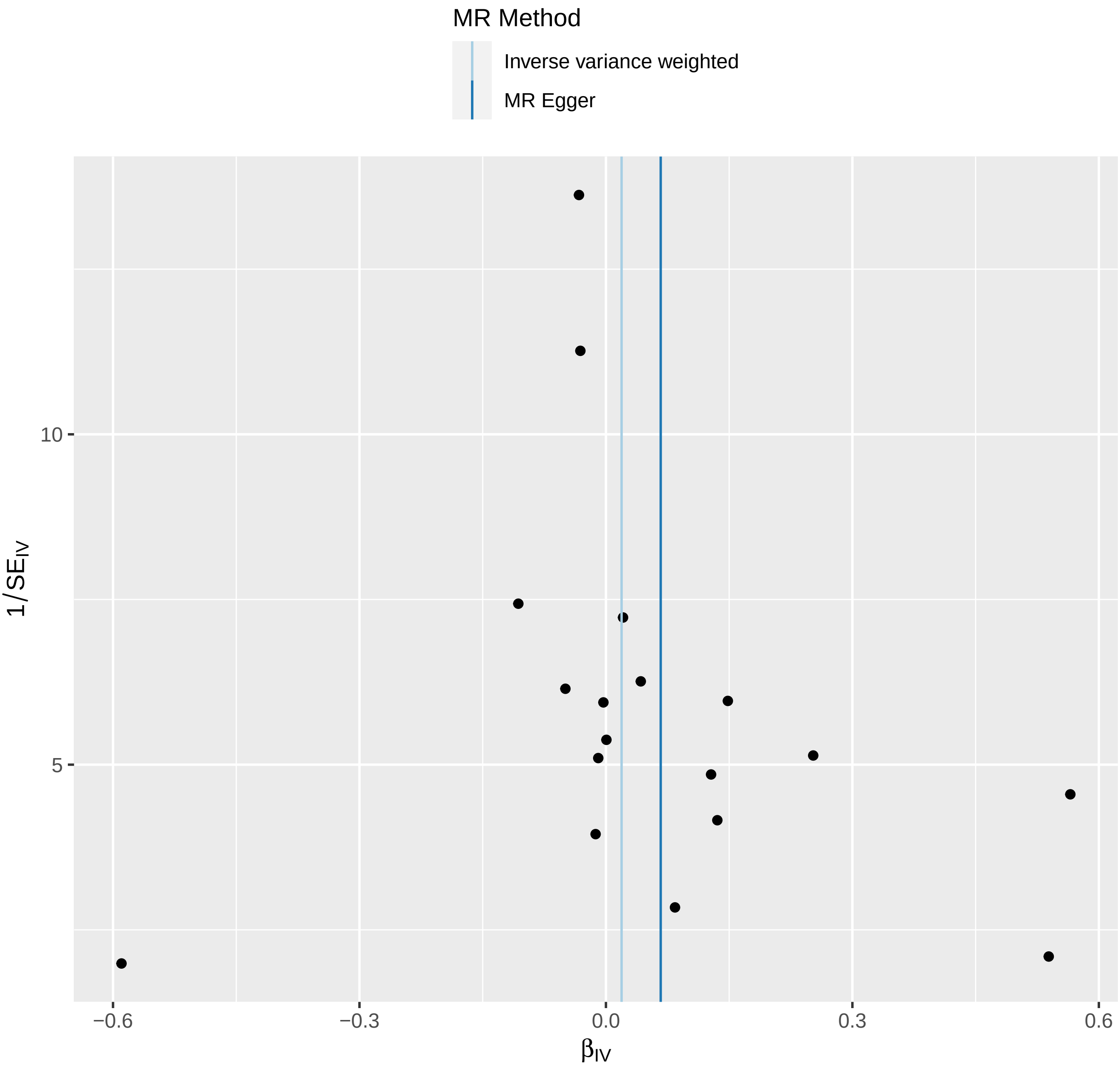

Batch 3−2471 : Ras−related protein Rab−14 on Diabetic nephropathy (more control exclusions)
with a threshold of 1e−05

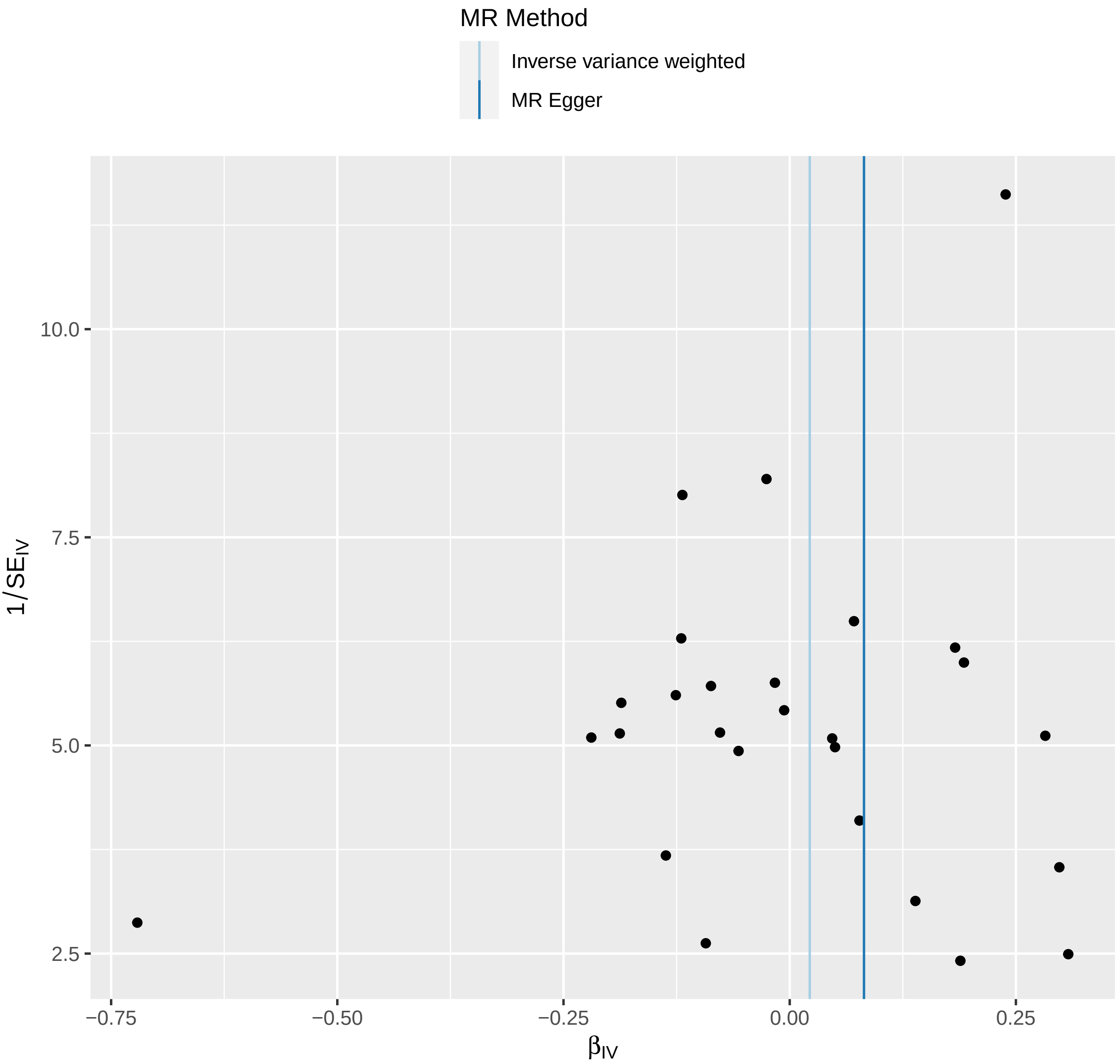

Batch 3−2472 : Ras−related protein Rab−22A on Diabetic nephropathy (more control exclusions)
with a threshold of 1e−05

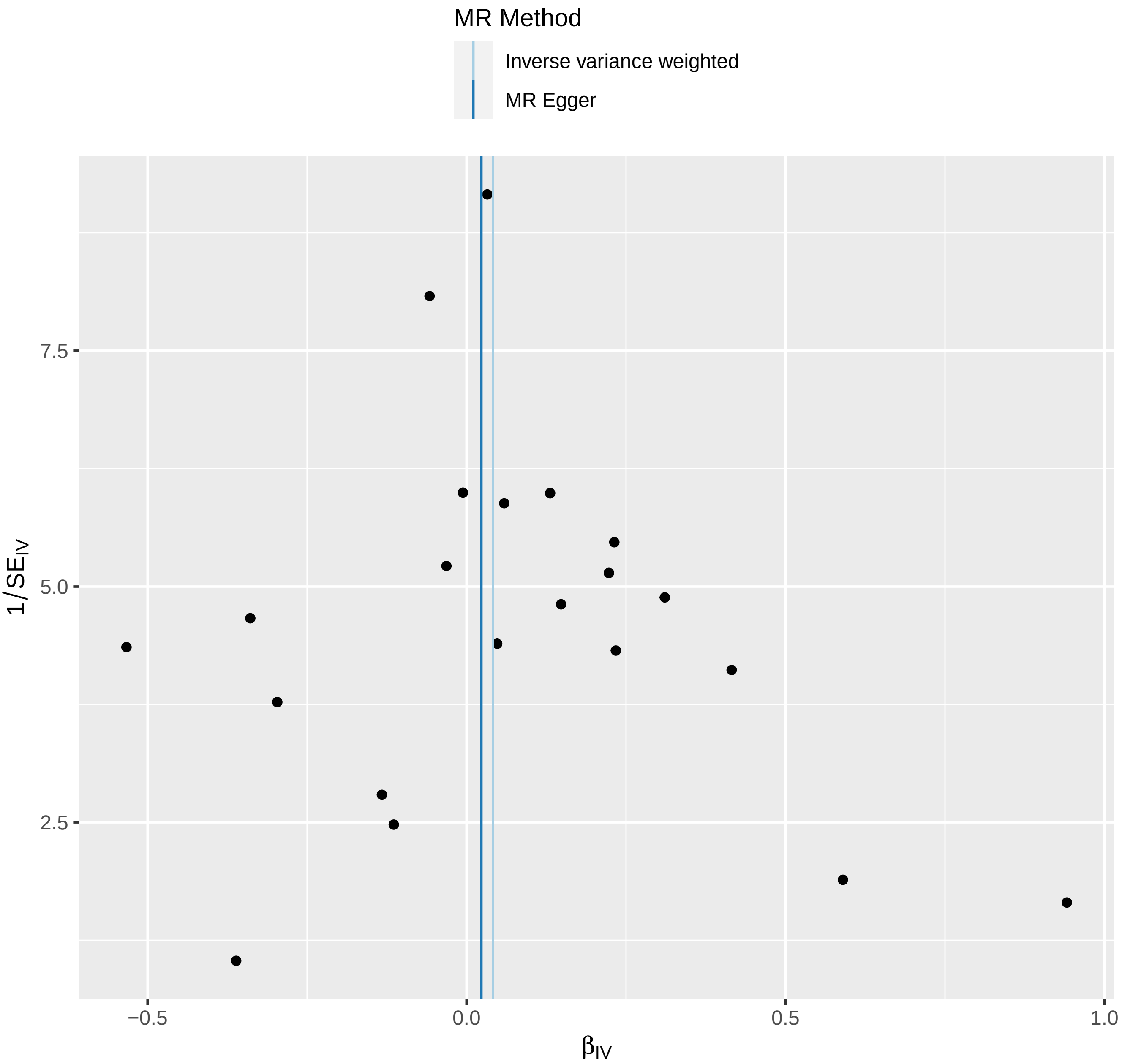

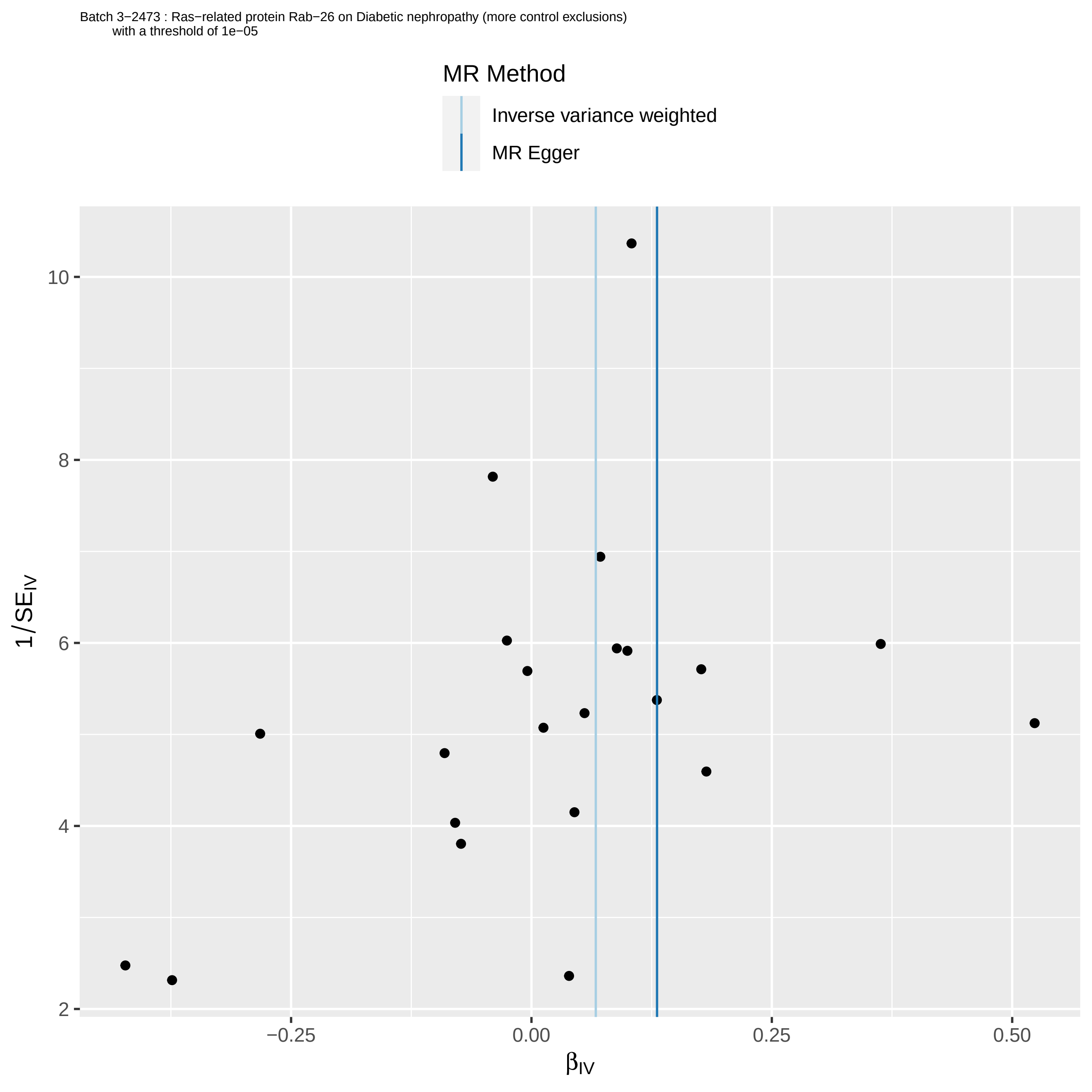

Batch 3−2473 : Ras−related protein Rab−26 on Diabetic nephropathy (more control exclusions)
with a threshold of 1e−05
MR Method
Inverse variance weighted
MR Egger
1/SE_IV
β_IV
10
8
6
4
2
−0.25
0.00
0.25
0.50

Batch 3−2474 : Ras−related protein Rab−27A on Diabetic nephropathy (more control exclusions) with a threshold of 1e−05

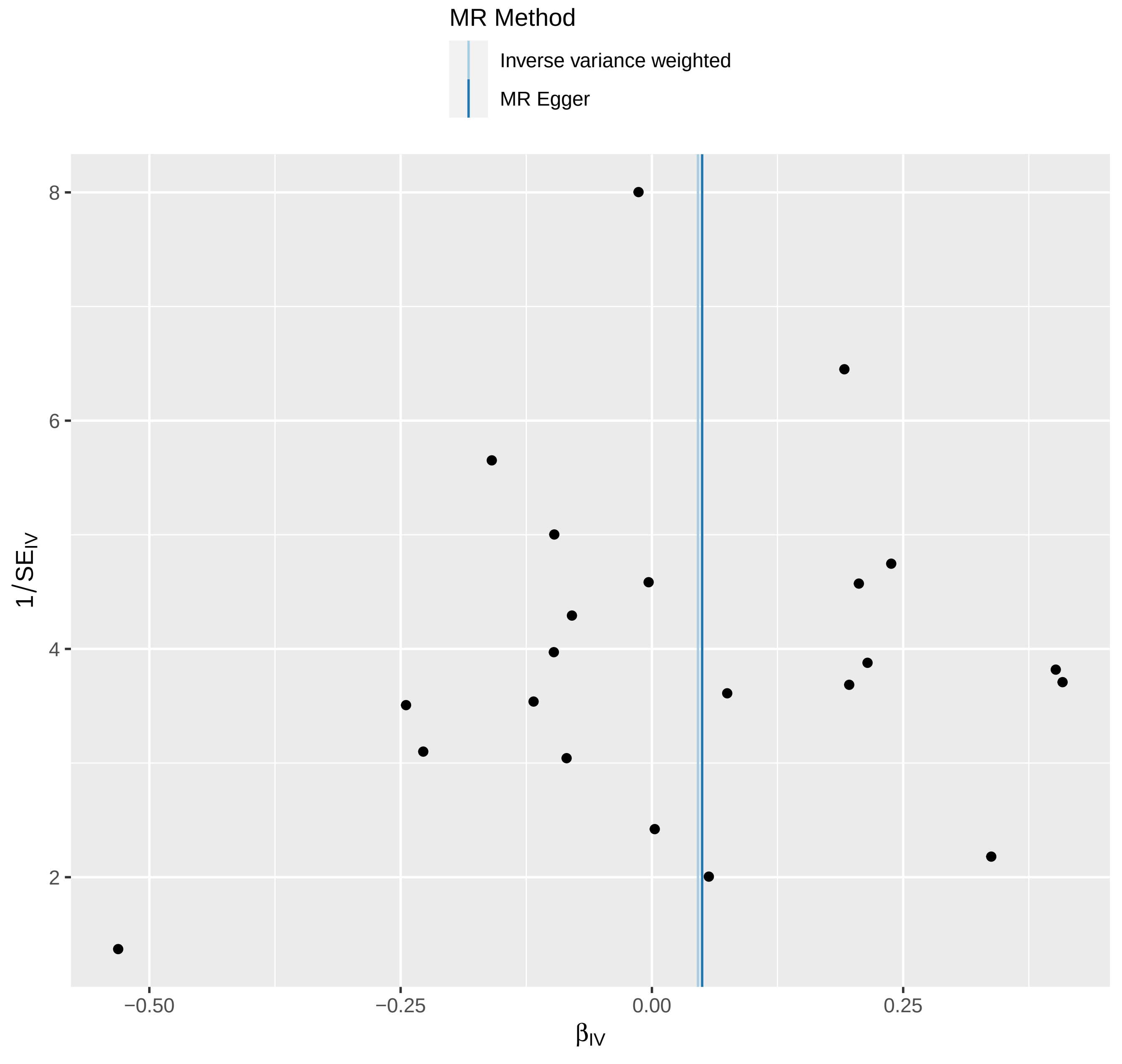

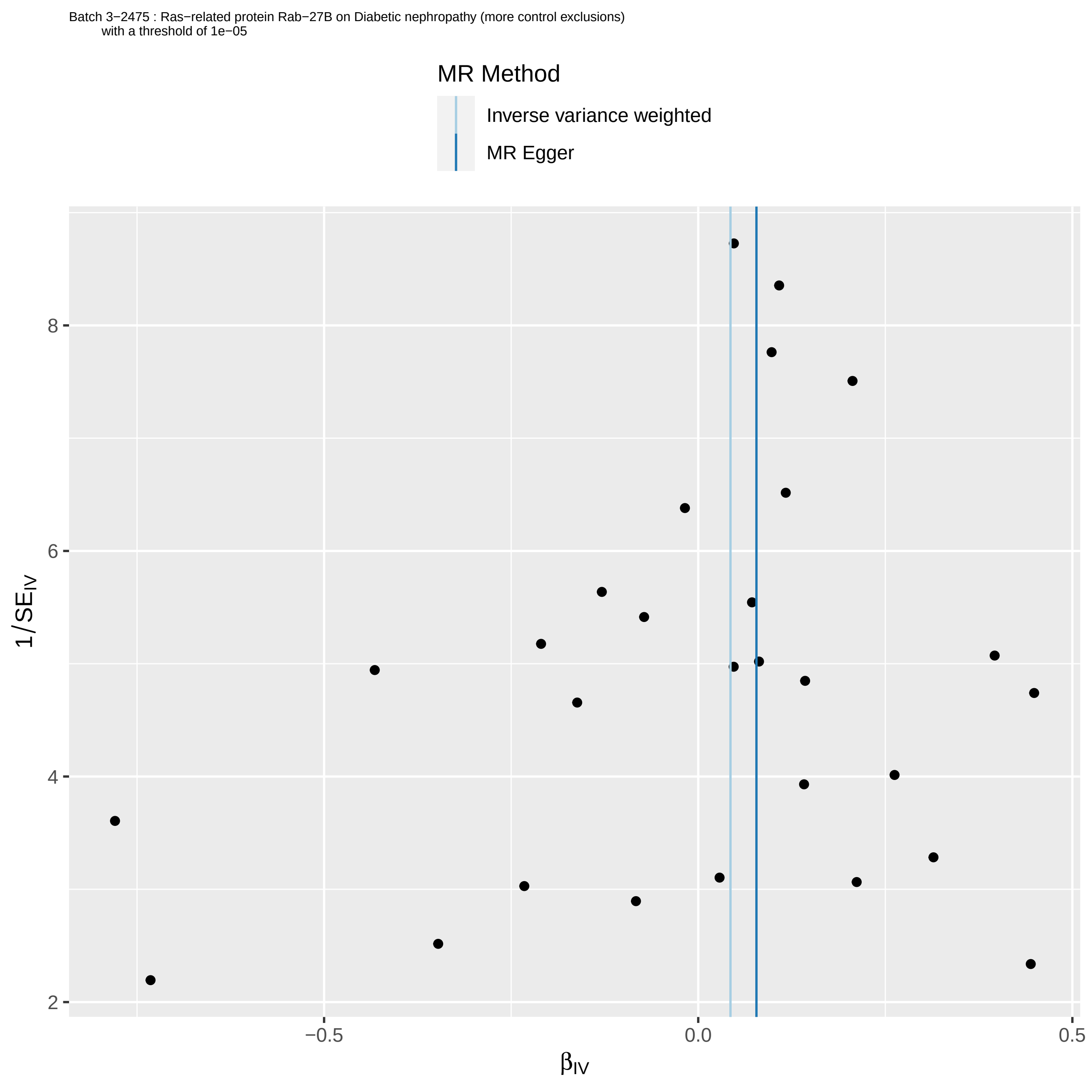
Batch 3−2475 : Ras−related protein Rab−27B on Diabetic nephropathy (more control exclusions)
with a threshold of 1e−05
MR Method
Inverse variance weighted
MR Egger
8
6
4
2
1/SE$_{IV}$
−0.5
0.0
0.5
β_{IV}

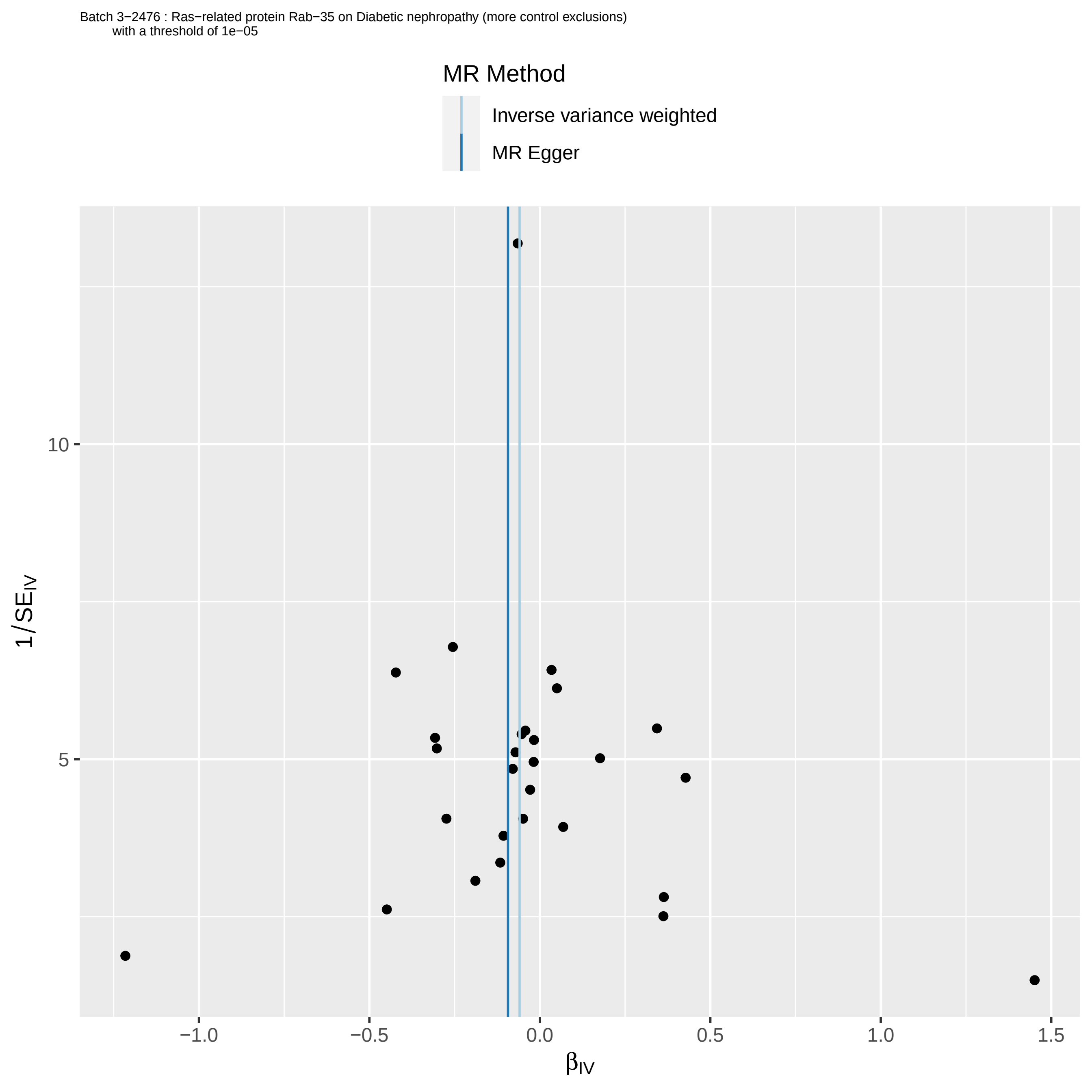

Batch 3−2476 : Ras−related protein Rab−35 on Diabetic nephropathy (more control exclusions)
with a threshold of 1e−05
MR Method
Inverse variance weighted
MR Egger
10
5
1/SE_IV
−1.0
−0.5
0.0
0.5
1.0
1.5
β_IV

Batch 3−2477 : Ras−related protein Rab−39B on Diabetic nephropathy (more control exclusions)
with a threshold of 1e−05

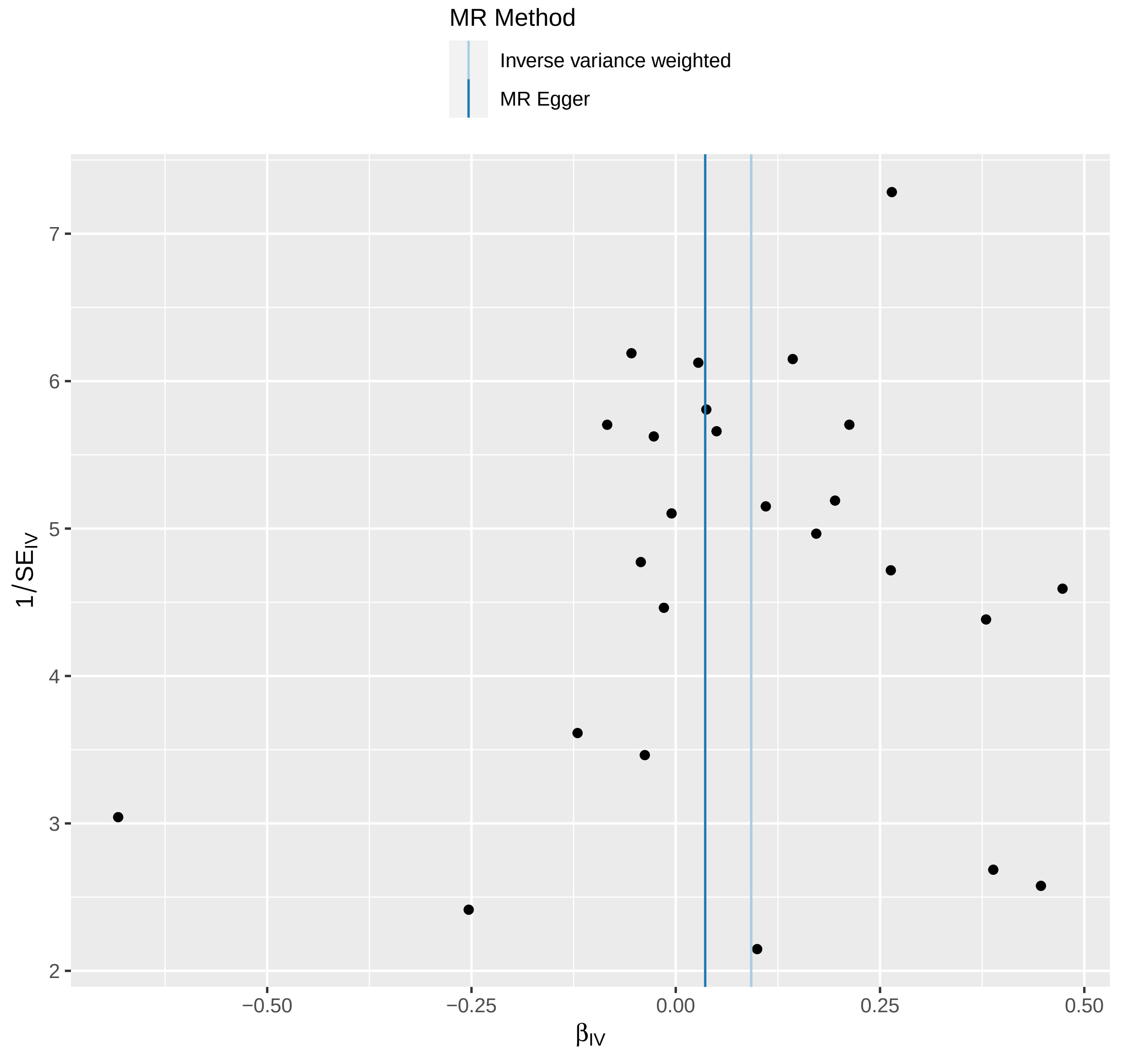

Batch 3−2478 : Ras−related protein Rab−7b on Diabetic nephropathy (more control exclusions)
with a threshold of 1e−05

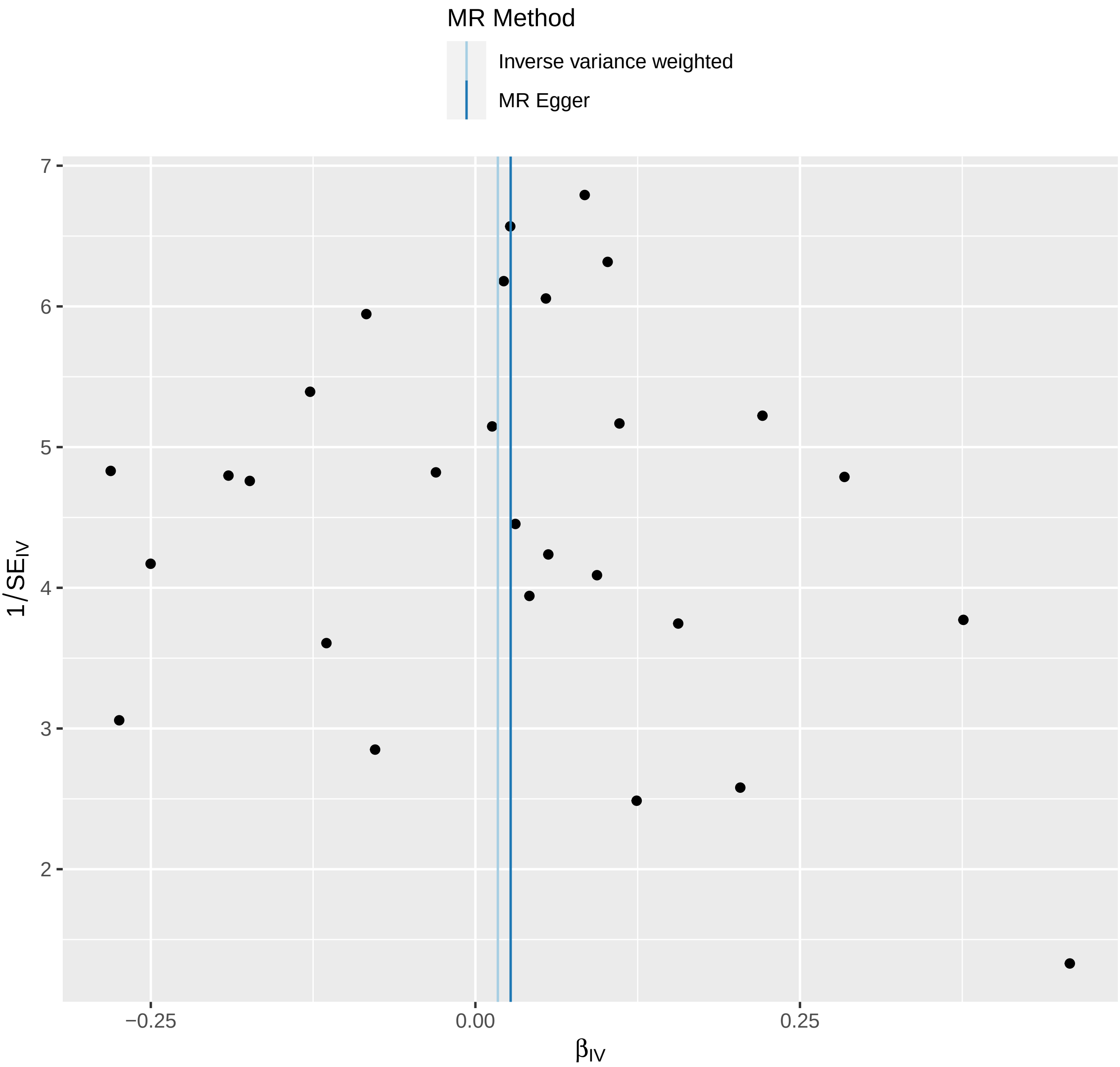

Batch 3−2479 : Rab9 effector protein with kelch motifs on Diabetic nephropathy (more control exclusions) with a threshold of 1e−05

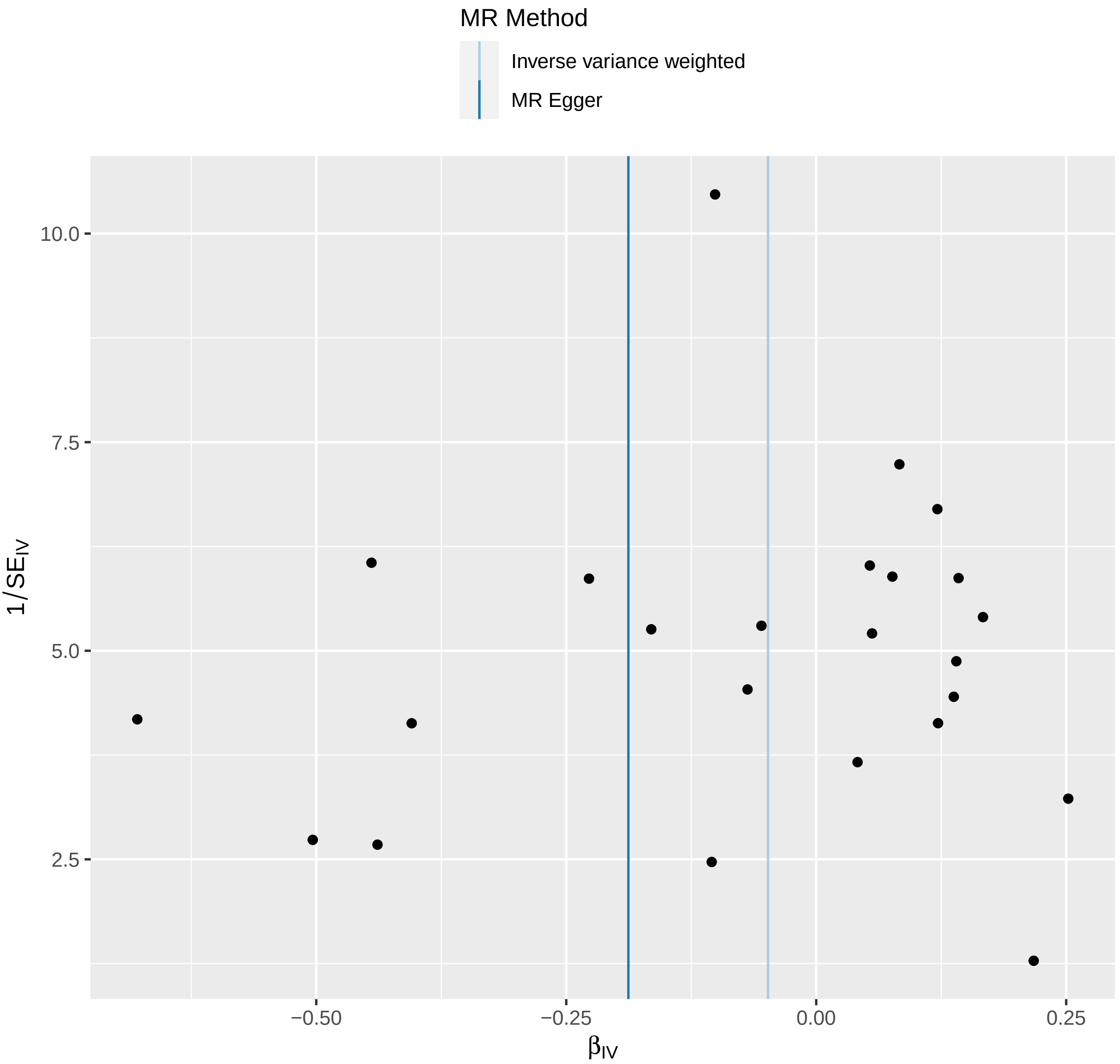

Batch 3−2480 : Ras−related C3 botulinum toxin substrate 3 on Diabetic nephropathy (more control exclusions) with a threshold of 1e−05

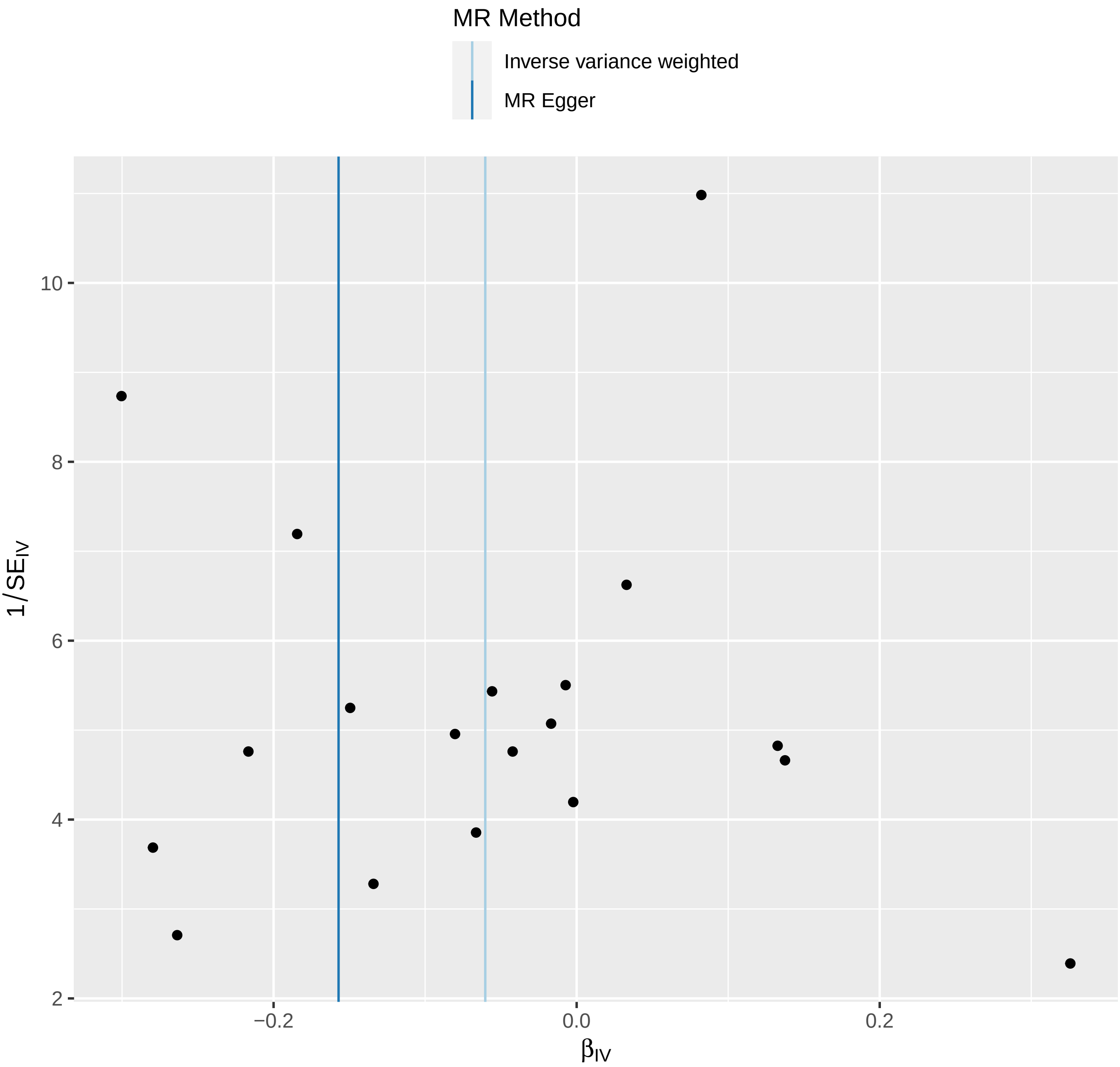

Batch 3−2481 : Rac GTPase−activating protein 1 on Diabetic nephropathy (more control exclusions)
with a threshold of 1e−05

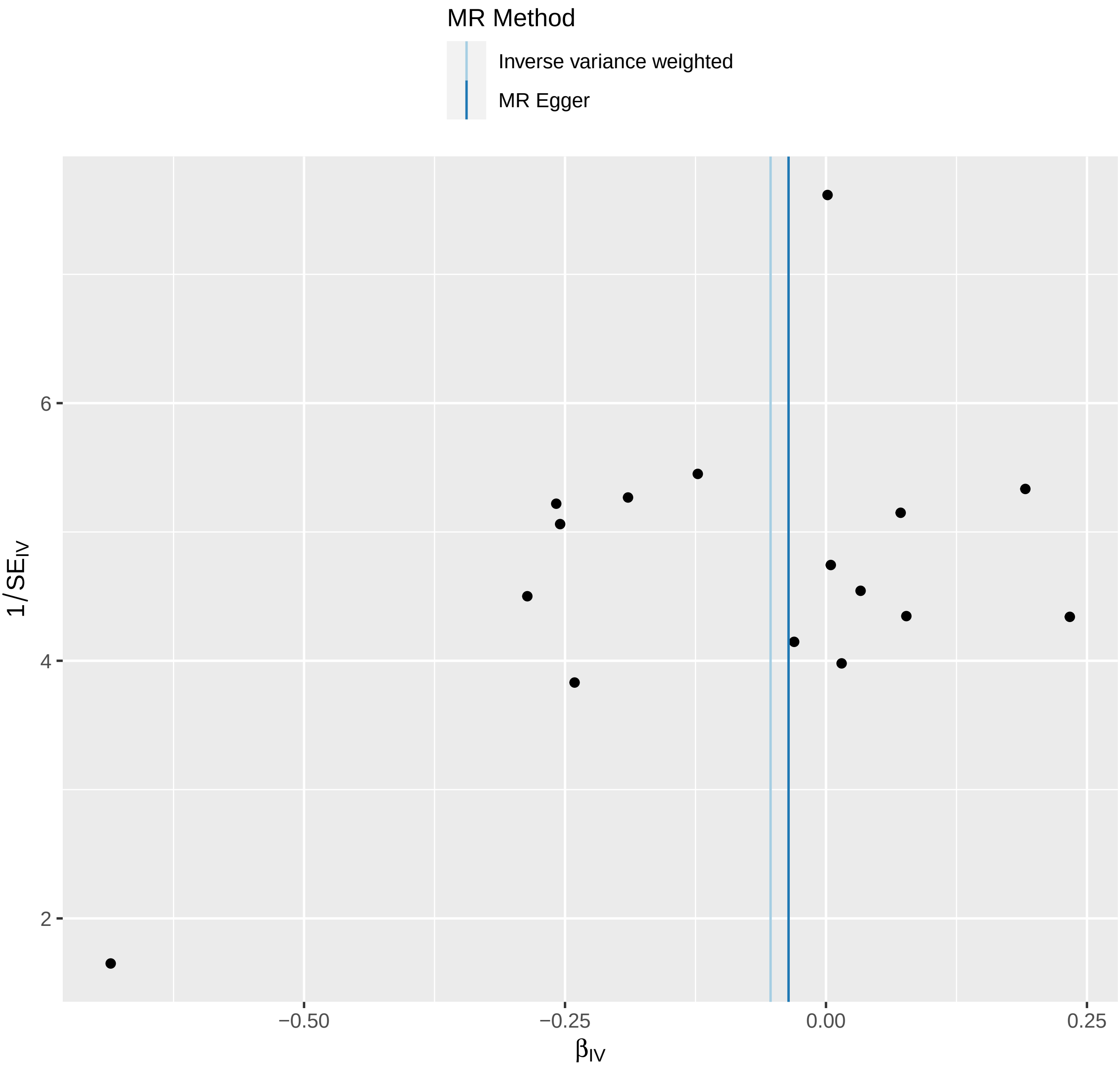

Batch 3−2482 : Cell cycle checkpoint protein RAD1 on Diabetic nephropathy (more control exclusions)
with a threshold of 1e−05

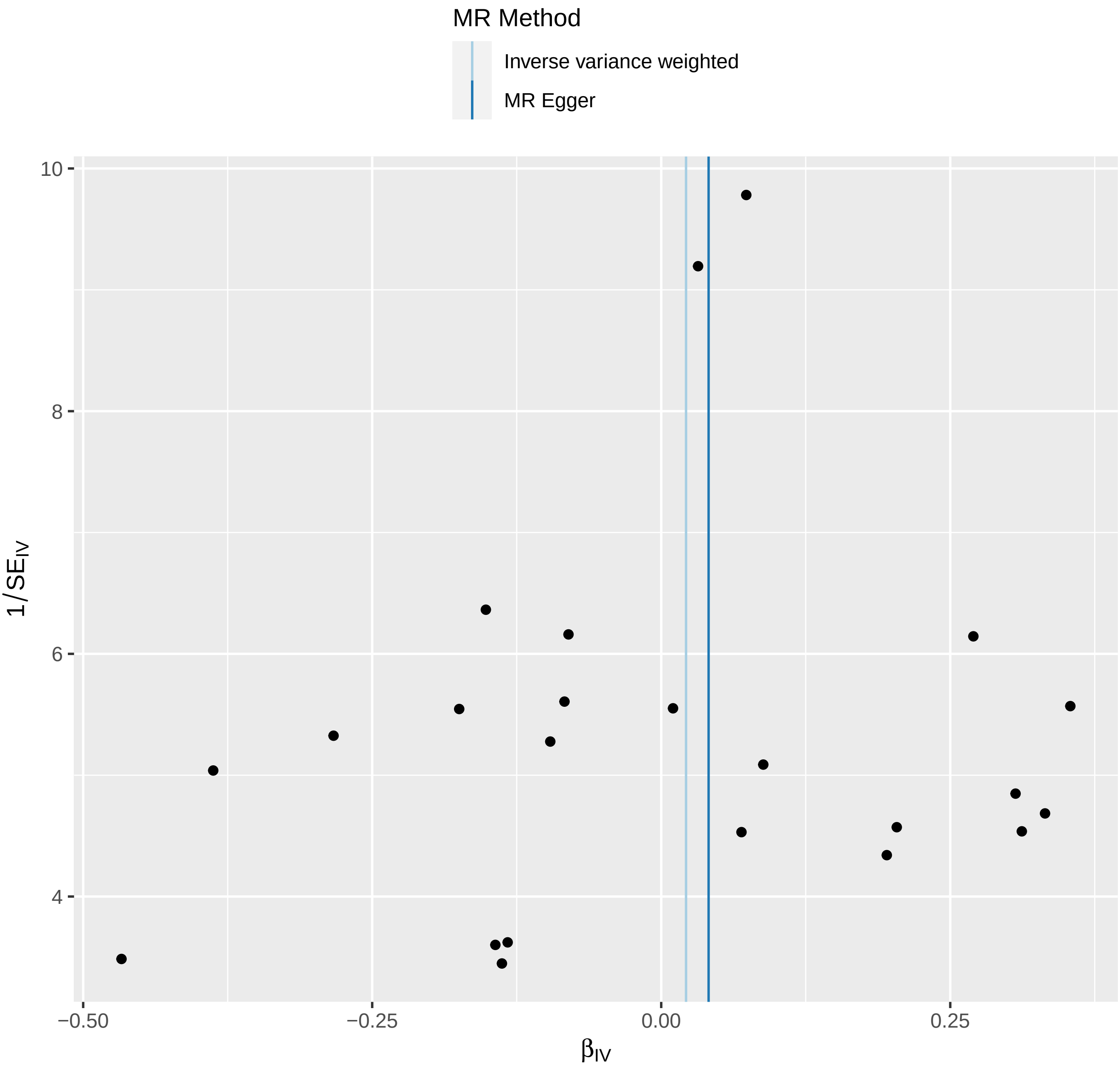

Batch 3−2483 : UV excision repair protein RAD23 homolog A on Diabetic nephropathy (more control exclusions) with a threshold of 1e−05

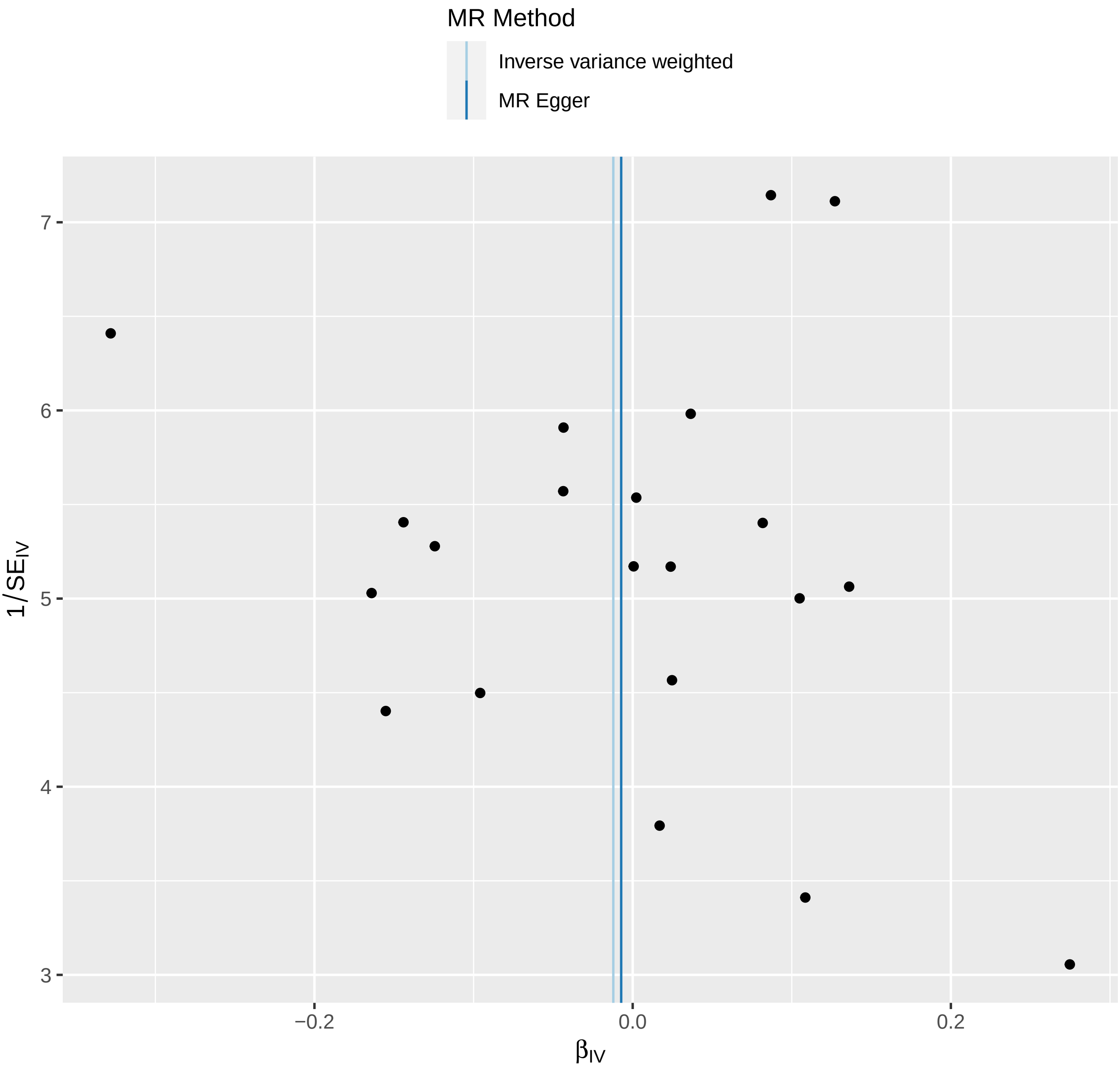

Batch 3−2484 : UV excision repair protein RAD23 homolog B on Diabetic nephropathy (more control exclusions) with a threshold of 1e−05

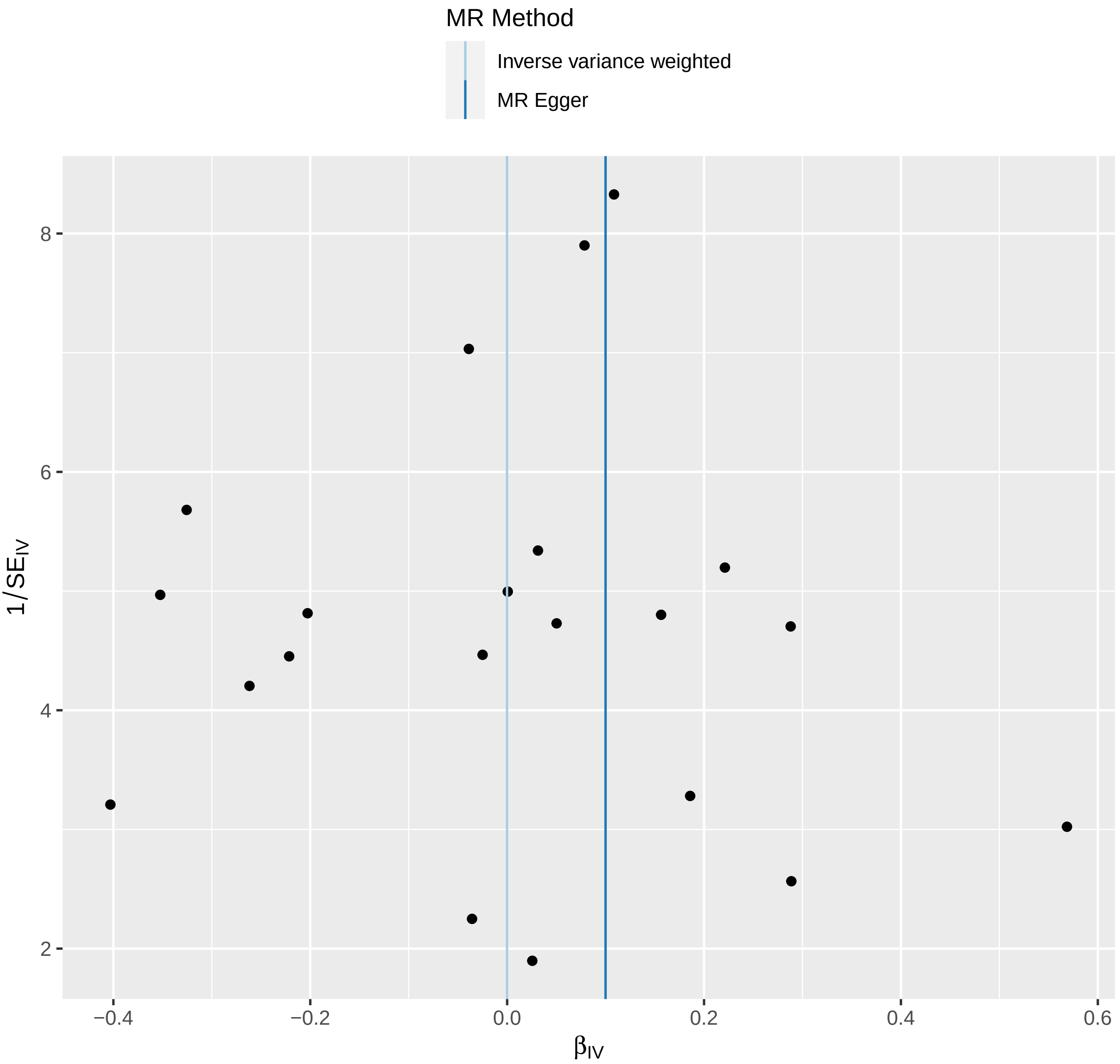

Batch 3−2485 : DNA repair protein RAD51 homolog 1 on Diabetic nephropathy (more control exclusions)
with a threshold of 1e−05

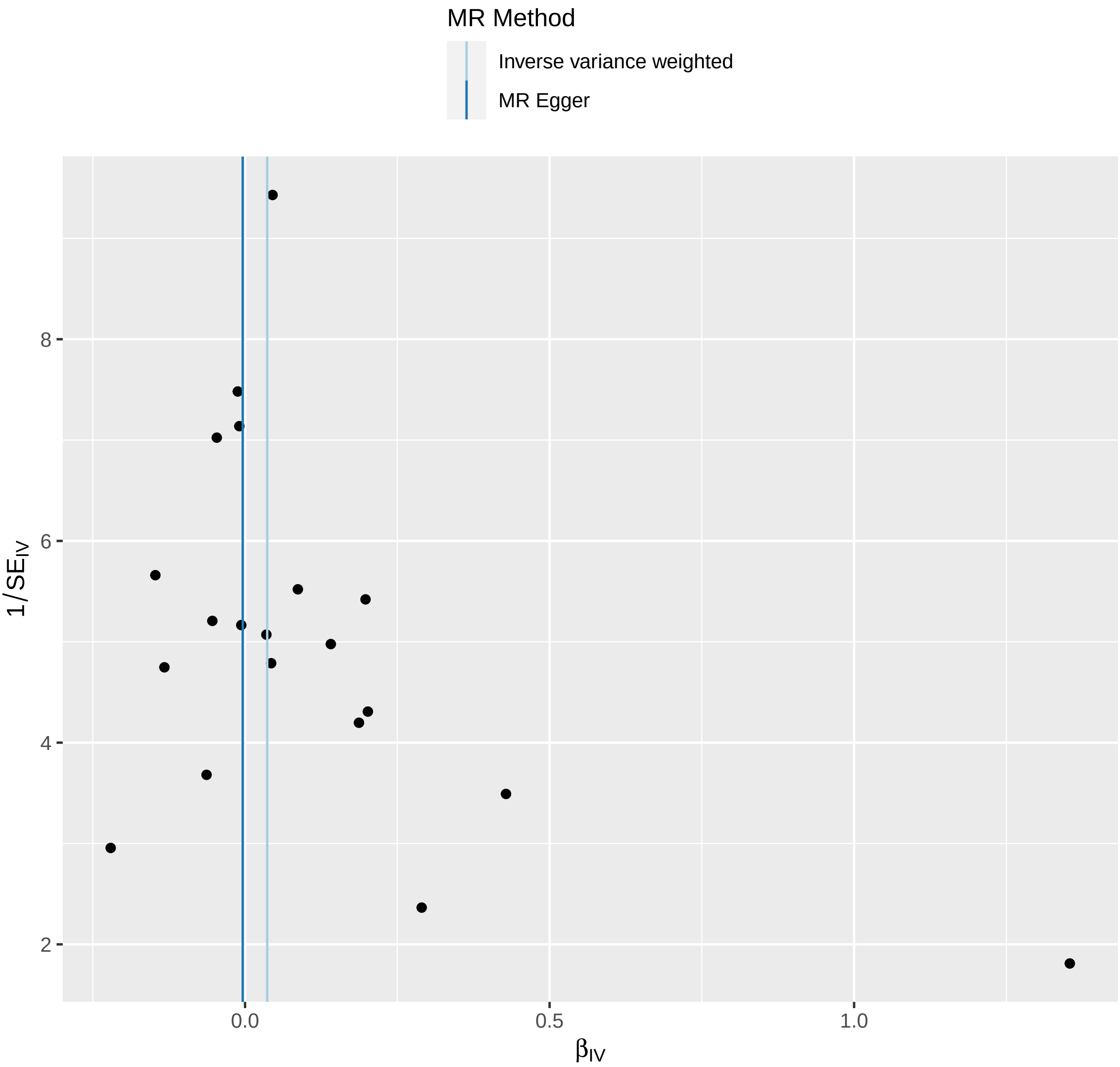

Batch 3−2486 : DNA repair protein RAD51 homolog 4 on Diabetic nephropathy (more control exclusions)
with a threshold of 1e−05

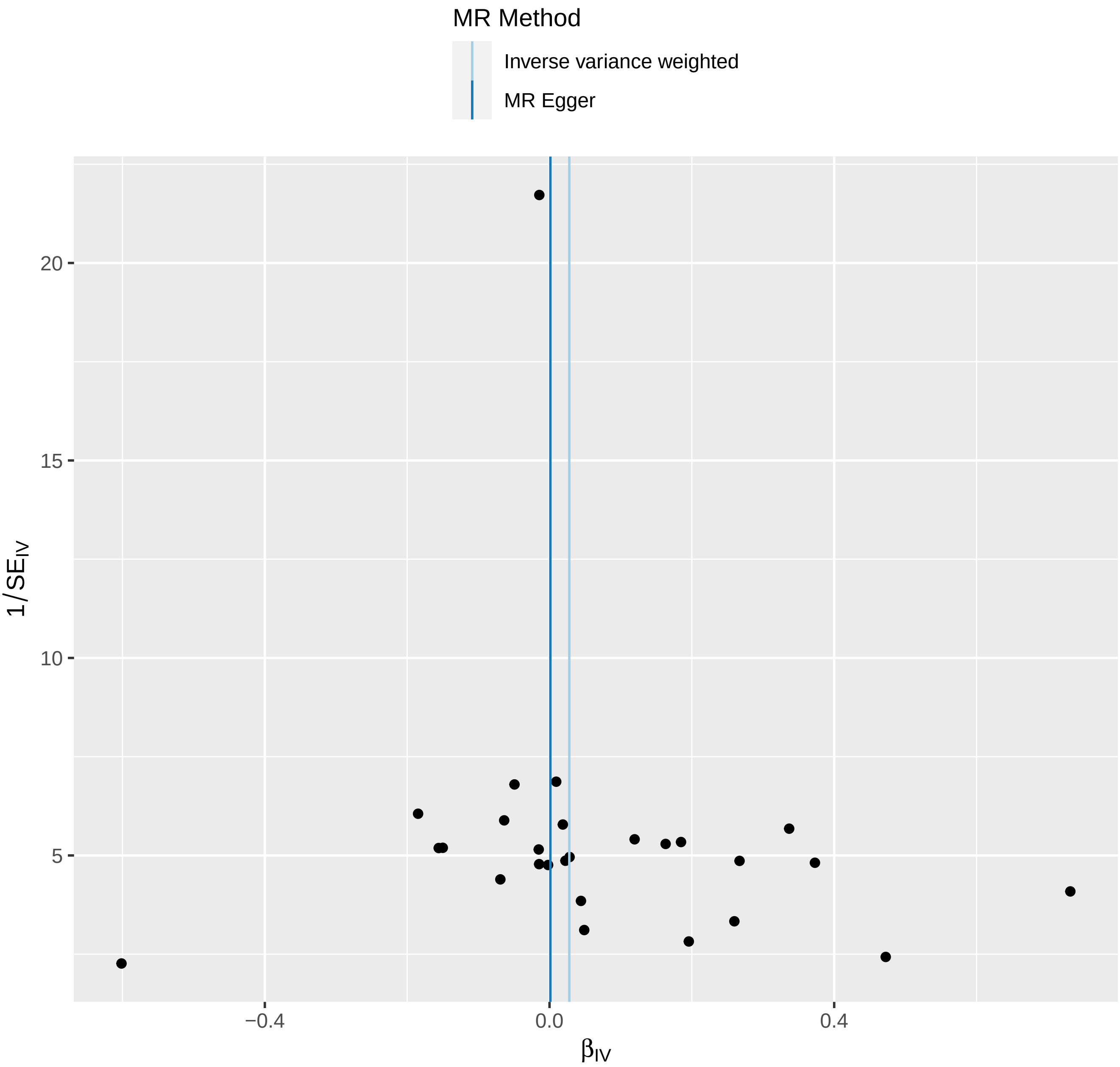

Batch 3−2487 : NKG2D ligand 4 on Diabetic nephropathy (more control exclusions)
with a threshold of 1e−05

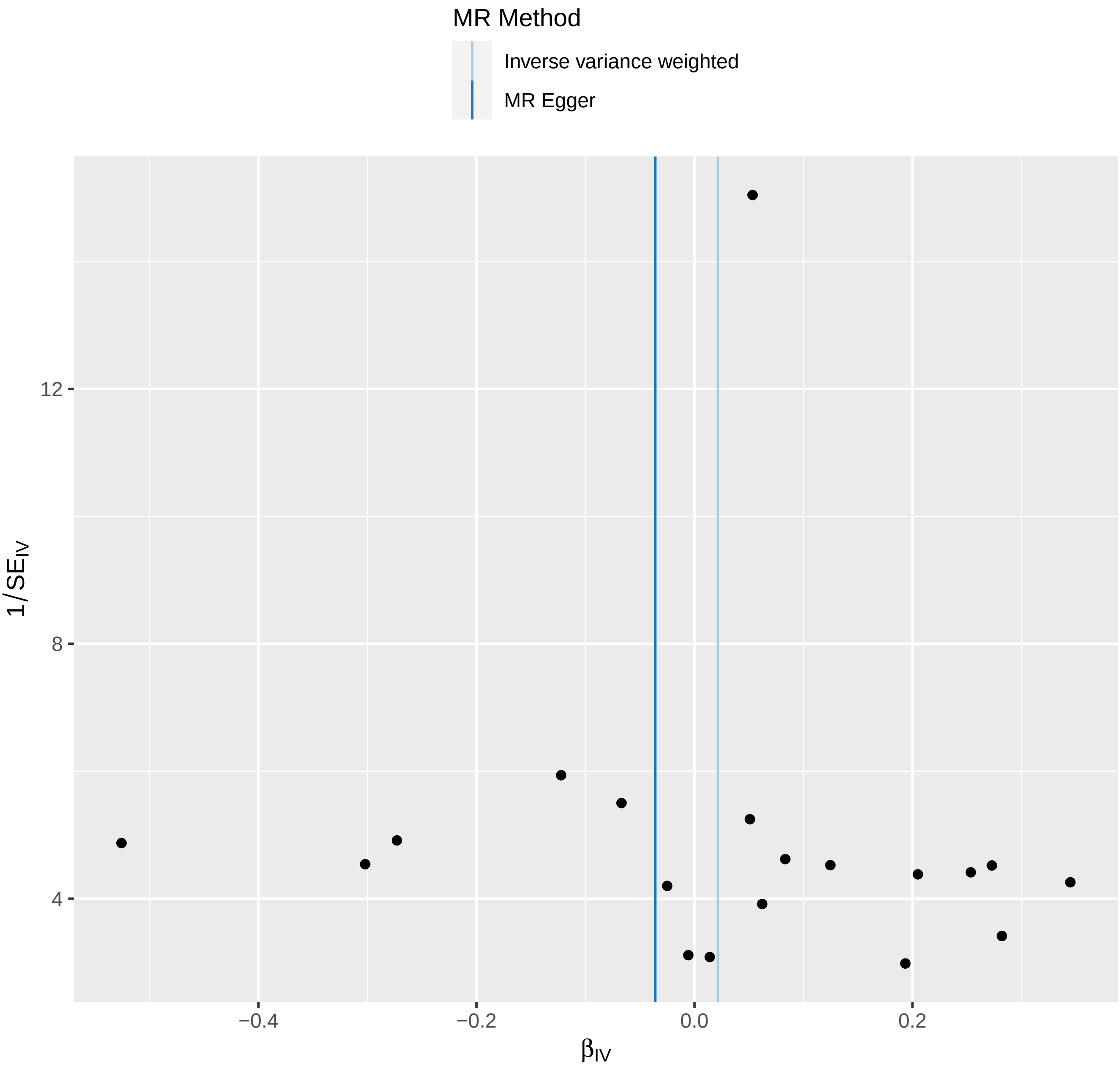

Batch 3−2488 : Ran−binding protein 3 on Diabetic nephropathy (more control exclusions)
with a threshold of 1e−05

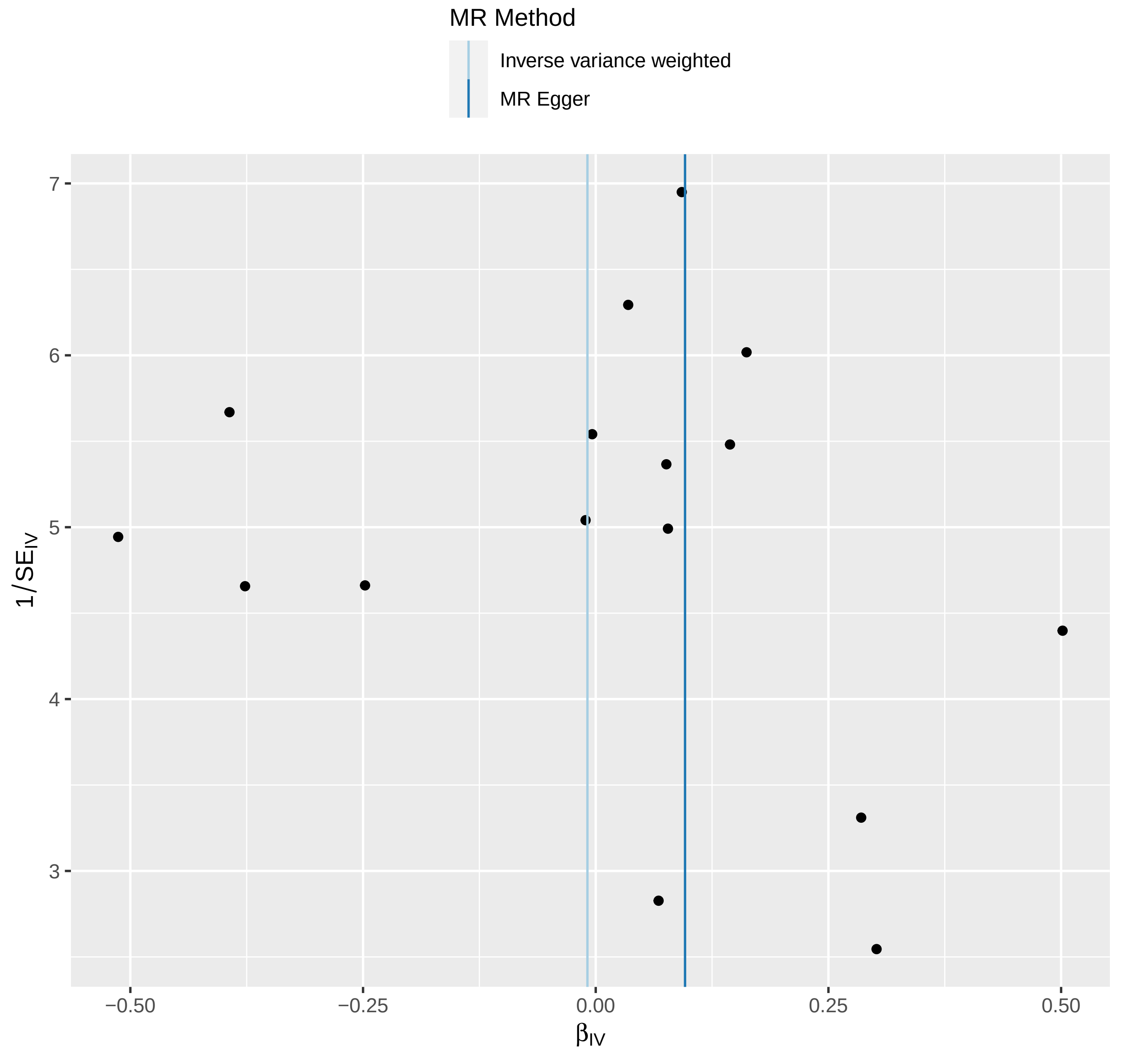

Batch 3−2489 : Rap1 GTPase−activating protein 1 on Diabetic nephropathy (more control exclusions)
with a threshold of 1e−05

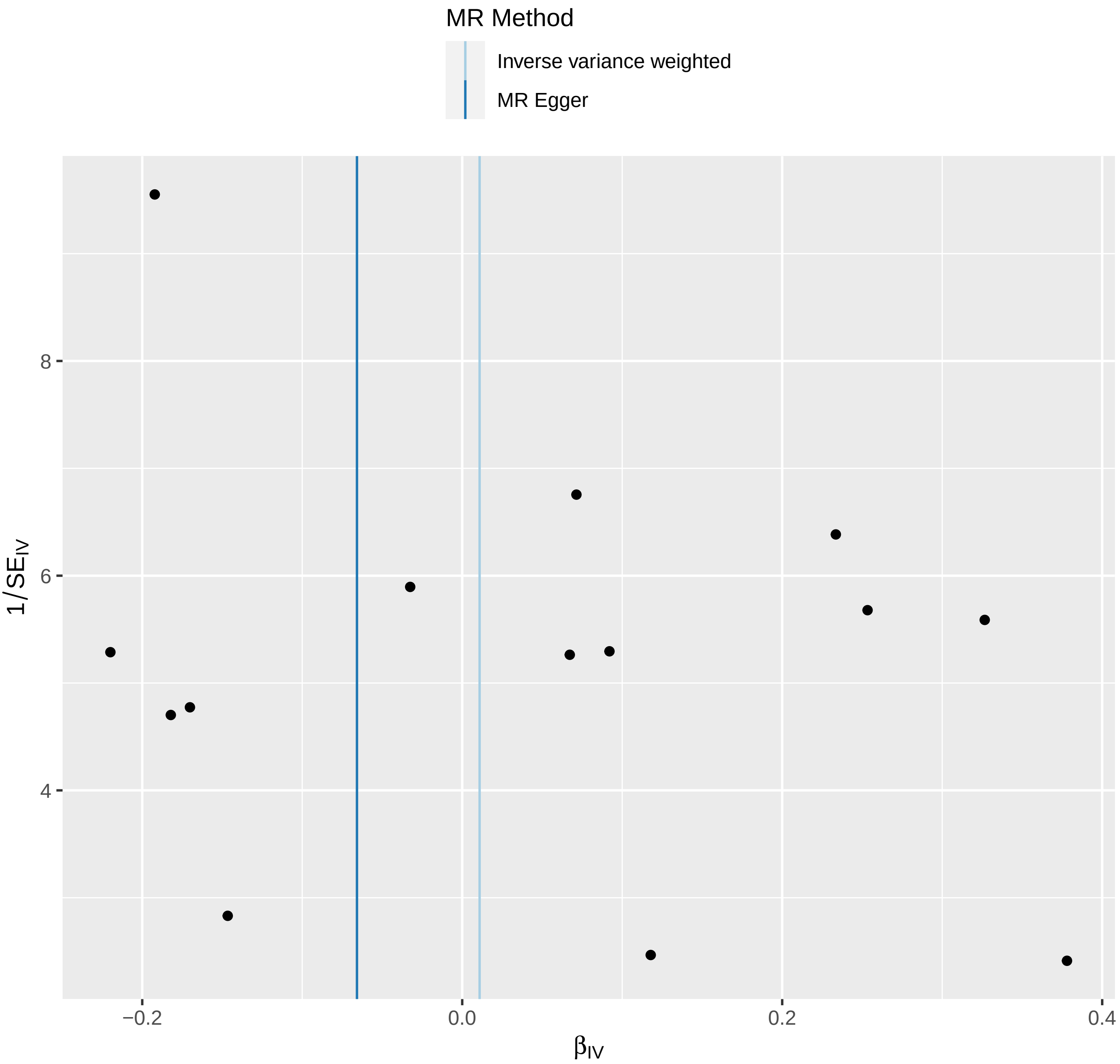

Batch 3−2490 : Rap1 GTPase−GDP dissociation stimulator 1 on Diabetic nephropathy (more control exclusions)
with a threshold of 1e−05

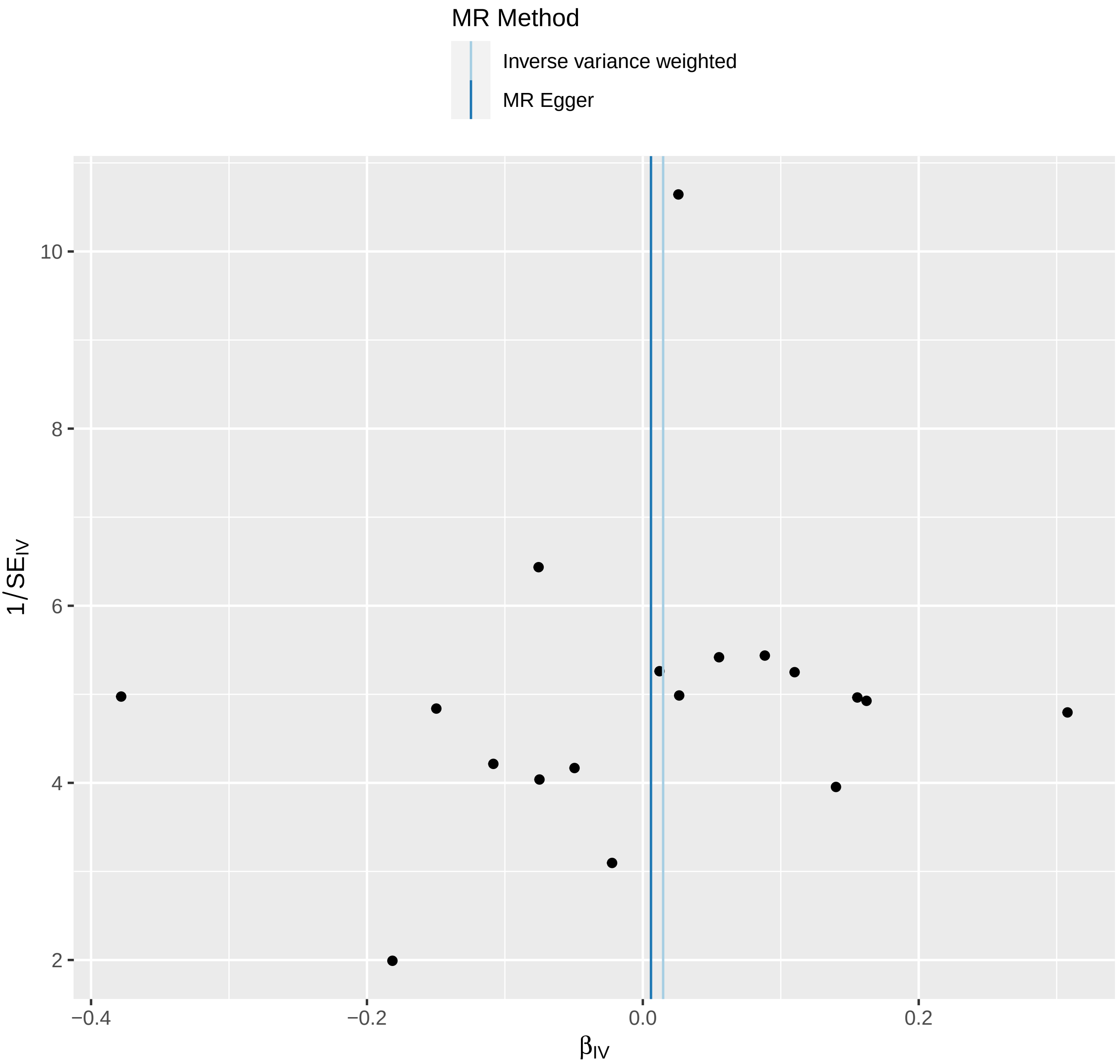

Batch 3−2491 : Ras−related protein Rap−2a on Diabetic nephropathy (more control exclusions)
with a threshold of 1e−05

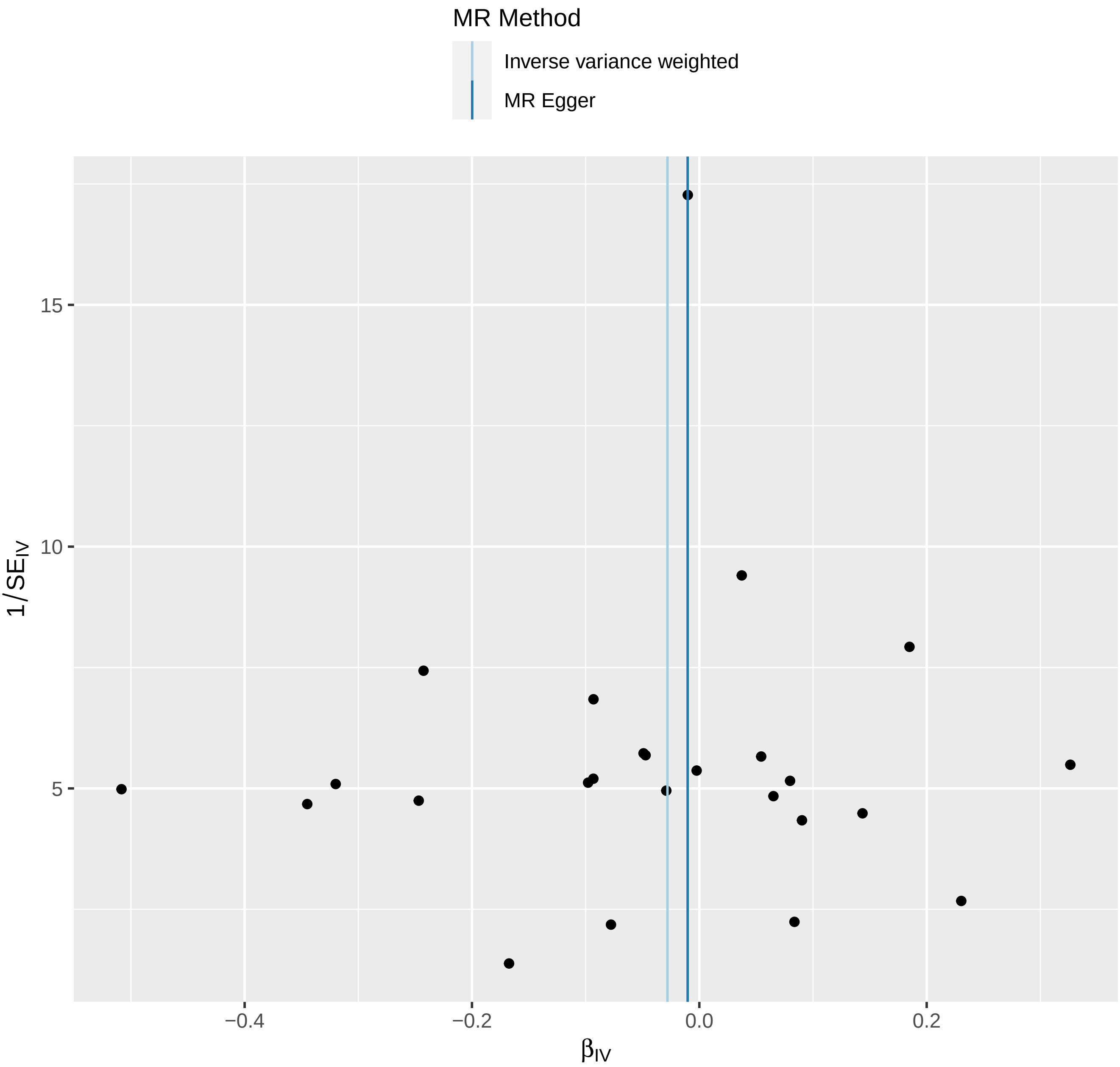

Batch 3−2492 : Rap guanine nucleotide exchange factor 5 on Diabetic nephropathy (more control exclusions)
with a threshold of 1e−05

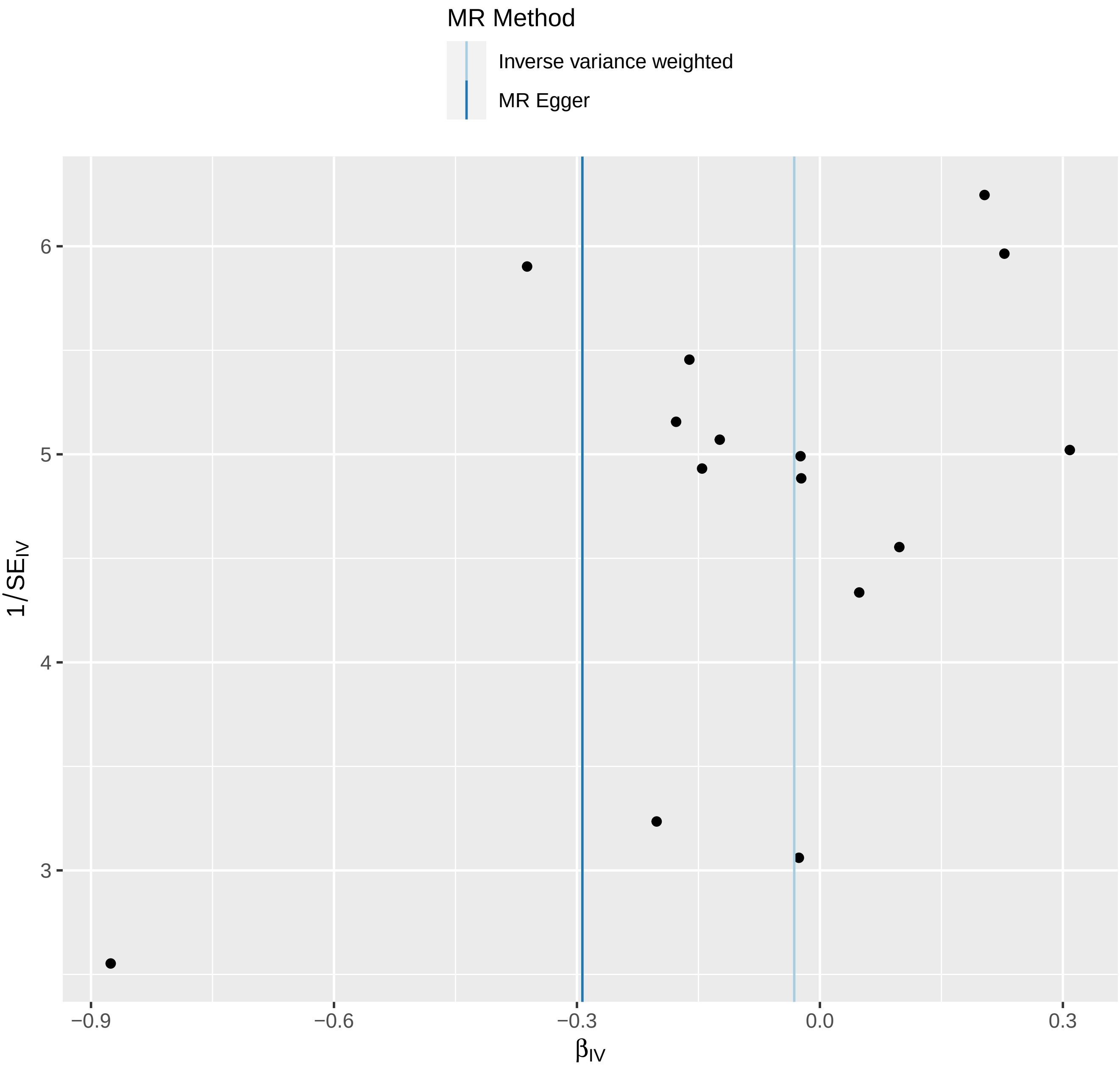

Batch 3−2493 : Retinoic acid receptor responder protein 1 on Diabetic nephropathy (more control exclusions)
with a threshold of 1e−05

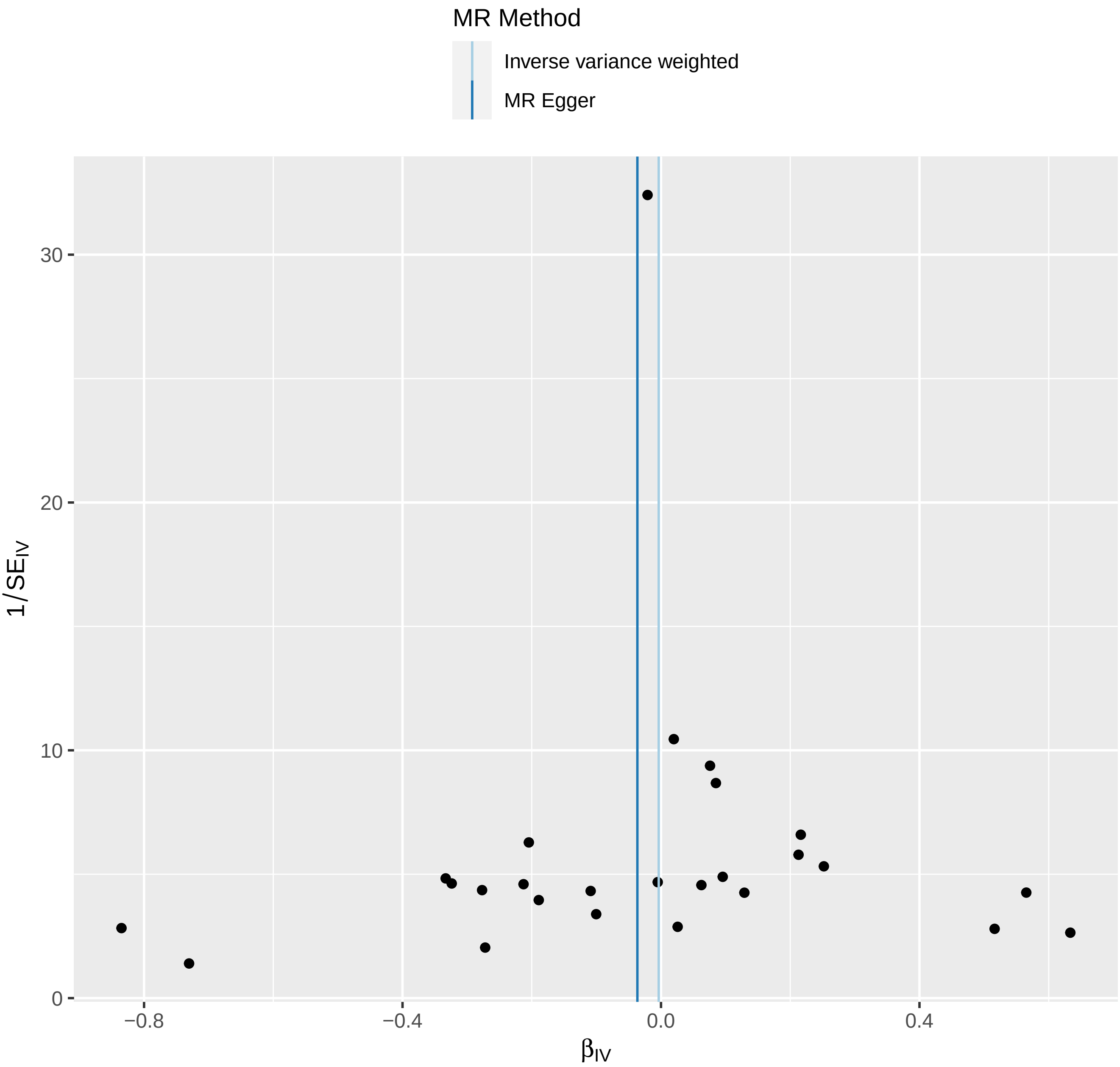

Batch 3−2494 : Retinoic acid receptor responder protein 3 on Diabetic nephropathy (more control exclusions) with a threshold of 1e−05

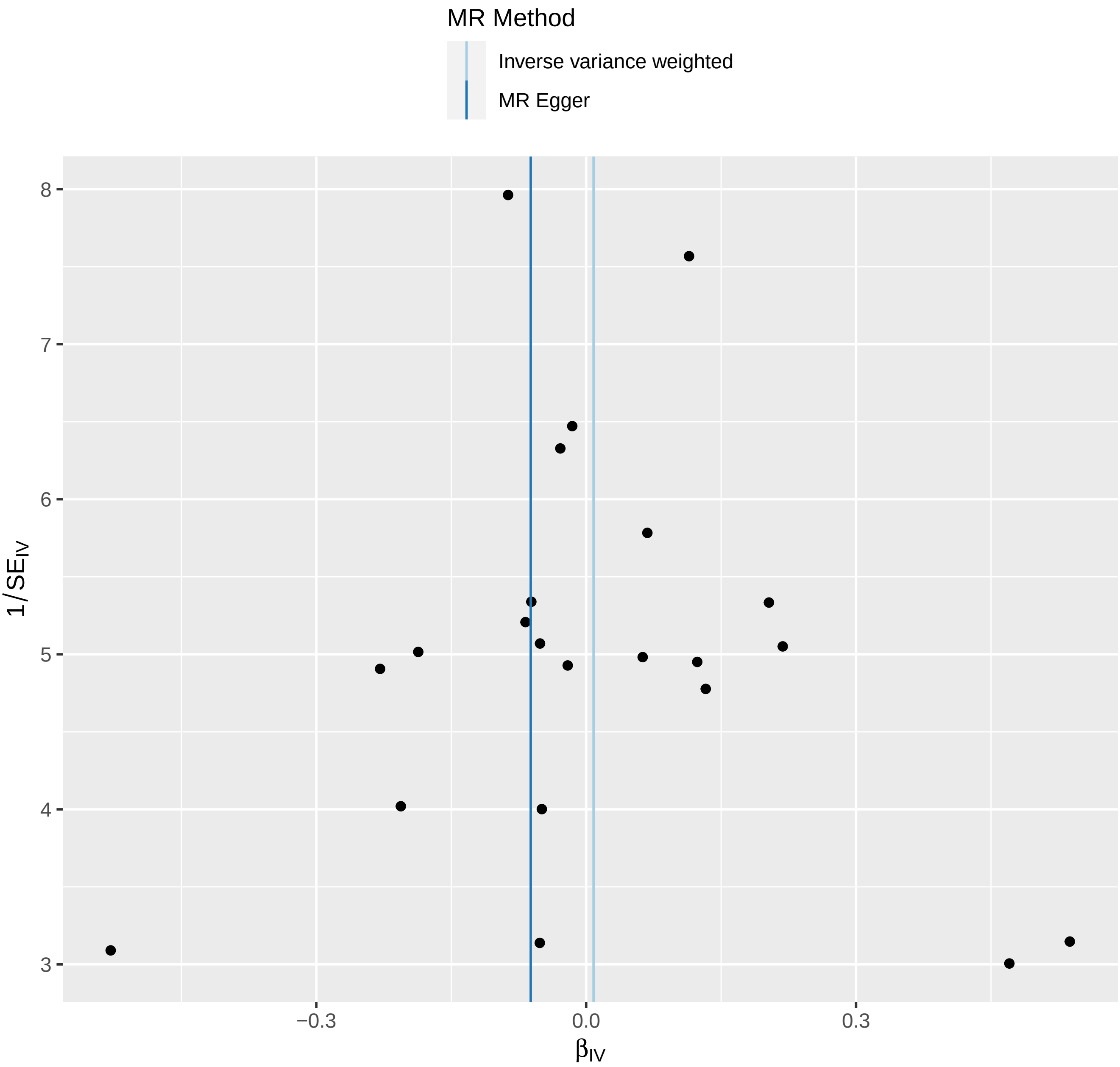

Batch 3−2495 : Ras GTPase−activating protein 1 on Diabetic nephropathy (more control exclusions)
with a threshold of 1e−05

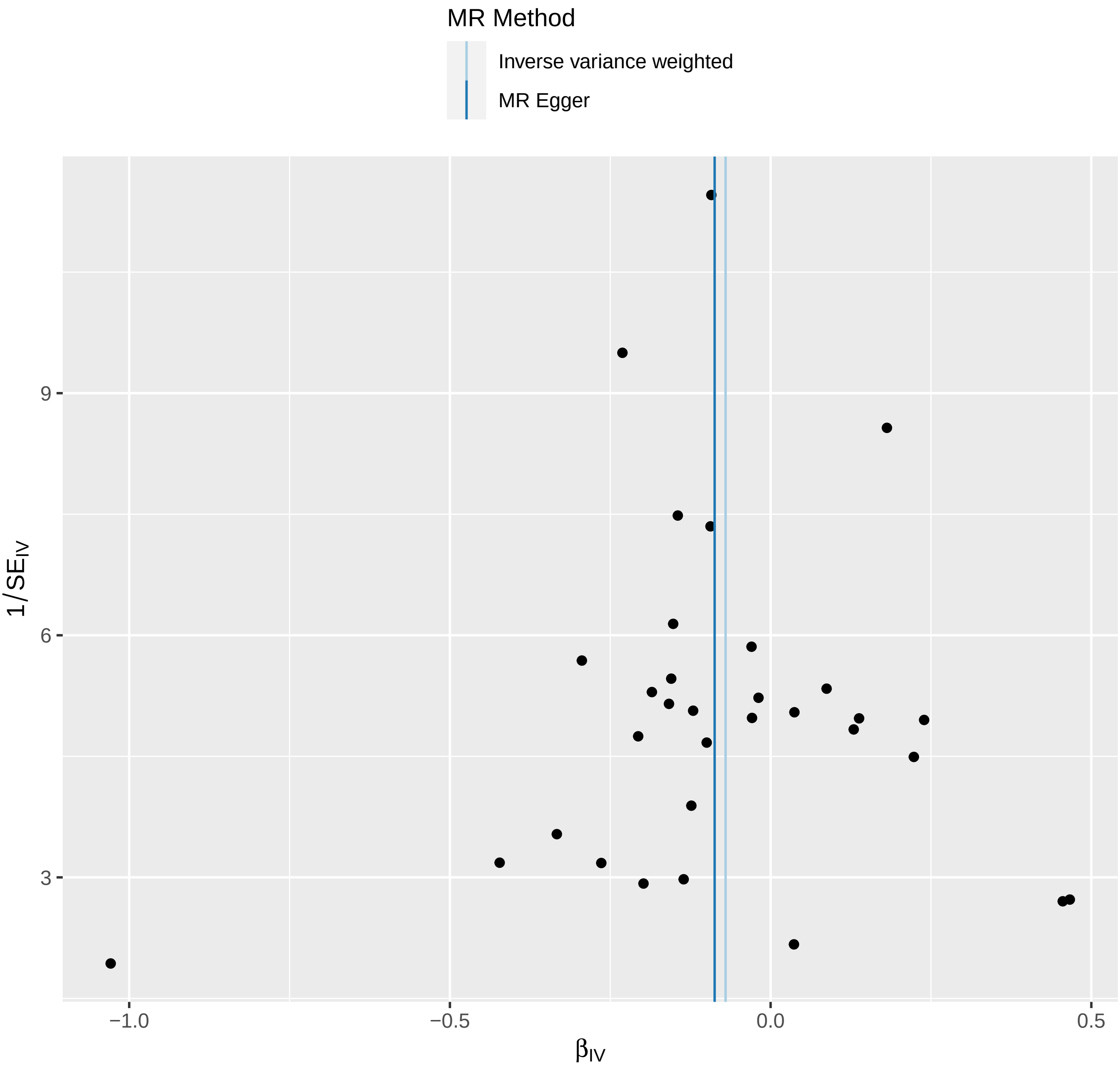

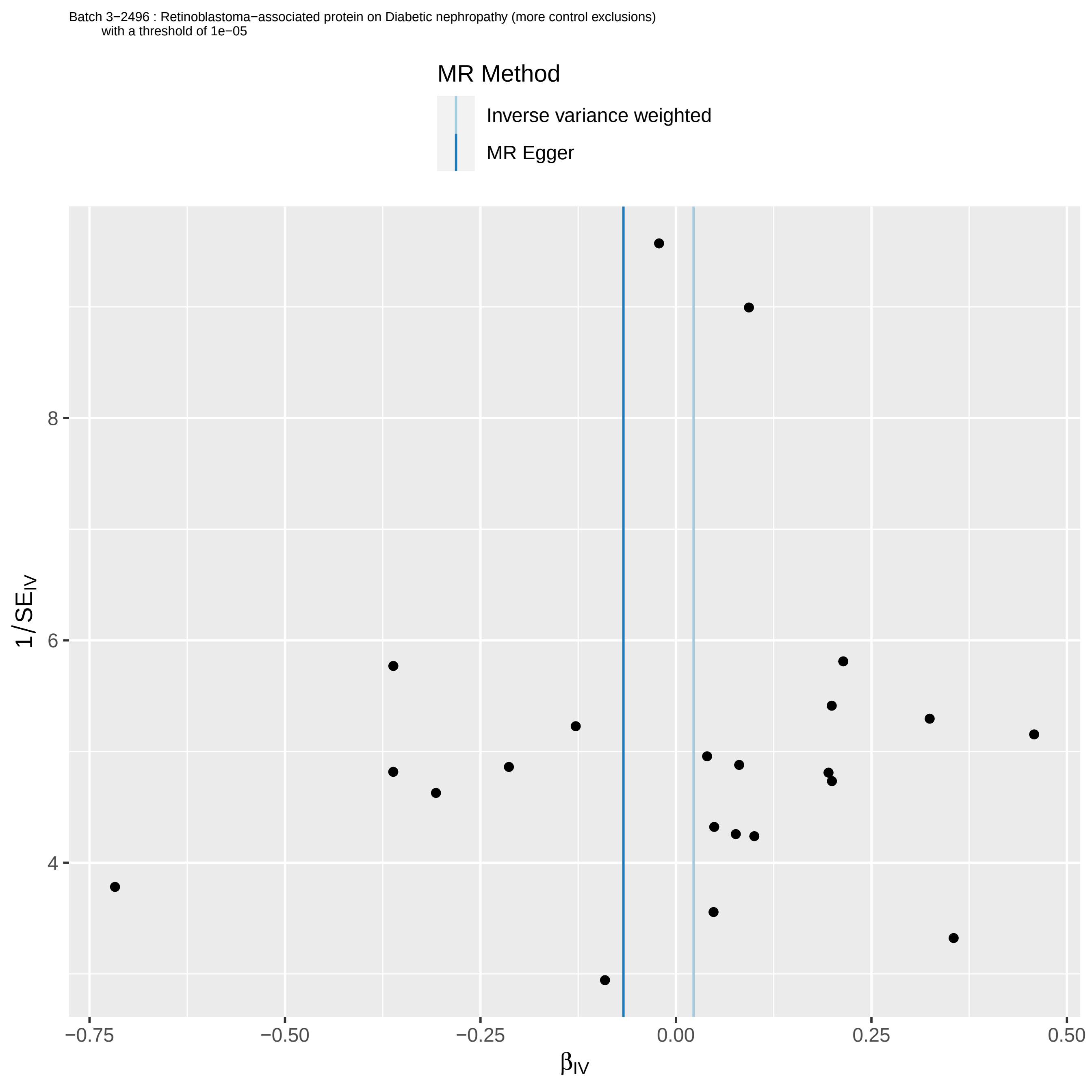
Batch 3−2496 : Retinoblastoma−associated protein on Diabetic nephropathy (more control exclusions)
with a threshold of 1e−05
MR Method
Inverse variance weighted
MR Egger
1/SE_IV
8
6
4
−0.75
−0.50
−0.25
0.00
0.25
0.50
β_IV

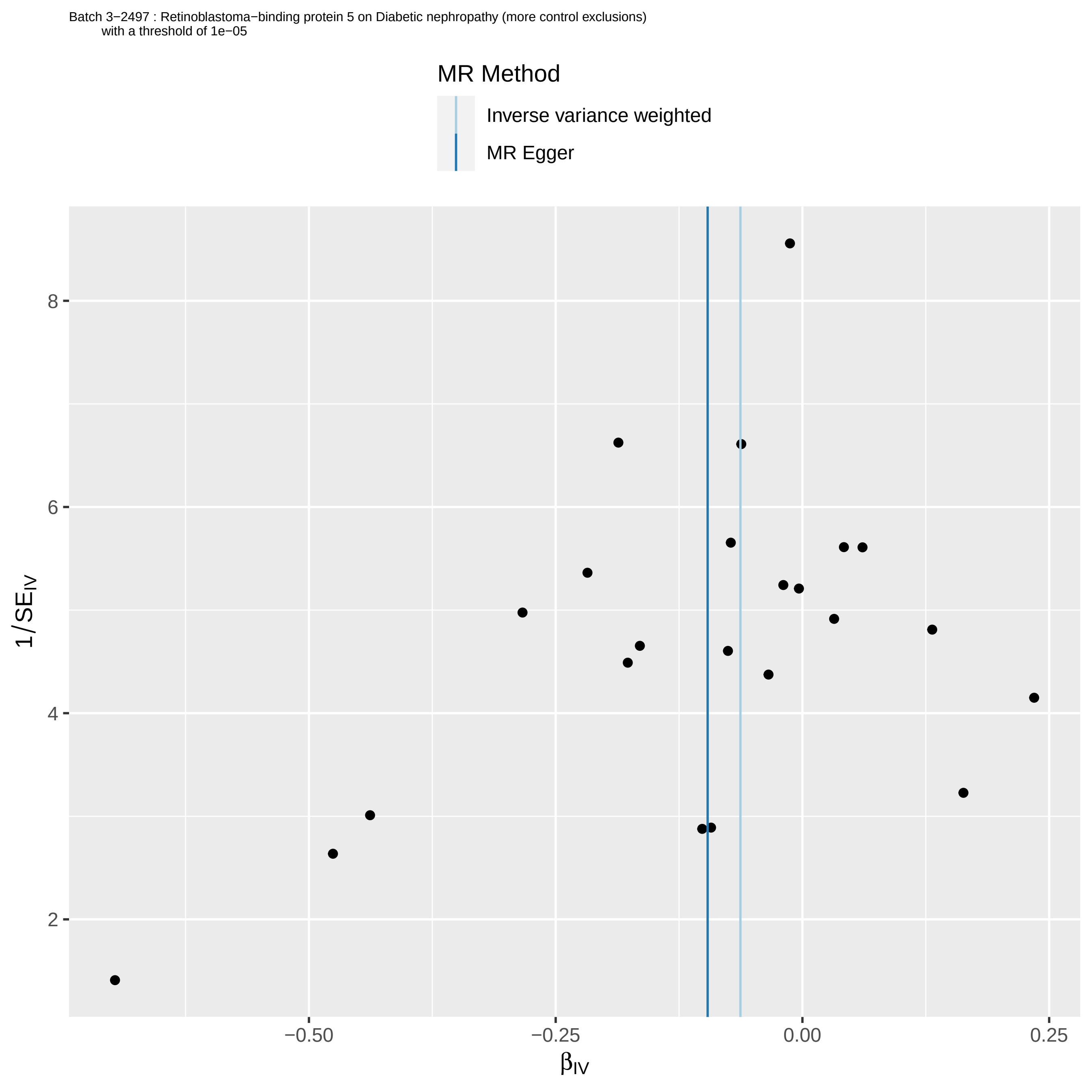
Batch 3−2497 : Retinoblastoma−binding protein 5 on Diabetic nephropathy (more control exclusions)
with a threshold of 1e−05
MR Method
Inverse variance weighted
MR Egger
1/SE_IV
2
4
6
8
−0.50
−0.25
0.00
0.25
β_IV

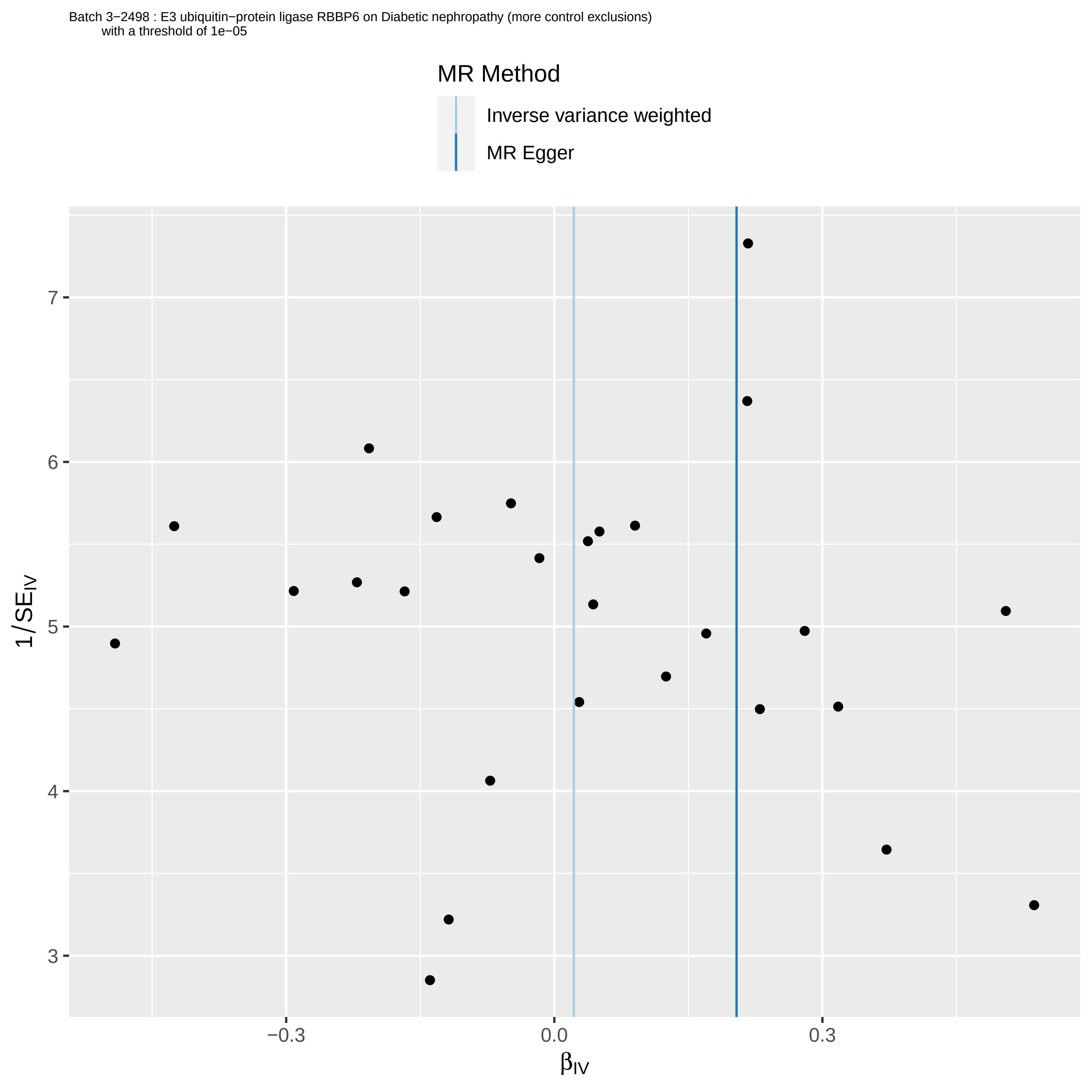
Batch 3−2498 : E3 ubiquitin−protein ligase RBBP6 on Diabetic nephropathy (more control exclusions)
with a threshold of 1e−05
MR Method
Inverse variance weighted
MR Egger
7
6
5
4
3
$1/SE_{IV}$
−0.3
0.0
0.3
β_{IV}

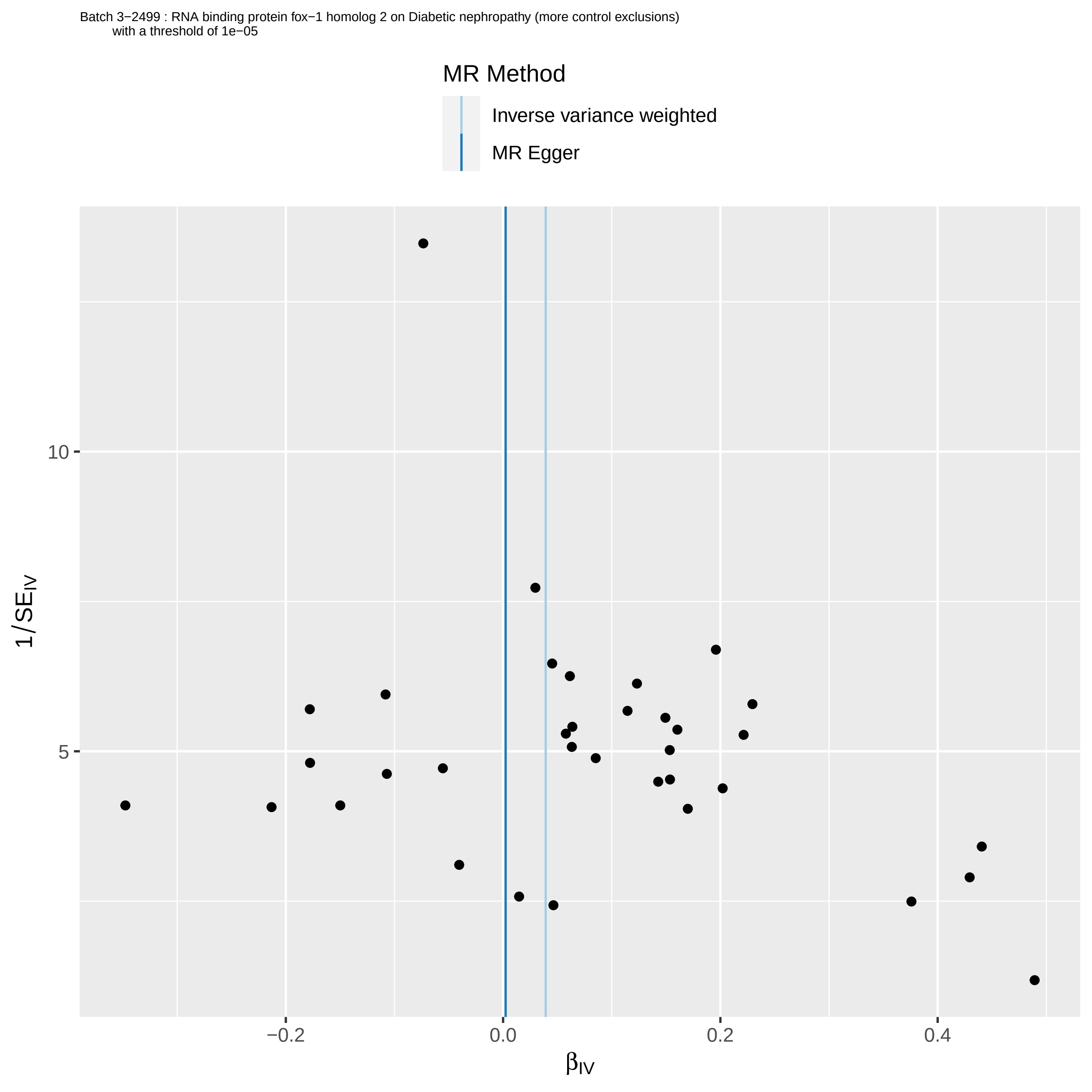

Batch 3−2499 : RNA binding protein fox−1 homolog 2 on Diabetic nephropathy (more control exclusions)
with a threshold of 1e−05
MR Method
Inverse variance weighted
MR Egger
10
5
1/SE_IV
−0.2
0.0
0.2
0.4
β_IV

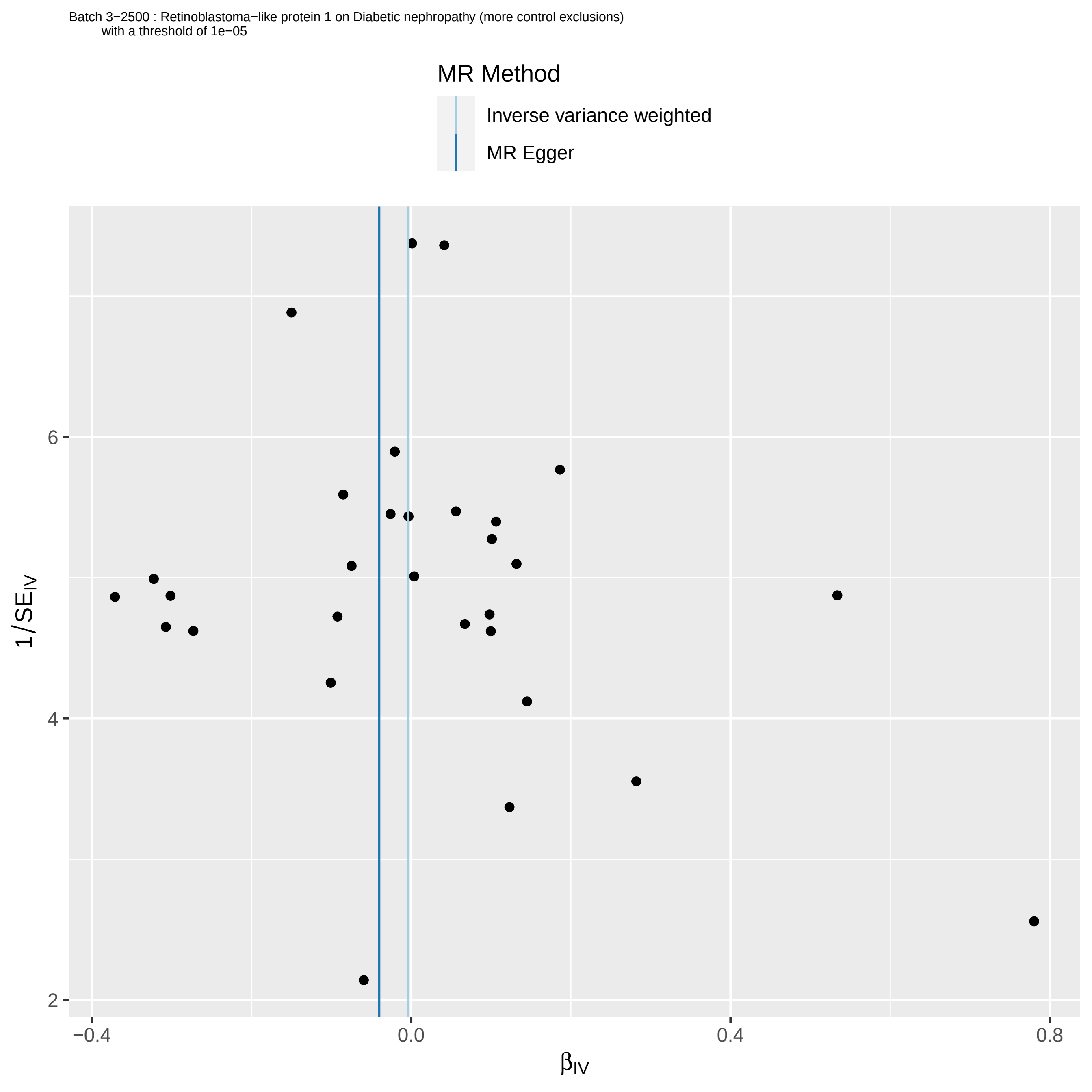
Batch 3−2500 : Retinoblastoma−like protein 1 on Diabetic nephropathy (more control exclusions)
with a threshold of 1e−05
MR Method
Inverse variance weighted
MR Egger
1/SE$_{IV}$
6
4
2
−0.4
0.0
0.4
0.8
β$_{IV}$

Batch 3−2501 : Probable RNA−binding protein 19 on Diabetic nephropathy (more control exclusions)
with a threshold of 1e−05

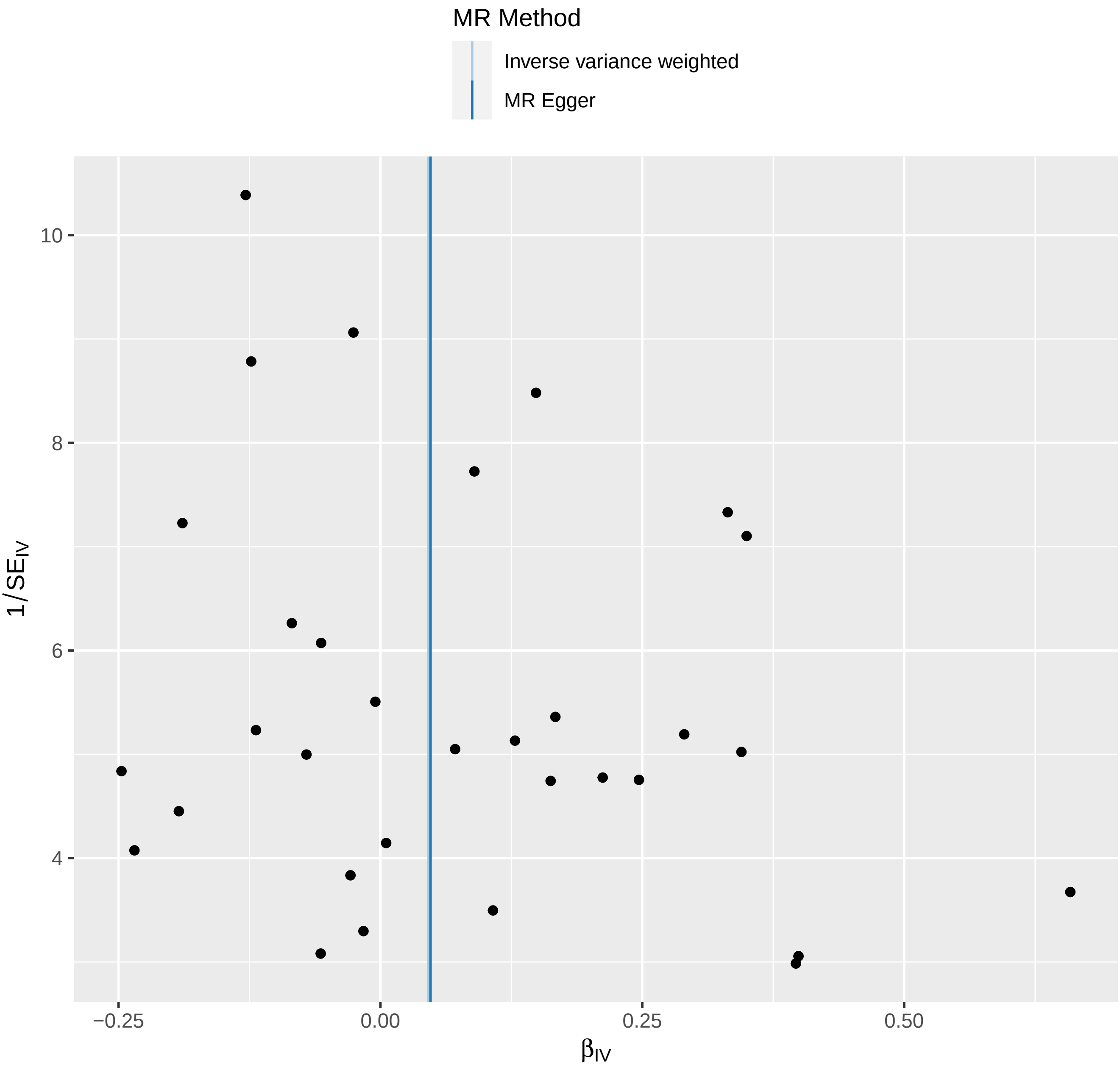

Batch 3−2502 : Probable RNA−binding protein 23 on Diabetic nephropathy (more control exclusions)
with a threshold of 1e−05

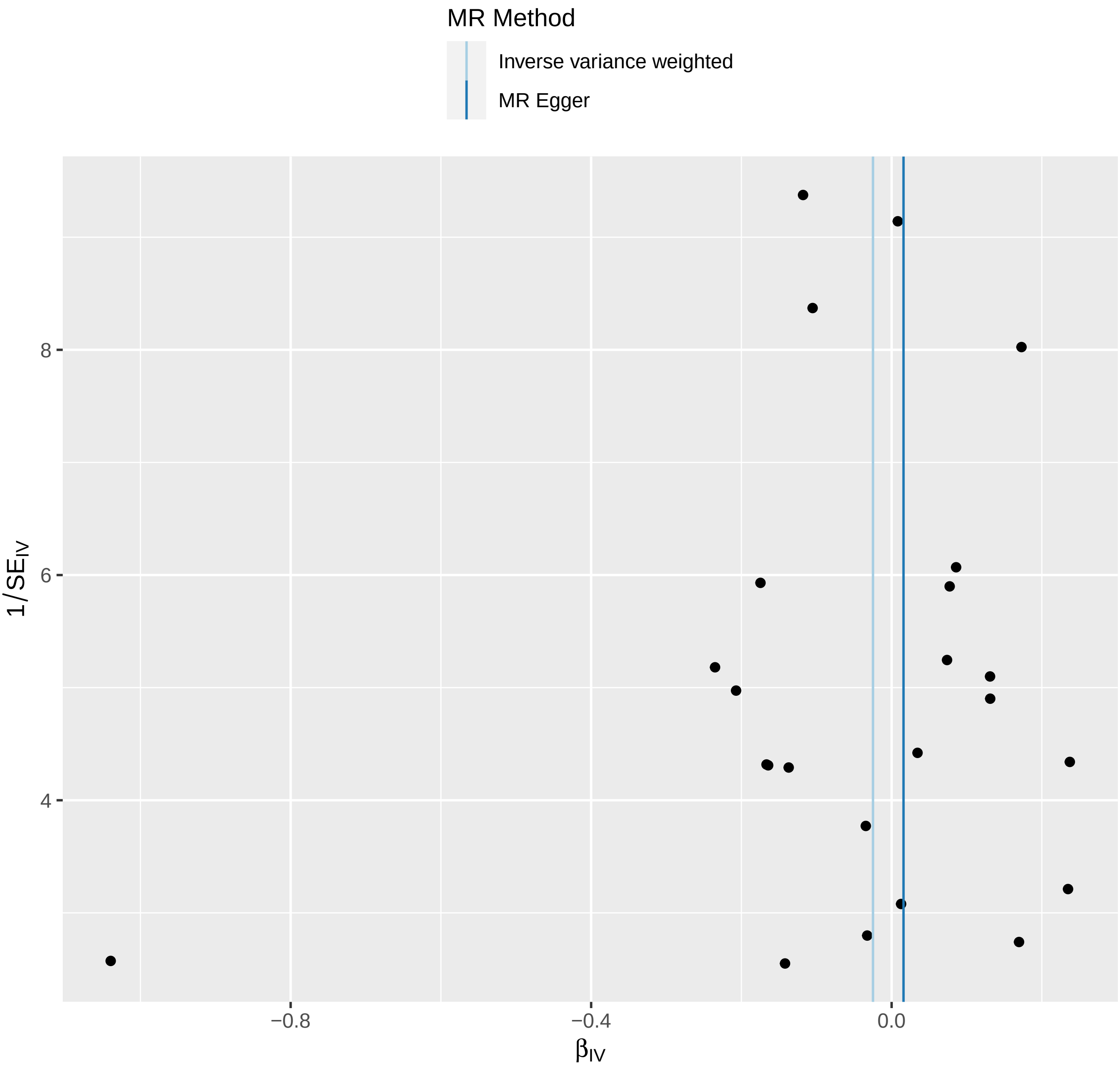

Batch 3−2503 : RNA−binding protein 24 on Diabetic nephropathy (more control exclusions)
with a threshold of 1e−05

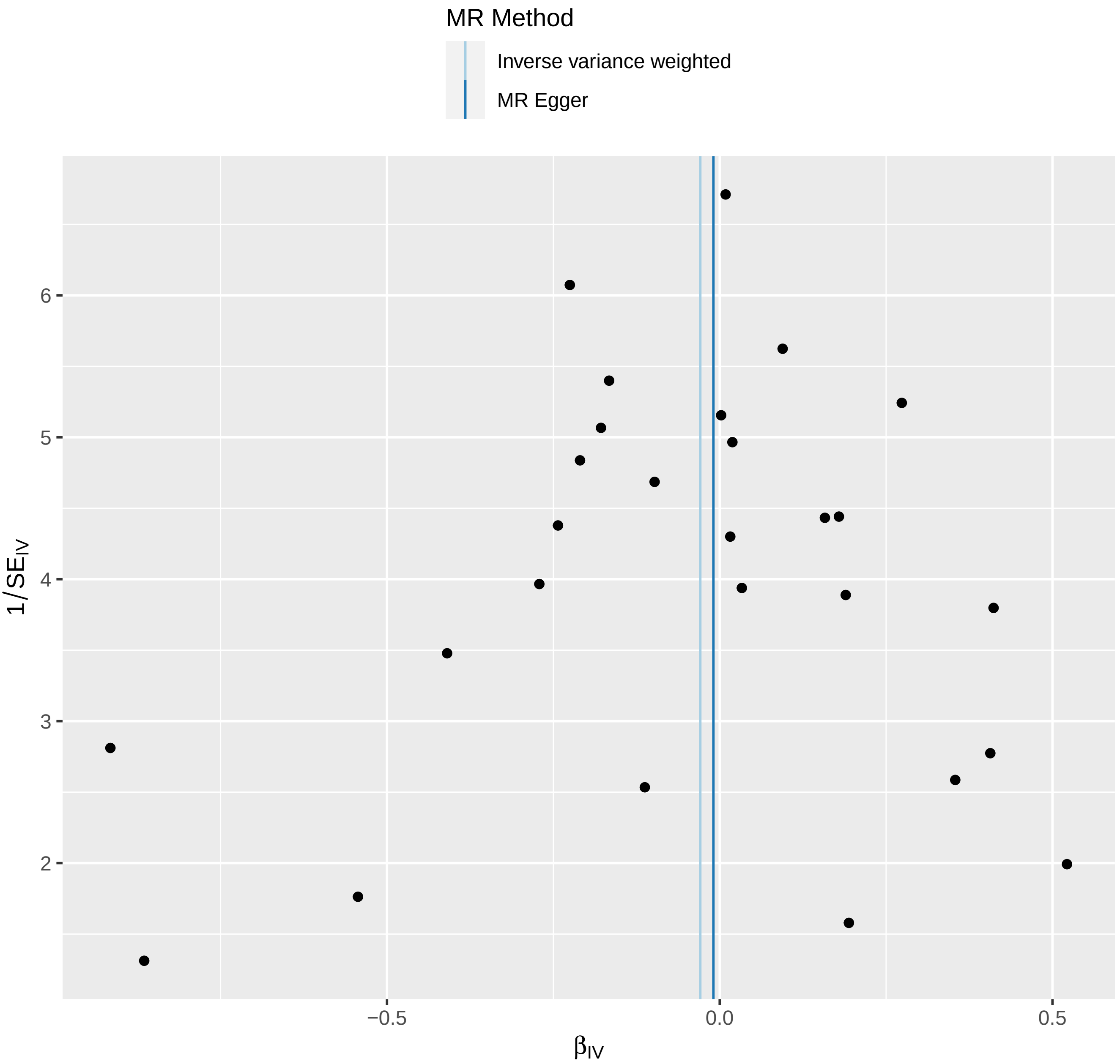

Batch 3−2504 : RNA−binding protein 28 on Diabetic nephropathy (more control exclusions)
with a threshold of 1e−05

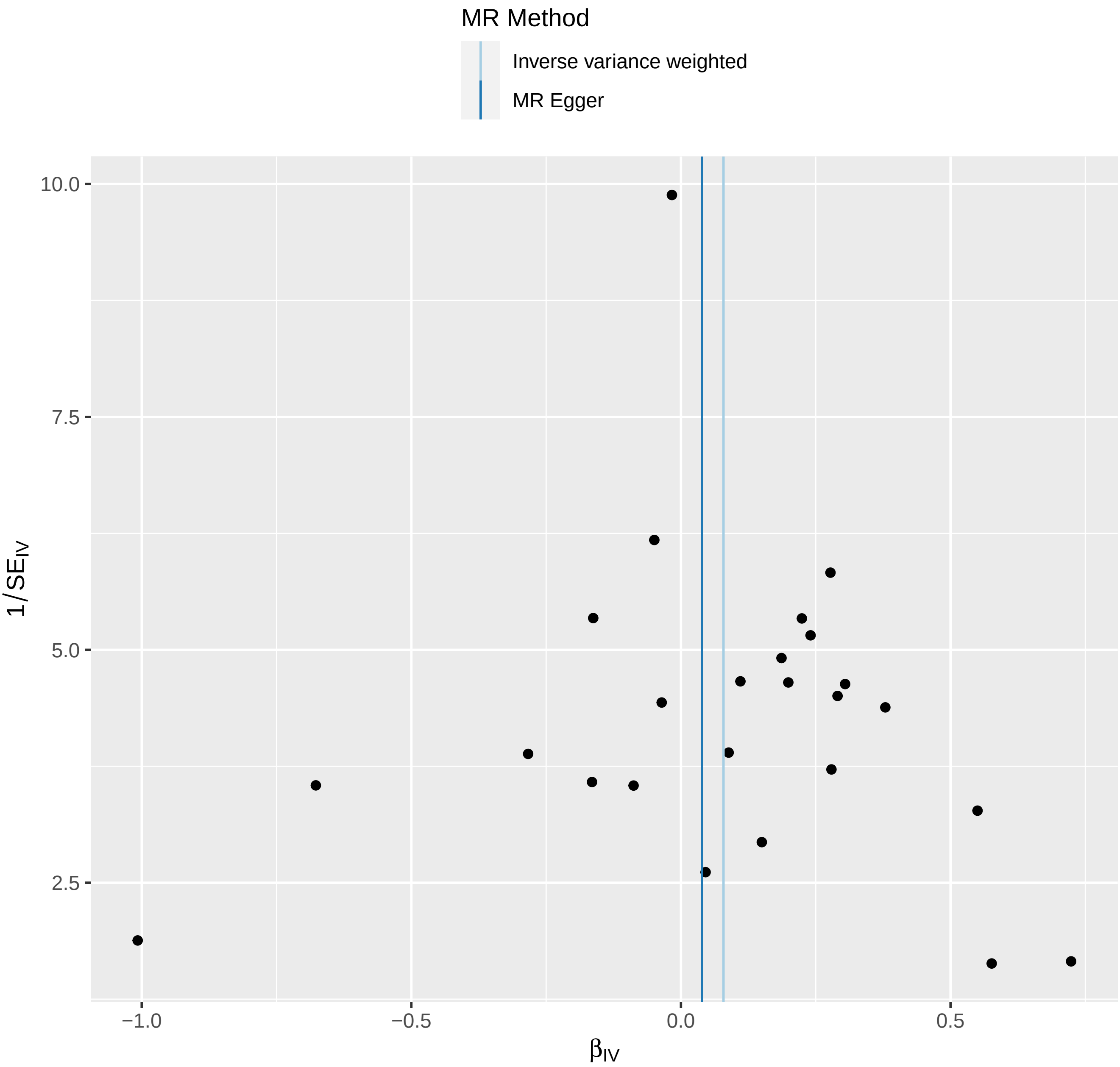

Batch 3−2505 : RNA−binding protein 3 on Diabetic nephropathy (more control exclusions)
with a threshold of 1e−05

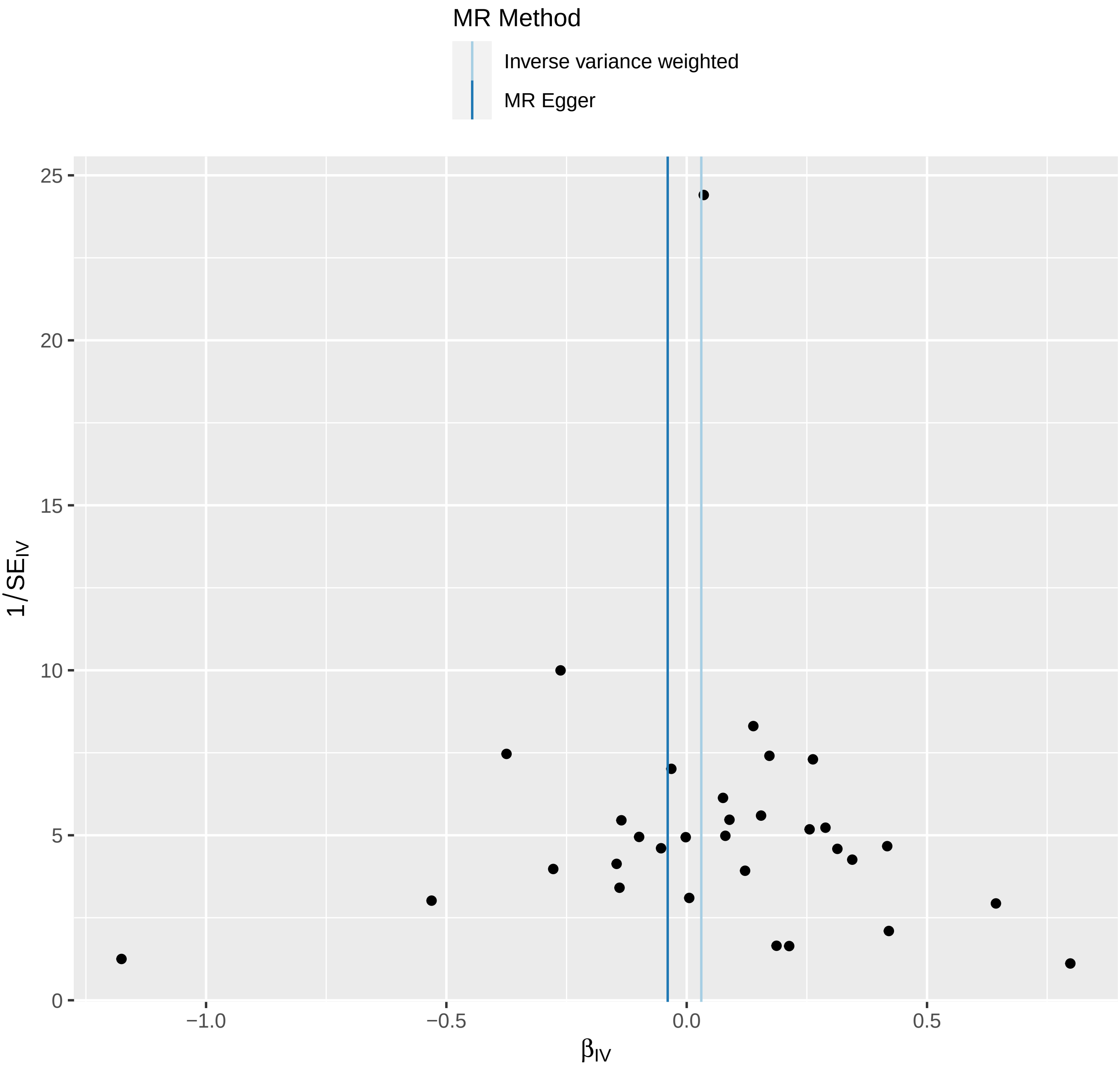

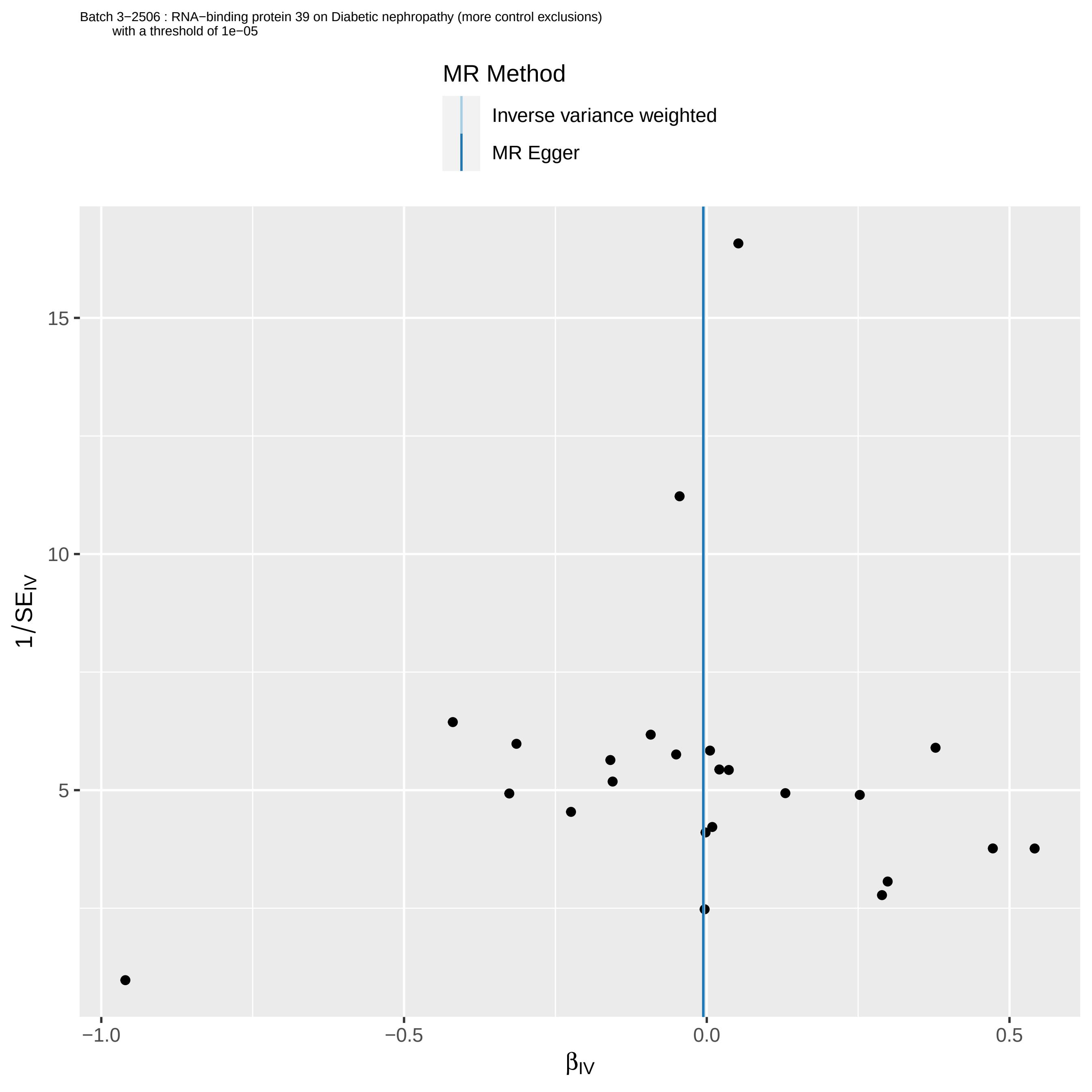
Batch 3−2506 : RNA−binding protein 39 on Diabetic nephropathy (more control exclusions)
with a threshold of 1e−05
MR Method
Inverse variance weighted
MR Egger
15
10
5
1/SE$_{IV}$
−1.0
−0.5
0.0
0.5
β$_{IV}$

Batch 3−2507 : Retinol−binding protein 4 on Diabetic nephropathy (more control exclusions)
with a threshold of 1e−05

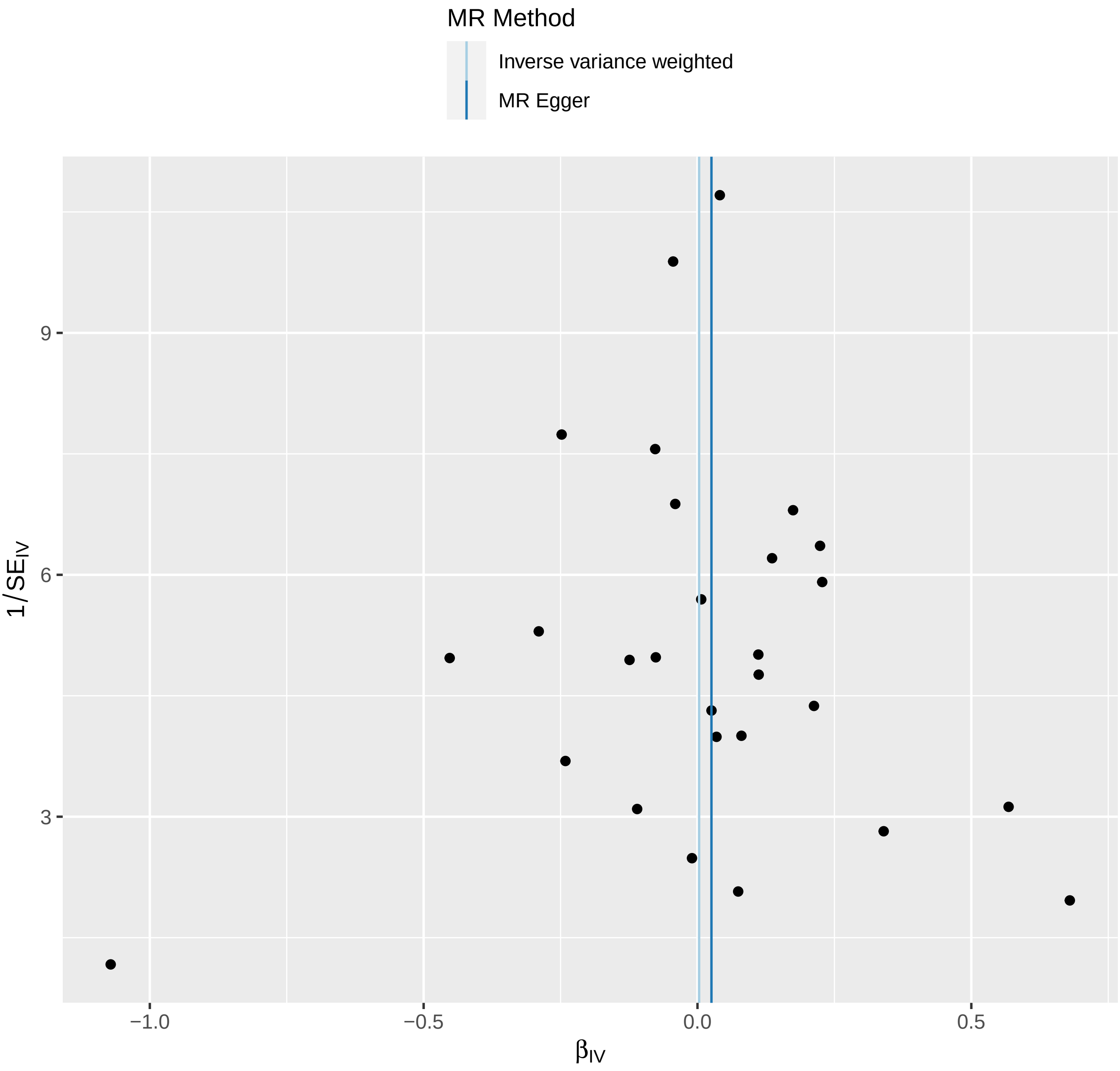

Batch 3−2508 : Retinoid−binding protein 7 on Diabetic nephropathy (more control exclusions)
with a threshold of 1e−05

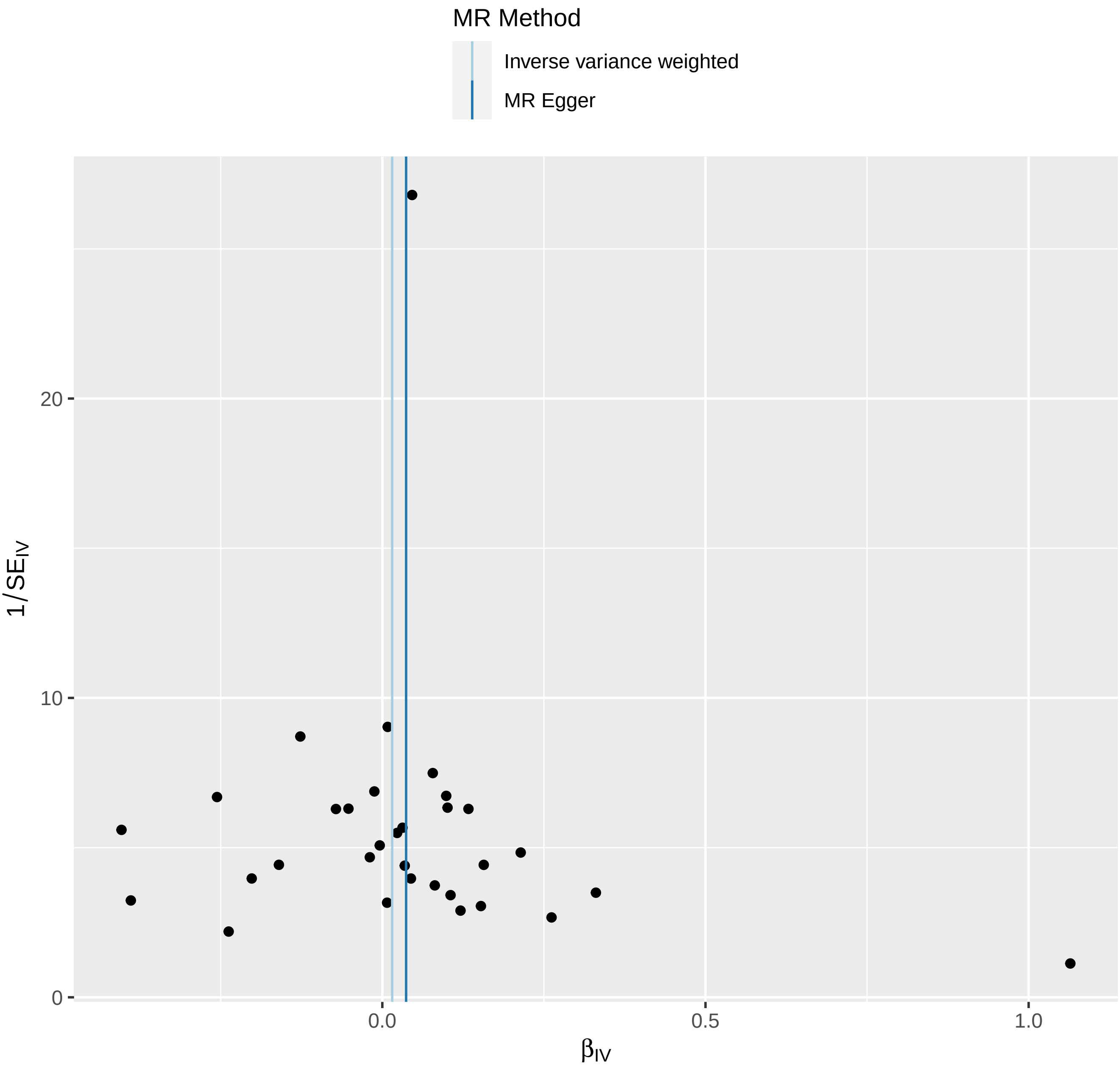

Batch 3−2509 : Calcipressin−1 on Diabetic nephropathy (more control exclusions)
with a threshold of 1e−05

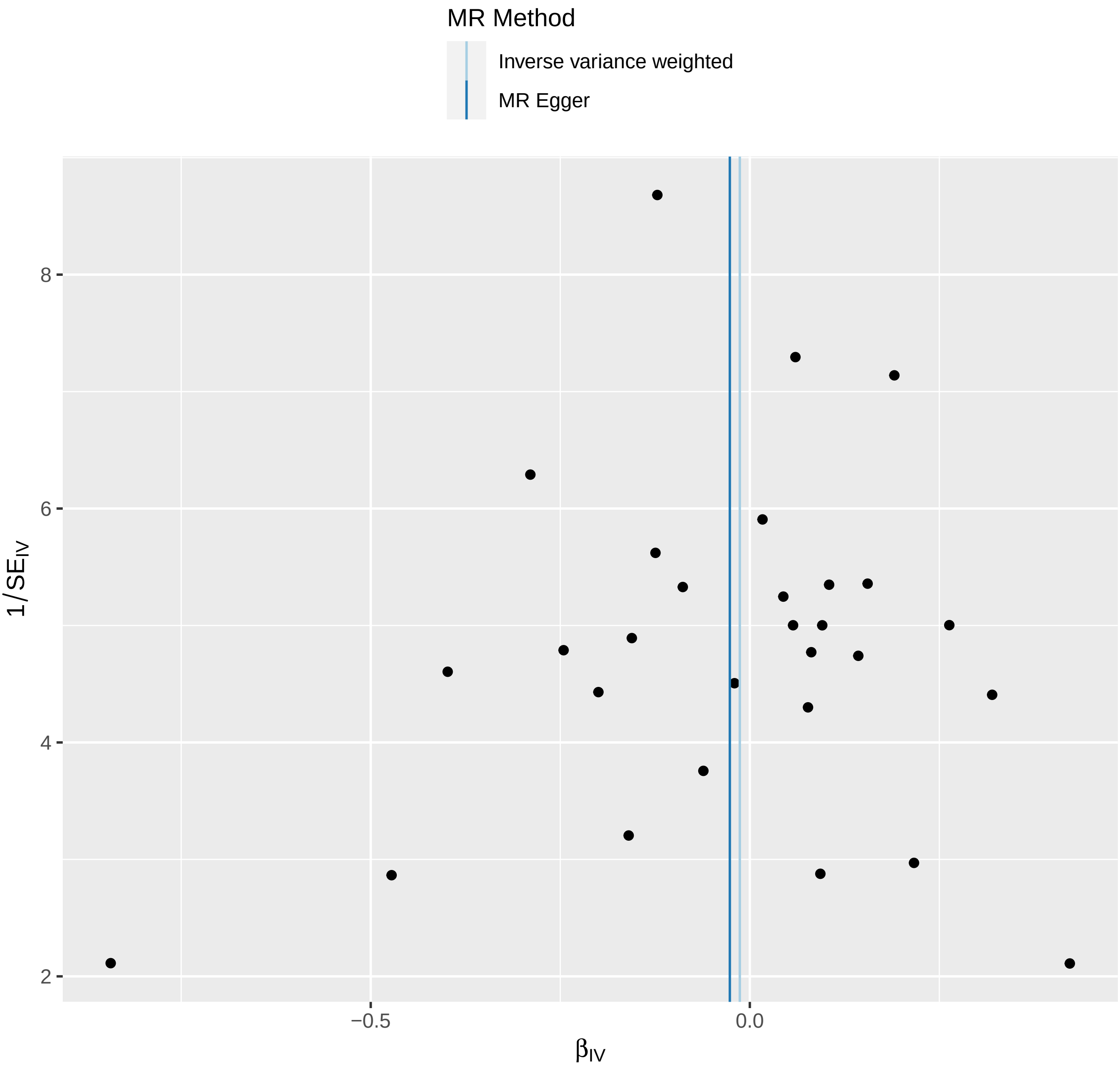

Batch 3−2510 : Reticulocalbin−2 on Diabetic nephropathy (more control exclusions)
with a threshold of 1e−05

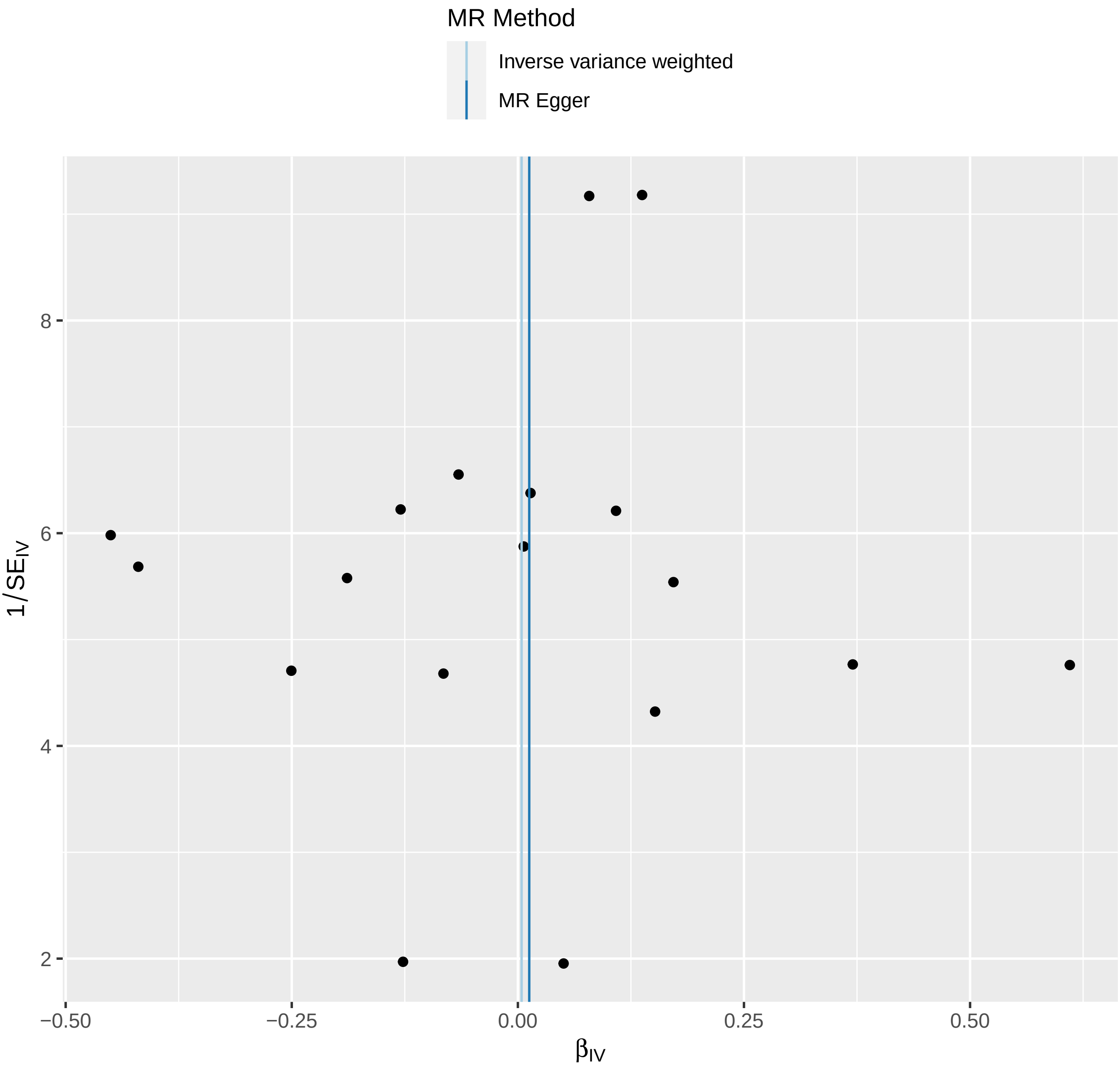

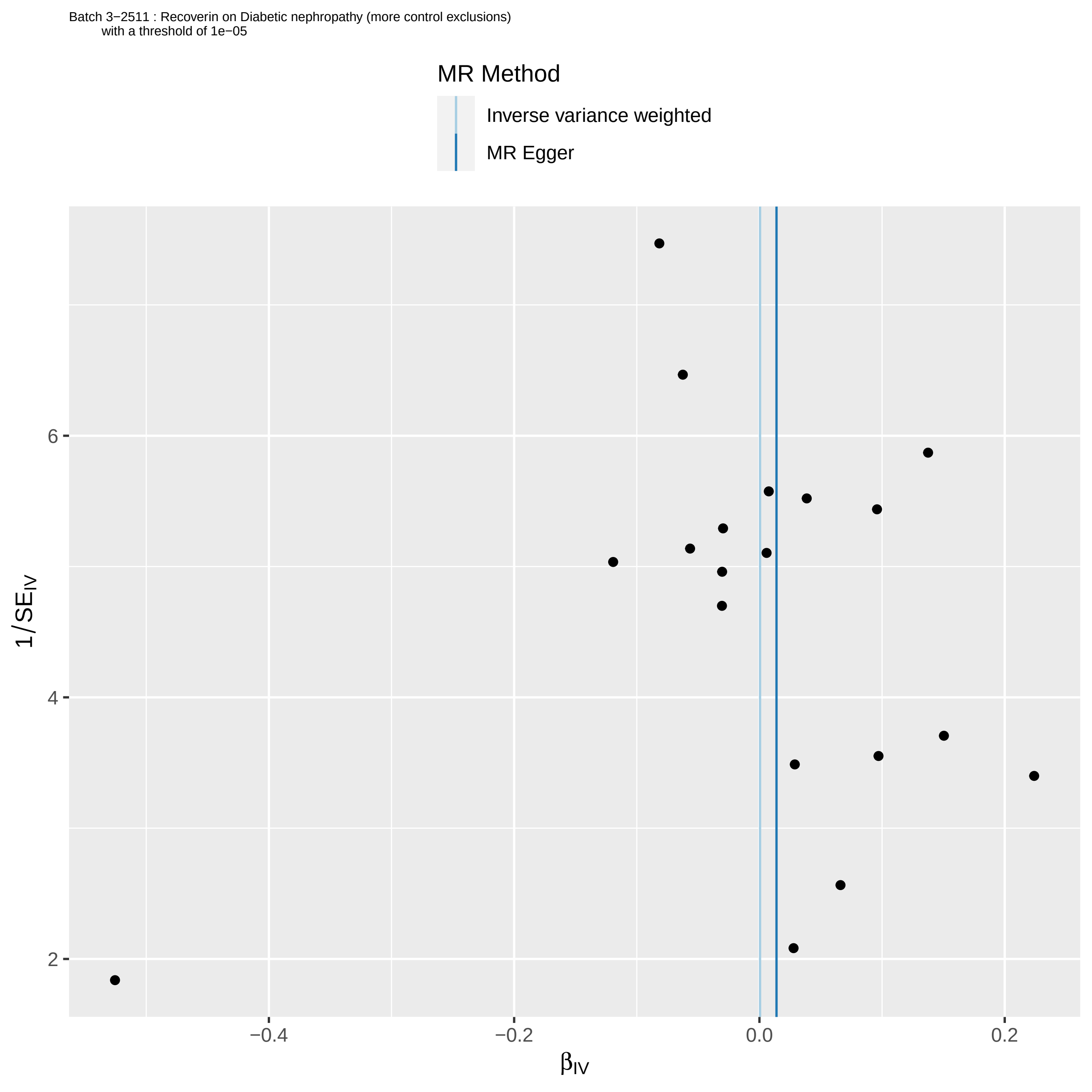

Batch 3−2511 : Recoverin on Diabetic nephropathy (more control exclusions)
with a threshold of 1e−05
MR Method
Inverse variance weighted
MR Egger
6
4
2
−0.4
−0.2
0.0
0.2
1/SE$_{IV}$
β$_{IV}$

Batch 3−2512 : Retinol dehydrogenase 16 on Diabetic nephropathy (more control exclusions)
with a threshold of 1e−05

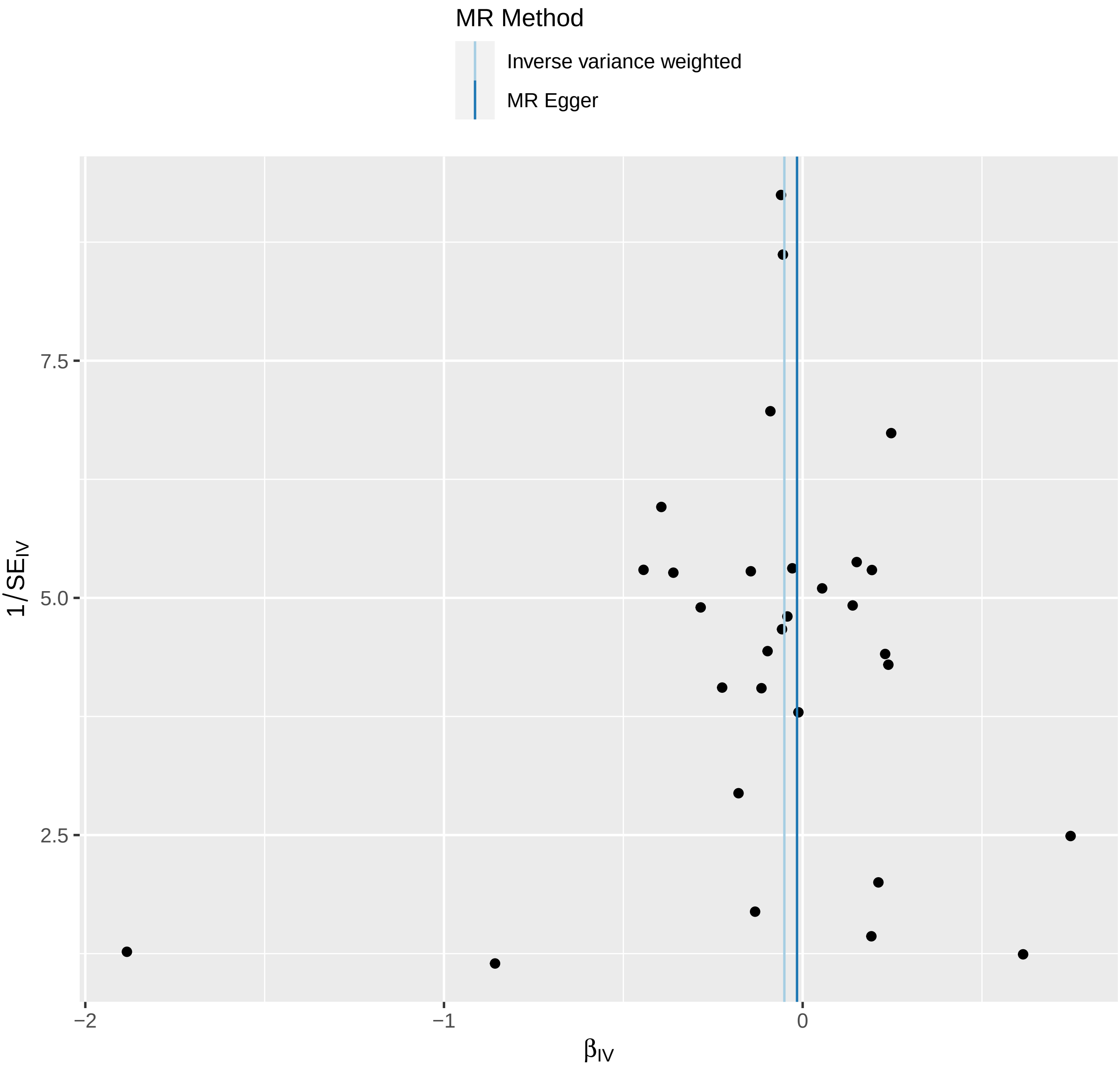

Batch 3−2513 : ATP−dependent DNA helicase Q1 on Diabetic nephropathy (more control exclusions) with a threshold of 1e−05

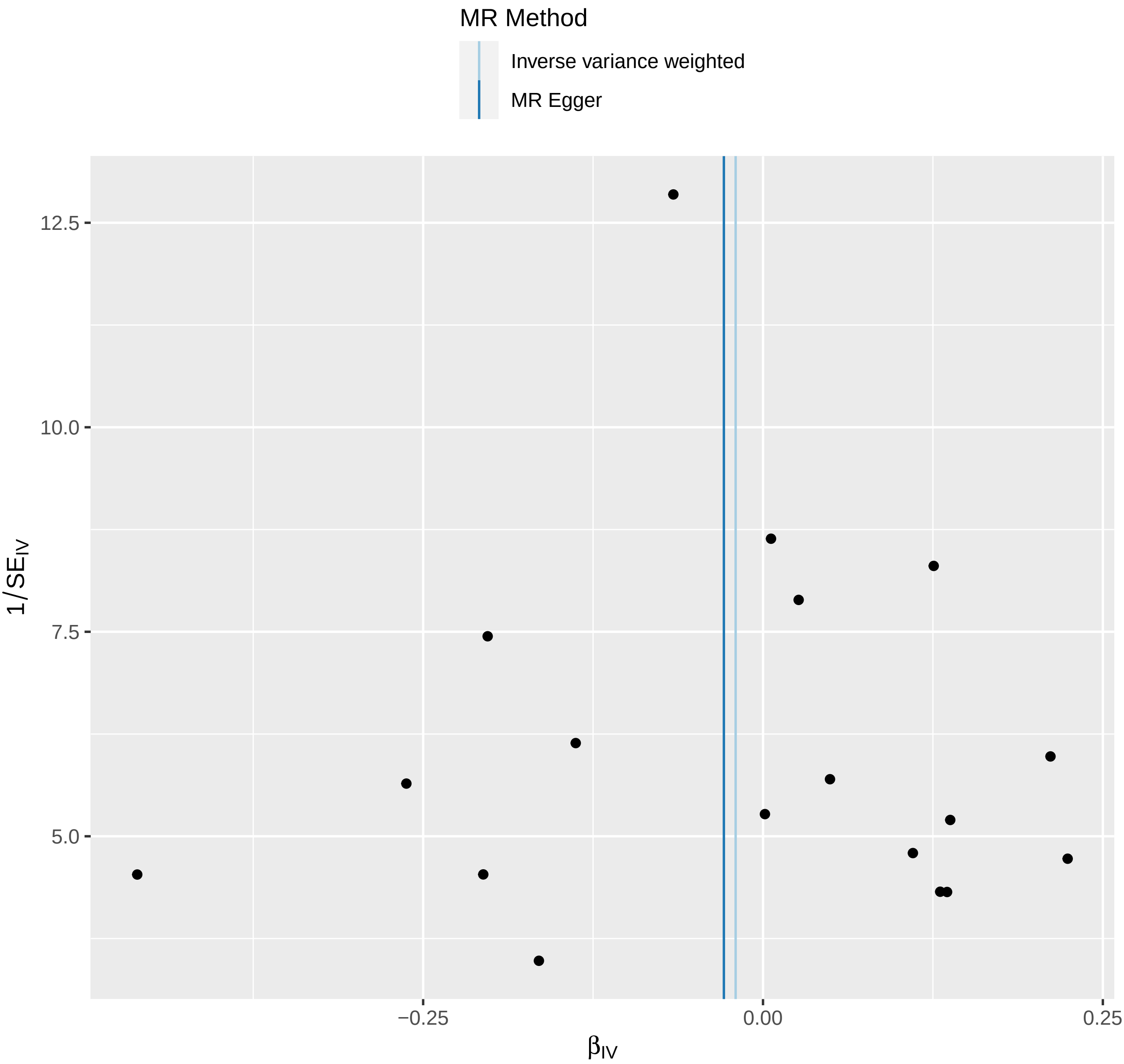

Batch 3−2514 : Regenerating islet−derived protein 3−alpha on Diabetic nephropathy (more control exclusions) with a threshold of 1e−05

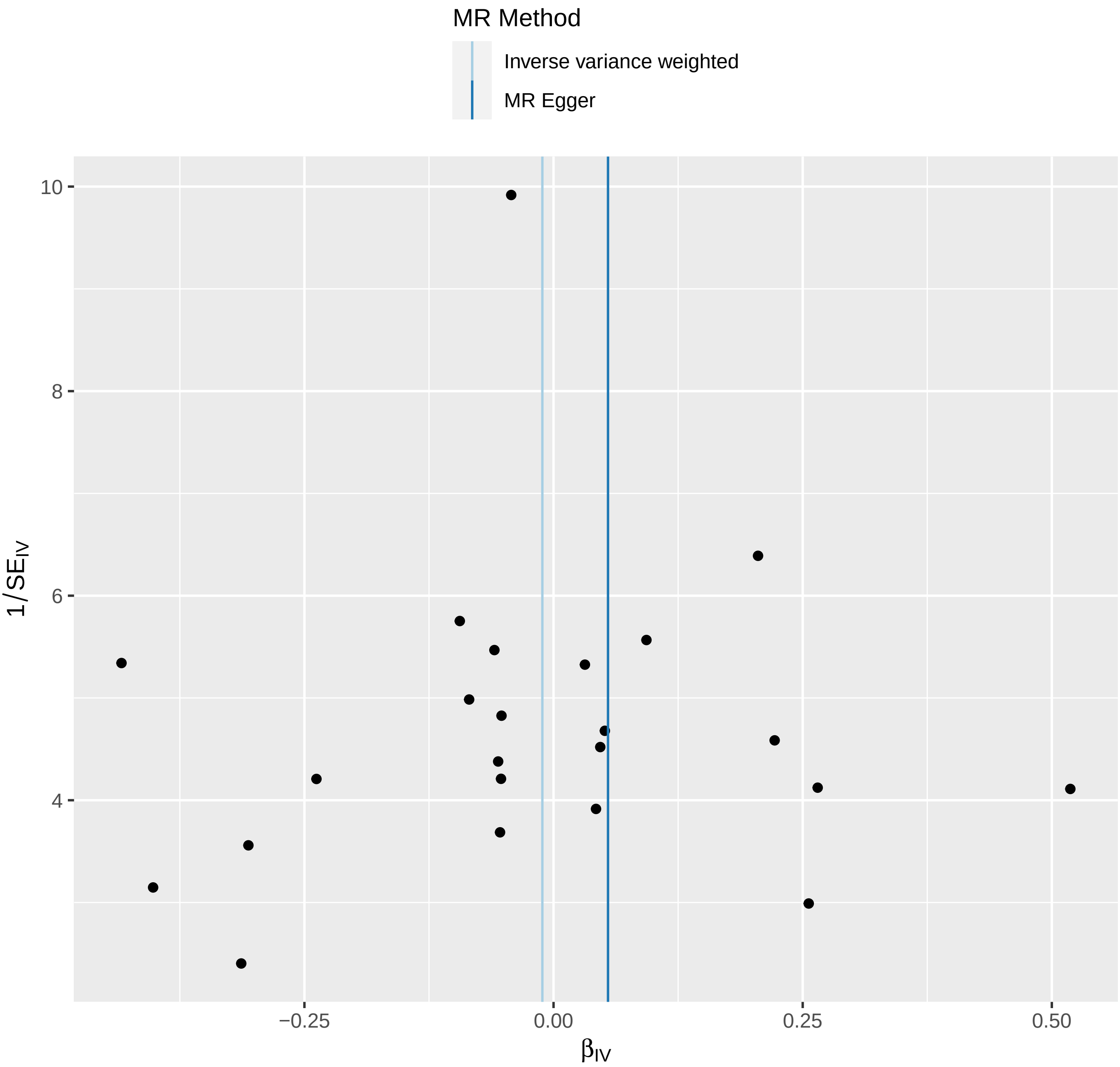

Batch 3−2515 : Regenerating islet−derived protein 4 on Diabetic nephropathy (more control exclusions)
with a threshold of 1e−05

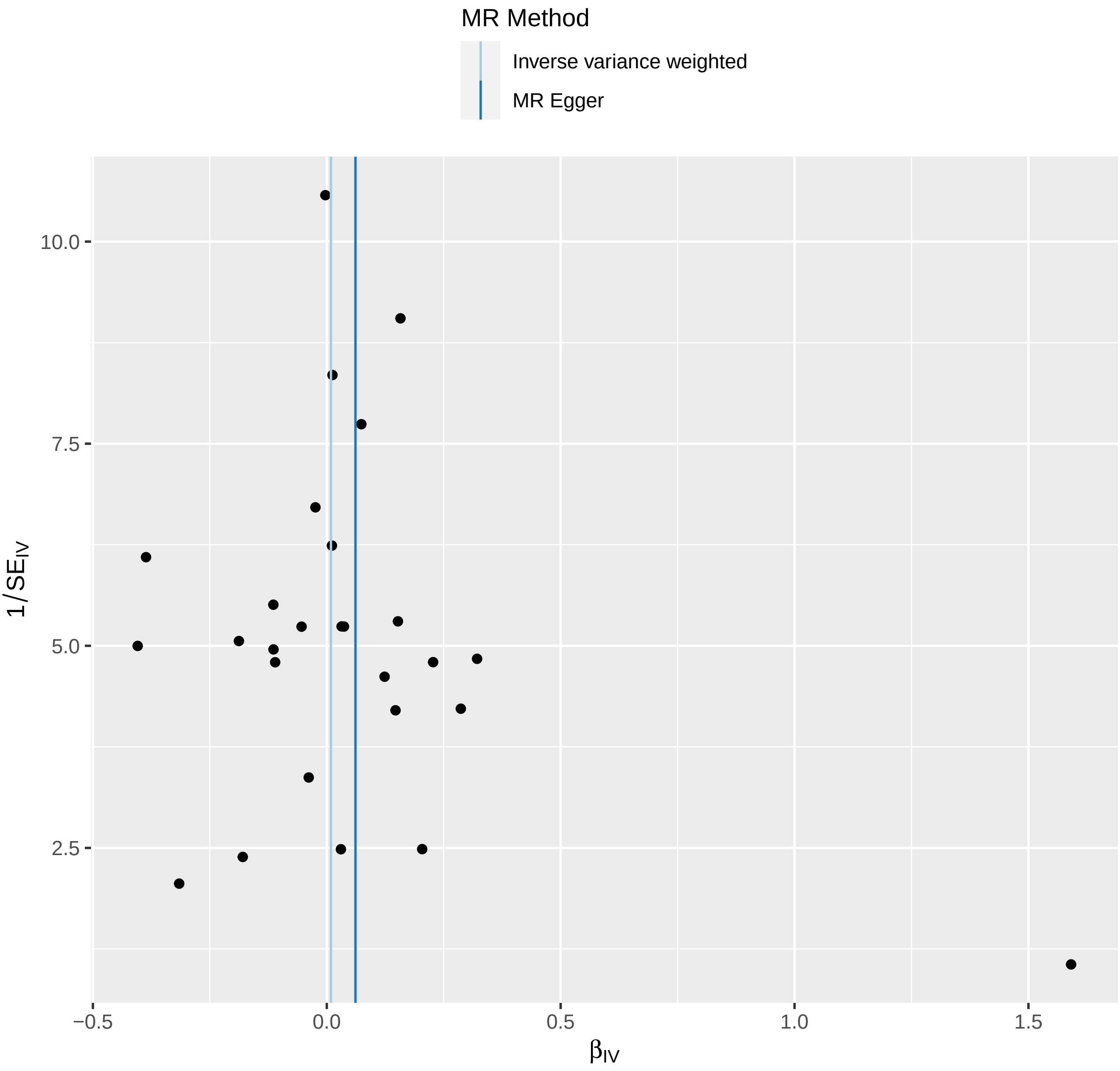

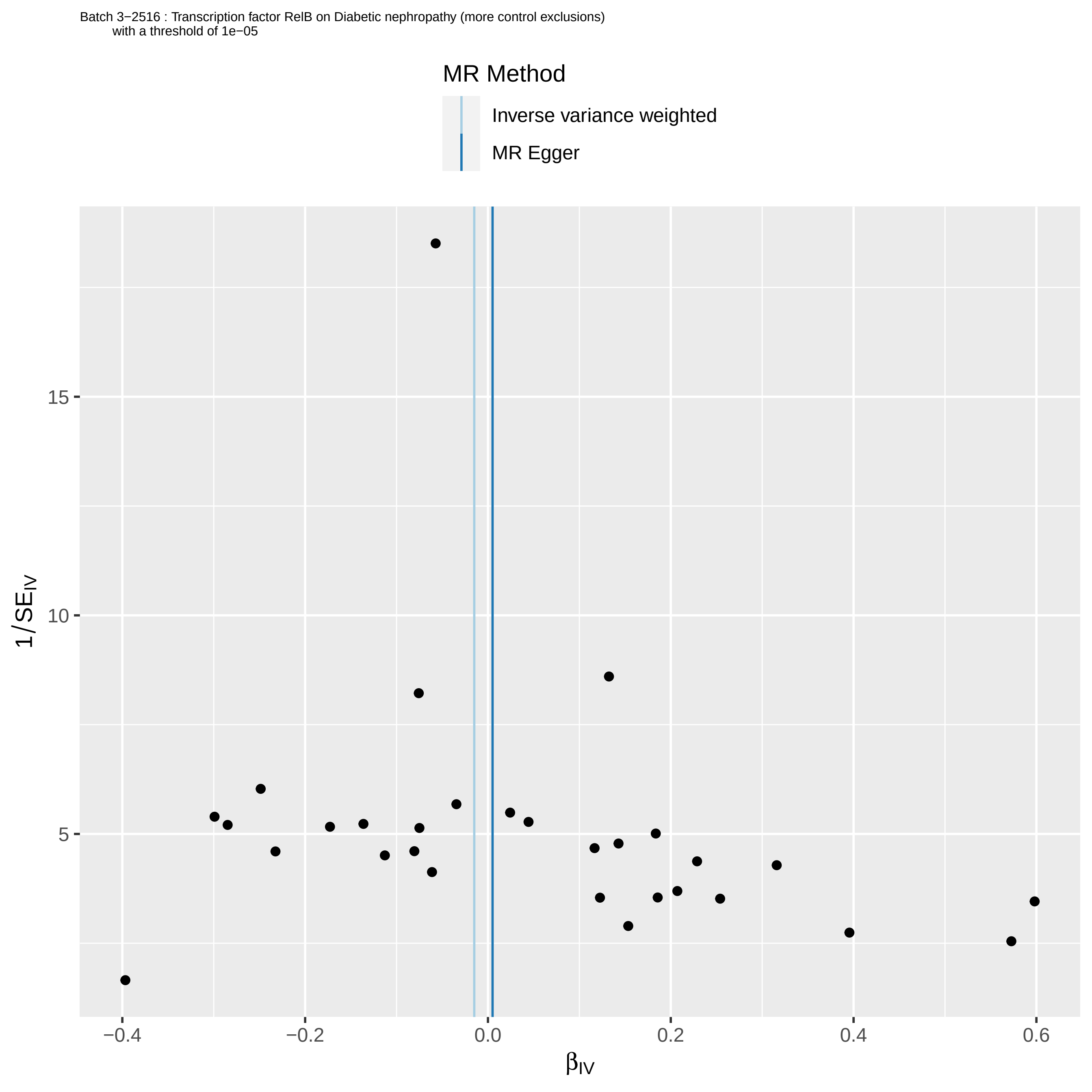

Batch 3−2516 : Transcription factor RelB on Diabetic nephropathy (more control exclusions)
with a threshold of 1e−05
MR Method
Inverse variance weighted
MR Egger
15
10
5
1/SE$_{IV}$
−0.4
−0.2
0.0
0.2
0.4
0.6
β$_{IV}$

Batch 3−2517 : RELT−like protein 1 on Diabetic nephropathy (more control exclusions)
with a threshold of 1e−05

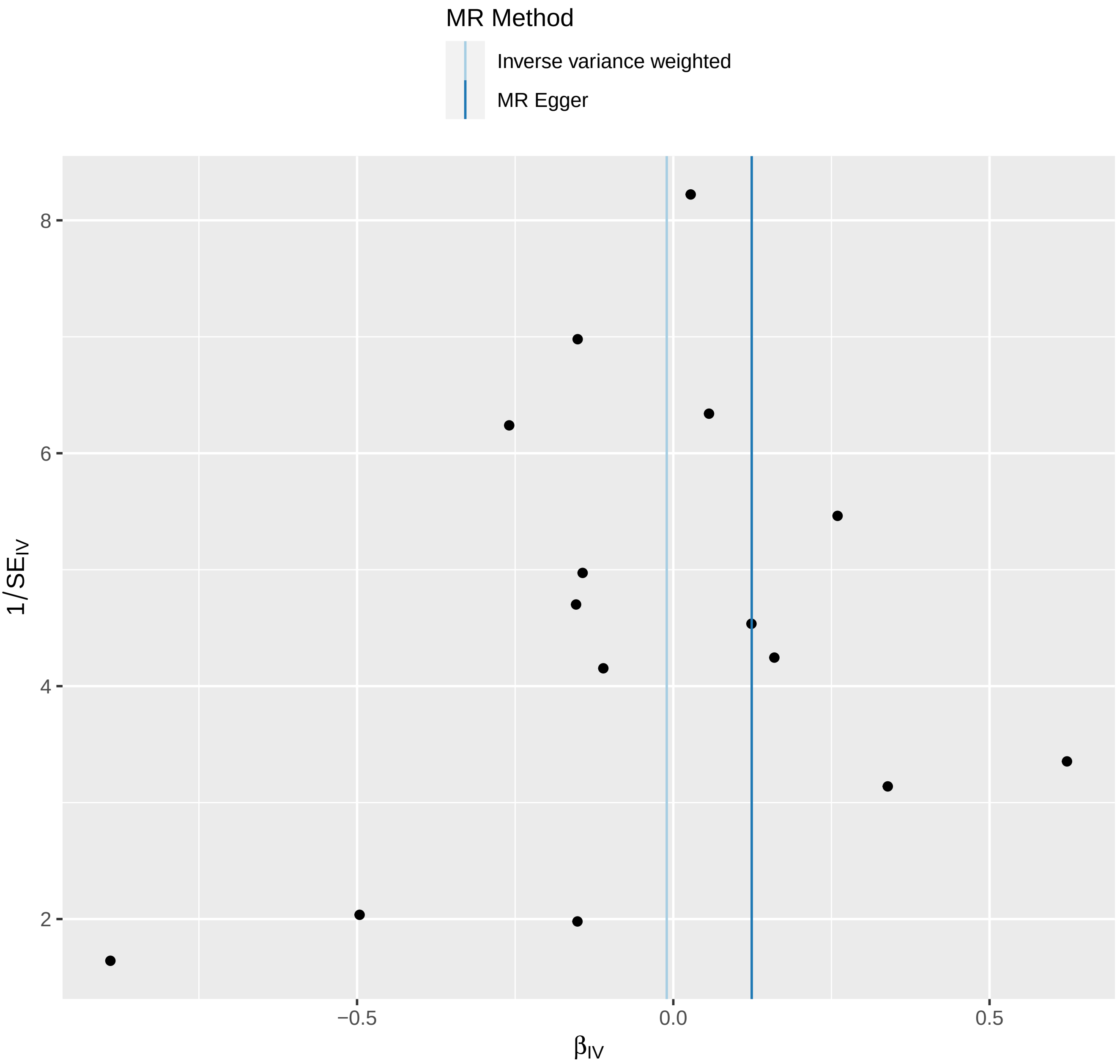

Batch 3−2518 : RELT−like protein 2 on Diabetic nephropathy (more control exclusions)
with a threshold of 1e−05

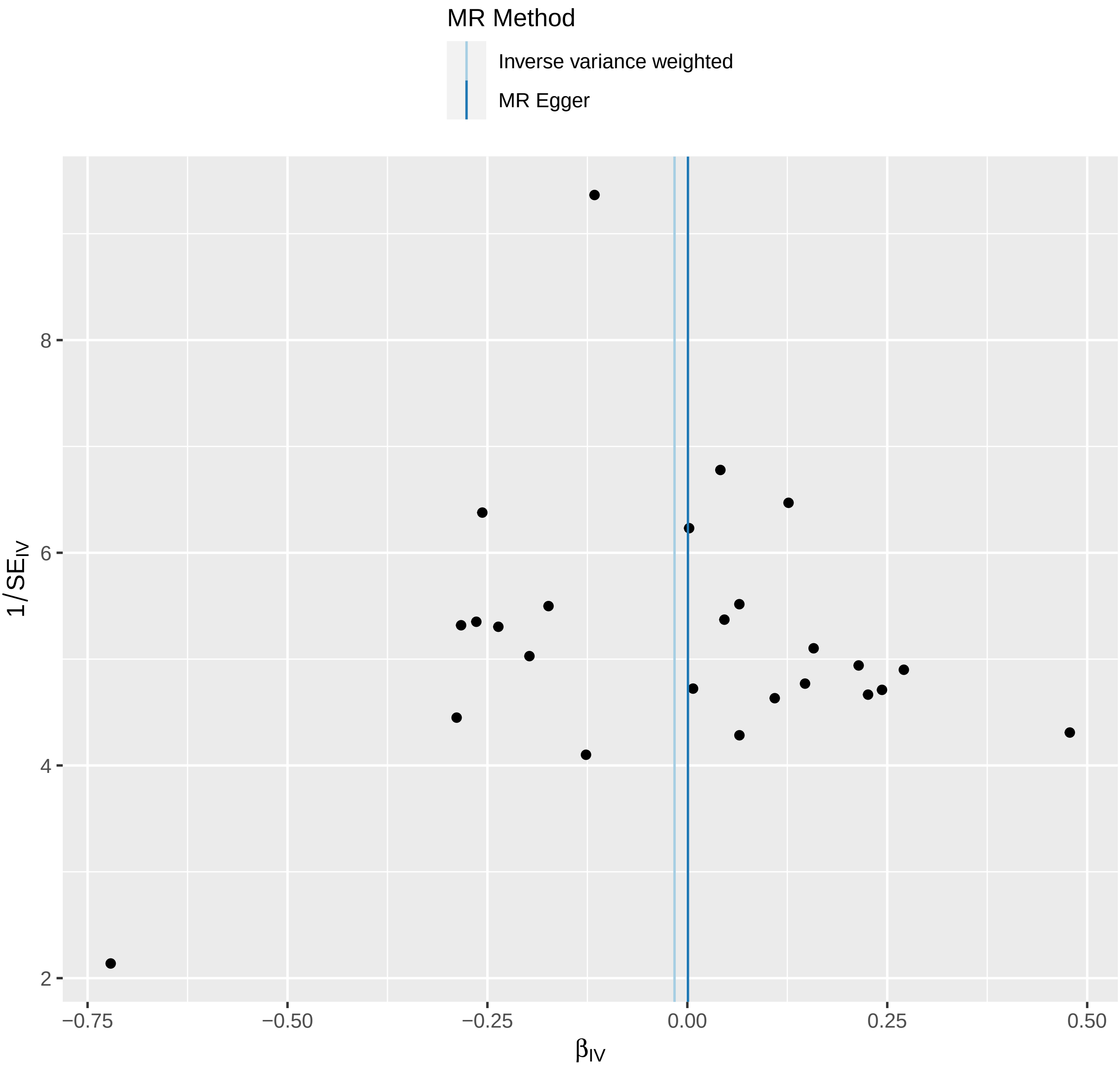

Batch 3−2519 : Tumor necrosis factor receptor superfamily member 19L on Diabetic nephropathy (more control exclusions)
with a threshold of 1e−05

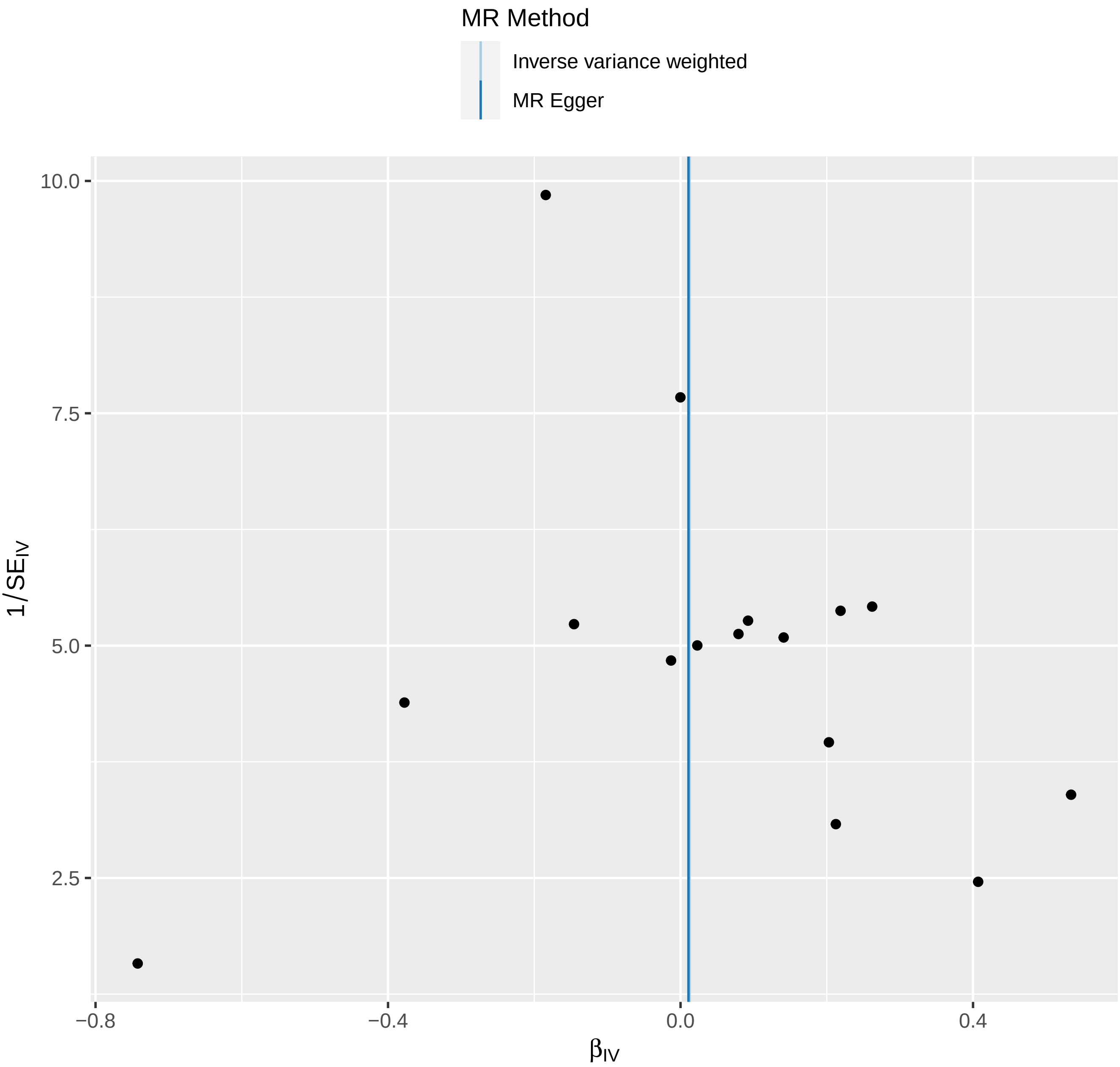

Batch 3−2520 : Tumor necrosis factor receptor superfamily member 19L on Diabetic nephropathy (more control exclusions) with a threshold of 1e−05

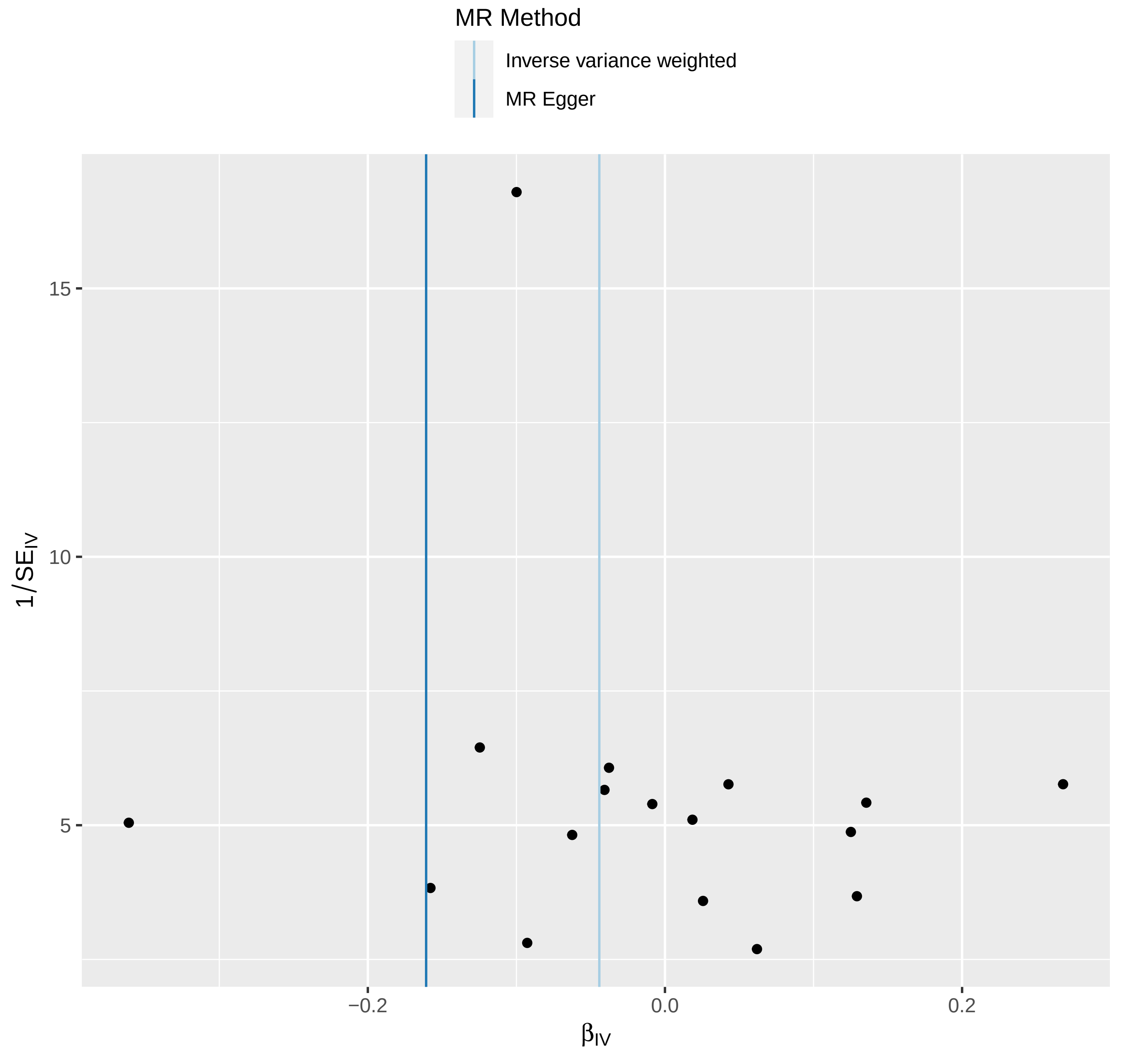

Batch 3−2521 : Renin on Diabetic nephropathy (more control exclusions)
with a threshold of 1e−05

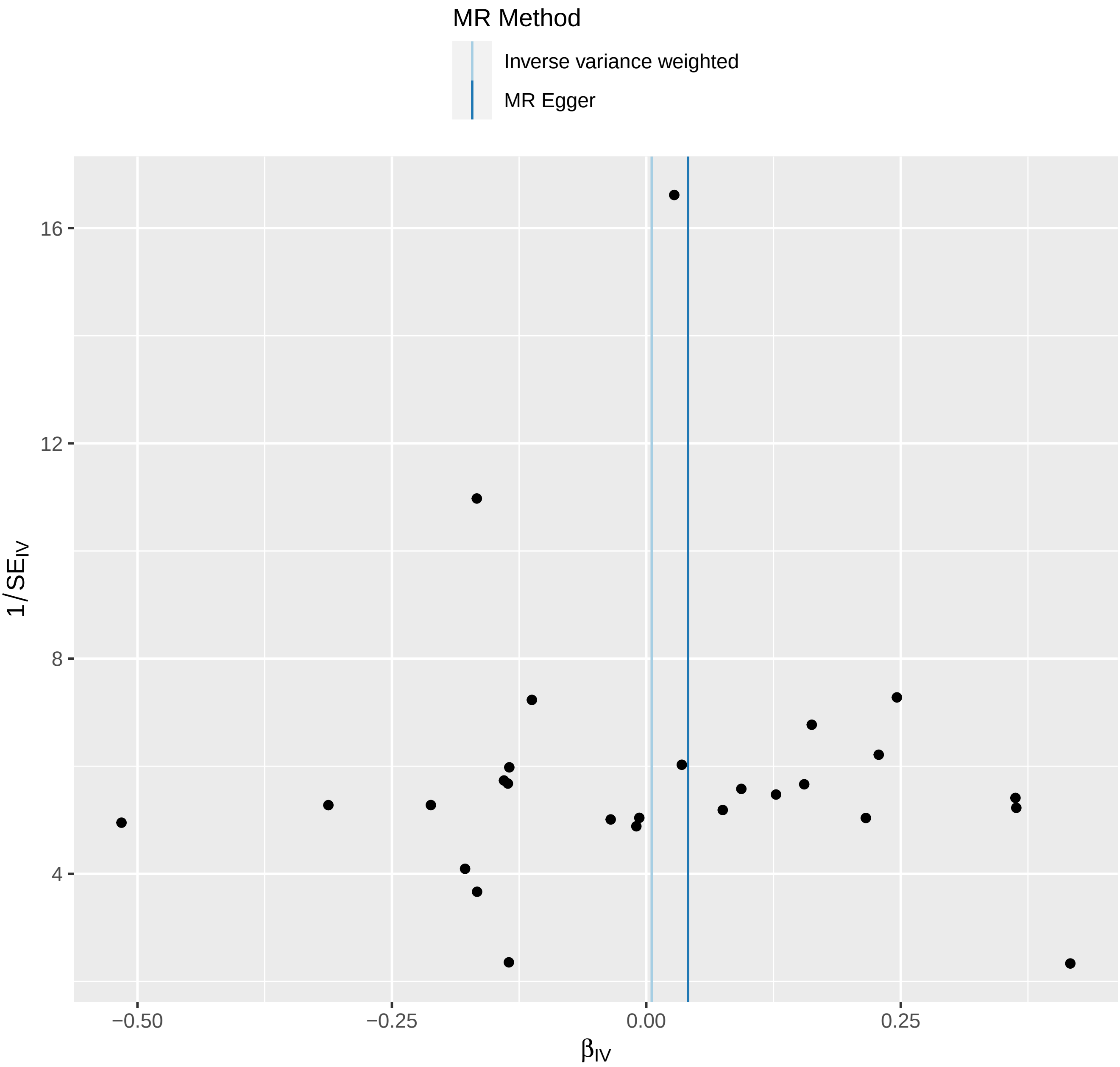

Batch 3−2522 : Replication initiator 1 on Diabetic nephropathy (more control exclusions) with a threshold of 1e−05

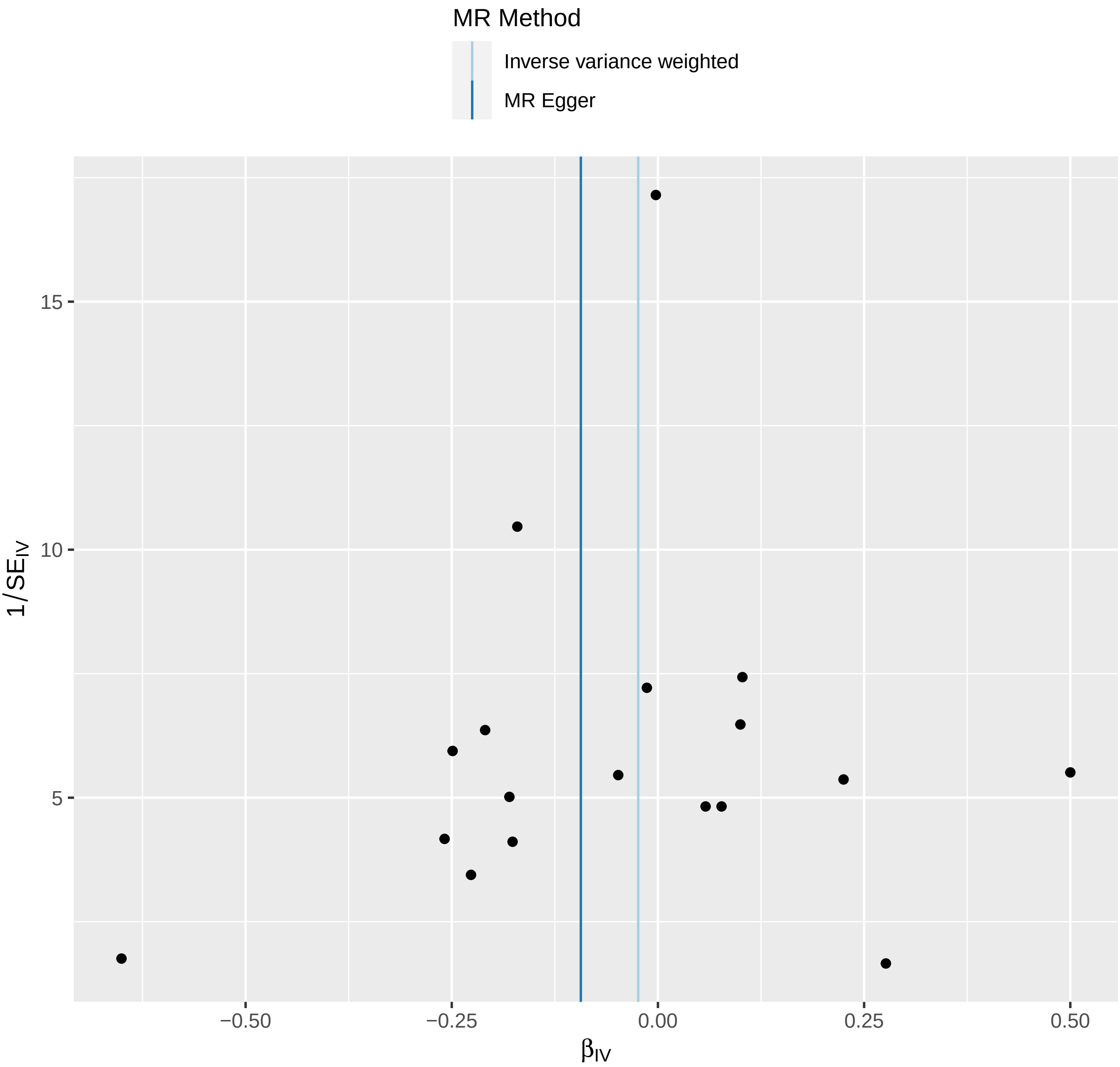

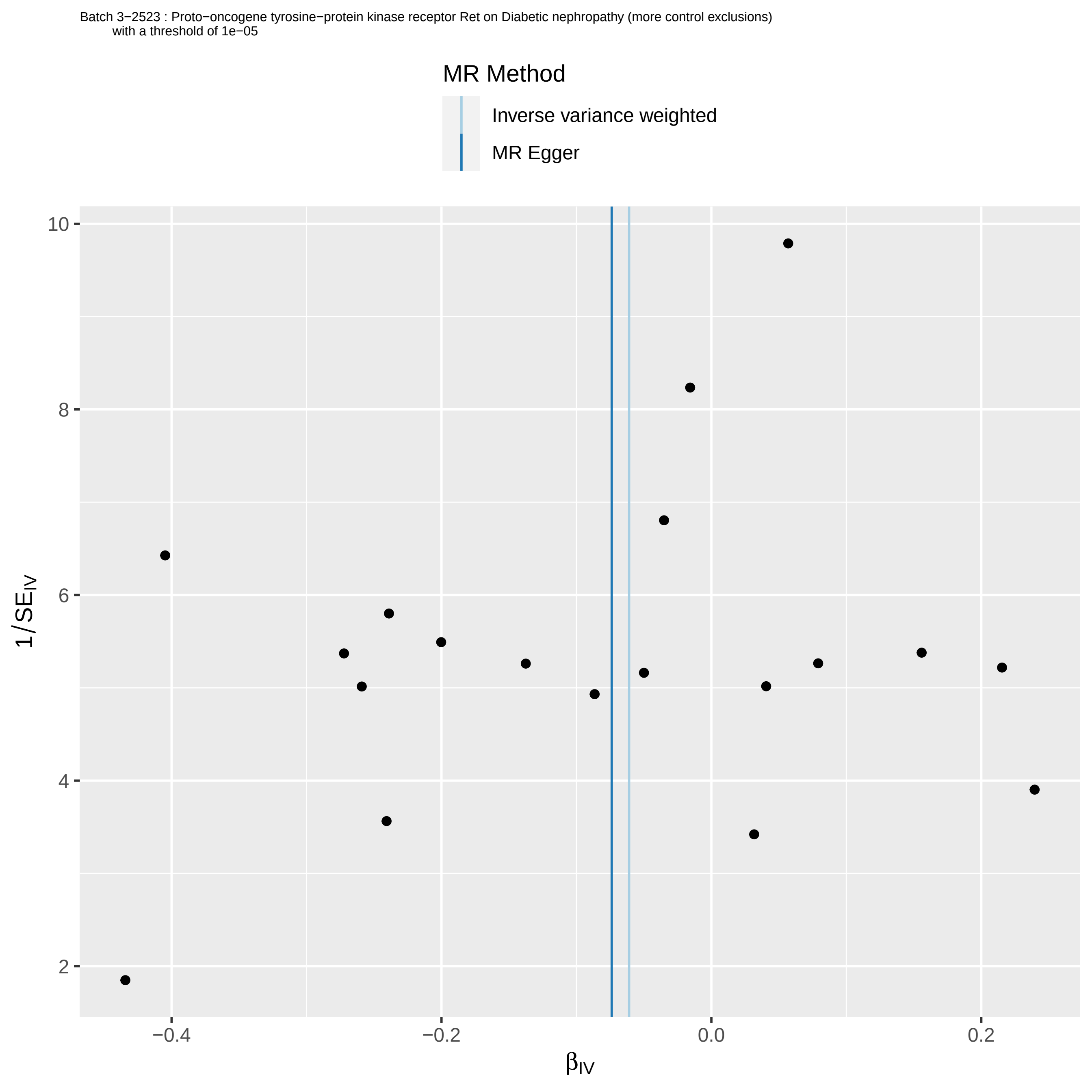
Batch 3−2523 : Proto−oncogene tyrosine−protein kinase receptor Ret on Diabetic nephropathy (more control exclusions)
with a threshold of 1e−05
MR Method
Inverse variance weighted
MR Egger
10
8
6
4
2
1/SE_IV
−0.4
−0.2
0.0
0.2
β_IV

Batch 3−2524 : Resistin on Diabetic nephropathy (more control exclusions)
with a threshold of 1e−05

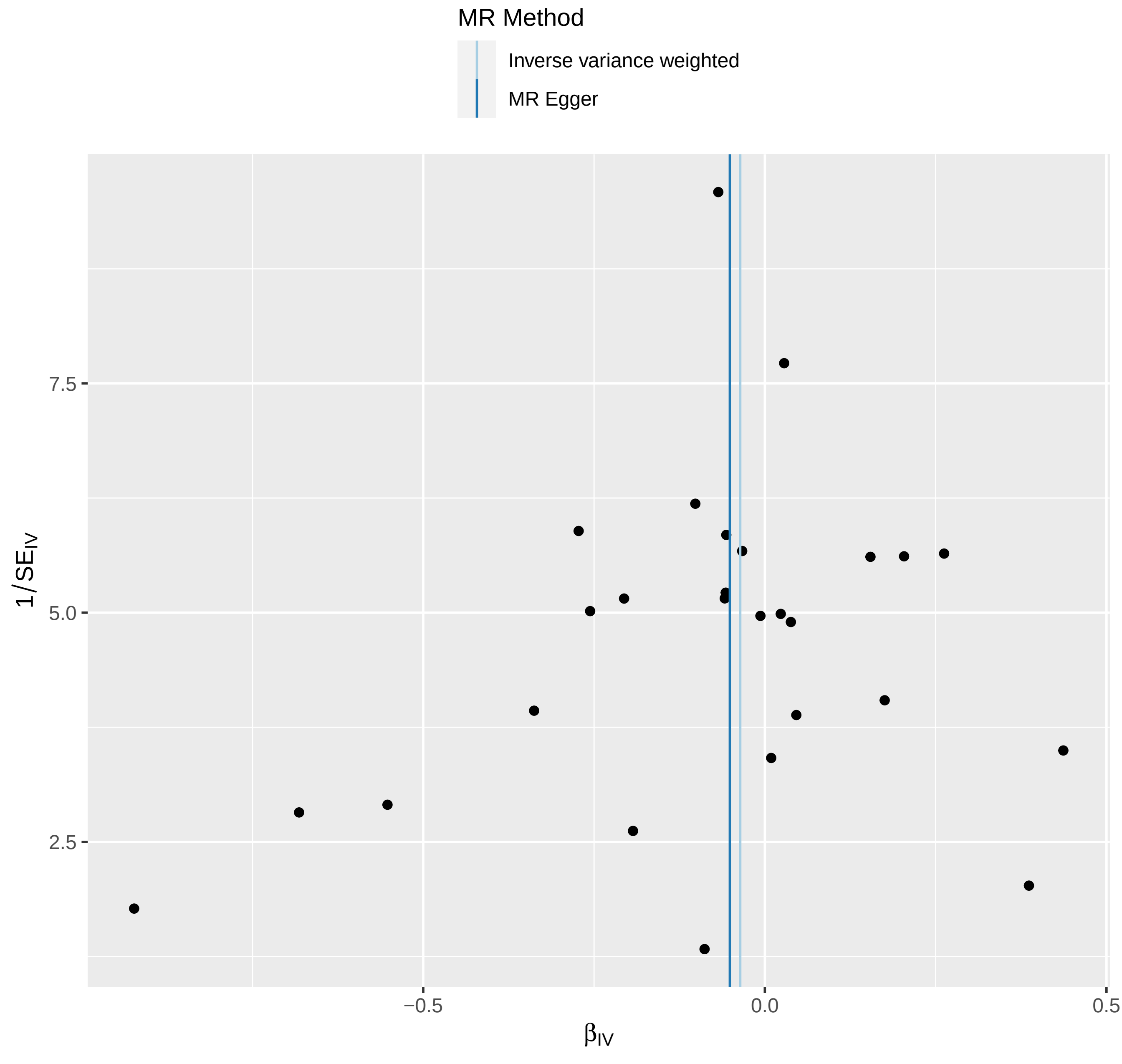

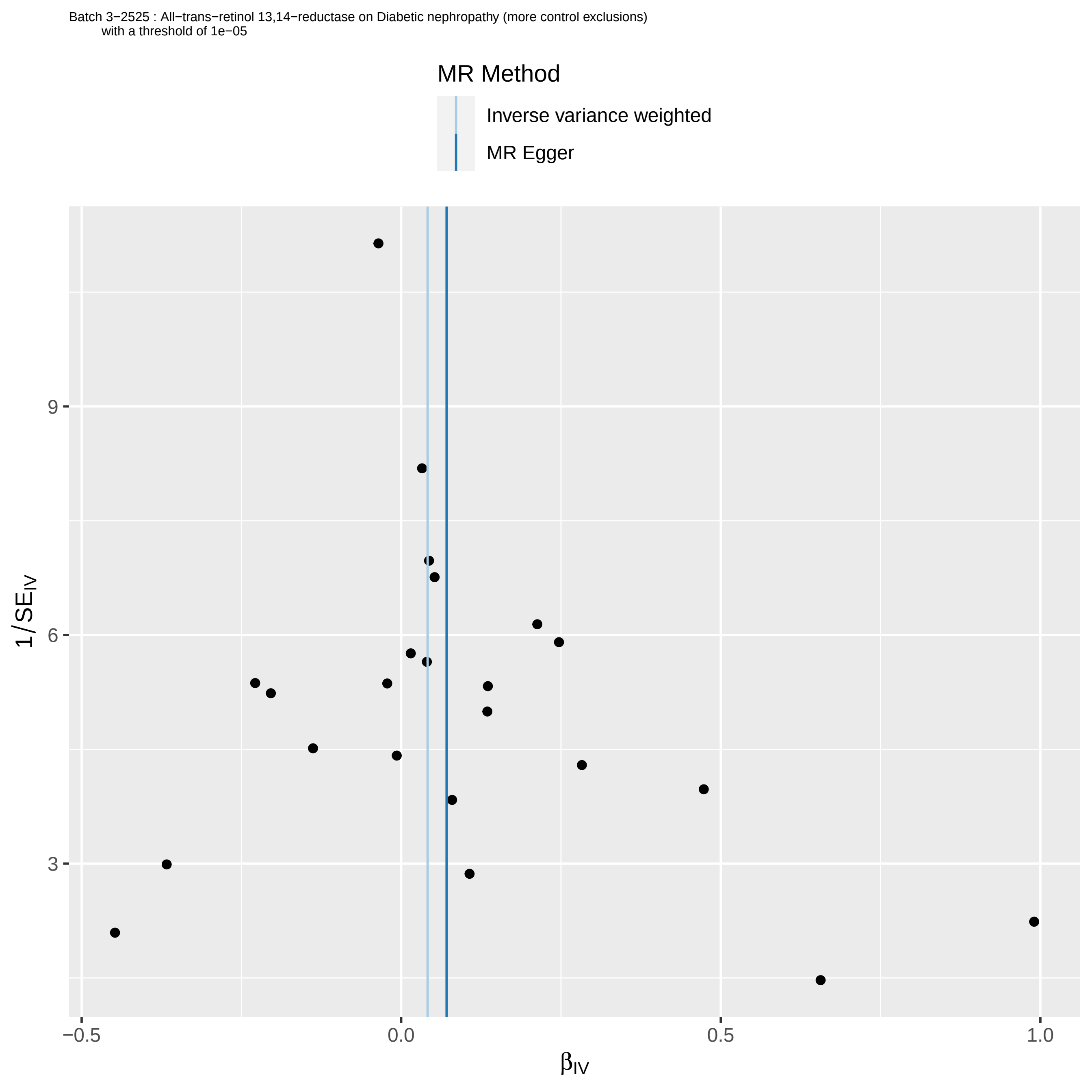

Batch 3−2525 : All−trans−retinol 13,14−reductase on Diabetic nephropathy (more control exclusions)
with a threshold of 1e−05
MR Method
Inverse variance weighted
MR Egger
9
6
3
1/SE_IV
−0.5
0.0
0.5
1.0
β_IV

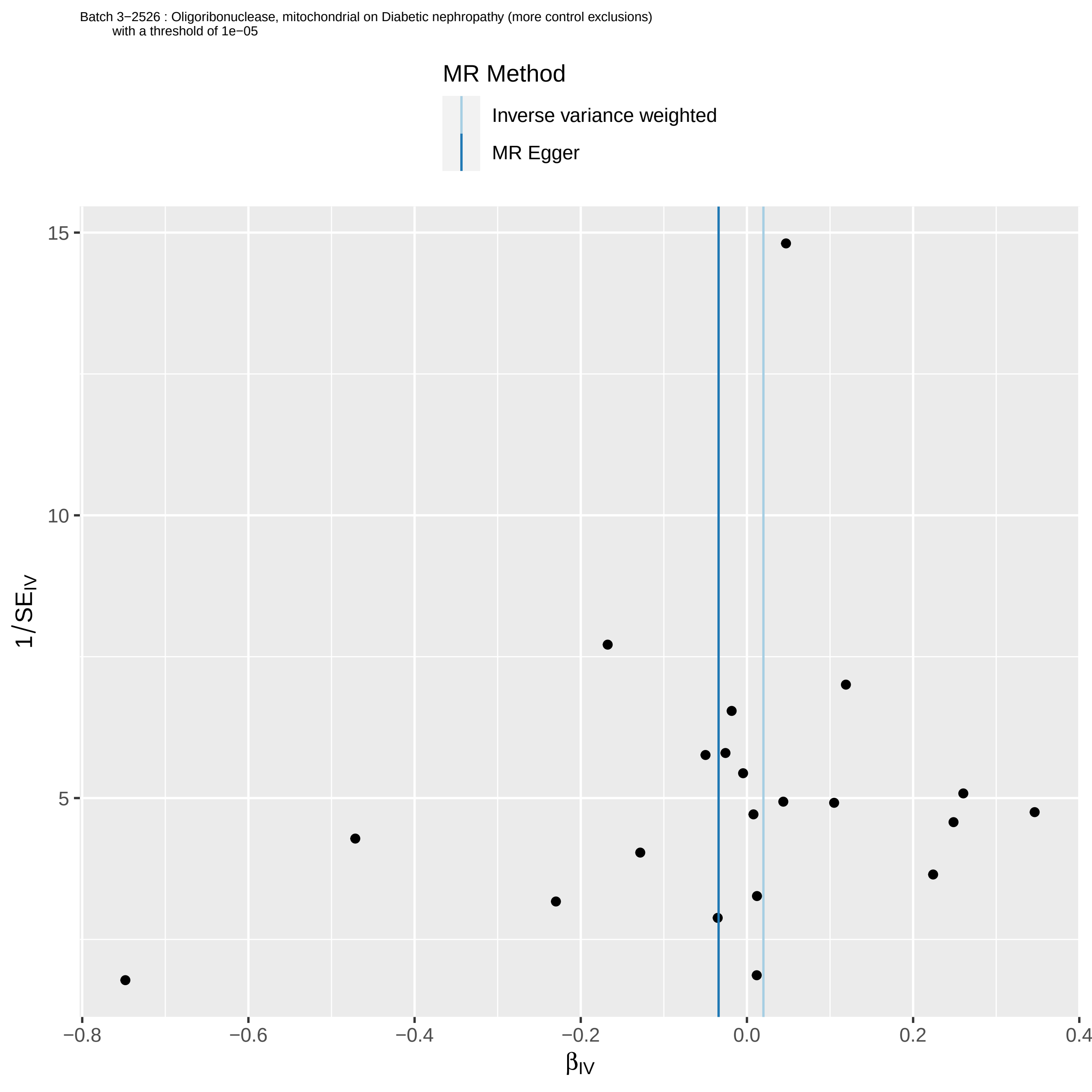
Batch 3−2526 : Oligoribonuclease, mitochondrial on Diabetic nephropathy (more control exclusions)
with a threshold of 1e−05
MR Method
Inverse variance weighted
MR Egger
15
10
5
1/SE$_{IV}$
−0.8
−0.6
−0.4
−0.2
0.0
0.2
0.4
β$_{IV}$

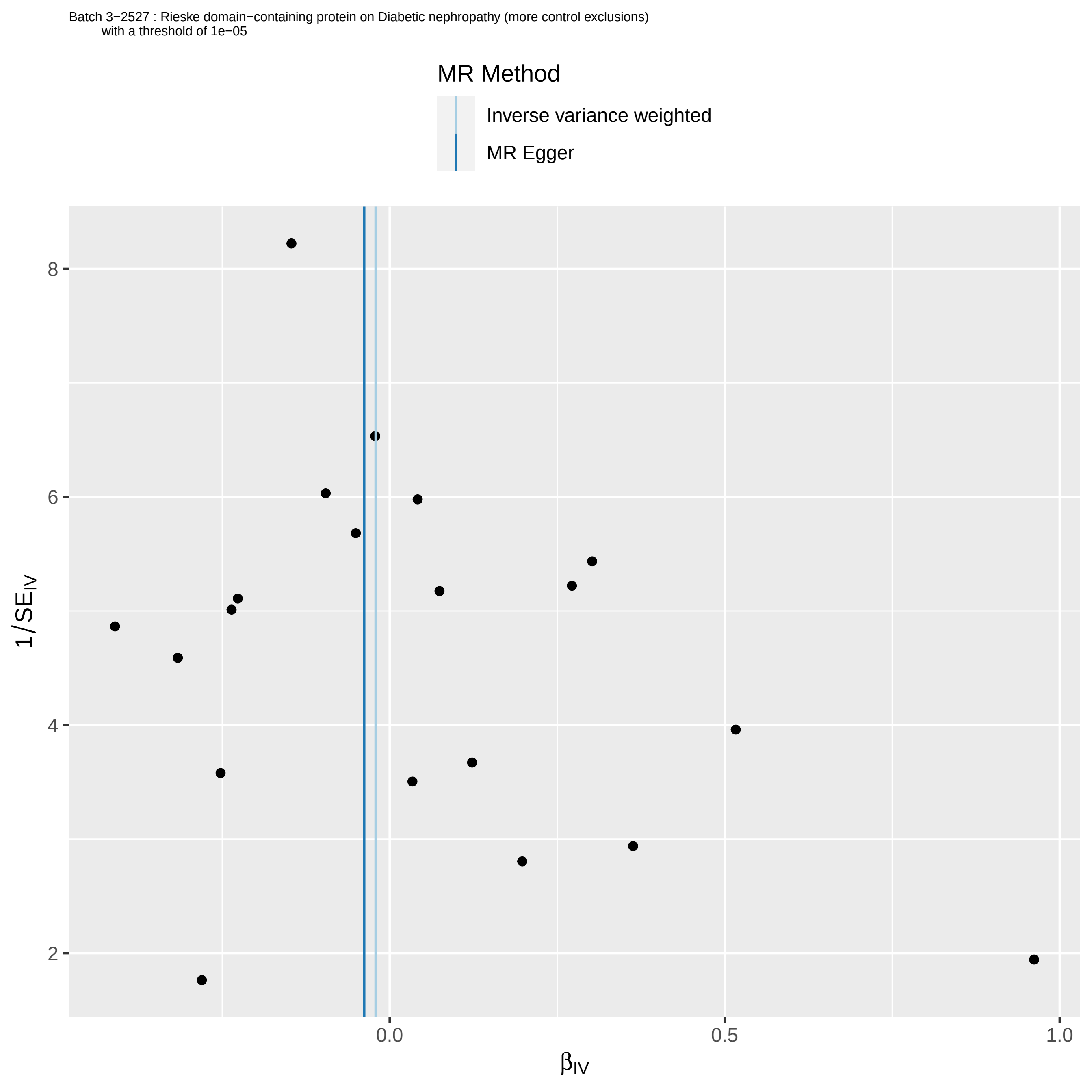
Batch 3−2527 : Rieske domain−containing protein on Diabetic nephropathy (more control exclusions)
with a threshold of 1e−05
MR Method
Inverse variance weighted
MR Egger
1/SE_IV
8
6
4
2
0.0
0.5
1.0
β_IV

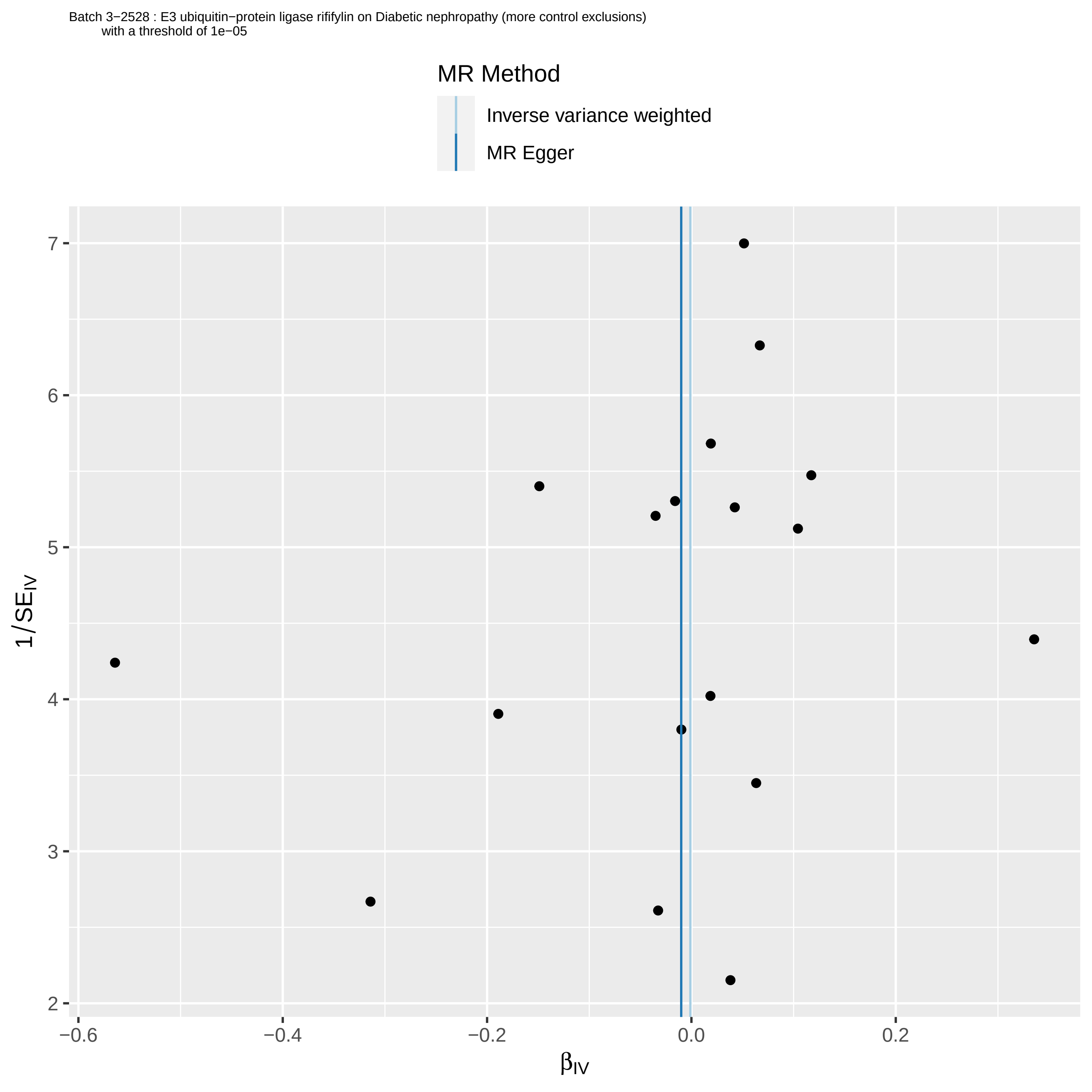

Batch 3−2528 : E3 ubiquitin−protein ligase rififylin on Diabetic nephropathy (more control exclusions)
with a threshold of 1e−05
MR Method
Inverse variance weighted
MR Egger
1/SE_IV
β_IV
7
6
5
4
3
2
−0.6
−0.4
−0.2
0.0
0.2

Batch 3−2529 : Riboflavin kinase on Diabetic nephropathy (more control exclusions)
with a threshold of 1e−05

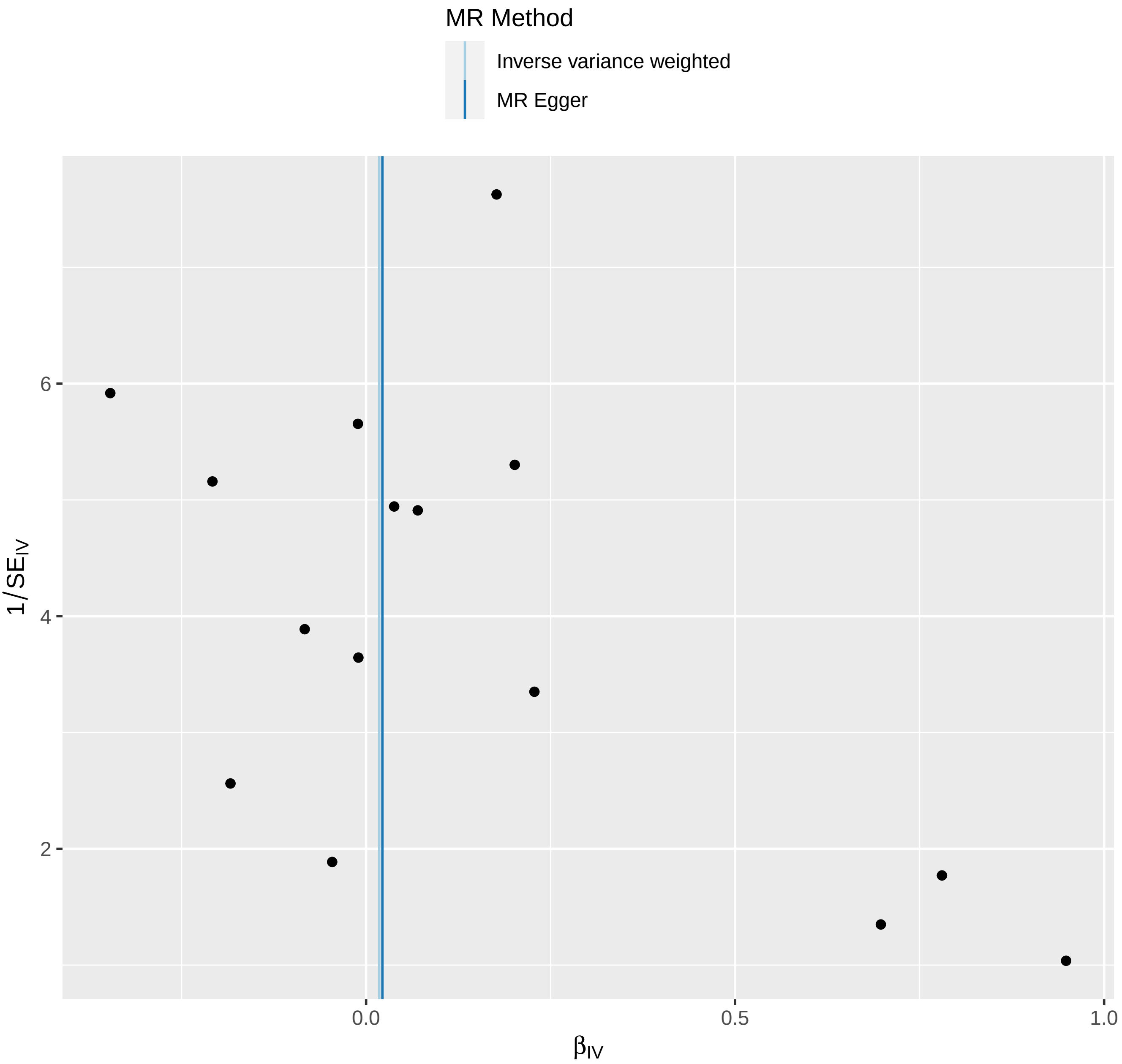

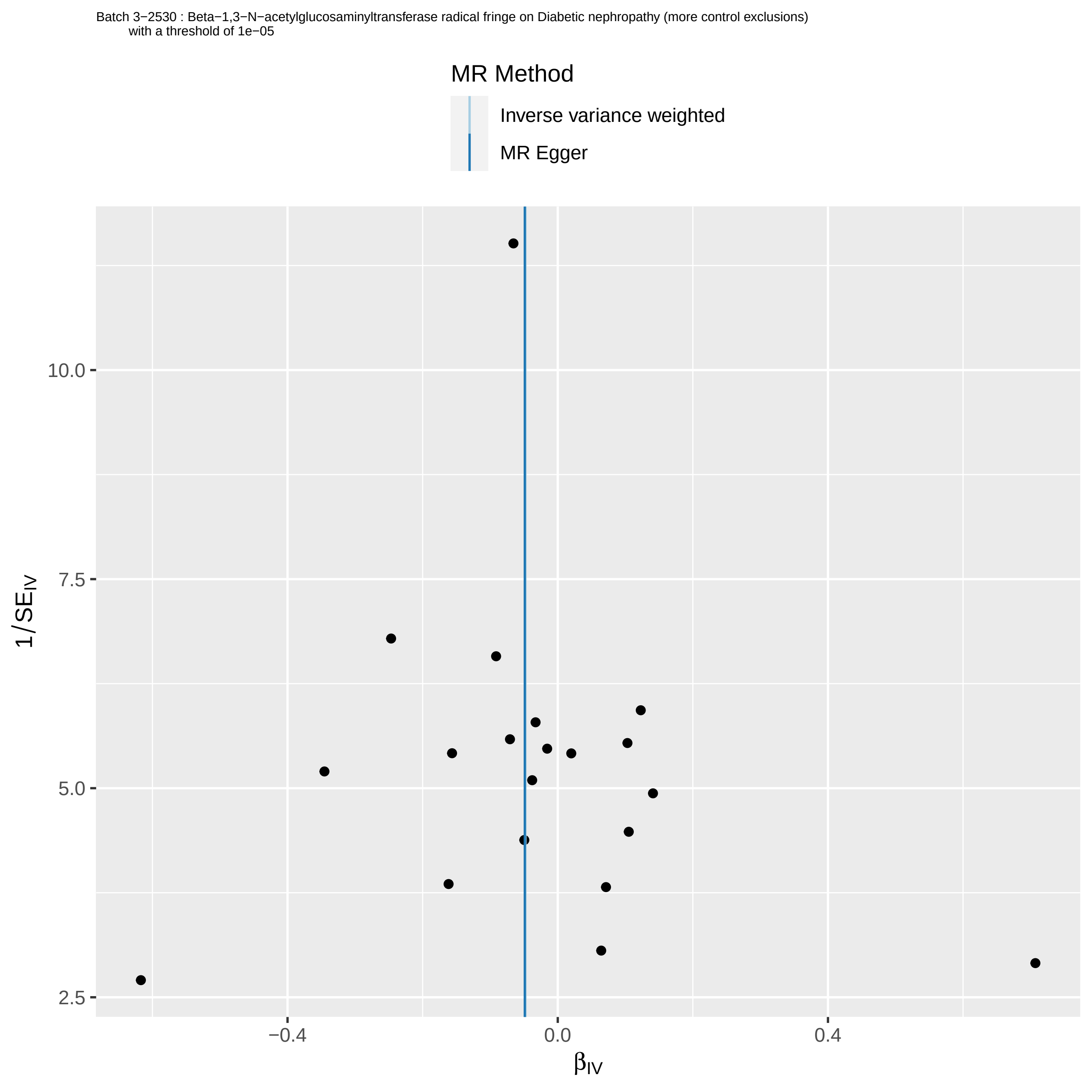
Batch 3−2530 : Beta−1,3−N−acetylglucosaminyltransferase radical fringe on Diabetic nephropathy (more control exclusions)
with a threshold of 1e−05
MR Method
Inverse variance weighted
MR Egger
10.0
7.5
5.0
2.5
1/SE_IV
−0.4
0.0
0.4
β_IV

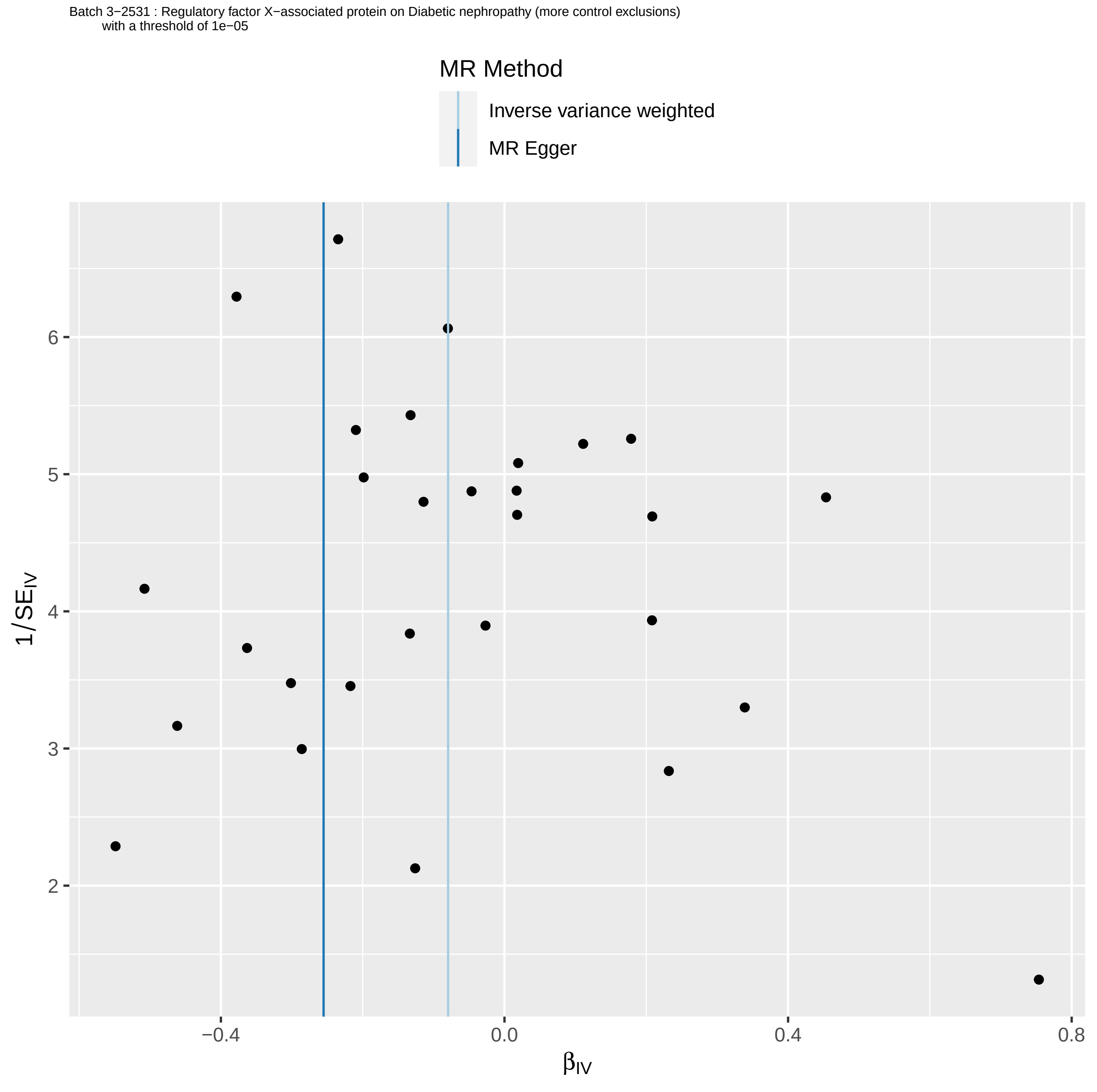
Batch 3−2531 : Regulatory factor X−associated protein on Diabetic nephropathy (more control exclusions)
with a threshold of 1e−05
MR Method
Inverse variance weighted
MR Egger
1/SE_IV
β_IV
−0.4
0.0
0.4
0.8
2
3
4
5
6

Batch 3−2532 : Repulsive guidance molecule A on Diabetic nephropathy (more control exclusions)
with a threshold of 1e−05

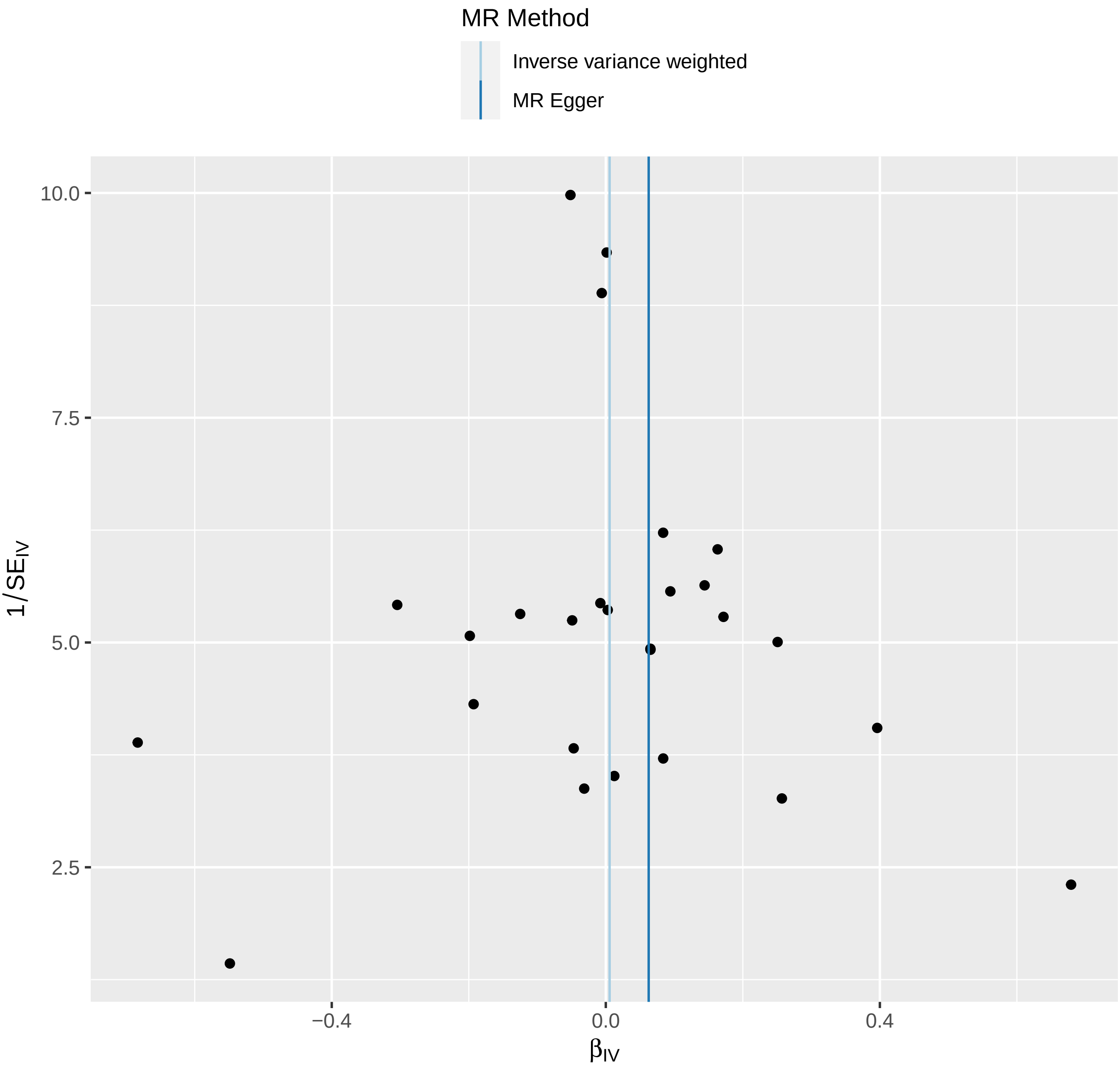

Batch 3−2533 : RGM domain family member B on Diabetic nephropathy (more control exclusions)
with a threshold of 1e−05

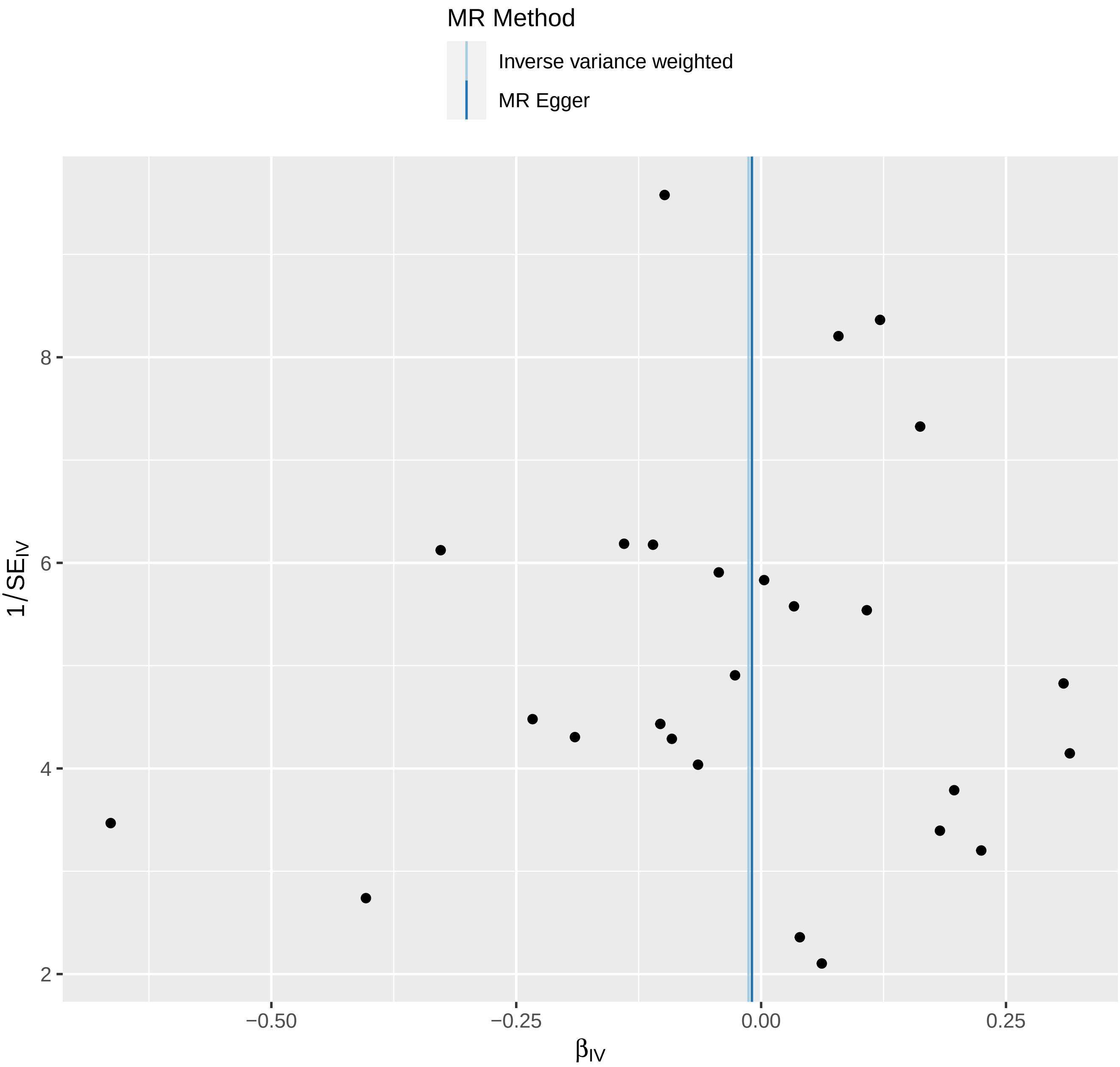

Batch 3−2534 : Regulator of G−protein signaling 10 on Diabetic nephropathy (more control exclusions)
with a threshold of 1e−05

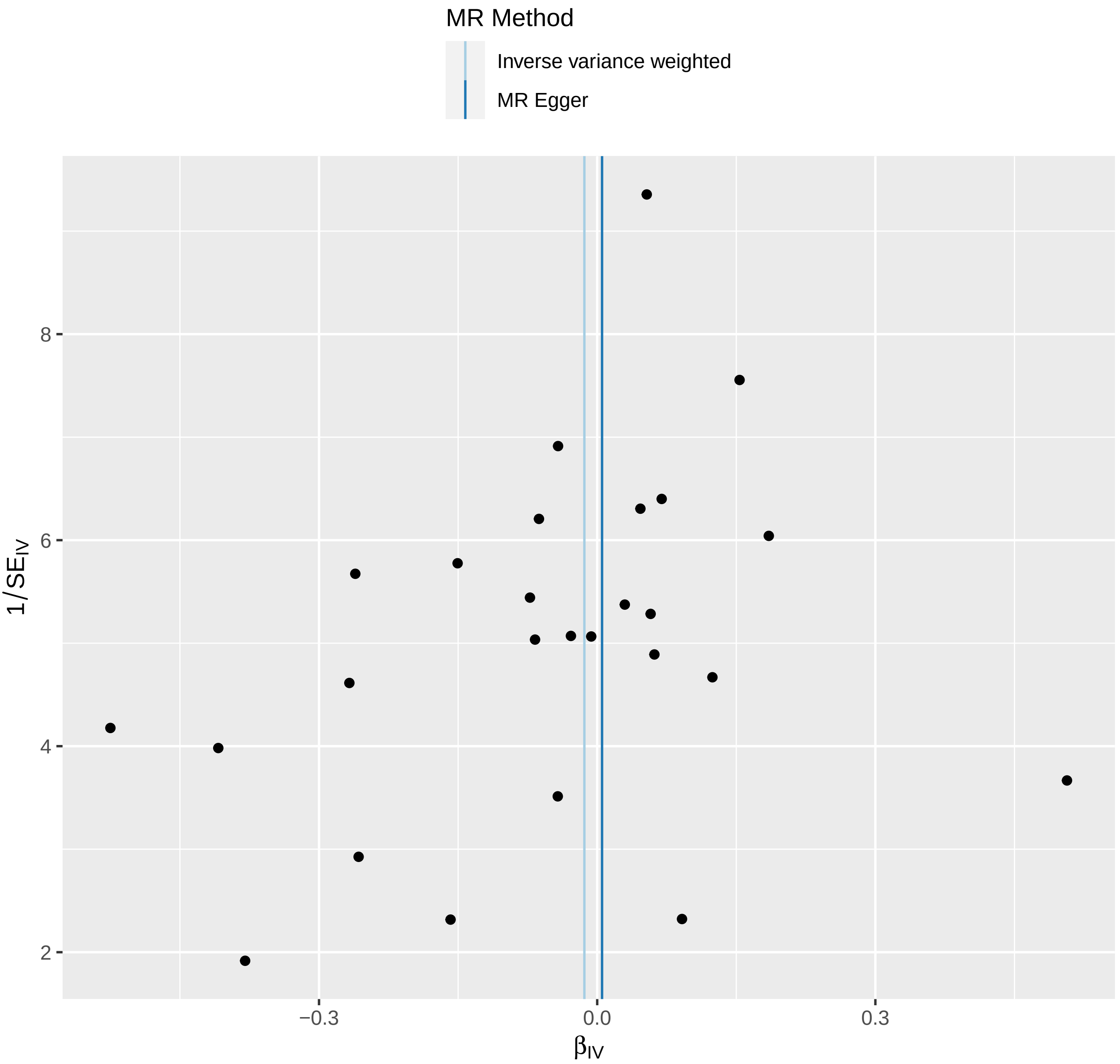

Batch 3−2535 : Regulator of G−protein signaling 18 on Diabetic nephropathy (more control exclusions)
with a threshold of 1e−05

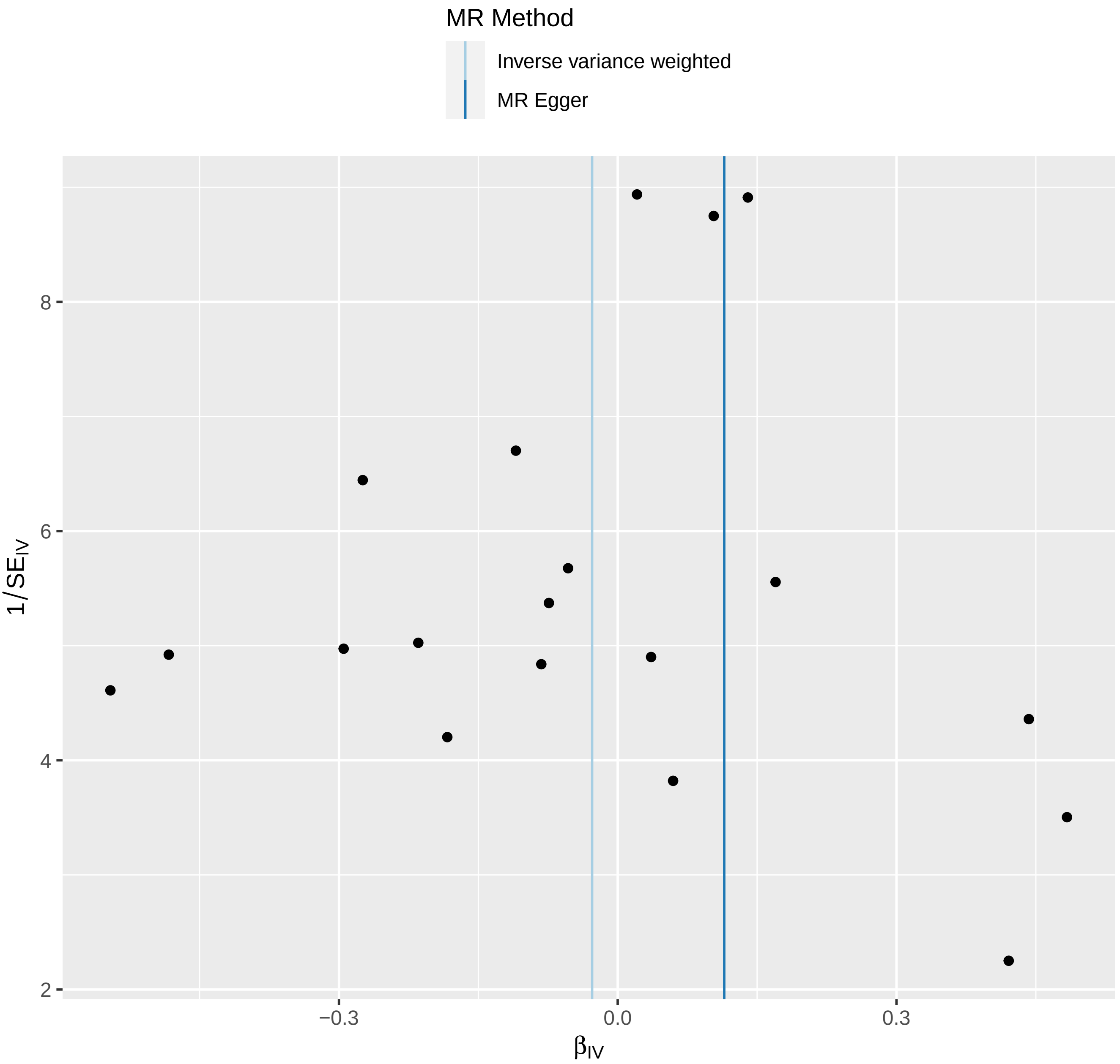

Batch 3−2536 : Regulator of G−protein signaling 19 on Diabetic nephropathy (more control exclusions)
with a threshold of 1e−05

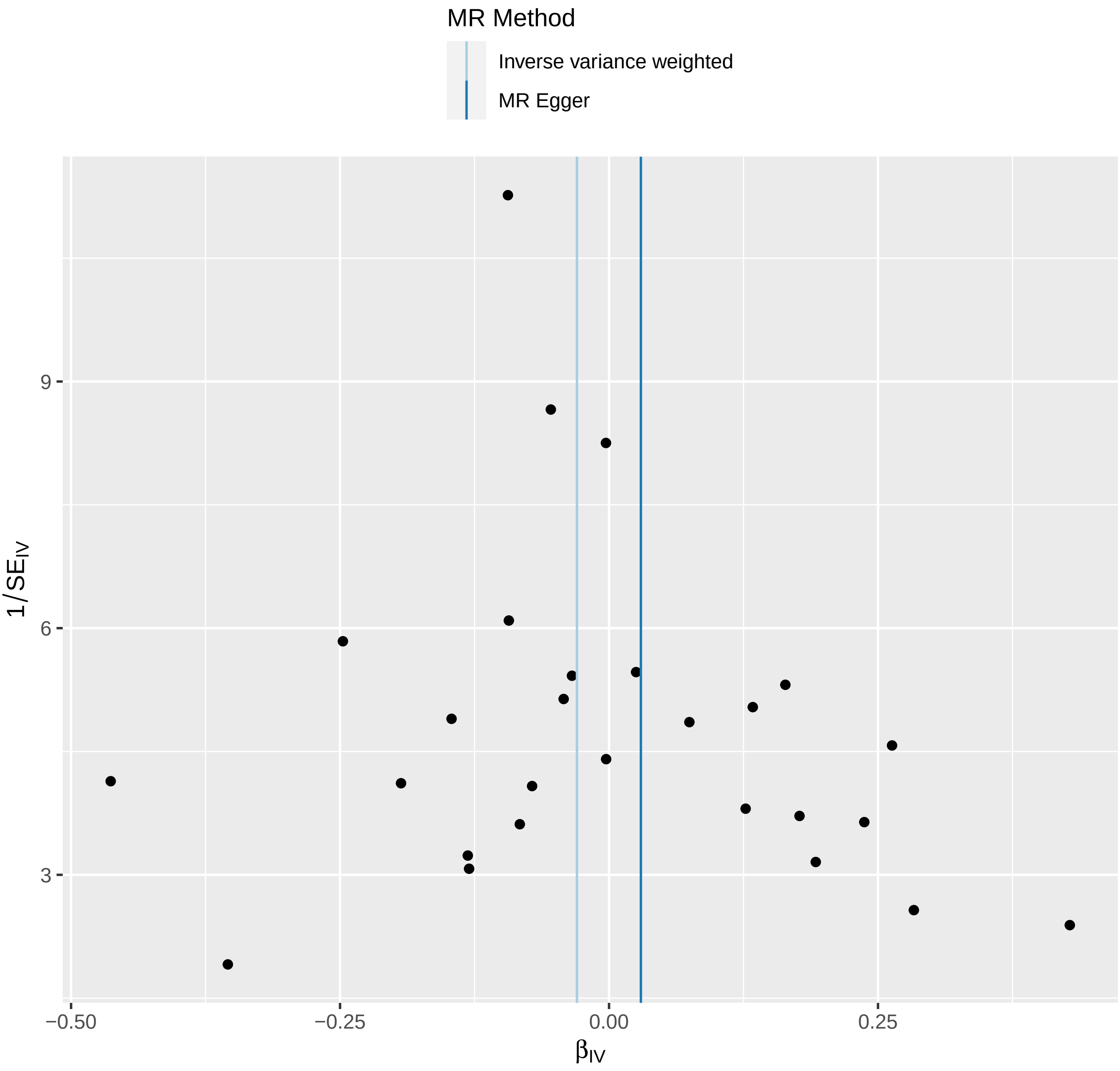

Batch 3−2537 : Regulator of G−protein signaling 3 on Diabetic nephropathy (more control exclusions)
with a threshold of 1e−05

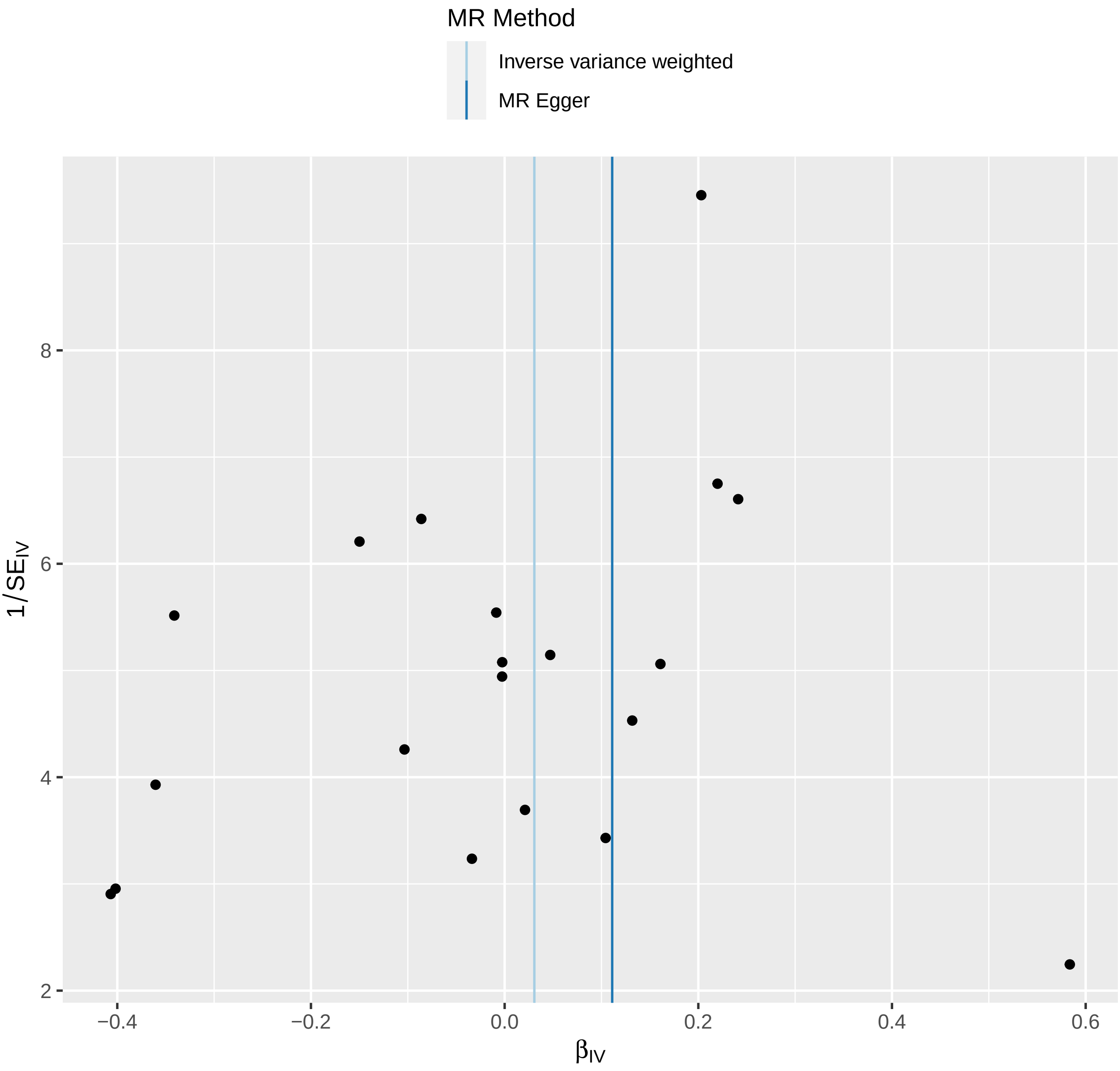

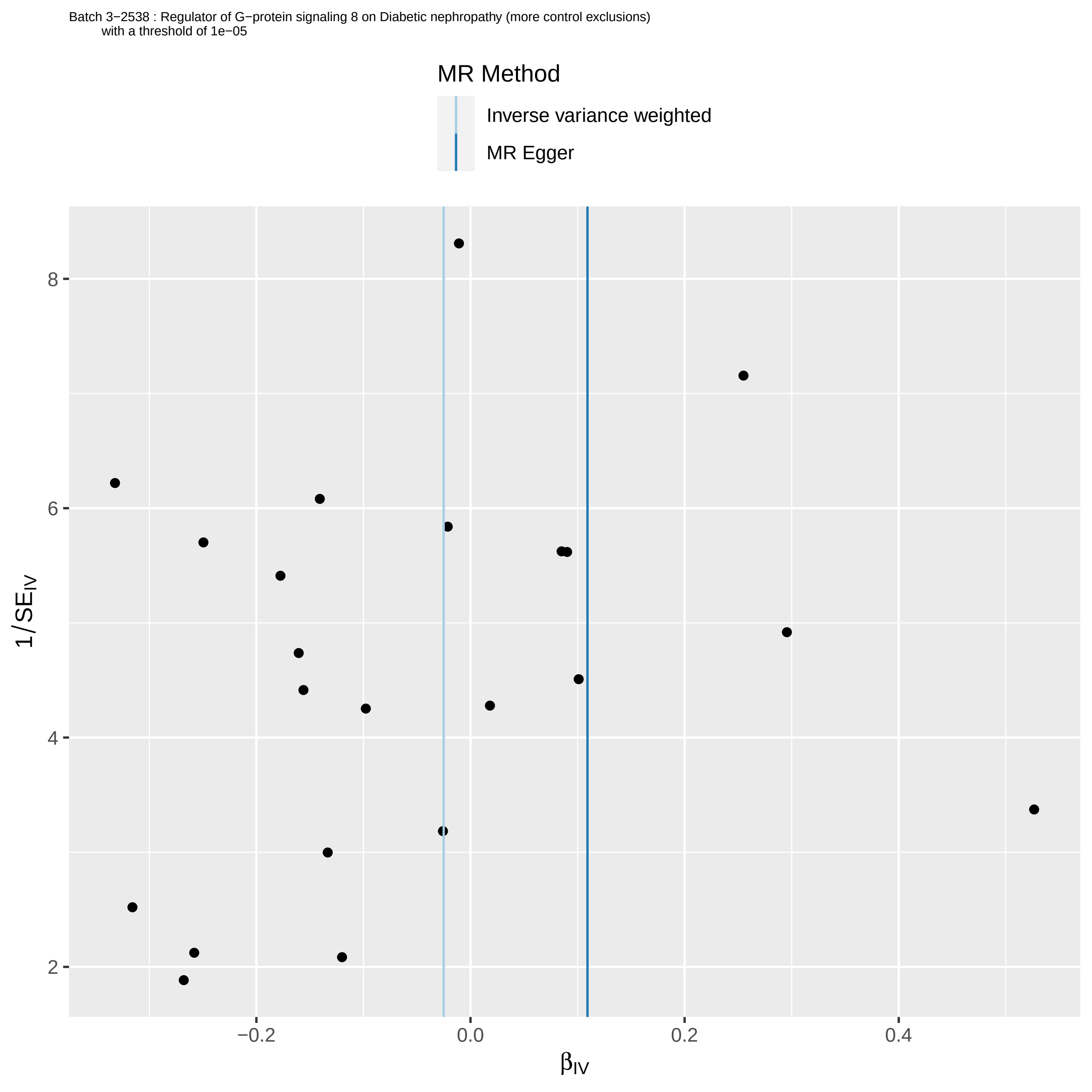
Batch 3−2538 : Regulator of G−protein signaling 8 on Diabetic nephropathy (more control exclusions)
with a threshold of 1e−05
MR Method
Inverse variance weighted
MR Egger
8
6
4
2
1/SE_IV
−0.2
0.0
0.2
0.4
β_IV

Batch 3−2539 : Transforming protein RhoA on Diabetic nephropathy (more control exclusions)
with a threshold of 1e−05

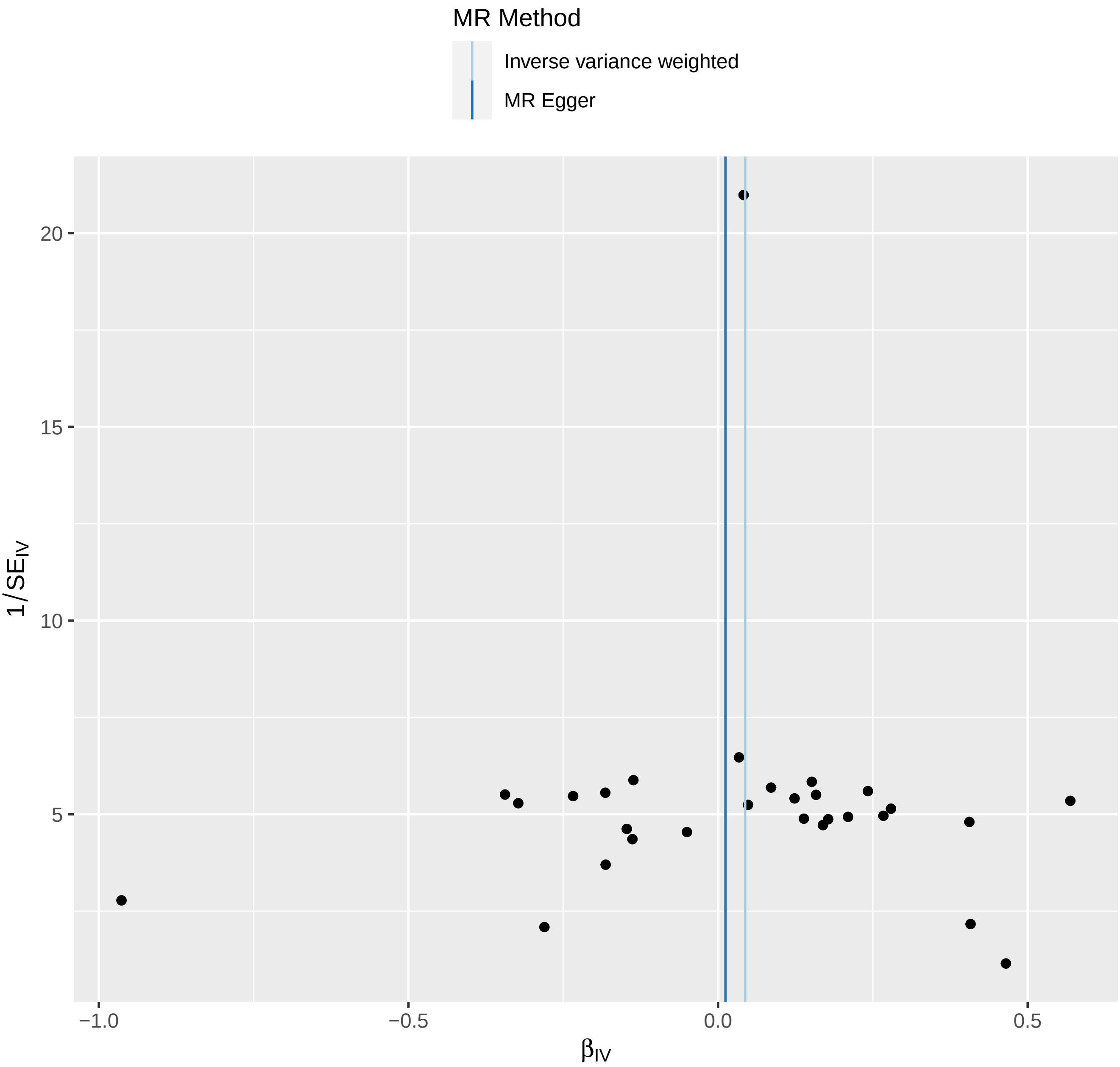

Batch 3−2540 : Rho−related GTP−binding protein RhoD on Diabetic nephropathy (more control exclusions)
with a threshold of 1e−05

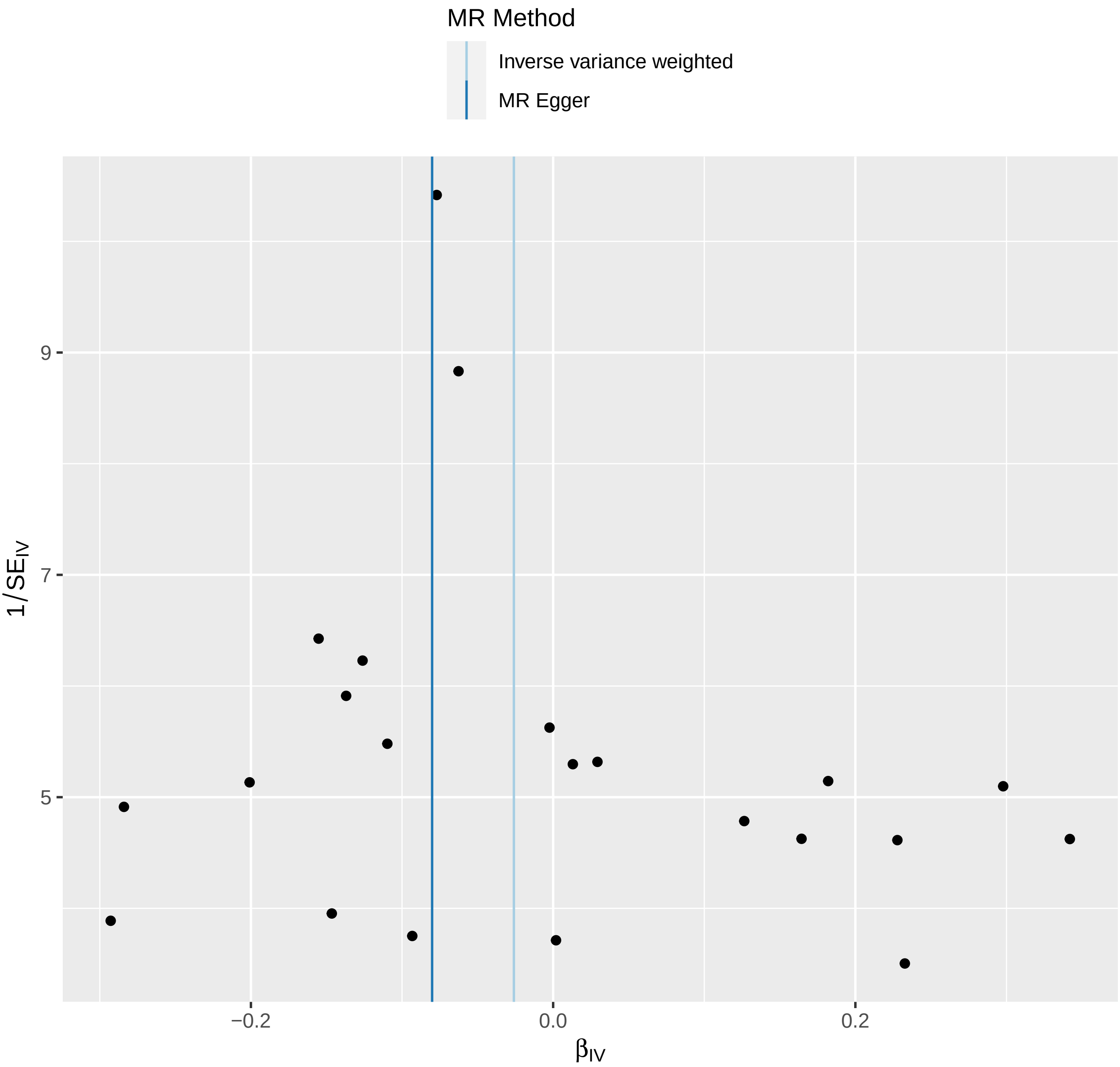

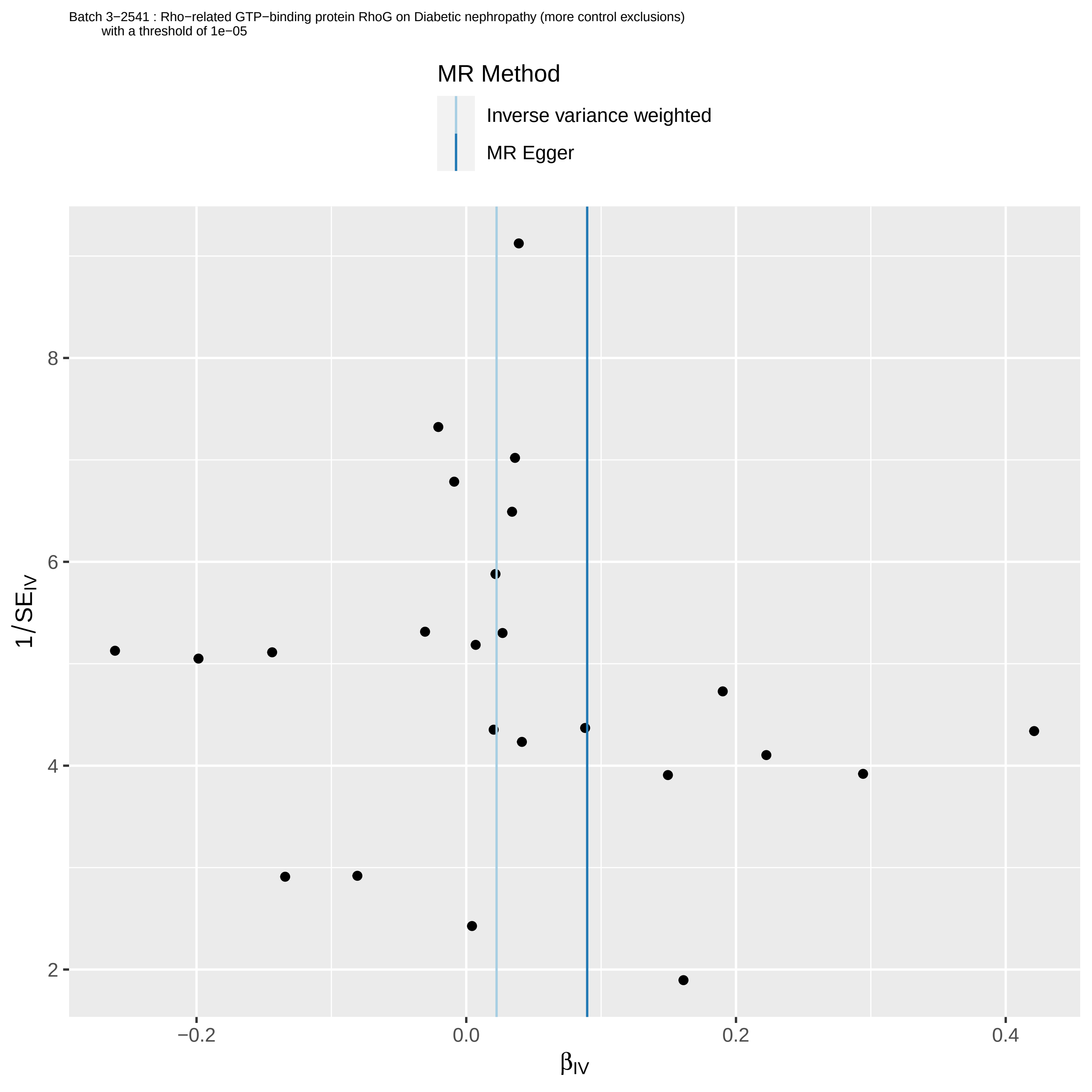

Batch 3−2541 : Rho−related GTP−binding protein RhoG on Diabetic nephropathy (more control exclusions)
with a threshold of 1e−05
MR Method
Inverse variance weighted
MR Egger
1/SE_IV
8
6
4
2
−0.2
0.0
0.2
0.4
β_IV

Batch 3−2542 : Rhophilin−2 on Diabetic nephropathy (more control exclusions)
with a threshold of 1e−05

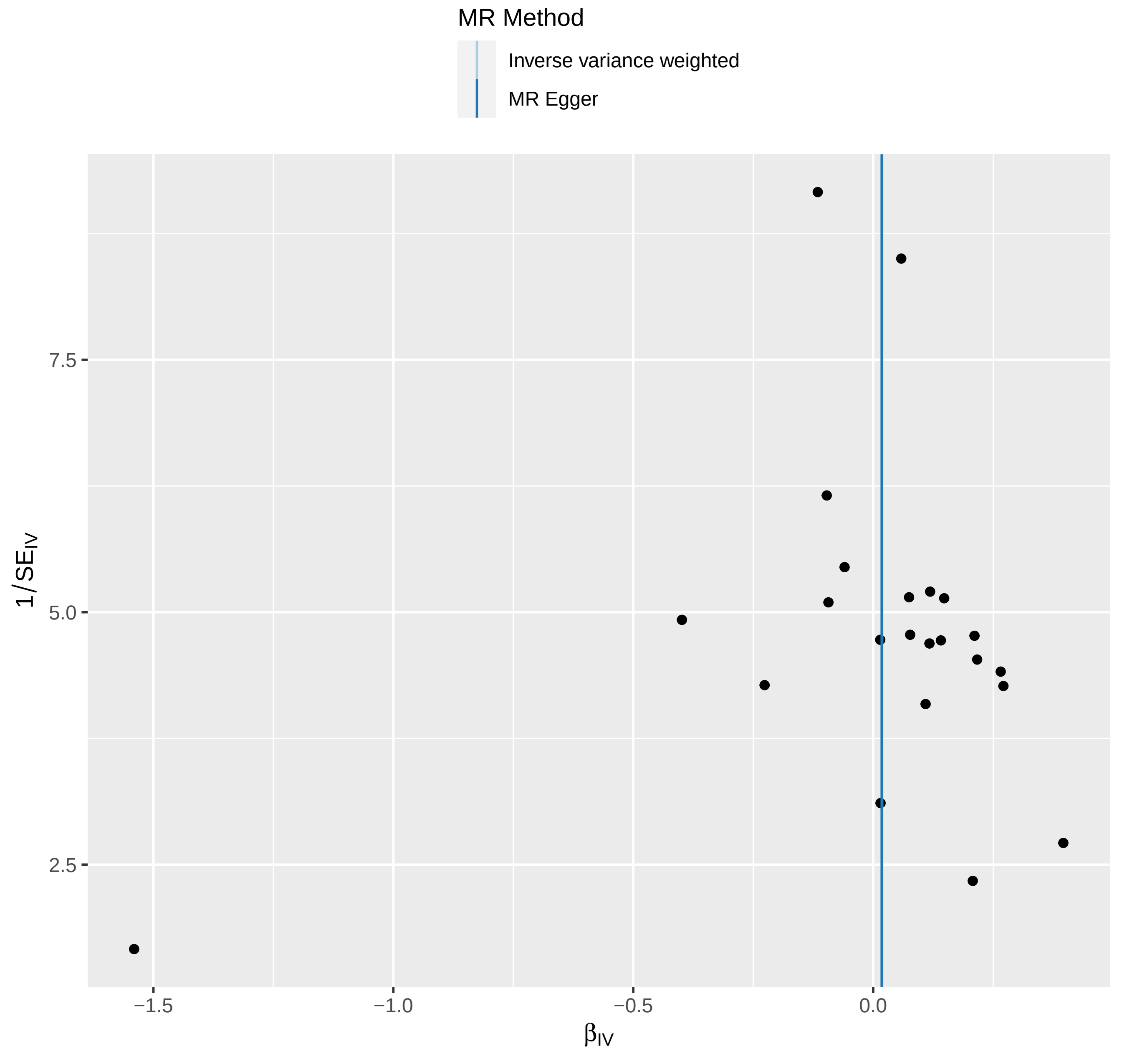

Batch 3−2543 : Protein RIC−3 on Diabetic nephropathy (more control exclusions)
with a threshold of 1e−05

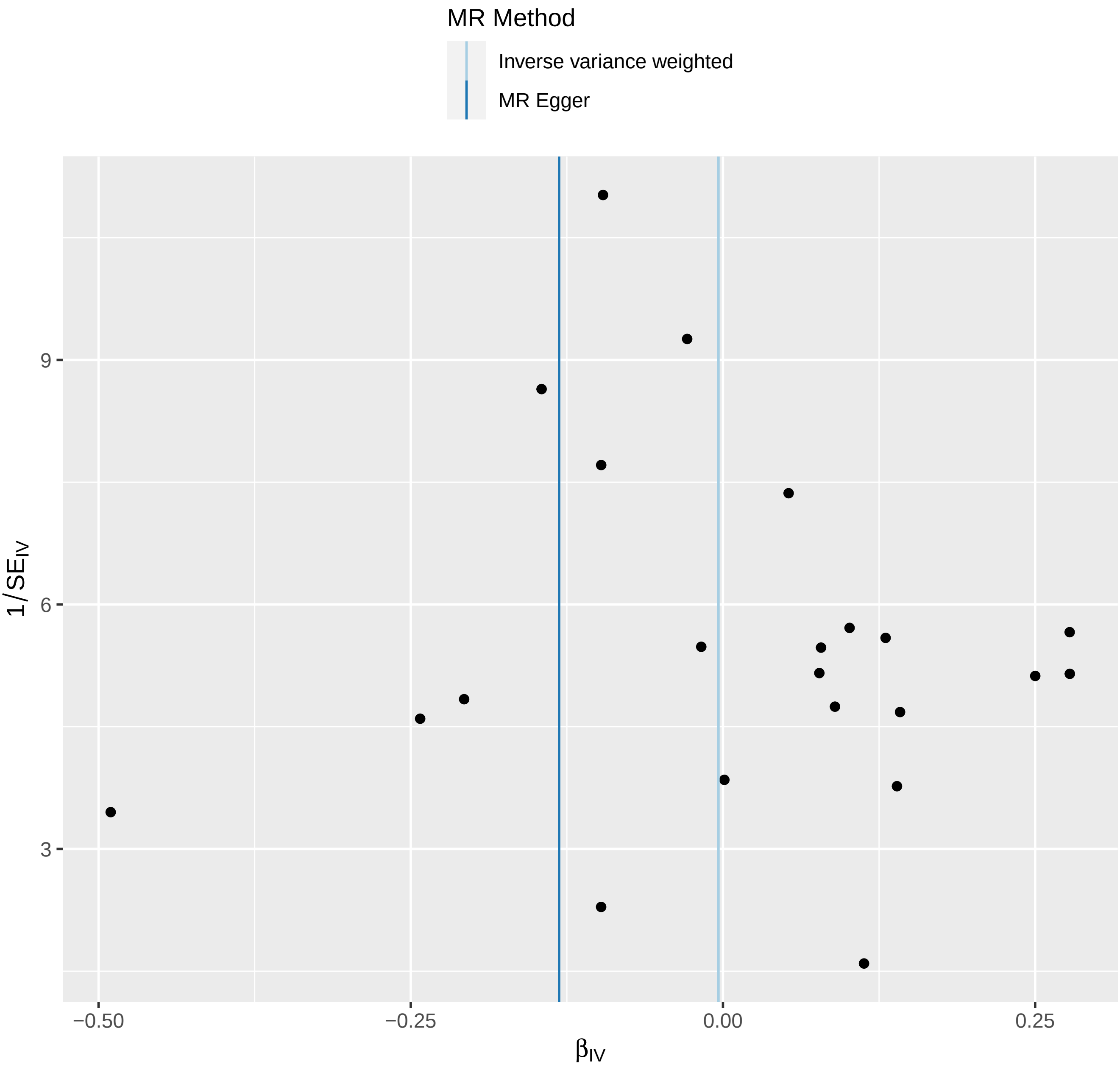

Batch 3−2544 : Protein RIC−3 on Diabetic nephropathy (more control exclusions)
with a threshold of 1e−05

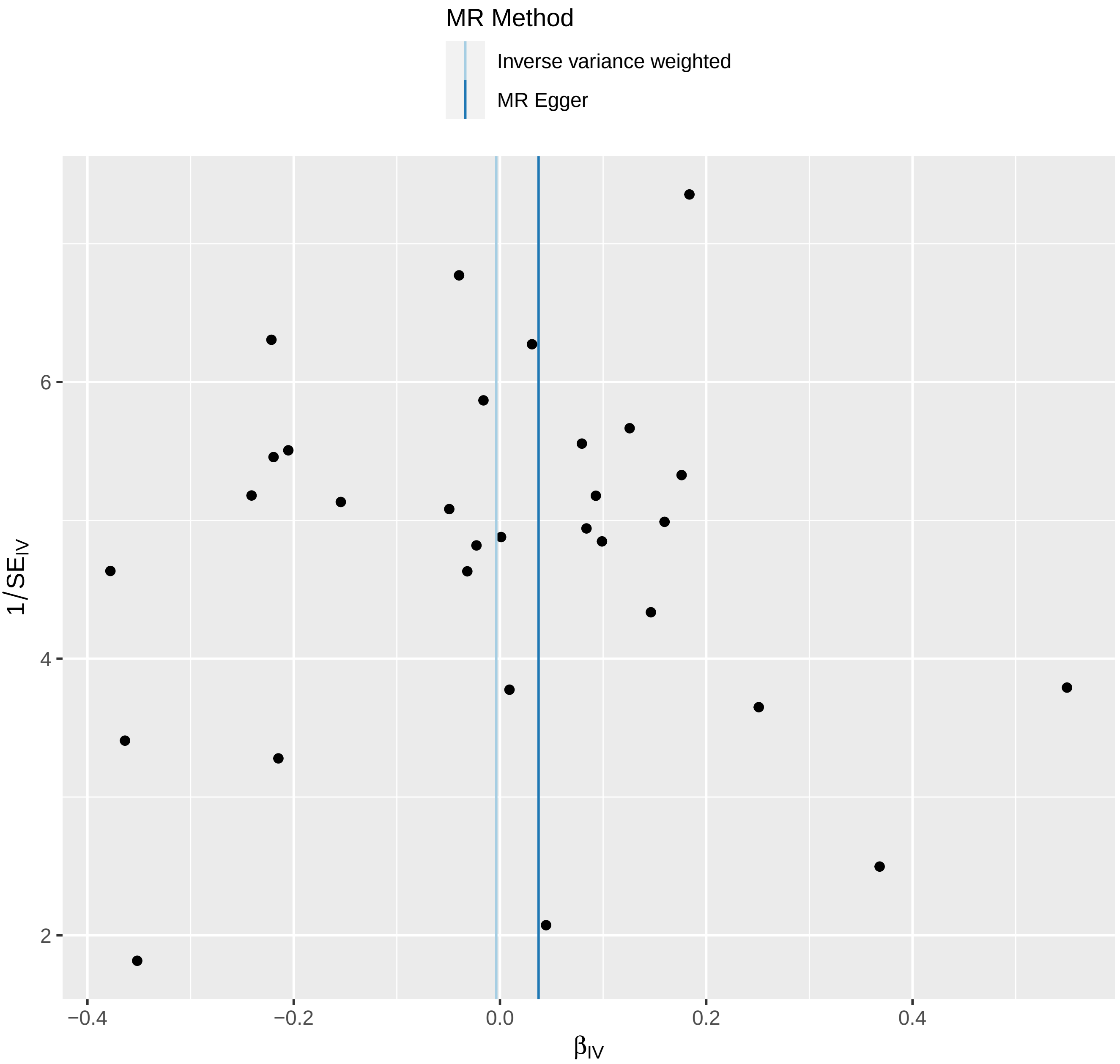

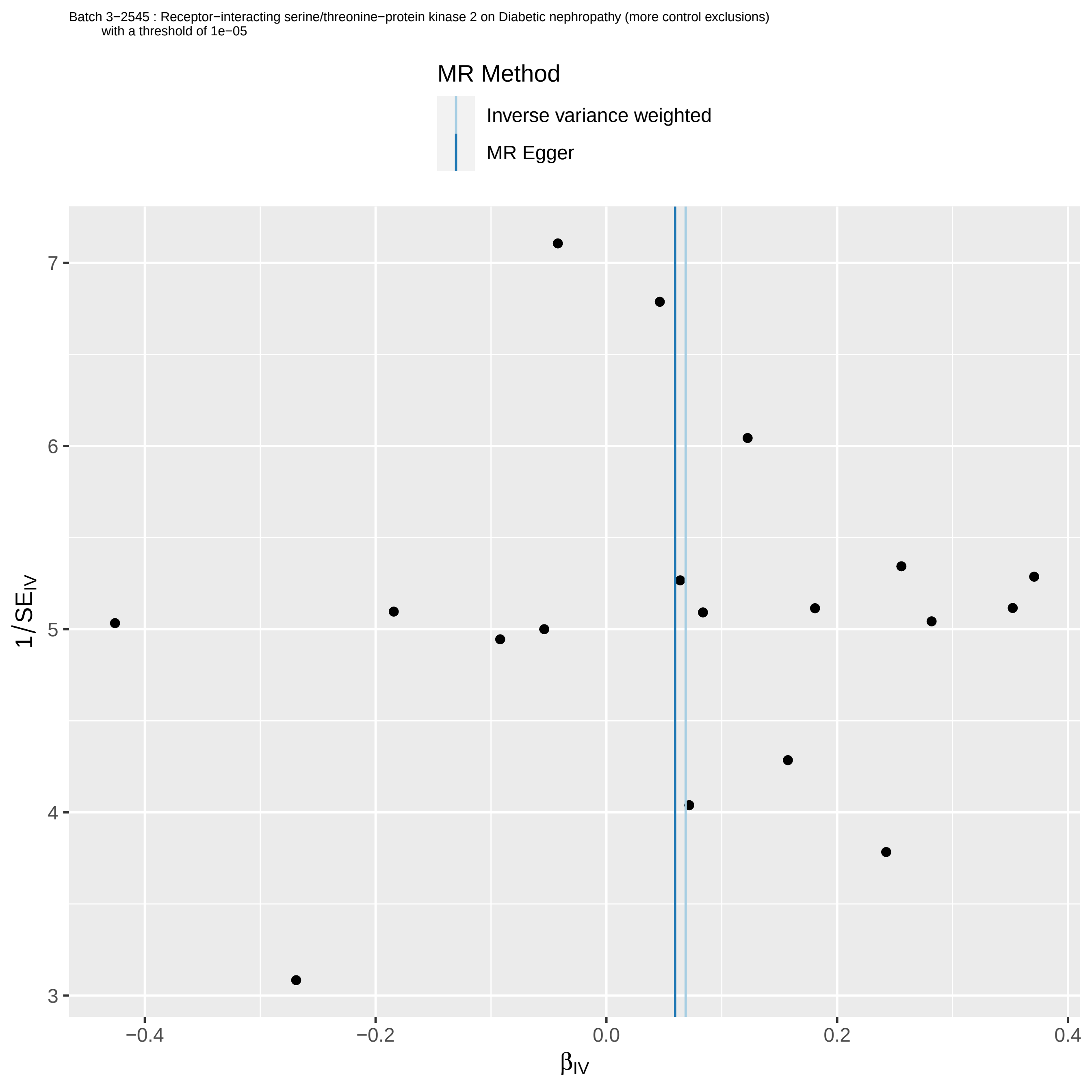
Batch 3−2545 : Receptor−interacting serine/threonine−protein kinase 2 on Diabetic nephropathy (more control exclusions)
with a threshold of 1e−05
MR Method
Inverse variance weighted
MR Egger
1/SE_IV
β_IV
7
6
5
4
3
−0.4
−0.2
0.0
0.2
0.4

Batch 3−2546 : Receptor−interacting serine/threonine−protein kinase 2 on Diabetic nephropathy (more control exclusions)
with a threshold of 1e−05

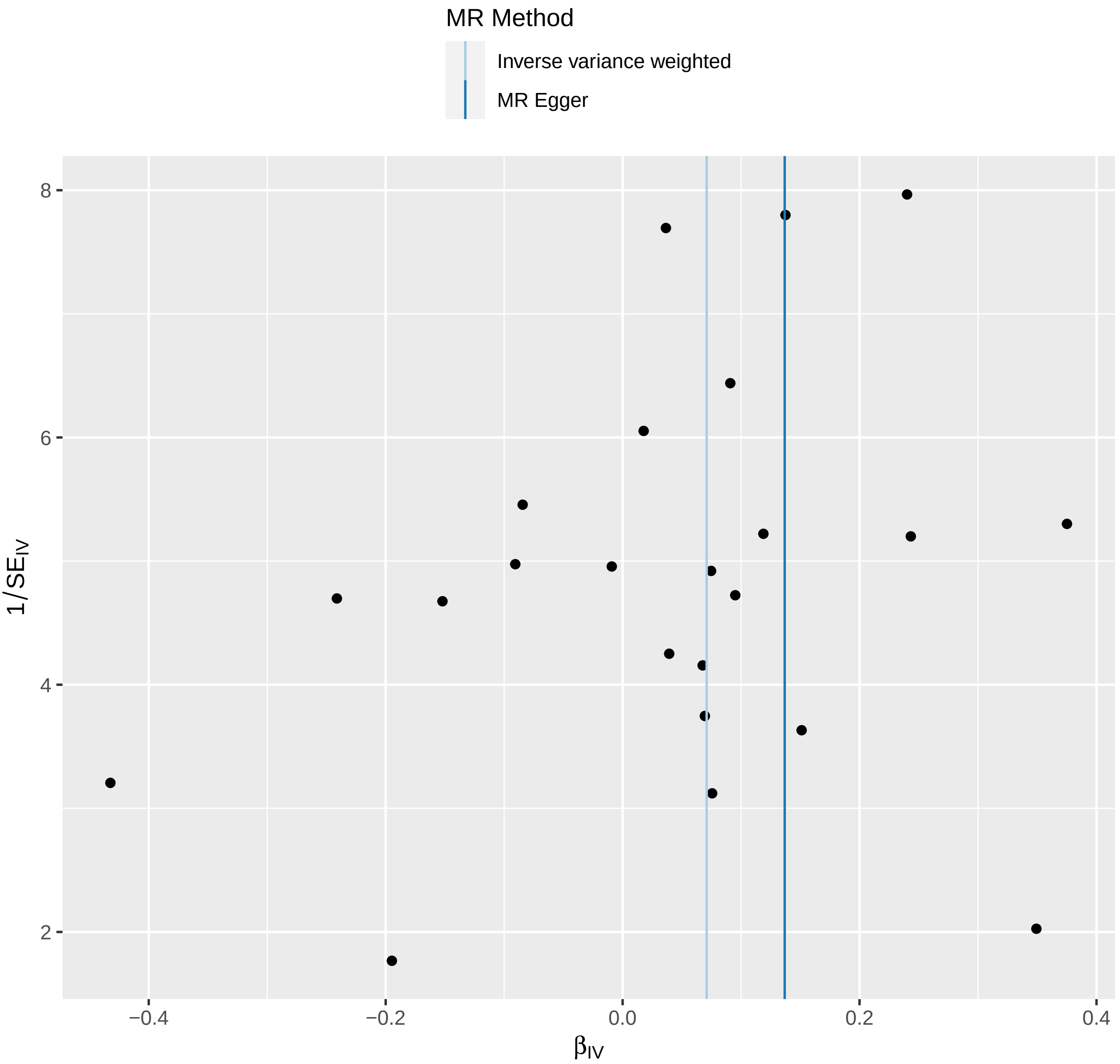

Batch 3−2547 : Protein ripply1 on Diabetic nephropathy (more control exclusions)
with a threshold of 1e−05

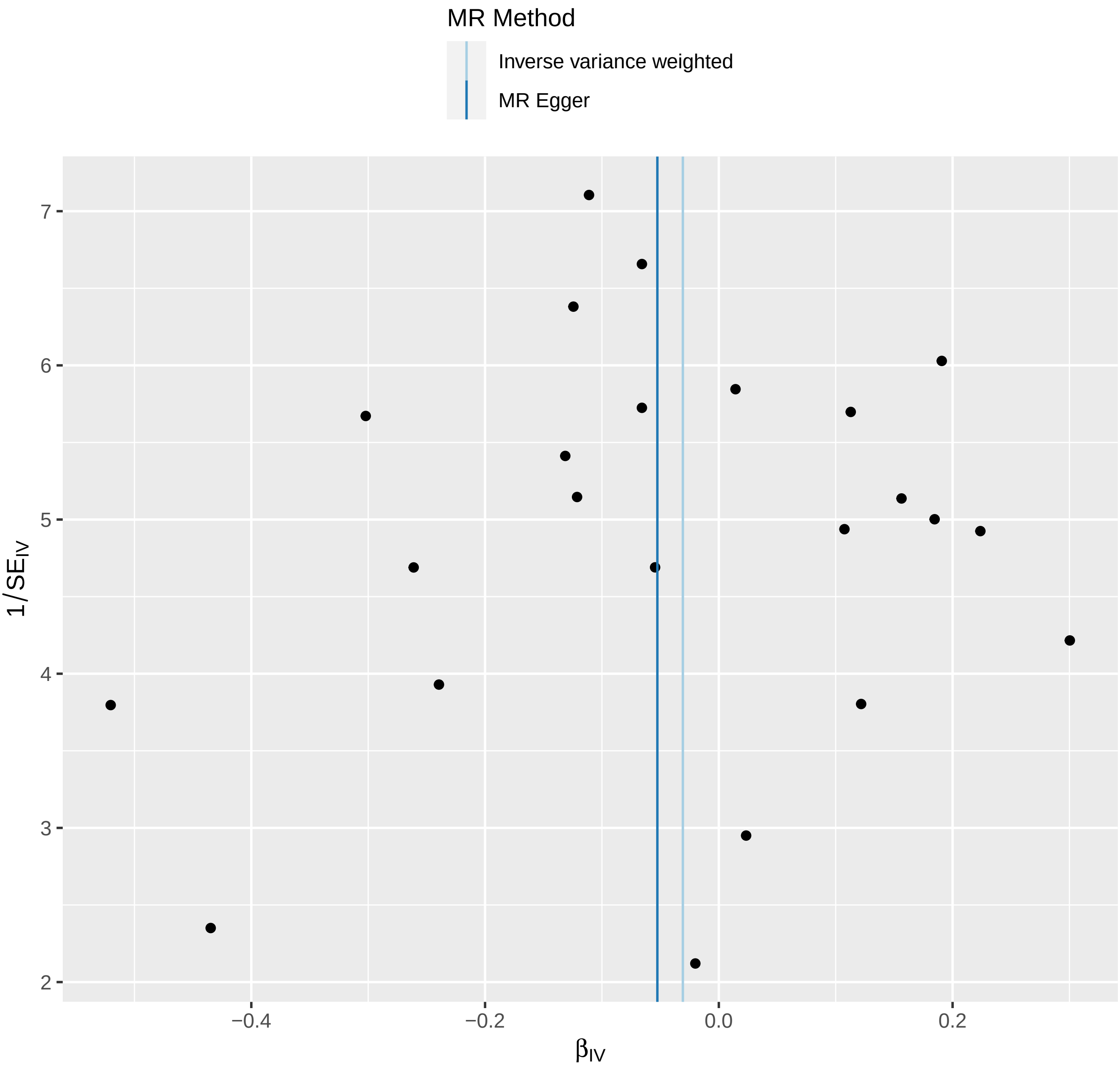

Batch 3−2548 : Retinaldehyde−binding protein 1 on Diabetic nephropathy (more control exclusions)
with a threshold of 1e−05

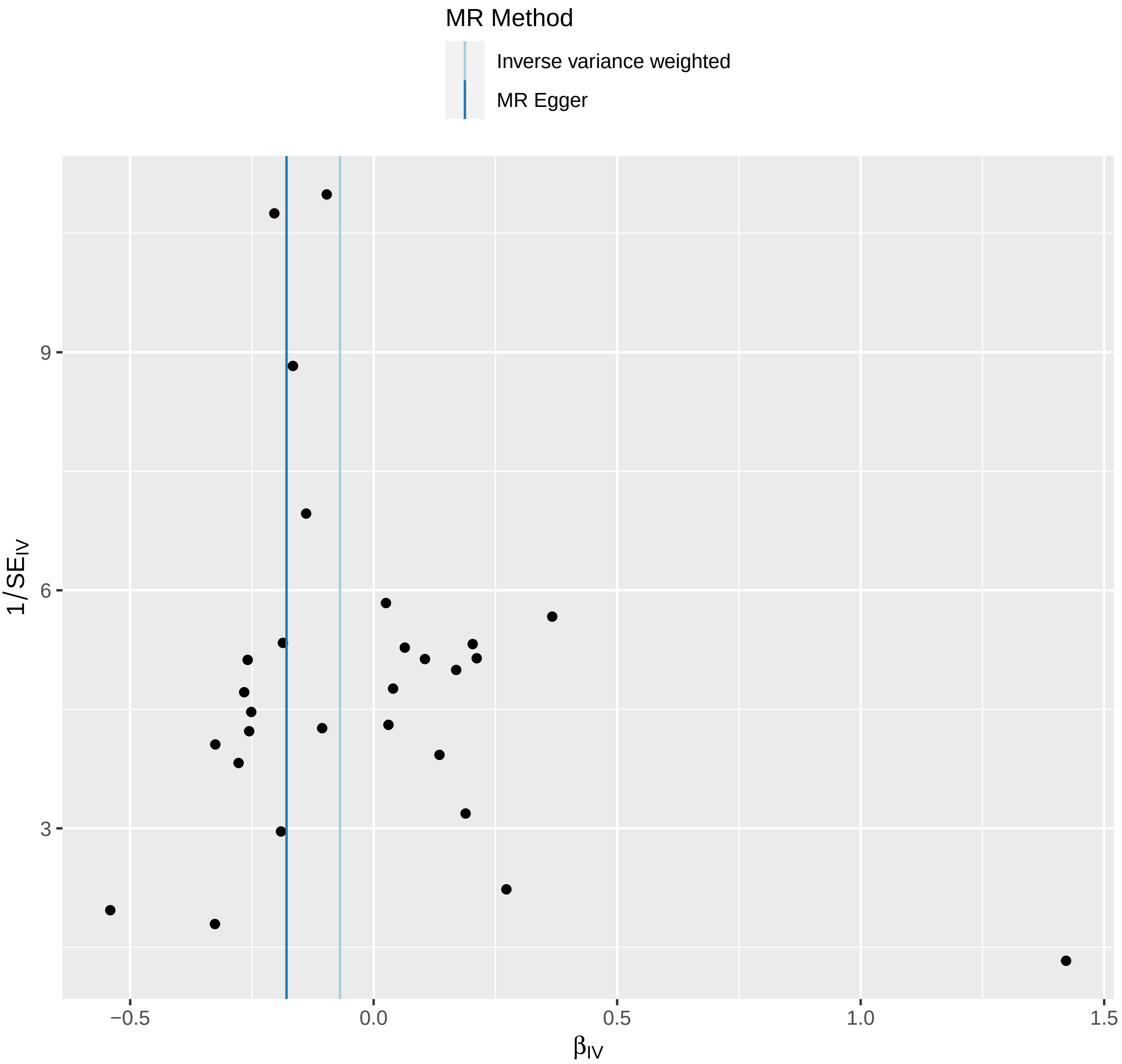

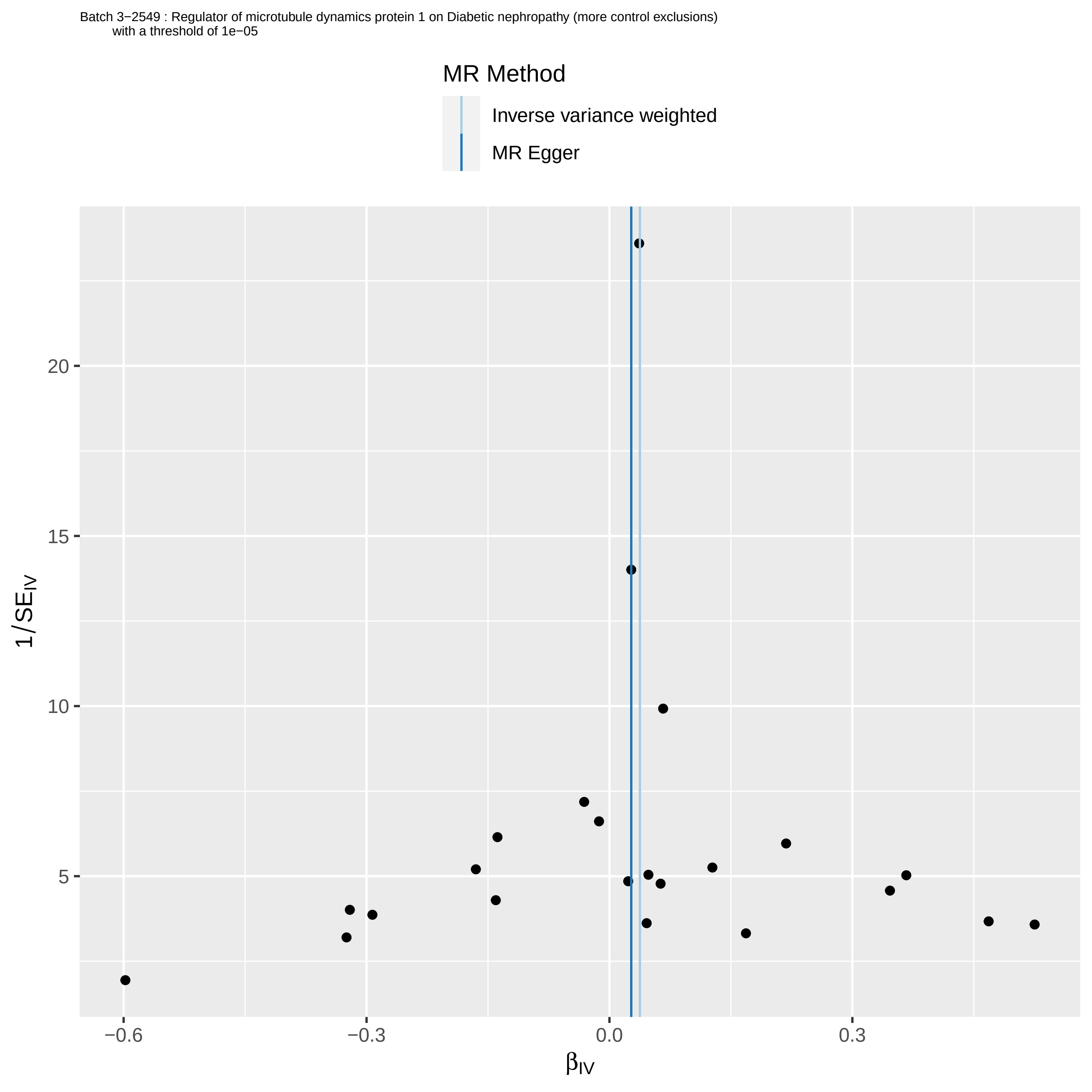
Batch 3−2549 : Regulator of microtubule dynamics protein 1 on Diabetic nephropathy (more control exclusions)
with a threshold of 1e−05
MR Method
Inverse variance weighted
MR Egger
20
15
10
5
1/SE_IV
−0.6
−0.3
0.0
0.3
β_IV

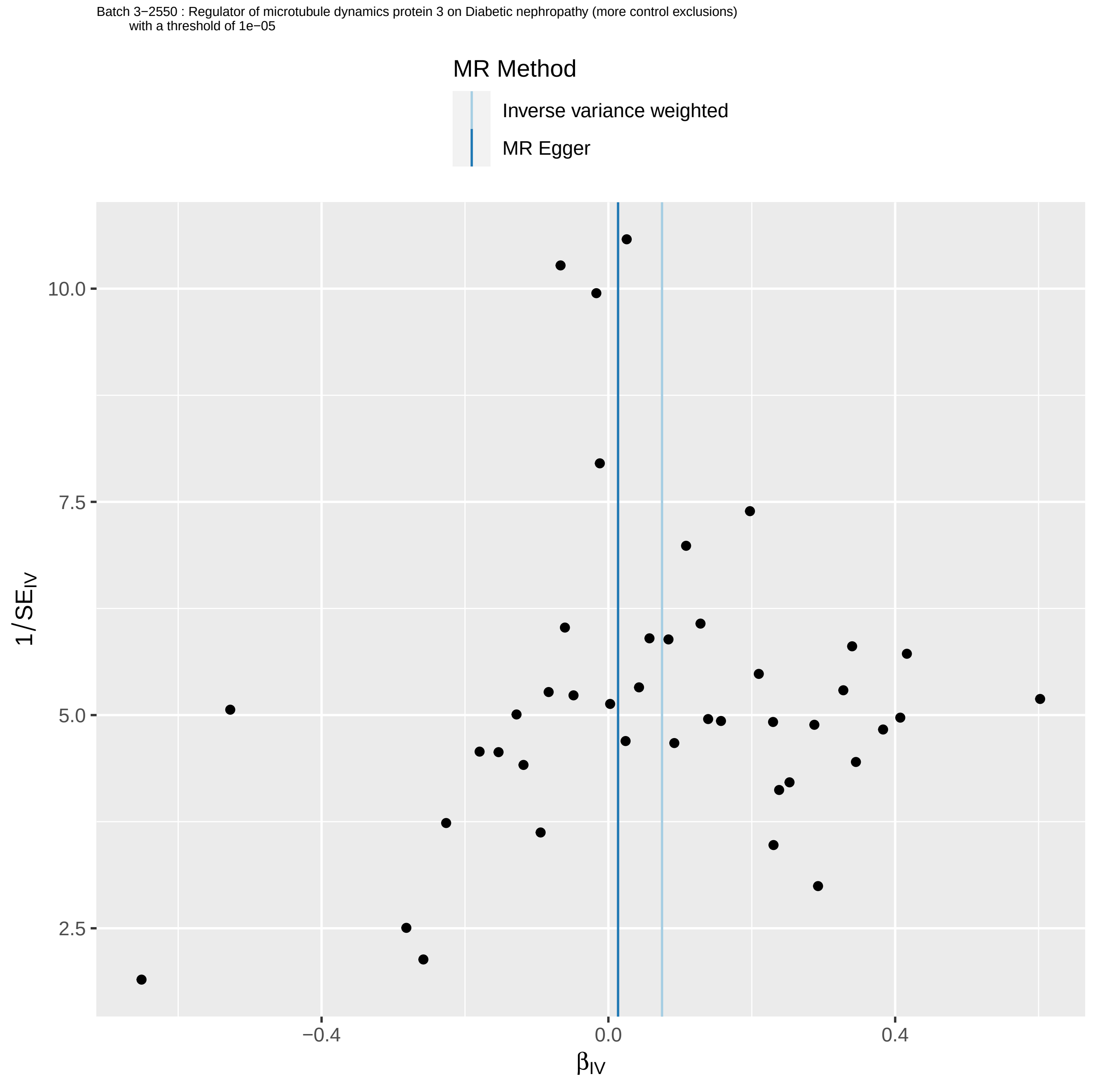

Batch 3−2550 : Regulator of microtubule dynamics protein 3 on Diabetic nephropathy (more control exclusions)
with a threshold of 1e−05
MR Method
Inverse variance weighted
MR Egger
10.0
7.5
5.0
2.5
1/SE_IV
−0.4
0.0
0.4
β_IV

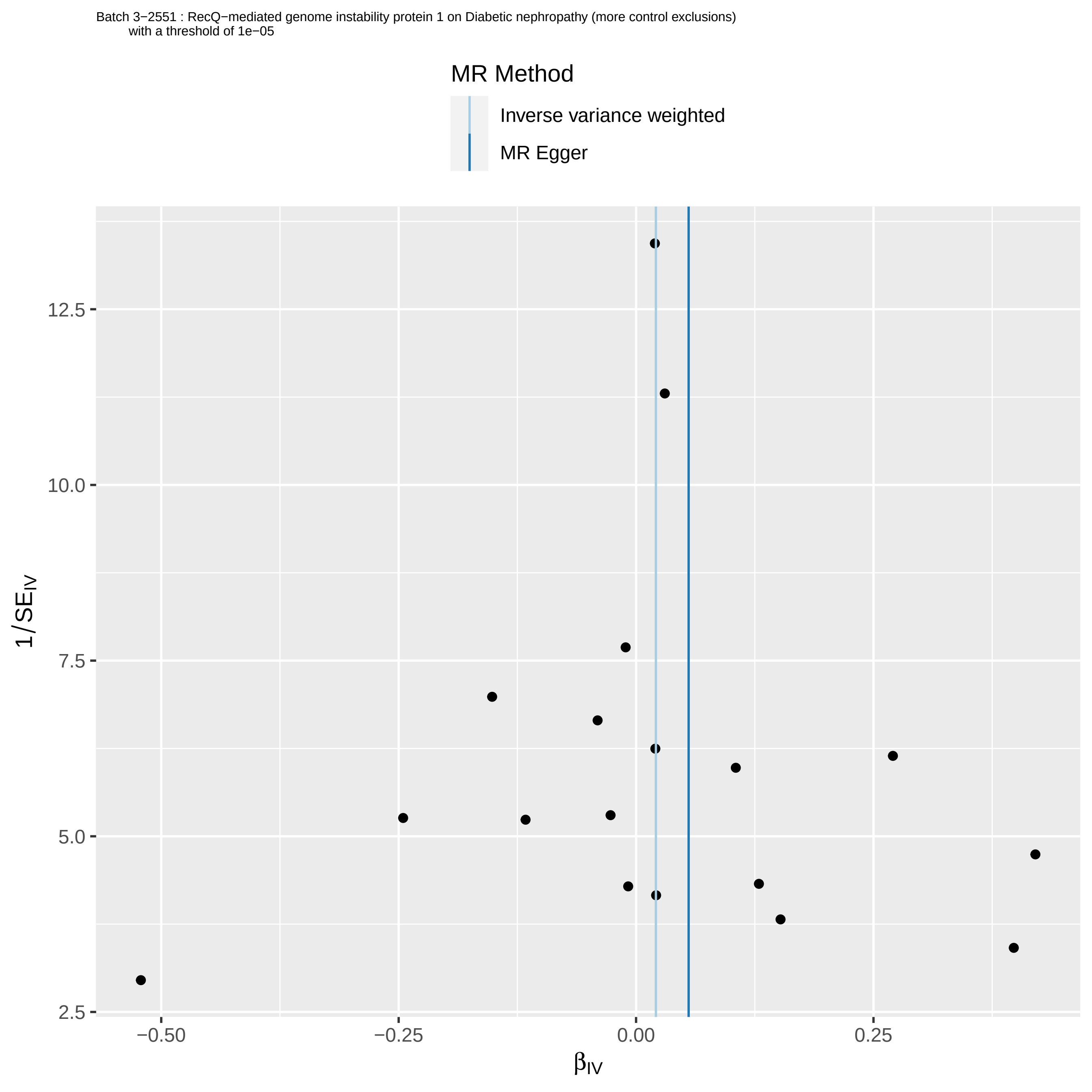
Batch 3−2551 : RecQ−mediated genome instability protein 1 on Diabetic nephropathy (more control exclusions)
with a threshold of 1e−05
MR Method
Inverse variance weighted
MR Egger
12.5
10.0
7.5
5.0
2.5
1/SE_IV
−0.50
−0.25
0.00
0.25
β_IV

Batch 3−2552 : Inactive ribonuclease−like protein 10 on Diabetic nephropathy (more control exclusions)
with a threshold of 1e−05

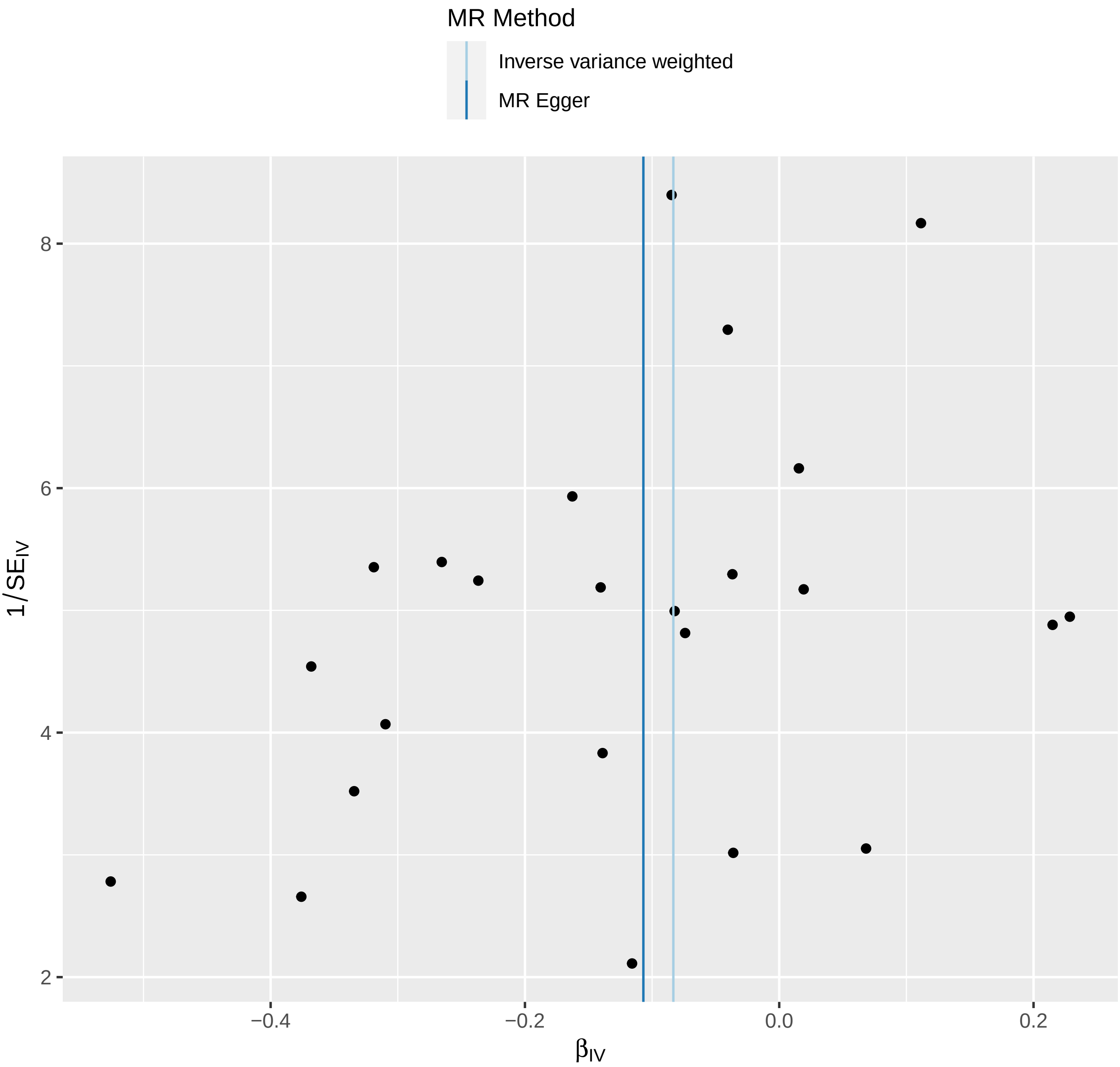

Batch 3−2553 : Probable inactive ribonuclease−like protein 13 on Diabetic nephropathy (more control exclusions)
with a threshold of 1e−05

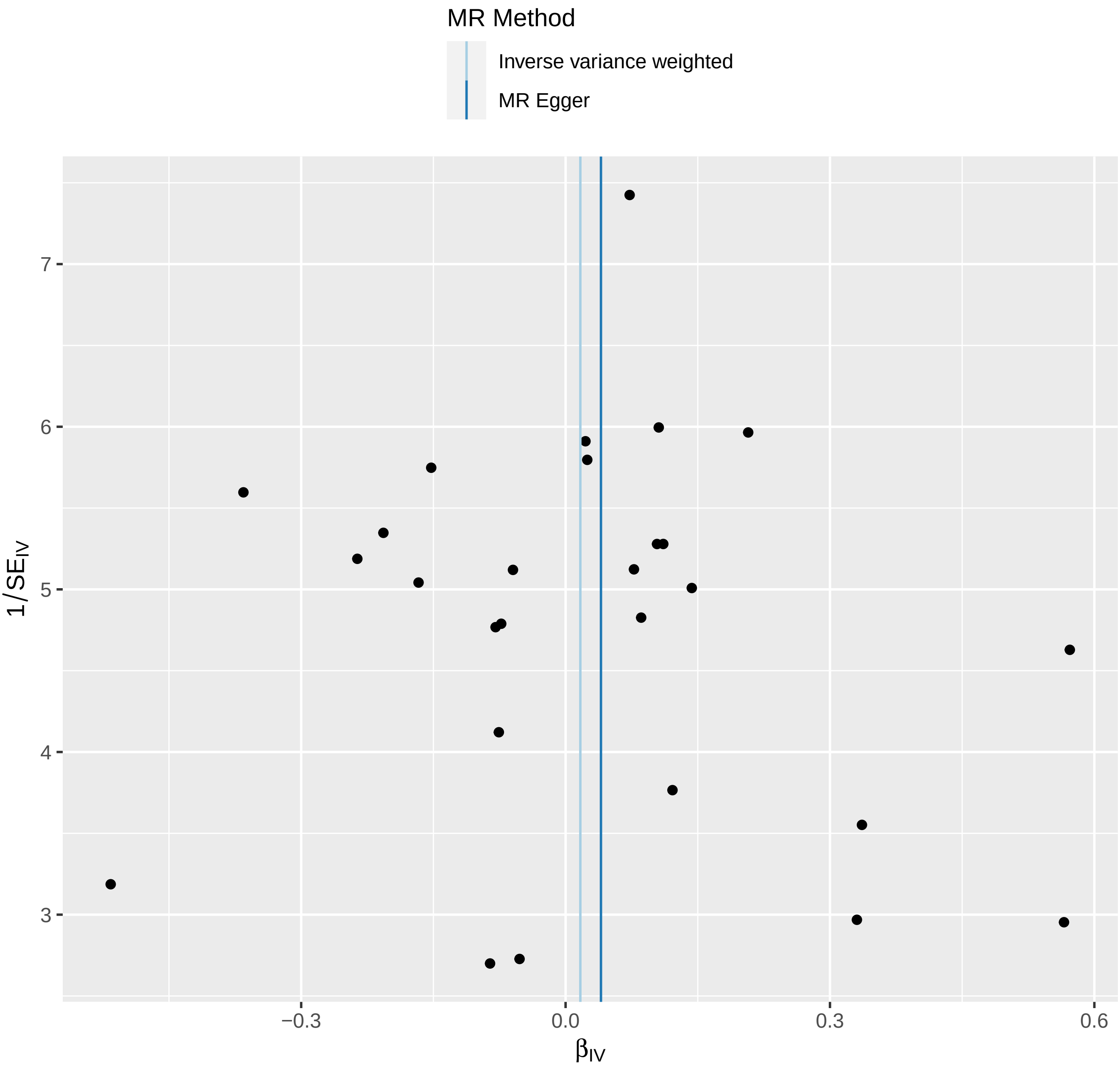

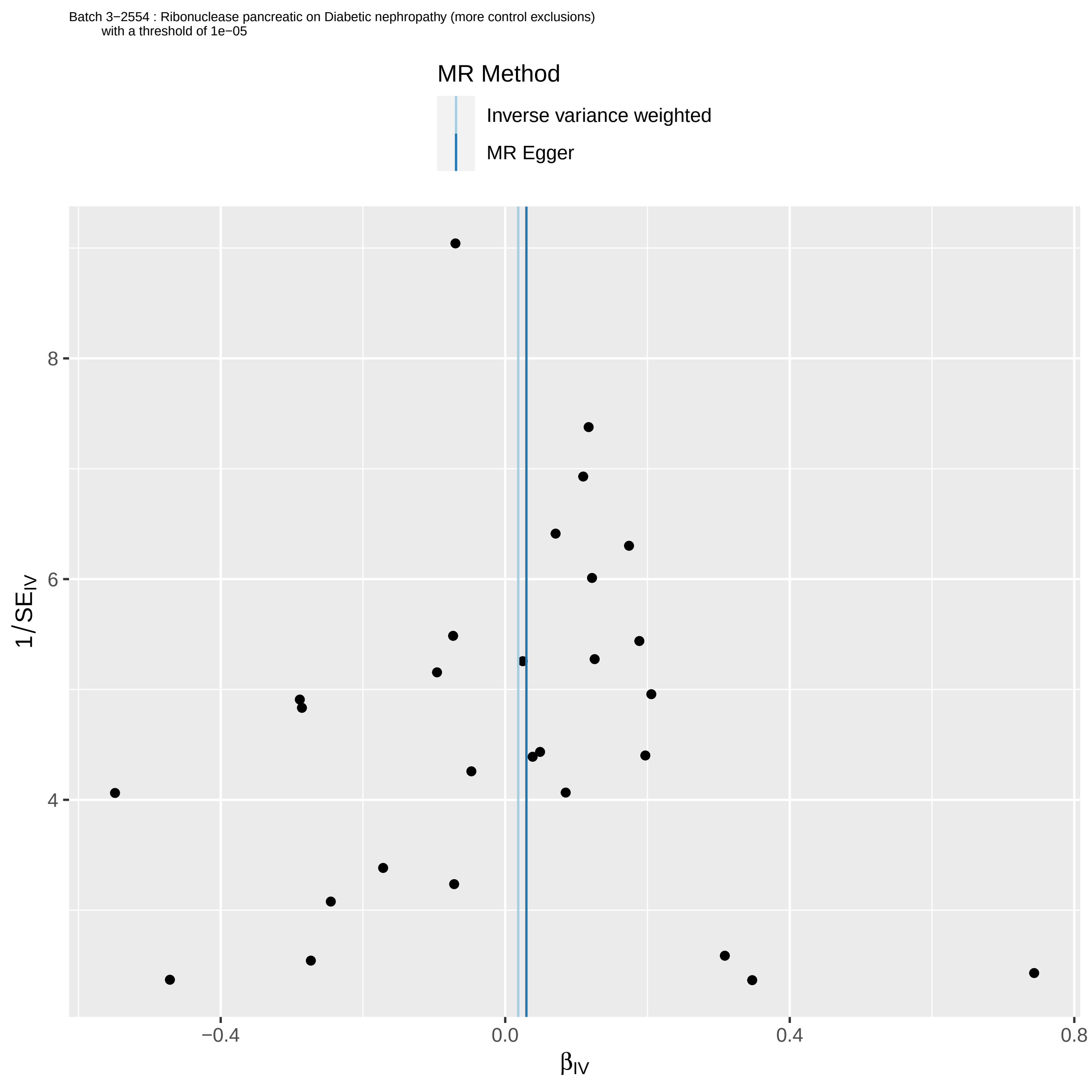

Batch 3−2554 : Ribonuclease pancreatic on Diabetic nephropathy (more control exclusions)
with a threshold of 1e−05
MR Method
Inverse variance weighted
MR Egger
8
6
4
1/SE$_{IV}$
−0.4
0.0
0.4
0.8
β_{IV}

Batch 3−2555 : Non−secretory ribonuclease on Diabetic nephropathy (more control exclusions)
with a threshold of 1e−05

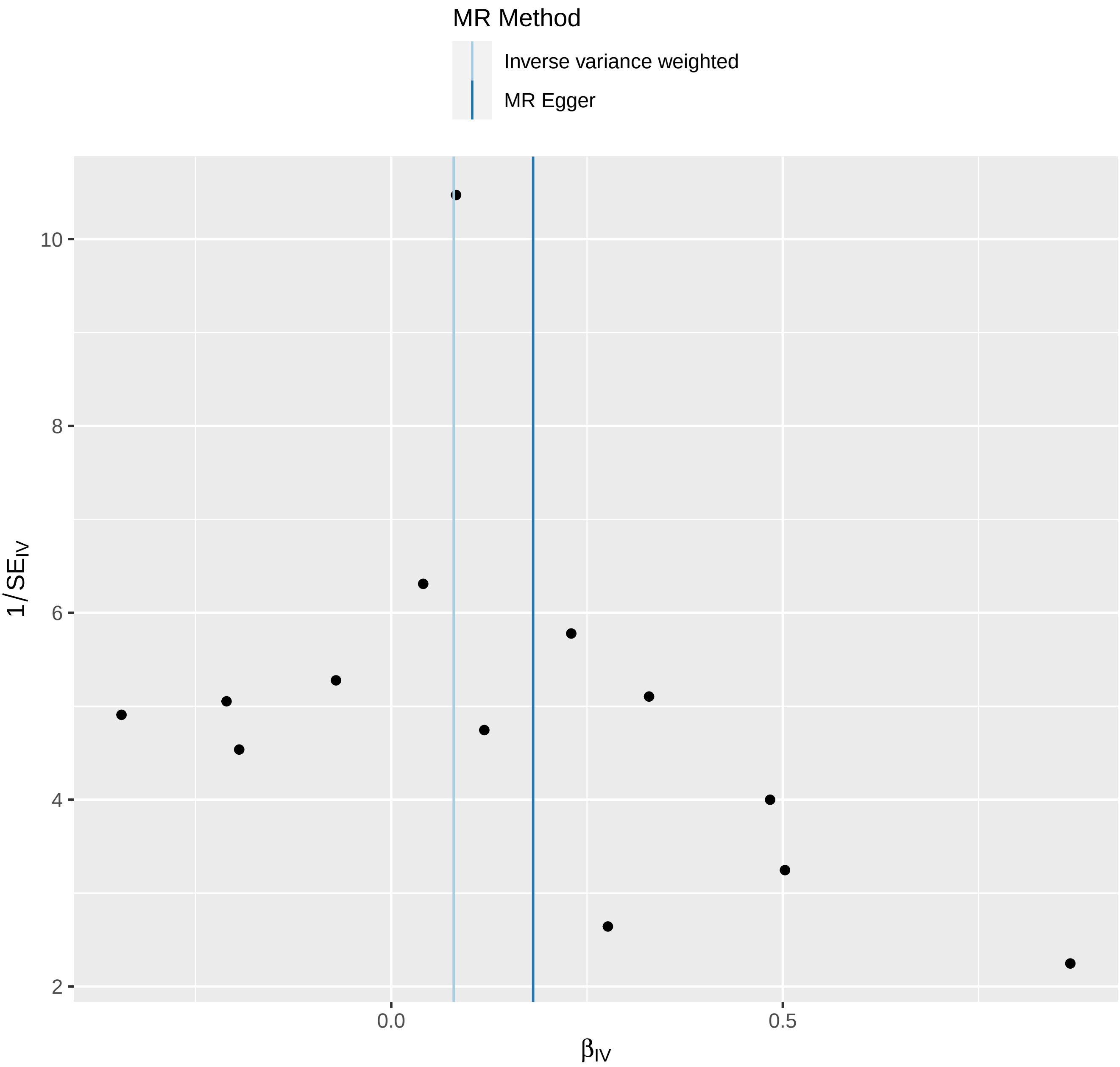

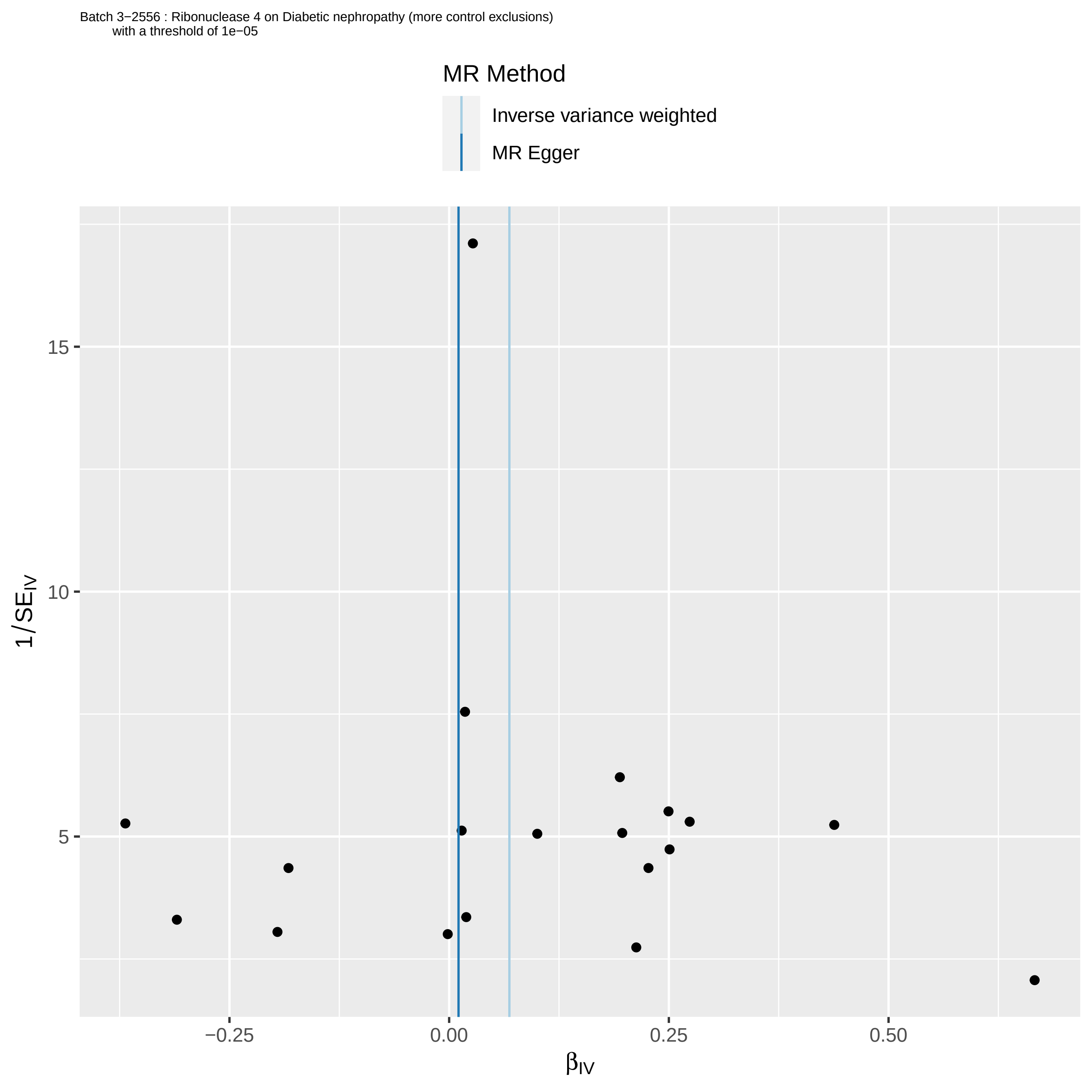
Batch 3−2556 : Ribonuclease 4 on Diabetic nephropathy (more control exclusions)
with a threshold of 1e−05
MR Method
Inverse variance weighted
MR Egger
15
10
5
1/SE_IV
−0.25
0.00
0.25
0.50
β_IV

Batch 3−2557 : Ribonuclease K6 on Diabetic nephropathy (more control exclusions)
with a threshold of 1e−05

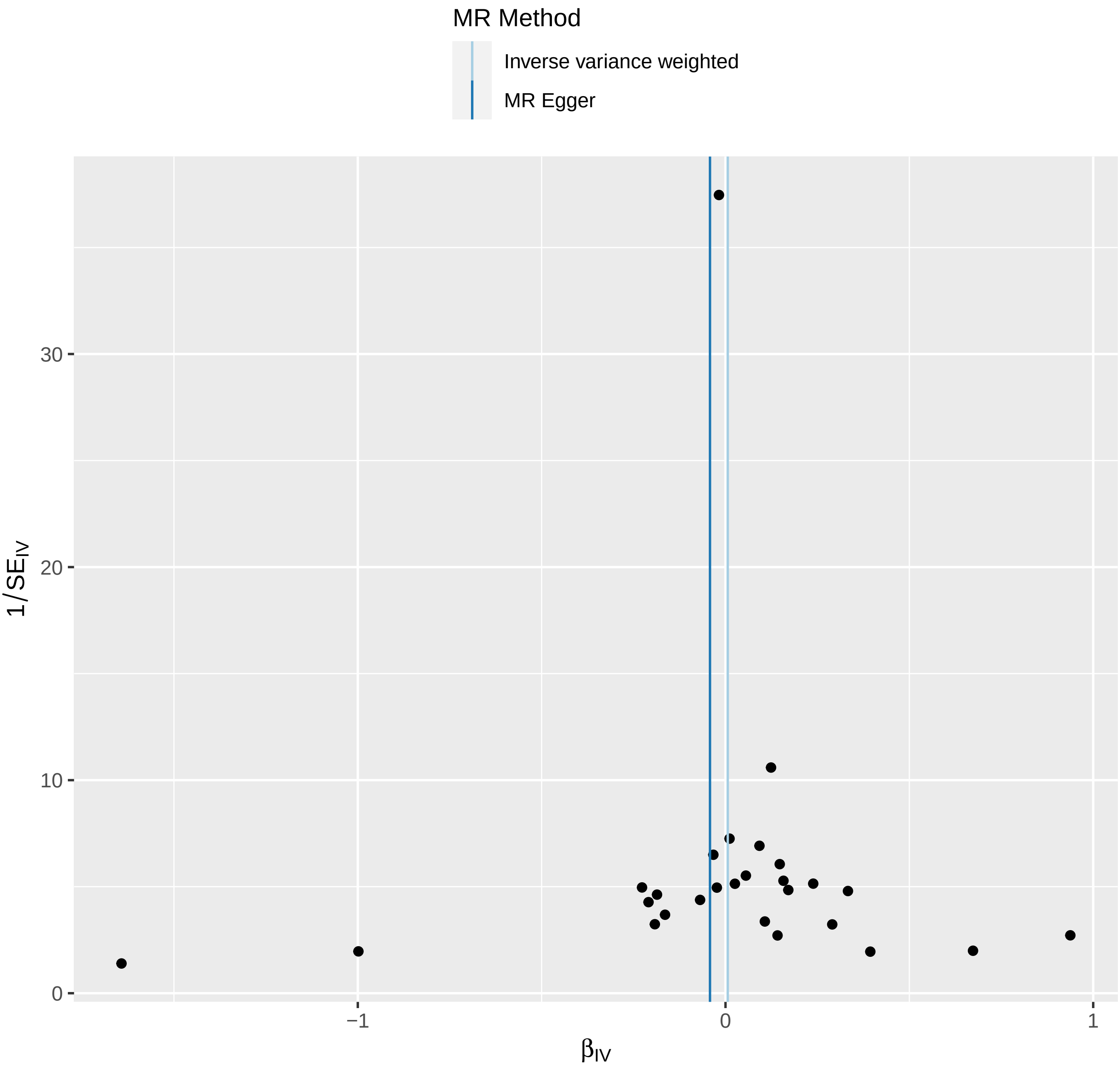

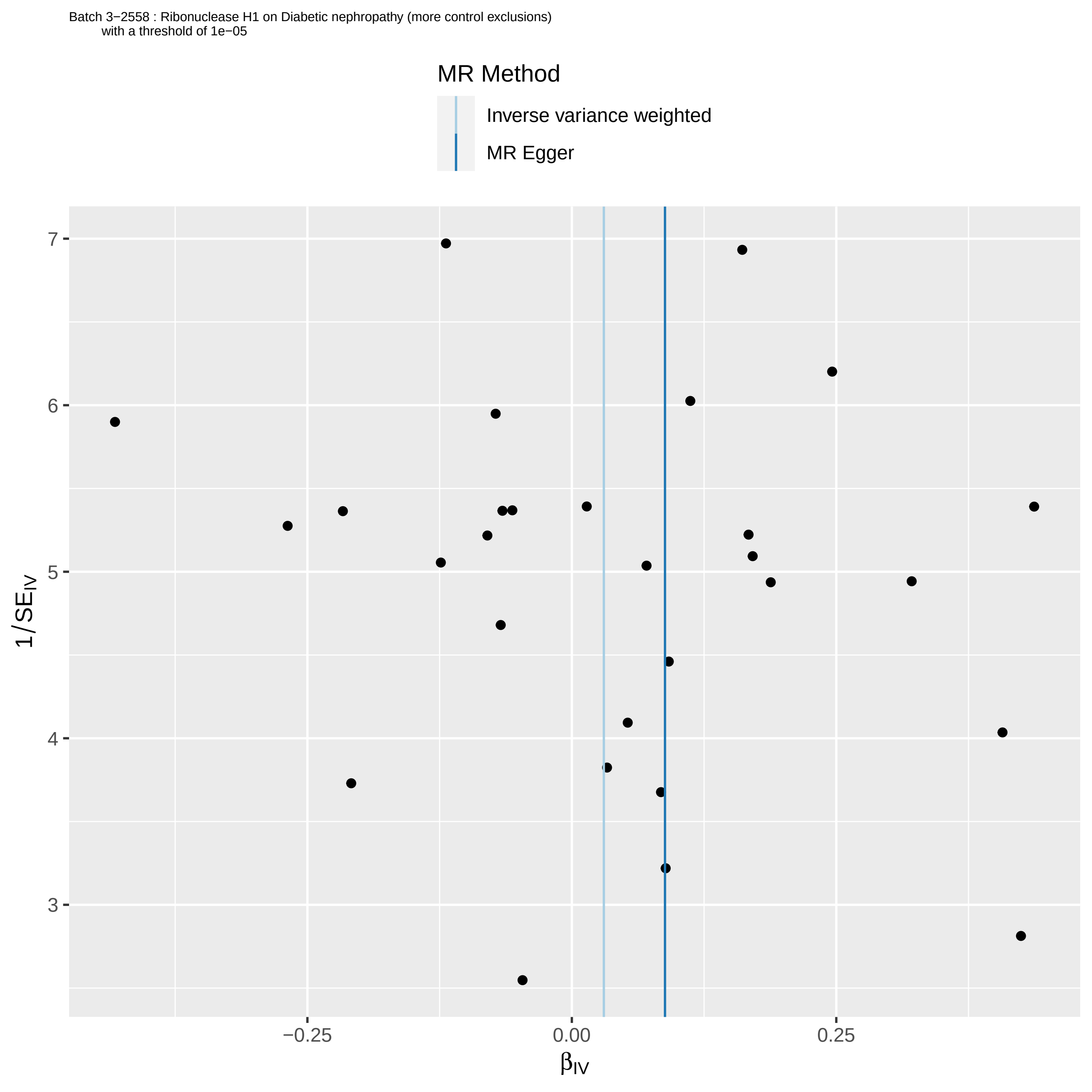

Batch 3−2558 : Ribonuclease H1 on Diabetic nephropathy (more control exclusions)
with a threshold of 1e−05
MR Method
Inverse variance weighted
MR Egger
7
6
5
4
3
1/SE_IV
−0.25
0.00
0.25
β_IV

Batch 3−2559 : Rho−related GTP−binding protein Rho6 on Diabetic nephropathy (more control exclusions)
with a threshold of 1e−05

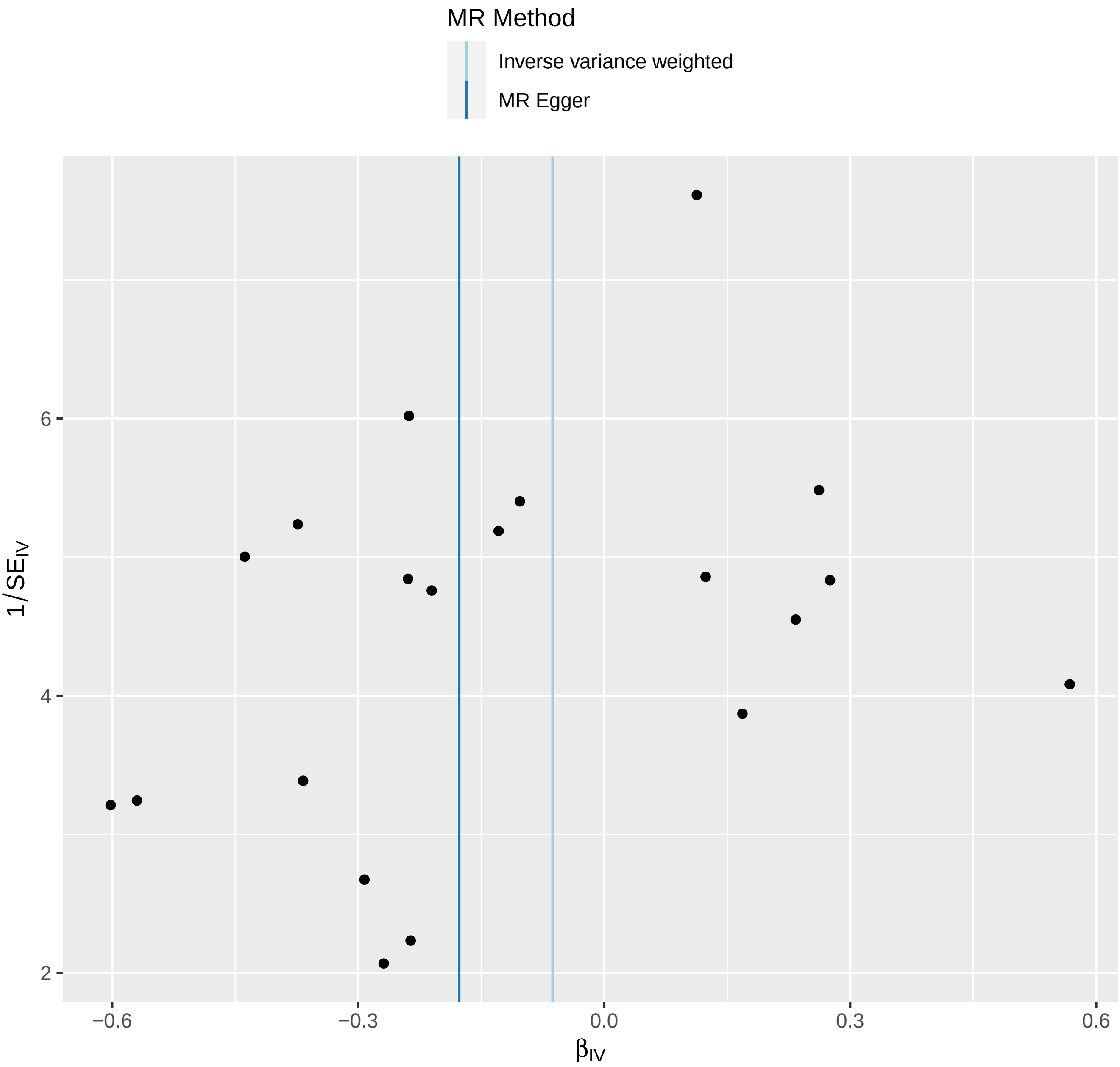

Batch 3−2560 : E3 ubiquitin−protein ligase RNF114 on Diabetic nephropathy (more control exclusions)
with a threshold of 1e−05

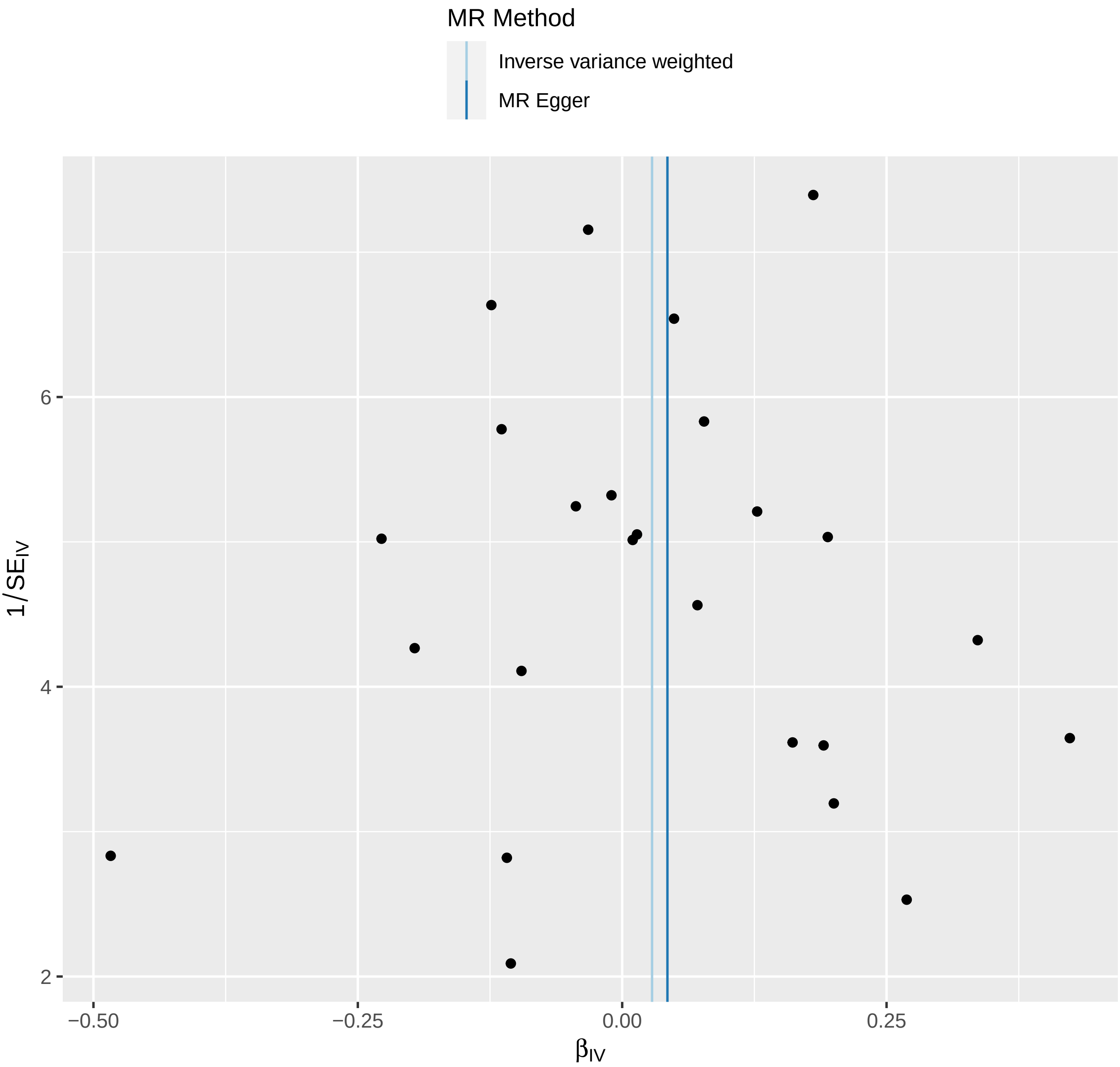

Batch 3−2561 : RING finger protein 122 on Diabetic nephropathy (more control exclusions)
with a threshold of 1e−05

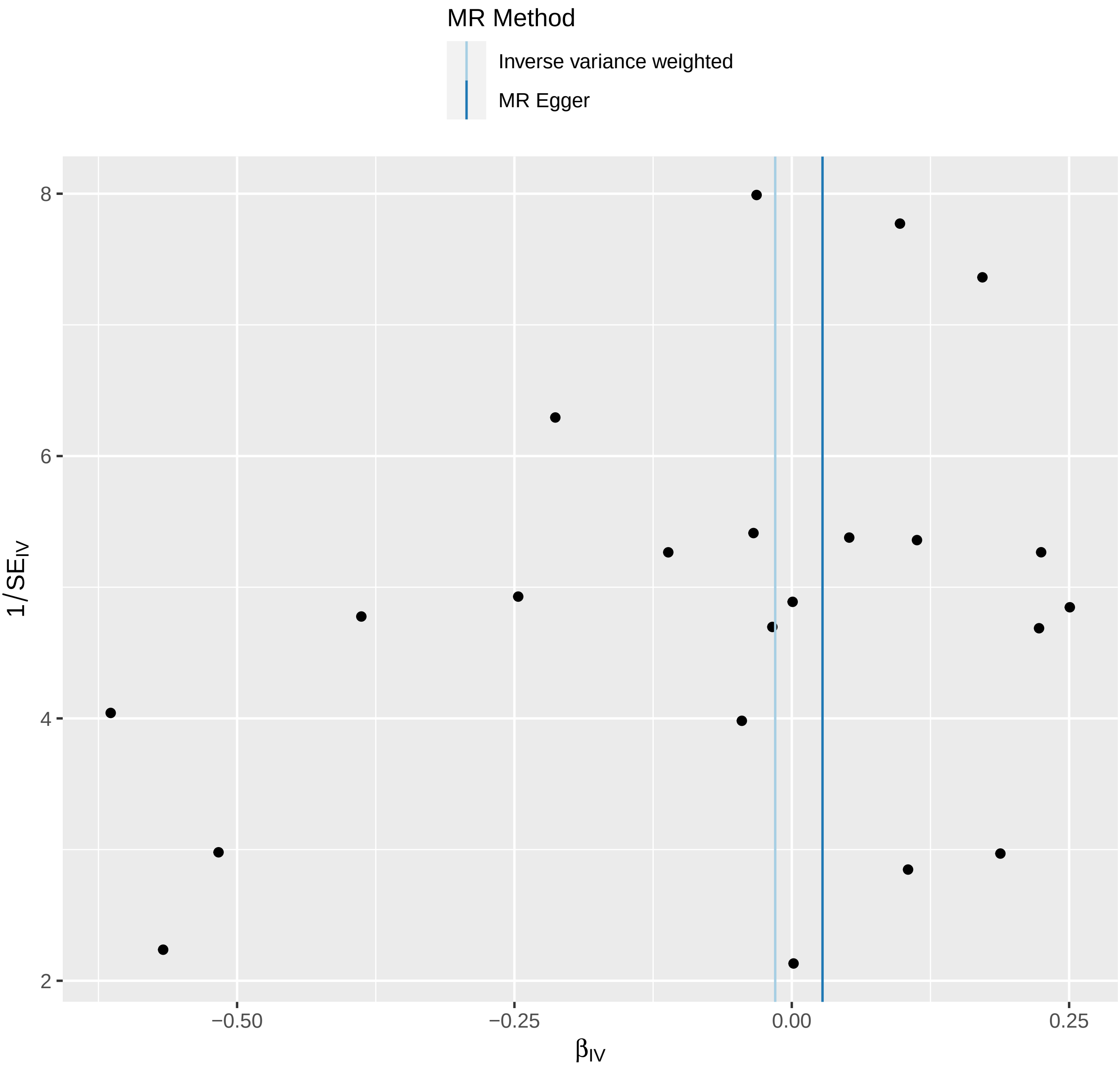

Batch 3−2562 : E3 ubiquitin−protein ligase RNF128 on Diabetic nephropathy (more control exclusions) with a threshold of 1e−05

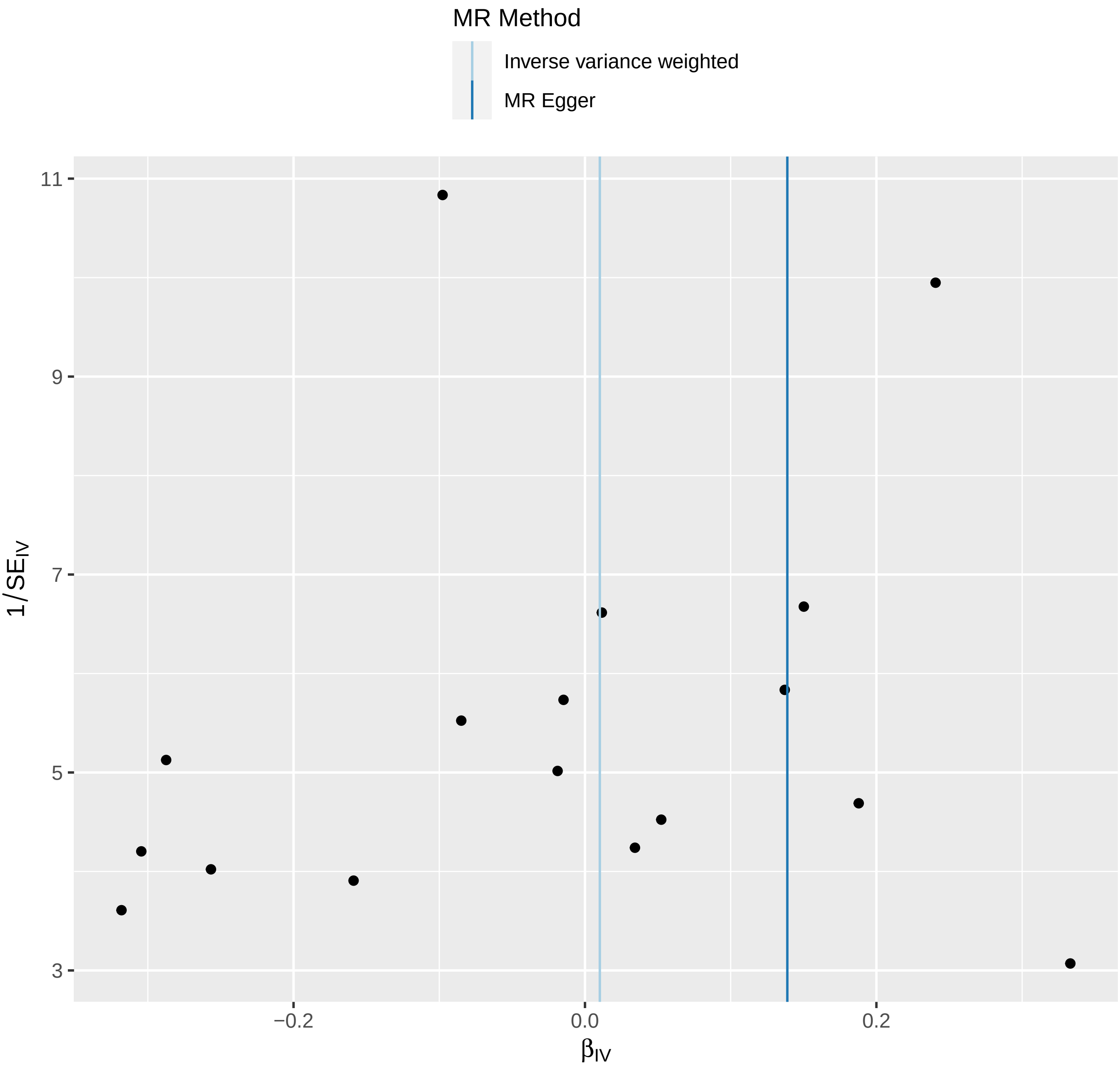

Batch 3−2563 : E3 ubiquitin−protein ligase RNF13 on Diabetic nephropathy (more control exclusions)
with a threshold of 1e−05

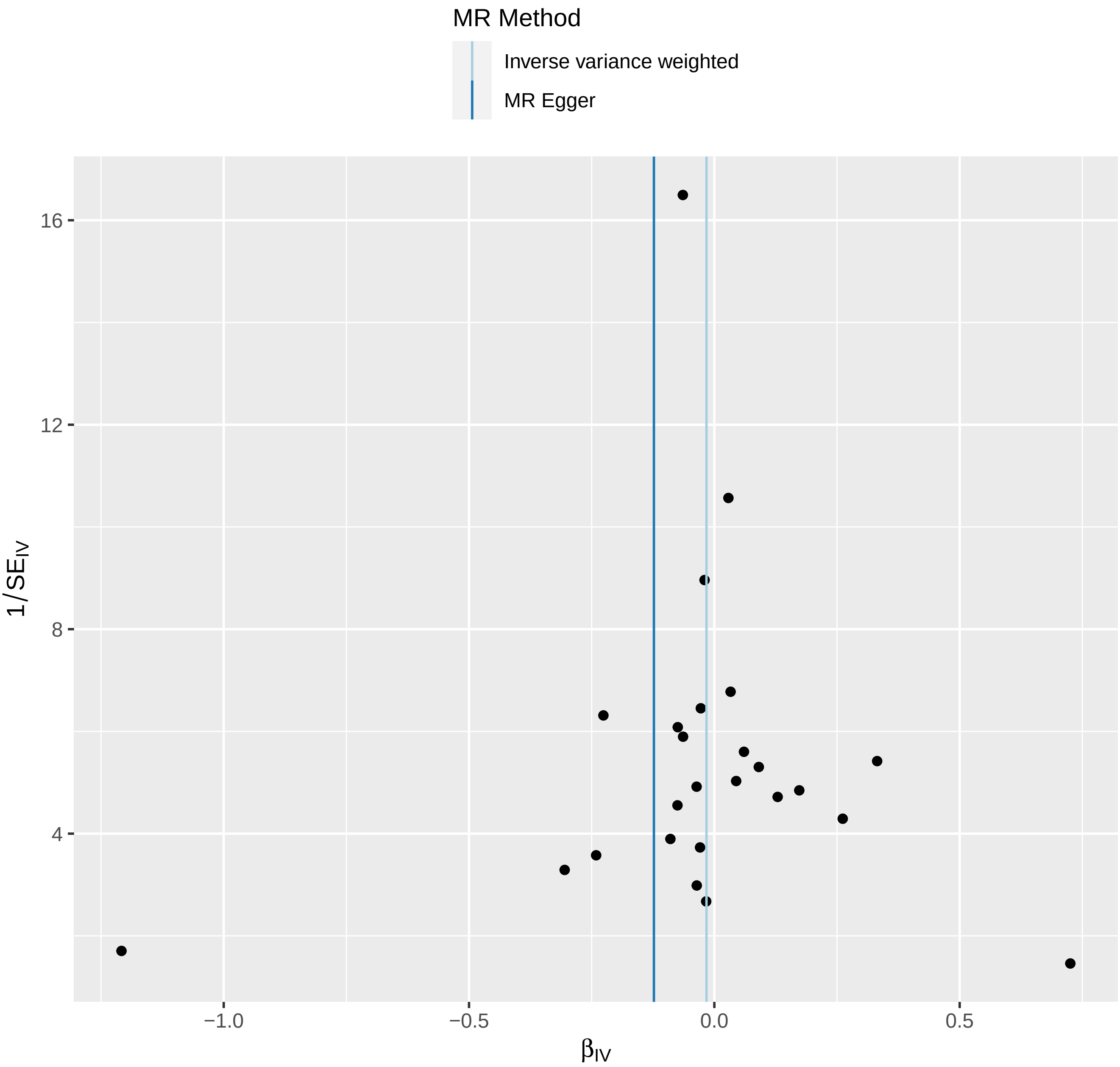

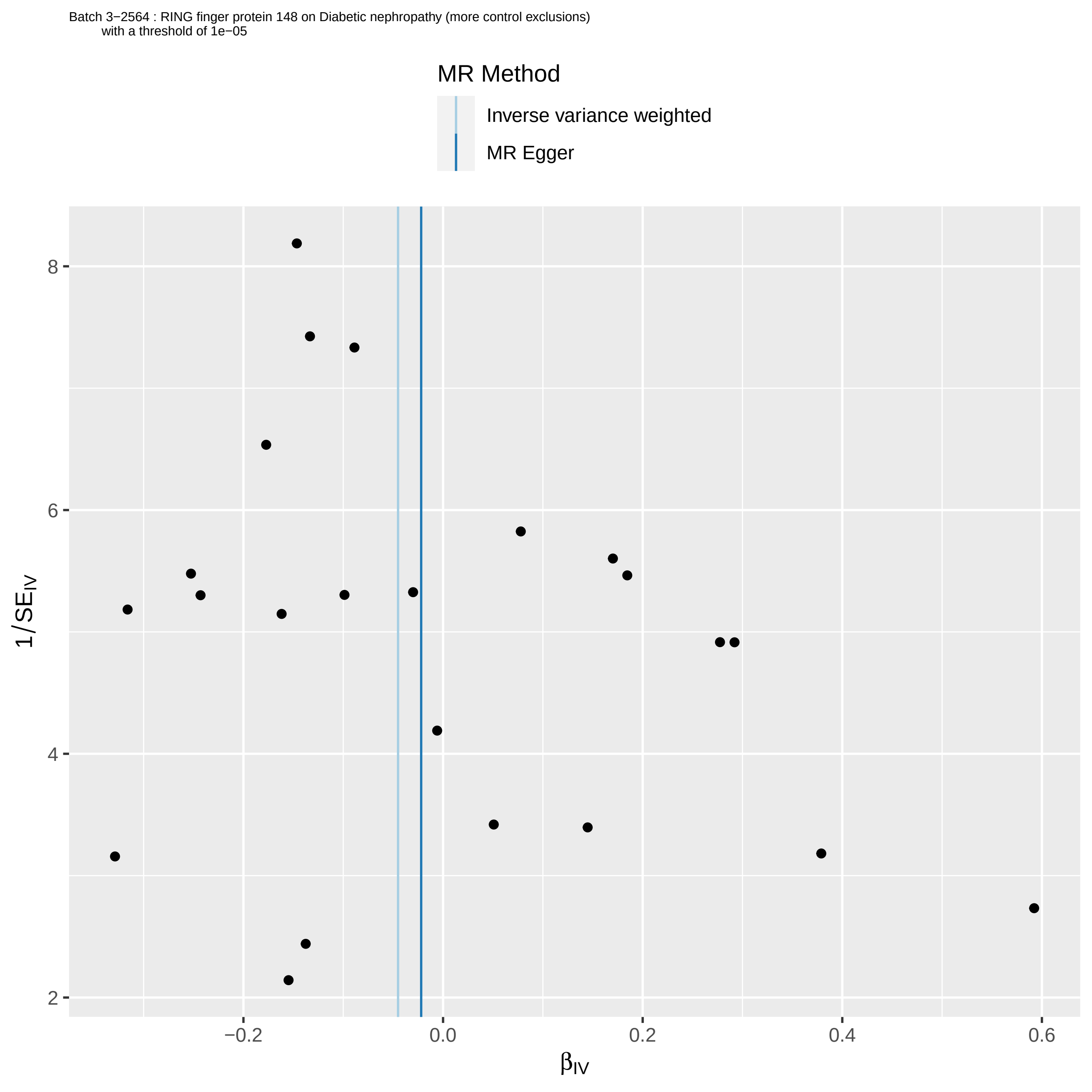

Batch 3−2564 : RING finger protein 148 on Diabetic nephropathy (more control exclusions)
with a threshold of 1e−05
MR Method
Inverse variance weighted
MR Egger
8
6
4
2
1/SE_IV
−0.2
0.0
0.2
0.4
0.6
β_IV

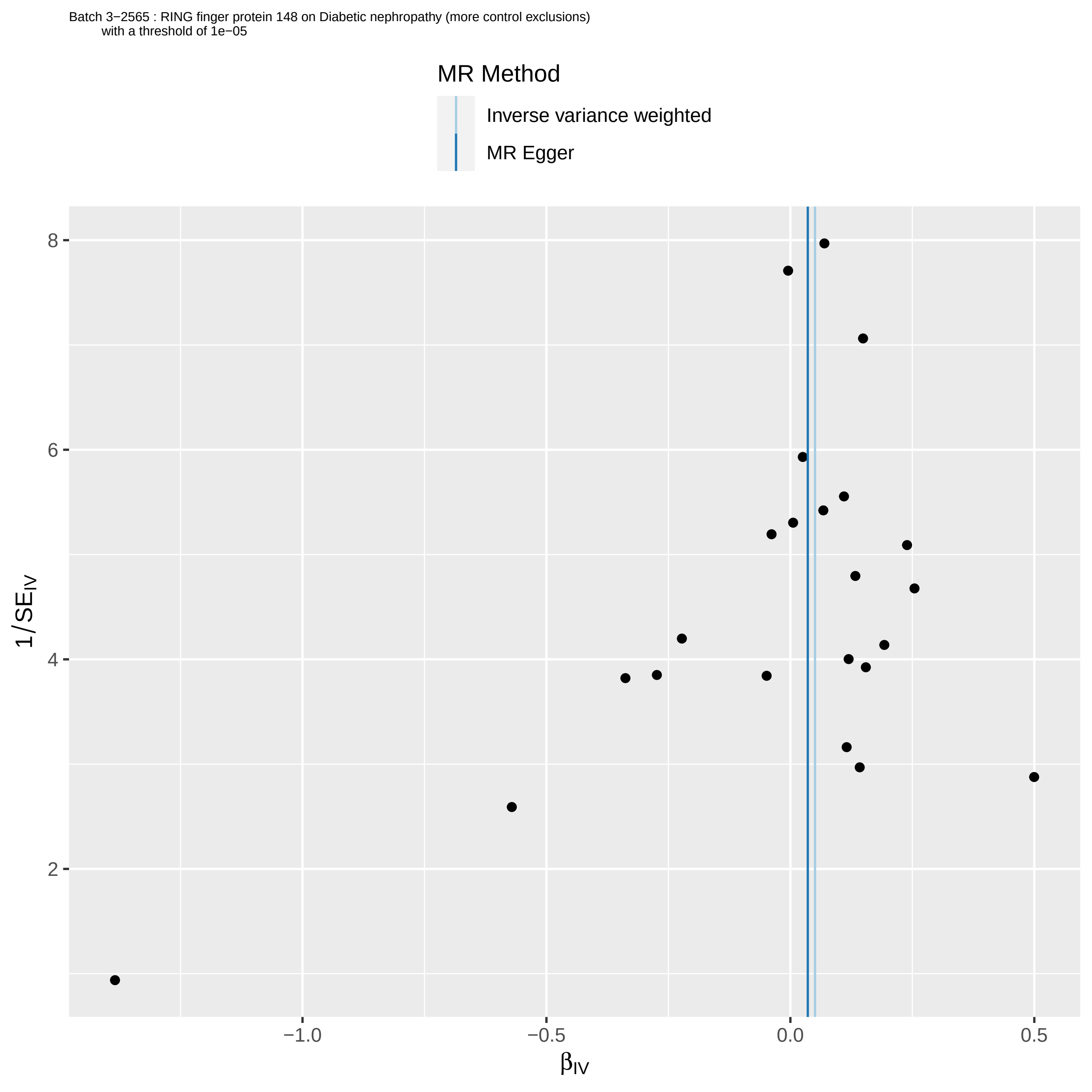

Batch 3−2565 : RING finger protein 148 on Diabetic nephropathy (more control exclusions)
with a threshold of 1e−05
MR Method
Inverse variance weighted
MR Egger
8
6
4
2
1/SE_IV
−1.0
−0.5
0.0
0.5
β_IV

Batch 3−2566 : E3 ubiquitin−protein ligase RNF149 on Diabetic nephropathy (more control exclusions)
with a threshold of 1e−05

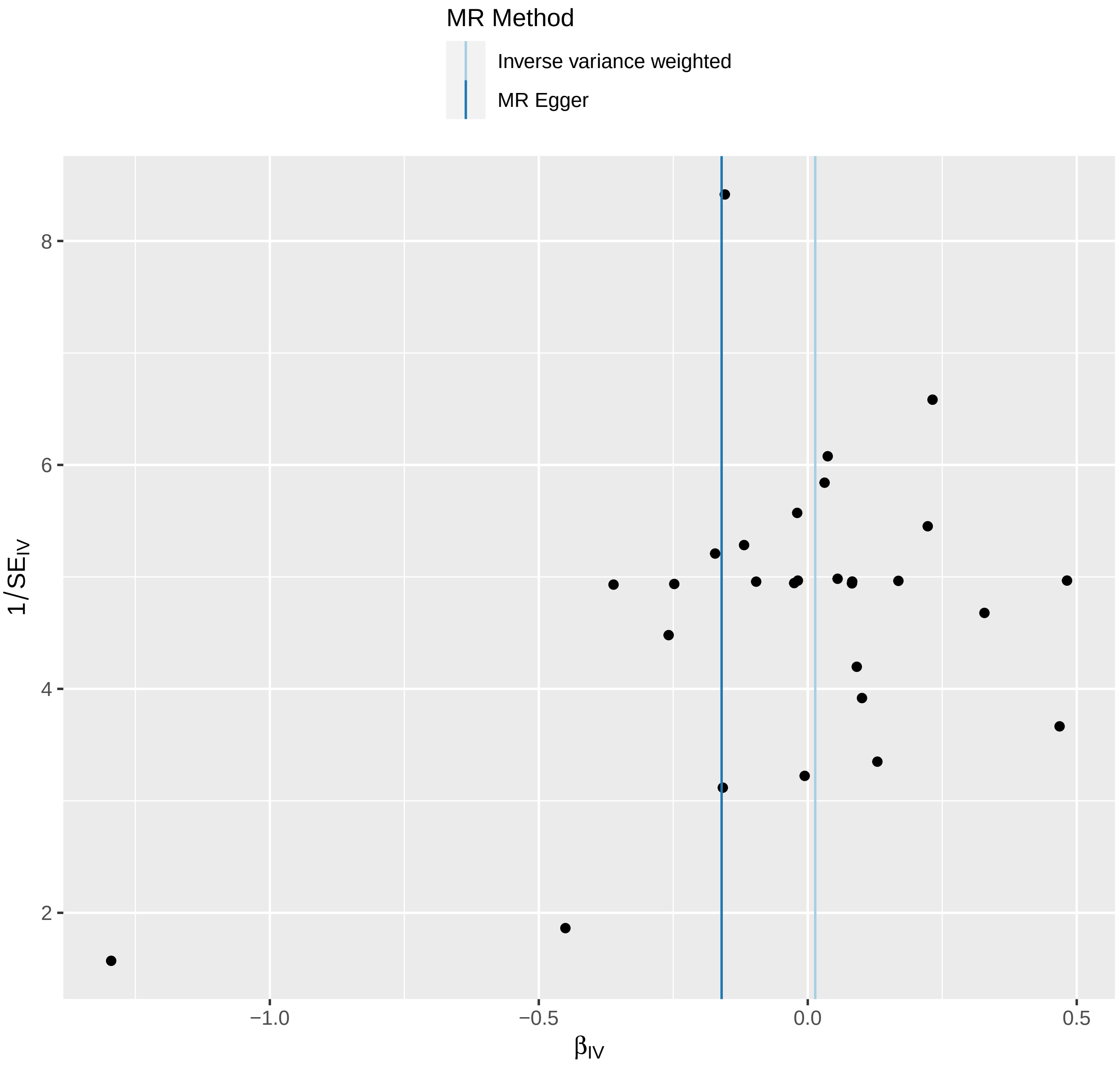

Batch 3−2567 : RING finger protein 150 on Diabetic nephropathy (more control exclusions)
with a threshold of 1e−05

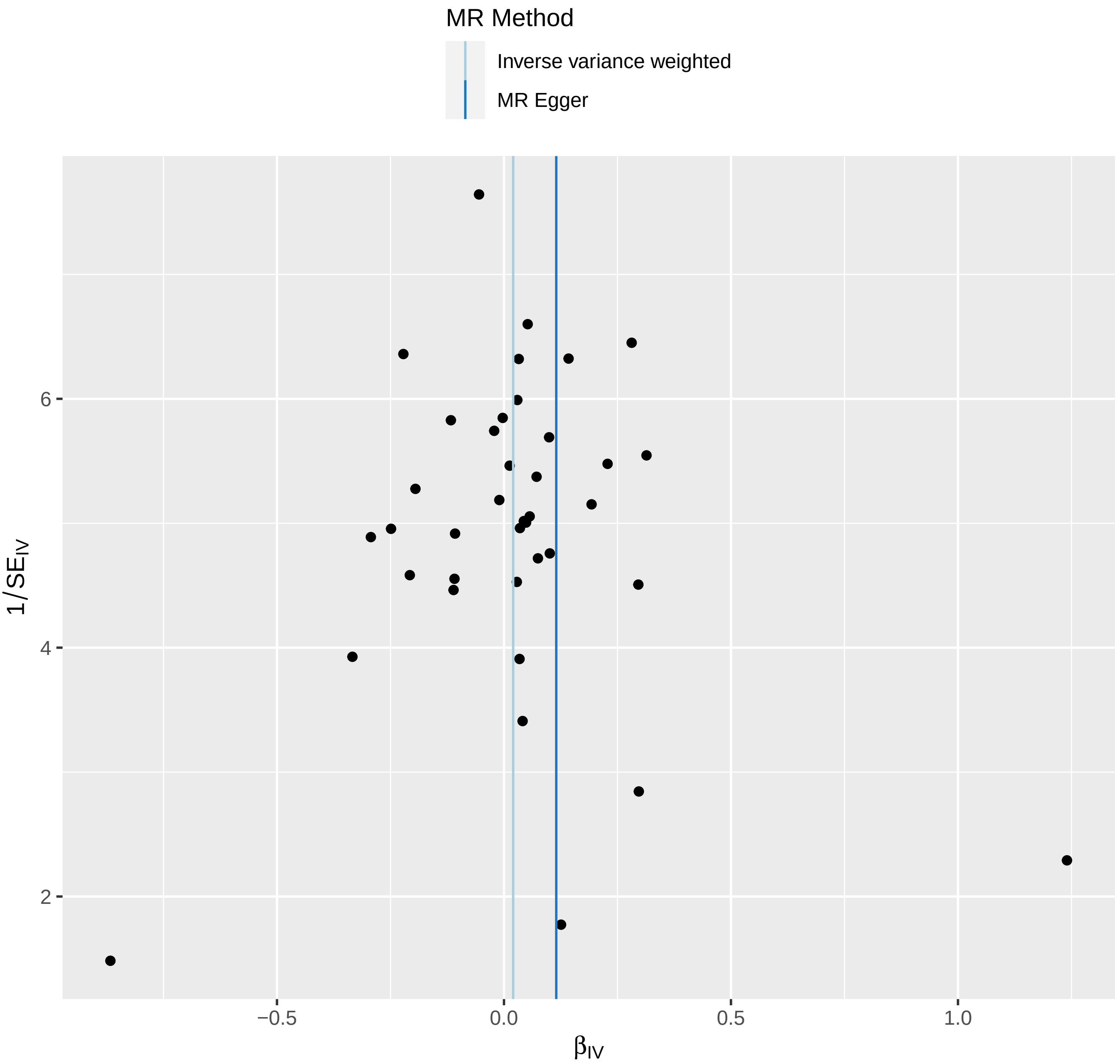

Batch 3−2568 : RING finger protein 165 on Diabetic nephropathy (more control exclusions)
with a threshold of 1e−05

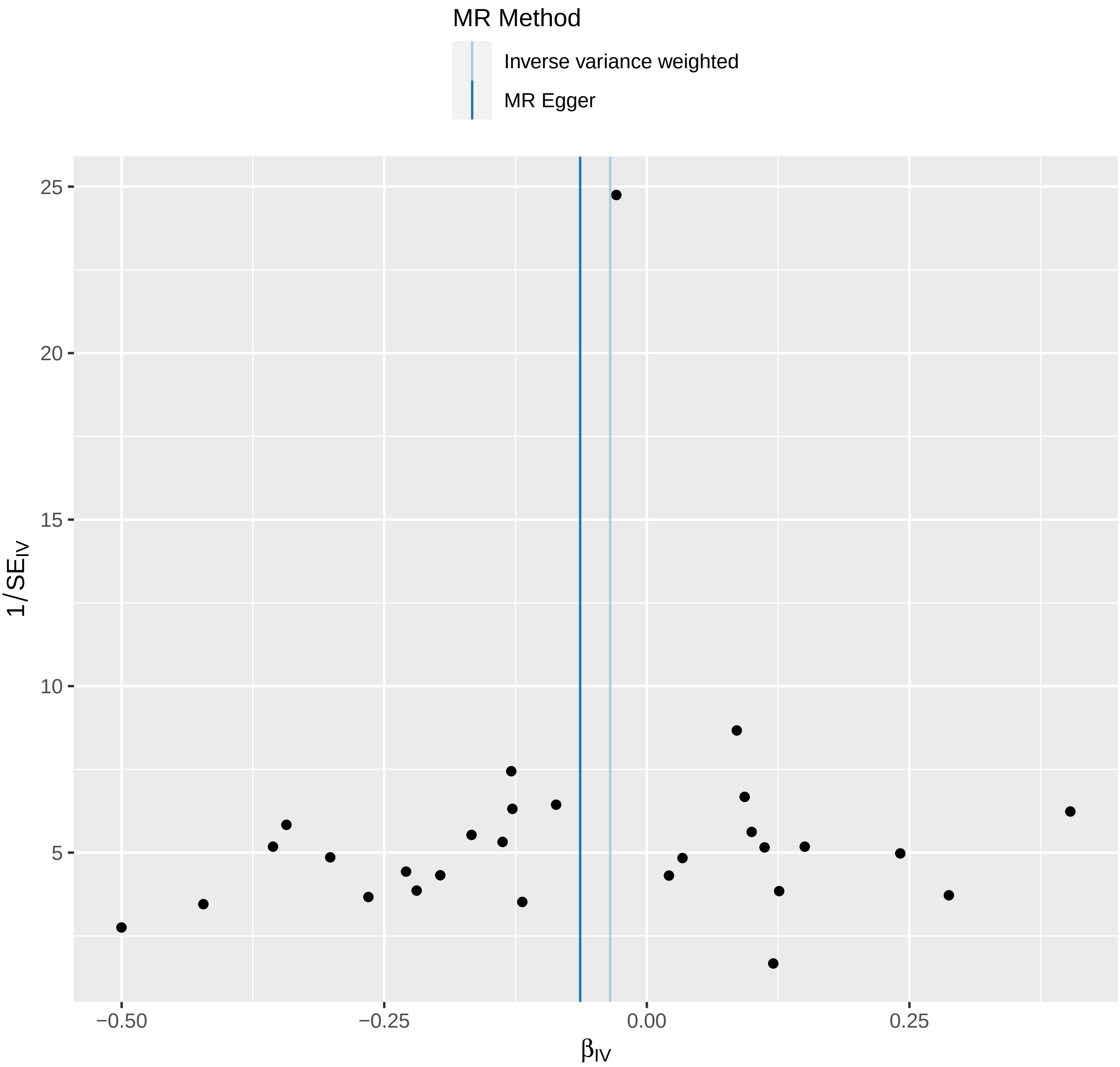

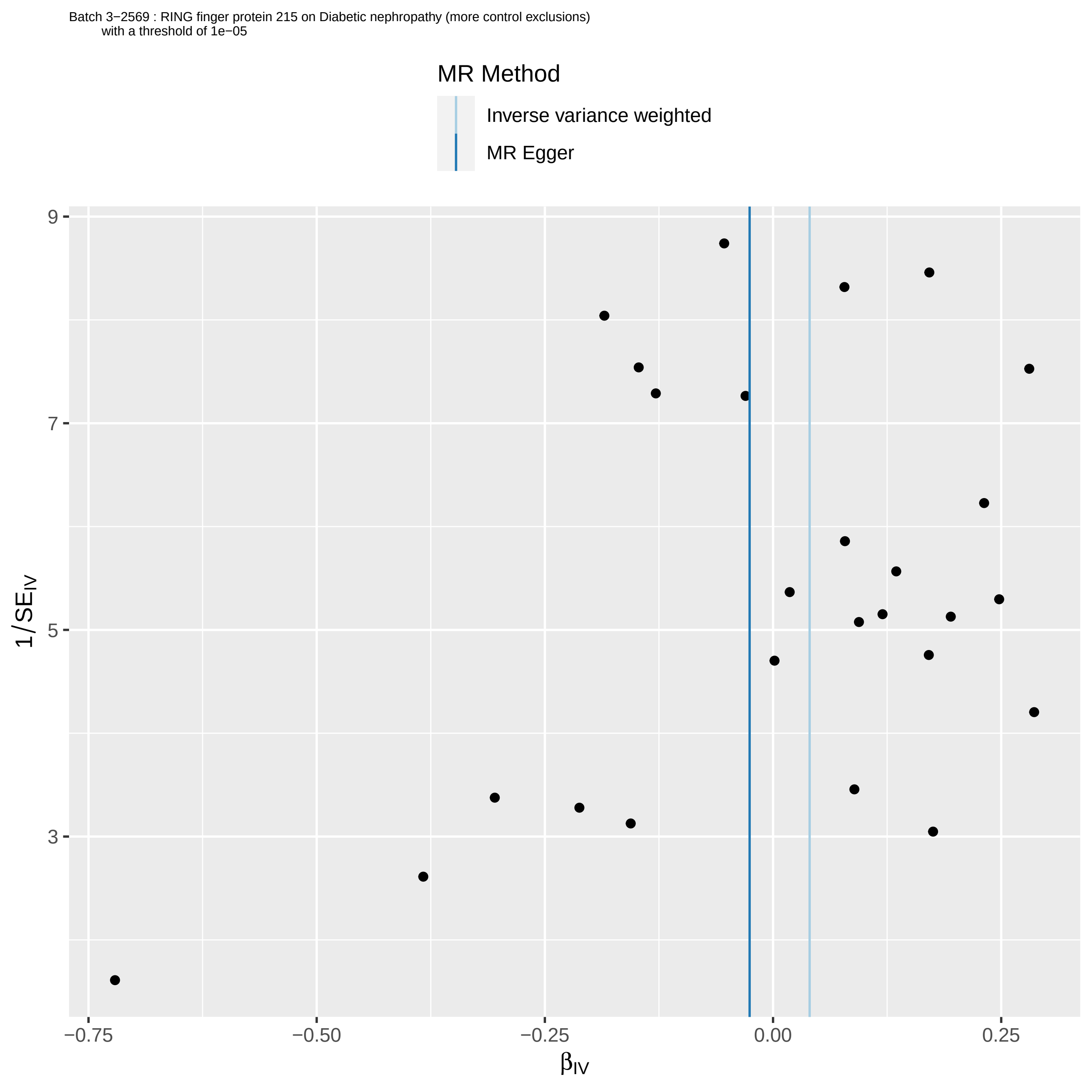
Batch 3−2569 : RING finger protein 215 on Diabetic nephropathy (more control exclusions)
with a threshold of 1e−05
MR Method
Inverse variance weighted
MR Egger
9
7
5
3
1/SE$_{IV}$
−0.75
−0.50
−0.25
0.00
0.25
β_{IV}

Batch 3−2570 : RING finger protein 219 on Diabetic nephropathy (more control exclusions)
with a threshold of 1e−05

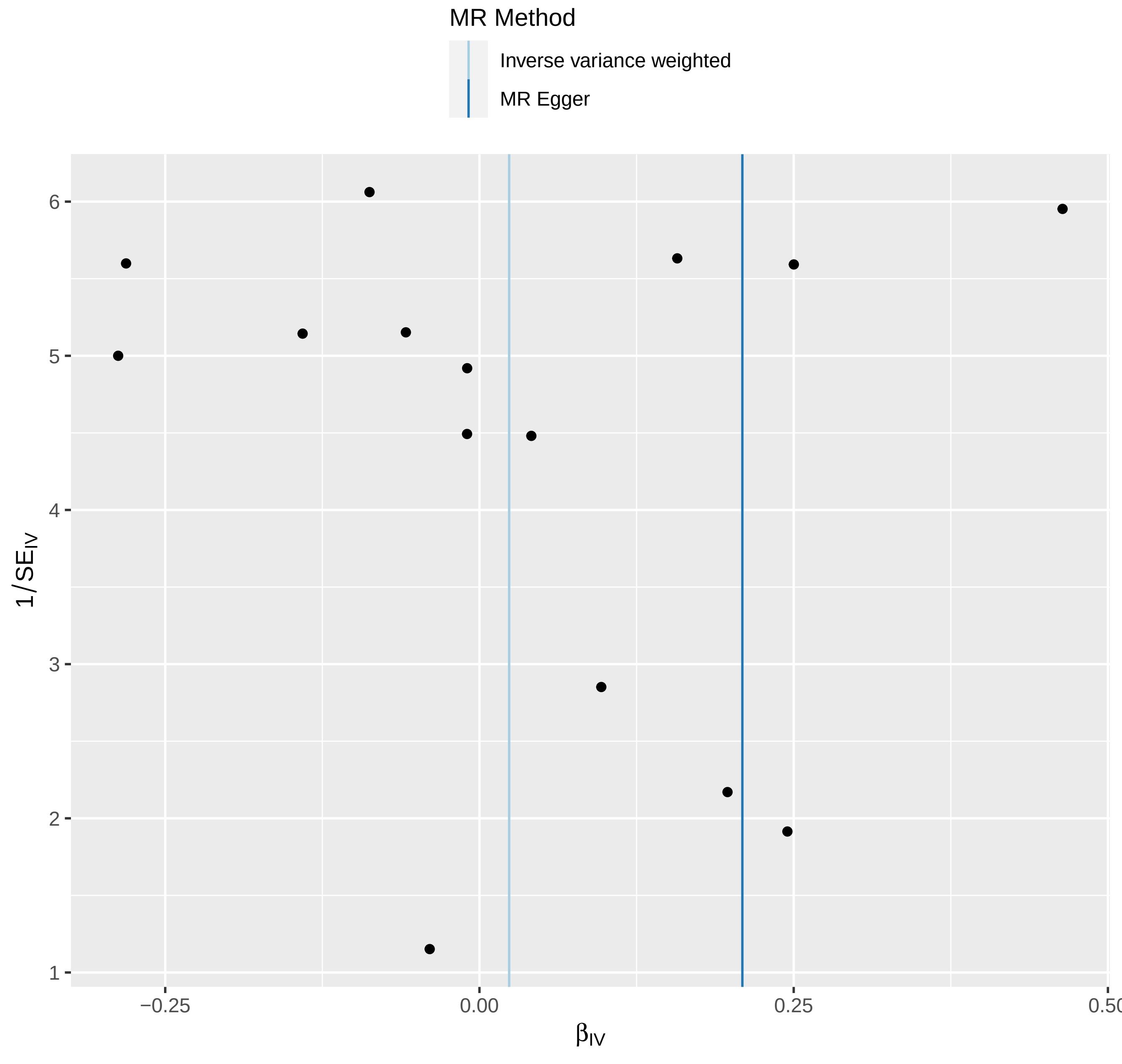

Batch 3−2571 : E3 ubiquitin−protein ligase RNF34 on Diabetic nephropathy (more control exclusions)
with a threshold of 1e−05

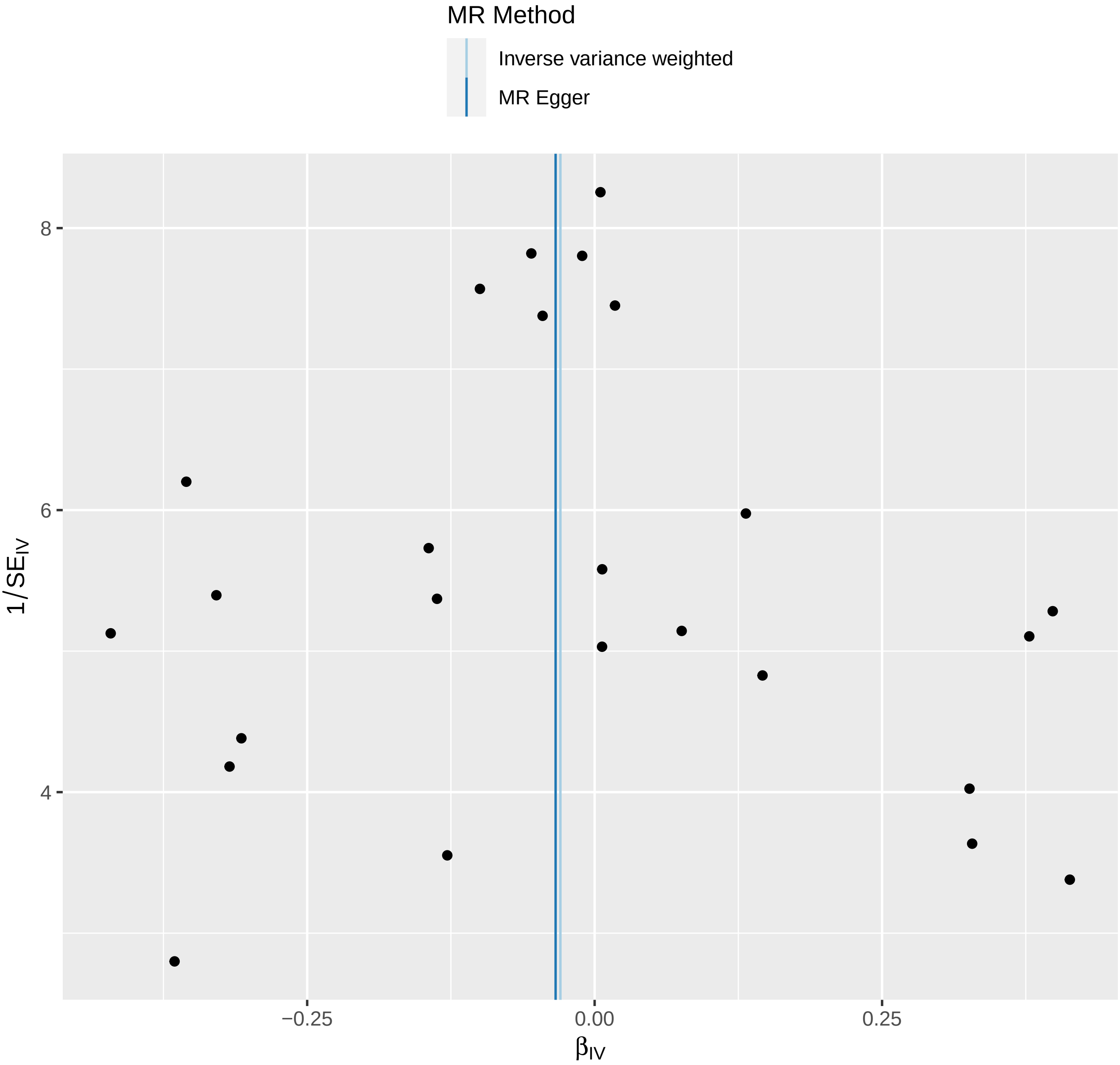

Batch 3−2572 : E3 ubiquitin−protein ligase RNF43 on Diabetic nephropathy (more control exclusions)
with a threshold of 1e−05

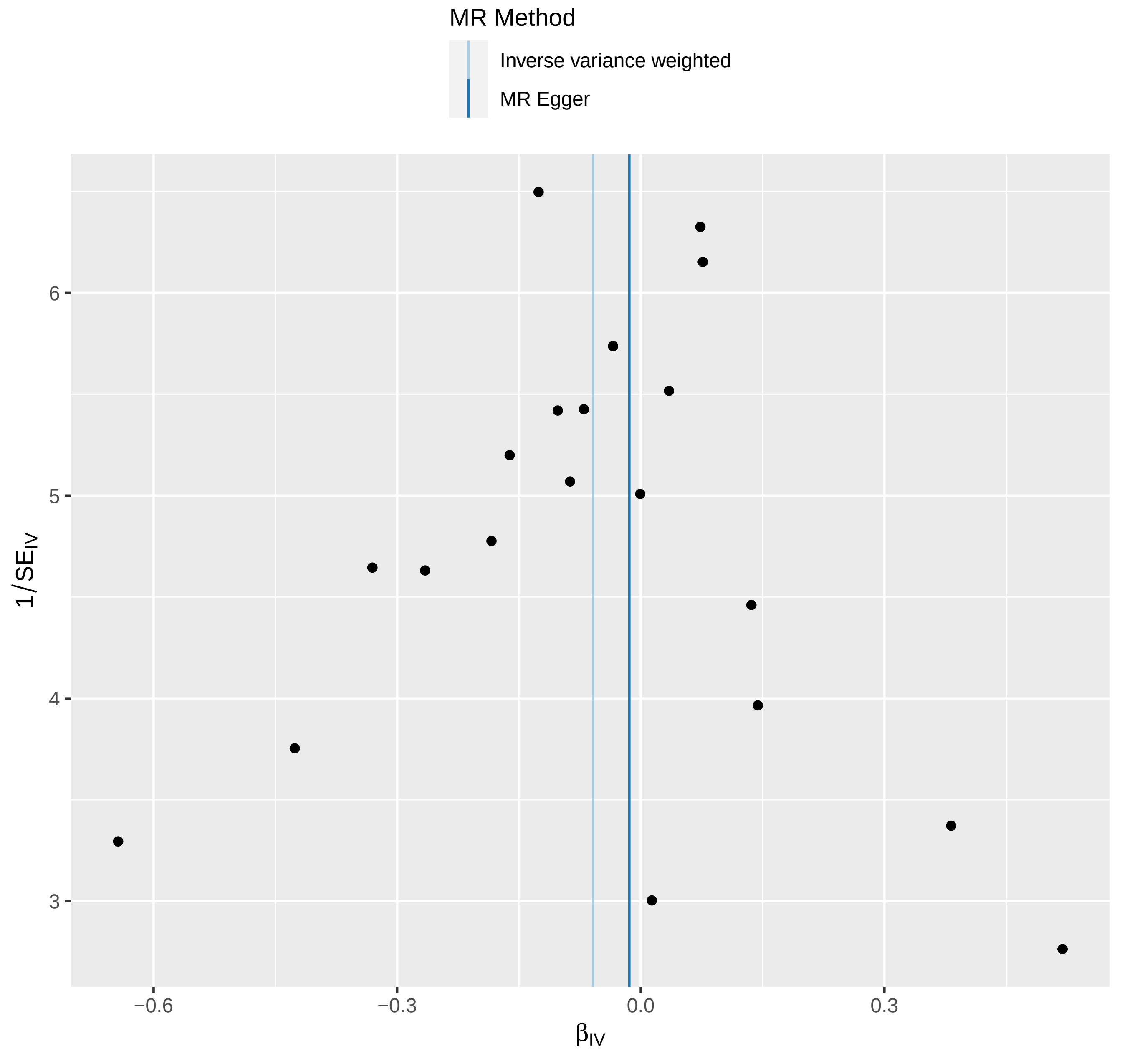

Batch 3−2573 : E3 ubiquitin−protein ligase RNF8 on Diabetic nephropathy (more control exclusions)
with a threshold of 1e−05

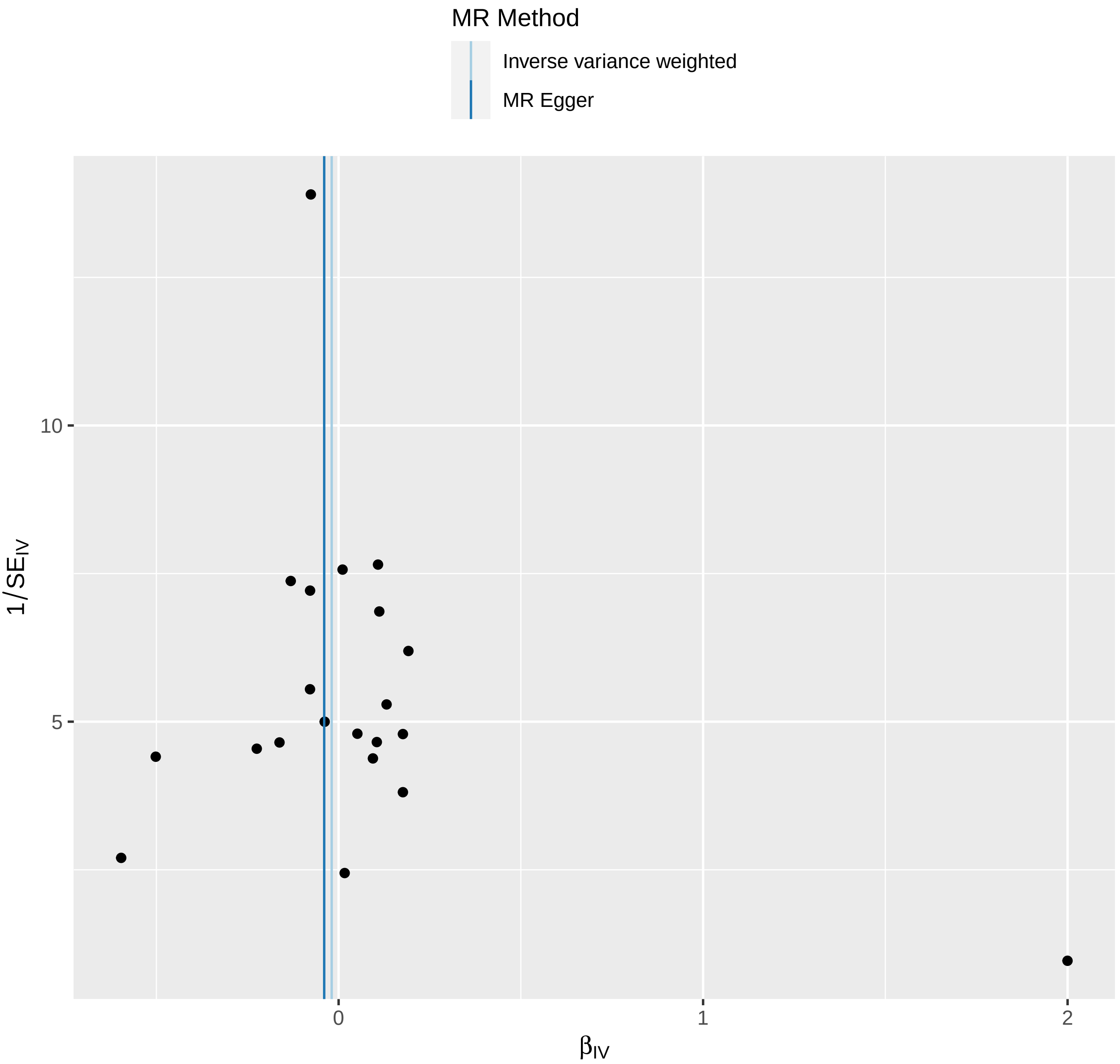

Batch 3−2574 : mRNA−capping enzyme on Diabetic nephropathy (more control exclusions)
with a threshold of 1e−05

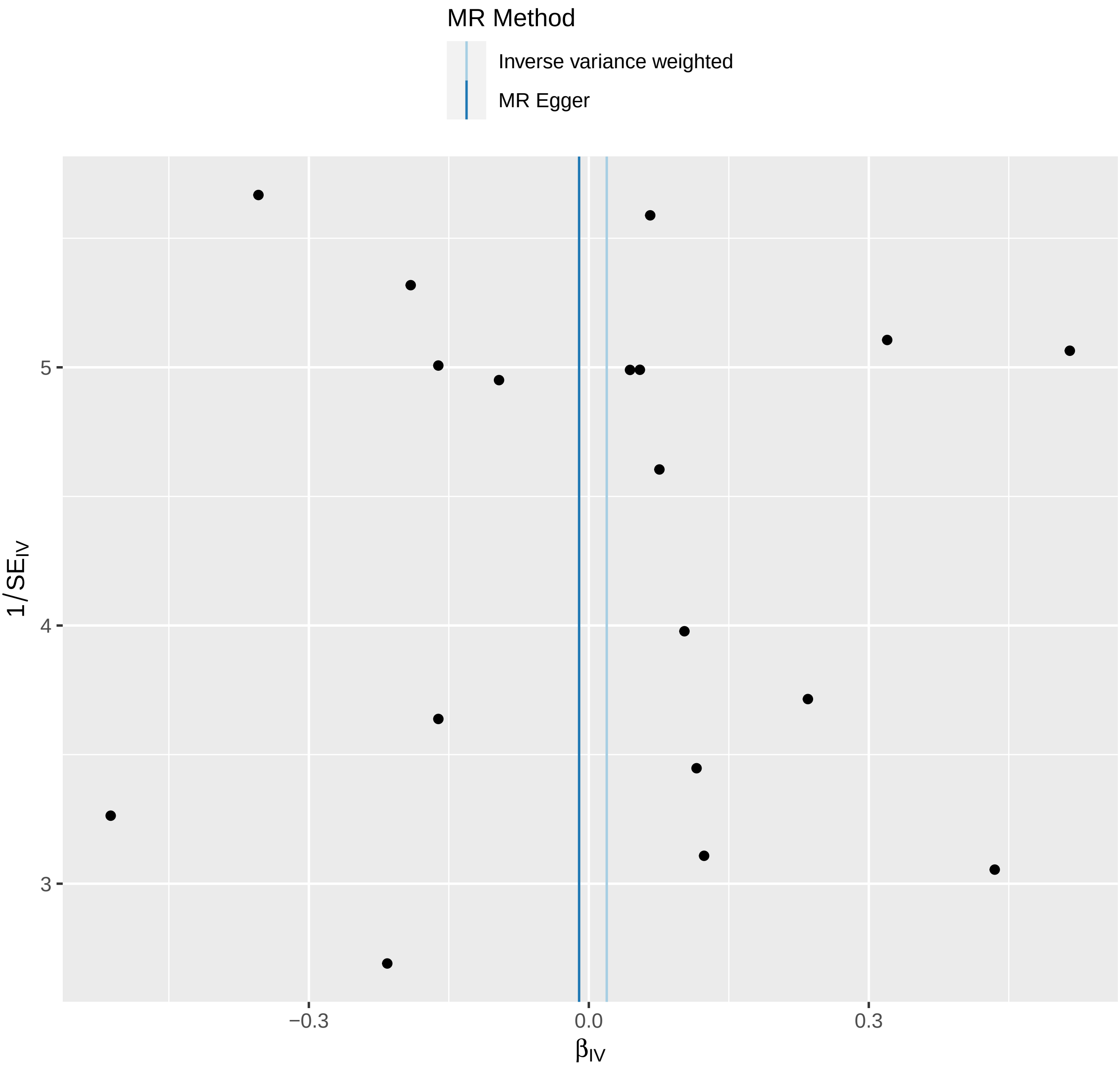

Batch 3−2575 : rRNA methyltransferase 3, mitochondrial on Diabetic nephropathy (more control exclusions)
with a threshold of 1e−05

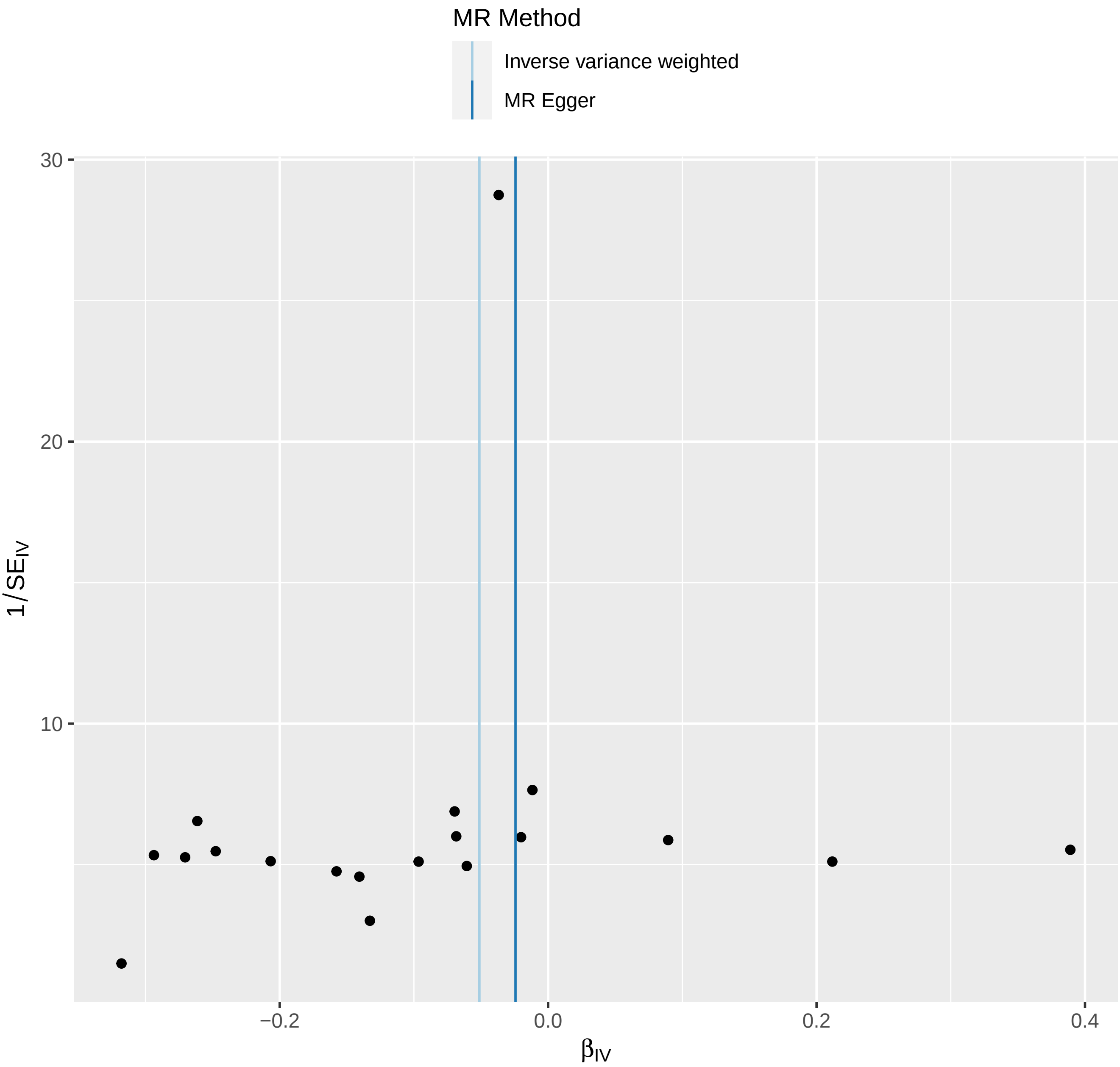

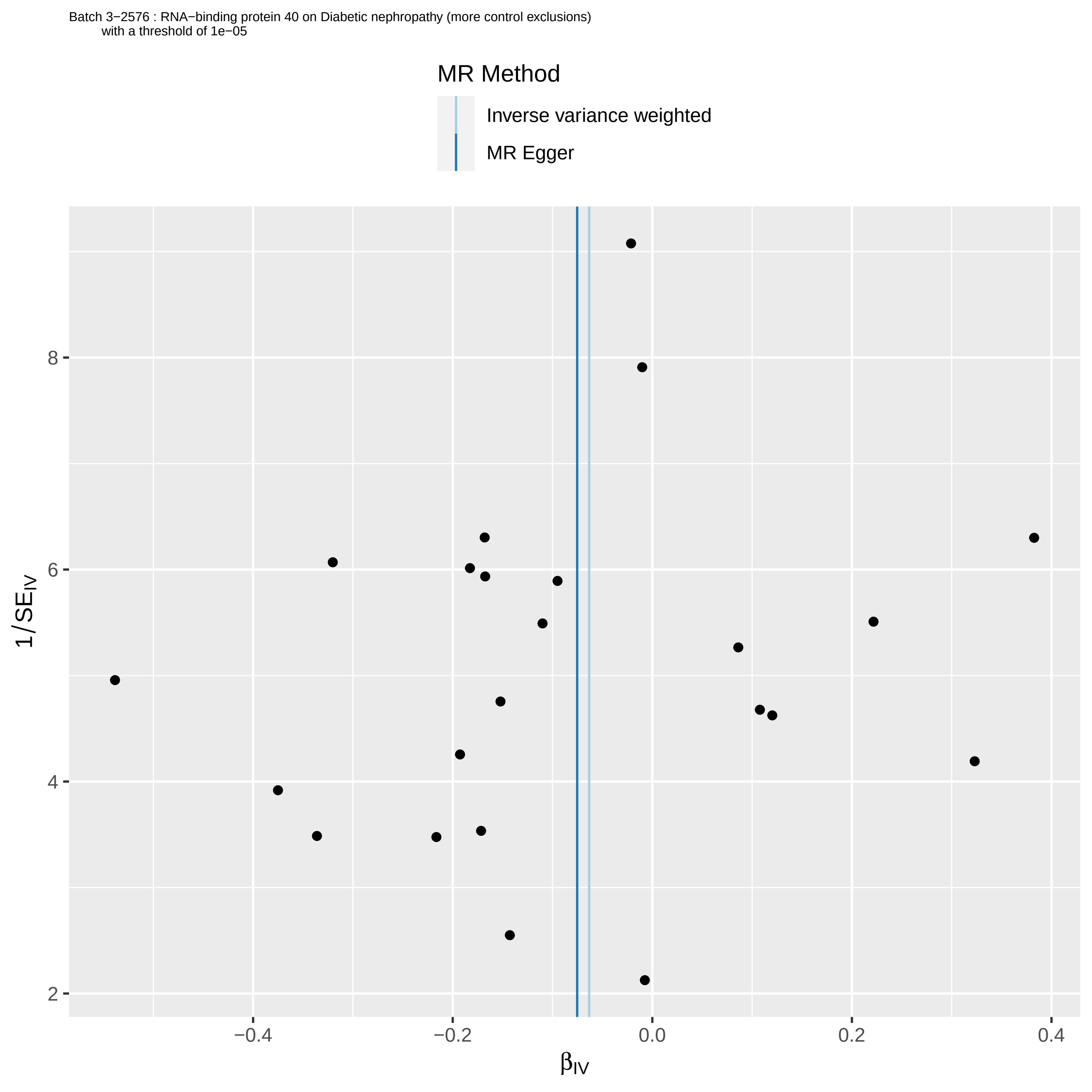
Batch 3−2576 : RNA−binding protein 40 on Diabetic nephropathy (more control exclusions)
with a threshold of 1e−05
MR Method
Inverse variance weighted
MR Egger
1/SE_IV
8
6
4
2
−0.4
−0.2
0.0
0.2
0.4
β_IV

Batch 3−2577 : Roundabout homolog 1 on Diabetic nephropathy (more control exclusions) with a threshold of 1e−05

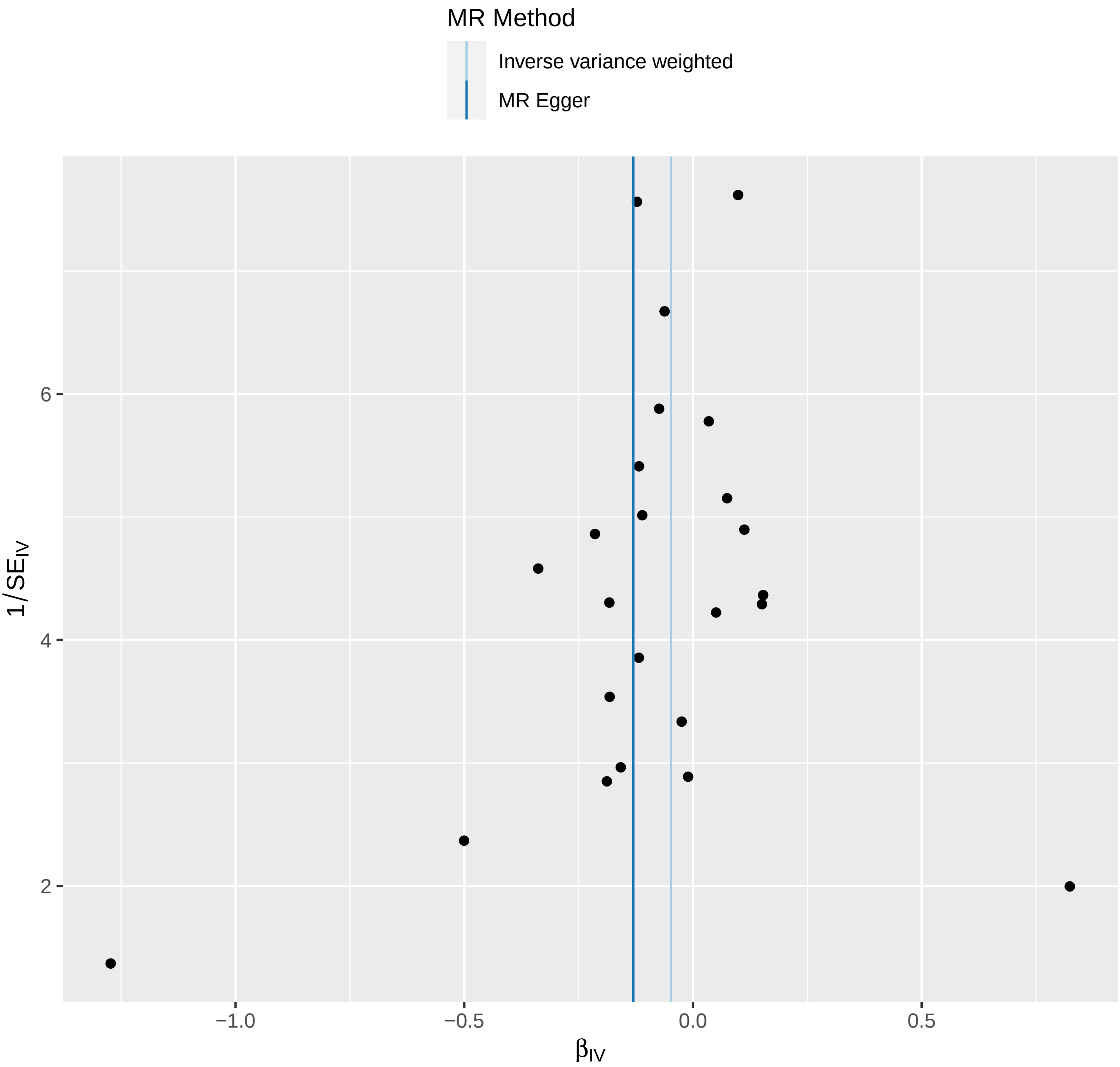

Batch 3−2578 : Roundabout homolog 2 on Diabetic nephropathy (more control exclusions)
with a threshold of 1e−05

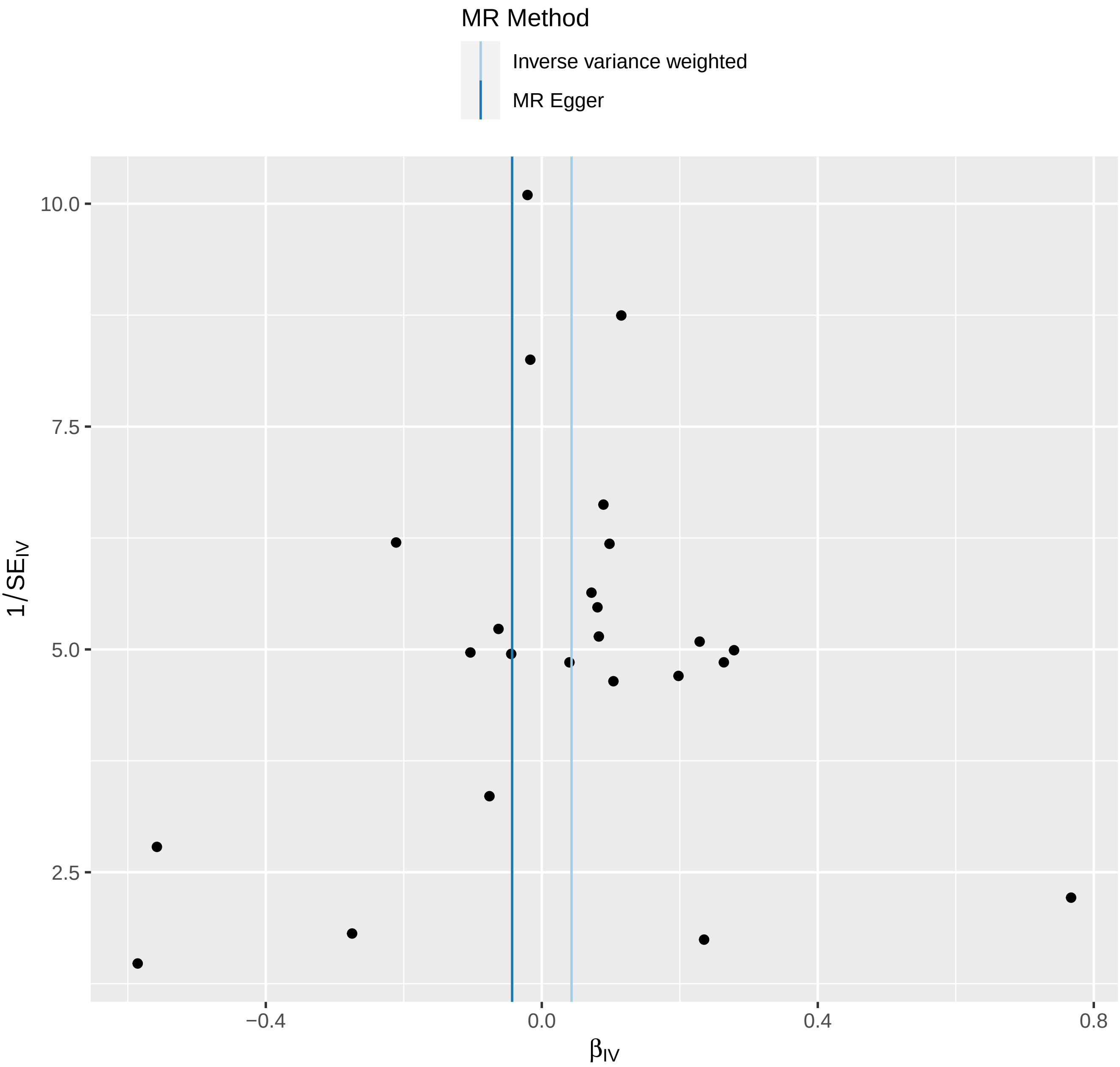

Batch 3−2579 : Roundabout homolog 3 on Diabetic nephropathy (more control exclusions)
with a threshold of 1e−05

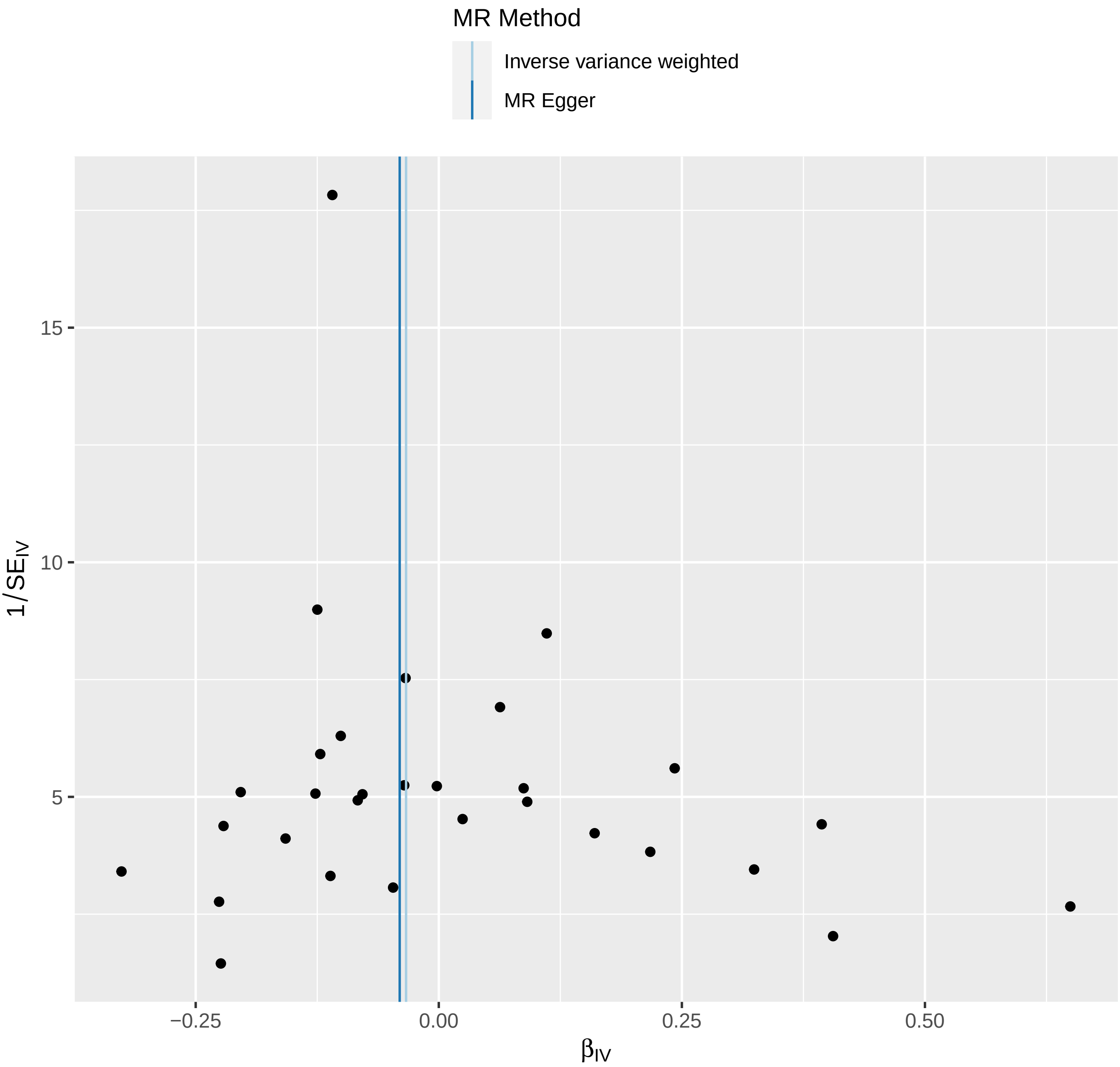

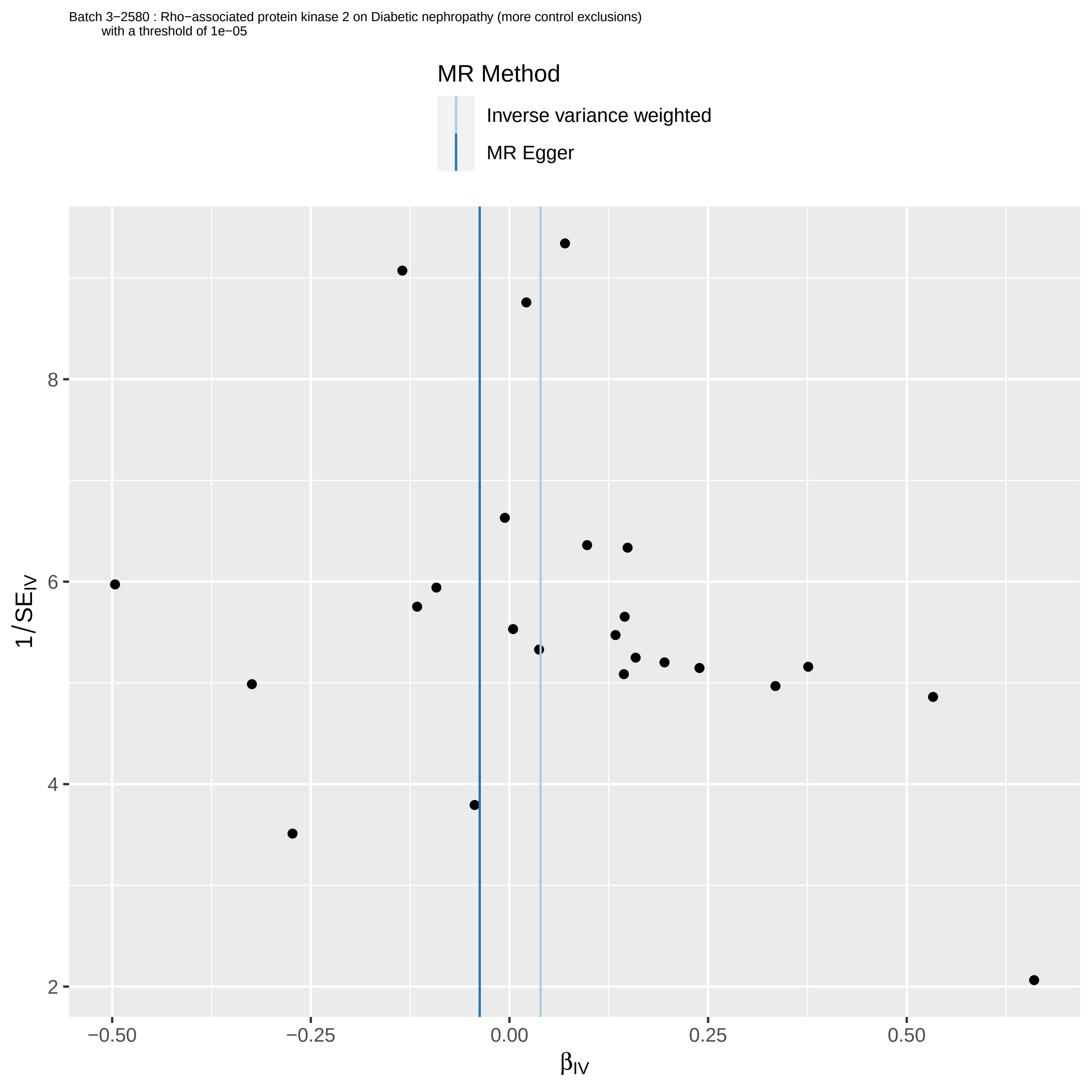

Batch 3−2580 : Rho−associated protein kinase 2 on Diabetic nephropathy (more control exclusions)
with a threshold of 1e−05
MR Method
Inverse variance weighted
MR Egger
8
6
4
2
1/SE$_{IV}$
−0.50
−0.25
0.00
0.25
0.50
β_{IV}

Batch 3−2581 : Tyrosine−protein kinase transmembrane receptor ROR1 on Diabetic nephropathy (more control exclusions)
with a threshold of 1e−05

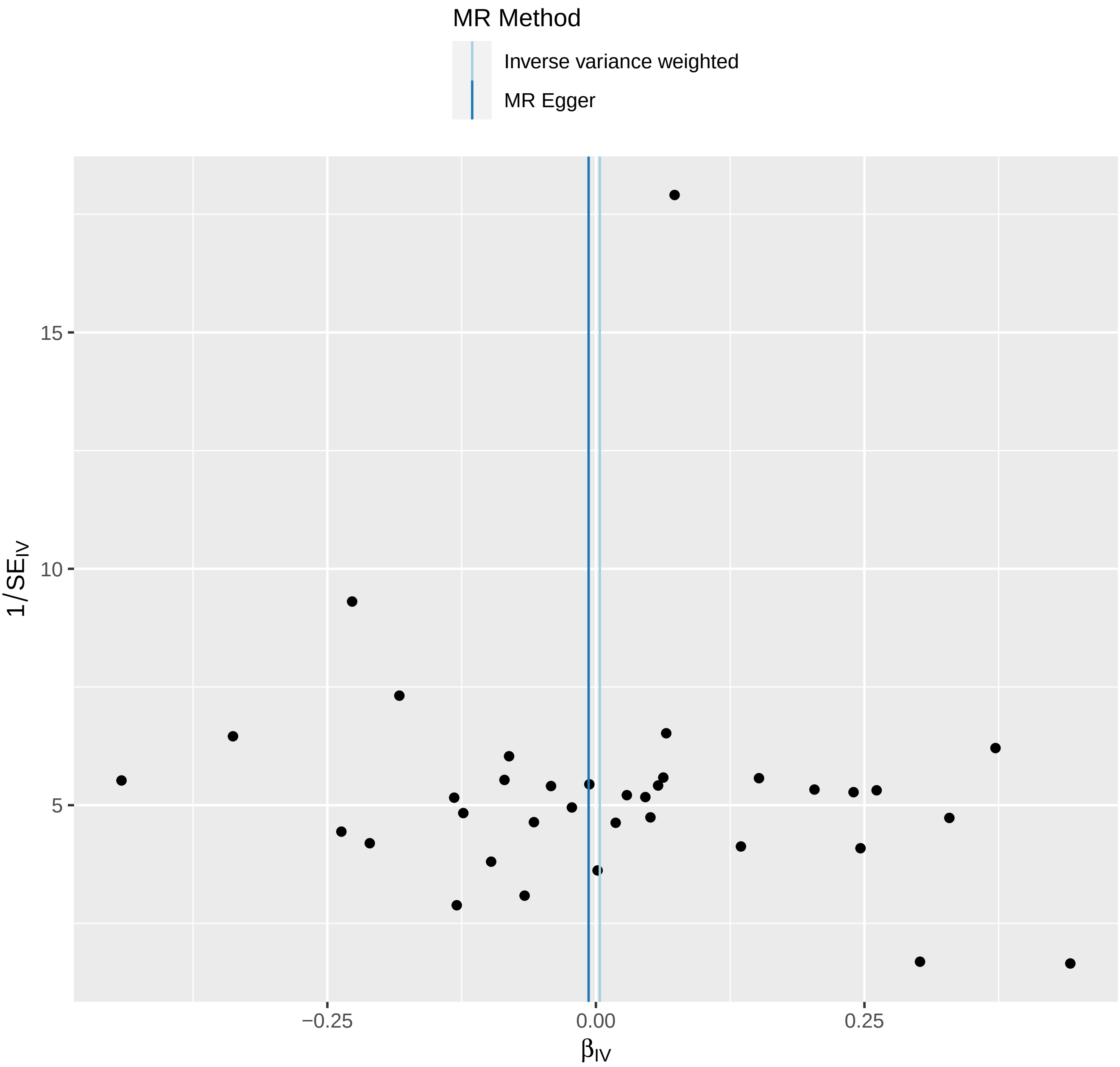

Batch 3−2582 : Tyrosine−protein kinase transmembrane receptor ROR2 on Diabetic nephropathy (more control exclusions) with a threshold of 1e−05

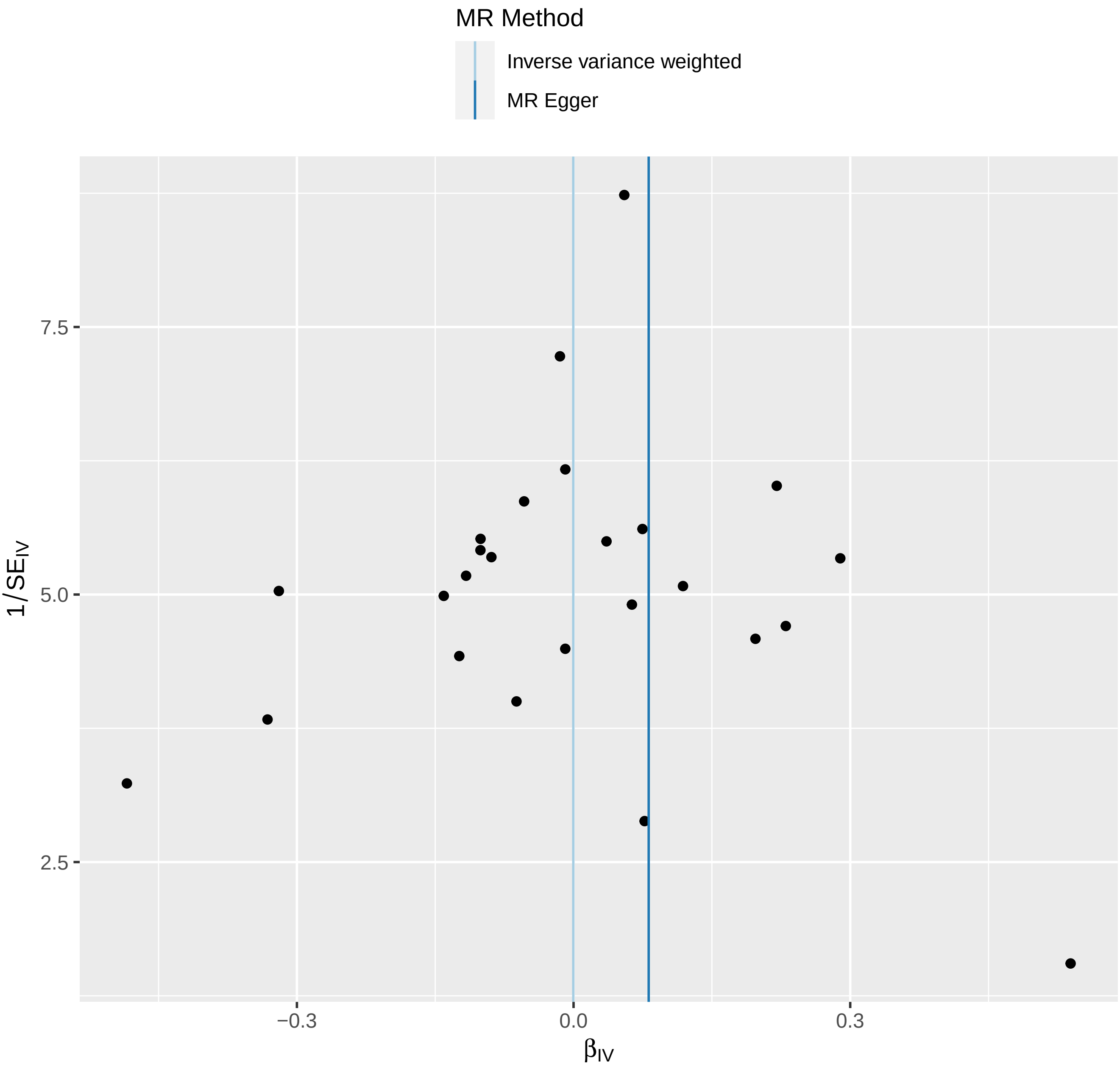

Batch 3−2583 : Nuclear receptor ROR−beta on Diabetic nephropathy (more control exclusions)
with a threshold of 1e−05

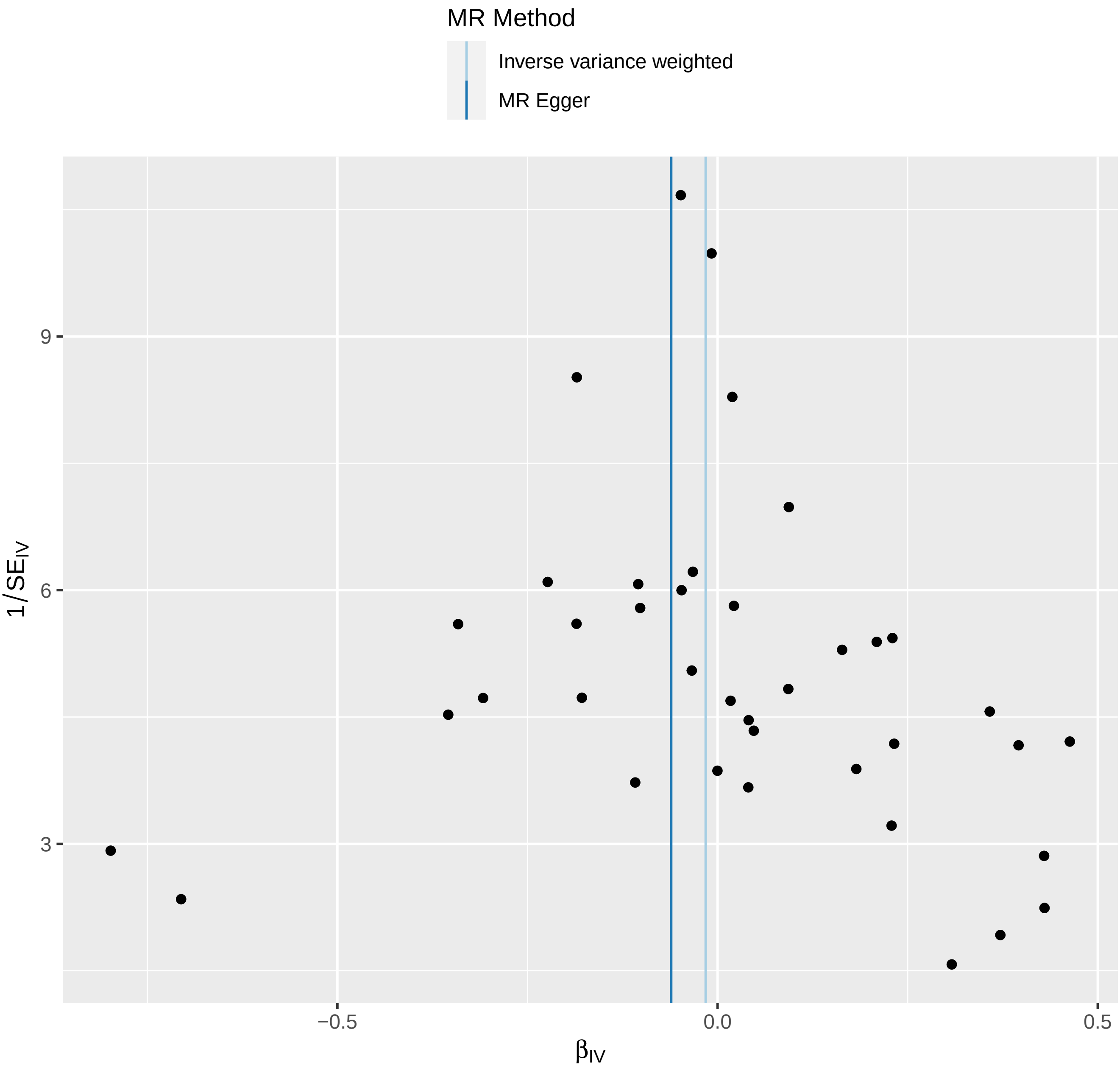

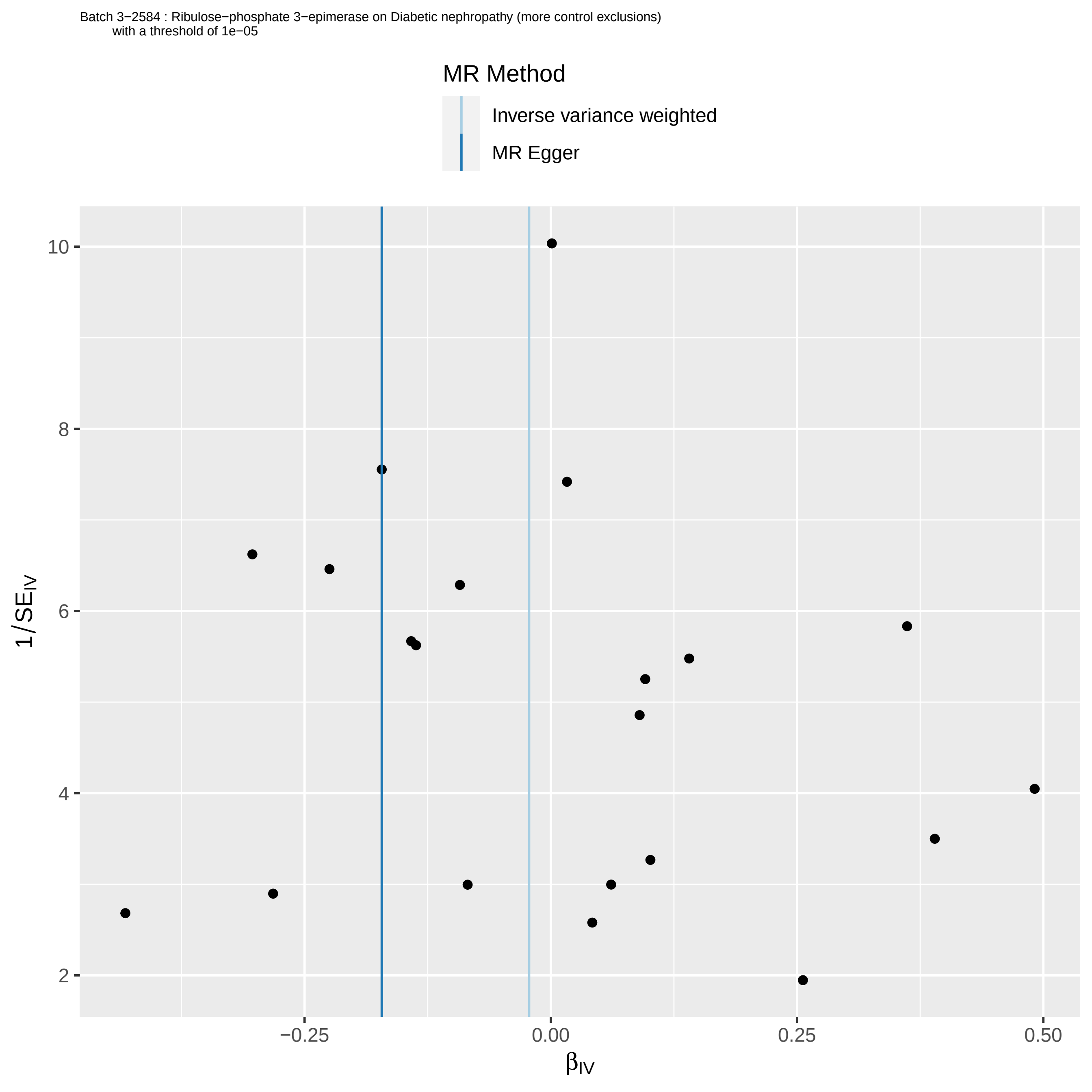

Batch 3−2584 : Ribulose−phosphate 3−epimerase on Diabetic nephropathy (more control exclusions)
with a threshold of 1e−05
MR Method
Inverse variance weighted
MR Egger
10
8
6
4
2
1/SE_IV
−0.25
0.00
0.25
0.50
β_IV

Batch 3−2585 : Ribose−5−phosphate isomerase on Diabetic nephropathy (more control exclusions)
with a threshold of 1e−05

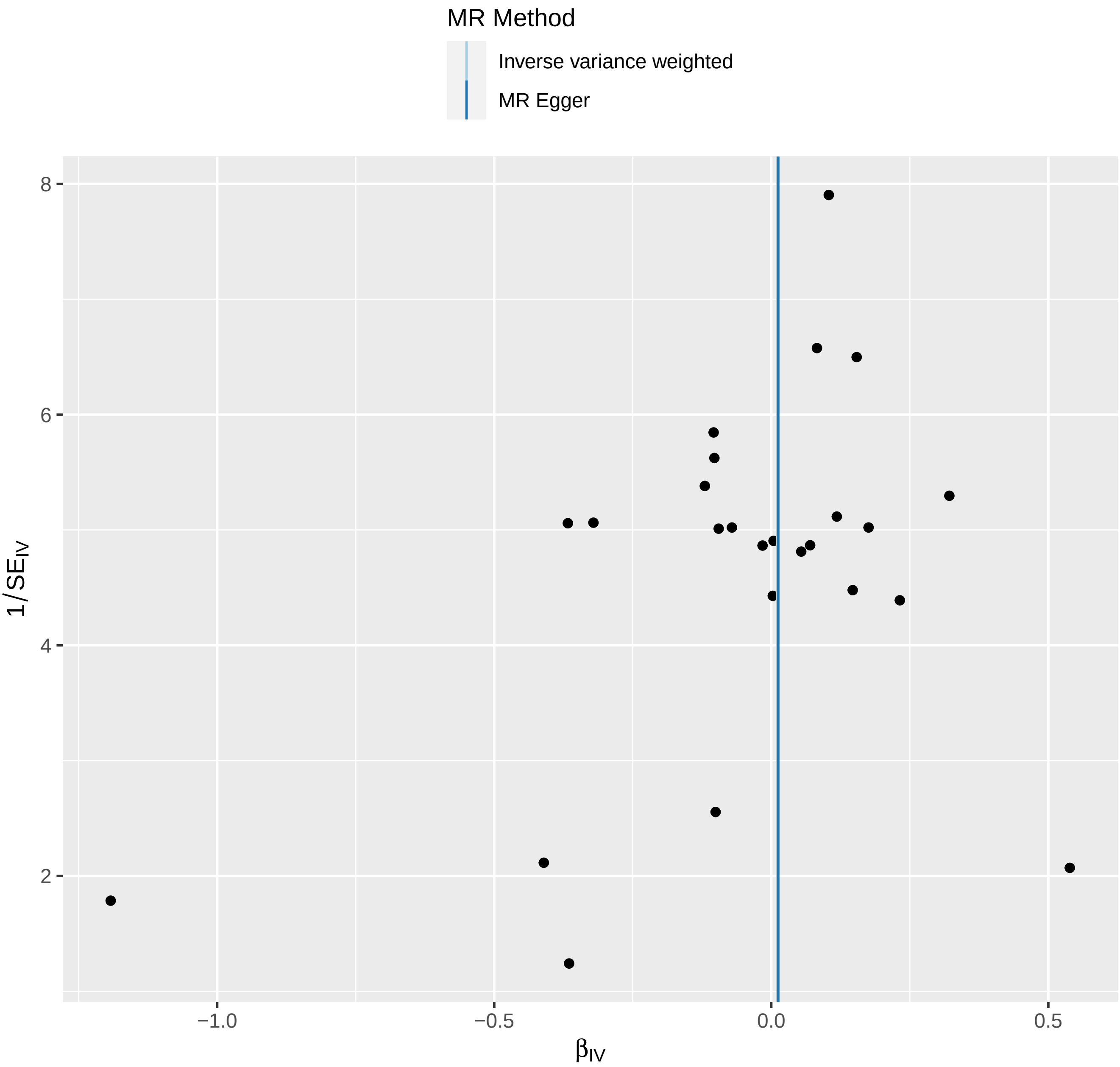

Batch 3−2586 : 60S ribosomal protein L30 on Diabetic nephropathy (more control exclusions)
with a threshold of 1e−05

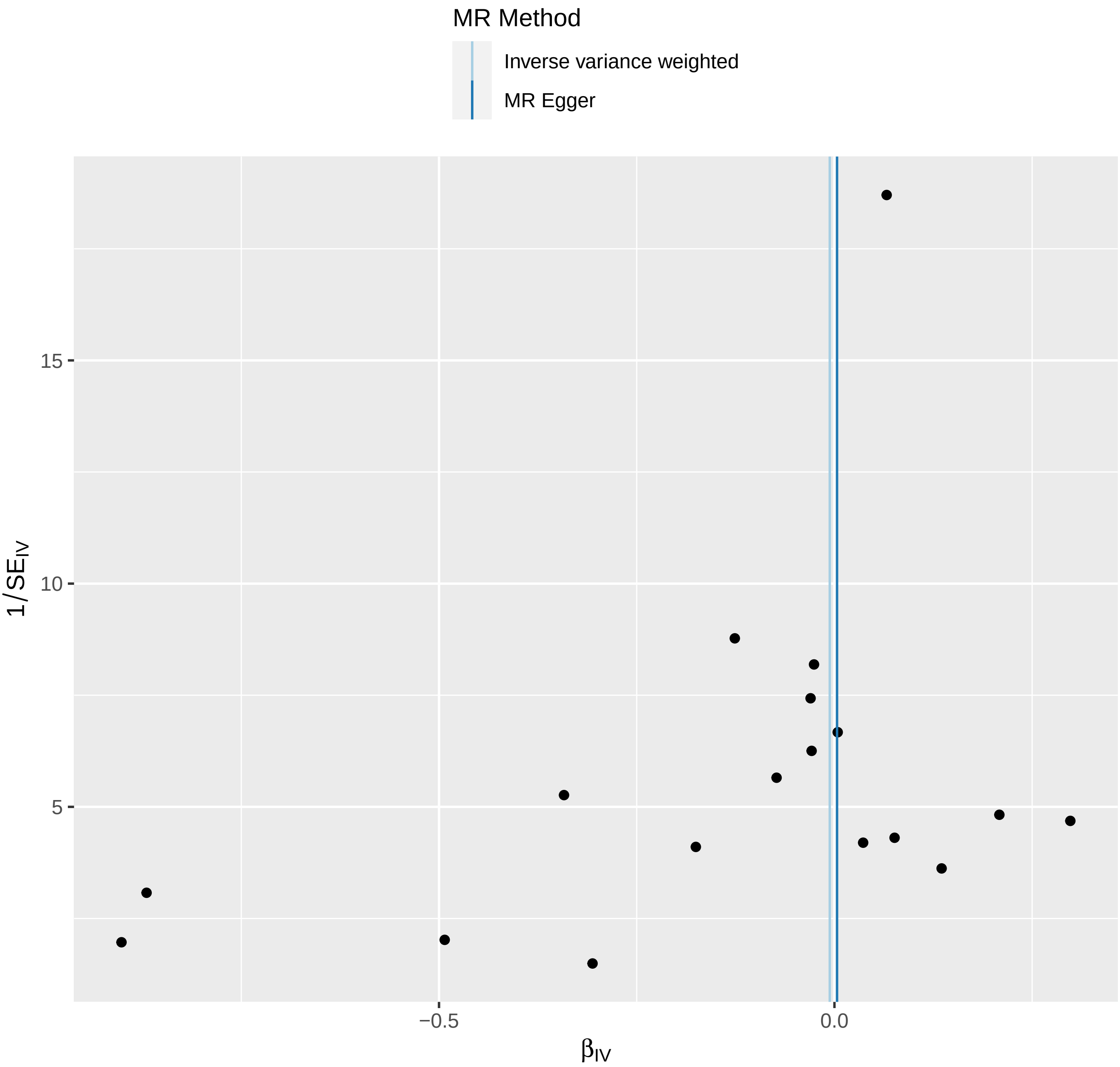

Batch 3−2587 : 60S acidic ribosomal protein P2 on Diabetic nephropathy (more control exclusions)
with a threshold of 1e−05

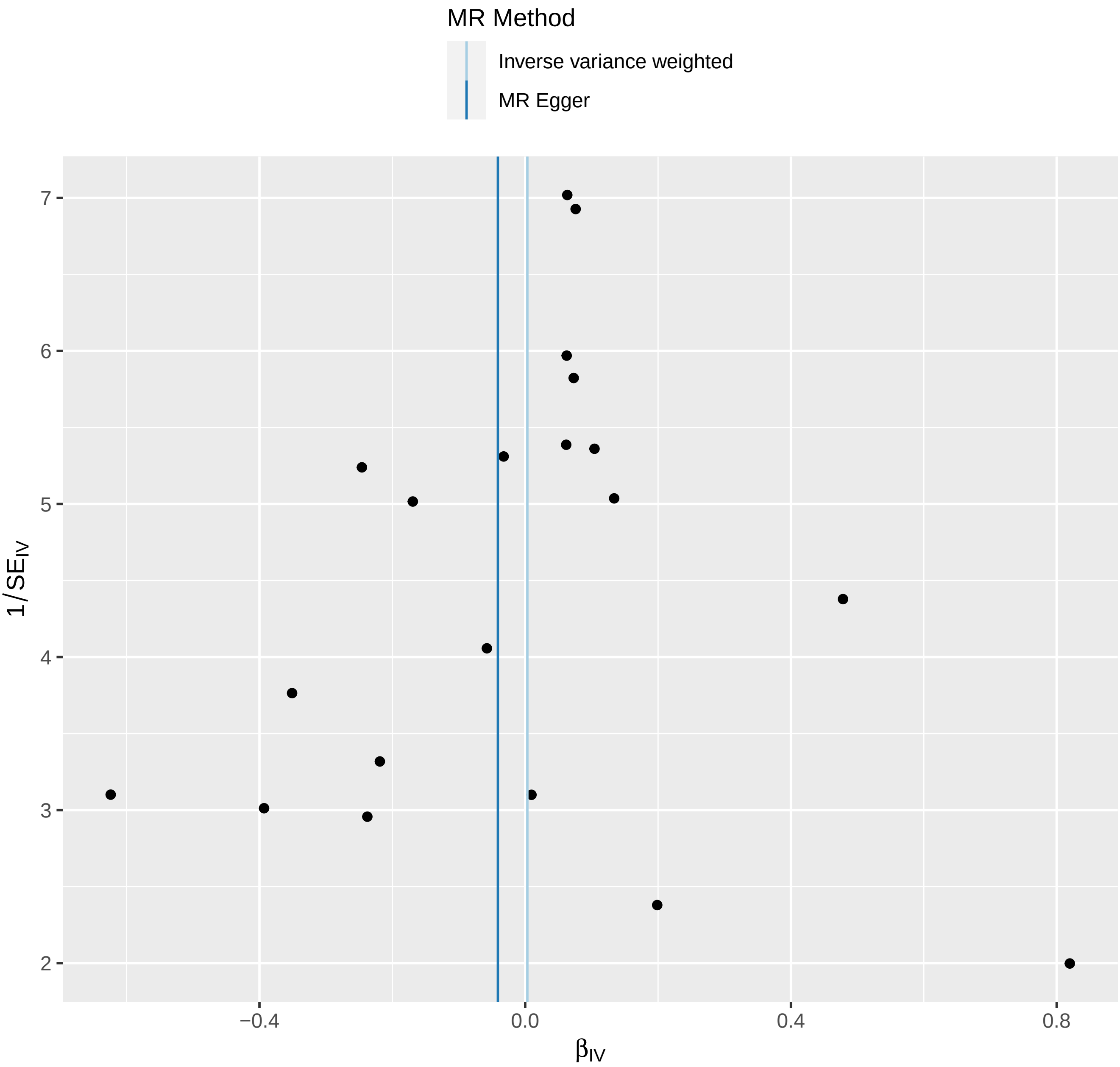

Batch 3−2588 : Dolichyl−diphosphooligosaccharide−−protein glycosyltransferase subunit 1 on Diabetic nephropathy (more control exclusions)
with a threshold of 1e−05

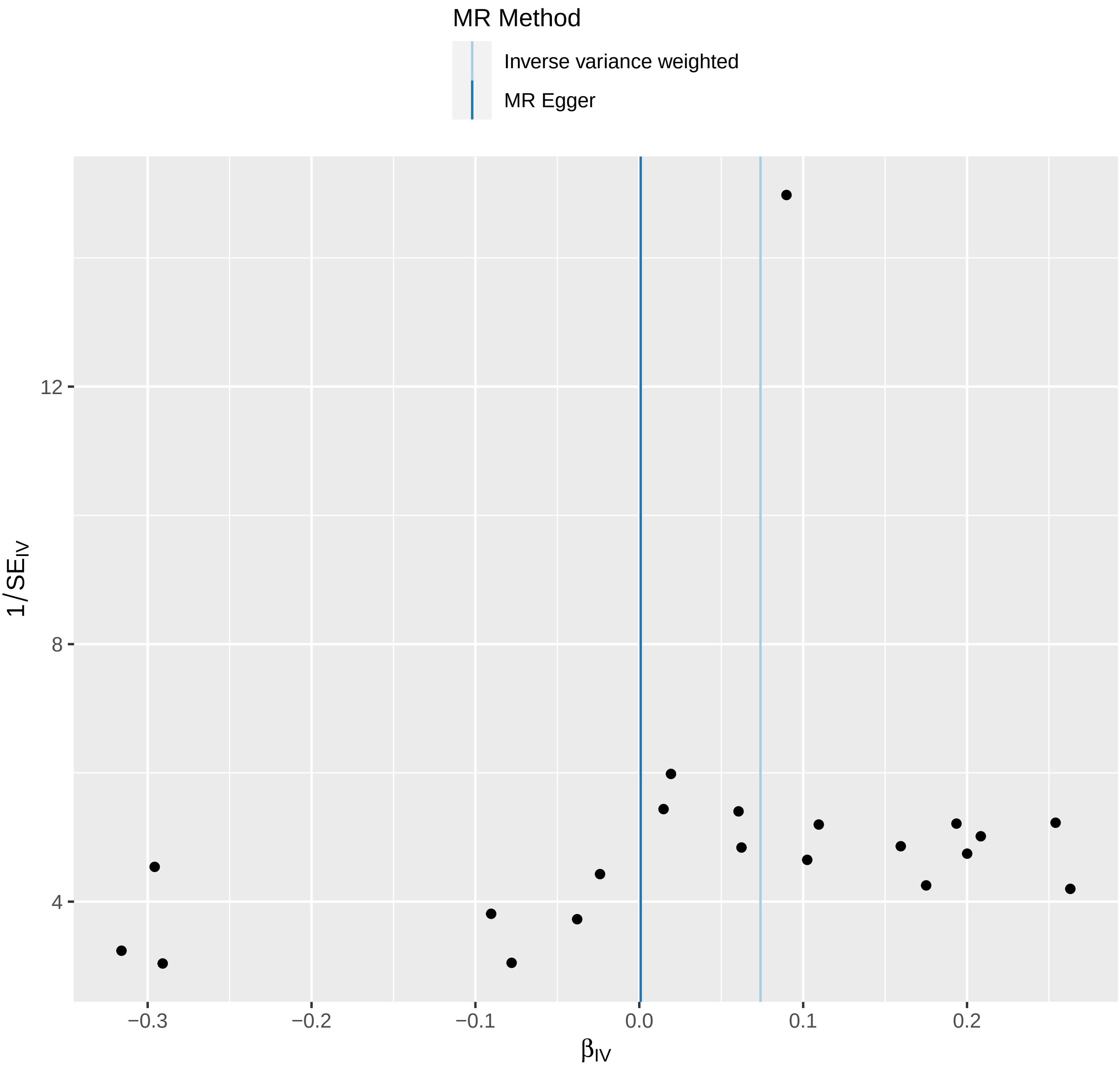

Batch 3−2589 : Dolichyl−diphosphooligosaccharide−−protein glycosyltransferase subunit 1 on Diabetic nephropathy (more control exclusions) with a threshold of 1e−05

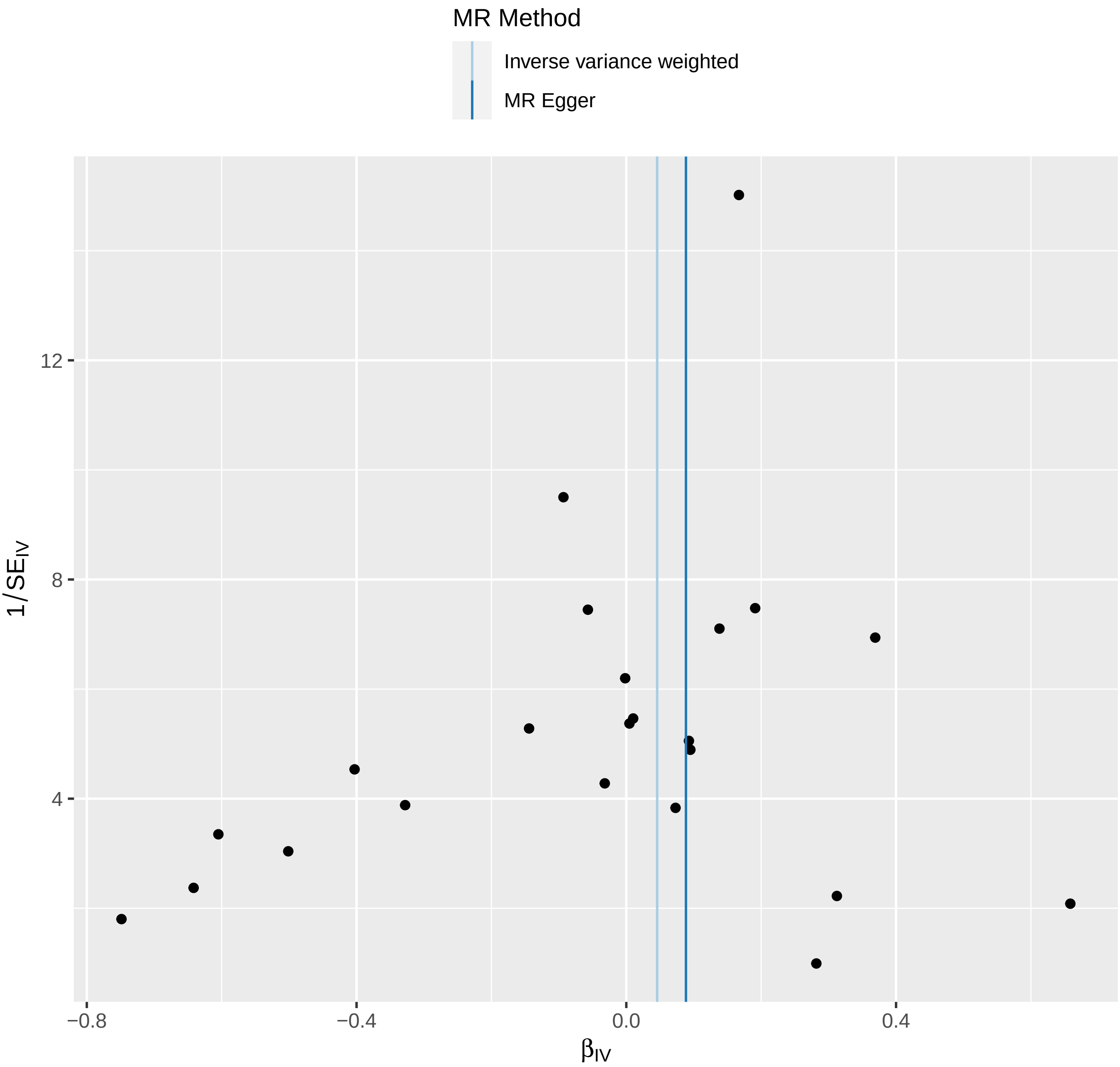

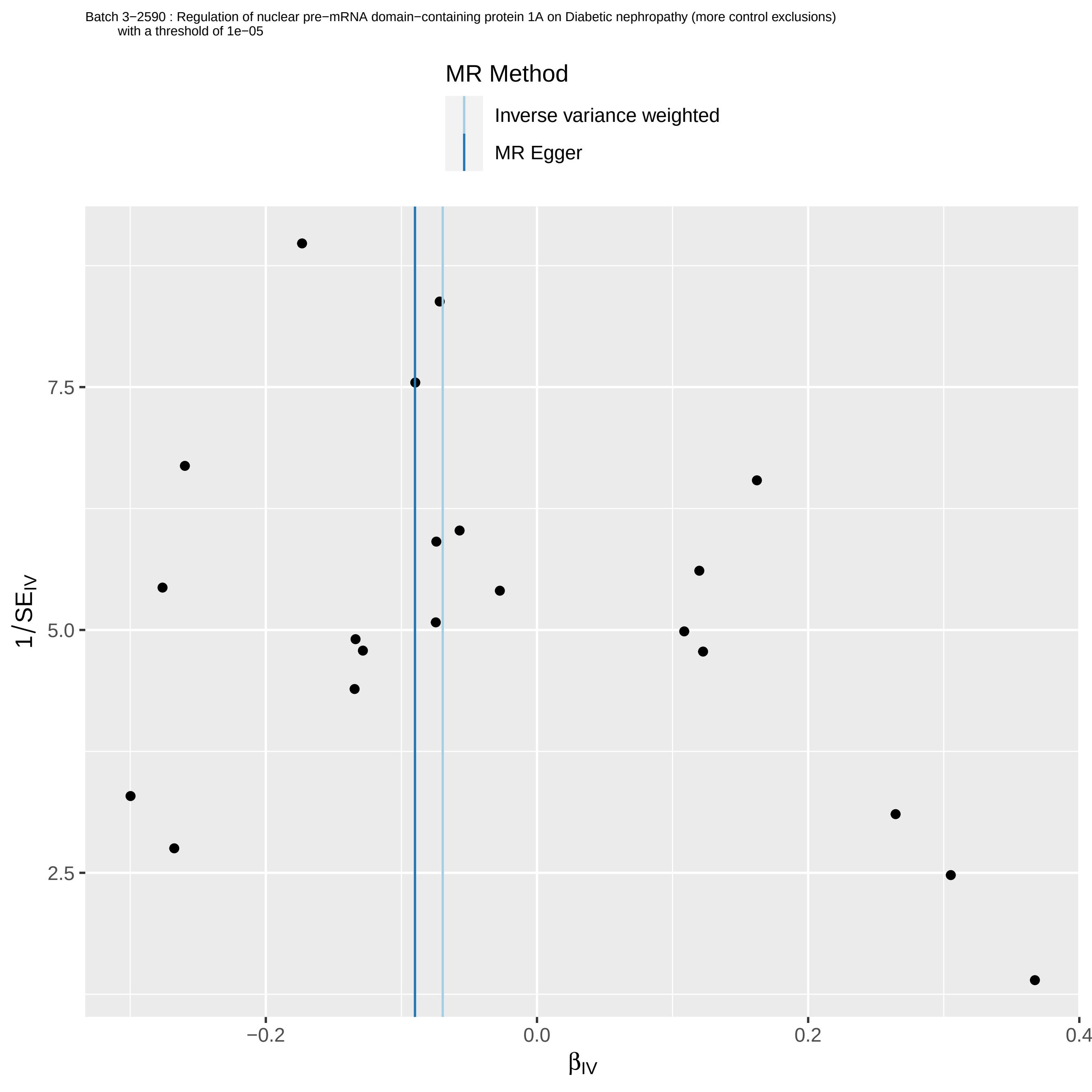
Batch 3−2590 : Regulation of nuclear pre−mRNA domain−containing protein 1A on Diabetic nephropathy (more control exclusions)
with a threshold of 1e−05
MR Method
Inverse variance weighted
MR Egger
7.5
5.0
2.5
1/SE_IV
−0.2
0.0
0.2
0.4
β_IV

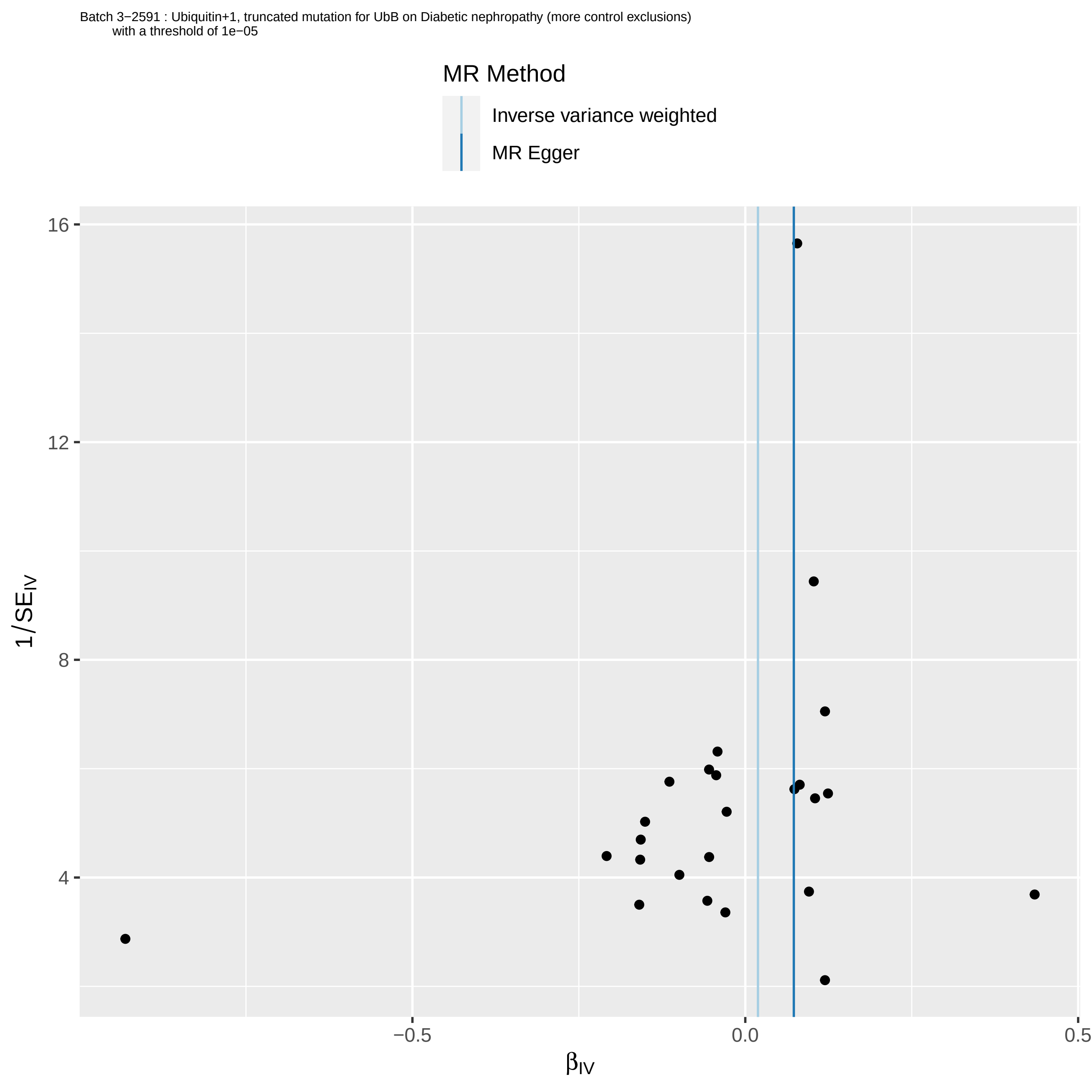
Batch 3−2591 : Ubiquitin+1, truncated mutation for UbB on Diabetic nephropathy (more control exclusions)
with a threshold of 1e−05
MR Method
Inverse variance weighted
MR Egger
16
12
8
4
1/SE$_{IV}$
−0.5
0.0
0.5
β$_{IV}$

Batch 3−2592 : Ubiquitin on Diabetic nephropathy (more control exclusions)
with a threshold of 1e−05

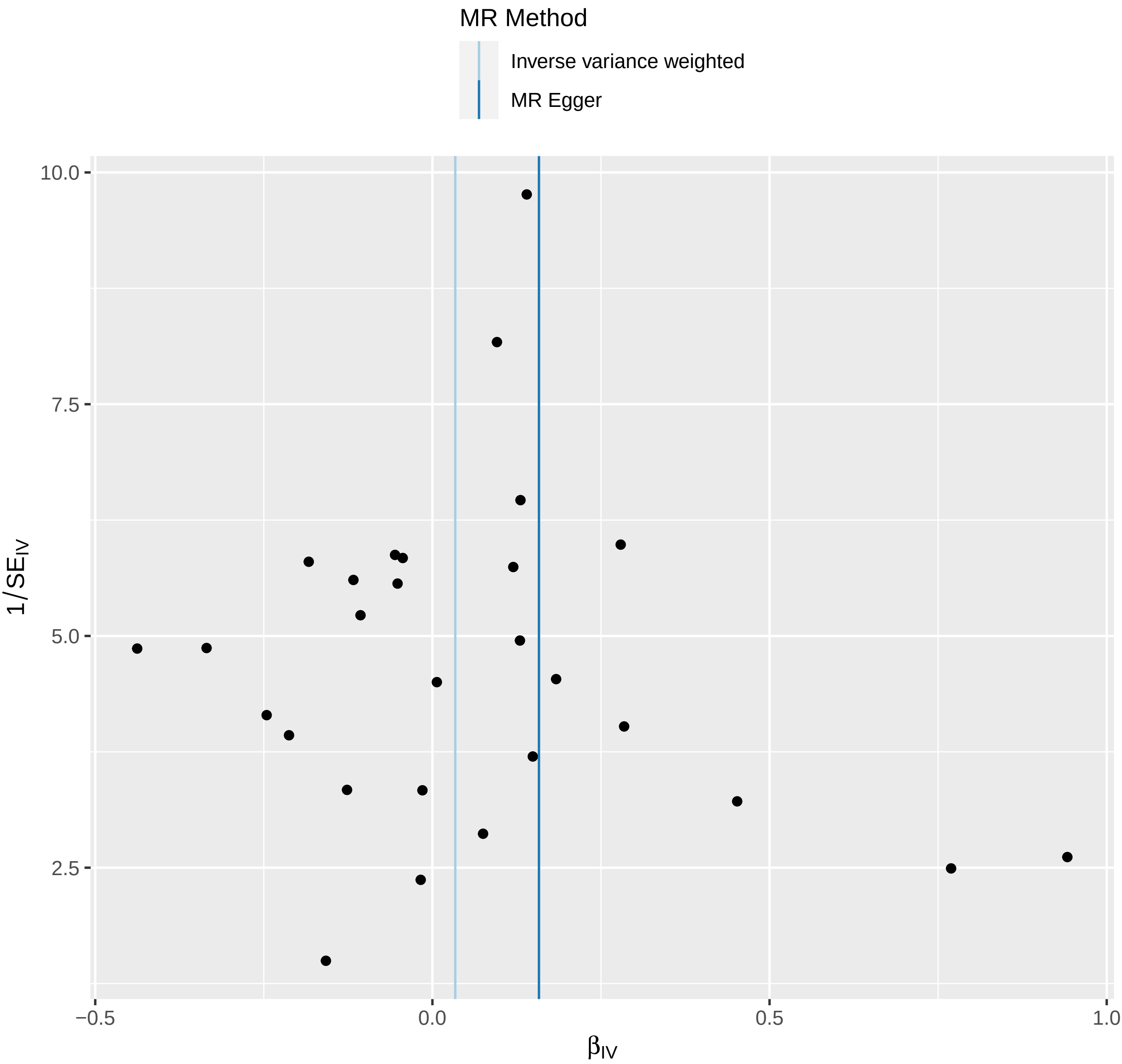

Batch 3−2593 : 40S ribosomal protein S3 on Diabetic nephropathy (more control exclusions)
with a threshold of 1e−05

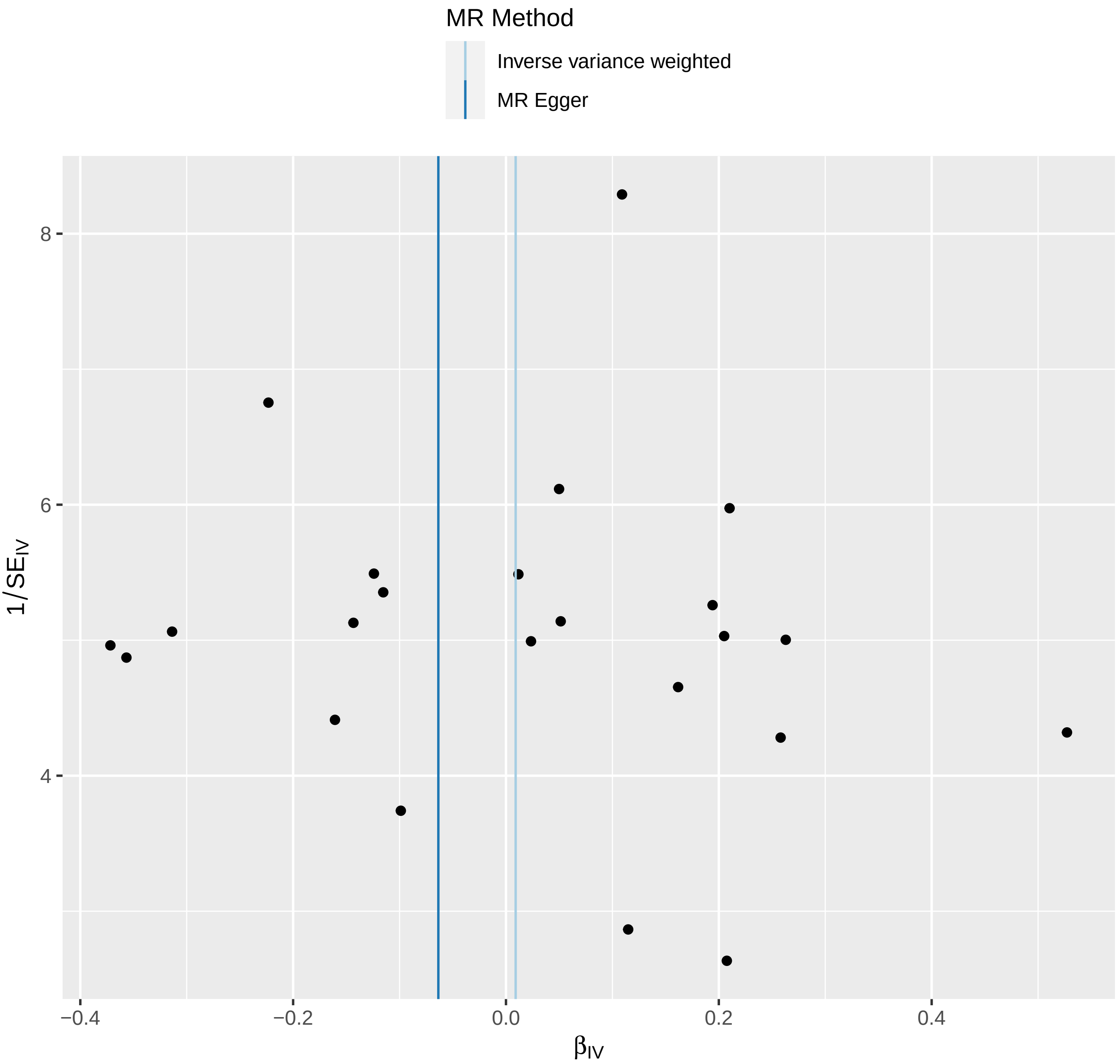

Batch 3−2594 : 40S ribosomal protein S3a on Diabetic nephropathy (more control exclusions)
with a threshold of 1e−05

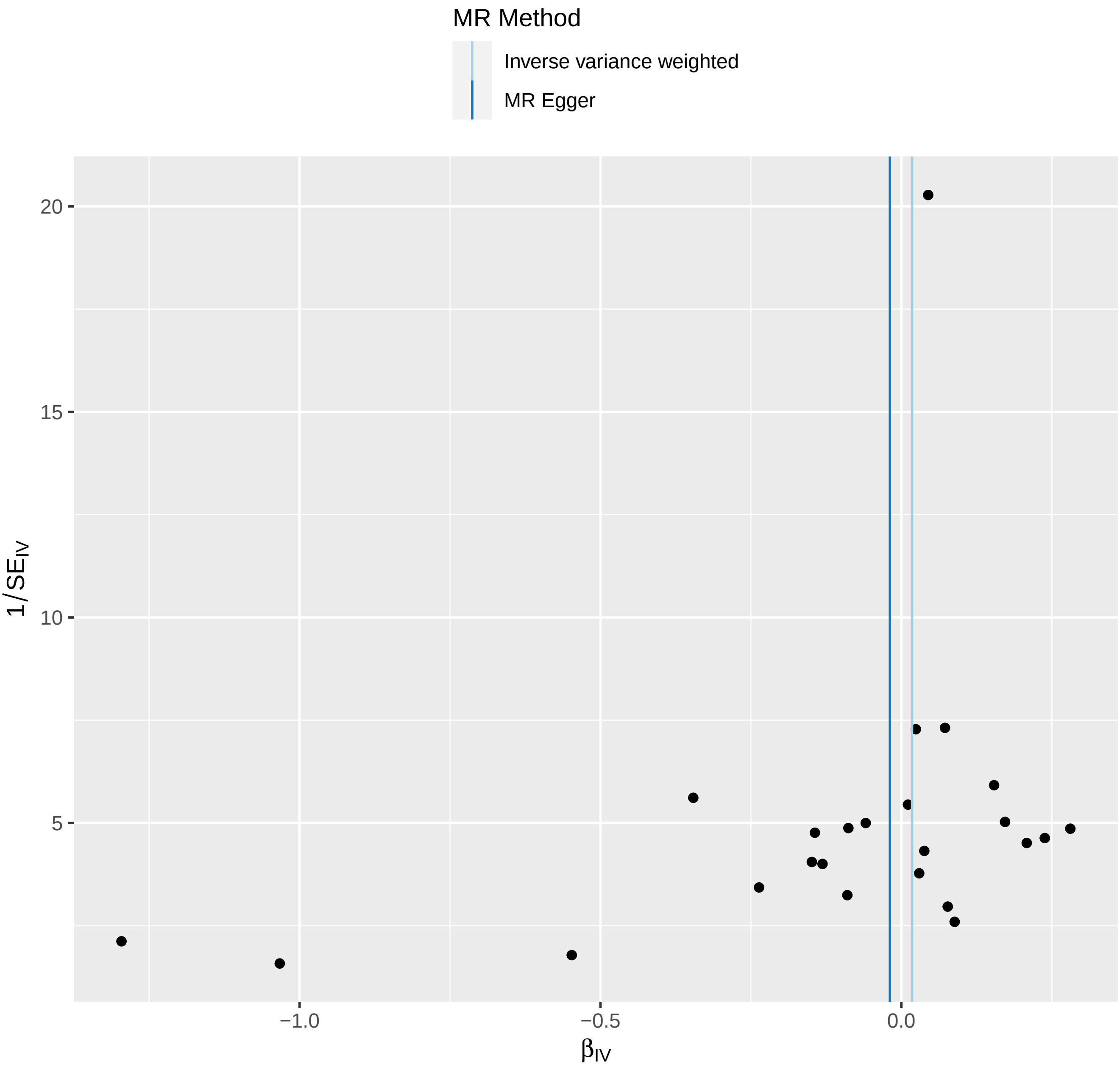

Batch 3−2595 : 40S ribosomal protein S4, X isoform on Diabetic nephropathy (more control exclusions)
with a threshold of 1e−05

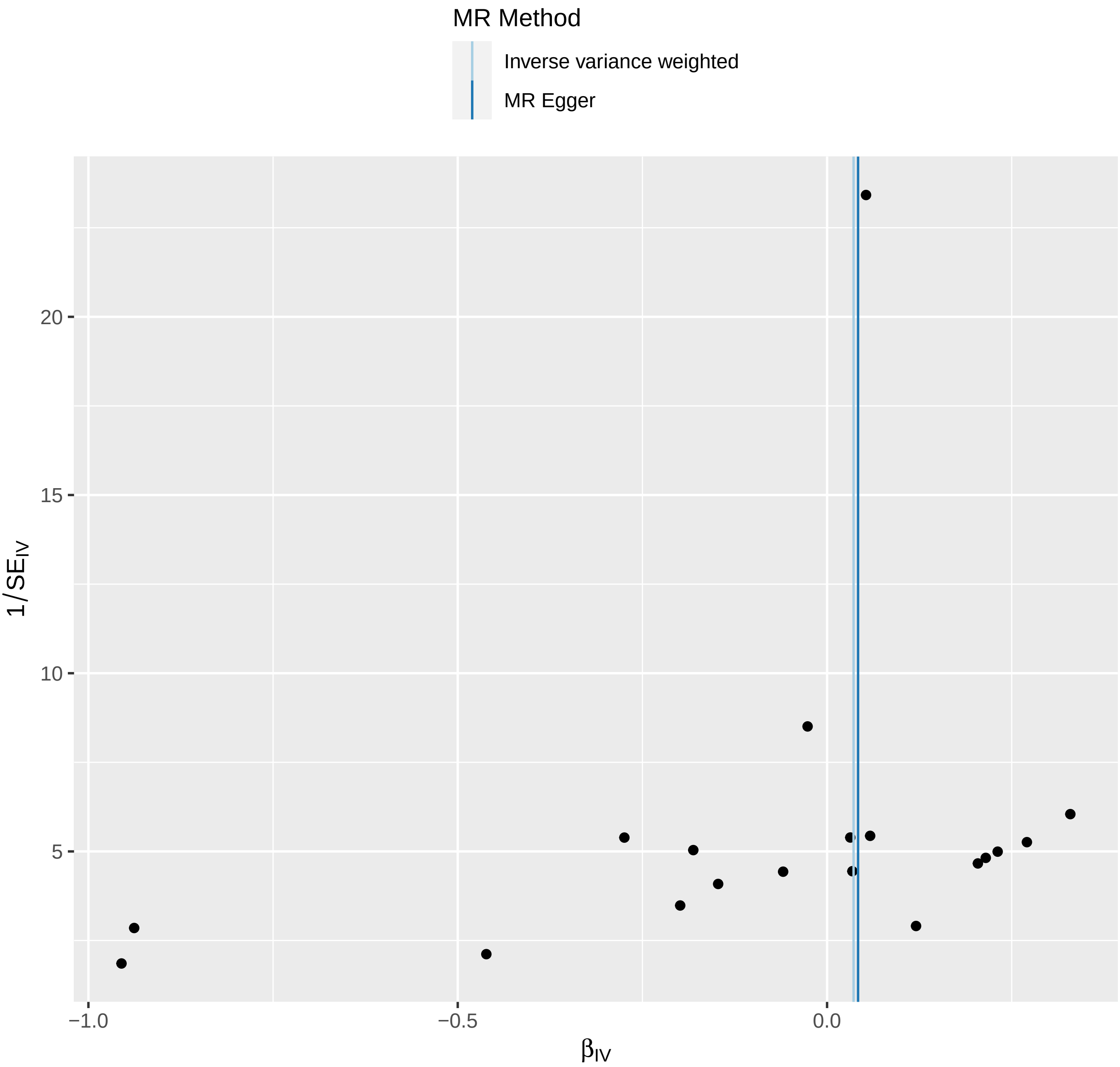

Batch 3−2596 : Ribosomal protein S6 kinase alpha−6 on Diabetic nephropathy (more control exclusions)
with a threshold of 1e−05

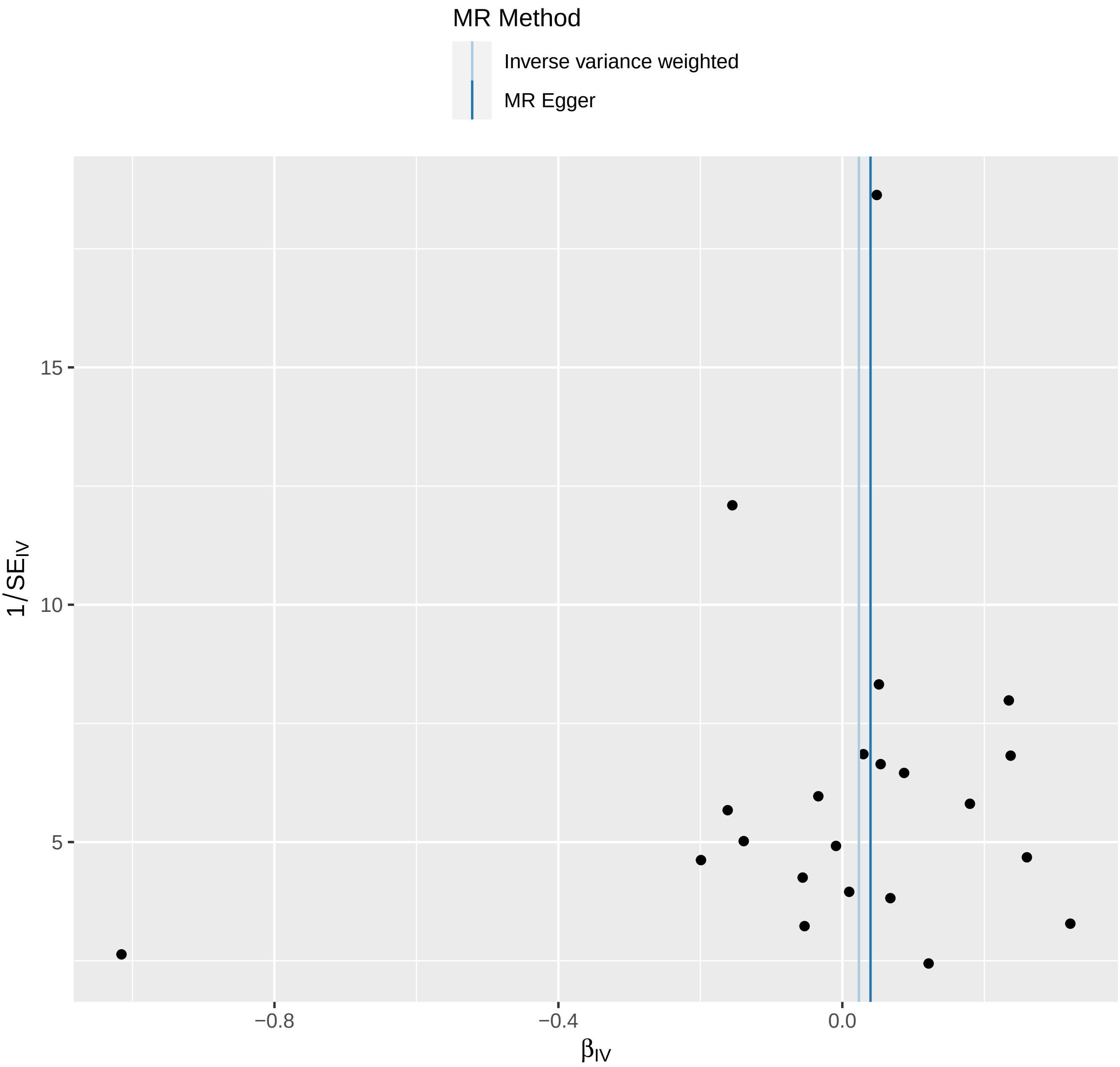

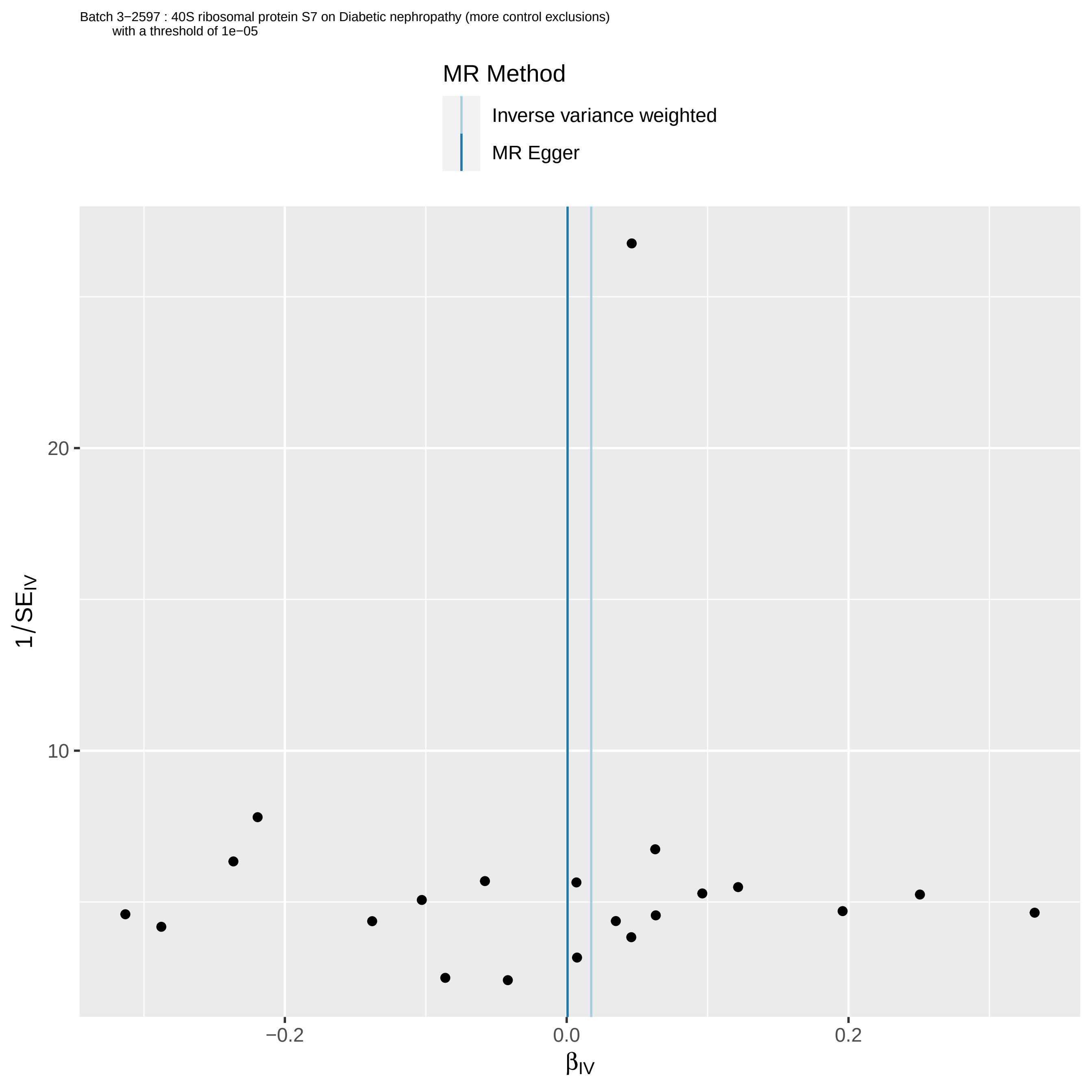
Batch 3−2597 : 40S ribosomal protein S7 on Diabetic nephropathy (more control exclusions)
with a threshold of 1e−05
MR Method
Inverse variance weighted
MR Egger
20
10
1/SE_IV
−0.2
0.0
0.2
β_IV

Batch 3−2598 : 40S ribosomal protein SA on Diabetic nephropathy (more control exclusions)
with a threshold of 1e−05

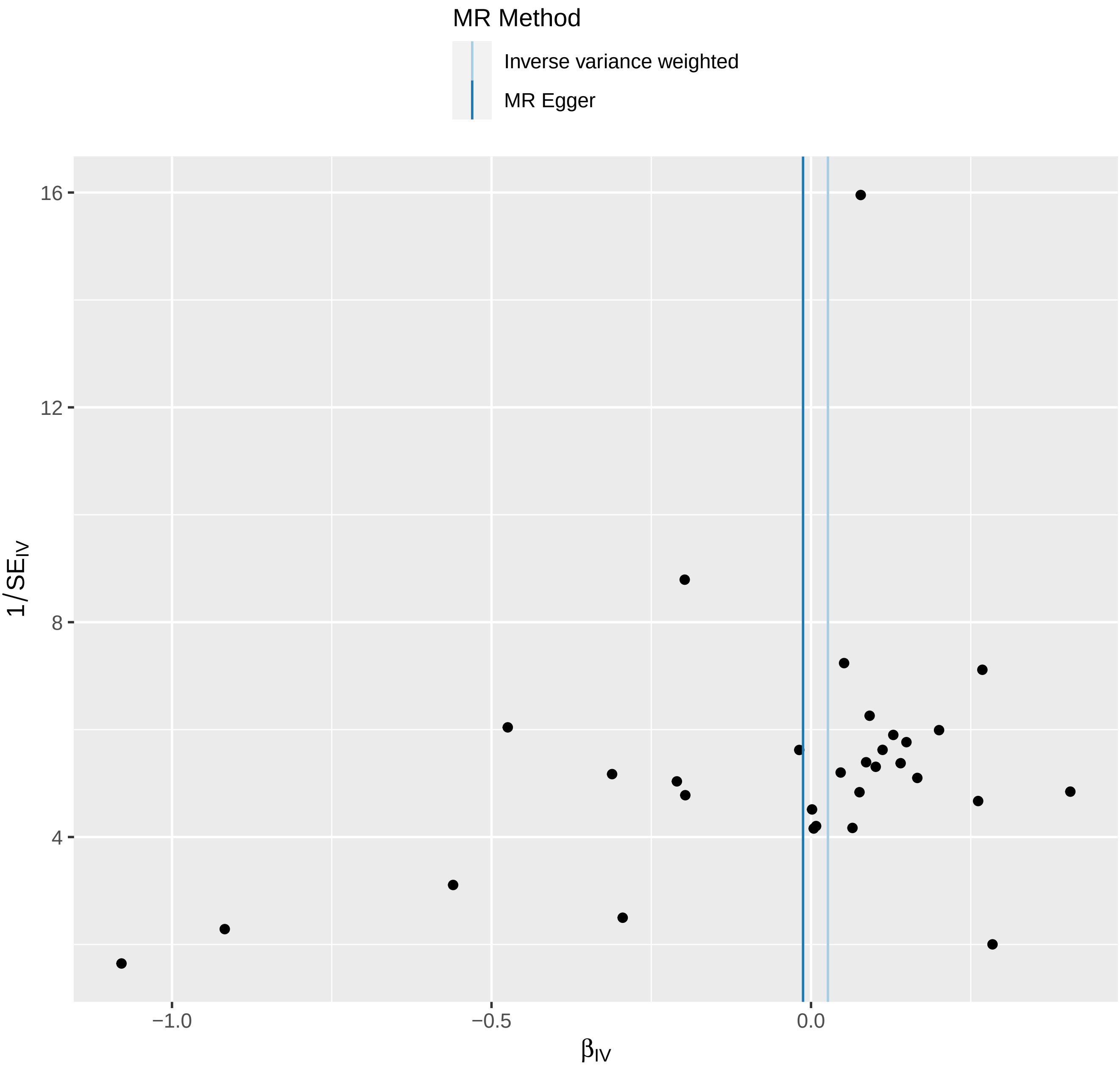

Batch 3−2599 : Cell differentiation protein RCD1 homolog on Diabetic nephropathy (more control exclusions)
with a threshold of 1e−05

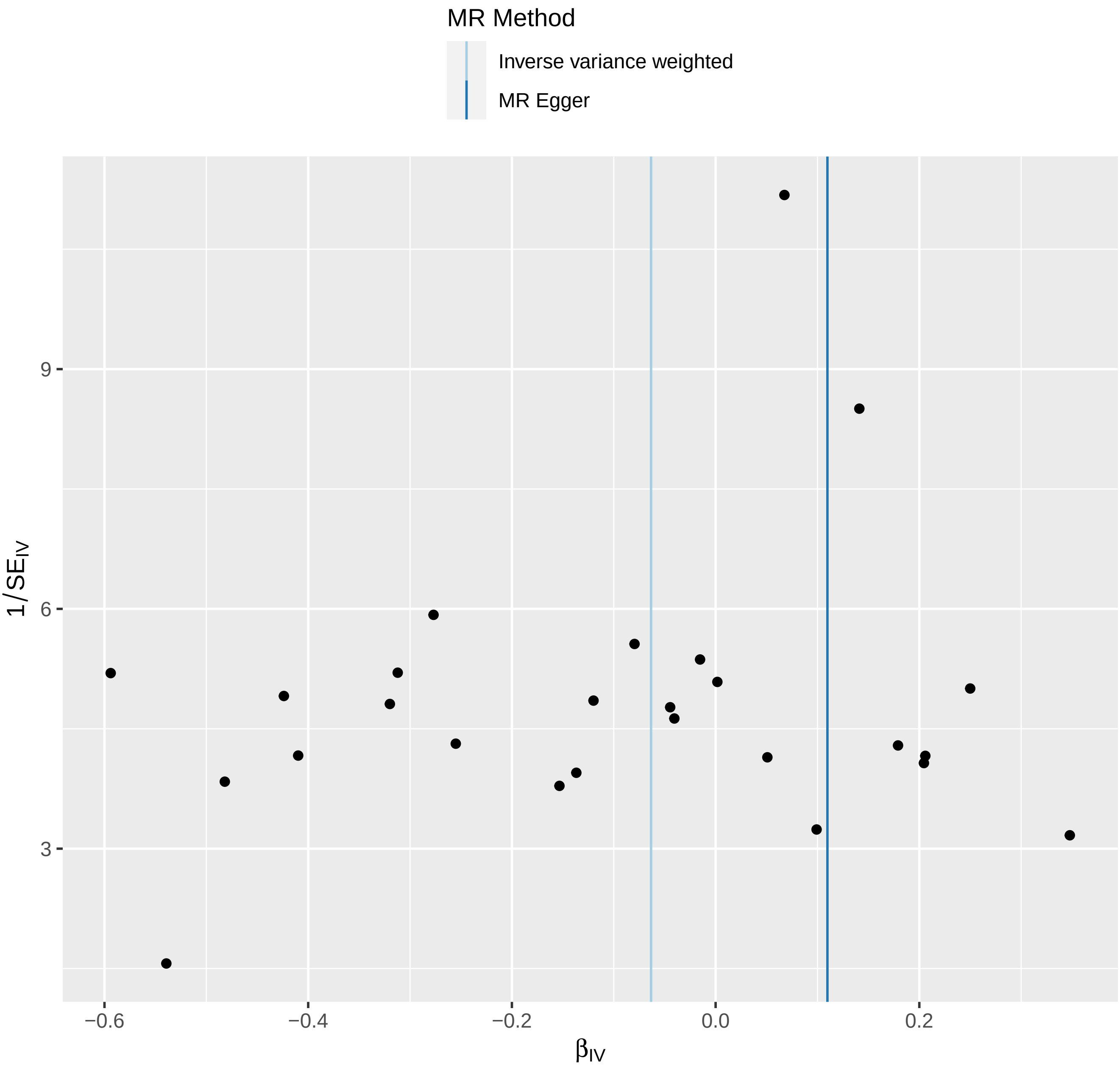

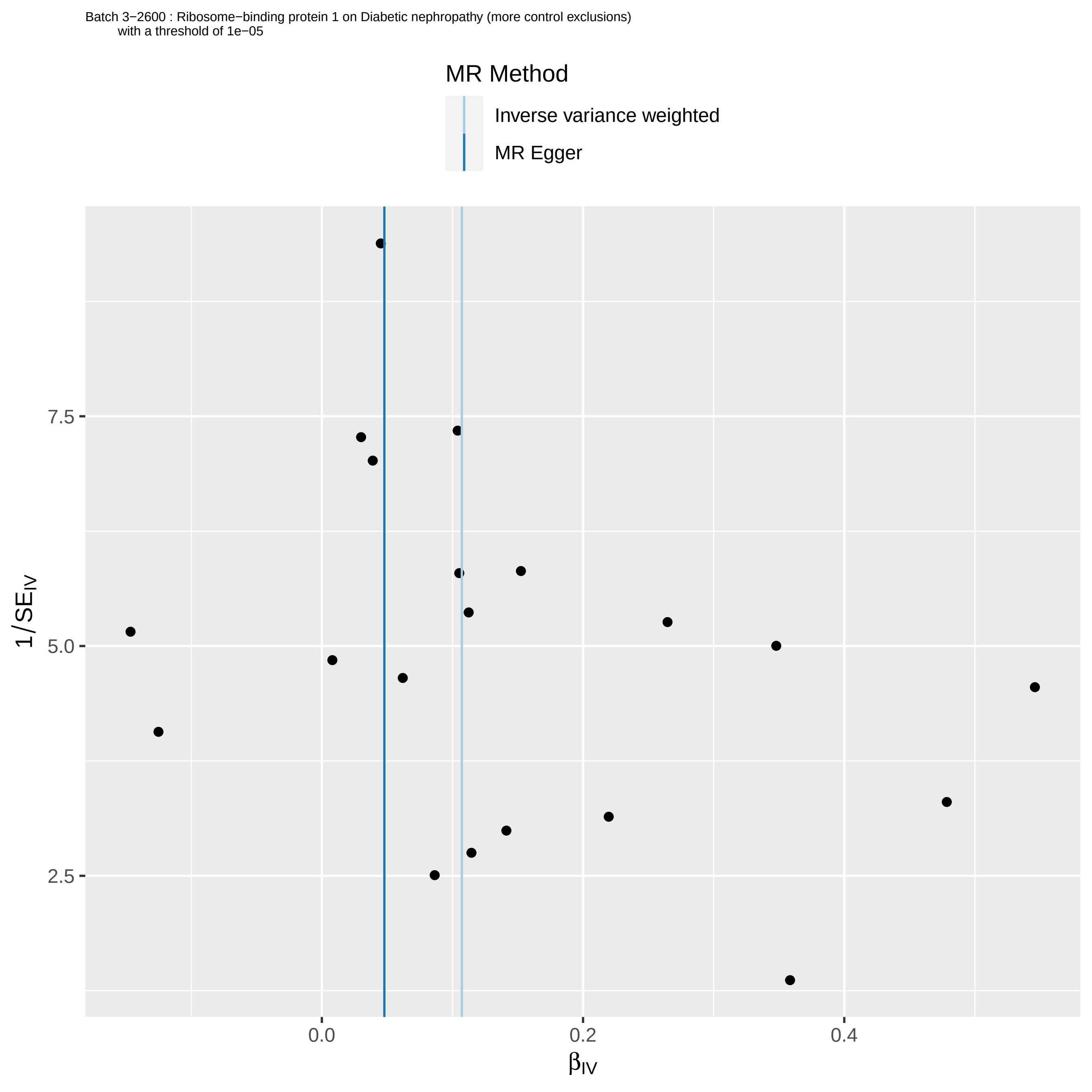

Batch 3−2600 : Ribosome−binding protein 1 on Diabetic nephropathy (more control exclusions)
with a threshold of 1e−05
MR Method
Inverse variance weighted
MR Egger
7.5
5.0
2.5
$1/SE_{IV}$
0.0
0.2
0.4
β_{IV}

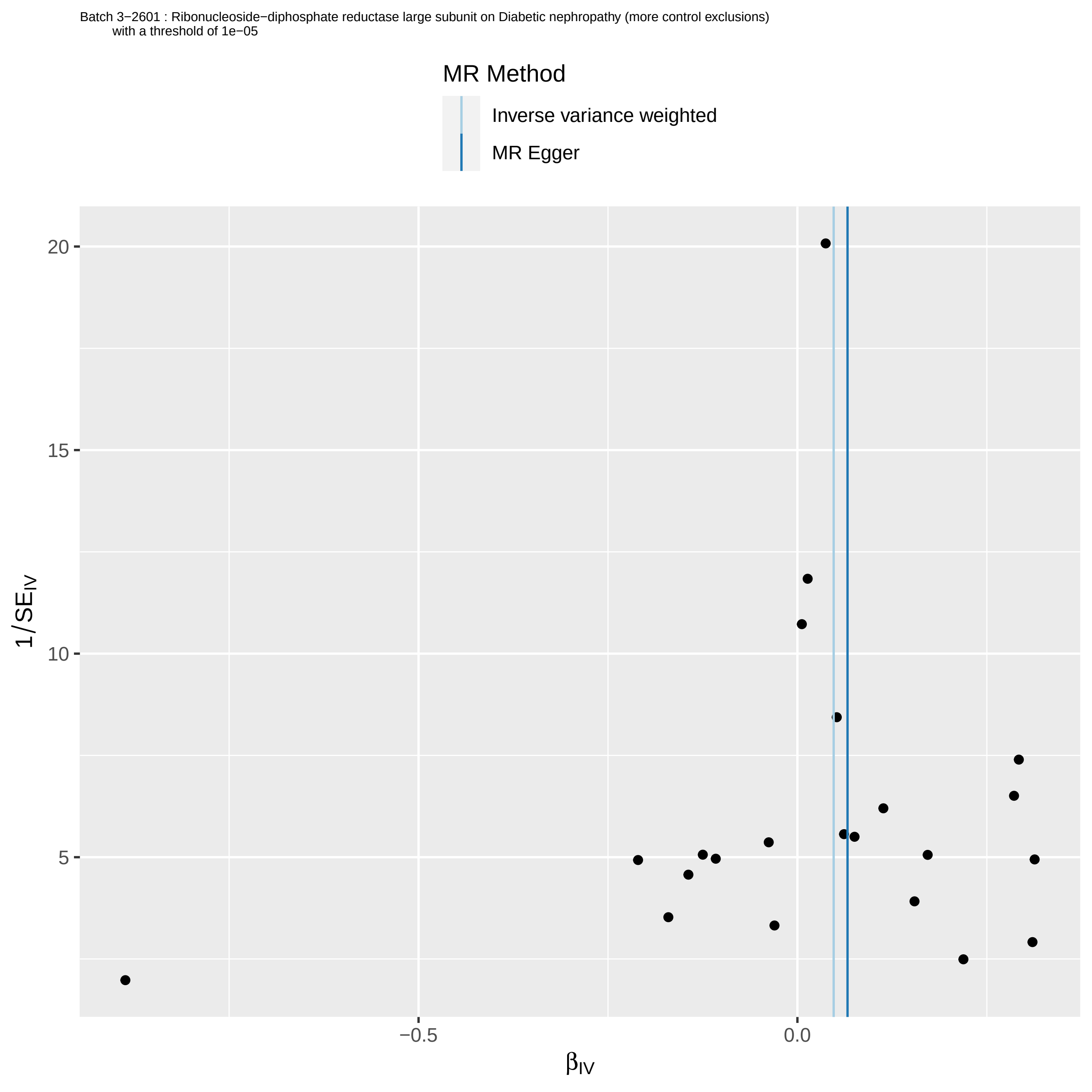

Batch 3−2601 : Ribonucleoside−diphosphate reductase large subunit on Diabetic nephropathy (more control exclusions)
with a threshold of 1e−05
MR Method
Inverse variance weighted
MR Egger
20
15
10
5
1/SE$_{IV}$
−0.5
0.0
β$_{IV}$

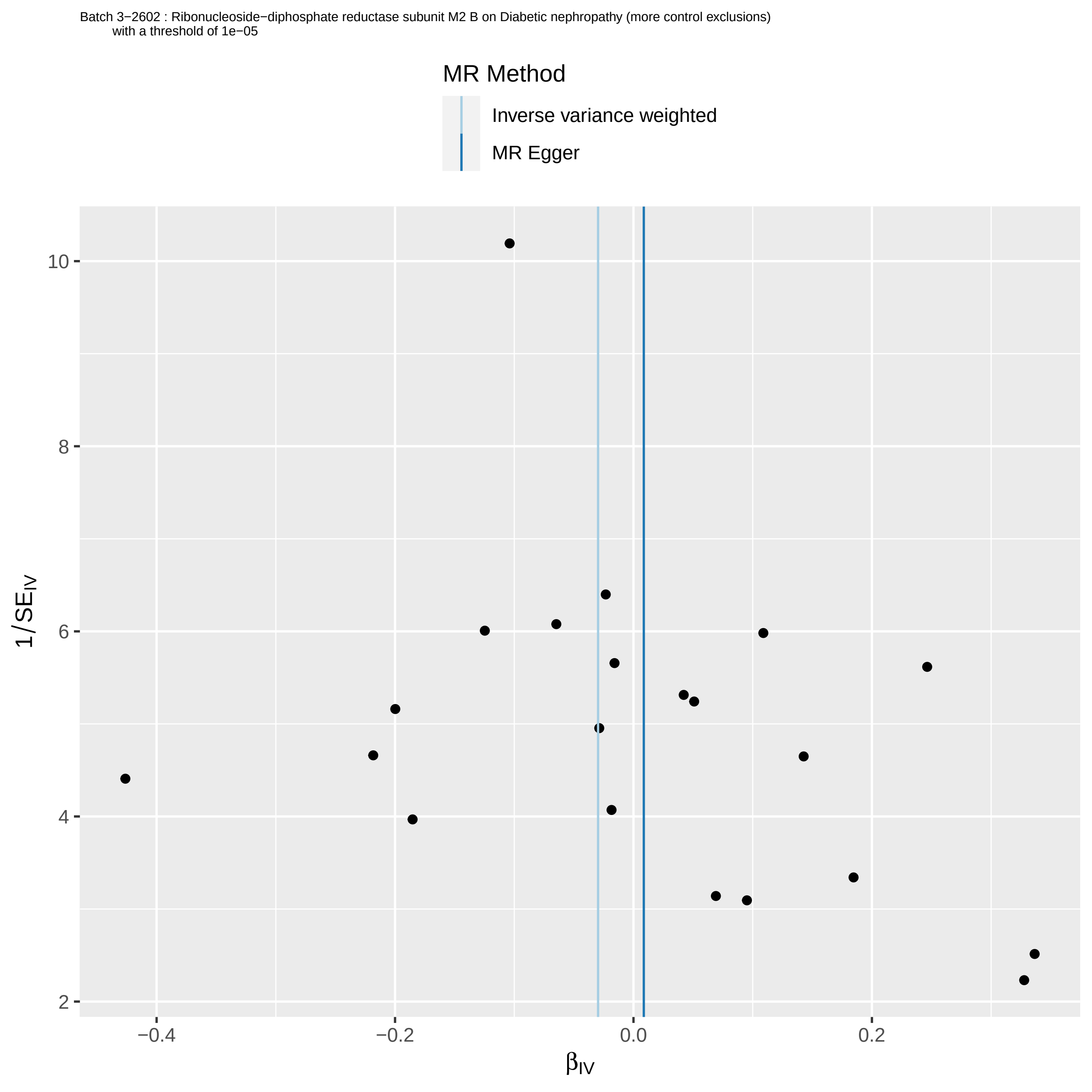
Batch 3−2602 : Ribonucleoside−diphosphate reductase subunit M2 B on Diabetic nephropathy (more control exclusions)
with a threshold of 1e−05
MR Method
Inverse variance weighted
MR Egger
10
8
6
4
2
$1/SE_{IV}$
−0.4
−0.2
0.0
0.2
β_{IV}

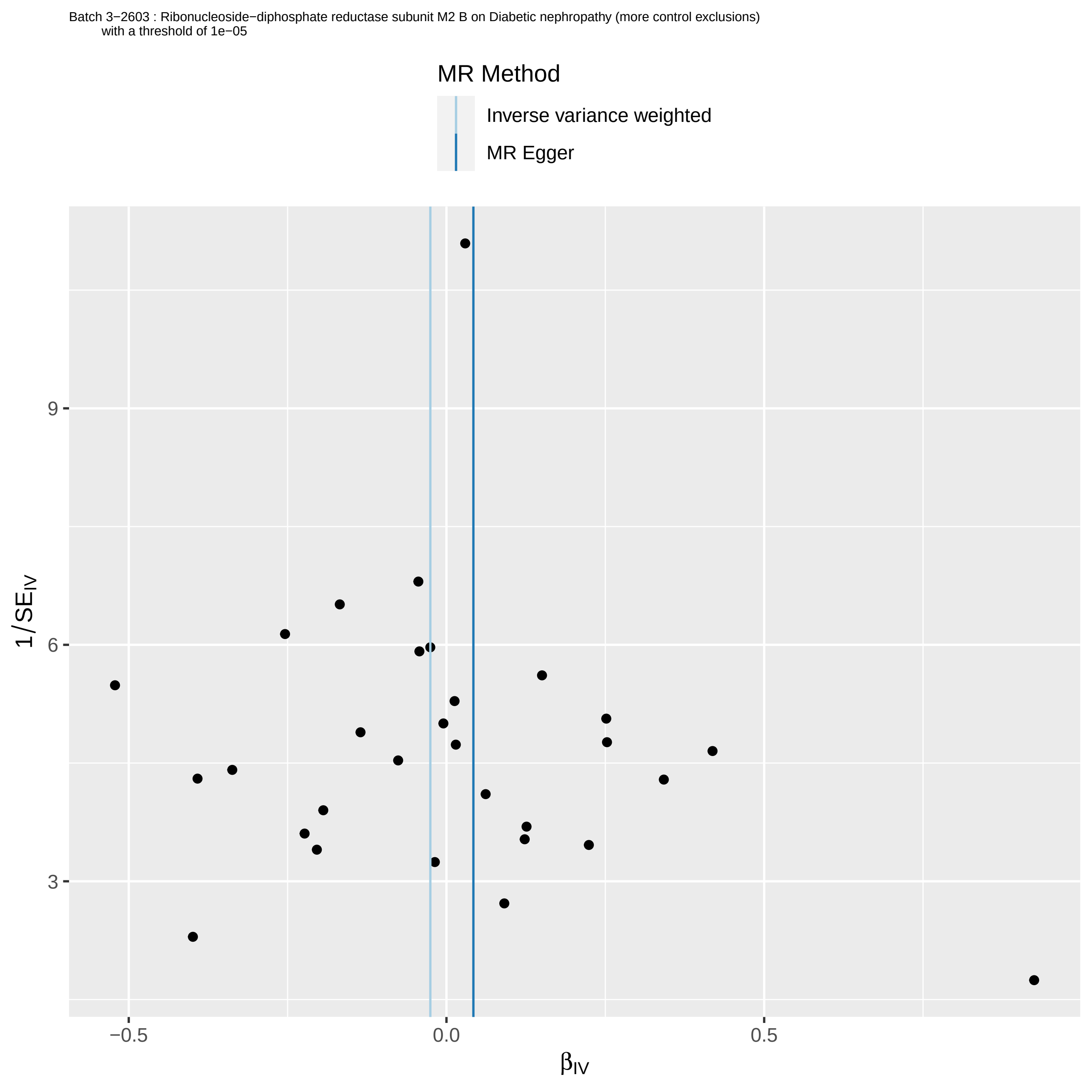
Batch 3−2603 : Ribonucleoside−diphosphate reductase subunit M2 B on Diabetic nephropathy (more control exclusions) with a threshold of 1e−05
MR Method
Inverse variance weighted
MR Egger
1/SE_IV
9
6
3
−0.5
0.0
0.5
β_IV

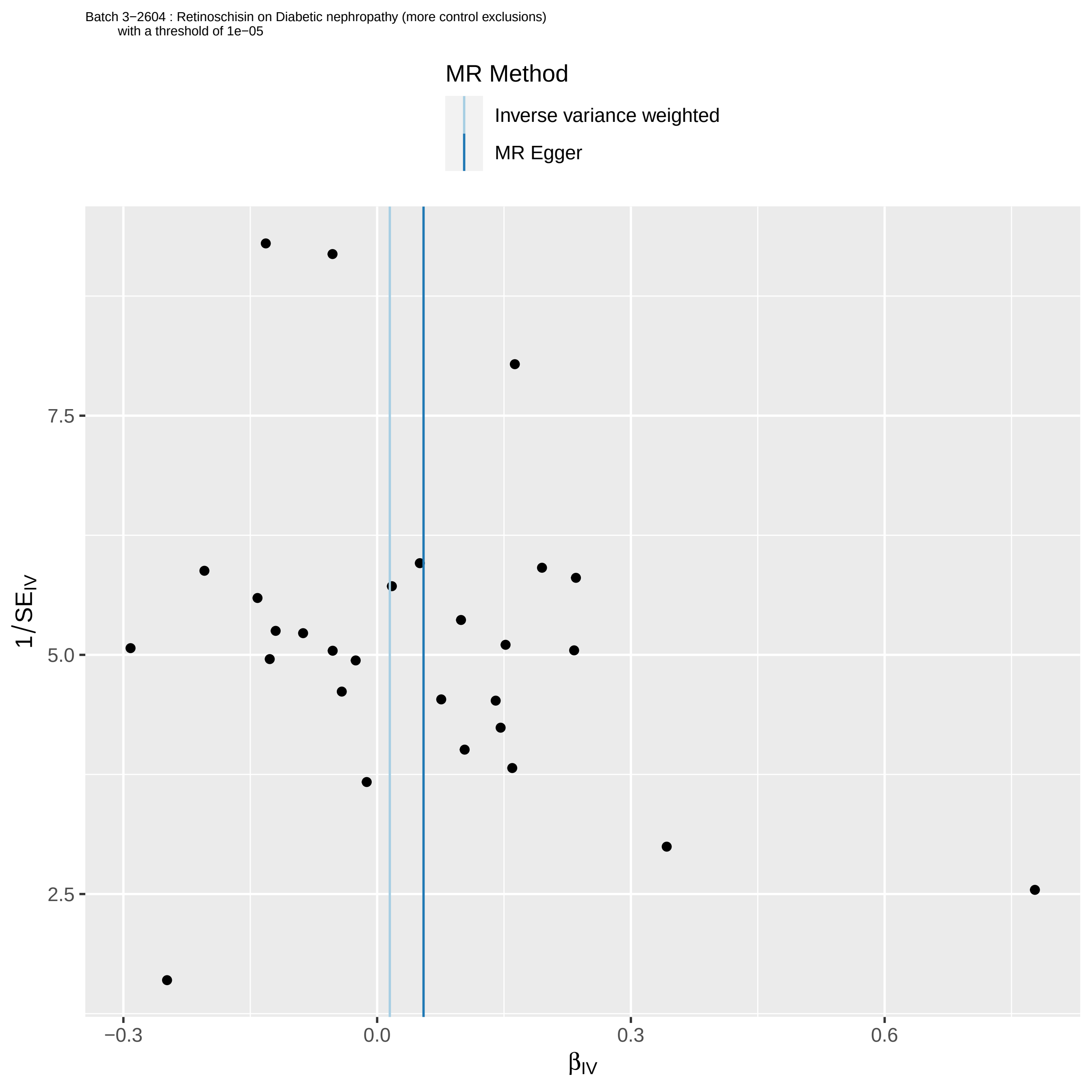

Batch 3−2604 : Retinoschisin on Diabetic nephropathy (more control exclusions)
with a threshold of 1e−05
MR Method
Inverse variance weighted
MR Egger
7.5
5.0
2.5
1/SE_IV
−0.3
0.0
0.3
0.6
β_IV

Batch 3−2605 : R−spondin−3 on Diabetic nephropathy (more control exclusions)
with a threshold of 1e−05

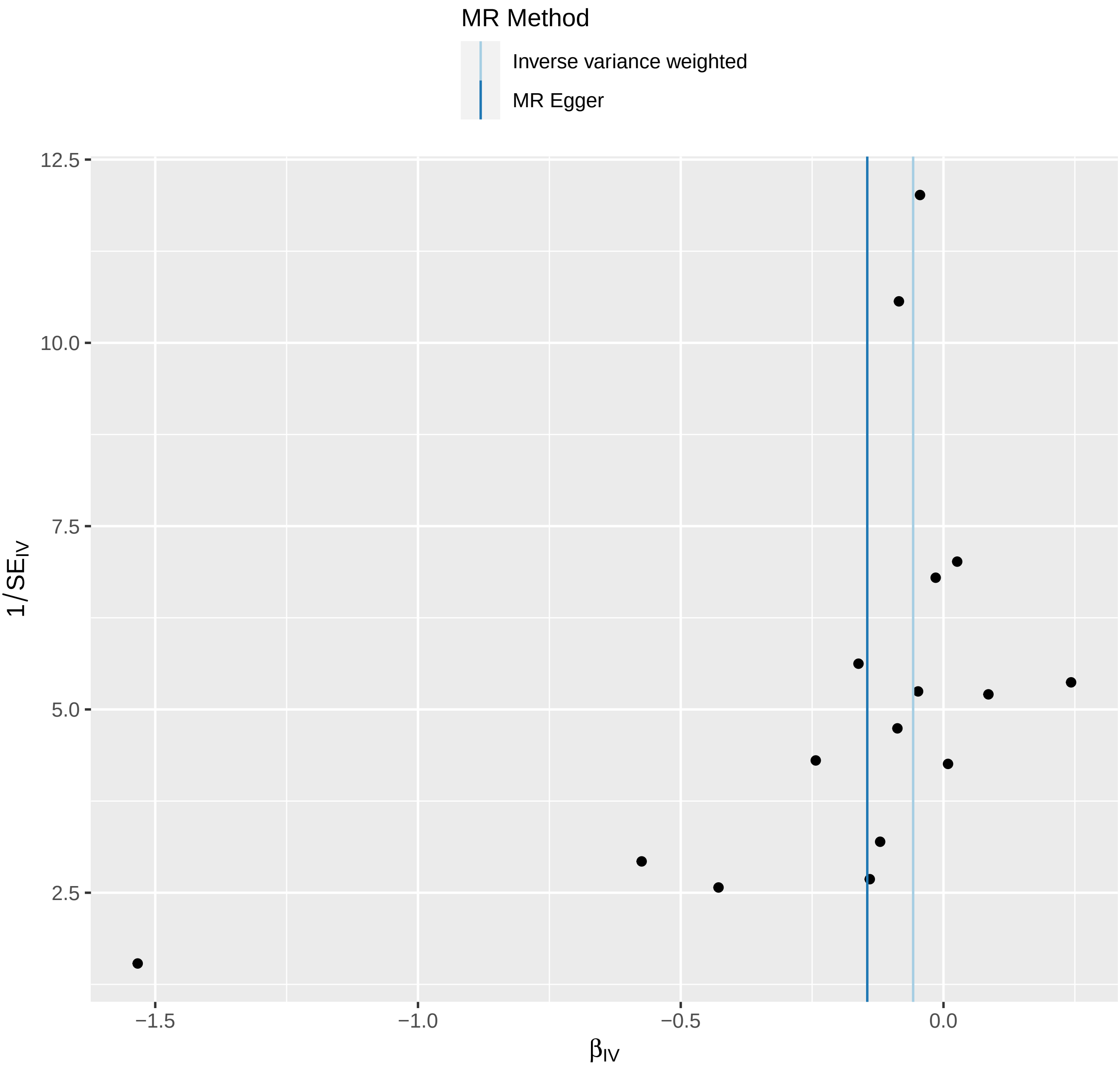

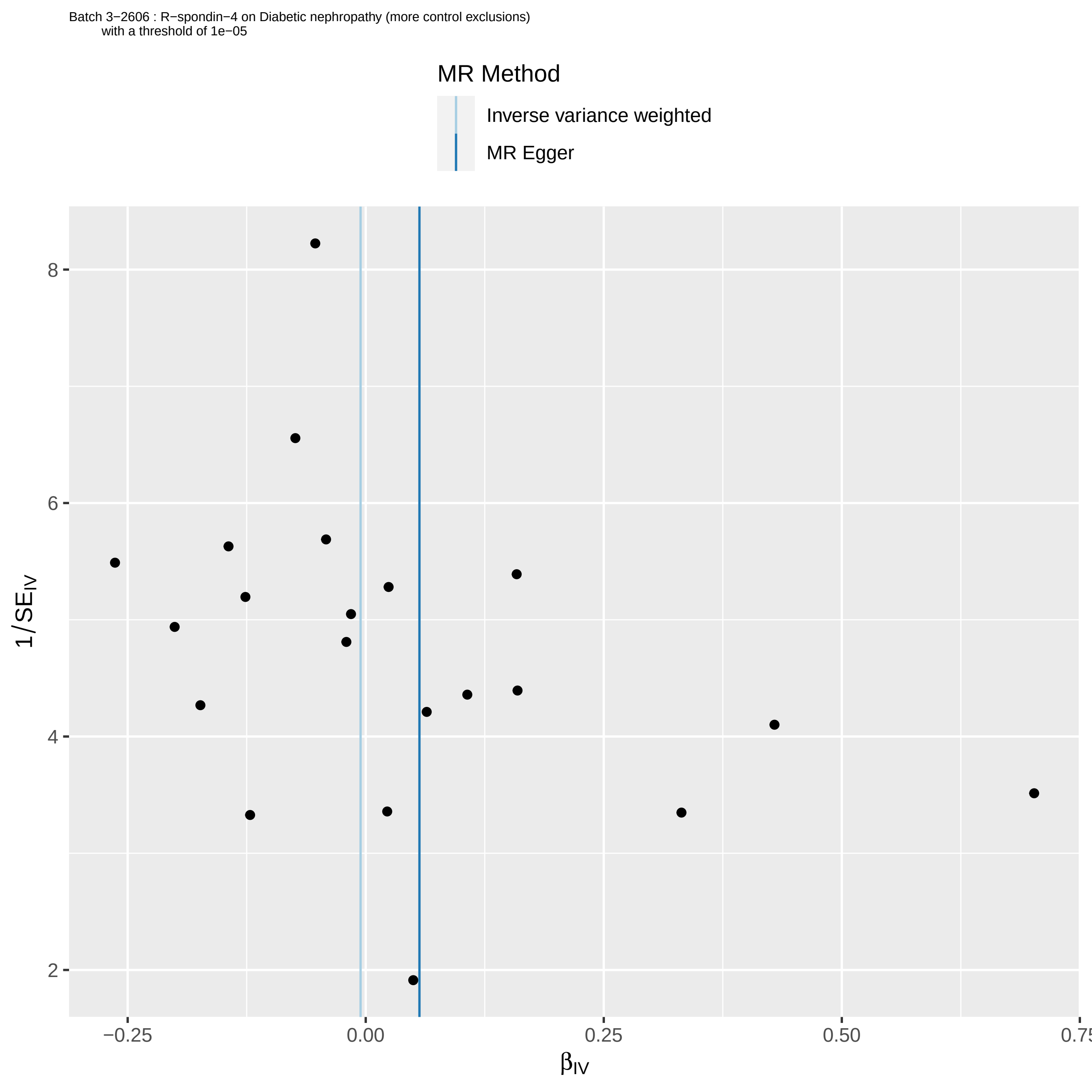

Batch 3−2606 : R−spondin−4 on Diabetic nephropathy (more control exclusions)
with a threshold of 1e−05
MR Method
Inverse variance weighted
MR Egger
8
6
4
2
1/SE_IV
−0.25
0.00
0.25
0.50
0.75
β_IV

Batch 3−2607 : Arginine/serine−rich protein 1 on Diabetic nephropathy (more control exclusions)
with a threshold of 1e−05

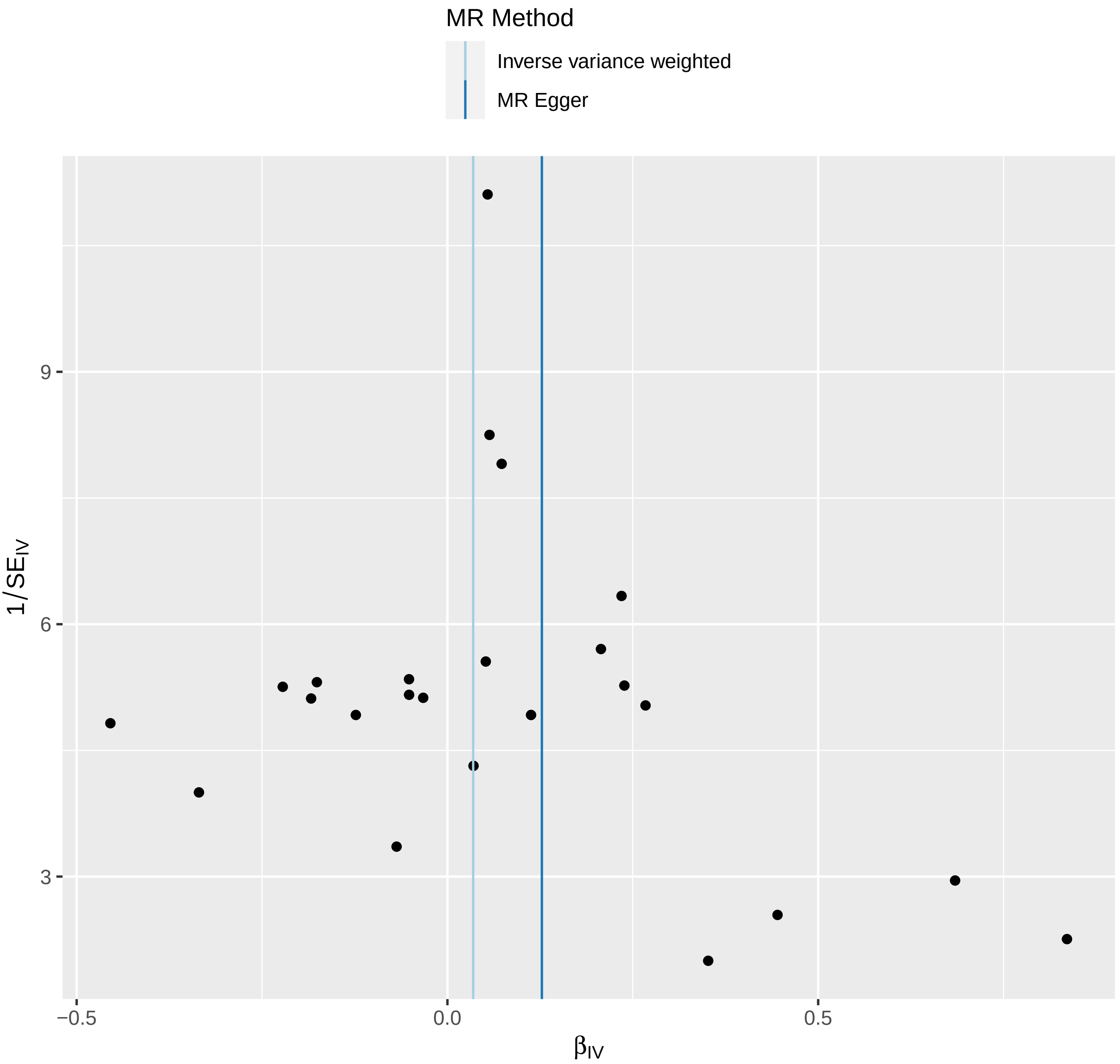

Batch 3−2608 : Retbindin on Diabetic nephropathy (more control exclusions)
with a threshold of 1e−05

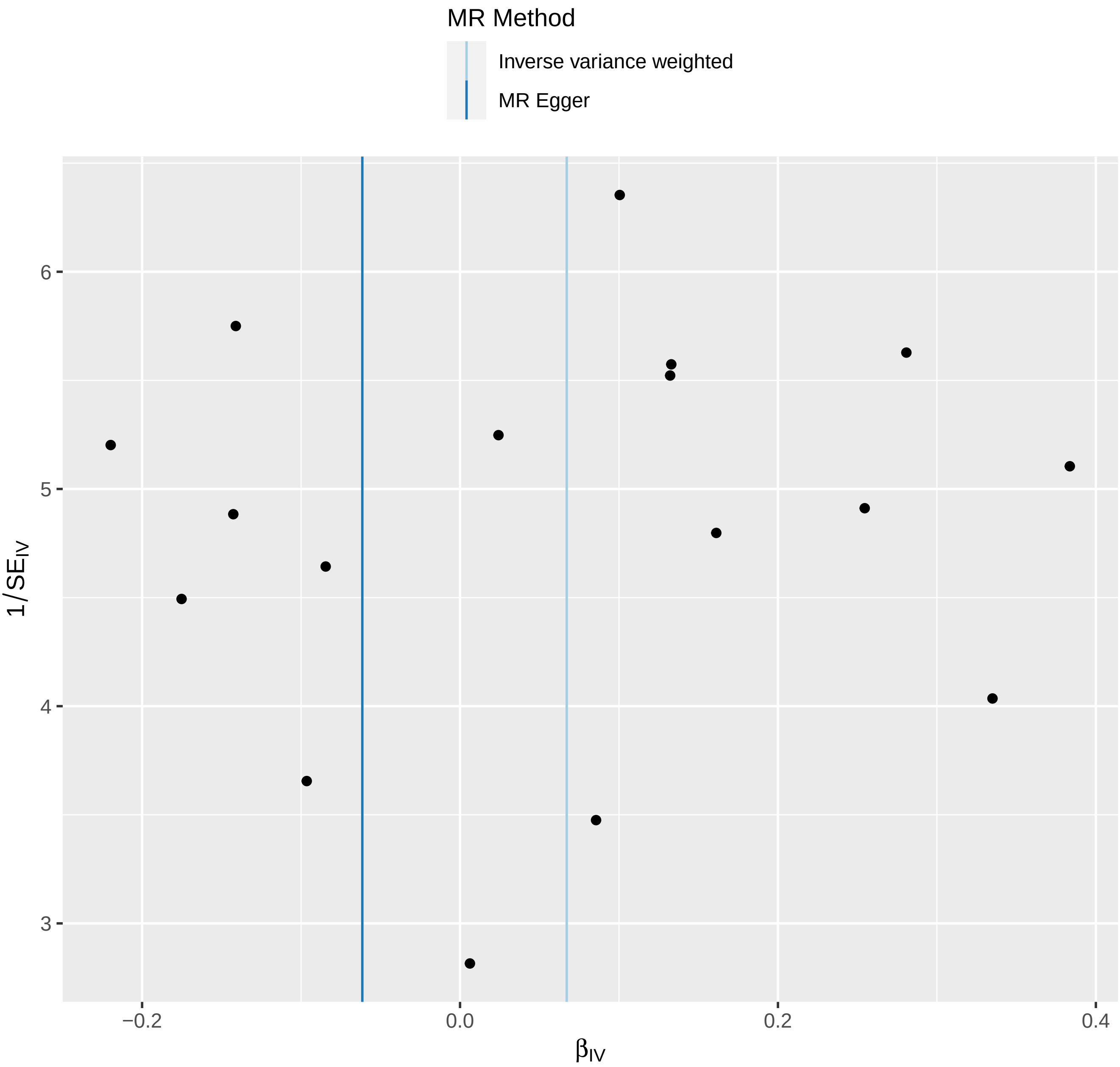

Batch 3−2609 : Reticulon−4 receptor on Diabetic nephropathy (more control exclusions)
with a threshold of 1e−05

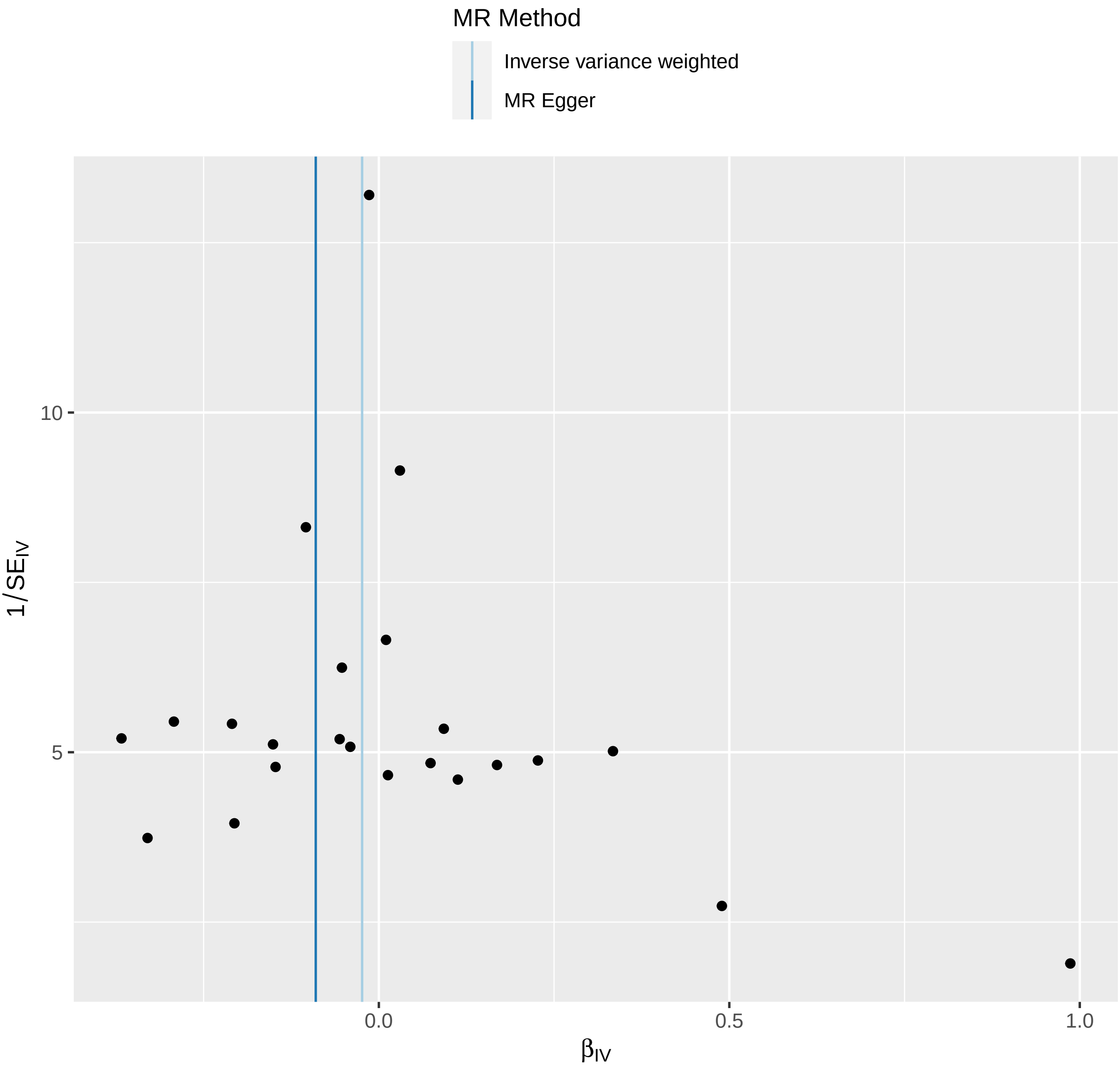

Batch 3−2610 : RUN and FYVE domain−containing protein 1 on Diabetic nephropathy (more control exclusions) with a threshold of 1e−05

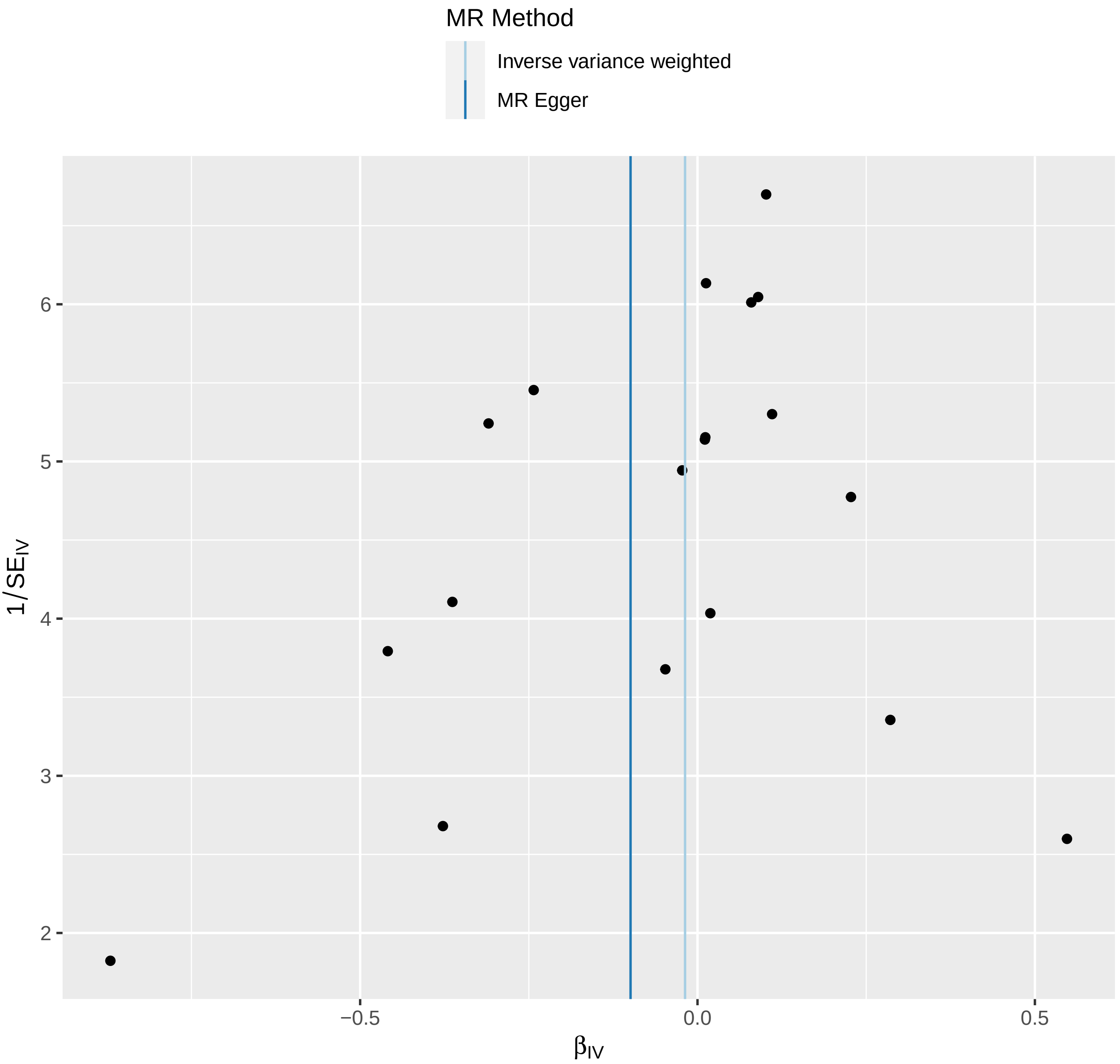

Batch 3−2611 : Runt−related transcription factor 3 on Diabetic nephropathy (more control exclusions)
with a threshold of 1e−05

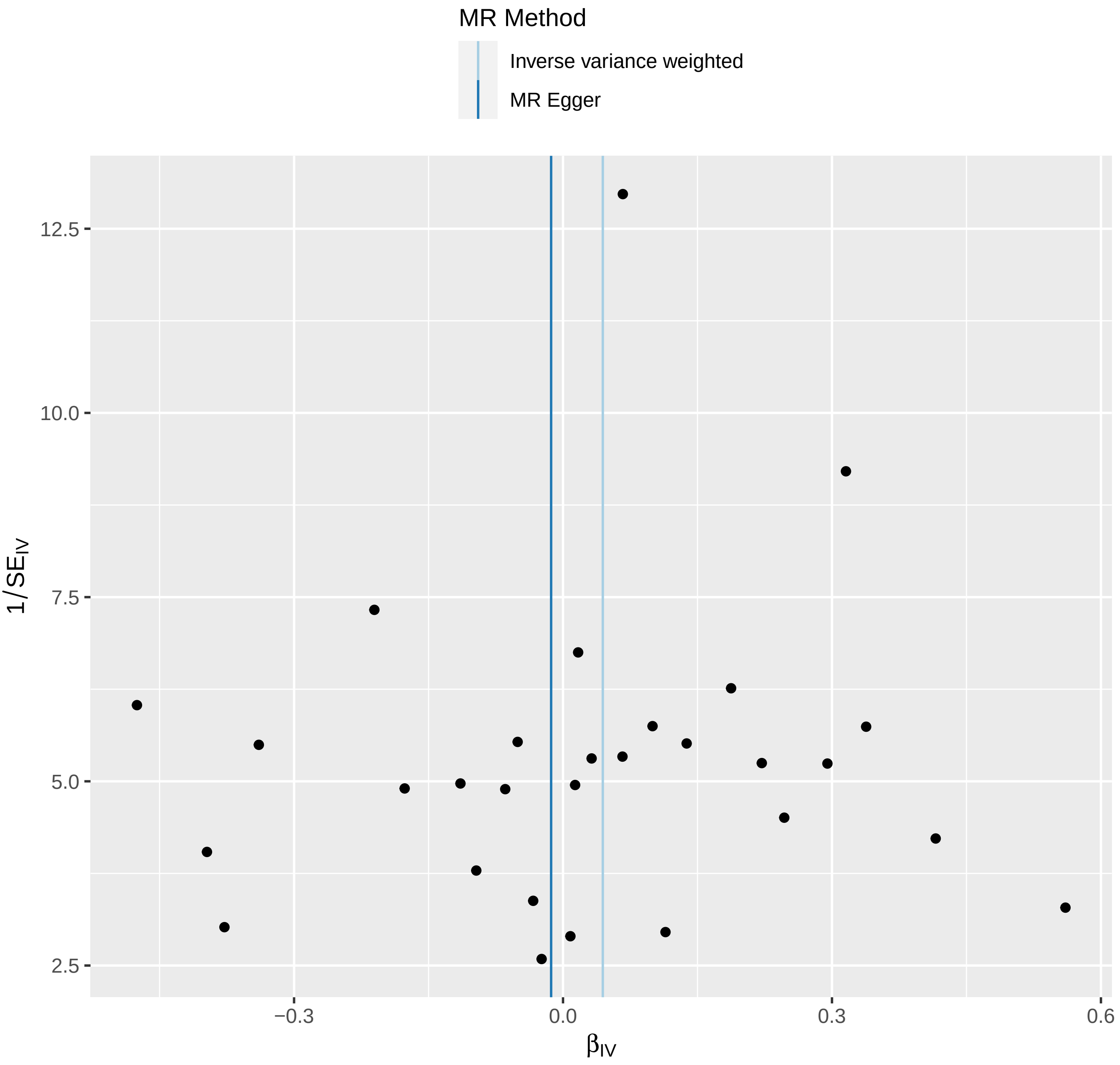

Batch 3−2612 : Relaxin receptor 1 on Diabetic nephropathy (more control exclusions) with a threshold of 1e−05

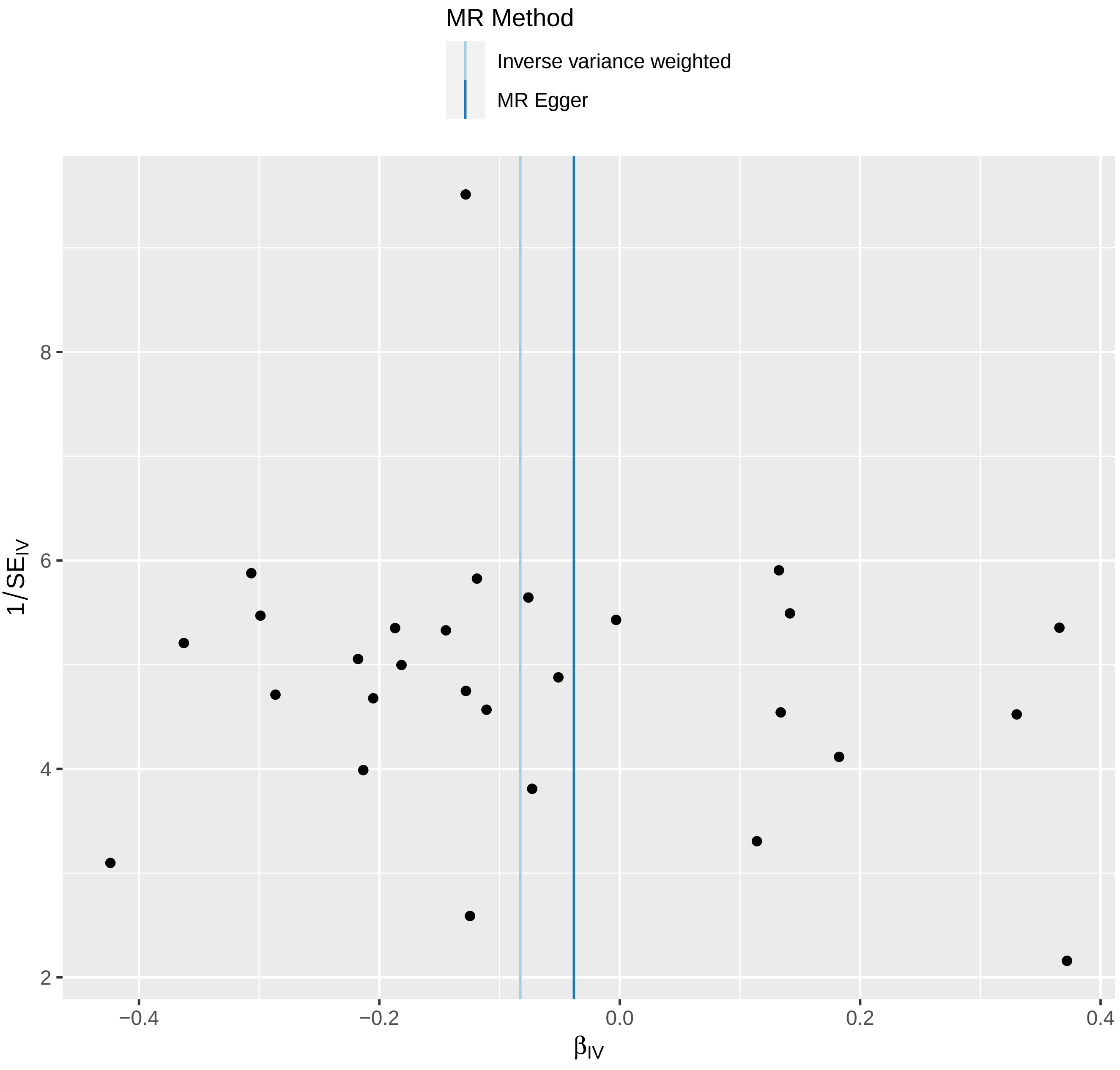

Batch 3−2613 : Tyrosine−protein kinase RYK on Diabetic nephropathy (more control exclusions)
with a threshold of 1e−05

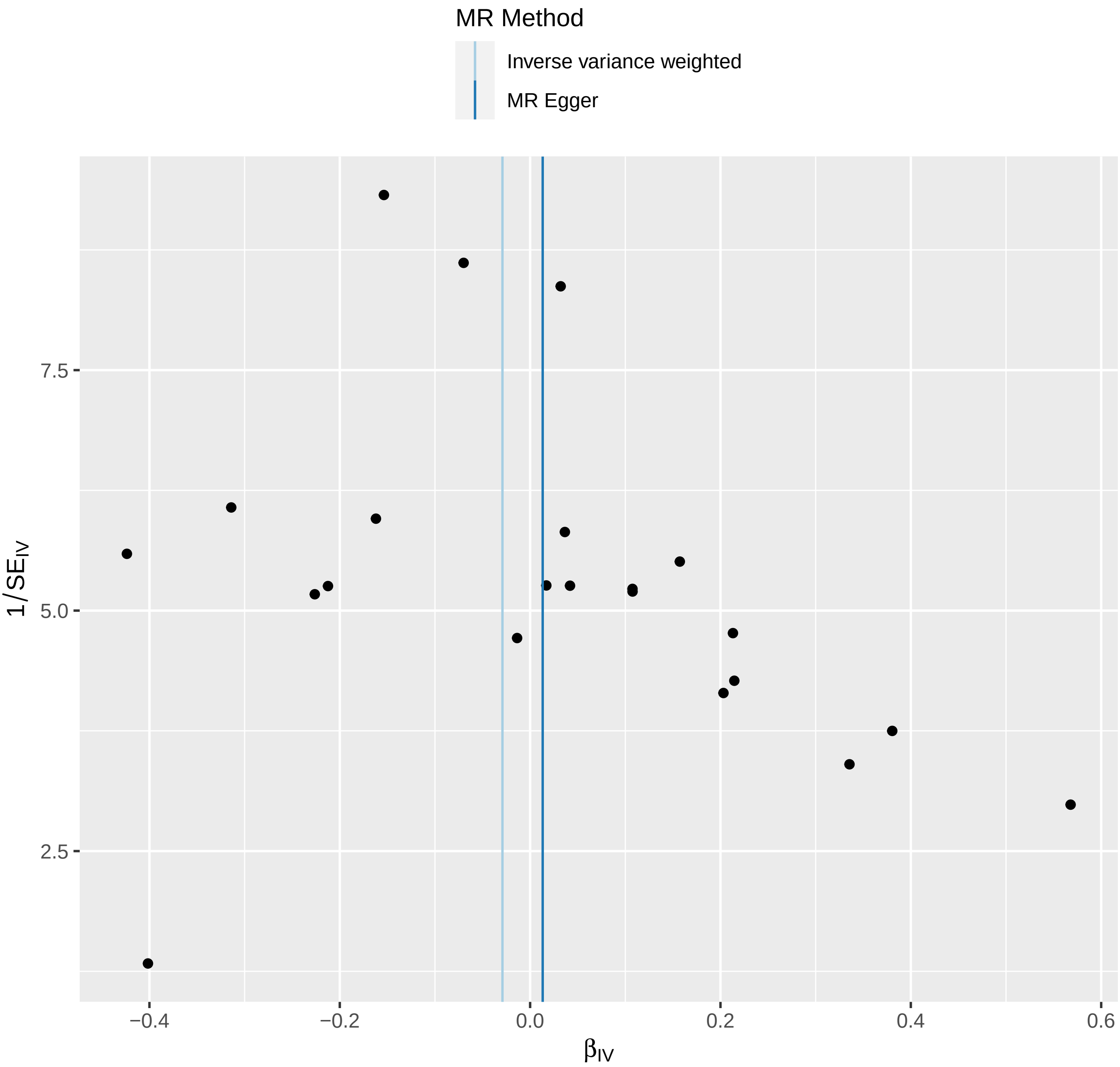

Batch 3−2614 : Protein S100−A11 on Diabetic nephropathy (more control exclusions)
with a threshold of 1e−05

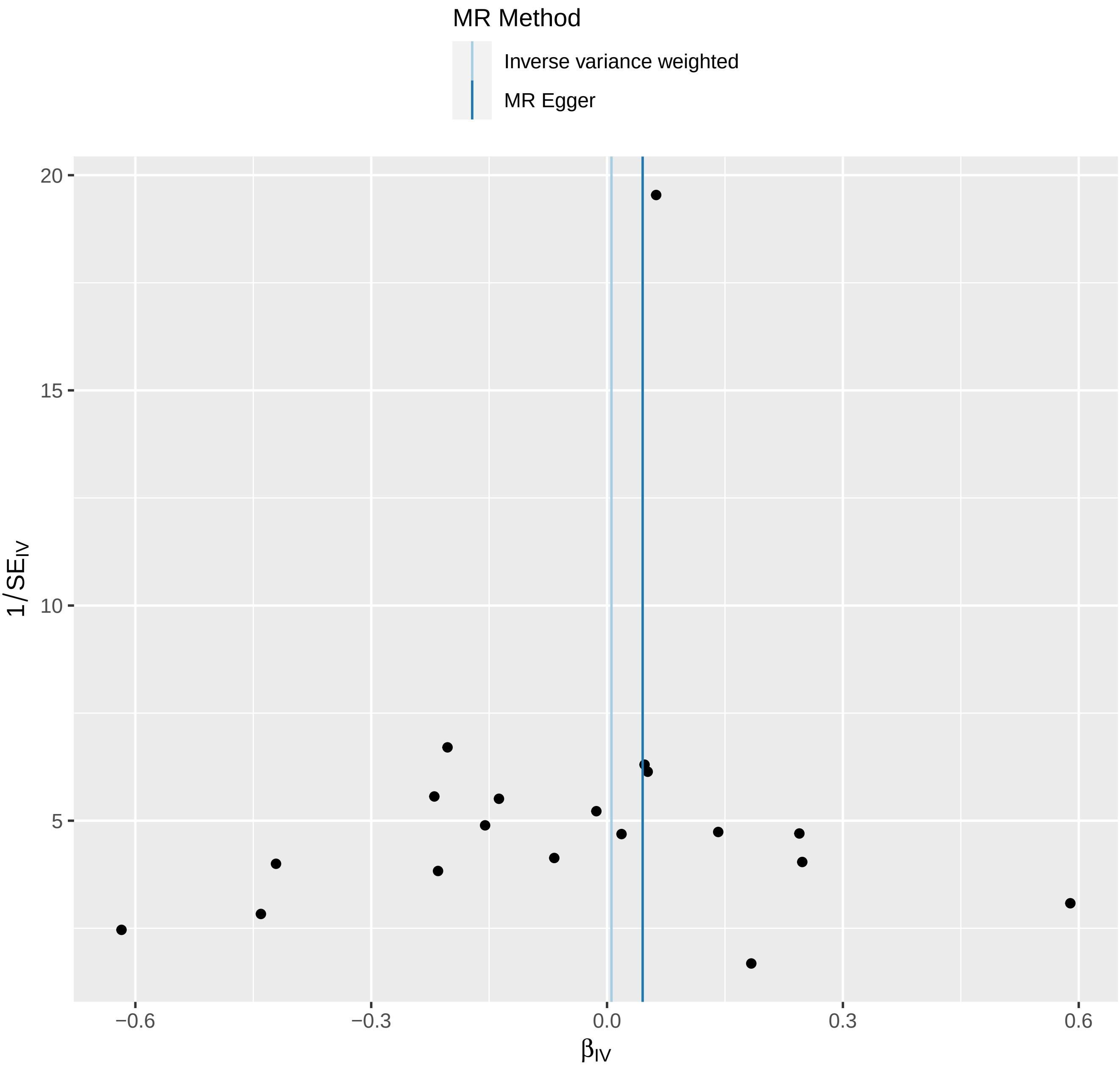

Batch 3−2615 : Protein S100−A12 on Diabetic nephropathy (more control exclusions)
with a threshold of 1e−05

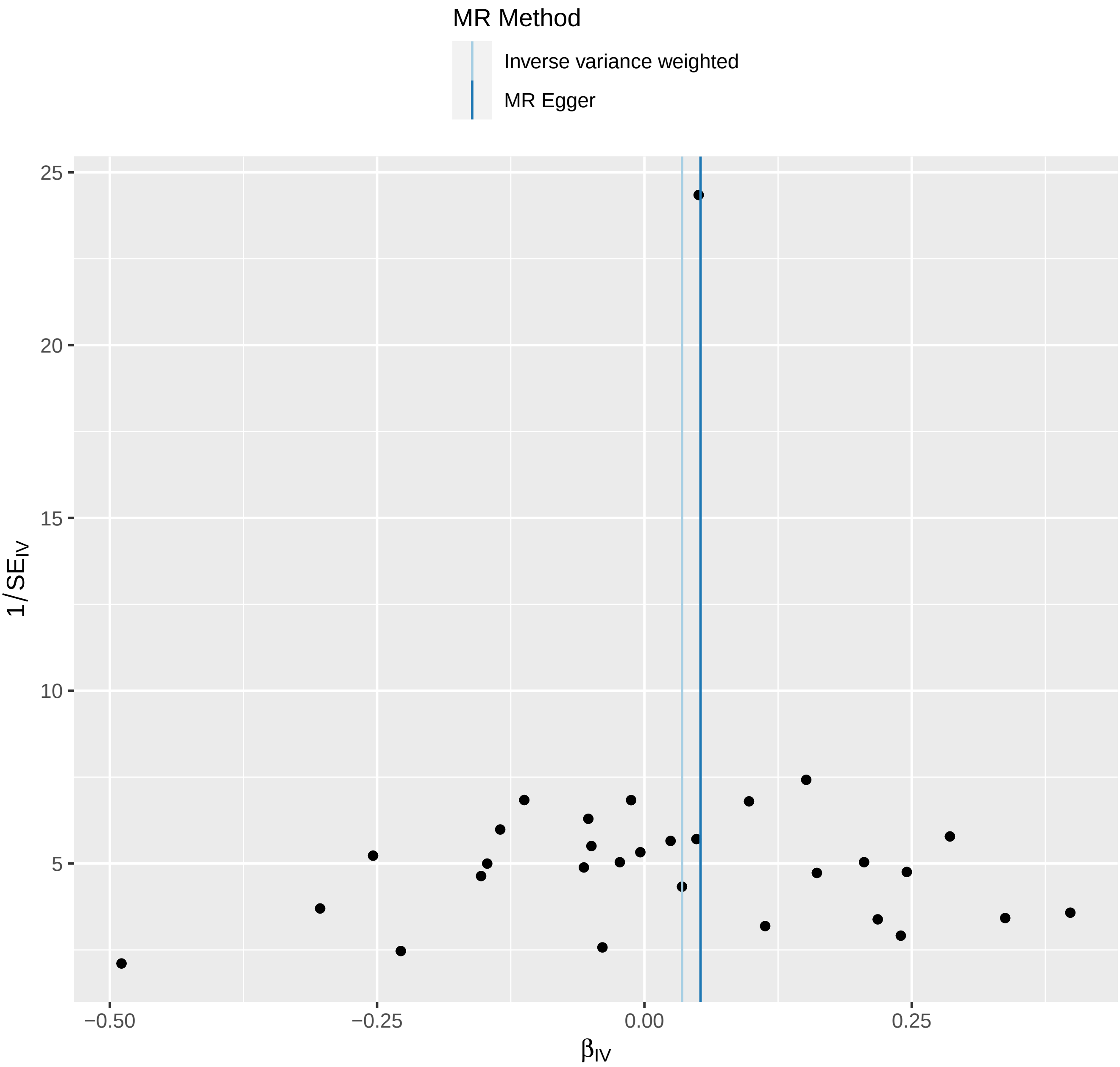

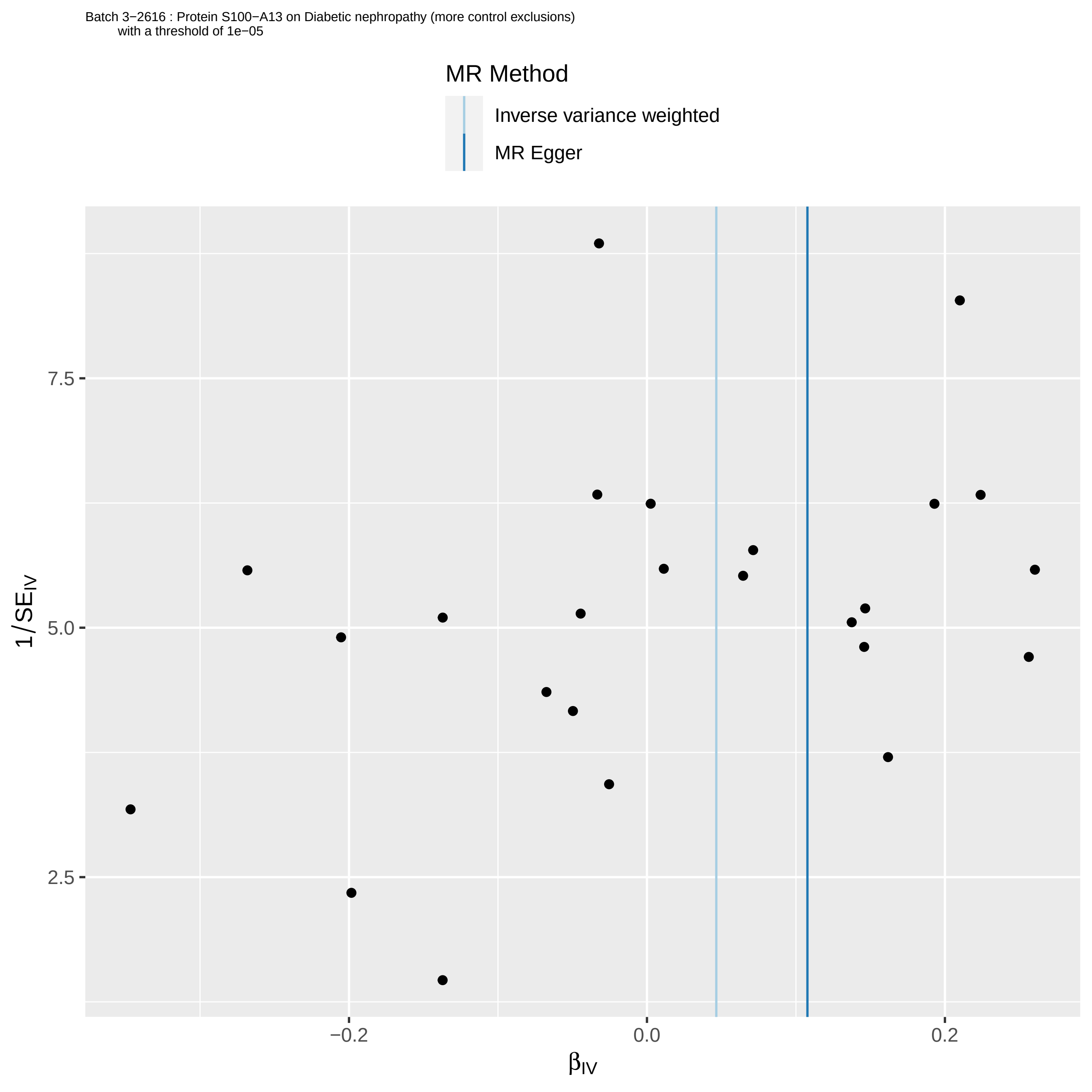

Batch 3−2616 : Protein S100−A13 on Diabetic nephropathy (more control exclusions)
with a threshold of 1e−05
MR Method
Inverse variance weighted
MR Egger
7.5
5.0
2.5
1/SE$_{IV}$
−0.2
0.0
0.2
β$_{IV}$

Batch 3−2617 : Protein S100−A2 on Diabetic nephropathy (more control exclusions)
with a threshold of 1e−05

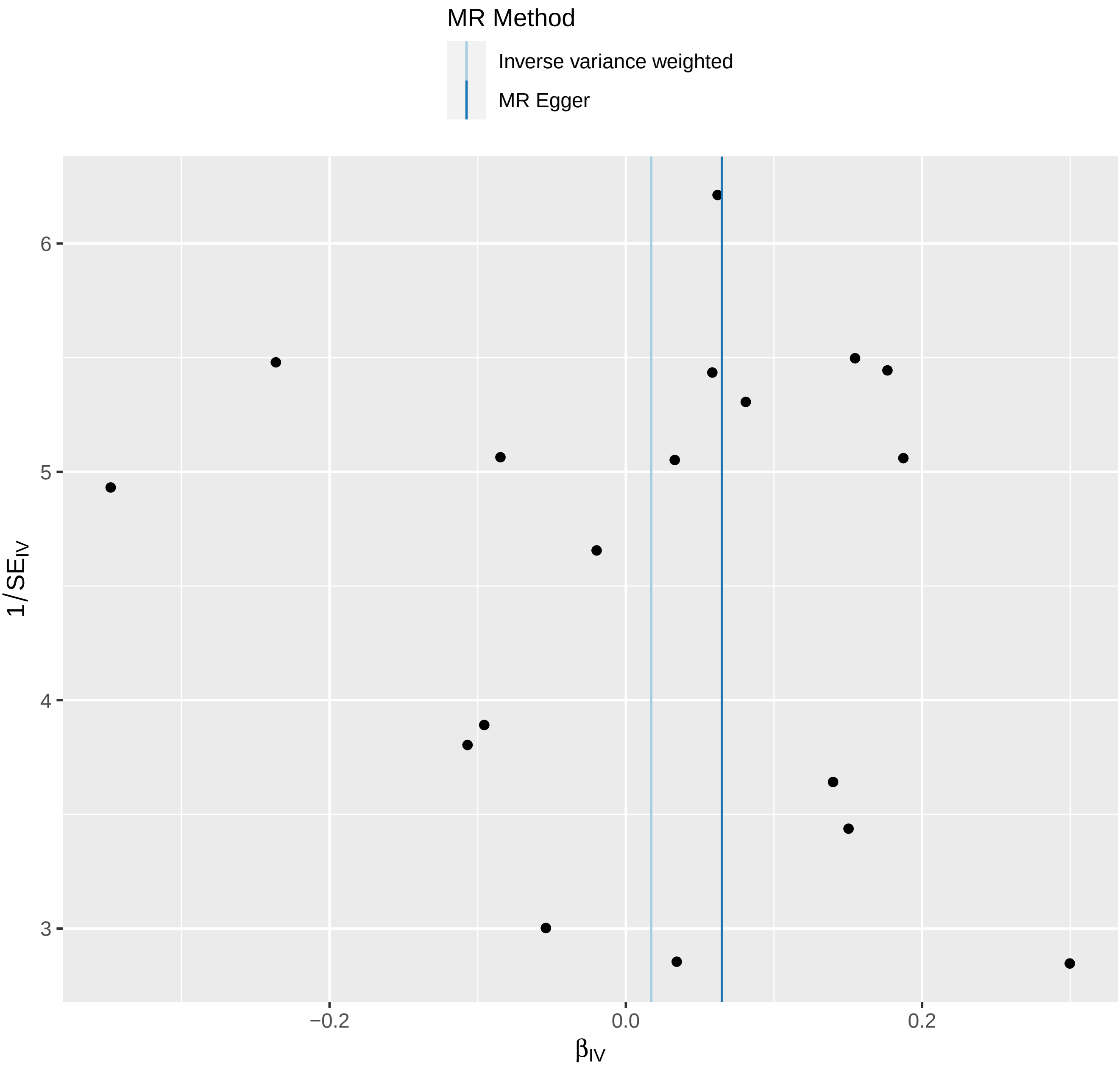

Batch 3−2618 : Protein S100−A4 on Diabetic nephropathy (more control exclusions)
with a threshold of 1e−05

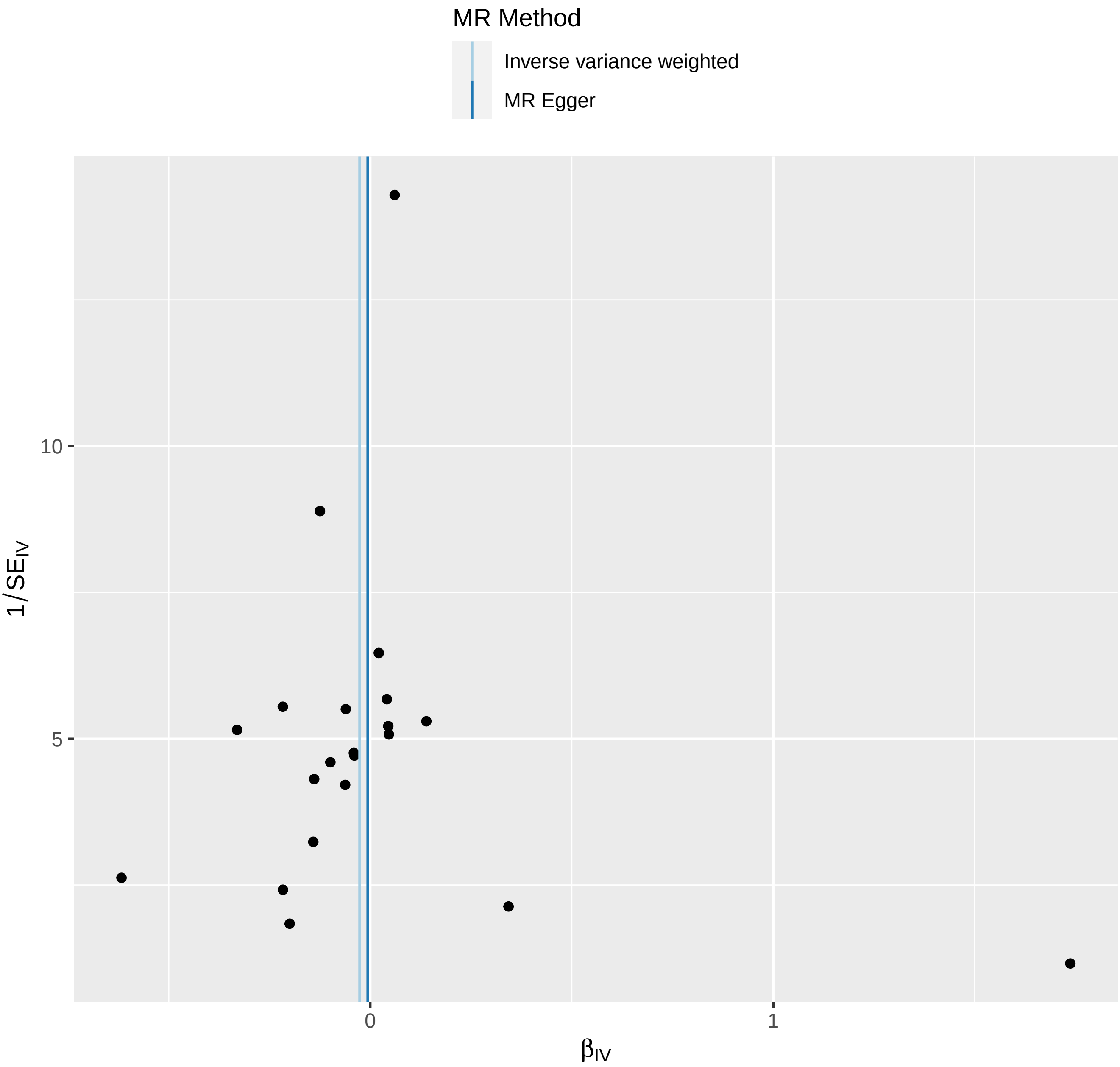

Batch 3−2619 : Protein S100−A5 on Diabetic nephropathy (more control exclusions)
with a threshold of 1e−05

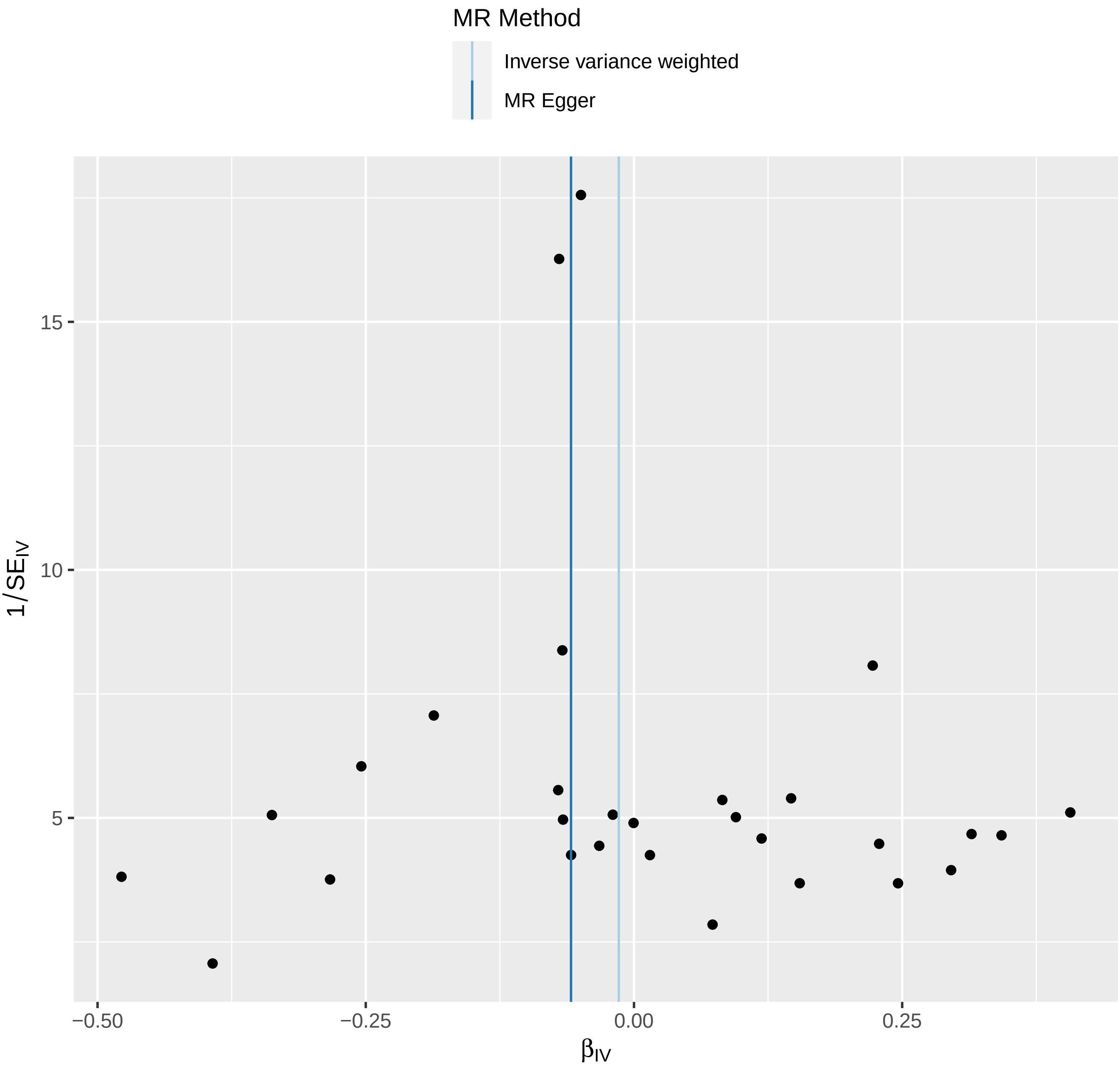

Batch 3−2620 : Protein S100−A6 on Diabetic nephropathy (more control exclusions)
with a threshold of 1e−05

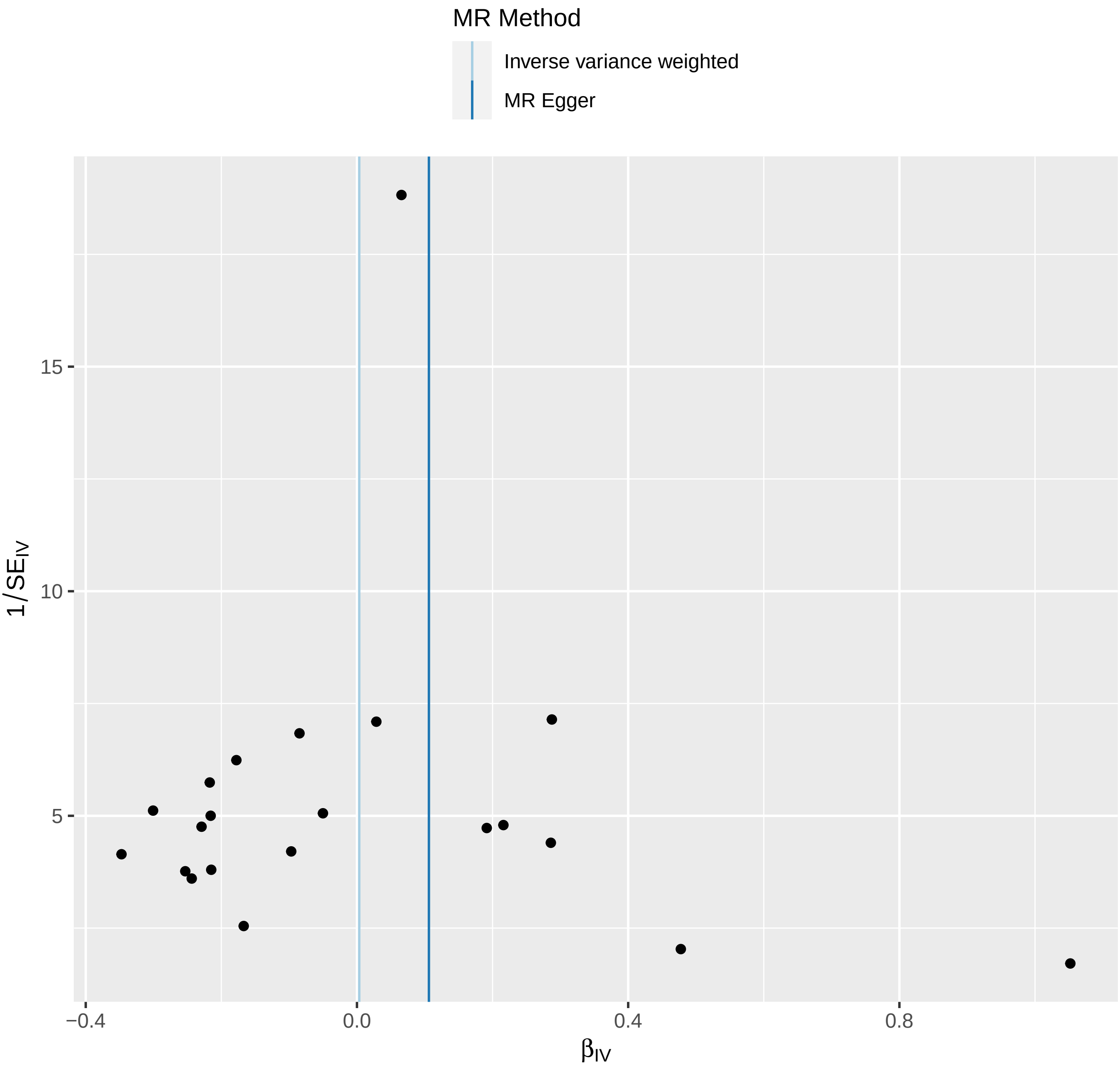

Batch 3−2621 : Protein S100−A7 on Diabetic nephropathy (more control exclusions)
with a threshold of 1e−05

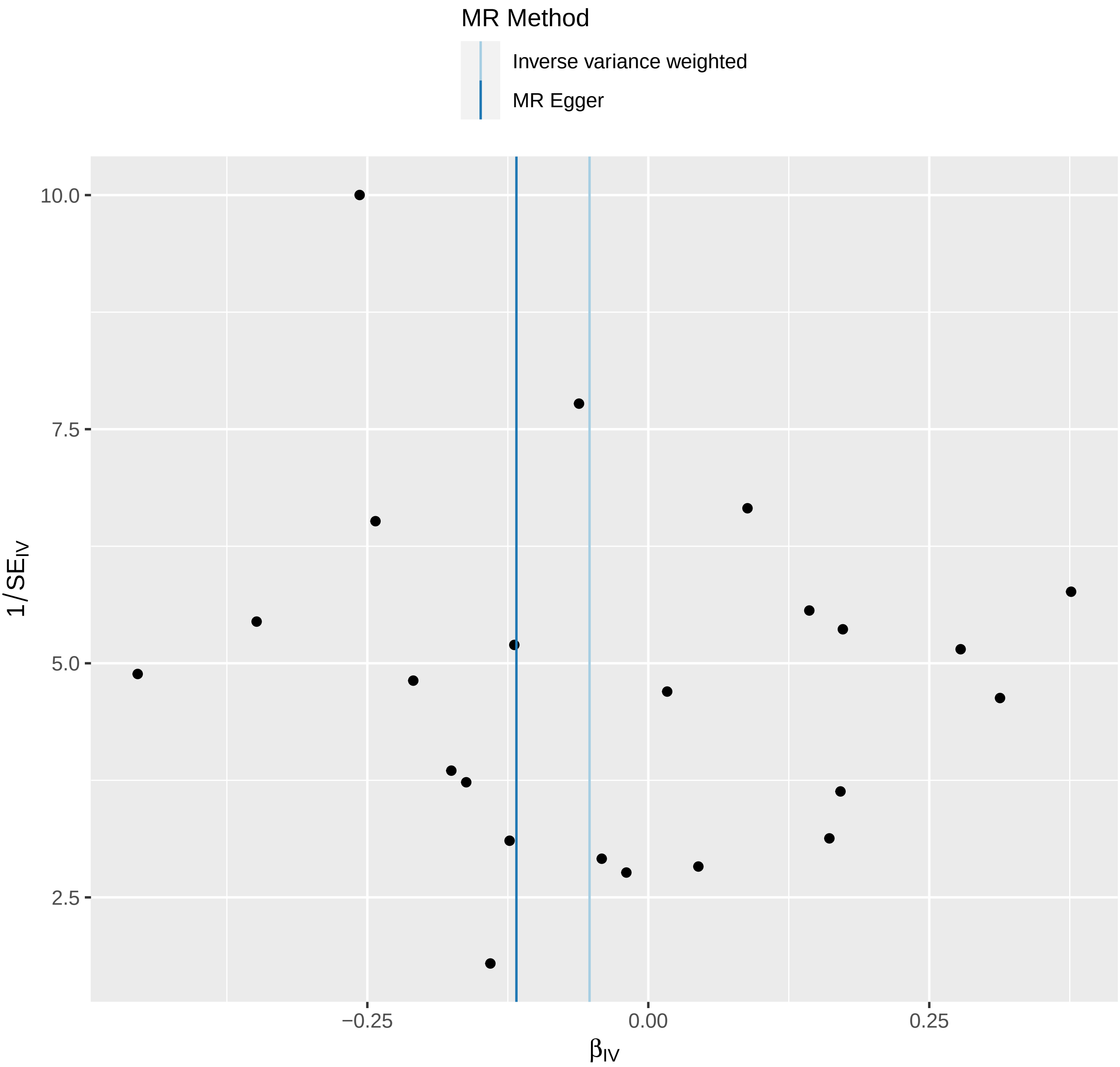

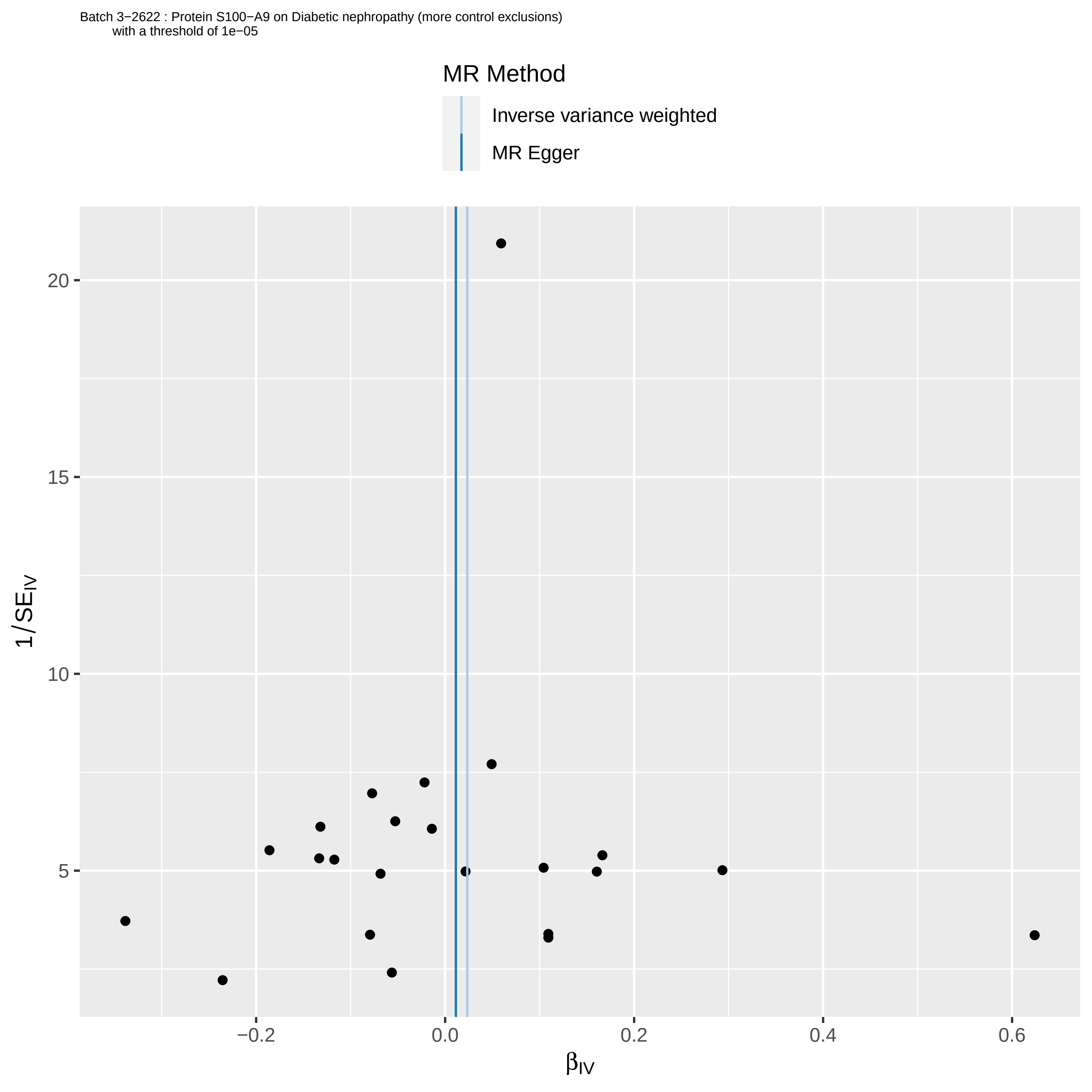
Batch 3−2622 : Protein S100−A9 on Diabetic nephropathy (more control exclusions)
with a threshold of 1e−05
MR Method
Inverse variance weighted
MR Egger
20
15
10
5
$1/SE_{IV}$
−0.2
0.0
0.2
0.4
0.6
β_{IV}

Batch 3−2623 : Serum amyloid A−1 protein on Diabetic nephropathy (more control exclusions)
with a threshold of 1e−05

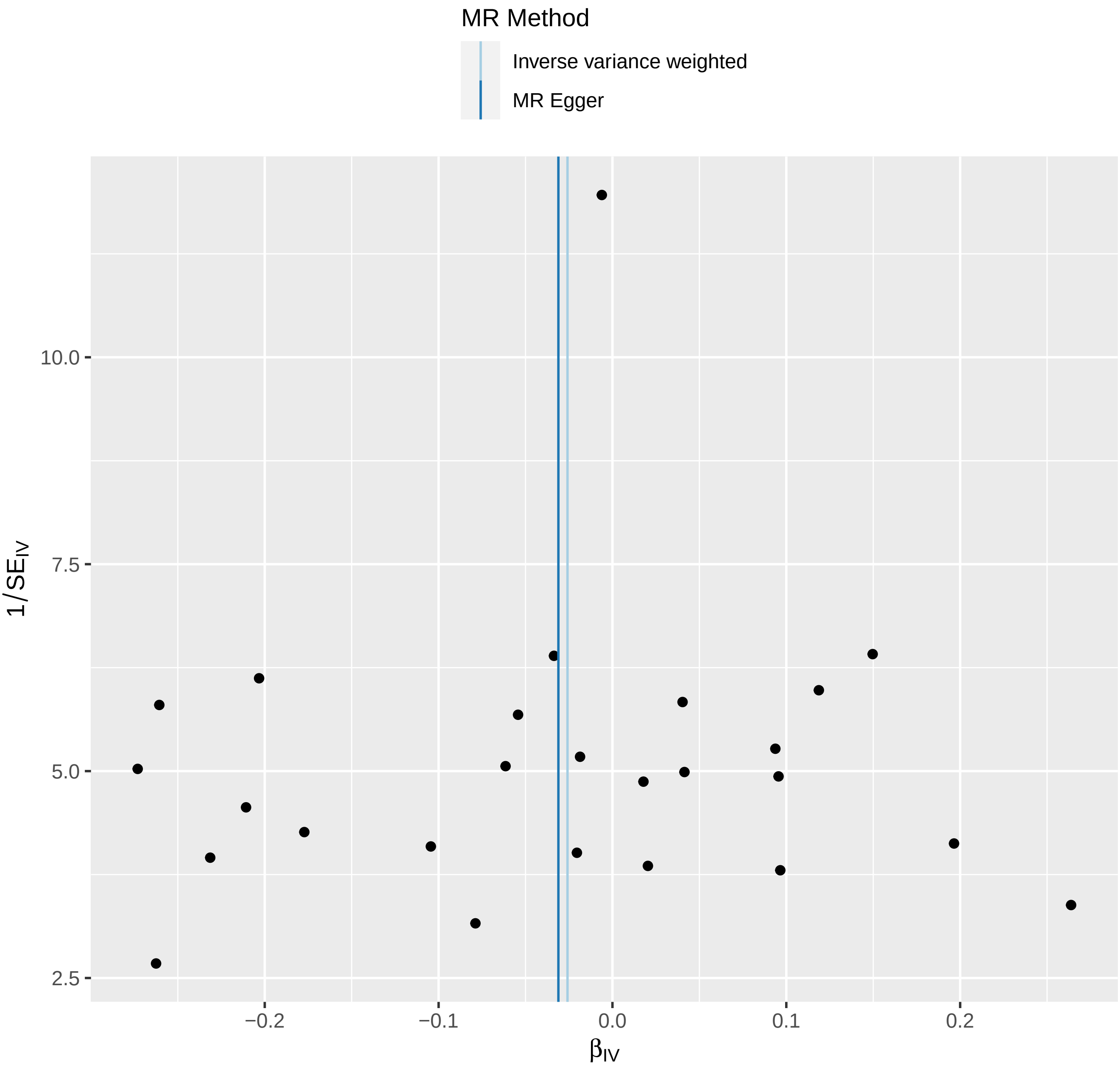

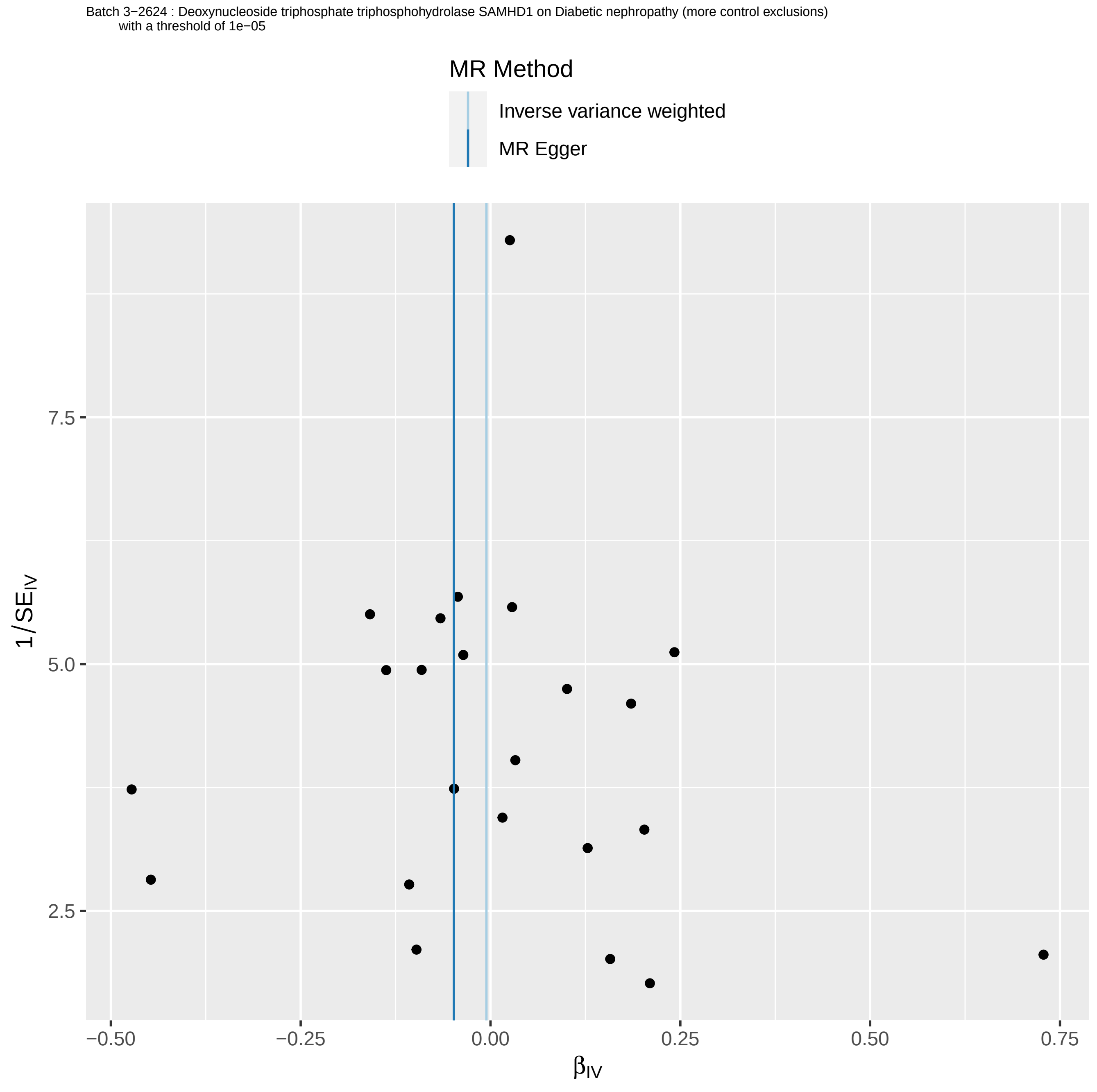
Batch 3−2624 : Deoxynucleoside triphosphate triphosphohydrolase SAMHD1 on Diabetic nephropathy (more control exclusions) with a threshold of 1e−05
MR Method
Inverse variance weighted
MR Egger
1/SE_IV
7.5
5.0
2.5
−0.50
−0.25
0.00
0.25
0.50
0.75
β_IV

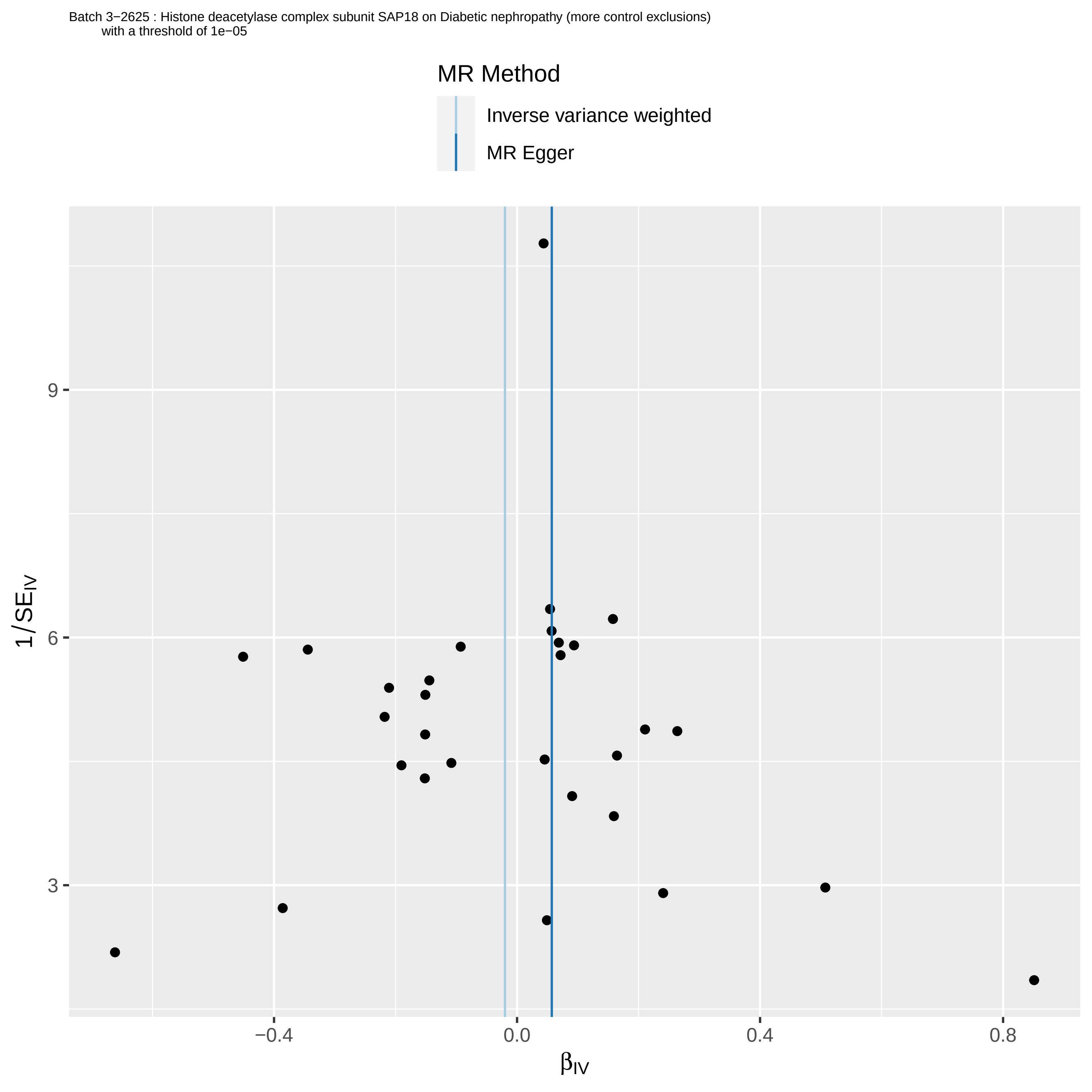

Batch 3−2625 : Histone deacetylase complex subunit SAP18 on Diabetic nephropathy (more control exclusions)
with a threshold of 1e−05
MR Method
Inverse variance weighted
MR Egger
9
6
3
1/SE_IV
−0.4
0.0
0.4
0.8
β_IV

Batch 3−2626 : Histone deacetylase complex subunit SAP30 on Diabetic nephropathy (more control exclusions)
with a threshold of 1e−05

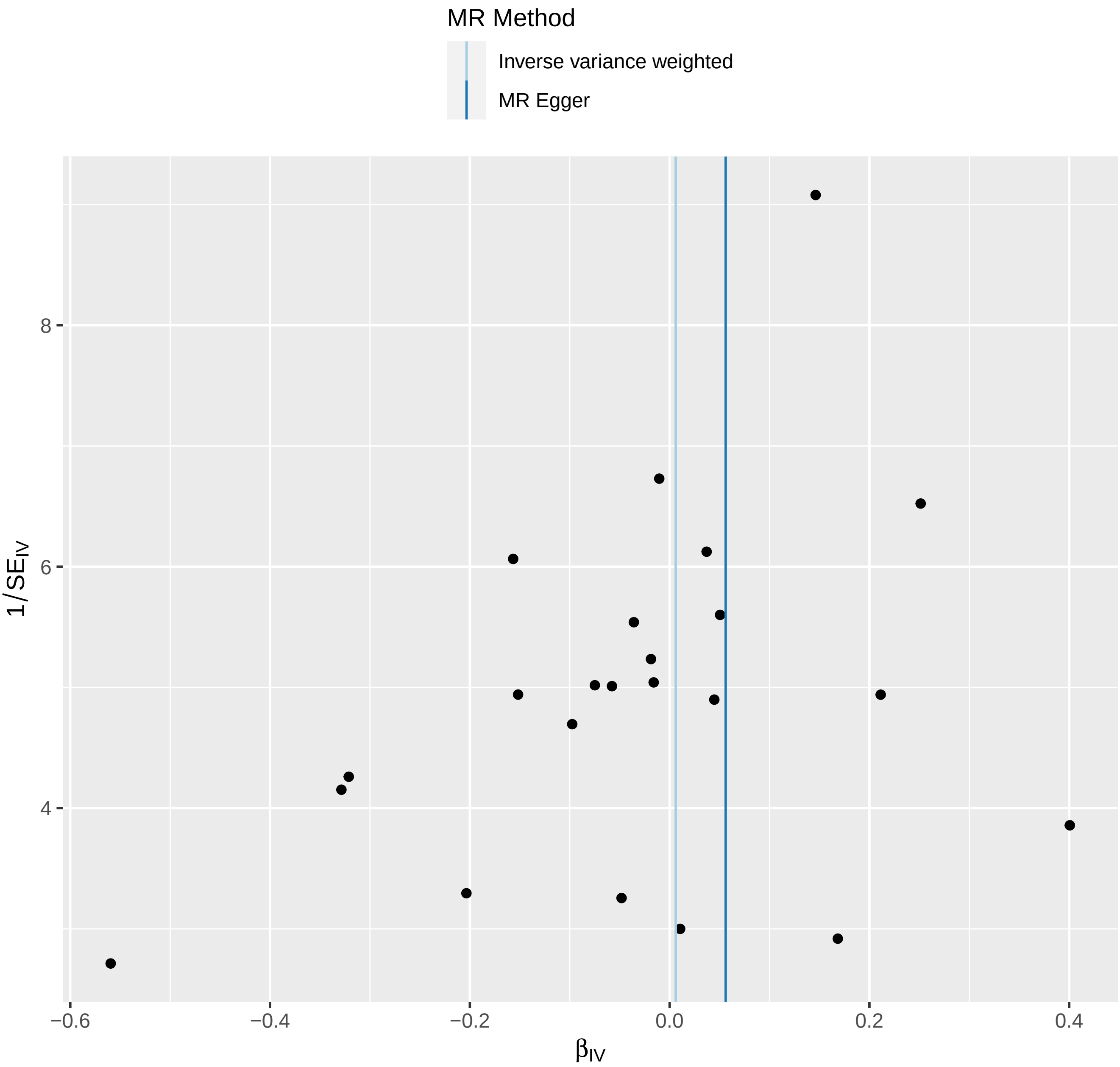

Batch 3−2627 : Serine−−tRNA ligase, mitochondrial on Diabetic nephropathy (more control exclusions)
with a threshold of 1e−05

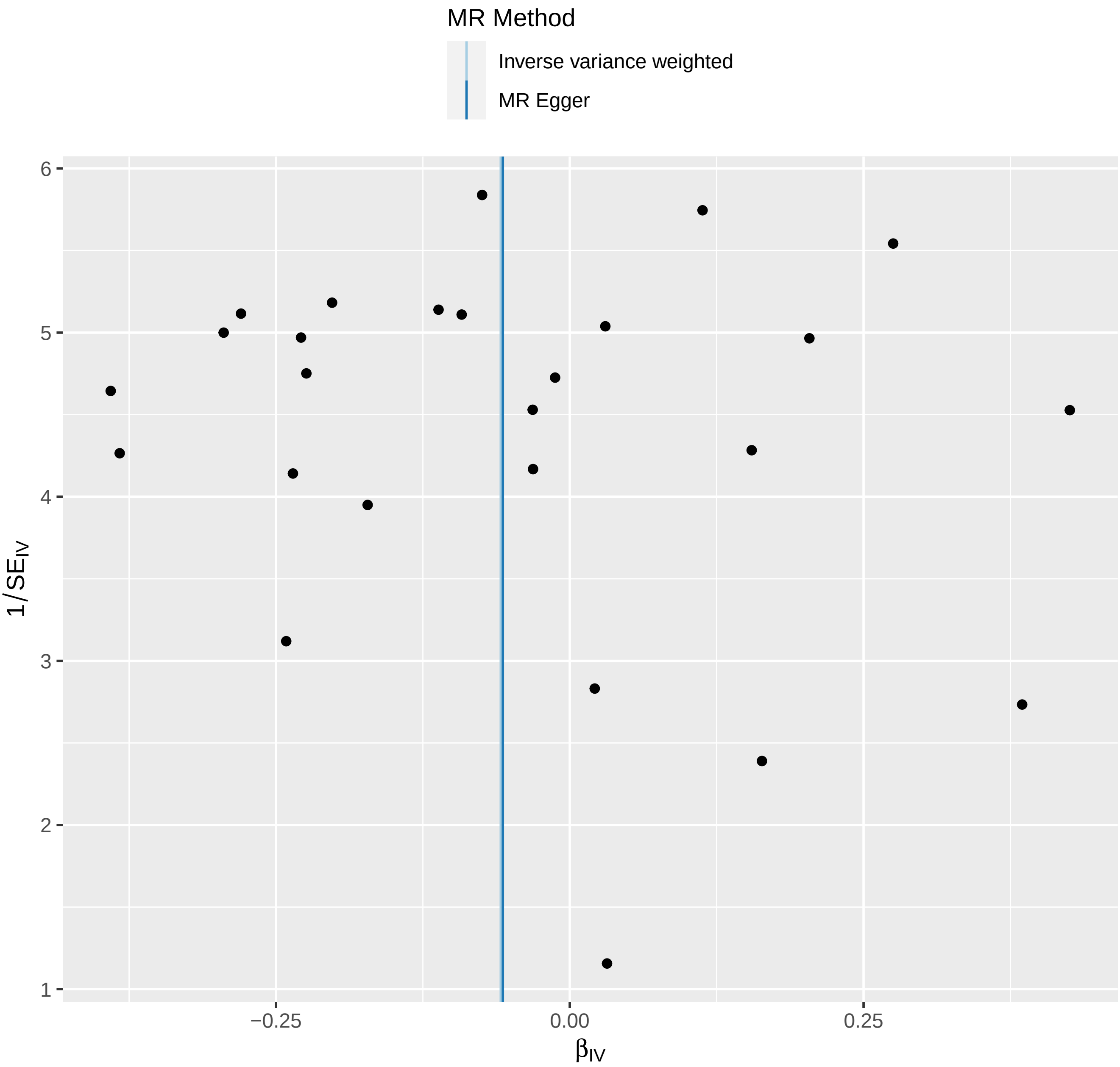

Batch 3−2628 : Diamine acetyltransferase 1 on Diabetic nephropathy (more control exclusions)
with a threshold of 1e−05

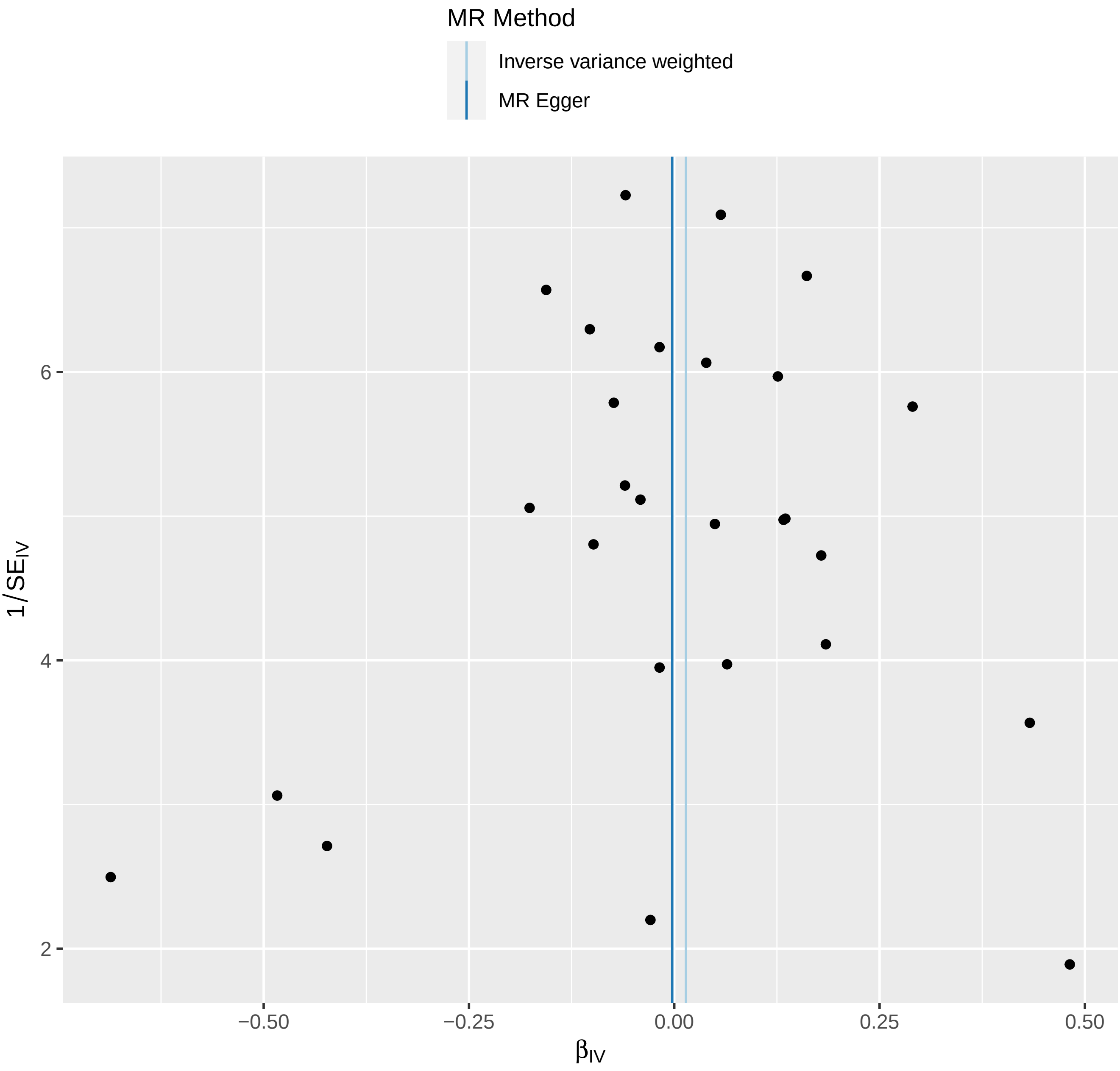

Batch 3−2629 : Diamine acetyltransferase 2 on Diabetic nephropathy (more control exclusions)
with a threshold of 1e−05

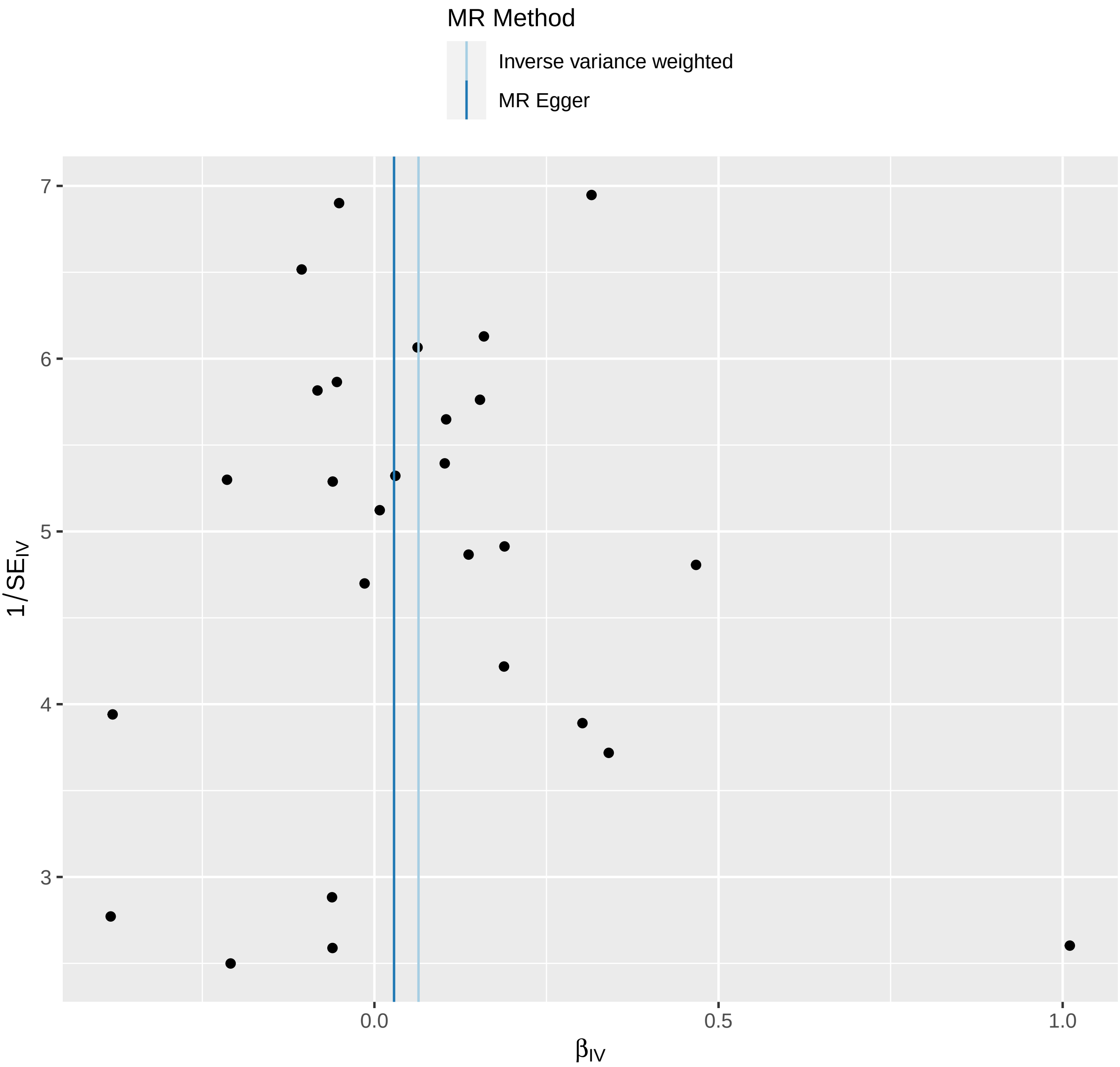

Batch 3−2630 : DNA−binding protein SATB1 on Diabetic nephropathy (more control exclusions)
with a threshold of 1e−05

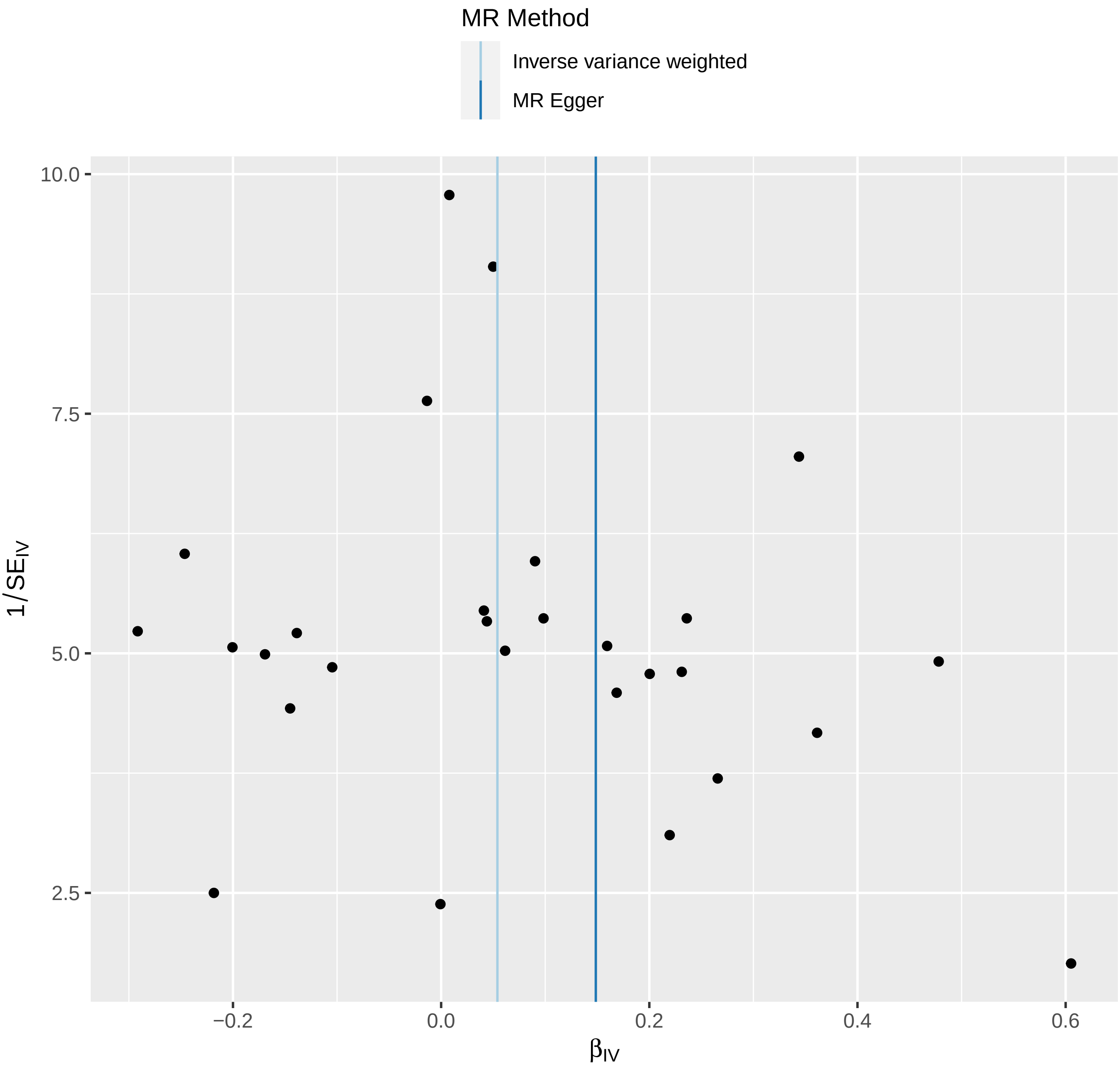

Batch 3−2631 : DNA−binding protein SATB2 on Diabetic nephropathy (more control exclusions)
with a threshold of 1e−05

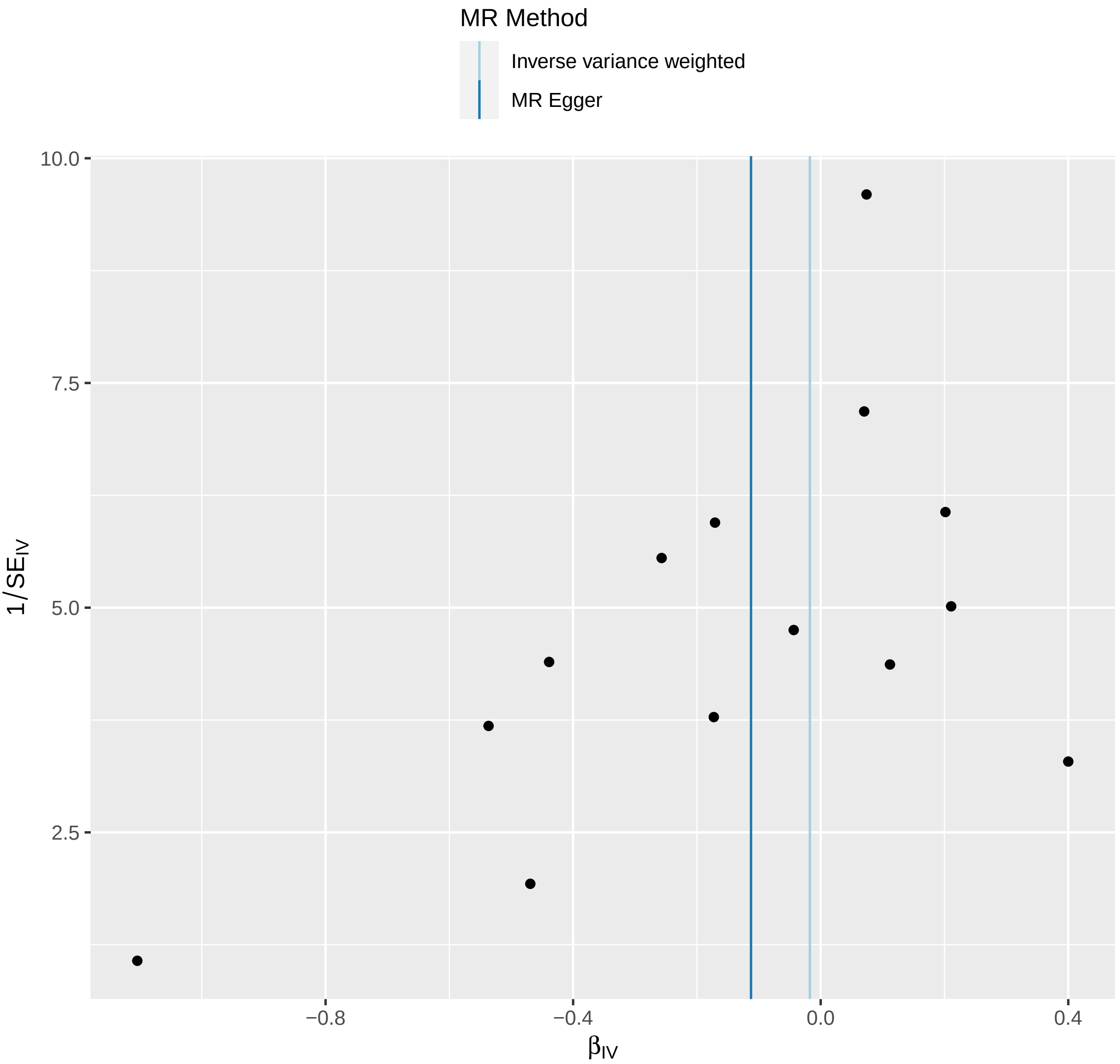

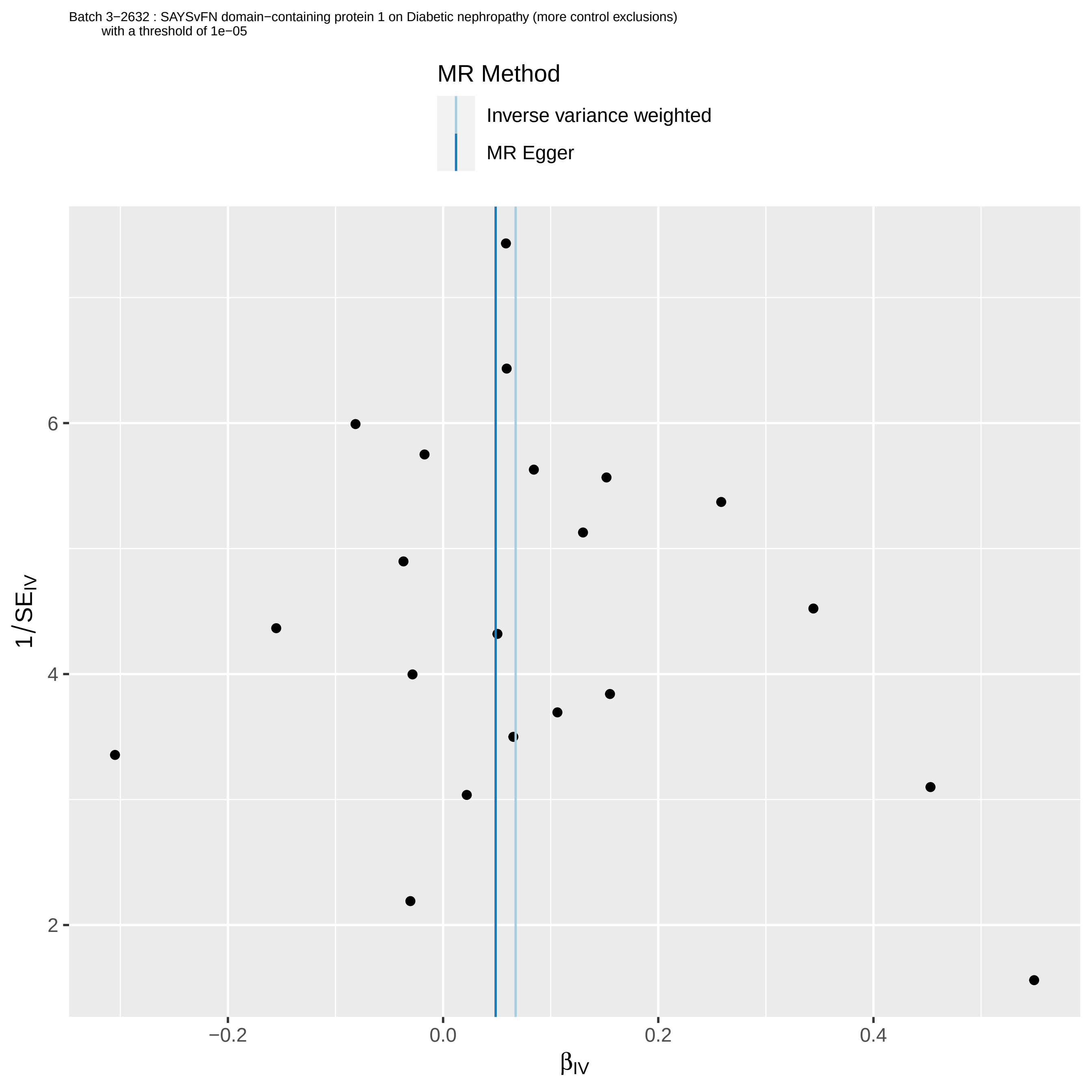
Batch 3−2632 : SAYSvFN domain−containing protein 1 on Diabetic nephropathy (more control exclusions)
with a threshold of 1e−05
MR Method
Inverse variance weighted
MR Egger
6
4
2
−0.2
0.0
0.2
0.4
$1/SE_{IV}$
β_{IV}

Batch 3−2633 : Suprabasin on Diabetic nephropathy (more control exclusions)
with a threshold of 1e−05

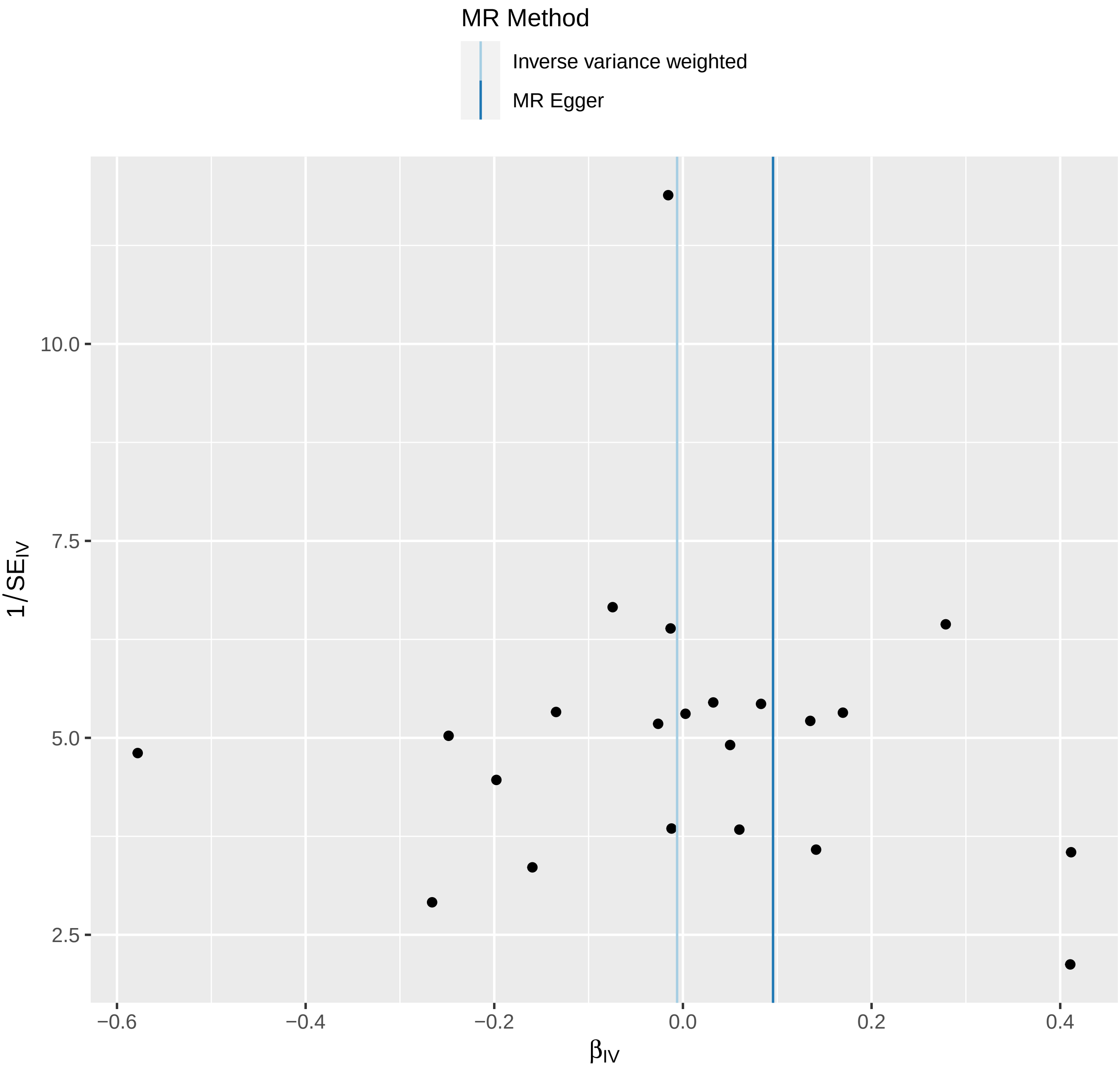

Batch 3−2634 : Secretory carrier−associated membrane protein 5 on Diabetic nephropathy (more control exclusions) with a threshold of 1e−05

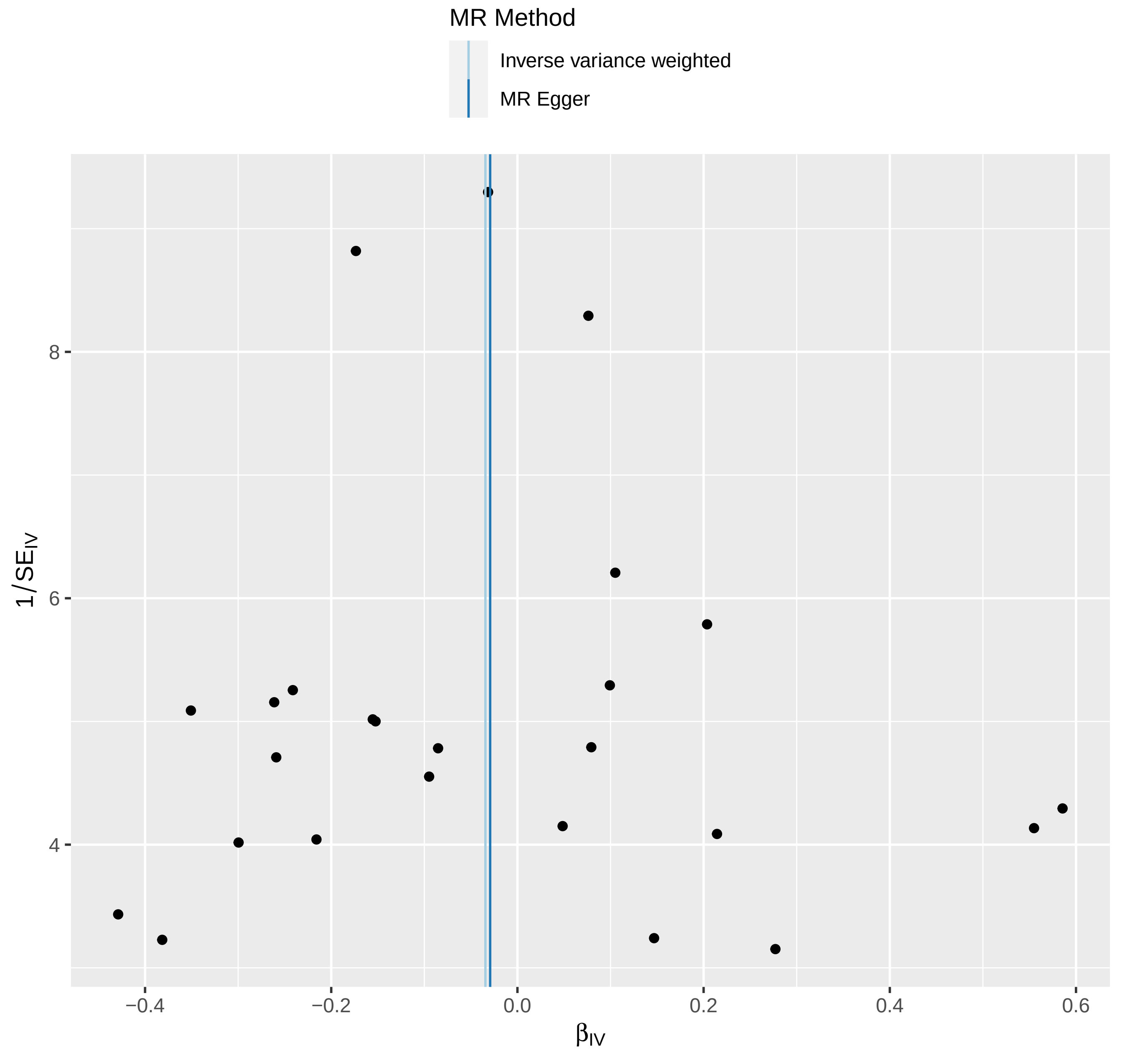

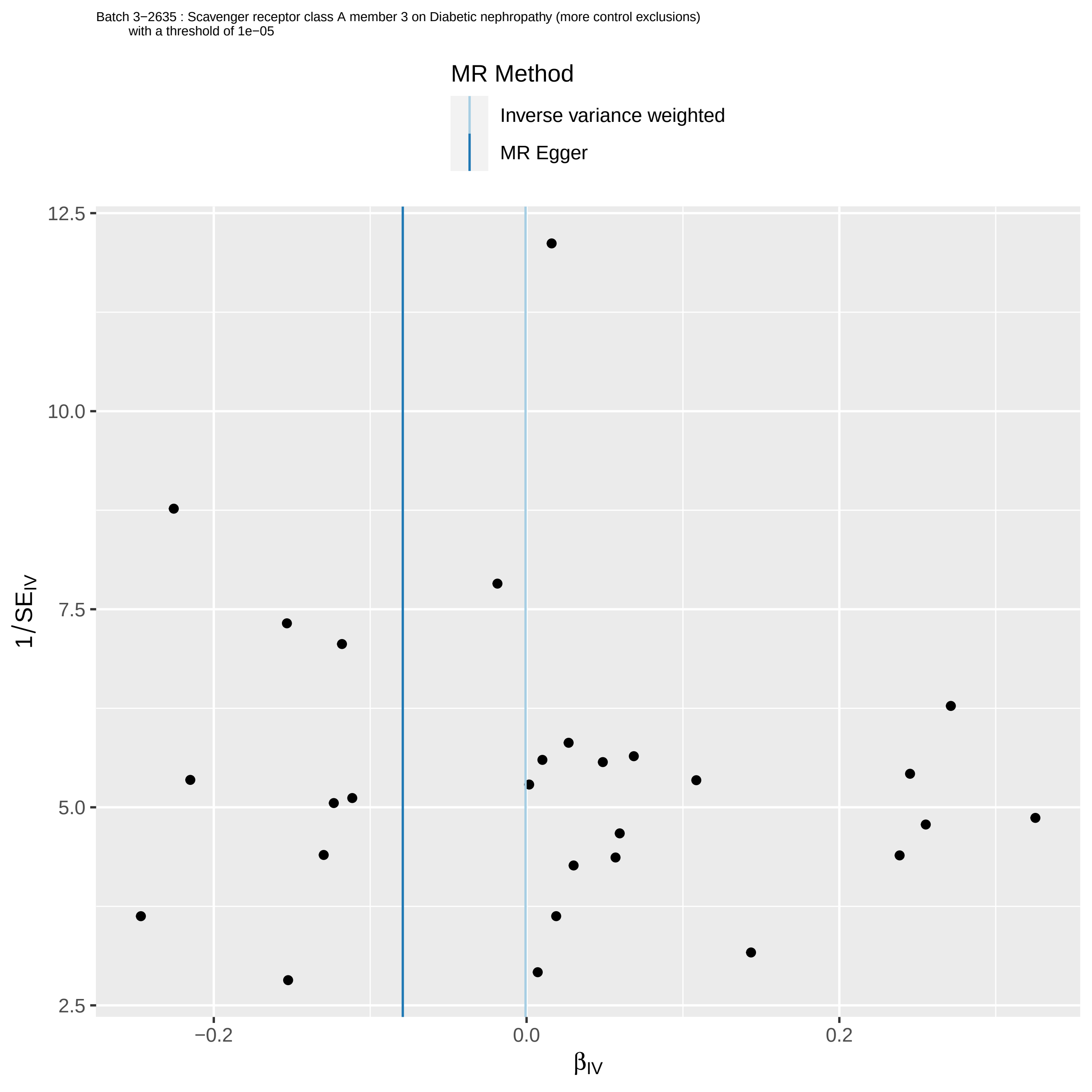

Batch 3−2635 : Scavenger receptor class A member 3 on Diabetic nephropathy (more control exclusions)
with a threshold of 1e−05
MR Method
Inverse variance weighted
MR Egger
12.5
10.0
7.5
5.0
2.5
1/SE_IV
−0.2
0.0
0.2
β_IV

Batch 3−2636 : Scavenger receptor class A member 3 on Diabetic nephropathy (more control exclusions)
with a threshold of 1e−05

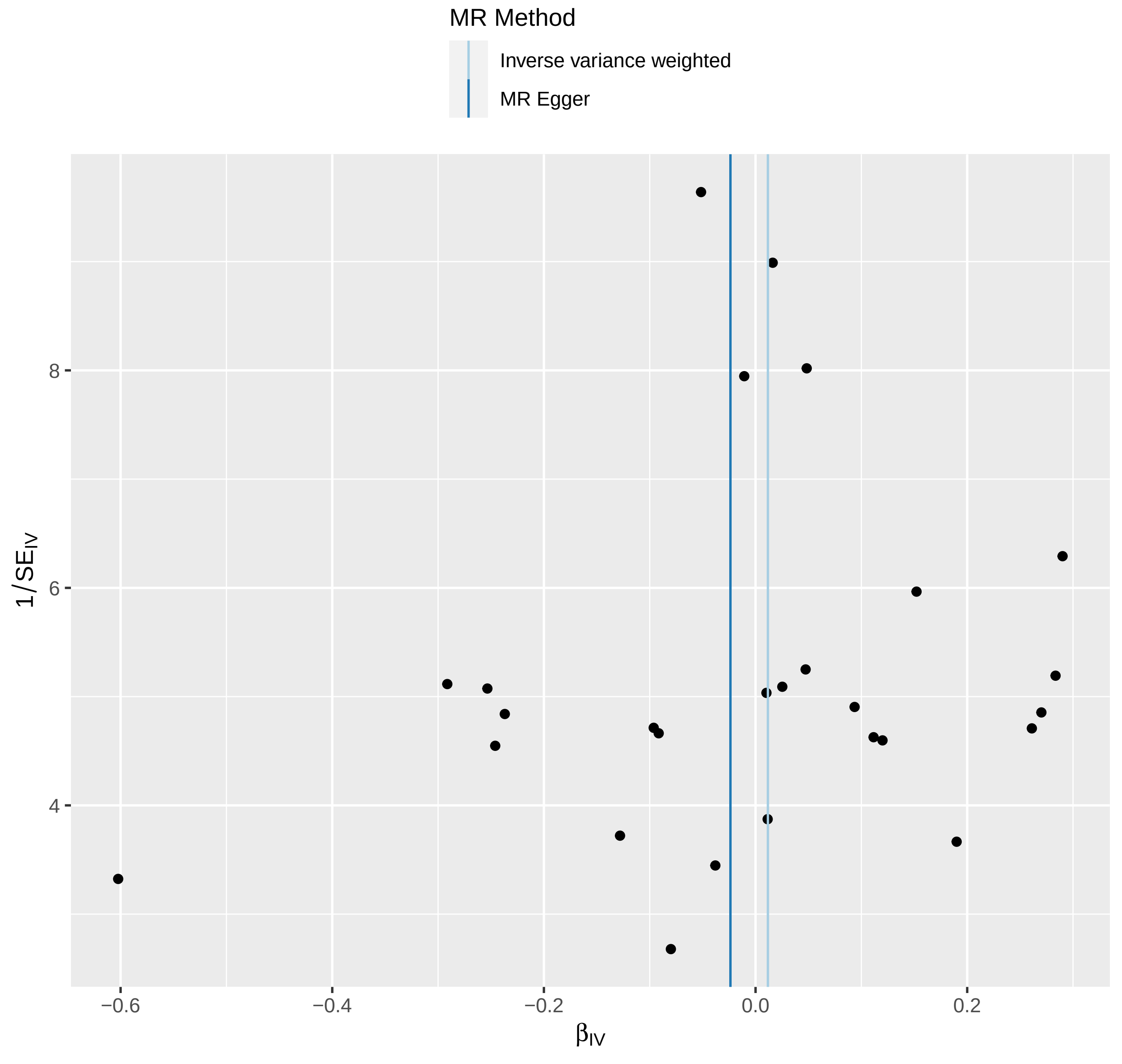

Batch 3−2637 : Scavenger receptor class A member 3 on Diabetic nephropathy (more control exclusions)
with a threshold of 1e−05

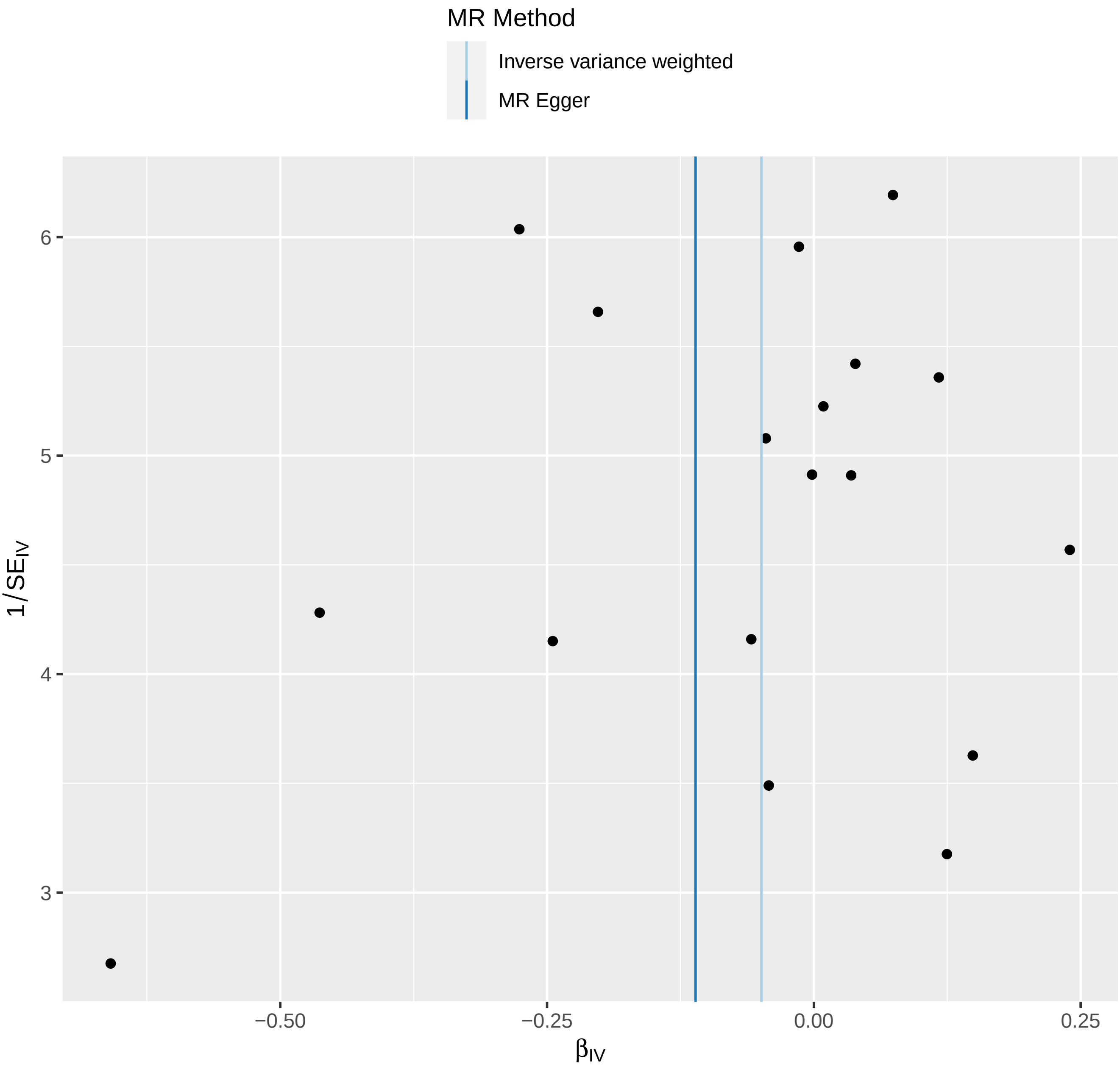

Batch 3−2638 : Scavenger receptor class A member 3 on Diabetic nephropathy (more control exclusions)
with a threshold of 1e−05

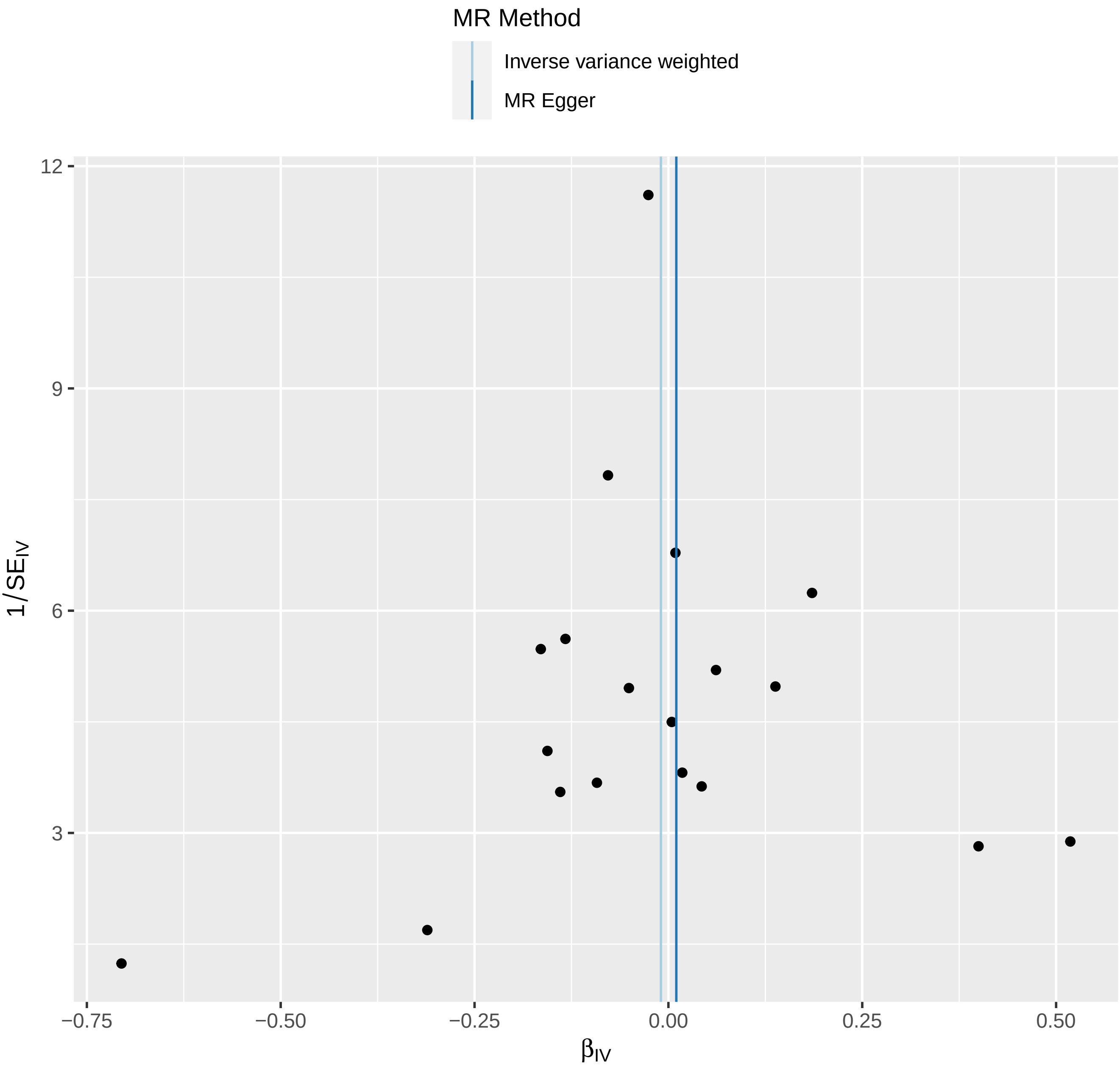

Batch 3−2639 : Scavenger receptor class A member 5 on Diabetic nephropathy (more control exclusions)
with a threshold of 1e−05

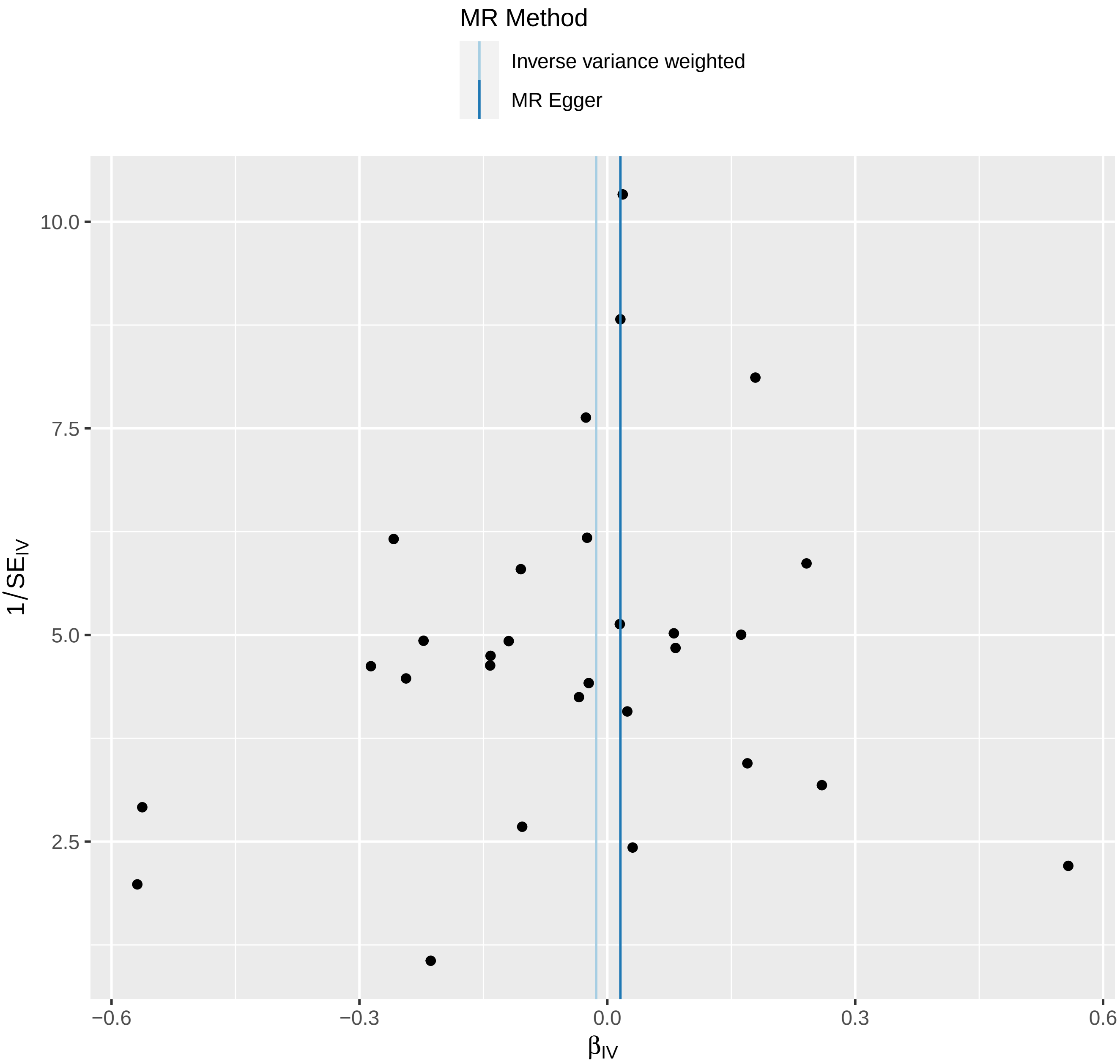

Batch 3−2640 : Lysosome membrane protein 2 on Diabetic nephropathy (more control exclusions)
with a threshold of 1e−05

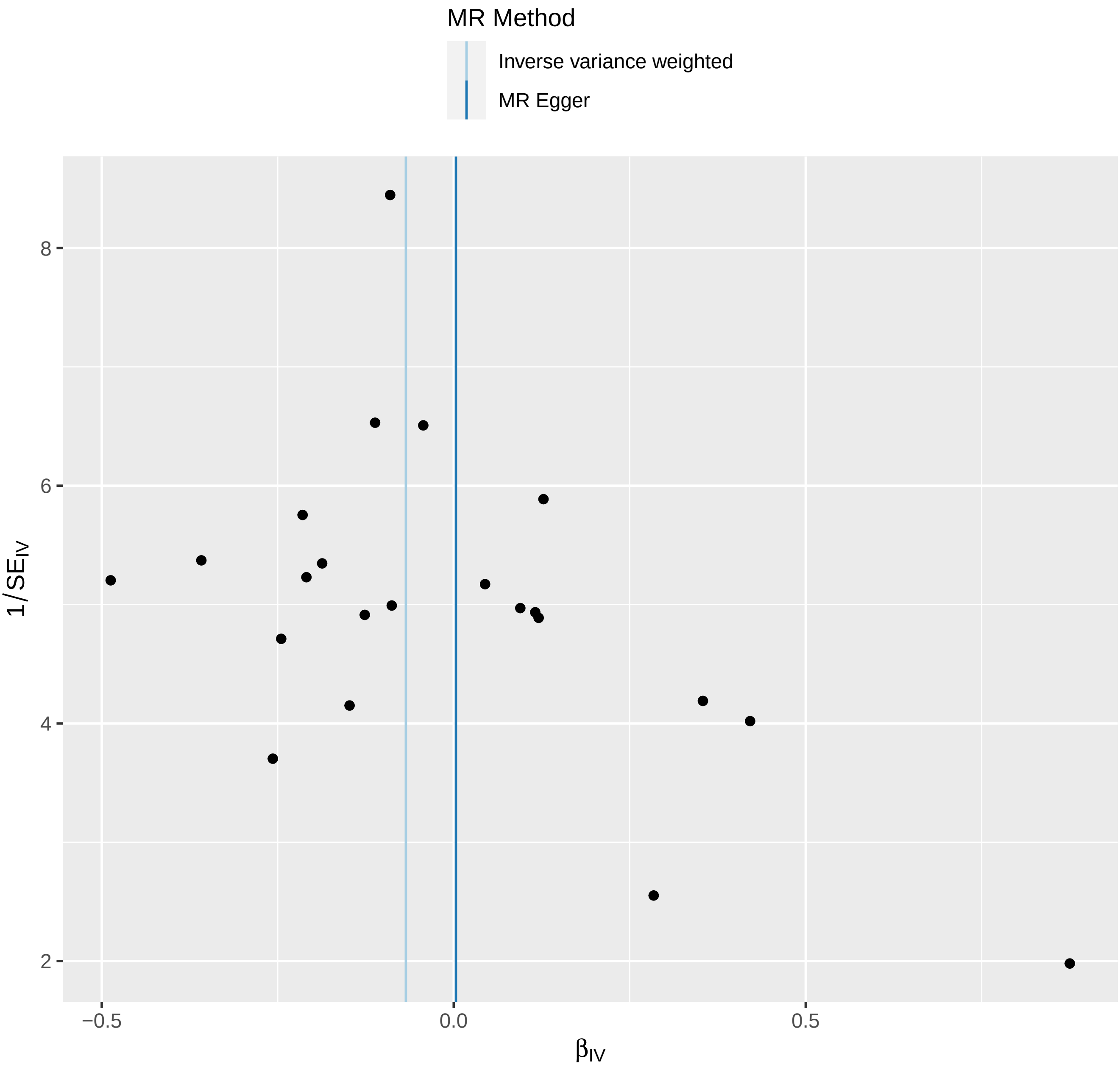

Batch 3−2641 : Scavenger receptor class F member 2 on Diabetic nephropathy (more control exclusions)
with a threshold of 1e−05

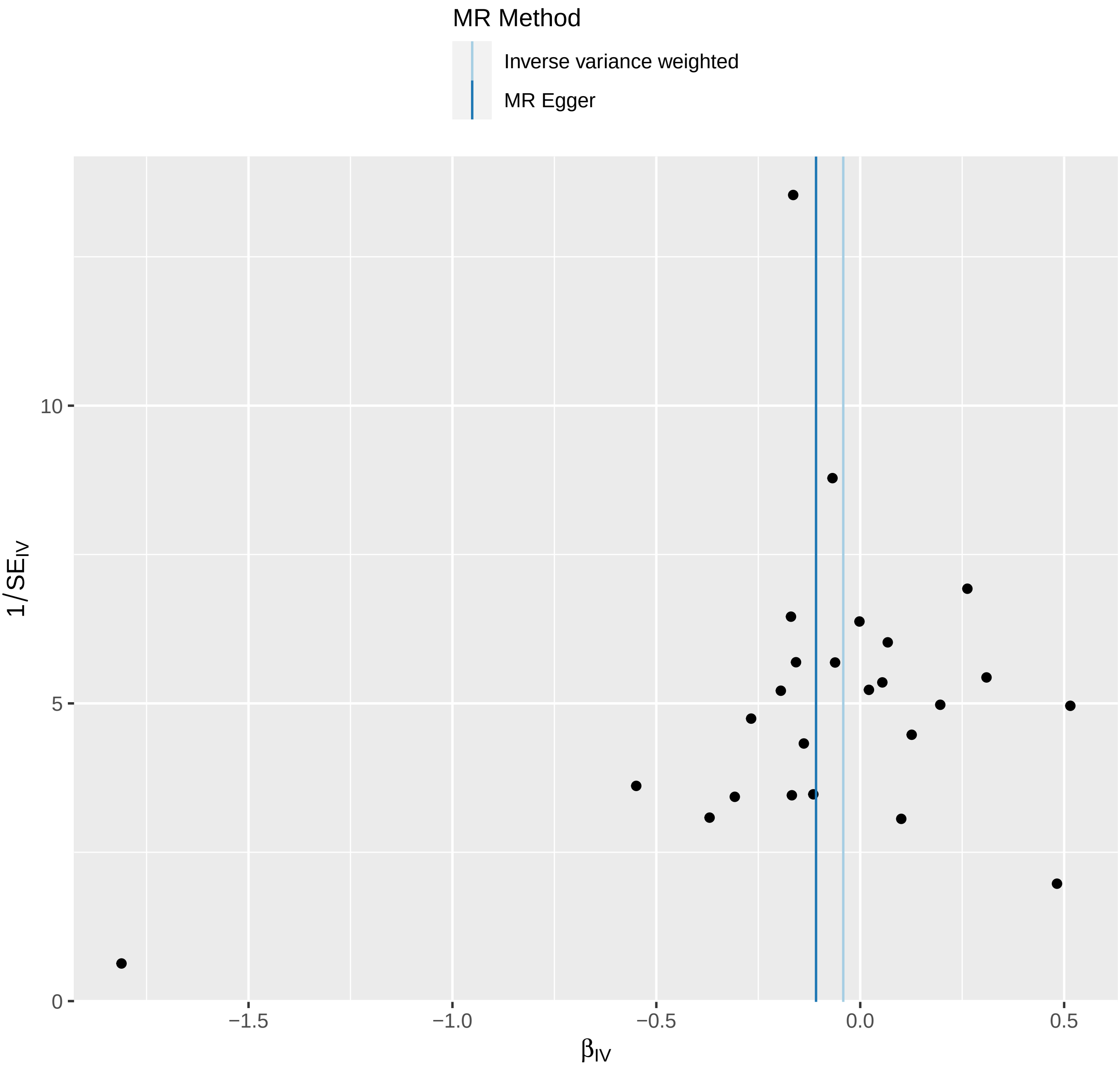

Batch 3−2642 : Scavenger receptor class F member 2 on Diabetic nephropathy (more control exclusions)
with a threshold of 1e−05

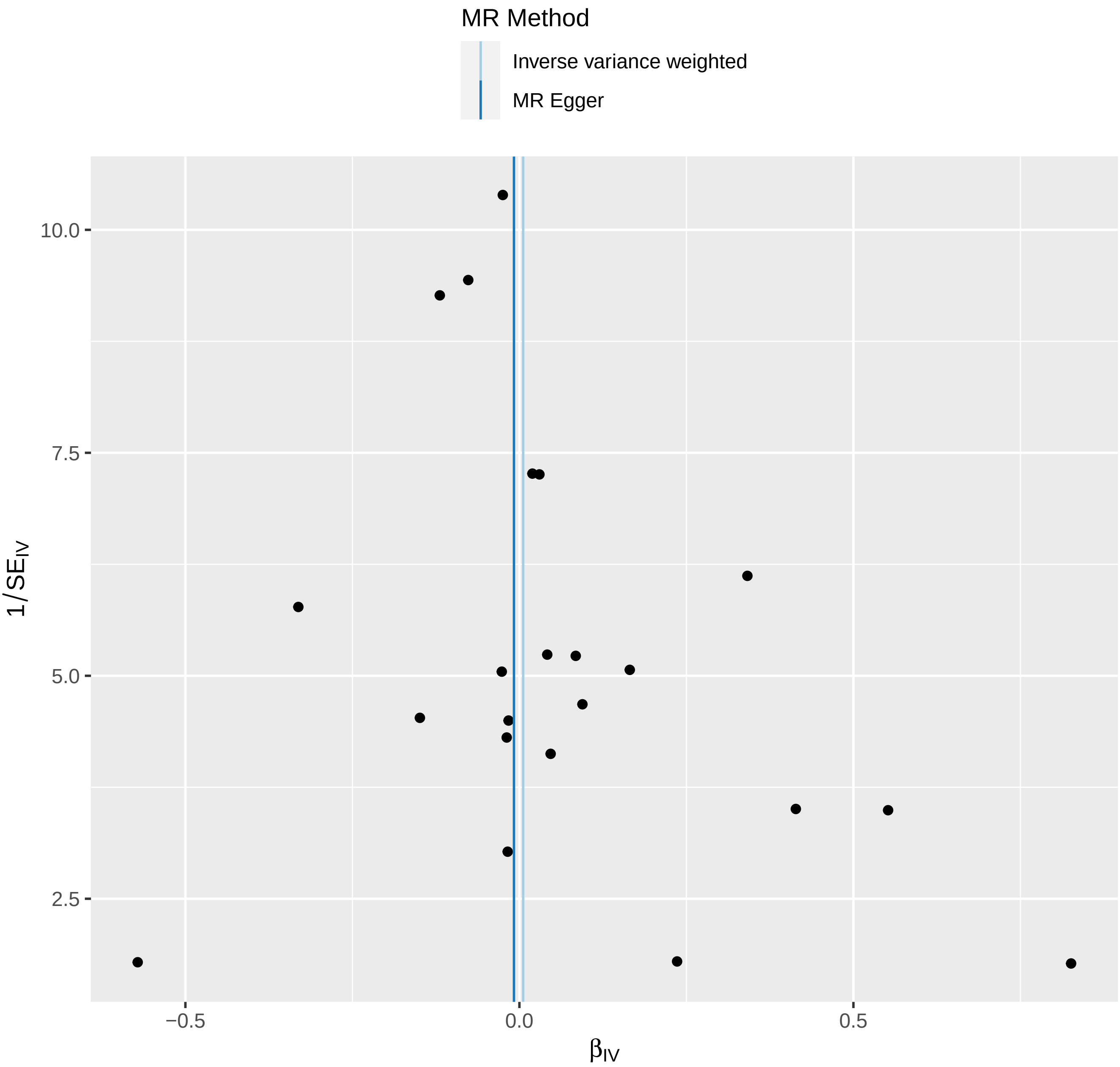

Batch 3−2643 : Scavenger receptor class F member 2 on Diabetic nephropathy (more control exclusions)
with a threshold of 1e−05

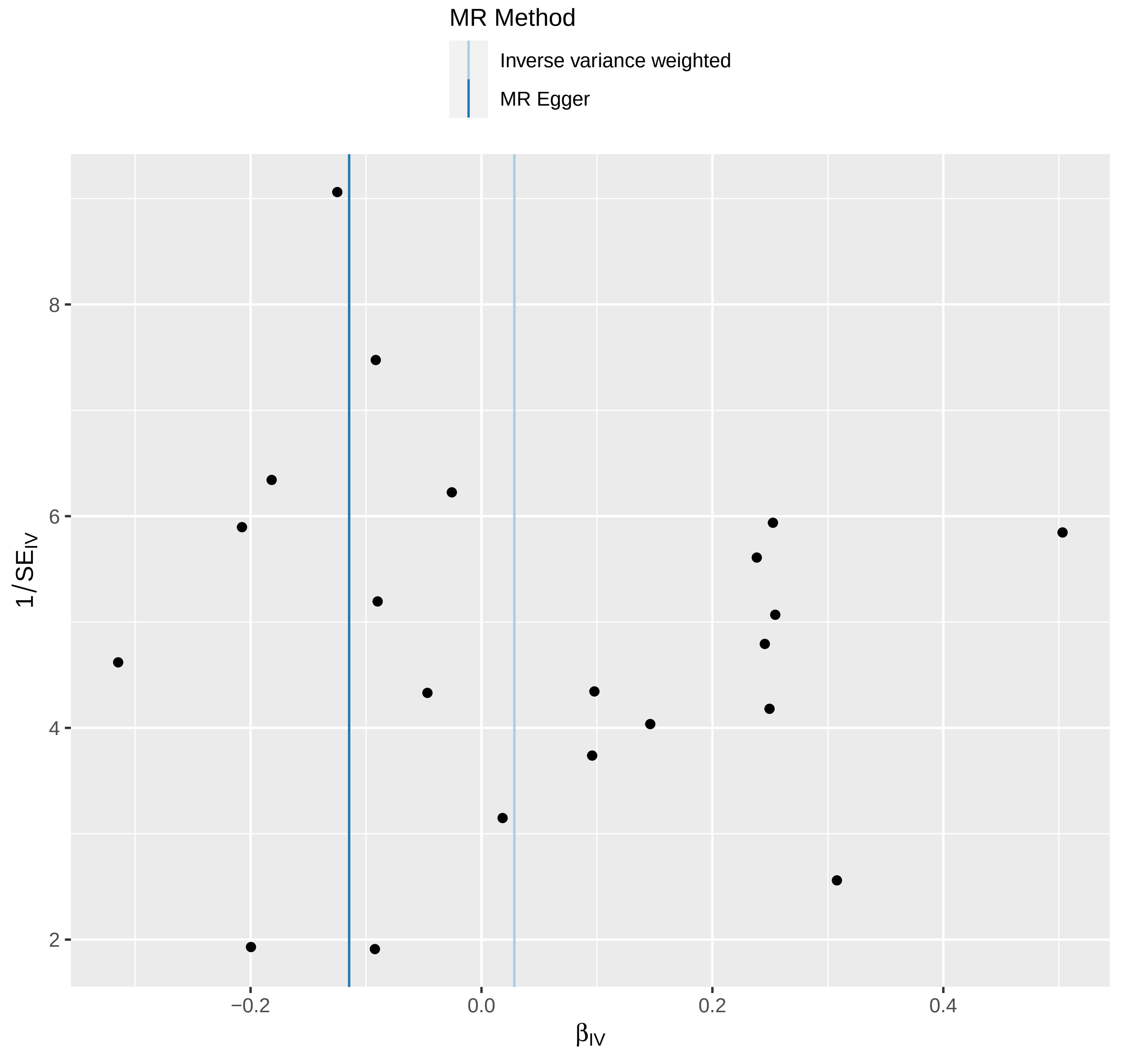

Batch 3−2644 : Secretogranin−2 on Diabetic nephropathy (more control exclusions)
with a threshold of 1e−05

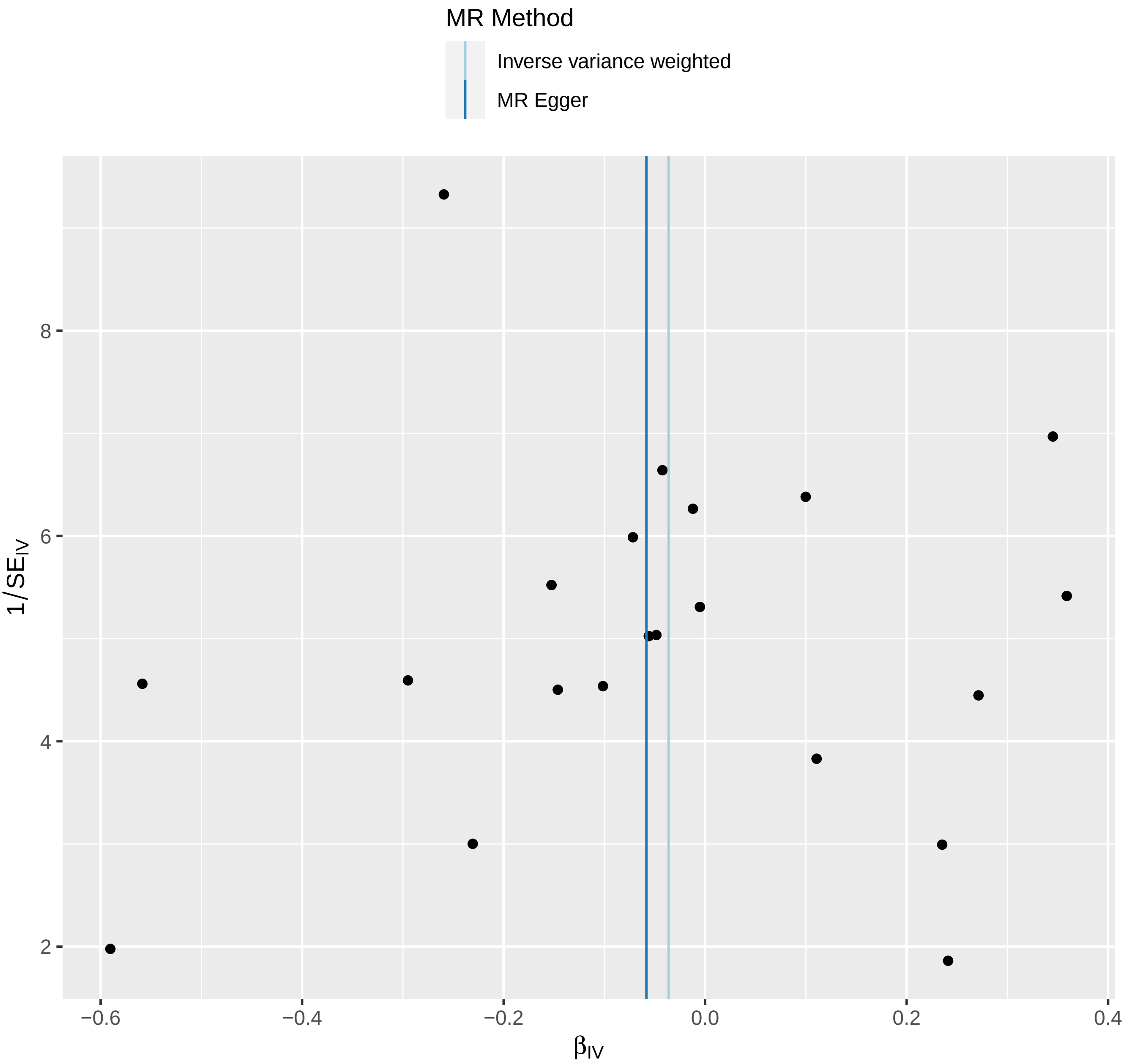

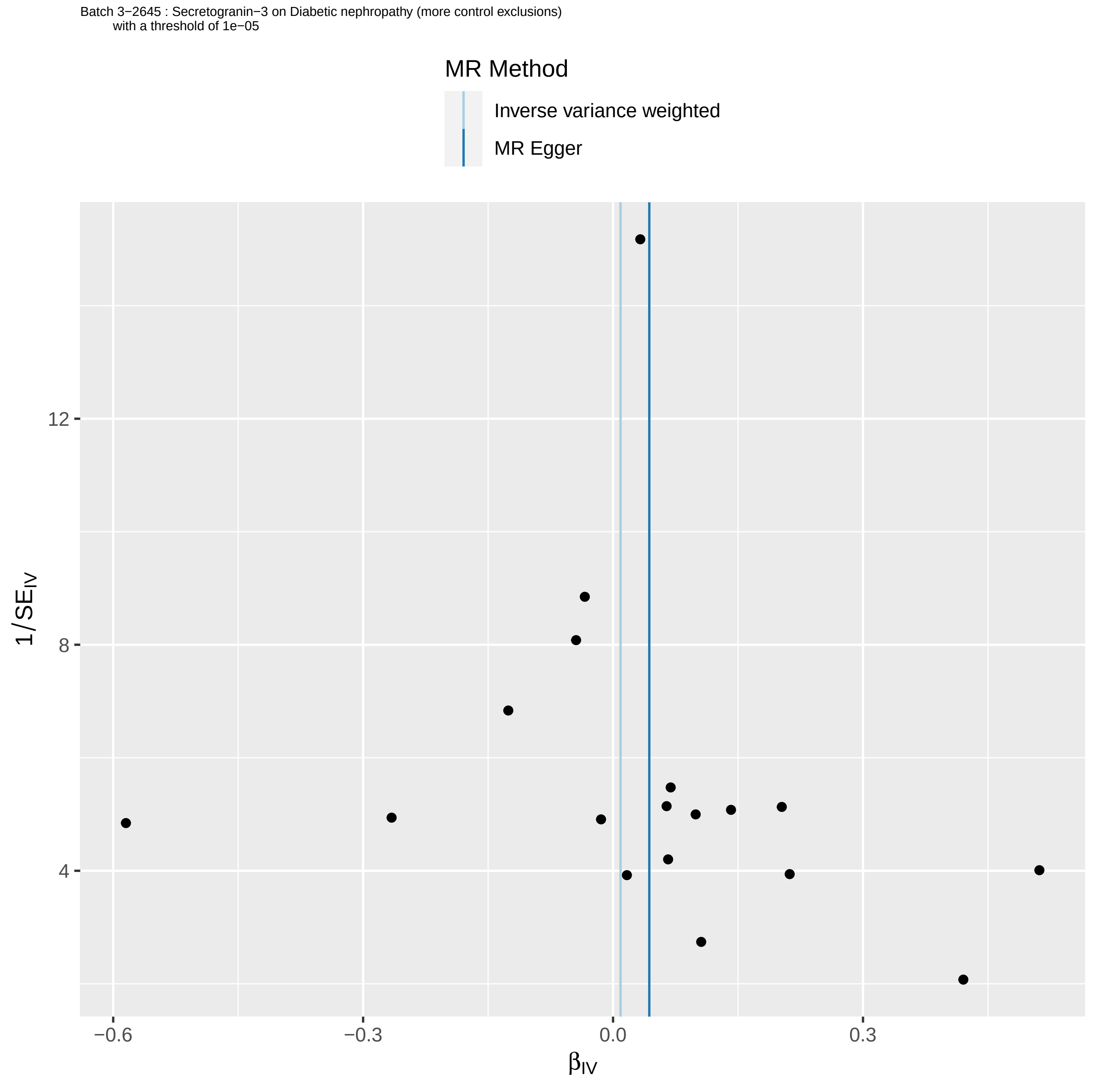

Batch 3−2645 : Secretogranin−3 on Diabetic nephropathy (more control exclusions)
with a threshold of 1e−05
MR Method
Inverse variance weighted
MR Egger
12
8
4
1/SE_IV
−0.6
−0.3
0.0
0.3
β_IV

Batch 3−2646 : Secretoglobin family 1C member 1 on Diabetic nephropathy (more control exclusions)
with a threshold of 1e−05

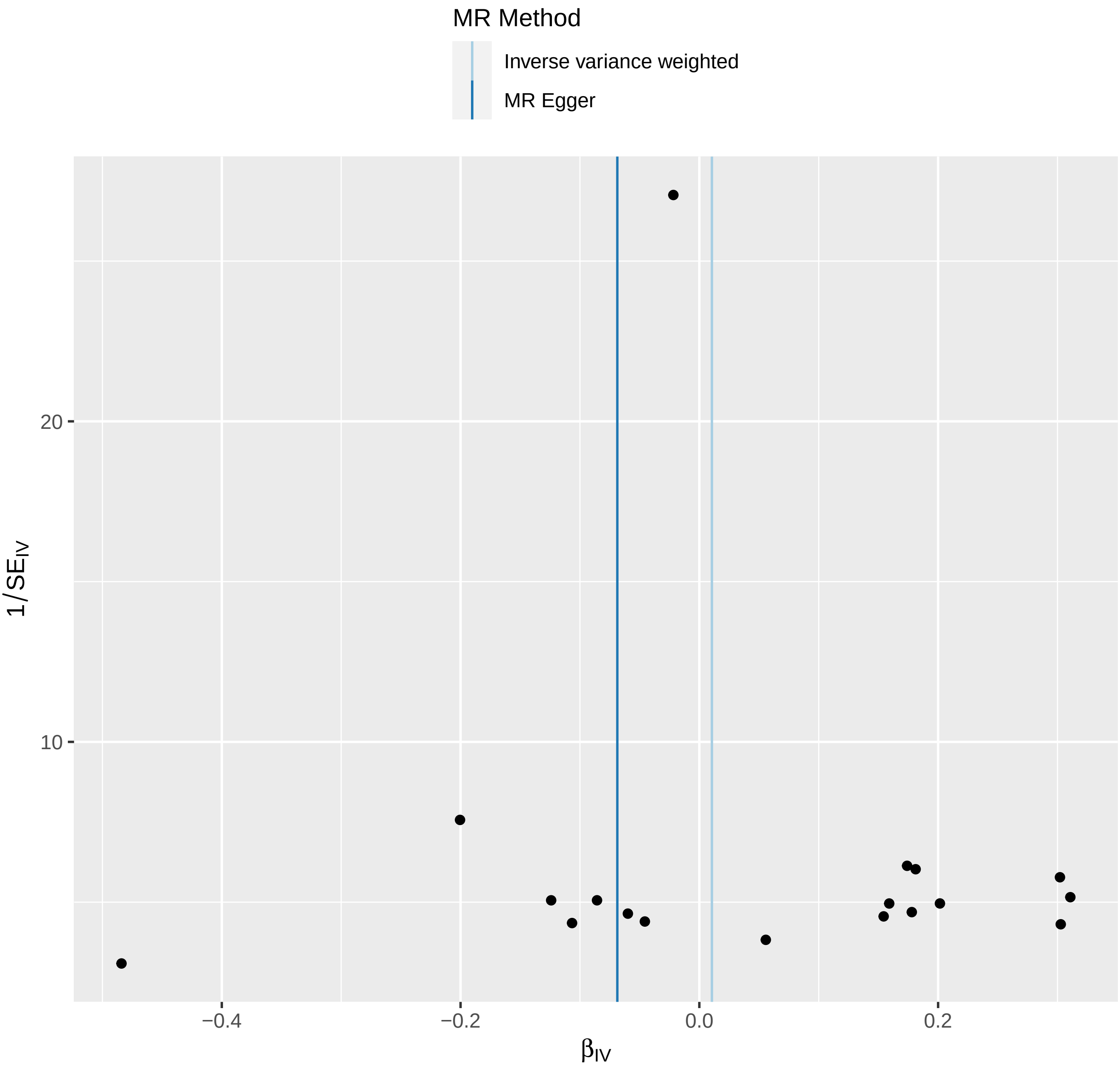

Batch 3−2647 : Secretoglobin family 1D member 2 on Diabetic nephropathy (more control exclusions)
with a threshold of 1e−05

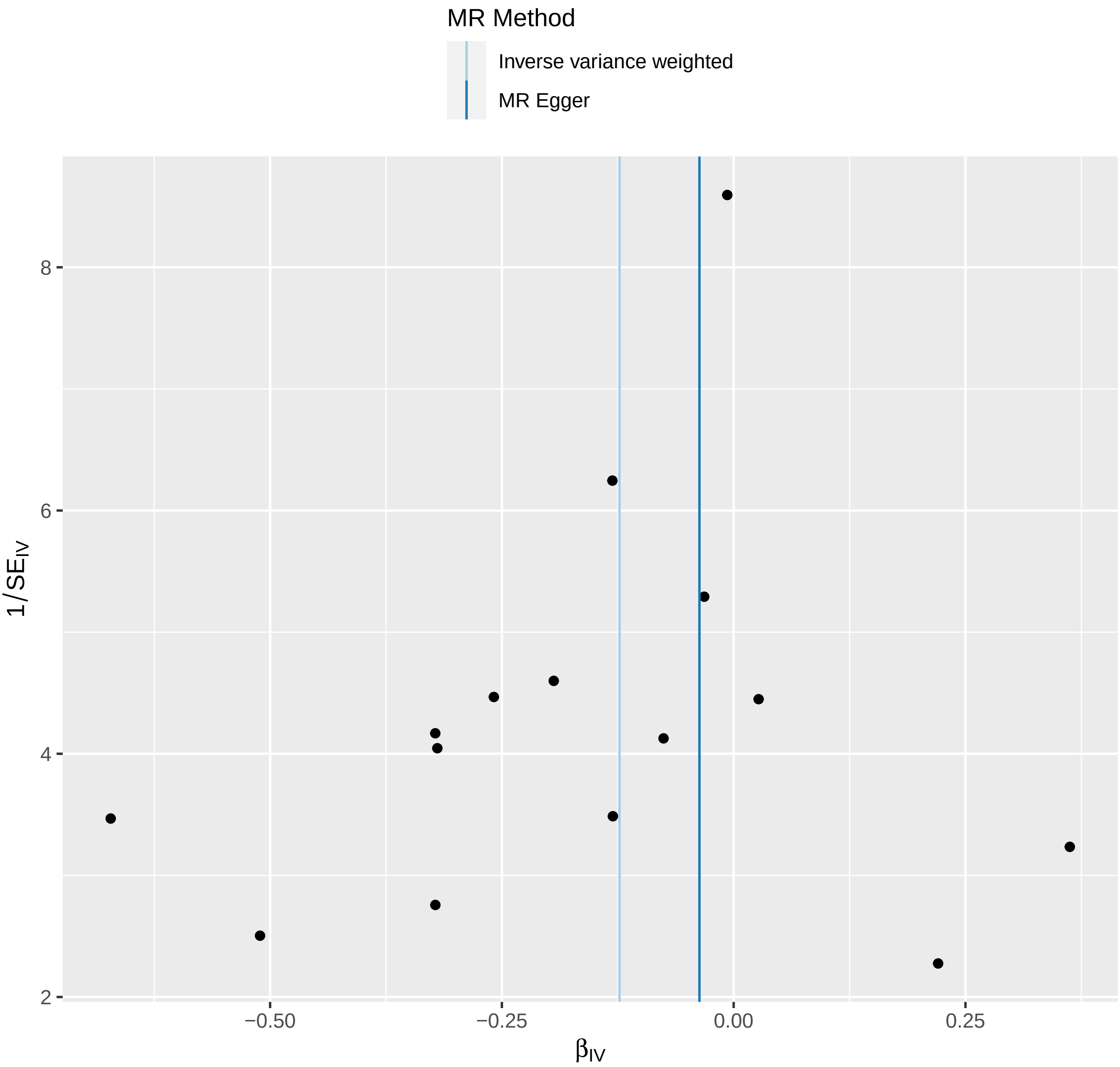

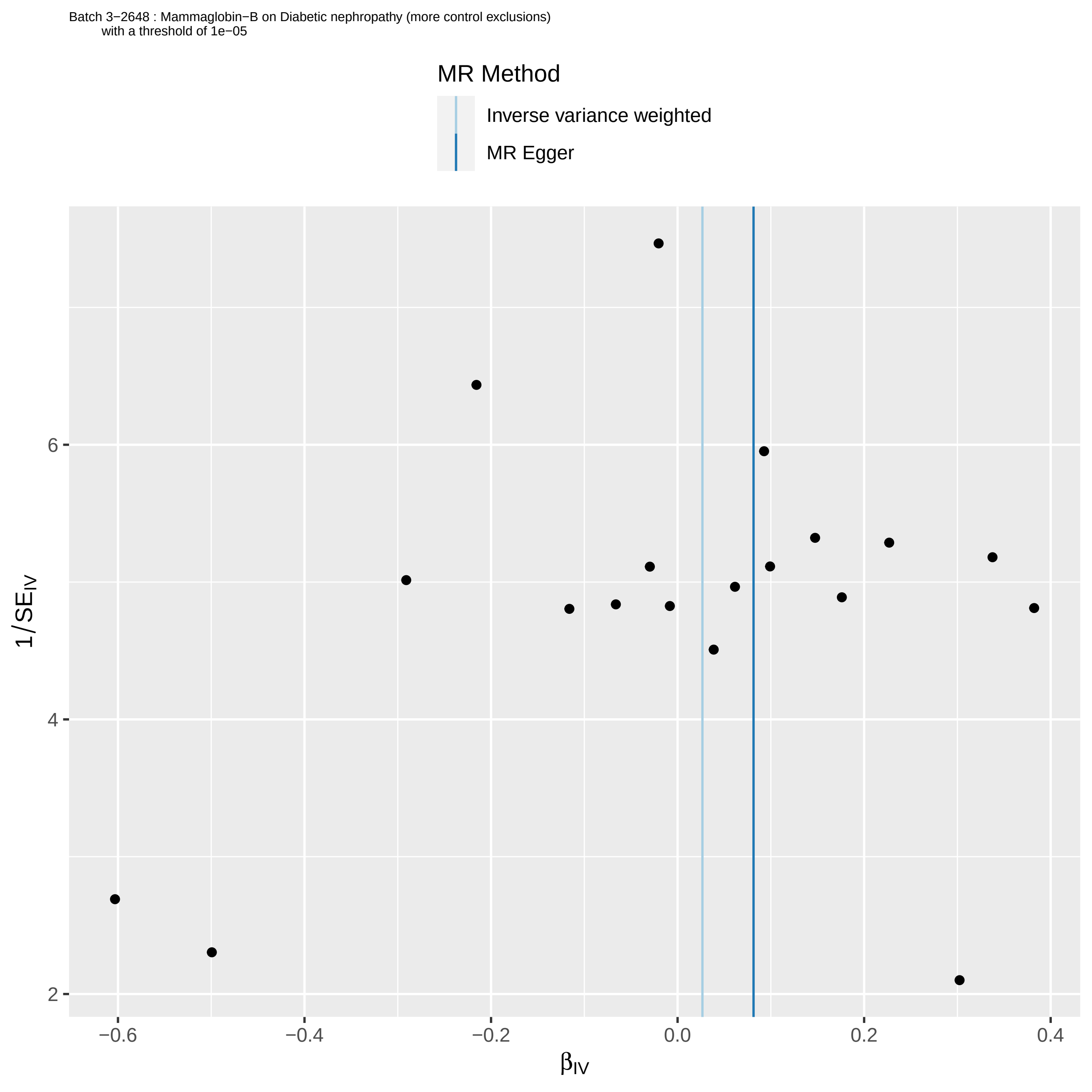

Batch 3−2648 : Mammaglobin−B on Diabetic nephropathy (more control exclusions)
with a threshold of 1e−05
MR Method
Inverse variance weighted
MR Egger
6
4
2
1/SE_IV
−0.6
−0.4
−0.2
0.0
0.2
0.4
β_IV

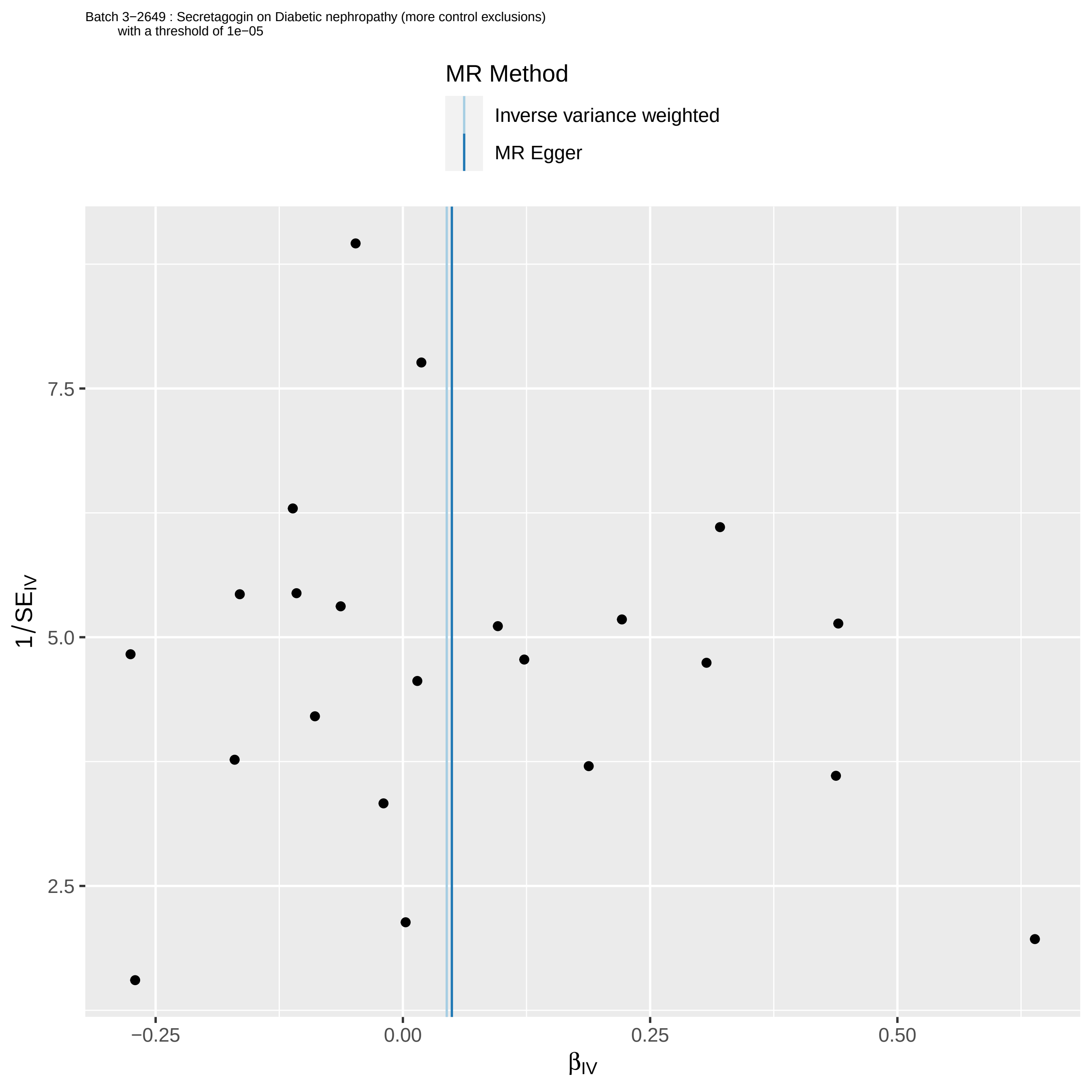

Batch 3−2649 : Secretagogin on Diabetic nephropathy (more control exclusions)
with a threshold of 1e−05
MR Method
Inverse variance weighted
MR Egger
7.5
5.0
2.5
1/SE$_{IV}$
−0.25
0.00
0.25
0.50
β$_{IV}$

Batch 3−2650 : Adseverin on Diabetic nephropathy (more control exclusions)
with a threshold of 1e−05

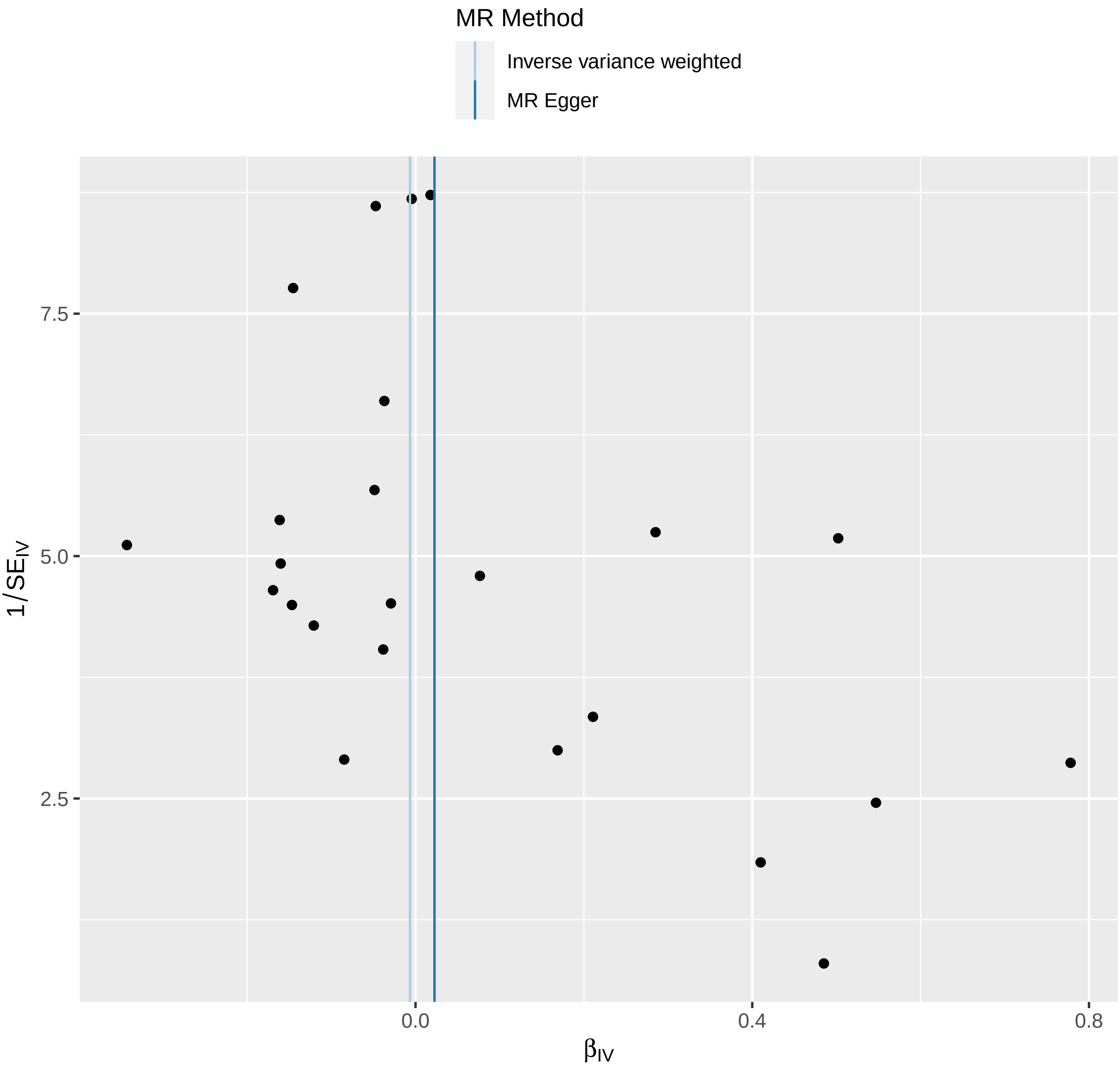

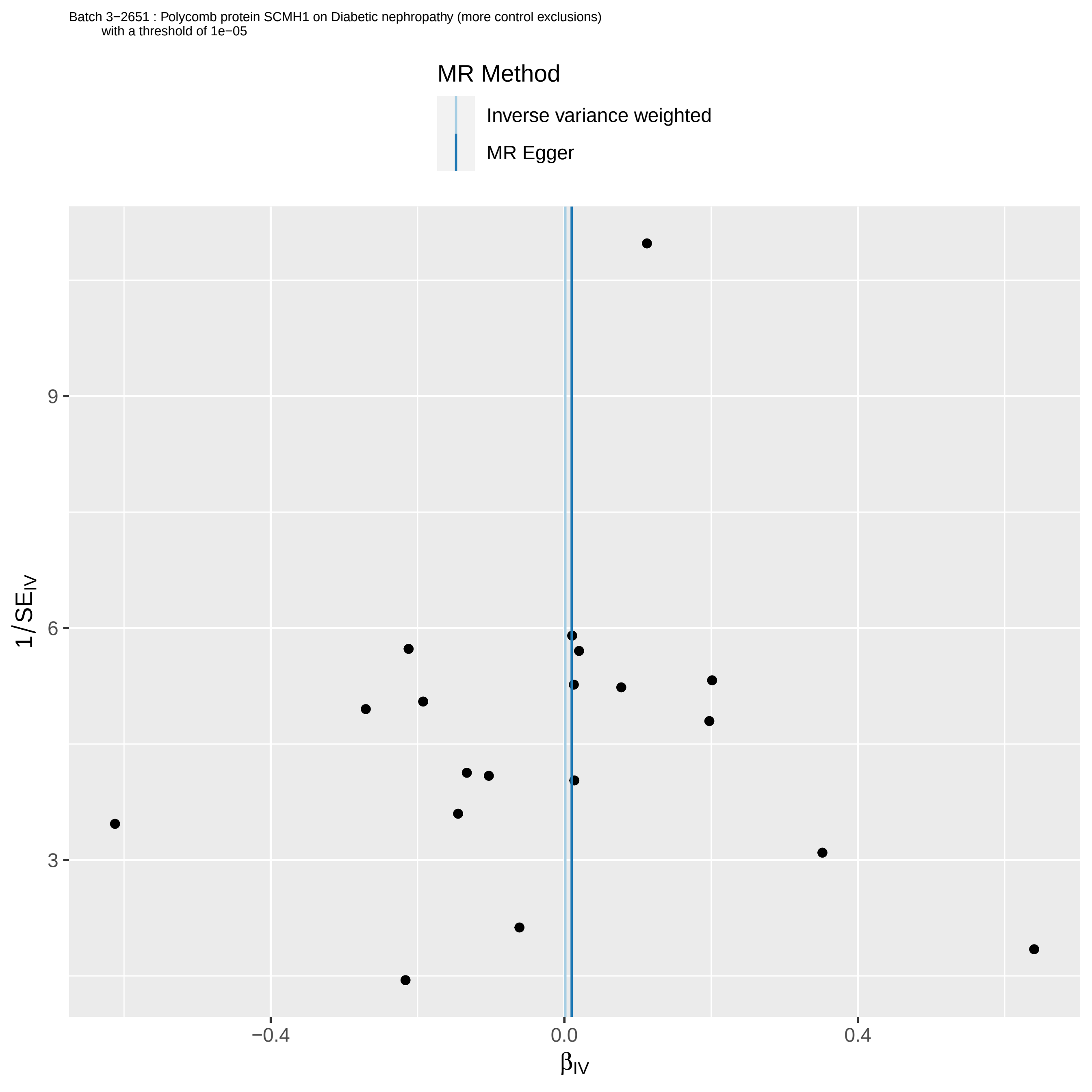

Batch 3−2651 : Polycomb protein SCMH1 on Diabetic nephropathy (more control exclusions)
with a threshold of 1e−05
MR Method
Inverse variance weighted
MR Egger
9
6
3
1/SE$_{IV}$
−0.4
0.0
0.4
β_{IV}

Batch 3−2652 : Sodium channel subunit beta−2 on Diabetic nephropathy (more control exclusions) with a threshold of 1e−05

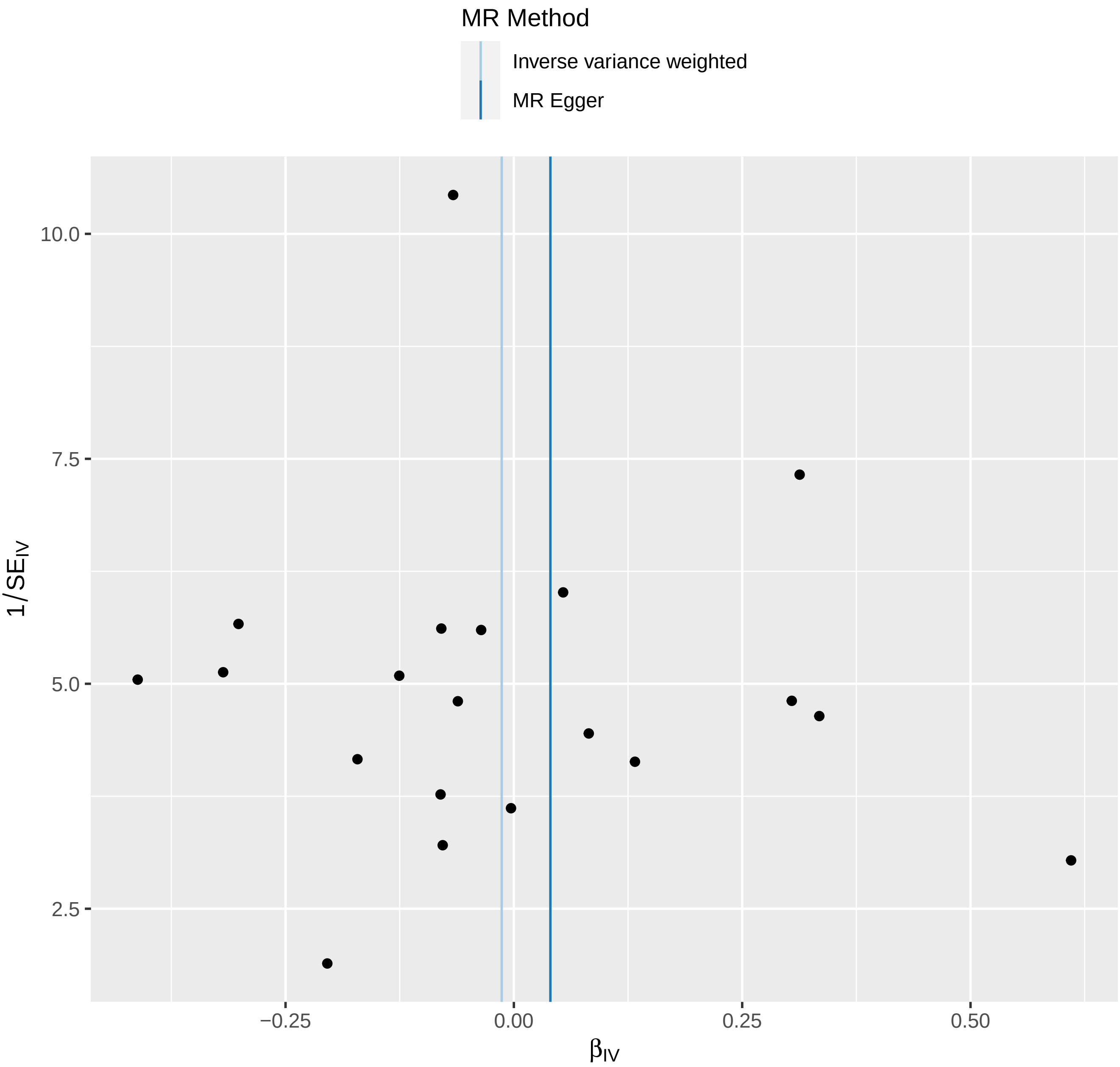

Batch 3−2653 : Protein SCO1 homolog, mitochondrial on Diabetic nephropathy (more control exclusions)
with a threshold of 1e−05

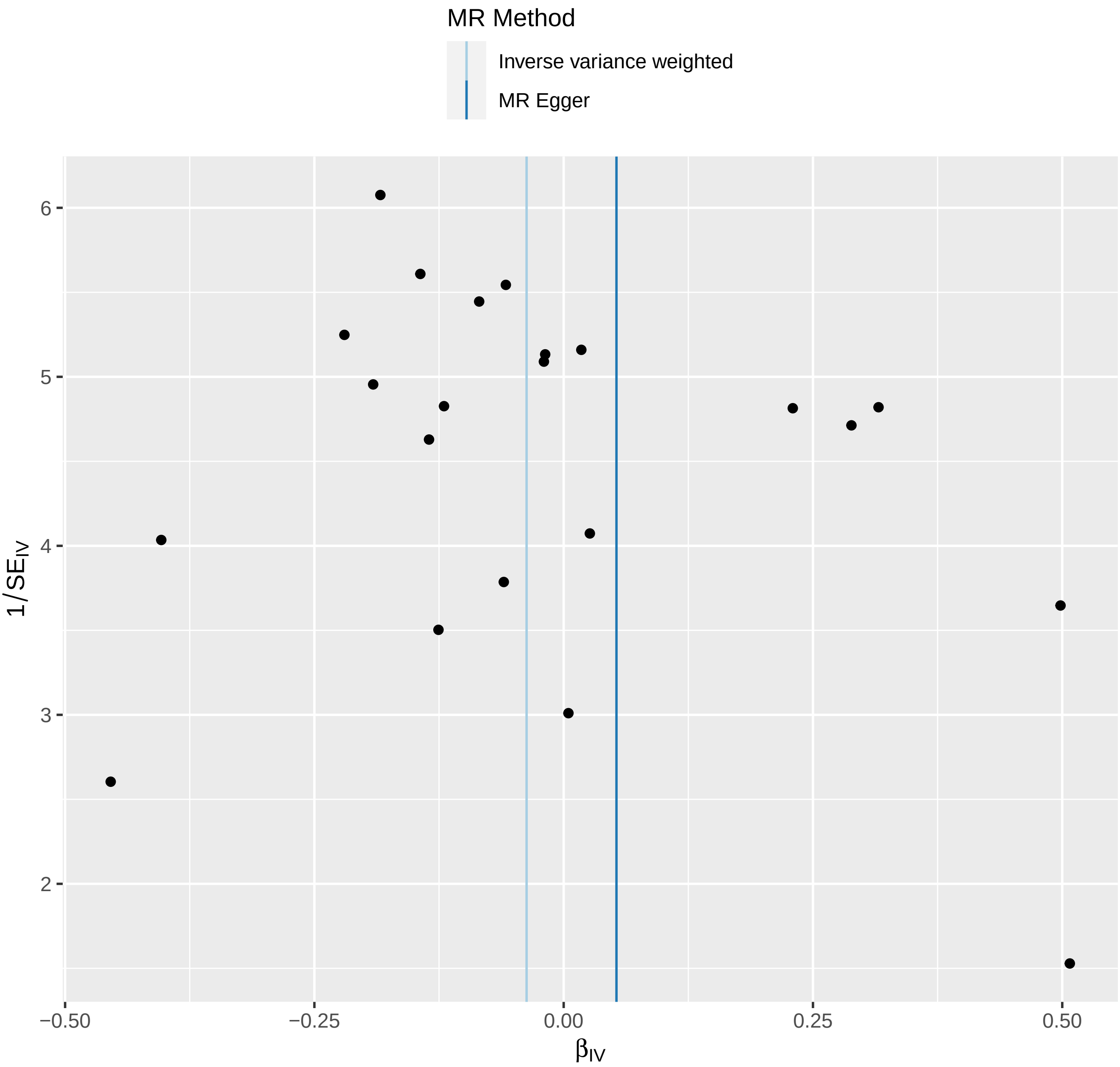

Batch 3−2654 : SCP2 sterol−binding domain−containing protein 1 on Diabetic nephropathy (more control exclusions)
with a threshold of 1e−05

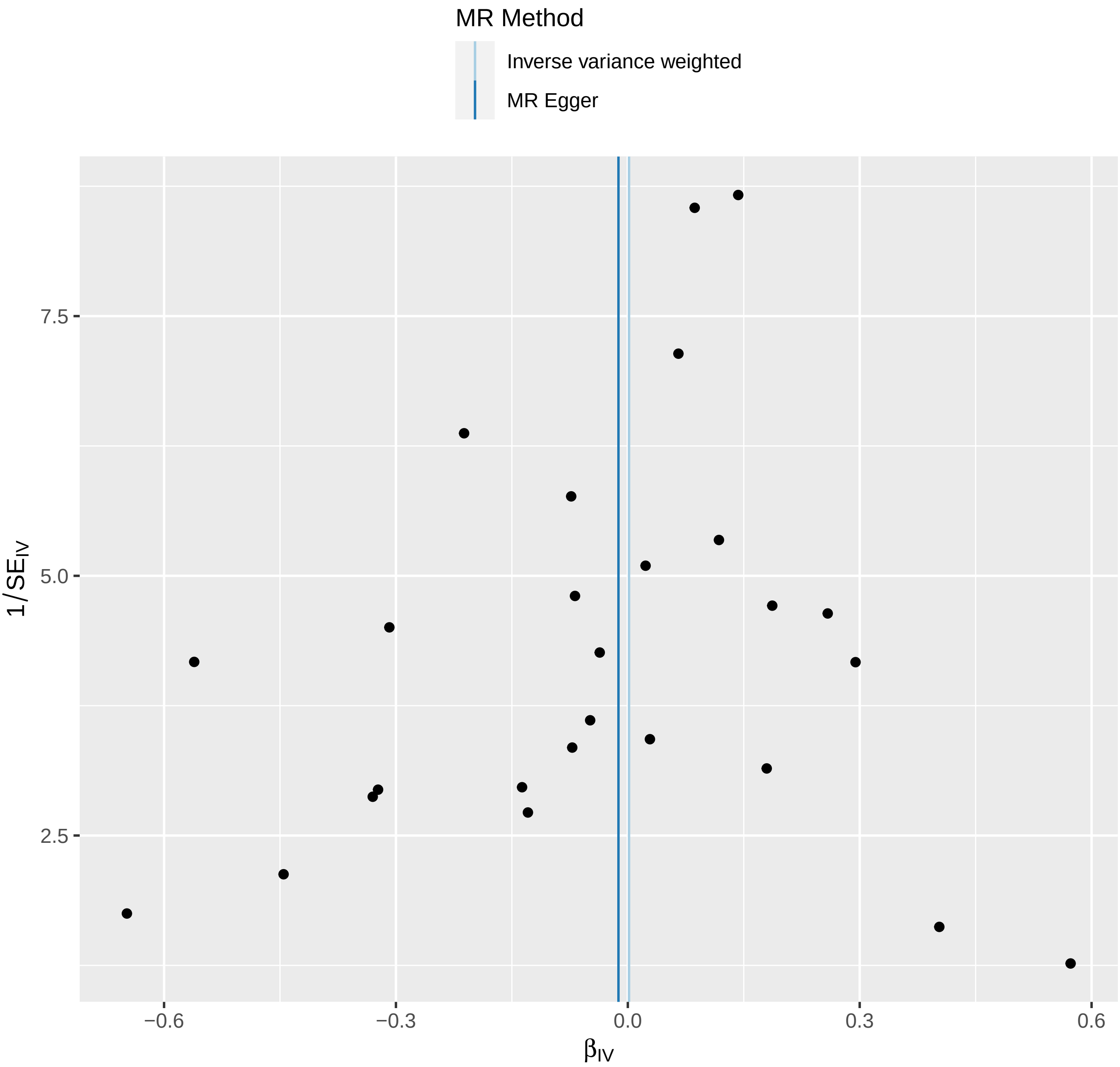

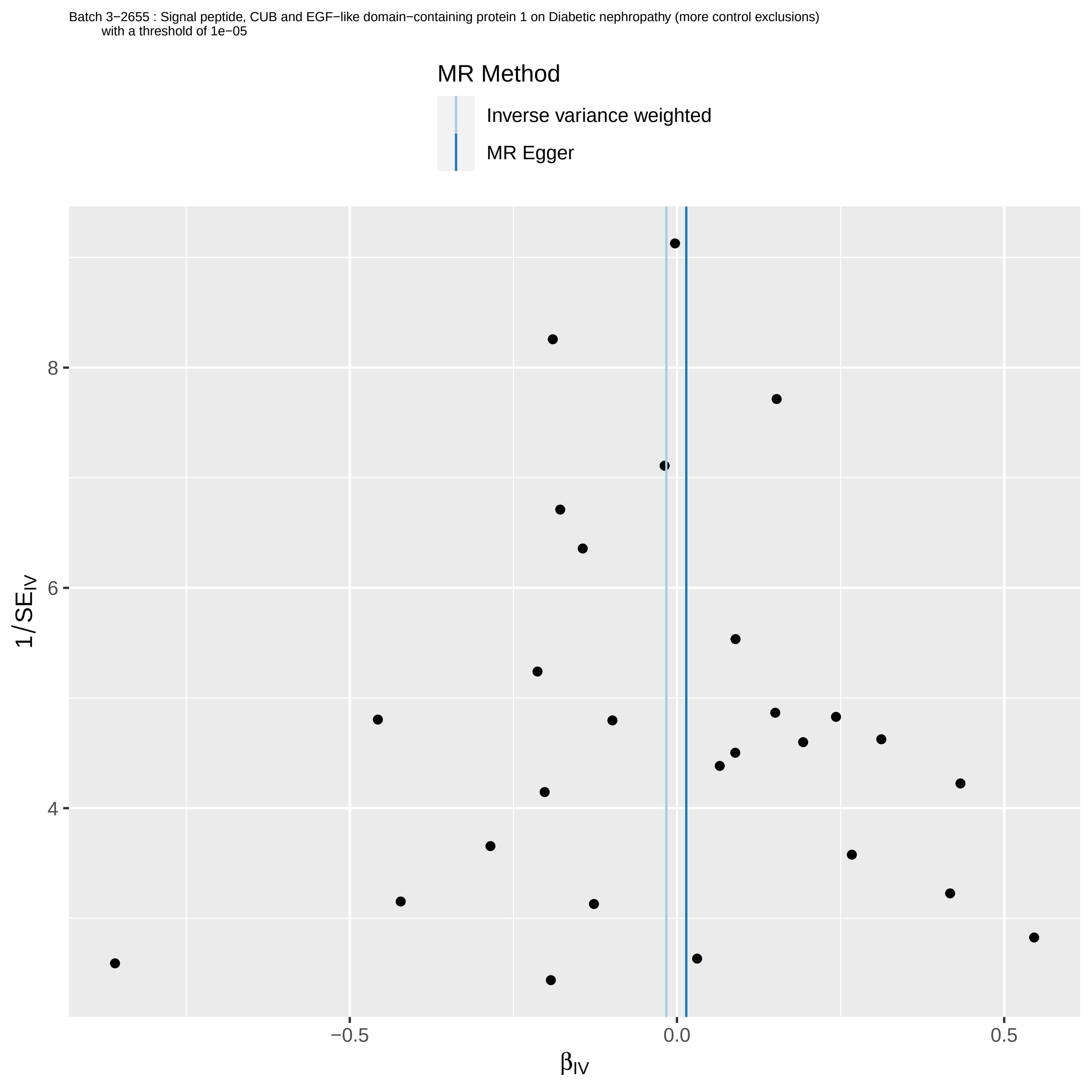
Batch 3−2655 : Signal peptide, CUB and EGF−like domain−containing protein 1 on Diabetic nephropathy (more control exclusions)
with a threshold of 1e−05
MR Method
Inverse variance weighted
MR Egger
8
6
4
1/SE_IV
−0.5
0.0
0.5
β_IV

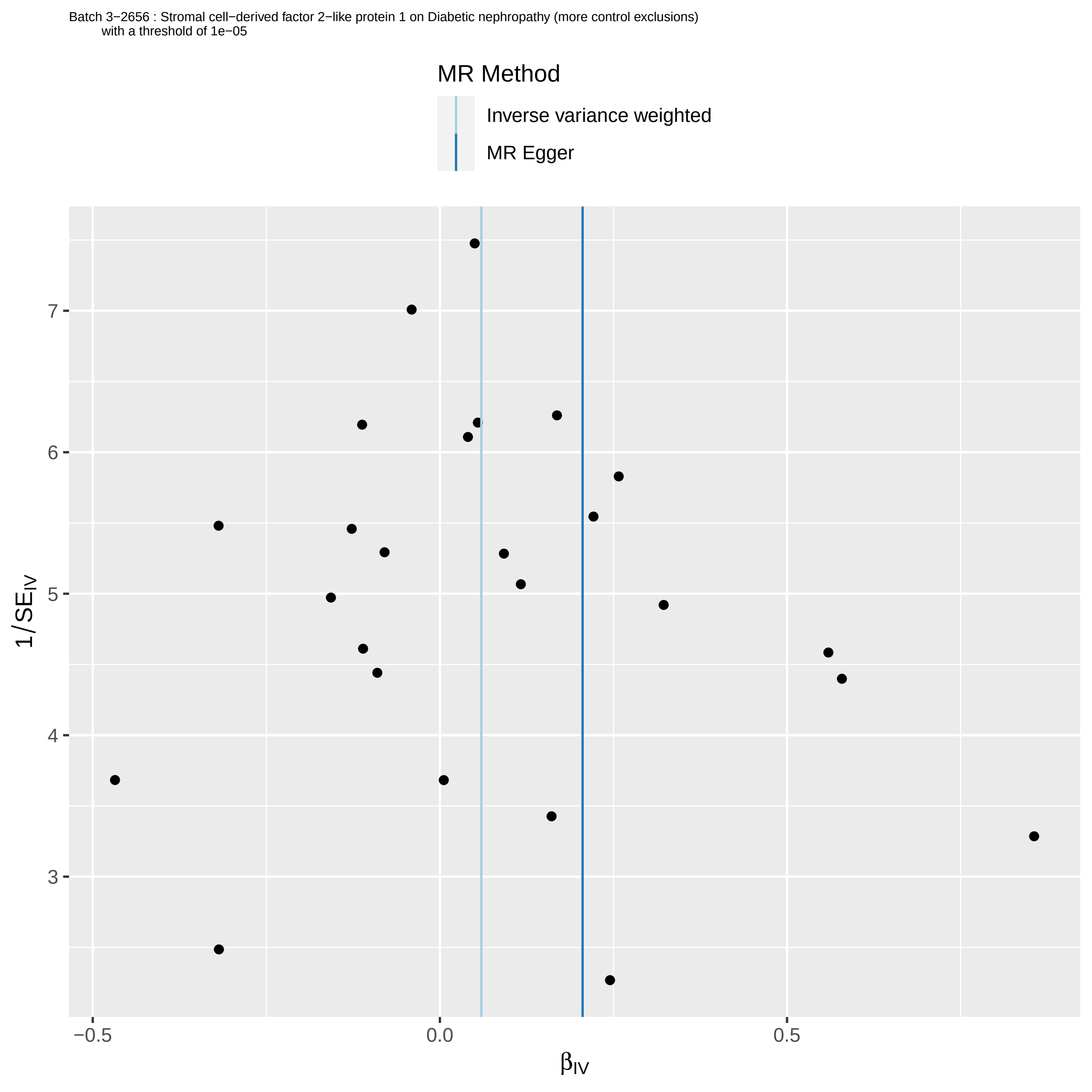

Batch 3−2656 : Stromal cell−derived factor 2−like protein 1 on Diabetic nephropathy (more control exclusions)
with a threshold of 1e−05
MR Method
Inverse variance weighted
MR Egger
7
6
5
4
3
1/SE$_{IV}$
−0.5
0.0
0.5
β_{IV}

Batch 3−2657 : Succinate dehydrogenase assembly factor 2, mitochondrial on Diabetic nephropathy (more control exclusions)
with a threshold of 1e−05

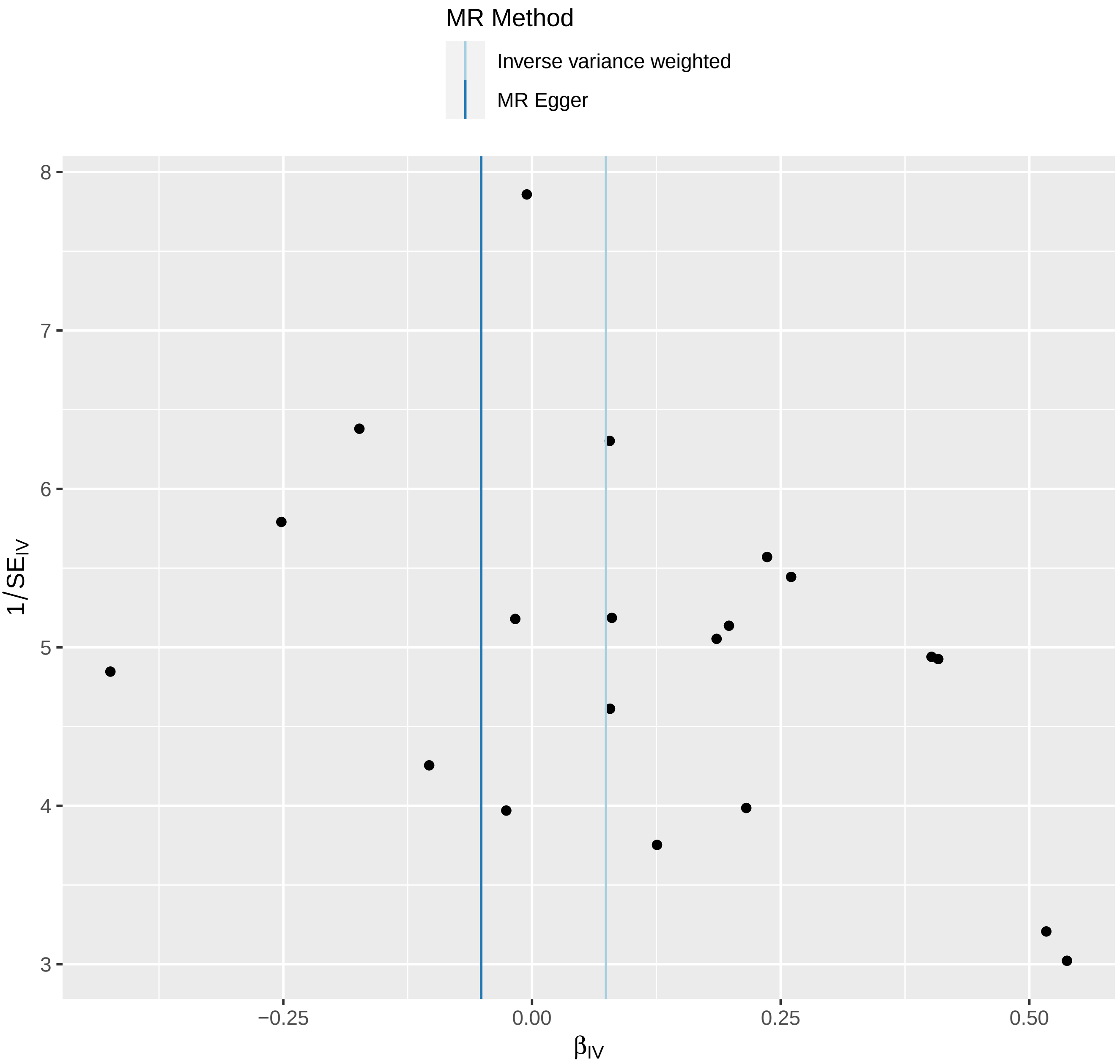

Batch 3−2658 : Signal peptidase complex catalytic subunit SEC11C on Diabetic nephropathy (more control exclusions)
with a threshold of 1e−05

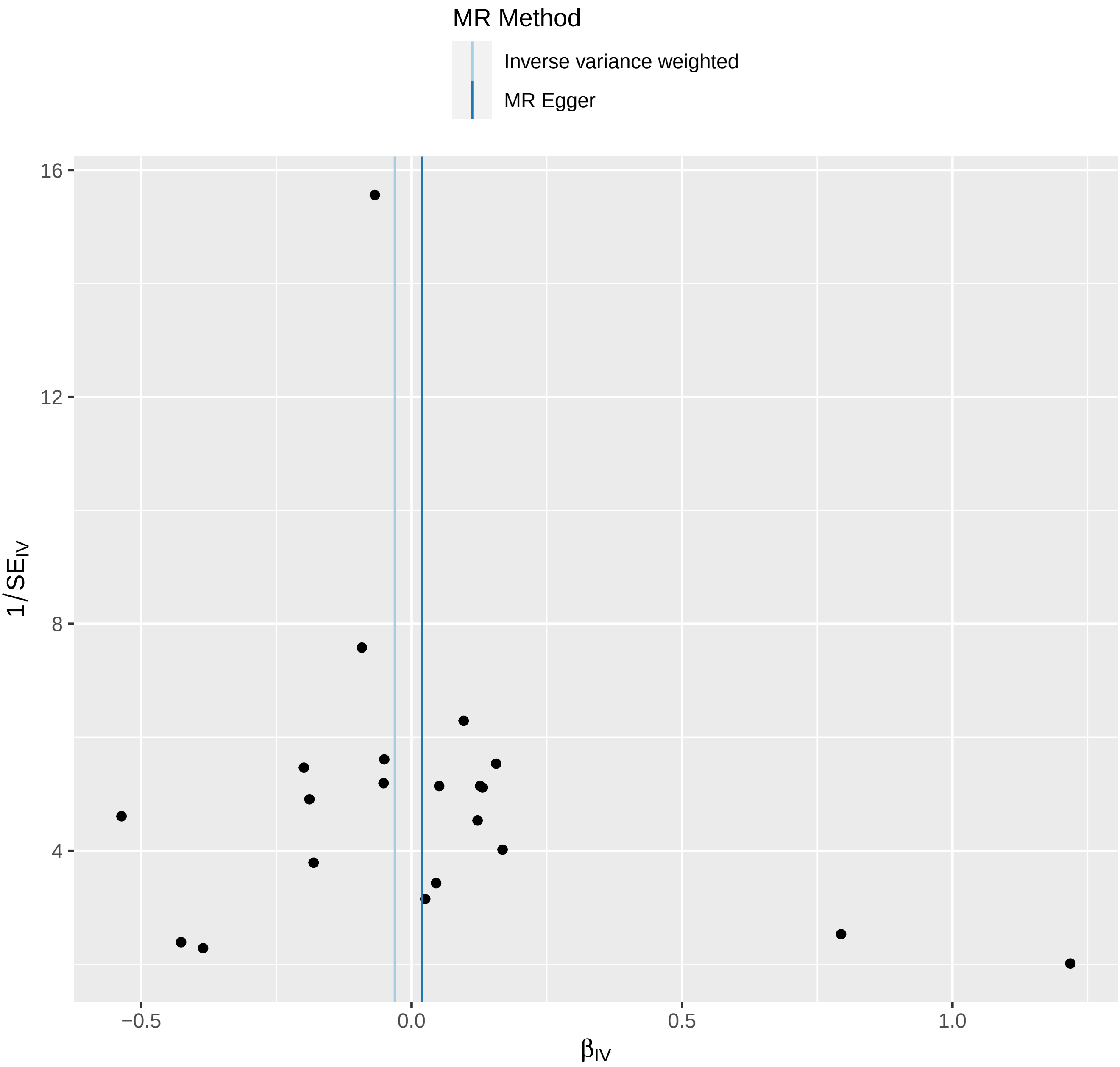

Batch 3−2659 : Protein SEC13 homolog on Diabetic nephropathy (more control exclusions)
with a threshold of 1e−05

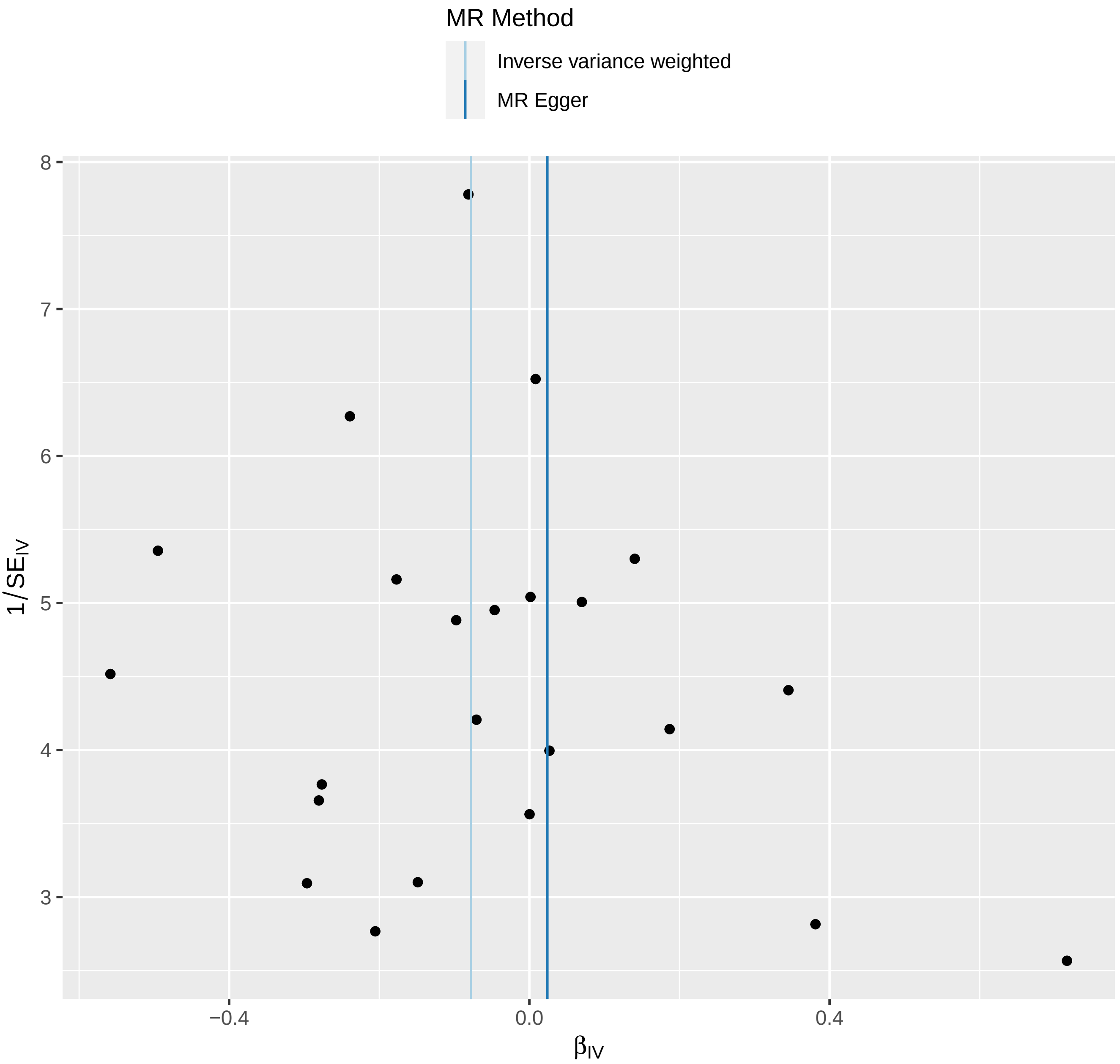

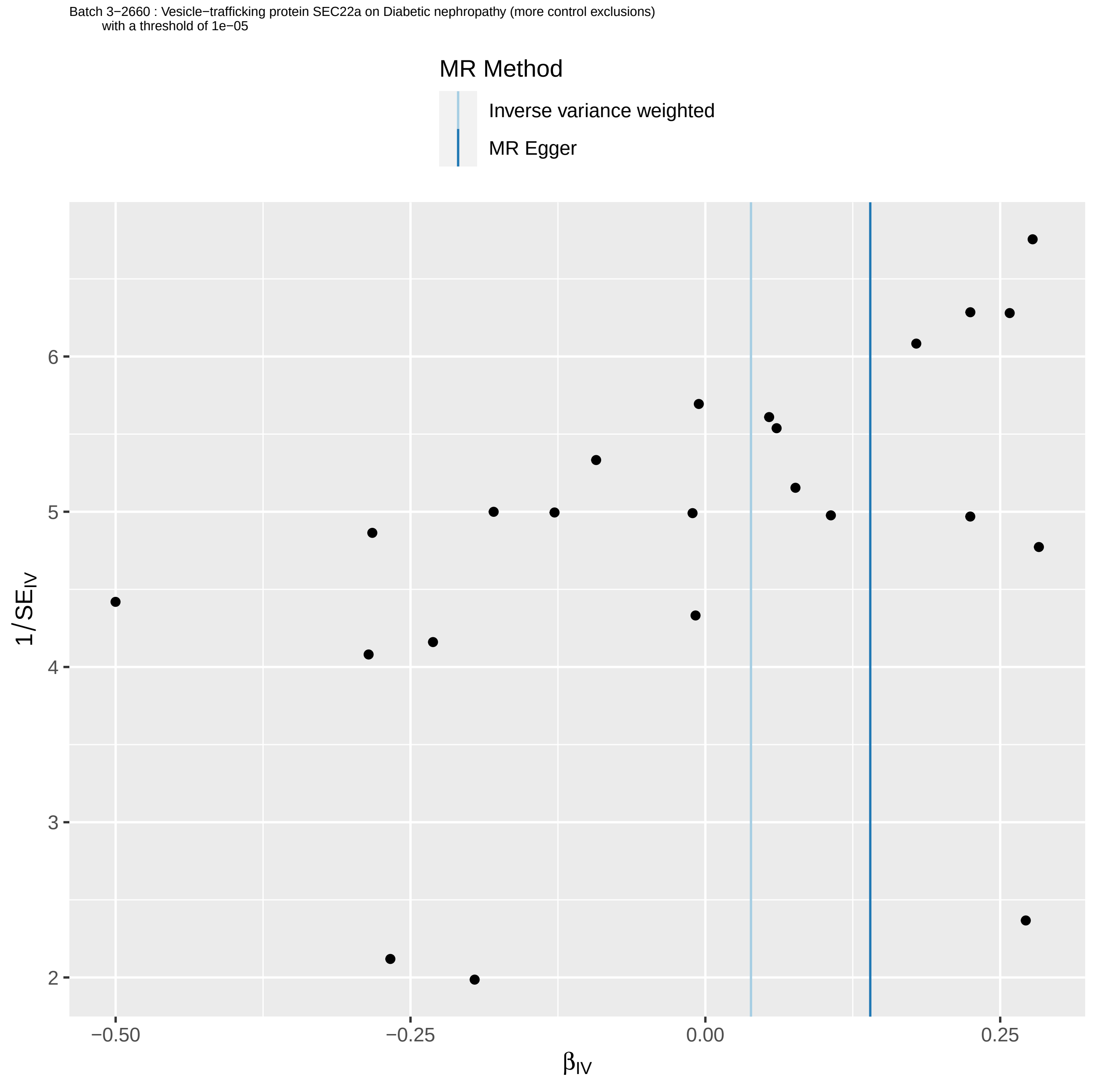

Batch 3−2660 : Vesicle−trafficking protein SEC22a on Diabetic nephropathy (more control exclusions)
with a threshold of 1e−05
MR Method
Inverse variance weighted
MR Egger
1/SE_IV
6
5
4
3
2
−0.50
−0.25
0.00
0.25
β_IV

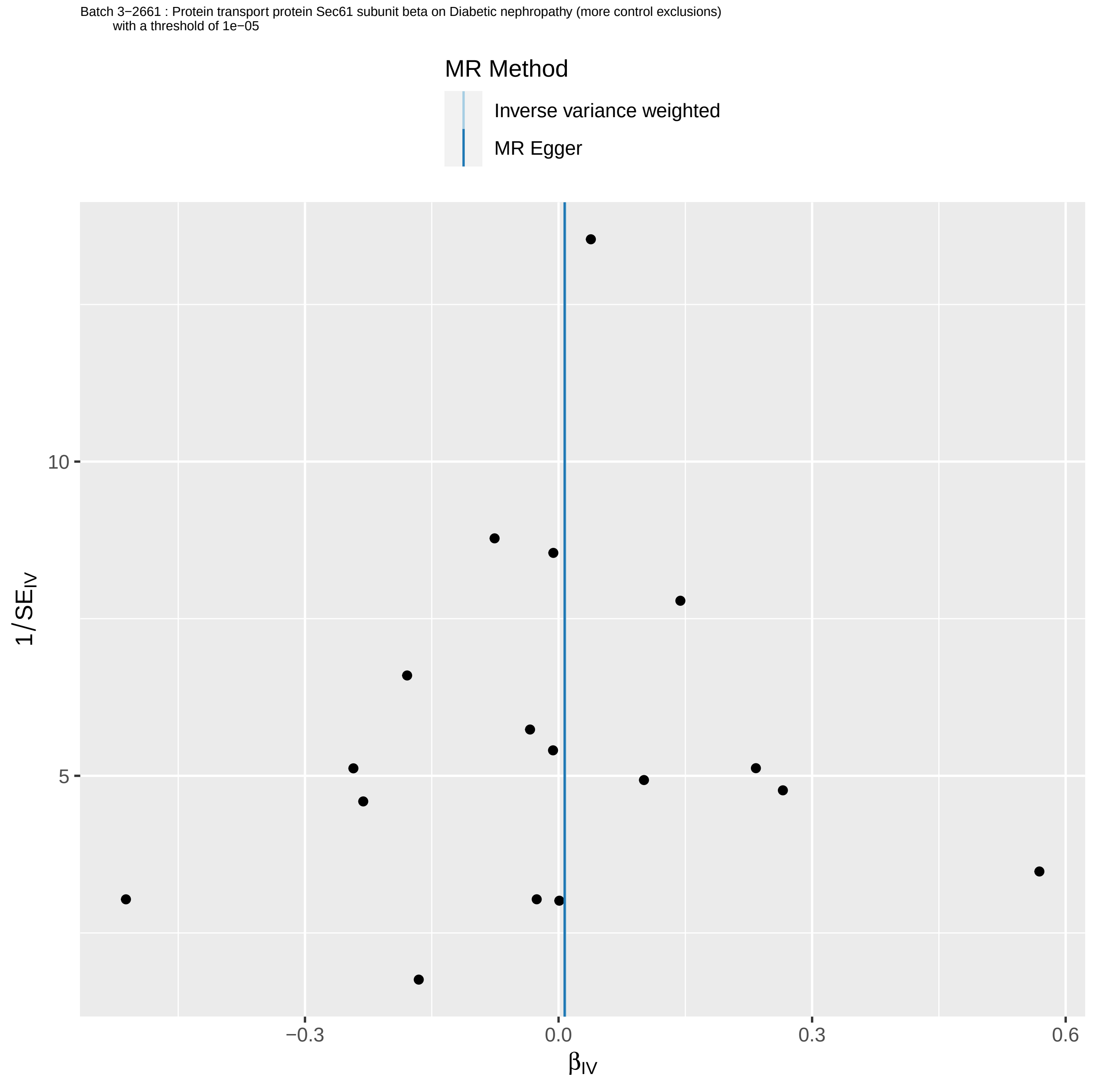
Batch 3−2661 : Protein transport protein Sec61 subunit beta on Diabetic nephropathy (more control exclusions) with a threshold of 1e−05
MR Method
Inverse variance weighted
MR Egger
10
5
1/SE_IV
−0.3
0.0
0.3
0.6
β_IV

Batch 3−2662 : Protein transport protein Sec61 subunit gamma on Diabetic nephropathy (more control exclusions)
with a threshold of 1e−05

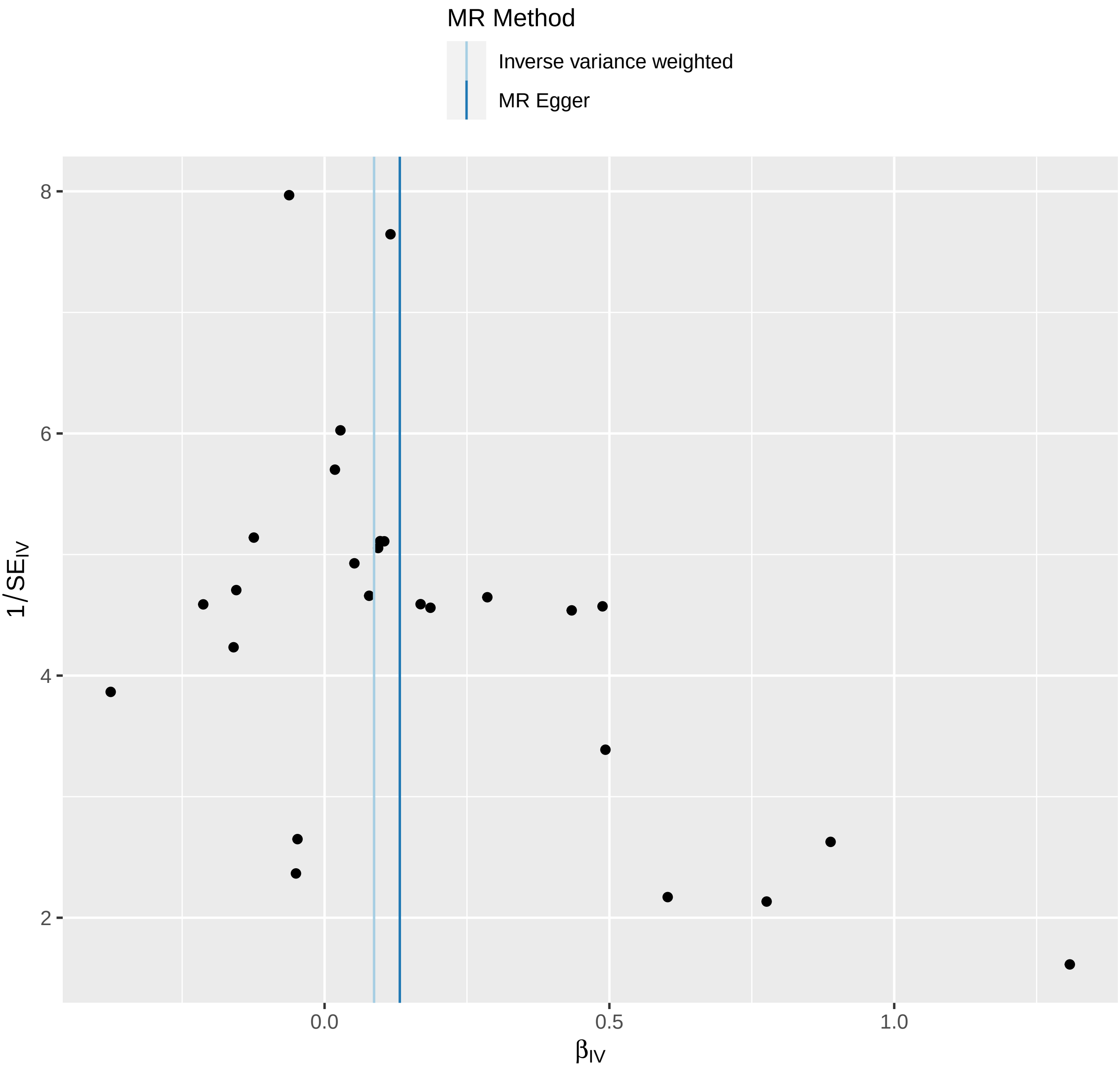

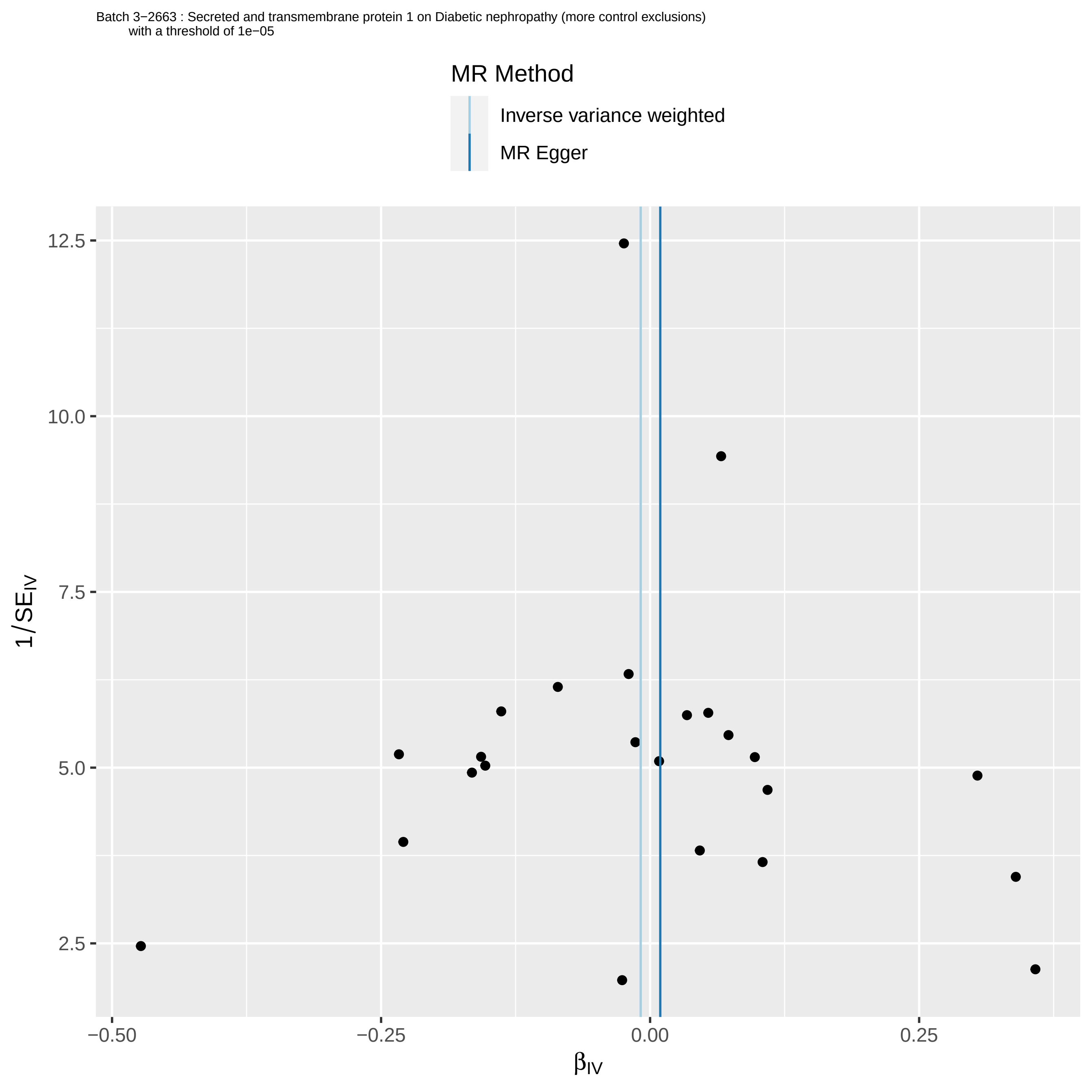
Batch 3−2663 : Secreted and transmembrane protein 1 on Diabetic nephropathy (more control exclusions)
with a threshold of 1e−05
MR Method
Inverse variance weighted
MR Egger
12.5
10.0
7.5
5.0
2.5
1/SE_IV
−0.50
−0.25
0.00
0.25
β_IV

Batch 3−2664 : Protein sel−1 homolog 2 on Diabetic nephropathy (more control exclusions)
with a threshold of 1e−05

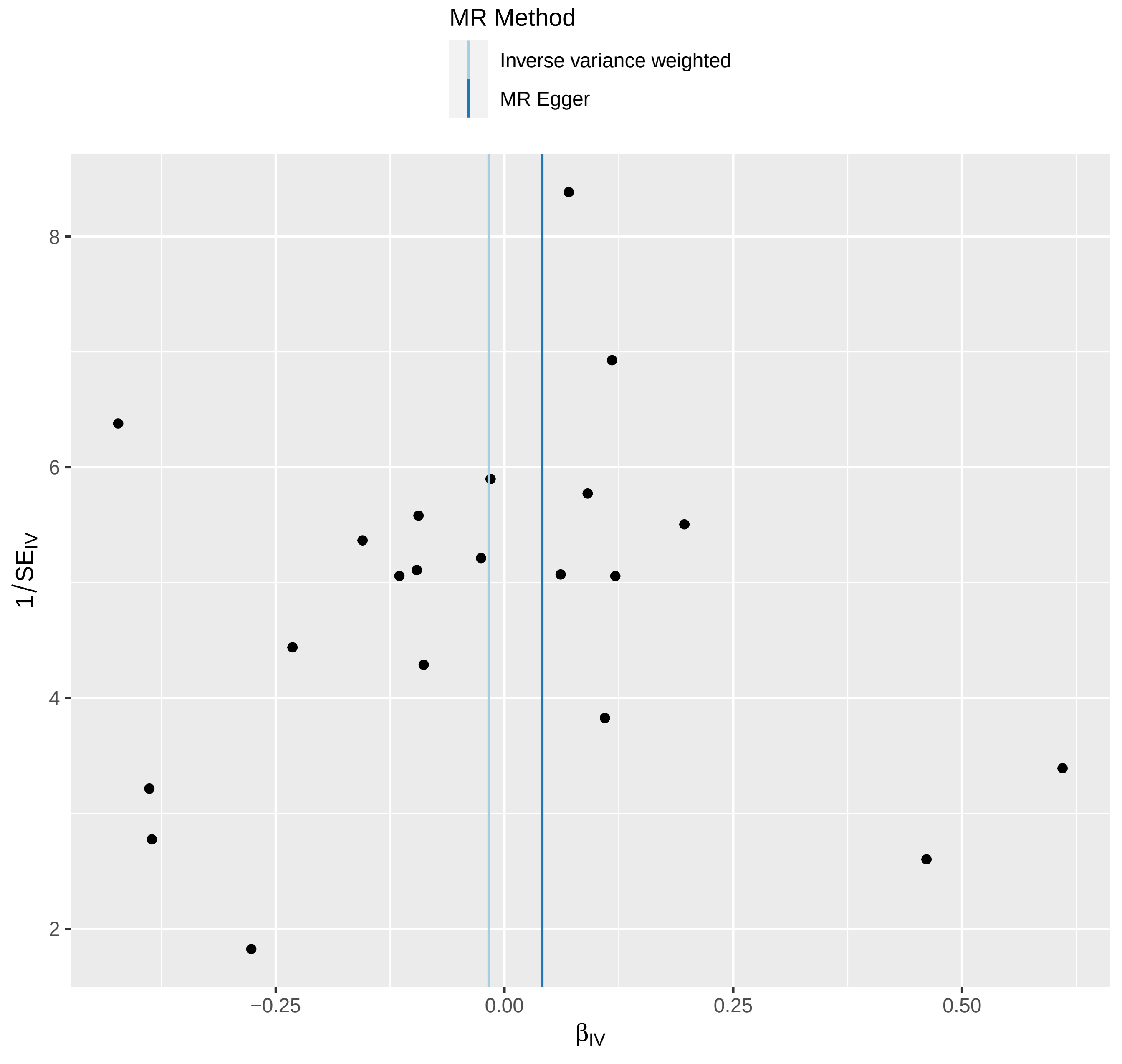

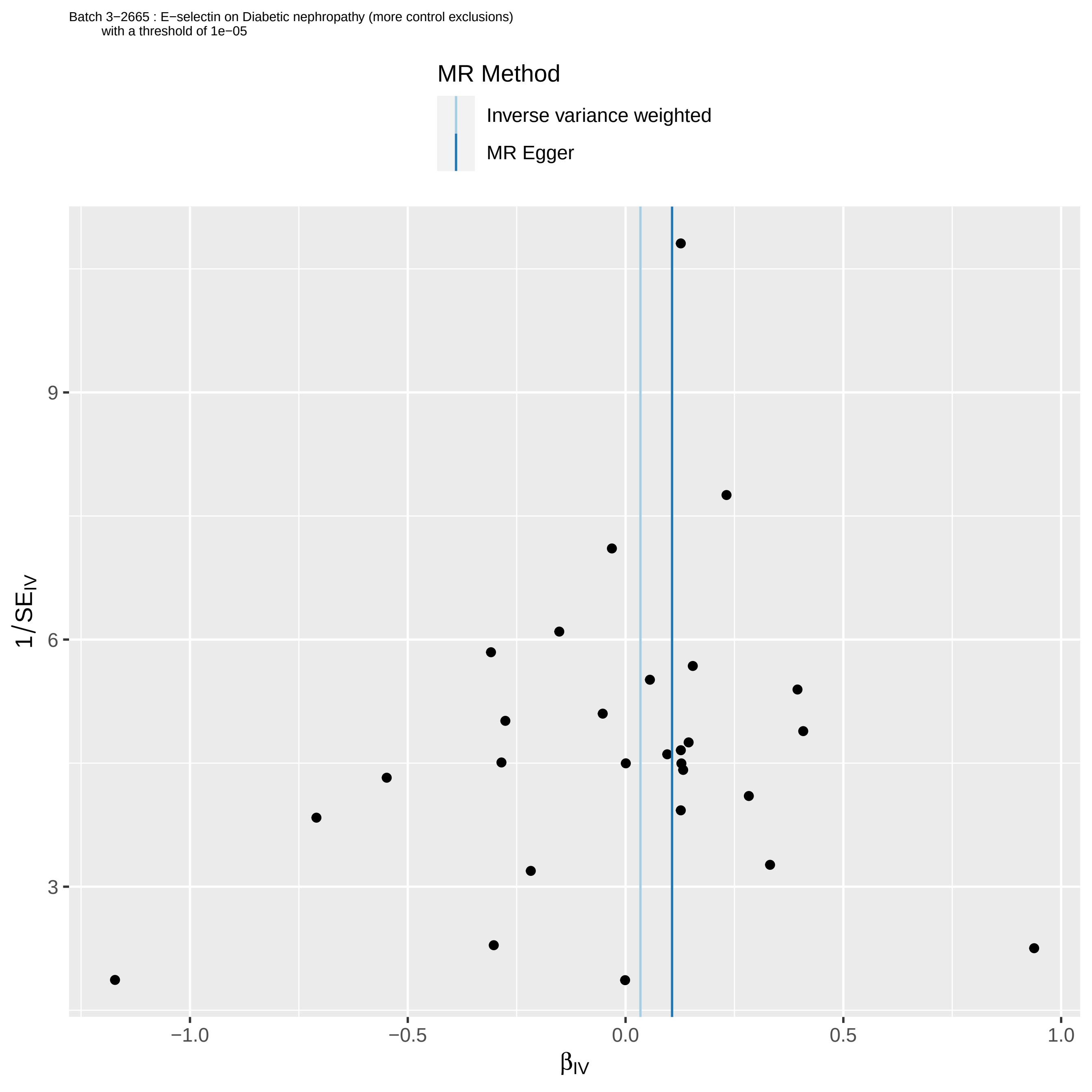

Batch 3−2665 : E−selectin on Diabetic nephropathy (more control exclusions)
with a threshold of 1e−05
MR Method
Inverse variance weighted
MR Egger
9
6
3
1/SE_IV
−1.0
−0.5
0.0
0.5
1.0
β_IV

Batch 3−2666 : L−Selectin on Diabetic nephropathy (more control exclusions)
with a threshold of 1e−05

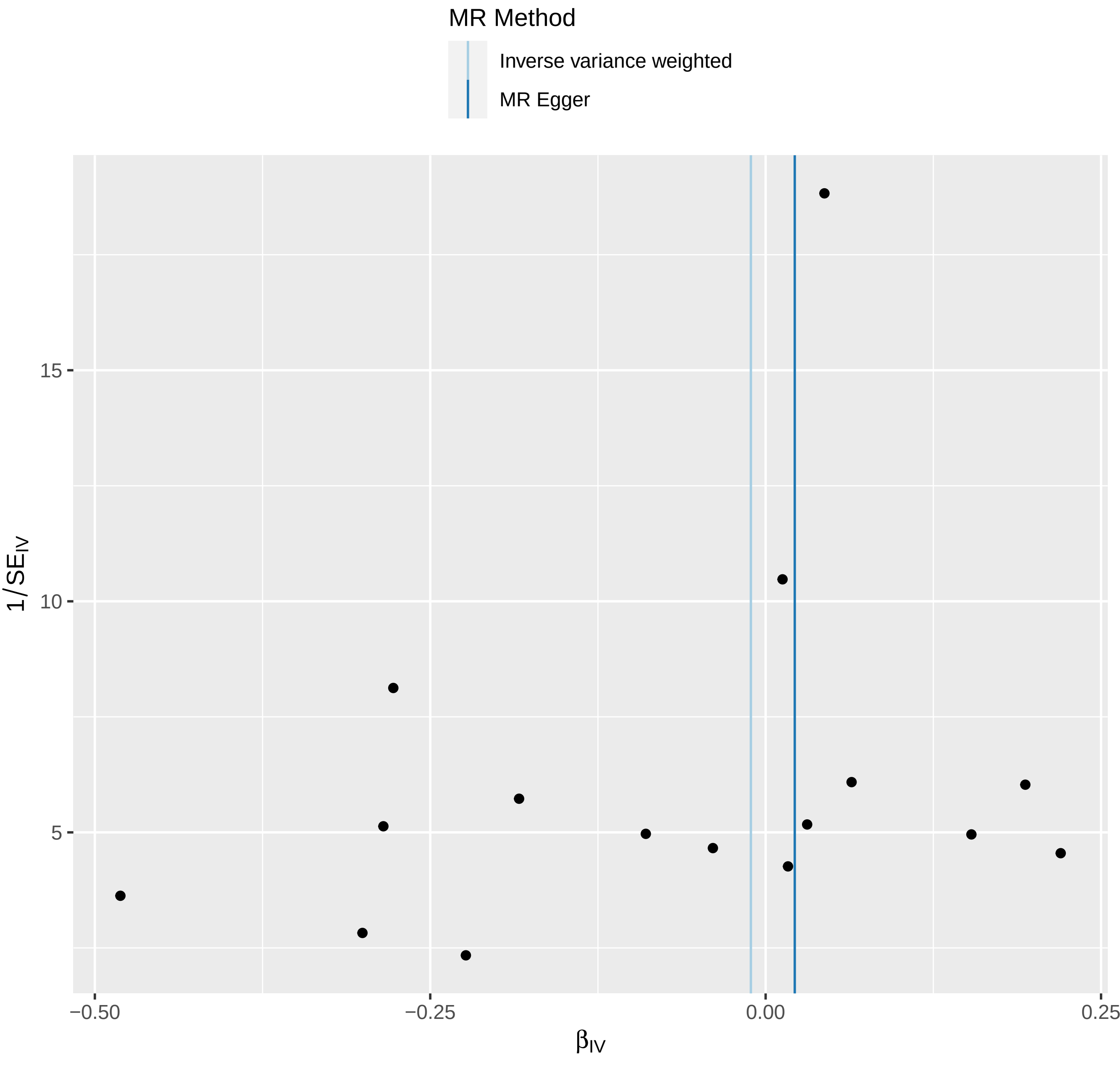

Batch 3−2667 : P−selectin on Diabetic nephropathy (more control exclusions)
with a threshold of 1e−05

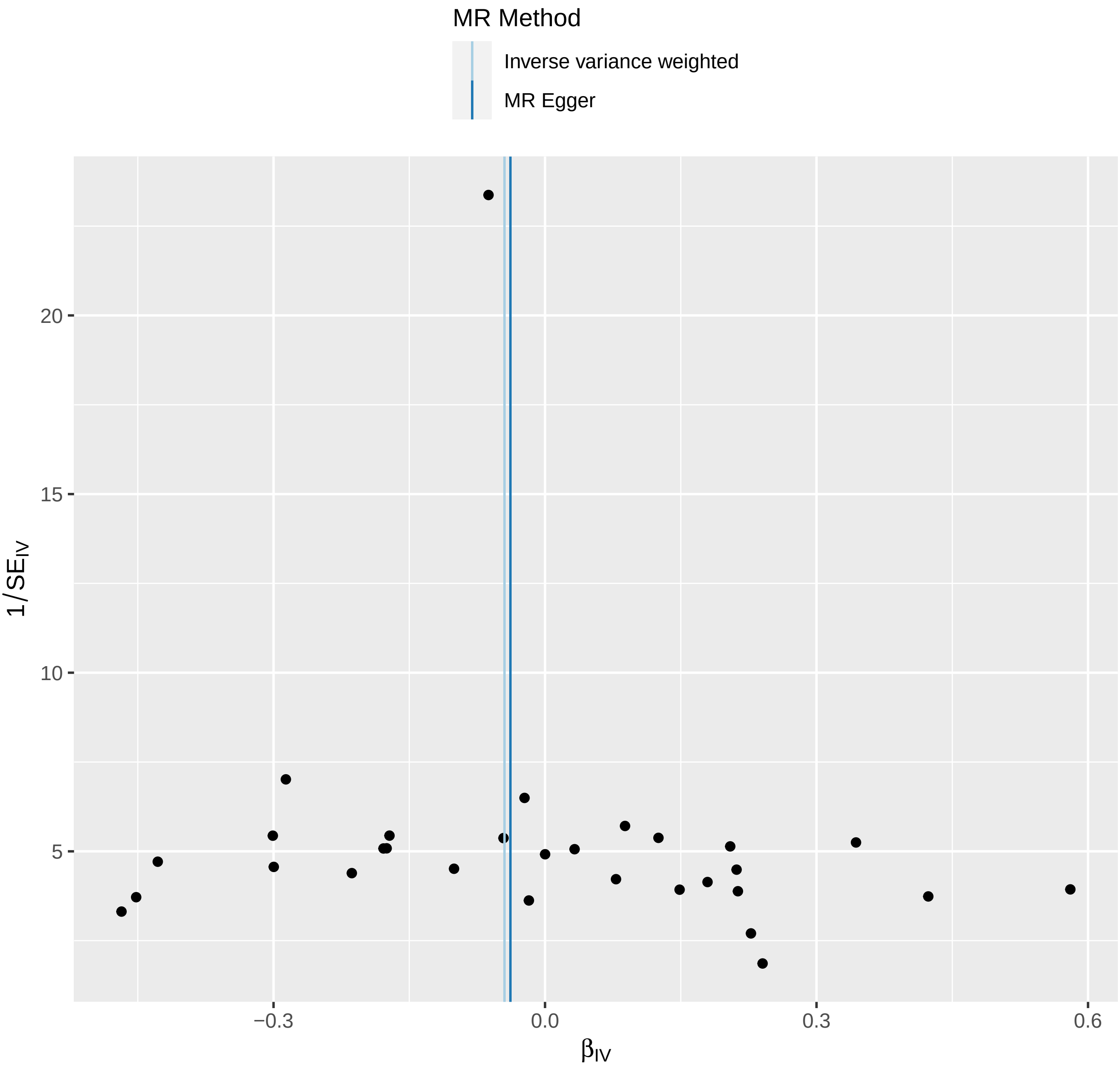

Batch 3−2668 : P−selectin glycoprotein ligand 1 on Diabetic nephropathy (more control exclusions) with a threshold of 1e−05

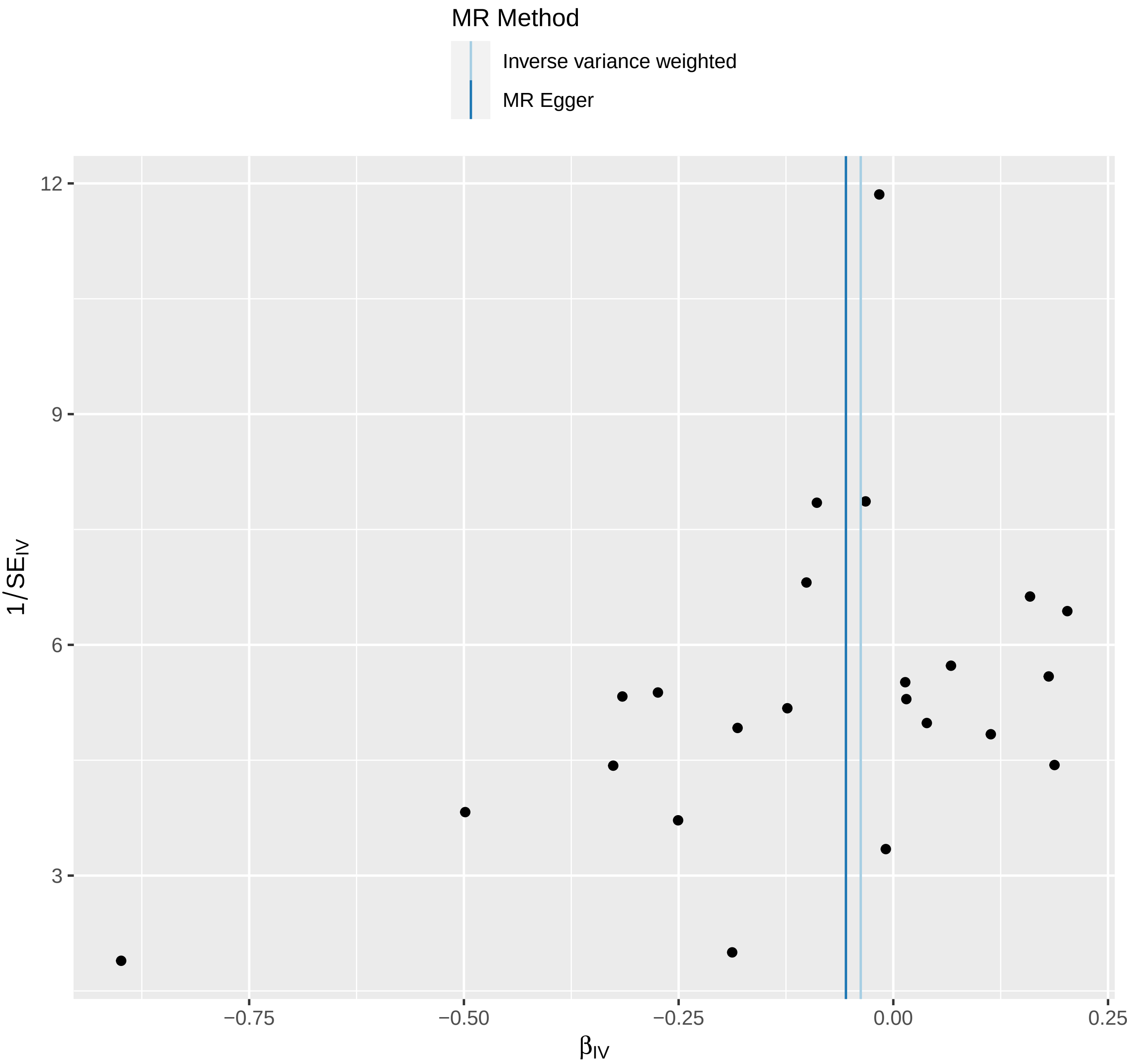

Batch 3−2669 : Semaphorin−3A on Diabetic nephropathy (more control exclusions)
with a threshold of 1e−05

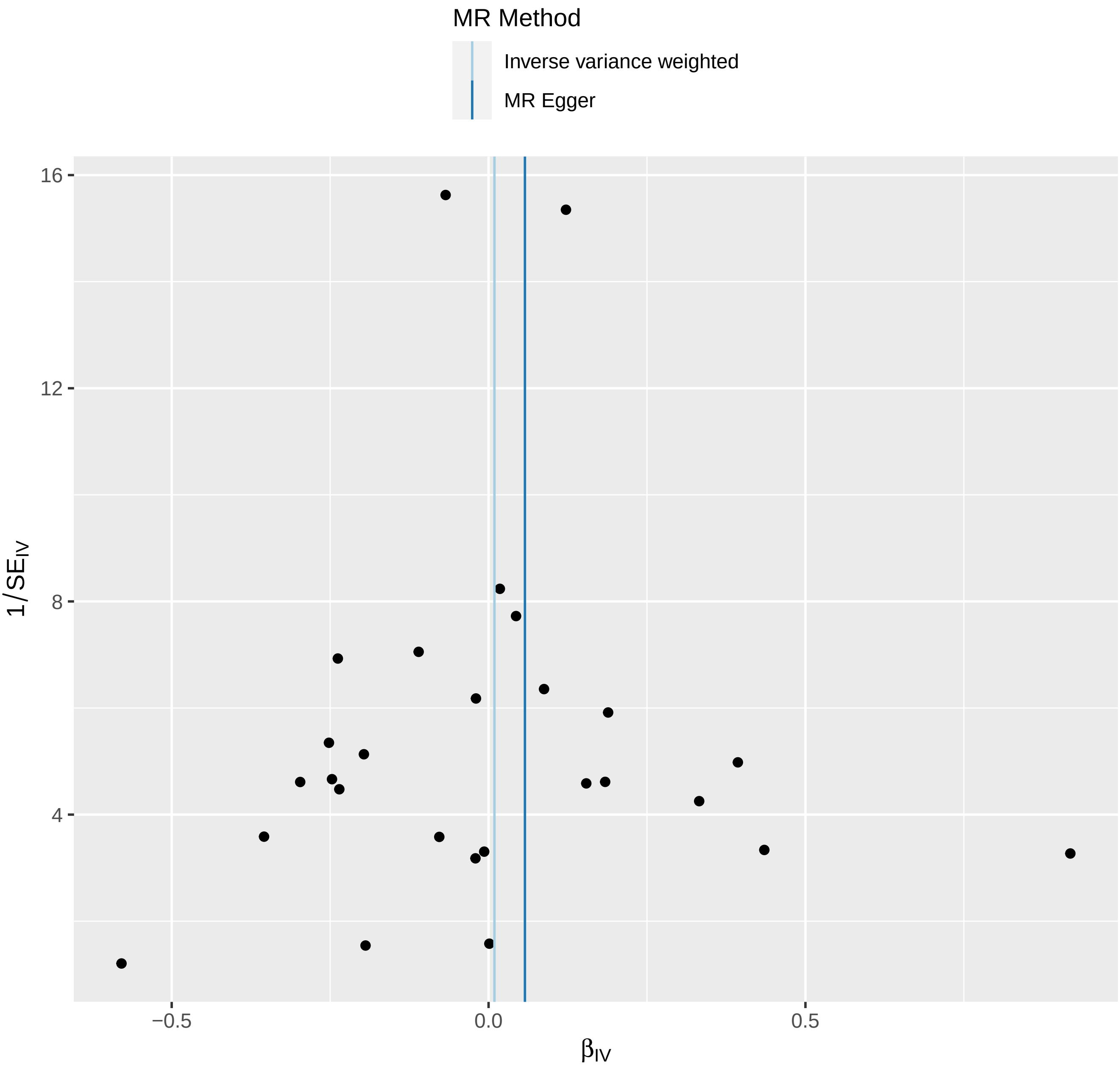

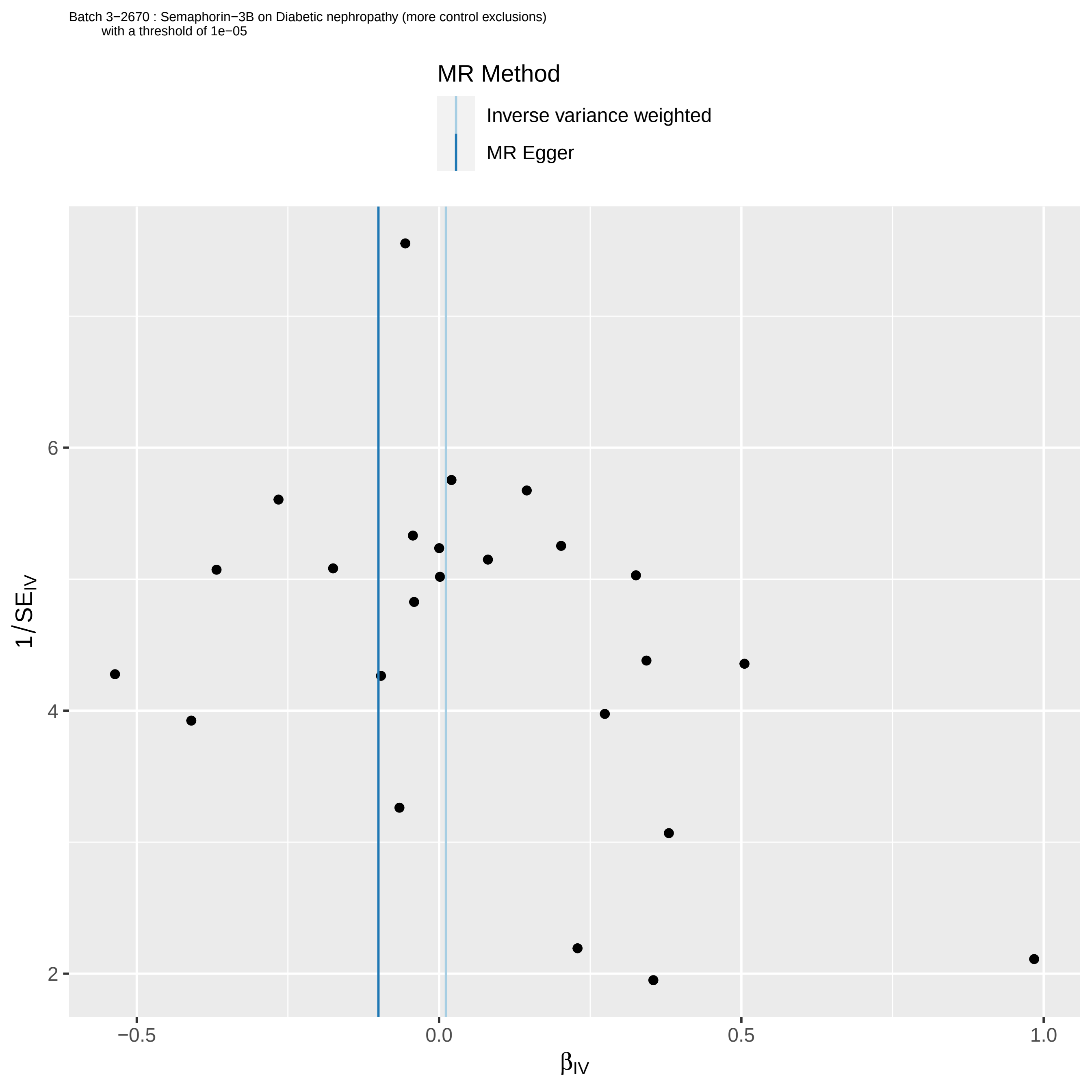
Batch 3−2670 : Semaphorin−3B on Diabetic nephropathy (more control exclusions)
with a threshold of 1e−05
MR Method
Inverse variance weighted
MR Egger
6
4
2
1/SE_IV
−0.5
0.0
0.5
1.0
β_IV

Batch 3−2671 : Semaphorin−3C on Diabetic nephropathy (more control exclusions)
with a threshold of 1e−05

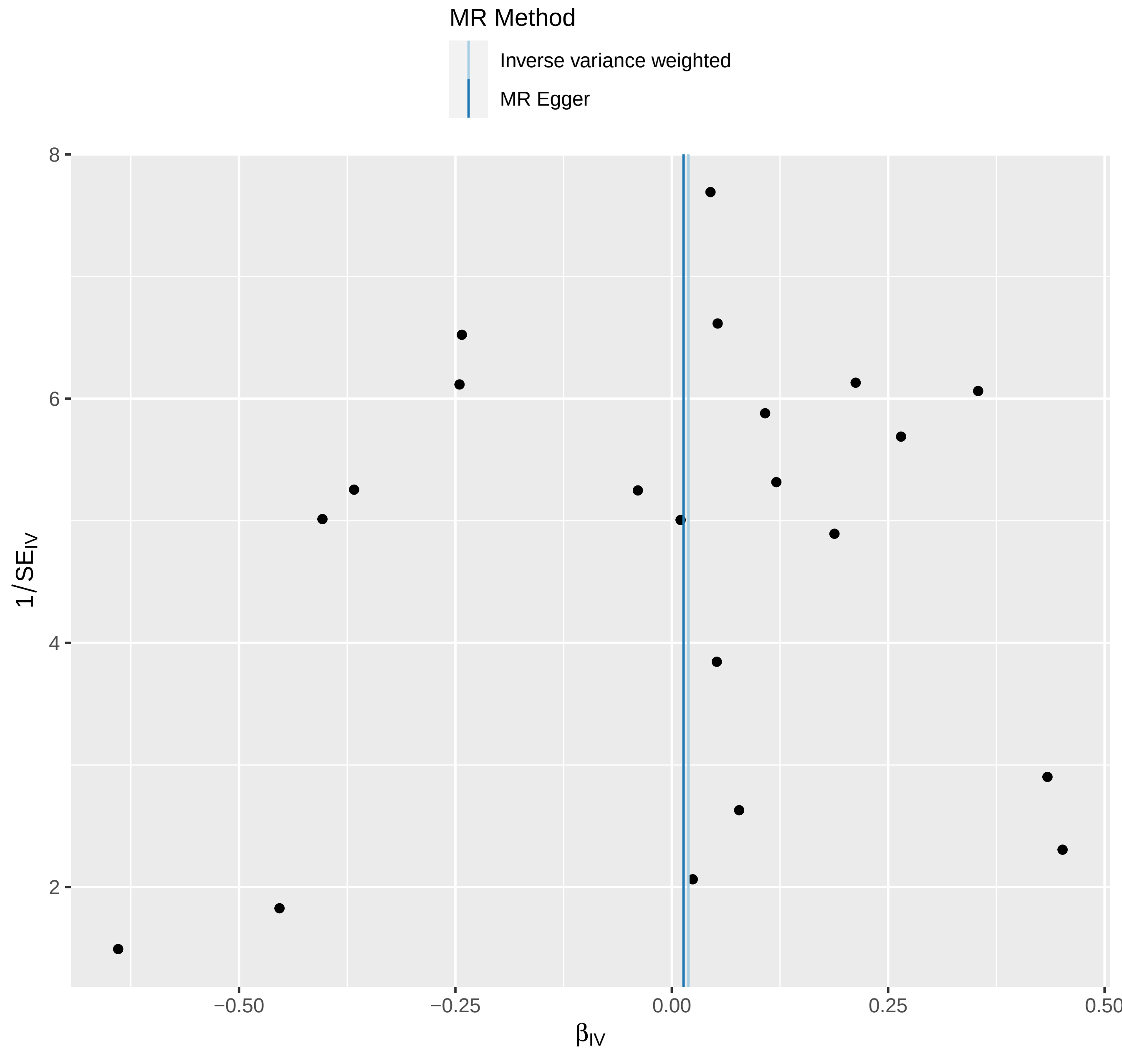

Batch 3−2672 : Semaphorin−3E on Diabetic nephropathy (more control exclusions)
with a threshold of 1e−05

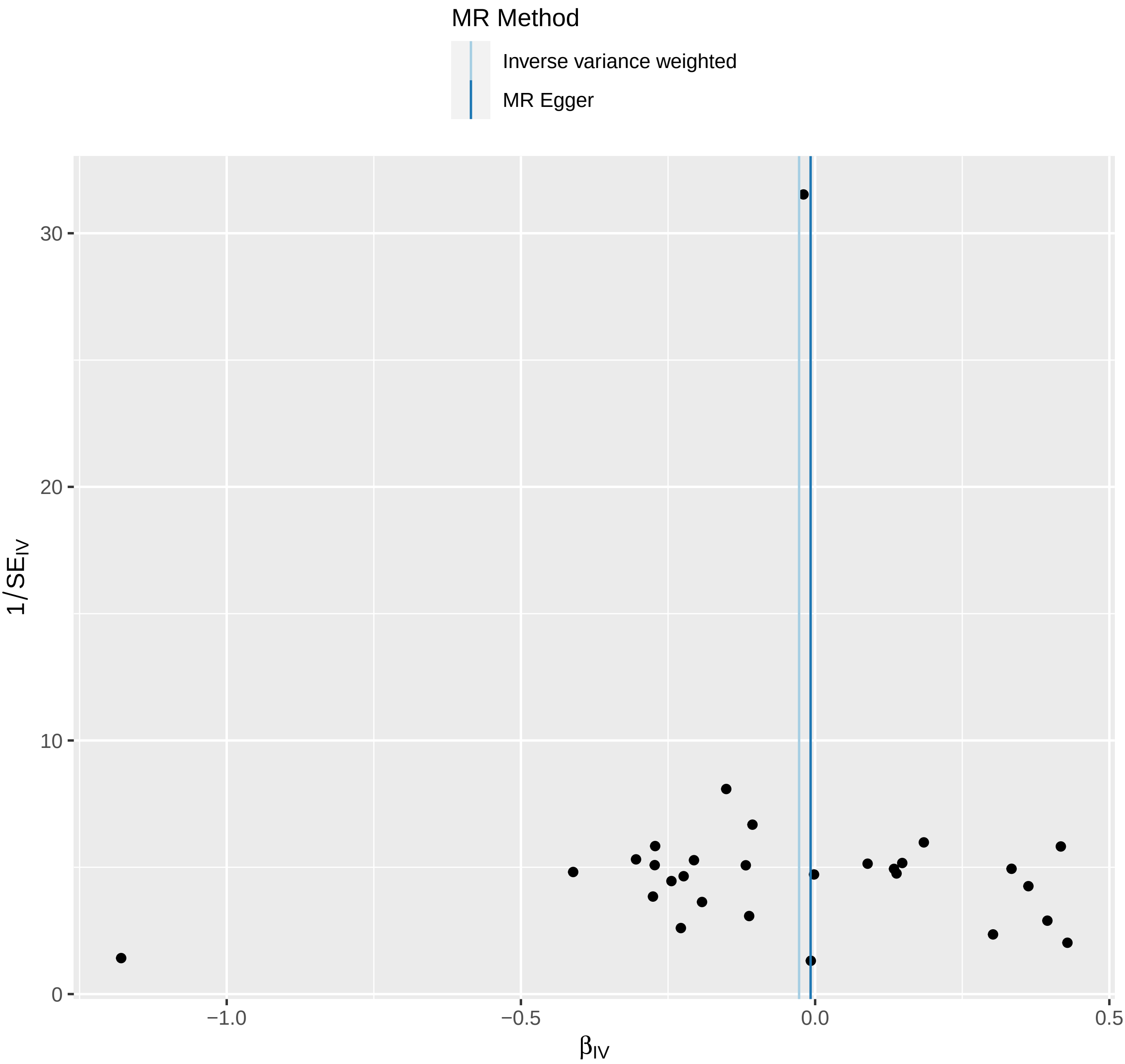

Batch 3−2673 : Semaphorin−3G on Diabetic nephropathy (more control exclusions)
with a threshold of 1e−05

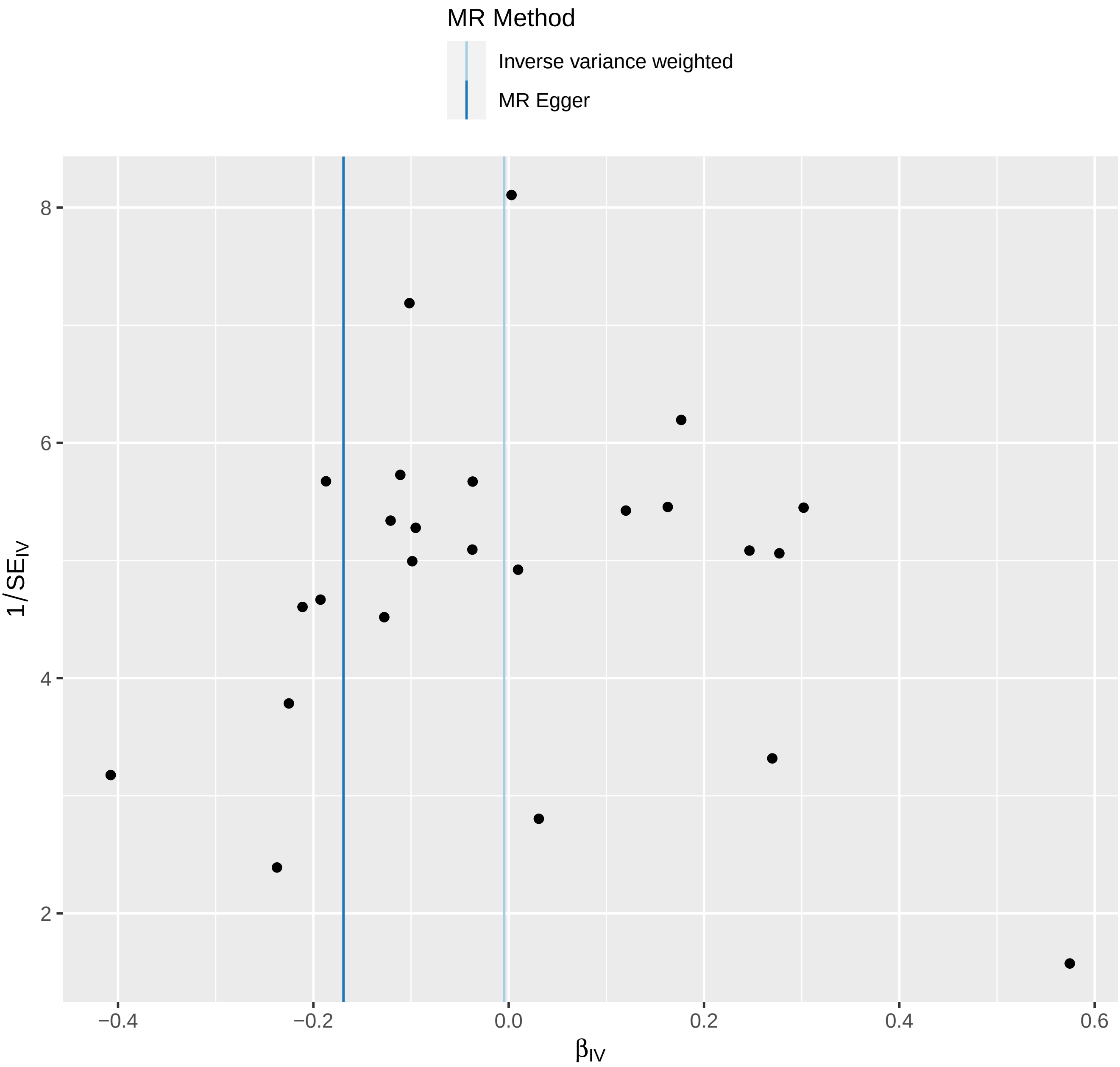

Batch 3−2674 : Semaphorin−4C on Diabetic nephropathy (more control exclusions)
with a threshold of 1e−05

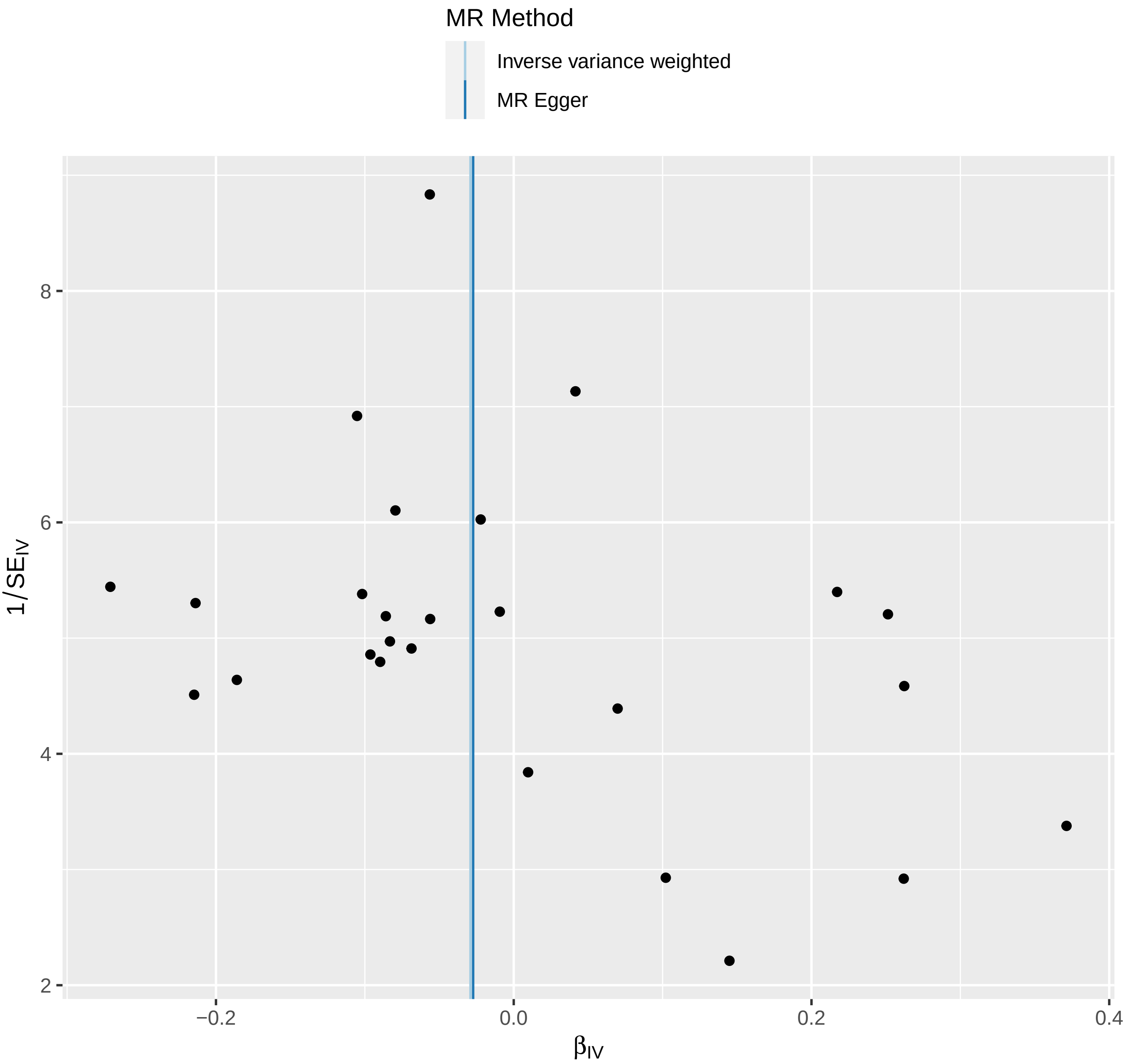

Batch 3−2675 : Semaphorin−4D on Diabetic nephropathy (more control exclusions)
with a threshold of 1e−05

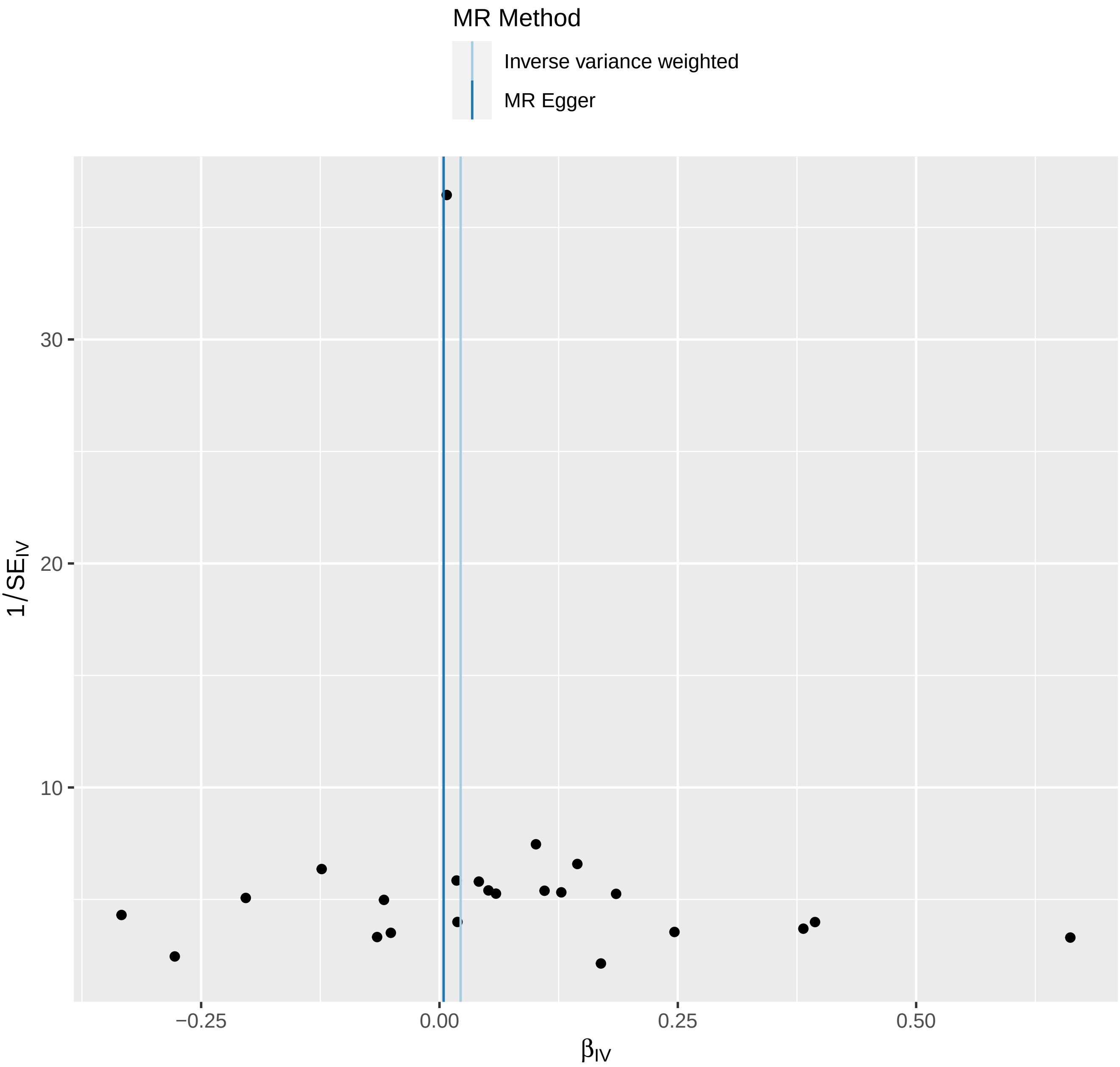

Batch 3−2676 : Semaphorin−5A on Diabetic nephropathy (more control exclusions)
with a threshold of 1e−05

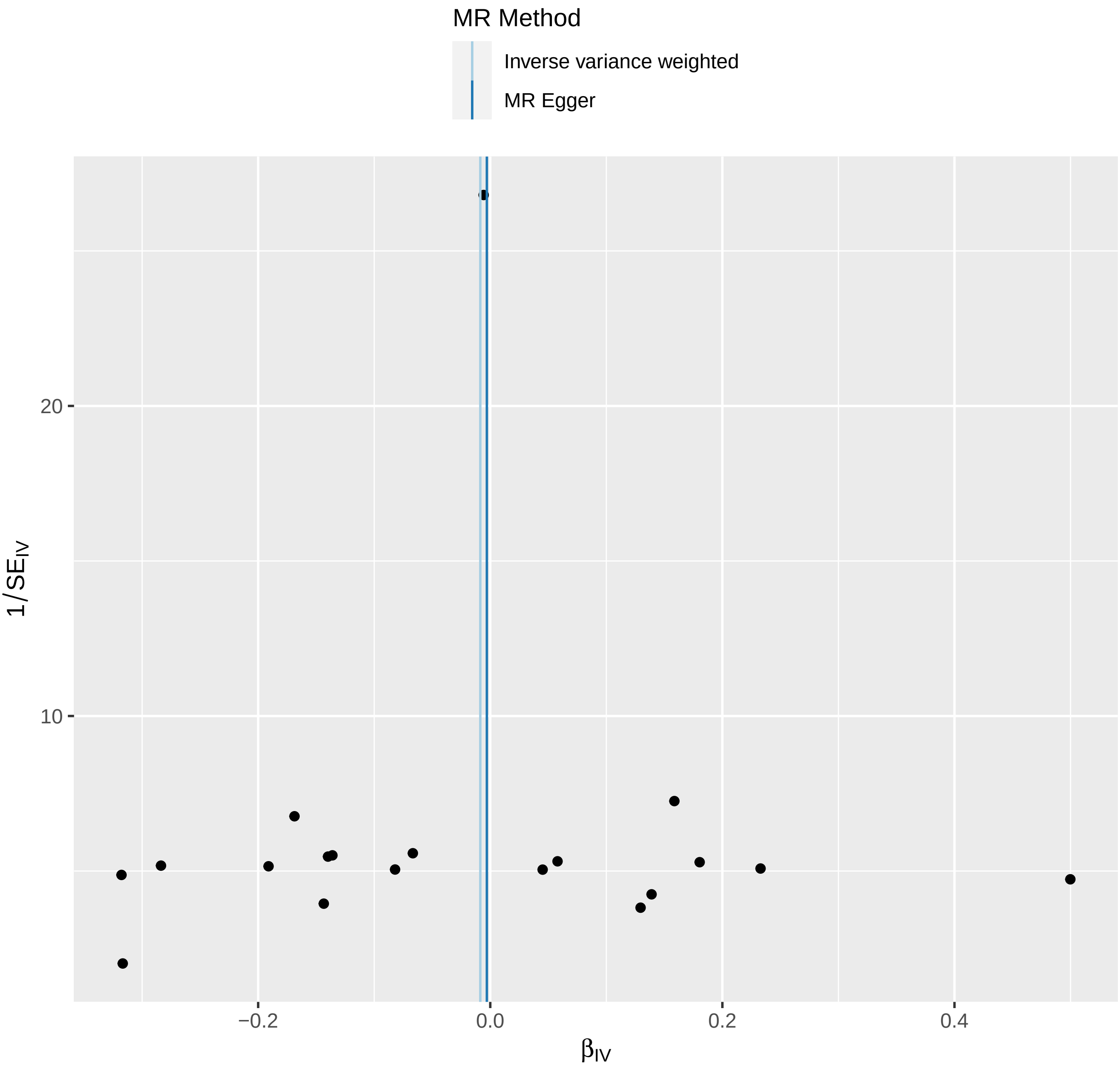

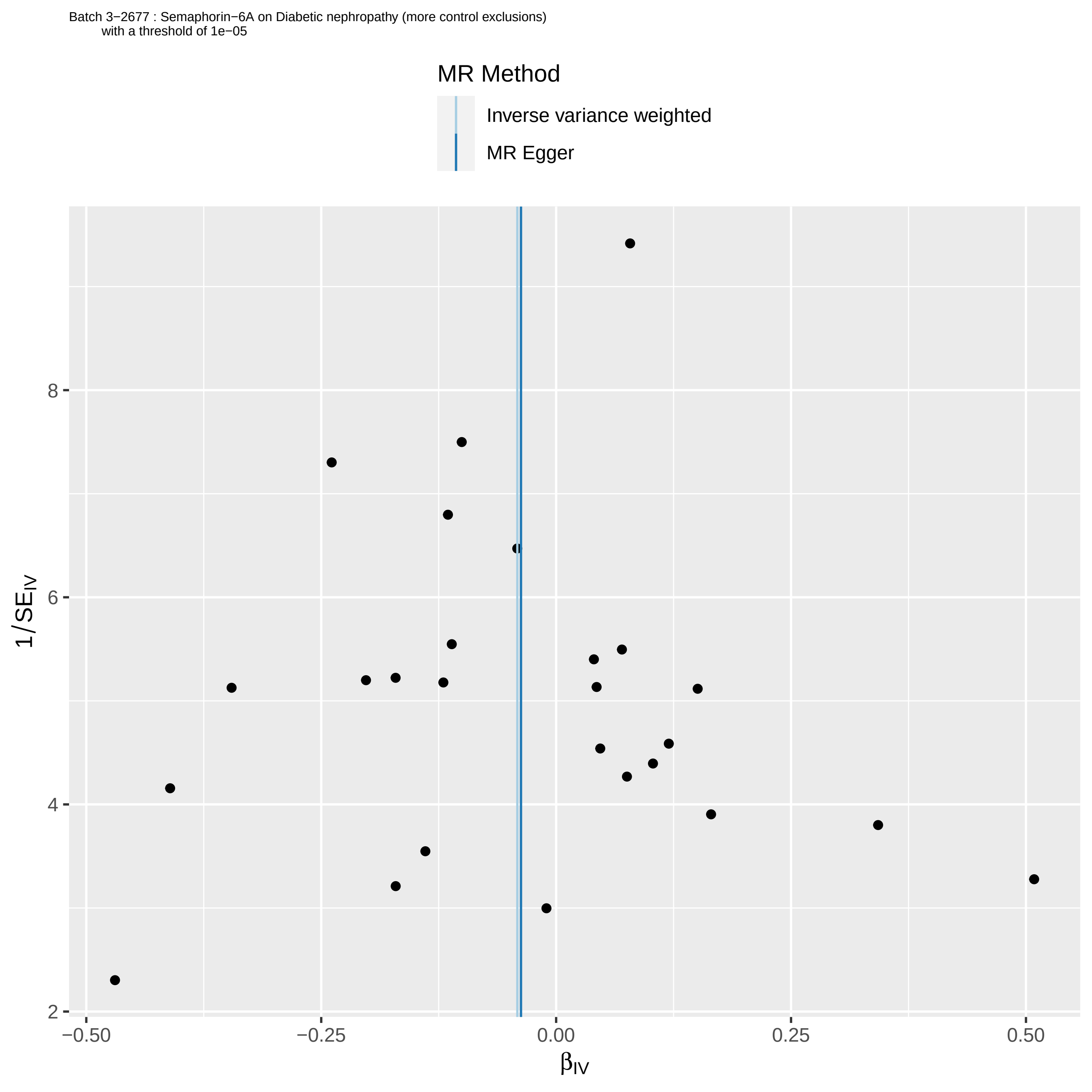
Batch 3−2677 : Semaphorin−6A on Diabetic nephropathy (more control exclusions)
with a threshold of 1e−05
MR Method
Inverse variance weighted
MR Egger
8
6
4
2
1/SE_IV
−0.50
−0.25
0.00
0.25
0.50
β_IV

Batch 3−2678 : Semaphorin−6A on Diabetic nephropathy (more control exclusions)
with a threshold of 1e−05

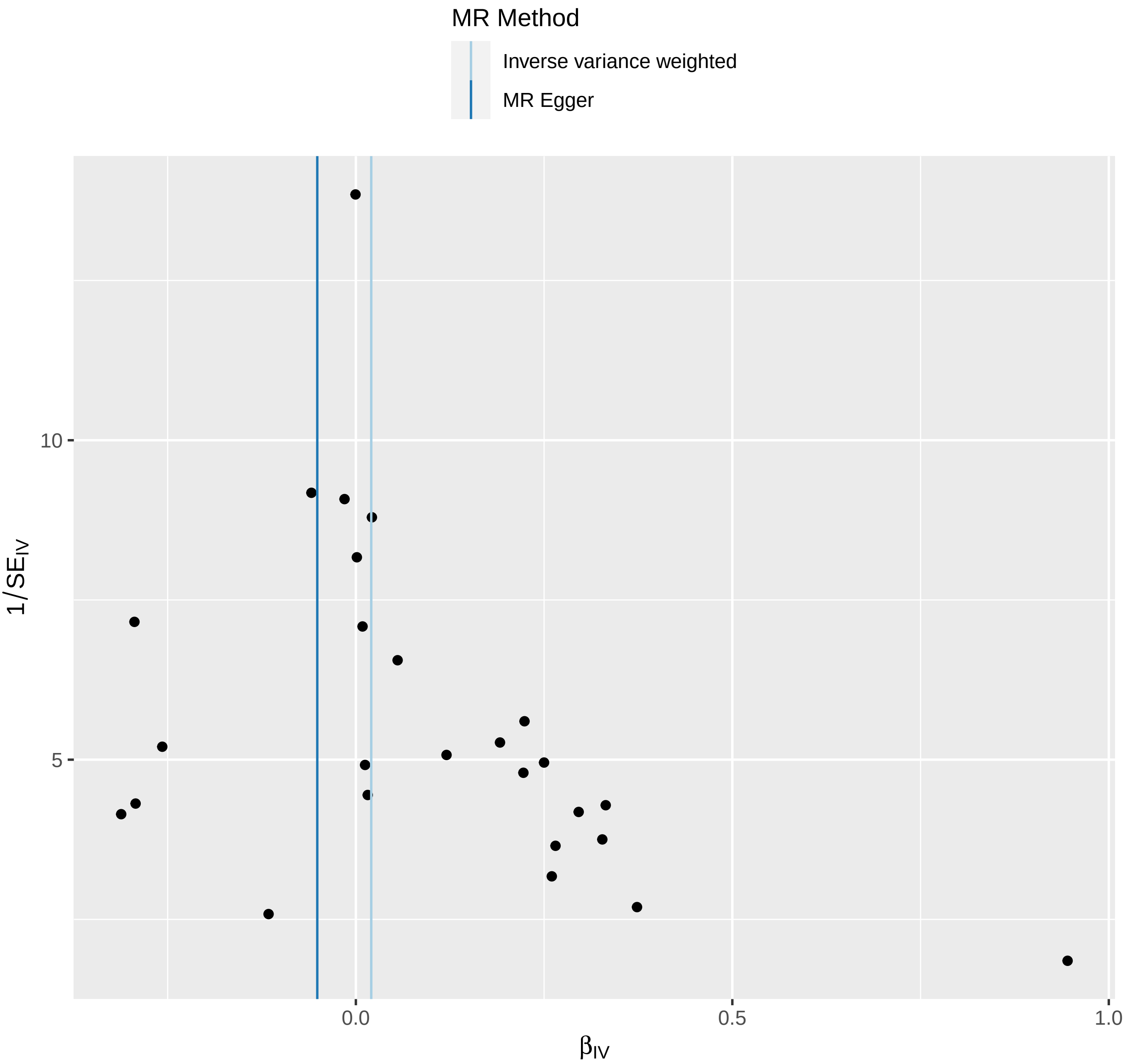

Batch 3−2679 : Semaphorin−6C on Diabetic nephropathy (more control exclusions)
with a threshold of 1e−05

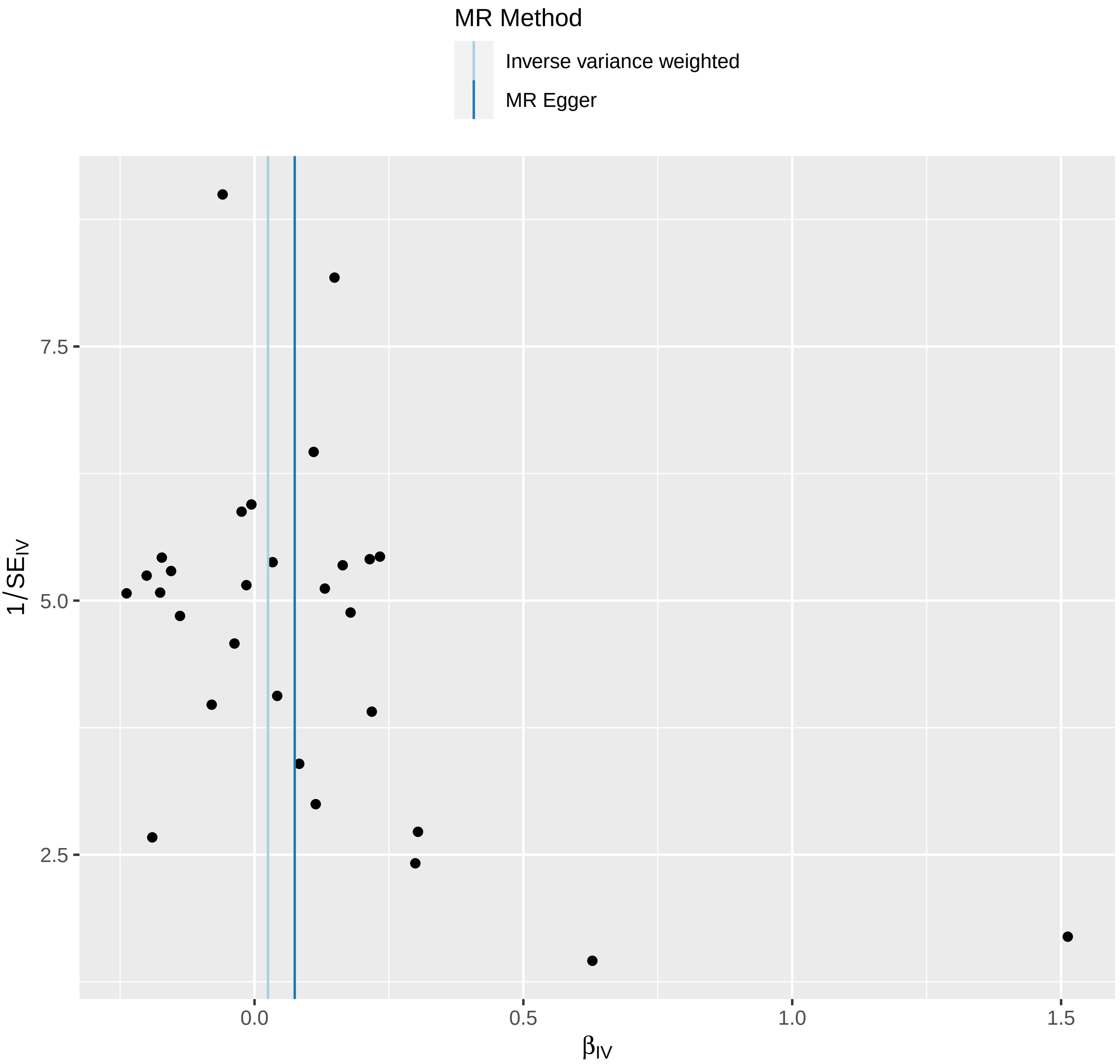

Batch 3−2680 : Semaphorin−7A on Diabetic nephropathy (more control exclusions)
with a threshold of 1e−05

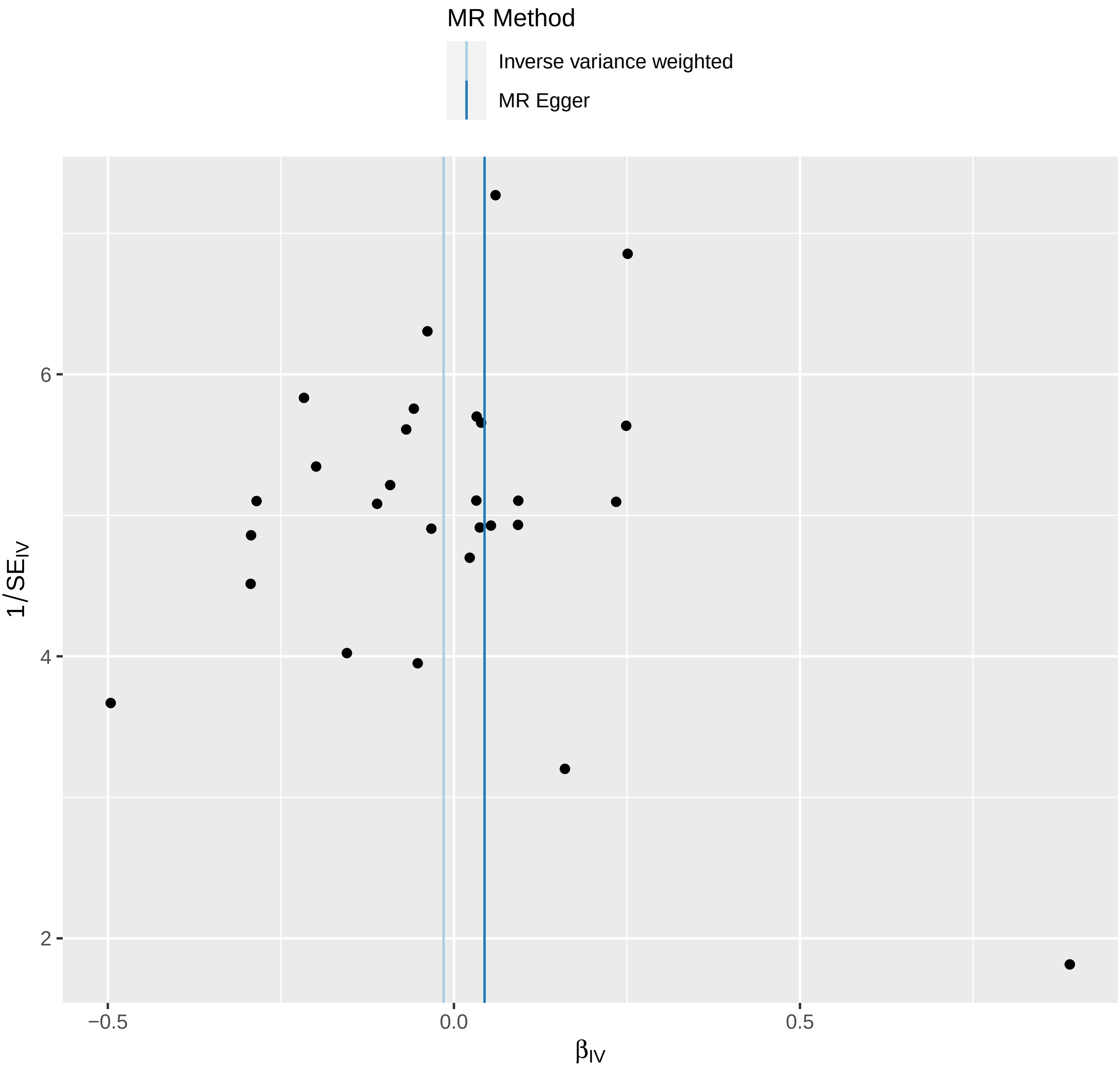

Batch 3−2681 : Semenogelin−1 on Diabetic nephropathy (more control exclusions)
with a threshold of 1e−05

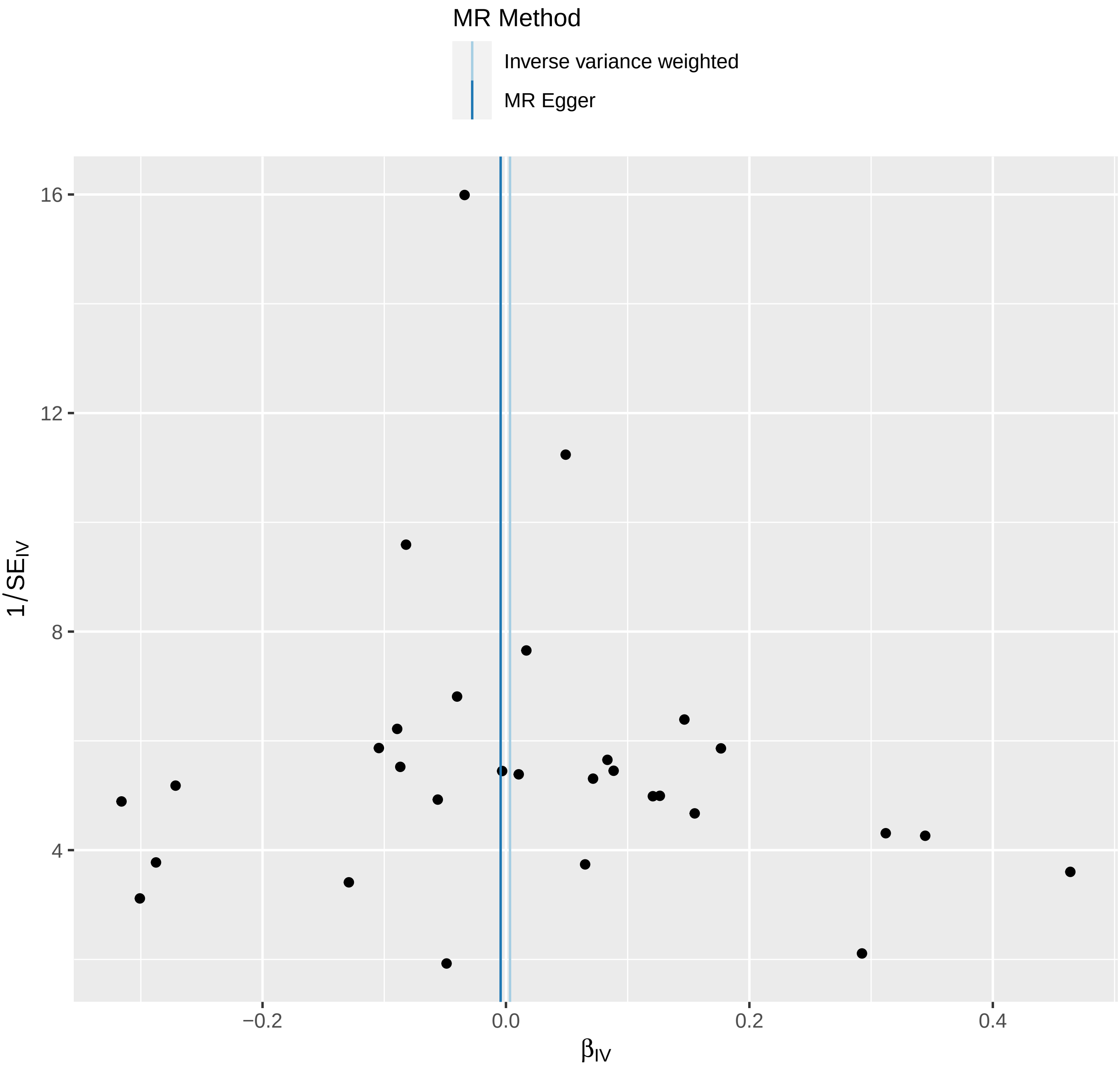

Batch 3−2682 : Semenogelin−2 on Diabetic nephropathy (more control exclusions)
with a threshold of 1e−05

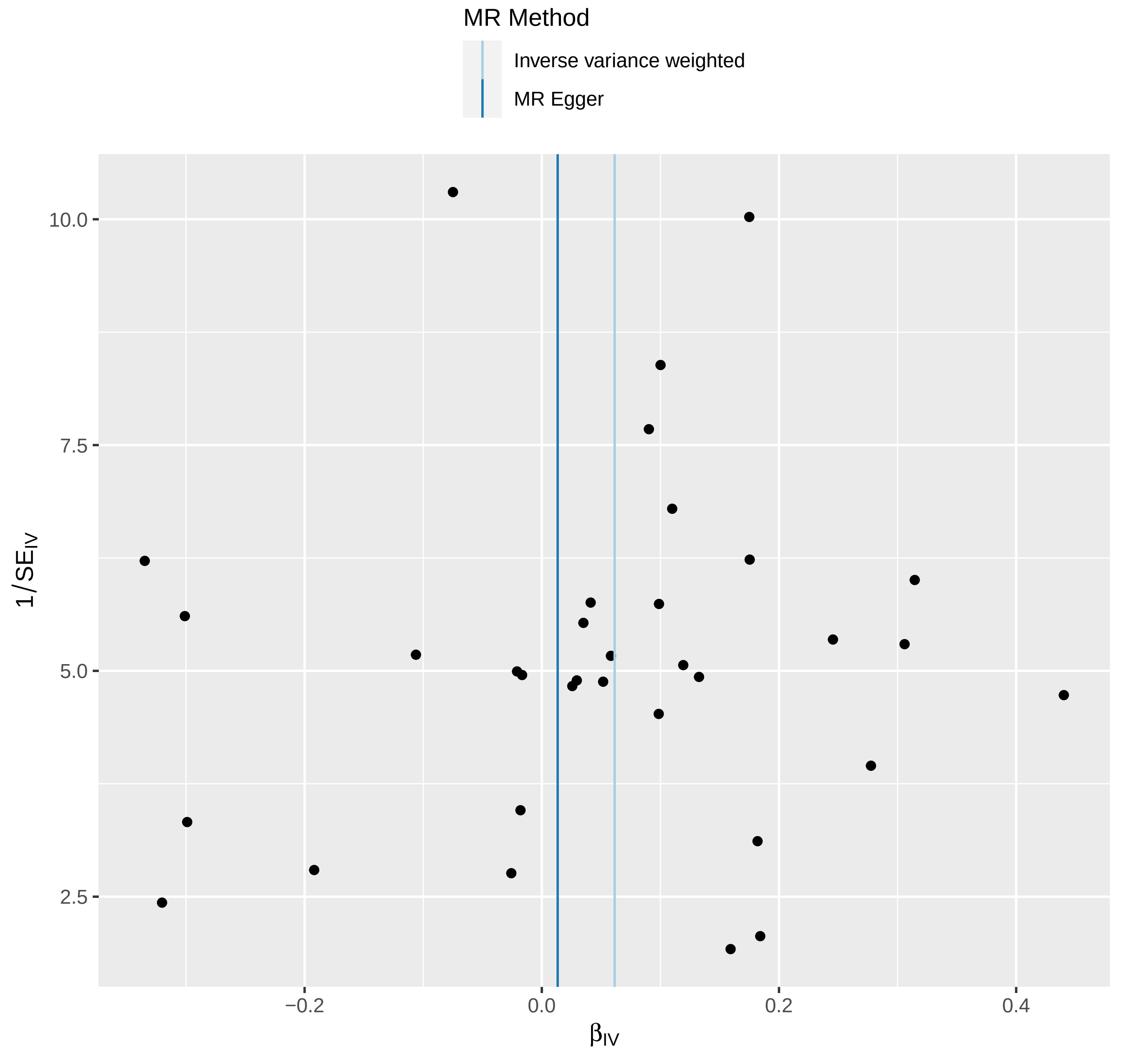

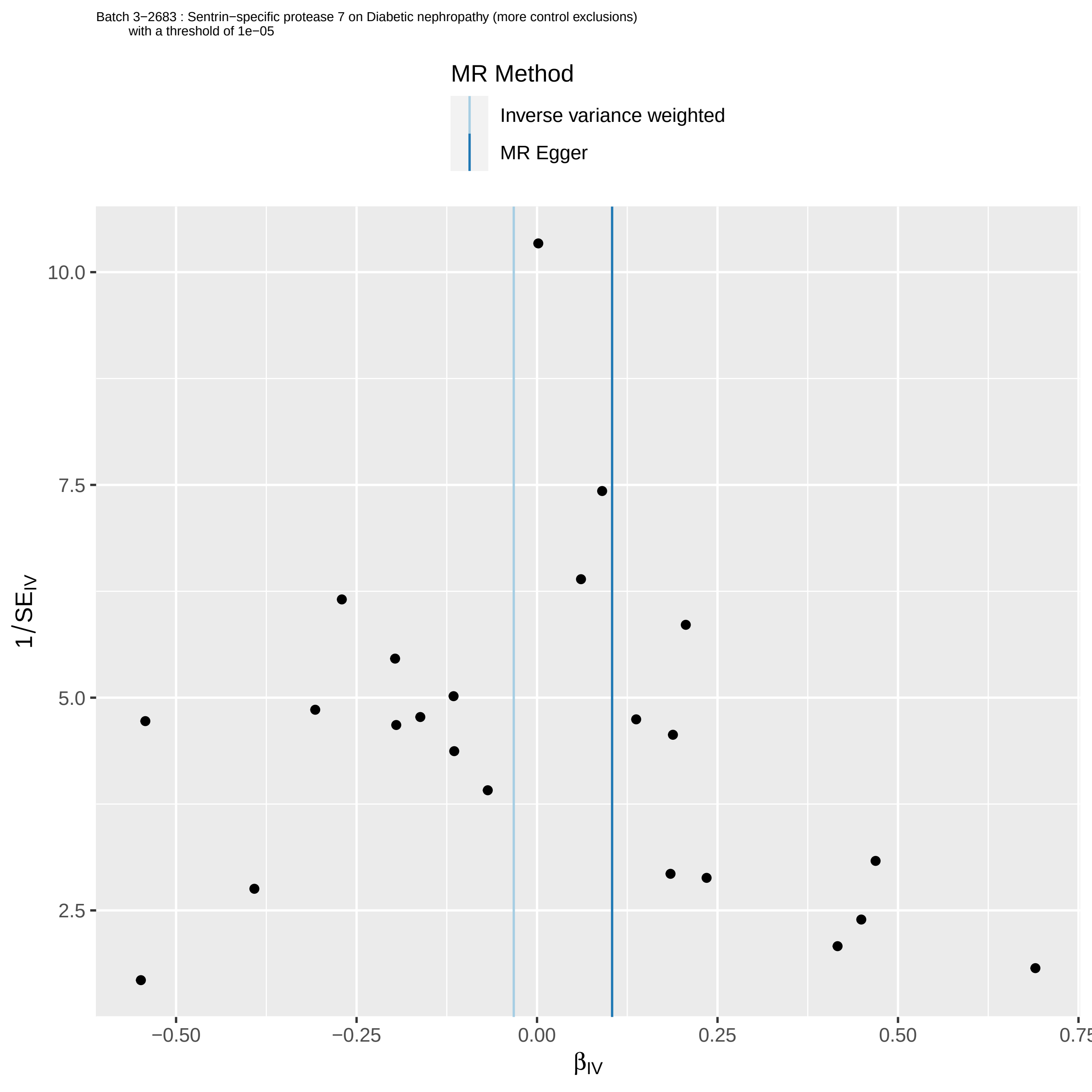

Batch 3−2683 : Sentrin−specific protease 7 on Diabetic nephropathy (more control exclusions)
with a threshold of 1e−05
MR Method
Inverse variance weighted
MR Egger
10.0
7.5
5.0
2.5
1/SE_IV
−0.50
−0.25
0.00
0.25
0.50
0.75
β_IV

Batch 3−2684 : Selenide, water dikinase 1 on Diabetic nephropathy (more control exclusions)
with a threshold of 1e−05

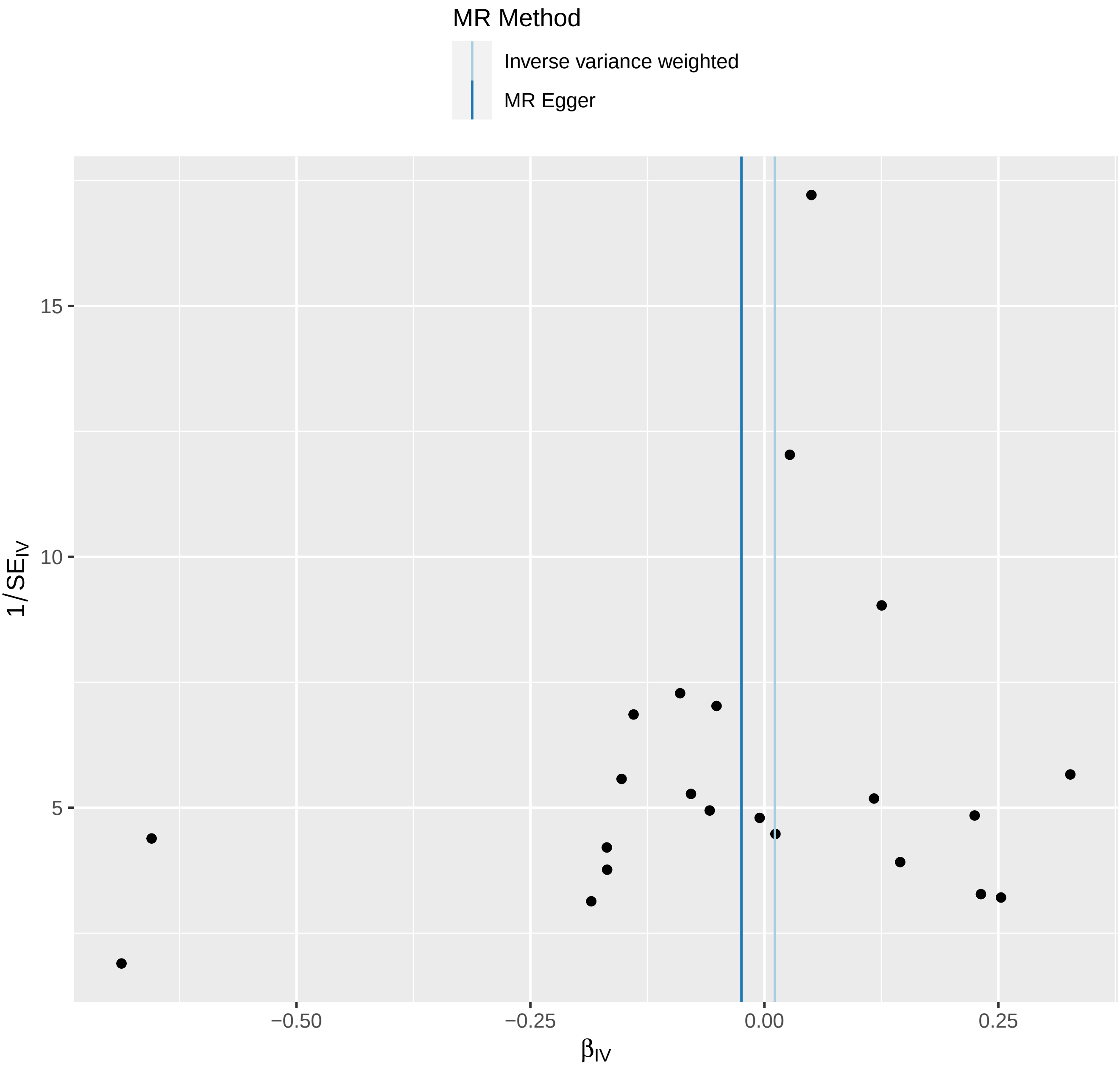

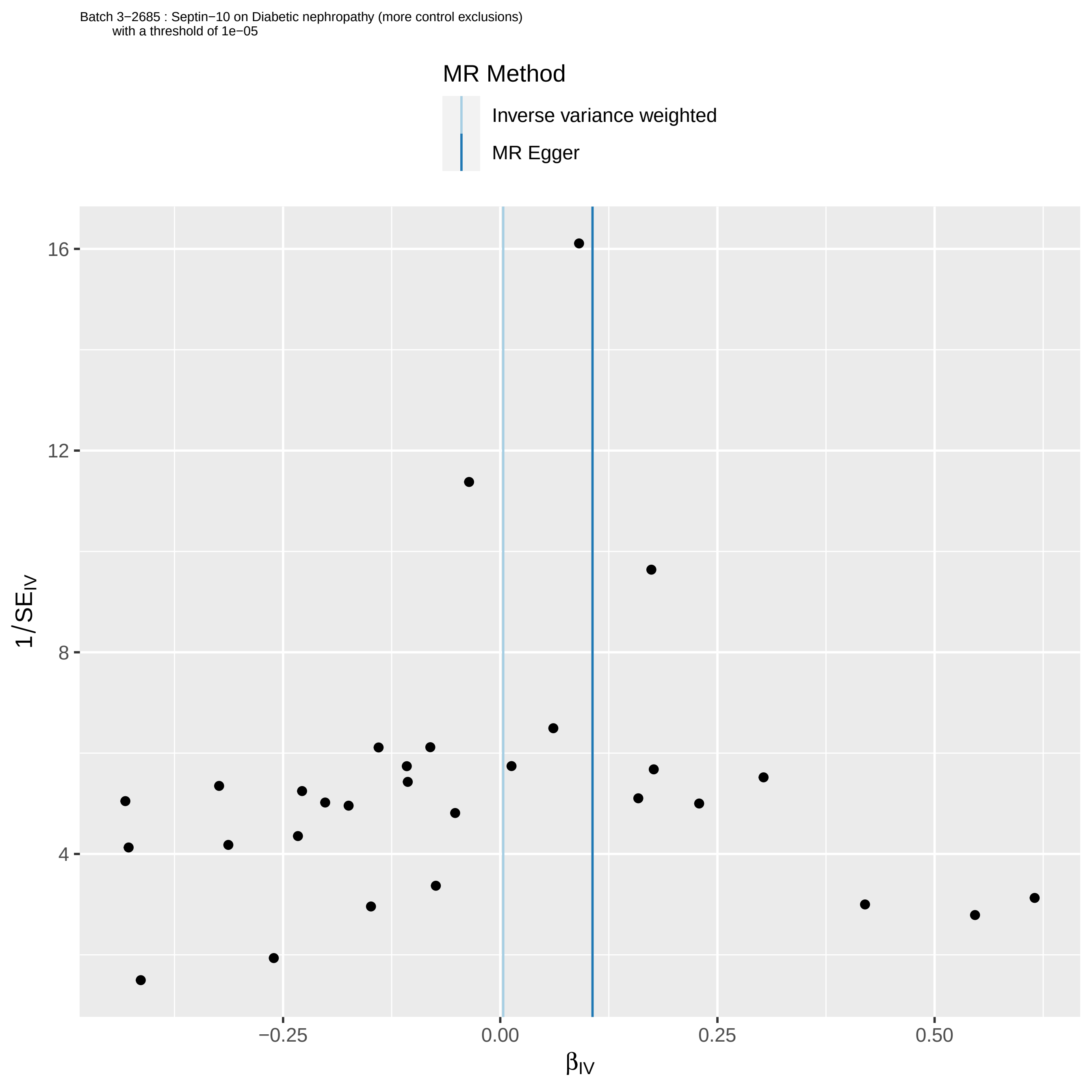
Batch 3−2685 : Septin−10 on Diabetic nephropathy (more control exclusions)
with a threshold of 1e−05
MR Method
Inverse variance weighted
MR Egger
16
12
8
4
1/SE$_{IV}$
−0.25
0.00
0.25
0.50
β_{IV}

Batch 3−2686 : Protein SERAC1 on Diabetic nephropathy (more control exclusions)
with a threshold of 1e−05

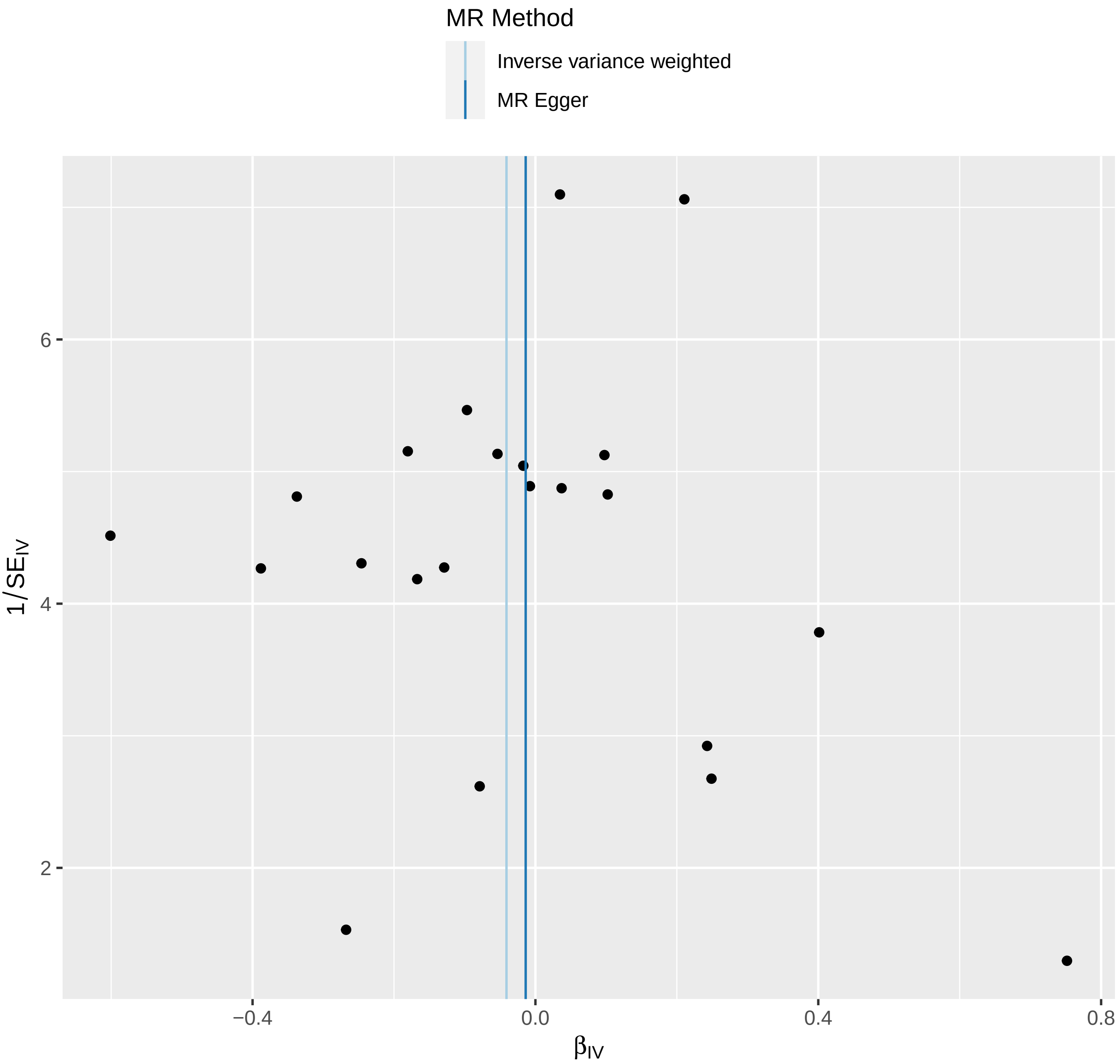

Batch 3−2687 : Small EDRK−rich factor 1 on Diabetic nephropathy (more control exclusions)
with a threshold of 1e−05

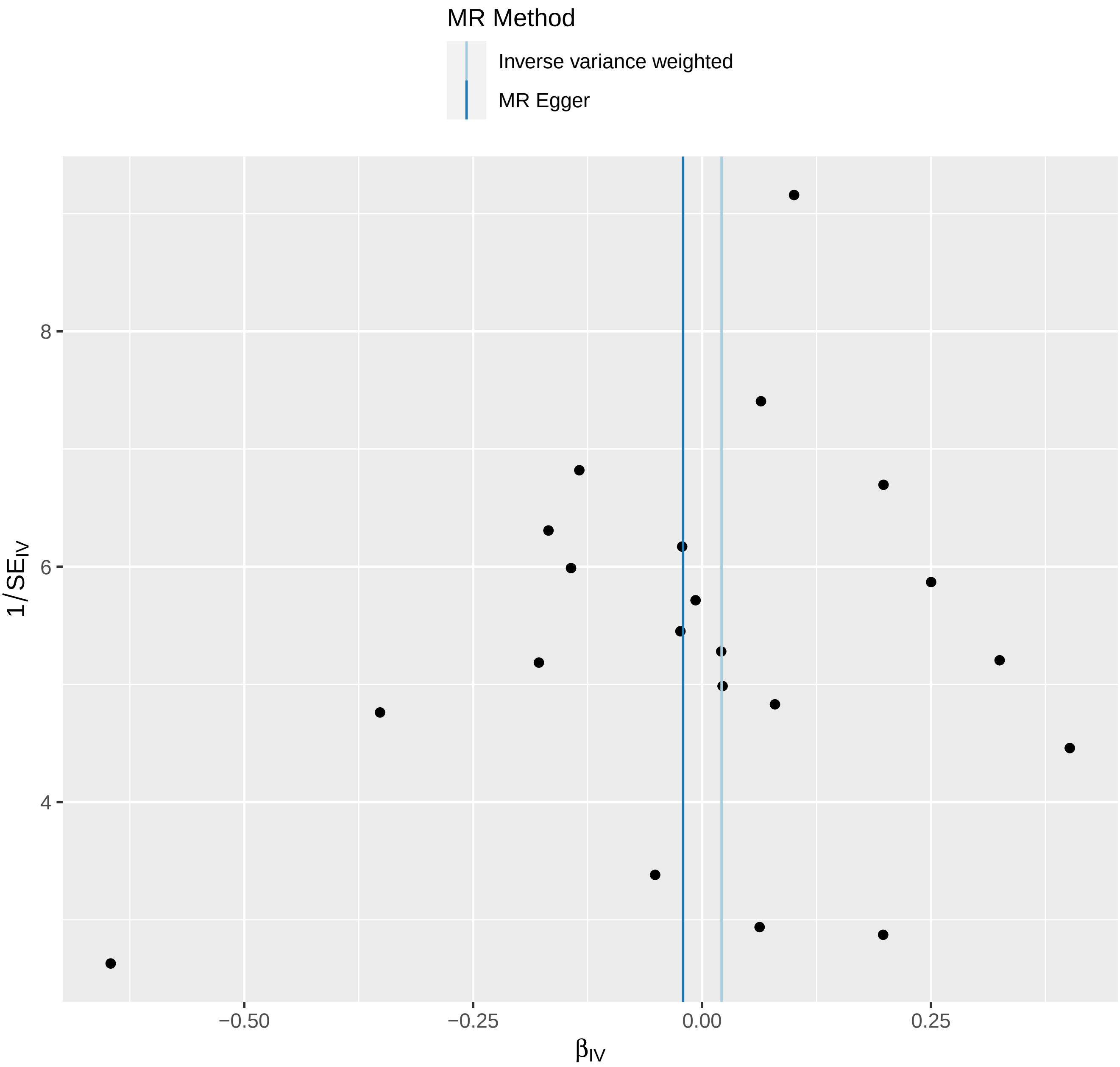

Batch 3−2688 : Protein Z−dependent protease inhibitor on Diabetic nephropathy (more control exclusions)
with a threshold of 1e−05

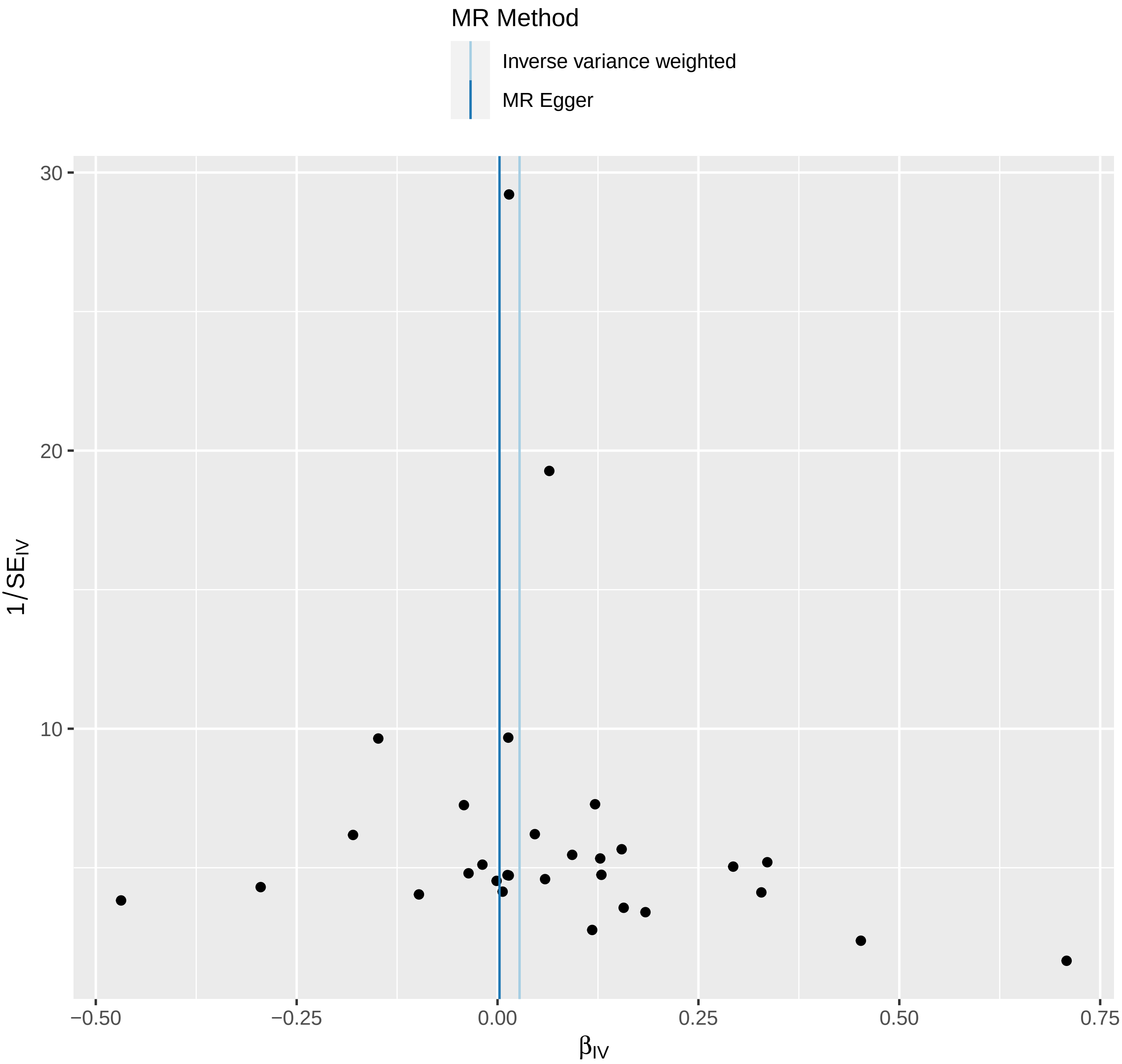

Batch 3−2689 : Protein Z−dependent protease inhibitor on Diabetic nephropathy (more control exclusions)
with a threshold of 1e−05

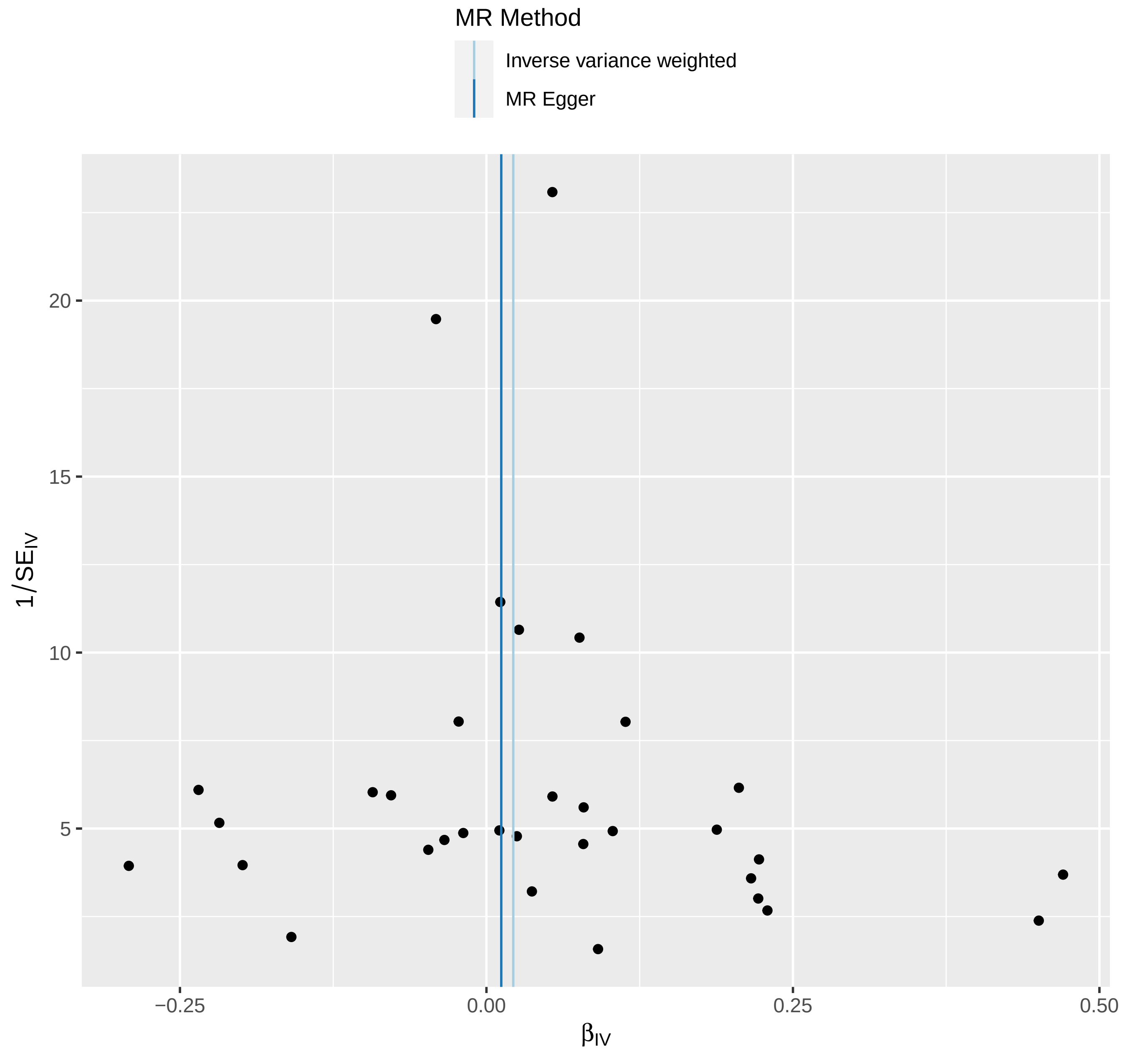

Batch 3−2690 : Serpin A12 on Diabetic nephropathy (more control exclusions)
with a threshold of 1e−05

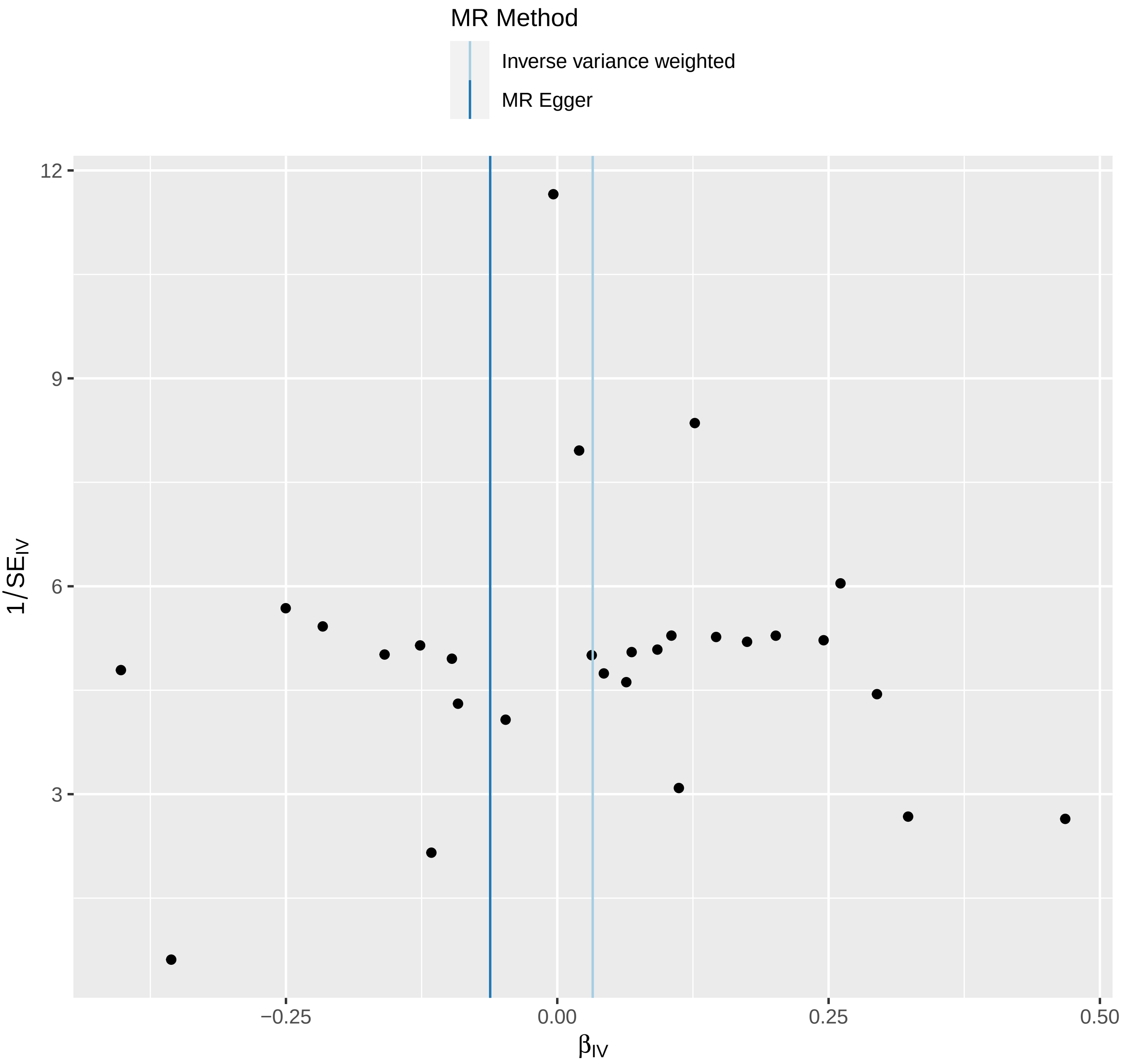

Batch 3−2691 : Kallistatin on Diabetic nephropathy (more control exclusions)
with a threshold of 1e−05

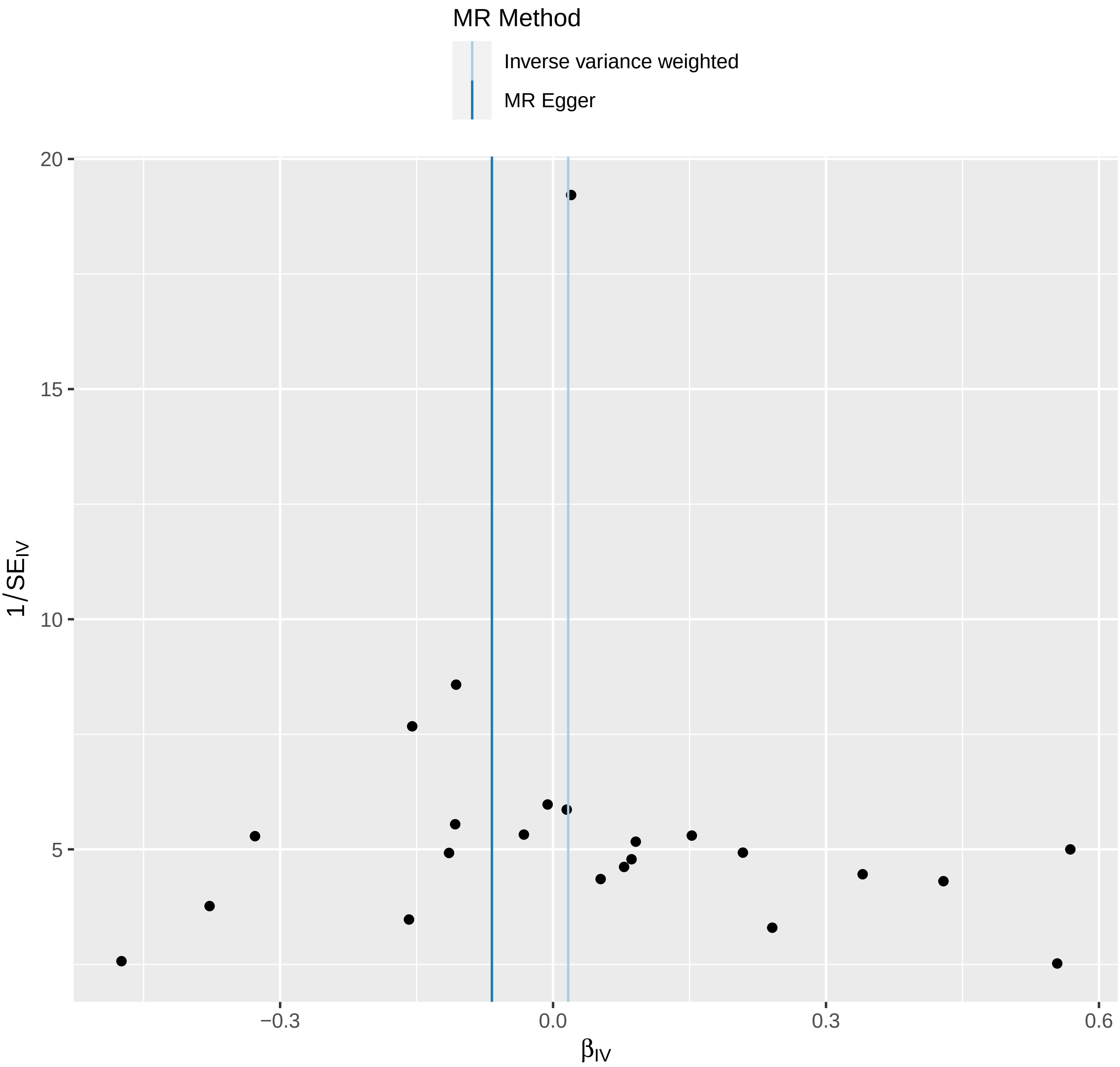

Batch 3−2692 : Kallistatin on Diabetic nephropathy (more control exclusions)
with a threshold of 1e−05

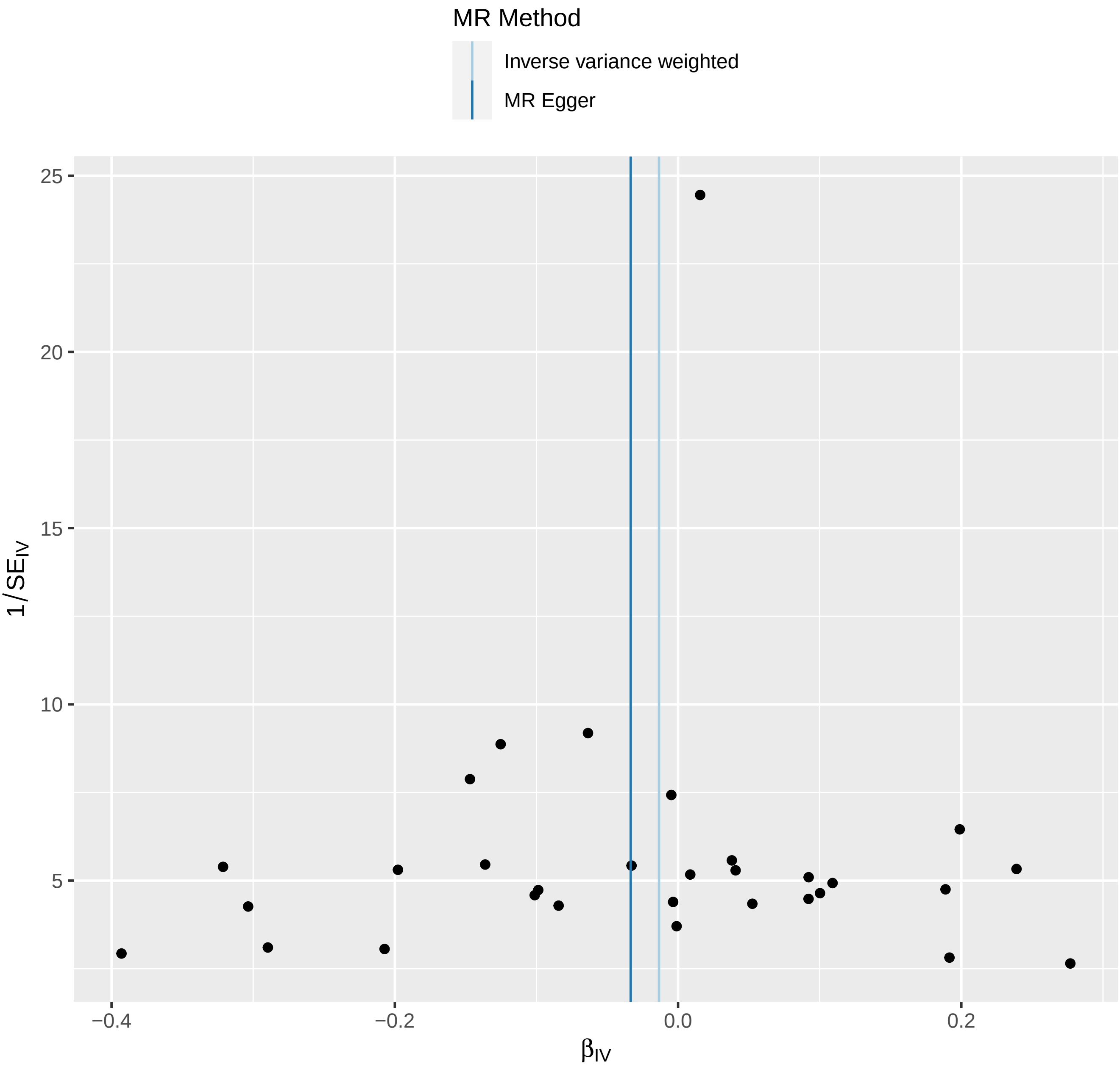

Batch 3−2693 : Corticosteroid−binding globulin on Diabetic nephropathy (more control exclusions)
with a threshold of 1e−05

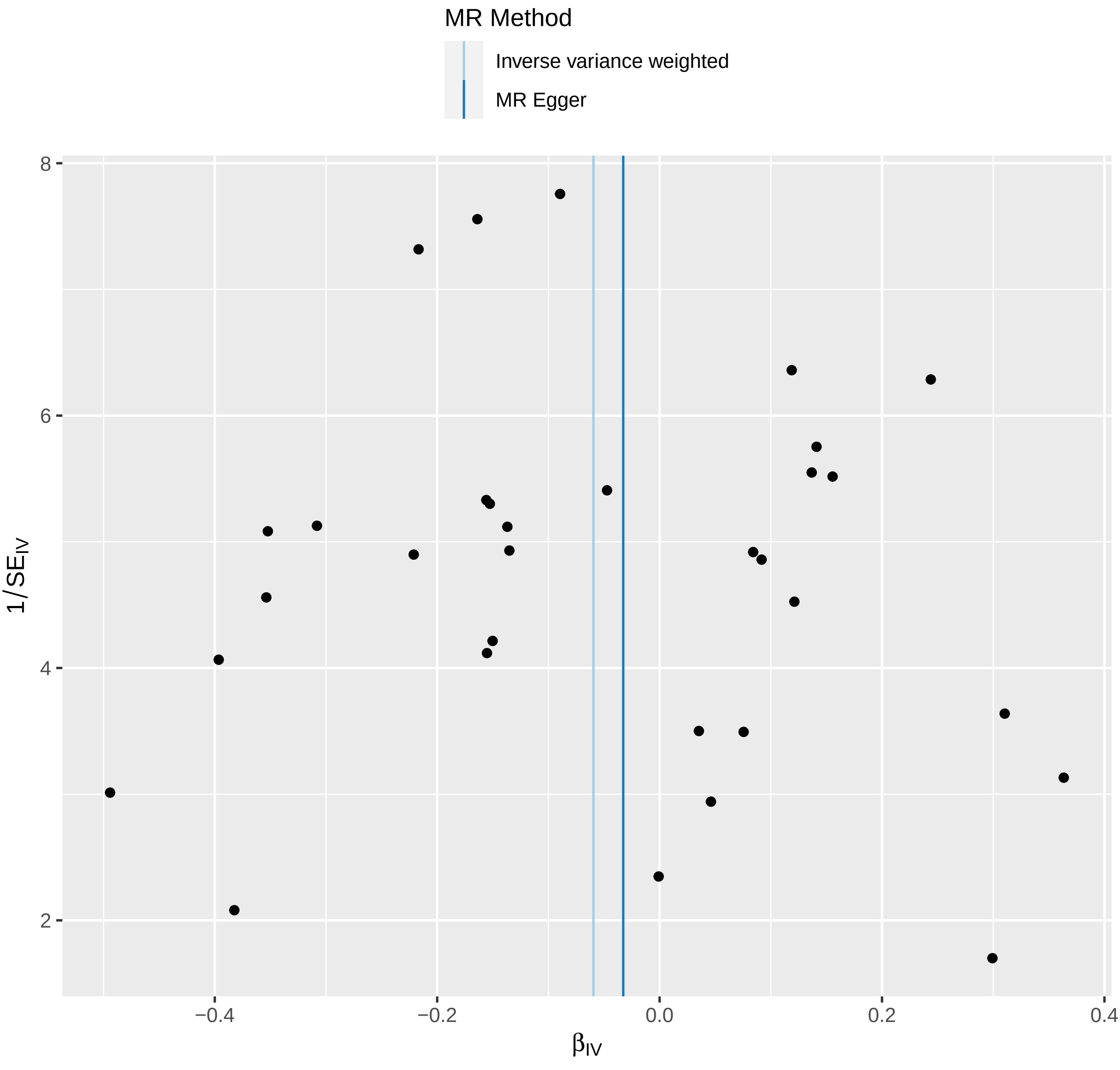

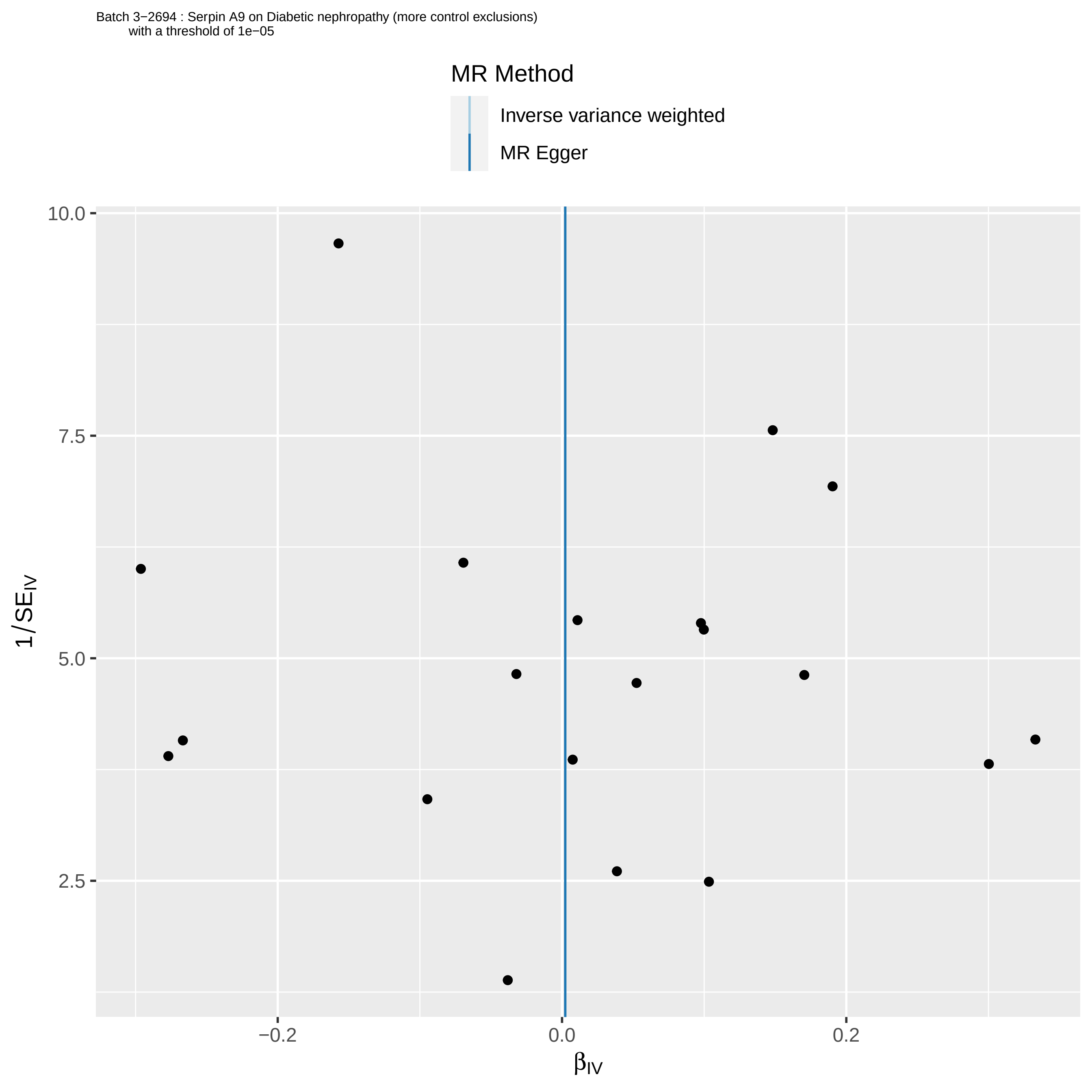
Batch 3−2694 : Serpin A9 on Diabetic nephropathy (more control exclusions)
with a threshold of 1e−05
MR Method
Inverse variance weighted
MR Egger
10.0
7.5
5.0
2.5
1/SE_IV
−0.2
0.0
0.2
β_IV

Batch 3−2695 : Antithrombin−III on Diabetic nephropathy (more control exclusions)
with a threshold of 1e−05

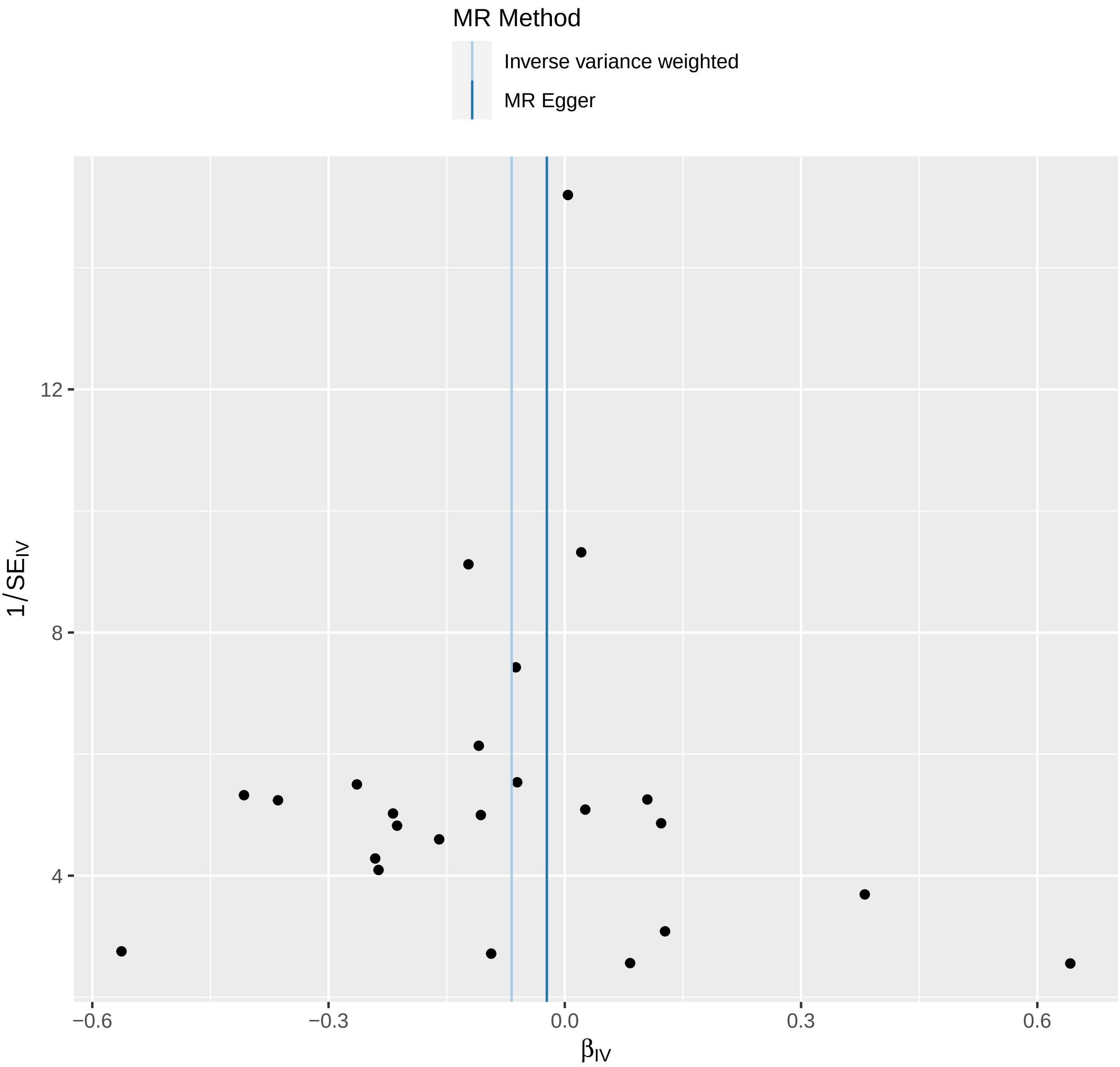

Batch 3−2696 : Plasminogen activator inhibitor 1 on Diabetic nephropathy (more control exclusions)
with a threshold of 1e−05

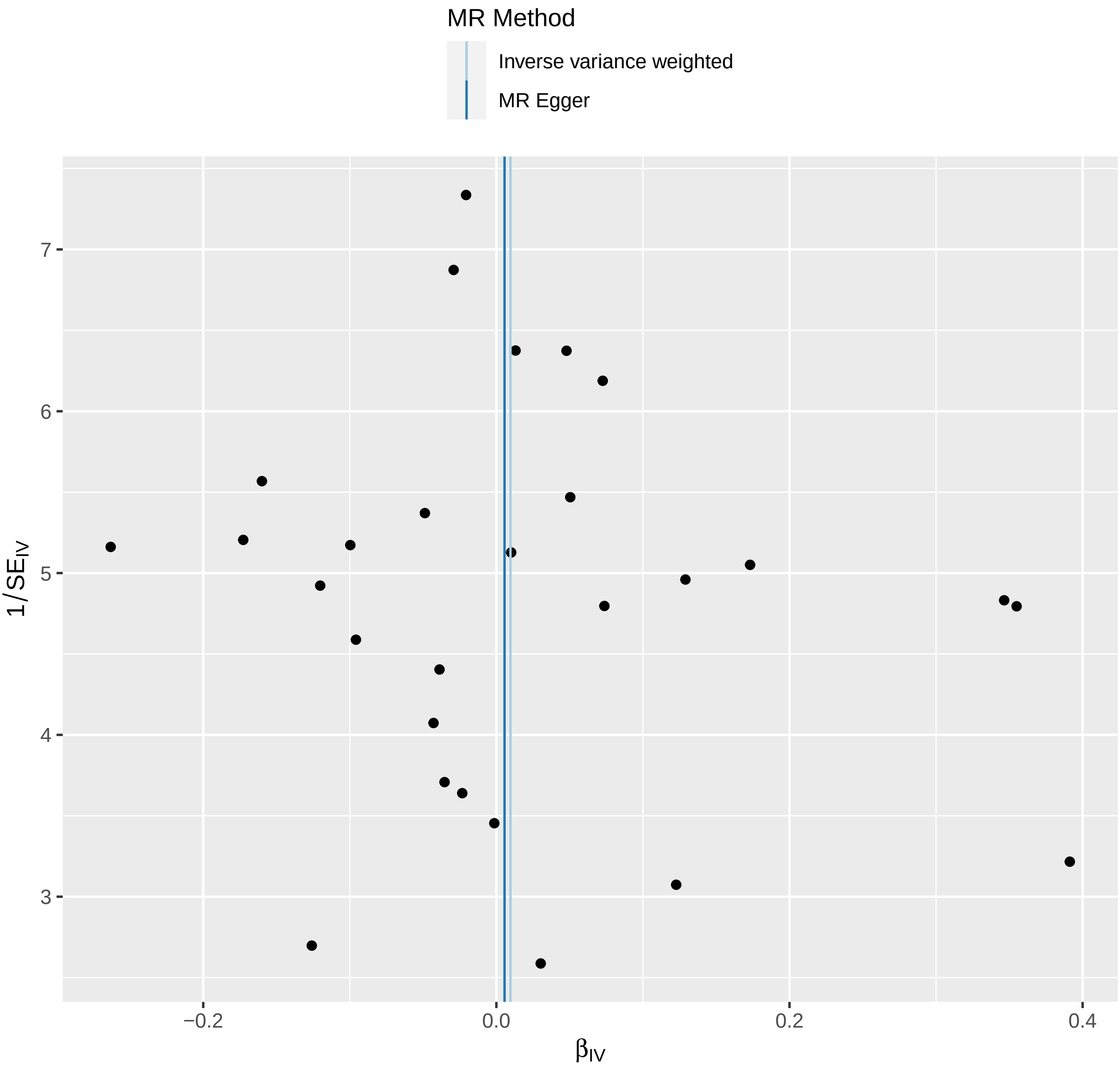

Batch 3−2697 : Glia−derived nexin on Diabetic nephropathy (more control exclusions)
with a threshold of 1e−05

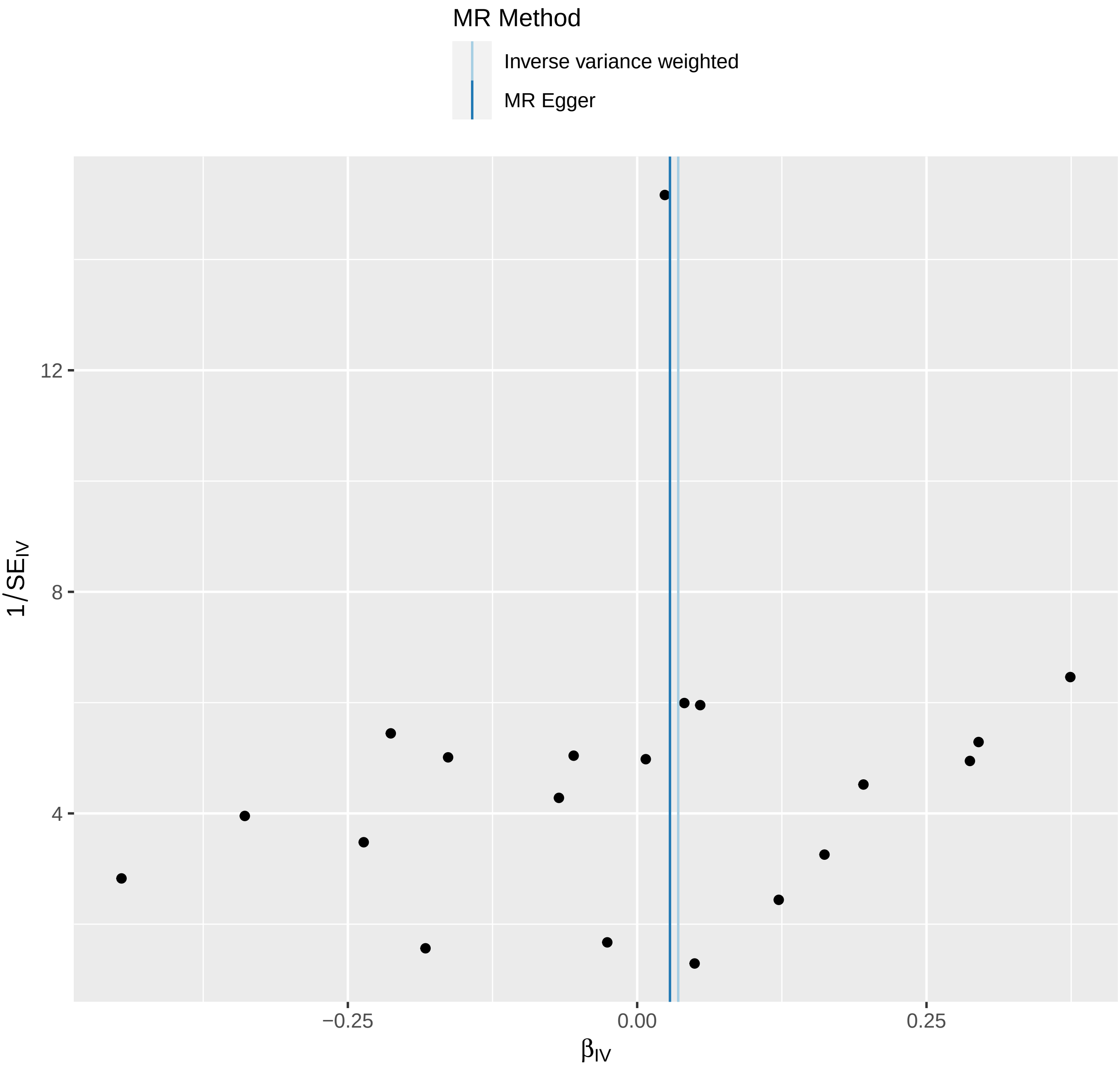

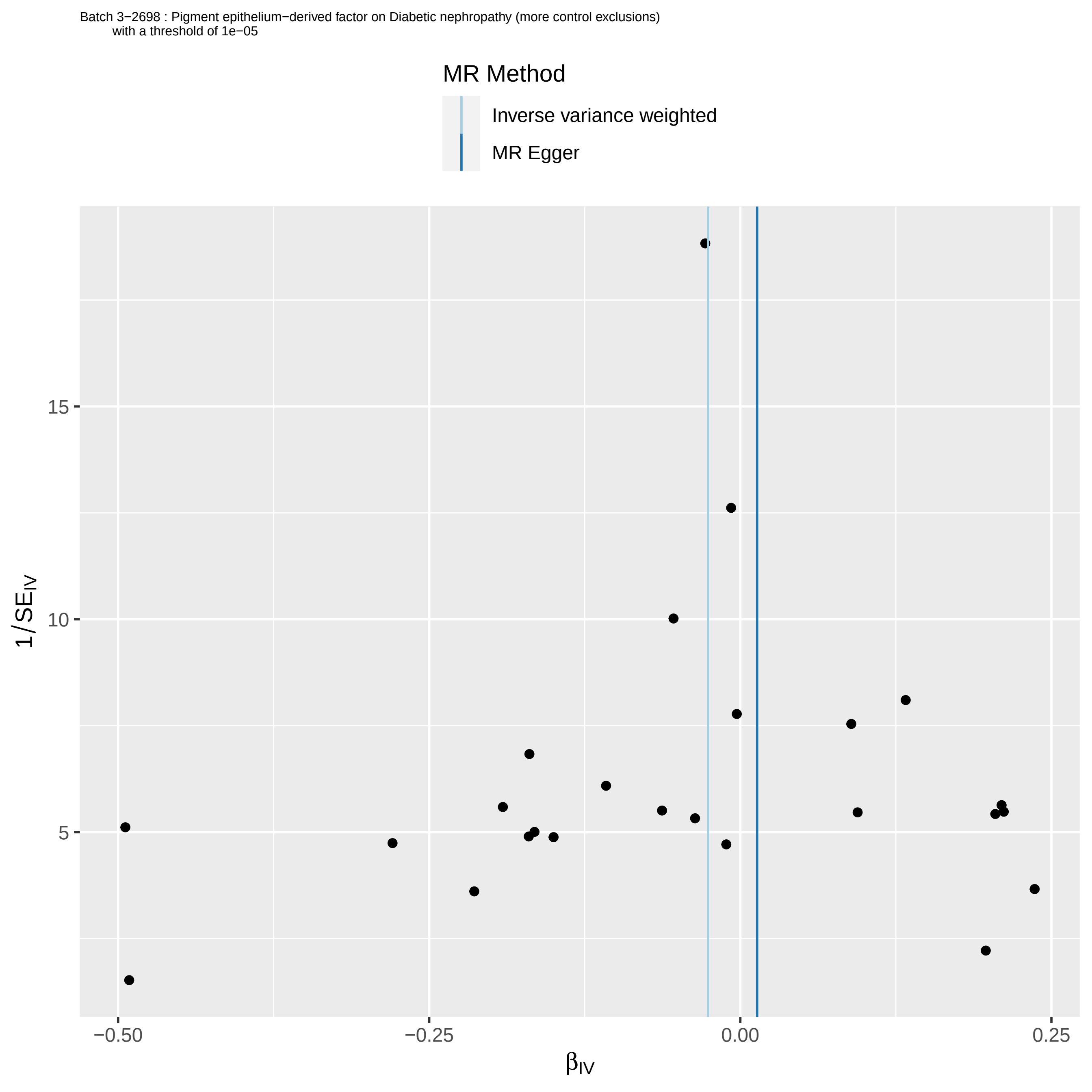

Batch 3−2698 : Pigment epithelium−derived factor on Diabetic nephropathy (more control exclusions)
with a threshold of 1e−05
MR Method
Inverse variance weighted
MR Egger
1/SE_IV
15
10
5
−0.50
−0.25
0.00
0.25
β_IV

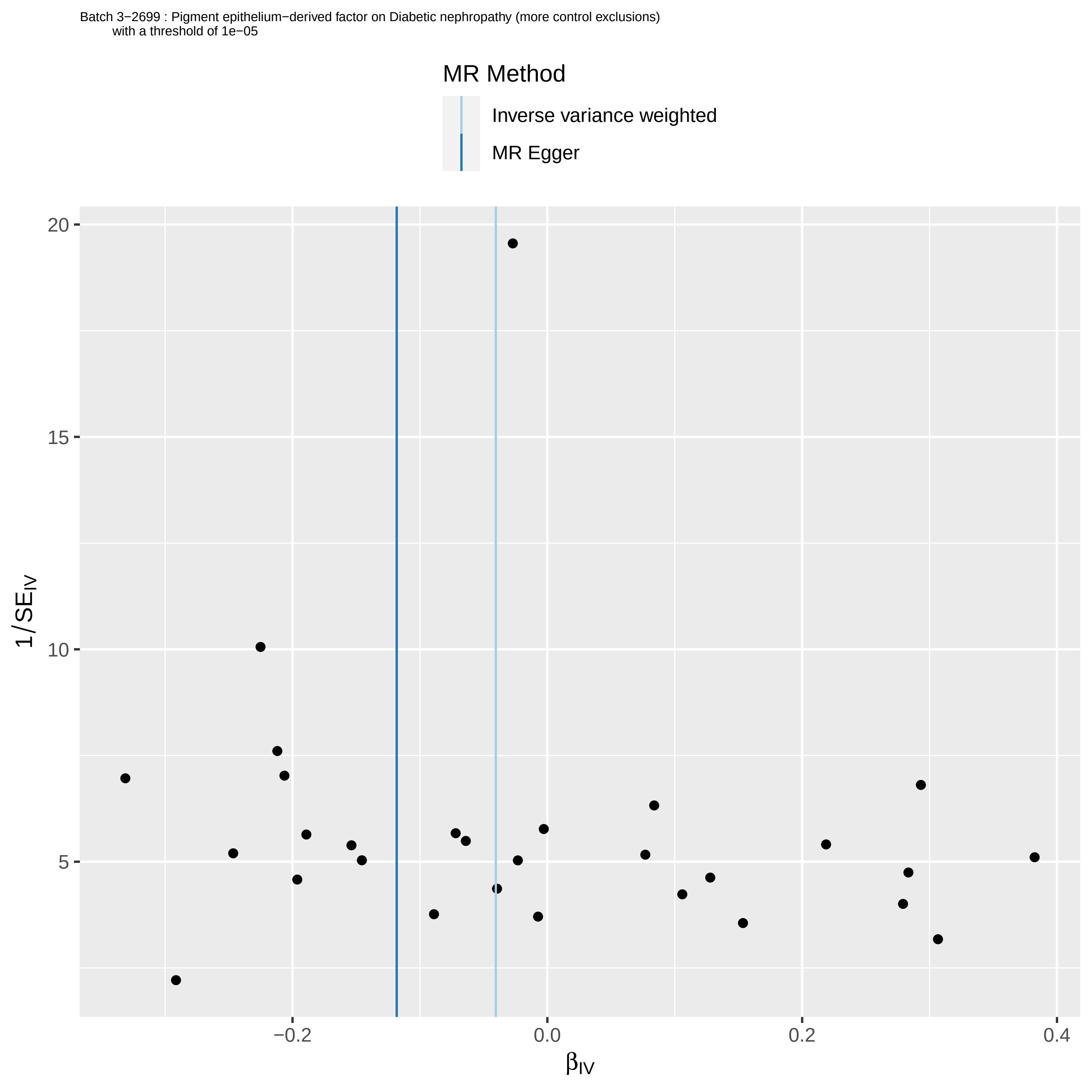
Batch 3−2699 : Pigment epithelium−derived factor on Diabetic nephropathy (more control exclusions)
with a threshold of 1e−05
MR Method
Inverse variance weighted
MR Egger
20
15
10
5
1/SE_IV
−0.2
0.0
0.2
0.4
β_IV

Batch 3−2700 : Alpha−2−antiplasmin on Diabetic nephropathy (more control exclusions)
with a threshold of 1e−05

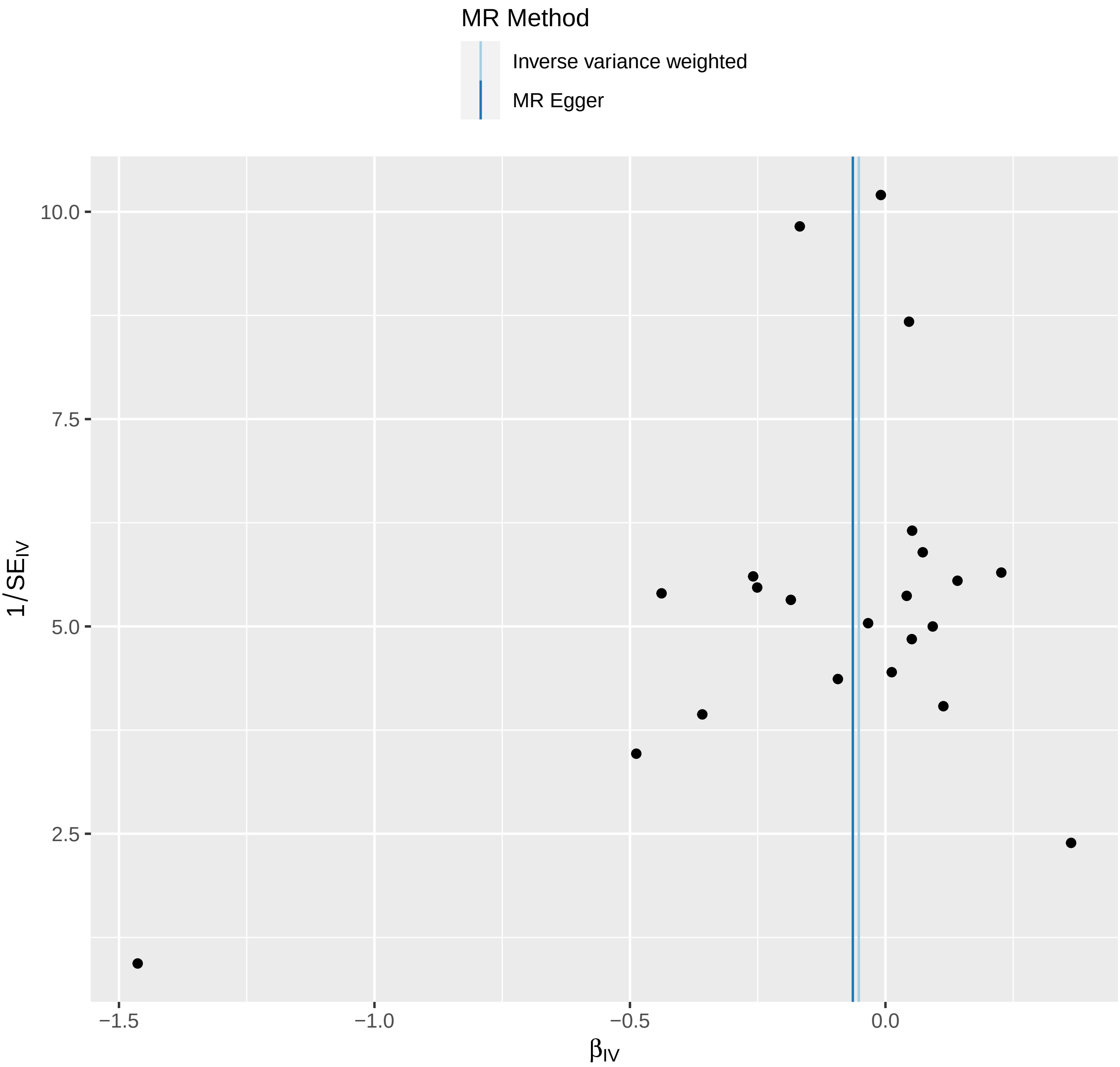

Batch 3−2701 : Plasma protease C1 inhibitor on Diabetic nephropathy (more control exclusions)
with a threshold of 1e−05

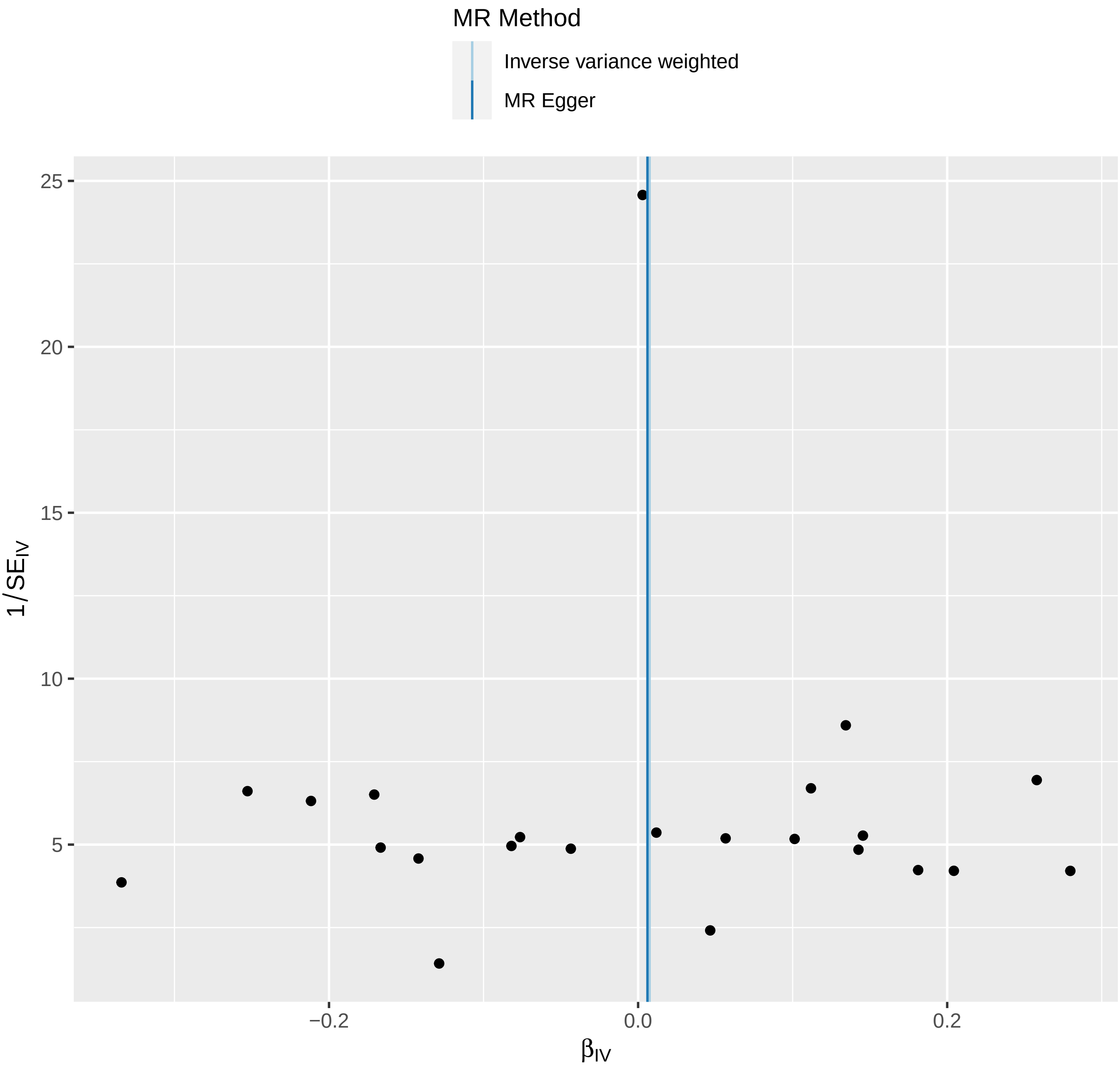

Batch 3−2702 : Serpin I2 on Diabetic nephropathy (more control exclusions)
with a threshold of 1e−05

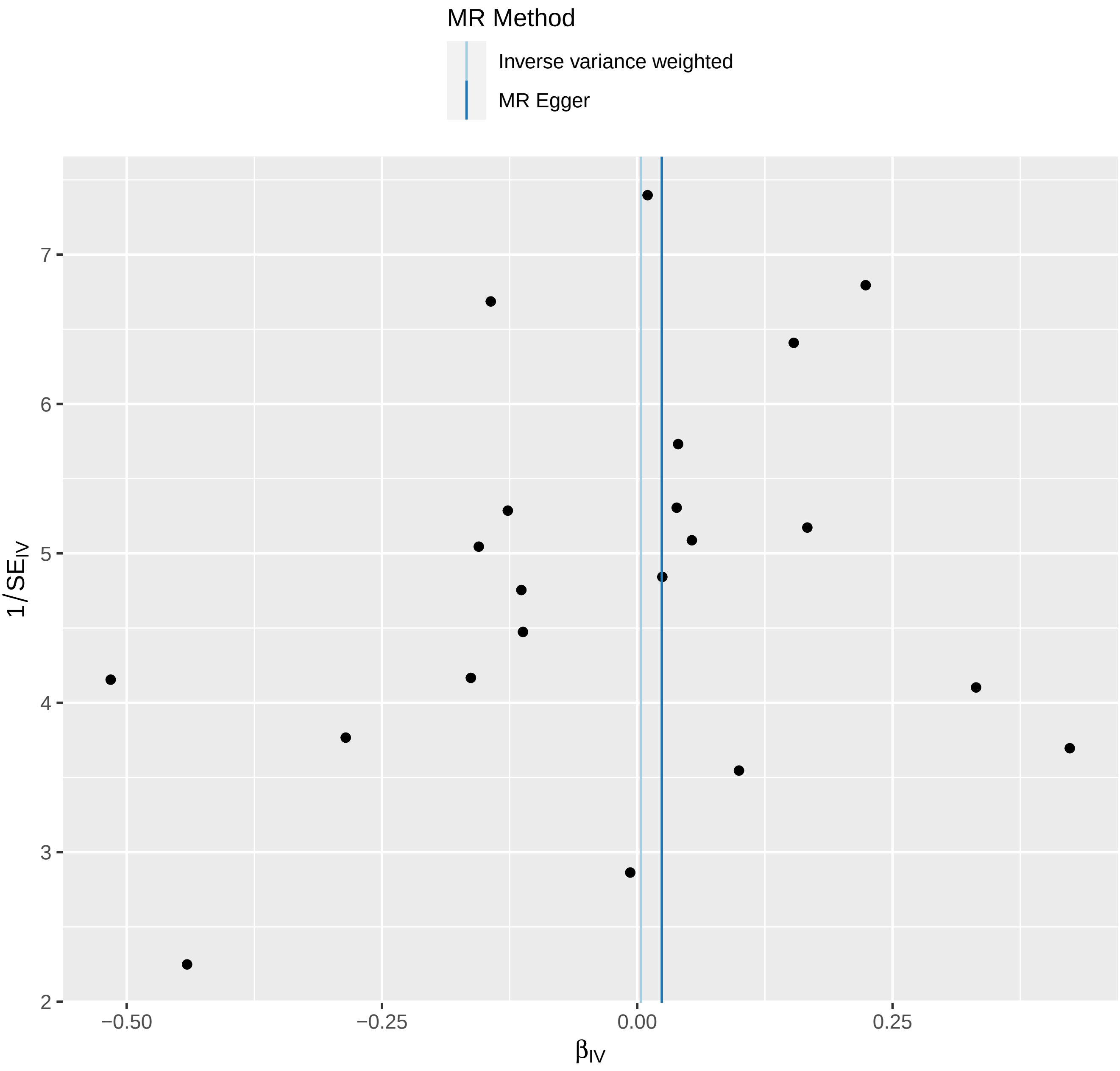

Batch 3−2703 : Histone−lysine N−methyltransferase SETD2 on Diabetic nephropathy (more control exclusions) with a threshold of 1e−05

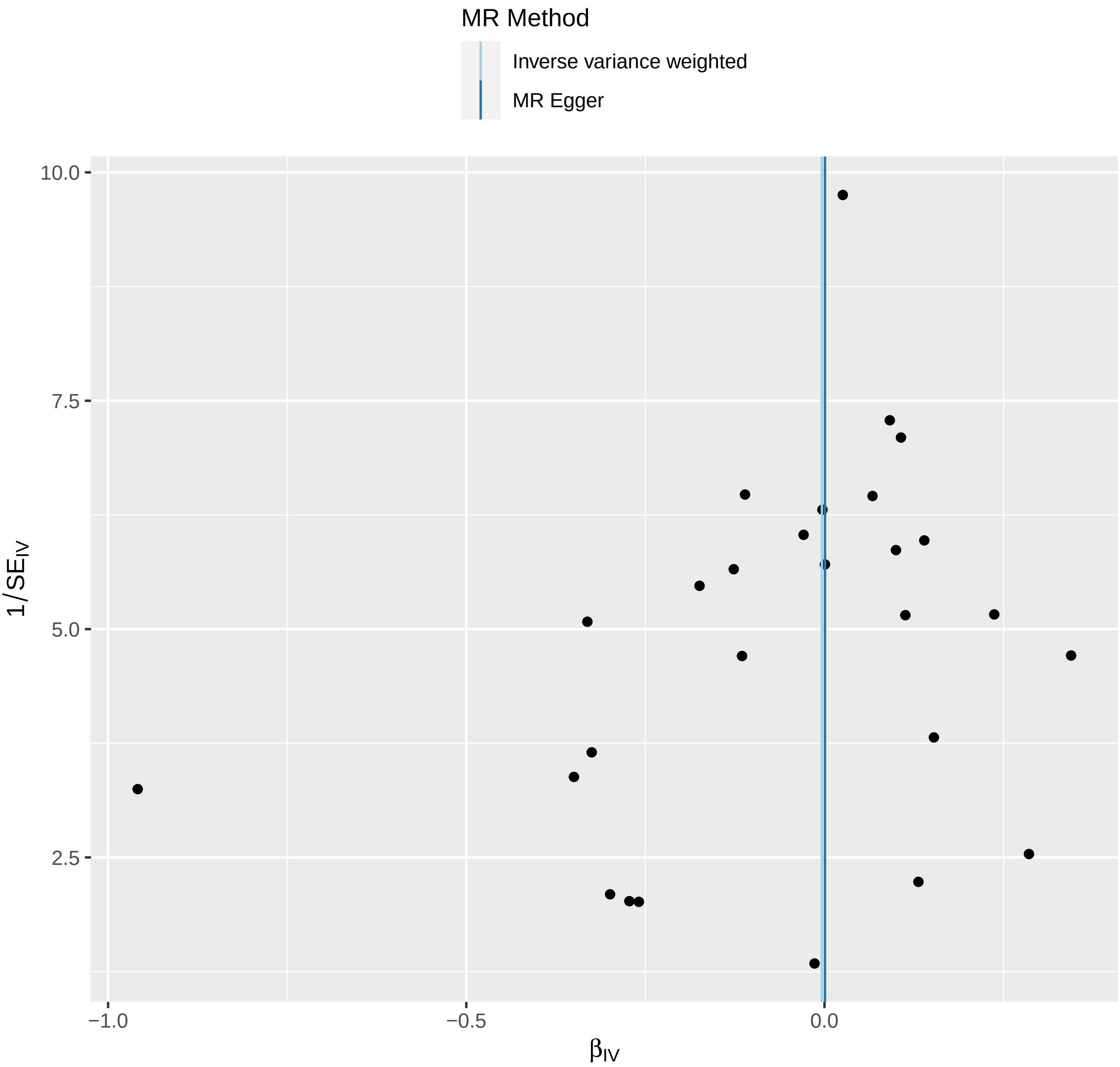

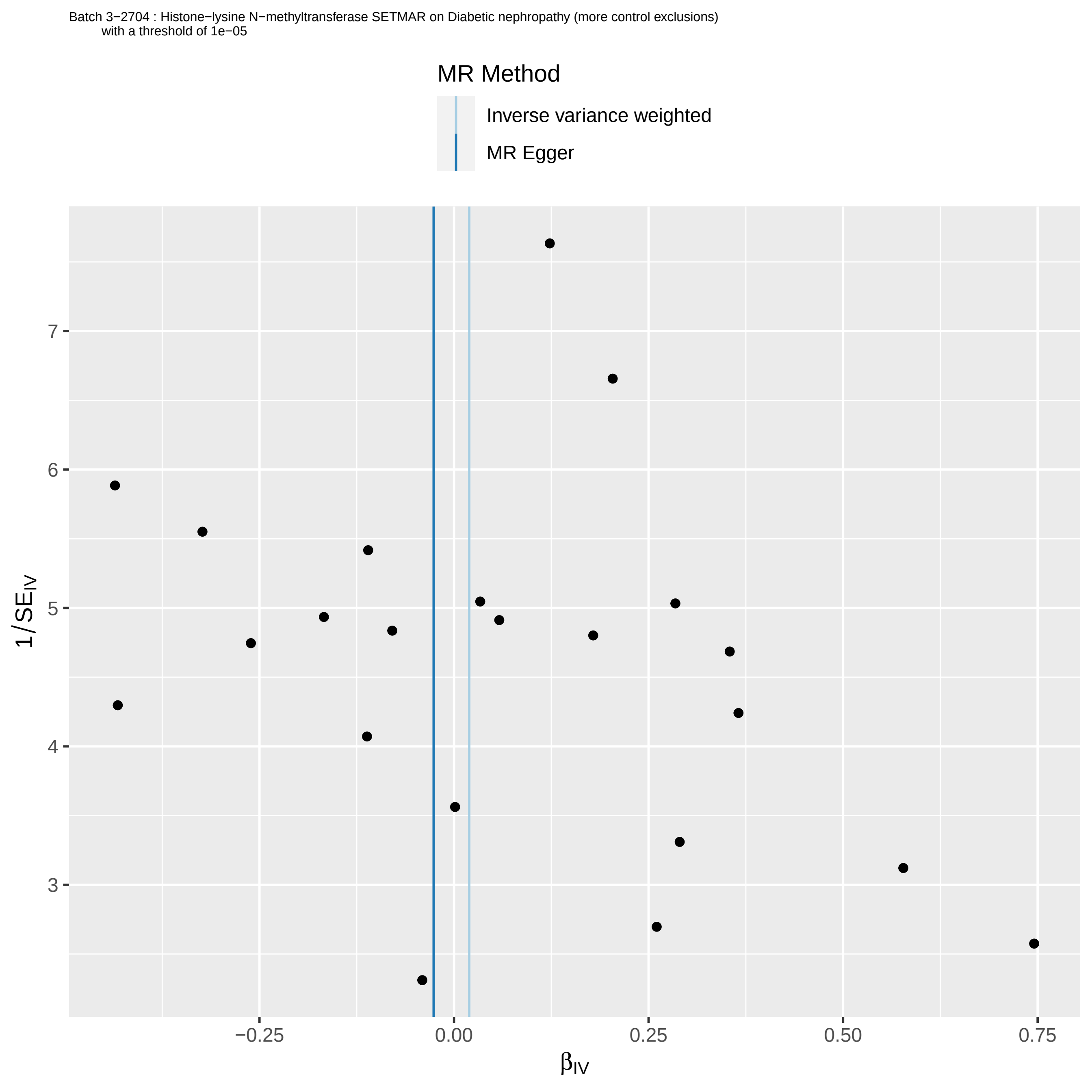

Batch 3−2704 : Histone−lysine N−methyltransferase SETMAR on Diabetic nephropathy (more control exclusions)
with a threshold of 1e−05
MR Method
Inverse variance weighted
MR Egger
7
6
5
4
3
$1/SE_{IV}$
−0.25
0.00
0.25
0.50
0.75
β_{IV}

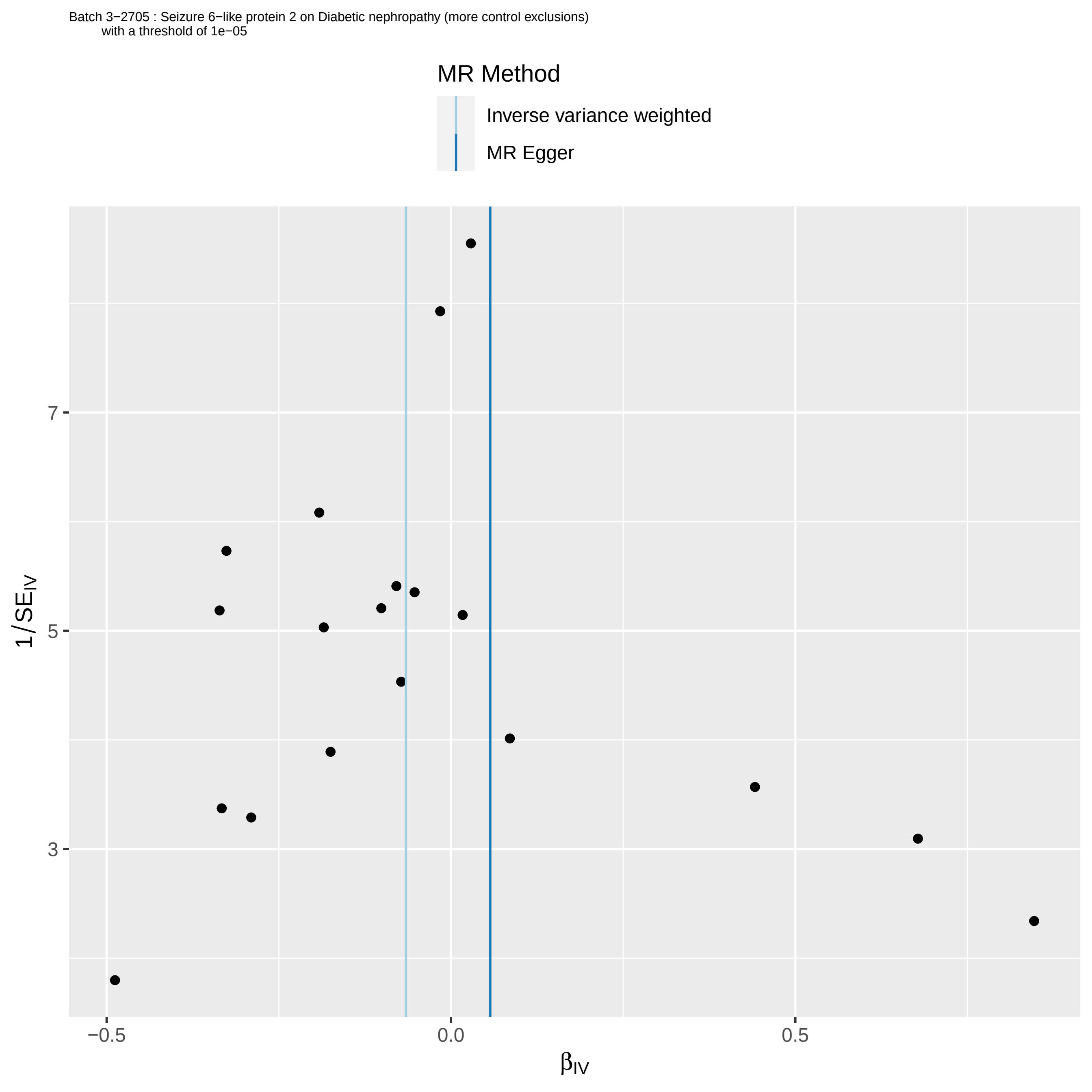
Batch 3−2705 : Seizure 6−like protein 2 on Diabetic nephropathy (more control exclusions)
with a threshold of 1e−05
MR Method
Inverse variance weighted
MR Egger
7
5
3
1/SE$_{IV}$
−0.5
0.0
0.5
β_{IV}

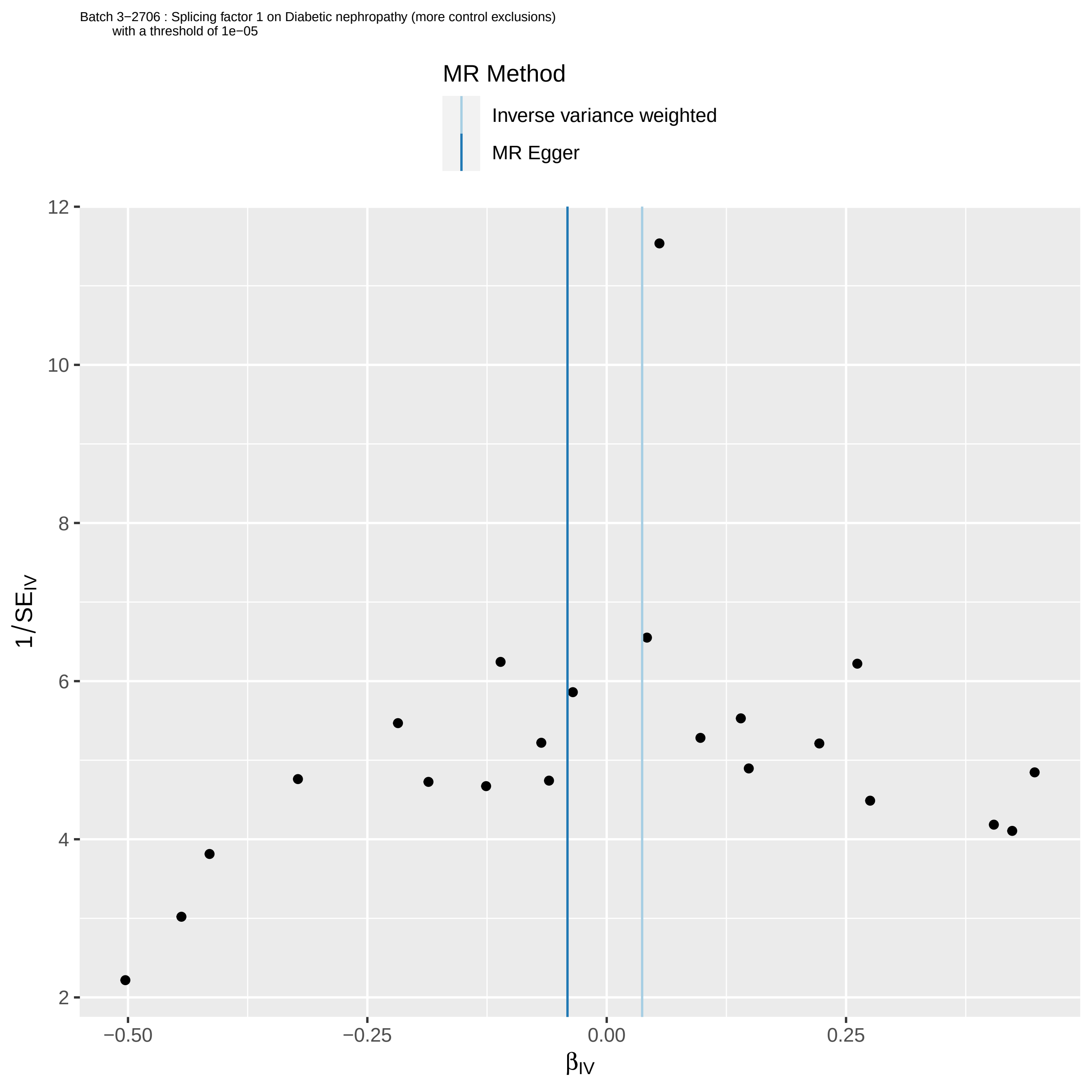
Batch 3−2706 : Splicing factor 1 on Diabetic nephropathy (more control exclusions)
with a threshold of 1e−05
MR Method
Inverse variance weighted
MR Egger
12
10
8
6
4
2
1/SE_IV
−0.50
−0.25
0.00
0.25
β_IV

Batch 3−2707 : Splicing factor 3B subunit 4 on Diabetic nephropathy (more control exclusions)
with a threshold of 1e−05

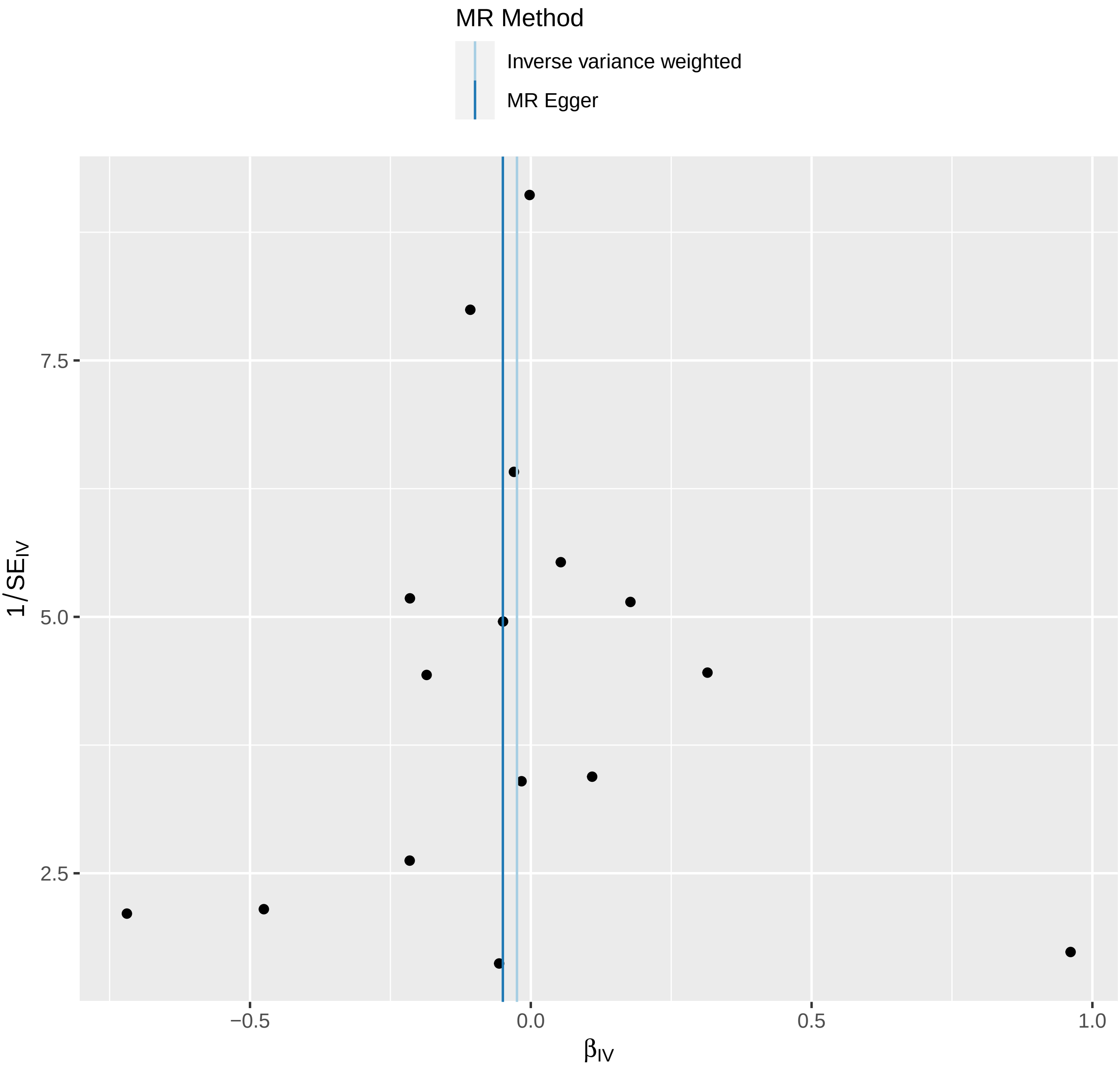

Batch 3−2708 : 14−3−3 protein sigma on Diabetic nephropathy (more control exclusions)
with a threshold of 1e−05

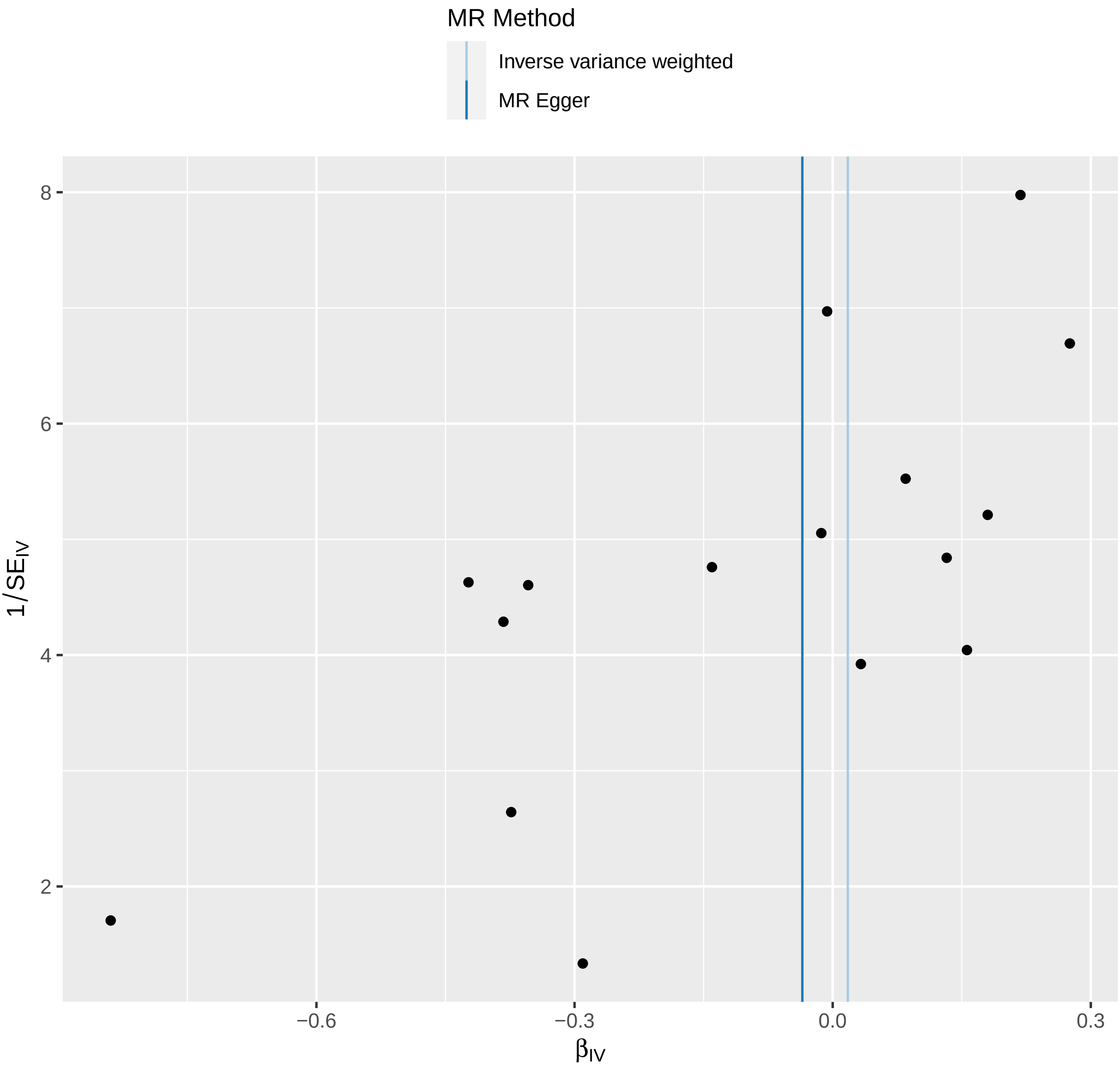

Batch 3−2709 : Secreted frizzled−related protein 1 on Diabetic nephropathy (more control exclusions)
with a threshold of 1e−05

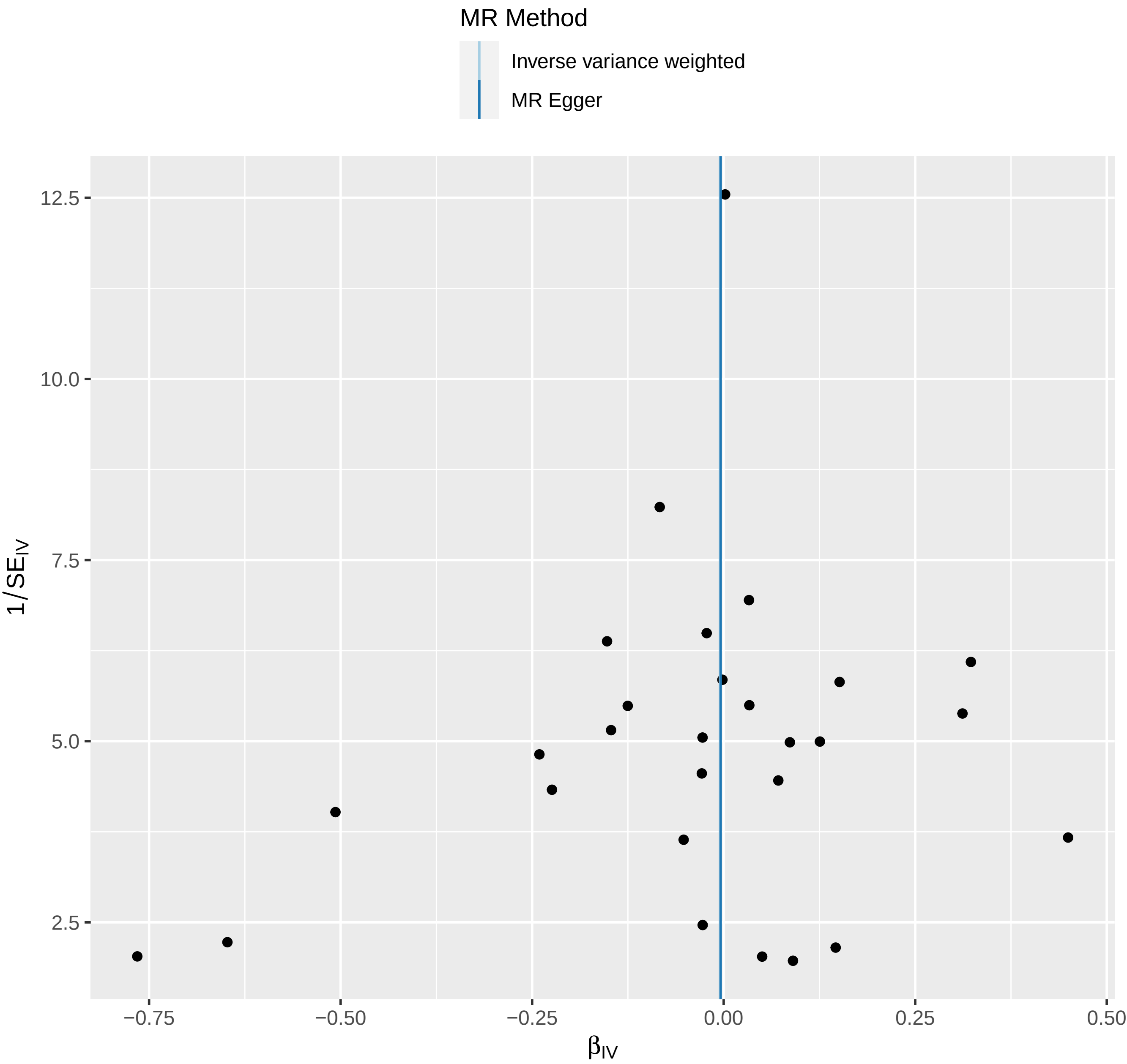

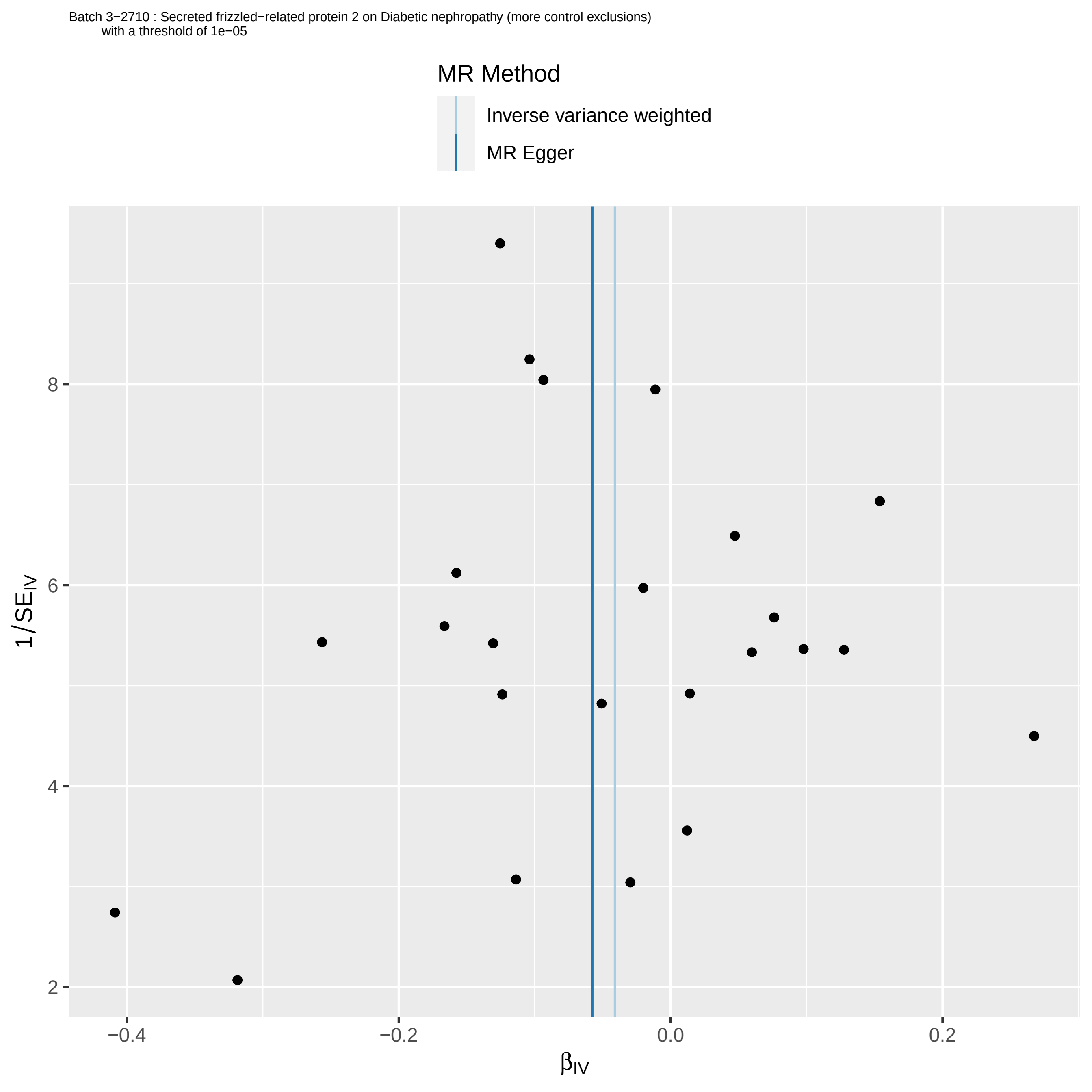

Batch 3−2710 : Secreted frizzled−related protein 2 on Diabetic nephropathy (more control exclusions)
with a threshold of 1e−05
MR Method
Inverse variance weighted
MR Egger
1/SE_IV
β_IV
8
6
4
2
−0.4
−0.2
0.0
0.2

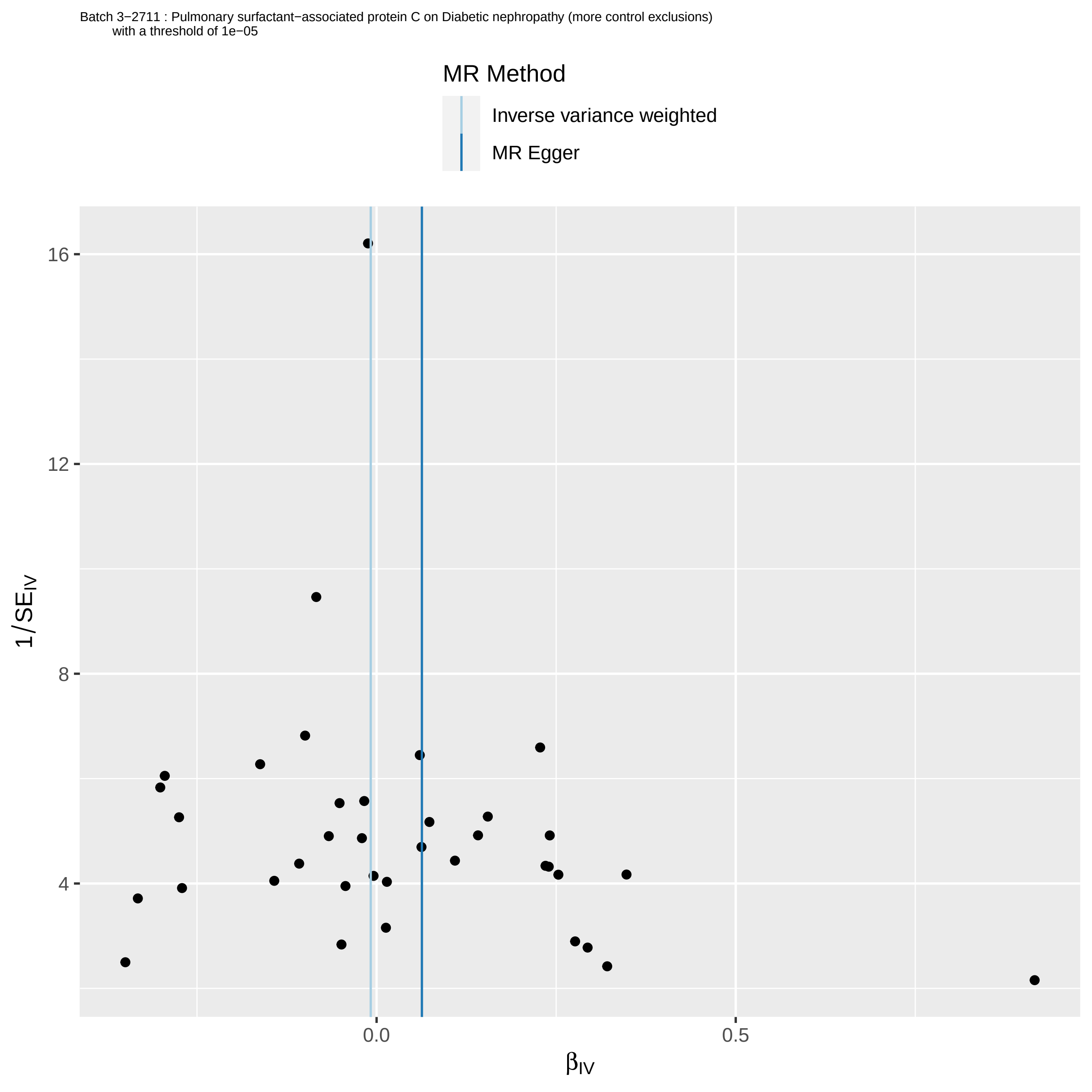
Batch 3−2711 : Pulmonary surfactant−associated protein C on Diabetic nephropathy (more control exclusions)
with a threshold of 1e−05
MR Method
Inverse variance weighted
MR Egger
16
12
8
4
1/SE_IV
0.0
0.5
β_IV

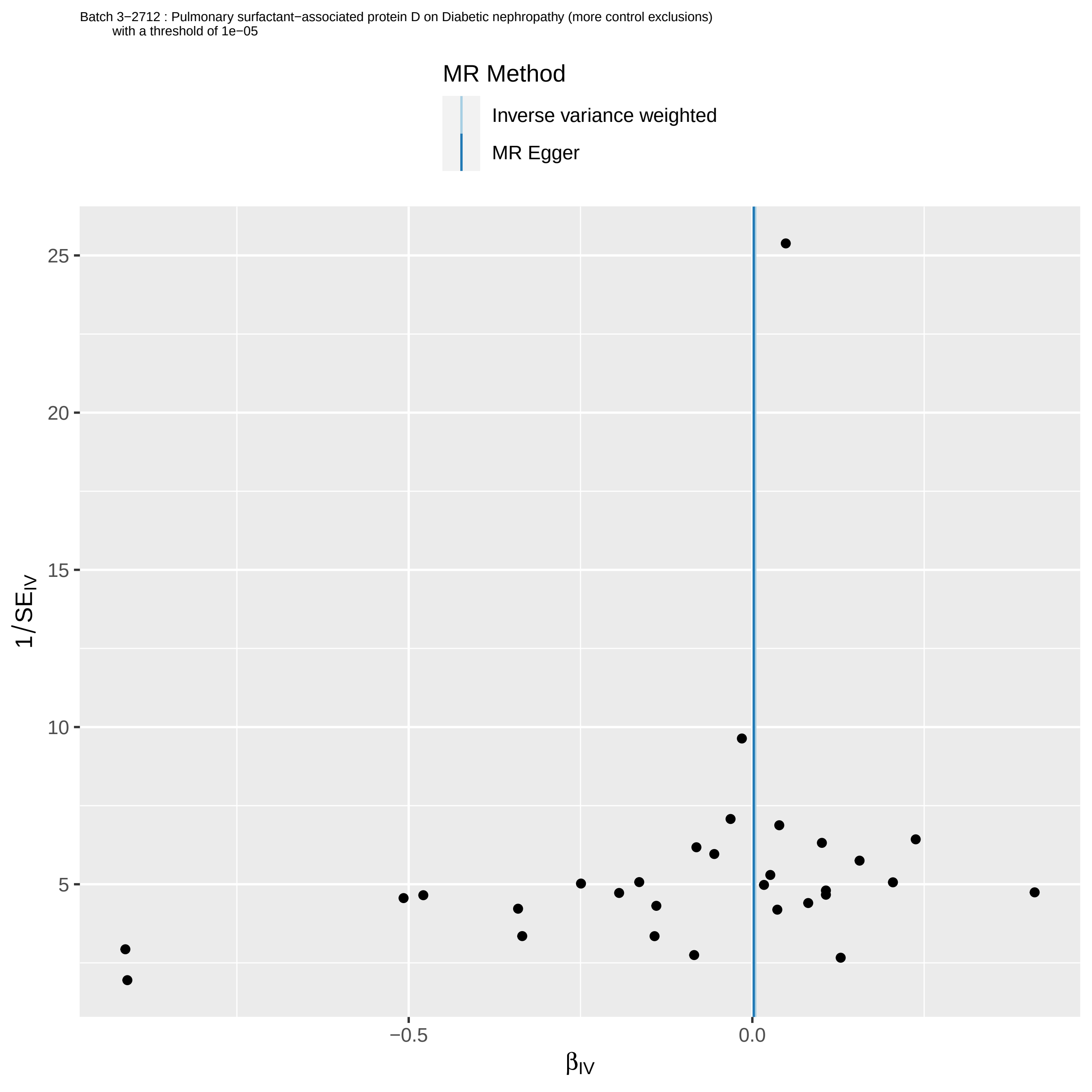

Batch 3−2712 : Pulmonary surfactant−associated protein D on Diabetic nephropathy (more control exclusions)
with a threshold of 1e−05
MR Method
Inverse variance weighted
MR Egger
25
20
15
10
5
1/SE_IV
−0.5
0.0
β_IV

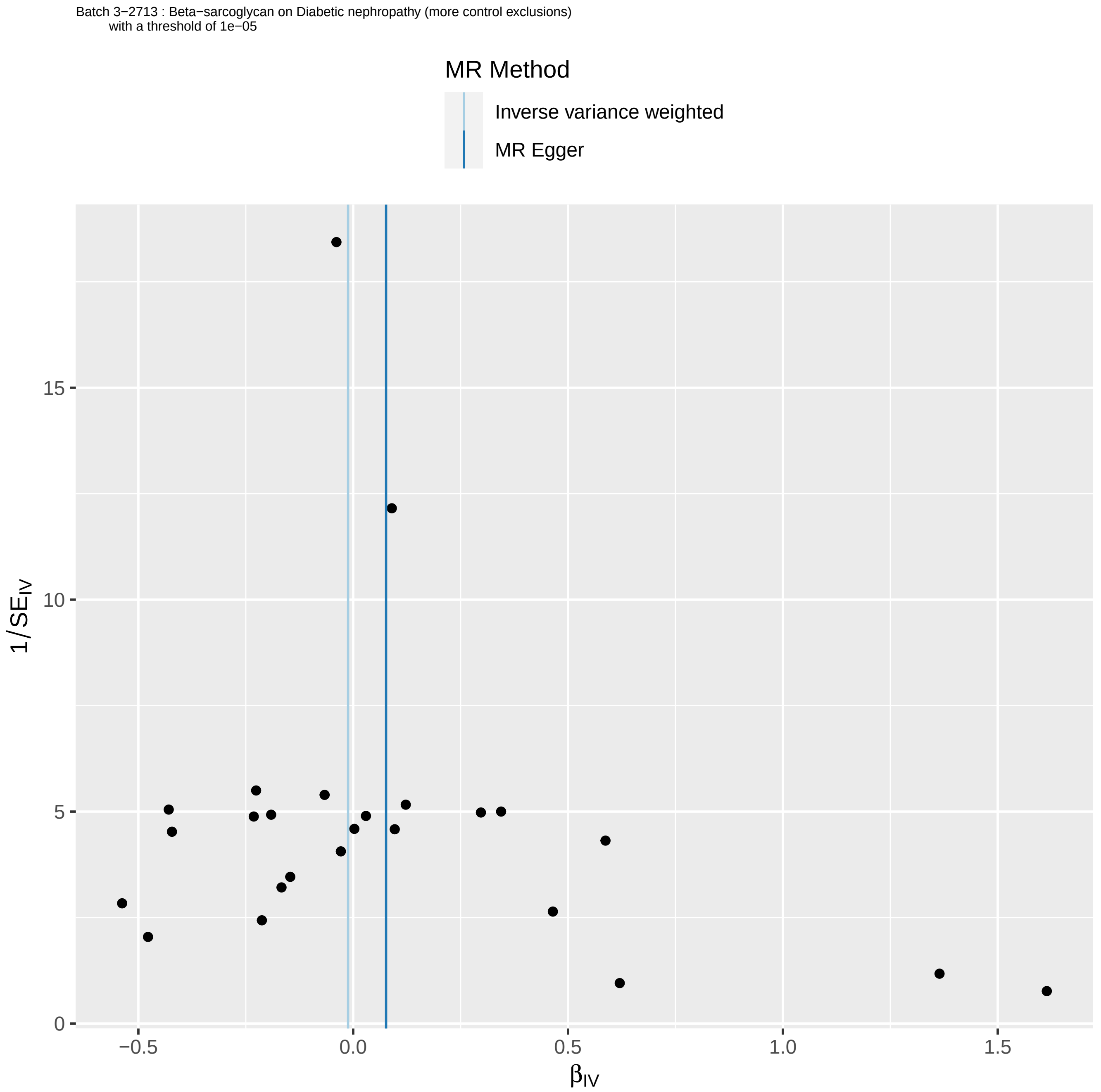
Batch 3−2713 : Beta−sarcoglycan on Diabetic nephropathy (more control exclusions)
with a threshold of 1e−05
MR Method
Inverse variance weighted
MR Egger
15
10
5
0
1/SE$_{IV}$
−0.5
0.0
0.5
1.0
1.5
β$_{IV}$

Batch 3−2714 : Small glutamine−rich tetratricopeptide repeat−containing protein alpha on Diabetic nephropathy (more control exclusions)
with a threshold of 1e−05

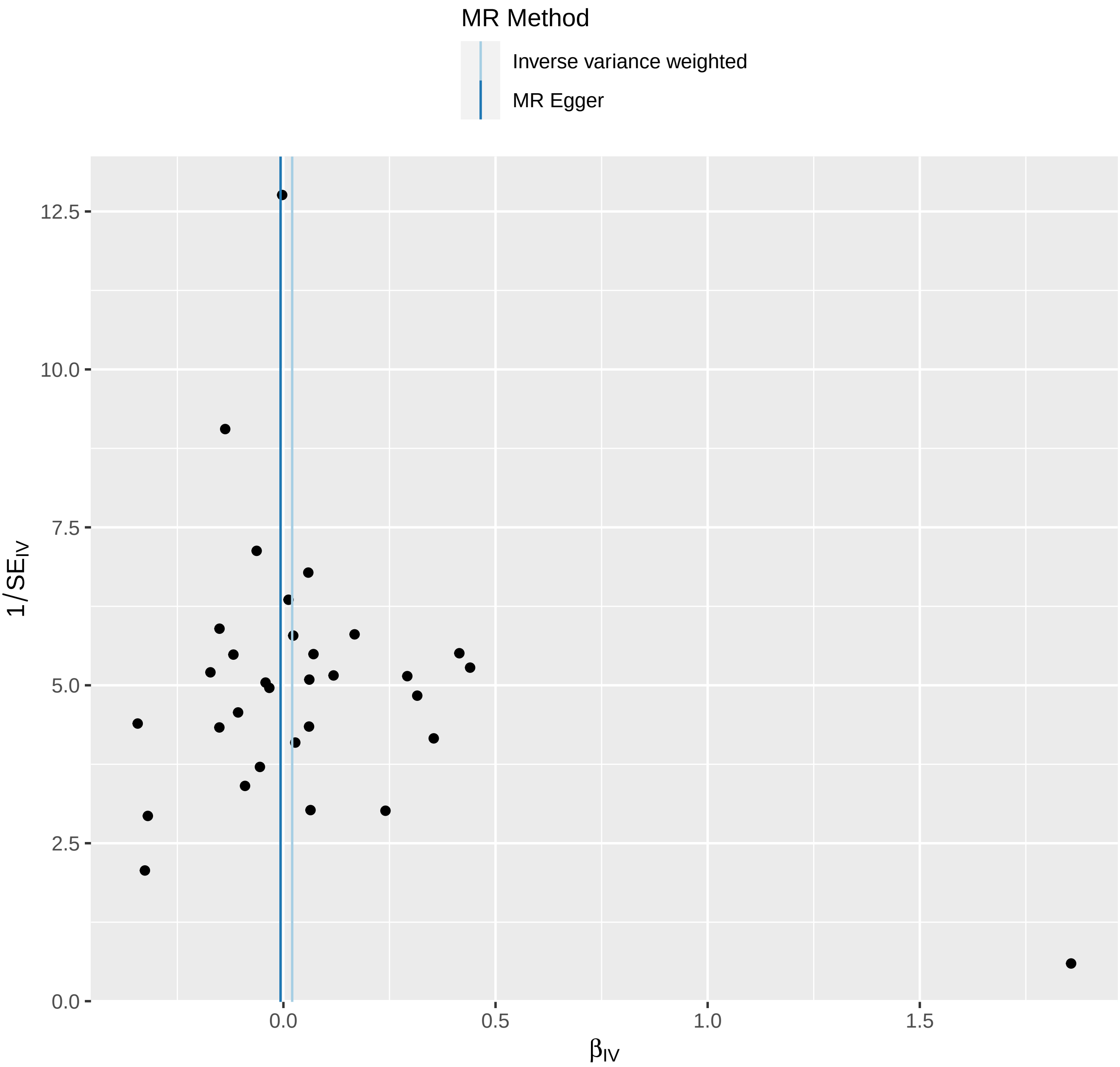

Batch 3−2715 : SH2 domain−containing protein 1A on Diabetic nephropathy (more control exclusions)
with a threshold of 1e−05

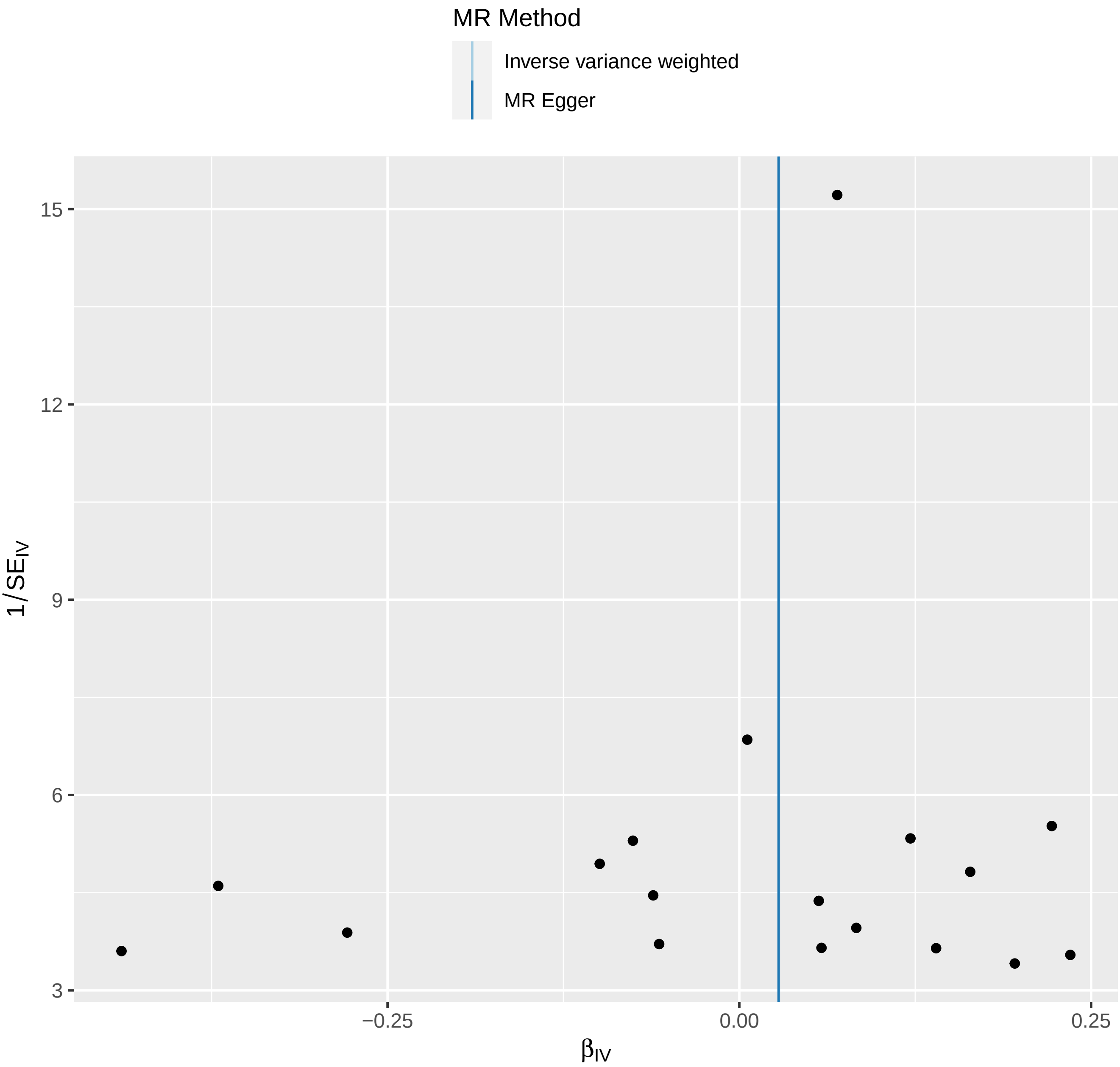

Batch 3−2716 : SH3 domain−binding protein 2 on Diabetic nephropathy (more control exclusions)
with a threshold of 1e−05

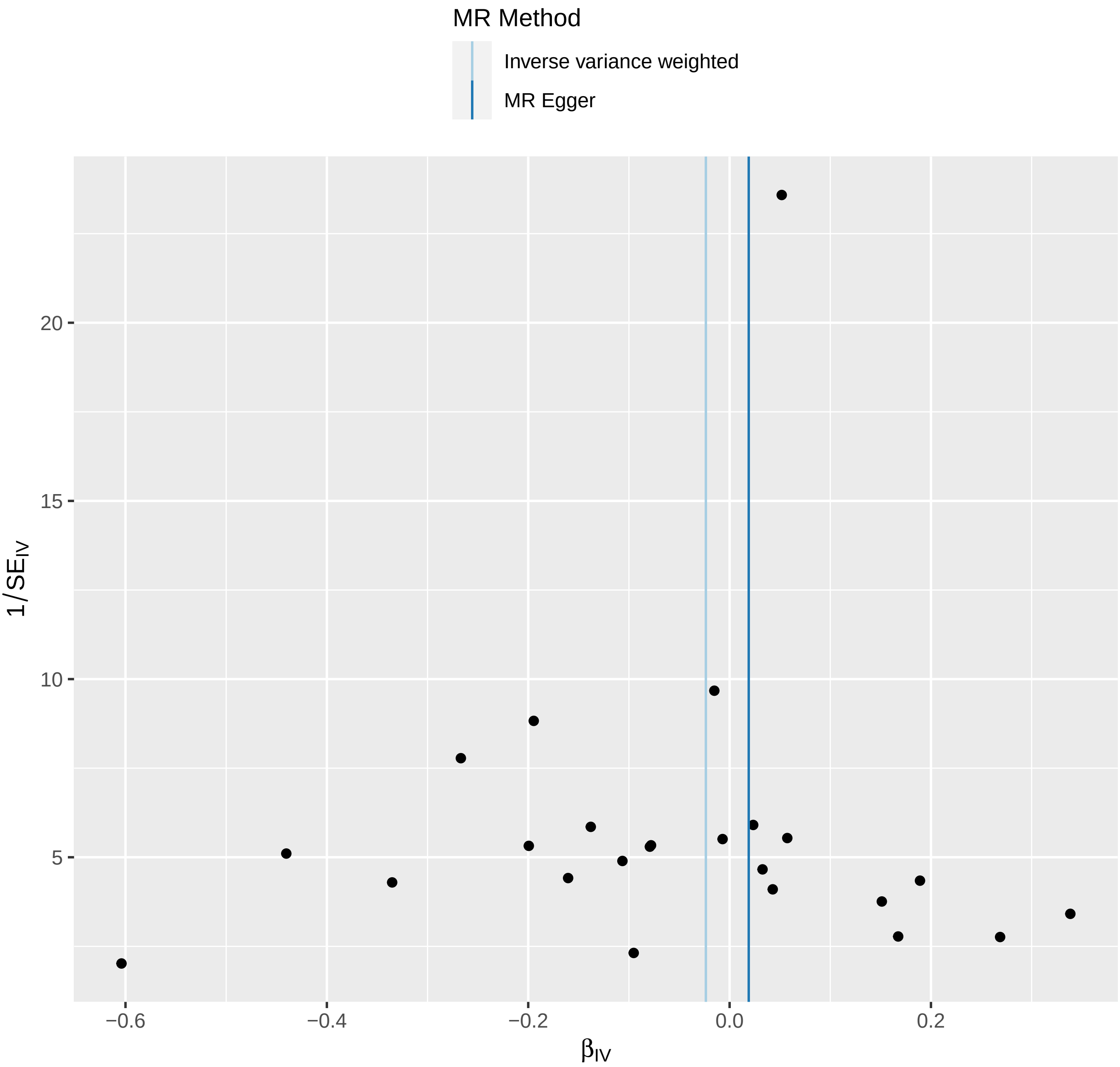

Batch 3−2717 : SHC−transforming protein 1 on Diabetic nephropathy (more control exclusions)
with a threshold of 1e−05

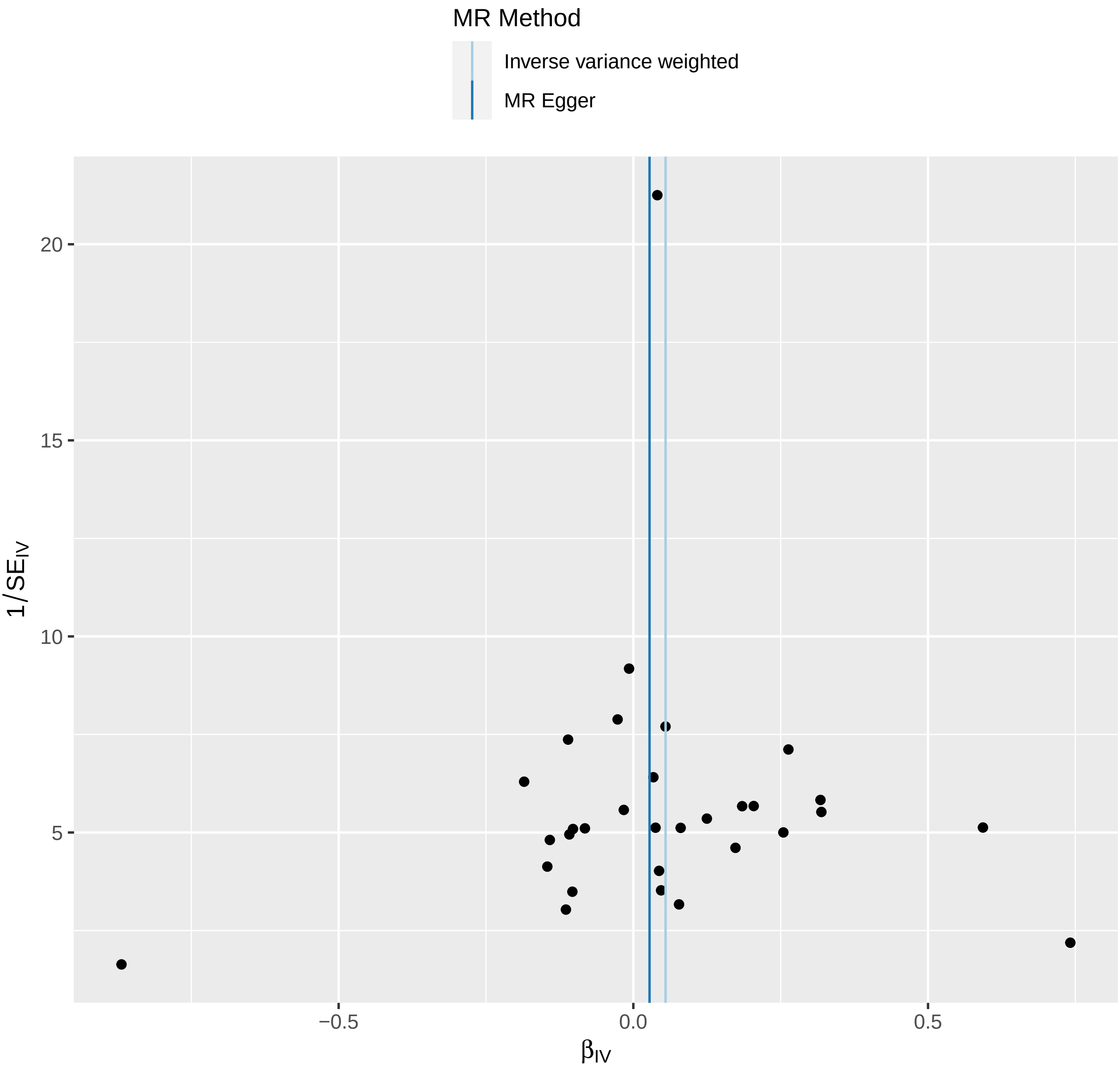

Batch 3−2718 : SHC−transforming protein 2 on Diabetic nephropathy (more control exclusions)
with a threshold of 1e−05

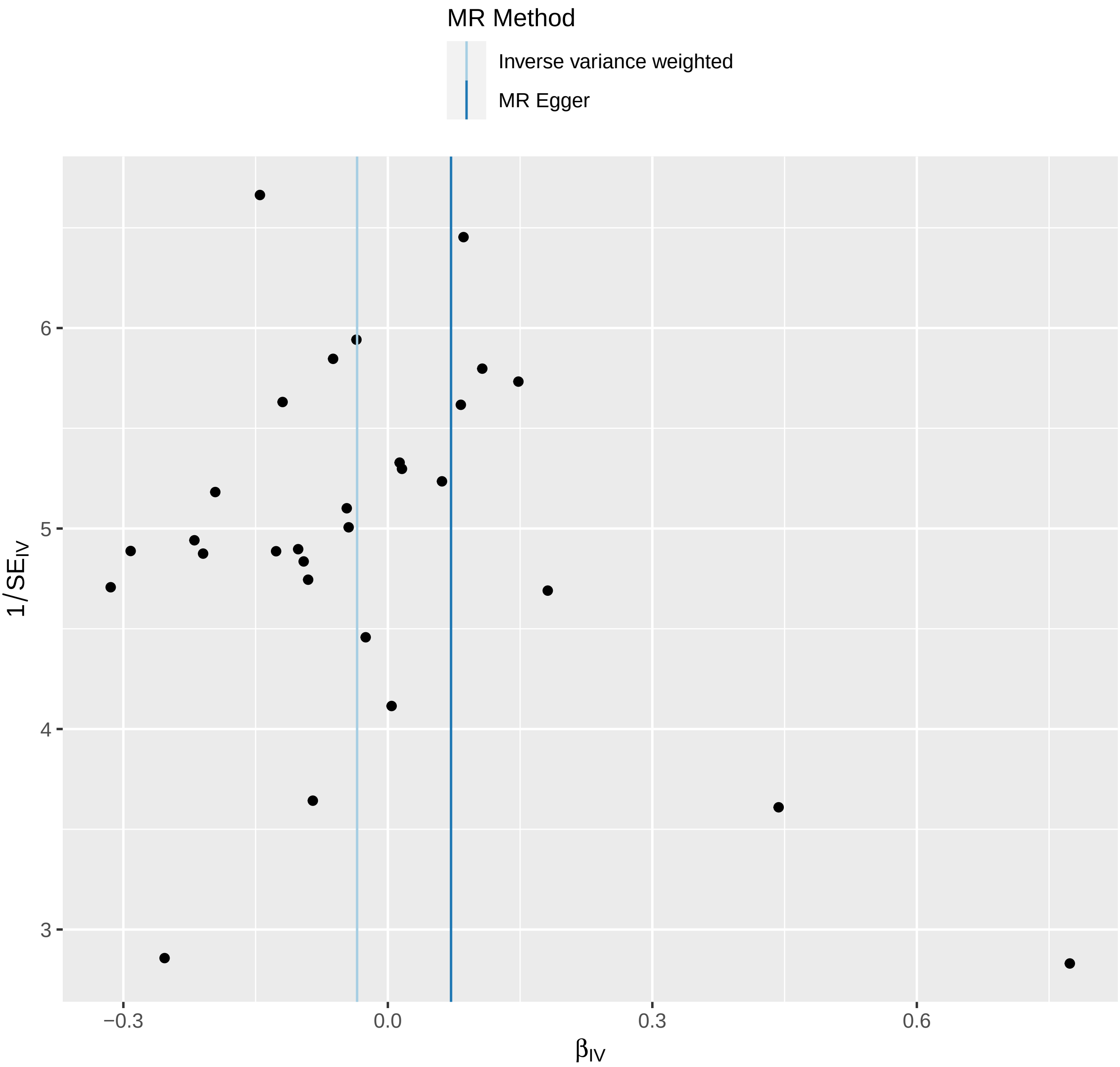

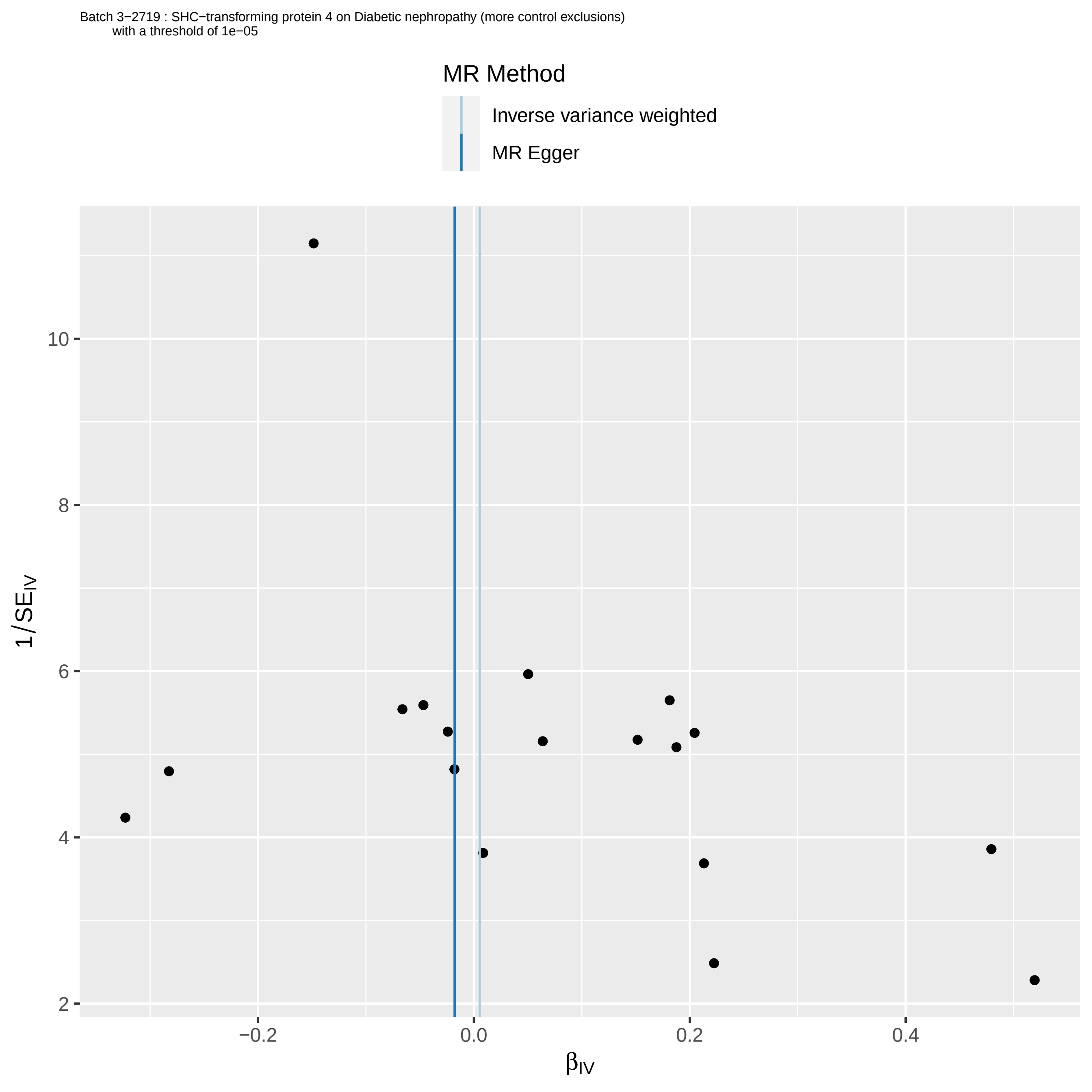
Batch 3−2719 : SHC−transforming protein 4 on Diabetic nephropathy (more control exclusions)
with a threshold of 1e−05
MR Method
Inverse variance weighted
MR Egger
1/SE_IV
β_IV
10
8
6
4
2
−0.2
0.0
0.2
0.4

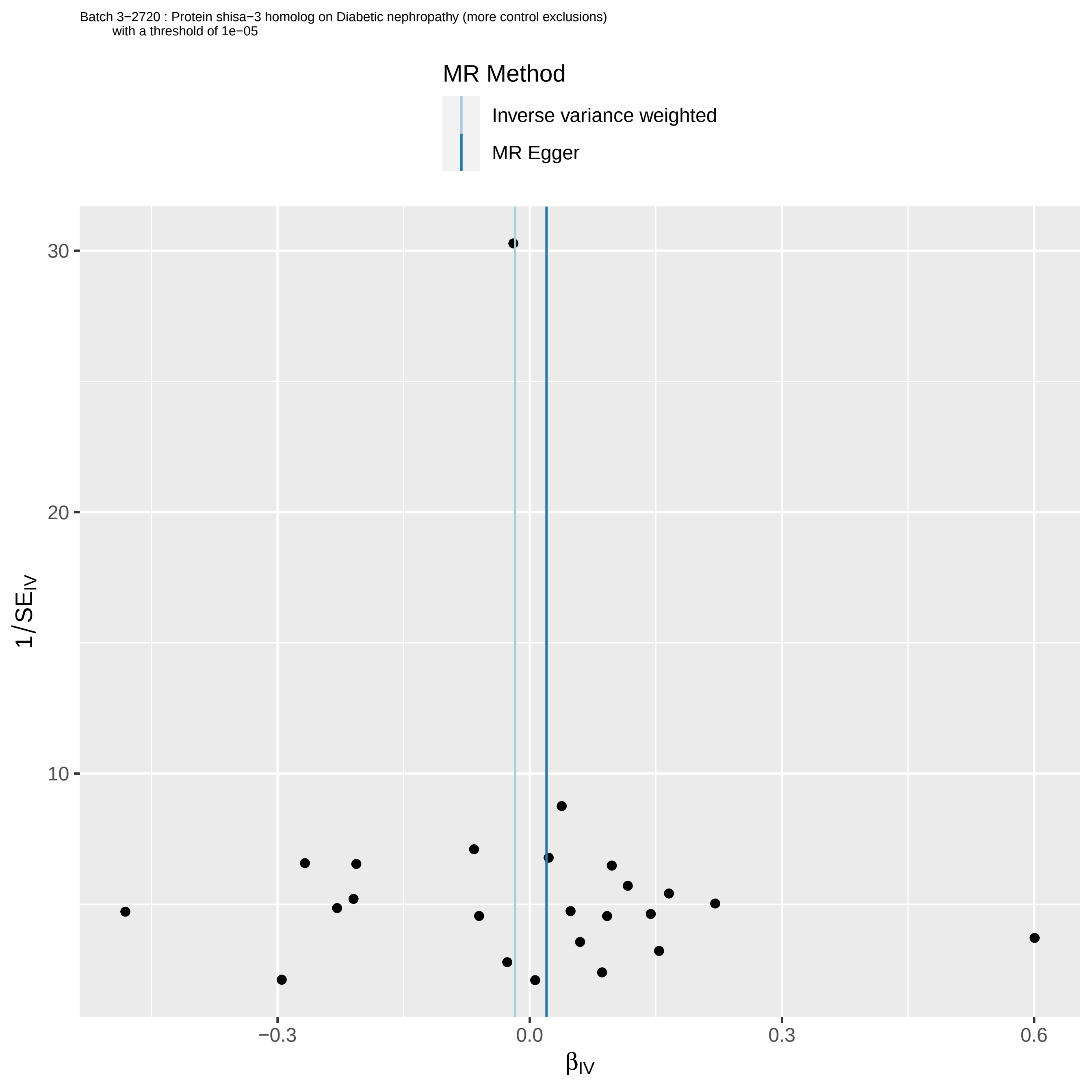
Batch 3−2720 : Protein shisa−3 homolog on Diabetic nephropathy (more control exclusions)
with a threshold of 1e−05
MR Method
Inverse variance weighted
MR Egger
30
20
10
1/SE_IV
−0.3
0.0
0.3
0.6
β_IV

Batch 3−2721 : Sialate O−acetylesterase on Diabetic nephropathy (more control exclusions)
with a threshold of 1e−05

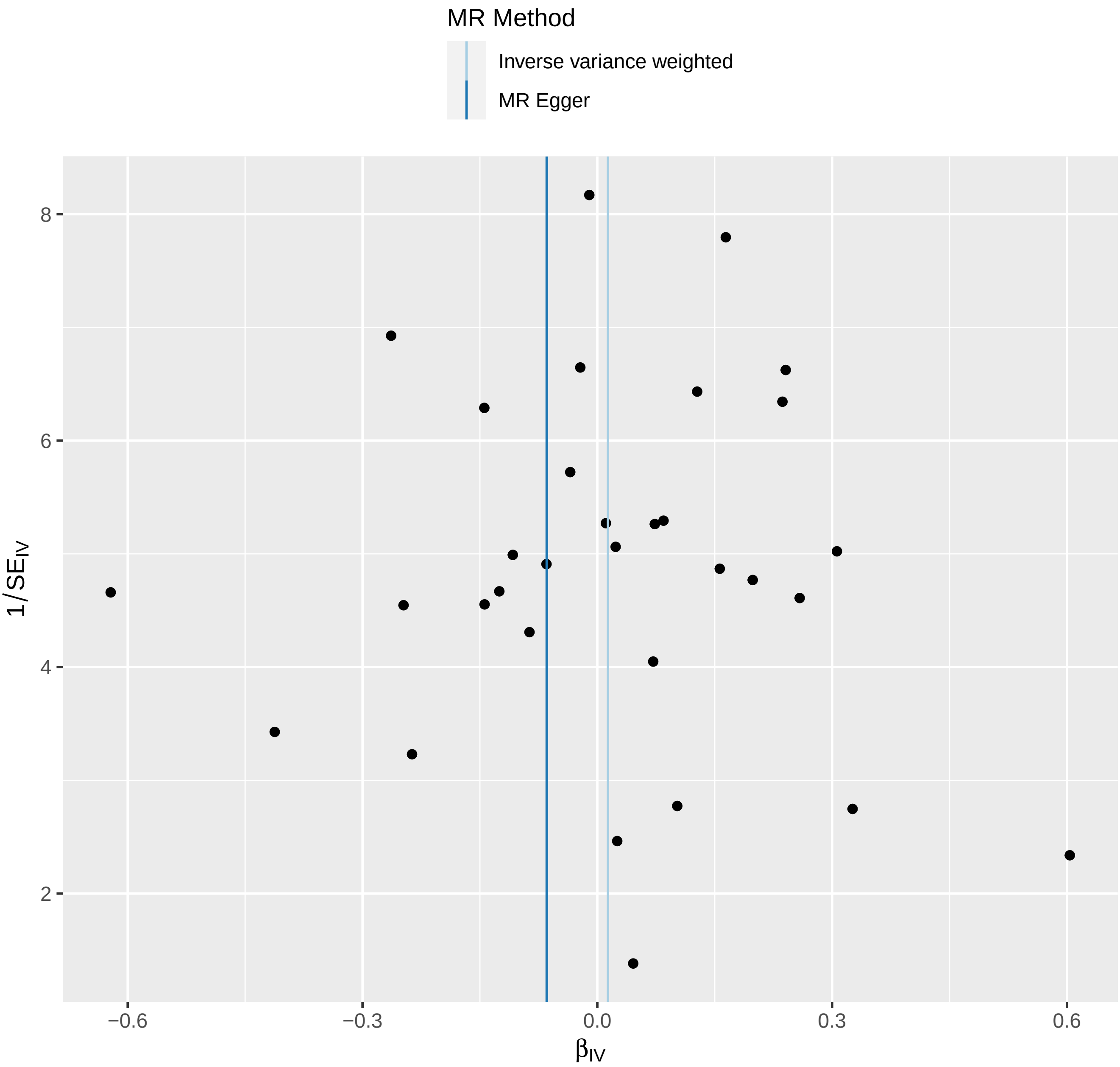

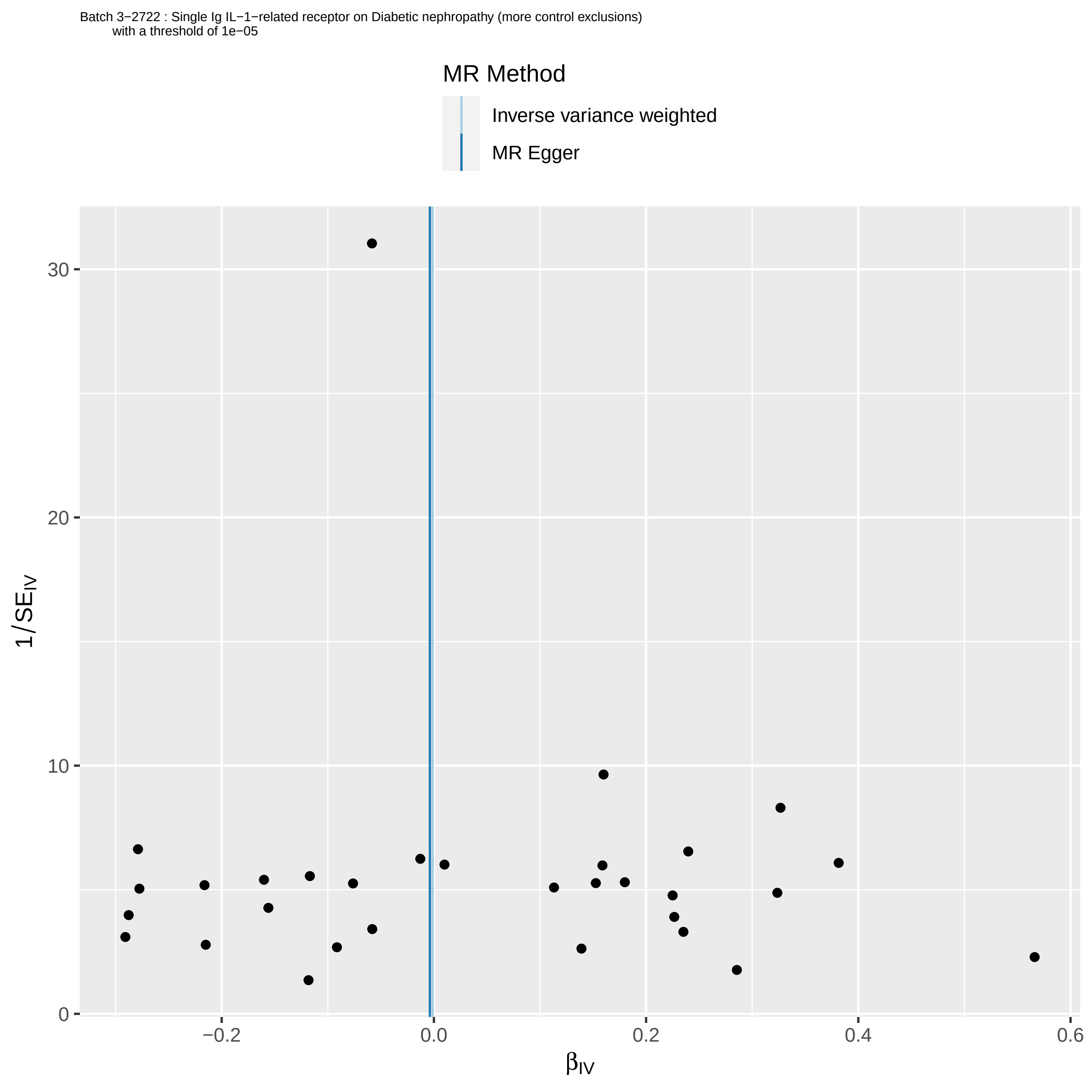
Batch 3−2722 : Single Ig IL−1−related receptor on Diabetic nephropathy (more control exclusions)
with a threshold of 1e−05
MR Method
Inverse variance weighted
MR Egger
30
20
10
0
−0.2
0.0
0.2
0.4
0.6
$1/SE_{IV}$
β_{IV}

Batch 3−2723 : Sialic acid−binding Ig−like lectin 10 on Diabetic nephropathy (more control exclusions)
with a threshold of 1e−05

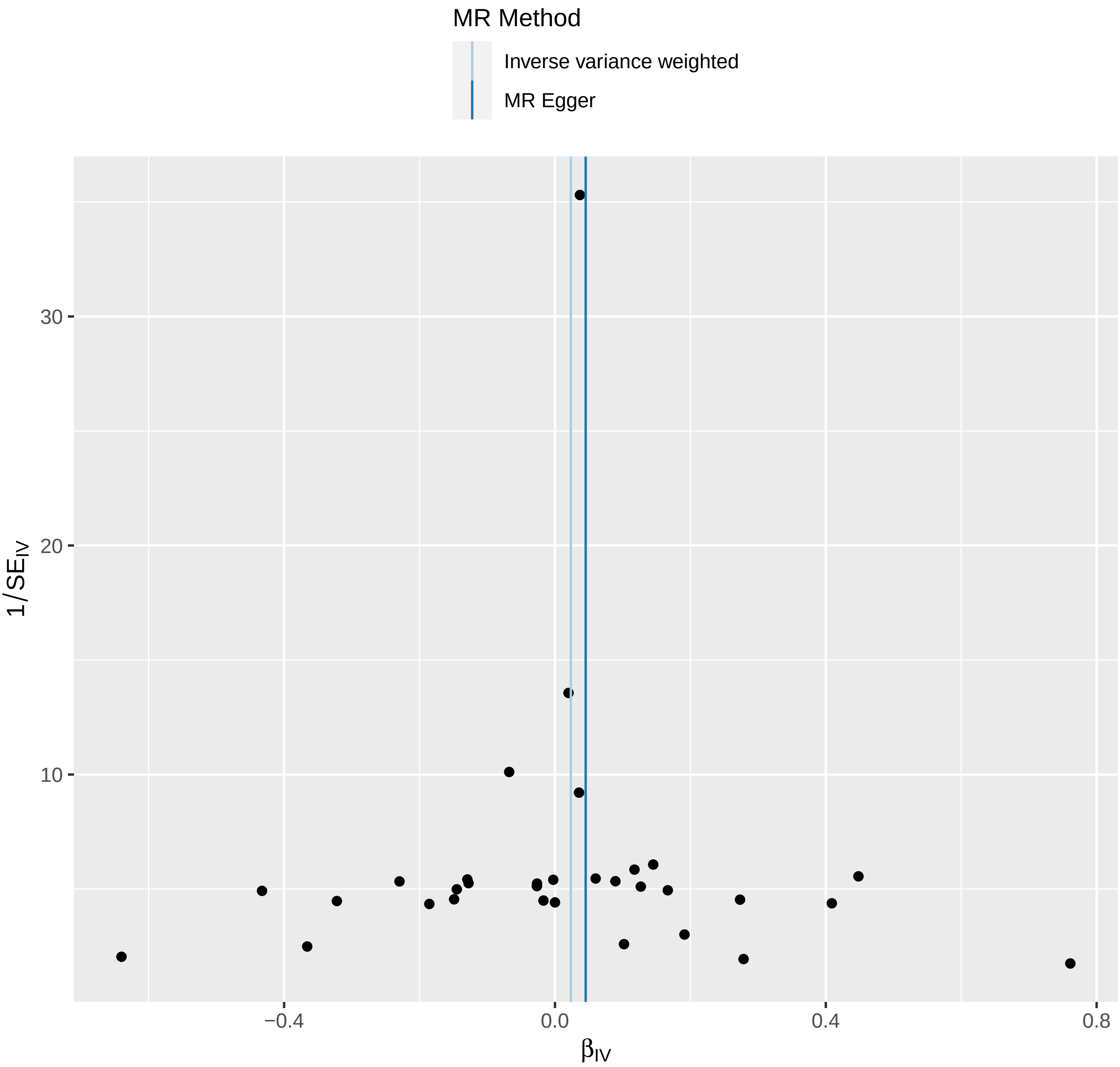

Batch 3−2724 : Sialic acid−binding Ig−like lectin 11 on Diabetic nephropathy (more control exclusions)
with a threshold of 1e−05

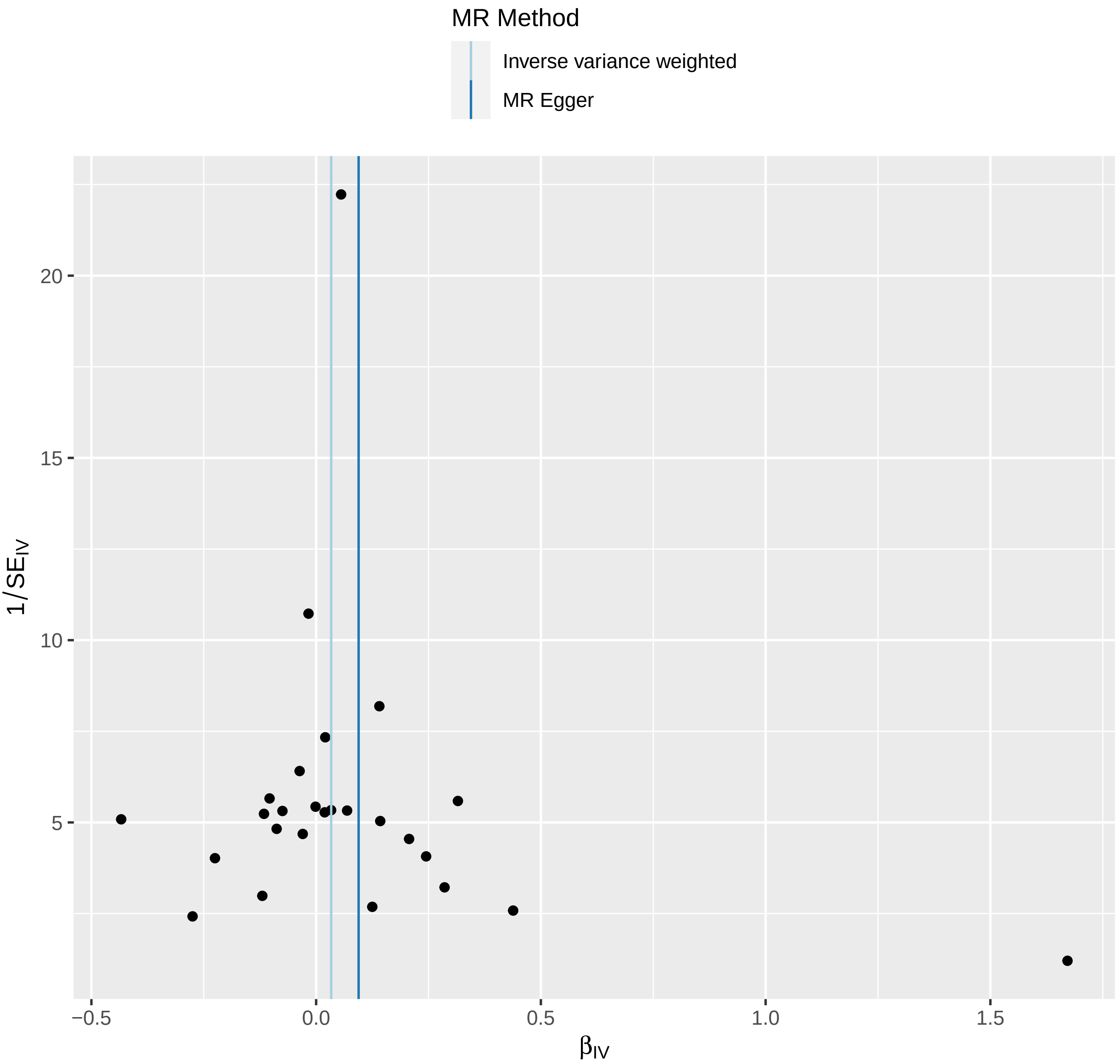

Batch 3−2725 : Sialic acid−binding Ig−like lectin 12 on Diabetic nephropathy (more control exclusions)
with a threshold of 1e−05

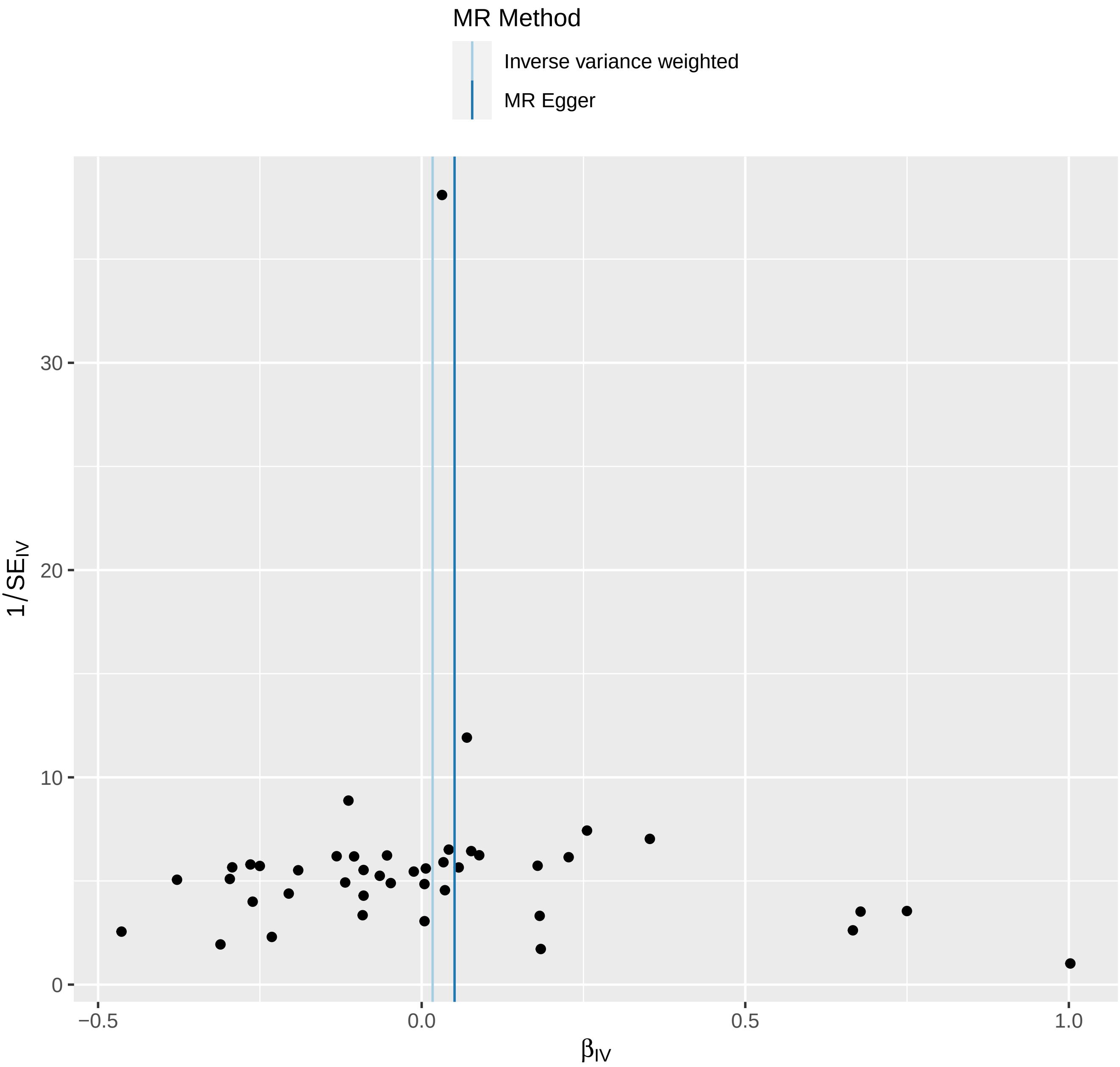

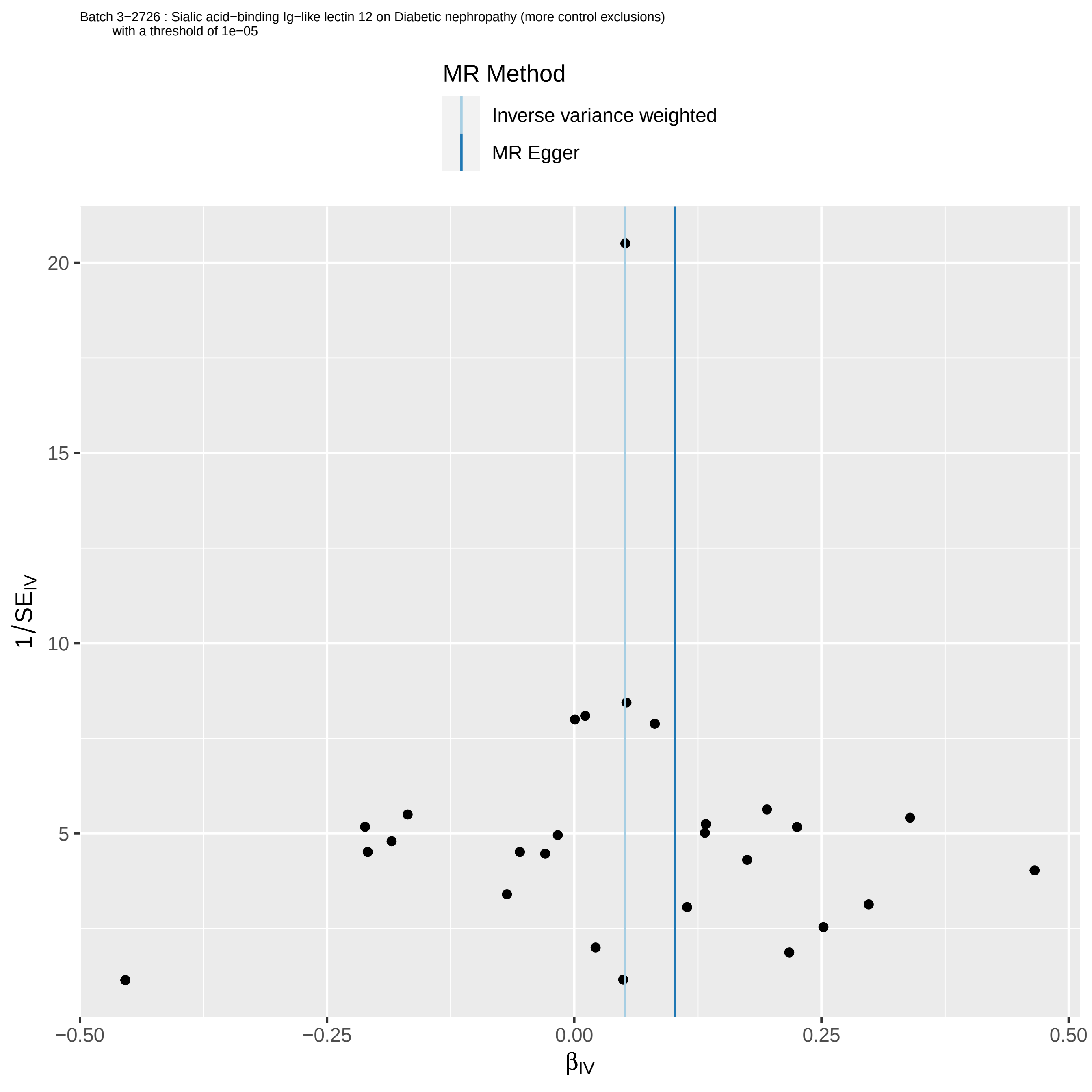
Batch 3−2726 : Sialic acid−binding Ig−like lectin 12 on Diabetic nephropathy (more control exclusions)
with a threshold of 1e−05
MR Method
Inverse variance weighted
MR Egger
1/SE_IV
β_IV
20
15
10
5
−0.50
−0.25
0.00
0.25
0.50

Batch 3−2727 : Sialic acid−binding Ig−like lectin 14 on Diabetic nephropathy (more control exclusions)
with a threshold of 1e−05

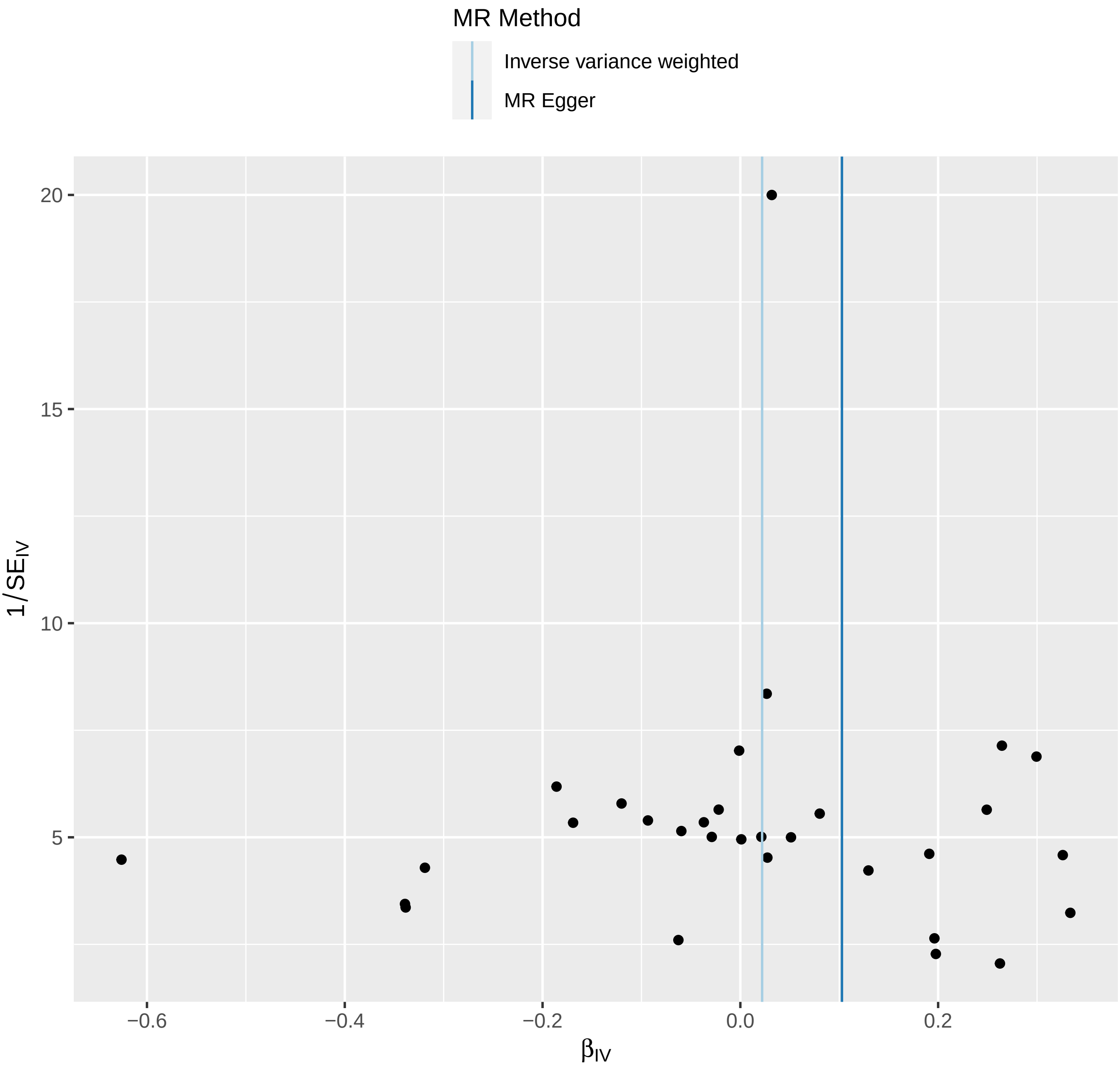

Batch 3−2728 : Sialic acid−binding Ig−like lectin 14 on Diabetic nephropathy (more control exclusions)
with a threshold of 1e−05

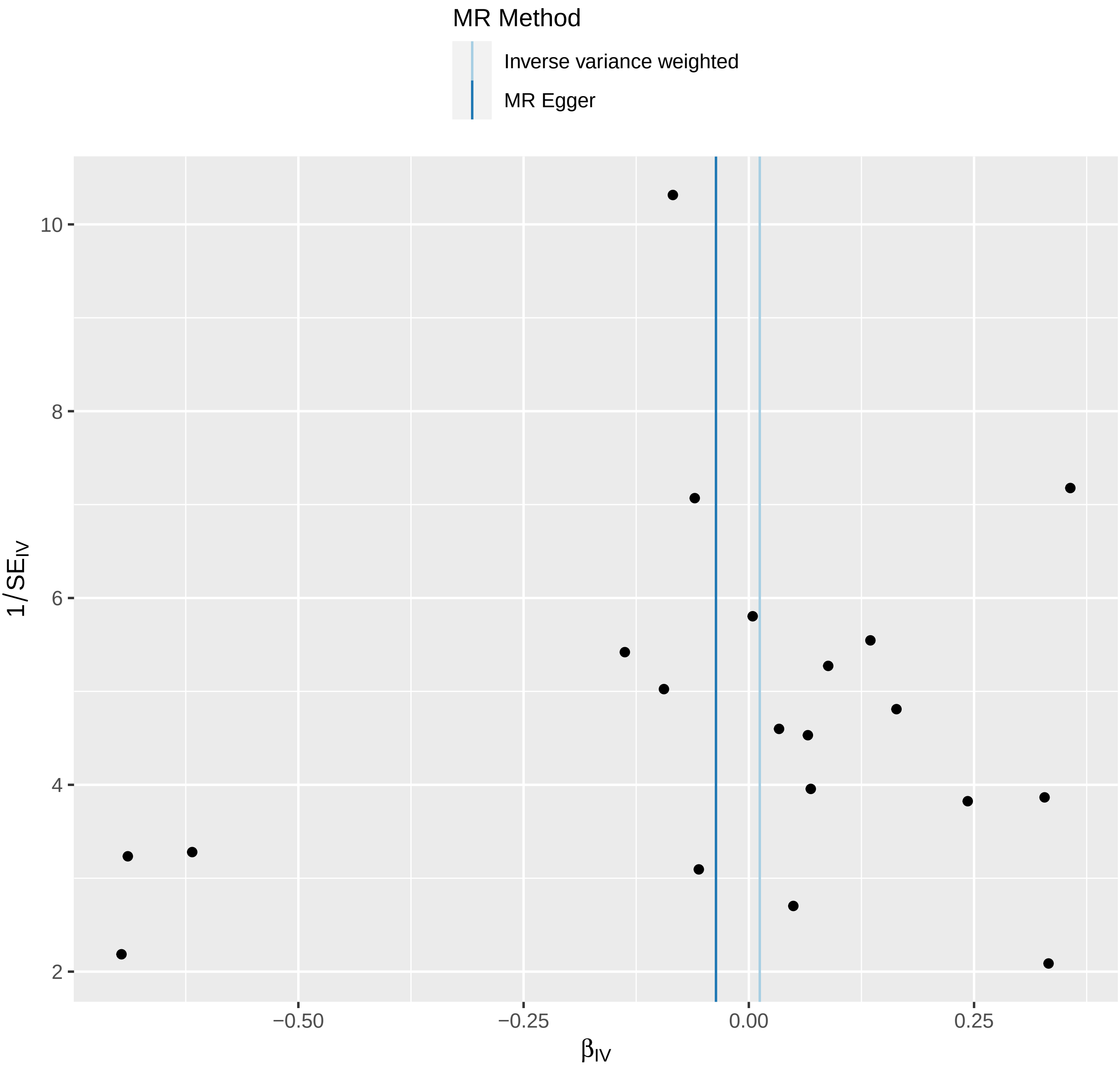

Batch 3−2729 : Sialic acid−binding Ig−like lectin 7 on Diabetic nephropathy (more control exclusions)
with a threshold of 1e−05

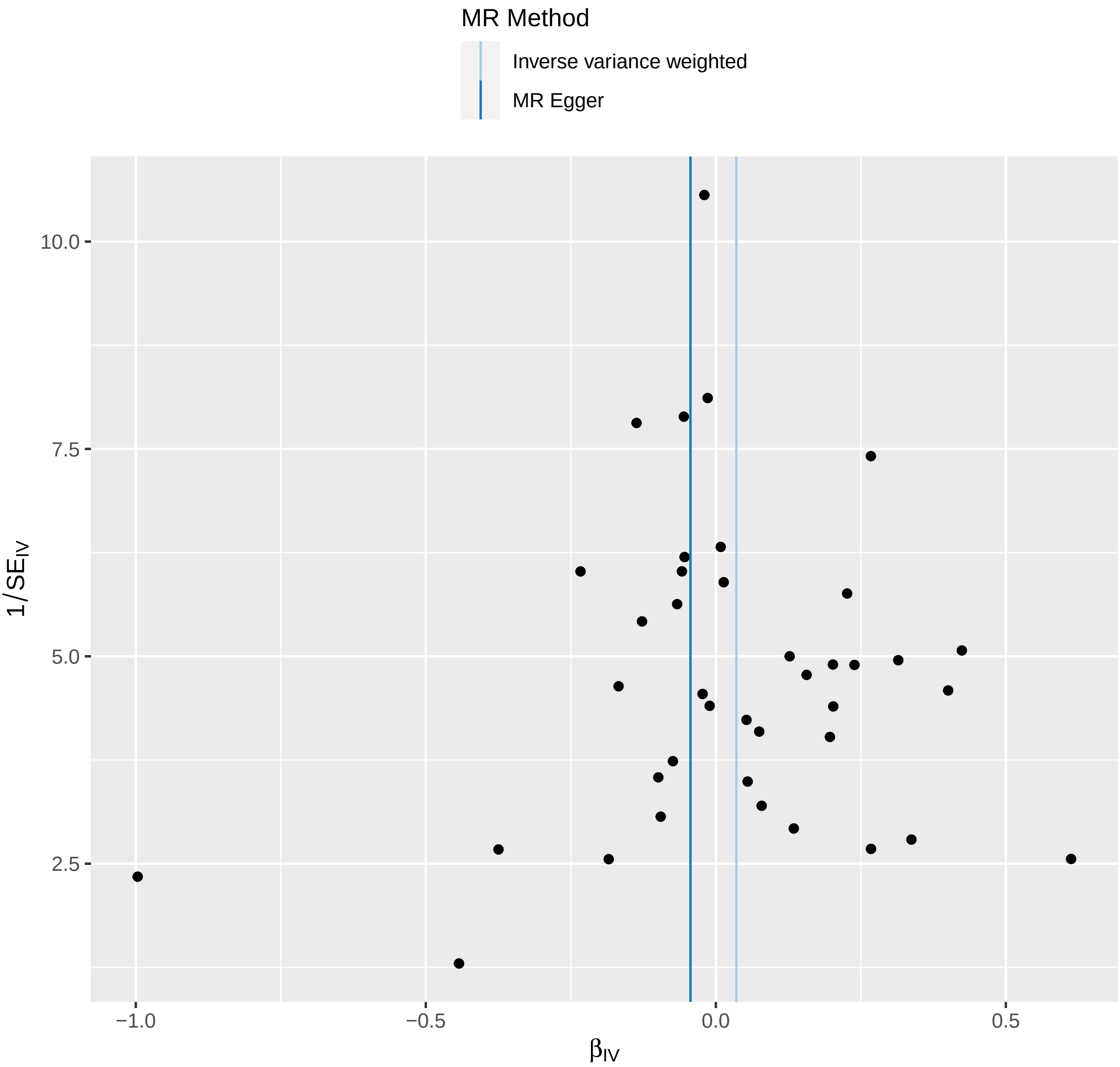

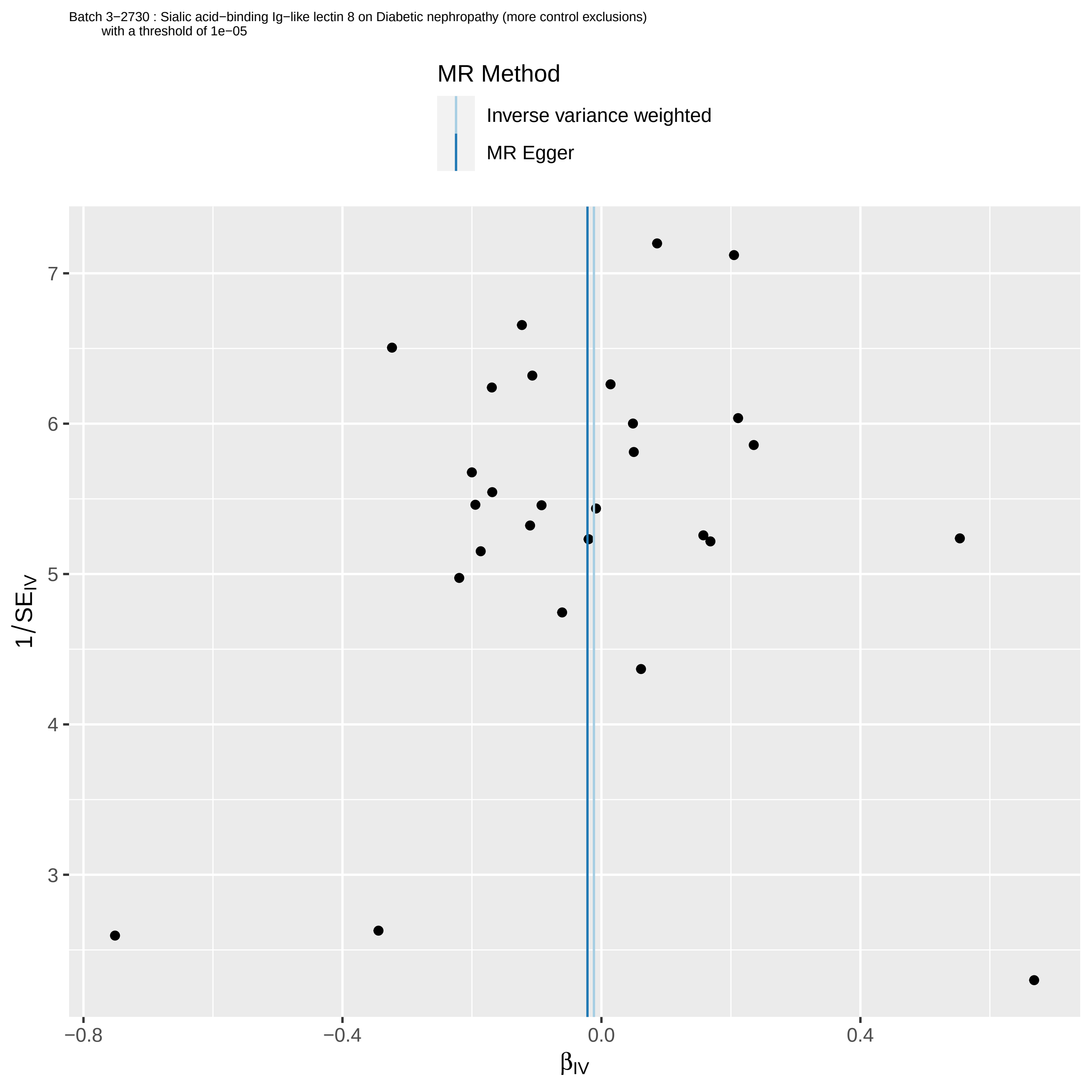
Batch 3−2730 : Sialic acid−binding Ig−like lectin 8 on Diabetic nephropathy (more control exclusions)
with a threshold of 1e−05
MR Method
Inverse variance weighted
MR Egger
7
6
5
4
3
−0.8
−0.4
0.0
0.4
$1/SE_{IV}$
β_{IV}

Batch 3−2731 : Sialic acid−binding Ig−like lectin 9 on Diabetic nephropathy (more control exclusions)
with a threshold of 1e−05

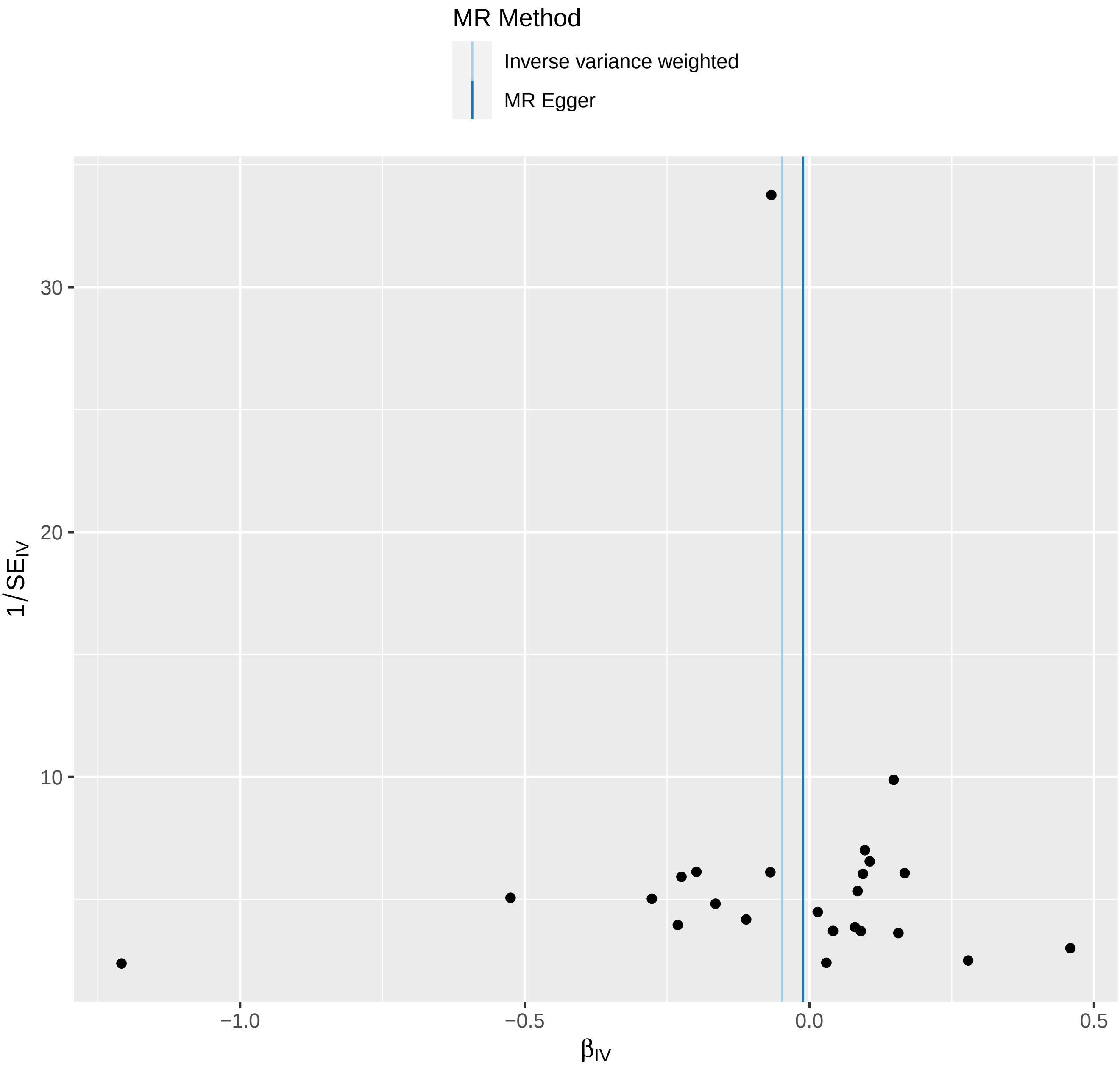

Batch 3−2732 : Tyrosine−protein phosphatase non−receptor type substrate 1 on Diabetic nephropathy (more control exclusions) with a threshold of 1e−05

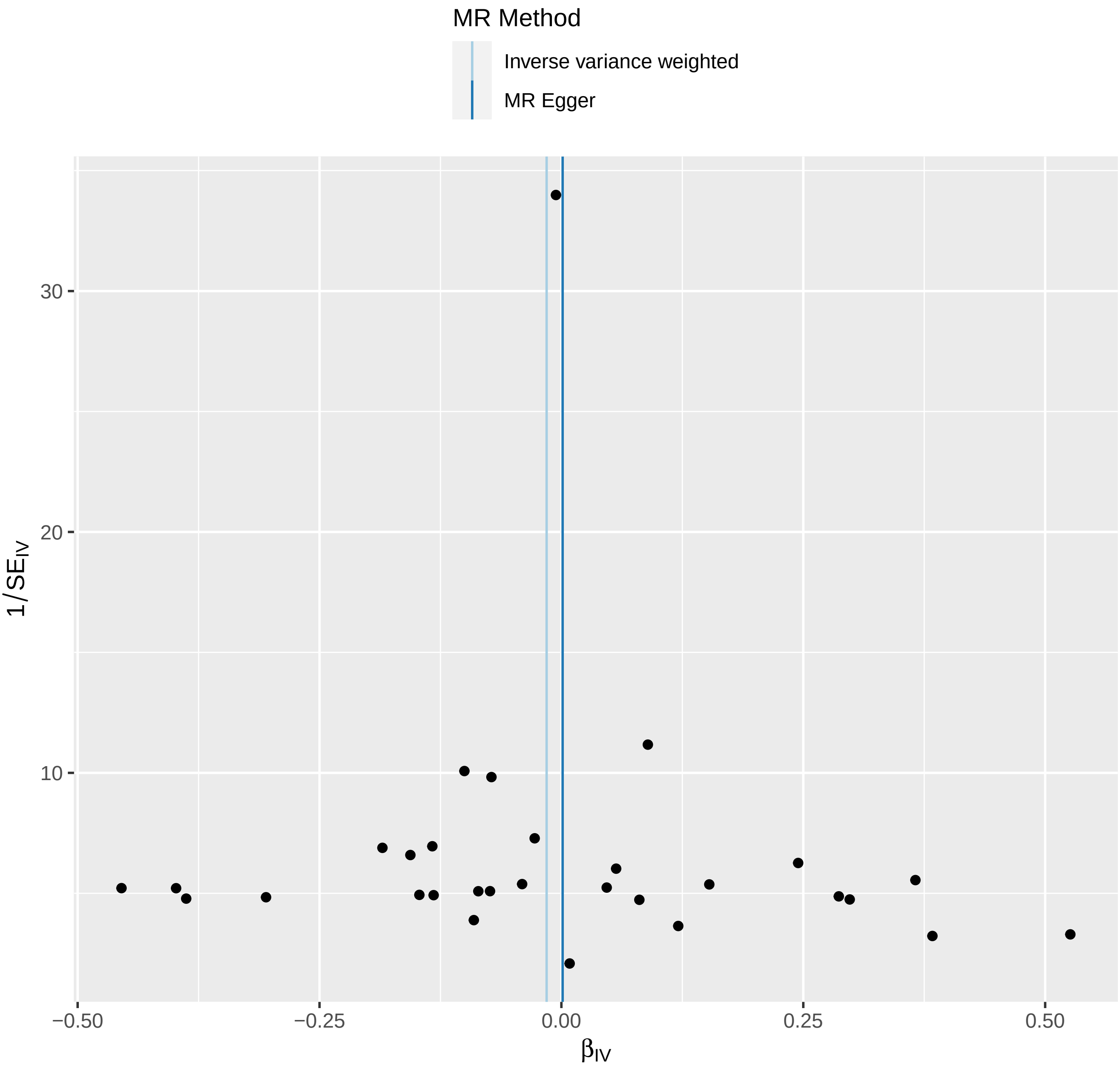

Batch 3−2733 : Signal−regulatory protein beta−1 on Diabetic nephropathy (more control exclusions)
with a threshold of 1e−05

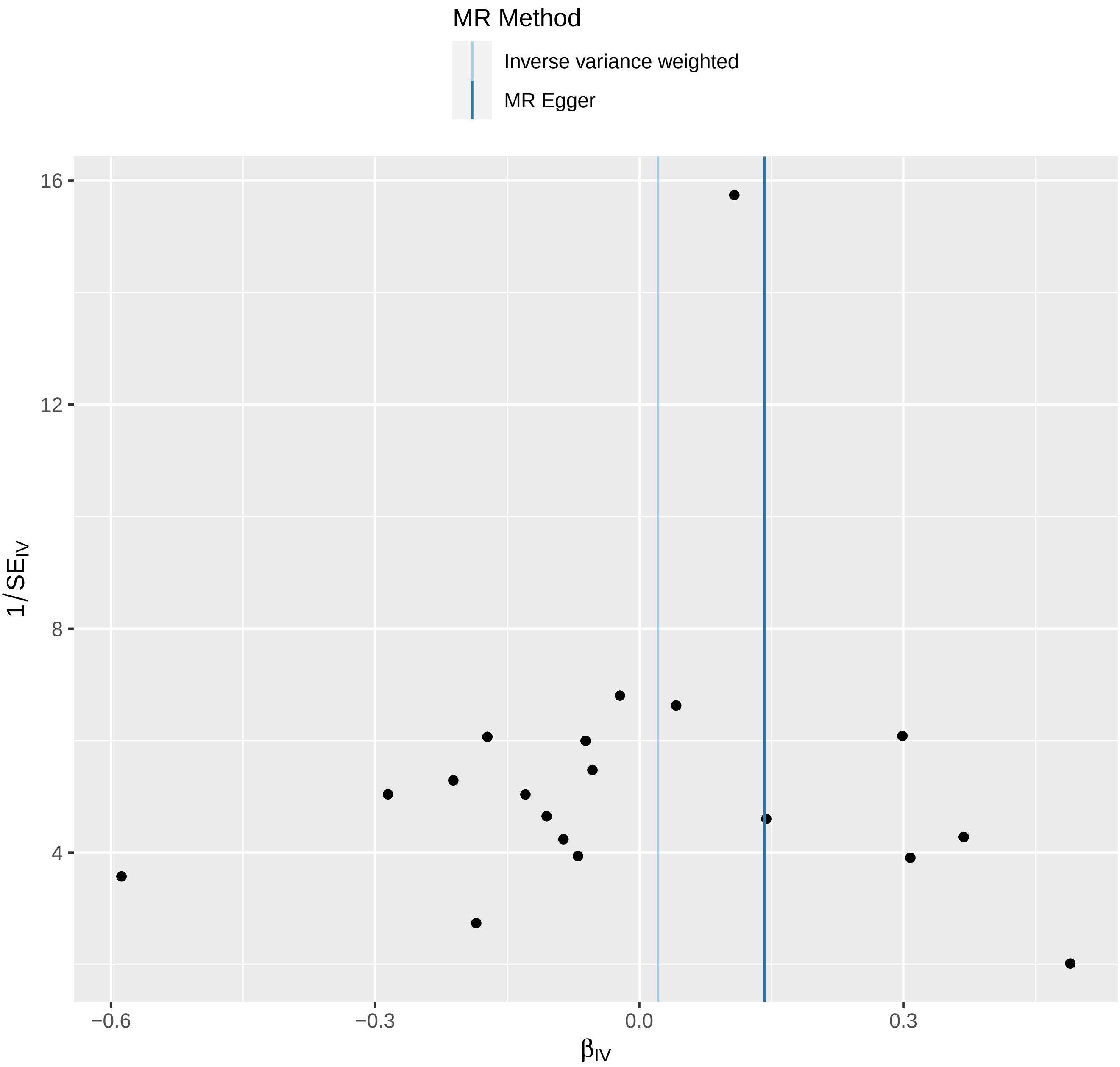

Batch 3−2734 : Signal−regulatory protein beta−2 on Diabetic nephropathy (more control exclusions)
with a threshold of 1e−05

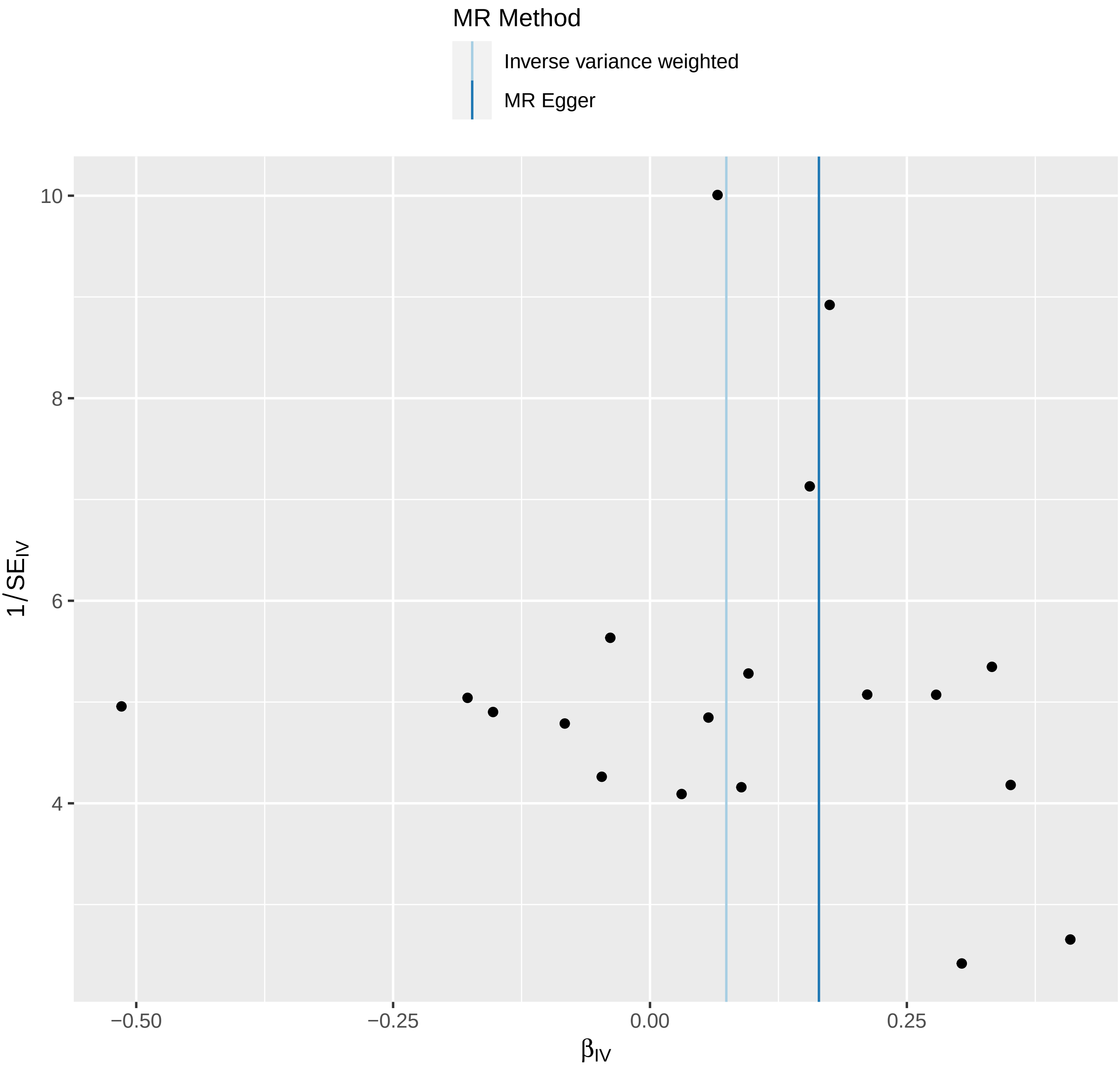

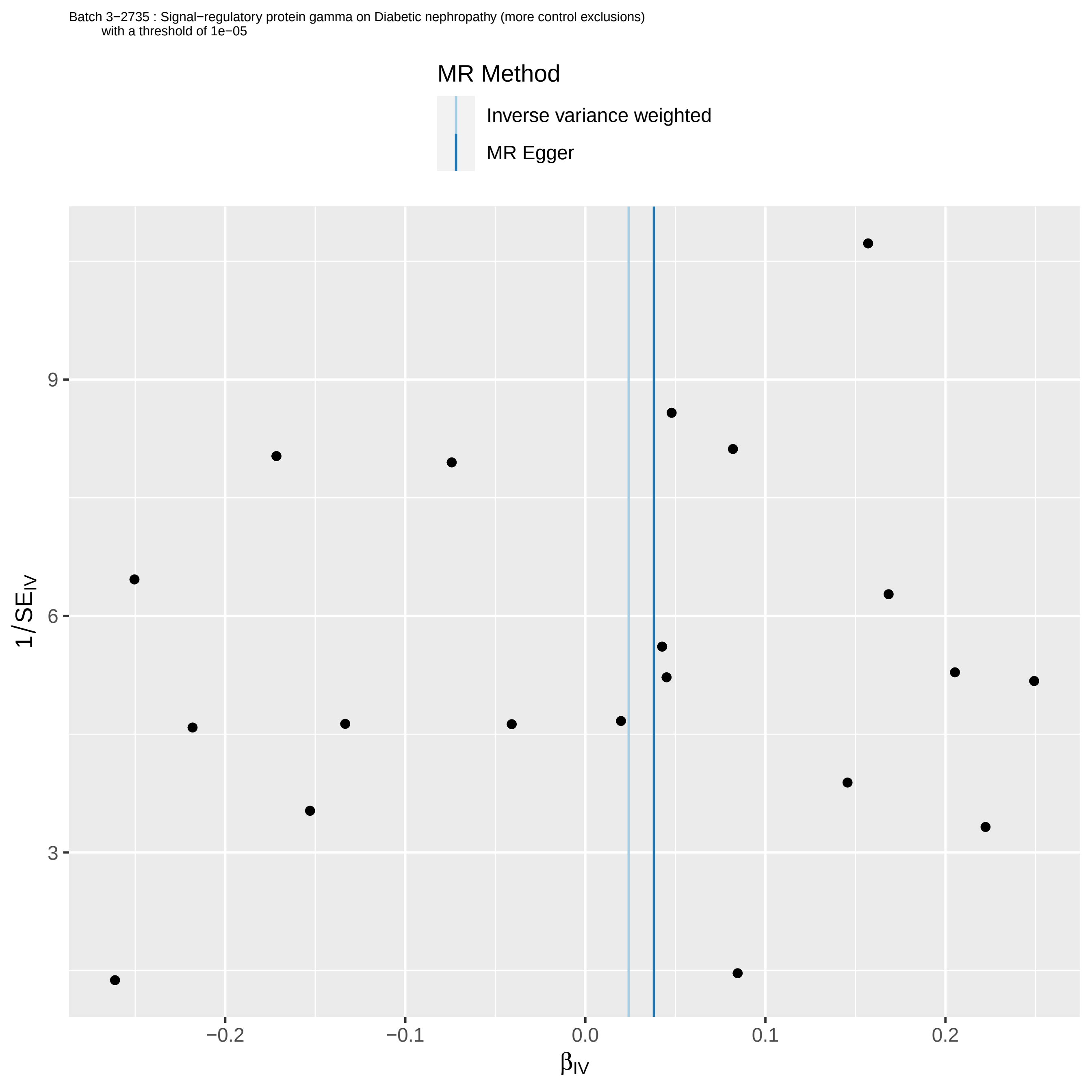
Batch 3−2735 : Signal−regulatory protein gamma on Diabetic nephropathy (more control exclusions)
with a threshold of 1e−05
MR Method
Inverse variance weighted
MR Egger
9
6
3
1/SE_IV
−0.2
−0.1
0.0
0.1
0.2
β_IV

Batch 3−2736 : NAD−dependent protein deacetylase sirtuin−2 on Diabetic nephropathy (more control exclusions)
with a threshold of 1e−05

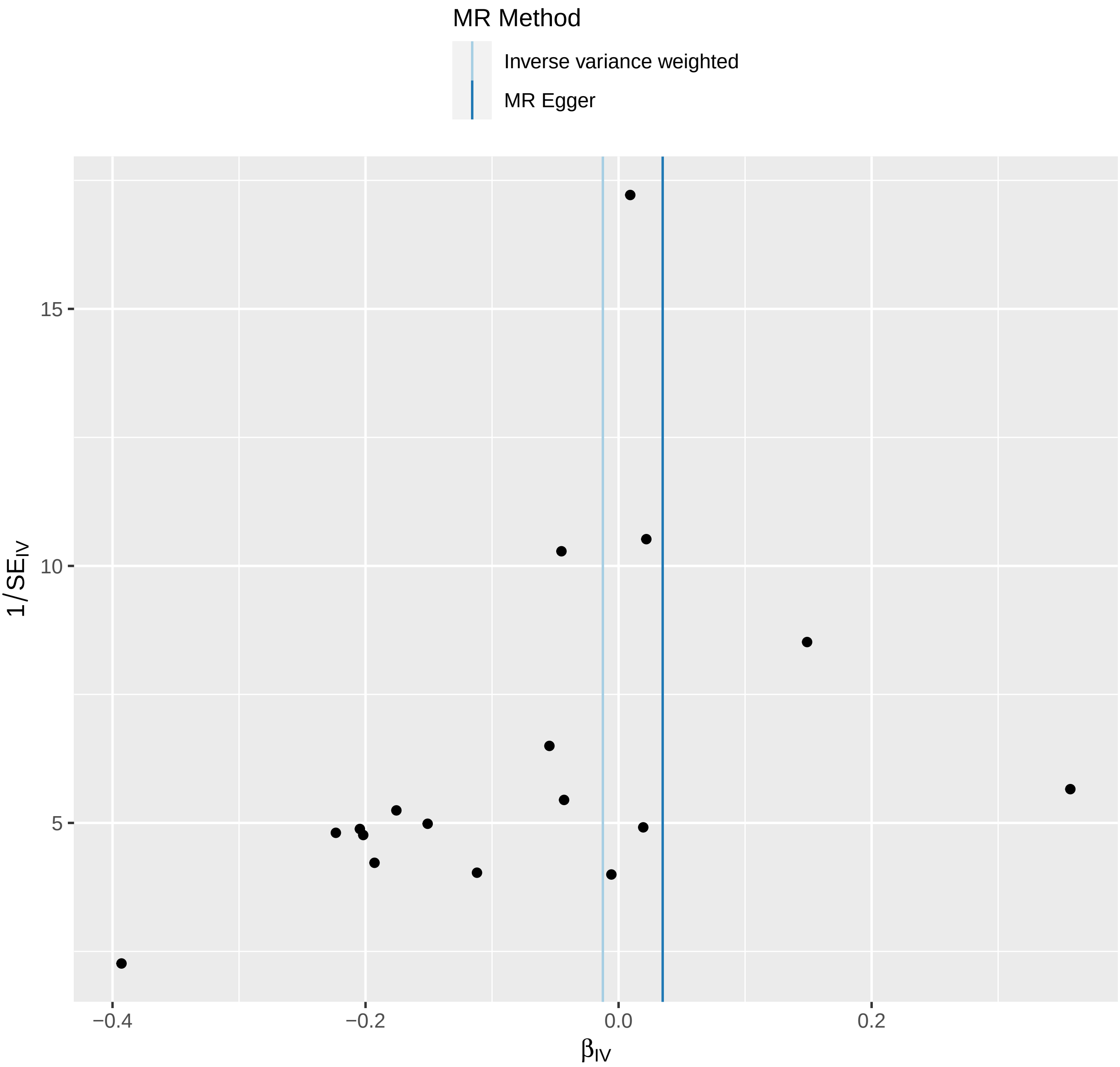

Batch 3−2737 : NAD−dependent protein deacylase sirtuin−5, mitochondrial on Diabetic nephropathy (more control exclusions)
with a threshold of 1e−05

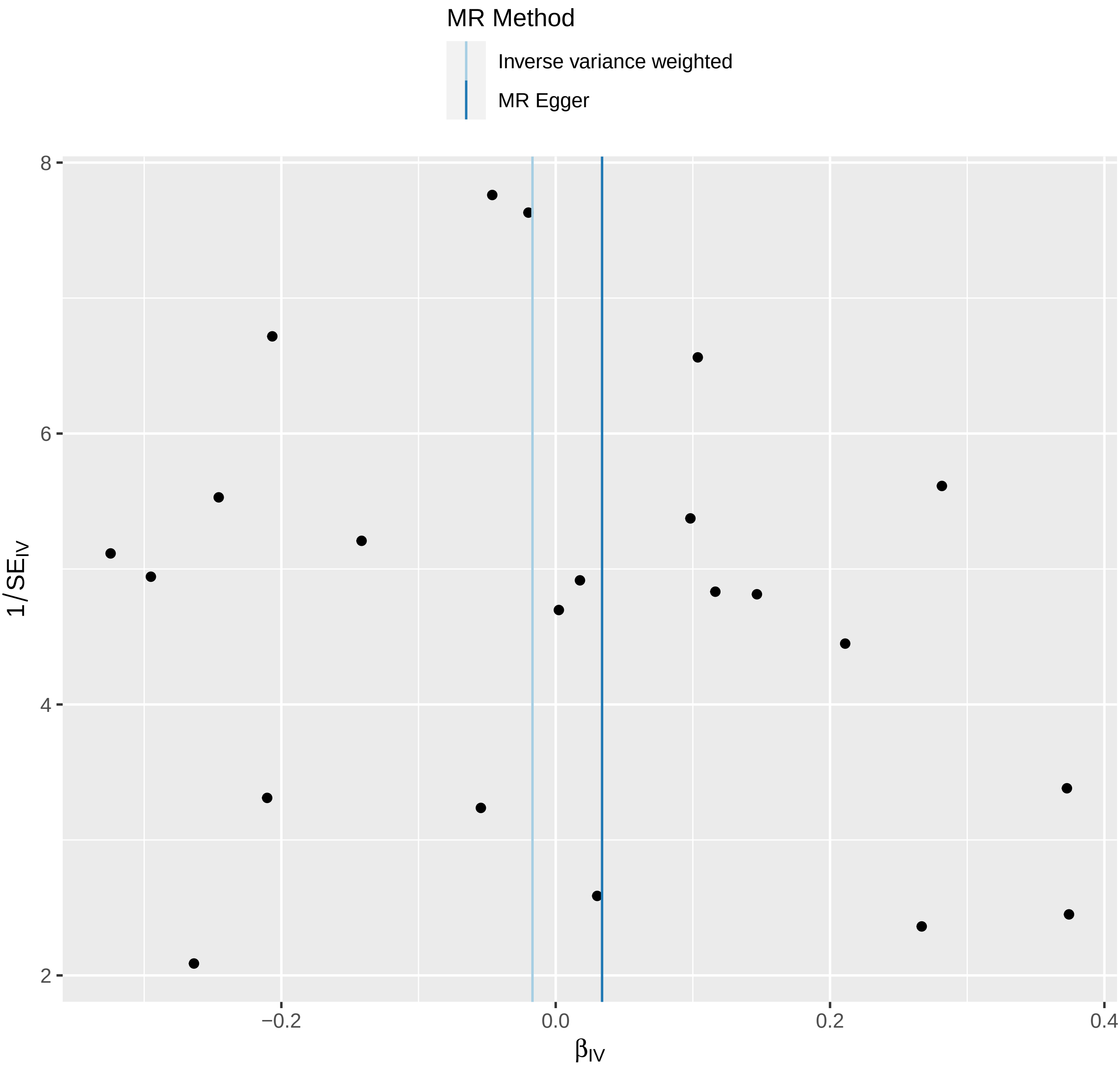

Batch 3−2738 : Signaling threshold−regulating transmembrane adapter 1 on Diabetic nephropathy (more control exclusions)
with a threshold of 1e−05

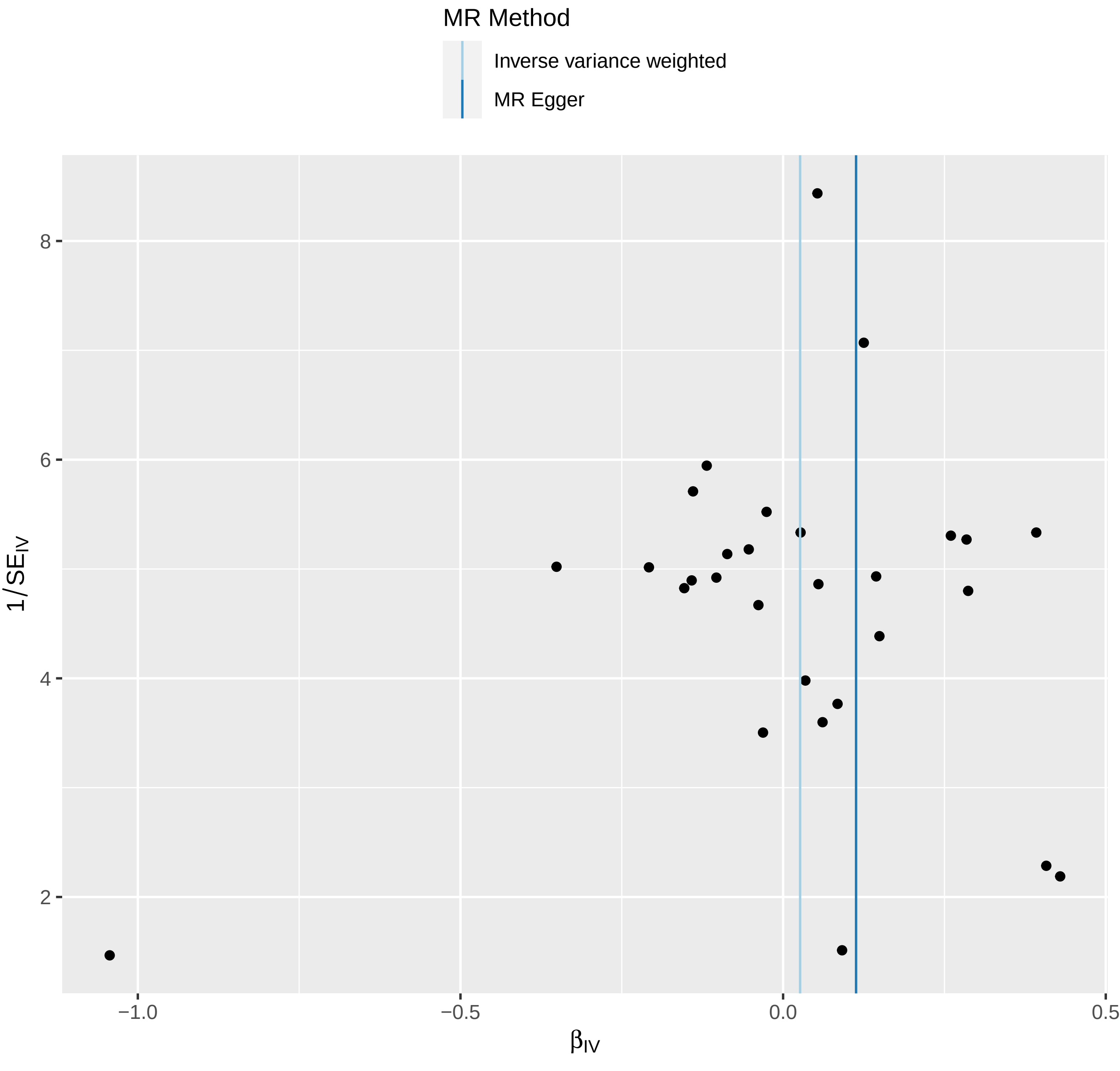

Batch 3−2739 : Ski−like protein on Diabetic nephropathy (more control exclusions)
with a threshold of 1e−05

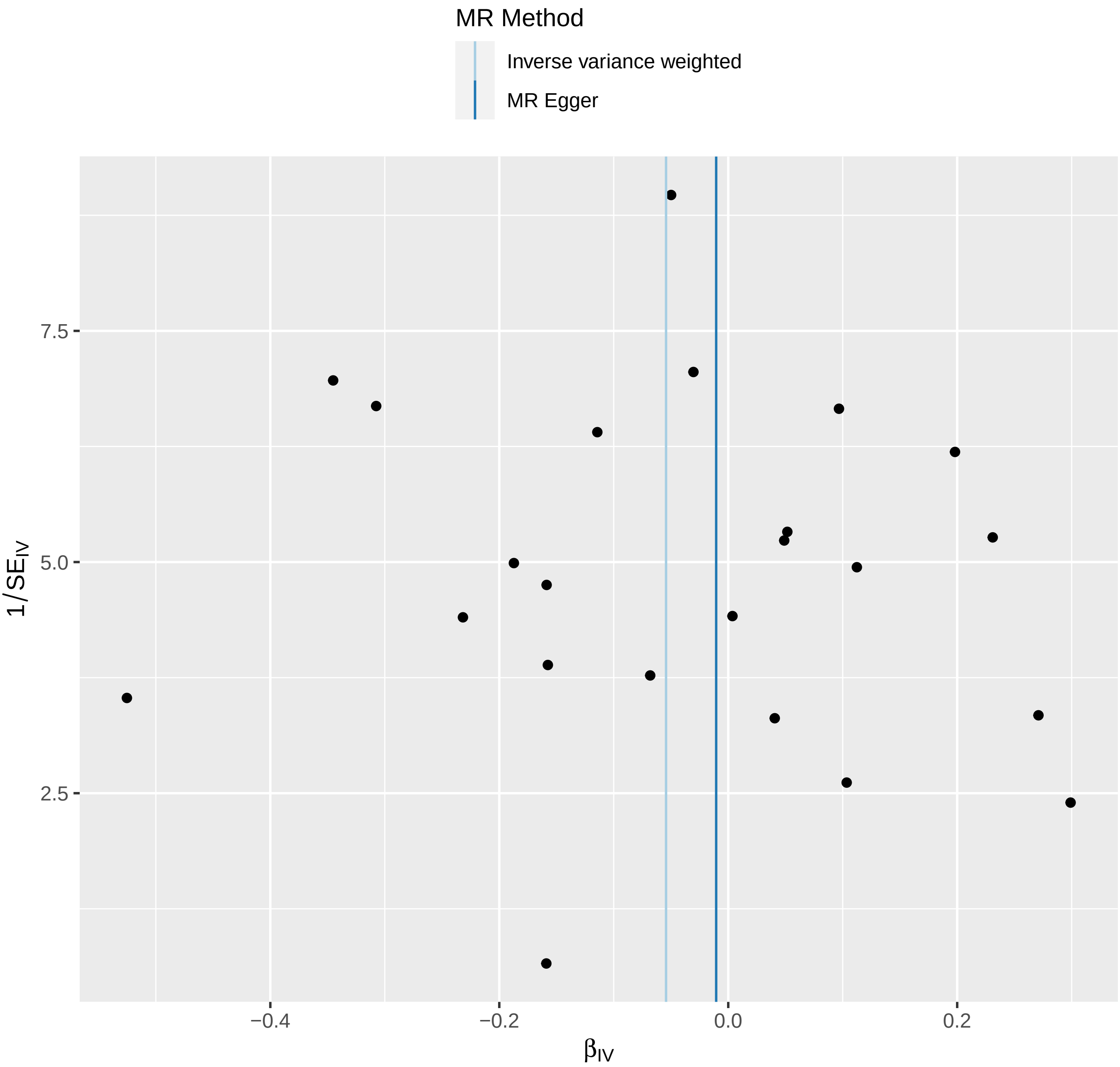

Batch 3−2740 : S−phase kinase−associated protein 1 on Diabetic nephropathy (more control exclusions) with a threshold of 1e−05

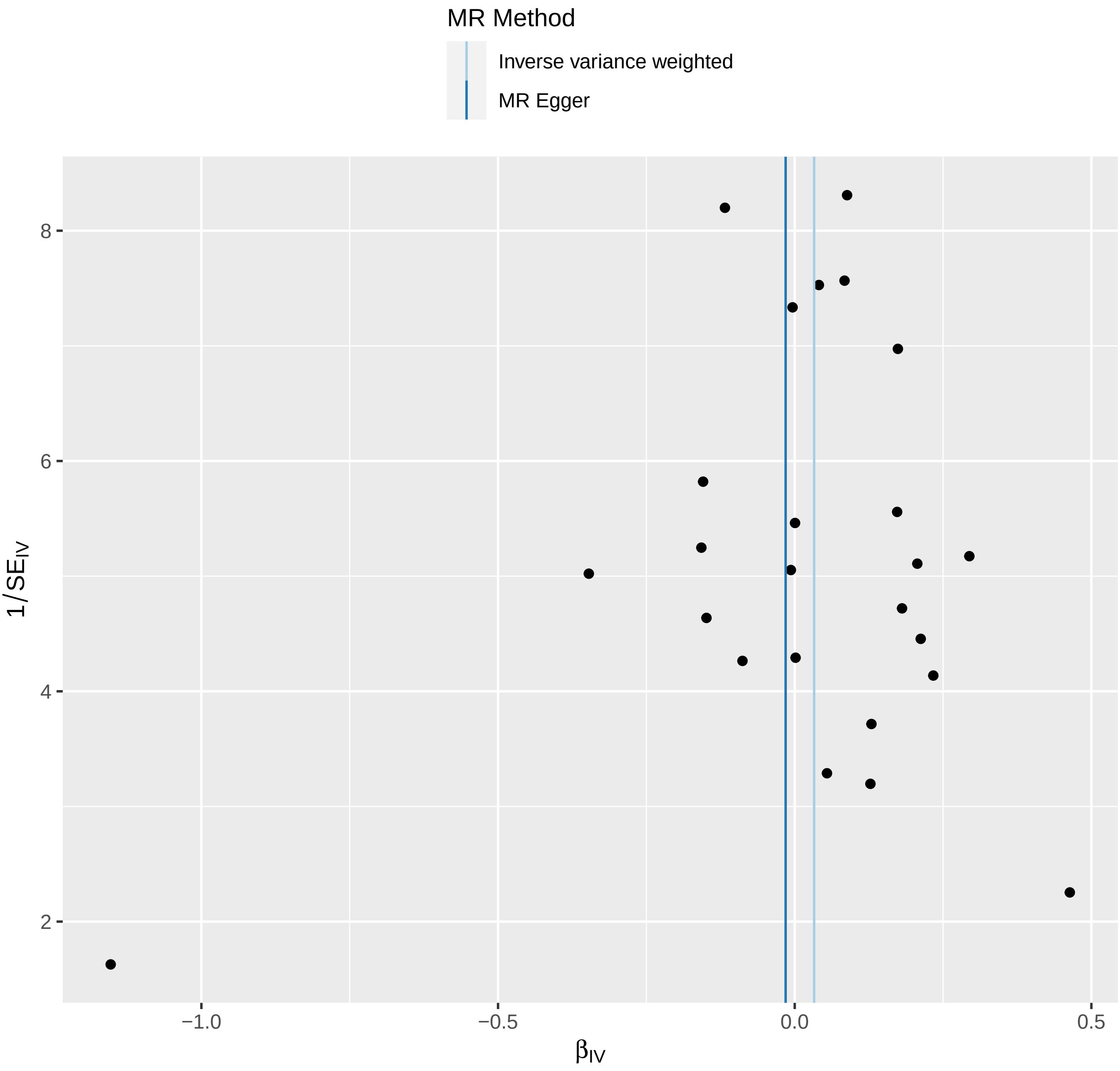

Batch 3−2741 : Signaling lymphocytic activation molecule on Diabetic nephropathy (more control exclusions)
with a threshold of 1e−05

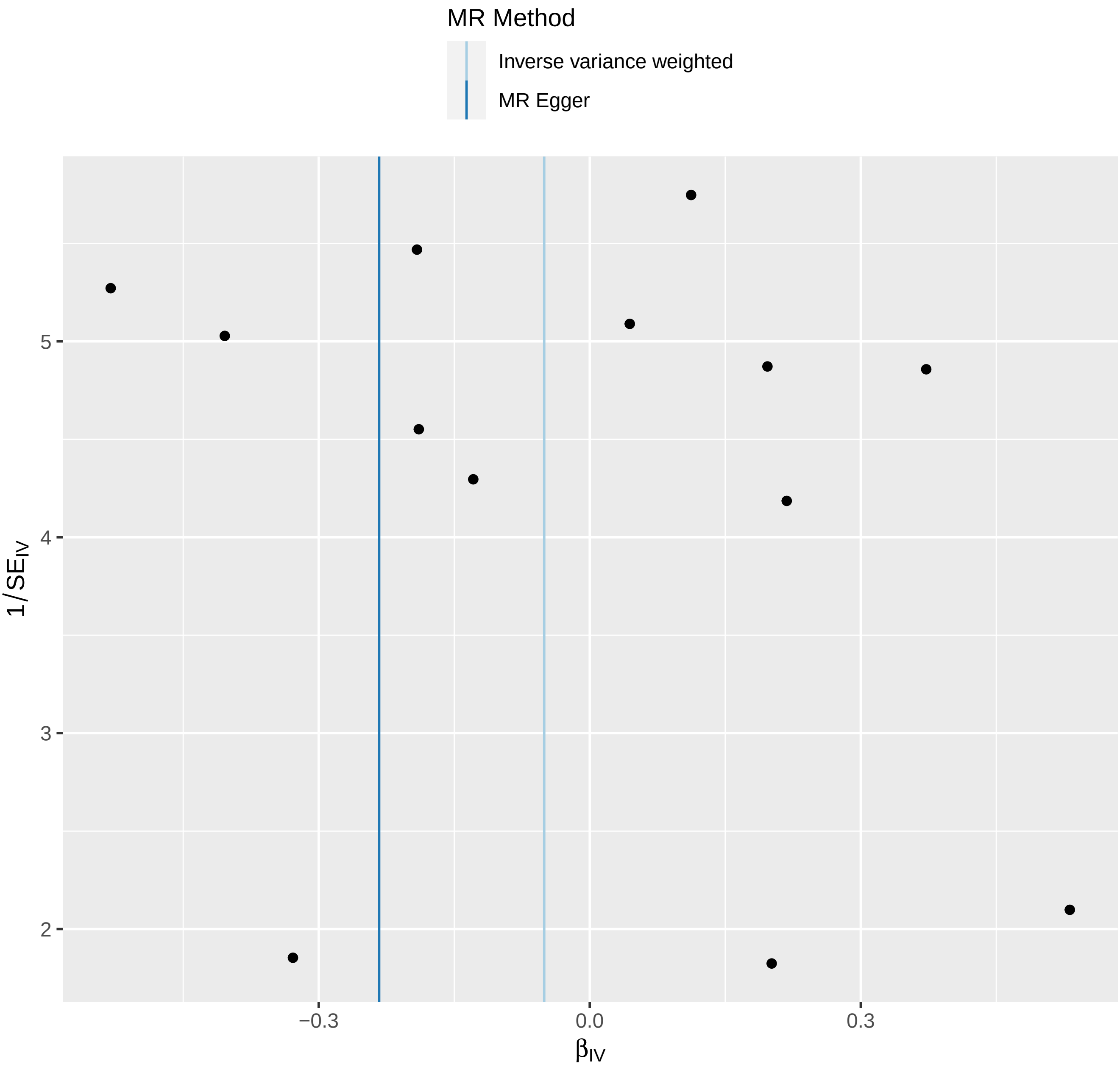

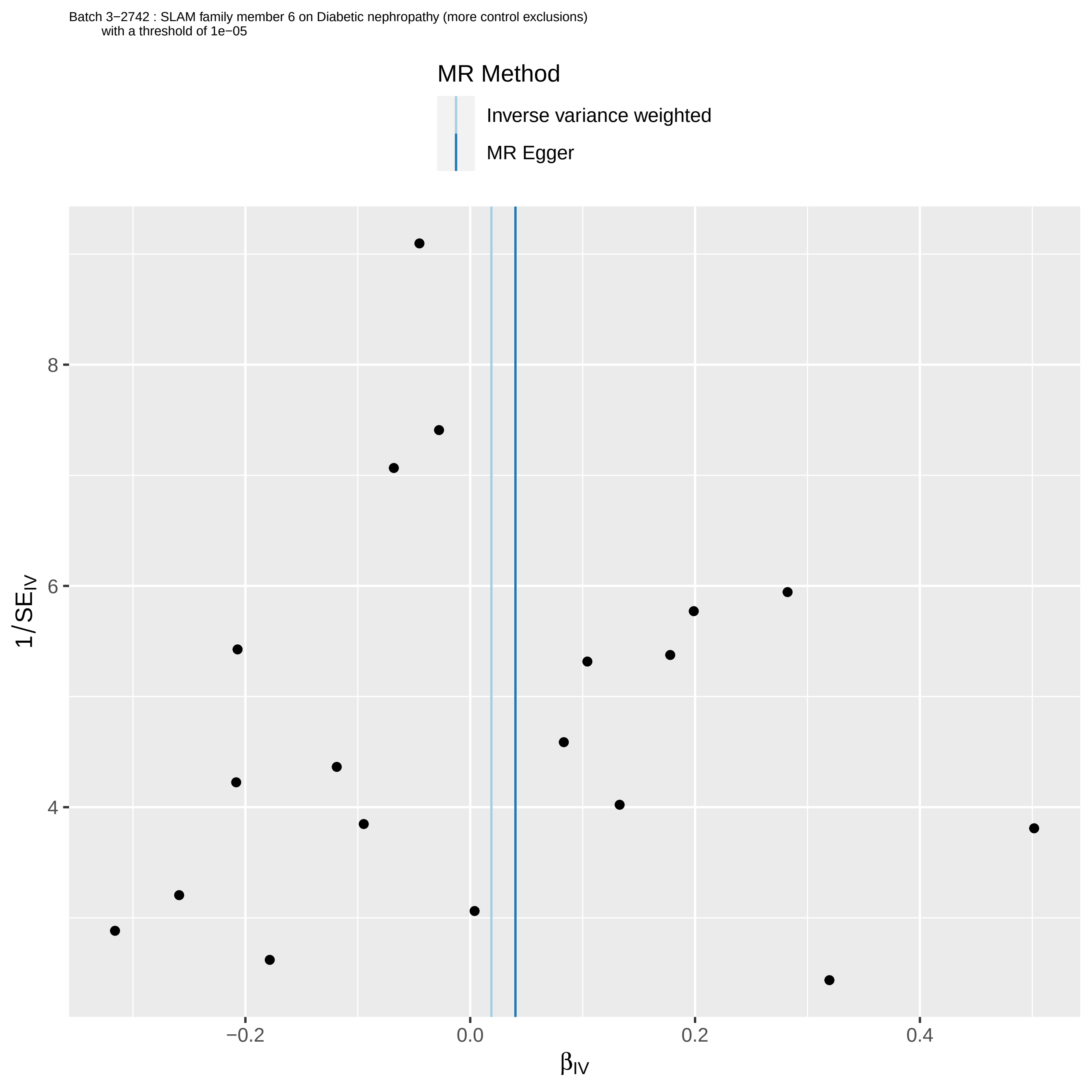
Batch 3−2742 : SLAM family member 6 on Diabetic nephropathy (more control exclusions)
with a threshold of 1e−05
MR Method
Inverse variance weighted
MR Egger
8
6
4
1/SE$_{IV}$
−0.2
0.0
0.2
0.4
β_{IV}

Batch 3−2743 : SLAM family member 7 on Diabetic nephropathy (more control exclusions)
with a threshold of 1e−05

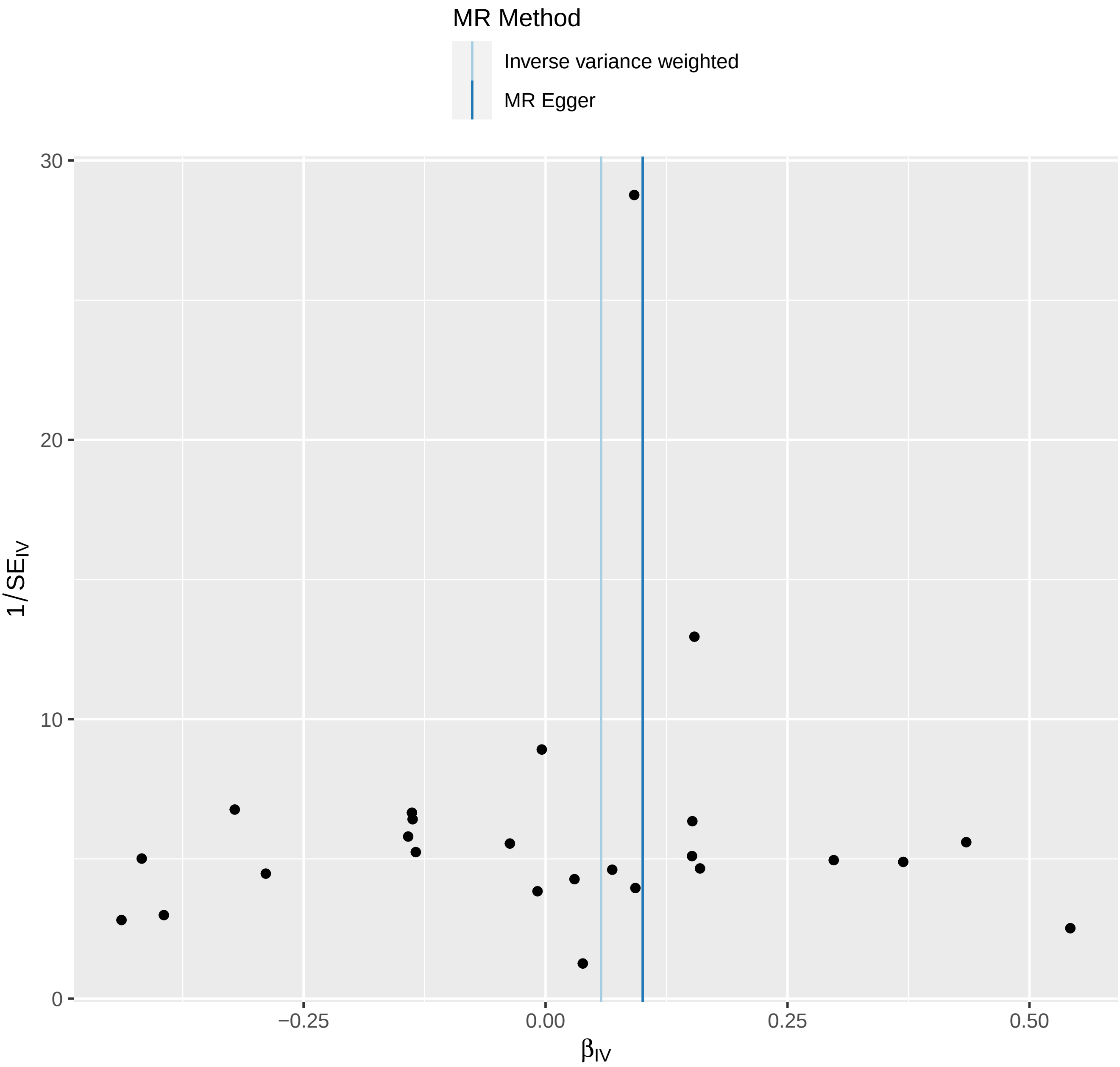

Batch 3−2744 : SLAM family member 7 on Diabetic nephropathy (more control exclusions)
with a threshold of 1e−05

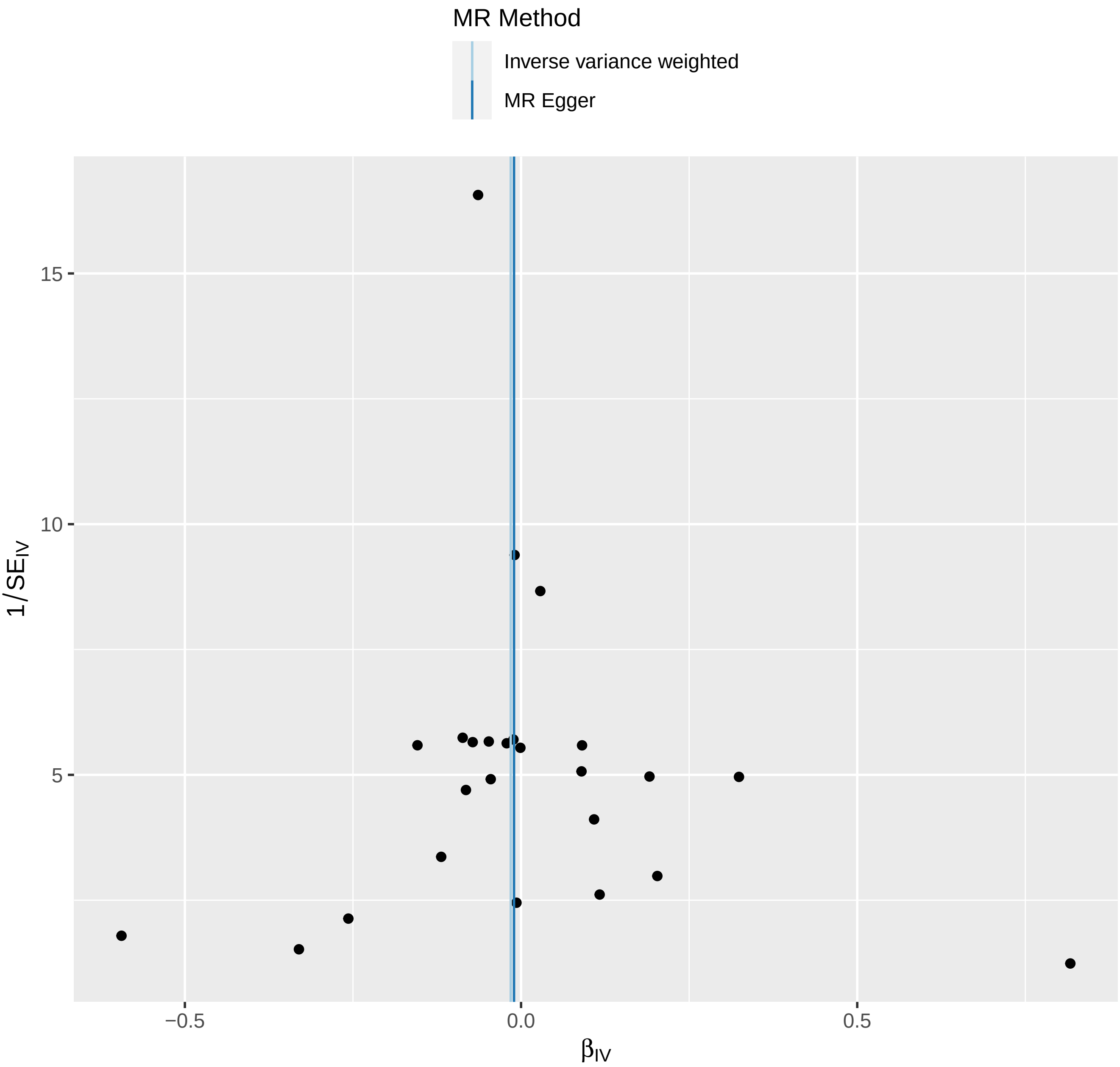

Batch 3−2745 : SLAM family member 8 on Diabetic nephropathy (more control exclusions)
with a threshold of 1e−05

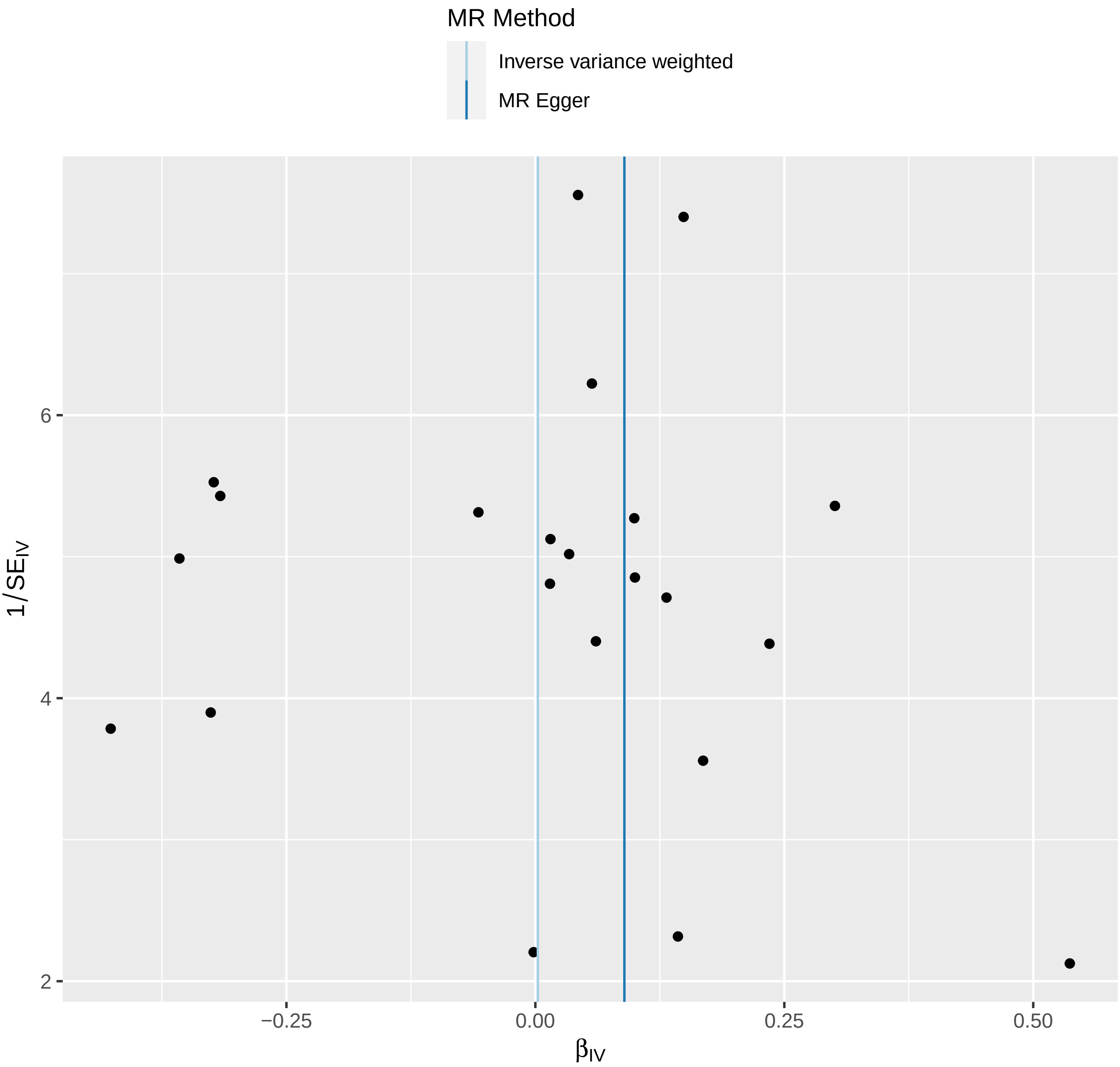

Batch 3−2746 : Urea transporter 1 on Diabetic nephropathy (more control exclusions)
with a threshold of 1e−05

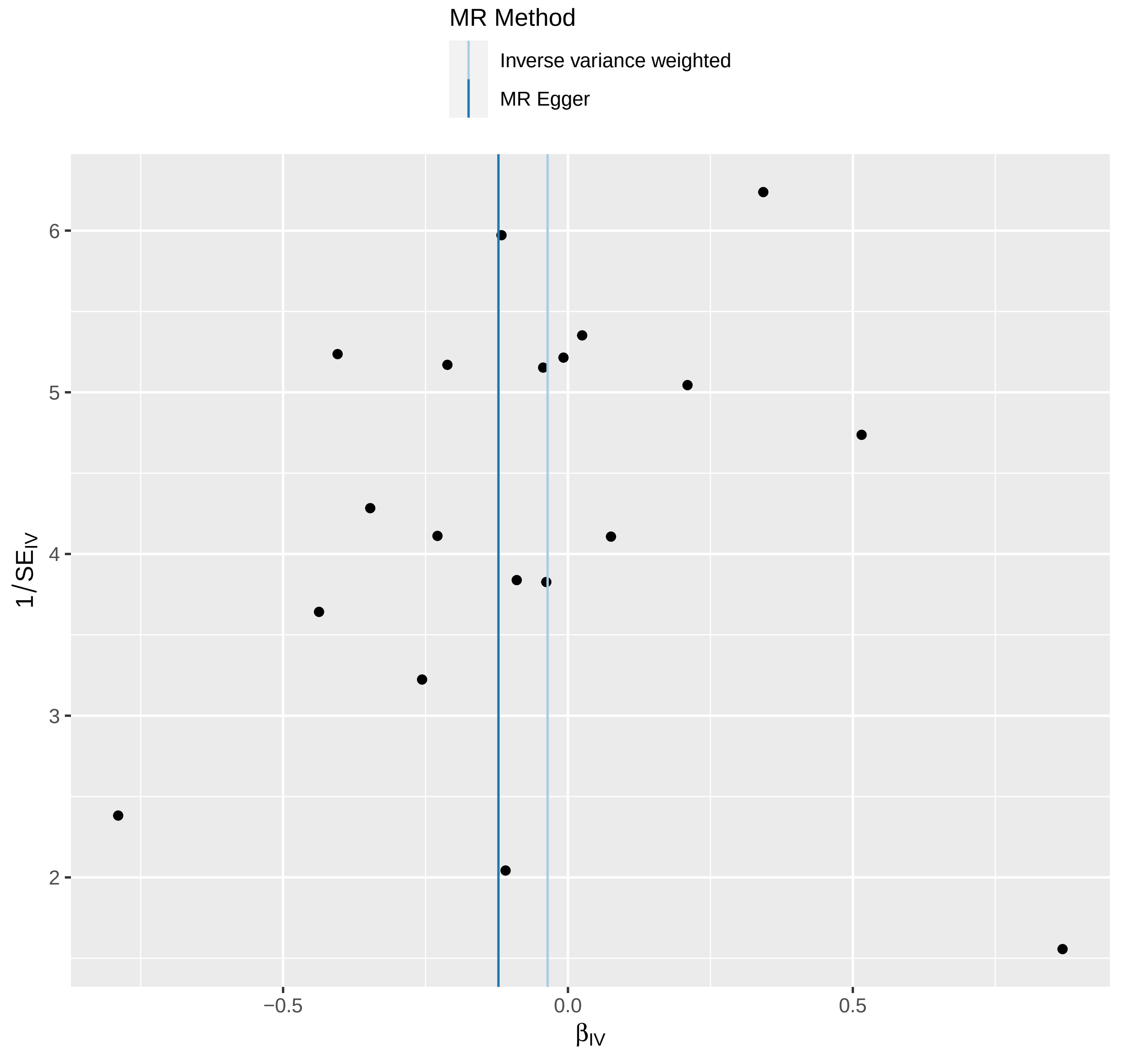

Batch 3−2747 : Urea transporter 2 on Diabetic nephropathy (more control exclusions)
with a threshold of 1e−05

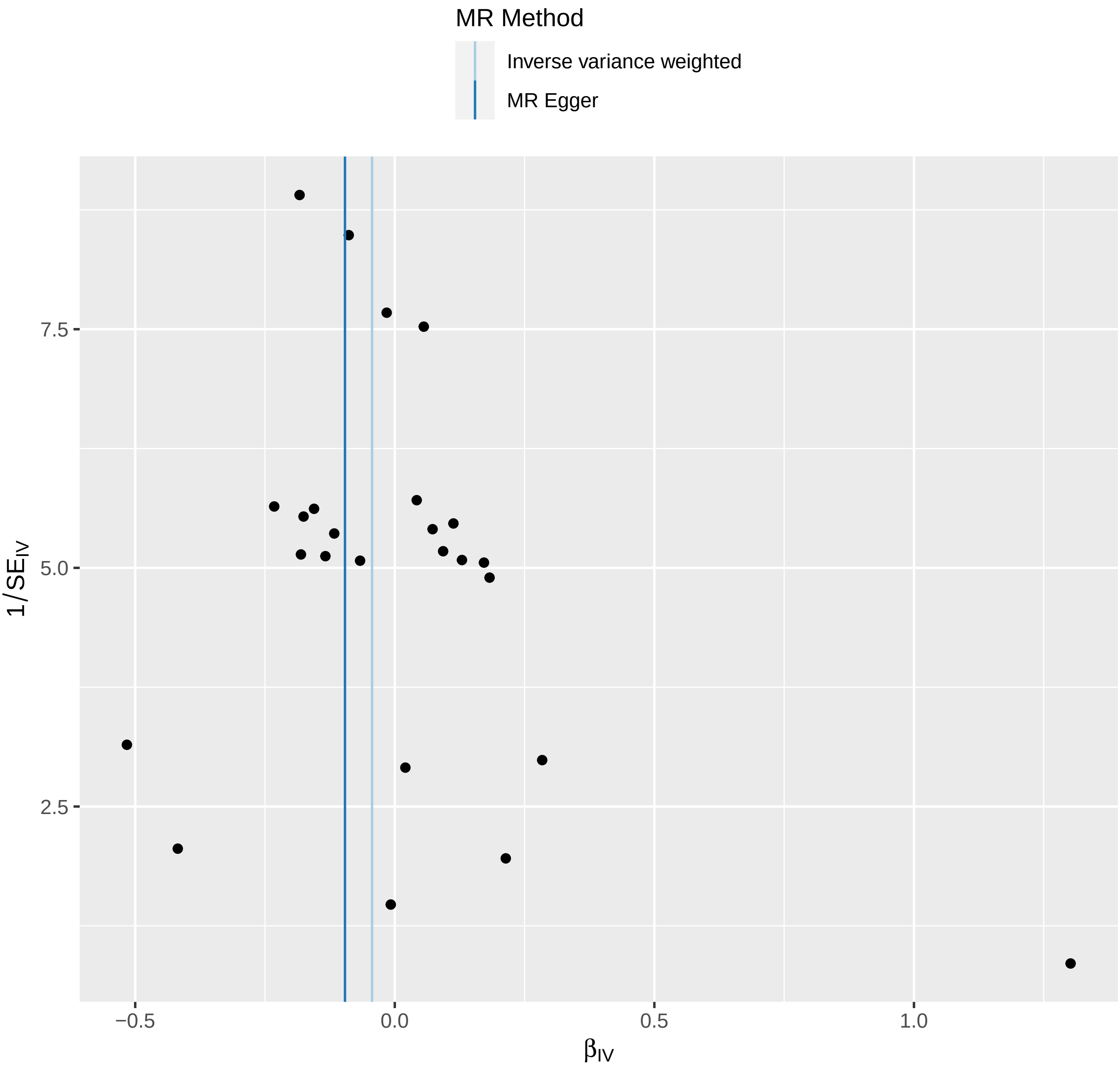

Batch 3−2748 : Solute carrier family 22 member 16 on Diabetic nephropathy (more control exclusions)
with a threshold of 1e−05

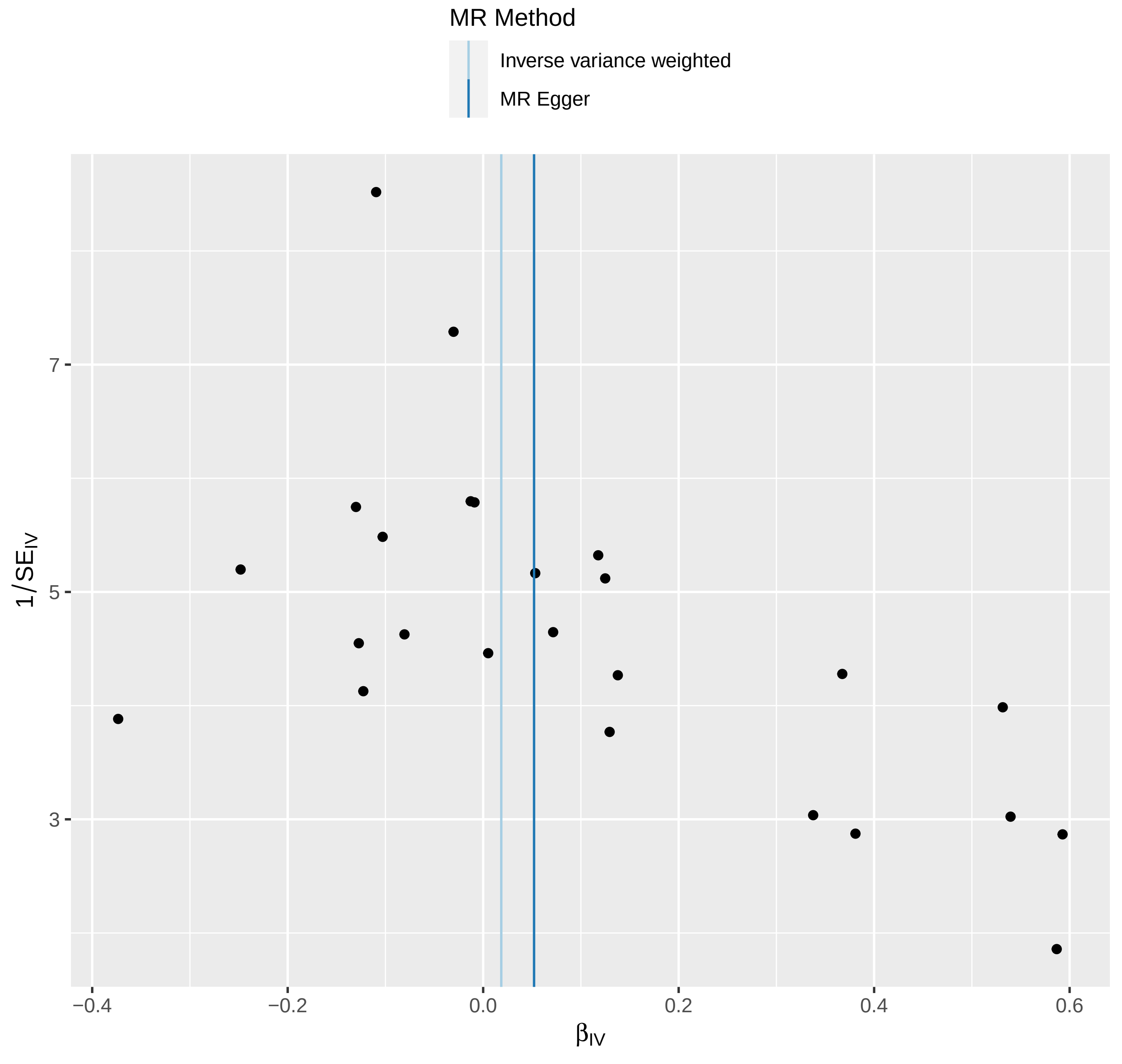

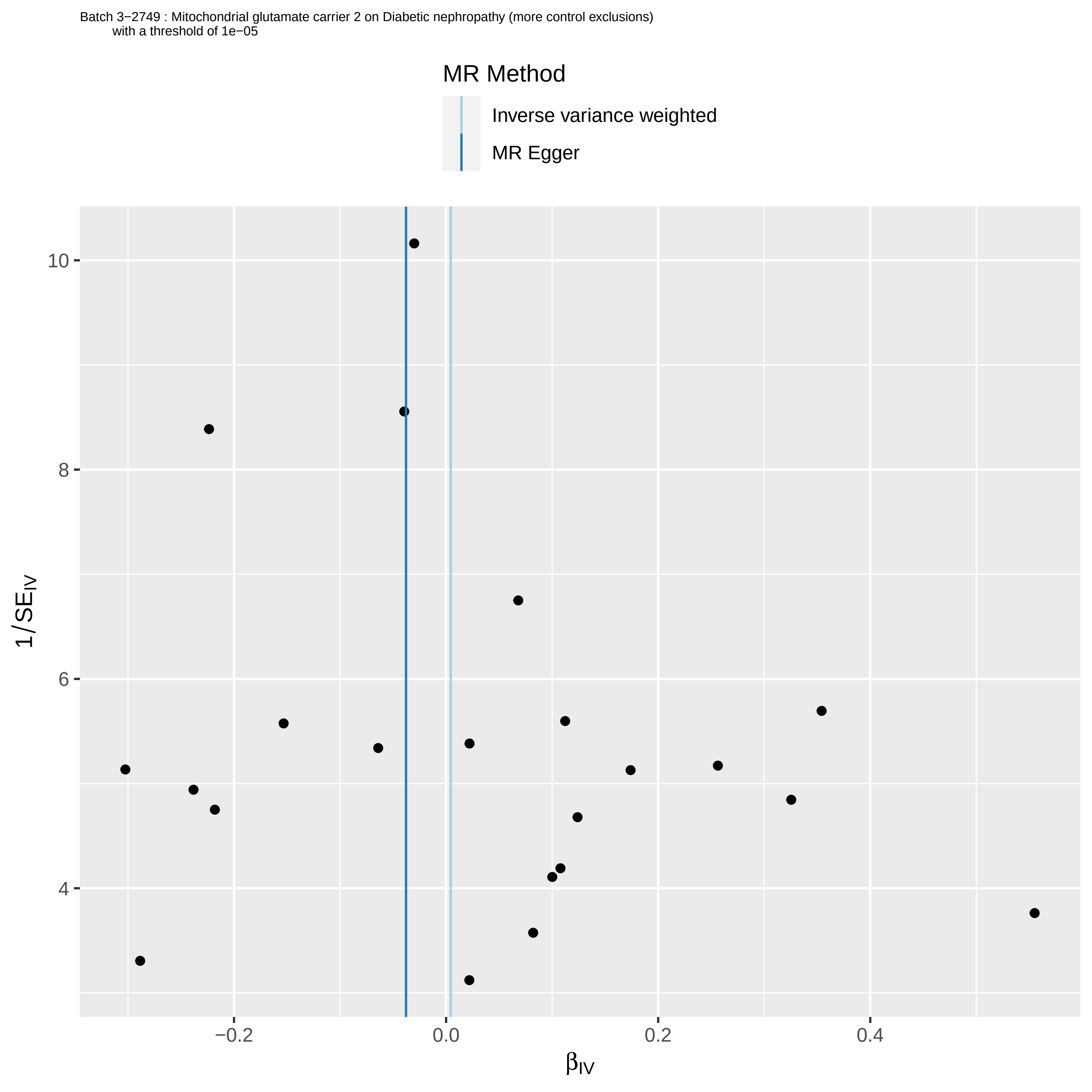
Batch 3−2749 : Mitochondrial glutamate carrier 2 on Diabetic nephropathy (more control exclusions)
with a threshold of 1e−05
MR Method
Inverse variance weighted
MR Egger
10
8
6
4
1/SE$_{IV}$
−0.2
0.0
0.2
0.4
β$_{IV}$

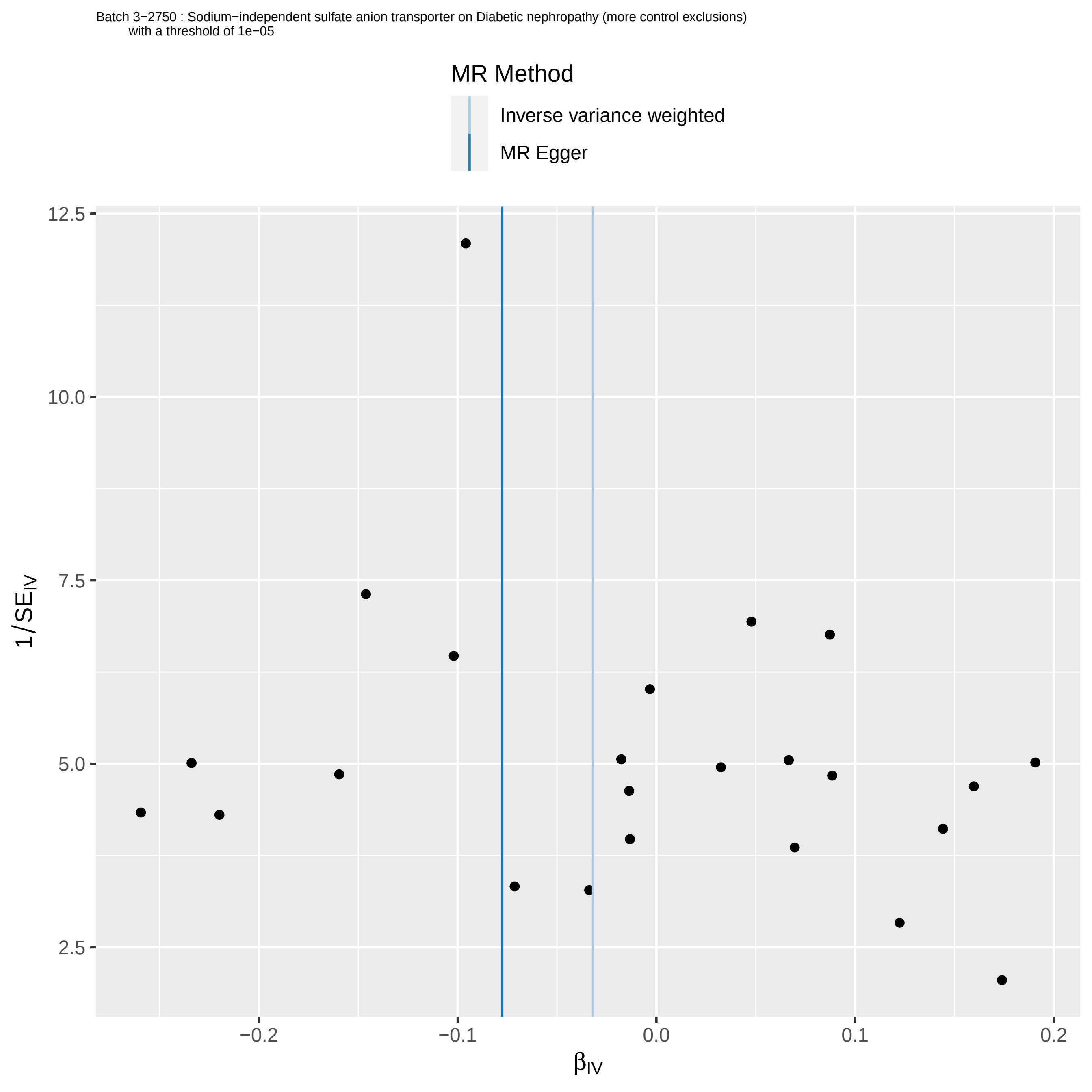

Batch 3−2750 : Sodium−independent sulfate anion transporter on Diabetic nephropathy (more control exclusions)
with a threshold of 1e−05
MR Method
Inverse variance weighted
MR Egger
12.5
10.0
7.5
5.0
2.5
1/SE_IV
−0.2
−0.1
0.0
0.1
0.2
β_IV

Batch 3−2751 : Prestin on Diabetic nephropathy (more control exclusions)
with a threshold of 1e−05

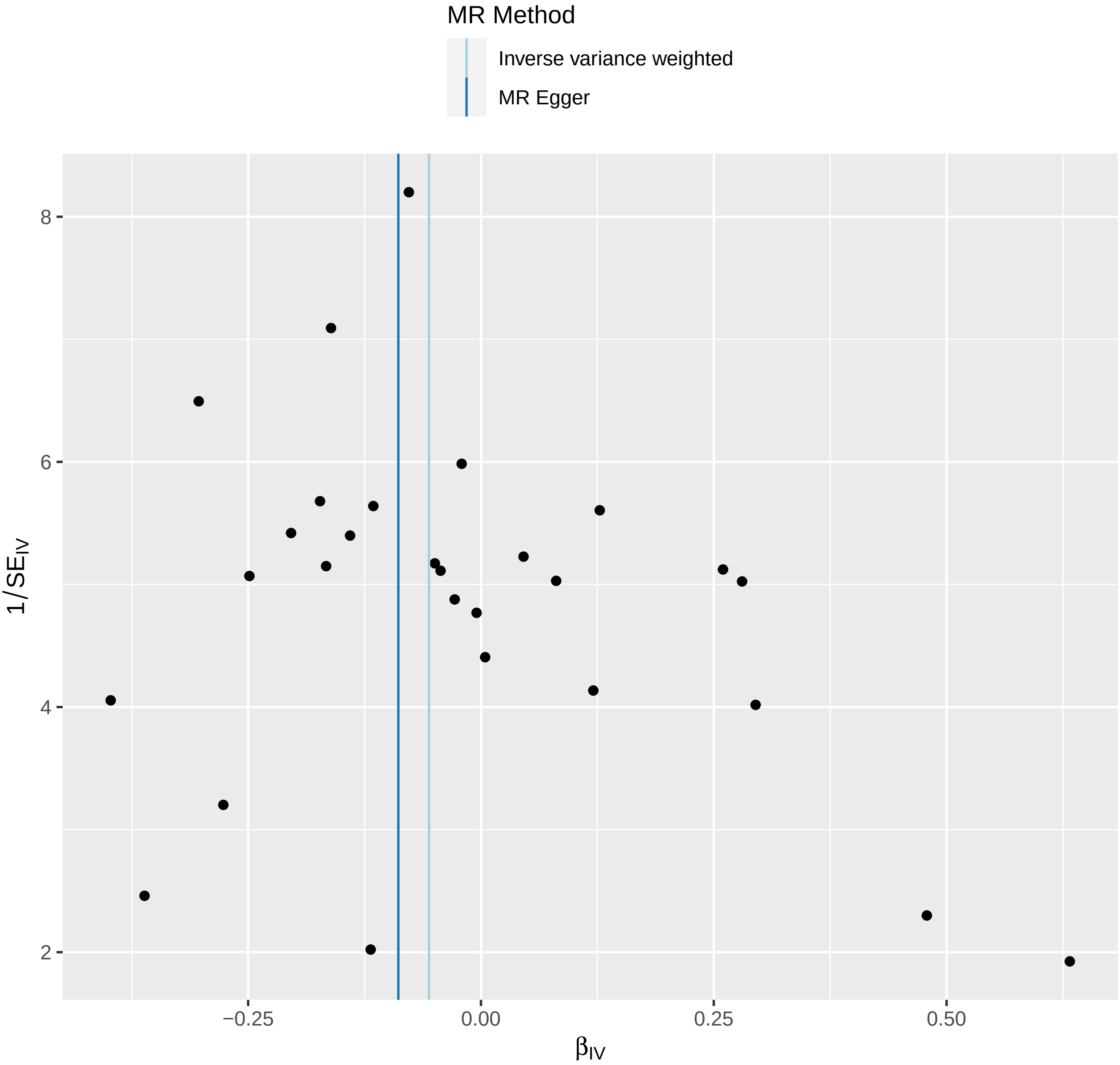

Batch 3−2752 : Very long−chain acyl−CoA synthetase on Diabetic nephropathy (more control exclusions)
with a threshold of 1e−05

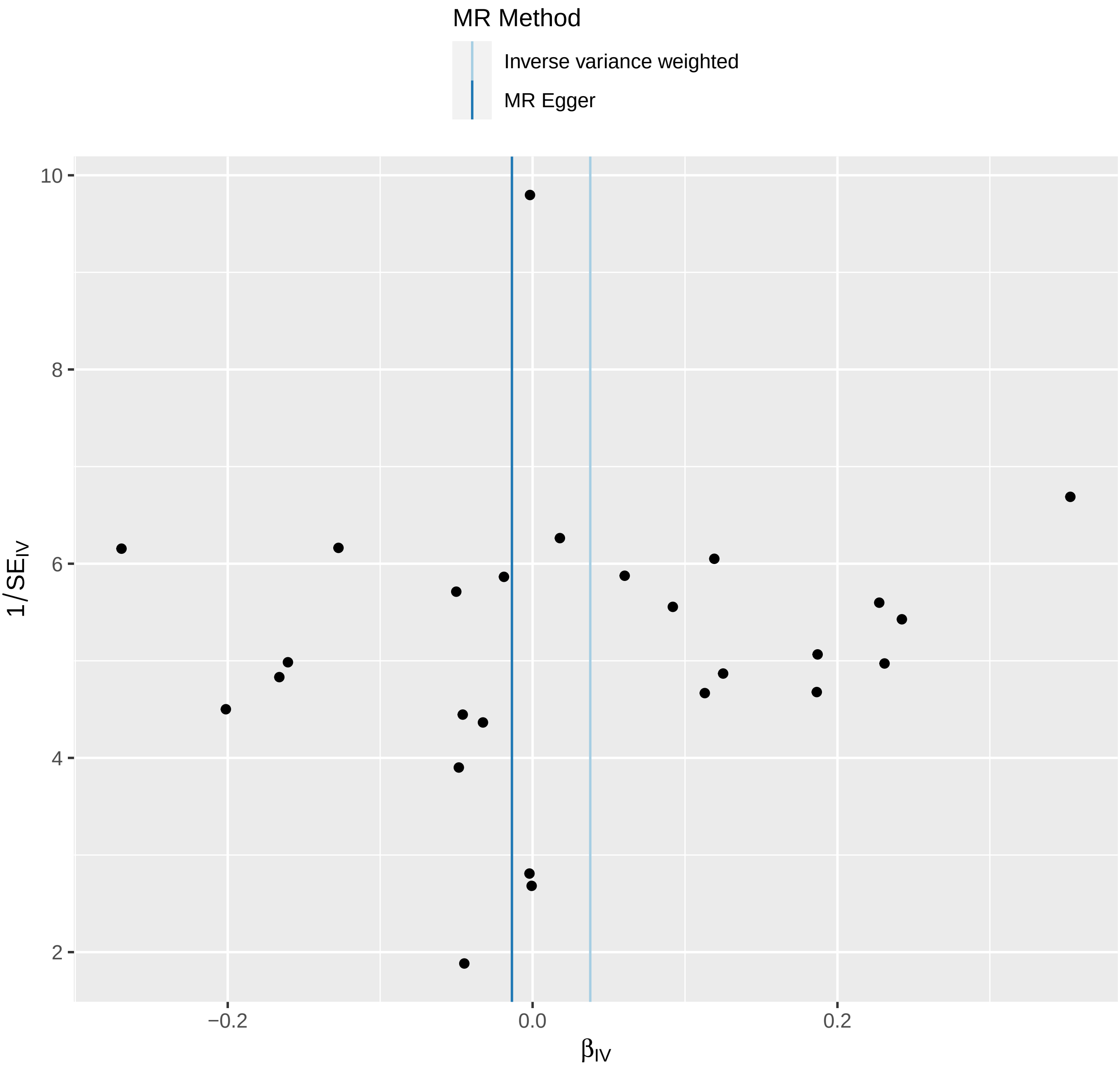

Batch 3−2753 : Zinc transporter 3 on Diabetic nephropathy (more control exclusions)
with a threshold of 1e−05

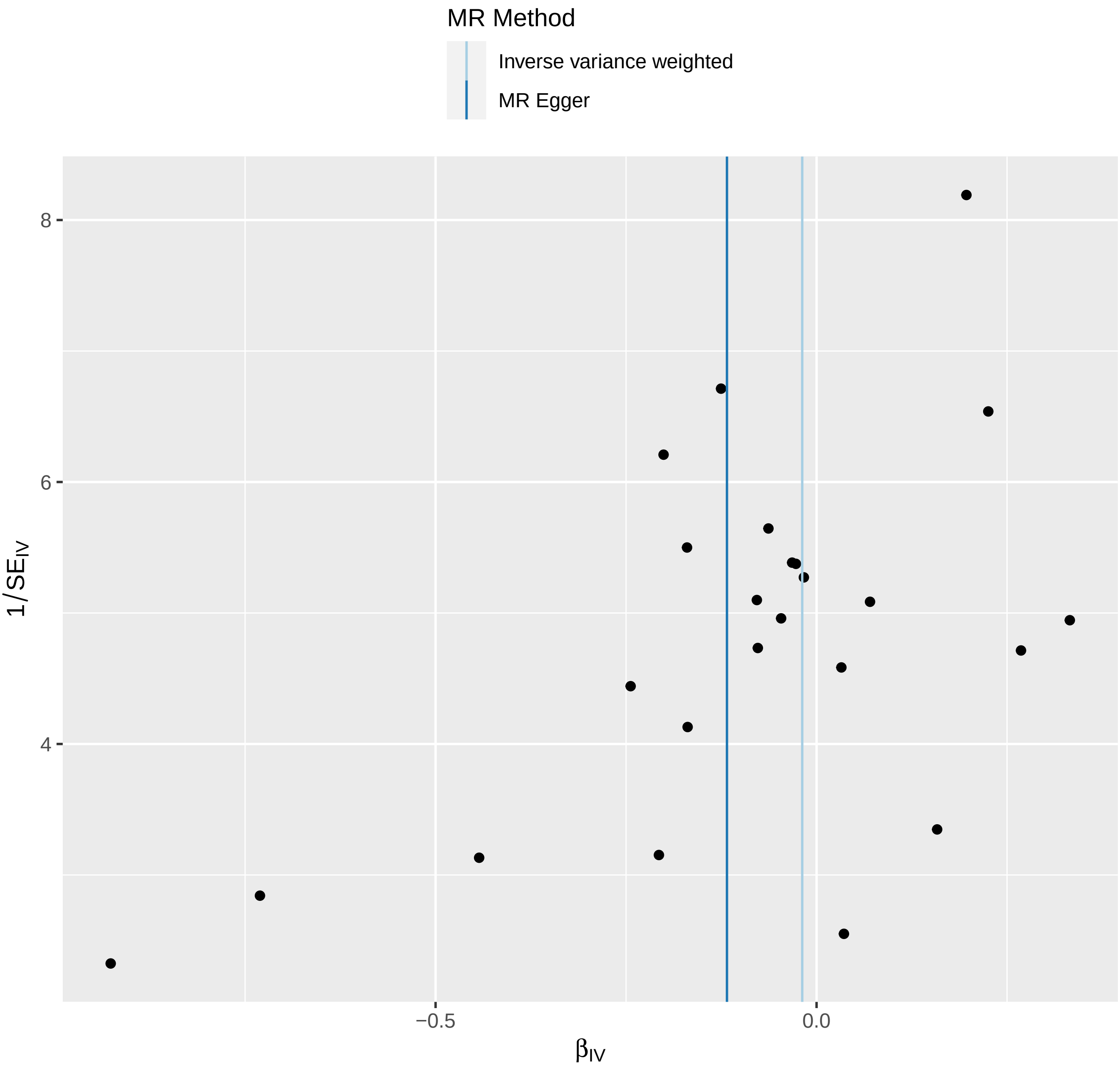

Batch 3−2754 : Zinc transporter 5 on Diabetic nephropathy (more control exclusions)
with a threshold of 1e−05

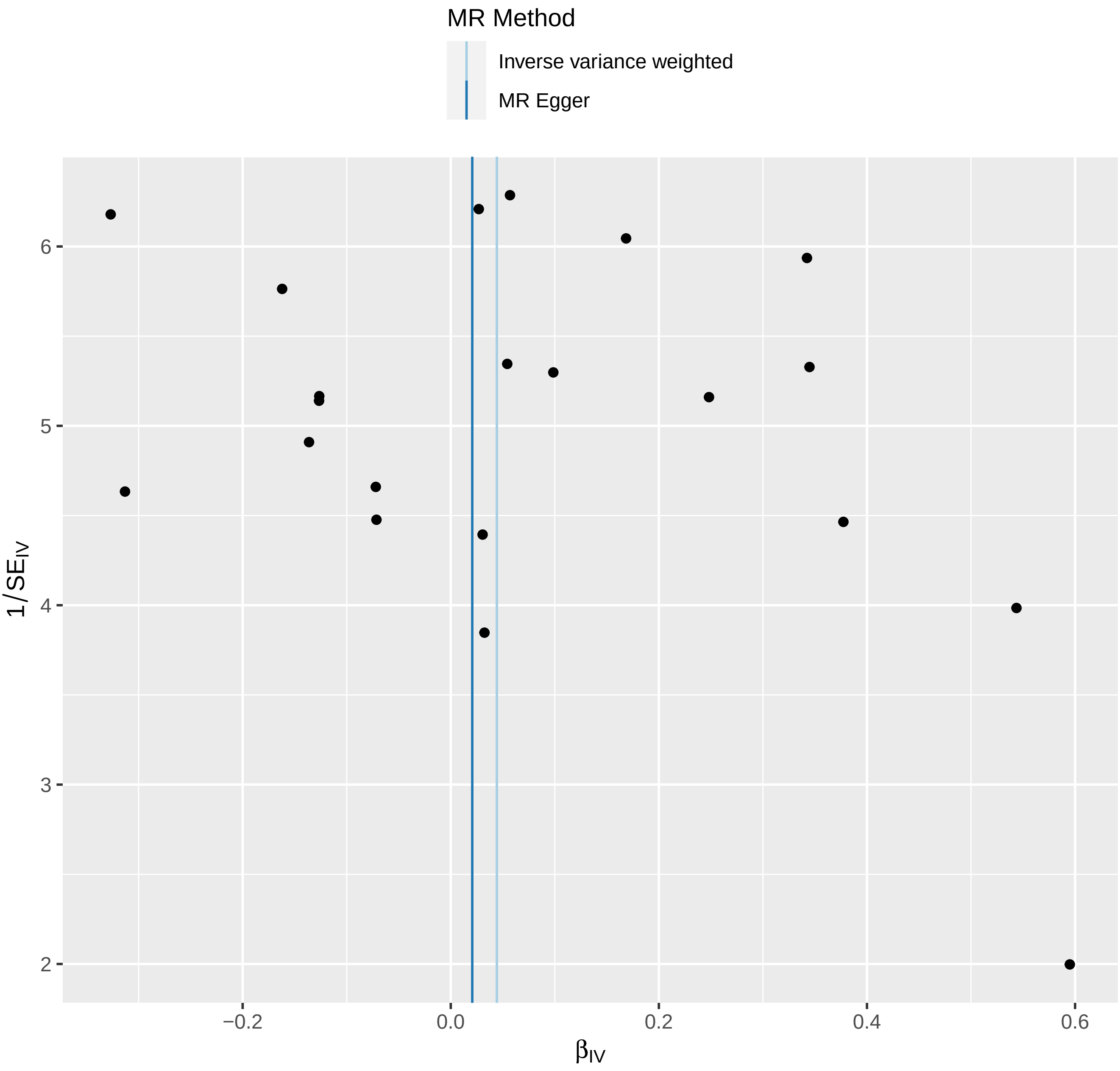

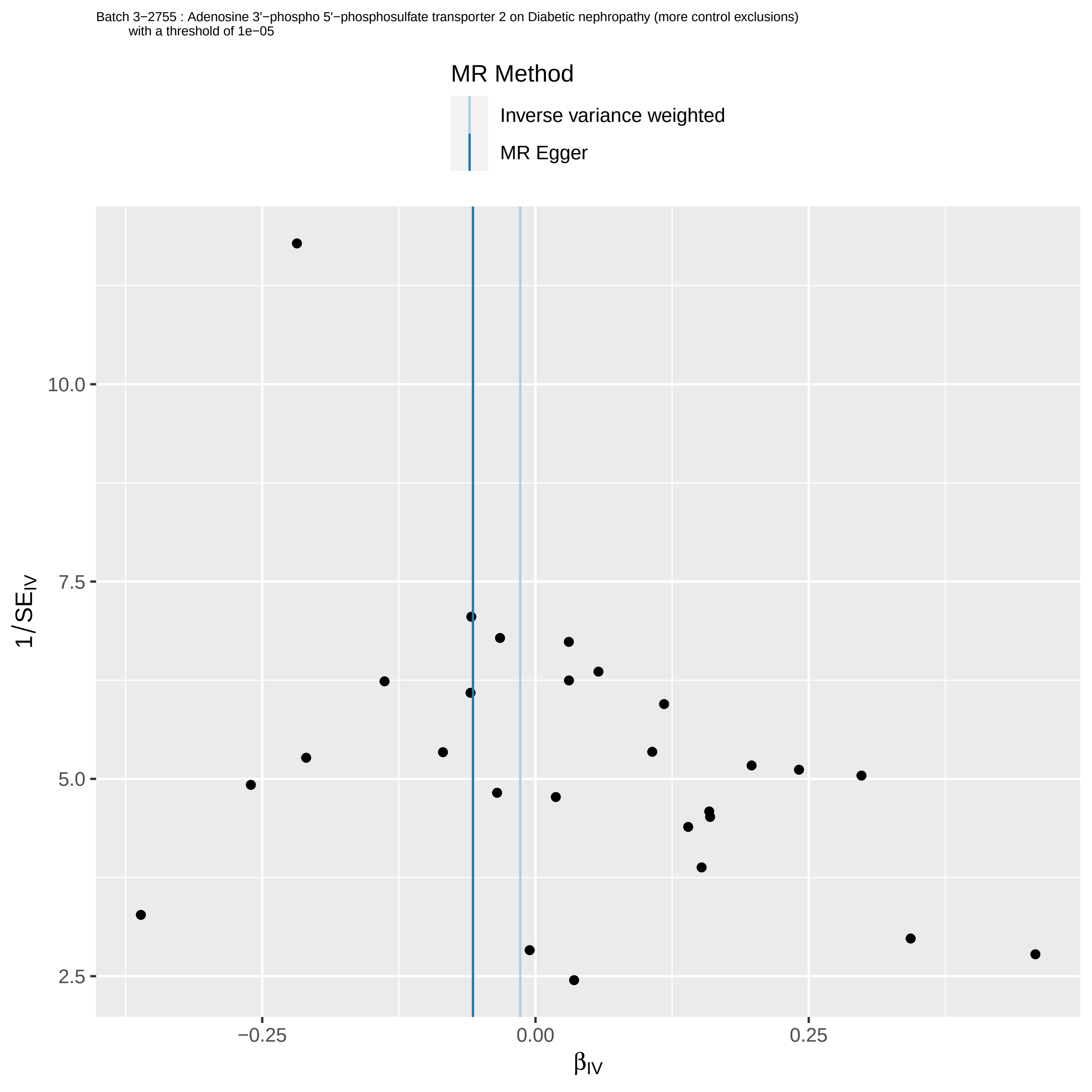
Batch 3−2755 : Adenosine 3'−phospho 5'−phosphosulfate transporter 2 on Diabetic nephropathy (more control exclusions)
with a threshold of 1e−05
MR Method
Inverse variance weighted
MR Egger
10.0
7.5
5.0
2.5
1/SE$_{IV}$
−0.25
0.00
0.25
β$_{IV}$

Batch 3−2756 : Solute carrier family 35 member G2 on Diabetic nephropathy (more control exclusions)
with a threshold of 1e−05

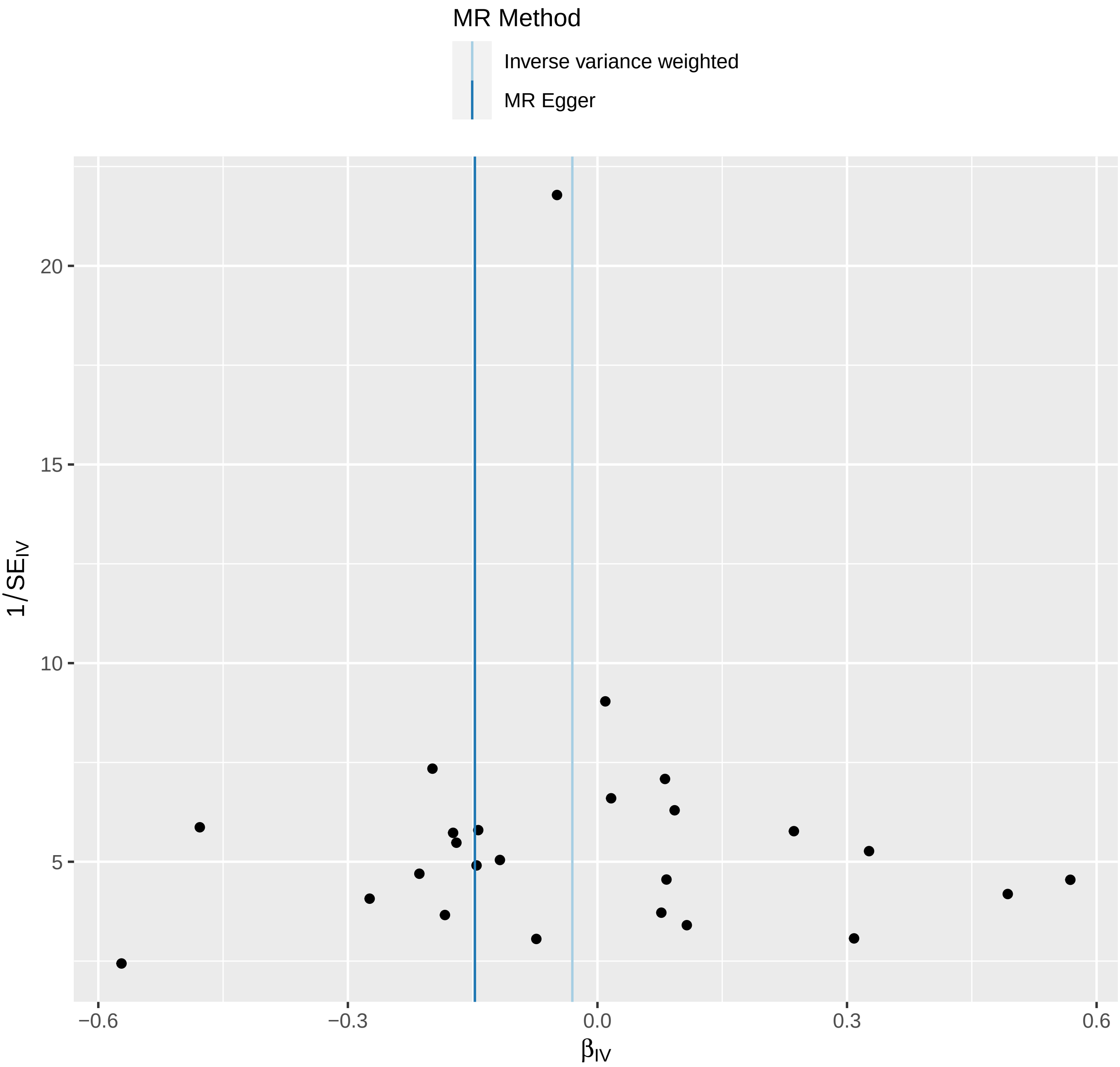

Batch 3−2757 : Solute carrier family 41 member 2 on Diabetic nephropathy (more control exclusions)
with a threshold of 1e−05

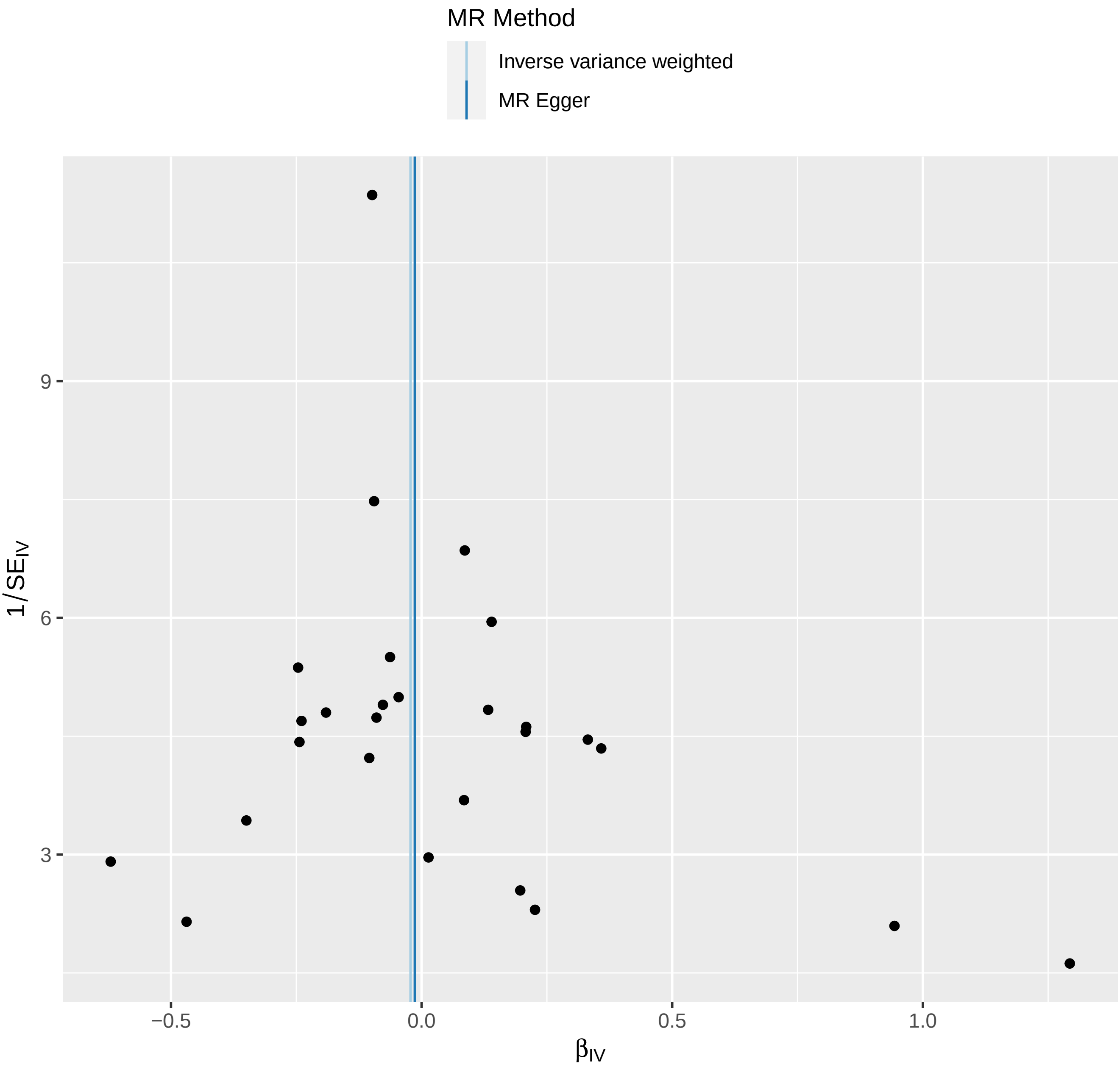

Batch 3−2758 : Electroneutral sodium bicarbonate exchanger 1 on Diabetic nephropathy (more control exclusions)
with a threshold of 1e−05

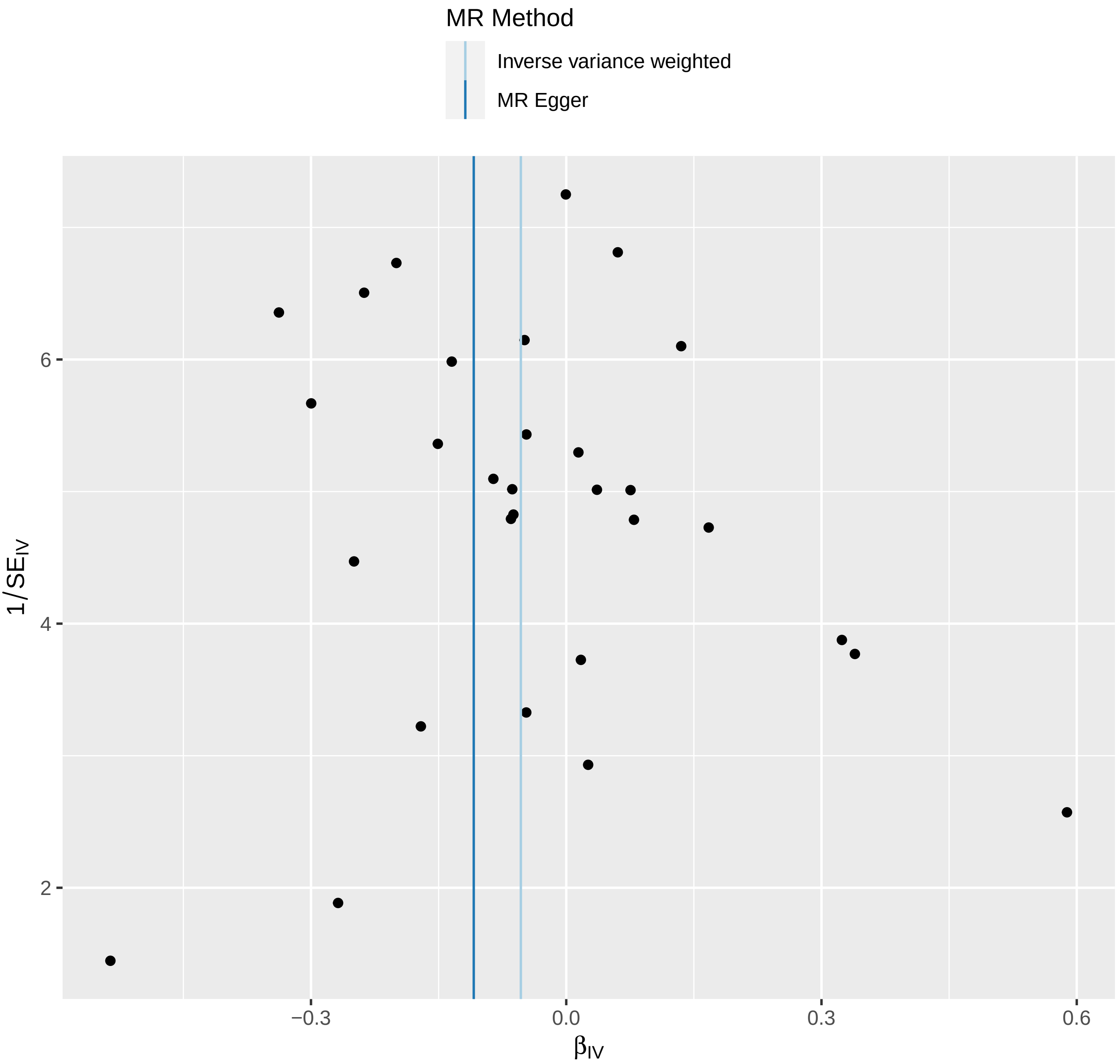

Batch 3−2759 : Sodium/iodide cotransporter on Diabetic nephropathy (more control exclusions)
with a threshold of 1e−05

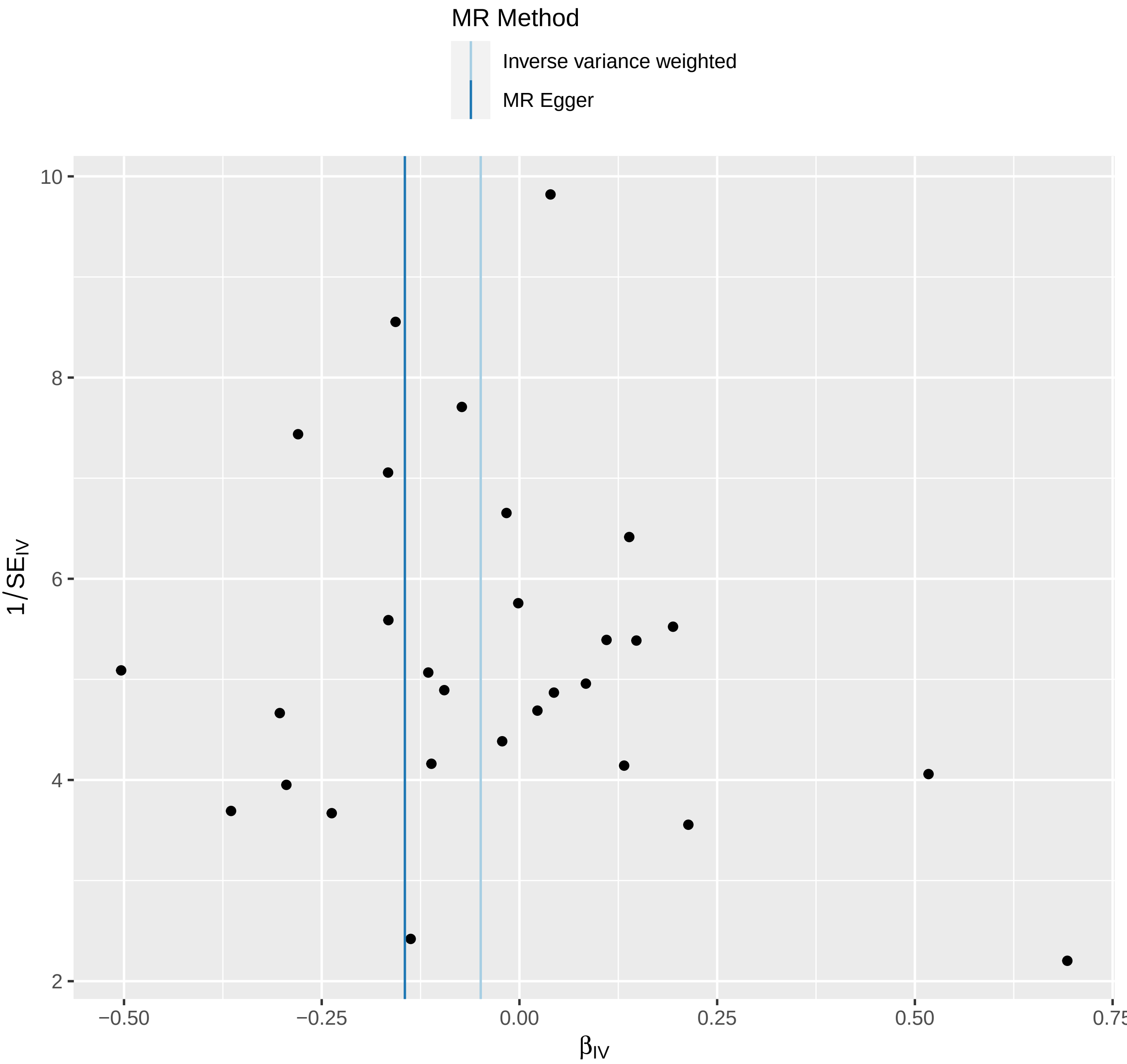

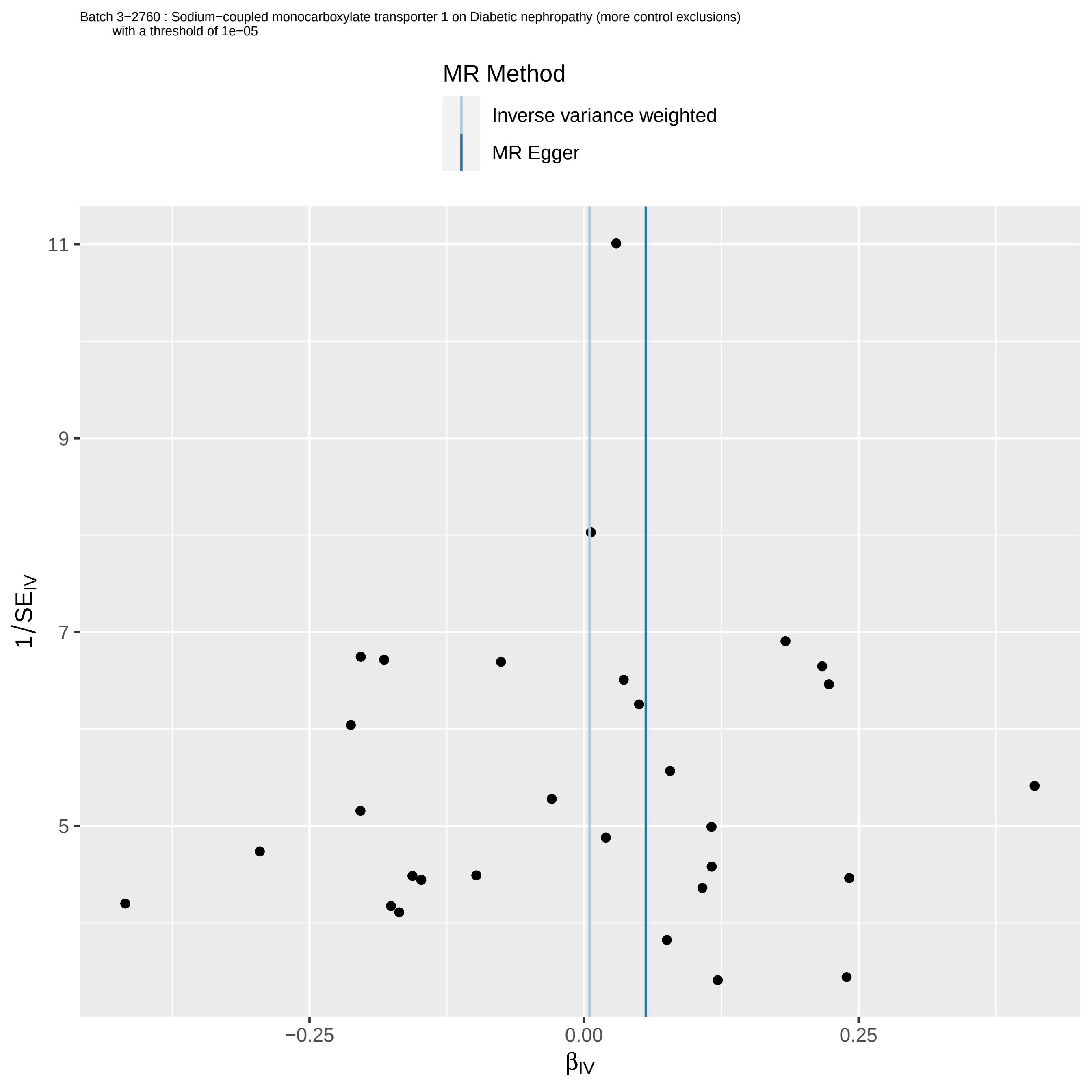

Batch 3−2760 : Sodium−coupled monocarboxylate transporter 1 on Diabetic nephropathy (more control exclusions)
with a threshold of 1e−05
MR Method
Inverse variance weighted
MR Egger
11
9
7
5
1/SE$_{IV}$
−0.25
0.00
0.25
β$_{IV}$

Batch 3−2761 : Sodium− and chloride−dependent neutral and basic amino acid transporter B(0+) on Diabetic nephropathy (more control exclusions) with a threshold of 1e−05

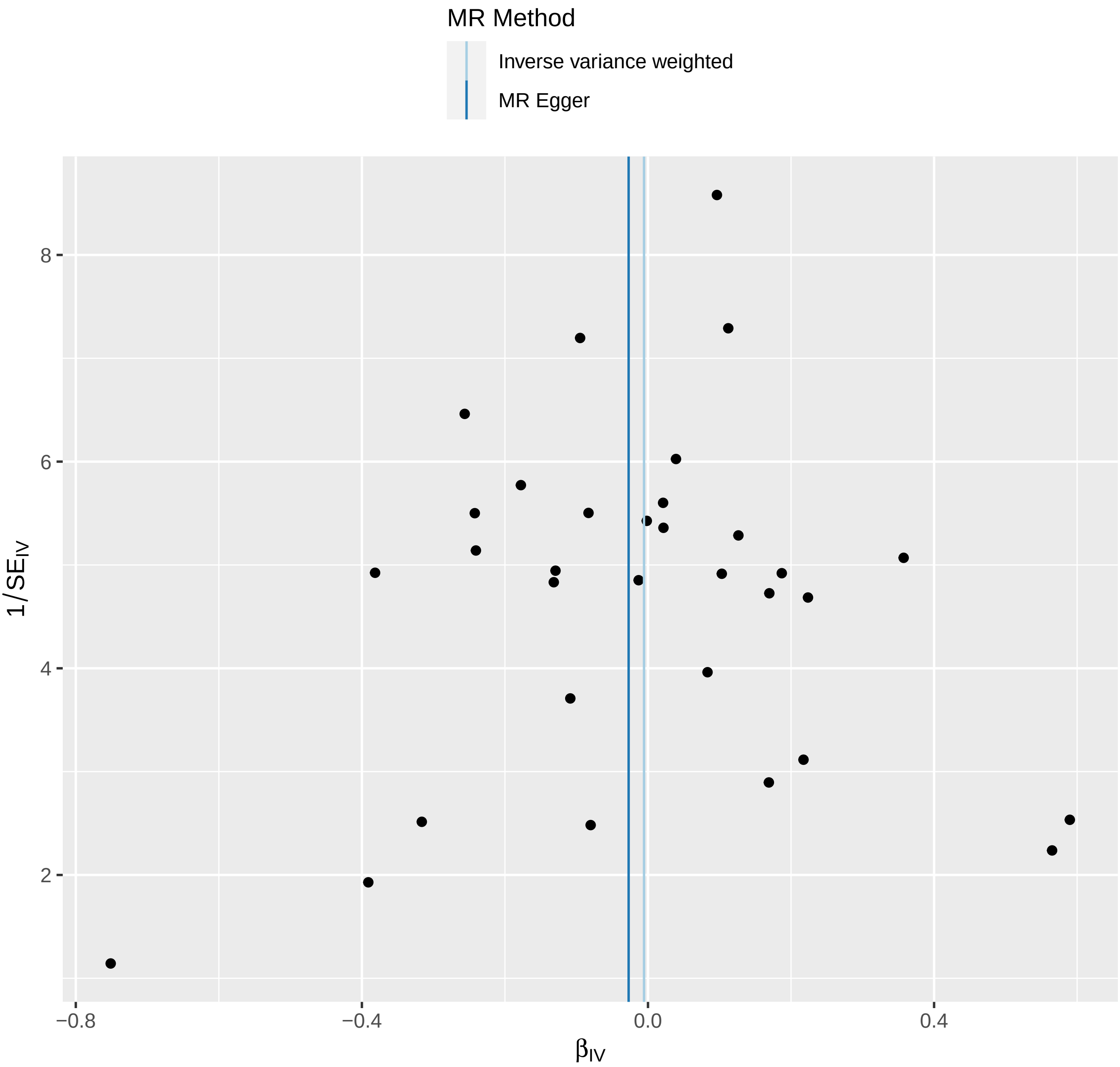

Batch 3−2762 : Orphan sodium− and chloride−dependent neurotransmitter transporter NTT5 on Diabetic nephropathy (more control exclusions) with a threshold of 1e−05

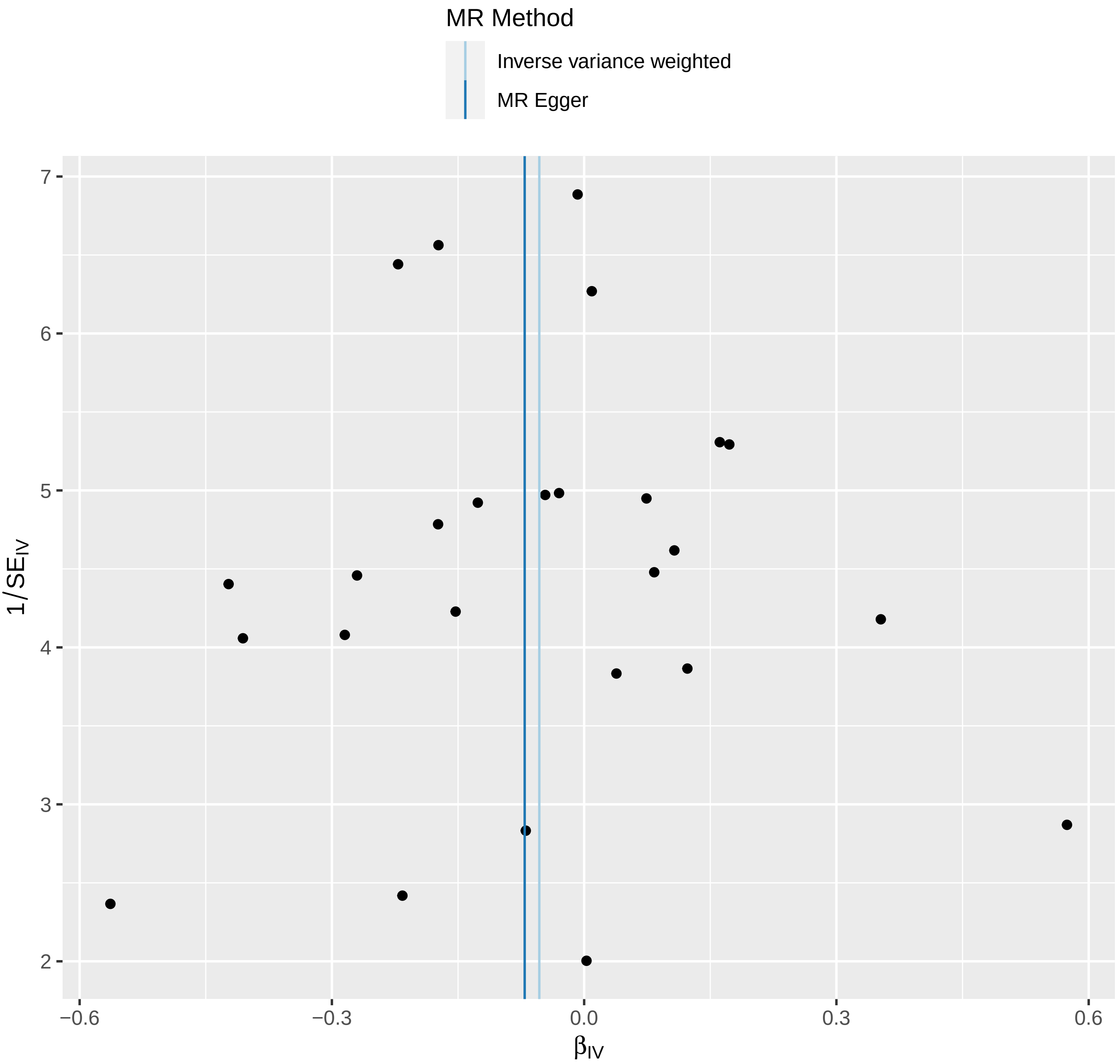

Batch 3−2763 : Sodium− and chloride−dependent glycine transporter 1 on Diabetic nephropathy (more control exclusions)
with a threshold of 1e−05

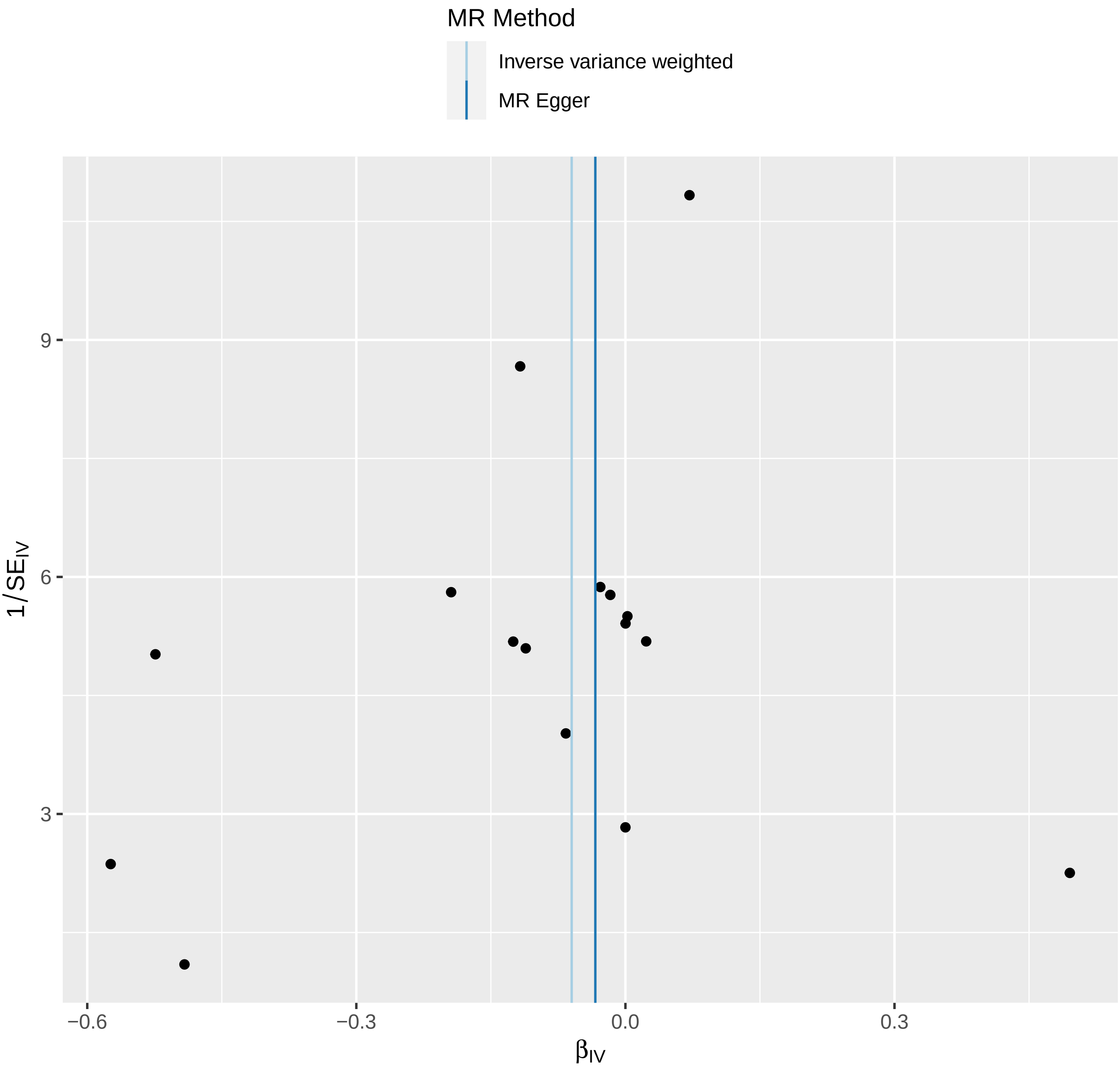

Batch 3−2764 : Mitochondrial sodium/hydrogen exchanger 9B2 on Diabetic nephropathy (more control exclusions)
with a threshold of 1e−05

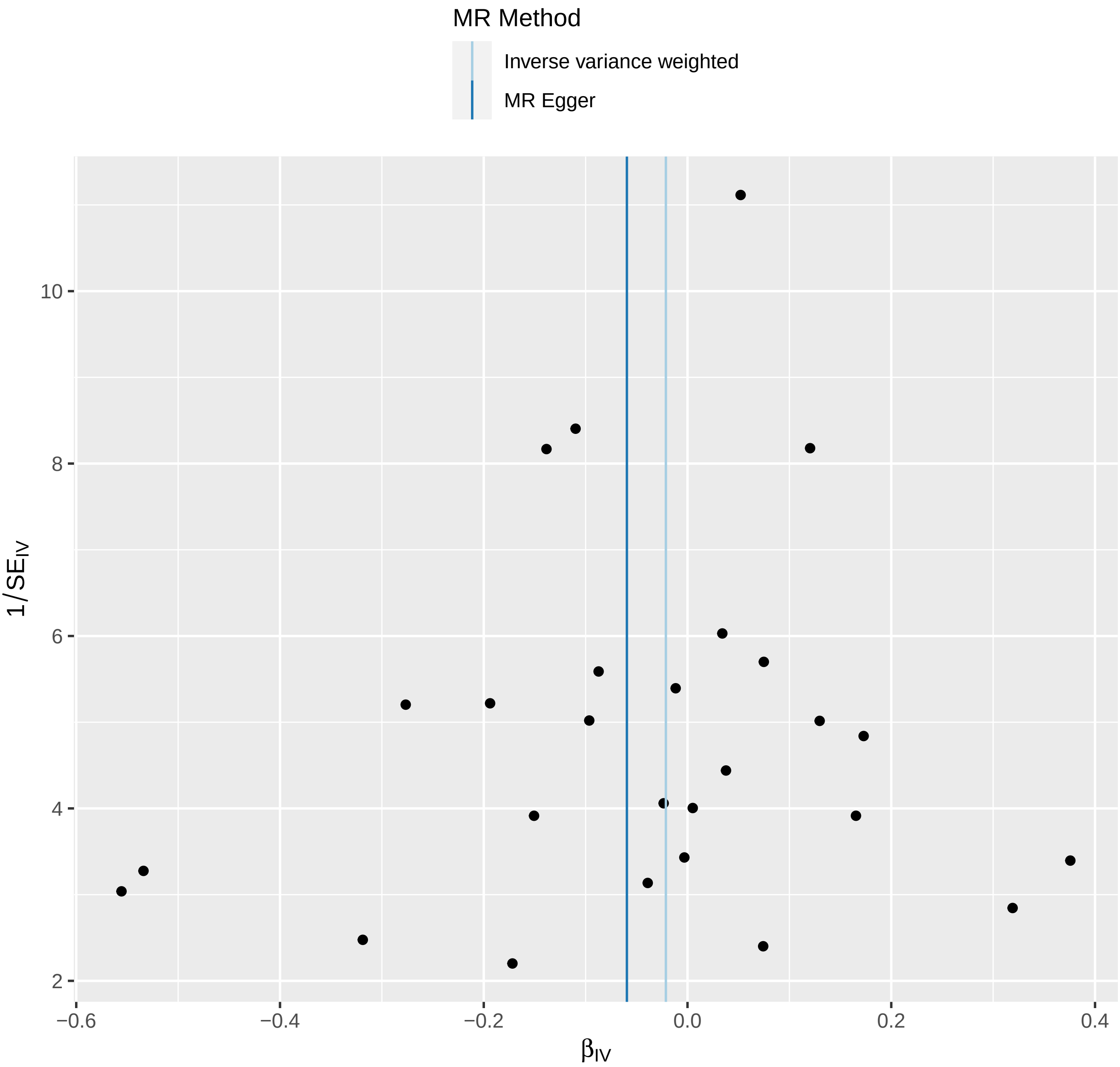

Batch 3−2765 : Solute carrier organic anion transporter family member 5A1 on Diabetic nephropathy (more control exclusions)
with a threshold of 1e−05

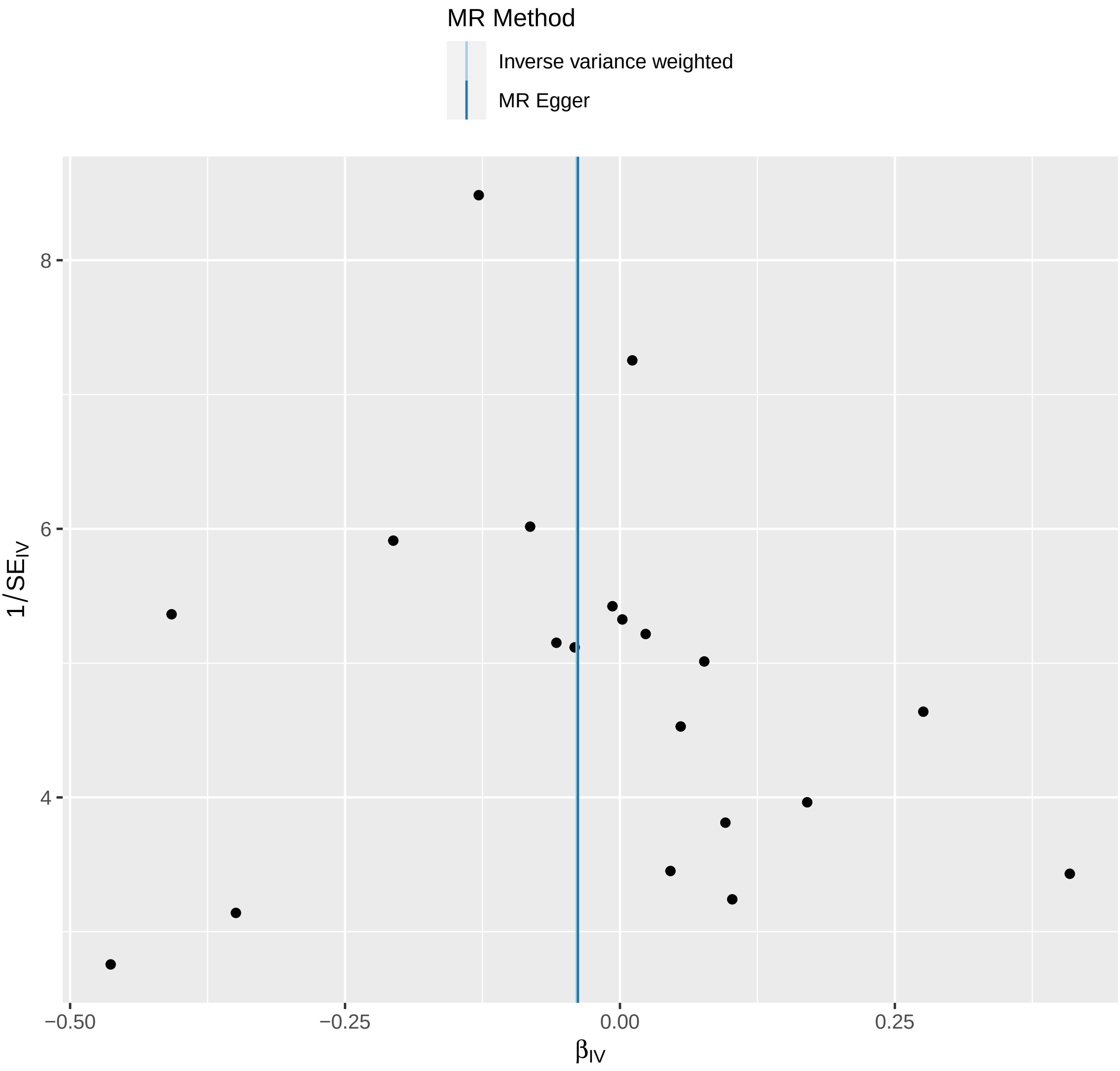

Batch 3−2766 : SLIT and NTRK−like protein 1 on Diabetic nephropathy (more control exclusions)
with a threshold of 1e−05

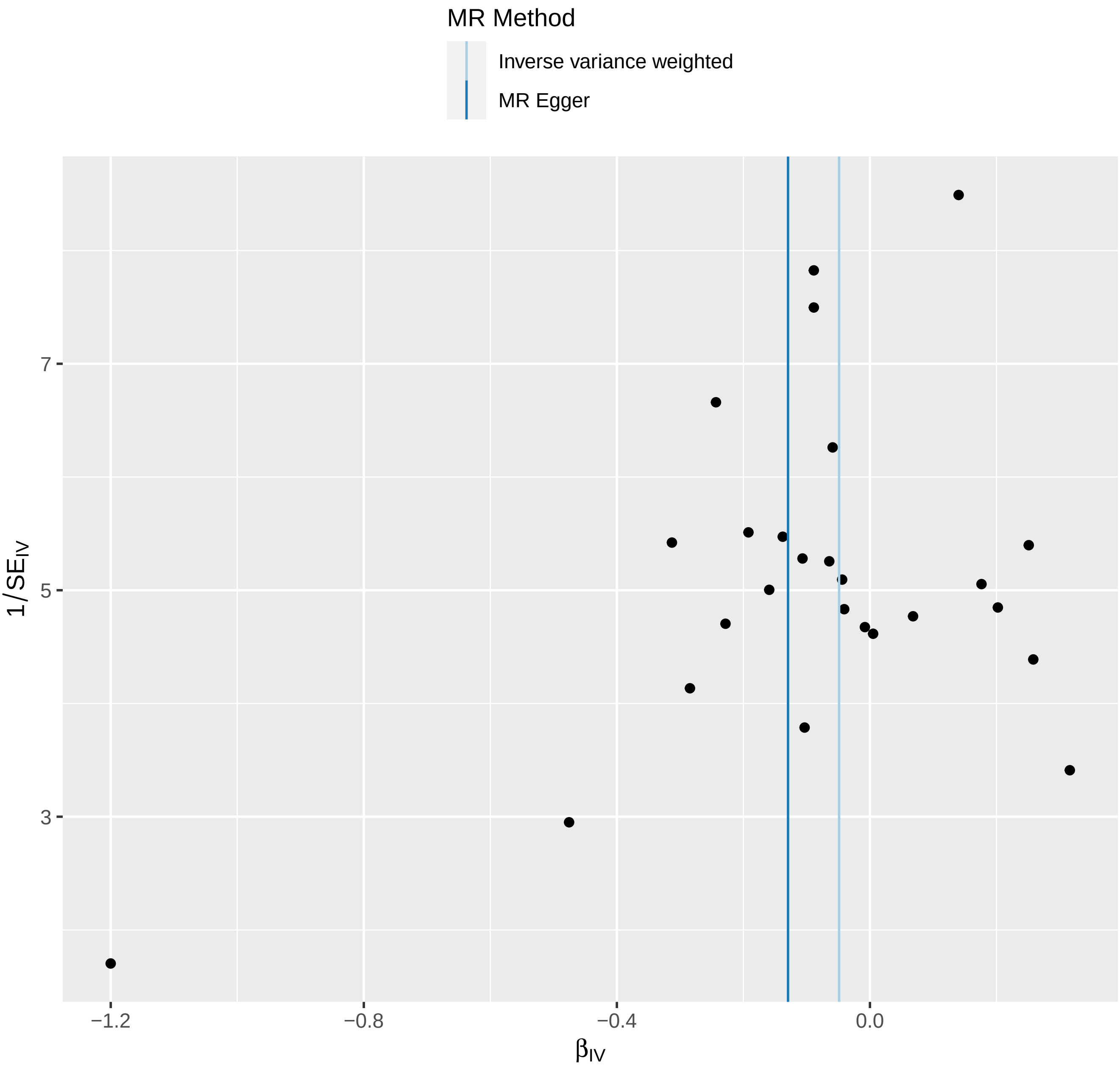

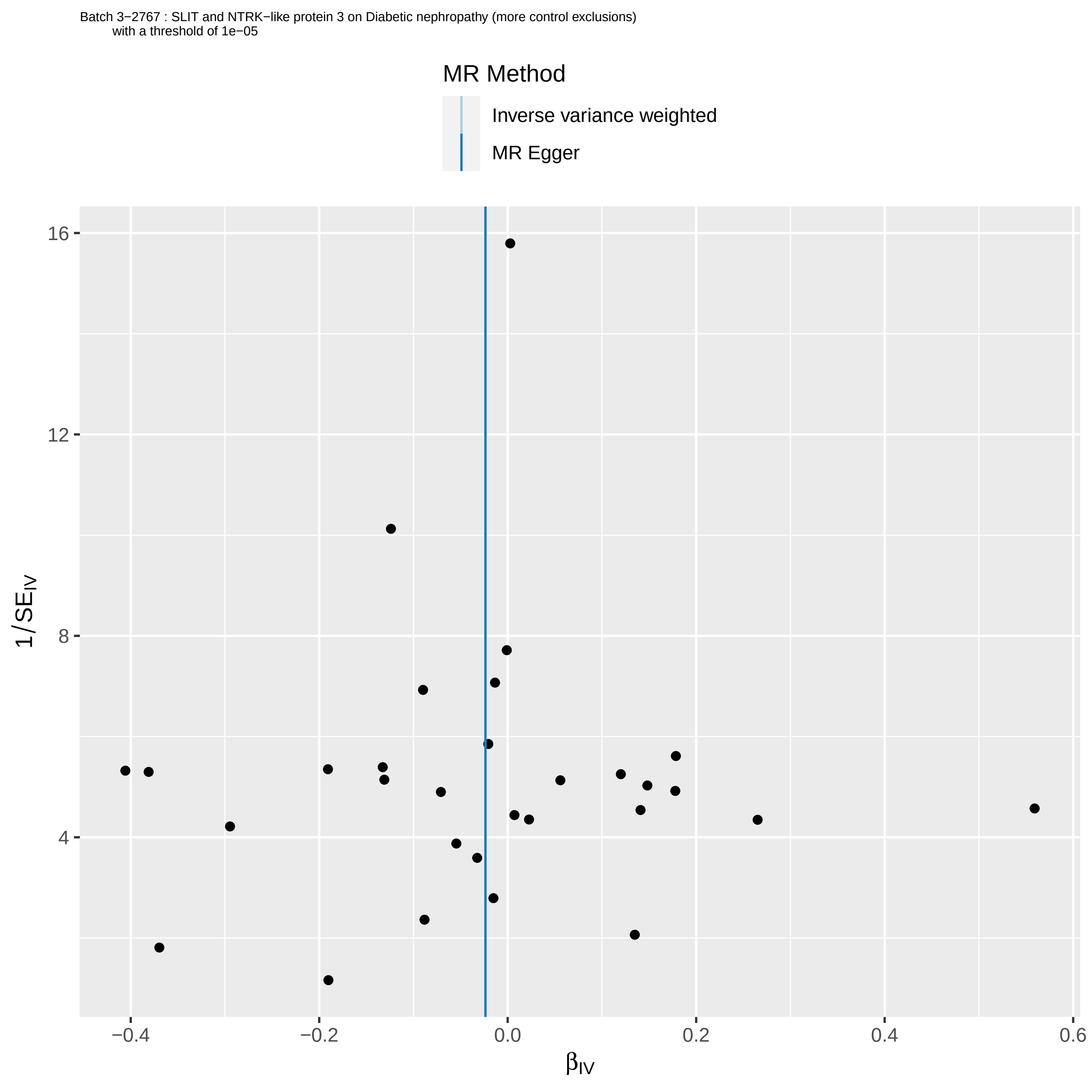
Batch 3−2767 : SLIT and NTRK−like protein 3 on Diabetic nephropathy (more control exclusions)
with a threshold of 1e−05
MR Method
Inverse variance weighted
MR Egger
16
12
8
4
1/SE_IV
−0.4
−0.2
0.0
0.2
0.4
0.6
β_IV

Batch 3−2768 : SLIT and NTRK−like protein 4 on Diabetic nephropathy (more control exclusions)
with a threshold of 1e−05

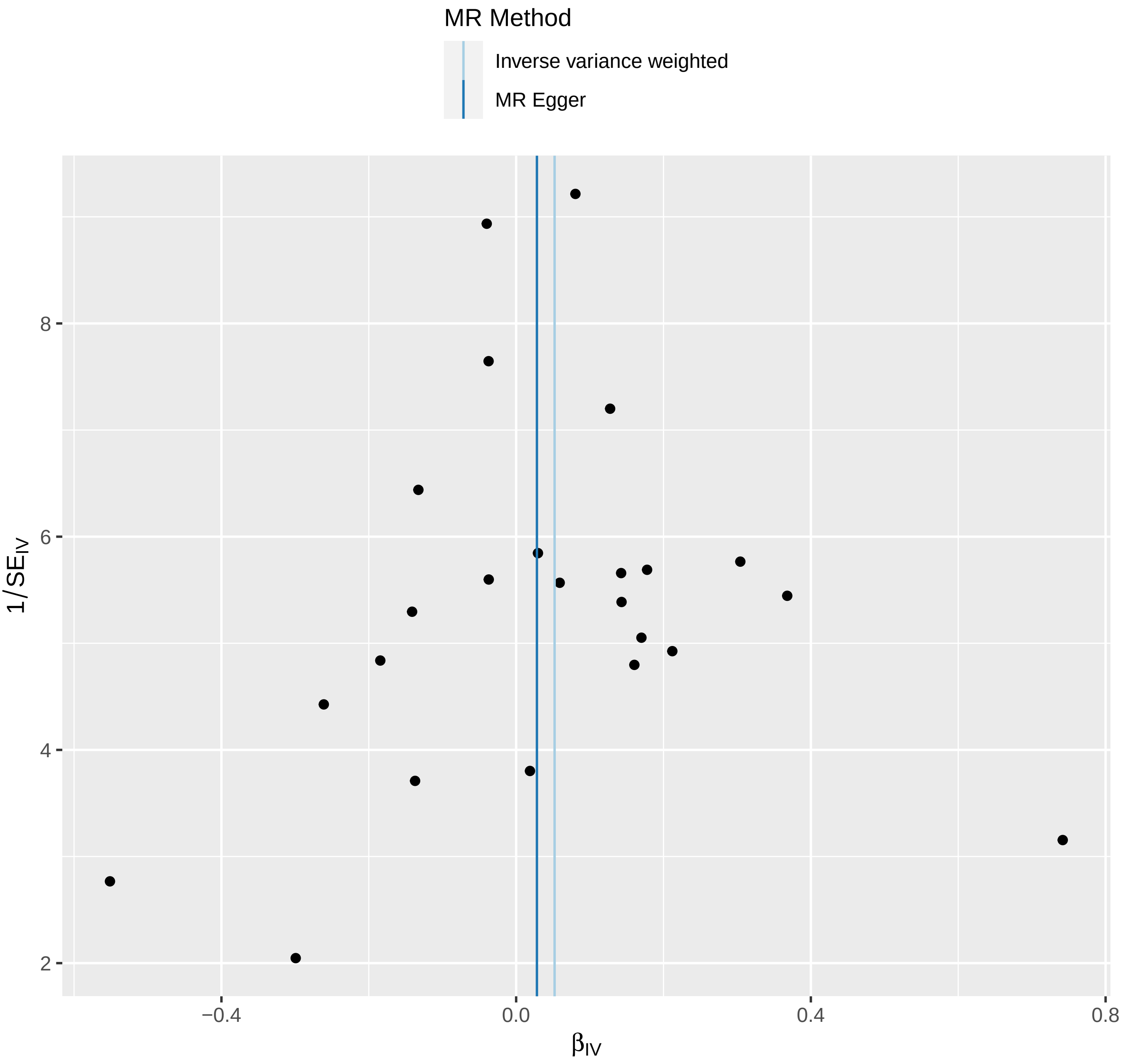

Batch 3−2769 : SLIT and NTRK−like protein 5 on Diabetic nephropathy (more control exclusions)
with a threshold of 1e−05

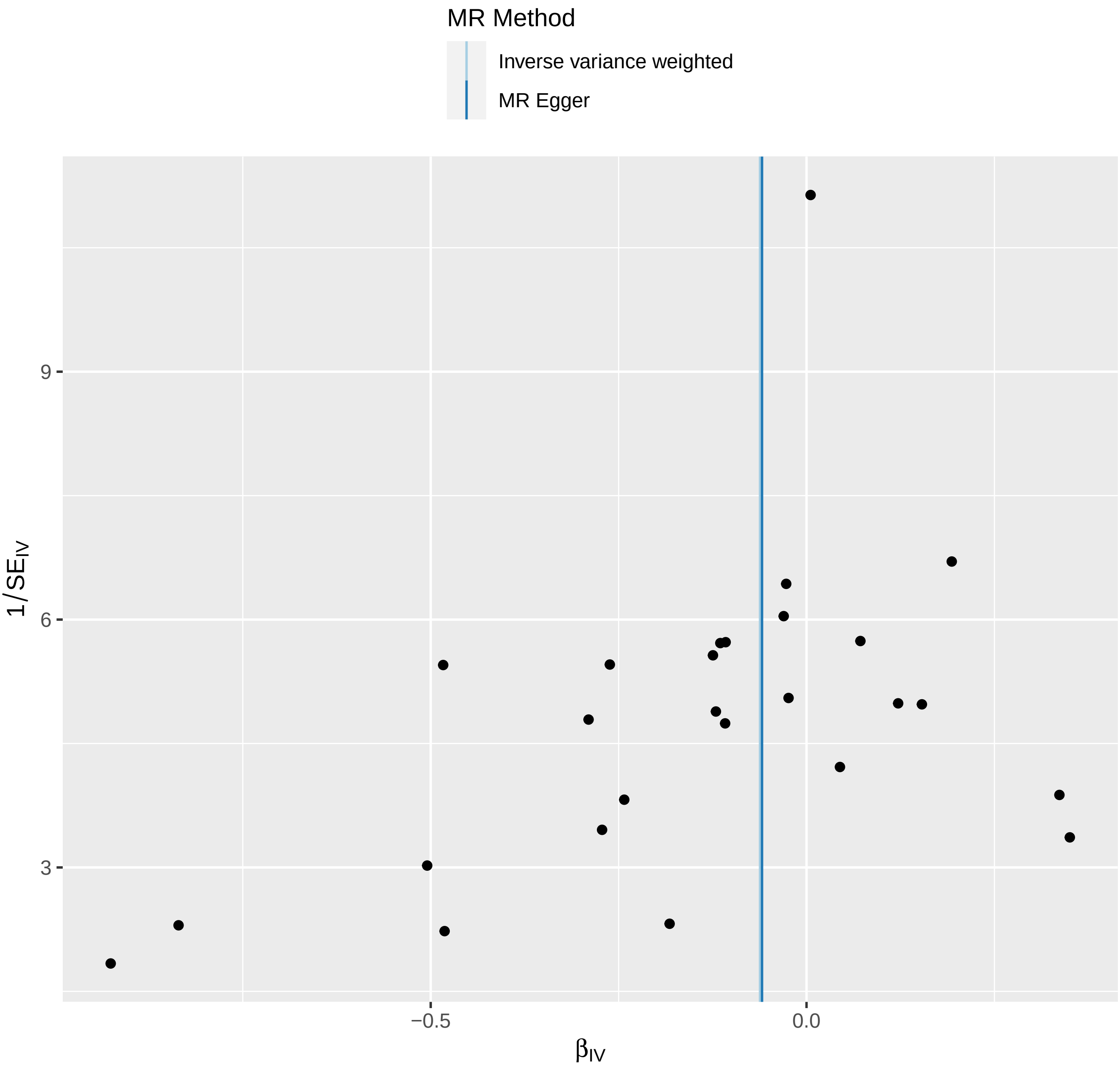

Batch 3−2770 : Secreted Ly−6/uPAR−related protein 1 on Diabetic nephropathy (more control exclusions) with a threshold of 1e−05

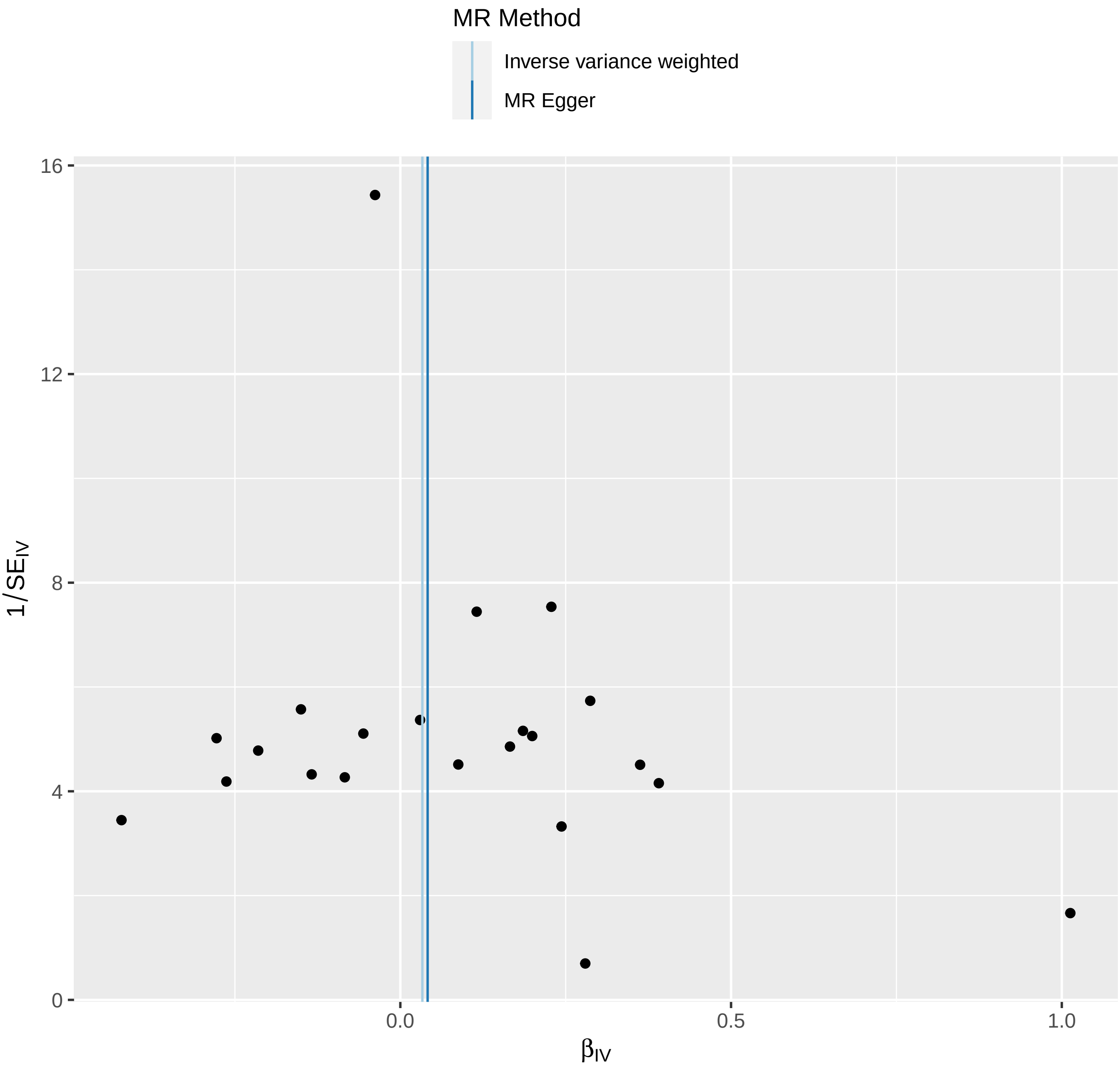

Batch 3−2771 : Mothers against decapentaplegic homolog 2 on Diabetic nephropathy (more control exclusions) with a threshold of 1e−05

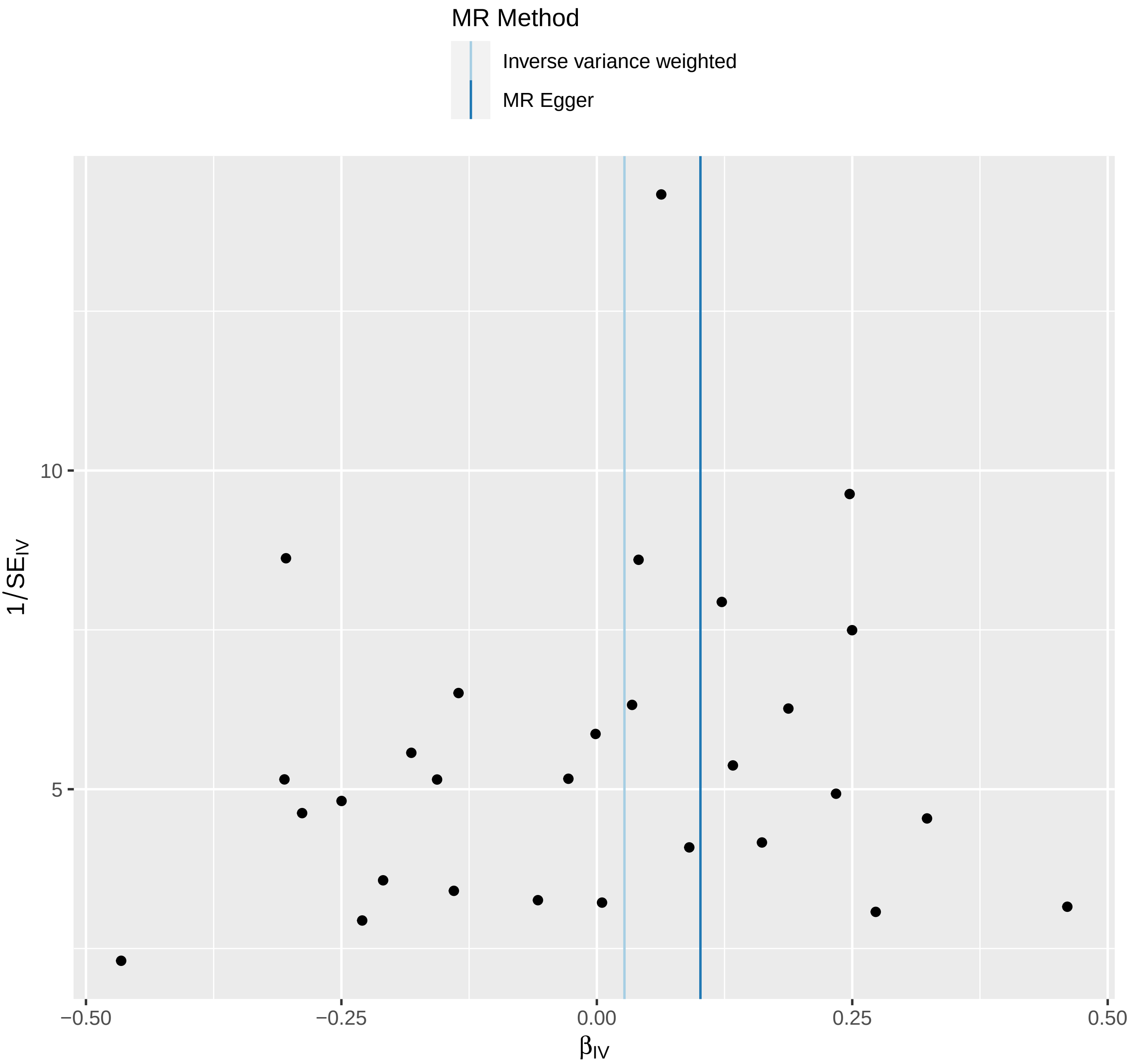

Batch 3−2772 : Mothers against decapentaplegic homolog 4 on Diabetic nephropathy (more control exclusions)
with a threshold of 1e−05

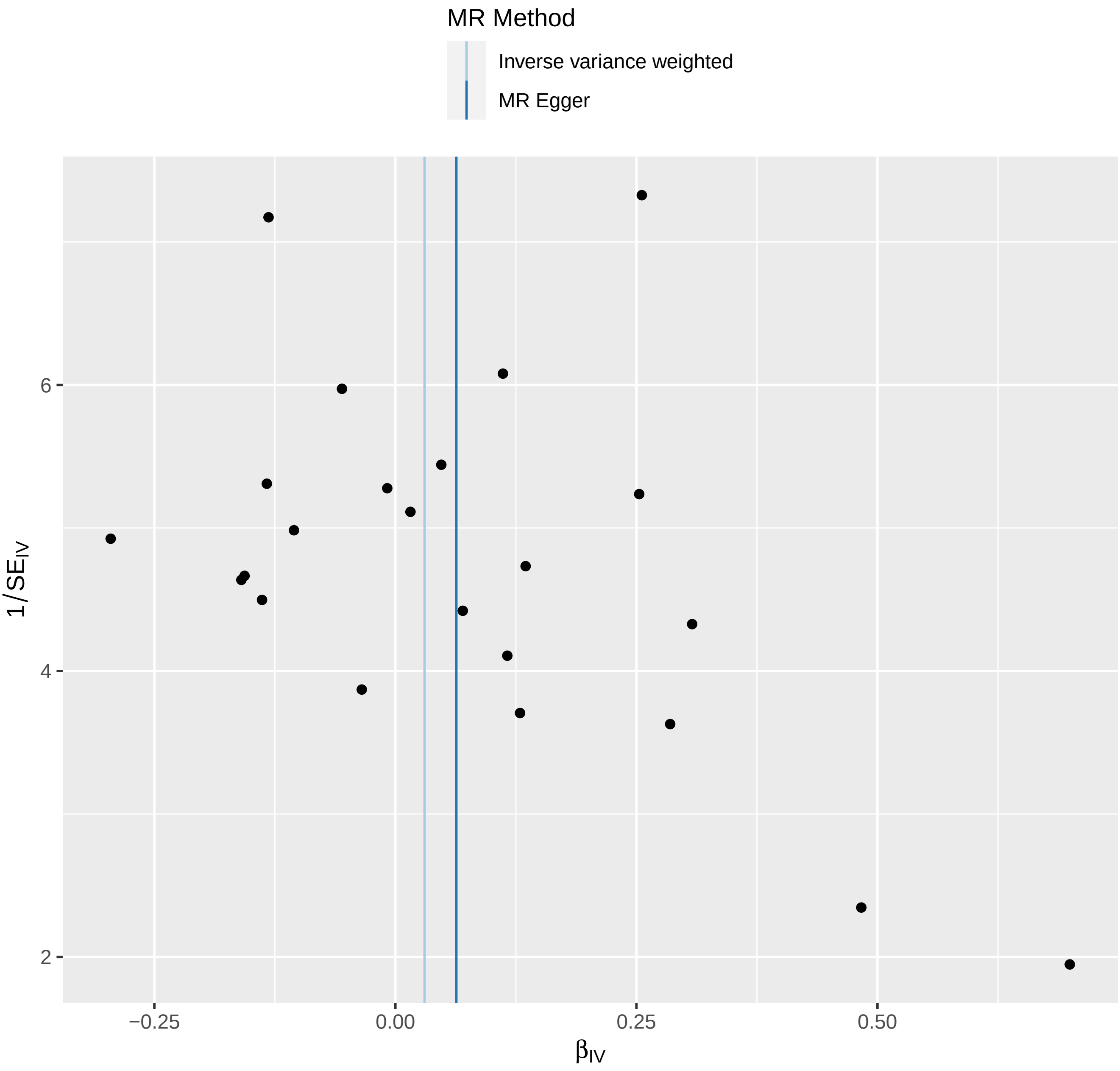

Batch 3−2773 : Stromal membrane−associated protein 1 on Diabetic nephropathy (more control exclusions)
with a threshold of 1e−05

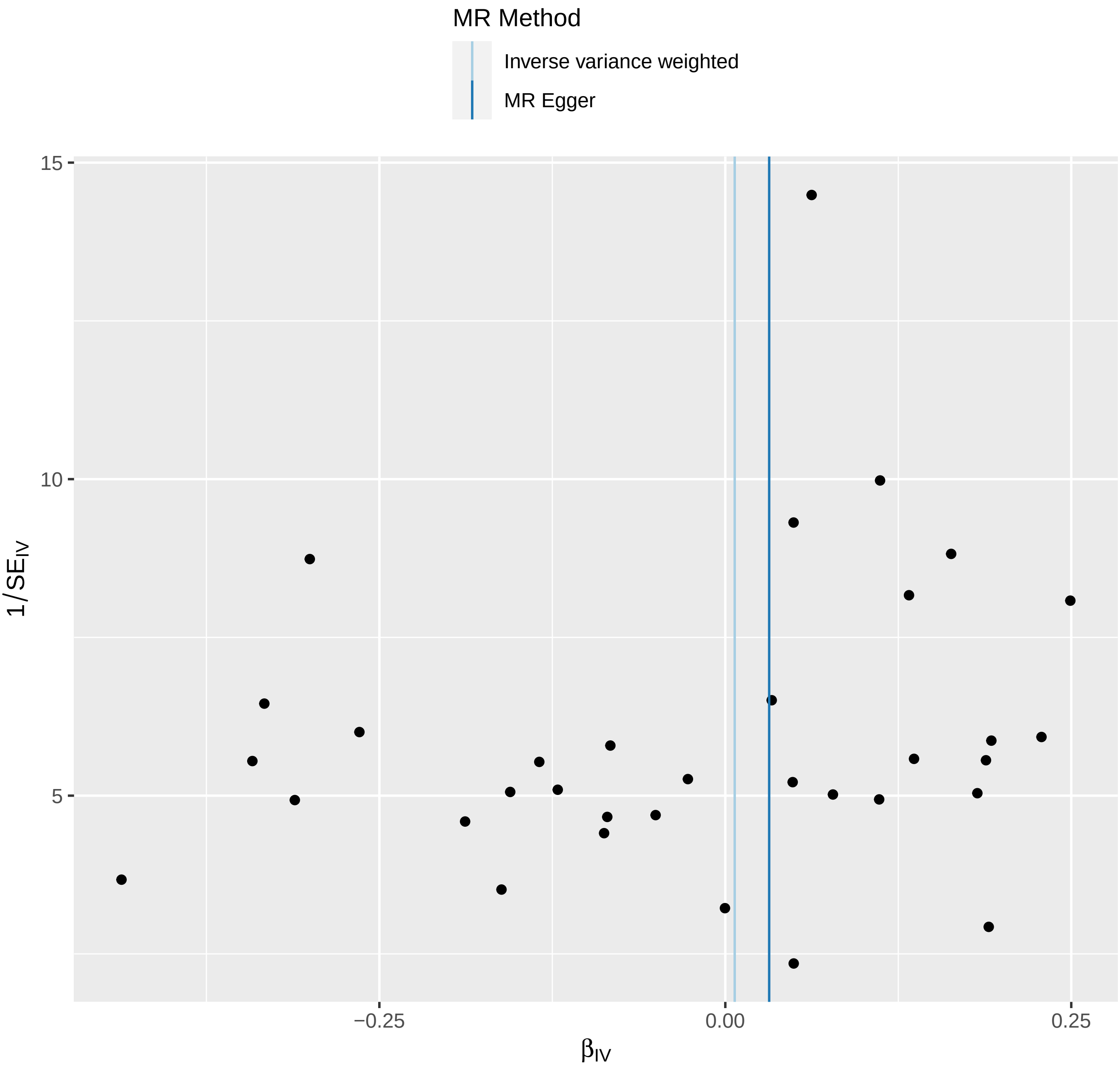

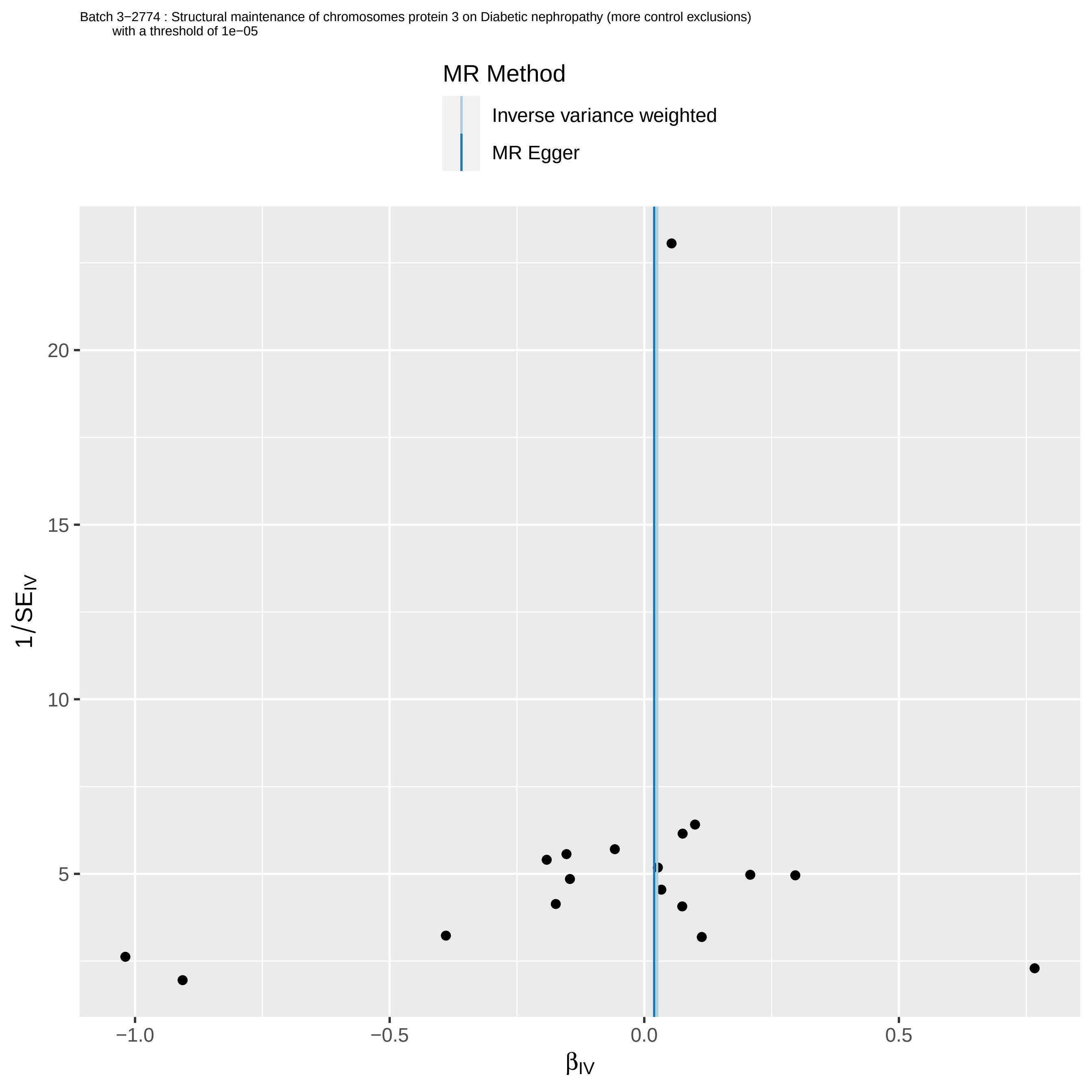
Batch 3−2774 : Structural maintenance of chromosomes protein 3 on Diabetic nephropathy (more control exclusions)
with a threshold of 1e−05
MR Method
Inverse variance weighted
MR Egger
1/SE_IV
20
15
10
5
−1.0
−0.5
0.0
0.5
β_IV

Batch 3−2775 : Single−pass membrane and coiled−coil domain−containing protein 2 on Diabetic nephropathy (more control exclusions) with a threshold of 1e−05

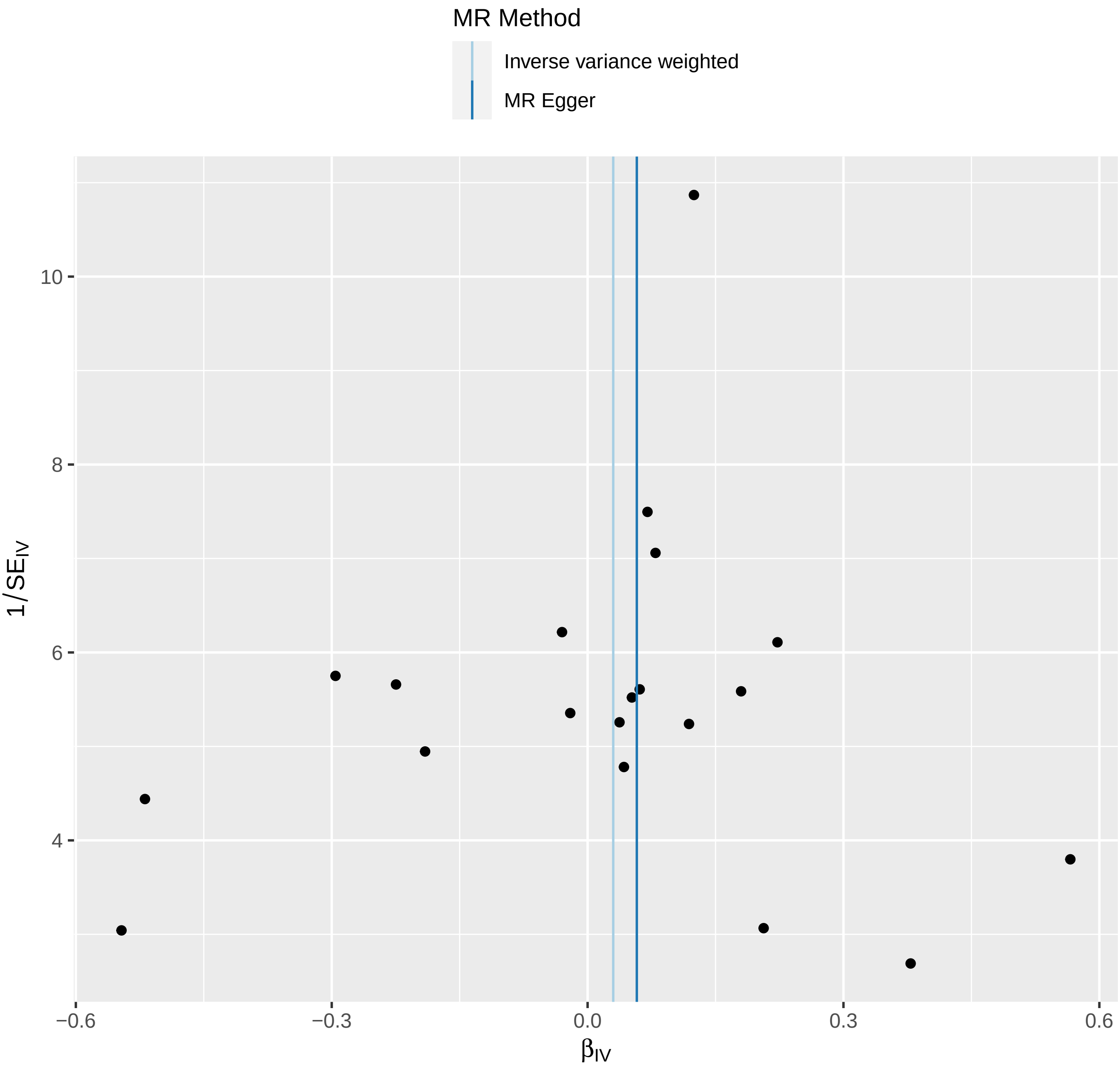

Batch 3−2776 : Essential MCU regulator, mitochondrial on Diabetic nephropathy (more control exclusions) with a threshold of 1e−05

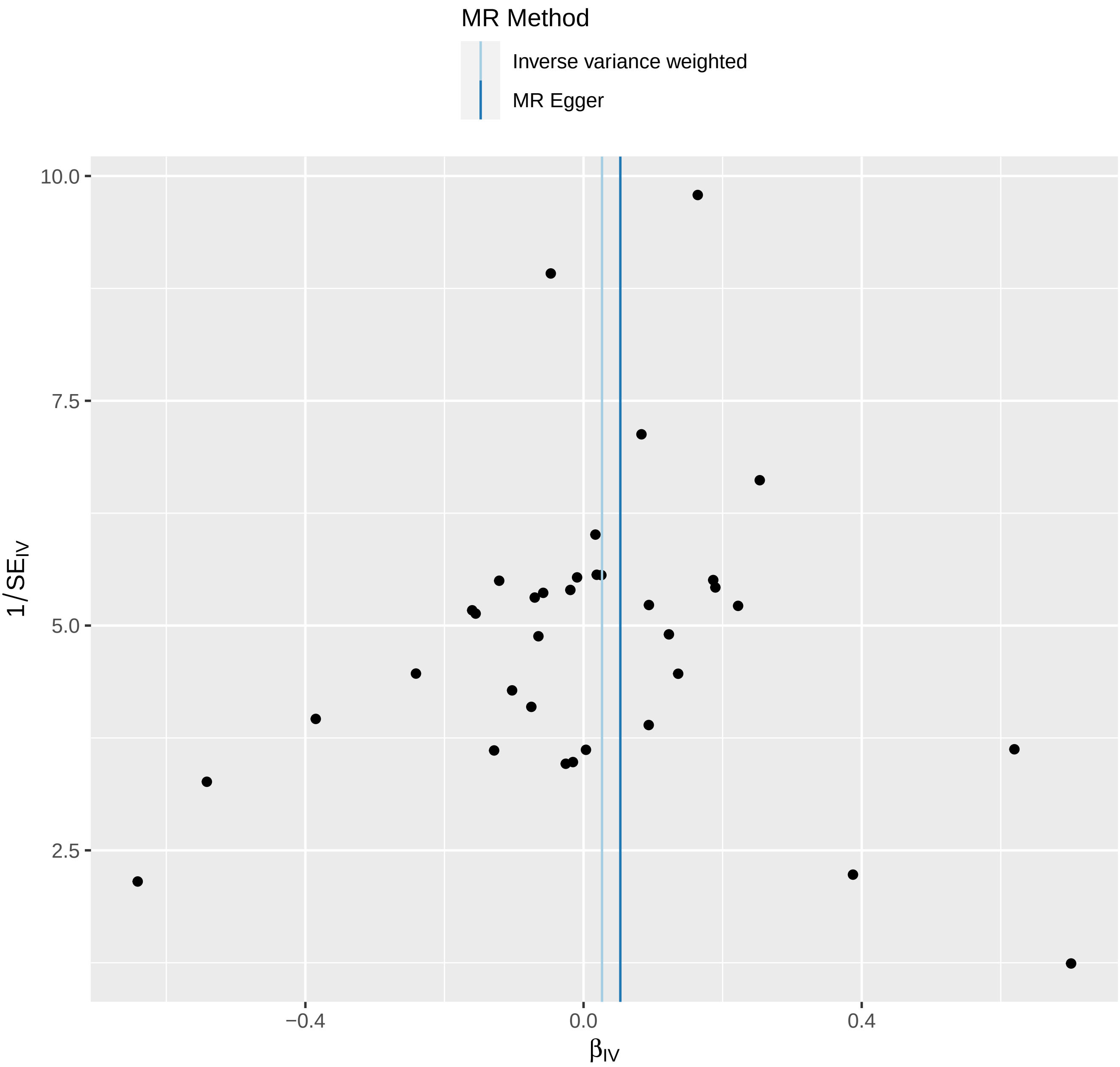

Batch 3−2777 : Small integral membrane protein 10 on Diabetic nephropathy (more control exclusions)
with a threshold of 1e−05

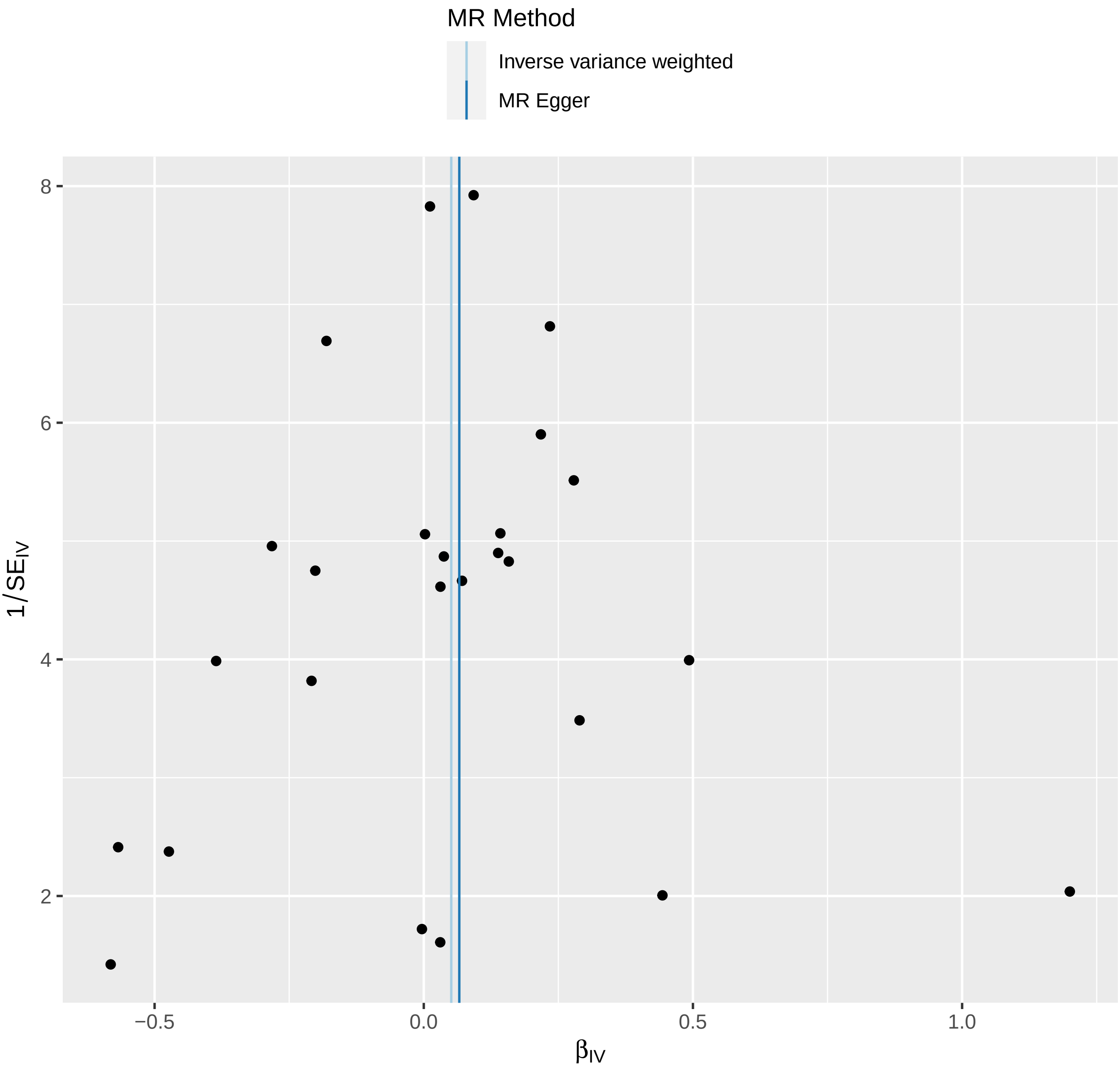

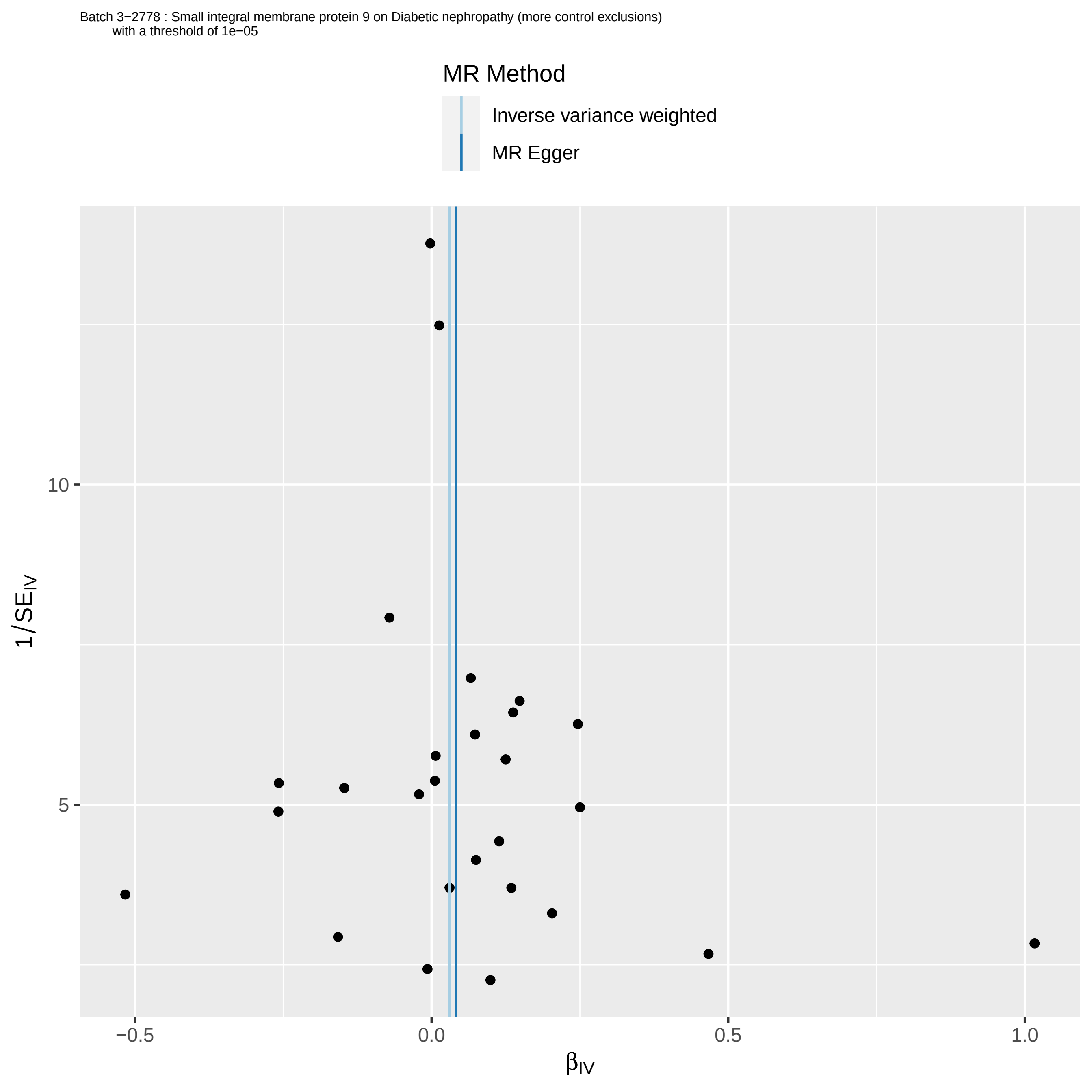
Batch 3−2778 : Small integral membrane protein 9 on Diabetic nephropathy (more control exclusions)
with a threshold of 1e−05
MR Method
Inverse variance weighted
MR Egger
10
5
1/SE_IV
−0.5
0.0
0.5
1.0
β_IV

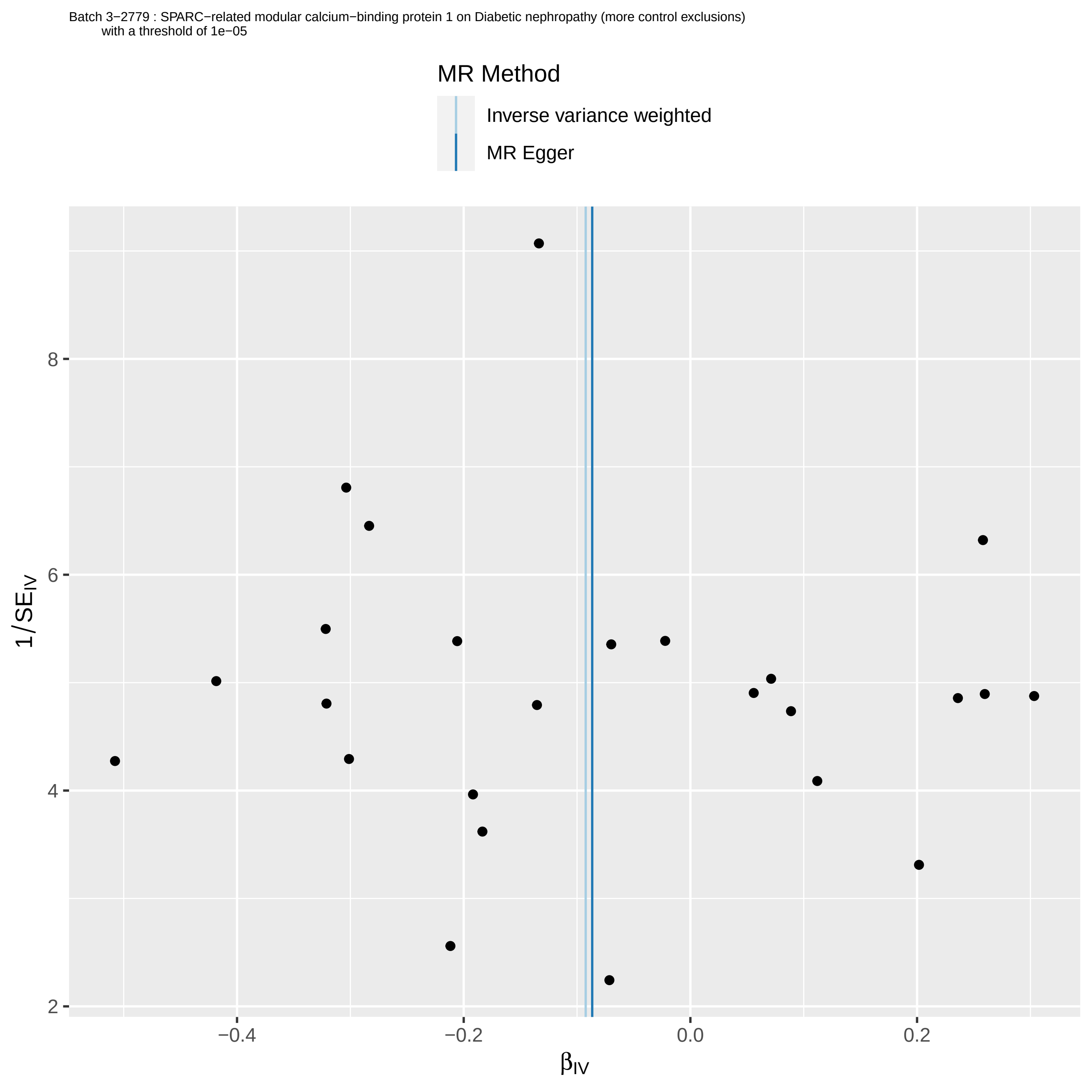

Batch 3−2779 : SPARC−related modular calcium−binding protein 1 on Diabetic nephropathy (more control exclusions)
with a threshold of 1e−05
MR Method
Inverse variance weighted
MR Egger
1/SE_IV
8
6
4
2
−0.4
−0.2
0.0
0.2
β_IV

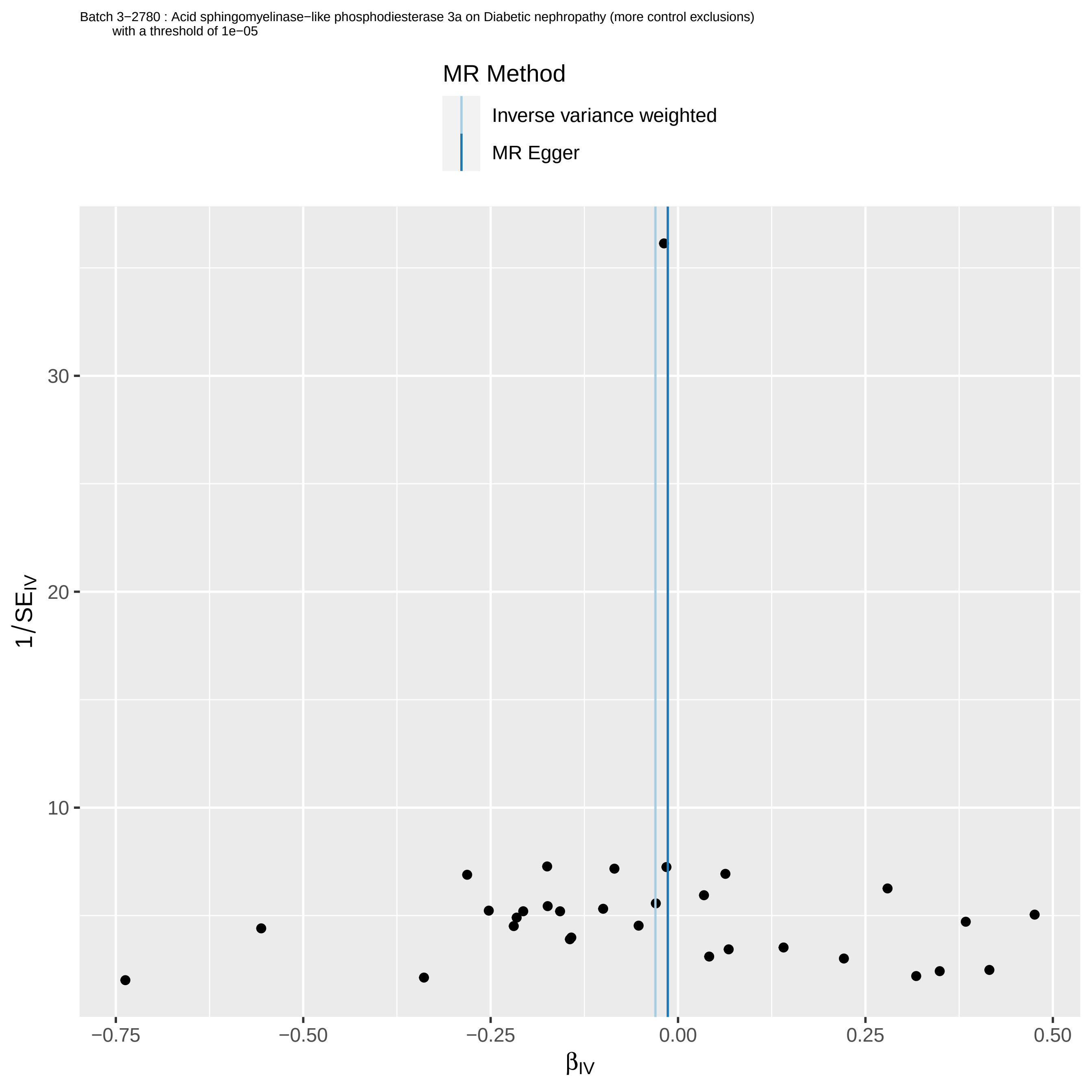

Batch 3−2780 : Acid sphingomyelinase−like phosphodiesterase 3a on Diabetic nephropathy (more control exclusions)
with a threshold of 1e−05
MR Method
Inverse variance weighted
MR Egger
30
20
10
1/SE$_{IV}$
−0.75
−0.50
−0.25
0.00
0.25
0.50
β$_{IV}$

Batch 3−2781 : Acid sphingomyelinase−like phosphodiesterase 3a on Diabetic nephropathy (more control exclusions)
with a threshold of 1e−05

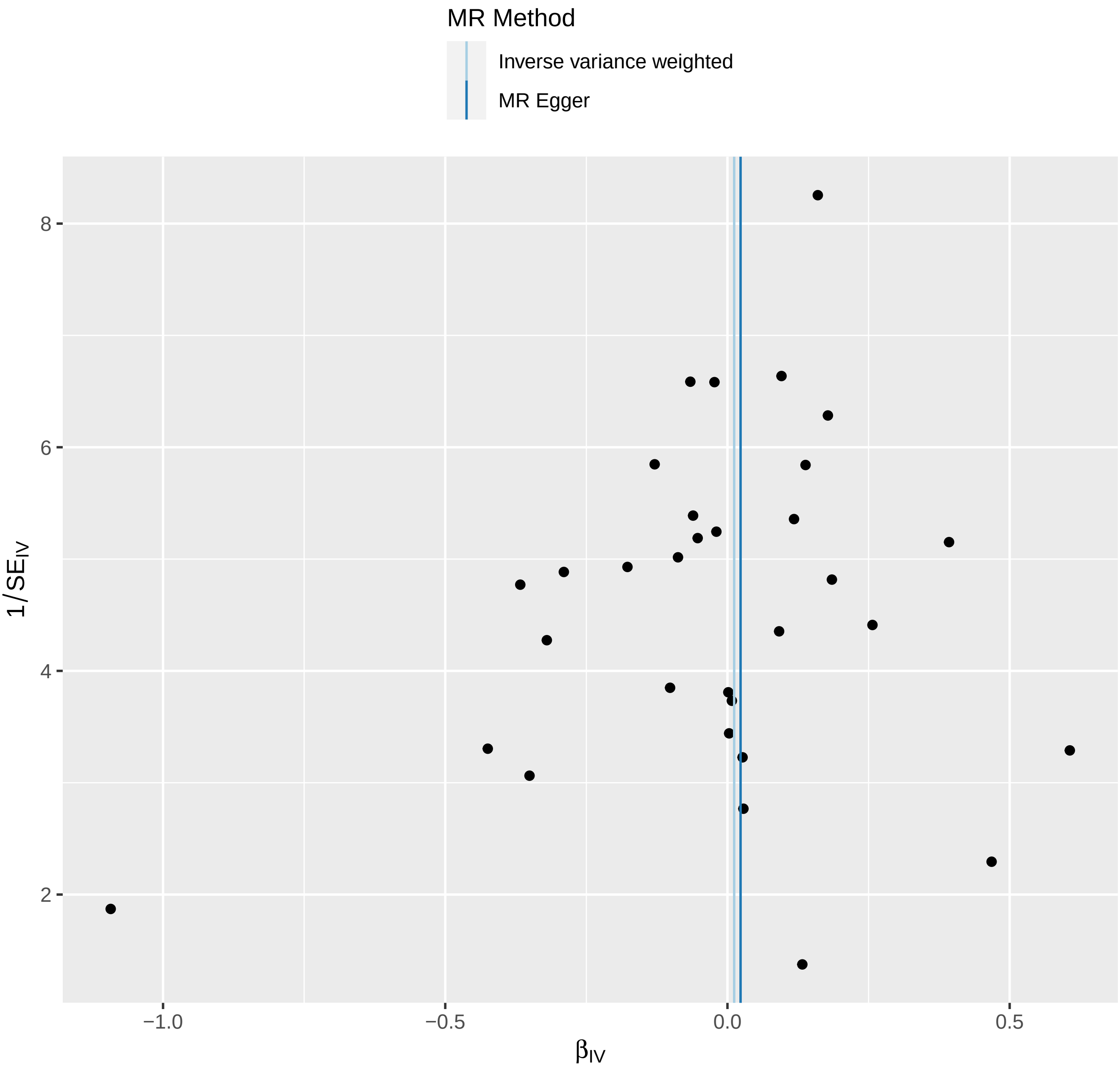

Batch 3−2782 : Submaxillary gland androgen−regulated protein 3A on Diabetic nephropathy (more control exclusions) with a threshold of 1e−05

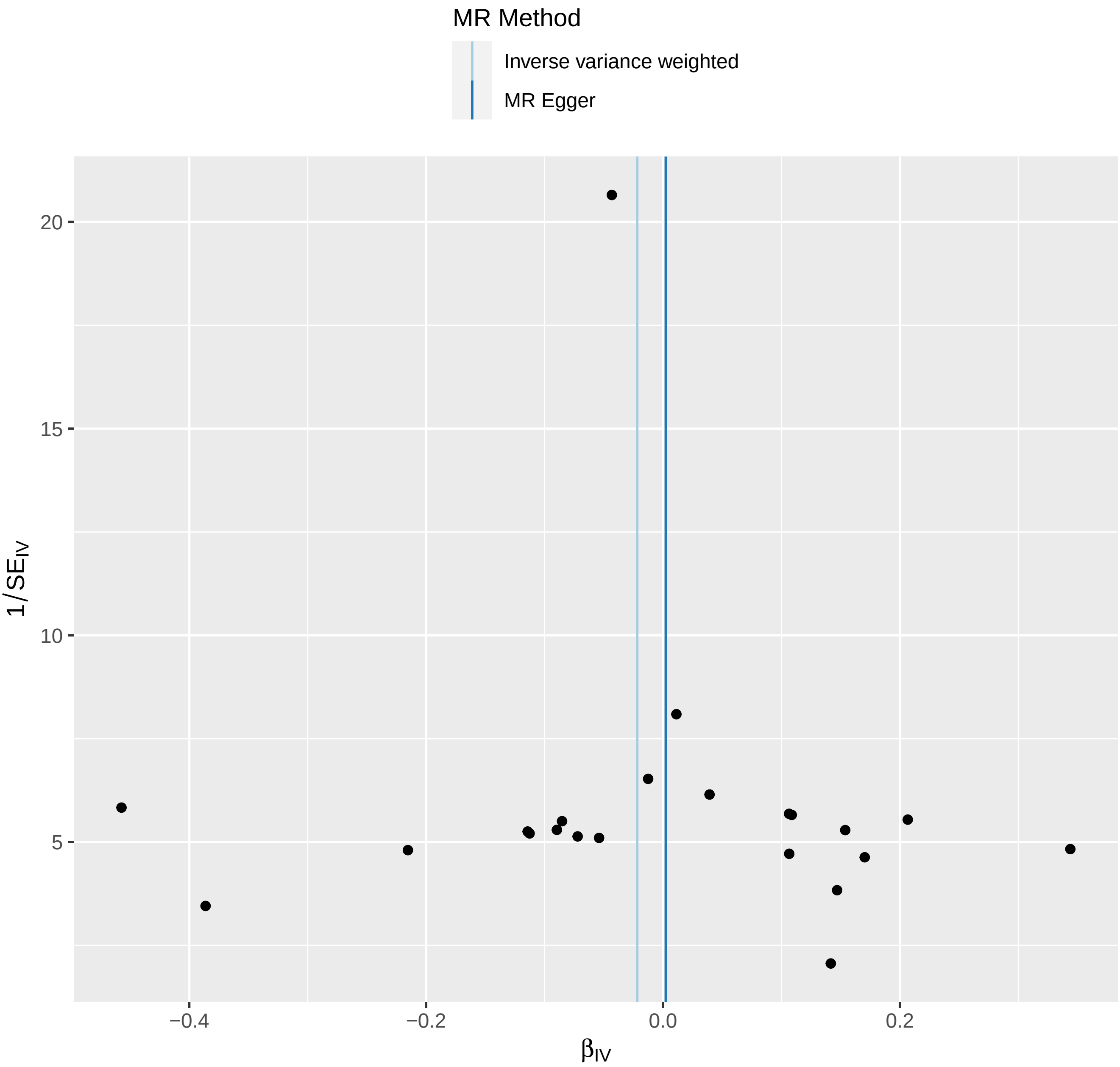

Batch 3−2783 : Submaxillary gland androgen−regulated protein 3B on Diabetic nephropathy (more control exclusions)
with a threshold of 1e−05

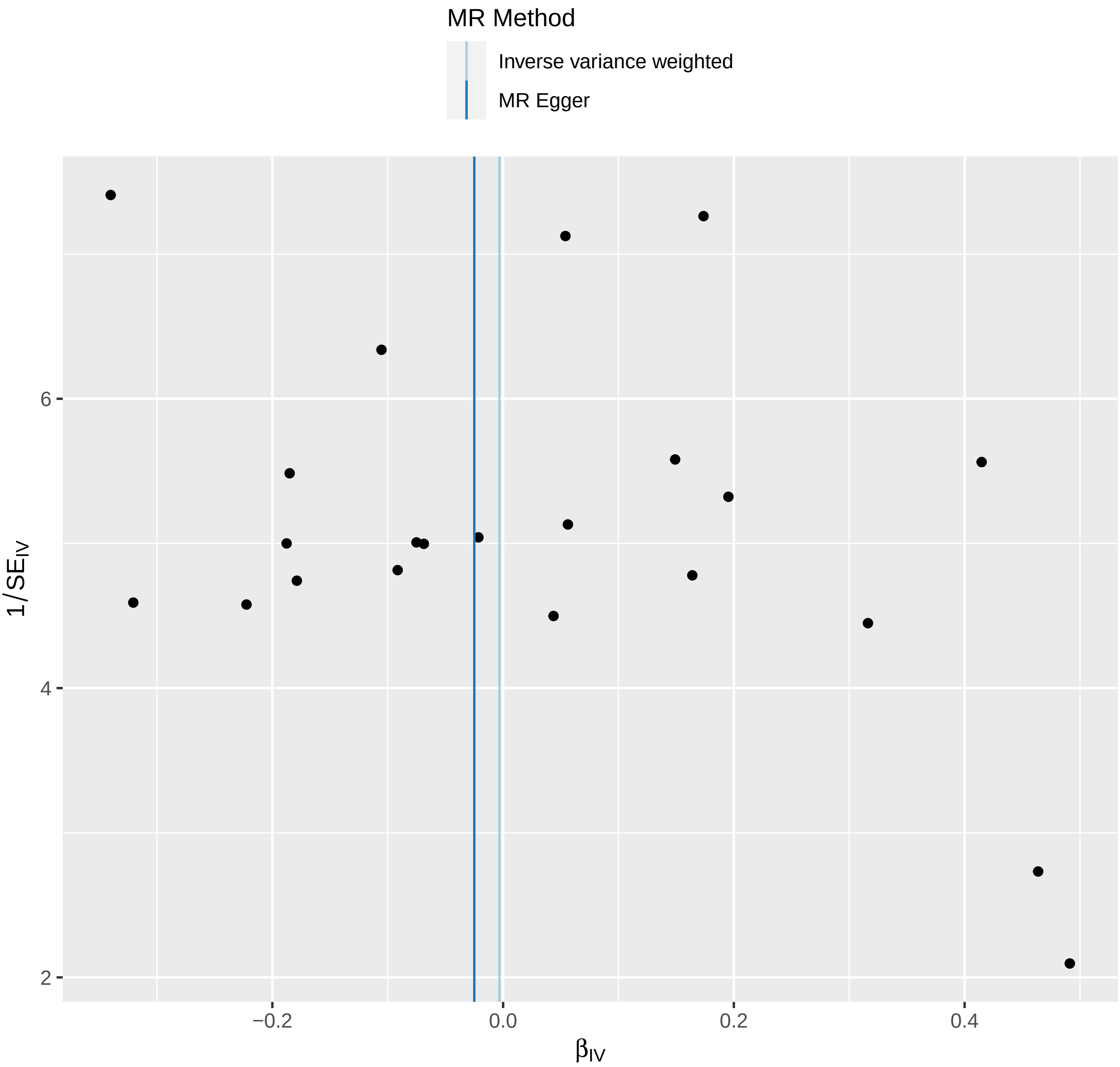

Batch 3−2784 : N−lysine methyltransferase SMYD2 on Diabetic nephropathy (more control exclusions) with a threshold of 1e−05

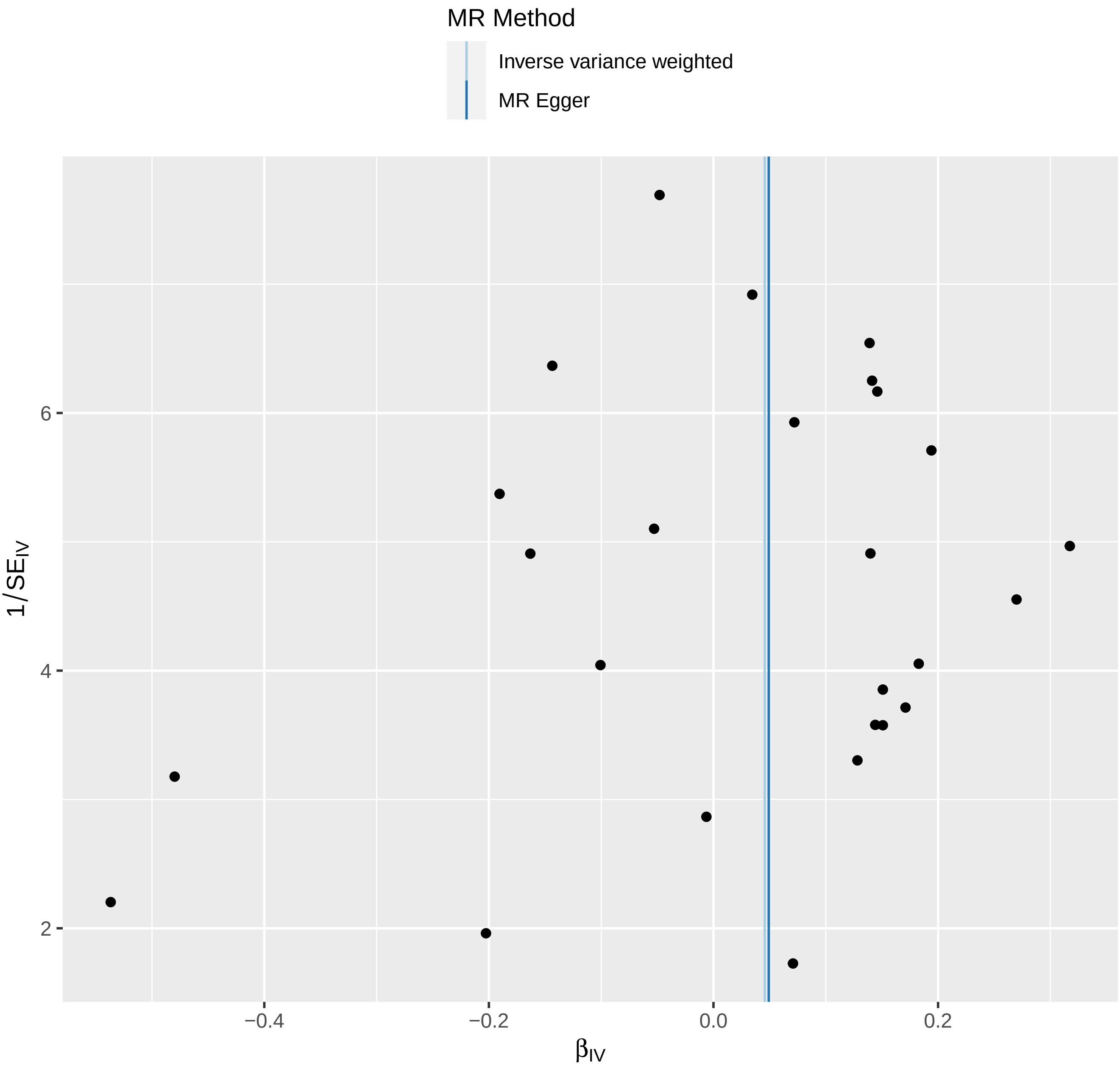

Batch 3−2785 : Zinc finger protein SNAI2 on Diabetic nephropathy (more control exclusions)
with a threshold of 1e−05

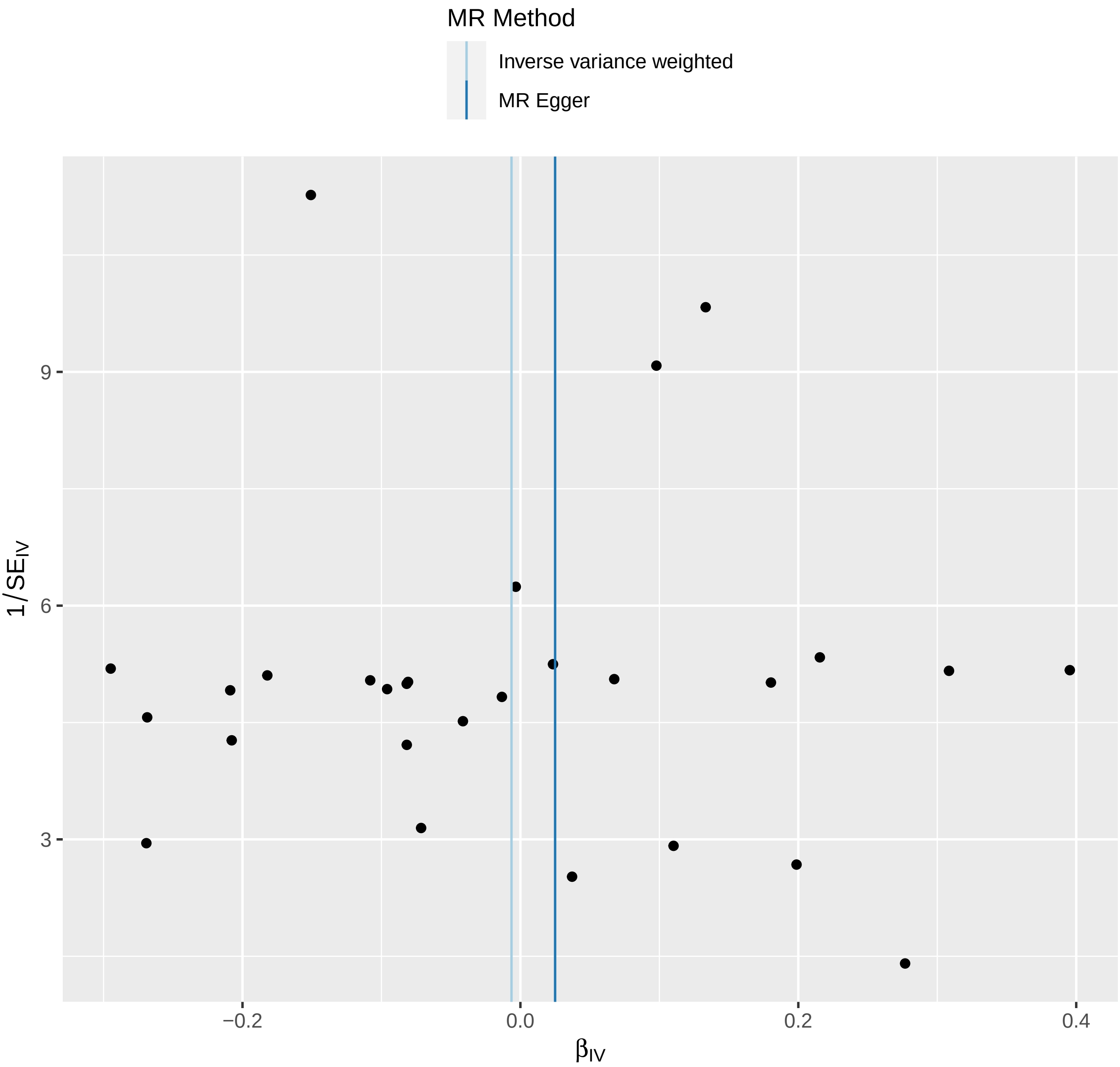

Batch 3−2786 : Synaptosomal−associated protein 25 on Diabetic nephropathy (more control exclusions)
with a threshold of 1e−05

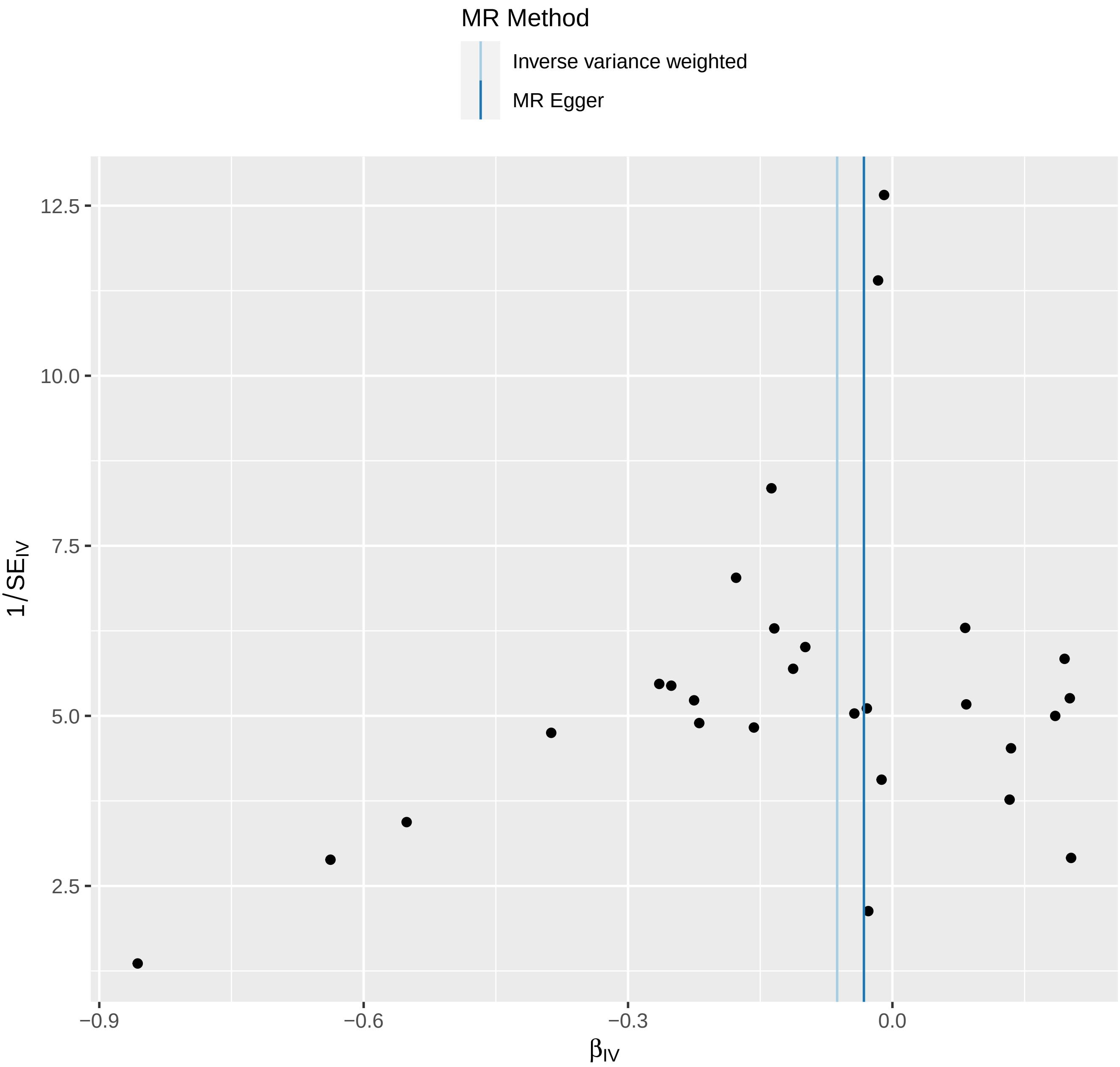

Batch 3−2787 : Synaptosomal−associated protein 29 on Diabetic nephropathy (more control exclusions) with a threshold of 1e−05

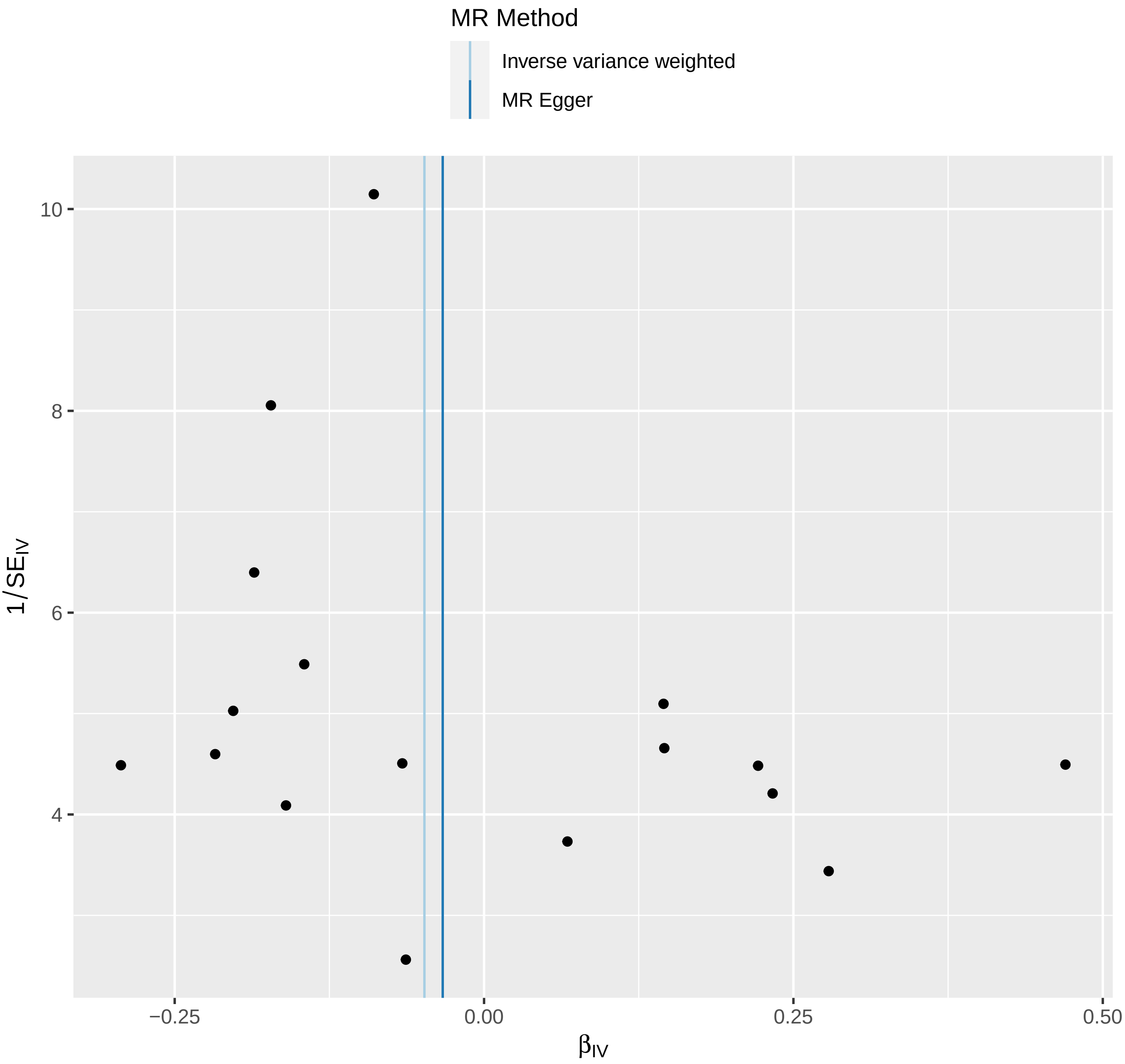

Batch 3−2788 : SNARE−associated protein Snapin on Diabetic nephropathy (more control exclusions)
with a threshold of 1e−05

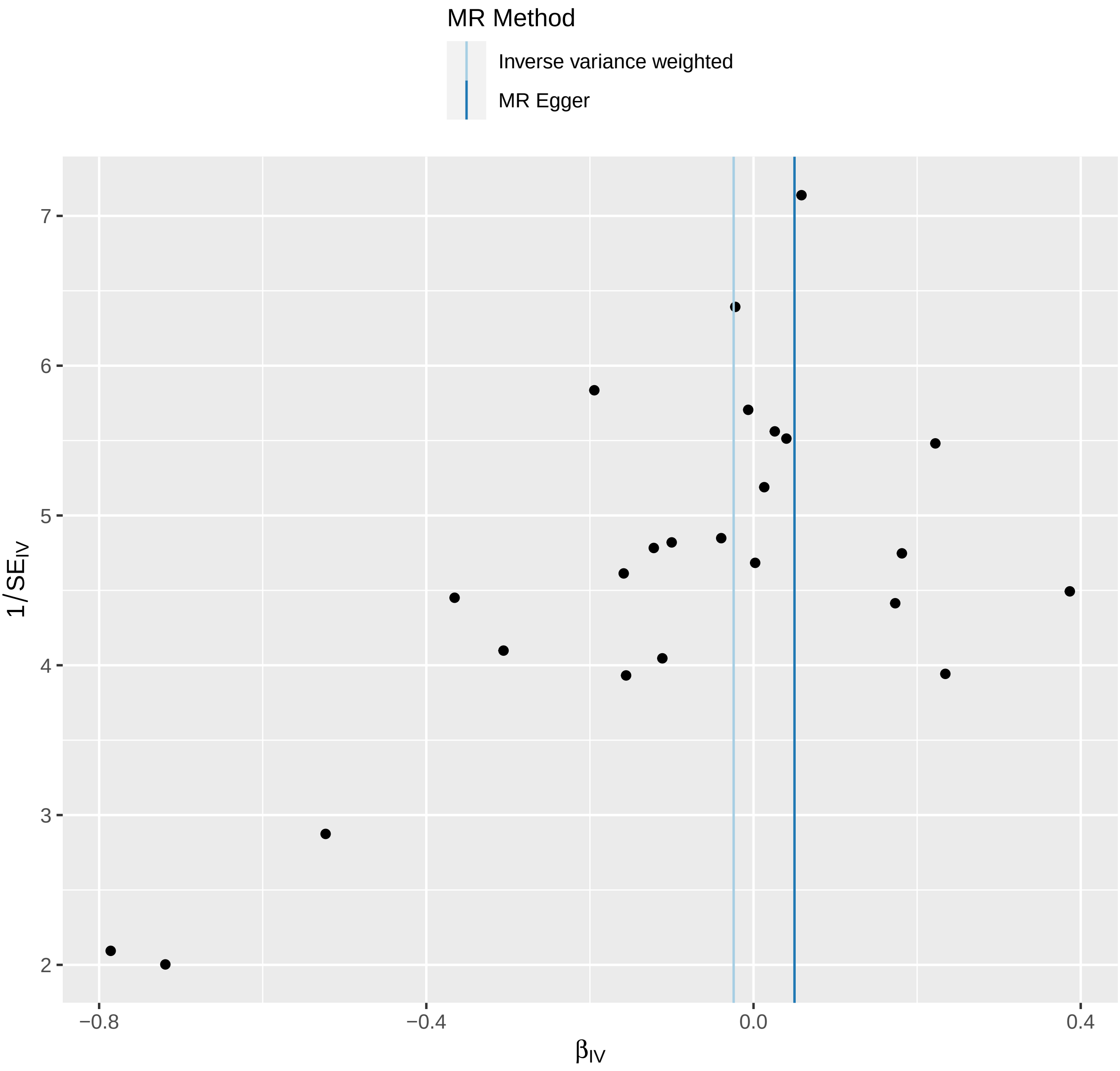

Batch 3−2789 : Alpha−synuclein on Diabetic nephropathy (more control exclusions)
with a threshold of 1e−05

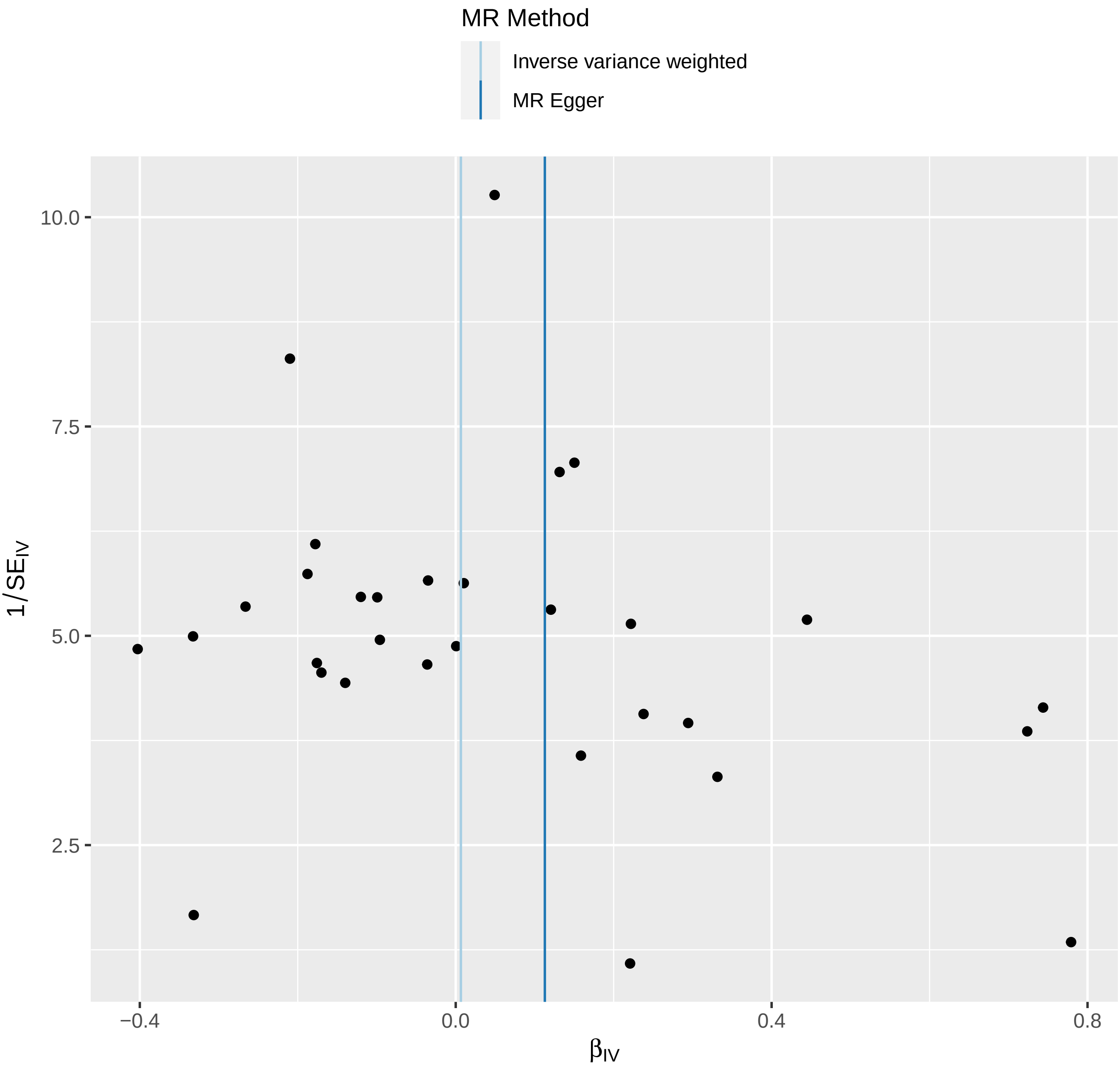

Batch 3−2790 : Alpha−synuclein on Diabetic nephropathy (more control exclusions)
with a threshold of 1e−05

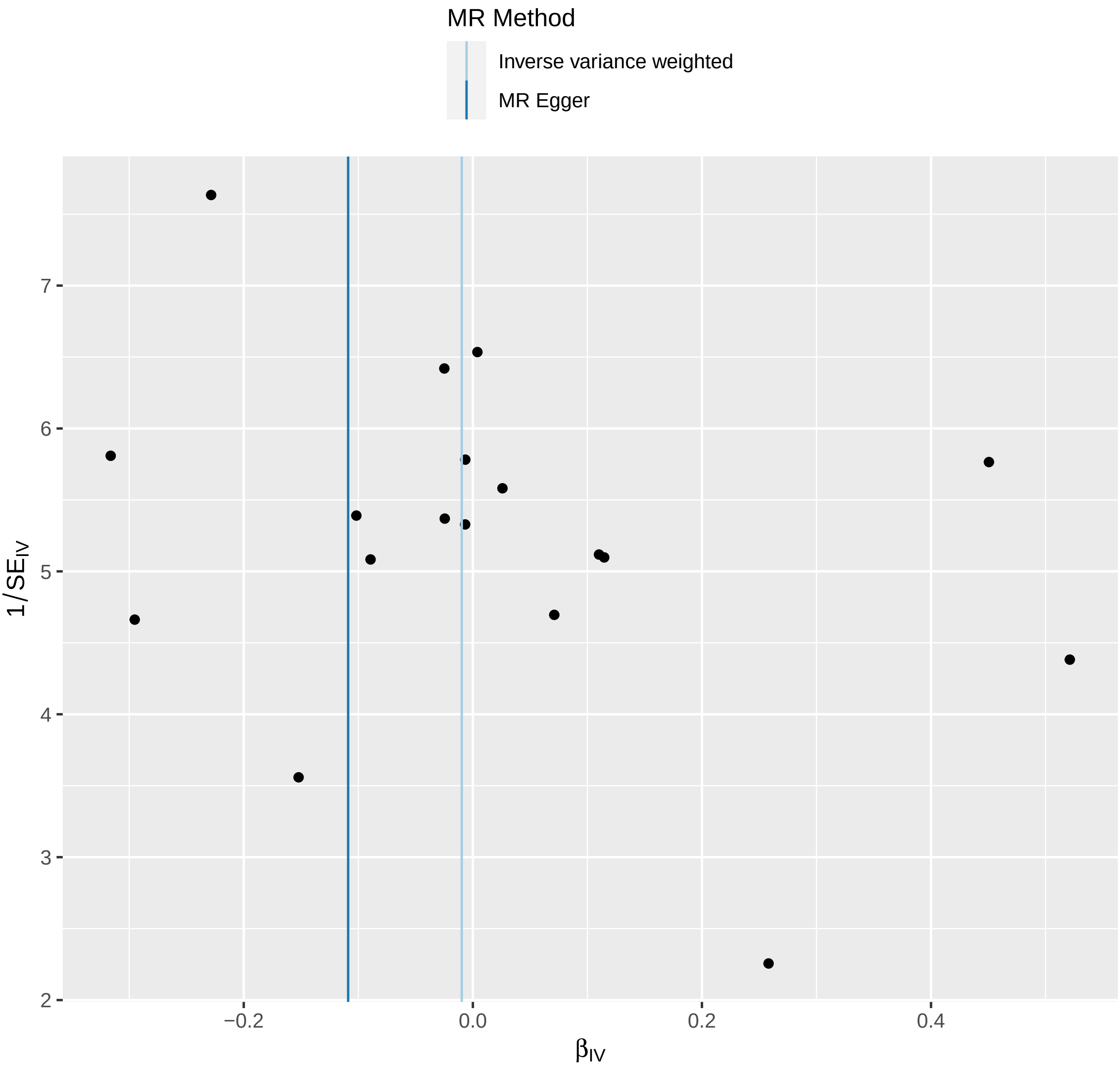

Batch 3−2791 : U1 small nuclear ribonucleoprotein A on Diabetic nephropathy (more control exclusions)
with a threshold of 1e−05

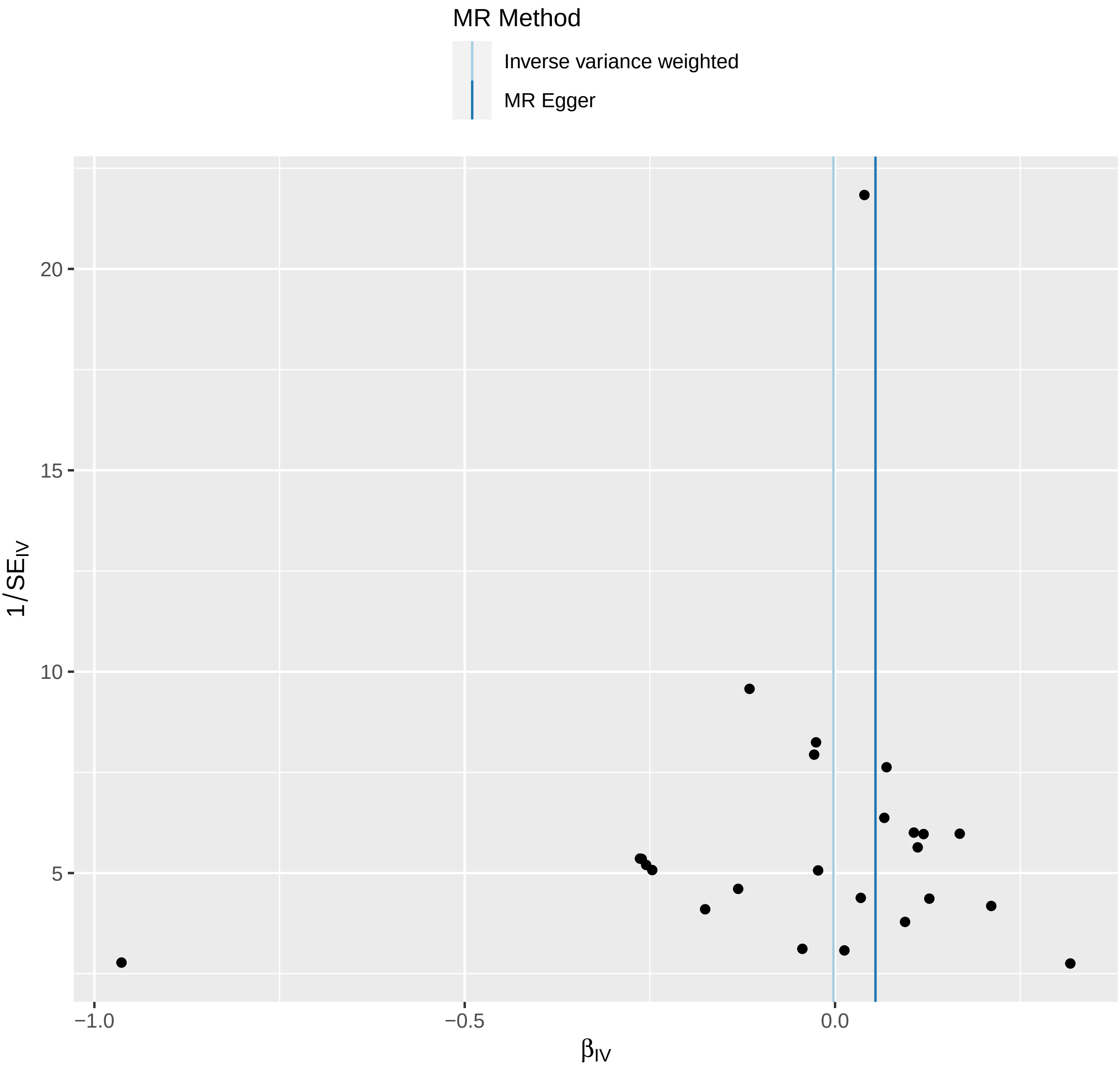

Batch 3−2792 : Sorting nexin−17 on Diabetic nephropathy (more control exclusions)
with a threshold of 1e−05

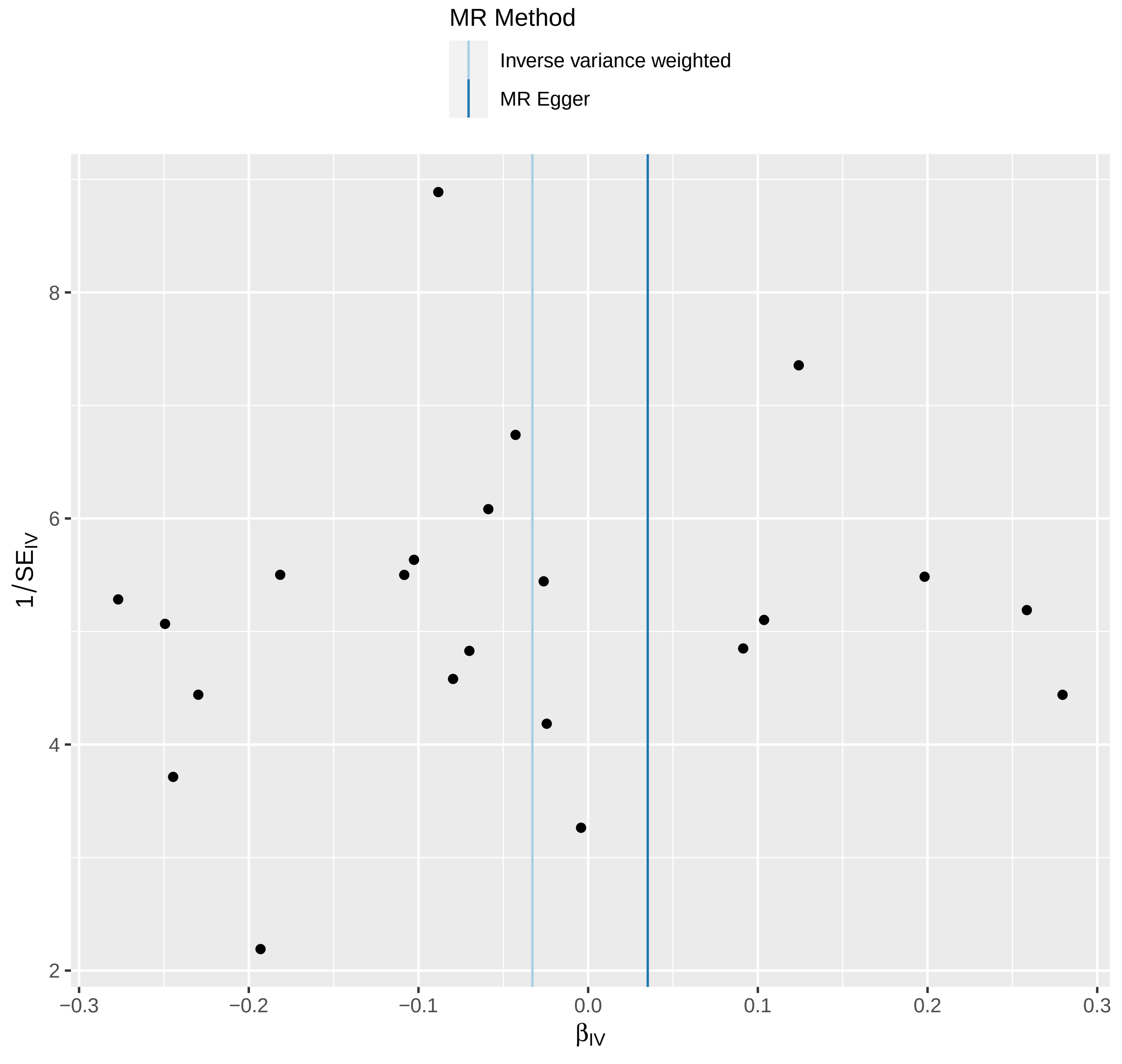

Batch 3−2793 : Sorting nexin−1 on Diabetic nephropathy (more control exclusions)
with a threshold of 1e−05

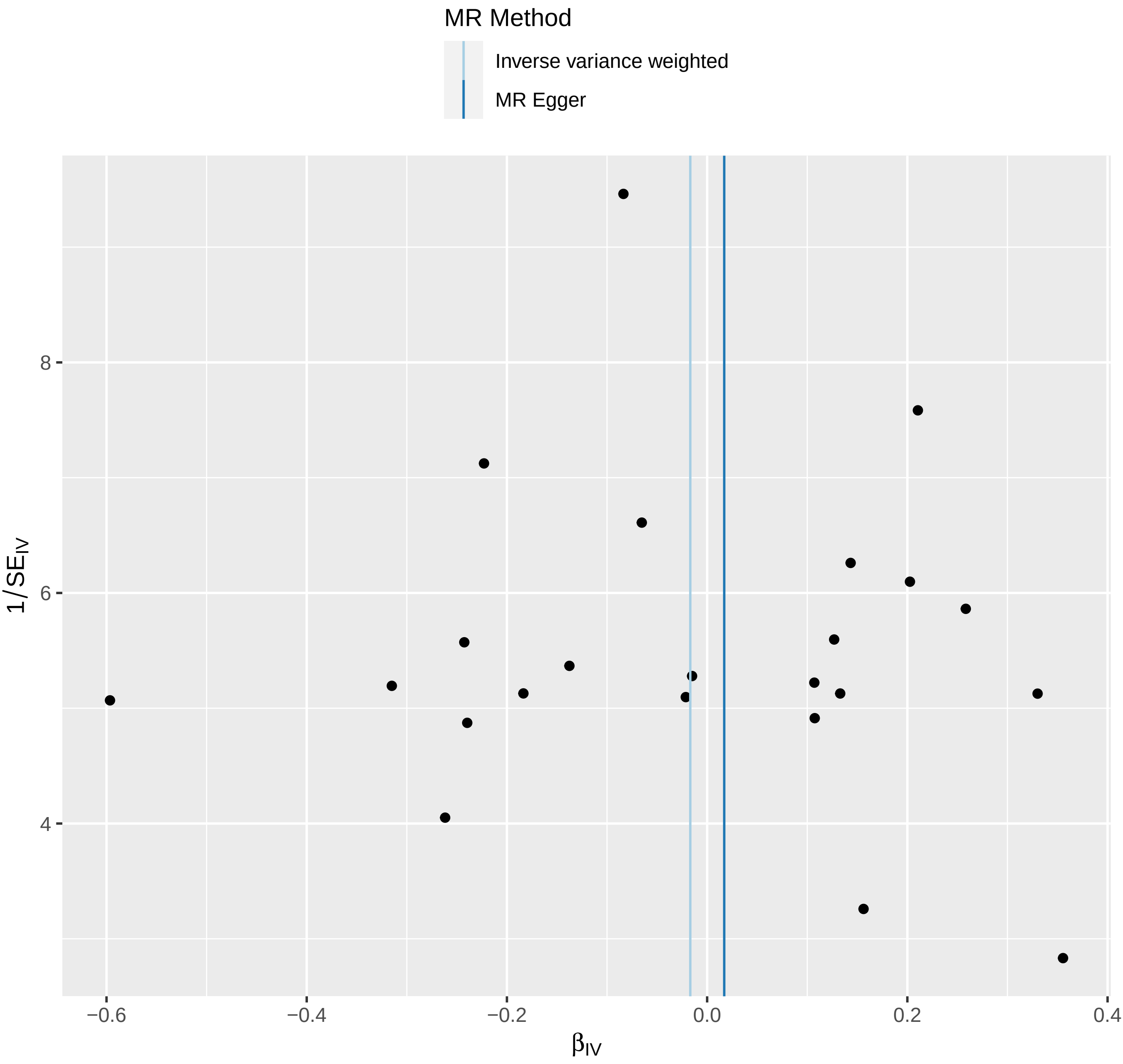

Batch 3−2794 : Sorting nexin−4 on Diabetic nephropathy (more control exclusions)
with a threshold of 1e−05

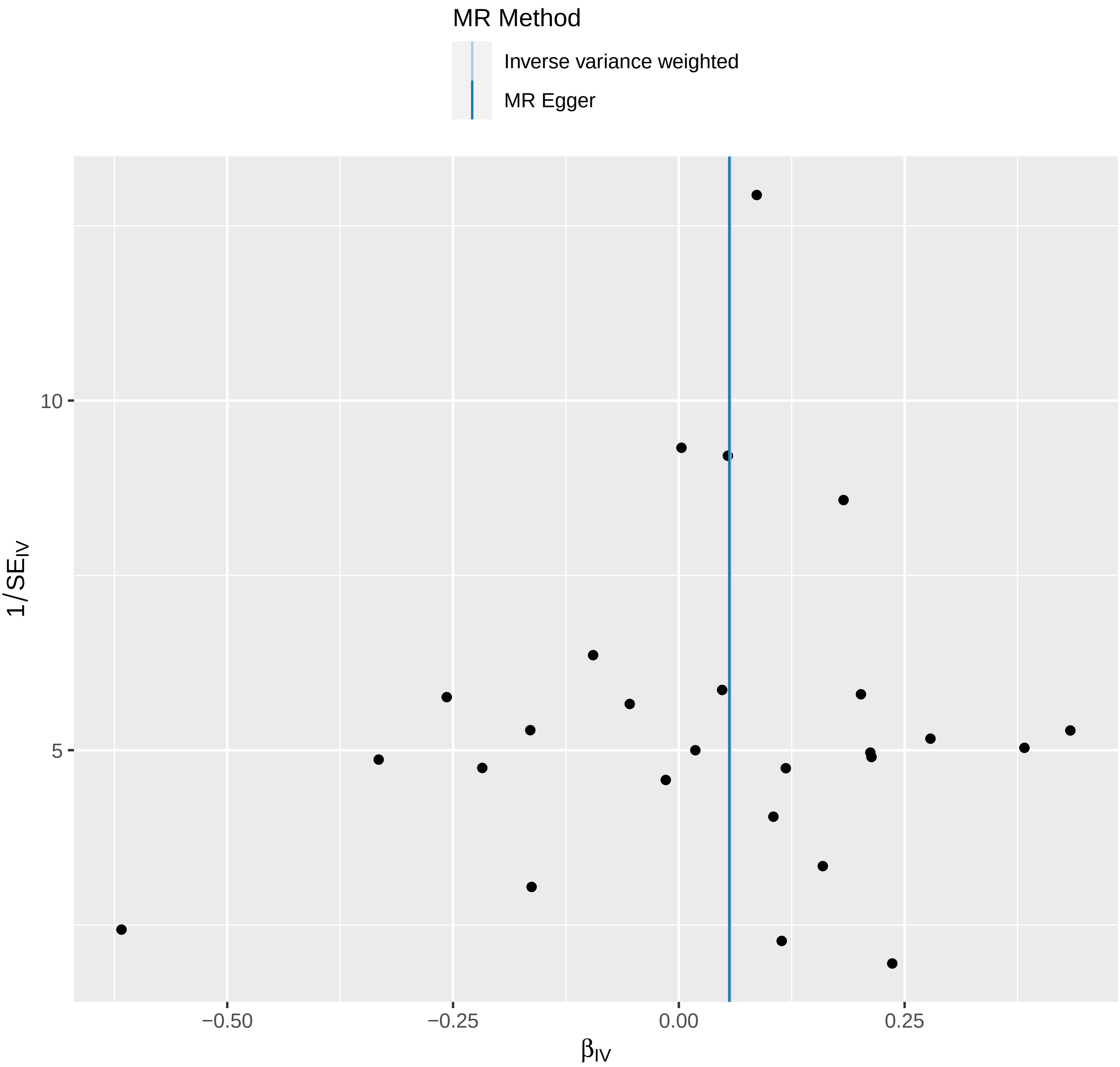

Batch 3−2795 : Sorting nexin−7 on Diabetic nephropathy (more control exclusions)
with a threshold of 1e−05

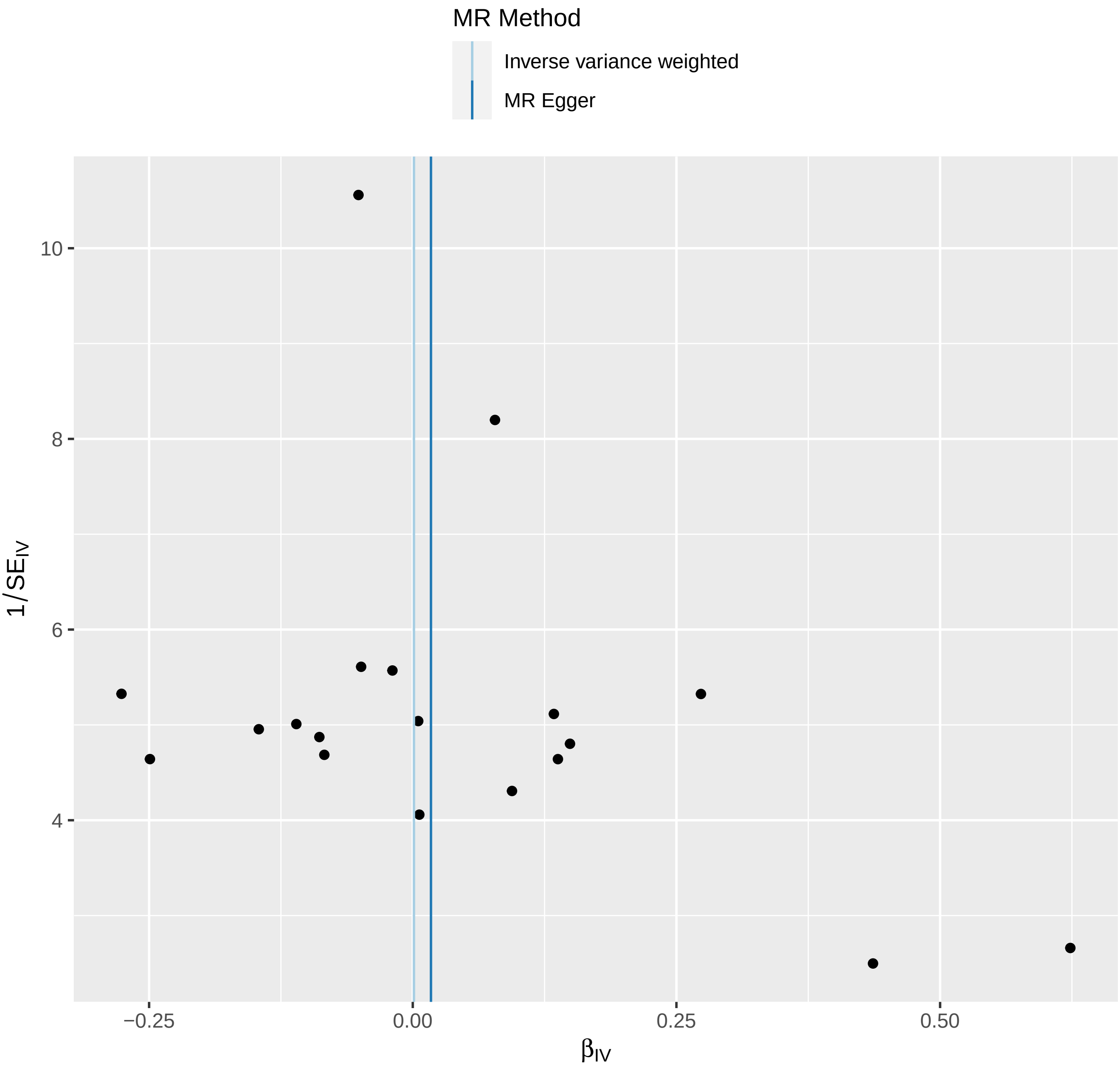

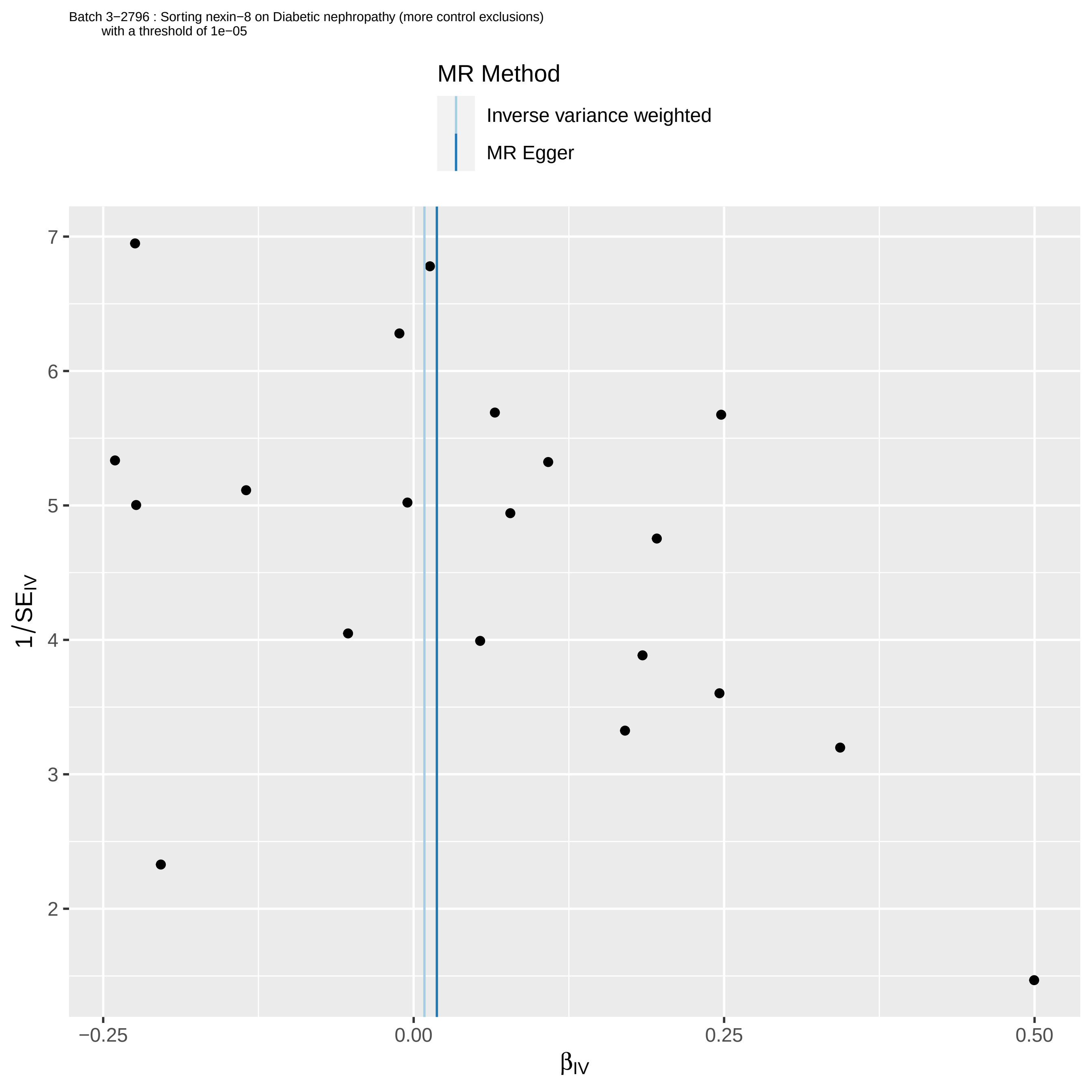

Batch 3−2796 : Sorting nexin−8 on Diabetic nephropathy (more control exclusions)
with a threshold of 1e−05
MR Method
Inverse variance weighted
MR Egger
$1/SE_{IV}$
β_{IV}
7
6
5
4
3
2
−0.25
0.00
0.25
0.50

Batch 3−2797 : Suppressor of cytokine signaling 3 on Diabetic nephropathy (more control exclusions)
with a threshold of 1e−05

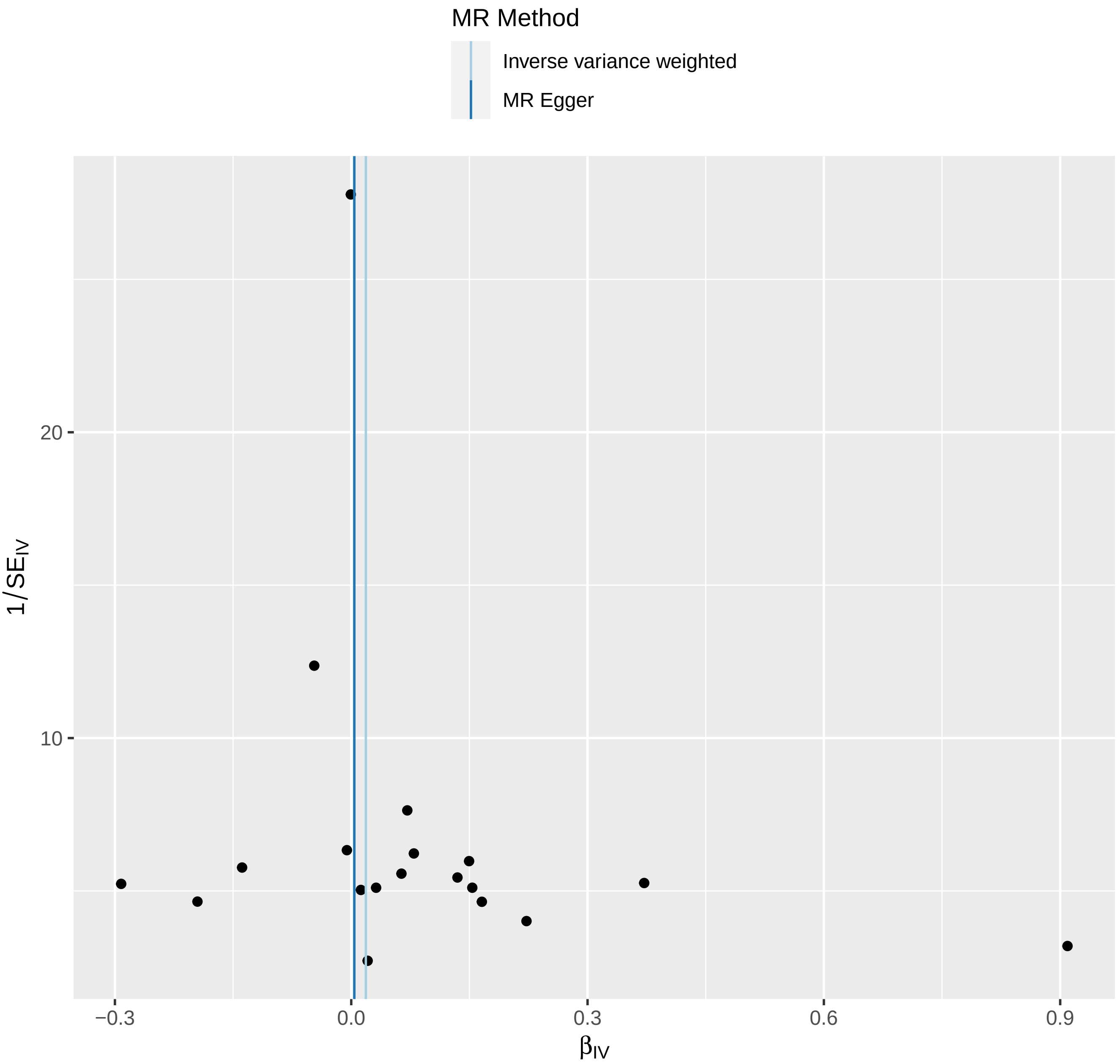

Batch 3−2798 : Suppressor of cytokine signaling 7 on Diabetic nephropathy (more control exclusions)
with a threshold of 1e−05

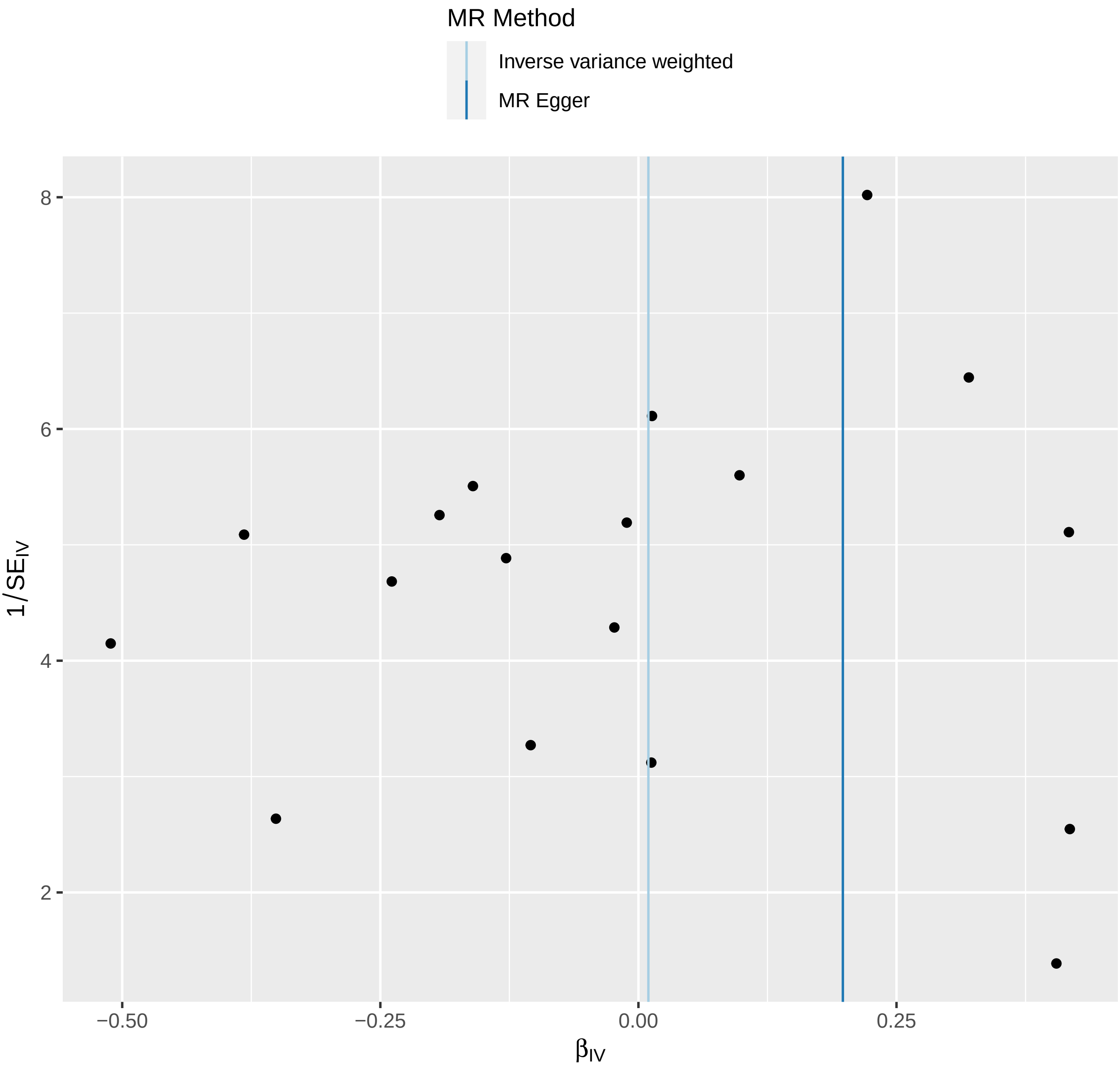

Batch 3−2799 : Superoxide dismutase [Mn], mitochondrial on Diabetic nephropathy (more control exclusions)
with a threshold of 1e−05

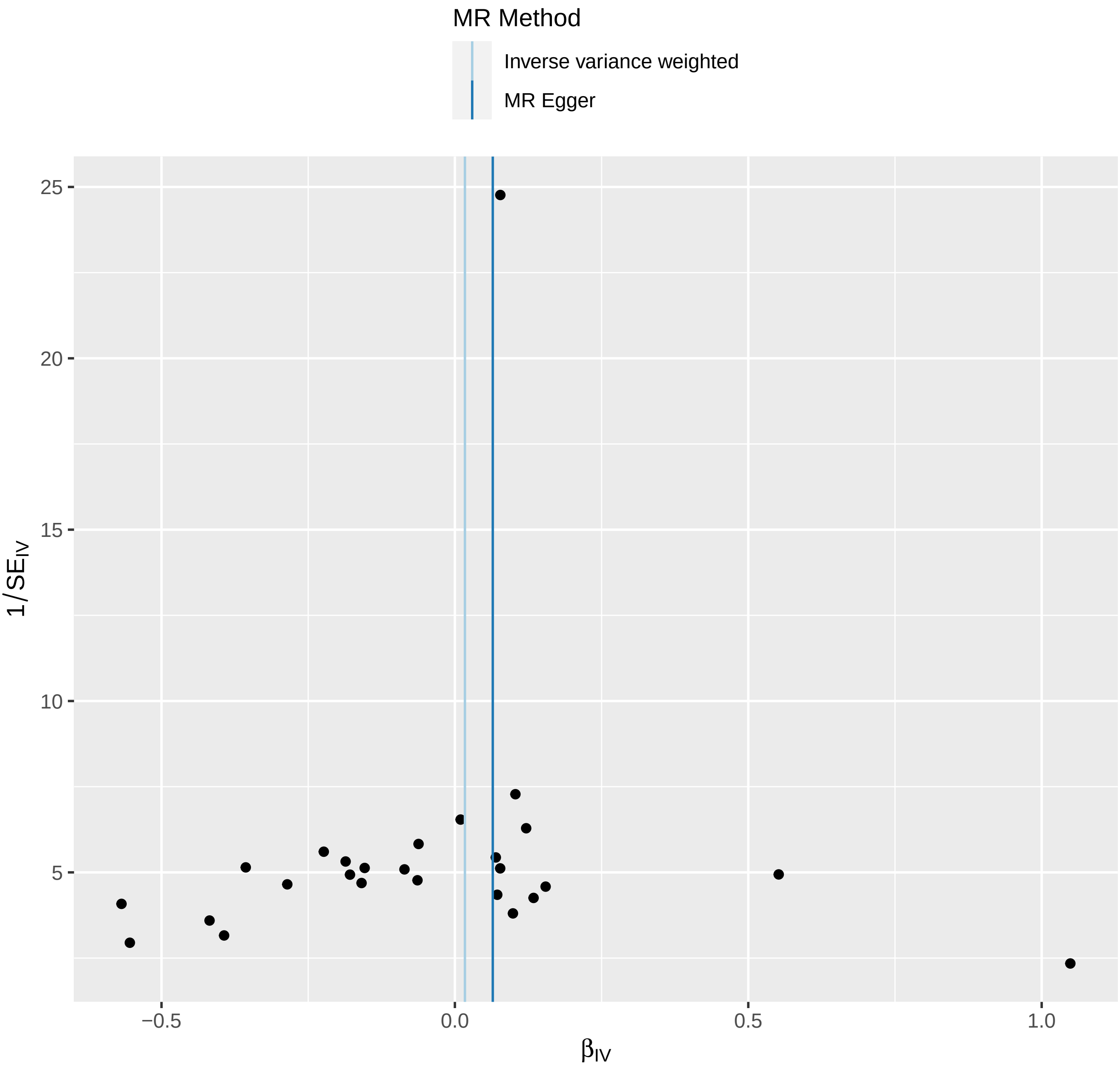

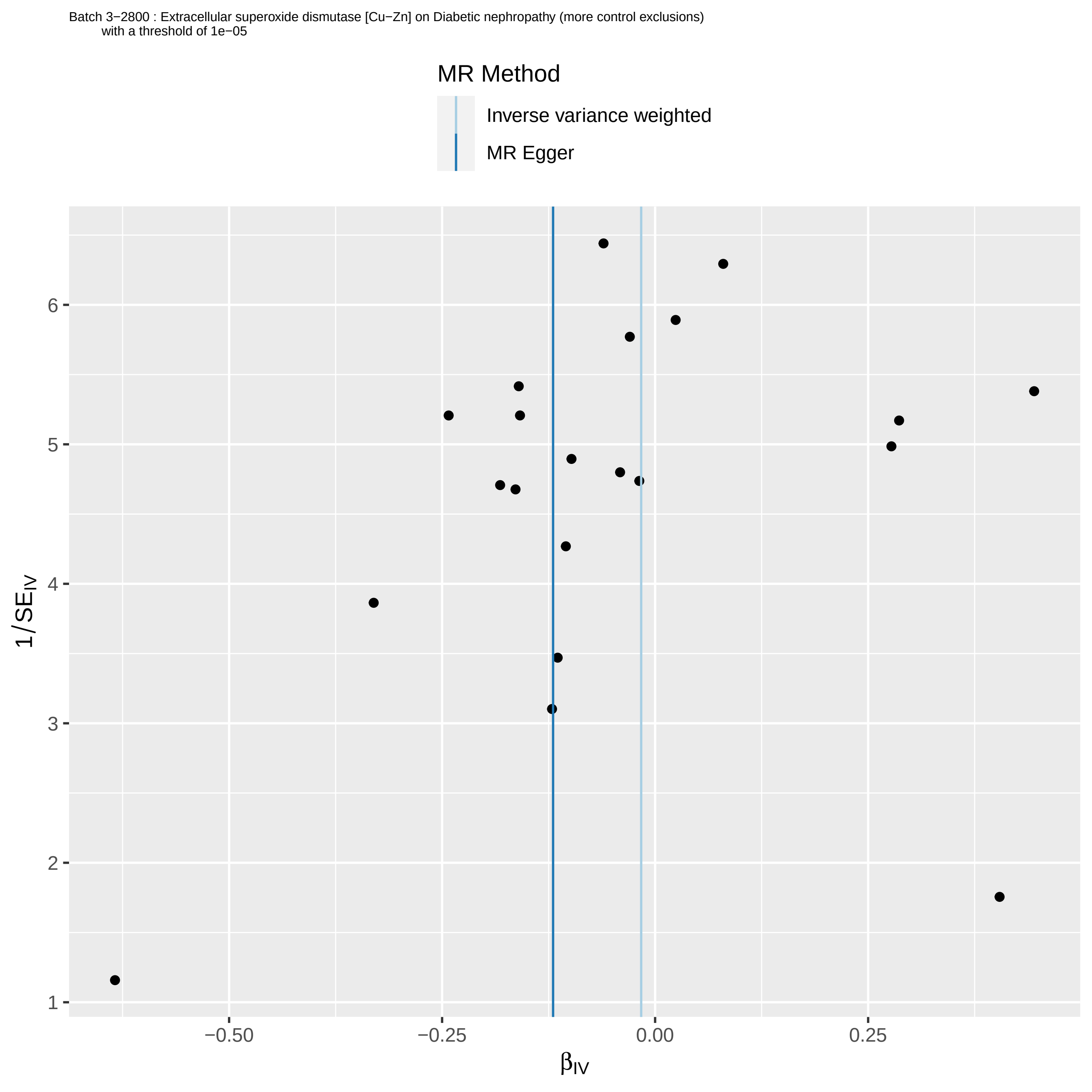
Batch 3−2800 : Extracellular superoxide dismutase [Cu−Zn] on Diabetic nephropathy (more control exclusions)
with a threshold of 1e−05
MR Method
Inverse variance weighted
MR Egger
1/SE_IV
β_IV
−0.50
−0.25
0.00
0.25
1
2
3
4
5
6

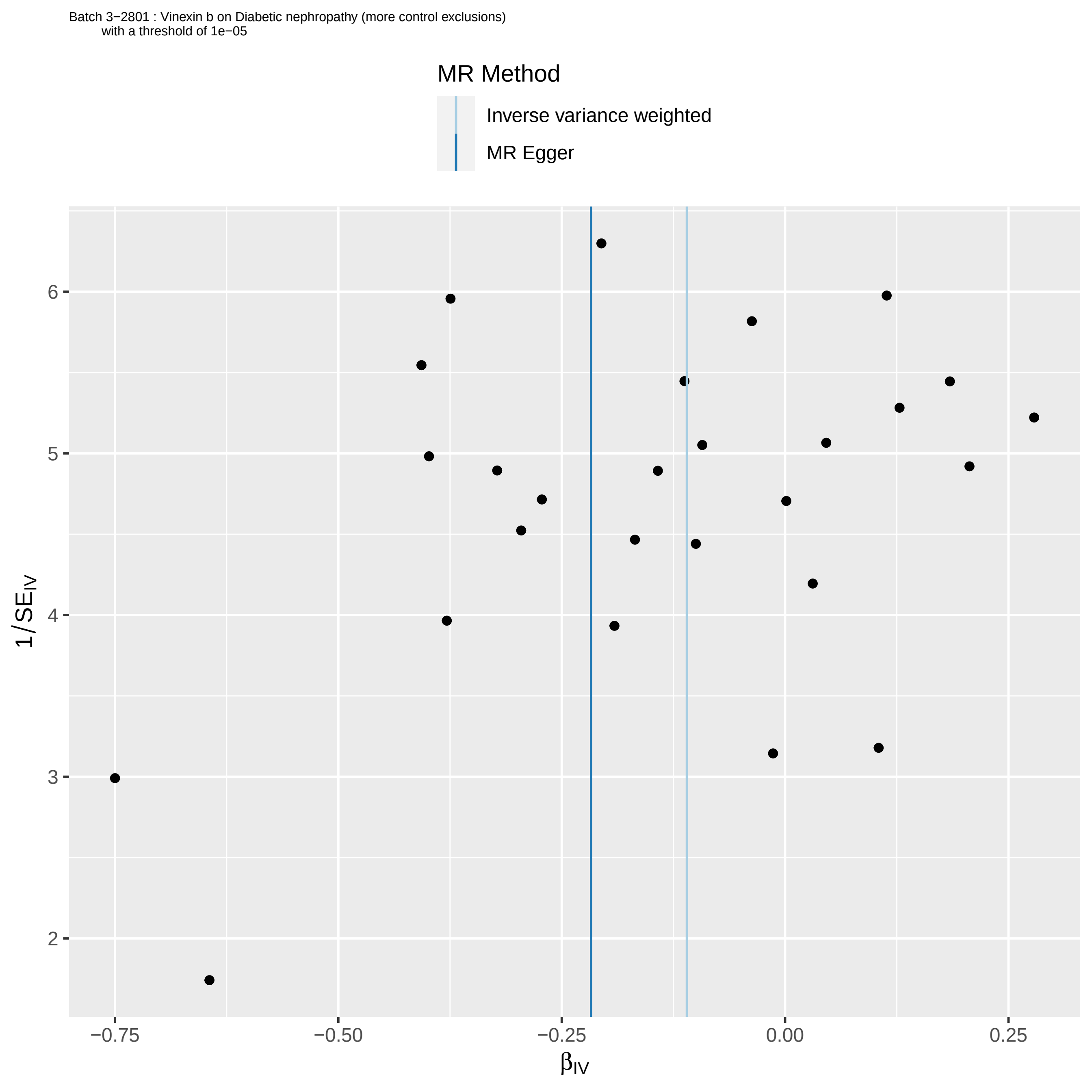

Batch 3−2801 : Vinexin b on Diabetic nephropathy (more control exclusions)
with a threshold of 1e−05
MR Method
Inverse variance weighted
MR Egger
6
5
4
3
2
$1/SE_{IV}$
−0.75
−0.50
−0.25
0.00
0.25
β_{IV}

Batch 3−2802 : Sortilin on Diabetic nephropathy (more control exclusions)
with a threshold of 1e−05

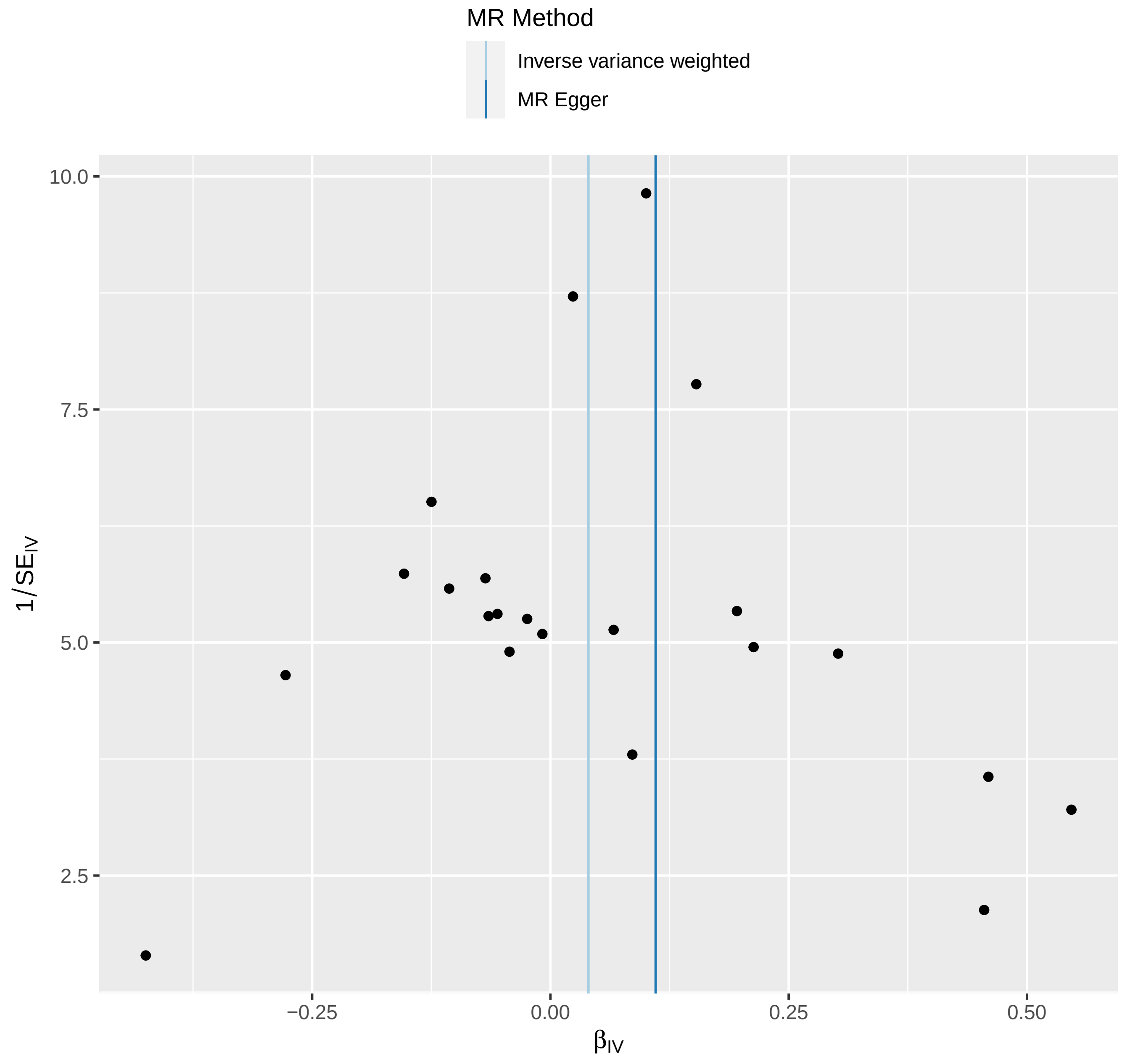

Batch 3−2803 : Sclerostin on Diabetic nephropathy (more control exclusions)
with a threshold of 1e−05

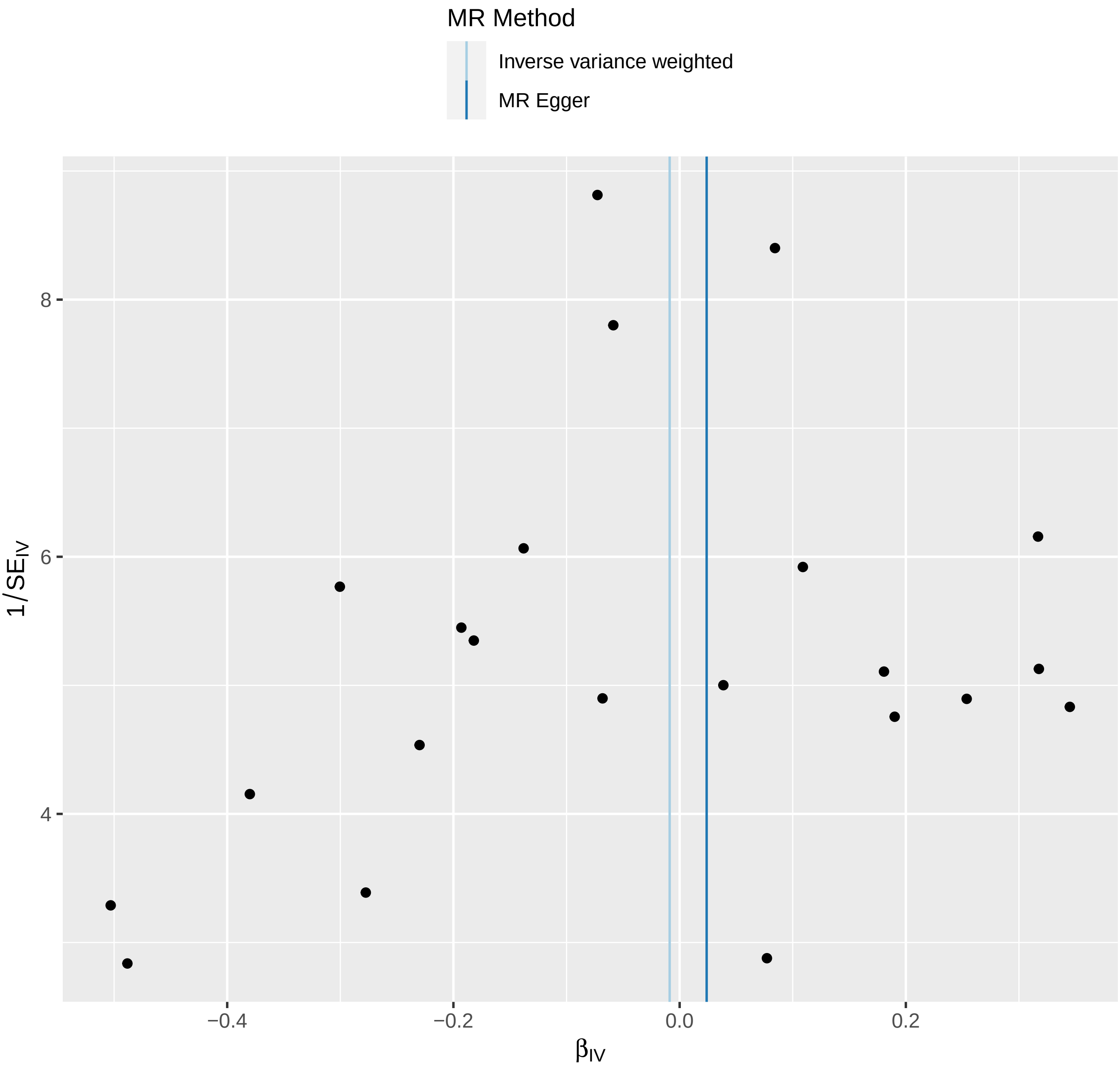

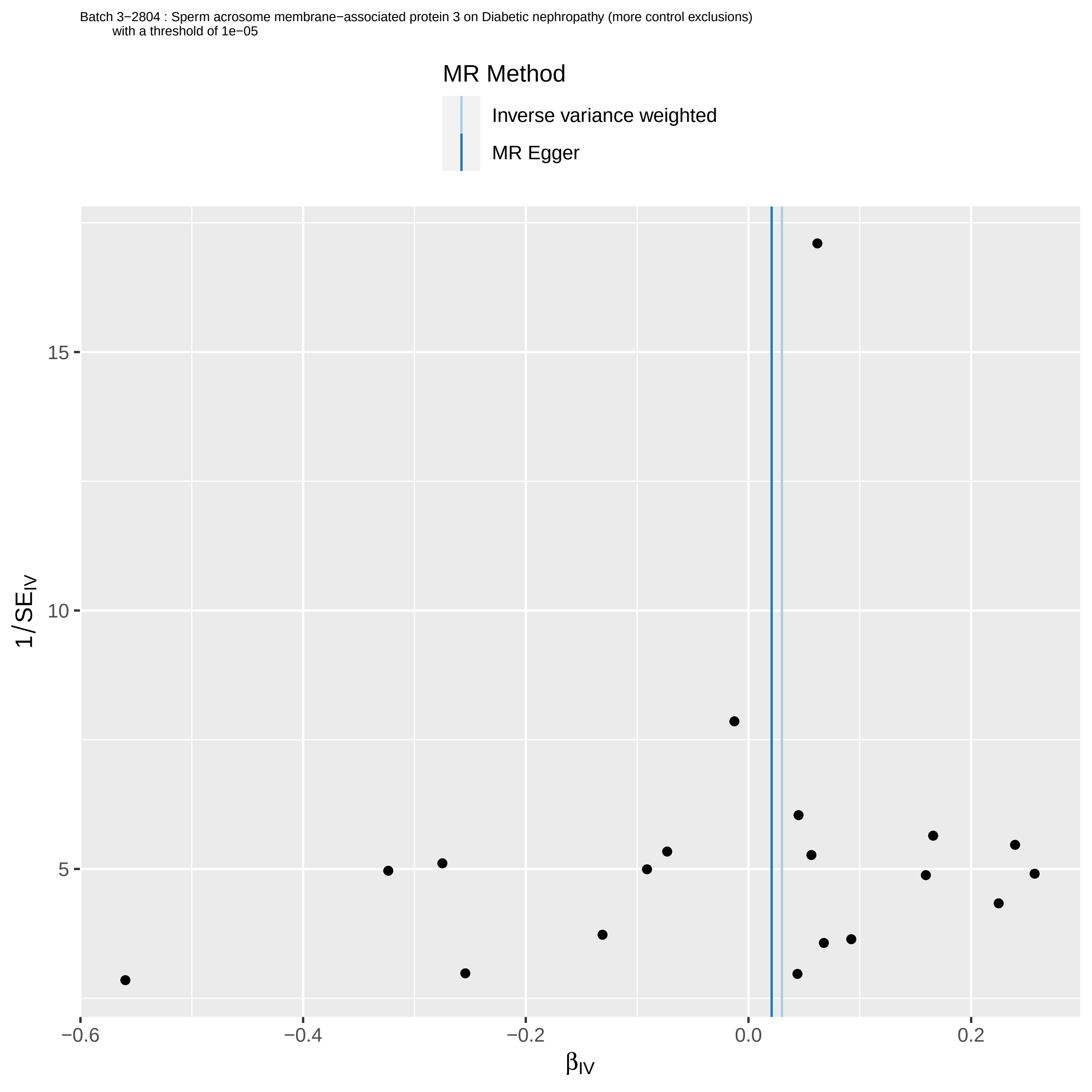
Batch 3−2804 : Sperm acrosome membrane−associated protein 3 on Diabetic nephropathy (more control exclusions)
with a threshold of 1e−05
MR Method
Inverse variance weighted
MR Egger
15
10
5
1/SE_IV
−0.6
−0.4
−0.2
0.0
0.2
β_IV

Batch 3−2805 : Protein SPACA7 on Diabetic nephropathy (more control exclusions)
with a threshold of 1e−05

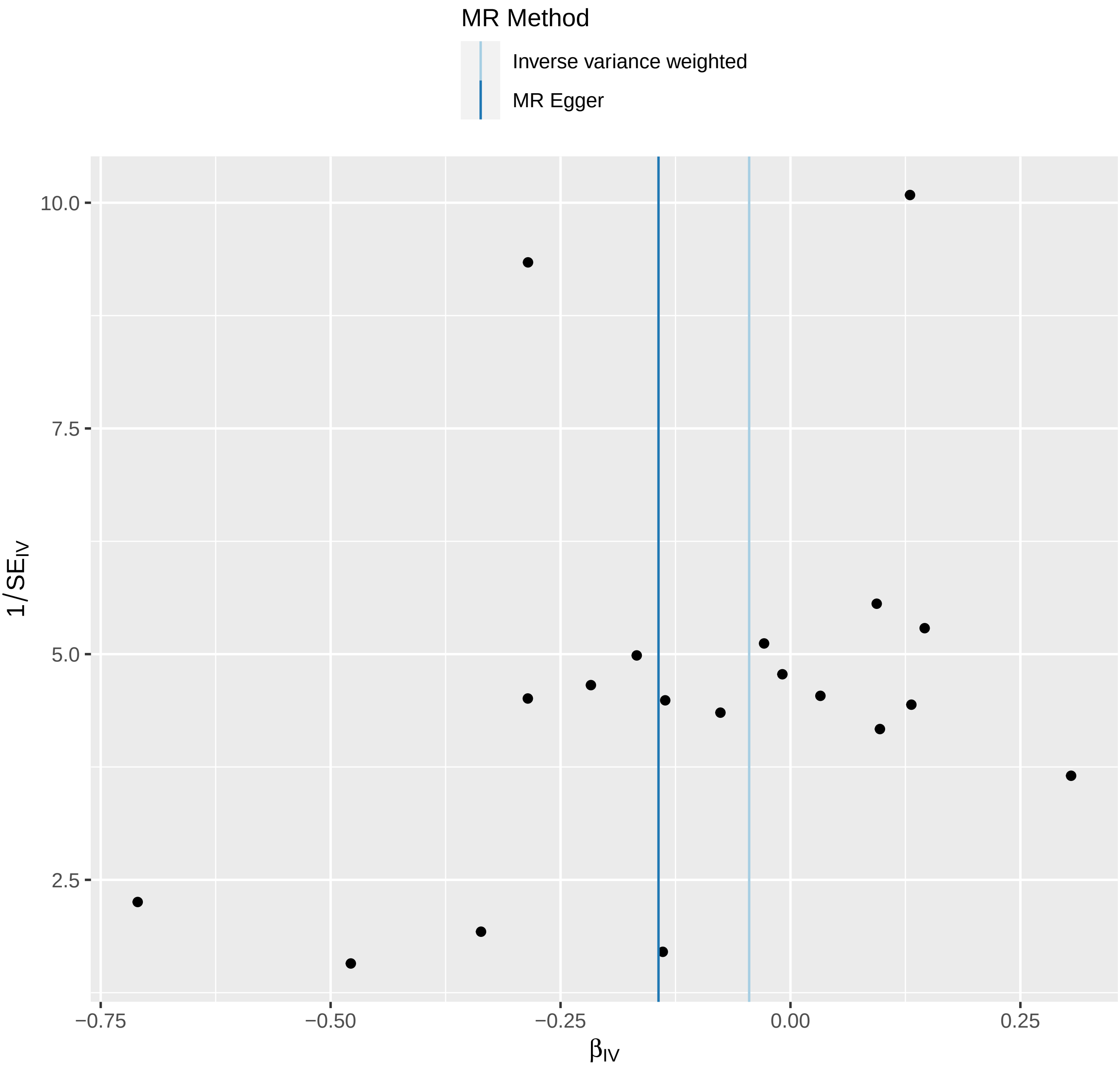

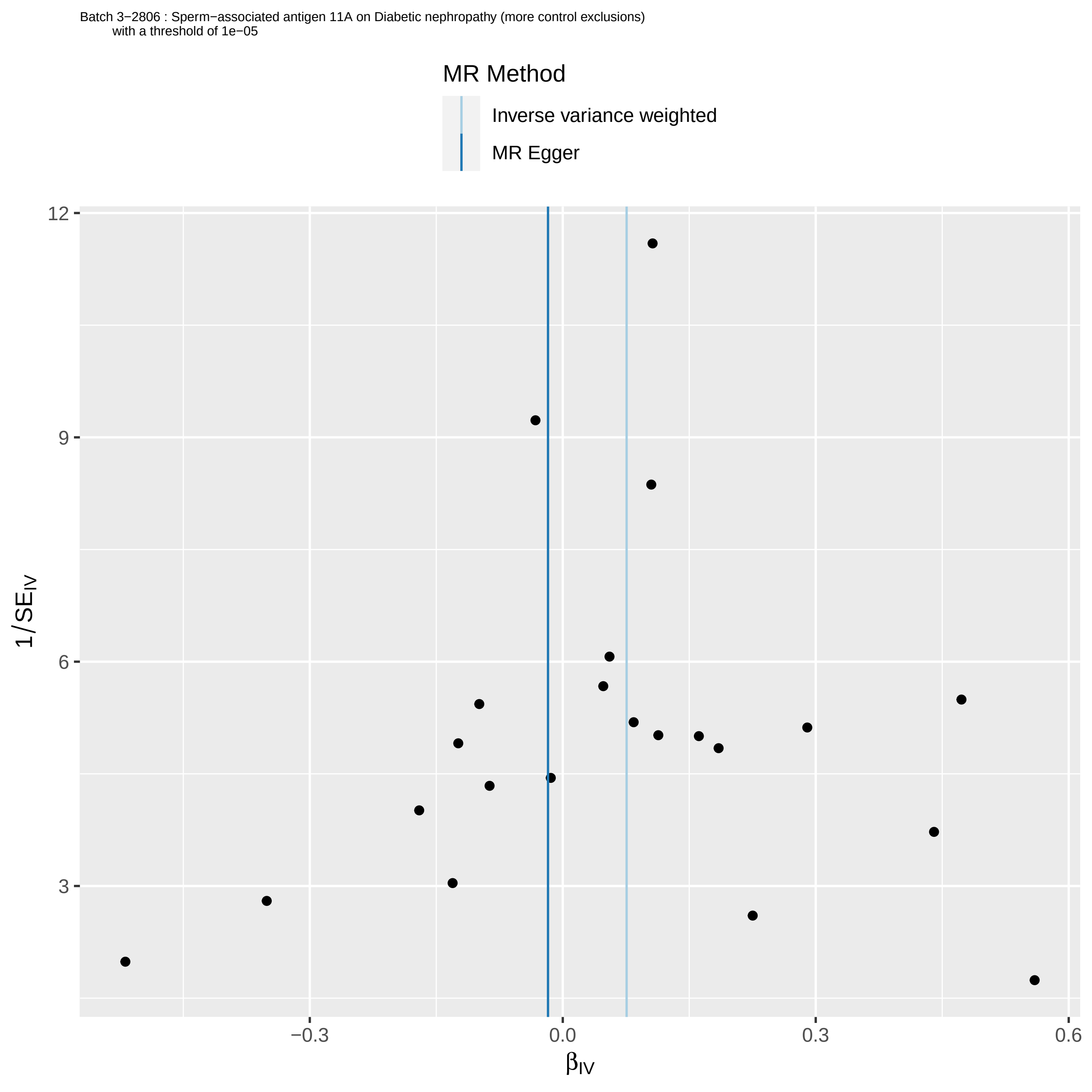

Batch 3−2806 : Sperm−associated antigen 11A on Diabetic nephropathy (more control exclusions)
with a threshold of 1e−05
MR Method
Inverse variance weighted
MR Egger
12
9
6
3
1/SE_IV
−0.3
0.0
0.3
0.6
β_IV

Batch 3−2807 : Sperm−associated antigen 11B on Diabetic nephropathy (more control exclusions)
with a threshold of 1e−05

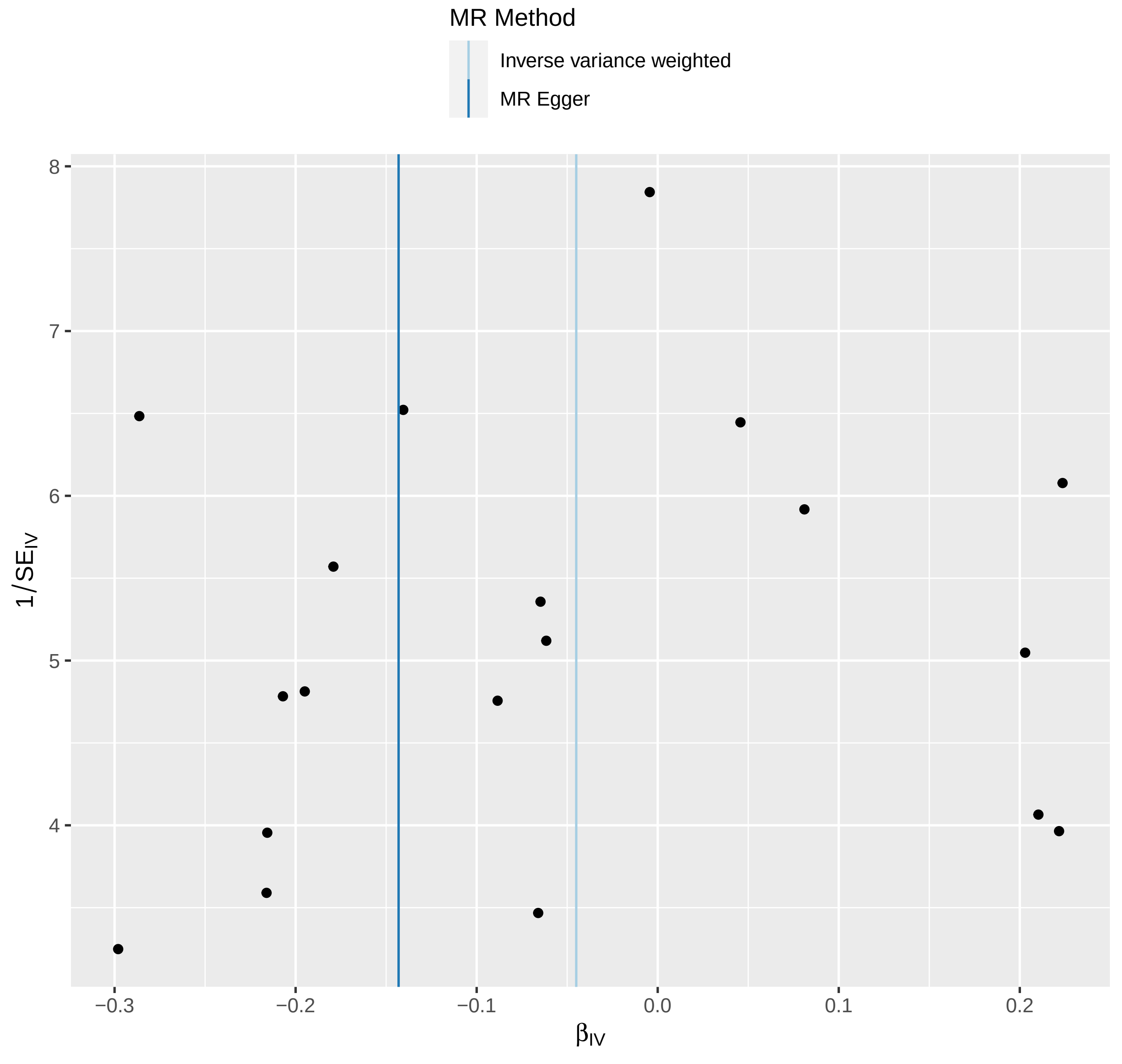

Batch 3−2808 : SPARC on Diabetic nephropathy (more control exclusions)
with a threshold of 1e−05

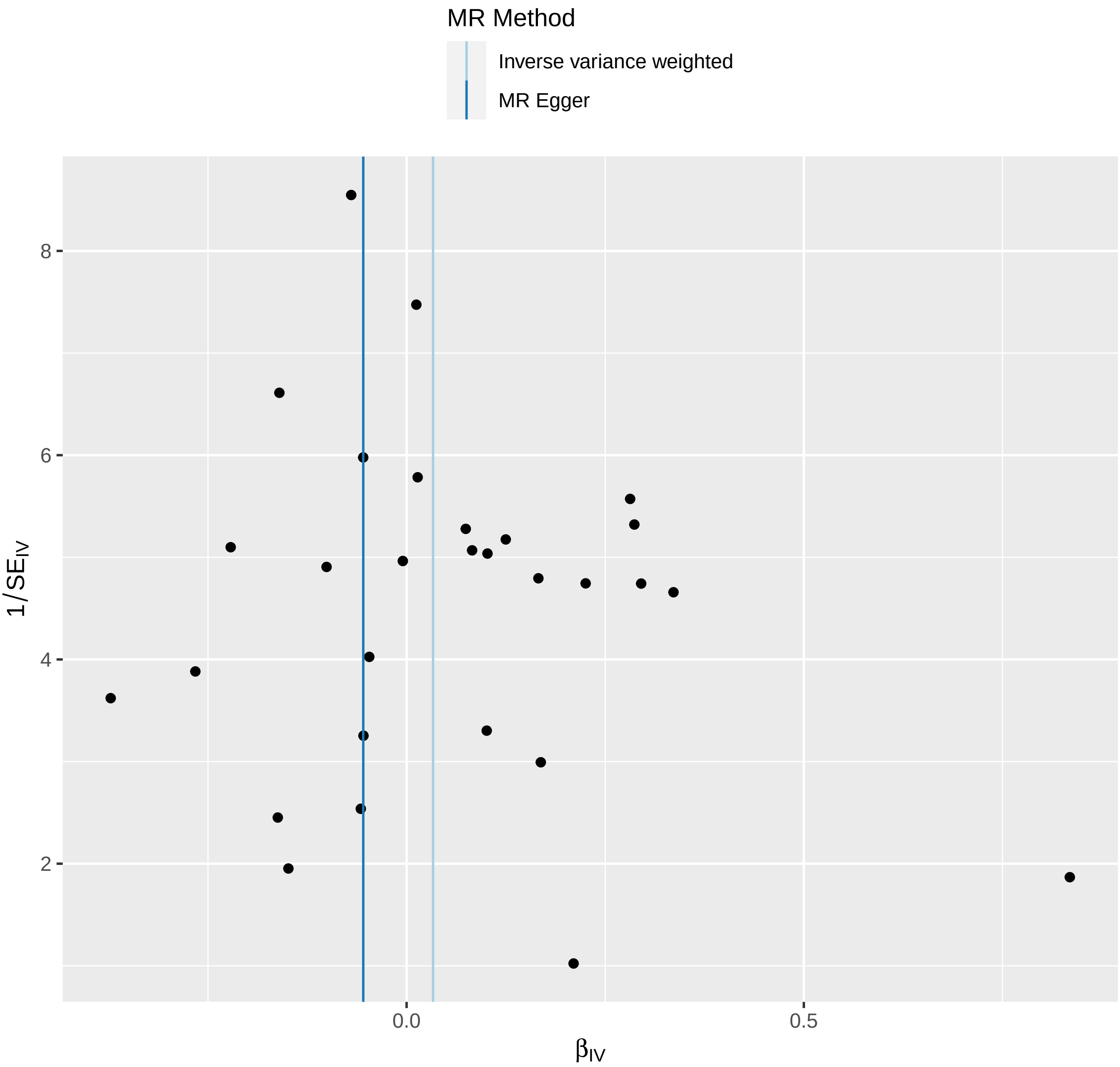

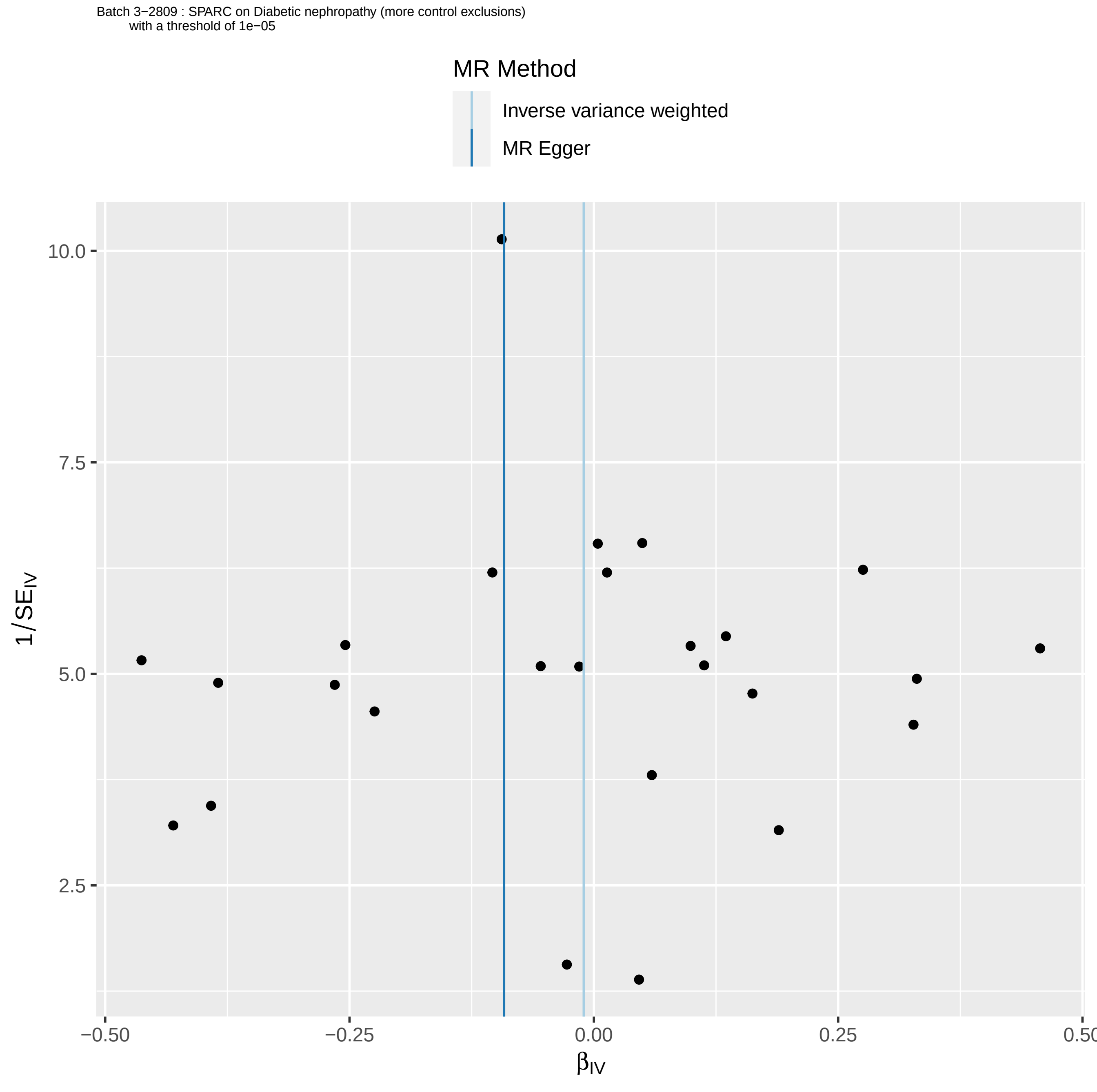
Batch 3−2809 : SPARC on Diabetic nephropathy (more control exclusions)
with a threshold of 1e−05
MR Method
Inverse variance weighted
MR Egger
10.0
7.5
5.0
2.5
1/SE$_{IV}$
−0.50
−0.25
0.00
0.25
0.50
β$_{IV}$

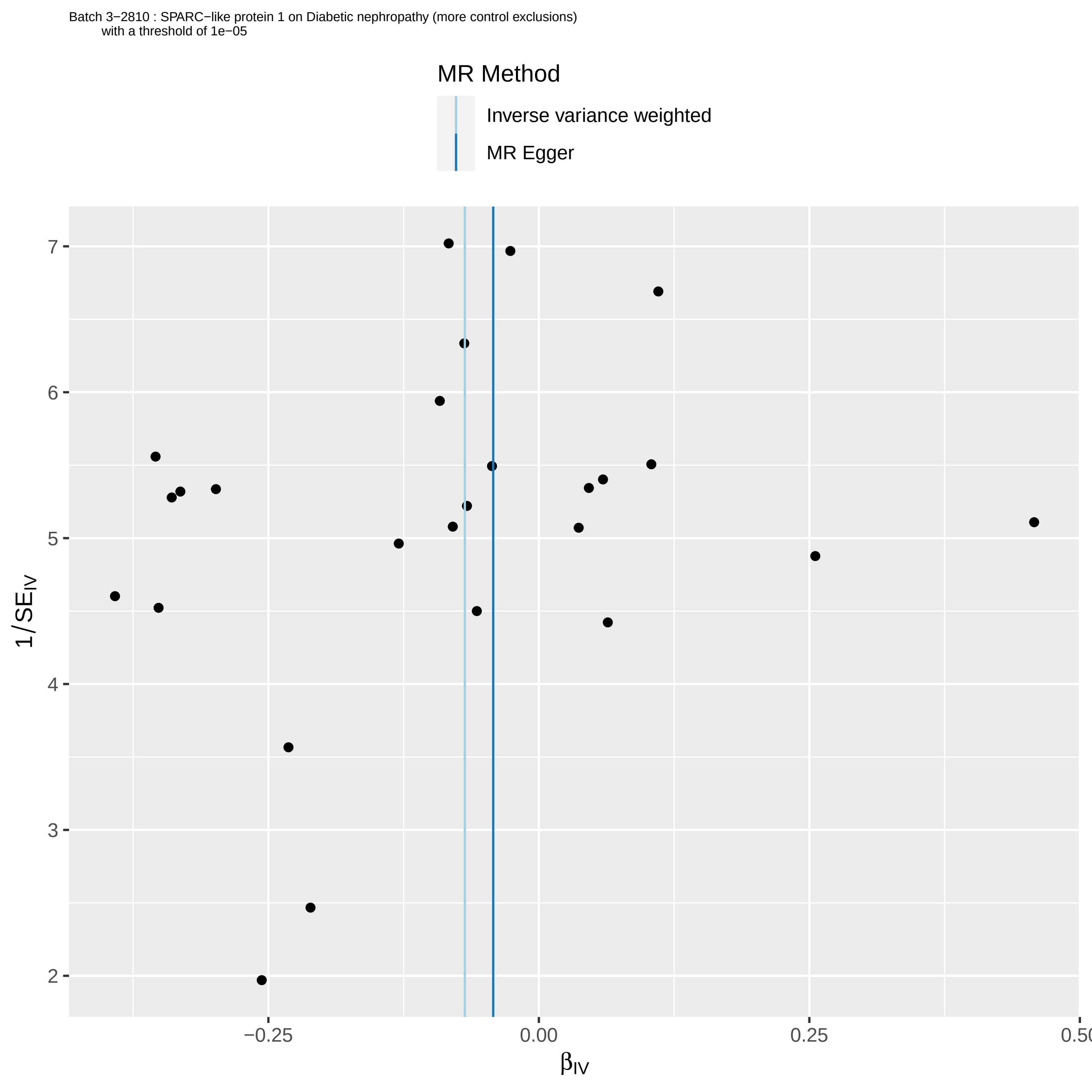

Batch 3−2810 : SPARC−like protein 1 on Diabetic nephropathy (more control exclusions)
with a threshold of 1e−05
MR Method
Inverse variance weighted
MR Egger
7
6
5
4
3
2
1/SE_IV
−0.25
0.00
0.25
0.50
β_IV

Batch 3−2811 : SPARC−like protein 1 on Diabetic nephropathy (more control exclusions) with a threshold of 1e−05

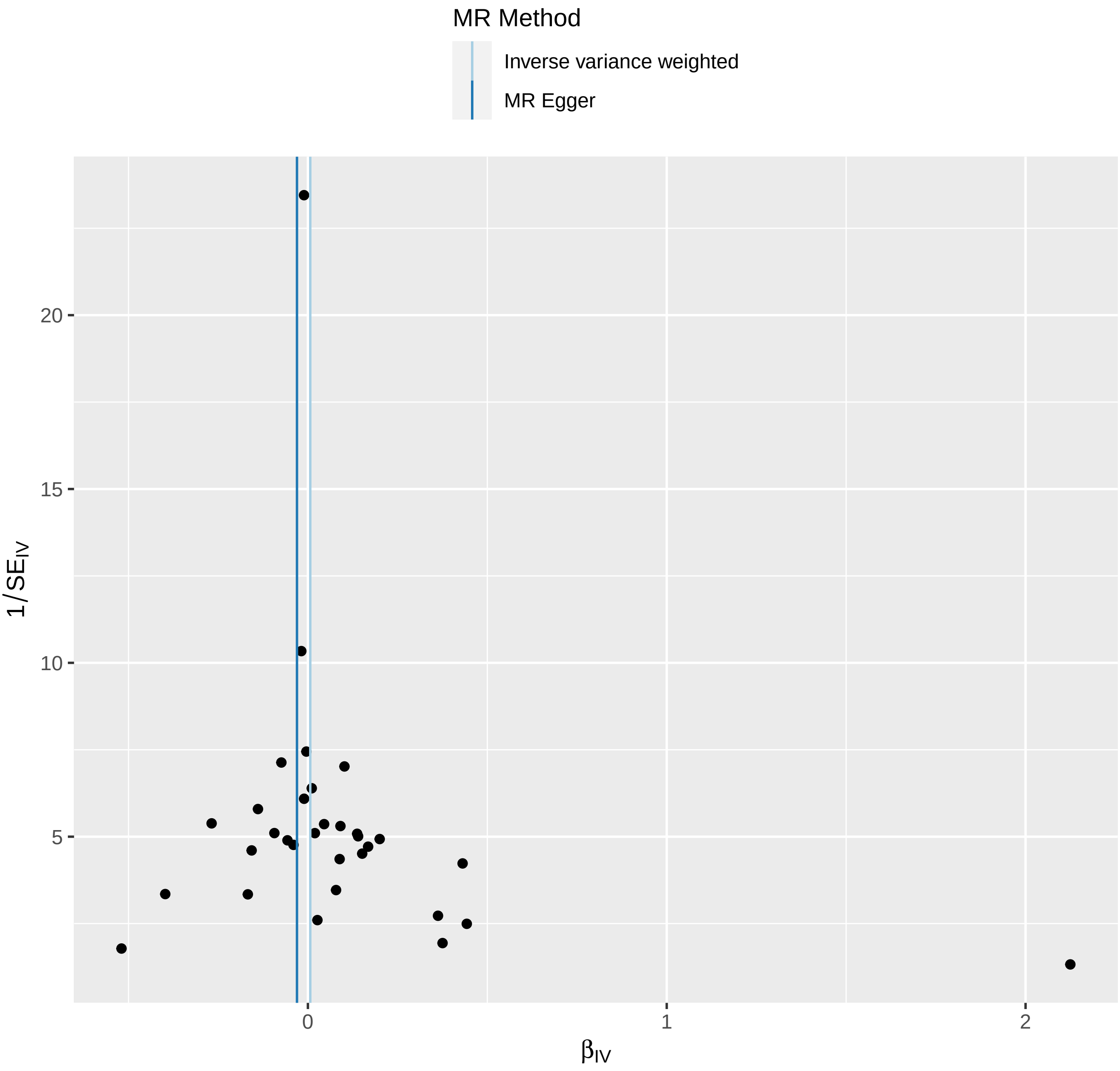

Batch 3−2812 : Spastin on Diabetic nephropathy (more control exclusions)
with a threshold of 1e−05

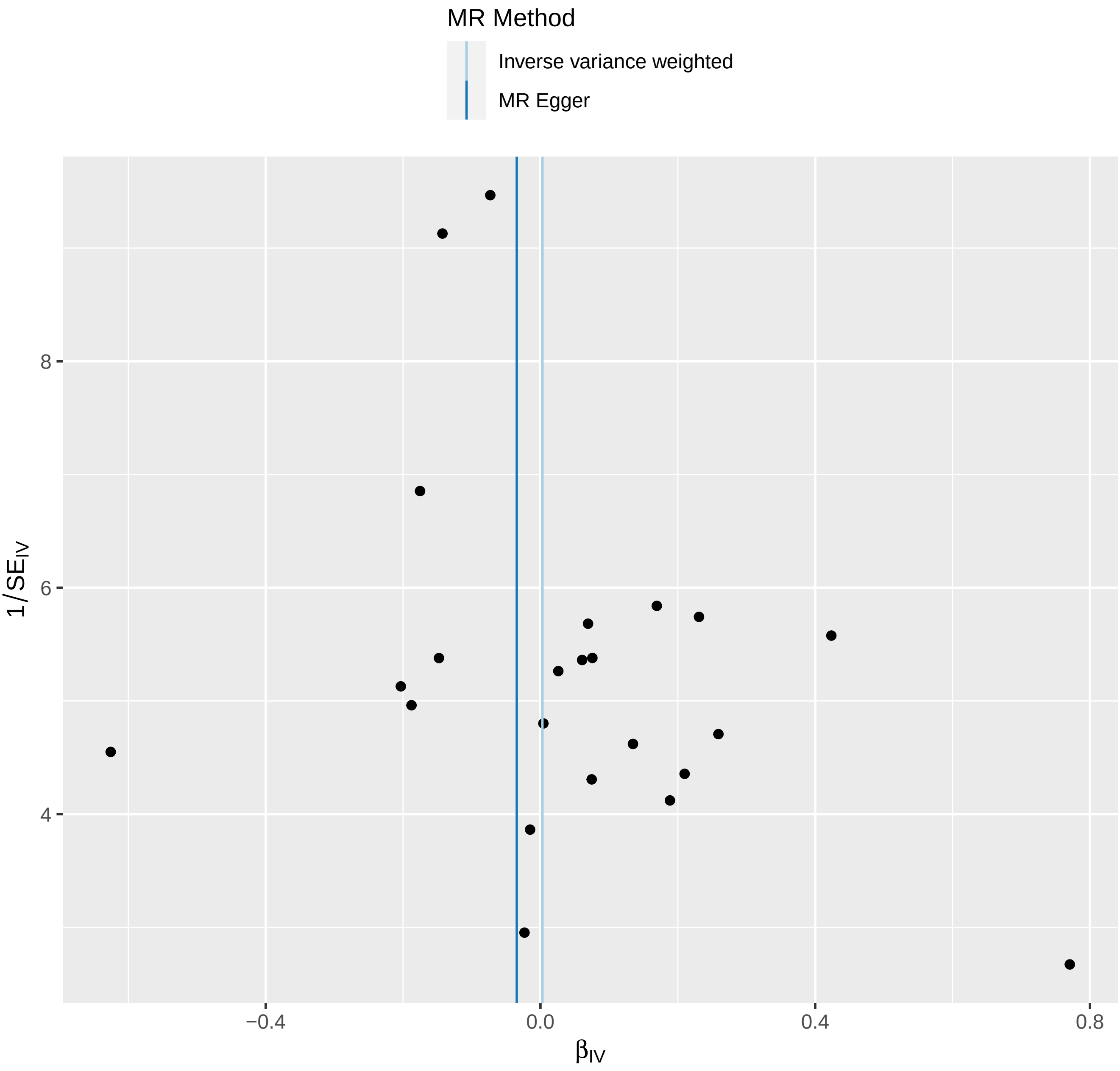

Batch 3−2813 : Spermatogenesis−associated protein 20 on Diabetic nephropathy (more control exclusions) with a threshold of 1e−05

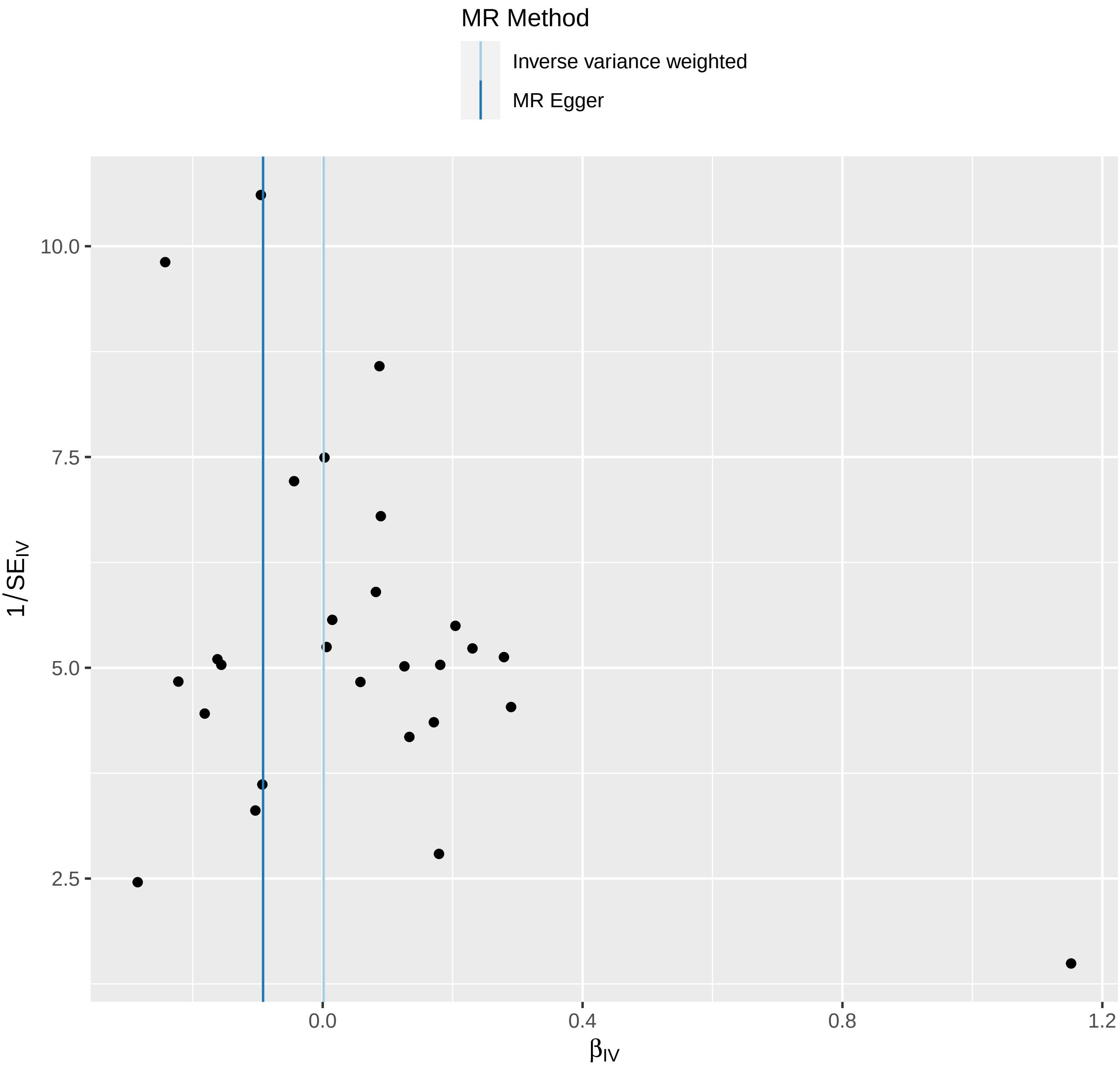

Batch 3−2814 : Spermatogenesis−associated protein 9 on Diabetic nephropathy (more control exclusions)
with a threshold of 1e−05

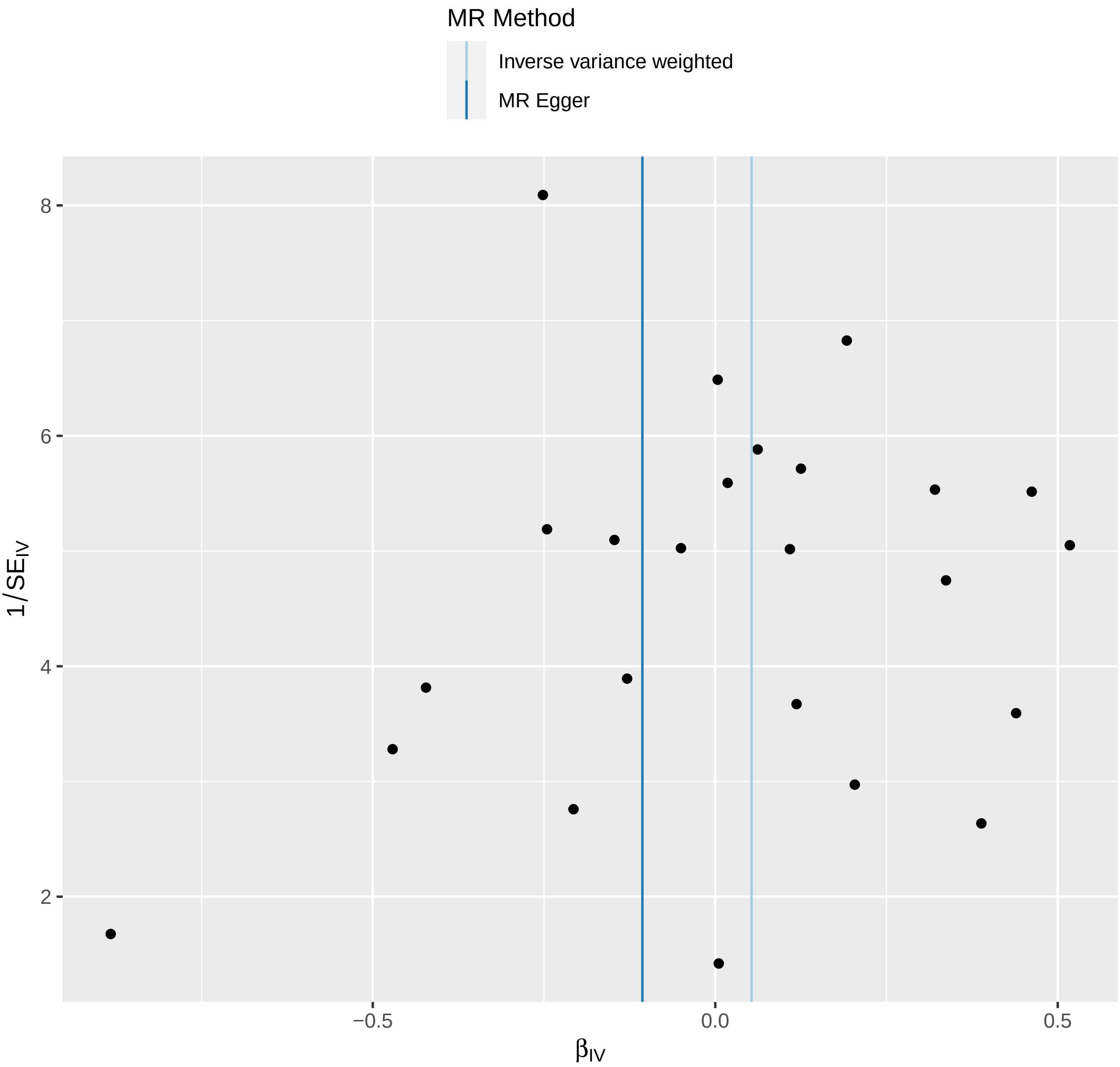

Batch 3−2815 : Maspardin on Diabetic nephropathy (more control exclusions)
with a threshold of 1e−05

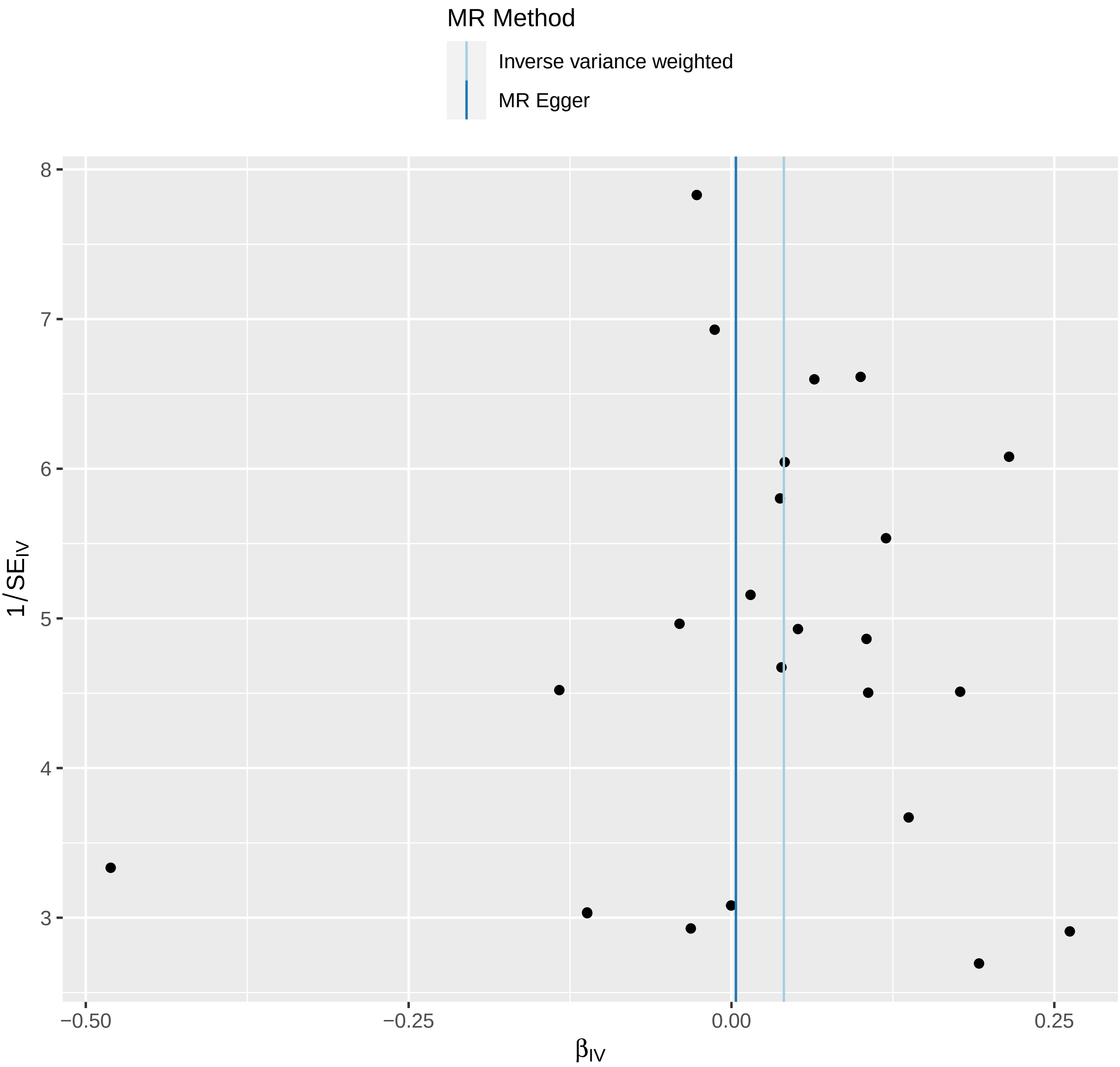

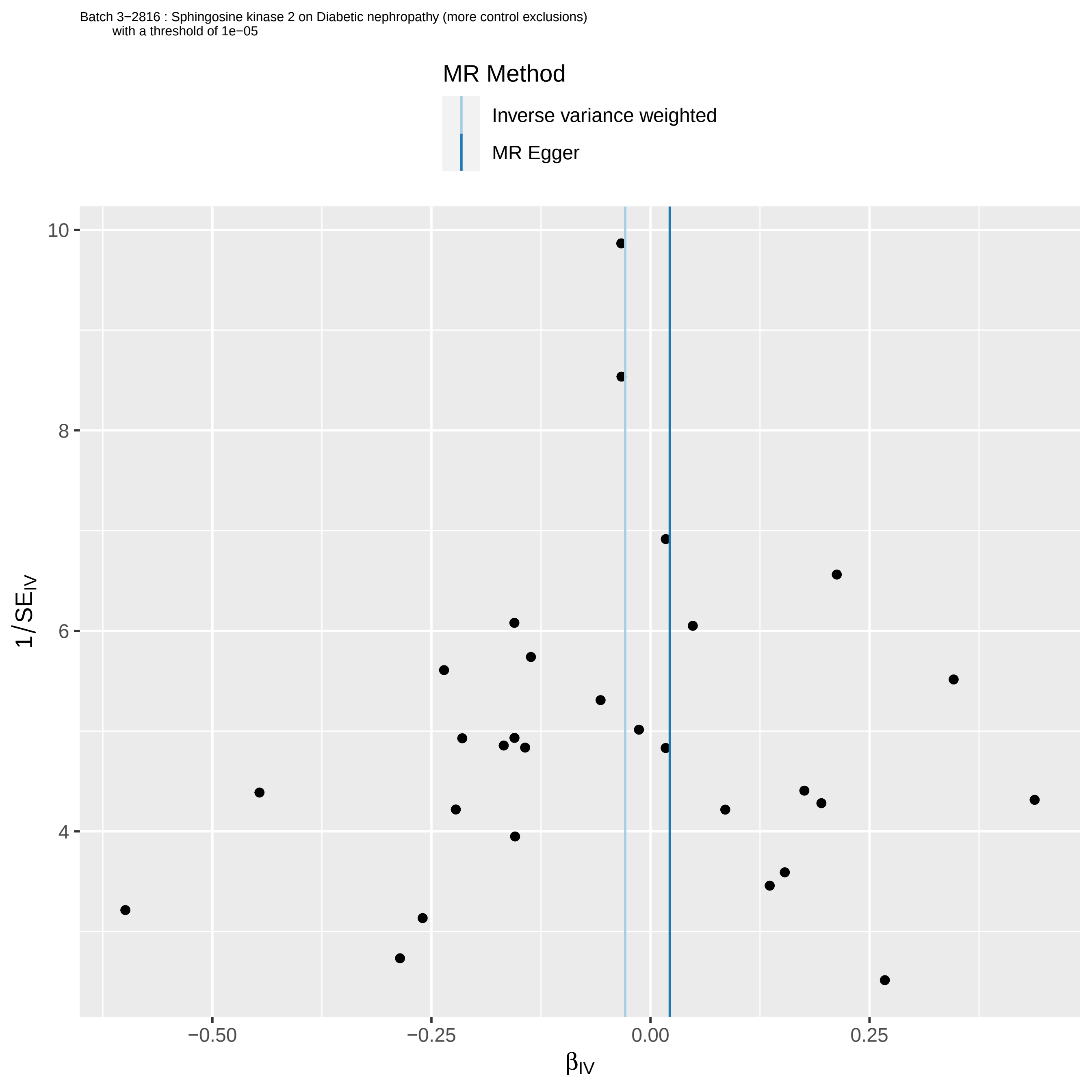

Batch 3−2816 : Sphingosine kinase 2 on Diabetic nephropathy (more control exclusions)
with a threshold of 1e−05
MR Method
Inverse variance weighted
MR Egger
10
8
6
4
1/SE_IV
−0.50
−0.25
0.00
0.25
β_IV

Batch 3−2817 : Serine protease inhibitor Kazal−type 13 on Diabetic nephropathy (more control exclusions)
with a threshold of 1e−05

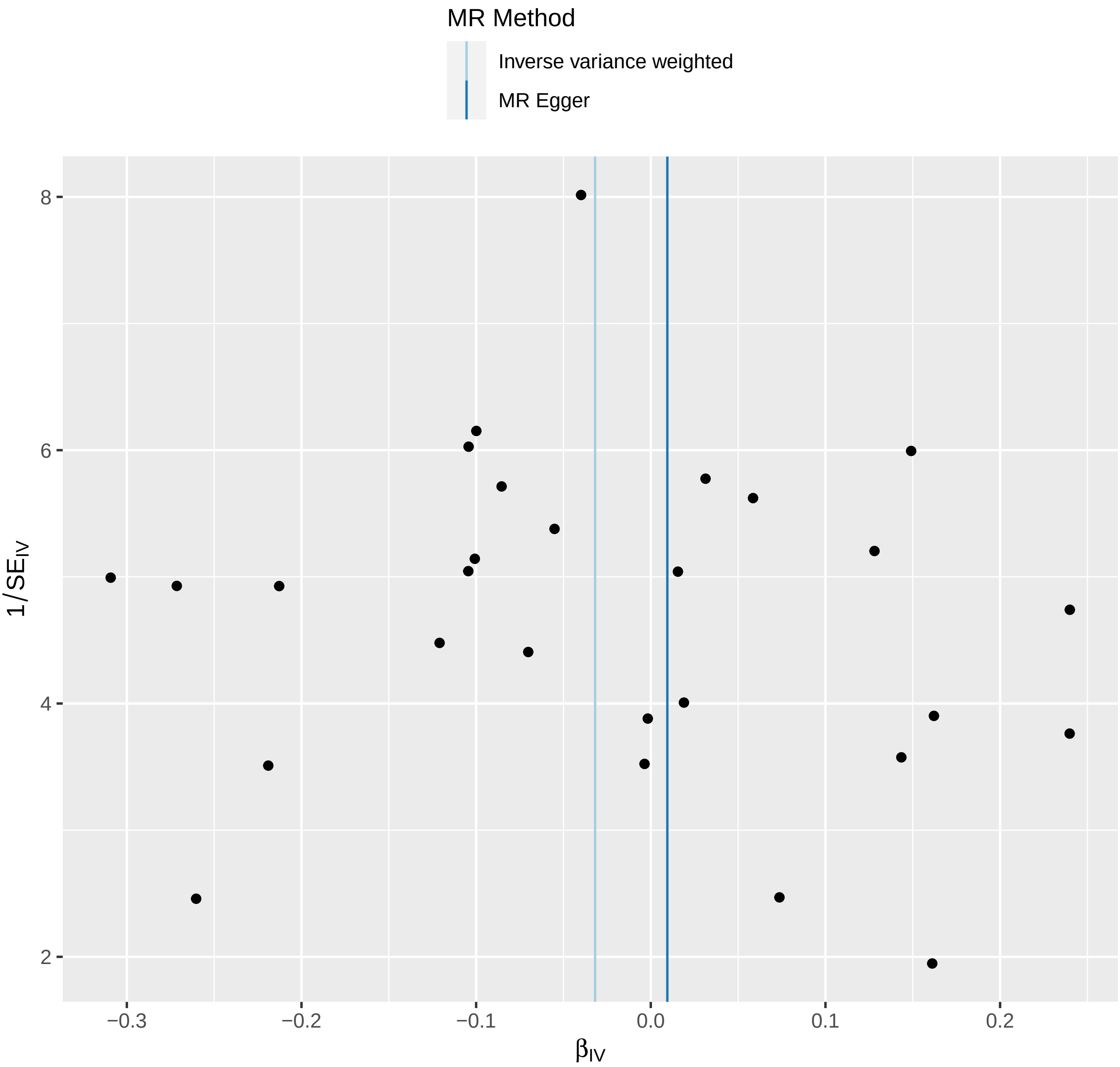

Batch 3−2818 : Serine protease inhibitor Kazal−type 2 on Diabetic nephropathy (more control exclusions)
with a threshold of 1e−05

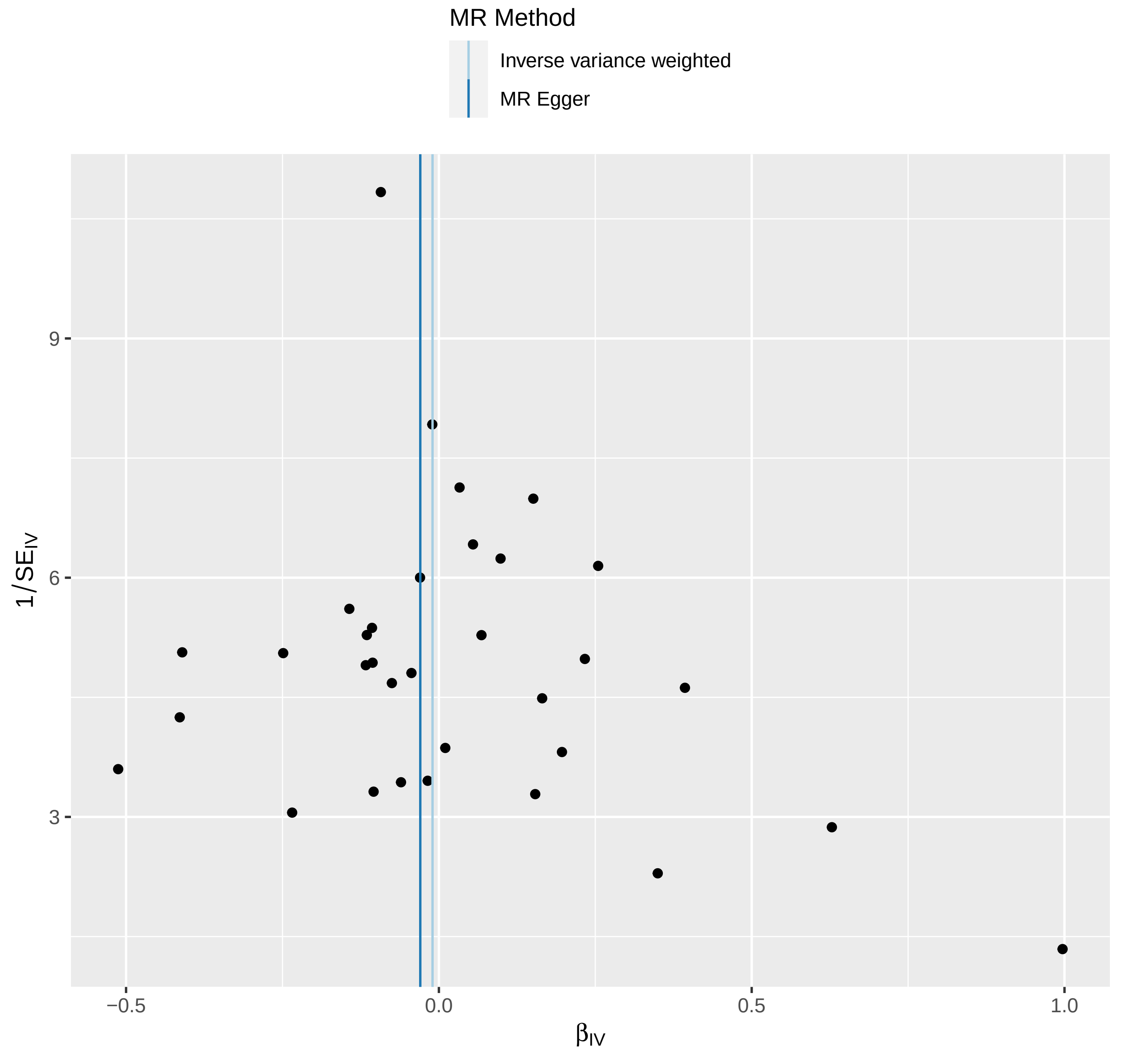

Batch 3−2819 : Serine protease inhibitor Kazal−type 5 on Diabetic nephropathy (more control exclusions)
with a threshold of 1e−05

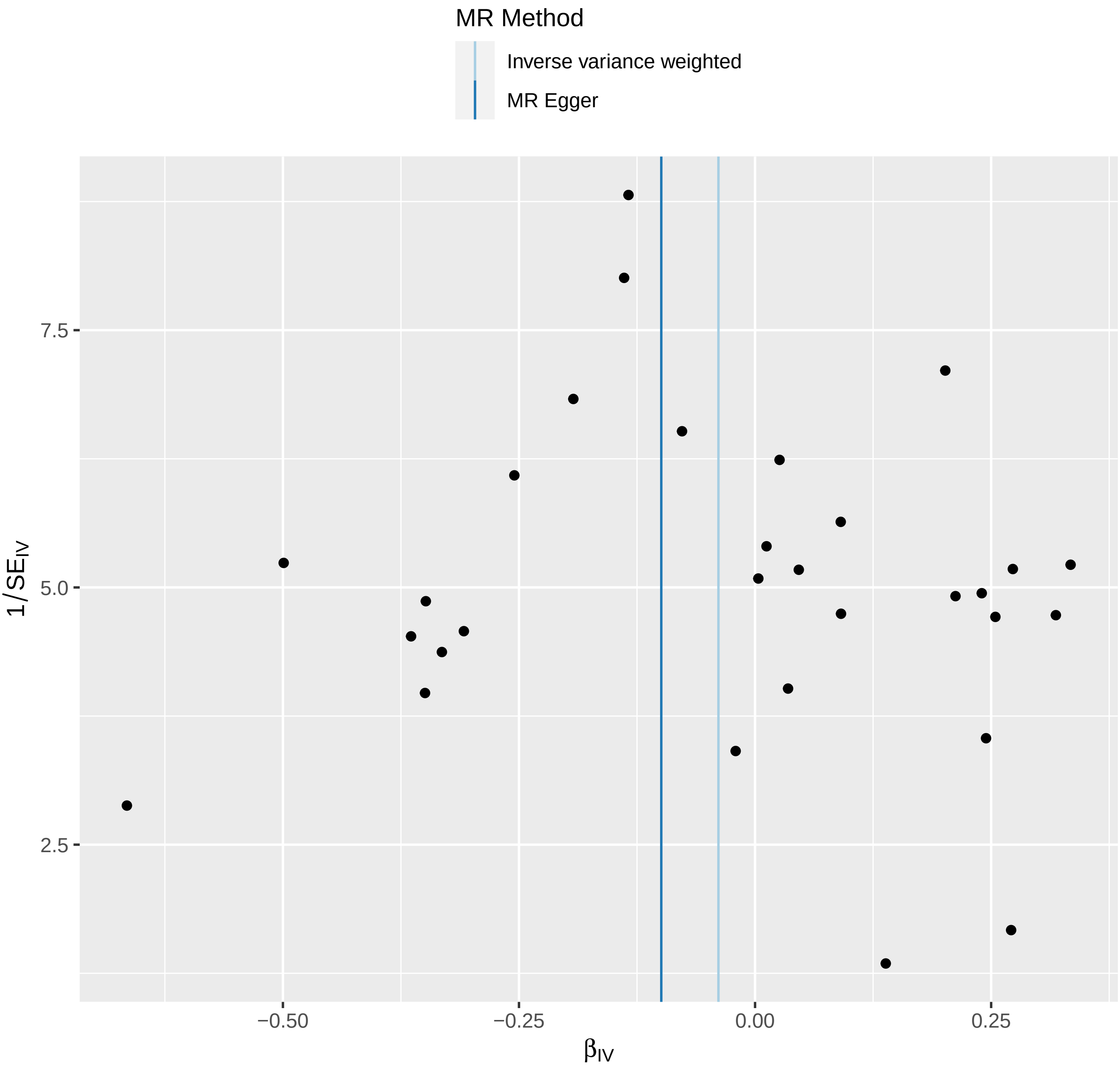

Batch 3−2820 : Serine protease inhibitor Kazal−type 6 on Diabetic nephropathy (more control exclusions)
with a threshold of 1e−05

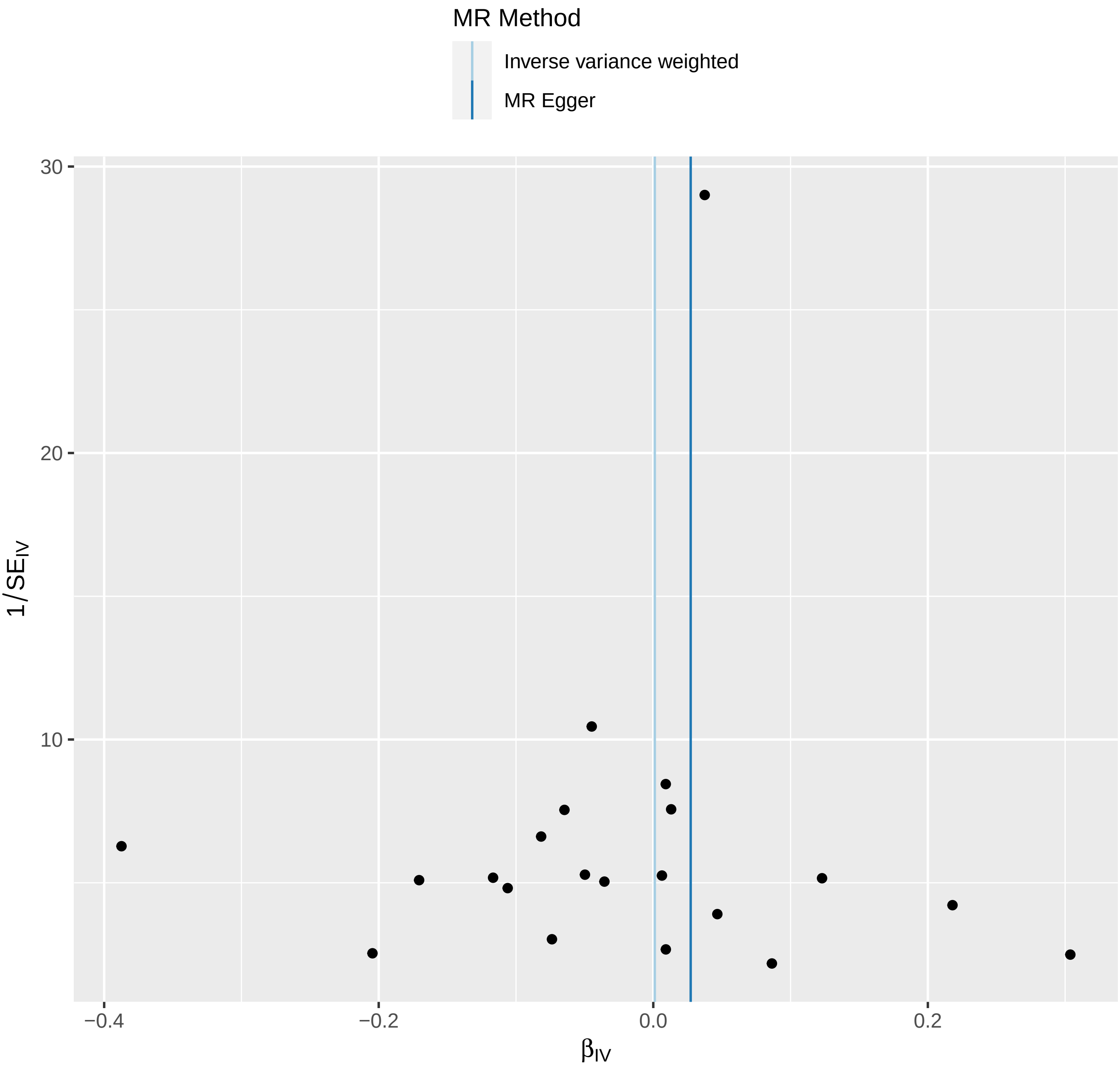

Batch 3−2821 : Serine protease inhibitor Kazal−type 8 on Diabetic nephropathy (more control exclusions)
with a threshold of 1e−05

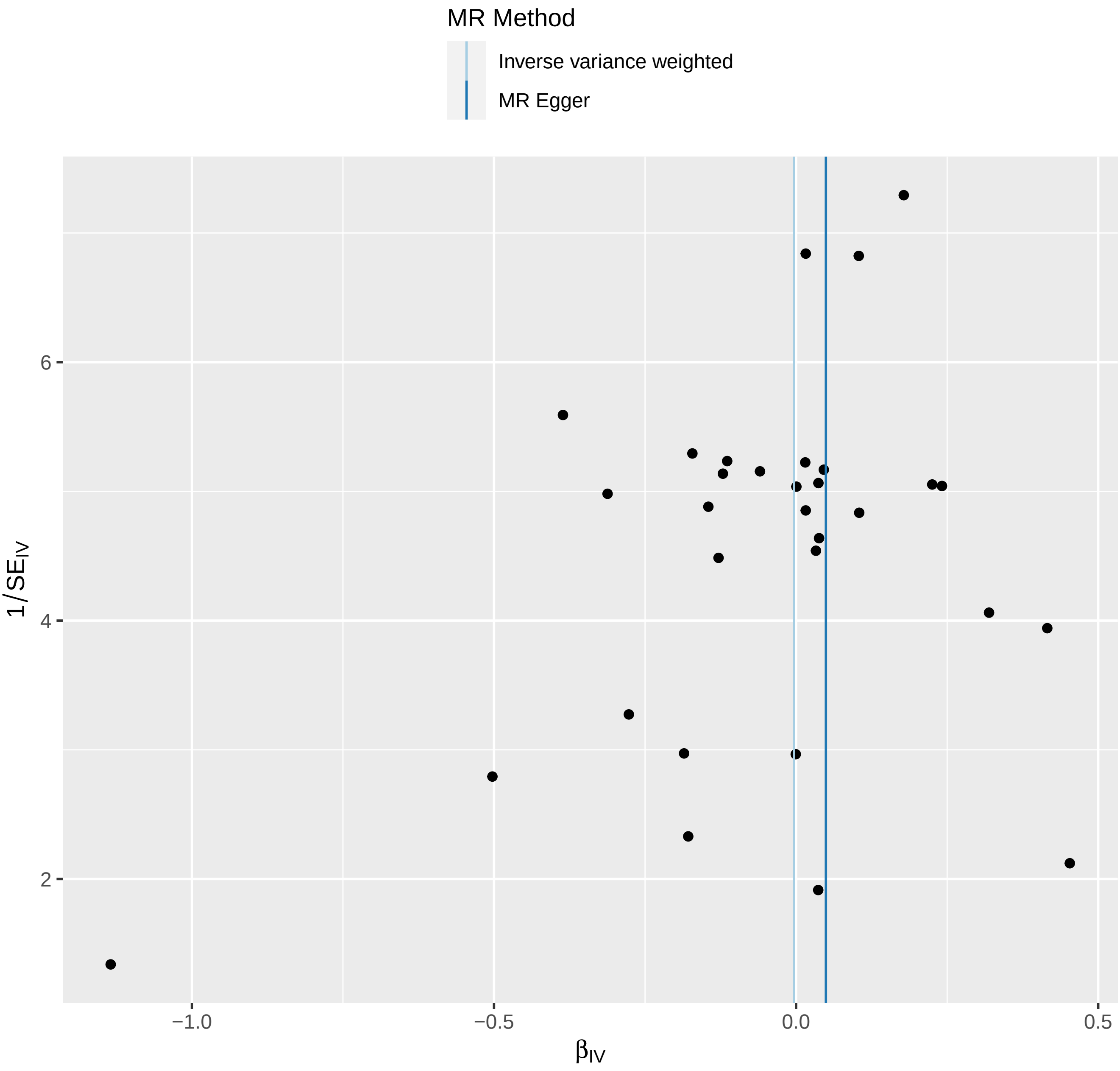

Batch 3−2822 : Serine protease inhibitor Kazal−type 9 on Diabetic nephropathy (more control exclusions)
with a threshold of 1e−05

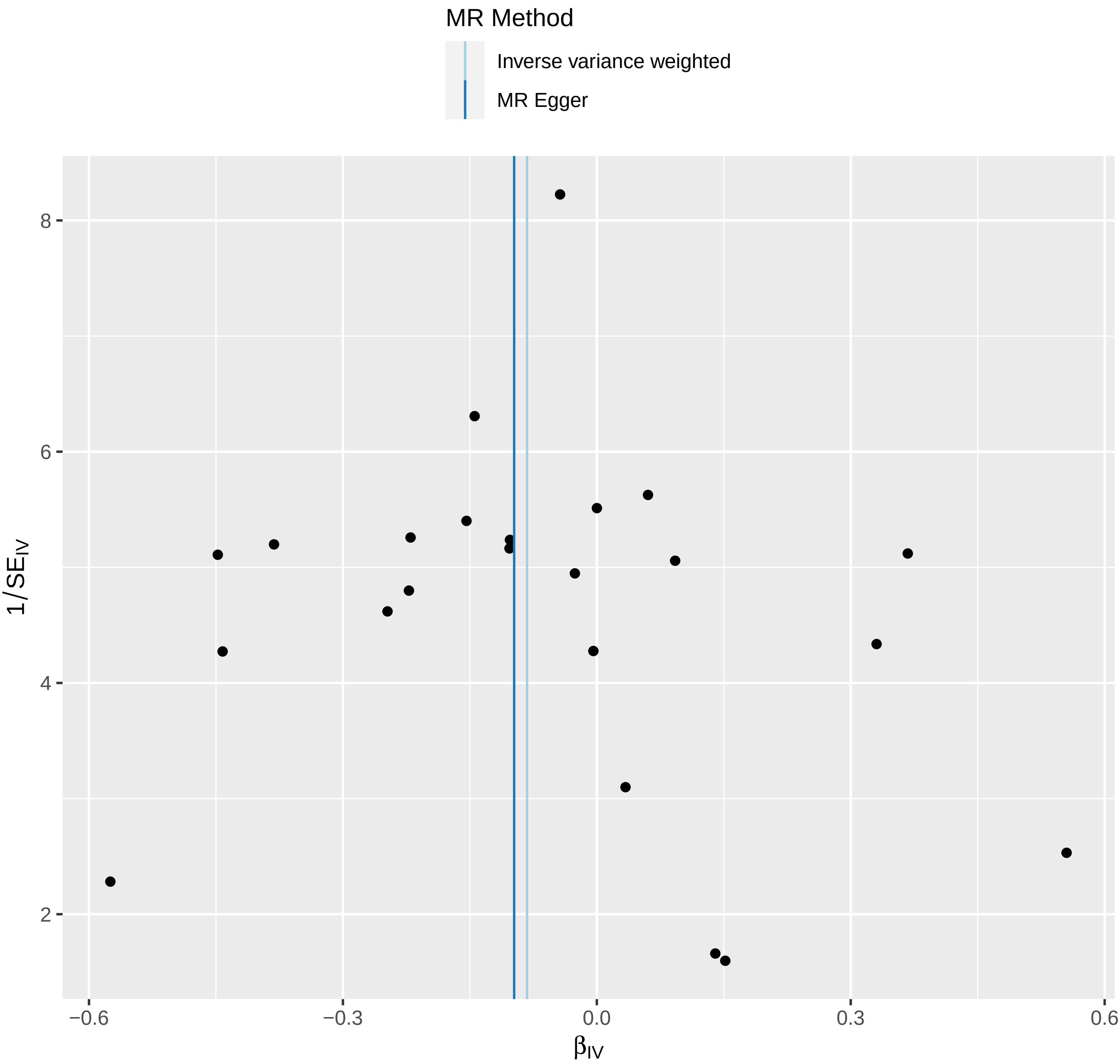

Batch 3−2823 : Kunitz−type protease inhibitor 1 on Diabetic nephropathy (more control exclusions)
with a threshold of 1e−05

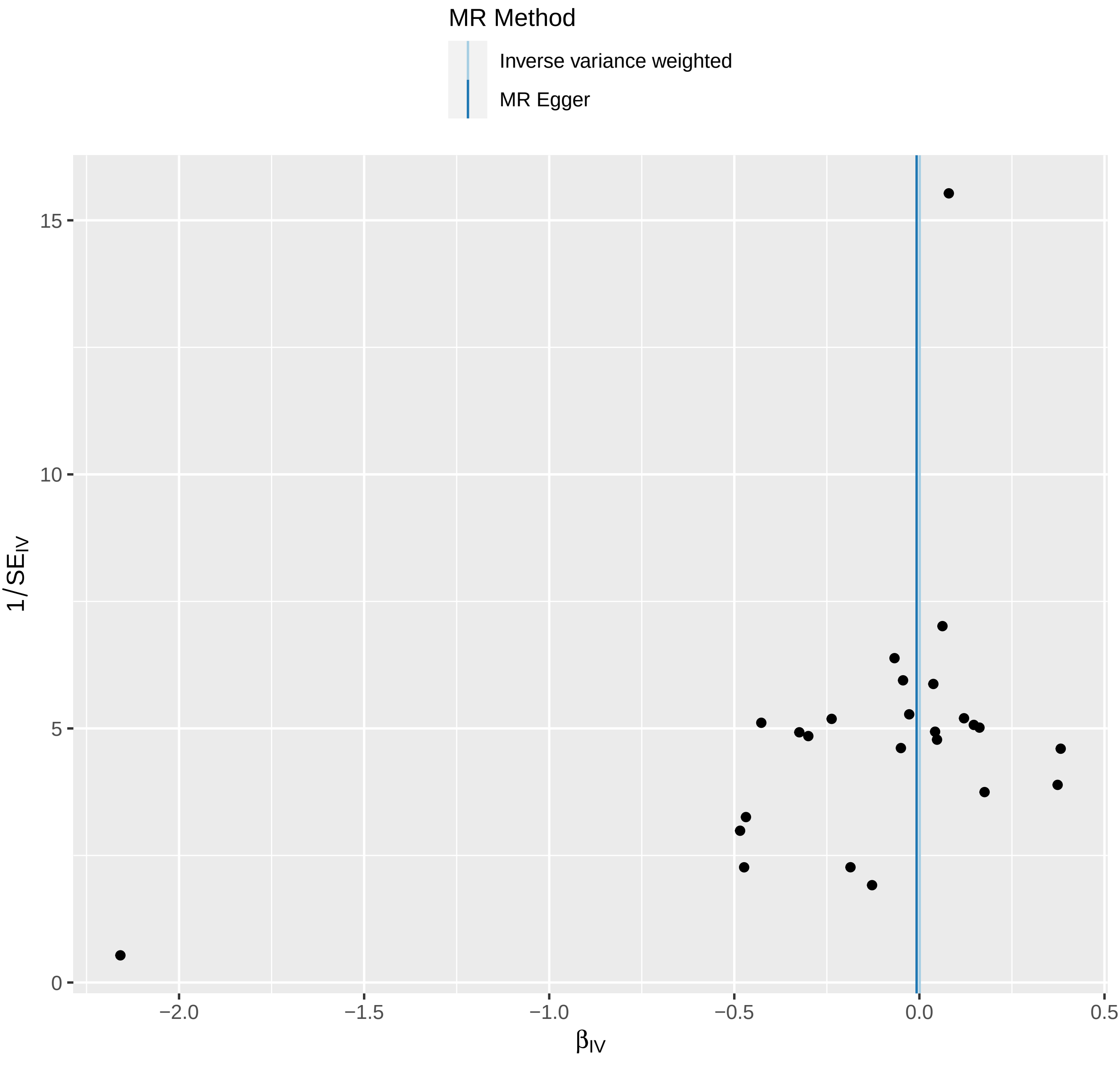

Batch 3−2824 : Kunitz−type protease inhibitor 2 on Diabetic nephropathy (more control exclusions)
with a threshold of 1e−05

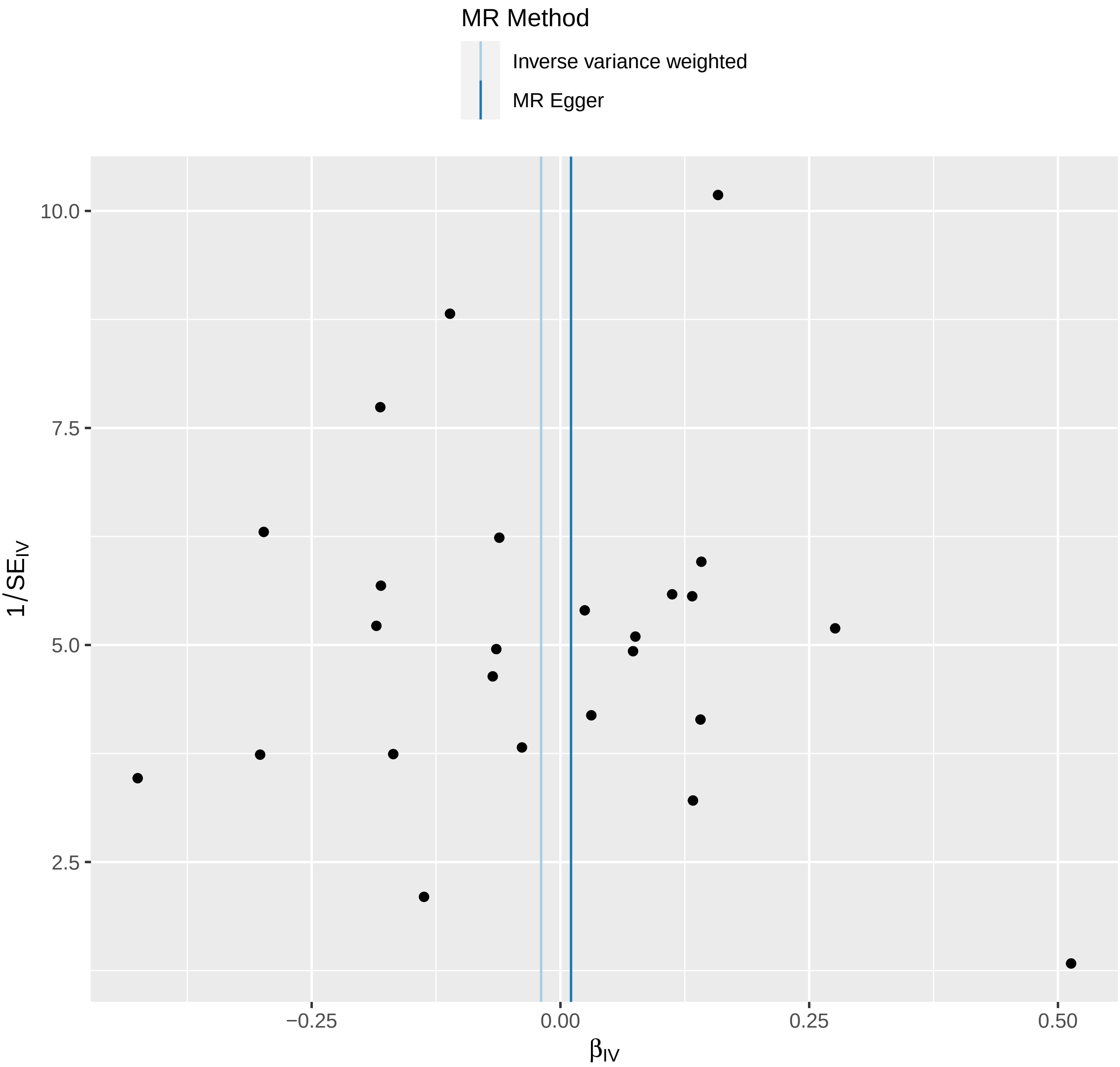

Batch 3−2825 : Kunitz−type protease inhibitor 3 on Diabetic nephropathy (more control exclusions)
with a threshold of 1e−05

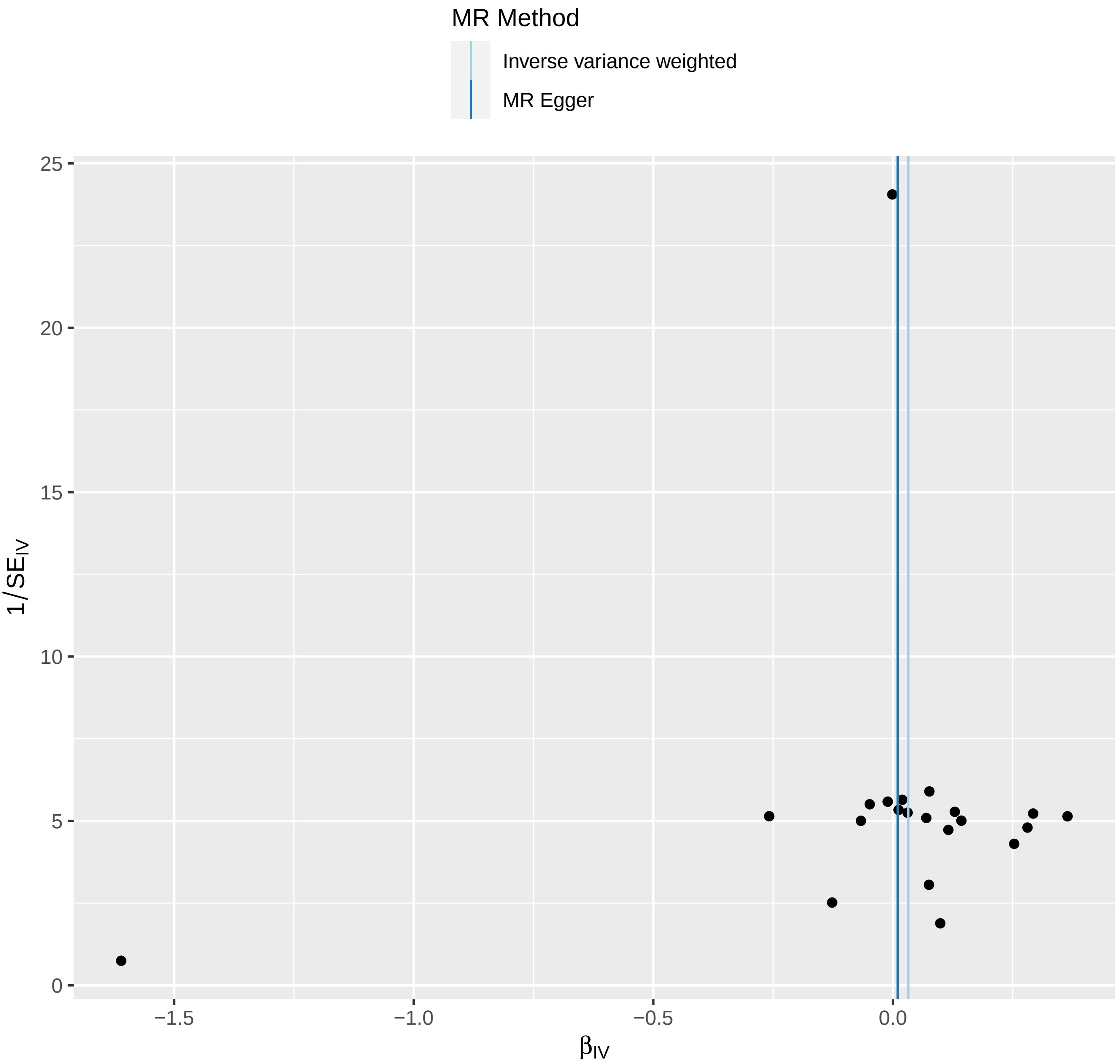

Batch 3−2826 : Testican−1 on Diabetic nephropathy (more control exclusions)
with a threshold of 1e−05

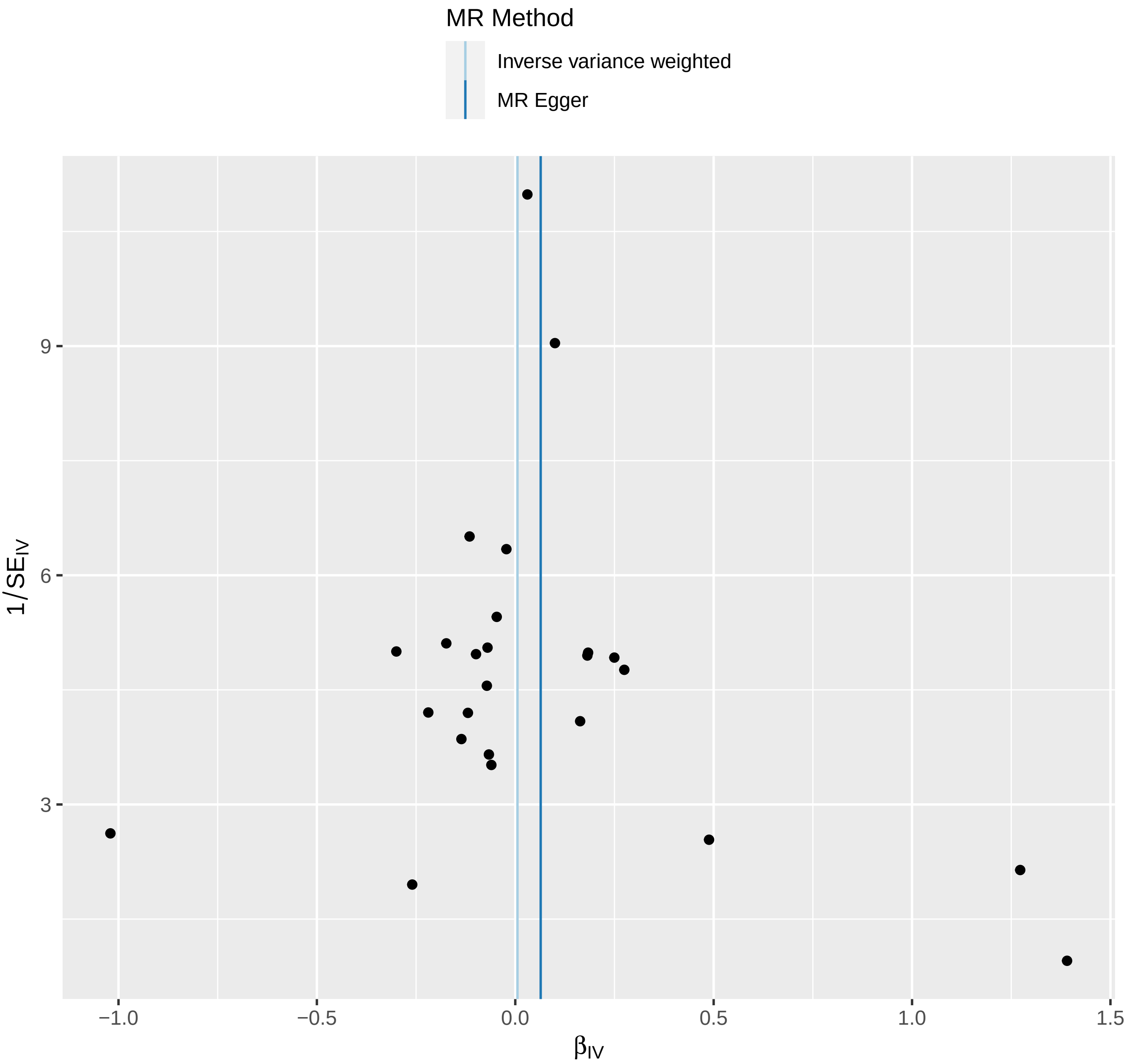

Batch 3−2827 : Testican−2 on Diabetic nephropathy (more control exclusions)
with a threshold of 1e−05

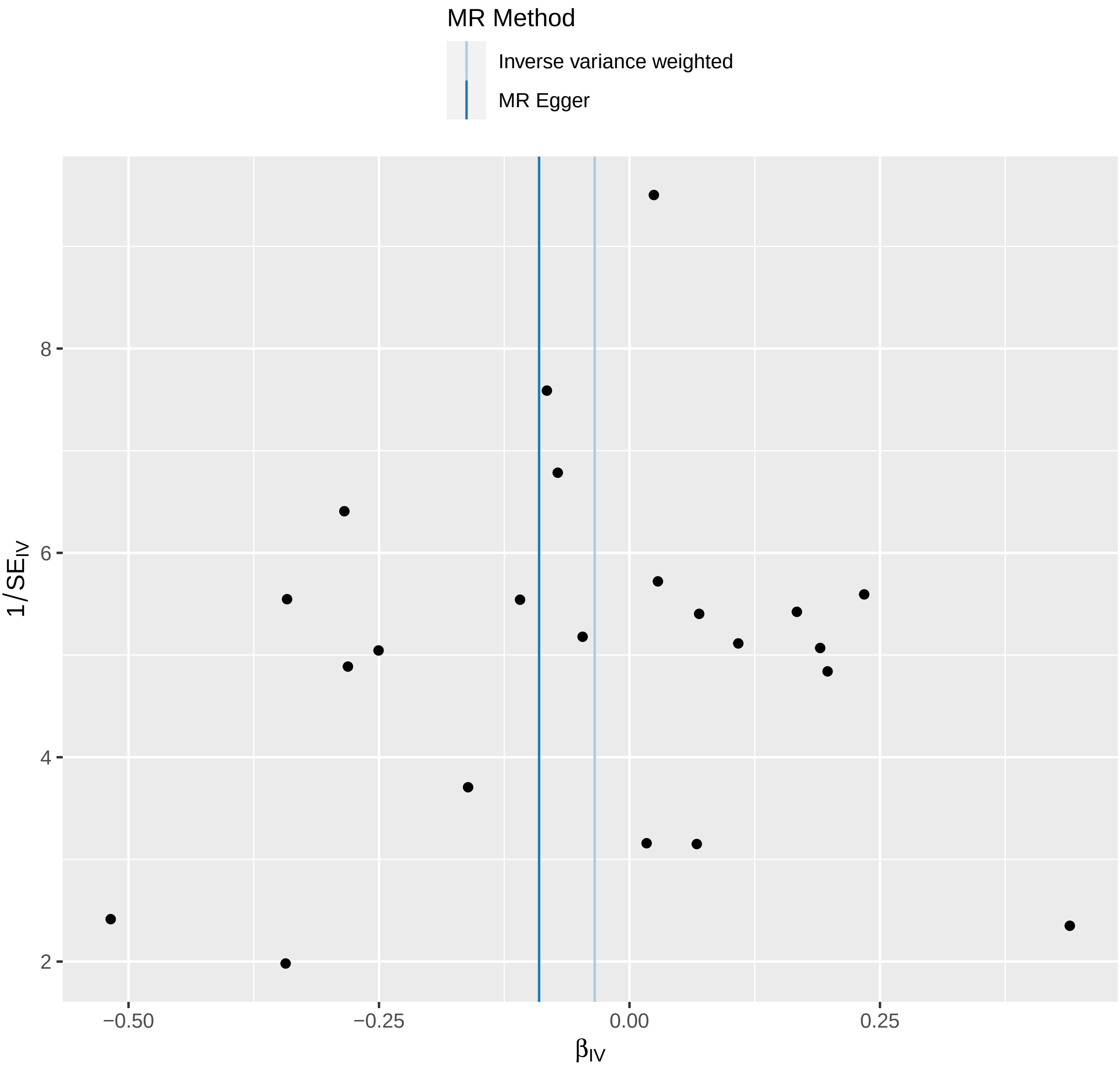

Batch 3−2828 : Testican−3 on Diabetic nephropathy (more control exclusions)
with a threshold of 1e−05

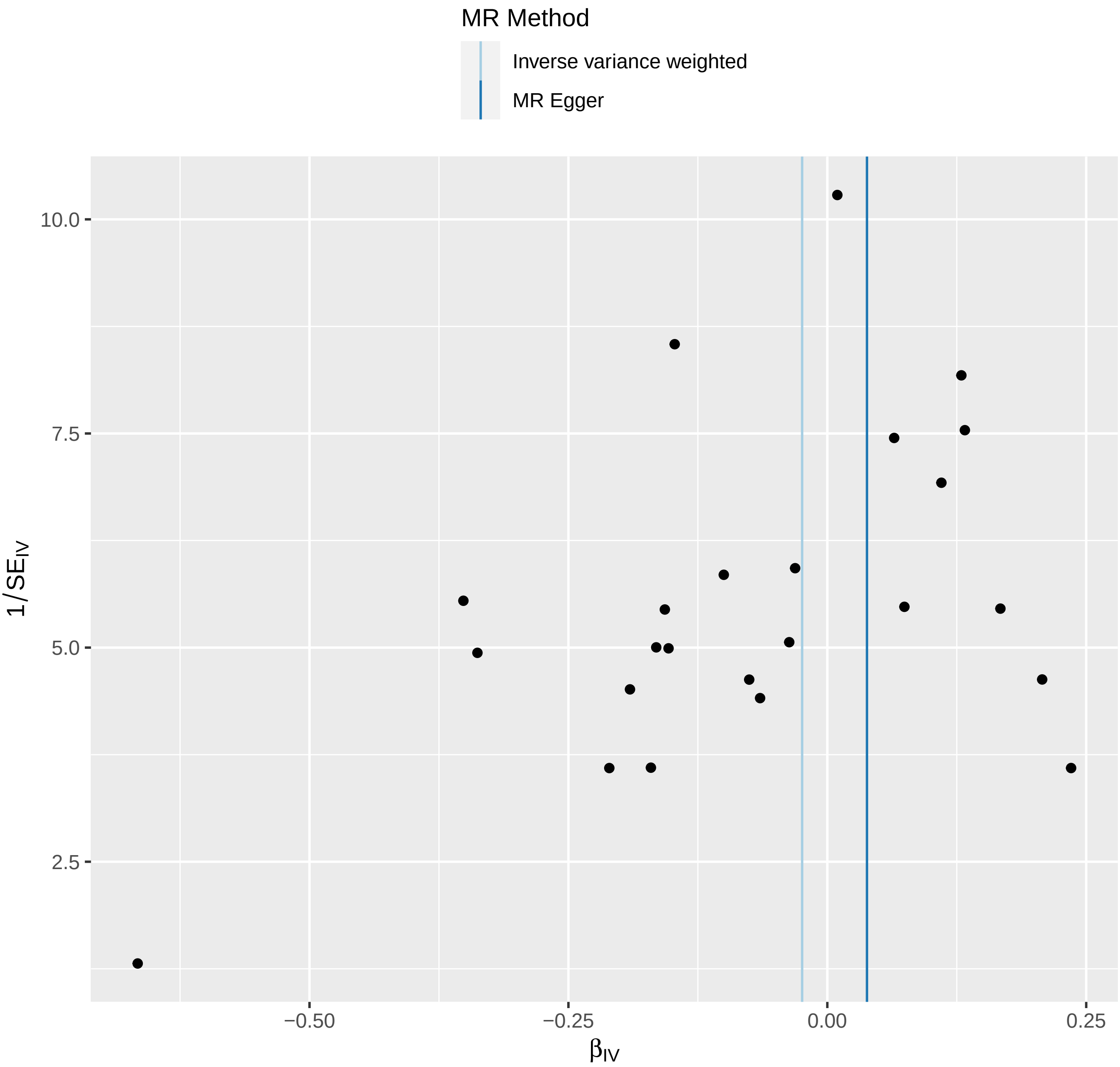

Batch 3−2829 : Spondin−1 on Diabetic nephropathy (more control exclusions)
with a threshold of 1e−05

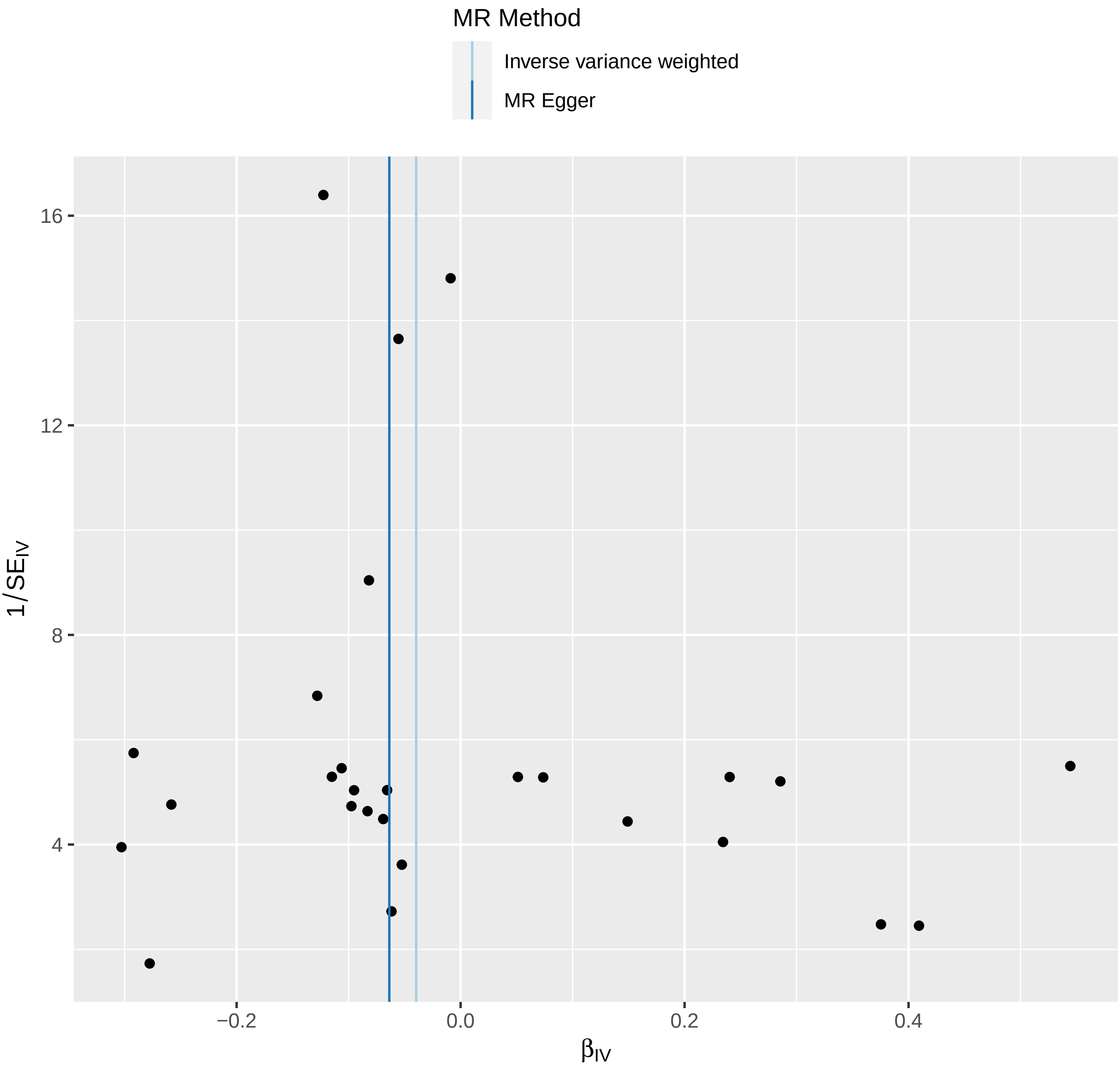

Batch 3−2830 : Speckle−type POZ protein on Diabetic nephropathy (more control exclusions)
with a threshold of 1e−05

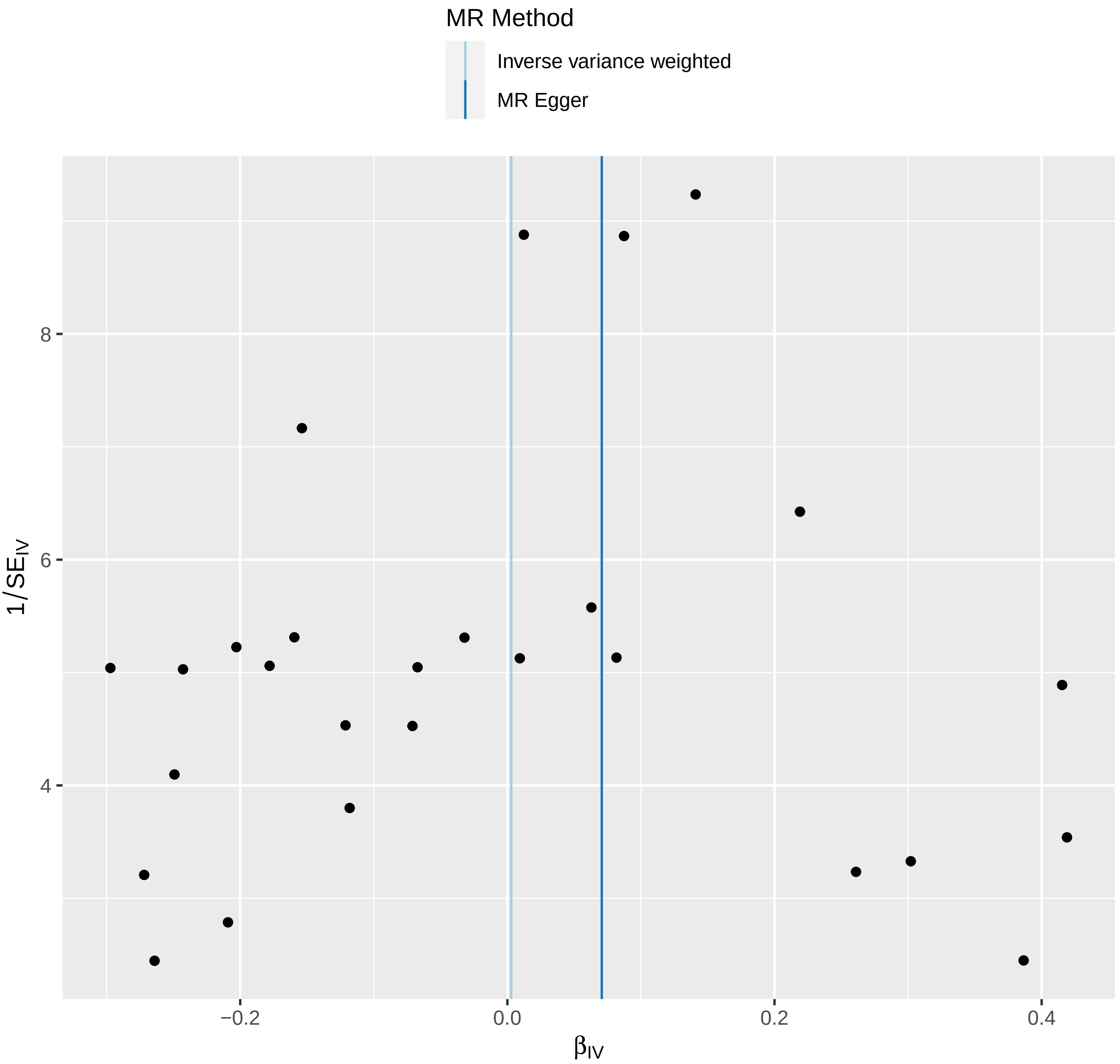

Batch 3−2831 : Sepiapterin reductase on Diabetic nephropathy (more control exclusions)
with a threshold of 1e−05

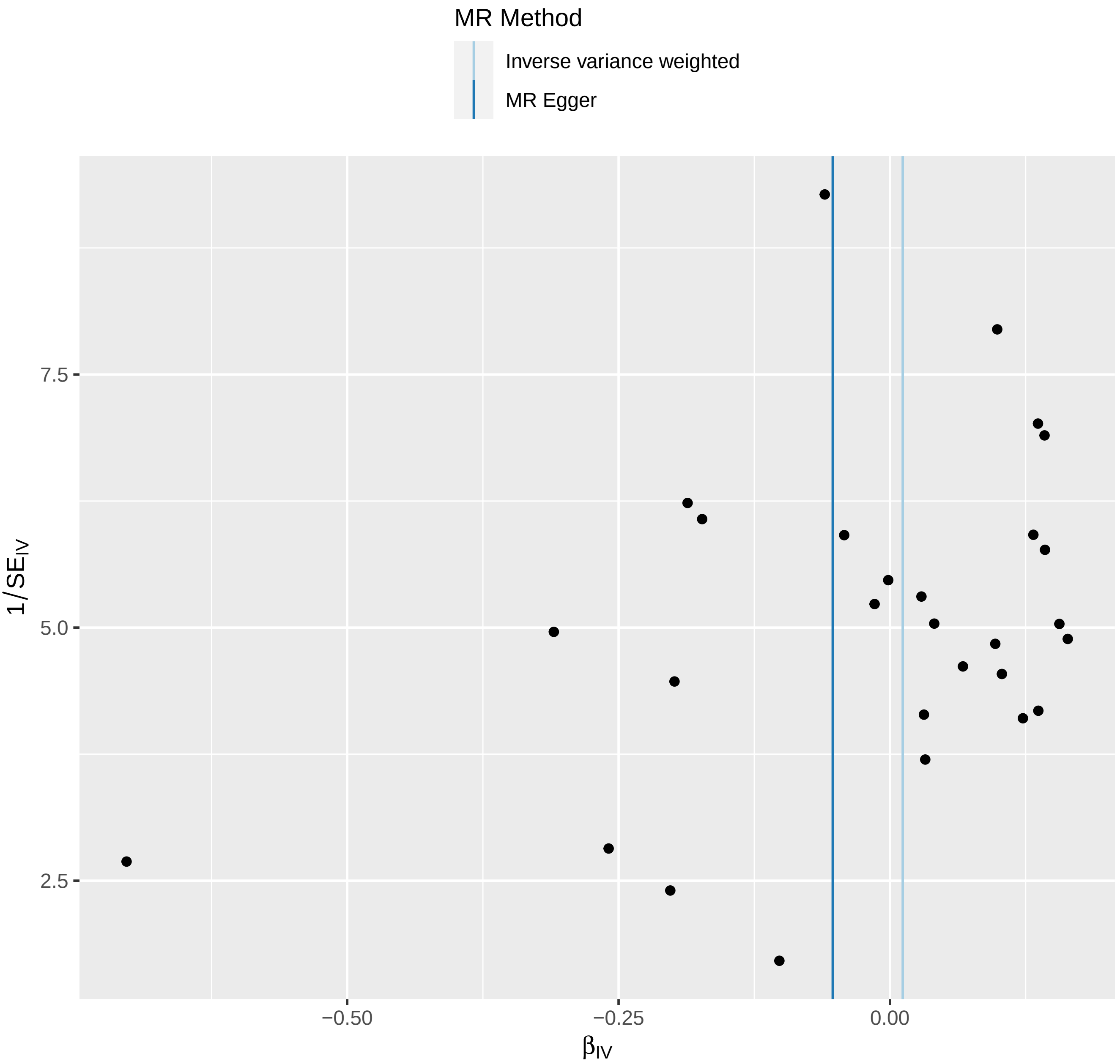

Batch 3−2832 : Sprouty−related, EVH1 domain−containing protein 1 on Diabetic nephropathy (more control exclusions) with a threshold of 1e−05

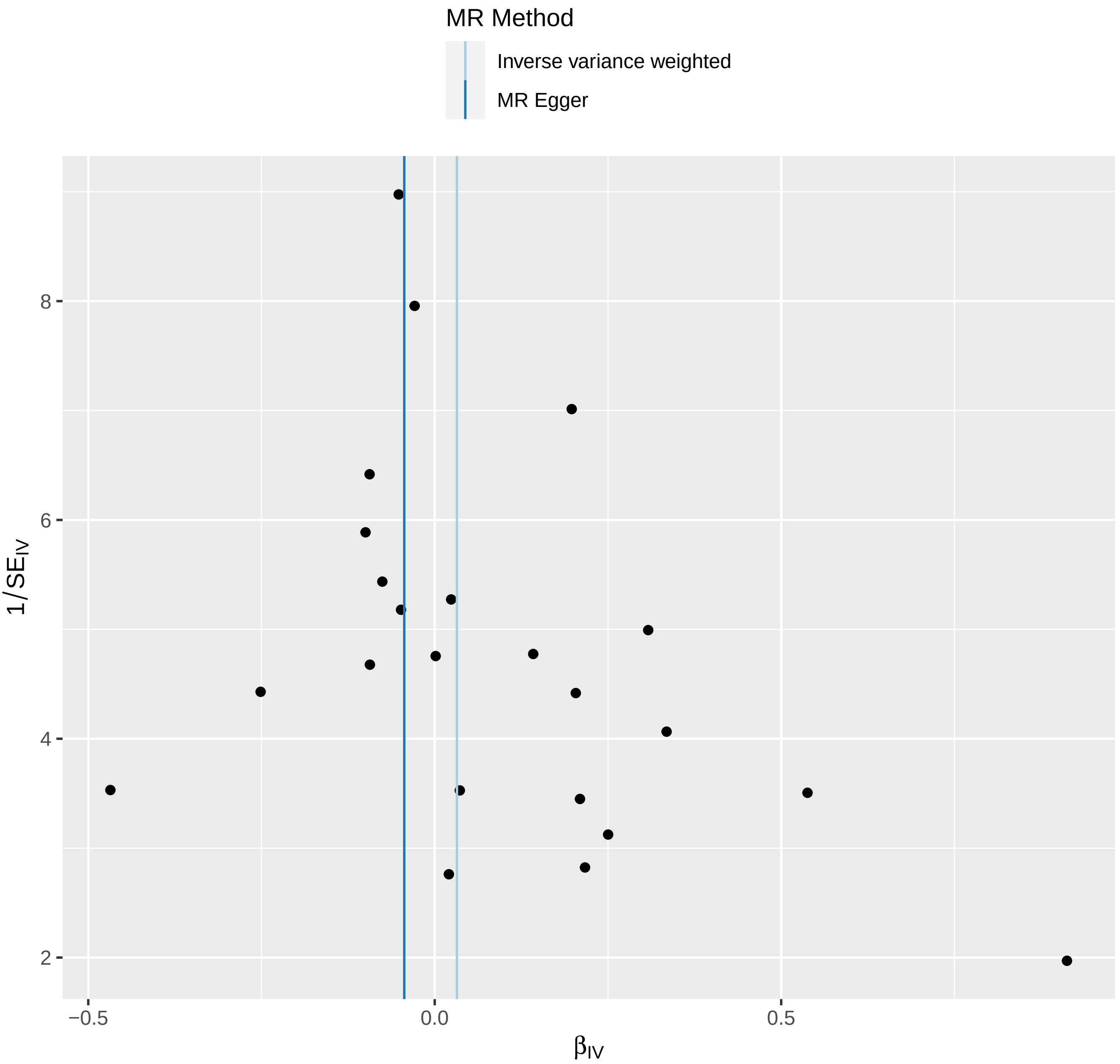

Batch 3−2833 : Protein sprouty homolog 1 on Diabetic nephropathy (more control exclusions)
with a threshold of 1e−05

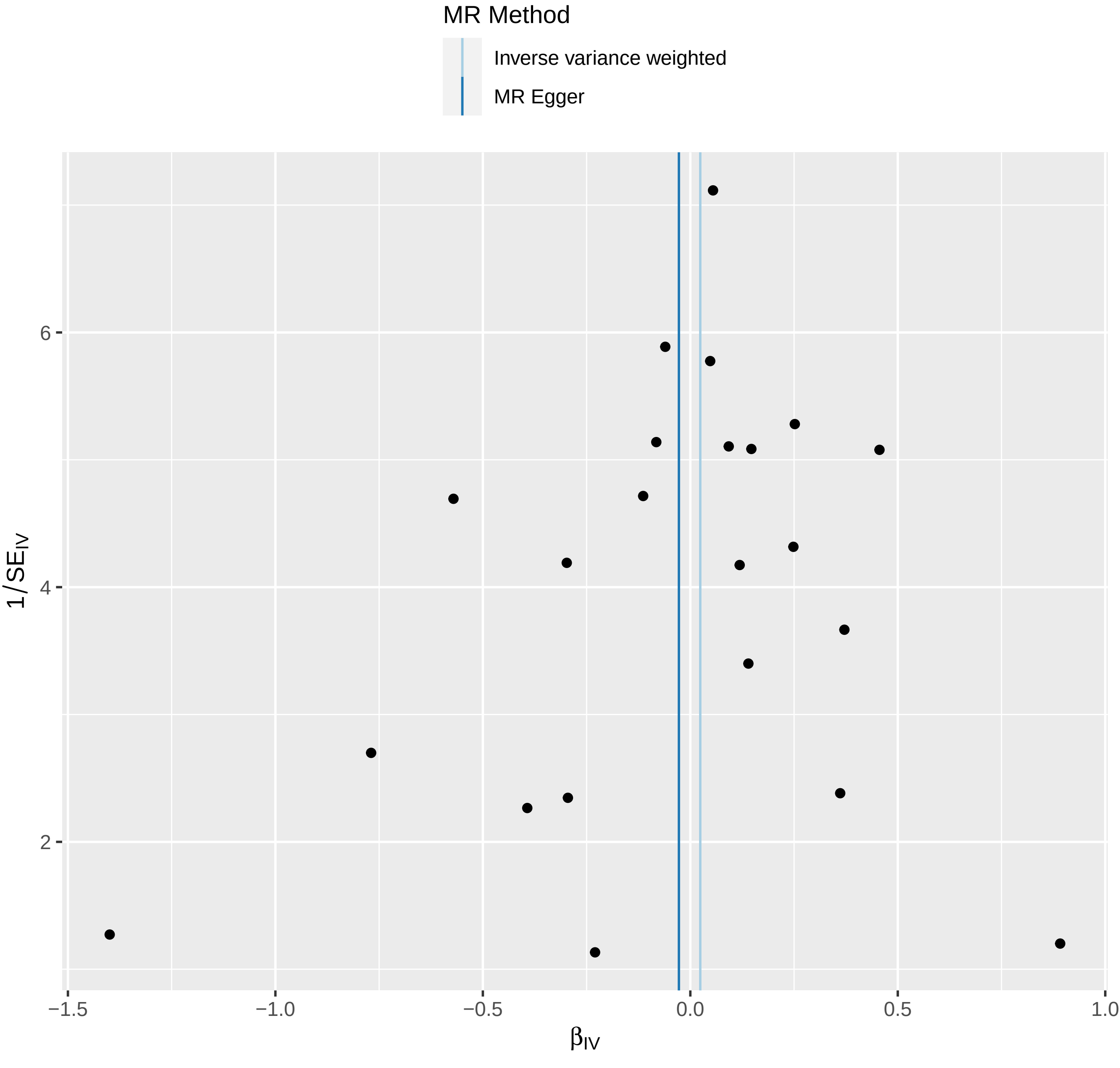

Batch 3−2834 : SPRY domain−containing SOCS box protein 1 on Diabetic nephropathy (more control exclusions)
with a threshold of 1e−05

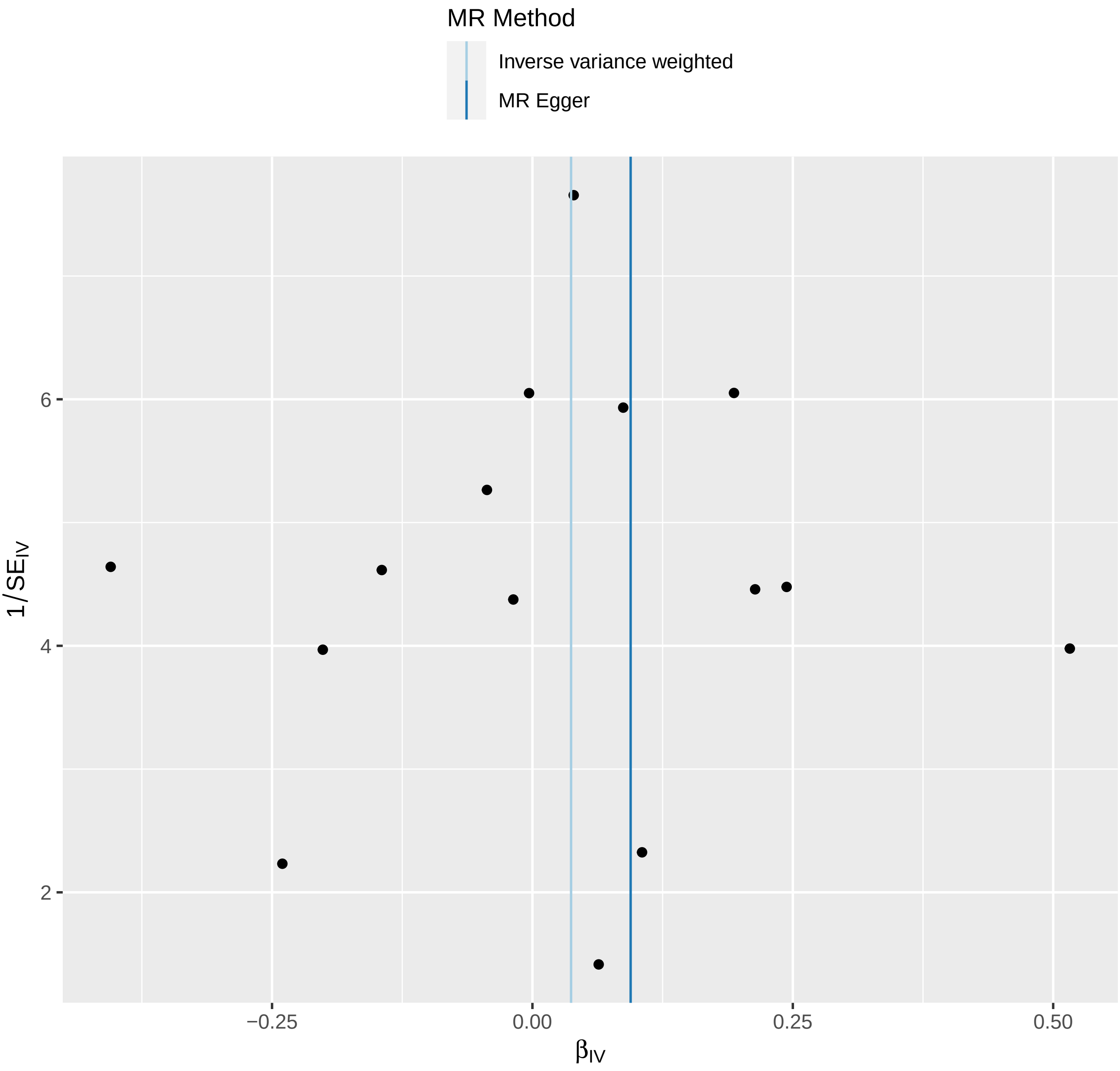

Batch 3−2835 : Spectrin alpha chain, non−erythrocytic 1 on Diabetic nephropathy (more control exclusions)
with a threshold of 1e−05

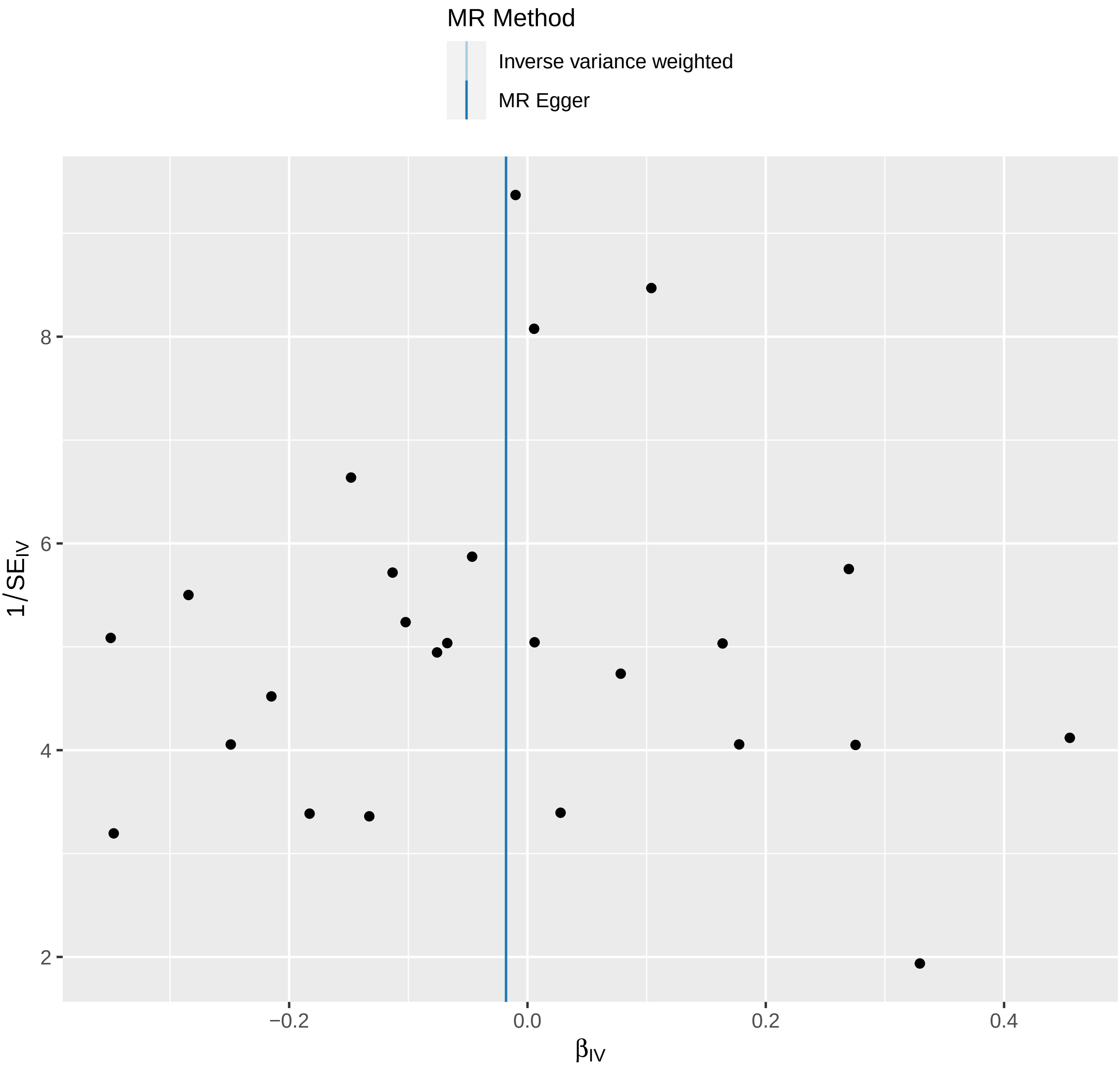

Batch 3−2836 : Serine palmitoyltransferase 1 on Diabetic nephropathy (more control exclusions)
with a threshold of 1e−05

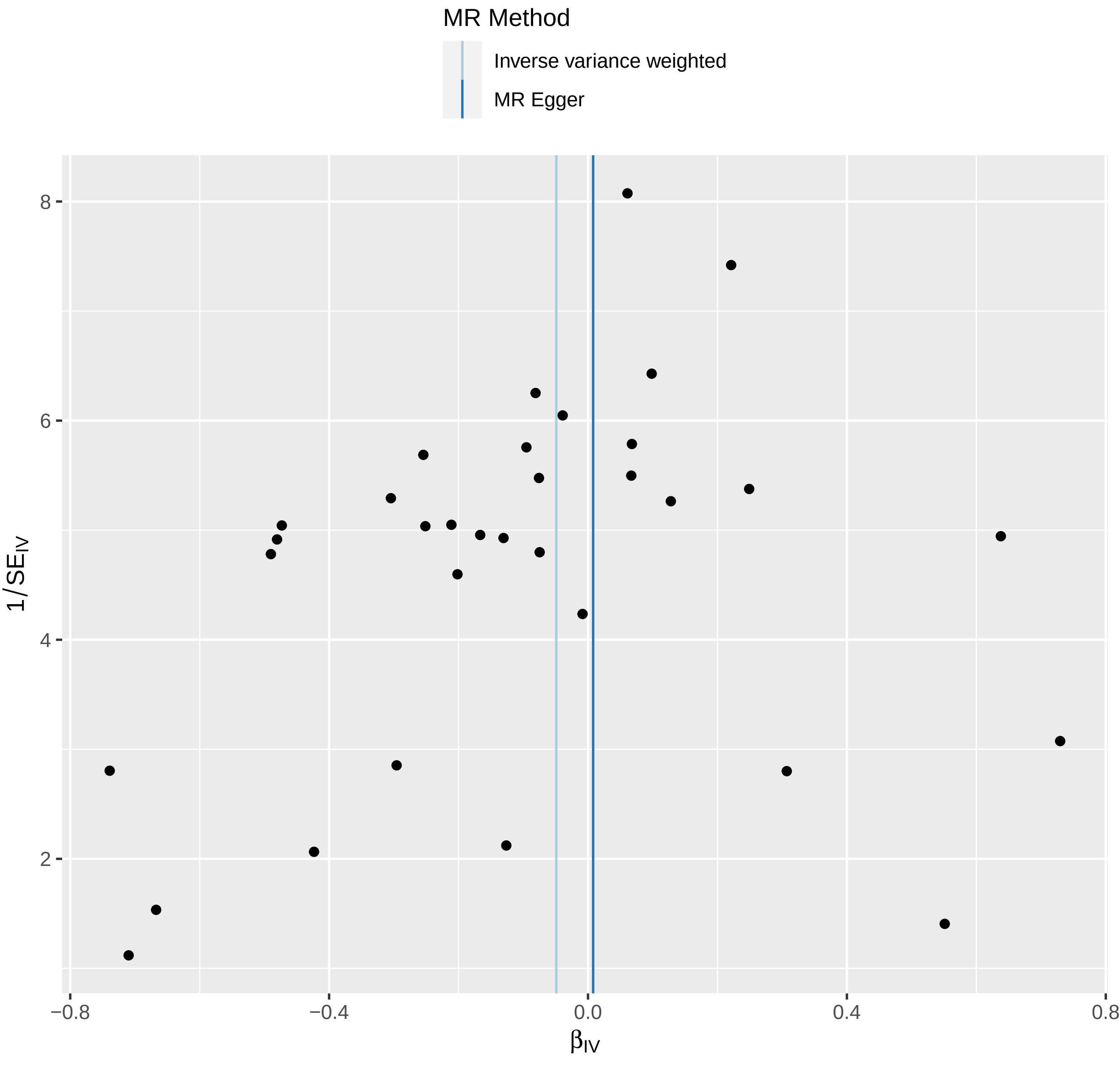

Batch 3−2837 : Serine palmitoyltransferase 2 on Diabetic nephropathy (more control exclusions)
with a threshold of 1e−05

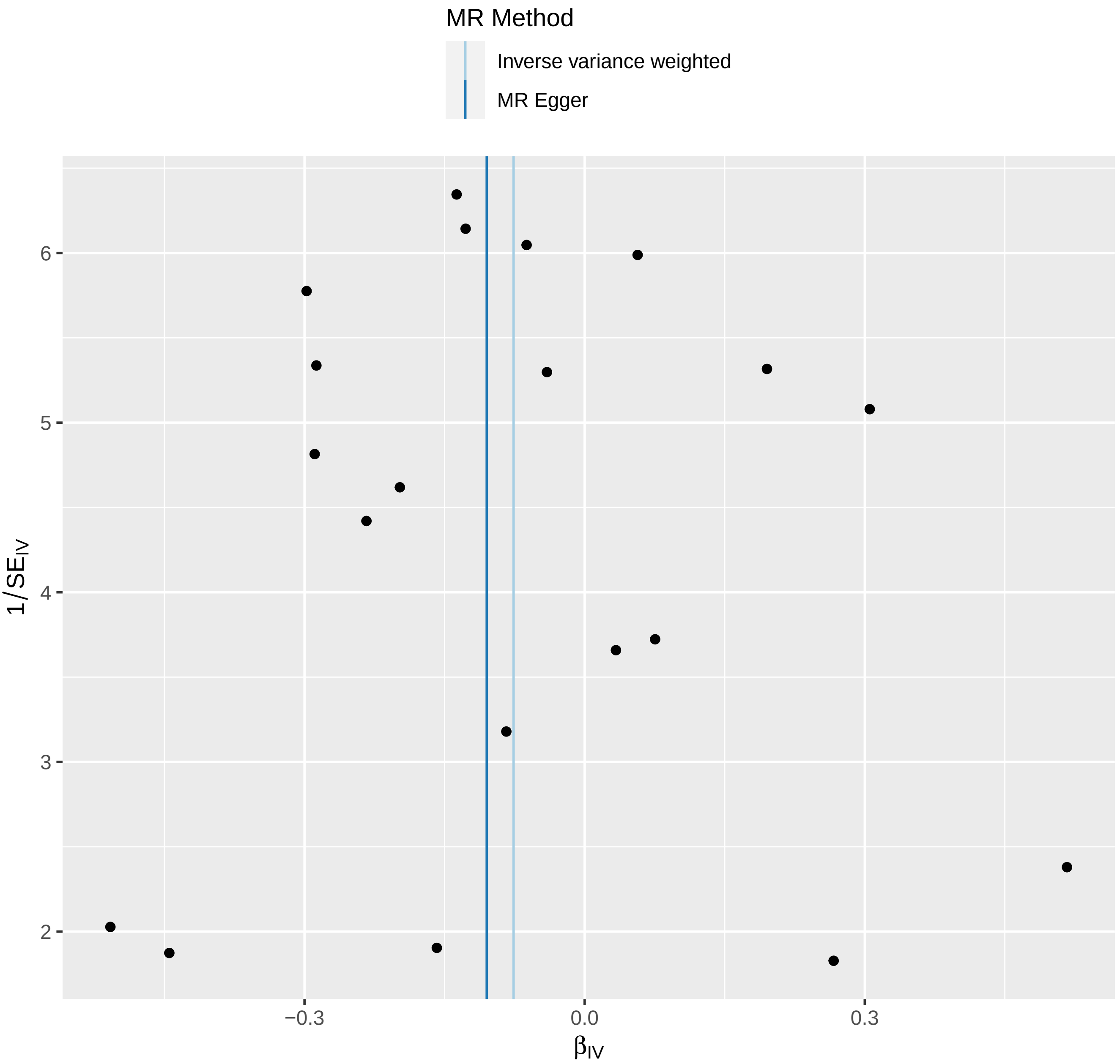

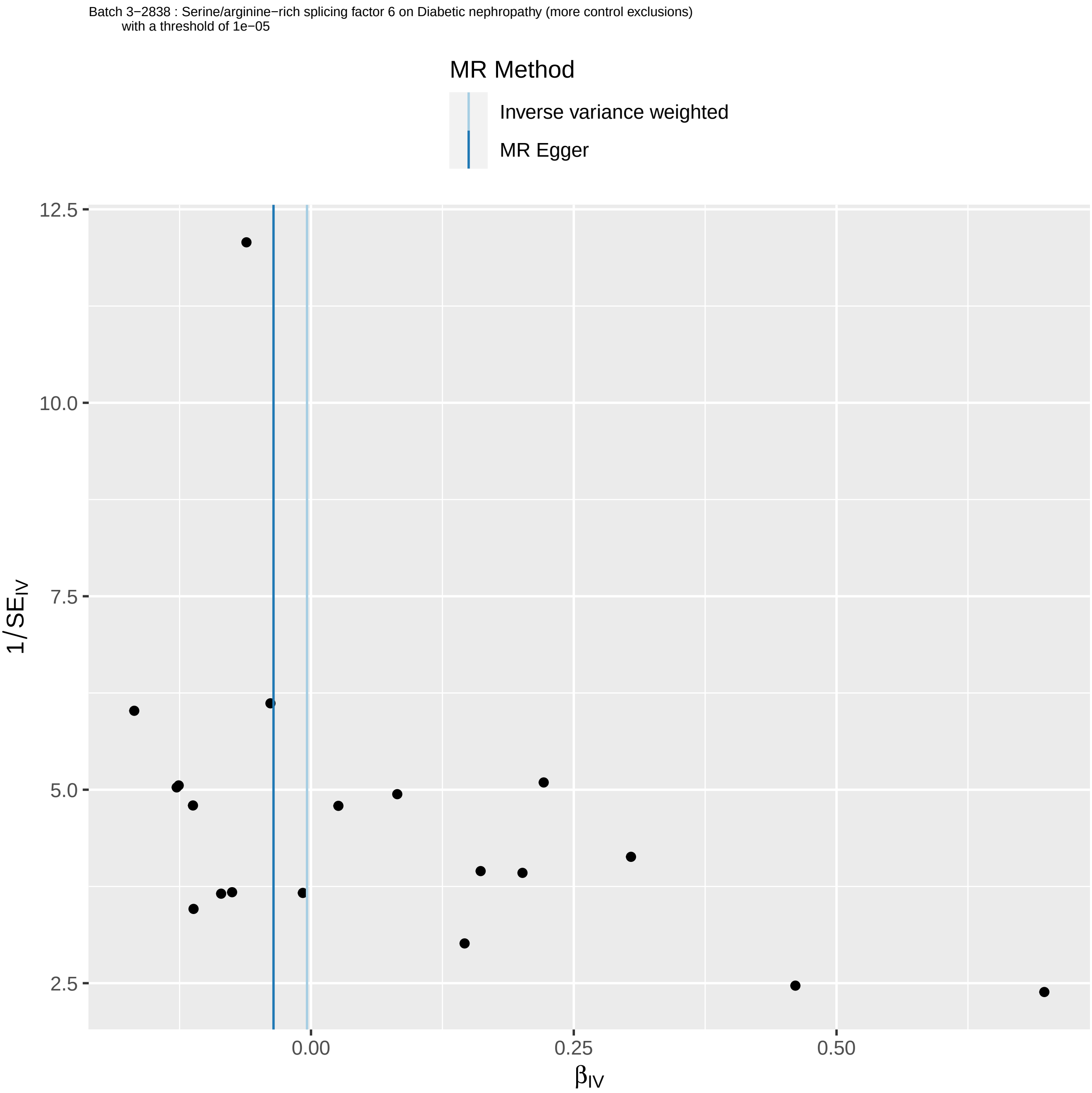

Batch 3−2838 : Serine/arginine−rich splicing factor 6 on Diabetic nephropathy (more control exclusions)
with a threshold of 1e−05
MR Method
Inverse variance weighted
MR Egger
12.5
10.0
7.5
5.0
2.5
1/SE$_{IV}$
0.00
0.25
0.50
β$_{IV}$

Batch 3−2839 : Serine/arginine−rich splicing factor 7 on Diabetic nephropathy (more control exclusions)
with a threshold of 1e−05

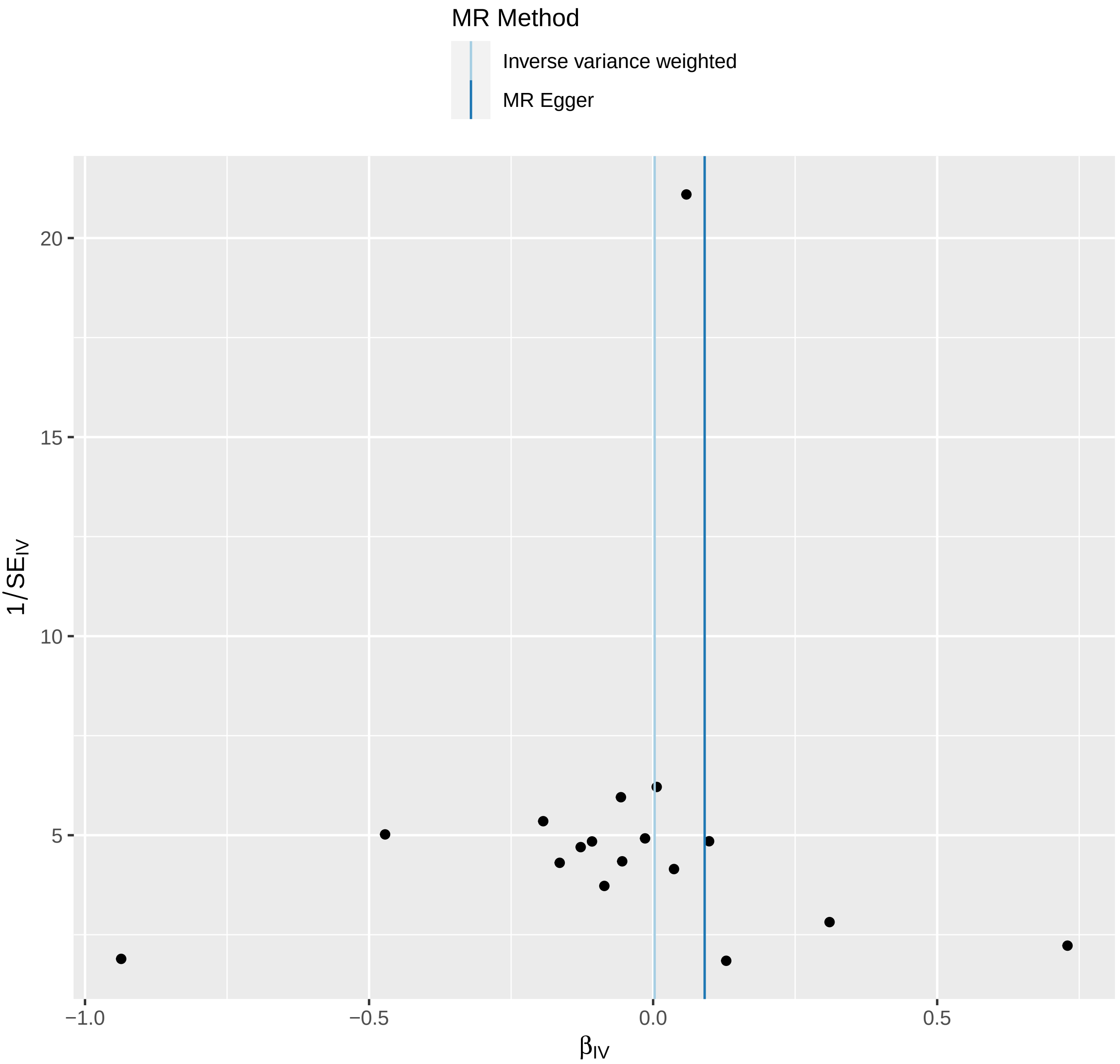

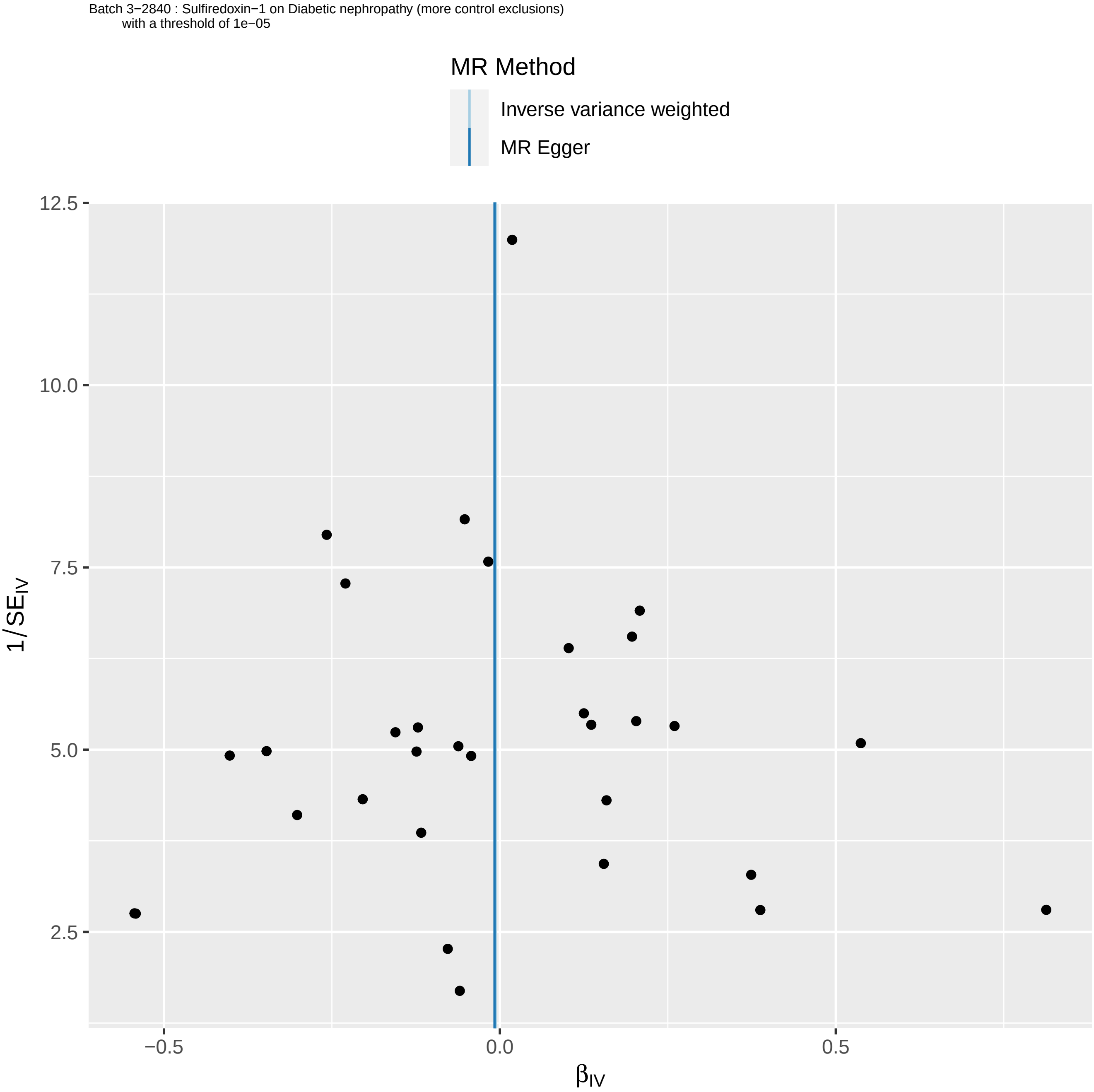
Batch 3−2840 : Sulfiredoxin−1 on Diabetic nephropathy (more control exclusions)
with a threshold of 1e−05
MR Method
Inverse variance weighted
MR Egger
12.5
10.0
7.5
5.0
2.5
1/SE_IV
−0.5
0.0
0.5
β_IV

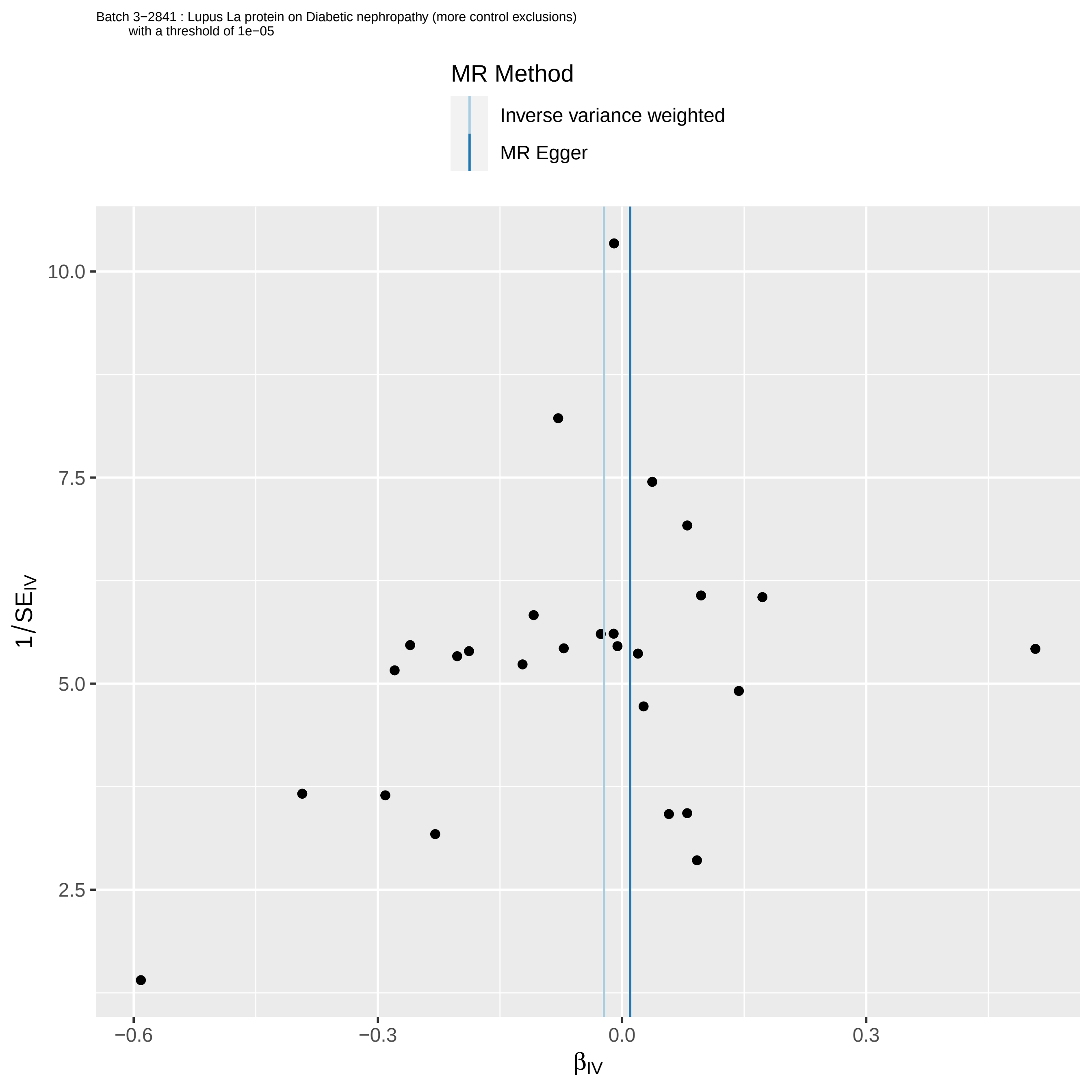

Batch 3−2841 : Lupus La protein on Diabetic nephropathy (more control exclusions)
with a threshold of 1e−05
MR Method
Inverse variance weighted
MR Egger
10.0
7.5
5.0
2.5
$1/SE_{IV}$
−0.6
−0.3
0.0
0.3
β_{IV}

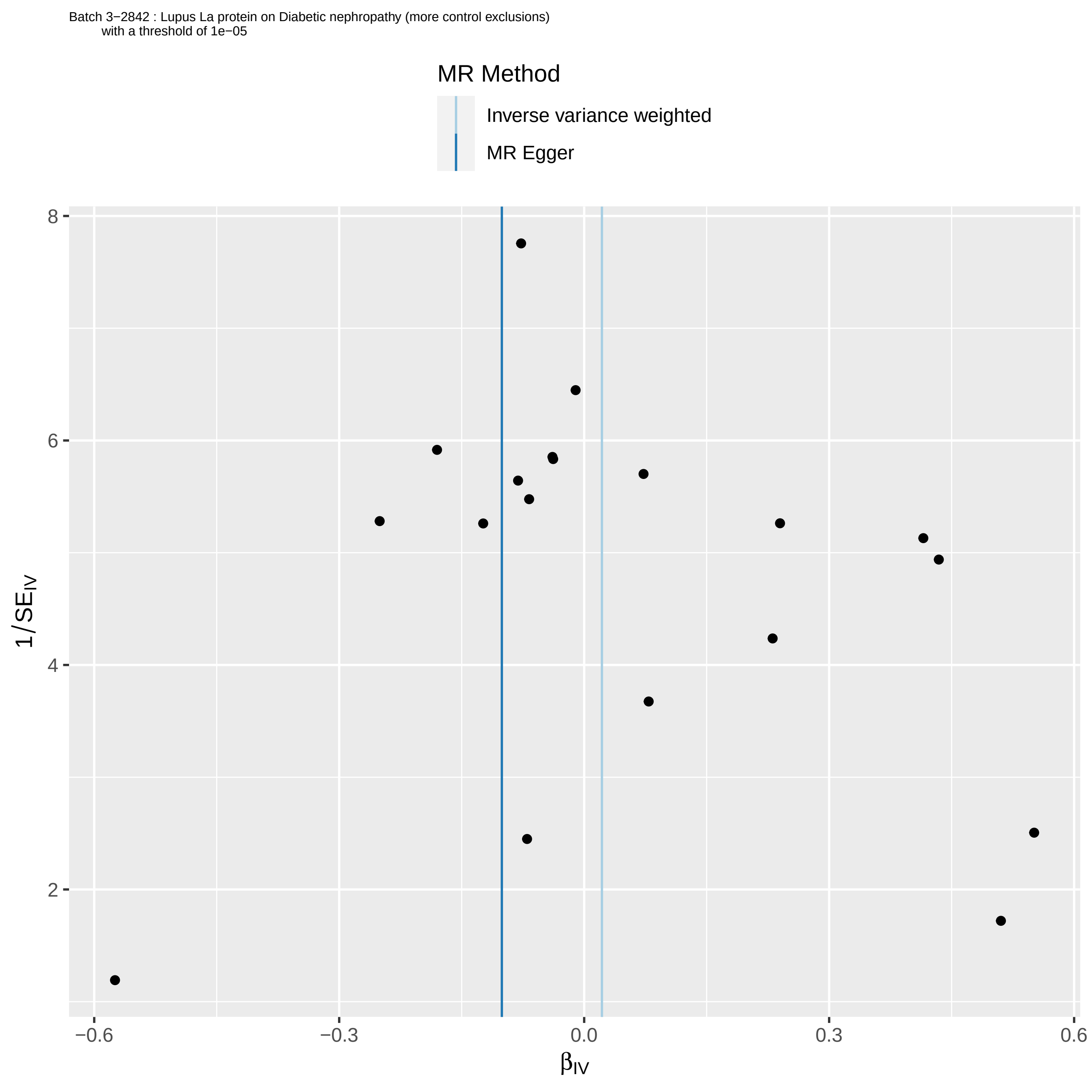

Batch 3−2842 : Lupus La protein on Diabetic nephropathy (more control exclusions)
with a threshold of 1e−05
MR Method
Inverse variance weighted
MR Egger
8
6
4
2
1/SE$_{IV}$
−0.6
−0.3
0.0
0.3
0.6
β_{IV}

Batch 3−2843 : Serine−rich single−pass membrane protein 1 on Diabetic nephropathy (more control exclusions)
with a threshold of 1e−05

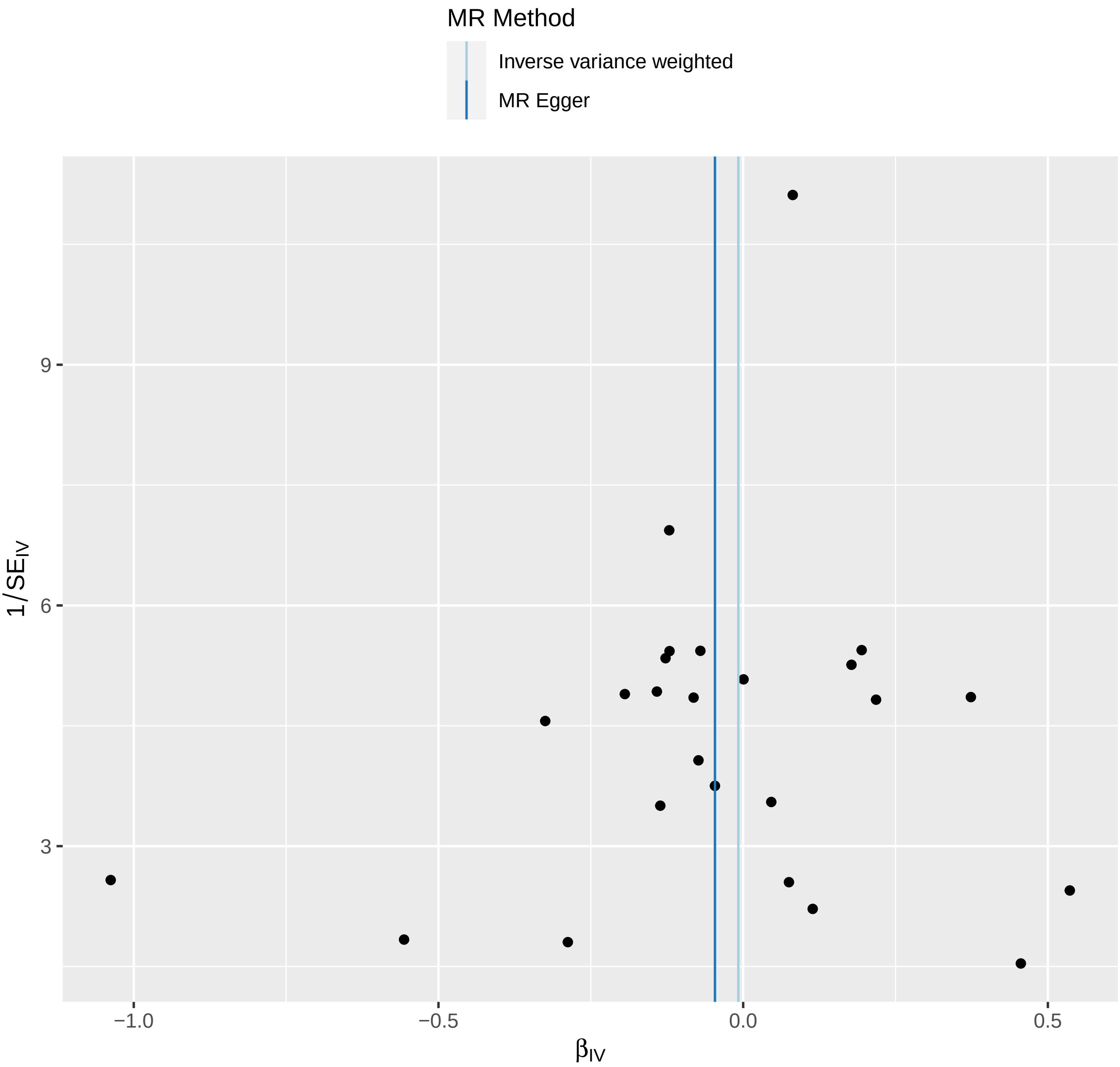

Batch 3−2844 : Translocon−associated protein subunit alpha on Diabetic nephropathy (more control exclusions)
with a threshold of 1e−05

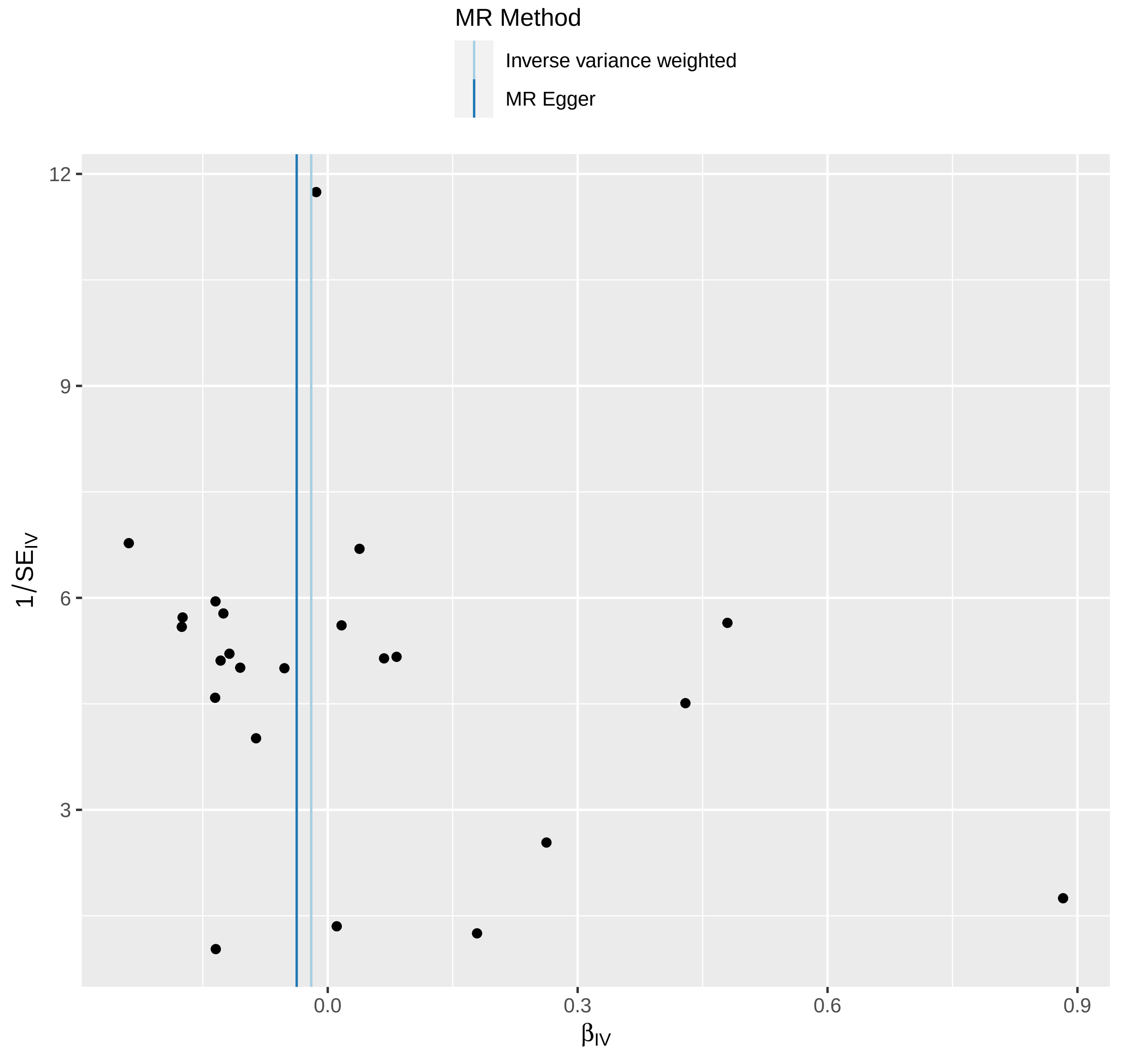

Batch 3−2845 : FACT complex subunit SSRP1 on Diabetic nephropathy (more control exclusions)
with a threshold of 1e−05

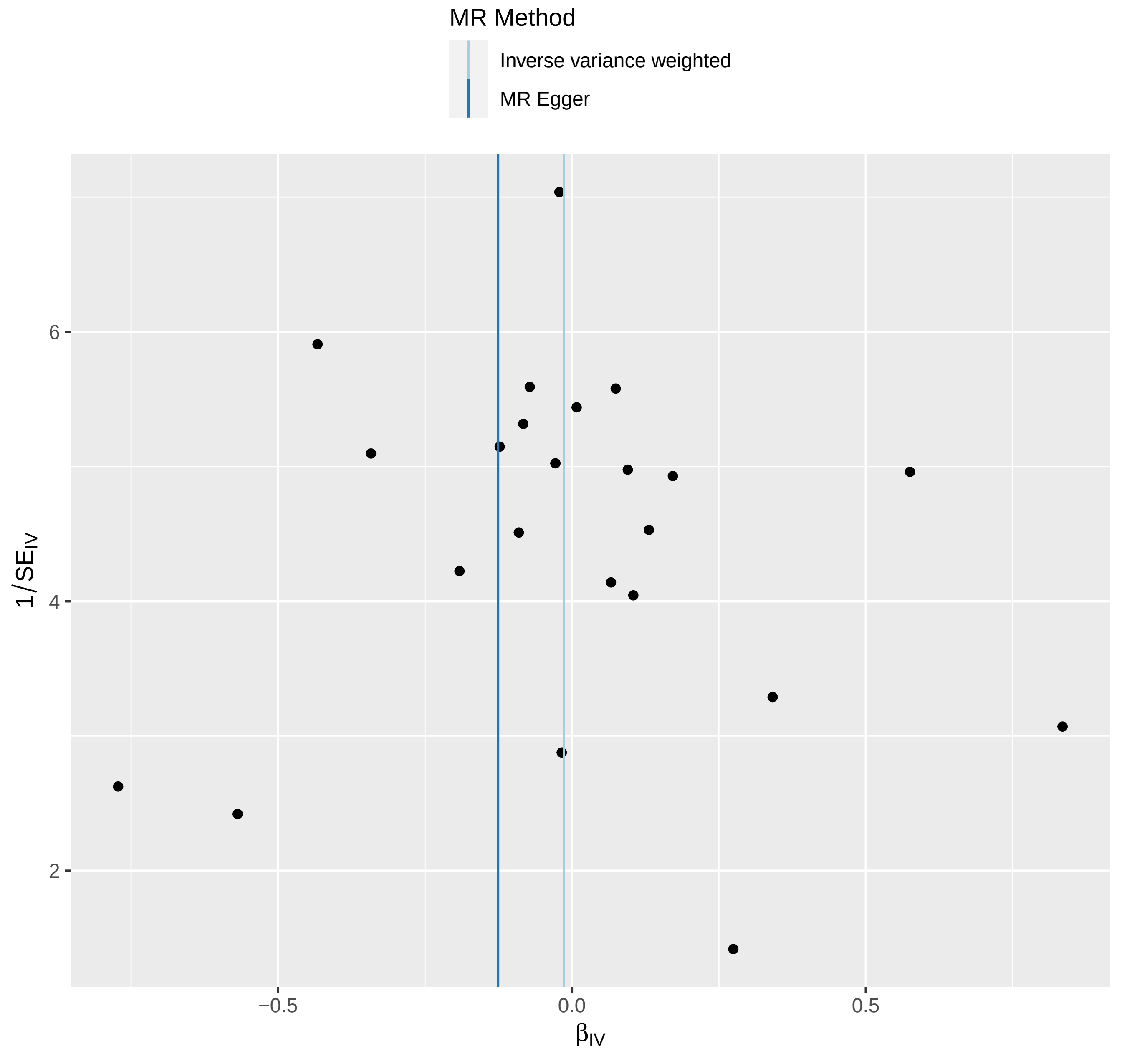

Batch 3−2846 : Somatostatin−28 on Diabetic nephropathy (more control exclusions)
with a threshold of 1e−05

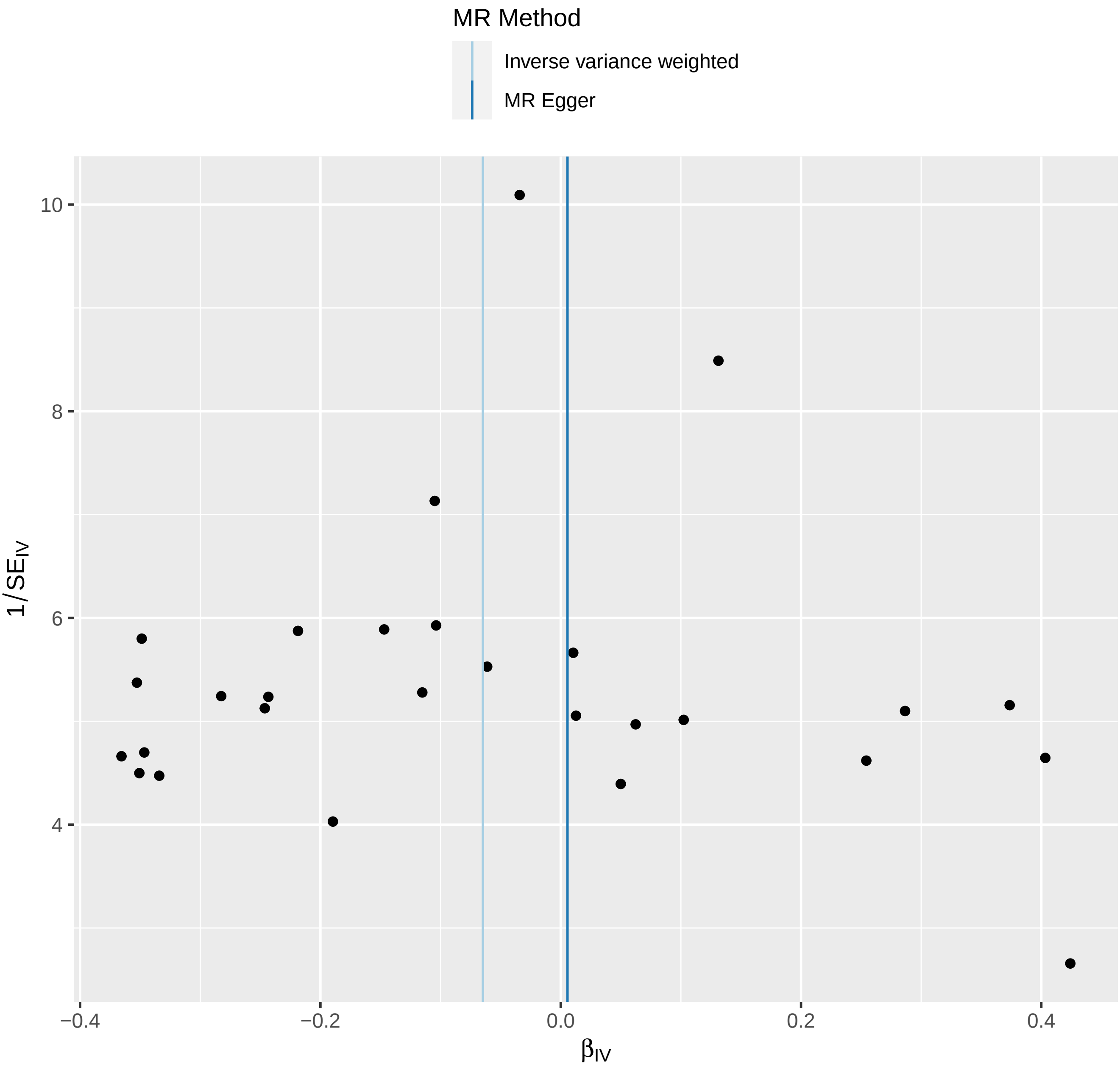

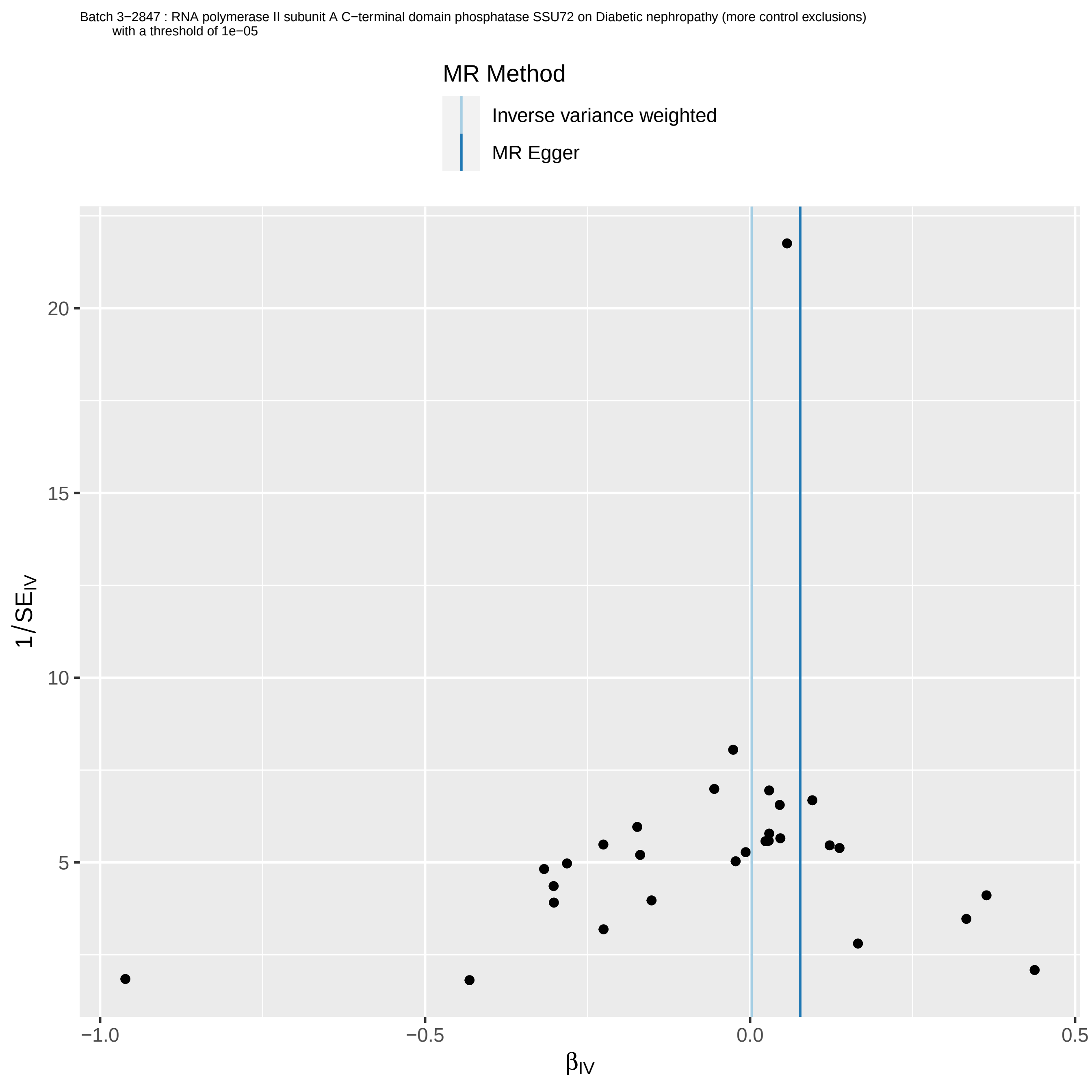
Batch 3−2847 : RNA polymerase II subunit A C−terminal domain phosphatase SSU72 on Diabetic nephropathy (more control exclusions) with a threshold of 1e−05
MR Method
Inverse variance weighted
MR Egger
20
15
10
5
1/SE_IV
−1.0
−0.5
0.0
0.5
β_IV

Batch 3−2848 : CMP−N−acetylneuraminate−beta−galactosamide−alpha−2,3−sialyltransferase 1 on Diabetic nephropathy (more control exclusions) with a threshold of 1e−05

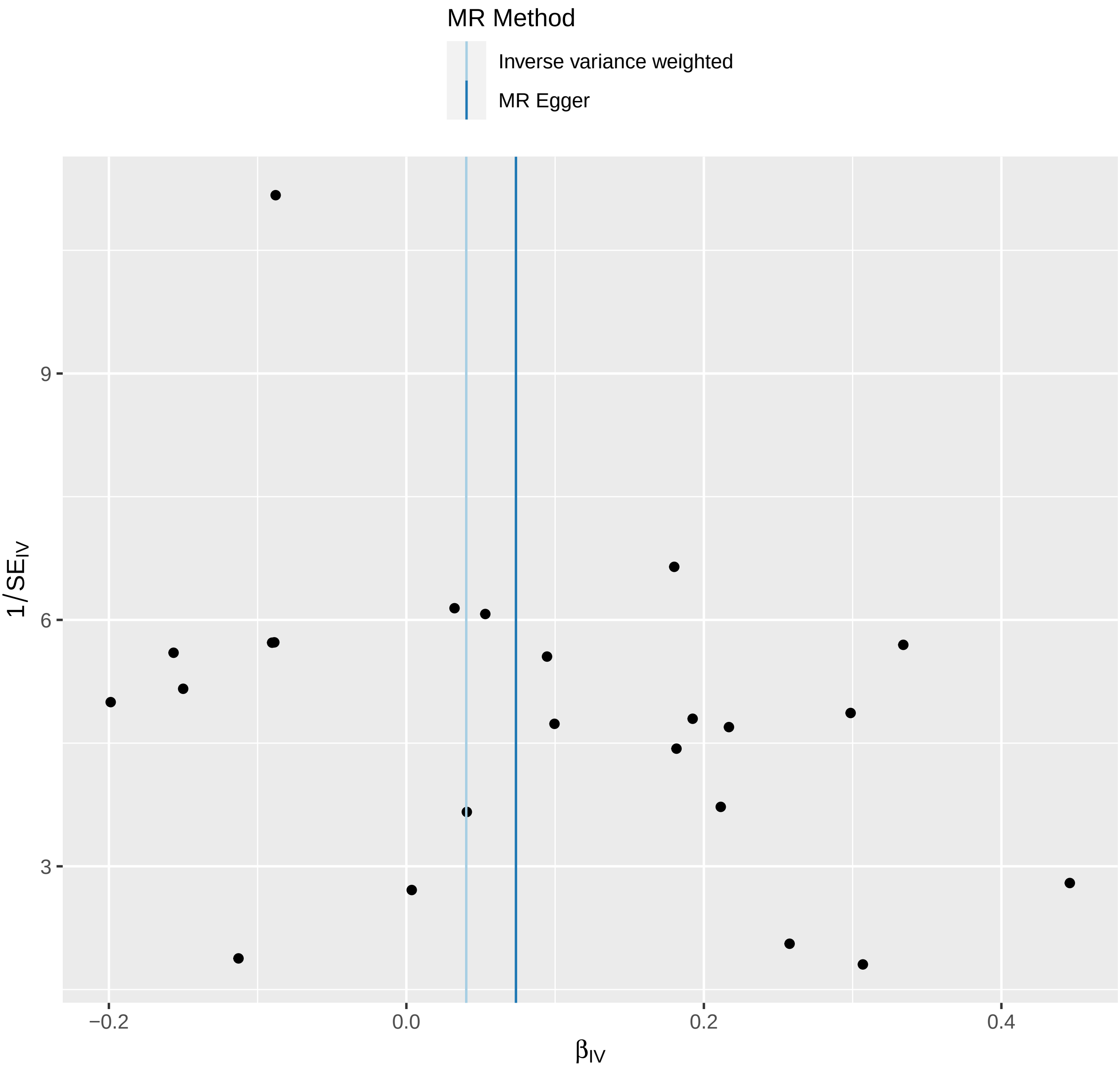

Batch 3−2849 : CMP−N−acetylneuraminate−beta−galactosamide−alpha−2,3−sialyltransferase 2 on Diabetic nephropathy (more control exclusions) with a threshold of 1e−05

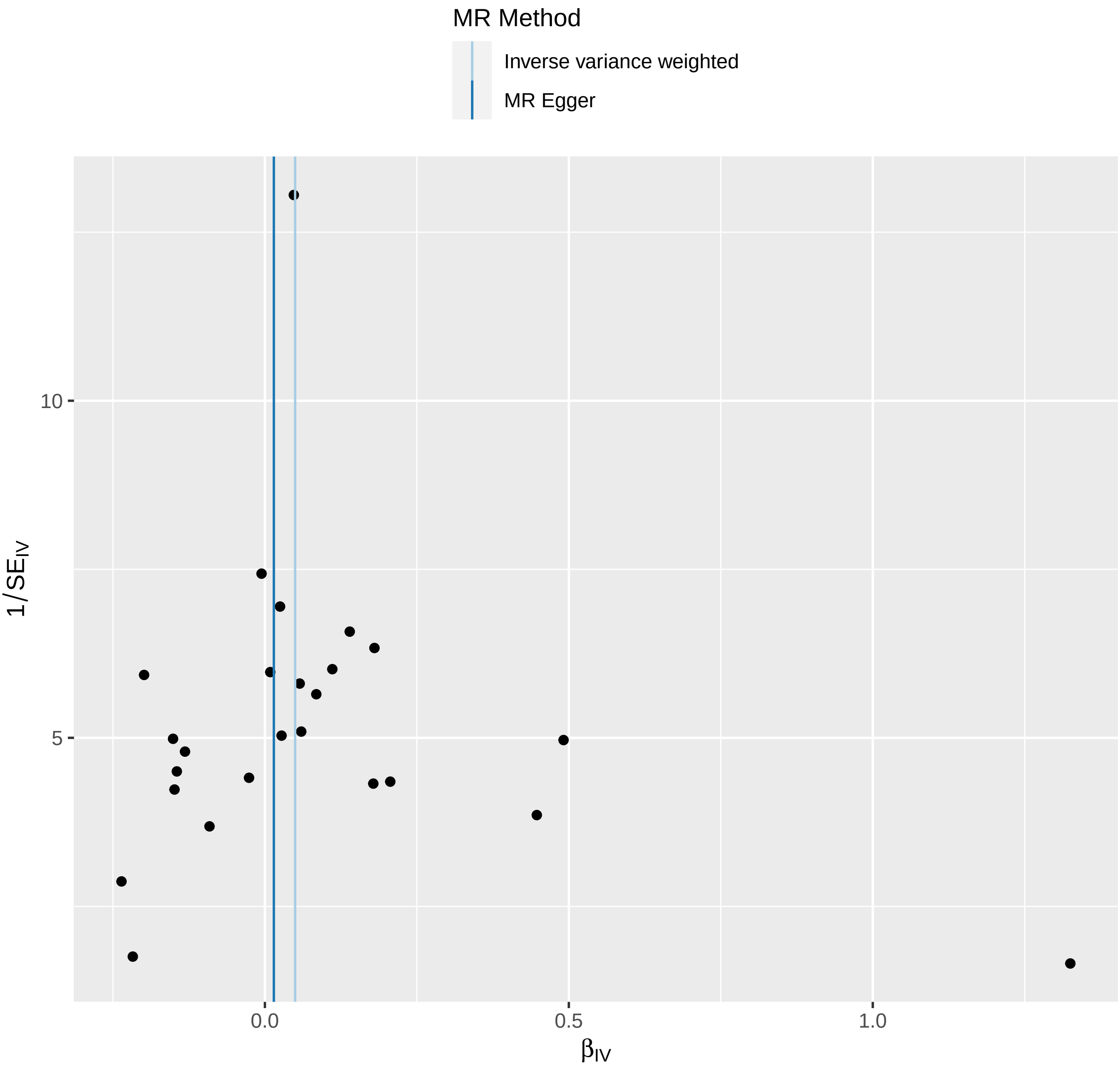

Batch 3−2850 : Type 2 lactosamine alpha−2,3−sialyltransferase on Diabetic nephropathy (more control exclusions) with a threshold of 1e−05

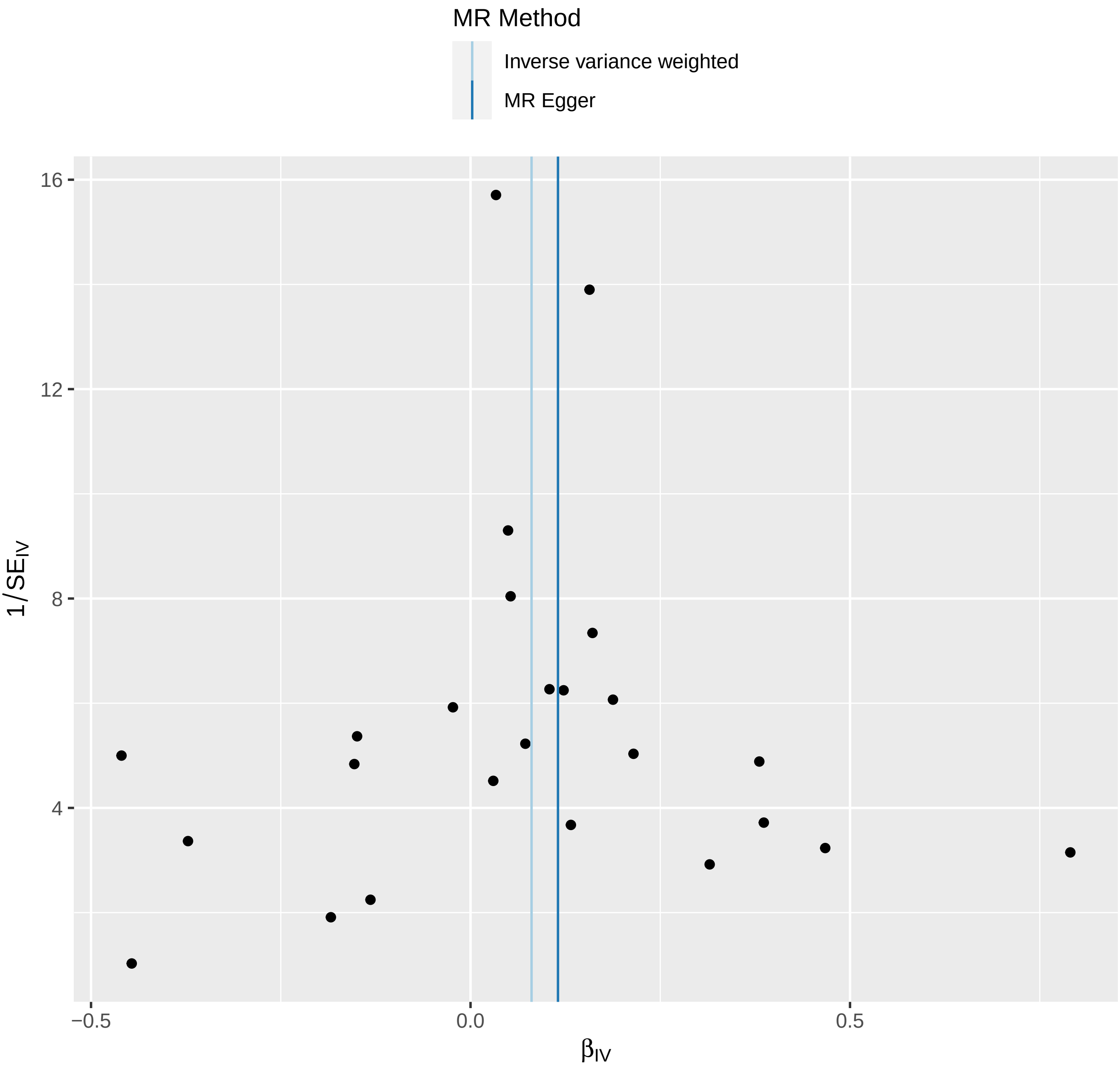

Batch 3−2851 : Beta−galactoside alpha−2,6−sialyltransferase 1 on Diabetic nephropathy (more control exclusions) with a threshold of 1e−05

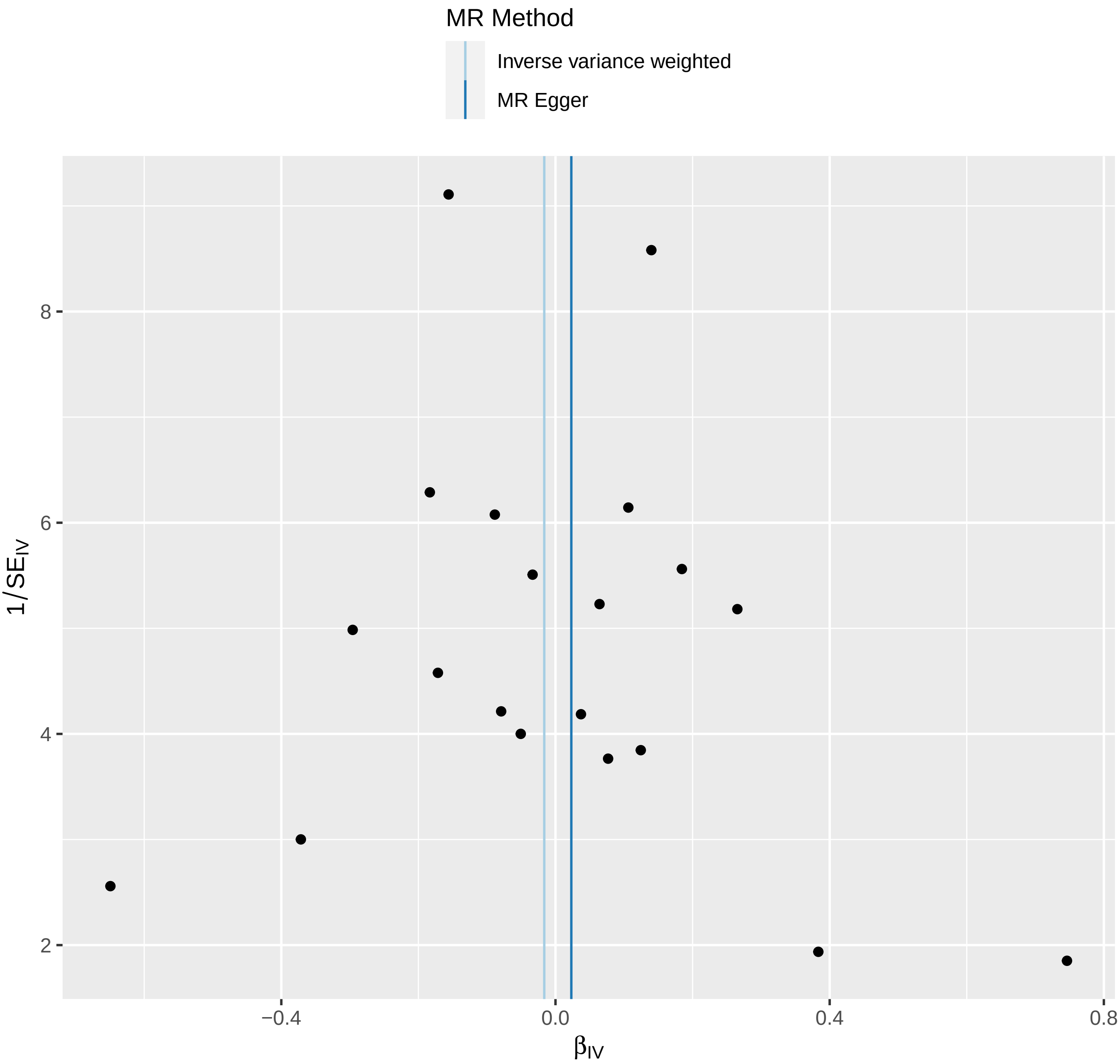

Batch 3−2852 : Alpha−N−acetylgalactosaminide alpha−2,6−sialyltransferase 1 on Diabetic nephropathy (more control exclusions) with a threshold of 1e−05

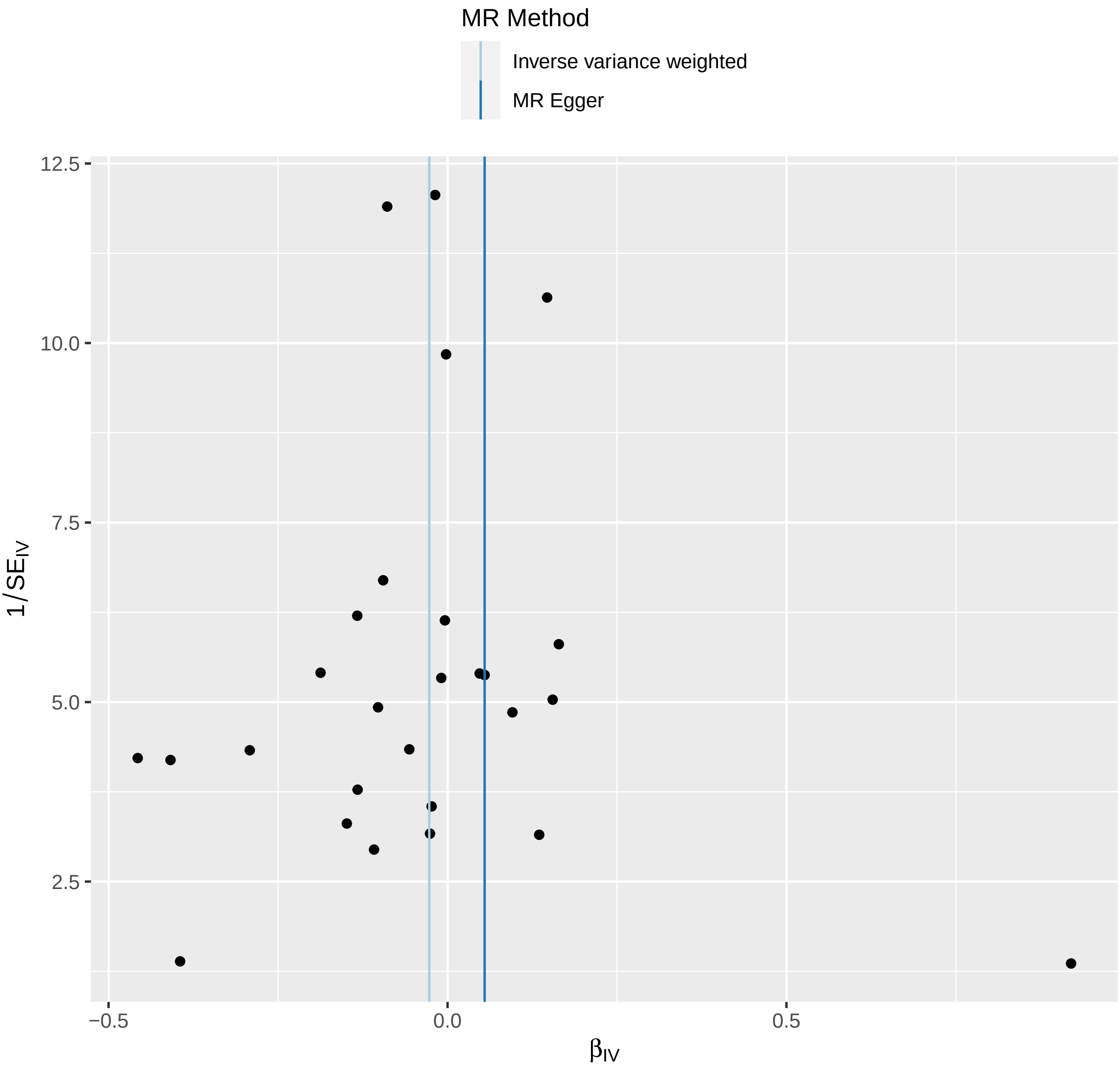

Batch 3−2853 : Alpha−N−acetylgalactosaminide alpha−2,6−sialyltransferase 2 on Diabetic nephropathy (more control exclusions)
with a threshold of 1e−05

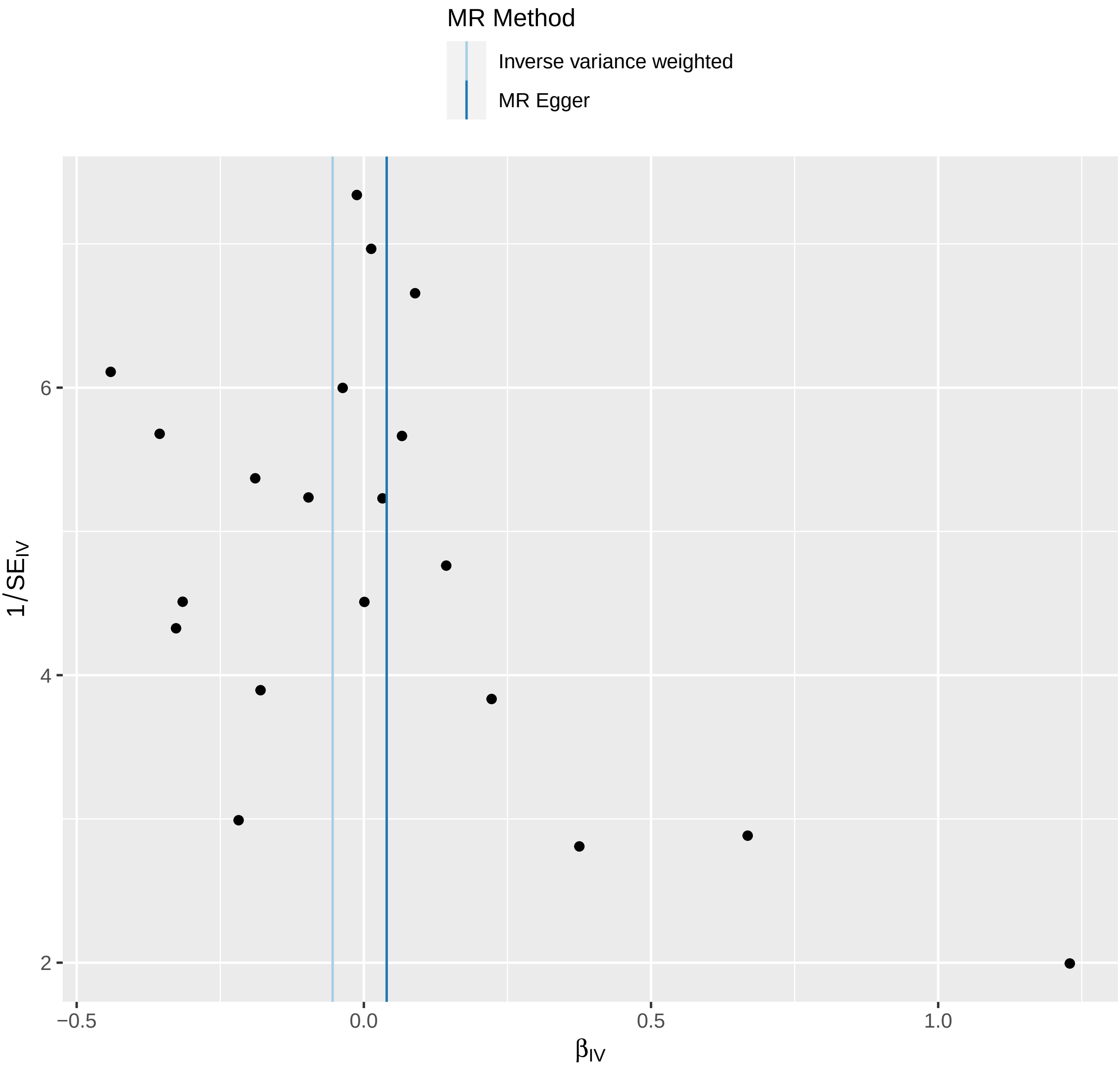

Batch 3−2854 : Alpha−N−acetylgalactosaminide alpha−2,6−sialyltransferase 3 on Diabetic nephropathy (more control exclusions)
with a threshold of 1e−05

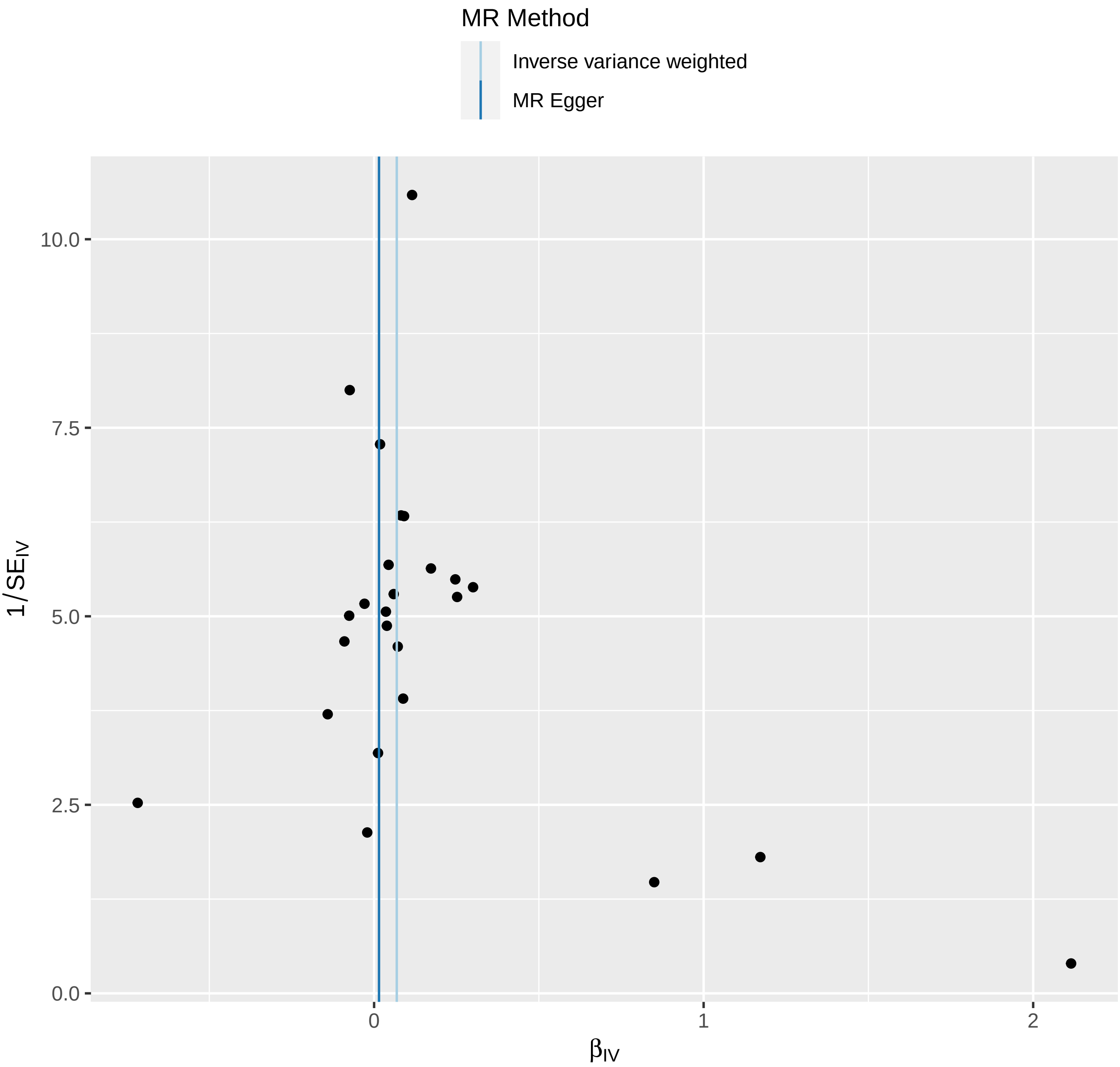

Batch 3−2855 : Alpha−N−acetylgalactosaminide alpha−2,6−sialyltransferase 3 on Diabetic nephropathy (more control exclusions)
with a threshold of 1e−05

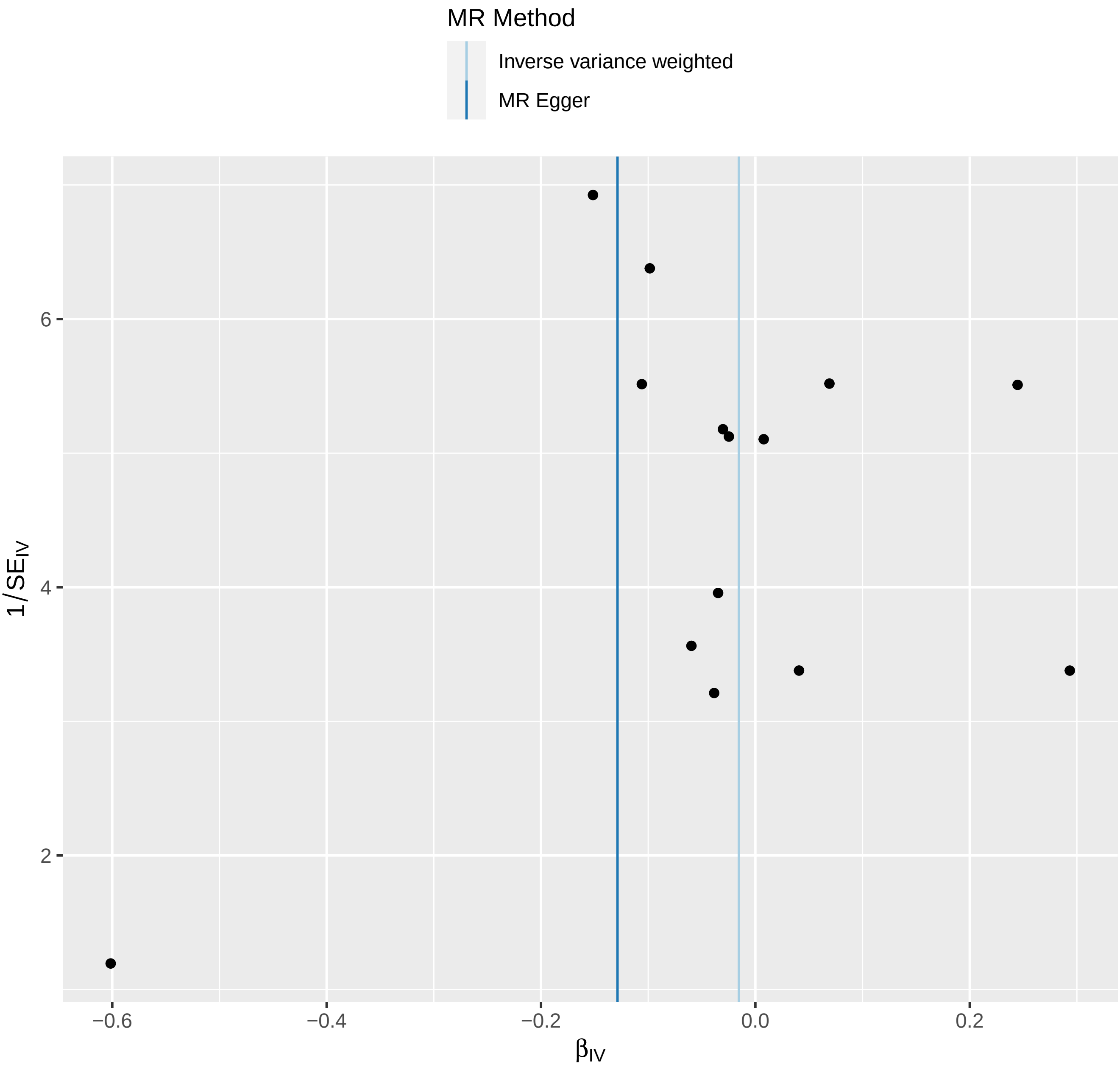

Batch 3−2856 : Alpha−N−acetylgalactosaminide alpha−2,6−sialyltransferase 5 on Diabetic nephropathy (more control exclusions) with a threshold of 1e−05

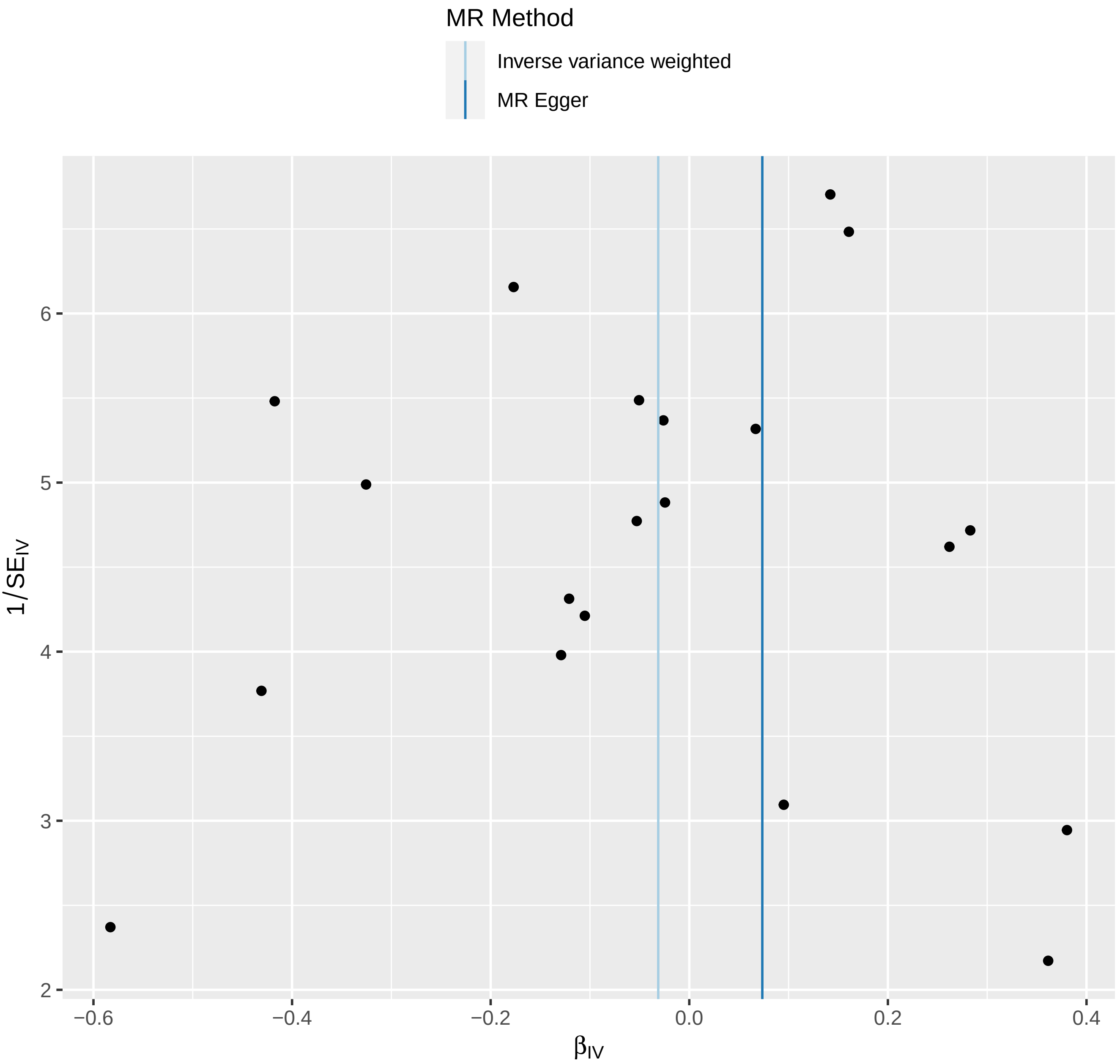

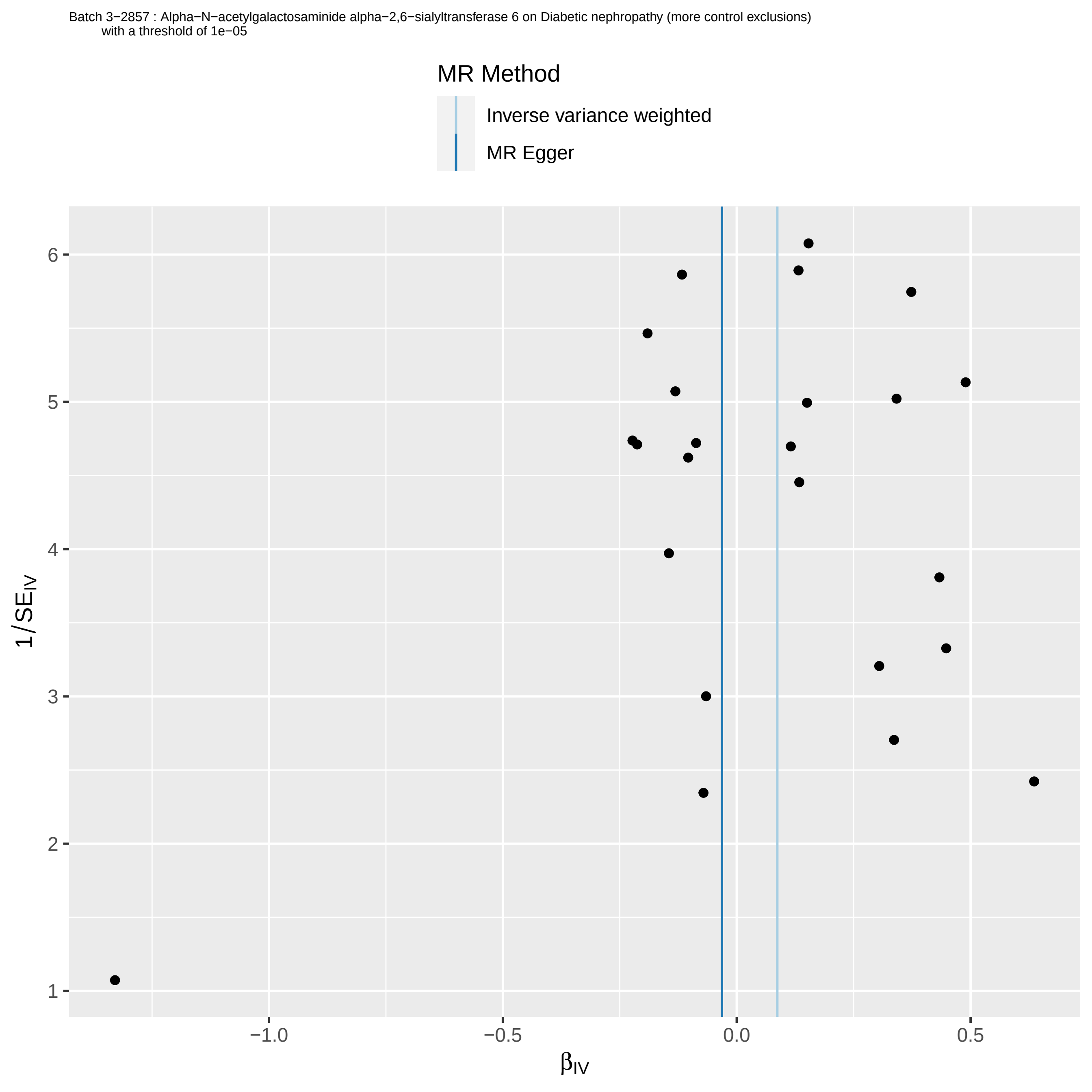

Batch 3−2857 : Alpha−N−acetylgalactosaminide alpha−2,6−sialyltransferase 6 on Diabetic nephropathy (more control exclusions)
with a threshold of 1e−05
MR Method
Inverse variance weighted
MR Egger
1/SE_IV
6
5
4
3
2
1
−1.0
−0.5
0.0
0.5
β_IV

Batch 3−2858 : Alpha−2,8−sialyltransferase 8B on Diabetic nephropathy (more control exclusions)
with a threshold of 1e−05

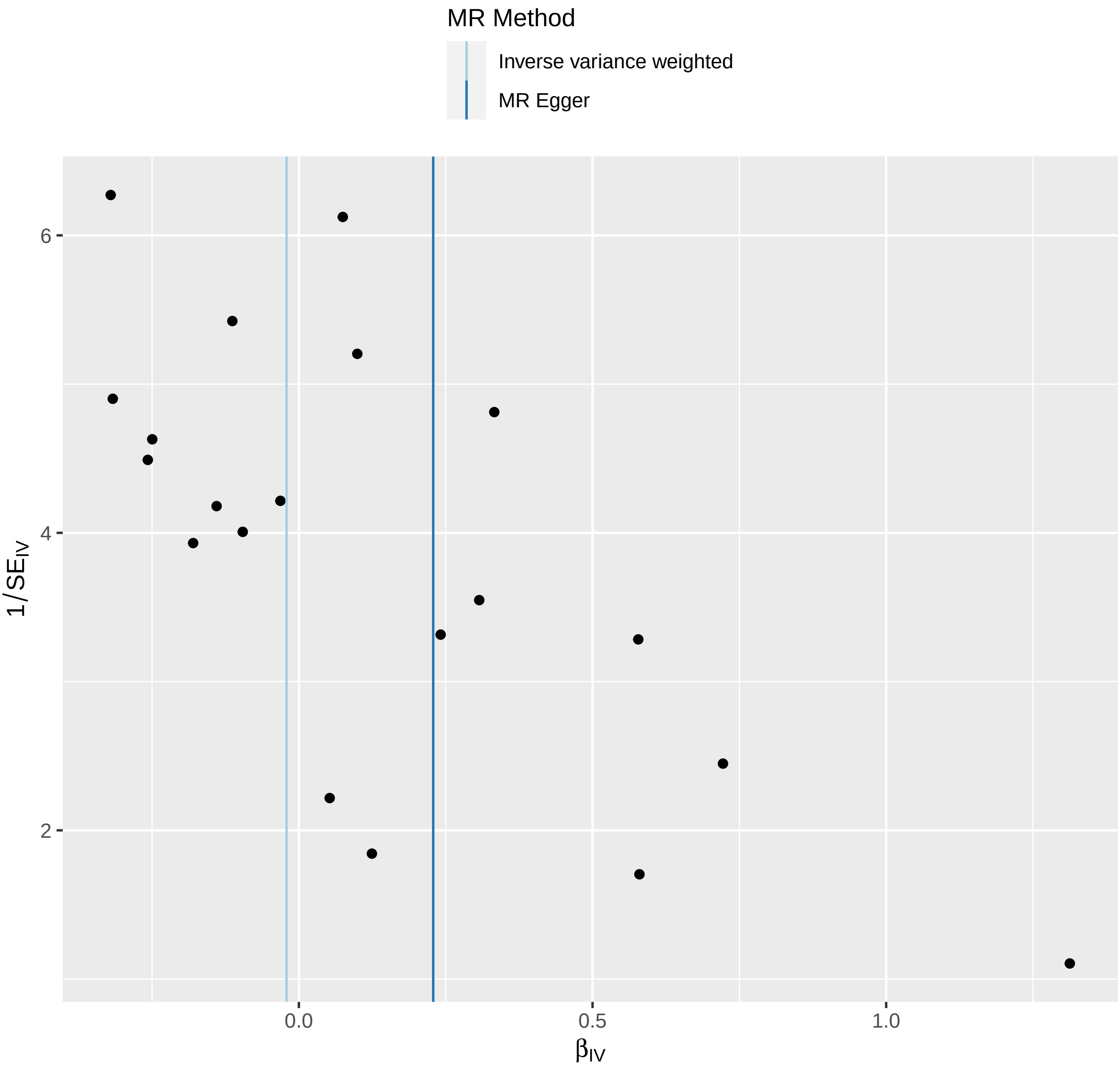

Batch 3−2859 : Sia−alpha−2,3−Gal−beta−1,4−GlcNAc−R:alpha 2,8−sialyltransferase on Diabetic nephropathy (more control exclusions) with a threshold of 1e−05

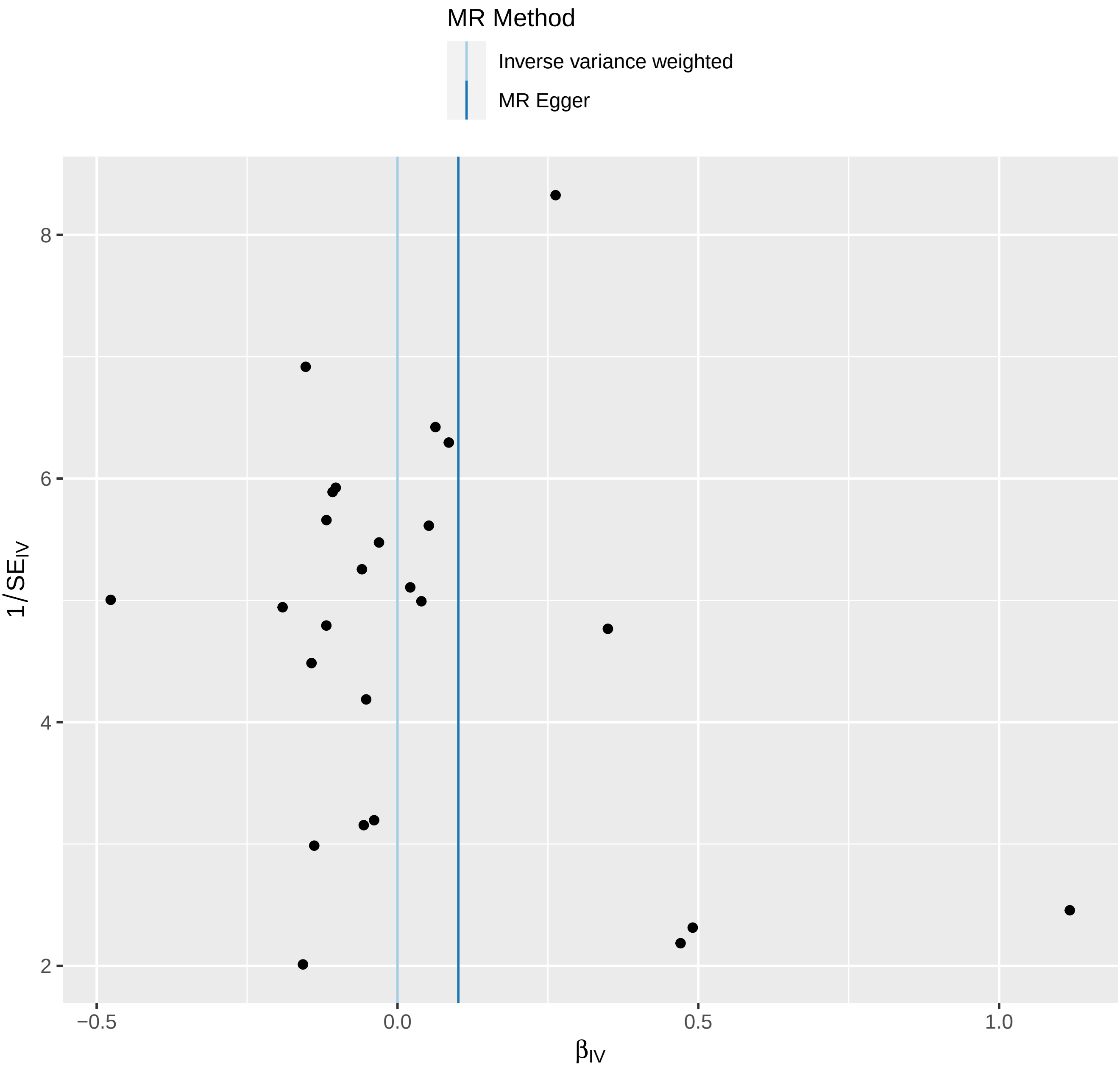

Batch 3−2860 : CMP−N−acetylneuraminate−poly−alpha−2,8−sialyltransferase on Diabetic nephropathy (more control exclusions) with a threshold of 1e−05

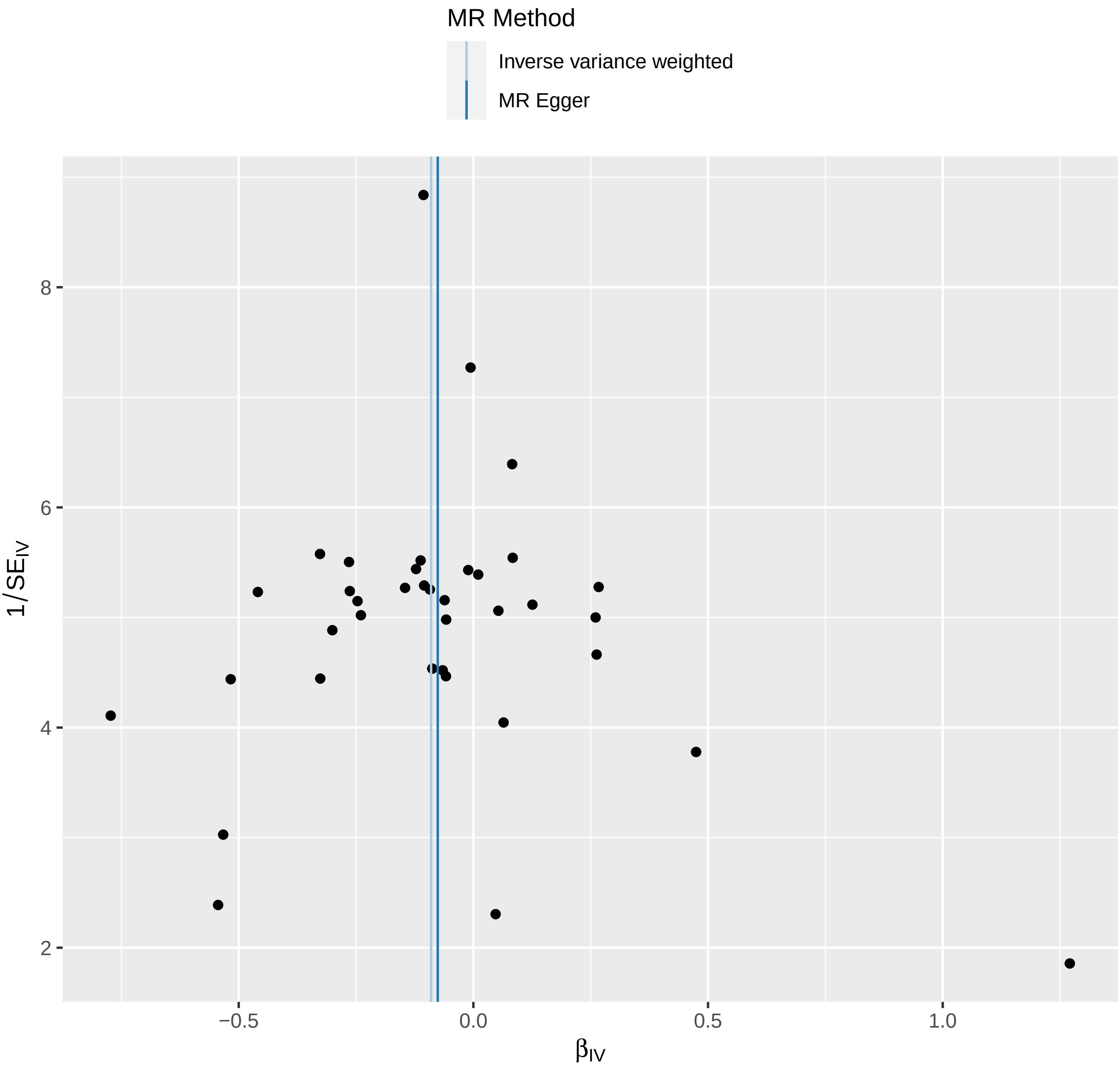

Batch 3−2861 : Alpha−2,8−sialyltransferase 8F on Diabetic nephropathy (more control exclusions)
with a threshold of 1e−05

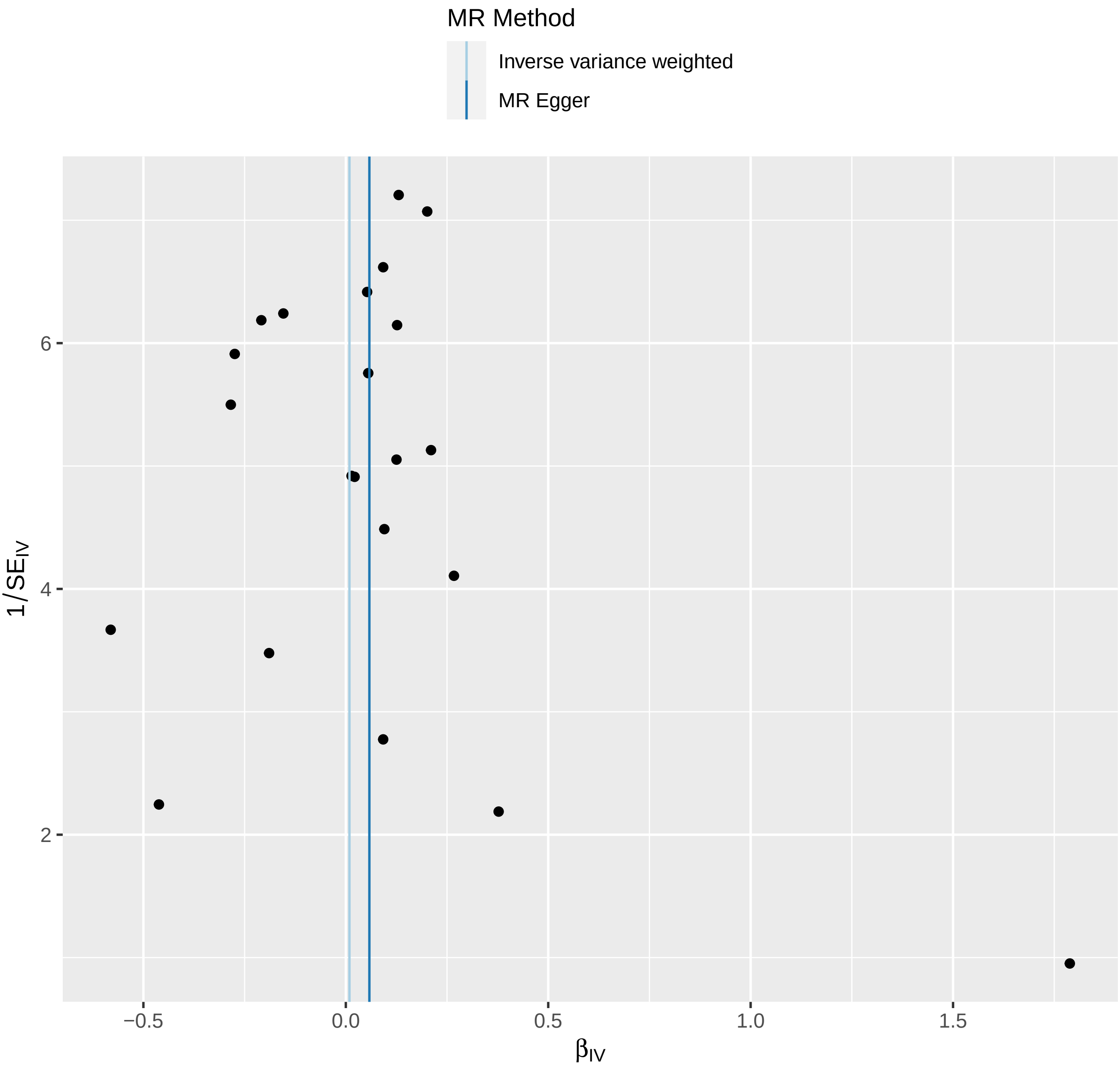

Batch 3−2862 : Stabilin−1 on Diabetic nephropathy (more control exclusions)
with a threshold of 1e−05

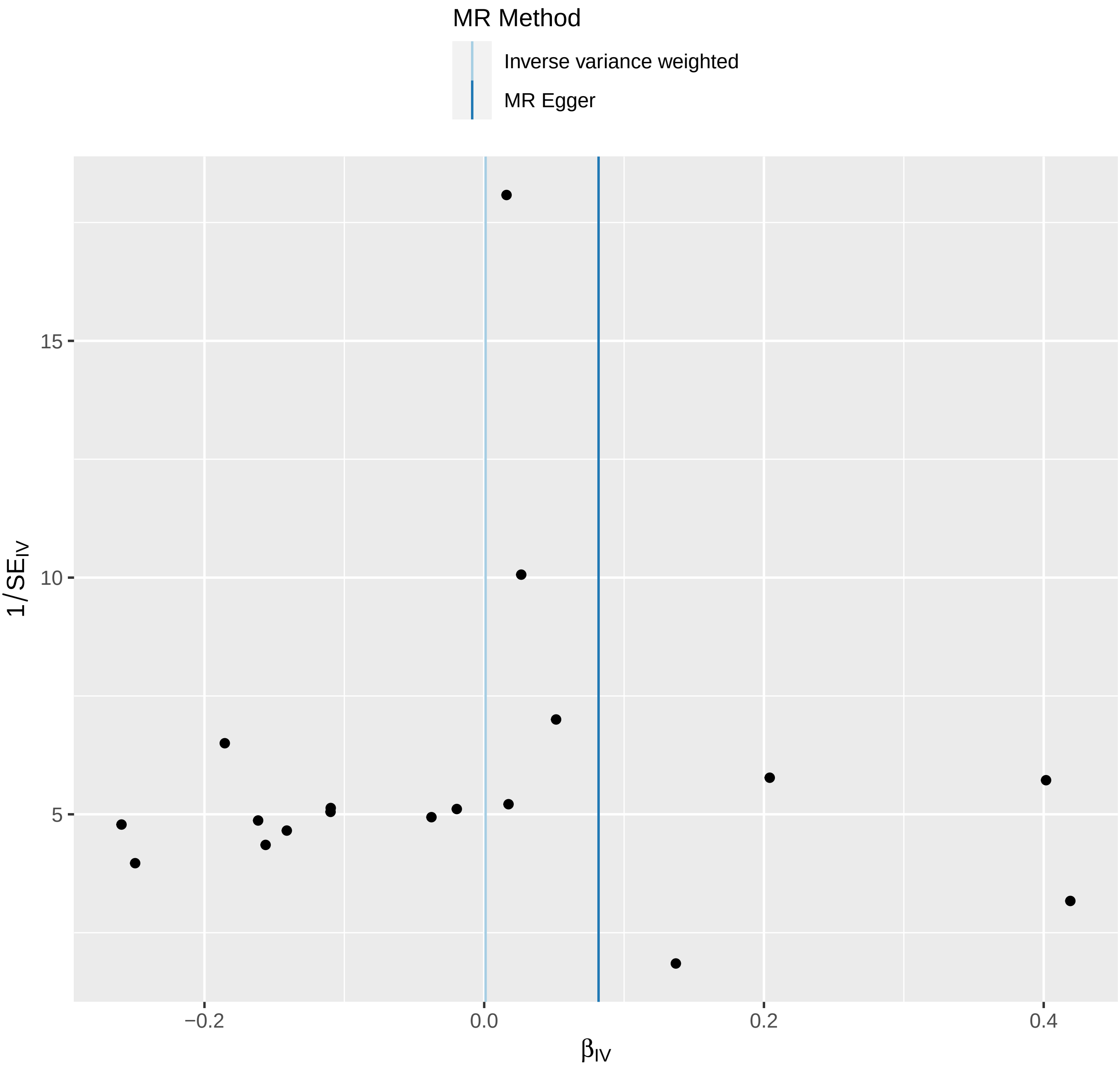

Batch 3−2863 : Stabilin−2 on Diabetic nephropathy (more control exclusions)
with a threshold of 1e−05

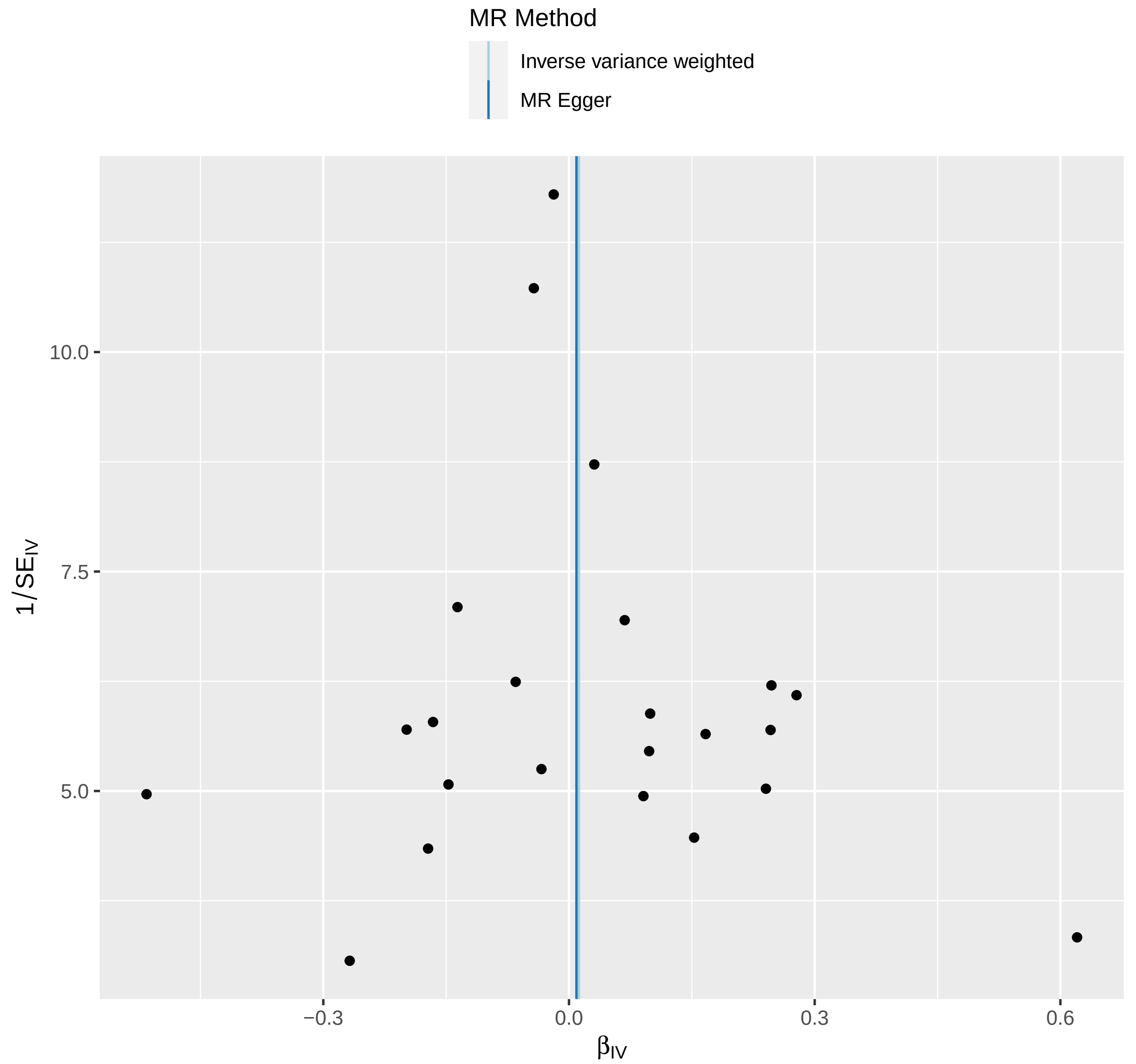

Batch 3−2864 : AMSH−like protease on Diabetic nephropathy (more control exclusions)
with a threshold of 1e−05

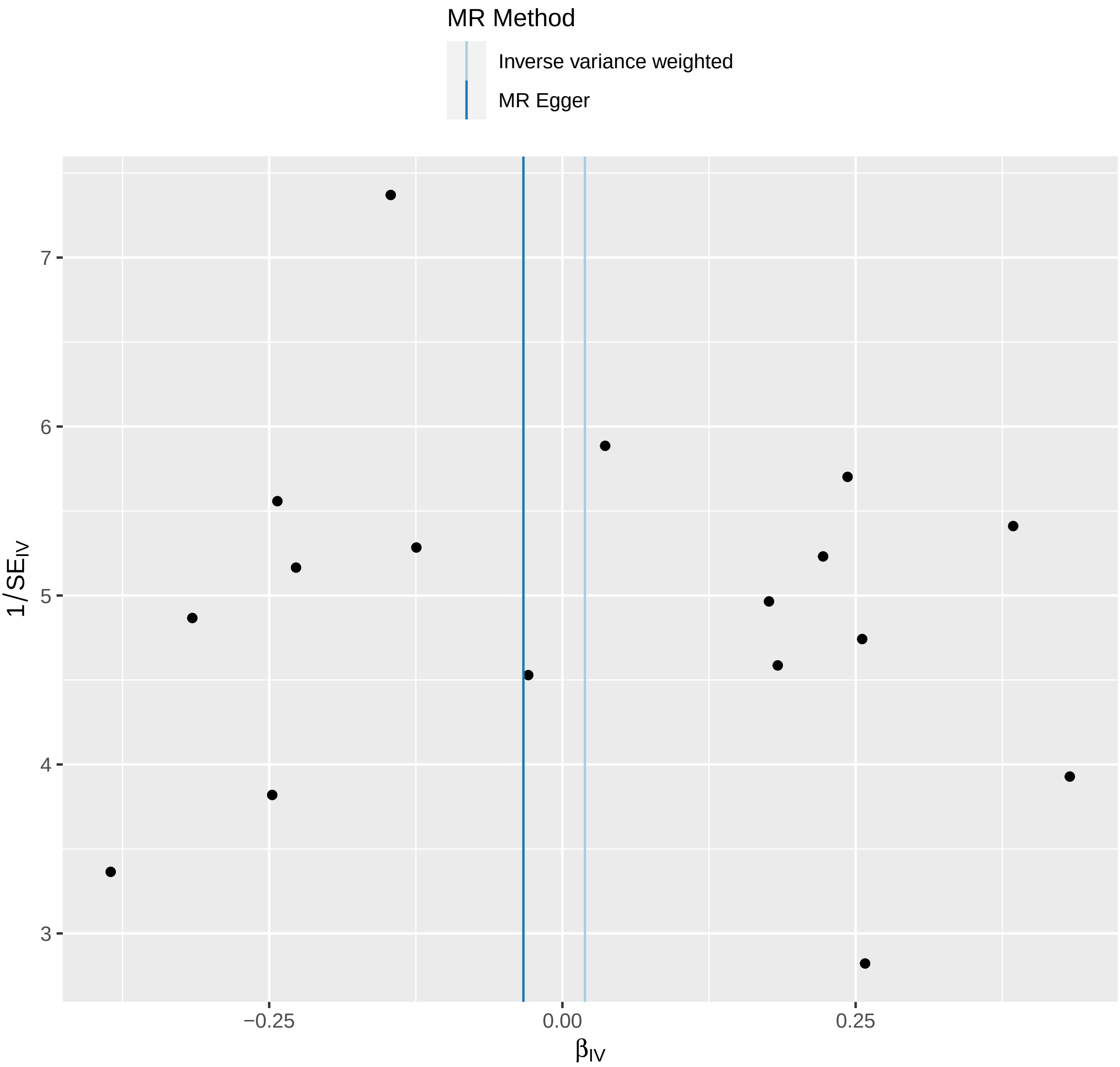

Batch 3−2865 : Signal−transducing adaptor protein 1 on Diabetic nephropathy (more control exclusions) with a threshold of 1e−05

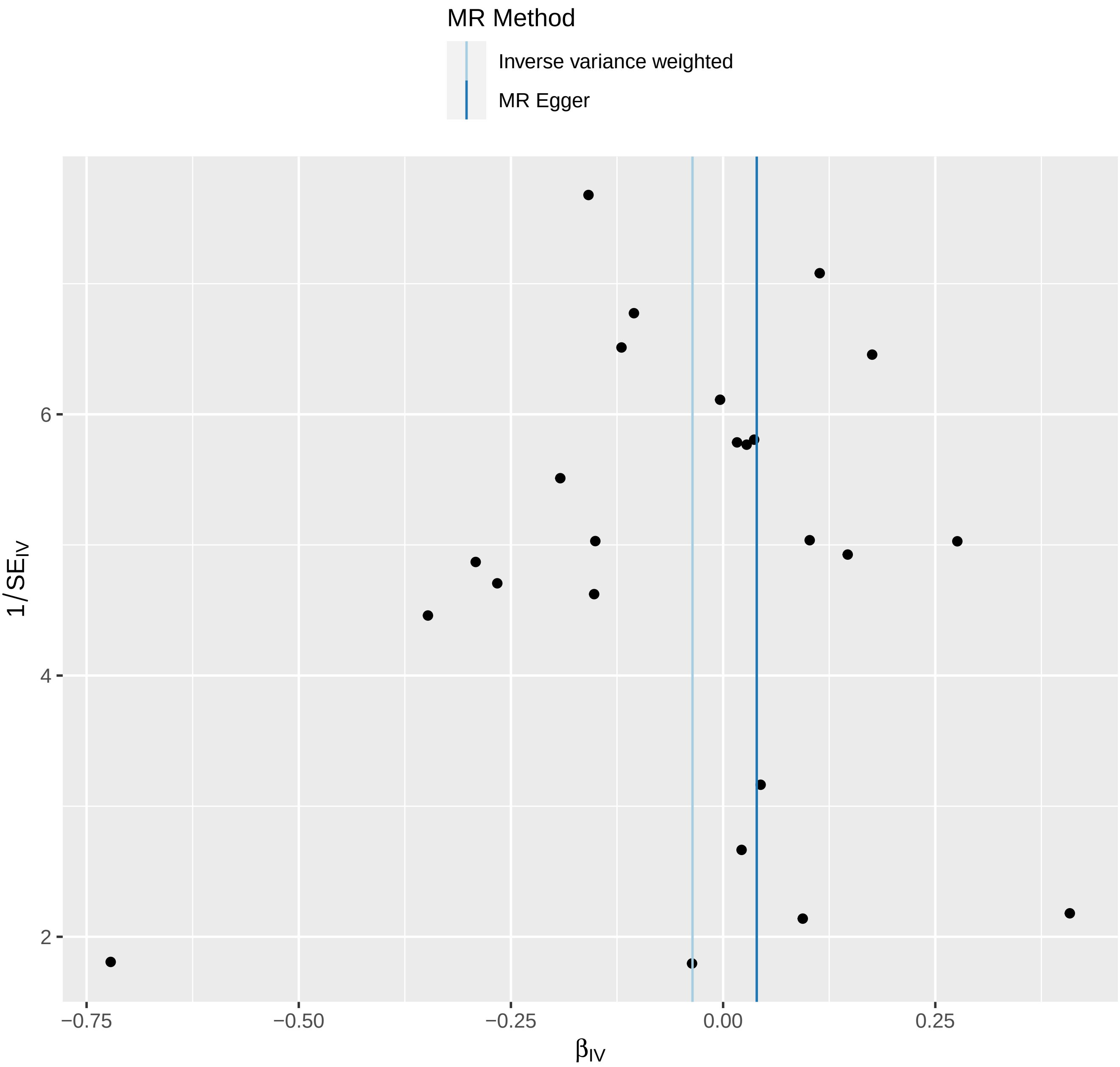

Batch 3−2866 : Steroidogenic acute regulatory protein, mitochondrial on Diabetic nephropathy (more control exclusions)
with a threshold of 1e−05

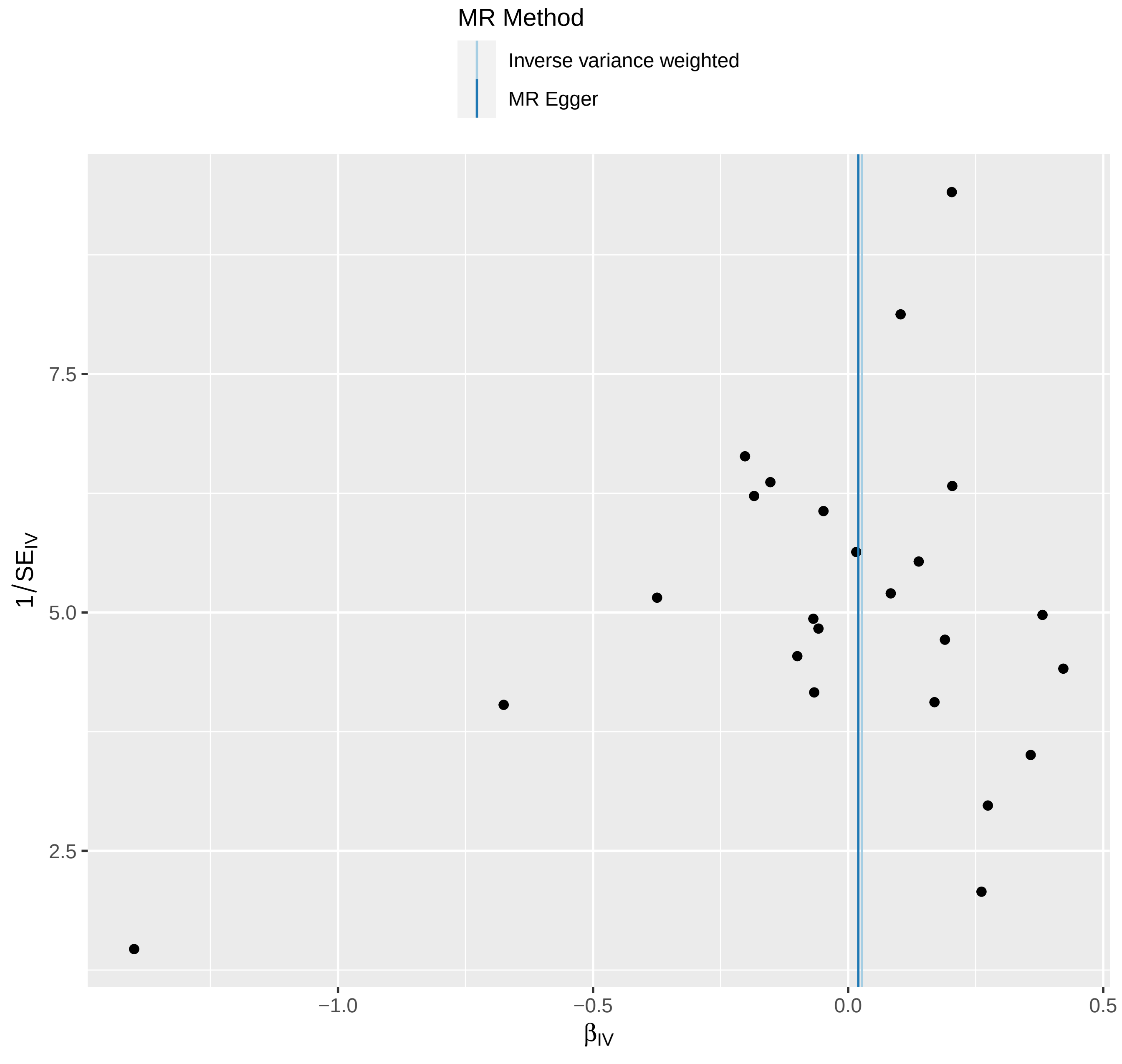

Batch 3−2867 : Signal transducer and activator of transcription 1−alpha/beta on Diabetic nephropathy (more control exclusions) with a threshold of 1e−05

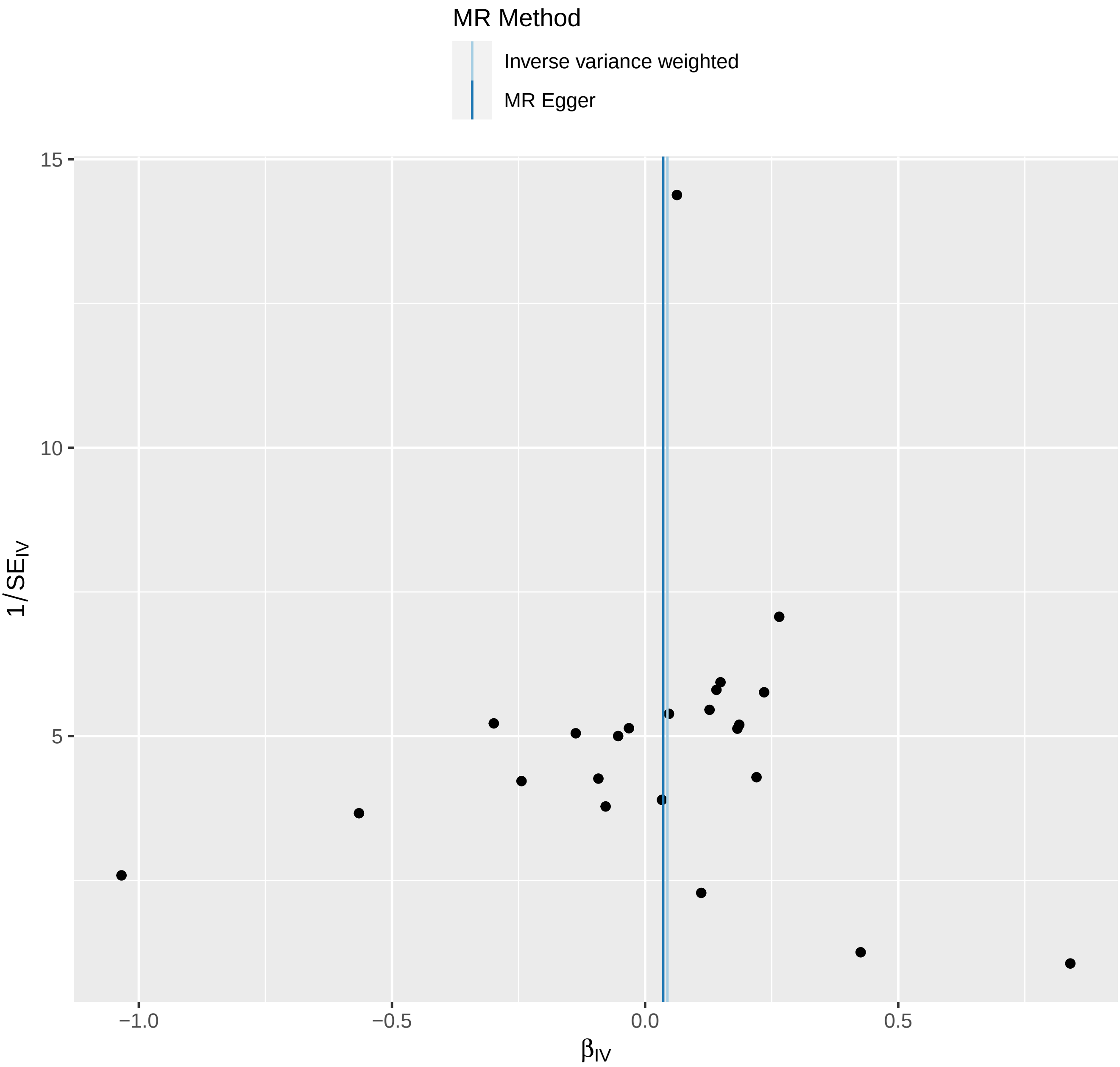

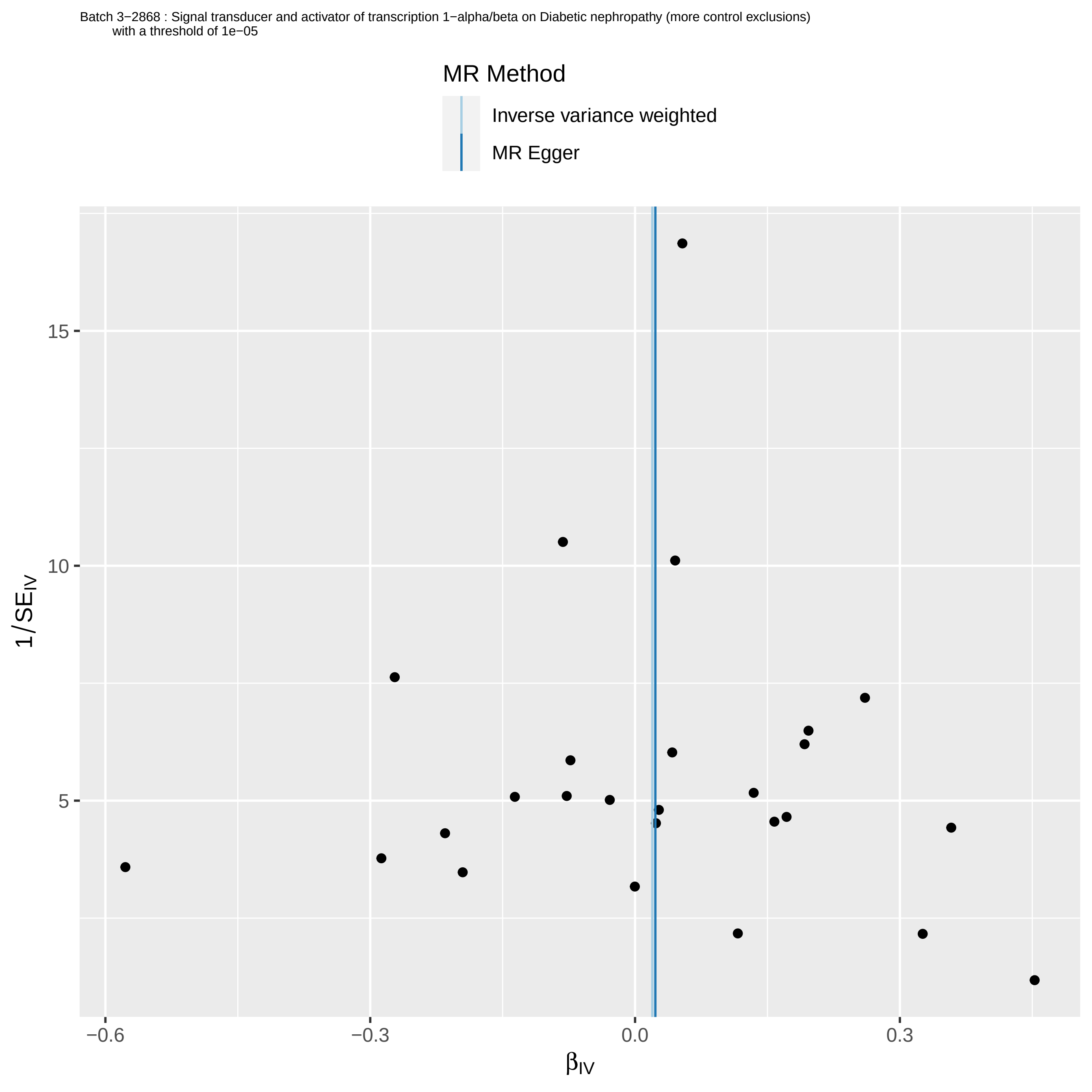
Batch 3−2868 : Signal transducer and activator of transcription 1−alpha/beta on Diabetic nephropathy (more control exclusions)
with a threshold of 1e−05
MR Method
Inverse variance weighted
MR Egger
15
10
5
1/SE_IV
−0.6
−0.3
0.0
0.3
β_IV

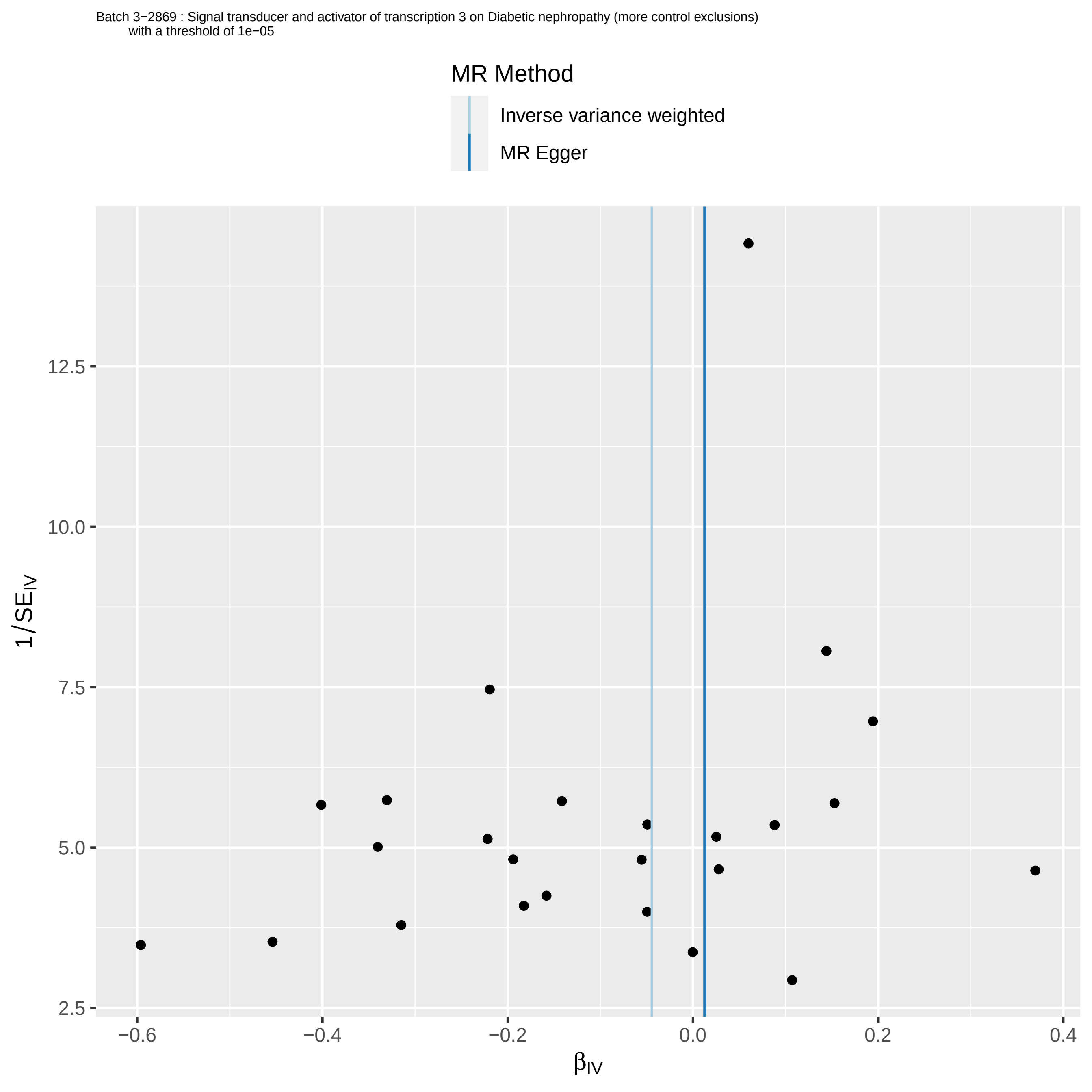

Batch 3−2869 : Signal transducer and activator of transcription 3 on Diabetic nephropathy (more control exclusions)
with a threshold of 1e−05
MR Method
Inverse variance weighted
MR Egger
1/SE$_{IV}$
12.5
10.0
7.5
5.0
2.5
−0.6
−0.4
−0.2
0.0
0.2
0.4
β$_{IV}$

Batch 3−2870 : Signal transducer and activator of transcription 6 on Diabetic nephropathy (more control exclusions)
with a threshold of 1e−05

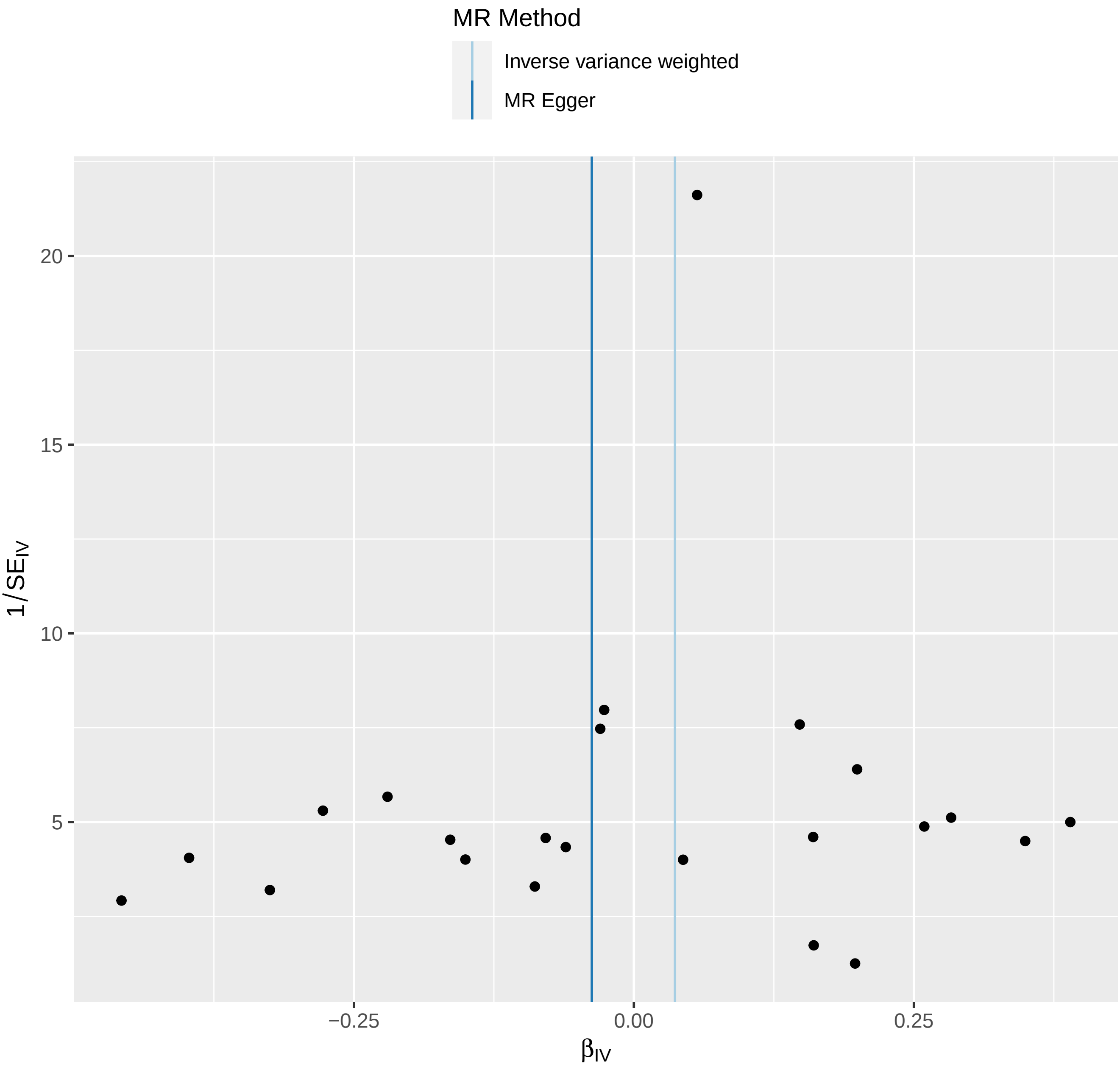

Batch 3−2871 : Double−stranded RNA−binding protein Staufen homolog 1 on Diabetic nephropathy (more control exclusions)
with a threshold of 1e−05

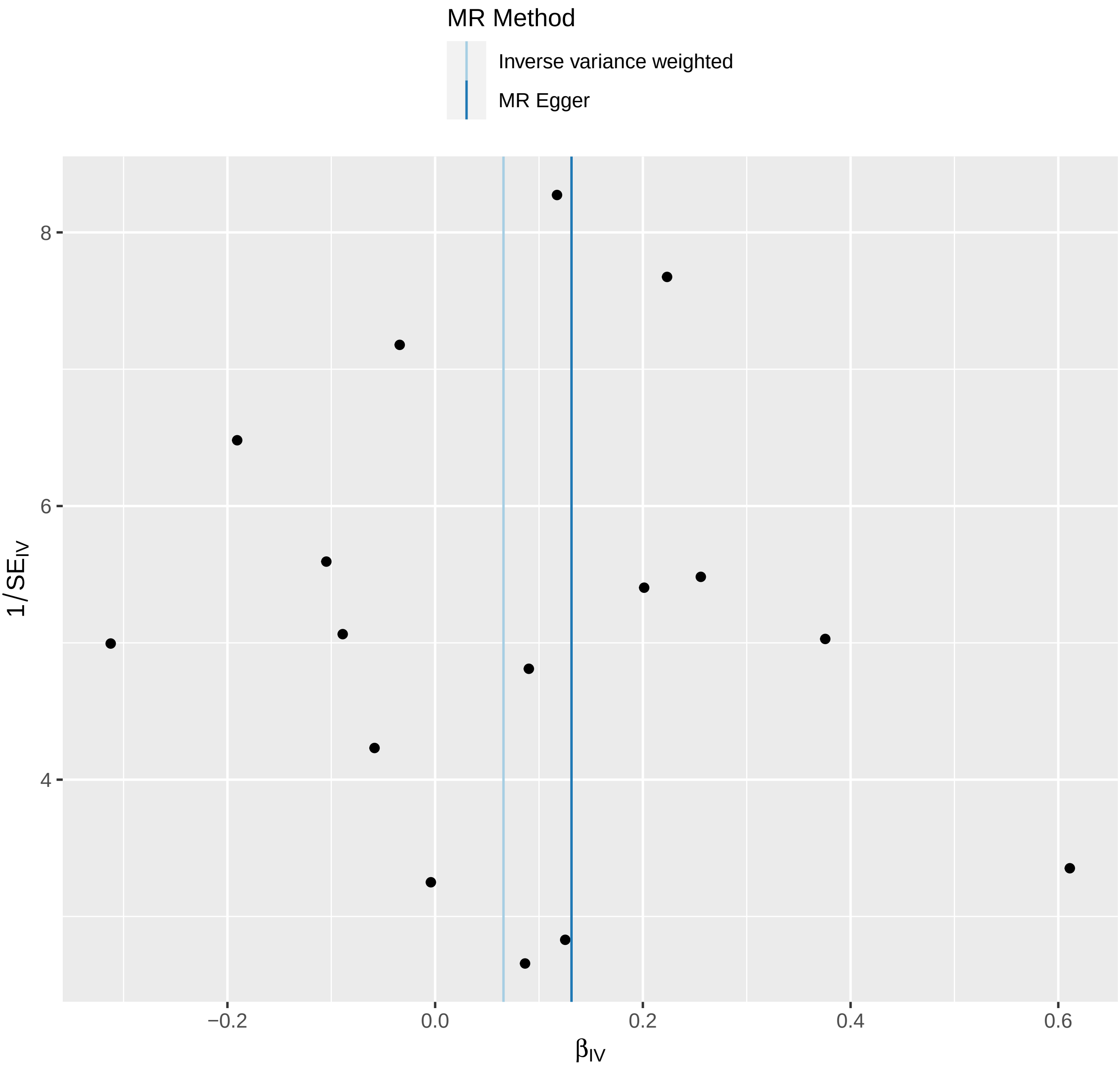

Batch 3−2872 : Double−stranded RNA−binding protein Staufen homolog 2 on Diabetic nephropathy (more control exclusions)
with a threshold of 1e−05

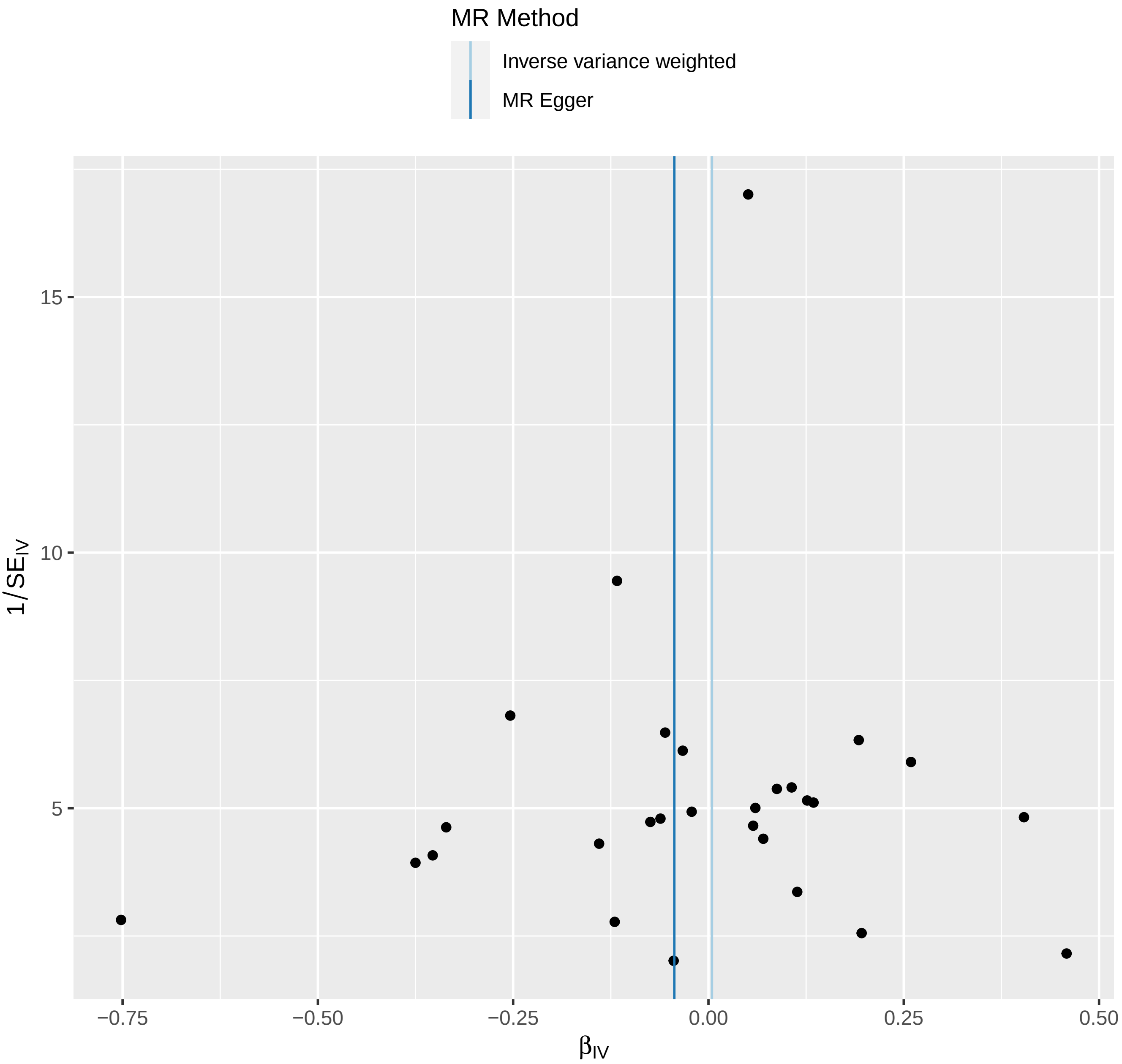

Batch 3−2873 : Stanniocalcin−1 on Diabetic nephropathy (more control exclusions)
with a threshold of 1e−05

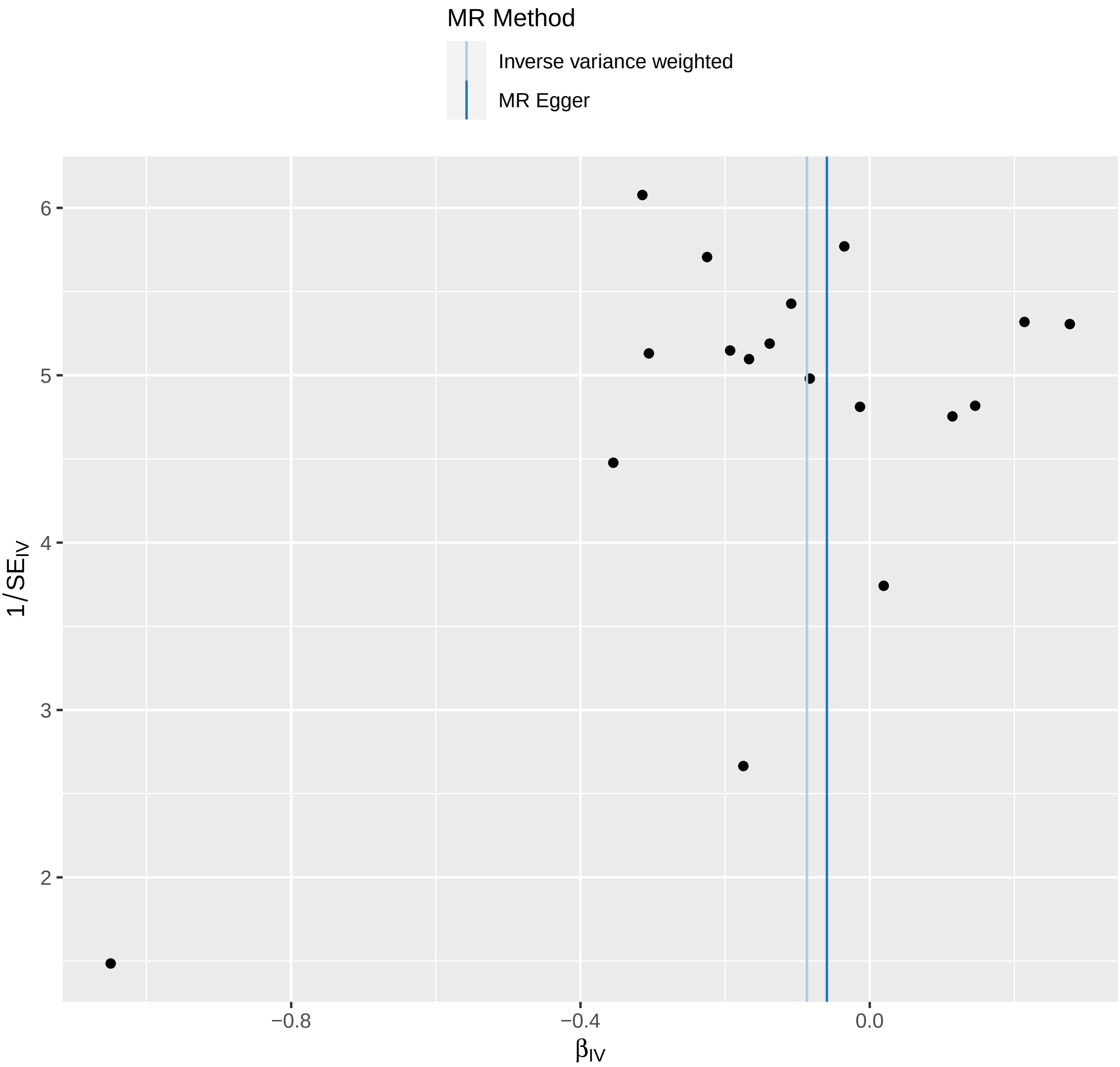

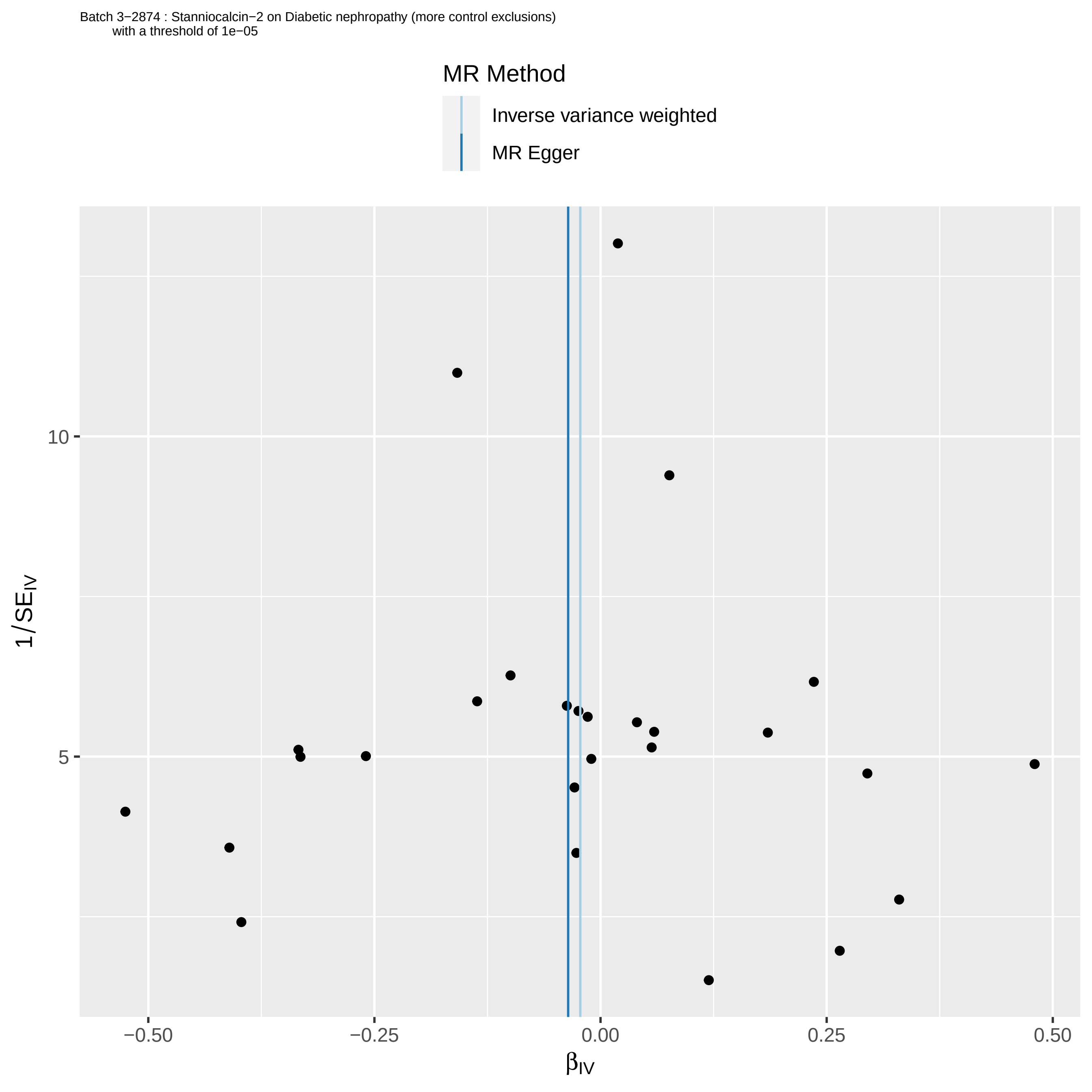
Batch 3−2874 : Stanniocalcin−2 on Diabetic nephropathy (more control exclusions)
with a threshold of 1e−05
MR Method
Inverse variance weighted
MR Egger
10
5
−0.50
−0.25
0.00
0.25
0.50
$1/SE_{IV}$
β_{IV}

Batch 3−2875 : Stromal interaction molecule 1 on Diabetic nephropathy (more control exclusions)
with a threshold of 1e−05

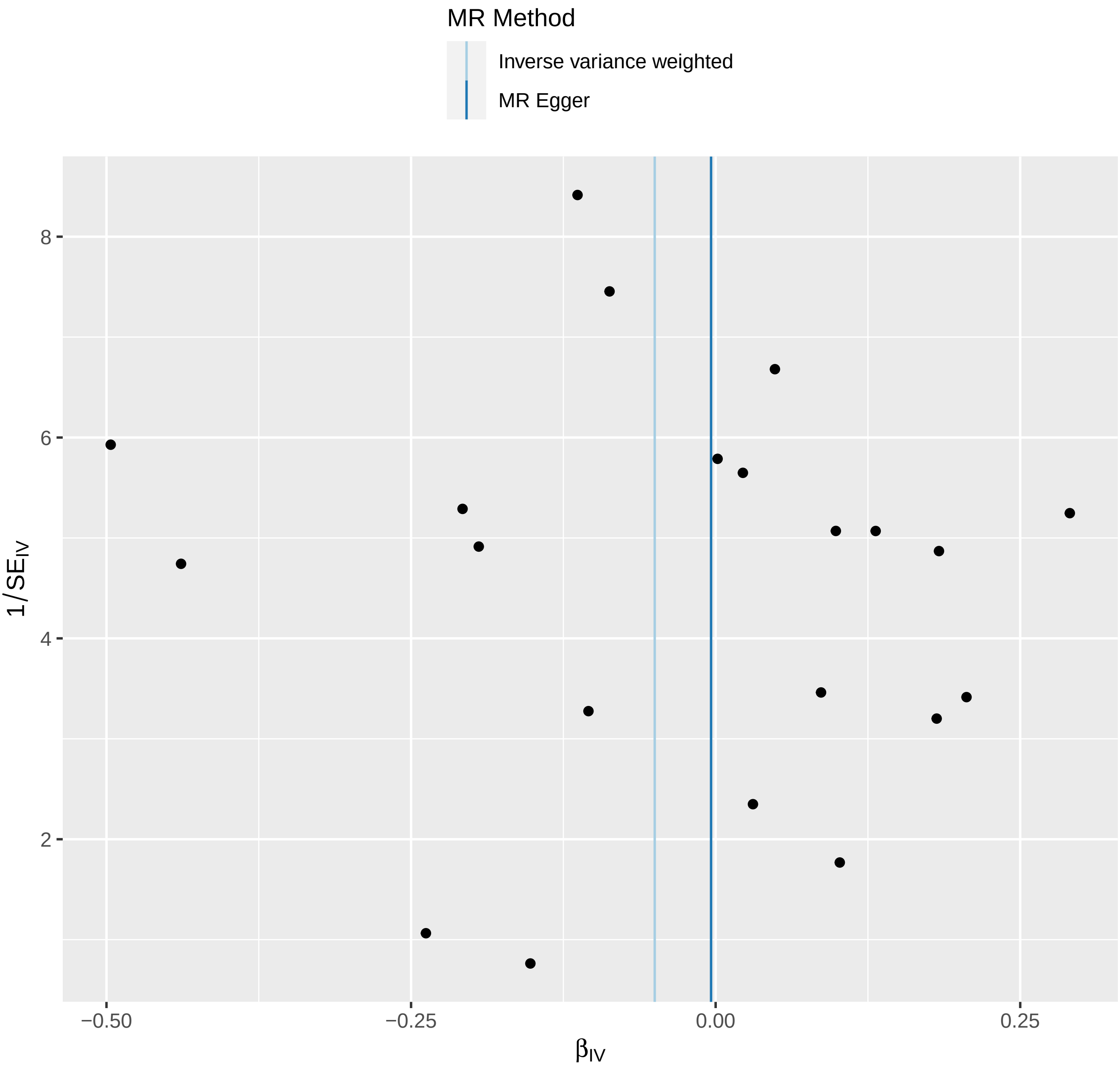

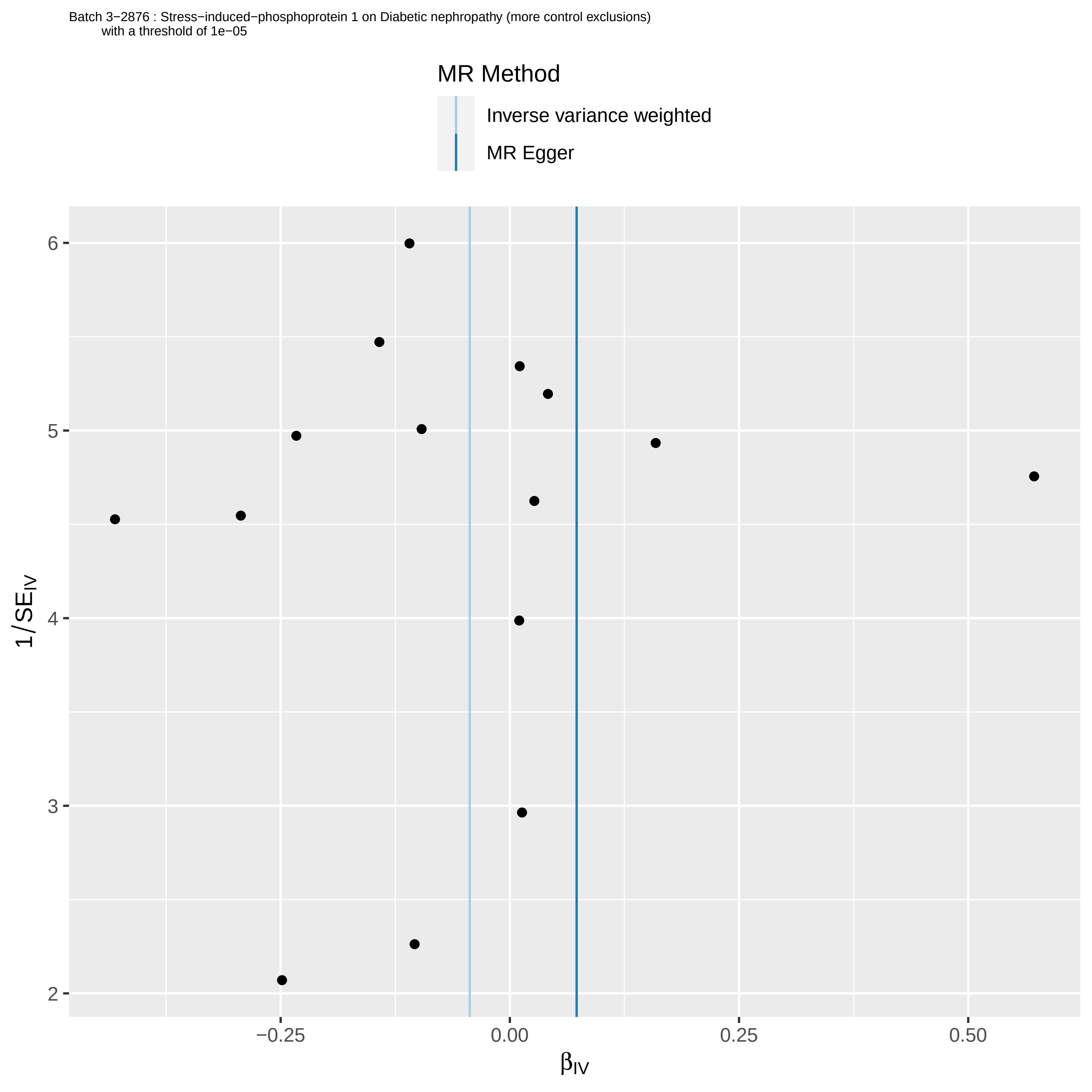

Batch 3−2876 : Stress−induced−phosphoprotein 1 on Diabetic nephropathy (more control exclusions)
with a threshold of 1e−05
MR Method
Inverse variance weighted
MR Egger
6
5
4
3
2
1/SE_IV
−0.25
0.00
0.25
0.50
β_IV

Batch 3−2877 : Serine/threonine−protein kinase 16 on Diabetic nephropathy (more control exclusions)
with a threshold of 1e−05

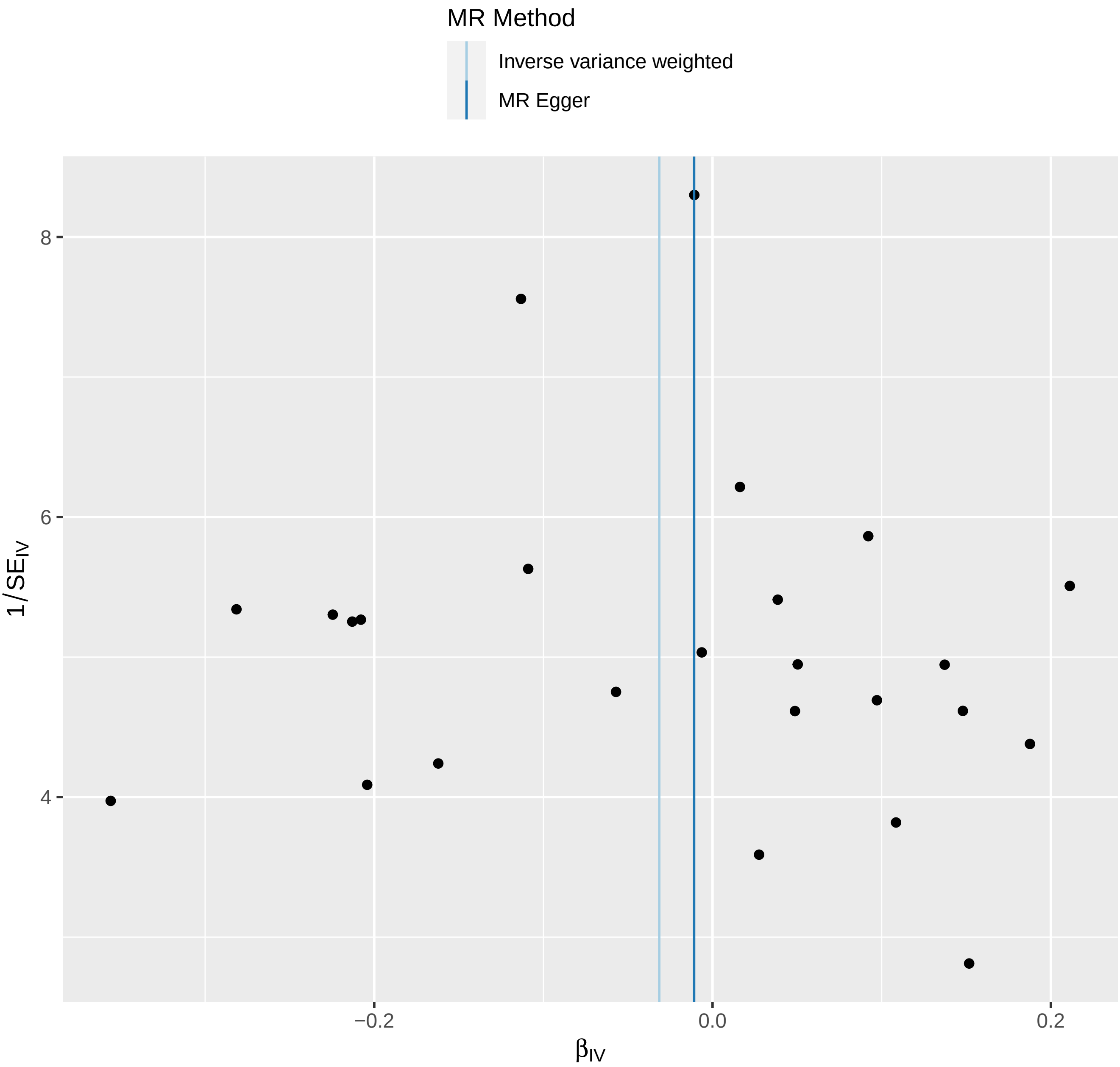

Batch 3−2878 : Serine/threonine−protein kinase 17B on Diabetic nephropathy (more control exclusions)
with a threshold of 1e−05

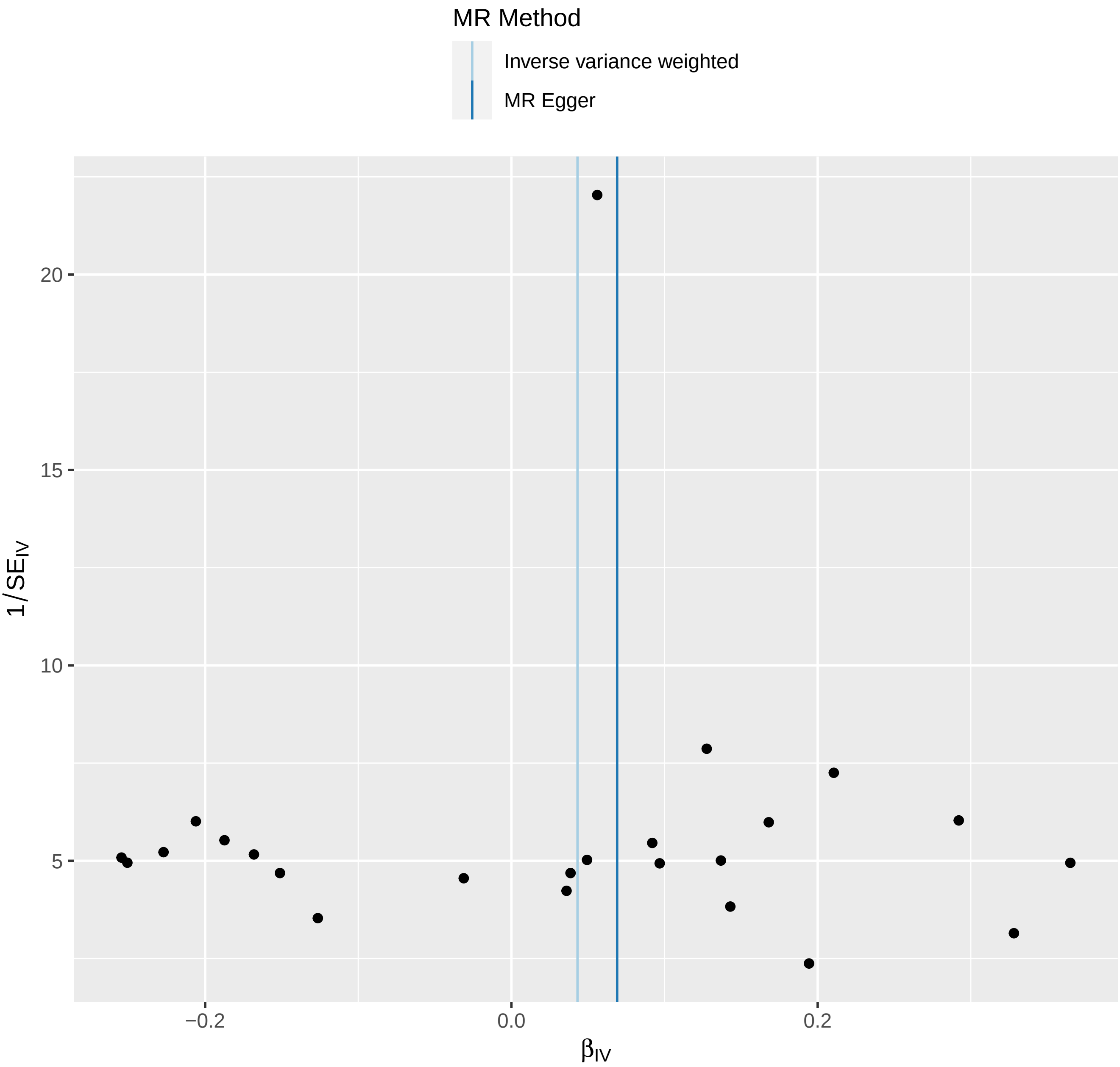

Batch 3−2879 : Stathmin−2 on Diabetic nephropathy (more control exclusions)
with a threshold of 1e−05

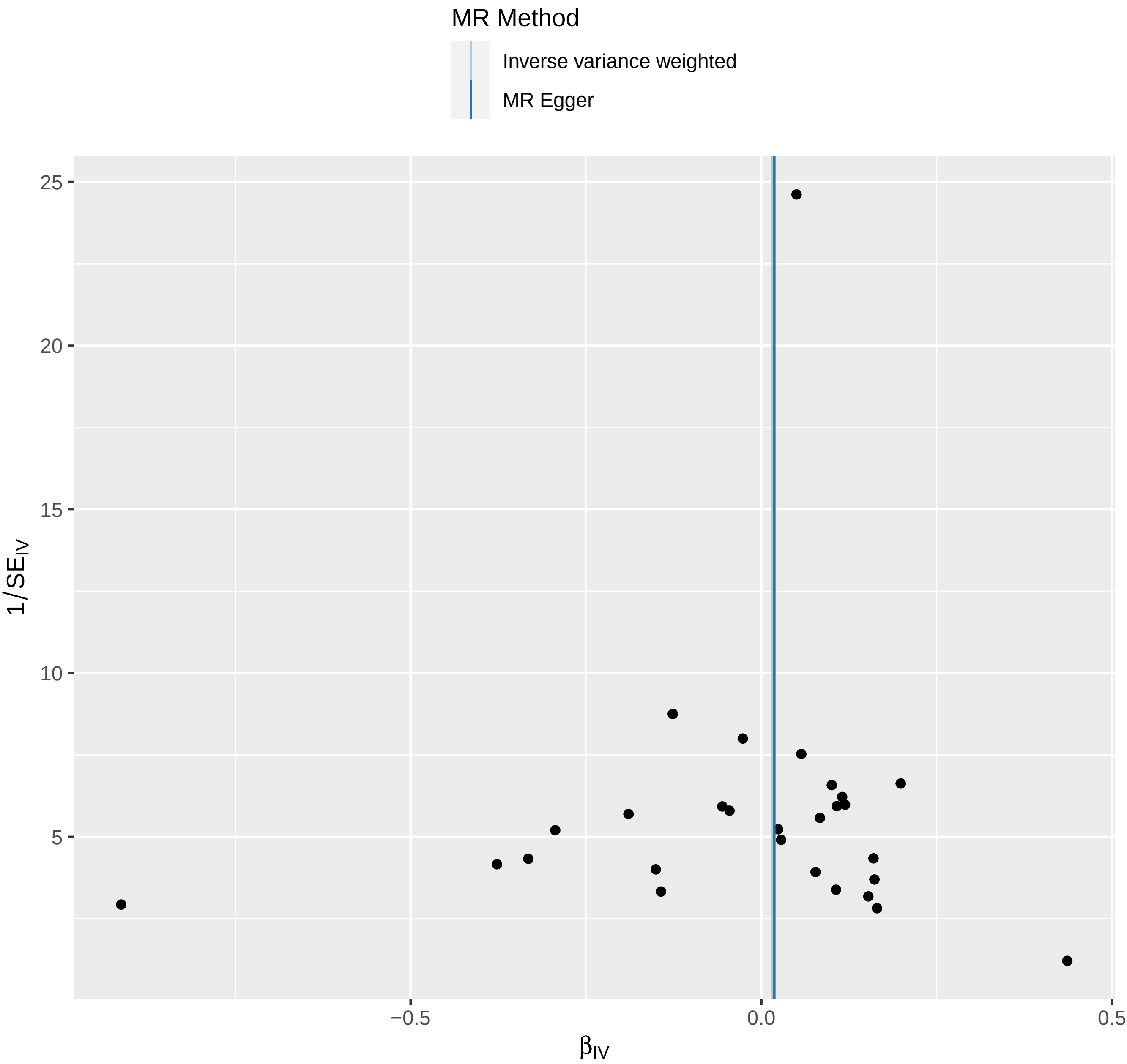

Batch 3−2880 : Stathmin−3 on Diabetic nephropathy (more control exclusions)
with a threshold of 1e−05

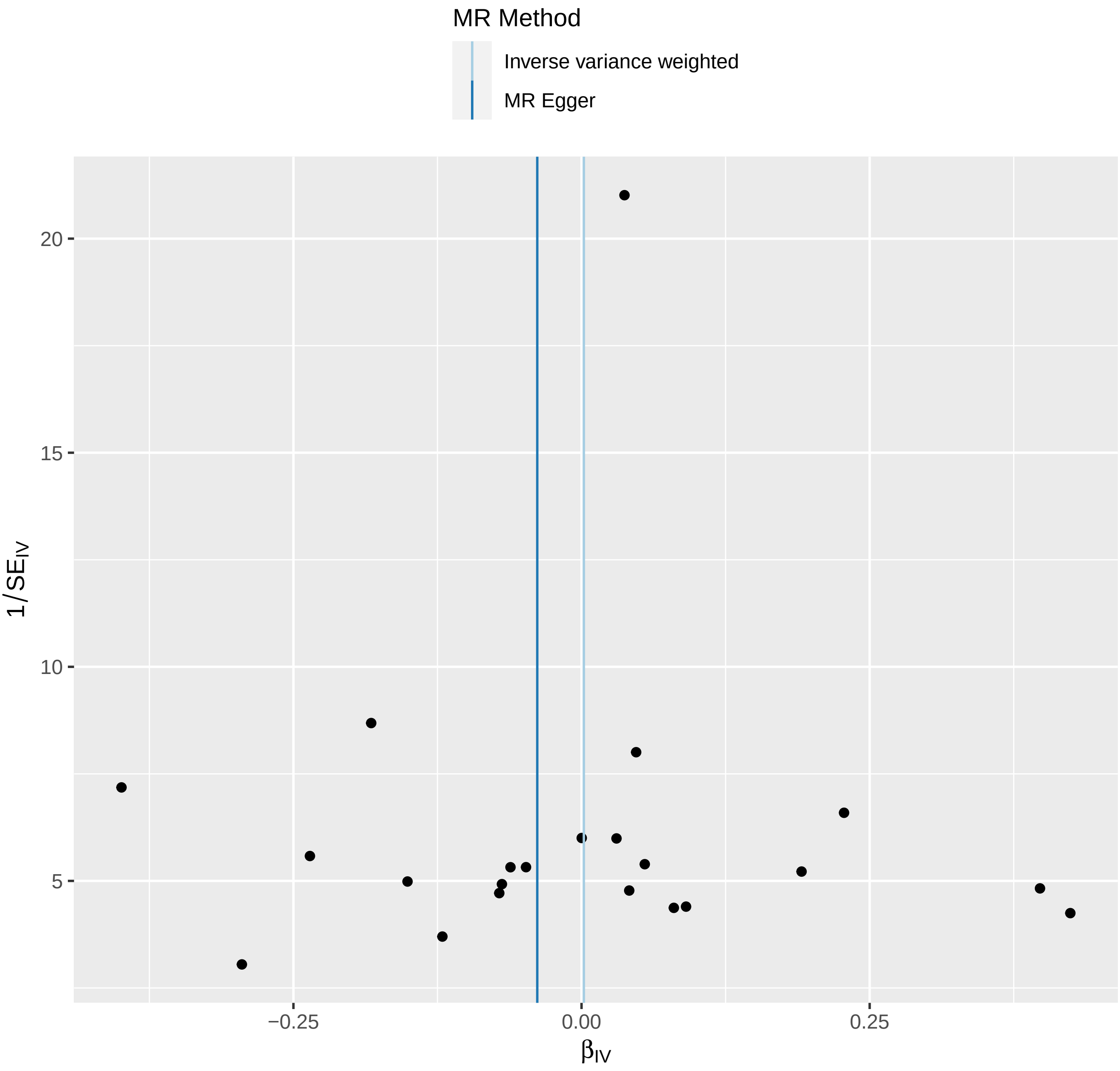

Batch 3−2881 : Stathmin−4 on Diabetic nephropathy (more control exclusions)
with a threshold of 1e−05

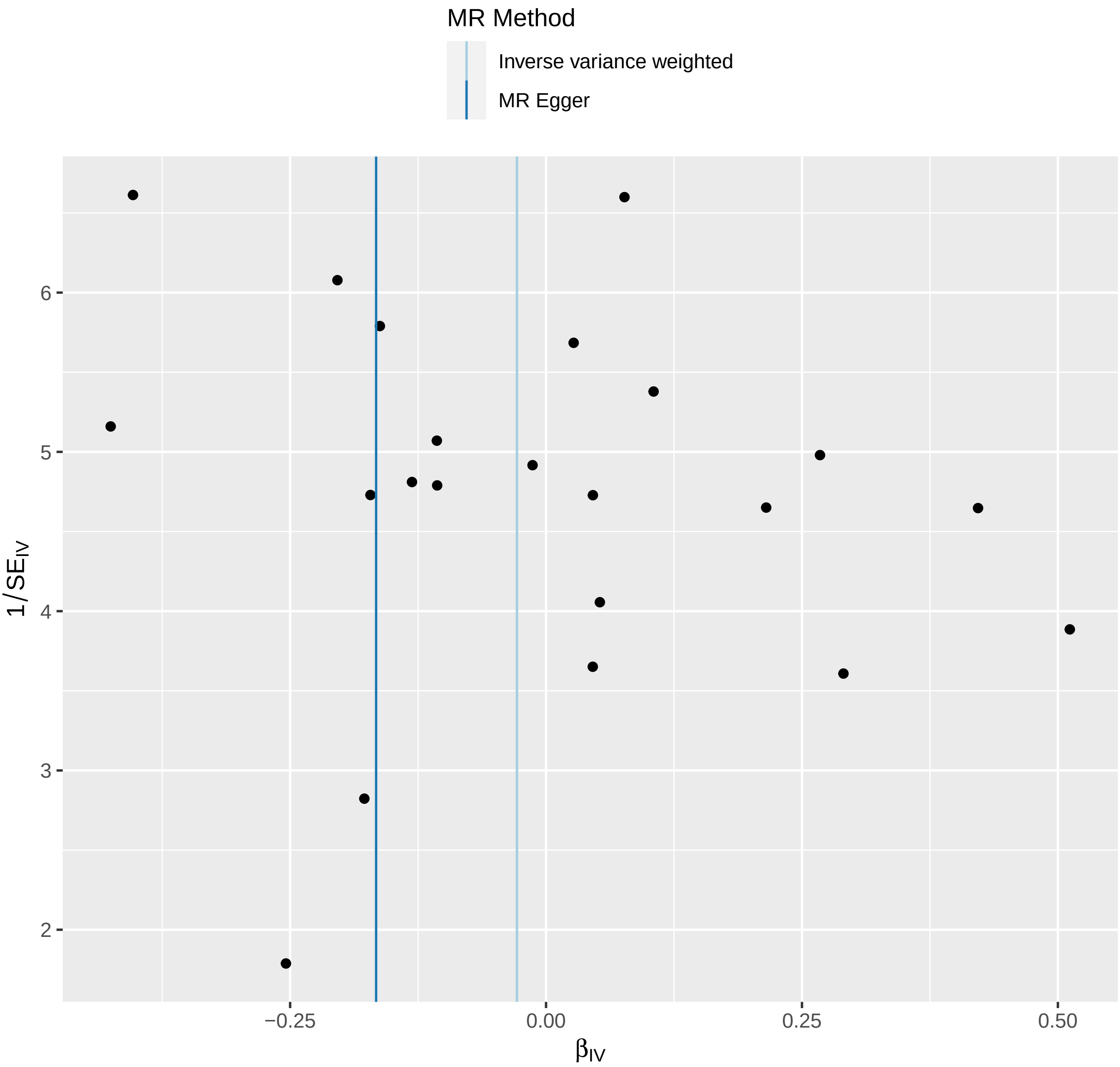

Batch 3−2882 : Erythrocyte band 7 integral membrane protein on Diabetic nephropathy (more control exclusions)
with a threshold of 1e−05

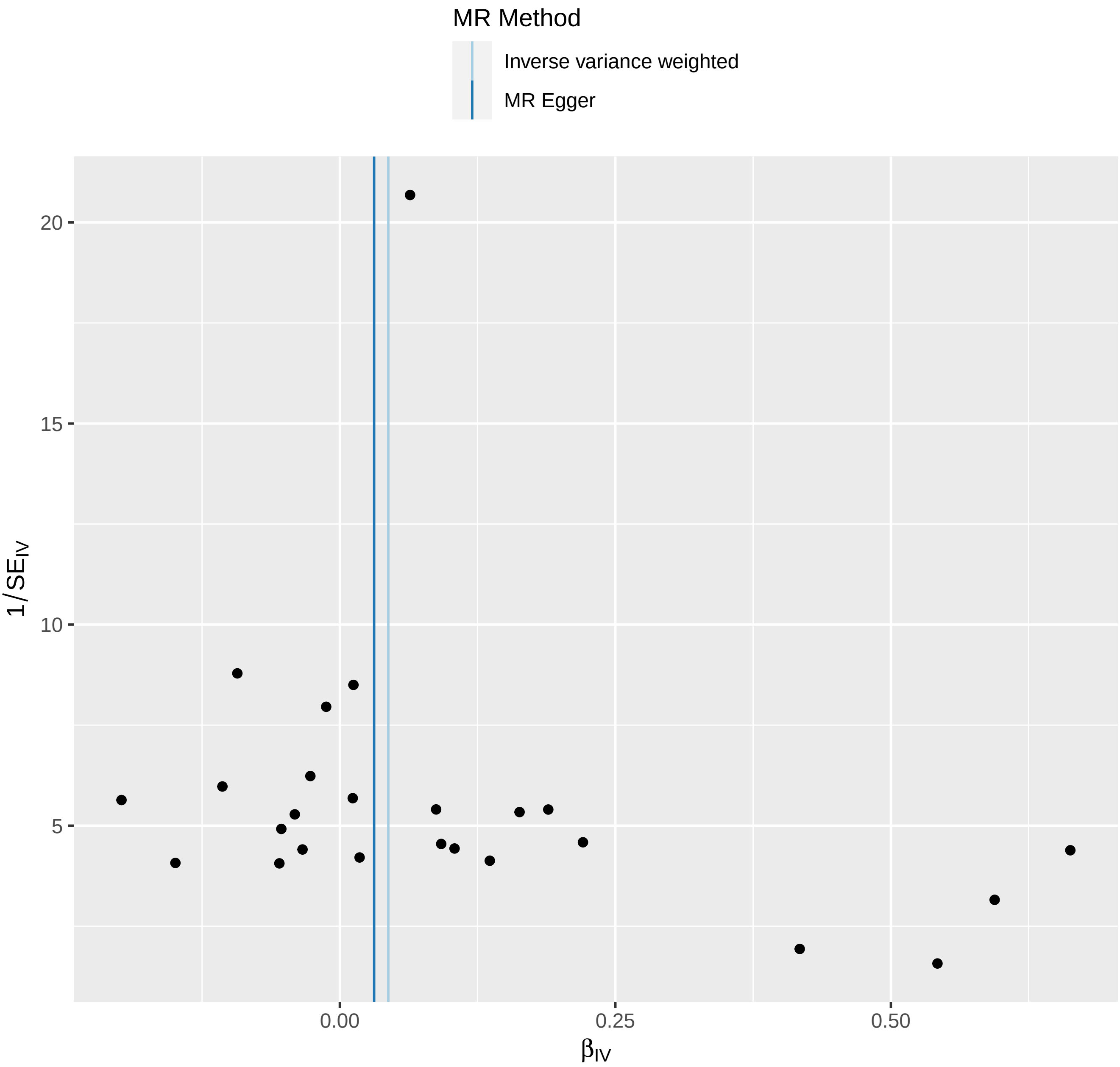

Batch 3−2883 : Syntaxin−10 on Diabetic nephropathy (more control exclusions)
with a threshold of 1e−05

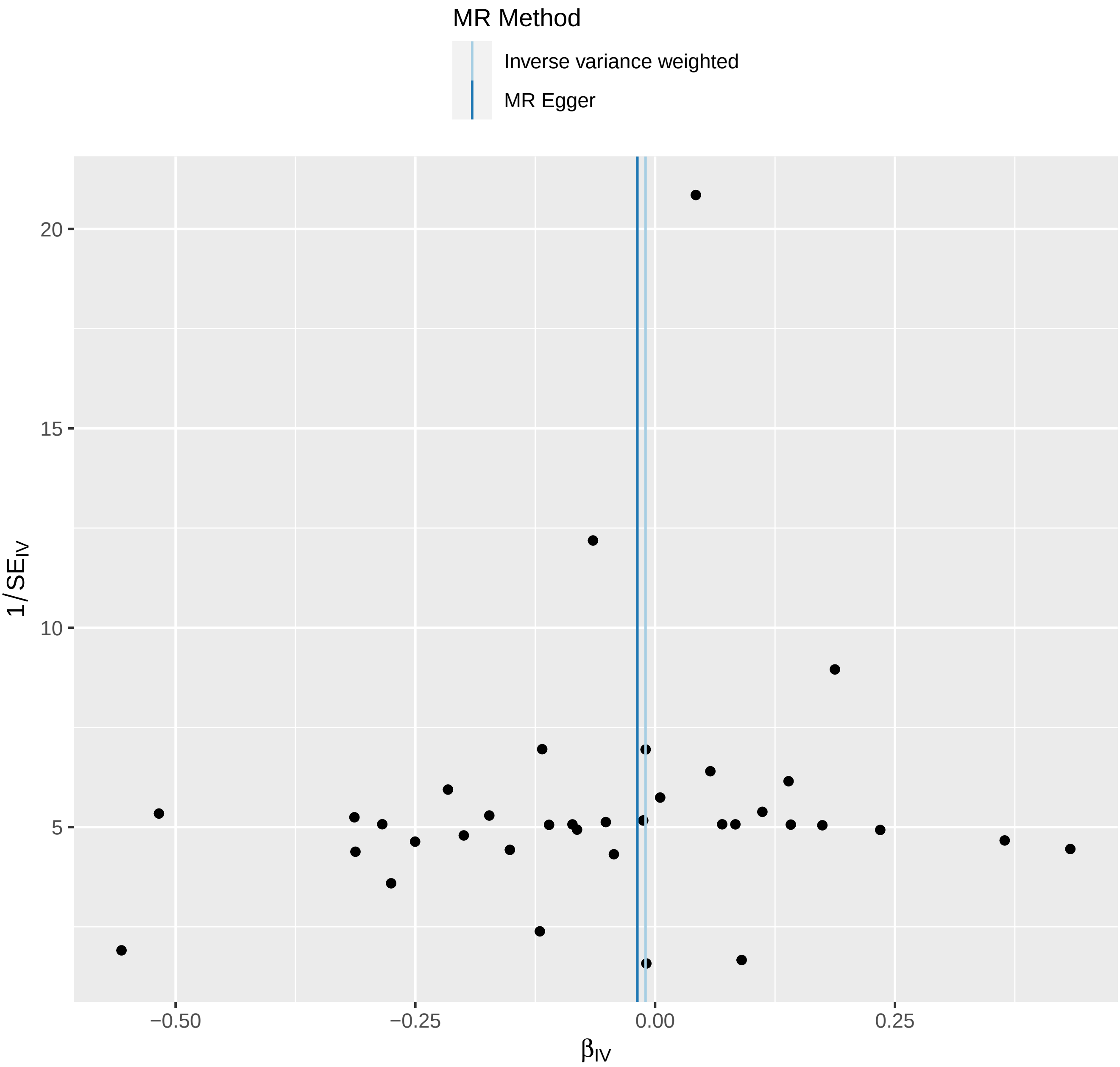

Batch 3−2884 : Syntaxin−18 on Diabetic nephropathy (more control exclusions)
with a threshold of 1e−05

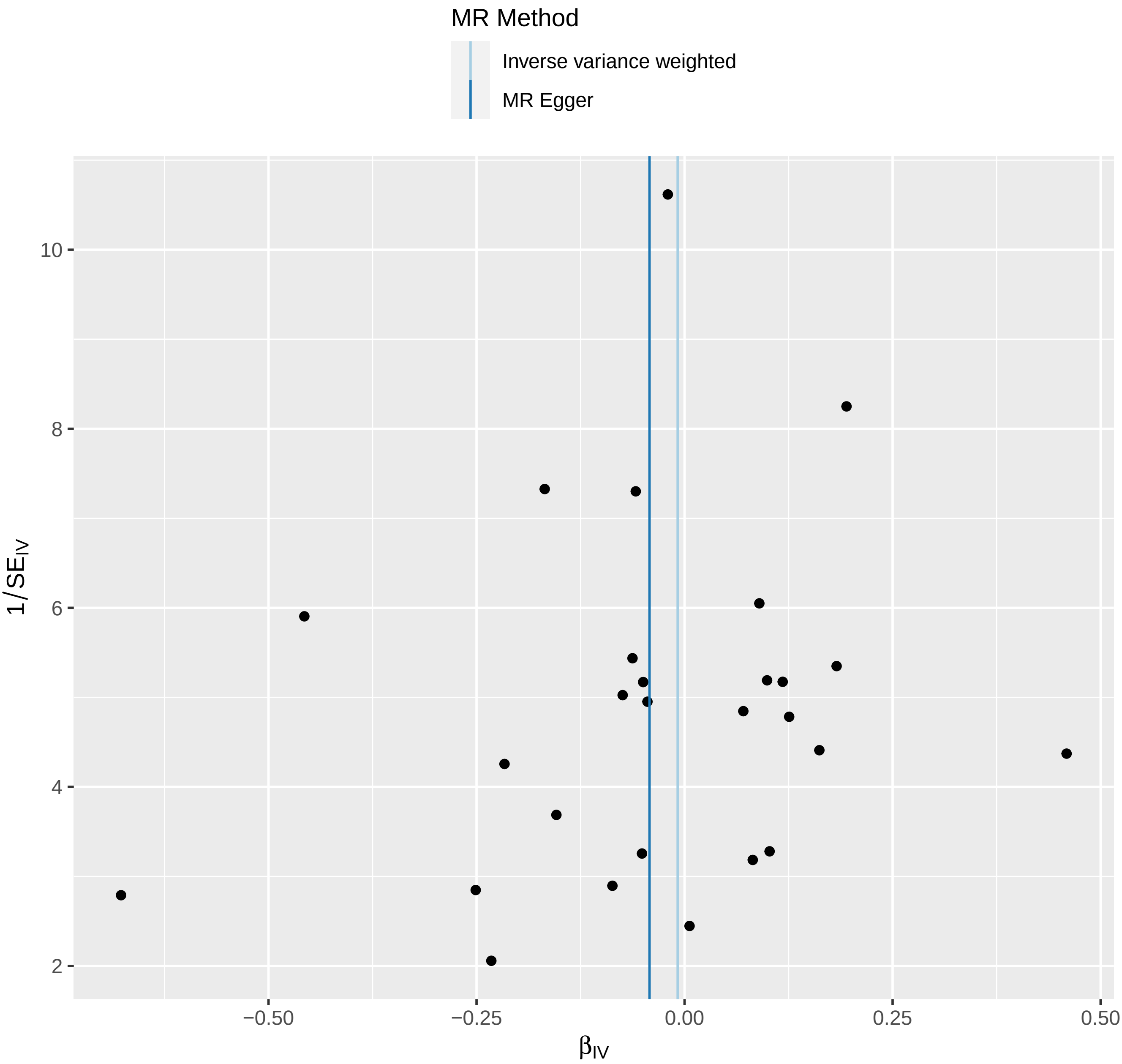

Batch 3−2885 : Syntaxin−1A on Diabetic nephropathy (more control exclusions)
with a threshold of 1e−05

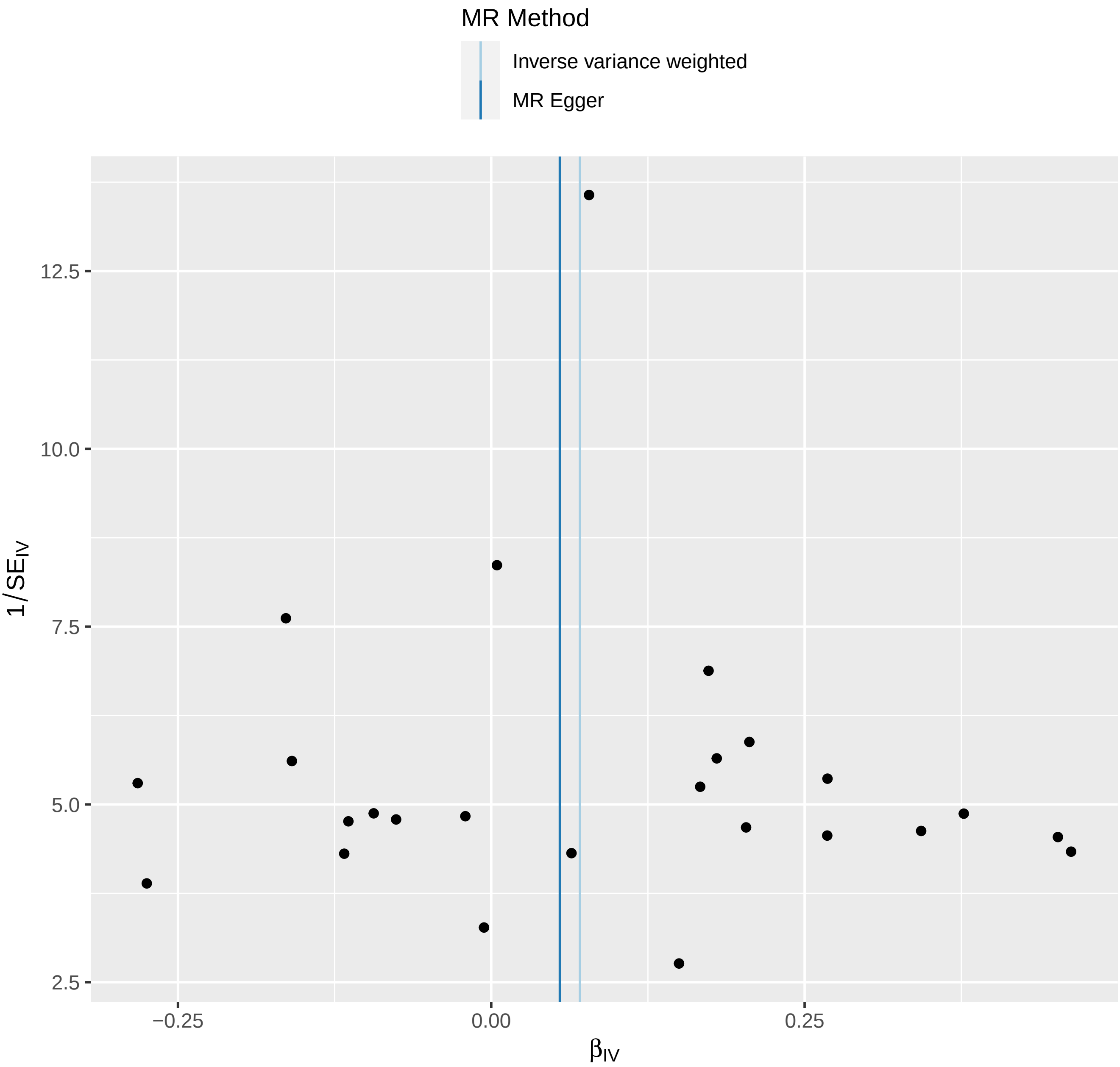

Batch 3−2886 : Syntaxin−1B on Diabetic nephropathy (more control exclusions)
with a threshold of 1e−05

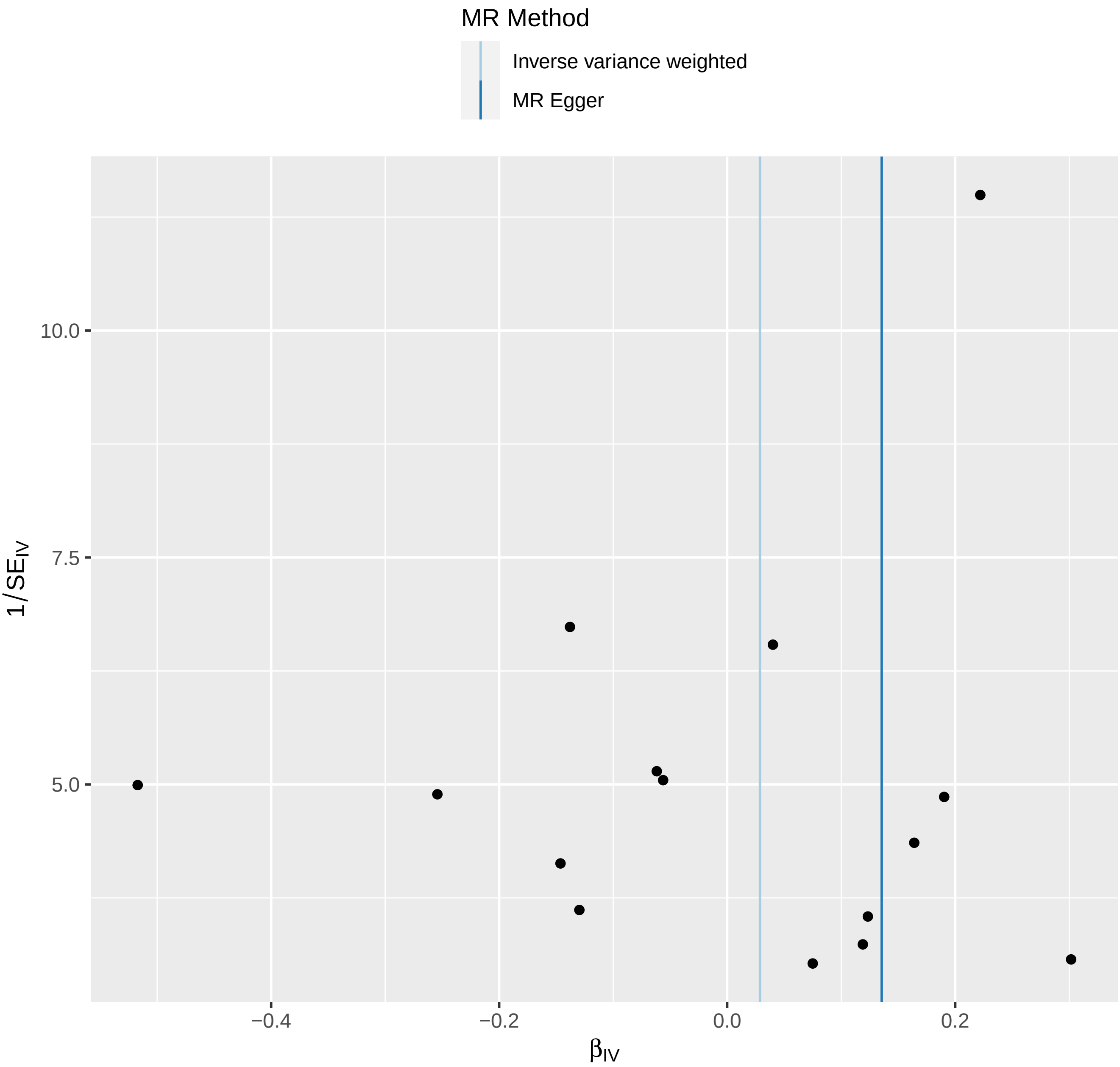

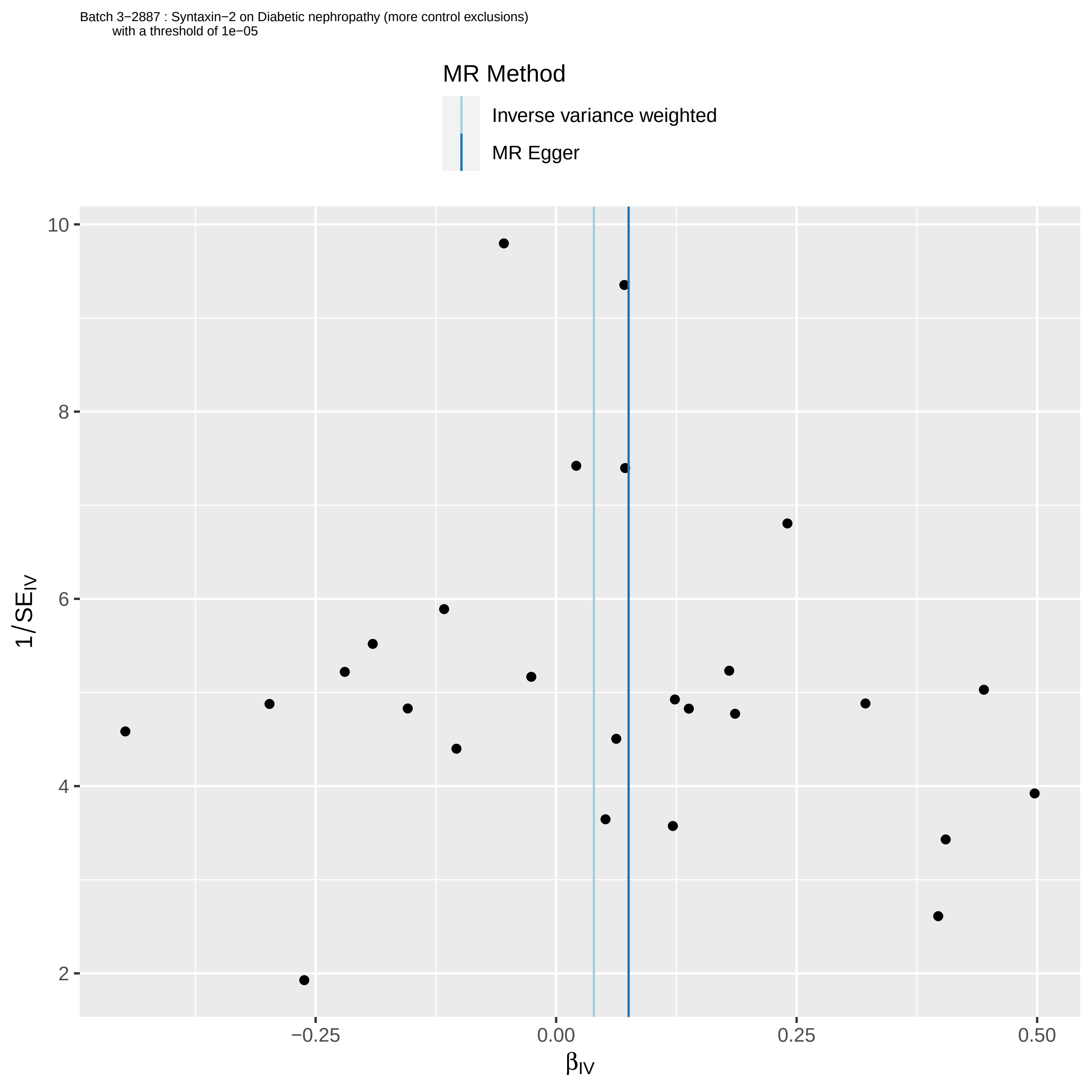

Batch 3−2887 : Syntaxin−2 on Diabetic nephropathy (more control exclusions)
with a threshold of 1e−05
MR Method
Inverse variance weighted
MR Egger
10
8
6
4
2
1/SE_IV
−0.25
0.00
0.25
0.50
β_IV

Batch 3−2888 : Syntaxin−6 on Diabetic nephropathy (more control exclusions)
with a threshold of 1e−05

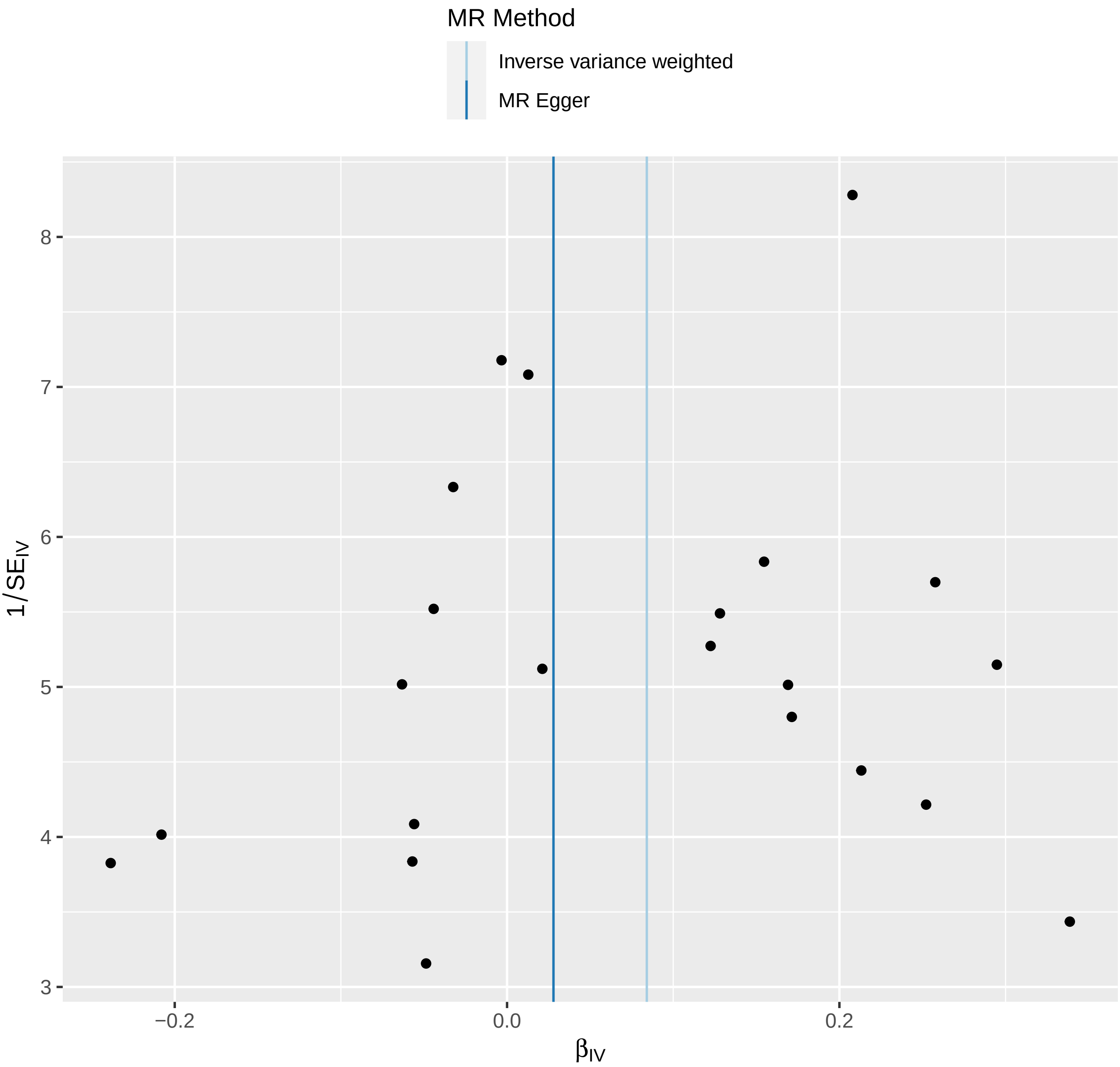

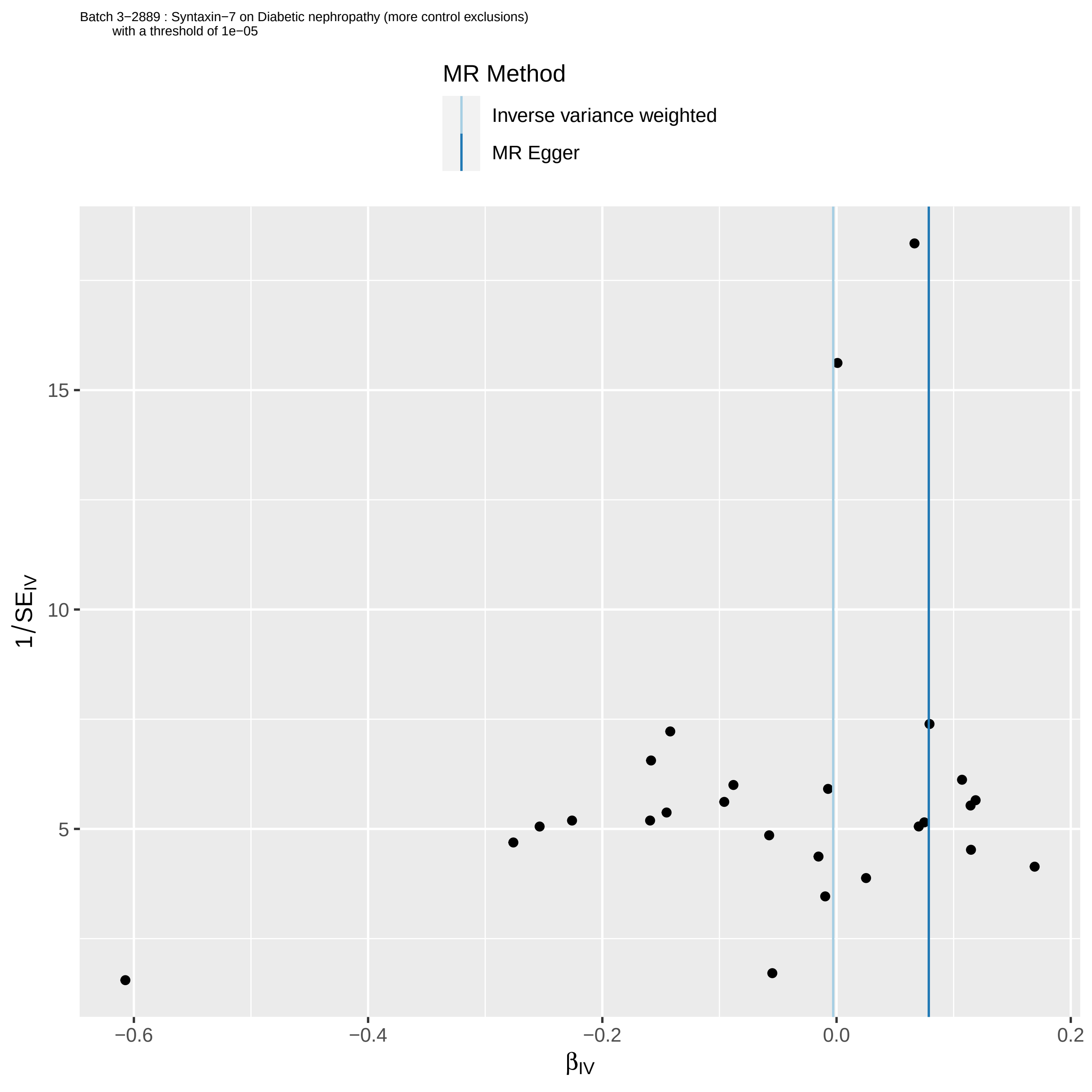

Batch 3−2889 : Syntaxin−7 on Diabetic nephropathy (more control exclusions)
with a threshold of 1e−05
MR Method
Inverse variance weighted
MR Egger
15
10
5
1/SE_IV
−0.6
−0.4
−0.2
0.0
0.2
β_IV

Batch 3−2890 : Syntaxin−8 on Diabetic nephropathy (more control exclusions)
with a threshold of 1e−05

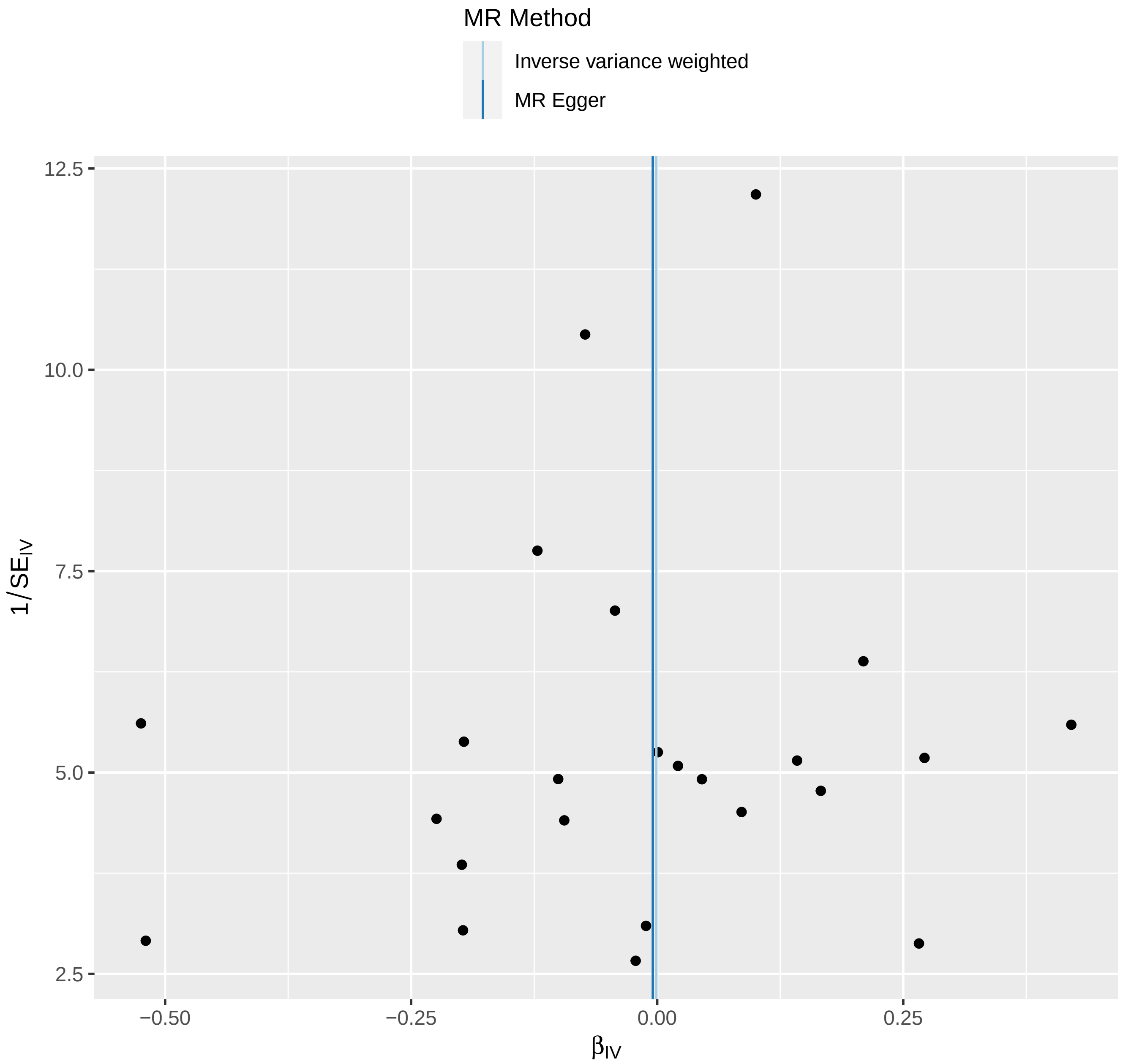

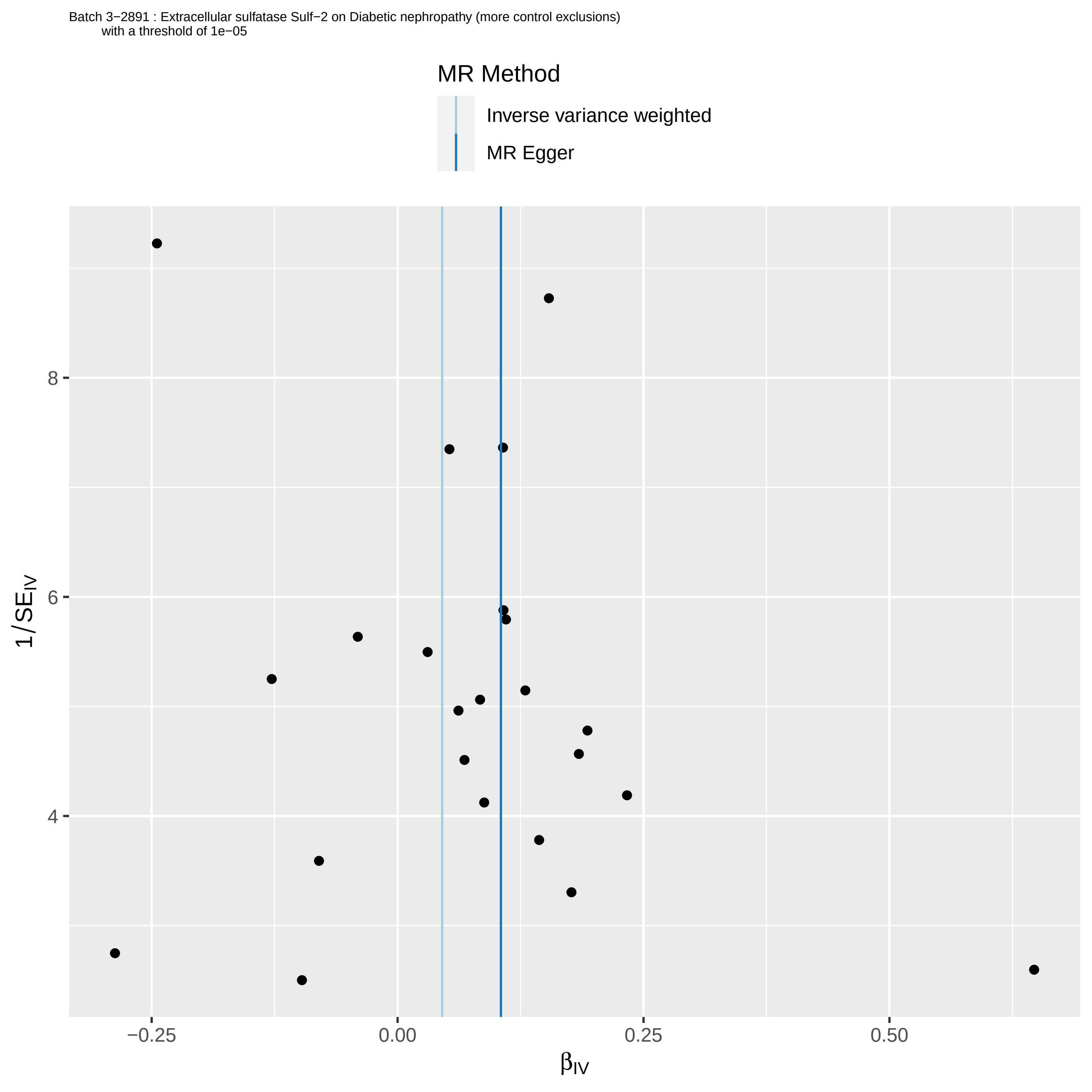
Batch 3−2891 : Extracellular sulfatase Sulf−2 on Diabetic nephropathy (more control exclusions)
with a threshold of 1e−05
MR Method
Inverse variance weighted
MR Egger
8
6
4
1/SE$_{IV}$
−0.25
0.00
0.25
0.50
β$_{IV}$

Batch 3−2892 : Estrogen sulfotransferase on Diabetic nephropathy (more control exclusions)
with a threshold of 1e−05

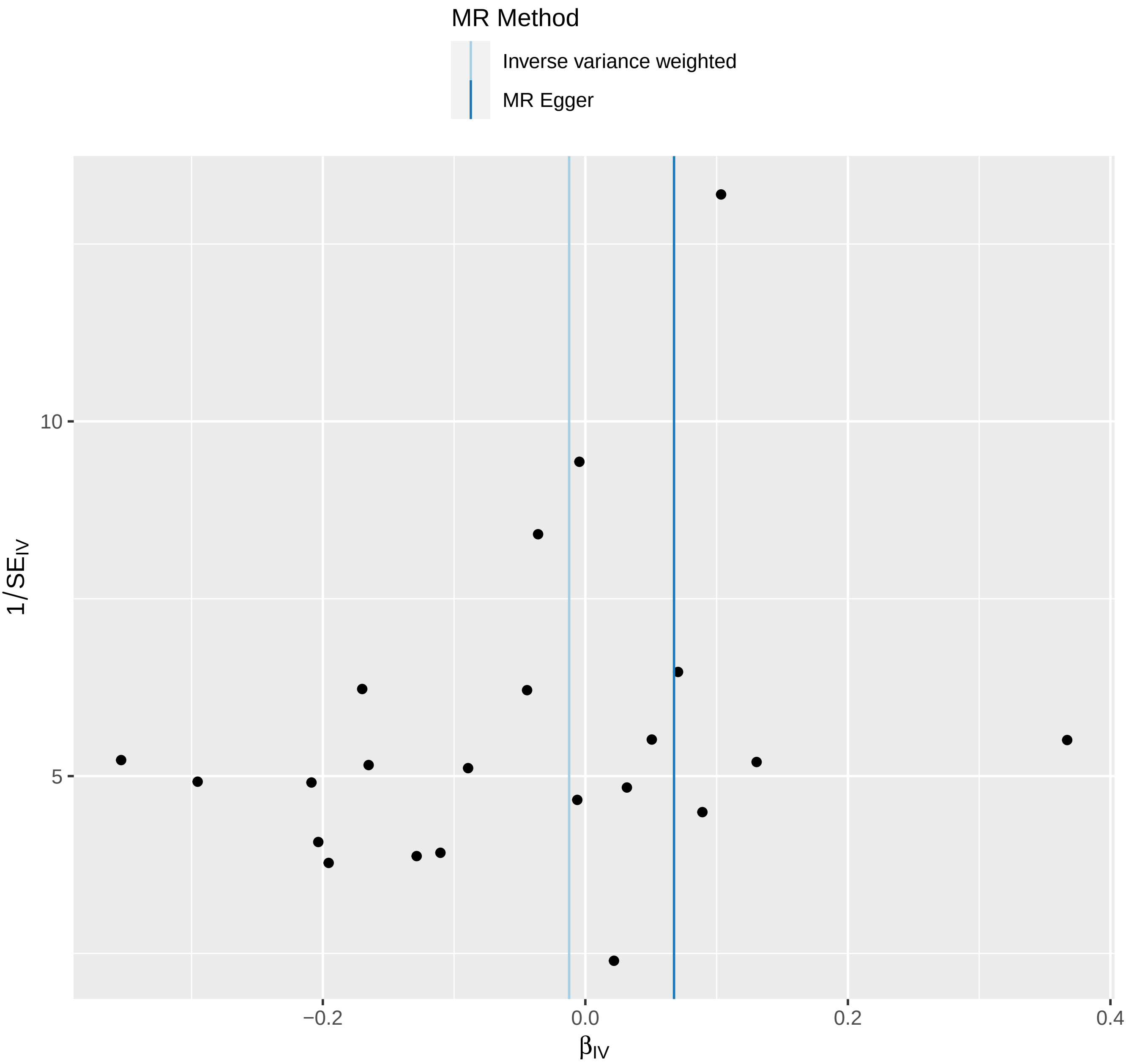

Batch 3−2893 : Bile salt sulfotransferase on Diabetic nephropathy (more control exclusions)
with a threshold of 1e−05

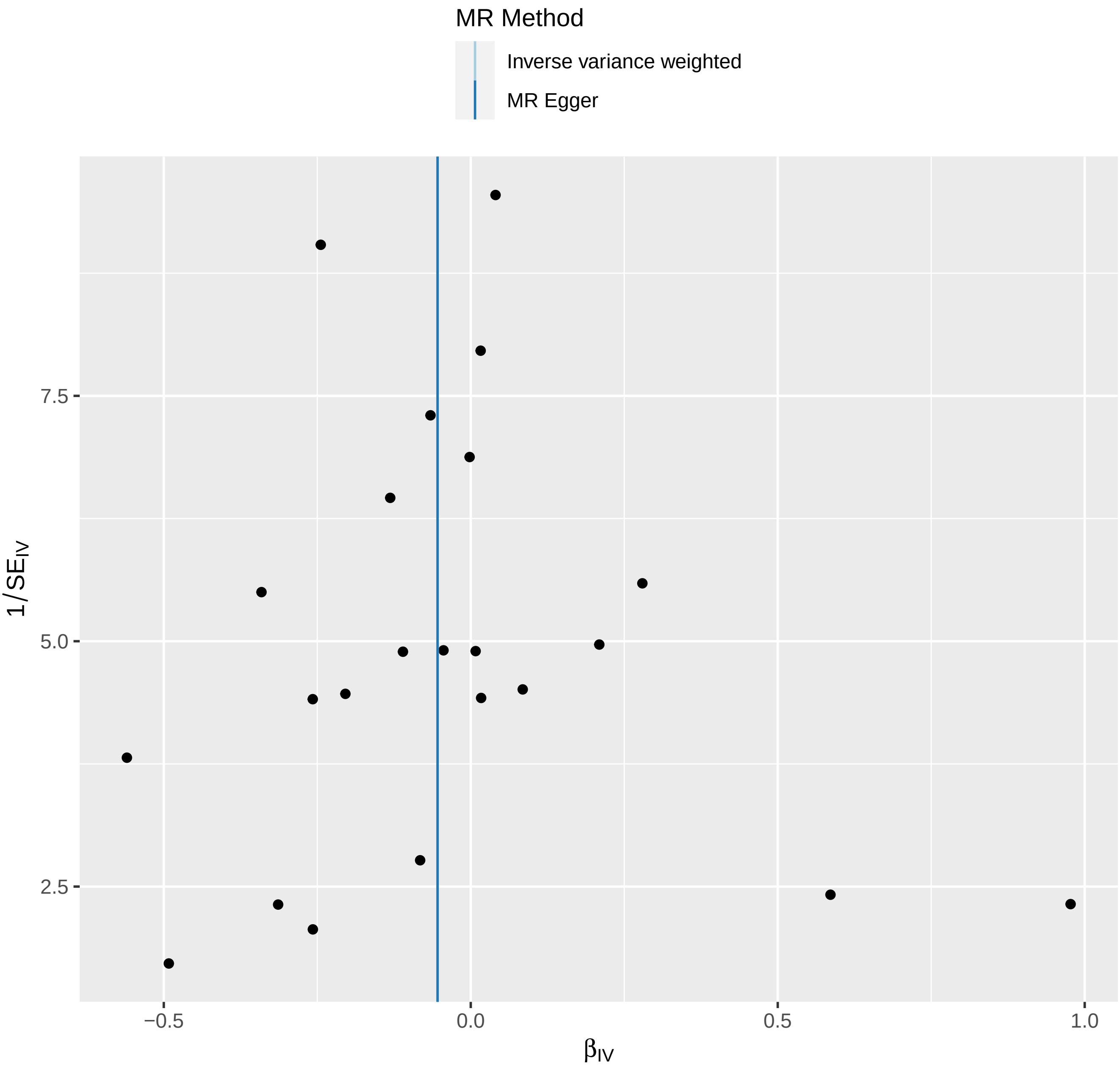

Batch 3−2894 : Sulfotransferase 4A1 on Diabetic nephropathy (more control exclusions)
with a threshold of 1e−05

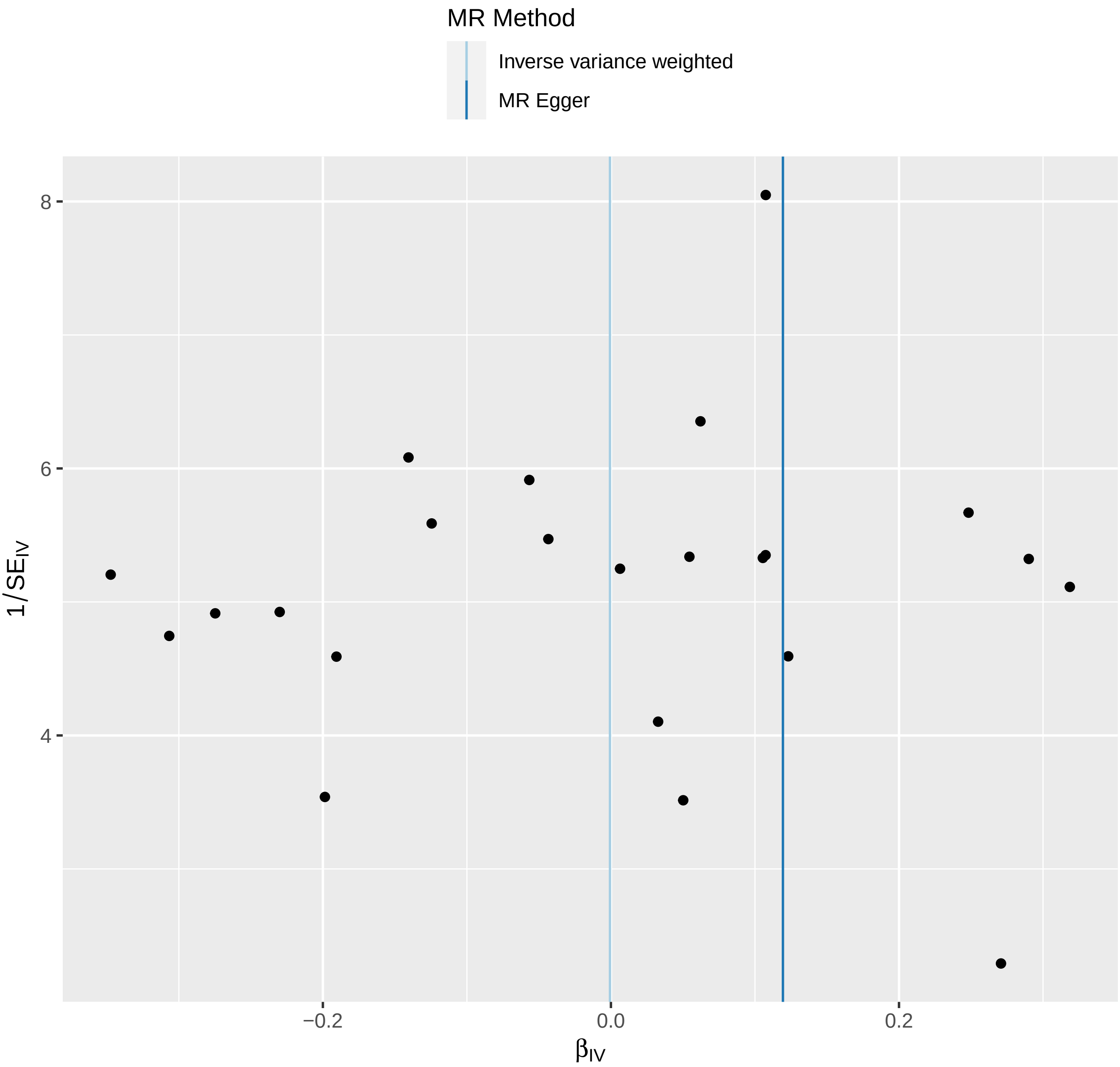

Batch 3−2895 : Sulfatase−modifying factor 1 on Diabetic nephropathy (more control exclusions)
with a threshold of 1e−05

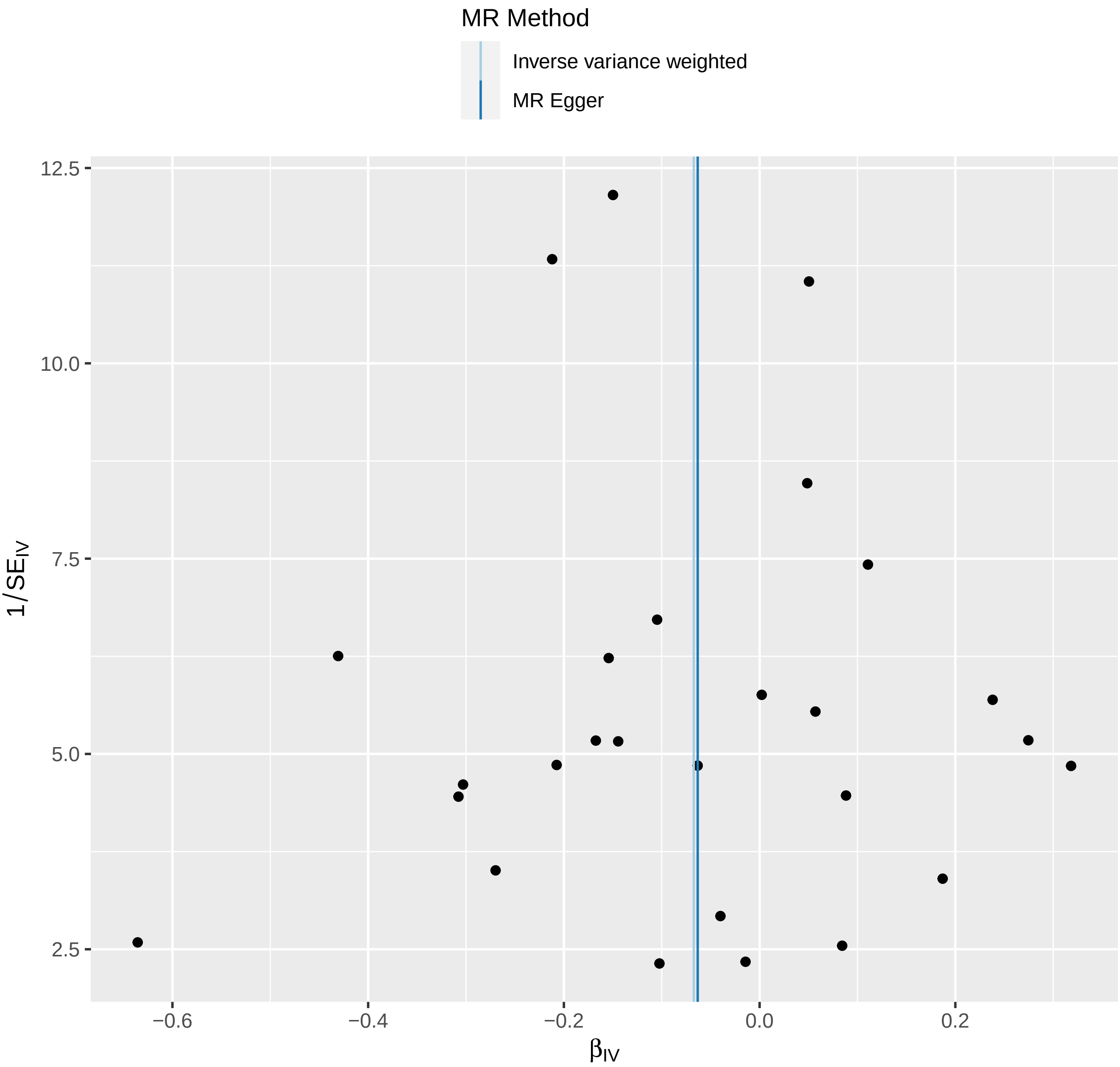

Batch 3−2896 : Small ubiquitin−related modifier 3 on Diabetic nephropathy (more control exclusions)
with a threshold of 1e−05

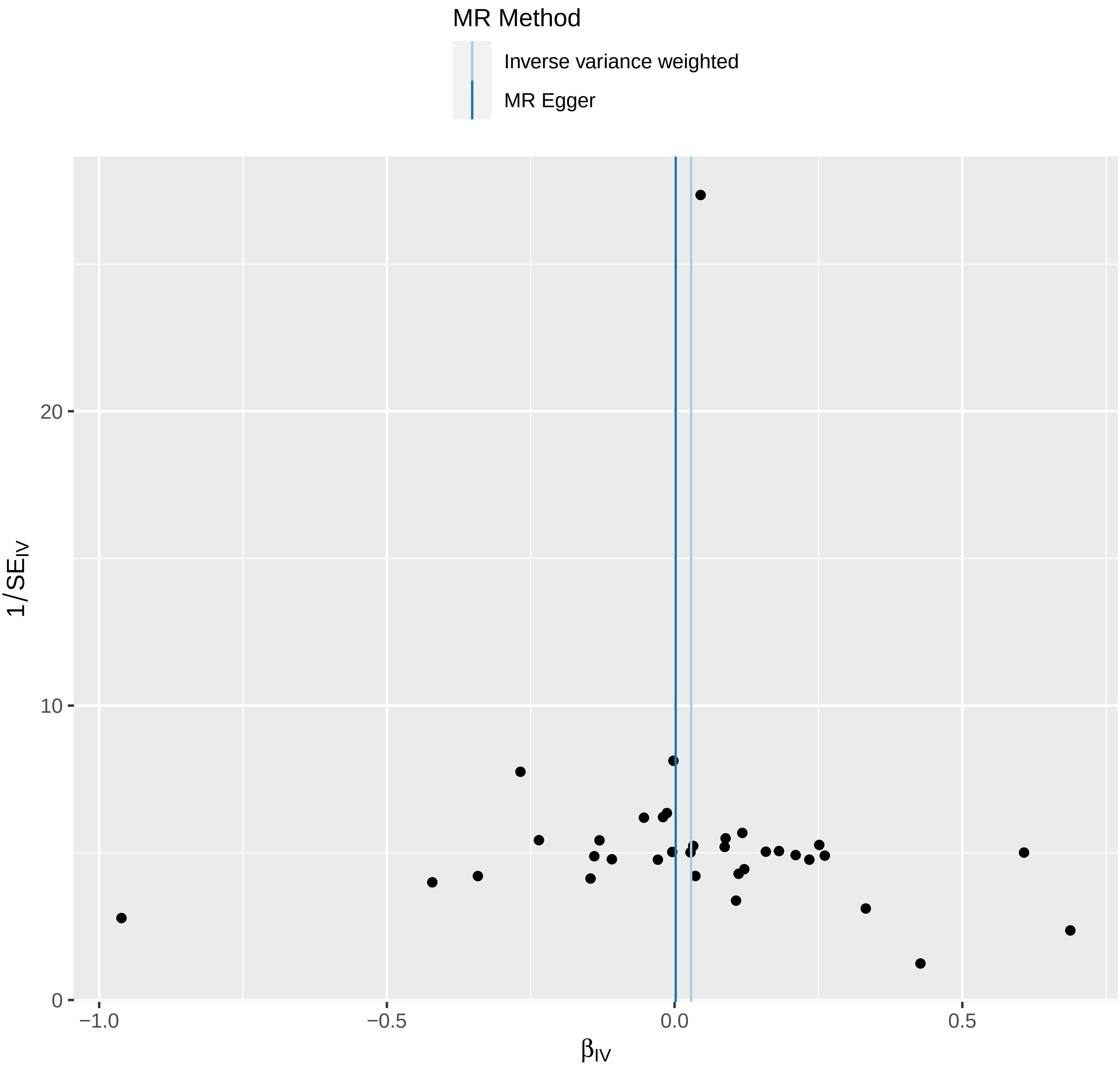

Batch 3−2897 : Small ubiquitin−related modifier 3 on Diabetic nephropathy (more control exclusions)
with a threshold of 1e−05

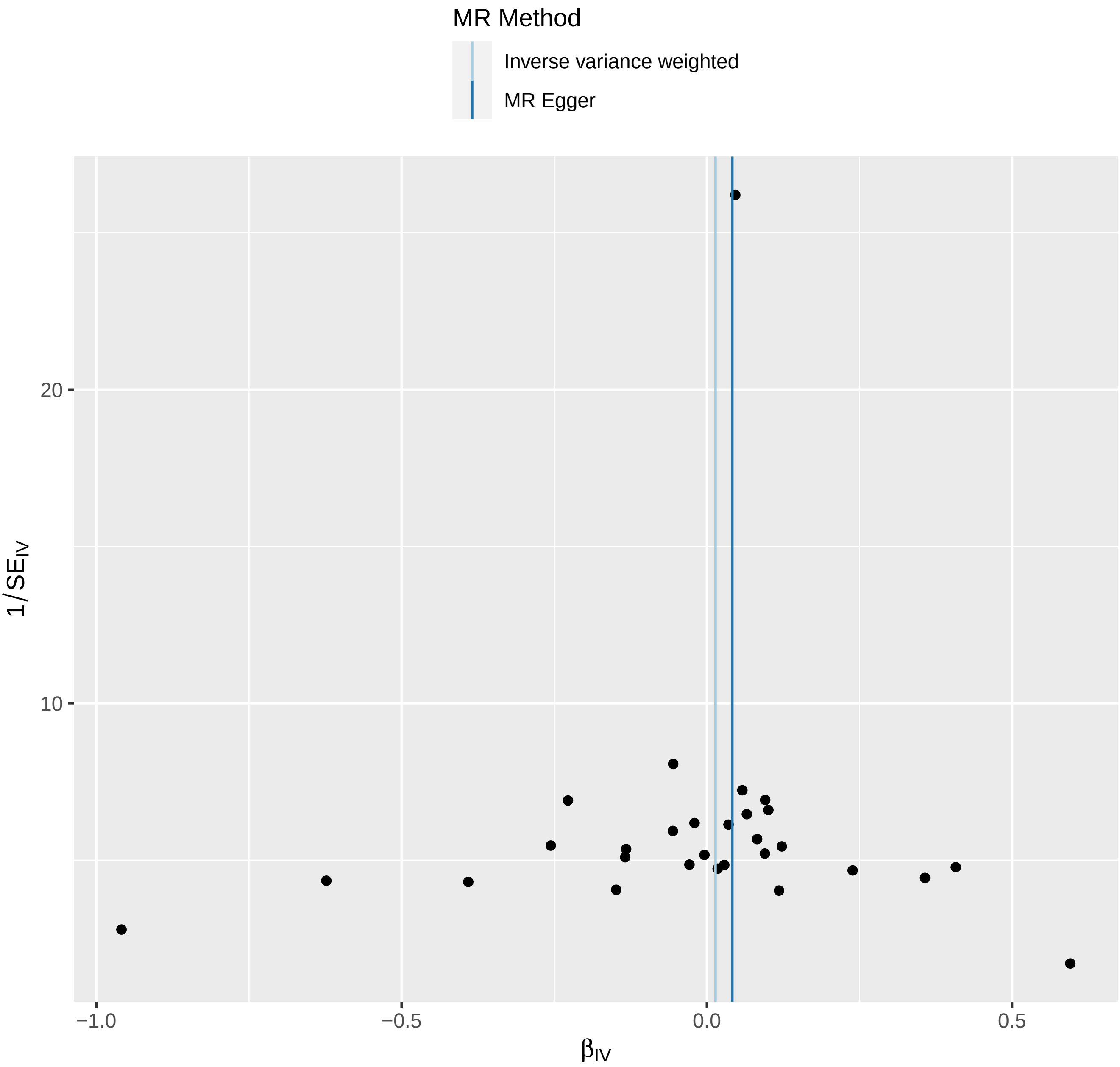

Batch 3−2898 : SUN domain−containing protein 3 on Diabetic nephropathy (more control exclusions)
with a threshold of 1e−05

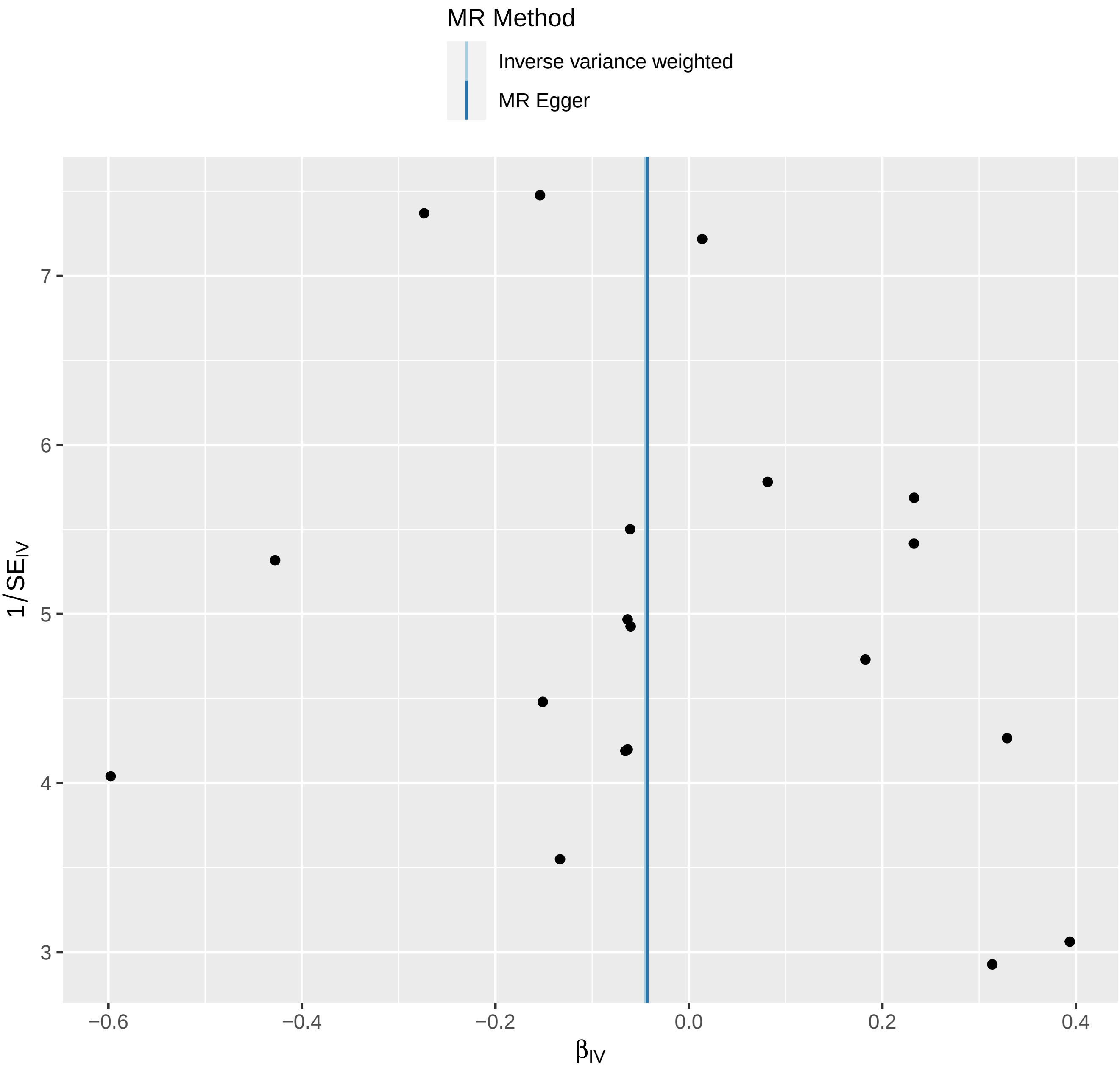

Batch 3−2899 : SUN domain−containing protein 5 on Diabetic nephropathy (more control exclusions)
with a threshold of 1e−05

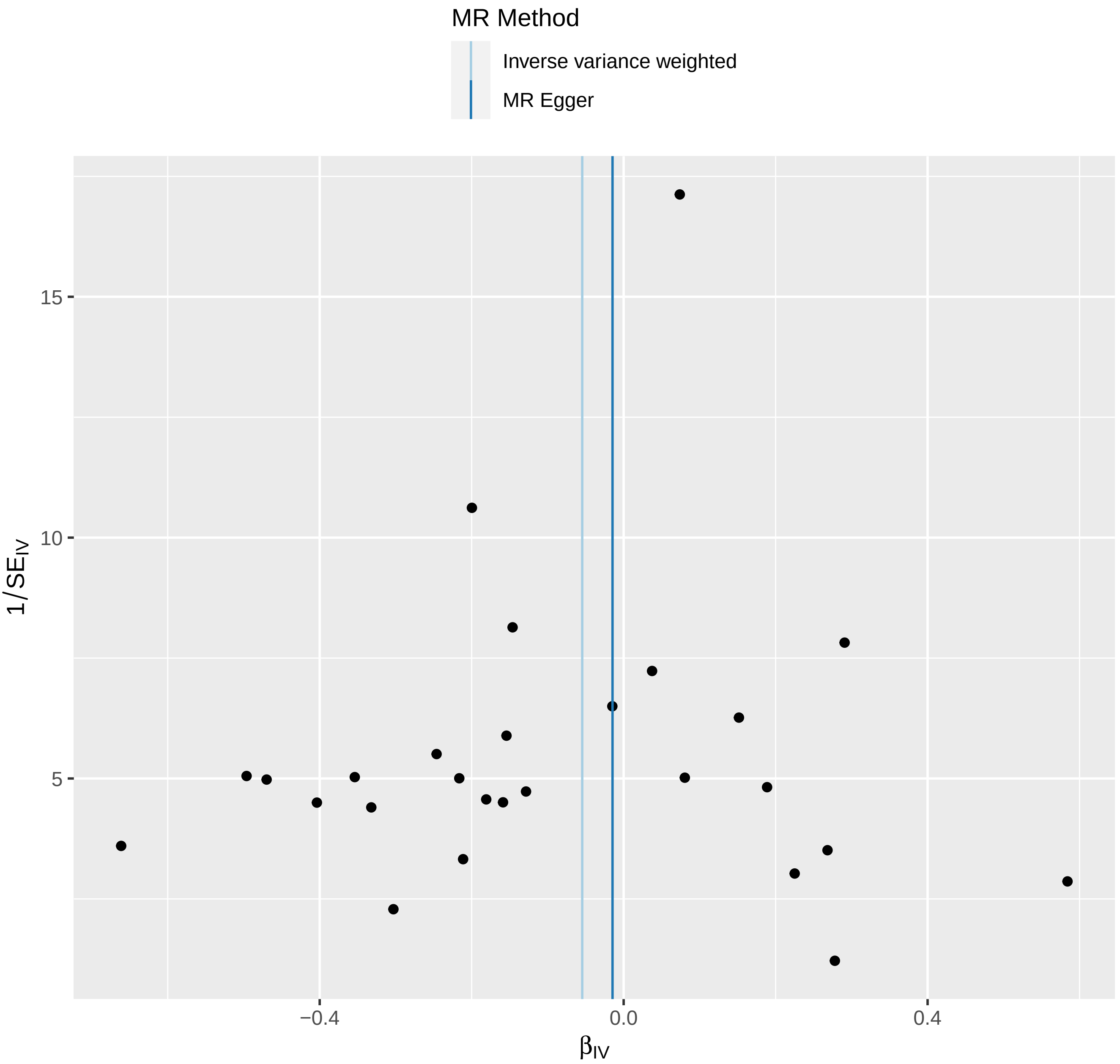

Batch 3−2900 : Surfeit locus protein 1 on Diabetic nephropathy (more control exclusions)
with a threshold of 1e−05

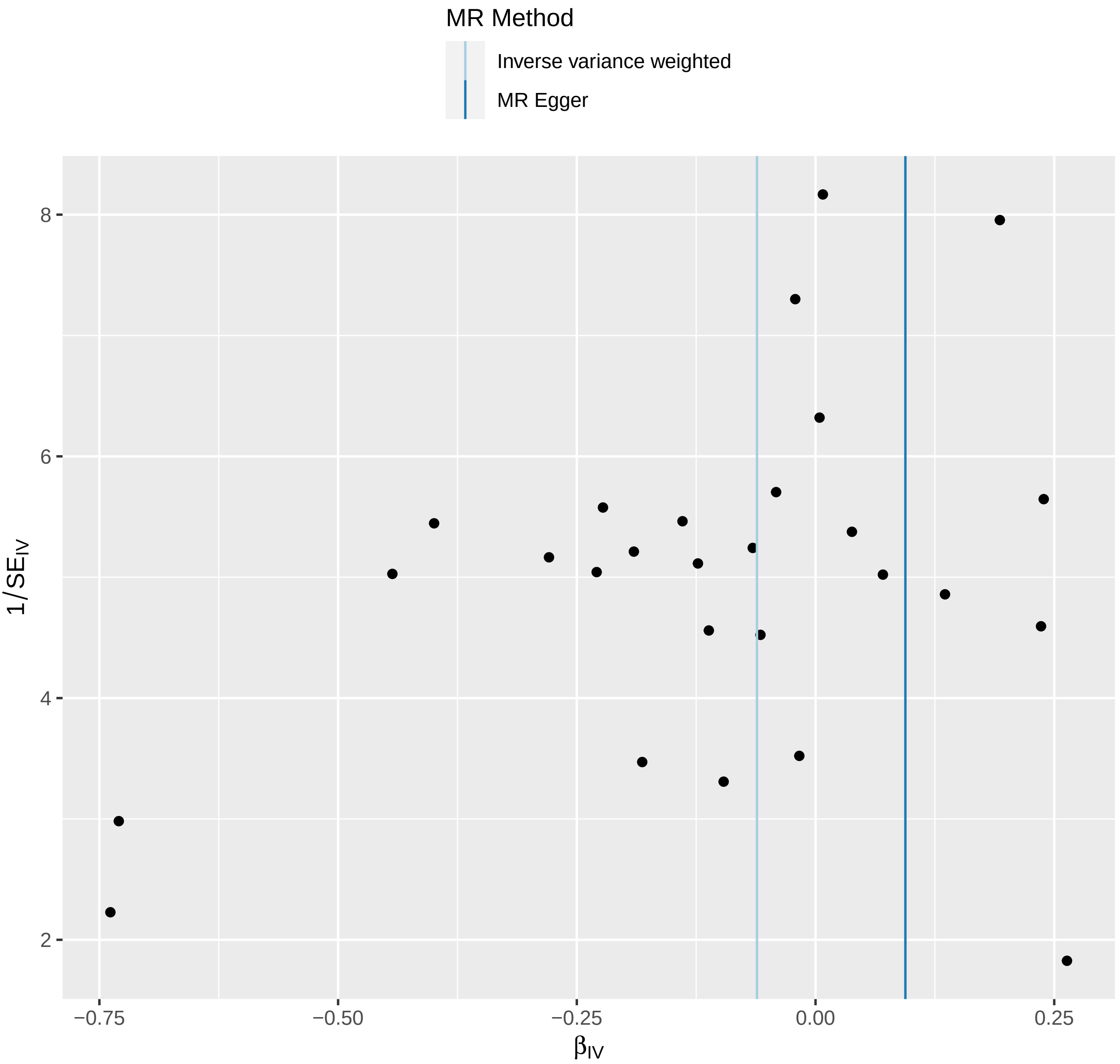

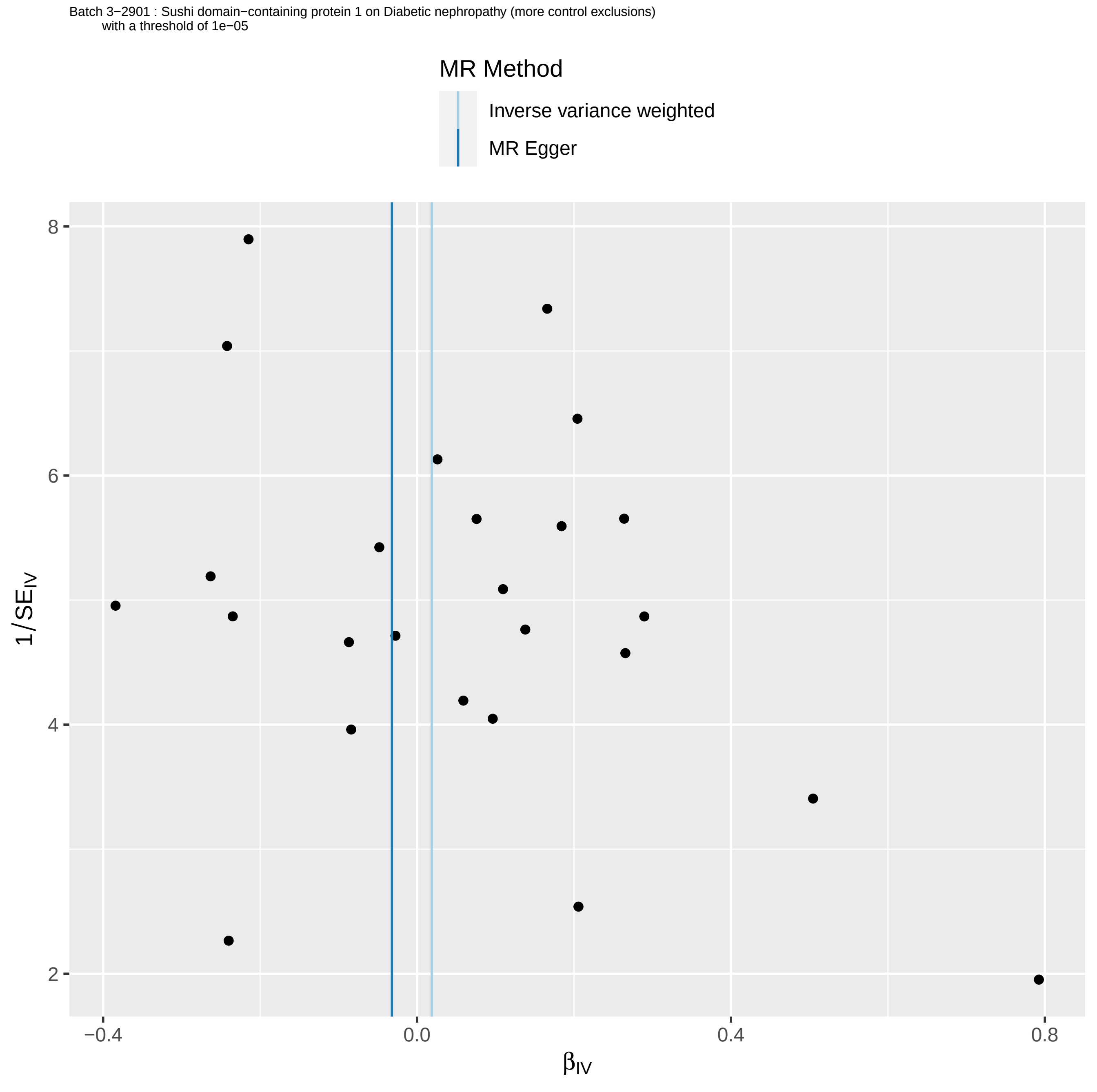

Batch 3−2901 : Sushi domain−containing protein 1 on Diabetic nephropathy (more control exclusions)
with a threshold of 1e−05
MR Method
Inverse variance weighted
MR Egger
8
6
4
2
−0.4
0.0
0.4
0.8
1/SE_IV
β_IV

Batch 3−2902 : Sushi domain−containing protein 2 on Diabetic nephropathy (more control exclusions)
with a threshold of 1e−05

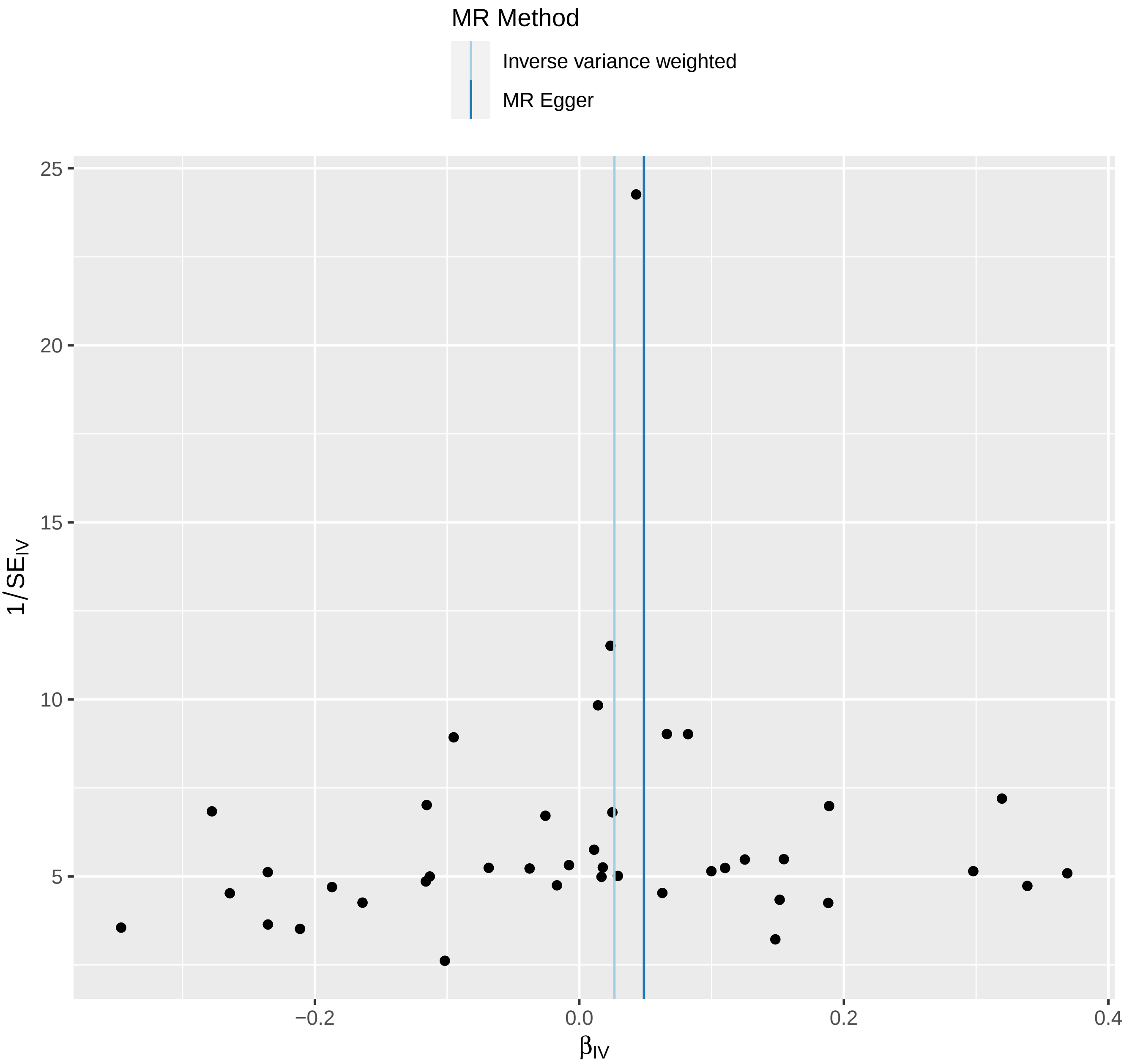

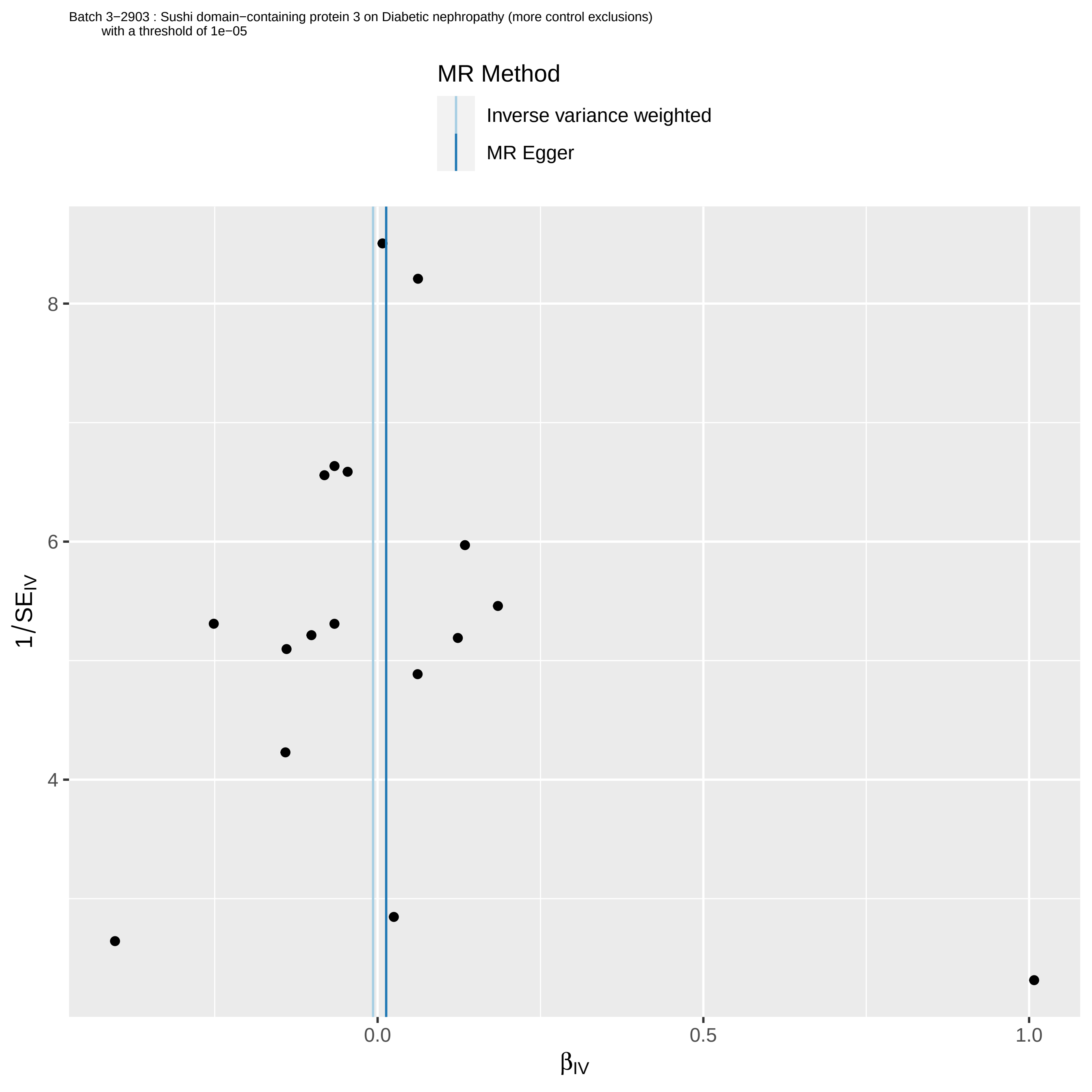

Batch 3−2903 : Sushi domain−containing protein 3 on Diabetic nephropathy (more control exclusions)
with a threshold of 1e−05
MR Method
Inverse variance weighted
MR Egger
8
6
4
1/SE$_{IV}$
0.0
0.5
1.0
β_{IV}

Batch 3−2904 : Histone−lysine N−methyltransferase SUV420H2 on Diabetic nephropathy (more control exclusions)
with a threshold of 1e−05

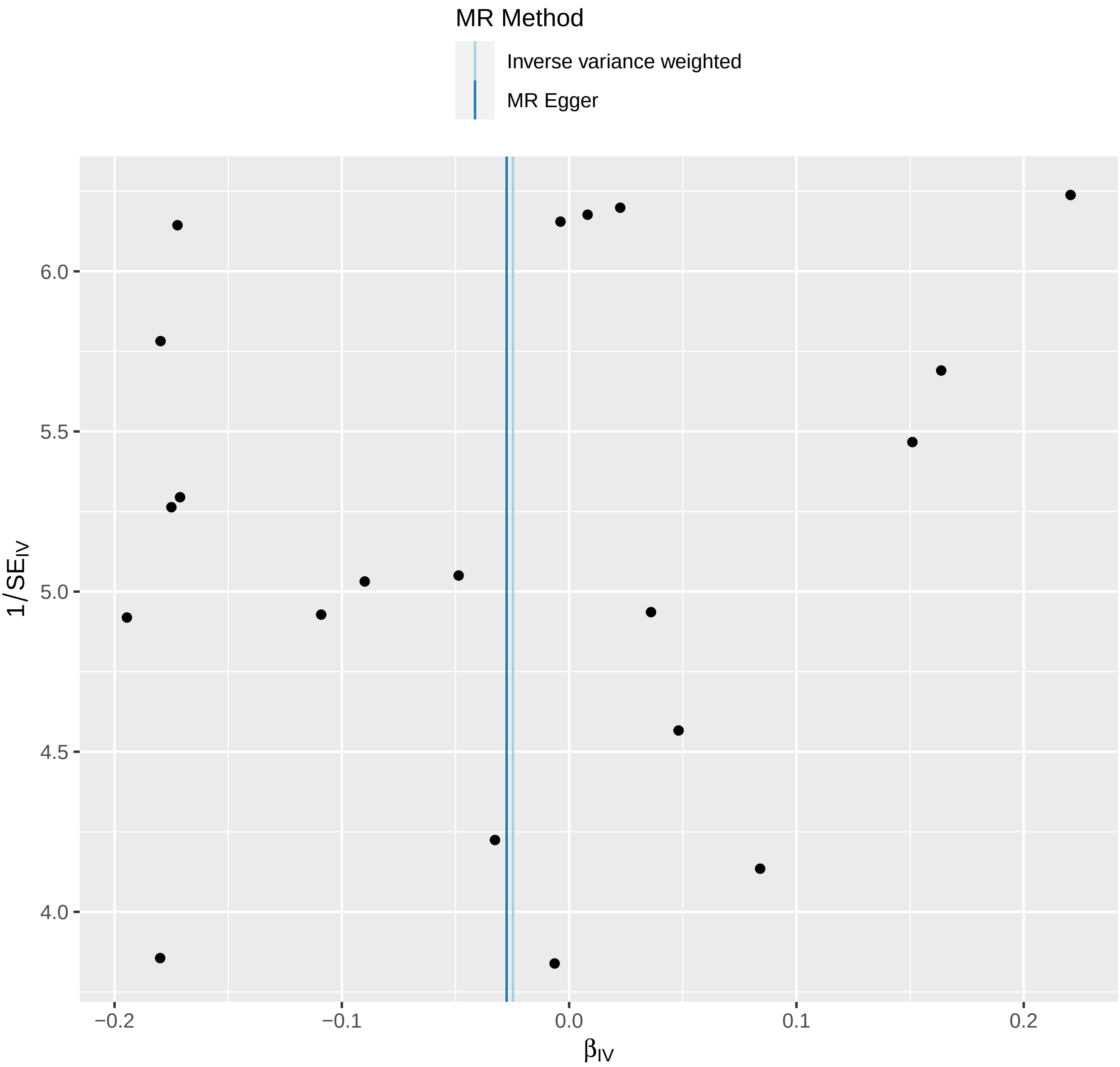

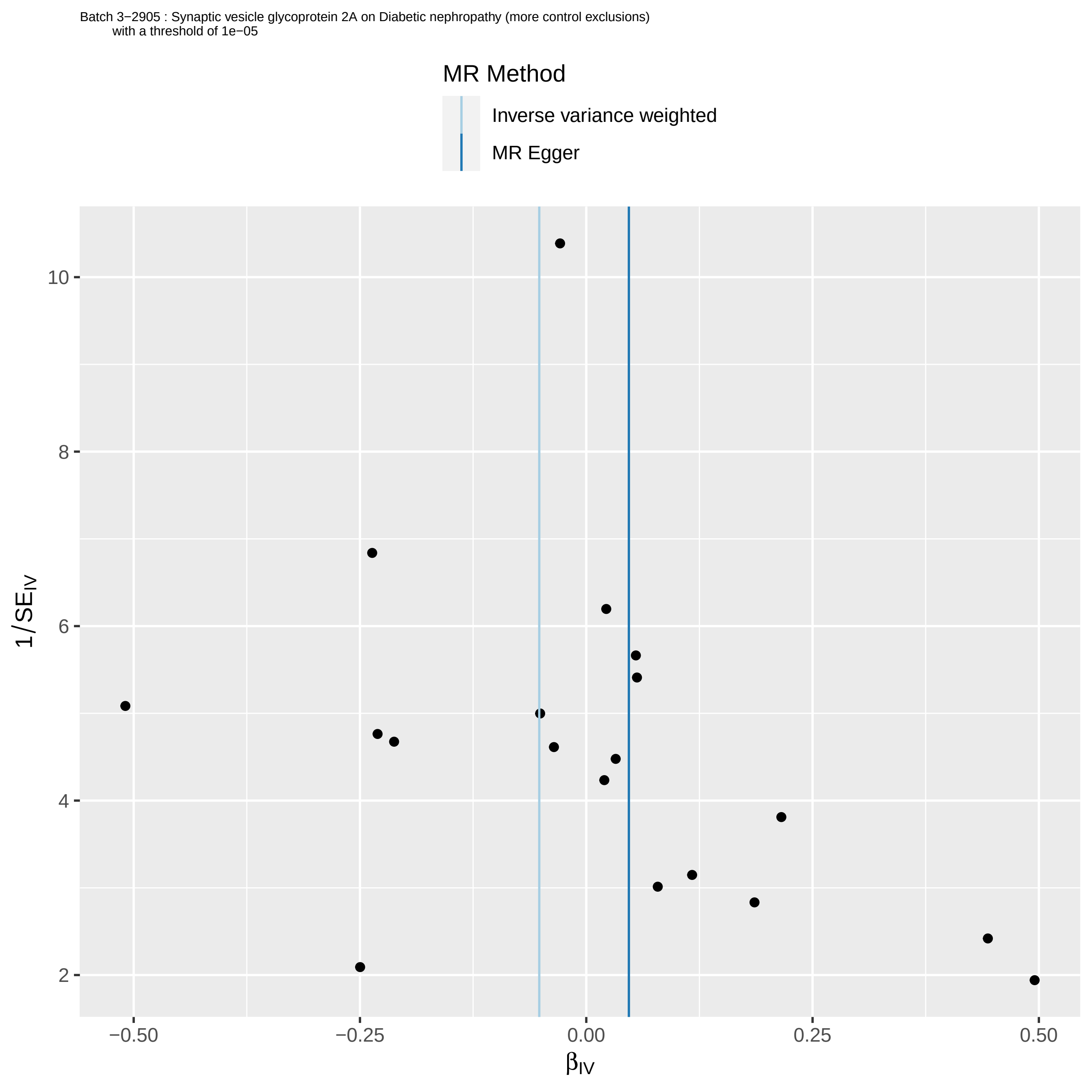
Batch 3−2905 : Synaptic vesicle glycoprotein 2A on Diabetic nephropathy (more control exclusions)
with a threshold of 1e−05
MR Method
Inverse variance weighted
MR Egger
10
8
6
4
2
1/SE_IV
−0.50
−0.25
0.00
0.25
0.50
β_IV

Batch 3−2906 : Sushi, von Willebrand factor type A, EGF and pentraxin domain−containing protein 1 on Diabetic nephropathy (more control exclusions) with a threshold of 1e−05

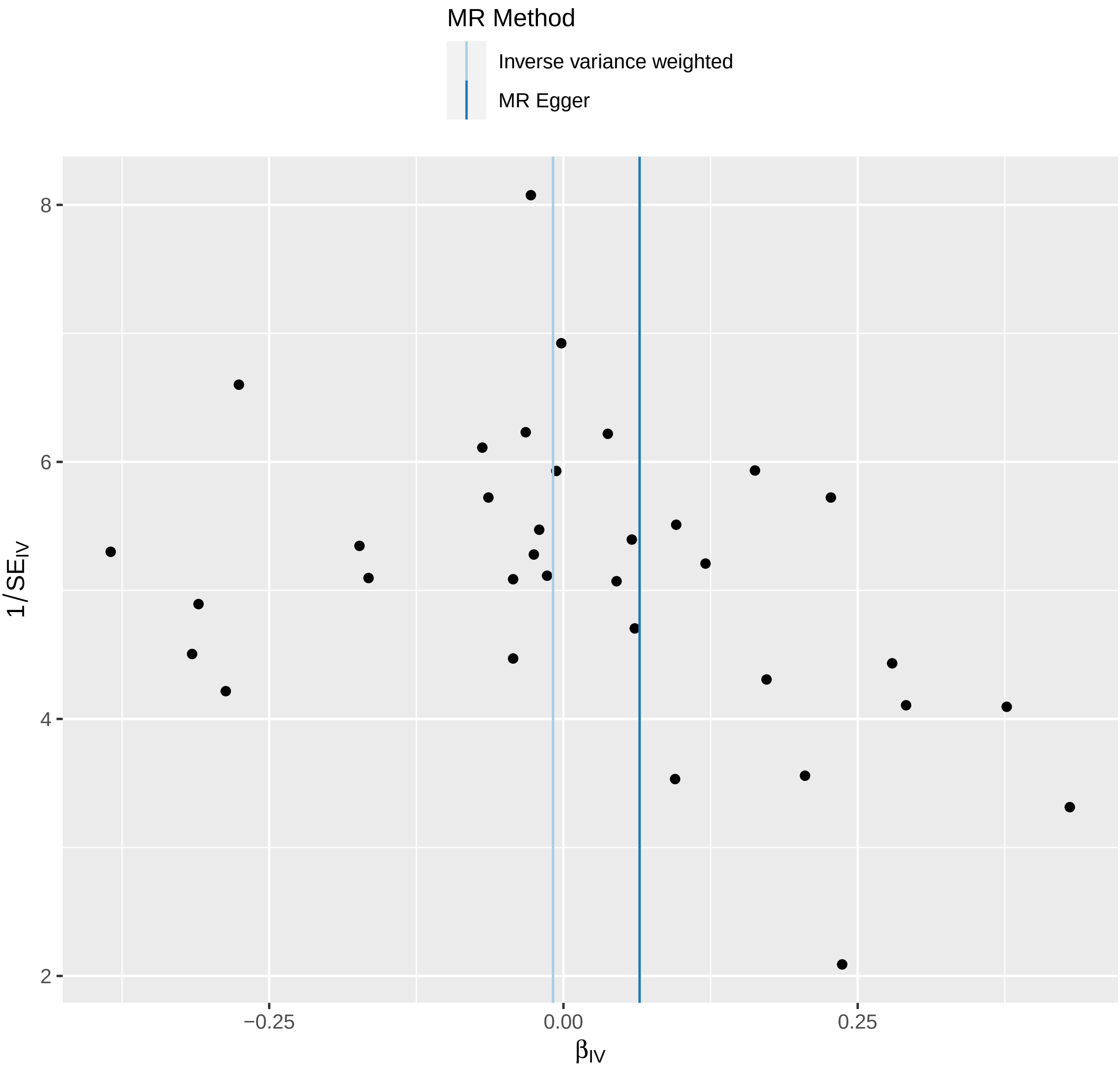

Batch 3−2907 : Sushi, von Willebrand factor type A, EGF and pentraxin domain−containing protein 1 on Diabetic nephropathy (more control exclusions) with a threshold of 1e−05

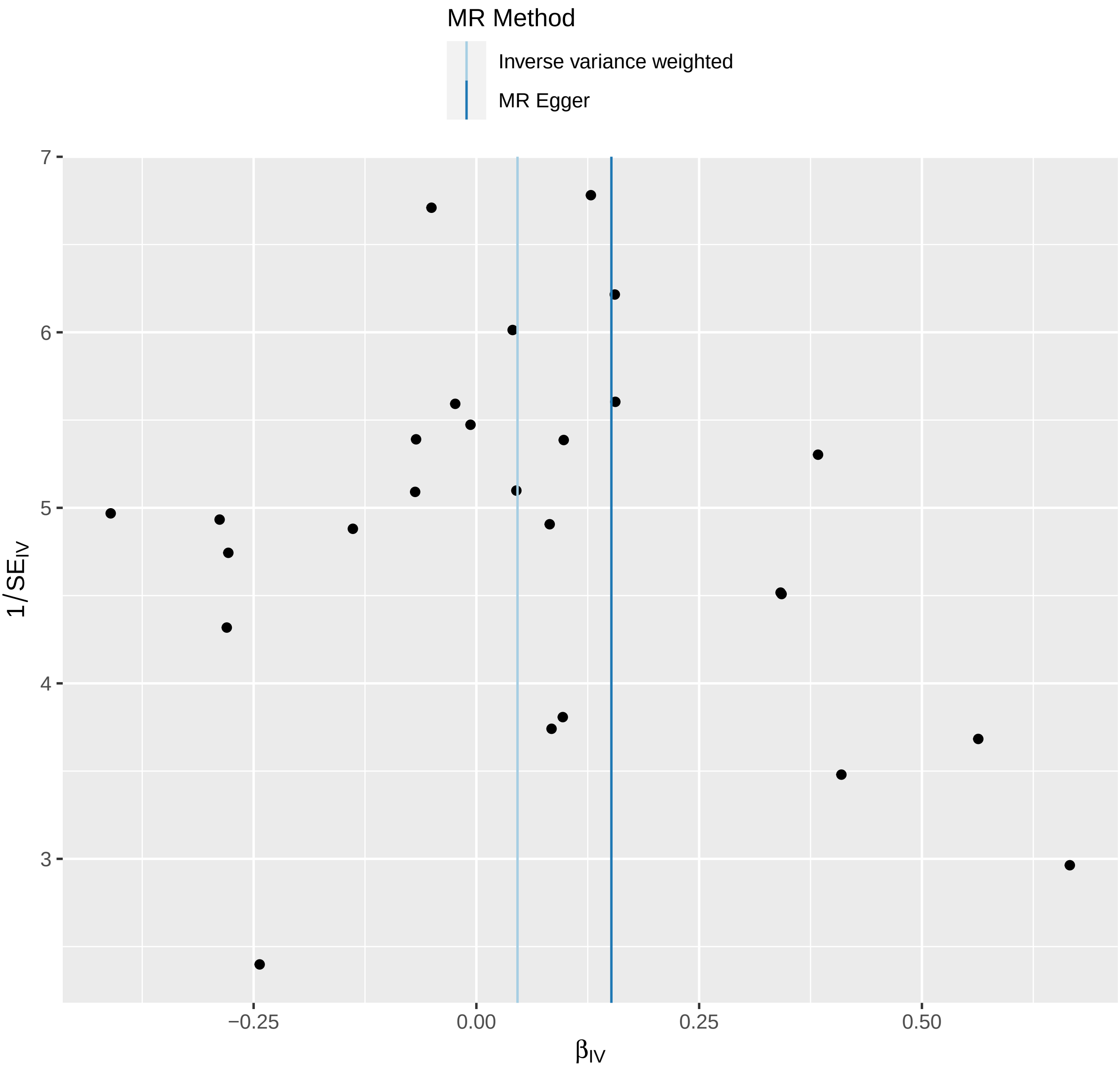

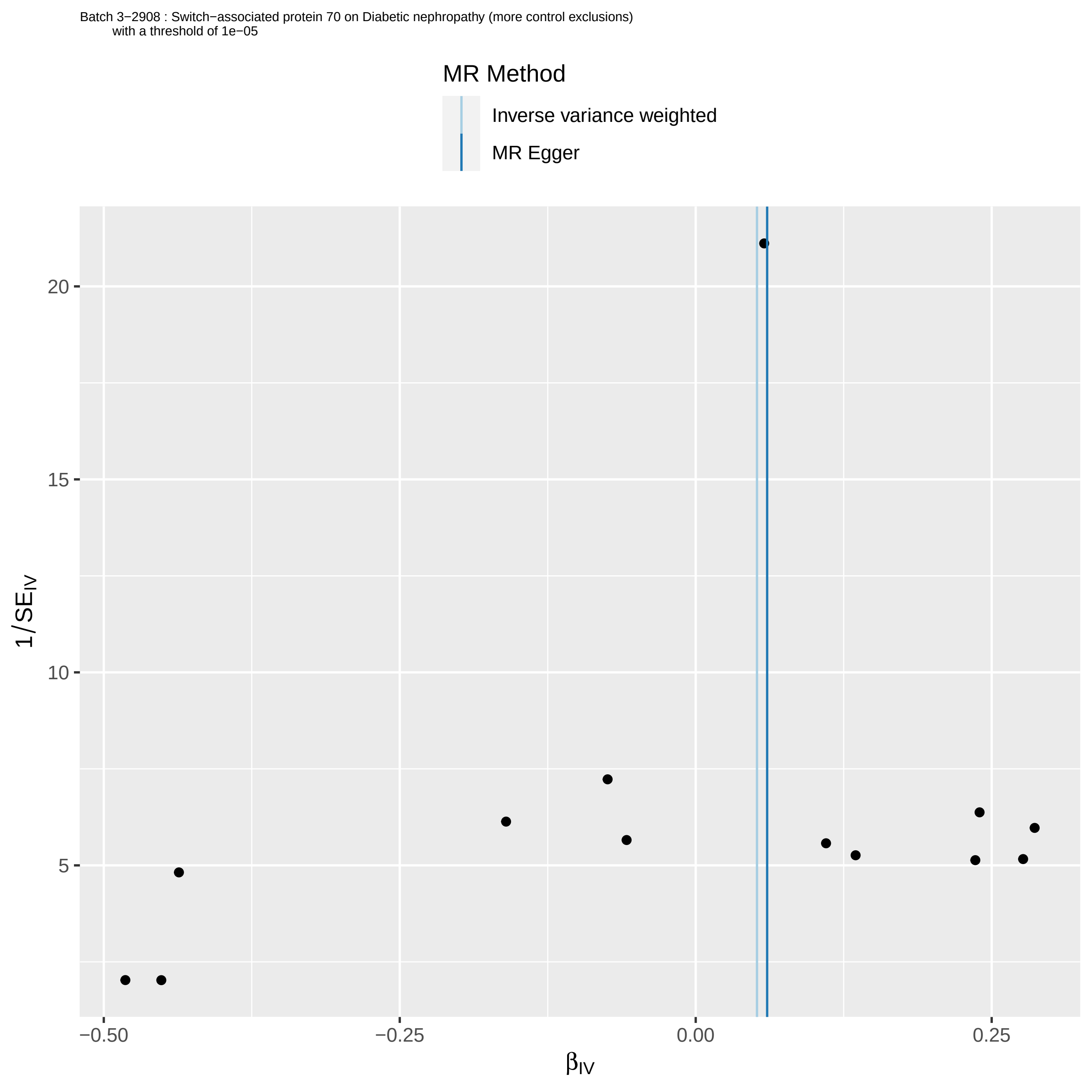
Batch 3−2908 : Switch−associated protein 70 on Diabetic nephropathy (more control exclusions)
with a threshold of 1e−05
MR Method
Inverse variance weighted
MR Egger
20
15
10
5
1/SE_IV
−0.50
−0.25
0.00
0.25
β_IV

Batch 3−2909 : Heterogeneous nuclear ribonucleoprotein Q on Diabetic nephropathy (more control exclusions)
with a threshold of 1e−05

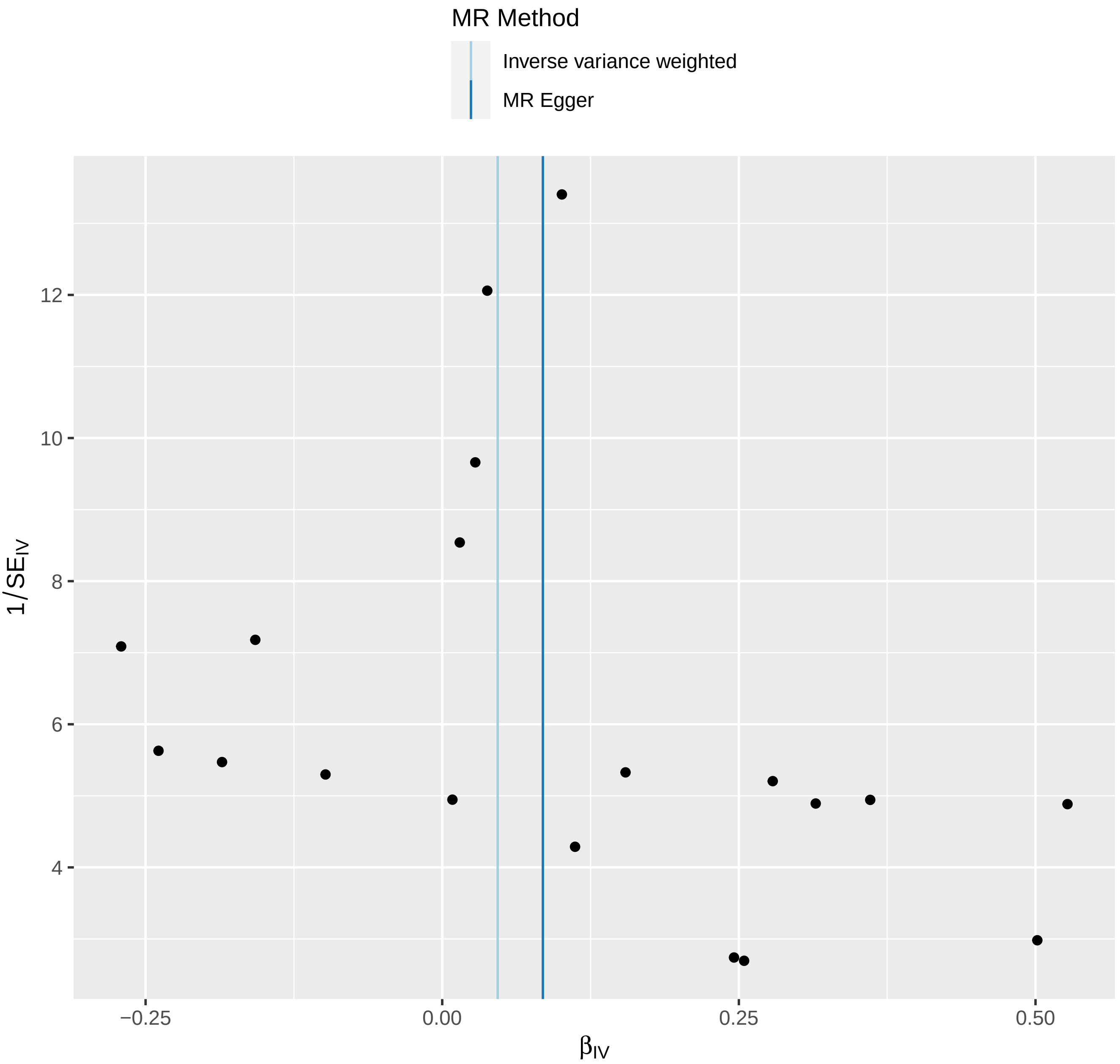

Batch 3−2910 : Nesprin−2 on Diabetic nephropathy (more control exclusions)
with a threshold of 1e−05

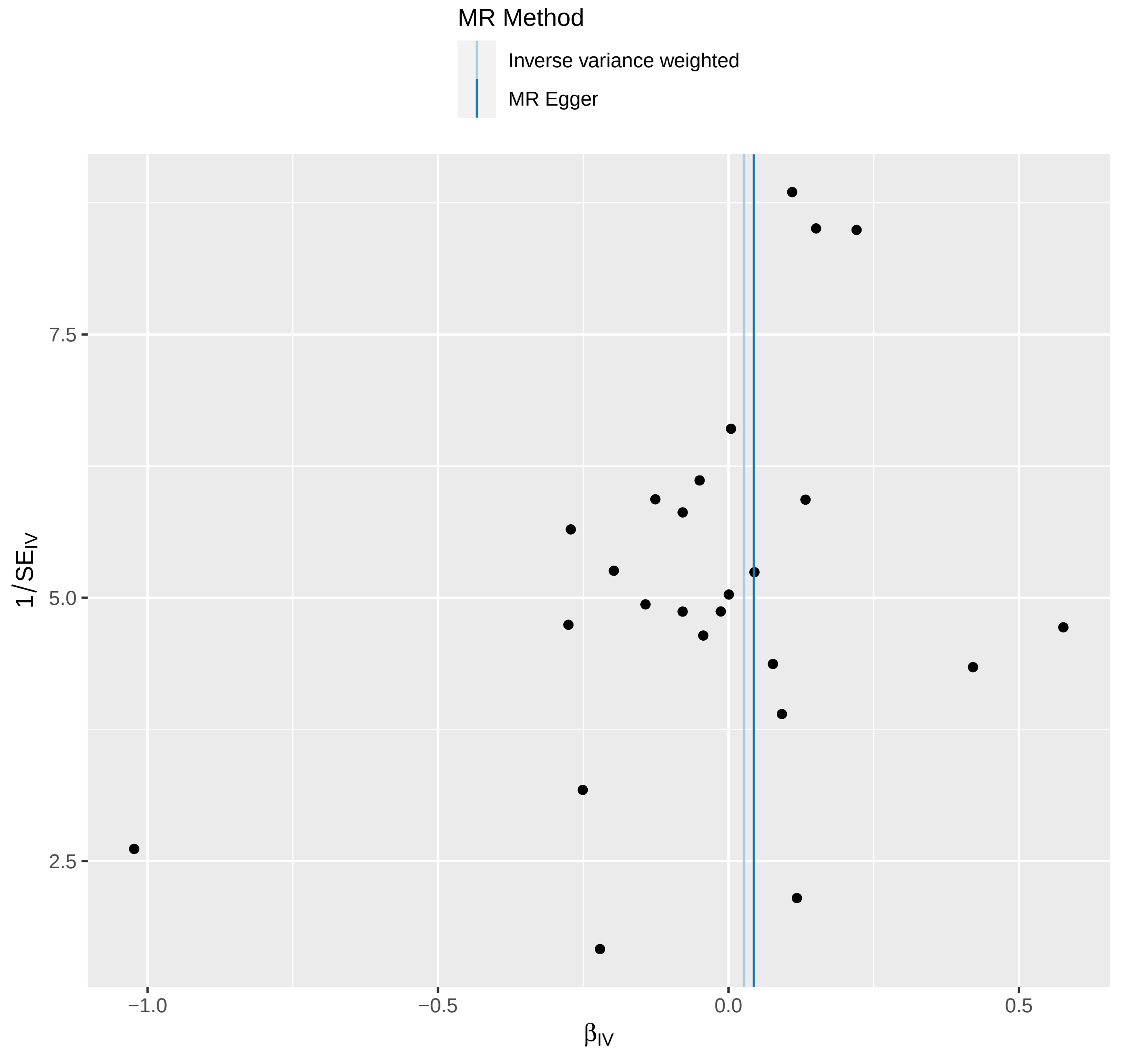

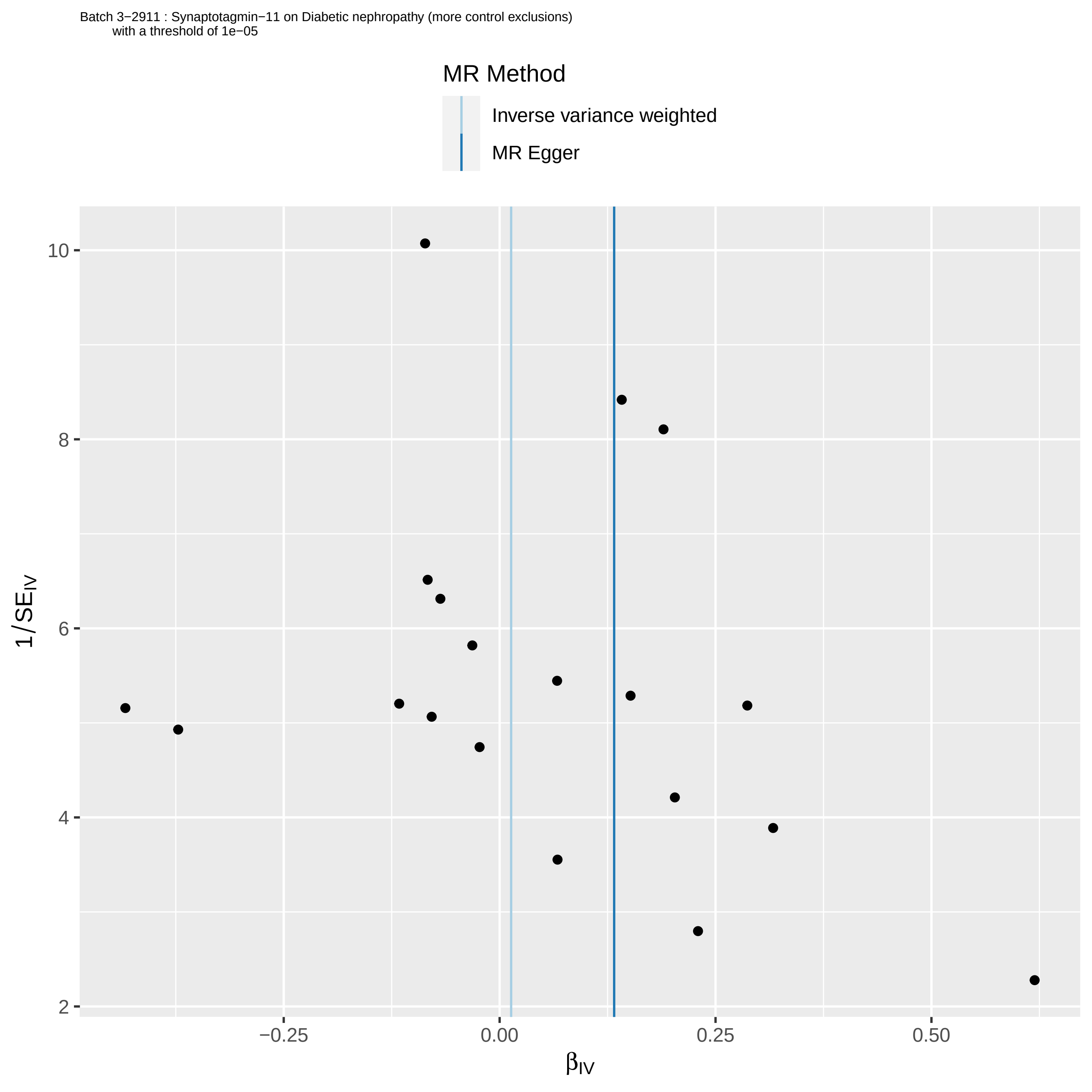

Batch 3−2911 : Synaptotagmin−11 on Diabetic nephropathy (more control exclusions)
with a threshold of 1e−05
MR Method
Inverse variance weighted
MR Egger
10
8
6
4
2
1/SE_IV
−0.25
0.00
0.25
0.50
β_IV

Batch 3−2912 : Synaptotagmin−17 on Diabetic nephropathy (more control exclusions)
with a threshold of 1e−05

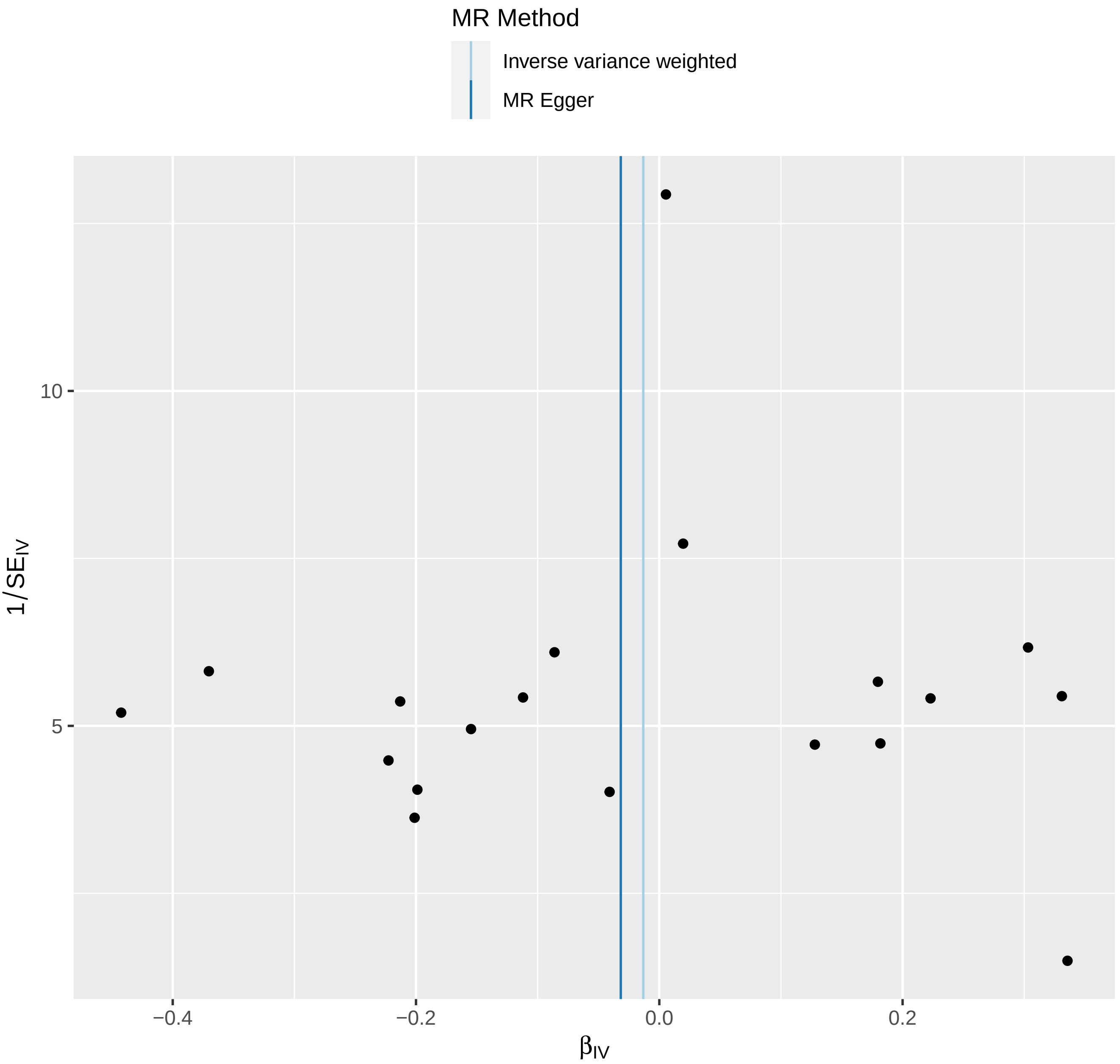

Batch 3−2913 : Synaptotagmin−2 on Diabetic nephropathy (more control exclusions)
with a threshold of 1e−05

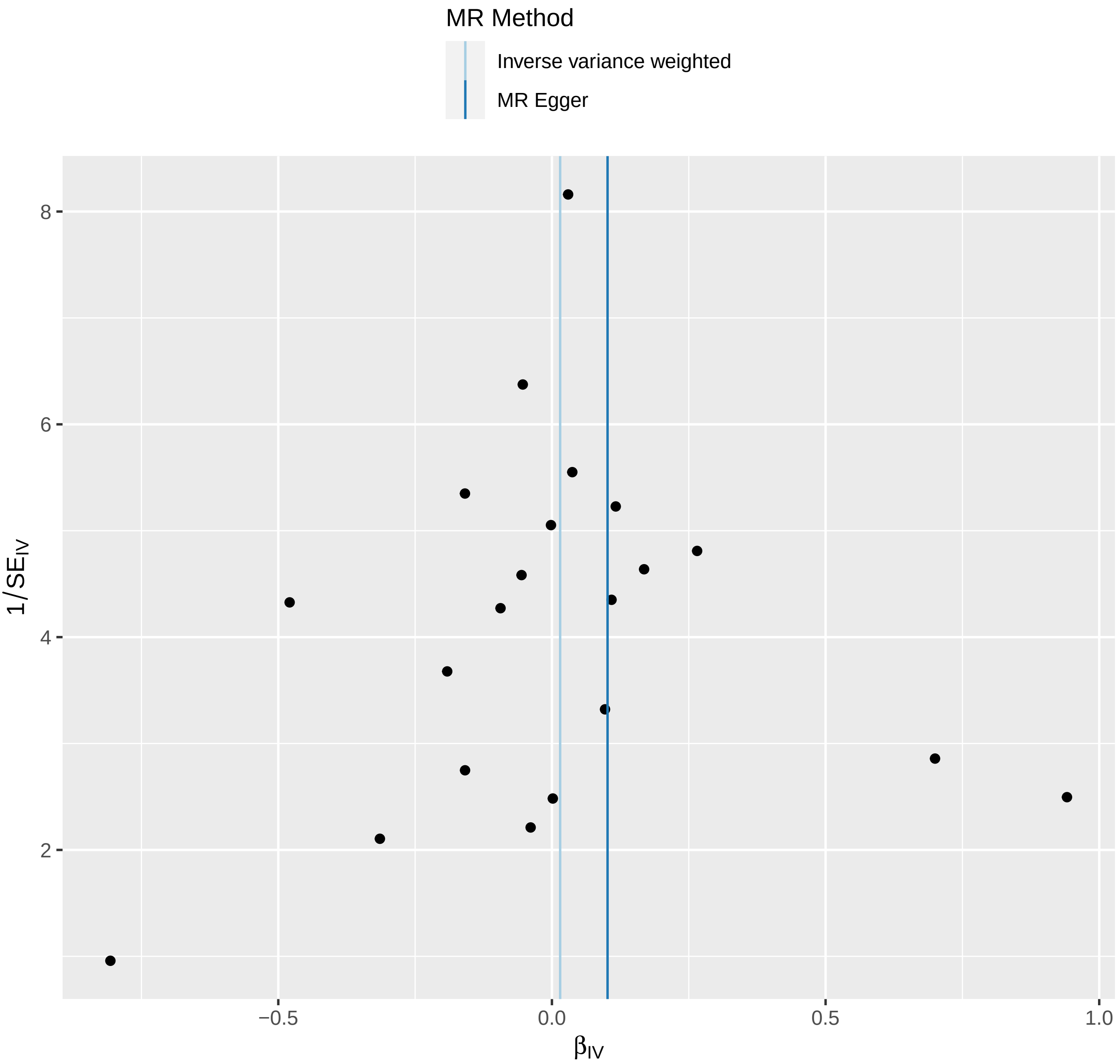

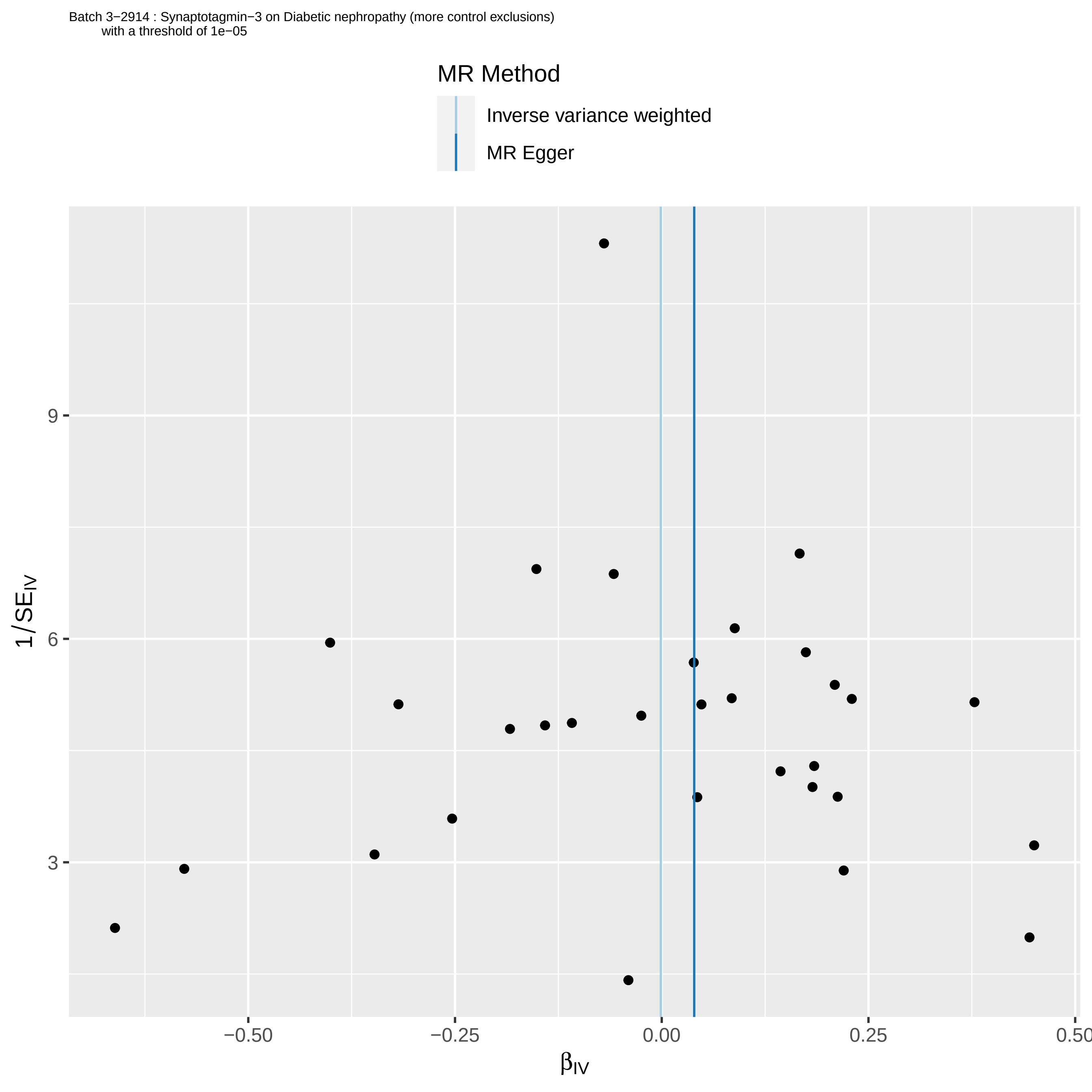
Batch 3−2914 : Synaptotagmin−3 on Diabetic nephropathy (more control exclusions)
with a threshold of 1e−05
MR Method
Inverse variance weighted
MR Egger
9
6
3
1/SE_IV
−0.50
−0.25
0.00
0.25
0.50
β_IV

Batch 3−2915 : Synaptotagmin−5 on Diabetic nephropathy (more control exclusions)
with a threshold of 1e−05

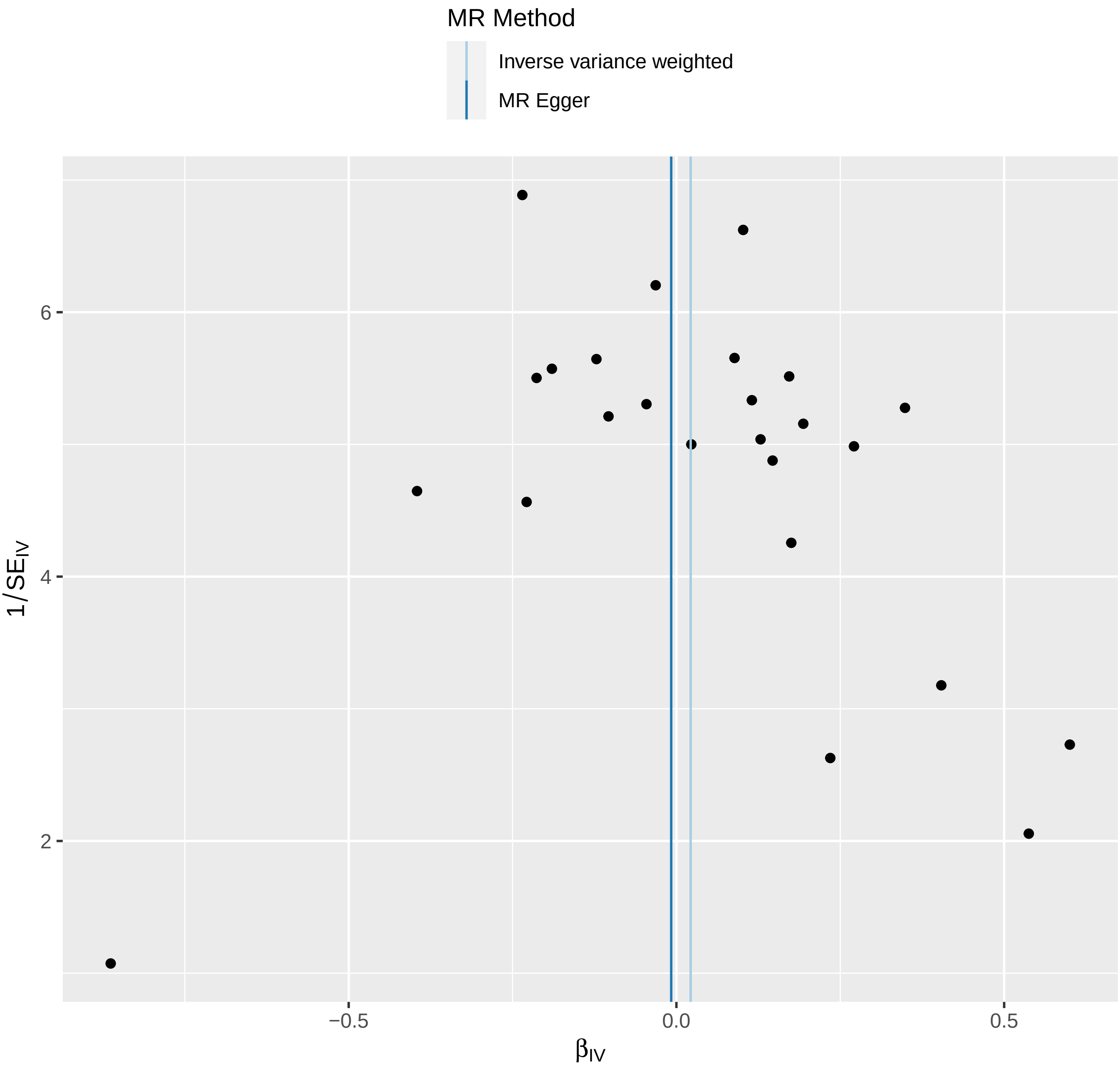

Batch 3−2916 : Synaptotagmin−7 on Diabetic nephropathy (more control exclusions)
with a threshold of 1e−05

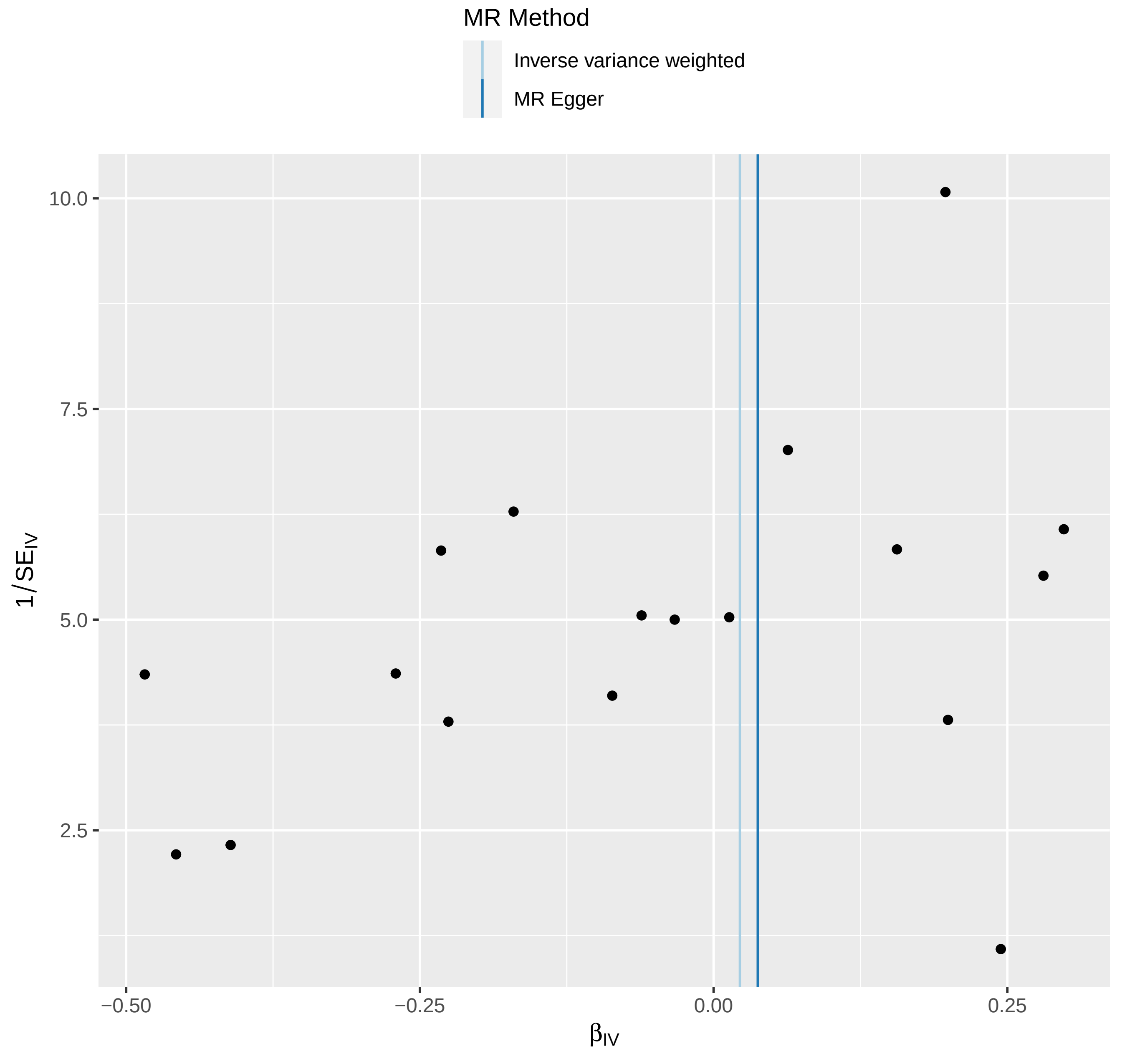

Batch 3−2917 : Synaptotagmin−8 on Diabetic nephropathy (more control exclusions)
with a threshold of 1e−05

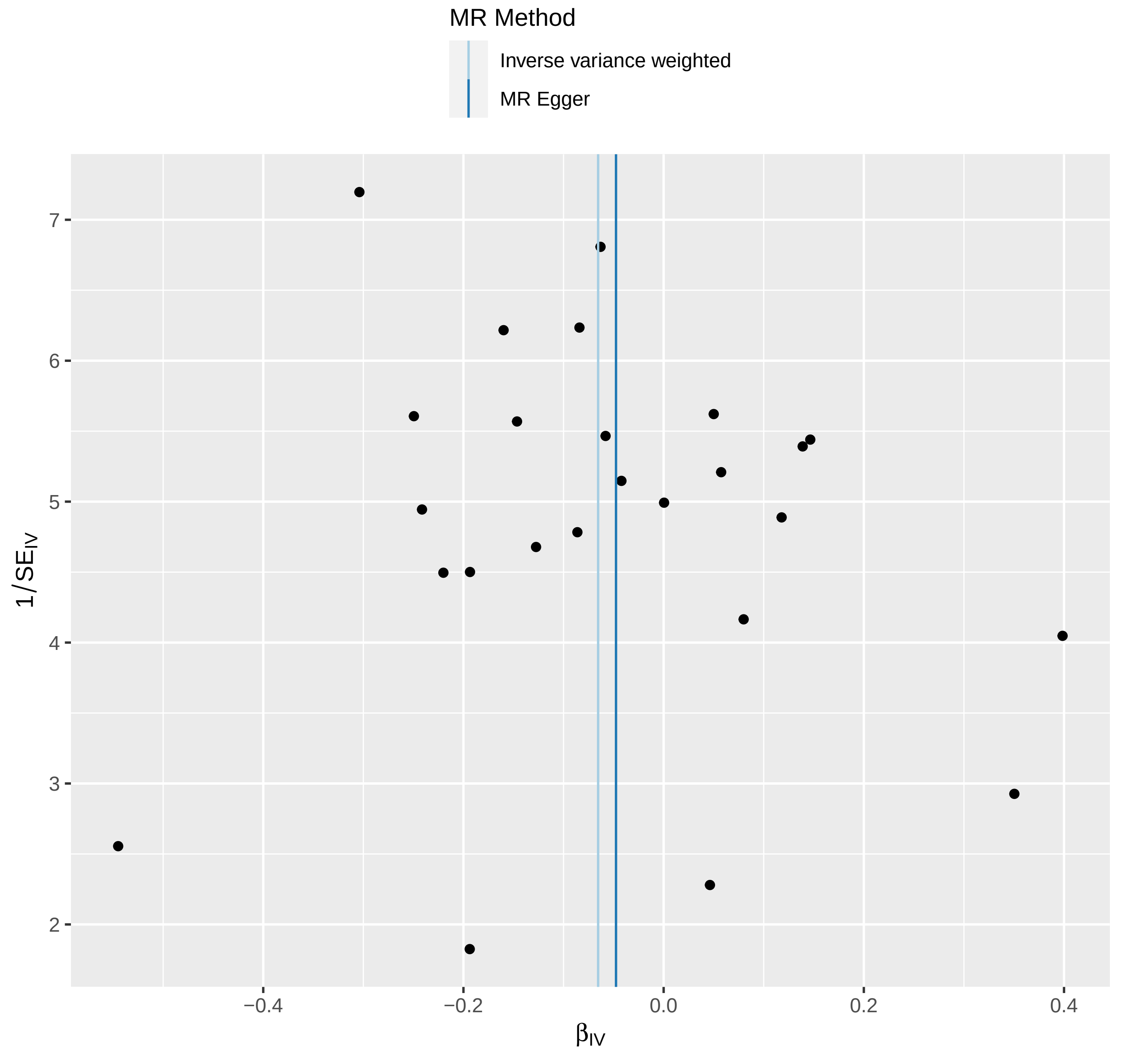

Batch 3−2918 : Synaptotagmin−like protein 1 on Diabetic nephropathy (more control exclusions)
with a threshold of 1e−05

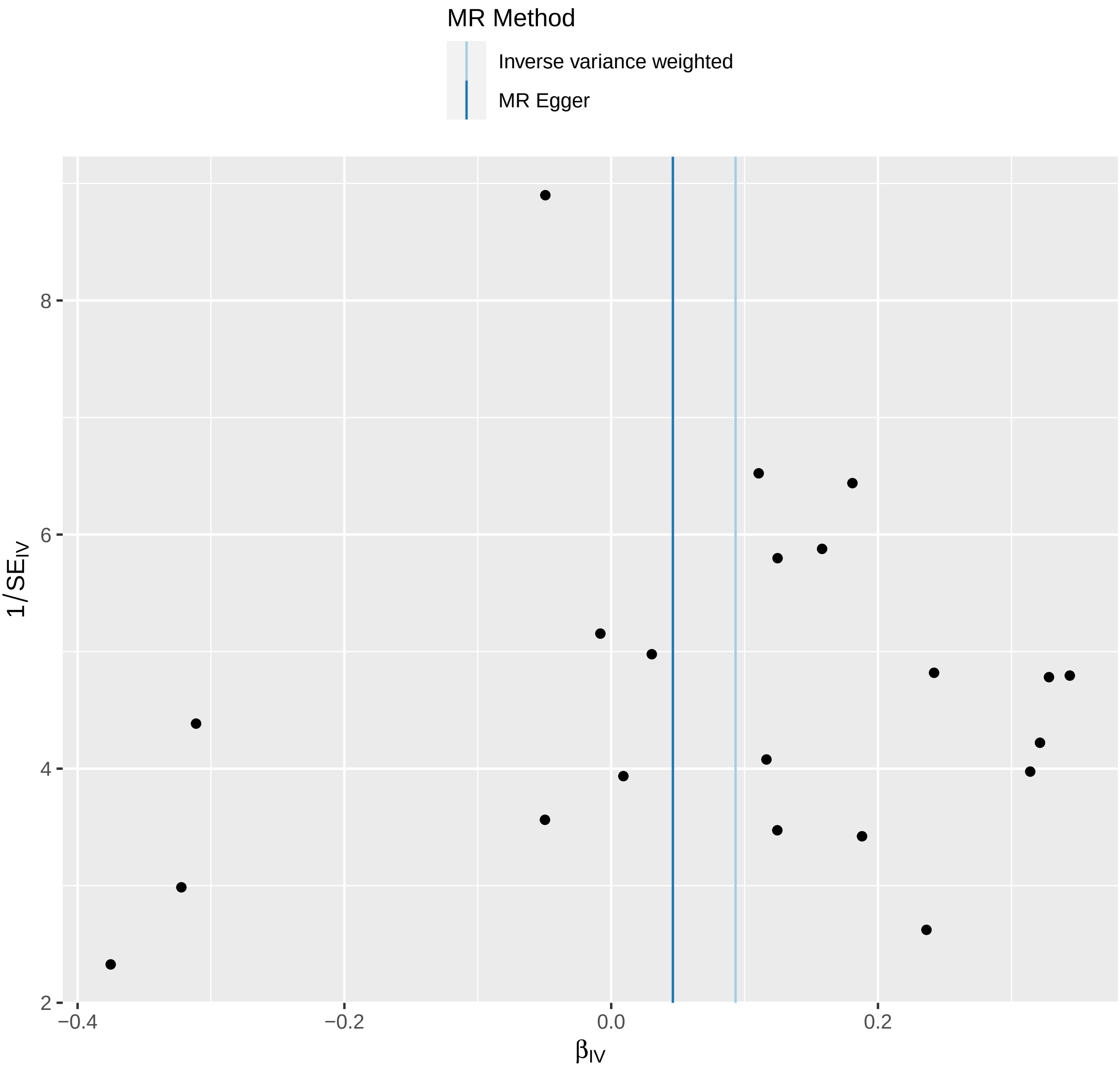

Batch 3−2919 : Synaptotagmin−like protein 4 on Diabetic nephropathy (more control exclusions)
with a threshold of 1e−05

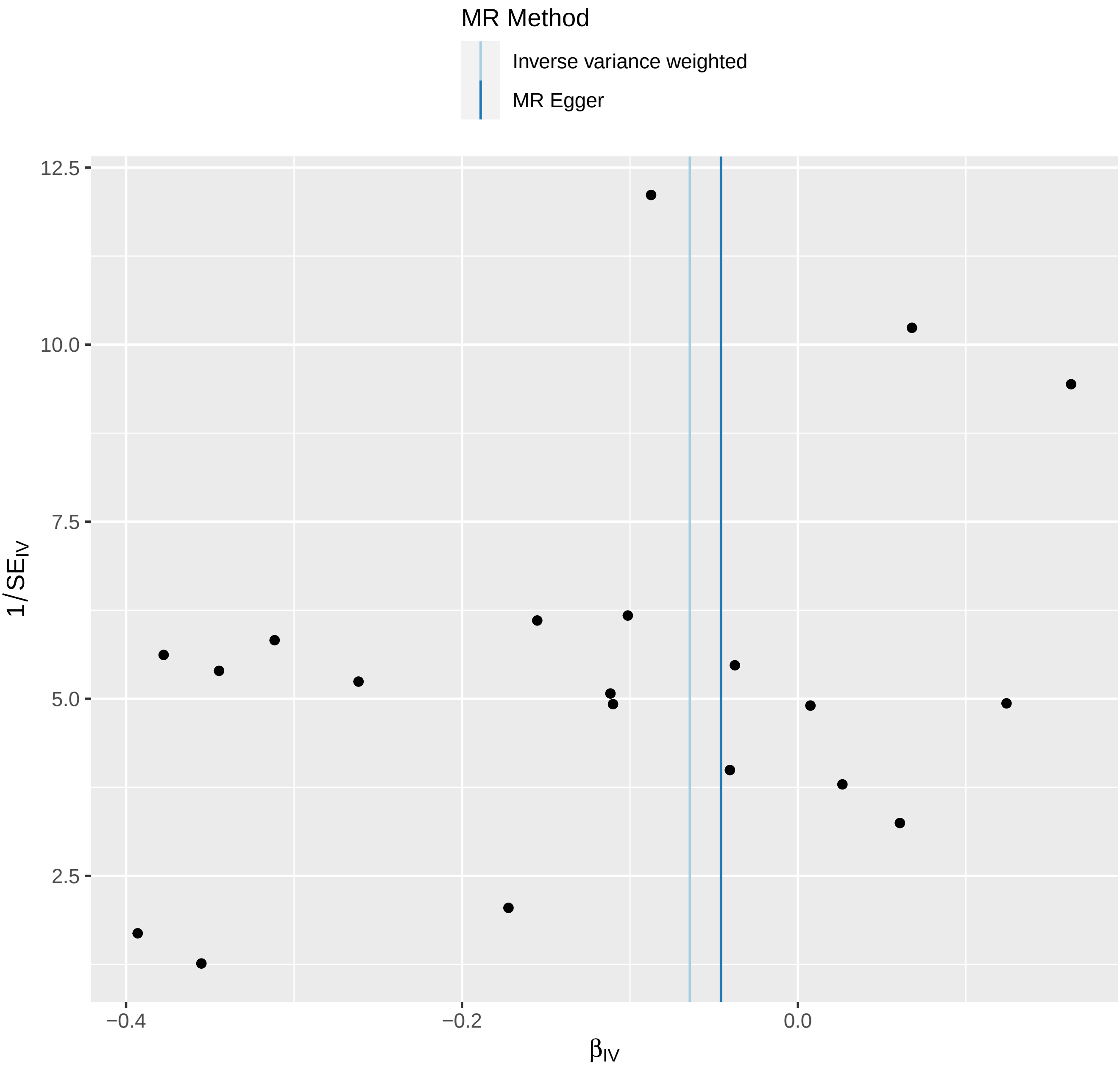

Batch 3−2920 : Protachykinin−1 on Diabetic nephropathy (more control exclusions)
with a threshold of 1e−05

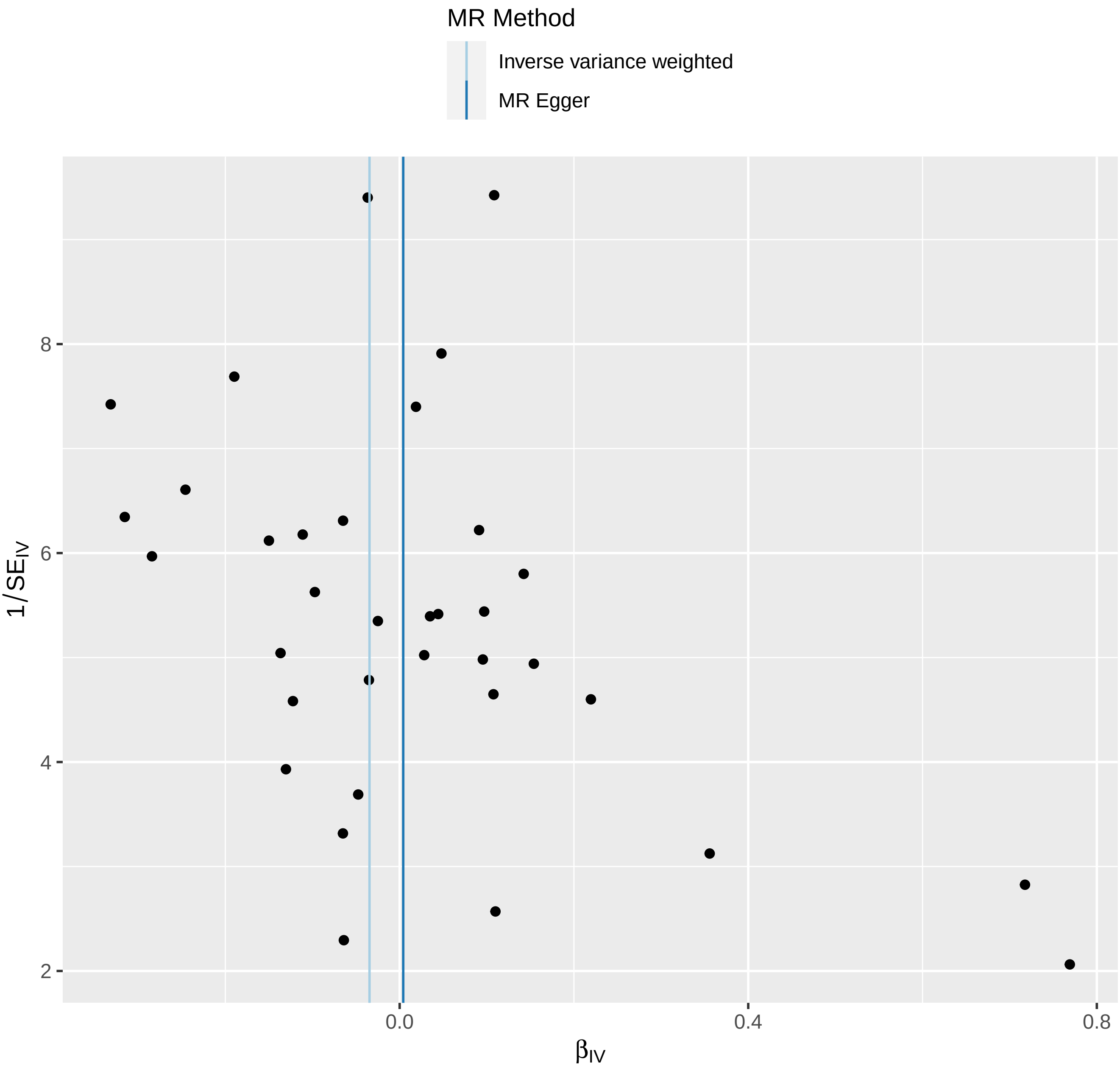

Batch 3−2921 : Tachykinin−3 on Diabetic nephropathy (more control exclusions)
with a threshold of 1e−05

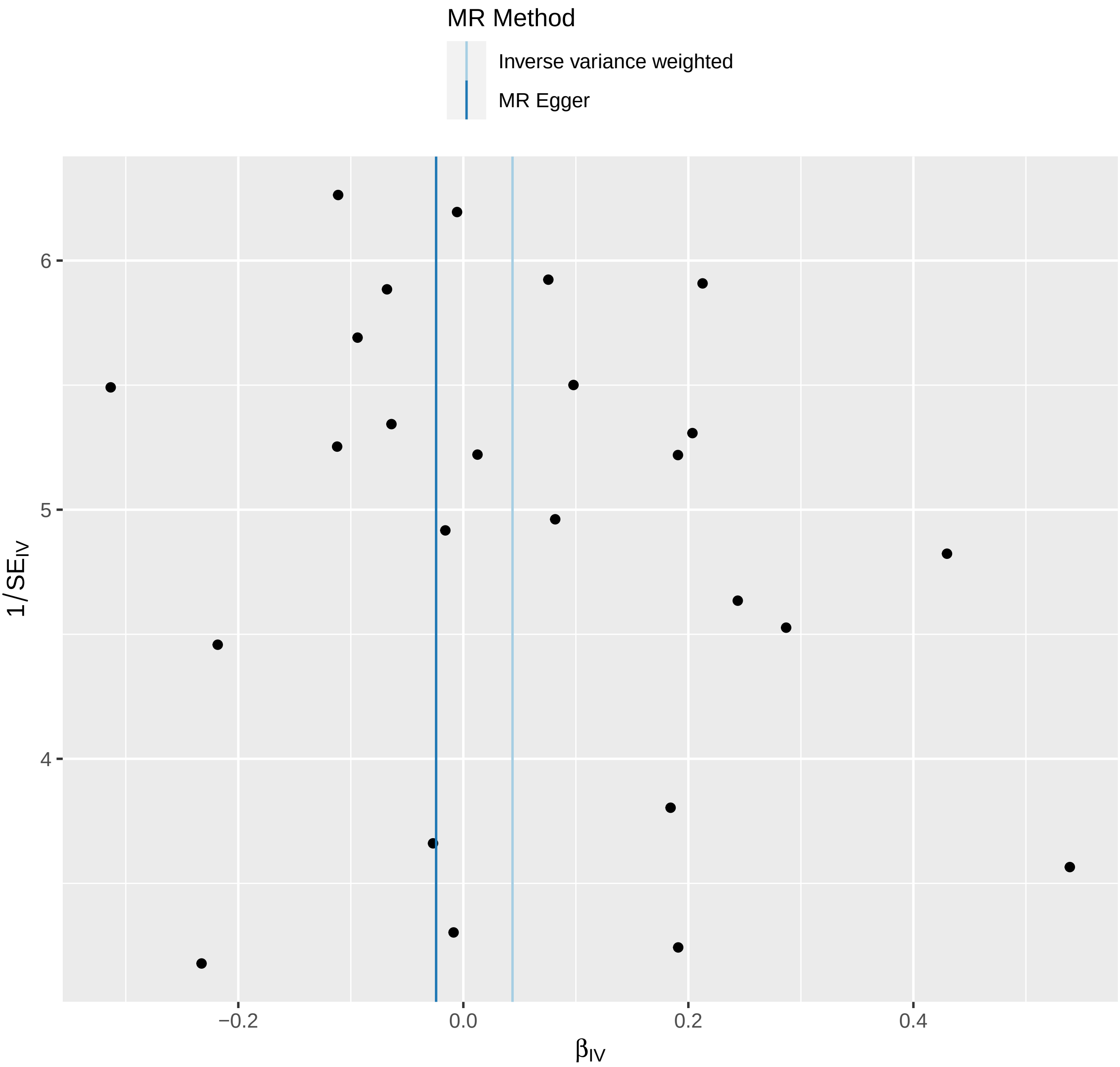

Batch 3−2922 : Tachykinin−4 on Diabetic nephropathy (more control exclusions)
with a threshold of 1e−05

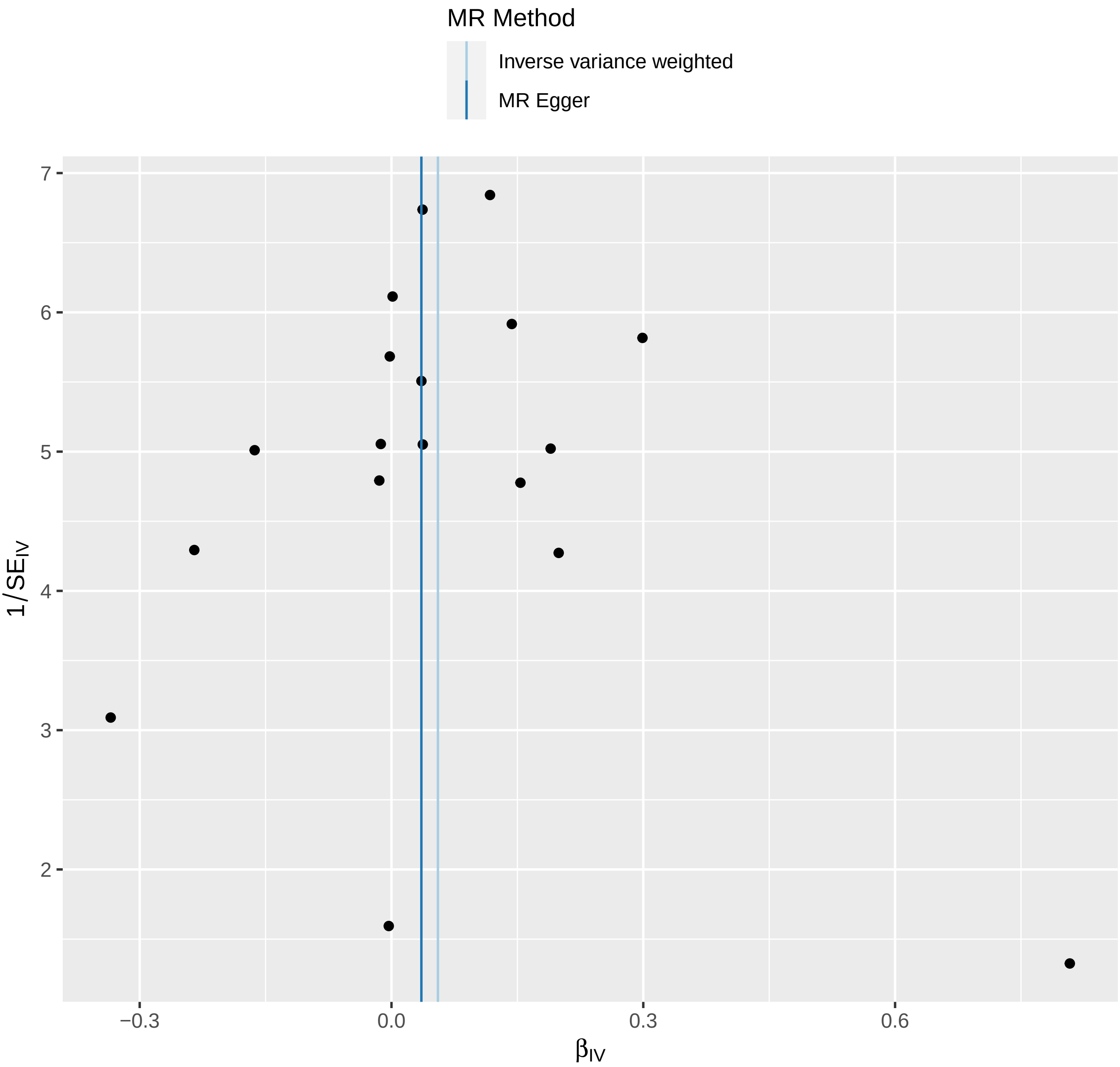

Batch 3−2923 : Tumor−associated calcium signal transducer 2 on Diabetic nephropathy (more control exclusions)
with a threshold of 1e−05

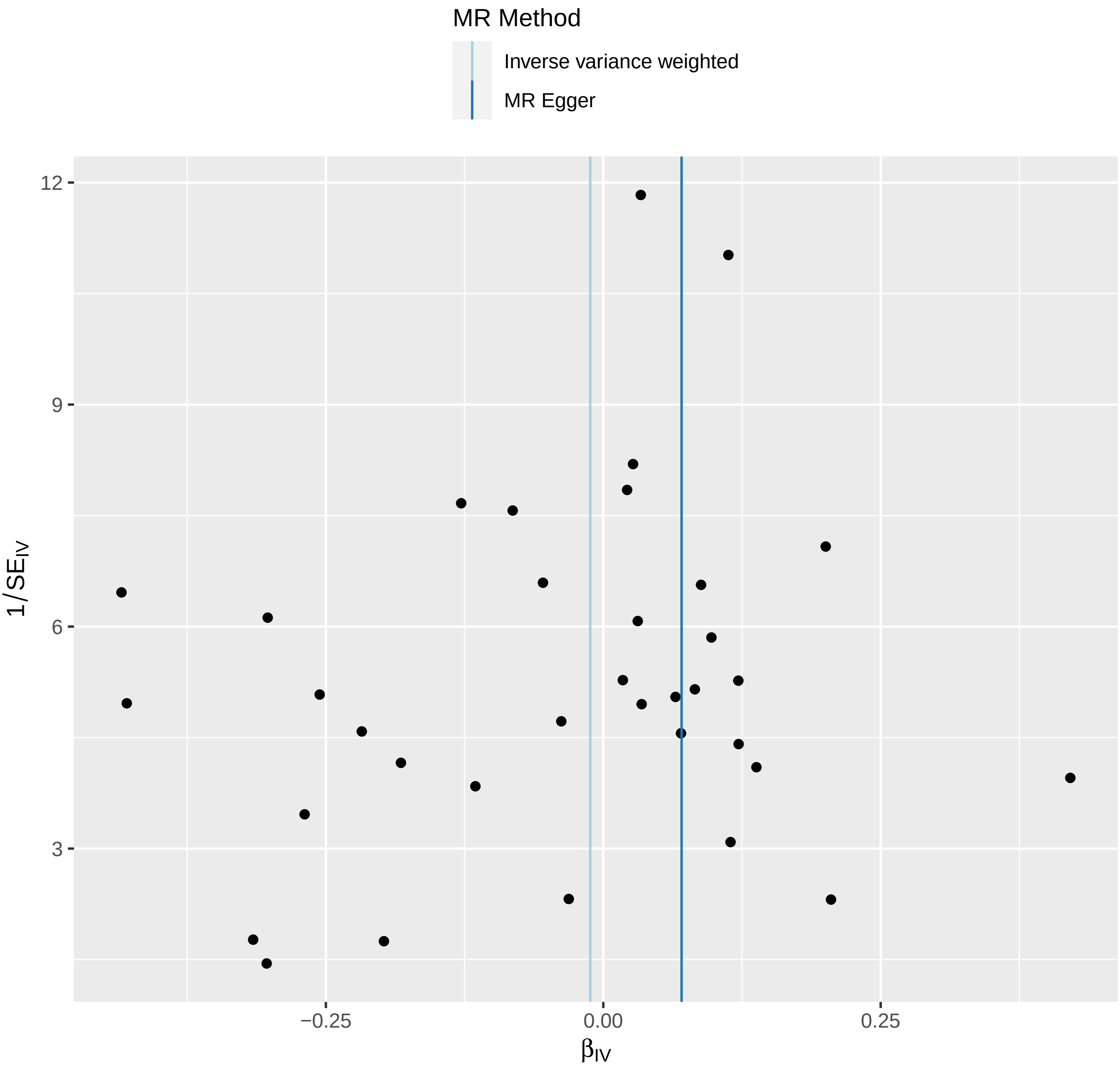

Batch 3−2924 : Tumor−associated calcium signal transducer 2 on Diabetic nephropathy (more control exclusions)
with a threshold of 1e−05

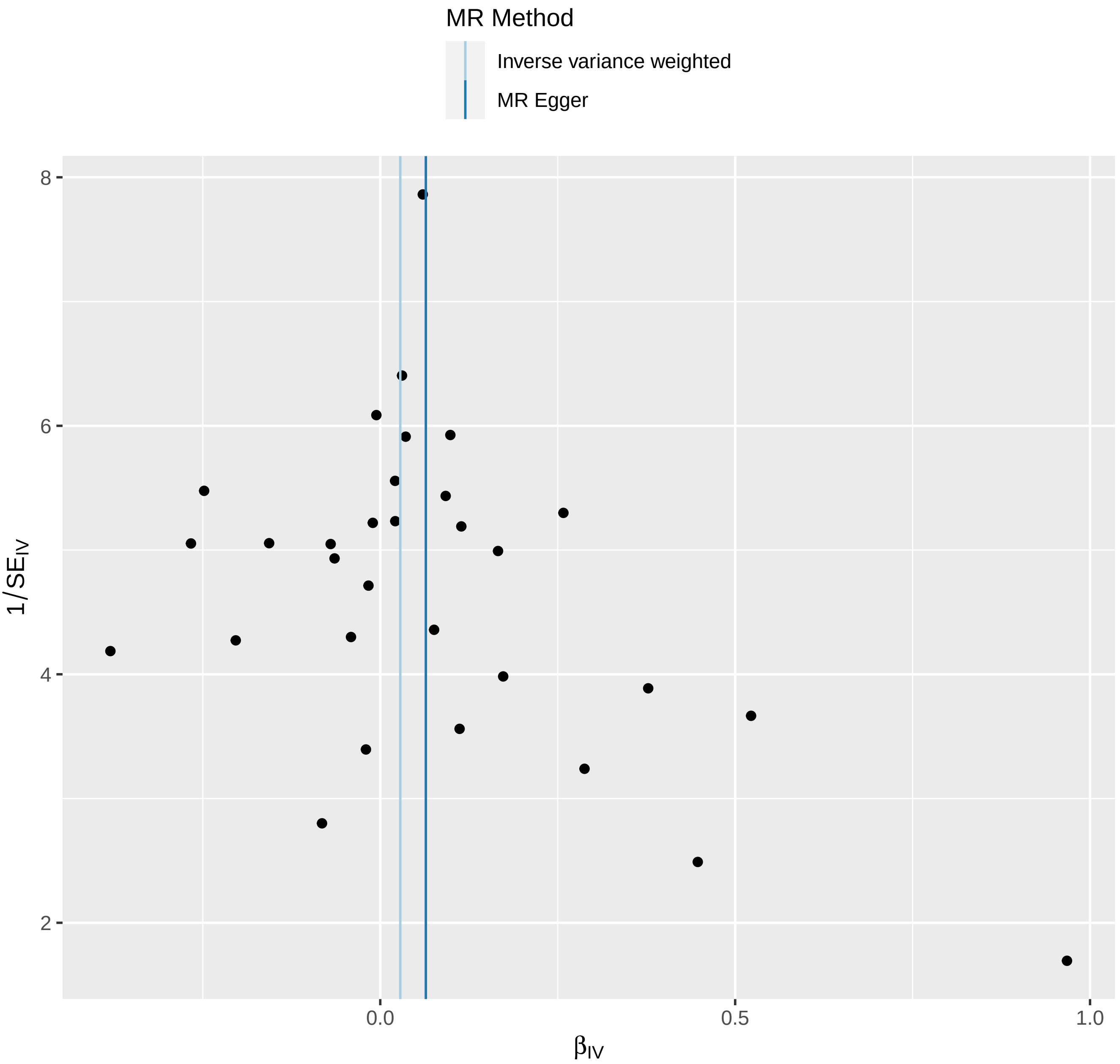

Batch 3−2925 : Transgelin on Diabetic nephropathy (more control exclusions)
with a threshold of 1e−05

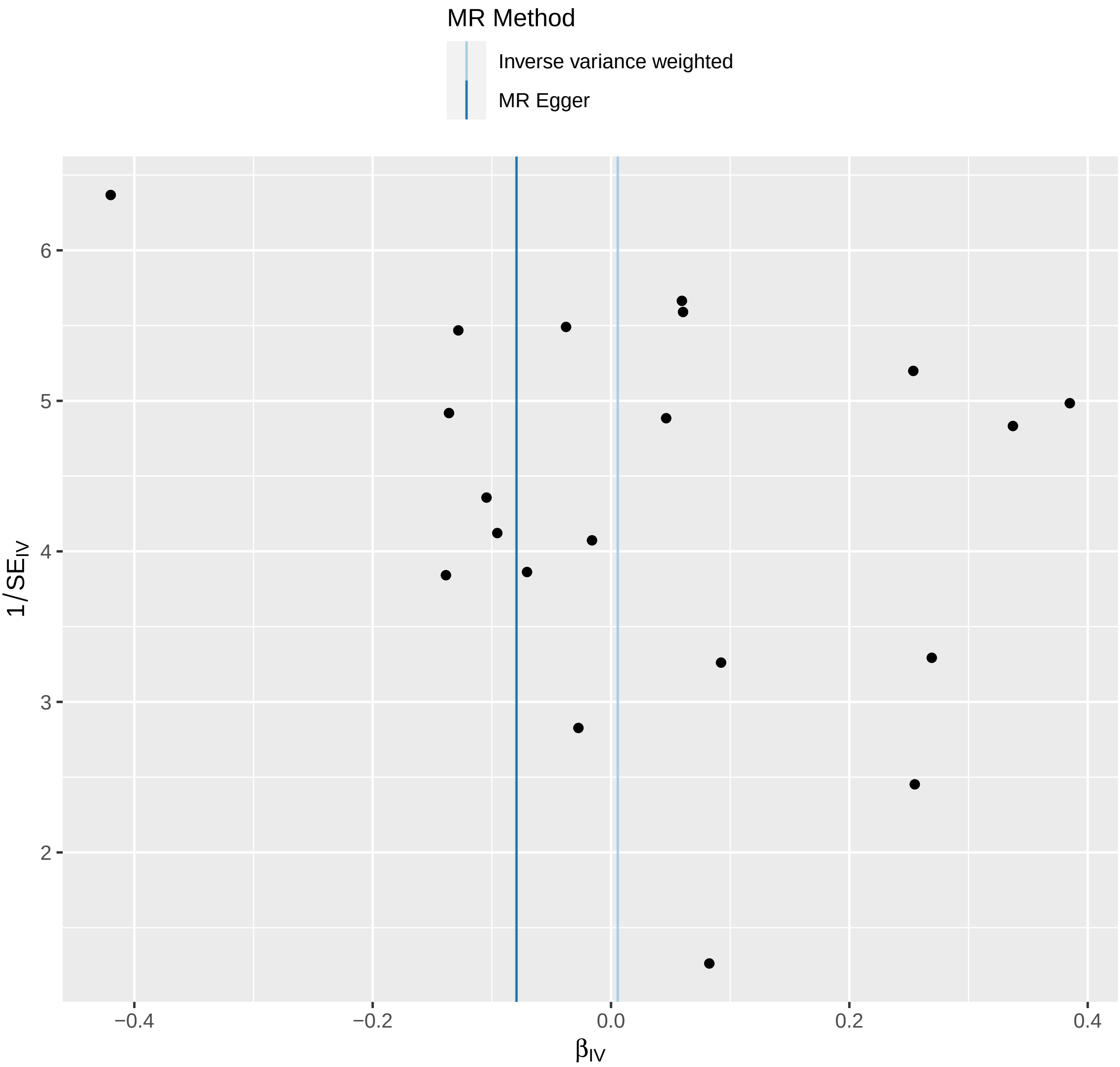

Batch 3−2926 : Tapasin on Diabetic nephropathy (more control exclusions)
with a threshold of 1e−05

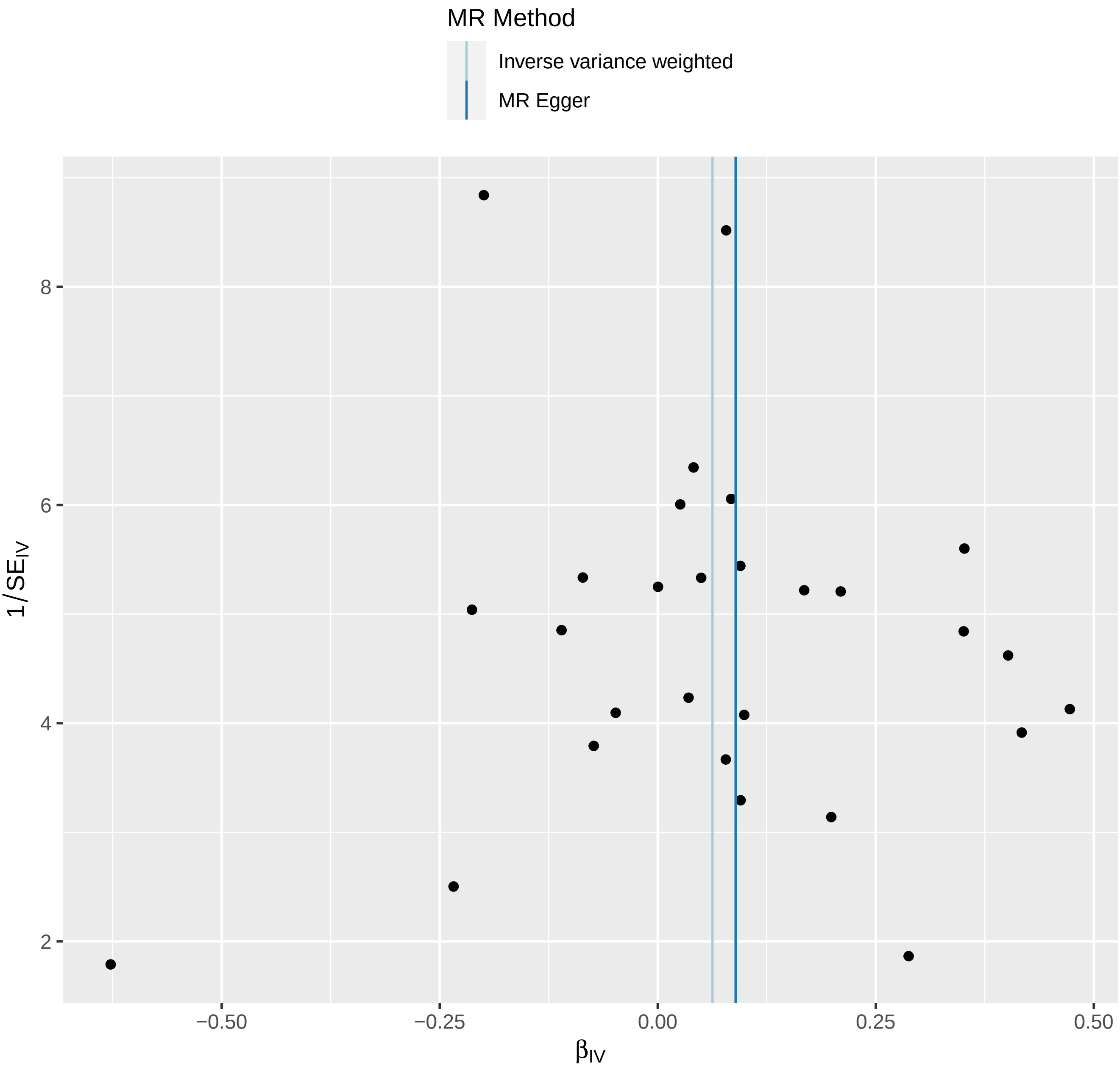

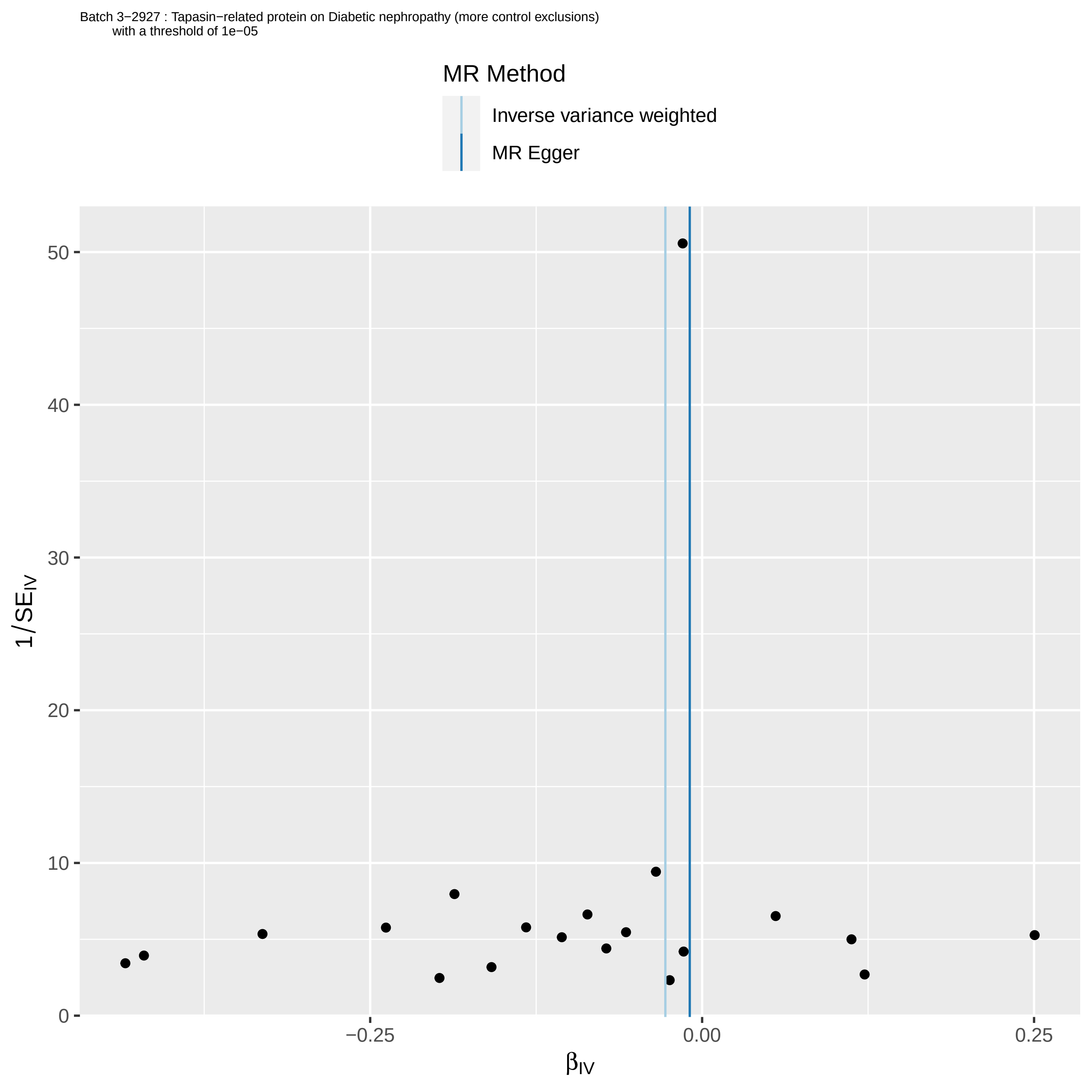
Batch 3−2927 : Tapasin−related protein on Diabetic nephropathy (more control exclusions)
with a threshold of 1e−05
MR Method
Inverse variance weighted
MR Egger
50
40
30
20
10
0
$1/SE_{IV}$
−0.25
0.00
0.25
β_{IV}

Batch 3−2928 : Tax1−binding protein 1 on Diabetic nephropathy (more control exclusions)
with a threshold of 1e−05

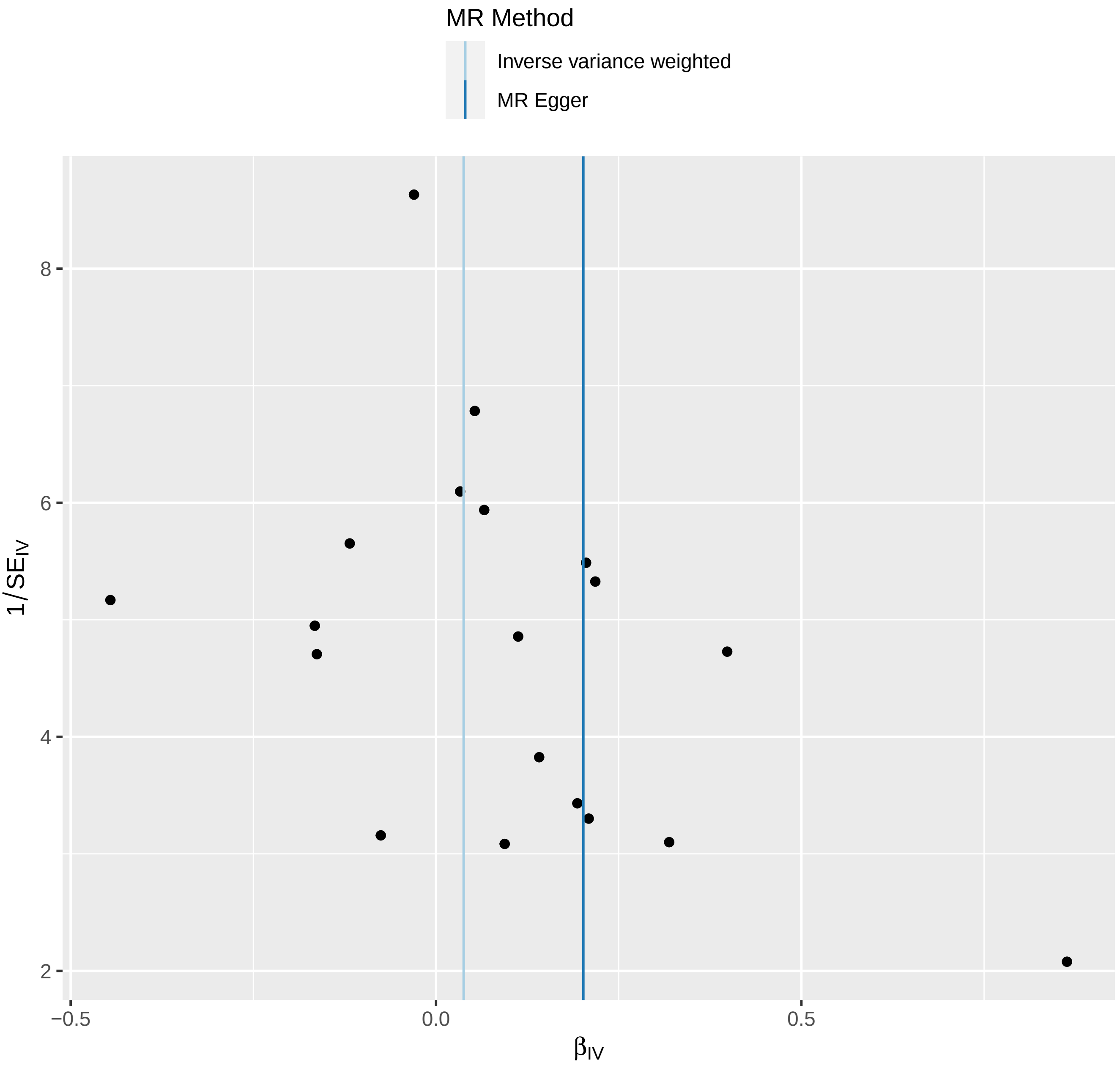

Batch 3−2929 : Tax1−binding protein 3 on Diabetic nephropathy (more control exclusions)
with a threshold of 1e−05

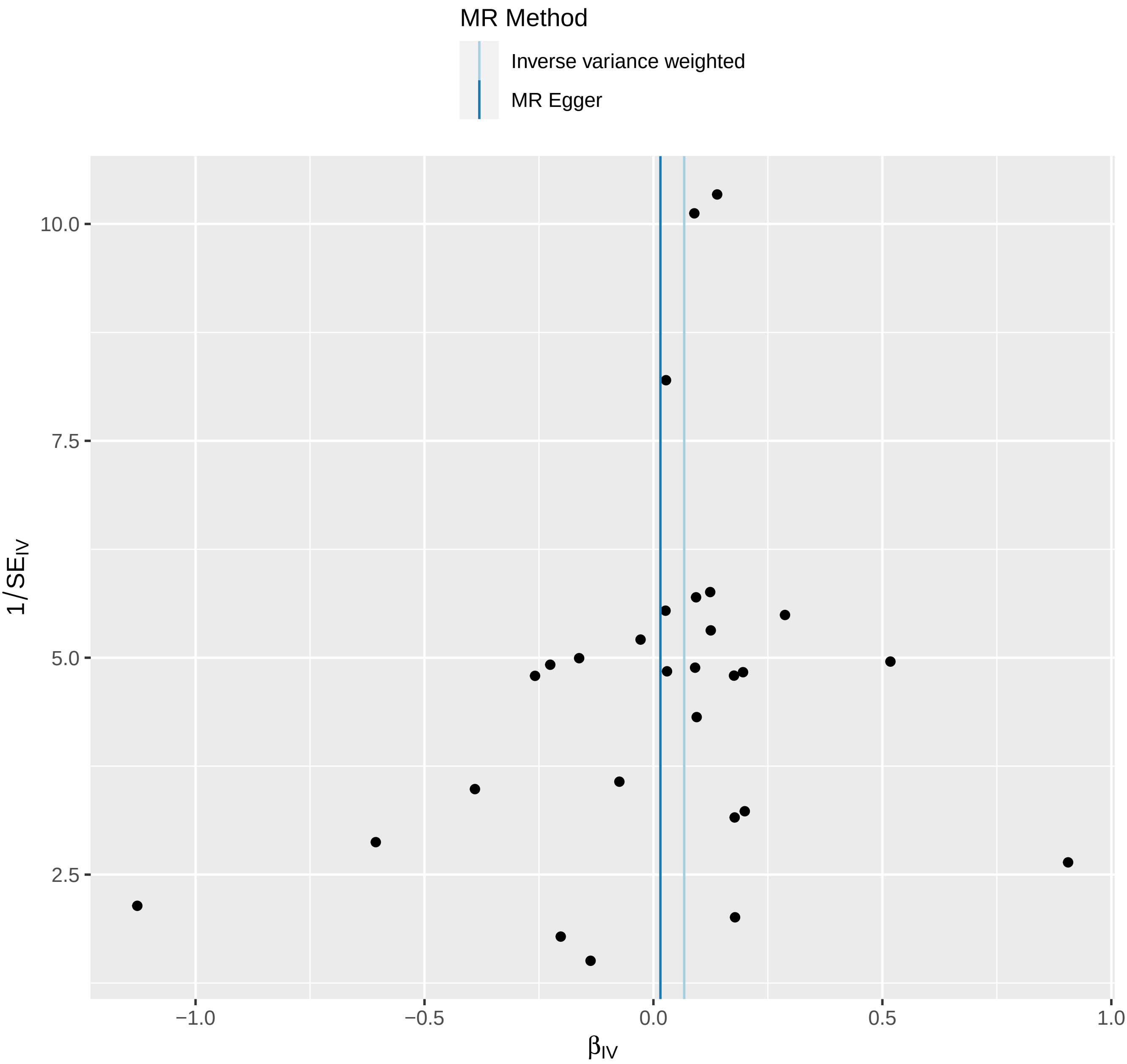

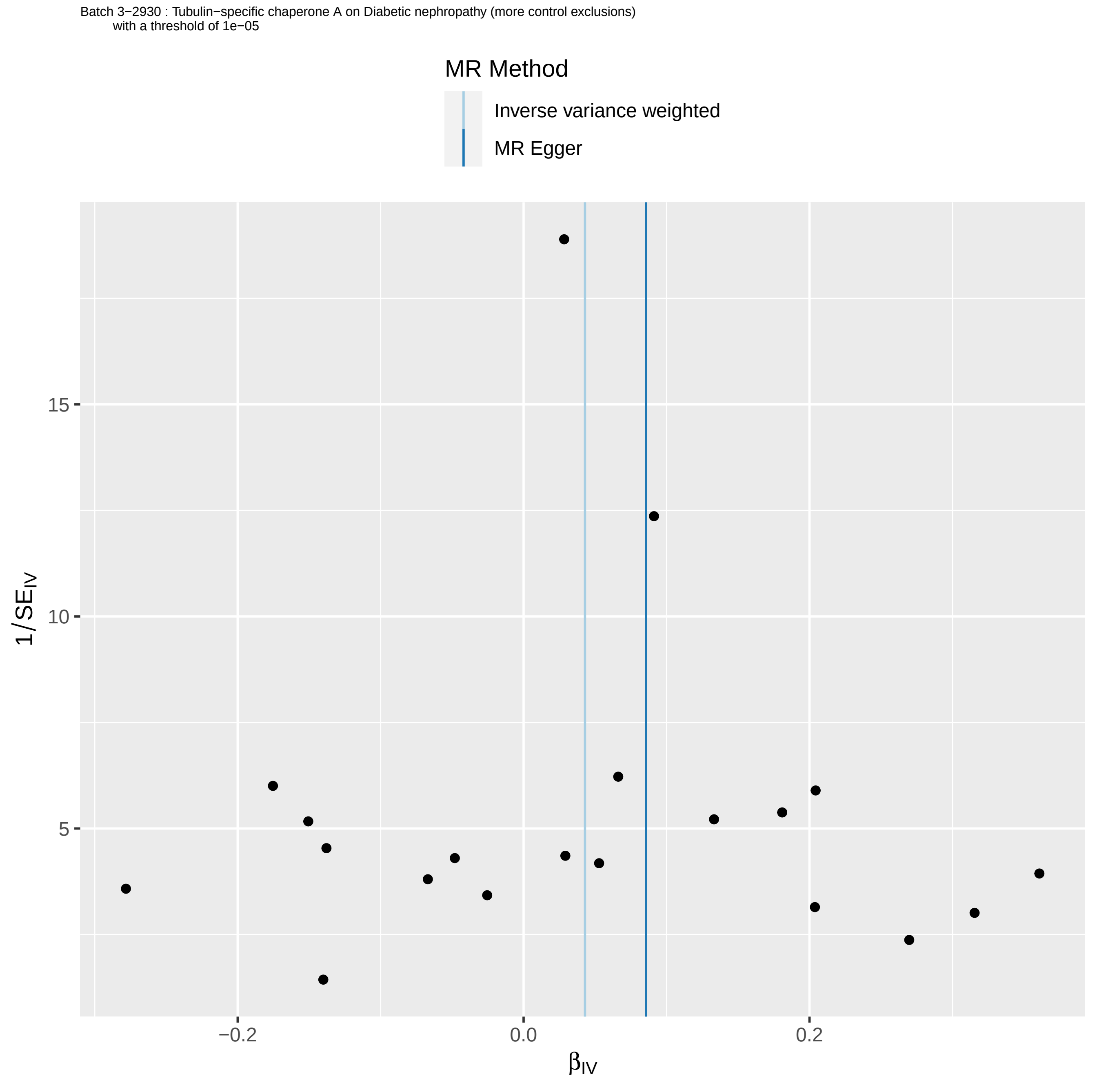

Batch 3−2930 : Tubulin−specific chaperone A on Diabetic nephropathy (more control exclusions)
with a threshold of 1e−05
MR Method
Inverse variance weighted
MR Egger
15
10
5
$1/SE_{IV}$
−0.2
0.0
0.2
β_{IV}

Batch 3−2931 : Tubulin−specific chaperone E on Diabetic nephropathy (more control exclusions)
with a threshold of 1e−05

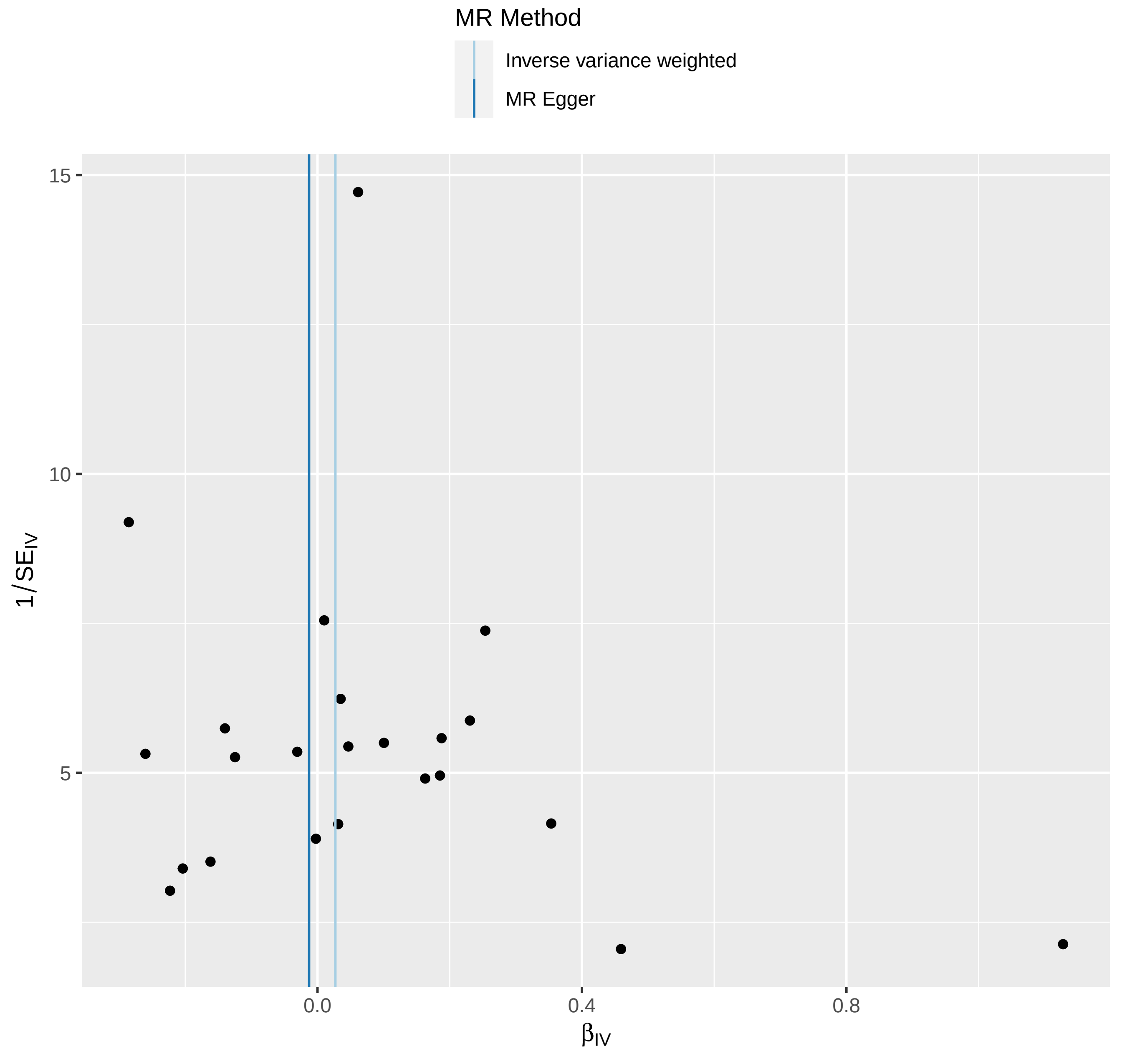

Batch 3−2932 : Serine/threonine−protein kinase TBK1 on Diabetic nephropathy (more control exclusions)
with a threshold of 1e−05

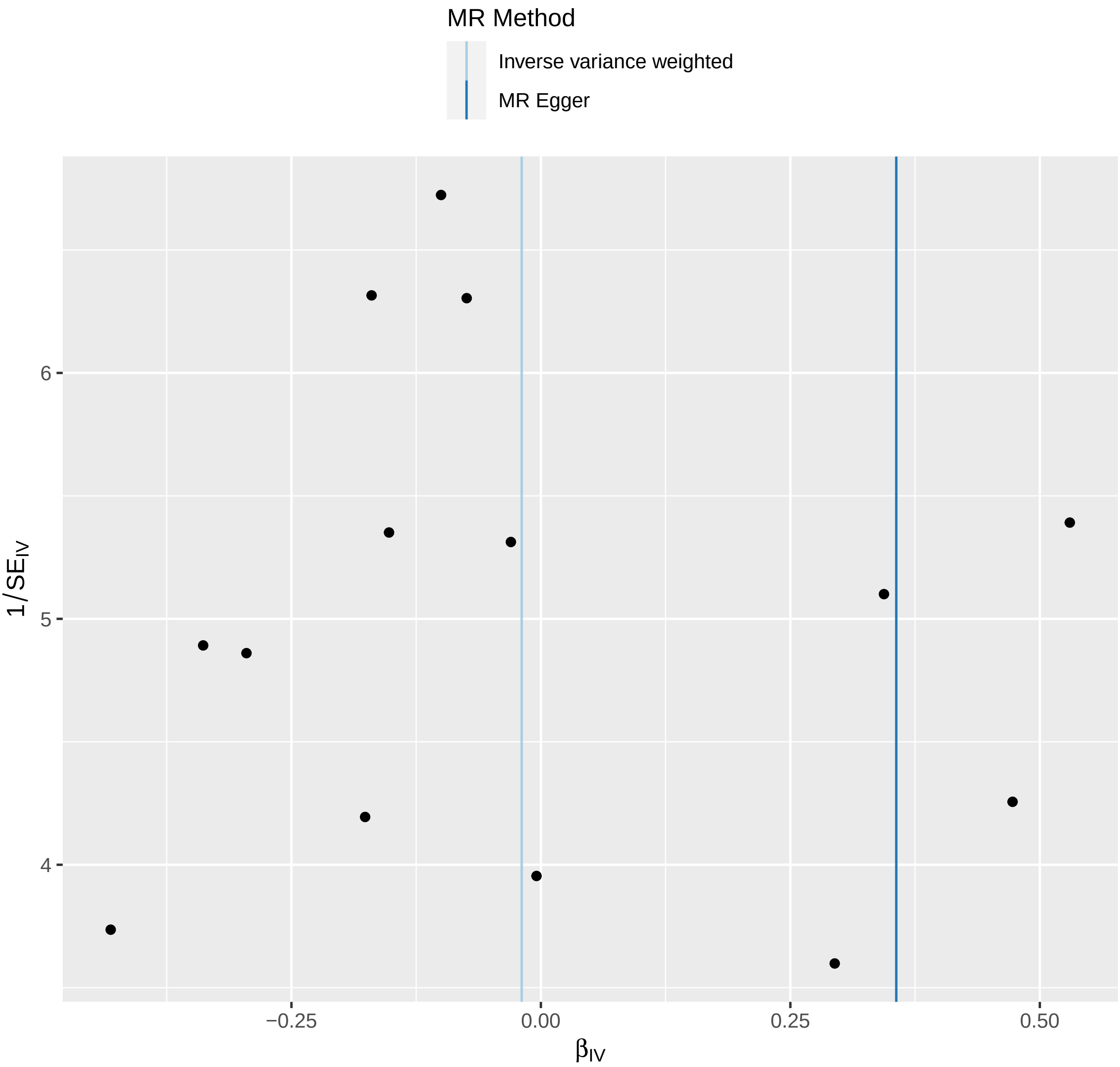

Batch 3−2933 : TATA−box−binding protein on Diabetic nephropathy (more control exclusions)
with a threshold of 1e−05

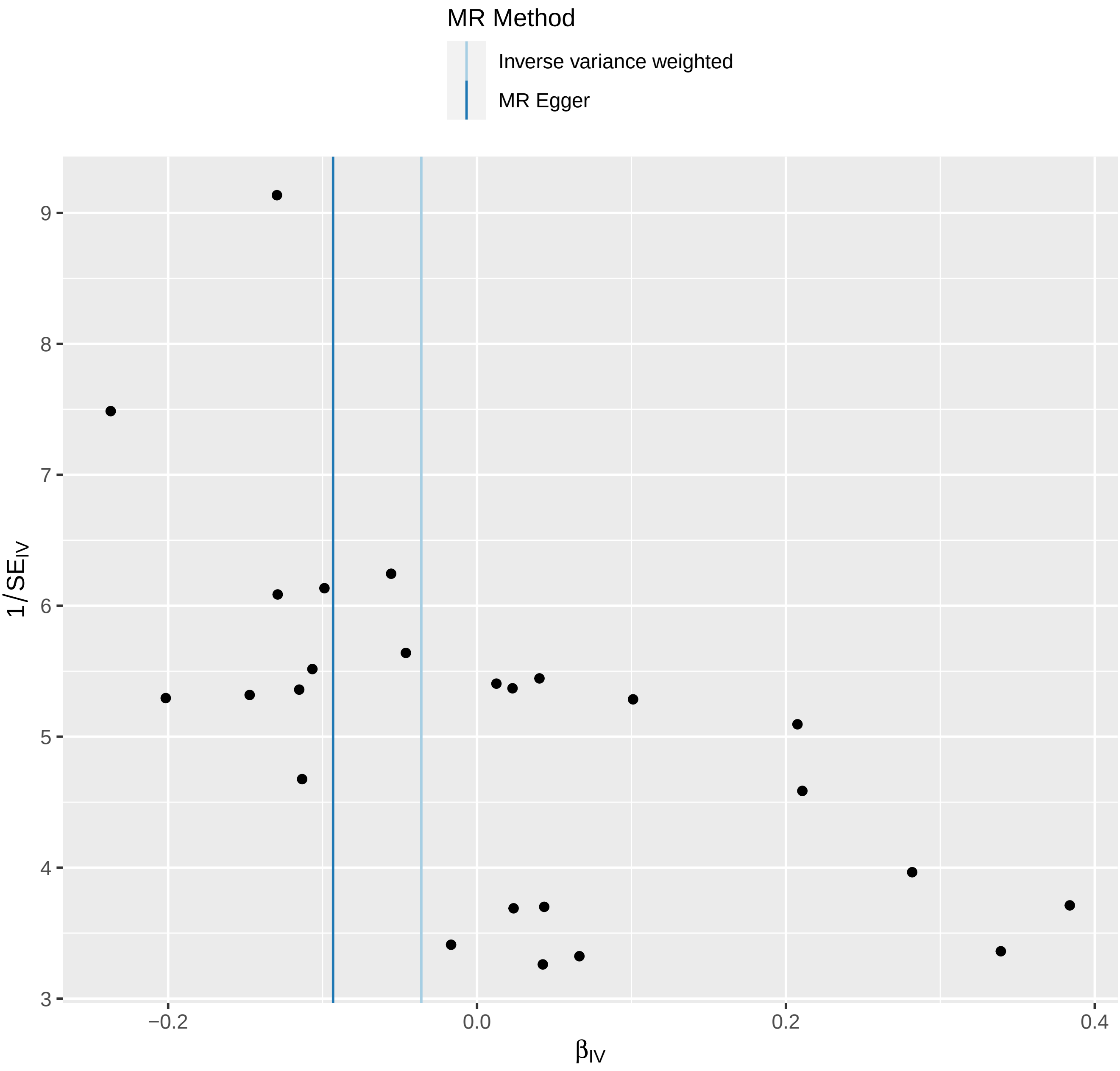

Batch 3−2934 : T−box transcription factor TBX22 on Diabetic nephropathy (more control exclusions)
with a threshold of 1e−05

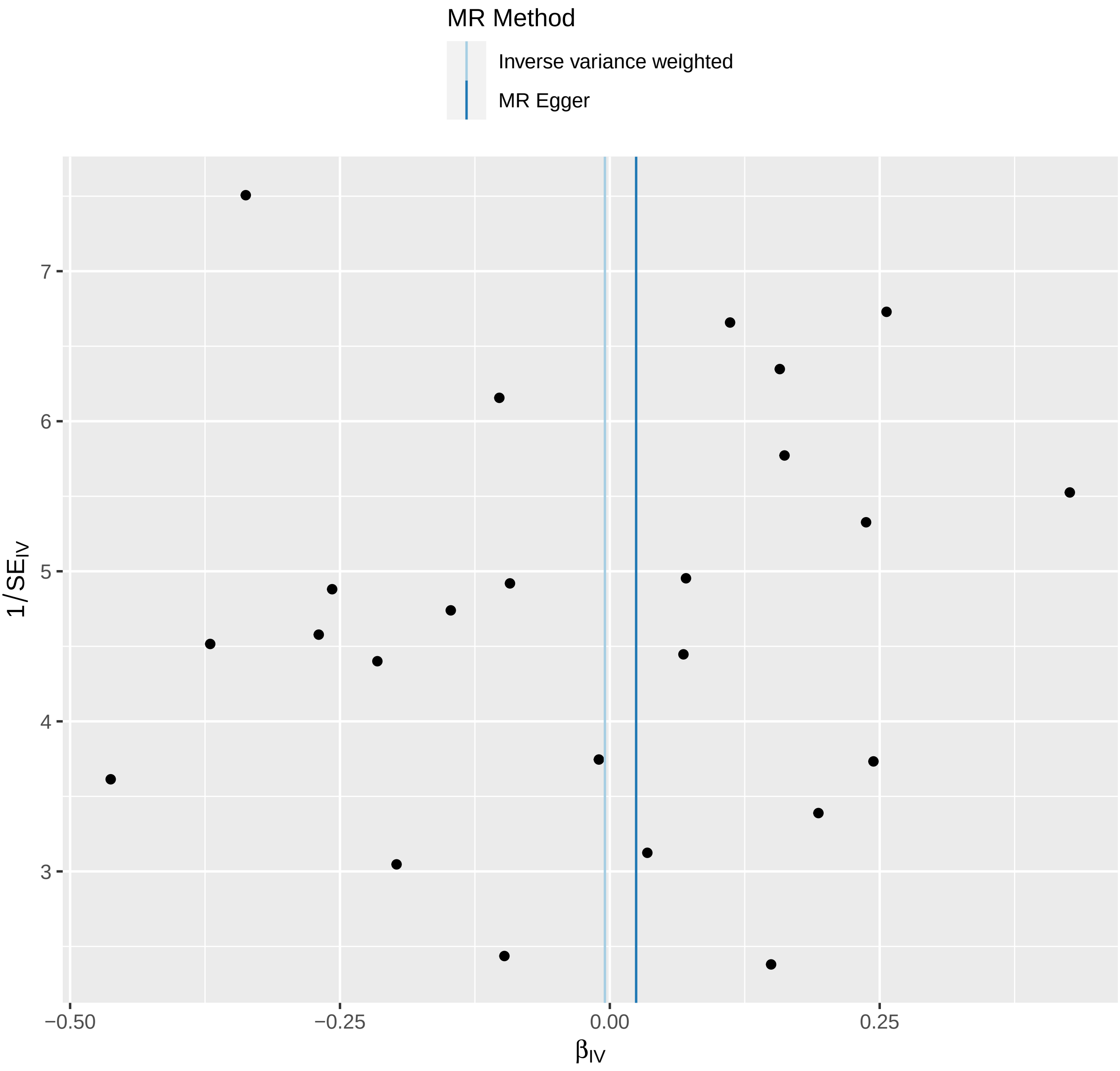

Batch 3−2935 : T−box transcription factor TBX3 on Diabetic nephropathy (more control exclusions)
with a threshold of 1e−05

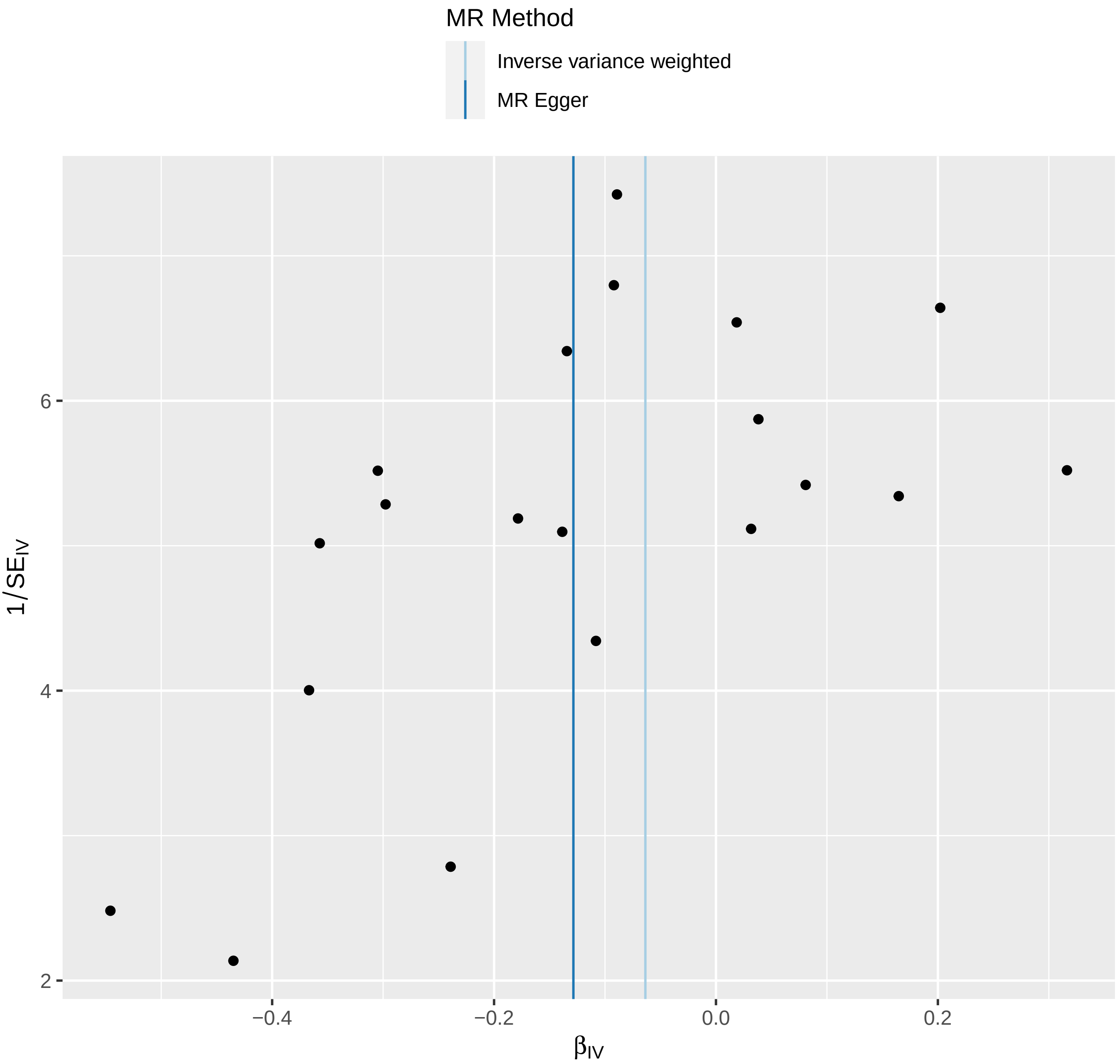

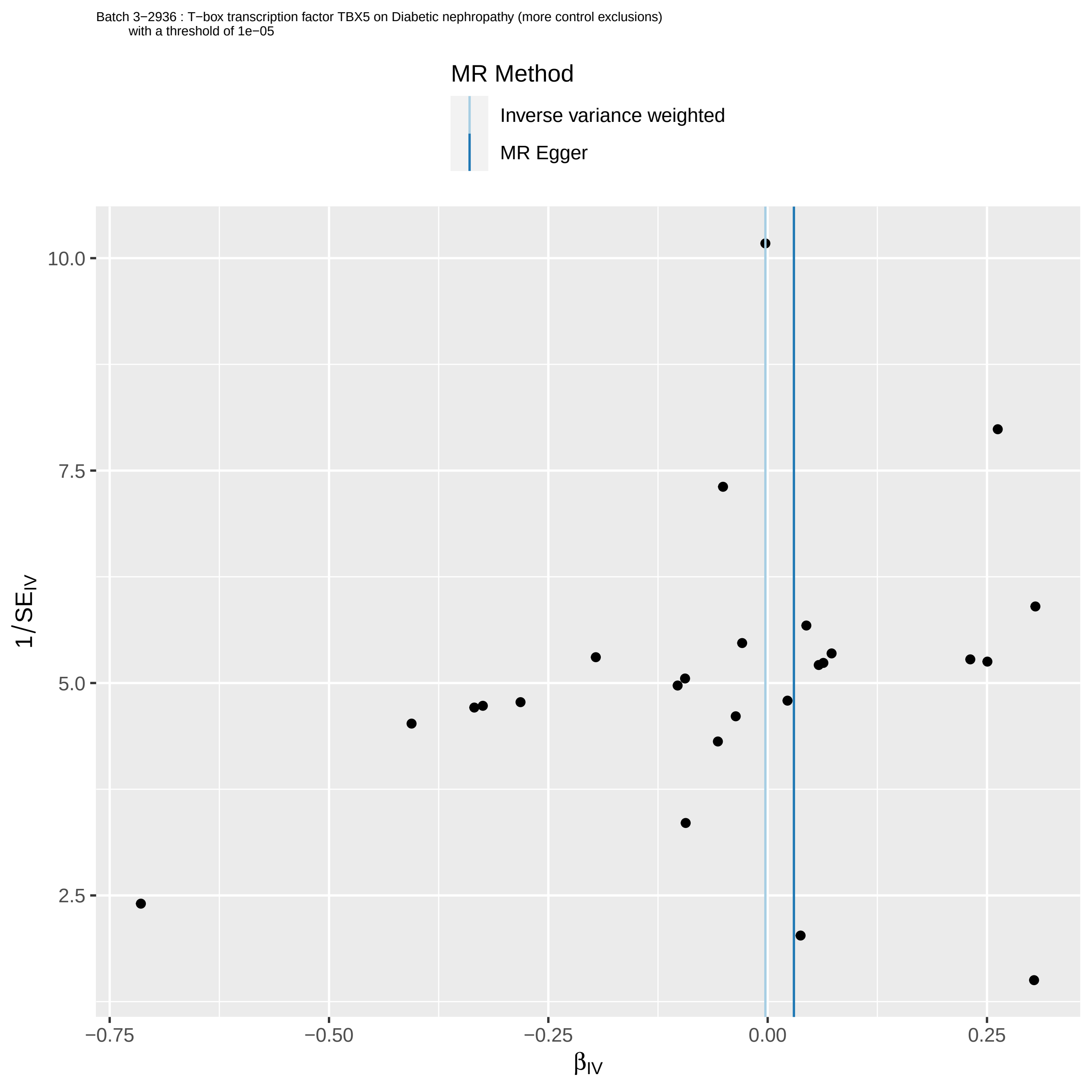

Batch 3−2936 : T−box transcription factor TBX5 on Diabetic nephropathy (more control exclusions)
with a threshold of 1e−05
MR Method
Inverse variance weighted
MR Egger
10.0
7.5
5.0
2.5
1/SE_IV
−0.75
−0.50
−0.25
0.00
0.25
β_IV

Batch 3−2937 : Thromboxane−A synthase on Diabetic nephropathy (more control exclusions)
with a threshold of 1e−05

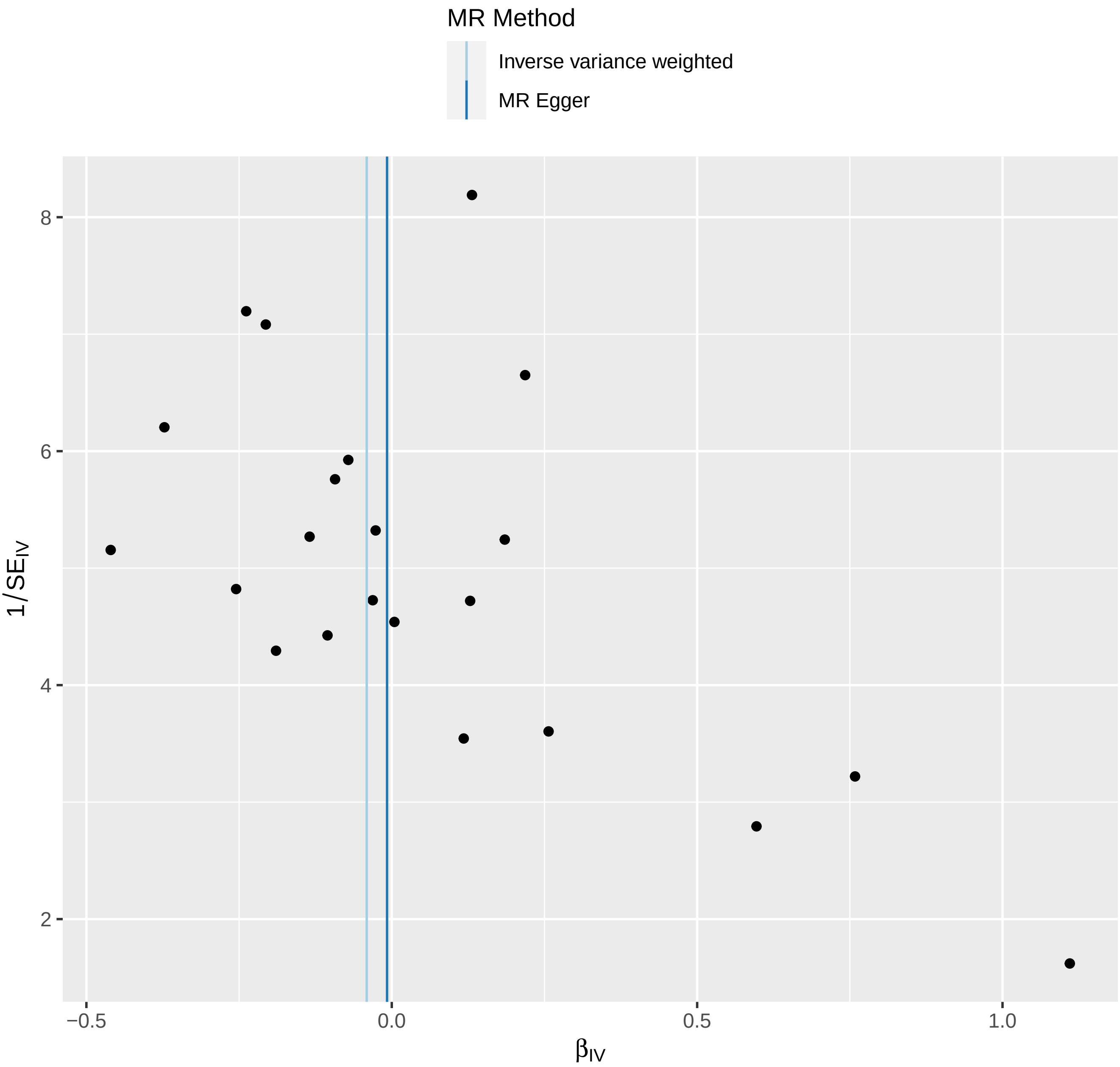

Batch 3−2938 : Transcobalamin−1 on Diabetic nephropathy (more control exclusions)
with a threshold of 1e−05

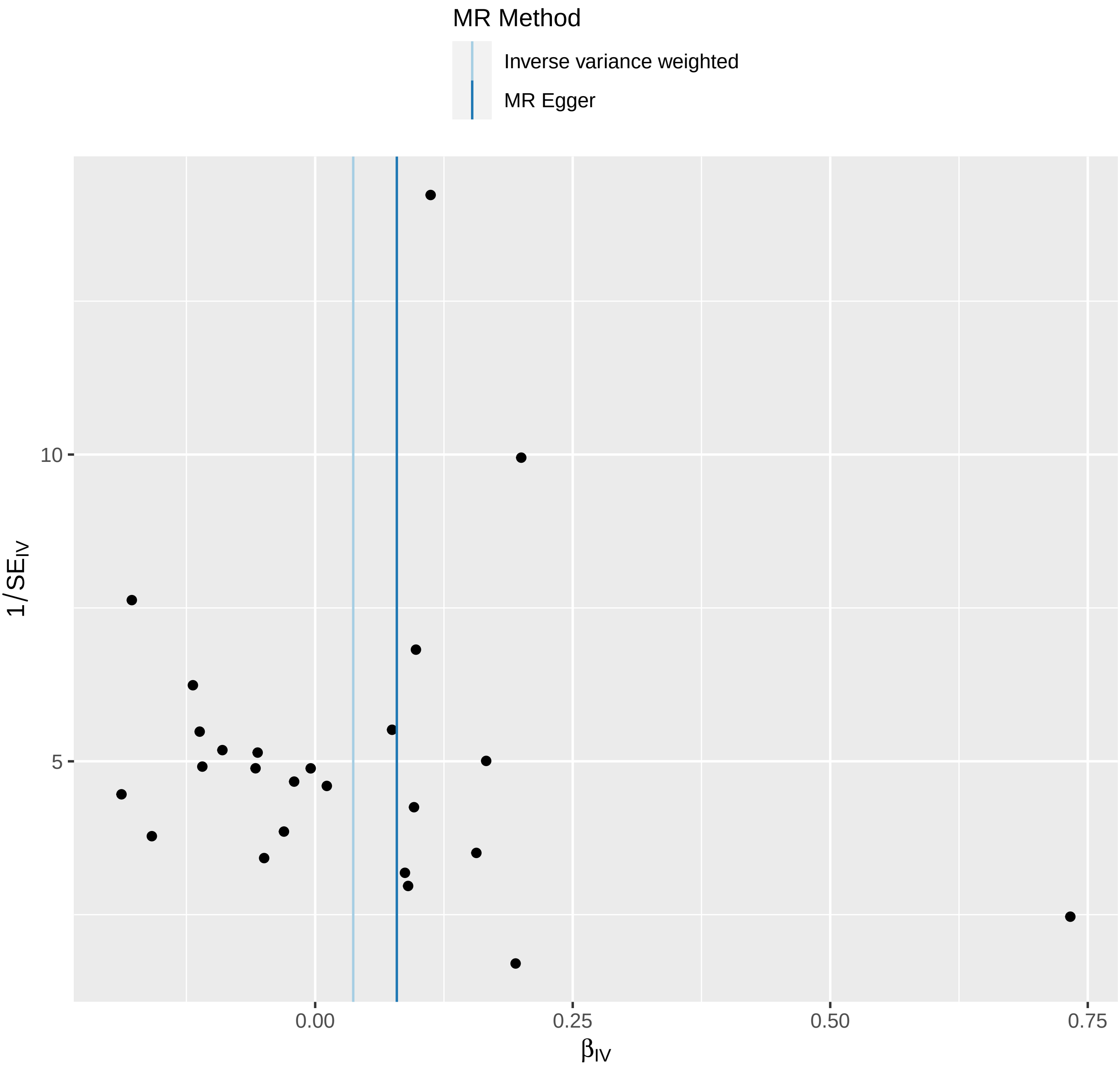

Batch 3−2939 : Holo−Transcobalamin−2 on Diabetic nephropathy (more control exclusions)
with a threshold of 1e−05

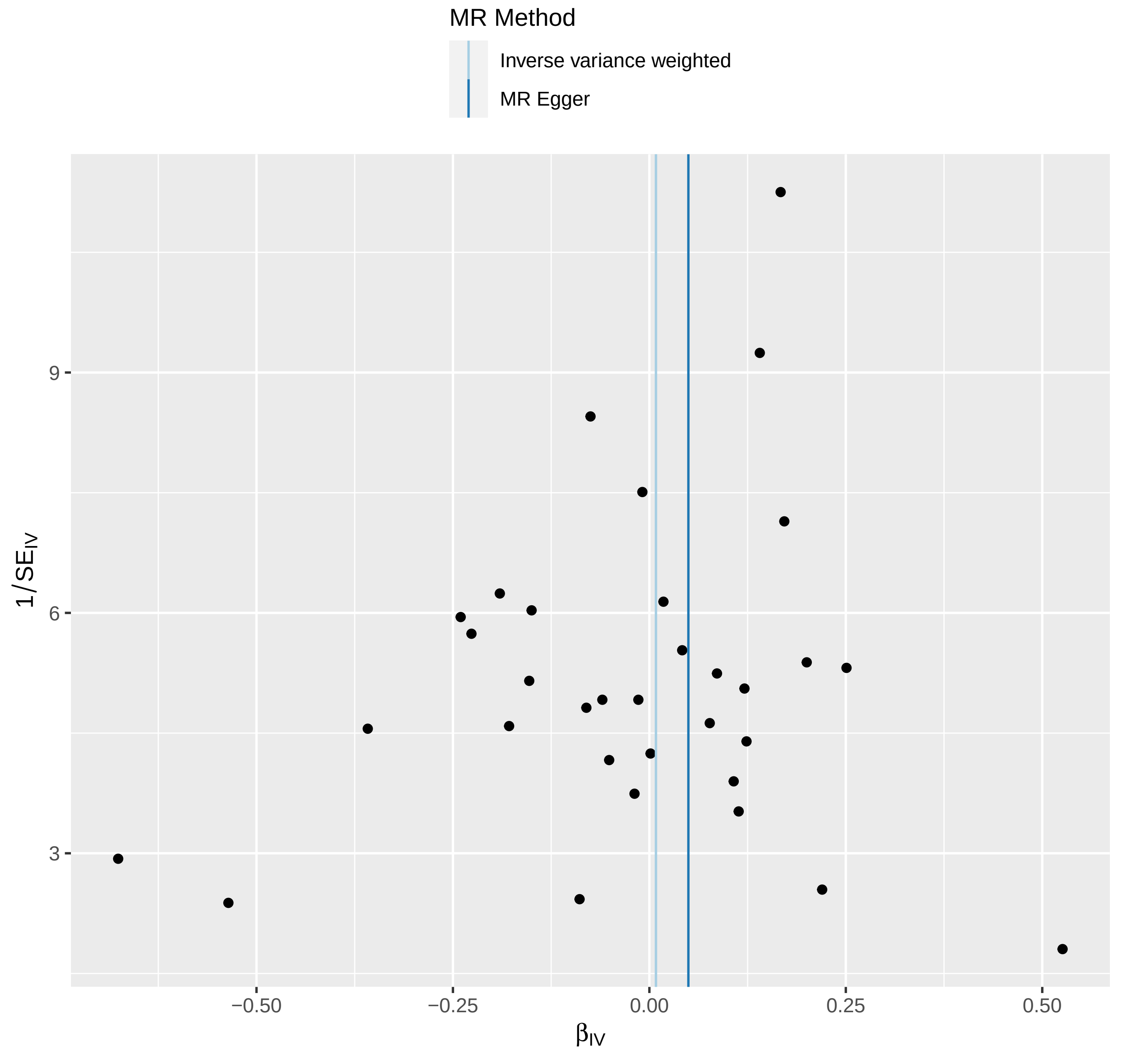

Batch 3−2940 : Teratocarcinoma−derived growth factor 1 on Diabetic nephropathy (more control exclusions)
with a threshold of 1e−05

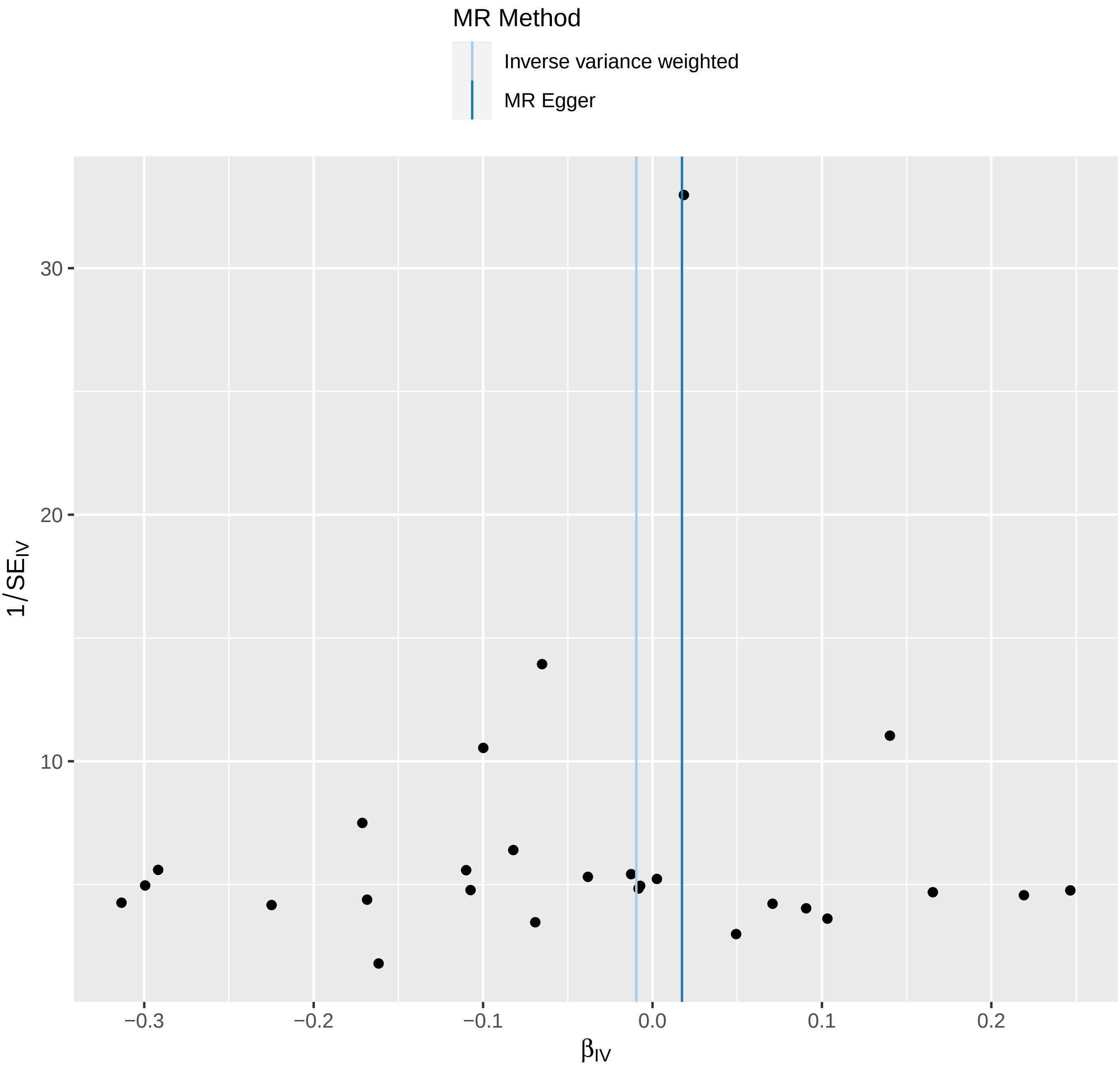

Batch 3−2941 : Tyrosyl−DNA phosphodiesterase 1 on Diabetic nephropathy (more control exclusions)
with a threshold of 1e−05

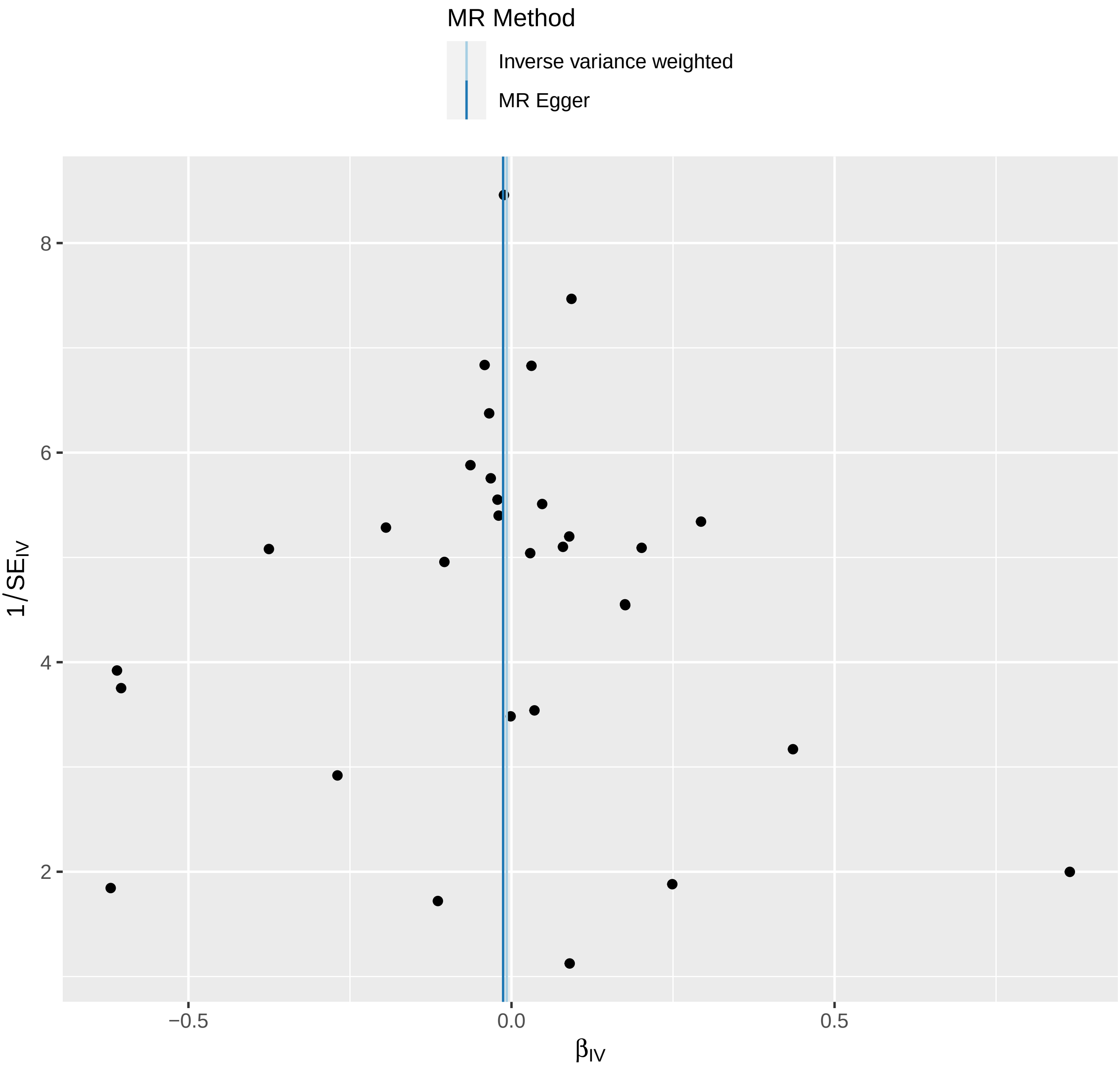

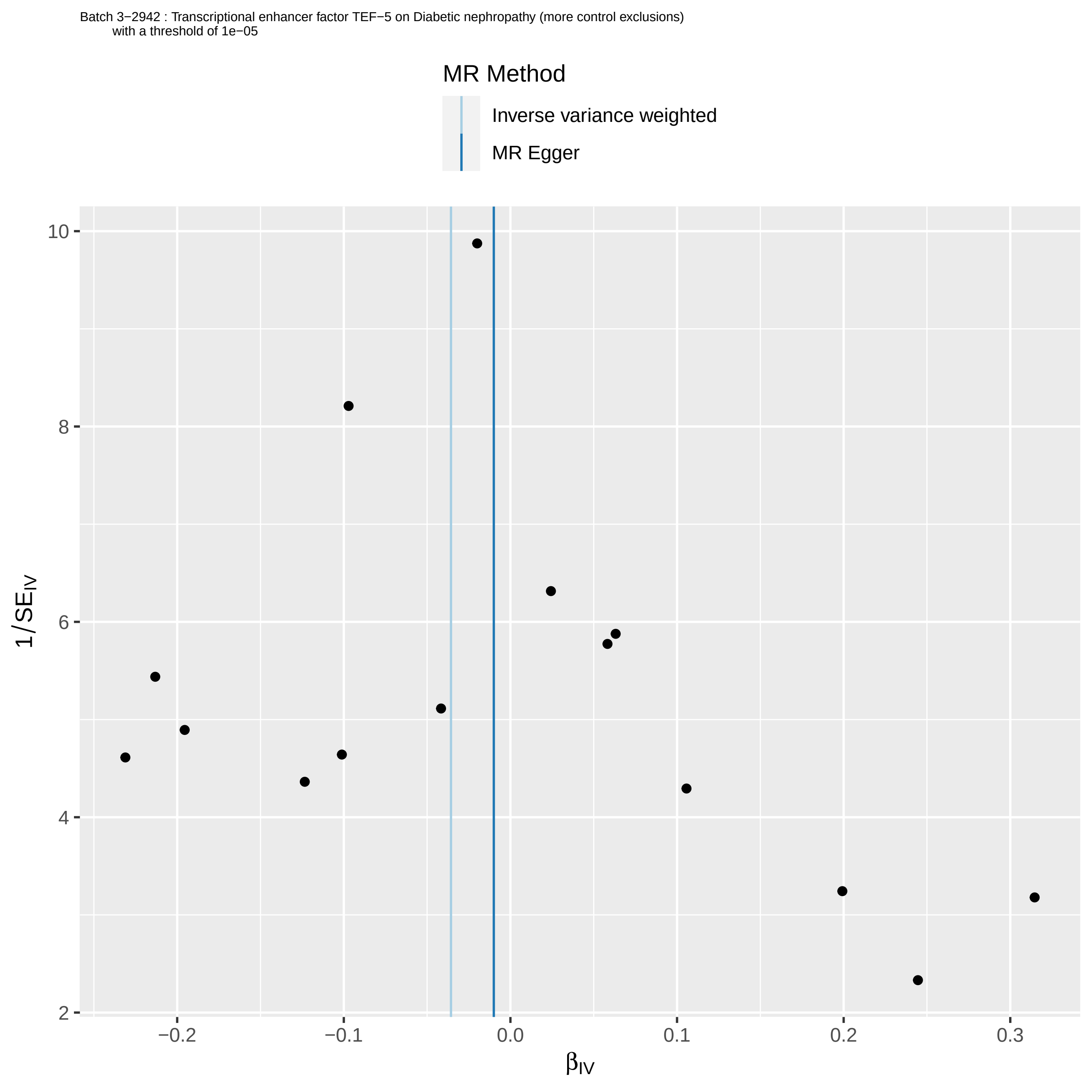

Batch 3−2942 : Transcriptional enhancer factor TEF−5 on Diabetic nephropathy (more control exclusions)
with a threshold of 1e−05
MR Method
Inverse variance weighted
MR Egger
10
8
6
4
2
1/SE_IV
−0.2
−0.1
0.0
0.1
0.2
0.3
β_IV

Batch 3−2943 : Transcriptional enhancer factor TEF−3 on Diabetic nephropathy (more control exclusions)
with a threshold of 1e−05

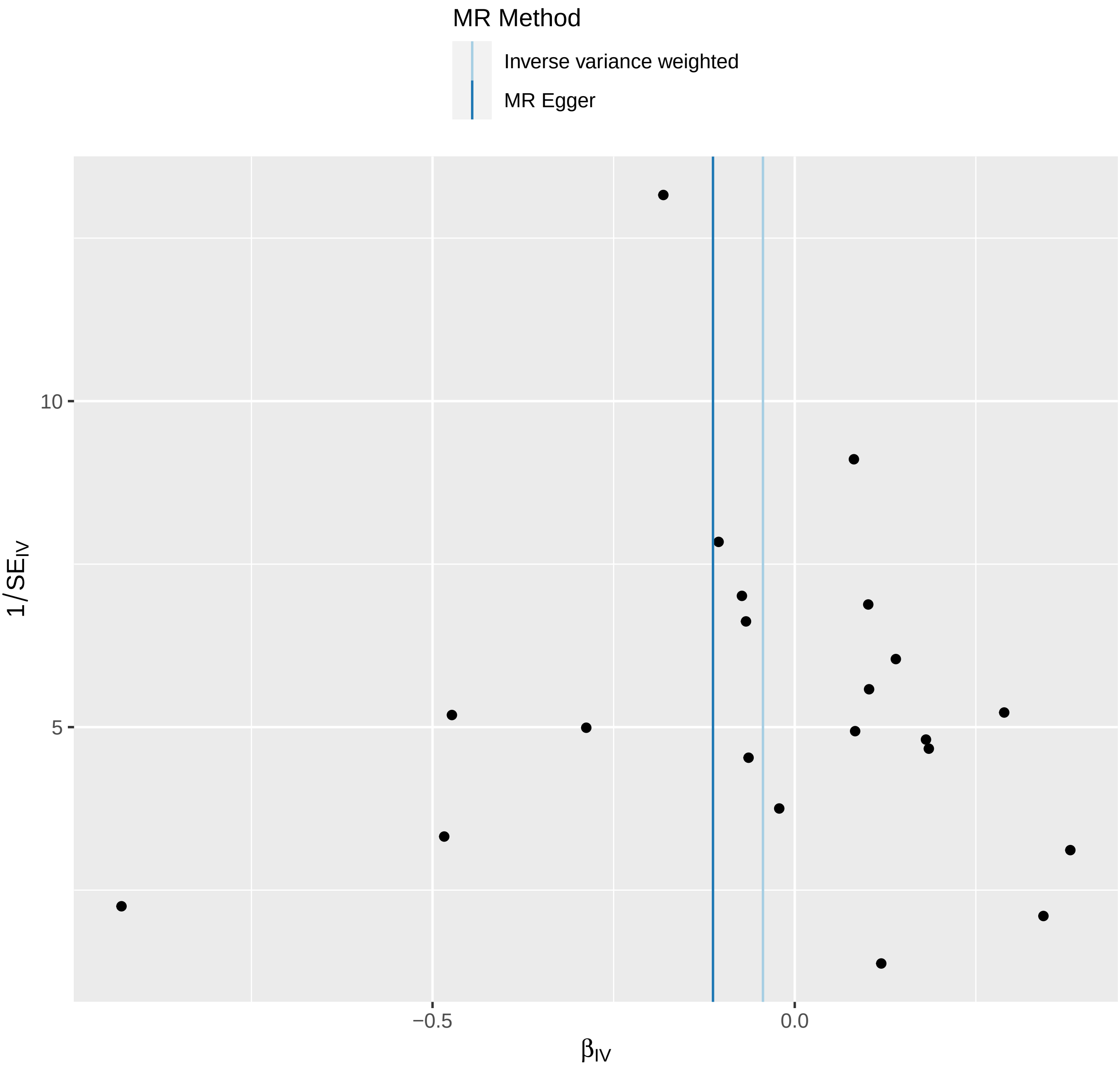

Batch 3−2944 : Angiopoietin−1 receptor, soluble on Diabetic nephropathy (more control exclusions)
with a threshold of 1e−05

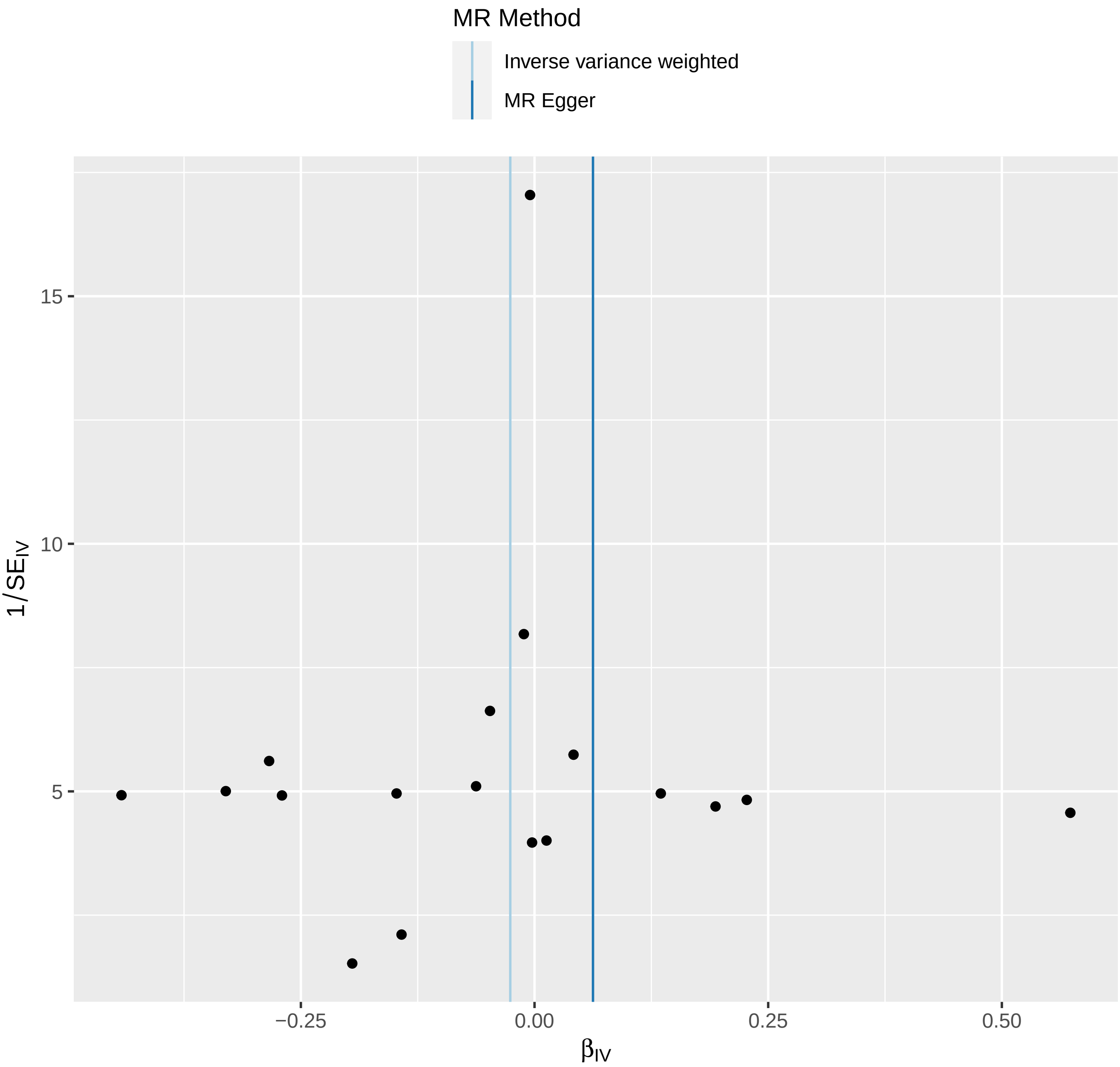

Batch 3−2945 : Teneurin−3 on Diabetic nephropathy (more control exclusions)
with a threshold of 1e−05

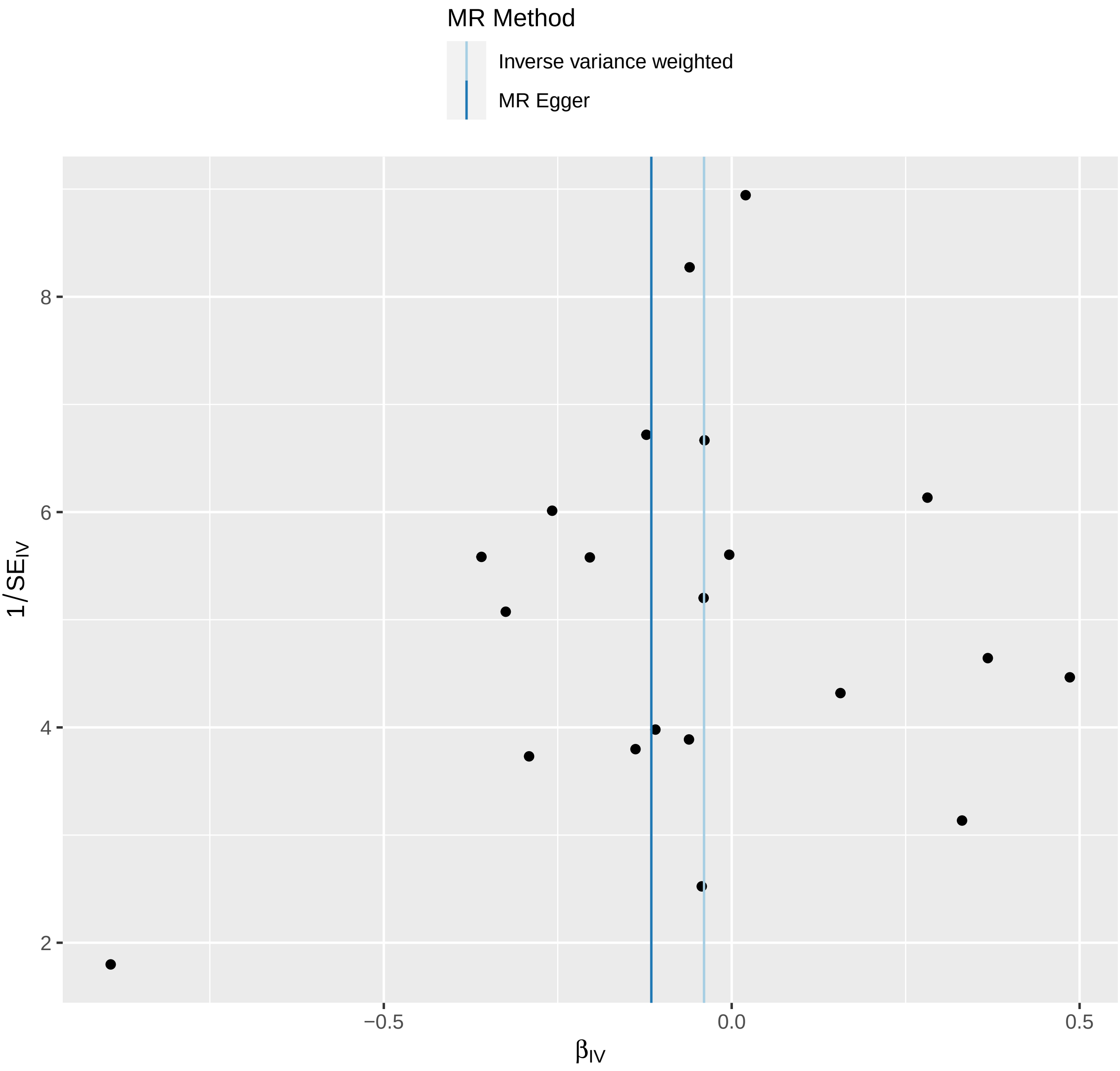

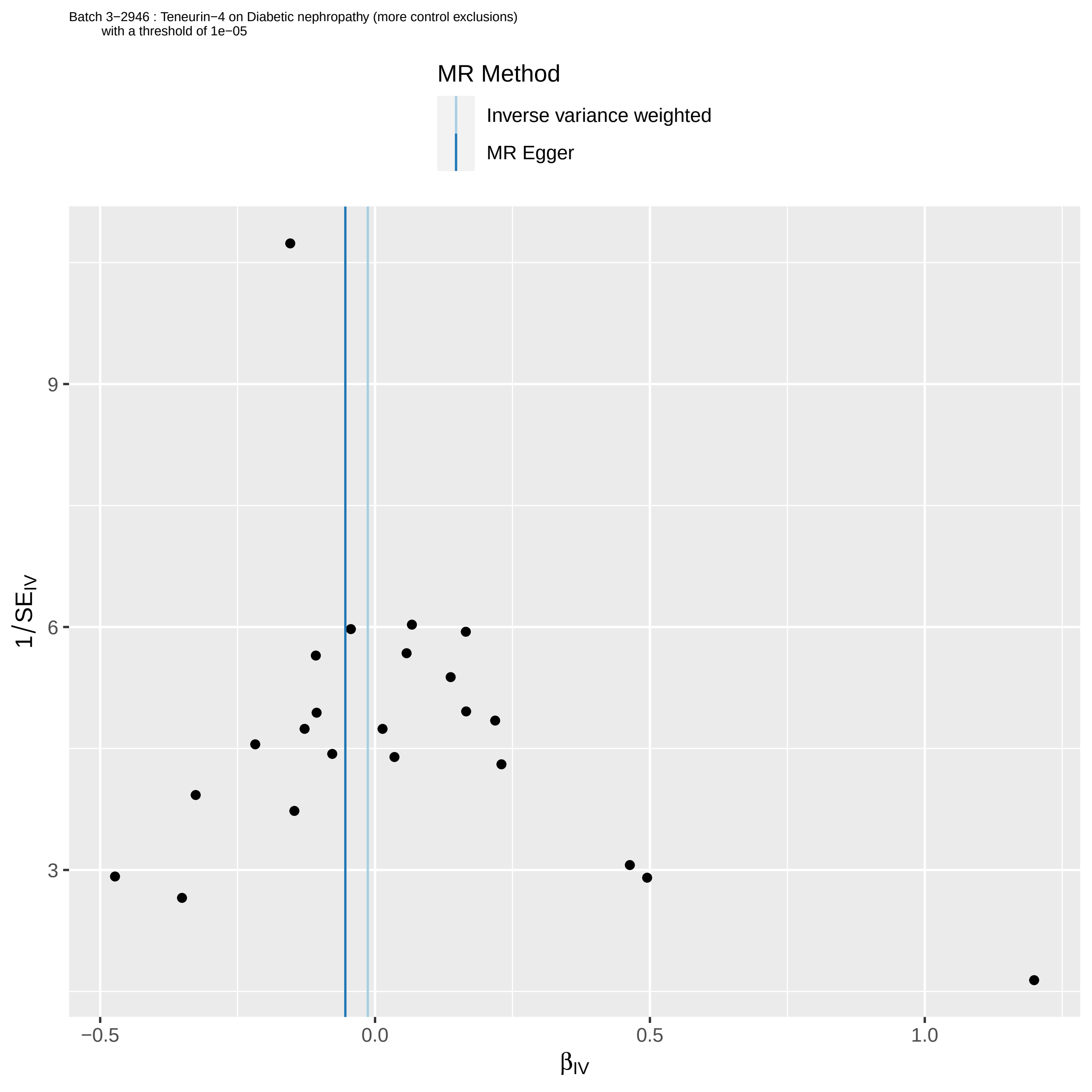

Batch 3−2946 : Teneurin−4 on Diabetic nephropathy (more control exclusions)
with a threshold of 1e−05
MR Method
Inverse variance weighted
MR Egger
9
6
3
$1/SE_{IV}$
−0.5
0.0
0.5
1.0
β_{IV}

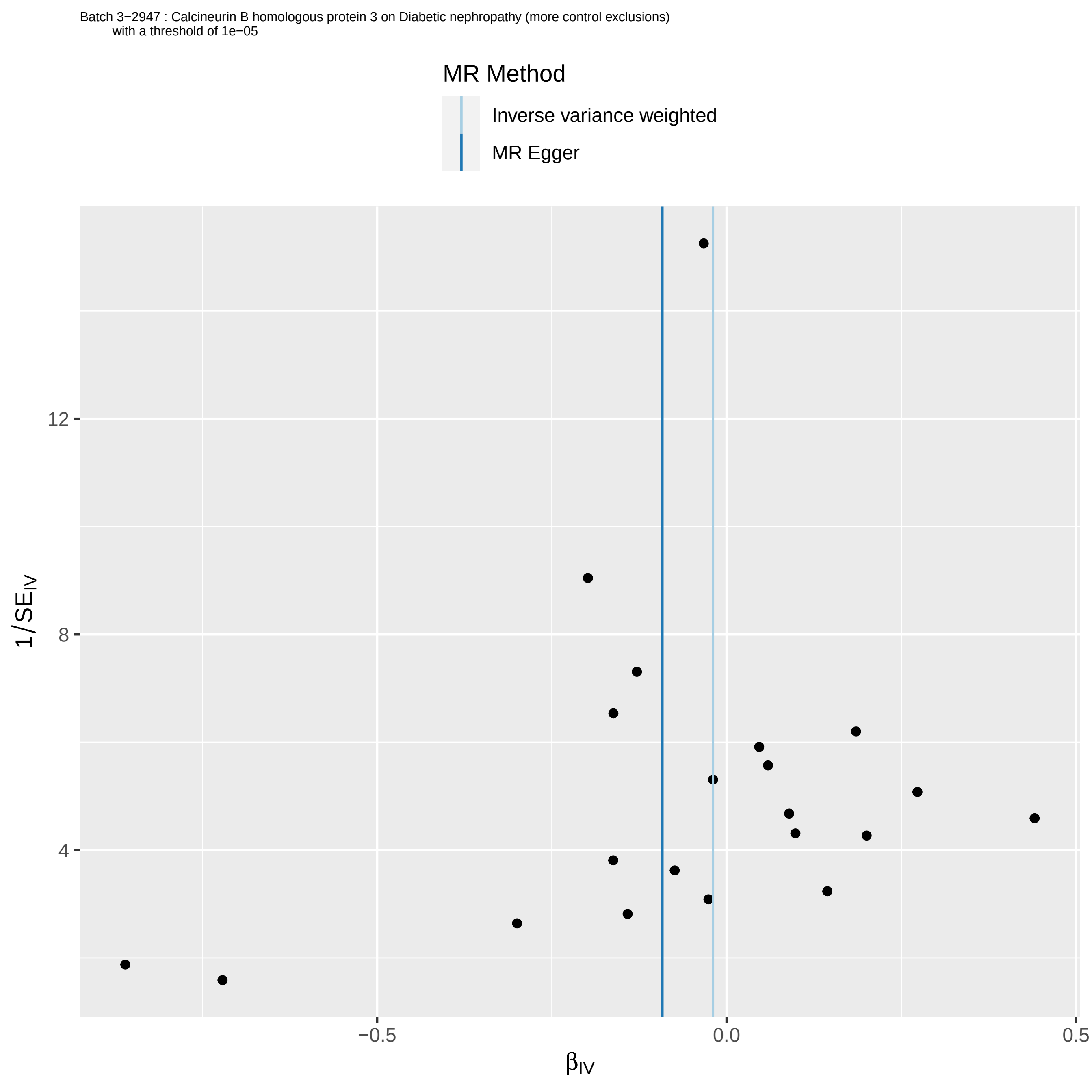

Batch 3−2947 : Calcineurin B homologous protein 3 on Diabetic nephropathy (more control exclusions)
with a threshold of 1e−05
MR Method
Inverse variance weighted
MR Egger
12
8
4
1/SE$_{IV}$
−0.5
0.0
0.5
β$_{IV}$

Batch 3−2948 : Testis−expressed sequence 29 protein on Diabetic nephropathy (more control exclusions)
with a threshold of 1e−05

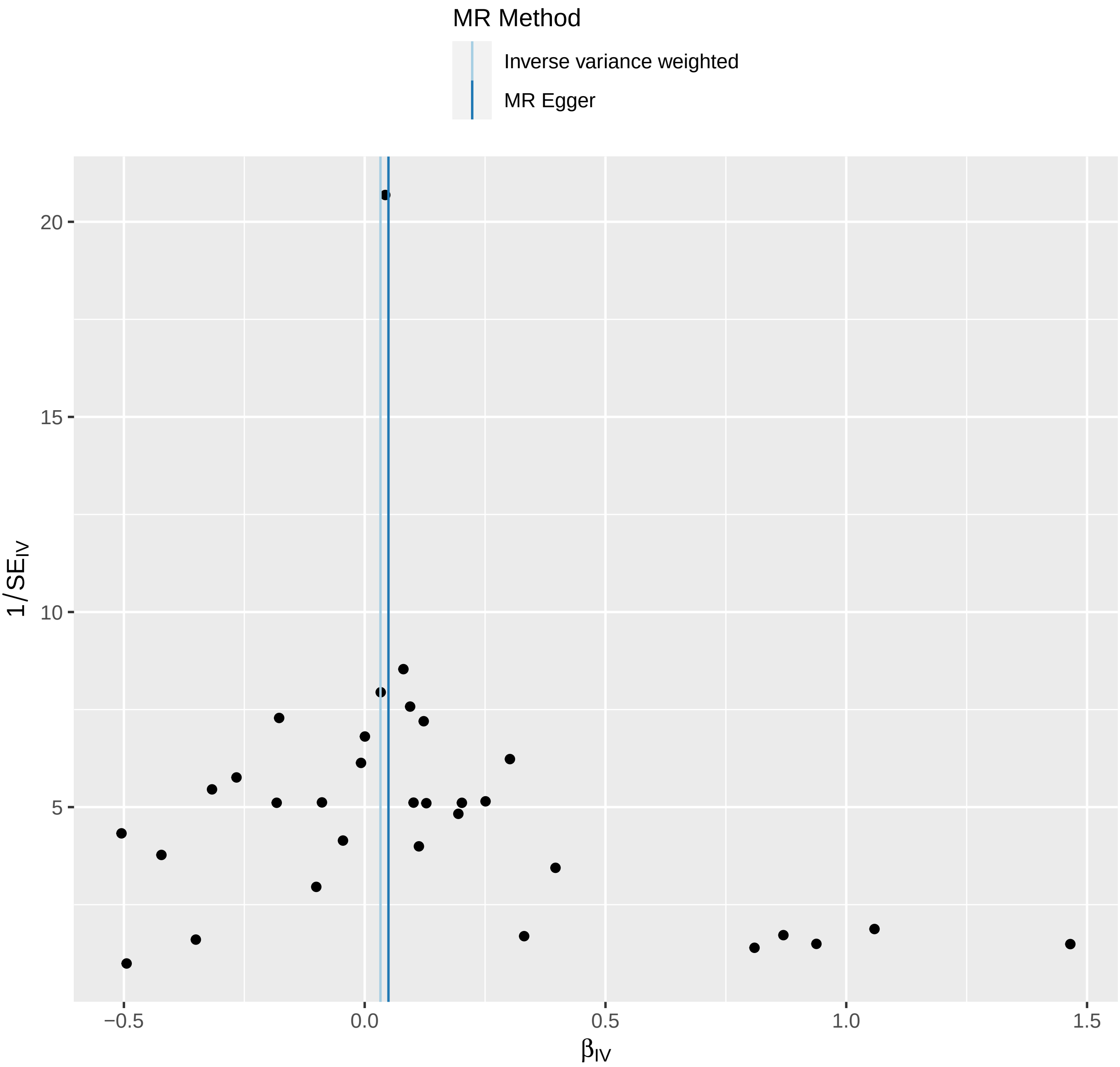

Batch 3−2949 : Testis−expressed sequence 29 protein on Diabetic nephropathy (more control exclusions)
with a threshold of 1e−05

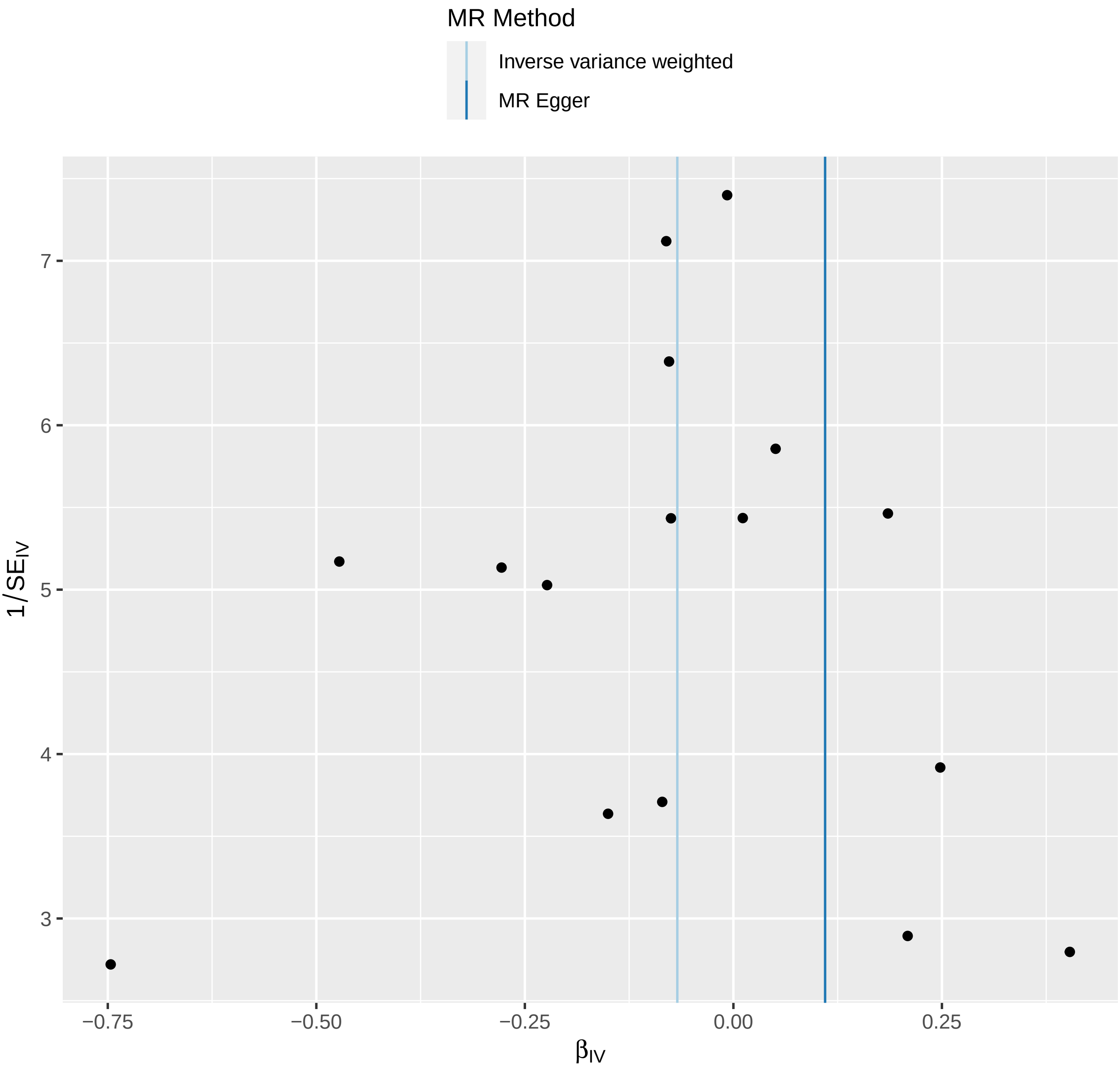

Batch 3−2950 : Serotransferrin on Diabetic nephropathy (more control exclusions)
with a threshold of 1e−05

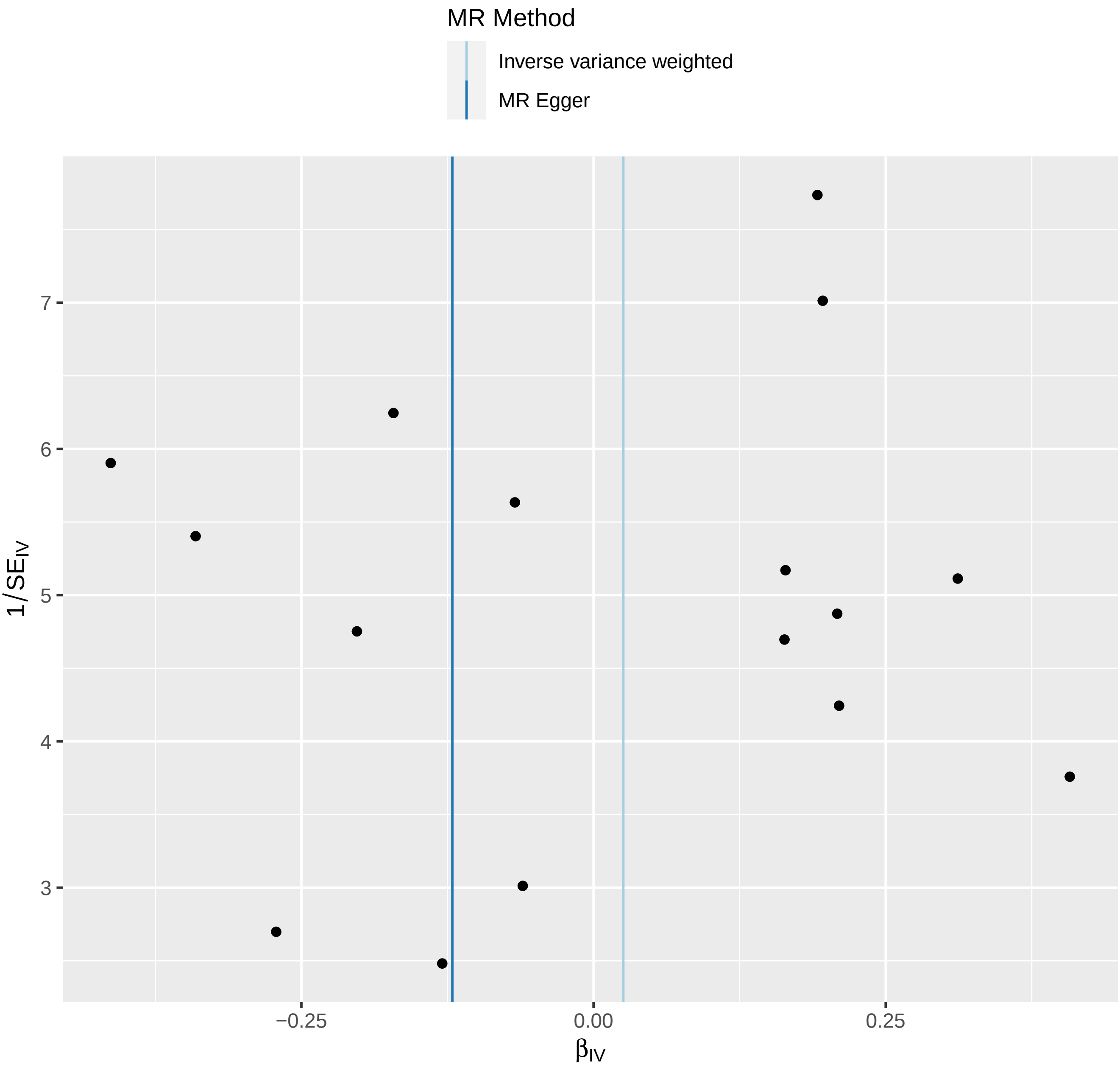

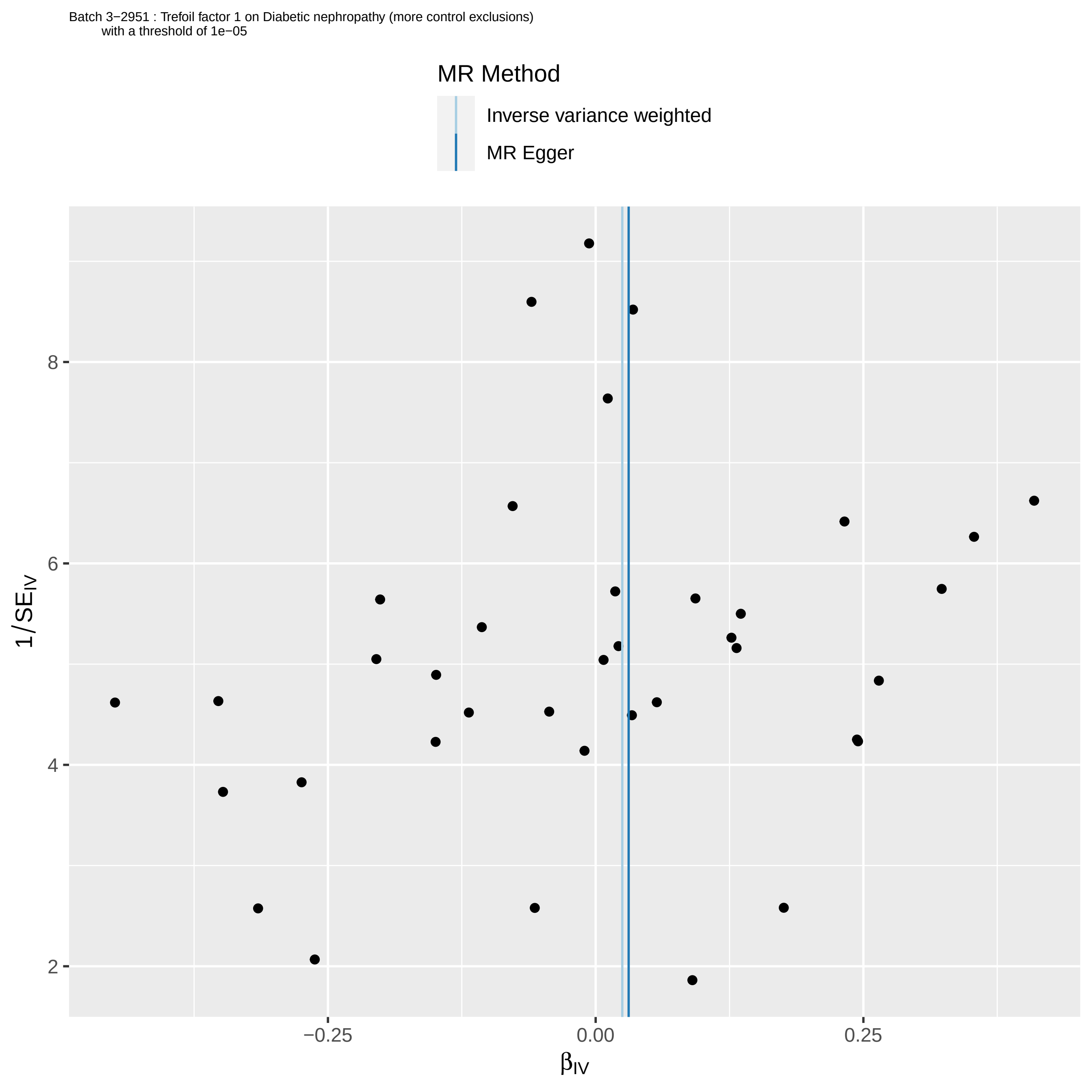

Batch 3−2951 : Trefoil factor 1 on Diabetic nephropathy (more control exclusions)
with a threshold of 1e−05
MR Method
Inverse variance weighted
MR Egger
8
6
4
2
1/SE$_{IV}$
−0.25
0.00
0.25
β$_{IV}$

Batch 3−2952 : Trefoil factor 2 on Diabetic nephropathy (more control exclusions)
with a threshold of 1e−05

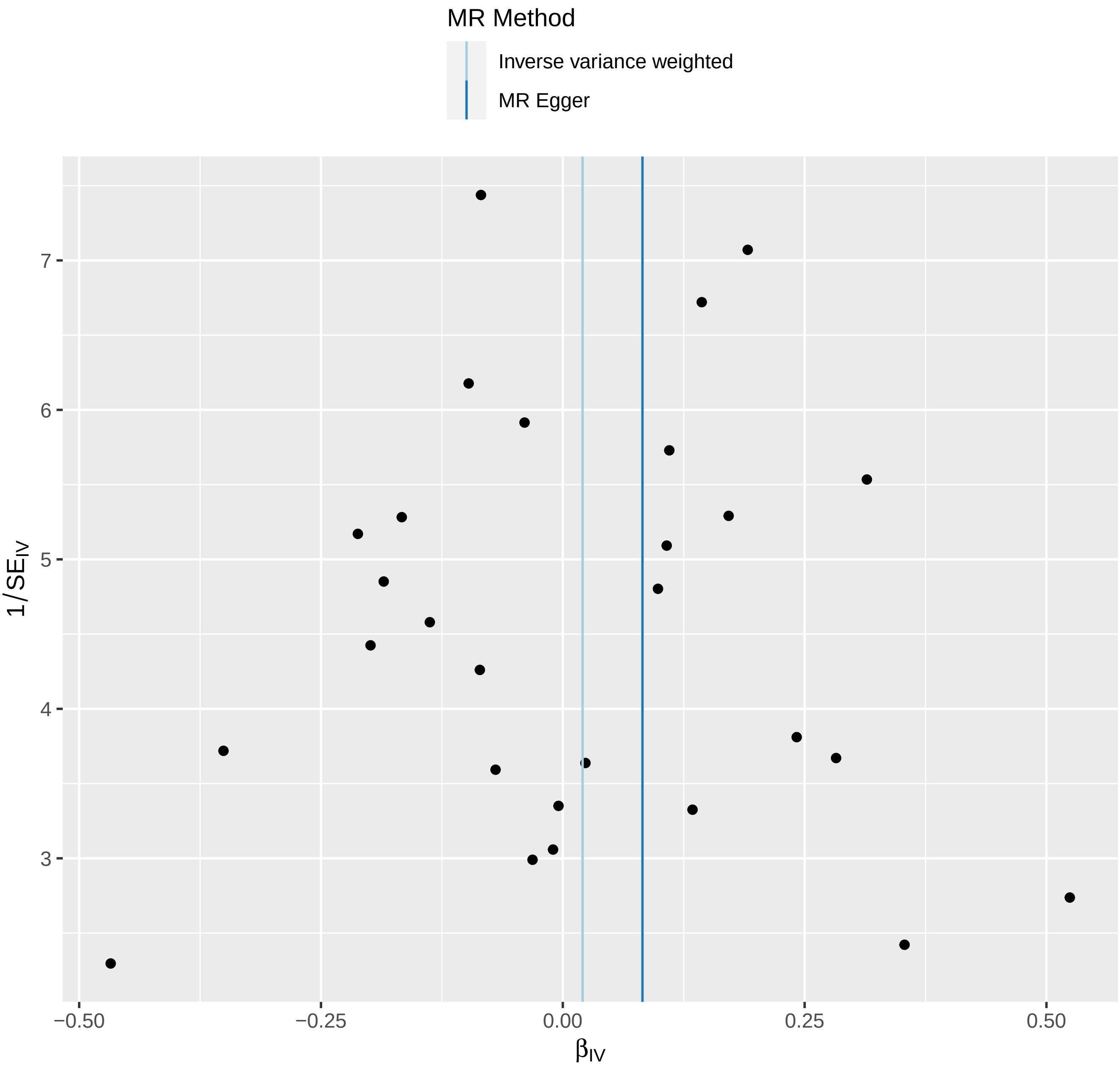

Batch 3−2953 : Trefoil factor 3 on Diabetic nephropathy (more control exclusions)
with a threshold of 1e−05

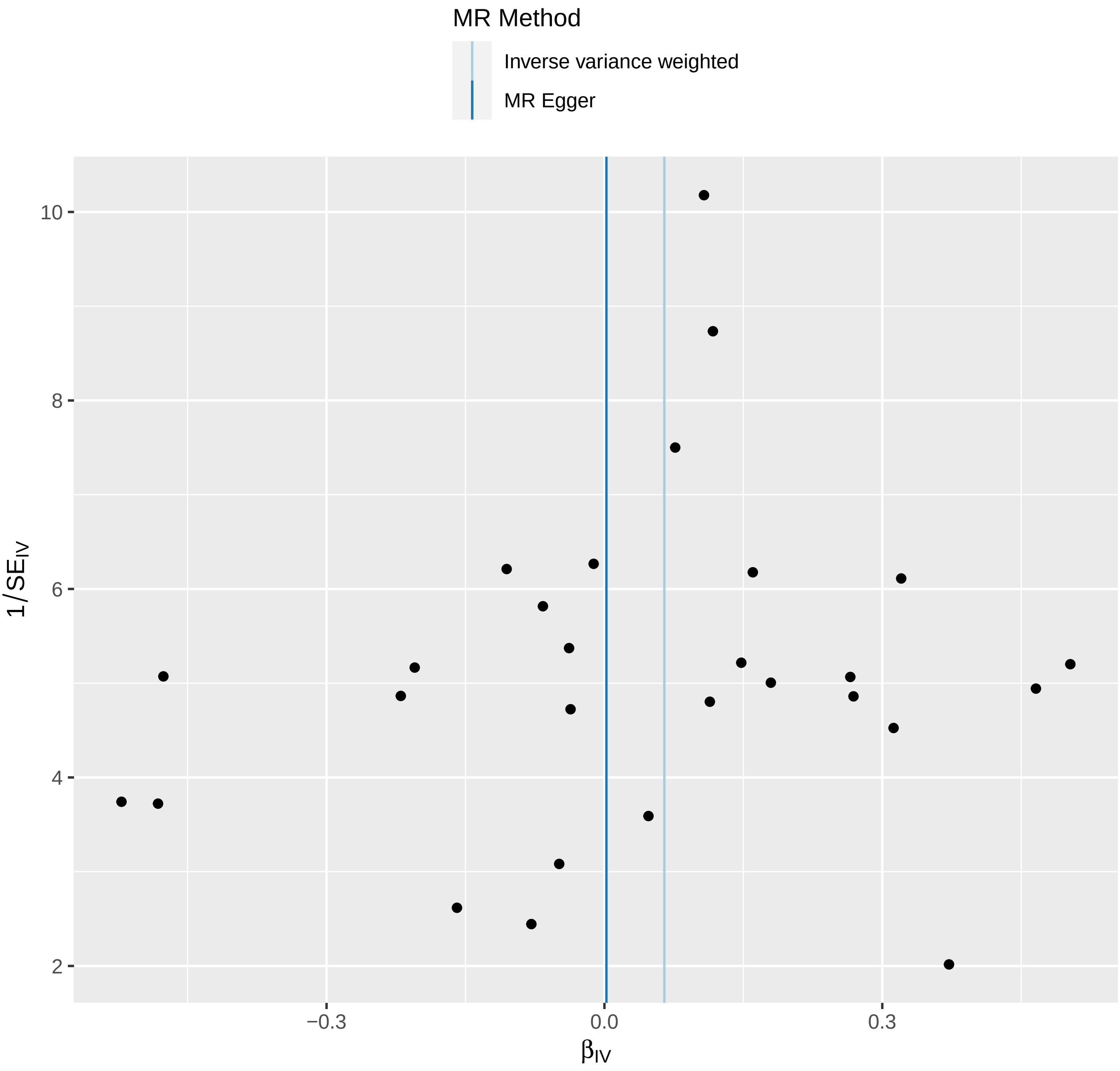

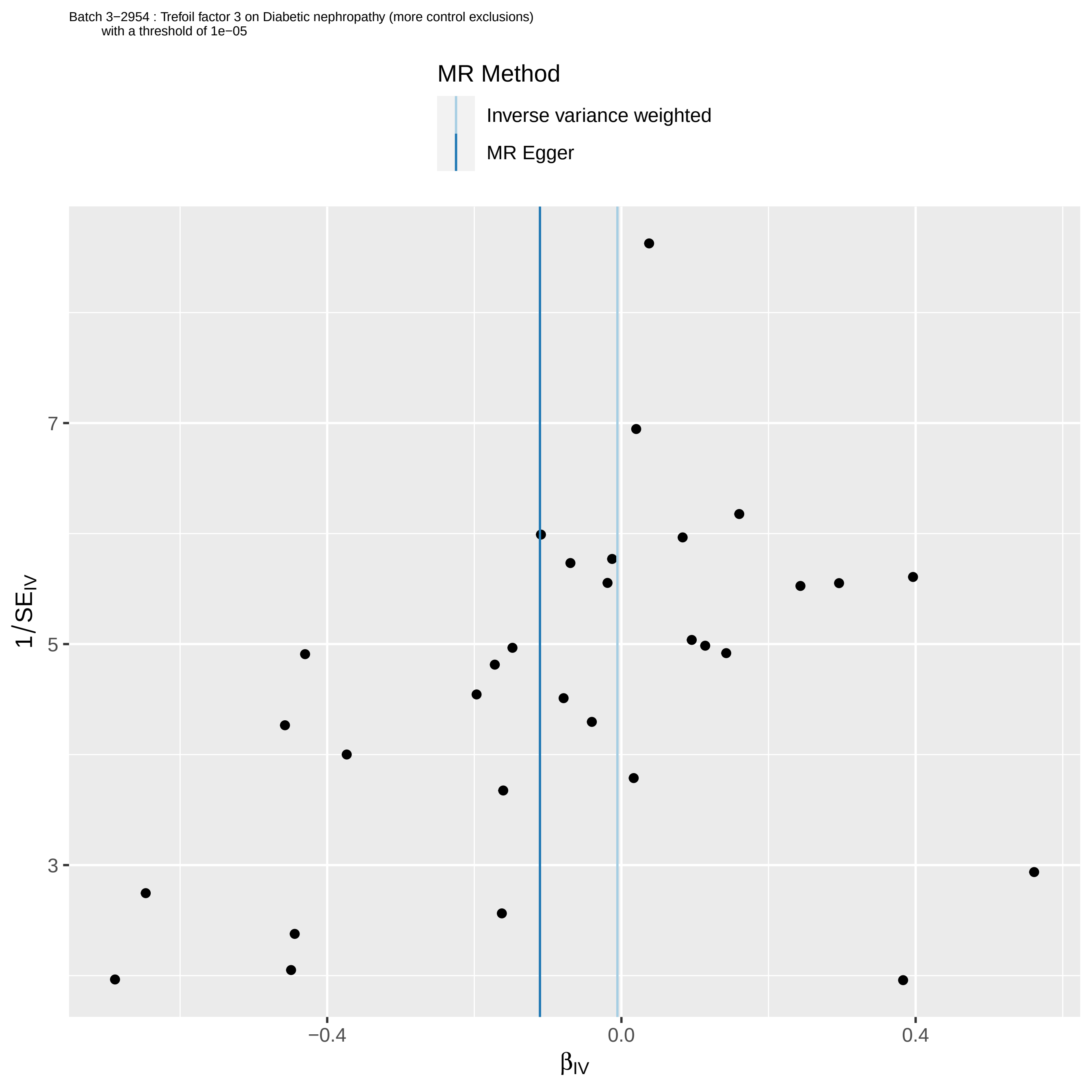
Batch 3−2954 : Trefoil factor 3 on Diabetic nephropathy (more control exclusions)
with a threshold of 1e−05
MR Method
Inverse variance weighted
MR Egger
7
5
3
$1/SE_{IV}$
−0.4
0.0
0.4
β_{IV}

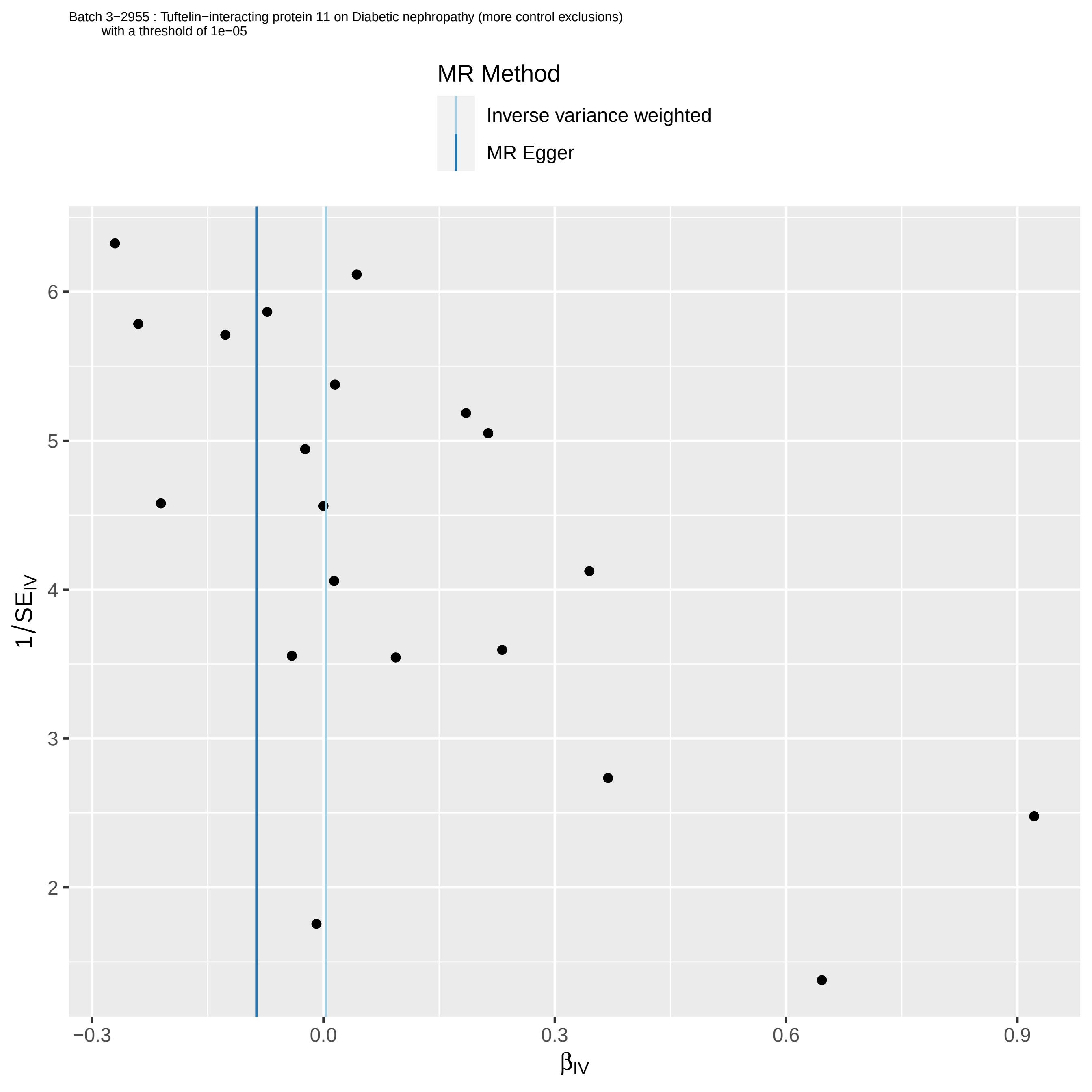
Batch 3−2955 : Tuftelin−interacting protein 11 on Diabetic nephropathy (more control exclusions)
with a threshold of 1e−05
MR Method
Inverse variance weighted
MR Egger
1/SEIV
βIV
−0.3
0.0
0.3
0.6
0.9
2
3
4
5
6

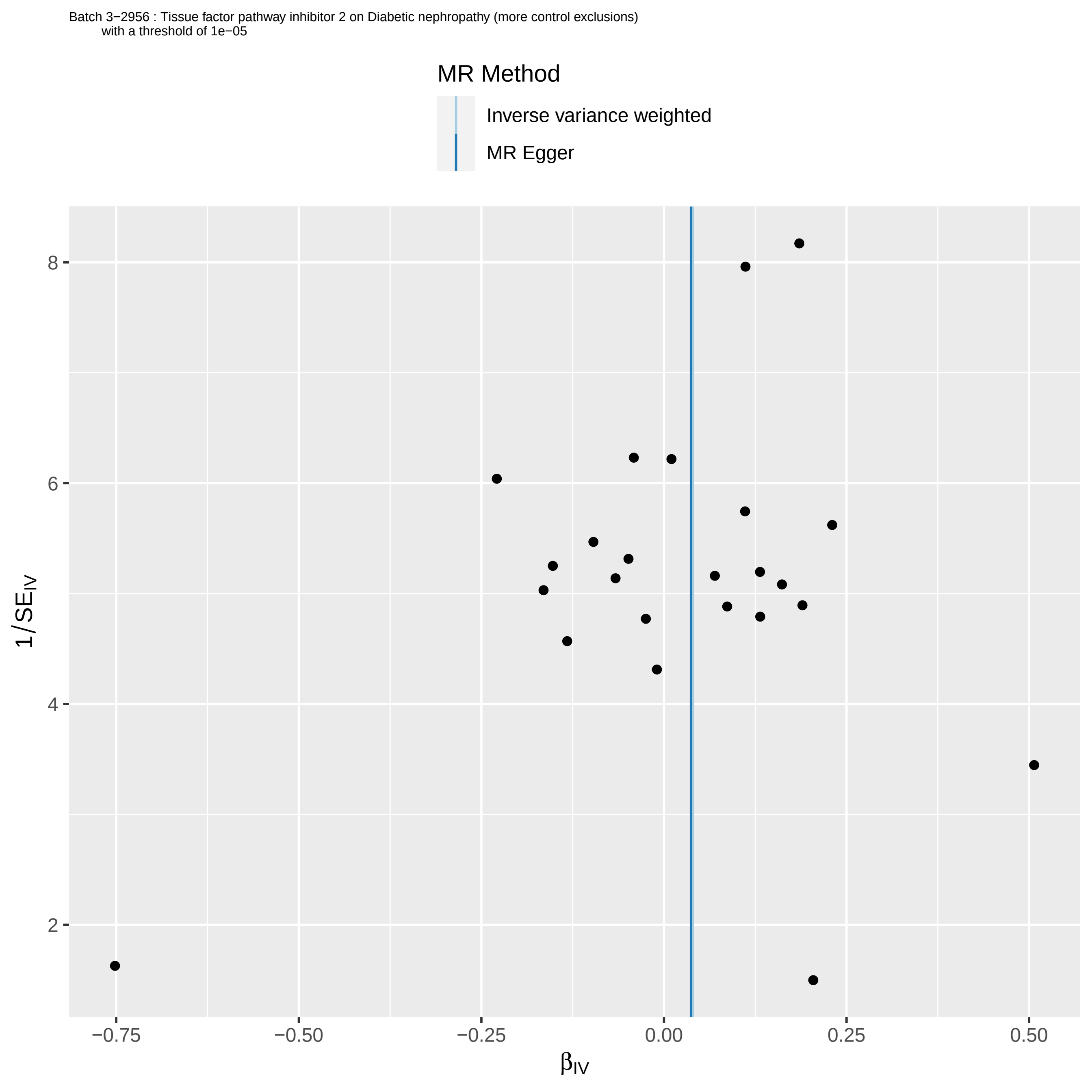

Batch 3−2956 : Tissue factor pathway inhibitor 2 on Diabetic nephropathy (more control exclusions)
with a threshold of 1e−05
MR Method
Inverse variance weighted
MR Egger
8
6
4
2
1/SE$_{IV}$
−0.75
−0.50
−0.25
0.00
0.25
0.50
β$_{IV}$

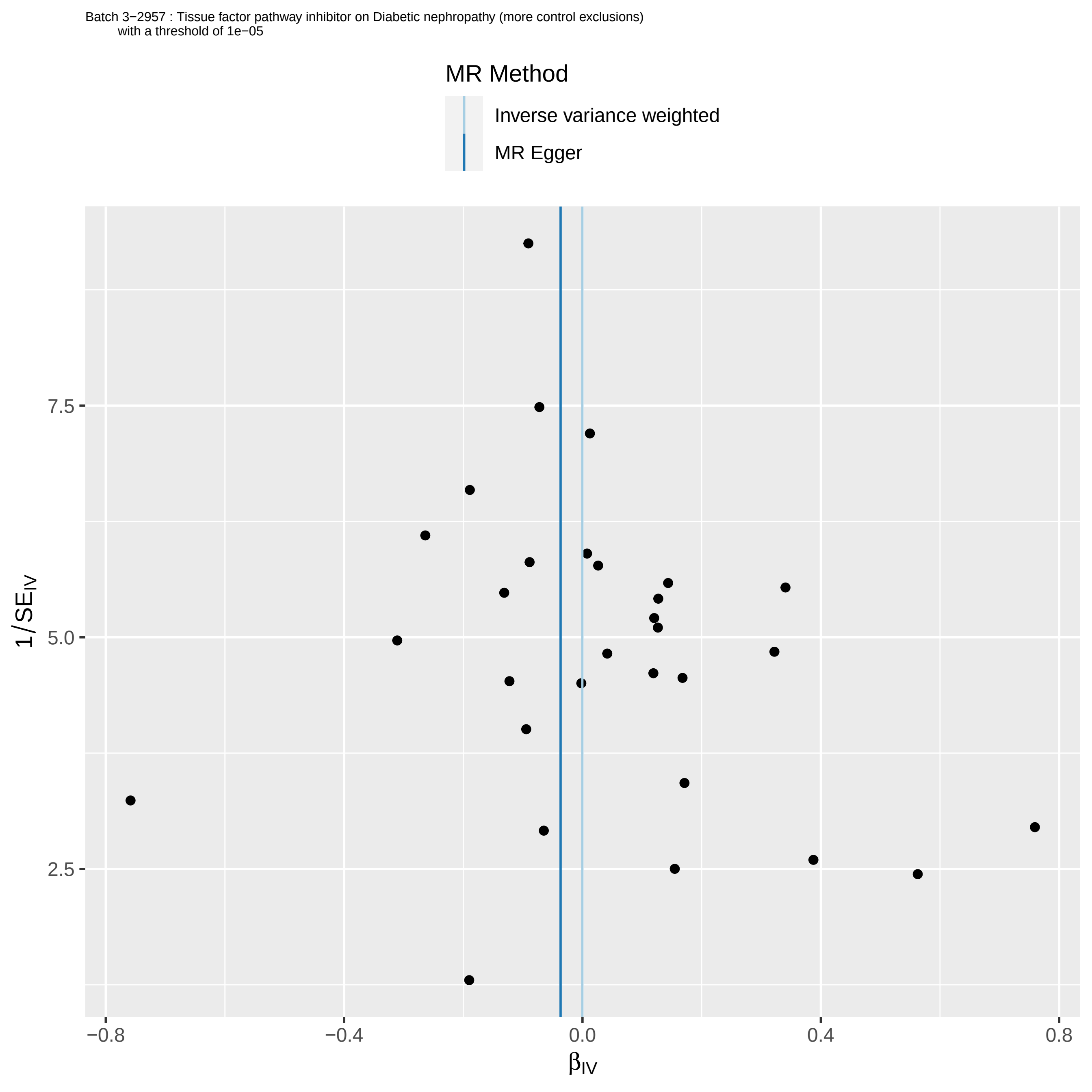
Batch 3−2957 : Tissue factor pathway inhibitor on Diabetic nephropathy (more control exclusions)
with a threshold of 1e−05
MR Method
Inverse variance weighted
MR Egger
7.5
5.0
2.5
1/SE_IV
−0.8
−0.4
0.0
0.4
0.8
β_IV

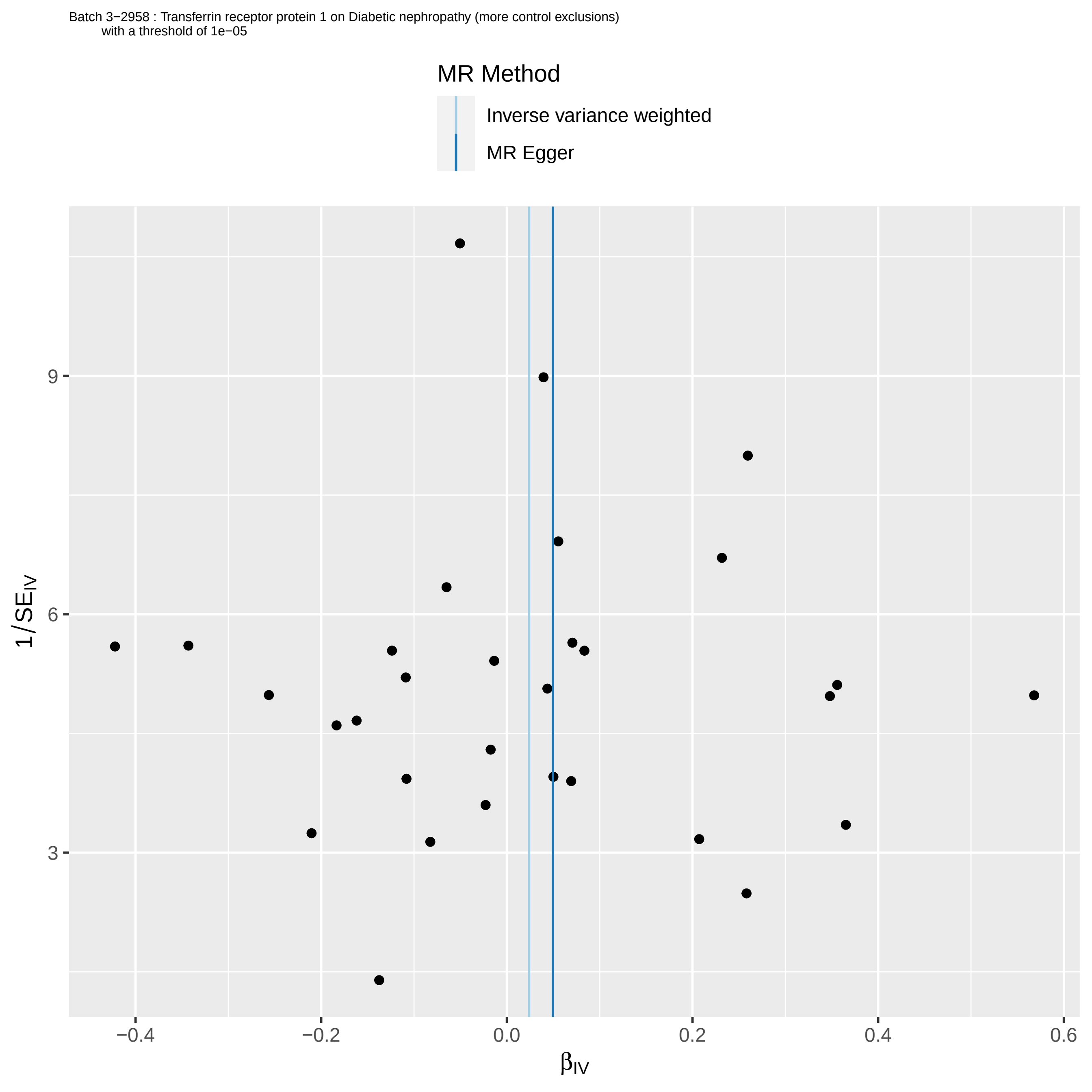
Batch 3−2958 : Transferrin receptor protein 1 on Diabetic nephropathy (more control exclusions)
with a threshold of 1e−05
MR Method
Inverse variance weighted
MR Egger
9
6
3
1/SE_IV
−0.4
−0.2
0.0
0.2
0.4
0.6
β_IV

Batch 3−2959 : Transferrin receptor protein 1 on Diabetic nephropathy (more control exclusions)
with a threshold of 1e−05

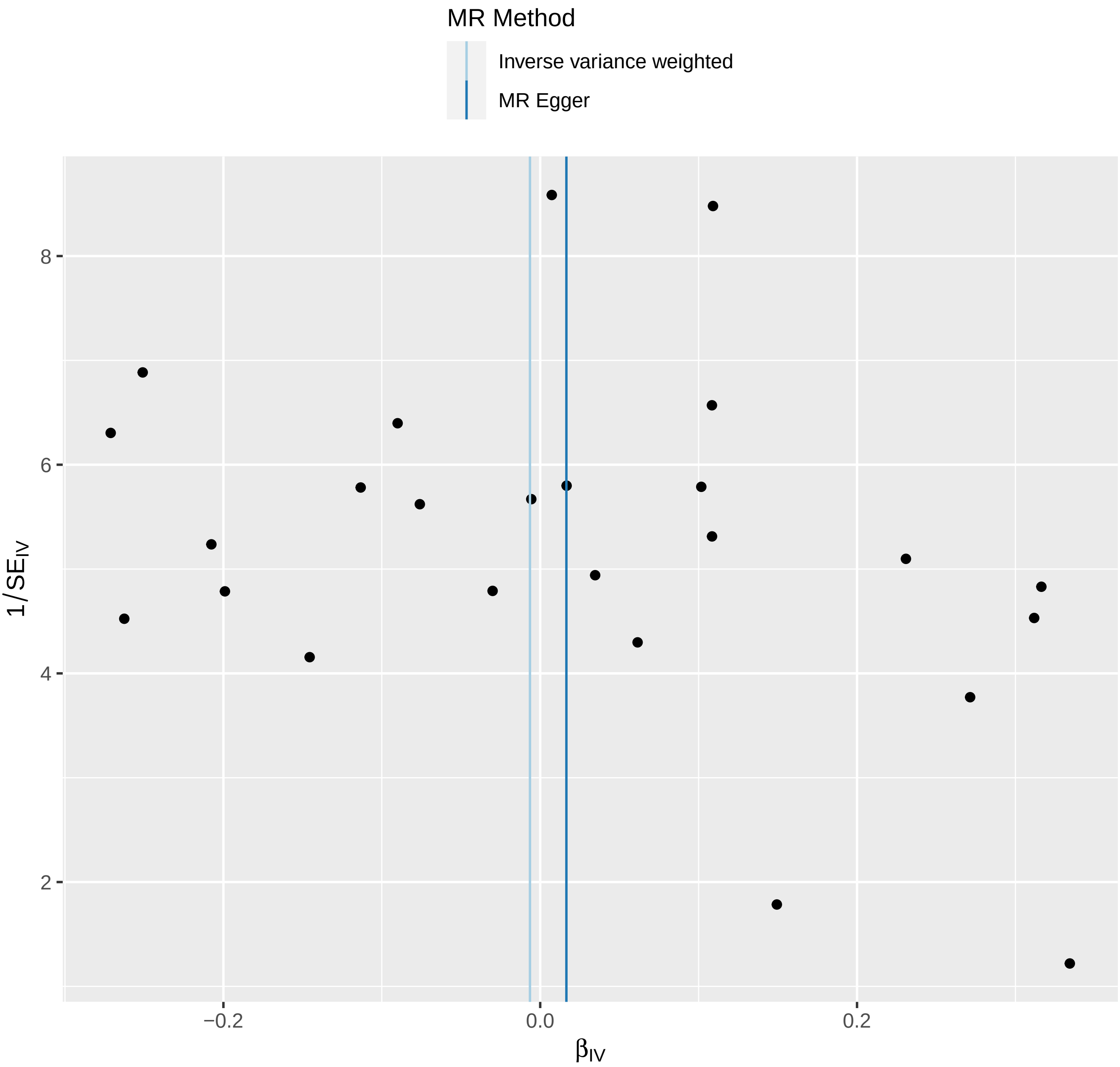

Batch 3−2960 : Thyroglobulin on Diabetic nephropathy (more control exclusions)
with a threshold of 1e−05

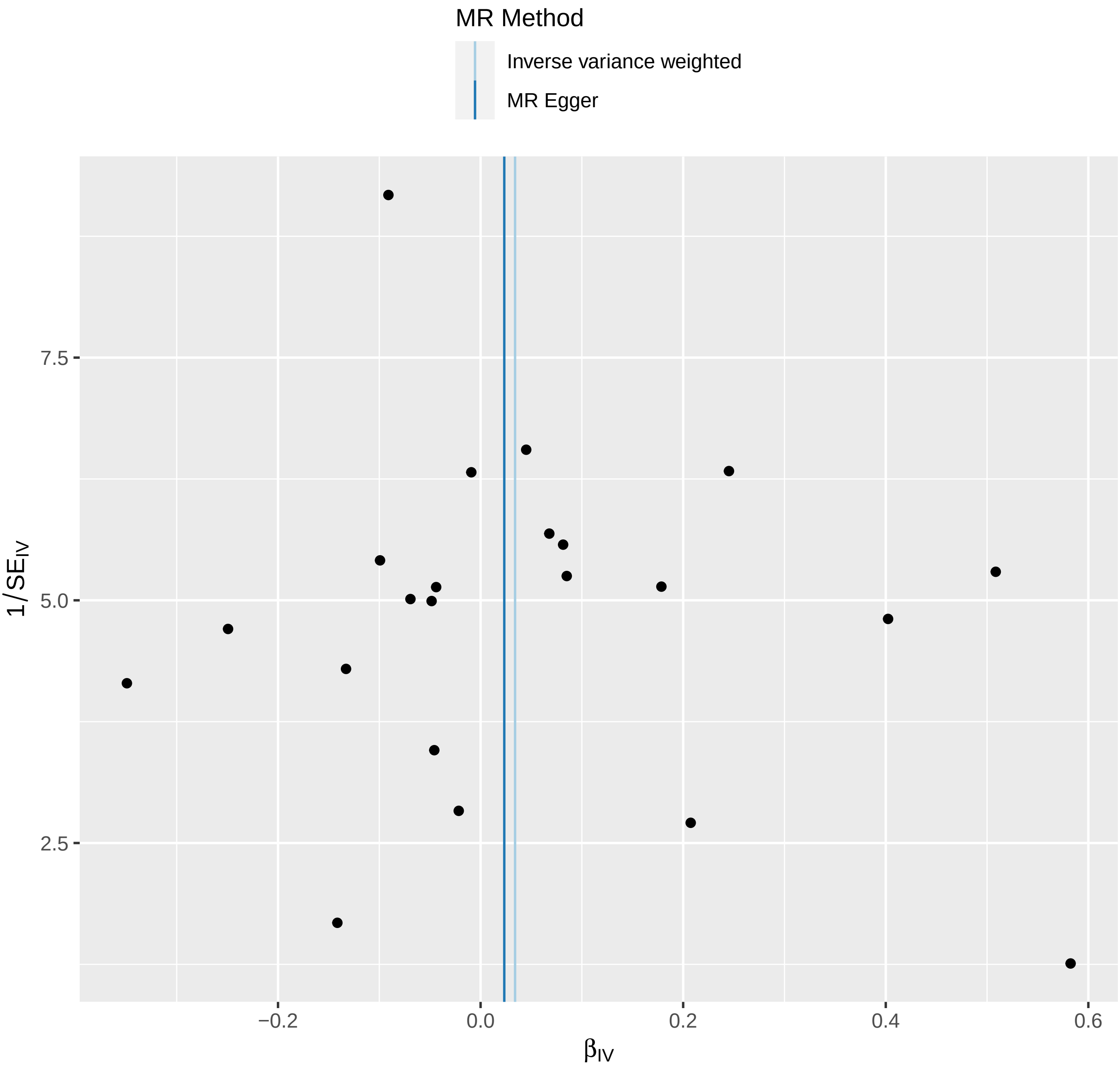

Batch 3−2961 : Transforming growth factor alpha on Diabetic nephropathy (more control exclusions)
with a threshold of 1e−05

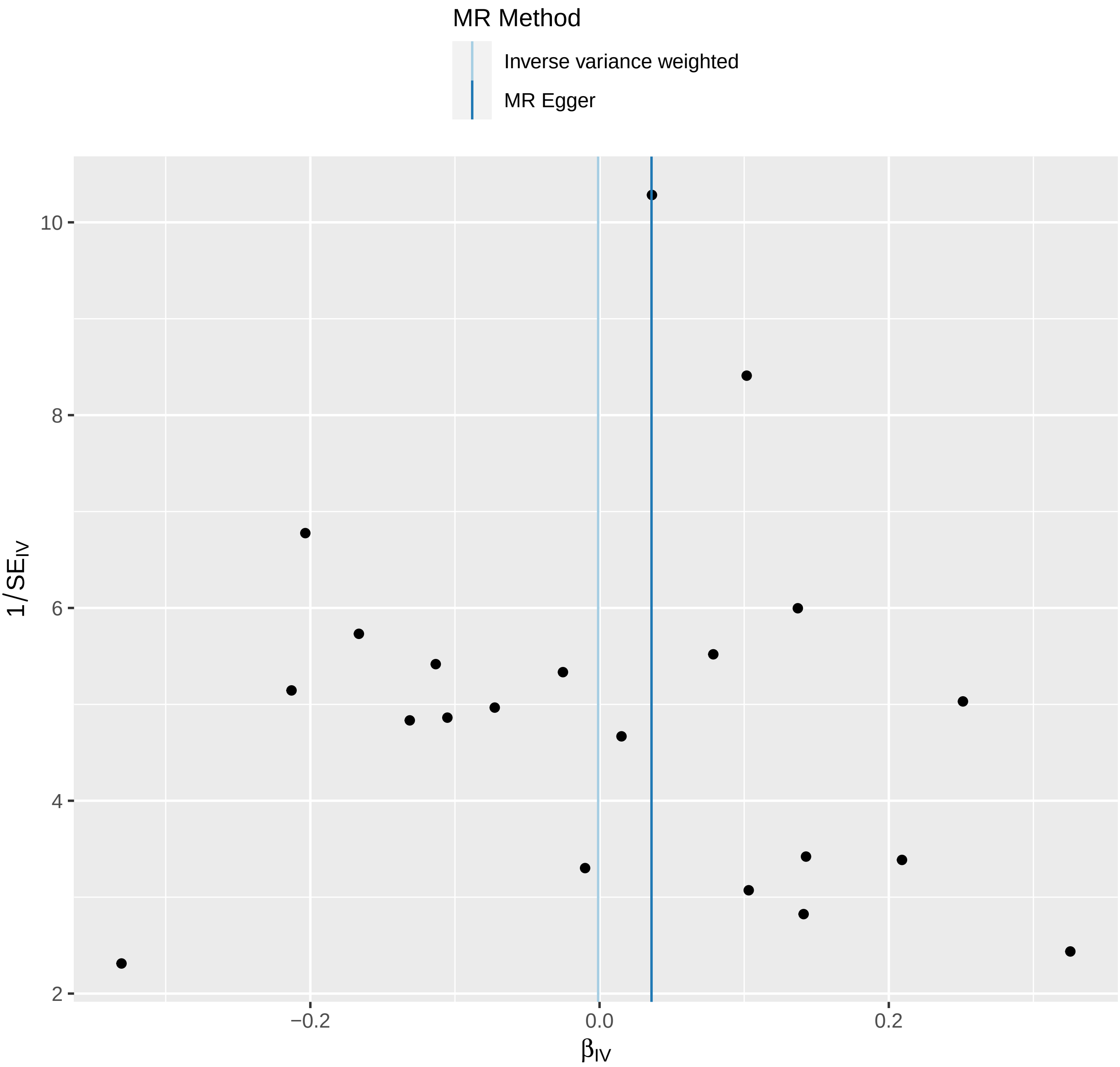

Batch 3−2962 : Transforming growth factor beta−1 on Diabetic nephropathy (more control exclusions)
with a threshold of 1e−05

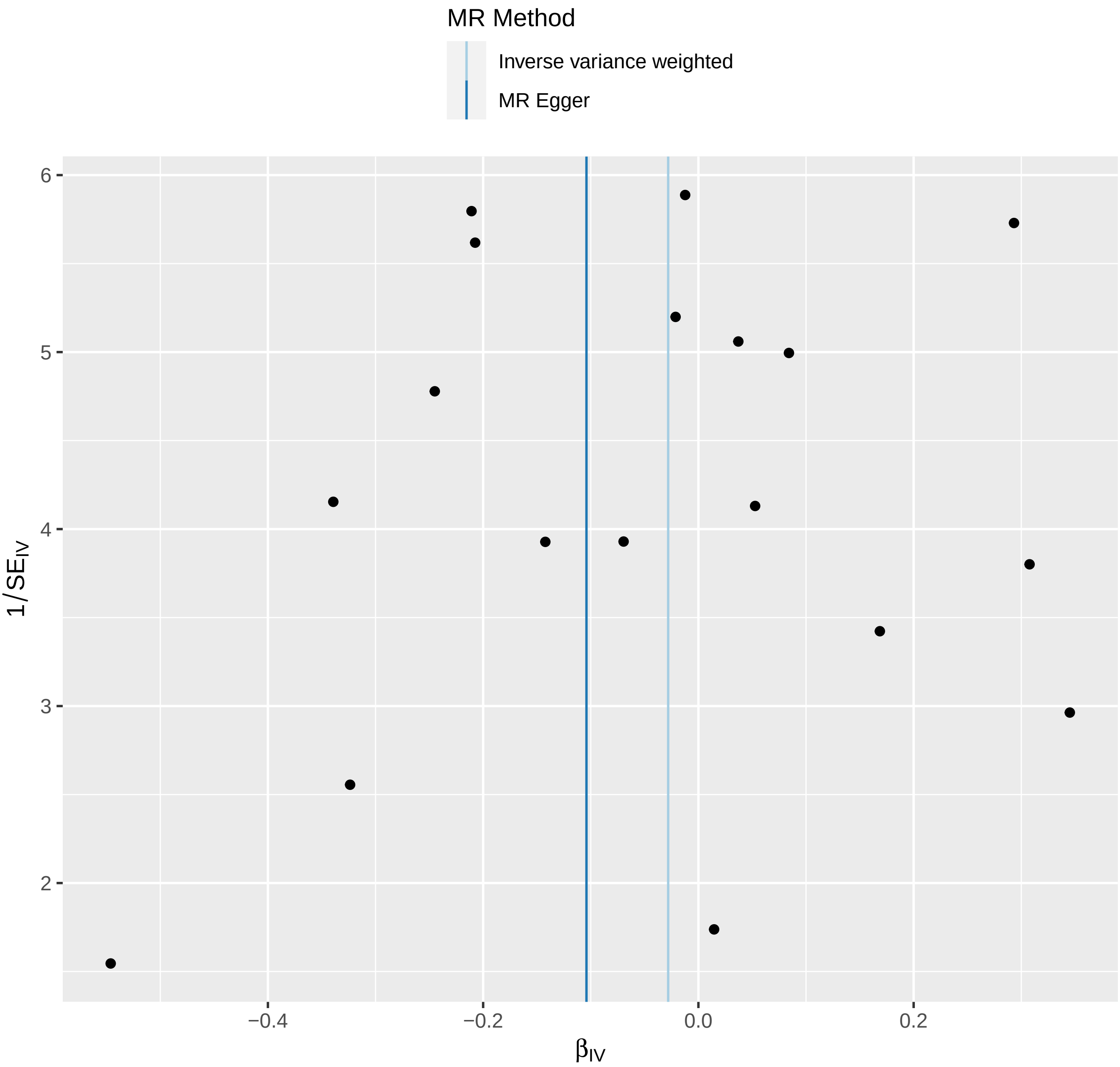

Batch 3−2963 : Transforming growth factor beta−1−induced transcript 1 protein on Diabetic nephropathy (more control exclusions)
with a threshold of 1e−05

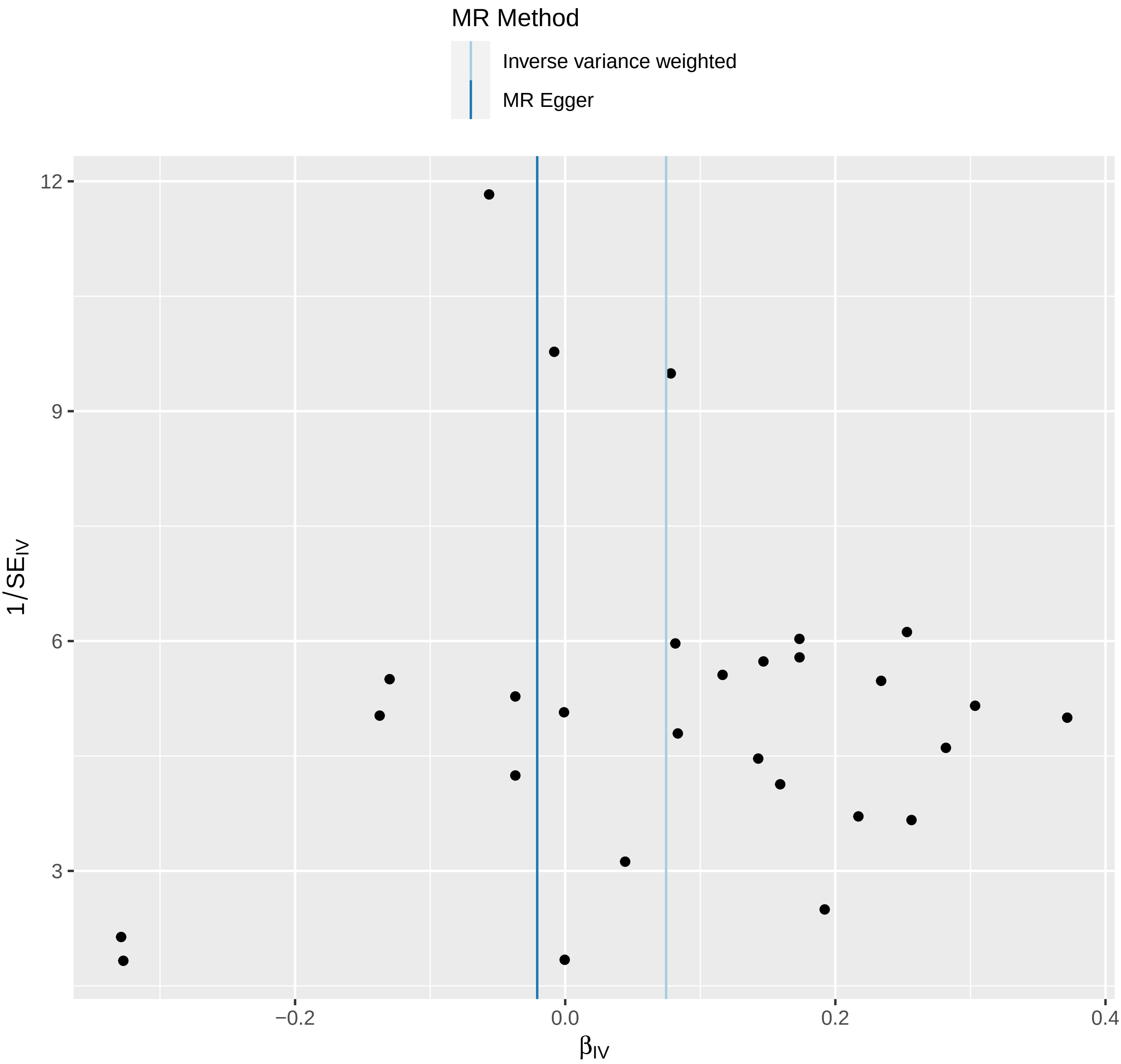

Batch 3−2964 : Transforming growth factor beta−2 on Diabetic nephropathy (more control exclusions)
with a threshold of 1e−05

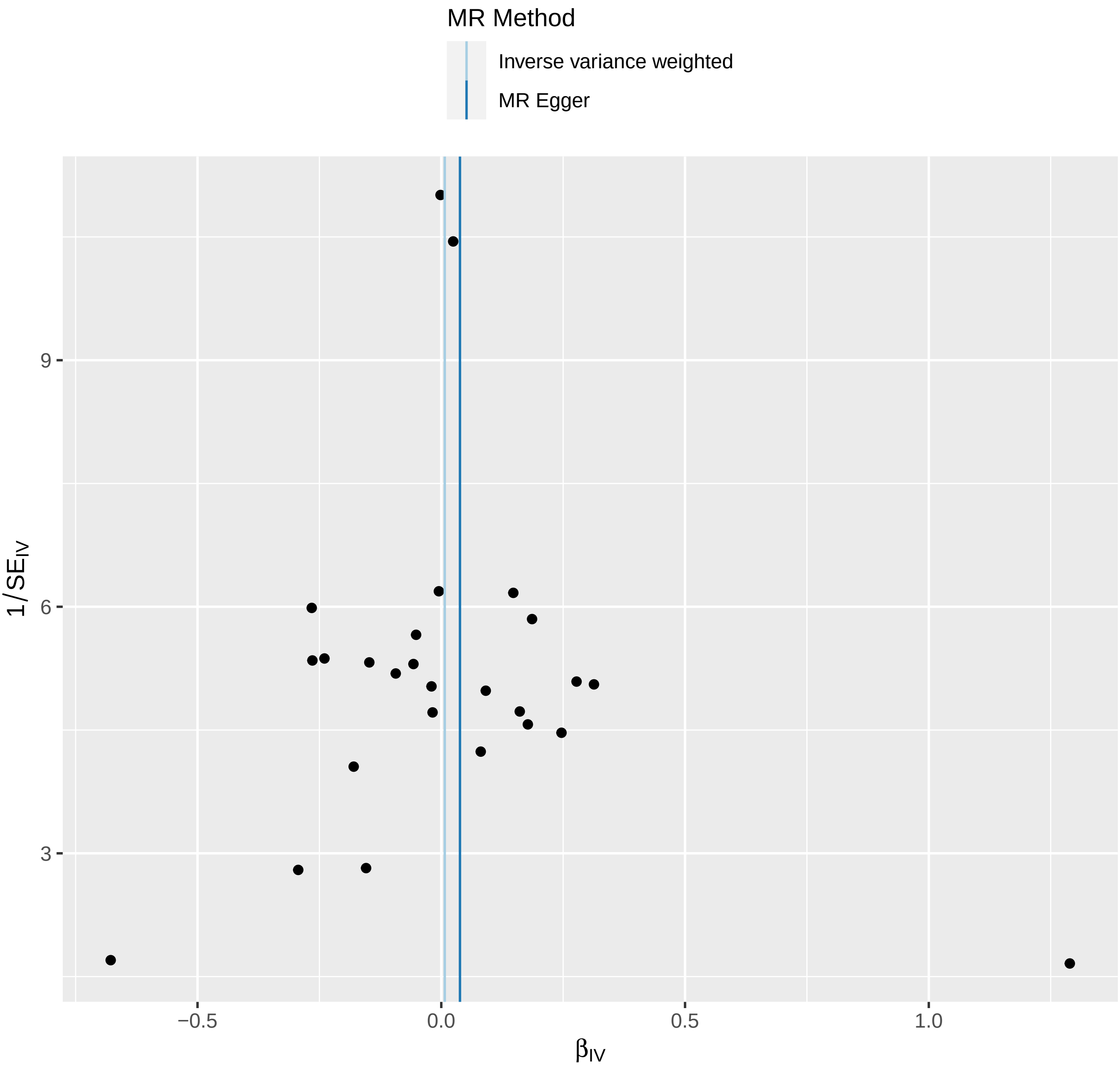

Batch 3−2965 : Transforming growth factor beta−3 on Diabetic nephropathy (more control exclusions)
with a threshold of 1e−05

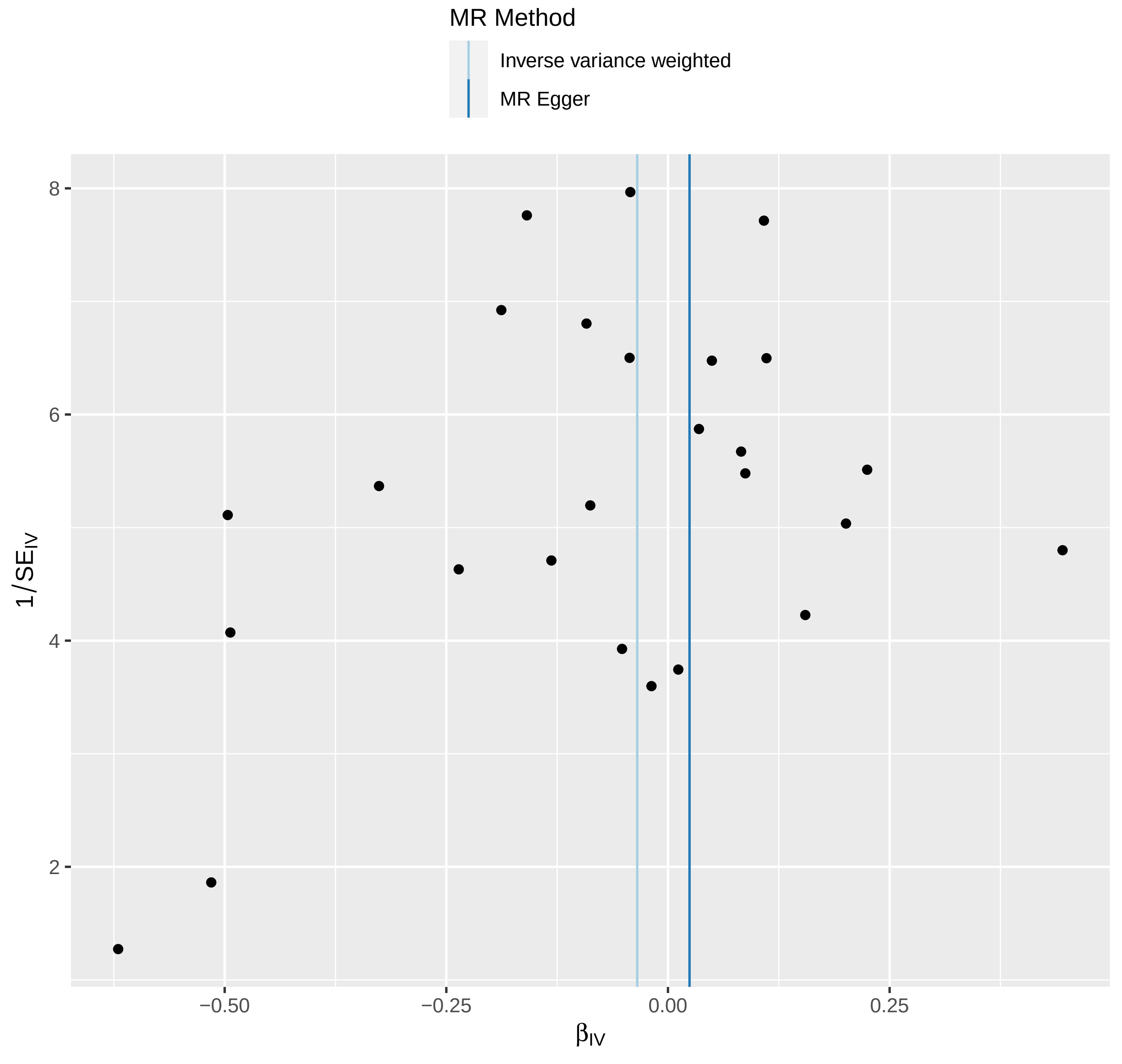

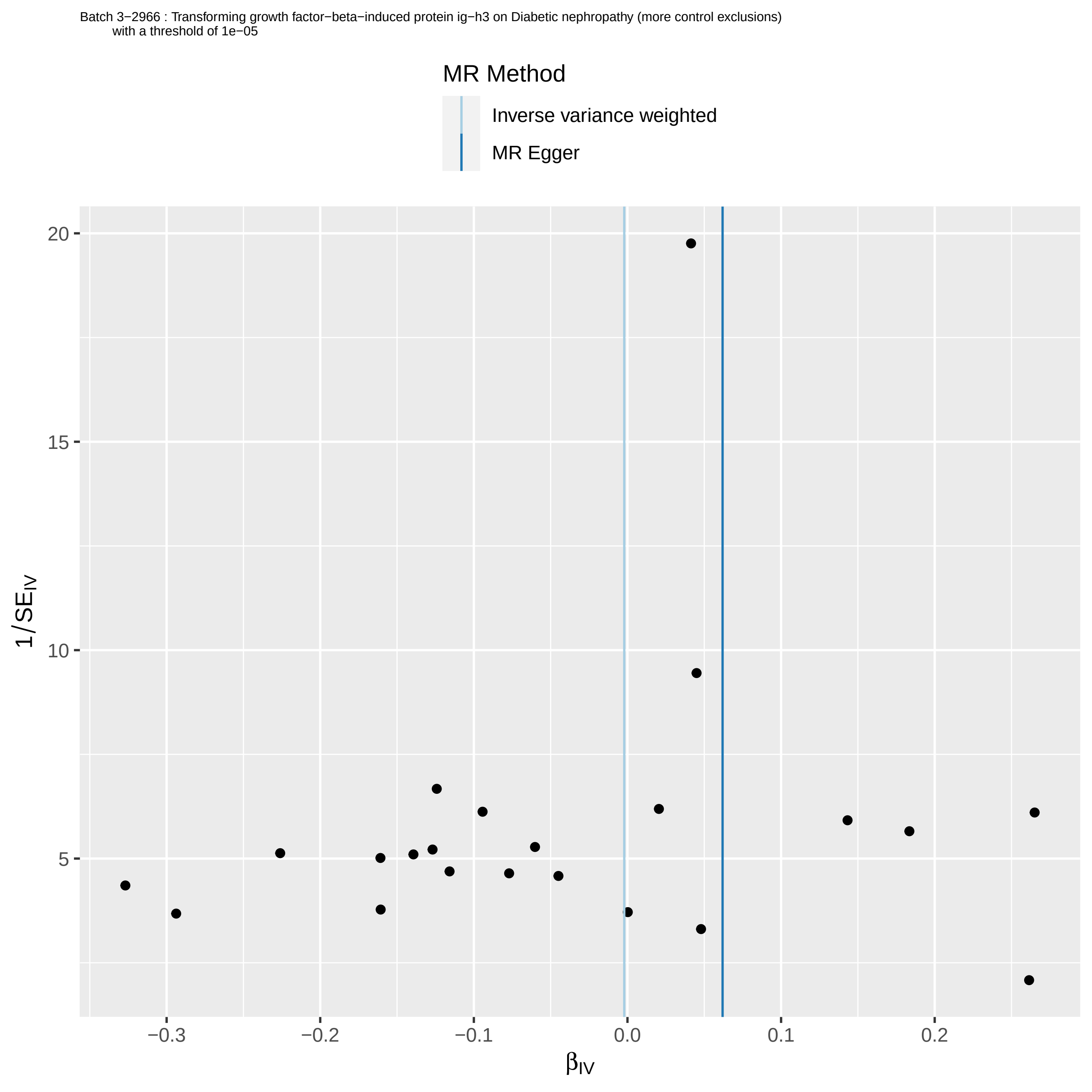

Batch 3−2966 : Transforming growth factor−beta−induced protein ig−h3 on Diabetic nephropathy (more control exclusions)
with a threshold of 1e−05
MR Method
Inverse variance weighted
MR Egger
20
15
10
5
1/SE_IV
−0.3
−0.2
−0.1
0.0
0.1
0.2
β_IV

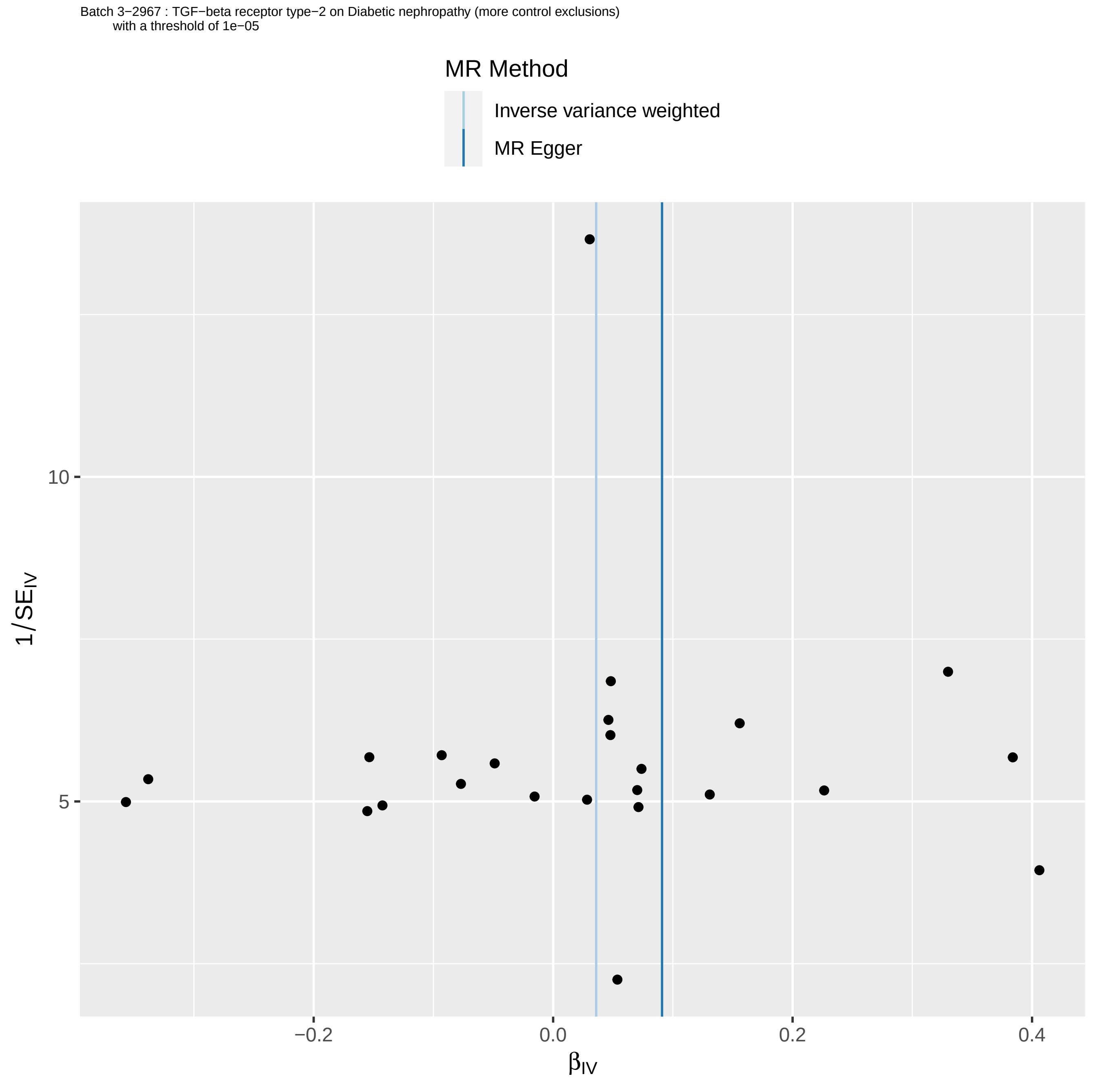

Batch 3−2967 : TGF−beta receptor type−2 on Diabetic nephropathy (more control exclusions)
with a threshold of 1e−05
MR Method
Inverse variance weighted
MR Egger
10
5
1/SE_IV
−0.2
0.0
0.2
0.4
β_IV

Batch 3−2968 : Transforming growth factor beta receptor type 3 on Diabetic nephropathy (more control exclusions)
with a threshold of 1e−05

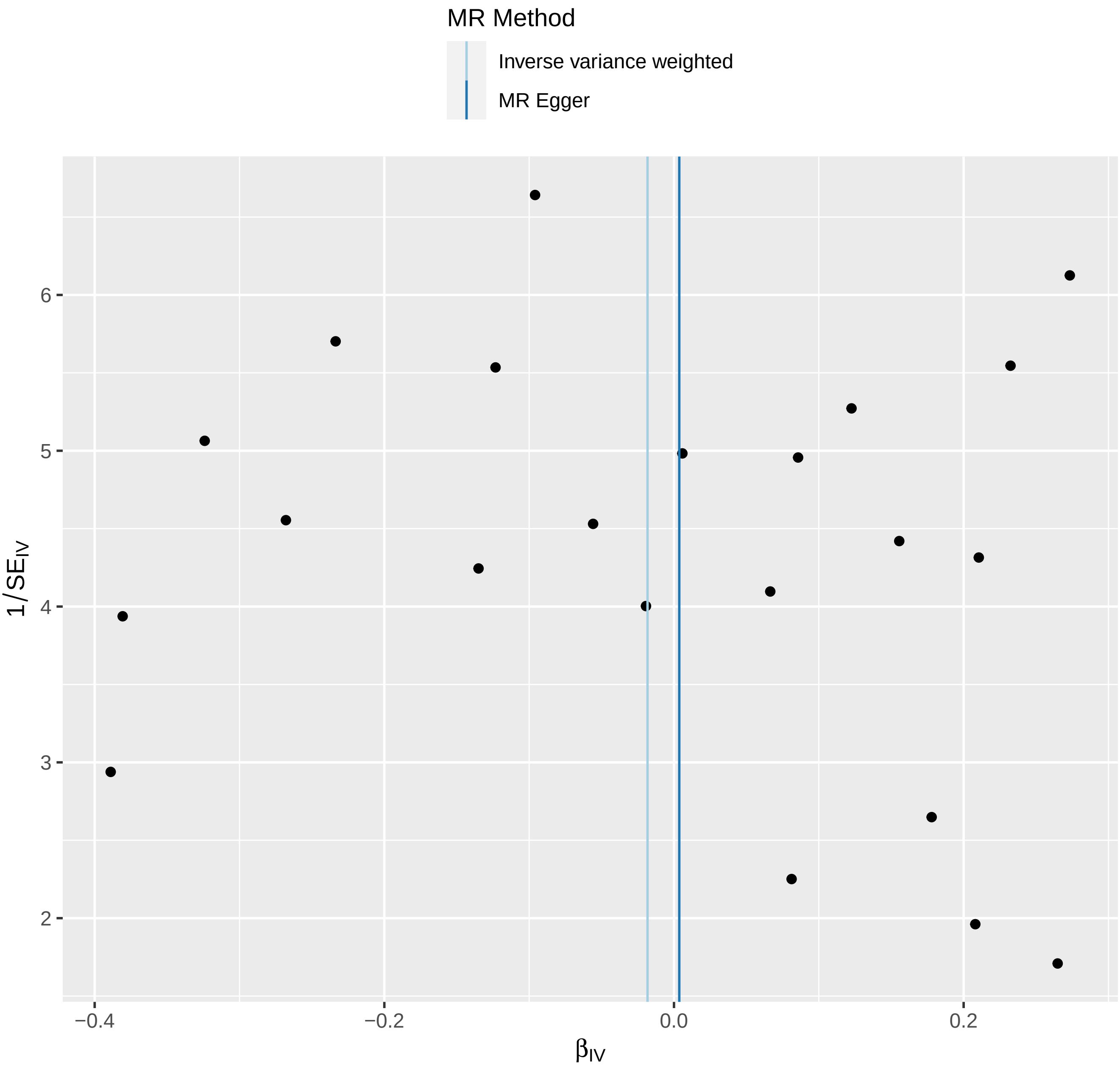

Batch 3−2969 : Homeobox protein TGIF2 on Diabetic nephropathy (more control exclusions)
with a threshold of 1e−05

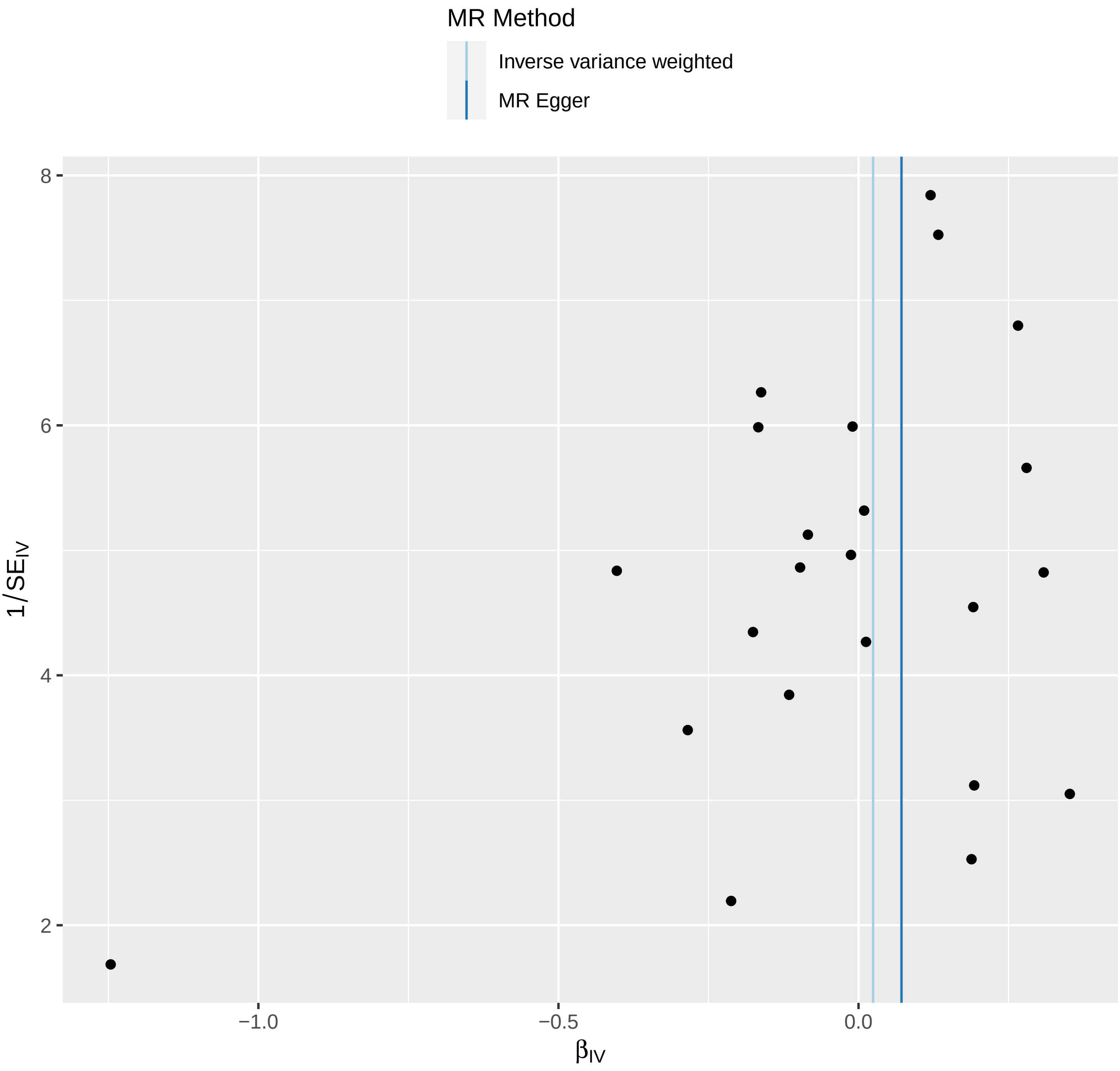

Batch 3−2970 : Thrombospondin−2 on Diabetic nephropathy (more control exclusions)
with a threshold of 1e−05

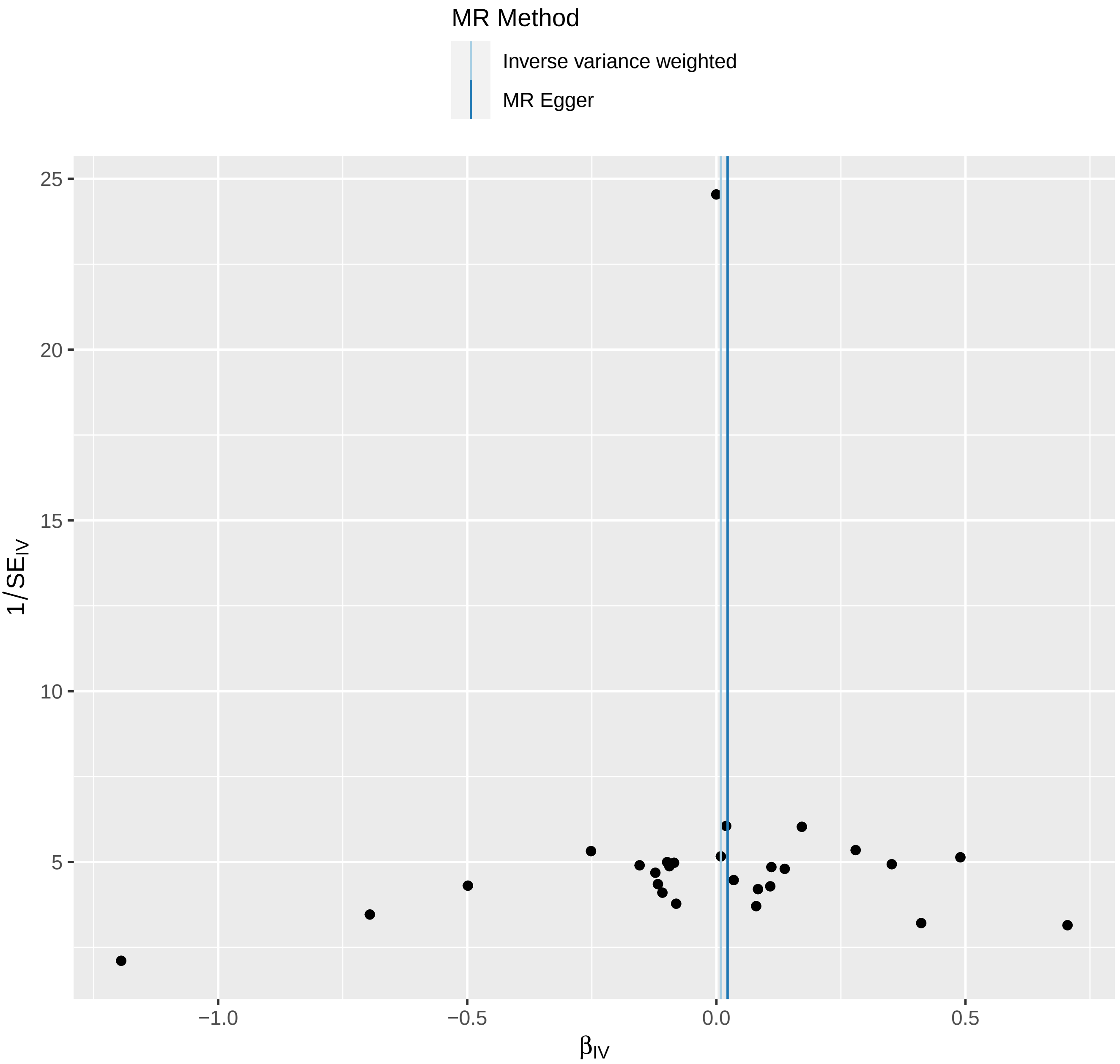

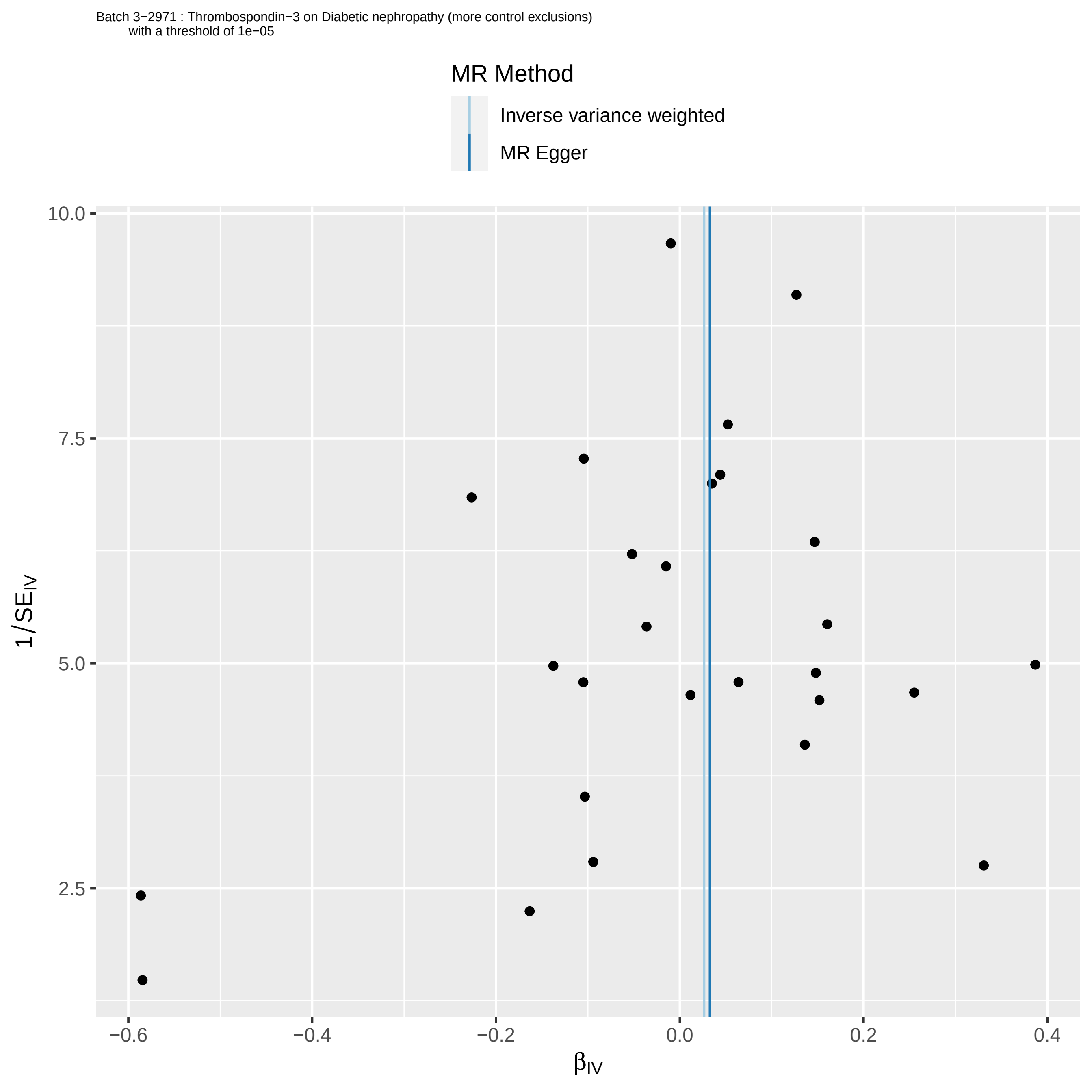

Batch 3−2971 : Thrombospondin−3 on Diabetic nephropathy (more control exclusions)
with a threshold of 1e−05
MR Method
Inverse variance weighted
MR Egger
10.0
7.5
5.0
2.5
1/SE_IV
−0.6
−0.4
−0.2
0.0
0.2
0.4
β_IV

Batch 3−2972 : Thrombopoietin on Diabetic nephropathy (more control exclusions)
with a threshold of 1e−05

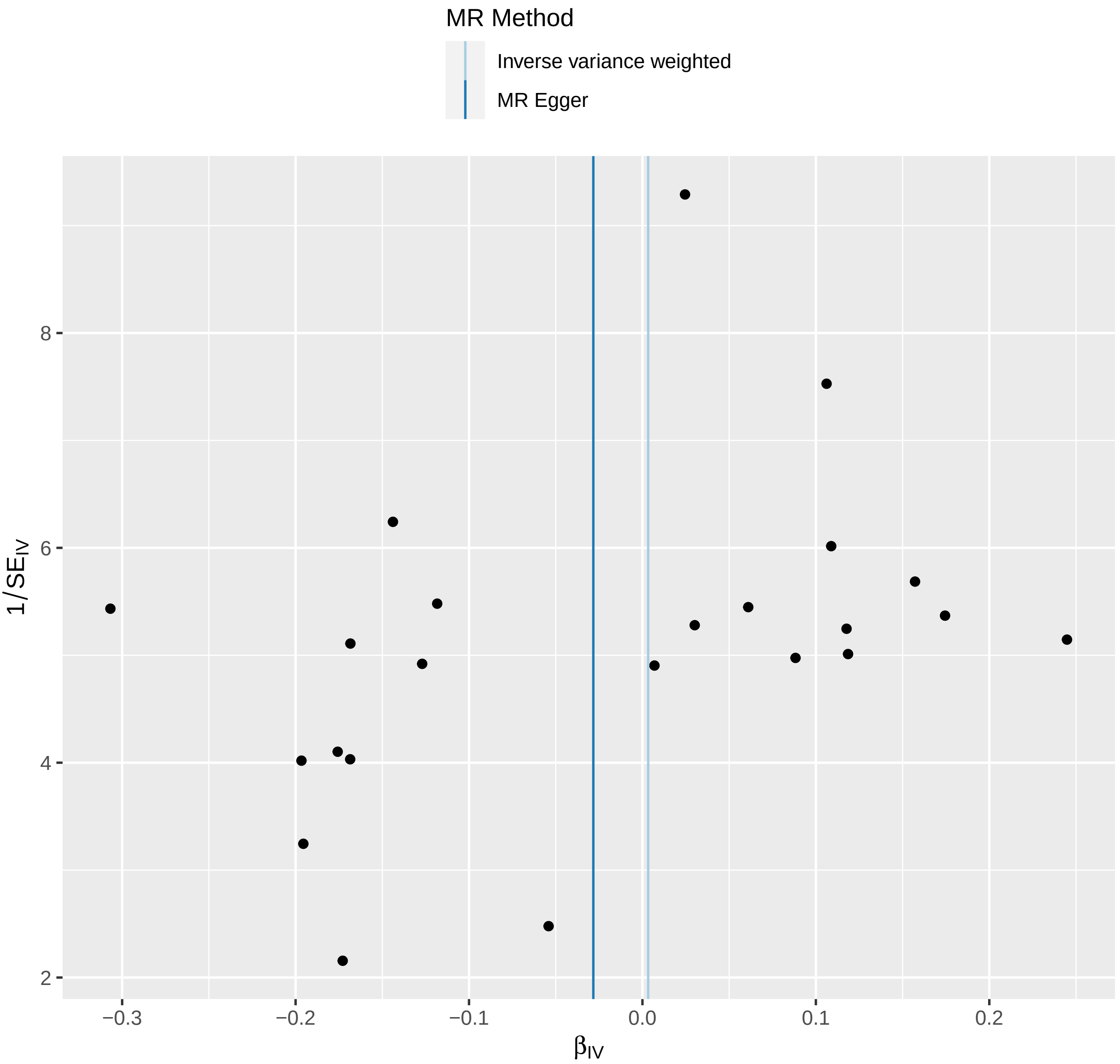

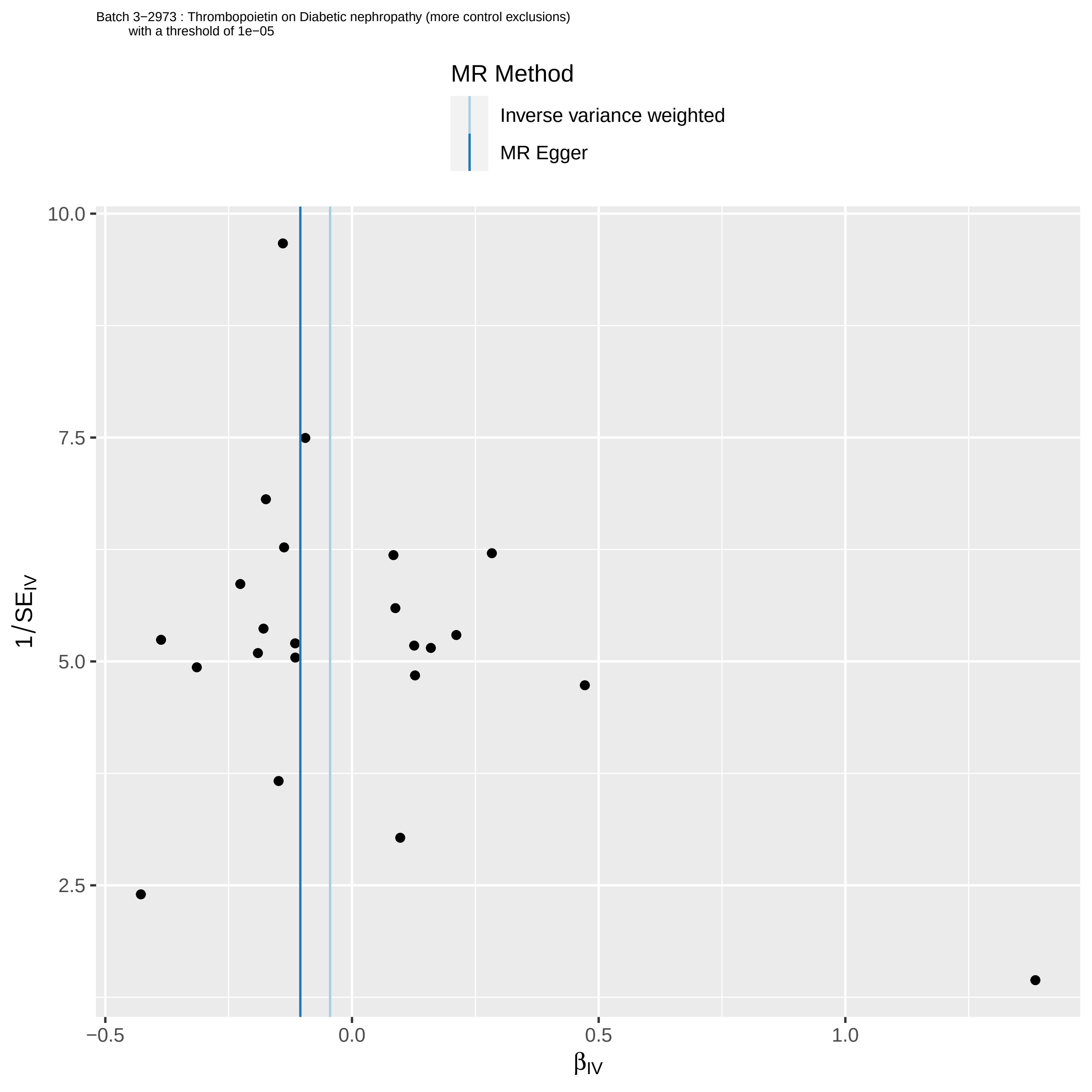

Batch 3−2973 : Thrombopoietin on Diabetic nephropathy (more control exclusions)
with a threshold of 1e−05
MR Method
Inverse variance weighted
MR Egger
10.0
7.5
5.0
2.5
1/SE_IV
−0.5
0.0
0.5
1.0
β_IV

Batch 3−2974 : Thyroid hormone receptor alpha on Diabetic nephropathy (more control exclusions)
with a threshold of 1e−05

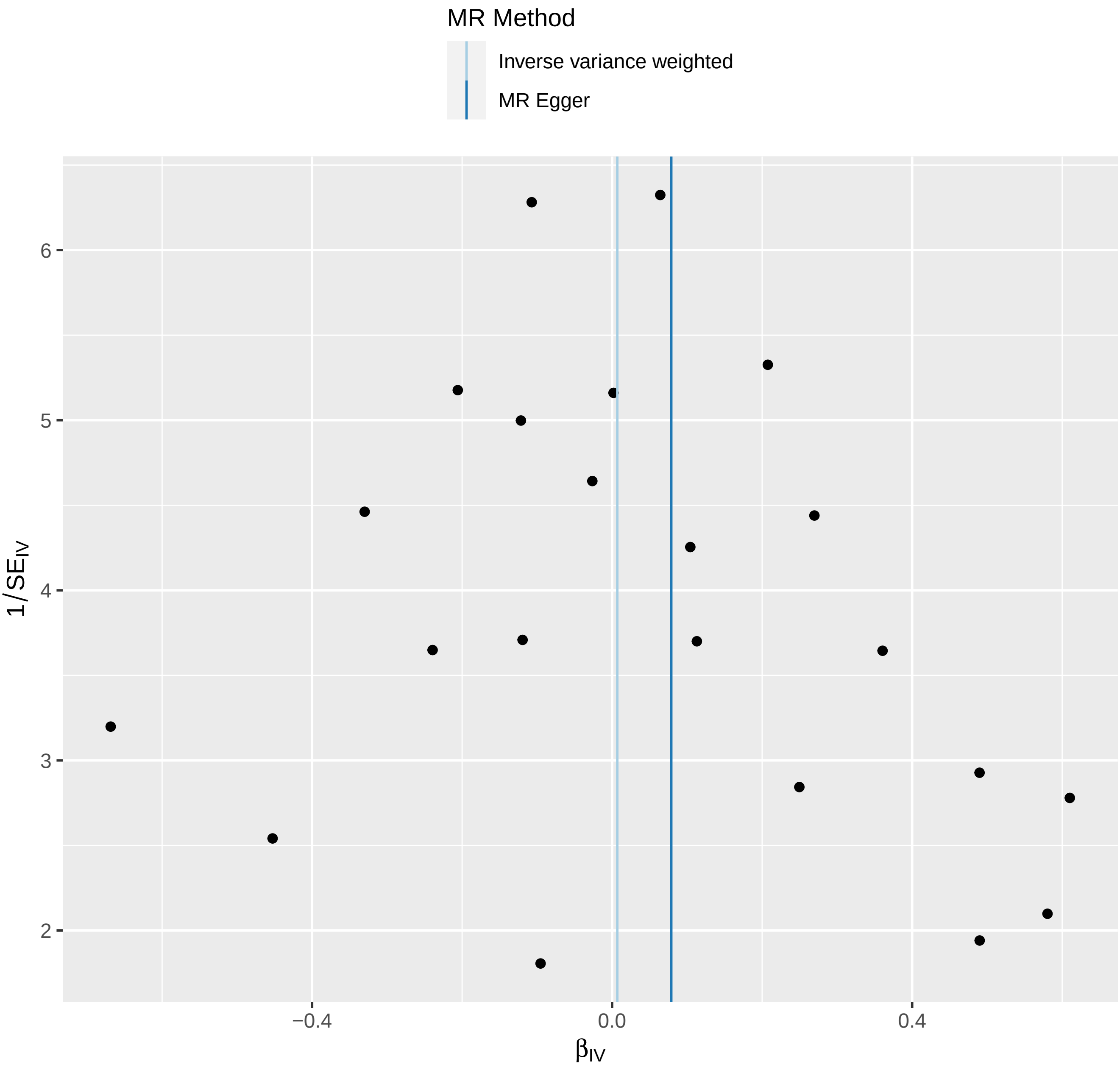

Batch 3−2975 : Thrombospondin type−1 domain−containing protein 1 on Diabetic nephropathy (more control exclusions)
with a threshold of 1e−05

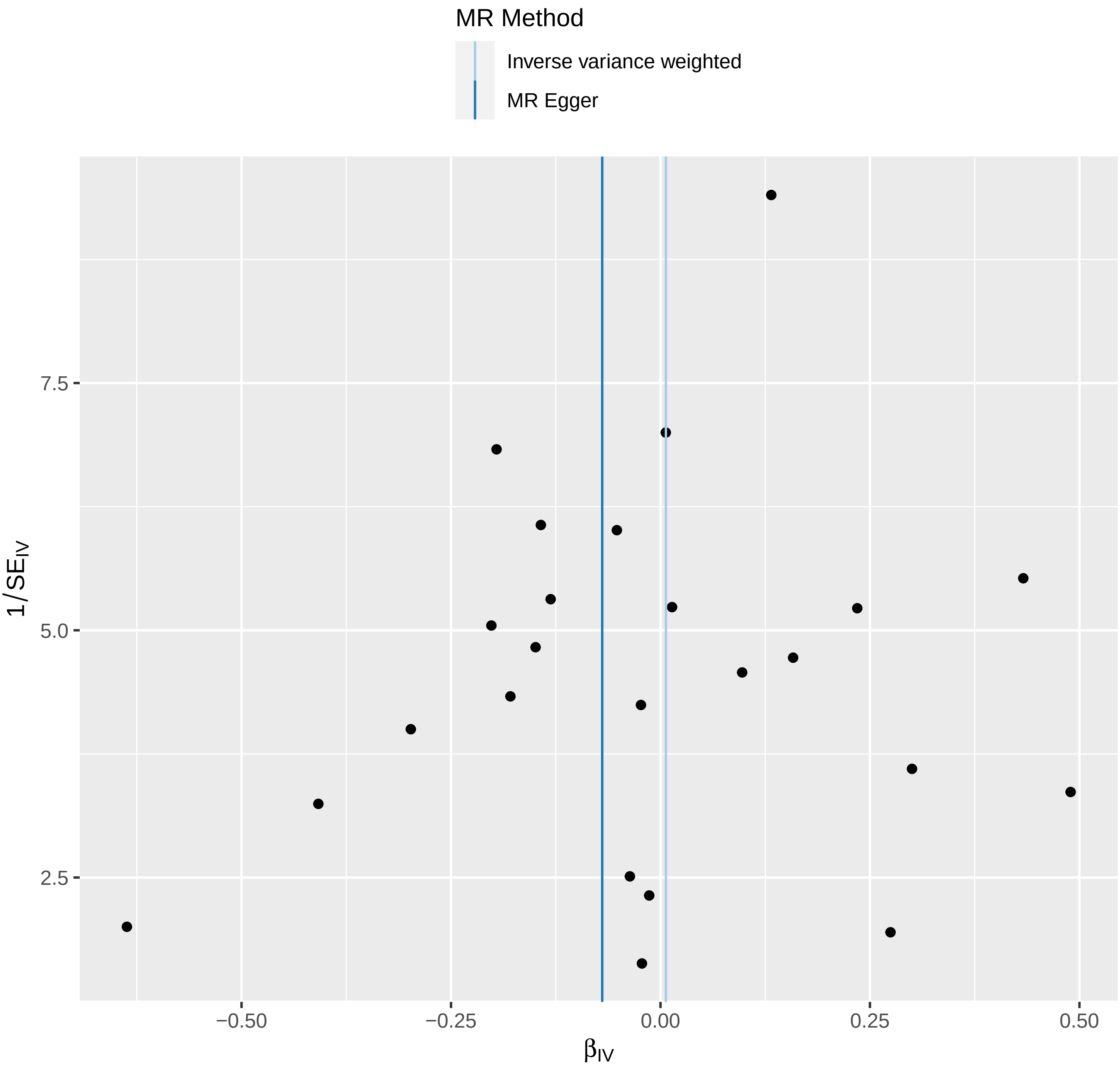

Batch 3−2976 : Thrombospondin type−1 domain−containing protein 7A on Diabetic nephropathy (more control exclusions)
with a threshold of 1e−05

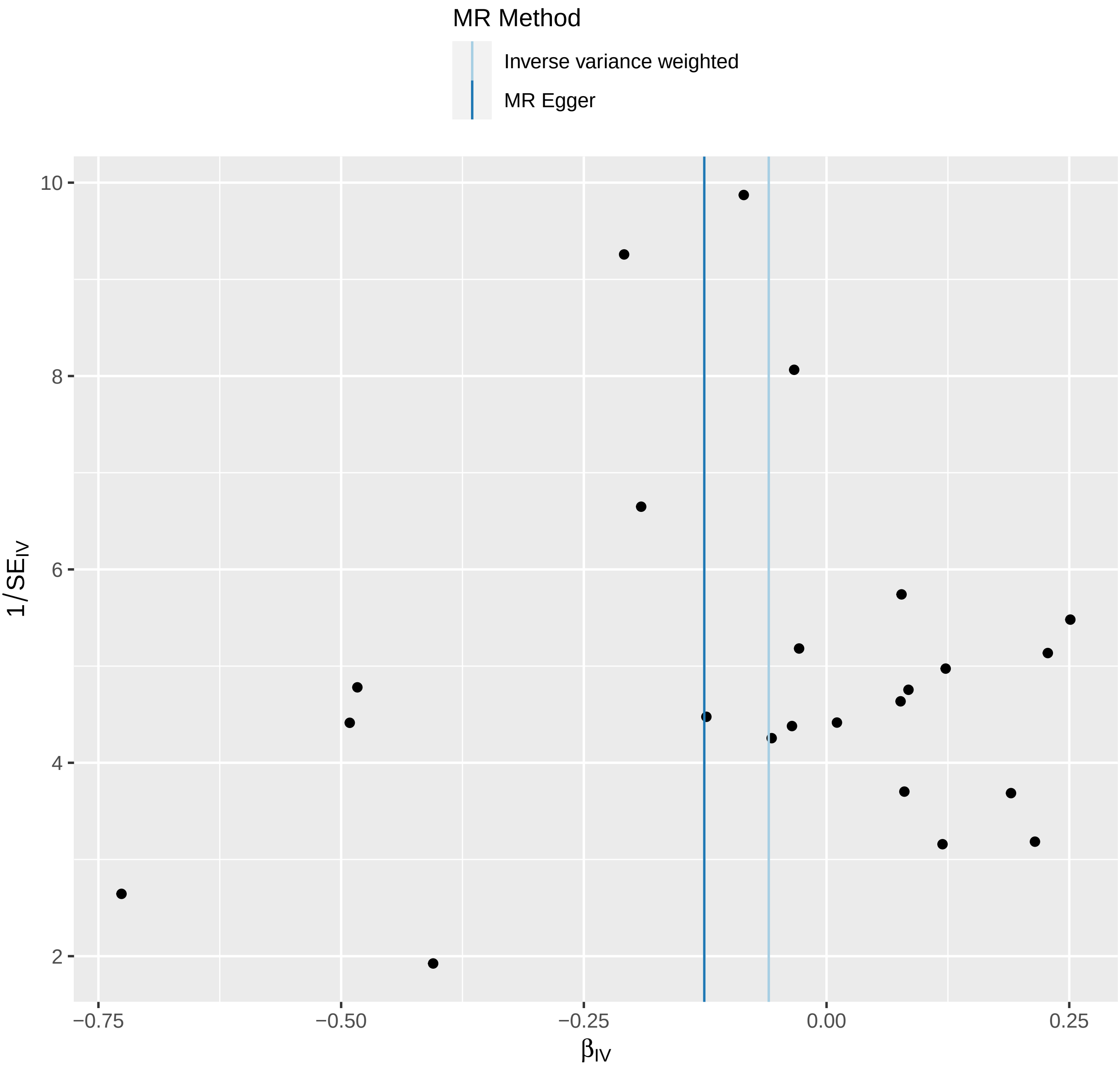

Batch 3−2977 : Thymocyte nuclear protein 1 on Diabetic nephropathy (more control exclusions)
with a threshold of 1e−05

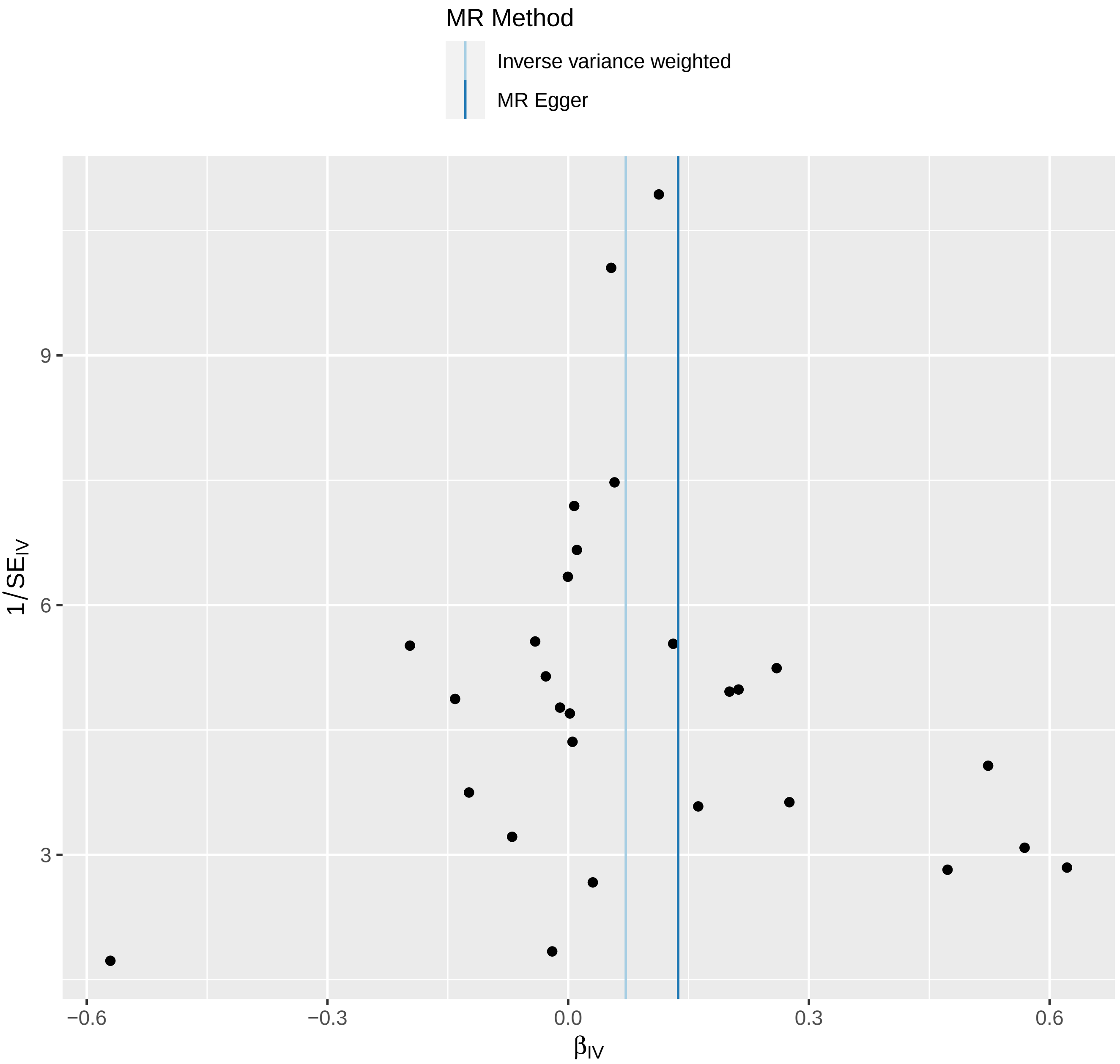

Batch 3−2978 : Tyrosine−protein kinase receptor Tie−1, soluble on Diabetic nephropathy (more control exclusions) with a threshold of 1e−05

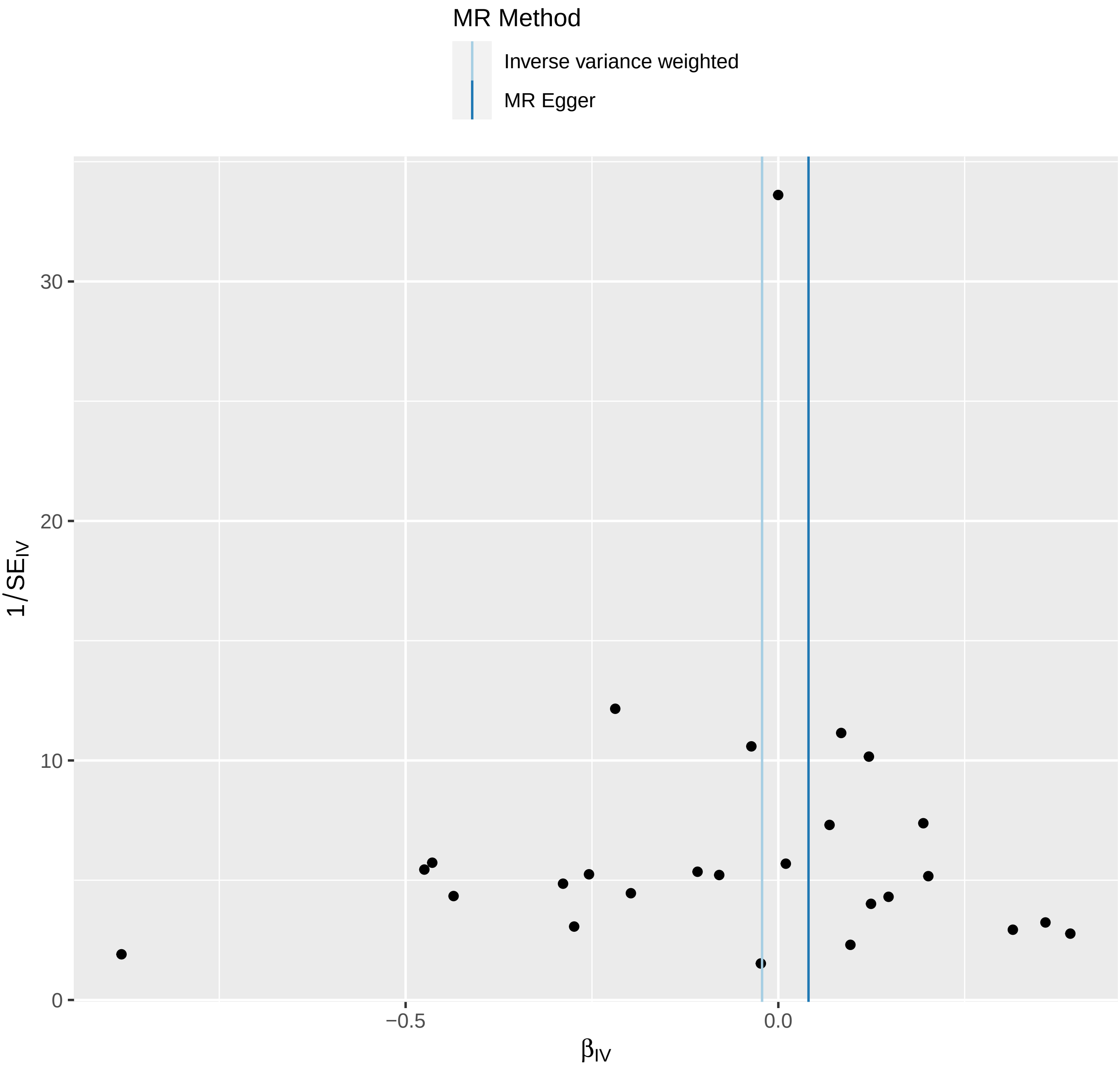

Batch 3−2979 : Fructose−2,6−bisphosphatase TIGAR on Diabetic nephropathy (more control exclusions)
with a threshold of 1e−05

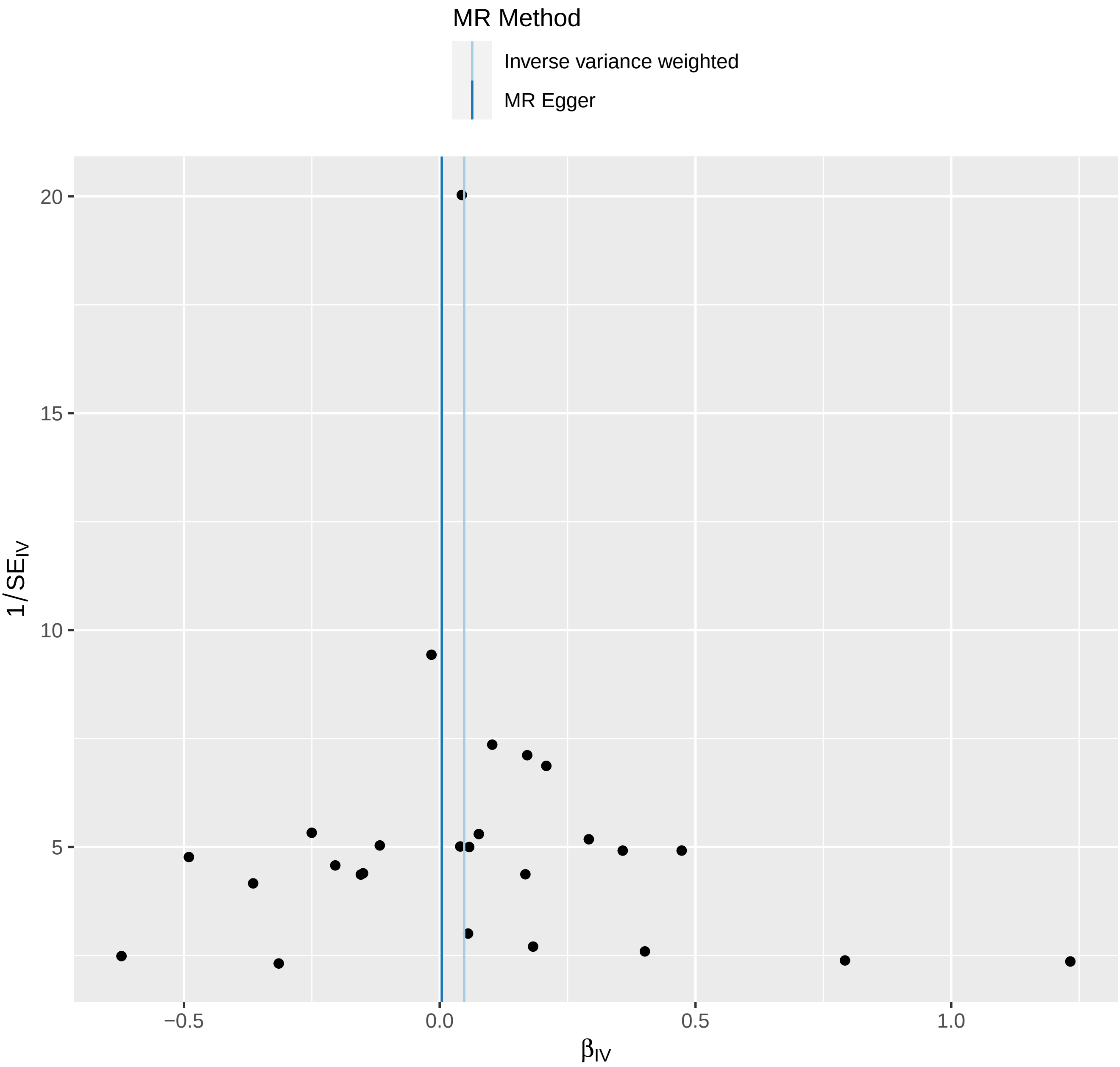

Batch 3−2980 : Metalloproteinase inhibitor 4 on Diabetic nephropathy (more control exclusions)
with a threshold of 1e−05

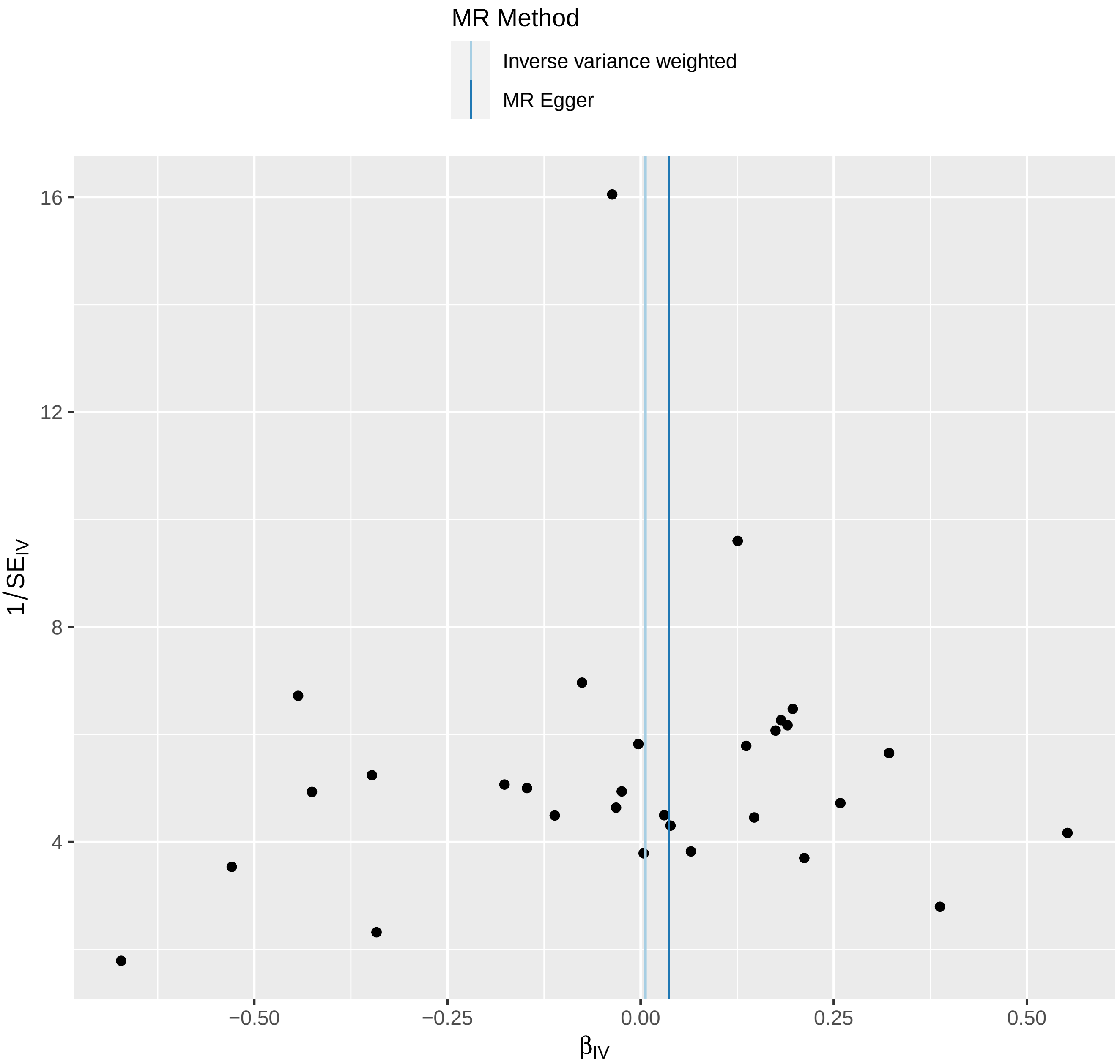

Batch 3−2981 : Tubulointerstitial nephritis antigen−like on Diabetic nephropathy (more control exclusions)
with a threshold of 1e−05

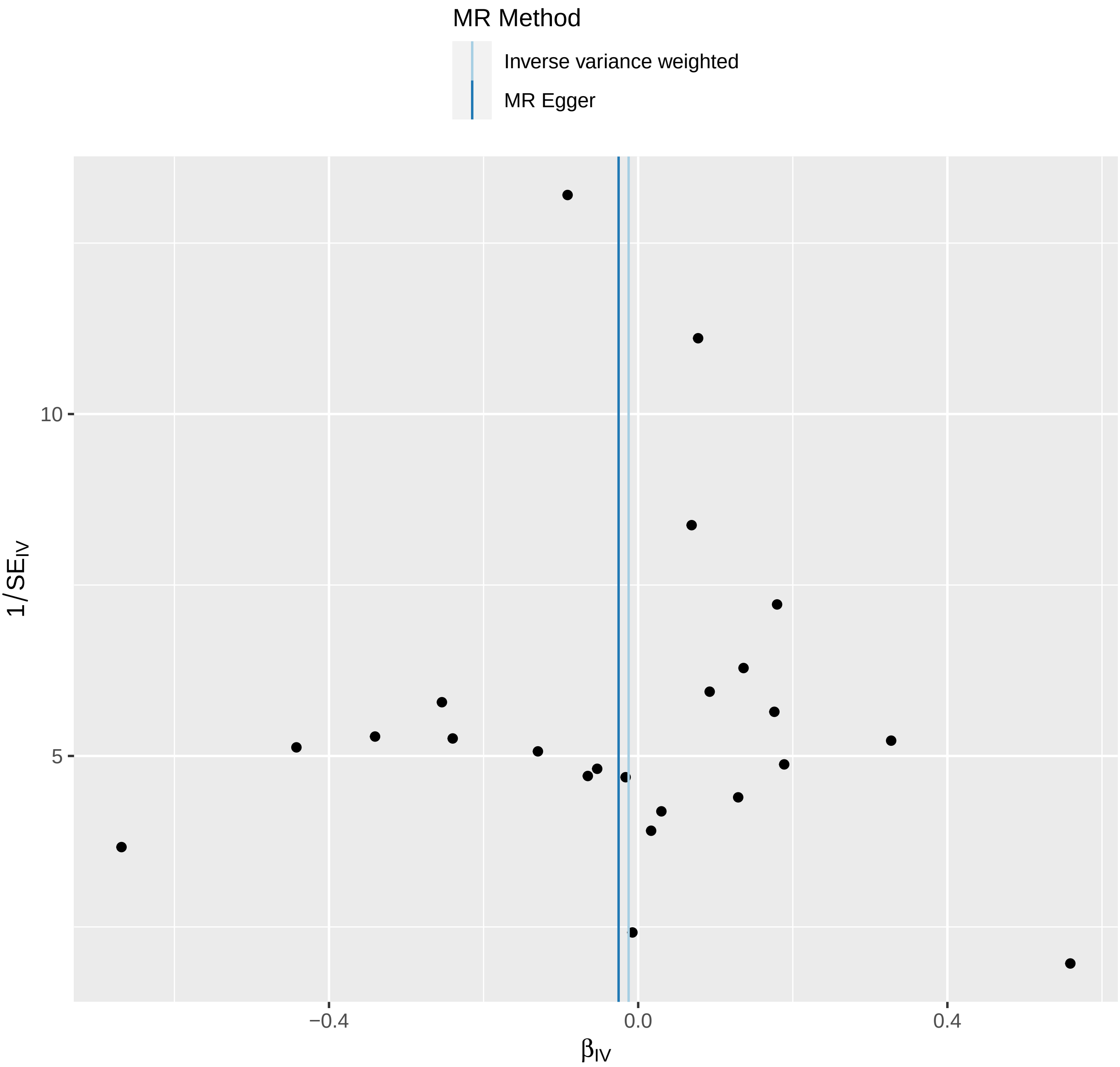

Batch 3−2982 : Toll/interleukin−1 receptor domain−containing adapter protein on Diabetic nephropathy (more control exclusions) with a threshold of 1e−05

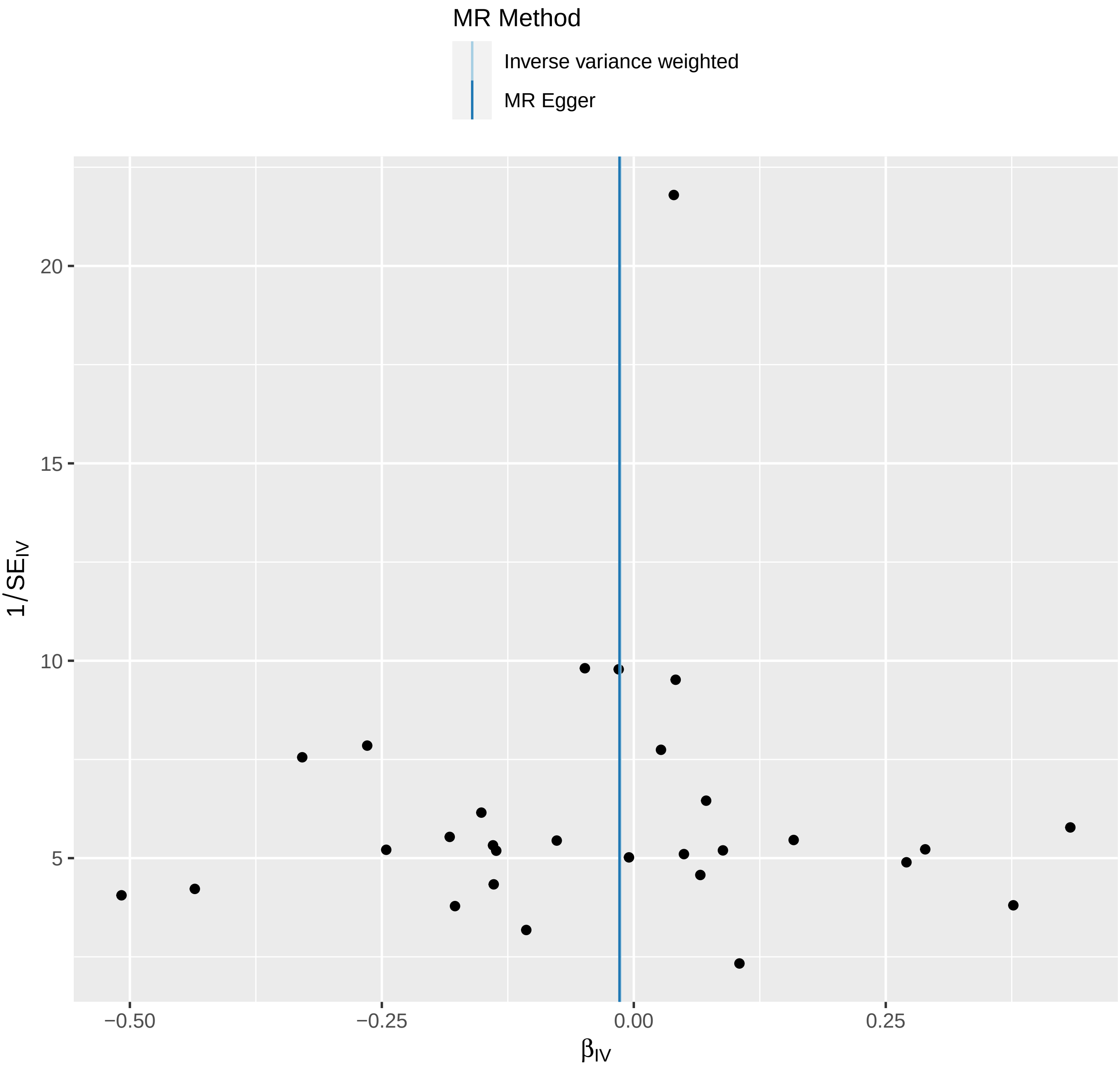

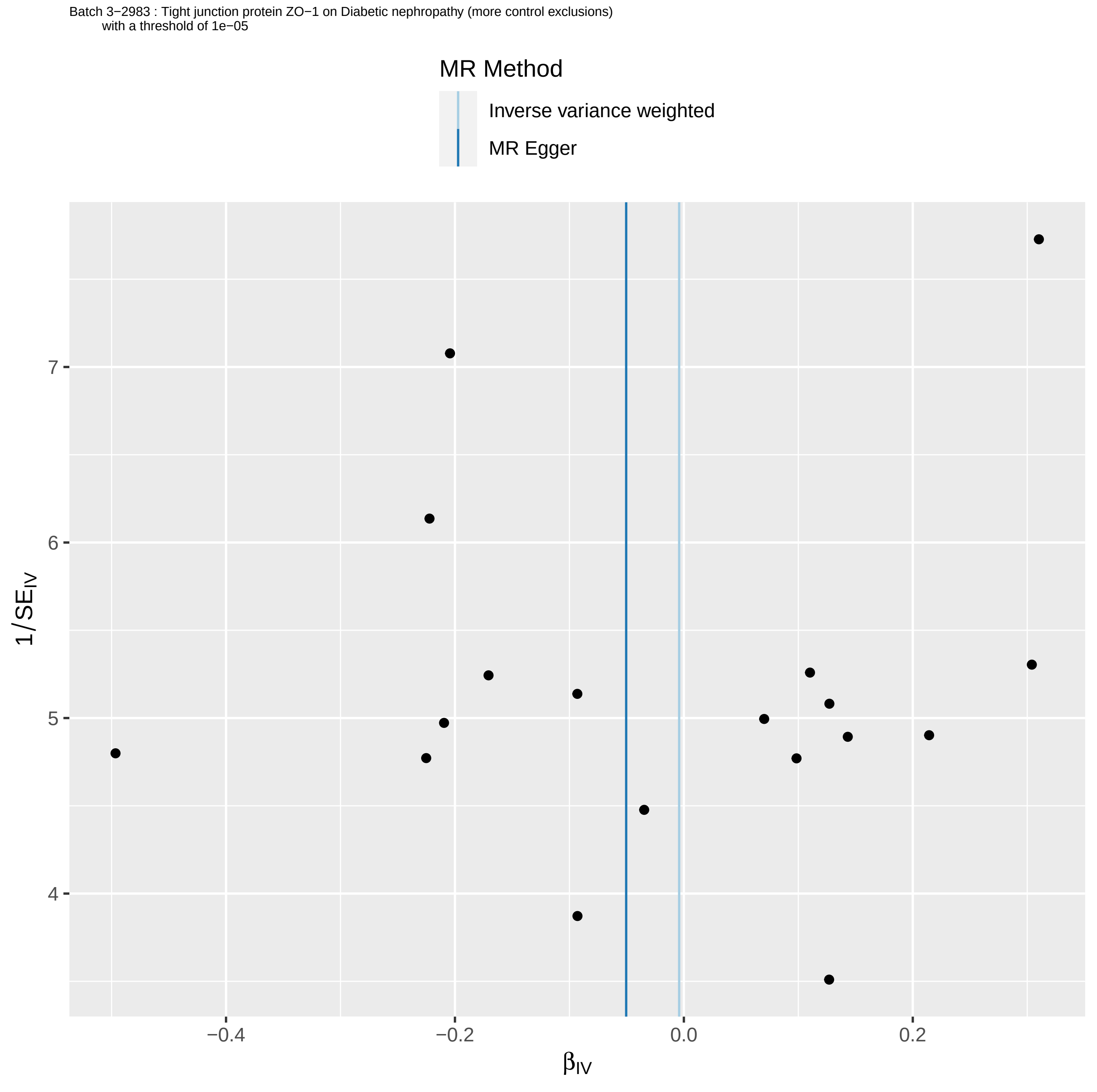
Batch 3−2983 : Tight junction protein ZO−1 on Diabetic nephropathy (more control exclusions)
with a threshold of 1e−05
MR Method
Inverse variance weighted
MR Egger
7
6
5
4
1/SE_IV
−0.4
−0.2
0.0
0.2
β_IV

Batch 3−2984 : Thymidine kinase, cytosolic on Diabetic nephropathy (more control exclusions)
with a threshold of 1e−05

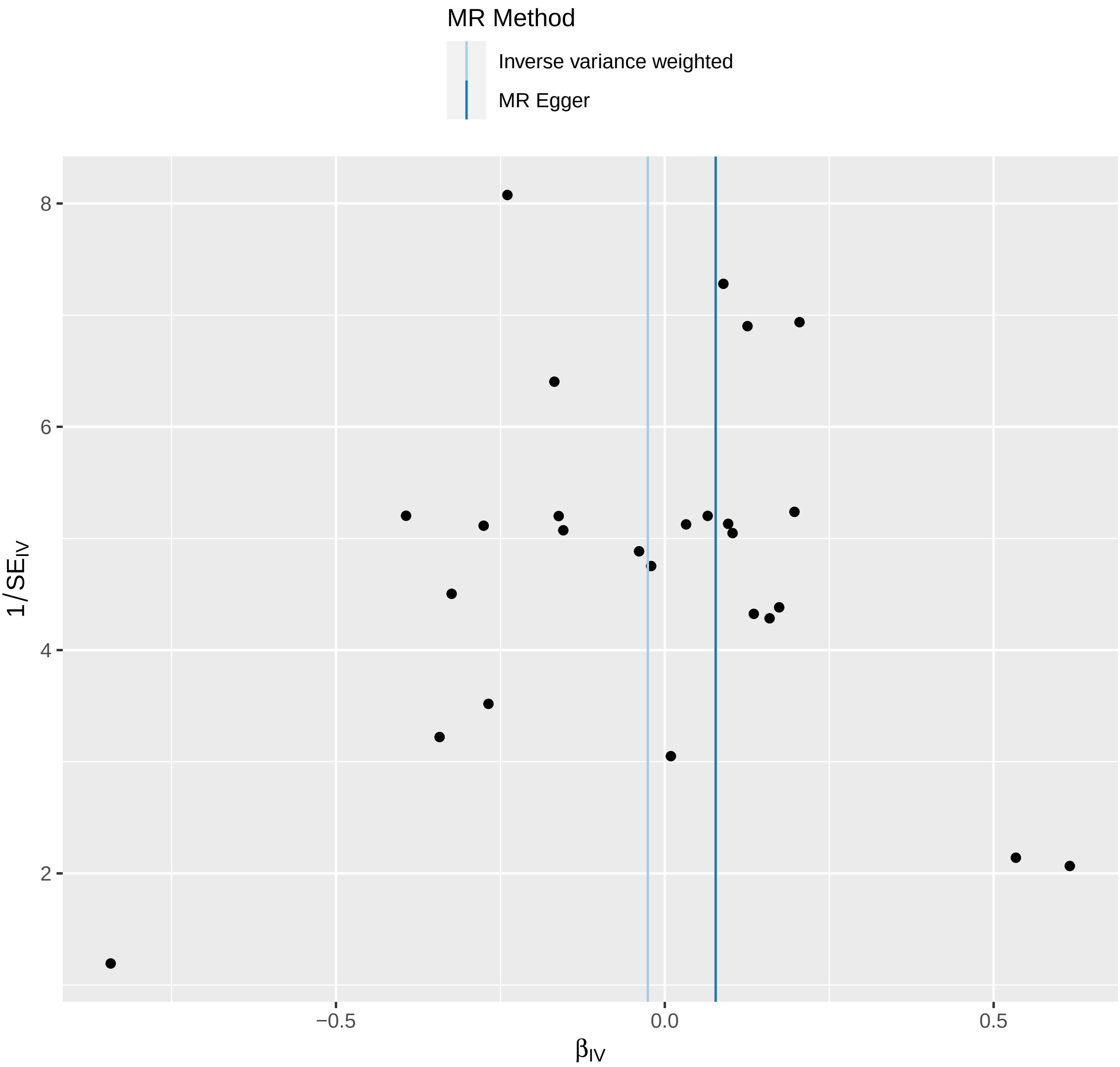

Batch 3−2985 : Transketolase on Diabetic nephropathy (more control exclusions)
with a threshold of 1e−05

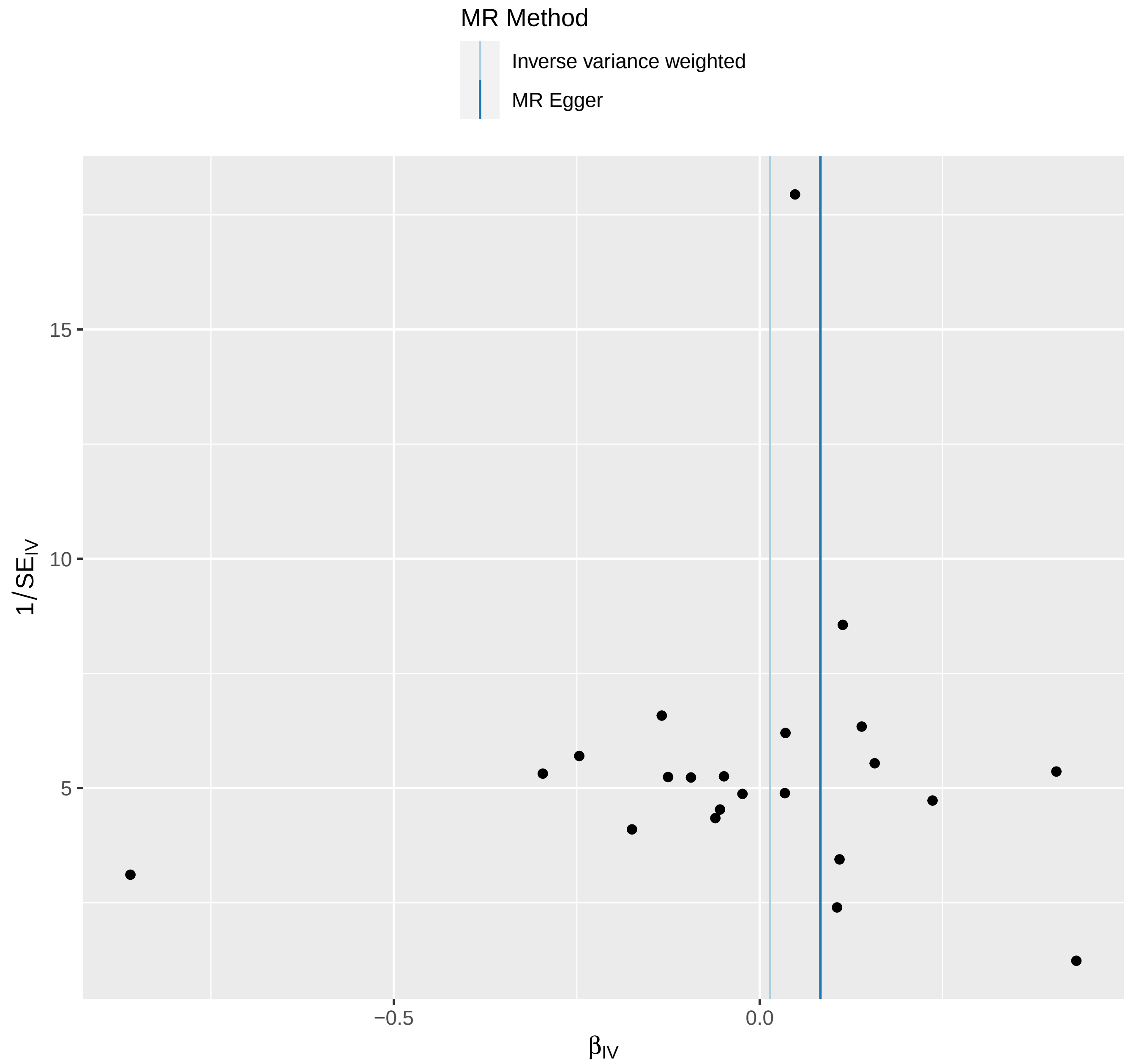

Batch 3−2986 : Tolloid−like protein 1 on Diabetic nephropathy (more control exclusions)
with a threshold of 1e−05

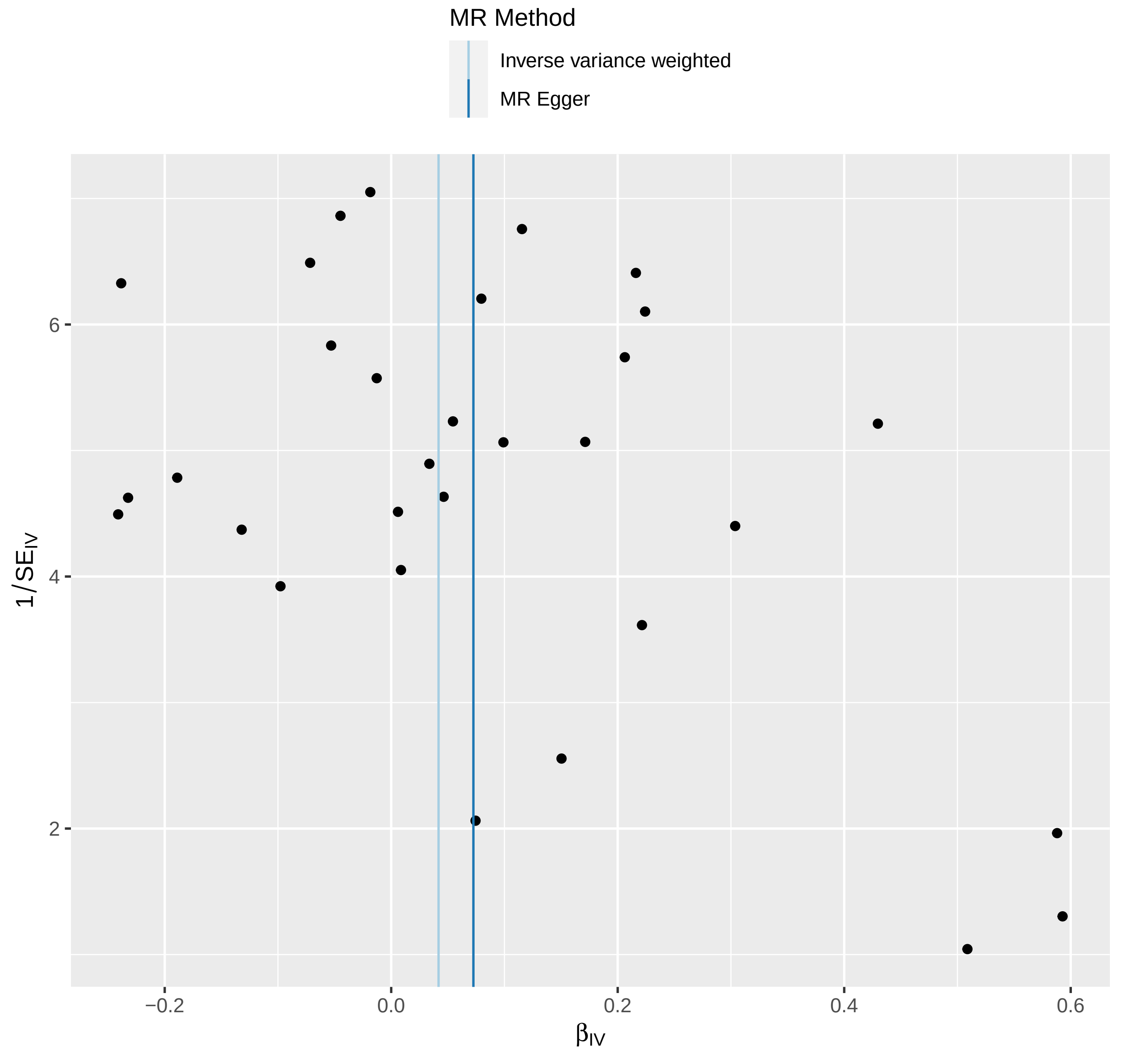

Batch 3−2987 : Talin−2 on Diabetic nephropathy (more control exclusions)
with a threshold of 1e−05

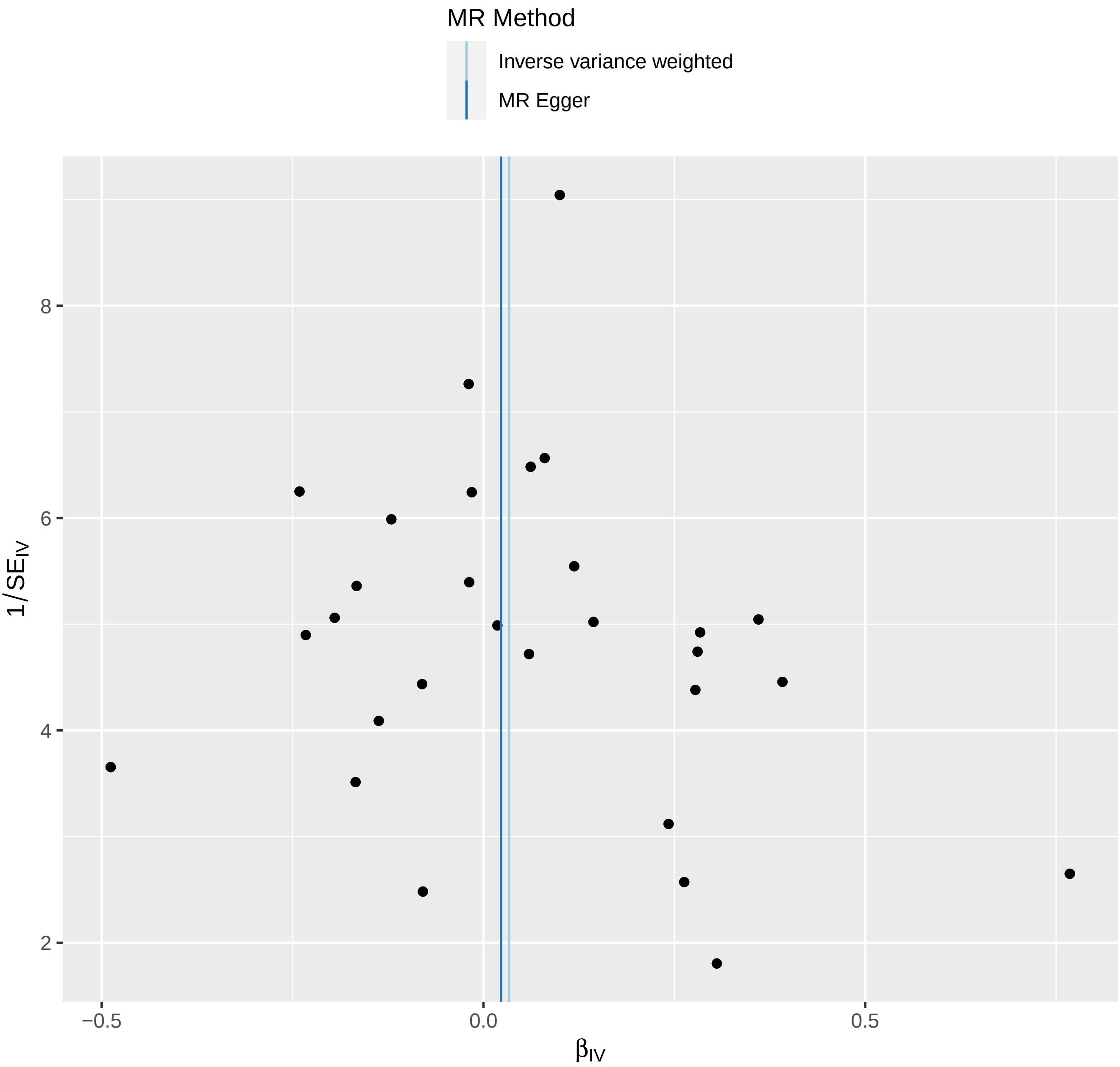

Batch 3−2988 : Toll−like receptor 1 on Diabetic nephropathy (more control exclusions)
with a threshold of 1e−05

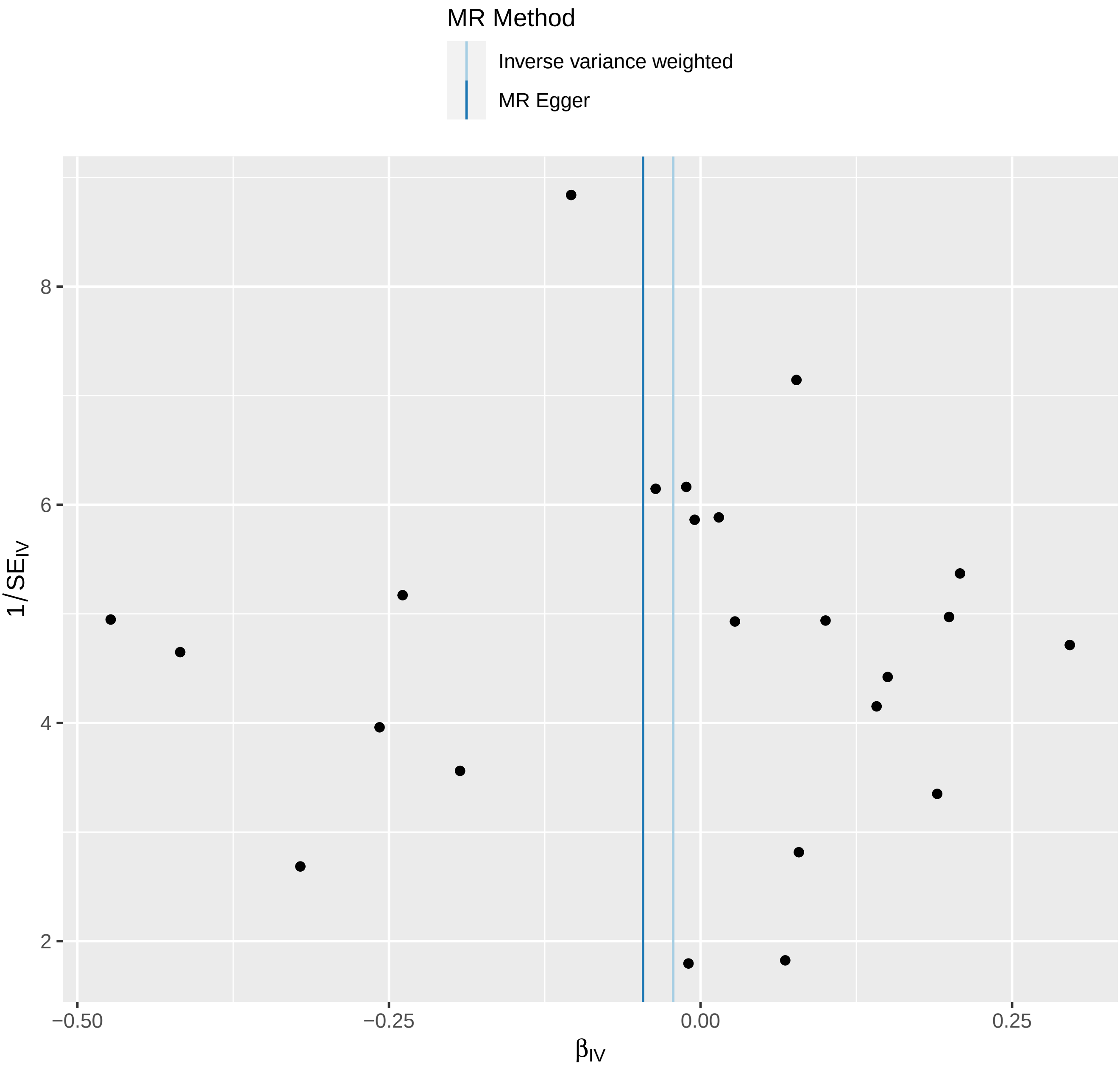

Batch 3−2989 : Toll−like receptor 2 on Diabetic nephropathy (more control exclusions)
with a threshold of 1e−05

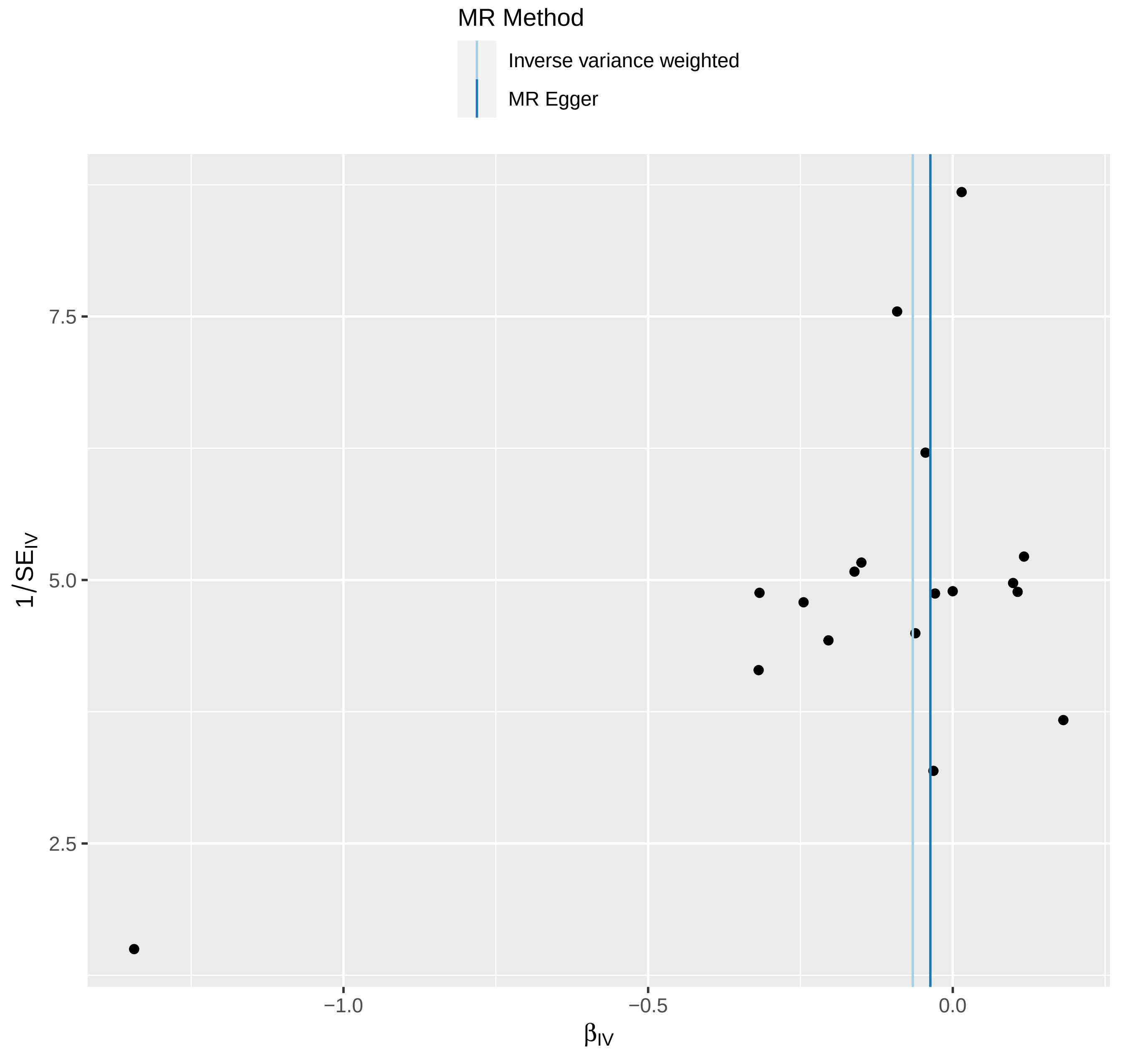

Batch 3−2990 : Toll−like receptor 4 on Diabetic nephropathy (more control exclusions)
with a threshold of 1e−05

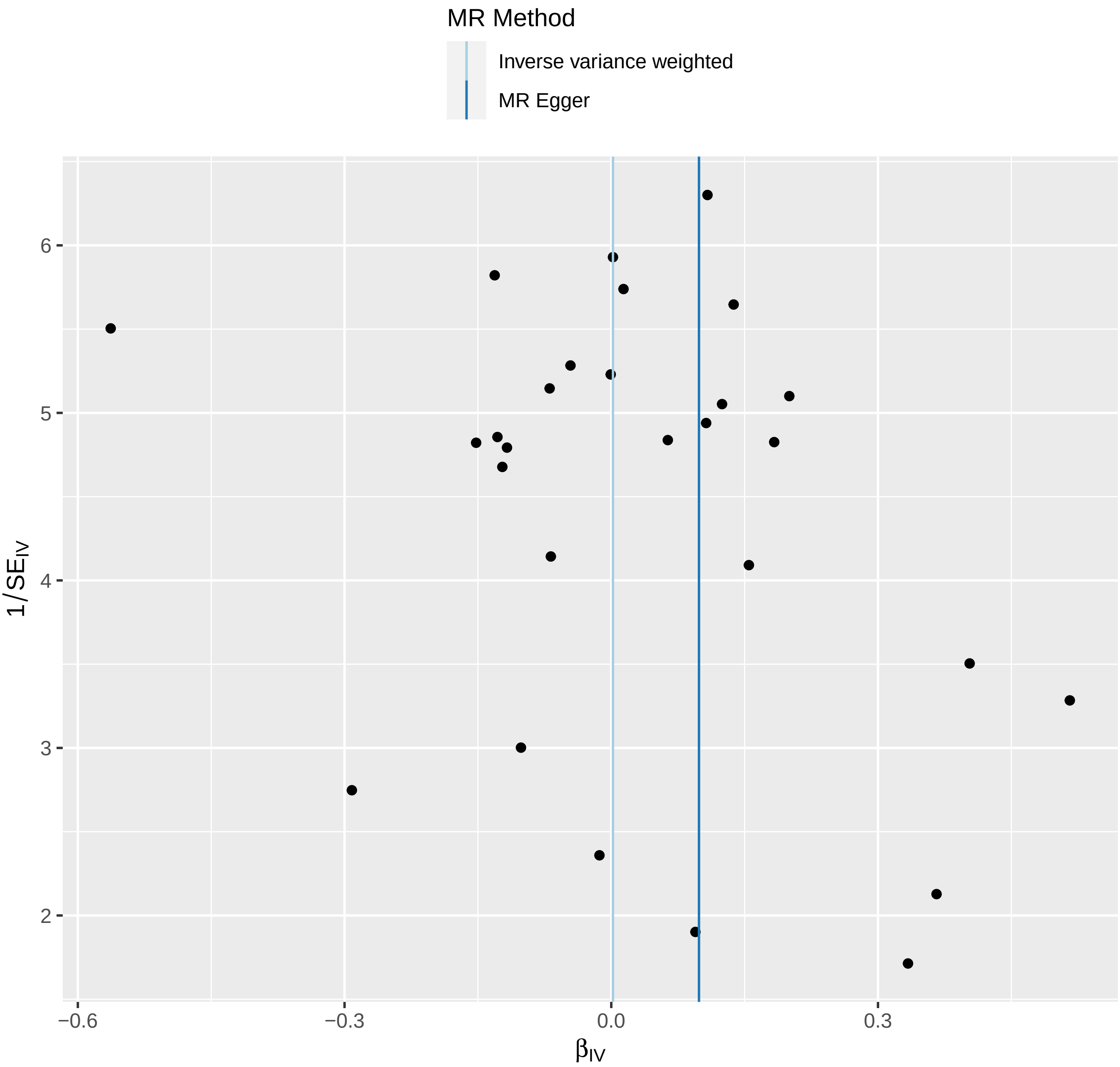

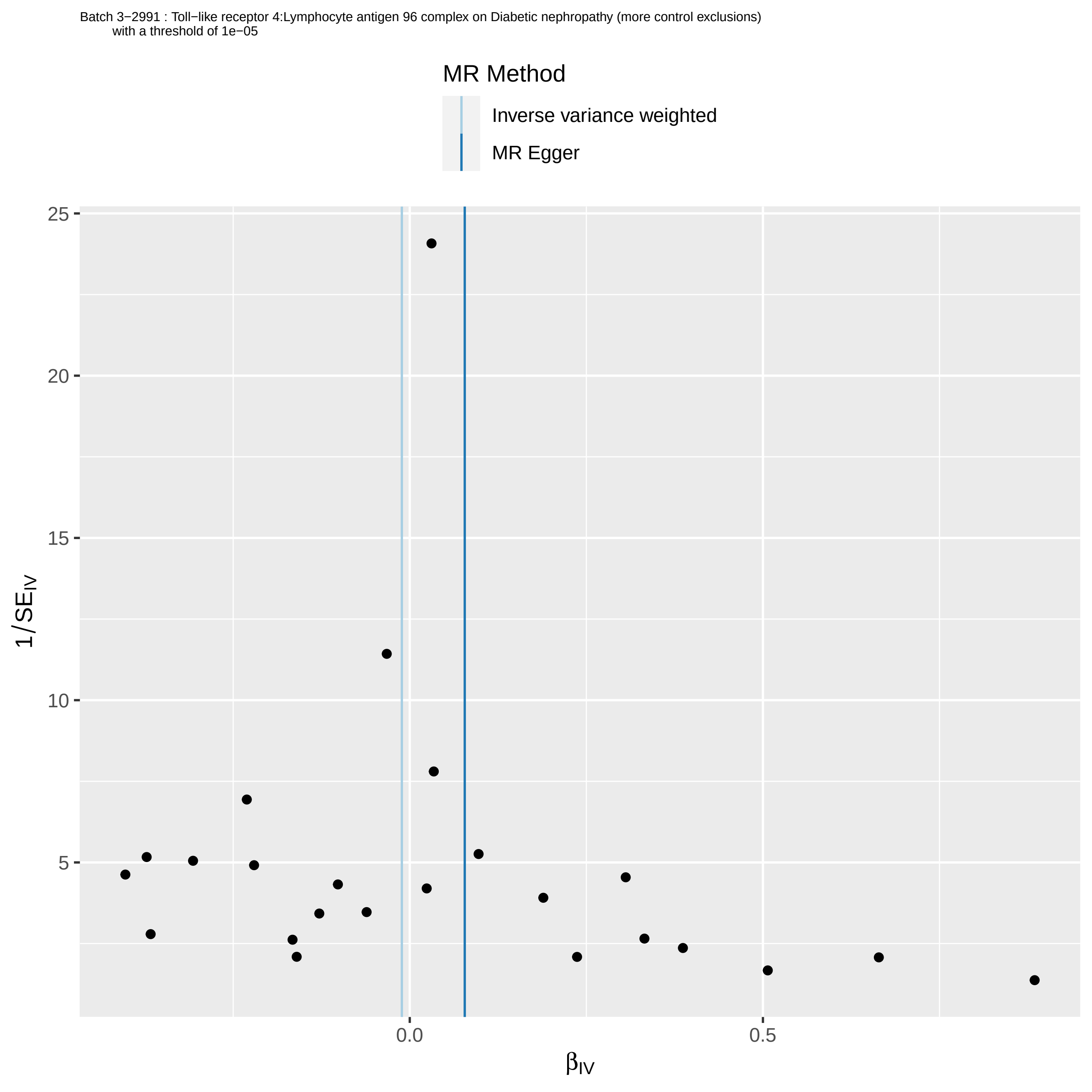

Batch 3−2991 : Toll−like receptor 4:Lymphocyte antigen 96 complex on Diabetic nephropathy (more control exclusions)
with a threshold of 1e−05
MR Method
Inverse variance weighted
MR Egger
25
20
15
10
5
0.0
0.5
1/SE_IV
β_IV

Batch 3−2992 : Transmembrane and coiled−coil domain−containing protein 5A on Diabetic nephropathy (more control exclusions) with a threshold of 1e−05

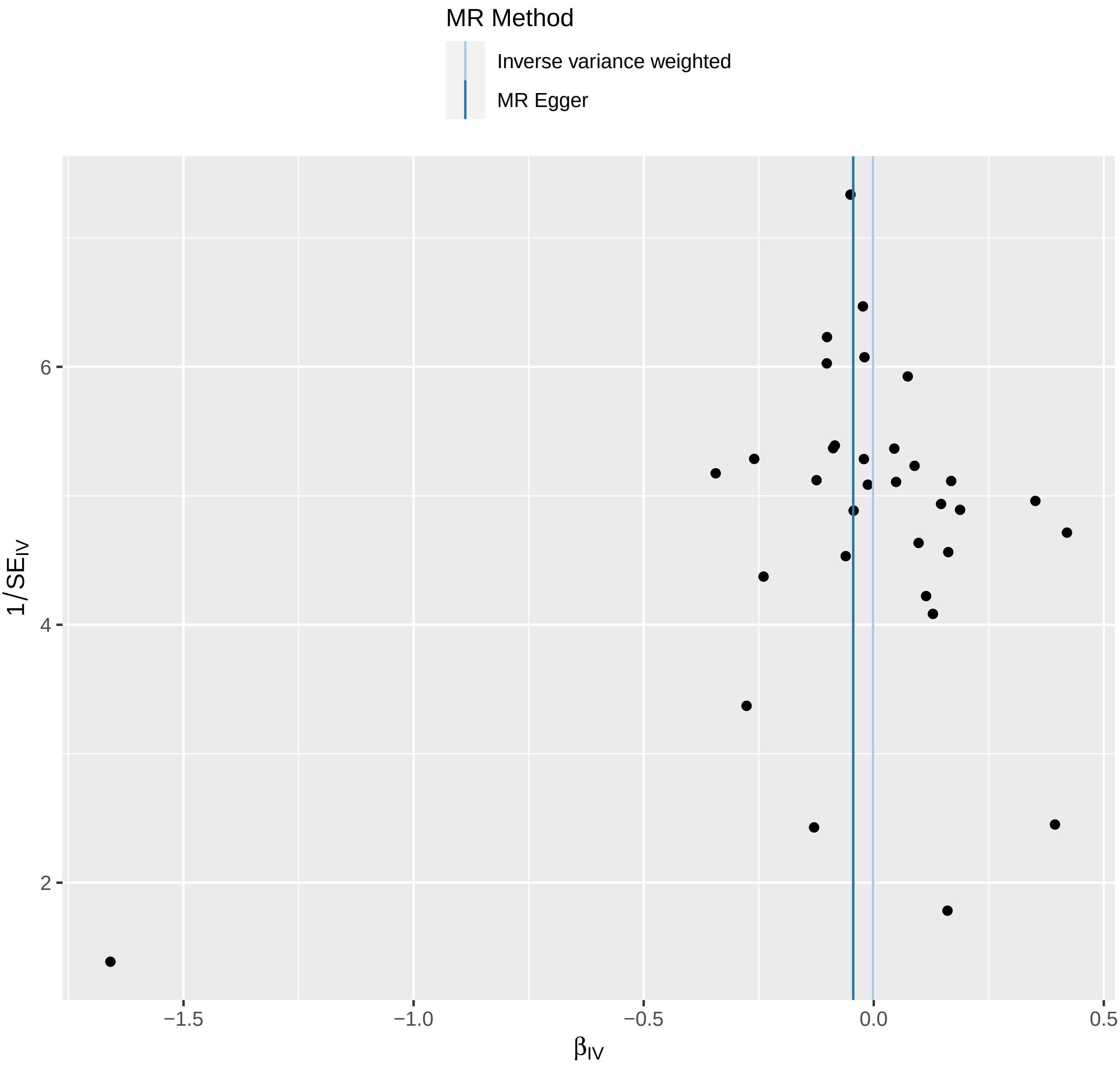

Batch 3−2993 : Transmembrane emp24 domain−containing protein 10 on Diabetic nephropathy (more control exclusions) with a threshold of 1e−05

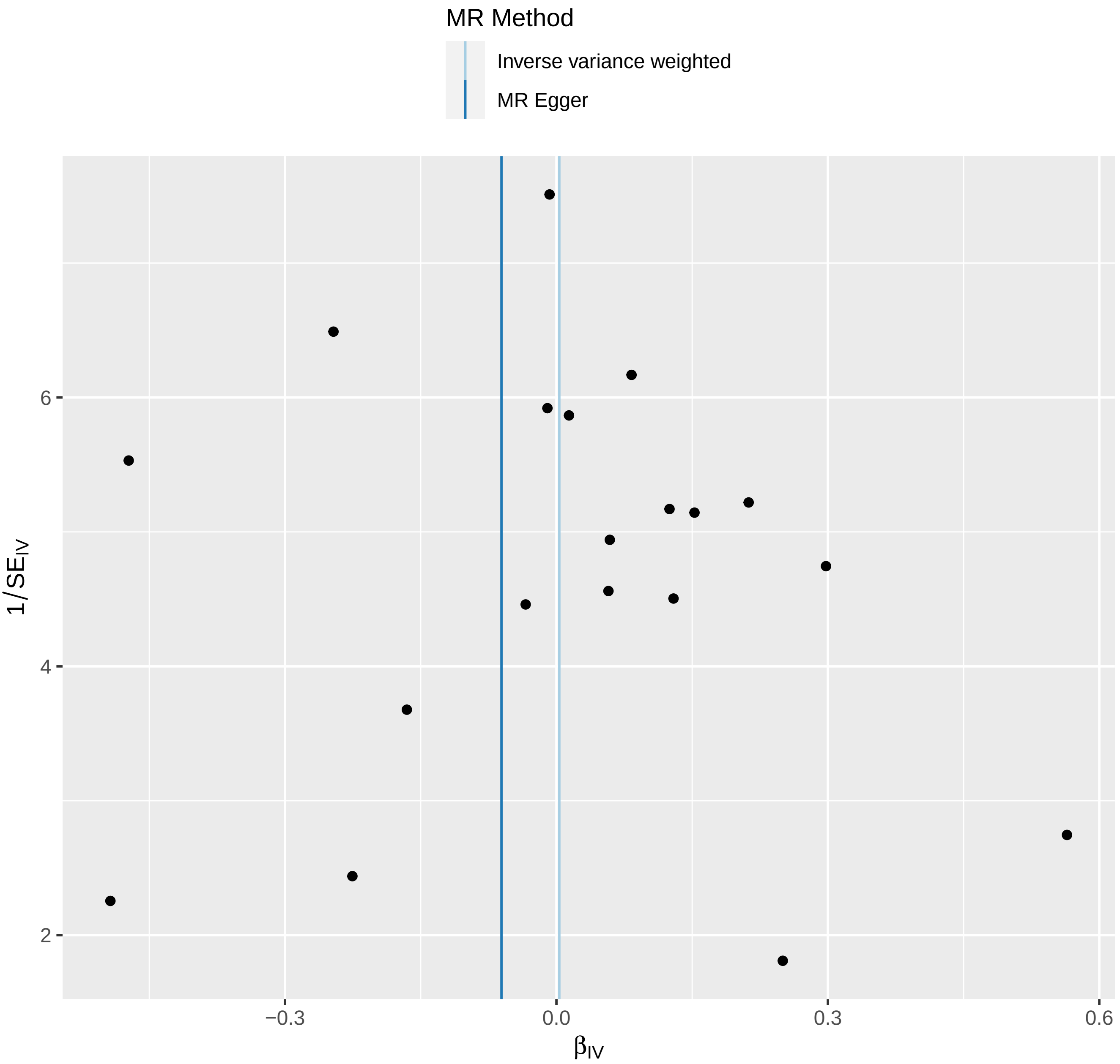

Batch 3−2994 : Transmembrane emp24 domain−containing protein 2 on Diabetic nephropathy (more control exclusions)
with a threshold of 1e−05

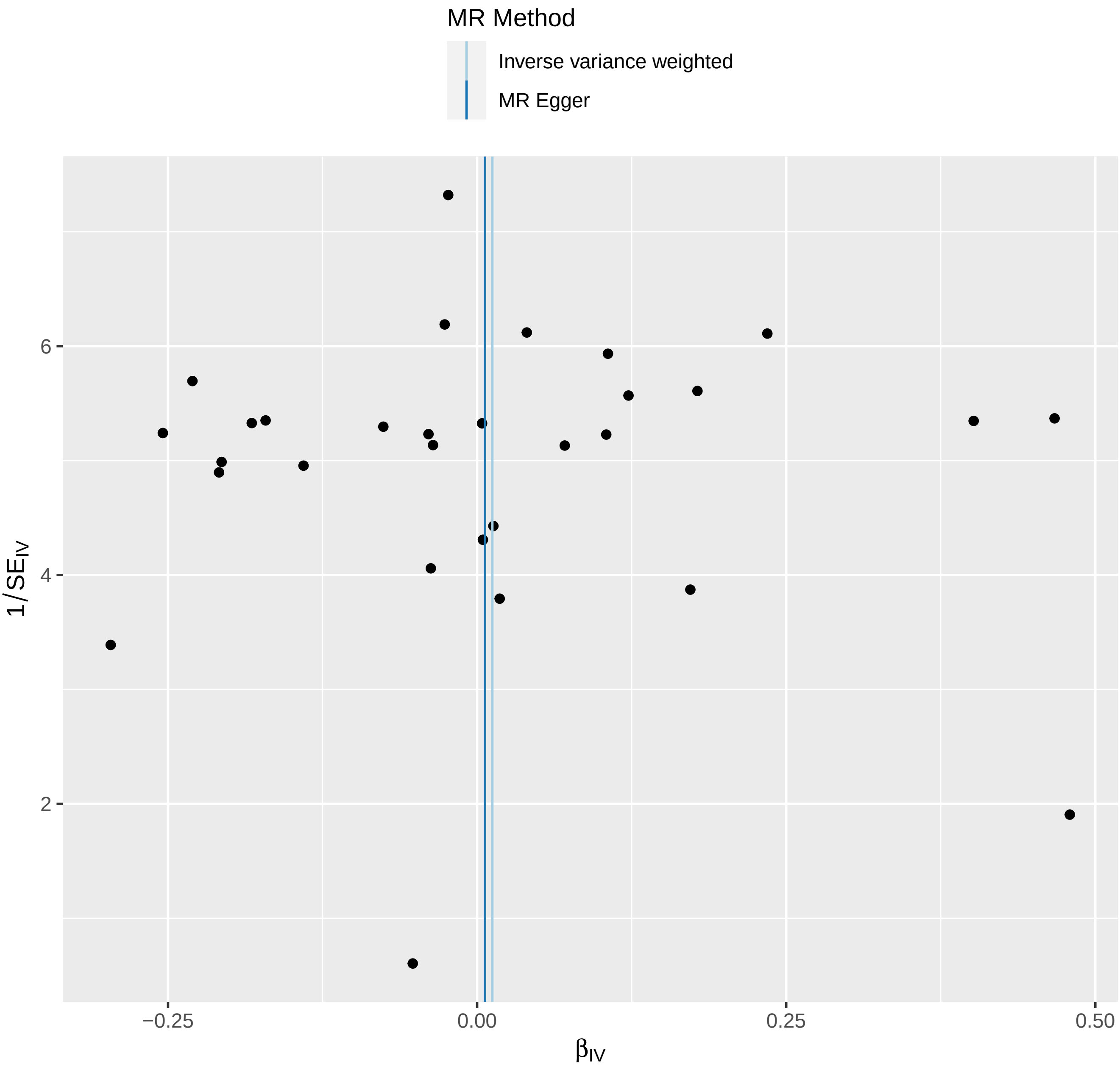

Batch 3−2995 : Transmembrane emp24 domain−containing protein 4 on Diabetic nephropathy (more control exclusions)
with a threshold of 1e−05

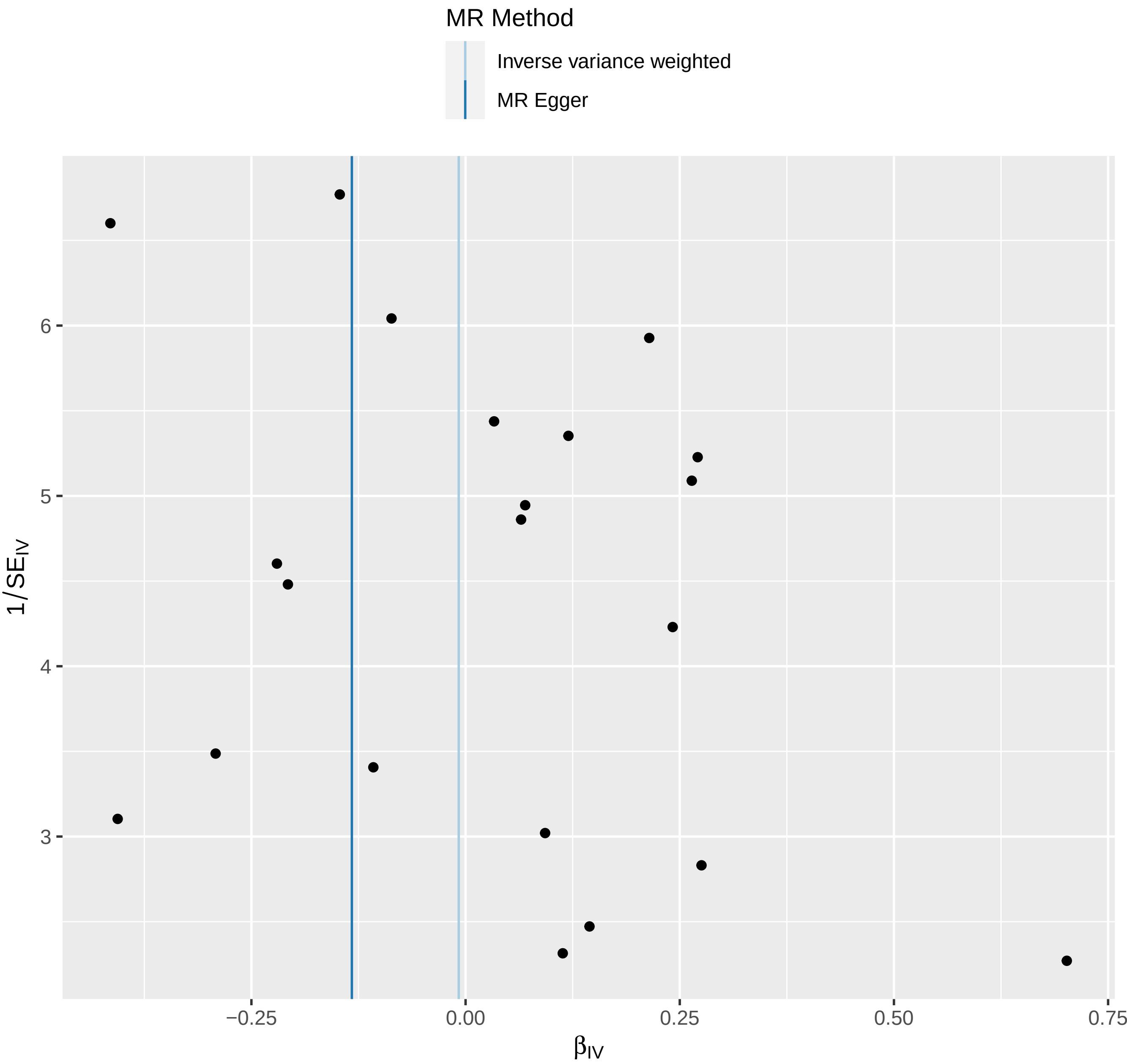

Batch 3−2996 : Transmembrane protein 119 on Diabetic nephropathy (more control exclusions)
with a threshold of 1e−05

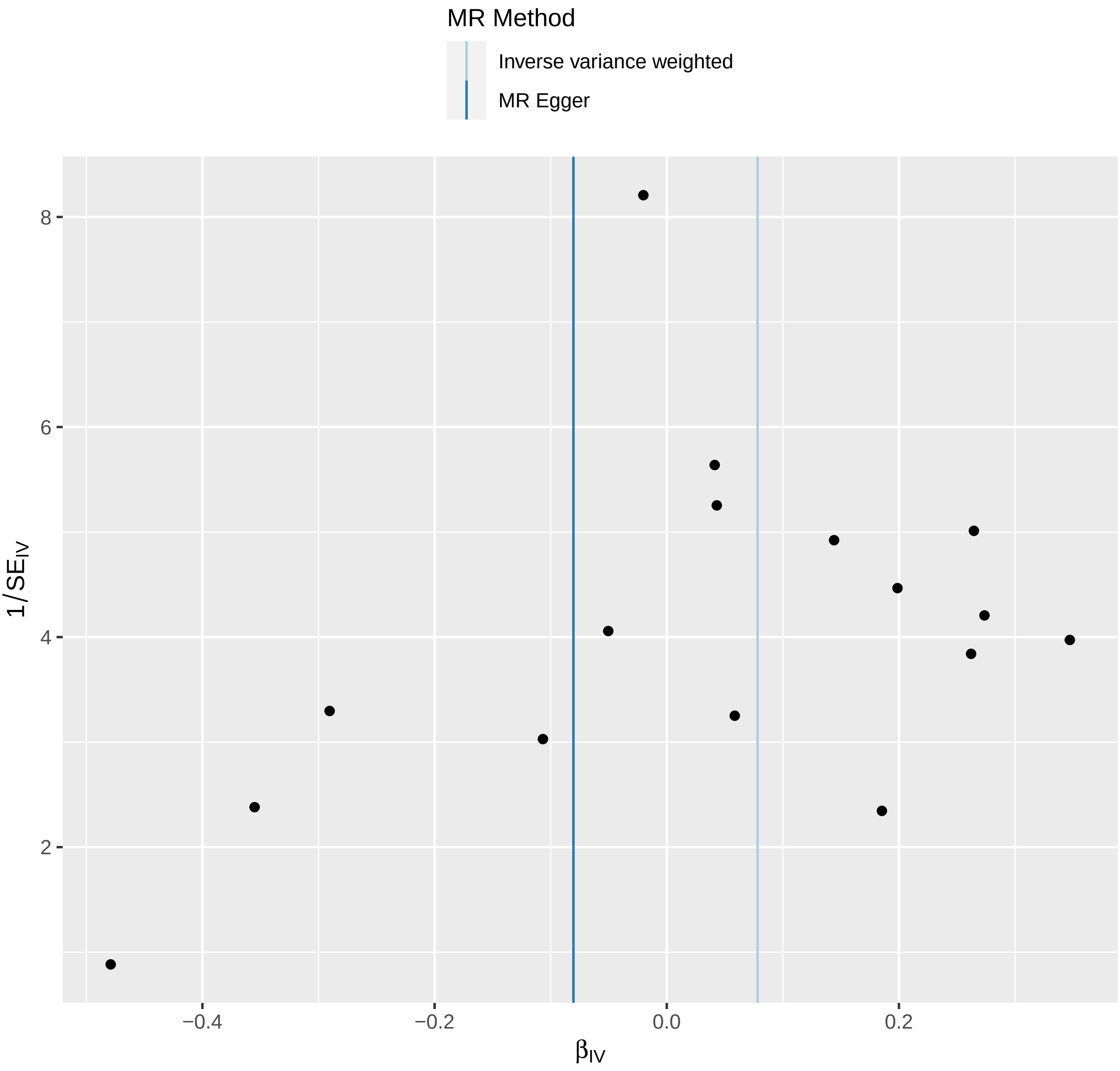

Batch 3−2997 : Transmembrane protein 132A on Diabetic nephropathy (more control exclusions)
with a threshold of 1e−05

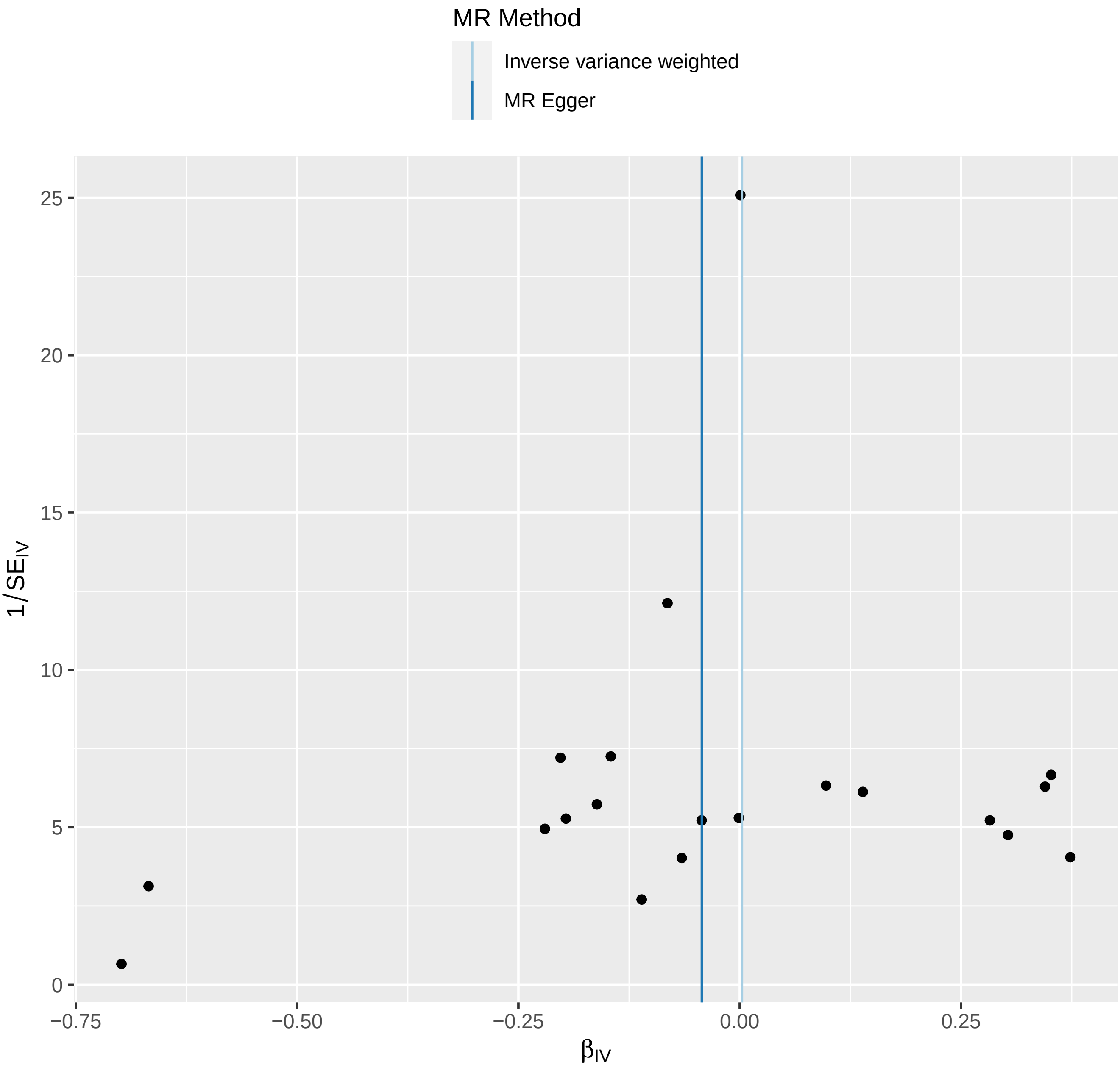

Batch 3−2998 : Transmembrane protein 132B on Diabetic nephropathy (more control exclusions)
with a threshold of 1e−05

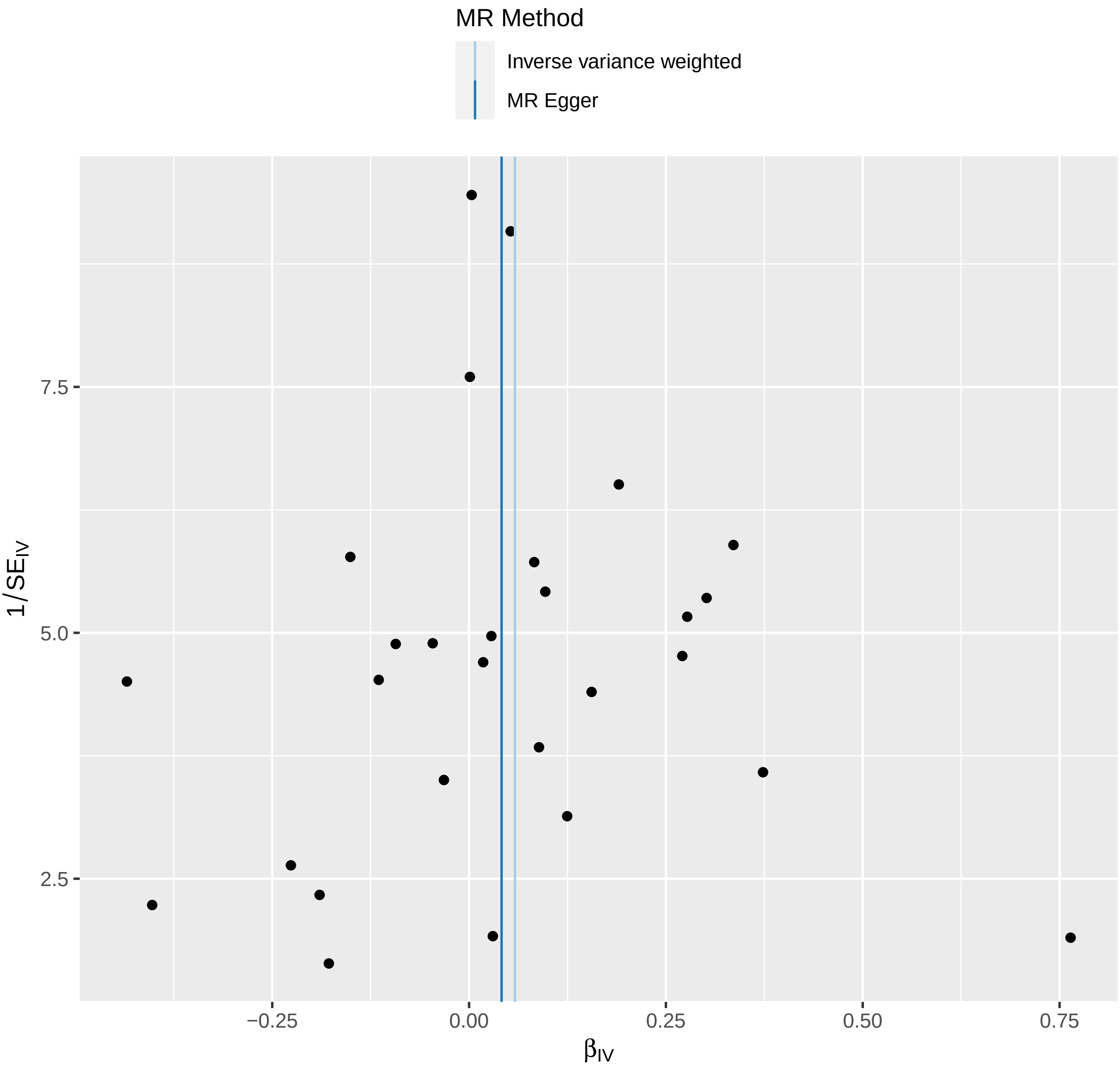

Batch 3−2999 : Transmembrane protein 132C on Diabetic nephropathy (more control exclusions)
with a threshold of 1e−05

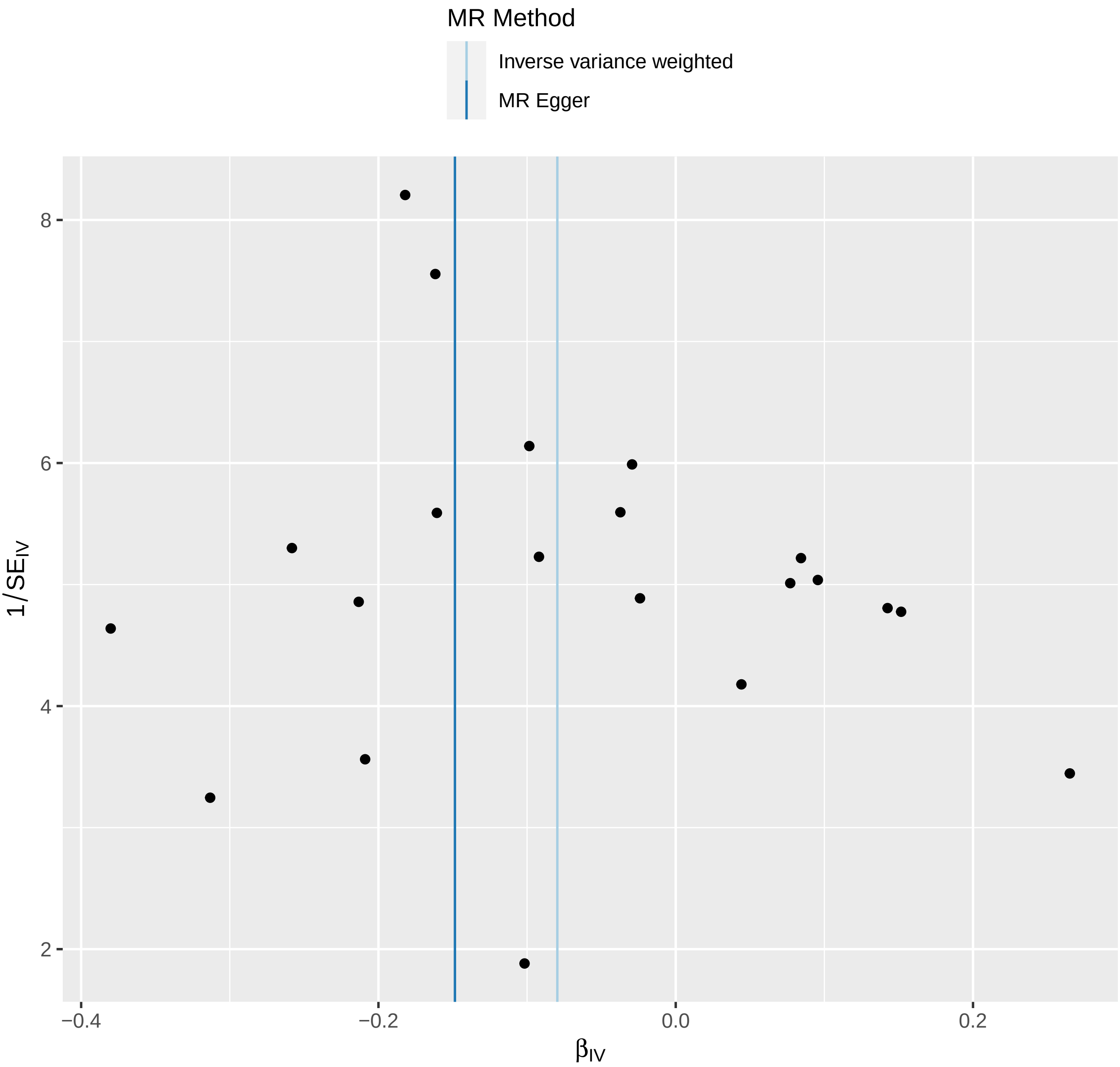

Batch 3−3000 : Transmembrane protein 132C on Diabetic nephropathy (more control exclusions)
with a threshold of 1e−05

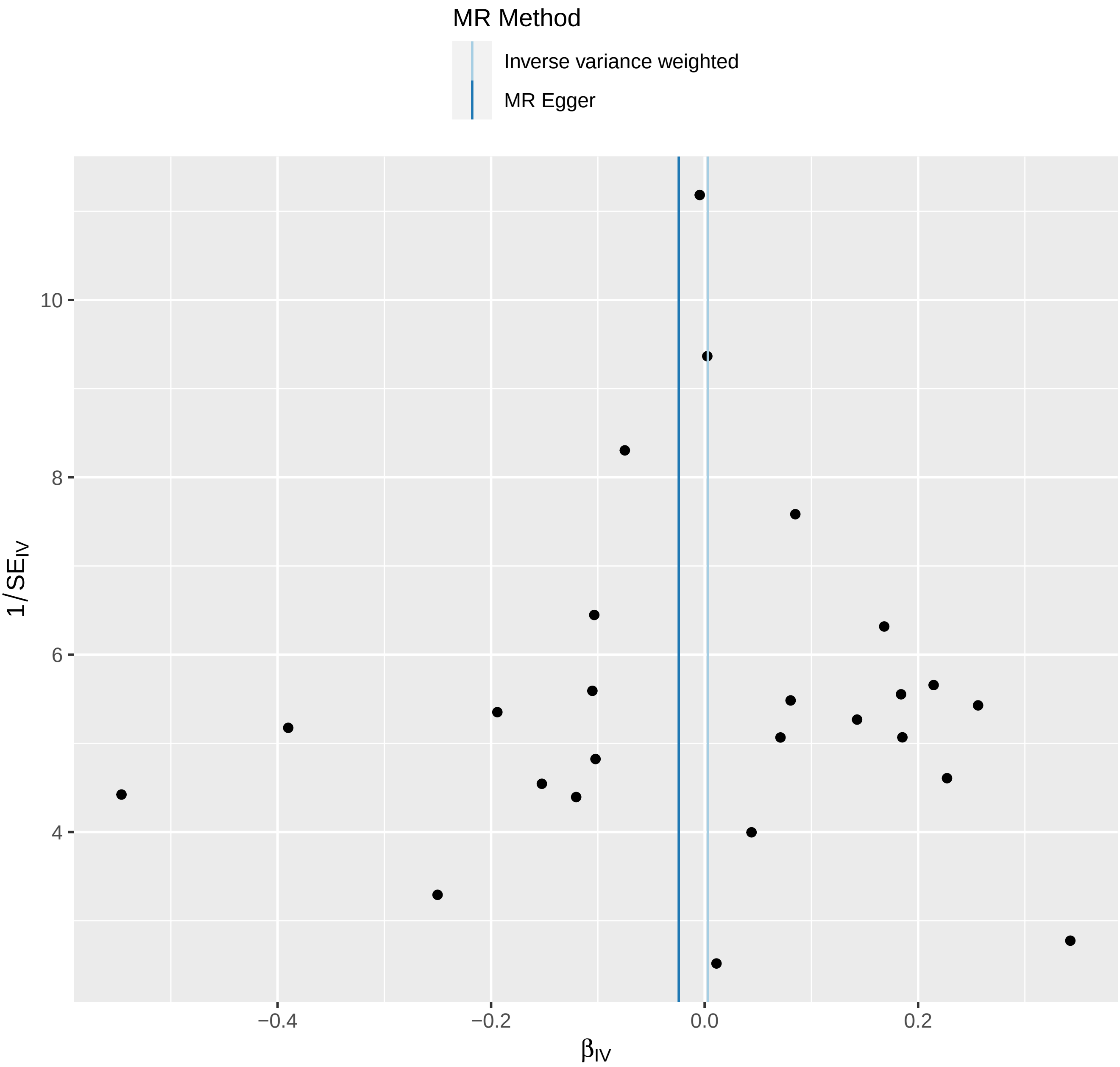

Batch 3−3001 : Transmembrane protein 132D on Diabetic nephropathy (more control exclusions)
with a threshold of 1e−05

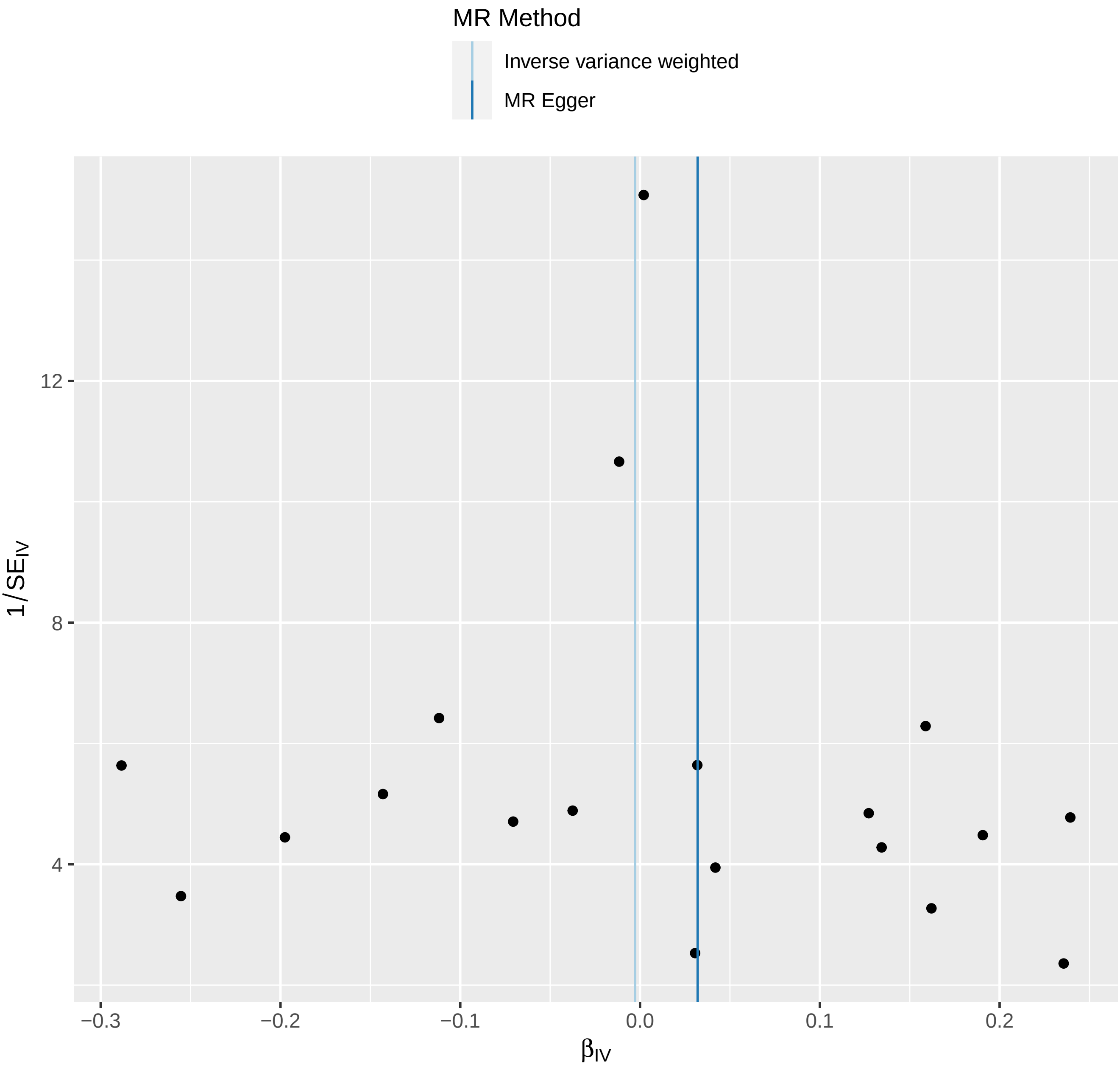

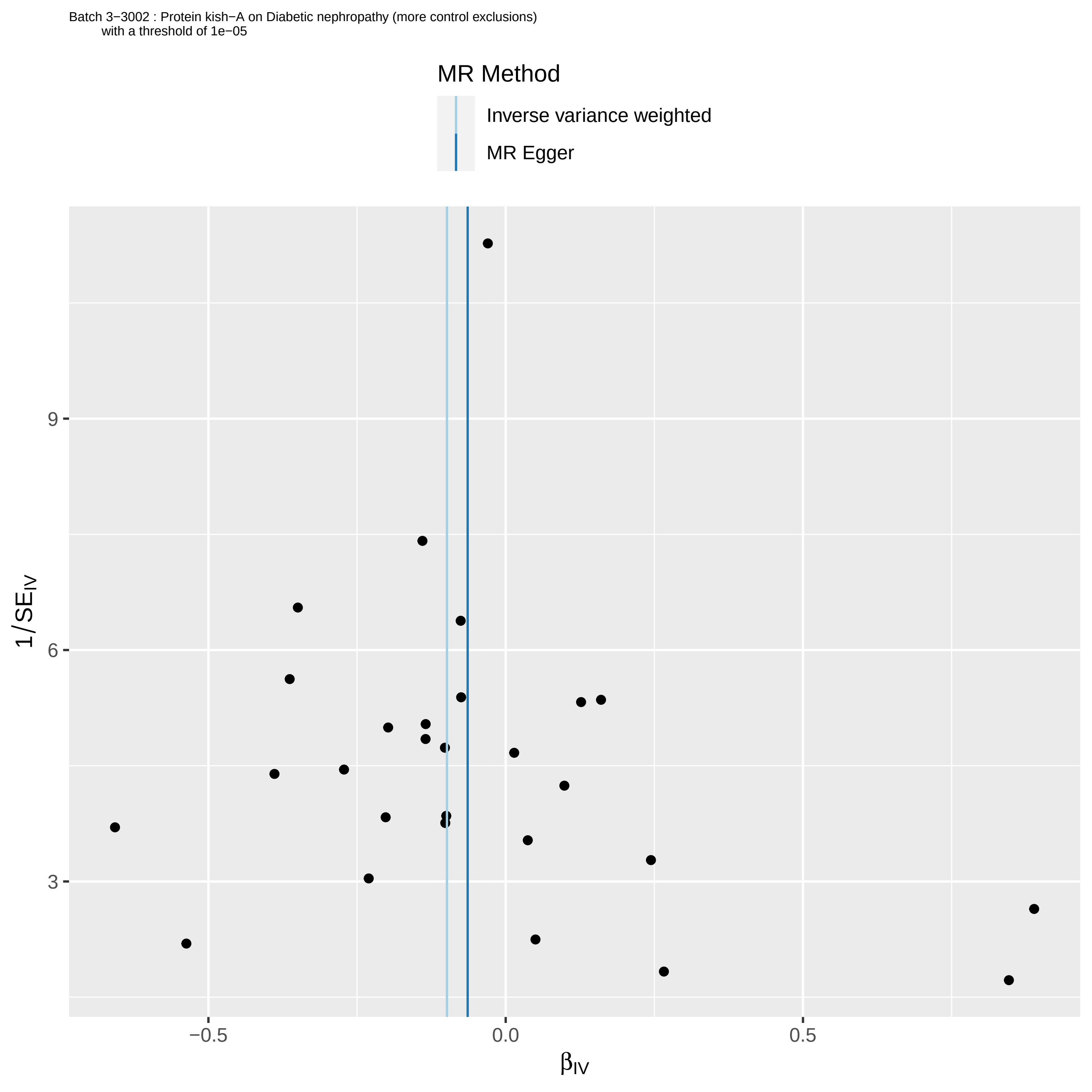
Batch 3−3002 : Protein kish−A on Diabetic nephropathy (more control exclusions)
with a threshold of 1e−05
MR Method
Inverse variance weighted
MR Egger
9
6
3
1/SE$_{IV}$
−0.5
0.0
0.5
β_{IV}

Batch 3−3003 : Protein kish−B on Diabetic nephropathy (more control exclusions)
with a threshold of 1e−05

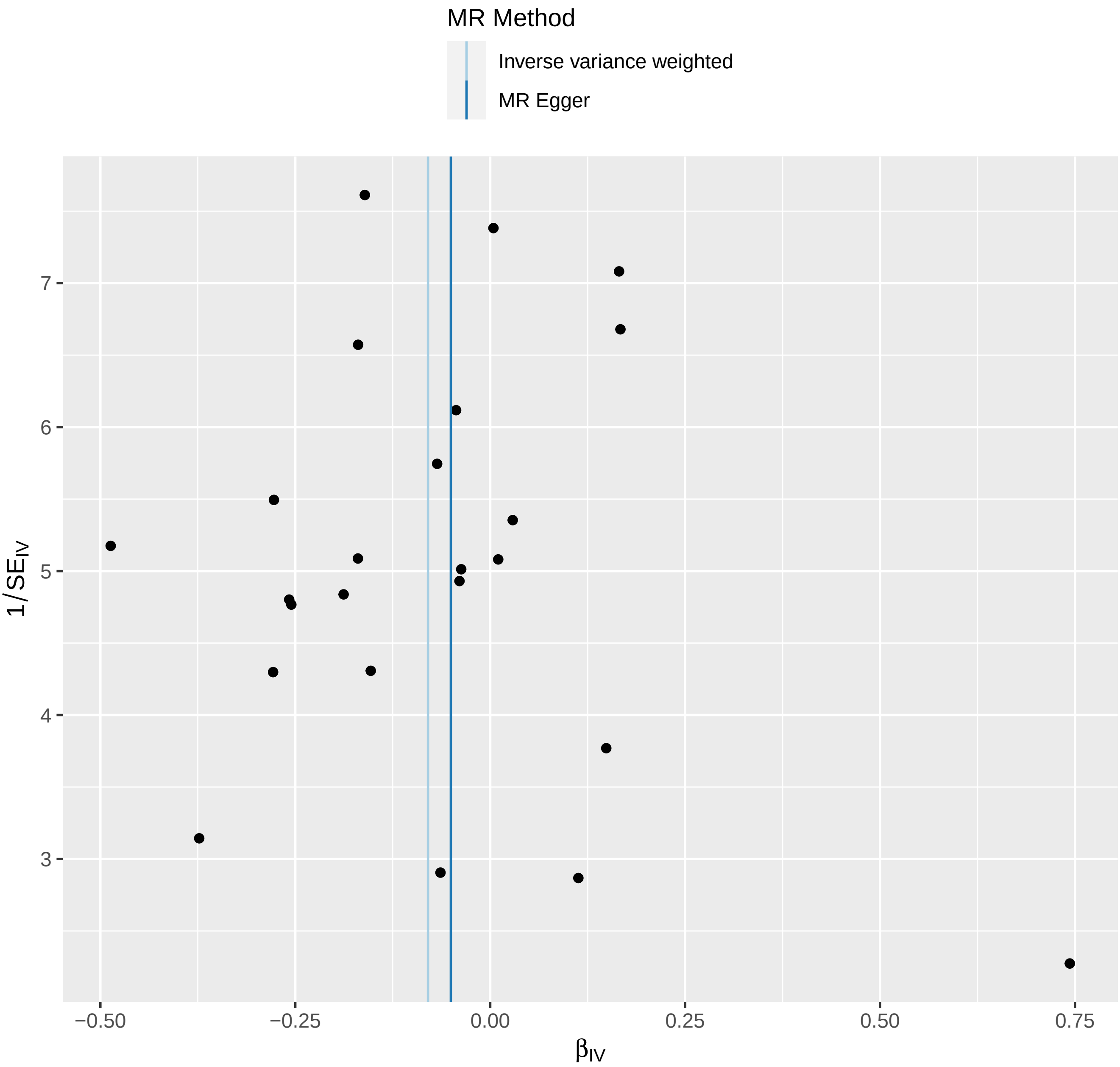

Batch 3−3004 : Transmembrane protein 190 on Diabetic nephropathy (more control exclusions)
with a threshold of 1e−05

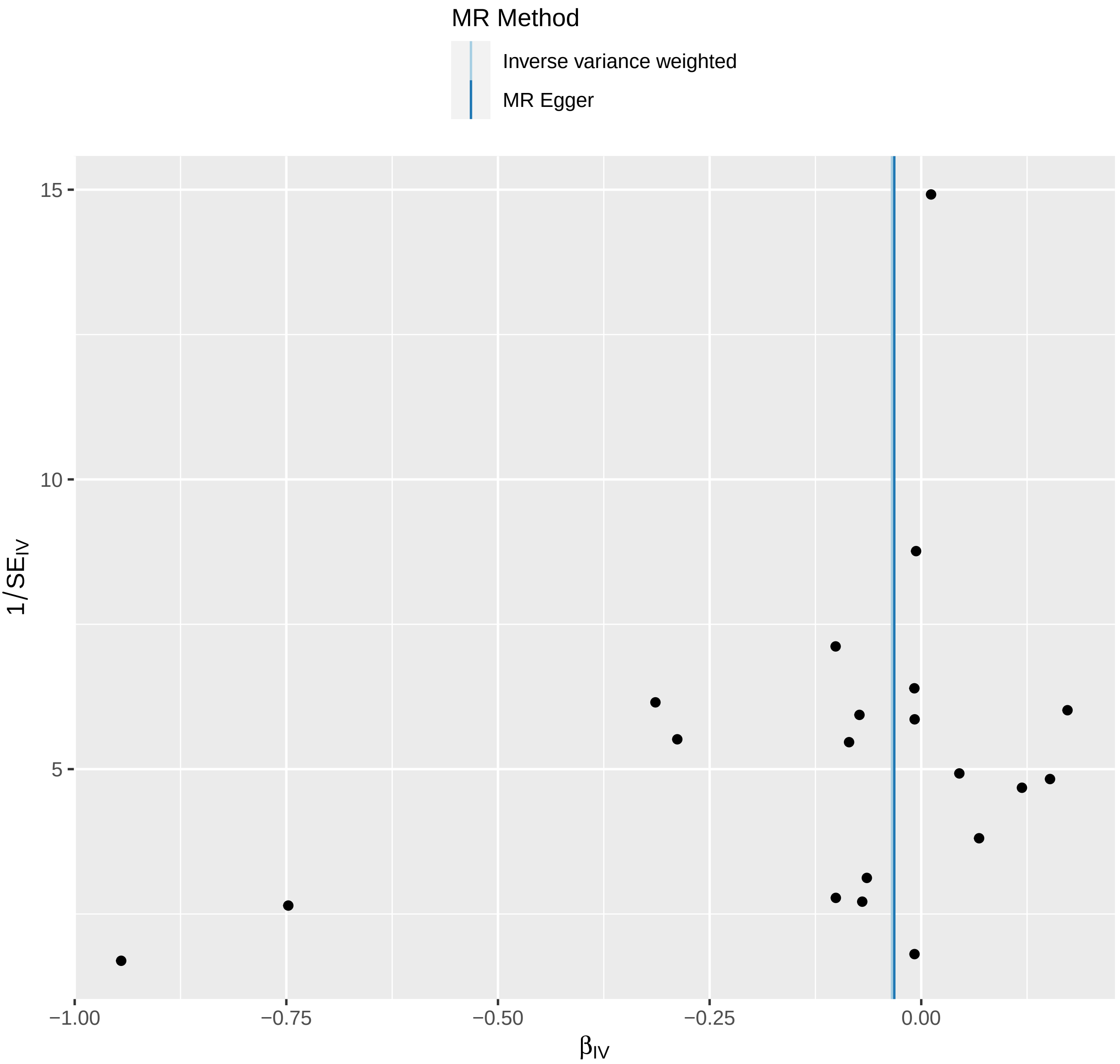

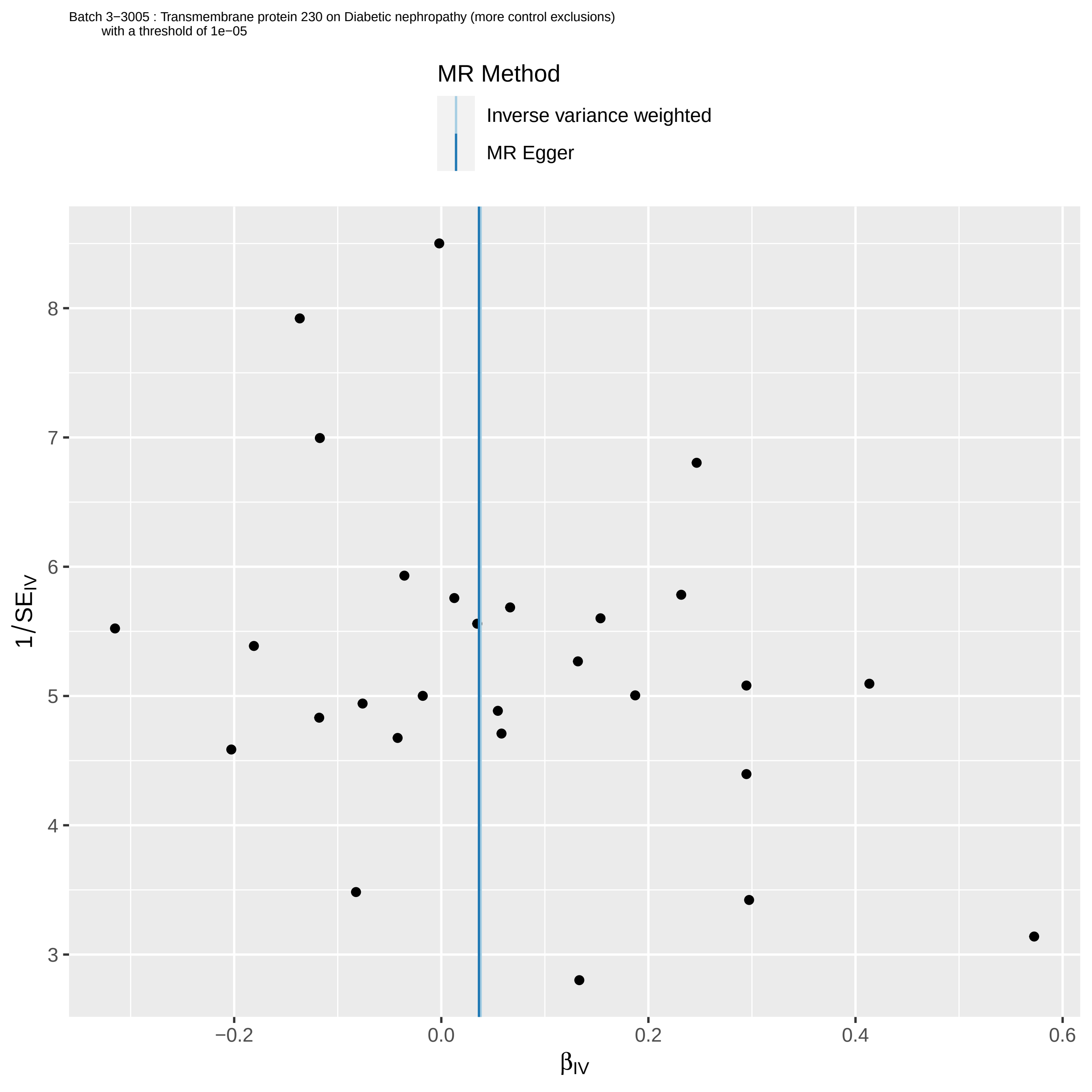

Batch 3−3005 : Transmembrane protein 230 on Diabetic nephropathy (more control exclusions)
with a threshold of 1e−05
MR Method
Inverse variance weighted
MR Egger
1/SE_IV
β_IV
8
7
6
5
4
3
−0.2
0.0
0.2
0.4
0.6

Batch 3−3006 : Transmembrane protein 234 on Diabetic nephropathy (more control exclusions)
with a threshold of 1e−05

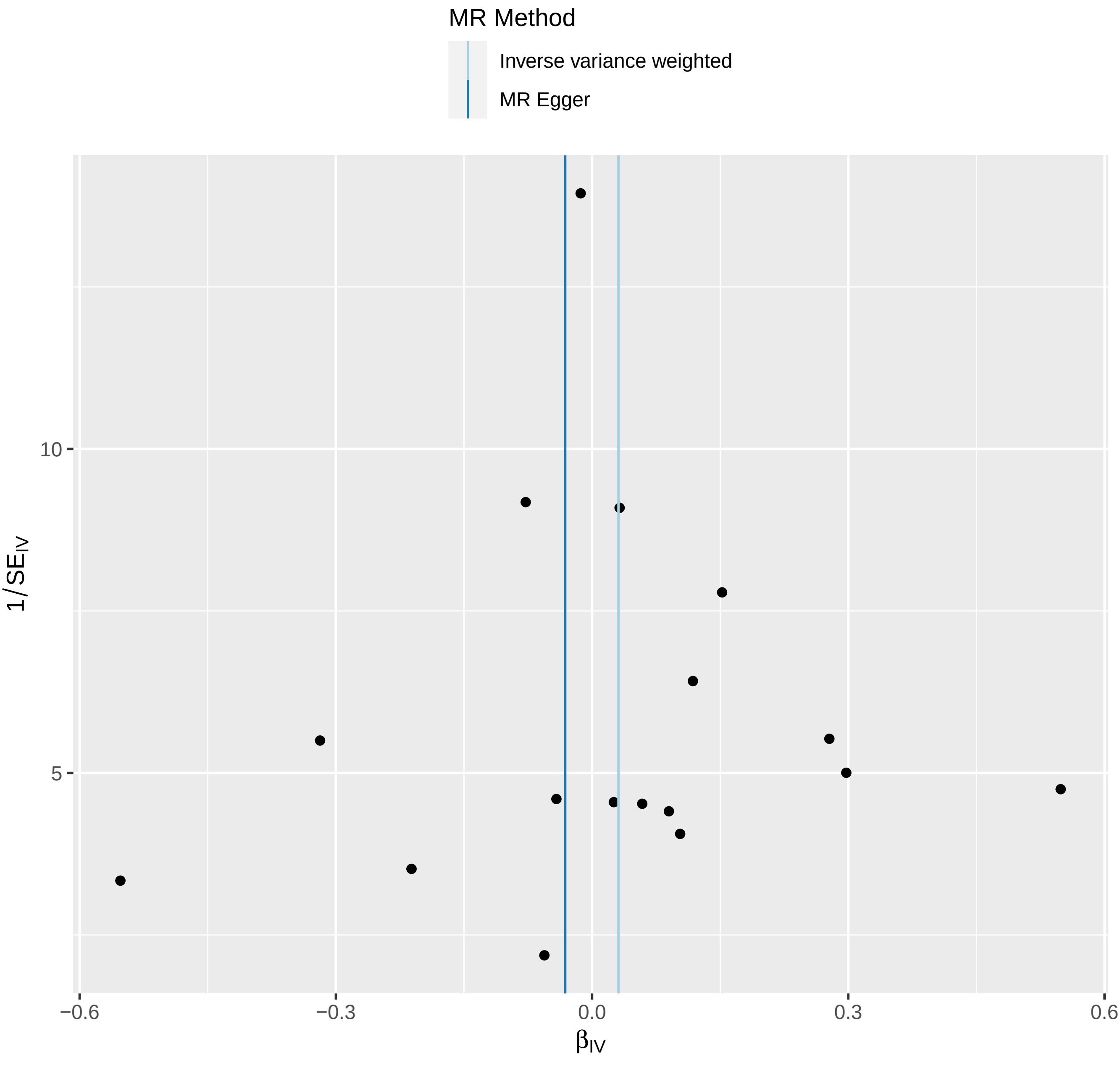

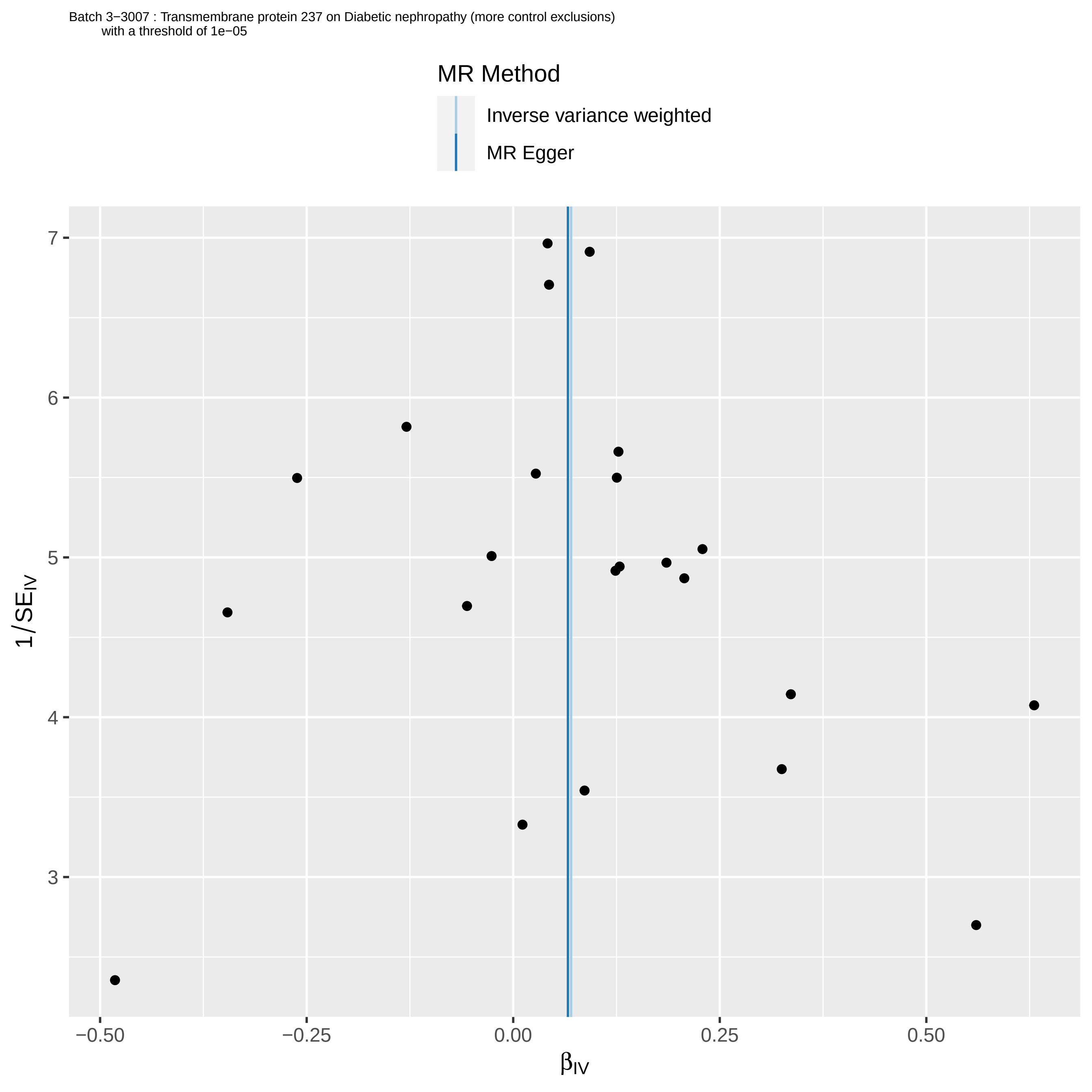
Batch 3−3007 : Transmembrane protein 237 on Diabetic nephropathy (more control exclusions)
with a threshold of 1e−05
MR Method
Inverse variance weighted
MR Egger
7
6
5
4
3
1/SE_IV
−0.50
−0.25
0.00
0.25
0.50
β_IV

Batch 3−3008 : Collectrin on Diabetic nephropathy (more control exclusions)
with a threshold of 1e−05

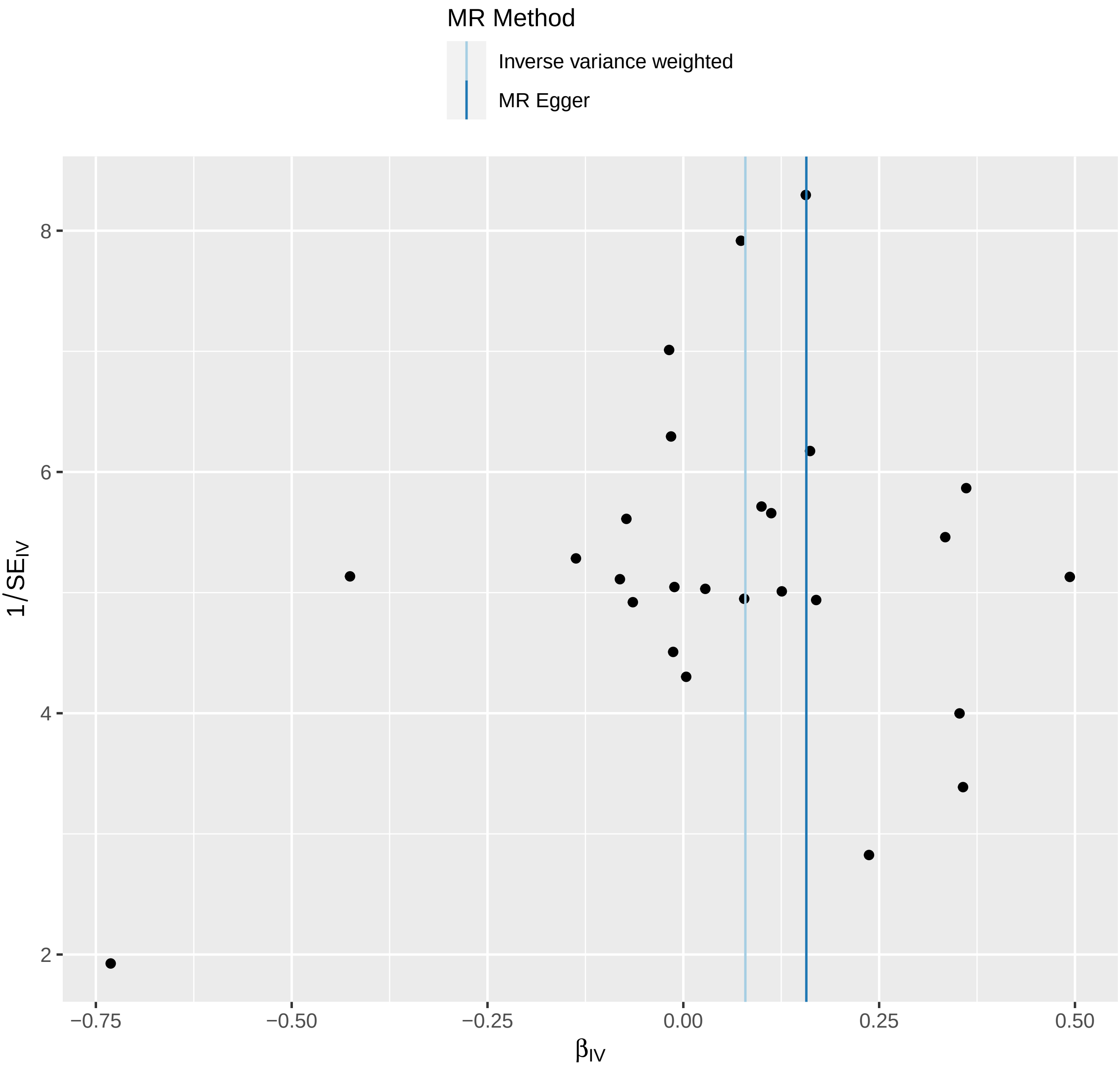

Batch 3−3009 : Transmembrane protein 2 on Diabetic nephropathy (more control exclusions)
with a threshold of 1e−05

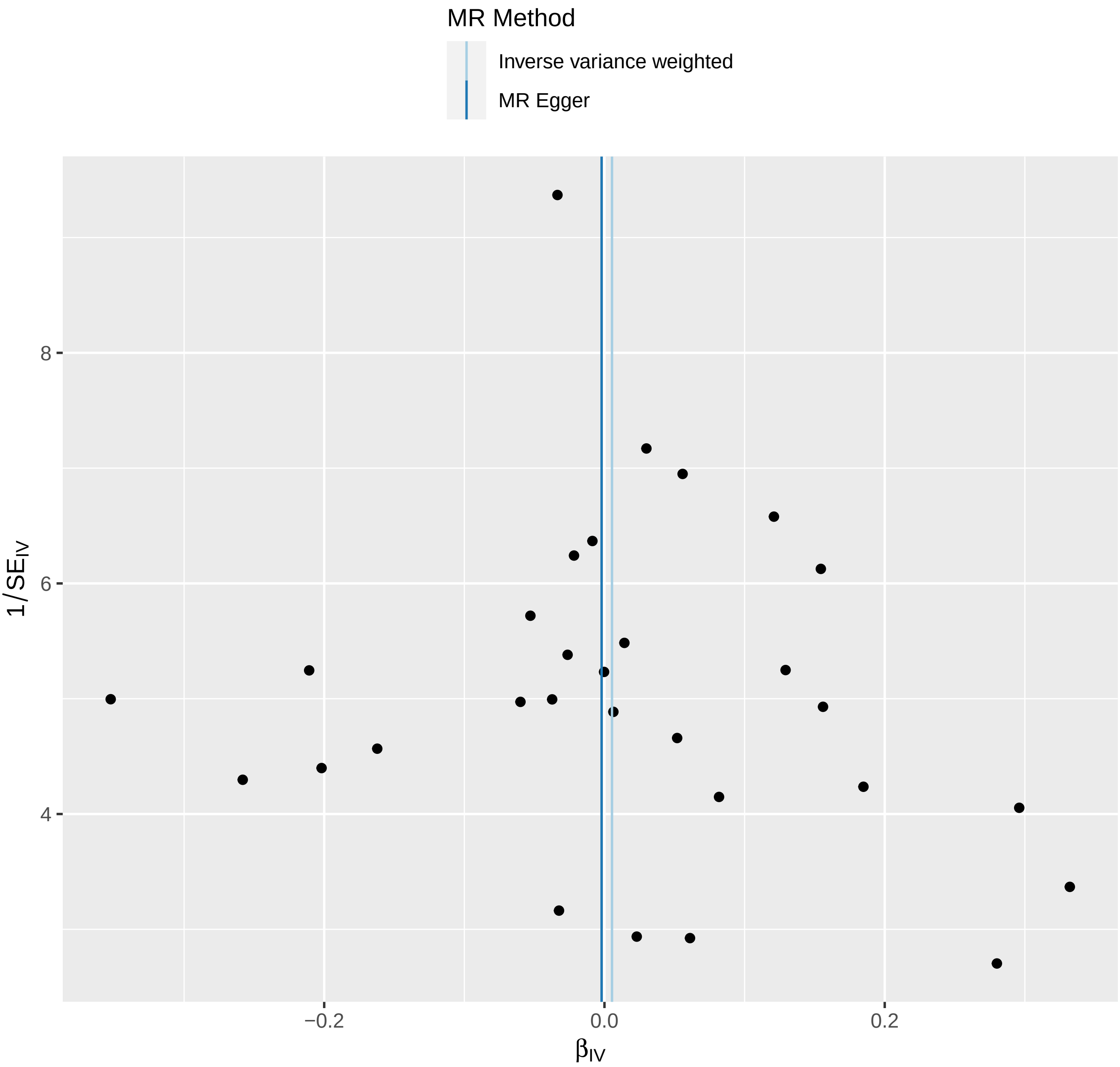

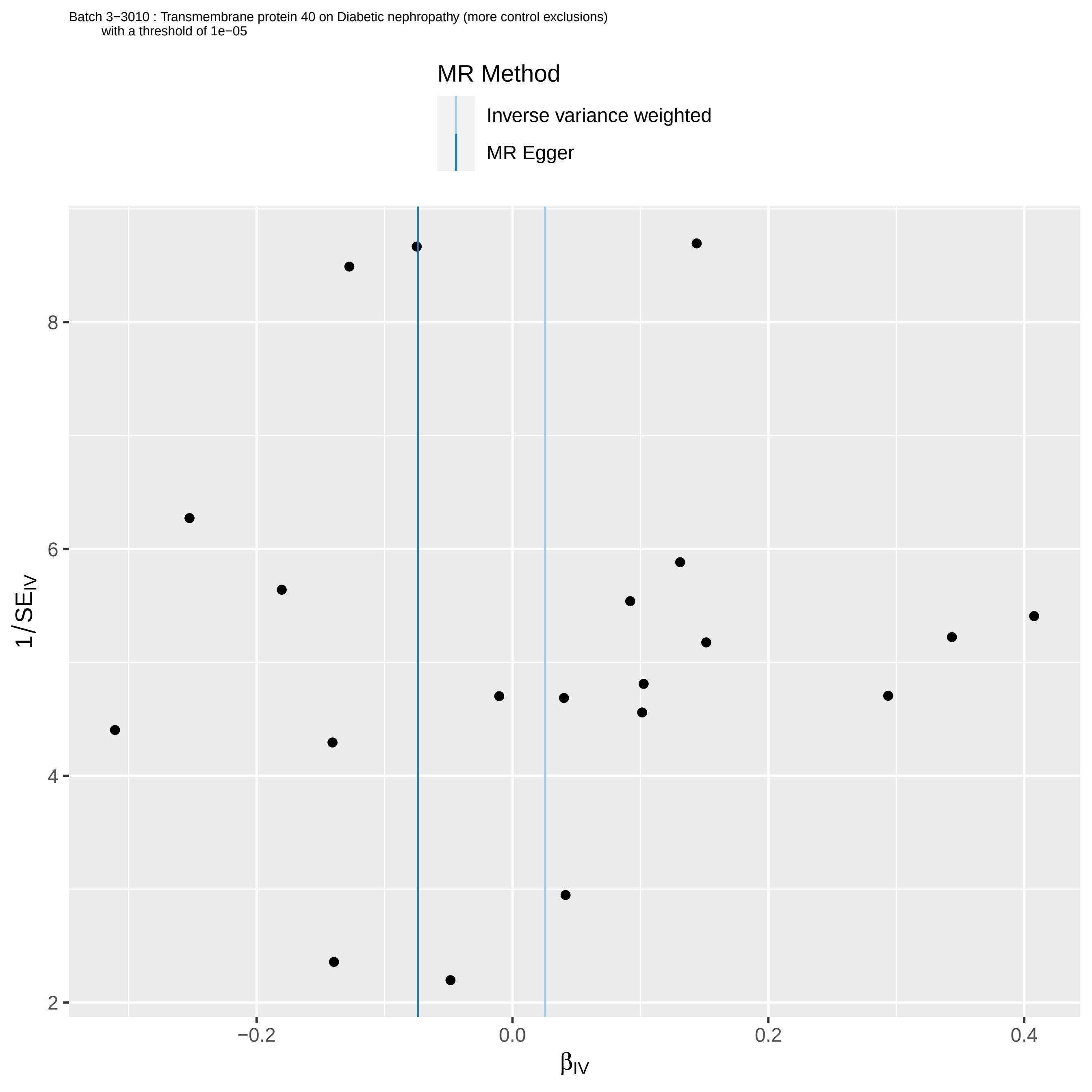
Batch 3−3010 : Transmembrane protein 40 on Diabetic nephropathy (more control exclusions)
with a threshold of 1e−05
MR Method
Inverse variance weighted
MR Egger
8
6
4
2
$1/SE_{IV}$
−0.2
0.0
0.2
0.4
β_{IV}

Batch 3−3011 : Transmembrane protein 41B on Diabetic nephropathy (more control exclusions)
with a threshold of 1e−05

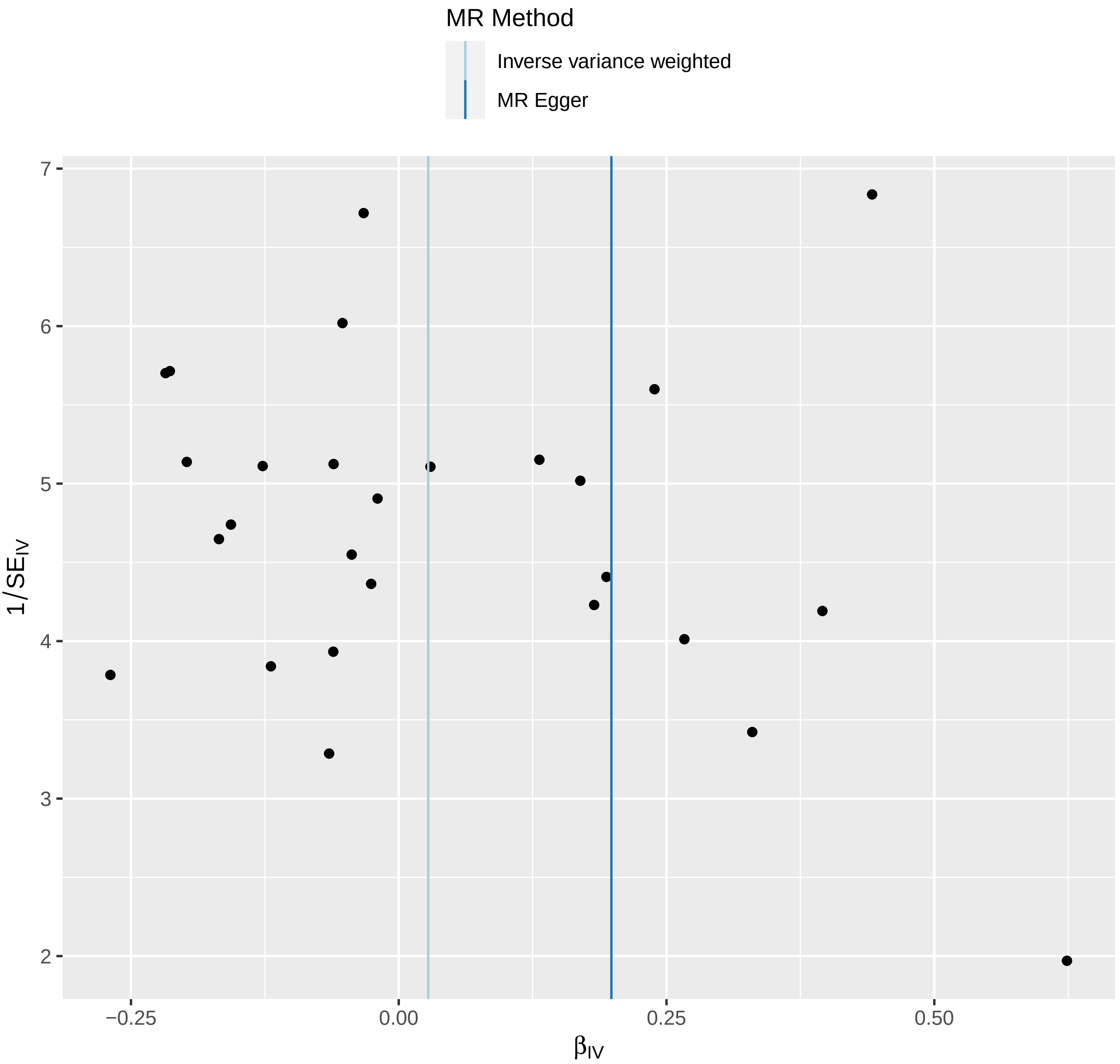

Batch 3−3012 : Transmembrane protein 52 on Diabetic nephropathy (more control exclusions)
with a threshold of 1e−05

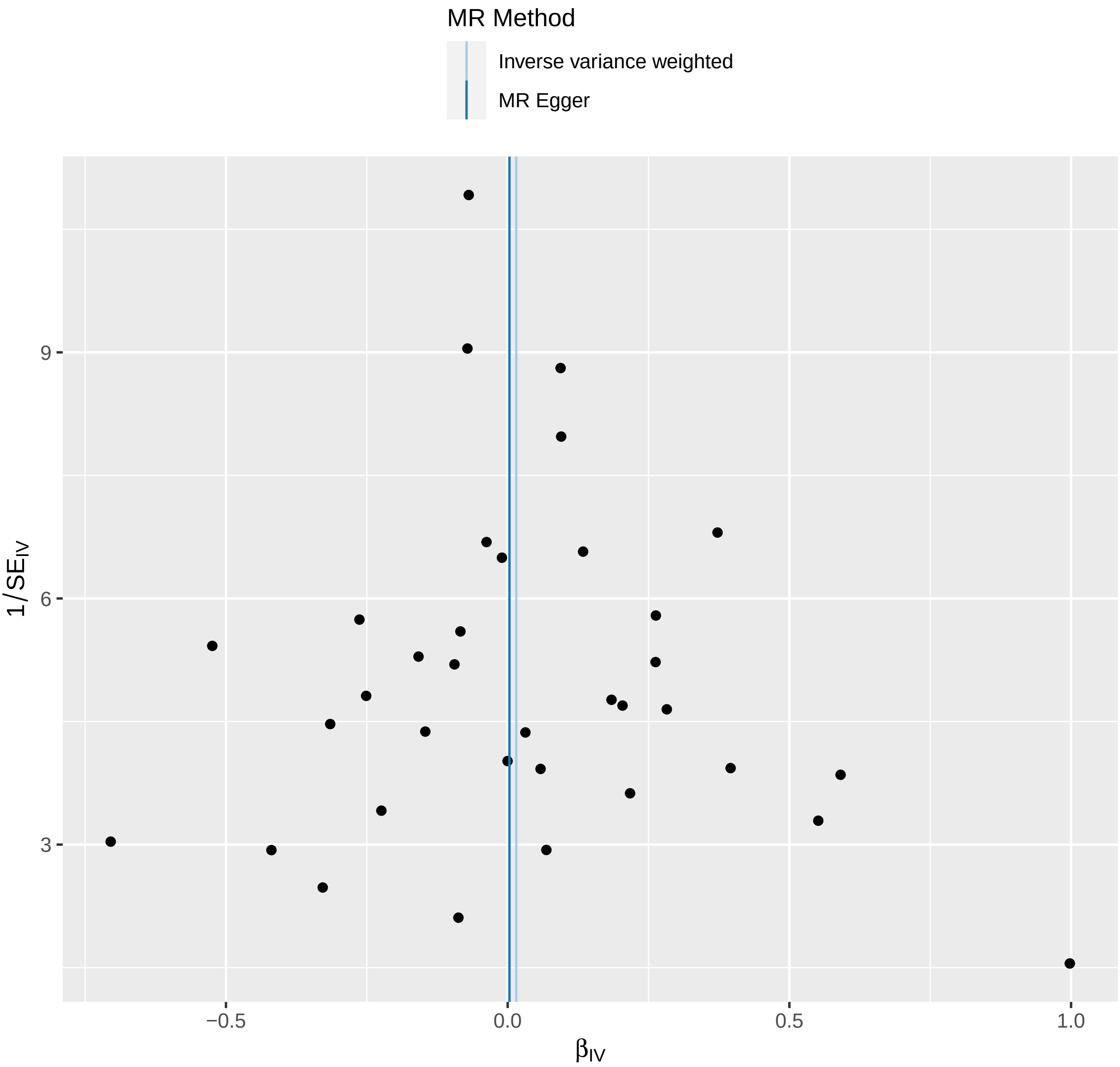

Batch 3−3013 : Macoilin on Diabetic nephropathy (more control exclusions)
with a threshold of 1e−05

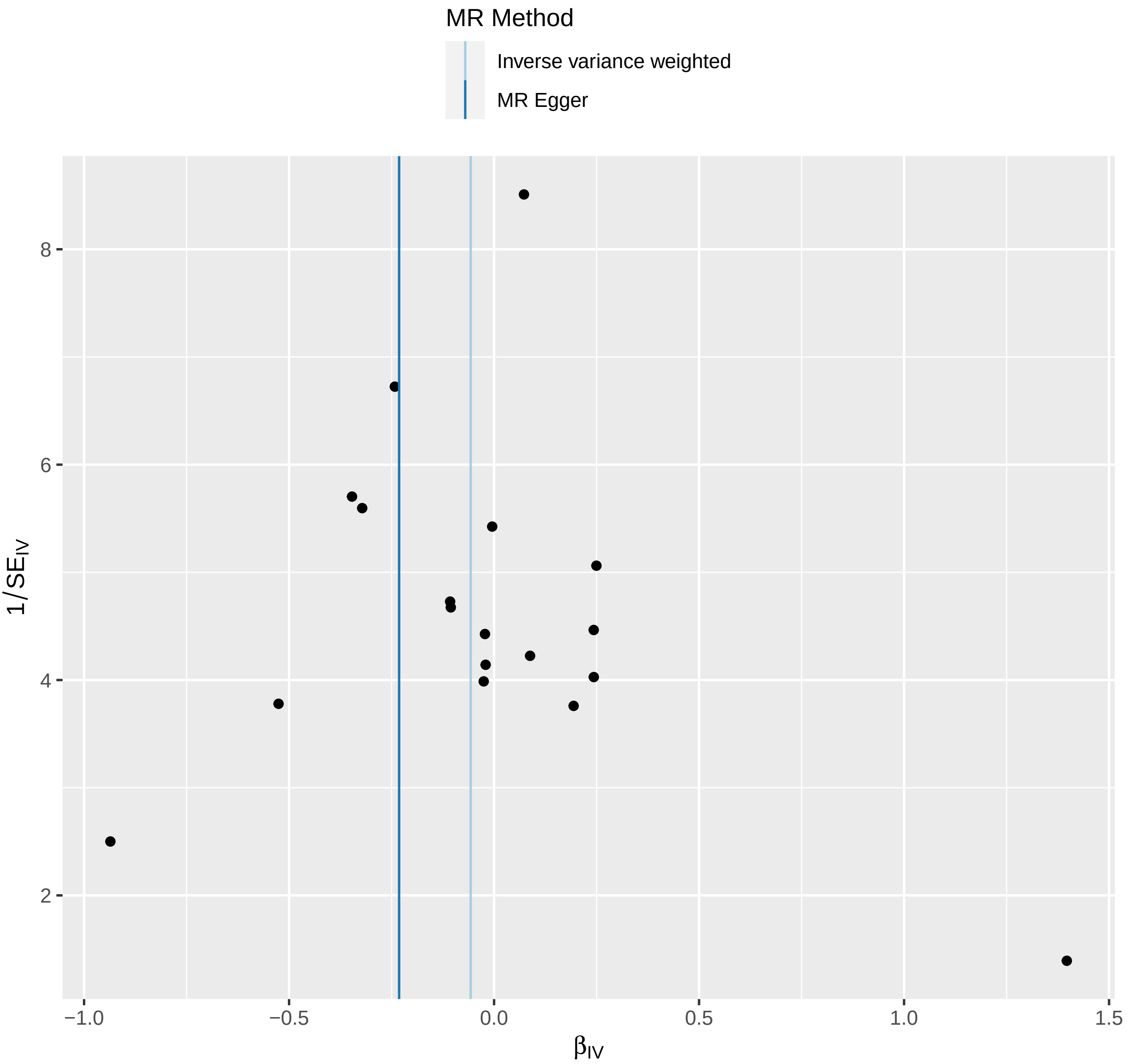

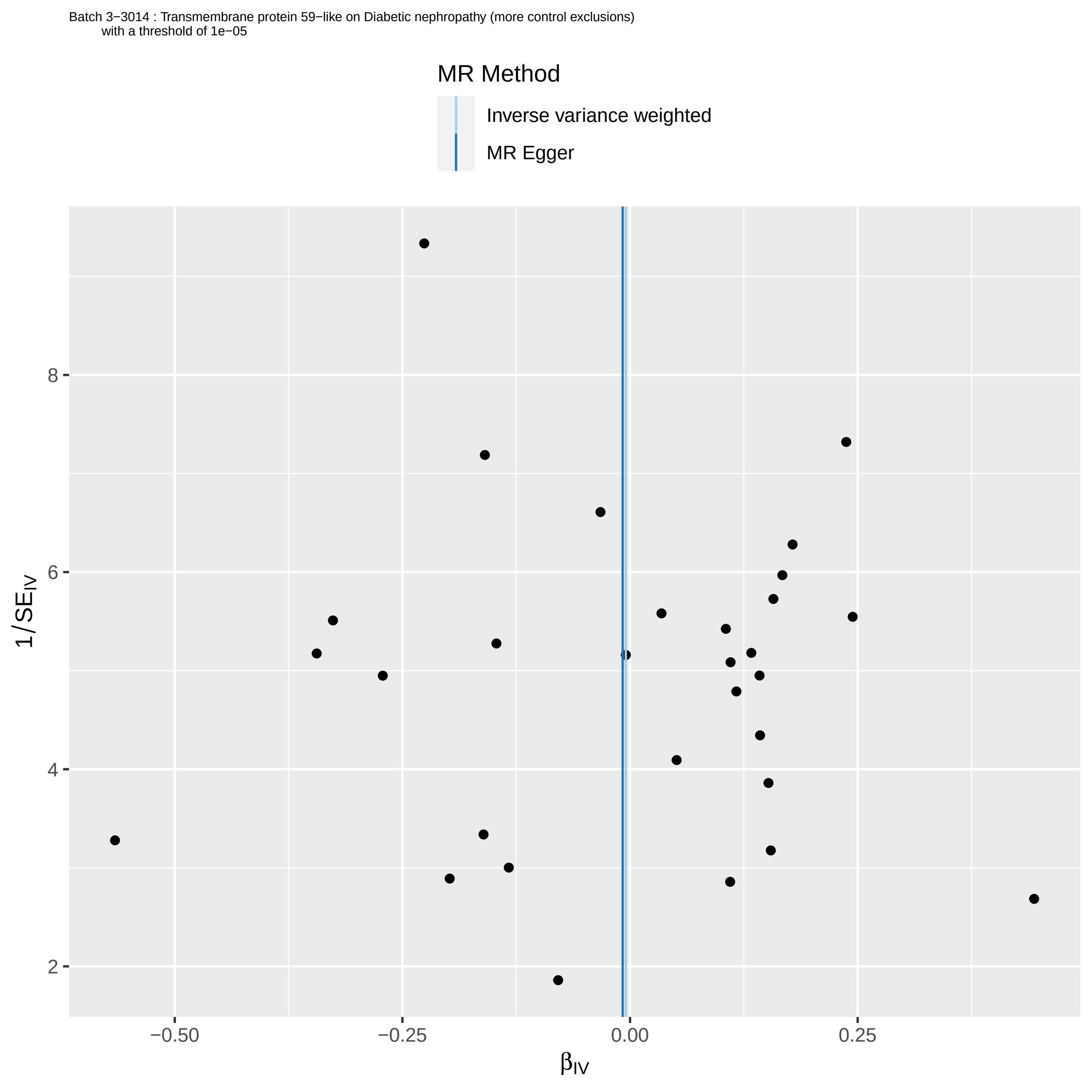

Batch 3−3014 : Transmembrane protein 59−like on Diabetic nephropathy (more control exclusions)
with a threshold of 1e−05
MR Method
Inverse variance weighted
MR Egger
8
6
4
2
1/SE$_{IV}$
−0.50
−0.25
0.00
0.25
β$_{IV}$

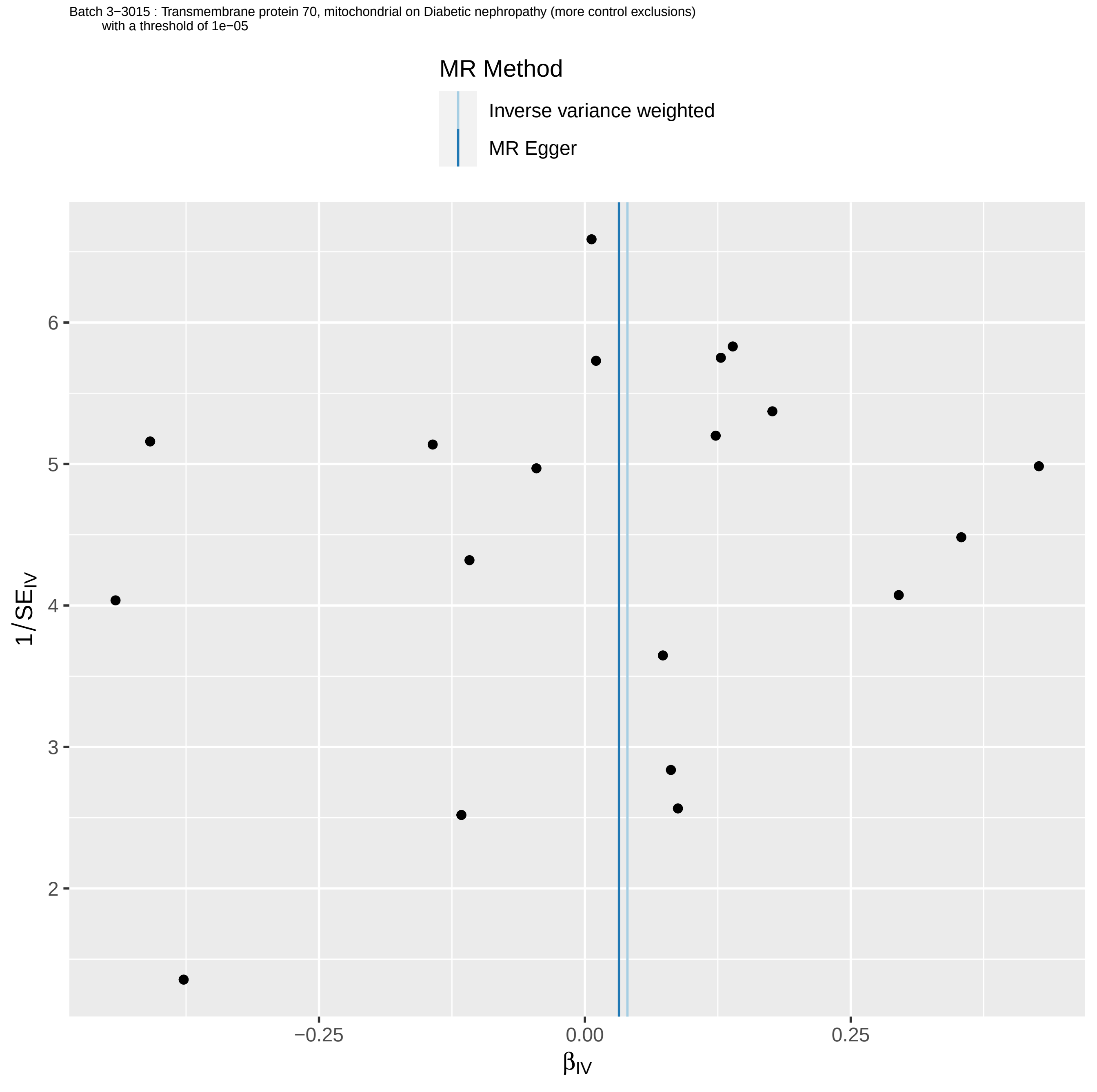
Batch 3−3015 : Transmembrane protein 70, mitochondrial on Diabetic nephropathy (more control exclusions)
with a threshold of 1e−05
MR Method
Inverse variance weighted
MR Egger
6
5
4
3
2
1/SE_IV
−0.25
0.00
0.25
β_IV

Batch 3−3016 : Transmembrane protein 87B on Diabetic nephropathy (more control exclusions)
with a threshold of 1e−05

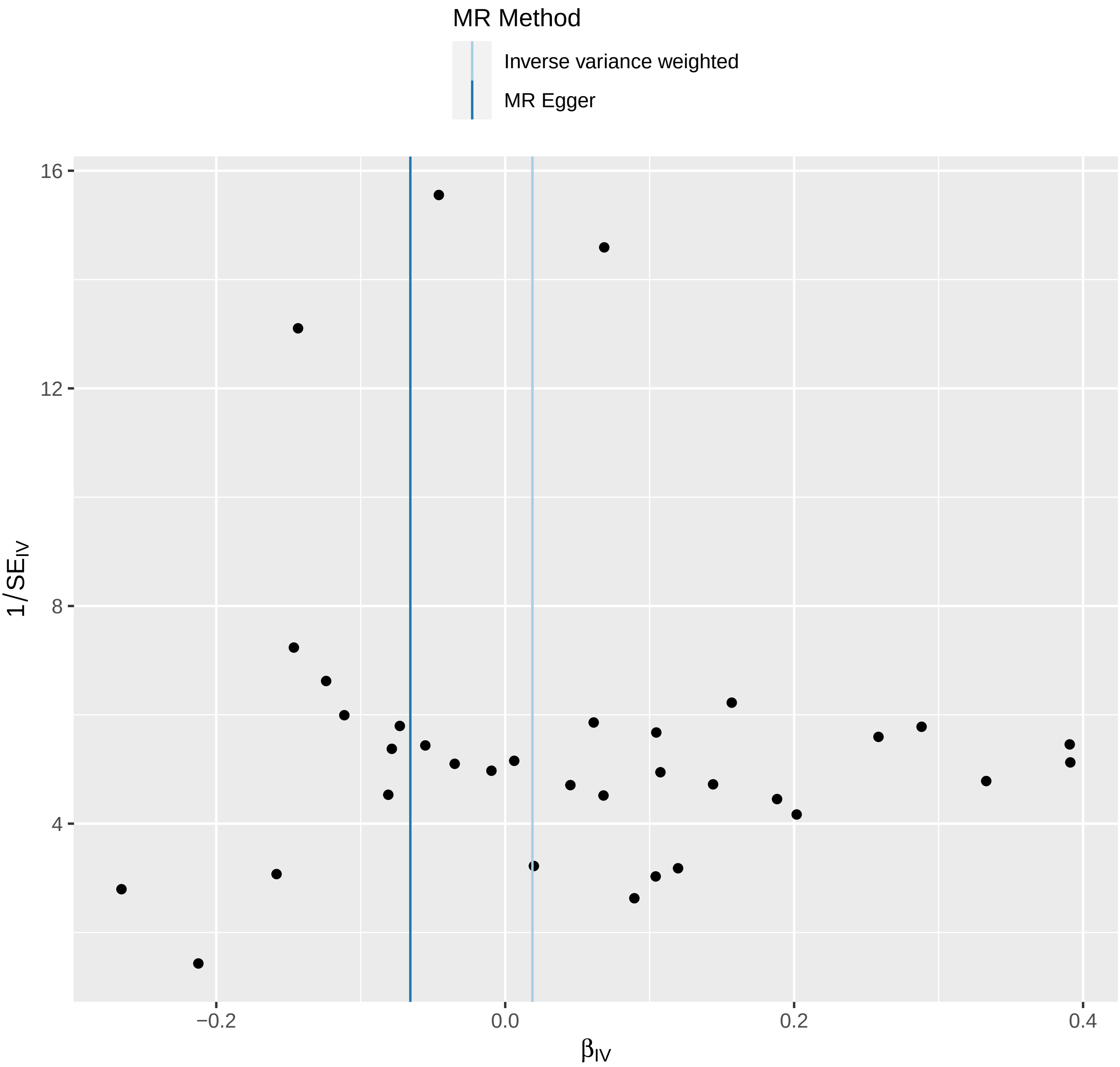

Batch 3−3017 : Transmembrane protein 8B on Diabetic nephropathy (more control exclusions)
with a threshold of 1e−05

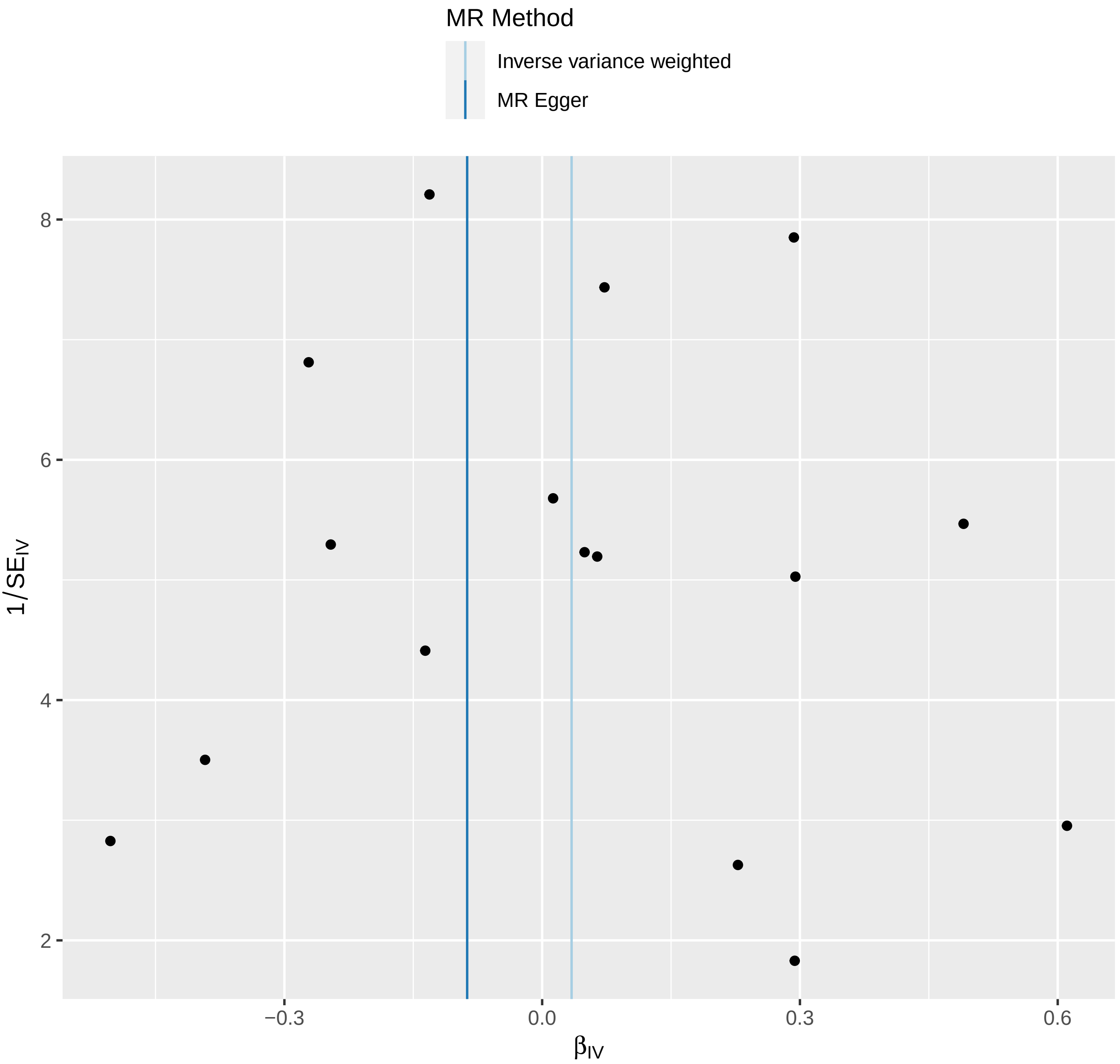

Batch 3−3018 : Transmembrane protein 9 on Diabetic nephropathy (more control exclusions)
with a threshold of 1e−05

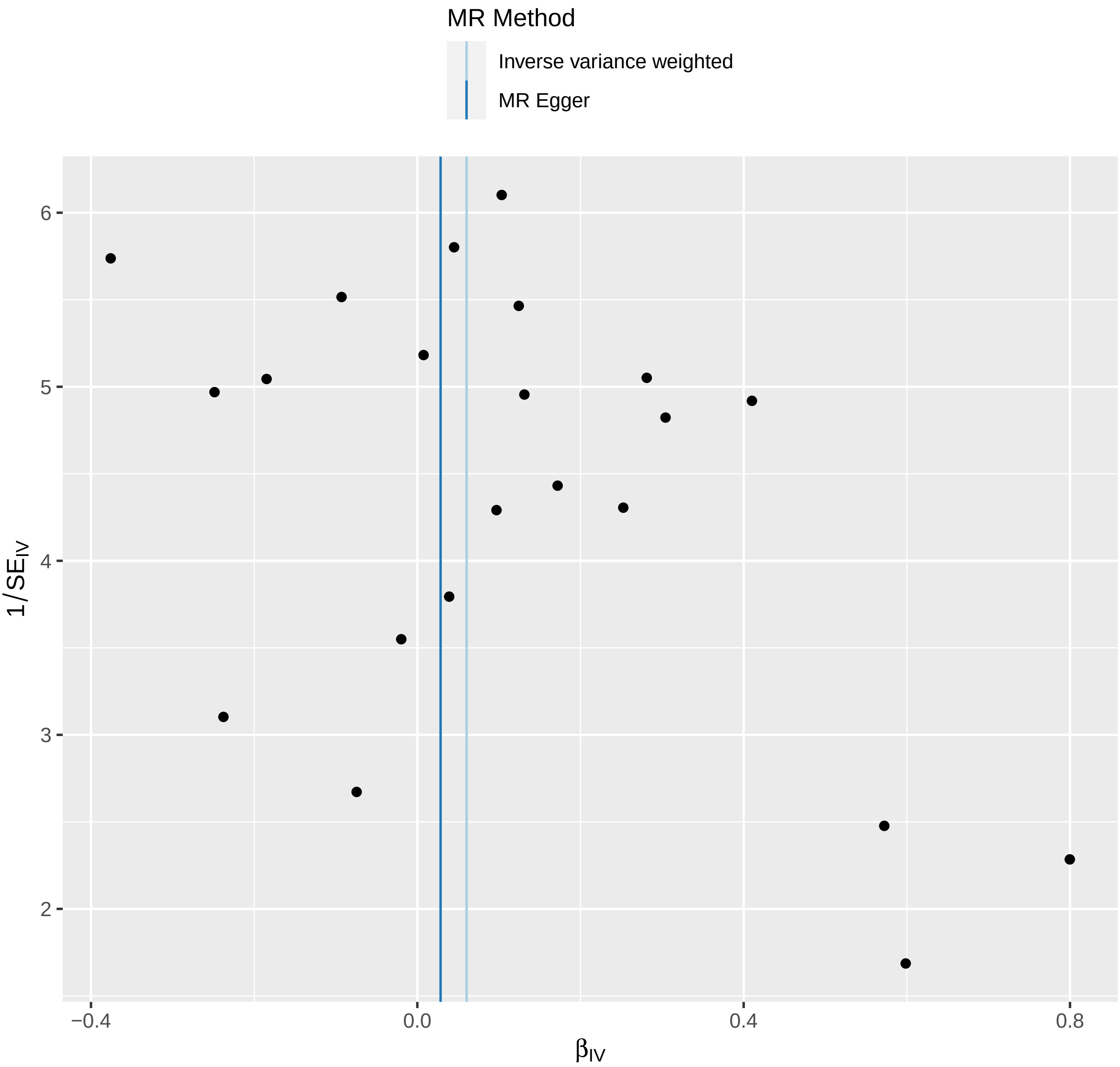

Batch 3−3019 : Transmembrane inner ear expressed protein on Diabetic nephropathy (more control exclusions) with a threshold of 1e−05

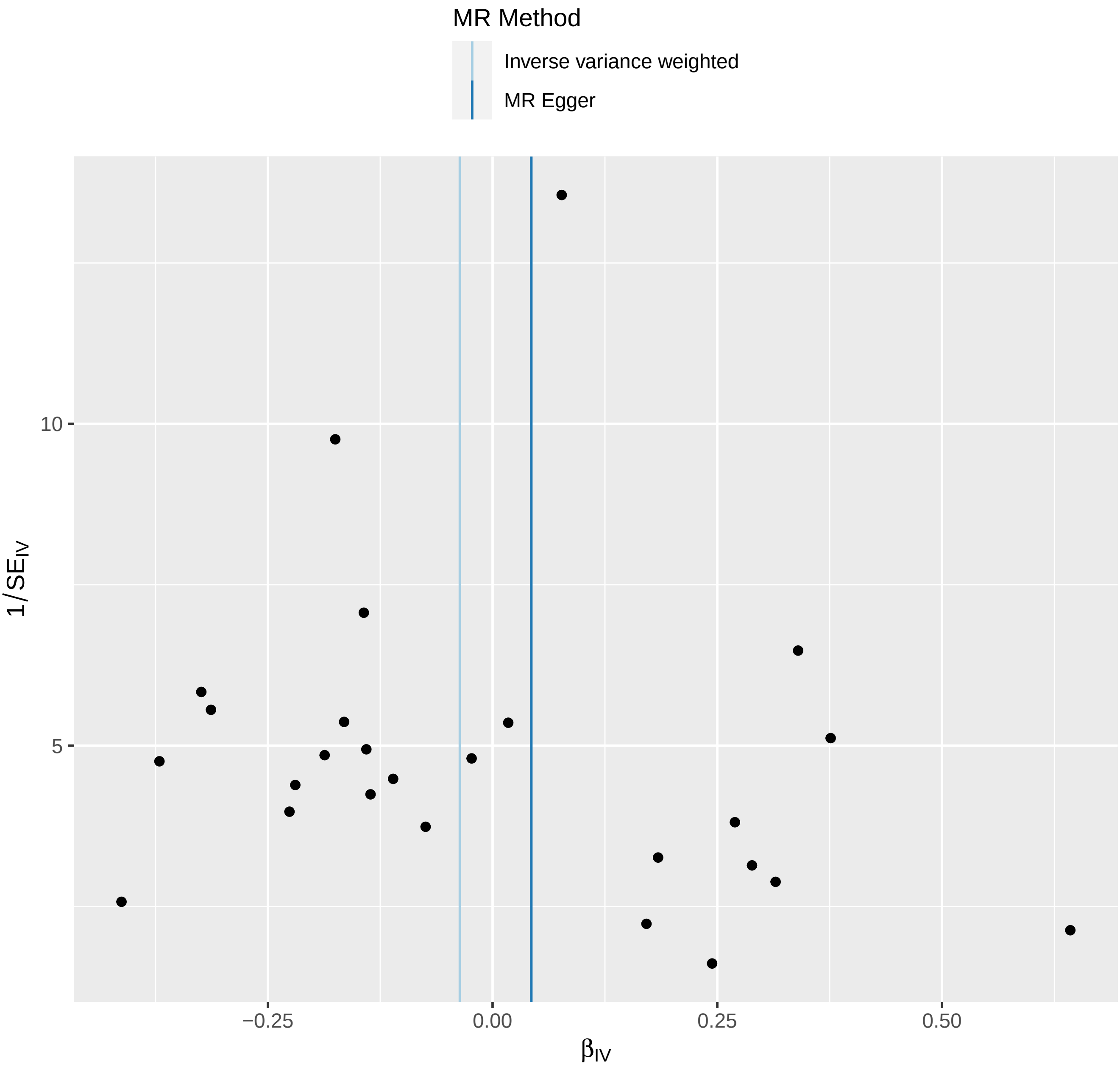

Batch 3−3020 : Tropomodulin−1 on Diabetic nephropathy (more control exclusions)
with a threshold of 1e−05

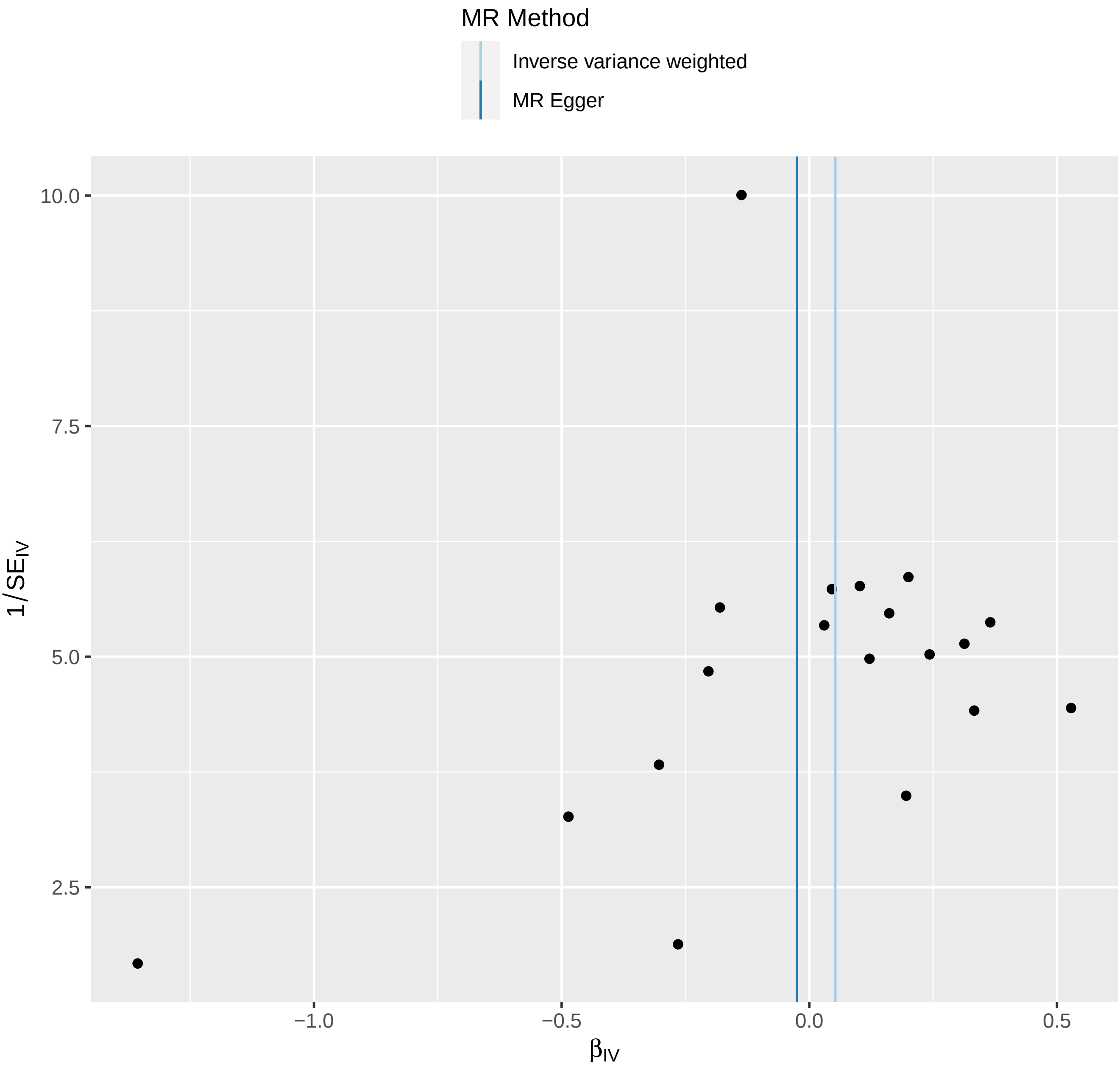

Batch 3−3021 : Lamina−associated polypeptide 2, isoforms beta/gamma on Diabetic nephropathy (more control exclusions) with a threshold of 1e−05

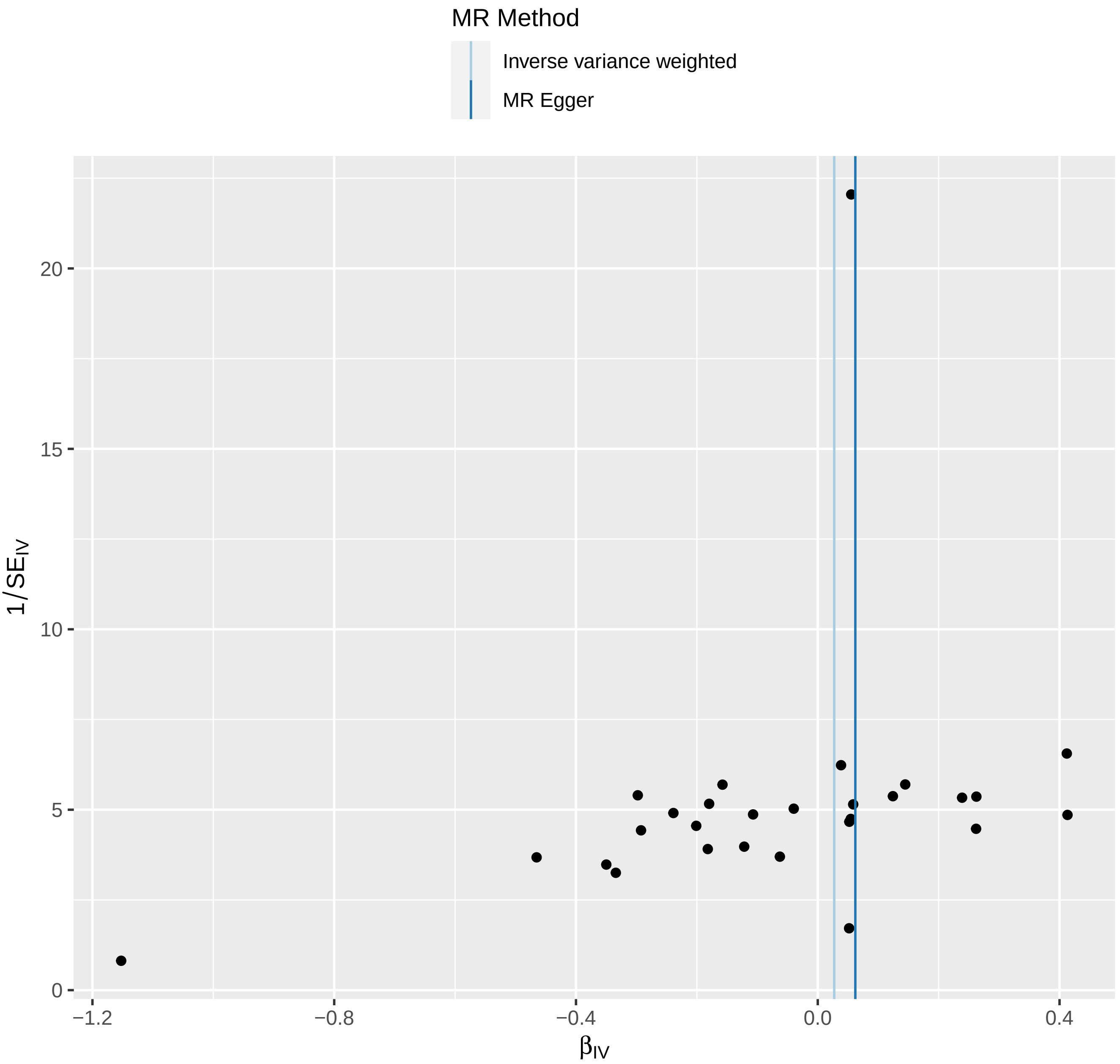

Batch 3−3022 : Transmembrane protease serine 11A on Diabetic nephropathy (more control exclusions)
with a threshold of 1e−05

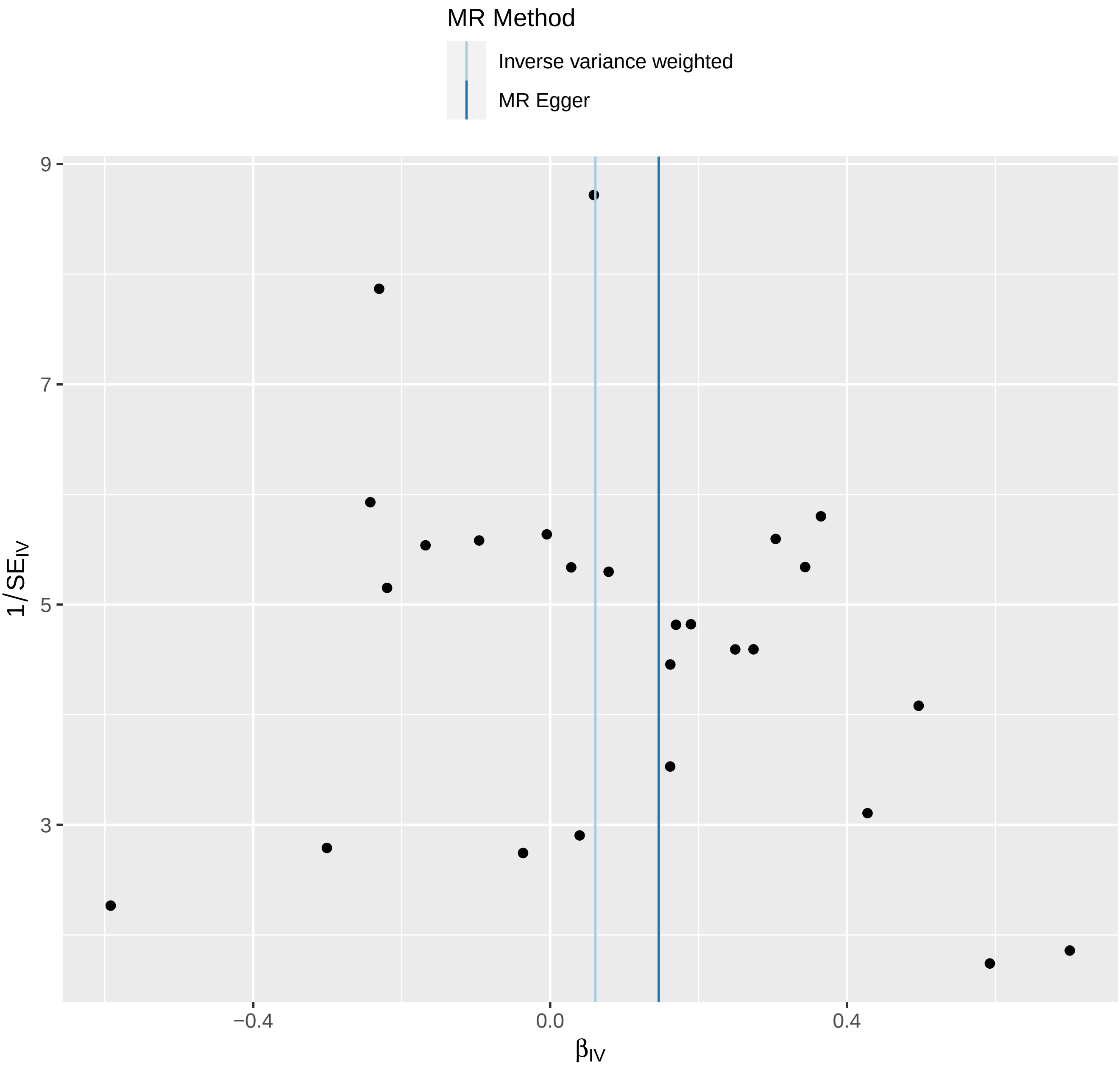

Batch 3−3023 : Transmembrane protease serine 11B on Diabetic nephropathy (more control exclusions)
with a threshold of 1e−05

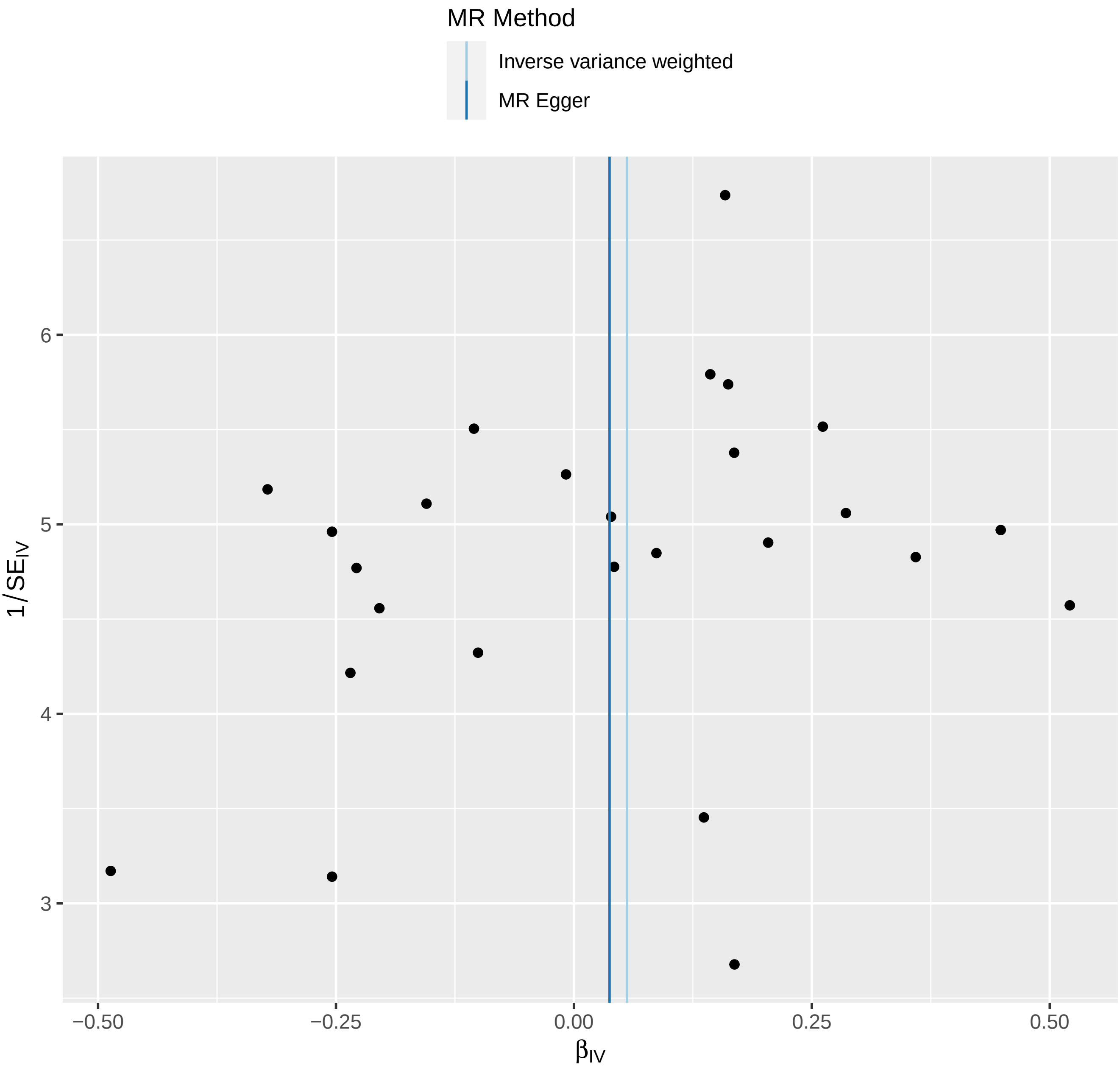

Batch 3−3024 : Transmembrane protease serine 11D on Diabetic nephropathy (more control exclusions)
with a threshold of 1e−05

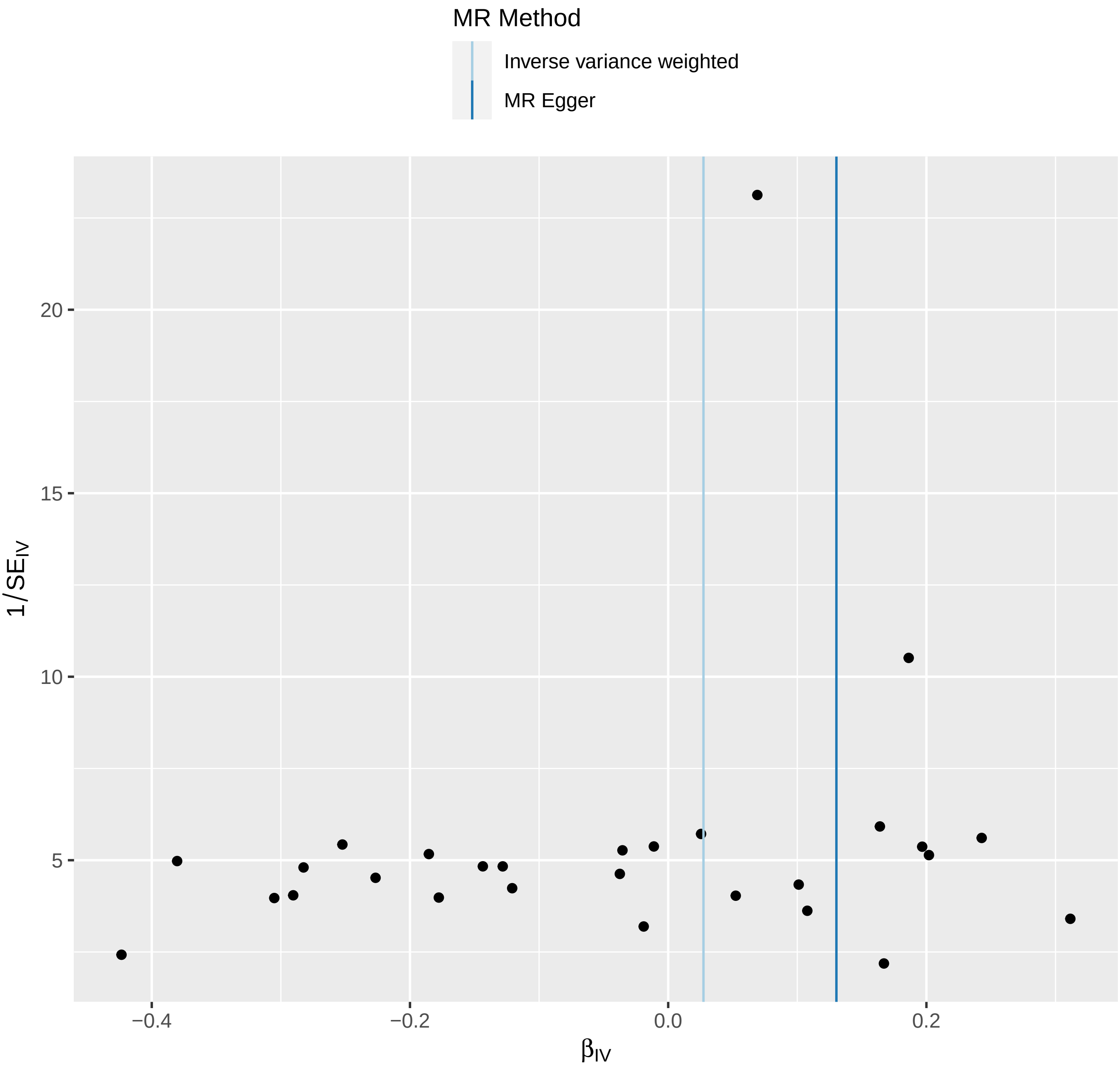

Batch 3−3025 : Enteropeptidase on Diabetic nephropathy (more control exclusions)
with a threshold of 1e−05

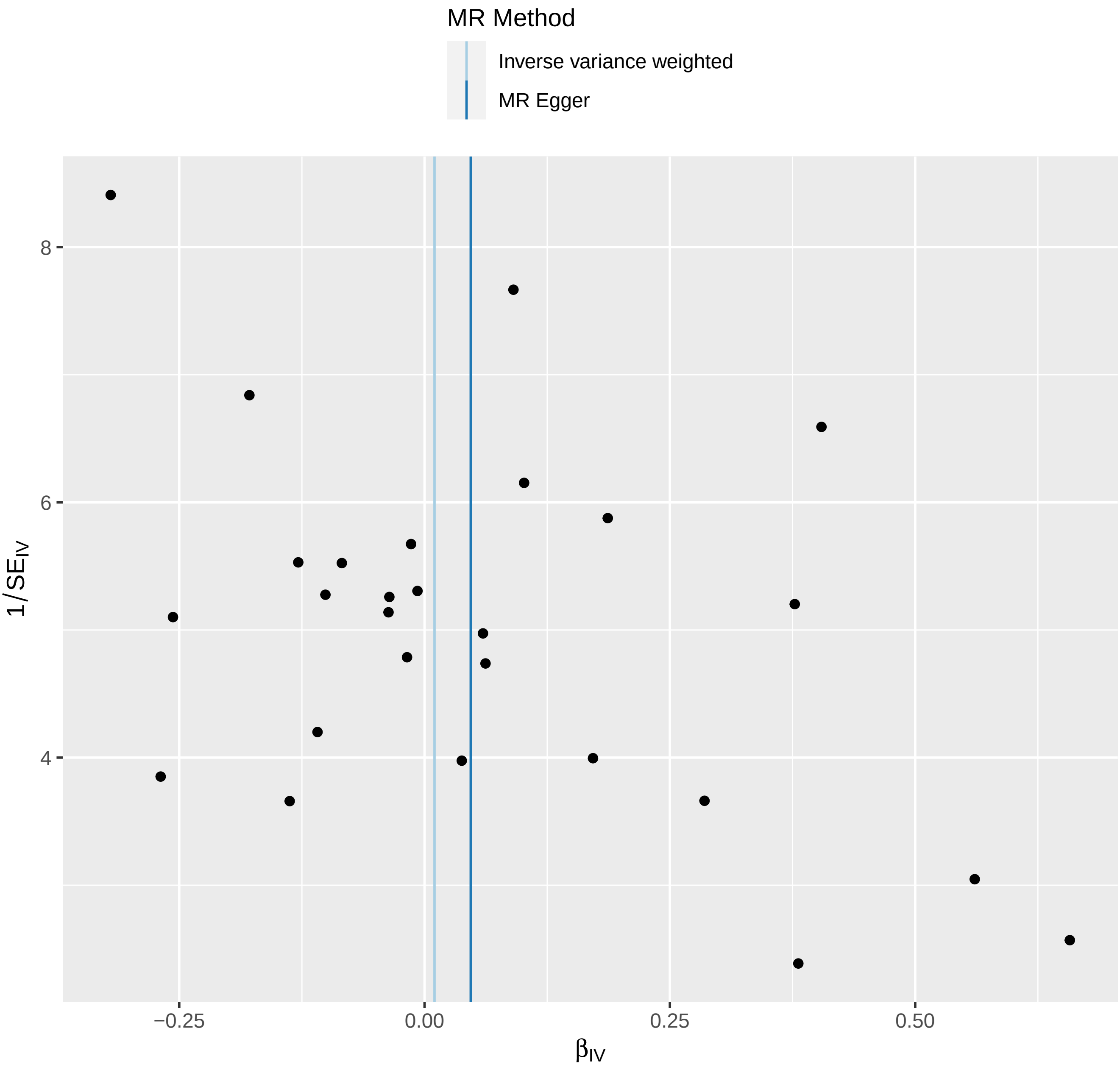

Batch 3−3026 : Transmembrane protease serine 5 on Diabetic nephropathy (more control exclusions)
with a threshold of 1e−05

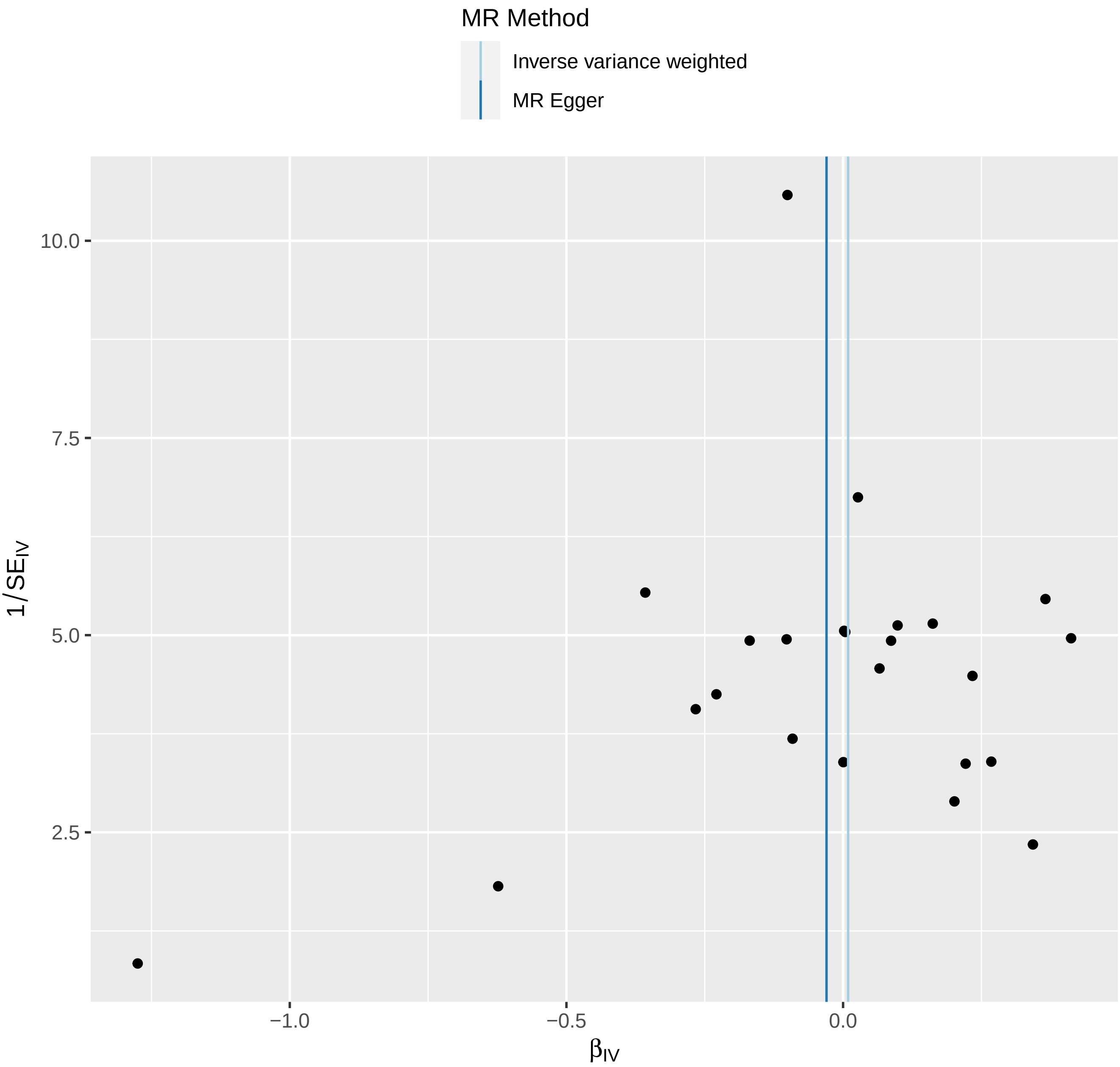

Batch 3−3027 : Transmembrane and ubiquitin−like domain−containing protein 2 on Diabetic nephropathy (more control exclusions) with a threshold of 1e−05

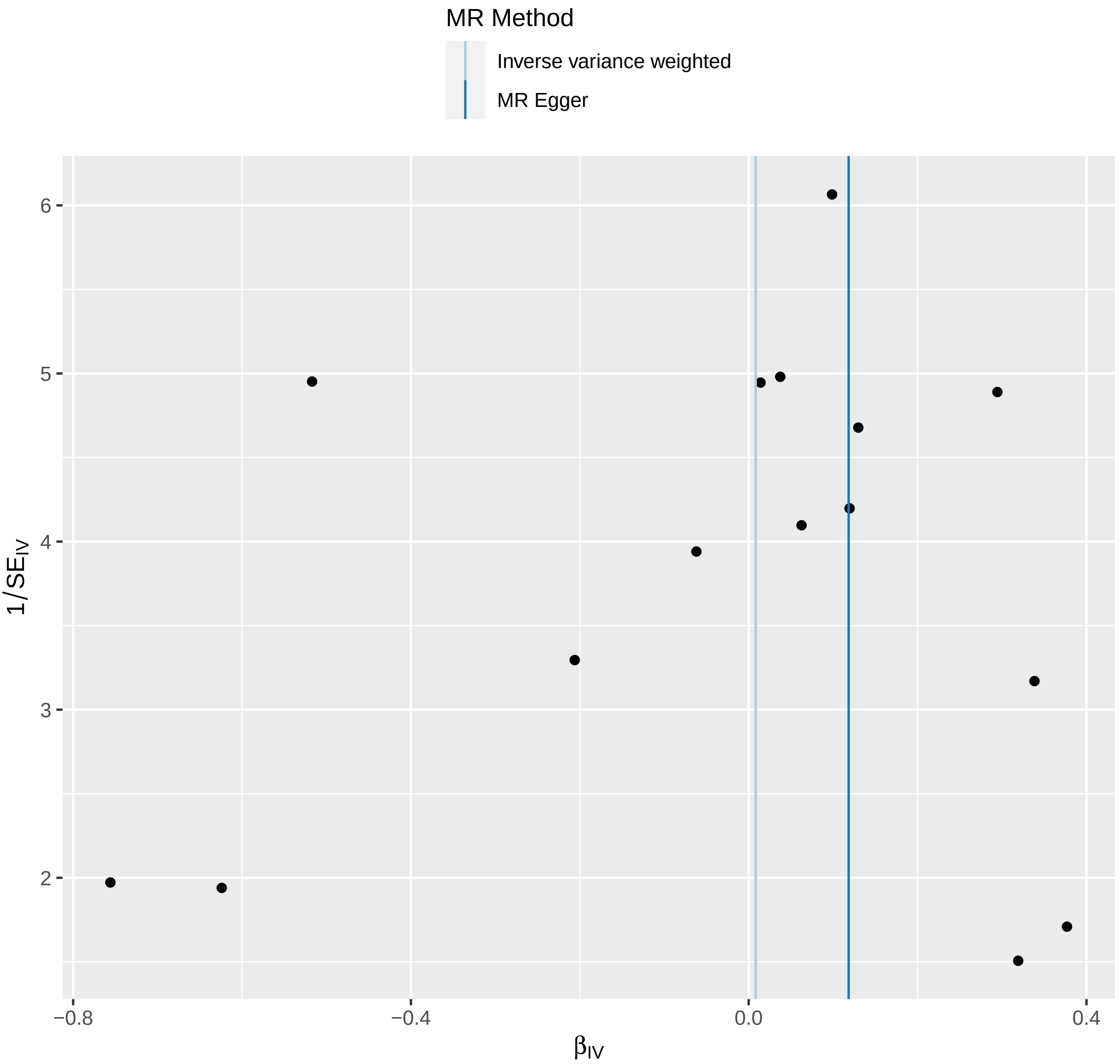

Batch 3−3028 : Tenascin on Diabetic nephropathy (more control exclusions)
with a threshold of 1e−05

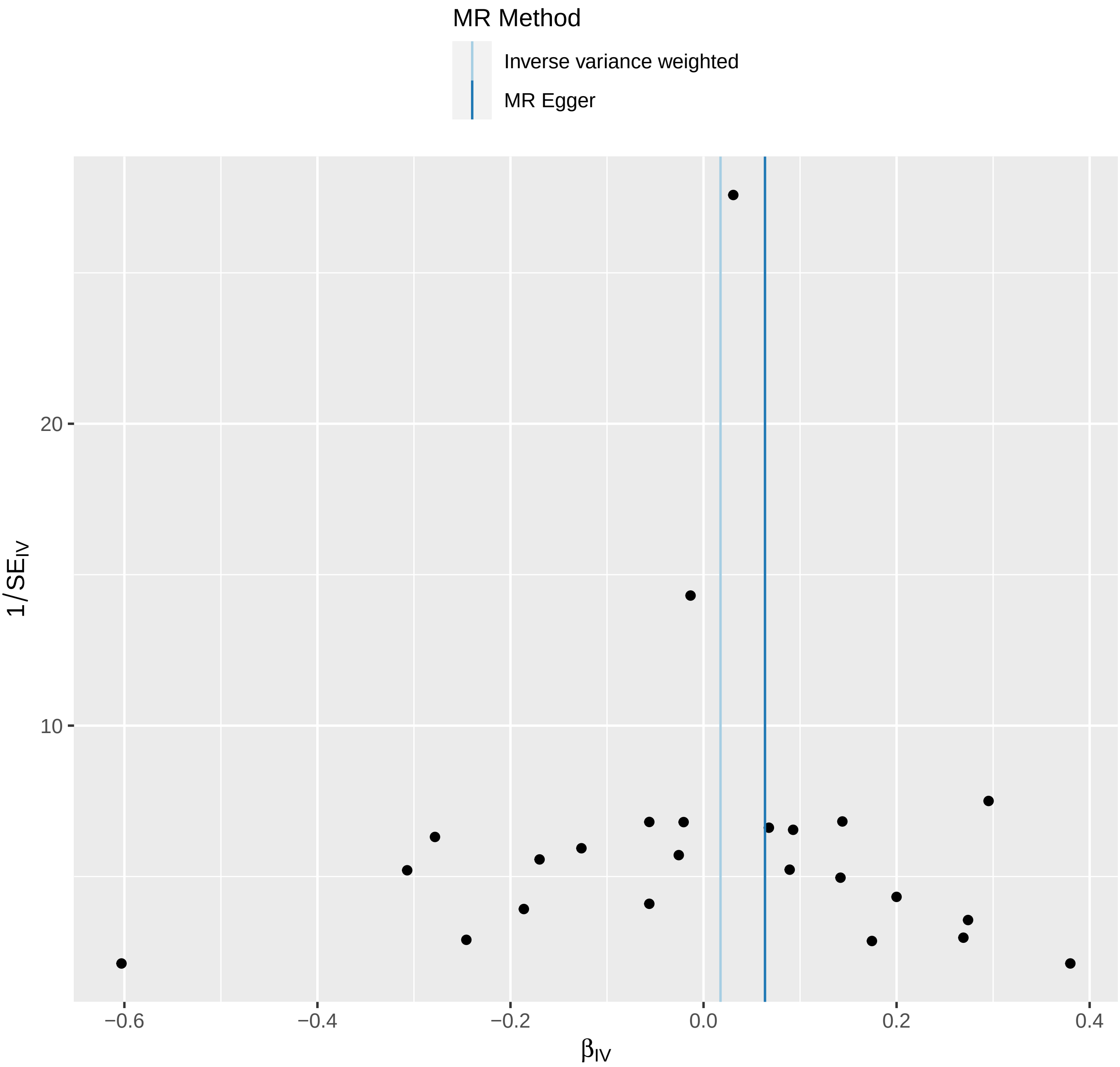

Batch 3−3029 : Tumor necrosis factor on Diabetic nephropathy (more control exclusions)
with a threshold of 1e−05

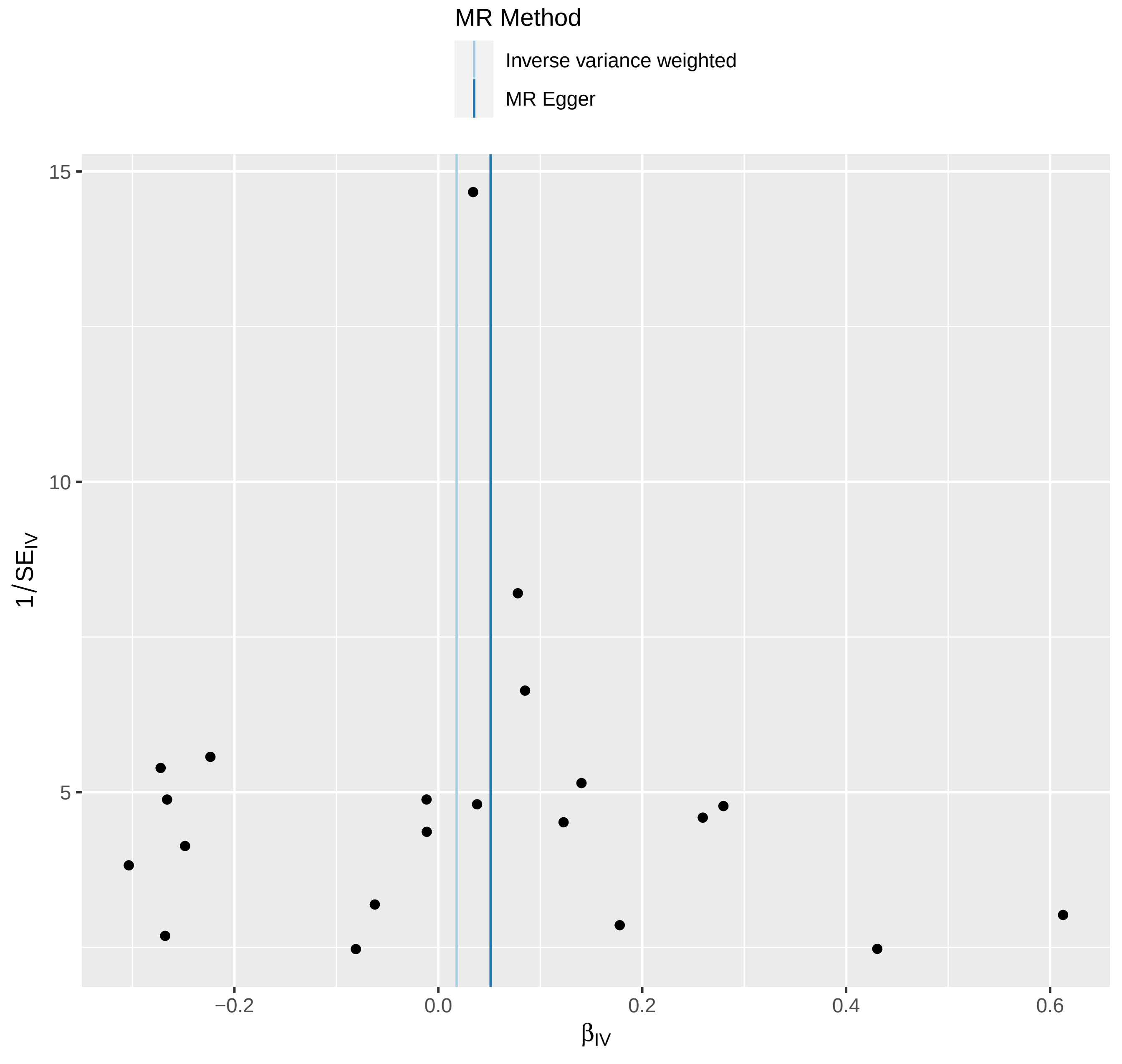

Batch 3−3030 : Tumor necrosis factor alpha−induced protein 3 on Diabetic nephropathy (more control exclusions)
with a threshold of 1e−05

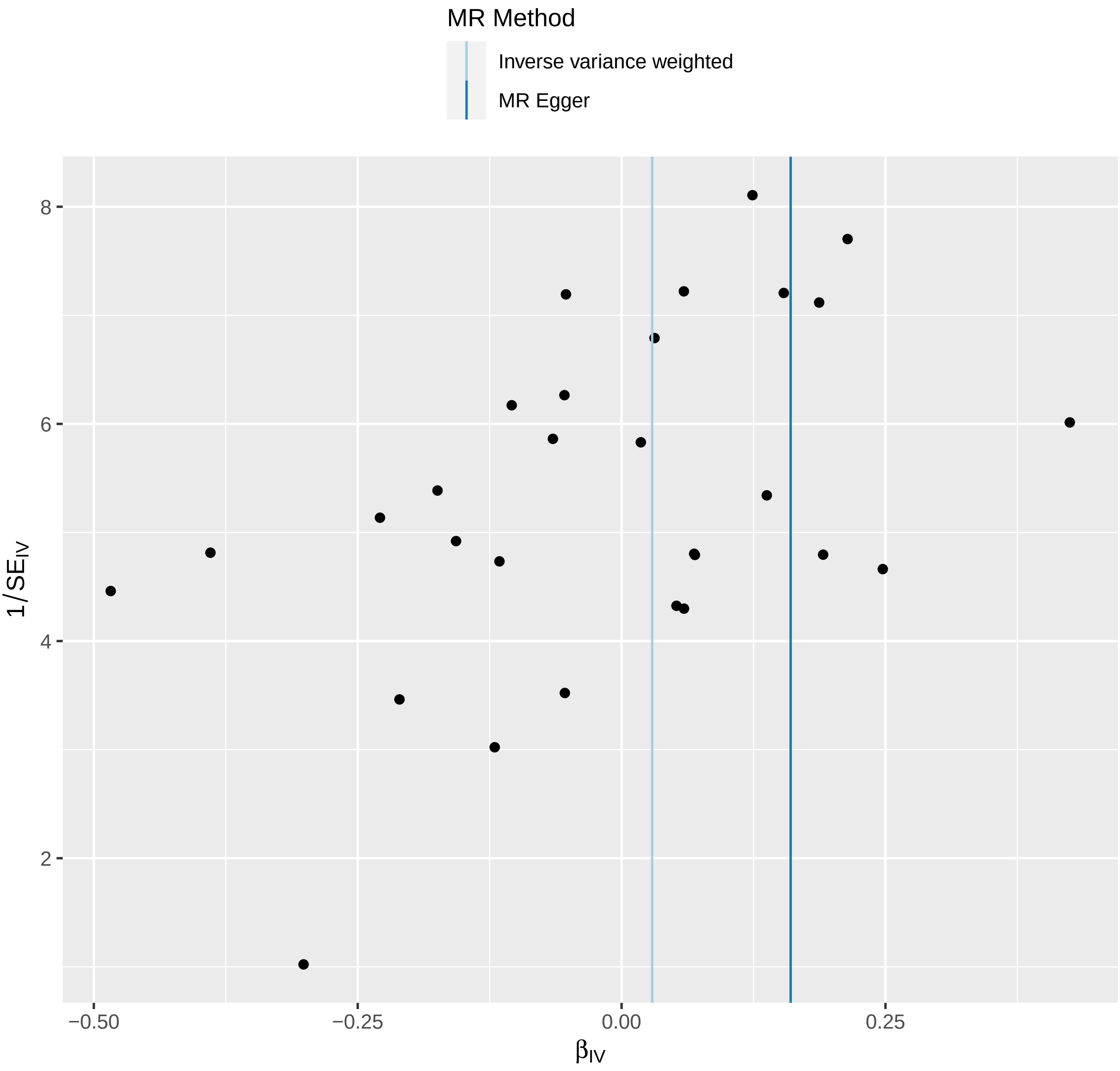

Batch 3−3031 : Tumor necrosis factor−inducible gene 6 protein on Diabetic nephropathy (more control exclusions)
with a threshold of 1e−05

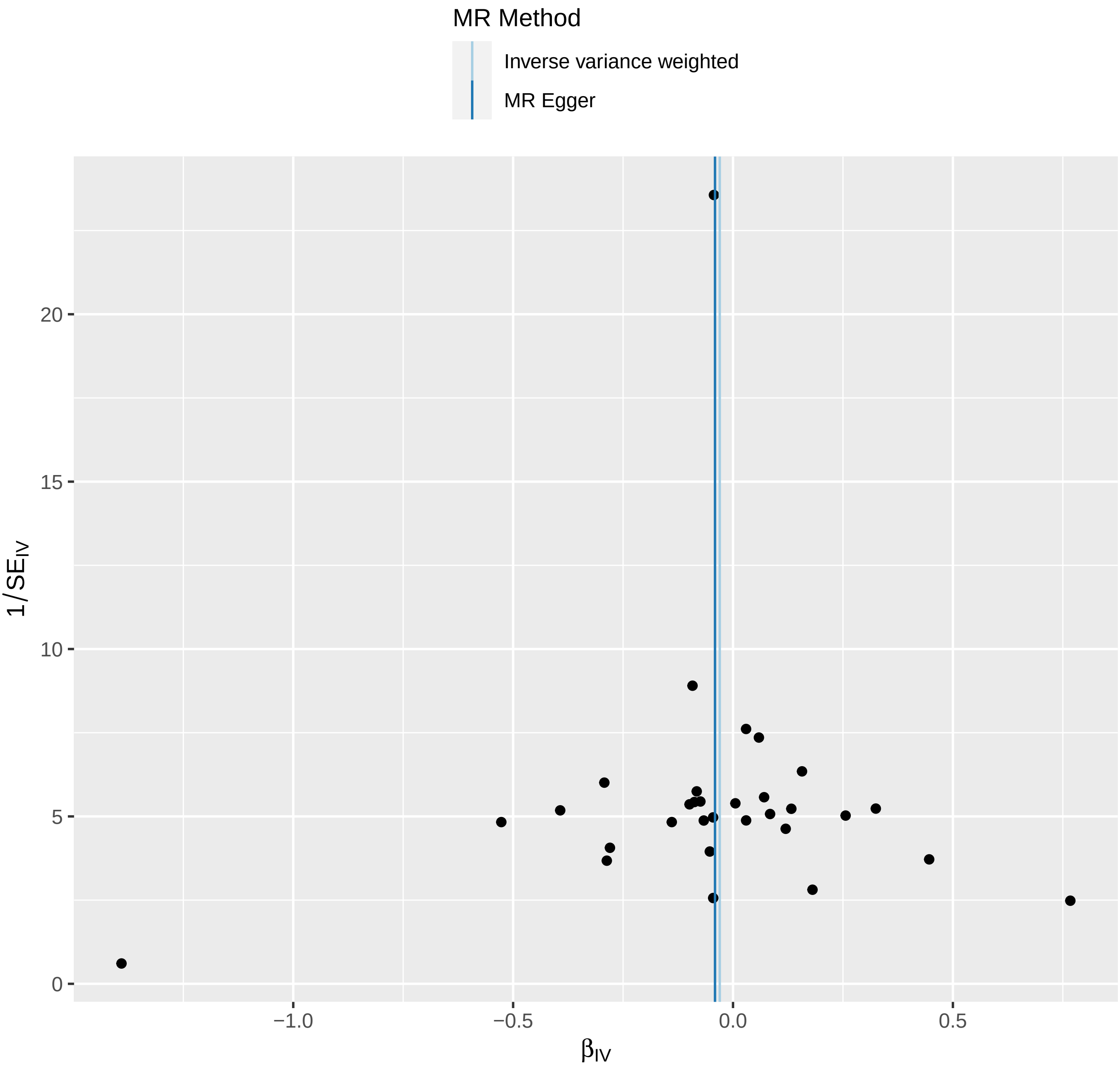

Batch 3−3032 : Tumor necrosis factor receptor superfamily member 10A on Diabetic nephropathy (more control exclusions) with a threshold of 1e−05

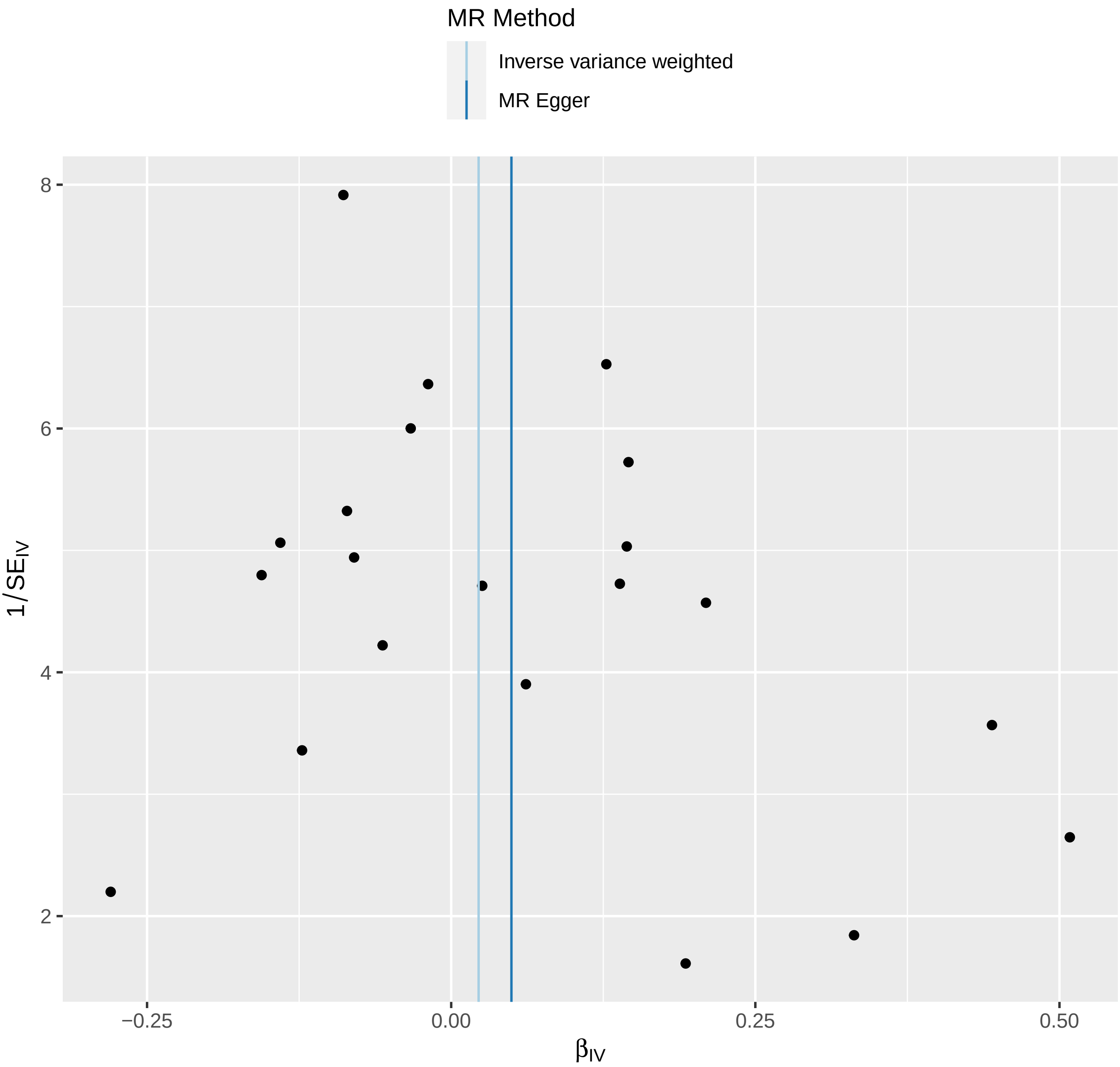

Batch 3−3033 : Tumor necrosis factor receptor superfamily member 10B on Diabetic nephropathy (more control exclusions)
with a threshold of 1e−05

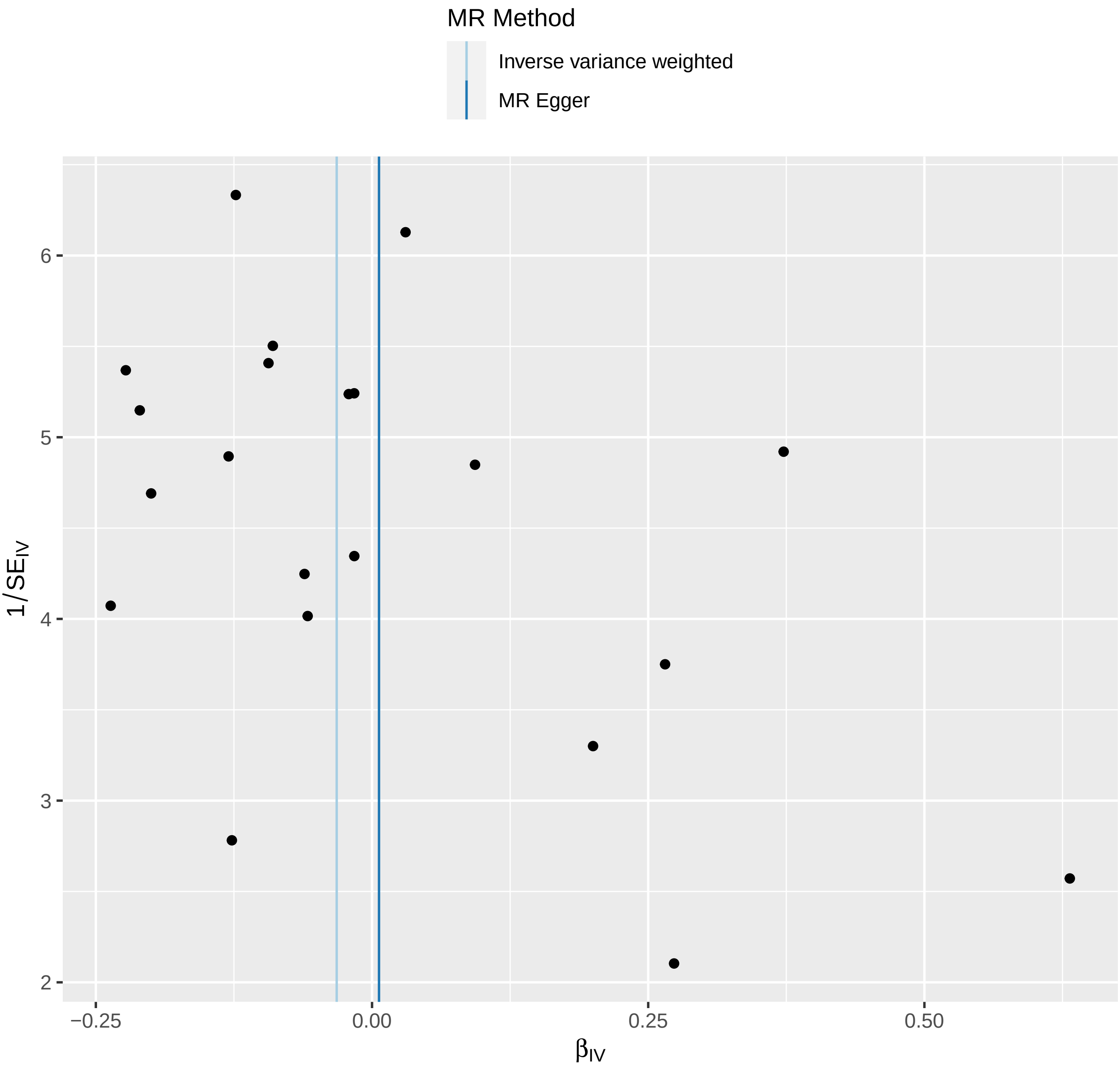

Batch 3−3034 : Tumor necrosis factor receptor superfamily member 10D on Diabetic nephropathy (more control exclusions)
with a threshold of 1e−05

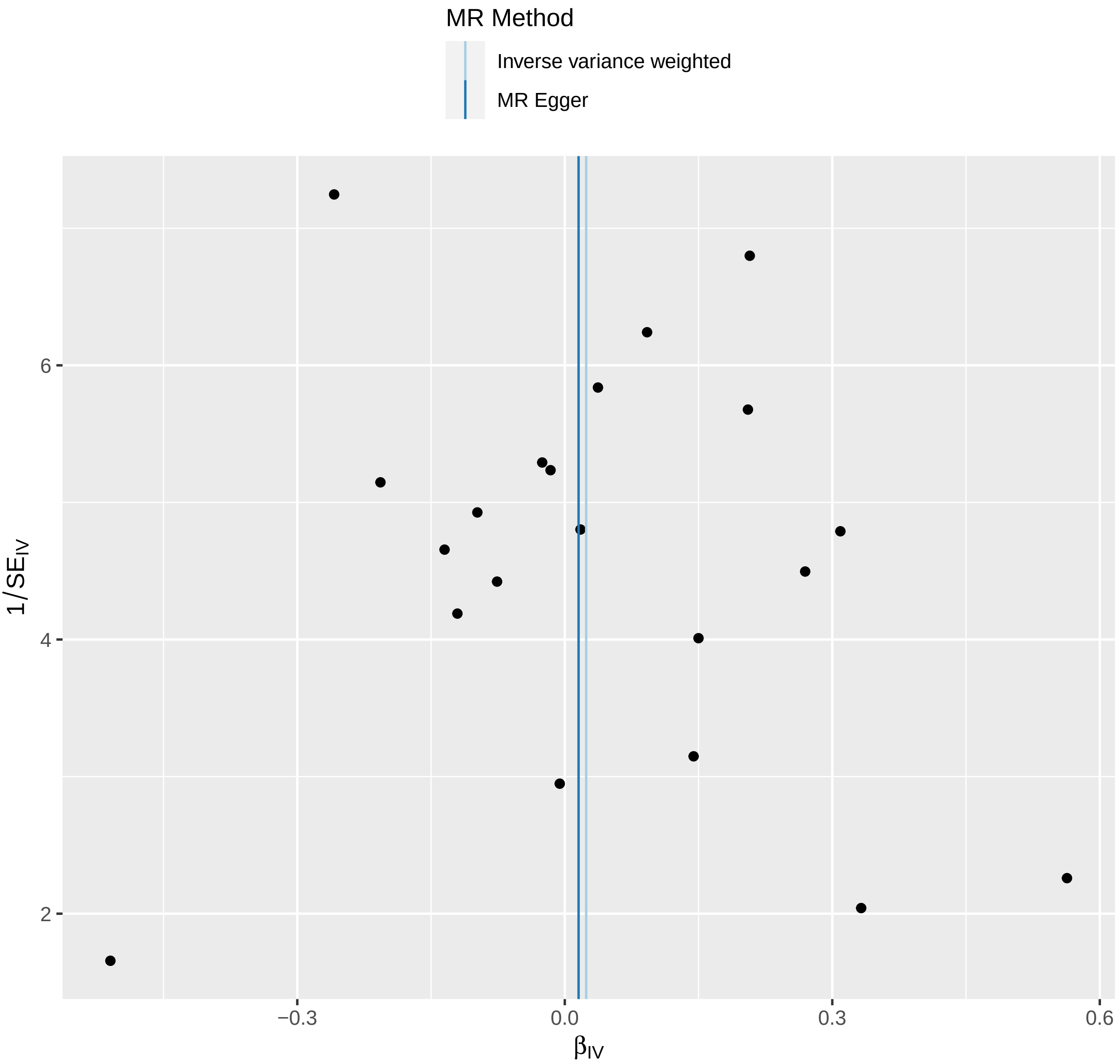

Batch 3−3035 : Tumor necrosis factor receptor superfamily member 11A on Diabetic nephropathy (more control exclusions)
with a threshold of 1e−05

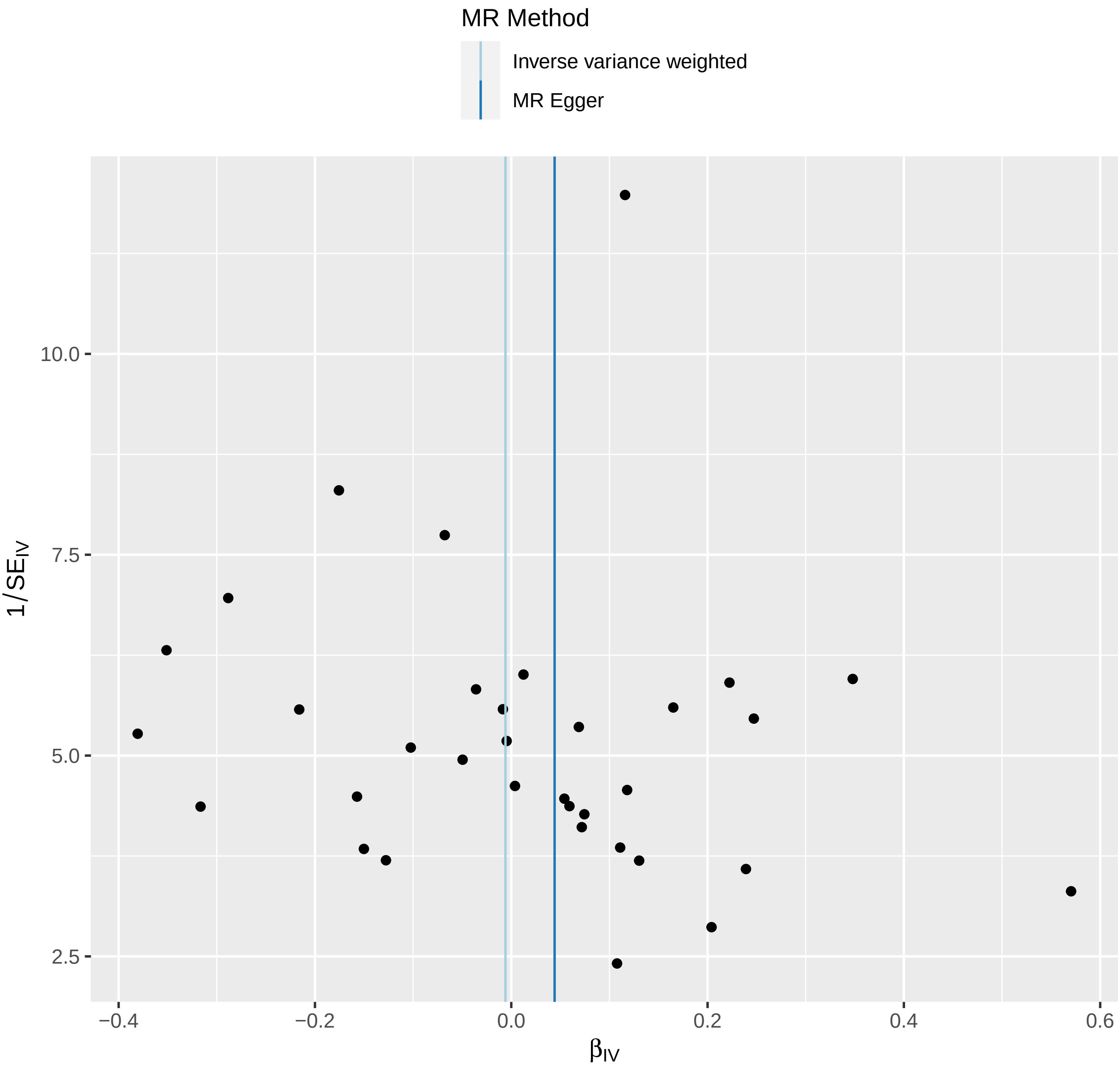

Batch 3−3036 : Tumor necrosis factor receptor superfamily member 11A on Diabetic nephropathy (more control exclusions) with a threshold of 1e−05

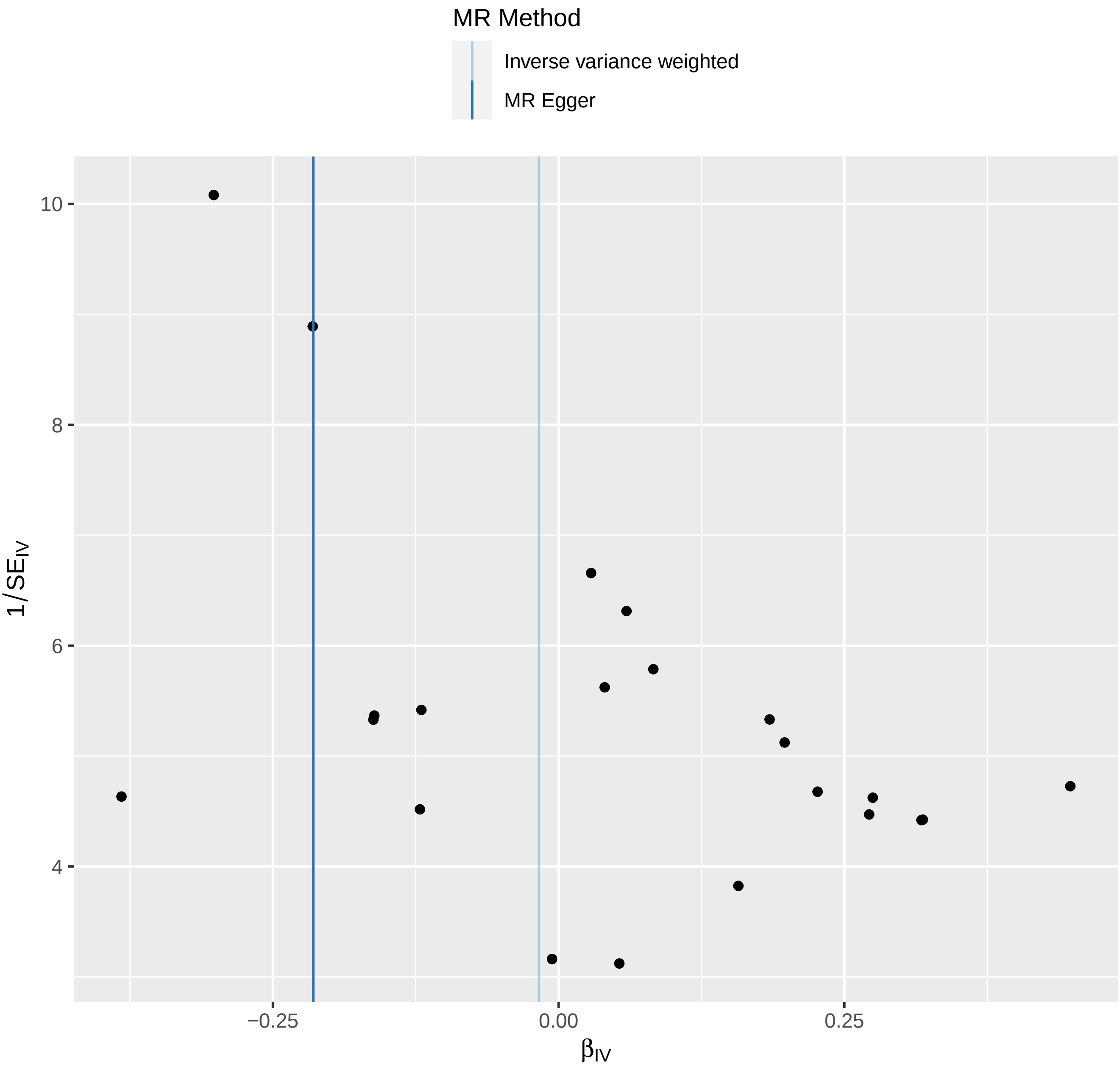

Batch 3−3037 : Tumor necrosis factor receptor superfamily member 11A on Diabetic nephropathy (more control exclusions) with a threshold of 1e−05

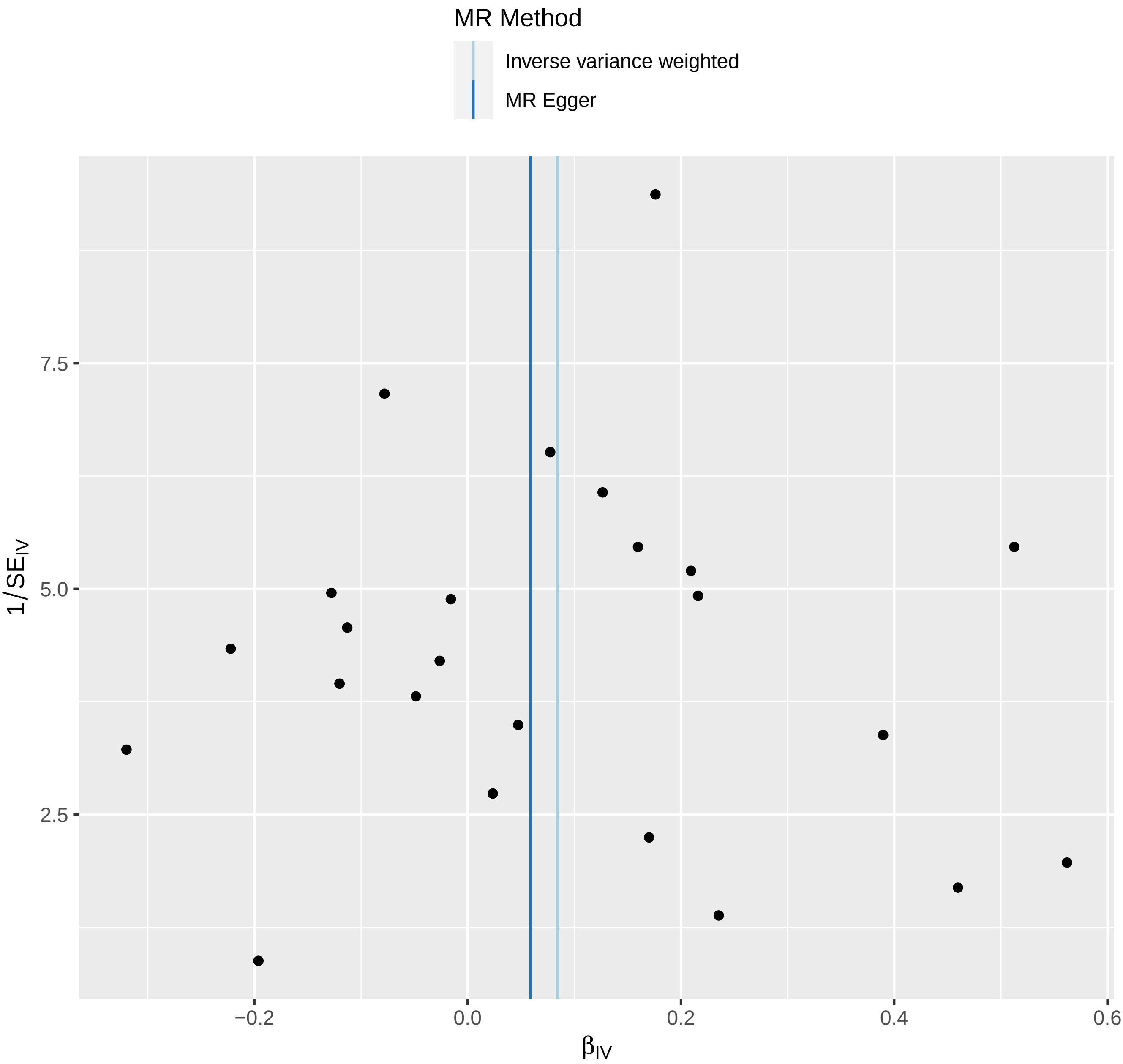

Batch 3−3038 : Tumor necrosis factor receptor superfamily member 11B on Diabetic nephropathy (more control exclusions)
with a threshold of 1e−05

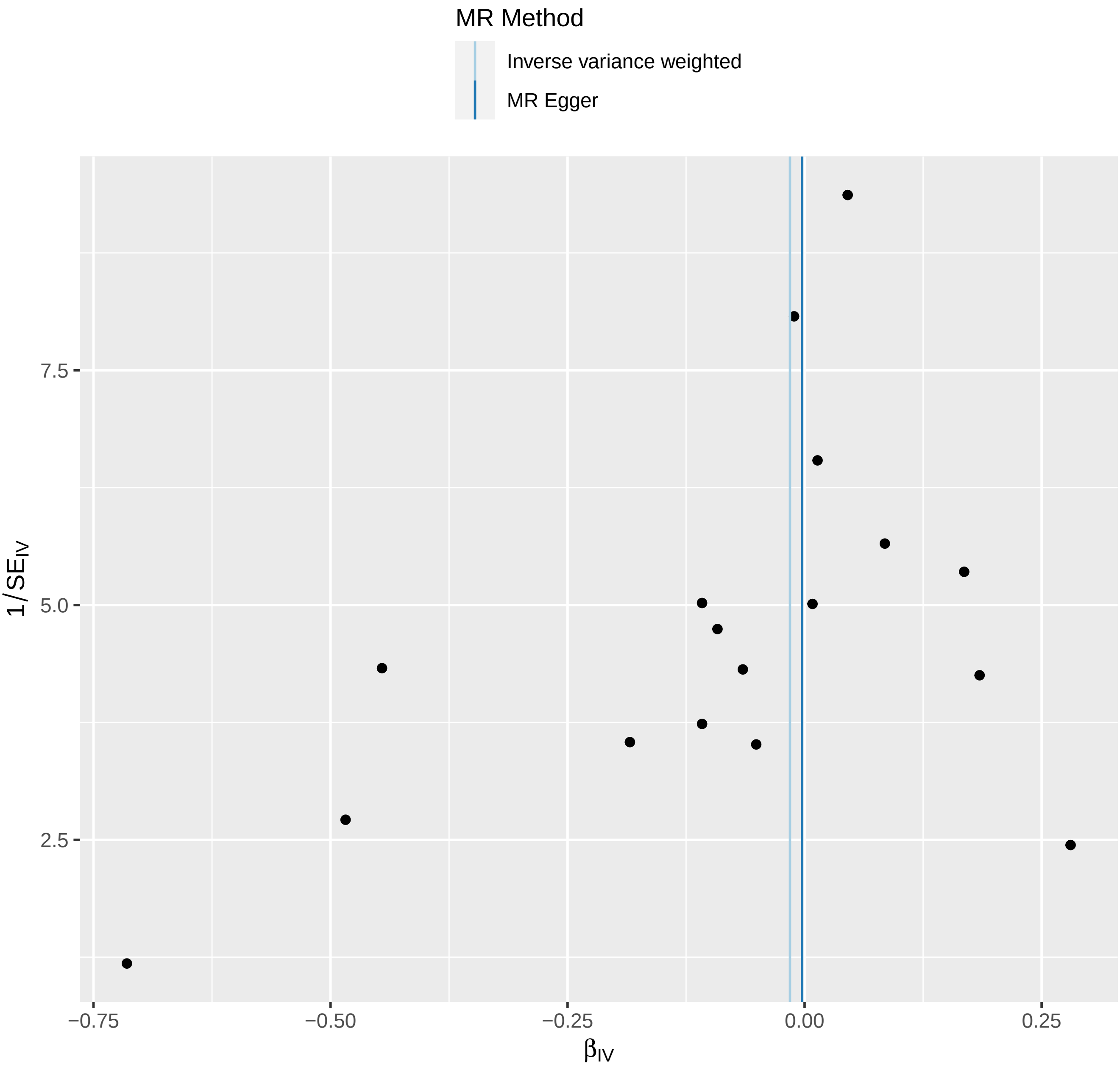

Batch 3−3039 : Tumor necrosis factor receptor superfamily member 11B on Diabetic nephropathy (more control exclusions) with a threshold of 1e−05

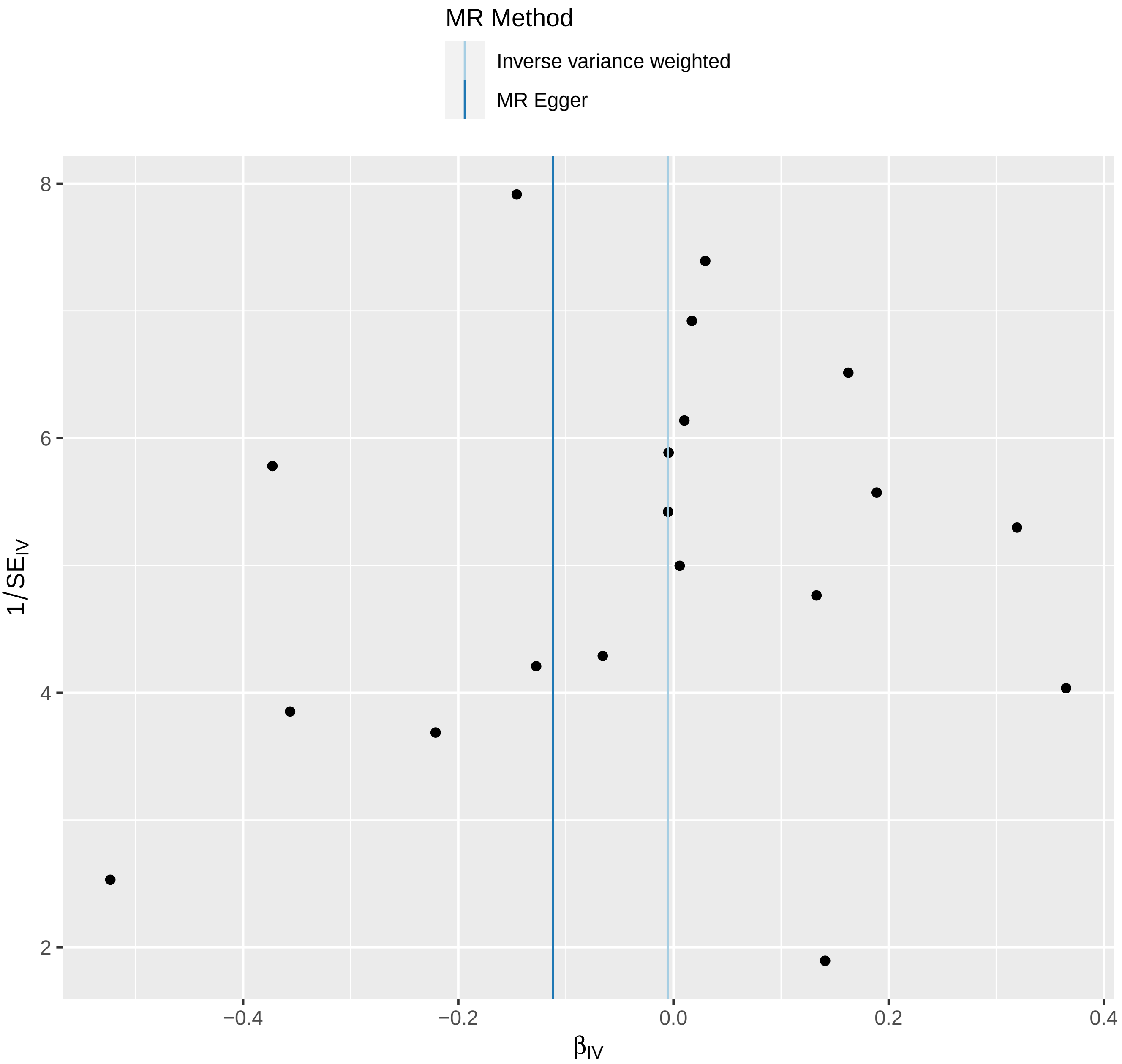

Batch 3−3040 : Tumor necrosis factor receptor superfamily member 12A on Diabetic nephropathy (more control exclusions)
with a threshold of 1e−05

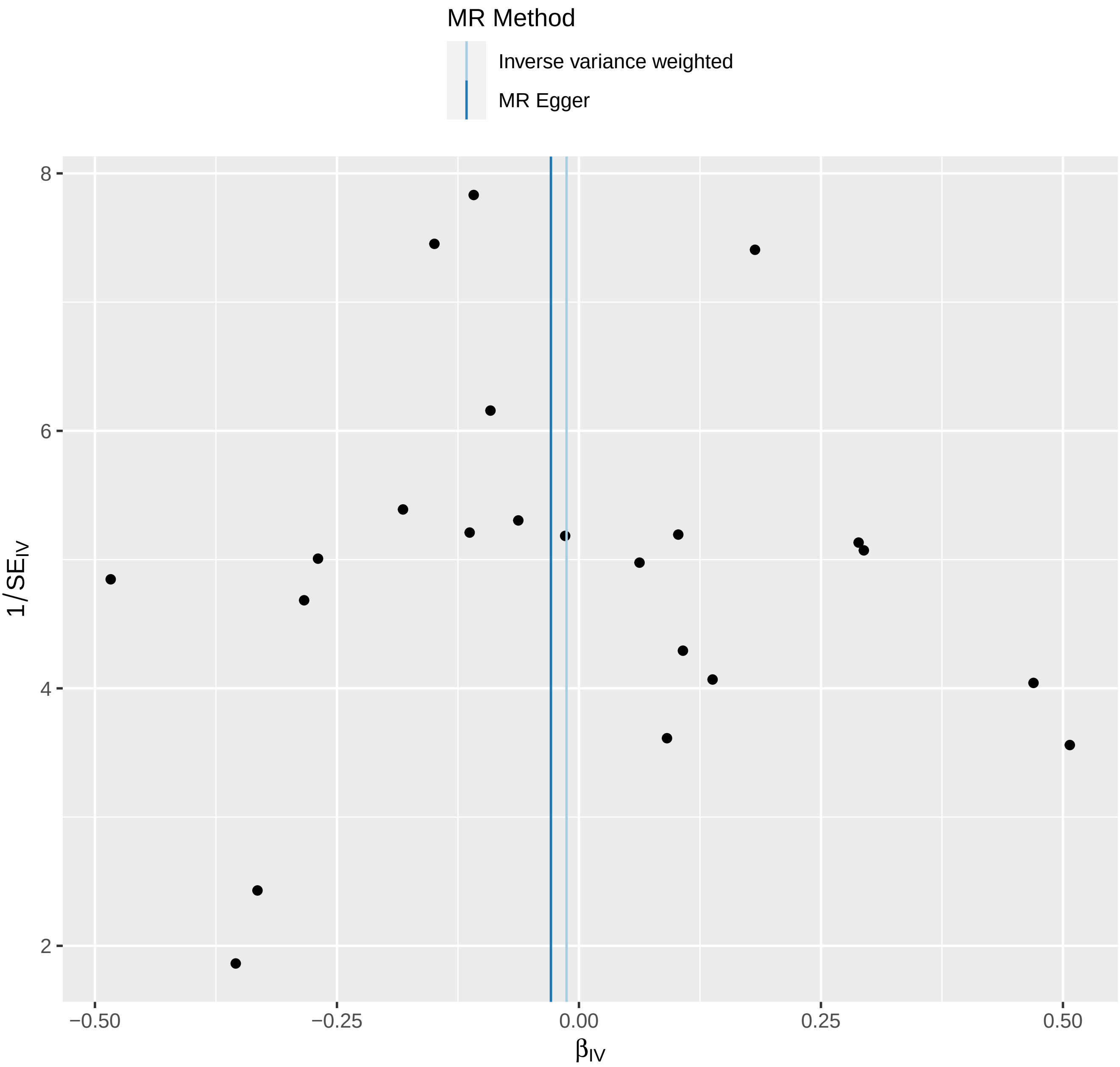

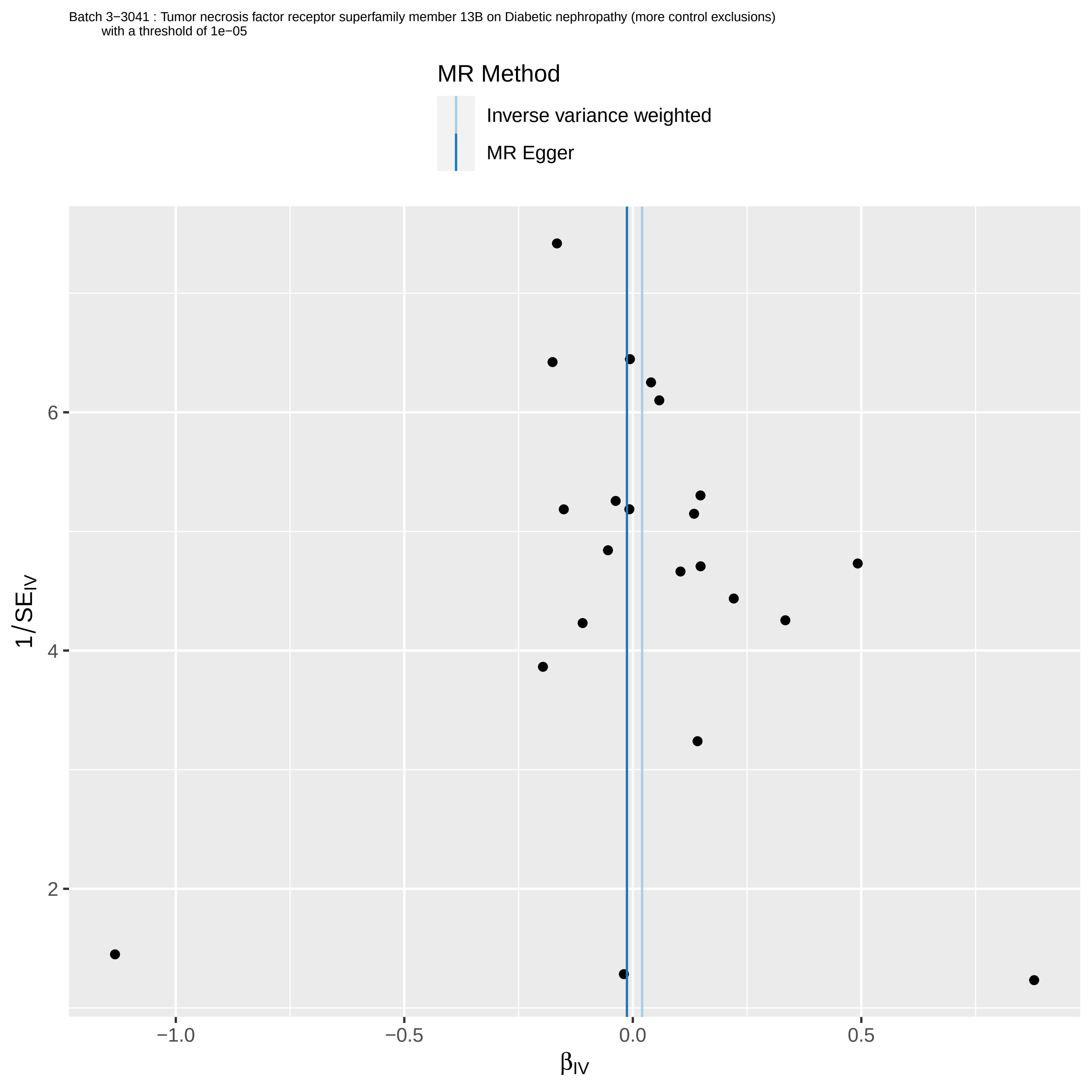
Batch 3−3041 : Tumor necrosis factor receptor superfamily member 13B on Diabetic nephropathy (more control exclusions)
with a threshold of 1e−05
MR Method
Inverse variance weighted
MR Egger
6
4
2
1/SE_IV
−1.0
−0.5
0.0
0.5
β_IV

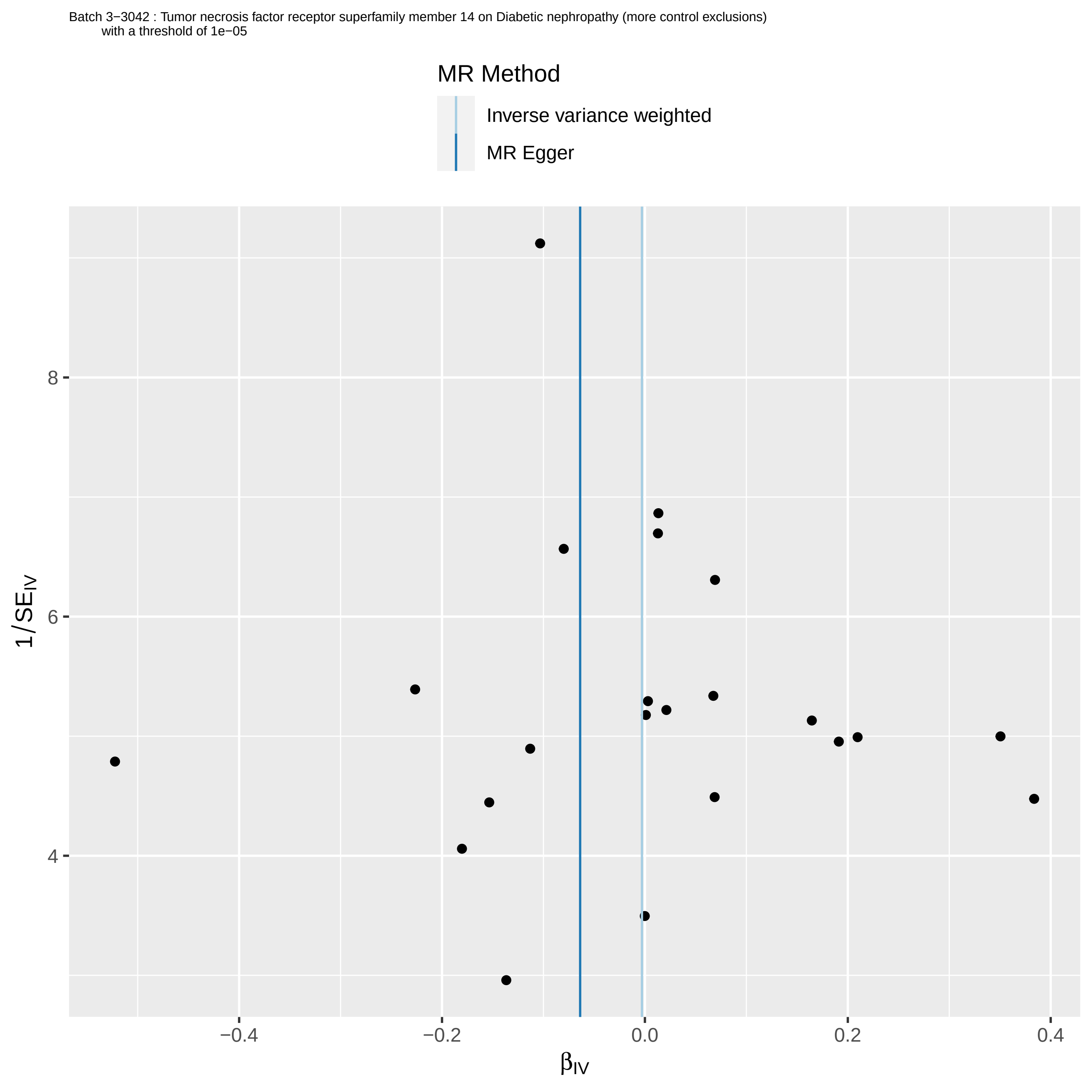
Batch 3−3042 : Tumor necrosis factor receptor superfamily member 14 on Diabetic nephropathy (more control exclusions)
with a threshold of 1e−05
MR Method
Inverse variance weighted
MR Egger
8
6
4
1/SE_IV
−0.4
−0.2
0.0
0.2
0.4
β_IV

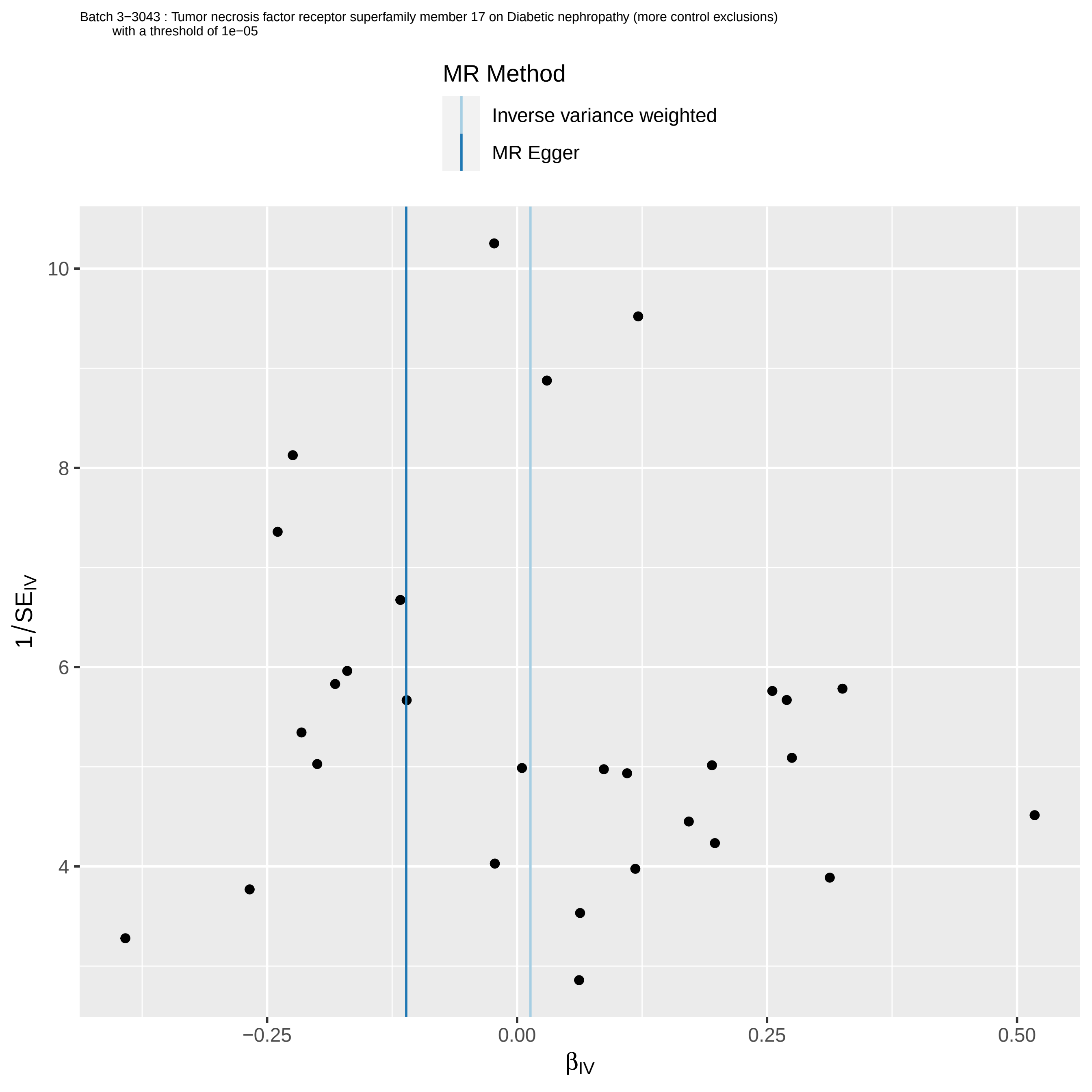

Batch 3−3043 : Tumor necrosis factor receptor superfamily member 17 on Diabetic nephropathy (more control exclusions)
with a threshold of 1e−05
MR Method
Inverse variance weighted
MR Egger
10
8
6
4
1/SE_IV
−0.25
0.00
0.25
0.50
β_IV

Batch 3−3044 : Tumor necrosis factor receptor superfamily member 18 on Diabetic nephropathy (more control exclusions)
with a threshold of 1e−05

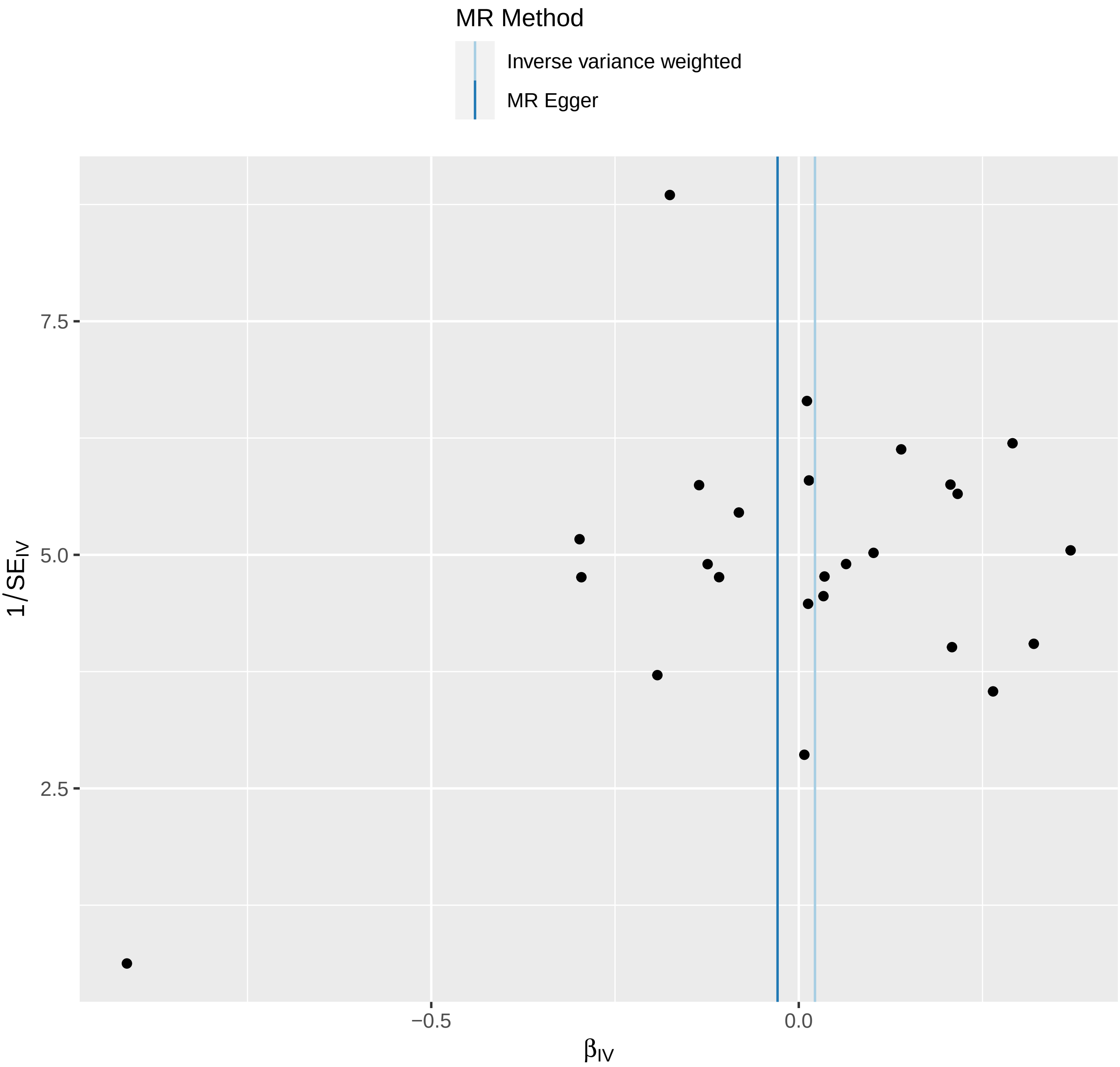

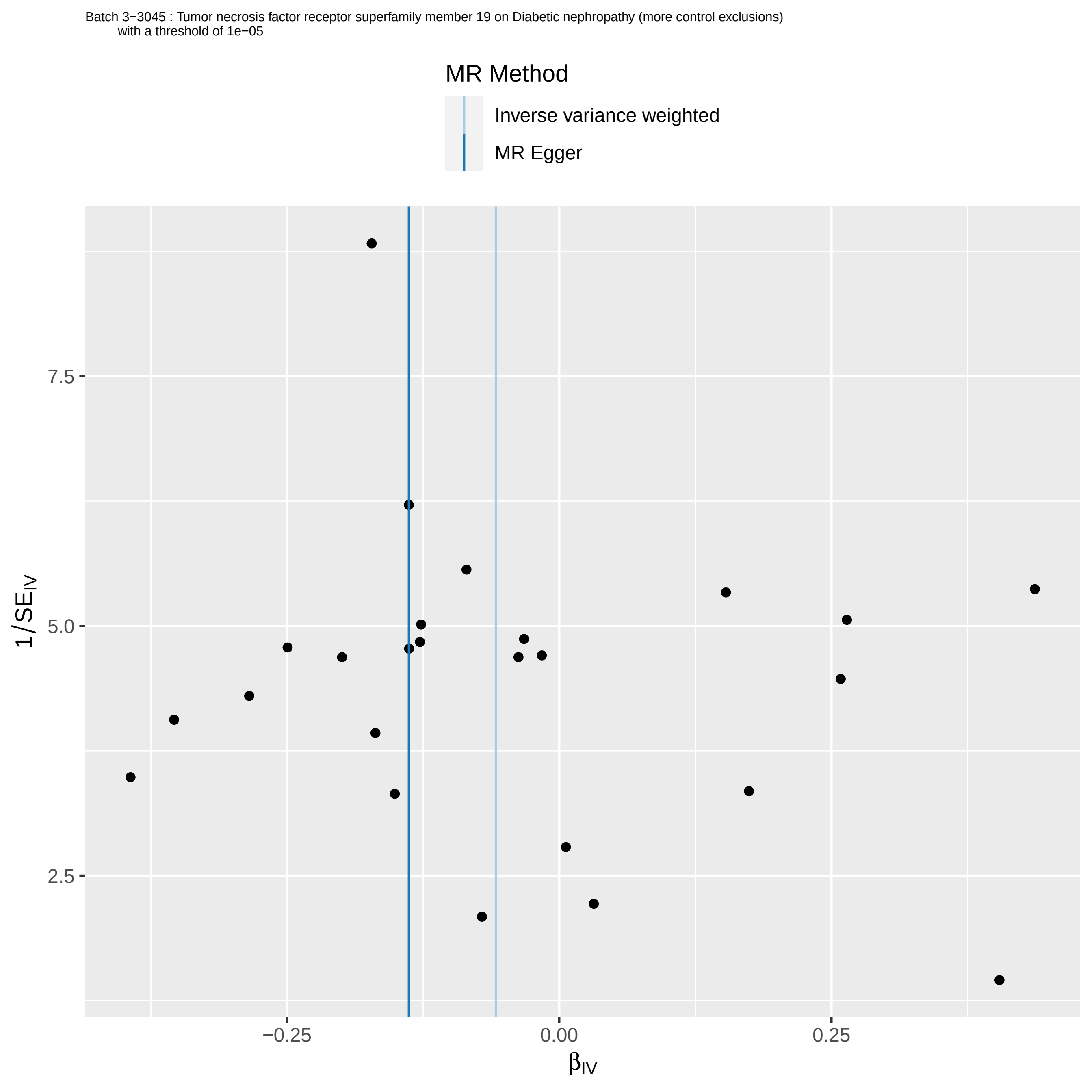

Batch 3−3045 : Tumor necrosis factor receptor superfamily member 19 on Diabetic nephropathy (more control exclusions)
with a threshold of 1e−05
MR Method
Inverse variance weighted
MR Egger
7.5
5.0
2.5
−0.25
0.00
0.25
1/SE_IV
β_IV

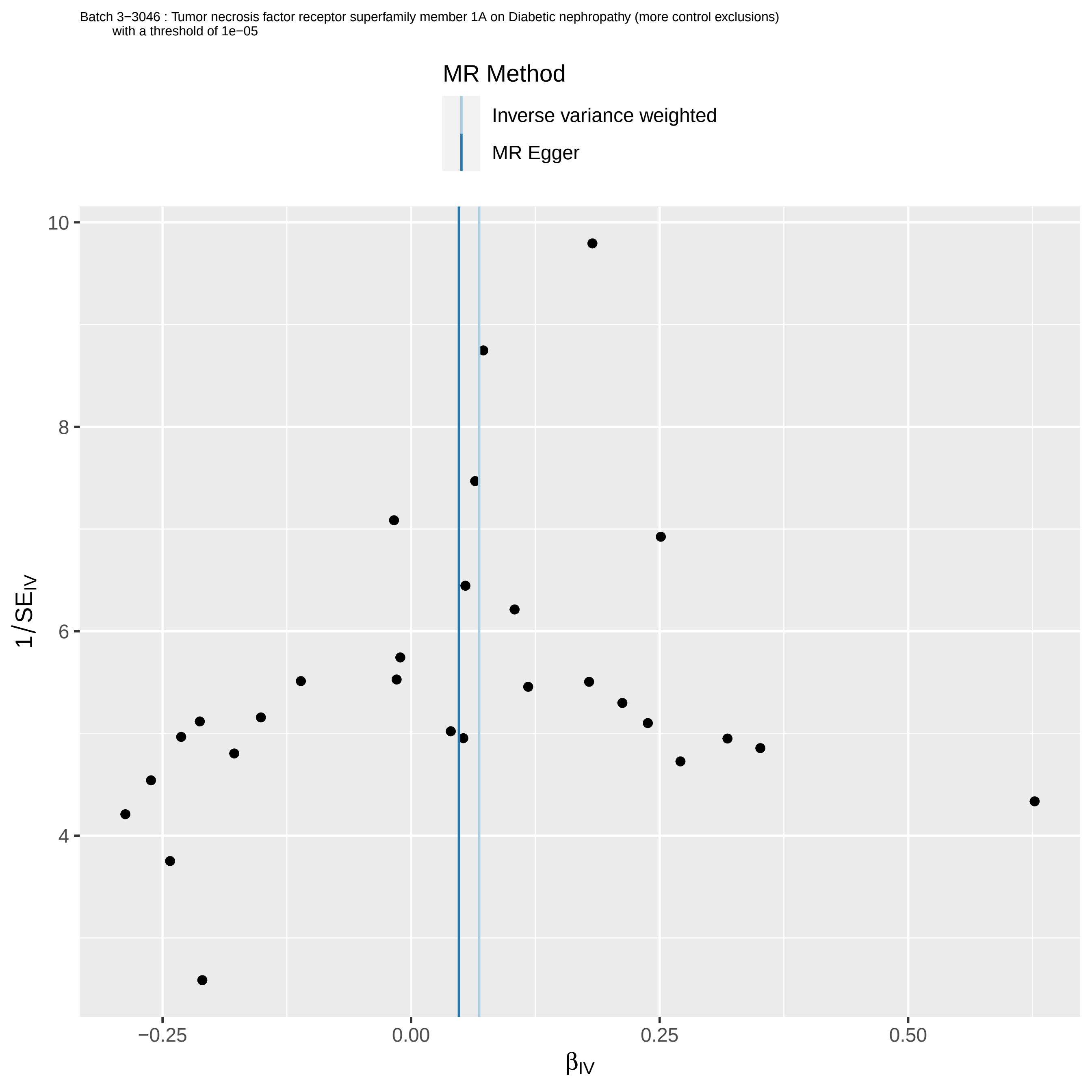
Batch 3−3046 : Tumor necrosis factor receptor superfamily member 1A on Diabetic nephropathy (more control exclusions)
with a threshold of 1e−05
MR Method
Inverse variance weighted
MR Egger
10
8
6
4
1/SE$_{IV}$
−0.25
0.00
0.25
0.50
β$_{IV}$

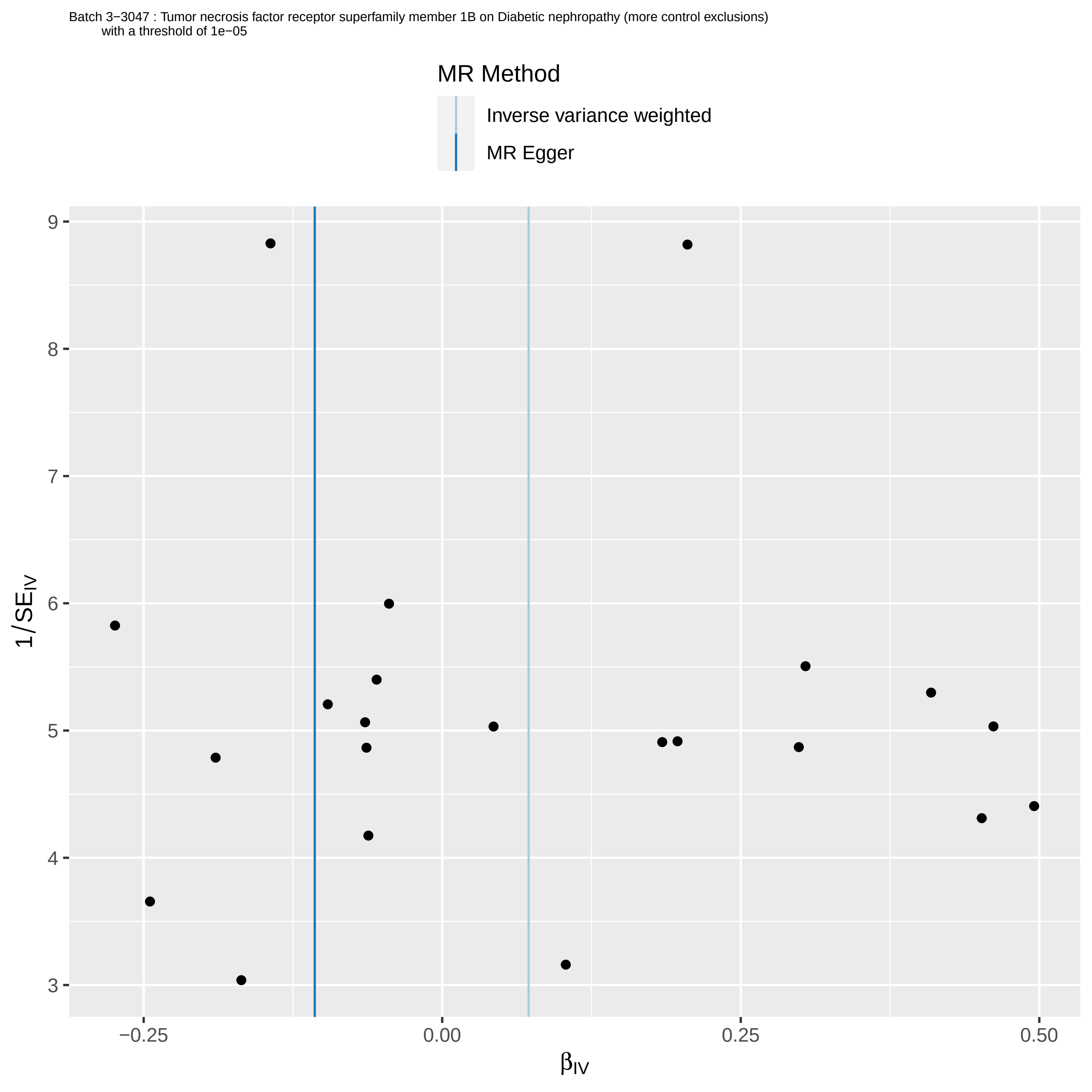
Batch 3−3047 : Tumor necrosis factor receptor superfamily member 1B on Diabetic nephropathy (more control exclusions)
with a threshold of 1e−05
MR Method
Inverse variance weighted
MR Egger
1/SE$_{IV}$
9
8
7
6
5
4
3
−0.25
0.00
0.25
0.50
β$_{IV}$

Batch 3−3048 : Tumor necrosis factor receptor superfamily member 1B on Diabetic nephropathy (more control exclusions)
with a threshold of 1e−05

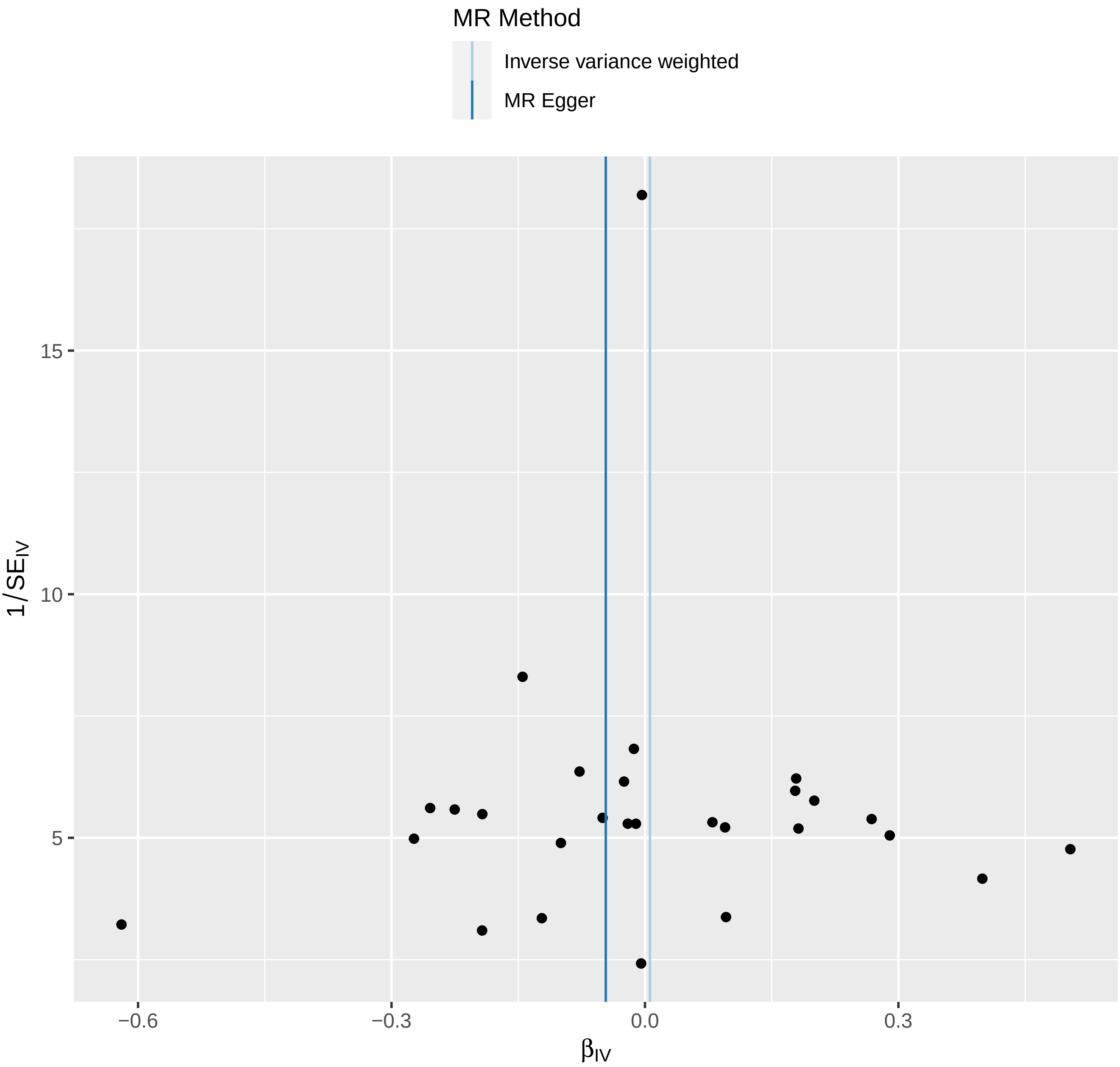

Batch 3−3049 : Tumor necrosis factor receptor superfamily member 21 on Diabetic nephropathy (more control exclusions)
with a threshold of 1e−05

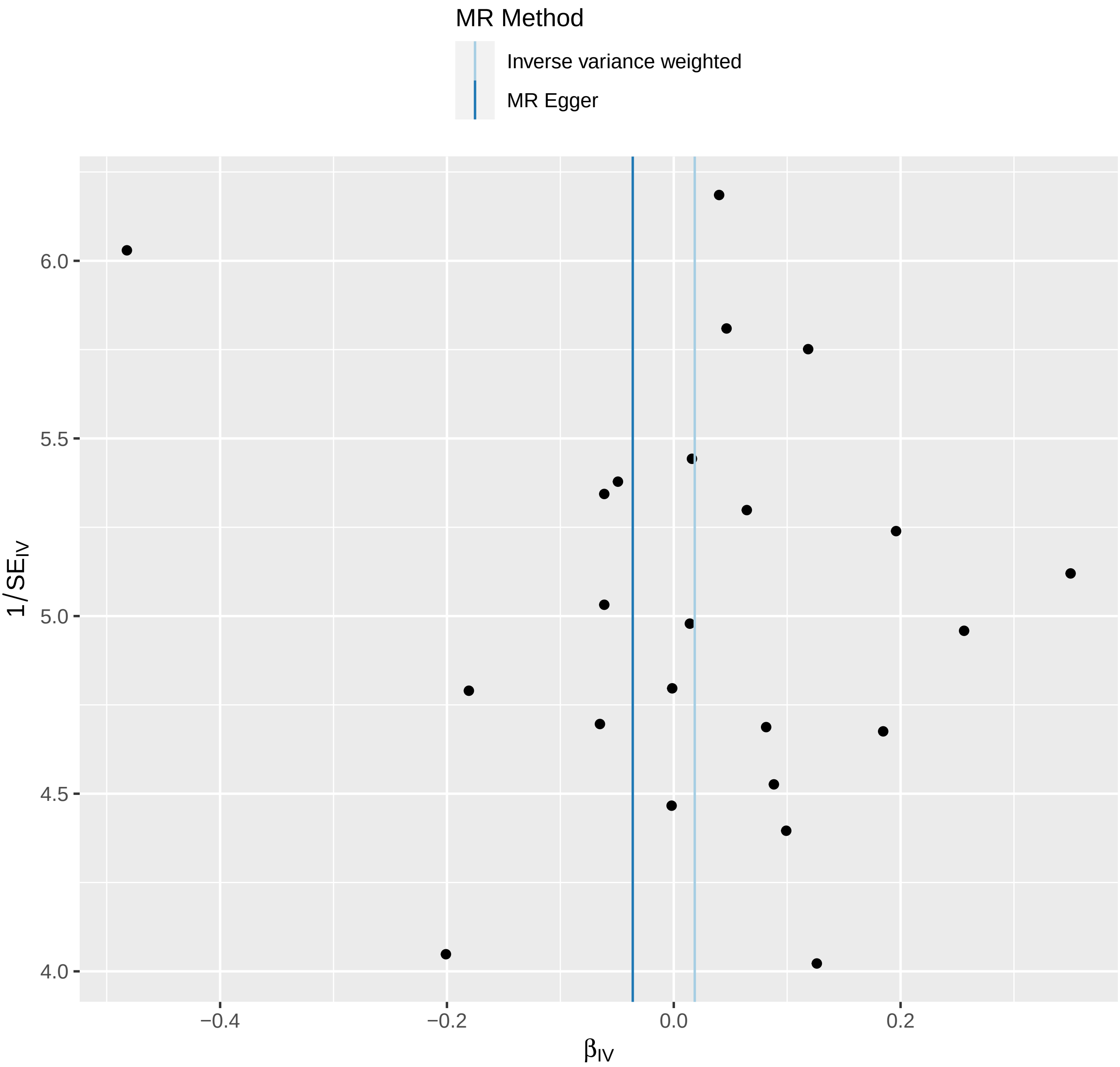

Batch 3−3050 : Tumor necrosis factor receptor superfamily member 21 on Diabetic nephropathy (more control exclusions) with a threshold of 1e−05

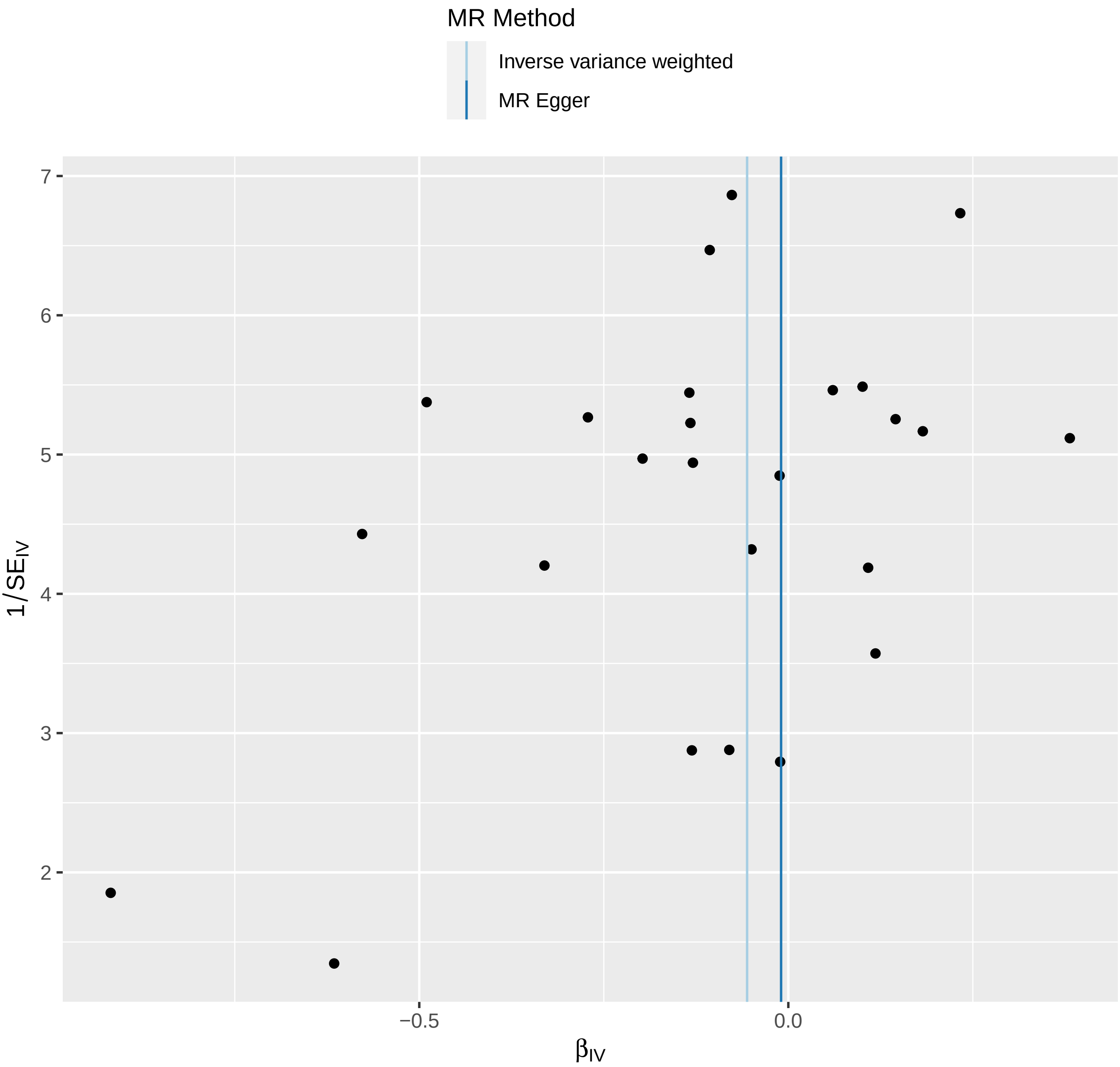

Batch 3−3051 : Tumor necrosis factor receptor superfamily member 4 on Diabetic nephropathy (more control exclusions) with a threshold of 1e−05

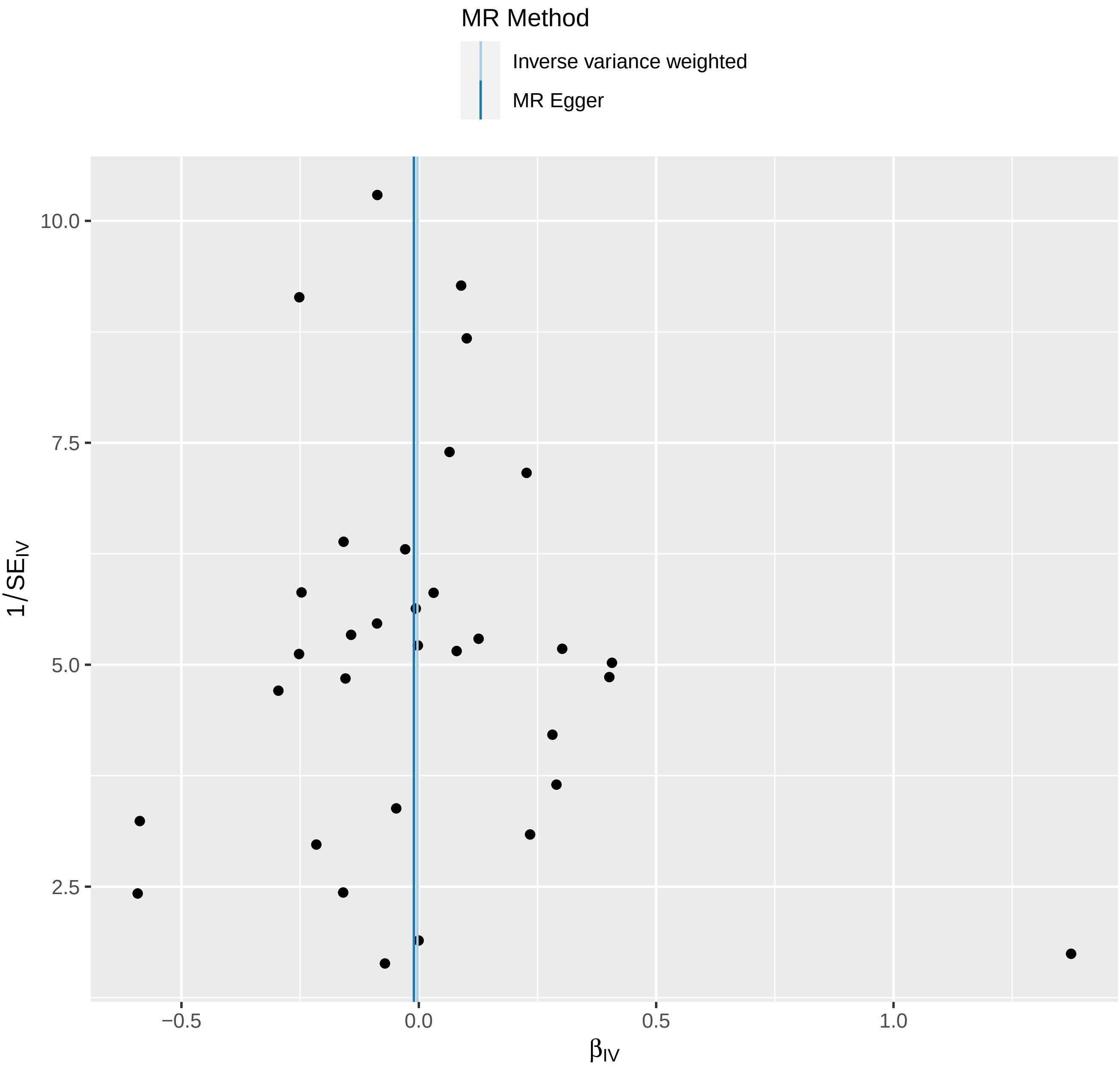

Batch 3−3052 : Tumor necrosis factor receptor superfamily member 6B on Diabetic nephropathy (more control exclusions)
with a threshold of 1e−05

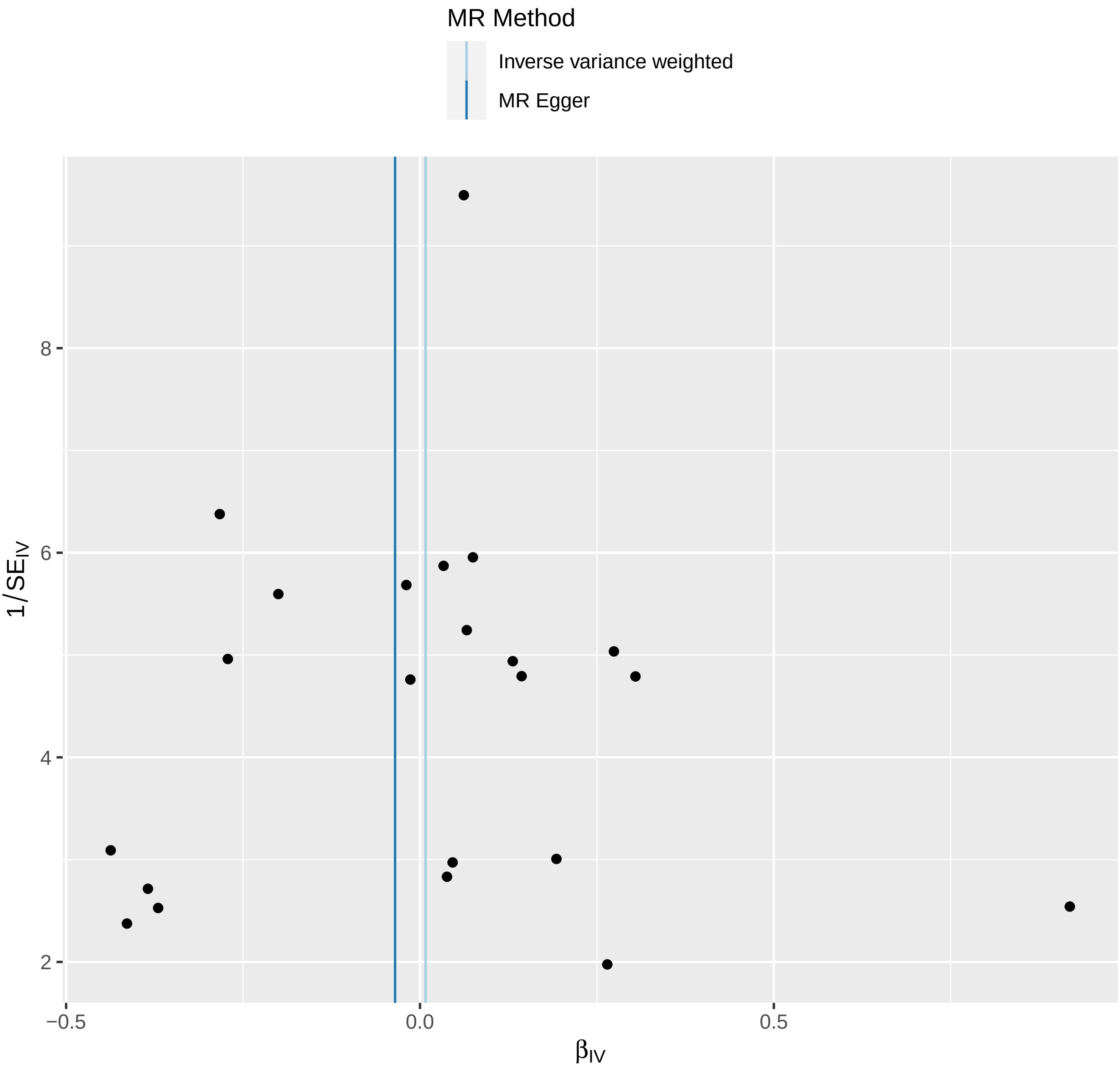

Batch 3−3053 : Tumor necrosis factor receptor superfamily member 8 on Diabetic nephropathy (more control exclusions) with a threshold of 1e−05

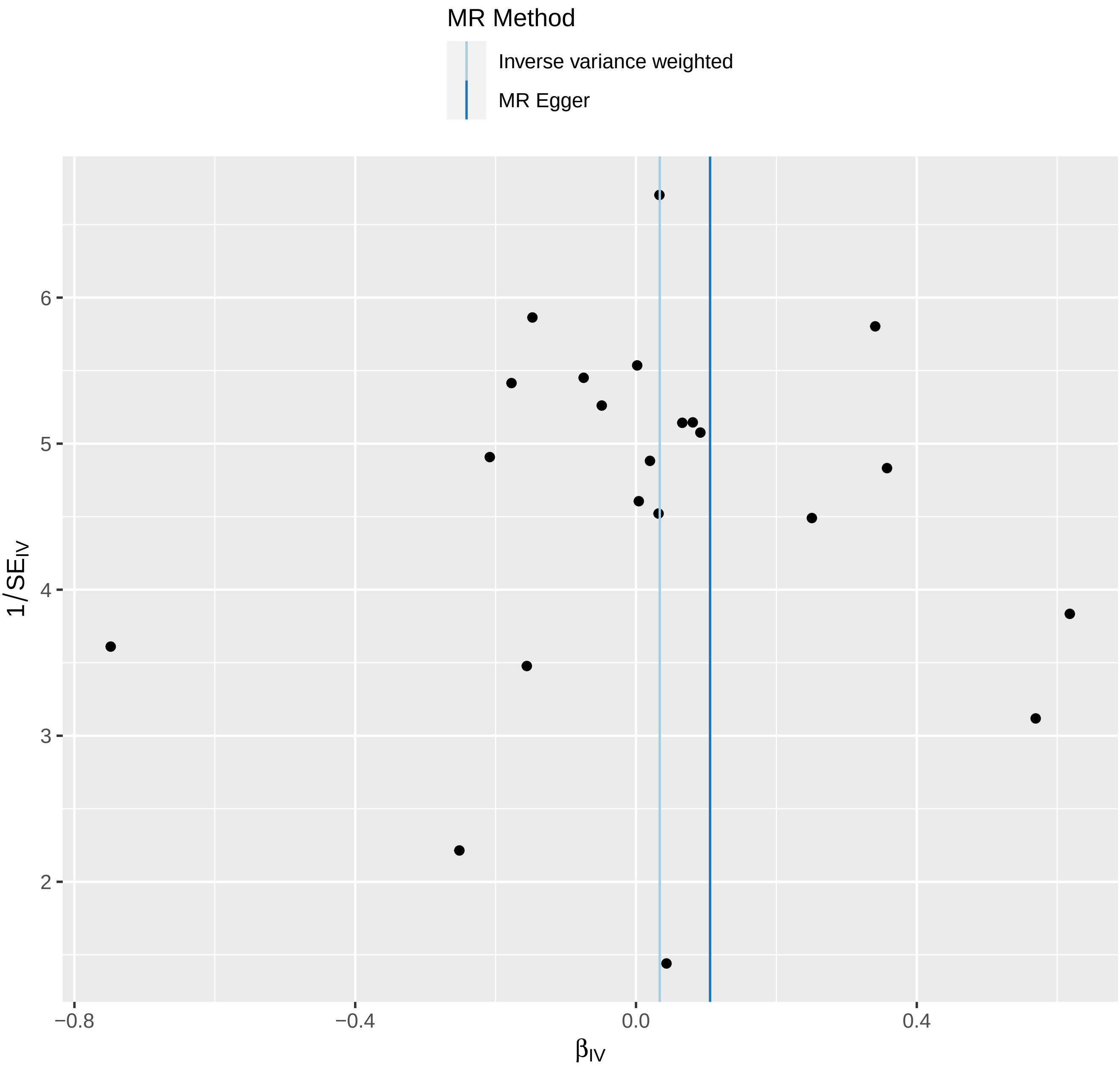

Batch 3−3054 : Tumor necrosis factor ligand superfamily member 11 on Diabetic nephropathy (more control exclusions) with a threshold of 1e−05

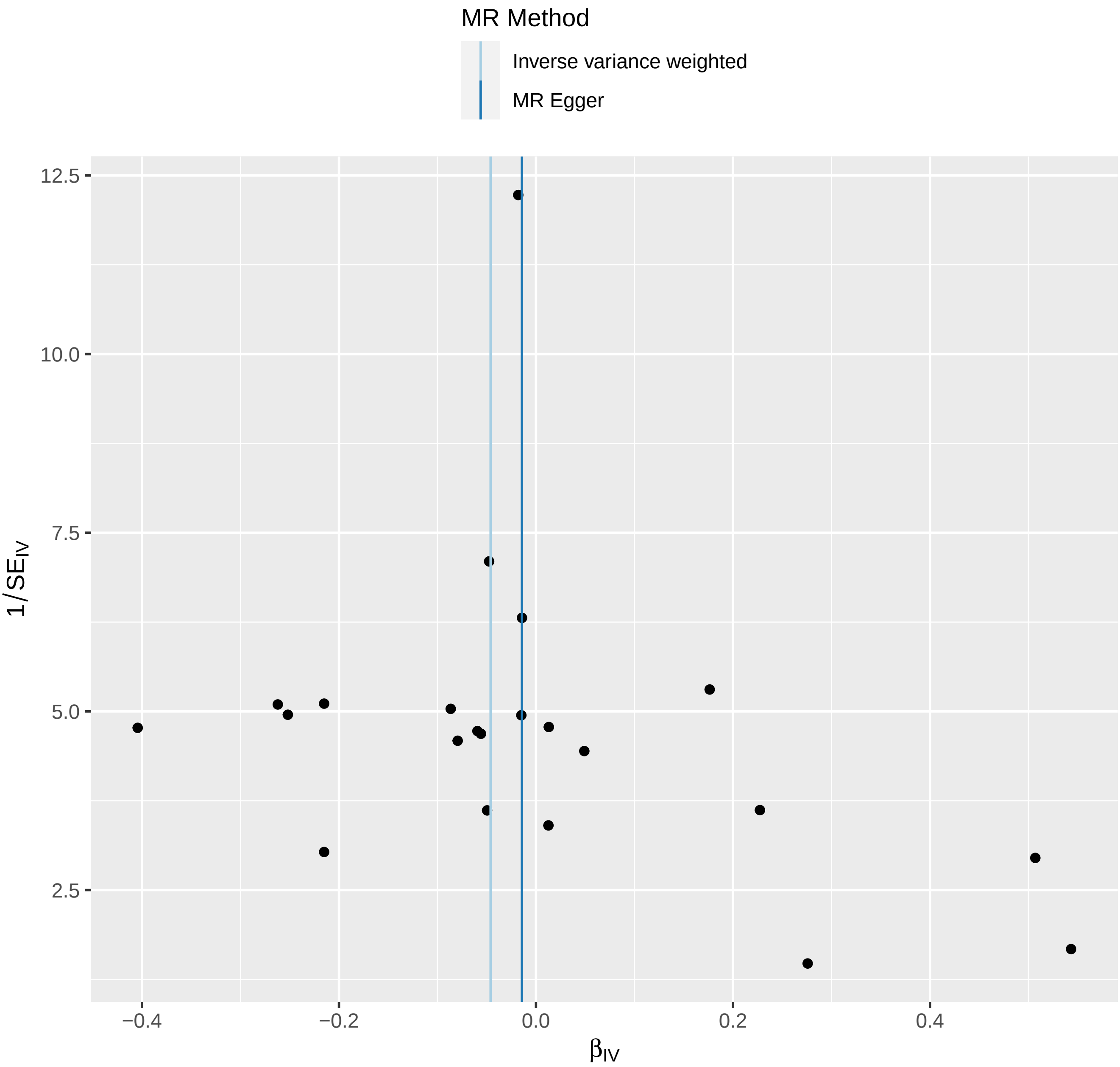

Batch 3−3055 : Tumor necrosis factor ligand superfamily member 12 on Diabetic nephropathy (more control exclusions)
with a threshold of 1e−05

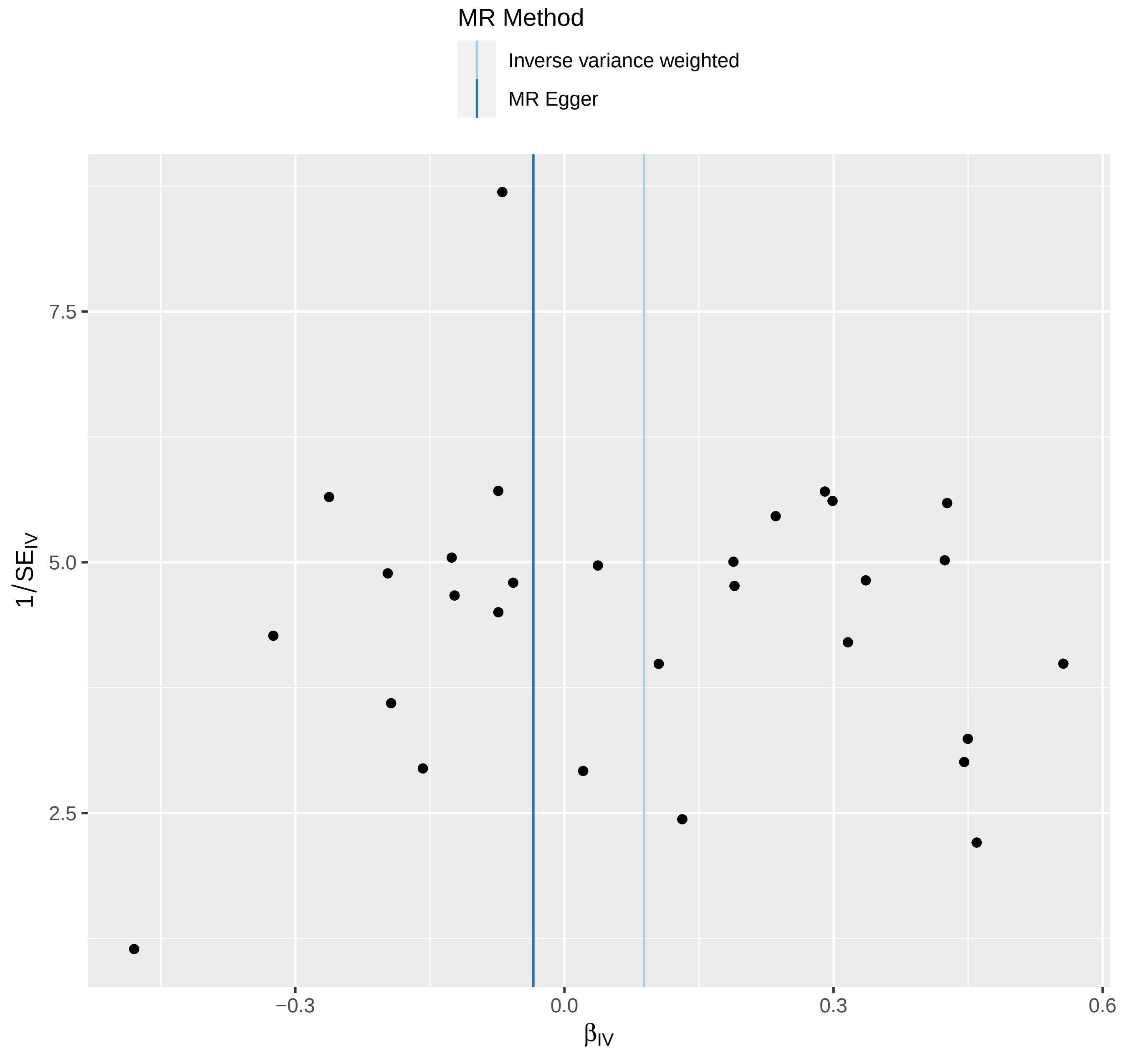

Batch 3−3056 : Tumor necrosis factor ligand superfamily member 13B on Diabetic nephropathy (more control exclusions)
with a threshold of 1e−05

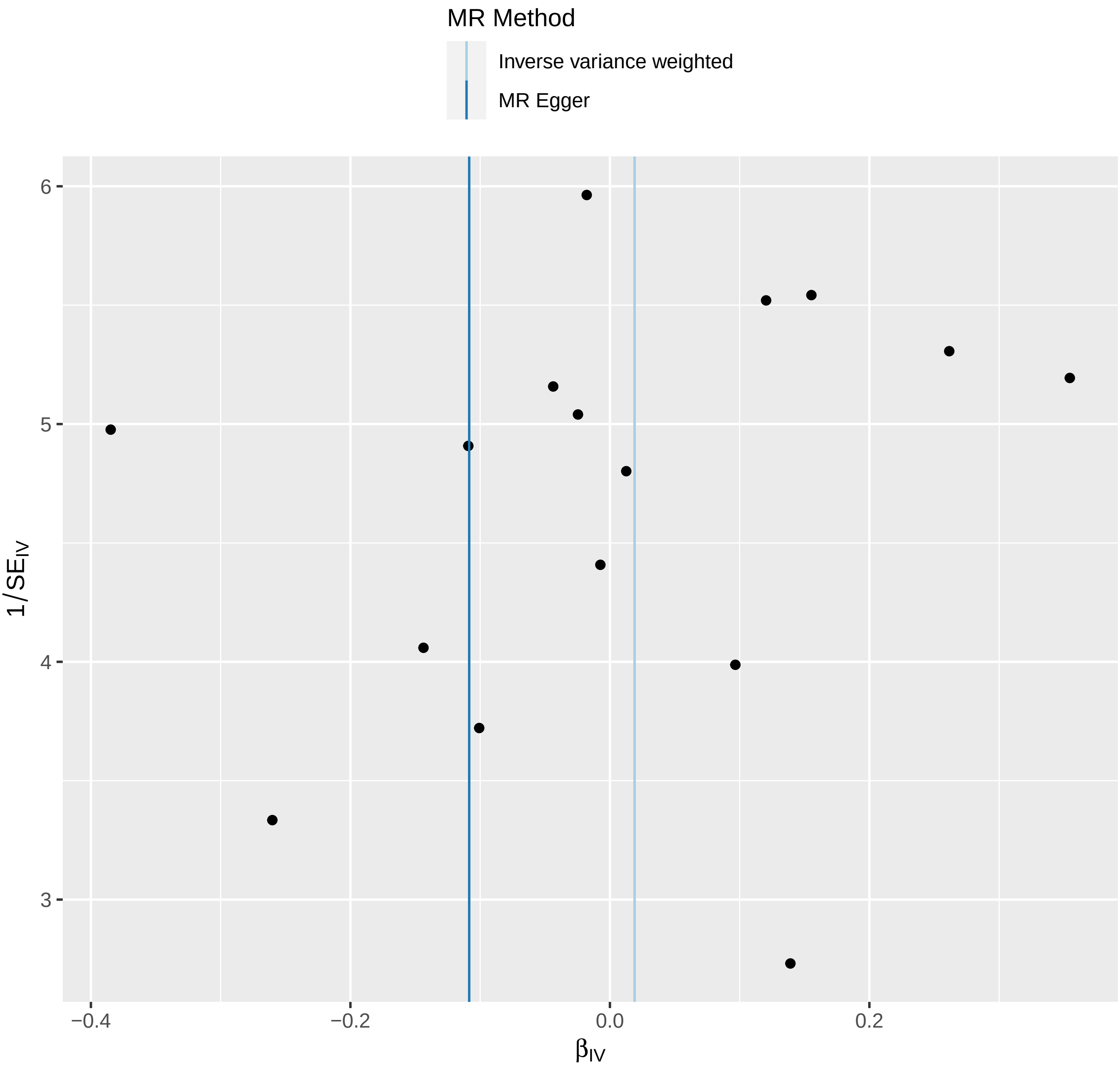

Batch 3−3057 : Tumor necrosis factor ligand superfamily member 14 on Diabetic nephropathy (more control exclusions)
with a threshold of 1e−05

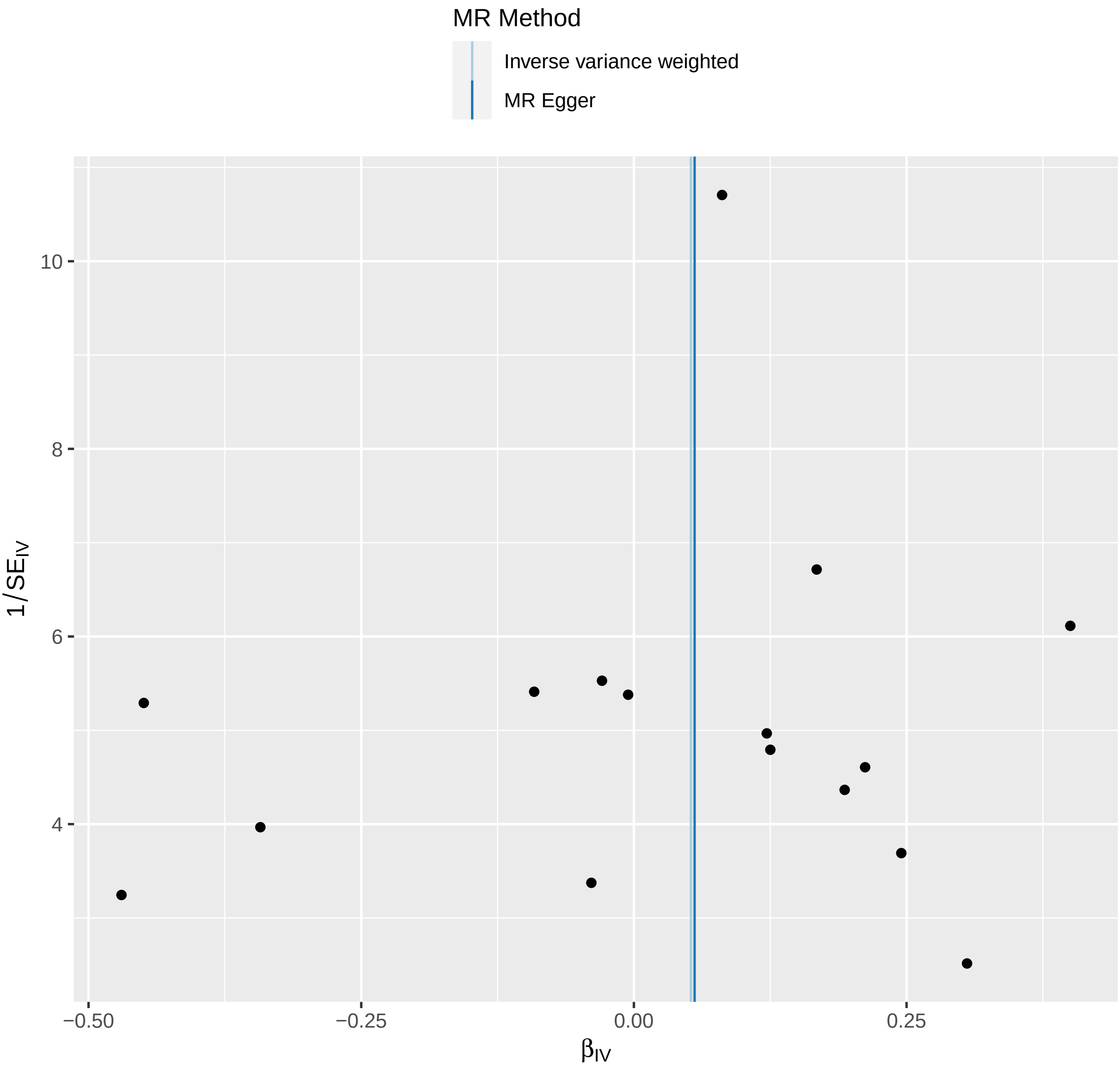

Batch 3−3058 : Tumor necrosis factor ligand superfamily member 15 on Diabetic nephropathy (more control exclusions) with a threshold of 1e−05

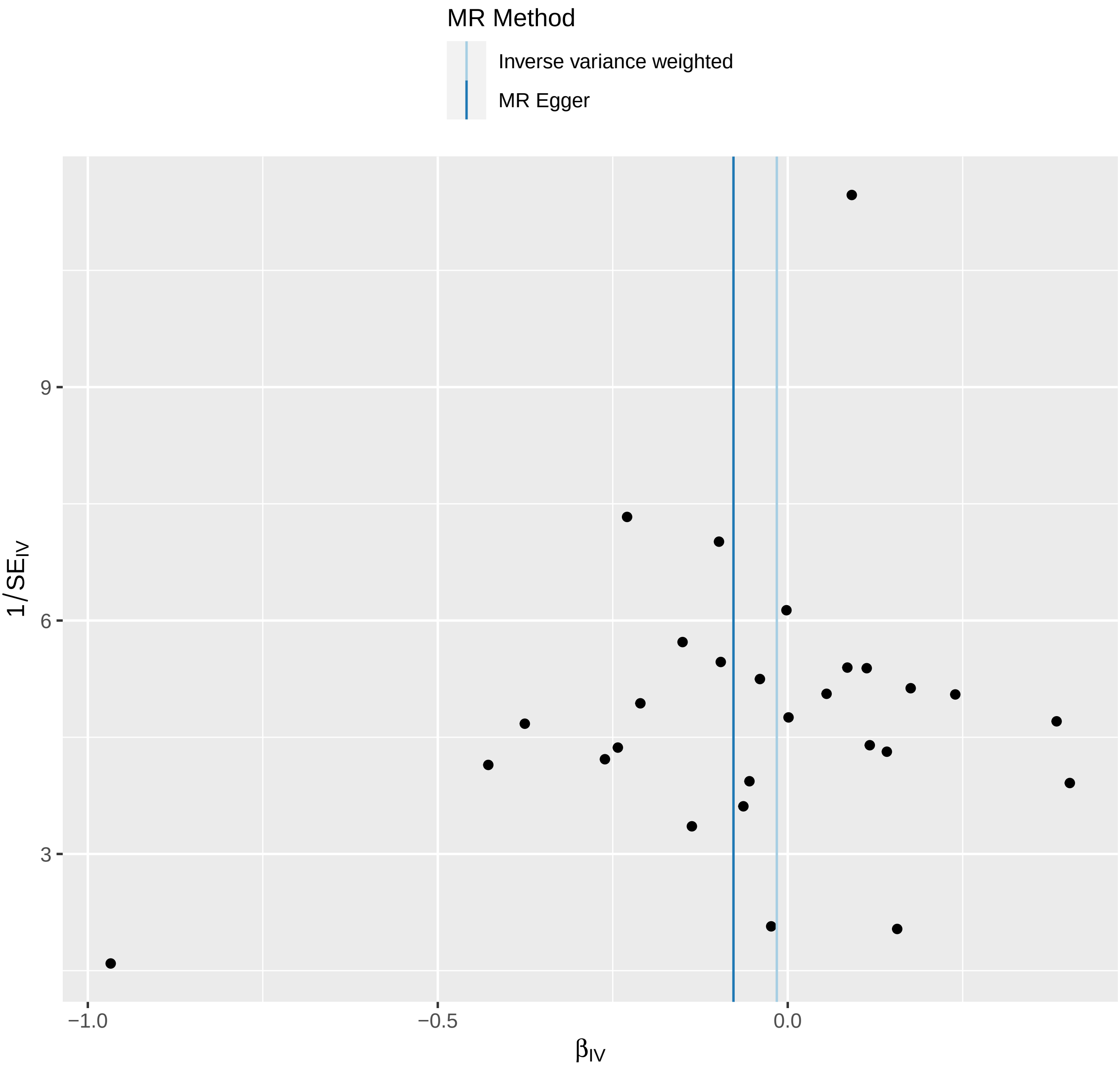

Batch 3−3059 : Tumor necrosis factor ligand superfamily member 15 on Diabetic nephropathy (more control exclusions)
with a threshold of 1e−05

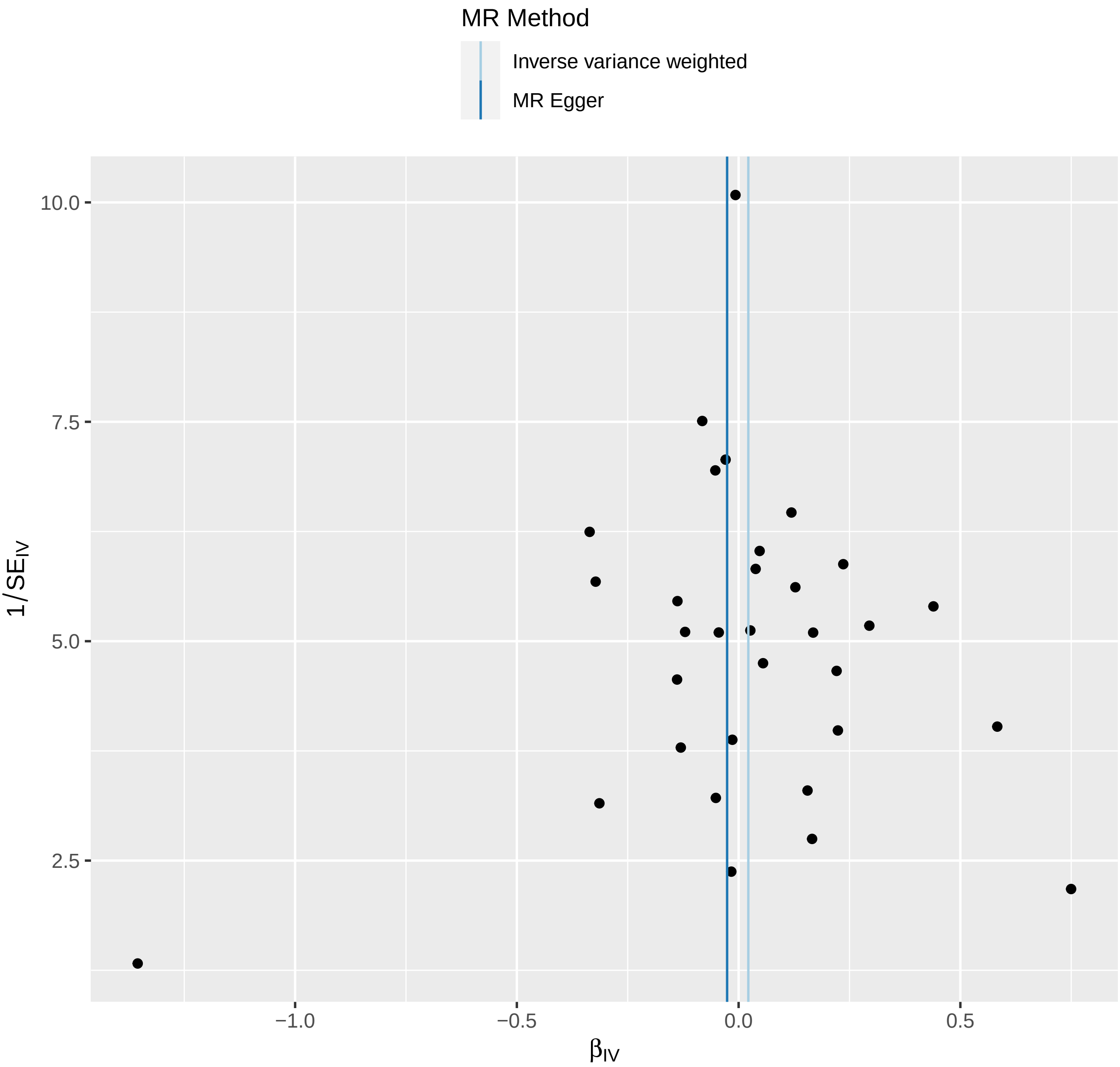

Batch 3−3060 : Tumor necrosis factor ligand superfamily member 18 on Diabetic nephropathy (more control exclusions) with a threshold of 1e−05

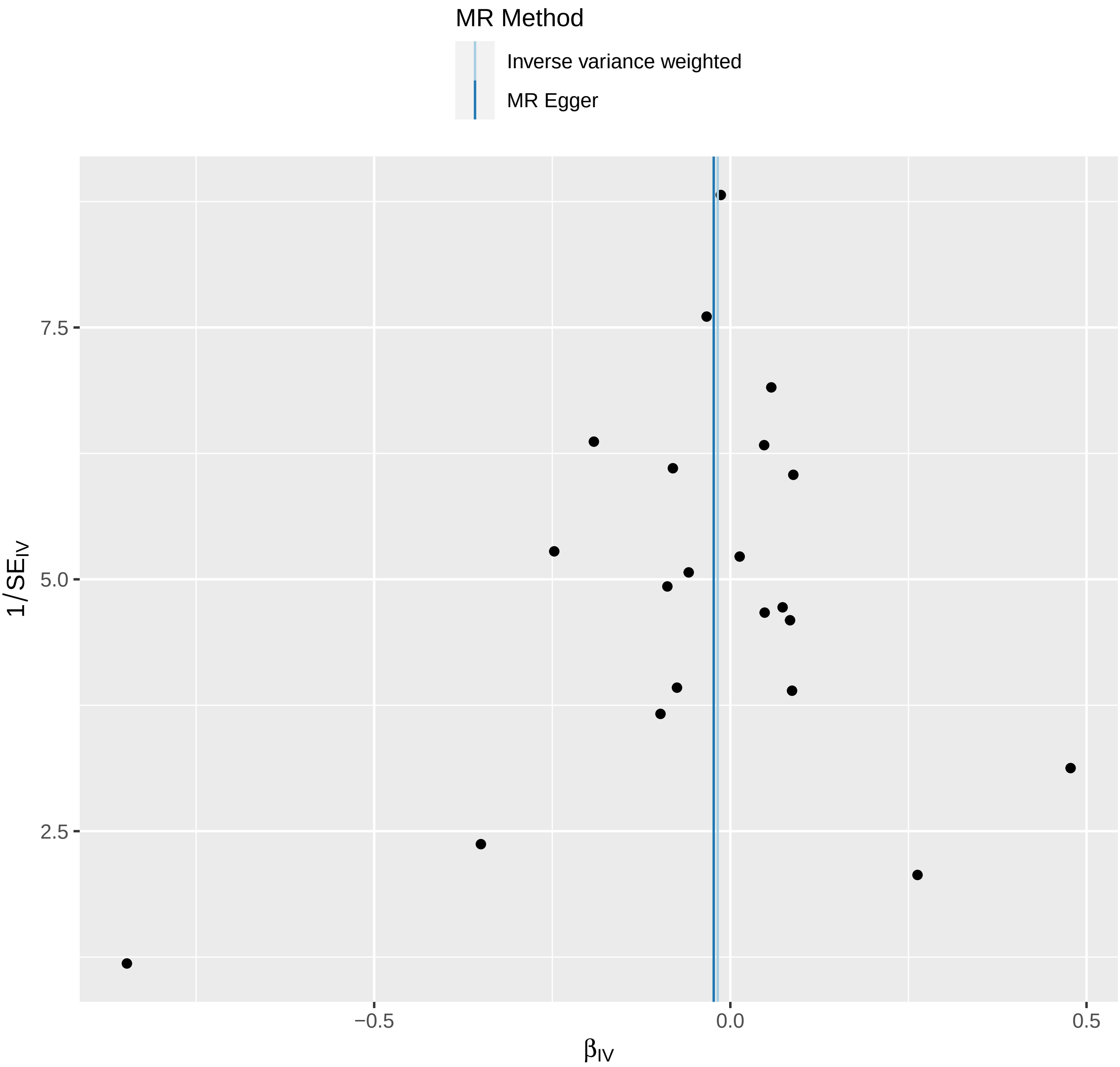

Batch 3−3061 : Tumor necrosis factor ligand superfamily member 4 on Diabetic nephropathy (more control exclusions)
with a threshold of 1e−05

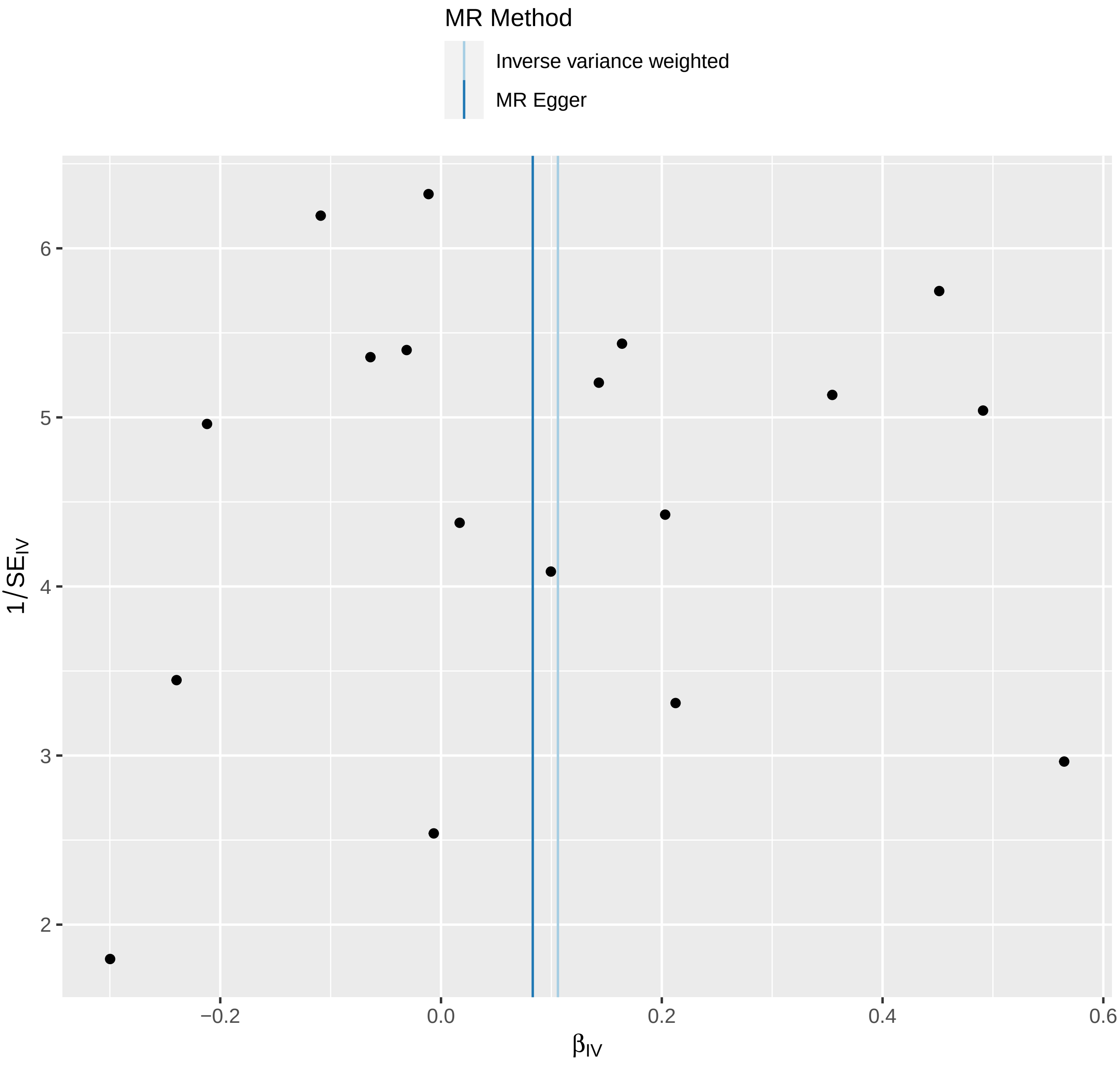

Batch 3−3062 : Tumor necrosis factor ligand superfamily member 4 on Diabetic nephropathy (more control exclusions) with a threshold of 1e−05

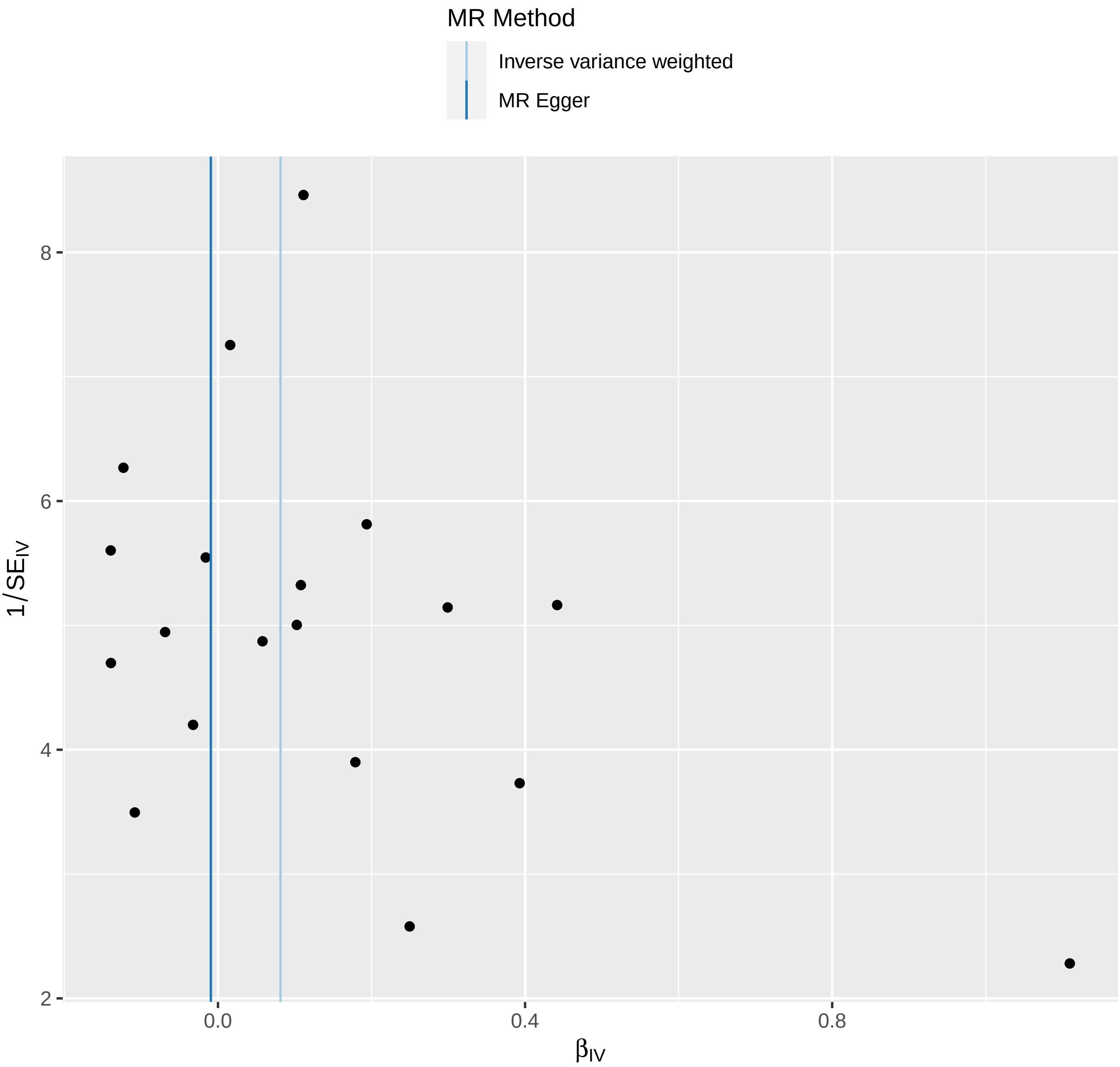

Batch 3−3063 : Tumor necrosis factor ligand superfamily member 8 on Diabetic nephropathy (more control exclusions)
with a threshold of 1e−05

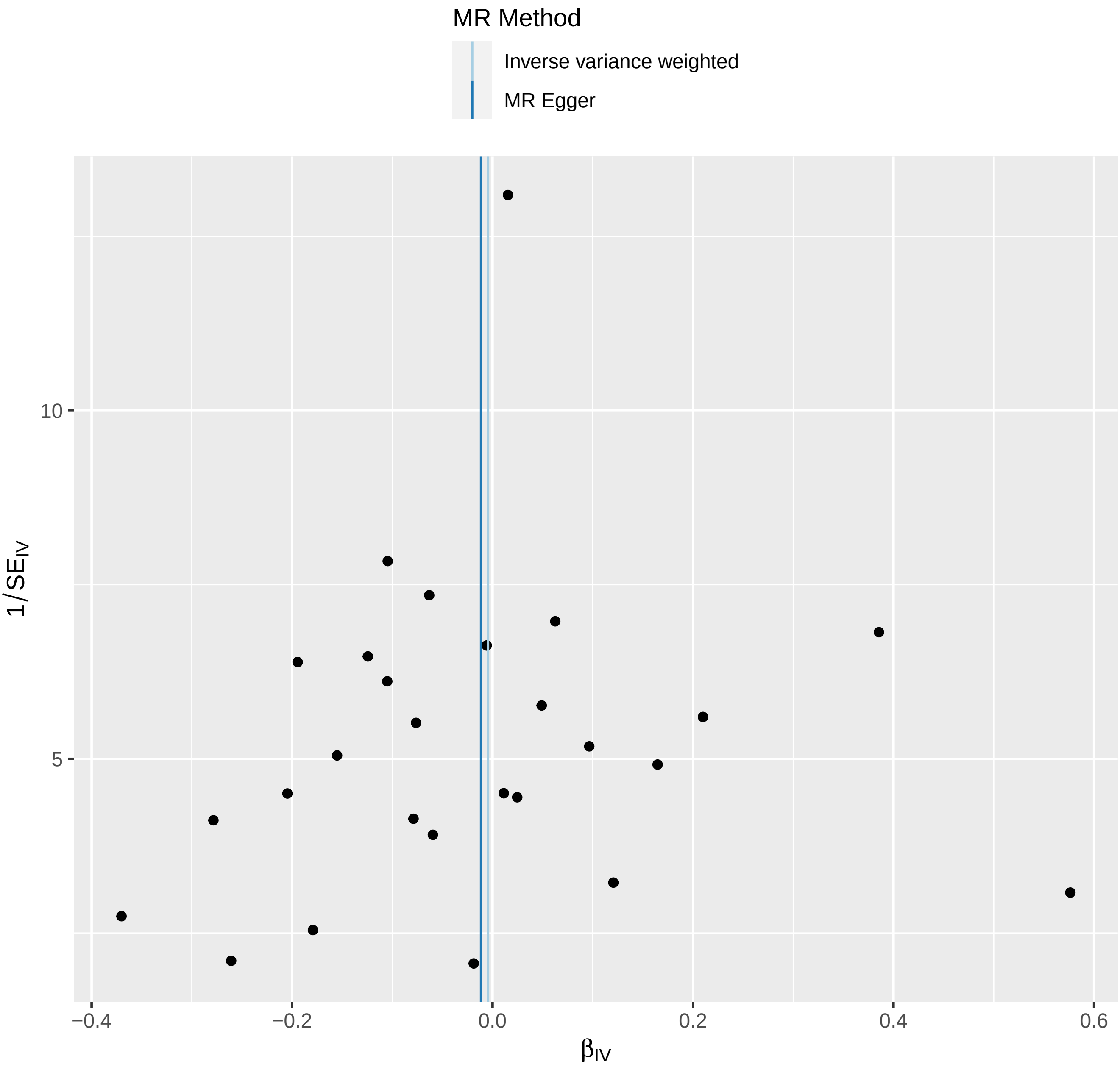

Batch 3−3064 : Tumor necrosis factor ligand superfamily member 9 on Diabetic nephropathy (more control exclusions)
with a threshold of 1e−05

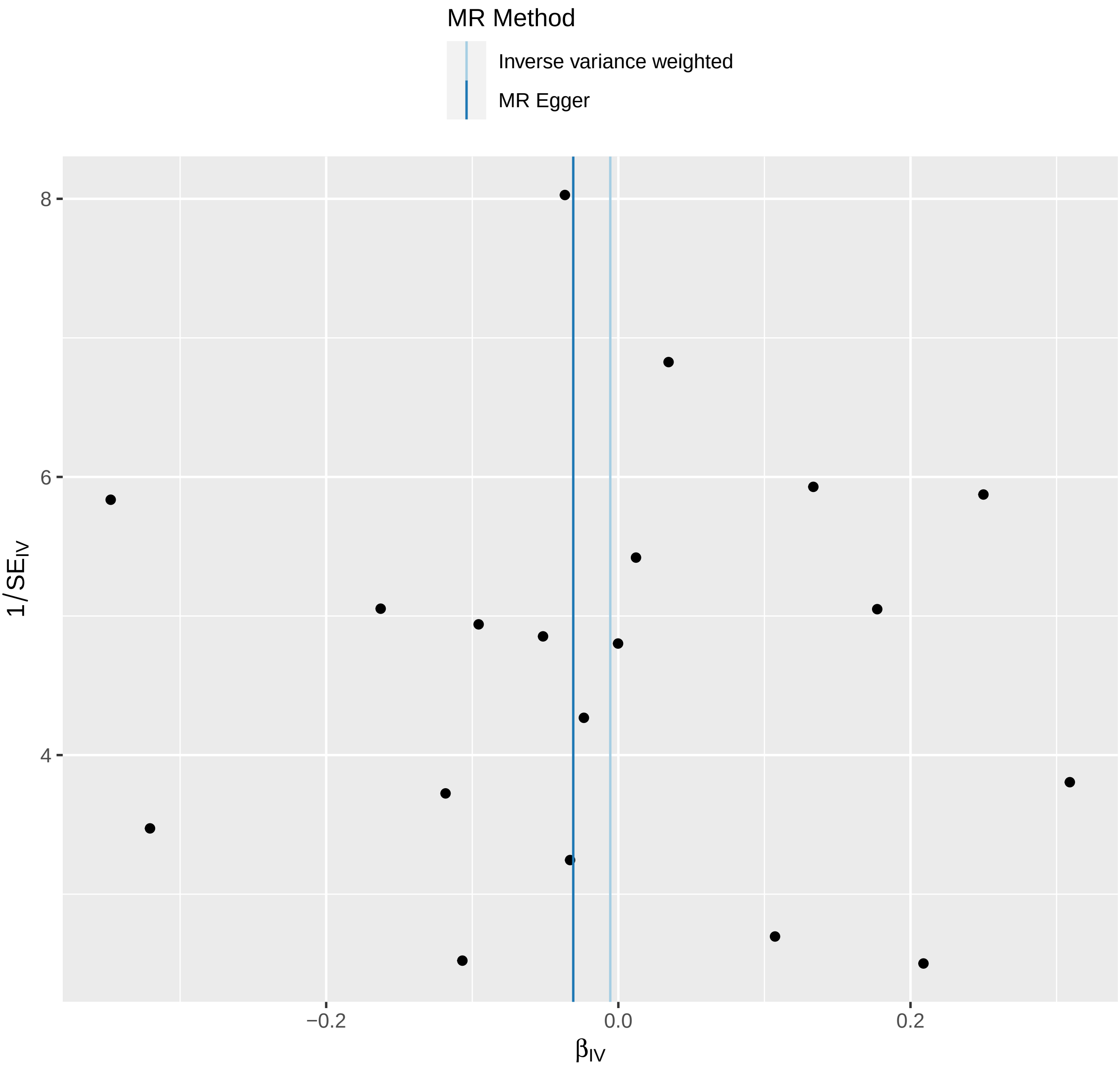

Batch 3−3065 : Tenomodulin on Diabetic nephropathy (more control exclusions)
with a threshold of 1e−05

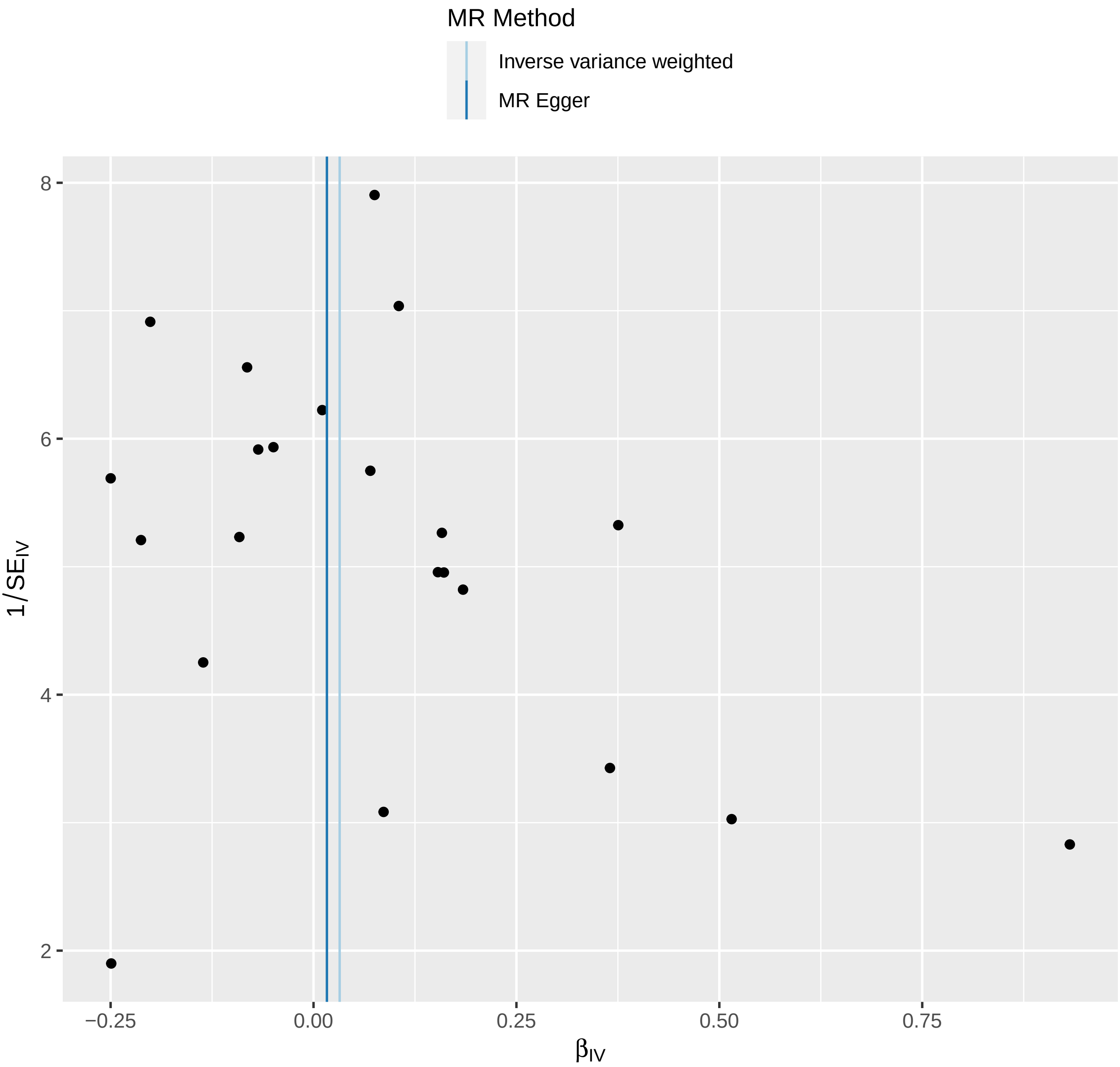

Batch 3−3066 : Troponin I, cardiac muscle on Diabetic nephropathy (more control exclusions)
with a threshold of 1e−05

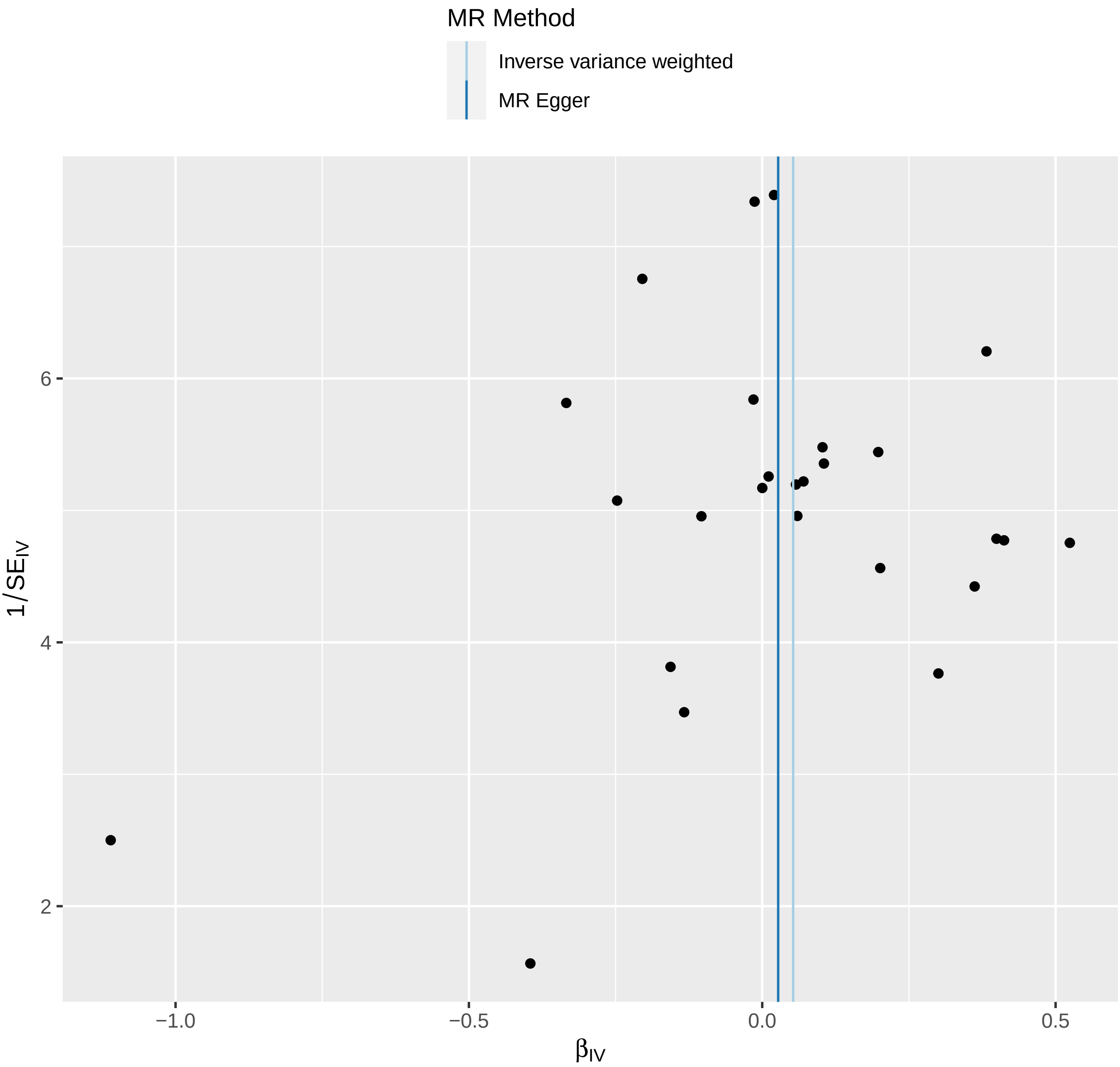

Batch 3−3067 : Troponin I, cardiac muscle on Diabetic nephropathy (more control exclusions)
with a threshold of 1e−05

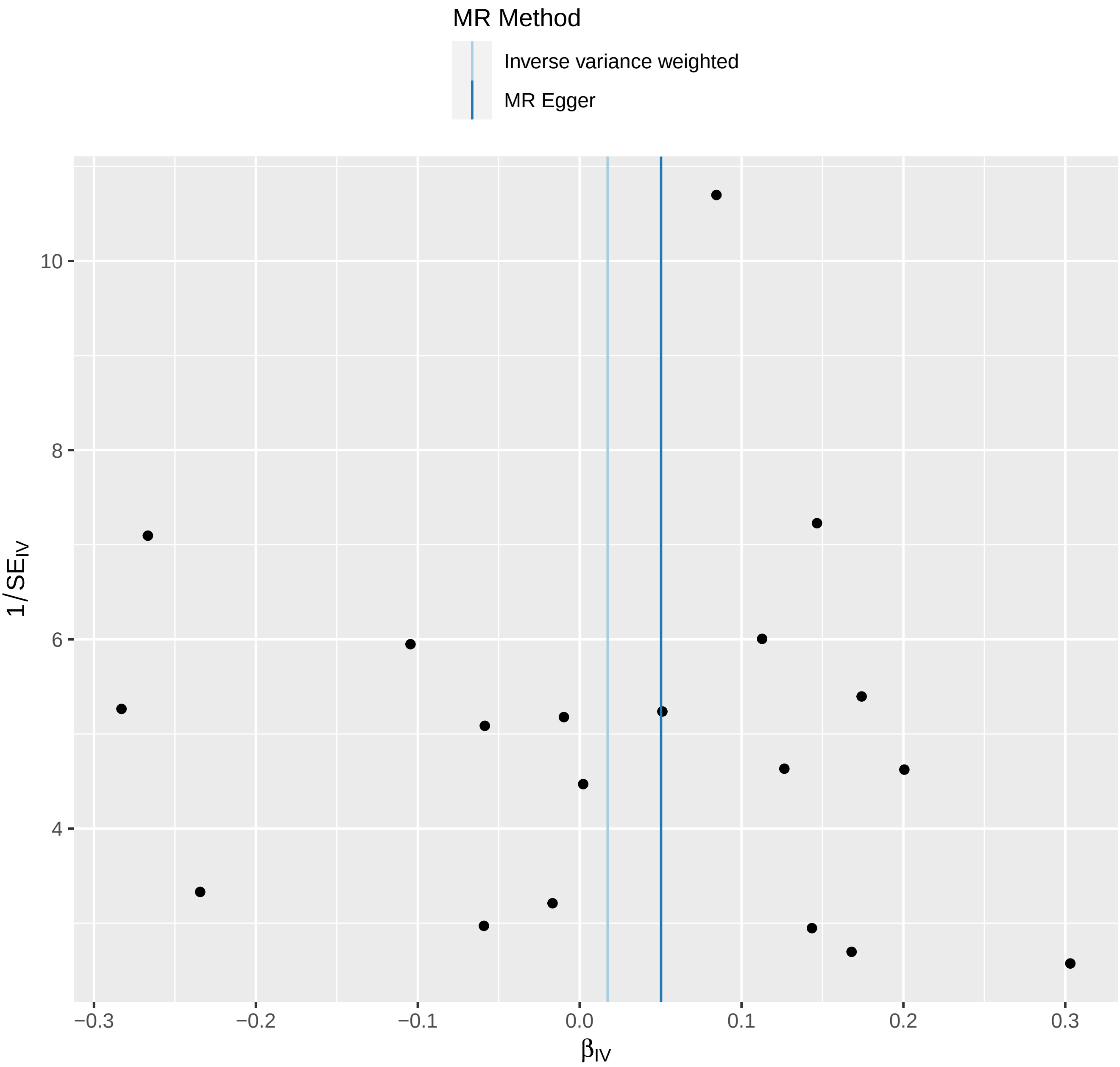

Batch 3−3068 : Tenascin−R on Diabetic nephropathy (more control exclusions)
with a threshold of 1e−05

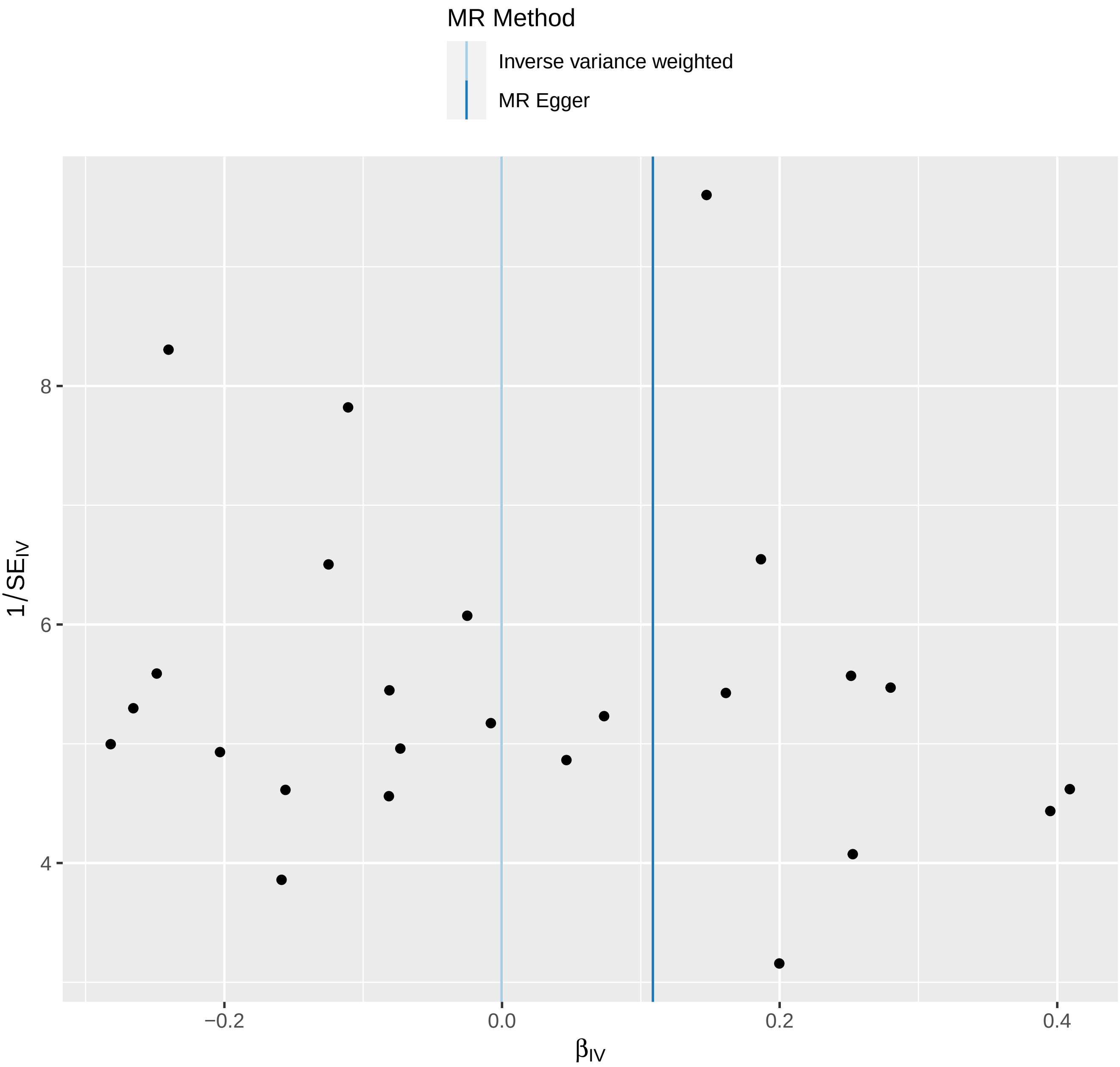

Batch 3−3069 : Tensin−2 on Diabetic nephropathy (more control exclusions)
with a threshold of 1e−05

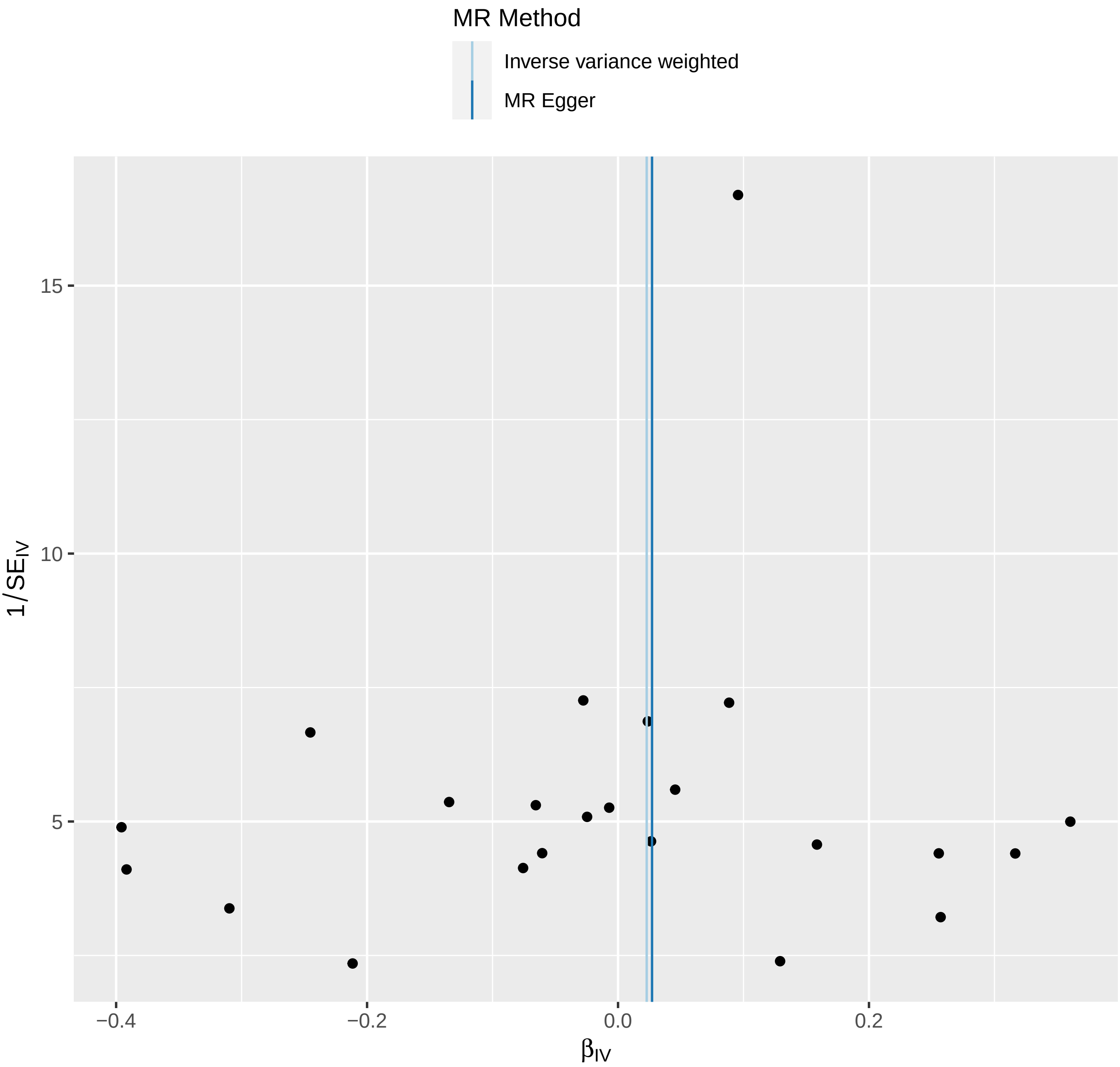

Batch 3−3070 : Tensin−4 on Diabetic nephropathy (more control exclusions)
with a threshold of 1e−05

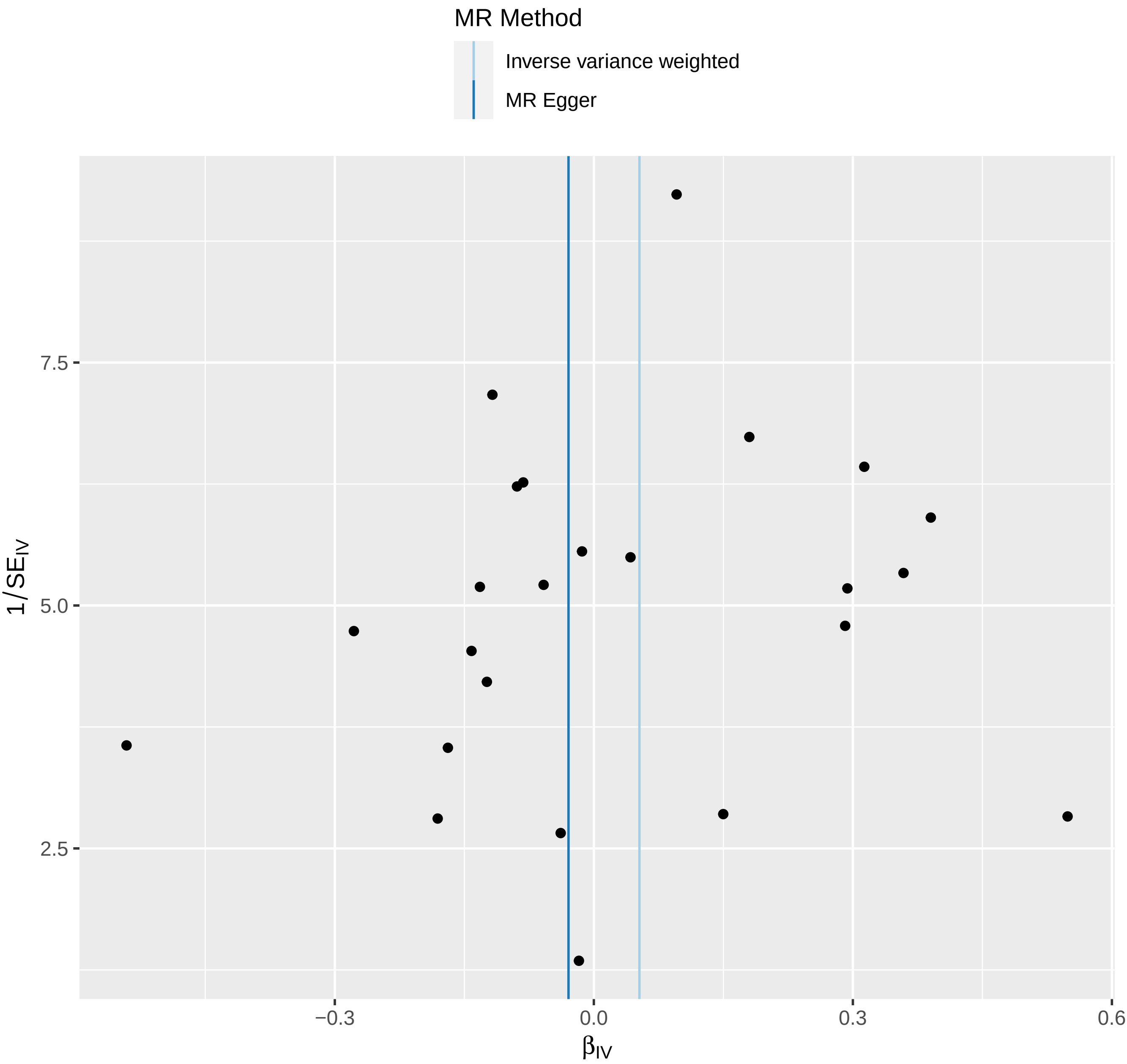

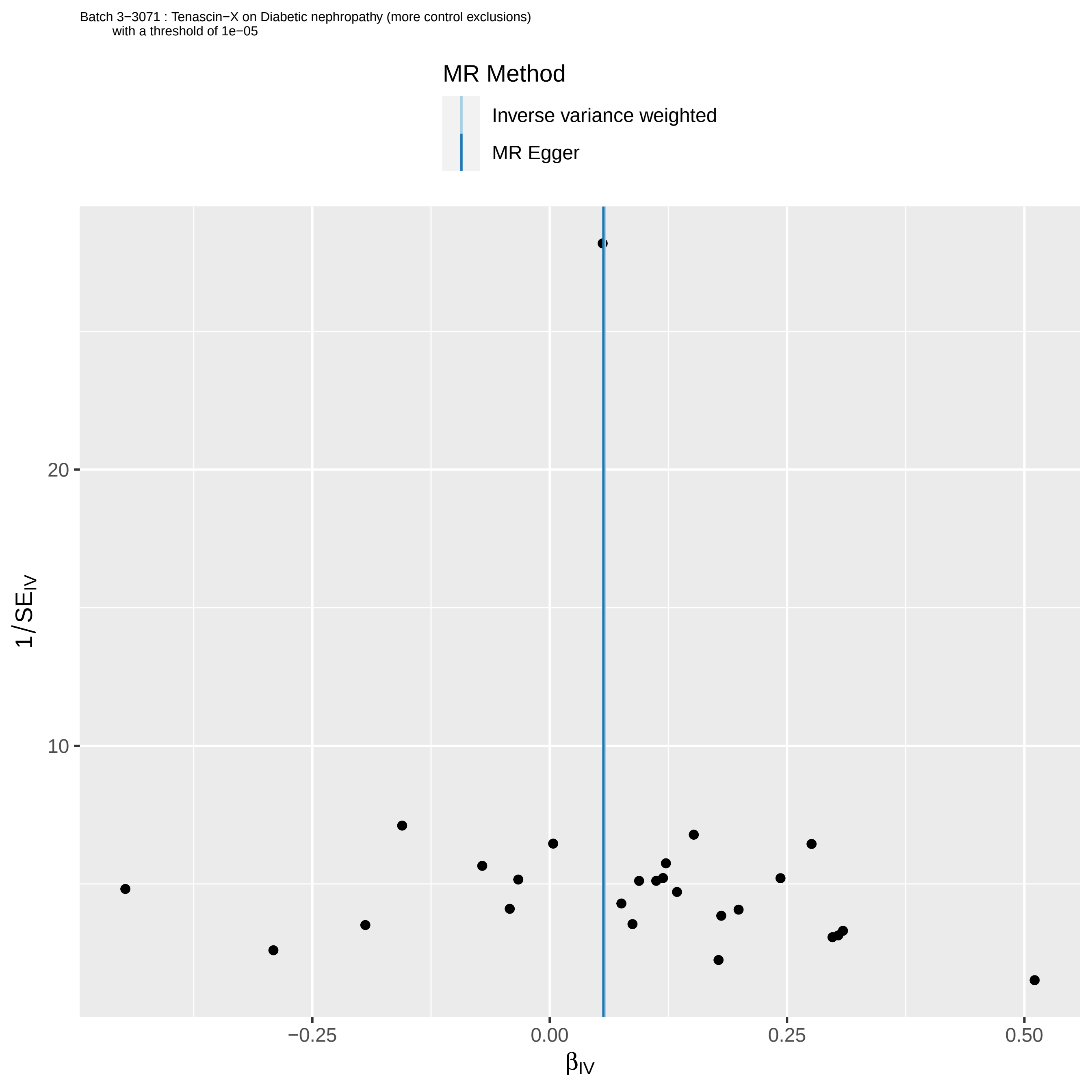

Batch 3−3071 : Tenascin−X on Diabetic nephropathy (more control exclusions)
with a threshold of 1e−05
MR Method
Inverse variance weighted
MR Egger
20
10
1/SE_IV
−0.25
0.00
0.25
0.50
β_IV

Batch 3−3072 : Toll−interacting protein on Diabetic nephropathy (more control exclusions)
with a threshold of 1e−05

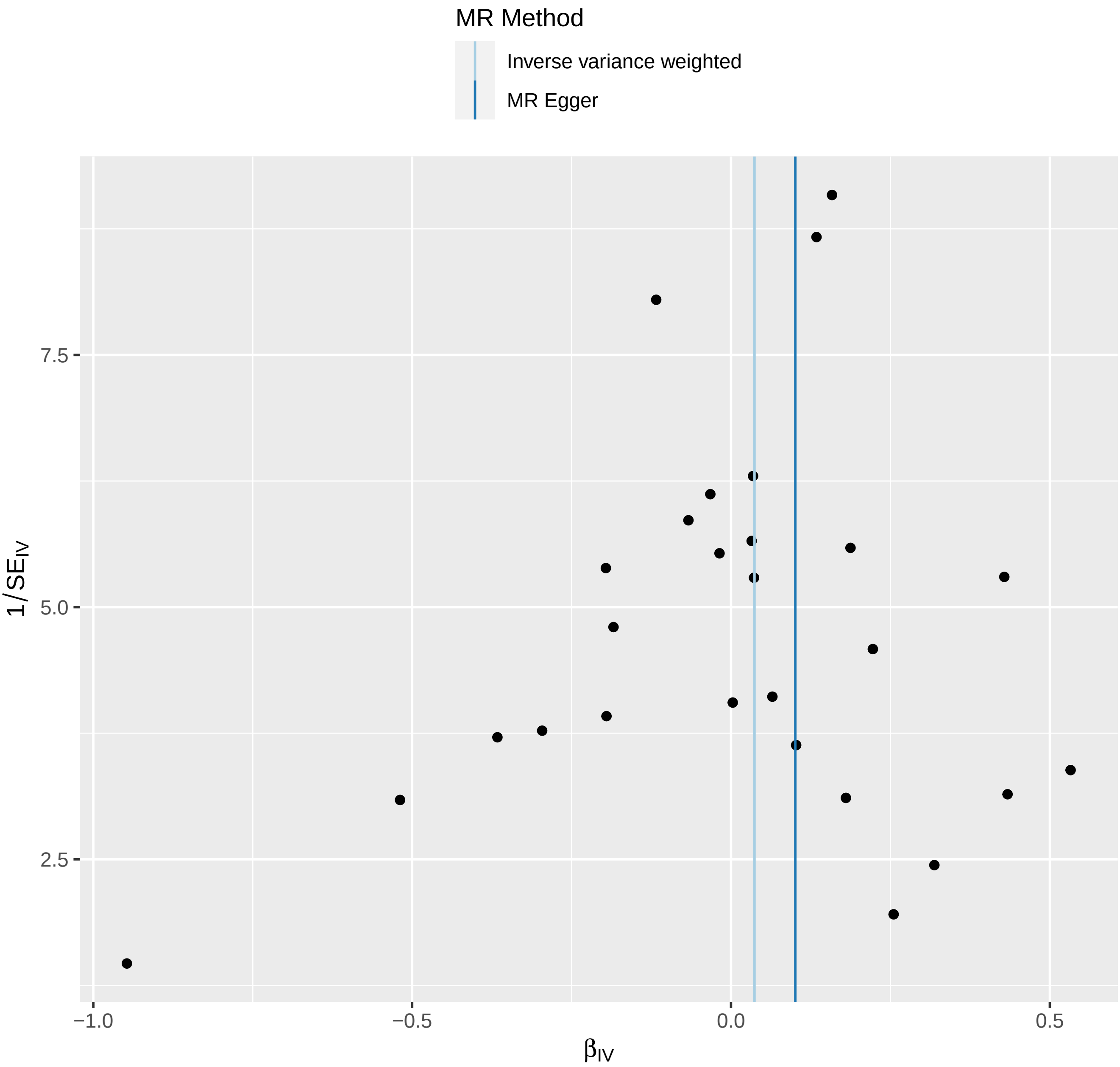

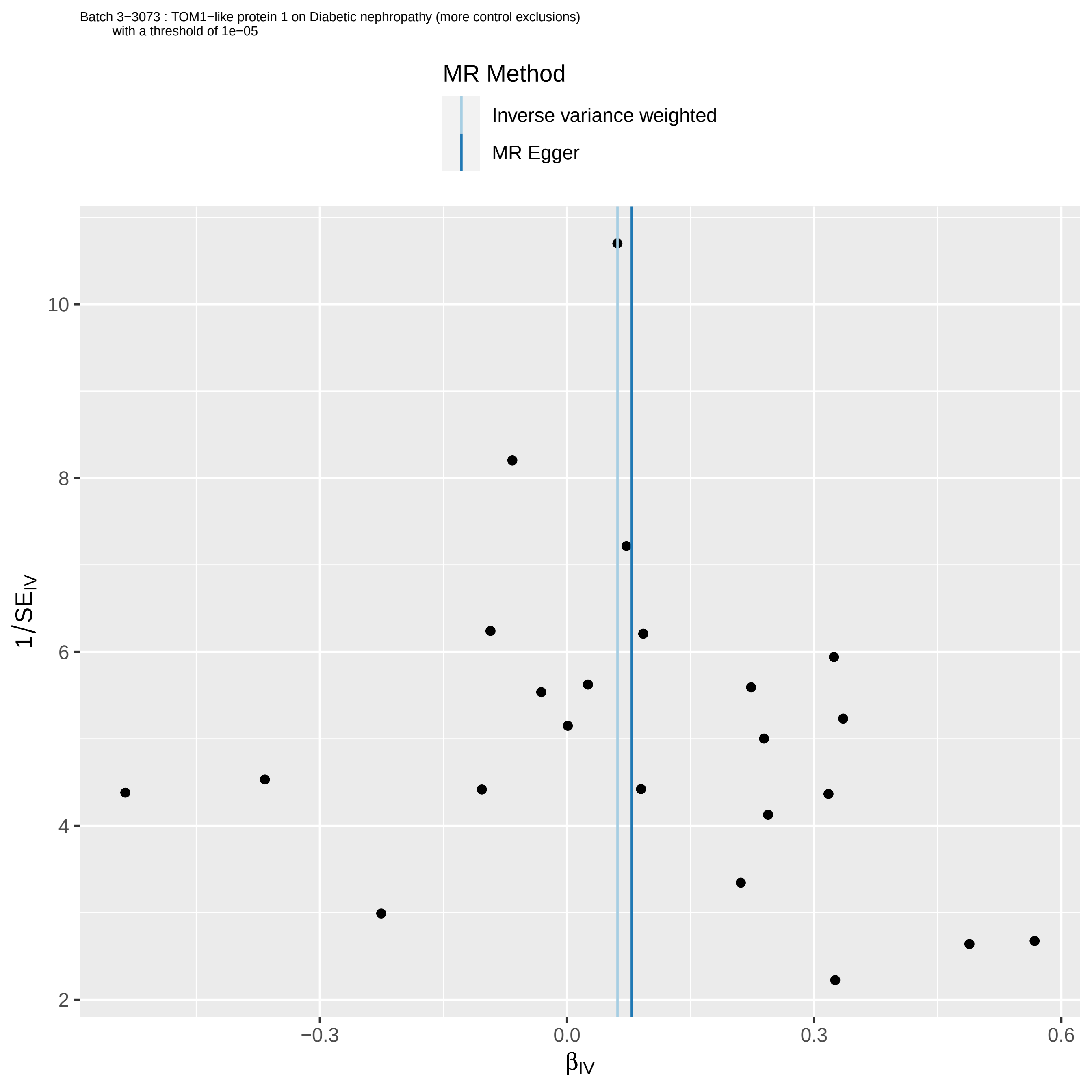

Batch 3−3073 : TOM1−like protein 1 on Diabetic nephropathy (more control exclusions)
with a threshold of 1e−05
MR Method
Inverse variance weighted
MR Egger
1/SE_IV
10
8
6
4
2
−0.3
0.0
0.3
0.6
β_IV

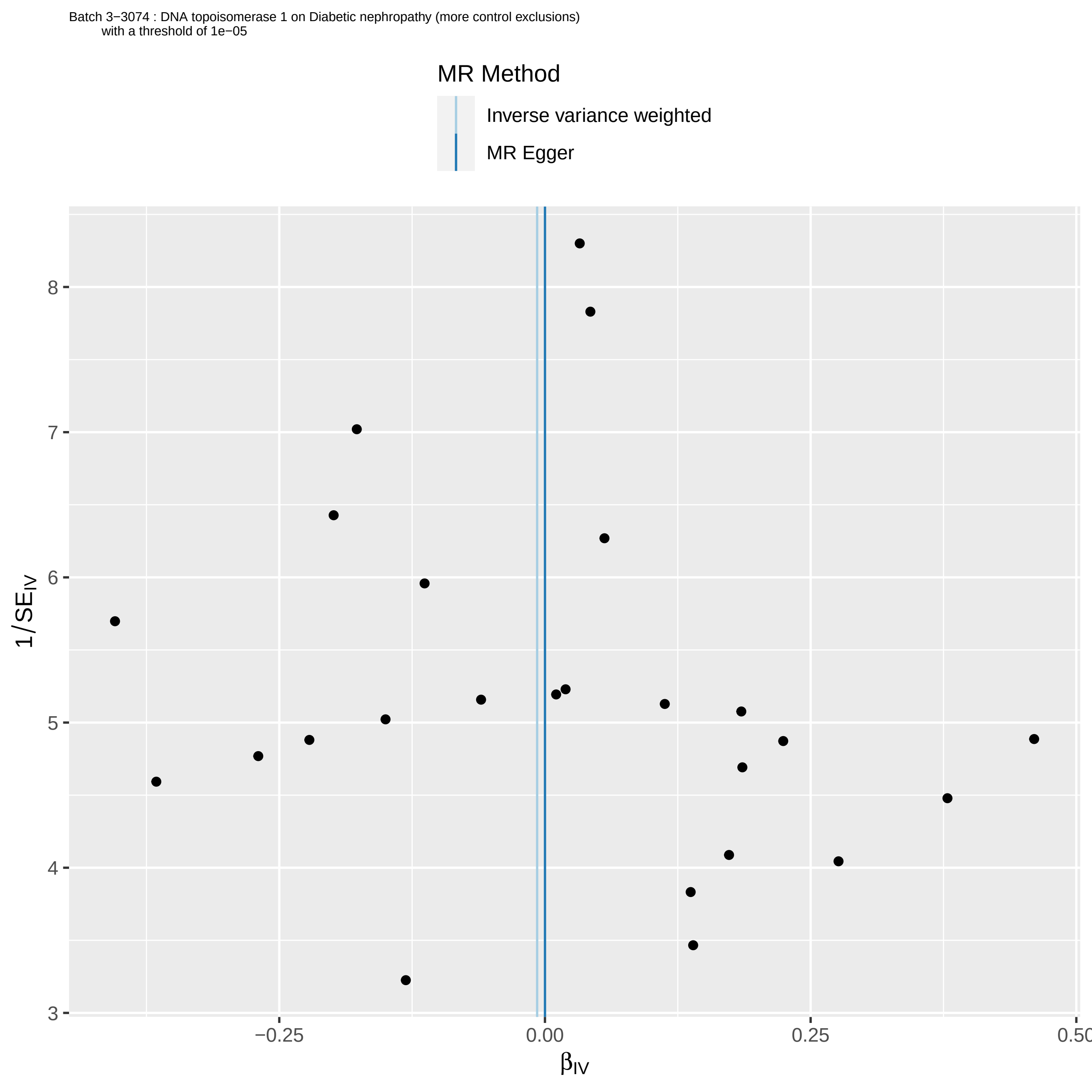
Batch 3−3074 : DNA topoisomerase 1 on Diabetic nephropathy (more control exclusions)
with a threshold of 1e−05
MR Method
Inverse variance weighted
MR Egger
8
7
6
5
4
3
1/SE$_{IV}$
−0.25
0.00
0.25
0.50
β_{IV}

Batch 3−3075 : DNA topoisomerase 2−binding protein 1 on Diabetic nephropathy (more control exclusions) with a threshold of 1e−05

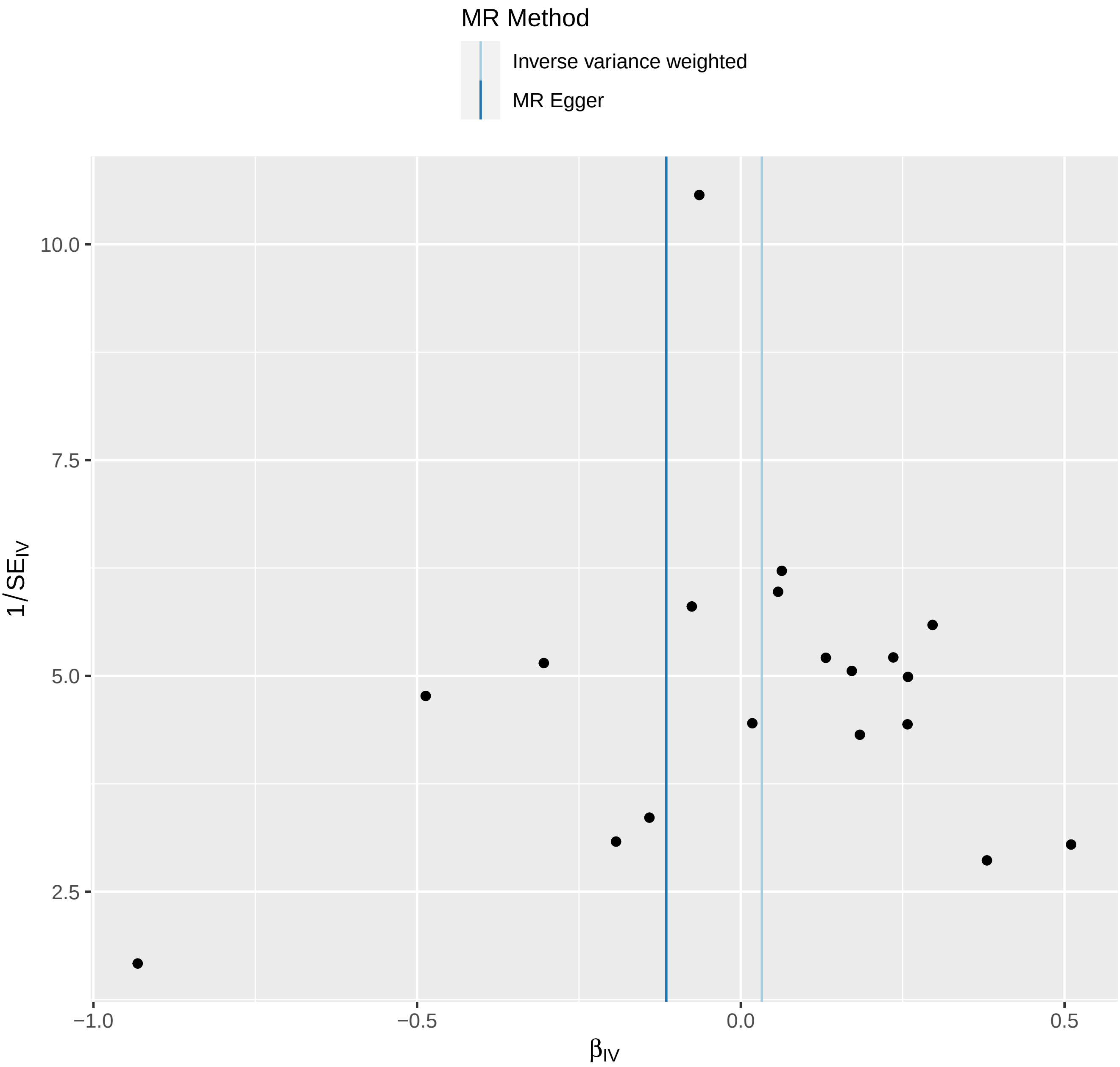

Batch 3−3076 : Torsin−1A−interacting protein 1 on Diabetic nephropathy (more control exclusions)
with a threshold of 1e−05

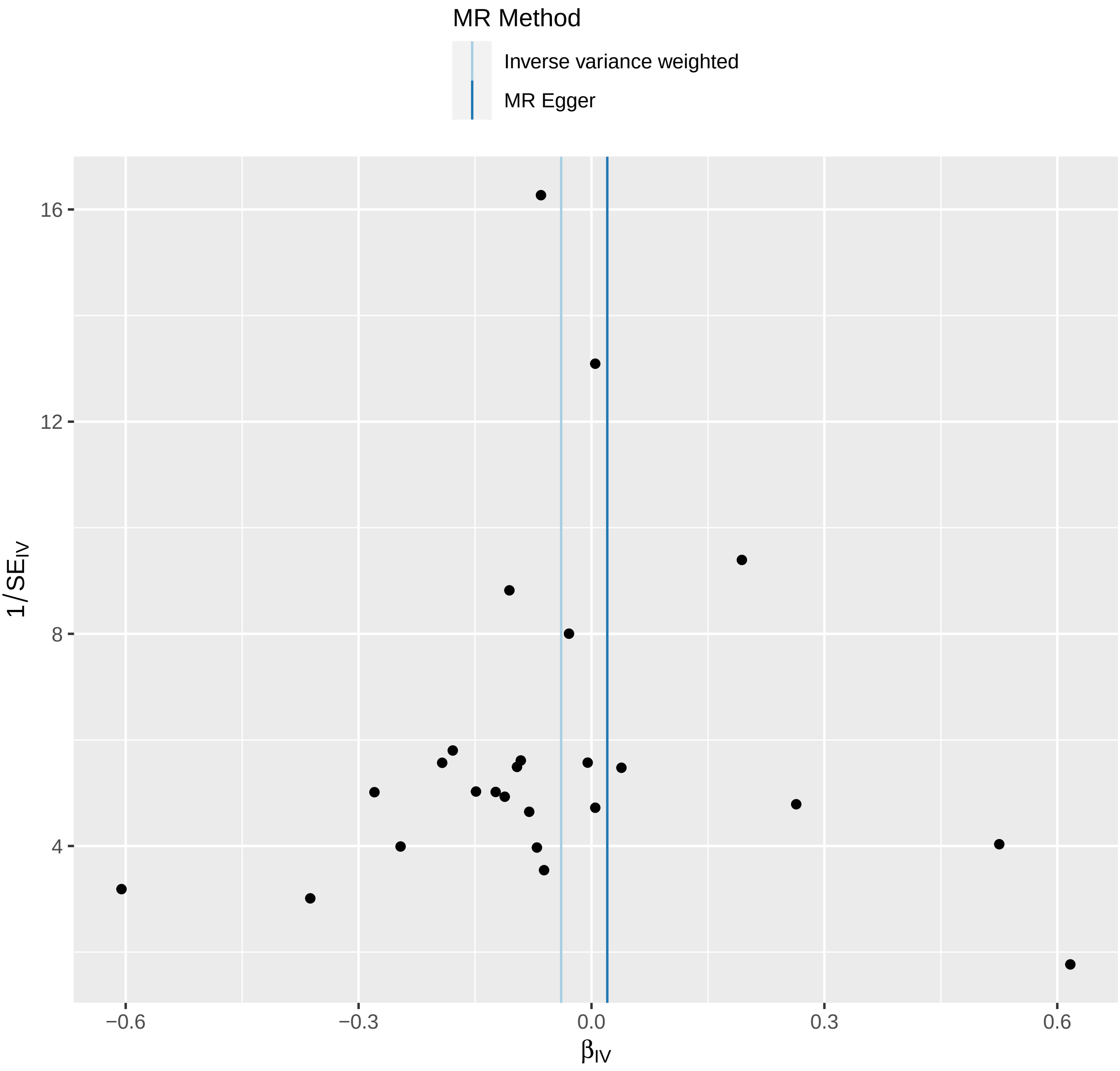

Batch 3−3077 : Torsin−1A−interacting protein 2 on Diabetic nephropathy (more control exclusions)
with a threshold of 1e−05

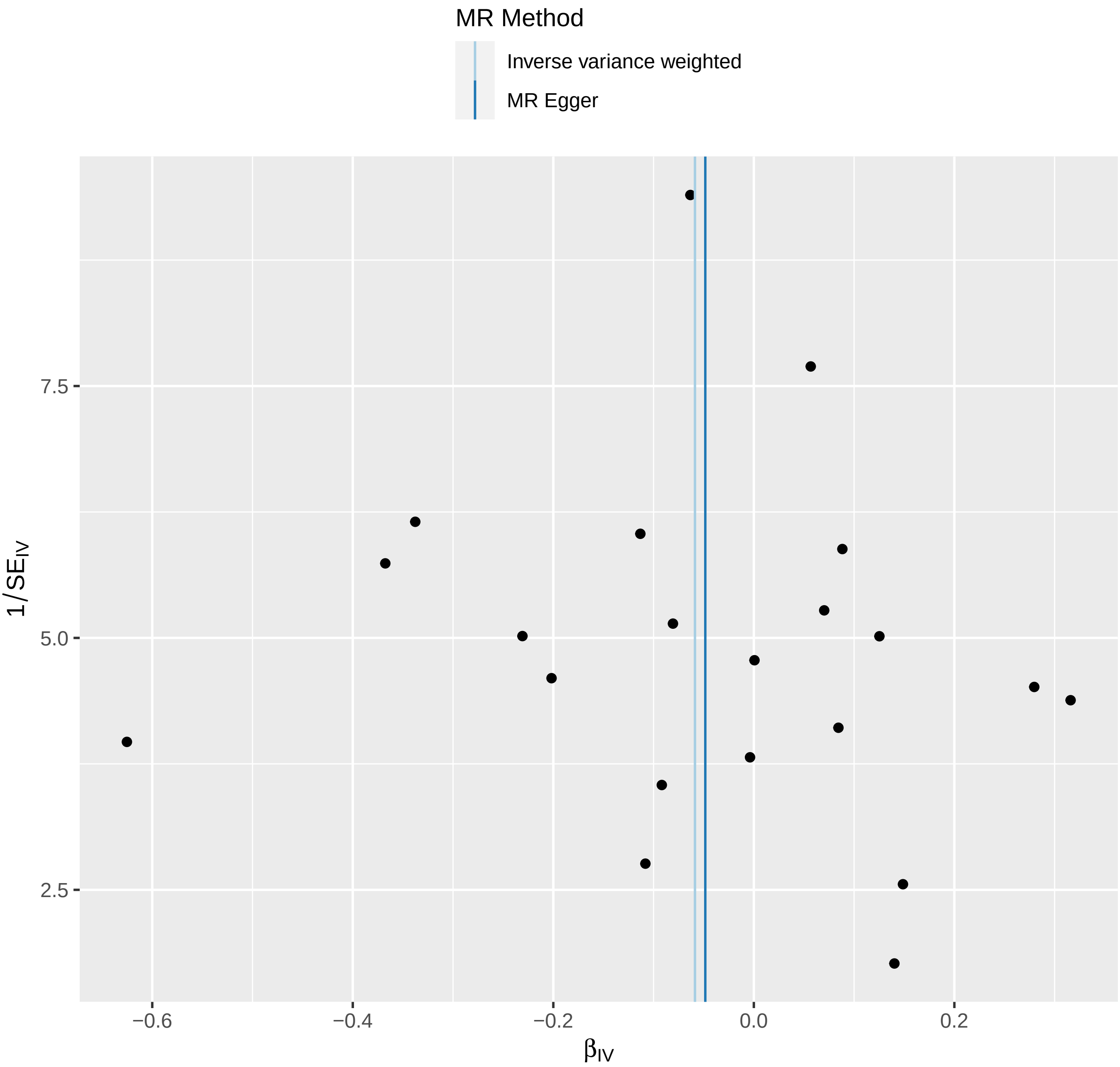

Batch 3−3078 : Torsin−1A−interacting protein 2 on Diabetic nephropathy (more control exclusions) with a threshold of 1e−05

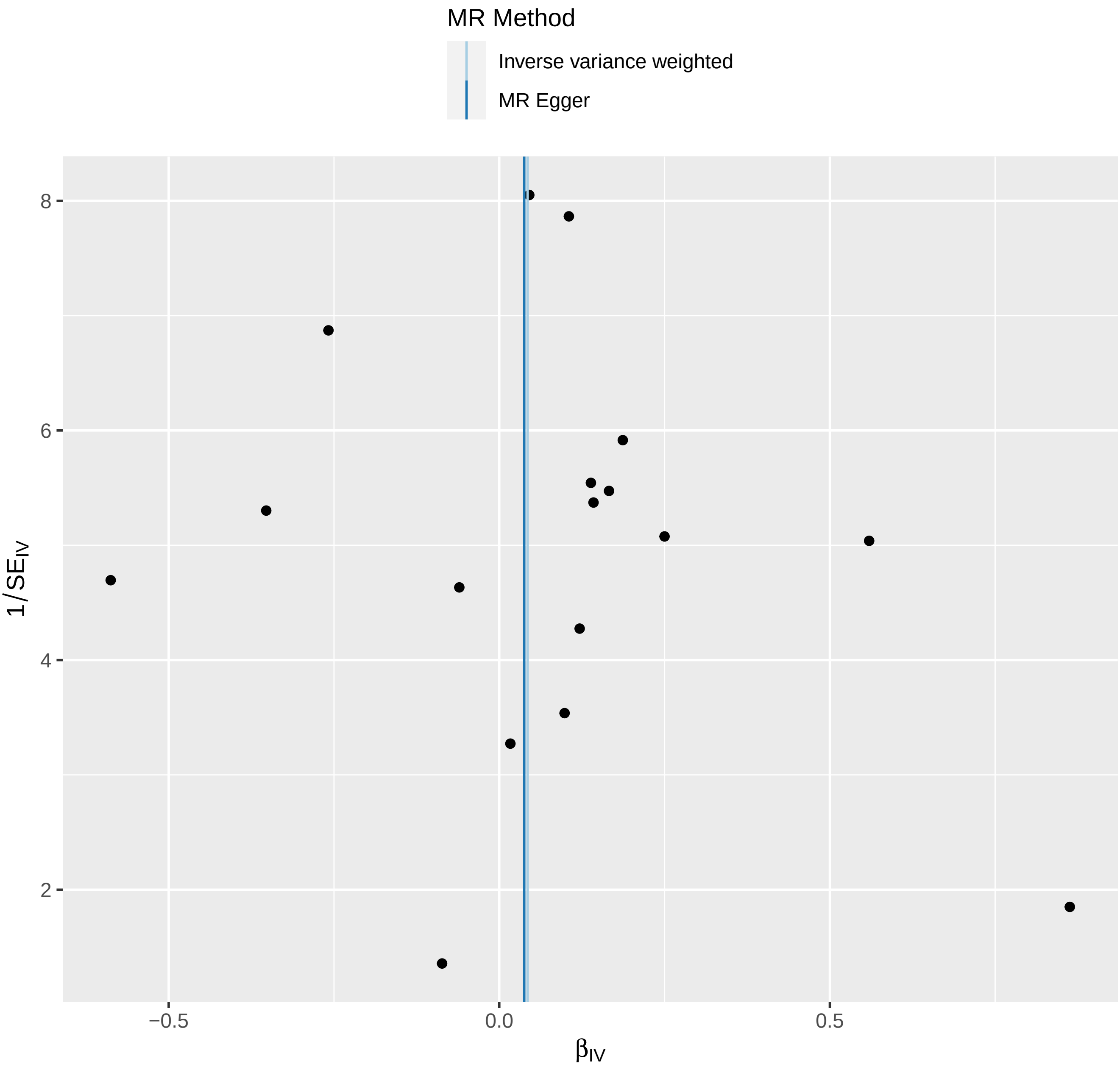

Batch 3−3079 : TOX high mobility group box family member 3 on Diabetic nephropathy (more control exclusions) with a threshold of 1e−05

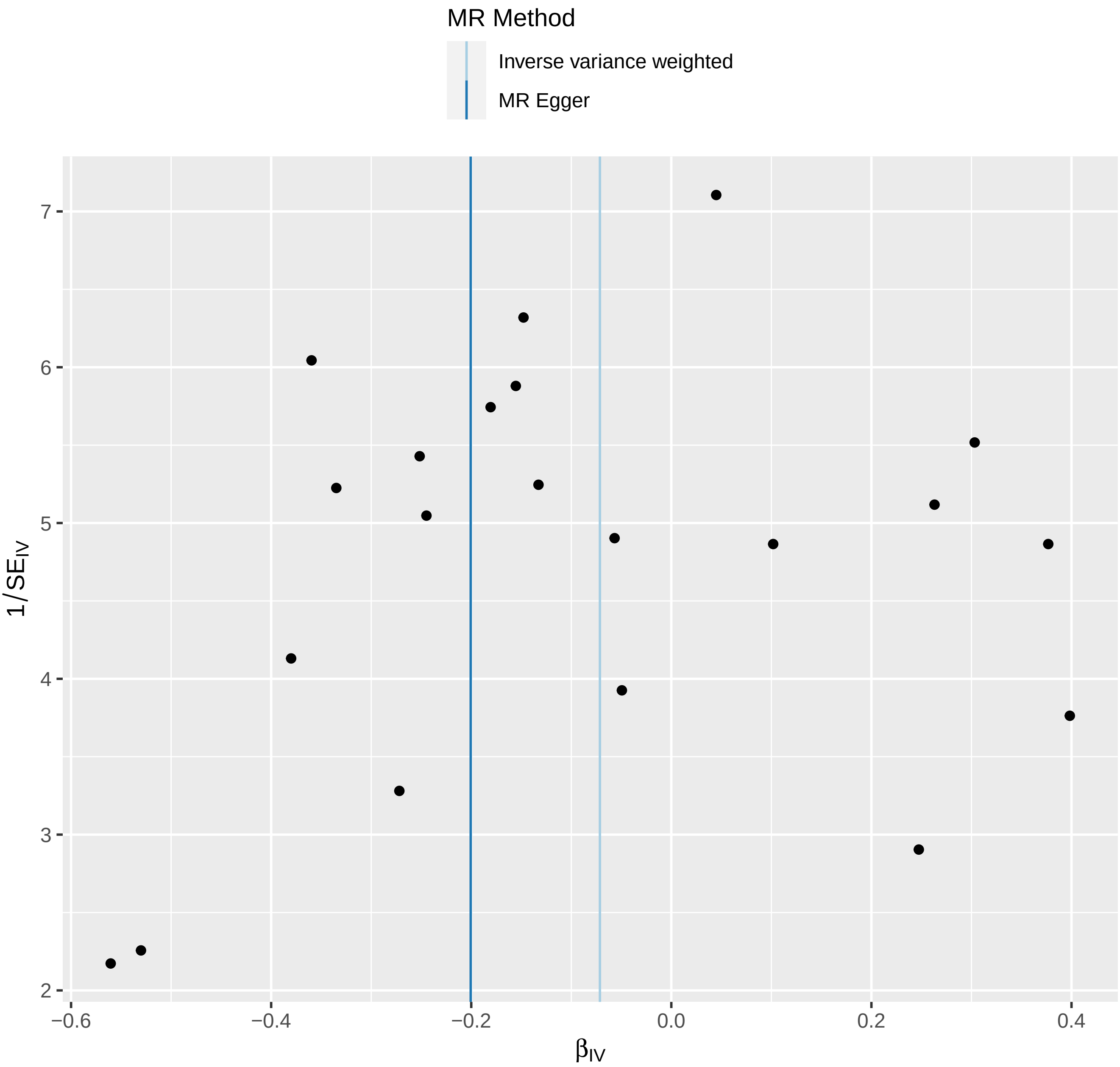

Batch 3−3080 : Cellular tumor antigen p53 on Diabetic nephropathy (more control exclusions)
with a threshold of 1e−05

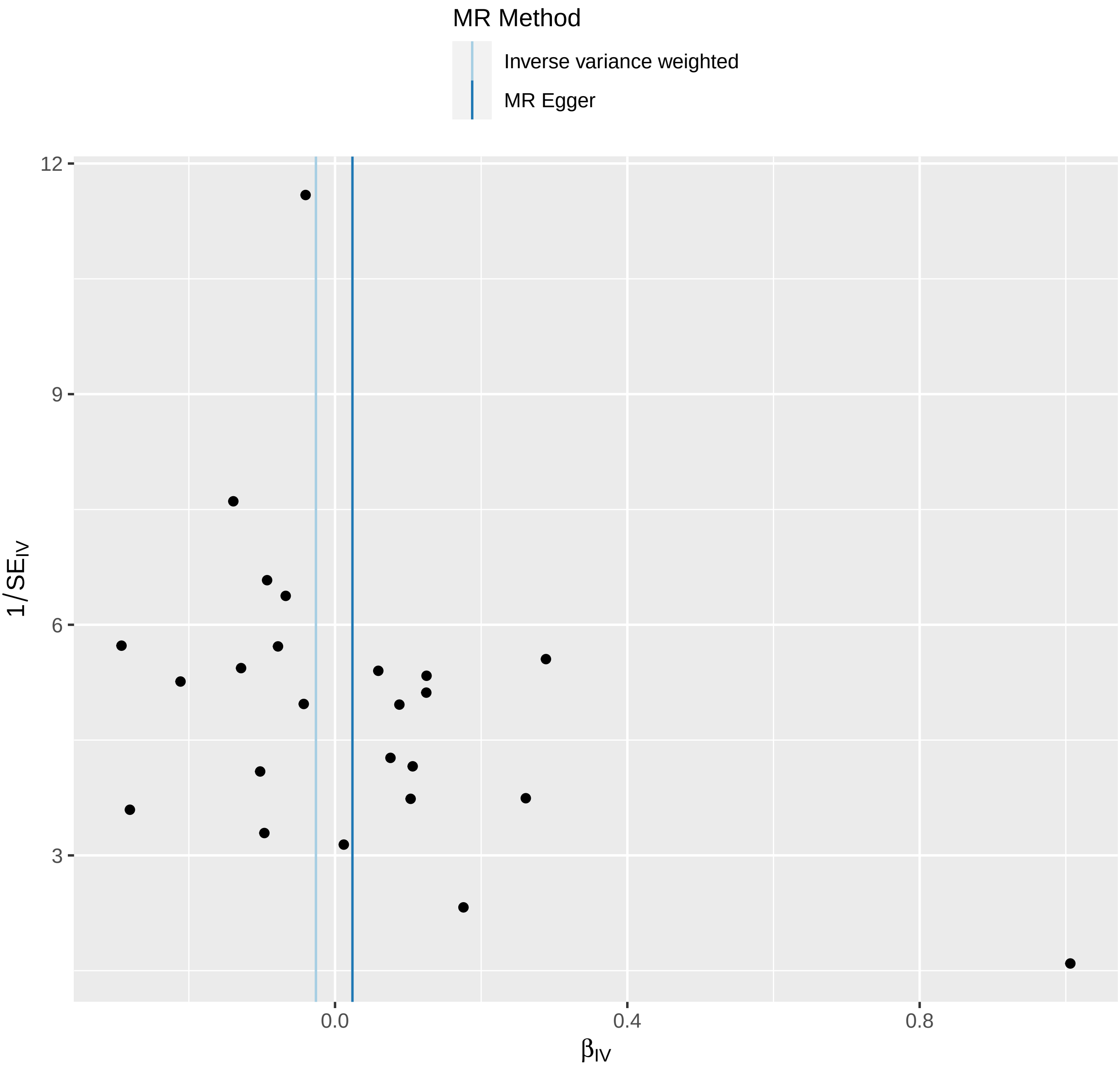

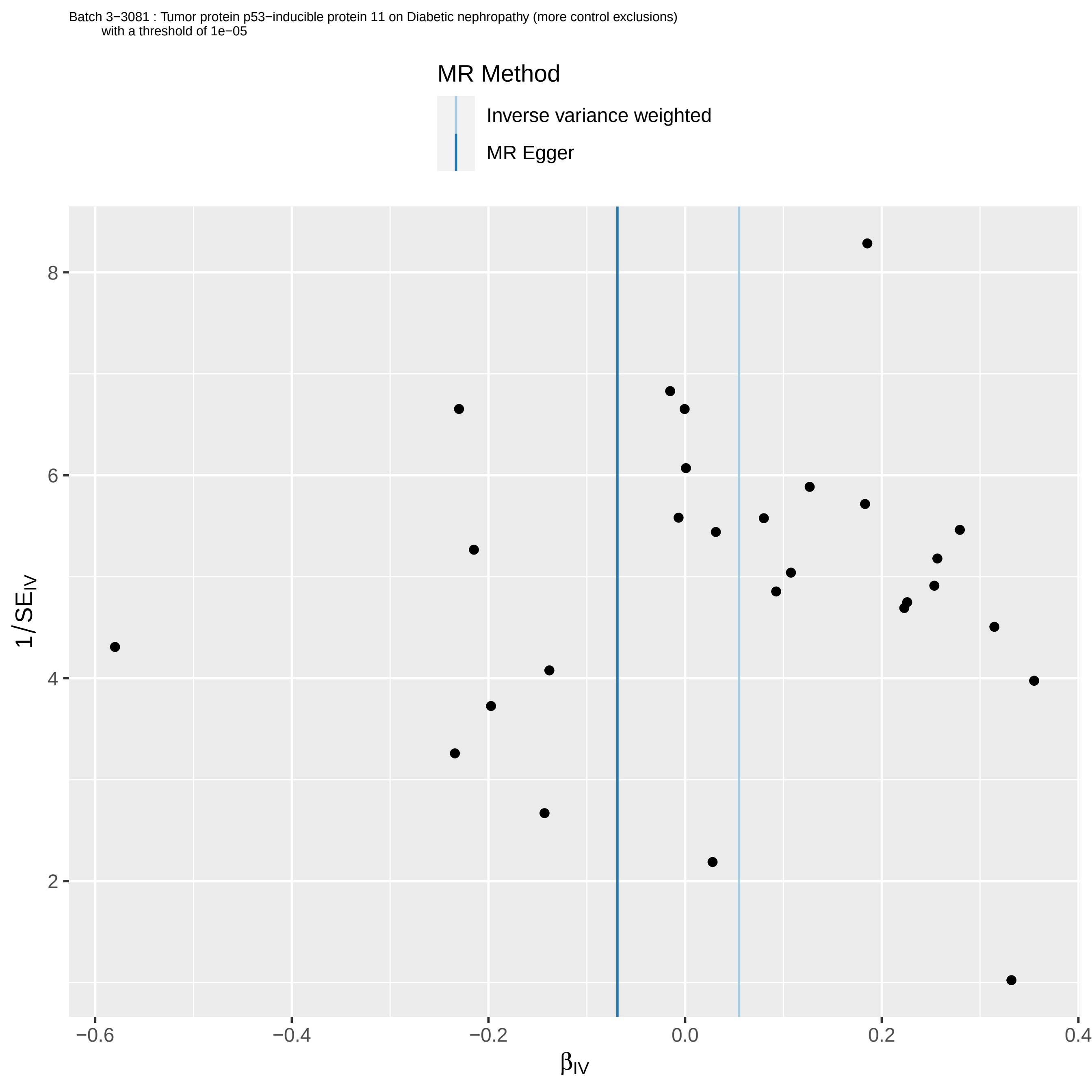
Batch 3−3081 : Tumor protein p53−inducible protein 11 on Diabetic nephropathy (more control exclusions)
with a threshold of 1e−05
MR Method
Inverse variance weighted
MR Egger
8
6
4
2
1/SE_IV
−0.6
−0.4
−0.2
0.0
0.2
0.4
β_IV

Batch 3−3082 : Tumor protein 63 on Diabetic nephropathy (more control exclusions)
with a threshold of 1e−05

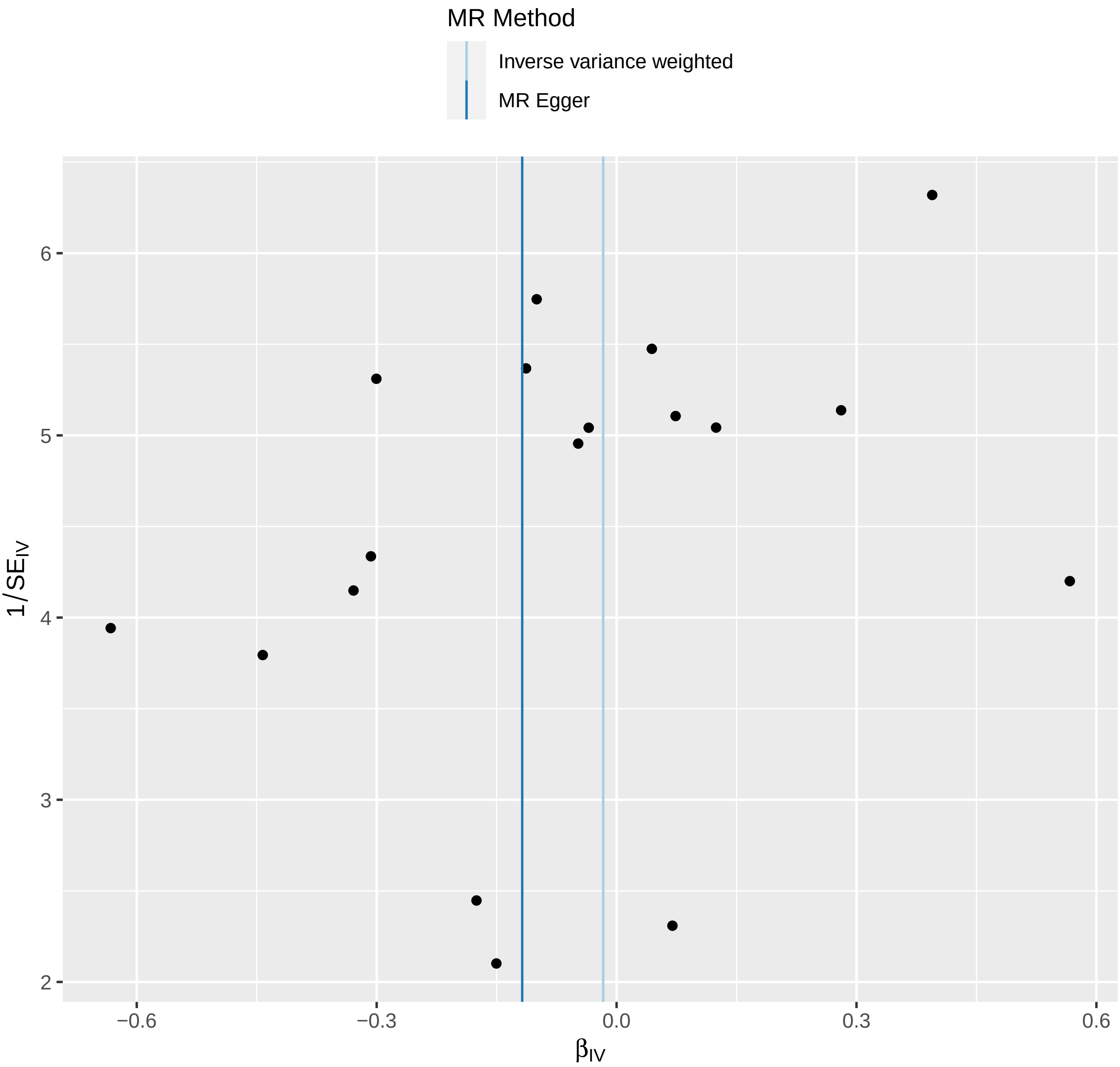

Batch 3−3083 : Triosephosphate isomerase on Diabetic nephropathy (more control exclusions)
with a threshold of 1e−05

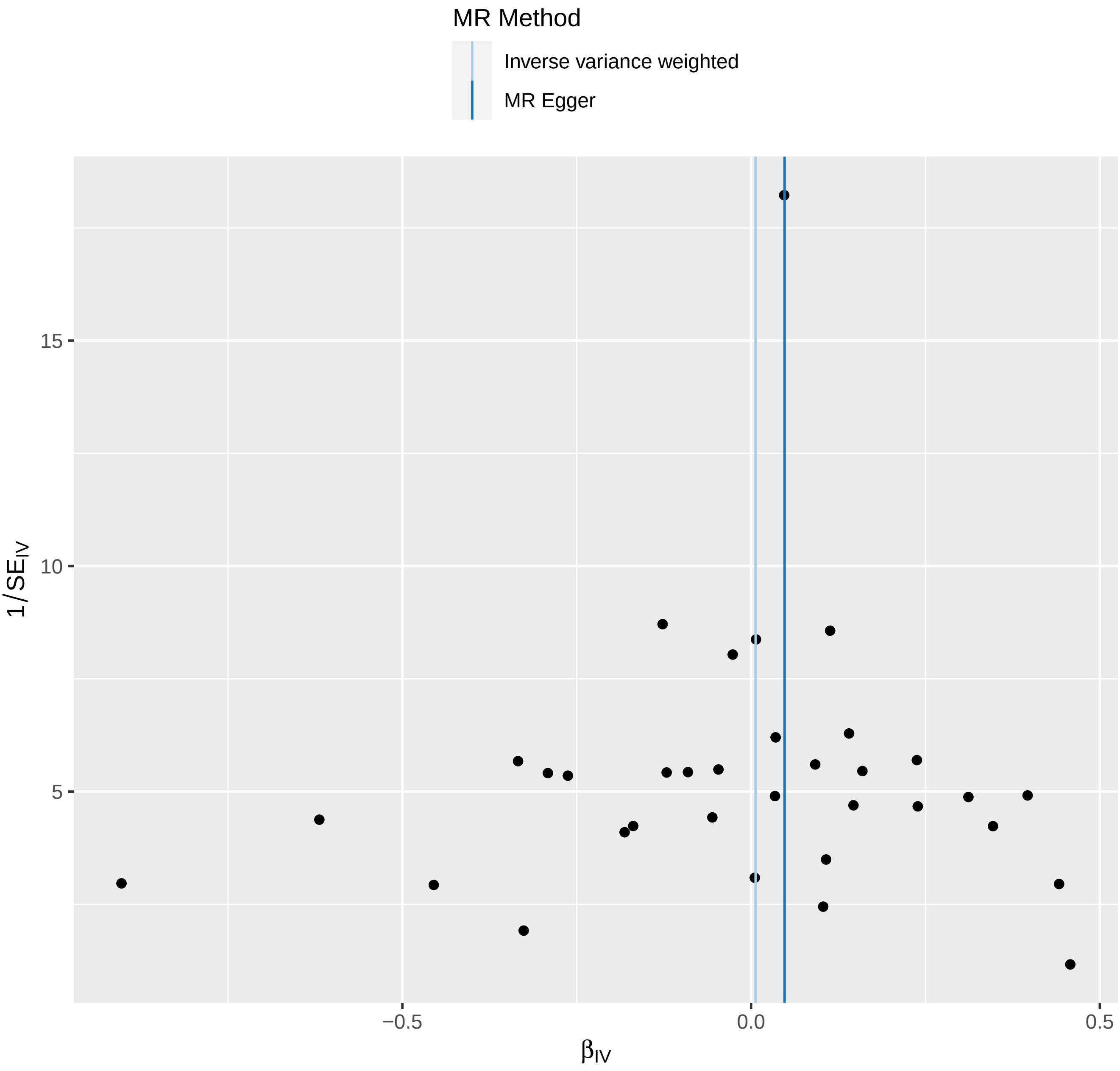

Batch 3−3084 : Thiamin pyrophosphokinase 1 on Diabetic nephropathy (more control exclusions)
with a threshold of 1e−05

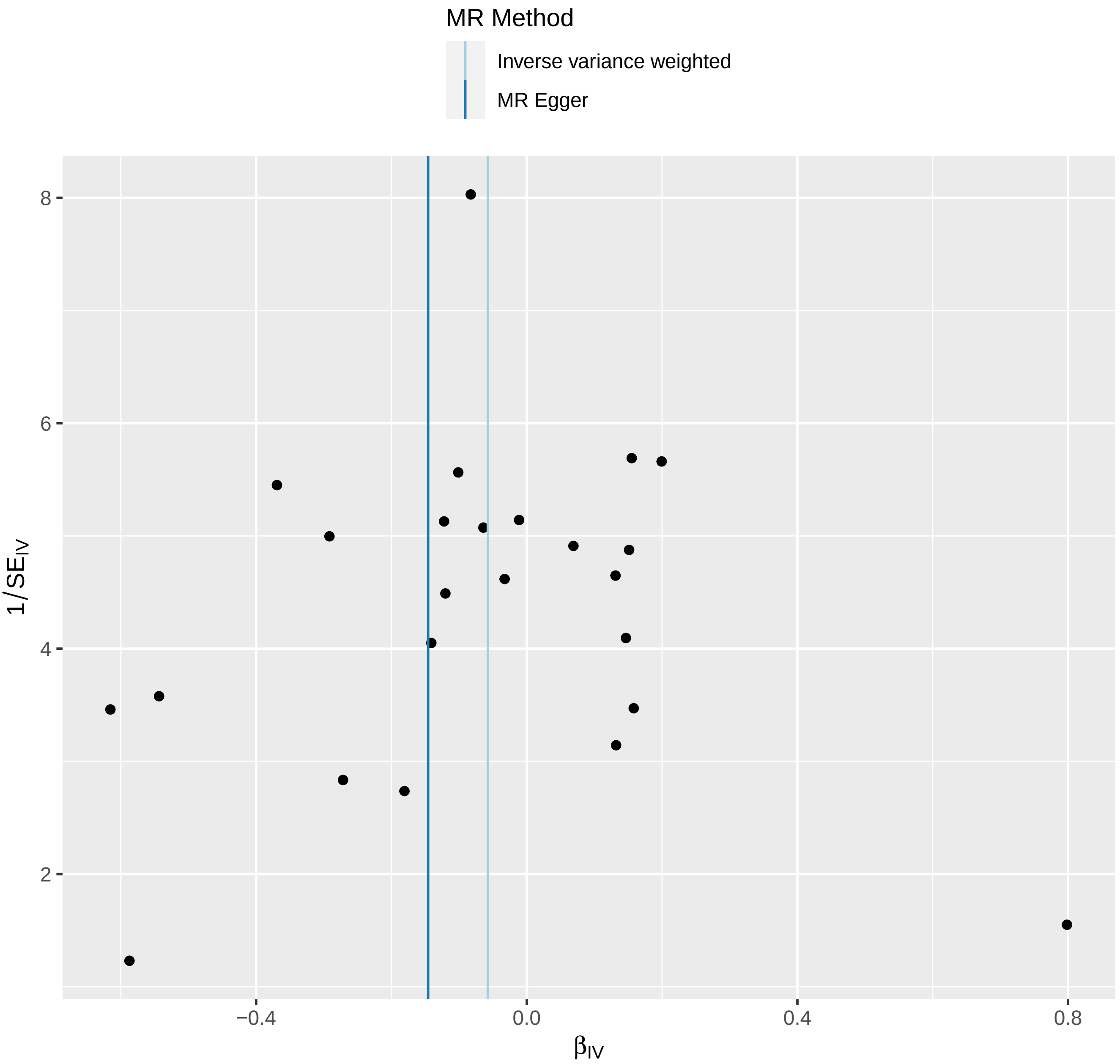

Batch 3−3085 : Tropomyosin alpha−1 chain on Diabetic nephropathy (more control exclusions)
with a threshold of 1e−05

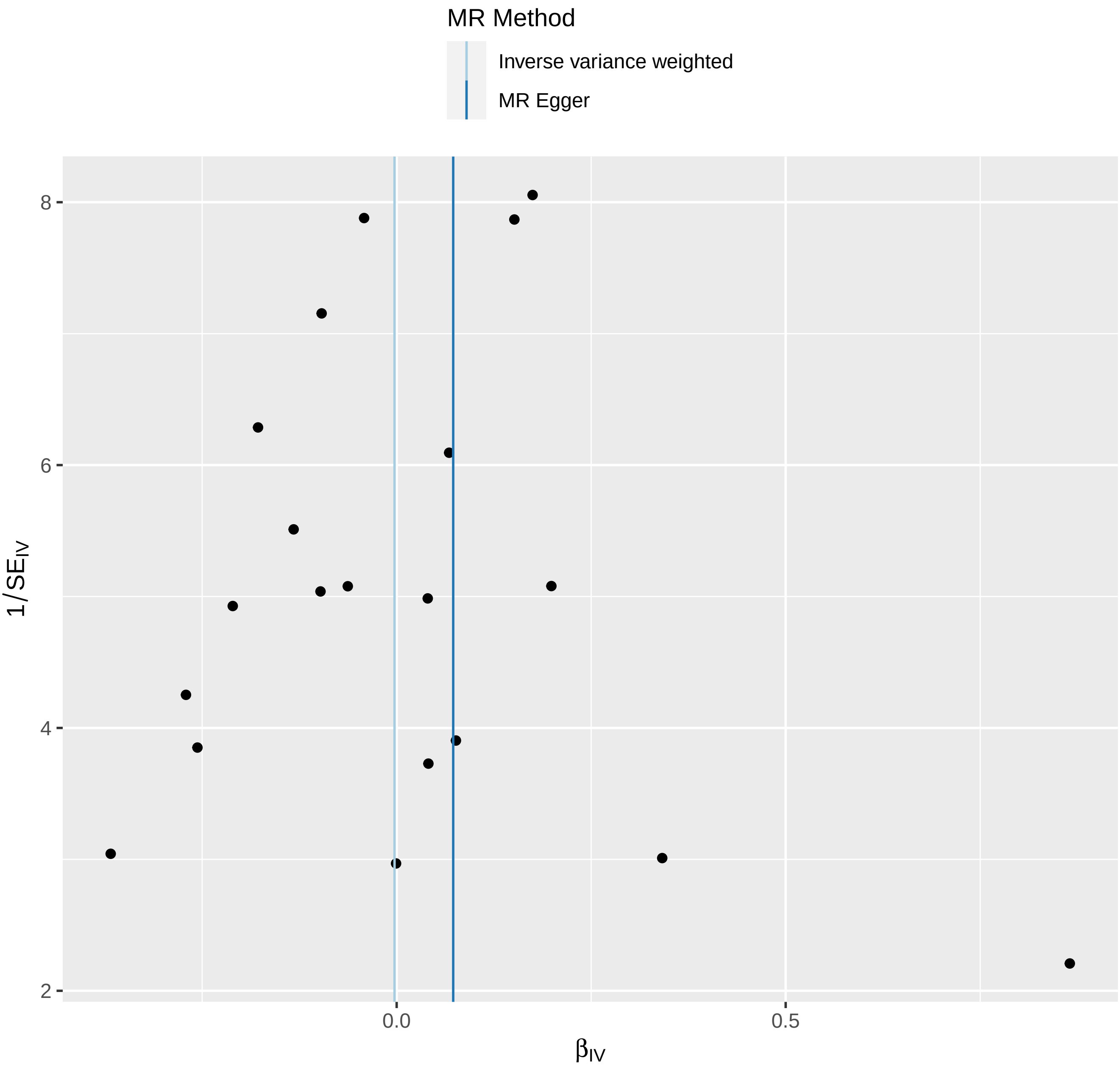

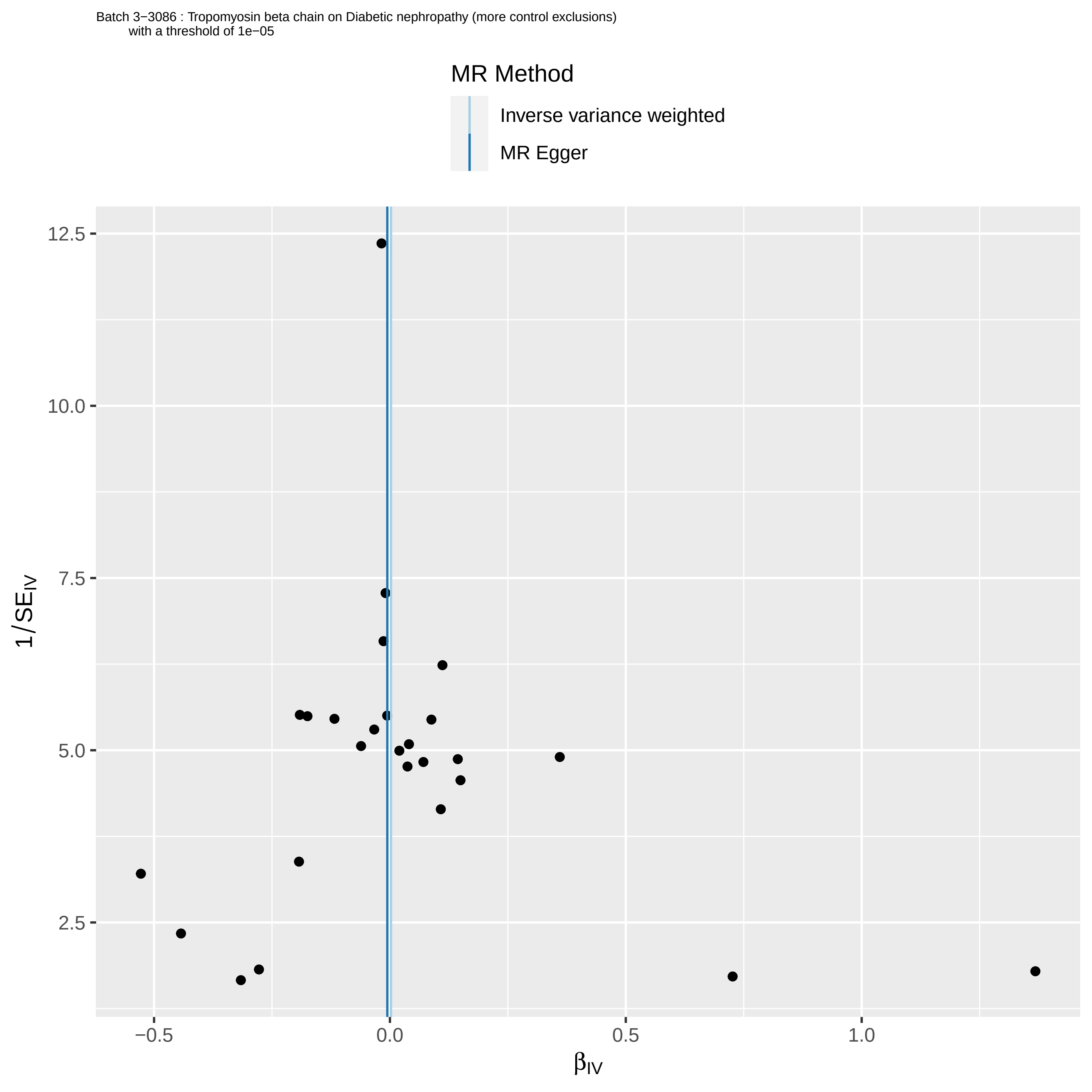

Batch 3−3086 : Tropomyosin beta chain on Diabetic nephropathy (more control exclusions)
with a threshold of 1e−05
MR Method
Inverse variance weighted
MR Egger
12.5
10.0
7.5
5.0
2.5
1/SE_IV
−0.5
0.0
0.5
1.0
β_IV

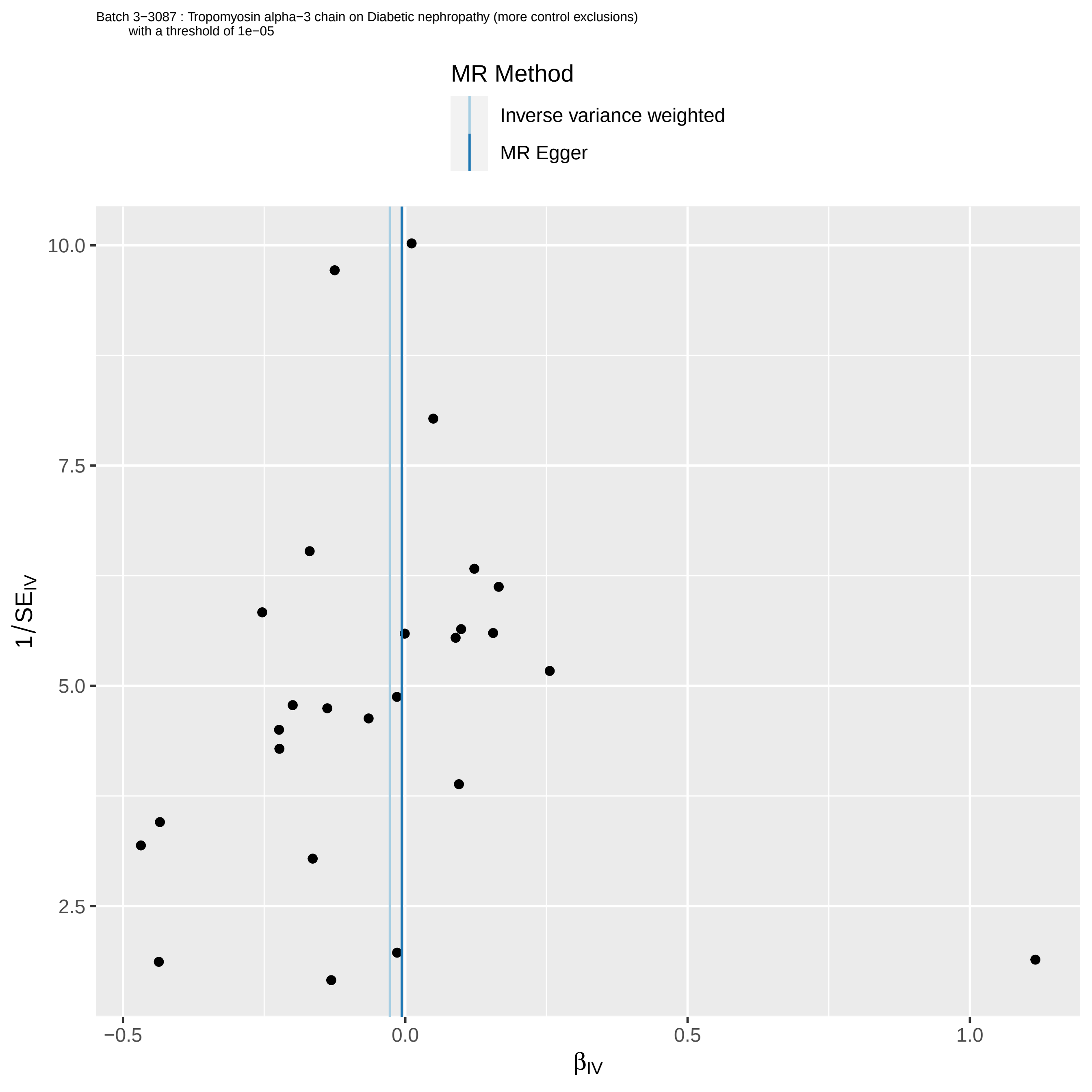
Batch 3−3087 : Tropomyosin alpha−3 chain on Diabetic nephropathy (more control exclusions)
with a threshold of 1e−05
MR Method
Inverse variance weighted
MR Egger
10.0
7.5
5.0
2.5
$1/SE_{IV}$
−0.5
0.0
0.5
1.0
β_{IV}

Batch 3−3088 : Thyroid peroxidase on Diabetic nephropathy (more control exclusions)
with a threshold of 1e−05

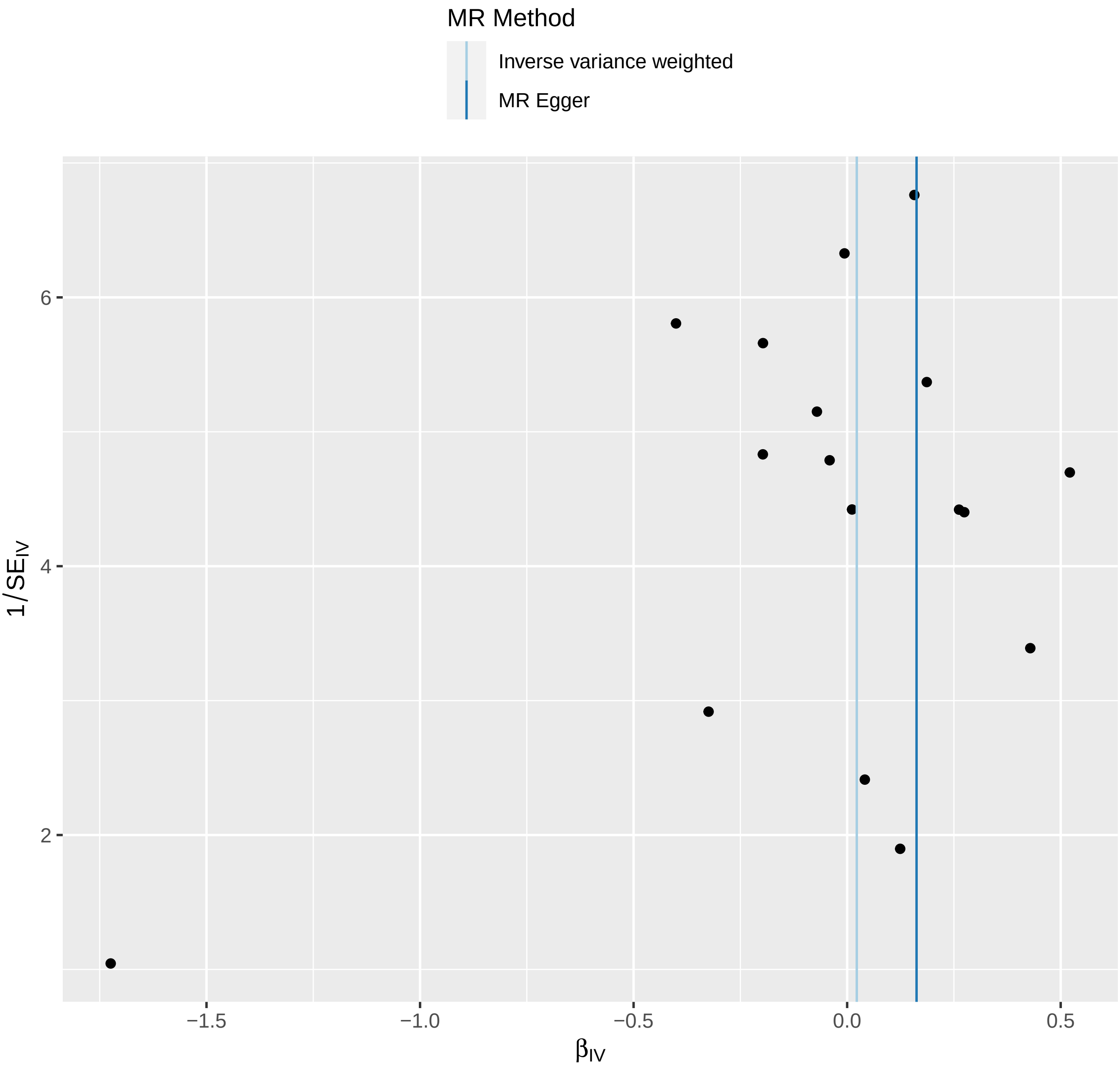

Batch 3−3089 : Tubulin polymerization−promoting protein family member 2 on Diabetic nephropathy (more control exclusions)
with a threshold of 1e−05

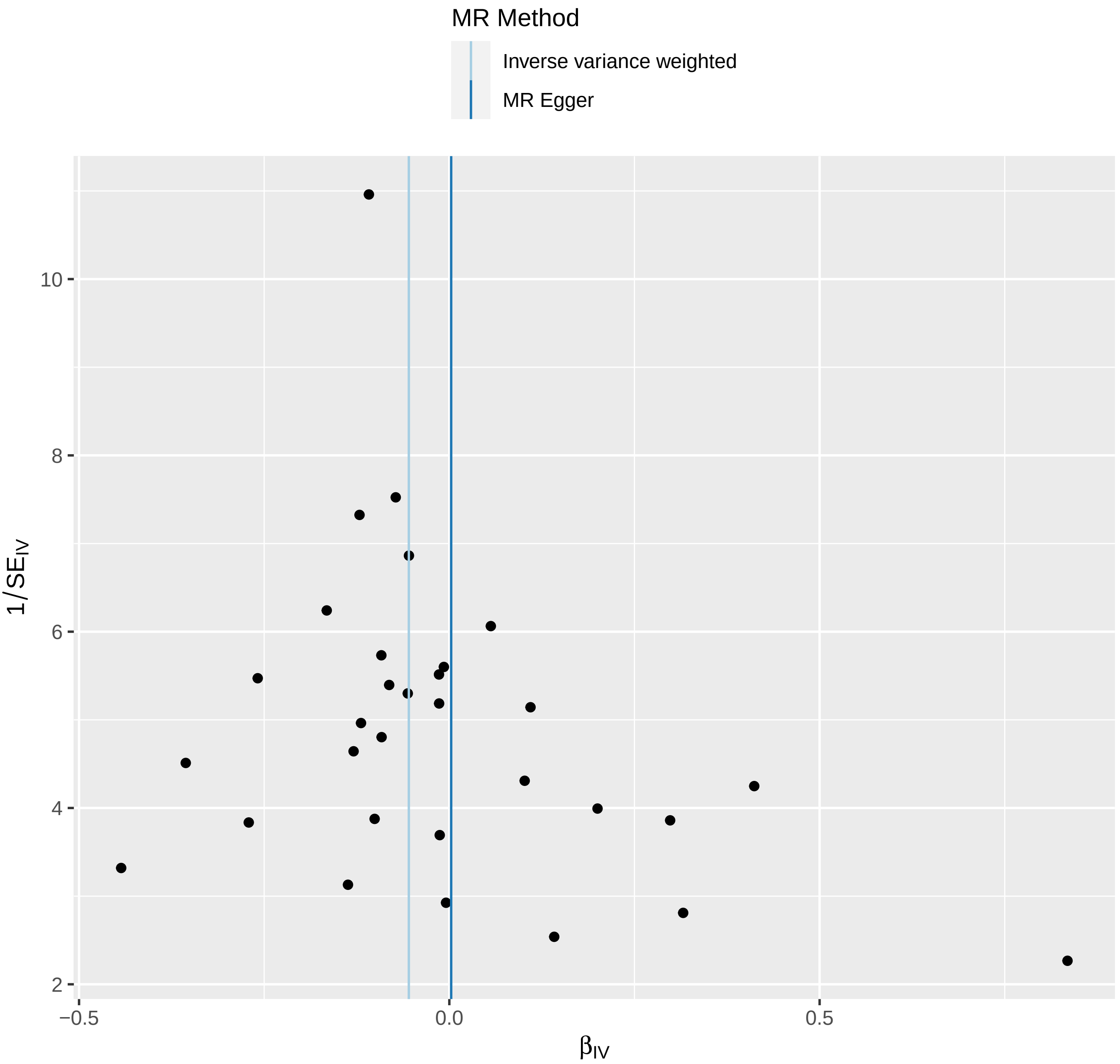

Batch 3−3090 : Tryptase beta−2 on Diabetic nephropathy (more control exclusions)
with a threshold of 1e−05

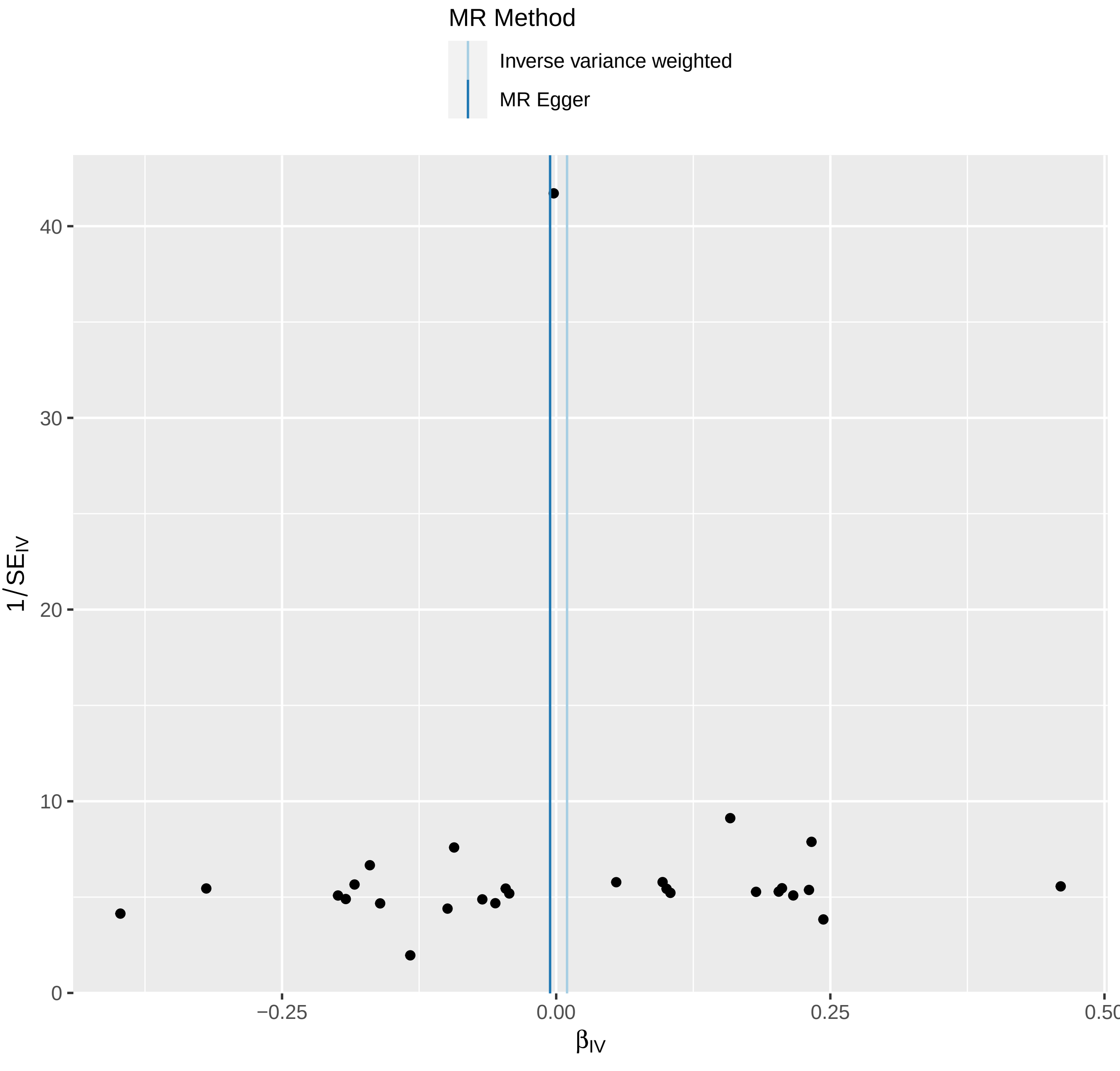

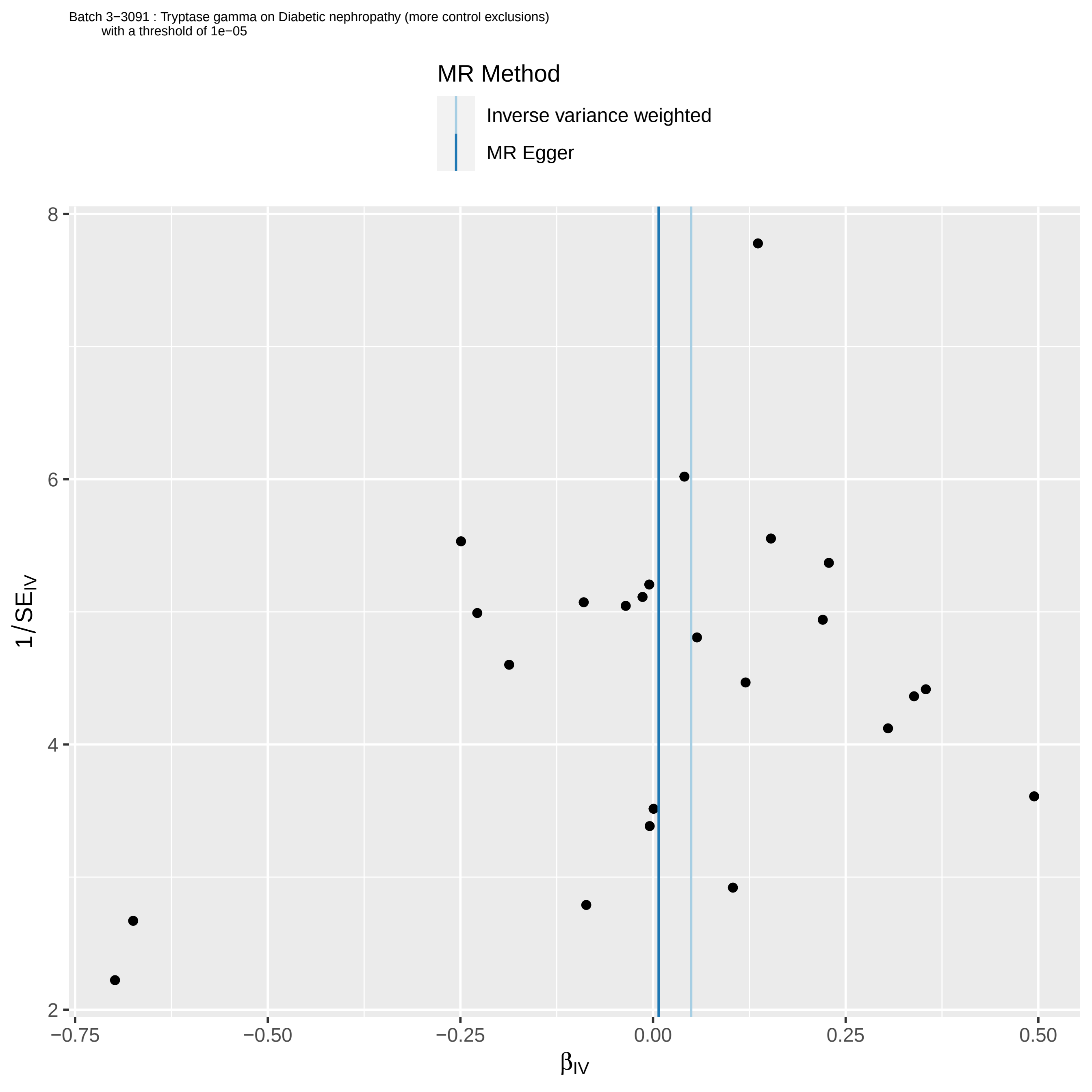

Batch 3−3091 : Tryptase gamma on Diabetic nephropathy (more control exclusions)
with a threshold of 1e−05
MR Method
Inverse variance weighted
MR Egger
8
6
4
2
1/SE_IV
−0.75
−0.50
−0.25
0.00
0.25
0.50
β_IV

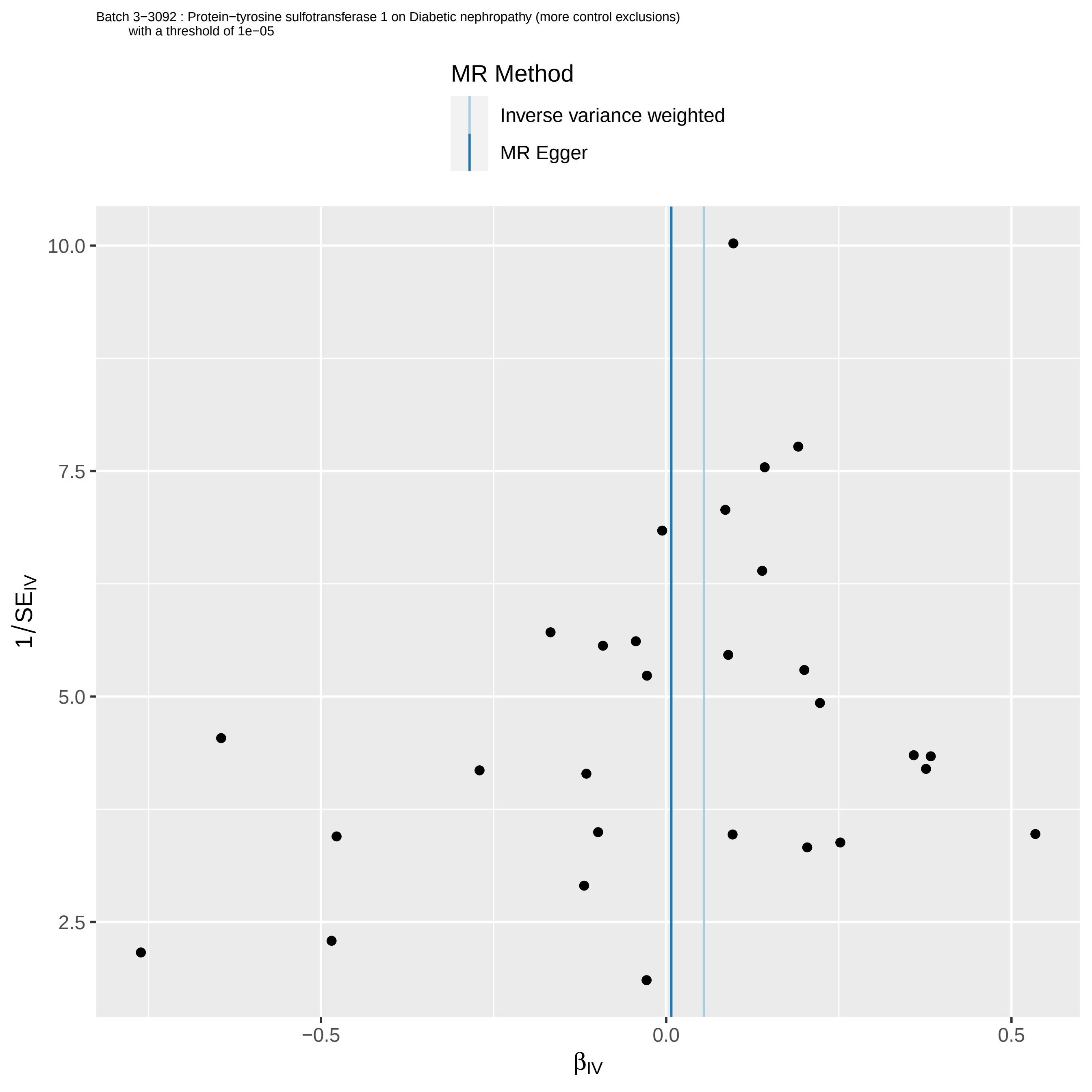

Batch 3−3092 : Protein−tyrosine sulfotransferase 1 on Diabetic nephropathy (more control exclusions)
with a threshold of 1e−05
MR Method
Inverse variance weighted
MR Egger
10.0
7.5
5.0
2.5
−0.5
0.0
0.5
$1/SE_{IV}$
β_{IV}

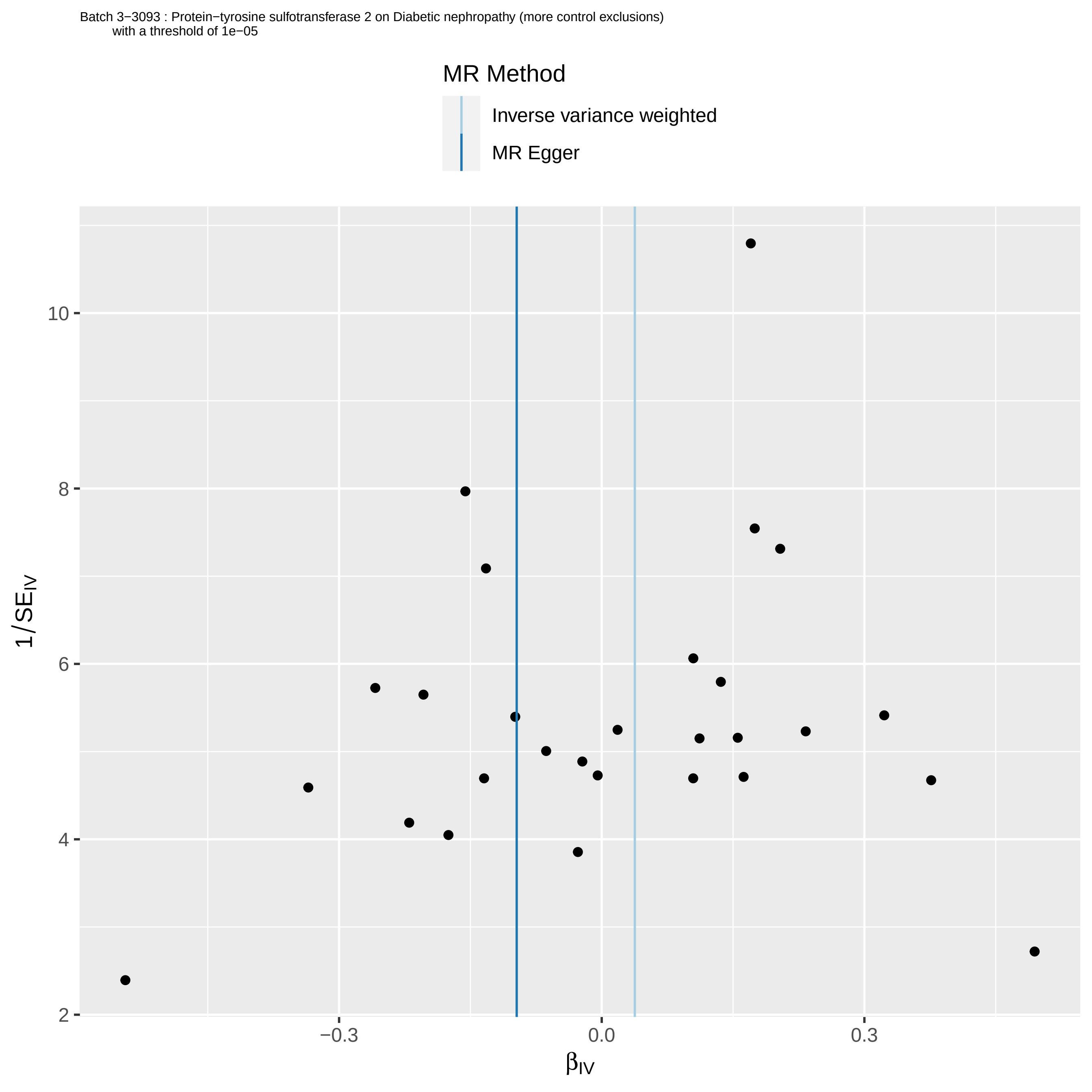

Batch 3−3093 : Protein−tyrosine sulfotransferase 2 on Diabetic nephropathy (more control exclusions)
with a threshold of 1e−05
MR Method
Inverse variance weighted
MR Egger
1/SE_IV
β_IV
10
8
6
4
2
−0.3
0.0
0.3

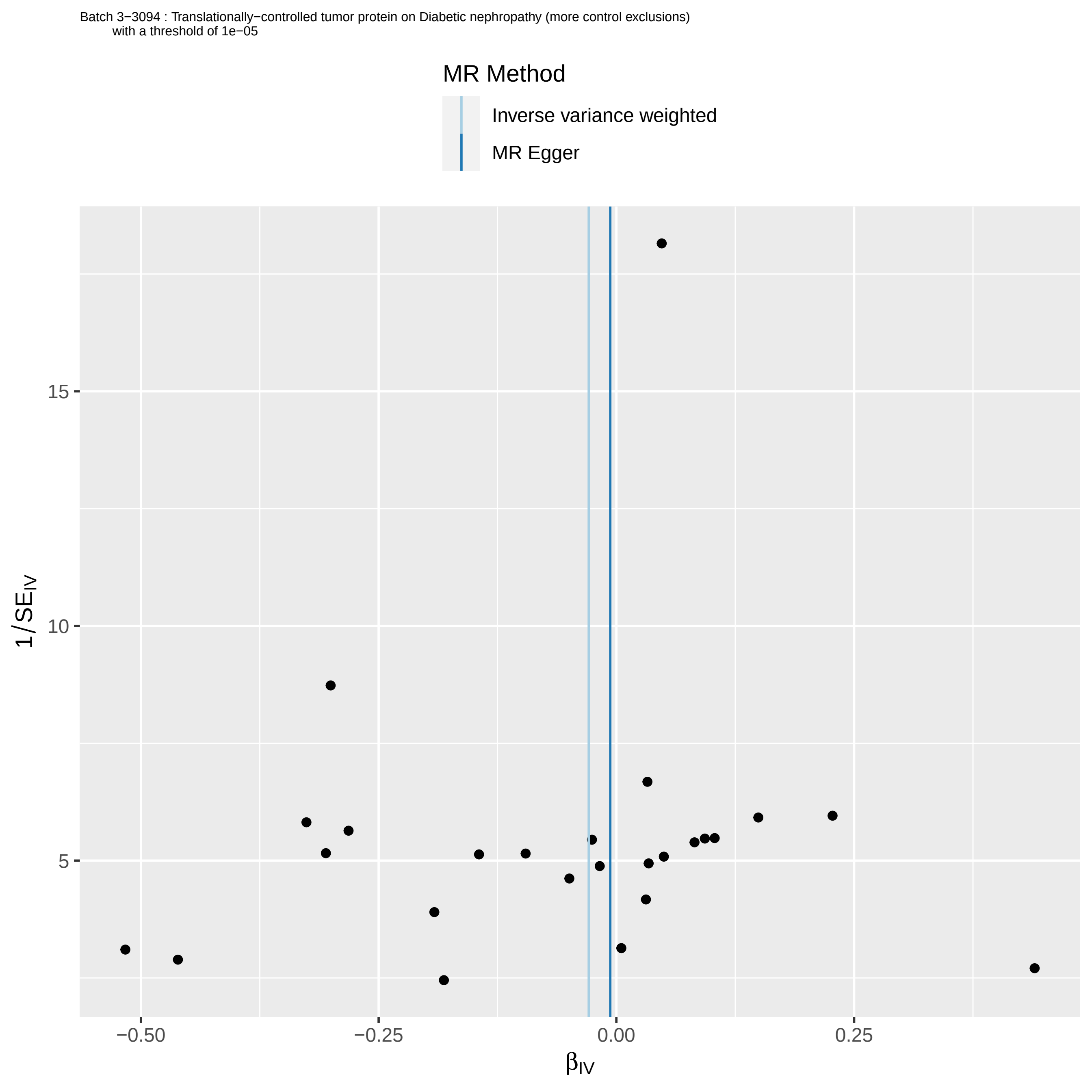
Batch 3−3094 : Translationally−controlled tumor protein on Diabetic nephropathy (more control exclusions)
with a threshold of 1e−05
MR Method
Inverse variance weighted
MR Egger
15
10
5
$1/SE_{IV}$
−0.50
−0.25
0.00
0.25
β_{IV}

Batch 3−3095 : Transformer−2 protein homolog beta on Diabetic nephropathy (more control exclusions) with a threshold of 1e−05

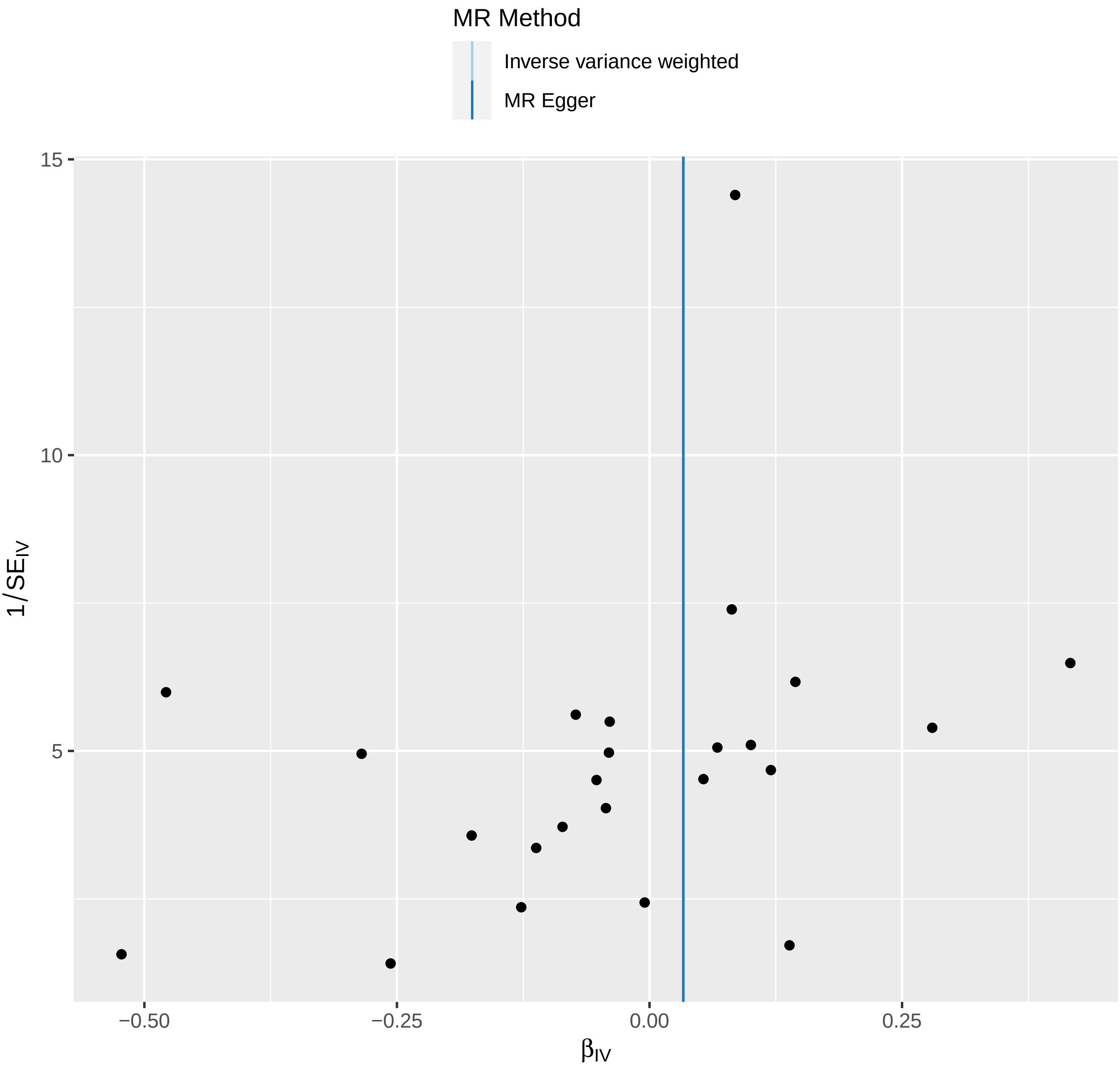

Batch 3−3096 : TNF receptor−associated factor 4 on Diabetic nephropathy (more control exclusions)
with a threshold of 1e−05

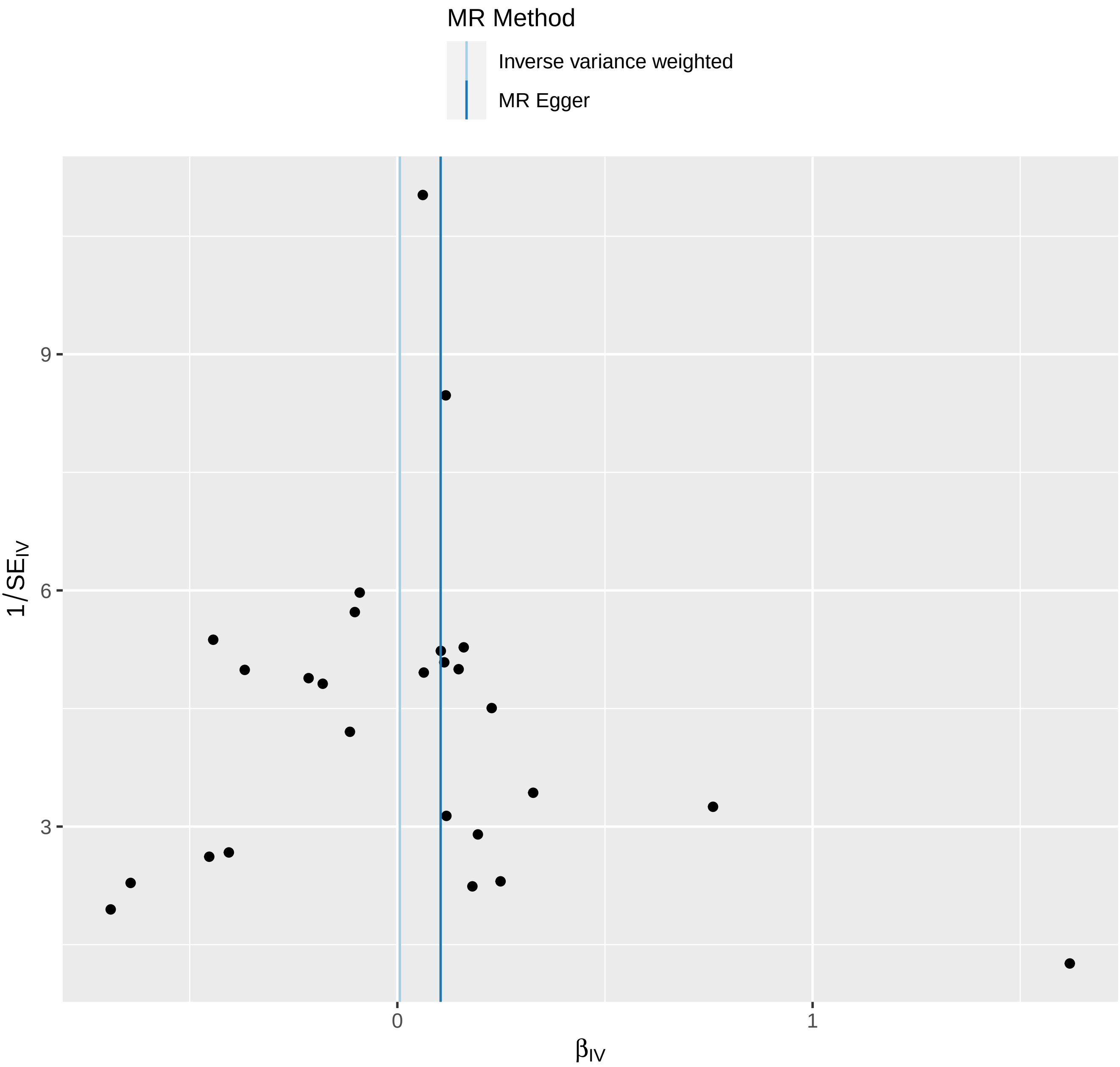

Batch 3−3097 : T−cell receptor−associated transmembrane adapter 1 on Diabetic nephropathy (more control exclusions) with a threshold of 1e−05

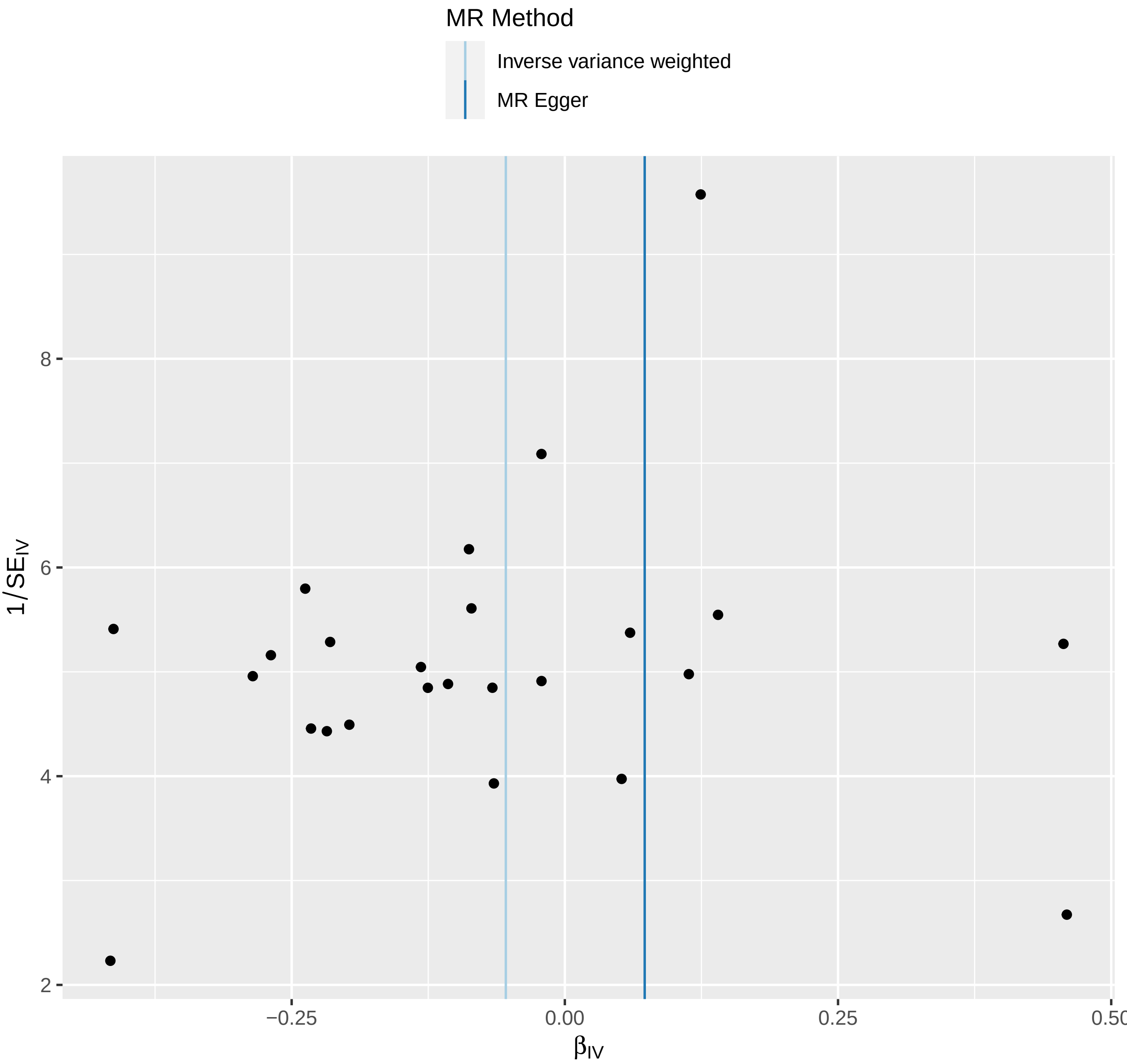

Batch 3−3098 : Triggering receptor expressed on myeloid cells 1 on Diabetic nephropathy (more control exclusions)
with a threshold of 1e−05

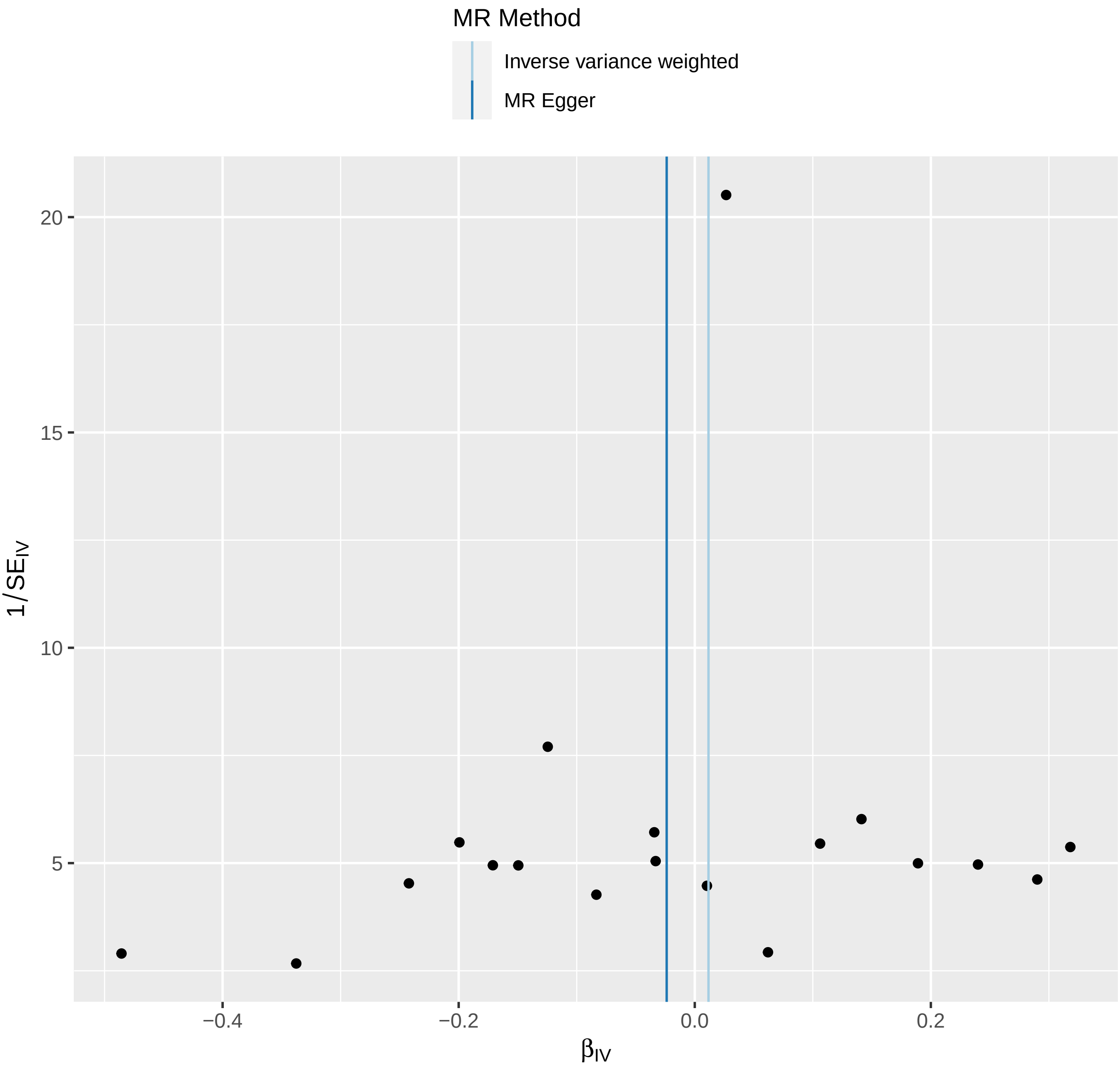

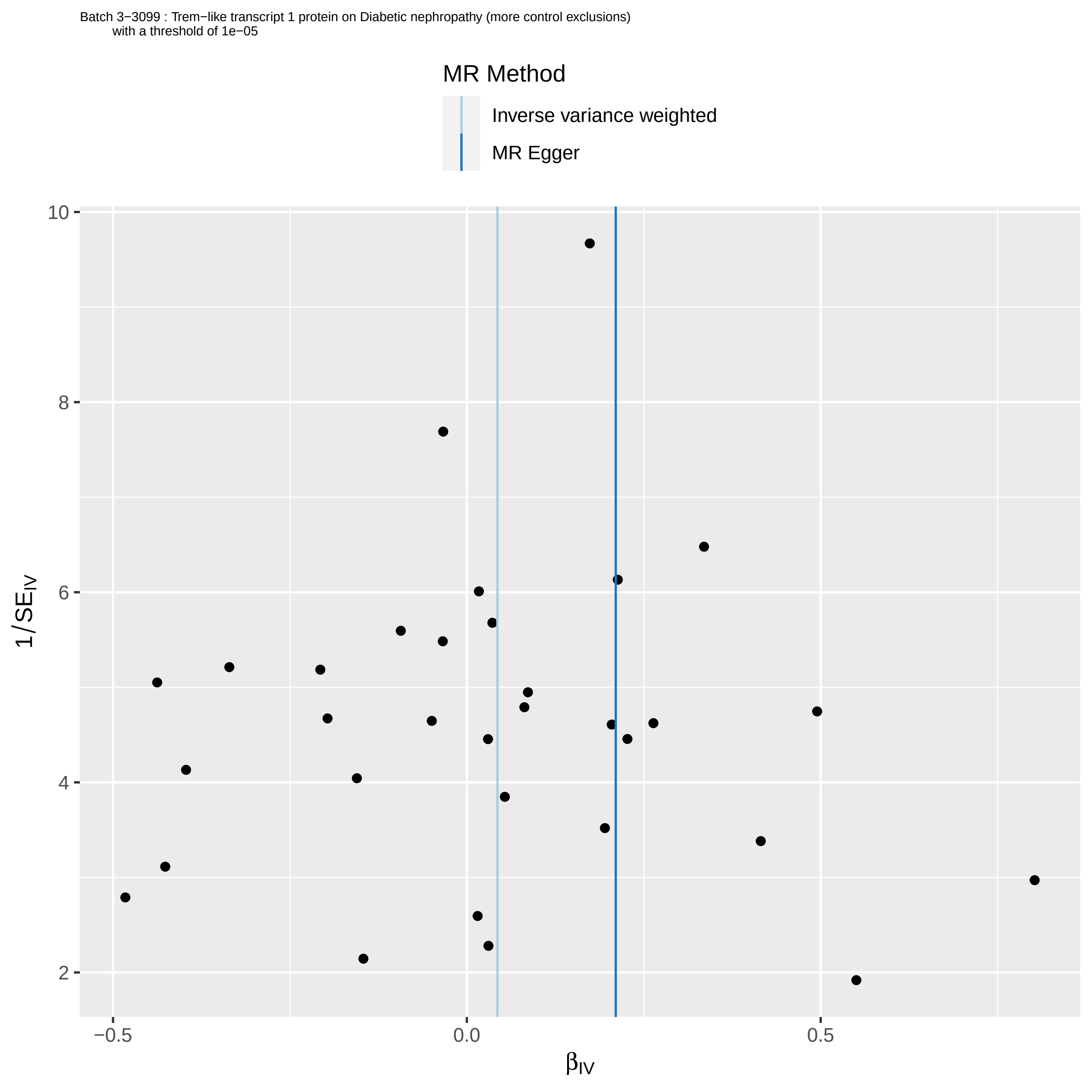
Batch 3−3099 : Trem−like transcript 1 protein on Diabetic nephropathy (more control exclusions)
with a threshold of 1e−05
MR Method
Inverse variance weighted
MR Egger
10
8
6
4
2
1/SE_IV
−0.5
0.0
0.5
β_IV

Batch 3−3100 : Trem−like transcript 2 protein on Diabetic nephropathy (more control exclusions)
with a threshold of 1e−05

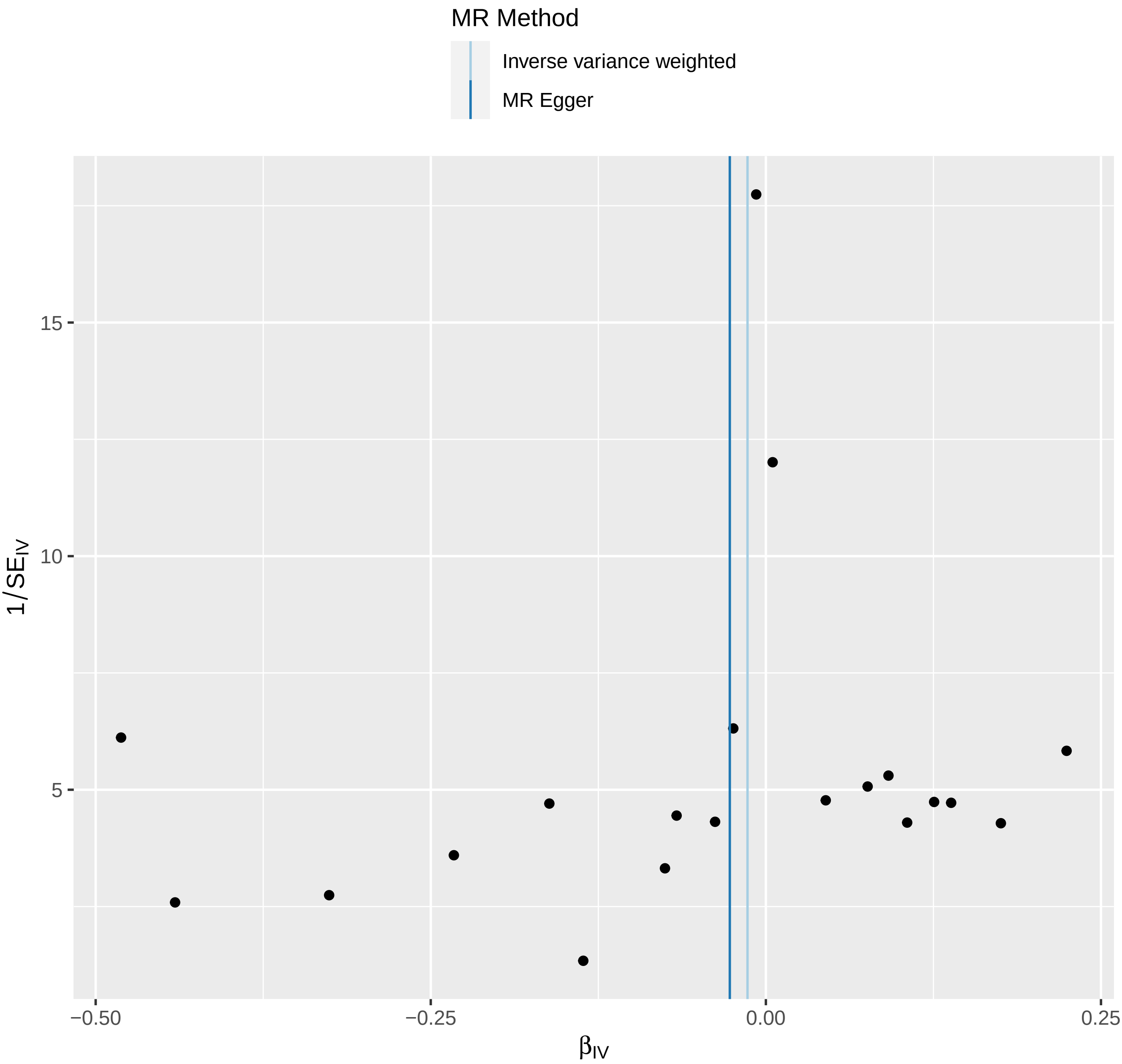

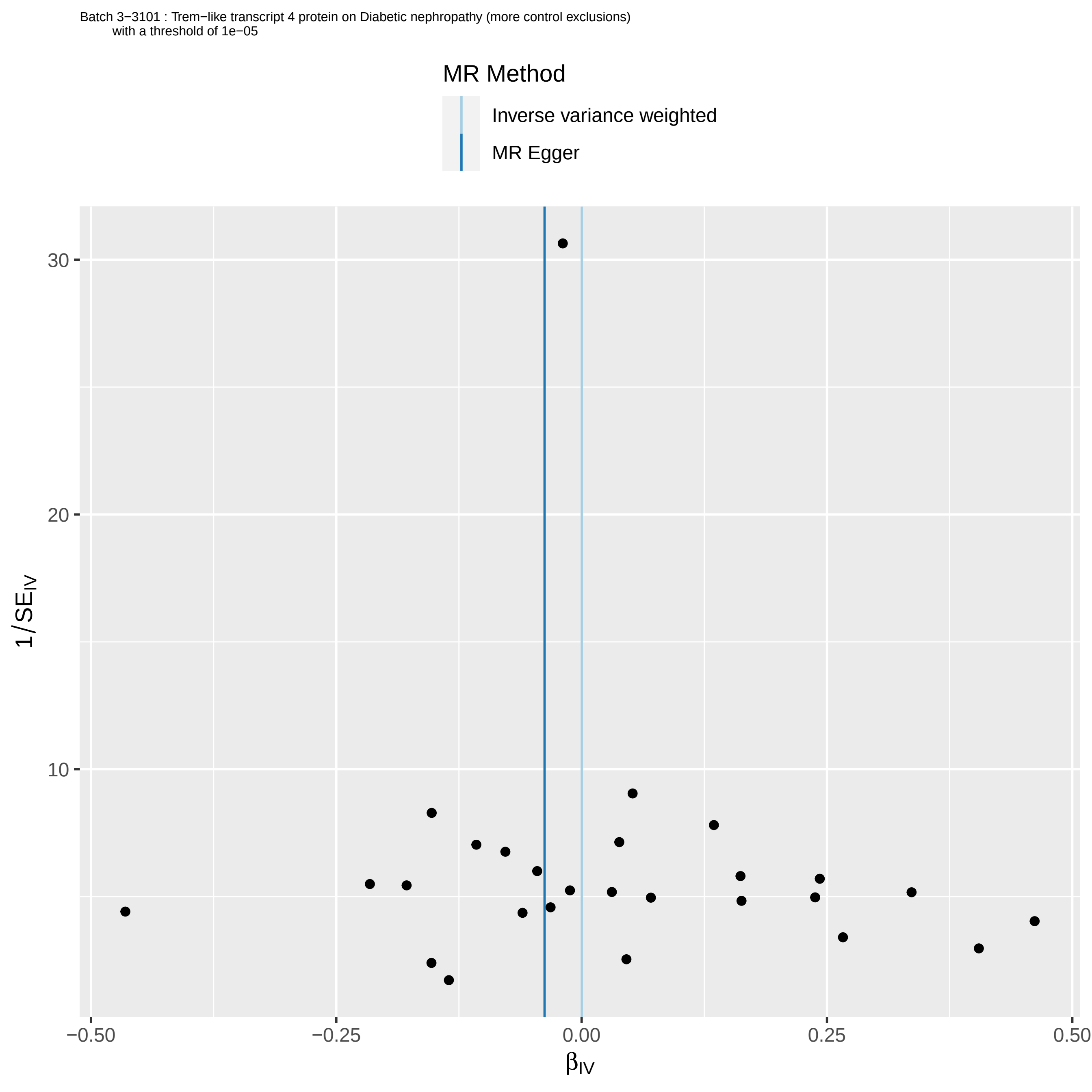
Batch 3−3101 : Trem−like transcript 4 protein on Diabetic nephropathy (more control exclusions)
with a threshold of 1e−05
MR Method
Inverse variance weighted
MR Egger
30
20
10
1/SE_IV
−0.50
−0.25
0.00
0.25
0.50
β_IV

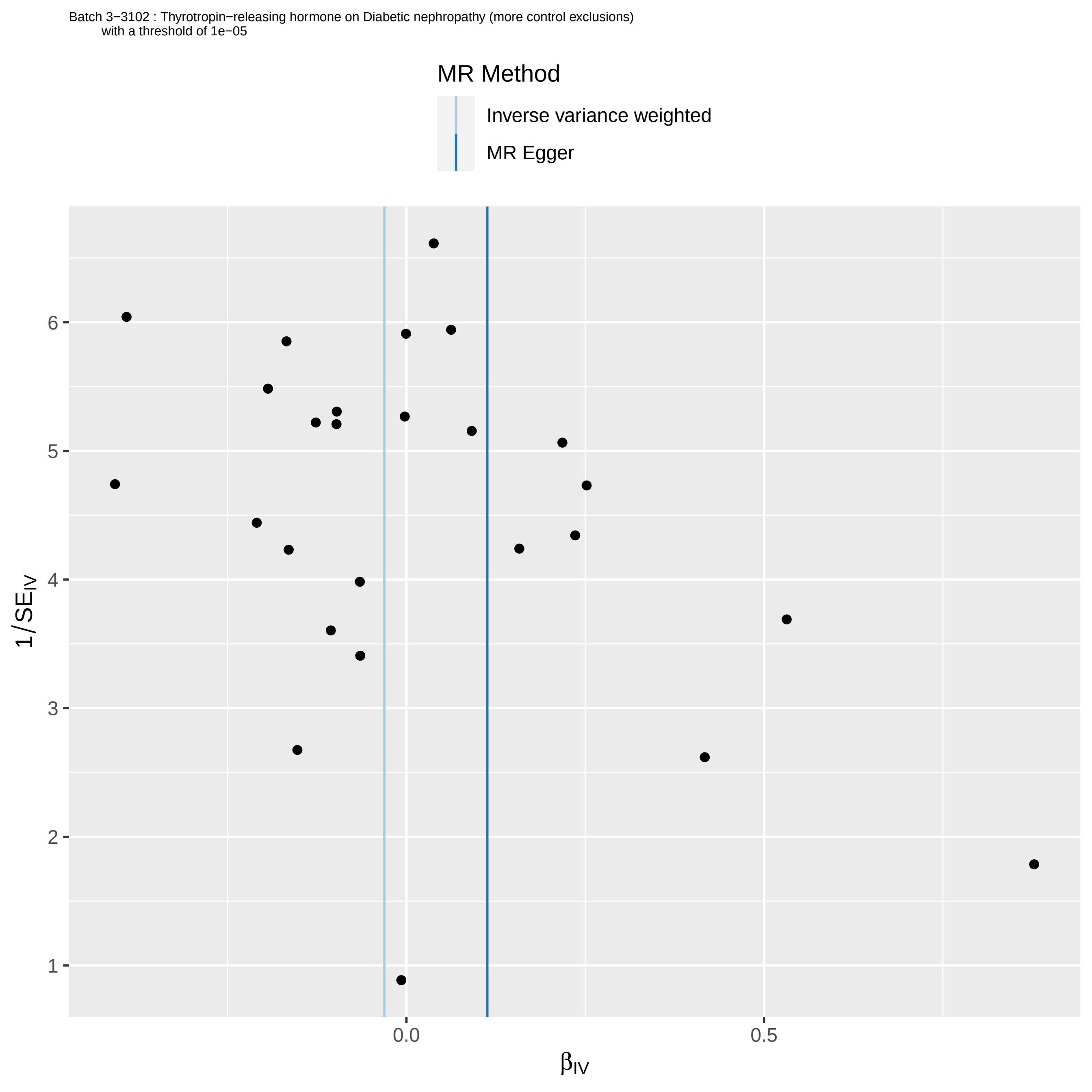
Batch 3−3102 : Thyrotropin−releasing hormone on Diabetic nephropathy (more control exclusions)
with a threshold of 1e−05
MR Method
Inverse variance weighted
MR Egger
1/SE$_{IV}$
6
5
4
3
2
1
0.0
0.5
β$_{IV}$

Batch 3−3103 : Tumor susceptibility gene 101 protein on Diabetic nephropathy (more control exclusions) with a threshold of 1e−05

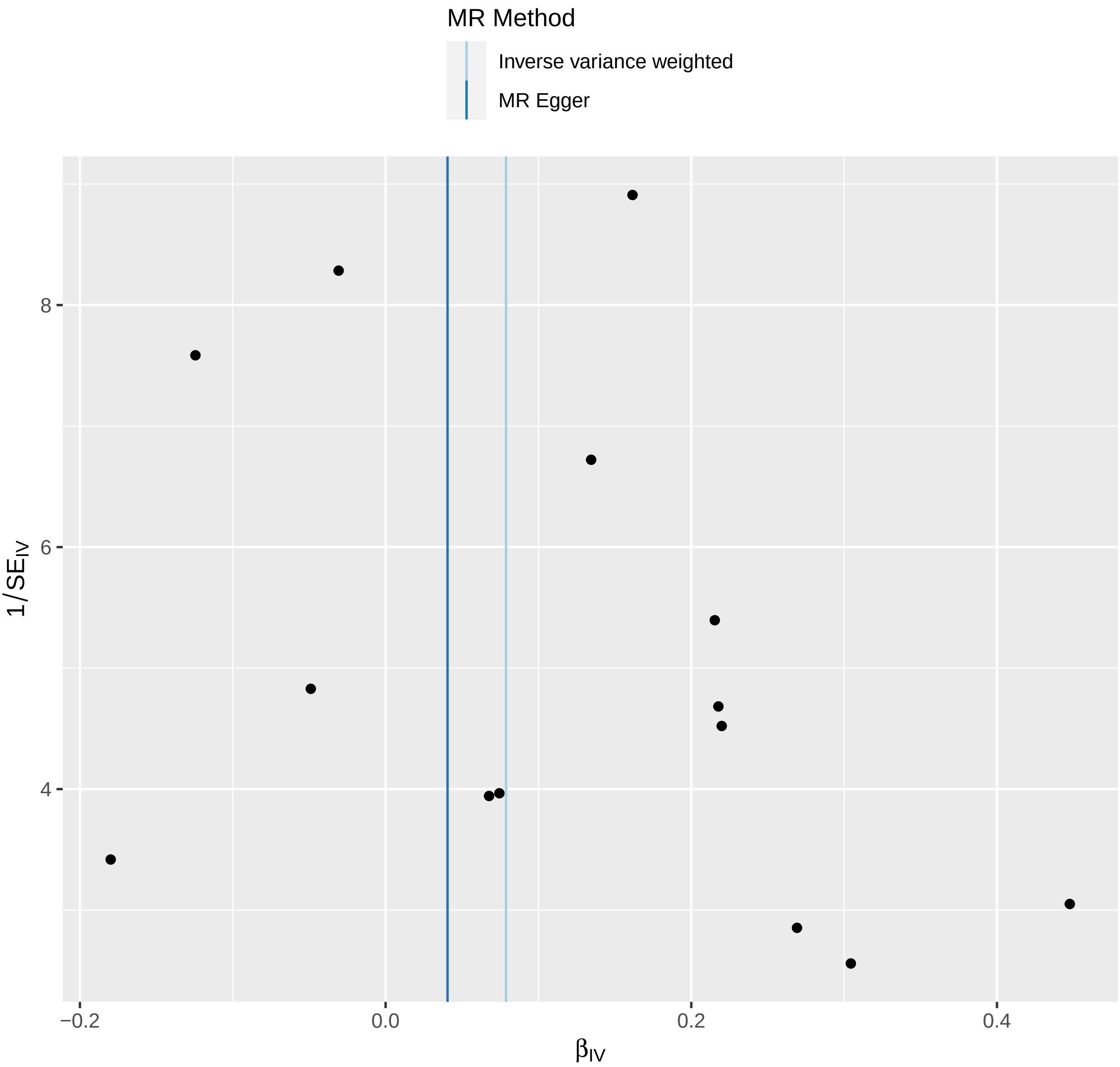

Batch 3−3104 : Thymic stromal lymphopoietin on Diabetic nephropathy (more control exclusions)
with a threshold of 1e−05

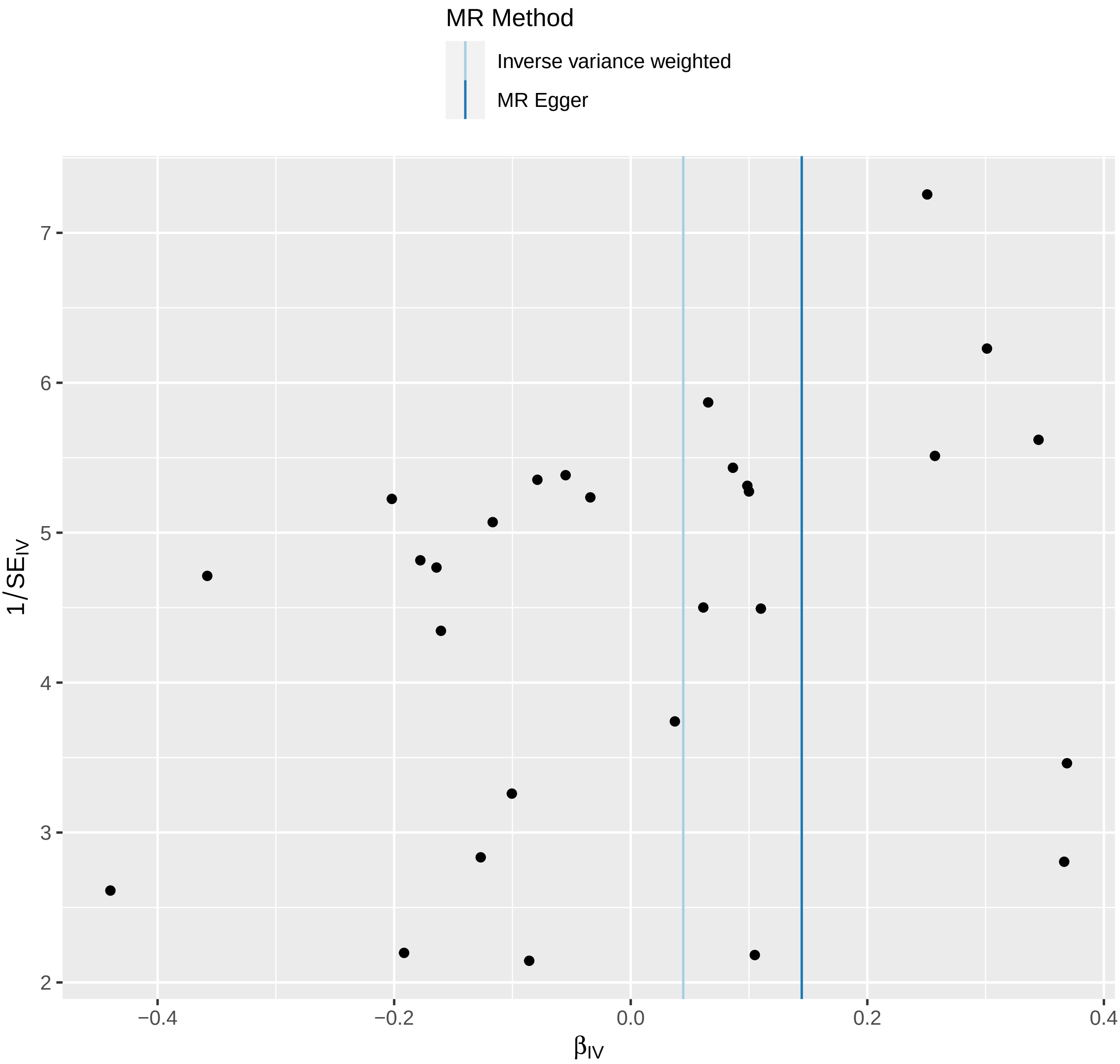

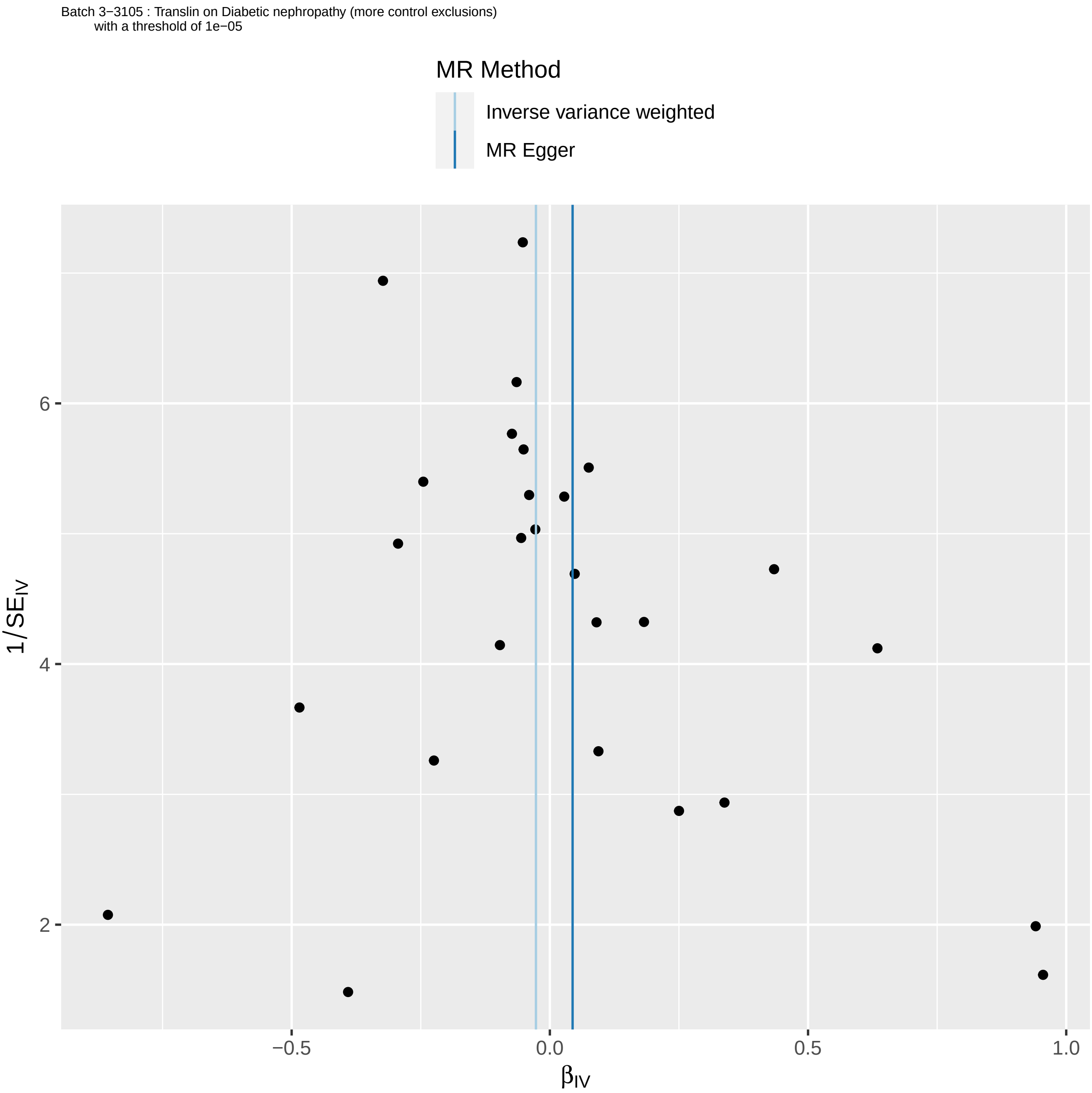
Batch 3−3105 : Translin on Diabetic nephropathy (more control exclusions)
with a threshold of 1e−05
MR Method
Inverse variance weighted
MR Egger
6
4
2
1/SE_IV
−0.5
0.0
0.5
1.0
β_IV

Batch 3−3106 : t−SNARE domain−containing protein 1 on Diabetic nephropathy (more control exclusions)
with a threshold of 1e−05

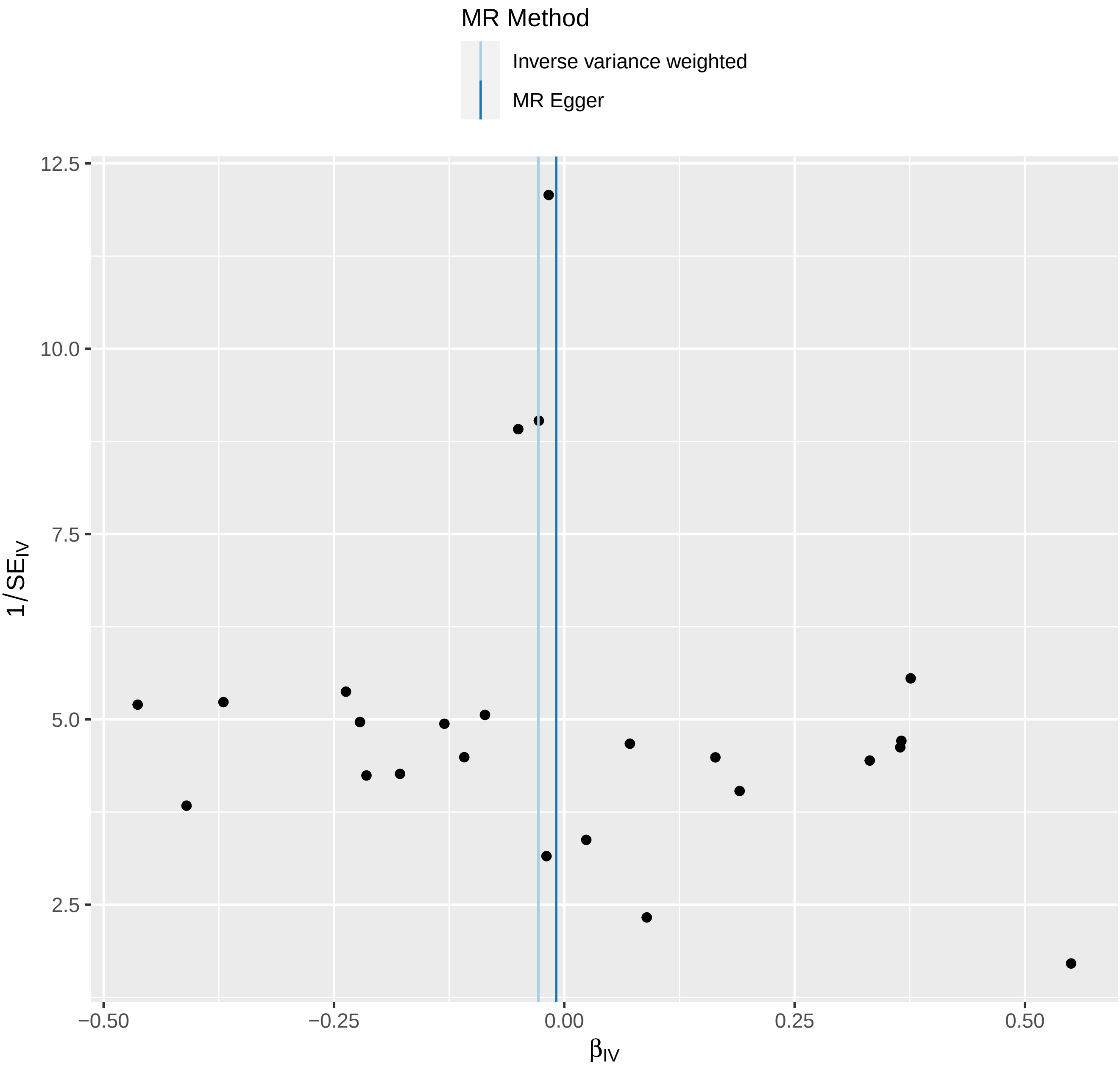

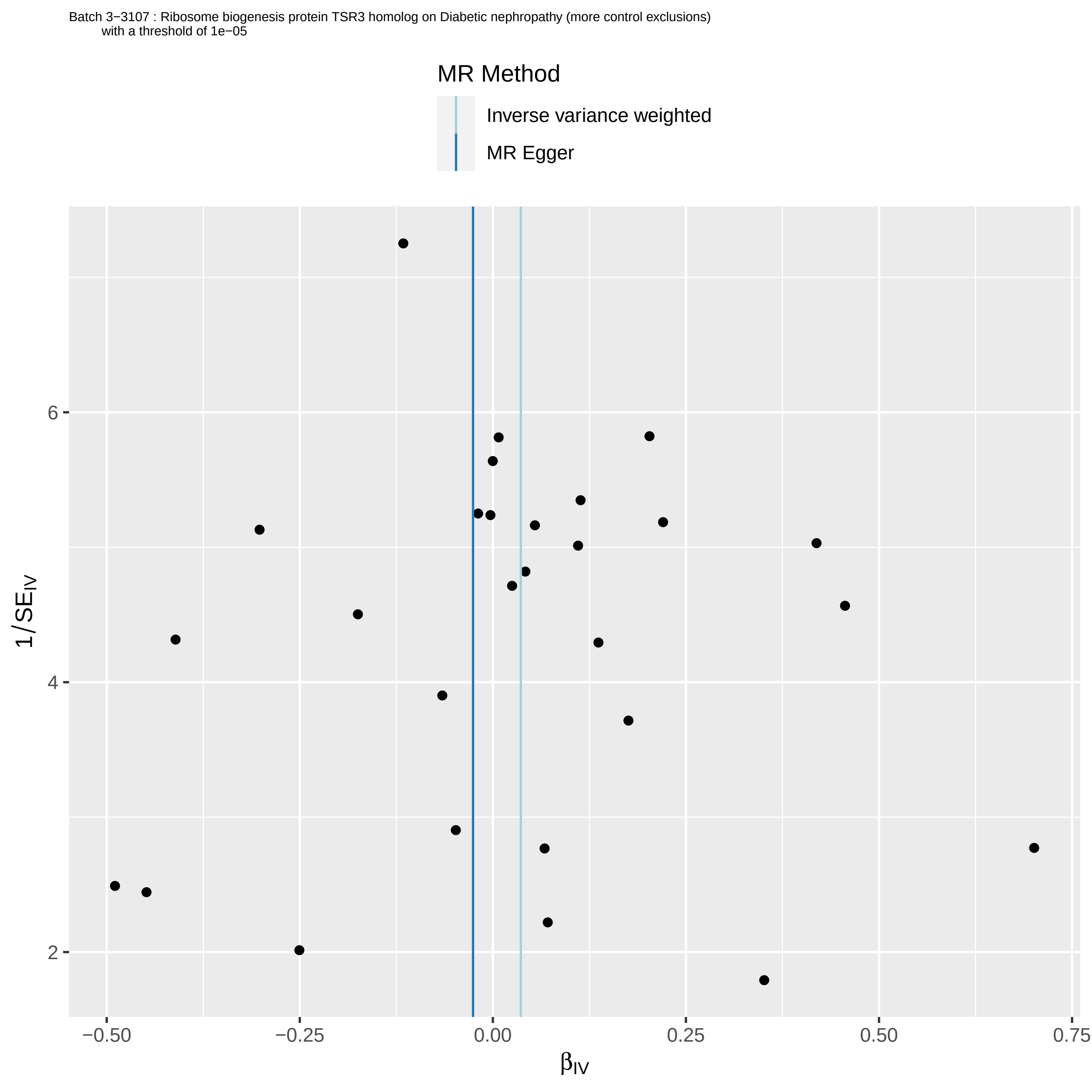

Batch 3−3107 : Ribosome biogenesis protein TSR3 homolog on Diabetic nephropathy (more control exclusions)
with a threshold of 1e−05
MR Method
Inverse variance weighted
MR Egger
6
4
2
1/SE$_{IV}$
−0.50
−0.25
0.00
0.25
0.50
0.75
β$_{IV}$

Batch 3−3108 : Testis−specific serine/threonine−protein kinase 2 on Diabetic nephropathy (more control exclusions) with a threshold of 1e−05

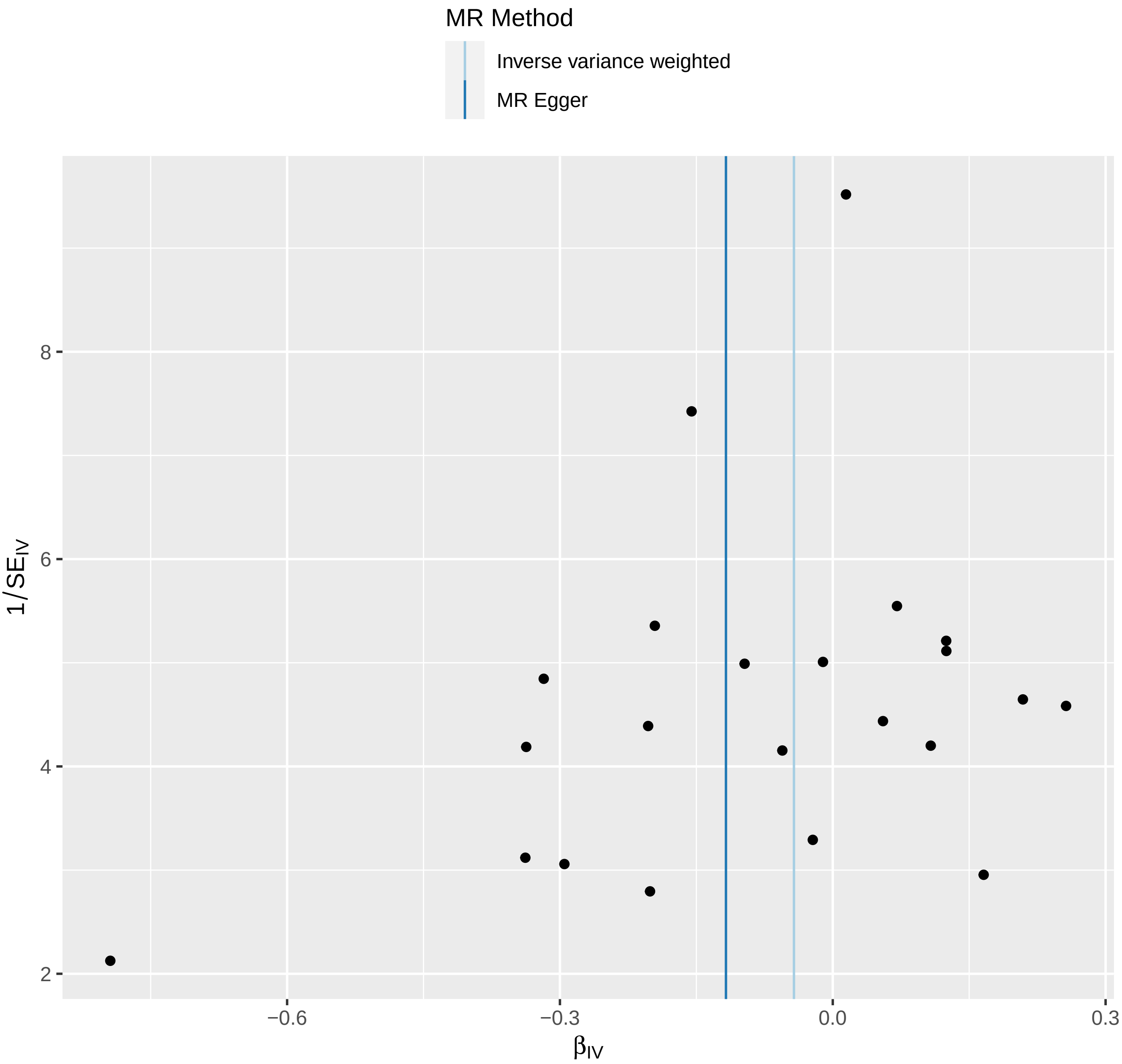

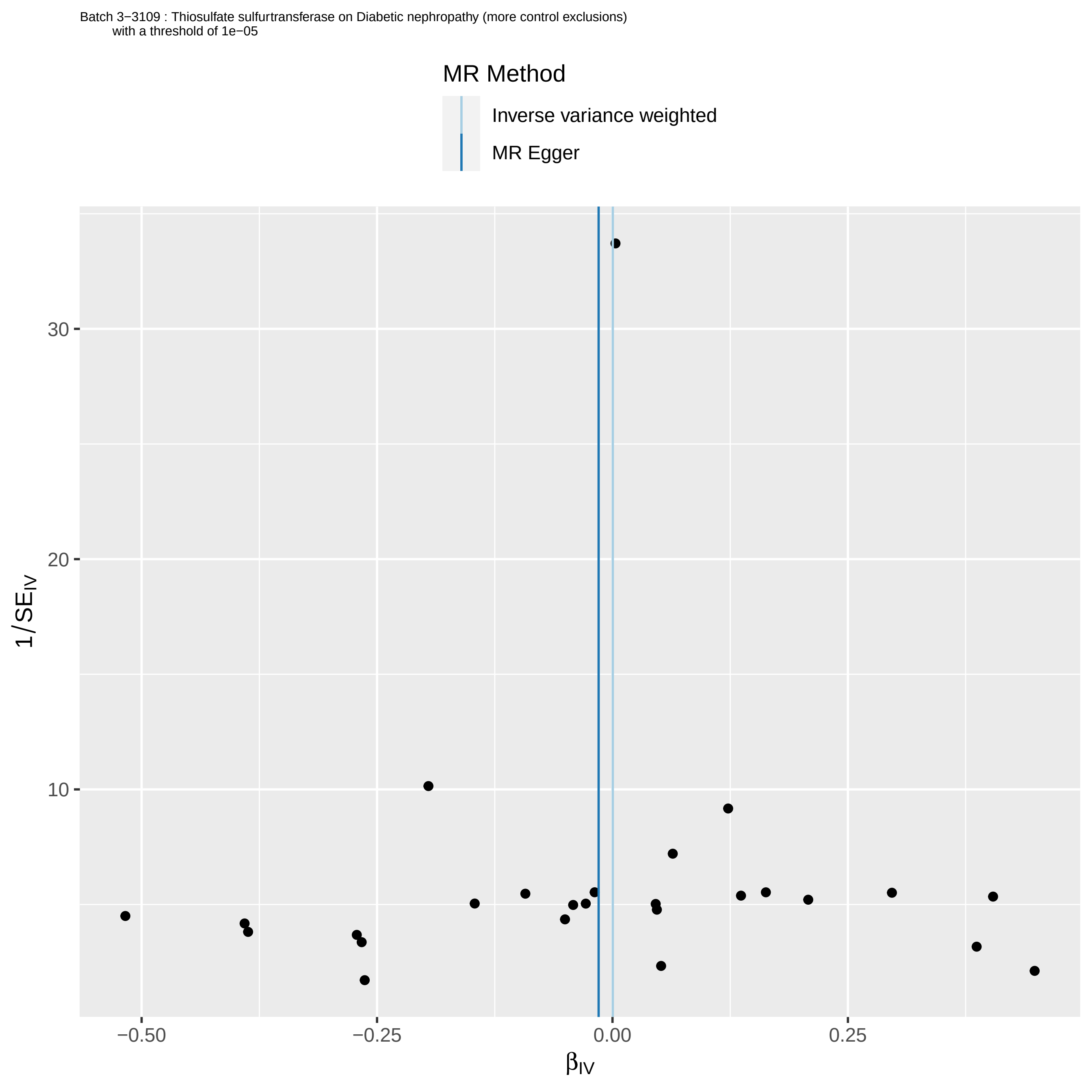

Batch 3−3109 : Thiosulfate sulfurtransferase on Diabetic nephropathy (more control exclusions)
with a threshold of 1e−05
MR Method
Inverse variance weighted
MR Egger
30
20
10
1/SE_IV
−0.50
−0.25
0.00
0.25
β_IV

Batch 3−3110 : GDP−L−fucose synthase on Diabetic nephropathy (more control exclusions)
with a threshold of 1e−05

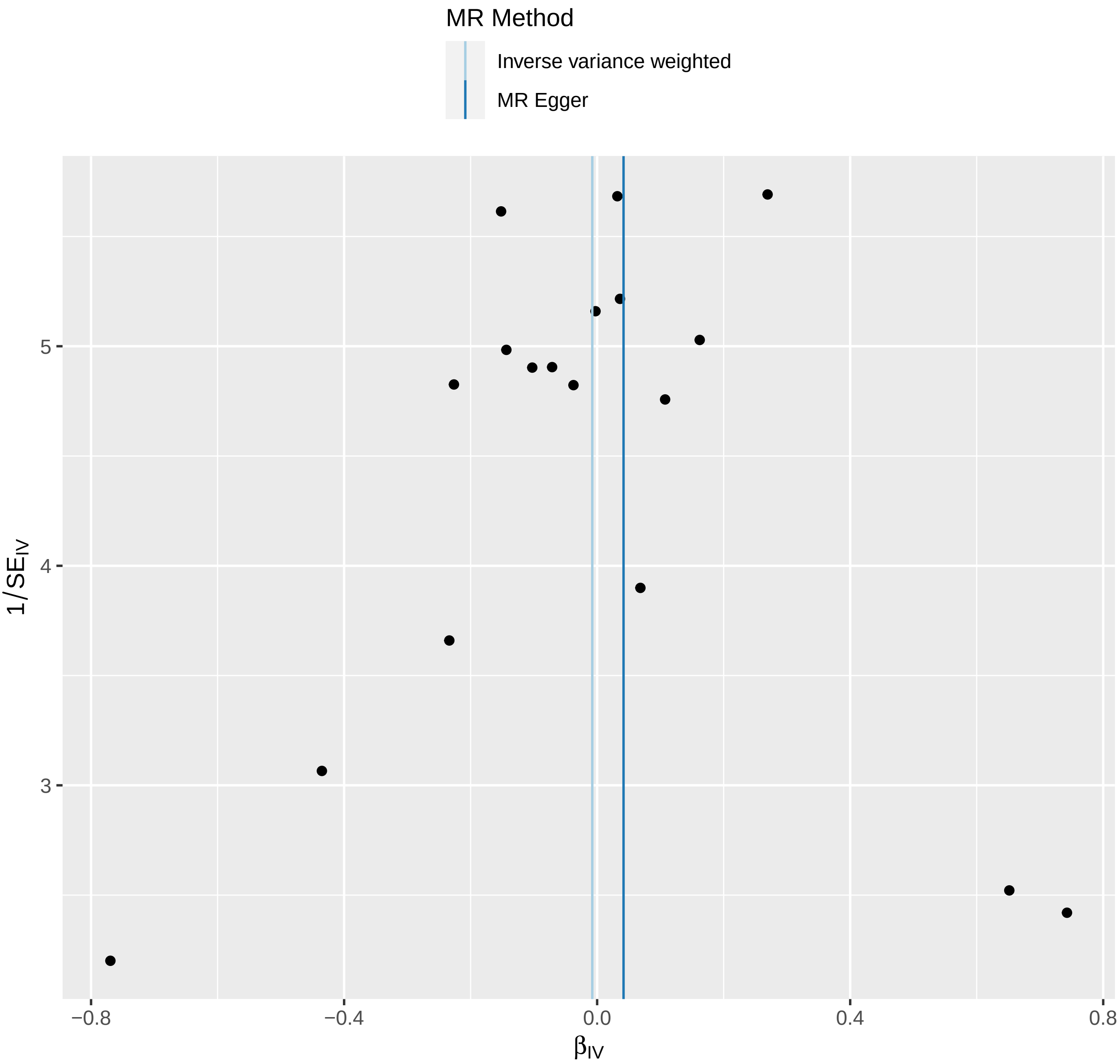

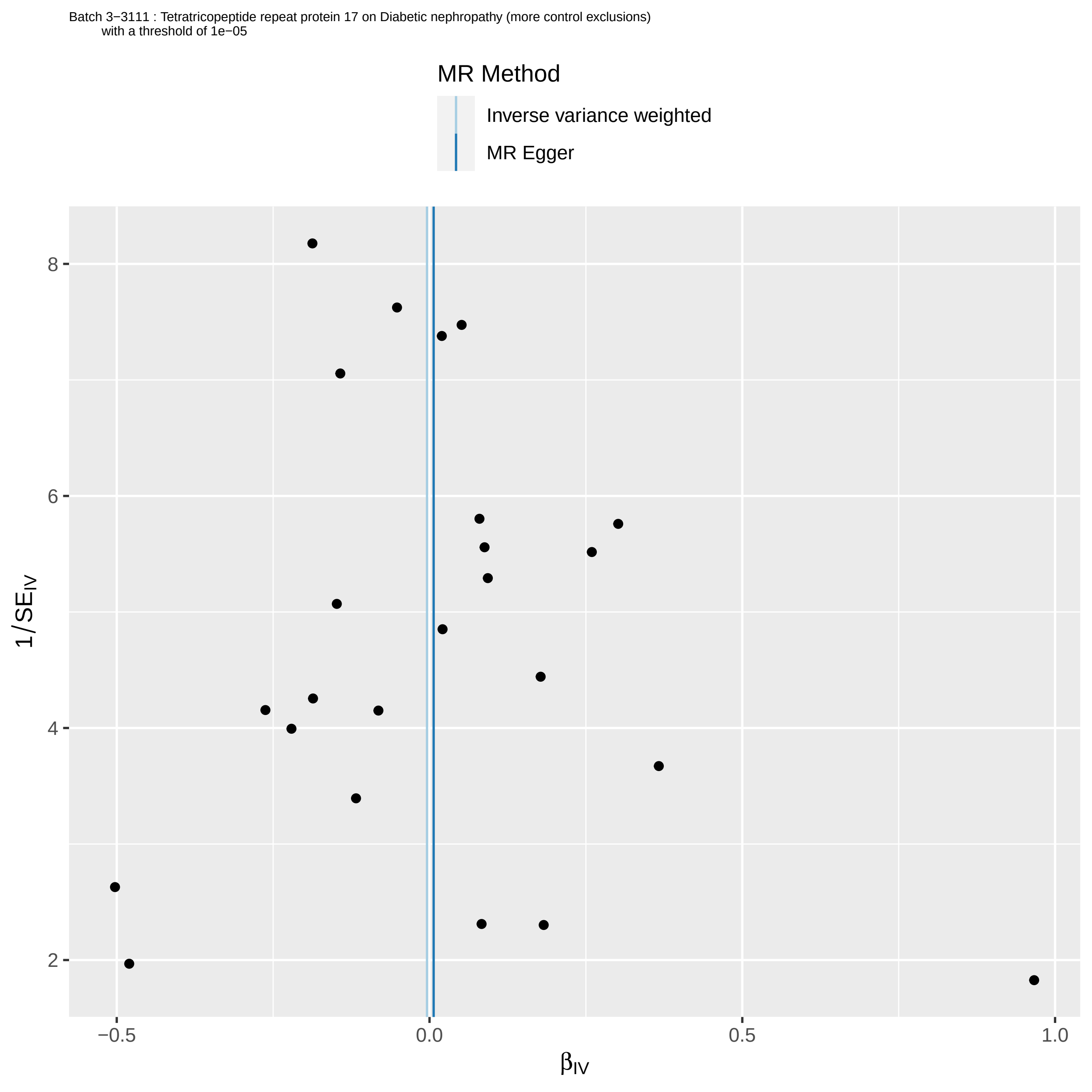
Batch 3−3111 : Tetratricopeptide repeat protein 17 on Diabetic nephropathy (more control exclusions)
with a threshold of 1e−05
MR Method
Inverse variance weighted
MR Egger
8
6
4
2
−0.5
0.0
0.5
1.0
$1/SE_{IV}$
β_{IV}

Batch 3−3112 : Tetratricopeptide repeat protein 17 on Diabetic nephropathy (more control exclusions)
with a threshold of 1e−05

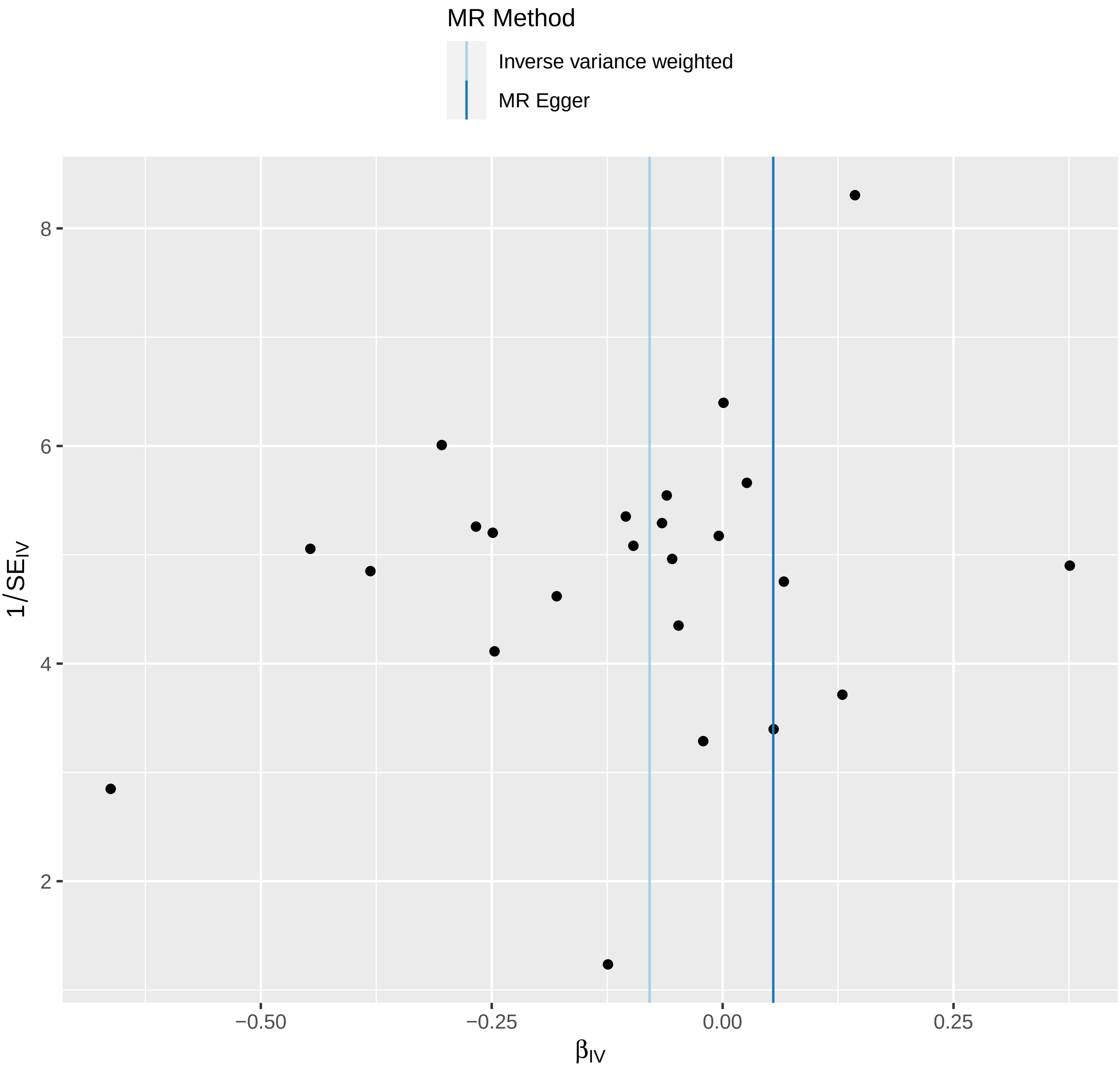

Batch 3−3113 : Tetratricopeptide repeat protein 9B on Diabetic nephropathy (more control exclusions)
with a threshold of 1e−05

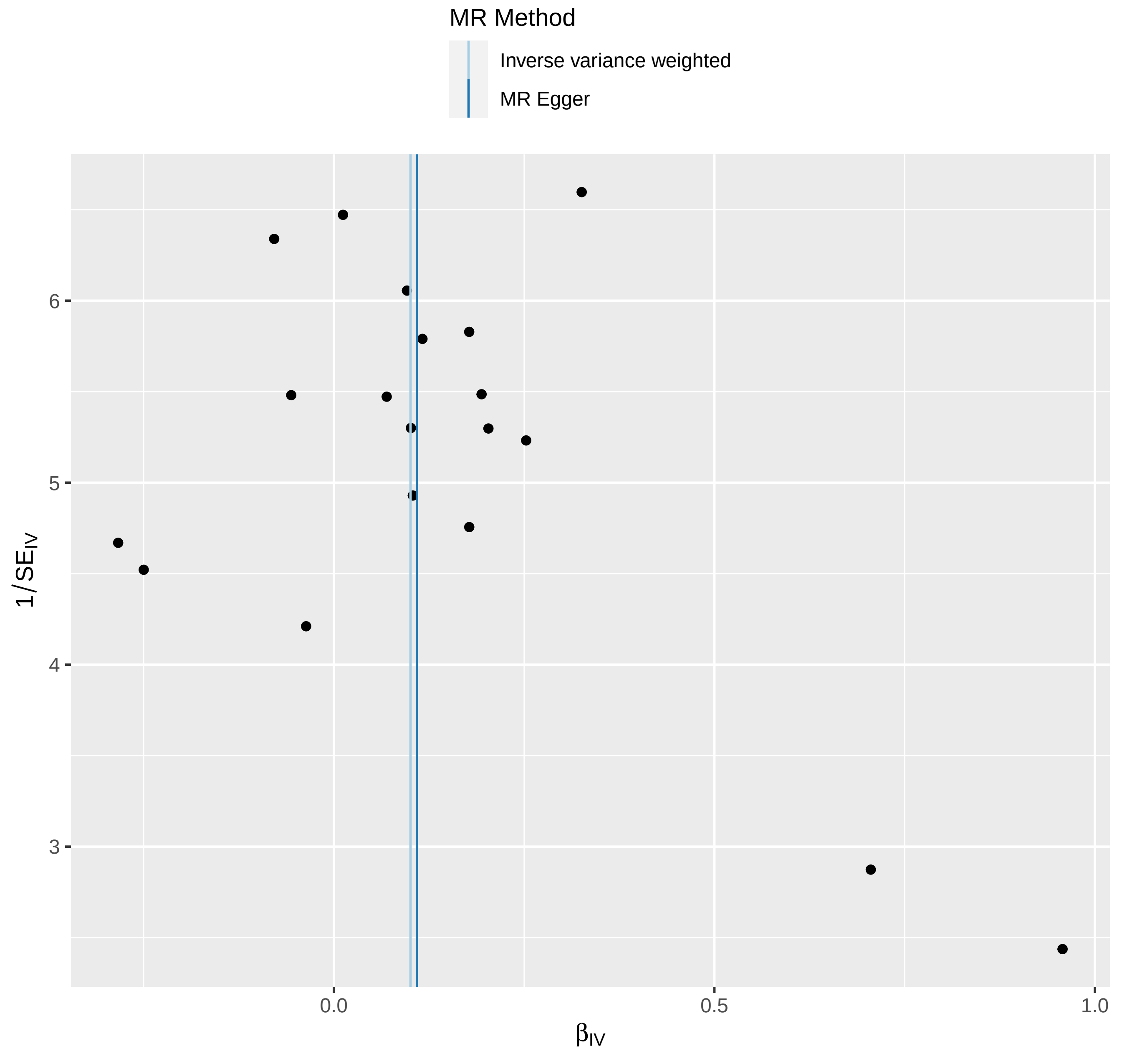

Batch 3−3114 : Tubulin−−tyrosine ligase on Diabetic nephropathy (more control exclusions)
with a threshold of 1e−05

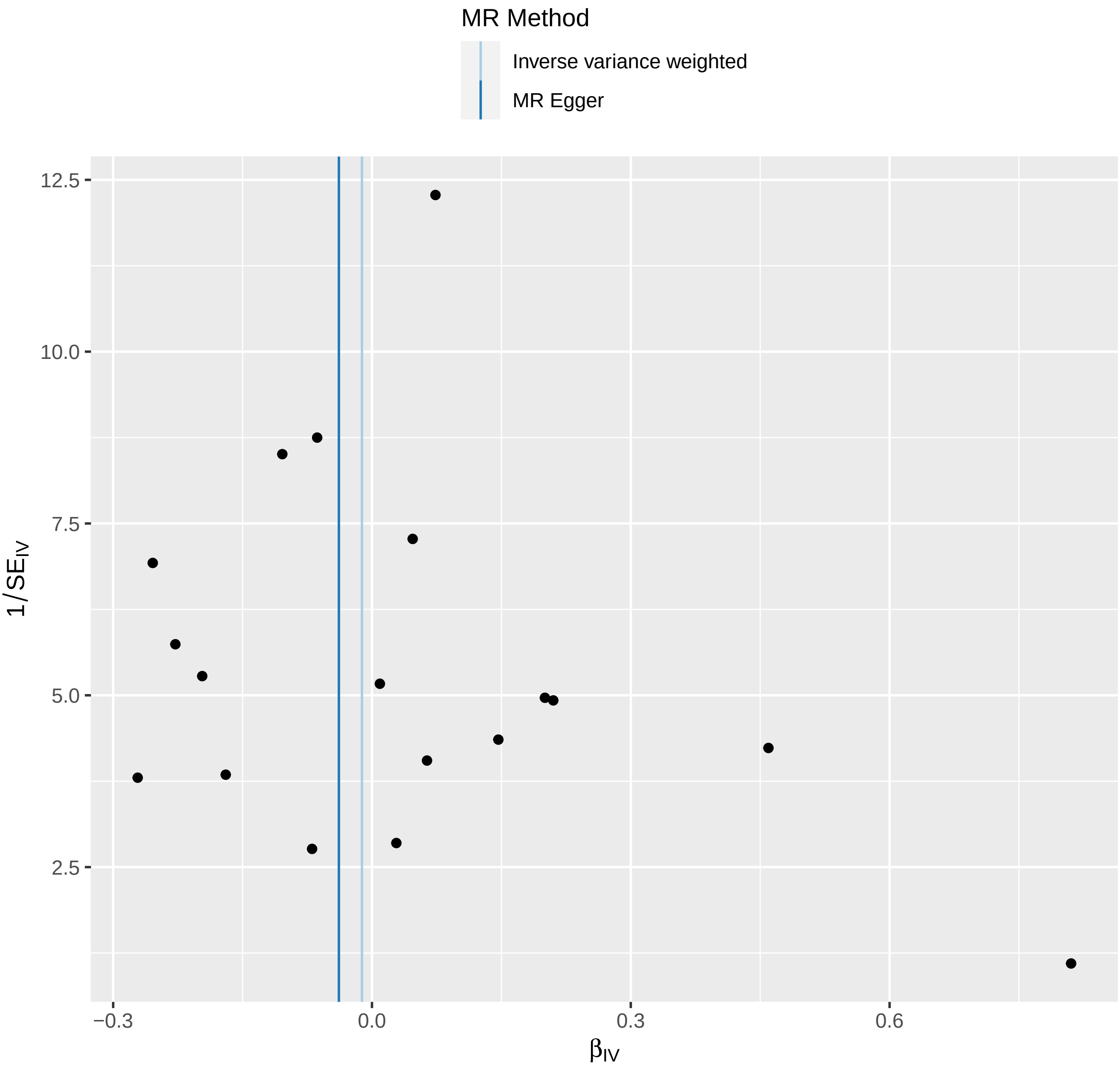

Batch 3−3115 : TPA−induced transmembrane protein on Diabetic nephropathy (more control exclusions)
with a threshold of 1e−05

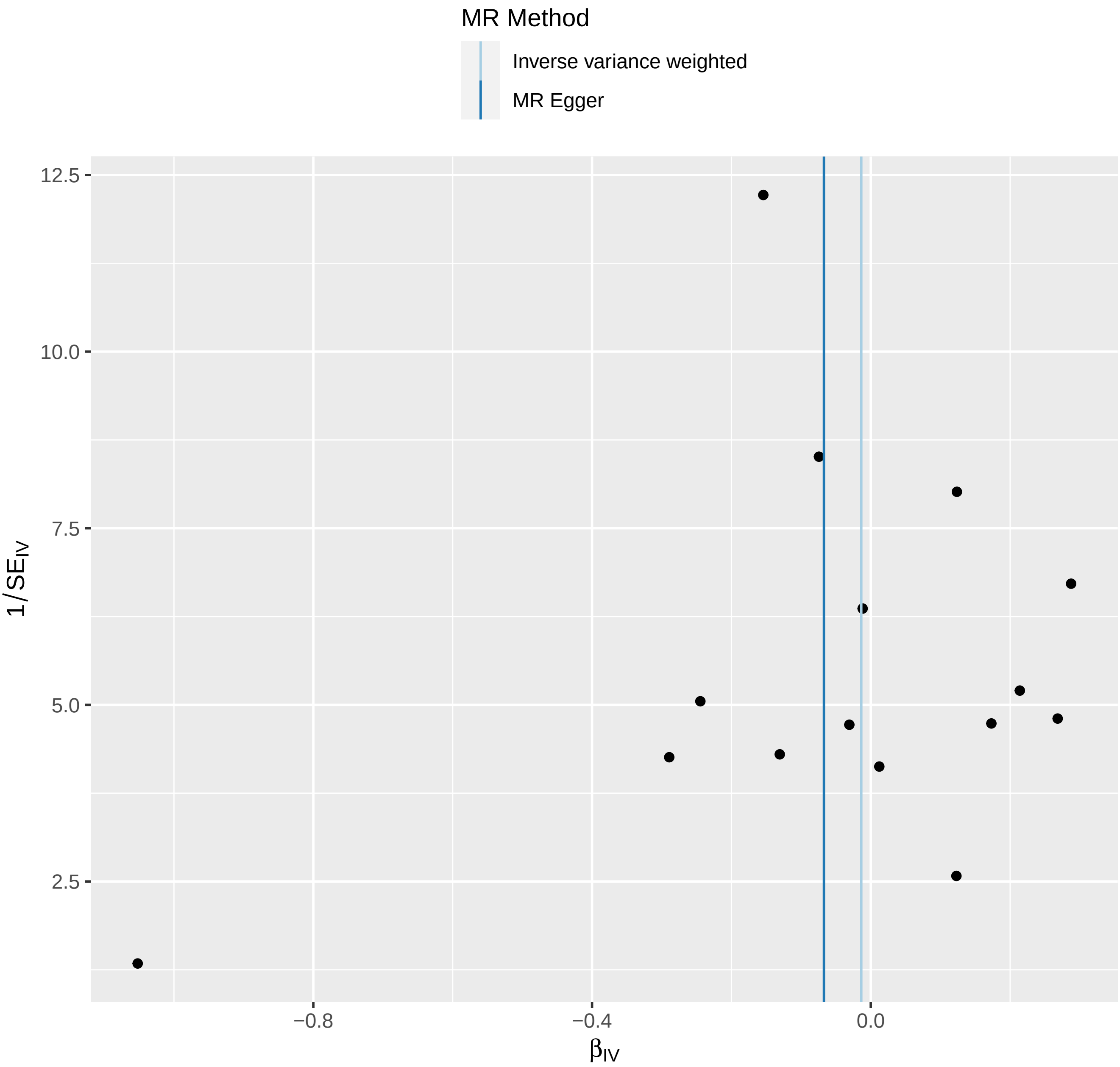

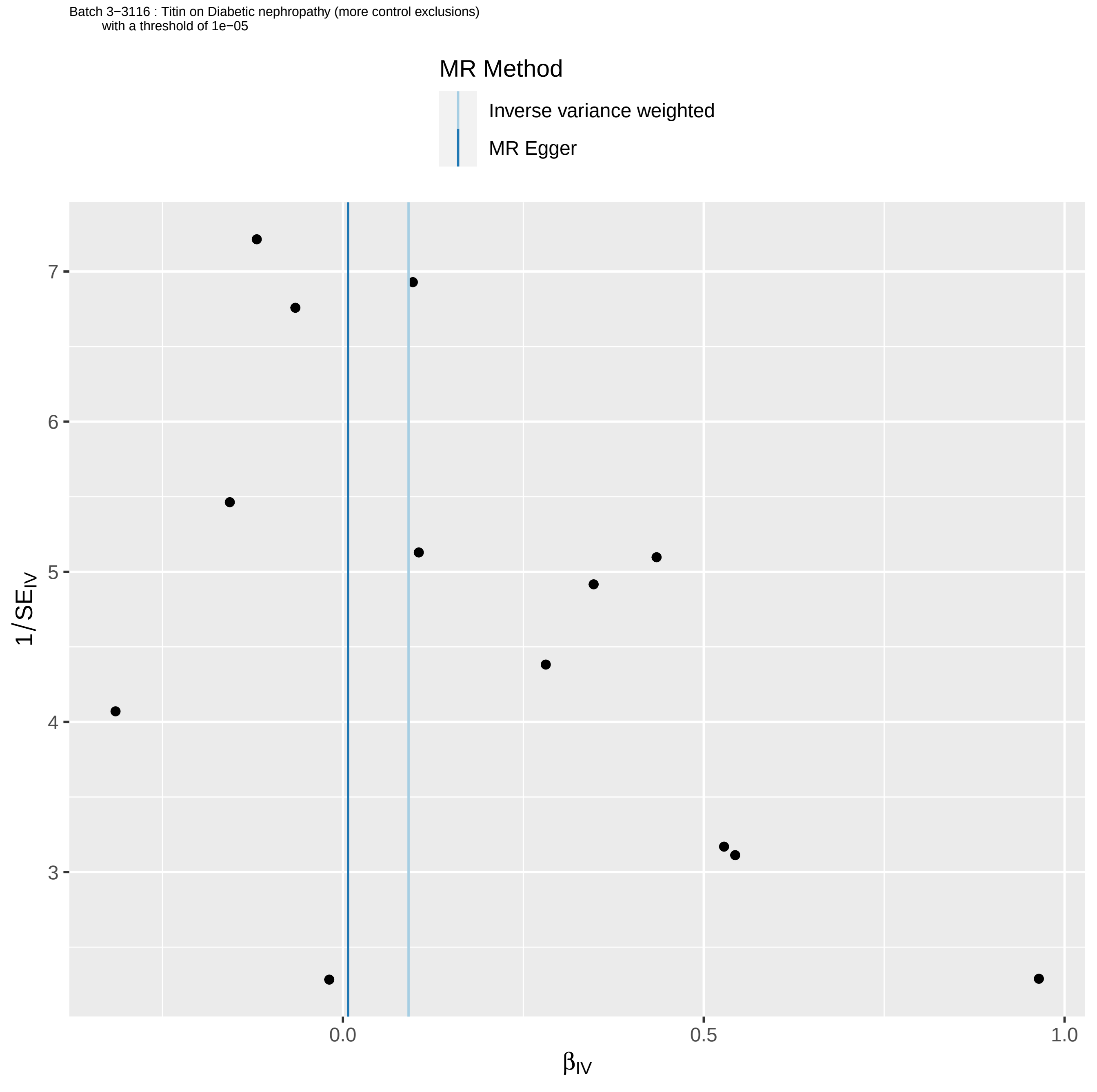

Batch 3−3116 : Titin on Diabetic nephropathy (more control exclusions)
with a threshold of 1e−05
MR Method
Inverse variance weighted
MR Egger
7
6
5
4
3
0.0
0.5
1.0
1/SE_IV
β_IV

Batch 3−3117 : Tuftelin on Diabetic nephropathy (more control exclusions)
with a threshold of 1e−05

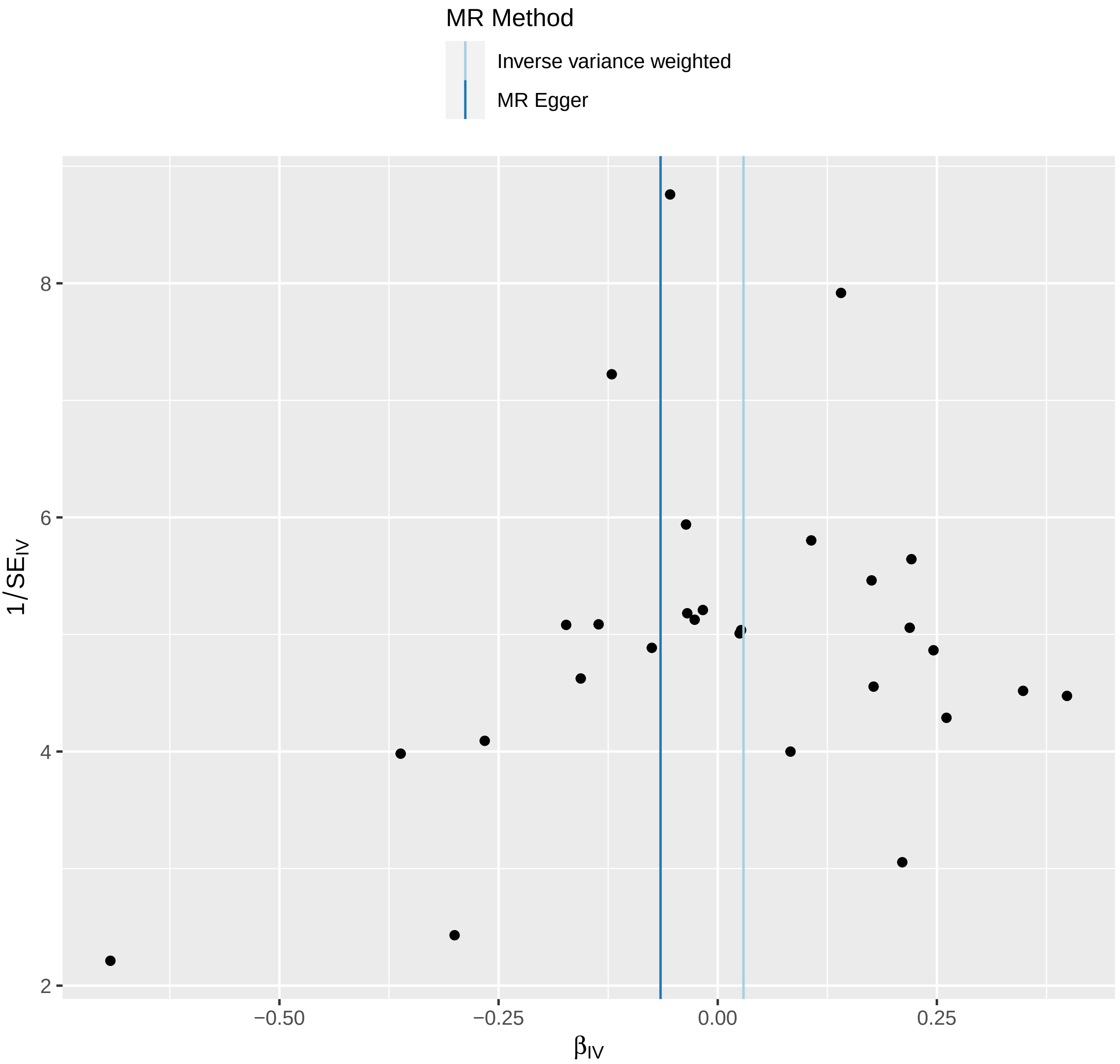

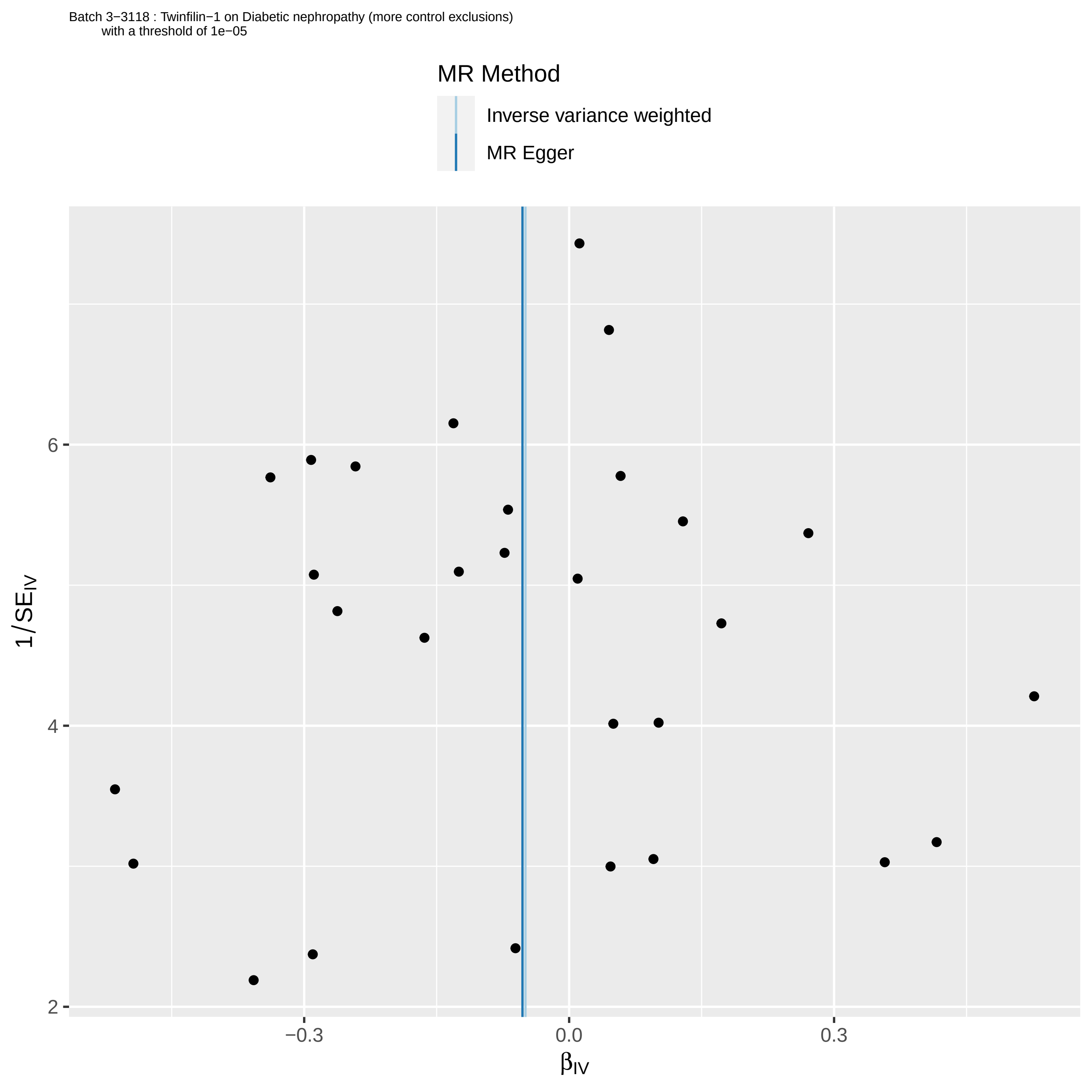

Batch 3−3118 : Twinfilin−1 on Diabetic nephropathy (more control exclusions)
with a threshold of 1e−05
MR Method
Inverse variance weighted
MR Egger
1/SE$_{IV}$
6
4
2
−0.3
0.0
0.3
β_{IV}

Batch 3−3119 : Twisted gastrulation protein homolog 1 on Diabetic nephropathy (more control exclusions)
with a threshold of 1e−05

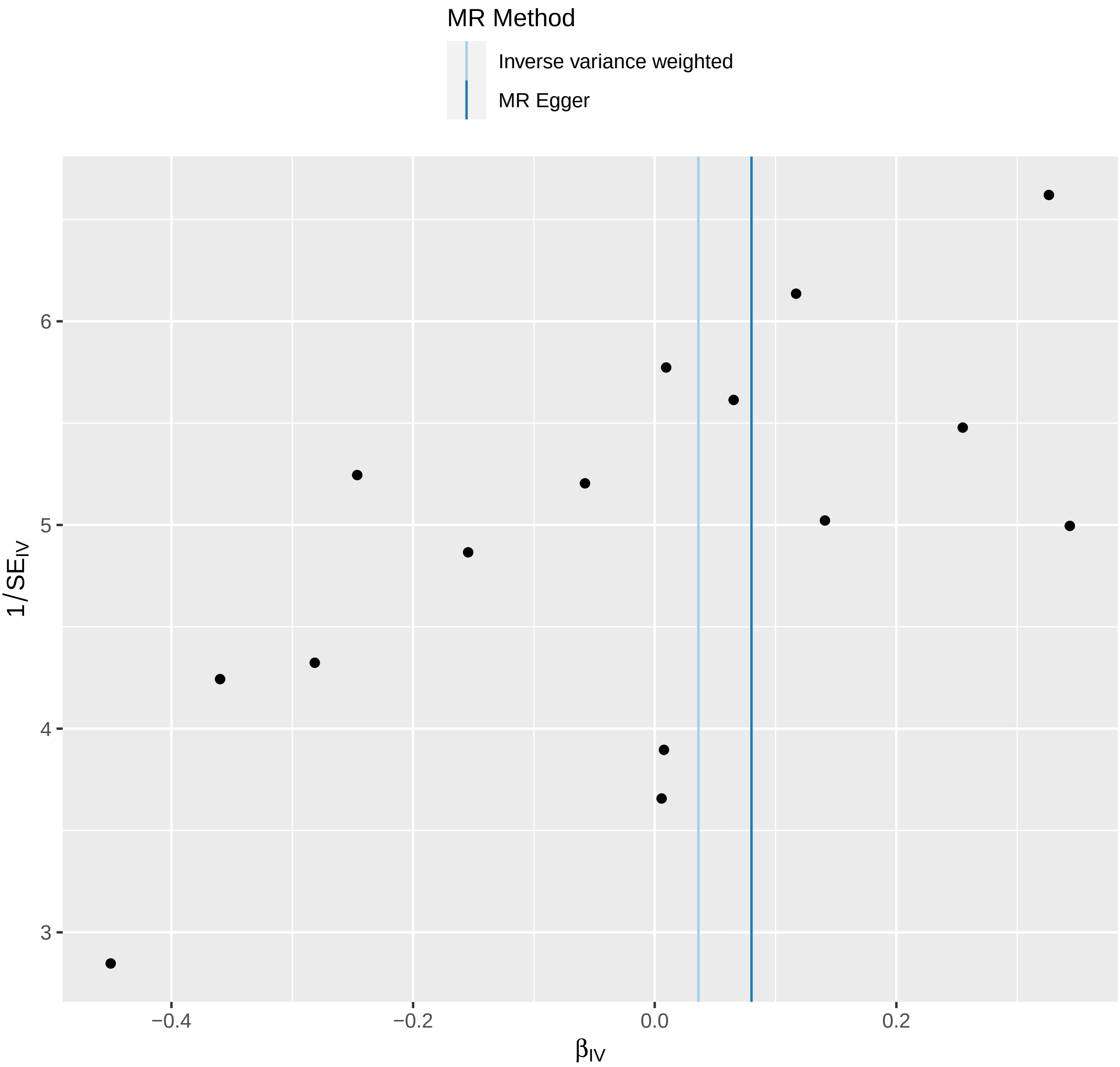

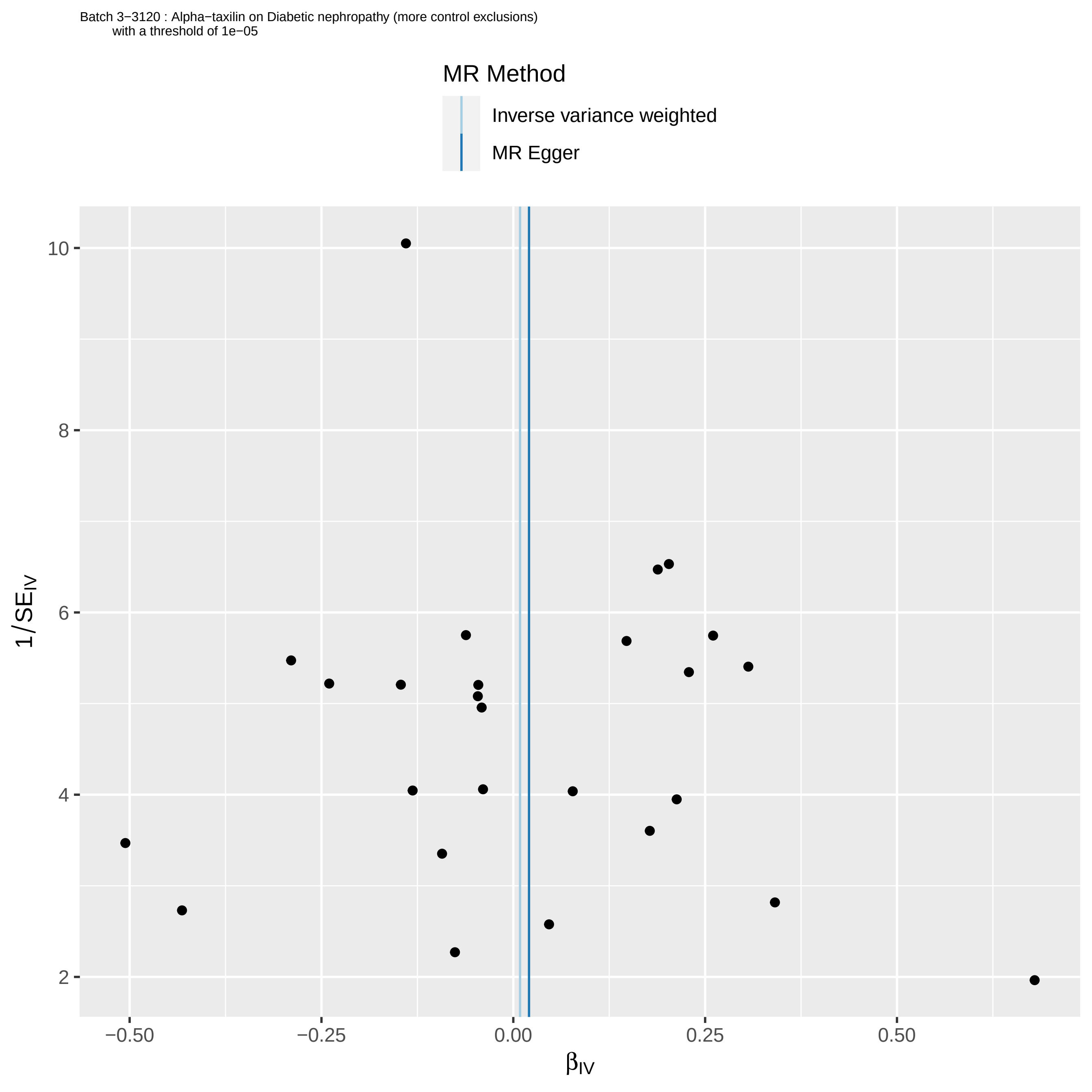
Batch 3−3120 : Alpha−taxilin on Diabetic nephropathy (more control exclusions)
with a threshold of 1e−05
MR Method
Inverse variance weighted
MR Egger
10
8
6
4
2
1/SE_IV
−0.50
−0.25
0.00
0.25
0.50
β_IV

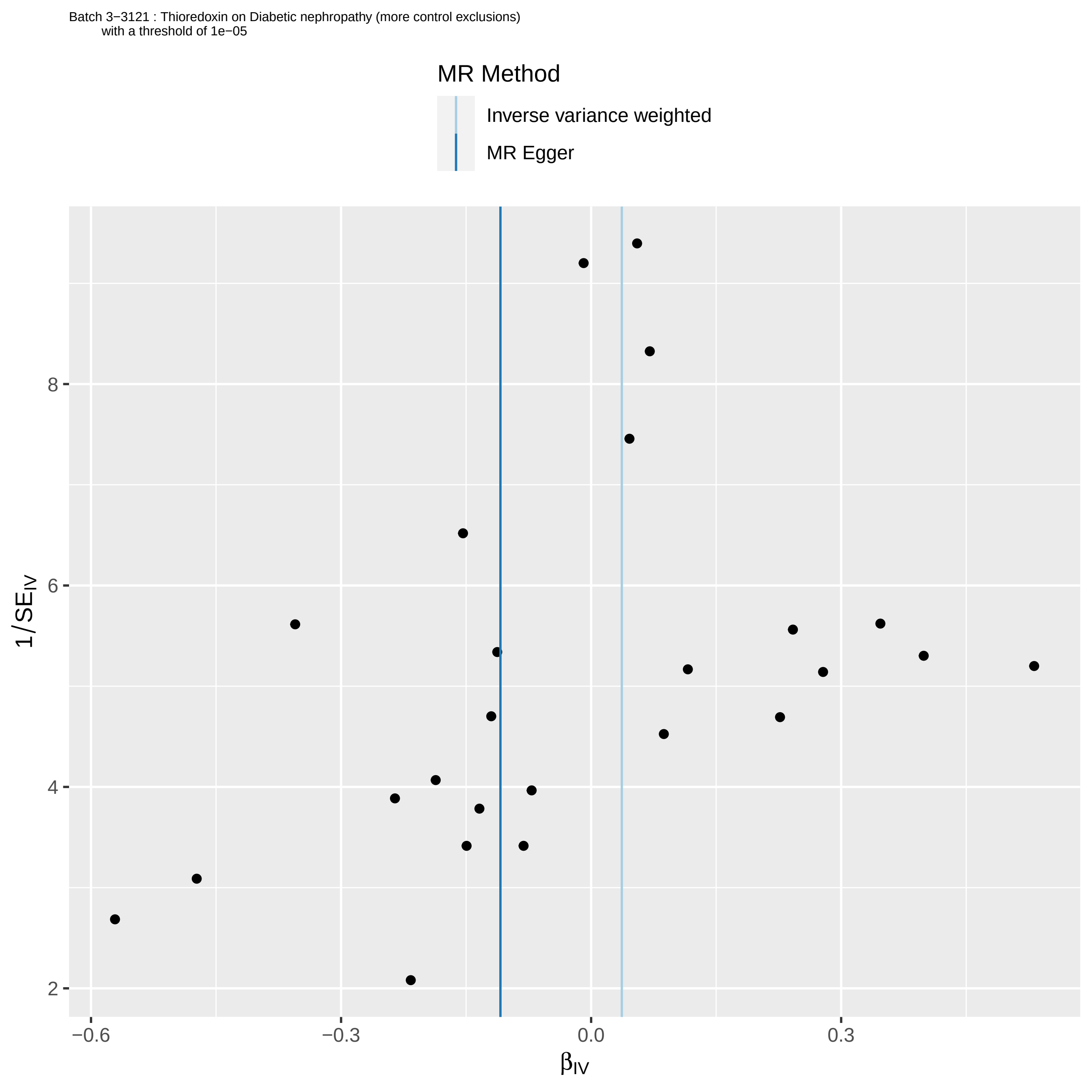
Batch 3−3121 : Thioredoxin on Diabetic nephropathy (more control exclusions)
with a threshold of 1e−05
MR Method
Inverse variance weighted
MR Egger
1/SE_IV
β_IV
8
6
4
2
−0.6
−0.3
0.0
0.3

Batch 3−3122 : Thioredoxin domain−containing protein 11 on Diabetic nephropathy (more control exclusions) with a threshold of 1e−05

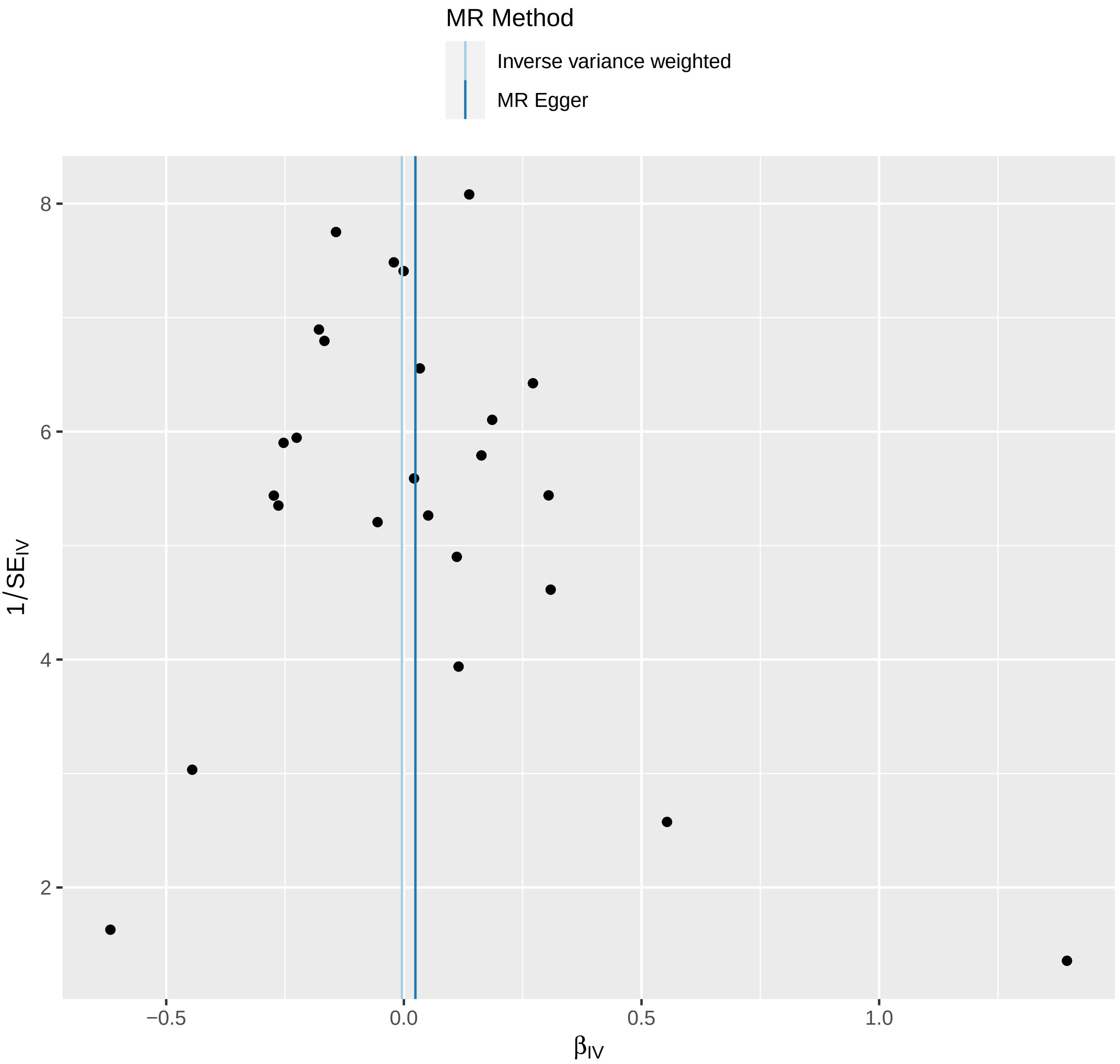

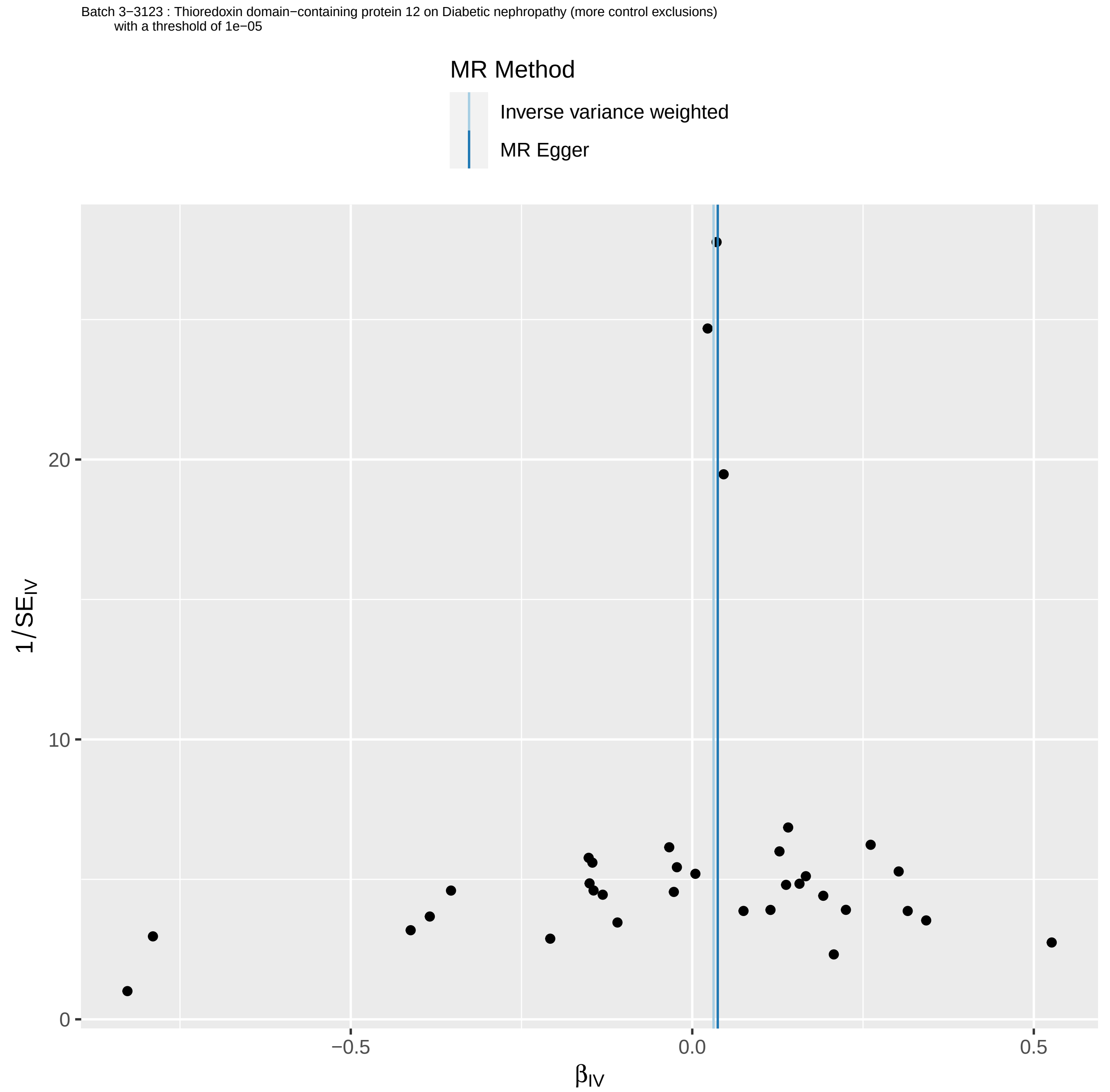
Batch 3−3123 : Thioredoxin domain−containing protein 12 on Diabetic nephropathy (more control exclusions)
with a threshold of 1e−05
MR Method
Inverse variance weighted
MR Egger
1/SE_IV
β_IV
0
10
20
−0.5
0.0
0.5

Batch 3−3124 : Thioredoxin domain−containing protein 5 on Diabetic nephropathy (more control exclusions) with a threshold of 1e−05

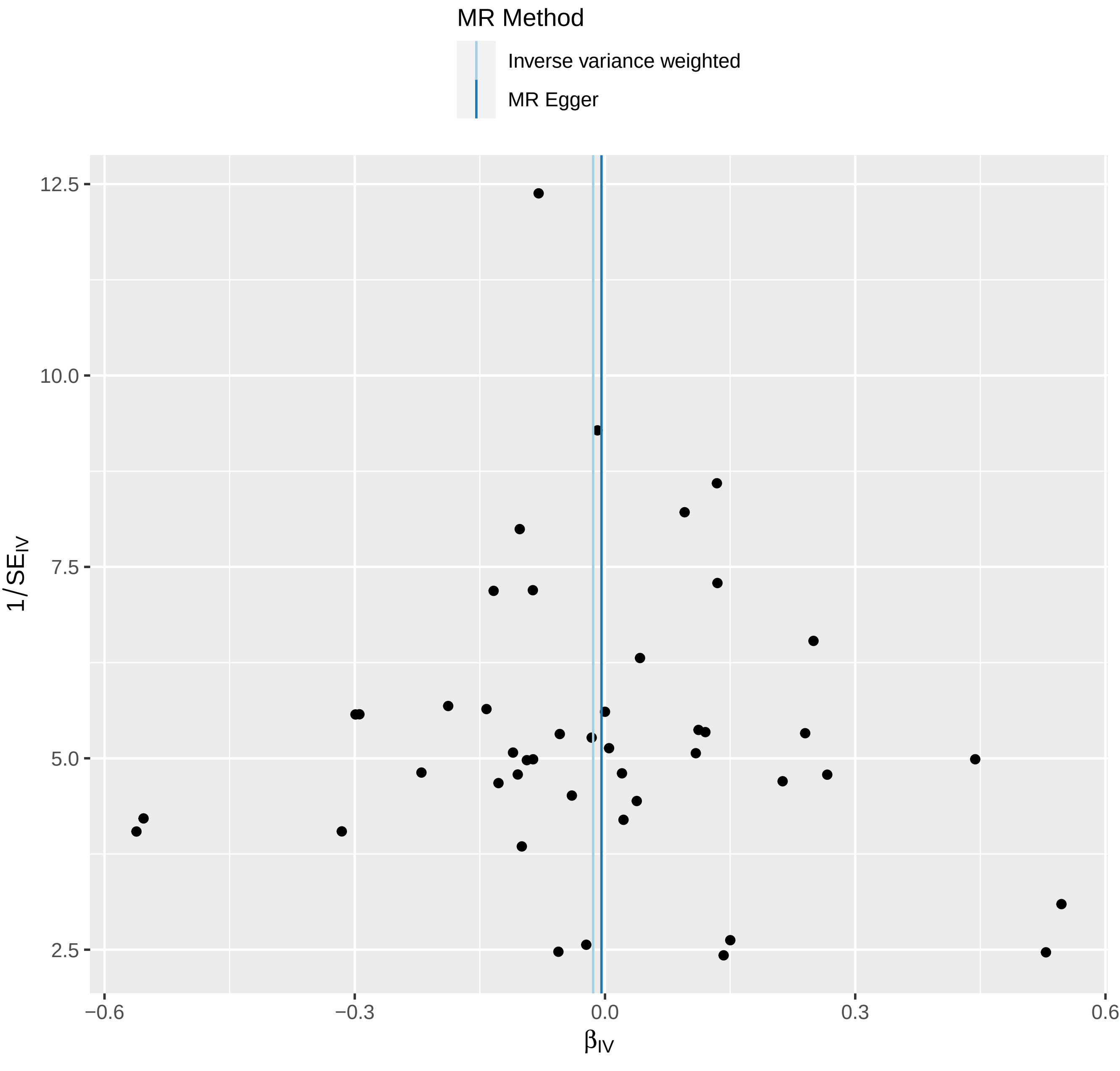

Batch 3−3125 : Thioredoxin−interacting protein on Diabetic nephropathy (more control exclusions)
with a threshold of 1e−05

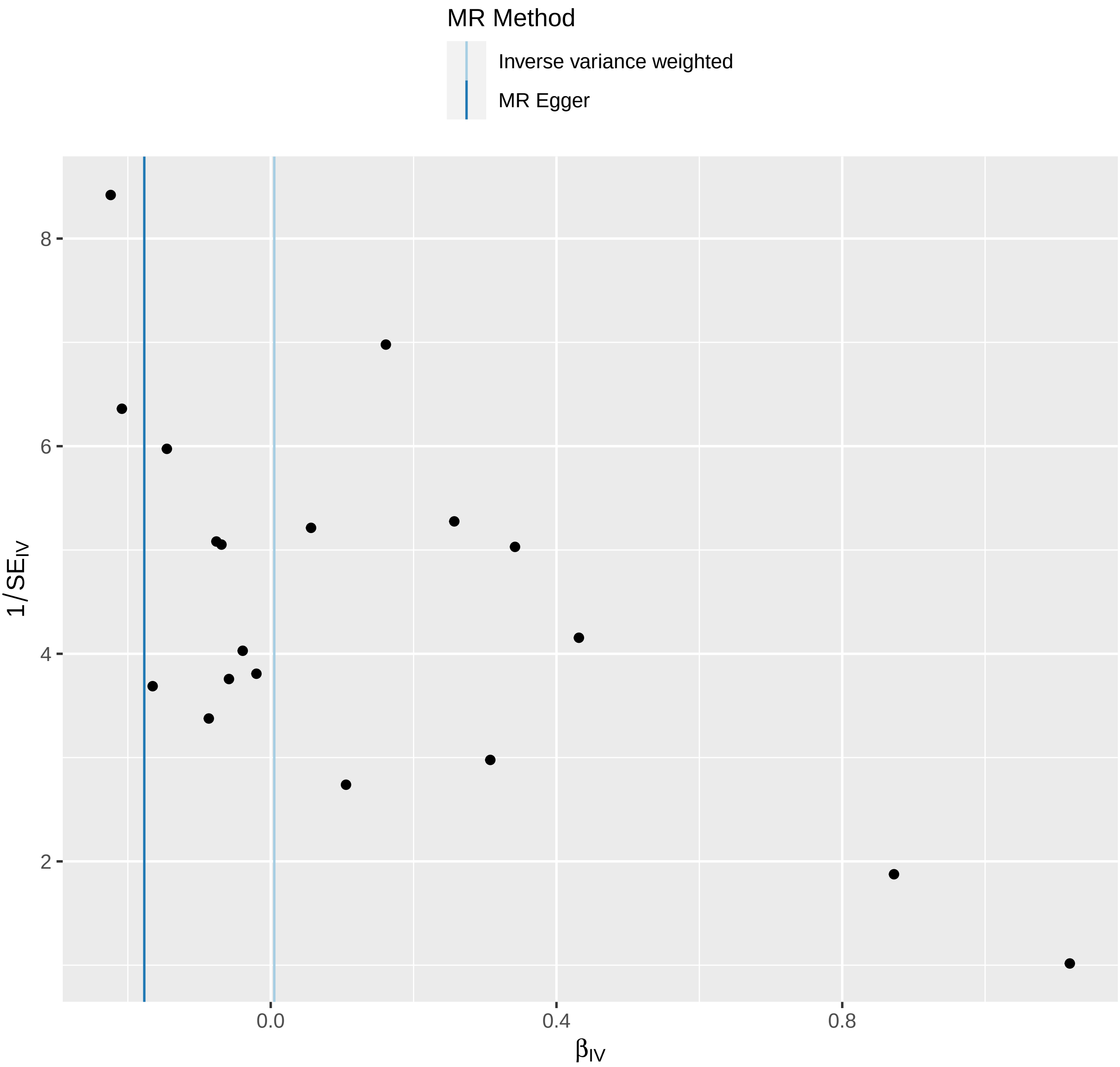

Batch 3−3126 : Thioredoxin reductase 1, cytoplasmic on Diabetic nephropathy (more control exclusions)
with a threshold of 1e−05

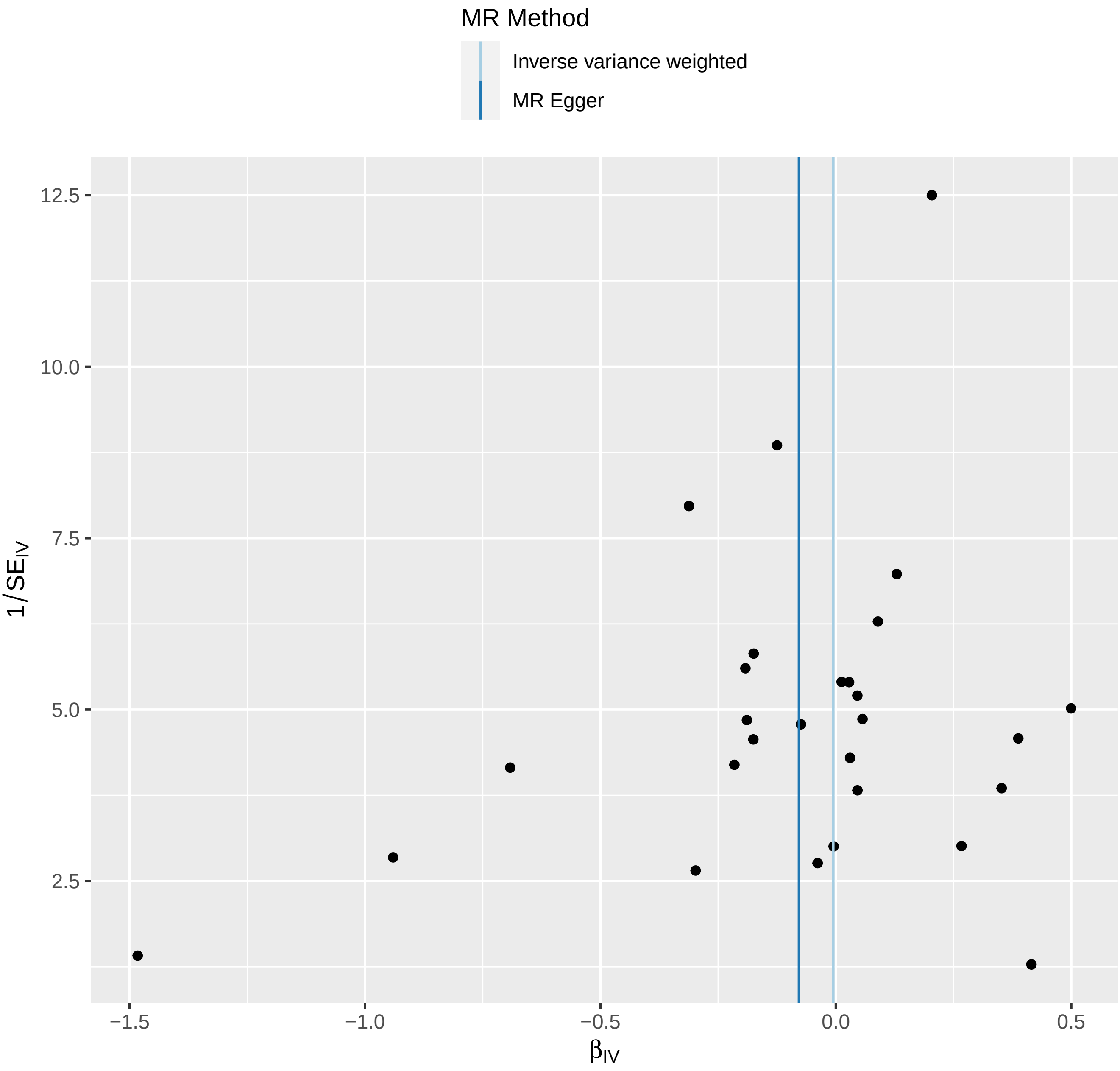

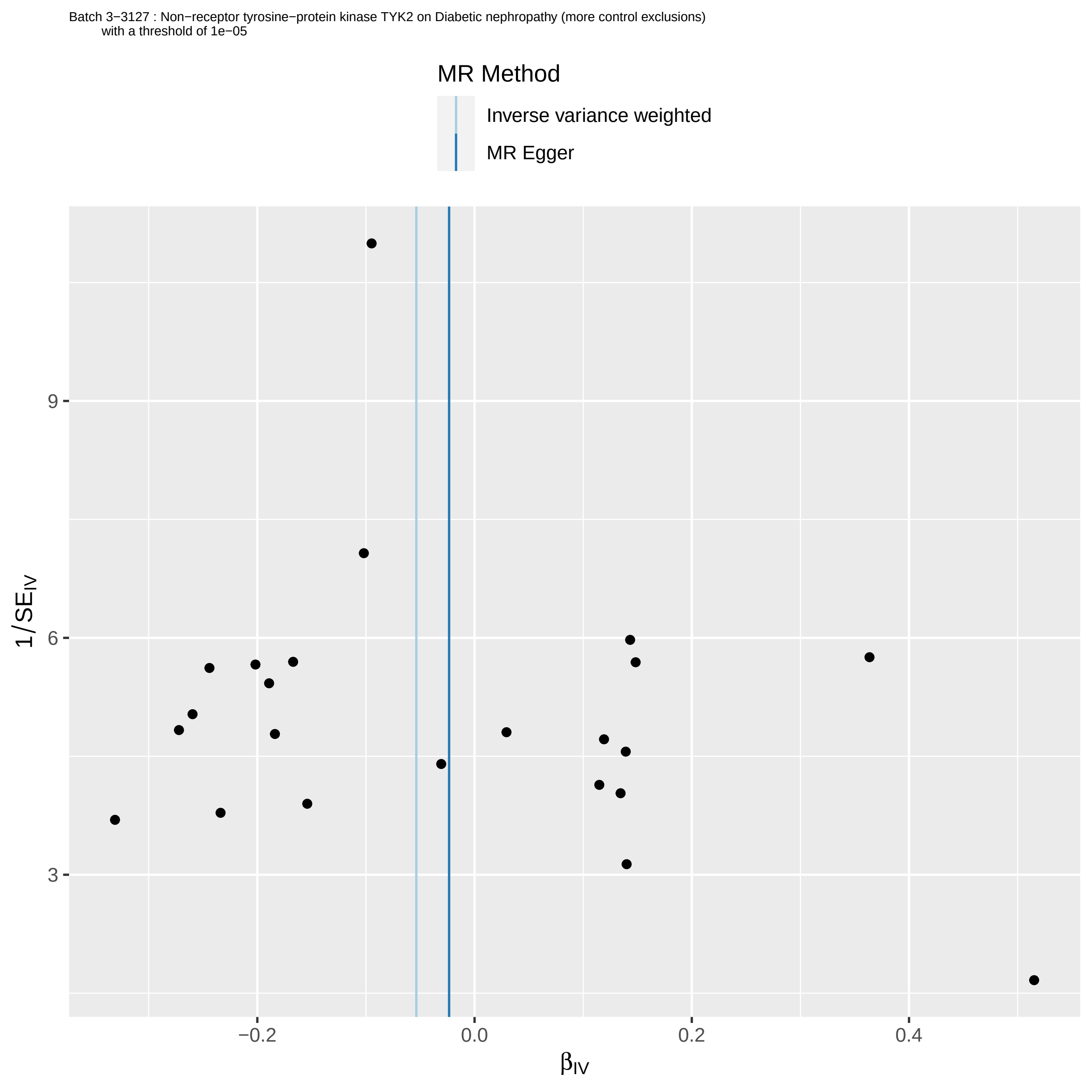

Batch 3−3127 : Non−receptor tyrosine−protein kinase TYK2 on Diabetic nephropathy (more control exclusions)
with a threshold of 1e−05
MR Method
Inverse variance weighted
MR Egger
9
6
3
1/SE_IV
−0.2
0.0
0.2
0.4
β_IV

Batch 3−3128 : TYMS opposite strand protein on Diabetic nephropathy (more control exclusions) with a threshold of 1e−05

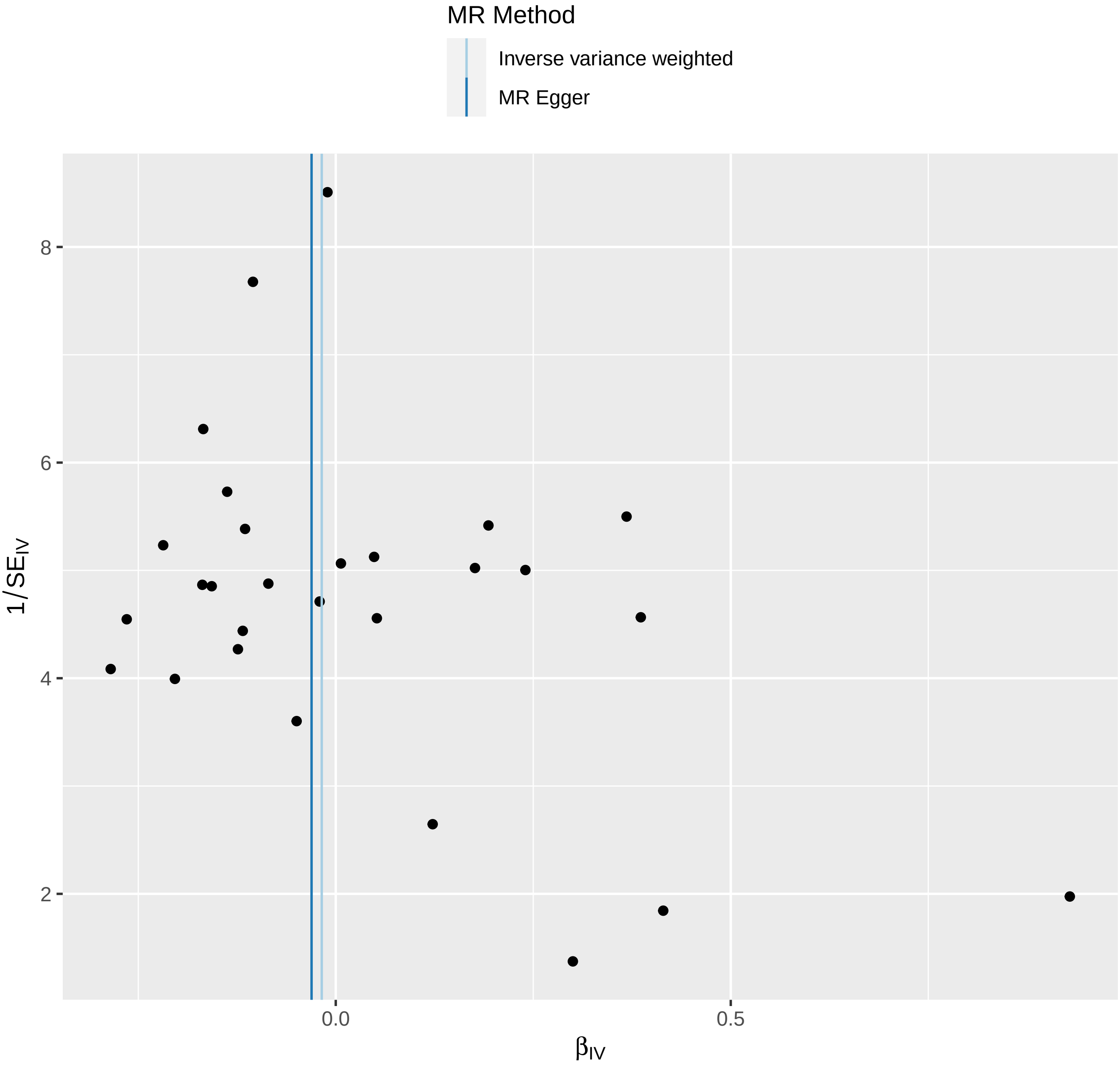

Batch 3−3129 : Tyrosine−protein kinase receptor TYRO3 on Diabetic nephropathy (more control exclusions)
with a threshold of 1e−05

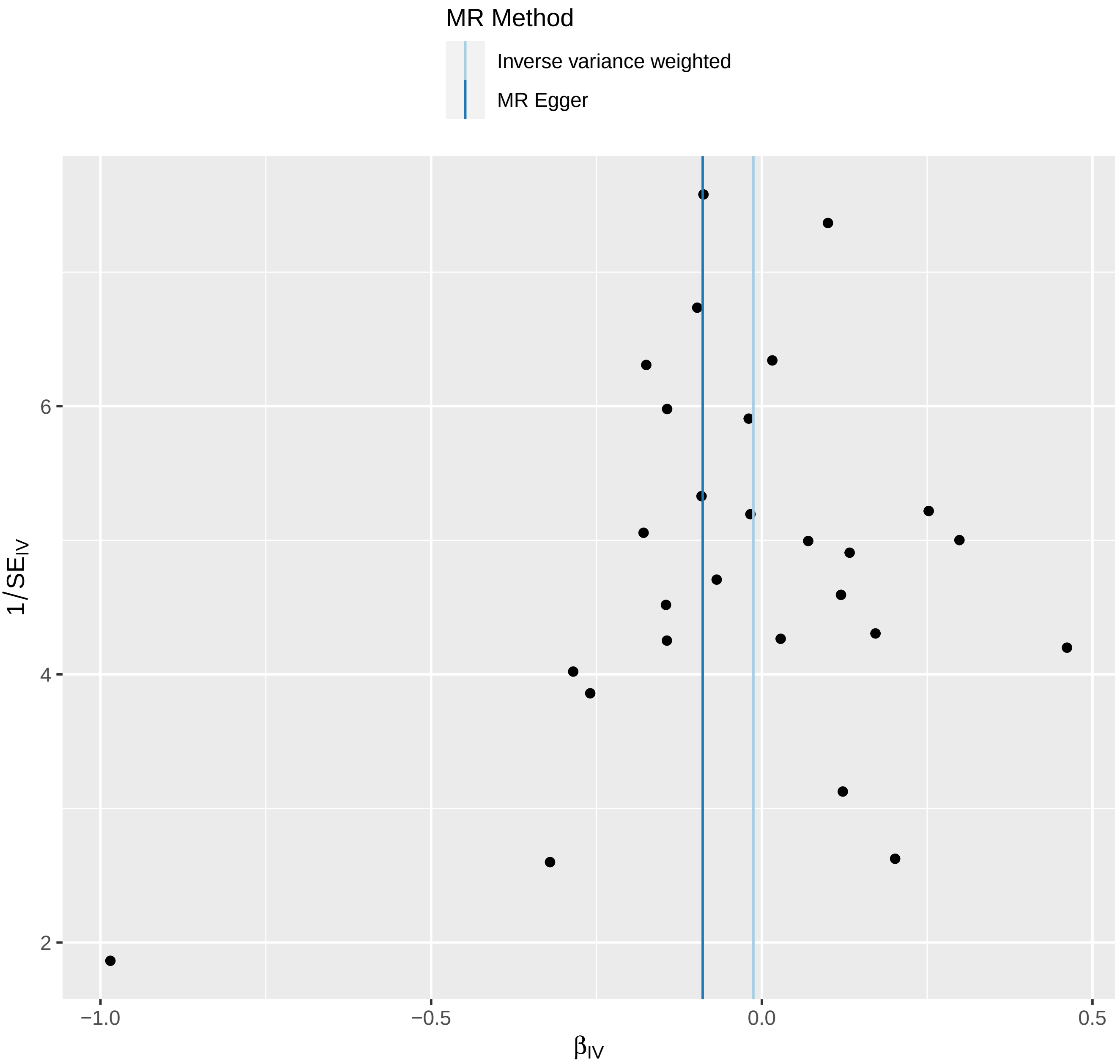

Batch 3−3130 : Splicing factor U2AF 65 kDa subunit on Diabetic nephropathy (more control exclusions)
with a threshold of 1e−05

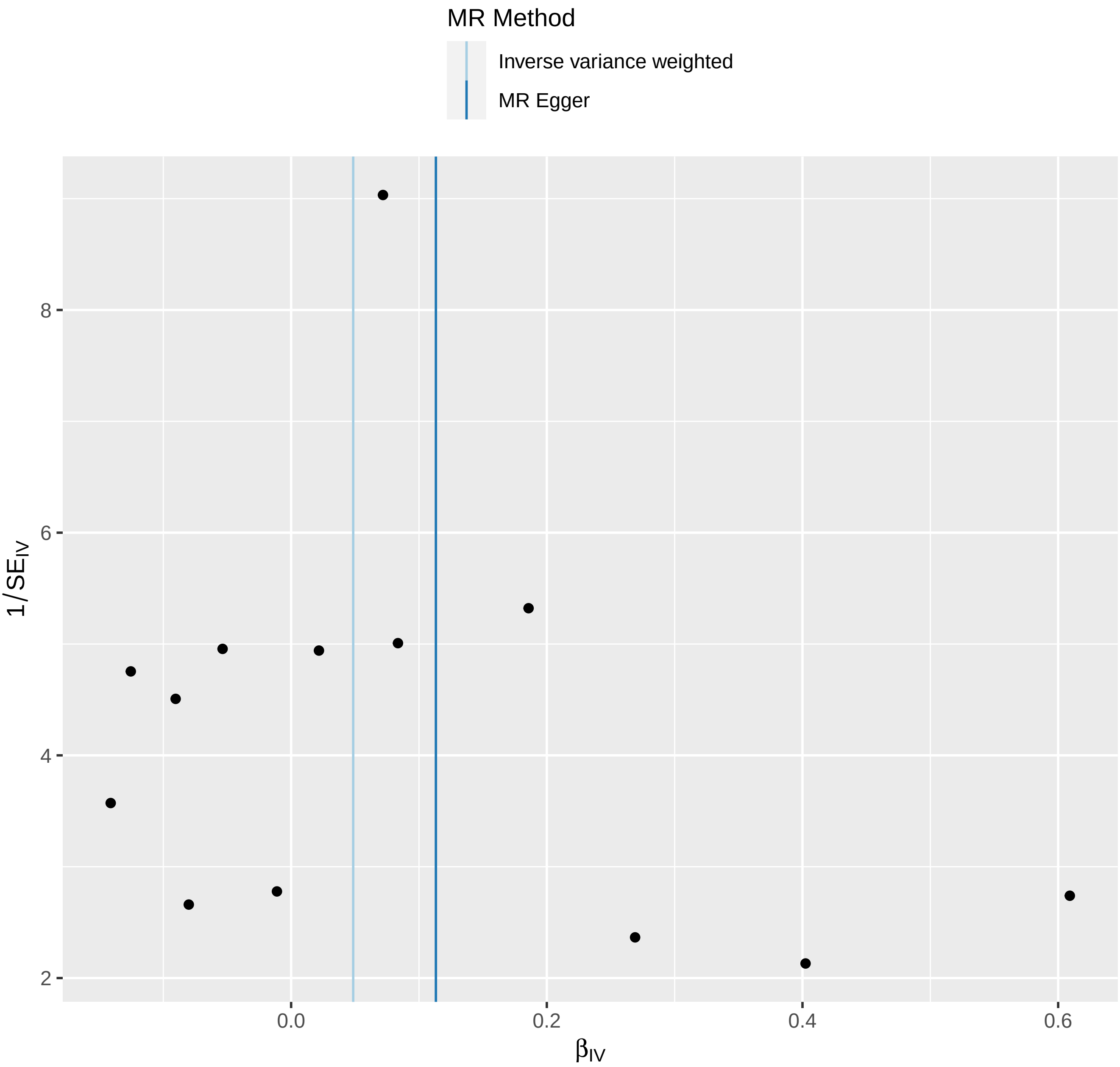

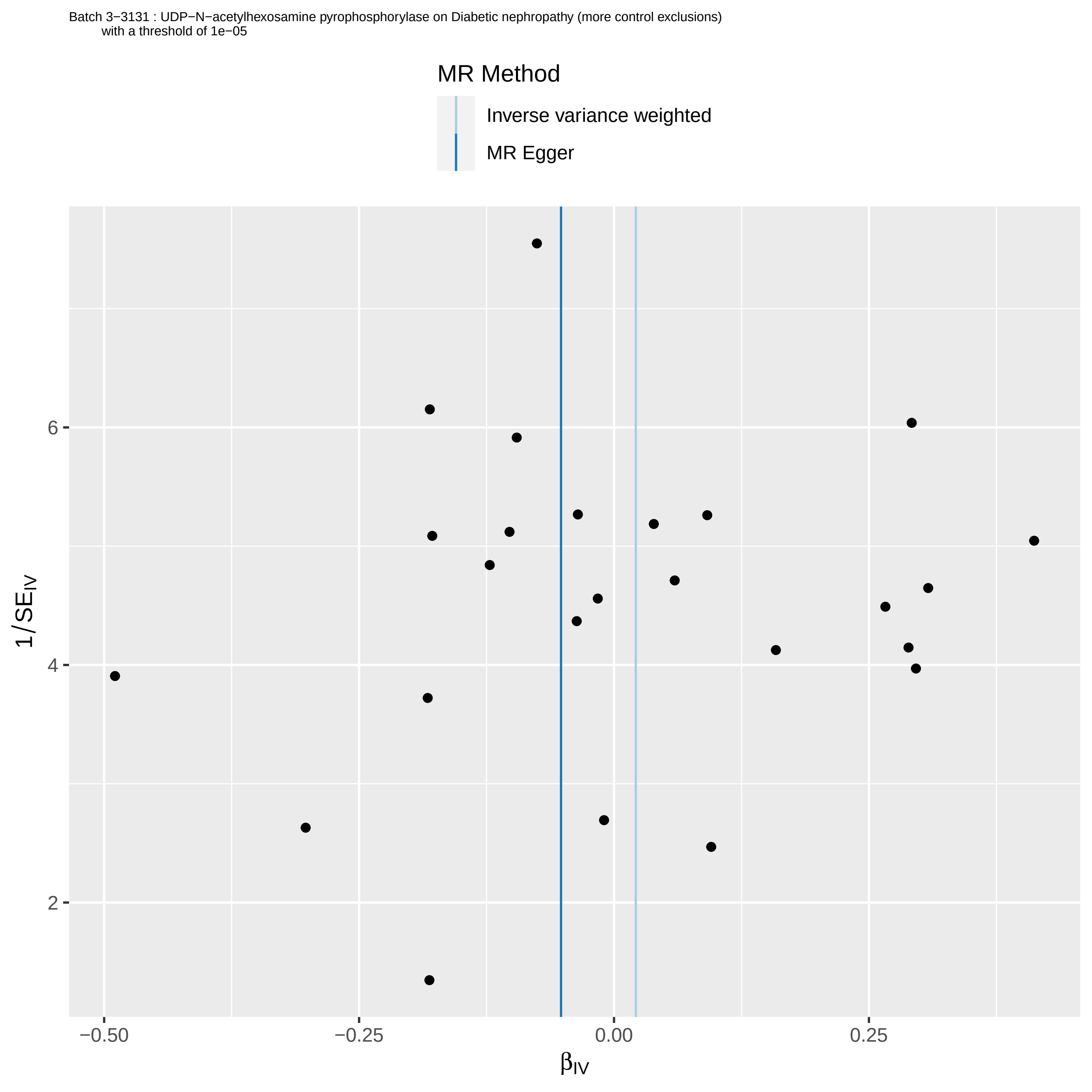

Batch 3−3131 : UDP−N−acetylhexosamine pyrophosphorylase on Diabetic nephropathy (more control exclusions)
with a threshold of 1e−05
MR Method
Inverse variance weighted
MR Egger
6
4
2
1/SE_IV
−0.50
−0.25
0.00
0.25
β_IV

Batch 3−3132 : PolyUbiquitin K48−linked on Diabetic nephropathy (more control exclusions)
with a threshold of 1e−05

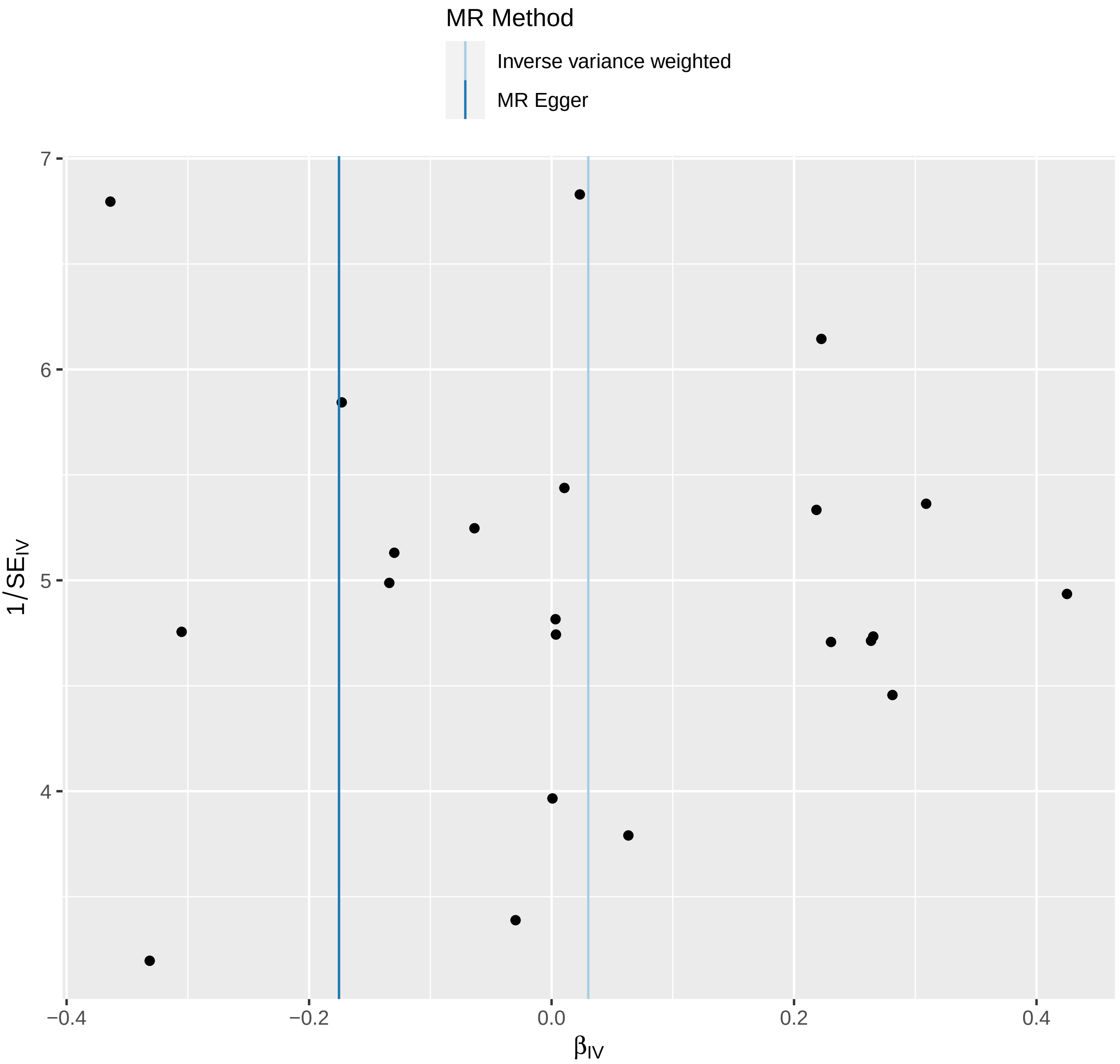

Batch 3−3133 : PolyUbiquitin K48−linked on Diabetic nephropathy (more control exclusions)
with a threshold of 1e−05

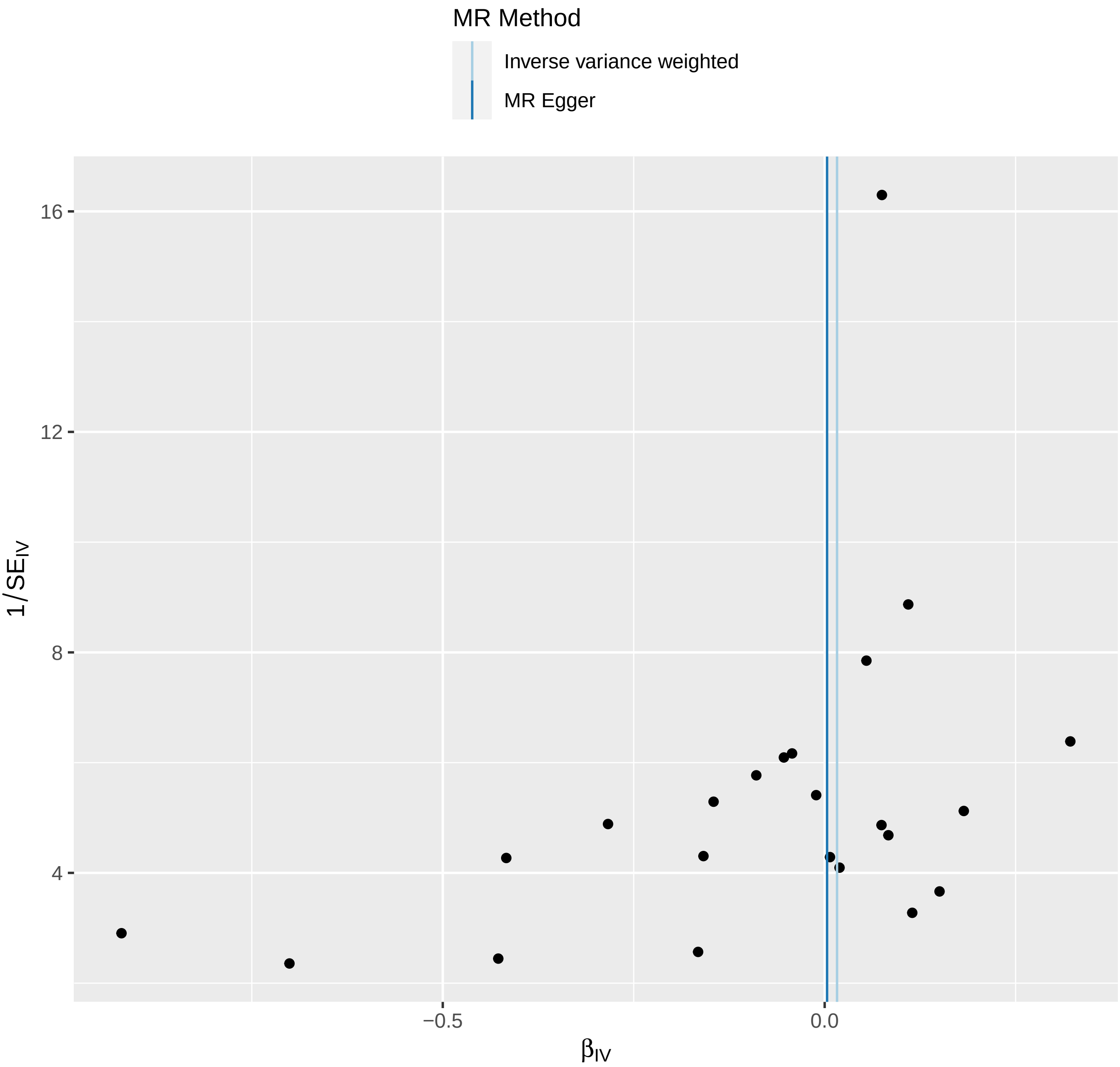

Batch 3−3134 : PolyUbiquitin K63−linked on Diabetic nephropathy (more control exclusions)
with a threshold of 1e−05

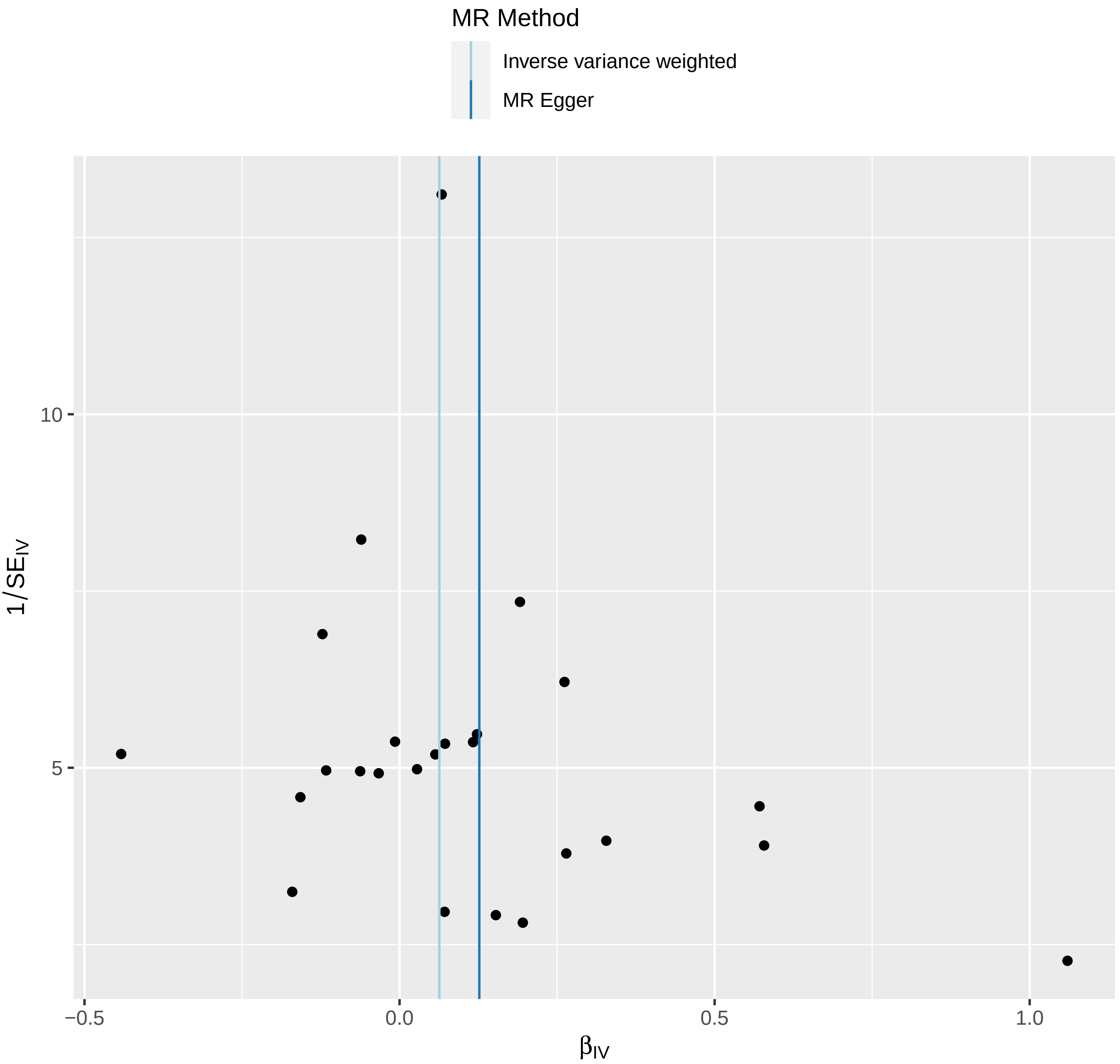

Batch 3−3135 : Ubiquitin−conjugating enzyme E2 B on Diabetic nephropathy (more control exclusions)
with a threshold of 1e−05

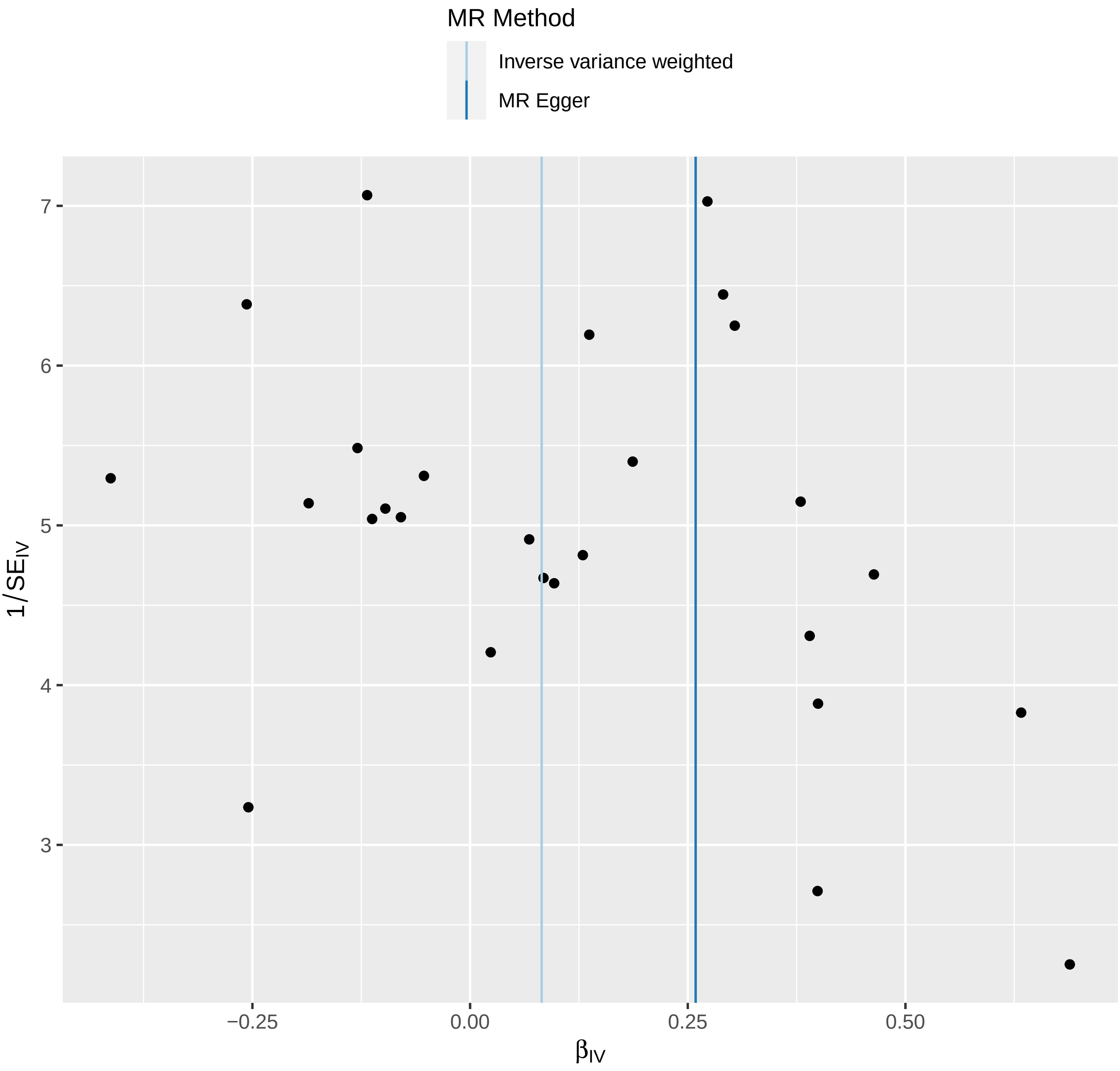

Batch 3−3136 : Ubiquitin−conjugating enzyme E2 D4 on Diabetic nephropathy (more control exclusions) with a threshold of 1e−05

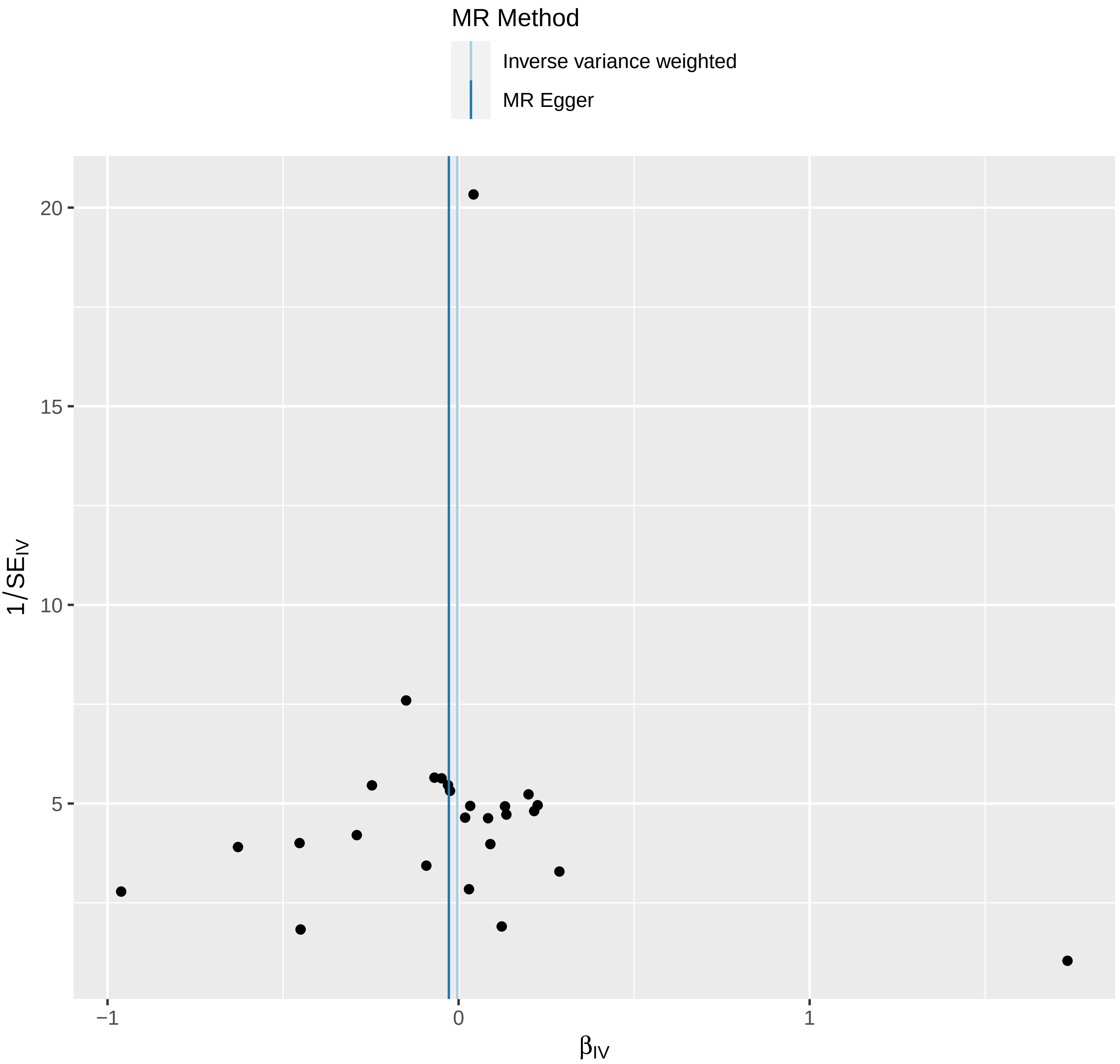

Batch 3−3137 : Ubiquitin−conjugating enzyme E2 G2 on Diabetic nephropathy (more control exclusions)
with a threshold of 1e−05

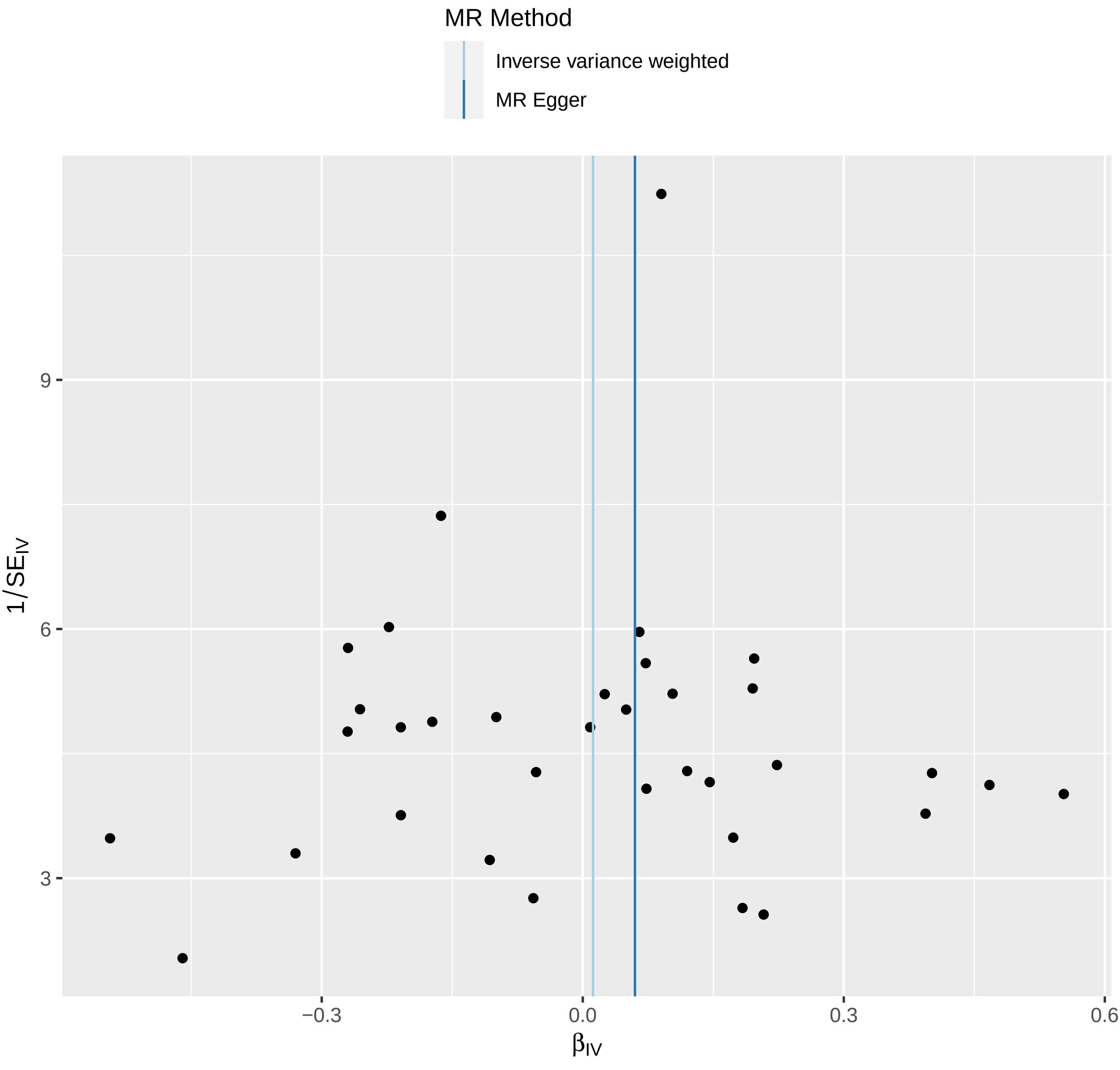

Batch 3−3138 : SUMO−conjugating enzyme UBC9 on Diabetic nephropathy (more control exclusions)
with a threshold of 1e−05

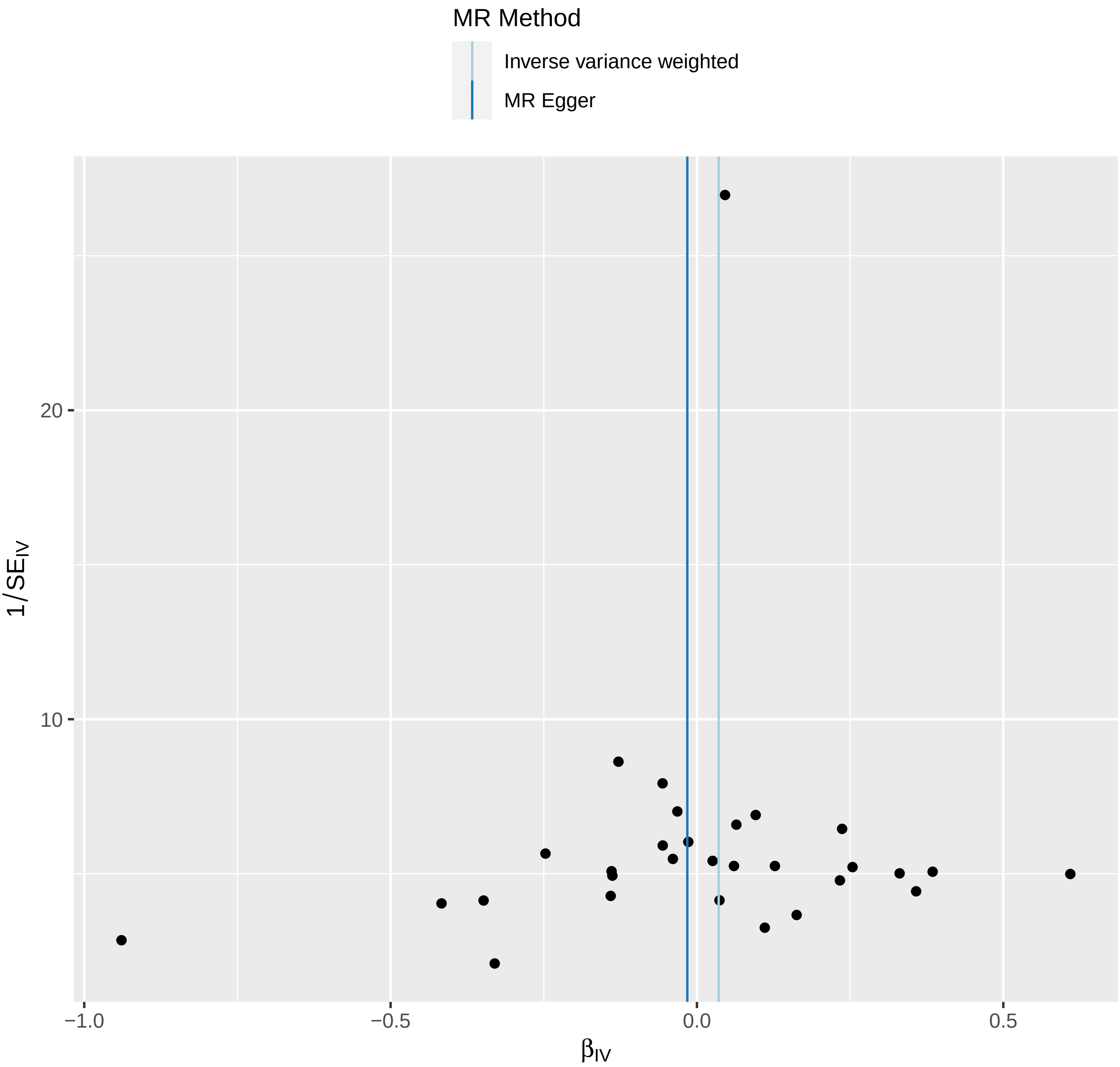

Batch 3−3139 : Ubiquitin−conjugating enzyme E2 J1 on Diabetic nephropathy (more control exclusions)
with a threshold of 1e−05

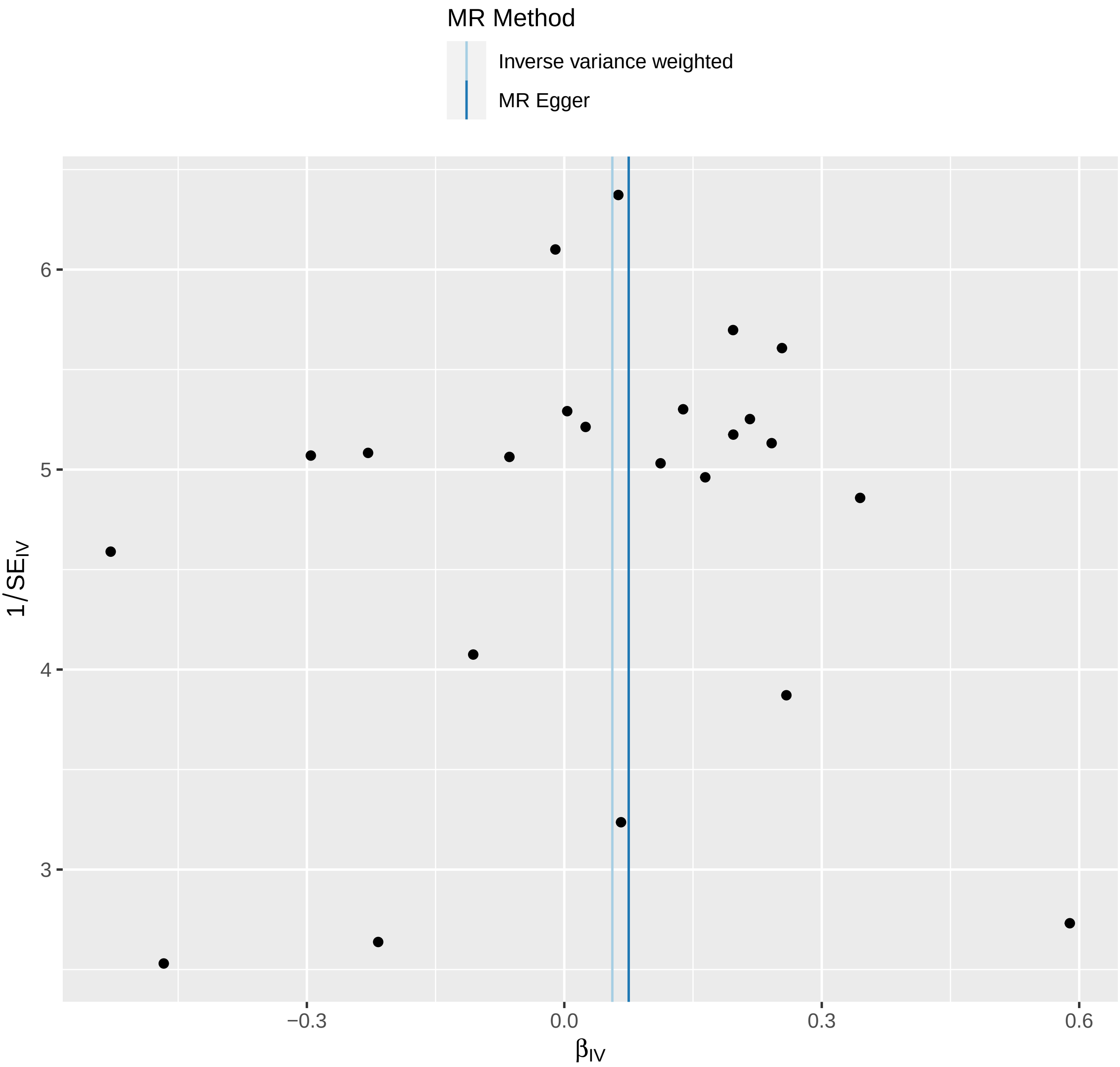

Batch 3−3140 : Ubiquitin−conjugating enzyme E2 J2 on Diabetic nephropathy (more control exclusions)
with a threshold of 1e−05

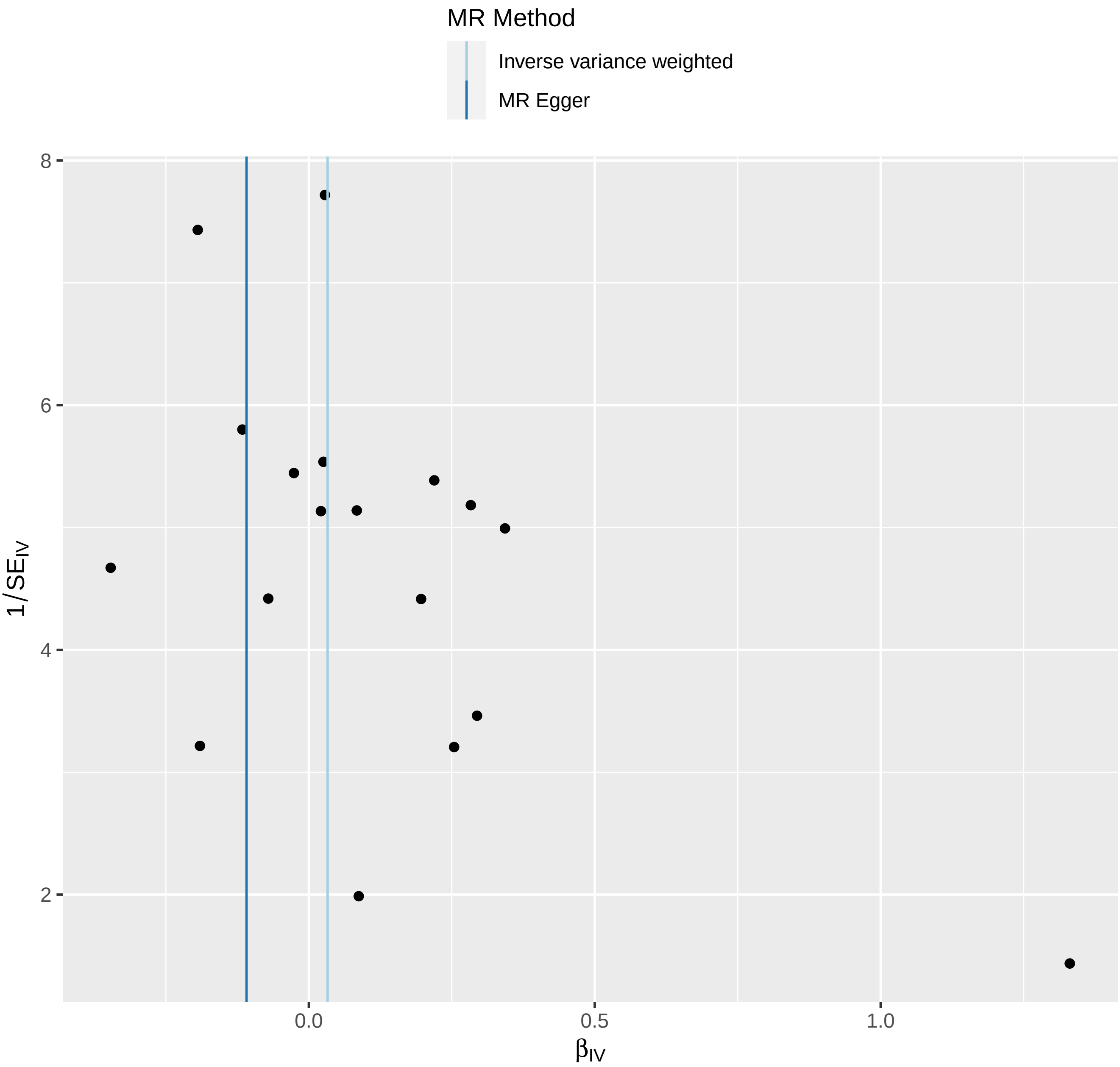

Batch 3−3141 : Ubiquitin−conjugating enzyme E2 J2 on Diabetic nephropathy (more control exclusions)
with a threshold of 1e−05

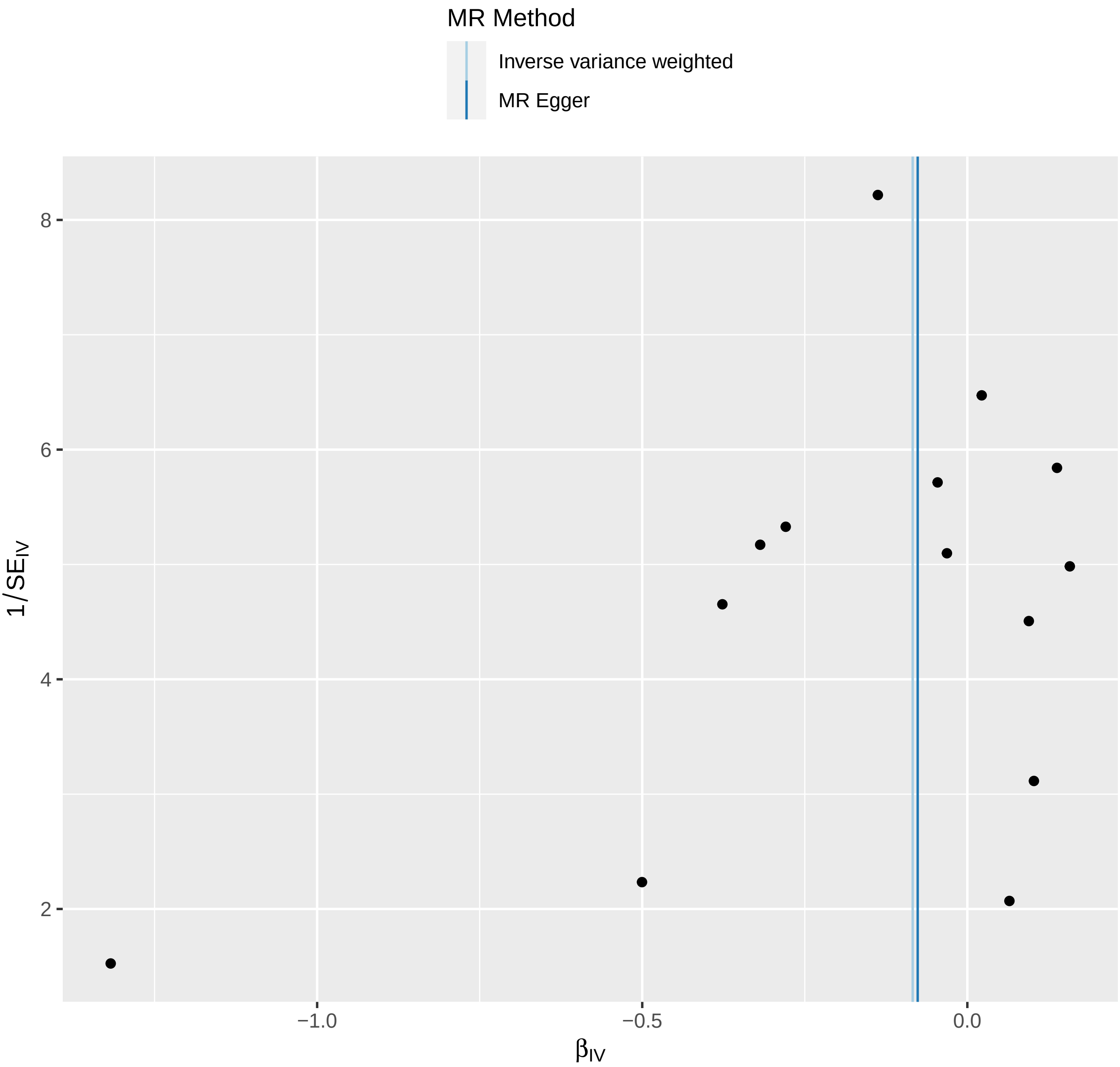

Batch 3−3142 : Ubiquitin−conjugating enzyme E2 L3 on Diabetic nephropathy (more control exclusions)
with a threshold of 1e−05

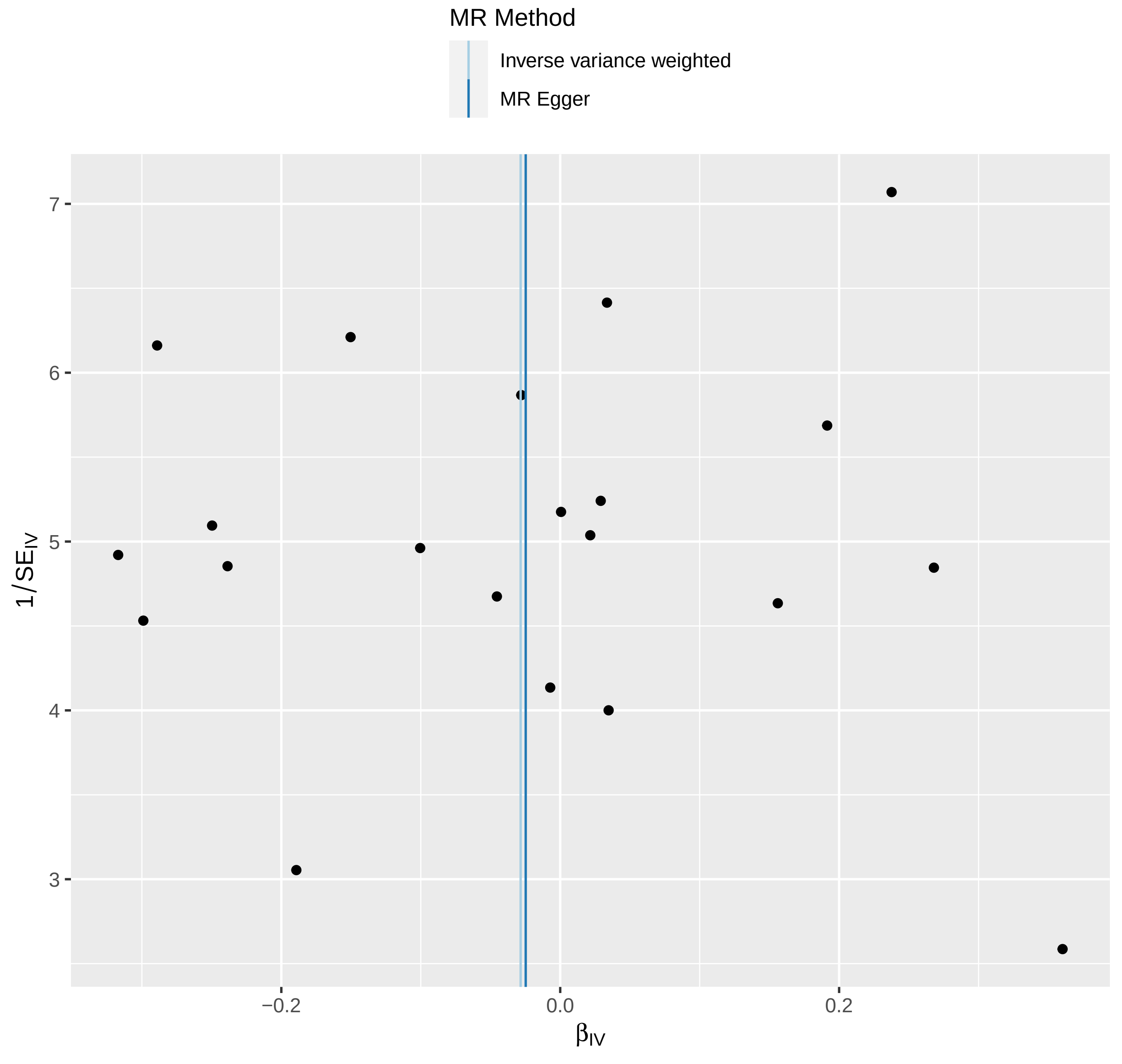

Batch 3−3143 : Ubiquitin−conjugating enzyme E2 N on Diabetic nephropathy (more control exclusions)
with a threshold of 1e−05

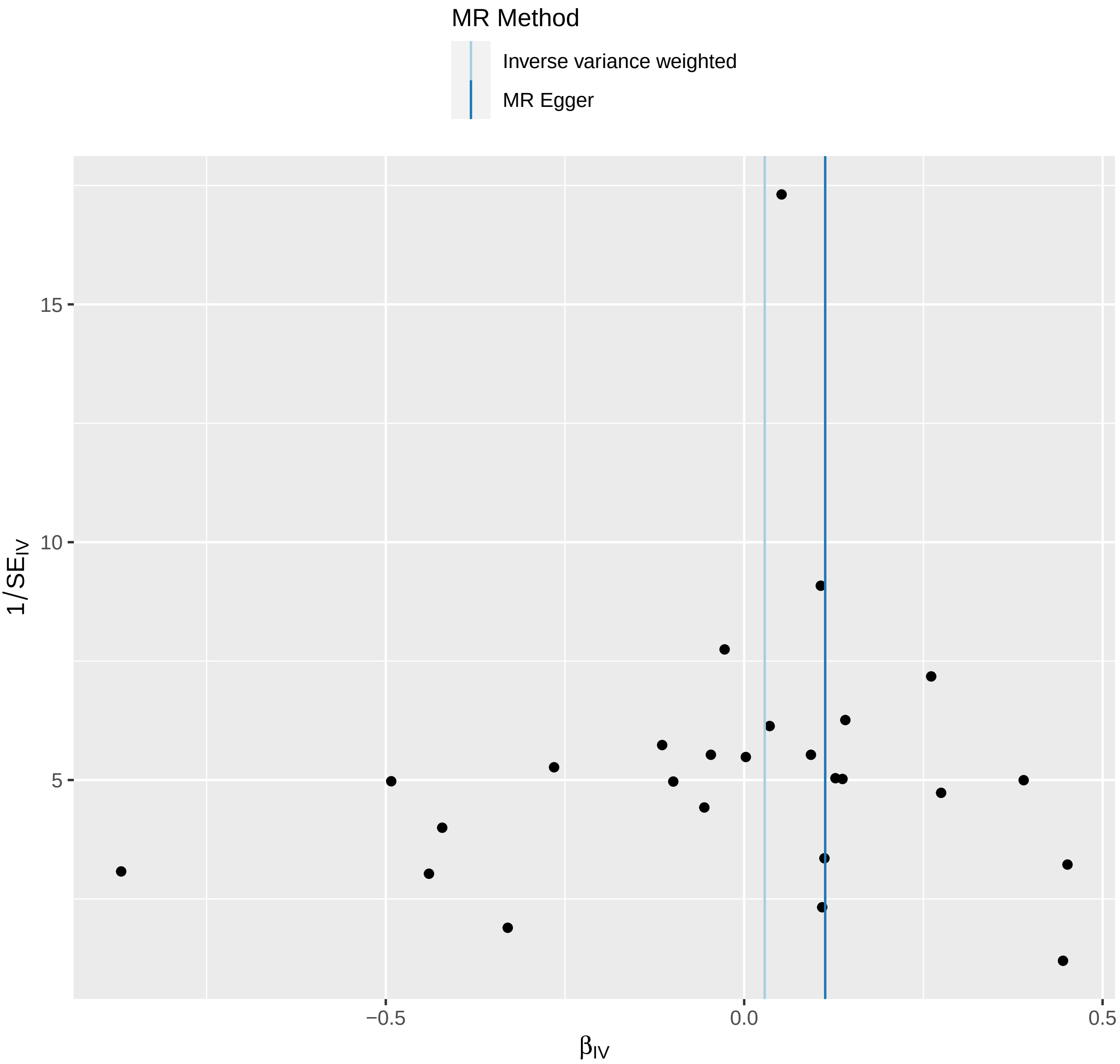

Batch 3−3144 : Ubiquitin−conjugating enzyme E2 T on Diabetic nephropathy (more control exclusions)
with a threshold of 1e−05

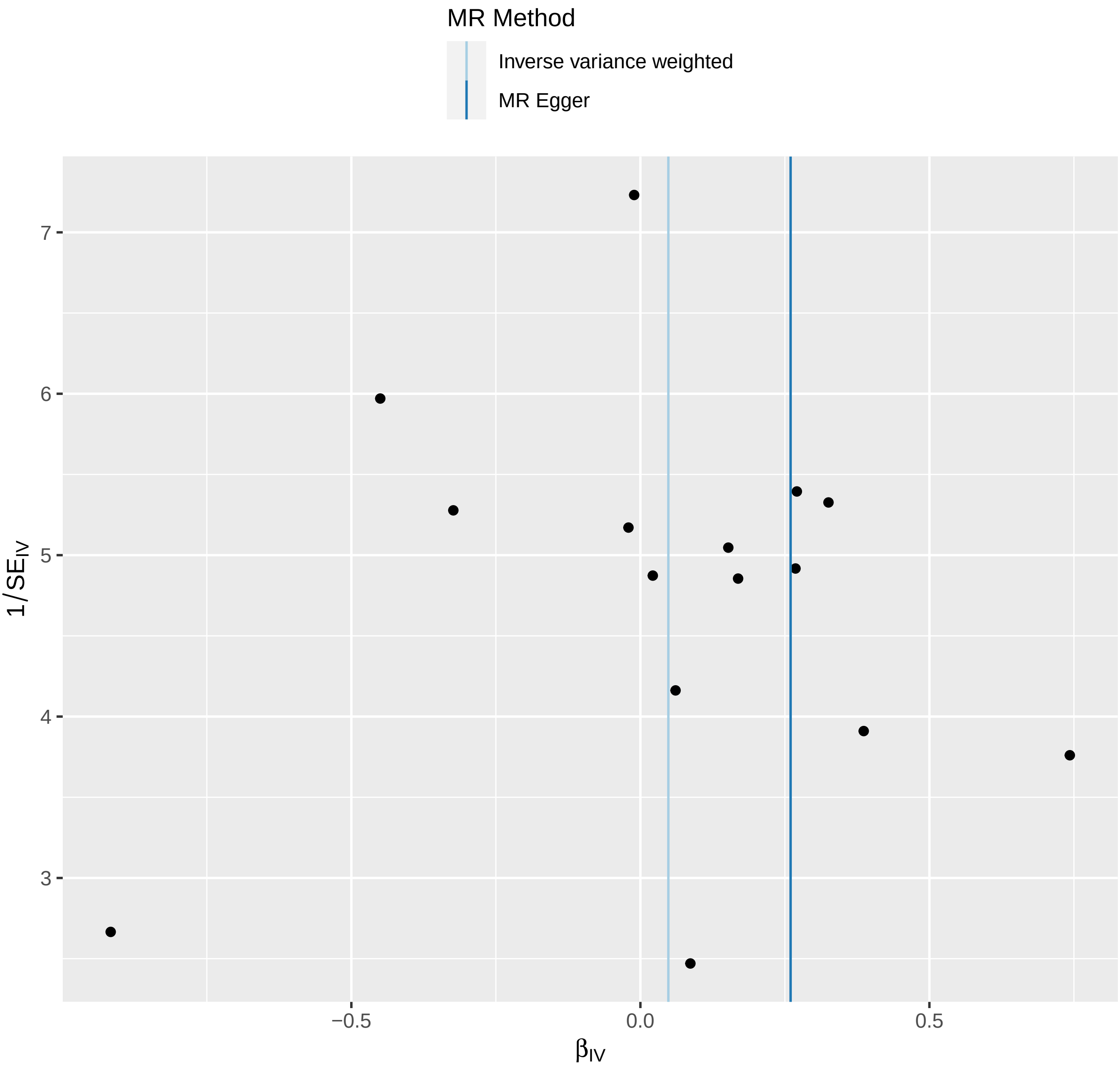

Batch 3−3145 : Ubiquitin−conjugating enzyme E2 variant 1 on Diabetic nephropathy (more control exclusions)
with a threshold of 1e−05

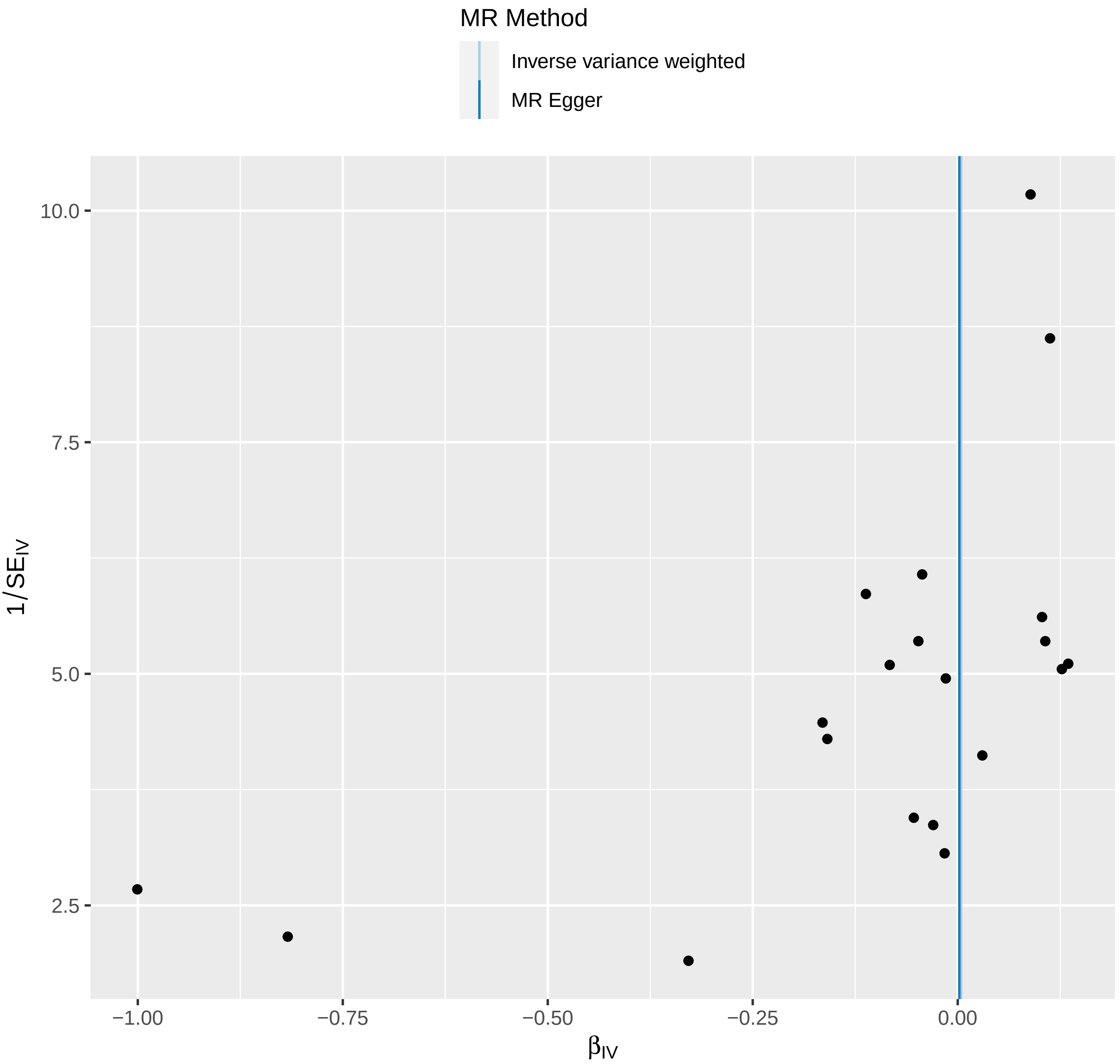

Batch 3−3146 : Ubiquitin−like protein 4A on Diabetic nephropathy (more control exclusions)
with a threshold of 1e−05

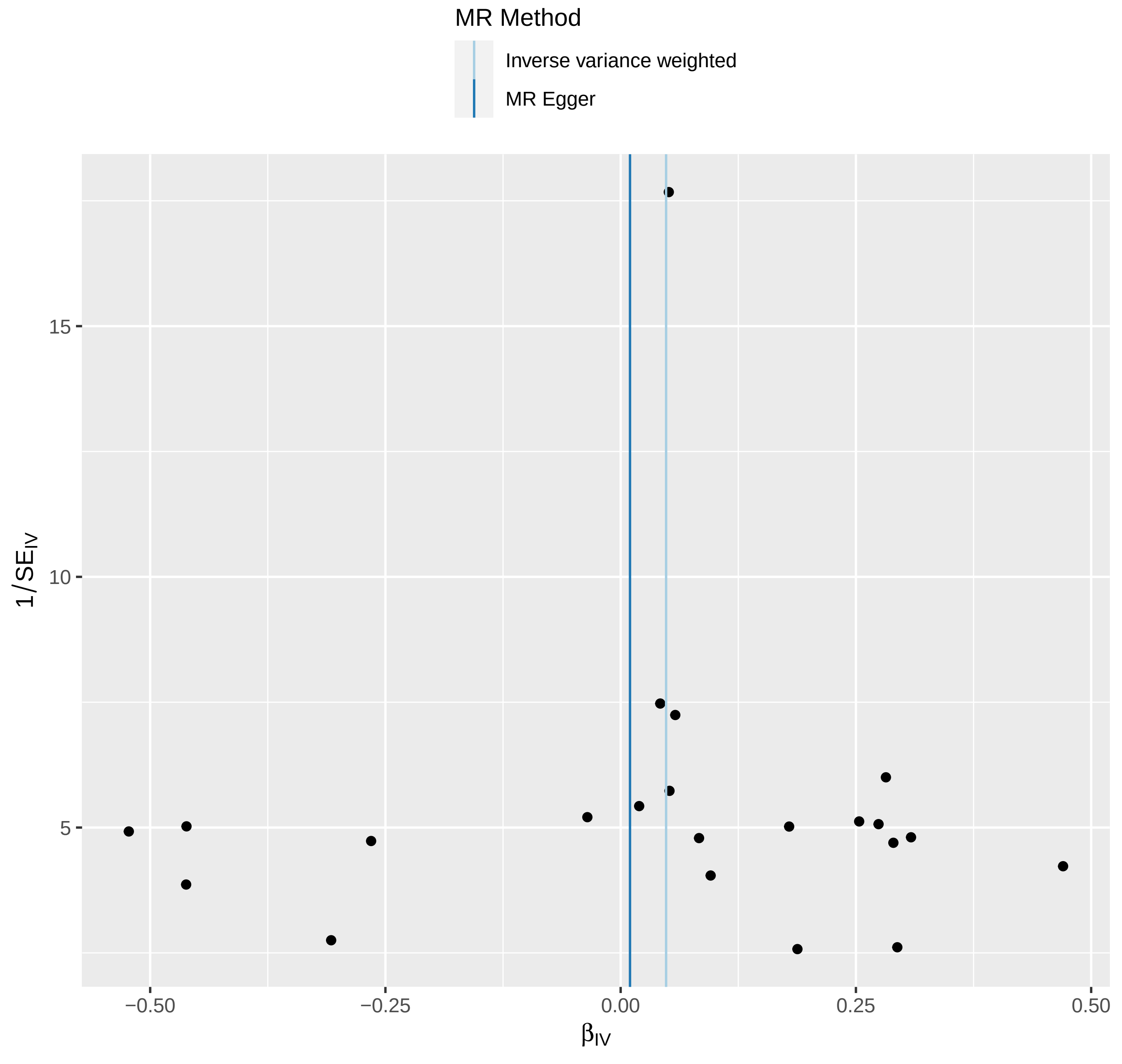

Batch 3−3147 : Ubiquilin−4 on Diabetic nephropathy (more control exclusions)
with a threshold of 1e−05

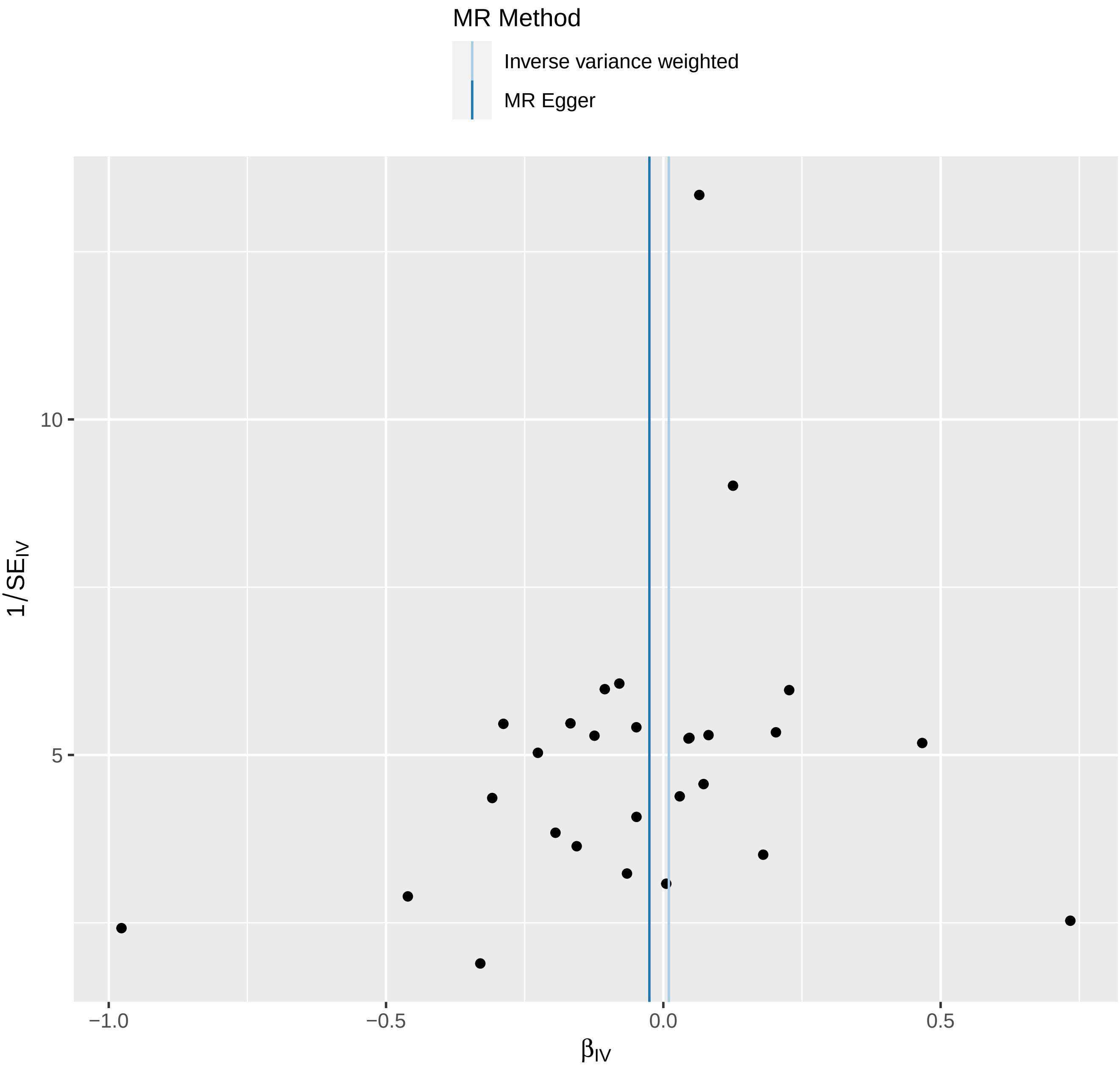

Batch 3−3148 : Ubiquitin domain−containing protein 2 on Diabetic nephropathy (more control exclusions)
with a threshold of 1e−05

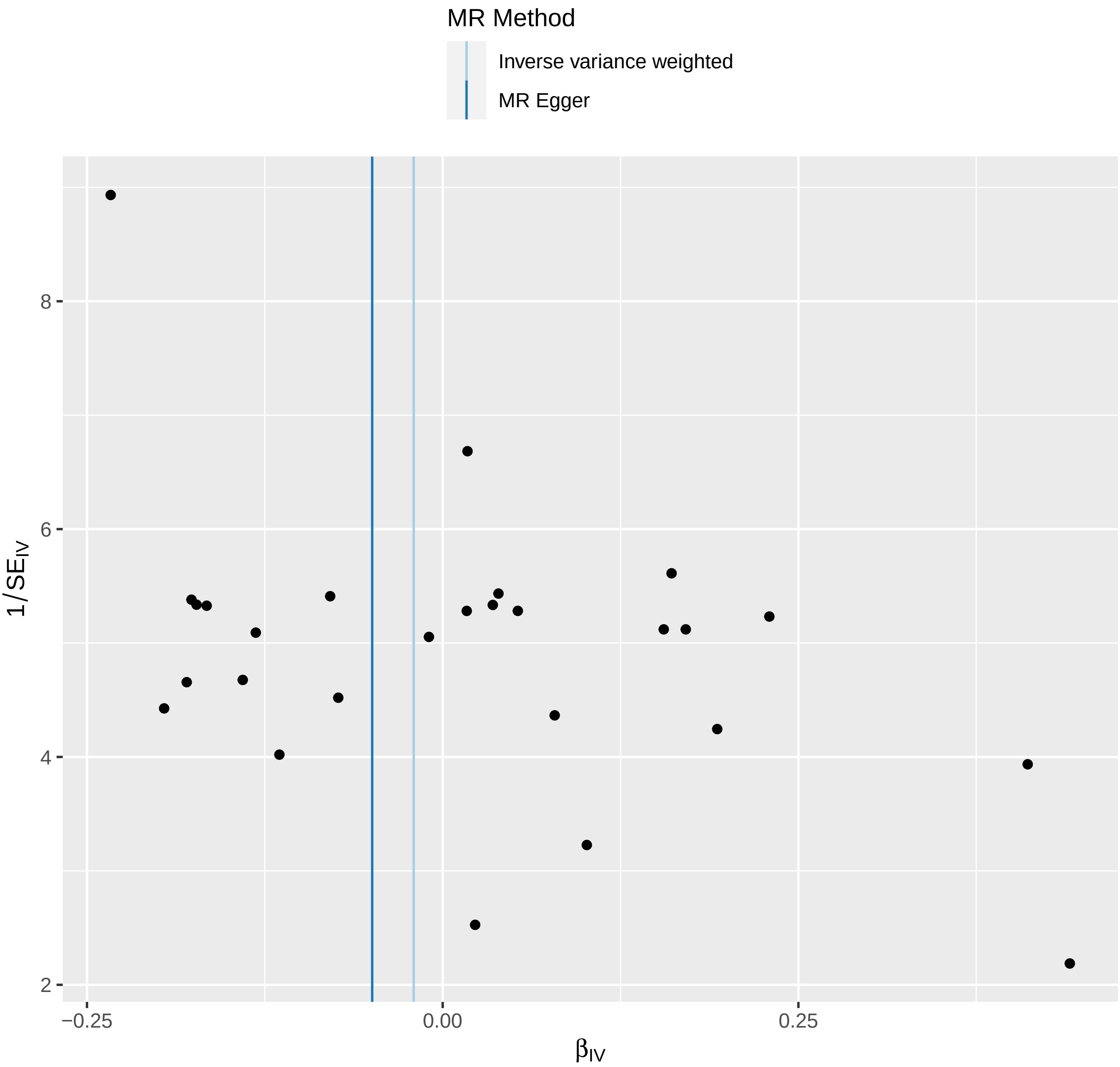

Batch 3−3149 : UBX domain−containing protein 4 on Diabetic nephropathy (more control exclusions)
with a threshold of 1e−05

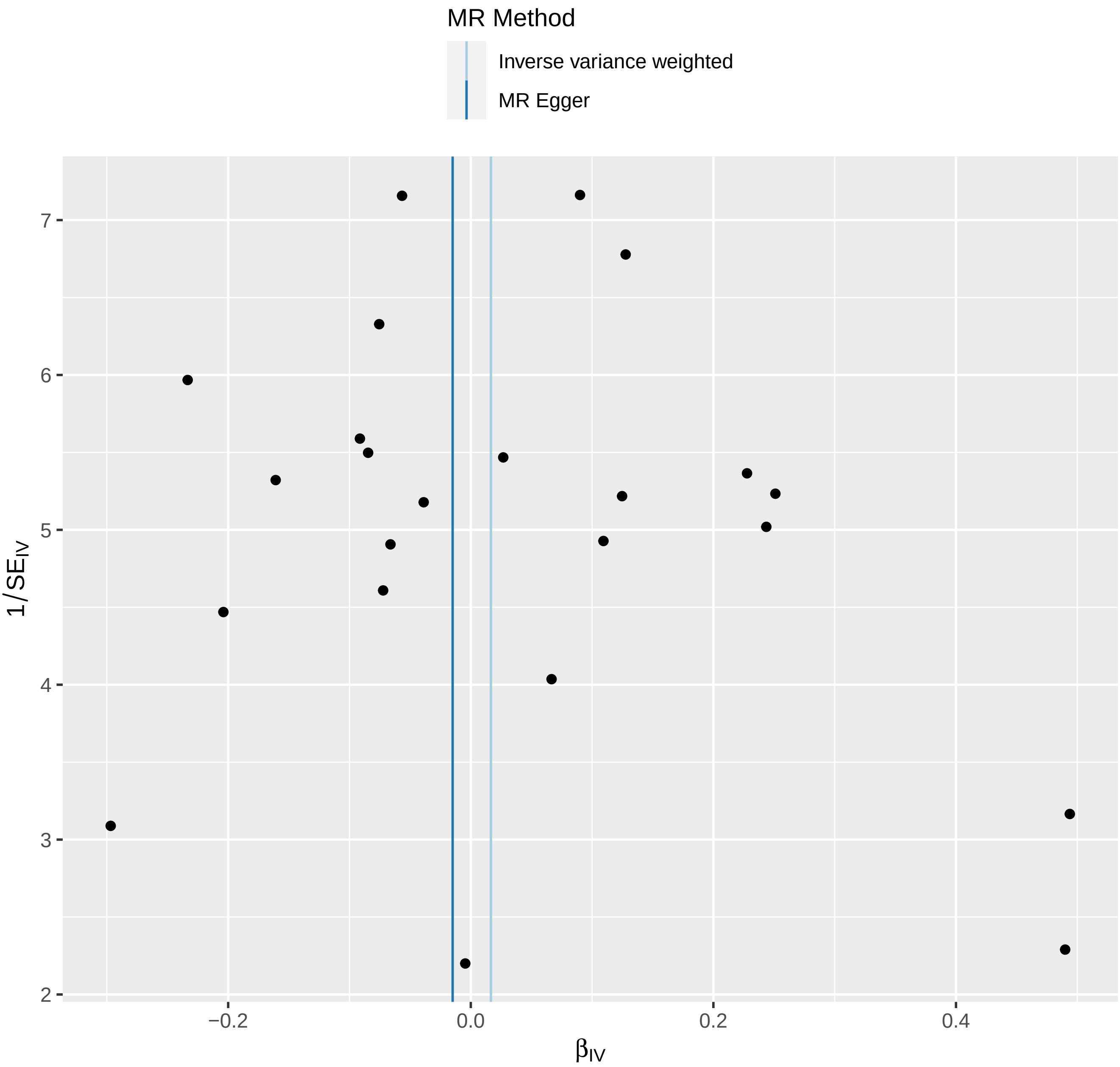

Batch 3−3150 : UBX domain−containing protein 4 on Diabetic nephropathy (more control exclusions)
with a threshold of 1e−05

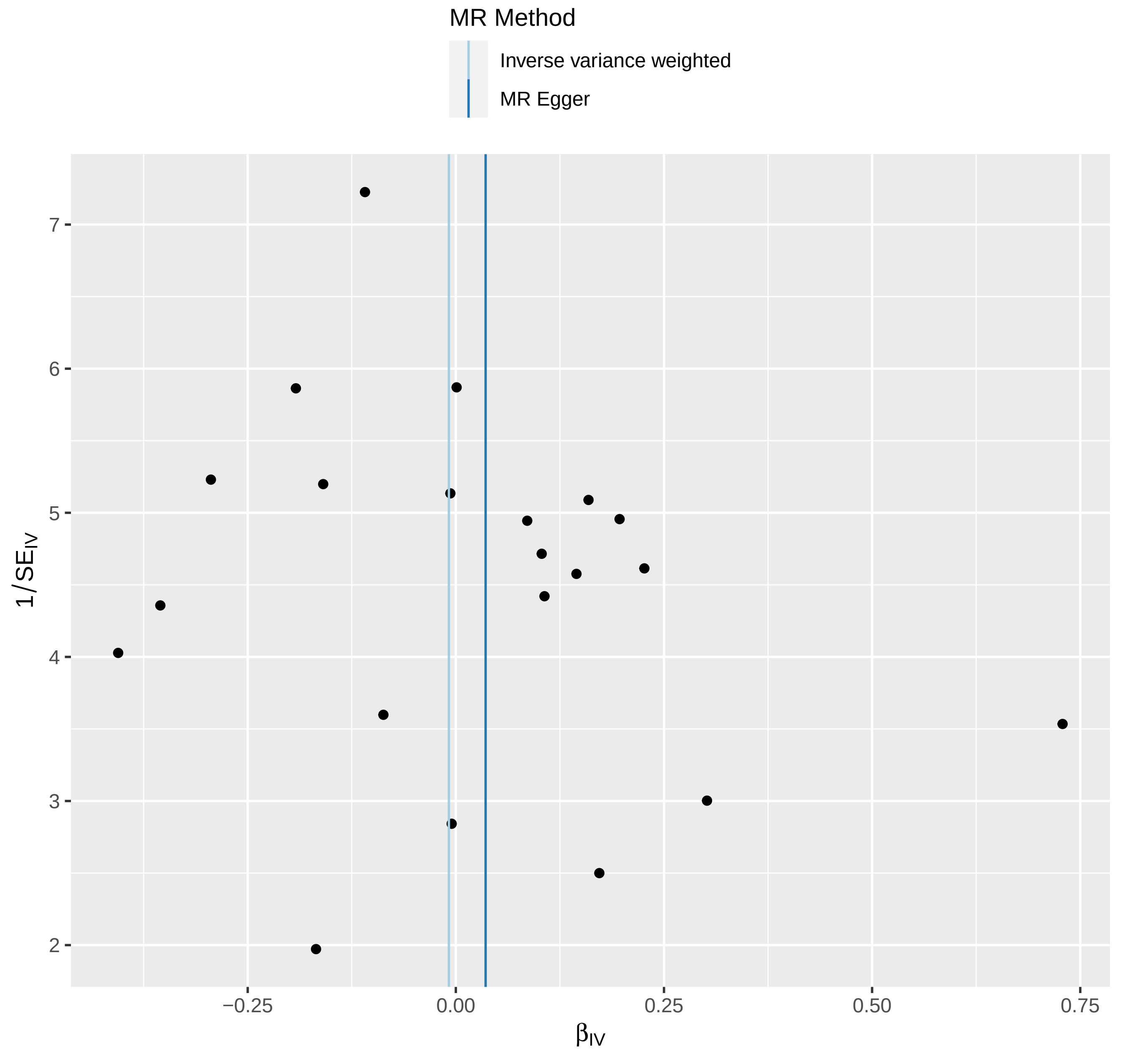

Batch 3−3151 : Uridine−cytidine kinase 2 on Diabetic nephropathy (more control exclusions)
with a threshold of 1e−05

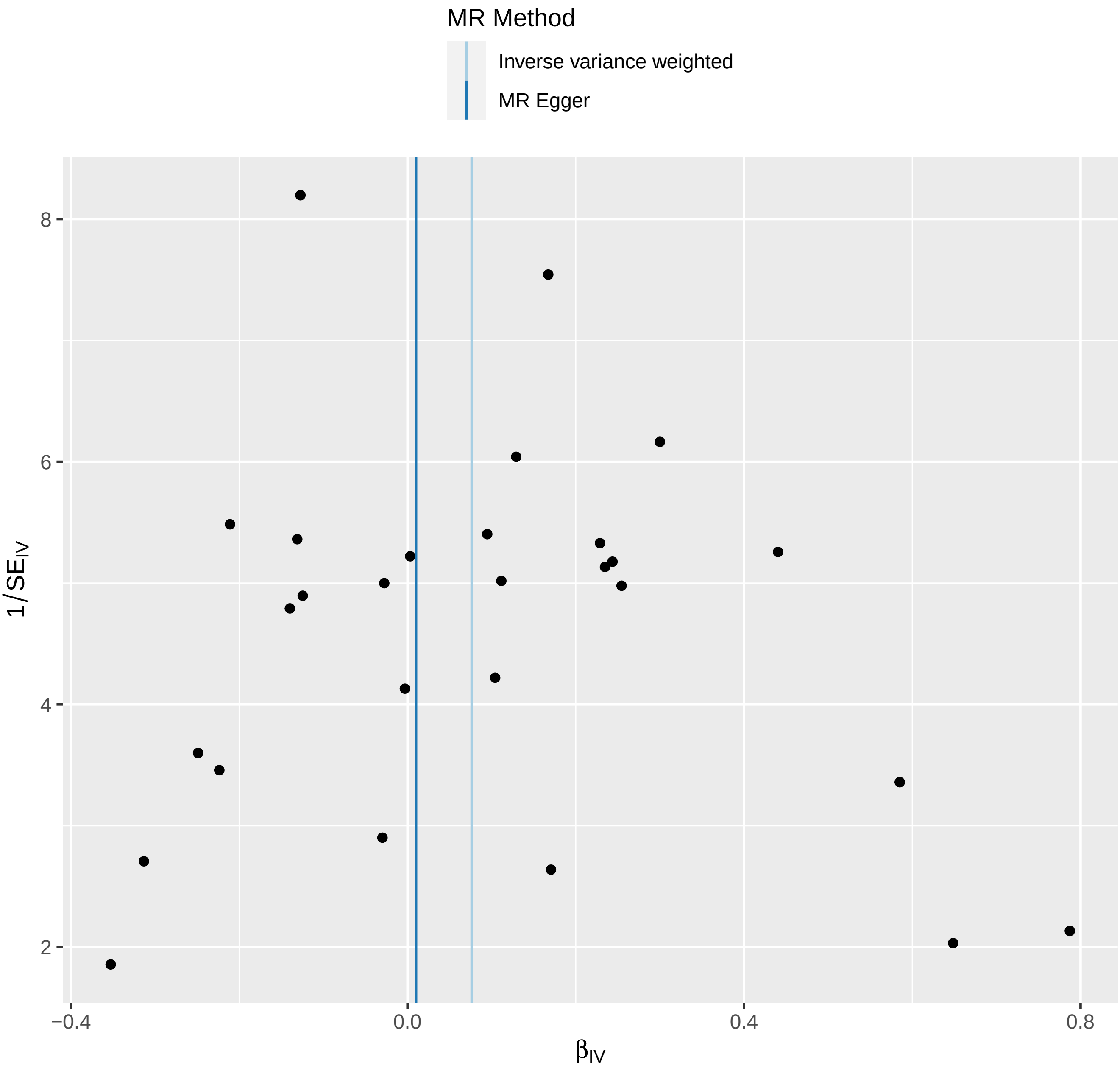

Batch 3−3152 : Unique cartilage matrix−associated protein on Diabetic nephropathy (more control exclusions)
with a threshold of 1e−05

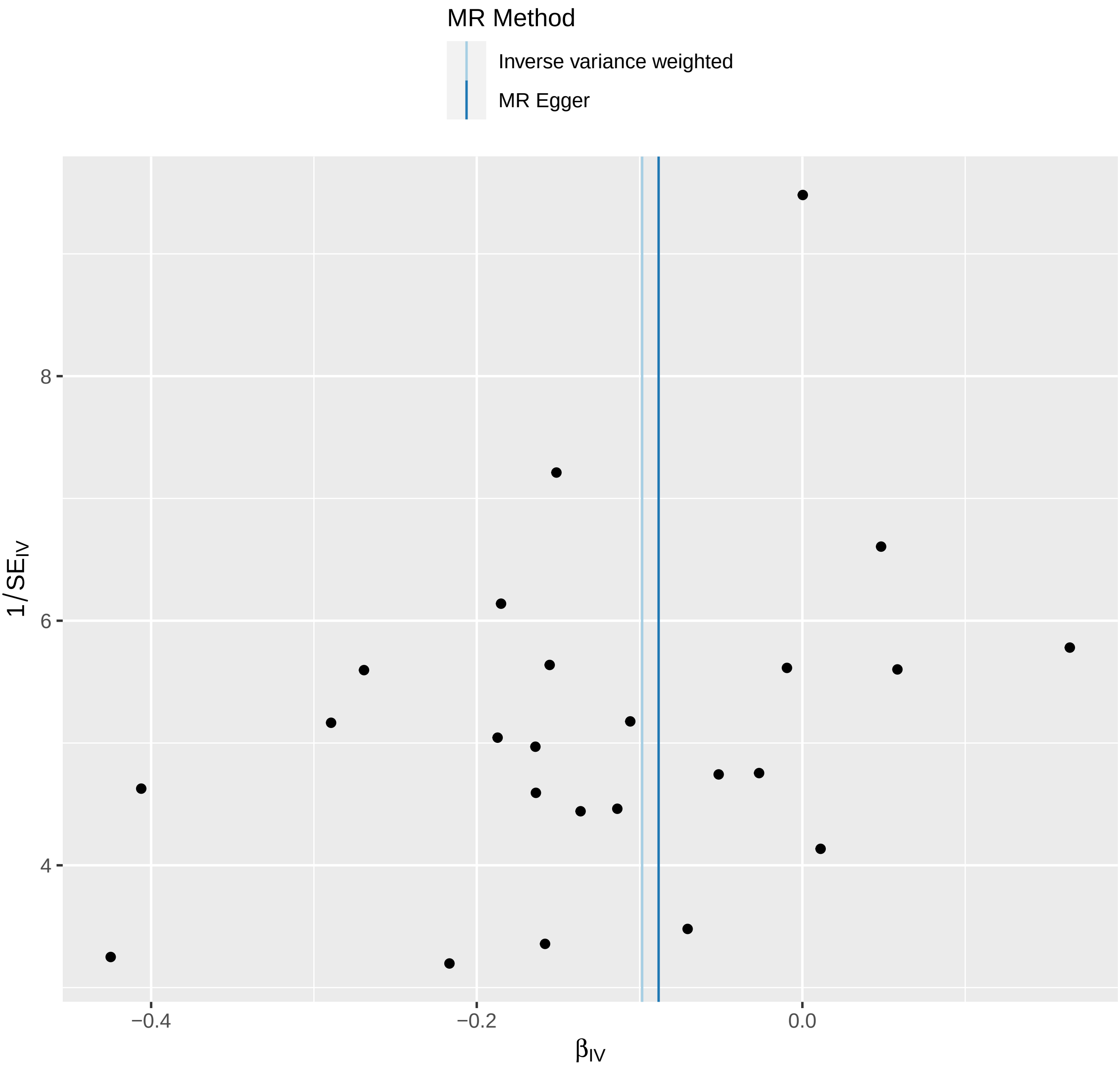

Batch 3−3153 : Ubiquitin−fold modifier−conjugating enzyme 1 on Diabetic nephropathy (more control exclusions) with a threshold of 1e−05

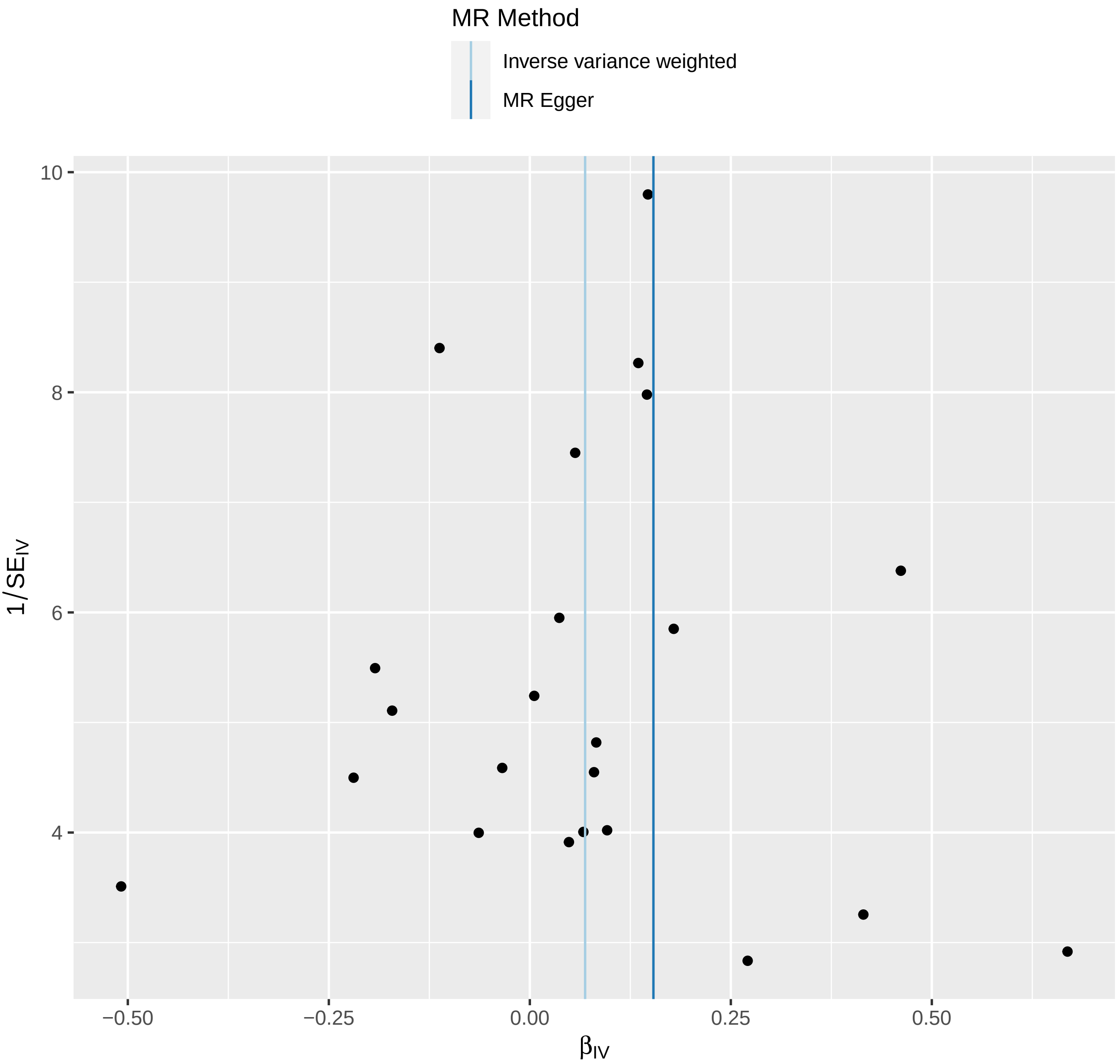

Batch 3−3154 : Ubiquitin−fold modifier 1 on Diabetic nephropathy (more control exclusions)
with a threshold of 1e−05

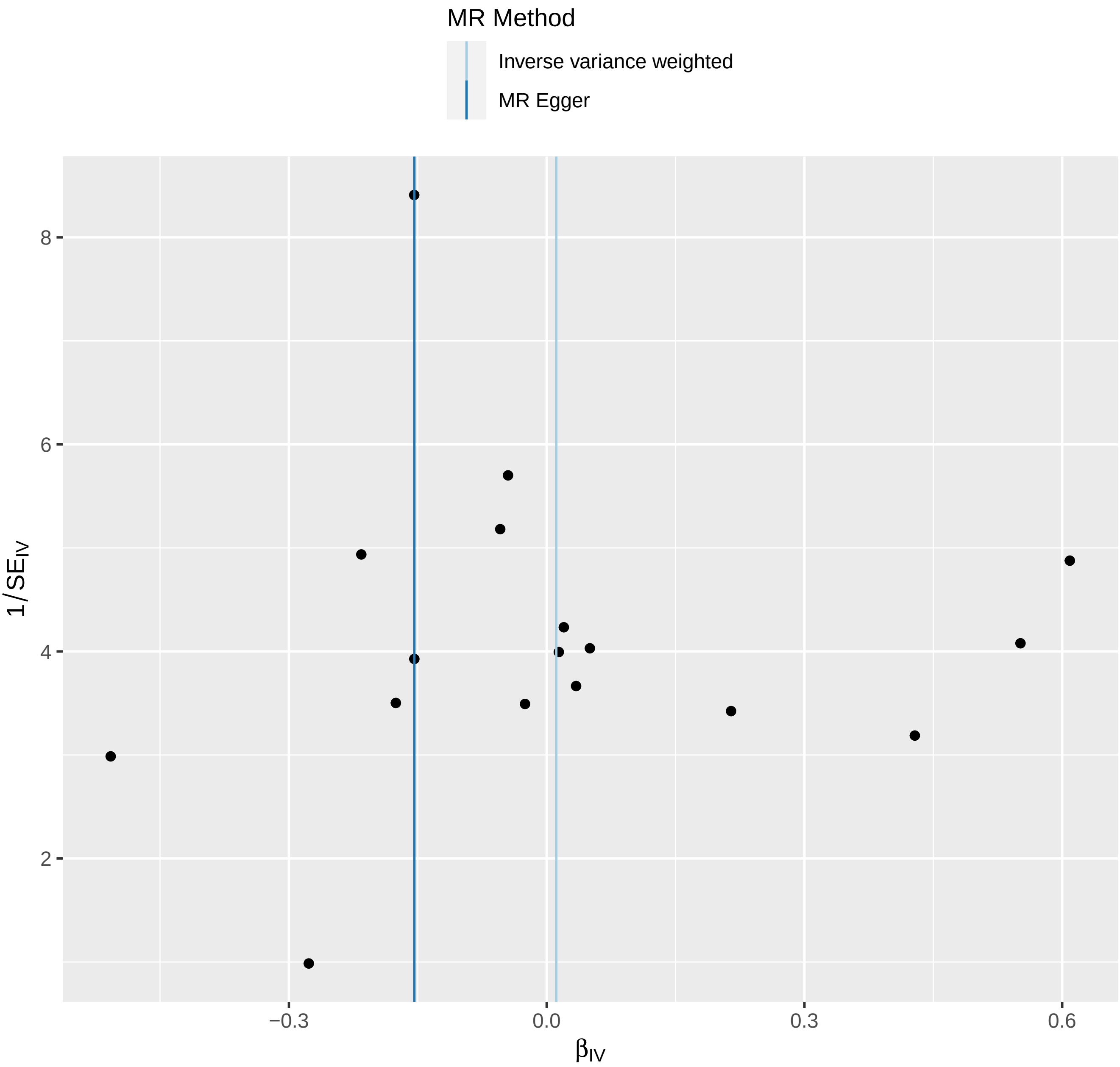

Batch 3−3155 : UTP−−glucose−1−phosphate uridylyltransferase on Diabetic nephropathy (more control exclusions) with a threshold of 1e−05

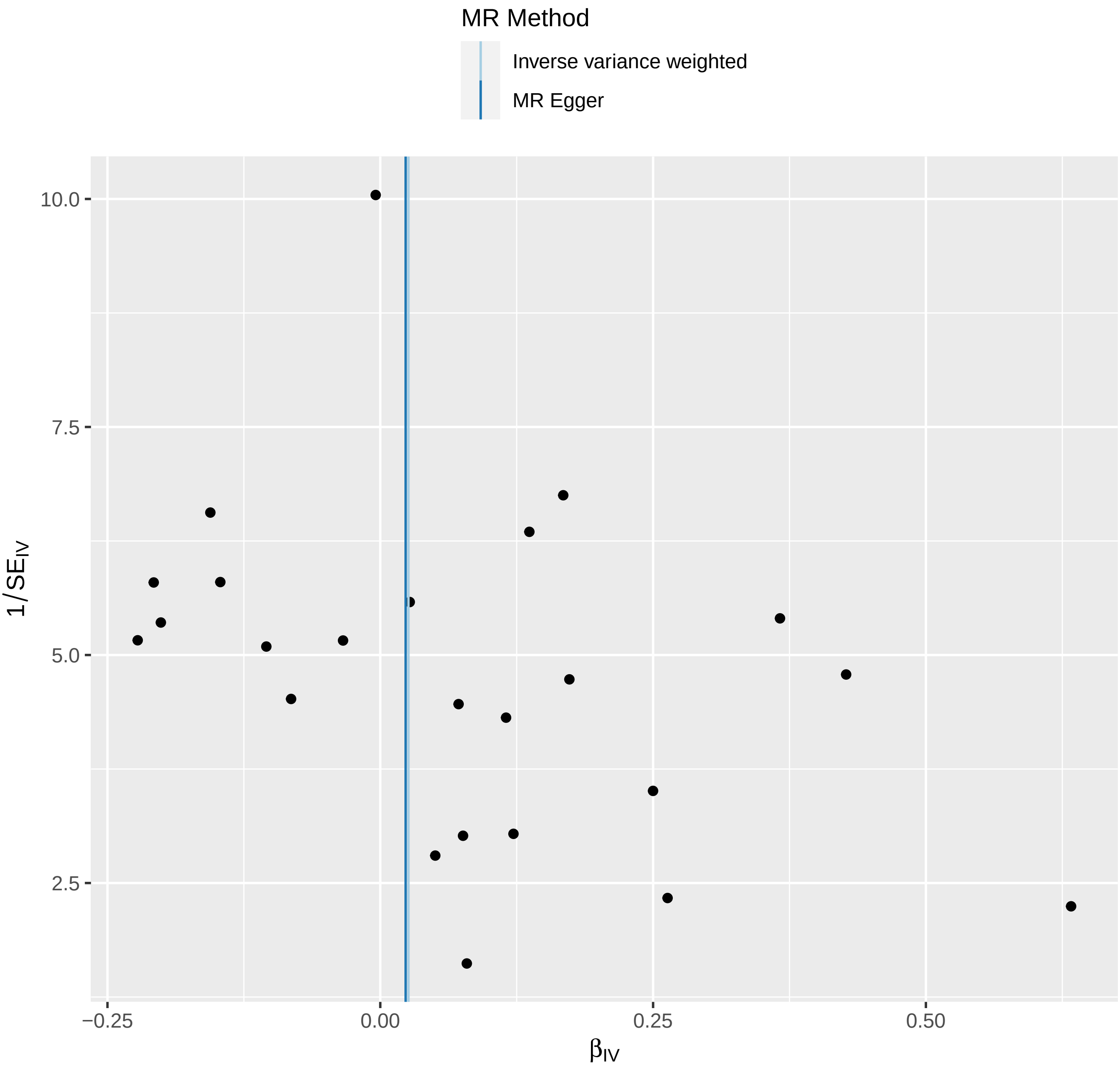

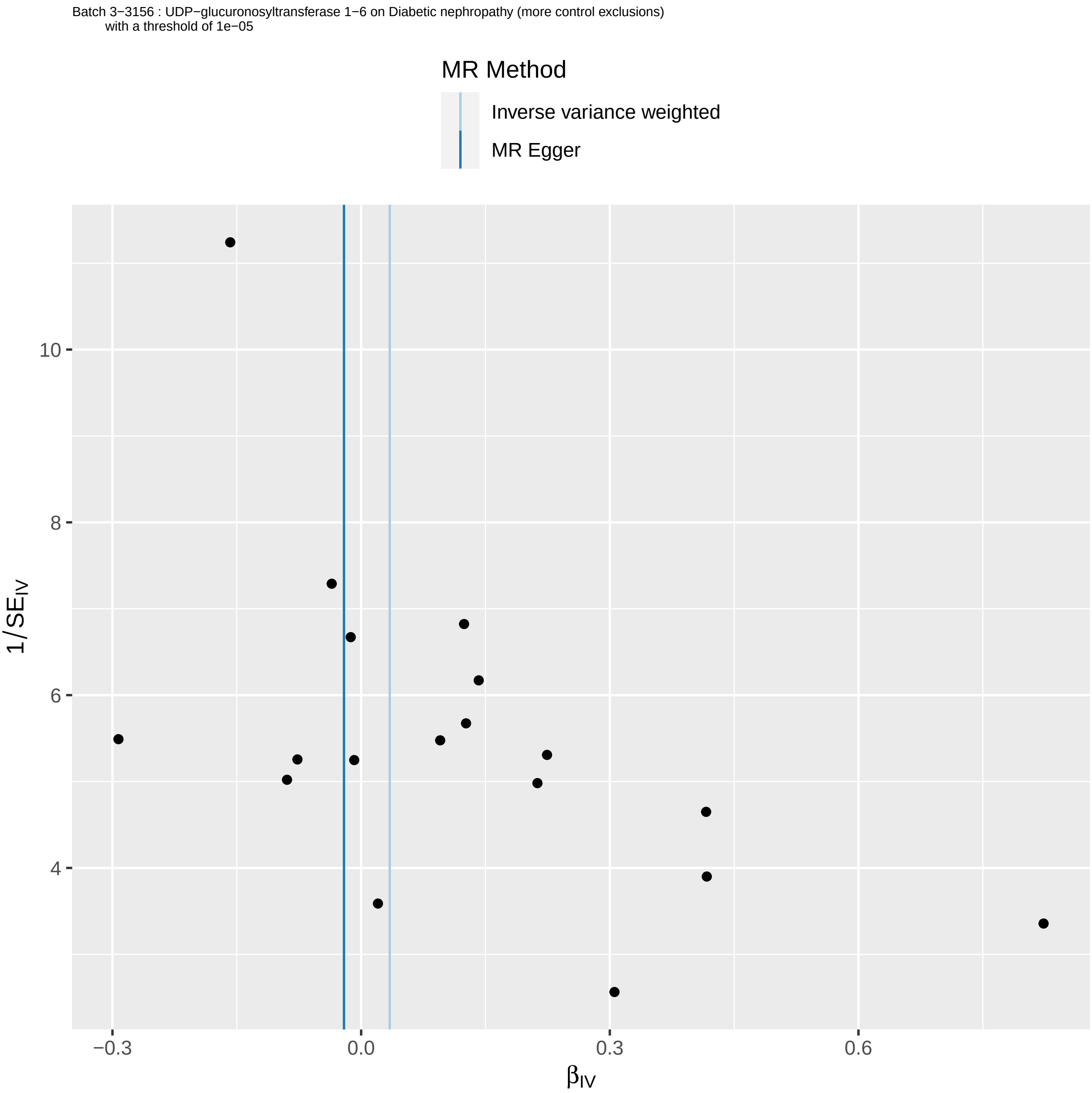

Batch 3−3156 : UDP−glucuronosyltransferase 1−6 on Diabetic nephropathy (more control exclusions)
with a threshold of 1e−05
MR Method
Inverse variance weighted
MR Egger
1/SE_IV
10
8
6
4
−0.3
0.0
0.3
0.6
β_IV

Batch 3−3157 : UDP−glucuronosyltransferase 1−8 on Diabetic nephropathy (more control exclusions)
with a threshold of 1e−05

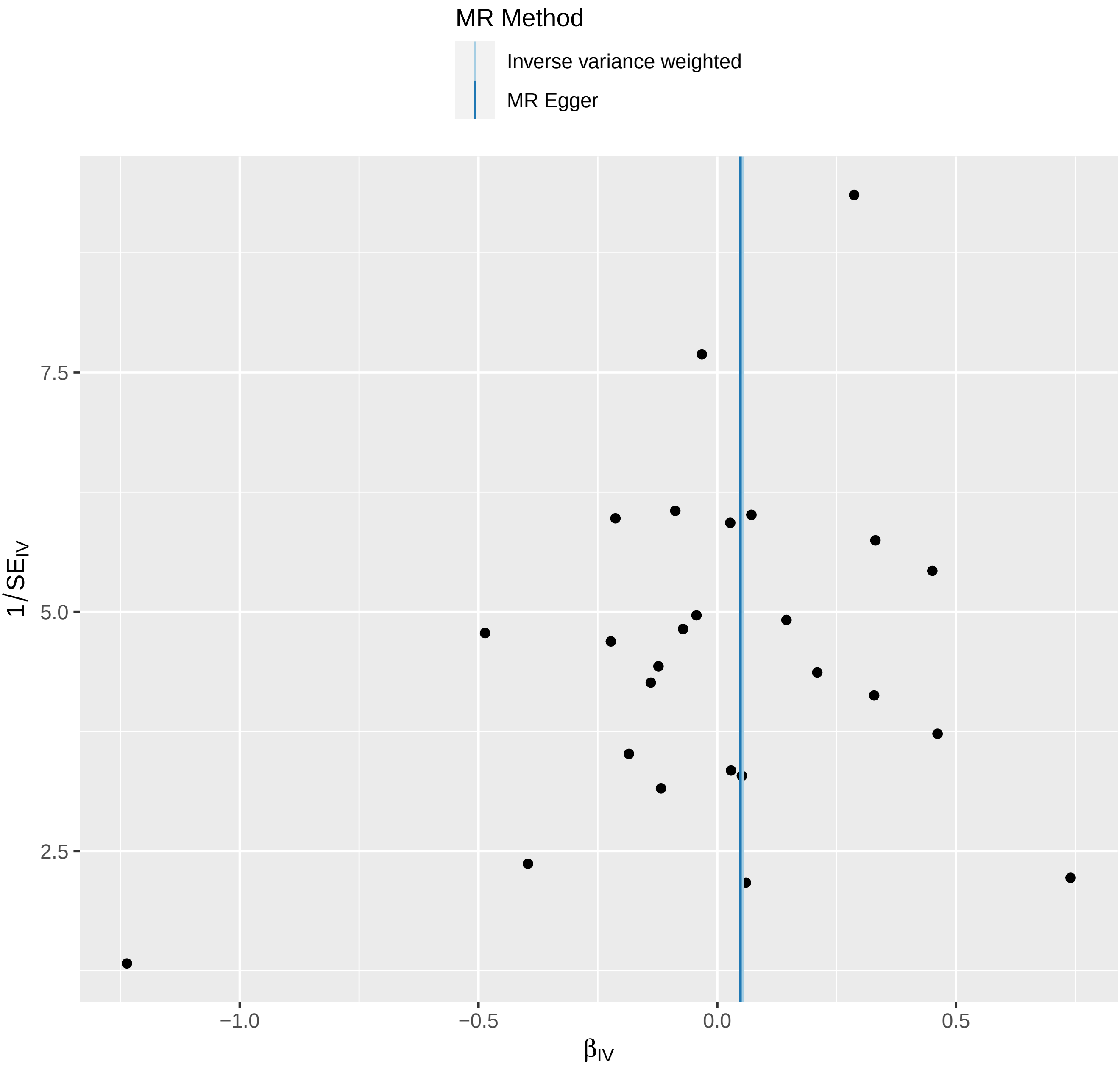

Batch 3−3158 : UDP−glucuronosyltransferase 2A1 on Diabetic nephropathy (more control exclusions)
with a threshold of 1e−05

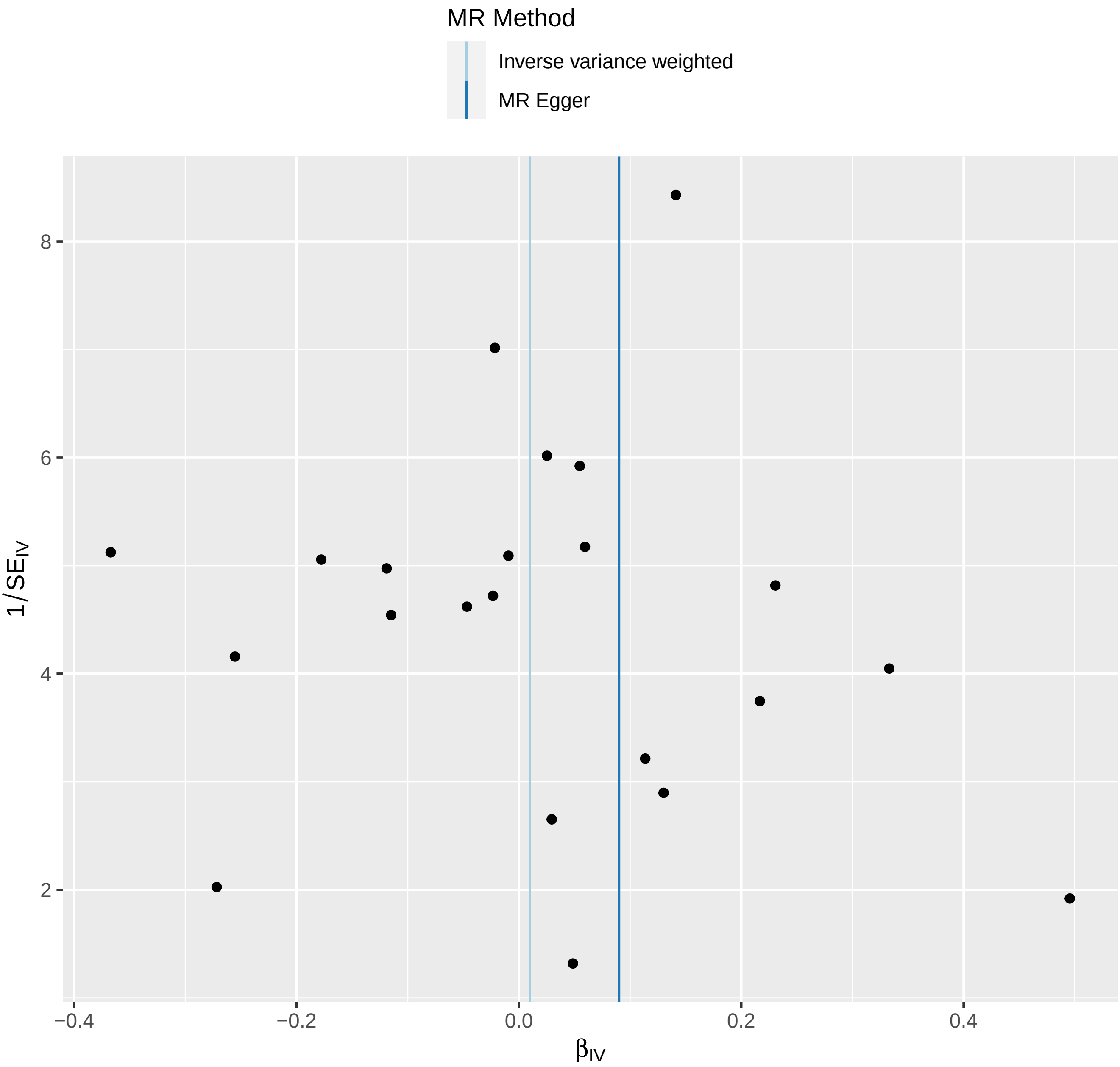

Batch 3−3159 : NKG2D ligand 1 on Diabetic nephropathy (more control exclusions)
with a threshold of 1e−05

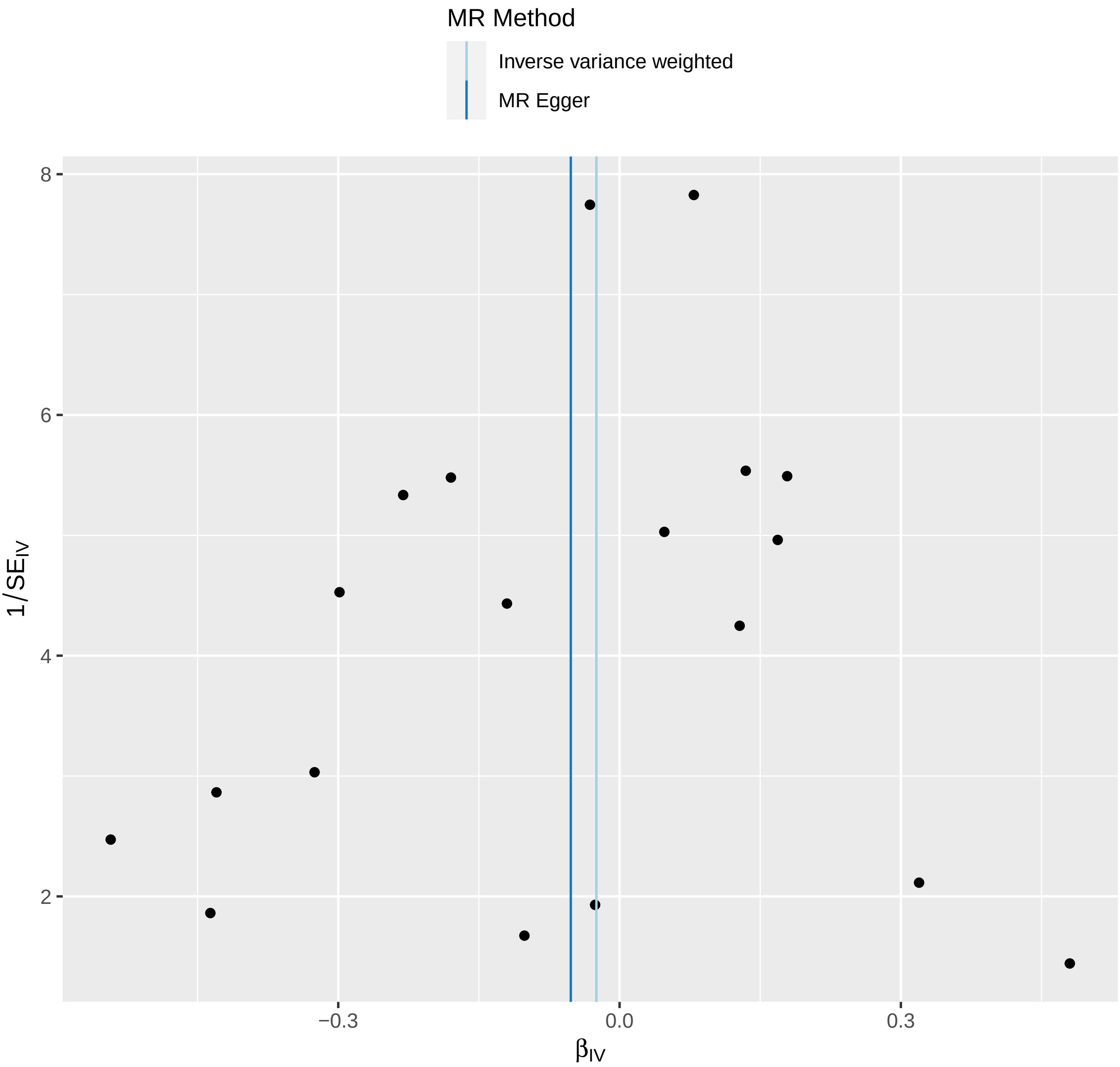

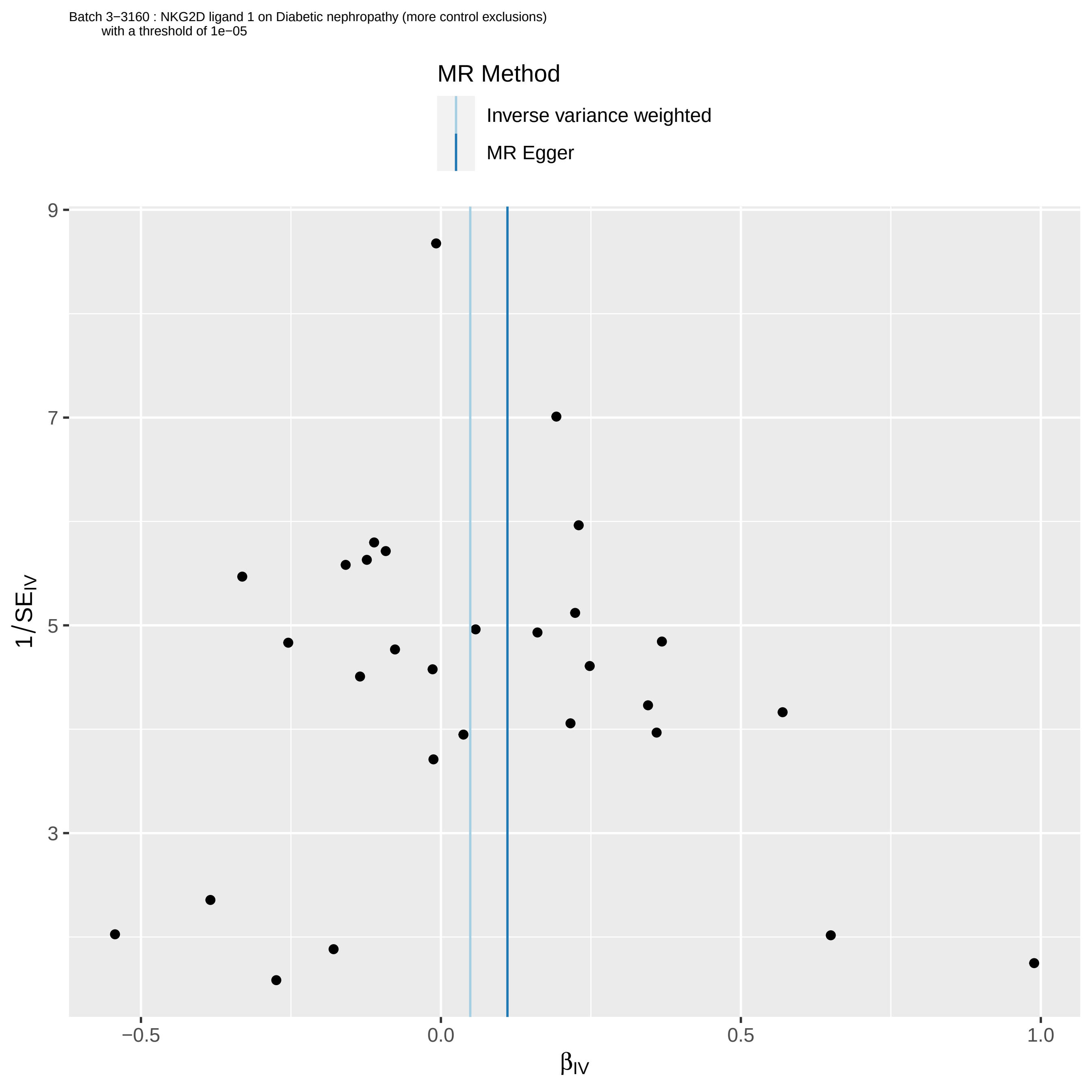
Batch 3−3160 : NKG2D ligand 1 on Diabetic nephropathy (more control exclusions)
with a threshold of 1e−05
MR Method
Inverse variance weighted
MR Egger
9
7
5
3
1/SE_IV
−0.5
0.0
0.5
1.0
β_IV

Batch 3−3161 : NKG2D ligand 2 on Diabetic nephropathy (more control exclusions)
with a threshold of 1e−05

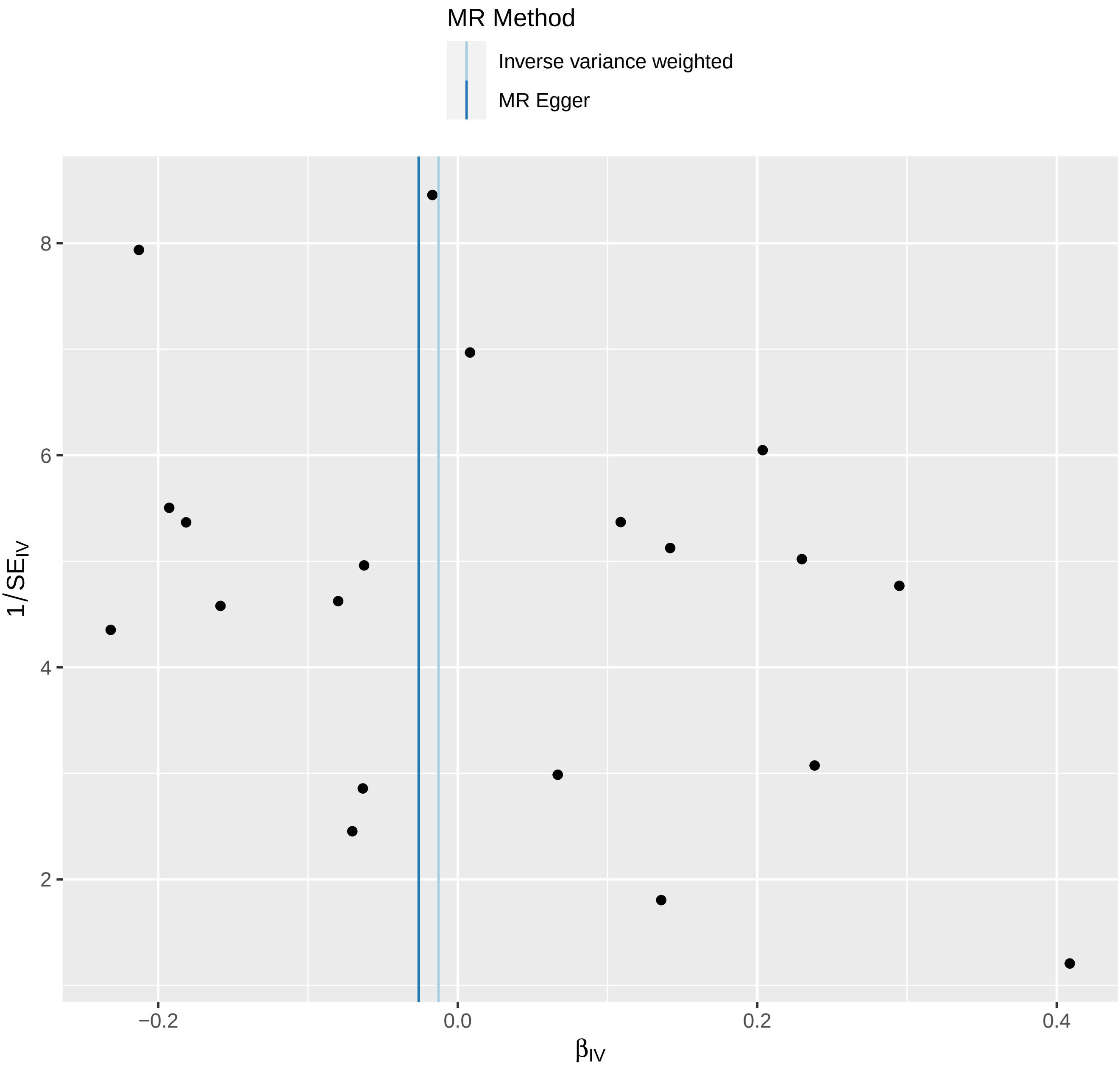

Batch 3−3162 : NKG2D ligand 3 on Diabetic nephropathy (more control exclusions)
with a threshold of 1e−05

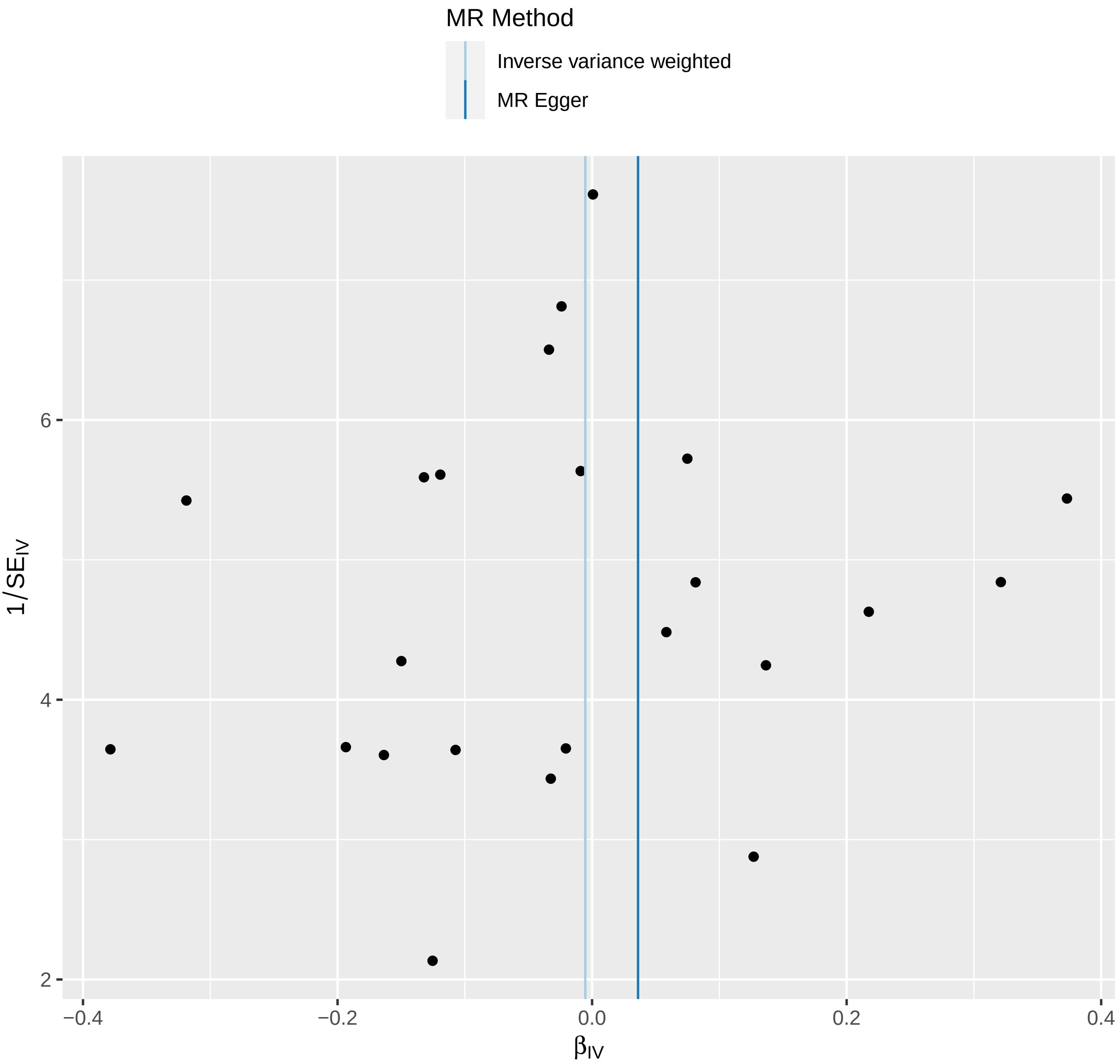

Batch 3−3163 : Serine/threonine−protein kinase ULK3 on Diabetic nephropathy (more control exclusions) with a threshold of 1e−05

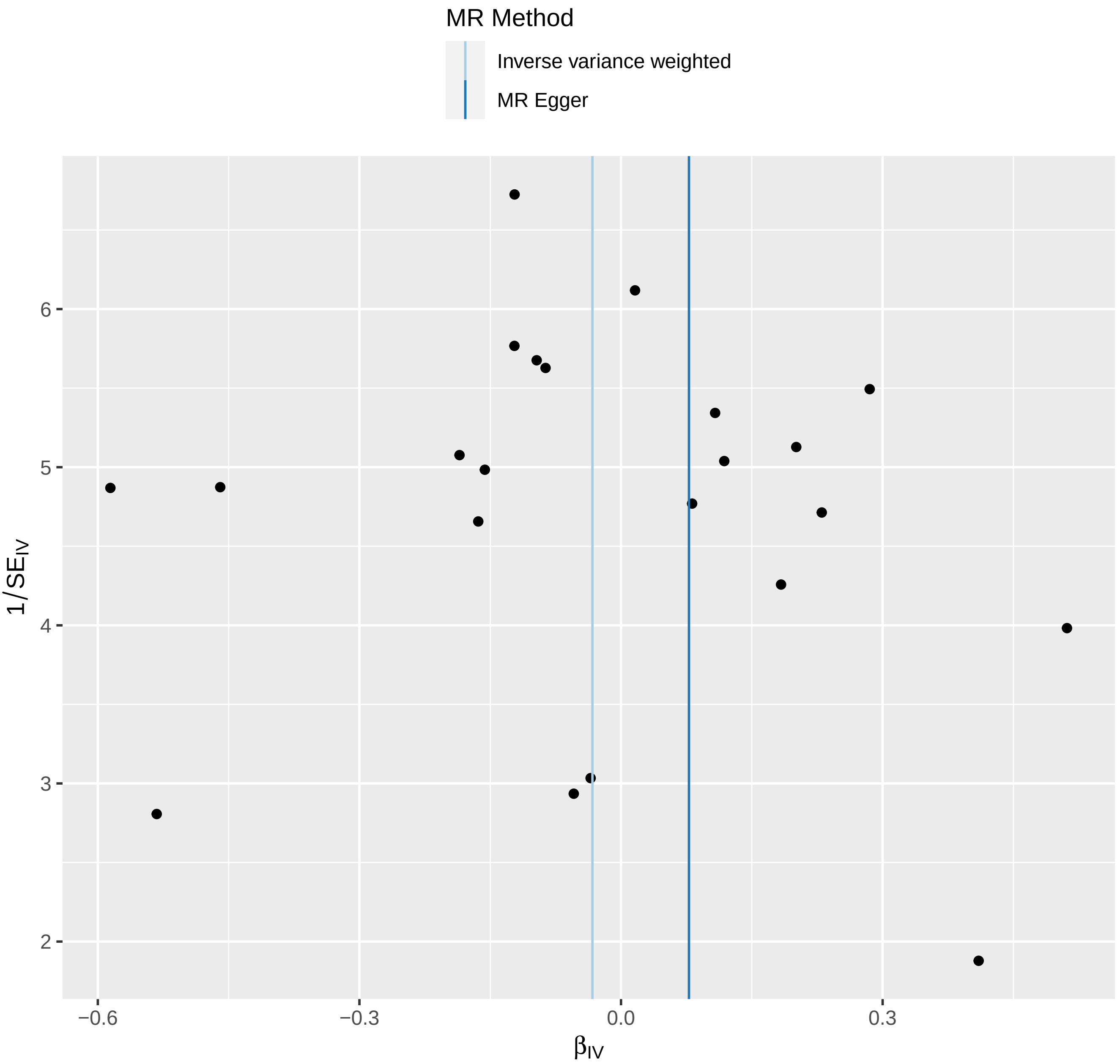

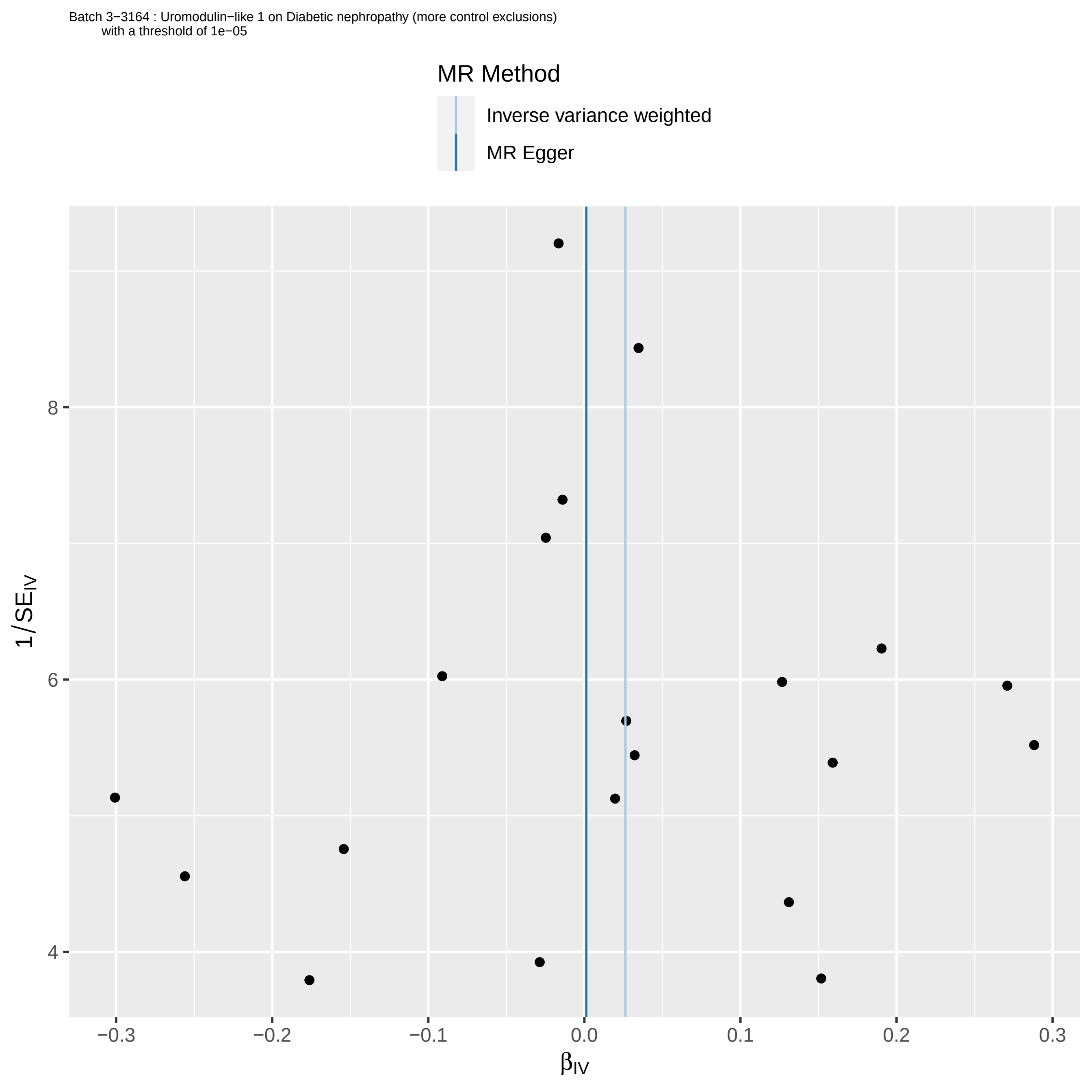

Batch 3−3164 : Uromodulin−like 1 on Diabetic nephropathy (more control exclusions)
with a threshold of 1e−05
MR Method
Inverse variance weighted
MR Egger
8
6
4
1/SE_IV
−0.3
−0.2
−0.1
0.0
0.1
0.2
0.3
β_IV

Batch 3−3165 : Protein unc−13 homolog A on Diabetic nephropathy (more control exclusions)
with a threshold of 1e−05

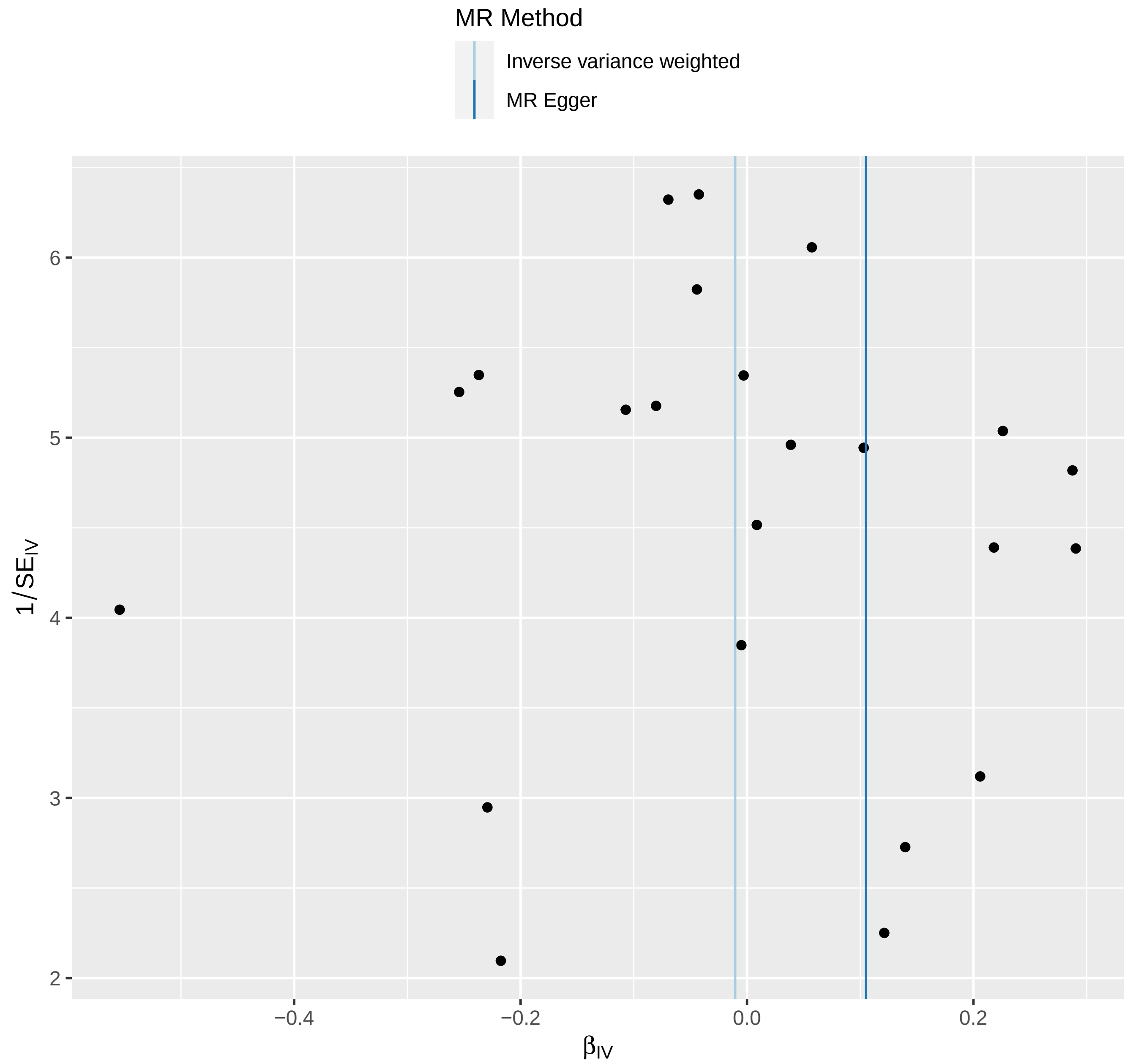

Batch 3−3166 : Netrin receptor UNC5A on Diabetic nephropathy (more control exclusions)
with a threshold of 1e−05

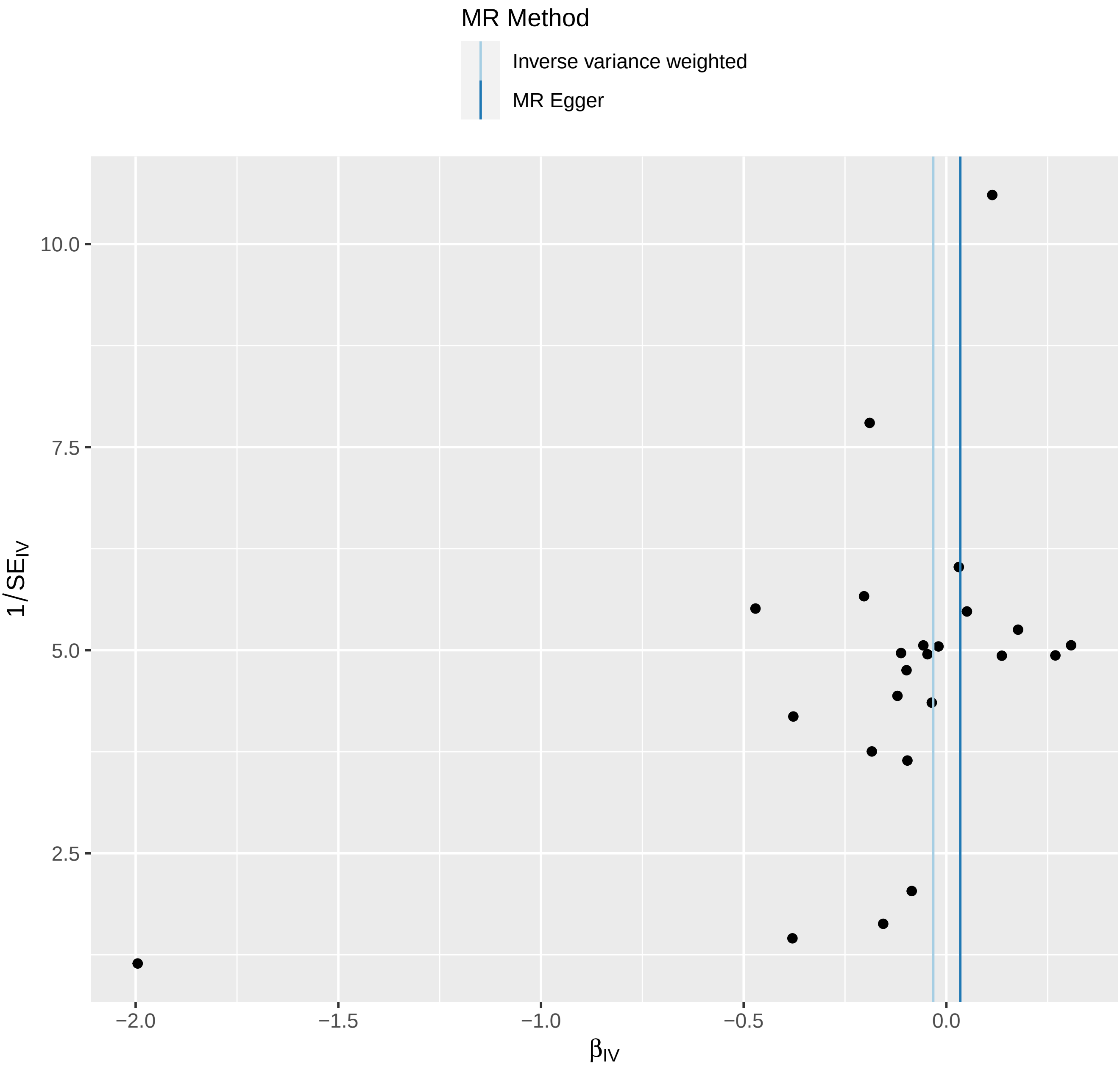

Batch 3−3167 : Netrin receptor UNC5B on Diabetic nephropathy (more control exclusions)
with a threshold of 1e−05

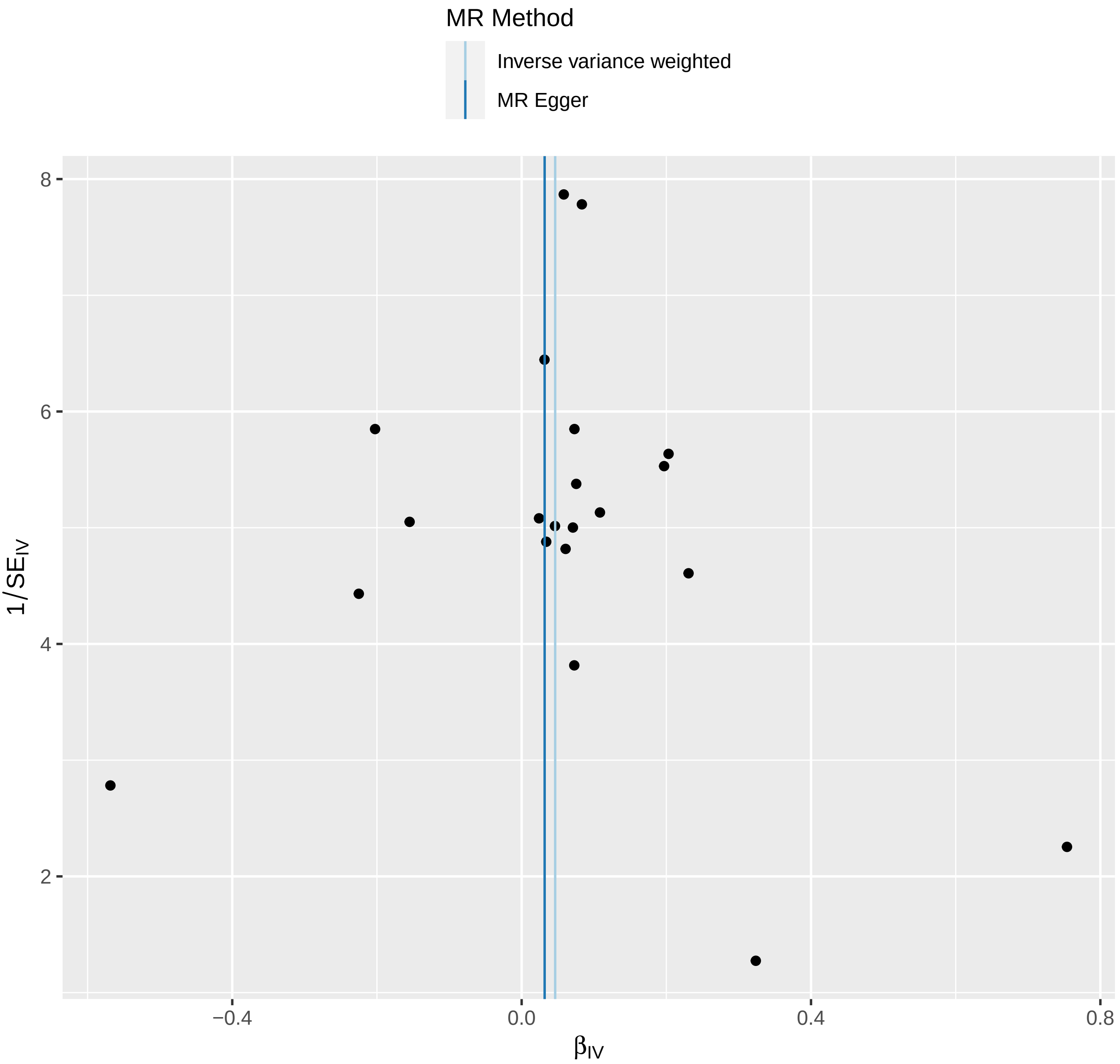

Batch 3−3168 : Netrin receptor UNC5C on Diabetic nephropathy (more control exclusions)
with a threshold of 1e−05

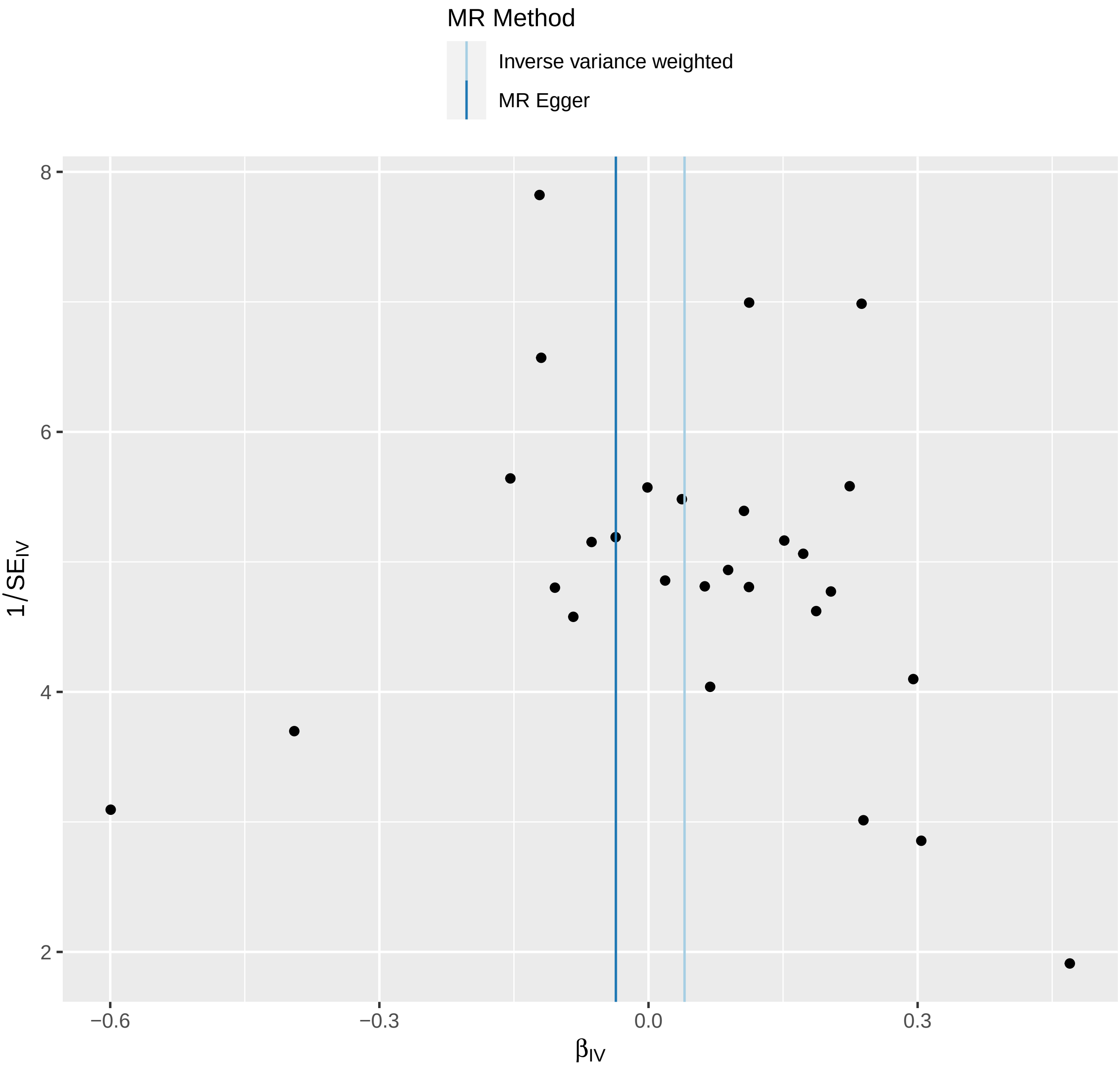

Batch 3−3169 : Netrin receptor UNC5D on Diabetic nephropathy (more control exclusions)
with a threshold of 1e−05

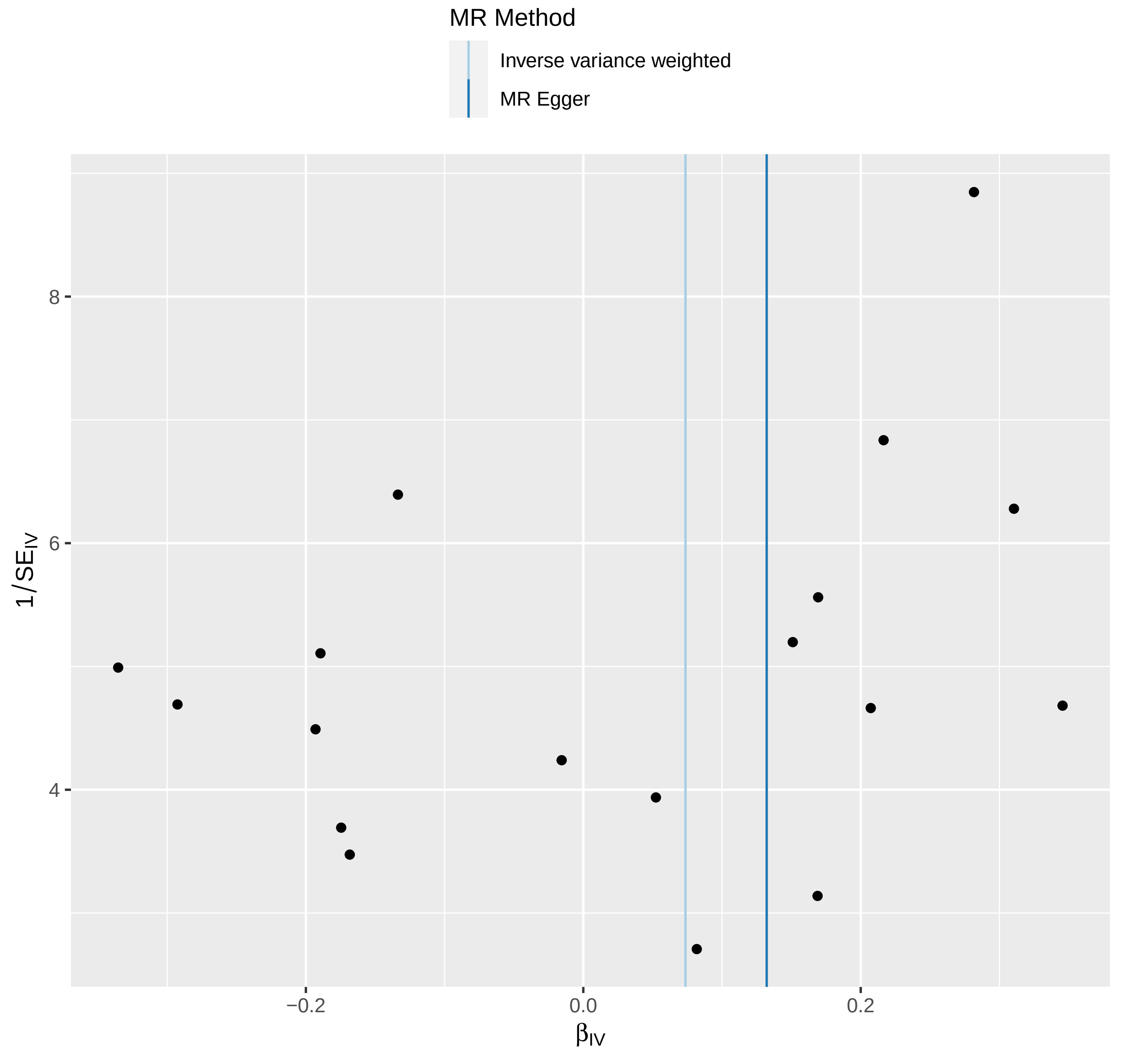

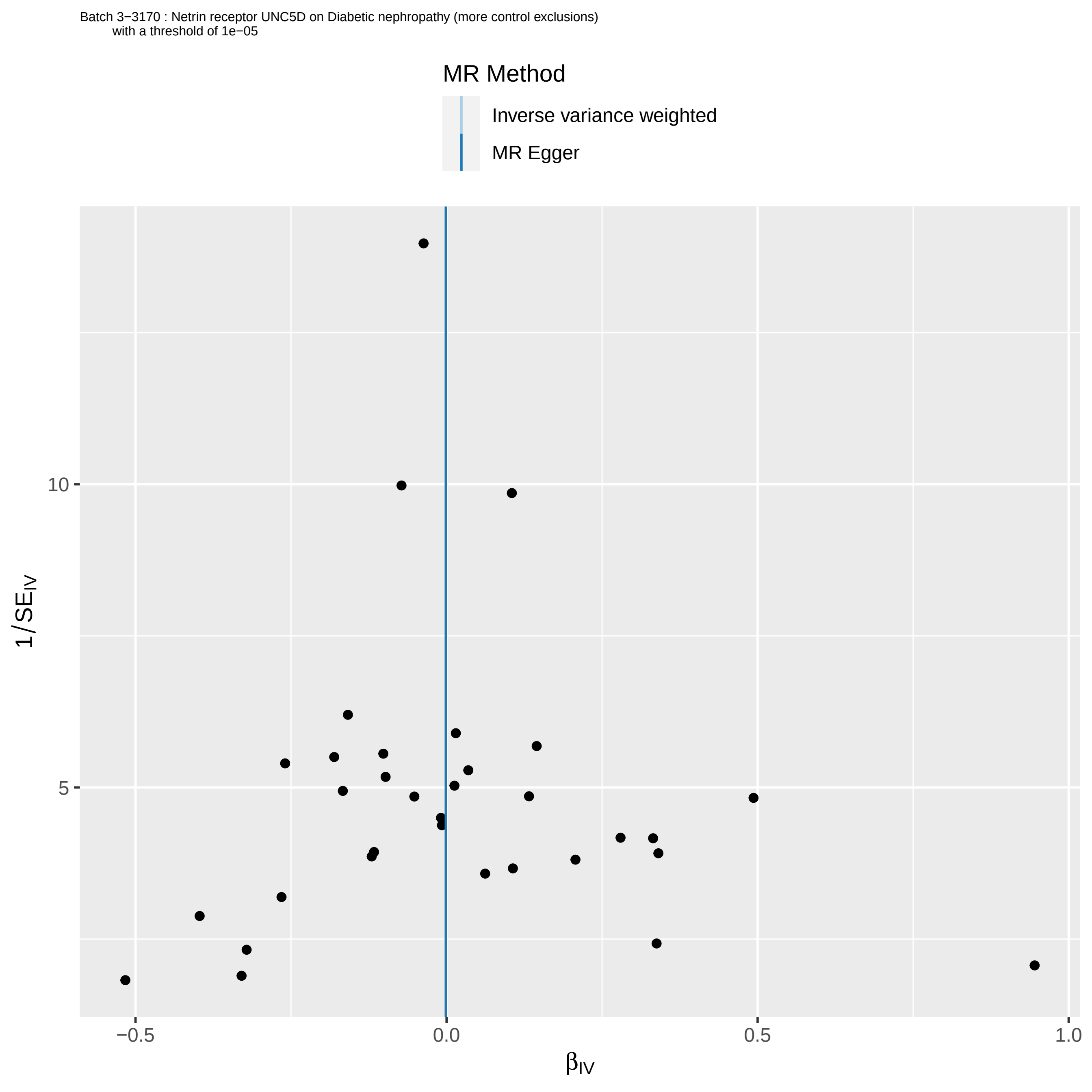
Batch 3−3170 : Netrin receptor UNC5D on Diabetic nephropathy (more control exclusions)
with a threshold of 1e−05
MR Method
Inverse variance weighted
MR Egger
1/SE_IV
10
5
−0.5
0.0
0.5
1.0
β_IV

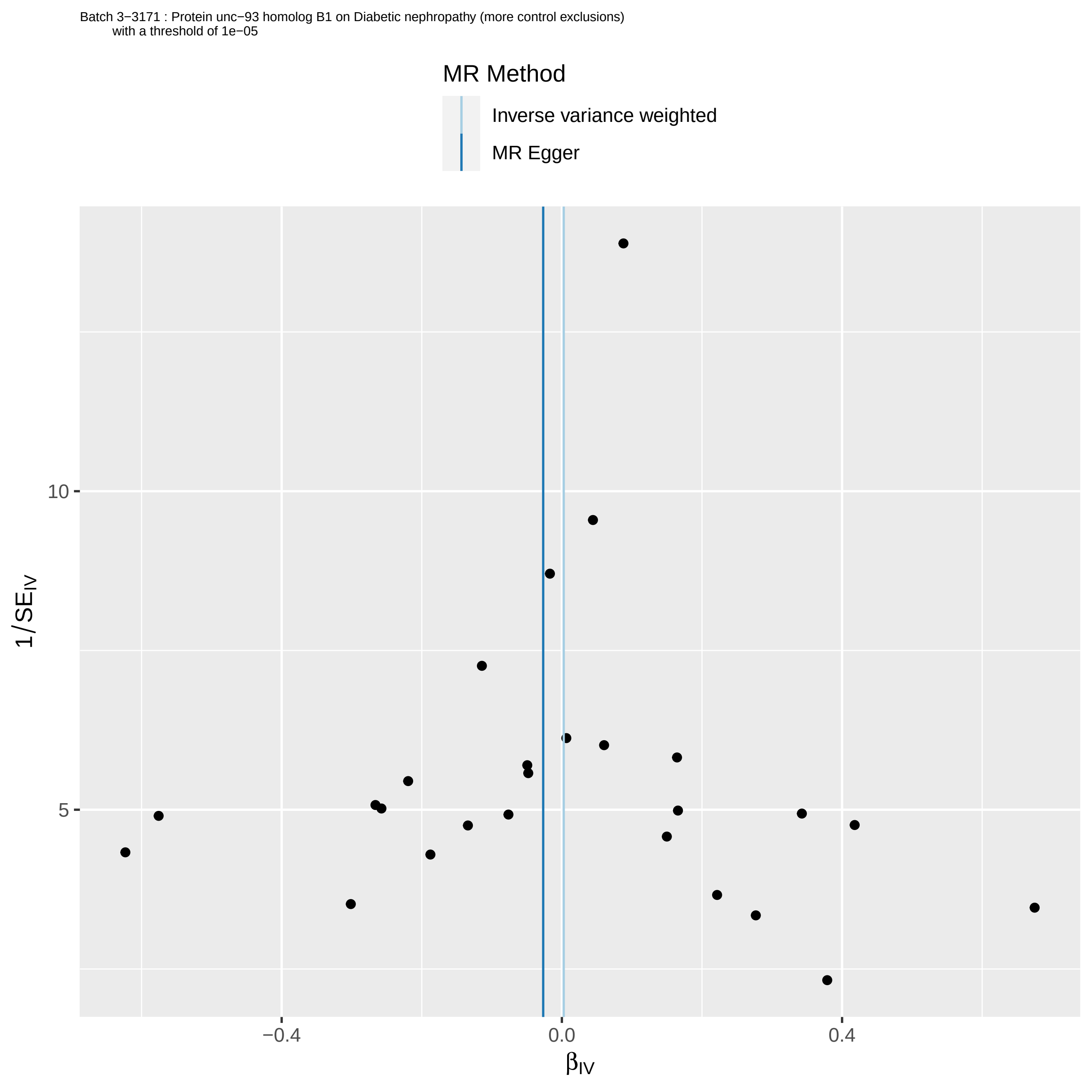

Batch 3−3171 : Protein unc−93 homolog B1 on Diabetic nephropathy (more control exclusions)
with a threshold of 1e−05
MR Method
Inverse variance weighted
MR Egger
10
5
1/SE$_{IV}$
−0.4
0.0
0.4
β$_{IV}$

Batch 3−3172 : Uroplakin−3b on Diabetic nephropathy (more control exclusions)
with a threshold of 1e−05

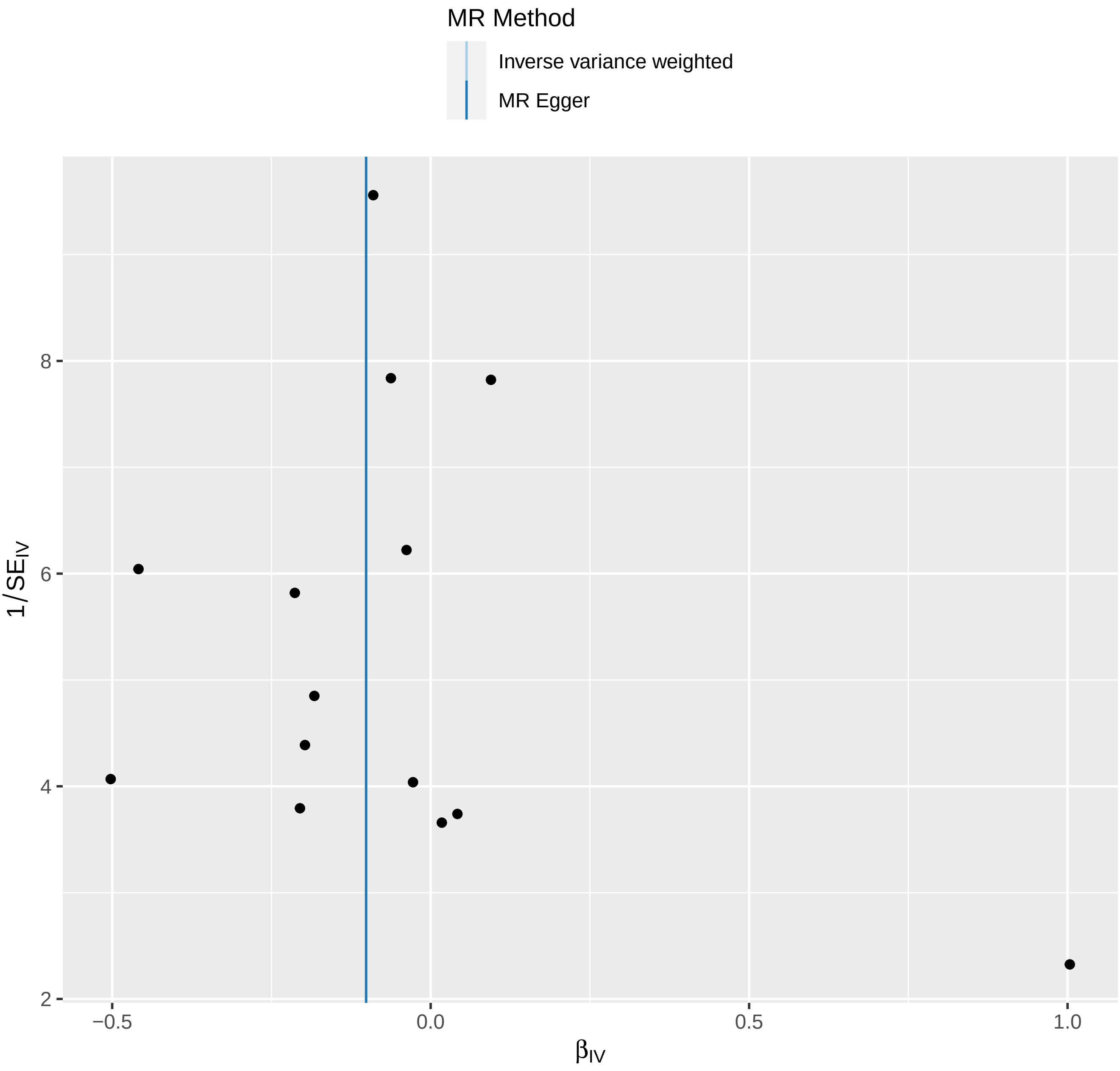

Batch 3−3173 : Uroplakin−3b−like protein on Diabetic nephropathy (more control exclusions)
with a threshold of 1e−05

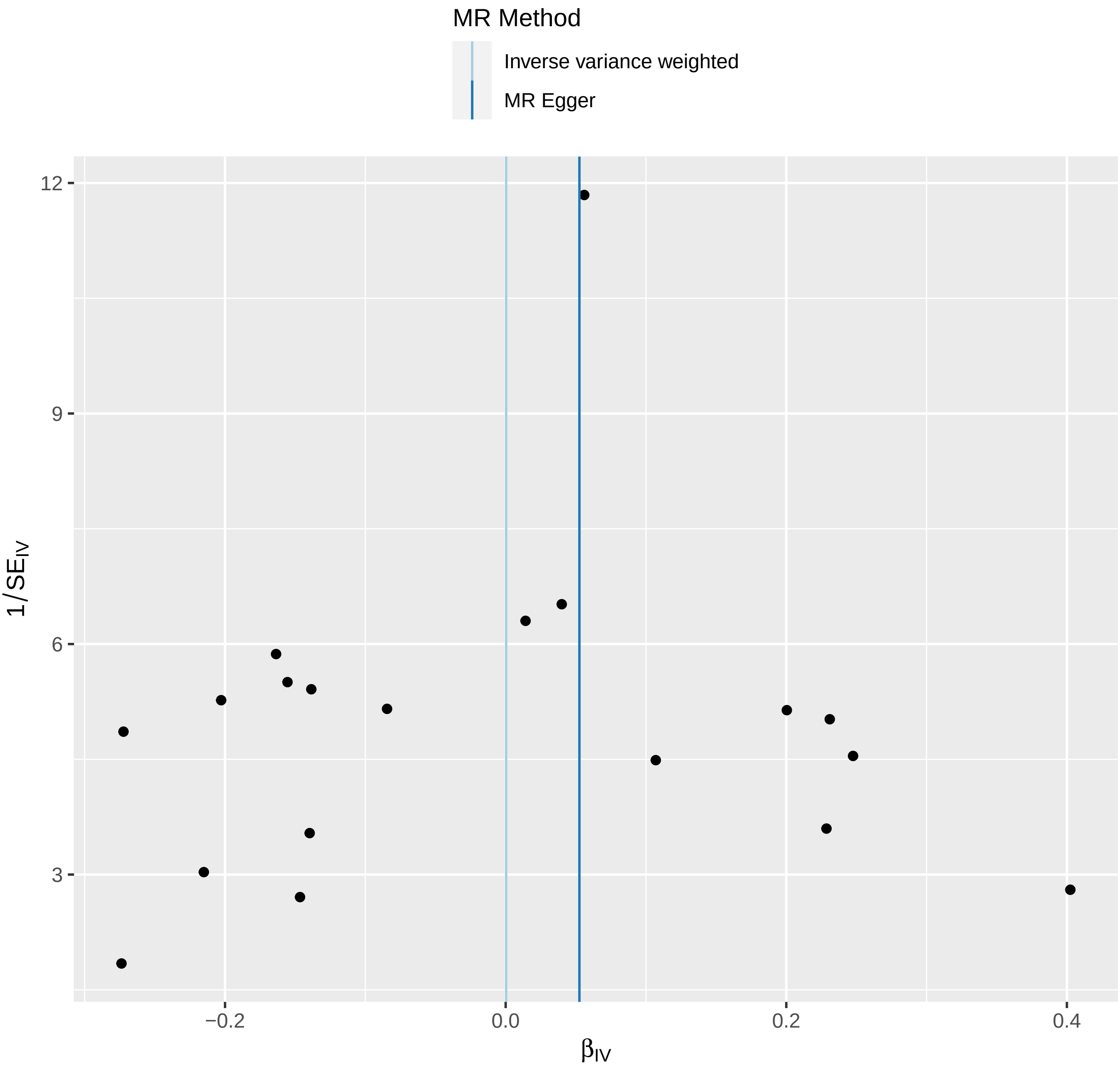

Batch 3−3174 : Ubiquinol−cytochrome−c reductase complex assembly factor 3 on Diabetic nephropathy (more control exclusions) with a threshold of 1e−05

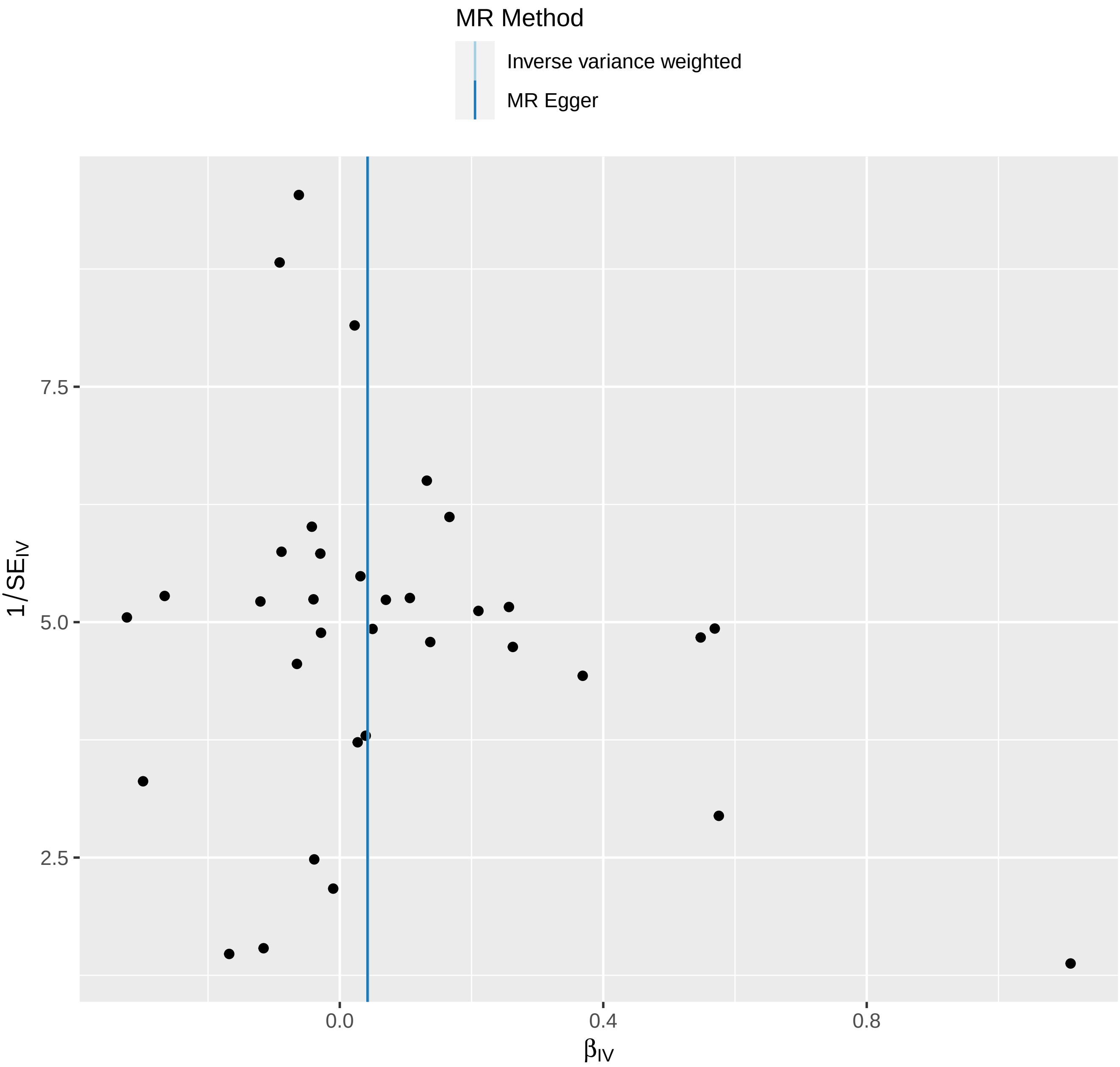

Batch 3−3175 : Cytochrome b−c1 complex subunit 7 on Diabetic nephropathy (more control exclusions)
with a threshold of 1e−05

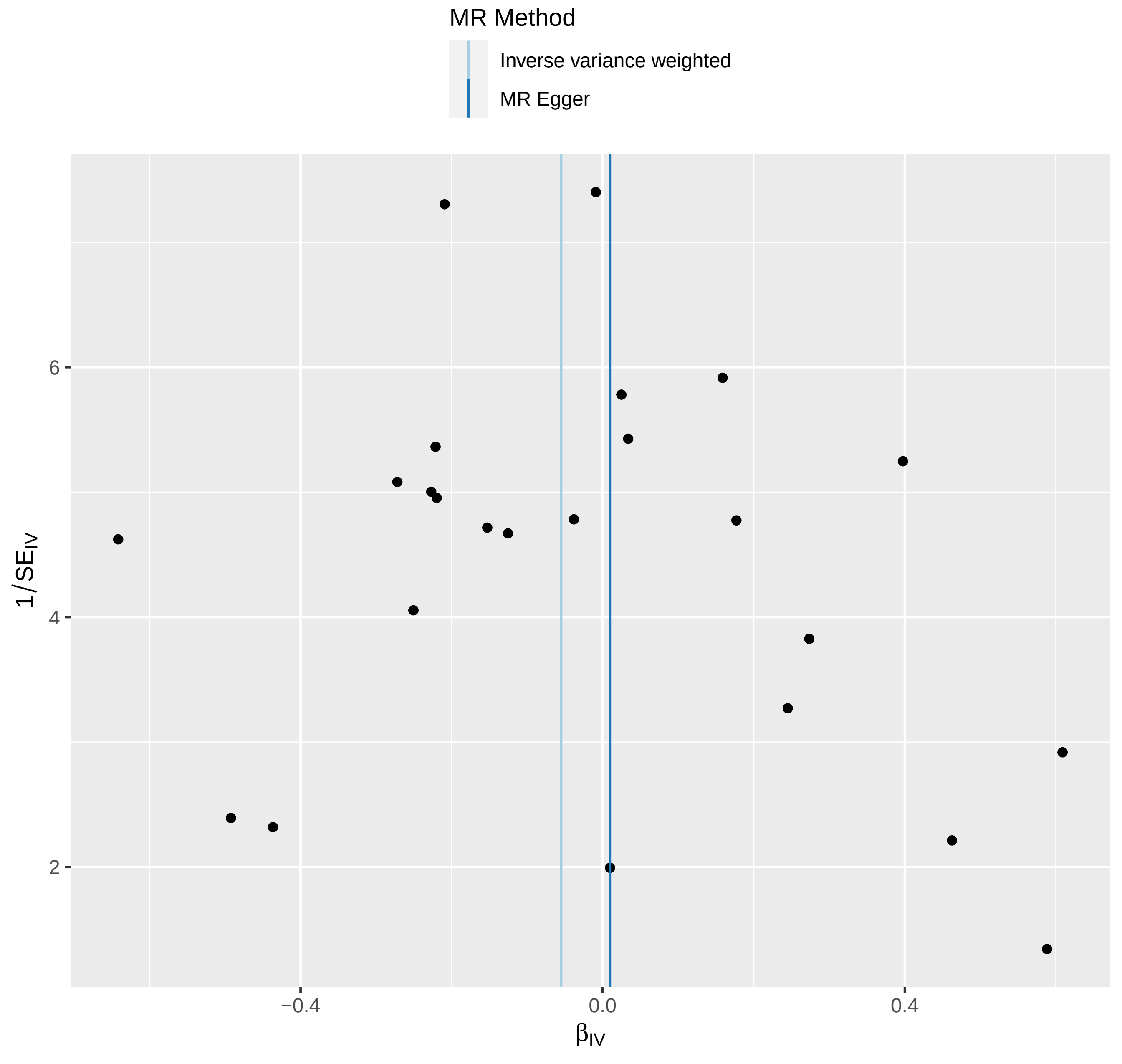

Batch 3−3176 : Uroporphyrinogen−III synthase on Diabetic nephropathy (more control exclusions)
with a threshold of 1e−05

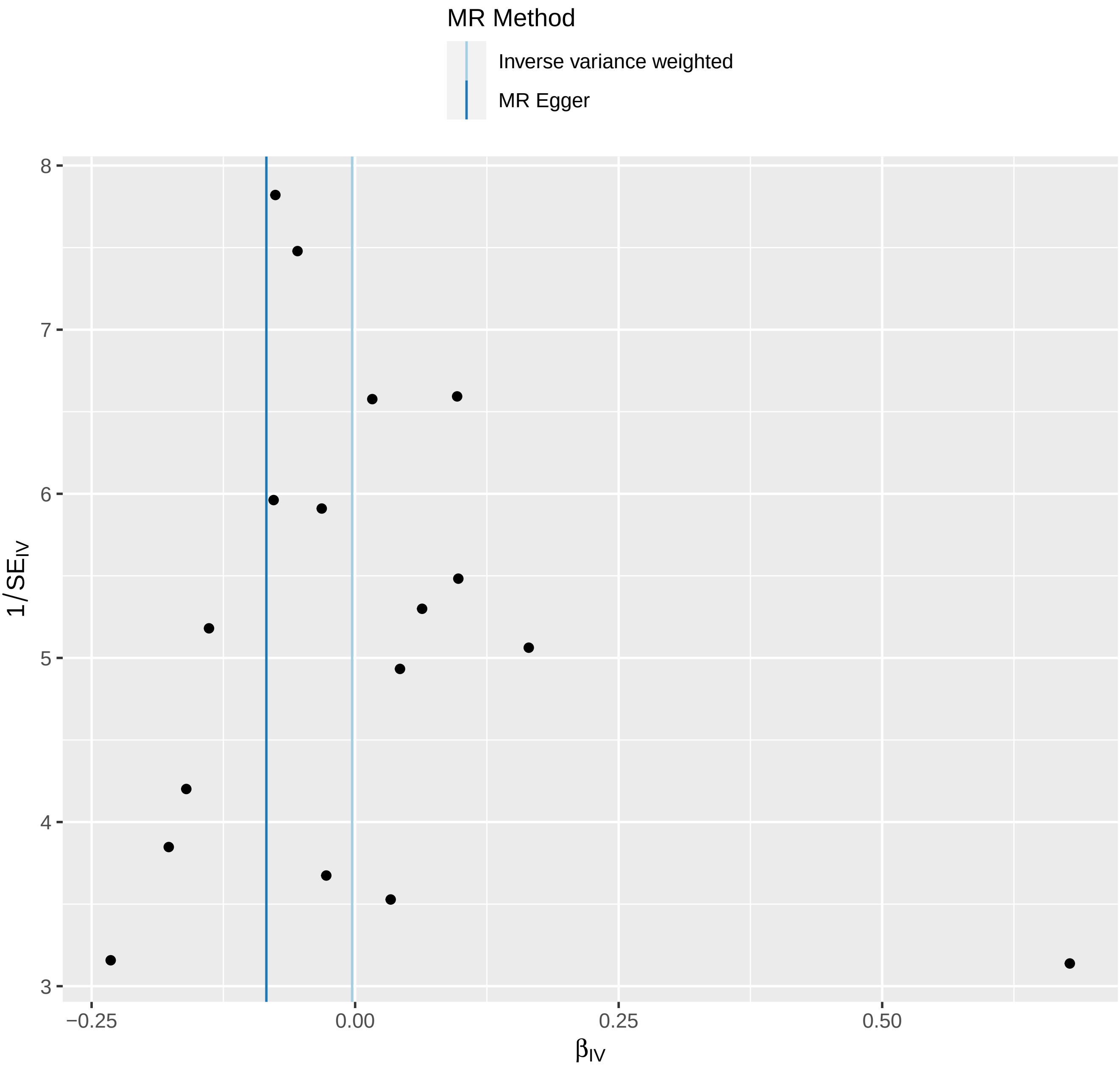

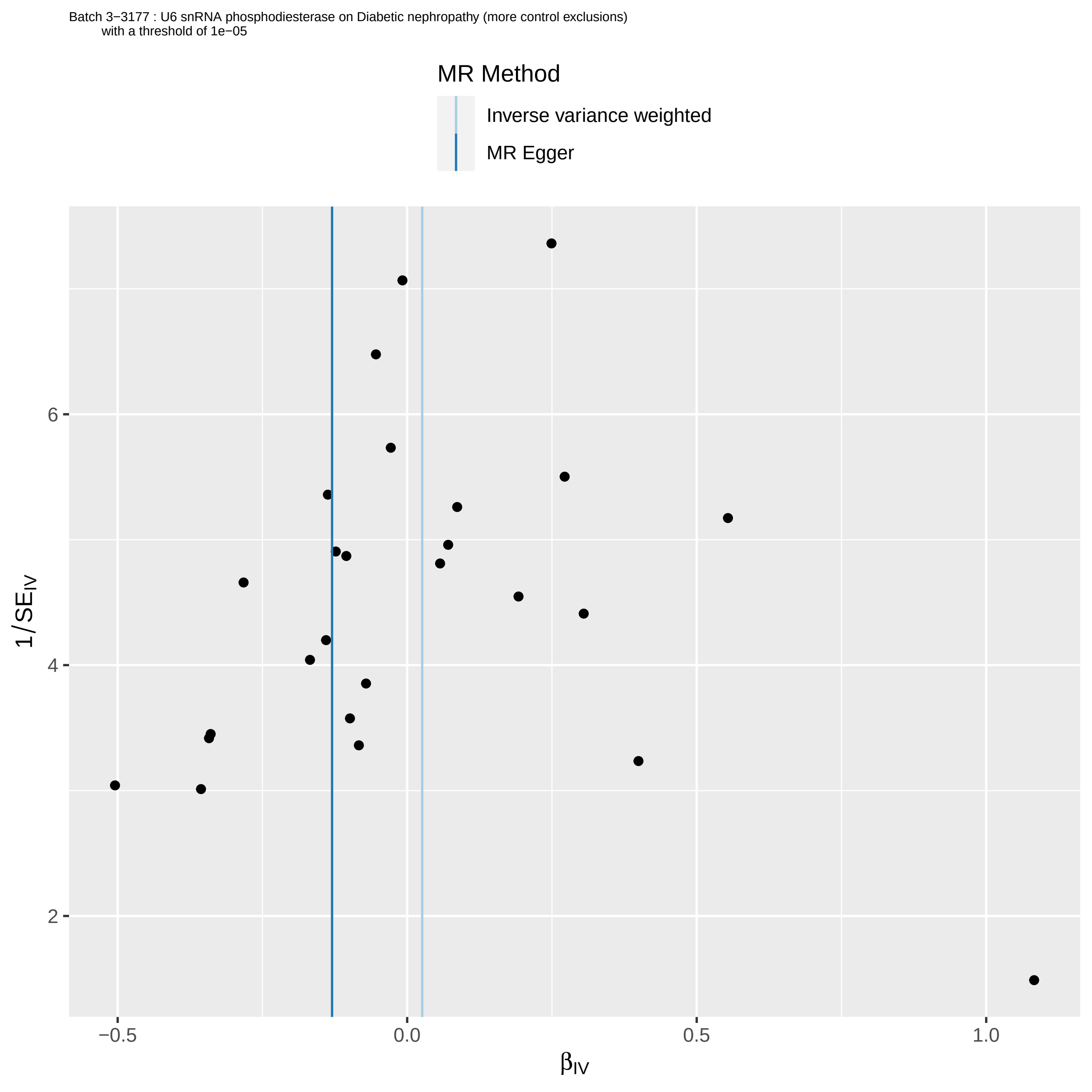
Batch 3−3177 : U6 snRNA phosphodiesterase on Diabetic nephropathy (more control exclusions)
with a threshold of 1e−05
MR Method
Inverse variance weighted
MR Egger
1/SE$_{IV}$
β_{IV}
6
4
2
−0.5
0.0
0.5
1.0

Batch 3−3178 : Vesicle transport protein USE1 on Diabetic nephropathy (more control exclusions)
with a threshold of 1e−05

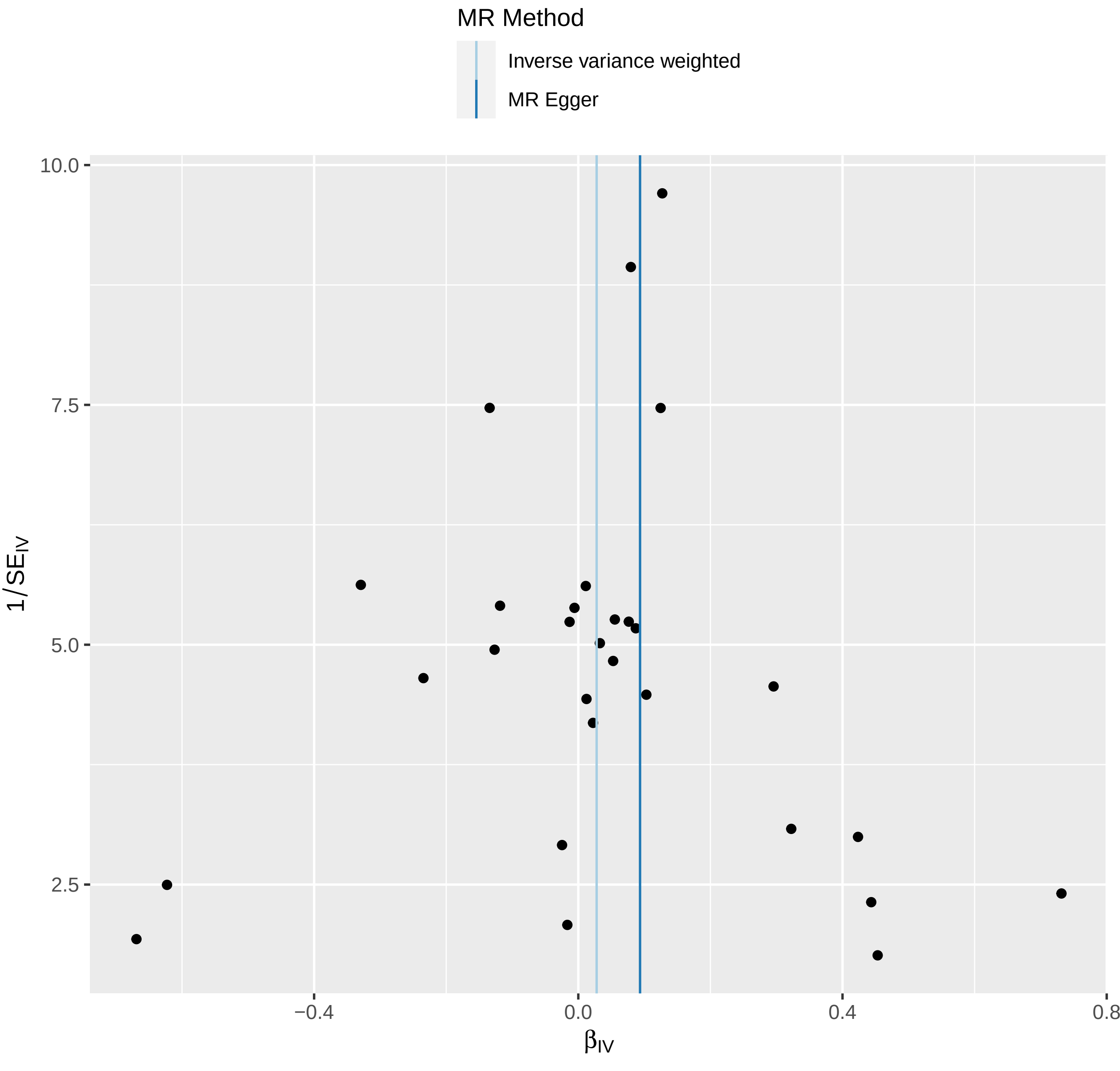

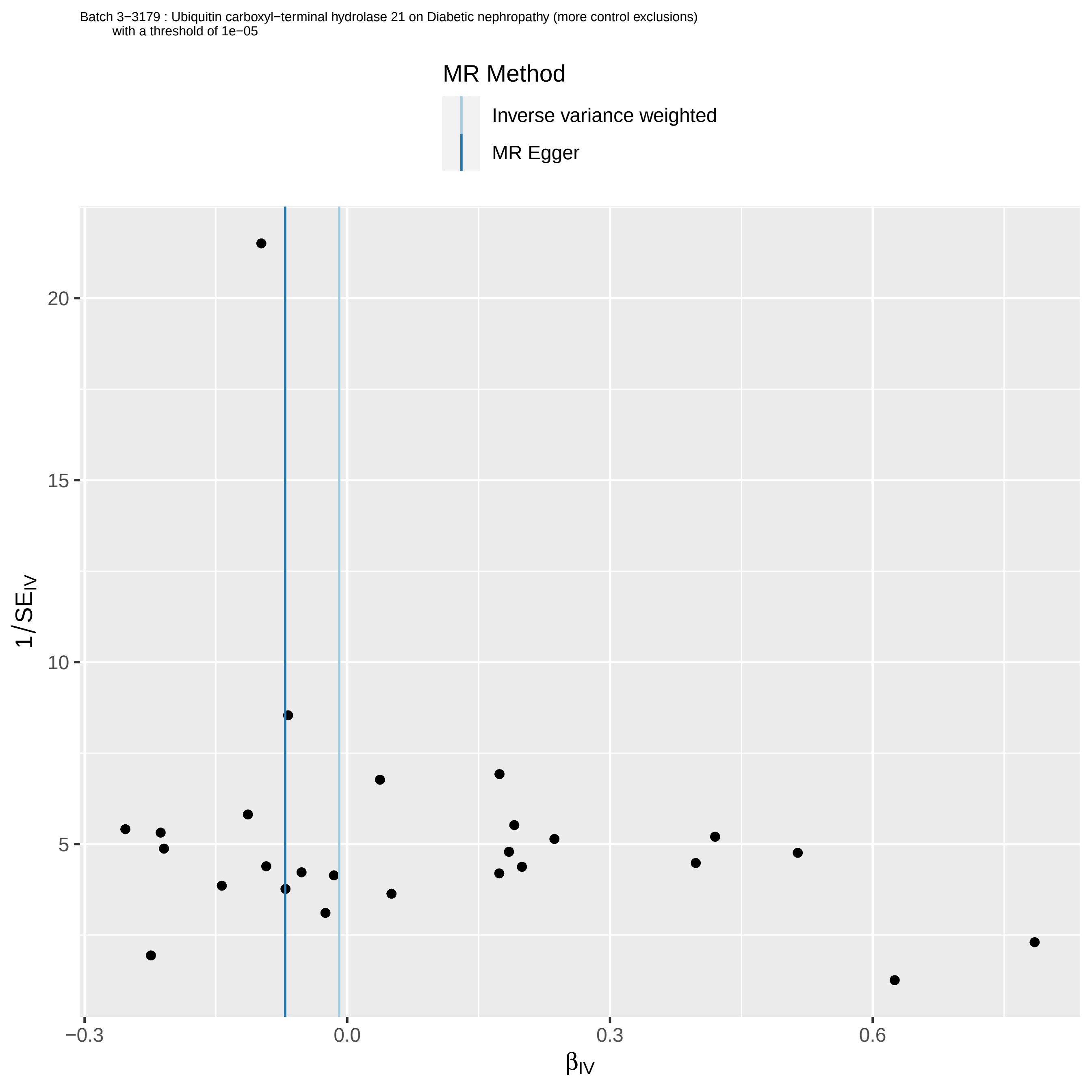
Batch 3−3179 : Ubiquitin carboxyl−terminal hydrolase 21 on Diabetic nephropathy (more control exclusions)
with a threshold of 1e−05
MR Method
Inverse variance weighted
MR Egger
20
15
10
5
1/SE_IV
−0.3
0.0
0.3
0.6
β_IV

Batch 3−3180 : Ubiquitin carboxyl−terminal hydrolase 25 on Diabetic nephropathy (more control exclusions)
with a threshold of 1e−05

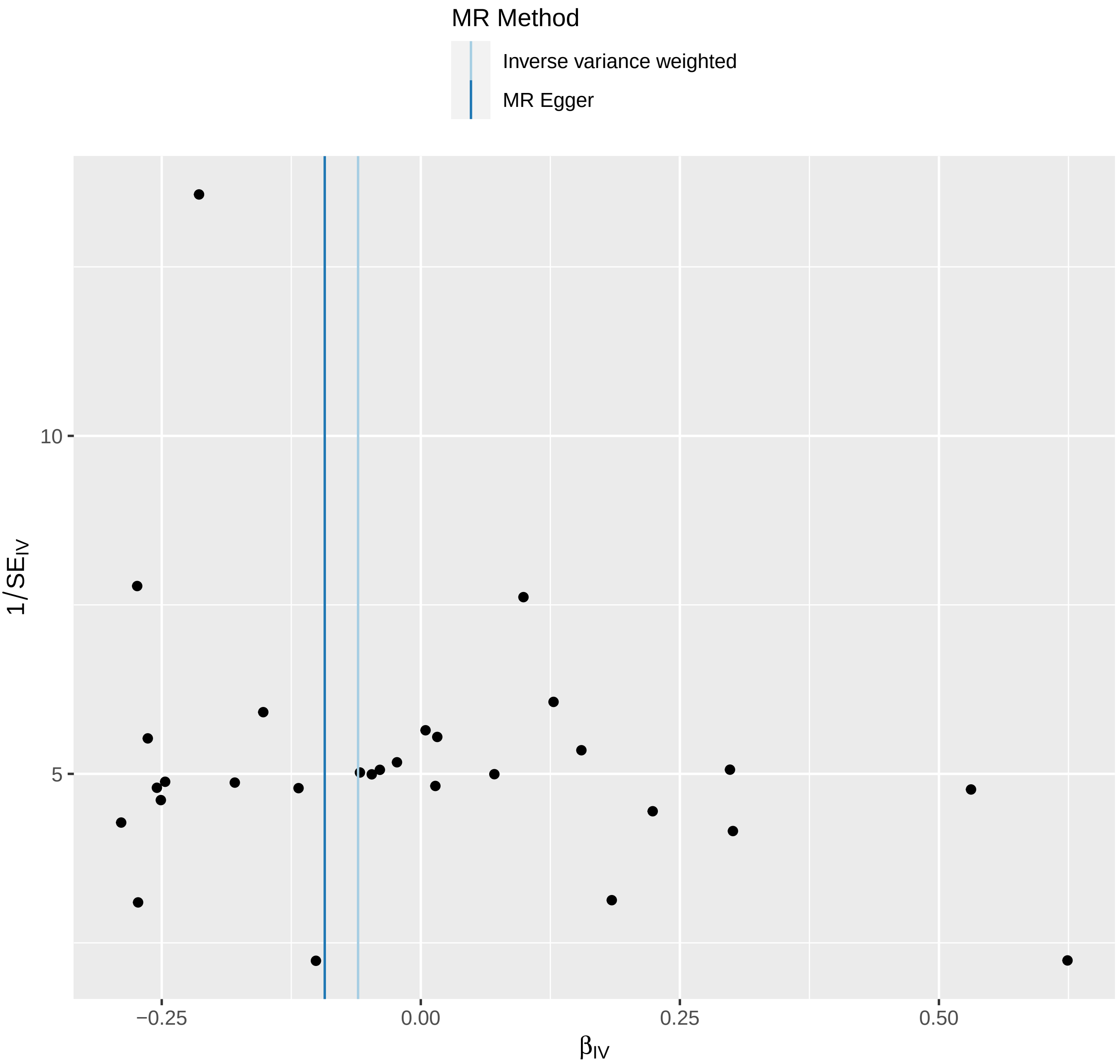

Batch 3−3181 : Ubiquitin carboxyl−terminal hydrolase 8 on Diabetic nephropathy (more control exclusions)
with a threshold of 1e−05

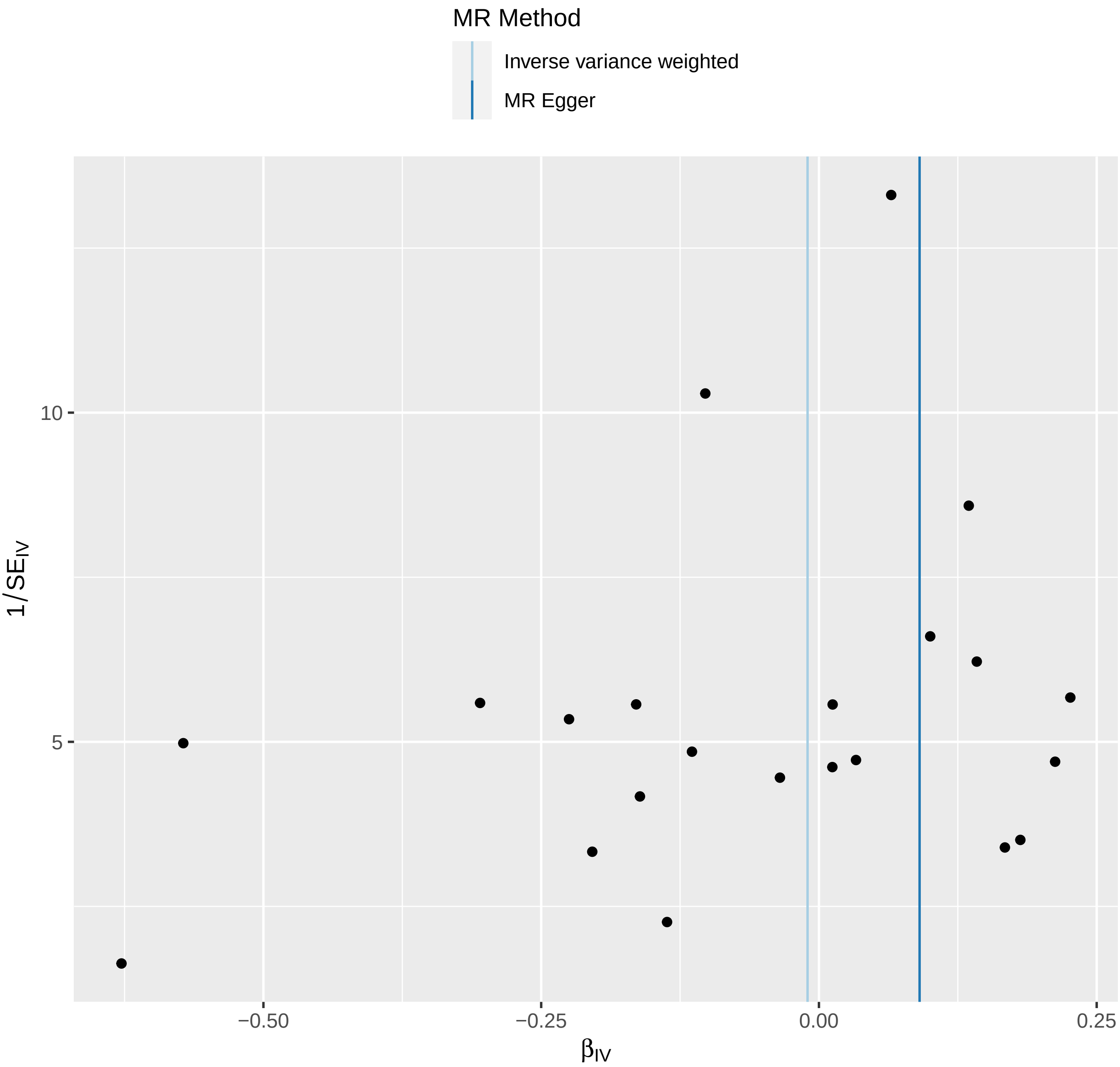

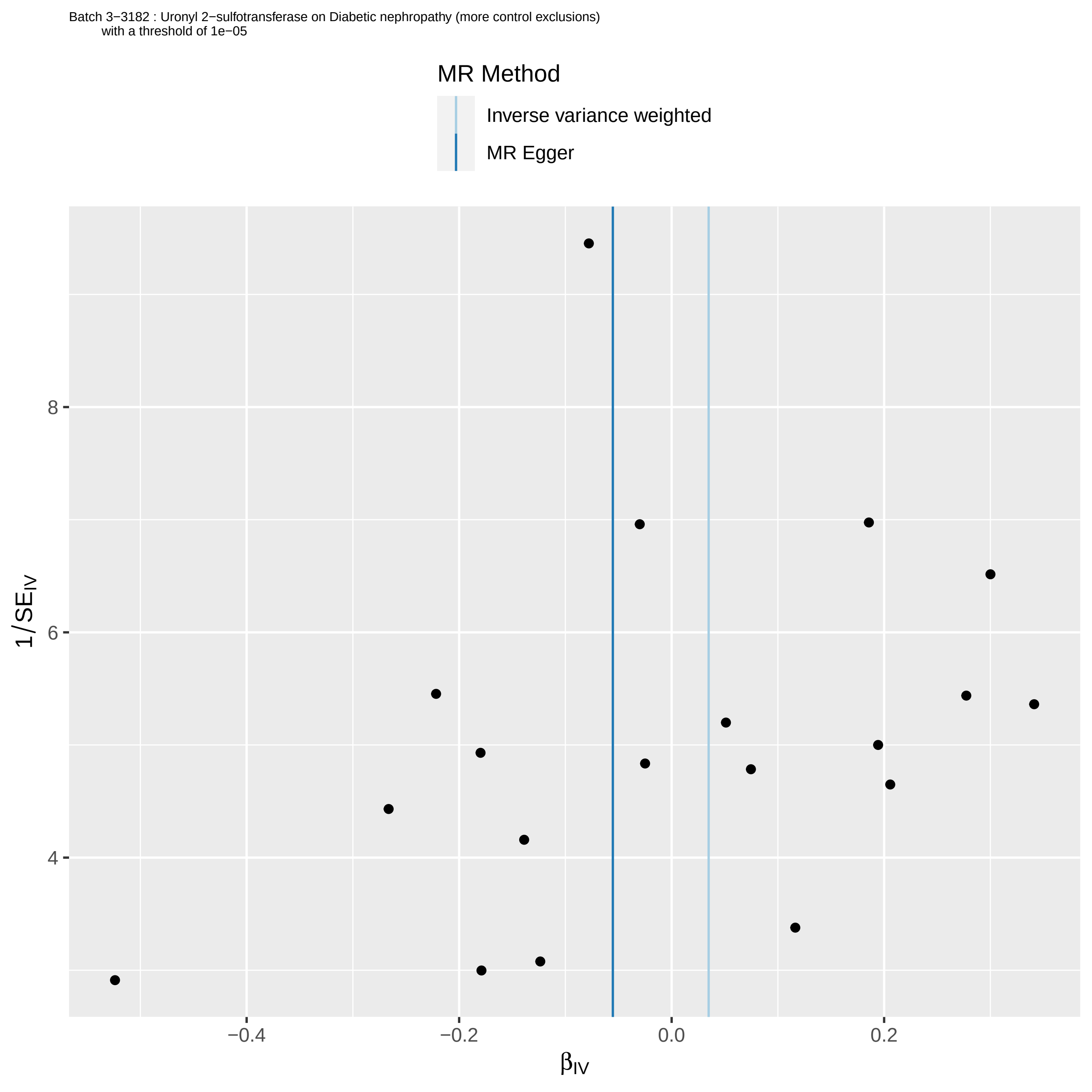
Batch 3−3182 : Uronyl 2−sulfotransferase on Diabetic nephropathy (more control exclusions)
with a threshold of 1e−05
MR Method
Inverse variance weighted
MR Egger
8
6
4
1/SE$_{IV}$
−0.4
−0.2
0.0
0.2
β_{IV}

Batch 3−3183 : Urotensin−2 receptor on Diabetic nephropathy (more control exclusions)
with a threshold of 1e−05

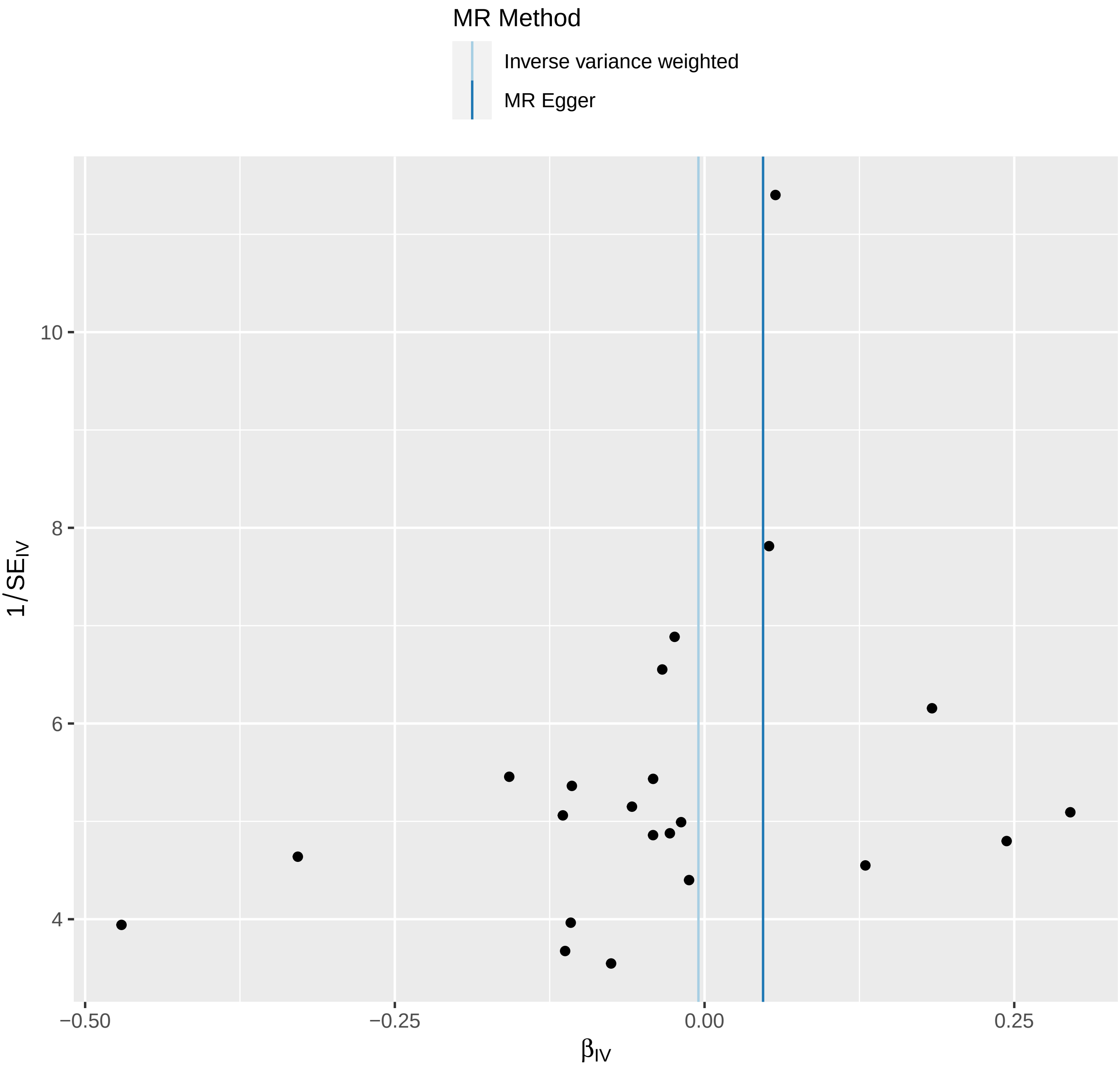

Batch 3−3184 : UDP−glucuronic acid decarboxylase 1 on Diabetic nephropathy (more control exclusions)
with a threshold of 1e−05

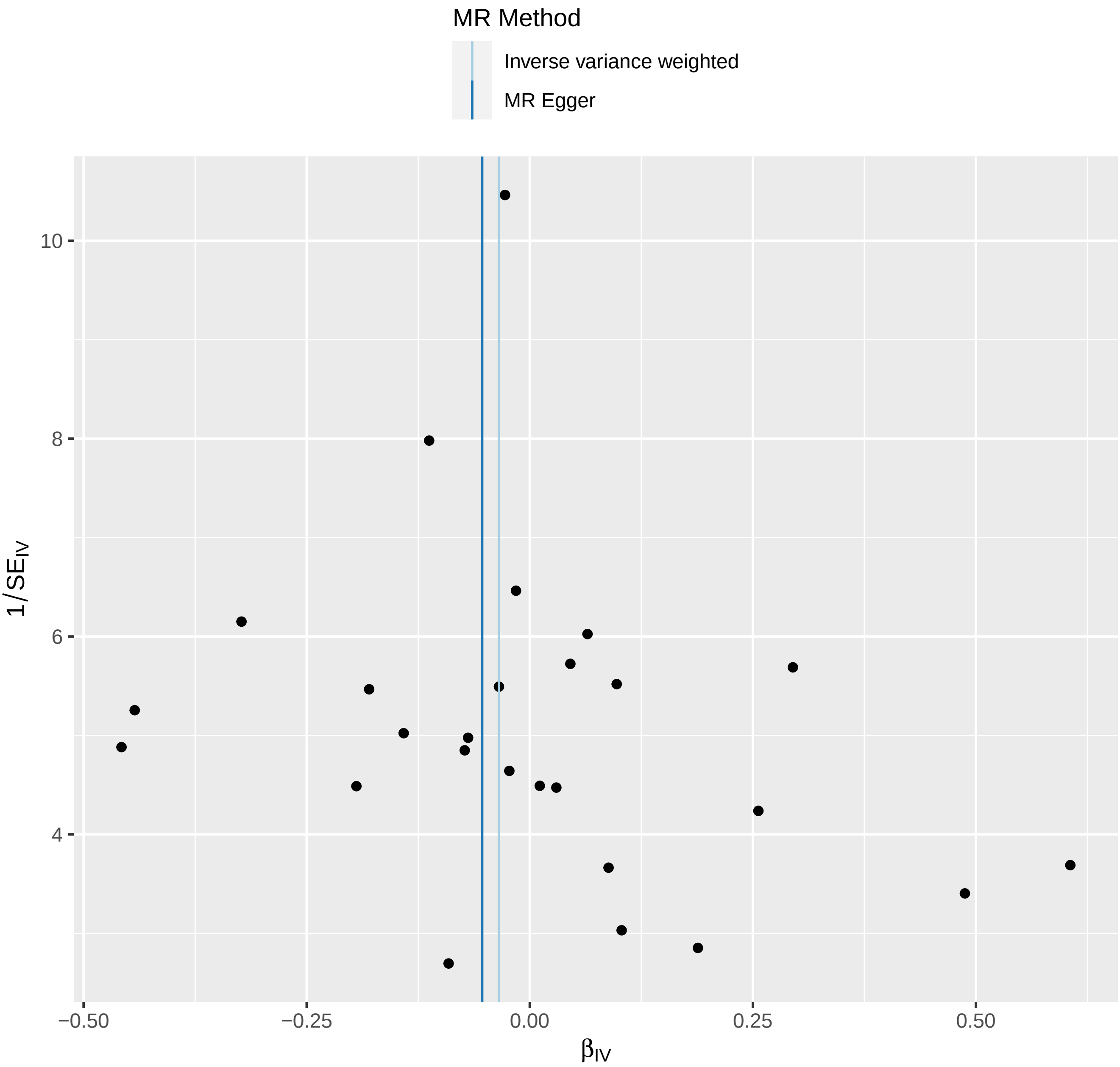

Batch 3−3185 : Vesicle−associated membrane protein 3 on Diabetic nephropathy (more control exclusions)
with a threshold of 1e−05

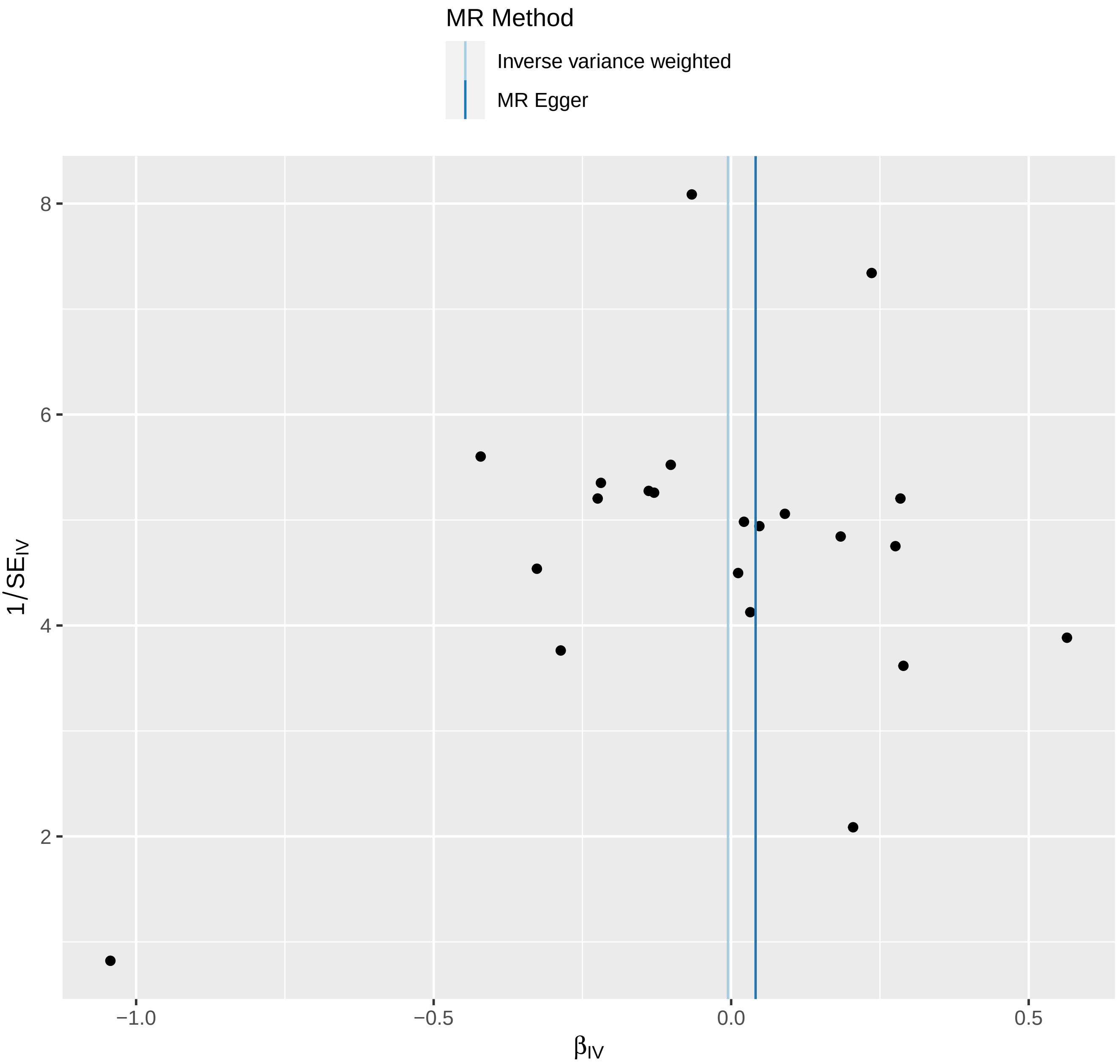

Batch 3−3186 : Vesicle−associated membrane protein 4 on Diabetic nephropathy (more control exclusions)
with a threshold of 1e−05

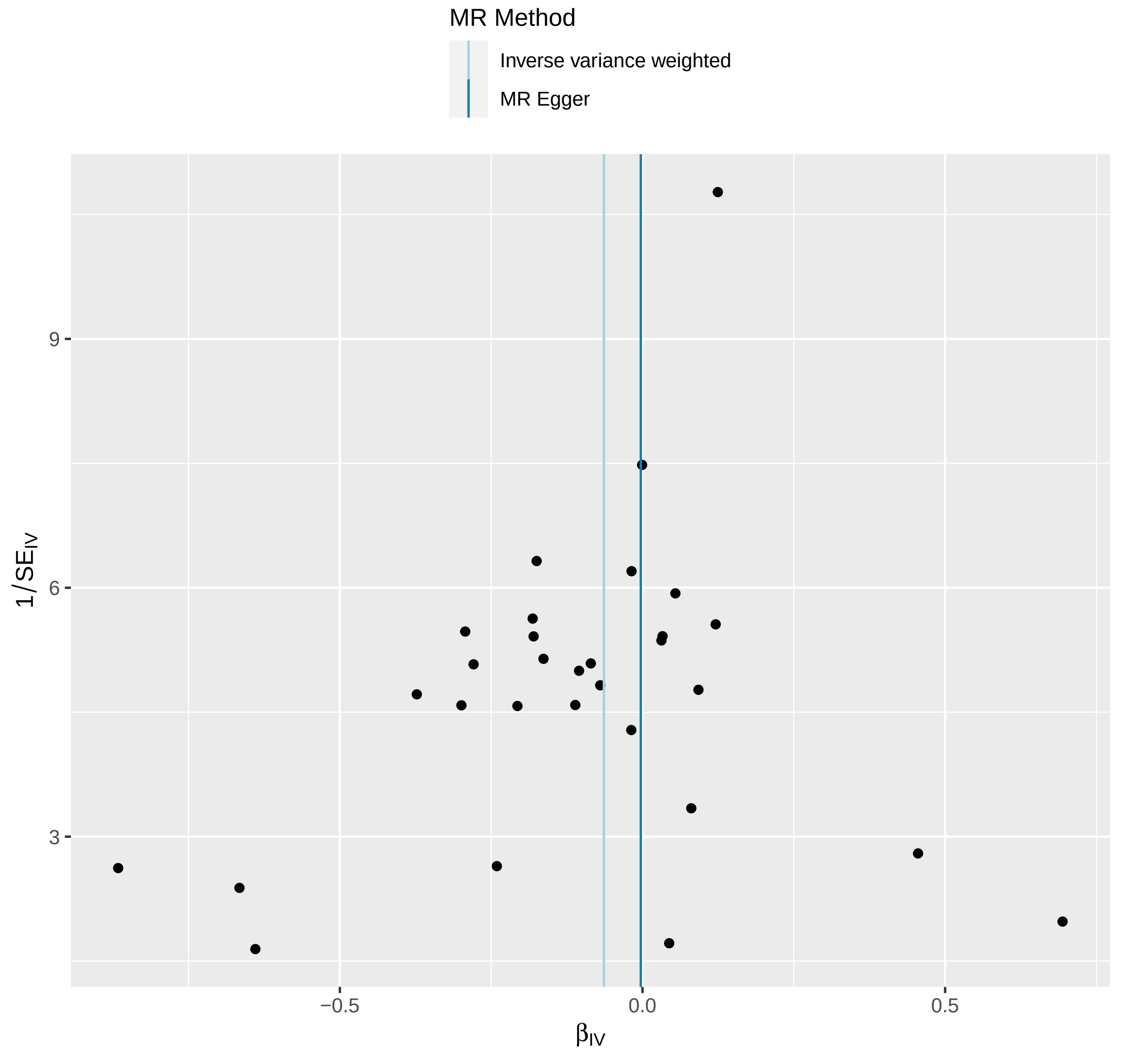

Batch 3−3187 : Vesicle−associated membrane protein 5 on Diabetic nephropathy (more control exclusions)
with a threshold of 1e−05

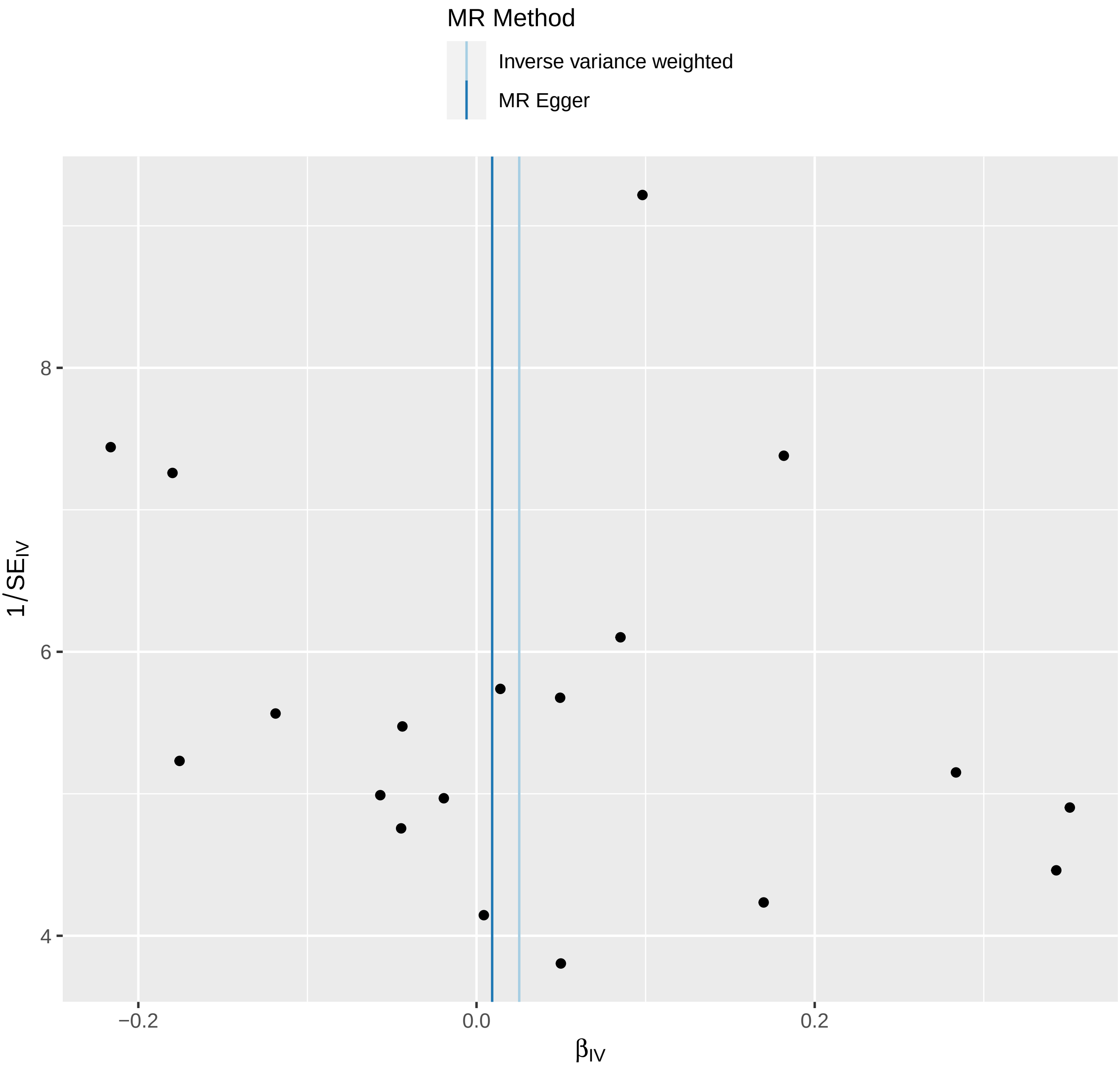

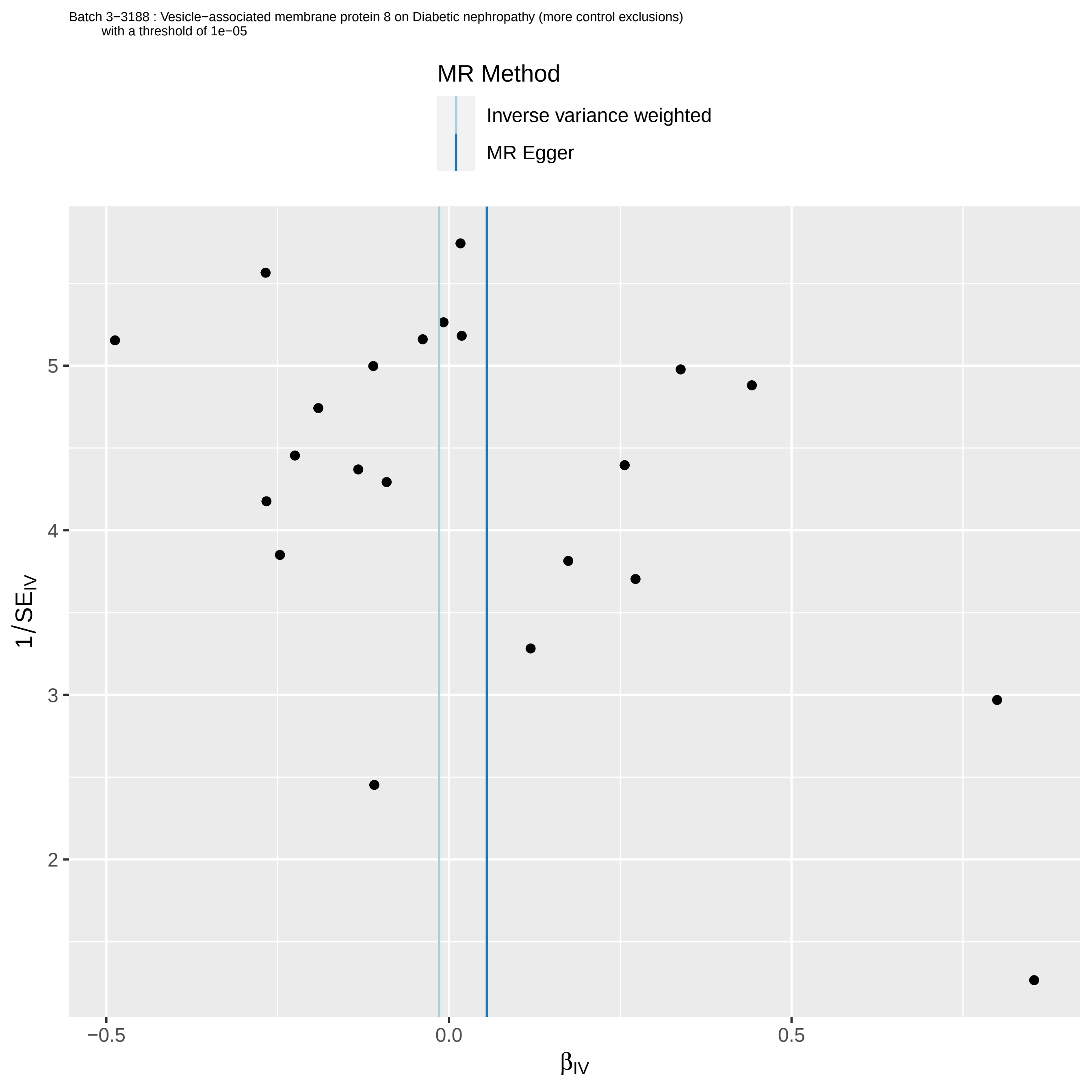

Batch 3−3188 : Vesicle−associated membrane protein 8 on Diabetic nephropathy (more control exclusions)
with a threshold of 1e−05
MR Method
Inverse variance weighted
MR Egger
5
4
3
2
1/SE_IV
−0.5
0.0
0.5
β_IV

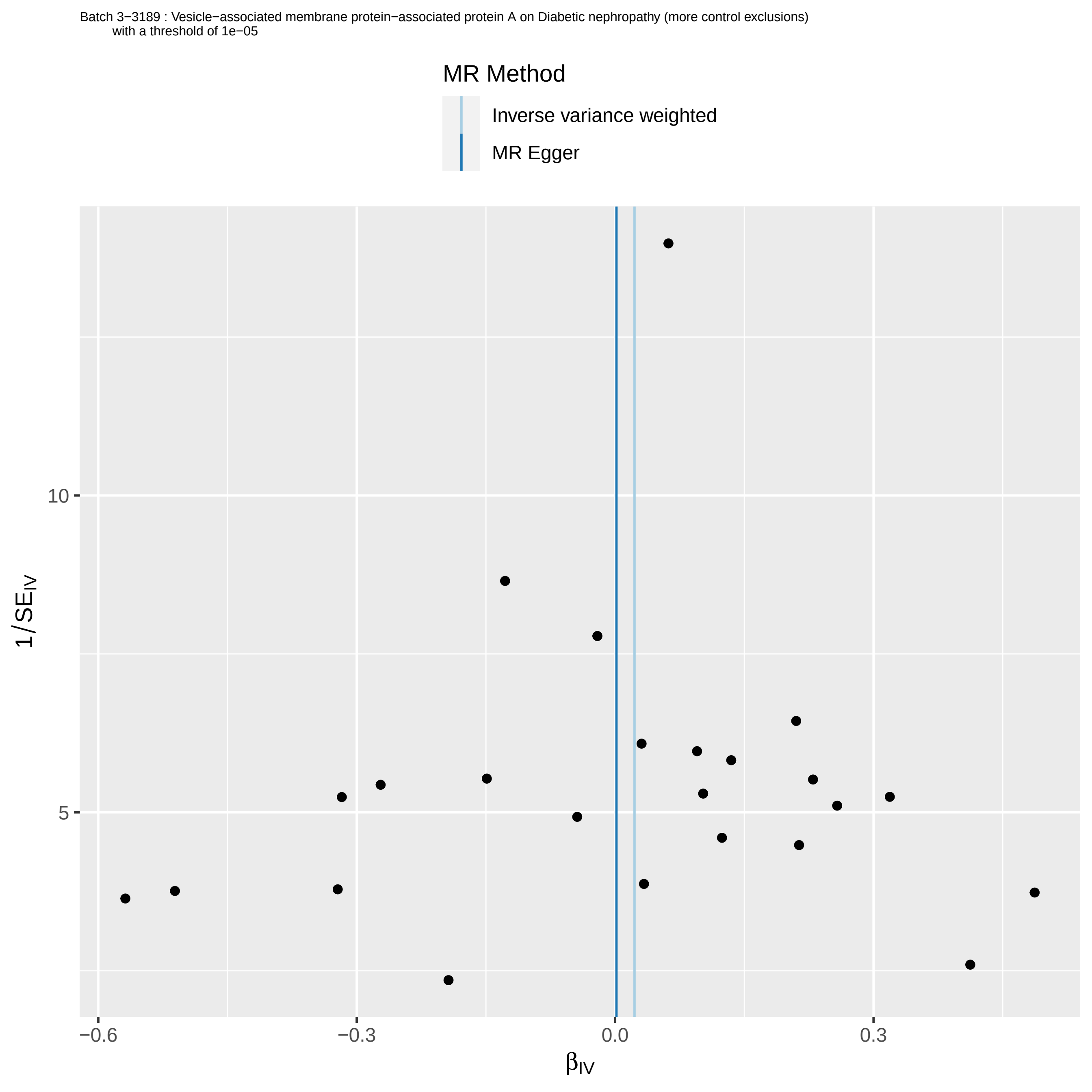

Batch 3−3189 : Vesicle−associated membrane protein−associated protein A on Diabetic nephropathy (more control exclusions)
with a threshold of 1e−05
MR Method
Inverse variance weighted
MR Egger
10
5
1/SE_IV
−0.6
−0.3
0.0
0.3
β_IV

Batch 3−3190 : Vesicle−associated membrane protein−associated protein B/C on Diabetic nephropathy (more control exclusions) with a threshold of 1e−05

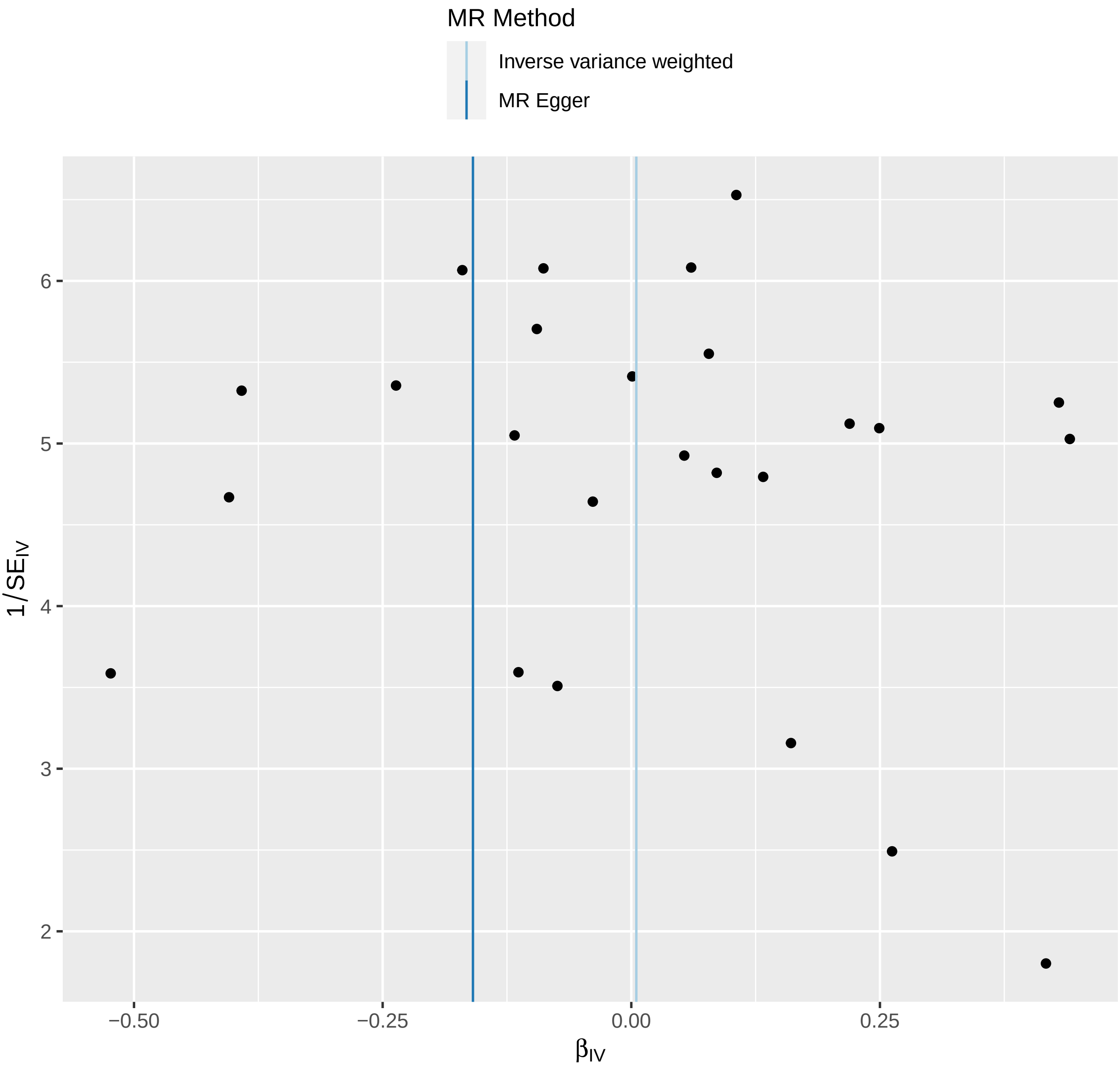

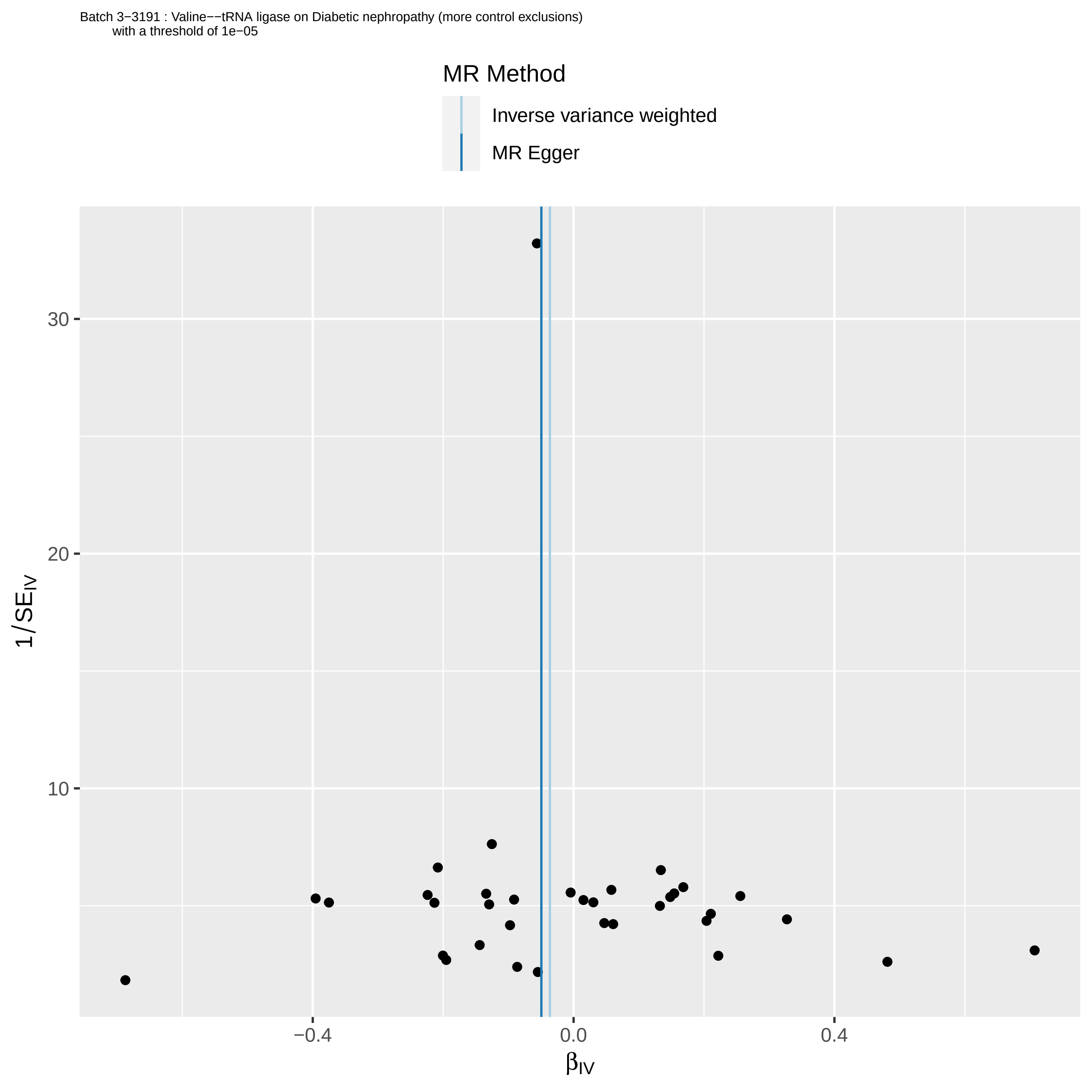

Batch 3−3191 : Valine−−tRNA ligase on Diabetic nephropathy (more control exclusions)
with a threshold of 1e−05
MR Method
Inverse variance weighted
MR Egger
30
20
10
1/SE_IV
−0.4
0.0
0.4
β_IV

Batch 3−3192 : Guanine nucleotide exchange factor VAV3 on Diabetic nephropathy (more control exclusions) with a threshold of 1e−05

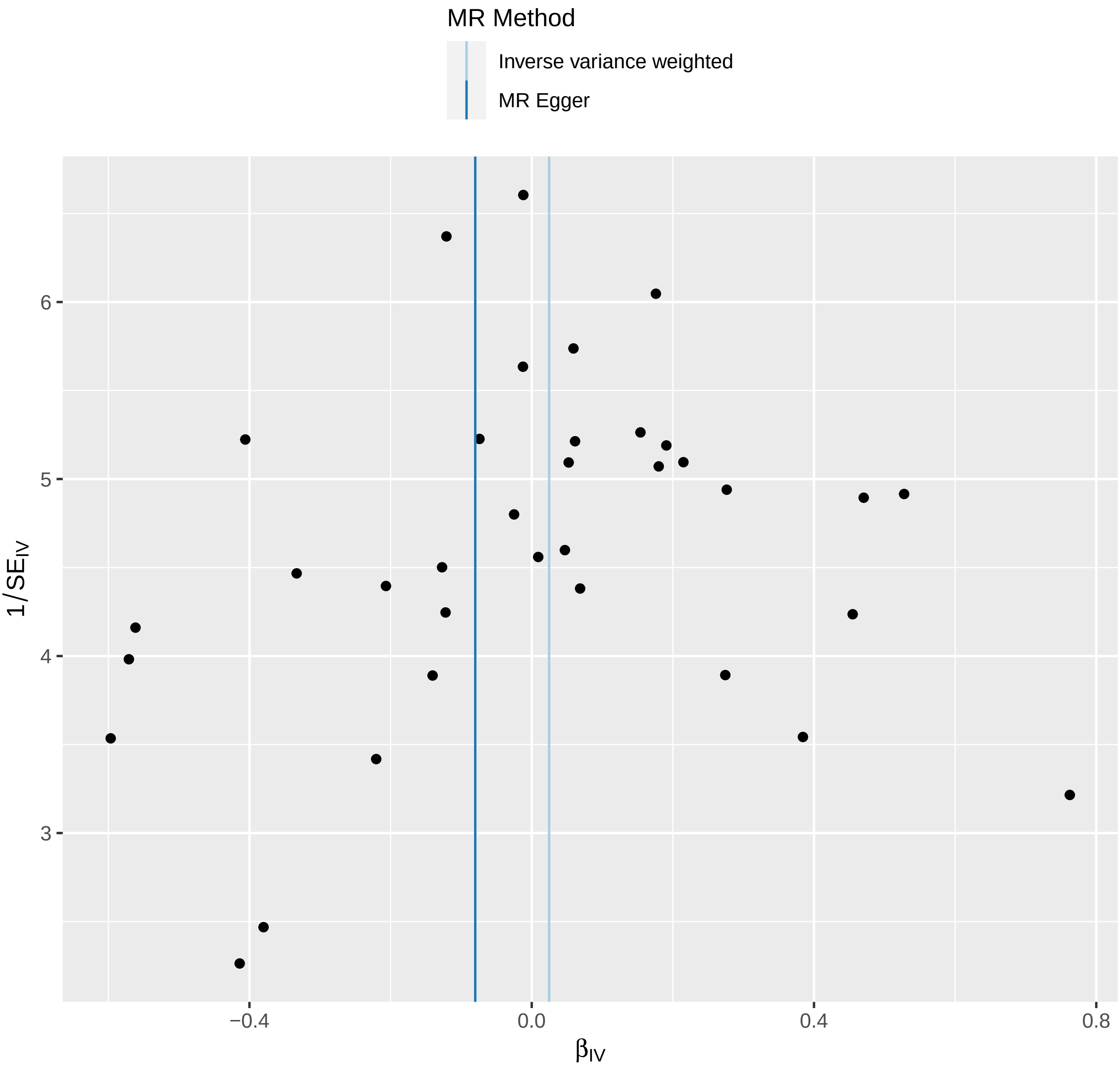

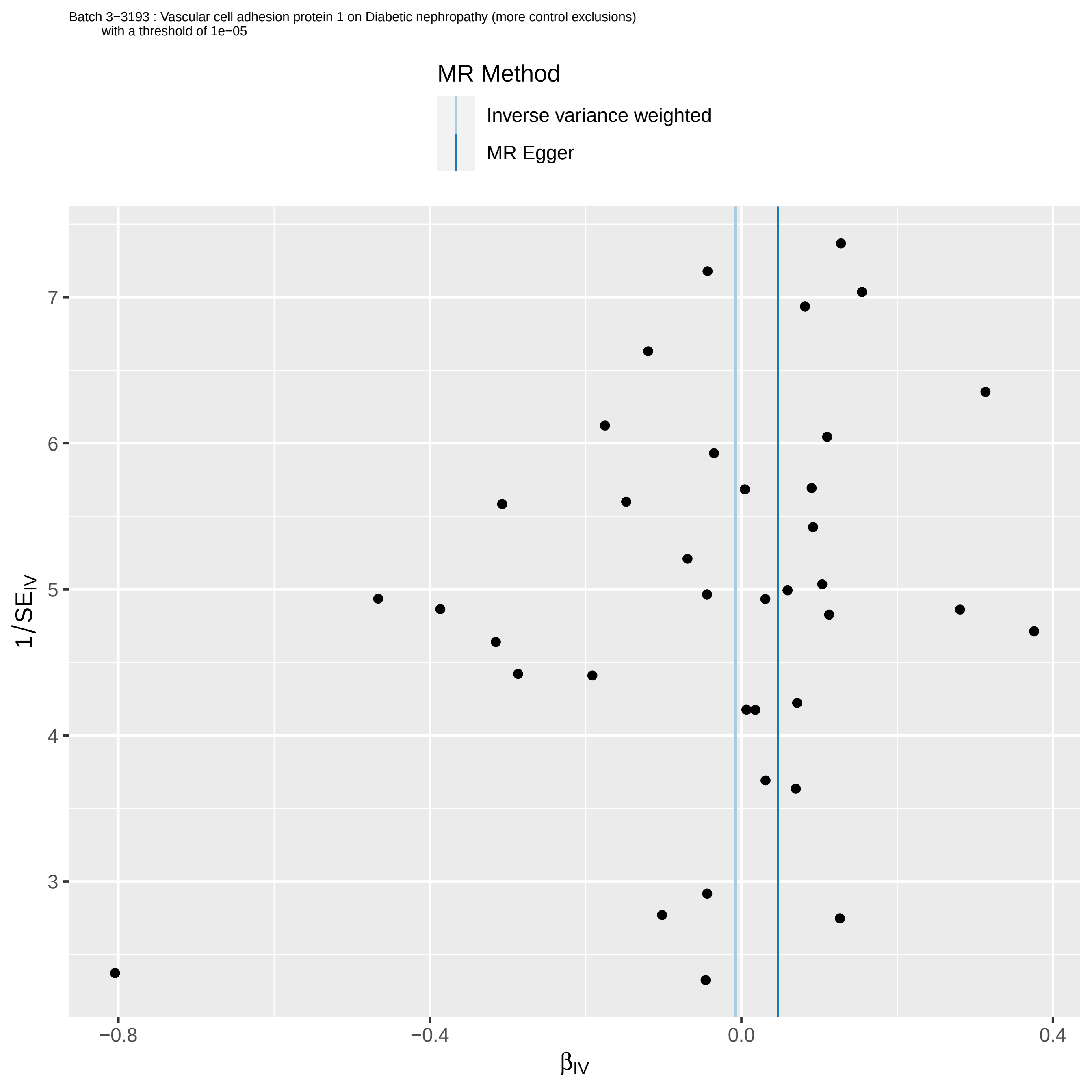

Batch 3−3193 : Vascular cell adhesion protein 1 on Diabetic nephropathy (more control exclusions)
with a threshold of 1e−05
MR Method
Inverse variance weighted
MR Egger
1/SE_IV
7
6
5
4
3
−0.8
−0.4
0.0
0.4
β_IV

Batch 3−3194 : Versican core protein on Diabetic nephropathy (more control exclusions)
with a threshold of 1e−05

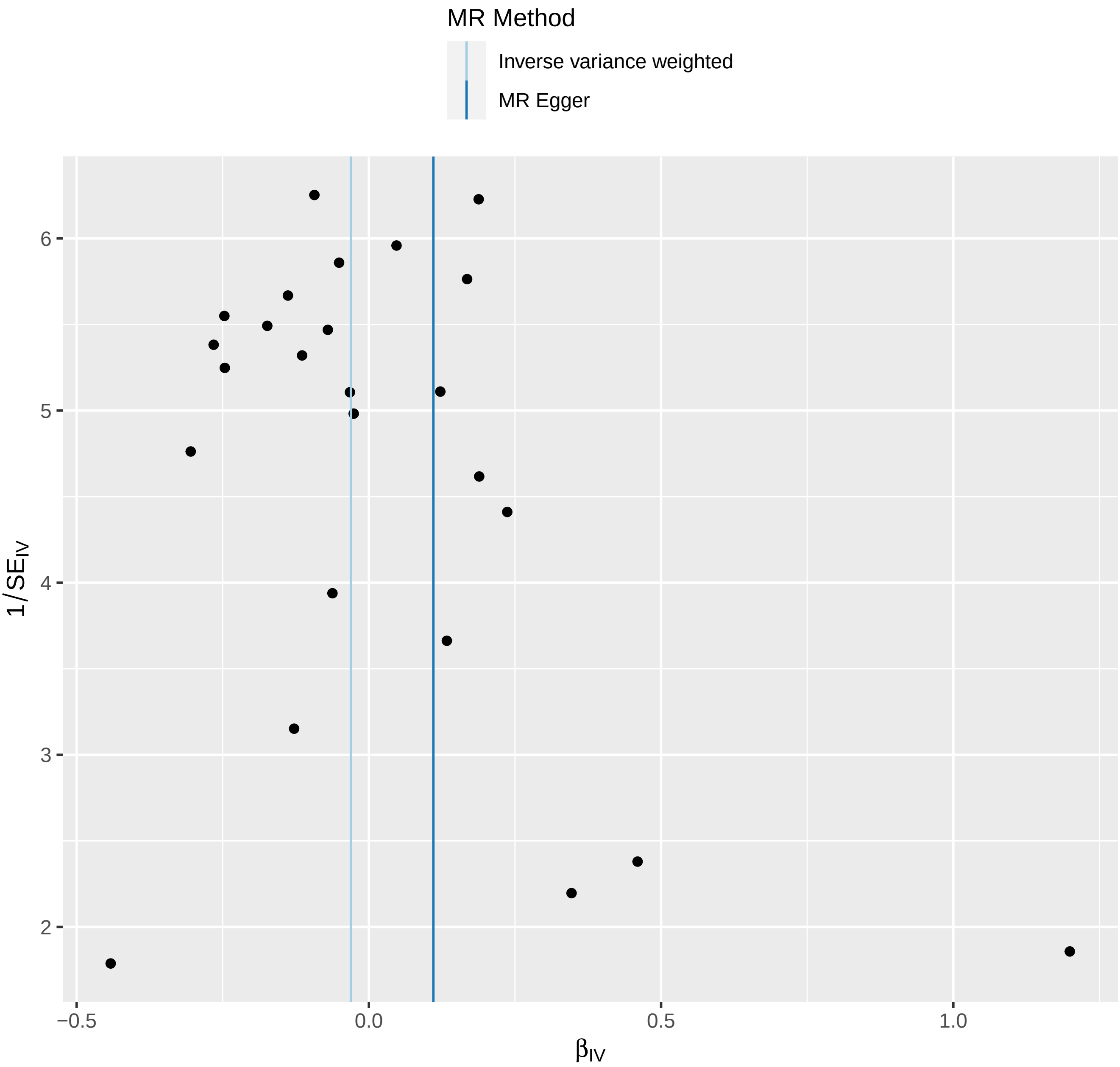

Batch 3−3195 : Vinculin on Diabetic nephropathy (more control exclusions)
with a threshold of 1e−05

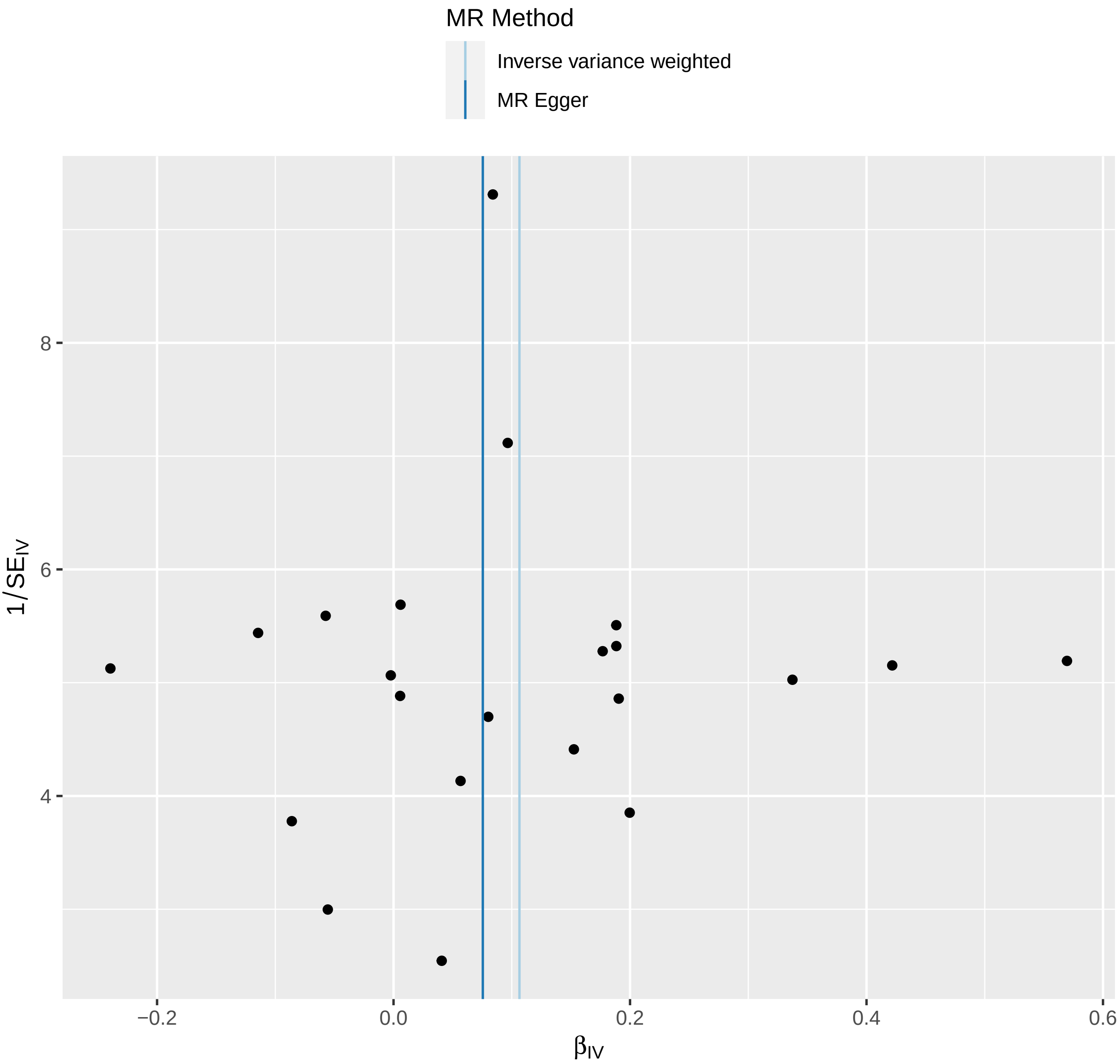

Batch 3−3196 : Testis−specific basic protein Y 1 on Diabetic nephropathy (more control exclusions)
with a threshold of 1e−05

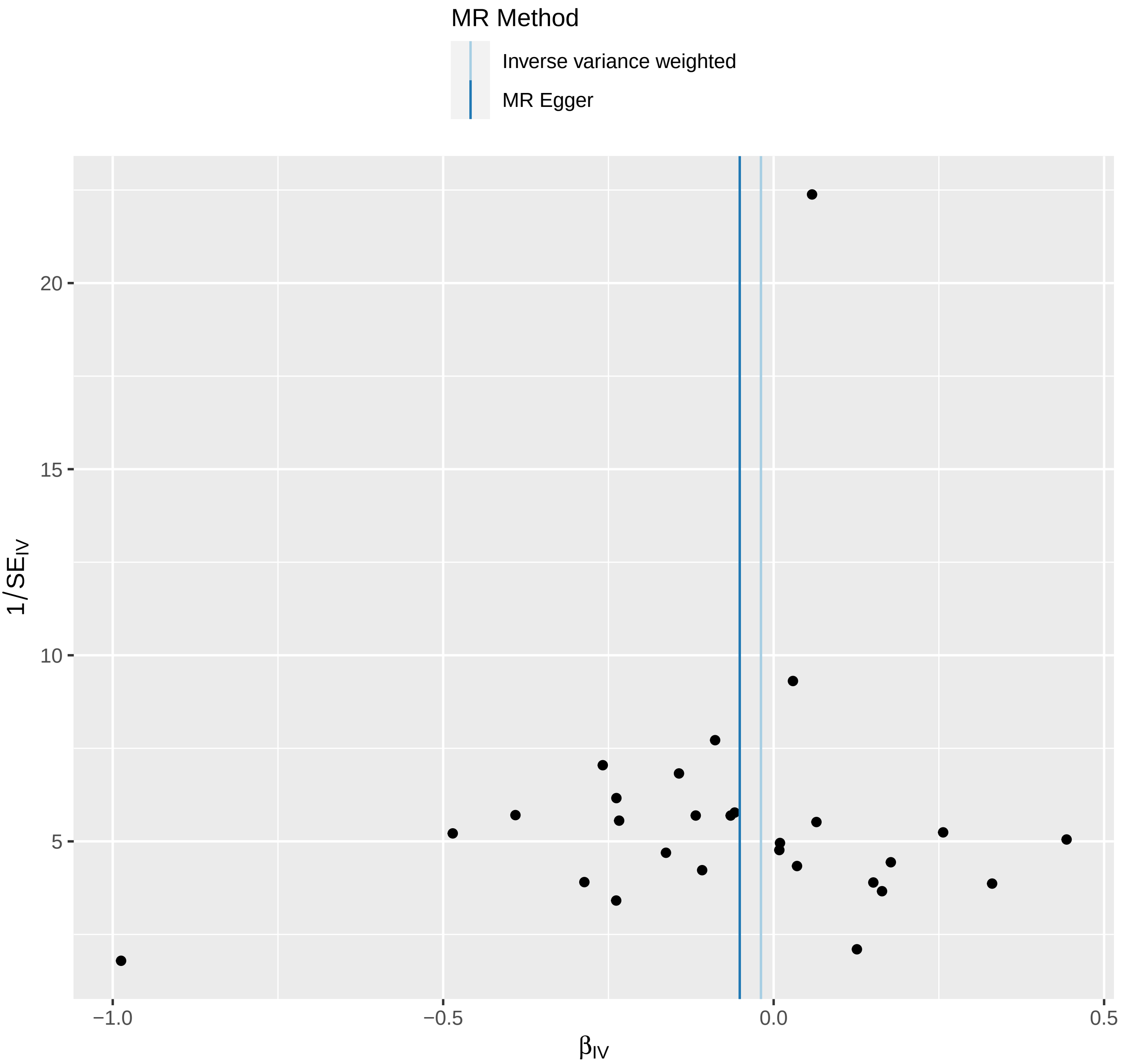

Batch 3−3197 : Vascular endothelial growth factor A, isoform 121 on Diabetic nephropathy (more control exclusions)
with a threshold of 1e−05

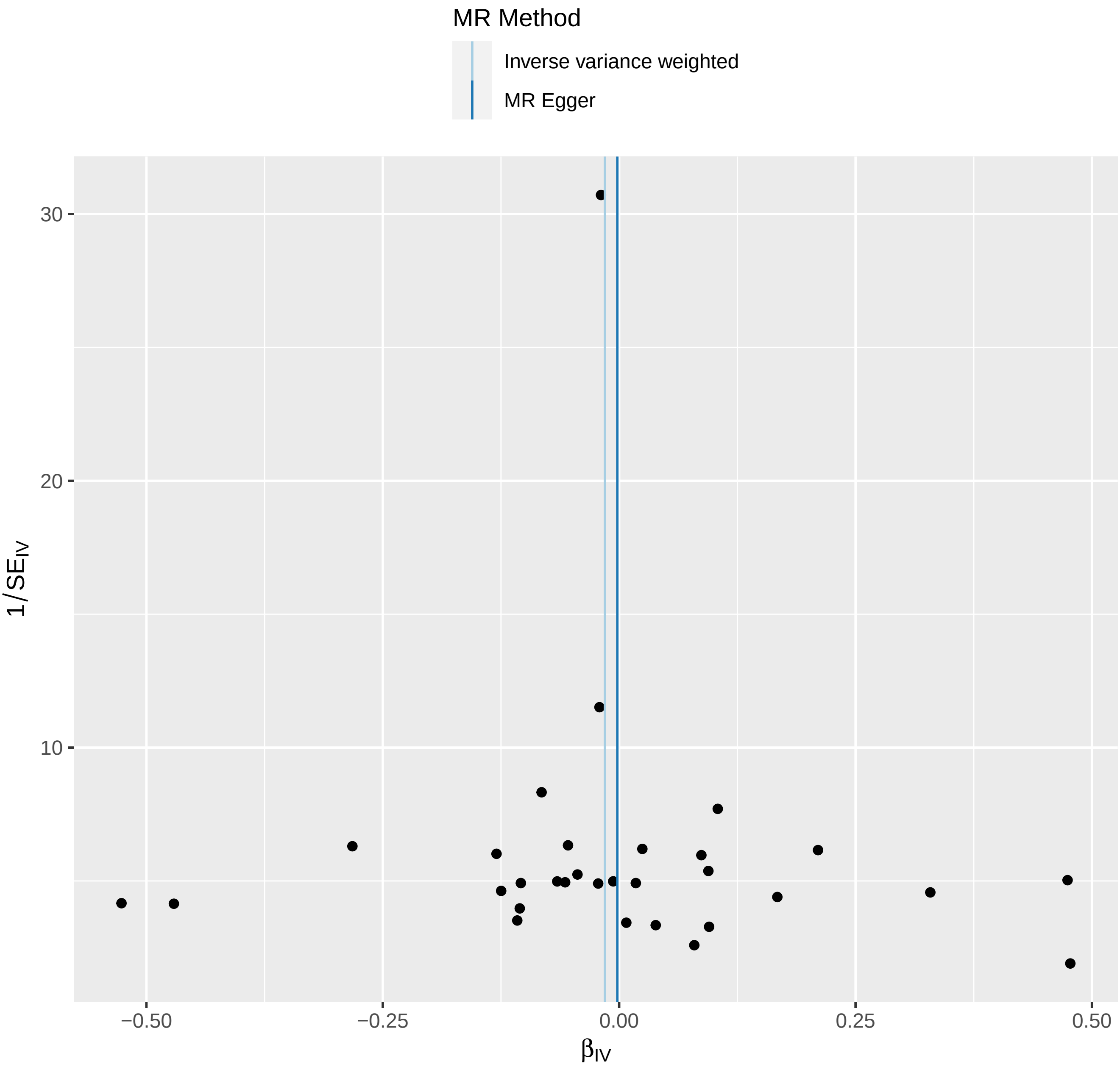

Batch 3−3198 : Vascular endothelial growth factor A on Diabetic nephropathy (more control exclusions)
with a threshold of 1e−05

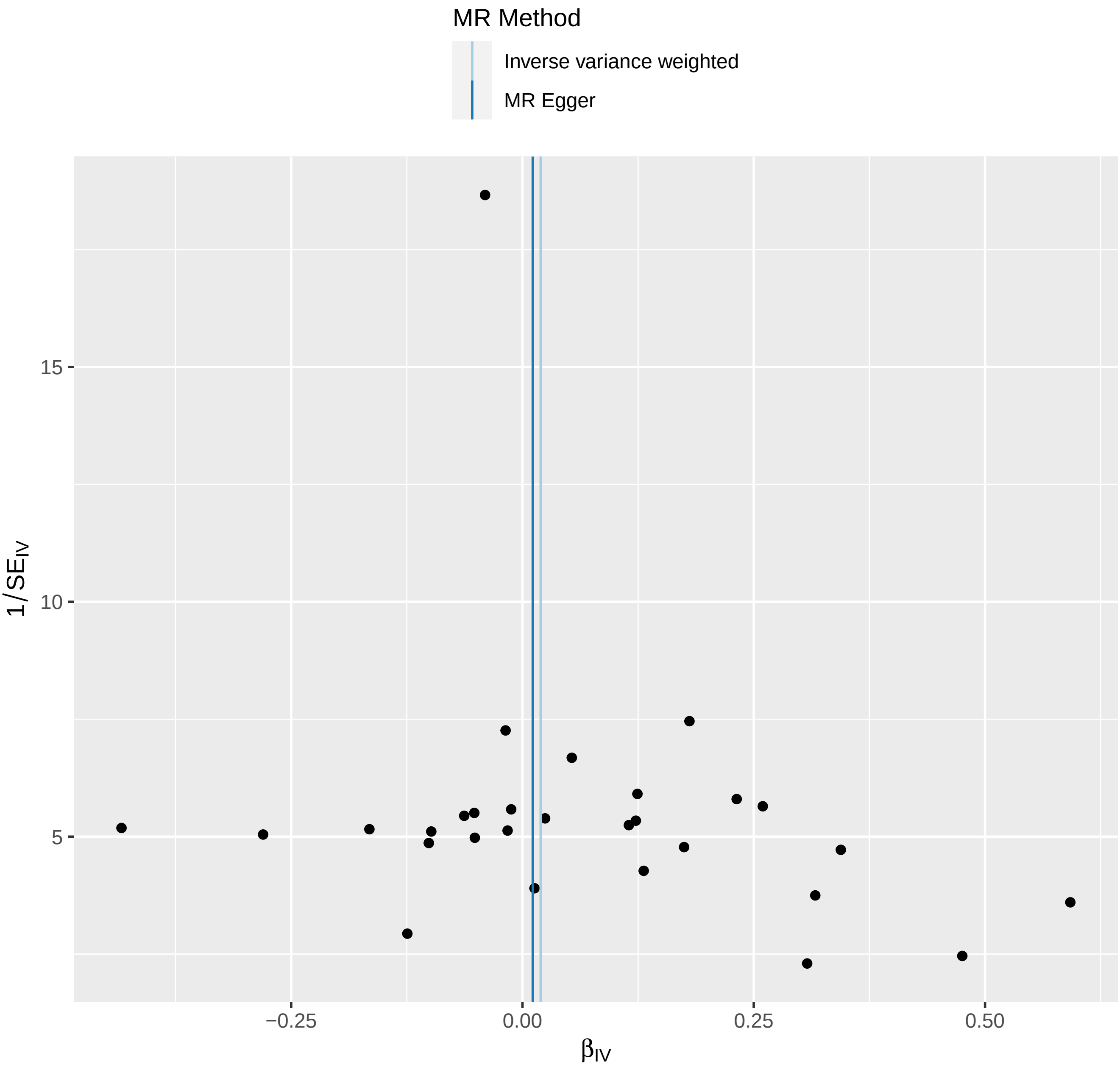

Batch 3−3199 : Vascular endothelial growth factor C on Diabetic nephropathy (more control exclusions)
with a threshold of 1e−05

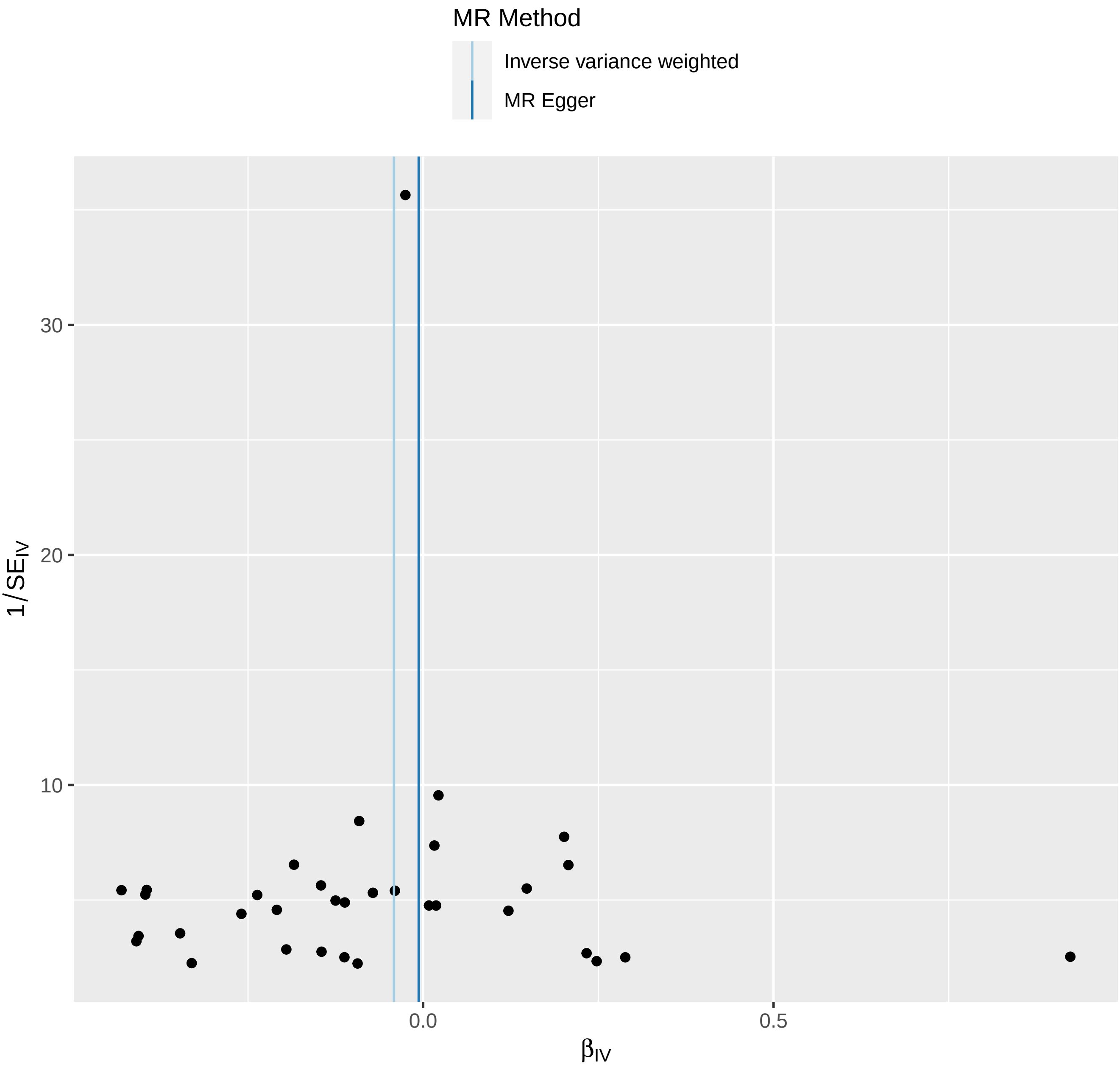

Batch 3−3200 : Selenoprotein S on Diabetic nephropathy (more control exclusions)
with a threshold of 1e−05

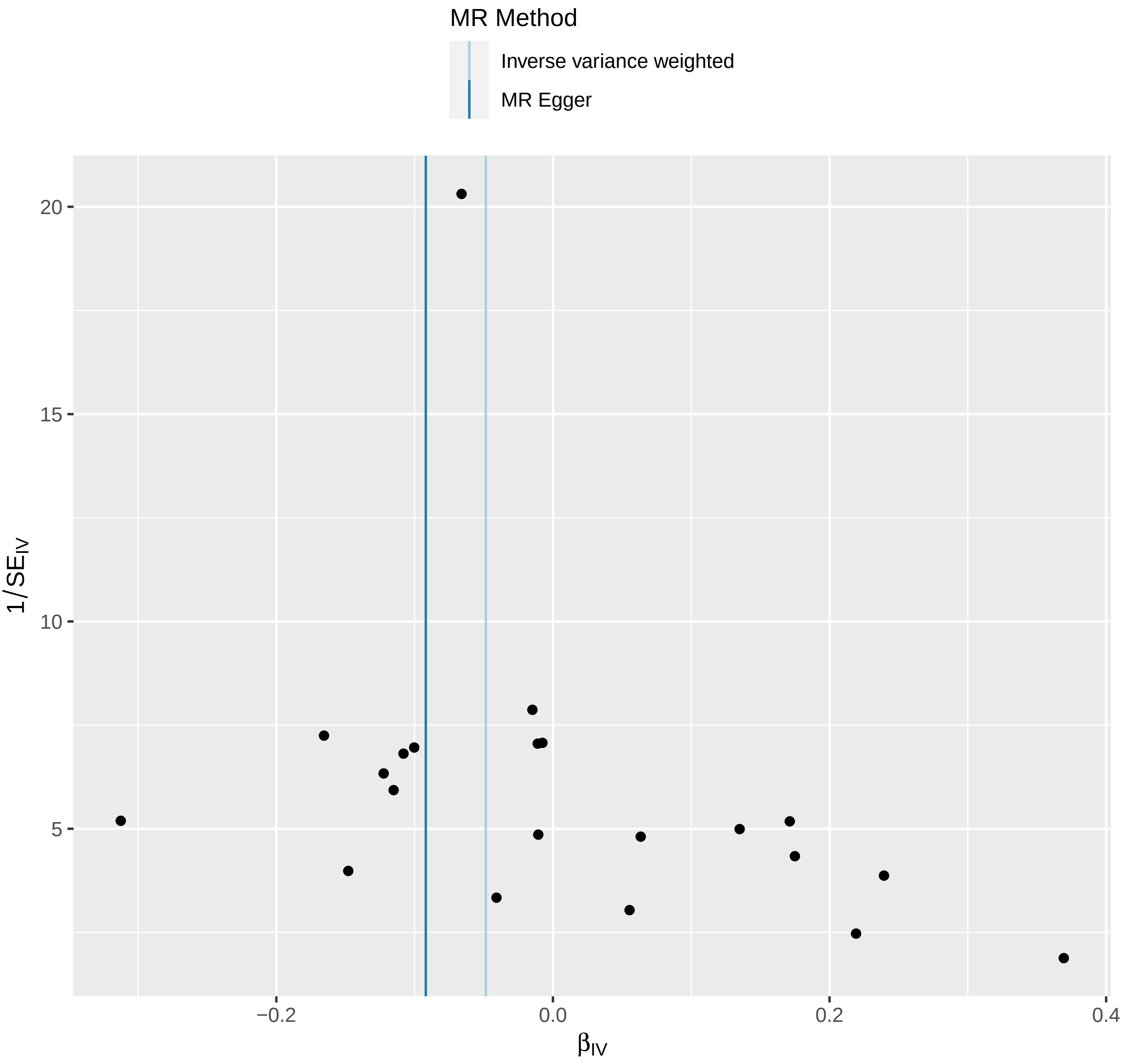

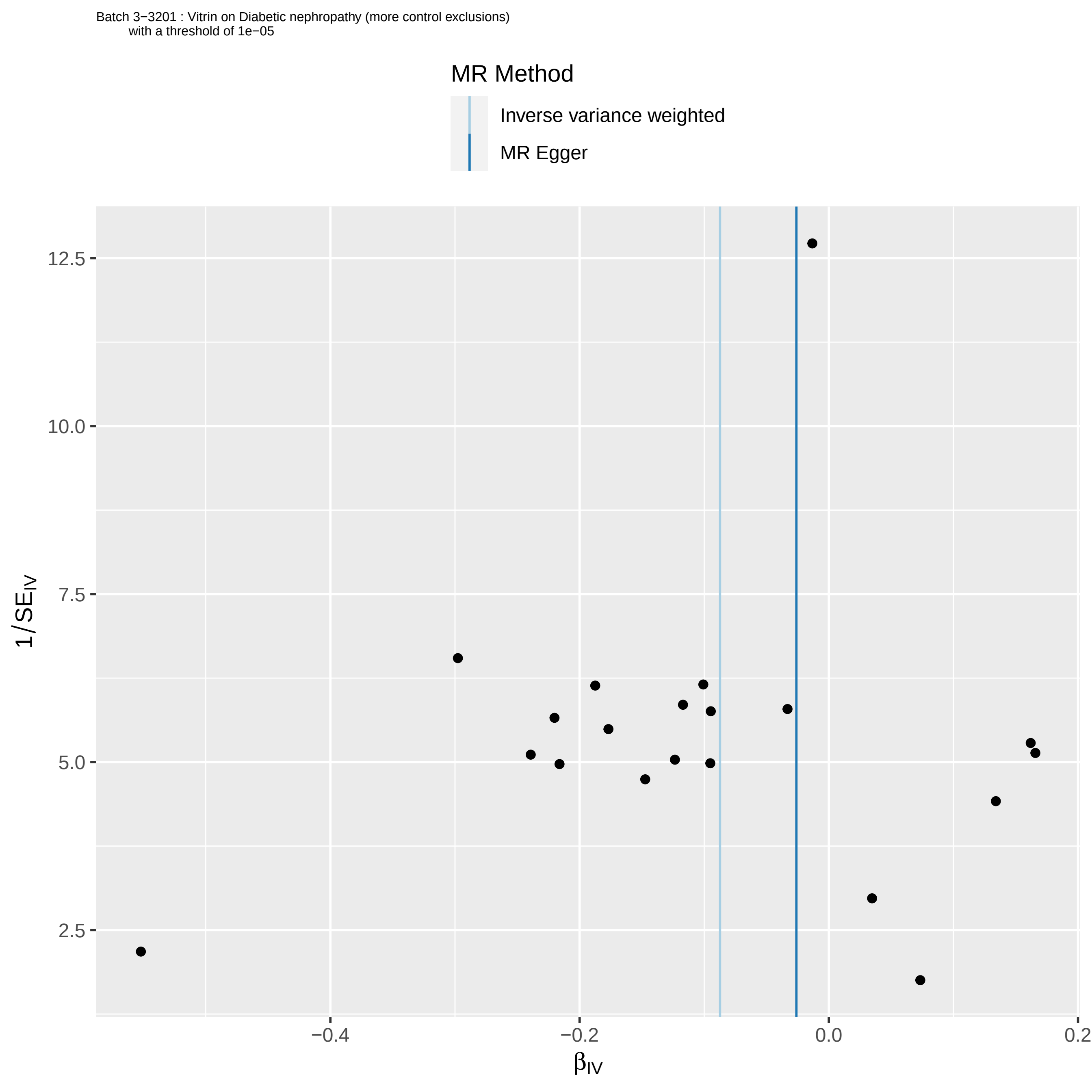
Batch 3−3201 : Vitrin on Diabetic nephropathy (more control exclusions)
with a threshold of 1e−05
MR Method
Inverse variance weighted
MR Egger
12.5
10.0
7.5
5.0
2.5
1/SE_IV
−0.4
−0.2
0.0
0.2
β_IV

Batch 3−3202 : Charged multivesicular body protein 3 on Diabetic nephropathy (more control exclusions)
with a threshold of 1e−05

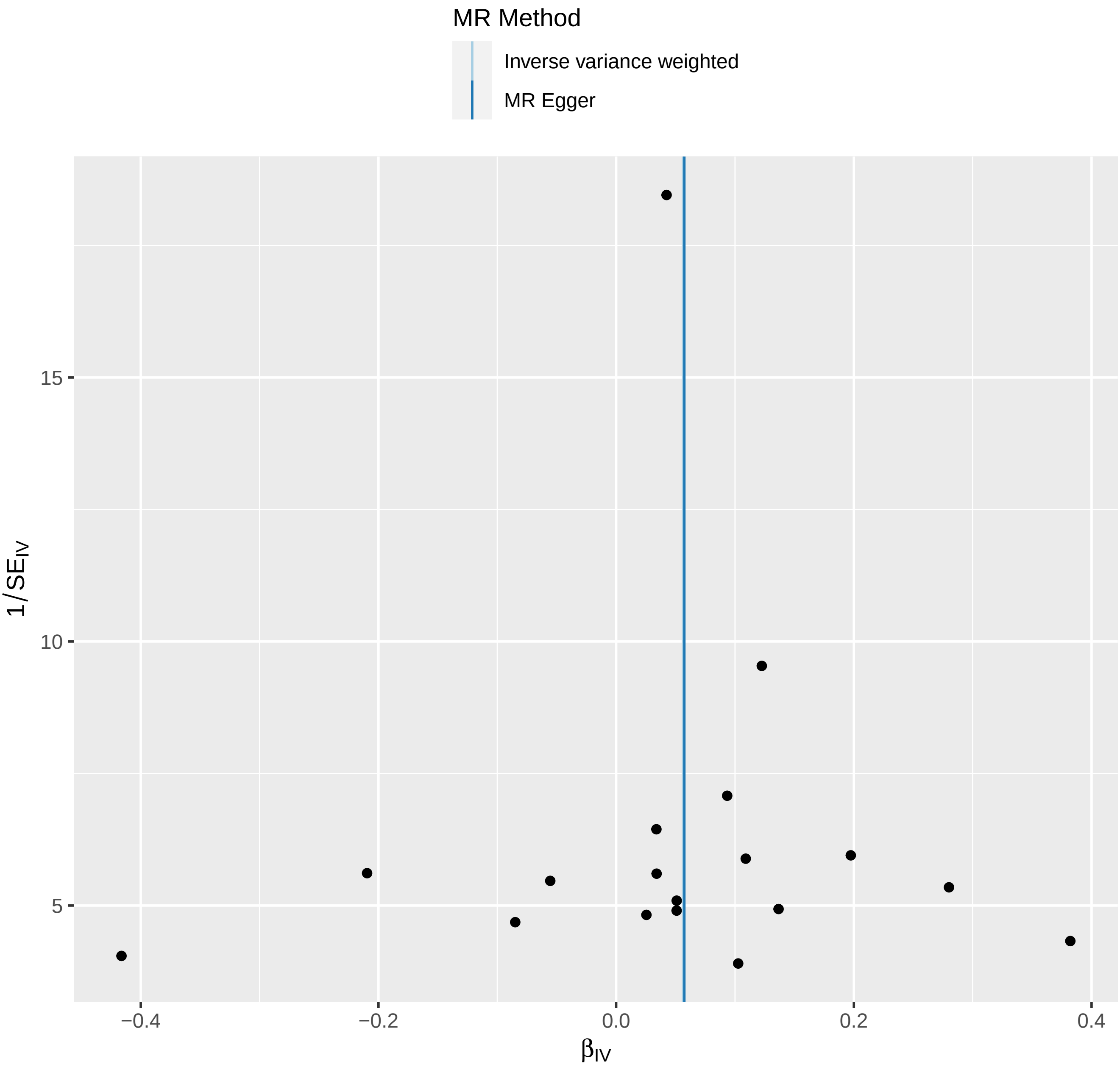

Batch 3−3203 : Vacuolar protein sorting−associated protein 29 on Diabetic nephropathy (more control exclusions) with a threshold of 1e−05

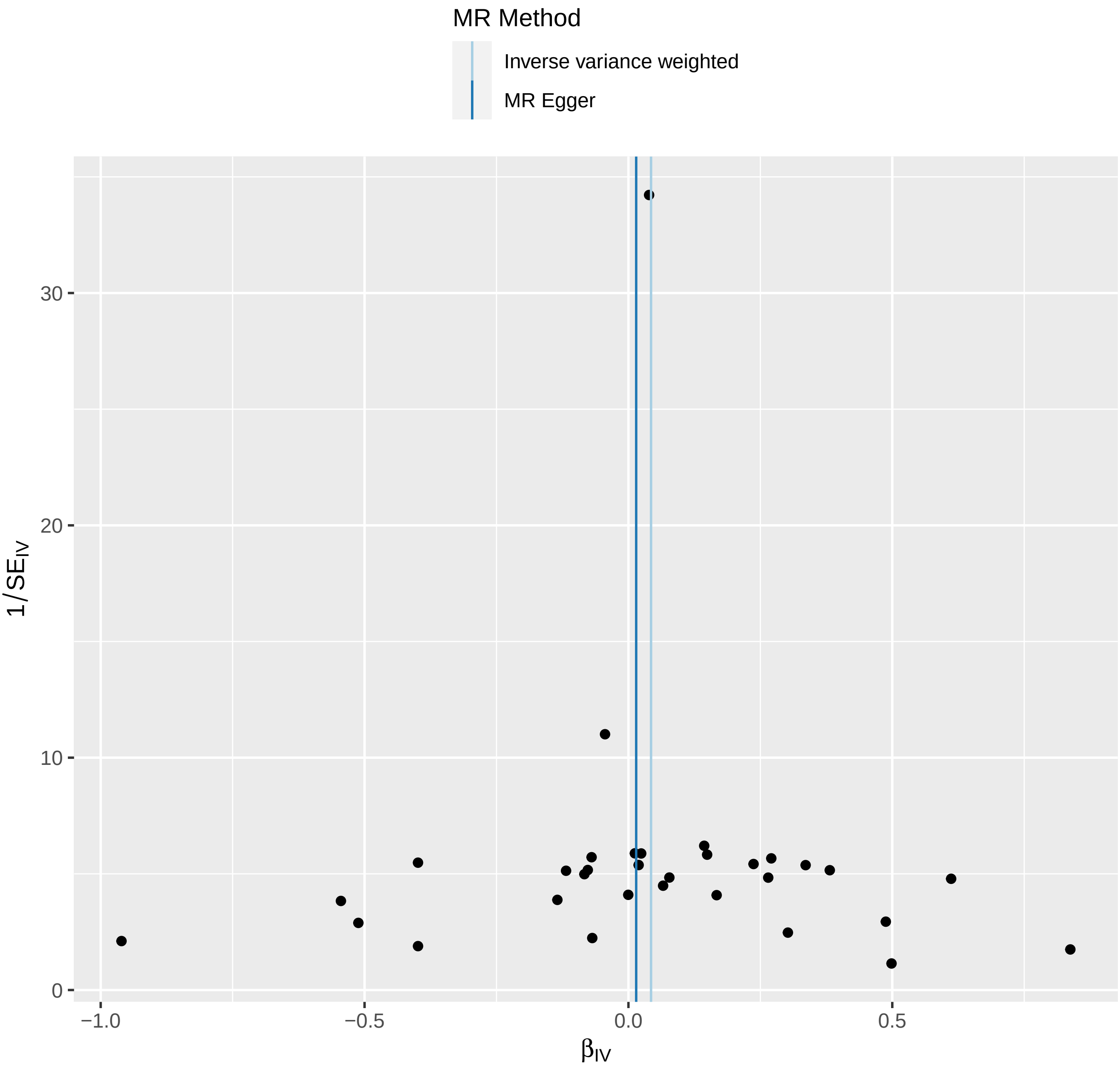

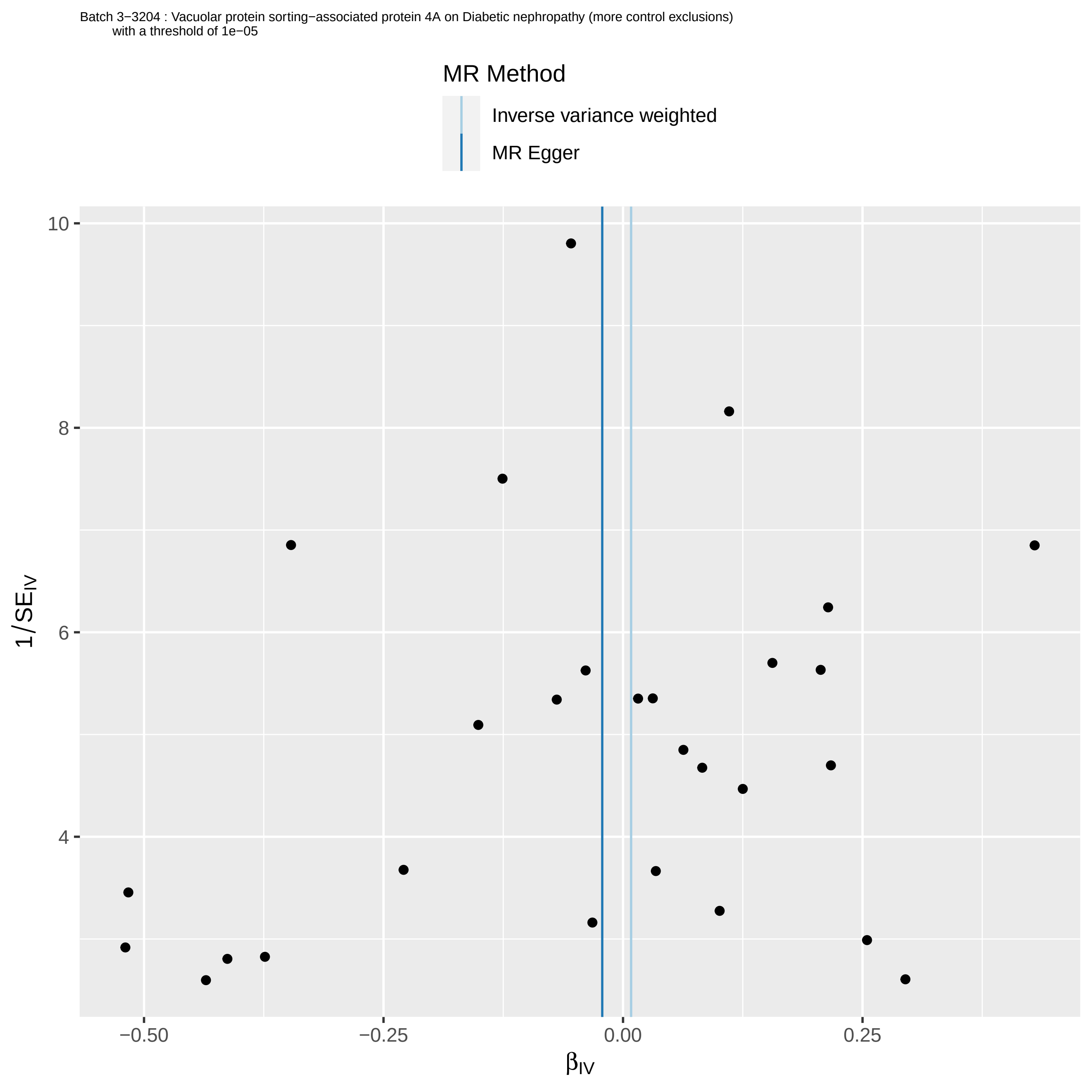

Batch 3−3204 : Vacuolar protein sorting−associated protein 4A on Diabetic nephropathy (more control exclusions)
with a threshold of 1e−05
MR Method
Inverse variance weighted
MR Egger
10
8
6
4
1/SE_IV
−0.50
−0.25
0.00
0.25
β_IV

Batch 3−3205 : Vacuolar protein sorting−associated protein 4B on Diabetic nephropathy (more control exclusions)
with a threshold of 1e−05

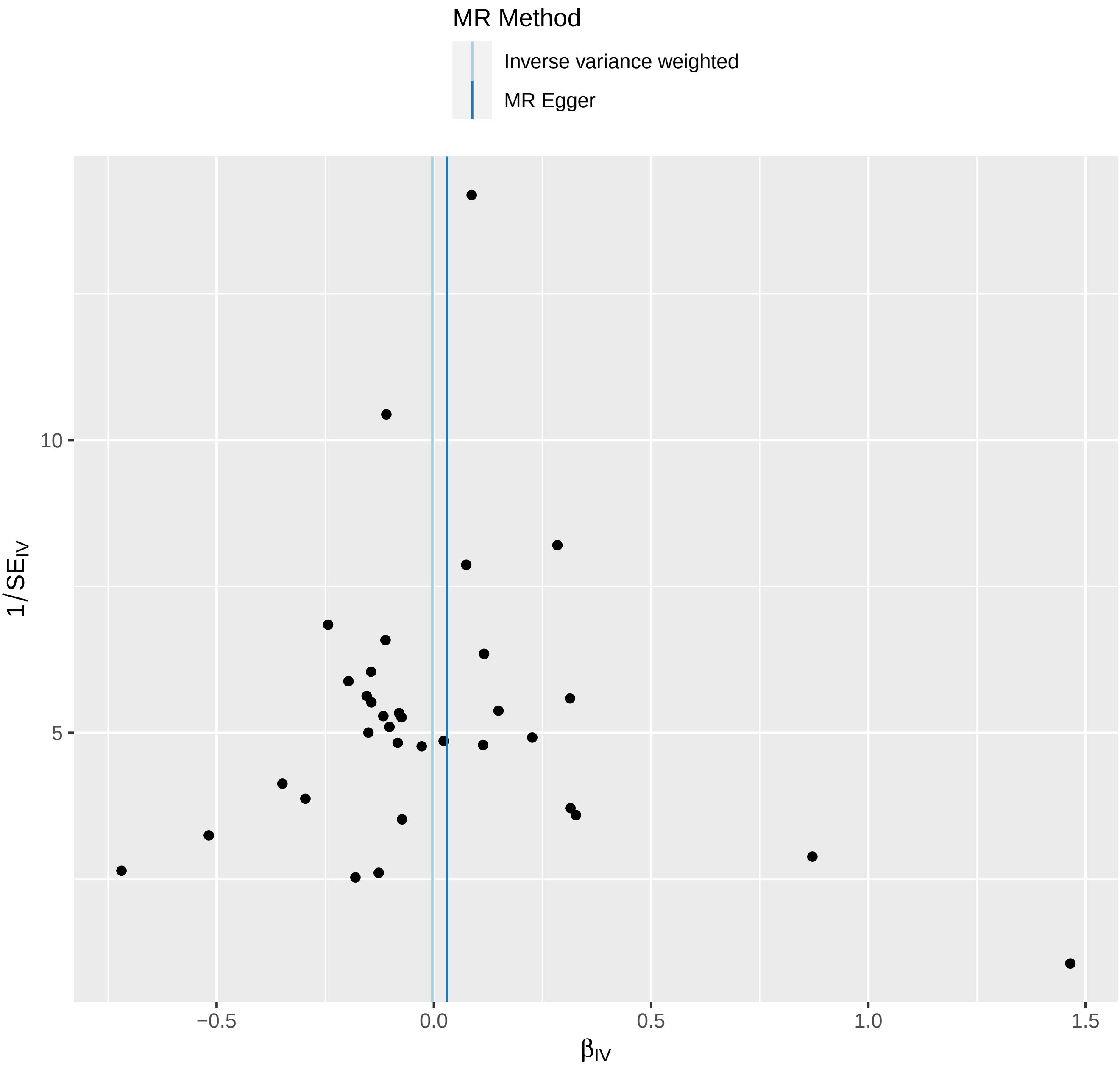

Batch 3−3206 : Serine/threonine−protein kinase VRK1 on Diabetic nephropathy (more control exclusions)
with a threshold of 1e−05

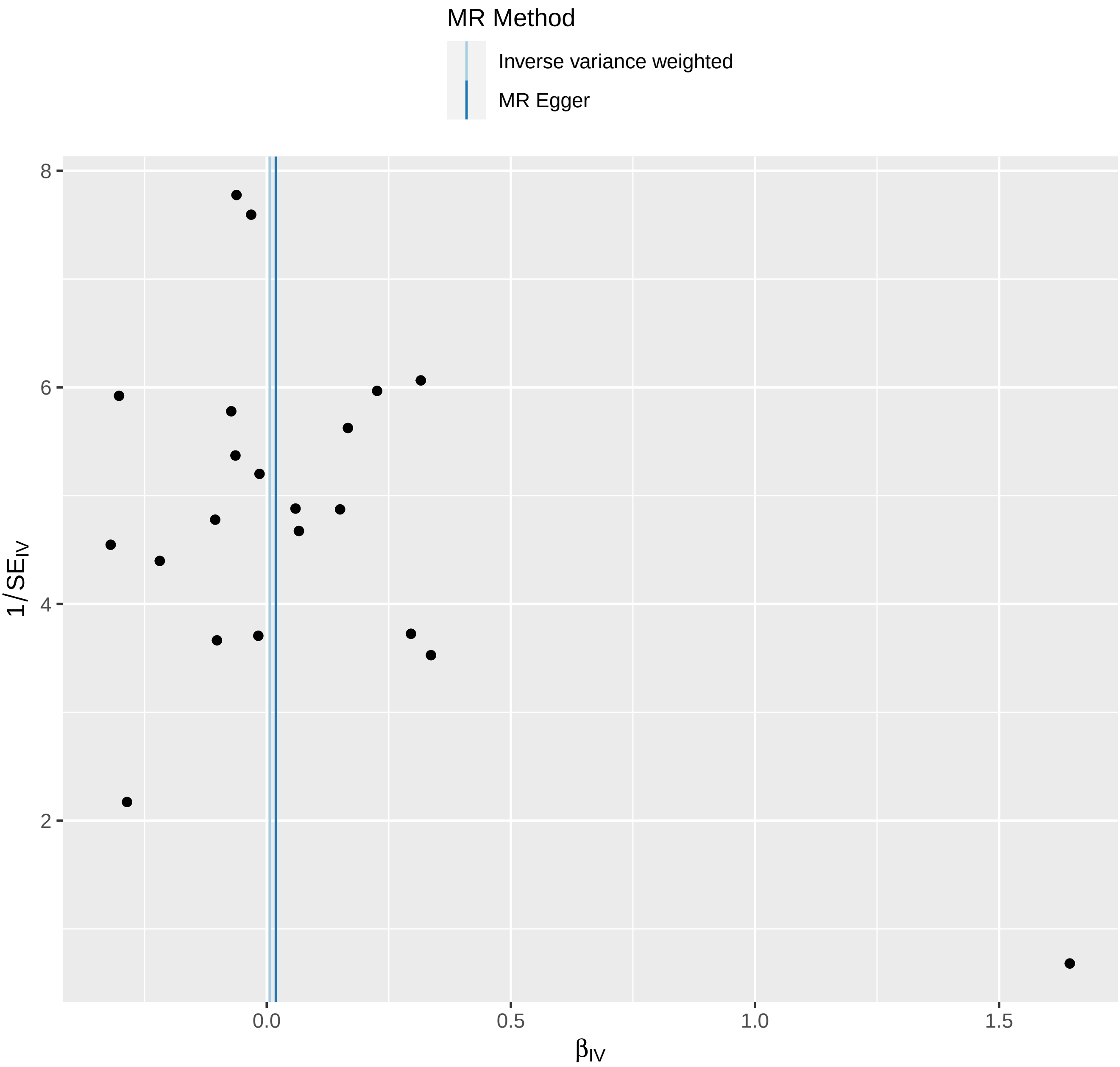

Batch 3−3207 : V−set and immunoglobulin domain−containing protein 1 on Diabetic nephropathy (more control exclusions)
with a threshold of 1e−05

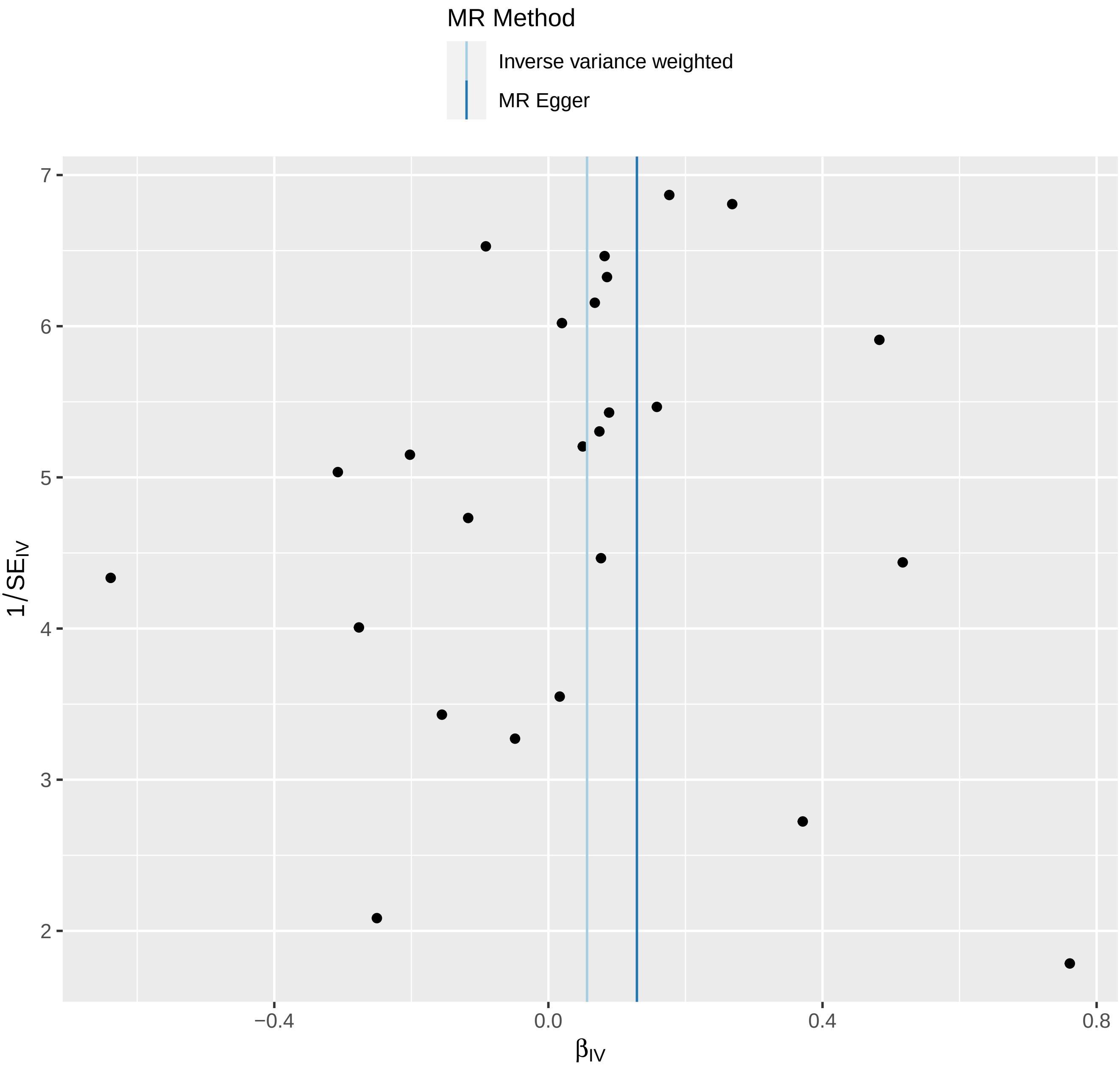

Batch 3−3208 : V−set and immunoglobulin domain−containing protein 2 on Diabetic nephropathy (more control exclusions) with a threshold of 1e−05

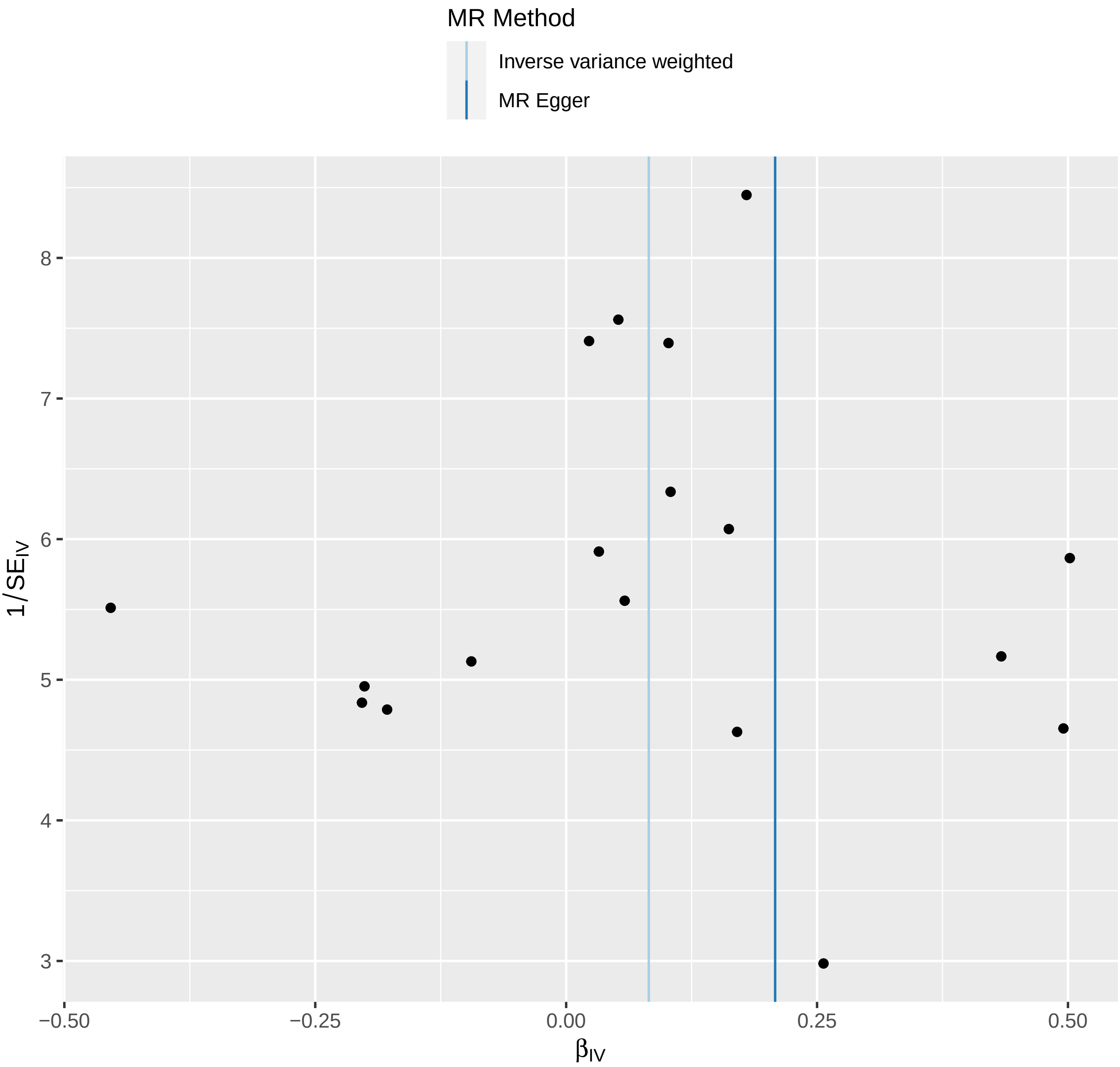

Batch 3−3209 : V−set and transmembrane domain−containing protein 2−like protein on Diabetic nephropathy (more control exclusions) with a threshold of 1e−05

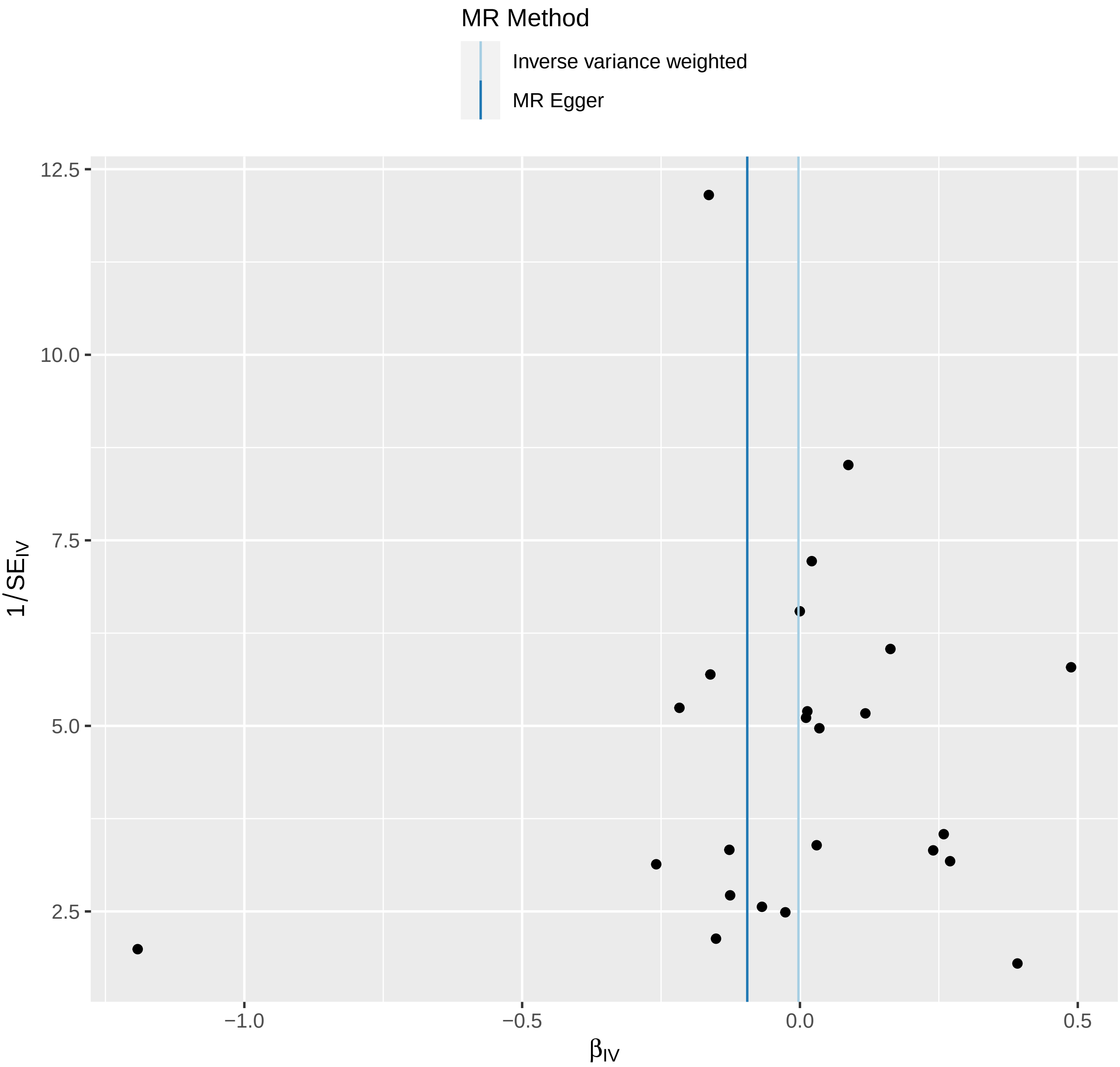

Batch 3−3210 : V−set and transmembrane domain−containing protein 4 on Diabetic nephropathy (more control exclusions)
with a threshold of 1e−05

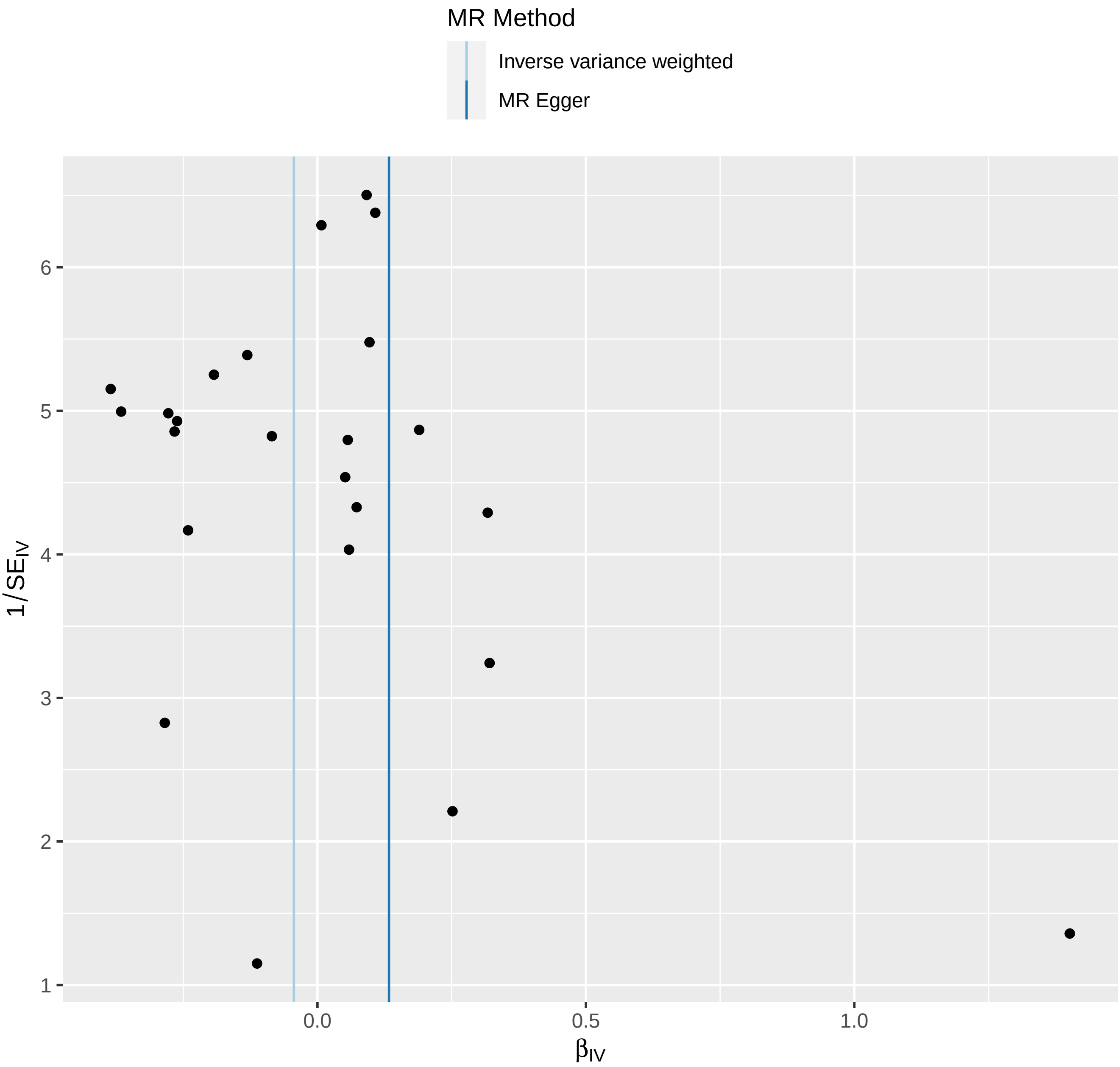

Batch 3−3211 : Vacuolar protein sorting−associated protein VTA1 homolog on Diabetic nephropathy (more control exclusions) with a threshold of 1e−05

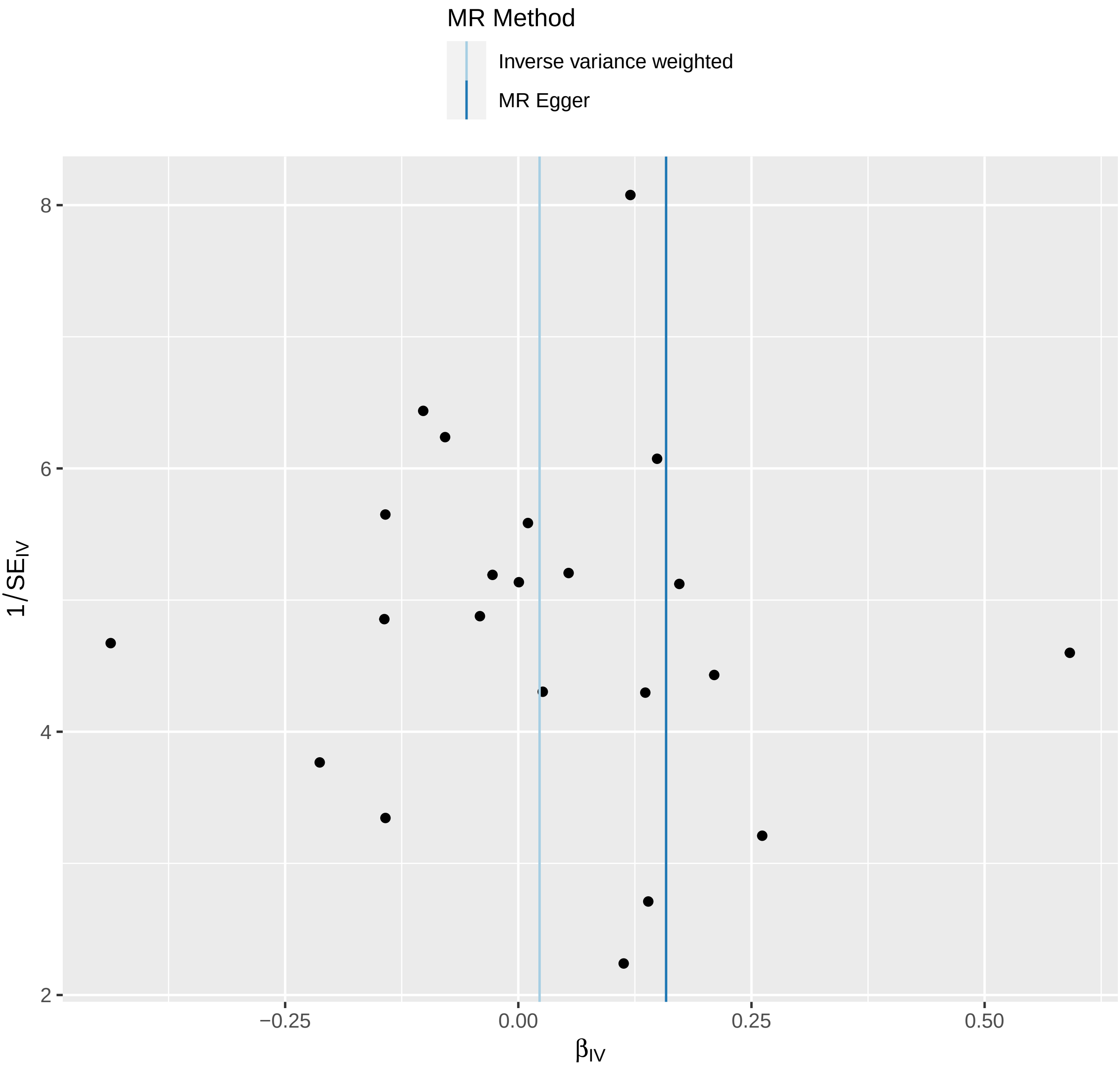

Batch 3−3212 : Vesicle transport through interaction with t−SNAREs homolog 1A on Diabetic nephropathy (more control exclusions)
with a threshold of 1e−05

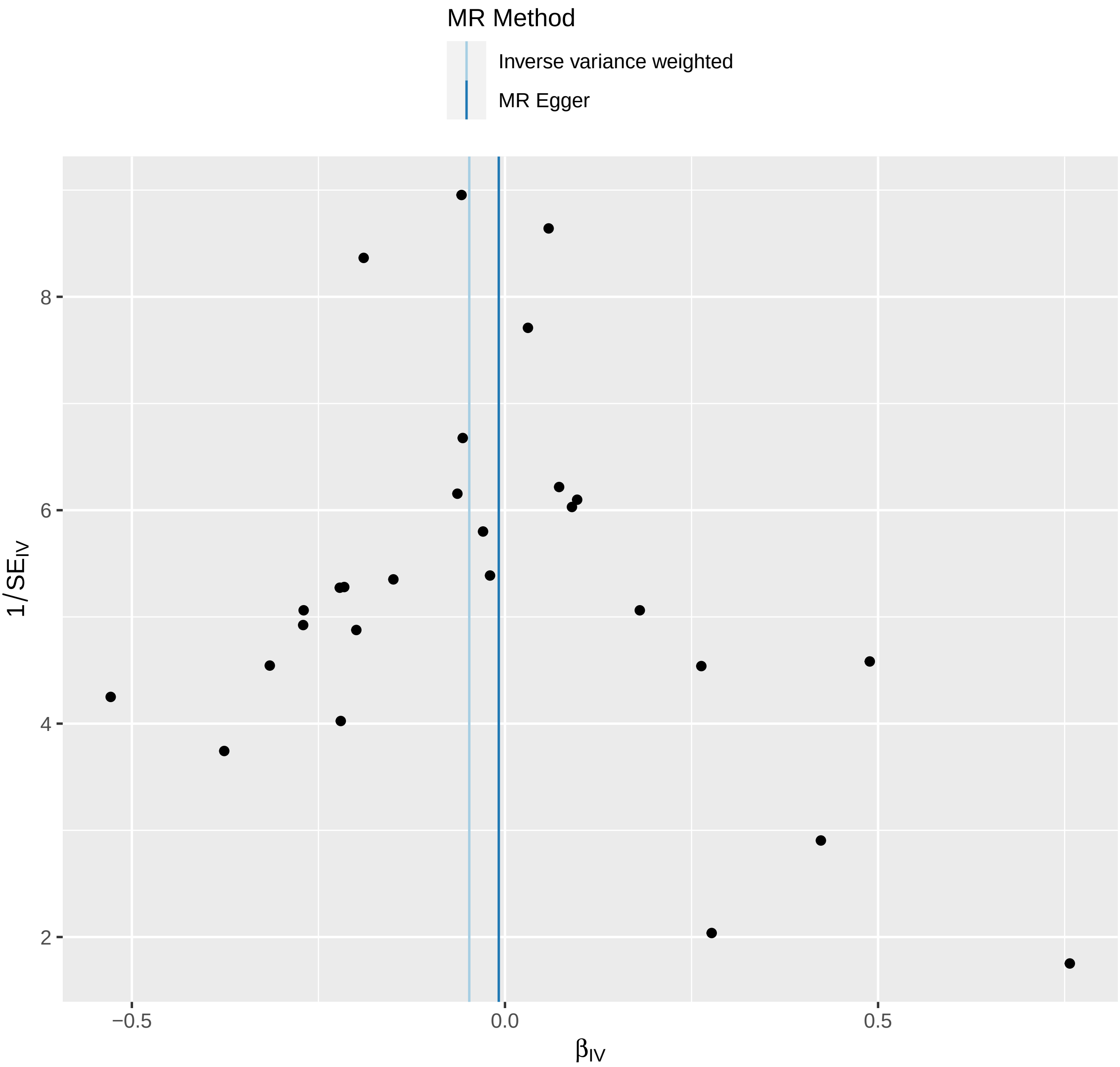

Batch 3−3213 : Vesicle transport through interaction with t−SNAREs homolog 1B on Diabetic nephropathy (more control exclusions) with a threshold of 1e−05

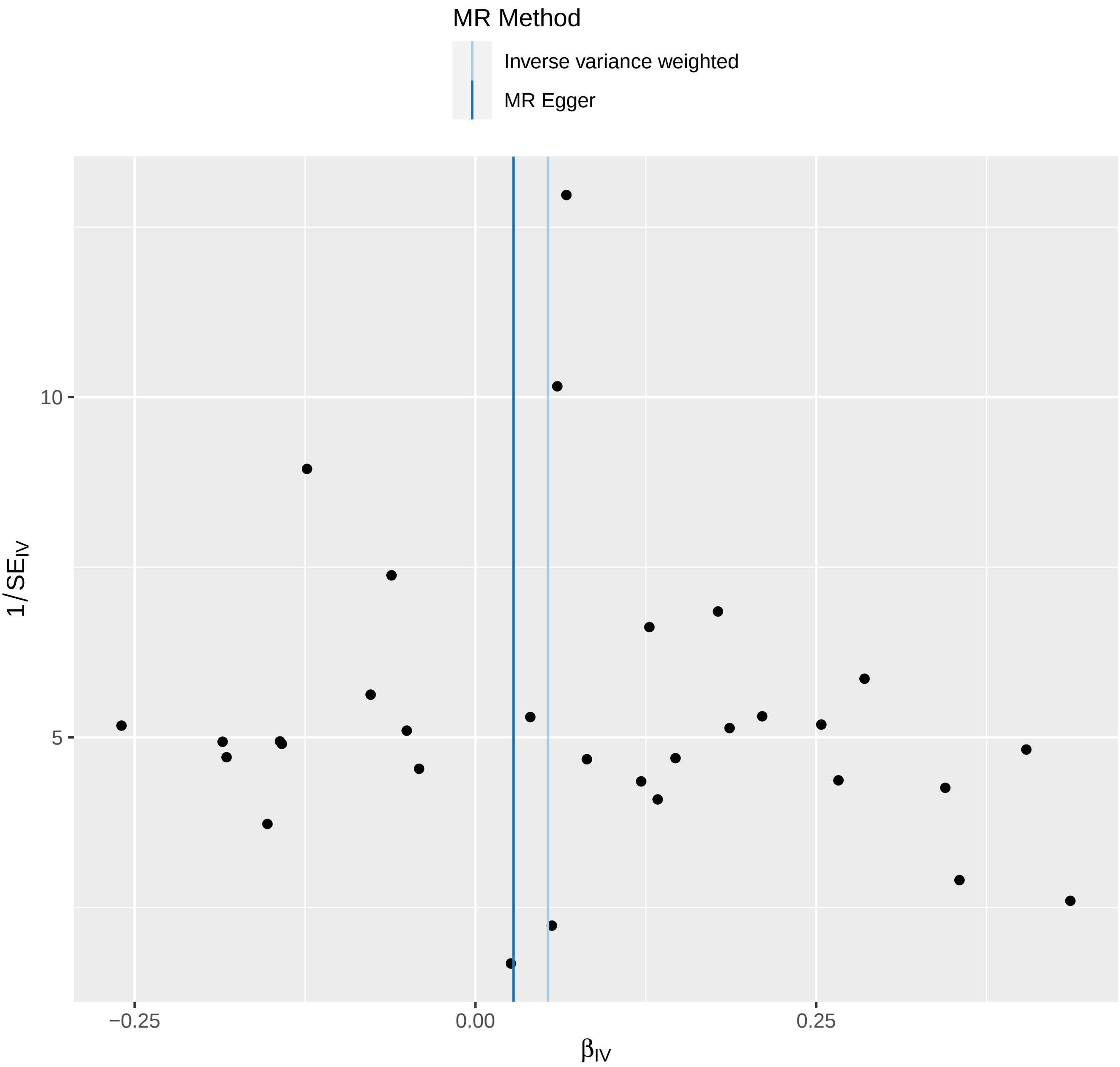

Batch 3−3214 : Vitronectin on Diabetic nephropathy (more control exclusions)
with a threshold of 1e−05

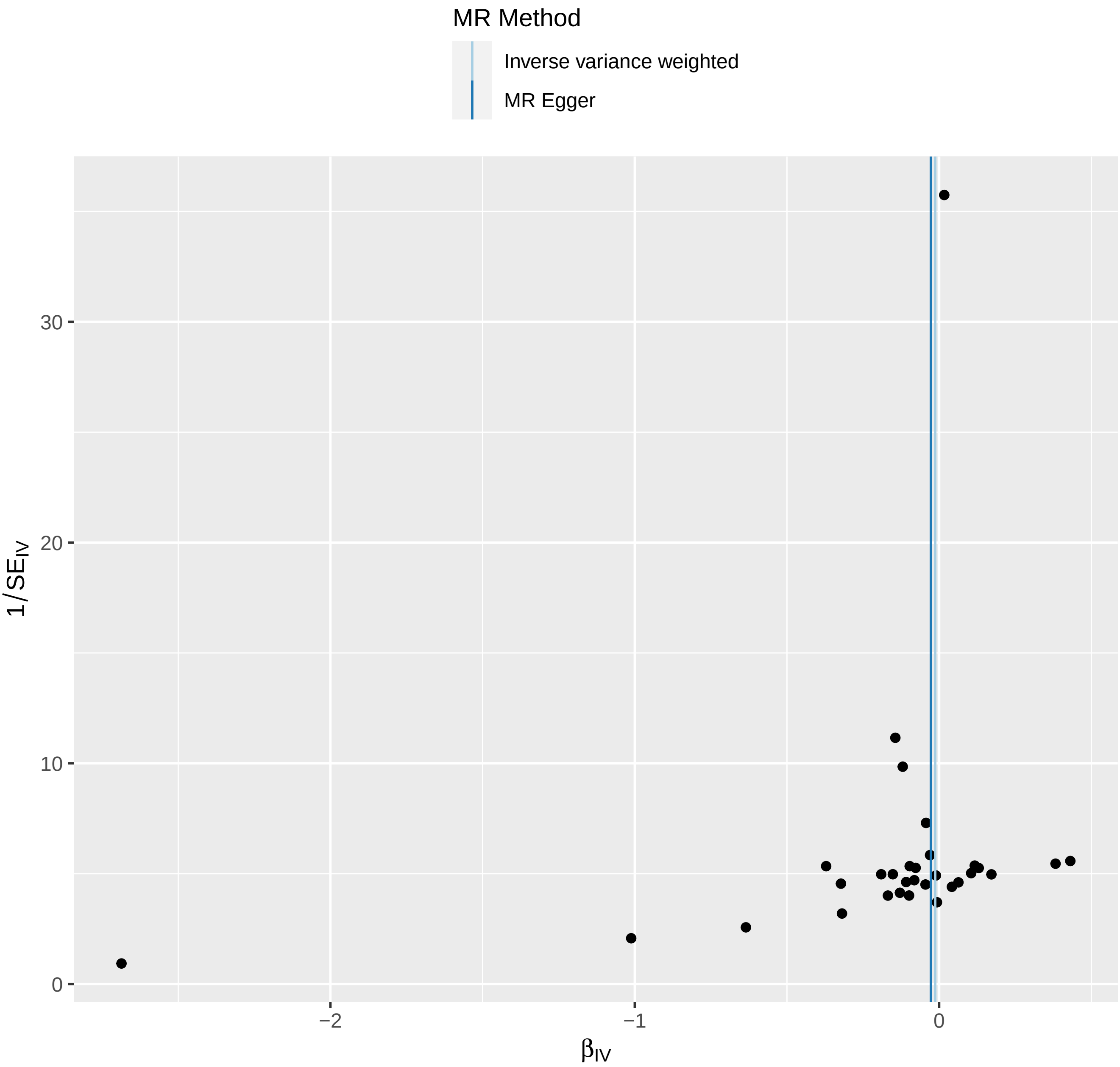

Batch 3−3215 : Vitronectin on Diabetic nephropathy (more control exclusions)
with a threshold of 1e−05

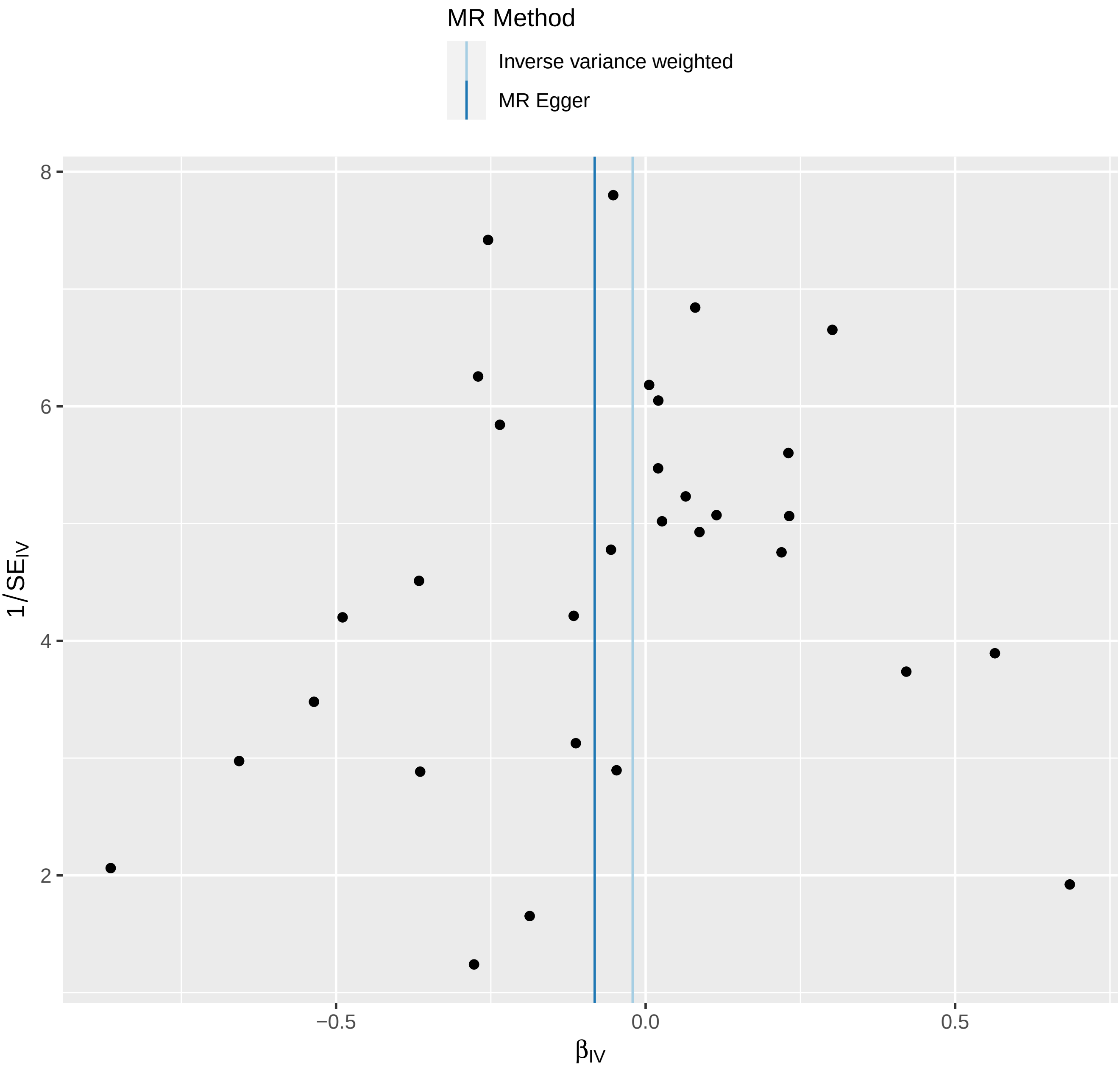

Batch 3−3216 : Brorin on Diabetic nephropathy (more control exclusions)
with a threshold of 1e−05

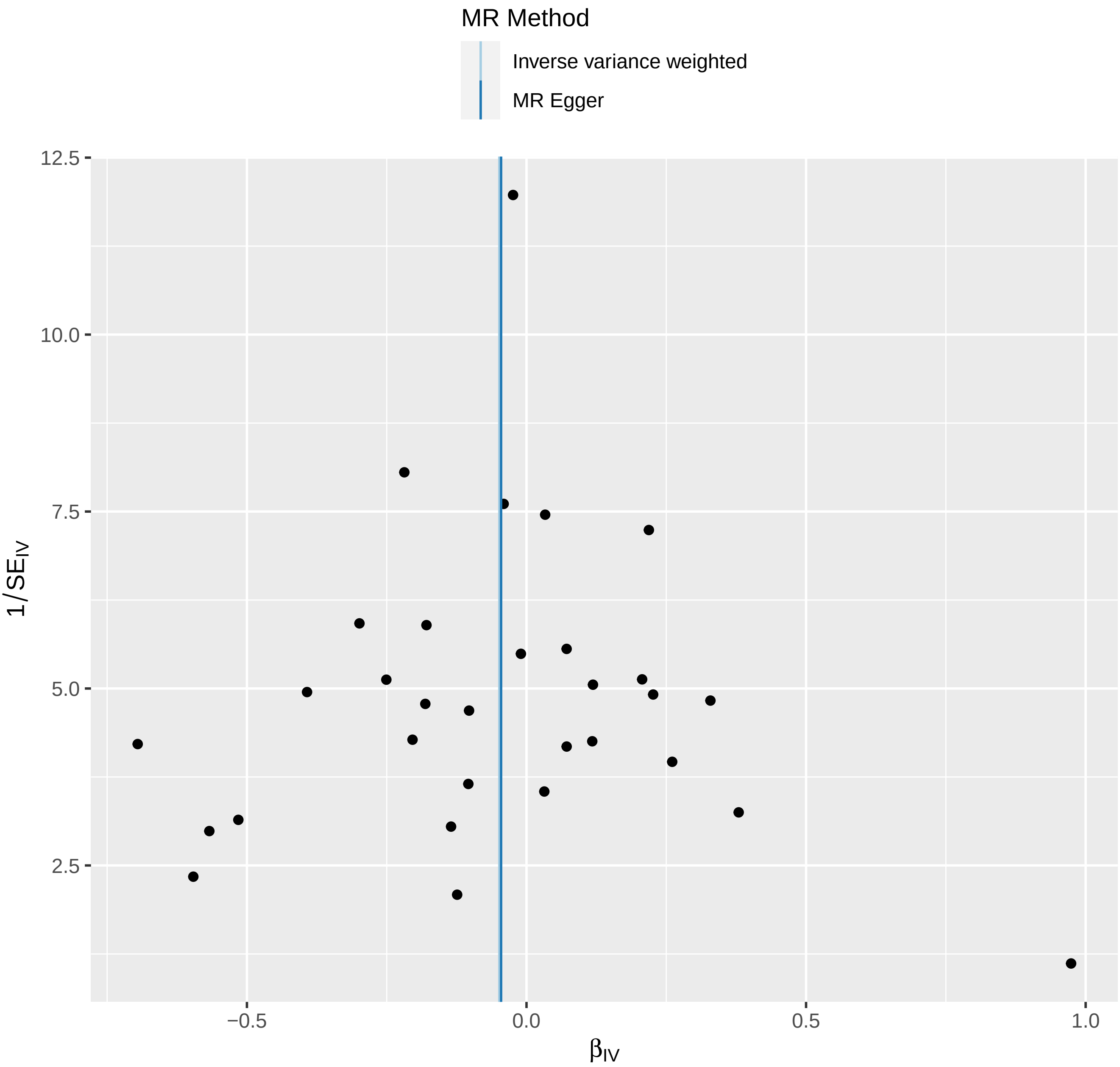

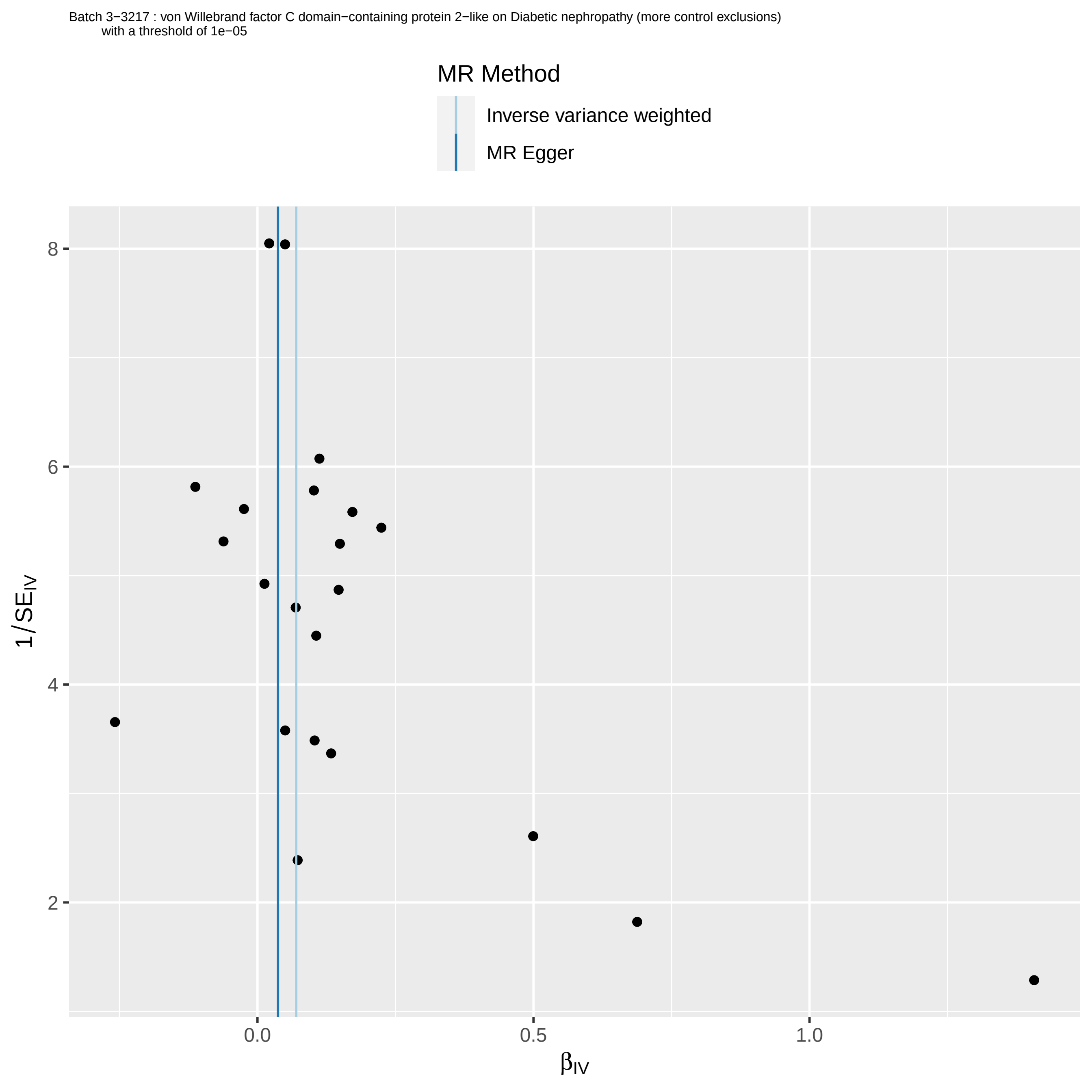
Batch 3−3217 : von Willebrand factor C domain−containing protein 2−like on Diabetic nephropathy (more control exclusions)
with a threshold of 1e−05
MR Method
Inverse variance weighted
MR Egger
8
6
4
2
1/SE$_{IV}$
0.0
0.5
1.0
β$_{IV}$

Batch 3−3218 : WW domain−binding protein 1 on Diabetic nephropathy (more control exclusions)
with a threshold of 1e−05

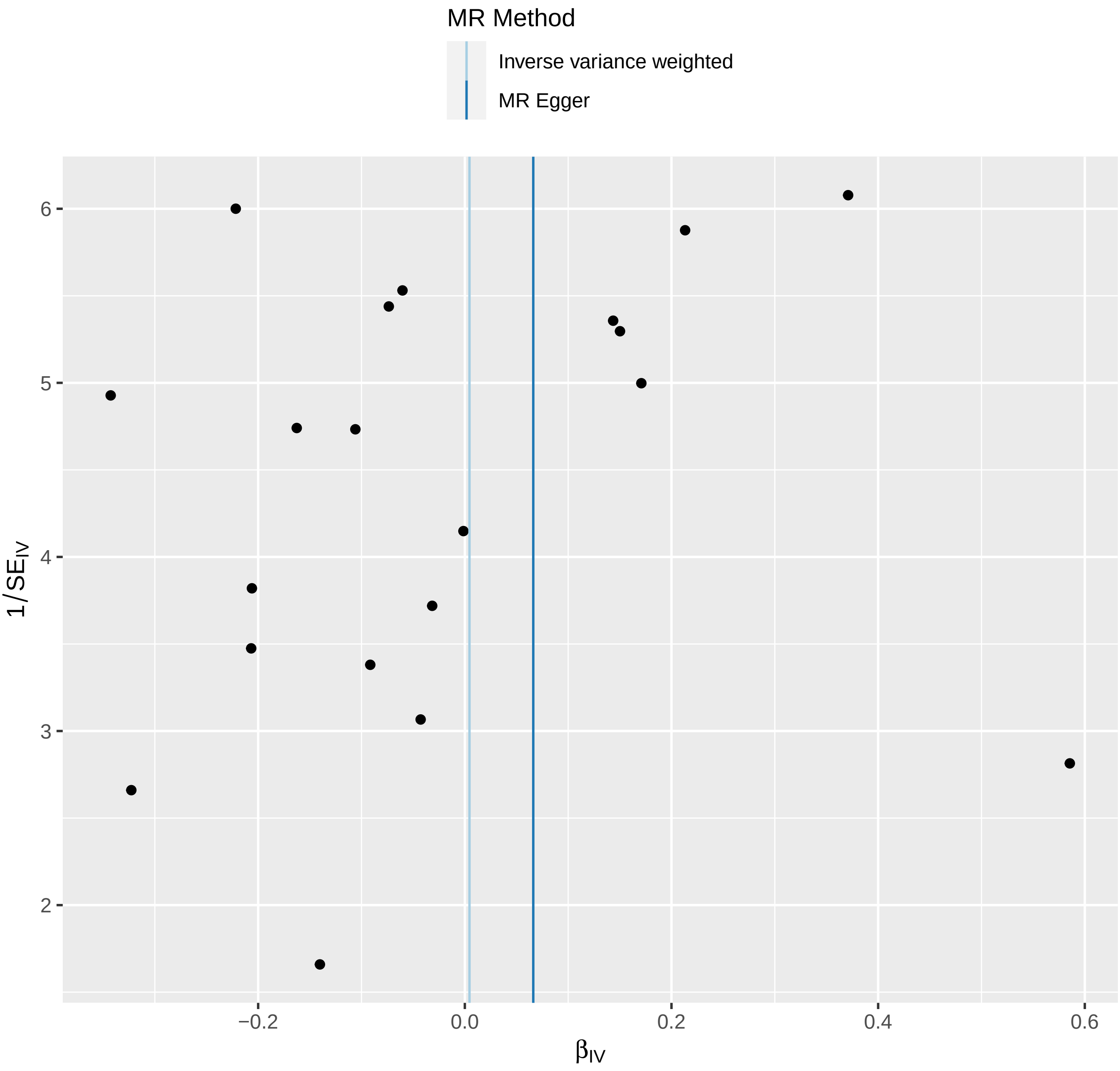

Batch 3−3219 : WW domain binding protein 1−like on Diabetic nephropathy (more control exclusions) with a threshold of 1e−05

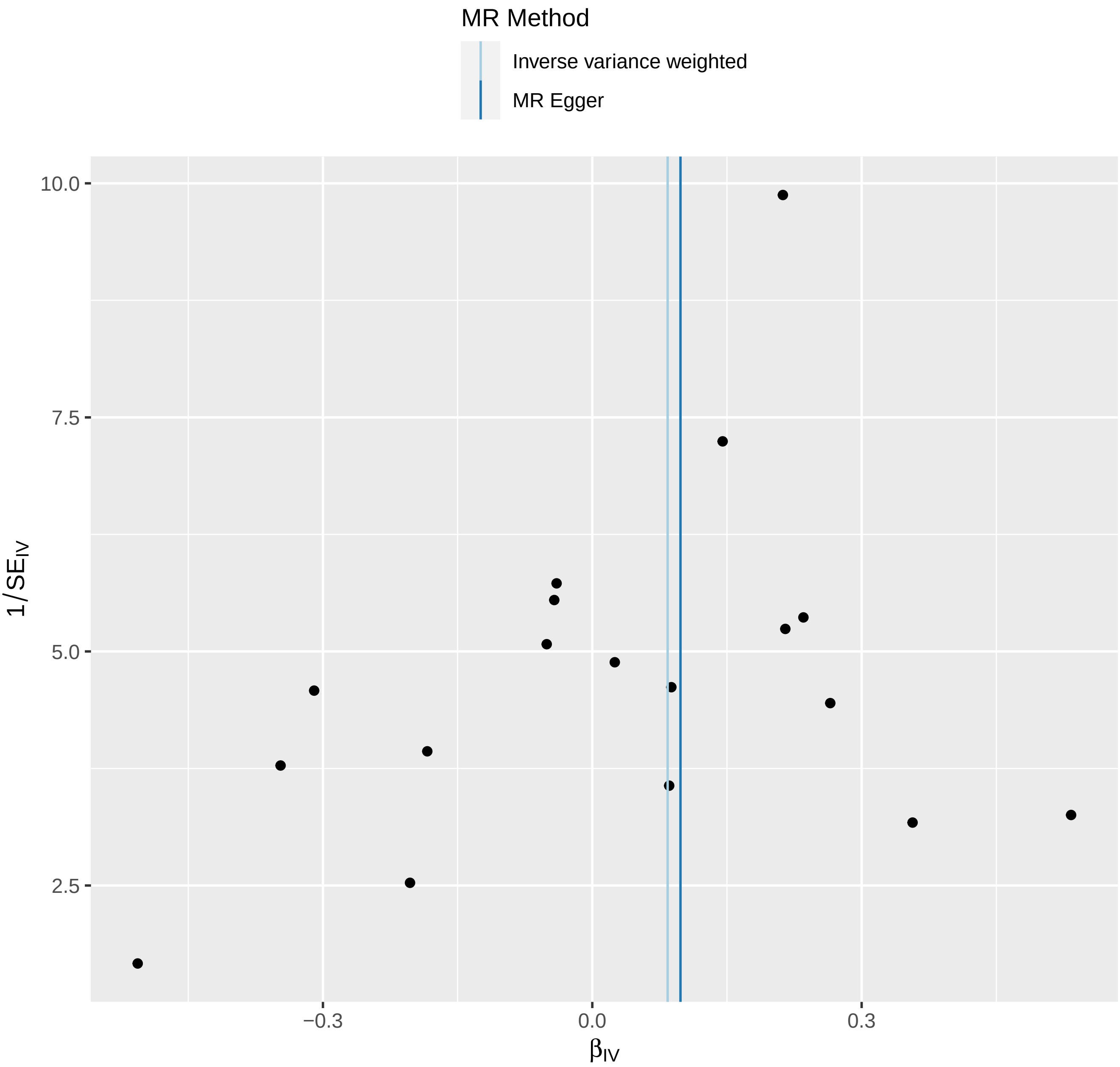

Batch 3−3220 : WD repeat−containing protein 1 on Diabetic nephropathy (more control exclusions)
with a threshold of 1e−05

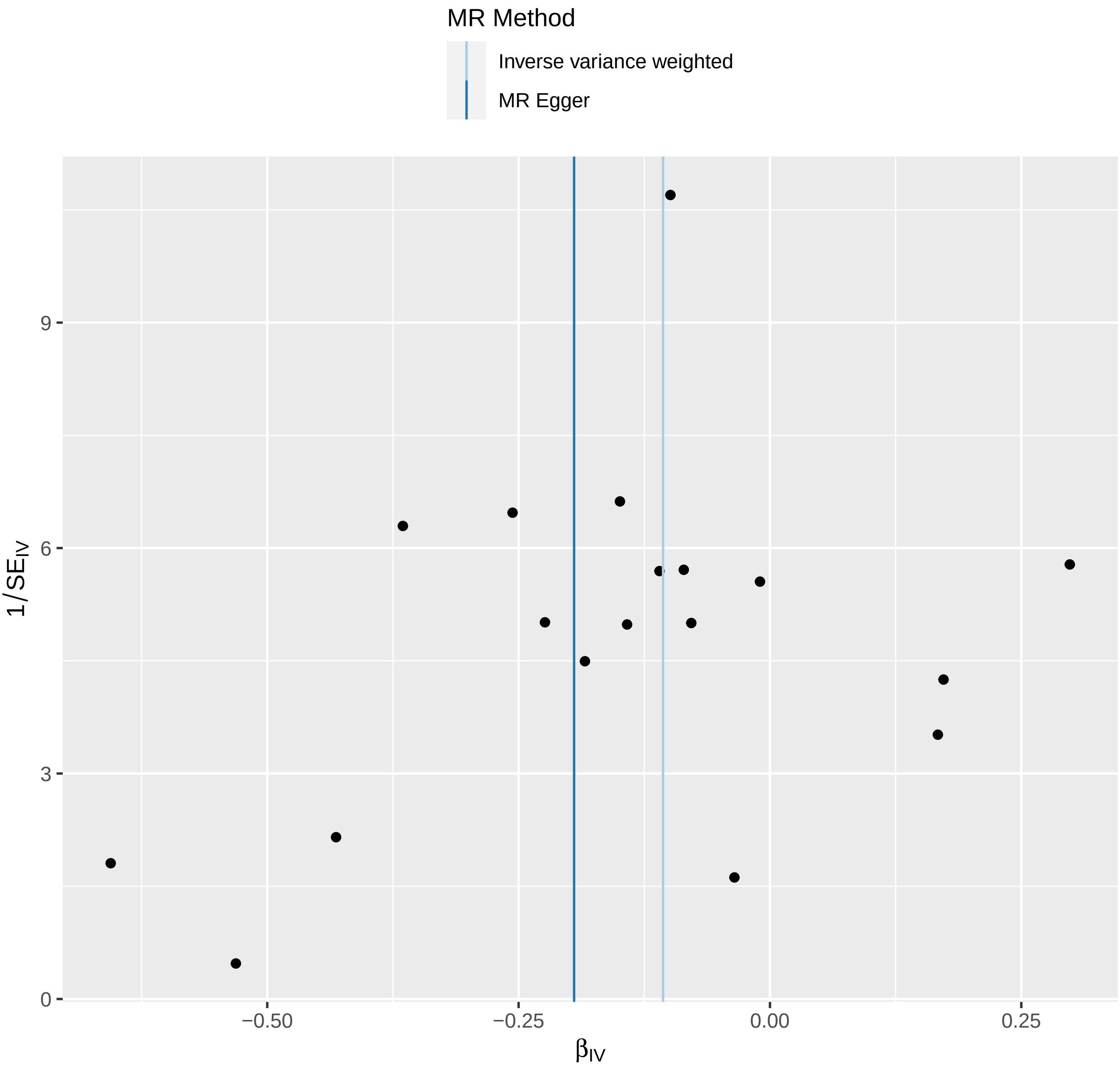

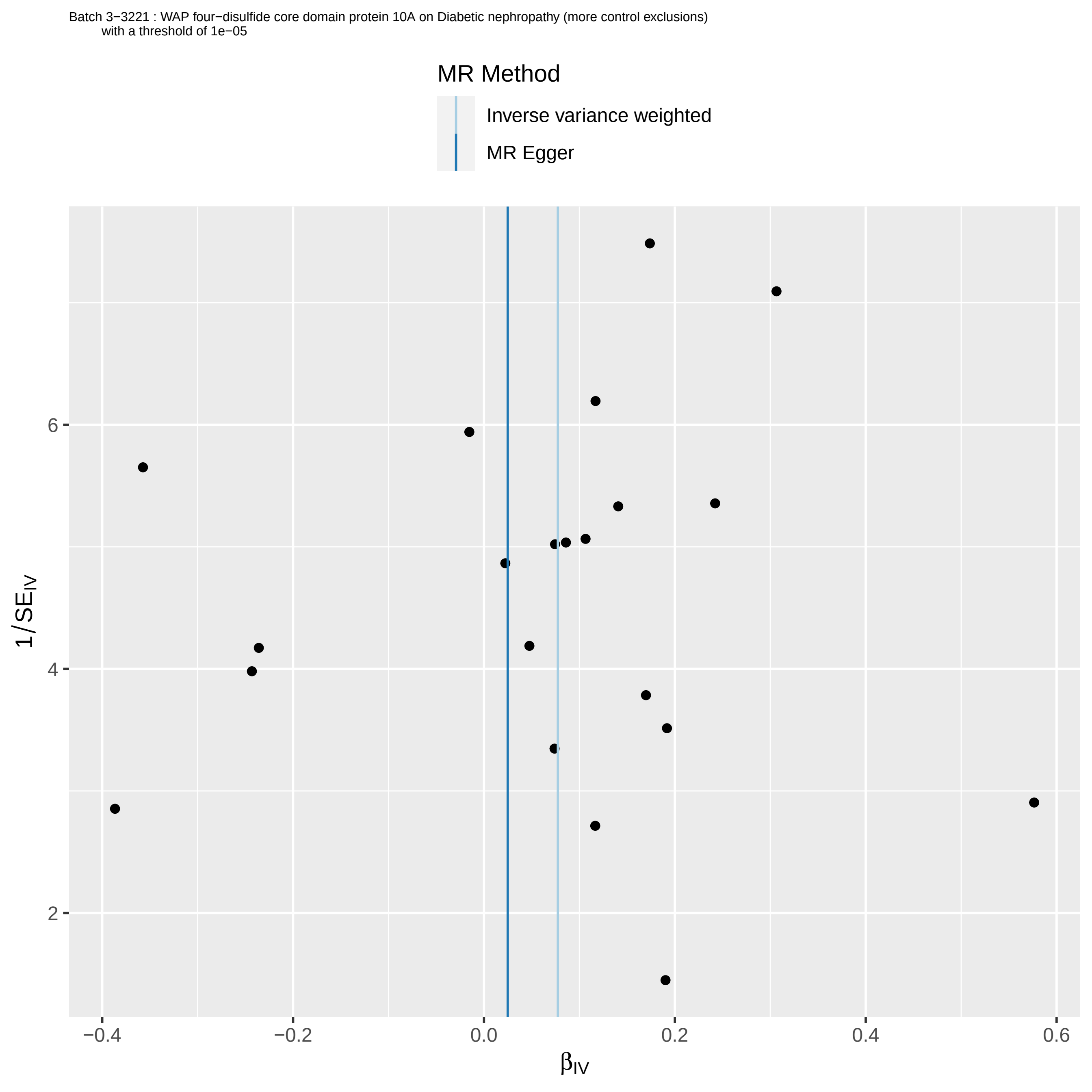

Batch 3−3221 : WAP four−disulfide core domain protein 10A on Diabetic nephropathy (more control exclusions)
with a threshold of 1e−05
MR Method
Inverse variance weighted
MR Egger
6
4
2
1/SE_IV
−0.4
−0.2
0.0
0.2
0.4
0.6
β_IV

Batch 3−3222 : WAP four−disulfide core domain protein 12 on Diabetic nephropathy (more control exclusions)
with a threshold of 1e−05

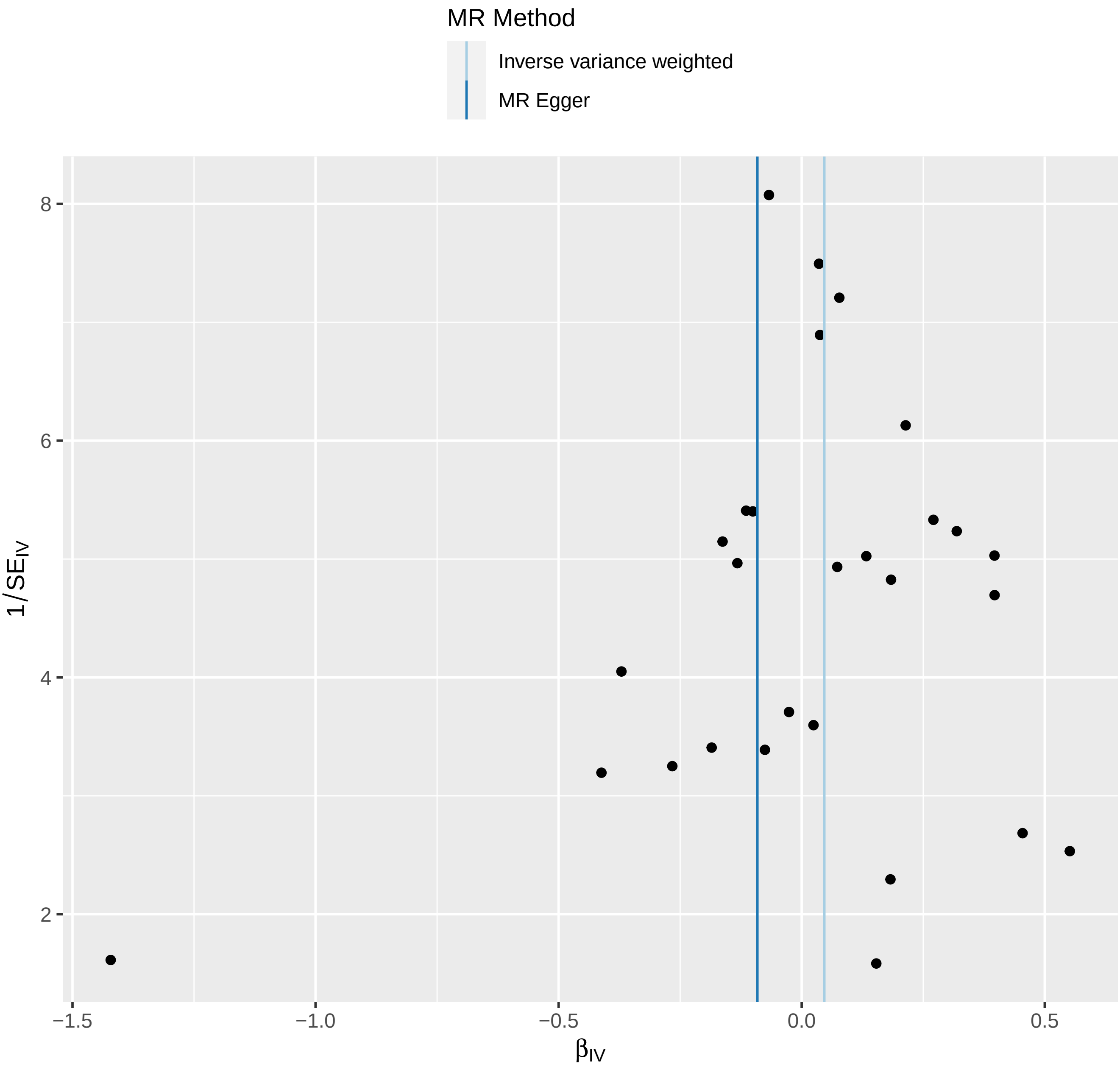

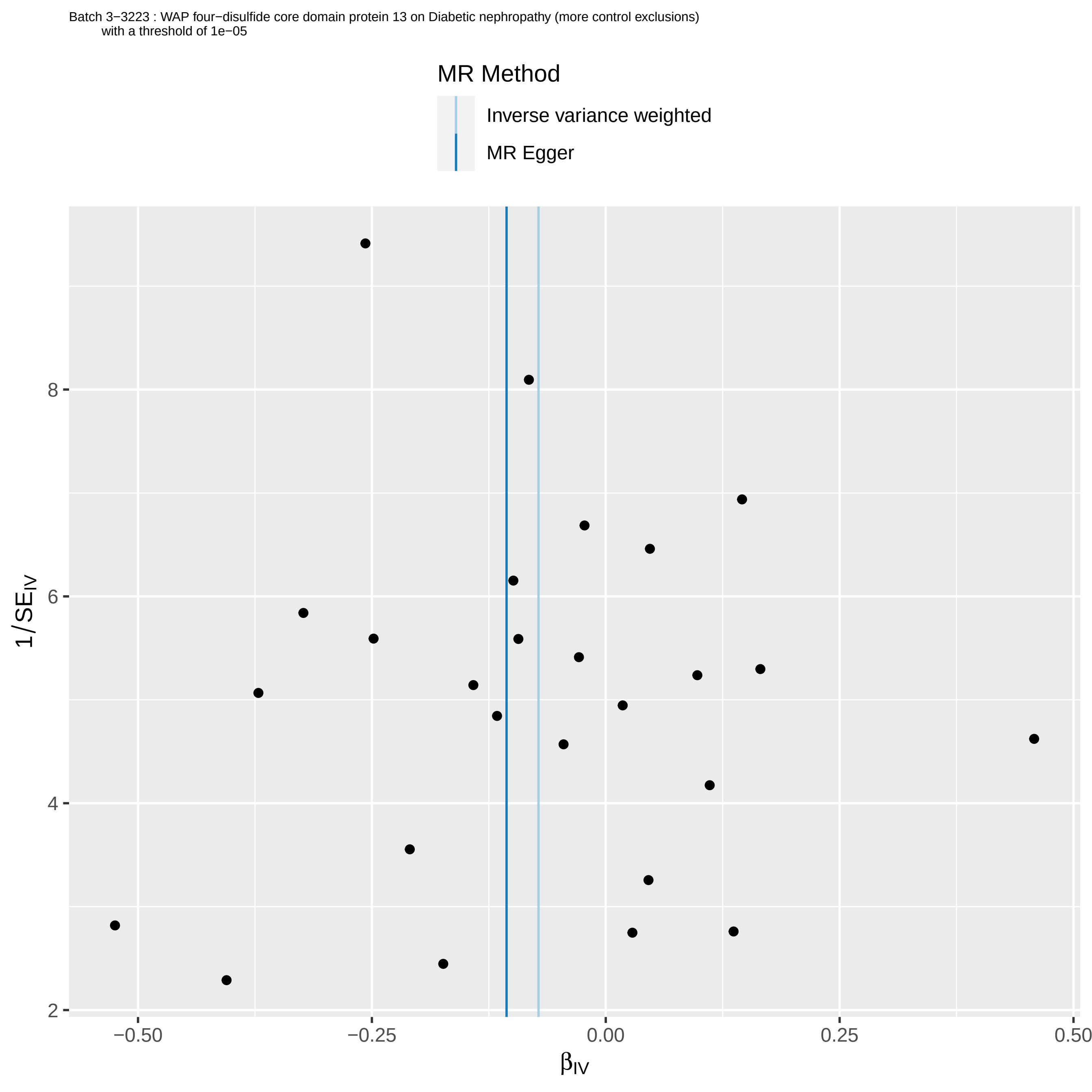
Batch 3−3223 : WAP four−disulfide core domain protein 13 on Diabetic nephropathy (more control exclusions)
with a threshold of 1e−05
MR Method
Inverse variance weighted
MR Egger
1/SE_IV
8
6
4
2
−0.50
−0.25
0.00
0.25
0.50
β_IV

Batch 3−3224 : WAP four−disulfide core domain protein 2 on Diabetic nephropathy (more control exclusions)
with a threshold of 1e−05

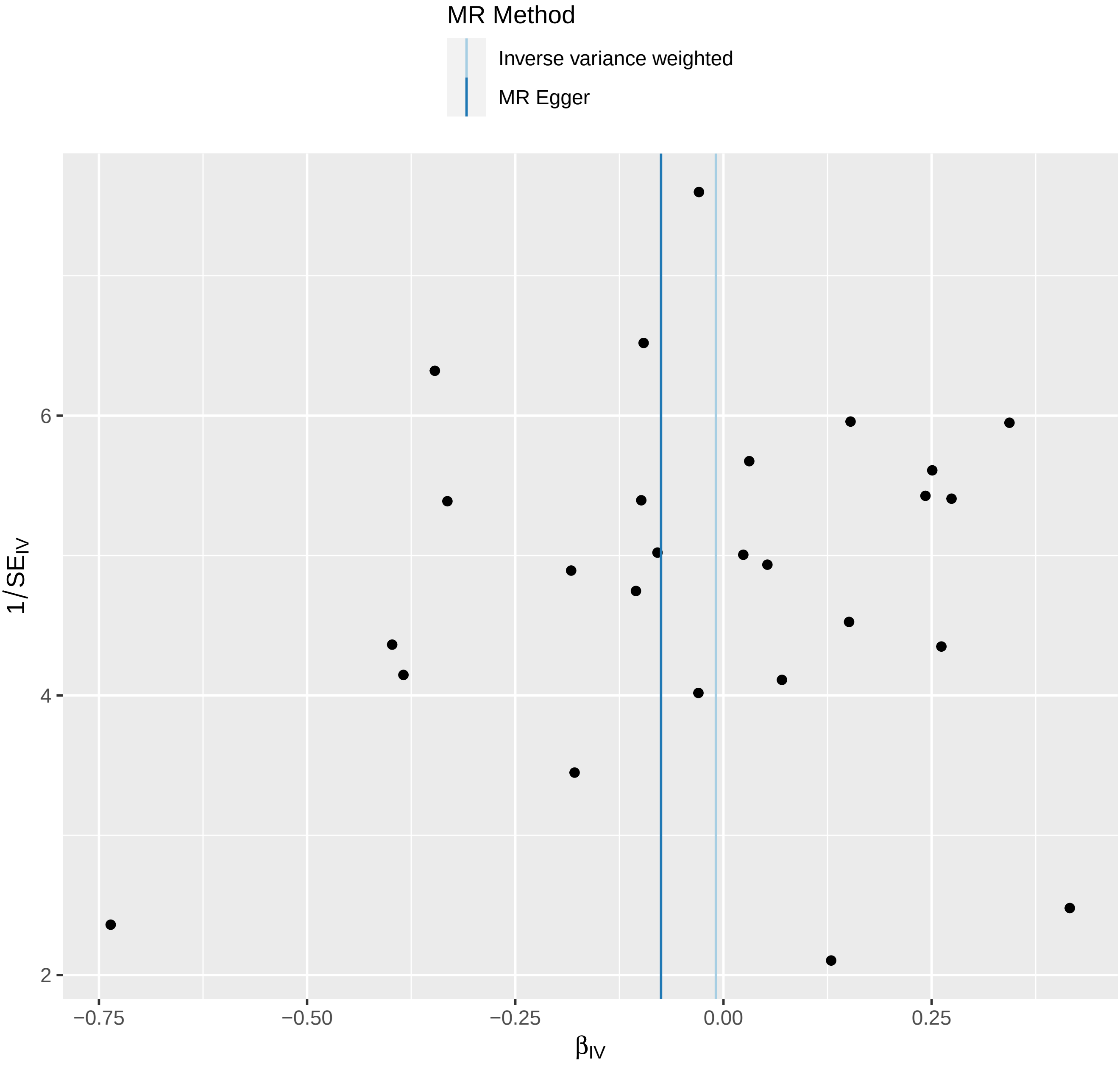

Batch 3−3225 : WAP four−disulfide core domain protein 3 on Diabetic nephropathy (more control exclusions)
with a threshold of 1e−05

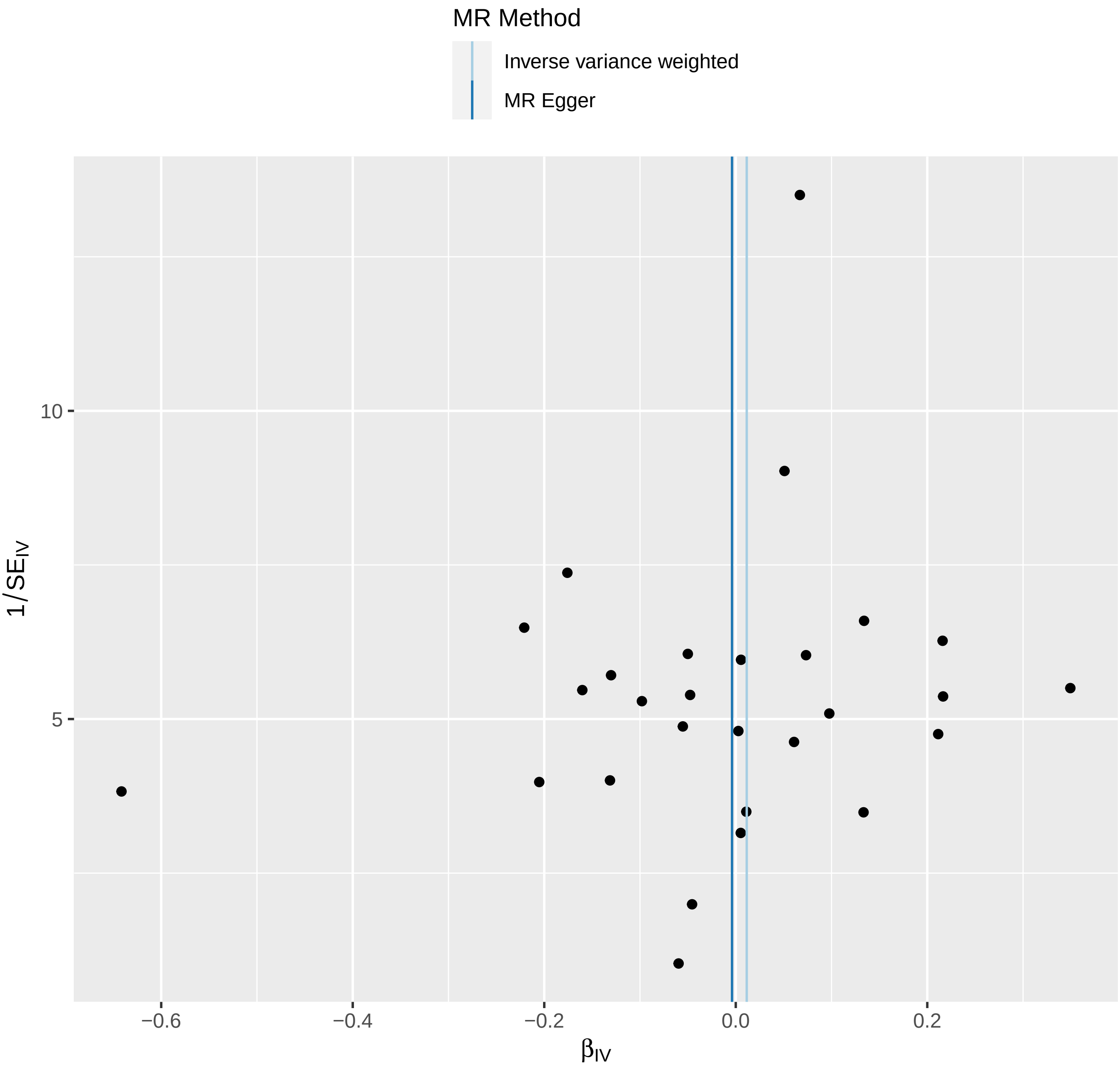

Batch 3−3226 : WAP four−disulfide core domain protein 5 on Diabetic nephropathy (more control exclusions)
with a threshold of 1e−05

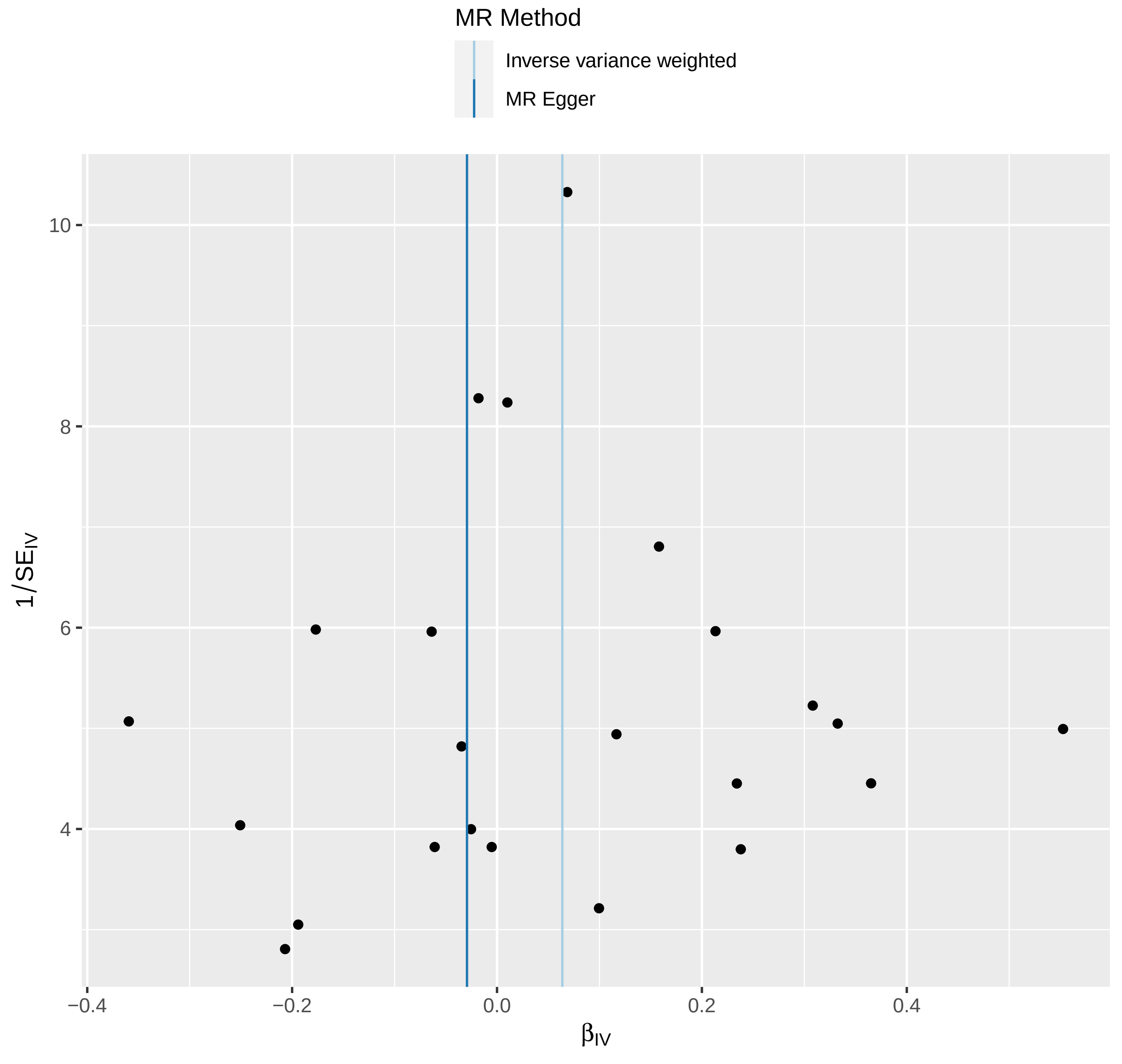

Batch 3−3227 : WAP, kazal, immunoglobulin, kunitz and NTR domain−containing protein 1 on Diabetic nephropathy (more control exclusions)
with a threshold of 1e−05

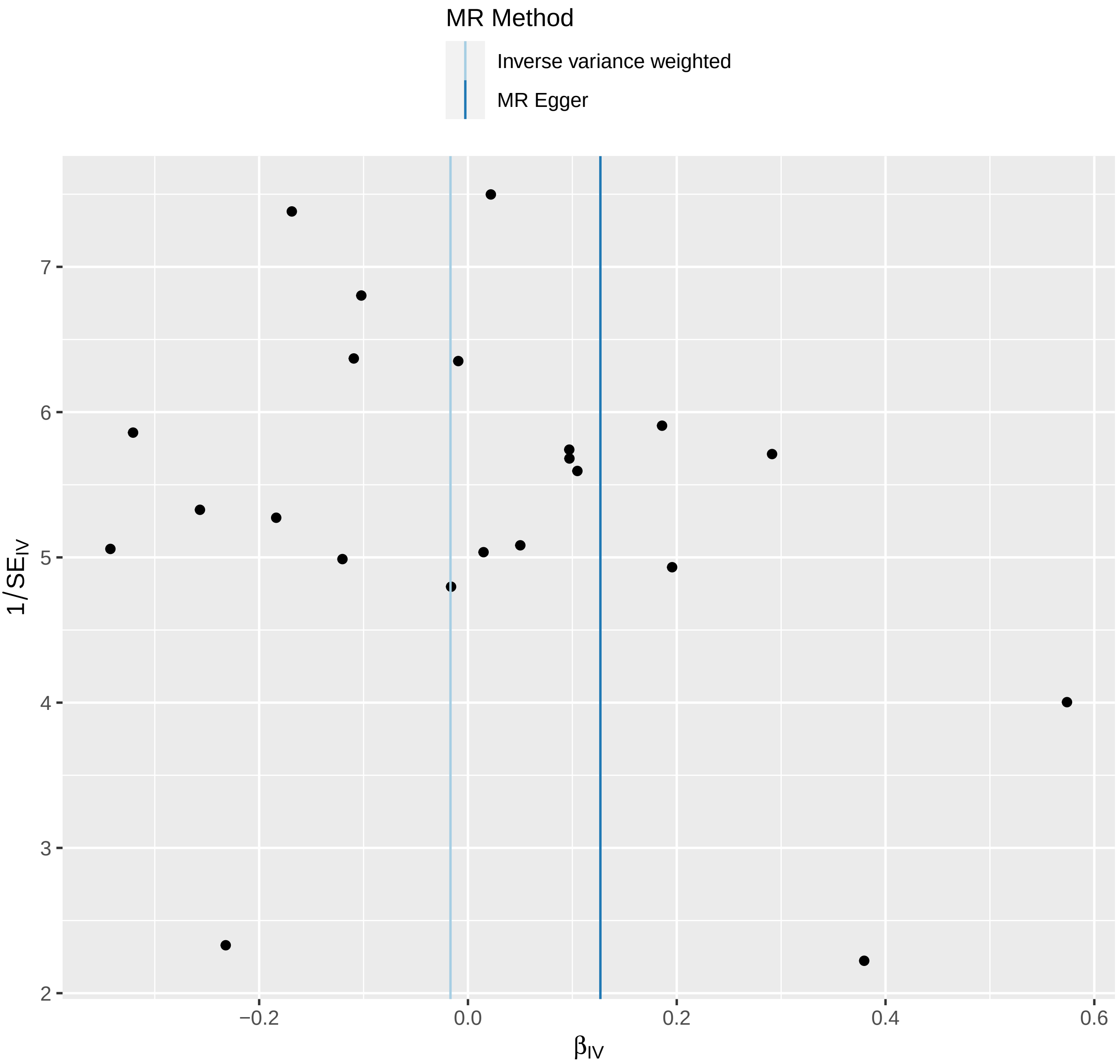

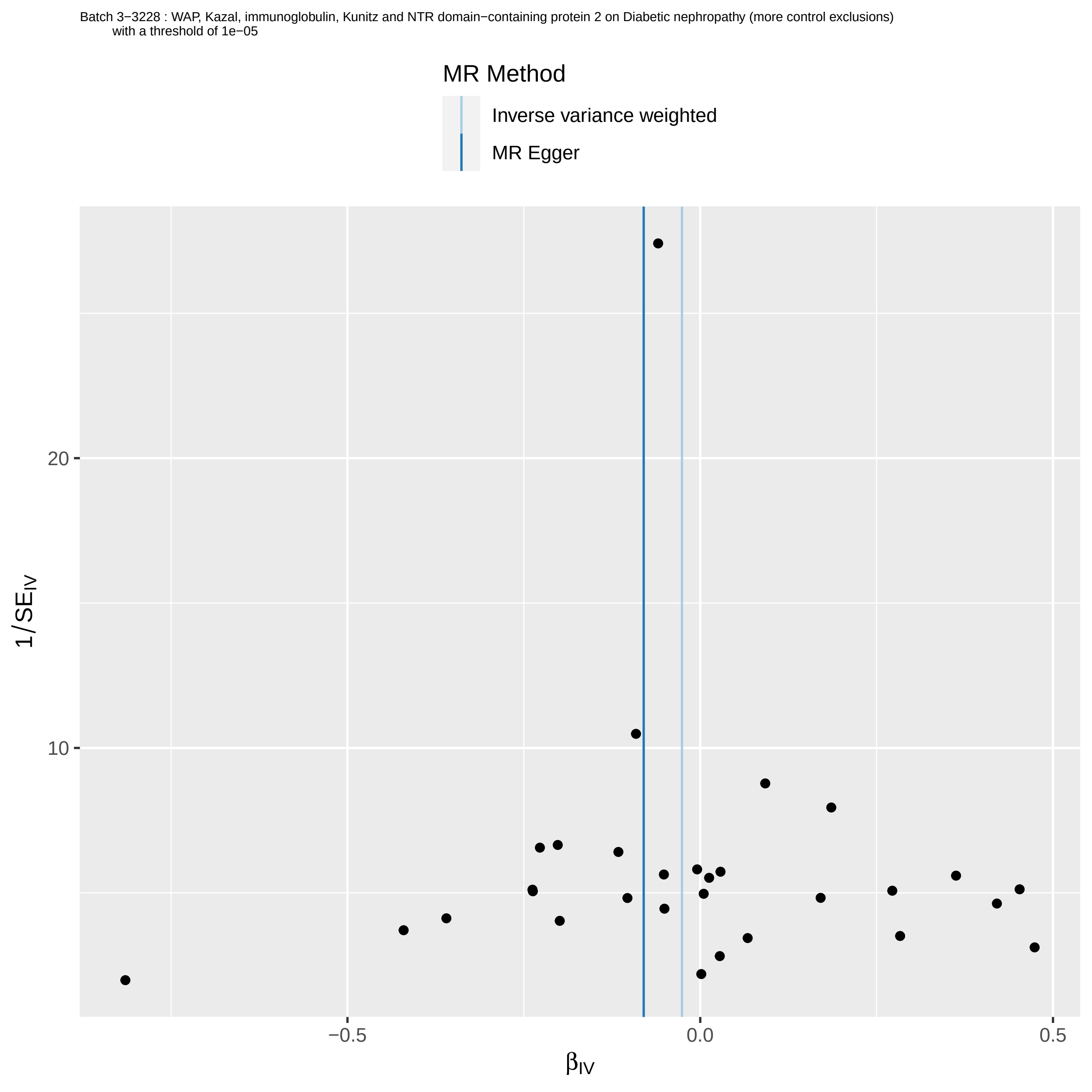
Batch 3−3228 : WAP, Kazal, immunoglobulin, Kunitz and NTR domain−containing protein 2 on Diabetic nephropathy (more control exclusions)
with a threshold of 1e−05
MR Method
Inverse variance weighted
MR Egger
20
10
1/SE$_{IV}$
−0.5
0.0
0.5
β$_{IV}$

Batch 3−3229 : WAP, Kazal, immunoglobulin, Kunitz and NTR domain−containing protein 2 on Diabetic nephropathy (more control exclusions)
with a threshold of 1e−05

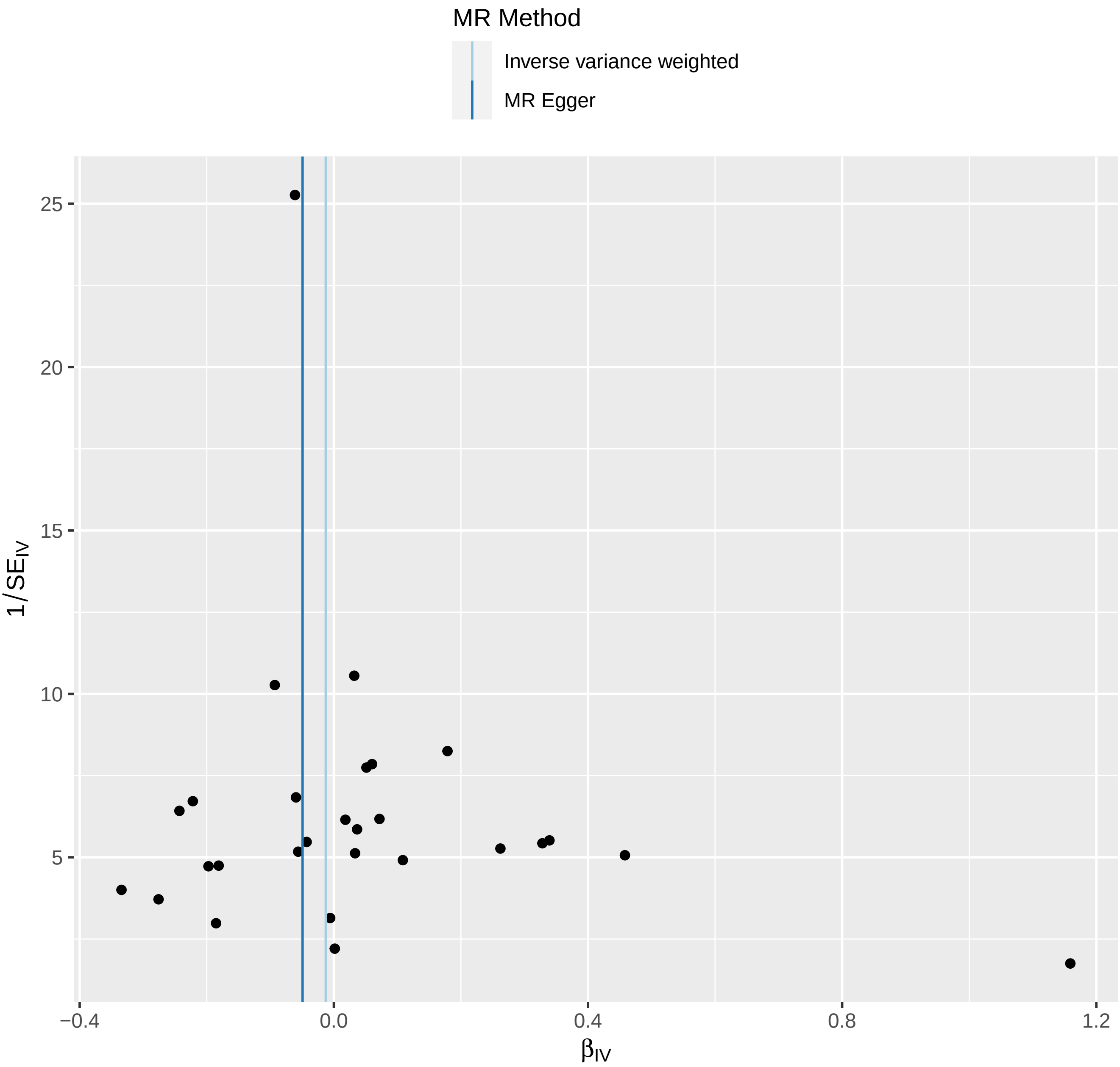

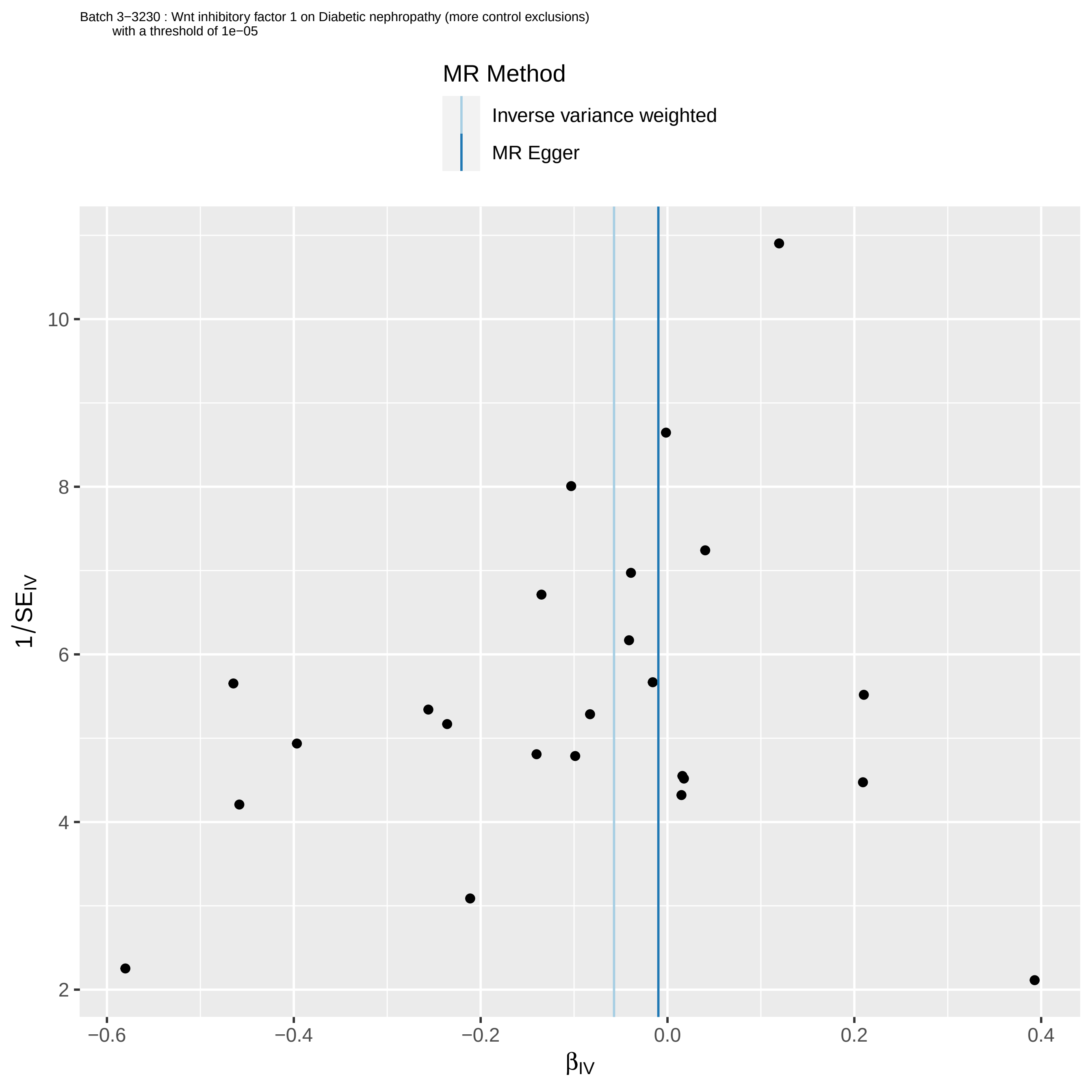

Batch 3−3230 : Wnt inhibitory factor 1 on Diabetic nephropathy (more control exclusions)
with a threshold of 1e−05
MR Method
Inverse variance weighted
MR Egger
10
8
6
4
2
1/SE_IV
−0.6
−0.4
−0.2
0.0
0.2
0.4
β_IV

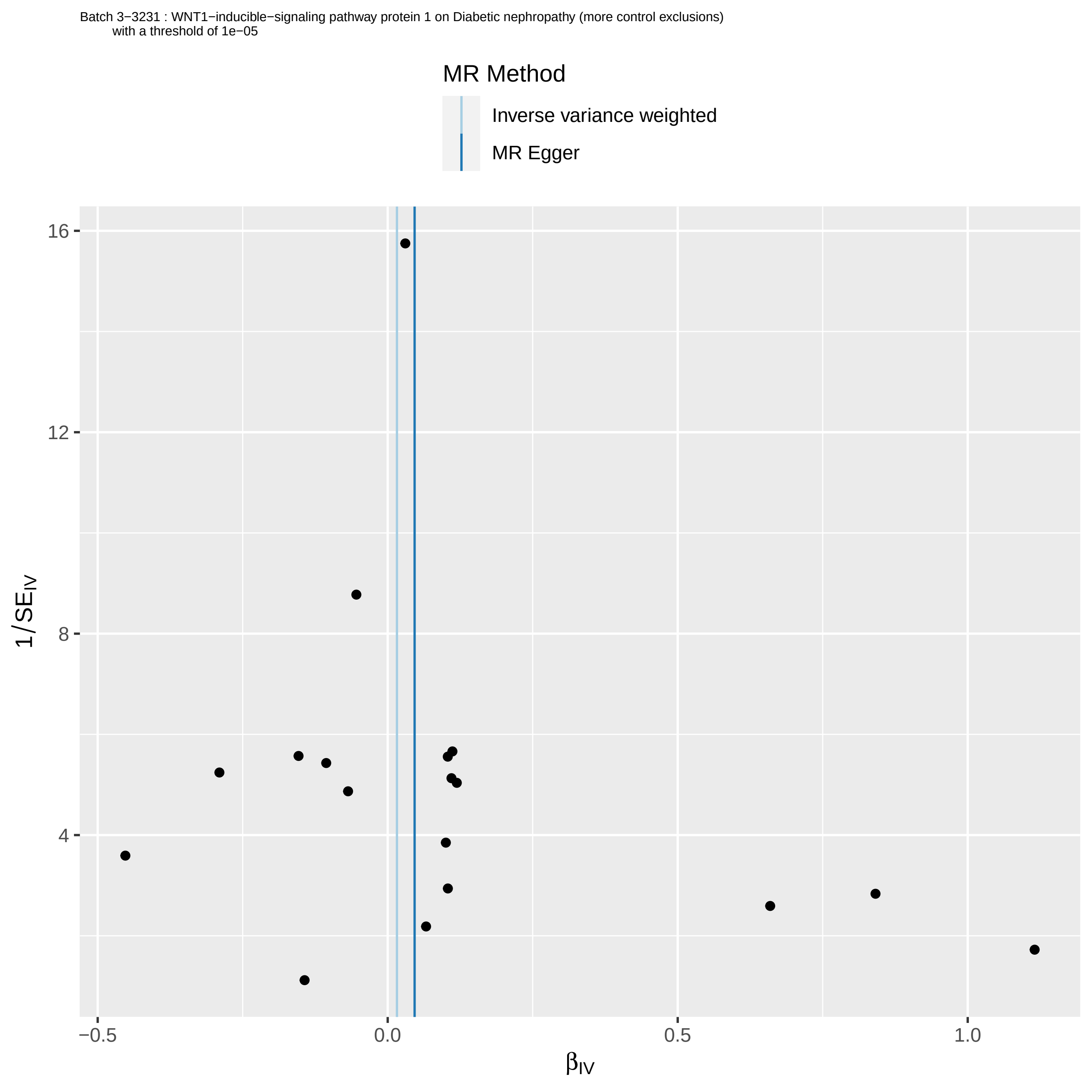
Batch 3−3231 : WNT1−inducible−signaling pathway protein 1 on Diabetic nephropathy (more control exclusions)
with a threshold of 1e−05
MR Method
Inverse variance weighted
MR Egger
16
12
8
4
1/SE_IV
−0.5
0.0
0.5
1.0
β_IV

Batch 3−3232 : WNT1−inducible−signaling pathway protein 3 on Diabetic nephropathy (more control exclusions)
with a threshold of 1e−05

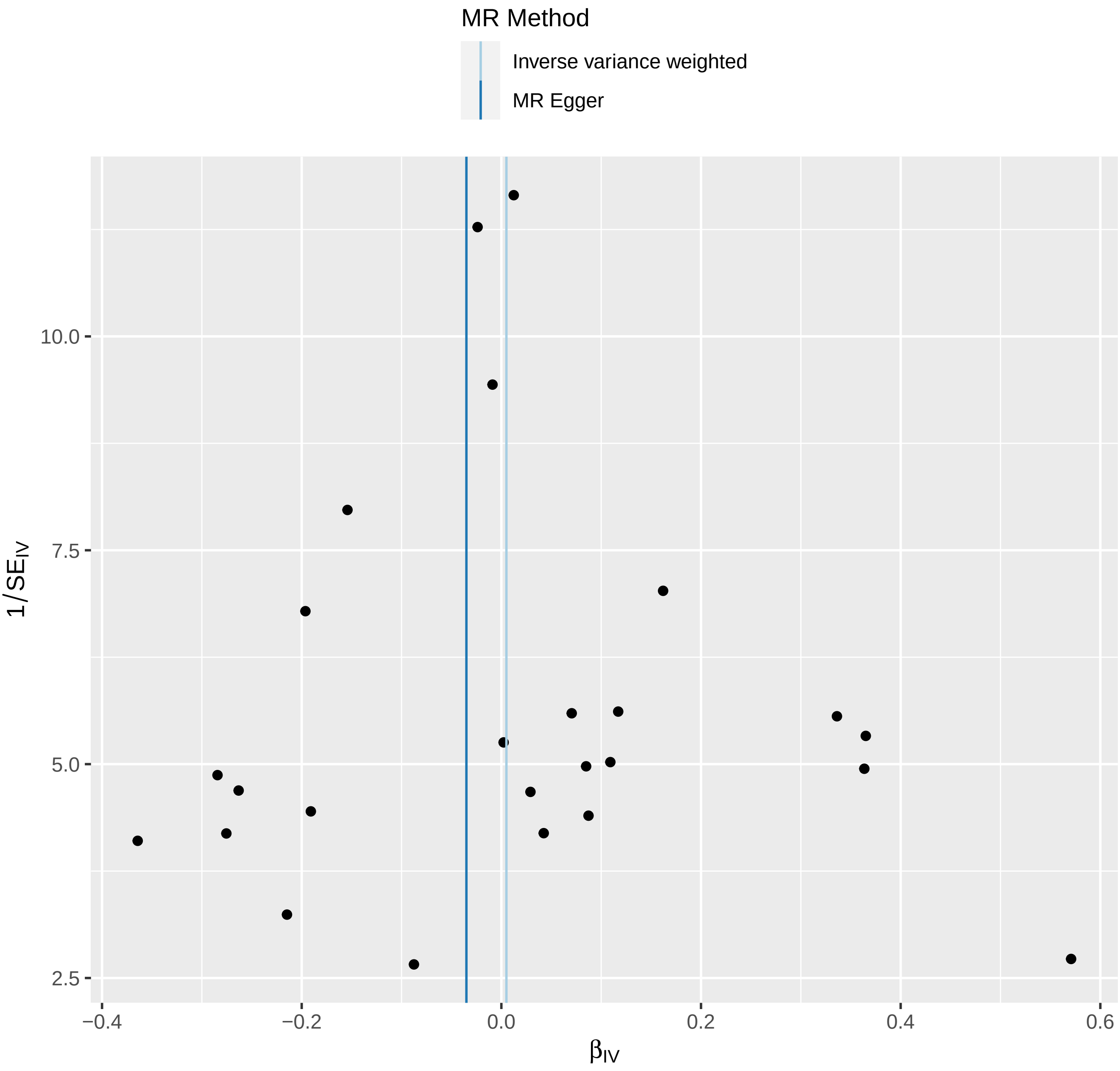

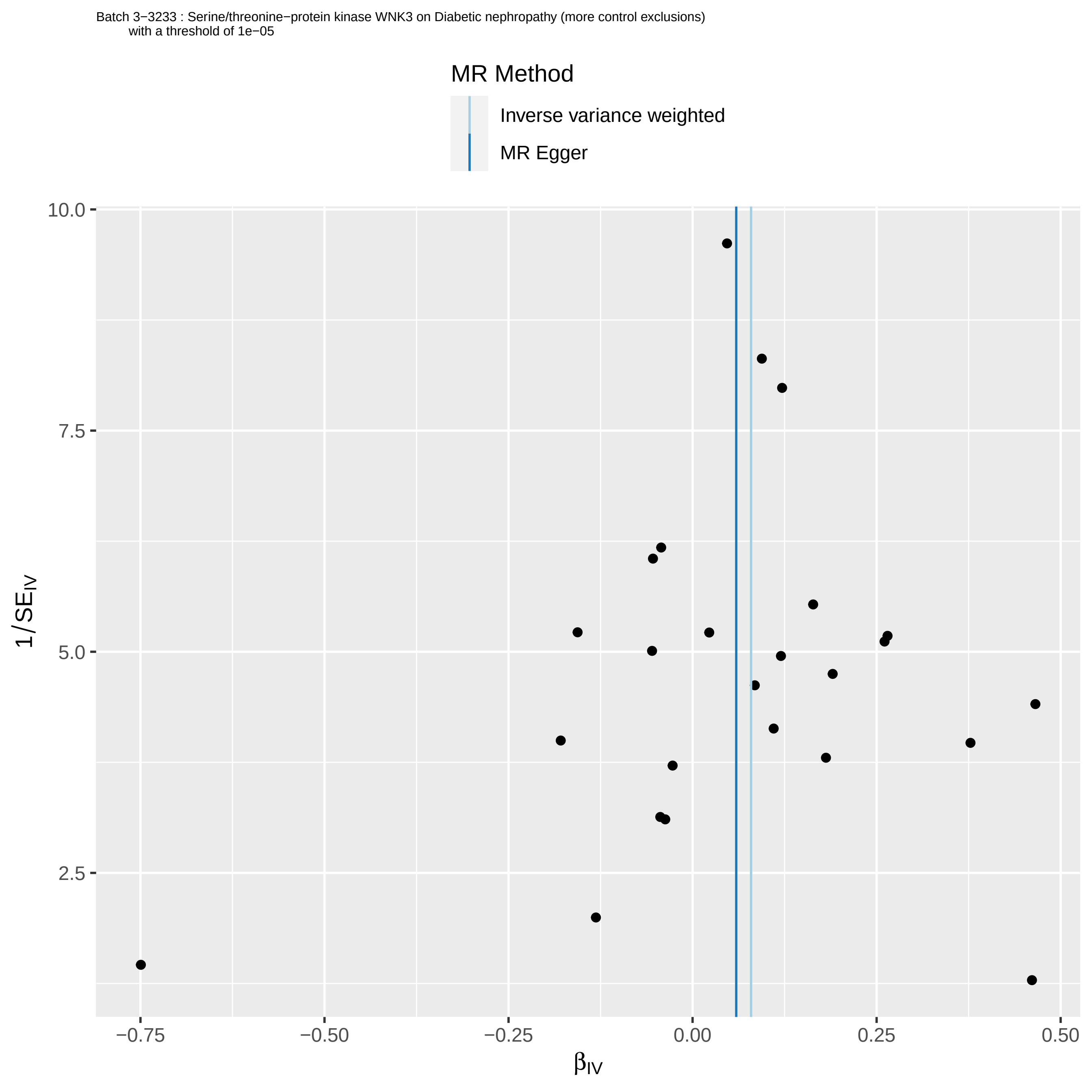

Batch 3−3233 : Serine/threonine−protein kinase WNK3 on Diabetic nephropathy (more control exclusions)
with a threshold of 1e−05
MR Method
Inverse variance weighted
MR Egger
10.0
7.5
5.0
2.5
1/SE_IV
−0.75
−0.50
−0.25
0.00
0.25
0.50
β_IV

Batch 3−3234 : Protein Wnt−7a on Diabetic nephropathy (more control exclusions)
with a threshold of 1e−05

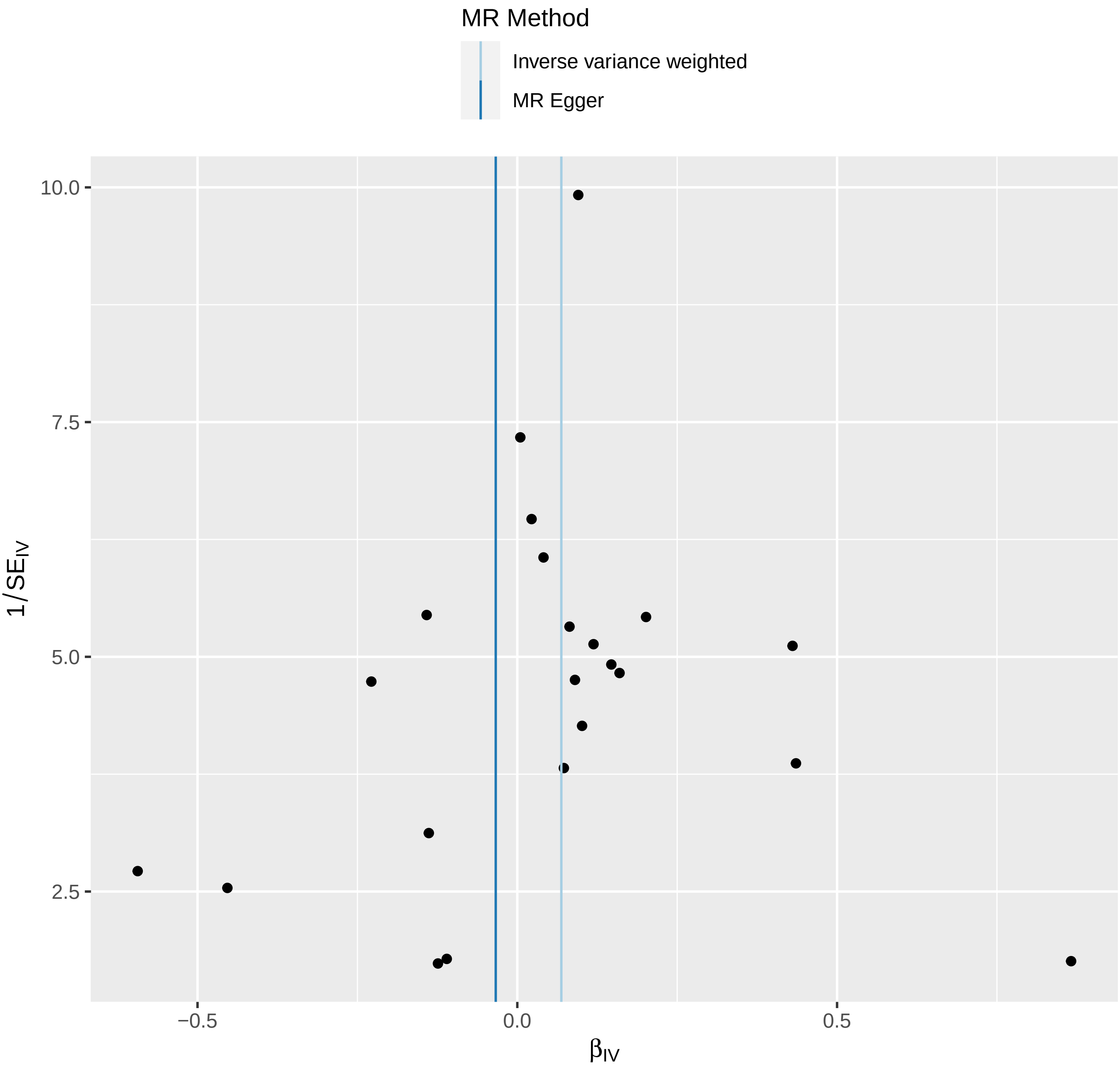

Batch 3−3235 : WSC domain−containing protein 2 on Diabetic nephropathy (more control exclusions)
with a threshold of 1e−05

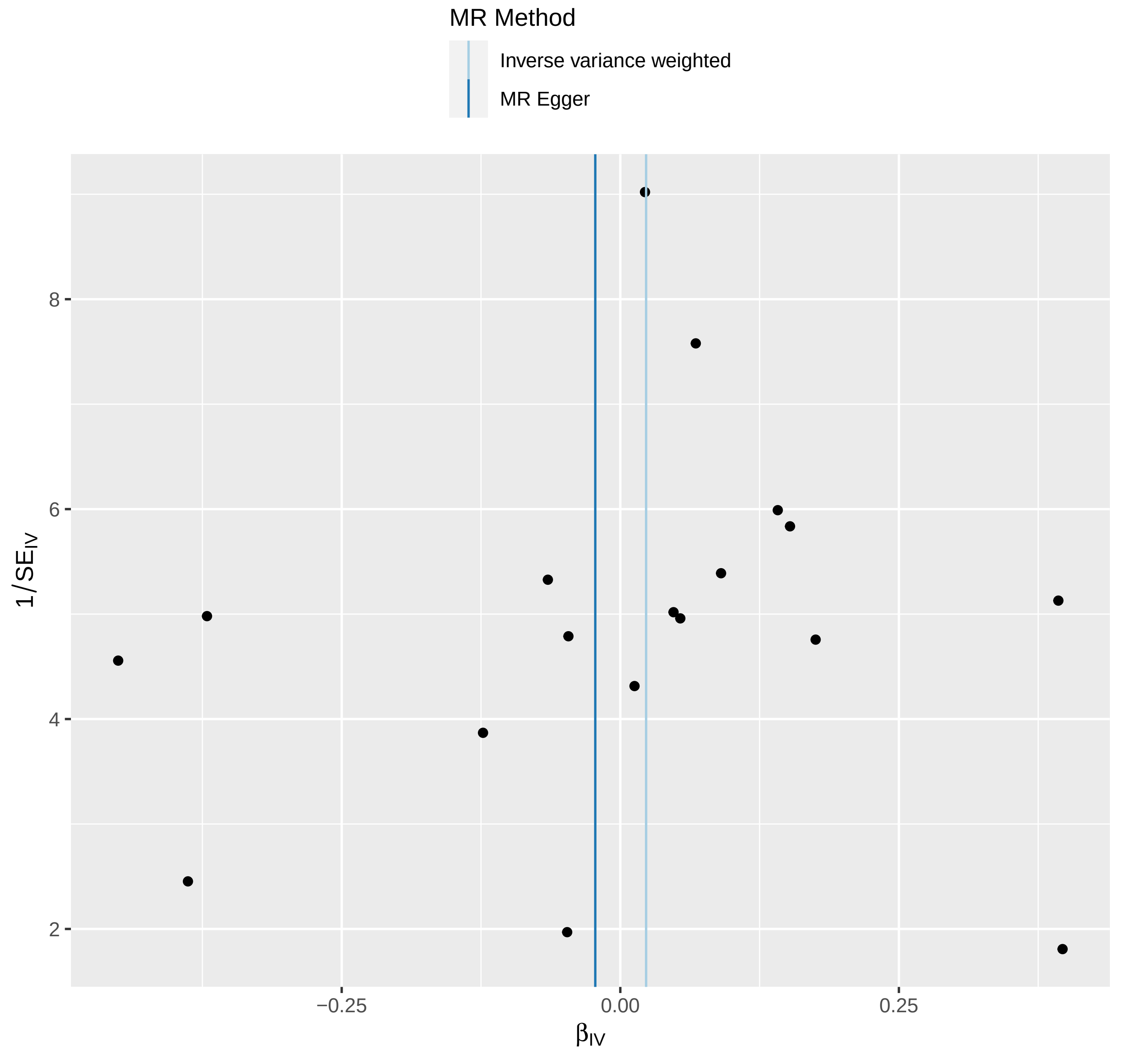

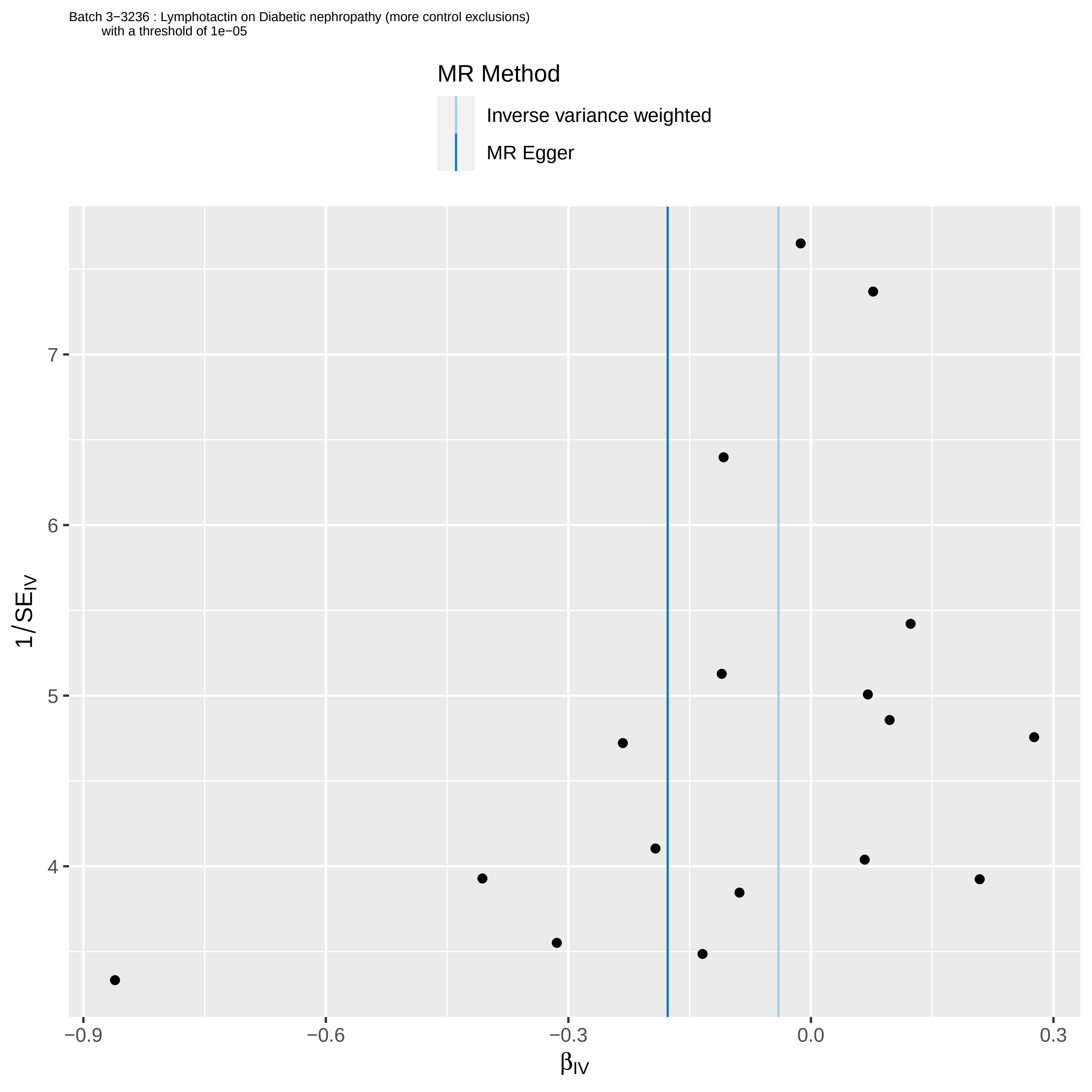

Batch 3−3236 : Lymphotactin on Diabetic nephropathy (more control exclusions)
with a threshold of 1e−05
MR Method
Inverse variance weighted
MR Egger
7
6
5
4
1/SE_IV
−0.9
−0.6
−0.3
0.0
0.3
β_IV

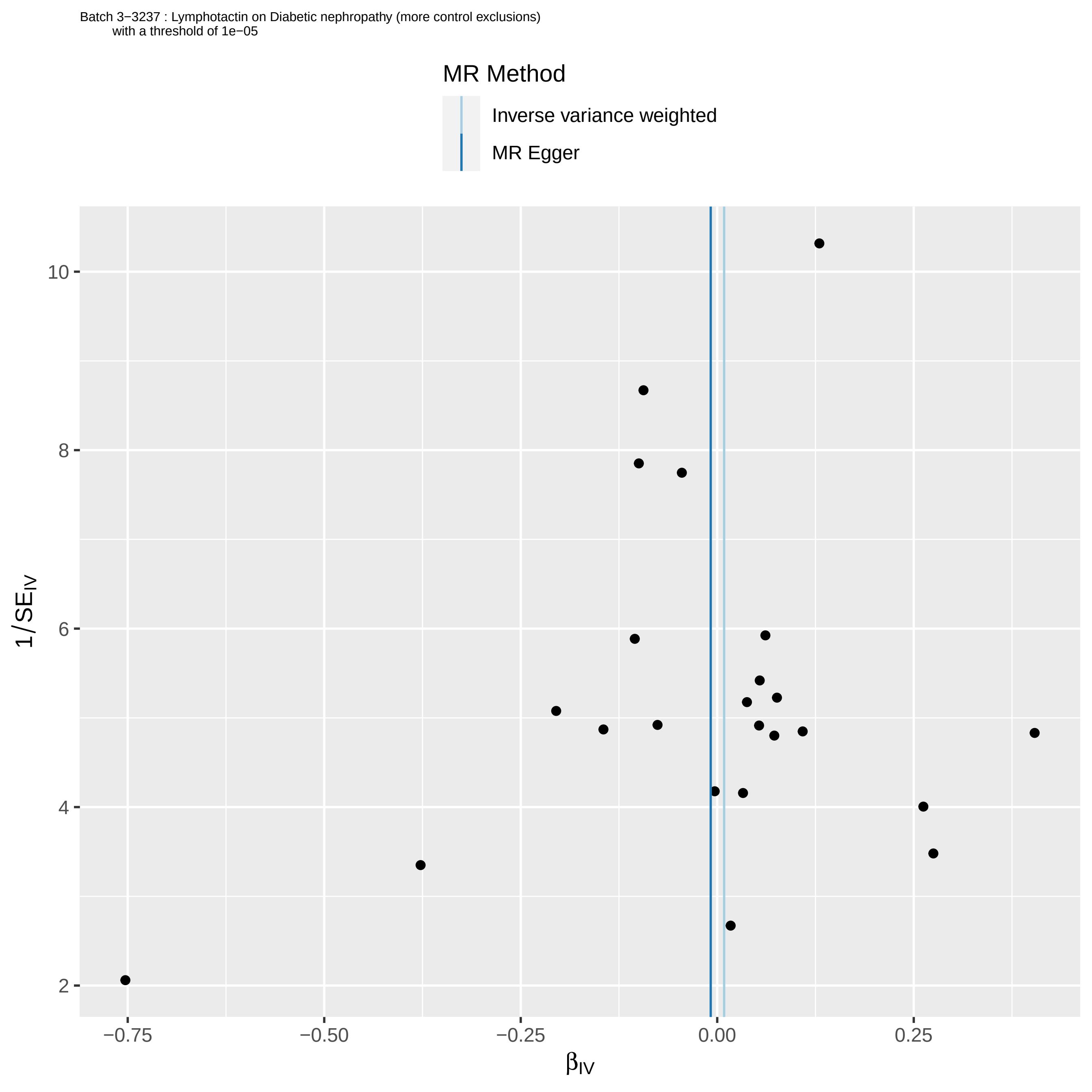

Batch 3−3237 : Lymphotactin on Diabetic nephropathy (more control exclusions)
with a threshold of 1e−05
MR Method
Inverse variance weighted
MR Egger
10
8
6
4
2
1/SE$_{IV}$
−0.75
−0.50
−0.25
0.00
0.25
β_{IV}

Batch 3−3238 : Xanthine dehydrogenase/oxidase on Diabetic nephropathy (more control exclusions)
with a threshold of 1e−05

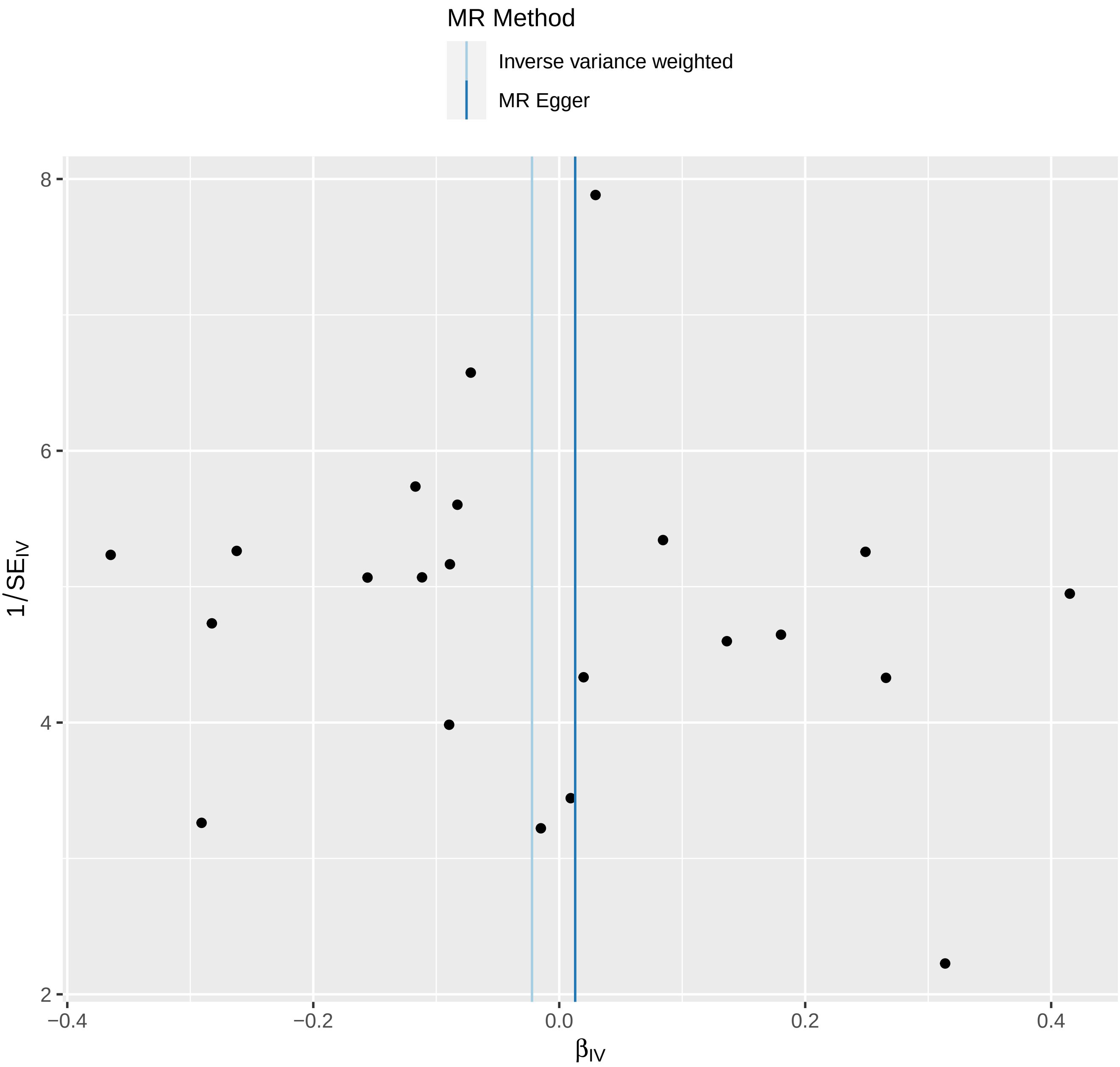

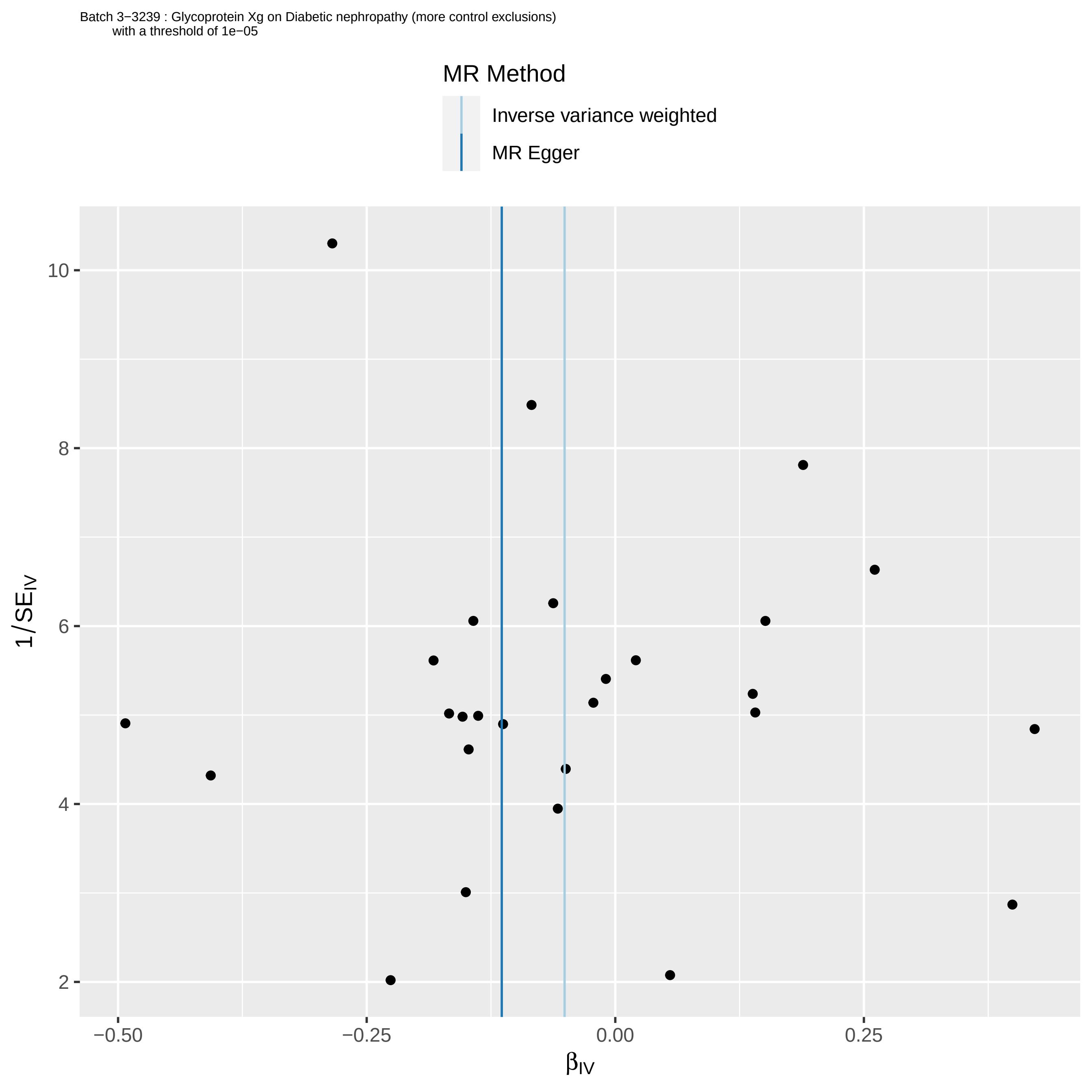
Batch 3−3239 : Glycoprotein Xg on Diabetic nephropathy (more control exclusions)
with a threshold of 1e−05
MR Method
Inverse variance weighted
MR Egger
1/SE_IV
β_IV
−0.50
−0.25
0.00
0.25
2
4
6
8
10

Batch 3−3240 : Xaa−Pro aminopeptidase 1 on Diabetic nephropathy (more control exclusions)
with a threshold of 1e−05

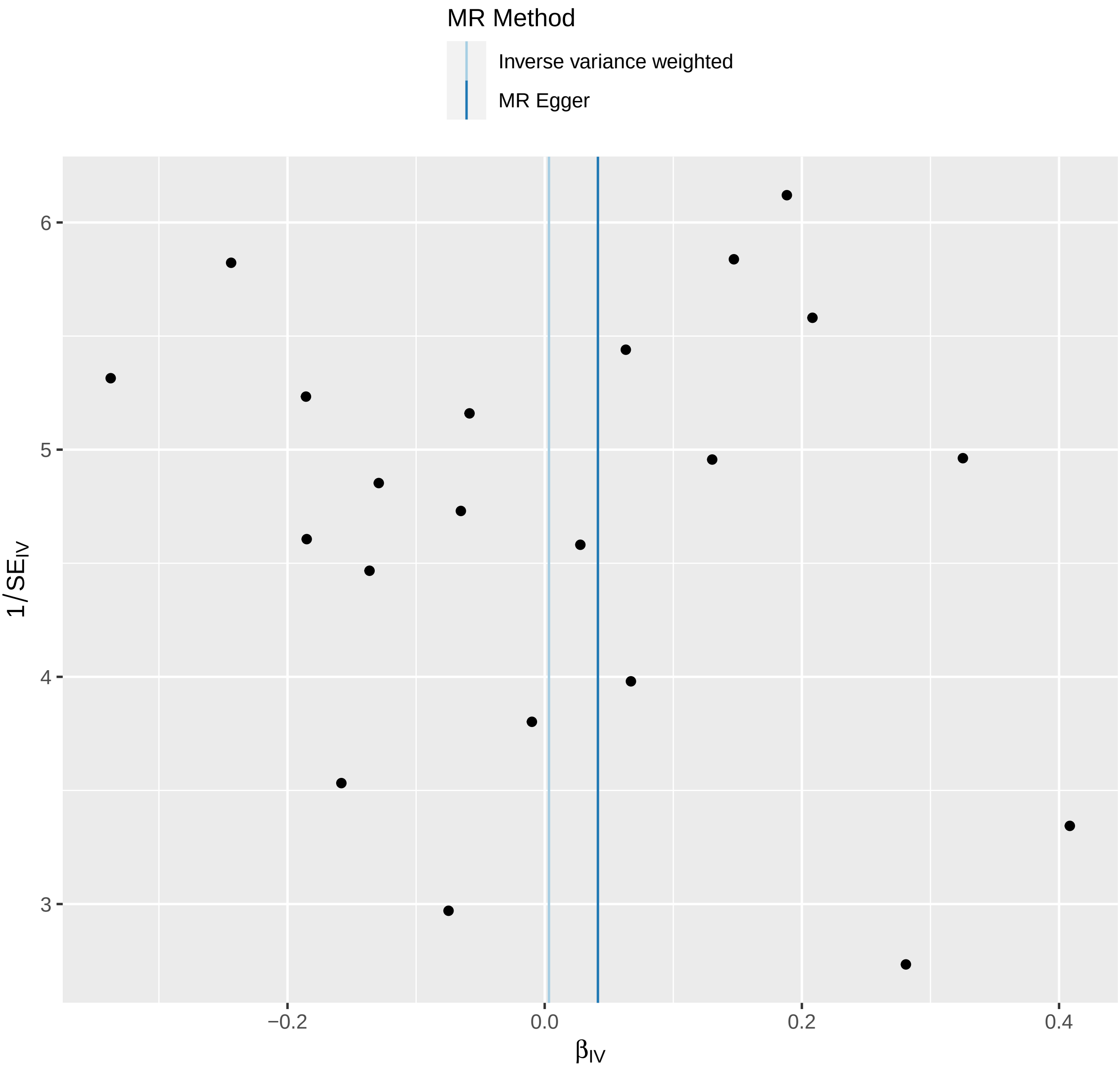

Batch 3−3241 : DNA repair protein XRCC1 on Diabetic nephropathy (more control exclusions)
with a threshold of 1e−05

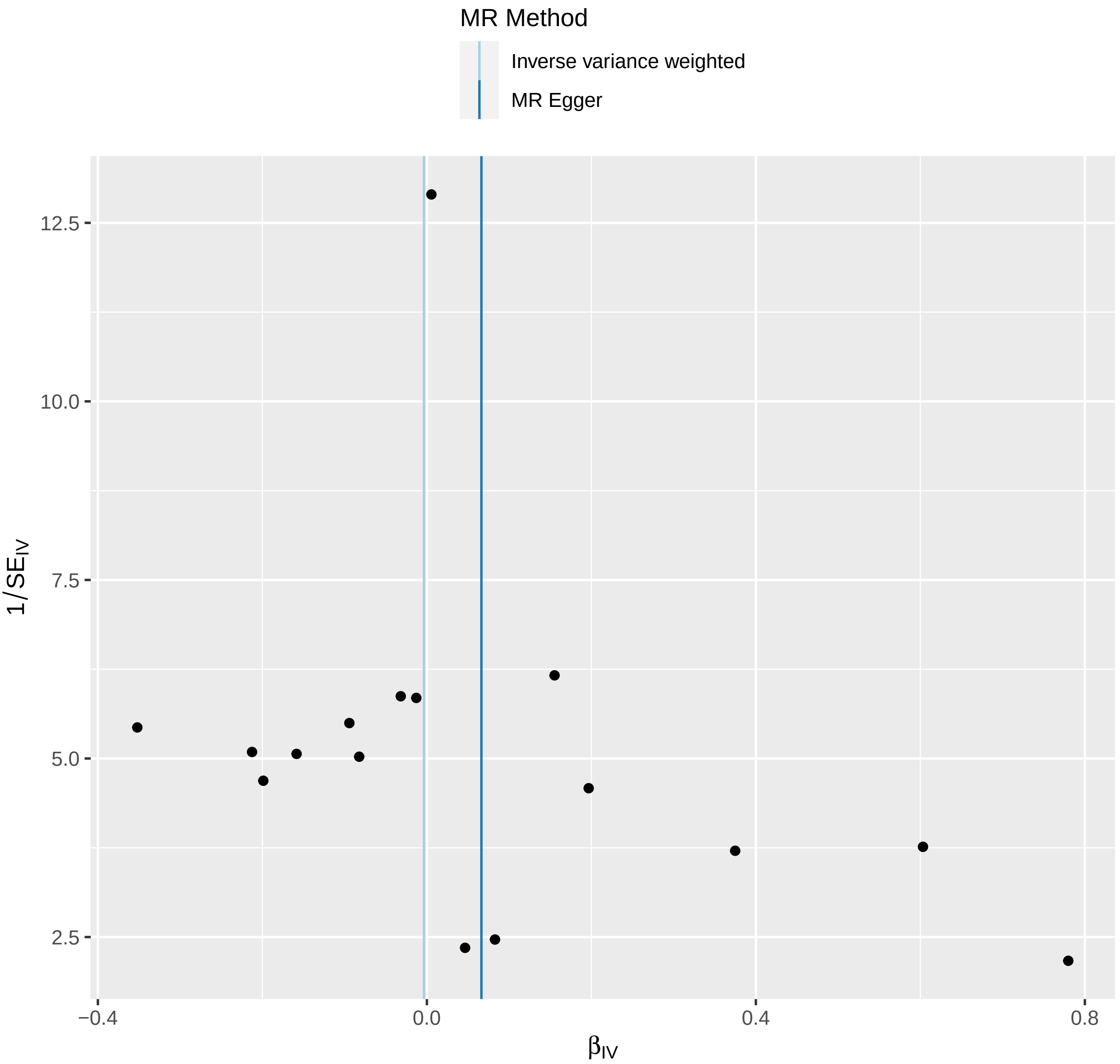

Batch 3−3242 : DNA repair protein XRCC4 on Diabetic nephropathy (more control exclusions)
with a threshold of 1e−05

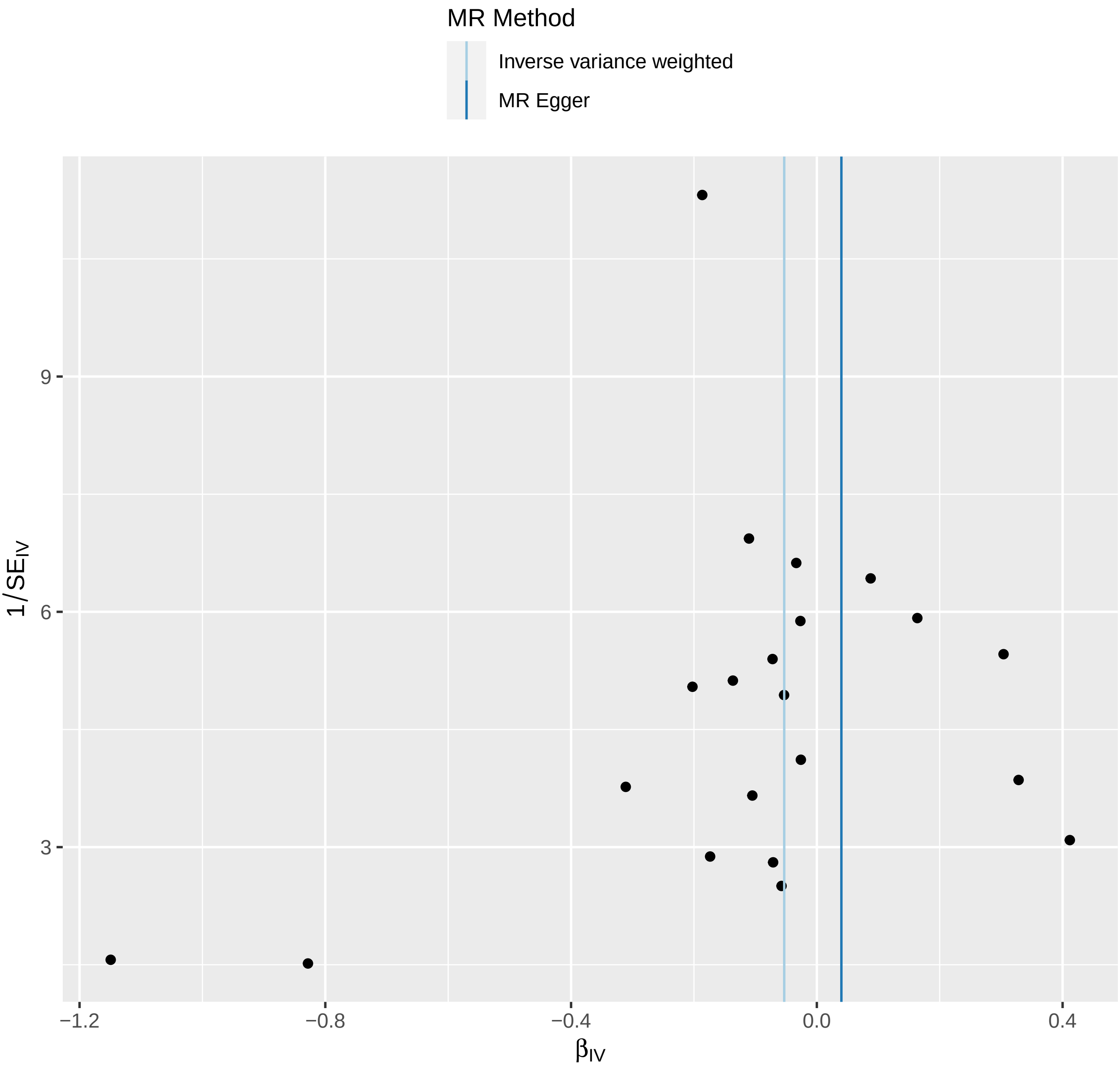

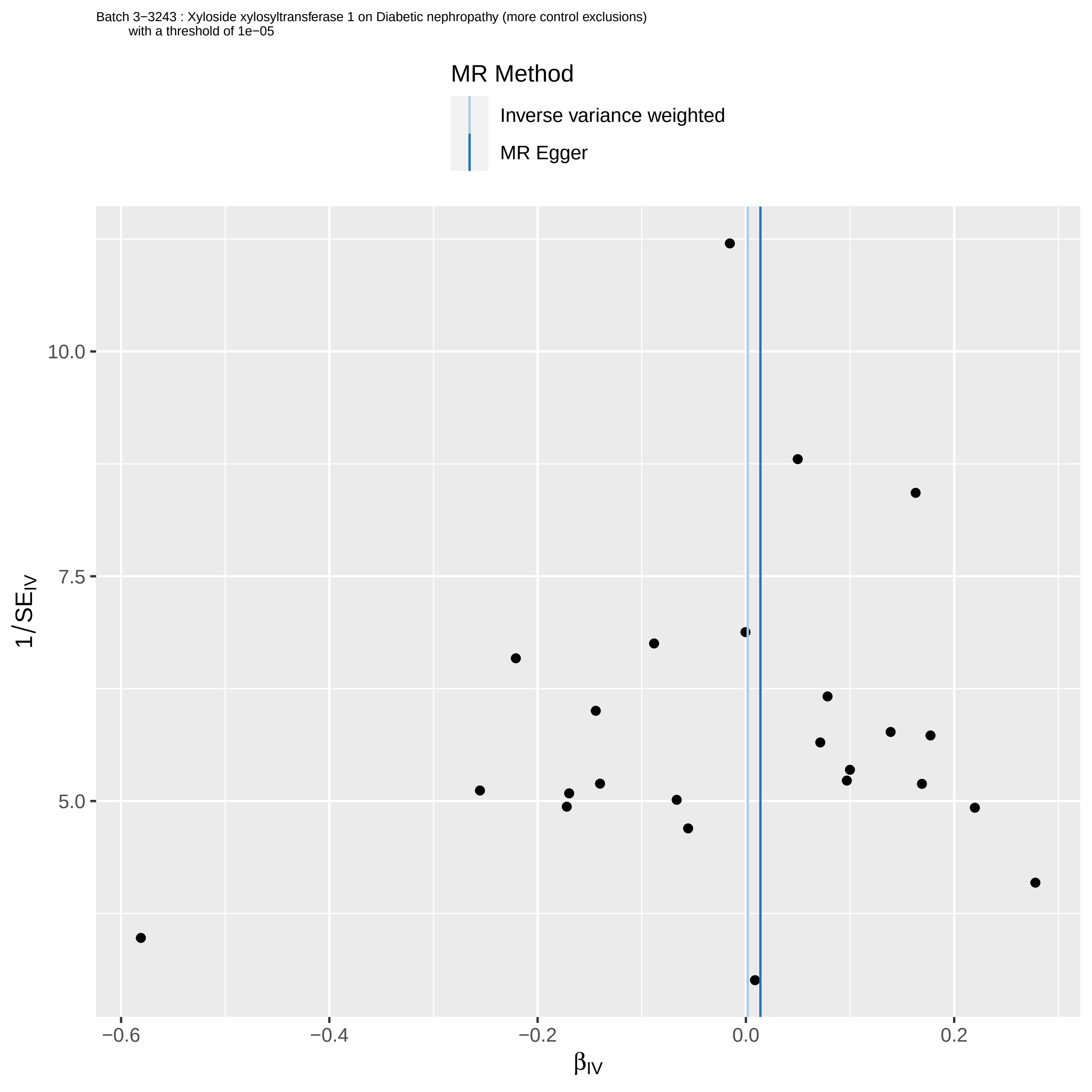

Batch 3−3243 : Xyloside xylosyltransferase 1 on Diabetic nephropathy (more control exclusions)
with a threshold of 1e−05
MR Method
Inverse variance weighted
MR Egger
10.0
7.5
5.0
1/SE_IV
−0.6
−0.4
−0.2
0.0
0.2
β_IV

Batch 3−3244 : Y−box−binding protein 2 on Diabetic nephropathy (more control exclusions)
with a threshold of 1e−05

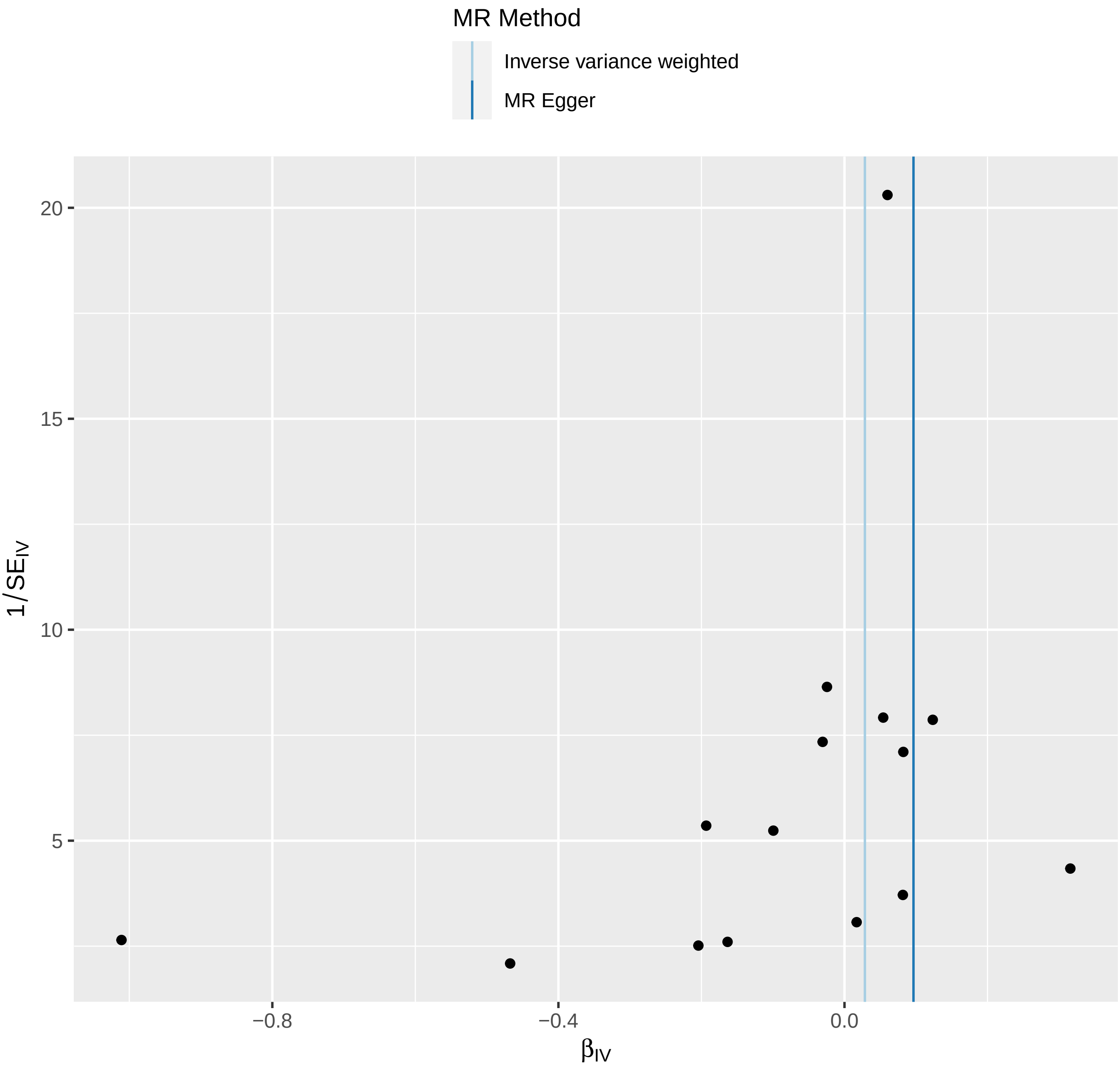

Batch 3−3245 : Tyrosine−protein kinase Yes on Diabetic nephropathy (more control exclusions)
with a threshold of 1e−05

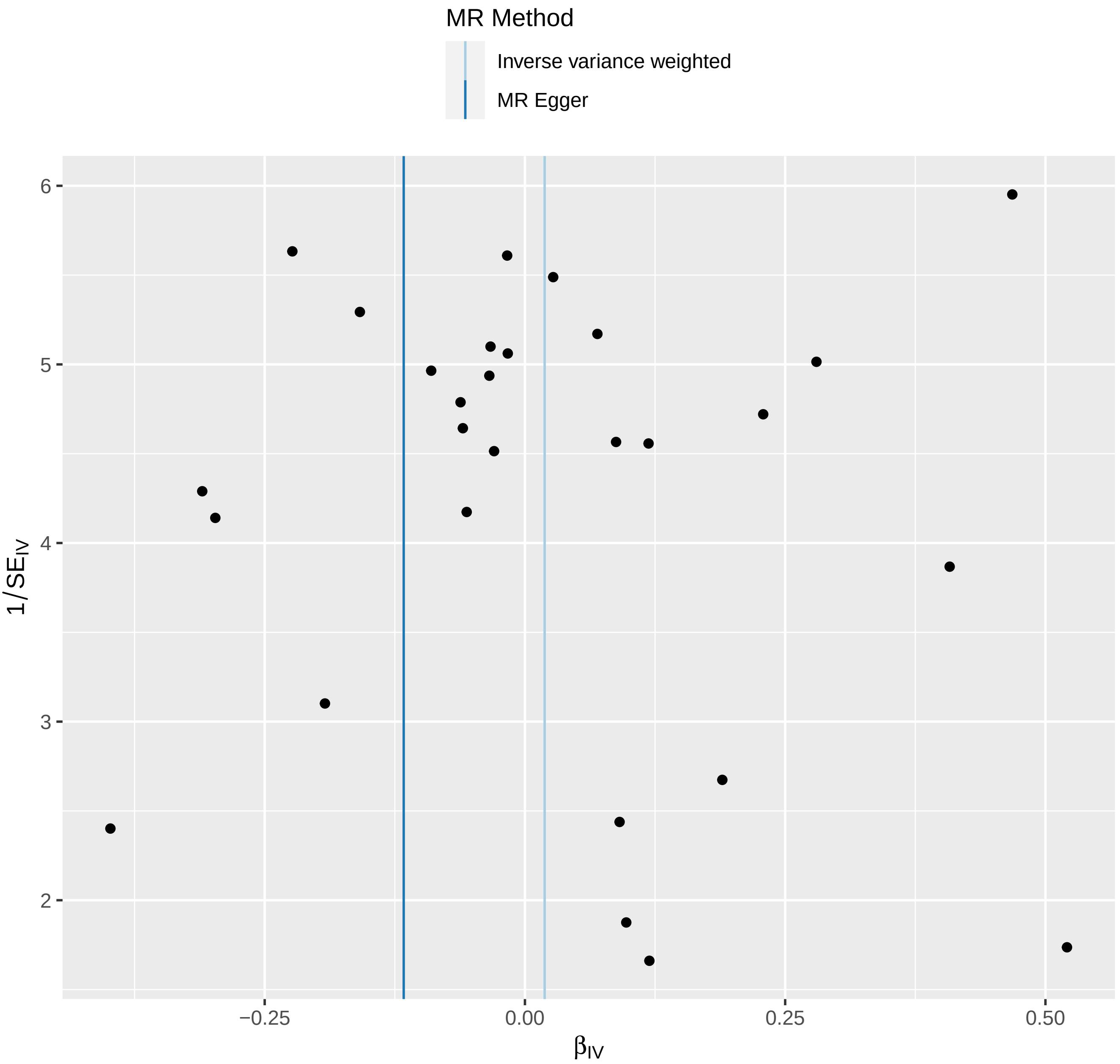

Batch 3−3246 : Protein YIPF6 on Diabetic nephropathy (more control exclusions)
with a threshold of 1e−05

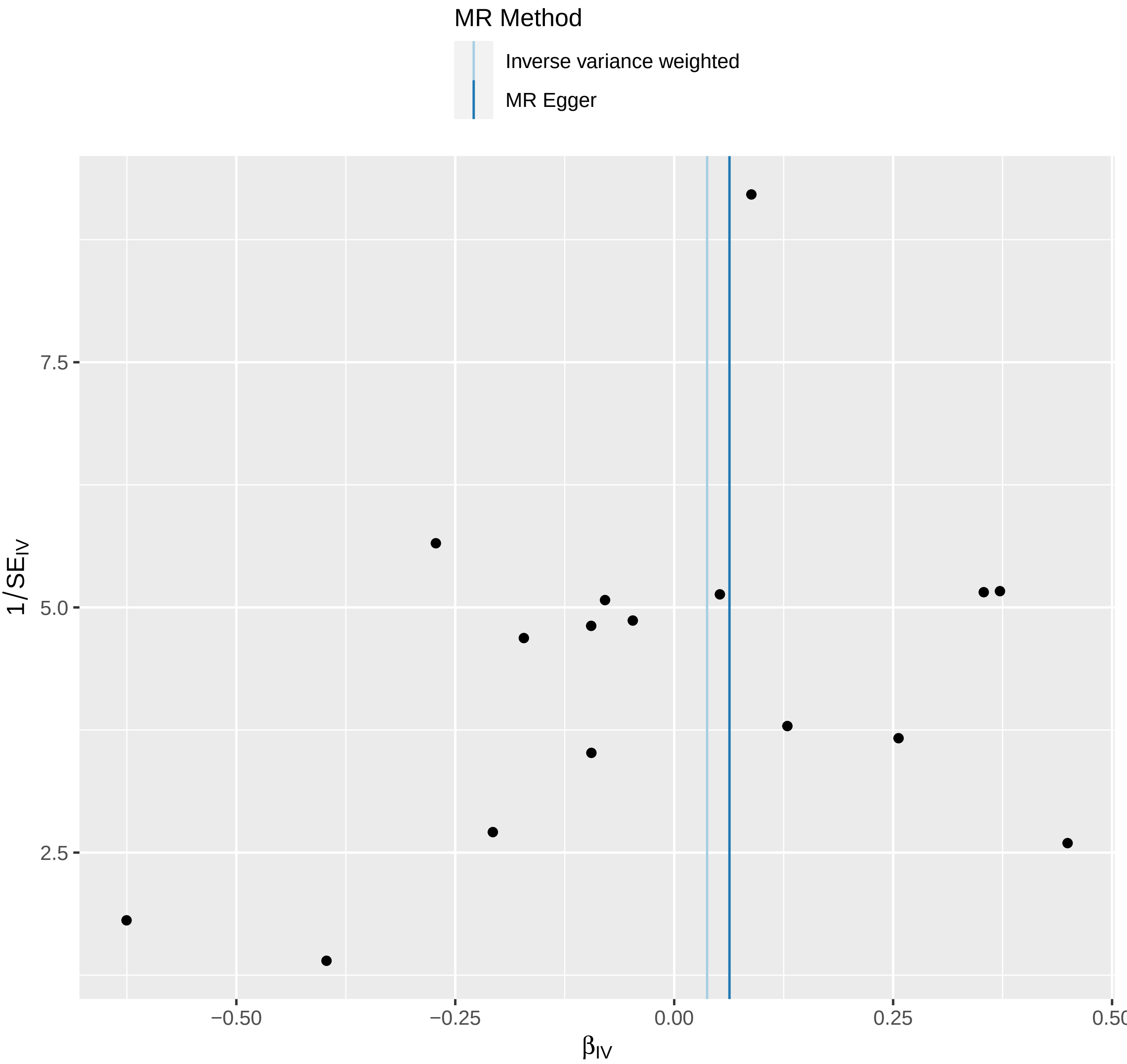

Batch 3−3247 : YTH domain−containing protein 1 on Diabetic nephropathy (more control exclusions)
with a threshold of 1e−05

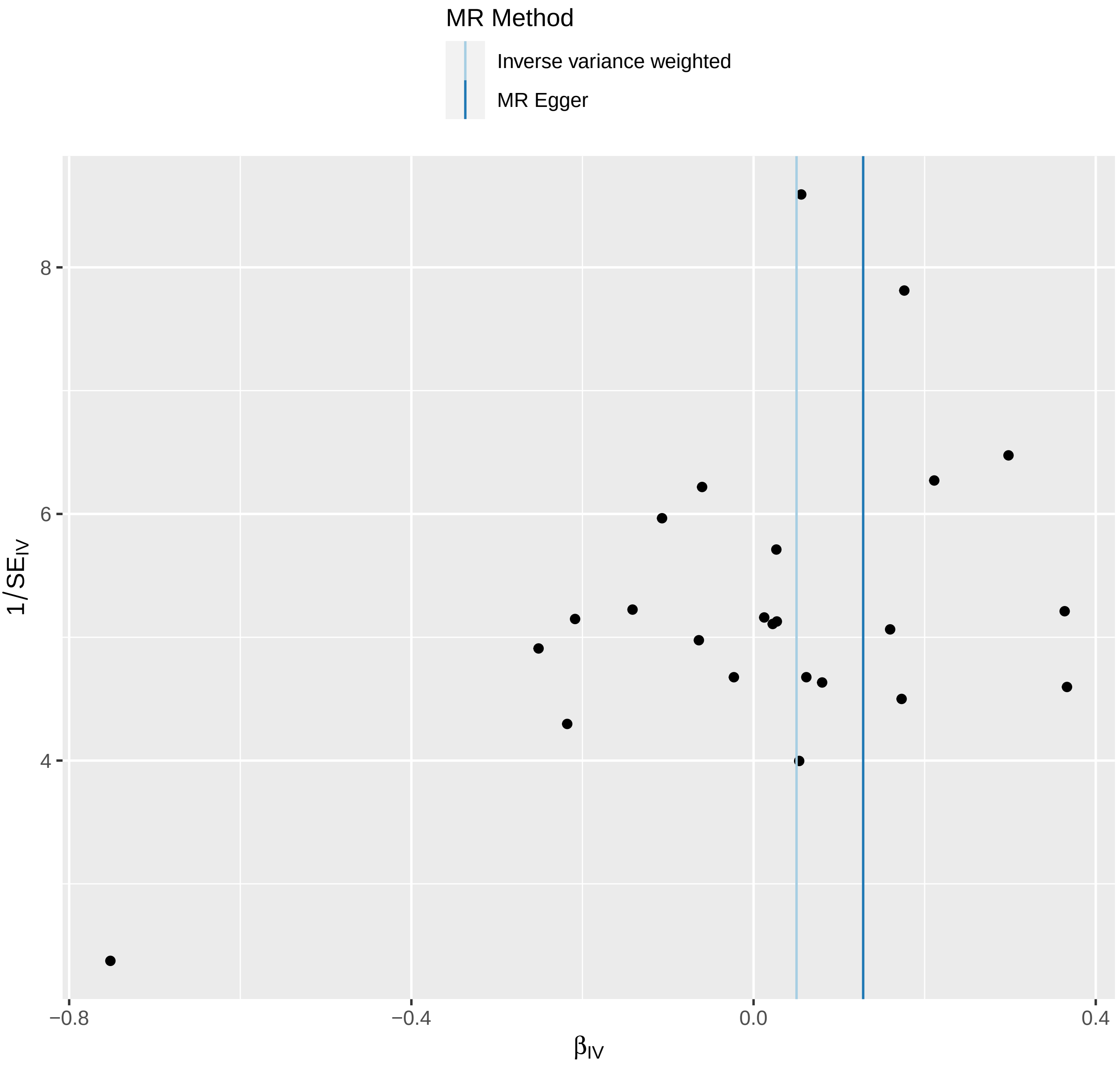

Batch 3−3248 : 14−3−3 protein family on Diabetic nephropathy (more control exclusions)
with a threshold of 1e−05

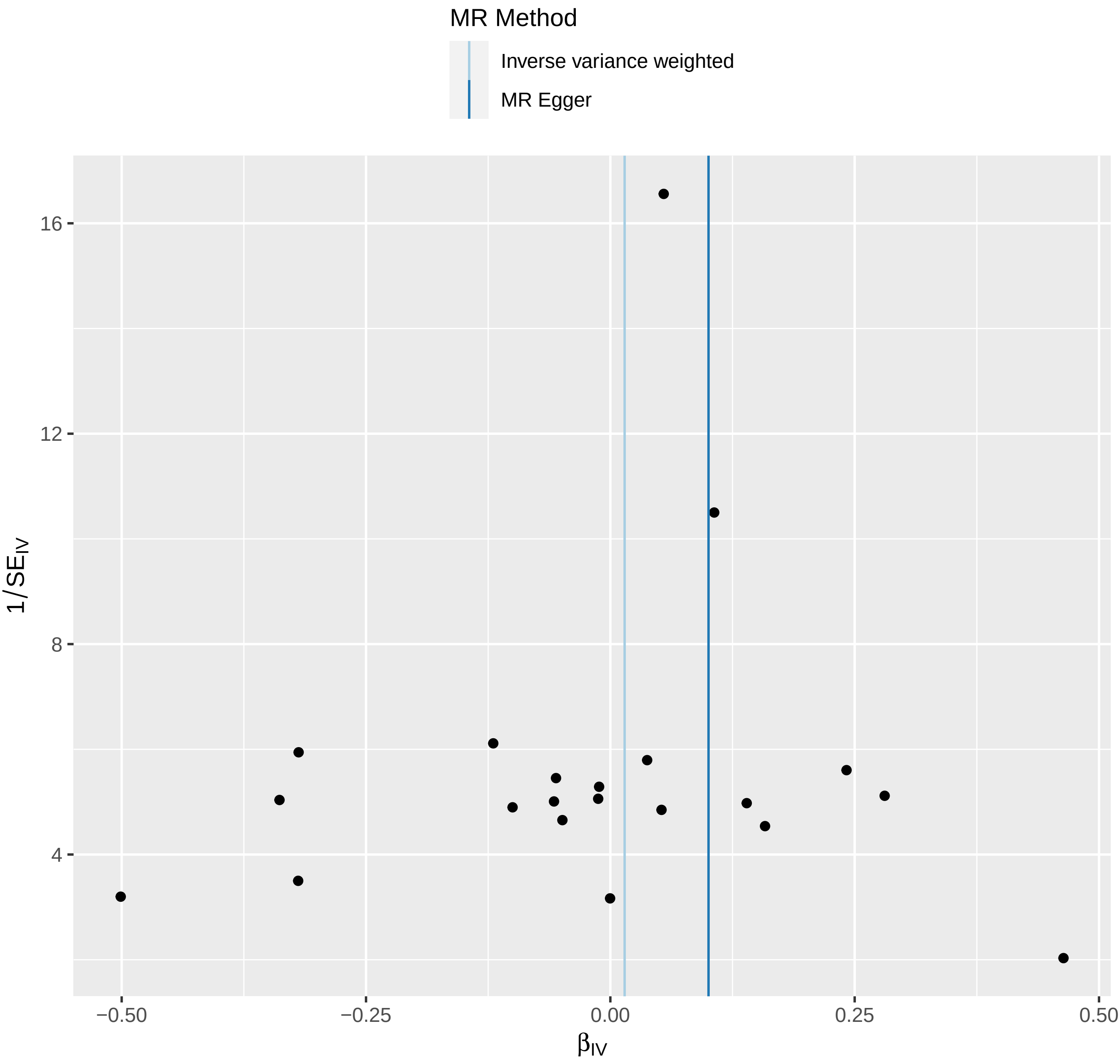

Batch 3−3249 : 14−3−3 protein epsilon on Diabetic nephropathy (more control exclusions)
with a threshold of 1e−05

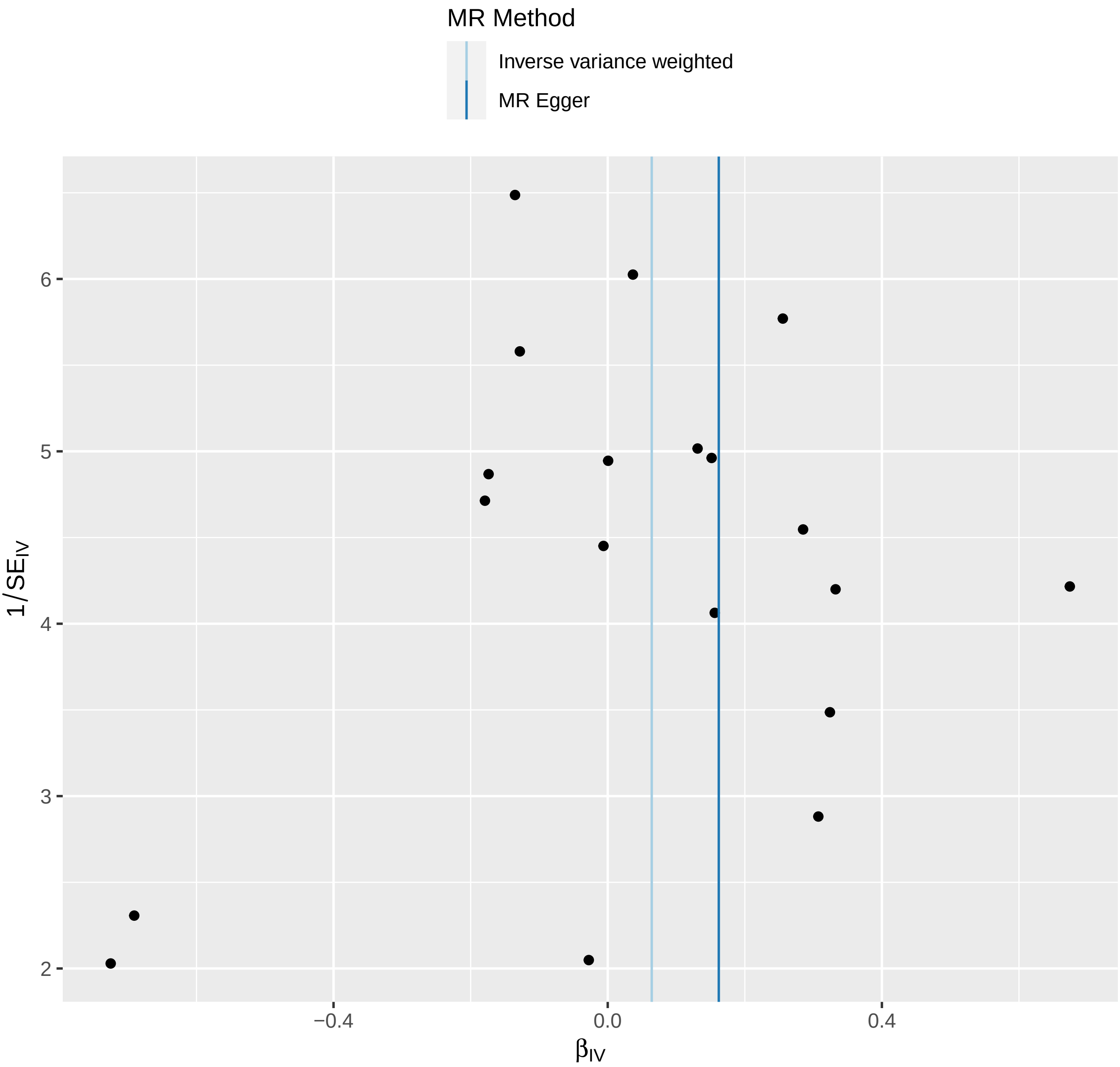

Batch 3−3250 : 14−3−3 protein theta on Diabetic nephropathy (more control exclusions)
with a threshold of 1e−05

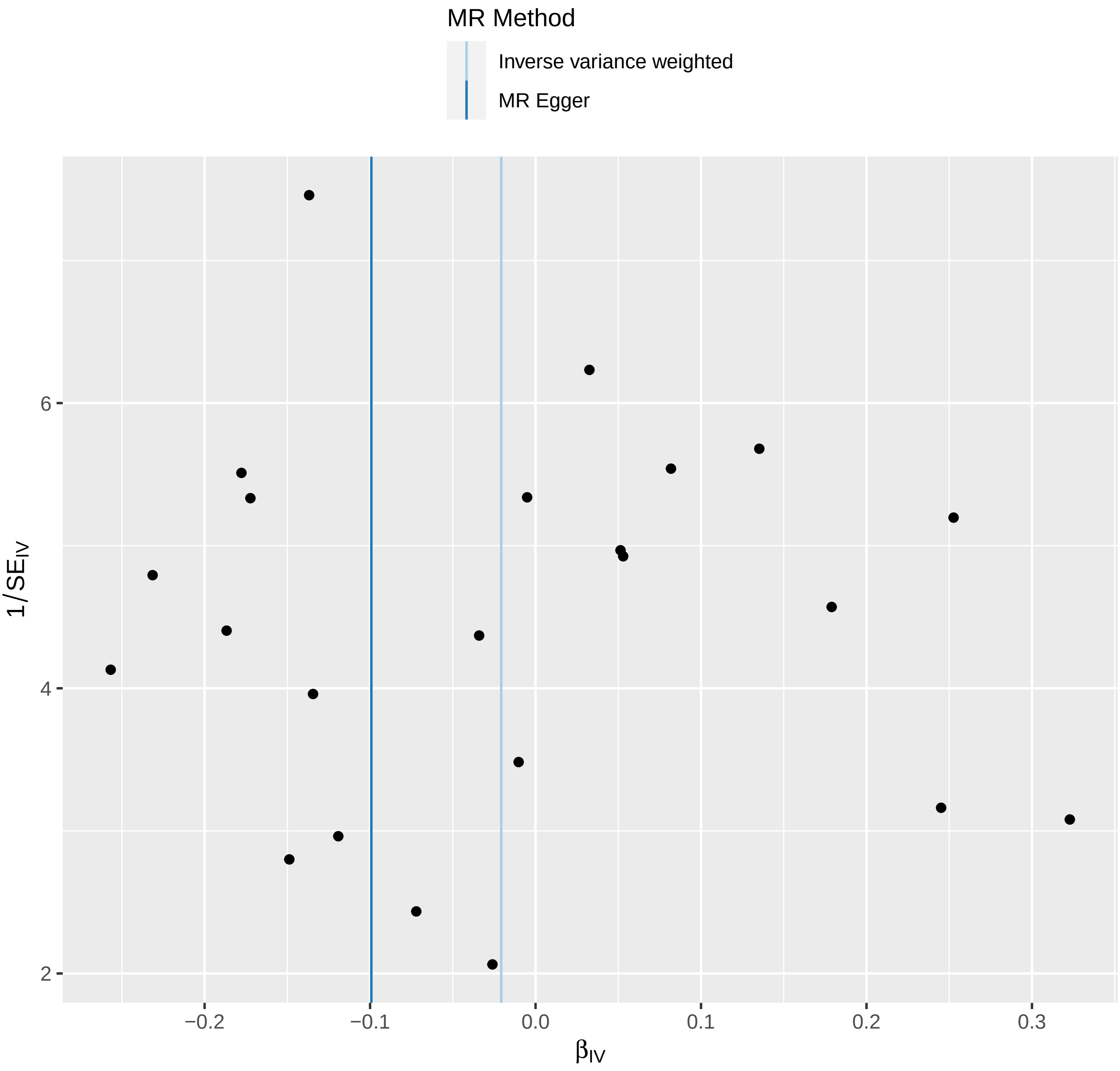

Batch 3−3251 : 14−3−3 protein zeta/delta on Diabetic nephropathy (more control exclusions)
with a threshold of 1e−05

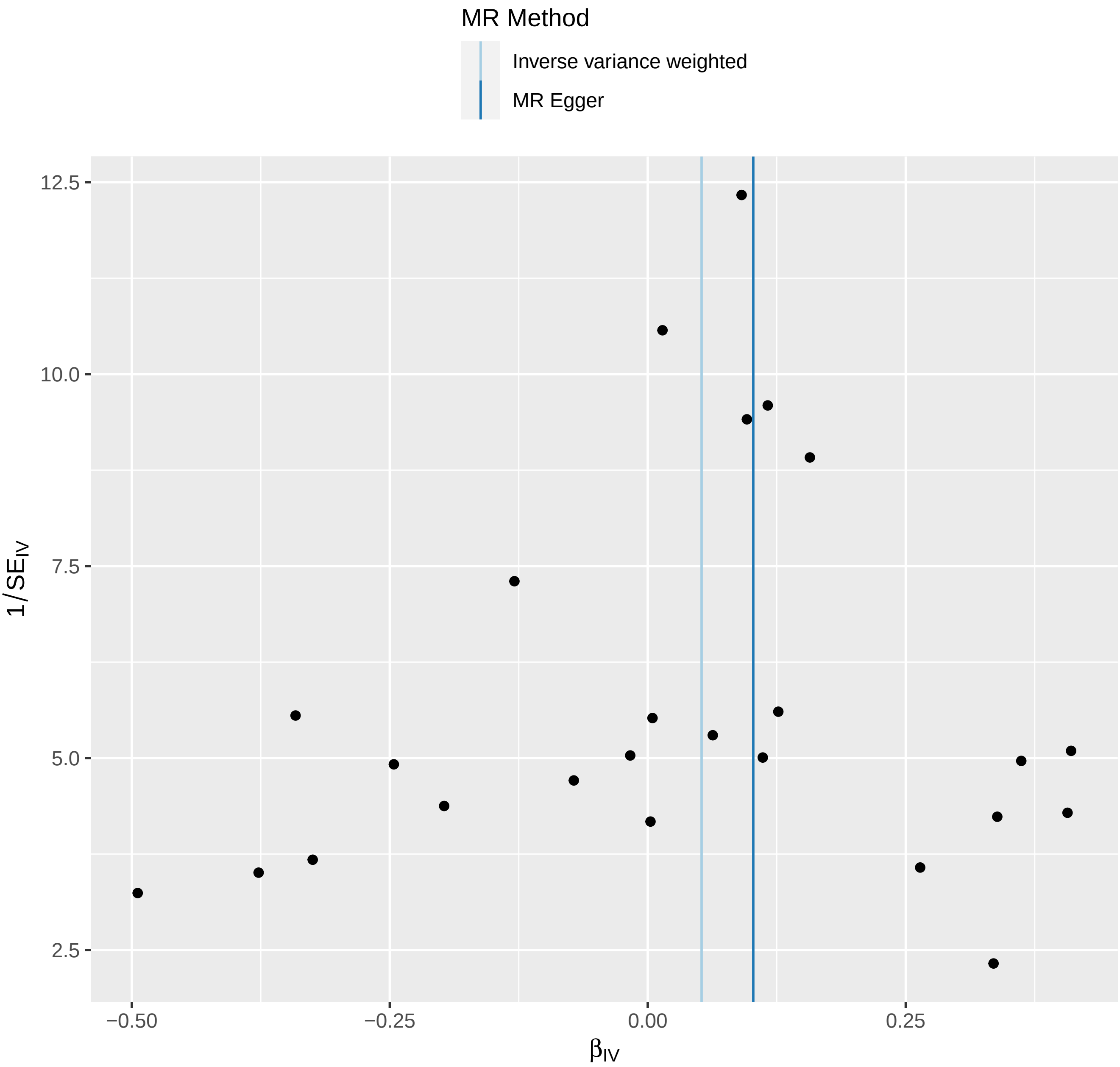

Batch 3−3252 : Tyrosine−protein kinase ZAP−70 on Diabetic nephropathy (more control exclusions)
with a threshold of 1e−05

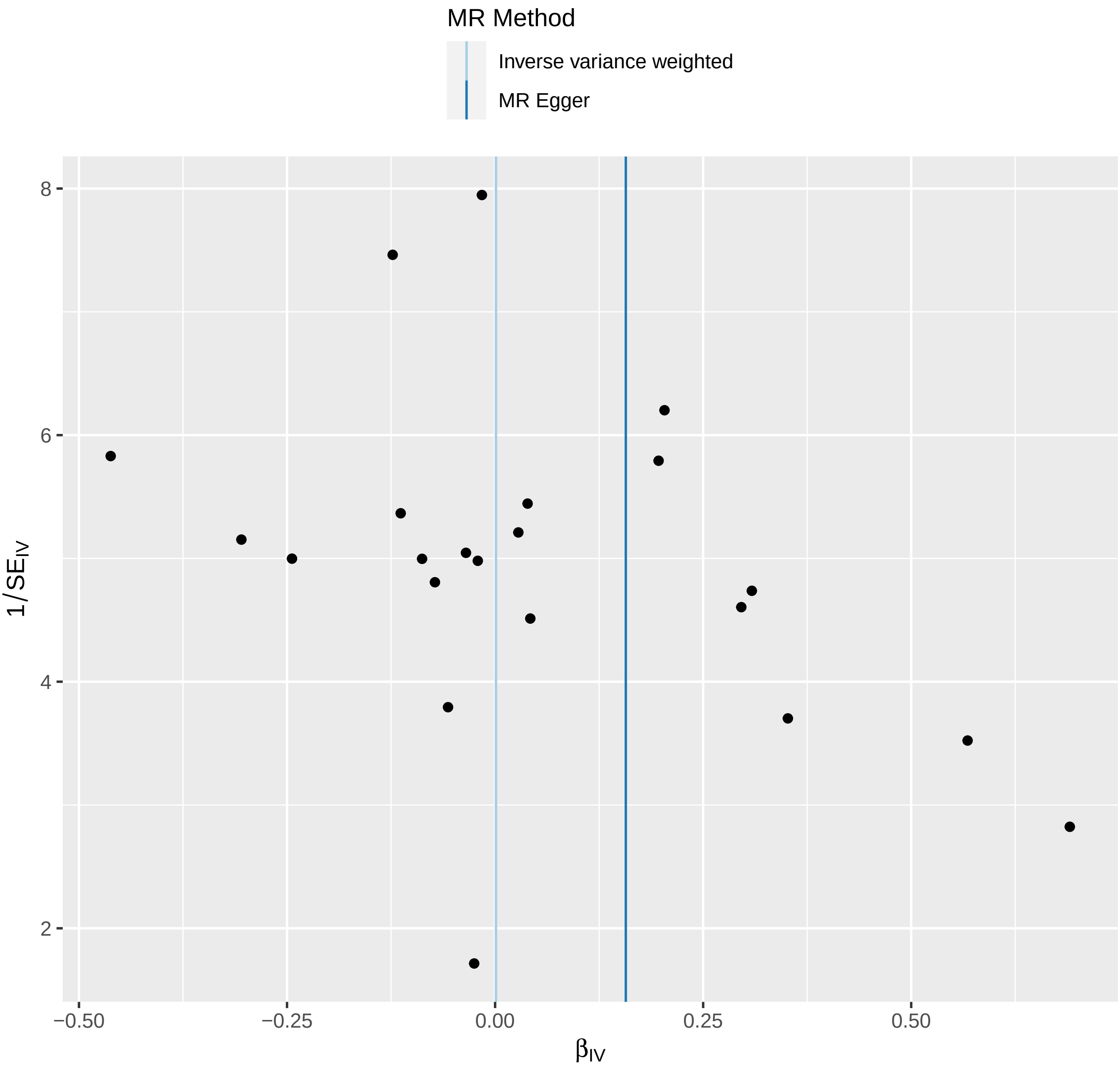

Batch 3−3253 : Tyrosine−protein kinase ZAP−70 on Diabetic nephropathy (more control exclusions)
with a threshold of 1e−05

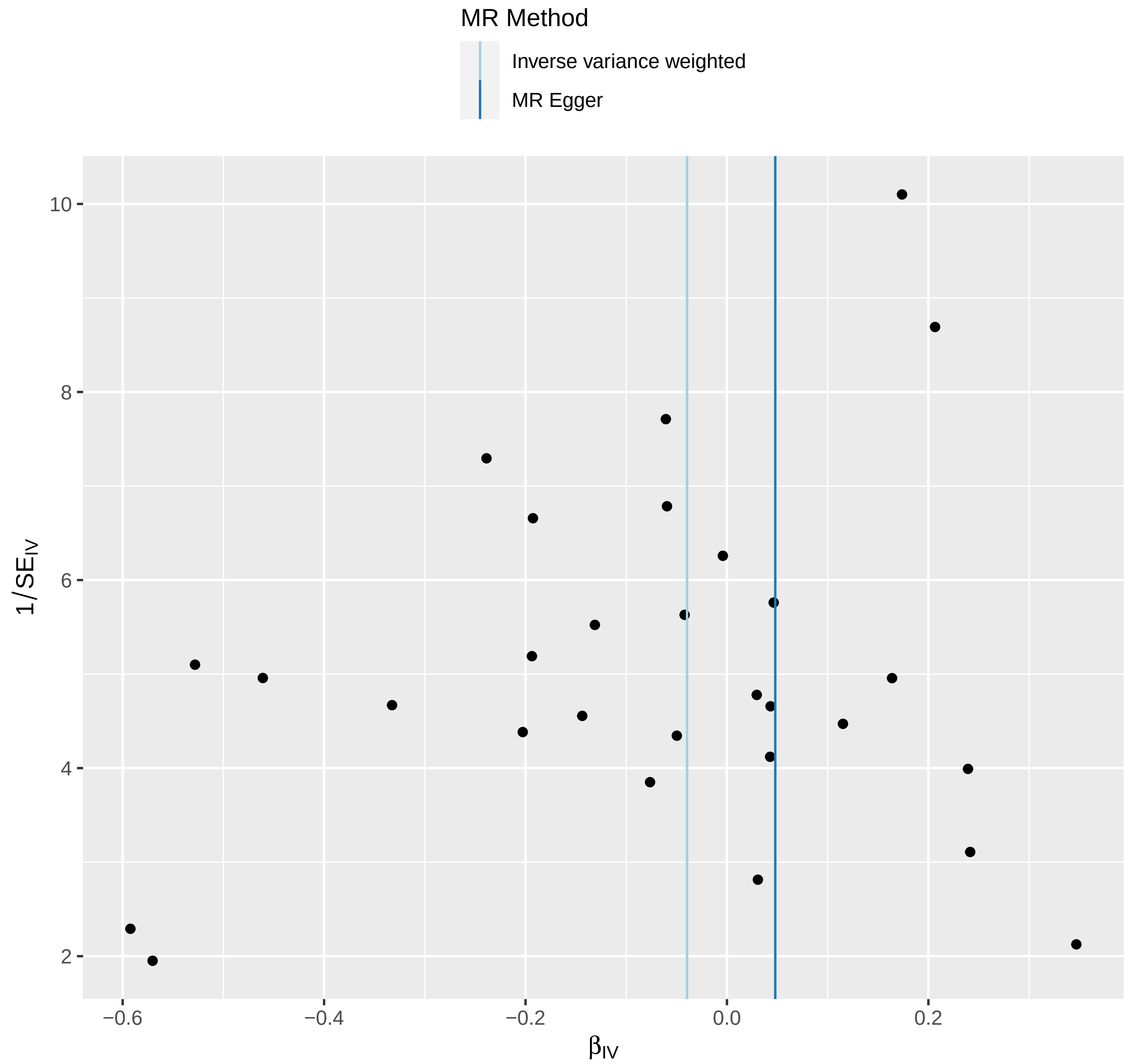

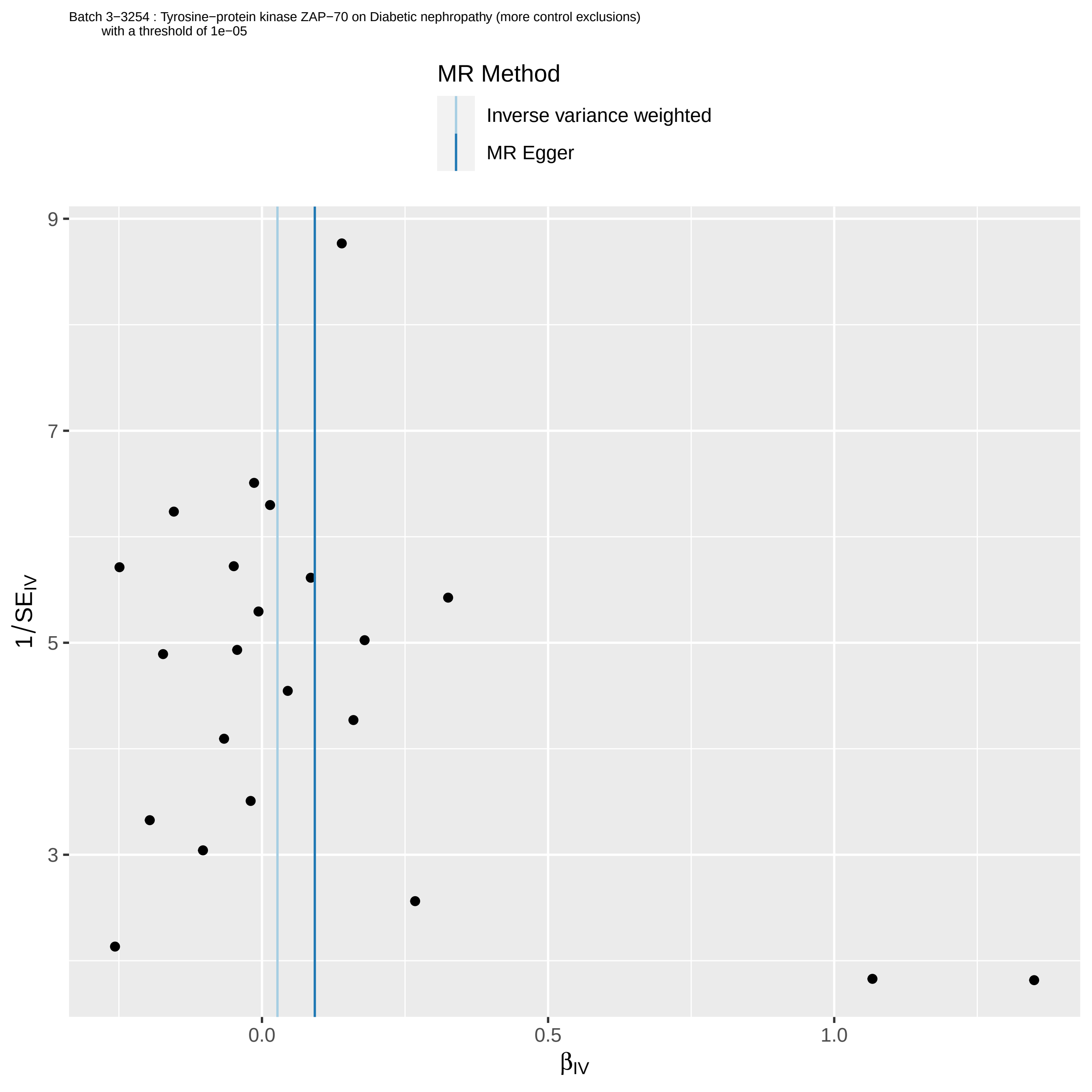

Batch 3−3254 : Tyrosine−protein kinase ZAP−70 on Diabetic nephropathy (more control exclusions)
with a threshold of 1e−05
MR Method
Inverse variance weighted
MR Egger
9
7
5
3
1/SE$_{IV}$
0.0
0.5
1.0
β$_{IV}$

Batch 3−3255 : Transcriptional regulator Kaiso on Diabetic nephropathy (more control exclusions)
with a threshold of 1e−05

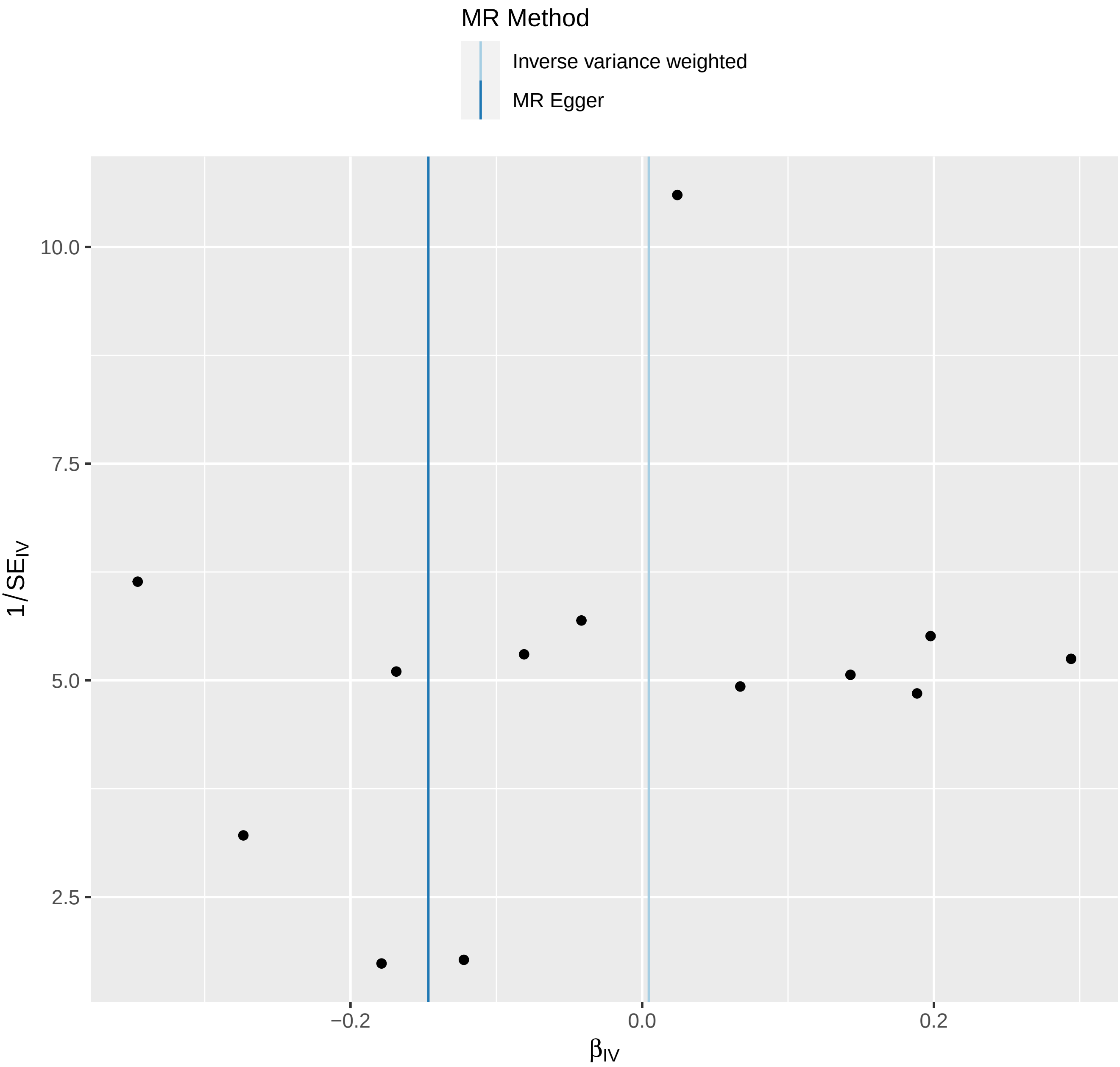

Batch 3−3256 : E3 ubiquitin−protein ligase ZFP91 on Diabetic nephropathy (more control exclusions)
with a threshold of 1e−05

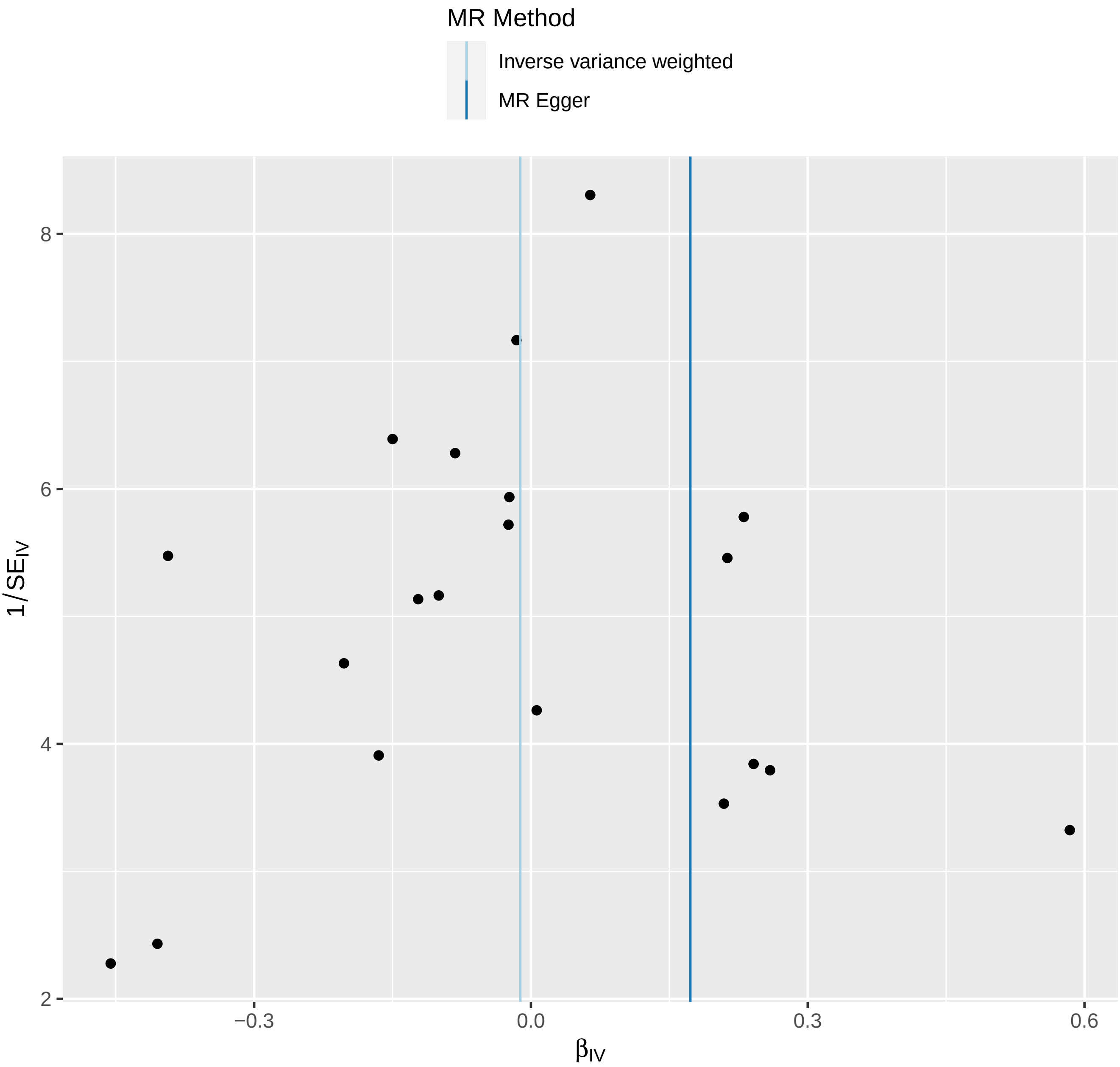

Batch 3−3257 : Protrudin on Diabetic nephropathy (more control exclusions)
with a threshold of 1e−05

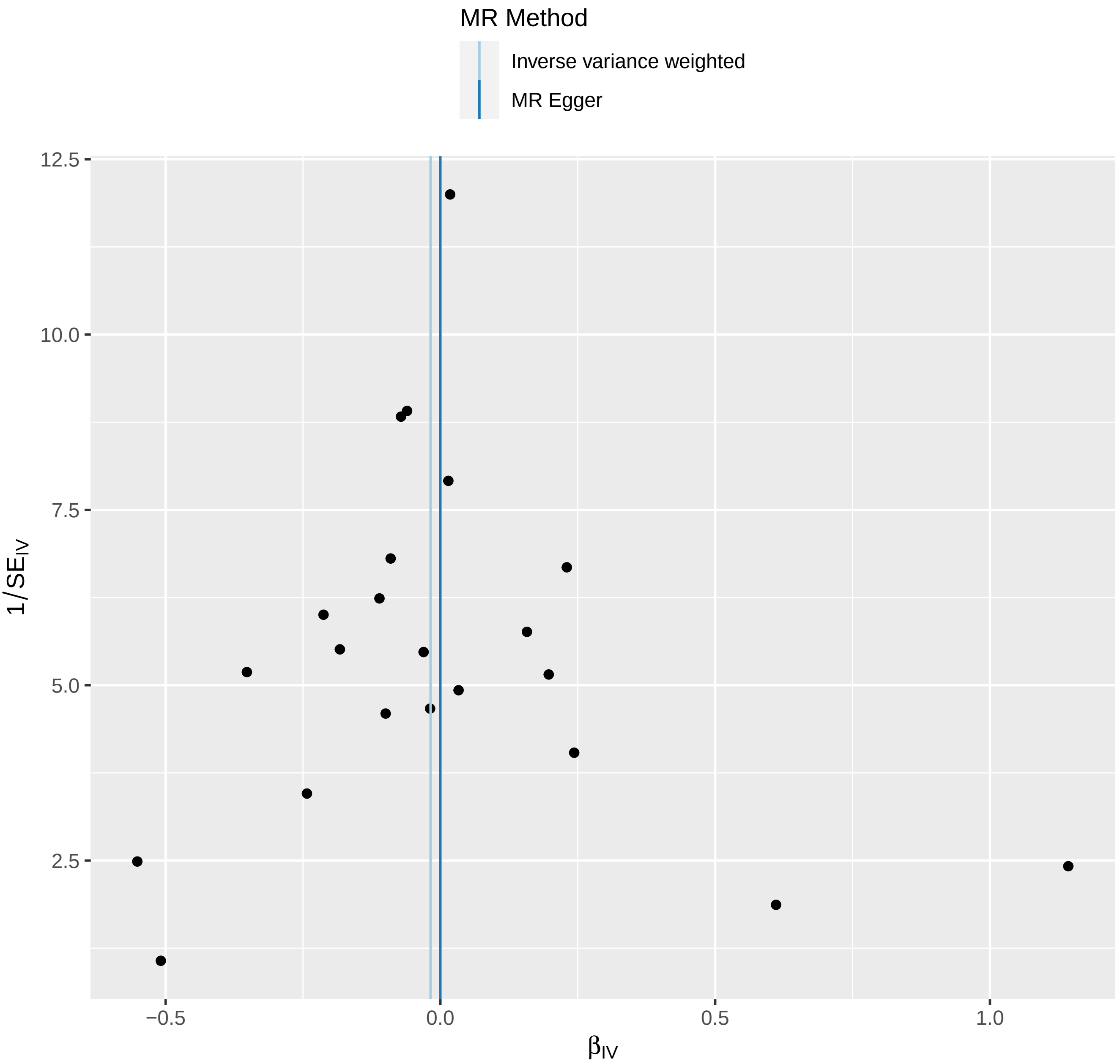

Batch 3−3258 : Zinc fingers and homeoboxes protein 3 on Diabetic nephropathy (more control exclusions) with a threshold of 1e−05

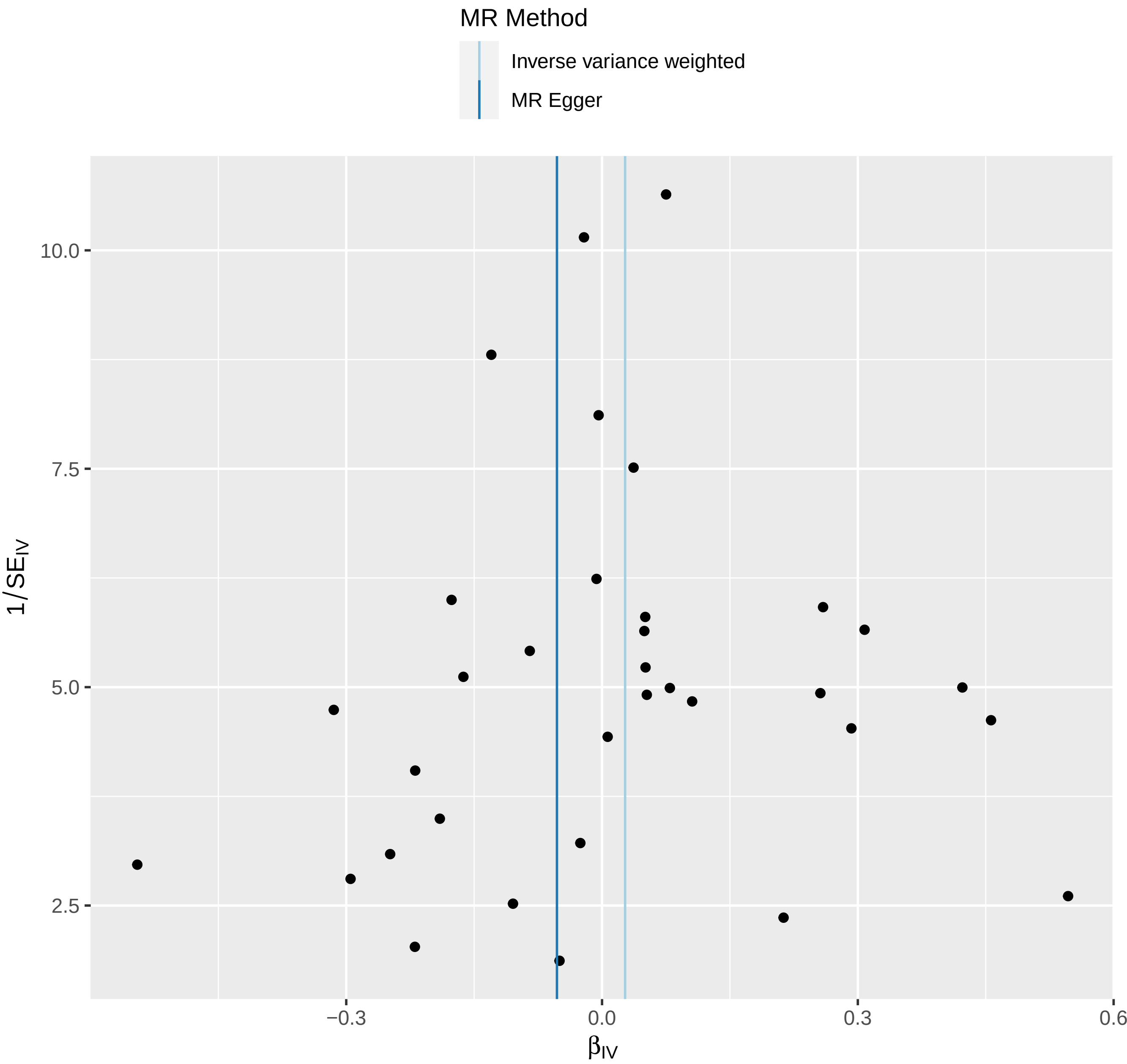

Batch 3−3259 : Zinc finger protein 10 on Diabetic nephropathy (more control exclusions)
with a threshold of 1e−05

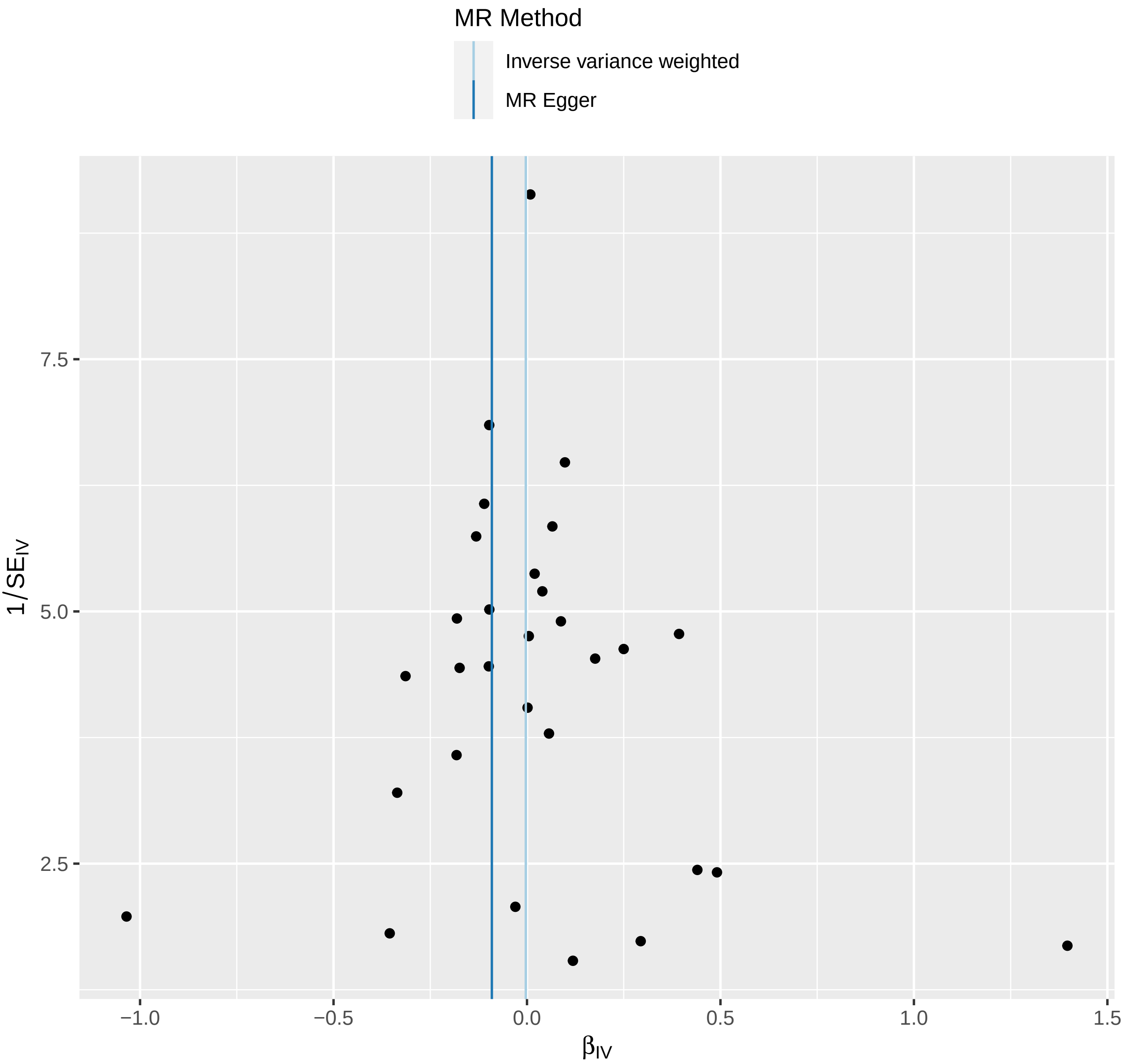

Batch 3−3260 : Zinc finger protein 134 on Diabetic nephropathy (more control exclusions)
with a threshold of 1e−05

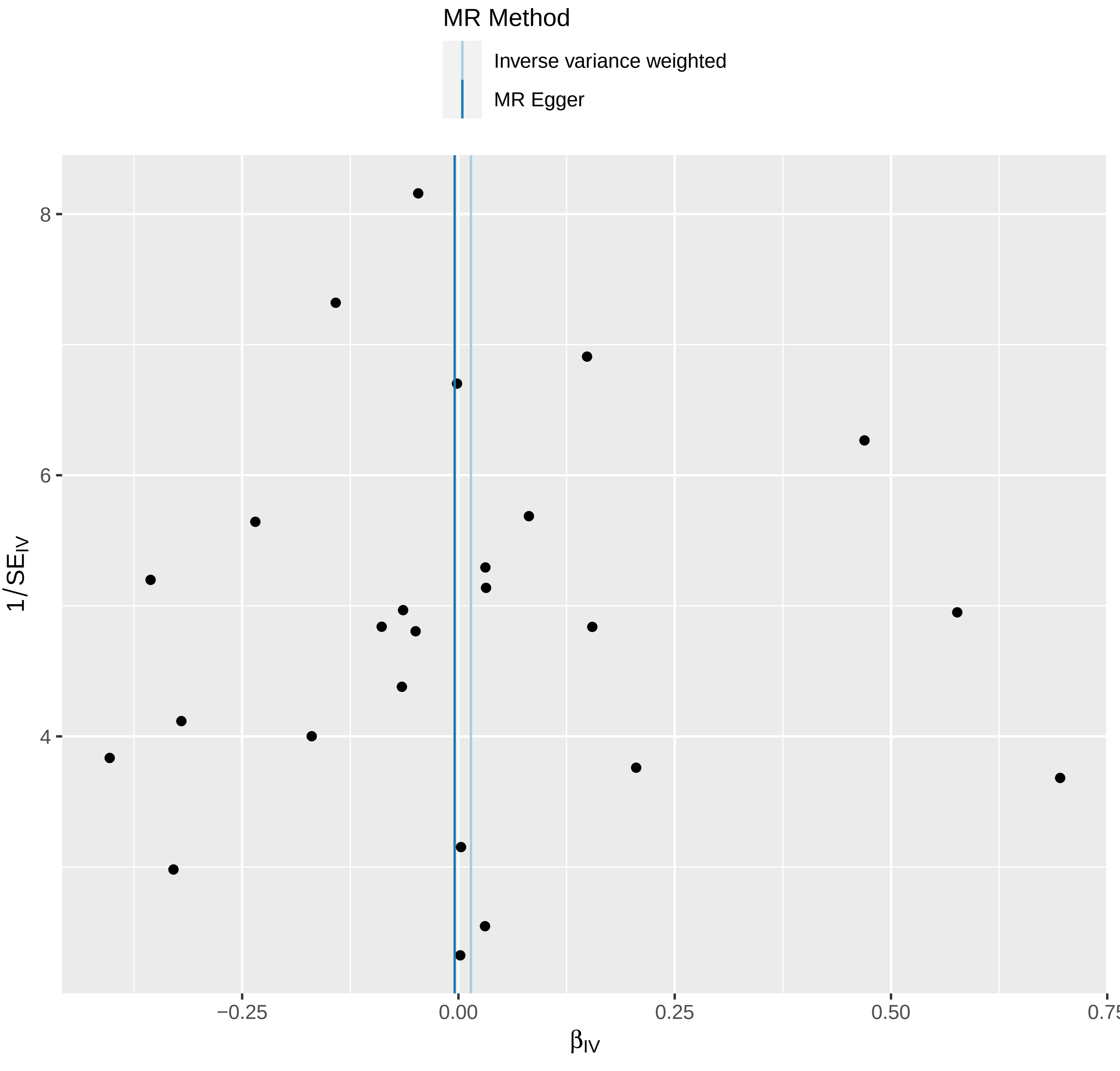

Batch 3−3261 : Zinc finger protein 174 on Diabetic nephropathy (more control exclusions)
with a threshold of 1e−05

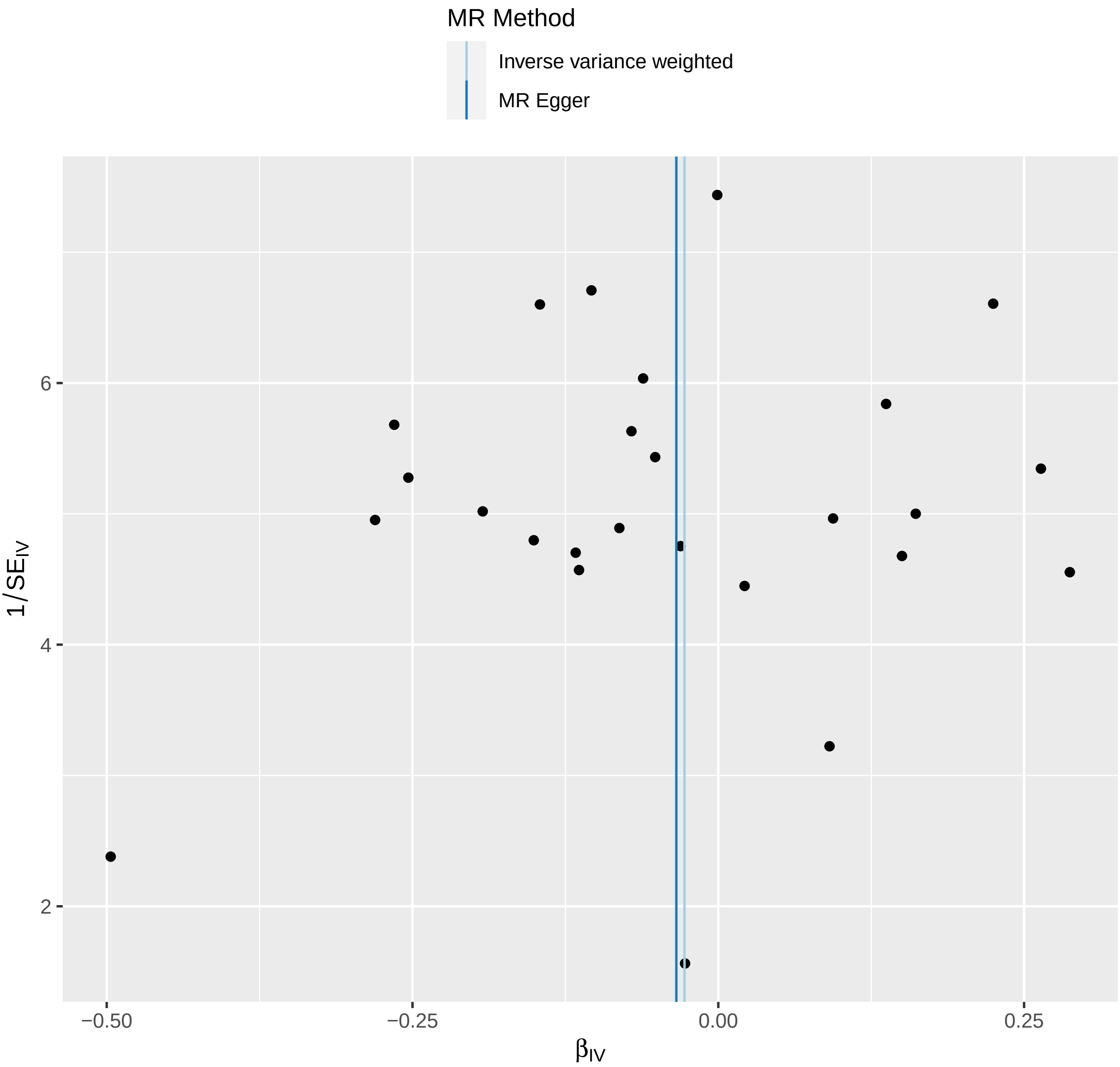

Batch 3−3262 : Zinc finger protein 175 on Diabetic nephropathy (more control exclusions)
with a threshold of 1e−05

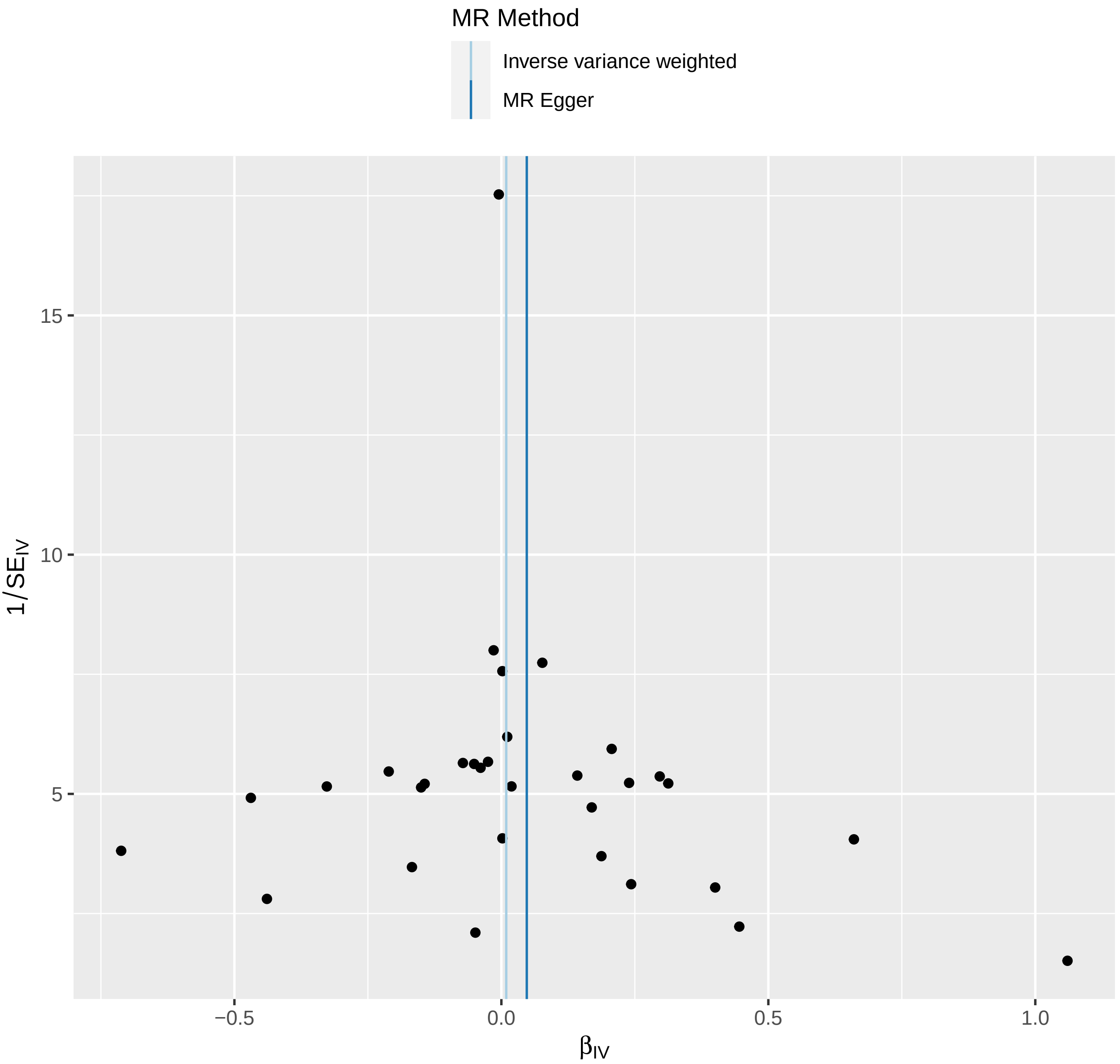

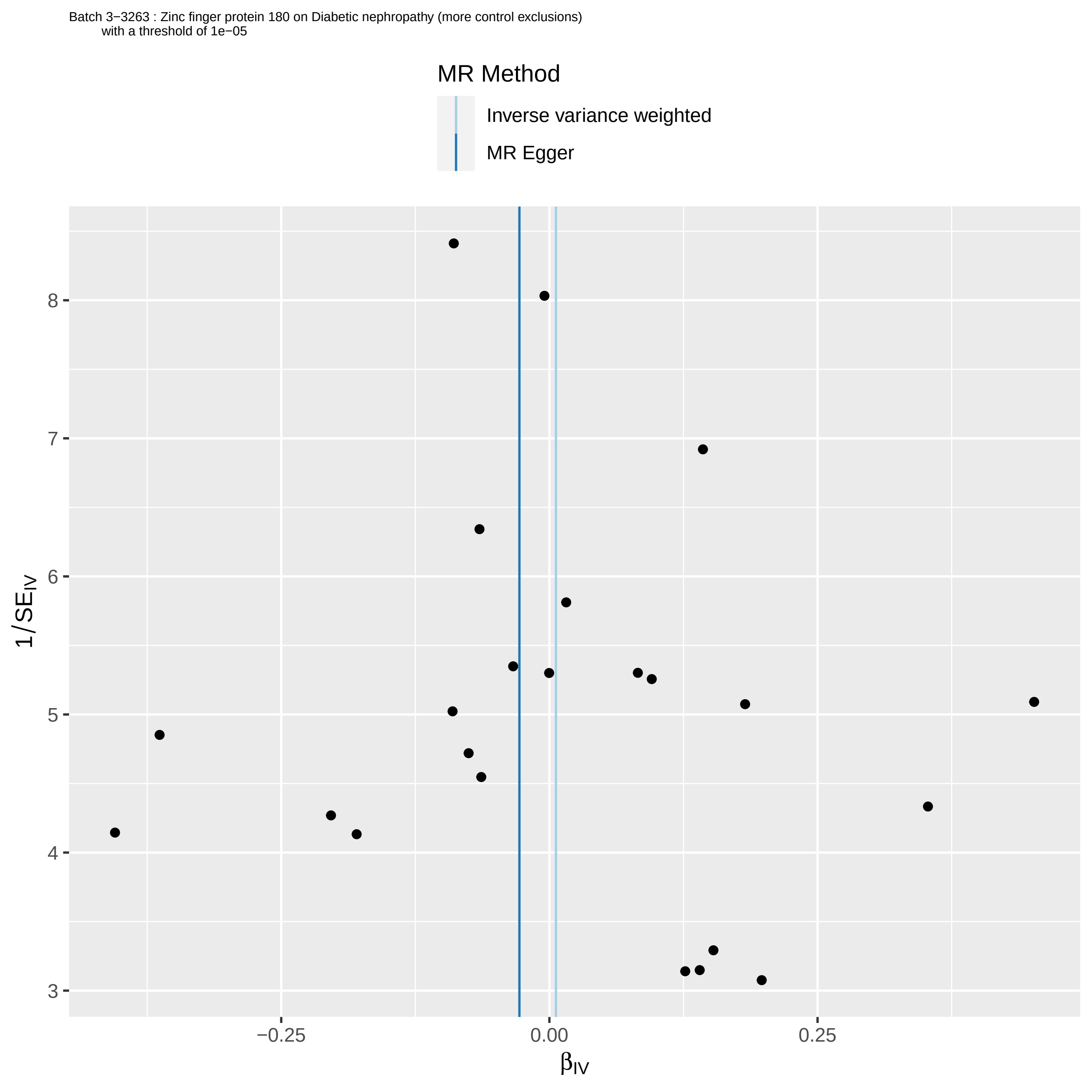
Batch 3−3263 : Zinc finger protein 180 on Diabetic nephropathy (more control exclusions)
with a threshold of 1e−05
MR Method
Inverse variance weighted
MR Egger
8
7
6
5
4
3
$1/SE_{IV}$
−0.25
0.00
0.25
β_{IV}

Batch 3−3264 : Zinc finger protein 18 on Diabetic nephropathy (more control exclusions)
with a threshold of 1e−05

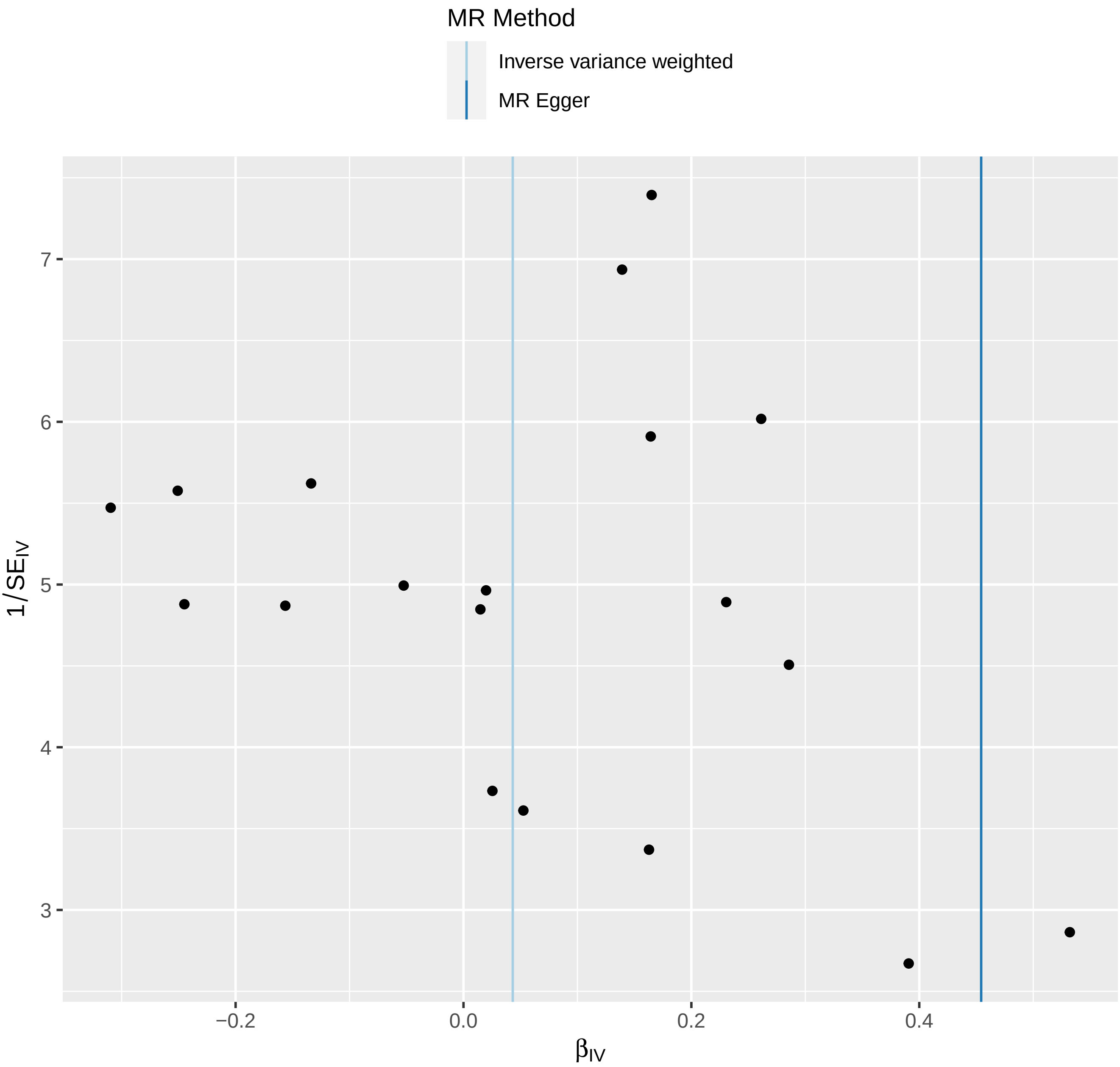

Batch 3−3265 : Zinc finger protein 23 on Diabetic nephropathy (more control exclusions)
with a threshold of 1e−05

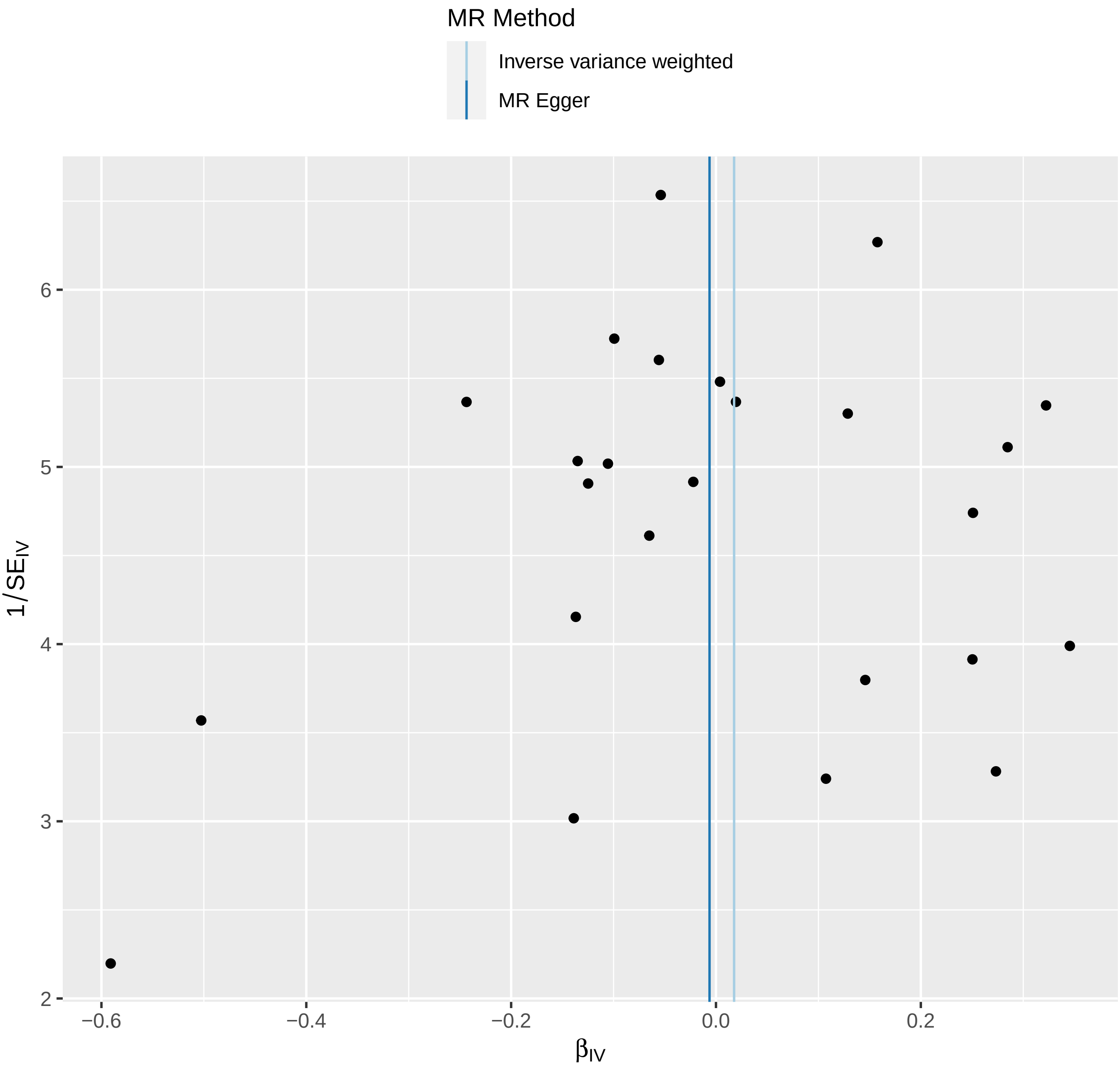

Batch 3−3266 : Zinc finger protein 264 on Diabetic nephropathy (more control exclusions)
with a threshold of 1e−05

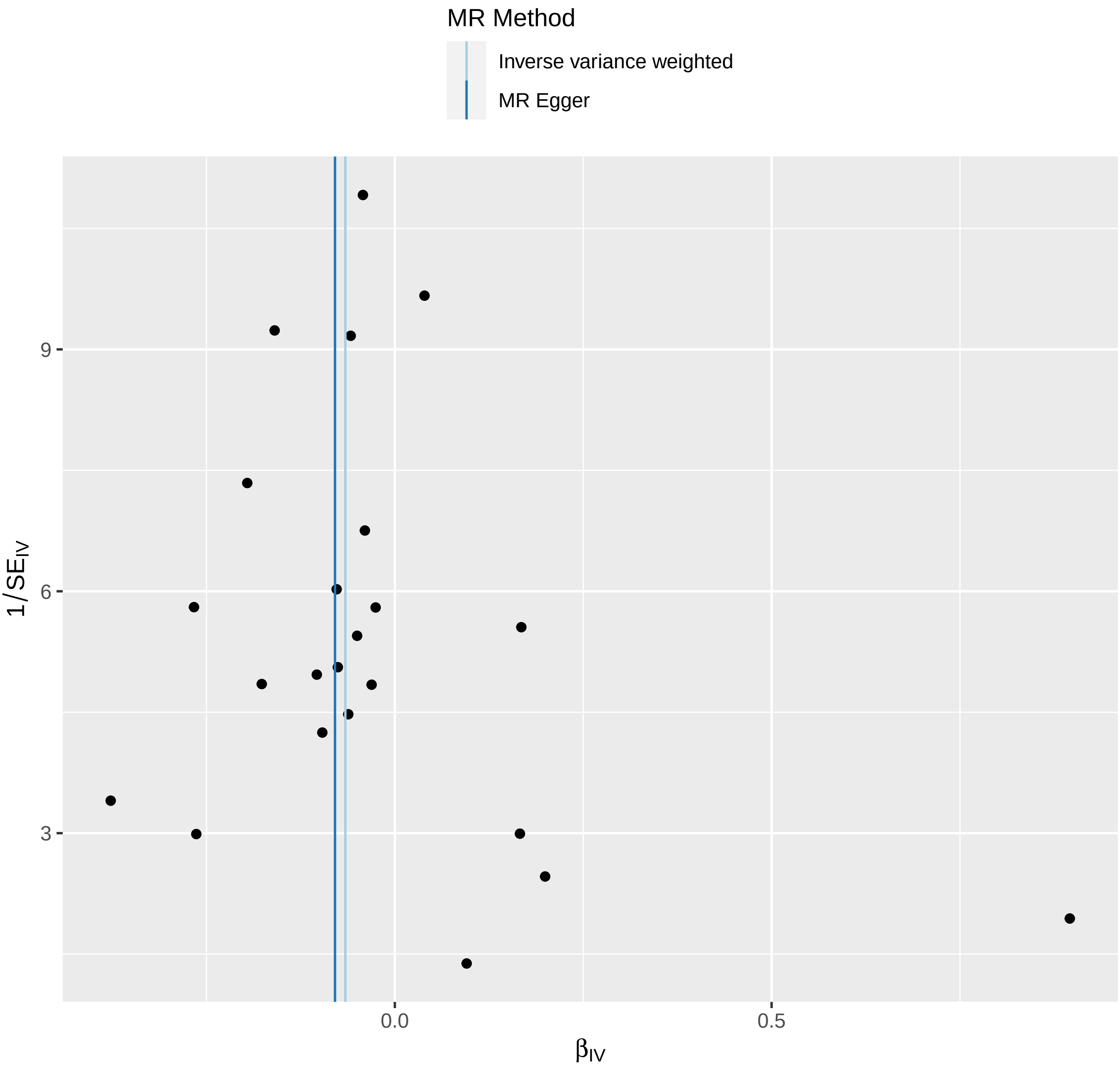

Batch 3−3267 : Zinc finger protein 276 on Diabetic nephropathy (more control exclusions)
with a threshold of 1e−05

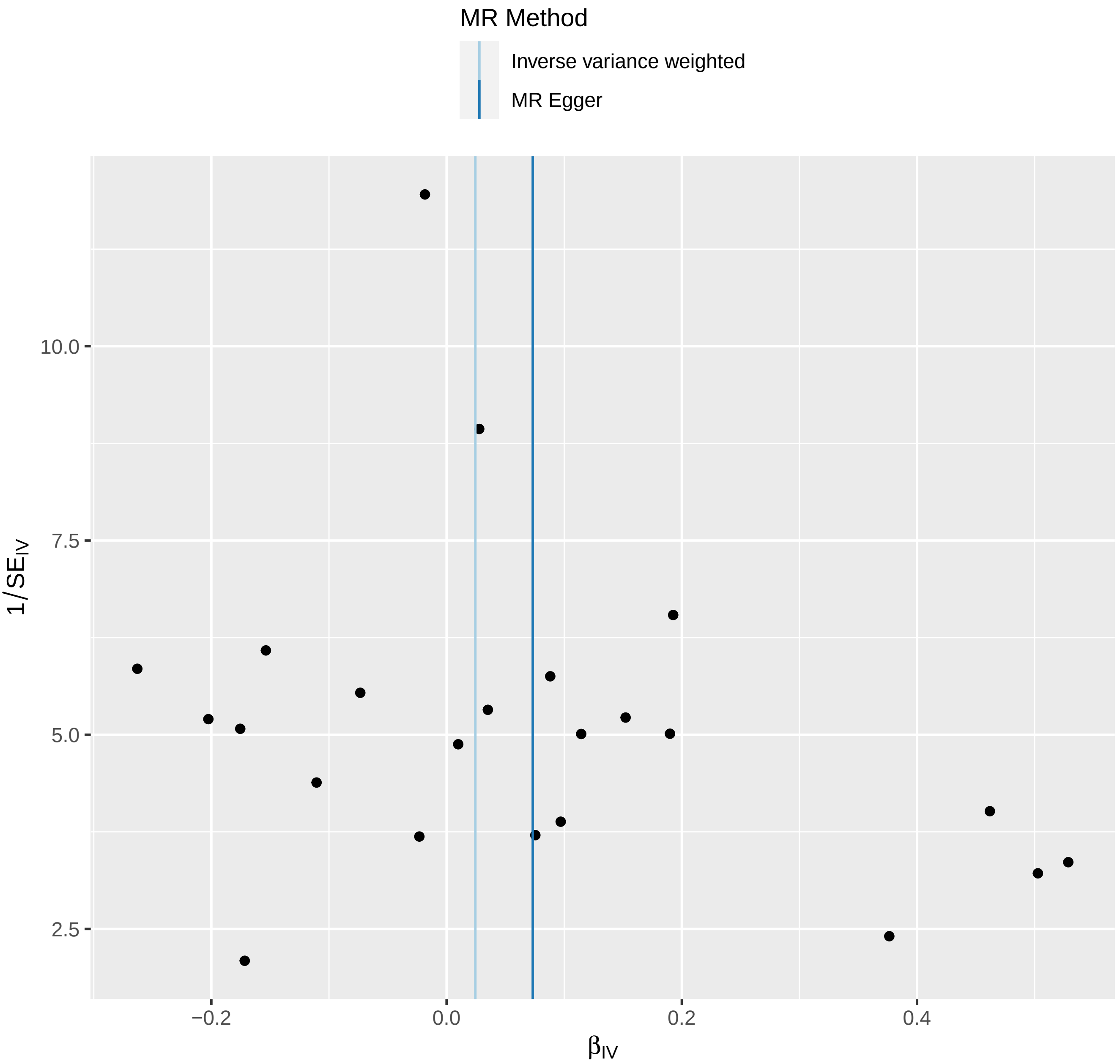

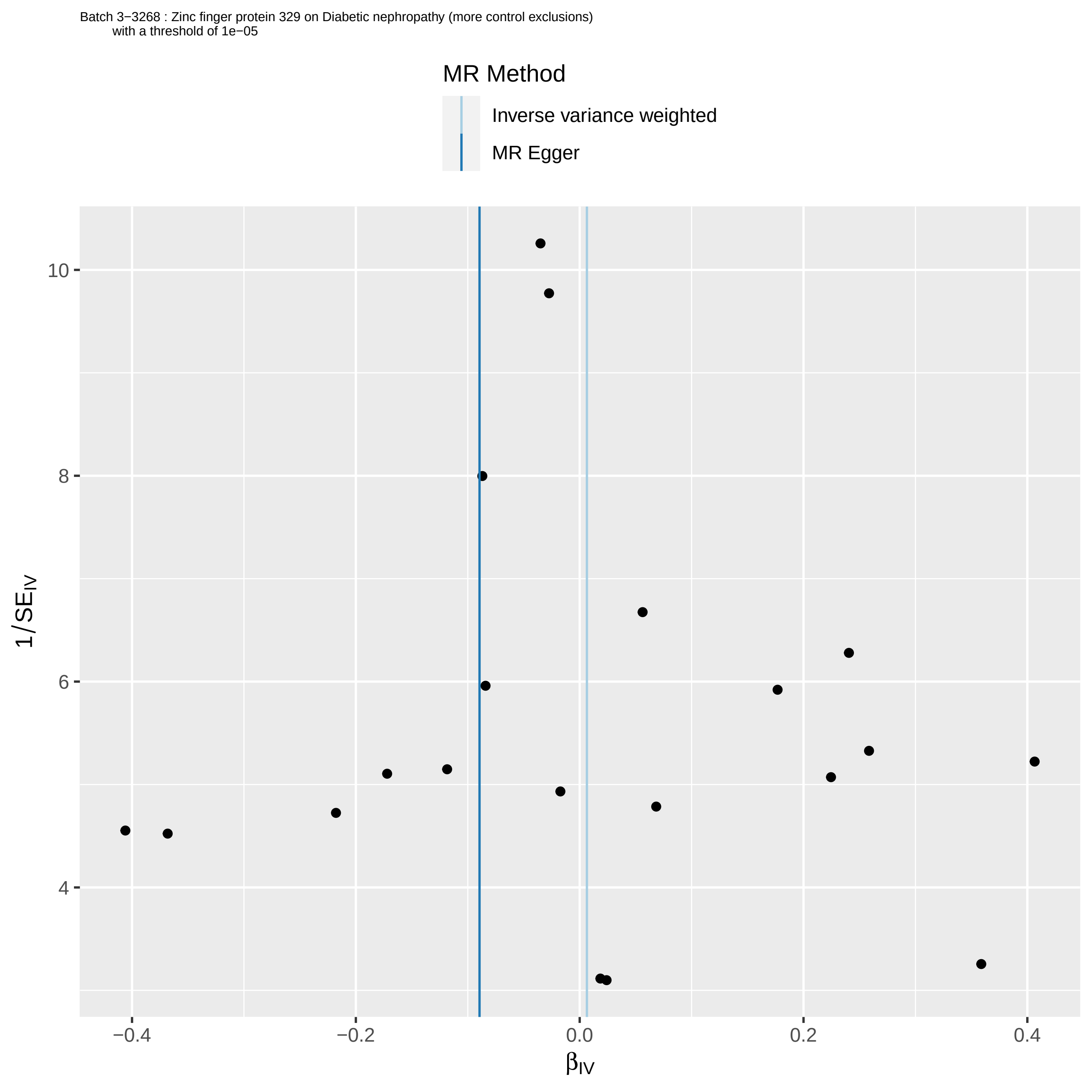
Batch 3−3268 : Zinc finger protein 329 on Diabetic nephropathy (more control exclusions)
with a threshold of 1e−05
MR Method
Inverse variance weighted
MR Egger
10
8
6
4
1/SE_IV
−0.4
−0.2
0.0
0.2
0.4
β_IV

Batch 3−3269 : Zinc finger protein 334 on Diabetic nephropathy (more control exclusions)
with a threshold of 1e−05

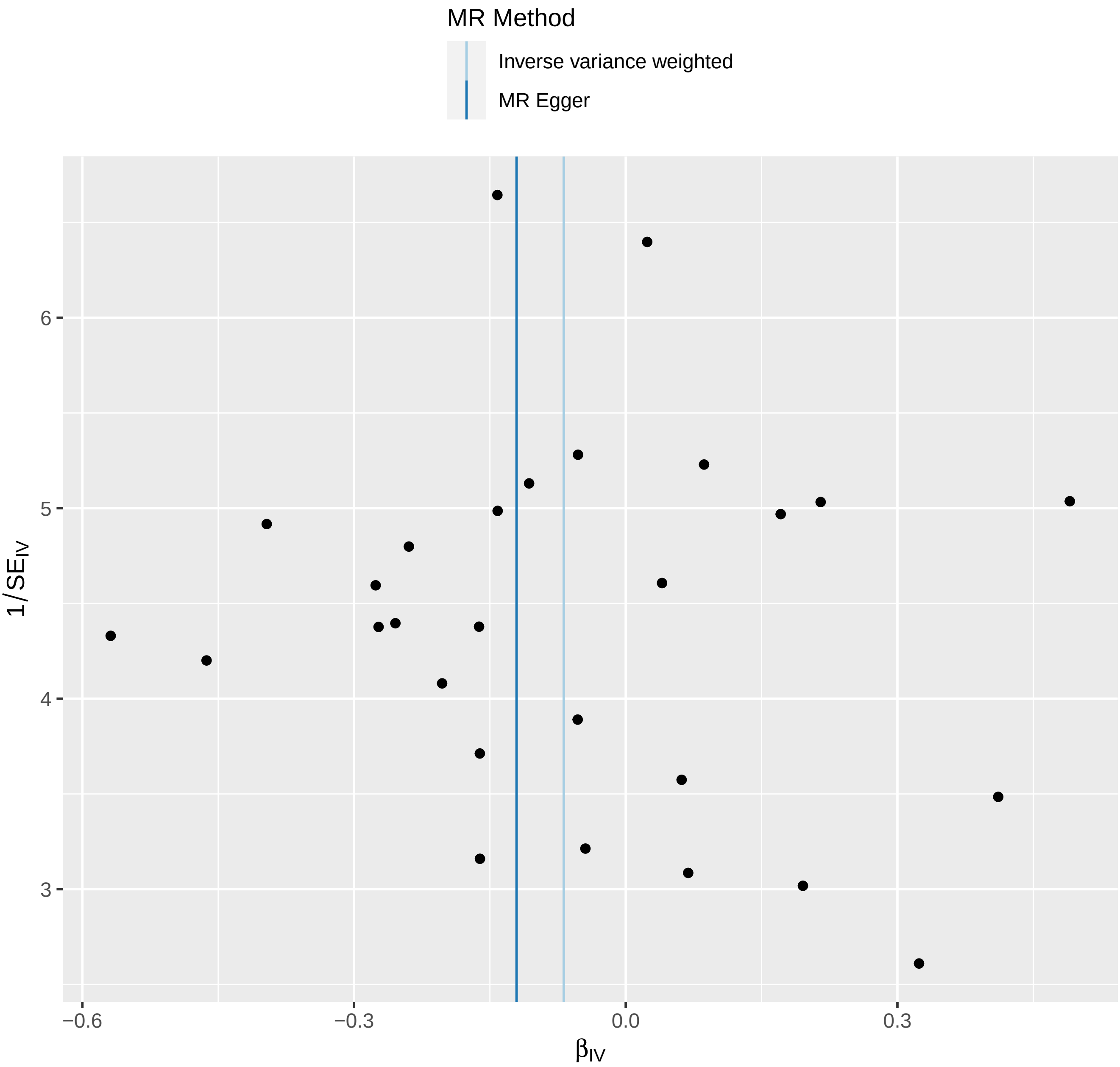

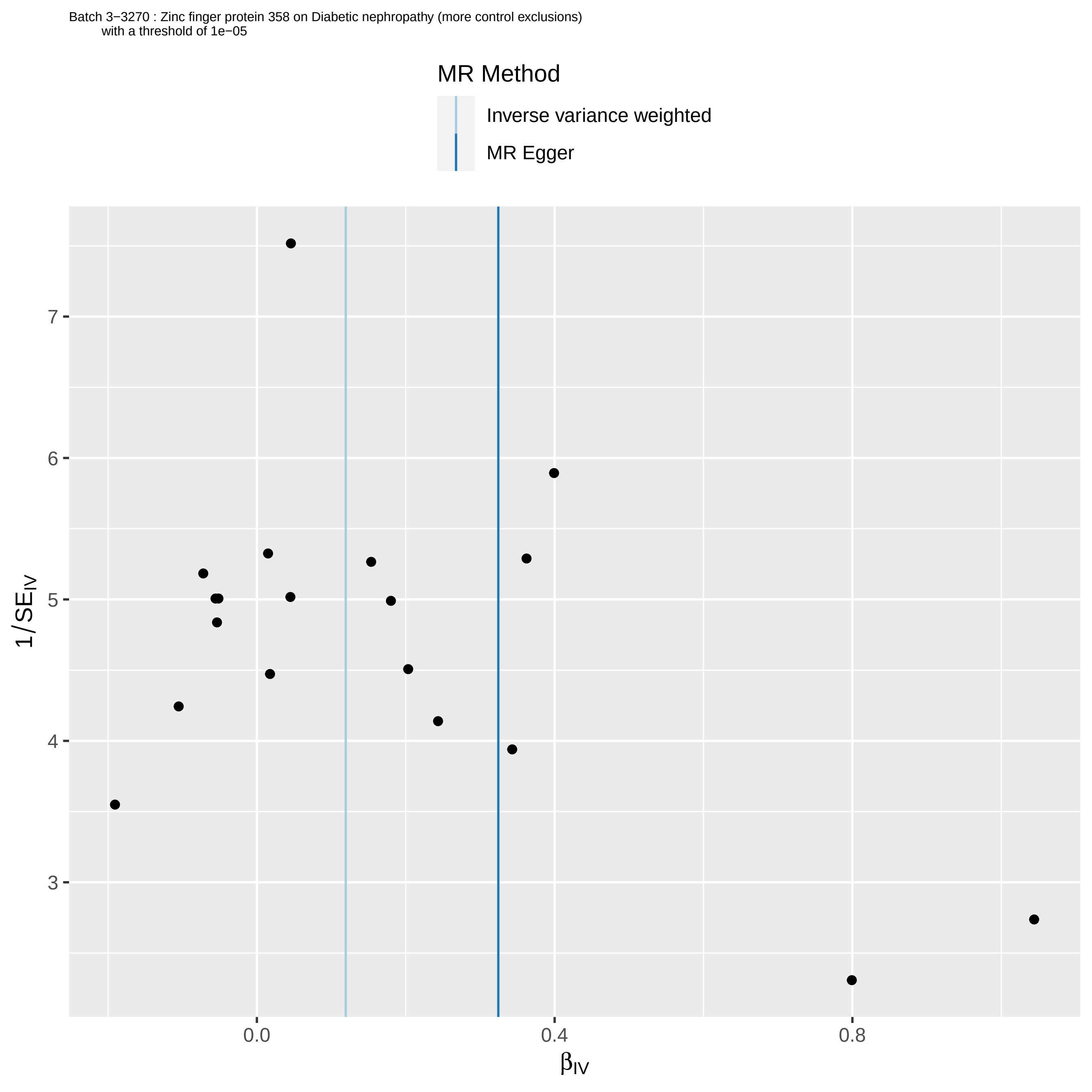
Batch 3−3270 : Zinc finger protein 358 on Diabetic nephropathy (more control exclusions)
with a threshold of 1e−05
MR Method
Inverse variance weighted
MR Egger
7
6
5
4
3
0.0
0.4
0.8
1/SE$_{IV}$
β_{IV}

Batch 3−3271 : Zinc finger protein 382 on Diabetic nephropathy (more control exclusions)
with a threshold of 1e−05

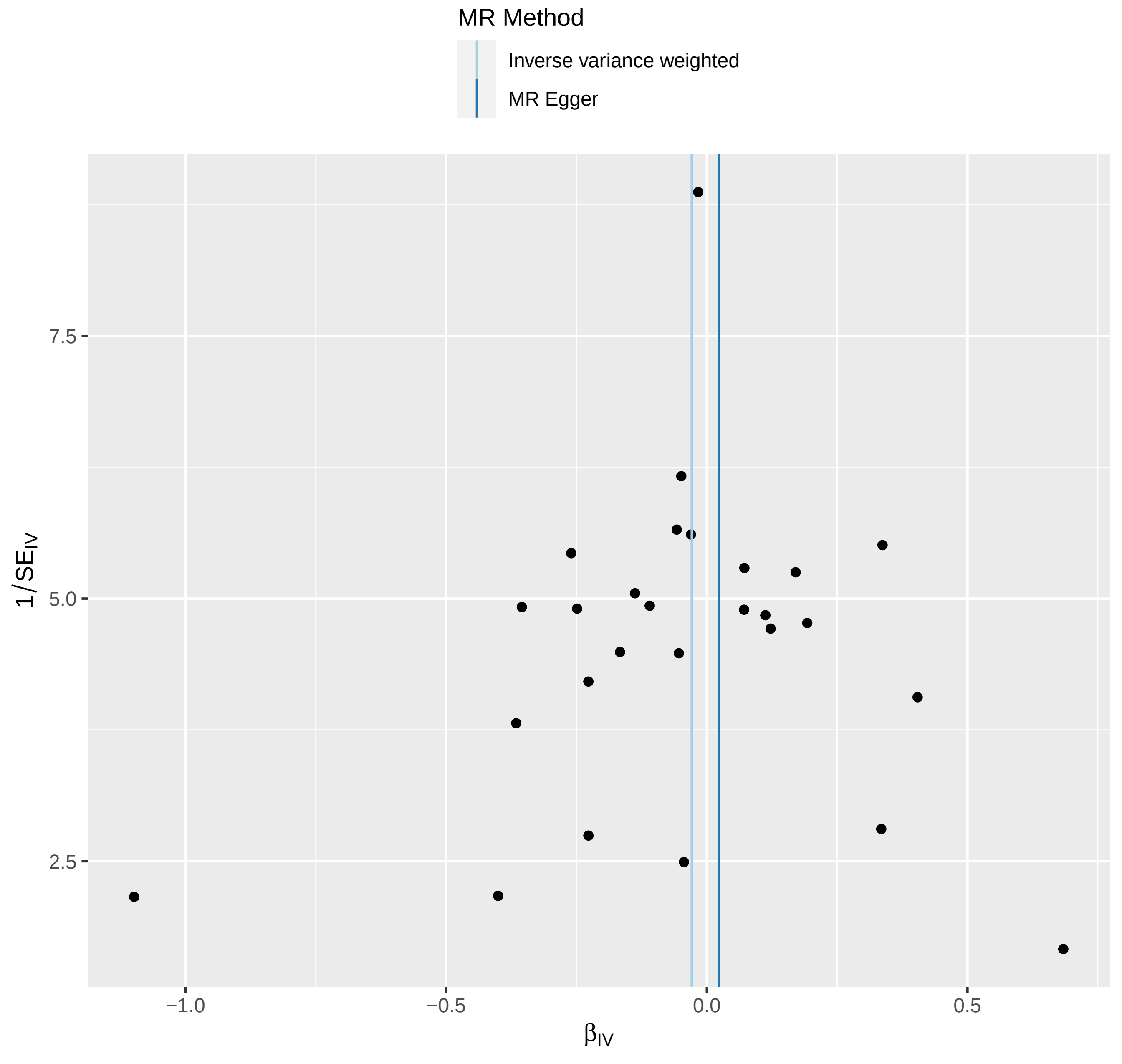

Batch 3−3272 : Zinc finger protein 410 on Diabetic nephropathy (more control exclusions)
with a threshold of 1e−05

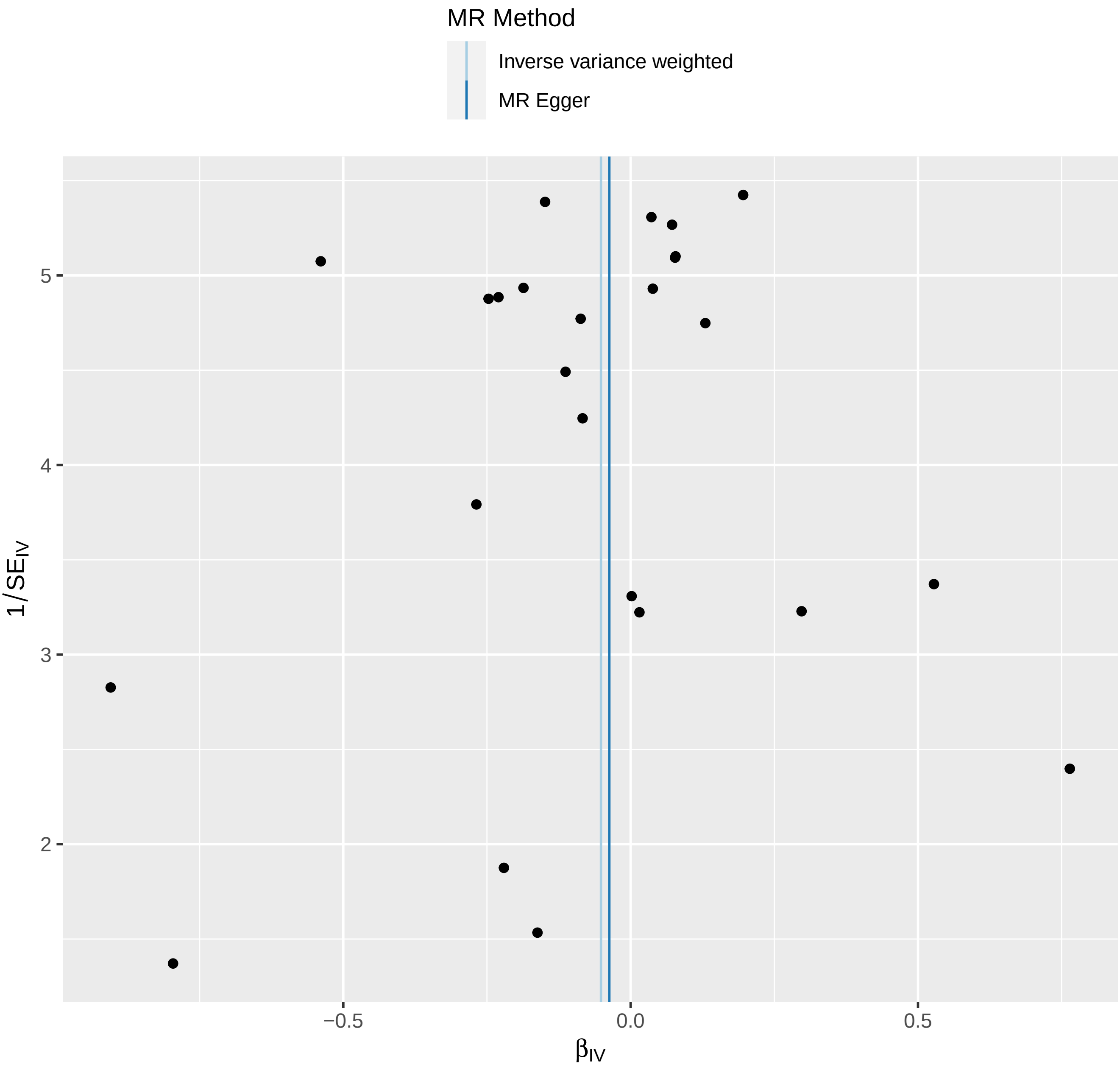

Batch 3−3273 : Zinc finger protein 415 on Diabetic nephropathy (more control exclusions)
with a threshold of 1e−05

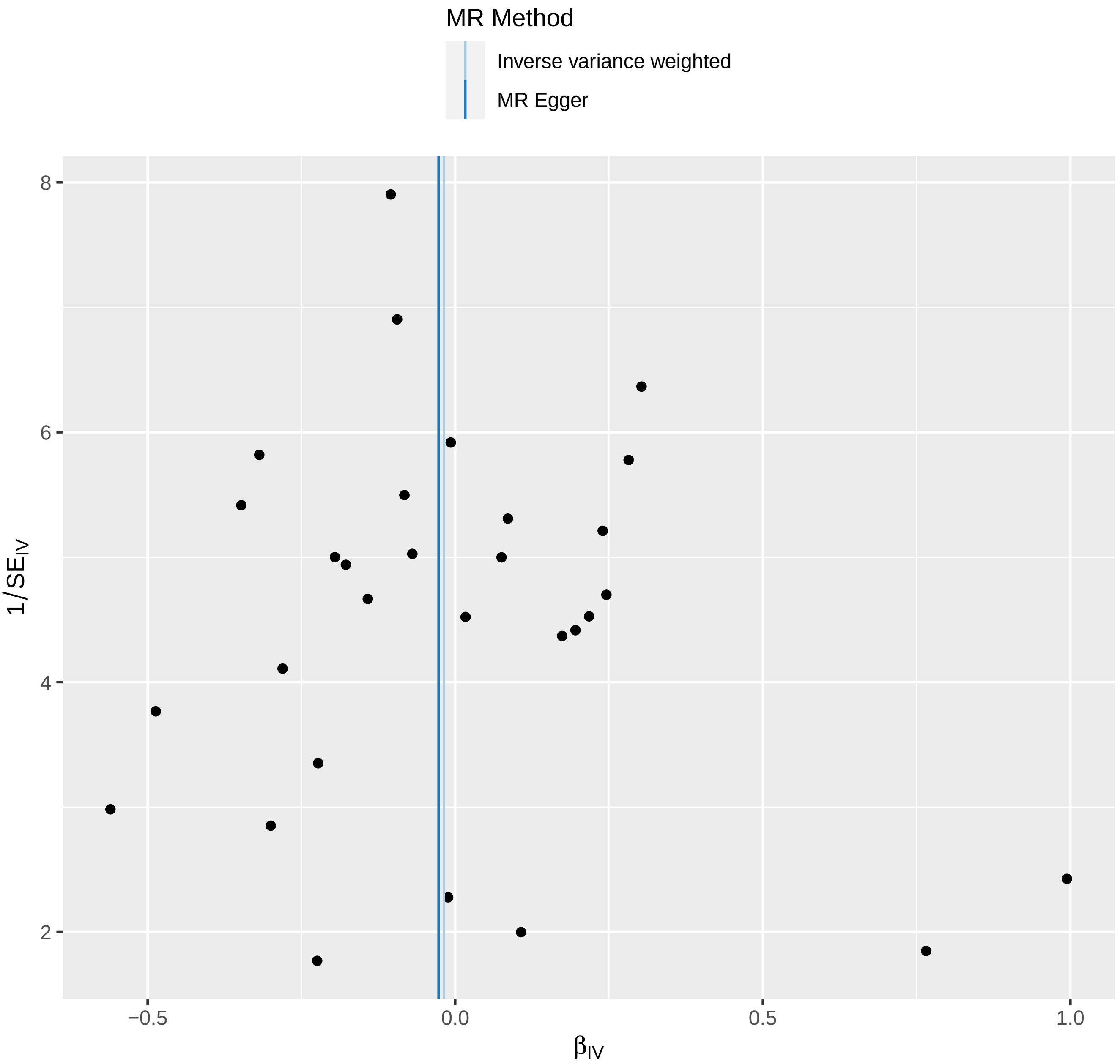

Batch 3−3274 : Zinc finger protein 566 on Diabetic nephropathy (more control exclusions)
with a threshold of 1e−05

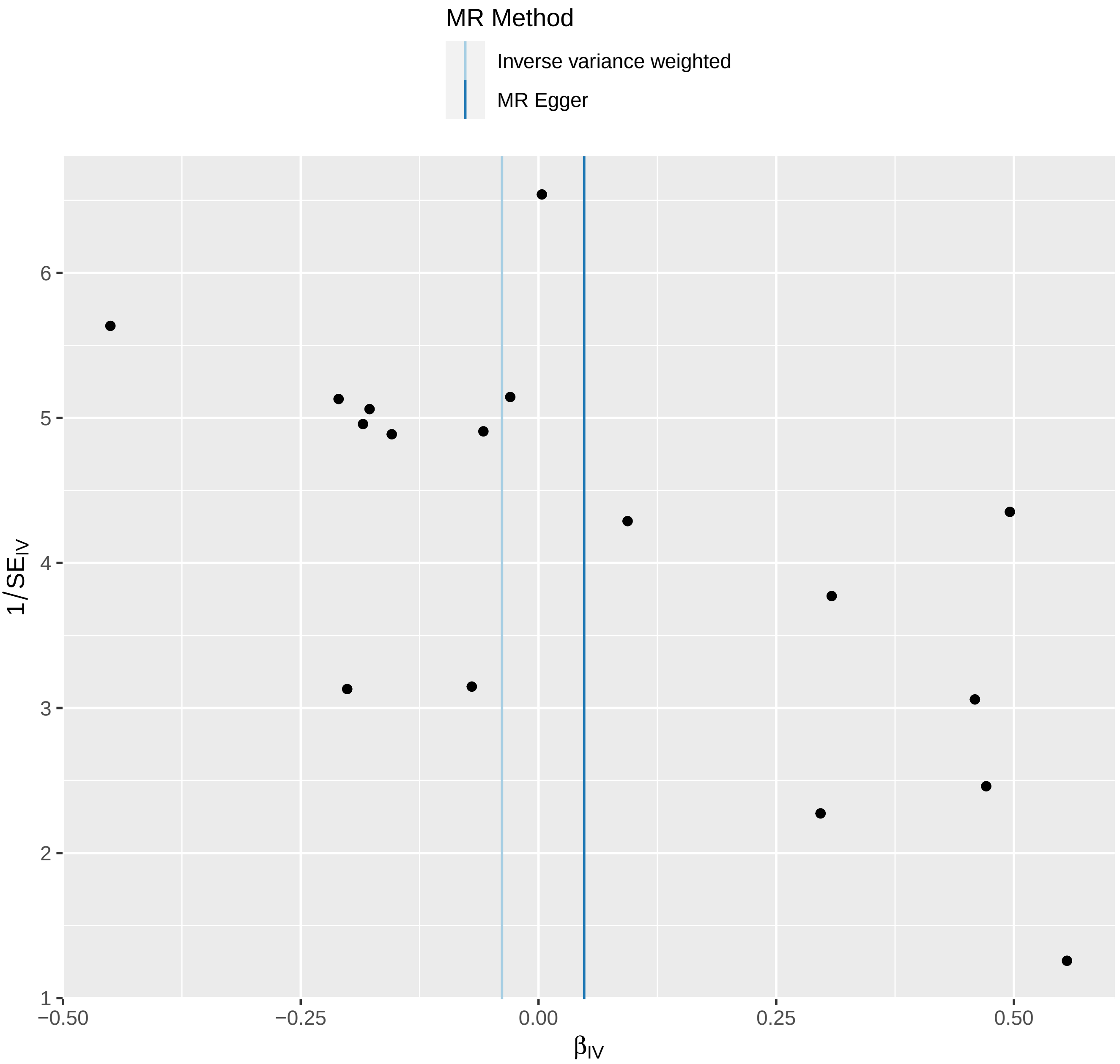

Batch 3−3275 : Zinc finger protein 75D on Diabetic nephropathy (more control exclusions)
with a threshold of 1e−05

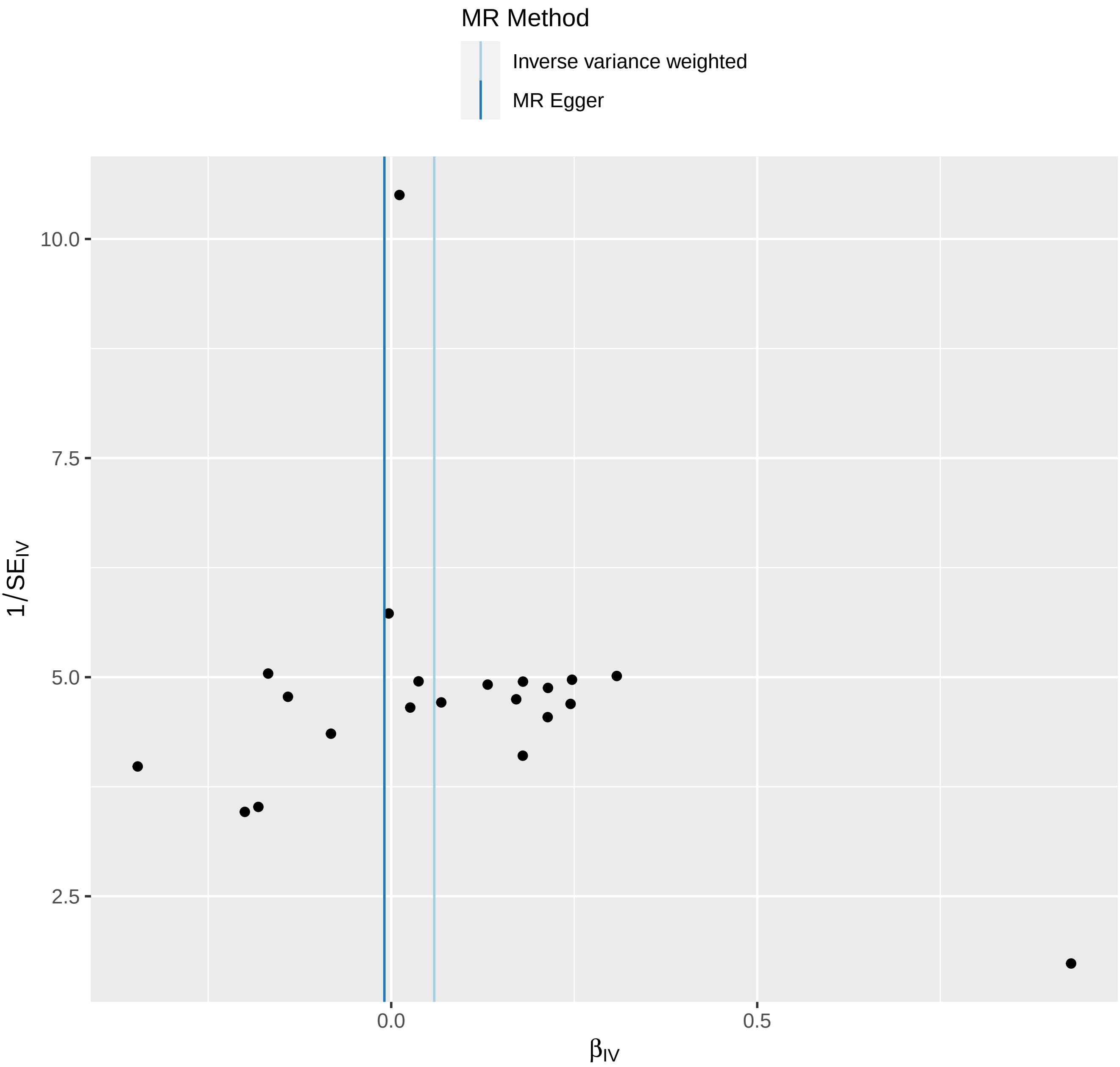

Batch 3−3276 : Zinc finger protein 774 on Diabetic nephropathy (more control exclusions)
with a threshold of 1e−05

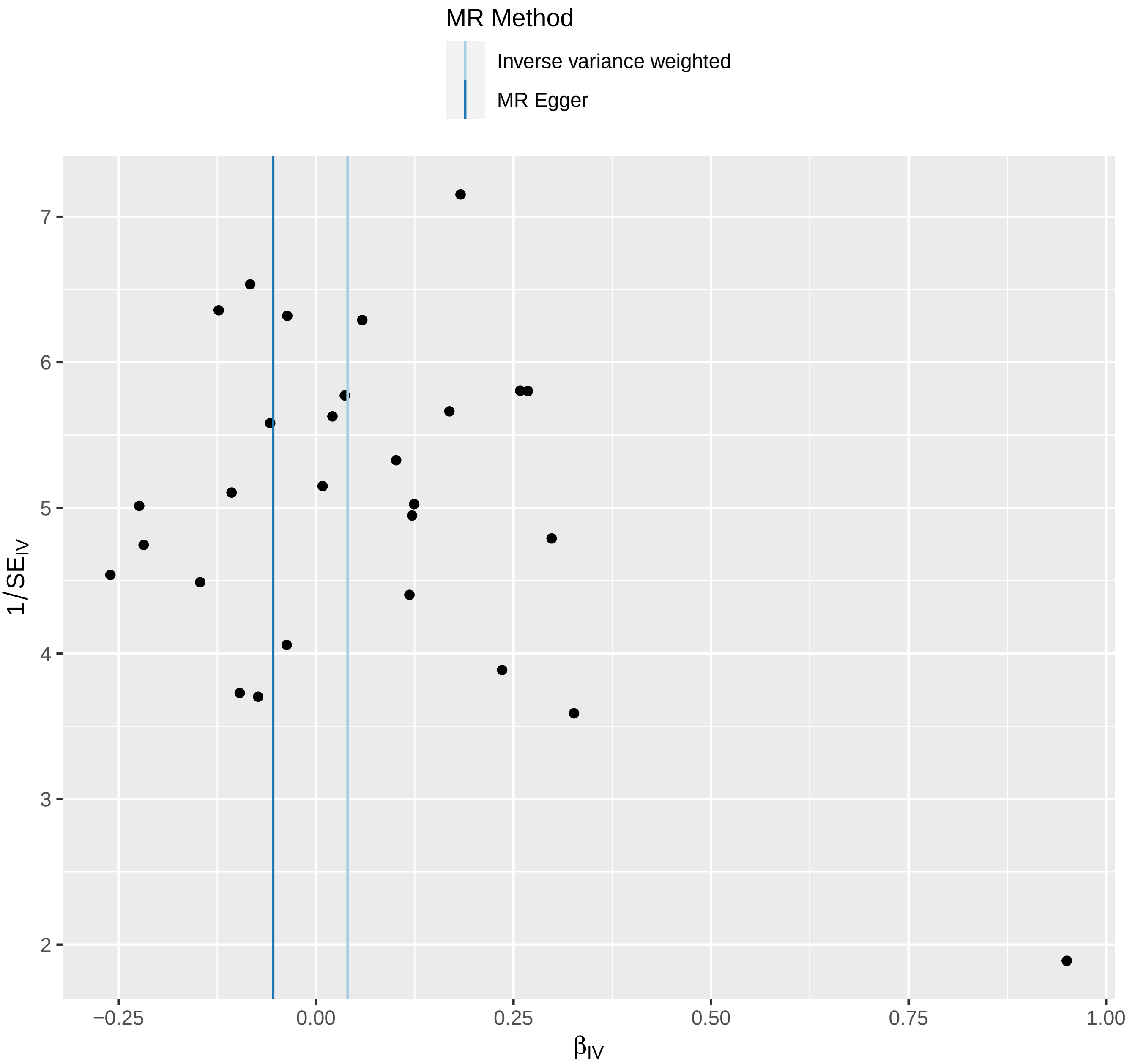

Batch 3−3277 : Zinc finger protein 843 on Diabetic nephropathy (more control exclusions)
with a threshold of 1e−05

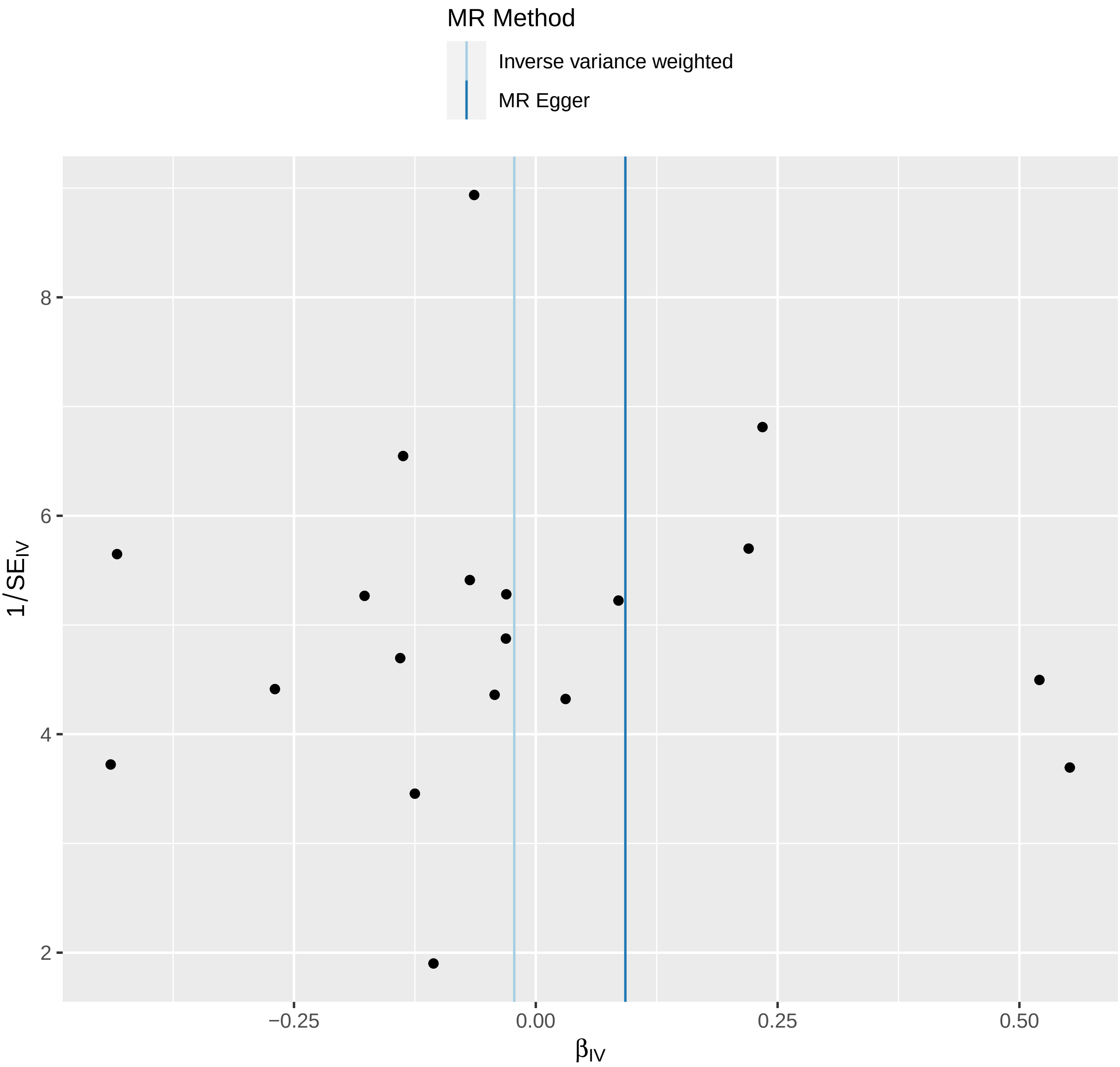

Batch 3−3278 : E3 ubiquitin−protein ligase ZNRF3 on Diabetic nephropathy (more control exclusions)
with a threshold of 1e−05

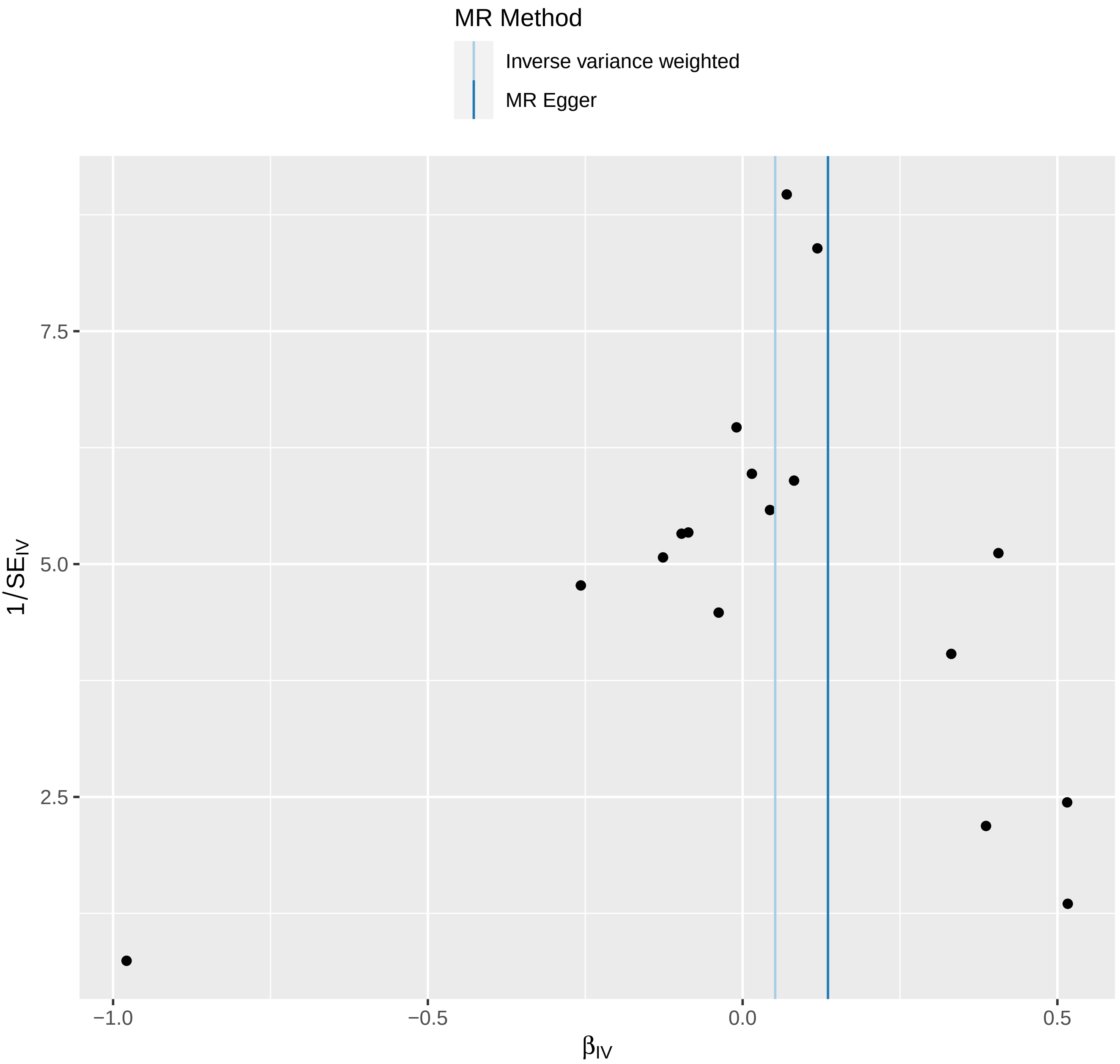

Batch 3−3279 : E3 ubiquitin−protein ligase ZNRF3 on Diabetic nephropathy (more control exclusions)
with a threshold of 1e−05

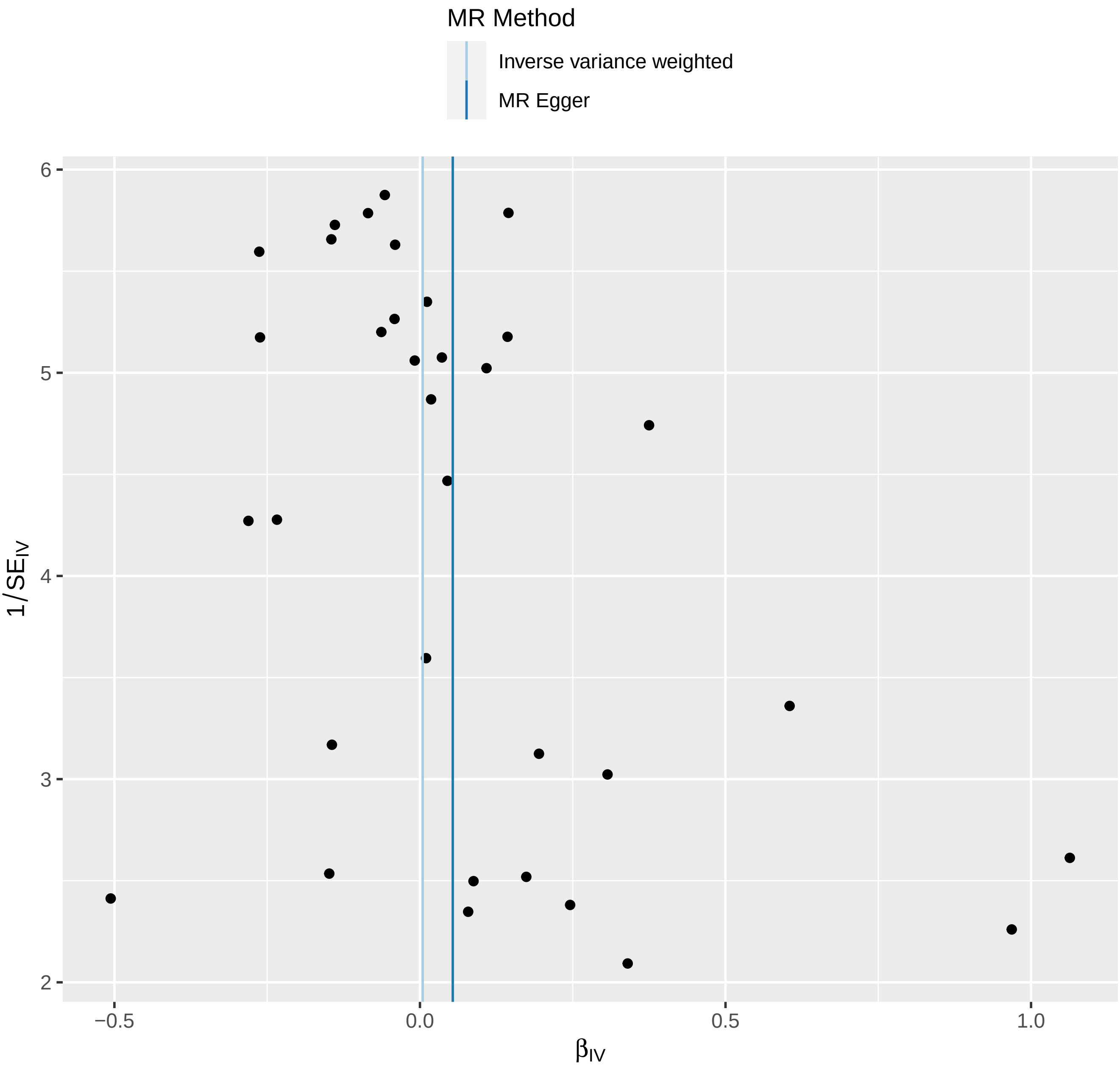

Batch 3−3280 : Zinc/RING finger protein 4 on Diabetic nephropathy (more control exclusions)
with a threshold of 1e−05

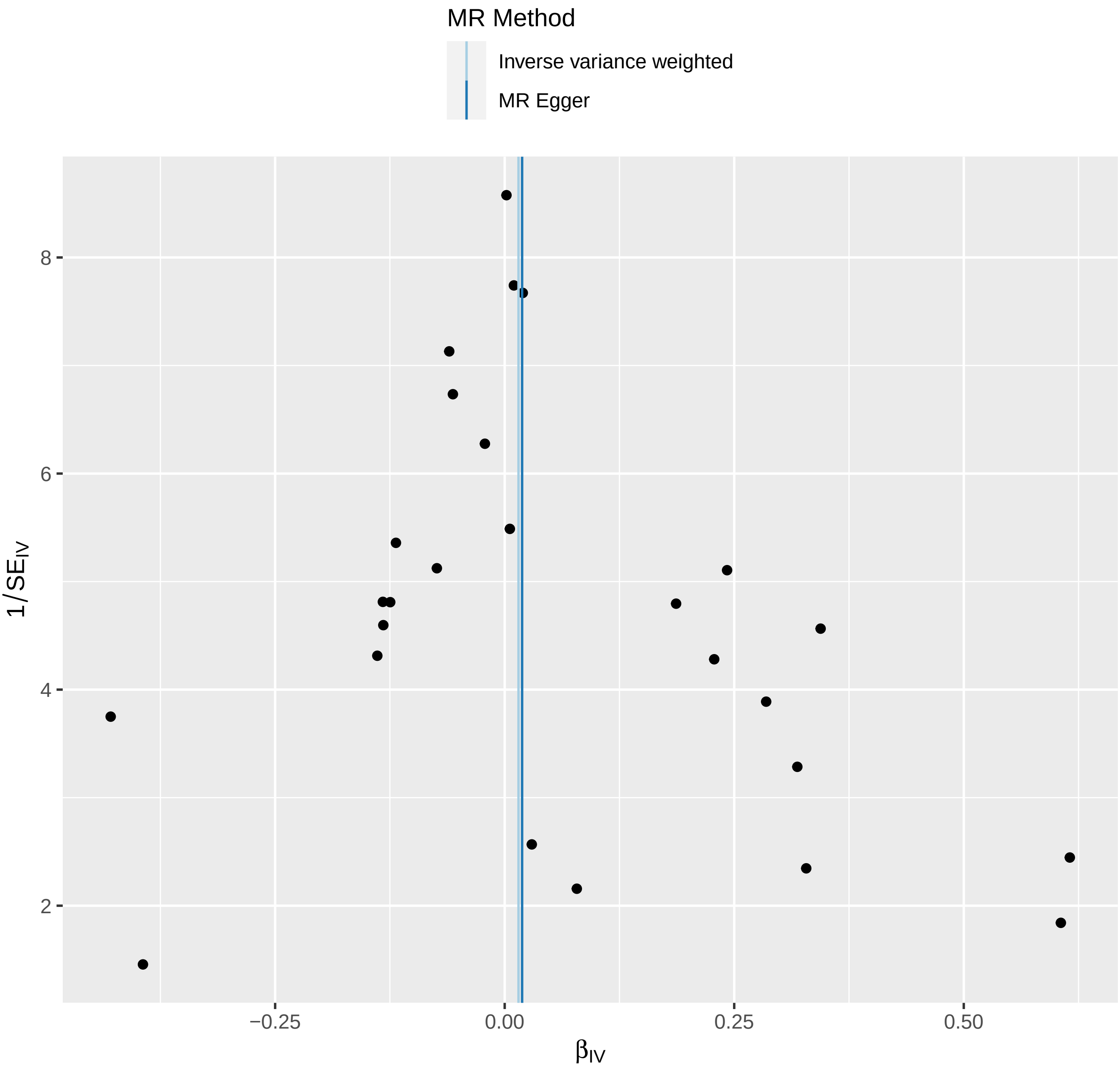

Batch 3−3281 : Zona pellucida sperm−binding protein 4 on Diabetic nephropathy (more control exclusions) with a threshold of 1e−05

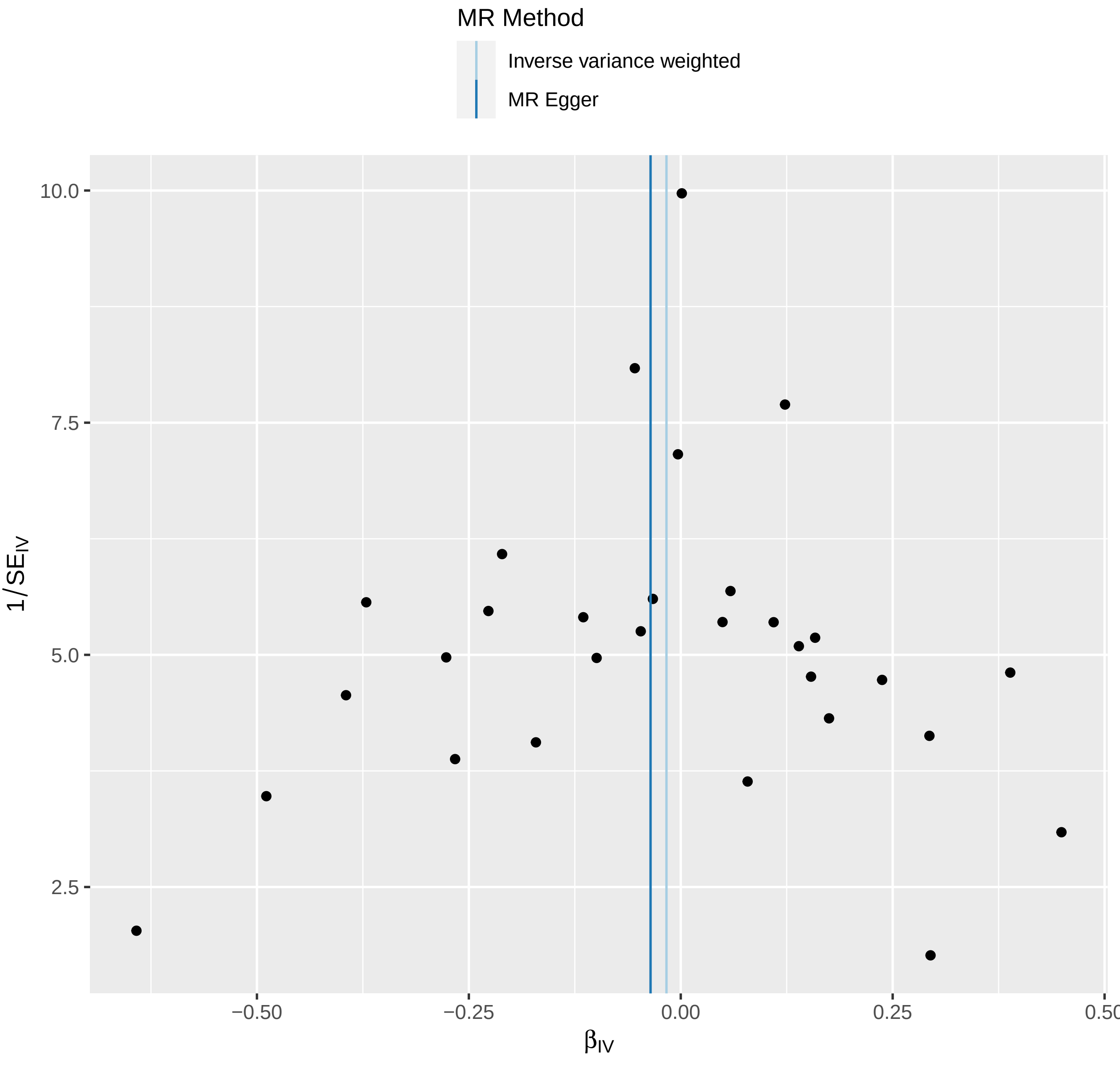

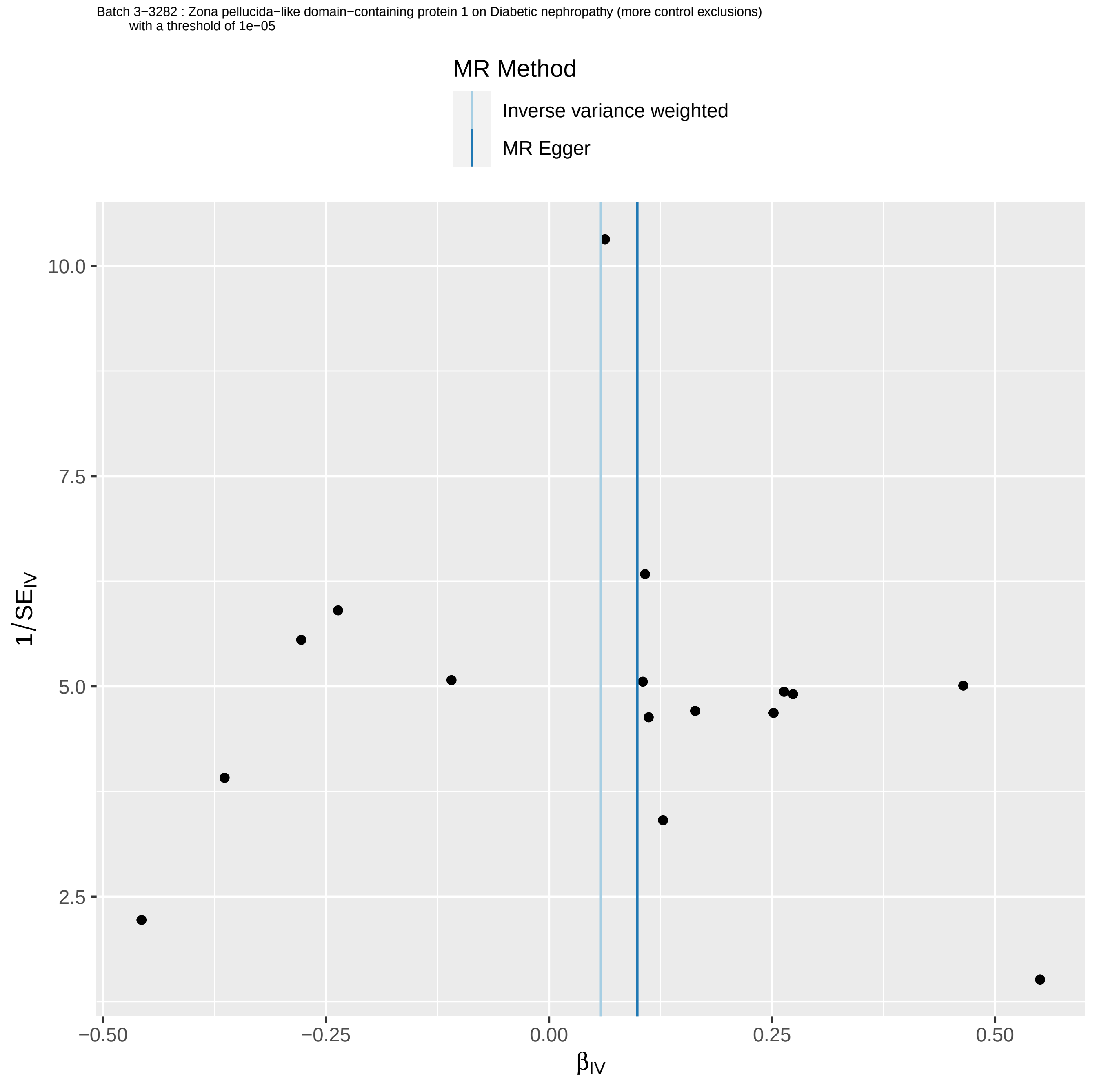

Batch 3−3282 : Zona pellucida−like domain−containing protein 1 on Diabetic nephropathy (more control exclusions)
with a threshold of 1e−05
MR Method
Inverse variance weighted
MR Egger
10.0
7.5
5.0
2.5
1/SE_IV
−0.50
−0.25
0.00
0.25
0.50
β_IV
